# Functional involvement of septal miR-132 in extinction and oxytocin-mediated reversal of social fear

**DOI:** 10.1038/s41380-023-02309-3

**Published:** 2023-11-08

**Authors:** Anna Bludau, Uwe Schwartz, Daniela M. Zeitler, Melanie Royer, Gunter Meister, Inga D. Neumann, Rohit Menon

**Affiliations:** 1 Department of Behavioral and Molecular Neurobiology, University of Regensburg, Regensburg, Germany; 2 NGS Analysis Center, Biology and Pre-Clinical Medicine, University of Regensburg, Regensburg, Germany; 3 Regensburg Center for Biochemistry, Laboratory of RNA Biology, University of Regensburg, Regensburg, Germany

**Keywords:** Neuroscience, Molecular biology

## Abstract

Social interactions are critical for mammalian survival and evolution. Dysregulation of social behavior often leads to psychopathologies such as social anxiety disorder, denoted by intense fear and avoidance of social situations. Using the social fear conditioning (SFC) paradigm, we analyzed expression levels of miR-132-3p and miR-124-3p within the septum, a brain region essential for social preference and avoidance behavior, after acquisition and extinction of social fear. Here, we found that SFC dynamically altered both microRNAs. Functional in vivo approaches using pharmacological strategies, inhibition of miR-132-3p, viral overexpression of miR-132-3p, and shRNA-mediated knockdown of miR-132-3p specifically within oxytocin receptor-positive neurons confirmed septal miR-132-3p to be critically involved not only in social fear extinction, but also in oxytocin-induced reversal of social fear. Moreover, Argonaute-RNA-co-immunoprecipitation-microarray analysis and further in vitro and in vivo quantification of target mRNA and protein, revealed growth differentiation factor-5 (Gdf-5) as a target of miR-132-3p. Septal application of GDF-5 impaired social fear extinction suggesting its functional involvement in the reversal of social fear. In summary, we show that septal miR-132-3p and its downstream target Gdf-5 regulate social fear expression and potentially mediate oxytocin-induced reversal of social fear.

## Introduction

Whether appetitive or aversive, social interactions are an indispensable component of our lives. Thus, dysregulation of social behavior often has tremendous consequences during an individual’s life and is a symptom of various psychopathologies. Social anxiety disorder (SAD) is characterized by intense fear and avoidance of social situations. With a lifetime prevalence of 8 to 15% [1] it is deemed a major health concern. The lack of appropriate animal models contributes to limited effective, efficient, and targeted treatment options for SAD [2]. The social fear conditioning (SFC) paradigm generates robust fear and avoidance of same-sex conspecifics in mice [3–5]. The neuropeptide oxytocin (OXT) was found to be essential for reversing SFC-induced social fear in male and female mice, with the observed effect being localized within the lateral septum (LS) [3, 6].

Although OXT attracts enormous scientific interest due to its capacity to modulate various socio-emotional behaviors [7–9], its mechanisms of action at the neuronal and molecular levels remain largely unknown. More specifically, the involvement of OXT in the stringent orchestration of transcriptional and post-transcriptional regulation of gene expression, which is required to shape behavioral adaptations, is still to be elucidated. Epigenetic mechanisms that intricately regulate gene expression are known to influence socio-emotional behaviors [10], including social fear [11]. The discovery of small non-coding RNAs, such as microRNAs (miRNAs), has extensively expanded our understanding of the cellular mechanisms used to regulate gene expression, which majorly contribute to socio-behavioral adaptations on the epigenetic level. miRNAs are small (~22 nucleotides) non-coding RNAs that bind to a member of the Argonaute protein family (Ago proteins) and post-transcriptionally regulate gene expression by binding to target mRNAs, thereby initiating mRNA decay or reducing translational efficiency [12].

Among miRNAs, miR-132-3p is highly expressed within neurons of the rodent brain and exhibits a rostro-caudal gradient, with the highest expression observed in the forebrain and the lowest in the cerebellum [13]. miR-132 has repeatedly been cited as a crucial regulator of synaptic plasticity and cognitive functions, such as learning and memory [14, 15]. For instance, hippocampal miR-132 increased after mice were exposed to the Barnes maze, novel object recognition test [16], or contextual [17] and trace [18] fear conditioning. Furthermore, miR-132 is required for fear memory acquisition, as its adeno-associated virus (AAV)-mediated inhibition led to impaired storage of temporally associated information [18]. Although miR-132 is the most-studied miRNA in the context of synaptic plasticity and higher cognitive functions, recent studies underscored the pivotal role of other miRNAs in these processes. For instance, the regulatory function of miR-124 in memory formation appears to be evolutionary highly conserved [19]. Reduction of hippocampal miR-124 via a locked nucleic acid (LNA) reversed the observed impairments in spatial memory, long-term potentiation, and social interaction in adult mice carrying a null mutation for the exchange protein directly activated by cAMP (EPAC) [20]. In contrast, the overexpression of hippocampal miR-124 mimicked the electrophysiological and behavioral phenotype of EPAC-deficient mice, highlighting a direct regulation of memory functions and social behavior by miR-124. In a frontotemporal dementia mouse model, socio-behavioral impairments have been associated with decreased miR-124 expression and concomitant increase of Gria2-4, leading to the imbalance of Ca^2+^-permeable and Ca^2+^-impermeable AMPA receptors in the prefrontal cortex [21]. Additionally, consistent alterations of miR-124 and AMPA receptor subunits have been found in the frontal cortex of frontotemporal dementia patients. Thus, different molecular and plasticity-related mechanisms are likely involved in the fine-tuned effects of miR-132 or miR-124 on social behavior, learning, and memory processes.

In this study, we demonstrate in male mice that expression of both miR-132 and miR-124 is dynamically altered in response to social fear acquisition (Acq) and extinction (Ext) in the septum – a brain region importantly involved in socio-emotional behavior regulation. We further show that septal miR-132 is functionally involved in social fear extinction and impairs the social fear-reversing effect of OXT. Expression analysis using Argonaute-RNA-immunoprecipitation microarray (Ago2-IP), PCR array, and in vitro inhibition of miR-132-3p revealed growth differentiation factor-5 (GDF-5) to be a target of miR-132-3p. Further pharmacological approaches demonstrated that GDF-5, possibly regulated by miR-132-3p, is involved in social fear extinction.

## Material and methods

### Animals and husbandry

Male CD1 (Charles River, Sulzfeld, Germany) or OXT receptor-Cre mice (OXTR-Cre; CD1 background; bred at University of Regensburg, Germany [22]), both 8–9 weeks of age at the start of the experiment, were group-housed under standard temperature- and humidity-controlled conditions with tap water and a standard laboratory diet provided *ad libitum*. Three days prior to behavioral testing or immediately after surgical procedures, animals were single-housed. Age and weight-matched unfamiliar male CD1 mice were used as social stimuli in the SFC paradigm. All behavioral procedures were performed between 08:00 and 12:00 in accordance with the Guide for the Care and Use of Laboratory Animals of the Local Government of Unterfranken, the ARRIVE guidelines [23], and recommendations from the NIH. Animals were randomly assigned to experimental groups.

### Social fear conditioning (SFC)

**Fig. 1:**
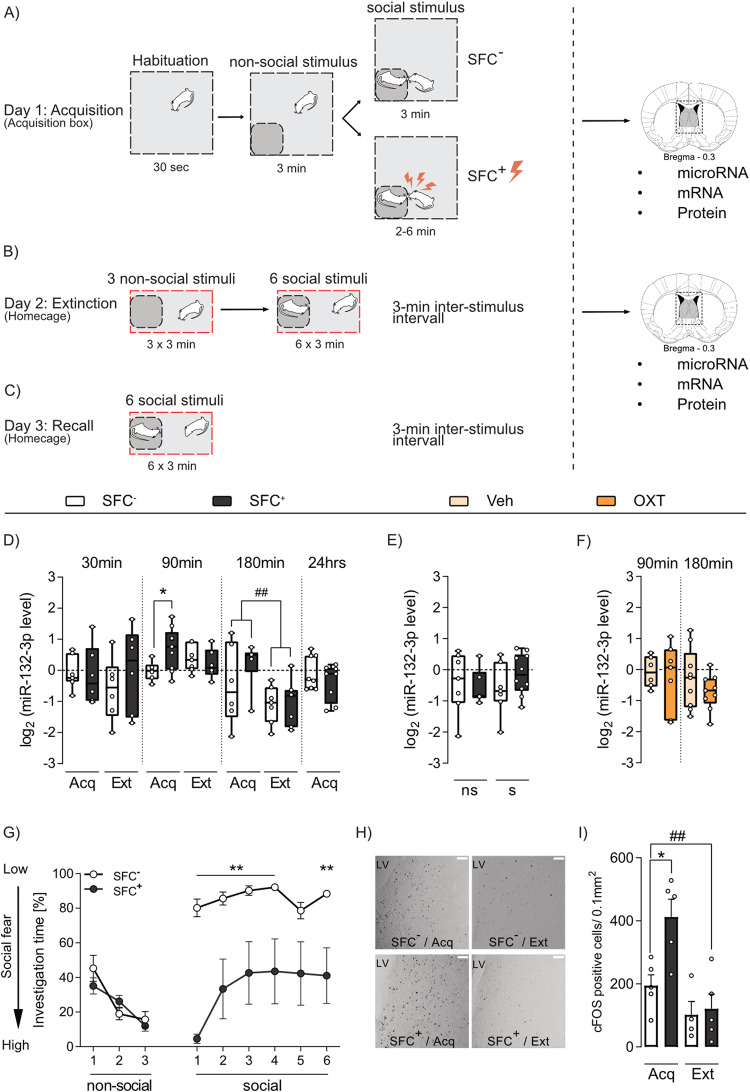
Social fear conditioning (SFC) results in dynamic changes of septal miR-132-3p and neuronal activation in male mice. **A**–**C** Schematic representation of the SFC paradigm. **A** During acquisition (Acq) on day 1, social fear-conditioned mice (SFC^+^) received a mild foot shock when sniffing an unknown same-sex, same-age conspecific to induce robust social fear, whereas unconditioned (SFC^−^) mice freely investigated the social stimulus. For the analysis of microRNA, mRNA and protein, brains were removed either 30 min, 90 min, 180 min or 24 h after Acq. **B** On the second day during extinction (Ext), SFC^+^ and SFC^-^ mice were exposed to 3 non-social stimuli (wire-mesh cages) and 6 unknown social stimuli (wire-mesh cages containing an unknown conspecific) for 3 min each with a 3-min inter-stimulus interval. Here, SFC^−^ mice displayed high investigation times of all six social stimuli, whereas SFC^+^ mice initially displayed low investigation times, which gradually increased from exposure of the first to the sixth social stimulus. In SFC^+^ mice, significant low social investigation during exposure to the first social stimulus indicated social fear, whereas high investigation of the sixth social stimulus reflected successful fear extinction. For the analysis of microRNA, mRNA and protein brains were removed at either 30 min, 90 min or 180 min after Ext. **C** During recall on day 3, mice were exposed to 6 unknown social stimuli. **D** Relative septal miR-132-3p level 30 min, 90 min, 180 min, and 24 h after Acq and Ext of social fear in SFC^−^ and SFC^+^ mice. ^##^*p* < 0.01; *p < 0.05. **E** Relative septal miR-132-3p transcript level of SFC^−^ and SFC^+^ mice 90 min after repeated exposure to non-social (ns) and social (s) stimuli. **F** Relative septal miR-132-3p transcript level 90 min and 180 min after icv application of oxytocin (OXT; 0.1 µg/2 µl) or vehicle (Veh; Ringer solution). **G** Percentage of time investigating the presented non-social and social stimuli during social fear extinction in SFC^−^ and SFC^+^ mice used for analysis of septal c-Fos expression. **p < 0.05 SFC^+^ vs SFC^−^. **H** Representative pictures of c-Fos immunohistochemistry in SFC^−^ and SFC^+^ male mice 90 min after Acq or Ext of social fear. Scale bar represents 100 µm; LV: lateral ventricle. **I** Number of c-Fos positive cells /0.1 mm^2^ within the dorsolateral septum of SFC^−^ and SFC^+^ mice after Acq or Ext of social fear. ^##^*p* < 0.01; *p < 0.05. **G**, **I** data represent mean + SEM; **A**–**C** *n* = 6–10/group; **D**, **F** *n* = 4–5/group.

The SFC paradigm was performed as previously described [3, 4]. During social fear acquisition (Acq; day 1), application of a mild foot shock (0.7mA, 1 sec) was the unconditioned stimulus (US), whereas the conspecific placed in a small wire-mesh cage was the conditioned stimulus (CS). For details, please refer to Fig. 1A–C. During social fear extinction training (Ext; day 2) and recall (day 3), the investigation of individual non-social and social stimuli (each placed in a small wire-mesh cage) by conditioned (SFC^+^) or unconditioned (SFC^−^) mice was manually scored by an observer blind to treatment and is shown as a percentage of time spent in direct contact. For convenience, the process of extinction training is termed as extinction also for SFC^−^ mice, which underwent the exposure to the 3 non-social and 6 social stimuli in the same manner although not being conditioned. Additional mice were exposed to 9 non-social stimuli to control for the effects of repeated social vs non-social interaction on miRNA levels.

### General anxiety-related behavior and locomotion

General anxiety-related behavior and locomotion were scored in the open field/novel object (OF/NO) test [24] using EthoVision XT (Noldus, Wageningen, The Netherlands).

### Stereotactic guide cannula implantation and substance infusion

Guide cannula implantation for subsequent intracerebroventricular (icv) or bilateral infusions into the LS [25] was performed as previously described (OXT: icv: 0.1µg/2µL, LS: 5ng/0.2µL/hemisphere; GDF-5: LS: 0.05µg/0.2µl/hemisphere) [6]. For details, see Supplementary Methods.

### Microinfusion to manipulate septal miR-132-3p activity or transcript level

To inhibit septal miR-132-3p function or to reduce its transcript level, locked nucleic acids (LNA) [26] or adeno-associated viruses (AAV) were used. For details, see Supplementary Methods.

Although microinfusions were performed directly within the LS, LNA and AAV transfection was found throughout the entire septum, i.e., within the LS and medial septum (Supplementary Fig. S1A).

### Cell culture

Mouse neuroblastoma Neuro-2a cells (Merck, Germany) were cultured according to the manufacturer. Cells were transfected with 3 nM of either a miR-132-3p mimic or miR-132-3p inhibitor (miRVana; Thermo Fischer) or the corresponding negative or positive (mimic: miR-1; inhibitor: let-7c) control 48 h prior to analysis or stimulation. For details on the used mirVana sequences, see Supplementary Table S8. Further, cells were stimulated with vehicle (growth medium) or OXT (250 nM) for 90 min prior to RNA isolation.

### Dissection of septal tissue

For molecular analysis, brains were dissected and flash-frozen in 2-methylbutane (Merck, Germany) either 30 min, 90 min, 180 min, or 24 h after Acq (Fig. 1A) or 30 min, 90 min or 180 min after Ext (Fig. 1B). Brains were cryo-cut, and septal tissue (approx. 1.0–0.2 mm from bregma) was punched for further RNA or protein quantification.

### RNA quantification

To quantify septal mRNA or miRNA in response to SFC Acq or Ext, RNA from individual tissue samples from the septum was isolated using peqGOLD TriFast (VWR, Radnor, USA) according to the manufacturers´ protocol. For miRNA analysis, 1000 ng of total RNA per sample was poly-A tailed (Invitrogen, Waltham, Massachusetts, USA) [27] and reverse-transcribed using Superscript IV First Strand Synthesis System (Invitrogen) according to the manufacturer´s protocols. For mRNA analysis, 1000 ng of total RNA per sample was reverse transcribed using Superscript IV First Strand System. Relative quantification of RNA levels was performed using PowerUp SYBR Green Master Mix (Thermo Fischer, Waltham, USA) and Gapdh (mRNA) or 18 S (miRNA) as a housekeeping gene (for primer sequences, see Supplementary Table S8). Primer efficiency was calculated by serial dilution using the Pfaffl method [28]. To highlight the dynamic alteration of miR-132-3p and miR-124-3p in response to SFC, their levels are additionally shown as fold-change calculated against SFC^−^/Acq samples in dependence of time (Supplementary Fig. S1E, F).

### RT^2^ Profiler PCR Array

To identify miR-132-3p target mRNAs, a customized RT^2^ Profiler PCR Array (Qiagen) was used according to the manufacturer´s protocol. mRNAs were normalized against the geometric mean of 5 housekeeping genes and are shown as fold-change calculated against SFC^−^/Acq samples. Raw data, fold-change (vs SFC^−^/Acq), p-values, and adjusted p-values can be found in Supplementary Table S4.

### c-Fos immunohistochemistry

To assess cellular activity specifically within the dorsal LS in response to SFC, c-Fos immunoreactivity was analyzed as described previously [3]. For details, see Supplementary Methods.

### Ago2-IP analysis

To determine miR-132-3p targets, septal miR-132-3p was inhibited (infusion of Inh-LNA or Scr-LNA) in mice, and tissue was punched 2 days thereafter. Septal Ago2 was pulled down by means of Argonaute-co-immunoprecipitation (6F4 hybridoma, anti-mAGO2 antibody [29]) using protein G beads. Further processing of associated mRNAs was performed at the Affymetrix Service Provider and Core Facility “KFB – Center of Excellence for Fluorescent Bioanalytics” (http://www.kfb-regensburg.de/; Regensburg, Germany) using the Affymetrix Clariome S mouse array. Microarray analysis was conducted as described in the Bioconductor tutorial [30] using the oligo package [31]. Briefly, probe intensities were normalized using the RMA function and only probes exhibiting intensities greater than 5 in at least two IP samples were kept for further analysis. Microarray data is shown as fold-change calculated against the respective input sample. Microarray data has been deposited in the GEO database (ID code GSE211449) and can be found in Supplementary Tables S12–S14; the used R code is available at Github (https://github.com/uschwartz/miR132_in_social_fear-).

### Protein quantification

To confirm septal GDF-5 alterations in response to SFC Acq and Ext, protein was isolated from septal tissue using RIPA Buffer (Sigma-Aldrich, St. Louis, Missouri, USA) according to the manufacturer´s protocol. 30 µg of protein was resolved on Criterion™ TGX Stain-Free™ Precast Gels (Bio-Rad, Feldkirchen, Germany) and transferred to a nitrocellulose membrane. Bands were visualized *via* chemiluminescent reaction using ECL western blot detection reagents (Bio-Rad; for antibodies and dilutions, see Supplementary Table S9). Images were acquired with the ChemiDoc XRS+ System (Bio-Rad), analyzed with Image Lab (Bio-Rad), and the abundance of target proteins was normalized to the total protein of the respective lane.

### Statistical analysis

SPSS 28 (IBM) was used for statistical analysis. Data were tested for normal distribution using the Kolmogorov-Smirnov test. For analysis of RNA, protein expression data, as well as cellular activation (c-Fos), the parametric two-way (factor conditioning x Ext/Acq or conditioning x s/ns) analysis of variance (ANOVA) followed by Bonferroni post hoc test, was performed. Whenever appropriate, separate parametric Student´s T-tests or non-parametric Mann–Whitney *U*-tests were performed to selectively compare groups. Similar analysis (two-way ANOVA, Bonferroni posthoc) was performed for data regarding anxiety-related behavior and locomotion (Supplementary Fig. S2; factor conditioning x treatment), as well as CS-US pairings after combinatorial treatment (Fig. 3B; factor LNA x local infusion). Social investigation time during extinction and recall of social fear were analyzed using a mixed model ANOVA (without treatment: conditioning x stimulus; with treatment: conditioning x treatment x stimulus) followed by Bonferroni posthoc test for non-social (ns1–ns3) and social (s1–s6) stimuli separately (Geisser–Greenhouse correction was applied when sphericity was violated; tested by Mauchly-test). Student’s *T*-tests or Mann–Whitney *U*-tests were performed to analyze CS-US-pairings as stated. False discovery rates (FDR) in the analysis of PCR Array data were corrected by the Benjamini, Krieger, and Yekutieli FDR correction (adjusted *p*-values in Supplementary Table S4). Statistical significance was accepted at *p* < 0.05. Statistical outliers were calculated by “mean ± 2x standard deviation”. Detailed reports for all statistical analyses and group sizes are available in Supplementary Table S6 and S7. Graphs were plotted using Prism 9 (GraphPad). RNA, microRNA, and protein quantifications are shown in log-transformed box plots (Minimum, maximum, median, first quartile, and third quartile). Other data is shown as stated in the respective figure description.

## Results

### Septal miR-132-3p and miR-124-3p expression is dynamically altered following social fear acquisition and extinction

Since miR-132 has frequently been reported as a dynamic regulator of neuronal plasticity, learning, and memory, we investigated its time-dependent expression within the septum of SFC^+^ and SFC^−^ mice, 30 min, 90 min, 180 min, and 24 h after social fear acquisition, or 30 min, 90 min, and 180 min after social fear extinction (Fig. 1D and Supplementary Fig. S1E). All SFC^+^ mice showed similar social fear learning, as they received an equal number of CS-US-pairings (foot shock when sniffing the conspecific) during  acquisition (Supplementary Table S1). During extinction, SFC^+^ and SFC^−^ mice showed the expected percentage of social investigation (Supplementary Table S2).

In septal tissue of SFC^+^ mice, miR-132-3p was found to be upregulated 90 min after acquisition and was not altered 180 min after acquisition compared to respective SFC^−^ mice (Fig. 1D). In response to extinction, miR-132-3p levels were significantly reduced 180 min after extinction independent of the animal´s conditioning status but remained unchanged at all other time-points in comparison to the respective post-acquisition levels. 30 min after acquisition and extinction as well as 24 h after acquisition, no changes in miR-132-3p were detected in SFC^+^ compared to SFC^−^ mice. In sum, this revealed a specific temporal dynamic of miR-132-3p transcript level in response to SFC.

Additionally, septal miR-124-3p levels were found to be upregulated in SFC^+^ compared to SFC^−^ mice 90 min after acquisition (Supplementary Fig. S1B, F). Moreover, miR-124-3p transcript levels in SFC^−^ mice were increased 90 min after extinction compared to respective SFC^−^ post-acquisition levels. However, septal miR-124-3p transcript levels remained unchanged in all groups at all earlier (30 min) or later (180 min, 24 h) time points.

Various forms of social encounters stimulate the central release of OXT and subsequent OXT receptor (OXTR)-mediated signaling [32, 33], and higher levels of intra-septal OXT release were associated with elevated investigation levels [6], which might be at least partly mediated by miRNAs. To investigate whether miR-132-3p or miR-124-3p transcription is altered in response to repeated social encounters, SFC^−^ and SFC^+^ mice were exposed to either the extinction protocol (3 non-social and 6 social stimuli) or to nine non-social stimuli only. Independent of the conditioning status, neither septal miR-132-3p (Fig. 1E) nor miR-124-3p (Supplementary Fig. S1C) levels were altered 90 min post-exposure. To further analyze whether these miRNAs are altered after OXTR activation, septal miRNA transcript levels were assessed 90 min and 180 min after icv infusion of OXT or Veh. Here, no significant alterations of septal miR-132-3p (Fig. 1F) and miR-124-3p (Supplementary Fig. S1D) were found.

Transcript levels of miR-132-3p are known to increase in response to neuronal activation [17]. Therefore, we assessed the cellular activation within the LS in response to SFC acquisition or extinction via c-Fos immunohistochemistry. SFC^+^ mice received equal CS-US pairings (Supplementary Table S1) during acquisition and displayed the expected initially reduced social investigation during extinction compared to SFC^−^ mice (Fig. 1G). c-Fos levels were increased 90 min after acquisition in SFC^+^ mice compared to respective SFC^−^ mice, whereas no alterations in response to social fear conditioning were observed after extinction (Fig. 1H-I). Whether the observed increase in septal miR-132-3p reflects local cellular activation needs further investigation.

In summary, the expression of both miR-132-3p and miR-124-3p in the septum is dynamically altered in response to SFC. As our preliminary data indicate an upregulation of miR-132-3p specifically within the hypothalamic paraventricular nucleus by icv OXT infusion in male rats [34], we decided to further decipher the functional involvement of miR-132-3p in SFC-related behavior and OXT-mediated social fear reversal.

### Septal miR-132-3p inhibition impairs, whereas its overexpression facilitates extinction of social fear

**Fig. 2:**
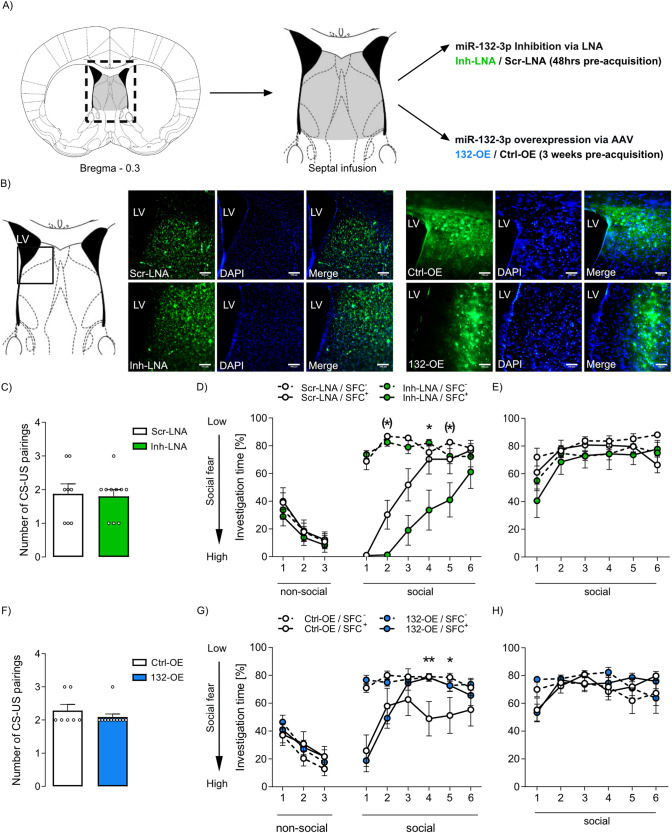
Inhibition of septal miR-132-3p impairs, whereas its overexpression facilitates extinction of social fear. **A** Schematic representation of septal treatments: local miR-132-3p inhibition was achieved by septal infusion of a locked nucleic acid (LNA) complementary to miR-132-3p (Inh-LNA; 0.5 nmol) or a scrambled sequence as control (Scr-LNA; 0.5 nmol). miR-132 overexpression (132-OE) was achieved by septal infusion of an adeno-associated virus (AAV; 4.9 × 10^12^ GC/ml) or control AAV (Ctrl-OE). Infusions were performed 48 h (LNA) or 3 weeks (AAV) prior to social fear acquisition. **B** Representative immunofluorescent verification of septal microinfusion placement and adequate intracellular localization of LNA and expression of AAV. LNA and AAV infusions into the lateral septum resulted in cellular transfection of the entire septum. Scale bar represents 100 µm; LV: lateral ventricle. **C** Number of CS-US pairings presented to Inh-LNA and Scr-LNA-infused conditioned (SFC^+^) mice during acquisition of social fear. **D** Percentage of time investigating the presented 3 non-social and 6 social stimuli during social fear extinction of Inh-LNA and Scr-LNA-treated SFC^+^ and unconditioned (SFC^−^) mice. *p < 0.05, (*) s2: *p* = 0.054, s5: *p* = 0.066 Inh-LNA/SFC^+^ vs Scr-LNA/SFC^+^. **E** Percentage of investigation time of 6 social stimuli presented during social fear recall. **F** Number of CS-US pairings presented to 132-OE or Ctrl-OE AAV-infused SFC^+^ mice during acquisition of social fear. **G** Percentage of nvestigation time of non-social and social stimuli presented during social fear extinction of 132-OE and Ctrl-OE SFC^+^ and SFC^−^ mice. **p < 0.01, *p < 0.05 132-OE/SFC^+^ vs Ctrl-OE/SFC^+^. **H** Percentage of investigation time of 6 social stimuli during social fear recall. Data represent mean ± SEM; *n* = 8–12/group.

The dynamic alterations of septal miR-132-3p in response to SFC (Fig. 1D and Supplementary Fig. S1E) prompted us to further analyze the involvement of septal miR-132-3p in social fear acquisition and extinction using LNA-induced inhibition or AVV-induced overexpression of miR-132-3p (Fig. 2A). Septal LNA and AAV infusion sites, as well as viral expression, were validated by fluorescence microscopy (Fig. 2B).

During acquisition, neither miR-132-3p inhibition (Inh-LNA; Fig. 2C) nor overexpression of miR-132-3p (132-OE; Fig. 2F) within the septum altered the number of CS-US pairings in SFC^+^ mice in comparison to their controls (LNA: Scr-LNA; AAV: Ctrl-OE). During extinction, no treatment effect was found on the investigation of the three non-social stimuli (Fig. 2D, G). In contrast, septal miR-132 inhibition differentially delayed extinction of social fear in SFC^+^ mice. First, Inh-LNA-treated SFC^+^ animals showed lower investigation of social stimuli 2 (by trend), 4, and 5 (by trend) compared to Scr-LNA-infused SFC^+^ mice (Fig. 2D). Second, Inh-LNA/SFC^+^ mice still showed lower social investigation times of stimuli 1 to 5 compared to Inh-LNA/SFC^−^ mice, indicating persistent social fear, whereas Scr-LNA/SFC^+^ controls displayed lower investigation only of stimuli 1 to 3 when compared to Scr-LNA/SFC^−^ mice (for statistical details see Supplementary Table S6).

In support of the functional involvement of miR-132-3p in extinction, AAV-induced overexpression of septal miR-132 (132-OE; Fig. 2G) facilitated fear extinction reflected by (i) higher investigation times of 132-OE-infused SFC^+^ mice during exposure to social stimulus 4 and 5 compared to Ctrl-OE-infused SFC^+^ animals, and (ii) reduced social investigation only during social stimuli 1–2 in 132-OE/SFC^+^ compared to 132-OE/SFC^−^ mice, whereas Ctrl-OE/SFC^+^ mice continued to avoid social stimuli 4 and 5 when compared to Ctrl-OE/SFC^−^ mice (for statistical details see Supplementary Table S6).

Importantly, neither inhibition nor overexpression of septal miR-132 altered social investigation of SFC^−^ mice indicating that septal miR-132 is not involved in general social behavior. During recall, LNA-mediated inhibition (Fig. 2E) and AAV-mediated overexpression (Fig. 2H) of septal miR-132 did not affect investigation times in all groups. Moreover, neither general anxiety-related behavior nor locomotion assessed in the OF/NO test was affected by miR-132-3p inhibition or overexpression (Supplementary Fig. S2). These data indicate that septal miR-132 contributes to the facilitation of social fear extinction.

Hypothalamic miR-132-3p inhibition has been shown to control water homeostasis and body weight gain [35]. However, we did not find alterations in body weight gain 24 h, 48 h, or 3 weeks after bilateral infusion of LNA or AAV into the LS (Supplementary Fig. S2I–J).

### Septal miR-132-3p is required for the OXT-induced reversal of social fear

**Fig. 3:**
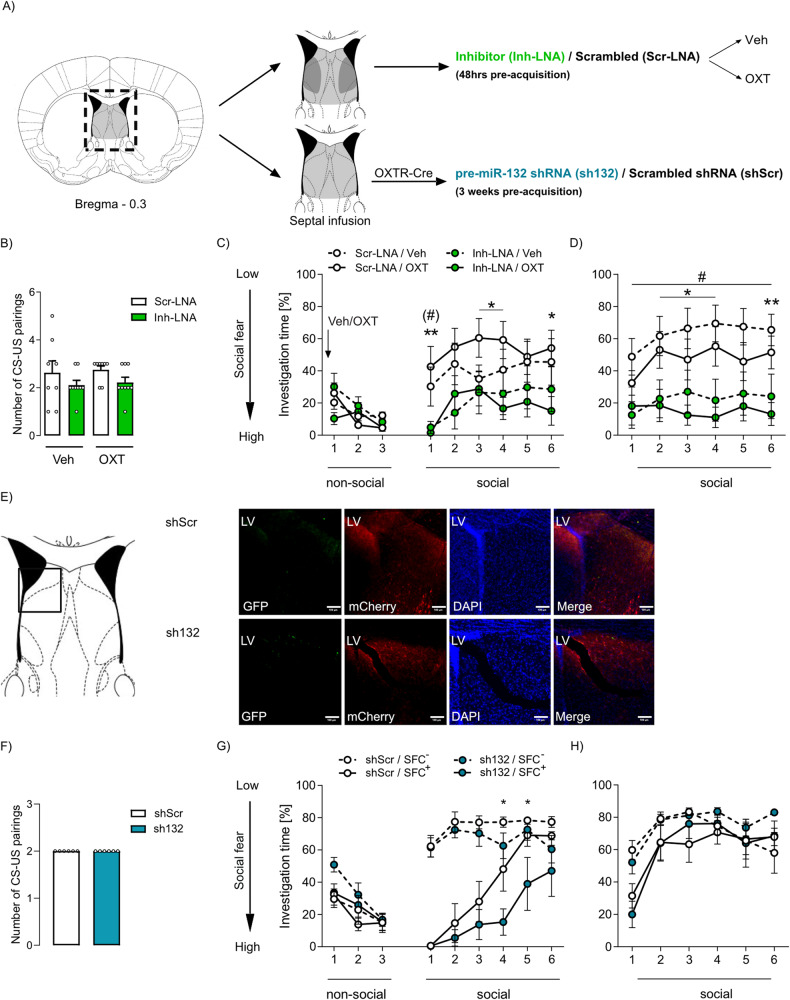
Septal miR-132-3p is required for the reversal of social fear mediated by oxytocin (OXT) receptor signaling within the lateral septum. **A** Schematic representation of septal treatments: 48 h prior to acquisition of social fear, mice were bilaterally infused with either a miR-132-3p inhibitor (Inh-LNA; 0.5 nmol) or scrambled (Scr-LNA; 0.5 nmol) locked nucleic acid (LNA; light gray) into the septum. 10 min prior to extinction of social fear they were locally infused (dark gray) with OXT (5 ng/0.2 µL/hemisphere) or vehicle (Veh; Ringer solution). Additionally, selective downregulation of miR-132 in OXT receptor-expressing neurons was achieved in OXT receptor (OXTR)-Cre mice, which were infused with a pre-miR-132 shRNA (sh132; 9.4 × 10^13^ GC/ml) or scrambled (shScr; 9.4 × 10^13^ GC/ml) adeno-associated virus into the septum 3 weeks prior to social fear acquisition. **B** Number of CS-US pairings during acquisition of social fear presented to conditioned (SFC^+^) Inh-LNA or Scr-LNA-treated mice that were further infused with OXT or Veh into the LS. **C** Percentage of investigation time of 3 non-social and 6 social stimuli during social fear extinction in Inh-LNA and Scr-LNA SFC^+^ mice locally treated with OXT or Veh. **p < 0.01; *p < 0.05 Inh-LNA/OXT vs Scr-LNA/OXT; (#) *p* = 0.061 Inh-LNA/Veh vs Scr-LNA/Veh. **D** Percentage of investigation time of  6 social stimuli during social fear recall. **p < 0.01, *p < 0.05 Inh-LNA/OXT vs Scr-LNA/OXT; ^#^*p* < 0.05 Inh-LNA/Veh vs Scr-LNA/Veh. **E** Representative immunofluorescent images of septal sh132 or shScr microinfusion placement. Viral shRNA expression in OXTR-Cre mice was turned on using a SICO vector, whereas GFP expression is turned off in the presence of Cre, while mCherry expression remains constitutive. AAV infusions into the lateral septum resulted in neuronal infection of the entire septum. Scale bar represents 100 µm; LV lateral ventricle. **F** Number of CS-US pairings presented to SFC^+^ sh132 or shScr-treated mice during social fear acquisition. **G** Investigation time of non-social and social stimuli during social fear extinction of SFC^+^ and unconditioned (SFC^−^) mice treated with septal sh132 or shScr. *p < 0.05 sh132/SFC^+^ vs shScr/SFC^+^. **H** Investigation time of social stimuli during social fear recall. Data represent mean ± SEM; *n* = 6–12/group.

Previous studies reported that OXT signaling within the LS promotes social fear extinction in male [6] and female [3] SFC^+^ mice. To analyze the involvement of miR-132-3p in OXT-mediated reversal of social fear, septal miR-132-3p of SFC^+^ mice was inhibited by Inh-LNA infusion and combined with local OXT infusion performed 10 min before extinction resulting in the following groups: Inh-LNA/OXT, Inh-LNA/Veh, Scr-LNA/OXT, and Scr-LNA/Veh (Fig. 3A).

In concordance with the previous experiment (Fig. 2C–E), septal miR-132-3p inhibition in SFC^+^ mice neither influenced acquisition of social fear nor the investigation of the 3 non-social stimuli during extinction (Fig. 3B, C). In support of our hypothesis that septal miR-132-3p inhibition prevents the social fear-reversing effect of OXT, significantly lower investigation times were displayed by Inh-LNA/OXT-treated compared to Scr-LNA/OXT-treated SFC^+^ mice during exposure to social stimuli 1, 3, 4, and 6 during extinction. Additionally, Inh-LNA/Veh treatment resulted in a decreased investigation time of social stimulus 1 compared to Scr-LNA/Veh. During recall (day 3), Inh-LNA/OXT-treated SFC^+^ mice showed a reduced investigation of social stimuli 2, 3, 4, and 6 compared to Scr-LNA/OXT-treated animals, whereas Inh-LNA/Veh treatment resulted in an overall decreased investigation of social stimuli 1 to 6, when compared to Scr-LNA/Veh-treated animals (Fig. 3D).

In this experiment, however, we could not confirm the reversing effect of OXT per se on extinction [6] in Scr-LNA/OXT-treated SFC^+^ mice, likely due to the essential pre-treatment septal surgeries and microinfusions. These procedures, together with a reduced post-surgical recovery period of only 72 h resulted in an overall decrease of social investigation times during extinction and recall independent of the treatment (e.g., Scr-LNA/Veh: approx. 60% (Fig. 3C) vs Scr-LNA/SFC+ : approx. 80% (Fig. 2D)). Moreover, lesions of septal tissue, i.e., via cannulation, is known to affect social behavior [36].

To further confirm the involvement of miR-132 in OXTR-mediated facilitation of social fear extinction, pre-miR-132, and concomitantly also mature miR-132-3p, was selectively down-regulated within septal OXTR-expressing neurons of OXTR-Cre mice using an AAV expressing a floxed shRNA against miR-132(sh132; Fig. 3A, E). pre-miR-132 knockdown in septal OXTR-expressing neurons neither affected the number of CS-US pairings during acquisition (Fig. 3F) nor investigation times of non-social stimuli independent of the conditioning status, nor social investigation times in SFC^−^ mice (Fig. 3G). However, pre-miR-132 knockdown, specifically in septal OXTR-expressing neurons, delayed the extinction of social fear in SFC^+^ mice. This was reflected by (1) reduced investigation of social stimuli 4 and 5 compared with shScr/SFC^+^ mice, and (2) lower investigation of stimulus 5 compared with sh132/SFC^−^ mice, whereas SFC+ mice treated with shScr showed reduced investigation of stimuli 1 to 4 only (shScr/SFC^+^ vs shScr/SFC^−^). This illustrated delayed extinction of social fear by knockdown of miR-132-3p in septal OXTR expressing neurons (for statistical details see Supplementary Table S6). During recall, no differences between the groups were found (Fig. 3H).

Taken together, septal miR-132-3p is significantly involved in signaling downstream of the OXTR and required for OXTR-mediated reversal of social fear, as its inhibition partly prevented the social fear-reversing effect of OXT, and its knockdown selectively in OXTR-expressing neurons impaired social fear extinction.

### Septal miR-132-3p influences social fear extinction by regulation of growth differentiation factor 5 (Gdf-5)

**Fig. 4:**
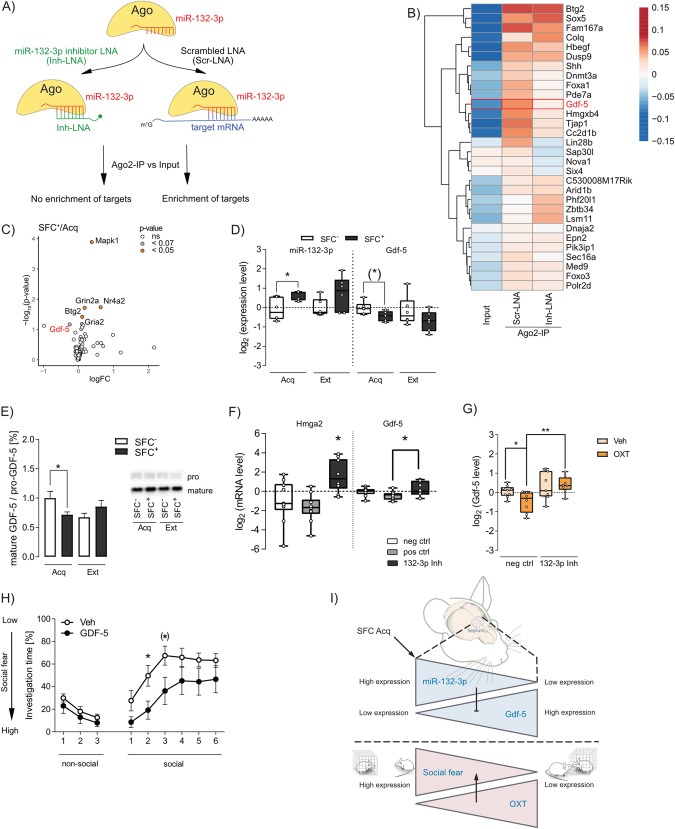
Septal miR-132-3p influences social fear extinction by regulation of growth differentiation factor 5 (Gdf-5). **A** Schematic representation of the performed Argonaute-RNA-co-immunoprecipitation (Ago2-IP) microarray analysis of septal tissue of mice infused with the miR-132-3p inhibitor (Inh-LNA; 0.5 nmol) or scrambled control (Scr-LNA; 0.5 nmol) locked nucleic acid (LNA). Target mRNAs regulated by miR-132-3p are not enriched or even de-riched in Inh-LNA samples after Ago2-IP, whereas Scr-LNA infusion is supposed to result in enrichment of miR-132-3p target mRNAs. **B** Heat-map showing the log fold-change to mean intensity of relevant genes after microarray analysis of Ago2-IP samples. Only predicted targets of miR-132-3p (congruent in MiRDB, miRMap, miRWalk2, and TargetScan Mouse 7.1), which show higher intensities in Scr-LNA compared to input samples are shown. **C** Volcano plot depicting the fold-change of conditioned (SFC^+^) mice after acquisition (Acq) of social fear (normalized to unconditioned mice (SFC^−^)/Acq) in dependence of the p-value of all genes detected via PCR Array analysis. **D** Relative miR-132-3p and Gdf-5 RNA expression in SFC^−^ and SFC^+^ mice 90 min after social fear Acq or extinction (Ext); *p < 0.05. **E** Ratio of septal mature GDF-5 to pro-GDF-5 protein level in SFC^−^ and SFC^+^ mice 90 min after Acq or Ext of social fear; *p < 0.05. **F** Relative Hmga2 and Gdf-5 mRNA level in Neuro-2a neuroblastoma cells 48 h after transfection with 3 nM of a negative control (neg ctrl), positive control (let-7c; pos ctrl), or miR-132-3p (132-3p Inh) inhibitor. *p < 0.05 vs all groups (Hmga2) or vs pos ctrl (Gdf-5). **G** Relative Gdf-5 level in Neuro-2a neuroblastoma cells transfected with neg ctrl or 132-3p Inh for 48 h and further stimulated with vehicle (Veh; growth medium) or oxytocin (OXT; 250 nmol) for 90 min. *p < 0.05; **p < 0.01. **H** Investigation time of non-social and social stimuli during social fear extinction in SFC^+^ mice locally infused with Veh (0.2 µl Ringer solution/hemisphere) or GDF-5 (0.05 µg/0.2 µl/hemisphere) 30 min prior to extinction. *p < 0.05; (*) *p* < 0.07 GDF-5 vs Veh. **I** In summary, in response to SFC, septal miR-132-3p was increased, which in turn downregulated Gdf-5. Septal upregulation of miR-132-3p, leading to decreased Gdf-5, resulted in high social fear, whereas inhibition of miR-132-3p, resulting in increased Gdf-5, led to low social fear. Moreover, miR-132-3p inhibition was found to prevent OXT-induced reversal of social fear. **E**, **H** data represent mean ± SEM; **B** *n* = 1–2/group; **C**–**E**, **H** *n* = 6–11/group; **F**, **G** *n* = 8–10/group.

To identify targets of septal miR-132-3p, which potentially mediate the observed effects of miR-132-3p on social fear extinction, Ago2-IP analysis was performed 48 h after septal infusion of Inh-LNA or Scr-LNA. In theory, only mRNAs, which are regulated by miRNAs, are enriched after Ago2-IP, when compared to the respective input samples since Ago proteins bind to miRNAs and tightly associate with the target mRNAs. Furthermore, mRNAs regulated by miR-132-3p are not enriched or even de-riched in Inh-LNA samples after Ago2-IP since the inhibitor binds to miR-132-3p, thereby dissociating miRNA-Ago complexes from miR-132-3p-specific target mRNAs (Fig. 4A). As expected, a principal component analysis (PCA) revealed the highest variation between input and Ago2-IP samples, confirming that Ago2 binds to distinct mRNAs during degradation (Supplementary Fig. S4A). Furthermore, the PCA provided evidence that the composition of mRNAs bound by Ago2 varies between Inh-LNA or Scr-LNA treatment, and the transcript abundance of 164 genes differed between both IP conditions (Supplementary Fig. 4B, used cutoff: *p* < 0.05; Supplementary Tables S12–S14). Further k-means clustering of the differentially enriched genes revealed three clusters (1, 4, 10), including 44 mRNAs, which were enriched in Scr-LNA-IP samples compared to the respective input sample and downregulated in Inh-LNA compared to Scr-LNA samples (Supplementary Fig. 4B). Additionally, 52 genes (clusters 3, 5, 6, 7, 8) were downregulated after Inh-LNA treatment compared to Scr-LNA, but not enriched after Scr-LNA-IP compared to its input. Selected septal mRNAs of these clusters were further analyzed 90 min after acquisition or extinction in SFC^−^ and SFC^+^ mice using a customized RT^2^ Profiler PCR Array (Qiagen; Supplementary Fig. 4C; Supplementary Table S4). SFC^+^ mice included in this analysis received equal CS-US pairings (Supplementary Table S4) during acquisition and showed initially low social investigation time during extinction, as expectced (Supplementary Fig. 4G). From all analyzed target mRNAs, including all annotated GDF-family members, Gdf-5 intensity was found to be increased in the Ago2-IP analysis after septal Inh-LNA treatment (Fig. 4B, C; Supplementary Fig. 4E, F; Supplementary Table S3). Further analysis by miRNA target prediction software (miRDB [37], miRMap [38], miRWalk2 [39], and TargetScan Mouse 7.1 [40]) revealed Gdf-5 as a putative target with an 8mer binding site within the miR-132-3p seed sequence. In confirmation of previous results (Fig. 1D), septal miR-132-3p was increased 90 min after acquisition in SFC^+^ mice compared to SFC^−^ mice, whereas septal Gdf-5 mRNA levels were decreased by trend (Fig. 4D). Analysis of septal GDF-5 protein levels revealed no alteration in the dimeric pro-form and monomeric mature form in response to acquisition or extinction (Supplementary Fig. 4H). However, the ratio of mature GDF-5 to pro-GDF-5 was decreased in SFC^+^ mice 90 min after acquisition compared to respective SFC^−^ animals (Fig. 4E), showing reduced septal mature GDF-5 in proportion to pro-GDF-5. This downregulation of septal GDF-5 might result from increased miR-132-3p in SFC^+^ mice after acquisition. Details of the behavior displayed by these mice during social fear acquisition and extinction are found in Supplementary Table S5.

To further prove whether miR-132-3p mediates the regulation of Gdf-5, Neuro-2a cells were transfected with either a miR-132-3p inhibitor (132-3p Inh) or negative (neg ctrl) or positive control (pos ctrl) inhibitor. Confirming the successful transfection, we revealed decreased miR-132-3p levels in 132-3p Inh cells compared to neg ctrl treatment (Supplementary Fig. S4I). Hmga2, which is a predicted (TargetScan Mouse 8.0) and validated [41] target of miR-132-3p, was increased after 132-3p Inh treatment compared to control inhibitors (Fig. 4F). Importantly, Gdf-5 was increased in 132-3p Inh-treated cells compared to positive control inhibitor-treated cells, suggesting that miR-132-3p indeed downregulates Gdf-5 expression. Further stimulation of 132-3p Inh-transfected Neuro-2a cells with OXT increased Gdf-5 levels in 132-3p Inh/OXT treated cells compared to neg ctrl/OXT treatment (Fig. 4G), suggesting that OXT leads to increased miR-132-3p, which regulated Gdf-5 expression. Additionally, OXT treatment decreased Gdf-5 levels in miR-132-3p neg ctrl-treated cells (neg ctrl/OXT vs neg ctrl/Veh), suggesting that miR-132-3p mediates the stimulatory effect of OXT on Gdf-5 expression.

Overexpression of miR-132-3p using a miR-132-3p mimic and corresponding negative and positive controls resulted in aberrant overexpression of miR-132-3p in Neuro-2a cells (Supplementary Fig. S4I), which might induce various counterbalancing regulatory effects. Therefore, analysis of Gdf-5 and effects of OXT treatment were not analyzed after miR-132-3p overexpression in Neuro-2a cells.

To further assess whether GDF-5 regulated by miR-132-3p is involved in the observed behavioral effect of septal miR-132-3p in social fear extinction, GDF-5 [42] or Veh was applied into the LS of SFC^+^ mice 30 min prior to extinction of SFC^+^ mice. The number of CS-US pairings during acquisition did not differ prior to treatment (Supplementary Fig. S4J). During extinction, no alterations in non-social investigation times were detected, whereas investigation times of social stimuli 2 and 3 (by trend) were reduced after local GDF-5 treatment indicating impaired extinction (Fig. 4H). The recall remained unaffected by local GDF-5 treatment (Supplementary Fig. S4K). These data further strengthen the suggestion of septal miR-132-3p mediating its effect on social fear extinction by post-transcriptional regulation of septal Gdf-5 abundance. However, viral Cre-dependent GDF-5 overexpression in septal OXTR-positive neurons did not alter acquisition, extinction, and recall in SFC^+^ mice compared to eGFP overexpression (Supplementary Fig. S3), suggesting that additional neuronal populations or target mRNAs might be involved in mediating the effect of miR-132-3p on social fear extinction.

Summarizing these molecular and behavioral analyses, we suggest septal miR-132-3p to influence social fear extinction via regulation of Gdf-5.

## Discussion

In this study, we observed a highly dynamic expression pattern of miR-132-3p and miR-124-3p within the mouse septum throughout SFC. We further focused on miR-132-3p and revealed a functional involvement of septal miR-132-3p in social fear extinction, as inhibition via LNA impaired, whereas AAV-mediated overexpression facilitated extinction. In addition, we showed that septal miR-132-3p at least partially mediates the OXT-induced facilitation of extinction previously reported [3, 6]. Both septal miR-132-3p inhibition and shRNA-mediated knockdown of pre-miR-132 in OXTR-expressing neurons prevented OXT-induced facilitation of extinction. Employing Ago2-IP in conjunction with target gene analysis, we confirmed Gdf-5 as target of septal miR-132-3p, with miR-132-3p exerting a negative regulatory effect on Gdf-5 expression. Moreover, we revealed increased Gdf-5 level 90 min after OXT treatment, which was found to be mediated by miR-132-3p, in vitro. We further pharmacologically validated the involvement of septal GDF-5 in extinction learning and found impaired extinction after local GDF-5 infusion. Thus, we suggest a pathway implicating septal miR-132-3p and its downstream target GDF-5 in mediating the facilitatory effect of OXT on social fear extinction.

Both, miR-132 and miR-124 are well-known to regulate neuronal development and plasticity [43] and have been suggested to contribute to the control of social and anxiety-related behavior [44]. As the septum, particularly the LS, is an essential integrative center for socio-emotional behavior [36] and social fear extinction [3, 6], we analyzed the dynamics of local expression patterns of miR-132-3p and miR-124-3p in response to SFC. Both miRNAs were increased in septal tissue of SFC^+^ mice after acquisition in a time-dependent manner, showing increased expression only 90 min after acquisition (Fig. 1A and Supplementary Fig. S1B). This suggests a putative function of both miRNAs in the regulation of fear learning processes. Moreover, independent of the conditioning status of the animal, miR-132-3p decreased 180 min after exposure to extinction training, i.e., to six individual conspecifics, compared to corresponding post-acquisition levels. Considering the fact that miR-132-3p is involved in associative learning, a post-extinction reduction in expression suggests that the memory trace formed by extinction learning is not robust [11].

Differing from miR-132-3p, miR-124-3p showed increased levels in SFC^−^ mice after extinction compared to the respective post-acquisition levels, whereas no alteration was seen, when comparing post-acquisition and post-extinction levels of SFC^+^ mice, suggesting septal miR-124-3p not to be involved in extinction learning. In support, miR-124-3p has been shown to regulate various social behaviors, as heterozygous knockdown leads to reduced social contact [45] and decreased cortical miR-124-3p levels result in reduced sociability in male mice via regulation of AMPAR subunit composition [21].

Various challenges, such as contextual fear conditioning, odor exposure, multimodal stress, and safety learning were described to induce neuronal miR-132-3p expression in brain regions explicitly activated by these paradigms [16–18, 46–49], suggesting that miR-132-3p expression is linked to neuronal activity. Indeed, acquisition of social fear was found to result in increased cellular activation in the LS of virgin female mice [3]. In confirmation, we observed increased c-Fos in the dorsal LS of male SFC^+^ mice 90 min after acquisition (Fig. 1I), which accompanies the observed increase in miR-132-3p expression in the septum.

miR-132-3p-mediated post-transcriptional regulation of neuronal genes results in orchestrated synaptic growth and increased dendrite length, branching, spine density and width [43]. As a result, strengthened synaptic transmission [50, 51] and enhanced long-term potentiation (LTP) [47], which is essential for associative learning, have been described. Inhibition or knockdown of miR-132-3p has already been shown to induce dysfunctions in non-social learning and memory [16, 18, 47]. Moreover, memory formation and retention are impaired in miR-132/212 knockout mice without altered general anxiety-related behavior and locomotion [52]. As we found septal miR-132-3p to be increased 90 min after social fear acquisition in SFC^+^ compared to SFC^−^ mice, we hypothesize that septal miR-132-3p is essential for learning of associative fear memories in a social context (Fig. 1A). However, downregulation of miR-132-3p 180 min after exposure to extinction training in both SFC^+^ and SFC^−^ mice suggests a general social exposure-dependent effect. Social investigation is known to stimulate septal OXT release [3, 6], which might enhance local GABAergic signaling and inhibition of neuronal activation. This effect might cause the downregulation of miR-132-3p 180 min post extinction. Alternatively, miR-132-3p might generally be involved in memory formation in a social context. Thus, post-acquisition upregulation of miR-132-3p indicates its role in acquisition memory formation, while its post-extinction downregulation rather suggests that the formed extinction memory might not be robust. Indeed, memory created by social fear extinction is prone to spontaneous recovery [11]. Thus, augmenting septal miR-132-3p actions might be crucial for the formation of robust extinction memory. The involvement of miR-132-3p in non-social fear extinction remains elusive. Corroborating this thought, inhibition of septal miR-132-3p impaired (Fig. 2C–E), while its overexpression facilitated extinction learning (Fig. 2F–H) without affecting fear acquisition and recall. Thus, we suggest that septal miR-132-3p critically regulates synaptic plasticity and the formation of LTP during associative learning, thereby modulating the formation of social fear extinction memory. The observed effects of septal miR-132-3p manipulation seemed specific for extinction learning, as septal miR-132-3p inhibition and overexpression neither altered social fear acquisition, anxiety-related behavior, nor locomotion in the OF/NO test (Supplementary Fig. S2).

During social fear extinction, we have previously reported an attenuated release of OXT within the septum of SFC^+^ mice, likely due to their low level of social investigation [6]. Therefore, we suggest that the reduced miR-132-3p expression during extinction in SFC^+^ mice might be causally linked either to reduced local OXT release or reduced social contact, or both. However, its expression remained unchanged 90 min and 180 min after icv OXT treatment or 90 min after repeated social compared to repeated object contact. These data suggest that miR-132-3p is involved in the formation of fear memory and requires the respective training processes to be activated. To evaluate, whether miR-132-3p is involved in OXT-mediated reversal of social fear [3, 6], we used two approaches, both leading to specific behavioral alterations during social fear extinction. First, inhibition of septal miR-132-3p prior to OXT infusions into the LS resulted in impaired extinction (Fig. 3B–D). Second, septal miR-132-3p inhibition prevented the OXT-mediated reversal of social fear. Although the fear-reversing effect of OXT has been localized within the LS [3, 6], we showed that inhibition of miR-132-3p within the entire septum prevented the effect of OXT infusion into the LS. Moreover, shRNA-mediated knockdown of pre-miR-132, specifically in septal OXTR-expressing neurons, also impaired extinction without affecting social fear acquisition or recall (Fig. 3F–H). Due to substantial LNA and AAV diffusion within the septum after bilateral infusion into the LS (Supplementary Fig. S1A), the specific contribution of miR-132-3p within the LS on the OXT-mediated reversal of social fear could not be selectively analyzed. However, the LS is reciprocally connected to the medial septum [53], and both subregions express the OXTR [7]. As shown, miR-132-3p shows highly dynamic expression in response to SFC. However, the presented manipulations of miR-132-3p (LNA and AAVs) lack temporal specificity as LNAs develop their full effect only 24–48 h after infusion [26], whereas AAVs need 2–3 weeks to be adequately expressed within the brain [3]. Consequently, we cannot provide an accurate time-window for the effects of miR-132-3p mimics or inhibitors.

OXT is known to signal via several cascades, including MEF2a- [54, 55] and CREB-mediated [9] activation of transcription. Interestingly, miR-132-3p transcription is known to be CREB-dependent [17], and its promoter includes multiple binding sites for these transcription factors. This suggests that miR-132-3p is regulated downstream of OXTR activation. However, the fact that icv OXT did not increase septal miR-132-3p, assessed at 90 min and 180 min post-infusion, could be either due to lack of spatial selectivity, as OXT was applied icv, or the chosen timepoints. Moreover, a training procedure might be required to induce miR-132-3p alterations in response to icv OXT treatment, as has already been shown for other miRNAs [56].

Employing Ago2-IP in conjunction with septal miR-132-3p inhibition, we detected numerous potential target mRNAs, which were subsequently validated in the context of SFC (Fig. 4, Supplementary Fig. 4). Here, we identified septal Gdf-5 as putative target of miR-132-3p both in vitro by inhibition of miR-132-3p and in vivo in response to acquisition and extinction of social fear. Moreover, we confirmed a reduction in mature GDF-5 protein level in proportion to pro-GDF-5 in the septum of SFC^+^ mice after acquisition compared to respective SFC^−^ animals. miR-132-3p-mediated regulation of GDF-5 expression has already been confirmed in ligament cells [57, 58] and intervertebral discs [59], which provides a functional relevance to this pathway. Thus, the pharmacological application of GDF-5 into the septum prior to extinction was performed and resulted in impaired extinction, which is in congruence with the expression pattern of septal miR-132-3p and its extinction-facilitating effect. However, GDF-5 overexpression in septal OXTR-expressing neurons did not alter acquisition, extinction, or recall in SFC^+^ mice (Supplementary Fig. S3). This lack of alteration in social fear expression could be caused by confounding behavioral effects of 3-week supraphysiological overexpression of GDF-5 in OXTR-expressing neurons. Moreover, miR-132-3p presumably targets additional mRNAs within OXTR-expressing neurons that might, in unison, lead to the observed social fear extinction impairment. However, to the best of our knowledge, this is the first study showing miR-132-3p-mediated regulation of GDF-5 in social behavior. GDF-5 is known to protect neurons against chemically-induced degeneration [60] and to regulate learning in the context of cued and contextual fear conditioning [42]. Similarly, in our experiments, septal GDF-5 application reduced social investigation during extinction, thereby increasing social fear expression.

In further support, septal miR-132-3p inhibition and pre-miR-132 knockdown in OXTR-expressing neurons, which should lead to increased septal GDF-5 level, resulted in increased social fear expression. Vice versa, miR-132-3p overexpression, possibly resulting in reduced GDF-5 level within the mouse septum, led to reduced social fear expression. Hence, we suggest that septal GDF-5 expression is regulated by miR-132-3p in response to SFC. The regulation of Gdf-5 by miR-132-3p was further confirmed in vitro by transfecting Neuro-2a cells with a miR-132-3p inhibitor, which led to increased Gdf-5 levels under basal conditions (Fig. 4F), but also to a more pronounced rise in response to OXT stimulation when compared to the respective control inhibitor (Fig. 4G). This supports our suggestion of OXT regulating miR-132-3p expression, and subsequently, miR-132-3p regulating Gdf-5. As abovementioned, a causal link could not be established in the context of SFC, hence we suggest that the observed behavioral effects might originate in two distinct regulatory mechanisms, i.e., both SFC as well as OXT regulating miR-132-3p levels in the septum, and miR-132-3p regulating Gdf-5.

In conclusion, our study revealed a putative novel mechanism involving septal miR-132-3p, which is dynamically altered in response to social fear acquisition and extinction, regulates local GDF-5 expression, and, thereby, fear expression during social fear extinction. Moreover, we showed that inhibition of septal miR-132-3p prevents the robust OXTR-mediated facilitation of social fear extinction, suggesting miR-132-3p to act downstream of septal OXTR activation.

## Supplementary information

Supplementary Fig. and Table Legends

Supplementary Fig. S1

Supplementary Fig. S2

Supplementary Fig. S3

Supplementary Fig. S4

Supplementary Methods

Supplementary Table S1

Supplementary Table S2

Supplementary Table S3

Supplementary Table S4

Supplementary Table S5

Supplementary Table S6

Supplementary Table S7

Supplementary Table S8

Supplementary Table S9

Supplementary Table S10

Supplementary Table S11

Supplementary Table S12

Supplementary Table S13

Supplementary Table S14
