# Supplementary material for: Functional involvement of septal miR-132 in extinction and oxytocin-mediated reversal of social fear
Source: Mol Psychiatry. 2023 Nov 8;29(6):1754–66. doi: 10.1038/s41380-023-02309-3 (PMC11371636; doi:10.1038/s41380-023-02309-3)
Supplement: Supplementary file 1 — Supplementary Fig. and Table Legends [file 41380_2023_2309_MOESM1_ESM.docx]

**LEGENDS OF SUPPLEMENTARY Figures**

**Supplementary Figure S1 – Septal microinfusion and dynamic alterations of septal miR-124-3p and miR-132-3p level in response to social fear conditioning, and miR-124-3p level after repeated stimulus exposure and intracerebroventricular (icv) oxytocin (OXT).** A) Representative brain slice of locked nucleic acid or adeno-associated virus microinfusion into the septum. Although the stereotaxic coordinates target the lateral septum, transfection and viral expression is found throughout the entire septum. Scalebar represents 500 µm. B) Relative septal miR-124-3p level 30min, 90min, 180min, and 24hrs after acquisition (Acq) and extinction (Ext) of social fear in conditioned (SFC^+^) and unconditioned (SFC^-^) male mice. ** p<0.01, * p=<0.05. C) Relative septal miR-124-3p transcript level of SFC^+^ and SFC^-^ mice 90min after repeated exposure to non-social (ns) and social (s) stimuli. D) Relative septal miR-124-3p transcript level 90min and 180min after icv application of oxytocin (OXT; 0.1 µg/2µl) or vehicle (Veh; 2 µl Ringer solution) in male mice. E) Fold-change of relative septal miR-132-3p level in dependence of the time (30min, 90min, and 180min) in relation to SFC^-^/Acq animals (corresponding to Figure 1D). F) Fold-change of relative septal miR-124-3p level in dependence of the time (30min, 90min, and 180min) in relation to SFC^-^/Acq animals (corresponding to Supplementary Figure S1B). B)-F) n=6-10/group.

**Supplementary Figure S2 – Locomotion, general anxiety-related behavior, and weight gain after septal miR-132-3p inhibition or overexpression.** A-B) Distance travelled (cm) and time spent in the center zone (s) of the open field test of miR-132-3p inhibitor (Inh-LNA) or scrambled (Scr-LNA) locked nucleic acid (LNA)-treated conditioned (SFC^+^) and unconditioned (SFC^-^) male mice. C-D) Distance travelled (cm) and time spent in the center zone (s) of the open field test of miR-132-3p overexpression (132-OE) or control (Ctrl-OE) adeno-associated virus-treated SFC^-^ and SFC^+^ animals. E-F) Distance travelled (cm) and time spent in the object zone (s) of the novel object investigation test of Inh-LNA or Scr-LNA-treated SFC^+^ and SFC^-^ mice. G-H) Distance travelled (cm) and time spent in the object zone (s) of the novel object investigation test of 132-OE or Ctrl-OE SFC^+^ and SFC^-^ animals. I) Weight gain (gram) of Inh-LNA and Scr-LNA-treated mice 24hrs and 48hrs after infusion. J) Weight gain (gram) of 132-OE and Ctrl-OE mice 24hrs, 48hrs, and 3 weeks after infusion. Data represent mean+SEM; A-G) n=6-10/group; I-J) n=15-20/group.

**Supplementary Figure S3 – Growth differentiation factor 5 (GDF-5) overexpression in septal oxytocin receptor (OXTR) expressing neurons.** A) Schematic representation of septal GDF-5 overexpression (GDF-5-OE; 2.0 x 10^13^ GC/ml) and control (eGFP-Ctrl; 2.0 x 10^13^ GC/ml) adeno-associated virus infusions that were performed 3 weeks prior to social fear acquisition. B) Number of CS-US pairings presented to GDF-5-OE or eGFP-infused conditioned (SFC^+^) mice during acquisition of social fear. C) Percentage of time investigating the presented non-social and social stimuli during social fear extinction of GDF-5-OE or eGFP-Ctrl-treated SFC^+^ and unconditioned (SFC^-^) mice. D) Investigation time during social fear recall. Data represent mean±SEM; n=12/group.

**Supplementary Figure S4 -** **Septal miR-132-3p regulates growth differentiation factor 5 (Gdf-5).** A) PCA plot of the calibrated, summarized data after Argonaute-RNA-co-immunoprecipitation (Ago2-IP) microarray analysis of septal tissue of mice infused with the miR-132-3p inhibitor (Inh-LNA; 0.5 nmol) or scrambled control (Scr-LNA; 0.5 nmol) locked nucleic acid. B) Volcano plot depicting the fold-change (normalized to respective input samples) in dependence of the p-value of all genes detected *via* microarray analysis after Ago2-IP of septal tissue of mice infused with Inh-LNA or Scr-LNA. Genes with p<0.05 are highlighted in orange. C) Heat-map illustrating the mRNA fold-change of conditioned (SFC^+^) and unconditioned (SFC^-^) mice 90 min post acquisition (Acq) or extinction (Ext) of social fear (in relation to SFC^-^/Acq mice) analyzed via PCR Array. D) K-means clustering of altered genes after microarray analysis (Scr-LNA vs Inh-LNA with p<0.05) of input or Ago2-IP samples of septal Inh-LNA or Scr-LNA-infused tissue. Clusters marked with ** represent those three target mRNA clusters, wherein genes follow the expected expression pattern: enrichment in Ago2-IP compared to input samples and downregulation in Inh-LNA-treated samples compared to Scr-LNA treatment, whereas * highlights clusters wherein mRNAs do not show an enrichment after Ago2-IP, but a downregulation after septal Inh-LNA compared to Scr-LNA treatment. E) Volcano plot depicting the fold-change of SFC^-^/Ext mice (normalized to SFC^-^/Acq mice) in dependence of the p-value of all genes detected via PCR Array analysis. F) Volcano plot depicting the fold-change of SFC^+^/Ext mice (normalized to SFC^-^/Acq mice) in dependence of the p-value of all genes detected via PCR Array analysis. G) Investigation time of non-social and social stimuli during social fear extinction in SFC^+^ and SFC^-^ mice that were used for RNA expression analysis via PCR Array; ** p<0.01, (*) p=0.057 SFC^+^ vs SFC^-^. H) Relative septal pro-GDF-5 and mature GDF-5 level in SFC^+^ and SFC^-^ mice 90 min post Acq or Ext of social fear. I) Relative miR-132-3p level 48hrs after transfecting Neuro-2a neuroblastoma cells with a negative control (neg ctrl), positive control (Mimic: miR-1; Inhibitor: let-7c; pos ctrl) or miR-132-3p (132-3p) mimic or inhibitor. ** p<0.01 vs all groups; * p<0.05 vs neg ctrl. J) Number of CS-US pairings presented to SFC^+^ mice during Acq of social fear, which were bilaterally infused into the lateral septum with either GDF-5 (0.05µg/0.2µl/hemisphere) or vehicle (Veh; 0.2µl Ringer solution/hemisphere) on the following day. K) Investigation time of social stimuli during social fear recall of mice infused with Veh or GDF-5 on the previous day (prior to extinction training). G), J), K) data represent mean±SEM; A)-B), D) n=1-2/group; C), E)-H) n=6-11/group; I) n=8-10/group; J) and K) n=6-11/group.

**LEGENDS OF SUPPLEMENTARY TABLES**

**Supplementary Table S1 -** Details and statistics on social fear acquisition corresponding to mice included in Figure 1 and Supplementary Figure S1.

**Supplementary Table S2 -** Details and statistics on investigation time [%] during social fear extinction corresponding to mice included in Figure 1 and Supplementary Figure S1.

**Supplementary Table S3 -** Ago2-IP analysis of annotated growth differentiation factors and target prediction analysis.

**Supplementary Table S4 -** CS-US pairings during acquisition of social fear, raw data, fold-change analysis and corresponding p-value of mRNA targets analyzed via the customized RT2 Profiler PCR Array for miR-132-3p target gene analysis.

**Supplementary Table S5 -** CS-US pairings, investigation time and statistics corresponding to animals used for GDF-5 protein analysis.

**Supplementary Table S6 -** Statistics corresponding to all Figures.

**Supplementary Table S7 -** Group sizes corresponding to all Figures.

**Supplementary Table S8 -** Primer sequence, accession ID, and amplicon size used for RT-qPCR analysis of mouse RNA from septal tissue as well as mirVana sequences used for transfection of Neuro-2a cells.

**Supplementary Table S9 -** Antibodies, dilution, method details, company, and reference number of antibodies used for protein analysis via Western Blot or immunohistochemistry.

**Supplementary Table S 10 -** Locked nucleic acid (LNA) sequences and concentrations as well as adeno-associated viruses (AAVs) and their genome copies per ml (GC/ml) used for manipulation of septal miR-132-3p levels.

**Supplementary Table S11 -** Company and reference number of further materials used.

**Supplementary Table S12 –** Microarray analysis of Argonaute-RNA-co-immunoprecipitation after septal infusion of a miR-132-3p inhibitor locked nucleic acid compared to the respective immunoprecipitation-input control.

**Supplementary Table S13 –** Microarray analysis of Argonaute-RNA-co-immunoprecipitation after septal infusion of a scrambled locked nucleic acid compared to the respective immunoprecipitation-input control.

**Supplementary Table S14 -** Microarray analysis of Argonaute-RNA-co-immunoprecipitation after septal infusion of a miR-132-3p inhibitor locked nucleic acid compared to infusion of a scrambled locked-nucleic acid.
