# Supplementary figures and images for: Functional involvement of septal miR-132 in extinction and oxytocin-mediated reversal of social fear
Source: Mol Psychiatry. 2023 Nov 8;29(6):1754–66. doi: 10.1038/s41380-023-02309-3 (PMC11371636; doi:10.1038/s41380-023-02309-3)

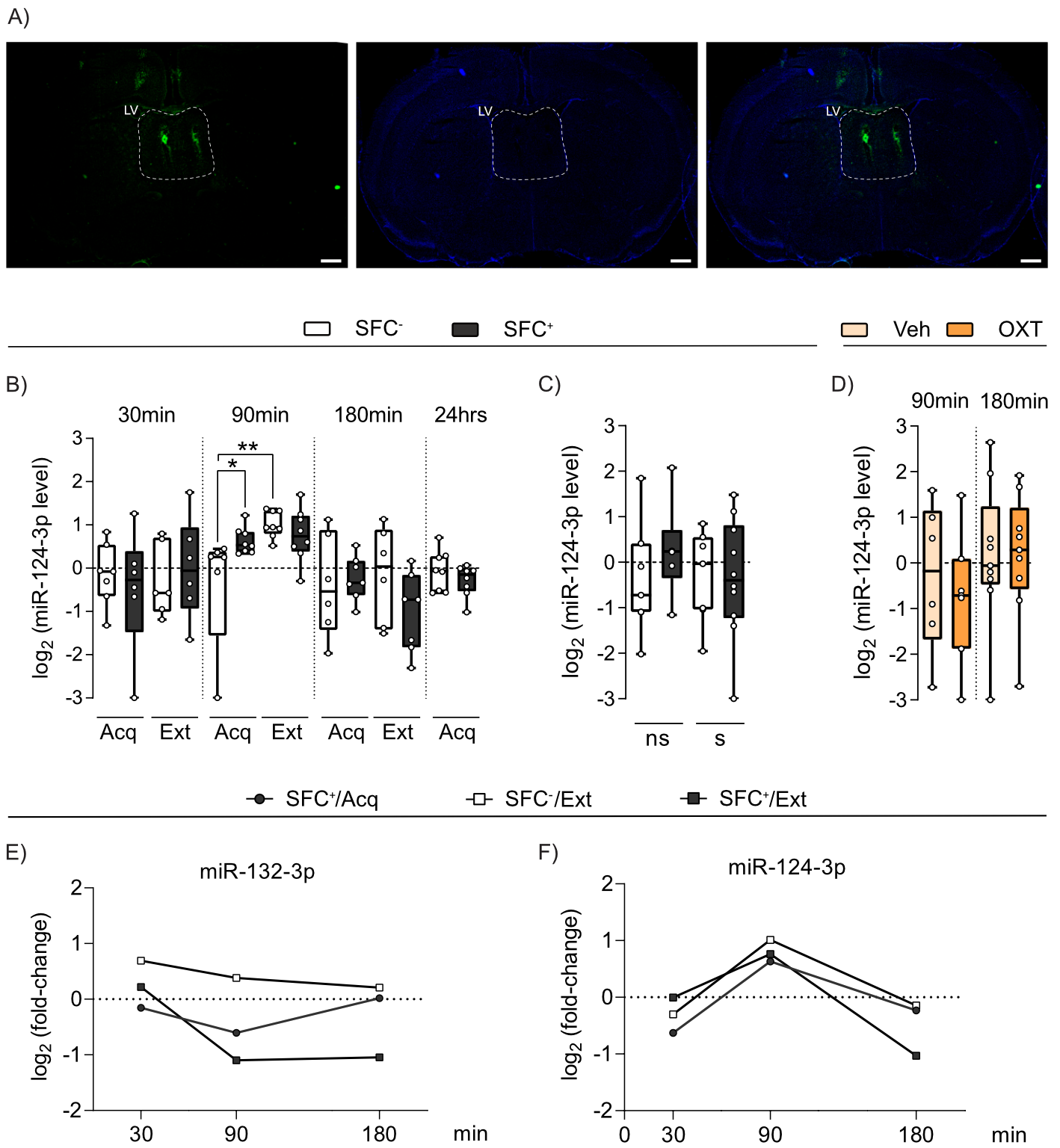

Supplement: Supplementary file 2 — Supplementary Fig. S1 [file 41380_2023_2309_MOESM2_ESM.tif]

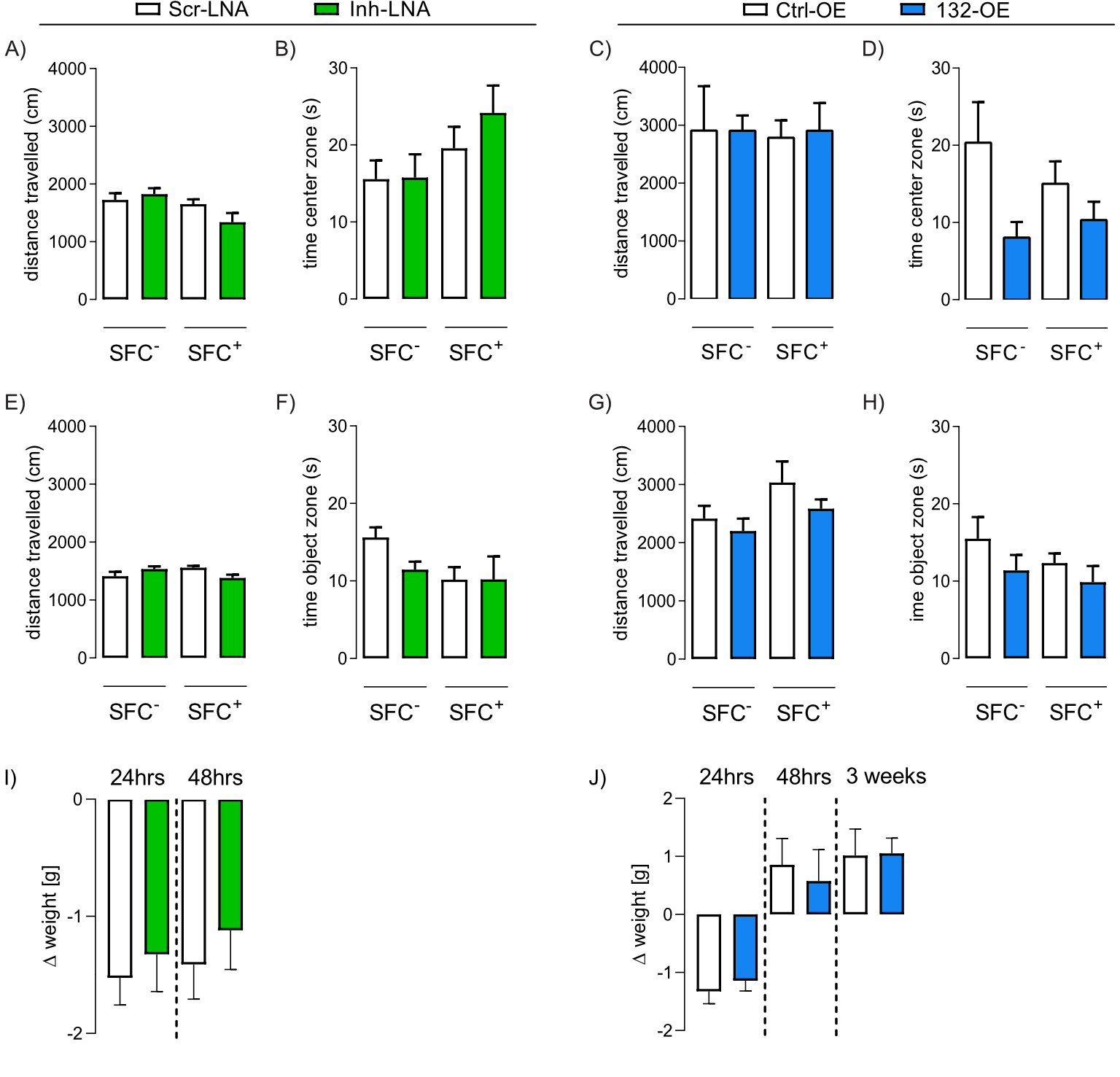

Supplement: Supplementary file 3 — Supplementary Fig. S2 [file 41380_2023_2309_MOESM3_ESM.tif]

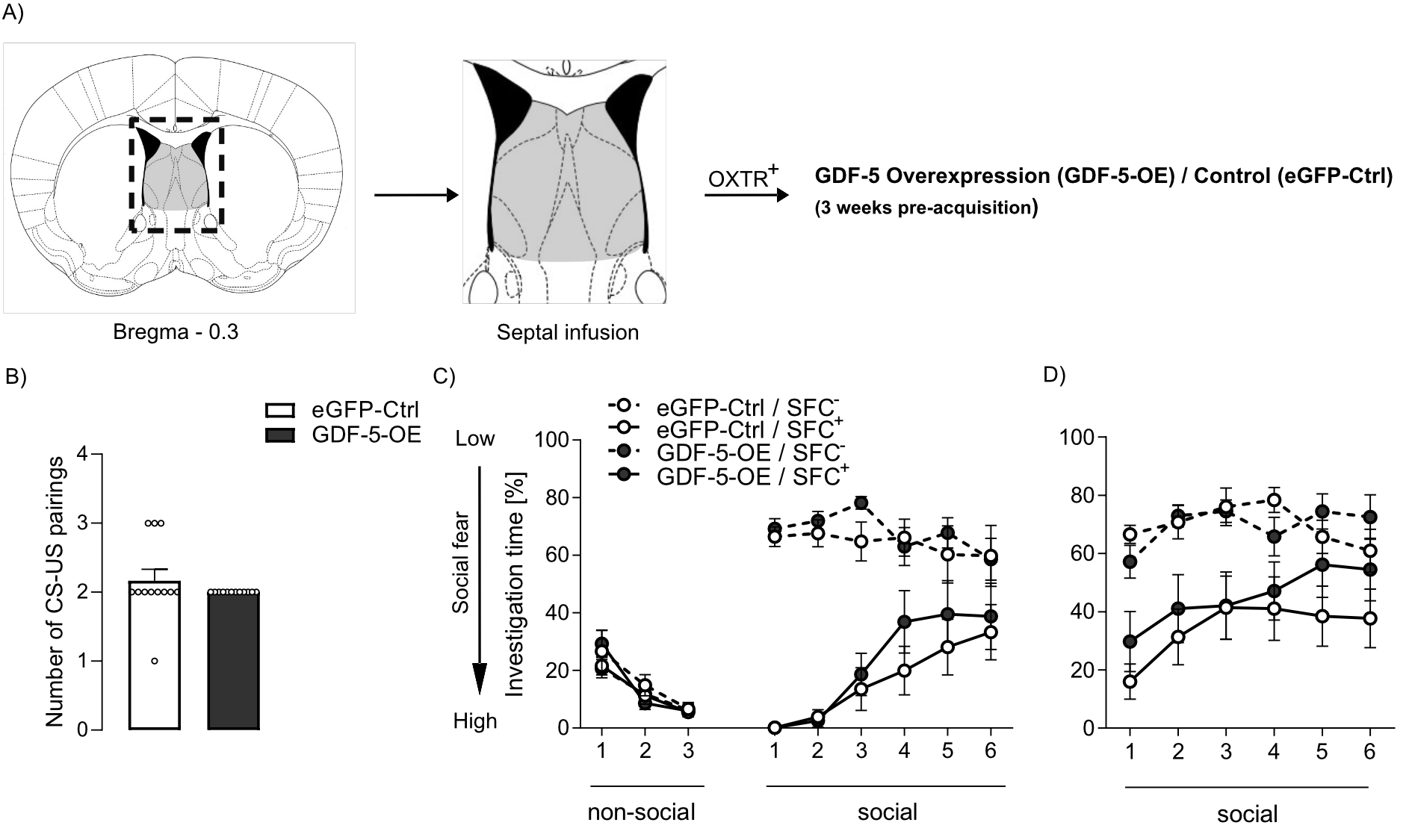

Supplement: Supplementary file 4 — Supplementary Fig. S3 [file 41380_2023_2309_MOESM4_ESM.tif]

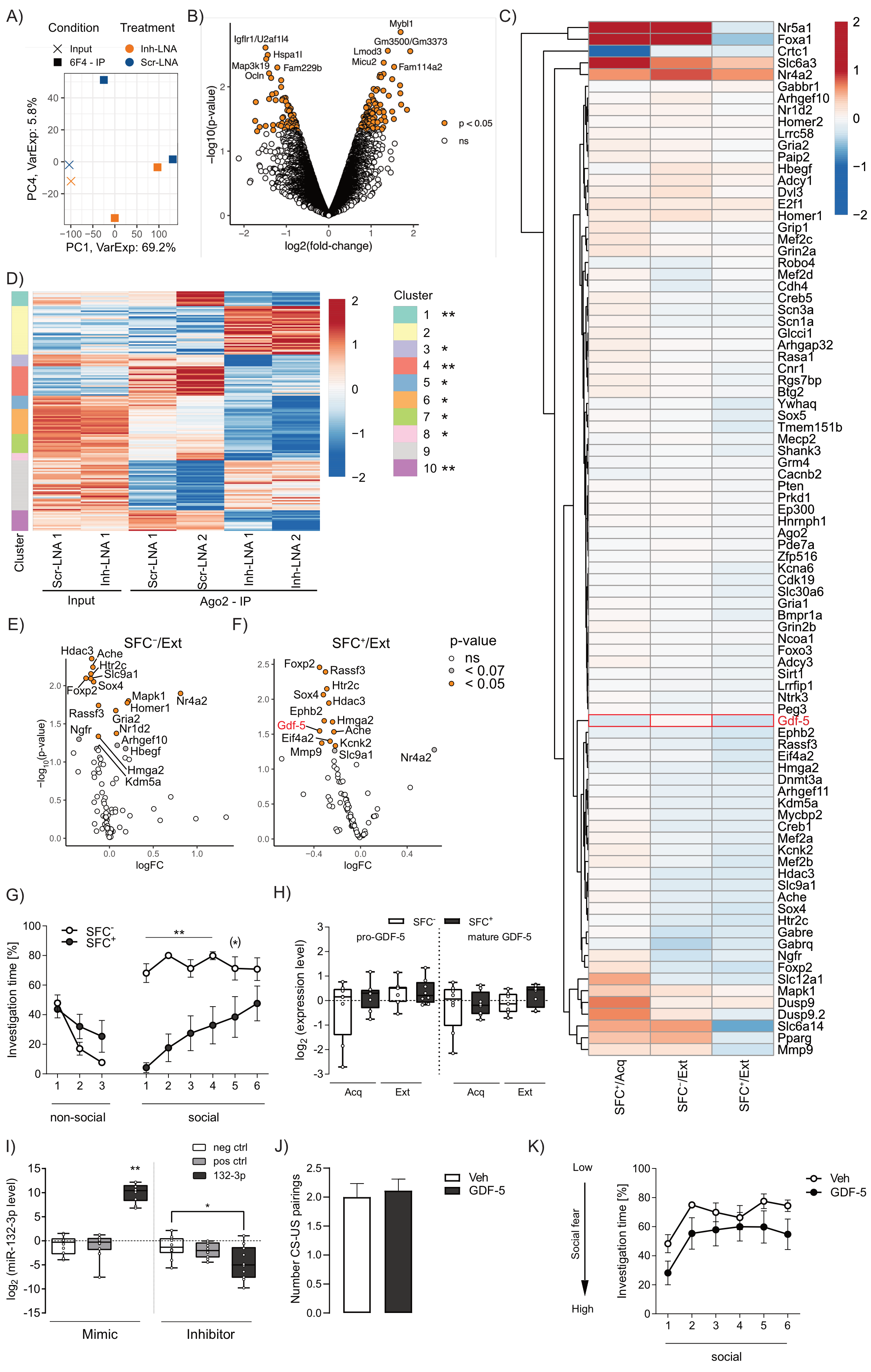

Supplement: Supplementary file 5 — Supplementary Fig. S4 [file 41380_2023_2309_MOESM5_ESM.tif]
