# Supplementary material for: Functional involvement of septal miR-132 in extinction and oxytocin-mediated reversal of social fear
Source: Mol Psychiatry. 2023 Nov 8;29(6):1754–66. doi: 10.1038/s41380-023-02309-3 (PMC11371636; doi:10.1038/s41380-023-02309-3)
Supplement: Supplementary file 6 — Supplementary Methods [file 41380_2023_2309_MOESM6_ESM.docx]

**SUPPLEMENTARY METHODS**

**Stereotactic guide cannula implantation and substance infusion:** For intracerebroventricular infusions (icv; from Bregma +0.20mm anteroposterior, +1.00mm mediolateral, -1.40mm dorsoventral [22]) or bilateral infusions into the LS (+0.30mm, ±0.50m, -1.60mm) guide cannulas (icv: 21G, 8mm; LS: 23G, 8mm) were stereotaxically implanted as described before [6]. Animals were allowed to recover for 2 (microinfusion to inhibit miR-132-3p prior to OXT infusion) or 7 (icv and intra-LS infusions) before behavioral experiments.

OXT (icv: 0.1µg/2µL, LS: 5ng/0.2µL/hemisphere; Bio-techne, Minneapolis, USA) or vehicle (Veh, sterile Ringer solution) was infused 10min prior to extinction of social fear [3, 6] or 30min or 90min before tissue harvesting. GDF-5 (0.05µg/0.2µl/hemisphere; Bio-techne) or Veh was infused 30min before extinction [37]. Correct cannula placement was histologically confirmed and, accordingly, animals with misplaced guide cannulas were removed from the statistical analysis.

**Microinfusion to manipulate septal miR-132-3p activity or transcript level:**

To inhibit miR-132-3p function within the mouse septum, locked nucleic acids (LNA; in artificial cerebrospinal fluid; Supplementary Table S10) were used as miR-132-3p inhibitor (Inh-LNA) or scrambled control LNA (Scr-LNA). Inh- or Scr-LNA was bilaterally microinfused into the LS (8x70nl per animal) at four dorsoventral positions per hemisphere (from Bregma +0.30mm anteroposterior, ±0.50mm mediolateral, and -3.80mm, -3.40mm, -3.10mm, -2.75mm dorsoventral). Animals were allowed to recover for 2 days to obtain the full LNA function, i.e., miR-132-3p inhibition, without generating compensatory effects [23]. LNAs were substituted with a 6-carboxyfluorescein (5’end). Correct microinfusion sites were analyzed by immunofluorescent microscopy.

Manipulations of miR-132 or GDF-5 in the mouse septum have also been performed using adeno-associated viruses (AAV; Supplementary Table S10): (i) Overexpression of miR-132 (132-OE) compared to expression of a scrambled miRNA (Ctrl-OE), both containing an eGFP reporter sequence (Figure 2F-H); (ii) shRNA-mediated knockdown of pre-miR-132 (sh132) specifically in OXTR expressing neurons using OXTR-Cre mice in comparison to a scrambled shRNA (shScr) as control (Figure 3E-H). Here, a SICO construct was used to turn on shRNA expression, whereas GFP expression was turned off in the presence of Cre; mCherry remained constitutively expressed under an EF1 promoter. (iii) Overexpression of Gdf-5 (GDF-5-OE) specifically in OXTR expressing neurons using OXTR-Cre mice compared to an eGFP-control (eGFP-Ctrl) (Supplementary Figure S3).

Animals were microinfused at the mentioned coordinates targeting the LS and allowed to recover for 3 weeks to establish adequate viral expression [3]. AAV reporter signals (eGFP, mCherry) were amplified by prior immunofluorescent staining (for antibodies and details see Supplementary Table S9). Animals with misplaced or unilateral microinfusion were removed from the statistical analysis.

Although microinfusions were performed directly within the LS, LNA and AAV transfection was found throughout the entire septum, i.e., within the LS and medial septum (Supplementary Figure S1A).

**c-Fos immunohistochemistry**

To assess cellular activity specifically within the dorsal LS in response to SFC, mice were transcardially perfused using 4% paraformaldehyde under deep anesthesia, brains were removed and processed for c-Fos immunoreactivity as previously described [3]. Details can be found in Supplementary Table S9. Briefly, an avidin-biotin-horseradish peroxidase procedure (Vectastain, Vector Laboratories, Burlingame, USA) with 3,30-diaminobenzidine (DAB) including nickel as the chromogen was used to visualize c-Fos-positive cells. Cells containing a nuclear brown-black reaction product were considered as c-Fos-positive. The anatomical localization of labeled cells within the LS was aided by a stereotaxic atlas [22]. The number of c-Fos-positive cells was counted bilaterally in 3 to 4 sections in a tissue area of 0.1mm^2^ by an observer blind to the experimental groups and averaged for each mouse.
