# Supplementary material for: Functional involvement of septal miR-132 in extinction and oxytocin-mediated reversal of social fear
Source: Mol Psychiatry. 2023 Nov 8;29(6):1754–66. doi: 10.1038/s41380-023-02309-3 (PMC11371636; doi:10.1038/s41380-023-02309-3)
Supplement: Supplementary file 10 — Supplementary Table S4 [file 41380_2023_2309_MOESM10_ESM.pdf]

Supplementary Table S4: CS-US pairings during acquisition of social fear, raw data, fold-change and corresponding p-value of mRNA targets analyzed via the customized RT2 Profiler PCR Array for miR-132-3p target gene analysis

	number ± SEM	
	SFC^+ / Acq	SFC^+ / Ext
CS-US pairings	2.17 ± 0.40	1.84 ± 0.17

Supplementary Table S4: CS-US pairings during acquisition of social fear, raw data, fold-change analysis and corresponding p-value of mRNA targets analyzed via the customized RT2 Profiler PCR Array for miR-132-3p target gene analysis

	Fold-change (over SFC$^-$ / Acq)			p-value (vs SFC$^-$ / Acq)			adjusted p-value (vs SFC$^-$ / Acq)		
	SFC$^+$ / Acq	SFC$^-$ / Ext	SFC$^+$ / Ext	SFC$^+$ / Acq	SFC$^-$ / Ext	SFC$^+$ / Ext	SFC$^+$ / Acq	SFC$^-$ / Ext	SFC$^+$ / Ext
Ache	0.04553	-0.18879	-0.22862	0.95000	0.41929	0.22151	0.89919	0.130248	0.289072
Adcy1	0.07899	0.17899	0.04171	0.59690	0.14074	0.22331	0.8534	0.339445	0.919765
Adcy3	0.05560	-0.03948	-0.08325	0.52559	0.65296	0.48469	0.89919	0.815519	0.797764
Ago2	-0.01053	-0.02010	-0.06473	0.35785	0.13753	0.15165	0.976156	0.896247	0.815565
Arhgap32	0.10842	0.00350	-0.03877	0.55905	0.75095	0.13336	0.89919	0.971473	0.875075
Arhgef10	0.00031	0.08846	-0.03482	0.45335	0.81409	0.65325	>0.99999	0.802911	0.885833
Arhgef11	-0.02584	-0.13990	-0.19876	0.52688	0.33692	0.83127	0.942085	0.326205	0.451338
Bmpr1a	0.01419	-0.03551	-0.12707	0.96211	0.00889	0.00861	0.976156	0.822384	0.704612
Btg2	0.10560	0.01974	-0.07751	0.41493	0.79085	0.15324	0.835114	0.896247	0.815565
Cacnb2	-0.08861	-0.04977	-0.06603	0.55543	0.57465	0.49657	0.89919	0.664313	0.815565
Cdh4	0.03787	-0.17617	-0.06947	0.21461	0.43179	0.46558	0.942085	0.350681	0.815565
Cdk19	-0.00918	-0.02272	-0.14483	0.53453	0.50917	0.03990	0.976156	0.896247	0.665766
Cnr1	0.05770	0.01170	-0.09902	0.17697	0.78246	0.34706	0.89919	0.896247	0.721763
Creb1	0.05742	-0.17772	-0.16731	0.21289	0.15868	0.02037	0.89919	0.398935	0.665766
Creb5	0.09919	-0.05373	-0.10053	0.84587	0.62896	0.39254	0.89919	0.896247	0.797764
Crtc1	-0.99079	-0.14770	-0.20956	0.06545	0.02120	0.94458	0.835114	0.896247	0.875075
Dnmt3a	-0.01086	-0.12873	-0.12533	0.01930	0.41722	0.89438	0.976156	0.389883	0.721763
Dusp9	0.70260	0.11745	0.14490	0.61714	0.52219	0.64404	0.89919	0.896247	0.880312
Dusp9	0.64657	0.18478	-0.17972	0.48852	0.89632	0.61767	0.89919	0.896247	0.721763
Dvl3	0.09165	0.22550	0.02803	0.54265	0.05029	0.30250	0.89919	0.339445	0.925248
E2f1	0.16218	0.15088	0.13239	0.95865	0.13444	0.34406	0.89919	0.802911	0.797764
Eif4a2	-0.07326	-0.07206	-0.26139	0.72834	0.07638	0.24936	0.89919	0.815519	0.342591
Ep300	0.01231	0.00550	-0.05532	0.27770	0.64727	0.27747	0.976156	0.969047	0.815565
Ephb2	-0.13768	-0.17196	-0.31194	0.15032	0.28679	0.18333	0.89919	0.435728	0.267869
Foxa1	2.205003	2.33195	0.50917	0.73045	0.00808	0.05450	0.89919	0.815519	0.665766
Foxo3	1.031816	0.972651	0.915342	0.67797	0.58245	0.07123	0.89919	0.853002	0.802449
Foxp2	0.20811	-0.26792	-0.35205	0.82393	0.50113	0.27097	0.89919	0.130248	0.180224
Gabbr1	-0.03316	0.02412	0.06239	0.20868	0.04217	0.95255	0.982897	0.800938	0.665766
Gabre	0.01373	-0.35736	-0.24080	0.01837	0.01263	0.05286	0.982897	0.398935	0.721763
Gabrq	-0.08630	-0.40987	-0.27656	0.39131	0.55380	0.33512	0.942085	0.335554	0.6926
Gdf5	-0.25379	0.01523	-0.35148	0.57790	0.19460	0.35803	0.835114	0.971473	0.289072
Glcci1	0.08506	-0.02404	-0.02720	0.49495	0.00699	0.00709	0.89919	0.896247	0.925248
Gria1	0.01064	-0.03445	-0.11051	0.35614	0.41154	0.09243	0.976156	0.896247	0.742326
Gria2	0.09832	0.07255	-0.00899	0.38065	0.28473	0.89527	0.835114	0.169339	0.962072
Grin2a	0.17391	0.06755	0.02102	0.20544	0.08726	0.44415	0.571918	0.800938	0.925248
Grin2b	0.04005	-0.04775	-0.05685	0.90082	0.46601	0.14946	0.914129	0.815519	0.815565
Grip1	0.18461	-0.06525	-0.03937	0.42273	0.09170	0.04636	0.89919	0.715793	0.880885
Grm4	-0.07092	-0.01314	-0.07038	0.54106	0.29485	0.64855	0.89919	0.93761	0.815565
Hbegf	-0.05268	0.18424	0.11747	0.37774	0.10620	0.37355	0.89919	0.326205	0.797764
Hdac3	0.03429	-0.20268	-0.27178	0.82658	0.54283	0.27463	0.89919	0.000145	0.201533
Hmga2	-0.08972	-0.12646	-0.24018	0.00013	0.01584	0.58622	0.89919	0.28966	0.267869
Hnrnph1	0.01744	0.00745	-0.06388	0.53566	0.50691	0.04240	0.942085	0.896247	0.815565
Homer1	0.13388	0.20375	0.11147	0.95801	0.10775	0.30316	0.835114	0.159391	0.797764
Homer2	0.02190	0.07754	0.02093	0.46795	0.00000	0.01134	0.942085	0.440729	0.925248
Htr2c	-0.03167	-0.21141	-0.28928	0.58444	0.00572	0.02927	0.89919	0.130248	0.191243
Kcna6	-0.00807	-0.03858	-0.15213	0.55350	0.19127	0.61078	0.976156	0.802911	0.5448
Kcnk2	0.08184	-0.09977	-0.21603	0.35558	0.71869	0.37117	0.89919	0.339445	0.343405
Kdm5a	0.02065	-0.14539	-0.19509	0.91158	0.68163	0.23221	0.944783	0.326205	0.538787

Lrrc58	0.05626	0.03142	0.03633	0.90406	0.81294	0.59698	0.89919	0.802911	0.885833
Lrrfip1	-0.01190	-0.05982	-0.08651	0.90051	0.12424	0.34471	0.976156	0.734948	0.797764
Mapk1	0.37611	0.21747	0.06883	0.27951	0.82154	0.74284	0.011297	0.159391	0.815565
Mecp2	-0.05791	0.01010	-0.11220	0.76454	0.06681	0.13099	0.89919	0.896247	0.721763
Mef2a	0.06322	-0.11696	-0.18380	0.56197	0.75365	0.62642	0.89919	0.398935	0.5448
Mef2b	0.08804	-0.08189	-0.27991	0.36638	0.82889	0.36579	0.89919	0.896247	0.538787
Mef2c	0.17418	0.03964	-0.07758	0.14026	0.32584	0.75323	0.89919	0.896247	0.815565
Mef2d	0.07998	-0.17421	-0.02751	0.72434	0.16552	0.87643	0.89919	0.722075	0.919765
Mmp9	0.20763	0.23406	-0.33300	0.07335	0.01678	0.47625	0.89919	0.815519	0.342591
Mycbp2	0.02942	-0.12766	-0.19462	0.55785	0.83819	0.09954	0.942085	0.326454	0.526453
Ncoa1	0.02259	-0.03774	-0.06404	0.12637	0.04615	0.02110	0.914129	0.800938	0.815565
Ngfr	0.14759	-0.34816	-0.17445	0.85314	0.93740	0.66067	0.89919	0.294608	0.721763
Nr1d2	0.05215	0.07954	-0.00706	0.29924	0.97291	0.72857	0.89919	0.285043	0.962072
Nr4a2	0.63013	0.81156	0.63285	0.62738	0.39594	0.59108	0.571918	0.158492	0.345982
Nr5a1	3.15671	0.98657	-0.26819	0.57326	0.52902	0.22929	0.89919	0.285043	0.721763
Ntrk3	0.03560	-0.08803	-0.11834	0.22038	0.78522	0.32130	0.89919	0.488568	0.721763
Paip2	0.09101	0.06796	-0.03329	0.16870	0.00799	0.00349	0.89919	0.802911	0.900248
Pde7a	-0.00874	0.02024	-0.06977	0.07517	0.80608	0.72194	0.976156	0.896247	0.815565
Peg3	0.02799	-0.08368	-0.07401	0.71404	0.07059	0.11846	0.944783	0.614716	0.815565
Pparg	0.42596	0.50600	-0.22559	0.99700	0.44299	0.77740	0.89919	0.800938	0.456417
Prkd1	0.03050	0.01588	-0.05779	0.58775	0.06682	0.47378	0.89919	0.896247	0.815565
Pten	0.02282	0.03860	-0.05740	0.74434	0.84551	0.54199	0.89919	0.488568	0.815565
Rasa1	0.09838	-0.00182	-0.05242	0.26607	0.45981	0.77210	0.840125	0.971473	0.815565
Rassf3	-0.04389	-0.12483	-0.30071	0.03819	0.75932	0.54235	0.89919	0.159391	0.180224
Rgs7bp	0.09096	0.02039	-0.10800	0.09602	0.08827	0.83822	0.89919	0.896247	0.721763
Robo4	-0.08771	-0.14040	-0.02159	0.45072	0.52762	0.48032	0.89919	0.663167	0.925248
Scn1a	0.07703	-0.10040	-0.11692	0.73137	0.10105	0.64897	0.89919	0.350681	0.721763
Scn3a	0.09135	-0.11025	-0.08474	0.86372	0.73135	0.63471	0.89919	0.339445	0.797764
Shank3	-0.05802	-0.01662	-0.13316	0.44973	0.09271	0.86288	0.89919	0.896247	0.665766
Sirt1	-0.00296	-0.06978	-0.11442	0.76535	0.25185	0.56334	0.982897	0.350681	0.721763
Slc12a1	0.53775	-0.16946	-0.26011	0.40847	0.76980	0.23143	0.89919	0.896247	0.704612
Slc30a6	-0.01078	-0.01644	-0.12038	0.84888	0.71619	0.07702	0.976156	0.815519	0.704612
Slc6a14	0.53889	0.57887	-0.67685	0.06788	0.95957	0.02841	0.942085	0.853002	0.422086
Slc6a3	0.98198	0.69997	0.42634	0.42090	0.73574	0.86690	0.89919	0.663167	0.626703
Slc9a1	0.02361	-0.21185	-0.22202	0.52760	0.01814	0.00406	0.942085	0.130248	0.345982
Sox4	0.00452	-0.18039	-0.31906	0.65861	0.84657	0.53369	0.982897	0.130248	0.191243
Sox5	0.03726	-0.01362	-0.16811	0.71066	0.06050	0.08633	0.89919	0.896247	0.5448
Tmem151b	0.01678	-0.03611	-0.22710	0.08507	0.96813	0.64611	0.976156	0.896247	0.427854
Ywhaq	-0.06220	-0.01380	-0.20276	0.36870	0.45207	0.80018	0.89919	0.896247	0.465649
Zfp516	-0.02799	0.01615	-0.07947	0.89786	0.35129	0.47804	0.944783	0.896247	0.815565

Housekeeper Actb
B2m
Gapdh
Gusb
Hsp90ab1

p-value vs SFC^- / Acq
Independent student t-test; two-tailed

adjusted p-value vs SFC- / Acq
Benjamini, Krieger and Yekutieli FDR correction

Supplementary Table S4: CS-US pairings during acquisition of social fear, raw data, fold-change and corresponding p-value of mRNA targets analyzed via the customized RT2 Profiler PCR Array for miR-132-3p target gene analysis

Metadata

**title**	PCR Array to determine septal target mRNAs of miR-132-3p in the context of social fear conditioning (SFC)
**raw data**	Ct values
**data processing**	2^deltaCt; Target gene signals normalized to geometric mean of housekeeping genes
**overall design**	unconditioned mice (SFC-), conditioned mice (SFC+); 90 min after acquisition (Acq) or extinction (Ext)

**Sample No**	**group**
**SAMPLE 1**	SFC^- / Acq
**SAMPLE 2**	SFC^+ / Acq
**SAMPLE 3**	SFC^- / Acq
**SAMPLE 4**	SFC^+ / Acq
**SAMPLE 5**	SFC^- / Acq
**SAMPLE 6**	SFC^+ / Acq
**SAMPLE 7**	SFC^- / Acq
**SAMPLE 8**	SFC^+ / Acq
**SAMPLE 9**	SFC^- / Acq
**SAMPLE 10**	SFC^+ / Acq
**SAMPLE 11**	SFC^- / Acq
**SAMPLE 12**	SFC^+ / Acq
**SAMPLE 13**	SFC^- / Ext
**SAMPLE 14**	SFC^+ / Ext
**SAMPLE 15**	SFC^- / Ext
**SAMPLE 16**	SFC^+ / Ext
**SAMPLE 17**	SFC^- / Ext
**SAMPLE 18**	SFC^+ / Ext
**SAMPLE 19**	SFC^- / Ext
**SAMPLE 20**	SFC^+ / Ext
**SAMPLE 21**	SFC^- / Ext
**SAMPLE 22**	SFC^+ / Ext
**SAMPLE 23**	SFC^- / Ext
**SAMPLE 24**	SFC^+ / Ext
**GDC**	genomic DNA control
**PPC**	positice PCR control
**RTC**	reverse transcription control

Non-normalized table of PCR-Array data

**Target Name**	**Sample No**	**raw Ct_Target**	**Sample No**	**raw Ct_Target**

Adcy1	1	22,60313225	3	22,64016342
Adcy1	2	22,50770187	4	22,68219376
Cnr1	1	22,21455574	3	22,37061501
Cnr1	2	22,43509865	4	22,31768227
Creb1	1	26,87860107	3	27,6257782
Creb1	2	27,36748886	4	27,43729591
Ephb2	1	25,87789154	3	25,89164543
Ephb2	2	25,91315269	4	26,71964645
Gria1	1	19,49512672	3	19,80296707
Gria1	2	19,51756477	4	19,94206619
Gria2	1	19,30721474	3	19,62005043
Gria2	2	19,47867012	4	19,554739
Grin2a	1	23,51861763	3	23,86934471
Grin2a	2	23,62645721	4	23,5430088
Grin2b	1	22,25593376	3	22,55309868
Grin2b	2	22,70895004	4	22,6084156
Grip1	1	25,26977539	3	25,65240479
Grip1	2	25,66571045	4	25,4587307
Grm4	1	23,14010048	3	23,56345749
Grm4	2	23,53558922	4	23,68627548
Homer1	1	20,69892311	3	21,19844627
Homer1	2	20,87158966	4	20,87627602
Mapk1	1	20,42567825	3	20,80108643
Mapk1	2	20,15254211	4	20,34255981
Adcy1	13	22,61764336	15	21,97721481
Adcy1	14	22,83632851	16	22,46899414
Cnr1	13	22,57167816	15	22,13408089
Cnr1	14	22,35305786	16	22,12803841
Creb1	13	27,59218979	15	27,74800301
Creb1	14	27,29895592	16	27,2403183
Ephb2	13	25,98864746	15	26,45176315
Ephb2	14	26,35908127	16	26,13243866
Gria1	13	19,6872673	15	19,80038452
Gria1	14	19,71111298	16	19,63800049
Gria2	13	19,66562462	15	19,31519699
Gria2	14	19,32646179	16	19,27871323
Grin2a	13	23,95016479	15	23,54948807
Grin2a	14	23,68696976	16	23,78759384
Grin2b	13	22,85006905	15	22,69384575
Grin2b	14	22,55730438	16	22,35817528
Grip1	13	25,77497101	15	25,45274353
Grip1	14	25,30652618	16	25,41705704
Grm4	13	23,40960693	15	23,36696815
Grm4	14	23,32842255	16	23,19249916
Homer1	13	20,97478294	15	20,54657936
Homer1	14	20,81409645	16	20,55944061
Mapk1	13	20,46769905	15	20,34952736
Mapk1	14	20,68777657	16	20,36749077
Mmp9	1	30,23545265	3	30,68392944

Mmp9	2	30,62952042	4	30,84763718
Ngfr	1	23,93907166	3	24,84211922
Ngfr	2	25,3010006	4	24,45618248
Sirt1	1	24,10849571	3	24,15803719
Sirt1	2	24,33201027	4	24,27349472
Ywhaq	1	19,17502975	3	19,24635887
Ywhaq	2	19,00156784	4	20,03708649
Gabbr1	1	20,39949799	3	20,58219528
Gabbr1	2	20,76397133	4	20,72983932
Mecp2	1	22,46500969	3	22,72017479
Mecp2	2	22,71932793	4	22,68053055
Hmga2	1	21,92411423	3	22,20012665
Hmga2	2	22,31921768	4	22,32571793
Pten	1	20,95732307	3	21,37368584
Pten	2	21,18527031	4	21,15897369
Hdac3	1	23,78162956	3	24,14916992
Hdac3	2	24,15114594	4	23,9392128
Creb5	1	26,97697639	3	26,66256523
Creb5	2	26,2928791	4	27,43504333
Ache	1	22,31913567	3	22,35377884
Ache	2	22,6626091	4	22,28874016
Sox4	1	26,41378212	3	26,56303787
Sox4	2	26,49588013	4	26,02110481
Mmp9	13	31,25043106	15	29,51606178
Mmp9	14	30,91051483	16	31,52794647
Ngfr	13	26,16044235	15	24,71814537
Ngfr	14	25,00818634	16	24,82550812
Sirt1	13	24,50445938	15	24,22304535
Sirt1	14	24,12970352	16	24,23969841
Ywhaq	13	19,30171585	15	19,09739304
Ywhaq	14	19,43861961	16	18,79016495
Gabbr1	13	20,69321632	15	20,83332253
Gabbr1	14	20,7442894	16	20,45461655
Mecp2	13	22,80127716	15	22,67102432
Mecp2	14	22,67151833	16	22,58944702
Hmga2	13	22,50993919	15	22,29167938
Hmga2	14	22,41773033	16	22,1623745
Pten	13	21,31332016	15	20,86840057
Pten	14	21,02616692	16	20,8943615
Hdac3	13	24,49591637	15	24,09211922
Hdac3	14	24,20083046	16	24,1634388
Creb5	13	27,21290207	15	27,20394897
Creb5	14	27,21764374	16	26,43035126
Ache	13	22,80541801	15	22,65284729
Ache	14	22,78302765	16	22,38155937
Sox4	13	26,80126381	15	26,680233
Sox4	14	27,1229229	16	26,78052139
Nr4a2	1	27,4864769	3	27,26628494
Nr4a2	2	26,45498085	4	26,99921989

Cdh4	1	25,43484879	3	25,6194725
Cdh4	2	25,92547035	4	25,95226479
Ntrk3	1	22,31648445	3	22,68035889
Ntrk3	2	22,64416122	4	22,7752533
Ep300	1	23,26369476	3	23,82826233
Ep300	2	23,71722031	4	23,62063408
Arhgap32	1	23,11282158	3	23,38860512
Arhgap32	2	23,50158501	4	22,82342148
Foxo3	1	22,98530579	3	23,34867477
Foxo3	2	23,26521111	4	23,18730545
Ago2	1	22,51002884	3	22,8534584
Ago2	2	22,80564117	4	22,90356827
Dvl3	1	26,76450539	3	26,88109207
Dvl3	2	26,8302536	4	26,92698479
Pparg	1	30,29424477	3	30,11195183
Pparg	2	30,52608871	4	29,79089165
Peg3	1	19,6127243	3	19,73936844
Peg3	2	20,17107582	4	19,65126419
Htr2c	1	21,79801369	3	21,99159813
Htr2c	2	21,72534752	4	22,00474167
Ncoa1	1	22,93412399	3	23,18703651
Ncoa1	2	23,18507385	4	23,0956974
Nr4a2	13	26,98557281	15	25,79033279
Nr4a2	14	26,4079113	16	26,55616379
Cdh4	13	26,39187241	15	25,74711037
Cdh4	14	25,84386635	16	25,6784687
Ntrk3	13	22,88476753	15	22,82449722
Ntrk3	14	22,63320541	16	22,62735367
Ep300	13	23,72899437	15	23,67329788
Ep300	14	23,59467316	16	23,53014755
Arhgap32	13	23,70693207	15	23,40431786
Arhgap32	14	23,50469017	16	23,1691246
Foxo3	13	23,31346893	15	23,28327751
Foxo3	14	23,35918808	16	23,21543884
Ago2	13	22,96305275	15	22,81316376
Ago2	14	22,7737999	16	22,75022125
Dvl3	13	26,83491135	15	26,51101112
Dvl3	14	26,5830574	16	26,19191742
Pparg	13	30,64071465	15	29,9197998
Pparg	14	30,77666092	16	30,35786247
Peg3	13	20,17735863	15	19,89980125
Peg3	14	19,88370323	16	19,74973488
Htr2c	13	22,24374199	15	22,4744854
Htr2c	14	22,40086174	16	22,00612831
Ncoa1	13	23,37648964	15	23,11210251
Ncoa1	14	22,95464706	16	22,91504097

Robo4	1	27,71628189	3	27,76289749
Robo4	2	27,89284515	4	27,63365364
Nr5a1	1	32,85213089	3	Undetermined

Nr5a1	2	34,40936279	4	Undetermined
E2f1	1	28,57919502	3	29,12886238
E2f1	2	28,49924278	4	28,48087311
Shank3	1	20,9576664	3	21,09319305
Shank3	2	21,42498016	4	21,12295532
Homer2	1	22,34321594	3	22,64711761
Homer2	2	22,42233276	4	22,61562538
Foxa1	1	Undetermined	3	33,33016205
Foxa1	2	34,47854614	4	34,26047897
Rgs7bp	1	21,56830978	3	21,92454147
Rgs7bp	2	21,91711998	4	21,65744591
Foxp2	1	25,4177494	3	25,73235512
Foxp2	2	25,95471954	4	24,99742126
Crtc1	1	24,43195724	3	34,82056427
Crtc1	2	32,34897614	4	32,86230087
Dusp9	1	30,89291763	3	30,58502007
Dusp9	2	31,54645157	4	31,75935745
Cdk19	1	26,65496063	3	26,5859108
Cdk19	2	26,40157509	4	26,56050491
Adcy3	1	23,10974312	3	23,57088852
Adcy3	2	23,51119995	4	23,32560921
Robo4	13	28,15414047	15	28,37934685
Robo4	14	27,69804001	16	27,69190216
Nr5a1	13	35,14324951	15	33,99924088
Nr5a1	14	34,12182617	16	Undetermined
E2f1	13	28,78486252	15	28,31724548
E2f1	14	28,361763	16	28,40419388
Shank3	13	21,46168709	15	21,00106049
Shank3	14	21,21392059	16	20,80018234
Homer2	13	22,79484367	15	22,39570045
Homer2	14	22,24295807	16	22,01105881
Foxa1	13	Undetermined	15	Undetermined
Foxa1	14	33,89967728	16	Undetermined
Rgs7bp	13	21,94160652	15	21,70601654
Rgs7bp	14	22,08183479	16	21,7515564
Foxp2	13	26,36029816	15	25,72725487
Foxp2	14	26,43147278	16	26,34769058
Crtc1	13	33,28149796	15	32,77161789
Crtc1	14	31,28914452	16	24,95004654
Dusp9	13	32,53790283	15	31,60126877
Dusp9	14	31,66410446	16	31,70464134
Cdk19	13	26,75950813	15	26,59130287
Cdk19	14	26,7953949	16	26,60353661
Adcy3	13	23,76861382	15	23,26993752
Adcy3	14	23,44133759	16	23,41282082

Pde7a	1	24,68088913	3	25,1026001
Pde7a	2	25,07896614	4	25,11025238
Mycbp2	1	21,78974533	3	22,38758659
Mycbp2	2	22,37874031	4	22,24432182

Scn3a	1	24,28834915	3	24,7153511
Scn3a	2	24,48769379	4	24,44368744
Arhgef11	1	23,37063599	3	23,50086784
Arhgef11	2	23,83224869	4	23,29520416
Tmem151b	1	24,39510727	3	24,54580498
Tmem151b	2	24,72169495	4	24,57380867
Kcnk2	1	22,94714165	3	22,8194046
Kcnk2	2	23,22964668	4	22,63492203
Hbegf	1	25,67870522	3	25,83310318
Hbegf	2	25,77183151	4	25,59204292
Eif4a2	1	20,68882179	3	20,4931488
Eif4a2	2	20,77025604	4	20,61893463
Gdf5	1	29,98099327	3	29,94593048
Gdf5	2	29,80574417	4	30,44621849
Dnmt3a	1	23,59198189	3	24,21209145
Dnmt3a	2	24,19030571	4	23,96704865
Slc6a14	1	34,24305725	3	Undetermined
Slc6a14	2	Undetermined	4	37,7348175
Cacnb2	1	23,3130455	3	23,77648735
Cacnb2	2	23,66346931	4	23,73039818
Pde7a	13	24,83080292	15	24,97231483
Pde7a	14	24,92265701	16	24,93251419
Mycbp2	13	22,52942848	15	22,28058434
Mycbp2	14	22,41100311	16	22,31472588
Scn3a	13	24,80576134	15	24,69096947
Scn3a	14	24,57016945	16	24,49866676
Arhgef11	13	23,73646736	15	23,69369698
Arhgef11	14	23,62965012	16	23,33213806
Tmem151b	13	24,89535141	15	24,31715965
Tmem151b	14	25,21389198	16	24,79171753
Kcnk2	13	23,287323	15	22,72416115
Kcnk2	14	23,26507378	16	22,8017025
Hbegf	13	25,65784836	15	25,38079643
Hbegf	14	25,25307083	16	25,40788078
Eif4a2	13	21,15953827	15	20,48412323
Eif4a2	14	20,97051811	16	20,69128609
Gdf5	13	30,56719017	15	30,44238663
Gdf5	14	29,88309288	16	30,12132072
Dnmt3a	13	24,31100273	15	24,29570007
Dnmt3a	14	24,02867508	16	24,09327126
Slc6a14	13	Undetermined	15	Undetermined
Slc6a14	14	34,33181381	16	35,13959503
Cacnb2	13	23,80604553	15	23,6248455
Cacnb2	14	23,33480644	16	23,68058586

Scn1a	1	23,32022667	3	23,46232986
Scn1a	2	23,47822189	4	23,29930878
Glcci1	1	24,59950829	3	25,07905769
Glcci1	2	25,09841156	4	24,65725327
Slc30a6	1	24,35136604	3	24,48217773

Slc30a6	2	24,59424591	4	24,44880104
Dusp9	1	31,52108002	3	30,81035233
Dusp9	2	32,42429733	4	31,4315834
Bmpr1a	1	23,22169495	3	23,42763901
Bmpr1a	2	22,93963242	4	23,29726601
Rassf3	1	26,19314575	3	26,34879303
Rassf3	2	26,71203041	4	26,2490139
Kdm5a	1	24,46816635	3	25,09194565
Kdm5a	2	24,84763908	4	24,96352005
Sox5	1	24,3379364	3	24,35262489
Sox5	2	24,33745193	4	24,5111351
Hnrnph1	1	20,9304142	3	21,29165459
Hnrnph1	2	21,14172745	4	21,16825485
Zfp516	1	26,12191582	3	26,5087471
Zfp516	2	26,41241646	4	26,13952446
Kcna6	1	23,34492493	3	23,40773392
Kcna6	2	23,59076881	4	23,54057884
Lrrc58	1	23,51761436	3	23,47279358
Lrrc58	2	23,24995995	4	23,42397881
Scn1a	13	23,77212906	15	23,43936157
Scn1a	14	23,47797394	16	23,44643021
Glcci1	13	25,27734375	15	24,9285717
Glcci1	14	25,19580078	16	25,02819061
Slc30a6	13	24,58840561	15	24,3512764
Slc30a6	14	24,43593407	16	24,26334381
Dusp9	13	33,70451355	15	31,98760223
Dusp9	14	32,44670486	16	30,81590271
Bmpr1a	13	23,51213074	15	23,15608597
Bmpr1a	14	23,4561882	16	22,98225784
Rassf3	13	26,75335121	15	26,23219299
Rassf3	14	26,92529678	16	26,38352013
Kdm5a	13	25,22026825	15	25,00978851
Kdm5a	14	25,19165421	16	24,90371513
Sox5	13	24,73464584	15	24,40404701
Sox5	14	24,5632534	16	24,34552956
Hnrnph1	13	21,27513695	15	21,13172531
Hnrnph1	14	21,15691376	16	20,90151978
Zfp516	13	26,2419281	15	26,32436752
Zfp516	14	26,29990768	16	25,87286949
Kcna6	13	23,64649963	15	23,37833786
Kcna6	14	23,66778374	16	23,22717857
Lrrc58	13	23,61486626	15	23,26446342
Lrrc58	14	23,26481056	16	22,95175743

Prkd1	1	25,36934471	3	25,43551636
Prkd1	2	25,52604485	4	25,54935265
Btg2	1	26,15592003	3	26,39699364
Btg2	2	25,96050644	4	26,27641487
Gabre	1	25,82963371	3	25,73768425
Gabre	2	26,70808411	4	26,52171516

Gabrq	1	24,58920479	3	24,52676582
Gabrq	2	26,18563652	4	25,22472954
Slc12a1	1	31,02320671	3	31,10155678
Slc12a1	2	30,21915817	4	30,92248344
Slc6a3	1	32,16132736	3	32,74918365
Slc6a3	2	31,11320305	4	30,98613358
Slc9a1	1	23,76258087	3	24,23332596
Slc9a1	2	24,20379829	4	24,23642349
Nr1d2	1	22,70645523	3	23,0127449
Nr1d2	2	22,71211433	4	22,9300251
Mef2a	1	22,25638771	3	22,72883224
Mef2a	2	22,57632828	4	22,6219902
Mef2b	1	29,59714508	3	28,77400398
Mef2b	2	29,06484413	4	29,52986145
Mef2c	1	24,53132439	3	24,60595703
Mef2c	2	24,87996292	4	23,80066299
Mef2d	1	25,52160645	3	25,76184464
Mef2d	2	25,88537216	4	25,33930969
Prkd1	13	25,57320213	15	25,48009109
Prkd1	14	25,33157158	16	25,10186958
Btg2	13	26,19299698	15	26,1711998
Btg2	14	26,4563427	16	26,29587173
Gabre	13	27,23010254	15	26,49300003
Gabre	14	27,09238625	16	26,14720535
Gabrq	13	26,32867622	15	25,20791435
Gabrq	14	26,08815384	16	24,86912346
Slc12a1	13	31,50789452	15	31,14557838
Slc12a1	14	30,12250328	16	30,93134689
Slc6a3	13	33,36841965	15	30,65831375
Slc6a3	14	30,66814804	16	31,70363426
Slc9a1	13	24,48241615	15	24,42732811
Slc9a1	14	24,40479851	16	24,26698875
Nr1d2	13	22,95513725	15	22,80324554
Nr1d2	14	22,69565964	16	22,60326767
Mef2a	13	22,74473	15	22,72287178
Mef2a	14	22,73931122	16	22,76980019
Mef2b	13	29,90411949	15	29,11018372
Mef2b	14	29,87910652	16	29,83575439
Mef2c	13	25,14657211	15	24,35681534
Mef2c	14	24,9342308	16	24,50574875
Mef2d	13	26,09375763	15	25,61637115
Mef2d	14	25,64409256	16	25,53603935

Arhgef10	1	24,12106895	3	23,94504738
Arhgef10	2	23,73613167	4	24,46830177
Rasa1	1	23,24930763	3	23,18561363
Rasa1	2	22,73834801	4	22,87964439
Paip2	1	21,7623291	3	21,20510674
Paip2	2	21,23226738	4	21,30455017
Lrrfip1	1	21,88721085	3	22,48818207

Lrrfip1	2	22,36584663	4	22,84971619
Actb	1	17,99102974	3	18,21930122
Actb	2	18,17956543	4	18,218153
B2m	1	22,2664299	3	22,46067238
B2m	2	22,15479469	4	22,40383148
Gapdh	1	17,98744011	3	18,2497406
Gapdh	2	18,08336639	4	17,97969818
Gusb	1	25,58551407	3	25,65475273
Gusb	2	25,27503014	4	25,94795799
Hsp90ab1	1	17,85575294	3	18,269907
Hsp90ab1	2	18,14155197	4	17,98318863
GDC	1	Undetermined	3	35,94869614
GDC	2	38,69247437	4	39,53117752
PPC	1	18,71460533	3	18,82476616
PPC	2	18,78368759	4	18,78552246
RTC	1	19,72883797	3	19,44197655
RTC	2	19,66075706	4	19,50373459
Arhgef10	13	23,68744659	15	23,85604477
Arhgef10	14	24,2700634	16	23,54295921
Rasa1	13	23,19468689	15	22,94867897
Rasa1	14	22,96776581	16	22,80944824
Paip2	13	21,47929001	15	20,95345497
Paip2	14	21,36314774	16	20,86252213
Lrrfip1	13	22,41015816	15	22,46687698
Lrrfip1	14	22,37047005	16	22,06505775
Actb	13	18,397789	15	17,90971565
Actb	14	18,25693703	16	17,85779762
B2m	13	22,48405838	15	21,97538948
B2m	14	22,5295372	16	21,4800415
Gapdh	13	18,43367577	15	17,99176788
Gapdh	14	18,29116821	16	18,06473732
Gusb	13	25,76580238	15	25,75156021
Gusb	14	25,74248505	16	25,57449341
Hsp90ab1	13	18,17626381	15	17,80265617
Hsp90ab1	14	18,22946358	16	17,49210358
GDC	13	38,02817917	15	39,45472717
GDC	14	35,35371017	16	35,42192841
PPC	13	18,92947769	15	18,72332191
PPC	14	18,95481491	16	18,67319107
RTC	13	20,80157471	15	19,42786598
RTC	14	19,86091423	16	19,43982506
Adcy1	5	23,41088867	7	22,64554596
Adcy1	6	23,2973671	8	22,44009399
Cnr1	5	23,11952972	7	22,24698257
Cnr1	6	22,93000412	8	21,9836235
Creb1	5	28,48388863	7	26,95604324
Creb1	6	27,84019089	8	26,71382713
Ephb2	5	26,71288681	7	26,38680267
Ephb2	6	26,68836212	8	26,46357918

Gria1	5	20,1614399	7	19,79848289
Gria1	6	20,23689079	8	19,4779129
Gria2	5	20,23644829	7	19,49056625
Gria2	6	19,90682602	8	18,9903698
Grin2a	5	24,28616714	7	23,83067894
Grin2a	6	24,16697884	8	23,36792374
Grin2b	5	23,42457771	7	22,60076523
Grin2b	6	23,41222382	8	22,1871357
Grip1	5	26,18626595	7	25,28714943
Grip1	6	25,8800621	8	24,7168808
Grm4	5	23,61112595	7	23,2641983
Grm4	6	23,80032158	8	23,21403694
Homer1	5	21,43468666	7	20,75909805
Homer1	6	21,23186874	8	20,49306488
Mapk1	5	21,48296165	7	20,71883774
Mapk1	6	21,00807381	8	20,17975044
Adcy1	17	23,40530396	19	22,34013557
Adcy1	18	22,51165199	20	21,71990395
Cnr1	17	23,04851723	19	22,09189224
Cnr1	18	22,7156868	20	22,07047653
Creb1	17	28,31810379	19	27,51477432
Creb1	18	27,74168777	20	27,14965248
Ephb2	17	26,78461838	19	26,54014778
Ephb2	18	26,87129402	20	26,06137848
Gria1	17	20,21892166	19	19,66811943
Gria1	18	19,91233063	20	19,40981293
Gria2	17	20,0382576	19	19,16348076
Gria2	18	19,65740585	20	19,04566002
Grin2a	17	24,37434769	19	23,29015541
Grin2a	18	23,6793232	20	23,20412445
Grin2b	17	23,50083923	19	22,39336777
Grin2b	18	22,72386169	20	22,34667969
Grip1	17	26,33611298	19	25,35403633
Grip1	18	25,69495964	20	24,92946625
Grm4	17	23,68238449	19	23,17637444
Grm4	18	23,51230812	20	22,7853508
Homer1	17	21,2801857	19	20,26477242
Homer1	18	20,72140503	20	20,31818962
Mapk1	17	21,15782166	19	20,31000137
Mapk1	18	20,85806274	20	20,15158463

Mmp9	5	32,06954193	7	29,93630409
Mmp9	6	29,66836166	8	30,70759773
Ngfr	5	25,90382195	7	24,54364204
Ngfr	6	25,14236641	8	24,08673859
Sirt1	5	24,75396538	7	24,12804604
Sirt1	6	24,5745163	8	23,80695534
Ywhaq	5	20,21367645	7	18,97256088
Ywhaq	6	19,82528496	8	18,96850586
Gabbr1	5	21,3835144	7	20,53588867

Gabbr1	6	21,16936684	8	20,44177055
Mecp2	5	23,45784187	7	22,6348114
Mecp2	6	23,38344955	8	22,51671982
Hmga2	5	22,98910332	7	21,97725105
Hmga2	6	22,84399986	8	22,21249199
Pten	5	21,87528419	7	20,97340393
Pten	6	21,82956123	8	20,87324142
Hdac3	5	24,70168877	7	23,88556862
Hdac3	6	24,57280731	8	23,62318611
Creb5	5	27,49355316	7	27,31540871
Creb5	6	27,27180099	8	26,97821808
Ache	5	22,99084282	7	22,22475243
Ache	6	23,11207008	8	21,93066406
Sox4	5	27,18214417	7	26,06272507
Sox4	6	27,19945717	8	26,54748726
Mmp9	17	31,35043335	19	30,70498657
Mmp9	18	30,99070358	20	30,9058609
Ngfr	17	25,96713066	19	25,25592995
Ngfr	18	25,25834656	20	24,90729141
Sirt1	17	24,92235374	19	24,17072487
Sirt1	18	24,52939987	20	23,83599091
Ywhaq	17	19,74172401	19	19,1566391
Ywhaq	18	19,77621841	20	18,9806366
Gabbr1	17	21,2198658	19	20,62200546
Gabbr1	18	21,02552795	20	20,44682884
Mecp2	17	23,3325901	19	22,53795052
Mecp2	18	23,01146889	20	22,44181061
Hmga2	17	22,93073845	19	22,30025864
Hmga2	18	22,84084892	20	22,28322029
Pten	17	21,73077202	19	20,91544342
Pten	18	21,45047569	20	20,78656197
Hdac3	17	24,88964081	19	24,22730064
Hdac3	18	24,73973083	20	23,91988182
Creb5	17	27,48719406	19	27,12343597
Creb5	18	27,28300476	20	26,61377144
Ache	17	23,30142784	19	22,72488976
Ache	18	22,91819954	20	22,43186188
Sox4	17	27,11518478	19	26,66174126
Sox4	18	27,12509727	20	26,25676918
Nr4a2	5	28,66213036	7	27,32459831
Nr4a2	6	27,22110367	8	26,29371071
Cdh4	5	26,74275398	7	25,80422211
Cdh4	6	26,3535881	8	25,53171539
Ntrk3	5	23,21306229	7	22,65721321
Ntrk3	6	23,15211105	8	22,23169518
Ep300	5	24,17783165	7	23,49701118
Ep300	6	24,16415977	8	23,39387321
Arhgap32	5	23,94089699	7	22,99440956
Arhgap32	6	23,86297035	8	22,85668182

Foxo3	5	23,67393684	7	23,2290287
Foxo3	6	23,60850143	8	22,97693443
Ago2	5	23,44530869	7	22,72644234
Ago2	6	23,3527832	8	22,62025452
Dvl3	5	27,27947235	7	27,73853493
Dvl3	6	27,46613693	8	26,43807602
Pparg	5	31,09003258	7	30,37253952
Pparg	6	28,97024536	8	29,63208771
Peg3	5	20,54067993	7	19,58359528
Peg3	6	20,36510277	8	19,47356224
Htr2c	5	22,48790741	7	21,91558838
Htr2c	6	22,71342468	8	21,83220673
Ncoa1	5	23,58252335	7	22,92183685
Ncoa1	6	23,55425835	8	22,78837395
Nr4a2	17	27,93433952	19	26,67875099
Nr4a2	18	27,28923416	20	25,79431152
Cdh4	17	26,76266479	19	26,17373657
Cdh4	18	25,96230507	20	25,70145798
Ntrk3	17	23,47549057	19	22,66505051
Ntrk3	18	22,83578682	20	22,51643181
Ep300	17	24,16342735	19	23,55583763
Ep300	18	23,86066628	20	23,26962852
Arhgap32	17	24,08736801	19	22,78595543
Arhgap32	18	23,53179359	20	22,75841141
Foxo3	17	23,73611641	19	23,10177422
Foxo3	18	23,29173088	20	22,81279945
Ago2	17	23,48372078	19	22,68460083
Ago2	18	22,98259544	20	22,46529961
Dvl3	17	27,10309219	19	26,58206749
Dvl3	18	27,23930359	20	26,5833931
Pparg	17	30,99945831	19	29,5401001
Pparg	18	30,56354904	20	29,78819656
Peg3	17	20,50787735	19	19,90316582
Peg3	18	19,99967575	20	19,65899086
Htr2c	17	22,52532387	19	22,16135979
Htr2c	18	22,52627945	20	21,86728668
Ncoa1	17	23,57342911	19	22,92710495
Ncoa1	18	23,27733994	20	22,73757553
Robo4	5	28,04331017	7	27,49763489
Robo4	6	28,15480614	8	28,12912178
Nr5a1	5	34,48907471	7	33,1889267
Nr5a1	6	30,02063942	8	32,30767059
E2f1	5	29,4101696	7	28,81966782
E2f1	6	28,78836441	8	28,660429
Shank3	5	21,53696442	7	20,84270859
Shank3	6	21,56303596	8	20,76537895
Homer2	5	23,01917076	7	22,47587395
Homer2	6	23,35466385	8	21,95399284
Foxa1	5	Undetermined	7	32,430233

Foxa1	6	30,10313034	8	Undetermined
Rgs7bp	5	22,46252251	7	21,79602051
Rgs7bp	6	22,29754257	8	21,56401062
Foxp2	5	26,36086655	7	25,30148888
Foxp2	6	26,00336075	8	25,24973679
Crtc1	5	25,44454002	7	31,4124012
Crtc1	6	25,2114048	8	31,69935417
Dusp9	5	32,78414917	7	34,36451721
Dusp9	6	30,41393661	8	30,69457626
Cdk19	5	27,3203373	7	26,45659256
Cdk19	6	27,11198616	8	26,80611229
Adcy3	5	23,83891869	7	23,56888771
Adcy3	6	23,77594566	8	23,15056229
Robo4	17	29,13241959	19	27,6452179
Robo4	18	27,53175545	20	27,49894524
Nr5a1	17	34,77231598	19	33,72655487
Nr5a1	18	33,93524551	20	34,22849274
E2f1	17	29,94663239	19	27,733778
E2f1	18	28,95689583	20	27,6835804
Shank3	17	21,52484894	19	20,66514206
Shank3	18	21,26821327	20	20,69750595
Homer2	17	22,8545208	19	22,26893806
Homer2	18	22,65990448	20	21,96338654
Foxa1	17	Undetermined	19	33,54711914
Foxa1	18	Undetermined	20	Undetermined
Rgs7bp	17	22,56970406	19	21,4717598
Rgs7bp	18	22,21087456	20	21,49828529
Foxp2	17	26,65085411	19	25,76575089
Foxp2	18	26,3496151	20	25,66610909
Crtc1	17	25,59202766	19	31,23986435
Crtc1	18	32,56523132	20	24,31992912
Dusp9	17	32,45857239	19	32,15820694
Dusp9	18	31,49571991	20	30,96417999
Cdk19	17	27,14437103	19	26,51541519
Cdk19	18	26,92076874	20	26,27040291
Adcy3	17	23,89311218	19	23,36783981
Adcy3	18	23,64341164	20	23,17500687

Pde7a	5	25,57213783	7	24,82208061
Pde7a	6	25,46831703	8	24,65474129
Mycbp2	5	22,9176178	7	22,09772873
Mycbp2	6	22,72132492	8	21,83504868
Scn3a	5	25,39595032	7	24,37878418
Scn3a	6	25,08702087	8	24,30463409
Arhgef11	5	23,95911217	7	23,2293148
Arhgef11	6	24,00339699	8	23,23162651
Tmem151b	5	25,68063164	7	24,71136665
Tmem151b	6	25,46285629	8	24,51000404
Kcnk2	5	23,34395981	7	22,64113235
Kcnk2	6	23,01314354	8	22,59494591

Hbegf	5	26,23715973	7	25,53815269
Hbegf	6	26,15790367	8	26,19011879
Eif4a2	5	21,80649185	7	20,40318108
Eif4a2	6	21,81715012	8	20,55063248
Gdf5	5	30,30647278	7	29,99500847
Gdf5	6	29,09852219	8	30,29527664
Dnmt3a	5	24,44575691	7	23,8613224
Dnmt3a	6	24,44494057	8	23,62493134
Slc6a14	5	34,2620163	7	33,80411911
Slc6a14	6	30,94737244	8	32,9514389
Cacnb2	5	24,25763702	7	23,6054287
Cacnb2	6	23,9815731	8	25,30043793
Pde7a	17	25,54036903	19	24,92382622
Pde7a	18	25,2102356	20	24,56604958
Mycbp2	17	23,09724236	19	22,21360588
Mycbp2	18	22,67154503	20	21,93761635
Scn3a	17	25,48125458	19	24,55450821
Scn3a	18	24,87765121	20	24,31404305
Arhgef11	17	24,40629768	19	23,61133385
Arhgef11	18	23,80918884	20	23,50961494
Tmem151b	17	25,64784813	19	24,80389595
Tmem151b	18	25,26658821	20	24,48758316
Kcnk2	17	23,47738838	19	22,70289993
Kcnk2	18	23,23790359	20	22,6607914
Hbegf	17	26,22727966	19	24,97127342
Hbegf	18	25,61994743	20	25,30673218
Eif4a2	17	21,62093544	19	20,26269531
Eif4a2	18	21,58765793	20	20,4375
Gdf5	17	30,97909927	19	29,83735466
Gdf5	18	31,55741692	20	29,75573921
Dnmt3a	17	24,83989525	19	23,93501091
Dnmt3a	18	24,25790596	20	23,66508293
Slc6a14	17	Undetermined	19	34,11717606
Slc6a14	18	34,12660217	20	34,49267197
Cacnb2	17	24,37076187	19	23,55320549
Cacnb2	18	23,77362823	20	23,52027321
Scn1a	5	23,85459328	7	23,45709801
Scn1a	6	23,84963989	8	22,91521263
Glcci1	5	25,58251953	7	24,92737198
Glcci1	6	25,45983696	8	24,45136261
Slc30a6	5	25,09524155	7	24,33346748
Slc30a6	6	24,88613701	8	24,29187584
Dusp9	5	31,93678284	7	31,37261963
Dusp9	6	29,7455883	8	30,70267487
Bmpr1a	5	23,9353981	7	23,19106865
Bmpr1a	6	23,66719246	8	23,21452332
Rassf3	5	27,07623672	7	26,26501083
Rassf3	6	26,81624222	8	26,32521057
Kdm5a	5	25,61043549	7	24,98600388

Kdm5a	6	25,51142311	8	24,69676018
Sox5	5	25,19954872	7	24,36746407
Sox5	6	24,97225952	8	24,11957741
Hnrnph1	5	21,78267097	7	20,95782661
Hnrnph1	6	21,65974998	8	20,8182888
Zfp516	5	26,72512627	7	25,77548218
Zfp516	6	26,65198898	8	25,95035553
Kcna6	5	24,24264908	7	23,46725464
Kcna6	6	24,26678276	8	23,23696136
Lrrc58	5	24,27023697	7	23,32139969
Lrrc58	6	24,23888206	8	23,21271706
Scn1a	17	24,23119354	19	23,43584633
Scn1a	18	23,62960243	20	23,30684471
Glcci1	17	25,74607086	19	24,79417801
Glcci1	18	24,84569931	20	24,49667549
Slc30a6	17	24,97408485	19	24,38440323
Slc30a6	18	24,7855854	20	24,36789894
Dusp9	17	31,82437897	19	31,39583397
Dusp9	18	31,66598129	20	31,59461403
Bmpr1a	17	23,77057838	19	23,11245346
Bmpr1a	18	23,49248695	20	22,98728371
Rassf3	17	27,13954544	19	26,37471771
Rassf3	18	26,89331627	20	26,55704498
Kdm5a	17	25,75864983	19	25,13221359
Kdm5a	18	25,37986755	20	24,79325104
Sox5	17	24,90232468	19	24,36441612
Sox5	18	24,76188087	20	24,26742744
Hnrnph1	17	21,69195366	19	21,00357246
Hnrnph1	18	21,3696003	20	20,98890305
Zfp516	17	26,89467049	19	25,98830223
Zfp516	18	26,55692673	20	25,71850586
Kcna6	17	24,23616791	19	23,57973671
Kcna6	18	24,07535744	20	23,25313187
Lrrc58	17	24,04422379	19	23,30942345
Lrrc58	18	23,74690437	20	22,959795
Prkd1	5	25,97136116	7	25,48557091
Prkd1	6	25,83917046	8	25,18821716
Btg2	5	26,69247437	7	26,23344612
Btg2	6	26,61061287	8	25,89470482
Gabre	5	28,19831276	7	26,46884727
Gabre	6	26,67969513	8	26,30168152
Gabrq	5	26,85860443	7	25,18842506
Gabrq	6	25,68513489	8	25,11847878
Slc12a1	5	30,33618164	7	30,65115547
Slc12a1	6	29,30998039	8	30,23386192
Slc6a3	5	33,44775772	7	31,21710968
Slc6a3	6	30,11645126	8	31,51838493
Slc9a1	5	24,90464401	7	24,09807396
Slc9a1	6	24,73707771	8	23,74206734

Nr1d2	5	23,53389359	7	22,91753197
Nr1d2	6	23,50605392	8	22,78400421
Mef2a	5	23,11927986	7	22,65328407
Mef2a	6	22,97920227	8	22,37837601
Mef2b	5	30,2800827	7	29,33145523
Mef2b	6	28,36404037	8	28,95657539
Mef2c	5	25,67318344	7	24,27619553
Mef2c	6	25,70511246	8	24,36181641
Mef2d	5	26,69399071	7	25,56415176
Mef2d	6	26,78330421	8	25,58067894
Prkd1	17	26,06828499	19	25,31868362
Prkd1	18	25,68118668	20	25,21637154
Btg2	17	26,64113998	19	26,20052528
Btg2	18	26,43374062	20	25,91164589
Gabre	17	27,68647957	19	27,45879173
Gabre	18	26,93546486	20	27,19216728
Gabrq	17	26,65141106	19	26,25551033
Gabrq	18	25,6753521	20	25,93997765
Slc12a1	17	32,57138824	19	31,11810112
Slc12a1	18	31,36252785	20	31,62786865
Slc6a3	17	32,49322128	19	31,25464058
Slc6a3	18	31,88201904	20	31,10157776
Slc9a1	17	25,06553459	19	24,37536812
Slc9a1	18	24,82708549	20	23,94478035
Nr1d2	17	23,40037346	19	22,68753052
Nr1d2	18	23,23376846	20	22,49811935
Mef2a	17	23,27640343	19	22,63477325
Mef2a	18	22,95079231	20	22,39154243
Mef2b	17	31,09438515	19	29,31267357
Mef2b	18	29,76702499	20	29,18309975
Mef2c	17	25,68758202	19	24,44569397
Mef2c	18	24,89299393	20	24,26178551
Mef2d	17	30,0686245	19	25,91353798
Mef2d	18	26,34573746	20	25,37110138

Arhgef10	5	24,6113987	7	24,19618416
Arhgef10	6	24,73408508	8	23,88690186
Rasa1	5	23,60292435	7	22,89851761
Rasa1	6	23,54431534	8	22,79471016
Paip2	5	21,95012093	7	21,18474388
Paip2	6	21,86670685	8	20,90310287
Lrrfip1	5	22,83359718	7	22,28160477
Lrrfip1	6	22,87606239	8	21,71949577
Actb	5	18,82847786	7	17,96167374
Actb	6	18,76325417	8	17,83637428
B2m	5	22,91904449	7	22,17103767
B2m	6	23,01099014	8	22,34776878
Gapdh	5	18,72020721	7	18,14811134
Gapdh	6	18,61503601	8	17,91106033
Gusb	5	26,42685127	7	25,75053787

Gusb		6	26,30603981	8	25,41301727
Hsp90ab1		5	18,52659416	7	17,87521172
Hsp90ab1		6	18,67261124	8	17,73571968
GDC		5	34,6476326	7	32,9123497
GDC		6	29,64113426	8	32,96495819
PPC		5	19,53227615	7	18,92616844
PPC		6	19,36554527	8	19,08332634
RTC		5	20,12062454	7	19,41349411
RTC		6	20,18565941	8	19,43272209
Arhgef10		17	24,54995537	19	24,18398285
Arhgef10		18	24,23966026	20	23,64472771
Rasa1		17	23,58958817	19	22,92790985
Rasa1		18	23,35025215	20	22,81122208
Paip2		17	21,64936066	19	21,17600441
Paip2		18	21,62468719	20	20,79297256
Lrrfip1		17	22,89705849	19	22,18696213
Lrrfip1		18	22,40820313	20	21,8615036
Actb		17	18,69861984	19	18,08626747
Actb		18	18,56289101	20	17,71236801
B2m		17	22,7052784	19	21,93767166
B2m		18	22,67054749	20	21,79989624
Gapdh		17	18,78032875	19	18,07737732
Gapdh		18	18,50679398	20	17,8894825
Gusb		17	26,40180779	19	25,72611618
Gusb		18	26,57988548	20	25,67431259
Hsp90ab1		17	18,5713253	19	17,69313812
Hsp90ab1		18	18,11228752	20	17,63834
GDC		17	37,75608063	19	Undetermined
GDC		18	Undetermined	20	36,17947006
PPC		17	19,28297806	19	18,84706497
PPC		18	19,41794586	20	18,87284088
RTC		17	20,04810905	19	19,20700645
RTC		18	19,91793251	20	19,43816757
Adcy1	9		22,67373848	11	23,13096046
Adcy1	10		22,61411858	12	22,63008118
Cnr1	9		22,33219528	11	22,68423653
Cnr1	10		22,17755127	12	22,38297844
Creb1	9		27,15514374	11	27,3680172
Creb1	10		27,10923958	12	27,14982033
Ephb2	9		26,2183094	11	26,15071297
Ephb2	10		26,0091629	12	26,51549149
Gria1	9		19,67268562	11	20,18009949
Gria1	10		19,68212318	12	19,88440323
Gria2	9		19,39522171	11	19,83069801
Gria2	10		19,4096489	12	19,49252701
Grin2a	9		23,64683723	11	24,46294022
Grin2a	10		23,58023834	12	23,61980629
Grin2b	9		22,56660843	11	23,12434006
Grin2b	10		22,49961662	12	22,49990845

Grip1	9	25,12946701	11	25,9903717
Grip1	10	25,16696548	12	25,01914787
Grm4	9	22,91591263	11	24,12091255
Grm4	10	23,17982483	12	23,46534348
Homer1	9	20,72051239	11	21,58054924
Homer1	10	20,58395195	12	20,93949699
Mapk1	9	20,74583054	11	21,38960266
Mapk1	10	20,35691643	12	20,44539642
Adcy1	21	22,69809914	23	22,62127876
Adcy1	22	22,54927635	24	23,19354439
Cnr1	21	22,39605713	23	22,51643944
Cnr1	22	22,13281822	24	22,29128265
Creb1	21	27,47323608	23	27,37128639
Creb1	22	26,95718002	24	27,37920761
Ephb2	21	26,37575531	23	26,70443344
Ephb2	22	26,32934189	24	26,58791924
Gria1	21	19,71875954	23	20,22904015
Gria1	22	19,5372467	24	19,87869644
Gria2	21	19,27689362	23	19,69480515
Gria2	22	19,10458565	24	19,51526451
Grin2a	21	23,88497734	23	23,87052536
Grin2a	22	23,34176636	24	23,70264626
Grin2b	21	22,60157394	23	22,77490425
Grin2b	22	22,4373436	24	22,36202812
Grip1	21	25,45450401	23	25,59272194
Grip1	22	25,10394669	24	25,21046257
Grm4	21	23,37032318	23	23,48801422
Grm4	22	22,93505096	24	23,19754028
Homer1	21	20,57394409	23	20,98453522
Homer1	22	20,24670792	24	20,7020092
Mapk1	21	20,55277634	23	20,87740517
Mapk1	22	20,22753716	24	20,43729019
Mmp9	9	30,46167946	11	30,51979256
Mmp9	10	30,34455681	12	30,41752243
Ngfr	9	25,26302719	11	25,20474625
Ngfr	10	25,41272736	12	24,06935692
Sirt1	9	24,17830086	11	24,40739059
Sirt1	10	24,25185394	12	24,2984066
Ywhaq	9	18,99442863	11	19,21893311
Ywhaq	10	19,2760849	12	19,13266373
Gabbr1	9	20,65826988	11	21,19706726
Gabbr1	10	20,56876945	12	20,8132
Mecp2	9	22,69424248	11	23,17654037
Mecp2	10	22,7689724	12	23,36995125
Hmga2	9	22,3575592	11	22,32421303
Hmga2	10	22,22634697	12	22,35505104
Pten	9	20,99624443	11	21,43171883
Pten	10	20,95228577	12	21,15530968
Hdac3	9	23,89764214	11	24,26506042

Hdac3	10	23,92903328	12	23,94483566
Creb5	9	27,25045013	11	26,93788147
Creb5	10	26,66186523	12	26,9434948
Ache	9	22,58887863	11	22,83547211
Ache	10	22,39315987	12	22,31683159
Sox4	9	26,41452599	11	26,72101021
Sox4	10	26,26331329	12	26,615448
Mmp9	21	30,62698746	23	29,35294151
Mmp9	22	30,46710968	24	30,10008621
Ngfr	21	25,18532181	23	25,7859478
Ngfr	22	24,26978111	24	24,34845161
Sirt1	21	23,9601326	23	24,47109604
Sirt1	22	23,81684303	24	24,08537292
Ywhaq	21	18,9407196	23	19,51735687
Ywhaq	22	19,27405357	24	19,34404755
Gabbr1	21	20,7061615	23	21,08448792
Gabbr1	22	20,49737358	24	20,81479263
Mecp2	21	22,52339363	23	23,06246185
Mecp2	22	22,53920174	24	22,87363052
Hmga2	21	22,28659439	23	22,46954346
Hmga2	22	21,95877075	24	22,18461418
Pten	21	21,00578308	23	21,31836128
Pten	22	20,75252342	24	21,07226372
Hdac3	21	24,26929855	23	24,54841995
Hdac3	22	23,98741341	24	24,31438828
Creb5	21	26,66907501	23	27,17672539
Creb5	22	26,77248764	24	26,84897614
Ache	21	22,60431099	23	22,88923454
Ache	22	22,27858162	24	22,49963951
Sox4	21	26,59752846	23	27,0939827
Sox4	22	26,3876667	24	26,94560432
Nr4a2	9	26,92061996	11	27,81565285
Nr4a2	10	27,74481392	12	26,48941803
Cdh4	9	25,91174889	11	26,58290482
Cdh4	10	25,94643974	12	25,68583107
Ntrk3	9	22,69421387	11	23,22053337
Ntrk3	10	22,59461403	12	22,78165817
Ep300	9	23,699646	11	24,27728462
Ep300	10	23,60431099	12	23,80689621
Arhgap32	9	23,22699738	11	23,89850998
Arhgap32	10	23,27527428	12	23,16283035
Foxo3	9	23,17362213	11	23,59945869
Foxo3	10	23,23893356	12	23,20947838
Ago2	9	22,92439461	11	23,40702629
Ago2	10	22,98550034	12	22,98761749
Dvl3	9	26,69408798	11	26,79754829
Dvl3	10	26,44043732	12	26,75914192
Pparg	9	29,66182327	11	30,35289383
Pparg	10	30,3673954	12	30,52216911

Peg3	9	19,96862984	11	20,48011208
Peg3	10	19,90215492	12	19,85589981
Htr2c	9	21,69824982	11	22,21278954
Htr2c	10	21,96051025	12	21,91075325
Ncoa1	9	22,9704113	11	23,57332802
Ncoa1	10	22,91184616	12	23,17381668
Nr4a2	21	26,51999664	23	26,49183273
Nr4a2	22	26,31328011	24	26,90189934
Cdh4	21	26,11751938	23	26,31989479
Cdh4	22	25,41603279	24	25,89213181
Ntrk3	21	22,68708229	23	22,89810753
Ntrk3	22	22,45581245	24	22,66727066
Ep300	21	23,49397087	23	23,90384102
Ep300	22	23,23455811	24	23,52606201
Arhgap32	21	23,3028183	23	23,22361374
Arhgap32	22	22,69429016	24	22,95545006
Foxo3	21	23,1268158	23	23,57281876
Foxo3	22	22,81347847	24	23,19906807
Ago2	21	22,71924591	23	23,21097374
Ago2	22	22,4710865	24	22,94935226
Dvl3	21	26,43276787	23	26,54297256
Dvl3	22	26,26745415	24	26,59902382
Pparg	21	30,88967896	23	28,31677818
Pparg	22	30,26329613	24	29,95858765
Peg3	21	19,73262215	23	20,25697327
Peg3	22	19,34304047	24	19,71110725
Htr2c	21	22,00154495	23	22,75174332
Htr2c	22	21,81142235	24	22,38693619
Ncoa1	21	22,99688339	23	23,38901901
Ncoa1	22	22,7296257	24	22,99633408
Robo4	9	27,72568893	11	28,30733681
Robo4	10	27,86109352	12	27,97148323
Nr5a1	9	32,91757202	11	32,76464462
Nr5a1	10	33,68848801	12	33,69872284
E2f1	9	28,09719658	11	28,95547295
E2f1	10	28,1660881	12	28,80560875
Shank3	9	20,7713623	11	21,40872765
Shank3	10	20,72908592	12	21,33499146
Homer2	9	22,39024734	11	22,8338089
Homer2	10	22,28752136	12	22,68408966
Foxa1	9	Undetermined	11	31,93803787
Foxa1	10	Undetermined	12	32,29665375
Rgs7bp	9	21,71848297	11	22,60497284
Rgs7bp	10	21,7231369	12	21,84394073
Foxp2	9	25,40008354	11	26,41843987
Foxp2	10	25,42749023	12	25,21440506
Crtc1	9	24,49619675	11	32,56607819
Crtc1	10	32,76280212	12	32,40594101
Dusp9	9	32,36912537	11	34,54212189

Dusp9	10	31,71351814	12	31,75358009
Cdk19	9	26,61278725	11	27,10087013
Cdk19	10	26,56955719	12	27,17703438
Adcy3	9	23,42773628	11	23,86283493
Adcy3	10	23,43782043	12	23,43033409
Robo4	21	27,47716331	23	27,73657608
Robo4	22	27,12148094	24	27,69607925
Nr5a1	21	32,98202515	23	30,13292122
Nr5a1	22	33,67443085	24	31,18619728
E2f1	21	28,74798203	23	28,43357849
E2f1	22	27,97335052	24	28,35563087
Shank3	21	20,75470352	23	21,25904655
Shank3	22	20,64038658	24	21,12706184
Homer2	21	22,18411064	23	22,48254967
Homer2	22	22,15052795	24	22,49879837
Foxa1	21	33,33207703	23	29,98440552
Foxa1	22	33,58815765	24	31,35134125
Rgs7bp	21	21,88621521	23	22,16107559
Rgs7bp	22	21,5114727	24	21,68299294
Foxp2	21	25,90046692	23	26,76097298
Foxp2	22	25,66767693	24	25,50280762
Crtc1	21	24,5627079	23	25,23949814
Crtc1	22	30,82974434	24	23,89095497
Dusp9	21	32,37878418	23	30,61921501
Dusp9	22	32,85469055	24	30,08695602
Cdk19	21	26,66859627	23	27,12327194
Cdk19	22	26,6772995	24	26,7021122
Adcy3	21	23,46154976	23	23,80582428
Adcy3	22	22,97560692	24	23,24439621
Pde7a	9	24,8620491	11	25,7335434
Pde7a	10	24,89980698	12	25,31964493
Mycbp2	9	22,29421425	11	22,75768852
Mycbp2	10	22,33987236	12	22,19706345
Scn3a	9	24,49201965	11	25,04186249
Scn3a	10	24,62144852	12	24,35739136
Arhgef11	9	23,48790359	11	23,90116882
Arhgef11	10	23,46234703	12	23,63431358
Tmem151b	9	24,7555809	11	25,21788216
Tmem151b	10	24,87138748	12	24,69970322
Kcnk2	9	22,68859482	11	23,21403122
Kcnk2	10	22,68527603	12	22,67700768
Hbegf	9	25,36684036	11	26,31655693
Hbegf	10	25,74267197	12	25,81702805
Eif4a2	9	20,63451576	11	20,56551933
Eif4a2	10	20,69032288	12	20,49902344
Gdf5	9	29,70499992	11	29,48303032
Gdf5	10	29,92968369	12	30,32670212
Dnmt3a	9	23,99997902	11	24,68570709
Dnmt3a	10	24,10383606	12	24,25031853

Slc6a14	9	32,74823761	11	32,01479721
Slc6a14	10	35,38624573	12	34,88579178
Cacnb2	9	23,52043152	11	24,1460247
Cacnb2	10	23,4539032	12	23,81957626
Pde7a	21	24,8245163	23	25,36622429
Pde7a	22	24,63527298	24	24,97085953
Mycbp2	21	22,44105148	23	22,69006729
Mycbp2	22	22,22816849	24	22,39865685
Scn3a	21	24,45982742	23	25,21496391
Scn3a	22	24,16254044	24	24,42970085
Arhgef11	21	23,63064384	23	23,60317802
Arhgef11	22	23,30355263	24	23,68624496
Tmem151b	21	24,90467072	23	24,92612267
Tmem151b	22	24,5428772	24	25,0475502
Kcnk2	21	22,7671051	23	23,47851563
Kcnk2	22	22,62758446	24	22,97881508
Hbegf	21	25,22603798	23	25,92750168
Hbegf	22	24,91174698	24	25,4157753
Eif4a2	21	20,57483292	23	20,92703247
Eif4a2	22	20,50232315	24	20,56153297
Gdf5	21	29,53639984	23	28,79994202
Gdf5	22	29,94034958	24	29,68390656
Dnmt3a	21	23,9498024	23	24,47304344
Dnmt3a	22	23,71542168	24	24,00160408
Slc6a14	21	34,3602066	23	31,26654434
Slc6a14	22	35,19781876	24	33,55014038
Cacnb2	21	23,57097816	23	23,9905529
Cacnb2	22	23,31995583	24	23,54984665
Scn1a	9	23,54929924	11	23,97742844
Scn1a	10	23,64352798	12	23,55298042
Glcci1	9	25,02013397	11	25,72702408
Glcci1	10	25,13625526	12	25,17202377
Slc30a6	9	24,39926147	11	24,68445778
Slc30a6	10	24,5374794	12	24,45994759
Dusp9	9	32,53365326	11	31,55378532
Dusp9	10	31,30296135	12	31,30929947
Bmpr1a	9	22,86768723	11	23,34194565
Bmpr1a	10	23,1653347	12	23,31695366
Rassf3	9	26,31591415	11	26,79120445
Rassf3	10	26,36687088	12	26,71330452
Kdm5a	9	25,0741806	11	25,40526962
Kdm5a	10	25,00395393	12	25,10148811
Sox5	9	24,33850288	11	24,75076294
Sox5	10	24,4237442	12	24,39698601
Hnrnph1	9	21,18586159	11	21,53956604
Hnrnph1	10	21,15917015	12	21,32315636
Zfp516	9	26,30843163	11	26,63705635
Zfp516	10	26,41176224	12	26,47628021
Kcna6	9	23,60751915	11	23,77088356

Kcna6	10	23,61203575	12	23,41400719
Lrrc58	9	23,35196495	11	23,68298721
Lrrc58	10	23,24139977	12	23,50878334
Scn1a	21	23,65478516	23	23,83696556
Scn1a	22	23,17721176	24	23,56796837
Glcci1	21	24,88584328	23	25,30472183
Glcci1	22	24,59869576	24	24,95566177
Slc30a6	21	24,35105515	23	24,72510338
Slc30a6	22	24,01215744	24	24,48417854
Dusp9	21	31,55744362	23	29,87864113
Dusp9	22	31,23872757	24	30,89312935
Bmpr1a	21	22,93330002	23	23,67862129
Bmpr1a	22	22,77815628	24	23,33958817
Rassf3	21	26,69727898	23	26,84010887
Rassf3	22	26,53802109	24	26,57813644
Kdm5a	21	25,05361176	23	25,64782524
Kdm5a	22	24,81777763	24	25,34902763
Sox5	21	24,40250397	23	24,54556084
Sox5	22	24,24689293	24	24,71732712
Hnrnph1	21	21,00530815	23	21,39091492
Hnrnph1	22	20,6924324	24	20,91016388
Zfp516	21	25,94418907	23	26,39569092
Zfp516	22	25,88871193	24	26,31820107
Kcna6	21	23,45299339	23	23,75436211
Kcna6	22	23,29091835	24	23,54687881
Lrrc58	21	23,18344879	23	23,79558372
Lrrc58	22	22,91927147	24	23,33574486
Prkd1	9	25,36698723	11	25,87921715
Prkd1	10	25,34944153	12	25,53672791
Btg2	9	26,1800251	11	26,3629837
Btg2	10	25,88142014	12	26,26573372
Gabre	9	27,38877106	11	26,83178711
Gabre	10	27,33038712	12	25,99458694
Gabrq	9	26,00782204	11	25,57562637
Gabrq	10	25,86095238	12	24,84807968
Slc12a1	9	30,50469589	11	30,7624588
Slc12a1	10	30,81645775	12	29,80633163
Slc6a3	9	30,91005516	11	31,87878609
Slc6a3	10	31,72063065	12	31,20524406
Slc9a1	9	24,14937401	11	24,68733788
Slc9a1	10	24,24148941	12	24,18651581
Nr1d2	9	22,90833473	11	23,3288784
Nr1d2	10	22,87962151	12	22,89781952
Mef2a	9	22,52617455	11	23,24997139
Mef2a	10	22,49489784	12	22,63469696
Mef2b	9	29,33003235	11	29,60757828
Mef2b	10	28,98882294	12	29,32816505
Mef2c	9	24,81170845	11	25,8694725
Mef2c	10	24,99557114	12	24,53067398

Mef2d	9	25,8947506	11	26,5619812
Mef2d	10	25,78938866	12	25,7216301
Prkd1	21	25,21772957	23	25,59693336
Prkd1	22	25,17311478	24	25,38237953
Btg2	21	25,8993454	23	26,67123413
Btg2	22	25,42476273	24	26,11039734
Gabre	21	26,8779583	23	27,54579735
Gabre	22	25,89360619	24	26,64826393
Gabrq	21	25,72812843	23	26,34518623
Gabrq	22	24,80502701	24	25,33378792
Slc12a1	21	31,58066177	23	29,53271675
Slc12a1	22	30,79935455	24	29,95260429
Slc6a3	21	31,3703289	23	29,90670395
Slc6a3	22	30,95885658	24	29,99257278
Slc9a1	21	24,64787292	23	24,76762009
Slc9a1	22	24,0076561	24	24,37034225
Nr1d2	21	22,6759491	23	23,10147858
Nr1d2	22	22,53856659	24	22,74769402
Mef2a	21	22,8268528	23	23,2641716
Mef2a	22	22,52398491	24	22,79328346
Mef2b	21	30,26788521	23	28,66999626
Mef2b	22	28,96404839	24	30,26386642
Mef2c	21	25,11413574	23	24,49273109
Mef2c	22	24,66956902	24	24,86618423
Mef2d	21	25,95510292	23	25,95703316
Mef2d	22	25,49260139	24	25,48438072
Arhgef10	9	24,18832016	11	24,24449348
Arhgef10	10	23,92555237	12	24,29633904
Rasa1	9	22,920578	11	23,3409214
Rasa1	10	23,01398468	12	23,13569641
Paip2	9	20,99333382	11	21,28202248
Paip2	10	20,9334259	12	21,01205444
Lrrfip1	9	22,14021301	11	22,70850182
Lrrfip1	10	22,15510941	12	22,31826782
Actb	9	18,1862812	11	18,47130966
Actb	10	17,97451973	12	18,14364433
B2m	9	21,89152336	11	21,90908432
B2m	10	22,13110161	12	22,35842323
Gapdh	9	18,10748672	11	18,57911301
Gapdh	10	18,10084915	12	18,12136459
Gusb	9	25,84763336	11	26,00189018
Gusb	10	25,7196064	12	26,40626717
Hsp90ab1	9	17,79516029	11	18,51688766
Hsp90ab1	10	17,98708916	12	18,16407013
GDC	9	34,4535408	11	33,2865181
GDC	10	35,37370682	12	33,31342697
PPC	9	19,02105904	11	19,13629532
PPC	10	18,89185143	12	19,18269348
RTC	9	19,36610222	11	19,61700821

RTC	10	19,3952961	12	19,53628731
Arhgef10	21	23,82025909	23	24,44053268
Arhgef10	22	23,76284599	24	23,93692589
Rasa1	21	22,94574165	23	23,46084404
Rasa1	22	22,5947361	24	22,93787575
Paip2	21	20,90211868	23	21,39106941
Paip2	22	20,5984211	24	21,09310341
Lrrfip1	21	22,29330063	23	22,5014286
Lrrfip1	22	22,02607155	24	22,25307083
Actb	21	18,13209724	23	18,49543762
Actb	22	17,66665459	24	18,13696098
B2m	21	22,13437653	23	22,29027748
B2m	22	21,90353394	24	22,22278214
Gapdh	21	18,13728142	23	18,49528885
Gapdh	22	17,75234795	24	18,22952461
Gusb	21	25,81367302	23	26,53092384
Gusb	22	25,52739143	24	Undetermined
Hsp90ab1	21	17,49835968	23	17,95516396
Hsp90ab1	22	17,34569168	24	17,78315544
GDC	21	38,0162468	23	31,08459473
GDC	22	Undetermined	24	31,71786308
PPC	21	18,79721832	23	19,2762413
PPC	22	18,86579323	24	19,01262665
RTC	21	19,37091446	23	19,83543587
RTC	22	19,4258461	24	19,51332664
