# Supplementary material for: Functional involvement of septal miR-132 in extinction and oxytocin-mediated reversal of social fear
Source: Mol Psychiatry. 2023 Nov 8;29(6):1754–66. doi: 10.1038/s41380-023-02309-3 (PMC11371636; doi:10.1038/s41380-023-02309-3)
Supplement: Supplementary file 12 — Supplementary Table S6 [file 41380_2023_2309_MOESM12_ESM.pdf]

Supplementary Table S6: Statistics corresponding to all Figures

Figure 1

**D: miR-132-3p** (Two way ANOVA followed by Bonferroni post-hoc or independent student t-test; two-tailed)			
	Interaction conditioning x Ext/Acq	Factor conditioning	Factor Ext/Acq
30min	F (1, 21) = 0.6713; p = 0.4218	F (1, 21) = 1.2920; p = 0.2684	F (1, 21) = 0.03241; p = 0.8589
	Bonferroni post-hoc		ns for all possible comparisons
90min	F (1, 24) = 4.5580; p = 0.0432*	F (1, 24) = 2.1870; p = 0.1522	F (1, 24) = 0.2580; p = 0.6161
	Bonferroni post-hoc		ns for all possible comparisons
	Status: SFC^{+}/Acq vs SFC^{-}/Acq (Independent student t-test; two-tailed)		T (13) = 2.2310; p = 0.0439*
	Status: SFC^{+}/Ext vs SFC^{-}/Ext (Independent student t-test; two-tailed)		T (11) = 0.6132; p = 0.5522
180min	F (1, 22) = 0.63472; p = 0.5617	F (1, 22) = 0.6500; p = 0.4287	F (1, 22) = 10.04; p = 0.0045**
	Bonferroni post-hoc		ns for all possible comparisons
	Status: SFC^{-}/Ext vs SFC^{-}/Acq (Independent Student´s T-test; two-tailed)		T (11) = 1.165; p = 0.2688
	Status: SFC^{+}/Ext vs SFC^{+}/Acq (Independent Student´s T-test; two-tailed)		T (11) = 3.5480; p = 0.0046##
24hrs			T(14) = 1.0540; p = 0.3099
**E: miR-132-3p** (Two way ANOVA followed by Bonferroni post-hoc)			
	Interaction conditioning x s/ns	Factor conditioning	Factor s/ns
	F (1, 26) = 1.7360; p = 0.1991	F (1, 26) = 0.1741; p = 0.6799	F (1, 26) = 0.0863; p = 0.7713
	Bonferroni post-hoc		ns for all possible comparisons
**F: miR-132-3p** (Independent Student´s T-test; two-tailed)			
90min			T (11) = 0.1593; p = 0.8763
180min			T (16) = 1.3880; p = 0.1842
**G: Extinction** (Mixed model ANOVA followed by Bonferroni post-hoc)			
ns1 - ns3	Interaction conditioning x stimulus	Factor conditioning	Factor stimulus
	F (2, 20) = 2.297; p = 0.126	F (1; 10) = 0.238; p = 0.636	F (2; 20) = 21.220; p < 0.001**
	Bonferroni post-hoc Factor conditioning		ns for all possible comparisons
	Bonferroni post-hoc Factor stimulus	Status SFC^{-}: ns1 vs ns2	p = 0.002**
		Status SFC^{-}: ns1 vs ns3	p = 0.008**
		Status SFC^{+}: ns1 vs ns3	p = 0.033*
		Status SFC^{+}: ns2 vs ns3	p = 0.015*
			ns for all other comparisons

s1 - s 6	Interaction conditioning x stimulus	Factor conditioning	Factor stimulus
	F (1.796, 17.963) = 2.330; p = 0.130	F (1; 10) = 12.283; p = 0.006**	F (1.796, 17.963) = 4.765; p = 0.025*
	Bonferroni post-hoc Factor conditioning	Status s1: SFC^+ vs SFC^-	p < 0.001**
		Status s2: SFC^+ vs SFC^-	p = 0.014*
		Status s3: SFC^+ vs SFC^-	p = 0.026*
		Status s4: SFC^+ vs SFC^-	p = 0.027*
		Status s5: SFC^+ vs SFC^-	p = 0.084
		Status s6: SFC^+ vs SFC^-	p = 0.015*
	Bonferroni post-hoc Factor stimulus	Status SFC^-: s3 vs s5	p = 0.004**
			ns for all other comparisons
**I: c-Fos** (Two way ANOVA followed by Bonferroni post-hoc)			
	Interaction conditioning x Ext/Acq	Factor conditioning	Factor Ext/Acq
	F (1, 15) = 4.548; p = 0.0499*	F (1, 15) = 6.463; p = 0.0225*	F (1, 15) = 17.02; p = 0.0009**
		Status SFC^+ / Acq vs SFC^- / Acq	p = 0.0234*
		Status SFC^+ / Acq vs SFC^+ / Ext	p = 0.0022**

Figure 2

**C: Acquisition** (Independent Student´s T-test; two-tailed)			
			T(16) = 0.2173; p = 0.8308
**D: Extinction** (Mixed model ANOVA followed by Bonferroni post-hoc)			
ns1 - ns3	Interaction conditioning x treatment x stimulus		F (1.601, 46.443) = 0.022; p = 0.958
	Interaction conditioning x stimulus		F (1.601, 46.443) = 0.024; p = 0.955
	Interaction treatment x stimulus		F (1.601, 46.443) = 0.794; p = 0.433
	Factor stimulus		F (1.601, 46.443) = 26.348; p < 0.001**
	Interaction conditioning x treatment	Factor conditioning	Factor treatment
	F (1, 29) = 0.252; p = 0.619	F (1, 29) = 0.101; p = 0.752	F (1, 29) = 0.566; p = 0.458
	Bonferroni post-hoc Factor conditioning		ns for all possible comparisons
	Bonferroni post-hoc Factor treatment		ns for all possible comparisons
	Bonferroni post-hoc Factor stimulus	Status Scr-LNA/SFC^-: ns1 vs ns2	p = 0.059(*)
		Status Inh-LNA/SFC^-: ns1 vs ns3	p = 0.008**
		Status Scr-LNA/SFC^+: ns1 vs ns2	p = 0.036*
		Status Scr-LNA/SFC^+: ns1 vs ns3	p = 0.003**
		Status Inh-LNA/SFC^+: ns1 vs ns3	p = 0.043*

s1 - s6	Interaction conditioning x treatment x stimulus		$F (5, 145) = 0.876; p = 0.499$
	Interaction conditioning x stimulus		$F (5, 145) = 18.267; p < 0.001$**
	Interaction treatment x stimulus		$F (5, 145) = 1.277; p = 0.277$
	Factor stimulus		$F (5, 145) = 19.280; p < 0.001$**
	Interaction conditioning x treatment	Factor conditioning	Factor treatment
	$F (1, 29) = 2.104; p = 0.158$	$F (1, 29) = 48.553; p < 0.001$**	$F (1, 29) = 3.715; p = 0.064$(*)
	Bonferroni post-hoc Factor conditioning	Status s1: Scr-LNA/SFC^{+} vs Scr-LNA/SFC^{-}	$p < 0.001$**
		Status s2: Scr-LNA/SFC^{+} vs Scr-LNA/SFC^{-}	$p < 0.001$**
		Status s3: Scr-LNA/SFC^{+} vs Scr-LNA/SFC^{-}	$p = 0.017$*
		Status s1: Inh-LNA/SFC^{+} vs Inh-LNA/SFC^{-}	$p < 0.001$**
		Status s2: Inh-LNA/SFC^{+} vs Inh-LNA/SFC^{-}	$p < 0.001$**
		Status s3: Inh-LNA/SFC^{+} vs Inh-LNA/SFC^{-}	$p < 0.001$**
		Status s4: Inh-LNA/SFC^{+} vs Inh-LNA/SFC^{-}	$p = 0.002$**
		Status s5: Inh-LNA/SFC^{+} vs Inh-LNA/SFC^{-}	$p = 0.036$*
			ns for all other comparisons
	Bonferroni post-hoc Factor treatment	Status s2: Inh-LNA/SFC^{+} vs Scr-LNA/SFC^{+}	$p = 0.054$(*)
		Status s4: Inh-LNA/SFC^{+} vs Scr-LNA/SFC^{+}	$p = 0.032$*
		Status s5: Inh-LNA/SFC^{+} vs Scr-LNA/SFC^{+}	$p = 0.066$(*)
			ns for all other comparisons
	Bonferroni post-hoc Factor stimulus	Status Scr-LNA/SFC^{+}: s1 vs s2	$p = 0.010$**
		Status Scr-LNA/SFC^{+}: s1 vs s3	$p < 0.001$**
		Status Scr-LNA/SFC^{+}: s1 vs s4	$p < 0.001$**
		Status Scr-LNA/SFC^{+}: s1 vs s5	$p < 0.001$**
		Status Scr-LNA/SFC^{+}: s1 vs s6	$p < 0.001$**
		Status Scr-LNA/SFC^{+}: s2 vs s3	$p = 0.036$*
		Status Scr-LNA/SFC^{+}: s2 vs s4	$p = 0.001$**
		Status Scr-LNA/SFC^{+}: s2 vs s5	$p = 0.002$**
		Status Scr-LNA/SFC^{+}: s2 vs s6	$p < 0.001$**
		Status Inh-LNA/SFC^{+}: s1 vs s4	$p = 0.024$*
		Status Inh-LNA/SFC^{+}: s1 vs s5	$p < 0.001$**
		Status Inh-LNA/SFC^{+}: s1 vs s6	$p < 0.001$**
		Status Inh-LNA/SFC^{+}: s2 vs s4	$p = 0.024$*
		Status Inh-LNA/SFC^{+}: s2 vs s5	$p = 0.005$**

		Status Inh-LNA/SFC^{+}: s2 vs s6	$p < 0.001$**
		Status Inh-LNA/SFC^{+}: s3 vs s6	$p = 0.019$*
			ns for all other comparisons
**E: Recall** (Mixed model ANOVA followed by Bonferroni post-hoc)			
s1 - s6	Interaction conditioning x treatment x stimulus		$F (1.898, 41.753) = 1.004; p = 0.372$
	Interaction conditioning x stimulus		$F (1.898, 41.753) = 0.711; p = 0.490$
	Interaction treatment x stimulus		$F (1.898, 41.753) = 1.155; p = 0.323$
	Factor stimulus		$F (1.898, 41.753) = 9.017; p < 0.001$**
	Interaction conditioning x treatment	Factor conditioning	Factor treatment
	$F (1, 22) = 0.135; p = 0.716$	$F (1, 22) = 1.596; p = 0.220$	$F (1, 22) = 3.288; p = 0.083$
	Bonferroni post-hoc Factor conditioning	Status s6: Scr-LNA/SFC^{+} vs Scr-LNA/SFC^{-}	$p = 0.025$*
			ns for all other comparisons
	Bonferroni post-hoc Factor treatment		ns for all possible comparisons
	Bonferroni post-hoc Factor stimulus	Status Scr-LNA/SFC^{+}: s3 vs s6	$p = 0.015$*
		Status Scr-LNA/SFC^{+}: s4 vs s6	$p = 0.036$*
		Status Inh-LNA/SFC^{+}: s1 vs s2	$p = 0.053$(*)
		Status Inh-LNA/SFC^{+}: s1 vs s6	$p = 0.041$*
			ns for all other comparisons
**F: Acquisition** (Mann Whitney U-test; two-tailed)			
		U = 31.00; n(Ctrl-OE) = 7; n(132-OE) = 11	$p = 0.5282$
**G: Extinction** (Mixed model ANOVA followed by Bonferroni post-hoc)			
ns1 - ns3	Interaction conditioning x treatment x stimulus		$F (2, 66) = 0.127; p = 0.881$
	Interaction conditioning x stimulus		$F (2, 66) = 2.790; p = 0.069$(*)
	Interaction treatment x stimulus		$F (2, 66) = 0.658; p = 0.521$
	Factor stimulus		$F (2, 66) = 55.219; p < 0.001$**
	Interaction conditioning x treatment	Factor conditioning	Factor treatment
	$F (1, 33) = 0.445; p = 0.509$	$F (1, 33) = 0.449; p = 0.507$	$F (1, 33) = 0.518; p = 0.477$
	Bonferroni post-hoc Factor conditioning		ns for all possible comparisons
	Bonferroni post-hoc Factor treatment		ns for all possible comparisons
	Bonferroni post-hoc Factor stimulus	Status Ctrl-OE/SFC^{-}: ns1 vs ns2	$p = 0.001$**
		Status Ctrl-OE/SFC^{-}: ns1 vs ns3	$p < 0.001$**
		Status 132-OE/SFC^{-}: ns1 vs ns2	$p < 0.001$**
		Status 132-OE/SFC^{-}: ns1 vs ns3	$p < 0.001$**

		Status 132-OE/SFC^{-}: ns2 vs ns3	$p = 0.038$*
		Status Ctrl-OE/SFC^{+}: ns1 vs ns3	$p = 0.019$*
		Status 132-OE/SFC^{+}: ns1 vs ns2	$p = 0.006$*
		Status 132-OE/SFC^{+}: ns1 vs ns3	$p < 0.001$**
			ns for all other comparisons
s1 - s6	Interaction conditioning x treatment x stimulus		$F (3.212, 105.997) = 2.807; p = 0.040$*
	Interaction conditioning x stimulus		$F (3.212, 105.997) = 9.793; p < 0.001$**
	Interaction treatment x stimulus		$F (3.212, 105.997) = 2.141; p = 0.095$
	Factor stimulus		$F (3.212, 105.997) = 12.917; p < 0.001$**
	Interaction conditioning x treatment	Factor conditioning	Factor treatment
	$F (1, 33) = 1.320; p = 0.259$	$F (1, 33) = 21.315; p < 0.001$**	$F (1, 33) = 0.912; p = 0.346$
	Bonferroni post-hoc Factor conditioning	Status s1: Ctrl-OE/SFC^{+} vs Ctrl-OE/SFC^{-}	$p < 0.001$**
		Status s2: Ctrl-OE/SFC^{+} vs Ctrl-OE/SFC^{-}	$p = 0.039$*
		Status s4: Ctrl-OE/SFC^{+} vs Ctrl-OE/SFC^{-}	$p < 0.001$**
		Status s6: Ctrl-OE/SFC^{+} vs Ctrl-OE/SFC^{-}	$p = 0.006$**
		Status s1: 132-OE/SFC^{+} vs 132-OE/SFC^{-}	$p < 0.001$**
		Status s2: 132-OE/SFC^{+} vs 132-OE/SFC^{-}	$p = 0.007$**
			ns for all other comparisons
	Bonferroni post-hoc Factor treatment	Status s4: 132-OE/SFC^{+} vs Ctrl-OE/SFC^{+}	$p < 0.001$**
		Status s5: 132-OE/SFC^{+} vs Ctrl-OE/SFC^{+}	$p = 0.021$*
			ns for all other comparisons
	Bonferroni post-hoc Factor stimulus	Status Ctrl-OE/SFC^{+}: s1 vs s2	$p = 0.009$**
		Status Ctrl-OE/SFC^{+}: s1 vs s3	$p = 0.014$*
		Status Ctrl-OE/SFC^{+}: s1 vs s4	$p = 0.051$(*)
		Status 132-OE/SFC^{+}: s1 vs s2	$p = 0.001$**
		Status 132-OE/SFC^{+}: s1 vs s3	$p < 0.001$**
		Status 132-OE/SFC^{+}: s1 vs s4	$p < 0.001$**
		Status 132-OE/SFC^{+}: s1 vs s5	$p < 0.001$**
		Status 132-OE/SFC^{+}: s1 vs s6	$p < 0.001$**
		Status 132-OE/SFC^{+}: s2 vs s3	$p = 0.006$**
		Status 132-OE/SFC^{+}: s2 vs s4	$p < 0.001$**
		Status 132-OE/SFC^{+}: s2 vs s5	$p = 0.006$**
			ns for all other comparisons

**H: Recall** (Mixed model ANOVA followed by Bonferroni post-hoc)			
s1 - s6	Interaction conditioning x treatment x stimulus		F (3.181, 104.975) = 0.310; p = 0.829
	Interaction conditioning x stimulus		F (3.181, 104.975) = 4.543; p = 0.004**
	Interaction treatment x stimulus		F (3.181, 104.975) = 1.086; p = 0.361
	Factor stimulus		F (3.181, 104.975) = 4.191; p = 0.007**
	Interaction conditioning x treatment	Factor conditioning	Factor treatment
	F (1, 33) = 0.095; p = 0.760	F (1, 33) = 0.705; p = 0.407	F (1, 33) = 0.304; p = 0.585
	Bonferroni post-hoc Factor conditioning	Status s1: Ctrl-OE/SFC^{+} vs Ctrl-OE/SFC^{-}	p = 0.065(*)
		Status s1: 132-OE/SFC^{+} vs 132-OE/SFC^{-}	p = 0.011*
			ns for all other comparisons
	Bonferroni post-hoc Factor treatment		ns for all possible comparisons
	Bonferroni post-hoc Factor stimulus	Status Ctrl-OE/SFC^{+}: s1 vs s3	p = 0.013*
		Status Ctrl-OE/SFC^{+}: s3 vs s4	p = 0.015*
		Status 132-OE/SFC^{+}: s1 vs s2	p = 0.006**
		Status 132-OE/SFC^{+}: s1 vs s3	p = 0.006**
		Status 132-OE/SFC^{+}: s1 vs s5	p = 0.068(*)
			ns for all other comparisons

Figure 3

**B: Acquisition** (Ordinary one way ANOVA followed by Bonferroni post-hoc)			
	Interaction LNA x local	Factor LNA	Faktor local
	F (1, 30) = 0.00056; p = 0.9812	F (1, 30) = 3.159; p = 0.0856	F (1, 30) = 0.1623; p = 0.6899
**C: Extinction** (Mixed model ANOVA followed by Bonferroni post-hoc)			
ns1 - ns3	Interaction LNA x local x stimulus		F (2, 60) = 5.817; p = 0.005**
	Interaction LNA x stimulus		F (2, 60) = 2.856; p = 0.065(*)
	Interaction local x stimulus		F (2, 60) = 0.117; p = 0.890
	Factor stimulus		F (2, 60) = 17.769; p < 0.001**
	Interaction LNA x local	Factor LNA	Factor local
	F (1, 30) = 0.976; p = 0.331	F (1, 30) = 0.061; p = 0.860	F (1, 30) = 2.950; p = 0.096
	Bonferroni post-hoc Factor LNA	Status ns1: Inh-LNA/OXT vs Scr-LNA/OXT	p = 0.057(*)
			ns for all other comparisons
	Bonferroni post-hoc Factor local	Status ns3: Scr-LNA/OXT vs Scr-LNA/Veh	p = 0.009**
		Status ns3: Inh-LNA/OXT vs Inh-LNA/Veh	p = 0.023*

			ns for all other comparisons
	Bonferroni post-hoc Factor stimulus	Status Scr-LNA/OXT: ns1 vs ns2	p = 0.001**
		Status Scr-LNA/OXT: ns1 vs ns3	p < 0.001**
		Status Inh-LNA/Veh: ns1 vs ns3	p = 0.001**
		Status Inh-LNA/Veh: ns2 vs ns3	p = 0.069(*)
		Status Inh-LNA/OXT: ns2 vs ns3	p = 0.054(*)
s1 - s6	Interaction LNA x local x stimulus		F (2.652, 79.571) = 0.701; p = 0.538
	Interaction LNA x stimulus		F (2.652, 79.571) = 0.649; p = 0.567
	Interaction local x stimulus		F (2.652, 79.571) = 1.231; p = 0.303
	Factor stimulus		F (2.652, 79.571) = 4.689; p = 0.006**
	Interaction LNA x local	Factor LNA	Factor local
	F (1, 30) = 0.741; p = 0.396	F (1, 30) = 7.823; p = 0.009**	F (1, 30) = 0.249; p = 0.622
	Bonferroni post-hoc Factor conditioning	Status s1: Inh-LNA/Veh vs Scr-LNA/Veh	p = 0.061(*)
		Status s1: Inh-LNA/OXT vs Scr-LNA/OXT	p = 0.002**
		Status s3: Inh-LNA/OXT vs Scr-LNA/OXT	p = 0.050*
		Status s4: Inh-LNA/OXT vs Scr-LNA/OXT	p = 0.010**
		Status s6: Inh-LNA/OXT vs Scr-LNA/OXT	p = 0.026*
			ns for all other comparisons
	Bonferroni post-hoc Factor local		ns for all possible comparisons
	Bonferroni post-hoc Factor stimulus	Status Inh-LNA/OXT: s1 vs s2	p = 0.058(*)
			ns for all other comparisons
**D: Recall** (Mixed model ANOVA followed by Bonferroni post-hoc)			
s1 - s6	Interaction LNA x local x stimulus		F (2.742, 82.262) = 0.782; p = 0.497
	Interaction LNA x stimulus		F (2.742, 82.262) = 2.020; p = 0.123
	Interaction local x stimulus		F (2.742, 82.262) = 1.029; p = 0.380
	Factor stimulus		F (2.742, 82.262) = 3.788; p = 0.016*
	Interaction LNA x local	Factor LNA	Factor local
	F (1, 30) = 0.159; p = 0.693	F (1, 30) = 13.106; p = 0.001**	F (1, 30) = 1.355; p = 0.254
	Bonferroni post-hoc Factor conditioning	Status s1: Inh-LNA/Veh vs Scr-LNA/Veh	p = 0.016*
		Status s2: Inh-LNA/Veh vs Scr-LNA/Veh	p = 0.025*
		Status s3: Inh-LNA/Veh vs Scr-LNA/Veh	p = 0.025*
		Status s4: Inh-LNA/Veh vs Scr-LNA/Veh	p = 0.006**
		Status s5: Inh-LNA/Veh vs Scr-LNA/Veh	p = 0.018*

		Status s6: Inh-LNA/Veh vs Scr-LNA/Veh	p = 0.016*
		Status s2: Inh-LNA/OXT vs Scr-LNA/OXT	p = 0.034*
		Status s3: Inh-LNA/OXT vs Scr-LNA/OXT	p = 0.036*
		Status s4: Inh-LNA/OXT vs Scr-LNA/OXT	p = 0.006**
		Status s6: Inh-LNA/OXT vs Scr-LNA/OXT	p = 0.010**
			ns for all other comparisons
	Bonferroni post-hoc Factor local		ns for all possible comparisons
	Bonferroni post-hoc Factor stimulus	Status Scr-LNA/OXT: s1 vs s2	p = 0.022*
			ns for all other comparisons
**F: Acquisition** (Mann Whitney U-test; two-tailed)			
		U = 18.00; n(shScr) = n(sh132) = 6	p > 0.9999
**G: Extinction** (Mixed model ANOVA followed by Bonferroni post-hoc)			
ns1 - ns3	Interaction conditioning x treatment x stimulus		F (1.647, 46.127) = 1.611; p = 0.213
	Interaction conditioning x stimulus		F (1.647, 46.127) = 0.653; p = 0.497
	Interaction treatment x stimulus		F (1.647, 46.127) = 1.358; p = 0.264
	Factor stimulus		F (1.647, 46.127) = 19.184; p < 0.001**
	Interaction conditioning x treatment	Factor conditioning	Factor treatment
	F (1, 28) = 0.386; p = 0.539	F (1, 28) = 1.094; p = 0.305	F (1, 28) = 2.344; p = 0.137
	Bonferroni post-hoc Factor conditioning	Status ns1: sh132/SFC^{+} vs sh132/SFC^{-}	p = 0.027*
			ns for all other comparisons
	Bonferroni post-hoc Factor treatment	Status ns1: sh132/SFC^{-} vs shScr/SFC^{-}	p = 0.003**
			ns for all other comparisons
	Bonferroni post-hoc Factor stimulus	Status shScr/SFC^{-}: ns1 vs ns3	p = 0.055(*)
		Status sh132/SFC^{-}: ns1 vs ns2	p = 0.041*
		Status sh132/SFC^{-}: ns1 vs ns3	p < 0.001**
		Status sh132/SFC^{-}: ns2 vs ns3	p = 0.008**
			ns for all other comparisons
s1 - s6	Interaction conditioning x treatment x stimulus		F (3.011, 84.317) = 0.600; p = 0.618
	Interaction conditioning x stimulus		F (3.011, 84.317) = 12.880; p <0.001**
	Interaction treatment x stimulus		F (3.011, 84.317) = 2.030; p = 0.116
	Factor stimulus		F (3.011, 84.317) = 17.396; p <0.001**
	Interaction conditioning x treatment	Factor conditioning	Factor treatment
	F (1, 28) = 0.820; p = 0.373	F (1, 28) = 59.802; p < 0.001**	F (1, 28) = 5.996; p = 0.021*

	Bonferroni post-hoc Factor conditioning	Status s1: shScr/SFC$^+$ vs shScr/SFC$^-$	$p < 0.001$**
		Status s2: shScr/SFC$^+$ vs shScr/SFC$^-$	$p < 0.001$**
		Status s3: shScr/SFC$^+$ vs shScr/SFC$^-$	$p < 0.001$**
		Status s4: shScr/SFC$^+$ vs shScr/SFC$^-$	$p = 0.018$*
		Status s1: sh132/SFC$^+$ vs sh132/SFC$^-$	$p < 0.001$**
		Status s2: sh132/SFC$^+$ vs sh132/SFC$^-$	$p < 0.001$**
		Status s3: sh132/SFC$^+$ vs sh132/SFC$^-$	$p < 0.001$**
		Status s4: sh132/SFC$^+$ vs sh132/SFC$^-$	$p < 0.001$**
		Status s5: sh132/SFC$^+$ vs sh132/SFC$^-$	$p = 0.006$**
			ns for all other comparisons
	Bonferroni post-hoc Factor treatment	Status s4: sh132/SFC$^+$ vs shScr/SFC$^+$	$p = 0.018$*
		Status s5: sh132/SFC$^+$ vs shScr/SFC$^+$	$p = 0.025$*
			ns for all other comparisons
	Bonferroni post-hoc Factor stimulus	Status shScr/SFC$^+$: s1 vs s4	$p = 0.005$**
		Status shScr/SFC$^+$: s1 vs s5	$p < 0.001$**
		Status shScr/SFC$^+$: s1 vs s6	$p < 0.001$**
		Status shScr/SFC$^+$: s2 vs s4	$p = 0.026$*
		Status shScr/SFC$^+$: s2 vs s5	$p < 0.001$**
		Status shScr/SFC$^+$: s2 vs s6	$p = 0.002$**
		Status shScr/SFC$^+$: s3 vs s4	$p = 0.061$(*)
		Status shScr/SFC$^+$: s3 vs s5	$p < 0.001$**
		Status shScr/SFC$^+$: s3 vs s6	$p = 0.011$*
		Status sh132/SFC$^+$: s1 vs s5	$p = 0.035$*
		Status sh132/SFC$^+$: s1 vs s6	$p = 0.012$*
		Status sh132/SFC$^+$: s2 vs s5	$p = 0.028$*
		Status sh132/SFC$^+$: s2 vs s6	$p = 0.027$*
		Status sh132/SFC$^+$: s3 vs s5	$p = 0.038$*
		Status sh132/SFC$^+$: s3 vs s6	$p = 0.064$(*)
		Status sh132/SFC$^+$: s4 vs s5	$p = 0.035$*
		Status sh132/SFC$^+$: s4 vs s6	$p = 0.014$*
			ns for all other comparisons
**H: Recall** (Mixed model ANOVA followed by Bonferroni post-hoc)			
s1 - s6	Interaction conditioning x treatment x stimulus		$F_{(2.645, 71.405)} = 0.971$; $p = 0.403$

Interaction conditioning x stimulus		F (2.645, 71.405) = 3.357; p = 0.028*
Interaction treatment x stimulus		F (2.645, 71.405) = 1.827; p = 0.156
Factor stimulus		F (2.645, 71.405) = 22.356; p <0.001**
Interaction conditioning x treatment	Factor conditioning	Factor treatment
F (1, 27) = 1.553; p = 0.223	F (1, 27) = 2.373; p = 0.135	F (1, 27) = 0.038; p = 0.847
Bonferroni post-hoc Factor conditioning	Status s1: shScr/SFC$^+$ vs shScr/SFC$^-$	p = 0.028*
	Status s1: sh132/SFC$^+$ vs sh132/SFC$^-$	p = 0.004**
		ns for all other comparisons
Bonferroni post-hoc Factor treatment	Status s6: sh132/SFC$^-$ vs shScr/SFC$^-$	p = 0.040*
		ns for all other comparisons
Bonferroni post-hoc Factor stimulus	Status shScr/SFC$^-$: s1 vs s2	p = 0.0573(*)
	Status shScr/SFC$^-$: s3 vs s6	p = 0.030*
	Status sh132/SFC$^-$: s1 vs s2	p = 0.004**
	Status sh132/SFC$^-$: s1 vs s3	p = 0.024*
	Status sh132/SFC$^-$: s1 vs s4	p = 0.006**
	Status shScr/SFC$^+$: s1 vs s2	p < 0.001**
	Status shScr/SFC$^+$: s1 vs s3	p = 0.016*
	Status shScr/SFC$^+$: s1 vs s4	p = 0.003**
	Status sh132/SFC$^+$: s1 vs s2	p < 0.001**
	Status sh132/SFC$^+$: s1 vs s3	p < 0.001**
	Status sh132/SFC$^+$: s1 vs s4	p < 0.001**
	Status sh132/SFC$^+$: s1 vs s5	p = 0.038*
	Status sh132/SFC$^+$: s1 vs s6	p = 0.009**
		ns for all other comparisons

Figure 4

**D: miR-132-3p** (Ordinary one way ANOVA followed by Bonferroni post-hoc)		
	F (3, 19) = 2.764	p = 0.0703
Status: SFC$^+$/Acq vs SFC$^-$/Acq (Independent Student´s T-test; two-tailed)		T (9) = 2.710; p = 0.0240*
**D: Gdf-5** (Ordinary one way ANOVA followed by Bonferroni post-hoc)		
	F (3, 19) = 1.289	p = 3070
Status: SFC$^+$/Acq vs SFC$^-$/Acq (Independent Student´s T-test; two-tailed)		T (9) = 2.074; p = 0.0679(*)
**E: Ratio mature-GDF-5 / pro-GDF-5** (Two way ANOVA followed by Bonferroni post-hoc)		

	Interaction conditioning x Ext/Acq	Factor conditioning	Factor Ext/Acq
	F (1, 27) = 6.793; p = 0.0147*	F (1, 27) = 0.3232; p = 0.5744	F (1, 27) = 1.127; p = 00.2977
	Bonferroni post-hoc	ns for all possible comparisons	
	Status SFC$^+$/Acq vs SFC$^-$/Acq (independent Student´s T-test, two-tailed)		T (14) = 2.294; p = 0.0379*
	Status SFC$^+$/Ext vs SFC$^-$/Ext (independent Student´s T-test, two-tailed)		T (13) = 1.411; p = 0.1819
**F: Hmga2** (Ordinary one way ANOVA followed by Bonferroni post-hoc)			
		F (2, 27) = 7.808	p = 0.002**
		Status 132-3p Inh vs neg ctrl	p = 0.010**
		Status 132-3p Inh vs pos ctrl	p = 0.003**
**F: Gdf-5** (Ordinary one way ANOVA followed by Bonferroni post-hoc)			
		F (2, 27) = 3.816	p = 0.034*
		Status 132-3p Inh vs pos ctrl	p = 0.031*
**G: Gdf-5** (Two way ANOVA followed by Bonferroni post-hoc)			
	Interaction 132-3p Inh/neg ctrl x OXT/Veh	Factor 132-3p Inh/neg ctrl	Factor OXT/Veh
	F (1, 32) = 0.904, p = 0.348	F (1, 32) = 6.686, p = 0.014*	F (1, 32) = 0.386, p = 0.539
			ns for all possible comparisons
	Status neg ctrl/OXT vs neg ctrl/Veh (independent Student´s T-test, two-tailed)		T (16) = 2.143; p = 0.047*
	Status 132-3p Inh/OXT vs neg ctrl/OXT (independent Student´s T-test, two-tailed)		T (15) = 3.072; p = 0.007**
**H: Extinction** (Mixed model ANOVA followed by Bonferroni post-hoc)			
ns1 - ns3	Interaction treatment x stimulus	Factor treatment	Factor stimulus
	F (2, 34) = 0.080; p = 0.923	F (1, 17) = 0.455; p = 0.509	F (2, 34) = 16.466; p < 0.001**
	Bonferroni post-hoc Factor treatment		ns for all possible comparisons
	Bonferroni post-hoc Factor stimulus	Status Veh: ns1 vs ns2	p = 0.010*
		Status Veh: ns1 vs ns3	p = 0.002**
		Status GDF-5: ns1 vs ns2	p = 0.029*
		Status GDF-5: ns1 vs ns3	p = 0.012*
			ns for all other comparisons
s1 - s6	Interaction treatment x stimulus	Factor treatment	Factor stimulus
	F (2.056, 34.950) = 0.391; p = 0.685	F (1, 17) = 4.862; p = 0.042*	F (2.056, 34.950) = 11.692; p < 0.001**
	Bonferroni post-hoc Factor treatment	Status s2: GDF-5 vs Veh	p = 0.042*
		Status s3: GDF-5 vs Veh	p = 0.057(*)
			ns for all other comparisons
	Bonferroni post-hoc Factor stimulus	Status Veh: s1 vs s3	p = 0.031*

		Status Veh: s1 vs s4	p = 0.046*
		Status Veh: s1 vs s5	p = 0.066(*)
			ns for all other comparisons

Supplementary Figure S1

**B: miR-124-3p** (Two way ANOVA followed by Bonferroni post-hoc or independent student t-test; two-tailed)			
	Interaction conditioning x Ext/Acq	Factor conditioning	Factor Ext/Acq
30min	F (1, 21) = 0.4234; p = 0.5223	F (1, 21) = 0.3198; p = 0.5777	F (1, 21) = 0.2066; p = 0.6541
	Bonferroni post-hoc	ns for all possible comparisons	
90min	F (1, 26) = 4.1370; p = 0.0523	F (1, 26) = 10.87; p = 0.0028**	F (1, 26) = 0.8069; p = 0.3773
	Bonferroni post-hoc	ns for all possible comparisons	
	Status: SFC^{+}/Acq vs SFC^{-}/Acq (Independent Student´s T-test; two-tailed)		T (12) = 2.5120; p = 0.0273*
	Status: SFC^{-}/Ext vs SFC^{-}/Acq (Independent Student´s T-test; two-tailed)		T (12) = 4.2400; p = 0.0011**
180min	F (1, 22) = 1.027; p = 0.3220	F (1, 22) = 0.1846; p = 0.6717	F (1, 22) = 2.1400; p = 0.1577
	Bonferroni post-hoc	ns for all possible comparisons	
24hrs			T(14) = 1.1600; p=0.2656
**C: miR-124-3p** (Two way ANOVA followed by Bonferroni post-hoc)			
	Interaction conditioning x Ext/Acq	Factor conditioning	Factor s/ns
	F (1, 27) = 0.1552; p = 0.6967	F (1, 27) = 0.0156; p = 0.9013	F (1, 27) = 0.0156; p = 0.9013
	Bonferroni post-hoc	ns for all possible comparisons	
**D: miR-124-3p** (Independent Student´s T-test; two-tailed)			
90min			T (11) = 0.3691; p = 0.7190
180min			T (16) = 0.9413; p = 0.3606

Supplementary Figure S2

**A: OF - Distance travelled** (Two way ANOVA followed by Bonferroni post-hoc)			
	Interaction conditioning x treatment	Factor conditioning	Factor treatment
	F (1, 32) = 2.7860; p = 0.1049	F (1, 32) = 5.1330; p = 0.0304*	F (1, 32) = 0.7412; p = 0.3957
	Bonferroni post-hoc	ns for all possible comparisons	
**B: OF - Time center zone** (Two way ANOVA followed by Bonferroni post-hoc)			
	Interaction conditioning x treatment	Factor conditioning	Factor treatment
	F (1, 32) = 0.5063; p = 0.14819	F (1, 32) = 4.0100; p = 0.0538	F (1, 32) = 0.6051; p = 0.4423

	Bonferroni post-hoc	ns for all possible comparisons	
**C: OF - Distance travelled** (Two way ANOVA followed by Bonferroni post-hoc)			
	Interaction conditioning x treatment	Factor conditioning	Factor treatment
	F (1, 28) = 0.7130; p = 0.4056	F (1, 28) = 0.4593; p = 0.5035	F (1, 28) = 0.7717; p = 0.3872
	Bonferroni post-hoc	ns for all possible comparisons	
**D: OF - Time center zone** (Two way ANOVA followed by Bonferroni post-hoc)			
	Interaction conditioning x treatment	Factor conditioning	Factor treatment
	F (1, 28) = 2.5710; p = 0.1201	F (1, 28) = 0.2621; p = 0.6127	F (1, 28) = 0.2205; p = 0.6423
	Bonferroni post-hoc	ns for all possible comparisons	
**E: NO - Distance travelled** (Two way ANOVA followed by Bonferroni post-hoc)			
	Interaction conditioning x treatment	Factor conditioning	Factor treatment
	F (1, 28) = 6.8290; p = 0.0143*	F (1, 28) = 0.007390; p = 0.9321	F (1, 28) = 0.2319; p = 0.6339
	Bonferroni post-hoc	ns for all possible comparisons	
**F: NO - Time object zone** (Two way ANOVA followed by Bonferroni post-hoc)			
	Interaction conditioning x treatment	Factor conditioning	Factor treatment
	F (1, 28) = 0.9023; p = 0.3503	F (1, 28) = 2.3490; p = 0.1366	F (1, 28) = 0.8707; p = 0.3587
	Bonferroni post-hoc	ns for all possible comparisons	
**G: NO - Distance travelled** (Two way ANOVA followed by Bonferroni post-hoc)			
	Interaction conditioning x treatment	Factor conditioning	Factor treatment
	F (1, 28) = 0.1727; p = 0.6809	F (1, 28) = 3.123; p = 0.0881	F (1, 28) = 1.361; p = 0.2532
	Bonferroni post-hoc	ns for all possible comparisons	
**H: NO - Time object zone** (Two way ANOVA followed by Bonferroni post-hoc)			
	Interaction conditioning x treatment	Factor conditioning	Factor treatment
	F (1, 28) = 0.1607; p = 0.6915	F (1, 28) = 1.293; p = 0.2652	F (1, 28) = 2.539; p = 0.1223
	Bonferroni post-hoc	ns for all possible comparisons	
**I: Weight gain** (Independent Student´s T-test; two-tailed)			
		24hrs	T (33) = 0.6746; p = 0.5046
		48hrs	T (33) = 0.6527; p = 0.5185
**J: Weight gain** (Independent Student´s T-test; two-tailed)			
		24hrs	T (33) = 0.5231; p = 0.6044
		48hrs	T (33) = 0.3779; p = 0.7079
		3 weeks	T (33) = 0.0774; p = 0.9388

Supplementary Figure S3

**B: Acquisition** (Mann-Whitney U; two-tailed)			
		U = 60.00; n(eGFP-Ctrl) = n(GDF-5-OE) = 12	p = 0.2174
**C: Extinction** (Mixed model ANOVA followed by Bonferroni post-hoc)			
ns1 - ns3	Interaction conditioning x treatment x stimulus		$F(1.626, 56.919) = 1.978$; $p = 0.155$
	Interaction conditioning x stimulus		$F(1.626, 56.919) = 0.935$; $p = 0.381$
	Interaction treatment x stimulus		$F(1.626, 56.919) = 0.504$; $p = 0.569$
	Factor stimulus		$F(1.626, 56.919) = 49.517$; $p < 0.001$**
	Interaction conditioning x treatment	Factor conditioning	Factor treatment
	$F(1, 35) = 0.938$; $p = 0.339$	$F(1, 35) = 0.080$; $p = 0.778$	$F(1, 35) = 0.070$; $p = 0.792$
	Bonferroni post-hoc Factor conditioning		ns for all possible comparisons
	Bonferroni post-hoc Factor treatment		ns for all possible comparisons
	Bonferroni post-hoc Factor stimulus	Status eGFP-OE/SFC$^-$: ns1 vs ns2	$p = 0.052$(*)
		Status eGFP-OE/SFC$^-$: ns1 vs ns3	$p < 0.001$**
		Status eGFP-OE/SFC$^-$: ns2 vs ns3	$p = 0.028$*
		Status GDF-5-OE/SFC$^-$: ns1 vs ns3	$p = 0.008$**
		Status eGFP-OE/SFC$^+$: ns1 vs ns2	$p = 0.033$*
		Status eGFP-OE/SFC$^+$: ns1 vs ns3	$p < 0.001$**
		Status GDF-5-OE/SFC$^+$: ns1 vs ns2	$p < 0.001$**
		Status GDF-5-OE/SFC$^+$: ns1 vs ns3	$p < 0.001$**
			ns for all other comparisons
s1 - s6	Interaction conditioning x treatment x stimulus		$F(2.306, 80.723) = 0.849$; $p = 0.466$
	Interaction conditioning x stimulus		$F(2.306, 80.723) = 11.667$; $p < 0.001$**
	Interaction treatment x stimulus		$F(2.306, 80.723) = 0.473$; $p = 0.652$
	Factor stimulus		$F(2.306, 80.723) = 4.881$; $p = 0.007$**
	Interaction conditioning x treatment	Factor conditioning	Factor treatment
	$F(1, 35) = 0.040$; $p = 0.843$	$F(1, 35) = 0.65.078$; $p <0.001$**	$F(1, 35) = 0.783$; $p = 0.382$
	Bonferroni post-hoc Factor conditioning	Status s1: eGFP-OE/SFC$^+$ vs eGFP-OE/SFC$^-$	$p < 0.001$**
		Status s2: eGFP-OE/SFC$^+$ vs eGFP-OE/SFC$^-$	$p < 0.001$**
		Status s3: eGFP-OE/SFC$^+$ vs eGFP-OE/SFC$^-$	$p < 0.001$**
		Status s4: eGFP-OE/SFC$^+$ vs eGFP-OE/SFC$^-$	$p < 0.001$**
		Status s5: eGFP-OE/SFC$^+$ vs eGFP-OE/SFC$^-$	$p = 0.031$*
		Status s1: GDF-5-OE/SFC$^+$ vs GDF-5-OE/SFC$^-$	$p < 0.001$**

		Status s2: GDF-5-OE/SFC^{+} vs GDF-5-OE/SFC^{-}	p < 0.001**
		Status s3: GDF-5-OE/SFC^{+} vs GDF-5-OE/SFC^{-}	p < 0.001**
		Status s4: GDF-5-OE/SFC^{+} vs GDF-5-OE/SFC^{-}	p = 0.0521(*)
		Status s5: GDF-5-OE/SFC^{+} vs GDF-5-OE/SFC^{-}	p = 0.061(*)
			ns for all other comparisons
	Bonferroni post-hoc Factor treatment		ns for all possible comparisons
	Bonferroni post-hoc Factor stimulus	Status eGFP-OE/SFC^{+}: s1 vs s5	p = 0.061(*)
		Status eGFP-OE/SFC^{+}: s1 vs s6	p = 0.022*
		Status eGFP-OE/SFC^{+}: s2 vs s6	p = 0.031*
		Status GDF-5-OE/SFC^{+}: s1 vs s4	p = 0.002**
		Status GDF-5-OE/SFC^{+}: s1 vs s5	p = 0.003**
		Status GDF-5-OE/SFC^{+}: s1 vs s6	p = 0.007**
		Status GDF-5-OE/SFC^{+}: s2 vs s3	p = 0.037*
		Status GDF-5-OE/SFC^{+}: s2 vs s4	p < 0.001**
		Status GDF-5-OE/SFC^{+}: s2 vs s5	p = 0.002**
		Status GDF-5-OE/SFC^{+}: s2 vs s6	p = 0.006**
		Status GDF-5-OE/SFC^{+}: s3 vs s4	p = 0.026*
		Status GDF-5-OE/SFC^{+}: s3 vs s5	p = 0.046*
			ns for all other comparisons
**D: Recall** (Mixed model ANOVA followed by Bonferroni post-hoc)			
s1 - s6	Interaction conditioning x treatment x stimulus		F (2.649, 92.720) = 0.704; p = 0.535
	Interaction conditioning x stimulus		F (2.649, 92.720) = 2.295; p = 0.091
	Interaction treatment x stimulus		F (2.649, 92.720) = 2.177; p = 0.104
	Factor stimulus		F (2.649, 92.720) = 6.713; p = < 0.001**
	Interaction conditioning x treatment	Factor conditioning	Factor treatment
	F (1, 35) = 0.429; p = 0.517	F (1, 35) = 12.858; p = 0.001**	F (1, 35) = 0.404; p = 0.529
	Bonferroni post-hoc Factor conditioning	Status s1: eGFP-OE/SFC^{+} vs eGFP-OE/SFC^{-}	p < 0.001**
		Status s2: eGFP-OE/SFC^{+} vs eGFP-OE/SFC^{-}	p = 0.005**
		Status s3: eGFP-OE/SFC^{+} vs eGFP-OE/SFC^{-}	p = 0.019*
		Status s4: eGFP-OE/SFC^{+} vs eGFP-OE/SFC^{-}	p = 0.009**
		Status s5: eGFP-OE/SFC^{+} vs eGFP-OE/SFC^{-}	p = 0.055(*)
		Status s1: GDF-5-OE/SFC^{+} vs GDF-5-OE/SFC^{-}	p = 0.016*
		Status s2: GDF-5-OE/SFC^{+} vs GDF-5-OE/SFC^{-}	p = 0.025*

		Status s3: GDF-5-OE/SFC$^+$ vs GDF-5-OE/SFC$^-$	p = 0.030*
			ns for all other comparisons
	Bonferroni post-hoc Factor treatment		ns for all possible comparisons
	Bonferroni post-hoc Factor stimulus	Status eGFP-OE/SFC$^+$: s1 vs s3	p = 0.003**
		Status eGFP-OE/SFC$^+$: s1 vs s4	p = 0.040*
		Status GDF-5-OE/SFC$^+$: s1 vs s5	p = 0.058(*)
			ns for all other comparisons

Supplementary Figure S4

**G: Extinction** (Mixed model ANOVA followed by Bonferroni post-hoc)			
ns1 - ns3	Interaction conditioning x stimulus	Factor conditioning	Factor stimulus
	F (2, 28) = 2.606; p = 0.092	F (1, 14) = 1.672; p = 0.217	F (2, 28) = 16.861; p <0.001**
	Bonferroni post-hoc Factor conditioning		ns for all possible comparisons
	Bonferroni post-hoc Factor stimulus	Status SFC-: ns1 vs ns2	p = 0.002**
		Status SFC-: ns1 vs ns3	p = 0.001**
			ns for all other comparisons
s1 - s6	Interaction conditioning x stimulus	Factor conditioning	Factor stimulus
	F (5, 70) = 3.605; p = 0.006**	F (1, 14) = 21.710; p < 0.001**	F (5, 70) = 3.787; p = 0.004**
	Bonferroni post-hoc Factor conditioning	Status s1: SFC$^+$ vs SFC$^-$	p < 0.001**
		Status s2: SFC$^+$ vs SFC$^-$	p < 0.001**
		Status s3: SFC$^+$ vs SFC$^-$	p = 0.005**
		Status s4: SFC$^+$ vs SFC$^-$	p = 0.003**
		Status s5: SFC$^+$ vs SFC$^-$	p = o.057(*)
			ns for all other comparisons
	Bonferroni post-hoc Factor stimulus	Status SFC+: s1 vs s6	p = 0.016*
			ns for all other comparisons
**H: Pro-GDF-5** (Two way ANOVA followed by Bonferroni post-hoc)			
	Interaction conditioning x Ext/Acq	Factor conditioning	Factor Ext/Acq
	F (1, 27) = 0.2835; p = 0.5988	F (1, 27) = 0.4329; p = 0.5161	F (1, 27) = 1.691; p = 0.2045
	Bonferroni post-hoc	ns for all possible comparisons	
**H: Mature-GDF-5** (Two way ANOVA followed by Bonferroni post-hoc)			
	Interaction conditioning x Ext/Acq	Factor conditioning	Factor Ext/Acq
	F (1, 27) = 1.191; p = 0.2847	F (1, 27) = 0.5886; p = 0.4496	F (1, 27) = 0.7190; p = 0.4039

	Bonferroni post-hoc	ns for all possible comparisons	
**I: miR-132-3p Mimic** (Ordinary one way ANOVA followed by Bonferroni post-hoc)			
		F (2, 27) = 12.54	$p < 0.001$**
		Status miR-132-3p mimic vs neg ctrl	$p < 0.001$**
		Status miR-132-3p mimic vs pos ctrl	$p < 0.001$**
**I: miR-132-3p Inhibitor** (Ordinary one way ANOVA followed by Bonferroni post-hoc)			
		F (2, 28) = 1.444	p = 0.253
	Bonferroni post-hoc		ns for all possible comparisons
	Status miR-132-3p inhibitor vs neg ctrl (independent Student´s T-test, two-tailed)		T (18) = 2.628; p = 0.017*
**J: Acquisition** (Mann-Whitney U; two-tailed)			
		U = 38.00; n(Veh) = 10; n(GDF-5) = 9	p = 0.6966
**K: Recall** (Mixed model ANOVA followed by Bonferroni post-hoc)			
s1 - s6	Interaction treatment x stimulus	Factor treatment	Factor stimulus
	F (1.749, 29.733) = 0.775; p = 0.454	F (1, 17) = 2.599; p = 0.125	F (1.749, 29.733) = 12.182; $p < 0.001$**
	Bonferroni post-hoc Factor treatment	Status s1: GDF-5 vs Veh	p = 0.063(*)
			ns for all other comparisons
	Bonferroni post-hoc Factor stimulus	Status Veh: s1 vs s2	p = 0.026*
		Status GDF-5: s1 vs s2	p = 0.034*
		Status GDF-5: s1 vs s5	p = 0.061(*)
			ns for all other comparisons
