# Supplementary material for: Functional involvement of septal miR-132 in extinction and oxytocin-mediated reversal of social fear
Source: Mol Psychiatry. 2023 Nov 8;29(6):1754–66. doi: 10.1038/s41380-023-02309-3 (PMC11371636; doi:10.1038/s41380-023-02309-3)
Supplement: Supplementary file 15 — Supplementary Table S9 [file 41380_2023_2309_MOESM15_ESM.pdf]

Supplementary Table S9: Antibodies, dilution, method details, company, and reference number of antibodies used for protein analysis via Western Blot or immunohistochemistry

Protein target	Method details	Dilution	Company	Ref. number
**Western Blot**	**Mol. weight (kDa)**			
rabbit-α-Gdf-5	100 (Proform)	1:1.000	abcam	ab93855
	25 (Mature form)			
goat-α-rabbit-HRP		1:1.000	Cell Signaling Technology	7074
**Immunohistochemistry**	**Incubation time**			
rabbit-α-GFP	12 hrs	1:1.000	abcam	ab290
chicken-α-mCherry	12 hrs	1:1.000	abcam	ab205402
goat-α-rabbit-Alexa Fluor 488	1 hr	1:1.000	Invitrogen	A-11008
goat-α-chicken-Alexa Fluor 594	1 hr	1:1.000	Invitrogen	A32759
rabbit-α-c-Fos	24 hrs	1:20.000	abcam	ab190289
Biotinylated goat-α-rabbit	2 hrs	1:500	Vektor Laboratories	PK-1000
