# Supplementary material for: Functional involvement of septal miR-132 in extinction and oxytocin-mediated reversal of social fear
Source: Mol Psychiatry. 2023 Nov 8;29(6):1754–66. doi: 10.1038/s41380-023-02309-3 (PMC11371636; doi:10.1038/s41380-023-02309-3)
Supplement: Supplementary file 19 — Supplementary Table S13 [file 41380_2023_2309_MOESM19_ESM.pdf]

PROBEID	SYMBOL	GENENAME	logFC	AveExpr	t	P.Value	adj.P.Val	B	P234 01 N Scr 1.CEL	P234 02 N Inh 3.CEL	P234 03 6F4 Scr 1.CEL	P234 04 6F4 Scr 3.CEL	P234 05 6F4 Inh 2.CEL	P234 06 6F4 Inh 3.CEL
TC1100001069.mm.2	Prpf8	pre-mRNA processing factor 8	3,05212E+14	6,5928E+13	9,96422E+14	3818049030	0.170747242106618	1,53766E+14	8,38078E+14	8,45677E+14	5,54834E+14	5,18498E+14	6,03514E+14	5,9508E+13
TC0500000096.mm.2	Pclo	piccolo (presynaptic cytomatrix protein)	3,04523E+14	7,85637E+14	8,78976E+14	8145244732	0.170747242106618	1,15818E+14	9,8049E+14	1,00368E+14	7,06161E+13	6,68958E+13	7,04185E+14	6,50352E+14
TC1700001633.mm.2	Tsc2	tuberous sclerosis 2	2,66484E+14	6,7373E+13	8,65193E+14	8953716761	0.170747242106618	1,10733E+14	8,32098E+14	8,47482E+14	5,85604E+14	5,61008E+13	6,21713E+14	5,94478E+14
TC0300002370.mm.2	Ints3	integrator complex subunit 3	2,4315E+14	6,85389E+14	7,96784E+14	0.000146114773434588	0.170747242106618	0.831866394370599	8,39864E+14	8,38667E+14	5,90616E+14	6,01614E+14	6,3837E+14	6,032E+14
TC1200002154.mm.2	Ston2	stonin 2	2,08657E+14	5,98807E+14	7,1842E+14	0.000267917144339513	0.170747242106618	0.462531278986636	7,13252E+14	7,11151E+14	4,97909E+14	5,09179E+14	5,8567E+14	5,75678E+14
TC1600001312.mm.2	Tango2	transport and golgi organization 2	2,09404E+14	6,93936E+14	6,95994E+14	0.000321844658756994	0.170747242106618	0.344750657466486	8,2252E+14	8,23331E+14	6,21307E+14	6,05736E+14	6,5934E+14	6,31385E+14
TC0100002827.mm.2	Kif1a	kinesin family member 1A	2,22088E+13	8,28921E+14	6,75679E+14	0.000381575575235045	0.170747242106618	0.232946665823408	9,996E+14	9,851E+14	7,93512E+14	7,47011E+14	7,39189E+13	7,09115E+14
TC1100004233.mm.2	Fasn	fatty acid synthase	1,99961E+14	7,45493E+14	6,73619E+14	0.000388304565333843	0.170747242106618	0.221332779204408	8,76359E+14	8,69743E+14	6,62878E+14	6,83303E+14	6,98309E+14	6,82365E+14
TC0200005502.mm.2	Ttbk2	tau tubulin kinase 2	2,57167E+14	6,93694E+14	6,73588E+14	0.000388409047642434	0.170747242106618	0.221153846301804	8,55537E+14	8,41414E+14	6,32834E+14	5,49782E+14	6,25237E+14	6,57361E+13
TC0400003234.mm.2	Scp2	sterol carrier protein 2, liver	2,44928E+14	5,9654E+13	6,53105E+14	0.000463242513951995	0.170747242106618	0.102722085707179	7,24958E+14	7,66556E+14	4,85507E+14	5,1615E+14	5,7768E+14	5,0839E+14
TC0100001447.mm.2	Tnr	tenascin R	2,21441E+14	6,93313E+14	6,5174E+14	0.000468780716280908	0.170747242106618	0.0946444194416109	8,23708E+14	8,37737E+14	6,40991E+14	5,77573E+14	6,38284E+14	6,41582E+14
TC1200001722.mm.2	Hectd1	HECT domain E3 ubiquitin protein ligase 1	2,15448E+13	6,64012E+14	6,44078E+14	0.00050132362702757	0.170747242106618	0.048816078627274	8,02684E+14	8,17002E+14	6,21498E+14	5,67293E+13	6,00347E+14	5,75246E+14
TC0100001605.mm.2	Copa	coatomer protein complex subunit alpha	2,26979E+14	7,09309E+14	6,43578E+14	0.000503534803861207	0.170747242106618	0.0457985939715693	8,72847E+14	8,61881E+14	6,69745E+14	6,11025E+13	6,41458E+14	5,98897E+14
TC0600001193.mm.2	Itpr1	inositol 1,4,5-trisphosphate receptor 1	2,28312E+14	8,32196E+14	6,33371E+14	0.000551186510132562	0.170747242106618	-0.016533849333122	9,56753E+14	9,90047E+14	7,63713E+14	7,26463E+14	8,0809E+14	7,48107E+14
TC1900000040.mm.2	Pcx	pyruvate carboxylase	2,01662E+14	5,94759E+14	6,29407E+14	0.000571057691969529	0.170747242106618	-0.0411231639010108	7,46733E+14	7,04425E+13	5,16641E+14	5,31193E+14	5,22705E+14	5,4686E+14
TC1600000918.mm.2	Ncam2	neural cell adhesion molecule 2	2,21363E+14	8,19312E+14	6,1737E+14	0.000636548213030813	0.170747242106618	-0.117106613304604	9,60772E+14	9,51232E+14	7,69648E+14	6,9963E+14	7,82491E+14	7,52099E+14
TC1000000100.mm.2	Hivep2	human immunodeficiency virus type I enhancer binding protein 2	1,97799E+14	7,01306E+14	6,10375E+14	0.000678506336378293	0.170747242106618	-0.162203846794577	8,42991E+14	8,34355E+14	6,65991E+14	6,15757E+14	6,32893E+14	6,15848E+14
TC1600002145.mm.2	Dscam	DS cell adhesion molecule	1,81013E+14	6,87544E+12	6,06545E+14	0.000702796120814702	0.170747242106618	-0.187185545726363	8,19642E+14	8,06077E+14	6,34966E+14	6,28727E+14	6,30314E+14	6,0554E+14
TC0200001838.mm.2	B2m	beta-2 microglobulin	1,91106E+14	9,12166E+14	6,03089E+14	0.000725568141365013	0.170747242106618	-0.209915168418953	1,0056E+14	1,03224E+14	8,40322E+14	8,15301E+14	8,74271E+14	9,05264E+14
TC0700001865.mm.2	Stx4a	syntaxin 4A (placental)	2,404E+14	6,51022E+14	6,02157E+14	0.00073185408222701	0.170747242106618	-0.216077022749533	7,9646E+14	8,01211E+14	6,0314E+14	5,13731E+14	5,74204E+14	6,17386E+14
TC1600001287.mm.2	Pi4ka	phosphatidylinositol 4-kinase alpha	1,78004E+14	7,64782E+14	6,00102E+14	0.000745923299168884	0.170747242106618	-0.229698708333741	8,70744E+14	8,85684E+14	7,01809E+14	6,9861E+14	7,26701E+14	7,05141E+13
TC0600002714.mm.2	Cfap100	cilia and flagella associated protein 100	-1,91999E+14	8,73004E+13	-5,95636E+14	0.000777575054172601	0.170747242106618	-0.25952189507924	7,47849E+14	7,35337E+14	9,14593E+14	9,52591E+14	9,26906E+14	9,60748E+14
TC0700000567.mm.2	Igflr1/U2af1l4	IGF-like family receptor 1/U2 small nuclear RNA auxiliary factor 1-like 4	1,83299E+14	7,18092E+14	5,90571E+13	0.000815327749608829	0.170747242106618	-0.29370271718673	7,99551E+14	7,81537E+14	6,2568E+14	5,88809E+14	7,518E+13	7,61176E+14
TC1000000191.mm.2	Slc18b1	solute carrier family 18, subfamily B, member 1	2,04632E+13	5,58976E+14	5,90557E+14	0.0008154307880025	0.170747242106618	-0.293794047714941	7,19467E+14	6,79305E+13	5,1376E+14	4,75747E+14	5,03804E+14	4,61775E+14
TC1000000830.mm.2	Wdr18	WD repeat domain 18	1,86597E+14	6,88197E+14	5,89324E+14	0.000824935816561849	0.170747242106618	-0.302174839260401	8,10848E+14	7,91073E+13	6,35023E+14	5,93704E+14	6,42599E+14	6,55932E+14
TC1400002473.mm.2	Akap11	A kinase (PRKA) anchor protein 11	2,09256E+13	7,91047E+14	5,87194E+14	0.000841648244291418	0.170747242106618	-0.316702578959096	9,33555E+13	9,21341E+14	7,53602E+14	6,82782E+14	7,39237E+14	7,15763E+14
TC1100003342.mm.2	5730455P16Rik	RIKEN cDNA 5730455P16 gene	2,28231E+14	5,93914E+14	5,86099E+14	0.000850386500413987	0.170747242106618	-0.324195714762111	7,40874E+14	7,23408E+14	5,35163E+14	4,72657E+14	5,79871E+14	5,11513E+14
TC0200000636.mm.2	Gsn	gelsolin	1,81183E+14	7,07E+14	5,85841E+14	0.000852463150916906	0.170747242106618	-0.325966286970051	8,00831E+14	8,27808E+14	6,38143E+14	6,28131E+14	6,58917E+14	6,8817E+13
TC1700001563.mm.2	Zfp942	zinc finger protein 942	1,73124E+14	5,94688E+13	5,85824E+14	0.000852598605350574	0.170747242106618	-0.326081642061891	6,70539E+12	6,74883E+14	5,08287E+14	4,90886E+14	6,03213E+14	6,20323E+14
TC0700003834.mm.2	Atg16l2	autophagy related 16-like 2 (S. cerevisiae)	-2,0415E+14	9,27567E+14	-5,84554E+14	0.000862894614095387	0.170747242106618	-0.334802052156445	7,84087E+14	7,79182E+14	9,50548E+14	1,02102E+14	1,01085E+14	1,01971E+14
TC1300000571.mm.2	Nol8	nucleolar protein 8	2,33295E+14	6,216E+13	5,83994E+14	0.000867478815296268	0.170747242106618	-0.338654683834299	7,81677E+14	7,76559E+14	5,93585E+14	4,98061E+14	5,26111E+14	5,53608E+14
TC0900000508.mm.2	Hyou1	hypoxia up-regulated 1	1,87619E+14	8,13991E+14	5,83579E+14	0.000870892244371169	0.170747242106618	-0.341511513360723	9,37054E+13	9,38092E+13	7,61522E+14	7,38386E+14	7,77572E+14	7,31319E+14
TC0200003109.mm.2	Hnmt	histamine N-methyltransferase	2,24886E+14	6,6554E+14	5,82931E+13	0.00087625649574551	0.170747242106618	-0.345980786837957	8,24463E+14	7,85382E+14	5,75751E+14	5,84322E+13	6,53264E+14	5,7006E+14
TC1100001345.mm.2	Mtmr4	myotubularin related protein 4	1,74721E+14	7,41135E+14	5,79325E+13	0.000906787732162847	0.170747242106618	-0.370959177278032	8,46856E+14	8,25315E+14	6,60543E+14	6,62187E+13	7,38342E+14	7,13564E+14
TC0600002293.mm.2	Hibadh	3-hydroxyisobutyrate dehydrogenase	2,22844E+13	6,71698E+14	5,77954E+14	0.000918715292140597	0.170747242106618	-0.380512289427365	8,2434E+14	8,1429E+13	5,90988E+14	6,01955E+14	6,44809E+14	5,53806E+14
TC1100003422.mm.2	Usp32	ubiquitin specific peptidase 32	1,98953E+14	6,32067E+14	5,75018E+14	0.000944850050587967	0.170747242106618	-0.401059915058093	7,36397E+14	7,37075E+14	5,15754E+14	5,59811E+14	6,48314E+14	5,95048E+14
TC0400000085.mm.2	Ccne2	cyclin E2	-1,72271E+14	6,25954E+14	-5,73667E+14	0.000957161802134757	0.170747242106618	-0.410562785531395	5,10286E+14	5,09094E+14	6,77794E+14	6,86129E+14	6,71132E+14	7,0129E+14
TC1600000199.mm.2	Top3b	topoisomerase (DNA) III beta	1,68356E+14	6,42988E+14	5,72898E+14	0.000964245958515073	0.170747242106618	-0.415980904391032	7,40029E+14	7,20127E+14	5,61481E+14	5,61963E+14	6,3235E+14	6,41977E+14
TC0X00000151.mm.2	Atp6ap2	ATPase, H+ transporting, lysosomal accessory protein 2	2,01028E+14	8,83989E+14	5,70553E+14	0.000986226602061819	0.170747242106618	-0.432566700275013	1,02641E+14	1,02411E+14	8,60781E+14	7,87687E+13	8,02841E+14	8,02098E+14
TC1500001646.mm.2	Kcnq3	potassium voltage-gated channel, subfamily Q, member 3	2,32638E+14	7,11787E+14	5,68805E+14	0.00100298085693977	0.170747242106618	-0.444986399858326	8,45771E+14	8,67729E+14	6,73363E+14	5,74862E+13	6,67834E+14	6,41161E+14
TC1300002520.mm.2	Map1b	microtubule-associated protein 1B	2,93759E+14	9,5519E+14	5,65036E+14	0.00104020111191742	0.170747242106618	-0.471920554757944	1,15727E+14	1,14228E+13	9,13021E+13	7,99011E+13	9,12034E+13	8,07526E+13
TC1200001233.mm.2	Amn	amnionless	-1,76782E+14	7,59187E+14	-5,64664E+14	0.00104396044462324	0.170747242106618	-0.474592364827319	6,44461E+14	6,43939E+13	7,98696E+14	8,43267E+14	8,118E+14	8,12961E+14
TC1900000042.mm.2	Sptbn2	spectrin beta, non-erythrocytic 2	1,97625E+14	8,05616E+14	5,62687E+13	0.00106419196801509	0.170747242106618	-0.488823621382461	9,34458E+14	9,41034E+14	7,68552E+14	7,11689E+14	7,61176E+14	7,16786E+14
TC0300002722.mm.2	Gstm7	glutathione S-transferase, mu 7	1,90339E+14	7,14493E+14	5,60305E+14	0.00108916510074356	0.170747242106618	-0.506056748765733	8,37105E+14	8,09779E+14	6,62239E+14	6,03966E+13	6,91455E+14	6,82413E+14
TC0600002237.mm.2	Igf2bp3	insulin-like growth factor 2 mRNA binding protein 3	-1,94868E+14	6,46194E+14	-5,5861E+14	0.00110733267063091	0.170747242106618	-0.518370442796732	5,41989E+14	5,08882E+14	6,89036E+14	7,51571E+14	6,90254E+14	6,9543E+13
TC0400001930.mm.2	Mtor	mechanistic target of rapamycin kinase	1,72705E+14	6,60884E+14	5,58428E+14	0.00110930429470815	0.170747242106618	-0.519695763239358	7,7471E+14	7,84793E+14	6,23106E+14	5,90987E+14	5,84121E+14	6,07584E+14
TC1100000585.mm.2	Cdkl3	cyclin-dependent kinase-like 3	1,7619E+14	6,42289E+14	5,5619E+14	0.00113386234590216	0.170747242106618	-0.536027371545062	7,59219E+14	7,3282E+14	5,85581E+14	5,54078E+14	5,98779E+14	6,23256E+14
TC1000001679.mm.2	Syne1	spectrin repeat containing, nuclear envelope 1	1,8288E+14	7,6986E+14	5,55362E+14	0.00114310891383724	0.170747242106618	-0.542093638349626	8,94125E+14	8,90145E+14	6,91965E+13	7,26546E+14	7,31195E+14	6,85185E+14
TC0800000955.mm.2	Cacna1a	calcium channel, voltage-dependent, P/Q type, alpha 1A subunit	1,60436E+14	7,22956E+14	5,55093E+14	0.00114612713433061	0.170747242106618	-0.544064141864737	8,42085E+14	8,40168E+14	6,83104E+14	6,78278E+14	6,52023E+14	6,42077E+14
TC0700000282.mm.2	Sympk	symplekin	1,74449E+14	6,99289E+14	5,53707E+14	0.0011618386225522	0.170747242106618	-0.554246388730181	8,24284E+14	8,05678E+14	6,30317E+14	6,50747E+14	6,6096E+14	6,23747E+14
TC1800000968.mm.2	Usp14	ubiquitin specific peptidase 14	2,01789E+14	6,59239E+14	5,5311E+14	0.00116868731945898	0.170747242106618	-0.558645875565001	8,0983E+14	8,00413E+14	5,68379E+14	6,38286E+14	5,88786E+14	5,49738E+14
TC0400003884.mm.2	Spen	spen family transcription repressor	1,89361E+13	5,86558E+14	5,47915E+14	0.00123017745894327	0.170747242106618	-0.597131745633932	7,19646E+13	6,95964E+14	5,35191E+14	5,01696E+14	5,6109E+14	5,05761E+14
TC0200003308.mm.2	Cdk9/Mir2861/Mir3960	cyclin-dependent kinase 9 (CDC2-related kinase)/microRNA 2861/microRNA 3960	1,8139E+14	6,1832E+13	5,44624E+14	0.00127102217550848	0.170747242106618	-0.621742302445937	7,17331E+14	7,17313E+14	5,65884E+14	5,0598E+14	6,05585E+14	5,97826E+14
TC1100000294.mm.2	Ccdc88a	coiled coil domain containing 88A	1,73214E+14	7,27883E+14	5,43121E+14	0.00129018057783162	0.170747242106618	-0.633039442095455	8,2262E+14	8,56517E+14	6,57901E+14	6,74808E+14	6,93765E+14	6,61688E+14
TC0700000165.mm.2	Cabp5	calcium binding protein 5	-2,07899E+13	6,95049E+13	-5,40847E+14	0.00131978276288532	0.170747242106618	-0.650198729471353	5,6299E+14	5,37448E+14	7,14283E+14	8,01952E+14	7,80263E+14	7,73359E+14
TC0600002275.mm.2	Hoxa1	homeobox A1	-1,8578E+14	5,80864E+14	-5,40384E+14	0.00132590783382904	0.170747242106618	-0.653705467873865	5,0129E+13	4,68207E+14	6,54076E+14	6,8698E+14	5,6279E+14	6,11844E+14
TC1400000298.mm.2	Selenok	selenoprotein K	1,72569E+14	8,52705E+14	5,40093E+14	0.00132976581117703	0.170747242106618	-0.655906691586488	9,432E+14	9,62503E+14	7,98177E+14	7,62388E+14	8,39633E+14	8,10326E+13
TC0500002254.mm.2	Sorcs2	sortilin-related VPS10 domain containing receptor 2	2,02336E+13	5,67606E+14	5,38889E+13	0.00134588960596256	0.170747242106618	-0.665043936724151	7,06973E+14	6,98039E+14	5,25661E+14	4,7468E+14	4,65374E+14	5,34906E+14
TC0300000272.mm.2	4932438A13Rik	RIKEN cDNA 4932438A13 gene	2,89749E+14	7,00888E+13	5,38652E+14	0.00134908731013388	0.170747242106618	-0.666844214244341	8,70418E+14	8,59188E+14	6,05936E+14	5,44172E+14	7,38068E+14	5,87549E+14
TC1100002028.mm.2	Hgs	HGF-regulated tyrosine kinase substrate	2,01537E+14	7,45664E+14	5,38275E+13	0.00135420715899837	0.170747242106618	-0.669718573420957	8,83484E+14	8,69137E+14	7,12346E+14	6,37201E+14	7,05651E+14	6,66165E+14
TC0700000936.mm.2	Herc2	HECT and RLD domain containing E3 ubiquitin protein ligase 2	1,99638E+14	6,49865E+13	5,38107E+14	0.00135648585931154	0.170747242106618	-0.670994691277374	7,60866E+14	8,01933E+14	6,18444E+14	5,45079E+14	5,88418E+14	5,84449E+14
TC0X00002441.mm.2	Hcfc1	host cell factor C1	1,75075E+14	6,53643E+14	5,37751E+14	0.00136133479073431	0.170747242106618	-0.673703711288382	7,84722E+14	7,70994E+14	6,1566E+14	5,89906E+14	5,57036E+14	6,03541E+13
TC0400003578.mm.2	Zfp362	zinc finger protein 362	-2,00504E+14	9,03675E+14	-5,37378E+14	0.00136644523947755	0.170747242106618	-0.676549349547988	7,75132E+14	7,85026E+14	1,02036E+14	9,40809E+14	9,65033E+14	9,35689E+14
TC0800000114.mm.2	F7	coagulation factor VII	-1,73374E+14	5,27934E+14	-5,3706E+14	0.00137080693513347	0.170747242106618	-0.67897041099624	4,40274E+14	4,44928E+14	5,89759E+14	6,4219E+14	5,27132E+14	5,23319E+14
TC1200000074.mm.2	Wdr35	WD repeat domain 35	1,56713E+14	5,8559E+14	5,36471E+14	0.00137893773048204	0.170747242106618	-0.683464941551002	6,75449E+14	6,88264E+14	5,28349E+14	5,21938E+14	5,43612E+13	5,55928E+14
TC1600000528.mm.2	Golgb1	golgi autoantigen, golgin subfamily b, macrogolgin 1	1,63776E+13	5,53599E+14	5,35072E+14	0.00139848392888075	0.170747242106618	-0.694171728243635	6,62158E+14	6,67683E+13	5,11207E+14	4,91081E+14	4,78952E+14	5,1051E+14
TC1700002597.mm.2	Slc8a1	solute carrier family 8 (sodium/calcium exchanger), member 1	2,22492E+14	7,41699E+14	5,34413E+14	0.00140779619885852	0.170747242106618	-0.699224849914637	8,88949E+14	8,8603E+14	7,15003E+13	6,14991E+14	6,93007E+14	6,52214E+14
TC0700000800.mm.2	Shank1	SH3 and multiple ankyrin repeat domains 1	1,67283E+14	8,50611E+14	5,33097E+14	0.00142659590293078	0.170747242106618	-0.70933403901763	9,4983E+14	9,47763E+14	8,02576E+14	7,60451E+14	8,297E+14	8,13344E+14
TC0800000736.mm.2	Hapln4	hyaluronan and proteoglycan link protein 4	1,7221E+14	7,00874E+14	5,33014E+14	0.00142779377694474	0.170747242106618	-0.709974052672676	7,9499E+14	8,14478E+14	6,55427E+14	6,09622E+14	6,56287E+14	6,74439E+13
TC1100000428.mm.2	Ccnjl	cyclin J-like	-1,63824E+14	7,26573E+14	-5,31866E+13	0.00144444427875616	0.170747242106618	-0.718819967319182	6,42518E+14	6,45993E+14	8,24009E+14	7,9215E+14	7,15582E+14	7,39188E+14
TC0400003605.mm.2	Dcdc2b	doublecortin domain containing 2b	-1,73696E+14	7,99593E+14	-5,31128E+14	0.00145526273566926	0.170747242106618	-0.724517814308737	6,68098E+14	6,94402E+14	8,30397E+14	8,79496E+14	8,65531E+14	8,59632E+14
TC0700001150.mm.2	Vps33b	vacuolar protein sorting 33B	1,59881E+14	5,7744E+14	5,29138E+14	0.00148490075036034	0.170747242106618	-0.739931959154345	7,02456E+14	6,8321E+14	5,4587E+14	5,20034E+14	5,11178E+14	5,01892E+14
TC0500000663.mm.2	Gabrb1	gamma-aminobutyric acid (GABA) A receptor, subunit beta 1	2,23316E+14	7,19679E+14	5,27316E+14	0.00151262878185303	0.170747242106618	-0.754100755252363	8,97083E+14	8,81743E+12	7,08271E+14	6,23923E+14	6,39228E+13	5,67823E+14
TC1100001628.mm.2	Cnp	2',3'-cyclic nucleotide 3' phosphodiesterase	2,11387E+14	8,58759E+14	5,26318E+14	0.00152806062335687	0.170747242106618	-0.761883964990926	9,94307E+14	9,709E+14	8,12488E+14	7,29944E+14	8,48762E+14	7,96156E+14
TC1200001348.mm.2	Efr3b	EFR3 homolog B	2,23134E+13	6,8167E+14	5,26147E+14	0.00153073072299044	0.170747242106618	-0.76322337147204	8,2555E+14	7,97916E+14	6,42782E+13	5,34416E+14	6,46006E+14	6,43348E+12
TC1000002008.mm.2	Marcks	myristoylated alanine rich protein kinase C substrate	1,80449E+13	9,30287E+14	5,26109E+14	0.00153132803910917	0.170747242106618	-0.763522712199769	1,04639E+14	1,05325E+14	9,01761E+14	8,36973E+14	8,63496E+14	8,79859E+14
TC0800002368.mm.2	Zfp868	zinc finger protein 868	1,75945E+14	5,92049E+14	5,25317E+14	0.00154373270679881	0.170747242106618	-0.769715251787723	7,00801E+14	6,87892E+14	5,30258E+14	5,06544E+14	5,91152E+14	5,35646E+14
TC1900000758.mm.2	Smc3	structural maintenance of chromosomes 3	1,74679E+14	6,5247E+13	5,25086E+14	0.00154735881057117	0.170747242106618	-0.771516859520837	7,52283E+14	7,53597E+14	5,90369E+13	5,66153E+14	6,53718E+14	5,98697E+14
TC0500000310.mm.2	Atraid	all-trans retinoic acid induced differentiation factor	2,15842E+14	7,78452E+14	5,22584E+14	0.00158741439784691	0.170747242106618	-0.791165729089045	9,02663E+12	9,04233E+14	7,30211E+14	6,45002E+14	7,76159E+14	7,12445E+14
TC1100001905.mm.2	Tmem94	transmembrane protein 94	1,82966E+14	6,15108E+14	5,21887E+14	0.00159877889567099	0.170747242106618	-0.79665797678358	7,31696E+14	7,32601E+14	5,84154E+14	5,1421E+14	5,72612E+14	5,55377E+14
TC0100003080.mm.2	Nfasc	neurofascin	1,54124E+14	8,41344E+14	5,21019E+14	0.00161305032499032	0.170747242106618	-0.803504772794569	9,46875E+14	9,47898E+14	7,97565E+14	7,88961E+14	7,95229E+14	7,71537E+14
TC1800000282.mm.2	Apc	APC, WNT signaling pathway regulator	2,20422E+14	7,4449E+14	5,19673E+14	0.00163548133397666	0.170747242106618	-0.814155053187687	8,88334E+14	8,98517E+14	6,98772E+13	6,47236E+14	7,1644E+14	6,17642E+14
TC0800003162.mm.2	Nup133	nucleoporin 133	1,56757E+14	6,30436E+14	5,17683E+14	0.00166926879234448	0.170747242106618	-0.829947892359964	7,30368E+14	7,5054E+14	5,72097E+13	5,95297E+14	5,59581E+14	5,74734E+14
TC0200000040.mm.2	Ucma	upper zone of growth plate and cartilage matrix associated	-1,67354E+14	7,29288E+14	-5,14201E+14	0.00173032143859852	0.170747242106618	-0.857757312110642	5,87579E+14	6,13516E+14	7,45743E+14	7,9006E+14	8,09812E+14	8,2902E+14
TC0500001868.mm.2	Pds5b	PDS5 cohesin associated factor B	2,20325E+14	7,52302E+14	5,13979E+14	0.00173429601064417	0.170747242106618	-0.859536422287026	9,01898E+14	8,97839E+14	7,14779E+14	6,44308E+13	7,22306E+14	6,3268E+14
TC0600000839.mm.2	Polr1a	polymerase (RNA) I polypeptide A	1,80646E+14	5,53012E+14	5,12745E+14	0.00175658911230739	0.170747242106618	-0.869446640420562	6,86222E+14	6,5093E+14	5,13597E+14	4,62262E+14	4,83391E+14	5,2167E+14
TC0800002039.mm.2	Tnks	tankyrase, TRF1-interacting ankyrin-related ADP-ribose polymerase	2,06101E+14	6,97542E+14	5,10464E+14	0.00179865193773223	0.170747242106618	-0.887835469805682	8,59585E+14	8,45476E+14	6,57119E+14	6,3574E+14	6,42889E+14	5,44441E+14
TC0600001093.mm.2	Ccdc174	coiled-coil domain containing 174	1,61856E+14	6,31018E+14	5,10437E+14	0.00179915995039958	0.170747242106618	-0.888055141434158	7,55727E+14	7,24296E+14	5,60981E+14	5,95329E+14	5,80934E+14	5,68841E+14
TC0X00003173.mm.2	Gnl3l	guanine nucleotide binding protein-like 3 (nucleolar)-like	1,67078E+14	7,02267E+14	5,10286E+14	0.00180197822306531	0.170747242106618	-0.889272769235785	8,29062E+13	7,95274E+14	6,22571E+14	6,67609E+14	6,4953E+14	6,49554E+14
TC1900000685.mm.2	Gbf1	golgi-specific brefeldin A-resistance factor 1	1,49497E+12	7,15045E+13	5,09229E+14	0.00182190301560724	0.170747242106618	-0.897831750031254	8,3588E+13	8,27994E+13	6,78629E+14	6,86251E+14	6,40067E+14	6,21451E+14
TC0400003530.mm.2	Thrap3	thyroid hormone receptor associated protein 3	1,68705E+14	7,02754E+14	5,09179E+14	0.00182283893761444	0.170747242106618	-0.898231674543289	8,32822E+14	8,27217E+13	6,81137E+14	6,41491E+14	5,95145E+13	6,38709E+14
TC1400002252.mm.2	Fzd3	frizzled class receptor 3	1,53887E+14	7,54407E+14	5,09061E+13	0.00182507803400246	0.170747242106618	-0.899187688789853	8,5293E+14	8,41407E+14	7,0905E+13	6,77512E+13	7,24167E+14	7,21377E+14
TC1100000053.mm.2	Nipsnap1	nipsnap homolog 1	1,48961E+14	8,08318E+14	5,08605E+14	0.0018337740561311	0.170747242106618	-0.902890406484435	8,99907E+14	8,91937E+13	7,38765E+14	7,55157E+14	7,77532E+14	7,86609E+14
TC0400002513.mm.2	Vcp	valosin containing protein	2,63867E+14	6,75854E+14	5,08537E+14	0.00183507062560243	0.170747242106618	-0.903441098254602	8,46228E+13	8,38586E+14	6,33681E+14	5,234E+14	6,609E+14	5,52325E+13
TC0X00002637.mm.2	Pdk3	pyruvate dehydrogenase kinase, isoenzyme 3	1,65118E+14	5,39833E+14	5,06888E+14	0.00186688060601268	0.170747242106618	-0.916841144658299	6,50468E+14	6,45956E+14	5,01647E+14	4,6454E+14	5,08393E+14	4,67991E+14
TC1100001176.mm.2	Nf1	neurofibromin 1	3,36903E+14	6,39123E+14	5,0687E+14	0.00186723483833956	0.170747242106618	-0.916989183016833	8,63883E+14	8,55205E+14	6,22355E+14	4,22927E+13	5,75044E+14	4,9532E+14
TC0200002228.mm.2	Pygb	brain glycogen phosphorylase	1,94939E+14	7,22852E+14	5,05906E+12	0.00188613803112694	0.170747242106618	-0.924851926283422	8,81661E+14	8,34461E+14	6,79271E+14	6,46972E+14	6,83454E+14	6,11293E+14
TC1200001758.mm.2	Baz1a	bromodomain adjacent to zinc finger domain 1A	-1,79326E+14	8,0621E+14	-5,05656E+14	0.00189105825473881	0.170747242106618	-0.926886617267924	6,69417E+14	6,85205E+14	8,33144E+14	8,8013E+14	8,56841E+14	9,12526E+14
TC0500002493.mm.2	Pds5a	PDS5 cohesin associated factor A	1,52492E+14	7,06608E+14	5,04812E+14	0.00190782769718383	0.170747242106618	-0.933785066719218	8,04013E+14	8,00916E+14	6,50647E+14	6,49299E+14	6,8402E+13	6,50754E+14
TC0600003321.mm.2	Grin2b	glutamate receptor, ionotropic, NMDA2B (epsilon 2)	1,97904E+14	8,25519E+14	5,04723E+14	0.00190961210614487	0.170747242106618	-0.934515838816844	9,25373E+14	9,51722E+14	7,62915E+14	7,18371E+14	8,37608E+14	7,57125E+13
TC1100001890.mm.2	Otop3	otopetrin 3	-1,87517E+14	5,0658E+14	-5,0387E+14	0.00192675186861594	0.170747242106618	-0.941503351847676	3,55685E+14	4,10566E+14	5,38978E+14	6,02307E+14	5,72538E+14	5,59409E+14
TC1600001764.mm.2	Col8a1	collagen, type VIII, alpha 1	-1,76459E+14	6,35875E+13	-5,03537E+14	0.00193348336327804	0.170747242106618	-0.944232033637289	4,79484E+14	5,2323E+14	6,49574E+14	7,06058E+14	7,24189E+14	7,32714E+14
TC1500000170.mm.2	Zfp622	zinc finger protein 622	1,58427E+13	5,98096E+14	5,02391E+14	0.00195686108622957	0.170747242106618	-0.953641132080275	7,38394E+14	6,9882E+14	5,68859E+14	5,51501E+14	5,06777E+13	5,24224E+14
TC0700001455.mm.2	Rrm1	ribonucleotide reductase M1	2,59168E+14	5,42837E+14	5,02318E+14	0.00195836603949076	0.170747242106618	-0.95424330357705	6,9729E+13	7,42085E+14	5,12082E+14	4,08957E+14	5,00692E+14	3,95918E+14
TC1300002258.mm.2	Zfp738	zinc finger protein 738	1,93909E+14	5,58572E+14	5,01626E+14	0.00197264181964576	0.170747242106618	-0.959934365849265	6,91151E+14	6,65915E+14	5,24193E+14	4,45055E+14	5,3318E+14	4,91937E+13
TC0200000620.mm.2	Hspa5	heat shock protein 5	2,23046E+14	9,21659E+14	5,01602E+14	0.00197314576991492	0.170747242106618	-0.960134573966264	1,04343E+14	1,0849E+14	8,98329E+14	7,83908E+14	8,59902E+14	8,59486E+14
TC0X00001922.mm.2	Slc9a7	solute carrier family 9 (sodium/hydrogen exchanger), member 7	1,55922E+14	6,77704E+14	5,01081E+14	0.00198397245999152	0.170747242106618	-0.964424462911193	7,92199E+14	7,5677E+14	6,29757E+14	6,07369E+14	6,44506E+14	6,3562E+14
TC0600002555.mm.2	Htra2	HtrA serine peptidase 2	1,59156E+14	6,87777E+14	5,00455E+14	0.00199709733050055	0.170747242106618	-0.969596203605586	8,12586E+14	7,97371E+14	6,54617E+14	6,37028E+14	6,34029E+14	5,9103E+14
TC1500002346.mm.2	Plec	plectin	1,53534E+14	6,54024E+14	5,00274E+14	0.00200090280955995	0.170747242106618	-0.971089876243084	7,54667E+14	7,73984E+14	6,13517E+14	6,08067E+14	6,03485E+14	5,70424E+14
TC0700001854.mm.2	Phkg2	phosphorylase kinase, gamma 2 (testis)	1,69324E+14	7,78224E+14	4,99813E+13	0.00201062328664415	0.170747242106618	-0.97489339573255	9,02414E+14	8,98788E+14	7,47654E+14	7,149E+14	7,30438E+14	6,75149E+14
TC1100003137.mm.2	Pitpnm3	PITPNM family member 3	1,62674E+14	5,86612E+14	4,99373E+14	0.00201997398800035	0.170747242106618	-0.978536303187719	6,548E+14	6,70958E+14	5,18212E+14	4,82197E+14	5,77569E+14	6,15937E+14
TC1700001160.mm.2	Birc6	baculoviral IAP repeat-containing 6	2,07584E+14	6,01385E+14	4,99277E+14	0.00202202114048503	0.170747242106618	-0.979331776626593	7,31614E+14	7,49142E+14	5,46987E+14	5,18601E+14	5,82221E+14	4,79743E+14

TC1100004062.mm.2	Grin2c	glutamate receptor, ionotropic, NMDA2C (epsilon 3)	1,55727E+14	6,26987E+14	4,99153E+14	0.00202466156713149	0.170747242106618	-0.980356688870448	7,35884E+14	7,10604E+11	5,58159E+14	5,76874E+14	5,75107E+14	6,05292E+14
TC0700000459.mm.2	Sertad3	SERTA domain containing 3	-1,86348E+14	7,89866E+14	-4,98707E+14	0.00203419783414305	0.170747242106618	-0.984048092249738	6,59038E+14	6,65997E+14	8,15519E+14	8,82211E+13	8,31552E+14	8,84875E+14
TC1000002484.mm.2	Med16	mediator complex subunit 16	1,67725E+14	7,17153E+14	4,9845E+14	0.00203973856021998	0.170747242106618	-0.986185553278157	8,42467E+14	8,25246E+14	6,62235E+14	6,70029E+14	6,81417E+14	6,21522E+14
TC0X00001282.mm.2	Rpl36a	ribosomal protein L36A	2,28752E+14	8,04187E+14	4,98289E+12	0.00204319779219621	0.170747242106618	-0.987517324063515	9,21772E+13	9,55776E+13	7,55323E+14	6,6472E+14	8,05964E+14	7,21569E+14
TC1200002002.mm.2	Erh	ERH mRNA splicing and mitosis factor	2,52539E+14	5,97385E+14	4,97837E+14	0.00205296157196742	0.170747242106618	-0.991265125479829	7,33807E+14	7,18901E+14	5,37149E+14	4,1048E+14	6,31389E+14	5,52582E+13
TC0200001552.mm.2	Cd59a	CD59a antigen	1,93749E+14	6,80817E+14	4,97618E+14	0.00205771877477616	0.170747242106618	-0.993085227581378	8,17311E+14	8,36306E+14	6,7704E+14	5,89078E+14	5,96558E+13	5,68607E+14
TC0900003064.mm.2	Wdr6	WD repeat domain 6	1,64088E+14	7,5089E+13	4,97448E+14	0.00206142349890005	0.170747242106618	-0.994499972560221	8,97551E+14	8,50744E+13	7,15405E+13	7,04714E+14	6,84826E+14	6,521E+14
TC0X00001526.mm.2	Huwe1	HECT, UBA and WWE domain containing 1	3,1784E+14	6,06156E+14	4,97129E+14	0.00206839052029001	0.170747242106618	-0.997154185154383	8,035E+14	7,99981E+14	5,46905E+14	4,20897E+14	6,1355E+14	4,52103E+14
TC0500002119.mm.2	Gm1979	predicted gene 1979	-1,70185E+14	5,96939E+14	-4,96578E+14	0.00208046066491819	0.170747242106618	-1,00173E+14	4,89559E+12	5,13742E+14	6,52555E+14	6,91115E+14	6,4191E+14	5,92751E+14
TC0200000501.mm.2	Setx	senataxin	1,8432E+14	5,76565E+14	4,95665E+14	0.00210065378287761	0.170747242106618	-1,00934E+14	6,77453E+14	6,69967E+14	4,66831E+14	5,11949E+14	6,02214E+14	5,30972E+13
TC0500000289.mm.2	Selenoi	selenoprotein I	1,50447E+14	7,64041E+14	4,9498E+14	0.00211595259206261	0.170747242106618	-1,01506E+14	8,61909E+14	8,59367E+14	7,27207E+14	6,93174E+13	7,16316E+13	7,26271E+13
TC1800001505.mm.2	Afg3l2	AFG3-like AAA ATPase 2	2,04224E+14	6,8993E+14	4,94275E+14	0.00213184262982472	0.170747242106618	-1,02096E+14	8,30393E+14	8,12503E+14	6,58014E+14	5,76434E+14	6,64146E+14	5,98091E+14
TC0200001378.mm.2	Ckap5/Snord67	cytoskeleton associated protein 5/small nucleolar RNA, C/D box 67	2,93282E+14	6,7911E+14	4,94184E+14	0.0021338915009851	0.170747242106618	-1,02171E+14	8,5932E+14	8,68193E+14	6,56374E+14	4,84574E+14	6,38758E+14	5,67441E+14
TC1100003790.mm.2	Stat3	signal transducer and activator of transcription 3	1,7526E+13	6,85036E+14	4,93066E+14	0.00215937342260171	0.170747242106618	-1,03108E+13	7,69454E+14	8,10602E+14	6,41336E+14	5,88201E+14	6,33614E+14	6,67009E+14
TC1700001366.mm.2	Tmem242	transmembrane protein 242	1,5706E+14	6,55541E+14	4,93017E+14	0.00216050728555315	0.170747242106618	-1,03149E+14	7,61592E+14	7,58174E+14	6,19713E+14	5,85933E+14	6,21817E+14	5,86015E+14
TC0900000922.mm.2	Bnip2	BCL2/adenovirus E1B interacting protein 2	1,6455E+14	6,12027E+14	4,92466E+14	0.00217318414030062	0.170747242106618	-1,03611E+13	6,72632E+14	6,98047E+14	5,22027E+14	5,19551E+14	6,57743E+14	6,02163E+13
TC1100003292.mm.2	Supt6	suppressor of Ty 6	1,75739E+14	7,44021E+14	4,9216E+14	0.00218028340738252	0.170747242106618	-1,03869E+14	8,68343E+14	8,55215E+14	7,07294E+14	6,64785E+14	7,15074E+14	6,53417E+14
TC0100001724.mm.2	Tmem63a	transmembrane protein 63a	2,42789E+14	6,669E+14	4,9167E+13	0.00219167860806415	0.170747242106618	-1,04281E+14	8,37199E+14	8,60331E+14	6,63211E+14	5,48741E+14	5,88106E+14	5,03814E+14
TC0200003135.mm.2	Tubb4b	tubulin, beta 4B class IVB	1,52287E+14	7,90182E+14	4,91437E+14	0.00219711597978312	0.170747242106618	-1,04477E+14	8,8612E+14	9,02436E+14	7,53374E+14	7,30607E+14	7,18882E+14	7,4967E+14
TC1400001545.mm.2	Myoz1	myozenin 1	-2,07542E+14	6,26145E+14	-4,914E+14	0.00219798777865249	0.170747242106618	-1,04508E+14	5,01165E+14	5,01191E+14	6,85153E+14	7,32287E+14	6,18282E+12	7,18792E+13
TC1700001308.mm.2	Cript	cysteine-rich PDZ-binding protein	2,00003E+13	7,04556E+14	4,90957E+14	0.00220838770695902	0.170747242106618	-1,04882E+14	8,67786E+14	8,55719E+14	6,94174E+13	6,29325E+14	6,30088E+14	5,50245E+14
TC0700001874.mm.2	Trim72	tripartite motif-containing 72	-1,76498E+14	6,0484E+14	-4,90928E+14	0.00220906884311231	0.170747242106618	-1,04906E+14	4,87836E+14	5,13337E+14	6,45754E+14	7,08414E+14	6,17945E+14	6,55751E+14
TC0800001740.mm.2	Rasa3	RAS p21 protein activator 3	1,60859E+14	6,06746E+14	4,90543E+14	0.00221814783700289	0.170747242106618	-1,0523E+14	6,91759E+14	6,97277E+14	5,46397E+14	5,20921E+14	6,17223E+14	5,66897E+14
TC0300002488.mm.2	Mrps21	mitochondrial ribosomal protein S21	1,5039E+13	6,6466E+14	4,89004E+13	0.00225486433797101	0.170747242106618	-1,0653E+14	7,70193E+14	7,54012E+14	6,10282E+14	6,13142E+14	6,38033E+13	6,02301E+14
TC1100002007.mm.2	Rptor	regulatory associated protein of MTOR, complex 1	1,69457E+14	6,28415E+14	4,87822E+14	0.00228352661057709	0.170747242106618	-1,07531E+14	7,55242E+14	7,29085E+14	5,71843E+14	5,73569E+14	6,02838E+14	5,37917E+14
TC0X00002789.mm.2	Rlim	ring finger protein, LIM domain interacting	2,35042E+14	6,28831E+14	4,87398E+14	0.00229392737201769	0.170747242106618	-1,07891E+14	7,89892E+14	7,61907E+14	5,97996E+14	4,83718E+14	6,06646E+14	5,32826E+13
TC0200004842.mm.2	Apmap	adipocyte plasma membrane associated protein	1,93411E+14	6,96982E+14	4,87391E+14	0.00229408775157668	0.170747242106618	-1,07897E+14	8,17692E+13	7,70167E+14	6,34388E+14	5,66647E+13	6,70097E+13	7,22899E+14
TC0700000870.mm.2	Nomo1	nodal modulator 1	1,54171E+14	7,33354E+14	4,8679E+14	0.00230890639327345	0.170747242106618	-1,08408E+14	8,20317E+14	8,30921E+14	6,93512E+14	6,49384E+14	7,10462E+13	6,95529E+14
TC0600001001.mm.2	Nfu1	NFU1 iron-sulfur cluster scaffold	1,89975E+14	7,23124E+14	4,86503E+14	0.00231599777981841	0.170747242106618	-1,08651E+14	8,45744E+14	8,1172E+14	6,80739E+14	5,96774E+14	7,159E+14	6,87868E+14
TC0900000081.mm.2	Ccdc82	coiled-coil domain containing 82	1,78474E+14	6,14089E+14	4,86233E+14	0.00232270957486683	0.170747242106618	-1,08881E+14	7,02333E+14	7,05825E+14	5,6504E+14	4,8617E+14	6,23508E+14	6,01658E+14
TC1300001273.mm.2	Dhx29	DEAH (Asp-Glu-Ala-His) box polypeptide 29	1,79813E+14	7,52344E+14	4,8607E+14	0.00232678878892626	0.170747242106618	-1,0902E+14	8,53906E+14	8,78616E+14	7,18002E+14	6,54894E+14	7,28766E+14	6,7988E+14
TC0900000173.mm.2	Zfp846	zinc finger protein 846	1,9085E+14	5,90578E+13	4,85793E+14	0.00233370965936994	0.170747242106618	-1,09256E+14	7,3673E+14	7,09203E+14	5,60543E+14	5,0369E+14	5,52853E+14	4,80448E+14
TC0200005114.mm.2	Sdc4	syndecan 4	1,41572E+14	7,89539E+14	4,85769E+14	0.00233429317274474	0.170747242106618	-1,09276E+13	8,65069E+14	8,74396E+14	7,26962E+14	7,2936E+14	7,78025E+14	7,63421E+14
TC0100002586.mm.2	Zfp142	zinc finger protein 142	2,02303E+14	5,92198E+14	4,85532E+14	0.00234024088683061	0.170747242106618	-1,09478E+14	7,22323E+14	6,93533E+14	5,56428E+14	4,54822E+14	5,51231E+14	5,74849E+14
TC1100000417.mm.2	Gabrb2	gamma-aminobutyric acid (GABA) A receptor, subunit beta 2	1,469E+14	8,44452E+14	4,85225E+14	0.00234796158020871	0.170747242106618	-1,0974E+14	9,39779E+14	9,13953E+14	7,85487E+14	7,74444E+13	8,36288E+14	8,16761E+14
TC1200000523.mm.2	Togaram1	TOG array regulator of axonemal microtubules 1	1,55716E+14	6,74104E+14	4,84827E+14	0.0023580332476579	0.170747242106618	-1,1008E+14	7,85729E+14	7,82712E+14	6,16249E+14	6,4076E+14	6,32168E+14	5,87006E+14
TC1000002612.mm.2	Rtcb	RNA 2',3'-cyclic phosphate and 5'-OH ligase	1,42832E+14	7,94113E+14	4,8435E+14	0.0023701244710713	0.170747242106618	-1,10487E+14	8,88613E+14	8,68971E+14	7,39027E+14	7,32893E+14	7,61605E+14	7,7357E+14
TC0700003434.mm.2	Lrrk1	leucine-rich repeat kinase 1	-1,81373E+14	7,25239E+14	-4,83871E+14	0.00238236390878706	0.170747242106618	-1,10896E+14	5,81012E+14	6,22261E+14	7,49675E+14	8,16345E+14	7,72997E+14	8,09143E+14
TC0200002830.mm.2	Prpf6	pre-mRNA splicing factor 6	2,63473E+14	6,58631E+14	4,83032E+14	0.00240397430593357	0.170747242106618	-1,11615E+14	8,56121E+13	8,25805E+14	6,48625E+14	5,06355E+14	5,97715E+14	5,17167E+14
TC1000002438.mm.2	Pfkl	phosphofructokinase, liver, B-type	1,63428E+14	7,58052E+14	4,82881E+14	0.00240786802452834	0.170747242106618	-1,11744E+14	8,63687E+14	8,76771E+14	7,28707E+14	6,84894E+14	7,19617E+14	6,74635E+13
TC0100002783.mm.2	Asb18	ankyrin repeat and SOCS box-containing 18	-1,92677E+14	6,1257E+14	-4,82495E+14	0.00241790981389233	0.170747242106618	-1,12075E+14	5,09192E+14	4,6211E+14	6,37866E+14	7,18789E+13	6,56948E+14	6,90513E+14
TC0700002444.mm.2	Ppp5c	protein phosphatase 5, catalytic subunit	1,8472E+14	8,20185E+14	4,82096E+14	0.00242833727054766	0.170747242106618	-1,12418E+14	9,52599E+14	9,25306E+14	7,90767E+14	7,17698E+14	7,90716E+14	7,44023E+14
TC0600002178.mm.2	Tcaf1	TRPM8 channel-associated factor 1	1,61441E+14	7,80249E+14	4,81117E+14	0.00245410687853476	0.170747242106618	-1,13259E+14	9,02848E+14	8,98128E+14	7,55719E+14	7,22375E+14	7,27336E+14	6,75091E+14
TC0800001955.mm.2	Adrb3	adrenergic receptor, beta 3	-1,62376E+14	4,64564E+14	-4,80915E+14	0.00245944202698727	0.170747242106618	-1,13432E+14	3,70975E+14	3,90642E+14	5,21021E+14	5,65349E+14	4,49131E+14	4,90268E+13
TC1300001781.mm.2	Tubb2b	tubulin, beta 2B class IIB	1,76508E+14	8,36723E+14	4,8084E+14	0.00246145244856614	0.170747242106618	-1,13497E+14	9,4077E+14	9,5259E+14	8,10673E+14	7,29671E+14	7,91598E+13	7,95034E+14
TC1600000491.mm.2	Adcy5	adenylate cyclase 5	2,01998E+14	7,98578E+14	4,80121E+14	0.00248063024287919	0.170747242106618	-1,14117E+13	9,32839E+14	9,2255E+14	7,80564E+14	6,7083E+14	7,4258E+14	7,42105E+14
TC1700002050.mm.2	Mog	myelin oligodendrocyte glycoprotein	1,91547E+14	6,93174E+13	4,79474E+13	0.00249802537371858	0.170747242106618	-1,14674E+14	8,40403E+14	8,12914E+14	6,6891E+14	6,01315E+14	6,51601E+14	5,83899E+14
TC0300001956.mm.2	Ndufc1	NADH:ubiquinone oxidoreductase subunit C1	2,17106E+14	9,31577E+13	4,78982E+14	0.0025113803250668	0.170747242106618	-1,151E+14	1,07131E+14	1,05239E+14	8,90727E+14	7,98767E+14	9,2994E+13	8,46327E+14
TC1200001353.mm.2	Ncoa1	nuclear receptor coactivator 1	1,48696E+14	7,45396E+14	4,77597E+14	0.0025493296175347	0.170747242106618	-1,16299E+14	8,84418E+14	8,60726E+14	7,32361E+14	7,15392E+14	6,55548E+14	6,23928E+14
TC1000000484.mm.2	Asf1a	anti-silencing function 1A histone chaperone	1,46296E+14	5,88752E+14	4,77457E+14	0.00255320127687294	0.170747242106618	-1,1642E+14	6,95116E+14	6,69944E+13	5,48114E+14	5,24354E+14	5,39449E+14	5,55537E+13
TC1100001291.mm.2	Ppm1d	protein phosphatase 1D magnesium-dependent, delta isoform	1,93511E+14	6,71938E+14	4,77207E+14	0.0025601260090189	0.170747242106618	-1,16636E+14	8,1534E+14	7,99519E+14	6,54714E+14	5,73122E+14	6,24563E+14	5,64368E+14
TC1800000503.mm.2	Dmxl1	Dmx-like 1	1,74851E+14	6,51465E+14	4,76365E+13	0.00258360743397681	0.170747242106618	-1,17367E+14	7,60905E+14	7,61285E+14	5,95638E+14	5,7685E+14	6,46922E+14	5,67193E+14
TC1500000660.mm.2	Triobp	TRIO and F-actin binding protein	1,41644E+14	6,37612E+14	4,75962E+14	0.00259494303196353	0.170747242106618	-1,17717E+14	7,31927E+14	7,54819E+14	6,09611E+13	5,93847E+14	5,67805E+14	5,67663E+14
TC1900000257.mm.2	Olfr1444	olfactory receptor 1444	-1,64365E+13	5,21829E+14	-4,75718E+14	0.00260184797576112	0.170747242106618	-1,1793E+14	4,36411E+14	4,06674E+14	5,56293E+14	6,15522E+14	5,48773E+14	5,673E+14
TC1000001543.mm.2	Avil	advillin	-2,22711E+14	7,2754E+14	-4,75701E+14	0.00260231436830356	0.170747242106618	-1,17945E+13	5,62219E+13	5,90916E+13	7,36331E+14	8,62227E+14	7,91779E+14	8,21769E+14
TC1800000370.mm.2	Pcdhb14	protocadherin beta 14	1,6655E+14	5,58368E+14	4,75204E+14	0.00261643176600918	0.170747242106618	-1,18378E+14	6,46663E+13	6,56016E+14	5,14235E+14	4,55344E+14	5,5913E+14	5,18817E+14
TC1500000409.mm.2	Wdyhv1	WDYHV motif containing 1	1,92347E+14	6,24132E+14	4,75085E+14	0.0026197953017557	0.170747242106618	-1,18481E+14	7,4356E+14	7,20007E+14	5,88061E+14	4,90811E+14	5,90547E+14	6,11804E+14
TC1900000165.mm.2	Ganab	alpha glucosidase 2 alpha neutral subunit	2,17711E+14	6,33262E+14	4,74223E+14	0.00264449187077245	0.170747242106618	-1,19233E+14	7,77445E+14	7,69865E+14	6,07988E+13	5,039E+14	6,07593E+14	5,32778E+14
TC1700000117.mm.2	Agpat4	1-acylglycerol-3-phosphate O-acyltransferase 4 (lysophosphatidic acid acyltransferase, delta)	1,70263E+14	8,27382E+13	4,74093E+13	0.00264822650523931	0.170747242106618	-1,19346E+14	9,33304E+14	9,33144E+14	7,72626E+14	7,53296E+14	8,23365E+14	7,48555E+14
TC1000000165.mm.2	Hbs1l	Hbs1-like (S. cerevisiae)	1,62714E+14	6,46818E+14	4,74019E+14	0.00265036331962056	0.170747242106618	-1,19411E+14	7,68769E+14	7,38965E+13	6,0899E+14	5,73316E+14	6,19806E+14	5,71064E+14
TC0700004166.mm.2	Smg1	SMG1 homolog, phosphatidylinositol 3-kinase-related kinase (C. elegans)	1,43627E+14	6,87551E+14	4,73168E+14	0.00267505425482774	0.170747242106618	-1,20155E+14	7,86554E+14	7,76922E+14	6,49529E+14	6,26694E+14	6,55235E+14	6,30373E+14
TC0400003349.mm.2	Mmachc	methylmalonic aciduria cblC type, with homocystinuria	1,6081E+14	7,15617E+14	4,72531E+13	0.0026937079982516	0.170747242106618	-1,20712E+14	8,24278E+14	8,15246E+14	6,69405E+14	6,485E+14	6,98791E+14	6,37481E+14
TC1200002273.mm.2	Serpina16	serine (or cysteine) peptidase inhibitor, clade A (alpha-1 antiproteinase, antitrypsin), member 16	-1,57162E+14	4,95978E+14	-4,72481E+13	0.00269517106565867	0.170747242106618	-1,20756E+14	3,79463E+14	4,00959E+14	5,2408E+14	5,70666E+14	5,66004E+14	5,34699E+14
TC0600001171.mm.2	Chl1	cell adhesion molecule L1-like	2,0608E+14	7,44304E+13	4,72333E+14	0.00269954429217262	0.170747242106618	-1,20886E+14	9,02521E+14	8,93852E+14	7,34803E+14	6,4941E+13	6,82923E+13	6,02313E+14
TC1200000576.mm.2	Dmac2l	distal membrane arm assembly complex 2 like	2,00995E+14	5,47037E+14	4,72147E+14	0.00270502850254178	0.170747242106618	-1,21049E+14	6,96721E+14	6,50092E+14	5,15481E+14	4,29341E+14	5,23113E+14	4,67473E+14
TC1400000410.mm.2	Dydc1	DPY30 domain containing 1	-1,84447E+14	5,33137E+14	-4,72033E+14	0.00270839579152584	0.170747242106618	-1,21149E+14	4,04798E+14	4,12155E+14	5,46806E+13	6,39041E+14	5,87305E+14	6,08714E+14
TC1300000161.mm.2	Trim27	tripartite motif-containing 27	1,73314E+14	5,57097E+14	4,71327E+14	0.00272935231990348	0.170747242106618	-1,21768E+14	6,74734E+13	6,52606E+14	5,29688E+14	4,51023E+14	5,22131E+14	5,12398E+13
TC0400003749.mm.2	Tmem50a	transmembrane protein 50A	1,59704E+14	7,45911E+14	4,71095E+14	0.00273628661761107	0.170747242106618	-1,21972E+14	8,42927E+14	8,32457E+14	6,80592E+14	6,75384E+14	6,90328E+14	7,53781E+14
TC0300000139.mm.2	Hltf	helicase-like transcription factor	1,42727E+12	6,21851E+14	4,71087E+14	0.00273653465384044	0.170747242106618	-1,21979E+14	6,9539E+14	7,21763E+14	5,65077E+14	5,66622E+14	5,80059E+14	6,02196E+14
TC0700004119.mm.2	Dkk3	dickkopf WNT signaling pathway inhibitor 3	1,73631E+14	7,87203E+14	4,70973E+14	0.00273994440765525	0.170747242106618	-1,22079E+13	8,46019E+14	9,22858E+14	7,201E+14	7,01514E+14	7,7874E+14	7,53986E+14
TC1100003908.mm.2	Cyb561	cytochrome b-561	1,51501E+14	6,20175E+14	4,70971E+14	0.00273999050247483	0.170747242106618	-1,22081E+14	6,94056E+14	6,99397E+14	5,70324E+14	5,20126E+14	6,12612E+14	6,24536E+13
TC0400003856.mm.2	Igsf21	immunoglobulin superfamily, member 21	1,68421E+14	7,56795E+14	4,70732E+14	0.00274715907242211	0.170747242106618	-1,22291E+14	8,52892E+12	8,65203E+13	7,2413E+14	6,57123E+14	7,38064E+14	7,03359E+14
TC1100000537.mm.2	Mrnip	MRN complex interacting protein	1,52934E+13	6,20554E+14	4,70469E+14	0.002755077861638	0.170747242106618	-1,22522E+14	7,40038E+14	7,2016E+14	6,0179E+14	5,5254E+14	5,6056E+14	5,48238E+14
TC0700002780.mm.2	Mag	myelin-associated glycoprotein	1,5443E+14	8,03886E+14	4,69649E+14	0.00277993059216421	0.170747242106618	-1,23245E+14	9,0038E+14	9,2606E+14	7,84421E+14	7,33158E+14	7,40121E+14	7,39178E+14
TC0700004565.mm.2	Ctsd/Ifitm10	cathepsin D/interferon induced transmembrane protein 10	2,10251E+14	9,69411E+14	4,69237E+14	0.00279250640897681	0.170747242106618	-1,23608E+14	1,06341E+14	1,12331E+14	9,35405E+14	8,30814E+14	9,18755E+13	9,44771E+14
TC0600000570.mm.2	Tmem176a	transmembrane protein 176A	1,3629E+14	7,52618E+14	4,68876E+14	0.00280358184797547	0.170747242106618	-1,23927E+14	8,21277E+14	8,36052E+14	6,89966E+14	6,94785E+14	7,38161E+14	7,35466E+14
TC1200001337.mm.2	Tmem196	transmembrane protein 196	1,97203E+14	7,3528E+13	4,68671E+14	0.00280989553358399	0.170747242106618	-1,24108E+13	8,61742E+14	8,42193E+14	7,08982E+14	6,00546E+14	7,00866E+14	6,97349E+14
TC0200001229.mm.2	Ssrp1	structure specific recognition protein 1	2,07928E+13	6,48673E+13	4,68429E+14	0.00281735333652873	0.170747242106618	-1,24321E+13	7,72814E+14	7,96148E+14	6,06031E+14	5,47075E+14	6,36714E+14	5,33258E+14
TC0800000619.mm.2	Glra3	glycine receptor, alpha 3 subunit	2,52062E+14	5,53438E+14	4,67592E+13	0.00284336269193497	0.170747242106618	-1,25062E+14	7,46872E+14	7,03873E+14	5,16176E+14	4,30444E+14	5,27885E+14	3,9538E+14
TC1700000912.mm.2	Mrps10	mitochondrial ribosomal protein S10	2,16141E+13	6,00324E+14	4,66959E+14	0.00286319319289447	0.170747242106618	-1,25622E+14	7,07701E+14	7,13396E+14	5,59411E+14	4,29405E+14	5,96941E+14	5,95087E+14
TC1700002556.mm.2	Heatr5b	HEAT repeat containing 5B	1,97617E+14	6,11906E+14	4,66653E+14	0.00287283451584654	0.170747242106618	-1,25893E+13	7,36467E+14	7,37402E+14	5,91316E+14	4,87318E+14	5,39278E+14	5,79656E+14
TC1600001722.mm.2	Alcam	activated leukocyte cell adhesion molecule	1,4738E+14	9,16455E+14	4,66443E+13	0.00287948124903476	0.170747242106618	-1,2608E+14	1,03949E+14	1,0229E+14	9,03063E+14	8,64562E+14	8,45139E+14	8,23584E+14
TC0200001810.mm.2	Ckmt1	creatine kinase, mitochondrial 1, ubiquitous	1,3849E+14	7,77141E+13	4,66404E+14	0.00288070367832513	0.170747242106618	-1,26114E+14	8,86481E+13	8,68954E+14	7,33557E+14	7,44899E+14	7,22932E+14	7,06024E+14
TC1200000009.mm.2	Asxl2	additional sex combs like 2, transcriptional regulator	1,57216E+14	7,239E+14	4,65934E+14	0.00289566578754446	0.170747242106618	-1,26532E+14	8,18314E+13	8,18323E+14	6,92381E+14	6,29825E+14	6,86989E+14	6,9757E+14
TC0900002944.mm.2	Ppp2r3a	protein phosphatase 2, regulatory subunit B'', alpha	1,47929E+14	6,117E+14	4,65806E+13	0.00289971880762163	0.170747242106618	-1,26645E+14	7,36797E+14	7,1824E+11	5,97273E+13	5,61906E+14	5,41758E+14	5,14224E+14
TC0900002113.mm.2	Sorl1	sortilin-related receptor, LDLR class A repeats-containing	2,18191E+14	7,77865E+14	4,65568E+14	0.00290734965389411	0.170747242106618	-1,26857E+14	9,11753E+14	9,095E+14	7,56305E+14	6,28566E+14	7,48619E+14	7,12446E+14
TC0800000105.mm.2	Sox1	SRY (sex determining region Y)-box 1	-1,42089E+14	9,16823E+14	-4,64934E+14	0.00292771061067823	0.170747242106618	-1,2742E+14	8,23766E+14	8,10374E+14	9,42395E+14	9,75922E+14	9,79006E+14	9,69473E+14
TC0800002169.mm.2	Stox2	storkhead box 2	2,13725E+14	8,04305E+14	4,64928E+14	0.00292792266636087	0.170747242106618	-1,27426E+14	9,23015E+14	9,28588E+14	7,76239E+14	6,47913E+14	7,71092E+14	7,78985E+14
TC0700002644.mm.2	Axl	AXL receptor tyrosine kinase	1,50463E+14	6,82764E+14	4,64731E+14	0.00293427497502876	0.170747242106618	-1,27601E+14	7,74412E+13	7,81935E+14	6,26415E+14	6,29005E+14	6,68869E+14	6,15948E+14
TC0600000250.mm.2	Strip2	striatin interacting protein 2	2,1603E+13	7,24886E+14	4,64678E+14	0.0029359700349919	0.170747242106618	-1,27648E+14	8,34325E+14	8,72193E+14	6,99913E+14	5,74546E+14	6,81475E+14	6,86866E+14
TC1000000498.mm.2	Gm9956	predicted gene 9956	-1,78107E+14	5,04606E+14	-4,64478E+14	0.00294245090061538	0.170747242106618	-1,27826E+14	3,98823E+14	3,90473E+14	5,30332E+13	6,15177E+14	5,3041E+14	5,62423E+13
TC0700003010.mm.2	Ppp1r15a	protein phosphatase 1, regulatory subunit 15A	-1,46453E+14	6,41807E+14	-4,63721E+14	0.0029671242207001	0.170747242106618	-1,28501E+14	5,62838E+14	5,29536E+14	6,7684E+14	7,0844E+14	6,91662E+14	6,81525E+14
TC1700002303.mm.2	Zfp119a	zinc finger protein 119a	1,39943E+14	5,18891E+14	4,63661E+14	0.00296909455711589	0.170747242106618	-1,28555E+14	5,69702E+14	6,01697E+14	4,49931E+13	4,41581E+14	5,24103E+14	5,26332E+14
TC0600002351.mm.2	Ppm1k	protein phosphatase 1K (PP2C domain containing)	1,56548E+14	7,17957E+14	4,63406E+14	0.00297745994554554	0.170747242106618	-1,28782E+14	8,24155E+13	8,15277E+14	6,74028E+14	6,52307E+14	7,00608E+14	6,41369E+13
TC0100003533.mm.2	Fcer1g	Fc receptor, IgE, high affinity I, gamma polypeptide	2,32677E+14	7,13325E+14	4,63401E+14	0.00297762416992758	0.170747242106618	-1,28787E+14	8,26166E+14	8,85426E+14	6,90637E+14	5,55601E+14	6,6496E+14	6,57159E+14
TC1100003819.mm.2	Mpp3	membrane protein, palmitoylated 3 (MAGUK p55 subfamily member 3)	2,02462E+13	5,66813E+14	4,6321E+14	0.00298392719106355	0.170747242106618	-1,28958E+14	6,63194E+14	6,80492E+14	5,2773E+14	4,11032E+14	5,62185E+14	5,56245E+14
TC1100003041.mm.2	Cyb5d1	cytochrome b5 domain containing 1	1,71514E+14	6,22198E+14	4,62921E+14	0.00299345191732572	0.170747242106618	-1,29215E+14	7,27979E+14	7,30345E+14	5,89783E+14	5,25513E+14	6,06716E+14	5,52855E+14
TC1300002237.mm.2	Mterf3	mitochondrial transcription termination factor 3	1,6013E+14	6,87438E+14	4,6205E+13	0.00302241395900309	0.170747242106618	-1,29995E+14	7,75738E+12	7,79385E+14	6,49293E+14	5,85569E+14	6,53579E+14	6,81063E+14
TC0400000272.mm.2	Lyrm2	LYR motif containing 2	1,54649E+14	6,50681E+14	4,61693E+14	0.00303439200396078	0.170747242106618	-1,30315E+13	7,45719E+14	7,21763E+14	6,07518E+13	5,50667E+14	6,40474E+13	6,37947E+14
TC1000000404.mm.2	F930017D23Rik	RIKEN cDNA F930017D23 gene	-1,67462E+14	6,17974E+14	-4,61176E+14	0.00305181248795625	0.170747242106618	-1,30778E+14	4,93799E+14	5,13967E+12	6,33013E+14	7,09677E+14	6,82451E+14	6,74937E+14
TC1900000102.mm.2	Batf2	basic leucine zipper transcription factor, ATF-like 2	-1,56795E+14	7,81123E+14	-4,60829E+14	0.00306357183255677	0.170747242106618	-1,3109E+14	6,82816E+14	6,75442E+14	8,06897E+14	8,64952E+14	8,13766E+14	8,42863E+14
TC1700002337.mm.2	Rfx2	regulatory factor X, 2 (influences HLA class II expression)	-1,71755E+14	7,24398E+14	-4,60178E+14	0.00308572538999298	0.170747242106618	-1,31674E+14	6,04372E+13	5,9851E+14	7,33084E+14	8,13307E+14	7,84166E+14	8,12951E+14
TC0200003473.mm.2	Zeb2/Mir5129	zinc finger E-box binding homeobox 2/microRNA 5129	1,52682E+14	8,11134E+14	4,59998E+14	0.00309190076596519	0.170747242106618	-1,31836E+14	8,66217E+14	9,25687E+14	7,41871E+14	7,4467E+14	7,92865E+14	7,95497E+14
TC1100001889.mm.2	Otop2	otopetrin 2	-1,80944E+14	8,56921E+14	-4,59969E+14	0.00309291104171897	0.170747242106618	-1,31863E+14	7,43499E+13	7,37303E+14	8,76818E+14	9,65874E+14	8,9088E+14	9,27155E+14
TC1700000048.mm.2	Gtf2h5	general transcription factor IIH, polypeptide 5	1,8764E+14	8,25295E+14	4,59798E+14	0.00309877883415284	0.170747242106618	-1,32016E+14	9,20353E+14	9,37515E+14	7,63061E+14	7,19528E+14	8,52055E+14	7,59255E+14
TC1100001704.mm.2	Nmt1	N-myristoyltransferase 1	2,09583E+14	8,02088E+14	4,59659E+13	0.00310354967159372	0.170747242106618	-1,32141E+13	9,27817E+14	9,36259E+14	7,84112E+14	6,60798E+14	7,37256E+14	7,66282E+14
TC0600001601.mm.2	Ddx47	DEAD (Asp-Glu-Ala-Asp) box polypeptide 47	1,33434E+14	7,33067E+13	4,59415E+13	0.0031119657080847З	0.170747242106618	-1,32361E+14	7,96651E+14	8,06222E+13	6,63051E+14	6,72953E+14	7,26522E+14	7,33004E+13
TC1600000439.mm.2	Ubxn7	UBX domain protein 7	1,44416E+14	7,26927E+14	4,59297E+14	0.00311605339666392	0.170747242106618	-1,32467E+14	8,15829E+14	8,21038E+14	6,95722E+14	6,52312E+13	6,95151E+14	6,81511E+14
TC0300000220.mm.2	4930429B21Rik	RIKEN cDNA 4930429B21 gene	2,48221E+14	5,33012E+14	4,59262E+14	0.00311726743015115	0.170747242106618	-1,32499E+13	6,34354E+14	6,47879E+14	4,74464E+14	3,11327E+13	5,58788E+14	5,71259E+13
TC1300000054.mm.2	Dip2c	disco interacting protein 2 homolog C	2,39946E+14	5,68736E+14	4,58873E+14	0.00313075268825835	0.170747242106618	-1,32849E+14	7,29392E+14	6,91015E+14	5,36691E+13	4,03824E+14	5,62567E+14	4,8893E+14
TC0200005314.mm.2	Ctsz	cathepsin Z	1,96914E+14	7,92025E+14	4,58471E+14	0.00314477808422807	0.170747242106618	-1,33212E+14	8,48403E+14	9,37361E+14	7,20176E+14	6,71761E+14	8,13919E+13	7,60532E+14
TC1600001758.mm.2	Tbc1d23	TBC1 domain family, member 23	1,77858E+14	6,83939E+14	4,57673E+14	0.00317277668411239	0.170747242106618	-1,33932E+14	7,89145E+14	7,71869E+14	6,37987E+14	5,67313E+14	6,9829E+14	6,39028E+14

TC0100002899.mm.2	Kdsr	3-ketodihydrosphingosine reductase	1,54094E+14	6,71824E+14	4,5767E+14	0.00317289929341285	0.170747242106618	-1,33935E+14	7,72292E+14	7,41447E+13	6,28996E+14	5,76554E+14	6,63898E+14	6,47754E+14
TC0700003526.mm.2	Sv2b	synaptic vesicle glycoprotein 2 b	2,21452E+14	7,39998E+14	4,57584E+14	0.00317592883291261	0.170747242106618	-1,34012E+14	8,61861E+14	9,35982E+14	7,15863E+14	6,39077E+14	6,88644E+13	5,98558E+14
TC0600003108.mm.2	C1s1	complement component 1, s subcomponent 1	-1,4487E+14	6,50199E+14	-4,57308E+14	0.00318569952742562	0.170747242106618	-1,34262E+14	5,60431E+14	5,76859E+14	6,92247E+14	7,34782E+14	6,61195E+14	6,75684E+14
TC1300001139.mm.2	Taf9/Ak6	TATA-box binding protein associated factor 9/adenylate kinase 6	1,42658E+14	8,10036E+14	4,57264E+14	0.00318726648884893	0.170747242106618	-1,34302E+14	8,91088E+14	8,93162E+14	7,64602E+14	7,34332E+14	8,04167E+14	7,72865E+14
TC0300002901.mm.2	Ank2	ankyrin 2, brain	1,38086E+14	7,27313E+14	4,55999E+14	0.00323248927192051	0.170747242106618	-1,35447E+14	8,27359E+14	8,07214E+14	6,93044E+12	6,65357E+14	6,86403E+14	6,845E+14
TC1500002139.mm.2	Scaf11	SR-related CTD-associated factor 11	1,85414E+14	6,21675E+14	4,55606E+14	0.00324666475397708	0.170747242106618	-1,35803E+14	7,30958E+14	7,34339E+14	5,7998E+14	5,1449E+14	6,25087E+14	5,45198E+14
TC0100003397.mm.2	Prrc2c	proline-rich coiled-coil 2C	2,1043E+14	8,41982E+14	4,55518E+14	0.00324986885604902	0.170747242106618	-1,35883E+14	9,88337E+14	9,80006E+14	7,81432E+14	7,66052E+14	8,32305E+13	7,03763E+14
TC0800001608.mm.2	Insr	insulin receptor	2,08941E+14	6,87262E+14	4,54754E+14	0.00327768911293814	0.170747242106618	-1,36577E+14	7,99642E+14	7,94974E+14	6,3715E+14	5,39584E+13	7,17921E+14	6,34304E+14
TC1100001047.mm.2	Cluh	clustered mitochondria (cluA/CLU1) homolog	1,40853E+14	7,5442E+14	4,5398E+14	0.00330617103489069	0.170747242106618	-1,37282E+14	8,59053E+14	8,57603E+14	7,03541E+14	7,31408E+14	7,03145E+14	6,71772E+14
TC1900000941.mm.2	Dpp3	dipeptidylpeptidase 3	1,54241E+14	6,34159E+14	4,53955E+14	0.00330710215654071	0.170747242106618	-1,37305E+14	7,38197E+13	7,48411E+13	6,12647E+13	5,65479E+14	5,91908E+13	5,4831E+14
TC0500001230.mm.2	Tchp	trichoplein, keratin filament binding	-1,36686E+14	1,04251E+14	-4,53796E+14	0.0033129856521526	0.170747242106618	-1,37449E+14	9,4231E+14	9,50723E+14	1,07004E+14	1,09637E+14	1,10628E+14	1,08935E+14
TC1000003007.mm.2	Cand1	cullin associated and neddylation disassociated 1	2,17922E+14	6,98983E+14	4,53424E+14	0.00332679538141901	0.170747242106618	-1,37788E+14	8,5885E+14	8,17415E+14	6,6923E+13	5,71191E+14	6,82657E+14	5,94555E+14
TC0100003079.mm.2	Cntn2	contactin 2	1,6441E+14	7,5985E+14	4,52959E+14	0.00334412594141979	0.170747242106618	-1,38212E+14	8,76205E+14	8,51621E+14	7,29871E+14	6,69136E+14	7,38679E+14	6,93586E+14
TC1400000946.mm.2	Stmn4	stathmin-like 4	1,60435E+13	8,22712E+14	4,52672E+14	0.00335487838508476	0.170747242106618	-1,38473E+14	9,03726E+14	9,04164E+14	7,74722E+14	7,12298E+14	8,40934E+14	8,0043E+14
TC0X00000618.mm.2	Aff2	AF4/FMR2 family, member 2	1,41095E+14	5,66035E+14	4,52662E+13	0.00335527152662341	0.170747242106618	-1,38483E+14	6,81114E+13	6,52572E+14	5,16892E+14	5,34605E+13	4,91827E+14	5,19203E+13
TC1200000806.mm.2	Ptgr2	prostaglandin reductase 2	1,34721E+14	5,89435E+14	4,52572E+14	0.00335864219197124	0.170747242106618	-1,38565E+14	6,84724E+14	6,67241E+14	5,4719E+14	5,35334E+14	5,6018E+14	5,41942E+14
TC1500002056.mm.2	Arsa	arylsulfatase A	1,44533E+13	5,33314E+14	4,52309E+14	0.00336857047602216	0.170747242106618	-1,38806E+14	6,71969E+14	6,29199E+14	5,12071E+12	5,0003E+14	4,31682E+14	4,54931E+14
TC0200000953.mm.2	Scn2a	sodium channel, voltage-gated, type II, alpha	2,29179E+14	7,99044E+14	4,52158E+14	0.00337426286309763	0.170747242106618	-1,38943E+14	9,29227E+14	9,25588E+13	7,73055E+14	6,23401E+12	7,73366E+14	7,69628E+14
TC0900002869.mm.2	Trpc1	transient receptor potential cation channel, subfamily C, member 1	1,36968E+14	7,03491E+14	4,52047E+14	0.0033784795036885	0.170747242106618	-1,39045E+14	7,98286E+14	8,03888E+14	6,8052E+14	6,47718E+14	6,49804E+14	6,40732E+14
TC1300002536.mm.2	Ocln	occludin	1,59099E+14	5,76481E+14	4,51976E+14	0.00338116790233824	0.170747242106618	-1,3911E+14	6,06081E+14	6,65147E+14	4,78604E+14	4,74426E+14	6,38598E+14	5,96026E+14
TC0600002562.mm.2	Wdr54	WD repeat domain 54	1,57285E+14	7,61305E+14	4,51959E+14	0.00338179416399748	0.170747242106618	-1,39125E+14	8,58219E+14	8,42433E+14	7,04596E+14	6,81485E+14	7,72856E+14	7,08241E+14
TC1700000835.mm.2	Pla2g7	phospholipase A2, group VII (platelet-activating factor acetylhydrolase, plasma)	2,25675E+14	7,32503E+14	4,51704E+14	0.00339148349048375	0.170747242106618	-1,39359E+14	9,14115E+14	8,76867E+14	7,18666E+14	6,20966E+14	6,83566E+14	5,80841E+14
TC0200003605.mm.2	Itgb6	integrin beta 6	-1,48417E+14	7,14874E+14	-4,51628E+14	0.00339434684043679	0.170747242106618	-1,39428E+14	6,02245E+14	6,09049E+14	7,29582E+14	7,78546E+14	7,70526E+14	7,993E+14
TC0500000611.mm.2	Lias	lipoic acid synthetase	1,50746E+14	6,01043E+14	4,51567E+13	0.00339666895662552	0.170747242106618	-1,39483E+14	7,1806E+14	6,77863E+14	5,39068E+14	5,55364E+14	5,79361E+14	5,36541E+14
TC0200002276.mm.2	Tcf15	transcription factor 15	-1,75801E+14	6,71515E+14	-4,51486E+14	0.00339979091370706	0.170747242106618	-1,39558E+13	5,3128E+14	5,6116E+14	6,81719E+14	7,62324E+14	7,26985E+14	7,65619E+14
TC0X00001128.mm.2	Zfp711	zinc finger protein 711	1,5565E+14	5,69685E+14	4,51457E+14	0.00340088501025787	0.170747242106618	-1,39585E+14	6,73836E+14	6,42746E+14	5,06845E+14	4,98437E+14	5,78258E+14	5,17984E+14
TC1100001273.mm.2	Synrg	synergin, gamma	2,66872E+13	6,41425E+14	4,51346E+14	0.0034051212372597	0.170747242106618	-1,39686E+14	8,28159E+14	8,18882E+14	6,2519E+14	4,88107E+14	6,06294E+14	4,81918E+14
TC0300002023.mm.2	Tm4sf1	transmembrane 4 superfamily member 1	2,0083E+14	6,23634E+14	4,51166E+14	0.00341198251688671	0.170747242106618	-1,3985E+14	7,27891E+14	8,0995E+14	5,68286E+14	5,67894E+14	5,78982E+14	4,88801E+14
TC0300002752.mm.2	Stxbp3	syntaxin binding protein 3	1,64399E+14	6,06171E+14	4,50688E+14	0.00343033710827118	0.170747242106618	-1,40289E+14	7,04959E+14	7,34335E+14	5,82207E+14	5,28289E+14	5,6972E+14	5,17516E+14
TC0600002800.mm.2	Lmod3	leiomodin 3 (fetal)	-1,33435E+14	4,28085E+14	-4,50271E+14	0.00344644974548179	0.170747242106618	-1,40671E+14	3,7776E+14	3,93445E+14	5,11161E+13	5,26913E+14	3,86374E+14	3,72858E+14
TC1400002267.mm.2	Ptk2b	PTK2 protein tyrosine kinase 2 beta	1,3606E+14	7,35256E+14	4,50247E+13	0.00344738497055962	0.170747242106618	-1,40693E+14	8,0804E+13	8,30855E+14	6,94521E+14	6,72254E+14	7,08191E+14	6,97673E+13
TC0700000798.mm.2	2410002F23Rik/Snord88a	RIKEN cDNA 2410002F23 gene/small nucleolar RNA, C/D box 88A	1,70002E+14	7,53375E+13	4,50093E+14	0.00345333956945075	0.170747242106618	-1,40834E+14	8,87284E+14	8,84706E+14	7,58344E+14	6,73641E+14	6,69852E+14	6,46421E+14
TC0100003576.mm.2	Fcrl6	Fc receptor-like 6	-1,42892E+14	4,257E+14	-4,49829E+14	0.0034635938791137	0.170747242106618	-1,41077E+14	3,05389E+14	3,21178E+14	4,34208E+14	4,78144E+14	5,00924E+14	5,14359E+13
TC0400002206.mm.2	Dpy19l4	dpy-19-like 4 (C. elegans)	-1,74479E+14	6,8519E+14	-4,49647E+14	0.00347071111987936	0.170747242106618	-1,41244E+14	6,05208E+14	5,54349E+14	7,16804E+14	7,9171E+13	7,33263E+14	7,09806E+13
TC0600003020.mm.2	Erc1	ELKS/RAB6-interacting/CAST family member 1	1,55779E+14	7,0073E+14	4,49271E+14	0.00348538743125024	0.170747242106618	-1,41589E+14	8,06152E+14	8,15616E+14	6,89093E+14	6,21116E+14	6,30403E+14	6,41999E+14
TC1400001314.mm.2	Ipo5	importin 5	1,32268E+14	7,88974E+14	4,48841E+14	0.00350228794453171	0.170747242106618	-1,41985E+14	8,7416E+14	8,81256E+14	7,54474E+14	7,36406E+14	7,50373E+14	7,37176E+13
TC1800001462.mm.2	Nars	asparaginyl-tRNA synthetase	1,53859E+14	7,57524E+14	4,4864E+14	0.00351024612208967	0.170747242106618	-1,4217E+14	8,69484E+14	8,71331E+14	7,47622E+14	6,85475E+13	6,98293E+14	6,72937E+14
TC0300000334.mm.2	Pcdh10	protocadherin 10	2,41667E+14	7,54804E+14	4,48581E+14	0.0035125502458148	0.170747242106618	-1,42224E+14	9,00263E+14	9,04392E+12	7,41646E+14	5,79675E+14	7,12136E+14	6,9071E+14
TC1500001183.mm.2	Slc1a3	solute carrier family 1 (glial high affinity glutamate transporter), member 3	1,46608E+14	8,02432E+12	4,48523E+14	0.00351485602918309	0.170747242106618	-1,42278E+14	9,02278E+13	8,84449E+14	7,71905E+14	7,21607E+13	7,7548E+13	7,58876E+14
TC0200002887.mm.2	Proser2	proline and serine rich 2	-1,6045E+14	6,92597E+14	-4,48434E+14	0.00351836598046148	0.170747242106618	-1,42359E+14	6,15762E+14	5,62415E+14	7,23942E+14	7,75135E+14	7,29408E+14	7,48921E+14
TC1600000205.mm.2	Ypel1	yippee like 1	-1,38473E+14	9,20426E+14	-4,48147E+14	0.00352976408422498	0.170747242106618	-1,42624E+14	8,24968E+14	8,35175E+14	9,51319E+14	9,85769E+14	9,53038E+13	9,72284E+14
TC1100003056.mm.2	Eif4a1	eukaryotic translation initiation factor 4A1	2,043E+14	8,67326E+14	4,47913E+14	0.0035390669192983	0.170747242106618	-1,42839E+14	9,81914E+14	1,00236E+14	8,35691E+14	7,39984E+14	8,62502E+14	7,81501E+14
TC1600001193.mm.2	Tekt5	tektin 5	1,59455E+14	6,35592E+14	4,47641E+14	0.00354995208161196	0.170747242106618	-1,43091E+14	7,57479E+14	7,32621E+14	6,21189E+14	5,50001E+14	5,78116E+14	5,74144E+13
TC1400000753.mm.2	Psmb11	proteasome (prosome, macropain) subunit, beta type, 11	-1,29741E+14	5,43641E+14	-4,47587E+14	0.00355209136564319	0.170747242106618	-1,4314E+14	4,66576E+14	4,63975E+14	5,95793E+14	5,9424E+14	5,63934E+14	5,7733E+14
TC1800000727.mm.2	Rnmt	RNA (guanine-7-) methyltransferase	1,57883E+14	6,53406E+14	4,47285E+14	0.00356420655039464	0.170747242106618	-1,43419E+14	7,24956E+14	7,3712E+14	6,07822E+13	5,38487E+14	6,66651E+14	6,45404E+14
TC0200001551.mm.2	Cd59b	CD59b antigen	1,40004E+14	5,68031E+14	4,47033E+14	0.00357435744651465	0.170747242106618	-1,43652E+14	6,80893E+14	6,5645E+14	5,46044E+14	5,11289E+14	5,13681E+14	4,9983E+13
TC1700001852.mm.2	Zfp799	zinc finger protein 799	1,67694E+13	5,60118E+14	4,46922E+14	0.00357883561122734	0.170747242106618	-1,43754E+14	6,74668E+14	6,75283E+14	5,48195E+14	4,66368E+14	5,12182E+14	4,84011E+14
TC0300003166.mm.2	5730460C07Rik	RIKEN cDNA 5730460C07 gene	-1,35553E+14	7,36004E+14	-4,46673E+14	0.00358888583710325	0.170747242106618	-1,43984E+14	6,3422E+14	6,41026E+13	7,61097E+14	7,85255E+14	8,09427E+14	7,84997E+14
TC1000000886.mm.2	Dapk3	death-associated protein kinase 3	2,57129E+14	6,74337E+14	4,46582E+14	0.00359260885087728	0.170747242106618	-1,44069E+14	8,70795E+14	8,28452E+14	6,76672E+14	5,08316E+14	6,02589E+14	5,592E+14
TC0700000849.mm.2	Tulp2	tubby-like protein 2	-1,49938E+14	5,59344E+14	-4,46508E+14	0.00359560316195381	0.170747242106618	-1,44137E+14	4,77233E+14	4,80628E+14	5,99022E+14	6,58715E+14	5,7904E+13	5,61425E+14
TC0700000019.mm.2	Rps9	ribosomal protein S9	1,35624E+14	7,83646E+14	4,46201E+14	0.00360807579167375	0.170747242106618	-1,44421E+14	8,56508E+14	8,62318E+14	7,30782E+14	7,16795E+14	7,51651E+14	7,83823E+14
TC1100003776.mm.2	Dnajc7	DnaJ heat shock protein family (Hsp40) member C7	2,06795E+14	7,67436E+14	4,45531E+14	0.00363546637700058	0.170747242106618	-1,45041E+14	9,08402E+14	9,03064E+14	7,62231E+14	6,35645E+14	7,13745E+14	6,81528E+14
TC0700002226.mm.2	Clcn4	chloride channel, voltage-sensitive 4	1,63184E+14	7,40105E+14	4,4522E+13	0.00364829252778018	0.170747242106618	-1,45329E+14	8,45367E+14	8,44576E+14	7,18948E+13	6,44627E+14	6,76706E+14	7,10408E+14
TC0700000006.mm.2	Prkcg	protein kinase C, gamma	1,89483E+14	7,71049E+14	4,44998E+14	0.00365745139885517	0.170747242106618	-1,45535E+14	8,83699E+14	9,16449E+14	7,54981E+14	6,66201E+14	7,33088E+14	6,71878E+14
TC0400002626.mm.2	Tex10	testis expressed gene 10	1,59755E+14	5,63969E+14	4,44328E+14	0.00368528673579372	0.170747242106618	-1,46157E+14	6,38407E+14	6,38432E+14	5,17139E+14	4,40191E+14	5,79491E+14	5,70157E+14
TC1300000998.mm.2	Edil3	EGF-like repeats and discoidin I-like domains 3	1,43242E+14	7,71371E+14	4,4431E+14	0.00368602268080789	0.170747242106618	-1,46173E+14	8,65894E+14	8,39692E+14	7,18215E+14	7,00888E+14	7,72761E+14	7,30774E+14
TC1100004051.mm.2	Cd300lb	CD300 molecule like family member B	-1,39528E+14	5,79376E+14	-4,43995E+14	0.00369919340324983	0.170747242106618	-1,46466E+14	4,98436E+14	4,683E+13	6,08112E+14	6,3768E+14	6,23188E+14	6,40543E+14
TC1600001117.mm.2	Crebbp	CREB binding protein	1,72884E+14	7,8406E+13	4,43876E+14	0.00370417894908563	0.170747242106618	-1,46577E+14	9,02694E+13	9,18928E+14	7,61414E+14	7,1444E+13	7,43512E+14	6,6337E+14
TC1500001065.mm.2	Grasp	GRP1 (general receptor for phosphoinositides 1)-associated scaffold protein	1,33256E+14	7,93128E+14	4,43819E+13	0.00370659514647753	0.170747242106618	-1,4663E+14	8,9116E+14	9,21255E+14	7,7479E+13	7,71112E+14	7,04011E+14	6,96441E+14
TC1900001203.mm.2	Psat1	phosphoserine aminotransferase 1	2,22589E+14	7,6293E+14	4,4341E+14	0.0037237843678212	0.170747242106618	-1,4701E+14	8,935E+14	9,08087E+14	7,46202E+14	6,10208E+14	7,37564E+14	6,82019E+14
TC0200000924.mm.2	Psmd14	proteasome (prosome, macropain) 26S subunit, non-ATPase, 14	1,39504E+14	7,27378E+14	4,43315E+14	0.00372779031800729	0.170747242106618	-1,47098E+13	8,09159E+14	8,41162E+14	6,95133E+13	6,7618E+14	6,84871E+14	6,57763E+14
TC0700001406.mm.2	C2cd3	C2 calcium-dependent domain containing 3	1,28548E+14	6,02162E+14	4,43292E+14	0.00372878871139759	0.170747242106618	-1,4712E+13	6,74323E+14	6,84748E+14	5,55231E+14	5,46743E+14	5,78008E+14	5,73919E+14
TC0600000016.mm.2	Ppp1r9a	protein phosphatase 1, regulatory subunit 9A	2,4951E+14	7,80139E+14	4,43139E+14	0.00373523358594858	0.170747242106618	-1,47262E+13	9,26046E+14	9,51899E+14	7,57037E+13	6,21889E+13	7,64949E+14	6,59012E+14
TC1100002920.mm.2	Prpsap2	phosphoribosyl pyrophosphate synthetase-associated protein 2	1,57999E+14	5,74109E+14	4,43002E+14	0.00374107215376945	0.170747242106618	-1,4739E+13	6,88825E+14	6,77609E+14	5,43204E+14	5,07233E+14	5,46763E+14	4,81018E+14
TC0200002772.mm.2	Cdh4	cadherin 4	1,659E+12	5,47271E+14	4,42772E+14	0.00375083898685425	0.170747242106618	-1,47604E+14	6,78423E+14	6,45568E+14	5,2207E+14	4,70121E+14	5,13971E+14	4,53474E+14
TC1400001057.mm.2	Zc3h13	zinc finger CCCH type containing 13	1,29377E+14	6,3923E+14	4,42173E+14	0.00377641632143305	0.170747242106618	-1,48163E+14	7,32388E+14	7,31984E+14	6,11388E+14	5,9423E+14	5,87653E+14	5,77739E+14
TC0X00001283.mm.2	Hnrnph2	heterogeneous nuclear ribonucleoprotein H2	1,83431E+14	7,82725E+14	4,42144E+14	0.00377763281903707	0.170747242106618	-1,48189E+14	9,03294E+14	8,88967E+14	7,60556E+14	6,64843E+14	7,62542E+14	7,16149E+14
TC1500001977.mm.2	Ttll1	tubulin tyrosine ligase-like 1	1,9684E+14	6,52763E+14	4,42072E+14	0.00378074834688162	0.170747242106618	-1,48257E+14	8,2014E+14	8,07473E+14	6,65696E+14	5,68237E+13	5,63651E+14	4,91383E+14
TC0X00002057.mm.2	Rpl39	ribosomal protein L39	1,38144E+13	8,39741E+13	4,4199E+14	0.00378424955001511	0.170747242106618	-1,48333E+14	9,40225E+14	9,42505E+14	8,14056E+14	7,92385E+14	7,55459E+14	7,93817E+14
TC0500002859.mm.2	Wdfy3	WD repeat and FYVE domain containing 3	1,93167E+14	6,47192E+14	4,41905E+14	0.00378789998239258	0.170747242106618	-1,48412E+14	7,95323E+14	7,49423E+14	6,31791E+14	5,26622E+13	6,04048E+14	5,75947E+14
TC1500001675.mm.2	Fam135b	family with sequence similarity 135, member B	2,16233E+13	6,23742E+13	4,41832E+14	0.0037910659330373	0.170747242106618	-1,48481E+14	7,50358E+14	7,51489E+14	6,02273E+14	4,67108E+14	5,63724E+14	6,07498E+14
TC1700001864.mm.2	Zfp955a	zinc finger protein 955A	1,59237E+14	7,64366E+14	4,41657E+14	0.00379857686853138	0.170747242106618	-1,48644E+14	8,51563E+14	8,58022E+14	7,3242E+14	6,58691E+14	7,55234E+14	7,30269E+14
TC1400000945.mm.2	Trim35	tripartite motif-containing 35	1,6866E+14	7,70392E+14	4,41511E+13	0.00380489108064794	0.170747242106618	-1,4878E+14	8,80783E+13	8,81485E+14	7,46176E+14	6,78772E+14	7,47597E+14	6,87542E+13
TC1100003047.mm.2	Wrap53	WD repeat containing, antisense to Trp53	1,31413E+14	5,86911E+13	4,41469E+12	0.00380670406476795	0.170747242106618	-1,48819E+14	6,76422E+13	6,92555E+14	5,58316E+13	5,47835E+14	5,33272E+14	5,13068E+14
TC0100000790.mm.2	Ptma	prothymosin alpha	1,44121E+14	9,95763E+14	4,41338E+14	0.00381236127108997	0.170747242106618	-1,48942E+14	1,08451E+14	1,09253E+14	9,71669E+14	9,17132E+14	9,58193E+14	9,50539E+13
TC0600001474.mm.2	D6Wsu163e	DNA segment, Chr 6, Wayne State University 163, expressed	1,78759E+14	7,34949E+14	4,41307E+14	0.00381374084130086	0.170747242106618	-1,48971E+13	8,44792E+13	8,29789E+14	6,97442E+14	6,19621E+13	7,41444E+14	6,76606E+14
TC1800000616.mm.2	Rps14	ribosomal protein S14	1,70119E+14	8,53738E+14	4,41262E+14	0.00381568611024265	0.170747242106618	-1,49013E+14	9,55517E+14	9,65087E+14	8,17173E+14	7,63194E+14	8,47786E+14	7,73672E+14
TC1100002064.mm.2	Fn3krp	fructosamine 3 kinase related protein	1,59693E+14	6,68224E+14	4,41156E+14	0.00382027480985753	0.170747242106618	-1,49112E+14	7,59098E+14	7,56182E+14	6,2564E+14	5,70254E+14	6,77188E+14	6,20984E+13
TC1100004196.mm.2	Slc38a10	solute carrier family 38, member 10	1,84875E+14	8,42551E+14	4,40991E+13	0.00382743323347426	0.170747242106618	-1,49266E+14	9,56888E+14	9,71537E+14	8,29078E+14	7,29597E+14	8,05754E+14	7,62451E+14
TC0600002745.mm.2	Prickle2	prickle planar cell polarity protein 2	1,89035E+14	6,95443E+14	4,40461E+14	0.00385056238320878	0.170747242106618	-1,49762E+14	8,1438E+14	8,30493E+14	6,29149E+14	6,37655E+14	6,86925E+14	5,74055E+14
TC1200002294.mm.2	Gsc	goosecoid homeobox	-1,40638E+14	5,87123E+14	-4,40317E+14	0.00385687957223088	0.170747242106618	-1,49897E+14	4,86978E+14	4,91577E+13	6,07575E+14	6,52256E+14	6,31159E+13	6,53192E+14
TC0400002480.mm.2	Ccl27a	chemokine (C-C motif) ligand 27A	1,44854E+14	9,5453E+14	4,40294E+14	0.00385789269515741	0.170747242106618	-1,49918E+14	1,0282E+14	1,04938E+13	9,1813E+13	8,6975E+14	9,42187E+14	9,19525E+14
TC0600000830.mm.2	Chmp3	charged multivesicular body protein 3	1,30793E+14	6,88104E+14	4,40049E+14	0.0038686572423578	0.170747242106618	-1,50148E+14	7,68285E+14	7,43178E+14	6,26631E+14	6,23246E+14	6,88696E+14	6,78587E+13
TC1100003918.mm.2	Smarcd2	SWI/SNF related, matrix associated, actin dependent regulator of chromatin, subfamily d, member 2	-2,09027E+14	7,08092E+14	-4,39698E+13	0.00388414412060575	0.170747242106618	-1,50477E+14	5,90699E+14	5,82772E+14	7,51616E+14	8,39909E+14	6,90325E+14	7,9323E+14
TC0900001939.mm.2	Glb1l2	galactosidase, beta 1-like 2	-1,58801E+14	4,90397E+14	-4,38407E+14	0.00394172970692227	0.170747242106618	-1,51689E+13	3,9046E+14	3,82696E+14	5,06998E+14	5,83761E+14	5,47957E+14	5,30511E+13
TC0200000868.mm.2	Gpd2	glycerol phosphate dehydrogenase 2, mitochondrial	1,6191E+14	6,76338E+14	4,38272E+14	0.00394780479865747	0.170747242106618	-1,51816E+14	7,73615E+14	7,94346E+14	6,5855E+14	5,85591E+14	6,40156E+14	6,05772E+14
TC1100000560.mm.2	Zfp354a	zinc finger protein 354A	-1,35464E+14	6,12755E+14	-4,3818E+14	0.00395192882907318	0.170747242106618	-1,51902E+14	5,26674E+14	5,41277E+14	6,52168E+14	6,8671E+13	6,26666E+14	6,43033E+13
TC0500003019.mm.2	Sez6l	seizure related 6 homolog like	2,27328E+14	7,79888E+14	4,38092E+14	0.00395592412222862	0.170747242106618	-1,51985E+14	9,47956E+14	9,26074E+14	7,85816E+14	6,33558E+14	6,83587E+14	7,02339E+14
TC1500001750.mm.2	Eef1d	eukaryotic translation elongation factor 1 delta (guanine nucleotide exchange protein)	2,20556E+14	6,98629E+14	4,37864E+14	0.00396623280889026	0.170747242106618	-1,522E+14	8,59585E+14	8,52577E+14	6,35238E+14	6,35811E+14	6,78384E+14	5,30178E+14
TC0800000783.mm.2	Use1	unconventional SNARE in the ER 1 homolog (S. cerevisiae)	1,33791E+14	8,08673E+14	4,37581E+14	0.00397904156069156	0.170747242106618	-1,52465E+14	8,97968E+14	8,81036E+14	7,66781E+14	7,4464E+14	7,68271E+14	7,9334E+14
TC0800002927.mm.2	Sf3b3	splicing factor 3b, subunit 3	1,91557E+14	6,53456E+14	4,37212E+14	0.00399584046838053	0.170747242106618	-1,52813E+14	7,83771E+14	7,70705E+14	6,44272E+14	5,27089E+14	6,03939E+14	5,90958E+14
TC1500001320.mm.2	Ankrd33b	ankyrin repeat domain 33B	-1,51041E+14	9,82905E+14	-4,37145E+14	0.00399892697168542	0.170747242106618	-1,52877E+14	8,72486E+14	8,66141E+14	9,8617E+14	1,05454E+14	1,06137E+14	1,05672E+14
TC1200002251.mm.2	Lgmn	legumain	1,75313E+13	9,32216E+14	4,36692E+14	0.00401966267689917	0.170747242106618	-1,53303E+14	1,00652E+13	1,06224E+14	9,00916E+13	8,17222E+14	9,03665E+14	9,02728E+14
TC1200001990.mm.2	Actn1	actinin, alpha 1	1,33362E+14	6,64983E+14	4,36684E+14	0.00402002884088989	0.170747242106618	-1,53311E+14	7,36764E+14	7,62068E+14	6,23322E+14	6,08786E+14	6,40946E+14	6,18011E+14
TC1100003856.mm.2	Gfap	glial fibrillary acidic protein	1,86781E+14	9,25644E+14	4,36633E+13	0.00402236915827457	0.170747242106618	-1,53359E+14	9,73304E+14	1,07493E+14	8,626E+14	8,12068E+13	9,12444E+14	9,1852E+14
TC0700002828.mm.2	Gpi1	glucose phosphate isomerase 1	1,6081E+14	1,0185E+13	4,36278E+14	0.00403872548610883	0.170747242106618	-1,53694E+14	1,13802E+14	1,1169E+14	1,00373E+14	9,29577E+14	9,76473E+14	9,46292E+13
TC0200005496.mm.2	Fam78a	family with sequence similarity 78, member A	-1,34794E+14	6,22416E+14	-4,36104E+14	0.00404677983013397	0.170747242106618	-1,53858E+14	5,11716E+13	5,40104E+14	6,52733E+14	6,68675E+14	6,68293E+14	6,92974E+13
TC0200005072.mm.2	Ptprt	protein tyrosine phosphatase, receptor type, T	1,41718E+14	6,3808E+14	4,36054E+14	0.00404909727888555	0.170747242106618	-1,53905E+14	7,05463E+14	7,55829E+14	5,84279E+14	5,93577E+13	5,85392E+14	6,03937E+14
TC0100001581.mm.2	Adamts4	a disintegrin-like and metallopeptidase (reprolysin type) with thrombospondin type 1 motif, 4	1,32843E+12	6,37144E+14	4,35922E+14	0.00405524570994007	0.170747242106618	-1,54031E+14	7,19323E+14	7,43388E+14	6,08003E+14	5,89022E+14	5,7173E+14	5,91394E+14
TC0X00003230.mm.2	Magea8	melanoma antigen, family A, 8	-1,53481E+14	5,12449E+13	-4,35842E+14	0.00405892005749228	0.170747242106618	-1,54105E+14	4,22283E+13	3,8547E+14	5,27834E+14	5,86881E+14	5,87177E+14	5,6505E+14
TC0100000228.mm.2	Amer3	APC membrane recruitment 3	2,07162E+14	5,4958E+14	4,35626E+14	0.00406896369572982	0.170747242106618	-1,5431E+14	7,00904E+14	6,70414E+14	5,3854E+14	4,18453E+14	5,12502E+14	4,5667E+13
TC0200002678.mm.2	Rbm38	RNA binding motif protein 38	-1,47072E+14	7,02909E+14	-4,35274E+14	0.00408542824469294	0.170747242106618	-1,54643E+14	6,19306E+14	6,003E+14	7,43063E+14	7,70686E+14	7,14933E+14	7,69164E+14
TC0400003705.mm.2	Nudc	nudC nuclear distribution protein	1,7909E+14	7,56359E+13	4,3512E+14	0.00409263881014035	0.170747242106618	-1,54789E+14	8,66364E+14	8,55367E+14	7,34307E+14	6,29244E+14	7,24799E+14	7,28072E+14
TC0200002667.mm.2	Cstf1	cleavage stimulation factor, 3' pre-RNA, subunit 1	1,86292E+14	6,36544E+14	4,34895E+14	0.00410319553224868	0.170747242106618	-1,55002E+14	7,61393E+13	7,68097E+14	6,3532E+14	5,21584E+14	5,65873E+14	5,66998E+14
TC0200003329.mm.2	Lmx1b	LIM homeobox transcription factor 1 beta	-1,40127E+14	8,10176E+14	-4,34423E+14	0.00412546666917527	0.170747242106618	-1,55449E+14	7,13087E+14	7,17928E+14	8,29478E+14	8,81792E+14	8,60326E+14	8,58443E+14
TC0100003566.mm.2	Atp1a2	ATPase, Na+/K+ transporting, alpha 2 polypeptide	1,82305E+14	8,40638E+13	4,3352E+14	0.0041683939168532	0.170747242106618	-1,56305E+14	9,74137E+13	9,47614E+14	8,22565E+14	7,34577E+14	8,12803E+14	7,52134E+14
TC1800001441.mm.2	Fbxo38	F-box protein 38	1,54062E+14	6,26083E+14	4,33411E+14	0.00417362682506224	0.170747242106618	-1,56408E+14	7,26686E+13	7,25167E+14	5,80929E+14	5,62798E+14	6,16867E+14	5,44052E+14
TC0300001999.mm.2	Nbea	neurobeachin	2,3355E+14	7,47362E+14	4,33289E+14	0.00417947319455309	0.170747242106618	-1,56524E+14	9,09319E+14	9,06406E+14	7,23481E+14	6,25143E+14	7,25239E+14	5,94585E+14

TC1600000955.mm.2	Usp16	ubiquitin specific peptidase 16	-1,42919E+14	9,60862E+13	-4,33123E+14	0.00418745190655314	0.170747242106618	-1,56682E+14	8,59885E+14	8,59454E+13	9,76359E+14	1,02882E+13	1,03291E+14	1,00774E+14
TC0200004763.mm.2	Rbbp9	retinoblastoma binding protein 9, serine hydrolase	2,79481E+14	5,74389E+14	4,32934E+14	0.00419655129733124	0.170747242106618	-1,56862E+14	7,52485E+14	7,2898E+14	5,40636E+14	3,81868E+14	5,86794E+14	4,55568E+14
TC0500002484.mm.2	Rpl9	ribosomal protein L9	1,8251E+13	9,90923E+13	4,32871E+14	0.0041995935043032	0.170747242106618	-1,56922E+12	1,11519E+14	1,10939E+14	9,76337E+14	8,83223E+14	9,60455E+14	9,00941E+13
TC0300003020.mm.2	Metap1	methionyl aminopeptidase 1	1,48263E+12	6,4956E+14	4,32849E+14	0.00420065320760436	0.170747242106618	-1,56942E+14	7,70864E+14	7,41167E+14	5,78122E+14	6,37382E+14	5,89333E+14	5,8049E+14
TC1400000170.mm.2	Sec24c	Sec24 related gene family, member C (S. cerevisiae)	1,59553E+14	6,35226E+14	4,32734E+14	0.00420618448400087	0.170747242106618	-1,57051E+14	7,26265E+14	7,41006E+14	5,80024E+14	5,68143E+14	6,38297E+14	5,57624E+13
TC1400000810.mm.2	Mphosph8	M-phase phosphoprotein 8	2,19851E+13	7,36159E+14	4,32532E+14	0.00421598463562278	0.170747242106618	-1,57244E+14	8,69537E+14	8,67489E+14	7,18701E+14	5,78623E+14	7,18617E+14	6,63989E+14
TC0500003728.mm.2	D5Ertd579e	DNA segment, Chr 5, ERATO Doi 579, expressed	1,46053E+14	6,56937E+14	4,31714E+14	0.00425578297315682	0.170747242106618	-1,58022E+14	7,74392E+14	7,66063E+14	6,26877E+14	6,21472E+14	6,08046E+13	5,4477E+14
TC0800001414.mm.2	Cdh13	cadherin 13	2,06089E+14	6,94205E+14	4,31603E+14	0.00426122218421825	0.170747242106618	-1,58128E+14	8,42269E+14	8,19169E+14	6,72975E+14	5,76286E+14	6,74233E+14	5,80298E+14
TC0100003840.mm.2	Camk1g	calcium/calmodulin-dependent protein kinase I gamma	1,53189E+13	7,27237E+14	4,30744E+14	0.00430360305450918	0.170747242106618	-1,58947E+13	8,26171E+14	8,35547E+14	7,05463E+14	6,49876E+14	6,48325E+14	6,98037E+14
TC1700000580.mm.2	Zfp955b	zinc finger protein 955B	1,54104E+14	7,23935E+14	4,30568E+14	0.00431230930409657	0.170747242106618	-1,59115E+12	8,05114E+14	8,15287E+14	6,91794E+14	6,20401E+14	7,1832E+13	6,92693E+14
TC0700001926.mm.2	Hmx2	H6 homeobox 2	-1,47068E+14	7,1498E+14	-4,30457E+14	0.00431782500072488	0.170747242106618	-1,59221E+14	6,16178E+14	6,14072E+14	7,39504E+14	7,84882E+14	7,43479E+14	7,91764E+14
TC0700002694.mm.2	Supt5	suppressor of Ty 5	1,39939E+14	6,46971E+14	4,30279E+14	0.00432673096867313	0.170747242106618	-1,59392E+14	7,75631E+14	7,46514E+14	6,36384E+14	6,05883E+13	5,41328E+14	5,76083E+14
TC0200002352.mm.2	Raly/a	hnRNP-associated with lethal yellow/nonagouti	1,80729E+14	6,83616E+14	4,30083E+14	0.00433649160048163	0.170747242106618	-1,59578E+14	7,83723E+14	7,72155E+14	6,51317E+14	5,43105E+14	6,83594E+14	6,67801E+14
TC0500003028.mm.2	Sgsm1	small G protein signaling modulator 1	1,63303E+14	7,53661E+14	4,29873E+14	0.00434700839438204	0.170747242106618	-1,59779E+12	8,81896E+14	8,68373E+14	7,55543E+14	6,6812E+14	6,686E+14	6,79433E+14
TC0800000763.mm.2	Pde4c	phosphodiesterase 4C, cAMP specific	-1,58646E+14	7,60404E+14	-4,29649E+14	0.00435827341737108	0.170747242106618	-1,59994E+14	6,69999E+14	6,50532E+14	7,81535E+14	8,56287E+14	7,86619E+14	8,17452E+14
TC0900001385.mm.2	Camkv	CaM kinase-like vesicle-associated	1,58113E+14	9,08113E+13	4,29235E+13	0.00437913385956435	0.170747242106618	-1,6039E+14	1,00259E+14	1,00686E+14	8,87881E+13	8,05338E+14	8,72319E+14	8,73696E+14
TC0100002997.mm.2	Lypd1	Ly6/Plaur domain containing 1	1,58961E+14	8,60987E+14	4,29023E+14	0.0043898728398485	0.170747242106618	-1,60593E+14	9,86961E+14	9,66912E+14	8,5708E+14	7,78871E+14	7,9945E+14	7,76646E+14
TC0700003827.mm.2	Arhgef17	Rho guanine nucleotide exchange factor (GEF) 17	1,50331E+14	7,52933E+14	4,28937E+14	0.004394236525123 5	0.170747242106618	-1,60676E+12	8,49939E+14	8,36055E+14	7,26412E+14	6,58919E+14	7,12743E+14	7,33527E+14
TC1700000341.mm.2	Caskin1	CASK interacting protein 1	1,35657E+14	7,13177E+14	4,28167E+14	0.00443349160005671	0.170747242106618	-1,61414E+14	8,50359E+14	8,05364E+14	6,96182E+13	6,88227E+14	6,26622E+14	6,12305E+14
TC0900001863.mm.2	Keap1	kelch-like ECH-associated protein 1	1,81068E+14	6,34077E+14	4,27707E+14	0.00445710094879951	0.170747242106618	-1,61855E+14	7,32134E+14	7,35004E+14	6,07128E+14	4,97874E+14	6,05118E+14	6,27205E+14
TC1700001877.mm.2	Angptl4	angiopoietin-like 4	-1,40649E+14	7,42995E+14	-4,27665E+14	0.00445927937321648	0.170747242106618	-1,61895E+14	6,59013E+14	6,31302E+14	7,90393E+14	7,81219E+14	7,7332E+14	8,22724E+14
TC0800001323.mm.2	Cog4	component of oligomeric golgi complex 4	1,38792E+14	5,68007E+14	4,27545E+13	0.00446548196342232	0.170747242106618	-1,62011E+14	6,48112E+14	6,32524E+13	4,76191E+14	5,26861E+14	5,67771E+14	5,56581E+14
TC1700002527.mm.2	Srd5a2	steroid 5 alpha-reductase 2	-1,32015E+14	5,4566E+14	-4,27528E+14	0.00446634830805593	0.170747242106618	-1,62027E+14	4,77618E+14	4,47836E+14	5,85979E+14	6,03505E+14	5,68891E+14	5,90133E+14
TC0200002741.mm.2	Gm14295	predicted gene 14295	2,58686E+14	7,14651E+13	4,27051E+14	0.00449107866867911	0.170747242106618	-1,62486E+14	8,12759E+13	8,82413E+14	6,71341E+14	5,06459E+13	7,41146E+14	6,73786E+14
TC0600003397.mm.2	Sox5	SRY (sex determining region Y)-box 5	-1,49127E+14	7,97795E+14	-4,25975E+14	0.00454736842778873	0.170747242106618	-1,63521E+13	6,82486E+14	7,05225E+14	8,41276E+14	8,44688E+14	8,90518E+13	8,22577E+14
TC0400000353.mm.2	Chmp5	charged multivesicular body protein 5	1,31023E+14	6,95333E+14	4,25844E+14	0.00455427259943498	0.170747242106618	-1,63647E+13	7,89356E+14	7,88843E+14	6,75598E+14	6,40556E+14	6,29752E+13	6,47895E+14
TC0300003211.mm.2	5830417I10Rik	gon-4-like pseudogene	1,81983E+14	6,10259E+14	4,25819E+14	0.00455559809468592	0.170747242106618	-1,63671E+14	7,24684E+14	7,40336E+13	6,02799E+14	4,98255E+14	5,27134E+14	5,68347E+14
TC1800001731.mm.2	Dok6	docking protein 6	2,64506E+14	5,70364E+14	4,2557E+14	0.00456876497935308	0.170747242106618	-1,63911E+14	7,25352E+14	7,30082E+14	5,4647E+14	3,79953E+14	5,7335E+14	4,66976E+14
TC1400002833.mm.2	Rnf31	ring finger protein 31	1,46879E+14	5,68723E+14	4,25437E+14	0.00457582582938919	0.170747242106618	-1,6404E+14	6,59341E+14	6,43727E+14	5,37317E+14	4,71993E+14	5,57067E+14	5,42895E+14
TC0500002649.mm.2	Spink2	serine peptidase inhibitor, Kazal type 2	-2,25277E+14	5,79589E+14	-4,254E+14	0.00457779381816774	0.170747242106618	-1,64076E+13	4,07763E+14	4,46323E+14	5,75221E+13	7,2942E+14	6,66229E+14	6,52579E+13
TC1600000484.mm.2	Ropn1	ropporin, rhophilin associated protein 1	-1,68625E+14	5,57302E+14	-4,25336E+14	0.00458118070832572	0.170747242106618	-1,64137E+14	4,59092E+14	4,41286E+13	5,82919E+14	6,5471E+14	5,70785E+14	6,35018E+14
TC1000002175.mm.2	Man1a	mannosidase 1, alpha	1,58212E+14	7,04272E+14	4,25123E+14	0.00459251701880116	0.170747242106618	-1,64343E+14	7,91509E+14	8,1939E+14	6,87269E+14	6,07206E+14	6,58743E+14	6,61514E+14
TC0600003000.mm.2	Bms1	BMS1, ribosome biogenesis factor	1,63918E+13	6,19988E+14	4,24757E+14	0.00461207222378588	0.170747242106618	-1,64696E+14	7,39787E+14	7,1492E+13	5,60778E+14	5,66093E+14	6,13587E+14	5,24765E+14
TC0200004530.mm.2	Secisbp2l	SECIS binding protein 2-like	1,69252E+14	7,61776E+14	4,24444E+14	0.00462888741120452	0.170747242106618	-1,64999E+14	8,74677E+14	8,94378E+14	7,23116E+14	7,07435E+14	7,33481E+14	6,37569E+14
TC1600001939.mm.2	Btg3/Gm7334	B cell translocation gene 3/B-cell translocation gene 3 pseudogene	1,33205E+14	6,45373E+13	4,24413E+14	0.00463054606186715	0.170747242106618	-1,65029E+14	7,27852E+14	7,03409E+14	5,75787E+14	5,89065E+14	6,20193E+14	6,55935E+14
TC0900002522.mm.2	Snx1	sorting nexin 1	1,38325E+14	6,20287E+14	4,24326E+14	0.00463521428374988	0.170747242106618	-1,65113E+14	7,14939E+14	7,05374E+14	5,98551E+14	5,45113E+14	5,83932E+14	5,73812E+14
TC0200003424.mm.2	Nr6a1	nuclear receptor subfamily 6, group A, member 1	-1,26795E+14	7,58057E+14	-4,24227E+14	0.00464055838234411	0.170747242106618	-1,65209E+14	6,85751E+14	6,60121E+14	8,02831E+14	7,96632E+14	7,94867E+14	8,08139E+14
TC0500003249.mm.2	Pitpnm2	phosphatidylinositol transfer protein, membrane-associated 2	1,25955E+14	7,17692E+14	4,24044E+14	0.00465045316227068	0.170747242106618	-1,65386E+14	7,91521E+14	8,06081E+14	6,6257E+12	6,83122E+14	6,86307E+14	6,76551E+14
TC1200000416.mm.2	Scfd1	Sec1 family domain containing 1	2,17391E+14	5,91416E+14	4,23702E+14	0.00466895739681725	0.170747242106618	-1,65717E+14	7,44851E+14	7,39615E+14	5,60741E+13	4,88942E+14	5,74361E+14	4,39985E+14
TC1000003218.mm.2	Atp5d	ATP synthase, H+ transporting, mitochondrial F1 complex, delta subunit	1,56154E+14	9,19079E+14	4,2345E+14	0.00468266234747921	0.170747242106618	-1,65961E+14	1,0248E+14	1,00958E+14	8,94796E+14	8,27279E+14	9,01823E+14	8,56194E+14
TC1700000050.mm.2	Tulp4	tubby like protein 4	1,39096E+14	6,9935E+13	4,23416E+14	0.00468451336480025	0.170747242106618	-1,65994E+14	8,2674E+14	8,15638E+14	6,87845E+14	6,76342E+14	6,22181E+14	5,67352E+14
TC0500000675.mm.2	Nipal1	NIPA-like domain containing 1	-1,44042E+14	6,03752E+14	-4,23248E+14	0.0046936477814023	0.170747242106618	-1,66156E+14	5,12378E+13	5,13173E+14	6,249E+14	6,88735E+14	6,48783E+14	6,34542E+14
TC0400000588.mm.2	Tal2	T cell acute lymphocytic leukemia 2	-1,683E+14	6,88779E+14	-4,23133E+14	0.00469993553486608	0.170747242106618	-1,66268E+14	5,73974E+14	5,84059E+14	7,1074E+14	7,83891E+13	7,07309E+14	7,72703E+14
TC0X00003399.mm.2	Sh3bgrl	SH3-binding domain glutamic acid-rich protein like	1,81084E+14	6,9419E+14	4,2305E+14	0.00470447958659942	0.170747242106618	-1,66348E+14	8,0941E+13	8,02388E+14	6,63215E+14	5,86417E+14	6,10024E+14	6,93687E+14
TC0X00002116.mm.2	Tenm1	teneurin transmembrane protein 1	1,90645E+14	6,42817E+12	4,22975E+14	0.0047086195710138	0.170747242106618	-1,66421E+13	8,08695E+14	7,69481E+14	6,38431E+14	5,58456E+14	5,84443E+14	4,974E+12
TC1000002440.mm.2	Aire	autoimmune regulator (autoimmune polyendocrinopathy candidiasis ectodermal dystrophy)	-1,57637E+14	7,07336E+14	-4,22883E+14	0.00471364423442428	0.170747242106618	-1,6651E+14	5,79428E+14	6,12965E+14	7,28668E+14	7,78999E+14	7,41997E+14	8,01962E+14
TC0100001222.mm.2	Chil1	chitinase-like 1	1,24844E+14	6,62615E+14	4,22578E+14	0.00473039008828403	0.170747242106618	-1,66806E+14	7,47189E+14	7,69756E+14	6,34042E+14	6,33215E+14	5,90803E+14	6,00687E+13
TC1600000212.mm.2	Hic2	hypermethylated in cancer 2	-1,42291E+14	7,29159E+14	-4,22E+14	0.00476234391337156	0.170747242106618	-1,67367E+14	6,48726E+13	6,29018E+13	7,51464E+14	8,10861E+13	7,62751E+14	7,72133E+14
TC1000001624.mm.2	Sarnp	SAP domain containing ribonucleoprotein	3,16931E+14	5,58726E+14	4,2175E+14	0.00477621596777034	0.170747242106618	-1,6761E+14	7,47887E+14	7,44706E+14	5,34913E+14	3,23818E+14	5,66545E+14	4,34488E+14
TC0400004177.mm.2	Akap2	A kinase (PRKA) anchor protein 2	1,24443E+14	6,09407E+14	4,21712E+13	0.004778374443713	0.170747242106618	-1,67647E+14	7,14942E+14	6,9274E+14	5,82079E+14	5,76717E+14	5,48851E+14	5,41111E+14
TC1600000329.mm.2	Eif4a2/Snord2/Snora81	eukaryotic translation initiation factor 4A2/small nucleolar RNA, C/D box 2/small nucleolar RNA, H/ACA box 81	1,33248E+14	7,90118E+14	4,20808E+14	0.00482896742048088	0.170747242106618	-1,68526E+14	8,9036E+14	8,51399E+13	7,48959E+14	7,26304E+14	7,69694E+14	7,53993E+14
TC0500003533.mm.2	Tnrc18	trinucleotide repeat containing 18	1,25704E+14	6,68865E+14	4,20583E+14	0.0048417039468796	0.170747242106618	-1,68746E+14	7,5903E+14	7,37941E+14	6,16146E+14	6,29418E+14	6,27418E+14	6,43234E+14
TC1000000817.mm.2	Fstl3	follistatin-like 3	-1,32288E+14	7,23682E+14	-4,20577E+14	0.00484203714192858	0.170747242106618	-1,68751E+14	6,22785E+14	6,48885E+13	7,86875E+14	7,4937E+14	7,69251E+14	7,64928E+13
TC0700001451.mm.2	Stim1	stromal interaction molecule 1	1,34736E+14	5,64362E+14	4,19849E+13	0.00488334212796091	0.170747242106618	-1,69461E+14	6,25566E+14	6,49758E+14	5,00985E+13	5,04866E+14	5,29986E+14	5,75012E+14
TC0100001252.mm.2	Csrp1	cysteine and glycine-rich protein 1	1,57273E+14	8,99559E+14	4,19636E+14	0.00489549600880522	0.170747242106618	-1,69668E+14	1,01614E+14	9,98909E+14	8,80111E+14	8,20395E+13	8,70833E+14	8,10961E+14
TC1100001006.mm.2	Zzef1	zinc finger, ZZ-type with EF hand domain 1	1,40081E+14	6,50095E+14	4,19523E+14	0.00490196933326007	0.170747242106618	-1,69779E+14	7,74794E+14	7,45071E+14	6,12396E+13	6,27308E+14	5,96073E+14	5,4493E+14
TC0400000459.mm.2	Ccin	calicin	-1,63283E+12	4,83753E+14	-4,19224E+14	0.00491914055667108	0.170747242106618	-1,70071E+14	3,92082E+14	4,29829E+14	5,3111E+14	6,17368E+14	4,63428E+14	4,68701E+14
TC0200004376.mm.2	Dph6	diphthamine biosynthesis 6	1,42556E+14	6,39539E+14	4,19207E+14	0.00492015785603129	0.170747242106618	-1,70088E+14	7,55302E+14	7,43296E+14	6,38451E+13	5,75035E+14	5,67306E+14	5,57843E+14
TC0500003399.mm.2	Ccl24	chemokine (C-C motif) ligand 24	-1,84768E+14	6,54583E+14	-4,18953E+14	0.0049347434485759	0.170747242106618	-1,70335E+14	5,66005E+14	4,96313E+14	6,72555E+14	7,593E+14	6,94214E+14	7,39113E+14
TC0600003016.mm.2	Lrtm2	leucine-rich repeats and transmembrane domains 2	1,58266E+14	6,59745E+14	4,18953E+14	0.00493476183216361	0.170747242106618	-1,70336E+14	7,73389E+14	7,47652E+14	6,35709E+14	5,68799E+14	6,41939E+14	5,90981E+13
TC0900000285.mm.2	Arhgap32	Rho GTPase activating protein 32	2,18875E+14	8,84074E+14	4,18906E+14	0.00493747356731758	0.170747242106618	-1,70381E+14	1,01403E+14	1,03929E+14	8,69225E+14	7,46346E+14	8,64562E+14	7,70989E+14
TC0700001948.mm.2	Bccip	BRCA2 and CDKN1A interacting protein	1,48659E+14	6,78105E+14	4,18363E+14	0.00496897506419581	0.170747242106618	-1,70913E+14	7,57879E+14	7,60924E+14	6,48139E+14	5,73346E+14	6,66289E+13	6,62052E+14
TC1700000275.mm.2	Zfp948	zinc finger protein 948	1,79962E+14	5,95364E+14	4,18075E+14	0.00498573690097054	0.170747242106618	-1,71194E+14	7,19349E+14	7,04008E+14	5,83183E+14	4,8025E+14	5,67102E+14	5,18292E+14
TC0800002382.mm.2	Ncan	neurocan	2,35405E+14	7,14764E+14	4,17983E+14	0.00499110889711341	0.170747242106618	-1,71284E+14	8,36943E+14	8,67004E+13	7,02101E+14	5,31036E+14	6,77314E+14	6,74188E+14
TC0X00001892.mm.2	Maob	monoamine oxidase B	1,4968E+14	6,02096E+14	4,17863E+14	0.00499814227211942	0.170747242106618	-1,71402E+14	7,51076E+14	6,8849E+14	5,81255E+14	5,58953E+14	5,35573E+14	4,97232E+14
TC0X00000888.mm.2	Pcyt1b	phosphate cytidylyltransferase 1, choline, beta isoform	1,78014E+14	5,31236E+14	4,17807E+12	0.00500140914775051	0.170747242106618	-1,71457E+14	6,31064E+14	6,76601E+14	5,09451E+14	4,42187E+14	5,0321E+14	4,24904E+14
TC1500001023.mm.2	Gpd1	glycerol-3-phosphate dehydrogenase 1 (soluble)	1,63902E+14	7,58541E+14	4,17793E+14	0.00500224264326195	0.170747242106618	-1,71471E+14	8,32322E+14	8,89116E+14	7,33302E+14	6,60331E+14	7,05579E+14	7,30593E+14
TC0300000507.mm.2	Gmps	guanine monophosphate synthetase	2,0276E+14	7,35762E+14	4,17514E+14	0.00501860431952275	0.170747242106618	-1,71744E+13	8,91427E+14	8,88346E+13	7,41161E+14	6,33092E+13	6,75068E+14	5,85478E+14
TC0800003233.mm.2	Trmt1	tRNA methyltransferase 1	1,8438E+14	5,97377E+14	4,17406E+14	0.00502497907144898	0.170747242106618	-1,7185E+14	7,33079E+14	7,41429E+13	6,04343E+14	5,01404E+14	5,32873E+14	4,71134E+14
TC1100004274.mm.2	Rpl19	ribosomal protein L19	1,34673E+14	9,64042E+14	4,16967E+14	0.00505089285572875	0.170747242106618	-1,7228E+14	1,06259E+14	1,03889E+14	8,92427E+14	9,39703E+14	9,26024E+14	9,2462E+14
TC1300002313.mm.2	Exoc3	exocyst complex component 3	1,43543E+12	6,74174E+14	4,16914E+14	0.00505400983144587	0.170747242106618	-1,72332E+14	7,97045E+14	7,76351E+14	6,67517E+14	6,18792E+14	6,14035E+14	5,71305E+11
TC0100000436.mm.2	Ndufb3	NADH:ubiquinone oxidoreductase subunit B3	1,20602E+14	6,97019E+14	4,16575E+14	0.00507414541714434	0.170747242106618	-1,72665E+14	7,86888E+13	7,8305E+14	6,62197E+14	6,66537E+14	6,47386E+14	6,36059E+14
TC1100002511.mm.2	Rars	arginyl-tRNA synthetase	1,69477E+14	7,02447E+13	4,16564E+14	0.00507482487748907	0.170747242106618	-1,72676E+14	8,06092E+13	8,1807E+14	6,92143E+14	5,93065E+14	6,65984E+14	6,39325E+14
TC0600001042.mm.2	Tpra1	transmembrane protein, adipocyte asscociated 1	1,54173E+14	6,46445E+14	4,16501E+14	0.00507853165308323	0.170747242106618	-1,72737E+14	7,38329E+14	7,42281E+14	6,27933E+14	5,44332E+14	6,12835E+14	6,12963E+14
TC0700004622.mm.2	Tm2d3	TM2 domain containing 3	1,40795E+14	7,63931E+13	4,16447E+14	0.0050818008062515	0.170747242106618	-1,72791E+14	8,68274E+14	8,25181E+14	7,26451E+14	6,85415E+14	7,50771E+14	7,27491E+14
TC0300002407.mm.2	Lce1g	late cornified envelope 1G	-1,38137E+14	6,26445E+14	-4,16335E+14	0.00508847198621361	0.170747242106618	-1,72901E+14	5,37882E+14	5,3198E+14	6,44198E+14	7,01938E+14	6,64776E+14	6,77896E+14
TC1100001153.mm.2	2610507B11Rik	RIKEN cDNA 2610507B11 gene	2,27965E+14	7,13566E+14	4,16165E+14	0.00509864509266805	0.170747242106618	-1,73068E+14	8,47189E+14	8,59739E+14	7,05046E+14	5,45952E+14	6,86763E+14	6,36708E+14
TC0X00001797.mm.2	Rbm3	RNA binding motif (RNP1, RRM) protein 3	1,57184E+14	9,65967E+13	4,1615E+14	0.00509951101359868	0.170747242106618	-1,73082E+14	1,04573E+13	1,07588E+14	9,37879E+14	8,69362E+14	9,56538E+14	9,10415E+14
TC1100000745.mm.2	Rai1	retinoic acid induced 1	-1,29477E+14	1,03489E+14	-4,15809E+14	0.00511996562699181	0.170747242106618	-1,73417E+14	9,51356E+14	9,41869E+14	1,05704E+13	1,09513E+14	1,0908E+14	1,07314E+14
TC0900003021.mm.2	Manf/Rbm15b	mesencephalic astrocyte-derived neurotrophic factor/RNA binding motif protein 15B	1,21892E+14	7,86711E+14	4,15758E+14	0.00512302333050704	0.170747242106618	-1,73467E+14	8,58897E+14	8,70742E+14	7,3989E+13	7,45964E+14	7,60447E+14	7,44326E+14
TC1900000024.mm.2	Doc2g/Nudt8	double C2, gamma/nudix (nucleoside diphosphate linked moiety X)-type motif 8	-1,88611E+14	7,62037E+14	-4,15694E+14	0.00512690561638098	0.170747242106618	-1,73531E+14	6,27031E+14	6,4159E+14	7,65245E+14	8,80598E+14	8,04687E+14	8,53073E+14
TC0500002077.mm.2	Cdk5	cyclin-dependent kinase 5	1,78668E+14	7,82074E+14	4,15642E+14	0.0051300339484597	0.170747242106618	-1,73582E+14	8,94112E+14	8,71632E+14	7,57865E+13	6,50543E+14	7,75909E+14	7,42383E+14
TC0200004729.mm.2	Esf1	ESF1 nucleolar pre-rRNA processing protein homolog	2,13167E+14	6,2302E+14	4,15416E+14	0.00514364343075073	0.170747242106618	-1,73804E+14	7,71512E+14	7,67697E+14	6,19183E+14	4,93691E+14	5,86277E+14	4,99761E+14
TC0100003096.mm.2	Atp2b4/Mir6903	ATPase, Ca++ transporting, plasma membrane 4/microRNA 6903	2,46259E+14	7,5036E+14	4,14796E+14	0.00518129586624361	0.170747242106618	-1,74415E+14	9,25164E+14	9,24922E+14	7,26511E+13	6,31058E+14	7,27256E+14	5,67248E+14
TC0500001746.mm.2	Lmtk2	lemur tyrosine kinase 2	1,22047E+14	7,668E+14	4,14694E+14	0.00518747857475775	0.170747242106618	-1,74515E+13	8,2709E+14	8,34343E+14	7,11947E+14	7,05392E+14	7,50687E+14	7,7134E+14
TC0500002559.mm.2	Gabra2	gamma-aminobutyric acid (GABA) A receptor, subunit alpha 2	2,0858E+14	8,81479E+14	4,14281E+14	0.00521276639325481	0.170747242106618	-1,74922E+14	1,03993E+14	1,02474E+14	8,8788E+14	7,59623E+13	8,24767E+14	7,51943E+14
TC0900001471.mm.2	Prss43	protease, serine 43	-1,20632E+14	6,00195E+13	-4,1413E+14	0.00522201345384553	0.170747242106618	-1,75071E+14	5,2258E+14	5,21343E+14	6,43864E+14	6,41323E+14	6,2756E+14	6,44501E+14
TC0700002980.mm.2	Prmt1	protein arginine N-methyltransferase 1	1,45305E+13	8,10218E+14	4,141E+14	0.00522384731867743	0.170747242106618	-1,751E+14	9,24897E+14	8,95192E+14	7,97536E+13	7,31942E+14	7,52997E+14	7,58741E+13
TC1100001777.mm.2	Psmc5	protease (prosome, macropain) 26S subunit, ATPase 5	2,4735E+14	7,26058E+14	4,13949E+14	0.00523316227005119	0.170747242106618	-1,7525E+14	8,48933E+14	8,72343E+14	6,88521E+14	5,38055E+13	7,59283E+14	6,49211E+14
TC1400000895.mm.2	Ctsb	cathepsin B	2,39479E+13	9,77444E+14	4,13616E+12	0.00525371536068892	0.170747242106618	-1,75578E+14	1,0997E+14	1,13476E+14	9,58698E+14	7,96796E+14	9,72912E+14	9,01809E+14
TC1600000998.mm.2	Olig2	oligodendrocyte transcription factor 2	1,25918E+14	8,15581E+14	4,1354E+14	0.00525843392456668	0.170747242106618	-1,75653E+14	8,81092E+14	9,12636E+14	7,71134E+13	7,70759E+14	7,8775E+14	7,70113E+13
TC1500001480.mm.2	Csmd3	CUB and Sushi multiple domains 3	1,43755E+14	5,50876E+14	4,13244E+14	0.00527678897944408	0.170747242106618	-1,75946E+14	6,59662E+14	6,26954E+14	4,75526E+14	5,23579E+14	5,29564E+14	4,89975E+14
TC1500000679.mm.2	Gtpbp1	GTP binding protein 1	1,47059E+14	6,40874E+14	4,12998E+14	0.00529207976712759	0.170747242106618	-1,76188E+14	7,67908E+14	7,2794E+14	6,32764E+14	5,68966E+14	5,76141E+14	5,71525E+14
TC0200004580.mm.2	Anapc1	anaphase promoting complex subunit 1	2,44201E+14	6,19813E+14	4,12902E+14	0.00529811873146015	0.170747242106618	-1,76284E+14	7,83279E+13	8,17E+14	6,03729E+14	5,08147E+14	5,80975E+14	4,25745E+14
TC0X00001286.mm.2	Armcx1	armadillo repeat containing, X-linked 1	1,57916E+14	6,67974E+14	4,12765E+14	0.00530664437722069	0.170747242106618	-1,76419E+14	7,68254E+14	7,42701E+14	6,37302E+14	5,5782E+14	6,68466E+14	6,333E+14
TC0X00001784.mm.2	Pqbp1	polyglutamine binding protein 1	1,93674E+14	7,7591E+14	4,12763E+14	0.00530678275818924	0.170747242106618	-1,76421E+13	9,17056E+14	8,98569E+14	7,79913E+14	6,48365E+14	7,09354E+14	7,02202E+14
TC1100002451.mm.2	Chac2	ChaC, cation transport regulator 2	1,24523E+14	6,01082E+14	4,12535E+14	0.00532104130889349	0.170747242106618	-1,76646E+14	6,59426E+14	6,83527E+14	5,54364E+14	5,39542E+14	5,93477E+14	5,76159E+14
TC0X00003396.mm.2	Nlgn3	neuroligin 3	2,29469E+14	7,82316E+14	4,12395E+14	0.00532983587088911	0.170747242106618	-1,76785E+12	9,52901E+14	9,22312E+14	7,79896E+14	6,36378E+14	7,44992E+14	6,57416E+14
TC0800001311.mm.2	Zfp612	zinc finger protein 612	1,48901E+14	7,36946E+14	4,11672E+13	0.00537553980555753	0.170747242106618	-1,77502E+14	8,36915E+14	8,21769E+14	7,1441E+14	6,46471E+14	7,19515E+14	6,82595E+13
TC1900001353.mm.2	Ranbp6	RAN binding protein 6	1,92083E+14	5,94402E+14	4,11642E+14	0.00537741117883457	0.170747242106618	-1,77531E+14	7,22367E+14	6,91572E+14	5,7242E+14	4,57353E+14	5,89626E+14	5,33076E+14
TC1600000415.mm.2	Dlg1	discs large MAGUK scaffold protein 1	1,7015E+14	7,91272E+13	4,11641E+14	0.00537751812654969	0.170747242106618	-1,77533E+14	8,95294E+14	8,8636E+14	7,67004E+14	6,7435E+14	7,88253E+14	7,3637E+14
TC0100000519.mm.2	Eef1b2	eukaryotic translation elongation factor 1 beta 2	1,20814E+14	9,51389E+14	4,11446E+14	0.00538991051911914	0.170747242106618	-1,77726E+14	1,04513E+14	1,03194E+14	9,15025E+14	9,20414E+14	8,89726E+14	9,06101E+14
TC0X00003401.mm.2	Gprasp2	G protein-coupled receptor associated sorting protein 2	2,30798E+14	8,44275E+13	4,11366E+14	0.00539500353775342	0.170747242106618	-1,77805E+14	1,01431E+14	9,7568E+14	8,42209E+14	6,86186E+14	8,05381E+14	7,41885E+14
TC0400003237.mm.2	Zyg11b	zyg-ll family member B, cell cycle regulator	1,51348E+14	8,60533E+14	4,10772E+14	0.00543300833411051	0.170747242106618	-1,78395E+14	9,75123E+14	9,62469E+14	8,31823E+14	8,03072E+14	8,33617E+14	7,57096E+14
TC1000002256.mm.2	Hk1	hexokinase 1	2,42709E+14	8,17284E+14	4,10465E+14	0.00545270891590712	0.170747242106618	-1,78699E+14	9,83337E+14	9,70364E+13	8,25699E+14	6,42583E+14	7,53615E+14	7,28107E+14
TC1200002120.mm.2	Vipas39	VPS33B interacting protein, apical-basolateral polarity regulator, spe-39 homolog	1,60834E+14	7,2564E+14	4,10423E+14	0.00545542166465635	0.170747242106618	-1,78741E+14	8,10695E+14	8,08576E+14	6,96538E+14	6,01066E+14	7,20309E+14	7,16655E+14
TC0700001615.mm.2	Akip1	A kinase (PRKA) interacting protein 1	-1,5835E+14	6,77911E+14	-4,10261E+14	0.00546591030877561	0.170747242106618	-1,78902E+14	5,87093E+14	5,47274E+14	7,51388E+14	6,99679E+14	7,73739E+14	7,08294E+14
TC0800000011.mm.2	Pnpla6	patatin-like phospholipase domain containing 6	1,43142E+14	6,38147E+14	4,10218E+14	0.00546870059052241	0.170747242106618	-1,78945E+14	7,5143E+14	7,14332E+14	6,18335E+14	5,61143E+14	6,01758E+14	5,81886E+14
TC1100001782.mm.2	Prr29	proline rich 29	-1,43554E+14	6,35441E+14	-4,10124E+14	0.00547478946072895	0.170747242106618	-1,79039E+14	5,57293E+12	5,3748E+14	6,77087E+14	7,04794E+14	6,36454E+14	6,99536E+14

TC1200002236.mm.2	Gpr68	G protein-coupled receptor 68	-1,63111E+14	6,97429E+14	-4,10075E+14	0.00547796316168222	0.170747242106618	-1,79087E+14	5,75611E+14	5,81406E+14	6,9543E+14	7,87811E+14	7,55108E+14	7,89207E+14
TC0900000892.mm.2	Rora	RAR-related orphan receptor alpha	1,46817E+14	6,26473E+14	4,09879E+14	0.00549062586251945	0.170747242106618	-1,79282E+14	7,30813E+14	7,27593E+13	6,03669E+14	5,61102E+14	5,99694E+14	5,35964E+14
TC1200002158.mm.2	Sel1l	sel-1 suppressor of lin-12-like (C. elegans)	1,4408E+14	7,26232E+14	4,09725E+14	0.0055006575685604	0.170747242106618	-1,79435E+14	8,06965E+14	8,0715E+14	6,94661E+14	6,31293E+14	7,26484E+13	6,90837E+14
TC0X00002166.mm.2	Zfp280c	zinc finger protein 280C	1,65721E+14	6,07622E+14	4,09108E+12	0.00554102512094767	0.170747242106618	-1,8005E+14	7,045E+14	7,25052E+14	5,7045E+14	5,2766E+14	6,04402E+14	5,13667E+14
TC0700004164.mm.2	Rps15a	ribosomal protein S15A	1,7205E+14	7,50776E+14	4,08792E+14	0.00556176664026745	0.170747242106618	-1,80364E+14	8,72112E+14	8,72029E+14	6,69337E+14	7,30705E+14	6,34415E+14	7,26056E+13
TC1600002015.mm.2	Krtap14	keratin associated protein 14	-1,70709E+14	5,25798E+14	-4,08704E+14	0.0055676057489794	0.170747242106618	-1,80452E+14	3,94195E+14	4,15085E+14	5,44205E+14	6,06492E+14	5,54094E+14	6,40719E+14
TC0700003609.mm.2	Ap3b2	adaptor-related protein complex 3, beta 2 subunit	1,7064E+14	6,77959E+13	4,08609E+14	0.00557388128374333	0.170747242106618	-1,80547E+14	8,12232E+14	8,02377E+14	6,49513E+14	6,23816E+14	6,42479E+14	5,37337E+14
TC0700000058.mm.2	Epn1	epsin 1	1,52528E+14	8,33632E+14	4,08534E+14	0.00557883244857999	0.170747242106618	-1,80622E+14	9,47578E+14	9,46175E+14	8,36819E+14	7,51877E+14	7,64493E+14	7,54847E+14
TC0100002589.mm.2	Prkag3	protein kinase, AMP-activated, gamma 3 non-catalytic subunit	-1,41385E+14	7,48722E+14	-4,08182E+14	0.00560215181835621	0.170747242106618	-1,80973E+14	6,63414E+14	6,62998E+14	7,69914E+14	8,39268E+13	7,77529E+14	7,7921E+14
TC0700002736.mm.2	Zfp27	zinc finger protein 27	1,19213E+14	5,74314E+14	4,07879E+14	0.00562228805876753	0.170747242106618	-1,81275E+14	6,59319E+14	6,68264E+14	5,37972E+13	5,51184E+14	5,09224E+14	5,19921E+14
TC1500002284.mm.2	Spryd3	SPRY domain containing 3	1,49618E+14	7,58158E+14	4,07874E+14	0.00562266879540869	0.170747242106618	-1,81281E+14	8,65933E+13	8,51067E+14	7,24581E+14	6,93183E+14	7,4411E+14	6,70072E+14
TC0500001860.mm.2	Fry	FRY microtubule binding protein	2,11549E+13	6,50908E+14	4,07747E+14	0.00563113623819944	0.170747242106618	-1,81407E+14	7,85634E+14	7,78339E+14	6,43096E+14	4,97779E+14	6,2691E+13	5,73693E+13
TC0100000280.mm.2	Pdcl3	phosducin-like 3	1,3573E+14	6,17643E+14	4,07703E+14	0.00563408352633375	0.170747242106618	-1,81451E+14	7,3596E+14	7,11253E+14	5,76363E+14	5,99389E+14	5,16467E+14	5,66427E+14
TC0200003674.mm.2	Scn1a	sodium channel, voltage-gated, type I, alpha	1.30953E+14	6,89278E+14	4,07699E+14	0.00563431994773392	0.170747242106618	-1,81455E+14	7,79697E+14	7,78047E+14	6,50132E+14	6,45706E+14	6,66678E+14	6,15406E+14
TC0400000280.mm.2	Gabrr1	gamma-aminobutyric acid (GABA) C receptor, subunit rho 1	-1,5653E+14	6,74479E+14	-4,07646E+14	0.00563785247860977	0.170747242106618	-1,81508E+14	5,78935E+14	5,89133E+14	7,04697E+14	7,76431E+14	6,71019E+14	7,26657E+14
TC0100002503.mm.2	Erbb4	erb-b2 receptor tyrosine kinase 4	1,3765E+14	5,82271E+14	4,07552E+14	0.00564412493056357	0.170747242106618	-1,81601E+14	7,09678E+14	6,95308E+14	5,67625E+14	5,62061E+14	5,10363E+14	4,48588E+14
TC1900001485.mm.2	Sorbs1	sorbin and SH3 domain containing 1	1,6184E+14	8,07707E+14	4,07507E+14	0.00564714781365233	0.170747242106618	-1,81646E+14	9,06664E+14	9,07366E+14	7,8898E+14	7,01371E+14	7,93246E+14	7,48615E+14
TC0600002668.mm.2	Cnbp	cellular nucleic acid binding protein	1,55791E+14	8,58339E+14	4,07425E+14	0.0056526640036503	0.170747242106618	-1,81729E+14	9,6944E+14	9,62145E+14	8,48468E+14	7,71534E+14	8,22756E+14	7,75688E+12
TC1400000517.mm.2	Ktn1	kinectin 1	2,11662E+14	6,89861E+14	4,07099E+14	0.00567457420814748	0.170747242106618	-1,82054E+14	8,14521E+14	8,16083E+14	6,80584E+13	5,26695E+14	6,61427E+14	6,3986E+14
TC1700002334.mm.2	Vmac	vimentin-type intermediate filament associated coiled-coil protein	-1,27152E+14	6,60868E+14	-4,0705E+14	0.00567792239746783	0.170747242106618	-1,82104E+14	6,00446E+13	5,65715E+14	7,1303E+14	7,07436E+14	6,76114E+14	7,02468E+14
TC1700000271.mm.2	Zfp51	zinc finger protein 51	1,23608E+14	5,60548E+14	4,06984E+13	0.00568236379938071	0.170747242106618	-1,8217E+14	6,47181E+14	6,3705E+13	5,2955E+14	5,07465E+14	5,08219E+14	5,33825E+14
TC1500001464.mm.2	Tmem74	transmembrane protein 74	1,54541E+14	6,01843E+14	4,06867E+14	0.00569023116536154	0.170747242106618	-1,82286E+14	6,76063E+14	7,1007E+14	5,73771E+14	5,0328E+14	5,95221E+13	5,52654E+14
TC0400000269.mm.2	Casp8ap2	caspase 8 associated protein 2	1,28488E+14	5,58998E+14	4,0681E+14	0.00569406721665601	0.170747242106618	-1,82343E+14	6,25348E+14	6,40262E+14	4,99095E+14	5,09539E+14	5,18189E+14	5,61556E+14
TC0300002295.mm.2	Bcan	brevican	1,2983E+14	7,3004E+14	4,06771E+14	0.00569675778744326	0.170747242106618	-1,82383E+14	8,41895E+14	8,11832E+14	7,04204E+14	6,89863E+13	6,47685E+14	6,84763E+14
TC1600001184.mm.2	Grin2a	glutamate receptor, ionotropic, NMDA2A (epsilon 1)	1,86728E+14	7,562E+14	4,06469E+14	0.00571718433984915	0.170747242106618	-1,82685E+14	8,38953E+14	8,77145E+14	7,16399E+14	6,26243E+14	7,80877E+14	6,97584E+13
TC0X00002333.mm.2	Slitrk4	SLIT and NTRK-like family, member 4	1,60107E+14	7,32843E+14	4,06284E+14	0.00572982573838785	0.170747242106618	-1,82871E+14	8,30702E+14	8,53124E+14	7,27468E+12	6,36144E+14	6,86128E+14	6,63491E+14
TC0400002530.mm.2	Tln1	talin 1	1,28219E+14	6,72697E+14	4,06267E+14	0.00573096454859383	0.170747242106618	-1,82887E+14	7,4645E+14	7,90683E+14	6,4576E+14	6,34934E+14	6,03724E+14	6,14631E+14
TC0600000651.mm.2	Wipf3	WAS/WASL interacting protein family, member 3	-1,278E+14	8,5025E+14	-4,06127E+14	0.00574047687378293	0.170747242106618	-1,83027E+14	7,7094E+13	7,79321E+14	9,17505E+14	8,88356E+14	8,55383E+14	8,89993E+13
TC0700000546.mm.2	Zfp260	zinc finger protein 260	1,42258E+14	6,05814E+14	4,06111E+14	0.005741612792215	0.170747242106618	-1,83044E+14	6,72853E+14	6,80378E+14	5,69686E+14	4,99028E+14	6,11821E+14	6,01116E+14
TC0600001366.mm.2	Pex26	peroxisomal biogenesis factor 26	1,38115E+13	5,43296E+14	4,06077E+14	0.00574389191350953	0.170747242106618	-1,83077E+14	6,13503E+14	6,1116E+14	4,52321E+13	4,96111E+14	5,19167E+14	5,67513E+14
TC0900000313.mm.2	Kirrel3	kirre like nephrin family adhesion molecule 3	1,63494E+14	6,71852E+14	4,06061E+14	0.00574498515859898	0.170747242106618	-1,83093E+14	7,92125E+14	7,76526E+12	6,31032E+14	6,10631E+14	6,59318E+14	5,61478E+14
TC0500000395.mm.2	Hmx1	H6 homeobox 1	-1,65961E+14	7,21758E+14	-4,05847E+14	0.00575967383030971	0.170747242106618	-1,83309E+14	6,01382E+14	6,11248E+14	7,27365E+14	8,17188E+14	7,60589E+14	8,12775E+14
TC0200001909.mm.2	Snrnp200	small nuclear ribonucleoprotein 200 (U5)	2,34454E+14	6,52073E+14	4,05824E+14	0.00576124691839168	0.170747242106618	-1,83332E+13	8,12127E+14	8,17901E+14	6,65411E+14	4,9571E+14	5,90127E+14	5,31162E+14
TC0700003798.mm.2	Rps3/Snord15b	ribosomal protein S3/small nucleolar RNA, C/D box 14B	1,76067E+14	9,48085E+14	4,05501E+14	0.00578342165119316	0.170747242106618	-1,83655E+14	1,06129E+14	1,0791E+14	9,2343E+14	8,64822E+14	9,29627E+14	8,30244E+14
TC0600003302.mm.2	Lrp6	low density lipoprotein receptor-related protein 6	1,25466E+14	6,76706E+14	4,05453E+14	0.00578669418940668	0.170747242106618	-1,83703E+14	7,71028E+14	7,73602E+14	6,57626E+14	6,36073E+14	6,28466E+14	5,93443E+14
TC0200003085.mm.2	Enkur	enkurin, TRPC channel interacting protein	1,41565E+14	5,81368E+14	4,05212E+14	0.00580330309071489	0.170747242106618	-1,83945E+14	7,10129E+14	6,80373E+14	5,84551E+14	5,22821E+14	5,04961E+14	4,85374E+14
TC0700002703.mm.2	Acp7	acid phosphatase 7, tartrate resistant	-1,40096E+14	6,50713E+14	-4,05037E+14	0.00581545380122354	0.170747242106618	-1,84121E+13	5,68283E+14	5,52535E+14	6,66905E+14	7,34106E+14	6,90097E+14	6,92354E+14
TC0400003747.mm.2	Maco1	macoilin 1	1,67736E+14	7,00506E+14	4,0498E+14	0.00581936005493171	0.170747242106618	-1,84178E+13	8,00206E+14	7,97237E+14	6,82108E+14	5,79863E+14	6,56138E+14	6,87482E+13
TC1200001953.mm.2	Sptb	spectrin beta, erythrocytic	1,26731E+14	7,07918E+14	4,04963E+14	0.00582055883331744	0.170747242106618	-1,84195E+14	8,18637E+13	8,08907E+14	6,86269E+14	6,87814E+14	6,44623E+14	6,01255E+14
TC0500003095.mm.2	Dynll1	dynein light chain LC8-type 1	1,26477E+14	9,1156E+14	4,04922E+14	0.00582341785043316	0.170747242106618	-1,84237E+14	9,80156E+14	9,8937E+14	8,74469E+14	8,42101E+14	9,0575E+14	8,77511E+14
TC1800000232.mm.2	Pik3c3	phosphatidylinositol 3-kinase catalytic subunit type 3	1,91146E+14	6,18821E+14	4,04703E+14	0.00583863664719479	0.170747242106618	-1,84457E+14	7,27712E+14	7,30284E+14	5,96881E+13	4,78821E+13	6,2174E+13	5,57487E+14
TC0900002271.mm.2	Sdhd	succinate dehydrogenase complex, subunit D, integral membrane protein	1,55079E+13	7,85813E+14	4,04453E+12	0.00585605124831587	0.170747242106618	-1,84708E+14	9,05642E+14	8,8282E+14	7,79207E+14	6,99097E+13	7,42444E+14	7,0567E+14
TC0600002027.mm.2	Chchd3	coiled-coil-helix-coiled-coil-helix domain containing 3	1,75439E+14	7,72757E+14	4,04421E+14	0.00585824984154586	0.170747242106618	-1,8474E+14	8,65015E+14	8,66123E+14	7,41269E+14	6,38992E+13	7,90516E+14	7,34627E+13
TC0700001435.mm.2	Clpb	ClpB caseinolytic peptidase B	1,92063E+14	6,2437E+14	4,04352E+14	0.00586308739746356	0.170747242106618	-1,84809E+14	7,4568E+14	7,59827E+13	6,23412E+14	4,97969E+14	5,8448E+14	5,3485E+14
TC1200000618.mm.2	Jkamp	JNK1/MAPK8-associated membrane protein	1,70129E+14	7,80534E+14	4,03887E+13	0.00589562915899032	0.170747242106618	-1,85276E+14	8,74857E+14	8,69118E+13	7,49115E+14	6,54602E+14	7,96203E+14	7,3931E+13
TC0700001432.mm.2	Pde2a	phosphodiesterase 2A, cGMP-stimulated	2,35284E+14	7,95029E+14	4,03856E+14	0.00589785917538705	0.170747242106618	-1,85308E+14	9,32419E+14	9,54334E+14	7,81512E+14	6,34673E+14	7,86043E+14	6,81193E+14
TC1700001689.mm.2	Nme4	NME/NM23 nucleoside diphosphate kinase 4	-1,26938E+14	6,65201E+14	-4,0369E+13	0.00590950004964125	0.170747242106618	-1,85475E+14	5,68338E+14	5,72258E+14	6,86459E+14	7,08014E+14	7,07951E+14	7,48186E+14
TC0X00001520.mm.2	Phf8	PHD finger protein 8	1,24945E+14	5,62907E+14	4,0367E+14	0.00591094042572922	0.170747242106618	-1,85495E+14	6,44982E+14	6,101E+14	4,93809E+14	5,11382E+14	5,51908E+12	5,65259E+14
TC0200001786.mm.2	Mapkbp1	mitogen-activated protein kinase binding protein 1	1,26879E+14	6,03383E+14	4,0356E+14	0.00591869101151945	0.170747242106618	-1,85606E+14	6,96261E+14	7,08732E+14	5,97194E+14	5,5404E+13	5,27634E+14	5,36435E+14
TC0200003281.mm.2	BC005624	cDNA sequence BC005624	1,16245E+14	8,08009E+14	4,03381E+11	0.00593135466624954	0.170747242106618	-1,85786E+14	8,9066E+14	8,96487E+14	7,77706E+13	7,76949E+14	7,55794E+14	7,50458E+13
TC1300002407.mm.2	Atg10	autophagy related 10	2,36392E+14	5,92396E+14	4,03286E+14	0.00593805412704197	0.170747242106618	-1,85882E+14	7,45742E+14	7,65934E+14	5,97729E+14	4,41162E+14	5,48412E+14	4,554E+13
TC0100003662.mm.2	Coq8a	coenzyme Q8A	1,48447E+14	6,54566E+14	4,03251E+14	0.0059405578576522	0.170747242106618	-1,85917E+14	7,22348E+14	7,26086E+14	6,1699E+14	5,34551E+14	6,64245E+14	6,63177E+14
TC1900001769.mm.2	Tmem179b	transmembrane protein 179B	-1,41572E+14	7,25119E+14	-4,03215E+14	0.00594312326917332	0.170747242106618	-1,85954E+13	6,28483E+14	6,65563E+14	8,18755E+14	7,58435E+14	7,47219E+13	7,3226E+14
TC1700000005.mm.2	Scaf8	SR-related CTD-associated factor 8	1,25808E+14	6,51229E+14	4,03069E+14	0.00595345790192995	0.170747242106618	-1,861E+14	7,32229E+14	7,51744E+14	6,26558E+14	6,058E+14	6,12106E+14	5,78938E+14
TC0500001715.mm.2	Foxk1	forkhead box K1	1,22212E+14	6,61936E+14	4,02974E+14	0.00596021886223923	0.170747242106618	-1,86196E+14	7,59522E+14	7,27172E+14	6,2134E+14	6,20929E+14	6,14908E+14	6,27748E+14
TC0500003518.mm.2	Card11	caspase recruitment domain family, member 11	-1,39894E+14	5,34331E+14	-4,02968E+14	0.00596062220981129	0.170747242106618	-1,86202E+14	4,29878E+14	4,58143E+14	5,51966E+14	6,15844E+14	5,74172E+14	5,75984E+14
TC0400000407.mm.2	Gm21953/Gm20878/Gm21586	predicted gene, 21953/predicted gene, 20878/predicted gene, 21586	1,53899E+14	9,09199E+14	4,02924E+14	0.0059637920928647	0.170747242106618	-1,86247E+14	9,8405E+14	1,00112E+14	8,61962E+13	8,15412E+14	9,34032E+14	8,58618E+14
TC0X00003432.mm.2	Mecp2	methyl CpG binding protein 2	1,35657E+14	7,10291E+14	4,02834E+14	0.00597022323368165	0.170747242106618	-1,86338E+14	8,17241E+14	7,94756E+14	6,89574E+14	6,51109E+14	6,76795E+14	6,3227E+14
TC0700004576.mm.2	Ascl2	achaete-scute family bHLH transcription factor 2	-1,58439E+14	5,12197E+13	-4,02806E+14	0.00597219847735675	0.170747242106618	-1,86366E+13	3,96215E+14	3,86931E+14	5,22106E+14	5,77919E+14	5,5602E+13	6,3399E+14
TC1000000839.mm.2	Stk11	serine/threonine kinase 11	1,17691E+14	7,96996E+14	4,02753E+14	0.0059759442342929	0.170747242106618	-1,86419E+14	8,89817E+14	8,80758E+14	7,6373E+14	7,71463E+14	7,30643E+13	7,45564E+14
TC0700000862.mm.2	Rpl18	ribosomal protein L18	1,31978E+14	9,6658E+14	4,02353E+14	0.00600457060213311	0.170747242106618	-1,86822E+14	1,05118E+14	1,04834E+14	9,4088E+14	8,94684E+14	9,48615E+14	9,1578E+13
TC0X00000650.mm.2	Mtmr1	myotubularin related protein 1	1,37445E+14	6,2956E+14	4,0225E+14	0.00601195422346263	0.170747242106618	-1,86926E+14	7,14061E+14	6,9339E+14	5,97781E+12	5,34781E+14	6,19315E+14	6,18034E+14
TC1000002264.mm.2	Ddx21	DEAD (Asp-Glu-Ala-Asp) box polypeptide 21	1,59303E+13	5,90616E+14	4,02163E+14	0.00601820107312697	0.170747242106618	-1,87014E+13	6,67497E+14	6,74828E+14	5,46451E+14	4,77268E+14	5,54466E+14	6,23187E+14
TC1000000517.mm.2	Ranbp2	RAN binding protein 2	2,13854E+14	6,1824E+14	4,01977E+14	0.00603160994541557	0.170747242106618	-1,87202E+14	7,49321E+13	7,646E+14	6,03271E+13	4,82942E+13	6,07263E+14	5,02044E+14
TC1100003855.mm.2	Eftud2	elongation factor Tu GTP binding domain containing 2	2,24662E+14	6,30856E+14	4,01844E+14	0.00604118712347142	0.170747242106618	-1,87336E+14	7,92354E+14	7,92578E+14	6,46593E+14	4,89015E+14	4,97833E+14	5,66764E+14
TC1100003797.mm.2	Ezh1	enhancer of zeste 1 polycomb repressive complex 2 subunit	1,48902E+14	6,80304E+14	4,0164E+14	0.00605595974608302	0.170747242106618	-1,87542E+14	8,2124E+14	7,59183E+14	6,69529E+14	6,1309E+14	6,06998E+13	6,11785E+14
TC1500000090.mm.2	Adamts12	a disintegrin-like and metallopeptidase (reprolysin type) with thrombospondin type 1 motif, 12	-1,28453E+13	7,24454E+13	-4,01505E+14	0.00606576682739618	0.170747242106618	-1,87679E+14	6,38511E+14	6,3604E+14	7,56985E+14	7,74472E+14	7,46722E+14	7,93991E+13
TC1300001997.mm.2	Shc3	src homology 2 domain-containing transforming protein C3	1,58863E+14	8,18589E+14	4,01325E+14	0.00607880835777925	0.170747242106618	-1,87861E+14	9,13992E+14	9,1576E+14	7,95314E+14	7,1671E+14	8,13833E+14	7,55924E+14
TC0X00003166.mm.2	Tro	trophinin	1,92885E+14	6,86035E+14	4,01309E+14	0.00608001660577653	0.170747242106618	-1,87878E+14	8,49699E+14	7,97826E+14	6,63617E+14	5,98138E+14	6,585E+14	5,48431E+13
TC1500002337.mm.2	Pfdn5	prefoldin 5	1,63329E+14	8,2897E+14	4,01048E+14	0.00609901463343686	0.170747242106618	-1,88141E+14	9,30601E+14	9,13578E+14	7,94609E+14	7,22913E+14	8,42403E+14	7,69716E+14
TC1700000611.mm.2	Rxrb	retinoid X receptor beta	1,49649E+14	6,09139E+14	4,00817E+14	0.00611588683834859	0.170747242106618	-1,88375E+14	7,00759E+14	7,27065E+14	6,0494E+14	5,23587E+13	5,49048E+14	5,49437E+14
TC0700002414.mm.2	Selenow	selenoprotein W	1,22094E+14	9,69391E+14	4,00414E+14	0.00614549499788072	0.170747242106618	-1,88783E+14	1,05851E+14	1,04E+14	9,15998E+14	9,38318E+13	9,42935E+14	9,20591E+14
TC1200001047.mm.2	Serpina3n	serine (or cysteine) peptidase inhibitor, clade A, member 3N	1,98313E+14	7,66112E+14	4,00284E+14	0.00615506584119919	0.170747242106618	-1,88915E+12	8,66106E+14	8,93375E+14	7,50771E+13	6,12082E+14	7,21575E+14	7,5276E+14
TC0500000710.mm.2	Kit	KIT proto-oncogene receptor tyrosine kinase	1,88787E+14	6,04407E+14	4,00228E+12	0.00615916831063712	0.170747242106618	-1,88971E+14	7,51934E+14	7,07081E+14	5,58578E+14	5,22863E+14	4,82347E+14	6,03637E+14
TC1200001272.mm.2	Pld4	phospholipase D family, member 4	1,39434E+14	6,45091E+14	3,99923E+14	0.00618170584785705	0.170747242106618	-1,8928E+14	7,01067E+14	7,30742E+14	6,07511E+14	5,4543E+14	6,34509E+14	6,51287E+14
TC0500001399.mm.2	P2rx7	purinergic receptor P2X, ligand-gated ion channel, 7	1,29879E+14	5,57035E+14	3,99716E+14	0.00619709746349093	0.170747242106618	-1,89491E+14	6,45146E+13	6,21348E+14	5,05017E+14	5,01719E+14	5,10079E+14	5,58903E+14
TC0700002748.mm.2	Ovol3	ovo like zinc finger 3	-1,19974E+14	6,46571E+14	-3,99509E+14	0.00621245010707538	0.170747242106618	-1,897E+14	5,83036E+14	5,53874E+14	6,83686E+14	6,93172E+13	6,79652E+14	6,86003E+13
TC0100001779.mm.2	Rab3gap2	RAB3 GTPase activating protein subunit 2	1,43055E+14	6,52961E+14	3,98725E+14	0.00627114152138075	0.170747242106618	-1,90496E+14	7,55804E+14	7,4014E+13	6,41722E+14	5,68114E+13	6,1427E+14	5,97714E+14
TC0200003299.mm.2	Swi5	SWI5 recombination repair homolog (yeast)	1,22078E+14	8,15023E+14	3,98654E+14	0.00627652760282052	0.170747242106618	-1,90569E+14	8,8102E+14	8,66992E+14	7,55929E+14	7,47928E+13	8,01943E+14	8,36323E+14
TC0700003485.mm.2	Mctp2	multiple C2 domains, transmembrane 2	-1,62125E+14	5,91954E+14	-3,98443E+14	0.00629242045934501	0.170747242106618	-1,90783E+14	4,94769E+14	4,99759E+14	6,28338E+14	6,90439E+14	5,77994E+14	6,60423E+14
TC0X00003402.mm.2	Bhlhb9	basic helix-loop-helix domain containing, class B9	1,16718E+14	7,10028E+14	3,98386E+14	0.0062967551272356	0.170747242106618	-1,90841E+14	7,92277E+14	7,84067E+14	6,62112E+13	6,80796E+14	6,71597E+14	6,69316E+14
TC1700001976.mm.2	Ddr1	discoidin domain receptor family, member 1	1,51359E+14	5,83922E+14	3,98328E+14	0.00630110205522779	0.170747242106618	-1,909E+14	6,54093E+14	6,85081E+14	5,4654E+14	4,89916E+14	5,33185E+14	5,94715E+14
TC0800002544.mm.2	Zfp330	zinc finger protein 330	1,76671E+14	5,9793E+14	3,97932E+14	0.0063311378147347	0.170747242106618	-1,91303E+14	7,24143E+14	7,09385E+14	5,92569E+14	4,87616E+14	5,66516E+14	5,07352E+13
TC0700001684.mm.2	Nucb2	nucleobindin 2	1,6544E+14	5,89363E+14	3,97551E+14	0.00636020280990108	0.170747242106618	-1,91691E+14	7,12675E+14	6,99059E+14	5,85238E+14	4,95616E+14	5,51113E+14	4,92478E+14
TC0100003864.mm.2	Psmd1	proteasome (prosome, macropain) 26S subunit, non-ATPase, 1	1,29436E+14	7,99079E+14	3,97518E+14	0.00636274485020891	0.170747242106618	-1,91725E+14	9,11131E+14	8,92446E+14	7,96314E+14	7,48391E+14	7,32744E+14	71345102689
TC1500001238.mm.2	Cdh6	cadherin 6	1,79986E+14	6,45677E+14	3,97461E+12	0.00636709915116634	0.170747242106618	-1,91783E+14	7,45305E+14	7,78862E+14	5,71146E+14	5,93049E+14	6,52328E+14	5,33373E+14
TC0100003620.mm.2	Cep170	centrosomal protein 170	2,32E+14	6,47785E+14	3,97401E+14	0.00637171451170897	0.170747242106618	-1,91844E+14	8,17447E+14	7,92226E+14	6,54226E+14	4,91447E+14	6,04679E+14	5,26688E+14
TC1900001493.mm.2	Tctn3	tectonic family member 3	1,20639E+14	6,67862E+13	3,97397E+14	0.00637201774242056	0.170747242106618	-1,91848E+14	7,41904E+14	7,2951E+14	6,27236E+14	6,02899E+14	6,63868E+14	6,41756E+14
TC1100000884.mm.2	Rpl26/Gm15772	ribosomal protein L26/ribosomal protein L26 pseudogene	1,34173E+14	9,83374E+14	3,97236E+14	0.00638433696185882	0.170747242106618	-1,92012E+14	1,07495E+14	1,07546E+14	9,64032E+14	9,18032E+14	9,55902E+14	9,11868E+14
TC1000000053.mm.2	Ppil4	peptidylprolyl isomerase (cyclophilin)-like 4	1,4682E+14	6,47152E+14	3,9717E+14	0.00638940512674818	0.170747242106618	-1,92079E+13	7,42921E+14	7,27914E+14	6,18623E+14	5,58571E+13	6,45396E+14	5,89485E+14
TC0600000556.mm.2	Atp6v0e2	ATPase, H+ transporting, lysosomal V0 subunit E2	1,50001E+14	8,71595E+14	3,96948E+14	0.00640650982962632	0.170747242106618	-1,92306E+13	9,83344E+14	9,76447E+14	8,57941E+14	8,01846E+14	8,38736E+14	7,71254E+14
TC0700002217.mm.2	Peg3	paternally expressed 3	1,71531E+14	7,6465E+14	3,96903E+14	0.00640999835730162	0.170747242106618	-1,92352E+13	9,5502E+14	8,88025E+14	7,67779E+14	7,32204E+14	6,66168E+14	5,78703E+14
TC1100001012.mm.2	Camkk1	calcium/calmodulin-dependent protein kinase kinase 1, alpha	1,23691E+14	7,30802E+14	3,96603E+14	0.00643311879332899	0.170747242106618	-1,92657E+14	8,11909E+13	8,18344E+14	7,09576E+14	6,73294E+14	6,97399E+14	6,74289E+13
TC0500001584.mm.2	Por	P450 (cytochrome) oxidoreductase	1,55041E+14	7,39044E+14	3,96583E+14	0.00643466388323697	0.170747242106618	-1,92677E+14	8,46243E+14	8,22805E+14	7,22372E+14	6,36594E+14	7,19944E+14	6,86306E+14
TC0100001237.mm.2	Syt2	synaptotagmin II	1,23843E+14	5,99839E+14	3,96358E+14	0.00645212024977698	0.170747242106618	-1,92907E+14	6,80385E+14	6,79832E+14	5,66705E+14	5,45826E+14	5,82521E+14	5,43766E+14
TC0700001913.mm.2	Htra1	HtrA serine peptidase 1	1,31506E+14	7,89302E+14	3,96336E+14	0.00645383987506768	0.170747242106618	-1,9293E+14	8,69112E+14	8,72504E+14	7,68148E+13	7,10455E+14	7,64898E+14	7,50692E+14
TC0500000316.mm.2	Snx17	sorting nexin 17	1,83549E+14	7,0804E+14	3,96194E+14	0.00646491331019969	0.170747242106618	-1,93075E+13	8,40497E+13	8,28395E+14	6,98516E+14	6,03278E+14	6,8285E+14	5,94703E+14
TC1100000028.mm.2	Pes1	pescadillo ribosomal biogenesis factor 1	1,43868E+14	7,87542E+14	3,96041E+14	0.00647683104705135	0.170747242106618	-1,93231E+14	8,6759E+14	8,74324E+14	7,19287E+14	7,34892E+14	7,25639E+14	8,03519E+13
TC0600002003.mm.2	Copg2	coatomer protein complex, subunit gamma 2	1,35151E+14	8,03315E+14	3,9565E+14	0.00650743196875078	0.170747242106618	-1,93631E+14	8,93312E+14	8,87658E+14	7,65225E+13	7,45443E+14	7,95614E+13	7,32637E+14
TC0700000537.mm.2	Zfp420	zinc finger protein 420	-1,31839E+14	8,40973E+14	-3,95535E+14	0.00651643481791071	0.170747242106618	-1,93748E+14	7,50087E+14	7,52923E+14	8,53086E+13	9,13603E+14	8,87602E+14	8,88535E+14
TC0600001833.mm.2	Dlx6os2	distal-less homeobox 6, opposite strand 2	-1,47168E+14	5,84061E+13	-3,95413E+14	0.00652604043452235	0.170747242106618	-1,93873E+14	4,72324E+14	4,90781E+14	5,91627E+14	6,65814E+14	6,23512E+14	6,60307E+14
TC1700000318.mm.2	Pkmyt1	protein kinase, membrane associated tyrosine/threonine 1	-1,46569E+14	6,57427E+14	-3,95091E+14	0.00655139478248873	0.170747242106618	-1,94203E+14	5,6558E+13	5,4151E+13	6,6025E+14	7,39979E+14	7,12571E+14	7,24669E+14
TC1000000708.mm.2	Bcr	breakpoint cluster region	1,37343E+14	7,49125E+14	3,95017E+14	0.00655726933512253	0.170747242106618	-1,94279E+14	8,35606E+14	8,34617E+13	7,29541E+14	6,65995E+14	7,29419E+14	6,99572E+14
TC1600001605.mm.2	Adprh	ADP-ribosylarginine hydrolase	1,16331E+14	6,95079E+13	3,9487E+14	0.00656887890309735	0.170747242106618	-1,94429E+14	7,67705E+14	7,45873E+14	6,37025E+14	6,4389E+14	6,85694E+14	6,90287E+14
TC1600000282.mm.2	Vwa5b2	von Willebrand factor A domain containing 5B2	-1,26653E+14	7,42174E+14	-3,94648E+14	0.00658651258110789	0.170747242106618	-1,94656E+14	6,43761E+14	6,57879E+14	7,6483E+14	7,90117E+14	7,77076E+14	8,19383E+14
TC1800000374.mm.2	Pcdhb17	protocadherin beta 17	1,96432E+14	5,97847E+14	3,94557E+14	0.0065937494135521	0.170747242106618	-1,9475E+14	7,47543E+13	6,94689E+14	5,85121E+14	4,64247E+14	5,78789E+14	5,16692E+14
TC0400004158.mm.2	Cptp	ceramide-1-phosphate transfer protein	1,37457E+14	5,66052E+14	3,94497E+14	0.00659846862568799	0.170747242106618	-1,9481E+14	6,68569E+14	6,42377E+14	4,86587E+14	5,49445E+14	5,34148E+14	5,15185E+14
TC0100000638.mm.2	Bcs1l	BCS1-like (yeast)	1,68765E+14	6,81985E+14	3,94426E+14	0.00660415316561113	0.170747242106618	-1,94883E+14	7,96209E+14	7,79976E+14	6,69485E+14	5,6917E+14	6,64039E+14	6,13033E+14
TC0100003294.mm.2	Cacna1e	calcium channel, voltage-dependent, R type, alpha 1E subunit	1,46835E+14	7,42386E+14	3,94413E+13	0.00660514677572996	0.170747242106618	-1,94896E+14	8,61406E+14	8,60021E+14	7,44443E+14	6,83313E+14	6,82016E+14	6,23118E+13
TC1600000376.mm.2	Il1rap	interleukin 1 receptor accessory protein	1,33032E+14	6,1491E+14	3,943E+14	0.00661423190452443	0.170747242106618	-1,95013E+14	6,72658E+14	7,11306E+13	5,73856E+14	5,44044E+14	6,14045E+14	5,73551E+14
TC0200004406.mm.2	Fsip1	fibrous sheath-interacting protein 1	-1,28397E+13	5,93821E+14	-3,9402E+13	0.00663658179689	0.170747242106618	-1,95299E+14	4,8235E+14	5,19115E+14	6,11171E+14	6,47089E+14	6,41633E+14	6,6157E+14

TC0400000404.mm.2	Gm21953/Gm20878/Gm21586	predicted gene, 21953/predicted gene, 20878/predicted gene, 21586	1,53982E+14	9,34709E+14	3,93694E+14	0.00666277500174528	0.170747242106618	-1,95634E+14	1,00808E+14	1,02679E+14	8,86459E+14	8,40447E+12	9,63827E+14	8,82649E+14
TC0600002936.mm.2	Atp2b2	ATPase, Ca++ transporting, plasma membrane 2	1,38932E+14	9,47753E+14	3,93603E+14	0.00667013038240519	0.170747242106618	-1,95728E+14	1,03418E+14	1,03654E+14	9,27785E+14	8,65066E+14	9,30628E+14	8,92326E+13
TC1500000603.mm.2	Kifc2	kinesin family member C2	1,3396E+13	6,91137E+14	3,93565E+14	0.00667315006367635	0.170747242106618	-1,95766E+14	7,76026E+14	7,68516E+14	6,6929E+14	6,07331E+14	6,5299E+14	6,72673E+13
TC0400001974.mm.2	Eno1/Eno1b	enolase 1, alpha non-neuron/enolase 1B, retrotransposed	2,11254E+14	1,02209E+14	3,93349E+14	0.00669061445563345	0.170747242106618	-1,95988E+14	1,14247E+14	1,1609E+14	1,0145E+14	8,6637E+14	1,00655E+14	9,4175E+14
TC0X00003360.mm.2	Tlr7	toll-like receptor 7	1,96216E+14	5,61314E+14	3,93332E+14	0.00669193373751832	0.170747242106618	-1,96005E+14	6,2321E+13	7,43867E+14	4,46207E+14	5,28438E+14	5,17216E+14	5,08943E+14
TC1500001430.mm.2	Dpys	dihydropyrimidinase	-1,88802E+14	6,35289E+14	-3,92946E+14	0.00672327249119513	0.170747242106618	-1,96402E+14	5,35495E+14	4,94636E+14	6,4397E+14	7,63765E+14	6,59306E+14	7,14562E+14
TC0X00001873.mm.2	Cask	calcium/calmodulin-dependent serine protein kinase (MAGUK family)	2,31115E+14	6,19534E+13	3,92788E+14	0.00673610735570944	0.170747242106618	-1,96564E+14	7,79454E+14	7,87185E+14	6,12663E+14	4,91747E+14	5,92412E+14	4,53743E+14
TSUnmapped00000025.mm.2	Gm21992	predicted gene 21992	1,1621E+14	7,10087E+14	3,92714E+14	0.00674211162763722	0.170747242106618	-1,9664E+14	7,84465E+14	7,77968E+14	6,70126E+13	6,59887E+14	6,95188E+14	6,72888E+14
TC0X00000912.mm.2	Zc3h12b	zinc finger CCCH-type containing 12B	1,25735E+14	5,55249E+14	3,92619E+14	0.00674987317078133	0.170747242106618	-1,96737E+13	6,25653E+14	6,4038E+14	5,31514E+14	4,83049E+14	5,2675E+14	5,24147E+14
TC1900000628.mm.2	Cnnm1	cyclin M1	1,31357E+14	7,35456E+14	3,92596E+14	0.0067517598302722	0.170747242106618	-1,96761E+14	8,27473E+14	8,09301E+14	7,15784E+14	6,58276E+14	7,06926E+14	6,94978E+14
TC0900000254.mm.2	Thyn1	thymocyte nuclear protein 1	1,27503E+14	7,0384E+13	3,9259E+14	0.0067522770304656	0.170747242106618	-1,96768E+14	7,62048E+14	7,85153E+14	6,37807E+14	6,54389E+14	7,14945E+14	6,68695E+13
TC0700000469.mm.2	Zfp60	zinc finger protein 60	1,62582E+14	7,22036E+14	3,9222E+14	0.00678254173439729	0.170747242106618	-1,97148E+14	8,3398E+14	8,19832E+14	6,99346E+14	6,29301E+14	7,148E+14	6,34958E+14
TC0900001009.mm.2	Myo5a	myosin VA	1,87662E+14	7,95572E+14	3,9221E+14	0.00678333646210565	0.170747242106618	-1,97158E+14	9,22226E+14	9,34696E+14	7,88599E+14	6,92999E+14	7,6627E+14	6,68639E+14
TC0500002777.mm.2	Gm16427	predicted gene 16427	-1,6603E+14	5,82398E+14	-3,92176E+14	0.00678618137990146	0.170747242106618	-1,97193E+14	4,4326E+14	5,01766E+13	5,9712E+13	6,79967E+13	6,12968E+14	6,59304E+14
TC1100002066.mm.2	Tbcd	tubulin-specific chaperone d	1,49716E+14	7,50207E+14	3,92059E+14	0.0067957509382826	0.170747242106618	-1,97313E+14	8,60704E+14	8,37897E+14	7,39368E+14	6,59802E+13	7,19669E+14	6,83803E+14
TC0700003727.mm.2	Tmem126b	transmembrane protein 126B	1,59416E+14	5,85655E+14	3,92038E+14	0.00679747691070062	0.170747242106618	-1,97335E+14	6,83913E+14	6,89188E+14	5,78604E+14	4,75665E+14	5,40824E+13	5,45738E+13
TC0100001351.mm.2	Ivns1abp	influenza virus NS1A binding protein	2,29289E+14	7,17202E+14	3,91927E+14	0.00680662009507747	0.170747242106618	-1,97449E+14	8,76515E+14	8,38464E+14	7,16141E+14	5,40259E+14	6,81879E+14	6,49953E+14
TC0X00000652.mm.2	Hmgb3	high mobility group box 3	1,6449E+13	6,24516E+14	3,91547E+14	0.00683803451471885	0.170747242106618	-1,97841E+14	7,29531E+14	7,25203E+14	6,10076E+14	5,15677E+14	6,11371E+14	5,55236E+14
TC1600001268.mm.2	Rpl31-ps12	ribosomal protein L31, pseudogene 1 2	1,23168E+14	9,41284E+14	3,91424E+14	0.00684822504463182	0.170747242106618	-1,97967E+14	1,02767E+14	1,01428E+14	9,12669E+14	8,82947E+14	9,213E+14	8,88834E+13
TC0900002325.mm.2	Wdr61	WD repeat domain 61	1,74048E+14	6,63614E+14	3,91379E+14	0.00685196134894005	0.170747242106618	-1,98014E+14	7,73057E+14	7,47308E+14	6,45472E+14	5,26797E+14	6,48721E+14	6,40332E+14
TC1300002318.mm.2	Sdha	succinate dehydrogenase complex, subunit A, flavoprotein (Fp)	2,15097E+14	7,05424E+14	3,91249E+14	0.00686277545954941	0.170747242106618	-1,98148E+14	8,44069E+14	8,23229E+14	6,97683E+14	5,39421E+14	6,90407E+14	6,37733E+14
TC0400003381.mm.2	Ipo13	importin 13	1,365E+14	5,65474E+14	3,90987E+14	0.00688460556332008	0.170747242106618	-1,98418E+14	6,69688E+14	6,71468E+14	5,46871E+14	5,21285E+14	4,586E+14	5,24934E+14
TC0100002353.mm.2	Sf3b1	splicing factor 3b, subunit 1	1,97344E+14	7,00305E+14	3,90929E+13	0.00688939709026855	0.170747242106618	-1,98477E+14	8,43929E+14	8,32448E+14	6,72046E+14	6,09643E+13	6,89208E+14	5,54558E+14
TC0900003344.mm.2	Trf	transferrin	1,49873E+14	9,15956E+14	3,90867E+13	0.00689457509979451	0.170747242106618	-1,98541E+14	1,0009E+14	1,04571E+14	9,13032E+12	8,33831E+14	8,49547E+14	8,52718E+14
TC0500002563.mm.2	Gabra4	gamma-aminobutyric acid (GABA) A receptor, subunit alpha 4	1,22101E+13	7,45848E+14	3,90855E+14	0.00689557437953364	0.170747242106618	-1,98554E+14	8,13138E+14	8,35198E+14	7,17766E+13	6,86368E+14	7,22045E+14	7,00574E+14
TC0900000751.mm.2	Pkm	pyruvate kinase, muscle	1,88321E+13	1,02708E+14	3,90656E+14	0.00691227813408206	0.170747242106618	-1,9876E+14	1,15018E+14	1,14738E+14	1,02788E+14	8,93042E+14	9,88462E+14	9,55569E+13
TC1500001085.mm.2	Eif4b	eukaryotic translation initiation factor 4B	2,31328E+14	8,4151E+14	3,90519E+14	0.00692379551935657	0.170747242106618	-1,98901E+13	9,87718E+14	9,8797E+14	8,4148E+14	6,71551E+14	8,17705E+14	7,42634E+14
TC0900002188.mm.2	Cd3g	CD3 antigen, gamma polypeptide	-1,73255E+14	4,73978E+14	-3,9028E+14	0.00694381342972002	0.170747242106618	-1,99147E+13	3,86794E+14	3,27203E+14	4,77476E+14	5,83032E+14	5,31736E+14	5,37627E+14
TC0200001608.mm.2	Kcna4	potassium voltage-gated channel, shaker-related subfamily, member 4	1,56929E+14	6,41069E+14	3,90181E+14	0.00695221835826598	0.170747242106618	-1,9925E+14	7,31918E+14	7,57828E+14	6,35251E+14	5,40638E+14	6,02261E+14	5,78515E+14
TC0200001096.mm.2	Hoxd8	homeobox D8	-1,38378E+14	6,42863E+14	-3,90161E+14	0.00695384430165966	0.170747242106618	-1,9927E+14	5,34295E+14	5,52262E+14	6,65655E+13	6,97658E+14	6,71199E+14	7,36109E+14
TC1500002191.mm.2	Prkag1	protein kinase, AMP-activated, gamma 1 non-catalytic subunit	1,72214E+14	7,13414E+14	3,90047E+14	0.00696349516856579	0.170747242106618	-1,99388E+14	8,26641E+14	8,07388E+14	6,67093E+14	6,22509E+14	7,33416E+14	6,23436E+14
TC0500001080.mm.2	Rpap2	RNA polymerase II associated protein 2	1,2562E+14	5,68181E+14	3,89897E+14	0.00697619209485401	0.170747242106618	-1,99543E+14	6,73279E+13	6,26848E+14	5,31654E+14	5,17234E+13	5,3964E+13	5,2043E+14
TC1000003156.mm.2	Ikzf4	IKAROS family zinc finger 4	-1,40265E+13	8,06354E+14	-3,89723E+14	0.00699091882759564	0.170747242106618	-1,99723E+14	7,17308E+14	7,28253E+14	8,53552E+14	8,72539E+14	7,95948E+14	8,70525E+14
TC0700001867.mm.2	Zfp646	zinc finger protein 646	1,25515E+14	6,04514E+14	3,89669E+13	0.00699553880085688	0.170747242106618	-1,99779E+14	6,63037E+13	6,96291E+14	5,72572E+14	5,35727E+14	5,87832E+14	5,71625E+14
TC1700002584.mm.2	Dhx57	DEAH (Asp-Glu-Ala-Asp/His) box polypeptide 57	1,13754E+14	6,49716E+14	3,89601E+14	0.00700129782129913	0.170747242106618	-1,99849E+14	7,29495E+14	7,18936E+14	6,16782E+14	6,04141E+14	6,10138E+14	6,18801E+14
TC1100000266.mm.2	Gm6899	predicted gene 6899	-1,60062E+14	6,68462E+14	-3,8938E+14	0.00702008295070734	0.170747242106618	-2,00077E+14	5,64546E+14	5,75085E+14	6,83159E+14	7,76596E+14	6,81854E+14	7,29533E+14
TC0900001236.mm.2	Copb2	coatomer protein complex, subunit beta 2 (beta prime)	1,77362E+13	7,02617E+14	3,89126E+14	0.00704178818691411	0.170747242106618	-2,0034E+14	8,16601E+13	8,05679E+14	6,68524E+14	5,99032E+14	7,15738E+14	6,1013E+14
TC0900002898.mm.2	Clstn2	calsyntenin 2	1,57523E+14	7,51596E+14	3,89094E+14	0.00704457883081477	0.170747242106618	-2,00374E+14	8,34789E+14	8,75579E+14	7,34979E+14	6,60343E+14	7,30348E+14	6,73537E+14
TC0100003102.mm.2	Btg2	B cell translocation gene 2, anti-proliferative	-1,4291E+14	6,10583E+14	-3,8902E+13	0.00705089565991355	0.170747242106618	-2,0045E+14	5,17184E+14	5,14698E+14	6,79263E+14	6,38441E+14	6,21367E+14	6,92545E+14
TC0600000416.mm.2	Mgam	maltase-glucoamylase	-1,34498E+14	4,68222E+13	-3,89014E+14	0.00705138975164653	0.170747242106618	-2,00456E+14	3,7794E+14	3,69533E+14	4,74329E+14	5,42139E+14	5,27367E+14	5,18026E+14
TC0300002714.mm.2	Strip1	striatin interacting protein 1	1,69819E+14	7,23216E+14	3,88999E+14	0.00705264565334016	0.170747242106618	-2,00471E+14	8,09148E+14	8,04141E+14	6,74828E+14	5,98823E+14	7,71153E+14	6,81201E+14
TC1600000988.mm.2	Mrap	melanocortin 2 receptor accessory protein	-1,17778E+14	6,96916E+14	-3,8885E+14	0.00706543523103158	0.170747242106618	-2,00626E+14	6,03486E+14	6,29696E+14	7,23426E+14	7,45313E+14	7,37836E+11	7,41738E+14
TC0700000832.mm.2	Slc17a7	solute carrier family 17 (sodium-dependent inorganic phosphate cotransporter), member 7	1,7511E+14	9,93945E+14	3,88814E+14	0.0070685672141713	0.170747242106618	-2,00663E+14	1,03379E+14	1,12791E+14	9,43346E+14	8,68135E+14	1,01069E+14	9,79794E+13
TC0300002828.mm.2	Ptbp2	polypyrimidine tract binding protein 2	1,83253E+14	8,28137E+14	3,88342E+14	0.00710920370636065	0.170747242106618	-2,01152E+13	9,48998E+14	9,30971E+14	8,06816E+14	7,06646E+14	8,31464E+14	7,4393E+13
TC1300001330.mm.2	Hcn1	hyperpolarization-activated, cyclic nucleotide-gated K+ 1	2,04989E+14	6,91384E+14	3,88332E+14	0.00711005289749719	0.170747242106618	-2,01162E+13	8,12252E+14	7,8984E+14	6,54521E+14	5,37594E+14	7,29599E+14	6,24499E+13
TC0300001152.mm.2	Clcc1	chloride channel CLIC-like 1	1,4265E+14	6,27843E+14	3,88301E+14	0.00711280529459807	0.170747242106618	-2,01195E+14	7,231E+13	7,01276E+14	5,30895E+14	6,08182E+14	5,93285E+14	6,10322E+14
TC1400001785.mm.2	Prxl2a	peroxiredoxin like 2A	2,3457E+14	8,407E+14	3,88206E+14	0.00712100968575224	0.170747242106618	-2,01293E+14	1,01974E+14	9,85277E+14	8,53922E+14	6,81959E+14	7,89E+14	7,14295E+14
TC0200004159.mm.2	Cd82	CD82 antigen	1,53258E+14	8,06541E+14	3,8803E+14	0.00713621217354657	0.170747242106618	-2,01475E+14	9,10661E+13	9,30348E+14	7,82618E+14	7,51875E+14	7,76908E+14	6,86839E+14
TC0300002815.mm.2	Palmd	palmdelphin	1,52022E+14	6,91997E+14	3,87839E+13	0.0071528972308417	0.170747242106618	-2,01674E+14	7,87762E+14	8,04884E+14	6,65749E+14	6,22855E+14	6,77228E+14	5,93506E+14
TC1000000508.mm.2	Fabp7	fatty acid binding protein 7, brain	3,05818E+14	8,38416E+14	3,8762E+14	0.00717190272084652	0.170747242106618	-2,019E+14	1,00909E+14	1,04533E+14	8,03535E+14	6,39251E+14	8,68056E+14	6,65233E+14
TC0X00002061.mm.2	Ndufa1	NADH:ubiquinone oxidoreductase subunit A1	1,62996E+13	7,11337E+14	3,87366E+14	0.00719418071580988	0.170747242106618	-2,02165E+14	8,16456E+12	8,06373E+14	7,03129E+14	5,93707E+14	6,78634E+14	6,69724E+14
TC0300002098.mm.2	4931440P22Rik	RIKEN cDNA 4931440P22 gene	-1,69159E+14	8,76493E+14	-3,87213E+14	0.00720754745466097	0.170747242106618	-2,02323E+14	7,563E+14	7,68479E+14	8,80229E+14	9,82869E+14	9,07058E+14	9,64021E+14
TC1100000956.mm.2	Pld2	phospholipase D2	1,27135E+14	5,68074E+14	3,87183E+14	0.00721020707369376	0.170747242106618	-2,02354E+14	6,78063E+14	6,55212E+14	5,5411E+14	5,24895E+14	5,19723E+14	4,76438E+14
TC0600003107.mm.2	Clstn3	calsyntenin 3	1,6109E+14	7,87324E+13	3,87095E+14	0.00721789410148027	0.170747242106618	-2,02445E+14	9,09338E+14	8,79185E+14	7,78025E+14	6,88318E+14	7,60514E+14	7,08562E+13
TC1300001395.mm.2	Pfkp	phosphofructokinase, platelet	1,99267E+13	7,9278E+14	3,86725E+14	0.00725052179496481	0.170747242106618	-2,0283E+14	8,94935E+14	9,27309E+13	7,71473E+13	6,52237E+14	8,00818E+14	7,09911E+14
TC0600000609.mm.2	Snx10	sorting nexin 10	1,51355E+14	8,12234E+14	3,86722E+14	0.00725080196153685	0.170747242106618	-2,02833E+14	9,13567E+14	9,09419E+14	7,85148E+14	7,35129E+14	7,24562E+14	8,05579E+14
TC0500000425.mm.2	Cytl1	cytokine-like 1	-1,52975E+14	6,53544E+14	-3,86574E+14	0.00726392340249026	0.170747242106618	-2,02988E+14	5,74633E+12	5,52042E+14	6,80613E+14	7,52012E+13	6,497E+13	7,12263E+14
TC1600000446.mm.2	Zdhhc19	zinc finger, DHHC domain containing 19	-1,36627E+14	6,48793E+14	-3,86535E+14	0.00726730869403623	0.170747242106618	-2,03027E+14	5,56903E+14	5,73906E+14	6,75386E+14	7,28676E+14	6,54852E+14	7,03036E+14
TC0800000088.mm.2	Col4a2	collagen, type IV, alpha 2	-1,58265E+14	6,7093E+14	-3,86425E+14	0.00727707145908869	0.170747242106618	-2,03142E+14	5,7228E+14	5,51968E+14	6,72626E+14	7,68151E+14	7,11446E+14	7,49106E+14
TC0600003123.mm.2	Gpr162	G protein-coupled receptor 162	1,12923E+14	7,59897E+12	3,86112E+13	0.00730491825758293	0.170747242106618	-2,03468E+14	8,18774E+14	8,32016E+14	7,13508E+14	7,11438E+14	7,34694E+14	7,4895E+13
TC1100000251.mm.2	Bcl11a	B cell CLL/lymphoma 11A (zinc finger protein)	1,32402E+14	8,01553E+14	3,859E+14	0.00732378658270952	0.170747242106618	-2,03688E+14	8,92154E+14	8,90881E+13	7,92726E+14	7,25504E+14	7,54689E+14	7,53365E+14
TC1100000786.mm.2	Fam83g	family with sequence similarity 83, member G	-1,33028E+14	6,62064E+14	-3,8588E+14	0.00732562974905747	0.170747242106618	-2,03709E+14	5,73425E+14	5,8811E+14	6,99241E+14	7,28351E+14	6,6161E+13	7,21646E+14
TC1100002288.mm.2	Actr2	ARP2 actin-related protein 2	1,1628E+14	9,63775E+14	3,85877E+14	0.00732585134737163	0.170747242106618	-2,03712E+14	1,02605E+14	1,04832E+14	9,32743E+14	9,09063E+14	9,32222E+14	9,34254E+14
TC0500000813.mm.2	Utp3	UTP3 small subunit processome component	1,16309E+14	7,08383E+14	3,85724E+13	0.00733958368273175	0.170747242106618	-2,03872E+14	7,98979E+14	7,74086E+14	6,78339E+14	6,62107E+13	6,75338E+14	6,61449E+14
TC0X00000321.mm.2	Gm6268	predicted gene 6268	-1,16923E+14	4,62093E+14	-3,8564E+14	0.00734707325723946	0.170747242106618	-2,03959E+14	3,89446E+14	3,74312E+14	4,83453E+14	5,1415E+14	5,08494E+14	5,02705E+14
TC0800000916.mm.2	Tbc1d9	TBC1 domain family, member 9	1,7437E+14	6,07759E+14	3,85546E+14	0.00735552725142694	0.170747242106618	-2,04057E+14	7,09895E+14	7,27165E+14	6,04084E+14	4,84236E+14	5,76616E+14	5,44556E+14
TC0500002229.mm.2	Poln/Haus3	DNA polymerase N/HAUS augmin-like complex, subunit 3	-1,33017E+14	5,58705E+14	-3,853E+14	0.0073776721362318	0.170747242106618	-2,04314E+14	4,46327E+14	4,92806E+14	5,91314E+14	6,13854E+14	5,81367E+14	6,26561E+14
TC1200001108.mm.2	Cyp46a1	cytochrome P450, family 46, subfamily a, polypeptide 1	1,15565E+14	8,10175E+13	3,85296E+14	0.00737800833097561	0.170747242106618	-2,04318E+14	8,76826E+14	8,81097E+14	7,77394E+14	7,49397E+14	7,94304E+14	7,82033E+14
TC1000000843.mm.2	Cirbp	cold inducible RNA binding protein	1,74191E+14	6,88253E+14	3,85268E+14	0.00738049292887364	0.170747242106618	-2,04346E+14	8,06173E+14	7,78873E+14	6,69787E+13	5,66878E+14	6,86836E+14	6,20974E+14
TC0900002728.mm.2	Slc17a5	solute carrier family 17 (anion/sugar transporter), member 5	1,20035E+14	6,8688E+14	3,85127E+14	0.00739319672901891	0.170747242106618	-2,04493E+14	7,7265E+13	7,90273E+14	6,73722E+14	6,49131E+14	6,02256E+14	6,33249E+14
TC0500003308.mm.2	Psph	phosphoserine phosphatase	-1,20402E+14	8,82658E+13	-3,85076E+14	0.00739785300382638	0.170747242106618	-2,04547E+14	7,80045E+13	8,00049E+14	8,9076E+14	9,30139E+14	9,46368E+14	9,48589E+14
TC0700004165.mm.2	Arl6ip1	ADP-ribosylation factor-like 6 interacting protein 1	2,14186E+13	8,83182E+14	3,85054E+14	0.00739983027628852	0.170747242106618	-2,0457E+14	1,01213E+14	1,01399E+14	8,62851E+14	7,34894E+14	8,94185E+14	7,81047E+14
TC0X00002479.mm.2	Rab39b	RAB39B, member RAS oncogene family	1,60376E+14	6,99368E+14	3,85034E+14	0.00740166389681283	0.170747242106618	-2,04591E+14	8,00153E+14	8,09826E+14	6,82633E+14	6,06593E+14	6,86645E+14	6,10356E+14
TC1700000202.mm.2	Riok2	RIO kinase 2	1,57229E+13	6,18931E+14	3,84903E+13	0.00741347498590833	0.170747242106618	-2,04727E+14	7,43462E+14	7,28803E+14	6,29421E+14	5,28386E+14	5,31509E+14	5,52009E+14
TC1800000509.mm.2	Hsd17b4	hydroxysteroid (17-beta) dehydrogenase 4	1,21191E+14	6,87924E+14	3,84838E+14	0.00741938916129979	0.170747242106618	-2,04795E+14	7,68748E+14	7,70619E+14	6,3449E+14	6,62494E+14	6,6396E+14	6,27231E+14
TC0900001570.mm.2	Vill	villin-like	-1,30557E+14	6,53942E+14	-3,84713E+14	0.00743065845850135	0.170747242106618	-2,04925E+14	5,8074E+14	5,58215E+14	6,86E+13	7,14068E+14	6,65394E+14	7,19235E+14
TC1200002296.mm.2	Clmn	calmin	1,39455E+14	6,68861E+13	3,84642E+14	0.00743710818390819	0.170747242106618	-2,04999E+14	7,72157E+14	7,72911E+14	6,49799E+14	6,16359E+14	6,3692E+14	5,65022E+14
TC1700001929.mm.2	Skiv2l/Mir6970	superkiller viralicidic activity 2-like (S. cerevisiae)/microRNA 6970	1,19576E+14	6,98255E+13	3,84428E+14	0.00745660480098043	0.170747242106618	-2,05222E+14	7,70091E+14	7,92184E+14	6,79738E+14	6,43385E+14	6,54061E+14	6,50068E+14
TC1000000884.mm.2	Eef2	eukaryotic translation elongation factor 2	1,34178E+14	1,05109E+13	3,84228E+14	0.00747487254298857	0.170747242106618	-2,05431E+14	1,14934E+14	1,14107E+14	1,04042E+14	9,81635E+14	1,01668E+14	9,77398E+14
TC1600000288.mm.2	Eif4g1	eukaryotic translation initiation factor 4, gamma 1	1,20951E+14	8,42038E+14	3,84216E+13	0.00747597047988363	0.170747242106618	-2,05444E+14	9,30169E+14	9,21168E+14	8,23554E+14	7,8588E+14	8,0825E+14	7,83204E+14
TC0800002596.mm.2	Rnaseh2a	ribonuclease H2, large subunit	1,24469E+14	5,99149E+14	3,84132E+13	0.00748368023170612	0.170747242106618	-2,05532E+14	6,77139E+14	6,653E+14	5,66317E+14	5,27184E+14	5,62056E+14	5,96901E+14
TC0800002843.mm.2	Lcat	lecithin cholesterol acyltransferase	1,18649E+14	6,73214E+14	3,83846E+14	0.0075098336522856	0.170747242106618	-2,0583E+14	7,54818E+14	7,32468E+14	6,29969E+13	6,20019E+14	6,67376E+13	6,34631E+14
TC1100000588.mm.2	Skp1a	S-phase kinase-associated protein 1A	2,54655E+14	9,23949E+14	3,83834E+14	0.00751096026751804	0.170747242106618	-2,05843E+14	1,07375E+14	1,0653E+14	8,88451E+12	7,41291E+14	9,65338E+14	8,09562E+14
TC0800001007.mm.2	Gm10638	predicted gene 10638	-1,45192E+14	7,3434E+14	-3,83735E+14	0.00752004555644459	0.170747242106618	-2,05946E+14	6,24477E+14	6,42634E+14	7,35583E+14	8,21912E+14	7,88981E+14	7,92454E+13
TC0200005490.mm.2	Gm14305	predicted gene 14305	2,75786E+14	7,44079E+14	3,83691E+14	0.00752406511798299	0.170747242106618	-2,05992E+14	8,51599E+14	9,12473E+14	7,08185E+14	5,04315E+14	7,95331E+14	6,92573E+13
TC0700001554.mm.2	Smpd1	sphingomyelin phosphodiesterase 1, acid lysosomal	1,57627E+14	7,86533E+14	3,83579E+14	0.00753443167136578	0.170747242106618	-2,0611E+14	8,86646E+14	8,61665E+14	7,65114E+14	6,67942E+14	7,85E+14	7,52831E+14
TC0100001586.mm.2	Pfdn2	prefoldin 2	1,34181E+14	8,0106E+14	3,83514E+14	0.00754039963125201	0.170747242106618	-2,06177E+14	9,05504E+14	8,8074E+14	7,70744E+14	7,47139E+14	7,82563E+14	7,19672E+14
TC0200003576.mm.2	Acvr1c	activin A receptor, type IC	1,29664E+14	6,50948E+14	3,83339E+14	0.00755661756354084	0.170747242106618	-2,06361E+14	7,01756E+14	7,48991E+14	5,98733E+14	5,92686E+14	6,53148E+14	6,10375E+14
TC0600000584.mm.2	Ccdc126	coiled-coil domain containing 126	1,38316E+14	5,41016E+14	3,83329E+14	0.00755752199466991	0.170747242106618	-2,06371E+14	6,15315E+14	6,04819E+13	5,08342E+14	4,3516E+14	5,54089E+14	5,28369E+14
TC1900000008.mm.2	Cpt1a	carnitine palmitoyltransferase 1a, liver	1,16255E+14	6,00035E+13	3,83321E+14	0.00755826417439939	0.170747242106618	-2,0638E+14	6,82733E+14	6,61089E+13	5,69159E+14	5,42153E+14	5,74436E+14	5,70643E+14
TC0800003248.mm.2	1190005I06Rik	RIKEN cDNA 1190005I06 gene	-1,16175E+14	7,65016E+14	-3,83285E+12	0.00756156573964093	0.170747242106618	-2,06417E+14	6,87028E+14	6,86945E+14	7,85866E+14	8,20455E+14	8,04275E+14	8,05528E+14
TC0200000503.mm.2	Med27	mediator complex subunit 27	2,03342E+14	7,26117E+14	3,83282E+14	0.00756181460094063	0.170747242106618	-2,0642E+14	8,93358E+14	8,50521E+14	7,32921E+14	6,04274E+13	6,8023E+14	5,95398E+13
TC1000002496.mm.2	Pcsk4	proprotein convertase subtilisin/kexin type 4	-1,21405E+13	6,46719E+14	-3,83148E+13	0.00757427790519286	0.170747242106618	-2,0656E+14	5,74512E+14	5,39171E+14	6,79396E+14	6,77098E+14	7,212E+14	6,88937E+13
TC0200002010.mm.2	Cdc25b	cell division cycle 25B	-1,51642E+13	5,48329E+14	-3,83067E+14	0.00758174026170336	0.170747242106618	-2,06645E+14	4,43899E+14	4,82904E+14	5,78353E+14	6,51734E+14	5,40898E+14	5,92187E+13
TC0Y00000434.mm.2	Ssty2	spermiogenesis specific transcript on the Y 2	-1,45617E+14	5,38958E+14	-3,83063E+13	0.00758211270835922	0.170747242106618	-2,06649E+14	4,21051E+14	4,51491E+14	5,45264E+14	6,18513E+14	5,78247E+14	6,1918E+14
TC0500003026.mm.2	Crybb3	crystallin, beta B3	-1,62423E+14	7,704E+14	-3,83027E+14	0.00758552944844317	0.170747242106618	-2,06687E+14	6,5828E+14	6,76671E+14	7,7666E+14	8,83138E+14	7,9939E+14	8,28262E+14
TC0200002693.mm.2	1700010B08Rik	RIKEN cDNA 1700010B08 gene	-1,47928E+14	5,12943E+14	-3,82911E+14	0.007596221842279	0.170747242106618	-2,06808E+14	4,17864E+14	4,16797E+14	5,3038E+14	6,00137E+14	5,25785E+14	5,86694E+14
TC0700003981.mm.2	Apbb1	amyloid beta (A4) precursor protein-binding, family B, member 1	1,88116E+14	8,32466E+14	3,82745E+14	0.00761171572642078	0.170747242106618	-2,06982E+14	9,41473E+14	9,3759E+14	8,22824E+14	6,80006E+14	8,09558E+14	8,03345E+14
TC1800000566.mm.2	Phax	phosphorylated adaptor for RNA export	2,07723E+14	6,59354E+14	3,82708E+14	0.00761520768551133	0.170747242106618	-2,07021E+14	7,90665E+14	7,79684E+12	6,35652E+14	5,19251E+14	6,73751E+14	5,57123E+14
TC0400000518.mm.2	Col15a1	collagen, type XV, alpha 1	-1,64886E+14	6,96381E+14	-3,82706E+14	0.00761534735507633	0.170747242106618	-2,07023E+14	6,18619E+13	5,67561E+14	7,06661E+14	8,09291E+14	7,32686E+14	7,43467E+14
TC0100000271.mm.2	Eif5b	eukaryotic translation initiation factor 5B	1,20056E+14	6,3291E+14	3,82438E+14	0.00764035405561925	0.170747242106618	-2,07303E+14	7,35931E+14	7,25114E+14	6,09015E+14	6,11919E+14	5,79714E+14	5,35769E+14
TC0400003685.mm.2	Stx12	syntaxin 12	1,25939E+14	7,92712E+14	3,82329E+14	0.0076505836498465	0.170747242106618	-2,07418E+14	8,60463E+14	8,53462E+14	7,57942E+13	7,04104E+14	8,00022E+14	7,80281E+14
TC1100003687.mm.2	Med24	mediator complex subunit 24	2,55698E+14	7,466E+14	3,82296E+14	0.00765364595187294	0.170747242106618	-2,07452E+14	9,23862E+14	9,38696E+14	7,3835E+14	6,12813E+14	7,20886E+14	5,44992E+14
TC0300000761.mm.2	Dcst2	DC-STAMP domain containing 2	-1,33862E+14	5,46161E+14	-3,82143E+14	0.00766803933647737	0.170747242106618	-2,07612E+14	4,52676E+14	4,73497E+14	5,83357E+14	6,10541E+13	5,46859E+14	6,10035E+14
TC0200005223.mm.2	Sall4	spalt like transcription factor 4	-2,04522E+14	7,81243E+14	-3,8212E+14	0.00767019740114327	0.170747242106618	-2,07637E+14	6,49547E+14	6,57416E+14	7,97758E+14	9,18248E+14	7,78284E+14	8,86203E+14
TC0100000558.mm.2	Unc80	unc-80, NALCN activator	2,04626E+13	7,44276E+14	3,81989E+14	0.00768257391347508	0.170747242106618	-2,07774E+14	9,1384E+14	8,73278E+14	7,2003E+13	6,57837E+14	7,22405E+14	5,78268E+14
TC1400000306.mm.2	Tkt/Mir3076	transketolase/microRNA 3076	1,11746E+14	8,26738E+14	3,81713E+14	0.00770853405079326	0.170747242106618	-2,08063E+14	9,28535E+14	9,16276E+14	8,08391E+14	8,1293E+14	7,39506E+14	7,54789E+14
TC0700004302.mm.2	Taok2	TAO kinase 2	1,55775E+14	8,4315E+14	3,81593E+14	0.00771989766022359	0.170747242106618	-2,08189E+14	9,49582E+14	9,55255E+14	8,37204E+14	7,56083E+14	8,12801E+14	7,47976E+14
TC1500002033.mm.2	Mlc1	megalencephalic leukoencephalopathy with subcortical cysts 1 homolog (human)	1,41211E+14	8,05866E+14	3,81452E+14	0.00773330303438376	0.170747242106618	-2,08338E+13	8,89361E+14	9,16012E+14	8,01031E+14	7,21919E+14	7,50324E+14	7,5655E+14
TC1100000093.mm.2	Ogdh	oxoglutarate (alpha-ketoglutarate) dehydrogenase (lipoamide)	1,37397E+14	8,71678E+14	3,81349E+14	0.007743060536063	0.170747242106618	-2,08445E+14	9,75198E+14	9,69001E+14	8,66714E+14	8,02691E+14	8,30255E+14	7,8621E+14
TC1900000520.mm.2	Hectd2	HECT domain E3 ubiquitin protein ligase 2	-1,11813E+14	7,87612E+14	-3,81138E+14	0.00776318032877844	0.170747242106618	-2,08667E+14	7,21332E+14	7,24599E+14	8,42625E+14	8,26933E+14	7,98121E+14	8,12058E+14

TC1100001442.mm.2	Lrrc59	leucine rich repeat containing 59	1,48173E+14	7,56815E+14	3,80995E+14	0.0077767715014236	0.170747242106618	-2,08817E+14	8,60696E+14	8,56244E+14	7,13766E+14	7,06827E+14	7,48495E+14	6,54861E+14
TC1700000356.mm.2	Spsb3	splA/ryanodine receptor domain and SOCS box containing 3	1,14144E+14	6,07787E+14	3,80923E+14	0.00778362253492944	0.170747242106618	-2,08893E+14	6,71015E+14	6,65464E+14	5,62093E+13	5,46098E+14	6,13713E+14	5,88341E+14
TC1700001939.mm.2	Sapcd1	suppressor APC domain containing 1	-1,33008E+14	6,44154E+14	-3,80922E+13	0.00778376359134061	0.170747242106618	-2,08894E+14	5,45888E+14	5,42288E+12	6,41573E+14	7,1262E+14	7,10514E+14	7,1204E+14
TC0400001957.mm.2	Clstn1	calsyntenin 1	1,8815E+14	9,047E+14	3,80881E+14	0.00778763482953089	0.170747242106618	-2,08937E+14	1,01567E+14	1,01412E+14	8,95071E+14	7,58417E+13	8,95321E+14	8,49603E+14
TC1300000267.mm.2	Slc17a1	solute carrier family 17 (sodium phosphate), member 1	-1,12541E+14	5,40255E+14	-3,8087E+14	0.00778867329625817	0.170747242106618	-2,08948E+14	4,27464E+14	4,51063E+14	5,49389E+14	5,5422E+14	6,32492E+14	6,26904E+14
TC1100001467.mm.2	Phb	prohibitin	1,4067E+14	8,49982E+14	3,80869E+14	0.00778877637465445	0.170747242106618	-2,08949E+13	9,36307E+14	9,28676E+14	8,24836E+14	7,58808E+14	8,50675E+11	8,0059E+14
TC0700004585.mm.2	Cdkn1c	cyclin-dependent kinase inhibitor 1C (P57)	-1,18642E+14	6,89607E+14	-3,80781E+14	0.00779718997175661	0.170747242106618	-2,09042E+14	6,13455E+13	6,05277E+14	7,06991E+14	7,49026E+14	7,34367E+14	7,28527E+14
TC1100002158.mm.2	Camk2b	calcium/calmodulin-dependent protein kinase II, beta	2,23871E+13	1,04676E+13	3,8072E+14	0.00780302142595674	0.170747242106618	-2,09106E+13	1,19492E+14	1,1995E+14	1,05887E+14	8,8781E+14	1,00352E+14	9,35914E+14
TC1400000833.mm.2	Rcbtb1	regulator of chromosome condensation (RCC1) and BTB (POZ) domain containing protein 1	2,41647E+14	5,3964E+13	3,80706E+14	0.00780439364506295	0.170747242106618	-2,09121E+14	6,84669E+14	6,79937E+14	5,41987E+14	3,39325E+14	4,90852E+14	5,01068E+13
TC0700002461.mm.2	Psg17	pregnancy specific glycoprotein 17	-1,72857E+14	5,3064E+14	-3,8067E+14	0.00780786742942863	0.170747242106618	-2,09159E+14	4,2607E+14	4,03624E+14	5,3265E+14	6,42758E+14	5,6076E+14	6,17978E+14
TC0300001112.mm.2	Kcna2	potassium voltage-gated channel, shaker-related subfamily, member 2	1,15036E+14	7,28071E+14	3,80422E+13	0.00783162949692643	0.170747242106618	-2,09419E+14	8,20356E+14	8,05293E+14	6,92827E+14	7,0275E+14	6,87893E+14	6,59305E+14
TC1100001247.mm.2	Ap2b1	adaptor-related protein complex 2, beta 1 subunit	2,56282E+14	7,25541E+14	3,80421E+14	0.00783179618760349	0.170747242106618	-2,09421E+13	8,77046E+14	8,82289E+14	7,31684E+14	5,15087E+14	6,8781E+14	6,5933E+14
TC1000002253.mm.2	Tspan15	tetraspanin 15	1,23174E+14	6,40637E+14	3,80375E+14	0.00783621160595	0.170747242106618	-2,09469E+13	7,14773E+14	7,04399E+14	6,10126E+14	5,62697E+14	6,37223E+14	6,14604E+14
TC0400001677.mm.2	Id3	inhibitor of DNA binding 3	1,09965E+14	6,04925E+14	3,80357E+14	0.00783789543718872	0.170747242106618	-2,09487E+14	6,63793E+14	6,72279E+14	5,54353E+13	5,61789E+14	5,89779E+14	5,87557E+14
TC1700000070.mm.2	Rps6ka2	ribosomal protein S6 kinase, polypeptide 2	1,26619E+14	7,71381E+14	3,80281E+14	0.00784523405079771	0.170747242106618	-2,09568E+14	8,73834E+13	8,65853E+14	7,68963E+14	7,17487E+14	7,163E+13	6,85851E+14
TC0800002471.mm.2	Large1	LARGE xylosyl- and glucuronyltransferase 1	2,78575E+14	6,82426E+14	3,80177E+14	0.00785521911110105	0.170747242106618	-2,09676E+14	8,4197E+14	8,68769E+14	6,89018E+14	4,6457E+14	6,57961E+14	5,7227E+14
TC0X00000363.mm.2	Zbtb33	zinc finger and BTB domain containing 33	2,63955E+14	5,46842E+13	3,80153E+14	0.00785756896756369	0.170747242106618	-2,09702E+14	6,97646E+14	7,11541E+14	5,51652E+14	3,29625E+13	5,17079E+14	4,73506E+14
TC0300002610.mm.2	Mab21l3	mab-21-like 3	-1,17095E+14	5,36411E+13	-3,80079E+14	0.00786472110154124	0.170747242106618	-2,0978E+14	4,5234E+14	4,71142E+14	5,79252E+14	5,7842E+14	5,51085E+14	5,86228E+14
TC1900001454.mm.2	Ide	insulin degrading enzyme	1,80596E+14	6,83278E+13	3,80041E+14	0.00786838850924279	0.170747242106618	-2,0982E+14	7,8222E+14	7,90967E+14	6,61916E+13	5,5008E+14	6,95457E+14	6,19028E+14
TC0700003045.mm.2	Uevld	UEV and lactate/malate dehyrogenase domains	2,25152E+14	5,20815E+14	3,80023E+14	0.00787012460642737	0.170747242106618	-2,09839E+14	6,54901E+14	6,45636E+13	5,09649E+14	3,40584E+14	5,25333E+14	4,48787E+14
TC0100000878.mm.2	Cops8	COP9 signalosome subunit 8	1,14423E+14	7,94616E+14	3,79949E+14	0.00787733747257042	0.170747242106618	-2,09917E+14	8,71404E+14	8,66769E+14	7,70414E+14	7,38912E+14	7,62957E+14	7,57239E+14
TC1000003183.mm.2	Olfr796	olfactory receptor 796	-1,12353E+14	4,82816E+14	-3,79947E+14	0.00787749915533095	0.170747242106618	-2,09919E+14	4,02067E+14	4,0758E+14	5,27047E+14	5,07307E+14	5,33665E+14	5,19228E+14
TC0700000220.mm.2	Kptn	kaptin	1,50868E+14	5,6947E+14	3,79928E+13	0.00787931787943207	0.170747242106618	-2,09939E+14	6,42066E+14	6,57346E+14	5,45811E+12	4,51864E+14	5,47585E+14	5,72147E+14
TC1200002089.mm.2	Pgf	placental growth factor	-1,28238E+13	5,52869E+13	-3,79825E+14	0.00788933882226666	0.170747242106618	-2,10048E+14	4,96749E+14	4,39984E+14	5,82459E+14	6,10749E+14	5,95884E+14	5,9139E+14
TC0700000933.mm.2	Cyfip1	cytoplasmic FMR1 interacting protein 1	1,45342E+14	7,92358E+14	3,79516E+13	0.00791935306191486	0.170747242106618	-2,10373E+14	8,91918E+14	9,06677E+13	7,95151E+14	7,12759E+14	7,06224E+14	7,41421E+14
TC0X00002211.mm.2	Gpc3	glypican 3	1,28217E+14	5,46653E+14	3,79508E+14	0.00792009547931663	0.170747242106618	-2,10381E+14	6,71241E+14	6,17437E+14	5,15604E+14	5,1664E+14	4,62421E+14	4,96575E+14
TC0200000409.mm.2	Grin1os	glutamate receptor, ionotropic, NMDA1 (zeta 1), opposite strand	-1,60973E+14	7,10153E+14	-3,79392E+14	0.00793139248850209	0.170747242106618	-2,10503E+14	6,02212E+14	6,08881E+14	7,12212E+14	8,20828E+14	7,45225E+14	7,71563E+14
TC0700002683.mm.2	Zfp780b	zinc finger protein 780B	1,49887E+14	6,1597E+13	3,79332E+14	0.00793731128901833	0.170747242106618	-2,10567E+14	7,28727E+14	6,86095E+14	5,85489E+14	5,29559E+14	6,16581E+14	5,49371E+14
TC1000001809.mm.2	Arfgef3	ARFGEF family member 3	1,27129E+14	6,50832E+14	3,79295E+14	0.00794091792036149	0.170747242106618	-2,10606E+14	7,47973E+14	7,27014E+13	6,20534E+14	6,00194E+14	6,31923E+13	5,77354E+14
TC0700003007.mm.2	Bax	BCL2-associated X protein	-1,18383E+14	8,33647E+14	-3,79293E+14	0.00794112017664377	0.170747242106618	-2,10608E+14	7,34919E+14	7,673E+14	8,55042E+13	8,83943E+14	8,77725E+14	8,82957E+14
TC0600000921.mm.2	Ccdc142/Mrpl53	coiled-coil domain containing 142/mitochondrial ribosomal protein L53	-1,56687E+14	8,60156E+14	-3,79232E+14	0.00794706825810816	0.170747242106618	-2,10672E+14	7,39053E+13	7,45465E+14	9,47028E+14	8,50863E+14	9,61895E+14	9,16631E+14
TC0700003724.mm.2	Ccdc83	coiled-coil domain containing 83	-1,13793E+14	5,2379E+14	-3,79088E+14	0.00796109994762033	0.170747242106618	-2,10823E+14	4,39874E+14	4,54904E+14	5,49455E+14	5,72909E+14	5,55819E+14	5,6978E+14
TC1100002557.mm.2	Gabra1	gamma-aminobutyric acid (GABA) A receptor, subunit alpha 1	1,14474E+14	8,57266E+14	3,79023E+14	0.00796751770235888	0.170747242106618	-2,10892E+14	9,44549E+13	9,18442E+14	8,12776E+14	8,21268E+14	8,1379E+14	8,32772E+14
TC1600001254.mm.2	Mcm4	minichromosome maintenance complex component 4	1,12409E+14	5,34393E+13	3,78971E+14	0.00797260919274738	0.170747242106618	-2,10947E+14	6,14708E+14	6,1197E+14	4,96801E+14	5,05059E+14	5,01379E+14	4,76438E+14
TC1300001471.mm.2	Tbce	tubulin-specific chaperone E	2,20093E+14	5,64208E+14	3,78953E+14	0.00797436986453429	0.170747242106618	-2,10966E+14	7,11681E+14	7,04249E+14	5,73473E+14	4,02271E+14	5,25945E+11	4,67628E+14
TC1100003966.mm.2	Prkca	protein kinase C, alpha	2,25768E+14	6,75677E+14	3,7895E+13	0.00797465325660349	0.170747242106618	-2,10969E+14	8,27152E+14	8,20484E+14	6,83163E+13	5,12938E+14	6,43929E+14	5,66393E+14
TC0900000055.mm.2	Tmem123	transmembrane protein 123	-1,21529E+14	7,59266E+13	-3,78869E+14	0.00798264284215086	0.170747242106618	-2,11055E+14	6,76345E+14	7,02639E+14	7,89143E+14	8,32899E+14	7,78819E+14	7,75752E+14
TC1100004288.mm.2	Urgcp	upregulator of cell proliferation	1,11531E+14	7,08569E+14	3,78702E+13	0.00799906884776245	0.170747242106618	-2,11231E+14	7,74811E+14	7,58285E+14	6,49516E+14	6,60517E+14	7,10129E+14	6,98156E+14
TC1100001644.mm.2	Cntnap1	contactin associated protein-like 1	2,16716E+14	7,71519E+14	3,78698E+14	0.00799938572574806	0.170747242106618	-2,11234E+14	9,07508E+14	9,06644E+14	7,69181E+14	6,11539E+14	6,76346E+14	7,57894E+14
TC0600000765.mm.2	Ndnf	neuron-derived neurotrophic factor	1,8893E+13	5,82967E+14	3,7858E+14	0.00801100715988524	0.170747242106618	-2,11359E+14	6,96367E+14	7,218E+14	5,70825E+13	4,69483E+14	5,70888E+14	4,68438E+14
TSUnmapped00000024.mm.2	Gm21992	predicted gene 21992	1,13649E+14	7,11438E+14	3,78458E+14	0.00802305989430555	0.170747242106618	-2,11488E+14	7,89854E+14	7,82309E+14	6,87581E+14	6,57284E+14	6,76946E+13	6,74656E+14
TC0500003313.mm.2	Gusb	glucuronidase, beta	1,17918E+14	6,50939E+14	3,78376E+14	0.00803121679337116	0.170747242106618	-2,11575E+14	7,12811E+14	7,38673E+14	6,245E+14	5,91149E+14	6,14454E+13	6,24045E+14
TC1100001443.mm.2	Mrpl27	mitochondrial ribosomal protein L27	1,22141E+14	8,25293E+14	3,78342E+14	0.00803452056275756	0.170747242106618	-2,1161E+14	9,10199E+14	8,71209E+14	7,57412E+13	7,79713E+14	8,0275E+14	8,30476E+14
TC0200003140.mm.2	Ndor1	NADPH dependent diflavin oxidoreductase 1	-1,29293E+14	7,00724E+14	-3,78302E+14	0.00803850553263308	0.170747242106618	-2,11653E+14	5,92636E+14	6,2584E+14	7,13948E+14	7,63115E+14	7,69279E+14	7,39524E+14
TC0800001228.mm.2	4933405L10Rik	RIKEN cDNA 4933405L10 gene	-1,40504E+13	5,18361E+14	-3,78297E+14	0.00803900409707228	0.170747242106618	-2,11658E+14	4,01897E+14	4,29428E+14	5,16378E+14	5,95954E+14	5,80353E+14	5,86156E+14
TC1300001851.mm.2	Tfap2a	transcription factor AP-2, alpha	-1,5786E+14	5,29725E+14	-3,78224E+14	0.00804622106864673	0.170747242106618	-2,11735E+14	4,4853E+13	3,76529E+14	5,30167E+14	6,10614E+14	6,00535E+13	6,11973E+14
TC1600000618.mm.2	Zbtb20/Mir568	zinc finger and BTB domain containing 20/microRNA 568	2,33114E+14	7,65805E+14	3,78192E+14	0.00804938677620913	0.170747242106618	-2,11769E+14	9,38958E+14	9,14261E+13	7,81635E+14	6,05354E+14	7,17815E+14	6,36806E+13
TC0200004137.mm.2	Large2	LARGE xylosyl- and glucuronyltransferase 2	-2,11763E+14	6,27618E+14	-3,77988E+14	0.00806969825817532	0.170747242106618	-2,11985E+14	5,07468E+13	4,80609E+14	6,29533E+14	7,8207E+14	6,45064E+14	7,20964E+14
TC0500002306.mm.2	Bod1l	biorientation of chromosomes in cell division 1-like	2,2668E+14	6,43016E+14	3,77818E+14	0.00808652003808546	0.170747242106618	-2,12163E+14	7,82224E+14	7,68378E+14	5,84931E+13	5,12311E+14	6,92034E+13	5,18215E+14
TC1000003109.mm.2	Mbd6	methyl-CpG binding domain protein 6	-1,38194E+14	7,00345E+14	-3,7764E+14	0.00810428901101995	0.170747242106618	-2,12351E+14	6,07173E+14	6,23073E+14	7,3988E+13	7,66754E+14	6,95079E+14	7,70112E+14
TC0700000498.mm.2	Paf1	Paf1, RNA polymerase II complex component	1,33526E+14	7,54454E+14	3,77496E+14	0.00811870598430733	0.170747242106618	-2,12504E+14	8,63037E+14	8,50047E+14	7,45724E+14	7,00307E+13	7,12274E+14	6,55333E+14
TC1100001250.mm.2	Rasl10b	RAS-like, family 10, member B	1,3602E+14	8,1623E+13	3,77484E+14	0.00811990857885272	0.170747242106618	-2,12517E+14	9,28184E+14	9,21926E+14	8,06769E+14	7,713E+14	7,69186E+14	7,00018E+14
TC1500001724.mm.2	Cyp11b2	cytochrome P450, family 11, subfamily b, polypeptide 2	-1,34138E+14	4,5893E+13	-3,77426E+14	0.00812571447867906	0.170747242106618	-2,12578E+14	3,73636E+14	3,9311E+14	5,14565E+14	5,20457E+14	4,39804E+14	5,12008E+14
TC0300002657.mm.2	Tafa3	TAFA chemokine like family member 3	-1,29812E+14	5,95403E+14	-3,77312E+14	0.00813715981517804	0.170747242106618	-2,12699E+14	5,22286E+14	5,35664E+14	6,25612E+14	6,91961E+14	5,95977E+14	6,00917E+14
TC1100002155.mm.2	Pold2	polymerase (DNA directed), delta 2, regulatory subunit	-1,5276E+13	9,89924E+14	-3,77251E+13	0.00814330351675164	0.170747242106618	-2,12763E+14	8,83235E+14	8,96419E+14	9,99137E+14	1,08604E+14	1,01116E+14	1,06355E+14
TC0300001478.mm.2	Znhit6	zinc finger, HIT type 6	1,60459E+14	5,99512E+14	3,77068E+14	0.00816170304006866	0.170747242106618	-2,12957E+14	7,09117E+14	6,96694E+14	5,80627E+14	5,04265E+14	5,94072E+14	5,12299E+14
TC0700000420.mm.2	Dmac2	distal membrane arm assembly complex 2	1,21112E+14	7,02923E+13	3,77057E+14	0.00816278457763027	0.170747242106618	-2,12968E+13	7,92564E+14	7,6404E+13	6,78402E+14	6,35979E+14	6,70195E+14	6,76357E+14
TC1000000837.mm.2	Gpx4	glutathione peroxidase 4	1,23614E+14	8,93114E+14	3,76868E+14	0.00818180790853386	0.170747242106618	-2,13168E+14	9,69692E+14	9,71624E+14	8,63776E+14	8,30312E+14	8,84529E+14	8,38752E+14
TC1300002498.mm.2	Arhgef28	Rho guanine nucleotide exchange factor (GEF) 28	1,32234E+14	6,40737E+14	3,76751E+14	0.00819366797260413	0.170747242106618	-2,13292E+14	7,40938E+14	7,22366E+14	6,34027E+14	5,6481E+13	5,86392E+12	5,95887E+14
TC1700001791.mm.2	Ccdc167	coiled-coil domain containing 167	-1,27532E+14	7,57494E+14	-3,76626E+14	0.00820629930732657	0.170747242106618	-2,13424E+14	6,88903E+14	6,77905E+14	7,83862E+14	8,38011E+14	7,61738E+13	7,94542E+14
TC0900002934.mm.2	Il20rb	interleukin 20 receptor beta	-1,16041E+14	5,12391E+14	-3,7658E+14	0.00821096940951074	0.170747242106618	-2,13473E+14	4,29247E+14	4,55067E+14	5,55637E+14	5,6076E+14	5,21807E+14	5,51831E+14
TC1900000439.mm.2	Jak2	Janus kinase 2	2,6035E+14	5,97081E+14	3,76523E+14	0.0082167202559881	0.170747242106618	-2,13533E+14	7,41996E+14	7,90426E+14	6,02302E+14	4,0942E+14	5,72384E+14	4,65961E+14
TC0500003709.mm.2	Babam2	BRISC and BRCA1 A complex member 2	1,6698E+13	8,21415E+14	3,76481E+14	0.00822096046708459	0.170747242106618	-2,13578E+14	9,21827E+14	9,13053E+14	7,92669E+14	7,08251E+14	8,39587E+14	7,53105E+13
TC0900000262.mm.2	Opcml	opioid binding protein/cell adhesion molecule-like	1,39042E+14	9,33741E+14	3,76413E+14	0.00822785201564293	0.170747242106618	-2,1365E+14	1,00778E+14	1,02601E+14	9,03636E+14	8,52073E+14	9,37856E+14	8,75086E+14
TC0900002594.mm.2	Rnf111	ring finger 111	-1,14603E+14	7,63665E+14	-3,76386E+14	0.00823064519812714	0.170747242106618	-2,13679E+14	7,07662E+14	7,09199E+14	8,40865E+14	8,05201E+14	7,62336E+14	7,56727E+14
TC0900001599.mm.2	Mobp	myelin-associated oligodendrocytic basic protein	1,09574E+13	8,36904E+14	3,76192E+14	0.00825039059724348	0.170747242106618	-2,13884E+14	9,00456E+14	9,13737E+14	7,99362E+14	7,95683E+14	8,01003E+14	8,11185E+14
TC0X00002668.mm.2	Asb12	ankyrin repeat and SOCS box-containing 12	-2,19253E+14	6,40618E+14	-3,75991E+14	0.00827085635221354	0.170747242106618	-2,14097E+14	4,69543E+14	5,31251E+14	6,36157E+14	8,03143E+14	6,82938E+13	7,20676E+14
TC1500001584.mm.2	Tatdn1	TatD DNase domain containing 1	1,20478E+14	6,82625E+14	3,75787E+14	0.00829171639310751	0.170747242106618	-2,14313E+14	7,78042E+14	7,58409E+14	6,69997E+14	6,25498E+14	6,39583E+13	6,2422E+14
TC0200000354.mm.2	Pdss1	prenyl (solanesyl) diphosphate synthase, subunit 1	-1,32242E+14	5,5612E+14	-3,75451E+14	0.00832625654773303	0.170747242106618	-2,1467E+14	4,87445E+14	4,47373E+14	6,10369E+13	5,88933E+13	6,29905E+14	5,72697E+14
TC0800000509.mm.2	Fat1	FAT atypical cadherin 1	1,23004E+14	6,69826E+14	3,75439E+14	0.00832746047370217	0.170747242106618	-2,14682E+14	7,66636E+14	7,54779E+14	6,14662E+14	6,60745E+14	6,26258E+14	5,95873E+14
TC0700000155.mm.2	Trim28	tripartite motif-containing 28	1,27424E+14	6,12932E+14	3,75375E+12	0.00833402215914152	0.170747242106618	-2,1475E+14	6,98939E+14	6,89832E+14	5,83523E+14	5,504E+14	5,49961E+14	6,04936E+14
TC1200000562.mm.2	Lrr1	leucine rich repeat protein 1	-1,1654E+14	4,74854E+13	-3,75279E+14	0.00834388073875482	0.170747242106618	-2,14852E+14	4,33316E+14	4,0149E+14	5,41489E+14	5,26397E+14	4,84882E+14	4,61548E+14
TC0100002392.mm.2	Trak2	trafficking protein, kinesin binding 2	2,13688E+14	7,43779E+14	3,75242E+14	0.0083477489131494	0.170747242106618	-2,14891E+14	8,58795E+14	8,89756E+14	7,17363E+14	6,03812E+14	7,62122E+14	6,30827E+14
TC1100003943.mm.2	Bptf	bromodomain PHD finger transcription factor	1,48885E+14	7,05695E+14	3,75136E+14	0.00835867353655225	0.170747242106618	-2,15004E+14	8,19458E+14	8,18467E+14	6,8028E+14	6,59874E+14	6,76066E+14	5,80028E+14
TC0100001317.mm.2	Uchl5	ubiquitin carboxyl-terminal esterase L5	2,46492E+13	7,01587E+14	3,75135E+14	0.00835880921621151	0.170747242106618	-2,15005E+14	8,34907E+14	8,38449E+14	6,91461E+14	4,88909E+14	7,09007E+14	6,46788E+14
TC1400000998.mm.2	Phyhip	phytanoyl-CoA hydroxylase interacting protein	1,4491E+14	9,40448E+14	3,75025E+14	0.00837013696276359	0.170747242106618	-2,15121E+14	1,03399E+14	1,03981E+14	9,35165E+14	8,48819E+14	9,09E+14	8,75904E+14
TC1600002084.mm.2	Cryzl1	crystallin, zeta (quinone reductase)-like 1	2,86423E+14	5,42099E+13	3,74983E+14	0.00837449143132757	0.170747242106618	-2,15166E+13	7,27212E+14	7,22943E+14	5,29357E+13	3,47952E+13	5,51297E+14	3,73832E+14
TC1100003692.mm.2	Gid3	gap junction protein, delta 3	-1,43118E+14	6,00773E+14	-3,74946E+14	0.00837834465363979	0.170747242106618	-2,15206E+14	5,0582E+14	5,03298E+14	6,0707E+14	6,88283E+14	6,30597E+14	6,6957E+14
TC0X00003146.mm.2	Lrch2	leucine-rich repeats and calponin homology (CH) domain containing 2	1,54534E+14	6,21824E+14	3,74933E+14	0.00837968188124142	0.170747242106618	-2,15219E+14	7,26435E+14	6,93272E+13	6,0022E+14	5,1042E+14	5,77565E+14	6,2303E+14
TC0700003971.mm.2	Olfr685	olfactory receptor 685	-1,40238E+14	5,41639E+14	-3,74867E+14	0.00838655668216068	0.170747242106618	-2,1529E+14	4,50969E+14	4,60654E+14	5,68284E+14	6,23815E+13	5,40636E+14	6,05476E+14
TC0X00000332.mm.2	Sowahd	sosondowah ankyrin repeat domain family member D	-1,21379E+14	5,31477E+14	-3,74846E+14	0.00838873671568374	0.170747242106618	-2,15312E+14	4,68871E+13	4,35172E+14	5,71781E+14	5,7502E+14	5,48263E+14	5,89754E+14
TC0500000297.mm.2	Slc35f6/Mir5625	solute carrier family 35, member F6/microRNA 5625	1,20306E+14	7,51926E+14	3,74792E+14	0.00839428607258655	0.170747242106618	-2,15369E+14	8,23681E+14	8,23486E+14	7,13924E+14	6,9263E+13	7,52266E+14	7,05569E+12
TC1000000813.mm.2	Hcn2	hyperpolarization-activated, cyclic nucleotide-gated K+ 2	1,78724E+14	7,33738E+14	3,74652E+14	0.00840883495458264	0.170747242106618	-2,15518E+14	8,543E+14	8,31986E+14	7,06622E+14	6,22217E+14	7,46158E+14	6,41143E+14
TC0300000307.mm.2	Hspa4l	heat shock protein 4 like	2,02122E+14	7,4917E+14	3,74525E+13	0.00842211804605355	0.170747242106618	-2,15653E+14	8,96677E+14	8,94141E+14	7,26171E+14	6,60404E+14	7,33828E+14	5,83799E+14
TC0X00003430.mm.2	Arhgap4	Rho GTPase activating protein 4	-1,37484E+14	5,92634E+14	-3,74486E+14	0.00842618563722223	0.170747242106618	-2,15695E+14	5,42804E+14	4,78878E+14	6,35739E+14	6,6091E+14	5,96392E+13	6,41077E+14
TC1000000661.mm.2	Ank3	ankyrin 3, epithelial	1,68535E+14	7,68505E+14	3,74311E+14	0.00844436311218729	0.170747242106618	-2,1588E+14	8,91381E+14	8,98216E+14	7,53572E+14	6,98954E+14	7,40189E+14	6,28717E+14
TC1800000373.mm.2	Pcdhb16	protocadherin beta 16	1,64462E+14	5,8074E+14	3,74221E+13	0.00845381811169346	0.170747242106618	-2,15976E+14	6,45491E+14	6,60323E+14	5,04976E+14	4,71915E+13	6,57678E+14	5,44057E+14
TC1500001944.mm.2	Cyp2d22	cytochrome P450, family 2, subfamily d, polypeptide 22	1,21825E+14	7,3033E+14	3,7419E+14	0.00845711065647515	0.170747242106618	-2,16009E+14	8,31009E+14	8,07472E+14	7,21905E+14	6,72926E+14	6,78264E+14	6,70404E+14
TC1300002095.mm.2	Ubqln1	ubiquilin 1	1,20247E+13	8,65951E+14	3,74177E+14	0.00845837442869685	0.170747242106618	-2,16022E+14	9,44265E+14	9,47393E+14	8,46246E+13	8,04918E+13	8,41828E+14	8,11055E+14
TC0400000439.mm.2	Creb3	cAMP responsive element binding protein 3	1,32285E+14	8,54315E+14	3,74167E+14	0.00845950870300945	0.170747242106618	-2,16034E+14	9,31108E+14	9,16812E+14	8,25452E+14	7,57898E+14	8,60448E+14	8,34175E+14
TC0500002420.mm.2	Sel1l3	sel-1 suppressor of lin-12-like 3 (C. elegans)	1,64041E+14	6,31325E+14	3,73845E+14	0.00849327250823868	0.170747242106618	-2,16376E+14	6,92948E+14	7,35496E+14	5,84383E+13	5,15979E+14	6,73321E+14	5,85821E+14
TC0800000732.mm.2	Sugp1	SURP and G patch domain containing 1	1,15264E+14	7,23595E+14	3,73782E+14	0.0084999276739478	0.170747242106618	-2,16443E+14	8,15952E+14	7,77629E+14	6,75643E+14	6,8741E+14	6,93631E+14	6,91305E+14
TC0500001134.mm.2	Golga3	golgi autoantigen, golgin subfamily a, 3	1,41327E+14	5,7459E+14	3,73764E+14	0.00850182038718895	0.170747242106618	-2,16463E+14	6,77767E+14	6,78755E+14	5,76872E+14	4,96995E+14	5,2721E+14	4,89938E+13
TC1500001520.mm.2	Samd12	sterile alpha motif domain containing 12	1,57989E+14	7,80857E+14	3,73707E+14	0.00850773695738674	0.170747242106618	-2,16522E+14	8,33107E+14	8,8106E+14	7,27602E+14	6,70586E+14	8,2768E+14	7,45105E+14
TC1300001069.mm.2	Wdr41	WD repeat domain 41	1,23046E+14	8,03573E+14	3,737E+13	0.00850847343138995	0.170747242106618	-2,1653E+14	8,94187E+14	8,71918E+14	7,86509E+14	7,33504E+14	7,69908E+14	7,65414E+14
TC0100000259.mm.2	Inpp4a	inositol polyphosphate-4-phosphatase, type I	1,71301E+13	7,81747E+14	3,73695E+14	0.0085090552381424	0.170747242106618	-2,16536E+13	8,89204E+14	8,88636E+14	7,72062E+14	6,63177E+14	7,72433E+14	7,04972E+14
TC1400002744.mm.2	Gpr18	G protein-coupled receptor 18	-1,37578E+13	4,97713E+14	-3,73651E+14	0.00851366703934041	0.170747242106618	-2,16582E+14	4,1231E+14	3,90223E+14	5,04554E+14	5,73135E+14	5,32859E+14	5,73197E+14
TC1000001131.mm.2	Lta4h	leukotriene A4 hydrolase	1,14137E+14	6,72395E+14	3,735E+14	0.00852963179143431	0.170747242106618	-2,16743E+14	7,38331E+13	7,25935E+14	6,06915E+14	6,29077E+14	6,53383E+14	6,80728E+14
TC0100000387.mm.2	Osgepl1	O-sialoglycoprotein endopeptidase-like 1	1,13902E+14	6,59937E+14	3,73432E+14	0.00853685515803398	0.170747242106618	-2,16816E+13	7,29971E+14	7,39898E+14	6,03376E+14	6,38689E+14	6,23155E+13	6,24534E+14
TC0300002306.mm.2	Tmem79	transmembrane protein 79	-1,25214E+14	6,4082E+14	-3,72809E+14	0.00860293356251208	0.170747242106618	-2,17479E+14	5,51543E+14	5,54881E+14	6,54201E+14	7,02651E+13	6,71252E+14	7,10392E+14
TC0700002763.mm.2	Arhgap33	Rho GTPase activating protein 33	1,60963E+14	7,23799E+14	3,72781E+14	0.00860593564976374	0.170747242106618	-2,17509E+14	8,40921E+14	8,54504E+14	7,37774E+14	6,35725E+13	6,10658E+14	6,63216E+14
TC0300001982.mm.2	Nhlrc3	NHL repeat containing 3	-1,14231E+14	6,10752E+14	-3,72719E+14	0.00861258536221433	0.170747242106618	-2,17575E+14	5,57961E+14	5,91998E+14	6,95598E+14	6,82824E+14	5,62125E+14	5,74005E+14
TC0100002409.mm.2	Ica1l	islet cell autoantigen 1-like	1,44828E+14	6,66271E+13	3,72664E+14	0.00861846516643253	0.170747242106618	-2,17634E+14	7,50472E+14	7,50022E+14	6,31627E+14	5,7921E+14	6,82069E+14	6,04228E+14
TC1000002017.mm.2	Fam229b	family with sequence similarity 229, member B	1,08244E+14	5,88835E+14	3,72548E+14	0.00863087209921052	0.170747242106618	-2,17758E+14	6,27021E+14	6,14074E+14	5,11203E+14	5,13405E+14	6,37751E+14	6,29553E+14
TC1500000775.mm.2	Bik	BCL2-interacting killer	-1,44041E+14	8,01453E+14	-3,72391E+14	0.00864771834232071	0.170747242106618	-2,17925E+14	6,84394E+13	7,21893E+14	8,07141E+14	8,87227E+14	8,39848E+14	8,68216E+14
TC0500001632.mm.2	Sap25/Lrch4/Gm20605	sin3 associated polypeptide/leucine-rich repeats and calponin homology (CH) domain containing 4/predicted gene 20605	-1,53358E+14	8,72472E+13	-3,72284E+14	0.00865910589490951	0.170747242106618	-2,18039E+14	7,60598E+14	7,77233E+14	9,62089E+14	8,8246E+14	9,60099E+14	8,92353E+14
TC0200003526.mm.2	Rbm43	RNA binding motif protein 43	1,27328E+14	5,72727E+14	3,72217E+14	0.00866632929183181	0.170747242106618	-2,1811E+14	6,89137E+14	6,5251E+14	5,67434E+14	5,19558E+14	5,17991E+14	4,89733E+14
TC0700000435.mm.2	Cyp2b9	cytochrome P450, family 2, subfamily b, polypeptide 9	-1,72921E+13	4,92869E+14	-3,72093E+14	0.00867969046186174	0.170747242106618	-2,18243E+14	4,04747E+14	3,94378E+14	5,14275E+14	6,30692E+14	4,76986E+14	5,36132E+14
TC0700003592.mm.2	Man2a2	mannosidase 2, alpha 2	1,19223E+14	7,92784E+14	3,72052E+14	0.00868409021644377	0.170747242106618	-2,18287E+14	8,73515E+14	8,94453E+14	7,84855E+14	7,44666E+14	7,41176E+14	7,18036E+14
TC0800002447.mm.2	Cyp4f18	cytochrome P450, family 4, subfamily f, polypeptide 18	-1,93357E+14	7,16894E+14	-3,71961E+14	0.00869391700649996	0.170747242106618	-2,18384E+14	5,63643E+14	6,18803E+14	7,20069E+14	8,49092E+14	7,41535E+13	8,08223E+14
TC0500002577.mm.2	Gm5868	predicted gene 5868	-1,3874E+14	6,46358E+14	-3,71894E+14	0.0087011354480371	0.170747242106618	-2,18455E+14	5,73637E+13	5,42857E+14	6,61185E+14	7,3279E+14	6,66464E+13	7,01214E+13
TC0900003050.mm.2	Rnf123	ring finger protein 123	1,24982E+14	6,54153E+14	3,71775E+14	0.00871402174949009	0.170747242106618	-2,18583E+14	7,6538E+14	7,36552E+14	6,17113E+13	6,34854E+14	6,11832E+14	5,59186E+14

TC0900000752.mm.2	Gramd2	GRAM domain containing 2	-1,5271E+14	5,9379E+14	-3,71754E+14	0.00871626600174821	0.170747242106618	-2,18605E+14	4,86415E+14	4,95195E+13	6,01586E+14	6,85444E+14	6,15523E+14	6,78575E+13
TC0500000534.mm.2	Anapc4	anaphase promoting complex subunit 4	2,47509E+14	6,35456E+14	3,71675E+14	0.00872484186231105	0.170747242106618	-2,18689E+14	7,89809E+14	7,7658E+14	6,22245E+14	4,49127E+14	6,5127E+14	5,23708E+14
TC0600001027.mm.2	Rpn1	ribophorin I	1,56916E+14	7,10206E+14	3,71658E+14	0.00872671104634502	0.170747242106618	-2,18708E+14	8,20434E+14	8,23994E+13	7,14652E+14	6,15944E+14	6,68369E+14	6,17846E+14
TC0400004193.mm.2	Emc1	ER membrane protein complex subunit 1	1,10752E+14	7,18576E+14	3,71623E+14	0.00873052414509412	0.170747242106618	-2,18745E+14	8,13816E+14	8,07758E+14	7,07739E+14	6,92332E+13	6,33382E+13	6,56427E+14
TC1700002719.mm.2	Lhcgr	luteinizing hormone/choriogonadotropin receptor	-1,66199E+14	4,35417E+14	-3,71589E+14	0.0087341949296589	0.170747242106618	-2,18781E+14	3,46985E+14	3,0051E+14	4,41298E+14	5,38595E+14	4,63044E+14	5,22071E+14
TC1400001107.mm.2	Sugt1	SGT1, suppressor of G2 allele of SKP1 (S. cerevisiae)	1,96443E+14	7,25647E+14	3,71243E+14	0.00877179860272679	0.170747242106618	-2,19151E+14	8,36532E+14	8,4298E+14	7,12114E+14	5,74511E+14	7,33927E+14	6,53817E+14
TC0200001809.mm.2	Map1a	microtubule-associated protein 1 A	1,67504E+14	8,20445E+14	3,71069E+14	0.00879077275726587	0.170747242106618	-2,19337E+14	9,55053E+14	9,22989E+14	7,6263E+13	7,80403E+14	8,10475E+14	6,91119E+13
TC1700002324.mm.2	Ptprs	protein tyrosine phosphatase, receptor type, S	1,42926E+14	9,52432E+14	3,70956E+14	0.00880311418271807	0.170747242106618	-2,19458E+14	1,04245E+14	1,05451E+14	9,48863E+14	8,62246E+14	9,17638E+14	8,88884E+14
TC1300001806.mm.2	Lyrm4	LYR motif containing 4	1,35109E+14	7,92066E+14	3,70696E+14	0.00883162636010369	0.170747242106618	-2,19736E+14	8,93445E+14	8,93164E+12	7,9441E+14	7,21982E+14	7,42171E+14	7,07224E+14
TC0700004475.mm.2	Stk32c	serine/threonine kinase 32C	1,23098E+13	7,17848E+14	3,70659E+14	0.00883561439062343	0.170747242106618	-2,19775E+14	7,75001E+14	8,0979E+14	6,68546E+14	6,70049E+14	7,16115E+14	6,67588E+14
TC0700003652.mm.2	Arnt2	aryl hydrocarbon receptor nuclear translocator 2	1,28522E+13	8,54696E+14	3,70558E+14	0.00884677866749389	0.170747242106618	-2,19884E+13	9,39825E+14	9,32617E+14	8,34126E+14	7,81271E+14	8,42575E+14	7,97764E+14
TC0900002159.mm.2	Vps11	VPS11, CORVET/HOPS core subunit	1,55991E+14	7,19242E+14	3,70485E+14	0.00885481704802841	0.170747242106618	-2,19962E+14	8,36238E+14	8,2863E+14	7,03315E+14	6,49573E+14	6,969E+14	6,00793E+12
TC1900000232.mm.2	Mrpl16	mitochondrial ribosomal protein L16	1,10892E+14	5,80302E+14	3,70463E+14	0.00885721006971641	0.170747242106618	-2,19985E+14	6,69179E+14	6,84587E+14	5,57053E+14	5,74929E+14	4,88691E+14	5,07374E+14
TC0700001721.mm.2	Tmem159	transmembrane protein 159	1,1273E+13	5,391E+14	3,7046E+13	0.00885753601174225	0.170747242106618	-2,19988E+14	5,87876E+13	6,16763E+14	4,96261E+14	4,82917E+14	5,3374E+14	5,17042E+14
TC0100001397.mm.2	Stx6	syntaxin 6	1,27257E+14	7,80186E+14	3,70446E+14	0.00885904236584	0.170747242106618	-2,20003E+14	8,82776E+14	8,59737E+14	7,75388E+14	7,12613E+14	7,2999E+14	7,20611E+14
TC0400004001.mm.2	Miip	migration and invasion inhibitory protein	-1,58933E+14	6,01243E+14	-3,7039E+13	0.00886528798607426	0.170747242106618	-2,20064E+14	4,80552E+14	5,0379E+14	6,05308E+14	6,96901E+14	6,28306E+14	6,926E+13
TC0300000935.mm.2	Hist2h2bb	histone cluster 2, H2bb	1,30328E+12	7,14975E+14	3,70382E+14	0.00886617473503309	0.170747242106618	-2,20072E+14	7,31771E+14	7,81342E+14	6,52777E+14	5,9968E+14	7,64875E+14	7,59408E+14
TC1900001621.mm.2	Atp5md	ATP synthase membrane subunit DAPIT	2,28336E+14	1,00429E+14	3,69968E+14	0.00891186188900251	0.170747242106618	-2,20515E+14	1,14352E+14	1,13107E+14	9,92697E+14	8,25218E+14	1,01702E+14	9,16189E+14
TC0400001212.mm.2	Gm12830	predicted gene 12830	-1,46912E+14	4,87353E+13	-3,69893E+14	0.00892021540984091	0.170747242106618	-2,20596E+14	4,10194E+14	4,08766E+14	5,09489E+14	6,03294E+14	4,81446E+14	5,10927E+14
TC1900000598.mm.2	Frat1	frequently rearranged in advanced T cell lymphomas	-1,48899E+14	8,10927E+14	-3,69886E+14	0.00892096490502644	0.170747242106618	-2,20603E+14	7,17928E+14	6,95404E+14	8,0628E+14	9,04851E+14	8,70874E+14	8,70228E+14
TC1100004207.mm.2	Oxld1	oxidoreductase like domain containing 1	-1,20681E+14	5,70131E+14	-3,69884E+14	0.00892118624934519	0.170747242106618	-2,20605E+14	5,16822E+13	5,12457E+13	6,08294E+14	6,62348E+14	5,6618E+14	5,54686E+14
TC0500002882.mm.2	Hsd17b11	hydroxysteroid (17-beta) dehydrogenase 11	1,19717E+14	7,73108E+14	3,69826E+14	0.00892763812738556	0.170747242106618	-2,20667E+14	8,38328E+14	8,53672E+14	7,24109E+14	7,28457E+14	7,72562E+14	7,21522E+14
TC0900001859.mm.2	Cdc37	cell division cycle 37	1,24896E+14	6,92881E+14	3,69775E+13	0.00893329811225861	0.170747242106618	-2,20722E+14	7,92656E+14	7,50665E+14	6,67691E+14	6,25838E+13	6,71833E+14	6,486E+14
TC0800002725.mm.2	Bbs2	Bardet-Biedl syndrome 2 (human)	1,60758E+14	6,01608E+14	3,69679E+14	0.00894395050442797	0.170747242106618	-2,20825E+14	6,72556E+13	7,25144E+14	5,85471E+14	4,90713E+14	5,45755E+14	5,90008E+14
TC1600001380.mm.2	Dgkg	diacylglycerol kinase, gamma	1,71238E+14	8,30522E+14	3,69602E+14	0.00895255540797758	0.170747242106618	-2,20907E+14	9,60126E+14	9,65632E+14	8,35449E+14	7,47833E+14	7,85218E+14	6,88874E+14
TC1400002027.mm.2	Chd8	chromodomain helicase DNA binding protein 8	1,51297E+14	5,4613E+14	3,69349E+14	0.0089807603277656	0.170747242106618	-2,21178E+14	6,28271E+14	6,32868E+14	5,30705E+14	4,27839E+13	5,38044E+14	5,19051E+14
TC0800002958.mm.2	Chst5	carbohydrate (N-acetylglucosamine 6-O) sulfotransferase 5	-1,29706E+14	4,56688E+14	-3,69287E+14	0.00898771398933587	0.170747242106618	-2,21245E+14	3,83422E+14	3,73651E+14	4,78055E+14	5,38429E+14	4,63869E+14	5,02701E+14
TC0X00001515.mm.2	Fam120c	family with sequence similarity 120, member C	1,43028E+14	7,02785E+14	3,69269E+14	0.00898978672842809	0.170747242106618	-2,21265E+13	7,99234E+14	7,94072E+14	6,98159E+14	6,09092E+14	6,71471E+14	6,44685E+14
TC1200001269.mm.2	Cep170b	centrosomal protein 170B	1,39352E+14	7,36199E+13	3,69205E+14	0.00899692427994107	0.170747242106618	-2,21333E+14	8,17643E+14	8,19586E+13	7,05839E+14	6,52685E+14	6,75808E+13	7,45633E+14
TC0300001382.mm.2	Ppp3ca	protein phosphatase 3, catalytic subunit, alpha isoform	1,98096E+14	1,07278E+14	3,69166E+14	0.00900134621190349	0.170747242106618	-2,21376E+14	1,18976E+13	1,20758E+14	1,07482E+14	9,26326E+14	1,0509E+13	9,87283E+14
TC1600001395.mm.2	Masp1	mannan-binding lectin serine peptidase 1	1,0831E+13	6,59241E+13	3,69143E+14	0.00900390670152375	0.170747242106618	-2,214E+14	7,09689E+14	7,26266E+14	6,12244E+14	6,07091E+14	6,56262E+14	6,43894E+14
TC0X00000990.mm.2	Pin4	protein (peptidyl-prolyl cis/trans isomerase) NIMA-interacting, 4 (parvulin)	1,84542E+14	7,16499E+14	3,6901E+14	0.00901878234443076	0.170747242106618	-2,21542E+14	8,44068E+14	8,28713E+14	6,84015E+14	6,19683E+13	7,26784E+14	5,95734E+14
TC1300000209.mm.2	Vmn1r201	vomeronasal 1 receptor 201	-1,79949E+14	4,32036E+12	-3,68992E+14	0.00902081004095005	0.170747242106618	-2,21562E+13	3,4032E+14	3,01336E+14	4,41126E+14	5,60429E+14	4,42032E+14	5,06972E+14
TC0600003116.mm.2	Eno2	enolase 2, gamma neuronal	1,90843E+13	8,23066E+14	3,68971E+13	0.00902322674322521	0.170747242106618	-2,21585E+14	9,51476E+14	9,46493E+14	8,30025E+14	6,86259E+14	7,89892E+14	7,3425E+14
TC1700001847.mm.2	Zfp871	zinc finger protein 871	3,25184E+14	6,37199E+14	3,6892E+14	0.0090289440754183	0.170747242106618	-2,21639E+14	7,69829E+14	8,27776E+14	6,15073E+14	3,32164E+14	6,77627E+14	6,00725E+14
TC0500001202.mm.2	Aym1	activator of yeast meiotic promoters 1	-1,6668E+14	6,12168E+14	-3,68862E+14	0.00903540907135174	0.170747242106618	-2,21701E+13	4,75746E+14	5,43835E+14	6,27183E+14	7,25759E+14	6,32281E+14	6,68206E+14
TC1600000738.mm.2	Rpl24/Zbtb11	ribosomal protein L24/zinc finger and BTB domain containing 11	1,18535E+14	9,2656E+14	3,68846E+14	0.00903729868291161	0.170747242106618	-2,21719E+14	9,98399E+14	9,82096E+14	8,95291E+14	8,48135E+14	9,111E+14	9,24342E+14
TC0200005476.mm.2	Nup35	nucleoporin 35	1,43252E+14	6,15884E+14	3,68836E+14	0.00903836492472938	0.170747242106618	-2,21729E+13	7,31354E+14	7,27819E+14	6,12747E+14	5,59922E+14	5,70394E+14	4,93064E+14
TC0600003124.mm.2	Cd4	CD4 antigen	1,99709E+13	6,99106E+14	3,68773E+12	0.00904548784810998	0.170747242106618	-2,21797E+14	8,24167E+14	8,63915E+14	7,16735E+14	5,7193E+14	6,42683E+14	5,75207E+14
TC0500001669.mm.2	Get4	golgi to ER traffic protein 4	1,51509E+14	6,81772E+14	3,68758E+13	0.00904713673787408	0.170747242106618	-2,21813E+14	7,5013E+14	7,89819E+14	6,52498E+14	5,84433E+14	6,91822E+14	6,2193E+14
TC0400001239.mm.2	Mknk1	MAP kinase-interacting serine/threonine kinase 1	1,49719E+13	5,83644E+13	3,68734E+14	0.00904981817126306	0.170747242106618	-2,21838E+14	6,51062E+14	6,56935E+14	5,46158E+14	4,624E+13	5,62924E+14	6,22387E+14
TC1200000563.mm.2	Mgat2	mannoside acetylglucosaminyltransferase 2	2,19986E+14	6,87713E+14	3,6871E+14	0.00905261377870517	0.170747242106618	-2,21865E+14	8,62787E+14	8,22719E+14	7,09252E+14	5,36282E+14	6,27791E+14	5,67446E+14
TC0600001536.mm.2	Gabarapl1	gamma-aminobutyric acid (GABA) A receptor-associated protein-like 1	1,19295E+14	1,01577E+14	3,68504E+14	0.00907584669143211	0.170747242106618	-2,22086E+14	1,09874E+14	1,0957E+14	9,96505E+14	9,5935E+14	9,91341E+12	9,52986E+14
TC0200003316.mm.2	Lrsam1	leucine rich repeat and sterile alpha motif containing 1	1,14868E+14	6,35826E+13	3,68348E+14	0.00909354688854673	0.170747242106618	-2,22254E+13	7,1076E+14	6,98858E+14	5,99424E+14	5,80457E+14	6,31357E+14	5,94098E+14
TC0200005512.mm.2	Nfs1	nitrogen fixation gene 1 (S. cerevisiae)	1,17647E+14	6,02791E+14	3,68328E+14	0.00909578750979881	0.170747242106618	-2,22275E+14	6,95481E+14	7,12039E+14	6,07445E+14	5,64781E+14	5,28615E+14	5,08387E+14
TC1100001143.mm.2	Fam222b	family with sequence similarity 222, member B	-1,22187E+14	8,6063E+13	-3,68276E+13	0.0091016625407451	0.170747242106618	-2,22331E+14	7,83619E+14	7,78779E+14	9,32996E+14	8,73776E+14	8,98838E+14	8,95771E+14
TC1100001527.mm.2	Scrn2	secernin 2	-2,11192E+13	7,55873E+14	-3,68273E+14	0.00910197542669402	0.170747242106618	-2,22334E+13	5,87525E+14	6,42926E+14	7,57229E+14	8,95606E+14	7,77341E+14	8,74608E+14
TC0X00002712.mm.2	Snx12	sorting nexin 12	2,16193E+13	6,58632E+14	3,68272E+14	0.00910207918795963	0.170747242106618	-2,22335E+14	7,9339E+13	8,08554E+14	6,41479E+14	5,28078E+14	6,61936E+14	5,18354E+14
TC0200001935.mm.2	Mertk	MER proto-oncogene tyrosine kinase	1,5906E+14	6,01443E+14	3,68046E+14	0.00912784711815045	0.170747242106618	-2,22578E+14	7,08402E+13	7,12836E+13	5,72717E+14	5,30401E+14	4,88314E+14	5,95989E+14
TC1300000281.mm.2	D130043K22Rik	RIKEN cDNA D130043K22 gene	1,29121E+14	6,00914E+14	3,67989E+14	0.00913426652877623	0.170747242106618	-2,22639E+14	6,70632E+14	6,93682E+14	5,87247E+13	5,18825E+14	5,68562E+14	5,66535E+14
TC1400002496.mm.2	Pcdh8	protocadherin 8	1,07607E+14	6,68888E+14	3,67908E+14	0.00914353911146022	0.170747242106618	-2,22727E+14	7,44613E+14	7,25983E+12	6,29454E+14	6,25928E+14	6,41172E+14	6,46178E+14
TC0200000057.mm.2	Upf2	UPF2 regulator of nonsense transcripts homolog (yeast)	1,50839E+14	7,16102E+14	3,6782E+14	0.00915351438230755	0.170747242106618	-2,22821E+14	8,09739E+14	8,17979E+14	6,63436E+12	6,62604E+14	7,2373E+14	6,19122E+14
TC0900000845.mm.2	Spg21	SPG21, maspardin	1,96602E+14	6,4226E+14	3,67751E+14	0.00916142835656355	0.170747242106618	-2,22895E+14	7,40648E+14	7,67759E+14	6,24657E+13	4,90546E+14	5,71907E+14	6,58044E+14
TC0800003242.mm.2	Trim61/Trim60	tripartite motif-containing 61/tripartite motif-containing 60	-1,49649E+14	6,59886E+14	-3,6767E+14	0.00917069528647442	0.170747242106618	-2,22982E+14	5,77371E+13	5,73685E+14	6,80246E+14	7,70108E+14	6,53457E+14	7,04449E+14
TC1100003775.mm.2	Acly	ATP citrate lyase	1,8358E+14	7,6319E+14	3,67661E+14	0.00917170040158508	0.170747242106618	-2,22992E+14	9,19713E+13	8,67352E+14	7,75738E+14	6,44167E+14	6,67409E+14	7,04761E+14
TC1700000913.mm.2	Guca1b	guanylate cyclase activator 1B	-1,09899E+14	6,77131E+14	-3,67447E+14	0.00919624569596852	0.170747242106618	-2,23222E+14	6,08571E+14	6,12991E+14	7,09503E+14	7,31856E+14	6,90345E+14	7,09523E+14
TC1300002510.mm.2	Tnpo1	transportin 1	1,13676E+14	7,44077E+14	3,67443E+14	0.00919671063990663	0.170747242106618	-2,23227E+14	8,13661E+13	8,17619E+14	7,17419E+14	6,86508E+14	7,28049E+14	7,01205E+14
TC0200004608.mm.2	Pdyn	prodynorphin	1,27231E+14	7,65132E+14	3,6738E+13	0.00920393658848335	0.170747242106618	-2,23294E+14	8,82043E+14	8,36123E+14	7,42321E+14	7,21383E+14	6,80689E+14	7,28235E+12
TC0300000948.mm.2	Hjv	hemojuvelin BMP co-receptor	-1,48633E+14	5,01141E+14	-3,6734E+13	0.00920851888253513	0.170747242106618	-2,23337E+14	4,18969E+14	4,05325E+14	5,11089E+14	6,10471E+14	5,40186E+14	5,20808E+14
TC1700000625.mm.2	H2-Ob	histocompatibility 2, O region beta locus	-1,28546E+14	5,76644E+14	-3,67236E+14	0.00922047445735036	0.170747242106618	-2,23449E+14	4,95557E+13	4,88802E+14	6,08991E+14	6,3246E+14	6,50733E+14	5,83323E+14
TC0100001452.mm.2	Mrps14	mitochondrial ribosomal protein S14	1,15104E+14	7,43013E+14	3,66926E+14	0.00925626769258667	0.170747242106618	-2,23783E+14	8,02241E+14	8,05497E+14	6,90219E+14	6,87311E+14	7,58874E+14	7,13936E+14
TC0500001307.mm.2	Hrk	harakiri, BCL2 interacting protein (contains only BH3 domain)	-1,21094E+14	8,01092E+14	-3,66846E+14	0.00926549470662946	0.170747242106618	-2,23869E+13	7,04216E+14	7,18658E+14	8,05714E+14	8,59349E+14	8,50491E+14	8,68126E+14
TC1200002434.mm.2	Akt1	thymoma viral proto-oncogene 1	1,63649E+14	8,59682E+14	3,66816E+14	0.00926892509946829	0.170747242106618	-2,23901E+14	9,58642E+14	9,43473E+14	8,48062E+14	7,26756E+14	8,43547E+14	8,3761E+14
TC0200005402.mm.2	Lama5	laminin, alpha 5	-1,96627E+14	6,5318E+13	-3,66778E+14	0.00927343431984381	0.170747242106618	-2,23943E+14	5,2455E+13	5,39346E+14	6,61035E+14	7,96115E+14	6,54632E+14	7,434E+13
TC0400000090.mm.2	Virma	vir like m6A methyltransferase associated	1,75906E+14	6,28051E+14	3,66694E+14	0.00928309455551008	0.170747242106618	-2,24033E+14	7,31523E+14	7,2988E+14	6,17588E+13	4,92003E+14	6,26959E+14	5,70353E+13
TC0800000965.mm.2	Farsa	phenylalanyl-tRNA synthetase, alpha subunit	1,12795E+14	6,37898E+14	3,66616E+14	0.00929220409418193	0.170747242106618	-2,24118E+14	7,44752E+14	7,31442E+14	6,10079E+14	6,40524E+14	5,39659E+14	5,6093E+14
TC0X00001798.mm.2	Tbc1d25	TBC1 domain family, member 25	1,24435E+14	5,69194E+12	3,66614E+14	0.00929234267158753	0.170747242106618	-2,24119E+14	6,81787E+14	6,38931E+14	5,26471E+14	5,45377E+14	4,8891E+13	5,33686E+13
TC1300000383.mm.2	Nqo2	N-ribosyldihydronicotinamide quinone reductase 2	1,53215E+14	5,5789E+13	3,66436E+14	0.00931308315525855	0.170747242106618	-2,24311E+14	6,48719E+13	6,58833E+13	5,45915E+14	4,55208E+14	4,89596E+14	5,49069E+14
TC0200004564.mm.2	Mall	mal, T cell differentiation protein-like	-1,29888E+14	7,5312E+14	-3,66406E+14	0.00931652268631792	0.170747242106618	-2,24343E+14	6,77863E+14	6,49139E+14	7,80826E+14	8,05951E+14	7,70503E+14	8,3444E+14
TC1500000053.mm.2	Nup155	nucleoporin 155	1,3693E+14	5,69158E+14	3,66404E+14	0.00931682754348685	0.170747242106618	-2,24346E+14	6,51315E+14	6,86325E+12	5,39235E+14	5,24546E+14	5,4518E+14	4,68349E+13
TC0200000856.mm.2	Kcnj3	potassium inwardly-rectifying channel, subfamily J, member 3	1,8103E+14	6,99957E+14	3,6633E+14	0.00932545747224377	0.170747242106618	-2,24426E+14	7,943E+14	8,19252E+14	6,8801E+14	5,63483E+14	7,01482E+14	6,33213E+14
TC0900002942.mm.2	Pccb	propionyl Coenzyme A carboxylase, beta polypeptide	1,42253E+14	6,5951E+14	3,66271E+13	0.00933233724705641	0.170747242106618	-2,24489E+14	7,68629E+14	7,60706E+14	6,54792E+13	5,90038E+14	6,25088E+14	5,5781E+13
TC0900003120.mm.2	Fam240a	family with sequence similarity 240 member A	-1,54475E+14	7,03866E+14	-3,66093E+14	0.00935309806700692	0.170747242106618	-2,24681E+14	5,99023E+14	5,93583E+14	7,03245E+14	7,98311E+13	7,3617E+14	7,92862E+14
TC1900001278.mm.2	Pip5k1b	phosphatidylinositol-4-phosphate 5-kinase, type 1 beta	-1,12666E+14	8,27719E+14	-3,65983E+14	0.0093659506538154	0.170747242106618	-2,248E+14	7,48254E+14	7,55249E+14	8,62599E+13	8,66236E+14	8,86406E+14	8,47568E+14
TC0900001459.mm.2	Scap	SREBF chaperone	2,45034E+14	7,33688E+14	3,65928E+14	0.00937243512142642	0.170747242106618	-2,2486E+14	8,80698E+14	8,64049E+14	7,24126E+14	5,30553E+14	7,4958E+14	6,5312E+14
TC0200003241.mm.2	Cfap77	cilia and flagella associated protein 77	-1,6007E+14	6,98876E+14	-3,65728E+14	0.00939588931821478	0.170747242106618	-2,25075E+14	5,85333E+14	5,75123E+14	6,9329E+14	7,87305E+13	7,40479E+14	8,11726E+14
TC0600002427.mm.2	Tacstd2	tumor-associated calcium signal transducer 2	-1,22155E+14	5,73264E+14	-3,65591E+14	0.00941200207307834	0.170747242106618	-2,25223E+13	5,10529E+13	4,94242E+14	6,00949E+14	6,48132E+14	5,75192E+14	6,10539E+14
TC0900002382.mm.2	Arid3b	AT rich interactive domain 3B (BRIGHT-like)	-1,17529E+14	5,7638E+14	-3,65351E+13	0.00944030875050668	0.170747242106618	-2,25482E+14	5,16889E+14	4,82033E+14	6,24681E+14	6,093E+14	6,30294E+14	5,9508E+14
TC0400001639.mm.2	Stmn1	stathmin 1	2,24389E+14	9,15482E+14	3,65237E+14	0.00945378083392697	0.170747242106618	-2,25606E+14	1,04579E+14	1,03617E+14	9,03182E+14	7,3E+14	9,33154E+14	8,44598E+14
TC1800000994.mm.2	Npc1	NPC intracellular cholesterol transporter 1	1,48465E+13	5,4705E+14	3,65114E+14	0.00946833225431615	0.170747242106618	-2,25738E+14	6,44159E+14	7,0485E+13	5,47787E+14	5,04291E+14	4,04994E+14	4,76216E+14
TC0400001177.mm.2	Dmrta2	doublesex and mab-3 related transcription factor like family A2	-1,09776E+14	6,26233E+14	-3,64903E+14	0.00949334509276226	0.170747242106618	-2,25966E+14	5,47873E+14	5,54793E+14	6,45999E+14	6,76219E+14	6,70155E+14	6,62357E+14
TC1700000523.mm.2	Glp1r	glucagon-like peptide 1 receptor	1,28862E+14	7,5194E+14	3,64859E+14	0.00949858981546575	0.170747242106618	-2,26014E+14	8,77754E+14	8,33034E+14	7,55794E+14	6,97269E+14	6,7365E+14	6,74141E+14
TC1700001716.mm.2	Uqcc2	ubiquinol-cytochrome c reductase complex assembly factor 2	-1,13102E+13	9,46731E+14	-3,64854E+13	0.00949921314932921	0.170747242106618	-2,2602E+14	8,83409E+14	8,61624E+14	9,93822E+14	9,77415E+14	9,9785E+14	9,66267E+14
TC1600000447.mm.2	Tfrc	transferrin receptor	1,73903E+14	6,0095E+14	3,64773E+14	0.00950883568717703	0.170747242106618	-2,26107E+14	7,199E+13	7,07653E+14	5,71695E+14	5,08052E+14	6,09229E+14	4,89173E+14
TC0200004789.mm.2	Nkx2-4	NK2 homeobox 4	-1,28644E+14	6,86365E+14	-3,64756E+14	0.00951087554839327	0.170747242106618	-2,26125E+14	5,99833E+13	6,1665E+14	7,04481E+14	7,69289E+14	6,98992E+14	7,28942E+14
TC0X00000226.mm.2	Agtr2	angiotensin II receptor, type 2	-1,08264E+14	4,73047E+14	-3,64754E+14	0.00951107588926475	0.170747242106618	-2,26127E+14	4,03899E+14	4,18907E+14	5,12364E+14	5,26969E+13	4,79794E+14	4,96351E+14
TC1100002301.mm.2	Slc1a4	solute carrier family 1 (glutamate/neutral amino acid transporter), member 4	1,78302E+14	6,1779E+13	3,64678E+14	0.00952014034648789	0.170747242106618	-2,2621E+14	7,25059E+14	7,79846E+13	6,30885E+14	5,17415E+13	5,59367E+14	4,94169E+14
TC0800002917.mm.2	Marveld3	MARVEL (membrane-associating) domain containing 3	-1,65903E+14	7,55835E+14	-3,64647E+14	0.00952383301546384	0.170747242106618	-2,26243E+14	6,39012E+14	6,4993E+14	7,58907E+14	8,61841E+14	7,76293E+14	8,49027E+14
TC1200000139.mm.2	Pdia6	protein disulfide isomerase associated 6	1,52544E+14	7,4016E+14	3,64609E+14	0.00952832178027015	0.170747242106618	-2,26284E+14	8,45518E+14	8,45741E+14	7,29731E+14	6,5644E+14	7,22147E+13	6,41382E+14
TC0400001686.mm.2	Htr1d	5-hydroxytryptamine (serotonin) receptor 1D	1,06649E+14	6,76122E+14	3,6454E+14	0.00953663804197127	0.170747242106618	-2,26359E+14	7,59106E+14	7,40697E+14	6,39688E+14	6,46818E+14	6,34901E+14	6,35523E+14
TC1800001637.mm.2	Slc14a1	solute carrier family 14 (urea transporter), member 1	2,14169E+14	6,53226E+14	3,64479E+14	0.00954390936595258	0.170747242106618	-2,26425E+14	7,69928E+13	8,19772E+14	6,68361E+14	4,93002E+14	5,95919E+13	5,72375E+14
TC1500000217.mm.2	Sema5a	sema domain, seven thrombospondin repeats (type 1 and type 1-like), transmembrane domain (TM) and short cytoplasmic domain, (semaphorin) 5A	1,45786E+14	6,91385E+14	3,64459E+14	0.00954625538414826	0.170747242106618	-2,26446E+14	7,92138E+14	7,82902E+14	6,8444E+14	5,99029E+14	6,70302E+14	6,19503E+14
TC1500001403.mm.2	Ubr5	ubiquitin protein ligase E3 component n-recognin 5	2,2685E+14	6,11711E+14	3,64448E+14	0.00954758628658075	0.170747242106618	-2,26458E+14	7,42285E+14	7,53213E+13	5,58772E+14	4,83025E+14	6,57805E+14	4,7517E+14
TC0500002406.mm.2	Dhx15	DEAH (Asp-Glu-Ala-His) box polypeptide 15	2,16549E+14	6,56301E+13	3,64417E+14	0.00955131653534154	0.170747242106618	-2,26492E+13	8,13192E+14	8,02778E+14	6,64188E+14	5,18683E+13	6,27614E+14	5,1135E+14
TC1900000502.mm.2	Ifit3b	interferon-induced protein with tetratricopeptide repeats 3B	1,63959E+14	6,07577E+14	3,64351E+14	0.00955920245075108	0.170747242106618	-2,26563E+14	6,86175E+14	7,36248E+14	4,94151E+14	6,00354E+14	5,84519E+14	5,44017E+14
TC0900002678.mm.2	Tmod3	tropomodulin 3	1,10076E+14	7,21793E+14	3,64272E+14	0.00956867717959387	0.170747242106618	-2,26649E+13	7,7432E+13	7,7776E+14	6,75996E+14	6,55931E+14	7,36717E+14	7,10035E+14
TC1300002093.mm.2	Hnrnpa0	heterogeneous nuclear ribonucleoprotein A0	1,62503E+14	7,07414E+14	3,64219E+14	0.009575002893596	0.170747242106618	-2,26706E+14	8,28445E+14	8,08788E+14	7,11769E+14	6,00458E+14	6,71008E+14	6,24016E+14
TC0400001921.mm.2	Nppb	natriuretic peptide type B	-1,36852E+14	5,20928E+14	-3,64184E+14	0.00957916890436354	0.170747242106618	-2,26744E+14	4,1705E+14	4,44391E+14	5,5137E+14	5,83775E+14	5,26591E+14	6,02391E+14
TC0300002549.mm.2	Phgdh	3-phosphoglycerate dehydrogenase	1,08409E+14	7,16529E+14	3,64117E+13	0.00958721636312371	0.170747242106618	-2,26816E+14	8,04366E+14	7,97522E+14	7,01235E+14	6,83834E+14	6,45701E+14	6,66517E+14
TC1000001570.mm.2	Myo1a	myosin IA	-1,7857E+14	6,61235E+14	-3,63982E+14	0.00960345024861347	0.170747242106618	-2,26962E+14	5,32433E+14	5,48578E+14	6,7473E+14	7,63422E+14	6,68871E+14	7,79375E+14
TC0800002844.mm.2	Slc12a4	solute carrier family 12, member 4	1,07162E+14	5,50825E+14	3,63911E+14	0.00961207836170236	0.170747242106618	-2,2704E+14	6,05628E+14	6,13045E+14	4,9502E+14	5,09329E+13	5,32671E+14	5,49256E+14
TC0400004146.mm.2	Mmp23	matrix metallopeptidase 23	-1,41228E+14	6,56649E+14	-3,63861E+14	0.00961804945953316	0.170747242106618	-2,27093E+14	5,87193E+14	5,28228E+14	6,6314E+13	7,34736E+14	7,1952E+14	7,07078E+14
TC0900002123.mm.2	Arhgef12	Rho guanine nucleotide exchange factor (GEF) 12	1,17564E+14	6,60062E+14	3,6386E+14	0.00961815852362372	0.170747242106618	-2,27094E+13	7,37618E+14	7,50221E+14	6,13898E+14	6,38813E+14	6,32434E+14	5,87388E+14
TC0200000037.mm.2	Phyh	phytanoyl-CoA hydroxylase	2,12279E+14	7,25457E+14	3,6365E+14	0.00964352800434414	0.170747242106618	-2,27322E+14	8,81104E+14	8,51517E+14	7,11518E+14	5,96545E+14	7,25157E+13	5,86902E+13
TC0200005175.mm.2	Prex1	phosphatidylinositol-3,4,5-trisphosphate-dependent Rac exchange factor 1	1,24919E+14	6,8442E+14	3,63647E+14	0.00964387117131381	0.170747242106618	-2,27325E+14	7,4352E+14	7,56589E+14	5,93202E+14	6,57069E+13	6,86862E+14	6,6928E+14
TC0300002054.mm.2	P2ry12	purinergic receptor P2Y, G-protein coupled 12	1,37968E+14	8,51581E+14	3,63461E+14	0.00966634041224874	0.170747242106618	-2,27526E+14	9,49958E+14	9,62947E+13	8,55146E+14	7,81824E+13	8,04145E+14	7,55468E+14
TC0800001739.mm.2	1700029H14Rik	RIKEN cDNA 1700029H14 gene	-1,15273E+14	7,49576E+14	-3,63345E+14	0.00968043855181846	0.170747242106618	-2,27652E+14	6,75131E+14	6,77674E+14	7,7099E+14	8,12362E+14	7,92987E+14	7,68309E+14
TC1200000786.mm.2	Dcaf4	DDB1 and CUL4 associated factor 4	1,3182E+14	7,12611E+14	3,63177E+13	0.00970089159274472	0.170747242106618	-2,27835E+14	8,05508E+14	7,75484E+13	6,92476E+14	6,24876E+14	6,74042E+13	7,03279E+14
TC1000000815.mm.2	Fgf22	fibroblast growth factor 22	-1,25999E+14	7,77916E+14	-3,63038E+13	0.00971782877602739	0.170747242106618	-2,27985E+14	6,92483E+14	6,91769E+14	7,9095E+14	8,45299E+14	8,01589E+14	8,45408E+14

TC0100003725.mm.2	Mia3	melanoma inhibitory activity 3	2,5566E+14	6,50563E+14	3,62982E+14	0.00972470110500293	0.170747242106618	-2,28047E+14	8,42247E+14	8,3134E+14	6,59284E+14	5,02984E+14	6,18374E+14	4,4915E+13
TC1100001049.mm.2	Mettl16	methyltransferase like 16	1,13568E+14	5,82968E+14	3,6292E+14	0.00973223632690001	0.170747242106618	-2,28114E+14	6,9027E+14	6,57546E+14	5,50738E+14	5,69942E+14	5,0307E+14	5,26241E+12
TC0300001530.mm.2	Usp33	ubiquitin specific peptidase 33	1,81057E+14	7,41511E+14	3,62861E+14	0.00973944145673582	0.170747242106618	-2,28177E+14	8,44271E+14	8,39487E+13	7,15723E+14	6,0592E+12	7,70114E+14	6,73553E+14
TC0900000599.mm.2	Il18	interleukin 18	1,55331E+14	6,961E+14	3,62861E+14	0.0097394750440904	0.170747242106618	-2,28178E+14	7,9691E+14	7,79422E+14	6,87553E+14	5,78117E+14	6,80222E+14	6,54373E+14
TC0200000929.mm.2	Slc4a10	solute carrier family 4, sodium bicarbonate cotransporter-like, member 10	1,98004E+14	8,59076E+14	3,62847E+14	0.00974113841465818	0.170747242106618	-2,28193E+14	9,7645E+14	1,00004E+14	8,67157E+13	7,13323E+14	8,28153E+14	7,69333E+14
TC0200000461.mm.2	Adamts13	a disintegrin-like and metallopeptidase (reprolysin type) with thrombospondin type 1 motif, 13	-1,8818E+14	5,05296E+14	-3,62741E+14	0.00975413189762656	0.170747242106618	-2,28308E+14	3,73308E+14	4,13633E+14	5,1446E+14	6,48841E+14	5,07938E+14	5,73596E+14
TC0400000278.mm.2	Gabrr2	gamma-aminobutyric acid (GABA) C receptor, subunit rho 2	-1,68382E+14	5,66252E+13	-3,62595E+14	0.0097720200197999	0.170747242106618	-2,28466E+14	4,54115E+14	4,56984E+12	5,74419E+14	6,73444E+13	5,76545E+14	6,62006E+14
TC0200004482.mm.2	Trp53bp1	transformation related protein 53 binding protein 1	2,45778E+14	6,58389E+14	3,62577E+14	0.00977425134646346	0.170747242106618	-2,28486E+14	8,22269E+14	7,88591E+14	6,60811E+14	4,58492E+14	6,50127E+12	5,70045E+14
TC0100001703.mm.2	Cdc42bpa	CDC42 binding protein kinase alpha	3,01681E+14	6,6767E+14	3,62445E+14	0.00979046115563149	0.170747242106618	-2,28629E+14	8,57807E+14	8,6813E+14	6,67965E+14	4,54611E+14	6,6939E+14	4,88117E+14
TC1300000969.mm.2	Cetn3	centrin 3	2,1609E+14	7,57786E+14	3,62274E+14	0.00981147013305477	0.170747242106618	-2,28815E+13	8,9449E+14	8,78945E+14	7,53121E+14	5,88133E+14	7,59829E+14	6,72196E+14
TC1200001018.mm.2	Chga	chromogranin A	1,76206E+14	8,48386E+14	3,62266E+14	0.00981237486249628	0.170747242106618	-2,28823E+14	9,74024E+14	9,44086E+14	8,51331E+14	7,14367E+14	8,09474E+14	7,97035E+14
TC0500003253.mm.2	Cdk2ap1	CDK2 (cyclin-dependent kinase 2)-associated protein 1	1,04787E+14	7,35103E+13	3,62205E+14	0.00981997080823692	0.170747242106618	-2,28889E+14	7,82406E+14	7,88703E+14	6,85907E+14	6,75627E+14	7,39562E+14	7,38416E+13
TC0200004906.mm.2	Defb19	defensin beta 19	-1,34022E+14	5,06717E+14	-3,62136E+14	0.00982849883827354	0.170747242106618	-2,28965E+14	4,2041E+14	4,06521E+14	5,07856E+14	5,8712E+14	5,47771E+14	5,70621E+14
TC0800001247.mm.2	Prmt7	protein arginine N-methyltransferase 7	1,10545E+14	7,42737E+14	3,61969E+14	0.00984906070265705	0.170747242106618	-2,29145E+14	8,31334E+14	8,2772E+14	7,31257E+14	7,06707E+14	6,93503E+14	6,65901E+14
TC0200005514.mm.2	Zhx3	zinc fingers and homeoboxes 3	2,26801E+14	5,75353E+14	3,61873E+13	0.00986103326974342	0.170747242106618	-2,2925E+14	7,09718E+14	7,10861E+14	5,72787E+13	3,9419E+13	5,77015E+14	4,87548E+14
TC0700003860.mm.2	Olfr544	olfactory receptor 544	-1,43338E+14	5,35023E+14	-3,61869E+14	0.00986143600992238	0.170747242106618	-2,29254E+14	4,67434E+14	4,4906E+14	5,57163E+14	6,46007E+13	5,26998E+14	5,63475E+12
TC1100000712.mm.2	Hist3h2ba	histone cluster 3, H2ba	-1,55485E+14	6,79442E+14	-3,61792E+14	0.00987100721044944	0.170747242106618	-2,29338E+14	6,04394E+14	5,735E+14	7,06427E+14	7,82437E+12	6,64934E+14	7,44962E+14
TC0900003098.mm.2	Camp	cathelicidin antimicrobial peptide	-1,19578E+14	6,12862E+14	-3,61743E+14	0.00987713272766988	0.170747242106618	-2,29391E+14	5,02901E+14	5,44236E+14	6,40378E+14	6,45915E+14	6,51277E+14	6,92465E+14
TC0500003410.mm.2	Ywhag	tyrosine 3-monooxygenase/tryptophan 5-monooxygenase activation protein, gamma polypeptide	1,78418E+14	9,4081E+14	3,61567E+14	0.00989892232910767	0.170747242106618	-2,29582E+14	1,06055E+14	1,04149E+14	9,40423E+13	8,0478E+14	9,20817E+14	8,76799E+14
TC1700000323.mm.2	Srrm2	serine/arginine repetitive matrix 2	2,38523E+13	9,04288E+14	3,61461E+14	0.00991215314826557	0.170747242106618	-2,29698E+14	1,07019E+14	1,07198E+14	8,94461E+14	7,70668E+14	8,95901E+14	7,22525E+14
TC1700001004.mm.2	Kdm4b	lysine (K)-specific demethylase 4B	1,09746E+14	6,53085E+14	3,61341E+14	0.00992711113476099	0.170747242106618	-2,29828E+14	7,19402E+14	7,03324E+14	5,86042E+14	6,17192E+14	6,44198E+14	6,48355E+14
TC0400001525.mm.2	Marcksl1	MARCKS-like 1	-1,19371E+14	7,65422E+14	-3,61321E+14	0.00992960639720838	0.170747242106618	-2,2985E+14	6,8911E+14	6,92469E+14	7,89247E+14	8,31073E+14	7,74951E+14	8,15683E+14
TC0100000375.mm.2	Stat1	signal transducer and activator of transcription 1	1,50552E+14	5,9967E+14	3,61179E+14	0.00994733859494465	0.170747242106618	-2,30004E+14	6,5704E+14	7,2133E+14	4,95326E+14	5,81941E+14	5,66353E+14	5,76027E+14
TC1900000563.mm.2	Cyp2c65	cytochrome P450, family 2, subfamily c, polypeptide 65	-1,28668E+14	4,62131E+13	-3,61078E+14	0.00995999537824054	0.170747242106618	-2,30114E+14	3,61106E+14	4,0369E+14	4,8709E+14	5,35042E+13	4,72924E+14	5,12936E+14
TC1700001996.mm.2	Gm11127	predicted gene 11127	-1,92267E+14	6,01449E+14	-3,61076E+14	0.00996028955580547	0.170747242106618	-2,30117E+13	4,58416E+14	4,79165E+14	5,82269E+14	7,39846E+14	6,62373E+14	6,86625E+14
TC0600000341.mm.2	Stra8	stimulated by retinoic acid gene 8	-1,34693E+14	4,68278E+14	-3,60963E+14	0.00997441474609911	0.170747242106618	-2,30239E+13	4,07247E+14	4,06832E+14	5,00163E+12	5,83303E+14	4,46341E+14	4,65783E+14
TC1000003170.mm.2	Nek7	NIMA (never in mitosis gene a)-related expressed kinase 7	1,3388E+14	6,64378E+14	3,60667E+14	0.0100116855371082	0.170747242106618	-2,30562E+13	7,41474E+14	7,39059E+14	6,26151E+14	5,86621E+14	6,83682E+14	6,09281E+14
TC1100003759.mm.2	Krt9	keratin 9	1,55914E+13	5,71401E+14	3,606E+14	0.010020070860541	0.170747242106618	-2,30635E+14	6,80986E+14	6,43448E+14	5,5918E+14	4,53427E+14	5,31248E+13	5,60114E+13
TC0300000530.mm.2	Htatsf1	HIV TAT specific factor 1	2,09941E+14	6,51345E+14	3,59975E+14	0.0100992216099806	0.170747242106618	-2,31316E+14	7,82073E+14	7,95236E+14	6,64198E+14	4,93229E+14	6,17179E+14	5,56155E+14
TC1700002787.mm.2	Ddx39b/Mir8094	DEAD (Asp-Glu-Ala-Asp) box polypeptide 39B/microRNA 8094	1,1584E+14	8,93347E+13	3,59845E+14	0.0101157845770449	0.170747242106618	-2,31458E+14	9,7362E+14	9,78935E+13	8,77047E+14	8,43827E+14	8,63193E+14	8,23462E+14
TC1600001641.mm.2	Atp6v1a	ATPase, H+ transporting, lysosomal V1 subunit A	1,77342E+14	8,31418E+14	3,59723E+14	0.0101313271330117	0.170747242106618	-2,31591E+14	9,49866E+14	9,45962E+14	8,29788E+14	7,11355E+13	8,16389E+14	7,35151E+14
TC0300002605.mm.2	Atp1a1	ATPase, Na+/K+ transporting, alpha 1 polypeptide	1,68185E+14	8,93163E+13	3,59557E+14	0.0101524833074845	0.170747242106618	-2,31771E+14	9,91922E+14	1,01713E+14	8,92191E+14	7,8049E+14	8,71826E+14	8,0542E+14
TC0200003873.mm.2	Tmx2	thioredoxin-related transmembrane protein 2	1,16446E+14	8,70429E+14	3,59478E+14	0.0101627009548223	0.170747242106618	-2,31858E+14	9,59418E+14	9,30107E+14	8,50164E+14	8,06469E+13	8,43444E+14	8,3298E+14
TC0900000216.mm.2	Zfp809	zinc finger protein 809	1,35029E+14	6,87687E+14	3,59442E+14	0.010167246894491	0.170747242106618	-2,31897E+14	7,74392E+14	7,71459E+14	6,1699E+14	6,58804E+14	6,90236E+14	6,14241E+14
TC0800001093.mm.2	Mt3	metallothionein 3	1,10515E+14	8,92669E+14	3,59359E+14	0.0101779093518285	0.170747242106618	-2,31988E+14	9,72544E+14	9,46184E+14	8,39083E+14	8,58614E+14	8,58958E+14	8,80631E+14
TC1800000462.mm.2	Ythdc2	YTH domain containing 2	1,09456E+14	5,8719E+14	3,59354E+14	0.0101785397044234	0.170747242106618	-2,31993E+14	6,81844E+14	6,54319E+14	5,51191E+14	5,66061E+14	5,44022E+14	5,25704E+14
TC0800000871.mm.2	Slc10a7	solute carrier family 10 (sodium/bile acid cotransporter family), member 7	-1,21693E+14	6,83795E+14	-3,59267E+14	0.0101897450312477	0.170747242106618	-2,32088E+14	5,89339E+14	6,19136E+14	6,97844E+14	7,54016E+14	7,16335E+14	7,26101E+14
TC1000001549.mm.2	Agap2	ArfGAP with GTPase domain, ankyrin repeat and PH domain 2	2,07509E+14	8,12436E+14	3,59231E+14	0.0101942925846374	0.170747242106618	-2,32127E+14	9,27878E+14	9,48398E+14	8,1777E+14	6,43488E+13	7,8792E+14	7,4916E+14
TC0600000912.mm.2	M1ap	meiosis 1 associated protein	-1,45435E+14	6,87247E+14	-3,59192E+14	0.0101993833941711	0.170747242106618	-2,3217E+14	5,73246E+14	6,28599E+14	7,06102E+13	7,86614E+13	6,99285E+14	7,29635E+14
TC0200001164.mm.2	Itprid2	ITPR interacting domain containing 2	1,28328E+13	5,76217E+14	3,59089E+14	0.0102125375076028	0.170747242106618	-2,32282E+14	6,28334E+14	6,4718E+14	5,29047E+14	4,89812E+14	6,12742E+14	5,50187E+14
TC1000002046.mm.2	Cdc40	cell division cycle 40	1,53935E+14	6,26134E+14	3,59025E+14	0.0102207779502406	0.170747242106618	-2,32352E+14	7,33049E+14	7,27714E+14	6,30412E+14	5,22481E+14	5,89931E+14	5,53216E+13
TC1100004182.mm.2	Eif4a3	eukaryotic translation initiation factor 4A3	1,6041E+14	6,78137E+14	3,59013E+14	0.010222346258314	0.170747242106618	-2,32365E+14	7,93527E+14	7,73536E+13	6,74942E+14	5,71301E+14	6,59051E+14	5,96465E+14
TC0800002821.mm.2	Kctd19	potassium channel tetramerisation domain containing 19	-1,19104E+14	5,23883E+14	-3,58957E+14	0.0102295815035009	0.170747242106618	-2,32426E+14	4,41807E+14	4,54693E+14	5,39047E+14	5,9566E+14	5,62579E+14	5,49513E+14
TC0500001944.mm.2	Grm3	glutamate receptor, metabotropic 3	1,9647E+14	8,11395E+14	3,58854E+14	0.010242938490137	0.170747242106618	-2,32539E+14	9,36546E+14	9,48827E+14	7,94159E+14	6,98275E+14	8,13454E+14	6,77112E+14
TC0500001355.mm.2	Hectd4	HECT domain E3 ubiquitin protein ligase 4	1,79626E+14	8,00051E+14	3,58833E+14	0.0102456168680883	0.170747242106618	-2,32562E+14	9,10123E+14	9,20968E+14	7,64177E+14	7,07661E+14	8,16325E+14	6,81051E+14
TC0X00000454.mm.2	Stk26	serine/threonine kinase 26	1,13009E+14	6,50447E+14	3,58762E+14	0.0102548048223365	0.170747242106618	-2,3264E+14	7,26625E+14	7,20221E+14	6,28346E+14	5,92482E+14	6,31787E+14	6,03218E+14
TC1400000174.mm.2	Zswim8	zinc finger SWIM-type containing 8	1,66598E+14	6,4228E+14	3,587E+14	0.010262807577533	0.170747242106618	-2,32707E+14	7,40717E+14	7,39488E+14	6,09592E+14	5,37417E+14	6,67811E+14	5,58654E+14
TC0300002681.mm.2	Atp5f1	ATP synthase, H+ transporting, mitochondrial F0 complex, subunit B1	2,10565E+14	8,51019E+14	3,5855E+14	0.0102822728437828	0.170747242106618	-2,32872E+14	9,54322E+14	9,58089E+13	8,27308E+14	6,63972E+14	8,93181E+14	8,0924E+14
TC0600003128.mm.2	Cops7a	COP9 signalosome subunit 7A	1,60003E+14	9,14689E+14	3,5854E+14	0.010283526658777	0.170747242106618	-2,32882E+14	1,02967E+14	1,00932E+14	9,11925E+14	8,07063E+14	8,95013E+14	8,35138E+14
TC1200000636.mm.2	Mnat1	menage a trois 1	1,26376E+14	5,43064E+14	3,58417E+14	0.0102994668502776	0.170747242106618	-2,33016E+14	6,37905E+13	6,15457E+14	5,05606E+14	4,95004E+14	5,36687E+13	4,67728E+14
TC1100003455.mm.2	Cltc	clathrin, heavy polypeptide (Hc)	2,26119E+14	9,49515E+14	3,58262E+14	0.0103196685565209	0.170747242106618	-2,33186E+14	1,11356E+14	1,09682E+14	9,55938E+14	8,02201E+14	9,28903E+14	7,99669E+14
TC1500000723.mm.2	Ep300	E1A binding protein p300	1,75252E+14	6,09685E+14	3,58248E+13	0.0103215105208169	0.170747242106618	-2,33202E+14	7,15347E+14	7,35233E+14	6,18221E+14	4,81855E+14	5,70826E+14	5,36626E+13
TC0900001487.mm.2	Trank1	tetratricopeptide repeat and ankyrin repeat containing 1	1,77911E+14	6,60183E+14	3,58229E+14	0.010323906019554	0.170747242106618	-2,33222E+14	7,78095E+14	7,63127E+14	6,64793E+14	5,20608E+14	6,2201E+14	6,12467E+14
TC0200005373.mm.2	Gm14325	predicted gene 14325	2,31519E+14	7,61029E+14	3,58219E+14	0.0103252193640007	0.170747242106618	-2,33233E+14	8,71683E+14	9,00609E+14	7,35464E+14	5,7379E+14	8,05815E+14	6,78811E+14
TC0200005437.mm.2	Srms	src-related kinase lacking C-terminal regulatory tyrosine and N-terminal myristylation sites	-1,69173E+14	5,83387E+14	-3,58121E+14	0.0103380247406278	0.170747242106618	-2,3334E+14	4,47971E+14	4,94386E+14	5,81914E+14	6,98789E+14	6,14816E+14	6,62445E+14
TC1100003438.mm.2	Med13	mediator complex subunit 13	1,69814E+13	6,23168E+14	3,58054E+14	0.0103468276990687	0.170747242106618	-2,33414E+14	7,28092E+14	7,53004E+14	6,32876E+14	5,08593E+13	5,81911E+14	5,34533E+14
TC1500000980.mm.2	4930415O20Rik	RIKEN cDNA 4930415O20 gene	-1,58021E+14	5,53812E+14	-3,5794E+14	0.0103617268376184	0.170747242106618	-2,33539E+13	4,50335E+14	4,35742E+14	5,76419E+14	6,257E+12	5,63008E+14	6,71669E+14
TC1700000603.mm.2	Wdr46	WD repeat domain 46	1,97869E+14	5,64391E+14	3,57932E+14	0.0103627542663216	0.170747242106618	-2,33547E+14	6,95383E+14	7,00859E+14	5,67002E+14	4,33502E+14	5,46843E+14	4,42754E+14
TC0100003721.mm.2	Disp1	dispatched RND transporter family member 1	-1,04459E+14	7,30231E+13	-3,57823E+14	0.0103770130071359	0.170747242106618	-2,33666E+14	6,76423E+14	6,60953E+14	7,7158E+14	7,74713E+14	7,44018E+14	7,53697E+14
TC0100003015.mm.2	Map3k19	mitogen-activated protein kinase kinase kinase 19	1,1879E+14	4,50779E+14	3,5759E+14	0.0104075109425258	0.170747242106618	-2,33921E+14	5,27539E+14	4,88047E+14	3,90723E+14	3,33282E+14	5,05504E+14	5,13576E+13
TC0400002535.mm.2	Spag8	sperm associated antigen 8	-1,12655E+14	7,68352E+14	-3,57391E+14	0.0104336902712768	0.170747242106618	-2,34138E+14	6,86764E+14	7,00172E+13	7,94545E+14	8,17701E+14	8,24474E+14	7,86458E+14
TC1200000068.mm.2	Pum2	pumilio RNA-binding family member 2	2,0073E+14	7,86884E+14	3,57246E+14	0.0104529199638828	0.170747242106618	-2,34298E+14	9,15277E+14	9,13829E+14	7,96209E+14	6,31436E+14	7,58898E+14	7,05653E+14
TC0X00002470.mm.2	Mpp1	membrane protein, palmitoylated	1,34928E+14	6,53742E+14	3,57207E+14	0.01045804777537	0.170747242106618	-2,34341E+14	7,61941E+14	7,48362E+14	6,34103E+14	6,06344E+14	6,27015E+14	5,44687E+14
TC1000000462.mm.2	Vgll2	vestigial like family member 2	-1,17835E+14	6,56955E+14	-3,57202E+14	0.0104587190581532	0.170747242106618	-2,34346E+14	5,79364E+14	5,94423E+14	6,88493E+14	7,20964E+13	6,56347E+14	7,02141E+14
TC0500001531.mm.2	Caln1	calneuron 1	1,5209E+14	7,60014E+14	3,57127E+14	0.0104685561651397	0.170747242106618	-2,34428E+14	8,606E+14	8,71638E+13	7,66066E+14	6,61992E+14	6,78948E+14	7,20841E+14
TC1200000765.mm.2	Pcnx	pecanex homolog	2,66826E+14	6,28693E+14	3,5684E+14	0.0105067079932644	0.170747242106618	-2,34743E+14	7,96827E+13	8,21785E+14	6,44402E+14	4,20559E+14	5,51694E+14	5,16889E+14
TC0200001747.mm.2	Disp2	dispatched RND transporter family member 2	2,3709E+14	7,48896E+14	3,56839E+14	0.0105067300156642	0.170747242106618	-2,34743E+14	9,29187E+14	8,90042E+14	7,73426E+13	5,71624E+13	6,9578E+14	6,33314E+14
TC0500002153.mm.2	3110082J24Rik	RIKEN cDNA 3110082J24 gene	-1,16068E+14	7,78857E+14	-3,5674E+14	0.0105199760587738	0.170747242106618	-2,34853E+14	6,98454E+14	6,8871E+14	7,85597E+14	8,33702E+14	8,21217E+14	8,45461E+14
TC0600003277.mm.2	Gm8882/Gm4736/Prb1/LOC100502592	predicted gene 8882/predicted gene 4736/proline-rich protein BstNI subfamily 1/proline-rich proteoglycan 2-like	-1,27583E+14	6,44417E+14	-3,56595E+14	0.0105392539926905	0.170747242106618	-2,35011E+14	5,5293E+14	5,56011E+14	6,49059E+14	7,15048E+14	6,77481E+14	7,15973E+14
TC0800000786.mm.2	Babam1	BRISC and BRCA1 A complex member 1	1,20446E+14	7,79254E+14	3,56442E+14	0.0105559630980335	0.170747242106618	-2,35179E+14	8,54313E+14	8,49755E+14	7,62583E+14	7,00593E+14	7,61289E+13	7,46991E+13
TC0400000314.mm.2	Cga	glycoprotein hormones, alpha subunit	-1,37804E+14	5,34468E+14	-3,56402E+14	0.0105650376452713	0.170747242106618	-2,35223E+14	4,36043E+14	4,39715E+14	5,29511E+14	6,21855E+14	5,85685E+14	5,93999E+14
TC1700000995.mm.2	Fsd1	fibronectin type 3 and SPRY domain-containing protein	1,423E+14	7,38736E+14	3,56397E+14	0.0105656233760969	0.170747242106618	-2,35228E+14	8,28753E+14	8,25803E+14	7,32265E+14	6,3769E+14	7,19222E+13	6,86682E+14
TC1200001314.mm.2	Rapgef5	Rap guanine nucleotide exchange factor (GEF) 5	1,65388E+14	7,11619E+14	3,56097E+14	0.0106058163989213	0.170747242106618	-2,35557E+14	7,97011E+14	8,30541E+14	7,00541E+14	5,96235E+14	7,08476E+14	6,36911E+13
TC1500000125.mm.2	Cdh10	cadherin 10	2,02117E+14	6,76427E+14	3,56023E+14	0.0106157578296139	0.170747242106618	-2,35639E+14	8,01285E+14	7,92347E+13	6,81714E+14	5,07683E+14	6,48568E+14	6,26967E+14
TC0800002580.mm.2	Ccdc130	coiled-coil domain containing 130	1,52742E+14	6,40138E+14	3,56022E+14	0.0106158358045568	0.170747242106618	-2,35639E+13	7,53796E+14	7,11808E+14	6,31361E+14	5,28759E+14	5,93726E+14	6,21381E+14
TC0800000747.mm.2	Comp	cartilage oligomeric matrix protein	-1,22704E+14	5,26234E+14	-3,55989E+14	0.0106202994381743	0.170747242106618	-2,35676E+14	4,61593E+14	4,53613E+14	5,51144E+14	6,09471E+14	5,23495E+14	5,5809E+14
TC1500002212.mm.2	Nckap5l	NCK-associated protein 5-like	-1,20261E+14	7,47109E+14	-3,55976E+14	0.010622037128113	0.170747242106618	-2,3569E+13	6,65182E+14	6,88385E+14	7,71645E+14	8,22444E+14	7,52151E+14	7,82846E+14
TC0900001357.mm.2	Dcaf1	DDB1 and CUL4 associated factor 1	3,04247E+14	6,10403E+14	3,55931E+14	0.0106281856373651	0.170747242106618	-2,3574E+14	7,83768E+14	7,84437E+14	6,15245E+14	3,44465E+14	6,17338E+14	5,17167E+14
TC1100001565.mm.2	Stard3	START domain containing 3	1,65817E+14	6,25992E+14	3,55924E+14	0.0106290292676236	0.170747242106618	-2,35747E+14	7,31116E+14	7,10243E+14	6,11853E+14	4,97873E+14	6,33536E+14	5,71332E+13
TC0500002592.mm.2	Ociad2	OCIA domain containing 2	1,36631E+14	7,22897E+14	3,55804E+14	0.0106451638012141	0.170747242106618	-2,35879E+14	8,0593E+14	8,19021E+14	7,16971E+14	6,34717E+14	6,99149E+14	6,61592E+14
TC0700003795.mm.2	Mogat2	monoacylglycerol O-acyltransferase 2	-1,54949E+14	8,37336E+13	-3,55752E+13	0.0106522299492893	0.170747242106618	-2,35936E+14	7,52323E+13	7,12383E+14	8,34555E+14	9,40048E+14	8,76098E+14	9,08608E+13
TC1900001105.mm.2	Tmem216	transmembrane protein 216	1,15042E+14	5,52883E+14	3,55701E+14	0.0106591331981853	0.170747242106618	-2,35992E+14	6,5198E+14	6,34176E+14	5,06481E+13	5,49592E+14	5,00379E+14	4,74691E+14
TC1600001375.mm.2	Igf2bp2	insulin-like growth factor 2 mRNA binding protein 2	-1,06324E+14	4,81443E+14	-3,55686E+14	0.0106611675708556	0.170747242106618	-2,36009E+14	4,31576E+14	4,04674E+14	5,18696E+14	5,30203E+14	5,03912E+14	4,99595E+13
TC0900001463.mm.2	Setd2	SET domain containing 2	1,10751E+14	6,26928E+14	3,55533E+14	0.0106818142633683	0.170747242106618	-2,36177E+14	6,98933E+13	7,10721E+14	6,08858E+14	5,79294E+14	5,96455E+14	5,6731E+14
TC0200001803.mm.2	Ccndbp1	cyclin D-type binding-protein 1	1,57213E+14	7,72717E+14	3,55525E+13	0.0106828762871498	0.170747242106618	-2,36186E+13	9,02333E+14	8,55932E+13	7,75125E+14	6,68714E+14	7,3334E+14	7,00859E+14
TC0400001539.mm.2	Col16a1	collagen, type XVI, alpha 1	-1,285E+14	7,98978E+14	-3,55522E+14	0.0106832211209713	0.170747242106618	-2,36188E+14	6,89676E+14	7,23925E+14	7,99907E+14	8,70694E+13	8,5465E+14	8,55015E+14
TC0500001992.mm.2	Ube2h	ubiquitin-conjugating enzyme E2H	1,59406E+14	7,42223E+14	3,55474E+14	0.0106897767180961	0.170747242106618	-2,36242E+14	8,74653E+14	8,38431E+14	7,03204E+14	6,51067E+14	7,6892E+14	6,5706E+14
TC1100003511.mm.2	Dgke	diacylglycerol kinase, epsilon	1,34645E+14	6,55657E+14	3,55389E+14	0.0107012885581073	0.170747242106618	-2,36335E+14	7,50573E+14	7,40456E+14	6,5287E+14	5,6887E+14	6,23671E+14	5,97499E+14
TC1000000566.mm.2	Lrrc20	leucine rich repeat containing 20	1,15664E+13	6,62002E+14	3,55338E+14	0.0107082365085116	0.170747242106618	-2,36391E+14	7,39564E+14	7,42147E+14	6,35143E+14	6,15241E+14	5,94432E+14	6,45484E+14
TC0800000587.mm.2	Vegfc	vascular endothelial growth factor C	-1,26984E+14	8,60109E+13	-3,55313E+14	0.0107115414148945	0.170747242106618	-2,36418E+14	7,71796E+13	7,69347E+14	8,62148E+14	9,32963E+14	8,98162E+14	9,26241E+14
TC1500002048.mm.2	Sbf1	SET binding factor 1	1,05325E+14	6,93842E+14	3,55311E+14	0.0107118222357413	0.170747242106618	-2,3642E+14	7,84341E+14	7,69265E+14	6,82069E+14	6,60887E+14	6,34612E+14	6,31877E+14
TC0300002882.mm.2	Gm13199	predicted gene 13199	-1,30886E+14	6,10862E+14	-3,55204E+14	0.0107264236436319	0.170747242106618	-2,36539E+14	4,89096E+14	5,29256E+14	6,0393E+14	6,76195E+14	6,8494E+14	6,81753E+14
TC1000002419.mm.2	Gm10941	predicted gene 10941	-1,64464E+14	4,35338E+14	-3,54961E+14	0.0107593905120679	0.170747242106618	-2,36805E+14	3,53782E+14	3,74613E+14	5,33343E+14	5,23979E+14	3,48986E+13	4,77323E+13
TSUnmapped00000045.mm.2	Aopep	aminopeptidase O	-1,24402E+14	6,58799E+14	-3,54896E+13	0.0107682932028834	0.170747242106618	-2,36877E+14	5,49789E+14	5,82348E+14	6,73643E+14	7,07297E+14	7,47211E+14	6,92508E+14
TC1200000427.mm.2	Arhgap5	Rho GTPase activating protein 5	2,91455E+14	7,33064E+14	3,54872E+14	0.0107716185436637	0.170747242106618	-2,36904E+14	9,11659E+14	9,20052E+14	7,35591E+14	5,13211E+14	7,40503E+14	5,77371E+14
TC1600001270.mm.2	Igll1	immunoglobulin lambda-like polypeptide 1	-1,44838E+14	5,51775E+14	-3,54767E+14	0.0107859711177939	0.170747242106618	-2,37019E+14	4,59726E+14	4,5765E+14	5,51645E+14	6,55408E+14	5,89635E+14	5,96586E+12
TC0600001007.mm.2	Gkn2	gastrokine 2	-1,46936E+14	6,12017E+13	-3,54741E+14	0.0107894539546706	0.170747242106618	-2,37047E+14	5,28329E+14	5,02765E+14	6,14602E+14	7,10365E+14	6,37869E+14	6,78169E+14
TC0300000521.mm.2	Kcnab1	potassium voltage-gated channel, shaker-related subfamily, beta member 1	2,01367E+14	8,14417E+14	3,54667E+14	0.0107995949600028	0.170747242106618	-2,37129E+14	9,48754E+14	9,55306E+14	8,30352E+14	6,70974E+14	7,7729E+14	7,03825E+14
TC1900000413.mm.2	Gm815	predicted gene 815	-1,19519E+14	6,83671E+14	-3,54636E+14	0.0108038368658607	0.170747242106618	-2,37163E+14	6,11929E+14	6,28154E+14	7,14874E+13	7,64248E+14	6,73457E+14	7,09364E+14
TC1000000512.mm.2	Gcc2	GRIP and coiled-coil domain containing 2	3,05771E+13	6,85242E+13	3,54444E+14	0.0108302308637887	0.170747242106618	-2,37375E+14	8,46633E+14	8,47432E+13	6,58245E+14	4,24278E+14	7,54549E+14	5,80316E+13
TC0200001994.mm.2	Itpa	inosine triphosphatase (nucleoside triphosphate pyrophosphatase)	1,10196E+14	6,8384E+13	3,54375E+14	0.0108396287284331	0.170747242106618	-2,3745E+13	7,44376E+14	7,69473E+14	6,37231E+13	6,56226E+14	6,62252E+14	6,33481E+14
TC0700001855.mm.2	Rnf40	ring finger protein 40	1,37676E+14	7,03208E+12	3,54316E+14	0.0108476836002821	0.170747242106618	-2,37514E+14	7,82853E+13	7,59352E+14	6,71552E+14	5,95301E+14	7,29765E+14	6,80424E+13
TC0100000591.mm.2	Apol7d	apolipoprotein L 7d	-1,84518E+14	5,67605E+14	-3,54263E+14	0.0108550768106143	0.170747242106618	-2,37574E+14	4,2092E+14	4,54864E+14	5,48403E+14	6,96417E+14	6,251E+14	6,59924E+14
TC0100001671.mm.2	Zbtb18	zinc finger and BTB domain containing 18	1,82744E+14	7,91395E+14	3,54252E+14	0.0108565116083196	0.170747242106618	-2,37585E+14	8,57279E+14	9,28105E+14	7,47937E+13	6,71958E+14	8,28155E+14	7,14937E+14
TC0900001422.mm.2	Ip6k2	inositol hexaphosphate kinase 2	1,11874E+14	8,7331E+14	3,54238E+14	0.0108584397718965	0.170747242106618	-2,376E+14	9,51931E+14	9,40571E+14	8,56541E+14	8,12122E+14	8,44683E+14	8,33919E+14
TC0200002844.mm.2	Rpp38	ribonuclease P/MRP 38 subunit	1,83149E+14	5,52271E+14	3,54194E+14	0.010864587821022	0.170747242106618	-2,3765E+14	6,89391E+14	6,47445E+14	5,53503E+13	4,17036E+14	4,74023E+14	5,32231E+13

TC0700000851.mm.2	Plekha4	pleckstrin homology domain containing, family A (phosphoinositide binding specific) member 4	-1,28439E+14	7,59159E+14	-3,54103E+14	0.0108770478850042	0.170747242106618	-2,37749E+14	6,88775E+14	6,59678E+14	7,74312E+14	8,31019E+14	7,76917E+14	8,24253E+14
TC0300003150.mm.2	St6galnac5	ST6 (alpha-N-acetyl-neuraminyl-2,3-beta-galactosyl-1,3)-N-acetylgalactosaminide alpha-2,6-sialyltransferase 5	1,70006E+14	8,341E+13	3,54046E+14	0.0108849957555086	0.170747242106618	-2,37813E+14	9,36827E+14	9,459E+14	8,37173E+14	7,05542E+14	8,09755E+14	7,69404E+14
TC0700000559.mm.2	Syne4	spectrin repeat containing, nuclear envelope family member 4	-1,30049E+13	6,49635E+14	-3,53989E+14	0.0108927687186174	0.170747242106618	-2,37875E+14	5,54727E+14	6,03227E+14	6,75923E+14	7,42129E+14	6,59732E+14	6,62071E+14
TC0200003573.mm.2	Ermn	ermin, ERM-like protein	1,89929E+14	8,25175E+14	3,53887E+14	0.0109069631035927	0.170747242106618	-2,37988E+14	9,42124E+14	9,66042E+14	8,04842E+14	7,23465E+14	8,2663E+14	6,87944E+14
TC0100001533.mm.2	Ildr2	immunoglobulin-like domain containing receptor 2	1,21331E+14	6,99751E+14	3,53867E+14	0.0109097570327621	0.170747242106618	-2,3801E+14	7,80094E+14	7,67526E+14	6,43166E+14	6,61792E+14	7,04574E+14	6,41351E+14
TC0700004215.mm.2	Usp31	ubiquitin specific peptidase 31	1,803E+14	7,13283E+14	3,53839E+14	0.0109135212804026	0.170747242106618	-2,3804E+14	8,34809E+14	8,55958E+14	7,06371E+14	6,23797E+14	6,91651E+14	5,67111E+14
TC1100000473.mm.2	Med7	mediator complex subunit 7	2,47495E+14	5,81227E+14	3,53822E+14	0.0109159217189657	0.170747242106618	-2,38059E+14	7,2979E+14	7,49626E+14	5,92279E+14	3,92147E+14	5,66222E+13	4,57296E+14
TC0600001129.mm.2	Arl6ip5	ADP-ribosylation factor-like 6 interacting protein 5	1,70005E+14	8,02422E+14	3,53758E+14	0.010924716615083	0.170747242106618	-2,38129E+14	9,0463E+14	9,28952E+13	8,03845E+14	6,89727E+14	7,80669E+14	7,06707E+13
TC0200003436.mm.2	Scai	suppressor of cancer cell invasion	1,50622E+14	7,1697E+14	3,53664E+13	0.0109377811186368	0.170747242106618	-2,38233E+14	8,04909E+14	8,23124E+14	6,7852E+14	6,48268E+14	7,27034E+14	6,19966E+14
TC0400002501.mm.2	Fam205a2	family with sequence similarity 205, member A2	1,33995E+14	5,78392E+14	3,53627E+14	0.0109430109464559	0.170747242106618	-2,38274E+14	6,45085E+14	6,57959E+14	5,09255E+14	5,25799E+14	6,09164E+14	5,2309E+13
TC0400003483.mm.2	D830031N03Rik/Macf1	RIKEN cDNA D830031N03 gene/microtubule-actin crosslinking factor 1	1,59722E+14	8,40836E+14	3,53561E+14	0.0109521052329328	0.170747242106618	-2,38346E+14	9,54559E+14	9,35357E+14	7,98962E+14	7,71511E+14	8,52536E+14	7,32089E+14
TC1300002506.mm.2	Tmem171	transmembrane protein 171	-1,39242E+14	7,30335E+14	-3,53396E+14	0.0109750765899315	0.170747242106618	-2,38528E+14	6,47662E+14	6,29648E+14	7,39341E+14	8,16452E+14	7,46713E+14	8,02194E+14
TC1100000722.mm.2	Jmjd4	jumonji domain containing 4	1,5559E+14	6,17599E+14	3,53288E+14	0.0109901397406899	0.170747242106618	-2,38647E+14	7,28254E+14	7,32888E+14	6,21172E+14	5,28789E+14	5,85231E+14	5,0926E+14
TC0300002474.mm.2	6330562C20Rik	RIKEN cDNA 6330562C20 gene	-1,43602E+13	6,96596E+14	-3,53222E+14	0.0109993557453795	0.170747242106618	-2,3872E+13	6,14352E+14	6,10862E+13	7,19569E+14	7,9285E+14	6,84719E+14	7,57227E+14
TC0200003656.mm.2	Cobll1	Cobl-like 1	-1,56001E+14	7,22216E+14	-3,53219E+14	0.0109997403743132	0.170747242106618	-2,38723E+14	6,38384E+13	6,04055E+14	7,20434E+13	8,34007E+14	7,58306E+14	7,78108E+14
TC0900001836.mm.2	Zfp426/Zfp26	zinc finger protein 426/zinc finger protein 26	2,51667E+14	6,51218E+14	3,53134E+14	0.0110115887399715	0.170747242106618	-2,38817E+14	8,06437E+14	7,82575E+14	6,58381E+14	4,27297E+13	6,29861E+14	6,02757E+14
TC1400001672.mm.2	Nisch	nischarin	2,2275E+14	8,70433E+14	3,53027E+14	0.0110266784288325	0.170747242106618	-2,38936E+14	1,02966E+14	1,01223E+14	8,81915E+14	7,14468E+14	8,47493E+14	7,36839E+14
TC1200000775.mm.2	Rgs6	regulator of G-protein signaling 6	1,40688E+13	6,46529E+14	3,52883E+14	0.0110467385078707	0.170747242106618	-2,39094E+14	7,25889E+14	7,30729E+13	6,2204E+13	5,53201E+13	6,59232E+13	5,8808E+14
TC0100001114.mm.2	Mgat5	mannoside acetylglucosaminyltransferase 5	1,03681E+14	7,75865E+14	3,52819E+14	0.0110557593243055	0.170747242106618	-2,39165E+14	8,39426E+14	8,32692E+14	7,38672E+14	7,26084E+14	7,50719E+14	7,67598E+14
TC1800001701.mm.2	Zfp407	zinc finger protein 407	2,25691E+14	5,34879E+14	3,52564E+13	0.0110916969499172	0.170747242106618	-2,39447E+14	6,69593E+14	6,55541E+12	5,30483E+14	3,43269E+14	5,46544E+14	4,63845E+14
TC0400000271.mm.2	Mdn1	midasin AAA ATPase 1	1,12335E+14	5,83356E+14	3,52532E+14	0.0110960976364982	0.170747242106618	-2,39481E+14	6,66407E+14	6,60698E+14	5,48634E+13	5,53801E+14	5,59702E+13	5,10896E+14
TC1100003562.mm.2	Cacna1g	calcium channel, voltage-dependent, T type, alpha 1G subunit	1,26466E+14	6,30248E+14	3,52416E+14	0.0111125710668575	0.170747242106618	-2,3961E+14	6,72011E+14	7,43924E+14	5,76413E+14	5,8659E+14	6,14212E+14	5,88336E+14
TC1100000058.mm.2	Ap1b1	adaptor protein complex AP-1, beta 1 subunit	2,24856E+14	6,04157E+14	3,52332E+14	0.0111244430129392	0.170747242106618	-2,39703E+14	8,07561E+13	7,84264E+14	6,45228E+14	4,96885E+14	5,14815E+14	3,76188E+14
TC0300000221.mm.2	Pik3ca	phosphatidylinositol-4,5-bisphosphate 3-kinase catalytic subunit alpha	1,85231E+14	7,26476E+14	3,52224E+14	0.0111396358118099	0.170747242106618	-2,39821E+14	8,62656E+14	8,56793E+14	7,15926E+14	6,33062E+14	7,12385E+14	5,78032E+14
TC0100003586.mm.2	Ackr1	atypical chemokine receptor 1 (Duffy blood group)	1,14368E+13	6,12071E+14	3,52176E+14	0.011146401629574	0.170747242106618	-2,39874E+14	6,76531E+14	6,95296E+14	5,89547E+14	5,53544E+14	5,96763E+14	5,60745E+14
TC1500000658.mm.2	Lgals1	lectin, galactose binding, soluble 1	2,04971E+14	7,32621E+14	3,52104E+14	0.011156669857693	0.170747242106618	-2,39954E+14	7,47956E+14	8,91377E+14	6,6997E+13	5,59421E+14	7,70129E+14	7,56872E+14
TC1100003774.mm.2	Klhl11	kelch-like 11	1,13493E+14	7,26334E+14	3,52066E+13	0.0111620935723456	0.170747242106618	-2,39996E+14	8,10541E+14	7,80758E+14	6,60832E+14	7,0348E+14	7,0442E+12	6,97976E+14
TC0300001582.mm.2	Lrrc40	leucine rich repeat containing 40	2,19429E+14	6,07408E+14	3,51886E+14	0.011187590757579	0.170747242106618	-2,40195E+12	7,70595E+14	7,64598E+13	5,85908E+14	5,10426E+14	5,98195E+14	4,14727E+14
TC0800001917.mm.2	Ddhd2	DDHD domain containing 2	1,25572E+14	7,53386E+14	3,51791E+14	0.011201097604222	0.170747242106618	-2,403E+14	8,47145E+13	8,37481E+14	7,50415E+14	6,83068E+14	7,17986E+14	6,84221E+13
TC0500003025.mm.2	Crybb2	crystallin, beta B2	-1,3588E+14	8,00708E+13	-3,51747E+14	0.0112074183680336	0.170747242106618	-2,40349E+14	6,96194E+14	7,15816E+14	8,09507E+13	8,74263E+14	8,22625E+14	8,85843E+13
TC0200002968.mm.2	Fbh1	F-box DNA helicase 1	1,32414E+13	6,78775E+14	3,51745E+14	0.0112076715485216	0.170747242106618	-2,40351E+14	7,86798E+14	7,62309E+14	6,83003E+13	6,01276E+14	6,28707E+14	6,10559E+14
TC1600001497.mm.2	Pak2	p21 (RAC1) activated kinase 2	1,21252E+14	6,57063E+14	3,51726E+14	0.0112103688638422	0.170747242106618	-2,40372E+14	7,26056E+14	7,34069E+14	6,14146E+14	6,03473E+14	6,65816E+14	5,98818E+14
TC0200005480.mm.2	Capn3	calpain 3	1,4563E+14	5,50194E+13	3,51705E+14	0.0112133577167549	0.170747242106618	-2,40395E+14	6,07813E+14	6,53867E+14	5,27332E+13	4,43088E+14	5,58066E+14	5,11E+14
TC1600002136.mm.2	Brwd1	bromodomain and WD repeat domain containing 1	1,4048E+14	5,73221E+14	3,51641E+13	0.0112225959921673	0.170747242106618	-2,40466E+14	6,49825E+13	6,40798E+14	5,47122E+14	4,6254E+14	5,95374E+14	5,43669E+14
TC1300002691.mm.2	Gzma	granzyme A	-1,19533E+14	5,36641E+14	-3,5155E+14	0.0112355813214207	0.170747242106618	-2,40567E+14	4,32923E+14	4,44098E+14	5,46231E+14	5,69855E+14	5,83502E+14	6,43238E+14
TC1200001813.mm.2	Sec23a	SEC23 homolog A, COPII coat complex component	2,84039E+14	6,44526E+14	3,51414E+14	0.0112549620722854	0.170747242106618	-2,40717E+14	7,95526E+14	8,14018E+14	6,41721E+14	3,99744E+14	6,68306E+14	5,47842E+14
TC0400001647.mm.2	Syf2	SYF2 homolog, RNA splicing factor (S. cerevisiae)	1,02153E+14	6,96848E+14	3,5141E+14	0.0112556146884608	0.170747242106618	-2,40722E+14	7,65662E+14	7,54615E+14	6,52443E+14	6,63529E+14	6,73583E+14	6,71256E+14
TC1100002854.mm.2	Zkscan17	zinc finger with KRAB and SCAN domains 17	-1,37688E+14	7,41896E+14	-3,51211E+14	0.0112841539057265	0.170747242106618	-2,40942E+14	6,53533E+14	6,5494E+14	7,45943E+14	8,37905E+14	7,66392E+14	7,92665E+14
TC0500002268.mm.2	Wfs1	wolframin ER transmembrane glycoprotein	1,3537E+14	8,51268E+14	3,51194E+14	0.0112864617142533	0.170747242106618	-2,4096E+14	9,59114E+14	9,48507E+14	8,61881E+14	7,74999E+14	7,67146E+14	7,95959E+14
TC0300002369.mm.2	Slc27a3	solute carrier family 27 (fatty acid transporter), member 3	-1,29037E+14	7,22301E+14	-3,5119E+14	0.011287116156391	0.170747242106618	-2,40965E+14	6,43282E+14	6,27132E+14	7,32867E+14	7,95622E+14	7,4222E+14	7,92682E+14
TC1200000990.mm.2	Psmc1	protease (prosome, macropain) 26S subunit, ATPase 1	1,20819E+14	8,4036E+14	3,51186E+14	0.0112876981408062	0.170747242106618	-2,40969E+14	9,19278E+14	9,04042E+14	8,17294E+14	7,64388E+14	8,38413E+14	7,98748E+14
TC0800001341.mm.2	Gabarapl2	gamma-aminobutyric acid (GABA) A receptor-associated protein-like 2	1,29489E+14	8,28833E+14	3,50996E+14	0.011315033838125	0.170747242106618	-2,4118E+14	9,11272E+14	9,0484E+13	8,01561E+14	7,55572E+14	8,34263E+14	7,65486E+14
TC0900001908.mm.2	Anln	anillin, actin binding protein	1,73496E+14	5,81493E+14	3,5089E+14	0.0113302694821496	0.170747242106618	-2,41297E+14	6,84066E+14	6,83657E+14	5,78668E+14	4,42064E+14	5,26992E+14	5,73512E+13
TC0400001445.mm.2	Grik3	glutamate receptor, ionotropic, kainate 3	1,76226E+14	6,66721E+14	3,50735E+14	0.0113527201830323	0.170747242106618	-2,41469E+14	8,0278E+14	7,90326E+14	6,80429E+14	5,60225E+14	5,4072E+13	6,2585E+14
TC1500000246.mm.2	Osr2	odd-skipped related 2	-1,17618E+14	5,11447E+14	-3,50517E+14	0.0113842100974563	0.170747242106618	-2,4171E+14	4,24439E+14	4,75586E+14	5,49985E+14	5,85275E+14	5,15967E+14	5,17429E+14
TC1100003437.mm.2	Ints2	integrator complex subunit 2	-1,1086E+14	5,90115E+14	-3,50301E+14	0.0114156157307198	0.170747242106618	-2,41949E+14	5,32926E+14	5,32887E+13	6,20056E+14	6,67477E+13	5,91756E+14	5,95588E+14
TC0200000545.mm.2	Tor1b	torsin family 1, member B	-1,00853E+14	7,73314E+14	-3,50276E+14	0.0114191927533244	0.170747242106618	-2,41977E+14	7,1538E+14	7,18586E+14	8,14929E+14	8,20744E+13	7,85353E+14	7,84891E+14
TC0700000369.mm.2	Zfp112	zinc finger protein 112	-1,5467E+14	5,85313E+14	-3,50275E+14	0.0114193325829423	0.170747242106618	-2,41978E+14	4,94542E+14	5,20349E+14	6,03982E+14	7,20249E+14	5,78075E+14	5,94679E+14
TC1700001950.mm.2	Prrc2a	proline-rich coiled-coil 2A	1,12398E+14	7,38497E+14	3,50272E+14	0.0114197284499269	0.170747242106618	-2,41981E+14	8,28322E+14	8,20717E+14	6,91745E+14	7,32498E+14	6,63795E+14	6,93907E+13
TC1800000827.mm.2	St8sia5	ST8 alpha-N-acetyl-neuraminide alpha-2,8-sialyltransferase 5	1,03136E+14	7,39324E+14	3,50074E+14	0.0114486316135932	0.170747242106618	-2,42201E+14	7,99543E+14	8,2064E+14	7,0834E+14	7,05572E+14	6,96118E+14	7,05733E+12
TC0X00001071.mm.2	Pgk1	phosphoglycerate kinase 1	1,54403E+14	8,65502E+14	3,50047E+14	0.0114525542417052	0.170747242106618	-2,4223E+14	9,62437E+14	9,56619E+14	8,44333E+14	7,65918E+14	8,77191E+14	7,86513E+14
TC0500001483.mm.2	Tmem132c	transmembrane protein 132C	1,38045E+14	5,48495E+14	3,50007E+14	0.0114584243430301	0.170747242106618	-2,42275E+14	6,70442E+14	6,16084E+14	5,06796E+14	5,0364E+14	5,3708E+14	4,5693E+14
TC0900002401.mm.2	Neo1	neogenin	2,58998E+14	7,10061E+14	3,49812E+14	0.0114869606554813	0.170747242106618	-2,42491E+14	9,05303E+14	8,9308E+14	7,51802E+14	5,28585E+14	6,40574E+14	5,41024E+14
TC0800002390.mm.2	Upf1	UPF1 regulator of nonsense transcripts homolog (yeast)	1,56543E+14	5,77339E+14	3,49722E+14	0.0115000805301256	0.170747242106618	-2,42591E+14	6,7347E+14	6,71599E+14	5,77442E+14	4,54543E+14	5,4017E+14	5,46809E+14
TC1600001917.mm.2	Hspa13	heat shock protein 70 family, member 13	1,71987E+14	8,07536E+14	3,49551E+14	0.0115252715329677	0.170747242106618	-2,42781E+14	9,20286E+14	9,04827E+14	8,04758E+14	6,76382E+13	8,00298E+14	7,38667E+14
TC0500000837.mm.2	5830473C10Rik	RIKEN cDNA 5830473C10 gene	-1,36338E+13	6,15171E+14	-3,49476E+14	0.011536197090196	0.170747242106618	-2,42864E+14	5,54339E+14	5,02246E+14	6,27894E+14	7,01366E+14	6,38058E+14	6,67121E+14
TC1600001054.mm.2	Ripply3	ripply transcriptional repressor 3	-1,74645E+14	7,08152E+14	-3,4937E+14	0.0115518117324406	0.170747242106618	-2,42981E+14	5,67219E+14	6,06596E+13	6,90945E+14	8,3216E+14	7,73325E+14	7,78667E+14
TC1000000473.mm.2	Slc35f1	solute carrier family 35, member F1	1,73373E+14	7,27017E+14	3,49283E+14	0.011564608163266	0.170747242106618	-2,43078E+14	8,56536E+14	8,57898E+14	7,33298E+14	6,34389E+14	6,93107E+14	5,86874E+14
TC1700002338.mm.2	Acsbg2	acyl-CoA synthetase bubblegum family member 2	-1,28826E+14	5,71354E+14	-3,49203E+14	0.0115764349406308	0.170747242106618	-2,43167E+14	4,75366E+14	4,9354E+14	5,72882E+14	6,53675E+14	6,13674E+14	6,18985E+13
TC1100000863.mm.2	Gsg1l2	GSG1-like 2	-1,73783E+14	7,17085E+14	-3,49181E+14	0.0115796453142198	0.170747242106618	-2,43191E+14	5,90827E+14	5,91148E+14	7,02551E+14	8,26989E+14	7,5757E+14	8,33423E+14
TC0300002802.mm.2	Vcam1	vascular cell adhesion molecule 1	1,46657E+14	5,84935E+14	3,49171E+14	0.0115811320539475	0.170747242106618	-2,43202E+14	6,32238E+12	7,04576E+14	5,08818E+14	5,34681E+14	5,25376E+14	6,03922E+14
TC0200000518.mm.2	Sptan1	spectrin alpha, non-erythrocytic 1	1,17214E+13	8,59225E+14	3,49152E+14	0.0115840386323471	0.170747242106618	-2,43224E+14	9,29275E+14	9,381E+14	8,42063E+14	7,90885E+13	8,44688E+14	8,10337E+13
TC1900000467.mm.2	Papss2	3'-phosphoadenosine 5'-phosphosulfate synthase 2	1,03349E+13	6,61594E+13	3,48992E+14	0.0116076340341578	0.170747242106618	-2,43401E+14	7,64819E+14	7,59206E+12	6,63854E+14	6,53474E+14	5,52684E+14	5,75527E+14
TC0X00001178.mm.2	Pcdh11x	protocadherin 11 X-linked	1,92166E+14	6,03485E+14	3,48979E+14	0.0116095841405545	0.170747242106618	-2,43416E+14	7,67928E+14	7,11427E+14	5,57724E+14	5,373E+14	6,01958E+12	4,44572E+14
TC0900002757.mm.2	Phip	pleckstrin homology domain interacting protein	2,45028E+14	6,38455E+14	3,48968E+14	0.0116111582503788	0.170747242106618	-2,43427E+14	7,98349E+14	7,88795E+13	6,14761E+14	4,82326E+14	6,67005E+14	4,79494E+14
TC1000001691.mm.2	Ipcef1	interaction protein for cytohesin exchange factors 1	1,26963E+14	6,53247E+14	3,48901E+14	0.0116211793412764	0.170747242106618	-2,43502E+14	7,19072E+14	7,64035E+14	6,16738E+14	6,12445E+14	6,36658E+14	5,70531E+14
TC0700004282.mm.2	Eif3c	eukaryotic translation initiation factor 3, subunit C	1,7703E+14	8,66428E+14	3,48882E+14	0.0116239887667616	0.170747242106618	-2,43524E+14	9,81676E+14	9,81647E+14	8,74556E+14	7,34707E+14	8,40101E+13	7,85884E+14
TC0200002435.mm.2	Adig	adipogenin	-1,20953E+14	5,47638E+13	-3,48765E+14	0.0116413441885388	0.170747242106618	-2,43653E+14	4,71128E+14	4,73799E+14	5,67395E+14	6,19439E+13	5,5393E+14	6,00137E+14
TC0700000789.mm.2	Klk1b21	kallikrein 1-related peptidase b21	-1,78147E+14	5,12956E+14	-3,48639E+14	0.0116601175366062	0.170747242106618	-2,43794E+14	3,98785E+14	3,85046E+14	5,14879E+14	6,25246E+14	5,25427E+14	6,28352E+14
TC1000001575.mm.2	Rdh19	retinol dehydrogenase 19	-1,74066E+14	5,87049E+14	-3,48578E+14	0.0116691828216543	0.170747242106618	-2,43861E+13	4,66162E+13	5,03385E+14	5,88517E+14	7,29161E+14	6,09064E+14	6,26007E+14
TC0X00001004.mm.2	Dmrtc1c2/Dmrtc1c1	DMRT-like family C1c2/DMRT-like family C1c1	-1,75158E+14	6,4724E+14	-3,48539E+14	0.0116750122235915	0.170747242106618	-2,43905E+14	5,16842E+14	5,29659E+13	6,43713E+14	7,53104E+14	6,7073E+14	7,6939E+13
TC0400000179.mm.2	Usp45	ubiquitin specific petidase 45	1,83067E+14	5,99318E+13	3,48537E+12	0.0116752575114736	0.170747242106618	-2,43907E+14	7,00375E+14	7,23982E+14	6,04792E+14	4,5343E+14	5,75353E+14	5,37979E+14
TC1300002535.mm.2	Gtf2h2	general transcription factor II H, polypeptide 2	1,0682E+13	5,83405E+14	3,48435E+14	0.0116904670640905	0.170747242106618	-2,4402E+14	6,67861E+14	6,3811E+14	5,4395E+14	5,48382E+14	5,39176E+14	5,62948E+14
TC0X00001054.mm.2	Pbdc1	polysaccharide biosynthesis domain containing 1	1,09389E+14	6,91724E+14	3,48418E+14	0.0116930736193256	0.170747242106618	-2,44039E+14	7,64539E+14	7,70817E+13	6,74851E+13	6,41727E+14	6,64393E+14	6,34014E+14
TC1300001135.mm.2	Serf1	small EDRK-rich factor 1	-1,2988E+13	6,65564E+14	-3,4815E+14	0.0117330971281191	0.170747242106618	-2,44337E+14	6,14494E+14	5,73028E+14	7,37704E+14	7,09578E+14	7,13826E+14	6,44752E+14
TC0X00001056.mm.2	Magee1	melanoma antigen, family E, 1	2,24666E+14	6,70925E+14	3,47894E+14	0.0117714574598258	0.170747242106618	-2,44621E+14	8,18666E+14	7,96006E+14	6,80429E+14	4,84912E+14	6,55475E+14	5,90063E+14
TC0700002057.mm.2	Ap2a2	adaptor-related protein complex 2, alpha 2 subunit	2,74913E+14	6,64363E+14	3,47881E+14	0.0117734860365134	0.170747242106618	-2,44636E+14	8,21043E+14	8,32842E+14	6,82471E+14	4,21587E+14	6,33493E+13	5,94742E+14
TC1700000009.mm.2	Tiam2	T cell lymphoma invasion and metastasis 2	1,68121E+13	7,46232E+13	3,47876E+14	0.0117742468037793	0.170747242106618	-2,44642E+14	8,25885E+14	8,68917E+14	7,34603E+14	6,23956E+14	7,48507E+14	6,75524E+14
TC1500002348.mm.2	Vps28	vacuolar protein sorting 28	1,5307E+13	9,07719E+14	3,4768E+13	0.0118037621425379	0.170747242106618	-2,4486E+14	1,01417E+14	9,88896E+14	9,05628E+14	7,913E+14	8,85424E+14	8,60897E+14
TC1800000625.mm.2	Csf1r	colony stimulating factor 1 receptor	1,80142E+14	7,6956E+12	3,47647E+14	0.0118086445195061	0.170747242106618	-2,44896E+14	8,69125E+14	8,96444E+14	7,73015E+14	6,32271E+14	6,94269E+14	7,52236E+14
TC0700000070.mm.2	Zfp444	zinc finger protein 444	-1,39427E+14	8,02762E+14	-3,47627E+14	0.0118116641987509	0.170747242106618	-2,44918E+13	7,17416E+14	6,91134E+14	8,11186E+13	8,76217E+14	8,24421E+14	8,96198E+14
TC0X00000343.mm.2	Rhox2b	reproductive homeobox 2B	-1,21857E+13	6,5654E+14	-3,47553E+14	0.0118228686638031	0.170747242106618	-2,45001E+14	5,84994E+14	5,87748E+14	6,73547E+14	7,42911E+14	6,84687E+14	6,65354E+13
TC0600002305.mm.2	Tril	TLR4 interactor with leucine-rich repeats	1,63036E+14	6,4438E+14	3,47535E+14	0.0118255783849166	0.170747242106618	-2,45021E+14	7,53207E+14	7,44639E+14	6,5166E+14	5,20114E+14	6,07119E+14	5,89541E+14
TC1700000335.mm.2	Abca3	ATP-binding cassette, sub-family A (ABC1), member 3	1,58025E+14	7,56397E+13	3,47495E+14	0.0118317423185366	0.170747242106618	-2,45066E+14	8,89144E+14	8,88987E+12	7,62564E+14	6,99518E+14	7,03869E+14	5,94303E+14
TC0200002797.mm.2	Col9a3	collagen, type IX, alpha 3	-1,01912E+13	7,58127E+14	-3,47342E+14	0.0118547639793062	0.170747242106618	-2,45235E+14	6,86836E+13	6,90361E+14	7,83127E+14	7,97893E+14	7,87798E+14	8,02748E+14
TC0600000236.mm.2	Irf5	interferon regulatory factor 5	-1,40423E+14	6,16325E+14	-3,47323E+14	0.0118576728053427	0.170747242106618	-2,45257E+14	5,43273E+14	5,12242E+13	6,21234E+14	7,15127E+14	6,40525E+14	6,65548E+14
TC1000000547.mm.2	Psap	prosaposin	1,33646E+14	1,05685E+14	3,47191E+14	0.0118778166080922	0.170747242106618	-2,45405E+14	1,1487E+14	1,1542E+14	1,06103E+14	9,74579E+14	1,01657E+13	9,85994E+14
TC1500001896.mm.2	Rpl3	ribosomal protein L3	1,33166E+14	1,01117E+14	3,47186E+14	0.0118785509507086	0.170747242106618	-2,4541E+14	1,10059E+13	1,10236E+14	1,00163E+14	9,34989E+14	9,94789E+14	9,32672E+14
TC1700000374.mm.2	Tpsb2	tryptase beta 2	-1,55468E+14	6,23776E+14	-3,47128E+14	0.0118873206632467	0.170747242106618	-2,45474E+14	5,3234E+14	5,28334E+14	6,30133E+14	7,41478E+14	6,28884E+14	6,81483E+14
TC1400000744.mm.2	Mrpl52	mitochondrial ribosomal protein L52	1,3673E+14	7,26525E+12	3,47052E+14	0.011898834095955	0.170747242106618	-2,45559E+14	7,97355E+13	7,99385E+14	7,01974E+14	6,21306E+14	7,46182E+14	6,92949E+14
TC1100003450.mm.2	Vmp1/Mir21a	vacuole membrane protein 1/microRNA 21a	1,59027E+13	7,63655E+14	3,46848E+14	0.011929929221391	0.170747242106618	-2,45786E+14	8,63682E+14	8,6767E+14	7,55586E+14	6,57711E+14	7,59943E+14	6,7734E+14
TC0300001955.mm.2	Mgarp	mitochondria localized glutamic acid rich protein	-1,26949E+14	4,68142E+13	-3,46817E+14	0.0119346341345714	0.170747242106618	-2,4582E+14	4,06353E+14	3,61417E+14	4,76954E+14	5,44714E+13	5,10669E+14	5,08745E+14
TC1100000170.mm.2	C1d	C1D nuclear receptor co-repressor	1,08633E+14	7,25125E+13	3,46744E+14	0.0119459080292737	0.170747242106618	-2,45903E+14	8,05871E+14	7,93219E+14	6,98193E+14	6,8363E+14	7,05129E+14	6,64707E+14
TC1900000111.mm.2	Ehd1	EH-domain containing 1	1,49509E+14	6,1808E+14	3,46676E+14	0.0119562046763817	0.170747242106618	-2,45978E+14	7,02252E+14	6,99326E+14	6,08826E+13	4,93733E+14	5,98715E+14	6,05631E+14
TC1900001779.mm.2	Itprip	inositol 1,4,5-triphosphate receptor interacting protein	-1,29639E+14	7,16907E+14	-3,46674E+13	0.0119566107835058	0.170747242106618	-2,45981E+14	6,2856E+14	6,14009E+14	7,08791E+14	7,93057E+14	7,7481E+14	7,82216E+14
TC1200000021.mm.2	Adcy3	adenylate cyclase 3	1,33376E+14	6,41504E+14	3,46665E+14	0.0119579656134748	0.170747242106618	-2,45991E+14	7,46315E+14	7,05415E+14	6,32313E+14	5,52665E+14	6,16204E+14	5,96113E+14
TC0100001527.mm.2	Cd247	CD247 antigen	-1,56201E+14	6,89322E+14	-3,46652E+14	0.0119598778712376	0.170747242106618	-2,46005E+14	5,94806E+14	5,91592E+14	6,8858E+14	8,10221E+14	7,12244E+14	7,3849E+14
TC0X00000212.mm.2	Uba1	ubiquitin-like modifier activating enzyme 1	1,68942E+13	8,88758E+14	3,46636E+13	0.0119623757609015	0.170747242106618	-2,46023E+14	1,00264E+14	1,01317E+14	9,02735E+13	7,75184E+14	8,49214E+14	7,89611E+12
TC0700002004.mm.2	Mtg1	mitochondrial ribosome-associated GTPase 1	1,05106E+14	6,06038E+14	3,46611E+14	0.0119661970766897	0.170747242106618	-2,46051E+14	6,91703E+14	6,89406E+14	5,70894E+14	6,00003E+14	5,32676E+14	5,51546E+14
TC0X00002805.mm.2	Magee2	melanoma antigen, family E, 2	1,96801E+14	5,58585E+14	3,46545E+14	0.0119763299680466	0.170747242106618	-2,46124E+14	7,11513E+14	6,46897E+14	5,63942E+14	4,00866E+13	5,09356E+14	5,18932E+14
TC0300000397.mm.2	Stoml3	stomatin (Epb7.2)-like 3	1,82557E+13	6,15052E+14	3,46492E+14	0.0119843850011583	0.170747242106618	-2,46183E+14	7,37801E+14	7,51844E+14	6,20704E+13	5,03827E+14	4,85088E+14	5,91046E+14
TC0600001964.mm.2	Zfp800	zinc finger protein 800	2,09366E+14	7,00171E+14	3,46479E+14	0.0119864414153761	0.170747242106618	-2,46198E+14	8,25139E+14	8,0311E+14	6,893E+14	5,20217E+14	7,24259E+14	6,38997E+14
TC1000002537.mm.2	Gipc3	GIPC PDZ domain containing family, member 3	-1,36287E+14	6,86005E+14	-3,46456E+14	0.0119899888384366	0.170747242106618	-2,46224E+14	5,93824E+14	5,9961E+14	6,87731E+14	7,78278E+14	7,12075E+13	7,44511E+14
TC0300000459.mm.2	Selenot	selenoprotein T	1,27026E+14	9,1695E+14	3,46406E+14	0.0119976058322134	0.170747242106618	-2,46279E+14	1,00524E+14	9,81714E+13	8,99483E+14	8,33416E+14	9,11893E+14	8,69961E+14
TC1100001011.mm.2	P2rx1	purinergic receptor P2X, ligand-gated ion channel, 1	-1,26694E+14	5,74235E+14	-3,46402E+14	0.0119982233389364	0.170747242106618	-2,46284E+14	4,70251E+14	4,94943E+14	5,72699E+14	6,45883E+13	6,18359E+14	6,43276E+14
TC0800002021.mm.2	Dctn6	dynactin 6	2,02332E+14	8,41816E+14	3,46188E+14	0.0120311329261934	0.170747242106618	-2,46522E+14	9,83172E+14	9,69743E+14	8,51998E+14	6,96253E+14	8,21978E+14	7,27751E+14
TC0700000461.mm.2	Prx	periaxin	-1,16781E+12	6,27554E+14	-3,4603E+14	0.0120554726262137	0.170747242106618	-2,46698E+14	5,49346E+14	5,42892E+14	6,41441E+14	6,84358E+14	6,50473E+14	6,96813E+14
TC1900001691.mm.2	Gpam	glycerol-3-phosphate acyltransferase, mitochondrial	2,10199E+14	7,25539E+14	3,45879E+14	0.0120787602927643	0.170747242106618	-2,46867E+14	8,5536E+14	8,51088E+14	7,38462E+14	5,47588E+14	6,90755E+14	6,69982E+14
TC0600002055.mm.2	Cyren	cell cycle regulator of NHEJ	-1,74718E+14	7,25711E+14	-3,45802E+14	0.0120907648079642	0.170747242106618	-2,46953E+13	5,84305E+14	6,2324E+14	7,0656E+14	8,50422E+14	7,94056E+14	7,95685E+14
TC0700000917.mm.2	Gas2	growth arrest specific 2	1,43381E+14	7,09129E+14	3,45649E+14	0.0121144228953943	0.170747242106618	-2,47124E+14	8,16674E+14	7,31733E+12	6,59307E+14	6,02338E+14	7,38583E+14	7,06137E+14
TC0800000353.mm.2	Eif4ebp1	eukaryotic translation initiation factor 4E binding protein 1	-1,60575E+14	6,2613E+14	-3,45568E+14	0.0121270380756566	0.170747242106618	-2,47214E+14	4,70966E+13	5,35971E+14	6,27493E+14	7,00595E+14	7,54415E+14	6,67337E+14
TC0200000274.mm.2	Plxdc2	plexin domain containing 2	1,98207E+14	8,18955E+13	3,45541E+12	0.0121311356269786	0.170747242106618	-2,47244E+14	9,57742E+14	9,5278E+14	8,35995E+14	6,78113E+14	7,85627E+13	7,03476E+14

TC1200000303.mm.2	Snx13	sorting nexin 13	1,93251E+14	7,07078E+14	3,45529E+14	0.0121330743597715	0.170747242106618	-2,47258E+14	8,2166E+13	8,17974E+14	6,82702E+14	5,70429E+14	7,40034E+14	6,09671E+14
TC1000001555.mm.2	Dctn2	dynactin 2	1,57663E+14	8,36791E+14	3,4552E+14	0.0121345374038565	0.170747242106618	-2,47268E+14	9,45641E+14	9,35498E+14	8,34017E+14	7,31795E+14	8,24347E+14	7,49451E+14
TC0900002105.mm.2	Crtam	cytotoxic and regulatory T cell molecule	-1,42032E+14	5,68888E+14	-3,45352E+14	0.0121605518489203	0.170747242106618	-2,47455E+13	4,86419E+13	4,75726E+14	5,7064E+14	6,75569E+14	6,04186E+14	6,00788E+14
TC1500000041.mm.2	Rictor	RPTOR independent companion of MTOR, complex 2	1,45034E+14	7,11511E+14	3,45232E+14	0.012179276438828	0.170747242106618	-2,47589E+14	7,88014E+14	8,00622E+14	7,02479E+14	5,96089E+14	7,03212E+14	6,7865E+14
TC0400001472.mm.2	AU040320	expressed sequence AU040320	1,11242E+14	7,19412E+14	3,45171E+14	0.0121888308323855	0.170747242106618	-2,47658E+14	7,99821E+14	7,96749E+14	7,07368E+14	6,66718E+14	6,8933E+14	6,56486E+14
TC1100003935.mm.2	Ddx5/Mir3064	DEAD (Asp-Glu-Ala-Asp) box polypeptide 5/microRNA 3064	1,15928E+14	1,00298E+13	3,45168E+14	0.0121893337301309	0.170747242106618	-2,47661E+14	1,07763E+14	1,07663E+14	9,72184E+14	9,50228E+14	9,99783E+14	9,41415E+14
TC1100003753.mm.2	Krt35	keratin 35	-1,62346E+14	4,57297E+14	-3,45152E+14	0.0121918899553983	0.170747242106618	-2,47679E+14	3,68766E+13	3,43928E+14	4,99351E+14	5,38035E+14	4,34155E+14	5,59547E+14
TC0300000831.mm.2	Sprr2k	small proline-rich protein 2K	-1,08863E+14	6,46494E+14	-3,45092E+14	0.0122012173457382	0.170747242106618	-2,47746E+14	5,69971E+14	5,71541E+14	6,63803E+14	6,95435E+14	6,71946E+14	7,0627E+14
TC1100003841.mm.2	Gpatch8	G patch domain containing 8	1,7641E+13	5,81845E+14	3,45056E+14	0.0122068587230347	0.170747242106618	-2,47786E+14	7,20414E+13	7,14638E+14	5,65976E+14	5,16256E+13	5,58472E+14	4,15317E+14
TC0400000390.mm.2	Gm21953/Gm21586/Gm20878	predicted gene, 21953/predicted gene, 21586/predicted gene, 20878	1,33456E+14	9,39378E+14	3,44953E+14	0.0122230021377691	0.170747242106618	-2,47902E+14	9,92889E+14	1,02789E+14	8,97952E+14	8,55911E+14	9,68365E+14	8,93262E+13
TC0X00002794.mm.2	Nexmif	neurite extension and migration factor	1,81933E+14	5,91343E+13	3,44859E+14	0.0122376511858685	0.170747242106618	-2,48006E+14	7,37058E+14	7,10388E+14	5,24743E+14	5,58837E+14	5,84844E+14	4,32188E+14
TC1500000022.mm.2	Ttc33	tetratricopeptide repeat domain 33	1,60875E+14	7,51233E+14	3,44859E+14	0.0122377530093505	0.170747242106618	-2,48007E+14	8,64404E+14	8,32856E+14	7,42597E+14	6,32912E+14	7,50273E+14	6,84359E+14
TC1600002135.mm.2	Psmg1	proteasome (prosome, macropain) assembly chaperone 1	1,03899E+14	6,12798E+14	3,44832E+14	0.0122418593805434	0.170747242106618	-2,48036E+14	6,62057E+14	6,70562E+14	5,65648E+13	5,59172E+14	5,94313E+14	6,25036E+14
TC1000002539.mm.2	4930404N11Rik	RIKEN cDNA 4930404N11 gene	-1,76145E+14	6,79204E+14	-3,44747E+14	0.0122553515098689	0.170747242106618	-2,48132E+14	5,74522E+14	5,39828E+14	6,6556E+14	8,0108E+14	7,18011E+14	7,76225E+14
TC1400001696.mm.2	Timm23	translocase of inner mitochondrial membrane 23	1,24644E+14	7,83038E+14	3,44685E+13	0.0122649481146791	0.170747242106618	-2,482E+14	8,72869E+14	8,54502E+14	7,56148E+14	7,21935E+14	7,80639E+14	7,12137E+14
TC0500003480.mm.2	1700123K08Rik	RIKEN cDNA 1700123K08 gene	-1,57232E+14	6,14279E+14	-3,44611E+14	0.0122766080677453	0.170747242106618	-2,48283E+14	5,08314E+14	4,91404E+14	6,00071E+14	7,14111E+14	6,59124E+14	7,1265E+14
TC1900001595.mm.2	Pitx3	paired-like homeodomain transcription factor 3	-1,2862E+14	6,08759E+14	-3,44497E+14	0.0122945665706686	0.170747242106618	-2,48411E+13	5,27828E+12	5,1998E+14	6,24463E+14	6,80585E+14	6,17837E+14	6,81859E+14
TC1800001252.mm.2	Grxcr2	glutaredoxin, cysteine rich 2	-1,37233E+14	6,25268E+14	-3,44433E+14	0.0123047599021604	0.170747242106618	-2,48483E+14	5,72067E+14	5,32881E+14	6,44346E+14	7,35067E+14	6,30739E+14	6,36505E+14
TC1600001071.mm.2	Ets2	E26 avian leukemia oncogene 2, 3' domain	1,78056E+14	6,80351E+13	3,44409E+14	0.0123085438193608	0.170747242106618	-2,4851E+13	8,05054E+14	7,89212E+14	6,91406E+11	5,46749E+14	6,50245E+14	5,99438E+14
TC1400001094.mm.2	Vwa8	von Willebrand factor A domain containing 8	1,02044E+14	6,32106E+14	3,44311E+14	0.0123239365449882	0.170747242106618	-2,48619E+14	7,07507E+14	7,05012E+14	6,09302E+14	5,99129E+14	5,97773E+14	5,73915E+13
TC1600002175.mm.2	Pcp4	Purkinje cell protein 4	1,10335E+14	9,74342E+14	3,442E+14	0.0123415397547708	0.170747242106618	-2,48743E+14	1,06397E+14	1,03722E+14	9,21946E+13	9,58577E+14	9,43792E+14	9,20543E+14
TC0X00000902.mm.2	Zxdb	zinc finger, X-linked, duplicated B	1,63572E+14	5,3554E+14	3,44199E+13	0.0123416770178044	0.170747242106618	-2,48744E+14	6,63481E+14	6,6307E+14	5,04148E+13	4,95258E+14	5,11372E+13	3,75913E+14
TC0X00001201.mm.2	Cldn34c1	claudin 34C1	-1,06926E+14	8,20078E+14	-3,44154E+14	0.0123488106543342	0.170747242106618	-2,48795E+14	7,49433E+14	7,40923E+14	8,32853E+13	8,71356E+14	8,55167E+13	8,70733E+14
TC1700000670.mm.2	Bag6	BCL2-associated athanogene 6	1,36759E+13	8,19225E+14	3,44044E+14	0.0123663297566601	0.170747242106618	-2,48919E+14	9,17562E+14	9,06412E+14	8,24078E+14	7,26379E+14	7,73413E+14	7,6751E+14
TC0500003116.mm.2	Hspb8	heat shock protein 8	1,39363E+14	5,39639E+14	3,43976E+14	0.0123770574793495	0.170747242106618	-2,48994E+14	6,33144E+14	6,52825E+14	4,78908E+14	5,28334E+14	5,16083E+14	4,28541E+14
TC1000000571.mm.2	Ppa1	pyrophosphatase (inorganic) 1	1,13597E+14	8,11283E+14	3,43873E+14	0.0123934132701162	0.170747242106618	-2,49109E+14	9,04863E+14	8,75282E+14	8,00989E+14	7,51963E+14	7,6141E+14	7,73194E+14
TC1500001683.mm.2	Kcnk9	potassium channel, subfamily K, member 9	1,1496E+14	6,49411E+14	3,43789E+13	0.0124068868833101	0.170747242106618	-2,49204E+14	7,43691E+14	7,01985E+14	5,86954E+12	6,28802E+14	6,09258E+14	6,25778E+14
TC1400001718.mm.2	A630023A22Rik	RIKEN cDNA A630023A22 gene	-1,31744E+13	5,52259E+14	-3,43646E+14	0.012429622000363	0.170747242106618	-2,49364E+14	5,06517E+14	4,36479E+14	5,9953E+14	6,06954E+14	5,53208E+14	6,10866E+14
TC1500001345.mm.2	Rpl30/Gm12191	ribosomal protein L30/ribosomal protein L30 pseudogene	1,79529E+14	8,62422E+14	3,43619E+14	0.012433917852784	0.170747242106618	-2,49394E+14	9,49167E+13	9,80138E+14	8,57327E+14	7,1292E+14	8,63361E+14	8,11618E+14
TC0900000368.mm.2	Esam	endothelial cell-specific adhesion molecule	-1,51268E+14	8,39991E+14	-3,43522E+14	0.0124494201303632	0.170747242106618	-2,49503E+14	7,47132E+14	7,48896E+14	8,43472E+13	9,5509E+14	8,51122E+14	8,94237E+13
TC0500001382.mm.2	Hvcn1	hydrogen voltage-gated channel 1	-1,17379E+14	5,42782E+14	-3,43485E+14	0.0124553987232163	0.170747242106618	-2,49545E+14	4,73933E+14	4,57801E+14	5,57278E+14	6,09214E+14	5,60233E+13	5,98234E+14
TC0800001872.mm.2	Polb	polymerase (DNA directed), beta	1,27725E+14	6,25359E+14	3,43415E+14	0.0124665240407269	0.170747242106618	-2,49623E+14	6,80449E+14	7,01596E+14	5,87809E+14	5,38786E+13	6,54632E+14	5,88884E+14
TC0X00002205.mm.2	1700080O16Rik	RIKEN cDNA 1700080O16 gene	-1,13907E+14	4,91908E+14	-3,4328E+14	0.0124881050681457	0.170747242106618	-2,49774E+14	3,97877E+14	4,45299E+14	5,19875E+14	5,51114E+14	5,09722E+14	5,27563E+14
TC0600002098.mm.2	Zc3hav1l	zinc finger CCCH-type, antiviral 1-like	-1,22473E+13	6,82055E+14	-3,43278E+14	0.0124885537327216	0.170747242106618	-2,49777E+14	6,13491E+13	6,01187E+14	7,61635E+14	6,97988E+14	6,89325E+14	7,28705E+14
TC0700003018.mm.2	Spaca4	sperm acrosome associated 4	-1,10296E+14	7,54506E+14	-3,43268E+14	0.0124901065845925	0.170747242106618	-2,49788E+14	7,03329E+14	6,56302E+14	7,8118E+14	7,99043E+14	7,99284E+14	7,87896E+14
TC1600000756.mm.2	Filip1l	filamin A interacting protein 1-like	-1,09915E+14	4,62582E+13	-3,43237E+14	0.0124950349304439	0.170747242106618	-2,49822E+14	4,0274E+14	3,6864E+14	4,89965E+14	5,01244E+14	4,88555E+14	5,24347E+14
TC1400000344.mm.2	Ogdhl	oxoglutarate dehydrogenase-like	1,46389E+14	7,31261E+14	3,43213E+14	0.0124988362582763	0.170747242106618	-2,49849E+14	8,60848E+14	8,14829E+14	7,15908E+14	6,66991E+14	7,09963E+14	6,19027E+14
TC0700001869.mm.2	Bckdk	branched chain ketoacid dehydrogenase kinase	1,33913E+14	6,76448E+14	3,43152E+13	0.0125087413962697	0.170747242106618	-2,49918E+14	7,61617E+14	7,41756E+14	6,44411E+14	5,91136E+14	6,22014E+14	6,97751E+14
TC0700001490.mm.2	Olfr598	olfactory receptor 598	-1,16564E+14	4,97816E+14	-3,4315E+14	0.0125090134918404	0.170747242106618	-2,4992E+14	4,07151E+14	4,39099E+14	5,23662E+13	5,55716E+14	5,07318E+14	5,53948E+14
TC0200000060.mm.2	Usp6nl	USP6 N-terminal like	1,12372E+14	6,79067E+14	3,43127E+14	0.0125127984248108	0.170747242106618	-2,49946E+14	7,55004E+14	7,64041E+14	6,7495E+14	6,1935E+14	6,29637E+14	6,31422E+14
TC0100001175.mm.2	Pm20d1	peptidase M20 domain containing 1	-1,58035E+14	5,90616E+14	-3,43076E+14	0.0125208550258856	0.170747242106618	-2,50002E+14	4,76748E+14	4,77574E+14	5,87165E+14	6,83227E+14	6,16352E+14	7,02631E+14
TC0X00000437.mm.2	Slc25a14	solute carrier family 25 (mitochondrial carrier, brain), member 14	1,86111E+14	6,4066E+14	3,43056E+14	0.0125241875242926	0.170747242106618	-2,50025E+14	7,8775E+14	7,72073E+14	6,21003E+14	5,66597E+14	6,25777E+14	4,70762E+14
TC1100003345.mm.2	Myo1d	myosin ID	1,0163E+14	6,02745E+14	3,43049E+14	0.0125252667031475	0.170747242106618	-2,50033E+14	6,80101E+14	6,63915E+14	5,6175E+14	5,79007E+13	5,71101E+14	5,60598E+13
TC1600000436.mm.2	Rnf168	ring finger protein 168	1,10311E+14	7,29608E+14	3,43029E+14	0.0125284978788697	0.170747242106618	-2,50055E+14	8,00526E+13	8,10722E+13	7,18707E+14	6,71919E+14	6,97755E+14	6,78019E+14
TC1900000516.mm.2	Rpp30	ribonuclease P/MRP 30 subunit	1,4828E+14	6,91457E+14	3,42979E+14	0.0125365609748887	0.170747242106618	-2,50112E+14	7,9241E+14	7,86834E+14	6,81864E+14	6,0082E+14	6,84705E+14	6,02109E+14
TC0600000998.mm.2	Aak1	AP2 associated kinase 1	1,6095E+14	8,98058E+14	3,42892E+14	0.0125505528991308	0.170747242106618	-2,50209E+14	1,00743E+14	9,91652E+14	8,97011E+14	7,80173E+14	8,86938E+14	8,2514E+13
TC0Y00000547.mm.2	Gm20871	Sycp3 like Y-linked pseudogene	-1,0691E+12	4,91483E+14	-3,42883E+14	0.0125520773585596	0.170747242106618	-2,5022E+13	4,10686E+14	3,9849E+13	5,25457E+14	4,97538E+14	5,42893E+14	5,73836E+14
TC0700003641.mm.2	Il16	interleukin 16	-1,24213E+14	5,65661E+14	-3,42789E+14	0.0125671059746699	0.170747242106618	-2,50324E+14	4,69939E+14	5,01353E+14	5,78252E+12	6,41467E+14	6,19414E+14	5,83543E+14
TC1900000703.mm.2	Trim8	tripartite motif-containing 8	1,67755E+14	7,03162E+13	3,42653E+14	0.0125891561977104	0.170747242106618	-2,50477E+14	7,88057E+14	8,03504E+14	6,90591E+14	5,65459E+14	7,18514E+13	6,52845E+14
TC0900000888.mm.2	Vps13c	vacuolar protein sorting 13C	2,60988E+14	5,86501E+14	3,42639E+13	0.0125913611593466	0.170747242106618	-2,50492E+14	7,53443E+14	7,85523E+14	5,70505E+14	4,46485E+14	5,90604E+14	3,72448E+14
TC0700004277.mm.2	Cd19	CD19 antigen	-1,34148E+14	4,73624E+14	-3,42638E+14	0.0125916359852103	0.170747242106618	-2,50494E+14	3,85963E+14	3,93288E+14	4,77484E+13	5,70064E+14	4,96608E+13	5,18336E+14
TC1300002037.mm.2	Cltb	clathrin, light polypeptide (Lcb)	1,43457E+14	8,51918E+14	3,42599E+14	0.0125979737206351	0.170747242106618	-2,50538E+14	9,56931E+14	9,44574E+14	8,55637E+14	7,58954E+14	8,22367E+14	7,73045E+13
TC1500001785.mm.2	Cyhr1	cysteine and histidine rich 1	1,23055E+14	6,311E+14	3,4251E+14	0.0126122718506413	0.170747242106618	-2,50637E+14	7,31304E+14	7,05357E+14	6,23964E+14	5,66587E+14	5,57236E+14	6,02155E+14
TC1900000205.mm.2	Ddb1	damage specific DNA binding protein 1	2,77545E+14	7,04566E+13	3,42444E+14	0.0126230217798988	0.170747242106618	-2,50712E+14	8,67552E+14	8,81159E+14	7,28272E+14	4,65349E+12	6,76269E+14	6,08796E+14
TC1200001281.mm.2	Crip2	cysteine rich protein 2	1,16665E+14	8,17164E+14	3,42401E+14	0.0126299454742787	0.170747242106618	-2,5076E+14	8,70889E+14	8,98282E+14	7,97641E+14	7,382E+13	7,96257E+14	8,01713E+14
TC1500001585.mm.2	Mtss1	MTSS I-BAR domain containing 1	1,15642E+12	6,25914E+14	3,42385E+14	0.0126326485433856	0.170747242106618	-2,50778E+14	7,25337E+14	6,7948E+14	6,08023E+14	5,6551E+14	5,94565E+14	5,82566E+14
TC0100003010.mm.2	Tmem163	transmembrane protein 163	1,40012E+14	6,88057E+14	3,42352E+14	0.012637992366046	0.170747242106618	-2,50815E+14	8,07708E+12	7,55864E+13	6,81572E+14	6,01976E+13	6,62236E+14	6,18988E+14
TC0300001888.mm.2	Ankrd50	ankyrin repeat domain 50	1,17682E+14	5,945E+14	3,42246E+14	0.0126551276249403	0.170747242106618	-2,50933E+14	6,55878E+14	6,52647E+14	5,68834E+14	5,04327E+14	6,02868E+14	5,82444E+14
TC1100003871.mm.2	Arhgap27	Rho GTPase activating protein 27	-1,25326E+13	6,38196E+14	-3,42208E+14	0.0126614450439429	0.170747242106618	-2,50977E+14	5,48643E+14	5,5755E+14	6,42104E+14	7,14741E+14	6,65204E+14	7,00934E+14
TC0X00002073.mm.2	Rhox8	reproductive homeobox 8	-1,63959E+14	6,1762E+14	-3,4207E+14	0.0126838094931774	0.170747242106618	-2,51131E+14	5,34631E+14	4,49674E+14	6,11527E+13	7,00696E+14	6,73932E+14	7,35261E+14
TC1100004294.mm.2	Glod4/Gemin4	glyoxalase domain containing 4/gem nuclear organelle associated protein 4	2,34674E+14	6,8917E+14	3,42012E+14	0.0126932896784871	0.170747242106618	-2,51196E+14	8,33417E+14	8,28236E+14	6,93353E+14	4,98952E+14	6,95569E+14	5,85495E+14
TC1900000067.mm.2	Fosl1	fos-like antigen 1	-1,19776E+14	6,9808E+13	-3,4186E+14	0.0127181319252293	0.170747242106618	-2,51367E+14	6,49414E+14	5,93347E+14	7,21712E+14	7,60601E+14	7,20363E+14	7,43041E+14
TC0500001586.mm.2	Mdh2	malate dehydrogenase 2, NAD (mitochondrial)	1,19287E+14	8,78901E+14	3,41598E+14	0.0127610932372946	0.170747242106618	-2,51662E+14	9,70807E+14	9,45557E+14	8,71179E+14	8,06611E+14	8,47673E+13	8,31582E+14
TC0200002669.mm.2	Rtf2	replication termination factor 2	1,12093E+14	6,55297E+14	3,41573E+14	0.0127652725479101	0.170747242106618	-2,5169E+14	7,27177E+14	7,39191E+14	6,07203E+13	6,34978E+14	5,87547E+14	6,35685E+14
TC0100001195.mm.2	Rbbp5	retinoblastoma binding protein 5, histone lysine methyltransferase complex subunit	1,45635E+14	7,00271E+14	3,41466E+14	0.0127828001518118	0.170747242106618	-2,5181E+14	8,00983E+14	7,95493E+14	7,08679E+14	5,96528E+14	6,56477E+14	6,43469E+14
TC0400000589.mm.2	Tmem38b	transmembrane protein 38B	-1,27295E+14	7,91946E+14	-3,41438E+14	0.0127874413876474	0.170747242106618	-2,51842E+14	7,06846E+14	7,04352E+14	7,90962E+14	8,74827E+14	8,29939E+14	8,4475E+14
TC1700000957.mm.2	Gm6934	predicted gene 6934	-1,44351E+14	7,7286E+14	-3,41396E+14	0.0127942820609541	0.170747242106618	-2,51888E+13	6,91844E+14	6,66911E+14	7,74429E+14	8,73028E+14	7,92933E+14	8,38015E+14
TC0200004913.mm.2	Bcl2l1	BCL2-like 1	1,71791E+14	7,02163E+13	3,41379E+14	0.012797164883237	0.170747242106618	-2,51908E+14	8,40294E+14	8,28947E+14	7,0373E+14	6,2193E+14	6,70544E+12	5,47533E+14
TC0600000633.mm.2	Tax1bp1	Tax1 (human T cell leukemia virus type I) binding protein 1	2,75387E+14	7,17724E+14	3,41377E+14	0.0127974096966292	0.170747242106618	-2,5191E+14	8,93451E+14	8,96695E+13	7,44482E+13	4,94891E+14	6,90308E+14	5,8652E+14
TC0700001253.mm.2	Grm5	glutamate receptor, metabotropic 5	2,03622E+13	7,63221E+14	3,41287E+14	0.0128122599338997	0.170747242106618	-2,52011E+14	9,05692E+14	9,04832E+14	7,45346E+14	6,57936E+13	7,65711E+14	5,99811E+14
TC0800002238.mm.2	Wdr17	WD repeat domain 17	1,65817E+14	6,34035E+14	3,41276E+14	0.0128141134957189	0.170747242106618	-2,52024E+13	7,61793E+14	7,66456E+14	5,76201E+14	6,20414E+14	6,06323E+14	4,73026E+14
TC0100000718.mm.2	Ccl20	chemokine (C-C motif) ligand 20	-1,05009E+14	6,3171E+14	-3,41267E+14	0.0128156543236772	0.170747242106618	-2,52034E+12	5,55365E+14	5,66103E+14	6,73505E+13	6,57983E+14	6,51308E+14	6,85997E+14
TC1900001592.mm.2	Armh3	armadillo-like helical domain containing 3	1,06075E+14	5,91857E+14	3,41238E+14	0.0128204023613649	0.170747242106618	-2,52067E+14	6,49562E+14	6,81841E+14	5,64827E+14	5,54426E+14	5,63088E+14	5,37398E+14
TC0800001133.mm.2	Mmp15	matrix metallopeptidase 15	1,15279E+14	6,8018E+14	3,41199E+13	0.0128268373557407	0.170747242106618	-2,5211E+14	7,69578E+14	7,49085E+14	6,23998E+13	6,64106E+14	6,59712E+14	6,146E+14
TC0900001486.mm.2	Epm2aip1	EPM2A (laforin) interacting protein 1	1,72365E+14	7,45117E+14	3,4114E+14	0.0128365075454243	0.170747242106618	-2,52176E+14	8,43079E+14	8,33143E+14	7,38995E+14	5,92497E+14	7,44292E+14	7,18694E+14
TC0300002242.mm.2	Trim2	tripartite motif-containing 2	1,84027E+14	9,04525E+14	3,41089E+14	0.0128449886022002	0.170747242106618	-2,52234E+14	1,01843E+14	1,01544E+14	9,07269E+14	7,58547E+14	8,97985E+14	8,29476E+14
TC1000002406.mm.2	Col6a1	collagen, type VI, alpha 1	-1,36438E+12	7,23014E+14	-3,41089E+14	0.0128450009210811	0.170747242106618	-2,52234E+14	6,22325E+14	6,35769E+14	7,34094E+13	7,96877E+14	7,36305E+13	8,12715E+13
TC1300000972.mm.2	Mef2c	myocyte enhancer factor 2C	2,14404E+14	9,12437E+14	3,40907E+14	0.0128751167006601	0.170747242106618	-2,52439E+14	1,00927E+14	1,06877E+14	9,00288E+14	7,48949E+14	9,32431E+14	8,14914E+14
TC0800003217.mm.2	2610044O15Rik8	RIKEN cDNA 2610044O15 gene	2,04135E+14	5,99792E+14	3,40703E+14	0.0129090247560263	0.170747242106618	-2,52668E+14	7,04963E+14	7,50325E+14	5,65251E+14	4,81767E+14	6,29657E+14	4,66789E+14
TC1900000325.mm.2	D030056L22Rik	RIKEN cDNA D030056L22 gene	-1,1639E+14	7,27226E+14	-3,40469E+14	0.0129480631998242	0.170747242106618	-2,52932E+14	6,79713E+14	6,34414E+13	7,5227E+13	7,94637E+14	7,39275E+14	7,63049E+14
TC0900003318.mm.2	Ppp2r3d	protein phosphatase 2 (formerly 2A), regulatory subunit B'', delta	-1,49807E+14	7,85612E+14	-3,40447E+14	0.0129516406869398	0.170747242106618	-2,52956E+14	6,87821E+14	6,86843E+14	7,87743E+13	8,86535E+14	7,98733E+12	8,65996E+14
TC0300002629.mm.2	Olfml3/LOC269472	olfactomedin-like 3/uncharacterized LOC269472	1,00811E+14	7,0017E+14	3,40395E+14	0.0129602904178492	0.170747242106618	-2,53015E+14	7,6768E+14	7,83421E+14	6,66711E+14	6,82767E+14	6,56491E+14	6,43948E+14
TC0100001330.mm.2	Brinp3	bone morphogenetic protein/retinoic acid inducible neural specific 3	1,89879E+14	6,39262E+13	3,40392E+14	0.0129608165714825	0.170747242106618	-2,53018E+14	7,51916E+14	7,40468E+14	6,21299E+14	4,91327E+14	6,7091E+14	5,5965E+14
TC0100000944.mm.2	Atg4b	autophagy related 4B, cysteine peptidase	1,02934E+14	7,57567E+14	3,40231E+14	0.0129877742933767	0.170747242106618	-2,532E+14	8,34836E+14	8,10671E+13	7,21814E+14	7,17825E+14	7,41901E+14	7,18354E+14
TC0200001524.mm.2	Slc1a2	solute carrier family 1 (glial high affinity glutamate transporter), member 2	2,19972E+14	9,52719E+14	3,40197E+14	0.0129934385494485	0.170747242106618	-2,53238E+13	1,09701E+14	1,10663E+14	9,42085E+14	8,21617E+14	9,58965E+14	7,89999E+14
TC1100001072.mm.2	Slc43a2	solute carrier family 43, member 2	1,12375E+14	6,60757E+14	3,40181E+14	0.0129962121363552	0.170747242106618	-2,53257E+14	7,72398E+14	7,43182E+13	6,70493E+13	6,20336E+14	5,75615E+14	5,82516E+13
TC0X00000204.mm.2	Rp2	retinitis pigmentosa 2 homolog	1,69365E+14	6,31139E+14	3,40086E+13	0.013012035459453	0.170747242106618	-2,53363E+14	7,05068E+14	7,3208E+13	6,05761E+14	4,92659E+14	6,69671E+14	5,81594E+14
TC0700002609.mm.2	Tex101	testis expressed gene 101	-2,0332E+14	6,11825E+14	-3,40038E+14	0.0130201684967193	0.170747242106618	-2,53418E+14	4,77475E+14	4,99301E+13	6,1579E+14	7,67626E+14	6,00989E+14	7,09773E+14
TC1800000071.mm.2	Thoc1	THO complex 1	1,49209E+14	6,18922E+14	3,40006E+14	0.0130255440528607	0.170747242106618	-2,53454E+14	7,3532E+14	6,96469E+14	6,10552E+14	5,22819E+14	6,09344E+14	5,39025E+14
TC0500000488.mm.2	Lap3	leucine aminopeptidase 3	2,18594E+14	7,30306E+14	3,40005E+14	0.0130256066920781	0.170747242106618	-2,53454E+14	8,32471E+14	8,7058E+14	7,3135E+14	5,34513E+14	7,30678E+14	6,82243E+14
TC1200002527.mm.2	Rpl36al	ribosomal protein L36A-like	1,48656E+14	6,91447E+14	3,39973E+14	0.0130310744051925	0.170747242106618	-2,53491E+14	8,03356E+14	7,71326E+13	6,50114E+14	6,27255E+14	7,04046E+14	5,92583E+14
TC0800001612.mm.2	Xab2	XPA binding protein 2	1,32906E+14	6,125E+14	3,39959E+14	0.0130333994025377	0.170747242106618	-2,53506E+14	7,36045E+14	7,02395E+14	5,53121E+14	6,19507E+13	5,61547E+14	5,02386E+14
TC0100001341.mm.2	Tpr	translocated promoter region, nuclear basket protein	2,84576E+14	6,81211E+14	3,39841E+14	0.013053239733119	0.170747242106618	-2,53639E+14	9,10554E+14	8,81633E+14	6,99658E+13	5,23377E+14	6,45173E+14	4,26872E+14
TC0700002185.mm.2	Rfpl4	ret finger protein-like 4	-1,35954E+14	6,88011E+14	-3,39739E+14	0.0130704975078044	0.170747242106618	-2,53755E+14	6,02857E+14	6,24129E+14	7,03625E+14	7,95269E+14	7,17877E+14	6,8431E+14
TC1000001490.mm.2	Wif1	Wnt inhibitory factor 1	-1,26889E+13	6,31563E+14	-3,39733E+14	0.0130714250305562	0.170747242106618	-2,53761E+14	5,50832E+14	5,45698E+14	6,41645E+14	7,08663E+14	6,44785E+13	6,97756E+14
TC0800001963.mm.2	Gm8096	3-phosphoglycerate dehydrogenase pseudogene	-1,15516E+14	6,64769E+14	-3,39709E+14	0.0130754518707671	0.170747242106618	-2,53788E+14	5,92048E+14	6,0391E+14	6,81671E+14	7,45318E+14	6,78899E+13	6,86769E+14
TC0800000403.mm.2	Saraf	store-operated calcium entry-associated regulatory factor	1,21459E+14	8,5561E+14	3,39683E+14	0.0130798468917411	0.170747242106618	-2,53817E+14	9,41829E+14	9,28813E+14	8,40766E+14	7,86958E+14	8,43922E+14	7,91375E+14
TC0300002418.mm.2	Crct1	cysteine-rich C-terminal 1	-1,04424E+14	5,48812E+14	-3,39625E+14	0.0130896665601767	0.170747242106618	-2,53883E+14	4,82814E+14	4,76278E+14	5,93819E+14	5,7412E+14	5,9958E+14	5,66263E+14
TC1800001000.mm.2	Osbpl1a	oxysterol binding protein-like 1A	2,12624E+13	8,32468E+14	3,3962E+14	0.0130904455300621	0.170747242106618	-2,53888E+14	9,11568E+14	9,38744E+14	7,93812E+14	6,31252E+14	9,15904E+14	8,03526E+14
TC0300001110.mm.2	AI504432	expressed sequence AI504432	2,18771E+14	6,13152E+14	3,39571E+14	0.0130987715444752	0.170747242106618	-2,53944E+14	7,81025E+14	7,44885E+13	5,89338E+14	4,9903E+14	6,23565E+14	4,41072E+14
TC0200004227.mm.2	Cat	catalase	1,81479E+14	8,46039E+14	3,39545E+14	0.0131031969798491	0.170747242106618	-2,53973E+14	9,81775E+13	9,85852E+14	8,79147E+14	7,25521E+13	7,76995E+14	7,26942E+13
TC0400001312.mm.2	Ebna1bp2/D4Ertd617e	EBNA1 binding protein 2/DNA segment, Chr 4, ERATO Doi 617, expressed	1,47192E+14	7,51493E+13	3,3946E+14	0.0131175151079648	0.170747242106618	-2,54069E+14	8,46444E+14	8,44304E+14	7,40678E+14	6,55687E+14	7,50719E+14	6,71124E+14
TC0700002846.mm.2	Lrp3	low density lipoprotein receptor-related protein 3	1,1621E+14	7,81886E+14	3,39398E+14	0.013128074470548	0.170747242106618	-2,54139E+14	8,63941E+14	8,6836E+13	7,27613E+14	7,72268E+14	7,54301E+14	7,04834E+14
TC0300002847.mm.2	Abcd3	ATP-binding cassette, sub-family D (ALD), member 3	1,35857E+14	6,51496E+14	3,39355E+14	0.0131352727535993	0.170747242106618	-2,54187E+14	7,41188E+14	7,49278E+14	6,26073E+14	5,92678E+14	6,46823E+14	5,52937E+14
TC1700002151.mm.2	Hsp90ab1	heat shock protein 90 alpha (cytosolic), class B member 1	1,65809E+14	9,43759E+14	3,39297E+14	0.0131451966506088	0.170747242106618	-2,54253E+14	1,05297E+14	1,04663E+14	9,5239E+14	8,15594E+14	9,15809E+14	8,79158E+14
TC0700002162.mm.2	Syt5	synaptotagmin V	2,16655E+14	9,32713E+14	3,39265E+13	0.0131506301335394	0.170747242106618	-2,54289E+14	1,06834E+14	1,05268E+14	9,4441E+14	7,43293E+14	9,10659E+13	8,76902E+13
TC0900002322.mm.2	Acsbg1	acyl-CoA synthetase bubblegum family member 1	1,10844E+14	9,00323E+14	3,39235E+14	0.0131556287983577	0.170747242106618	-2,54322E+14	9,82194E+13	9,79178E+14	8,64381E+14	8,75302E+14	8,7776E+14	8,23124E+14
TC0600001867.mm.2	Thsd7a	thrombospondin, type I, domain containing 7A	1,36819E+14	7,65027E+14	3,39219E+14	0.0131584266144815	0.170747242106618	-2,54341E+14	8,34705E+14	8,34909E+14	7,33239E+14	6,62738E+14	7,25766E+14	7,98803E+14
TC0200000404.mm.2	Tmem203	transmembrane protein 203	1,18158E+14	6,6331E+13	3,39116E+13	0.0131758140508219	0.170747242106618	-2,54456E+14	7,11015E+14	7,26166E+14	6,30115E+14	5,70751E+12	6,88531E+14	6,53282E+14
TC0200001049.mm.2	Rapgef4	Rap guanine nucleotide exchange factor (GEF) 4	1,3939E+14	8,93693E+14	3,39076E+14	0.0131827055645407	0.170747242106618	-2,54502E+14	9,48195E+14	9,91006E+14	8,57203E+14	8,03218E+14	9,21174E+14	8,41362E+14

TC1200002267.mm.2	Ddx24	DEAD (Asp-Glu-Ala-Asp) box polypeptide 24	1,52095E+13	8,49656E+13	3,38972E+14	0.0132003813512105	0.170747242106618	-2,54619E+14	9,60854E+14	9,58201E+14	8,49813E+14	7,65053E+13	8,26964E+14	7,37052E+14
TC0900000532.mm.2	Mpzl3	myelin protein zero-like 3	-1,02683E+14	5,47322E+14	-3,38957E+14	0.0132029128038618	0.170747242106618	-2,54636E+14	4,97052E+14	4,95581E+14	6,13379E+14	5,8462E+14	5,37217E+14	5,56085E+14
TC0200004364.mm.2	Scg5	secretogranin V	1,65834E+14	9,74935E+14	3,38857E+14	0.0132200758218574	0.170747242106618	-2,5475E+14	1,07486E+13	1,05696E+14	9,59163E+14	8,40993E+14	9,97225E+14	9,20405E+14
TC0X00003364.mm.2	Frmpd4	FERM and PDZ domain containing 4	2,31505E+14	7,16166E+14	3,38679E+14	0.0132503782650053	0.170747242106618	-2,5495E+14	8,84053E+14	8,96535E+14	7,17409E+14	6,00167E+14	6,93723E+14	5,0511E+14
TC1600001047.mm.2	Dop1b	DOP1 leucine zipper like protein B	1,26789E+14	6,66835E+14	3,38655E+14	0.0132545023470906	0.170747242106618	-2,54977E+14	7,57895E+14	7,39534E+13	6,32394E+13	6,11458E+14	5,8914E+14	6,7059E+14
TC0600002924.mm.2	Emc3	ER membrane protein complex subunit 3	1,01536E+14	7,51772E+14	3,38475E+14	0.0132853865782579	0.170747242106618	-2,55181E+14	8,13449E+13	8,14522E+14	7,26599E+14	6,98301E+14	7,2268E+14	7,35085E+14
TC0600002225.mm.2	Rarres2	retinoic acid receptor responder (tazarotene induced) 2	-1,51548E+14	5,71712E+14	-3,3837E+13	0.0133034621686788	0.170747242106618	-2,553E+14	4,61234E+13	4,89389E+14	5,73789E+14	6,7993E+14	5,85024E+14	6,40904E+14
TC1100004206.mm.2	Pde6g	phosphodiesterase 6G, cGMP-specific, rod, gamma	-1,34633E+12	4,5179E+14	-3,38369E+14	0.0133036393602888	0.170747242106618	-2,55301E+14	3,11816E+14	3,74455E+14	4,42426E+14	5,13111E+14	5,20116E+14	5,48818E+14
TC0400001683.mm.2	Hnrnpr	heterogeneous nuclear ribonucleoprotein R	1,26024E+14	7,1003E+14	3,38339E+14	0.0133087558297116	0.170747242106618	-2,55334E+14	8,0759E+14	7,85455E+13	7,10253E+13	6,30744E+14	6,73248E+14	6,52888E+14
TC1700002286.mm.2	Sgo1	shugoshin 1	-1,36845E+14	5,46251E+14	-3,38336E+14	0.0133093127198698	0.170747242106618	-2,55338E+14	4,31434E+14	4,83111E+14	5,63497E+13	6,24739E+14	5,55734E+13	6,18989E+14
TC1100004040.mm.2	Sdk2	sidekick cell adhesion molecule 2	1,26722E+14	5,93255E+14	3,38325E+14	0.0133112020543746	0.170747242106618	-2,5535E+14	6,85732E+14	6,73787E+14	5,45513E+14	5,60562E+14	5,04965E+14	5,88969E+14
TC1300002766.mm.2	Nsun2	NOL1/NOP2/Sun domain family member 2	1,91832E+14	6,00493E+14	3,38232E+14	0.0133271327516862	0.170747242106618	-2,55455E+14	7,15373E+14	7,02165E+14	5,92568E+13	4,41306E+14	6,19646E+14	5,31901E+14
TC0700001008.mm.2	Igf1r	insulin-like growth factor I receptor	1,00507E+14	6,28279E+13	3,38221E+14	0.0133290204746899	0.170747242106618	-2,55467E+13	6,86738E+14	6,92455E+14	5,81821E+14	5,96356E+14	6,17282E+13	5,95019E+14
TC0400000488.mm.2	Dcaf10	DDB1 and CUL4 associated factor 10	1,48842E+13	5,87258E+14	3,38147E+14	0.0133417750797731	0.170747242106618	-2,55551E+14	6,7711E+14	6,68781E+14	5,83155E+14	4,65052E+14	5,73137E+14	5,56314E+14
TC0800001124.mm.2	Polr2c	polymerase (RNA) II (DNA directed) polypeptide C	1,16576E+14	7,84548E+14	3,38041E+13	0.0133601304059927	0.170747242106618	-2,55671E+14	8,76373E+14	8,55287E+13	7,81732E+14	7,16776E+14	7,40416E+14	7,36706E+14
TC0100003310.mm.2	Cep350	centrosomal protein 350	2,11933E+14	5,49188E+14	3,37958E+14	0.0133743694295899	0.170747242106618	-2,55765E+13	7,01066E+14	6,90903E+13	5,75886E+14	3,92217E+14	5,06649E+14	4,28404E+14
TC1100003095.mm.2	Pelp1	proline, glutamic acid and leucine rich protein 1	1,69783E+14	5,34517E+14	3,37901E+14	0.0133842739900904	0.170747242106618	-2,55829E+14	6,32918E+14	6,24225E+13	5,25486E+12	3,9209E+14	4,84634E+14	5,47747E+13
TC1700002199.mm.2	Ubr2	ubiquitin protein ligase E3 component n-recognin 2	1,22915E+14	6,25938E+14	3,37819E+14	0.0133985144970454	0.170747242106618	-2,55922E+13	6,89302E+14	7,16445E+14	6,1404E+14	5,45877E+13	6,10998E+14	5,78968E+14
TC0700001321.mm.2	Prcp	prolylcarboxypeptidase (angiotensinase C)	1,44131E+14	5,82378E+13	3,37767E+13	0.0134073875109502	0.170747242106618	-2,5598E+14	6,72818E+14	6,83571E+14	4,79937E+14	5,8819E+14	5,5042E+14	5,19329E+14
TC0400003016.mm.2	Klhl9	kelch-like 9	1,48055E+14	7,47384E+14	3,37757E+12	0.0134092159499055	0.170747242106618	-2,55992E+14	8,71521E+14	8,57862E+14	7,57897E+14	6,75375E+14	7,02953E+14	6,18698E+13
TC0300000766.mm.2	Pmvk	phosphomevalonate kinase	1,0404E+13	6,87241E+14	3,37702E+14	0.0134186794679268	0.170747242106618	-2,56054E+14	7,56869E+14	7,49021E+14	6,63307E+14	6,34504E+14	6,73082E+14	6,4666E+13
TC0600001953.mm.2	Gpr37	G protein-coupled receptor 37	1,41503E+14	7,82465E+14	3,37683E+14	0.013422039741956	0.170747242106618	-2,56076E+14	8,75691E+14	8,96112E+14	7,71606E+14	7,17191E+14	7,6343E+14	6,70758E+14
TC0100002685.mm.2	Sphkap	SPHK1 interactor, AKAP domain containing	1,75196E+14	7,2314E+14	3,37665E+13	0.0134251918282658	0.170747242106618	-2,56096E+14	8,31135E+14	8,29117E+13	7,19004E+14	5,90857E+14	7,27892E+14	6,40833E+14
TC1500001470.mm.2	Kcnv1	potassium channel, subfamily V, member 1	1,39722E+14	7,56159E+14	3,37661E+14	0.0134258962341603	0.170747242106618	-2,56101E+14	8,01814E+14	8,36363E+13	7,2981E+13	6,28923E+14	7,72861E+14	7,67183E+14
TC0700003793.mm.2	Dgat2	diacylglycerol O-acyltransferase 2	1,25955E+14	8,23935E+14	3,37529E+14	0.0134487121858604	0.170747242106618	-2,56249E+13	9,07137E+14	9,13037E+14	8,13297E+14	7,54966E+14	8,08629E+14	7,46541E+13
TC0800003161.mm.2	Acta1	actin, alpha 1, skeletal muscle	-1,04481E+14	6,25618E+14	-3,37509E+14	0.0134523189691884	0.170747242106618	-2,56273E+14	5,37294E+14	5,68498E+14	6,46041E+14	6,68712E+14	6,73988E+14	6,59173E+14
TC1100003995.mm.2	1700012B07Rik	RIKEN cDNA 1700012B07 gene	-1,41151E+14	5,80072E+14	-3,37475E+14	0.0134581912760907	0.170747242106618	-2,56311E+14	4,65696E+14	5,0912E+14	5,78602E+14	6,78517E+13	6,22456E+14	6,26042E+14
TC0500001087.mm.2	Rpl5	ribosomal protein L5	1,94145E+14	8,83573E+14	3,37267E+14	0.013494464668142	0.170747242106618	-2,56547E+14	9,96473E+14	1,00038E+14	8,76332E+14	7,32233E+14	9,00973E+14	7,95047E+14
TC1100001632.mm.2	Stat5a	signal transducer and activator of transcription 5A	-1,22401E+13	6,96468E+14	-3,37226E+14	0.0135016238714245	0.170747242106618	-2,56593E+14	6,38619E+14	6,19966E+14	7,18801E+14	7,84587E+14	6,88105E+14	7,2873E+14
TC0200001366.mm.2	Psmc3	proteasome (prosome, macropain) 26S subunit, ATPase 3	1,47625E+14	8,62437E+14	3,37199E+14	0.0135062682037881	0.170747242106618	-2,56623E+14	9,41423E+14	9,5208E+14	8,56785E+14	7,41468E+14	8,53899E+13	8,28966E+14
TC1000003220.mm.2	Nt5dc3	5'-nucleotidase domain containing 3	1,16138E+14	7,40333E+14	3,37145E+14	0.0135156496038766	0.170747242106618	-2,56684E+14	7,88987E+14	8,27899E+14	6,71994E+14	7,12616E+14	7,39481E+14	7,01019E+14
TC1700000277.mm.2	Gm10509	predicted gene 10509	2,12932E+14	5,76879E+14	3,37111E+14	0.0135216479148144	0.170747242106618	-2,56723E+14	6,8507E+14	6,71906E+14	5,56655E+14	3,74456E+14	6,29385E+14	5,438E+13
TC0600000678.mm.2	Fkbp9	FK506 binding protein 9	2,38982E+14	6,90609E+13	3,37092E+14	0.0135250188524398	0.170747242106618	-2,56745E+13	8,20708E+14	8,34943E+14	6,6344E+14	5,14247E+14	7,43988E+13	5,66325E+14
TC1400000975.mm.2	Entpd4	ectonucleoside triphosphate diphosphohydrolase 4	1,01367E+14	8,77667E+14	3,37084E+14	0.0135264539268208	0.170747242106618	-2,56754E+14	9,55949E+14	9,56319E+14	8,62999E+14	8,46536E+14	8,08483E+14	8,35715E+14
TC1200001030.mm.2	Ifi27	interferon, alpha-inducible protein 27	1,42225E+14	5,40948E+14	3,37063E+14	0.0135299787595178	0.170747242106618	-2,56777E+14	5,90741E+14	6,79888E+14	4,64262E+14	5,21917E+12	5,1033E+14	4,78552E+14
TC1500000543.mm.2	Them6	thioesterase superfamily member 6	-1,13157E+14	8,46686E+14	-3,36943E+14	0.0135510105137091	0.170747242106618	-2,56913E+14	7,81167E+14	7,50603E+14	9,03212E+13	8,54872E+14	9,07549E+13	8,82714E+14
TC1300000336.mm.2	Dusp22	dual specificity phosphatase 22	-1,30864E+14	8,83674E+12	-3,36874E+14	0.0135632240792177	0.170747242106618	-2,56991E+14	8,03614E+13	7,86708E+14	9,70922E+14	8,81128E+14	9,40345E+14	9,19328E+14
TC0300001362.mm.2	Tacr3	tachykinin receptor 3	1,50695E+14	5,91261E+14	3,36859E+14	0.013565887475218	0.170747242106618	-2,57009E+14	7,32069E+14	6,34533E+14	5,28112E+14	5,37099E+14	5,95048E+14	5,20704E+14
TC0800000297.mm.2	Sfrp1	secreted frizzled-related protein 1	2,18683E+14	6,09699E+14	3,36845E+14	0.0135683175565342	0.170747242106618	-2,57024E+14	7,86066E+13	7,6241E+14	6,5413E+14	4,56981E+14	5,31558E+14	4,67046E+14
TC0700002033.mm.2	Pgghg	protein glucosylgalactosylhydroxylysine glucosidase	-1,41329E+14	6,67986E+14	-3,36811E+14	0.0135741546416521	0.170747242106618	-2,57062E+14	5,7508E+14	5,72221E+14	6,61657E+14	7,68302E+14	7,02622E+14	7,28038E+14
TC1800000266.mm.2	Camk4	calcium/calmodulin-dependent protein kinase IV	1,55872E+14	7,27889E+14	3,36803E+14	0.0135756388370284	0.170747242106618	-2,57071E+14	8,05525E+14	8,63489E+14	6,69864E+14	6,87405E+14	7,28106E+14	6,12947E+14
TC1900001066.mm.2	Slc3a2	solute carrier family 3 (activators of dibasic and neutral amino acid transport), member 2	1,66006E+14	9,29861E+14	3,36706E+14	0.0135927709225545	0.170747242106618	-2,57182E+14	1,0215E+14	1,04136E+14	9,22064E+14	8,08787E+14	9,35796E+14	8,49659E+14
TC0800001074.mm.2	Irx5	Iroquois homeobox 5	-1,35867E+13	6,20904E+14	-3,36687E+14	0.013596057959025	0.170747242106618	-2,57203E+14	5,53033E+14	4,97327E+14	6,41044E+14	6,81048E+13	6,38992E+14	7,13977E+14
TC1200002536.mm.2	Serpina9	serine (or cysteine) peptidase inhibitor, clade A (alpha-1 antiproteinase, antitrypsin), member 9	1,28352E+14	6,02796E+14	3,36684E+14	0.013596652053792	0.170747242106618	-2,57207E+14	7,06457E+14	6,80221E+13	6,05558E+14	5,24417E+14	5,35906E+14	5,64216E+13
TC0100000021.mm.2	Rb1cc1	RB1-inducible coiled-coil 1	1,87899E+14	5,63771E+14	3,3661E+14	0.0136095410219361	0.170747242106618	-2,5729E+14	6,83761E+14	7,06953E+14	5,47184E+14	4,67733E+14	5,63676E+14	4,13318E+14
TC0900002054.mm.2	Tbrg1	transforming growth factor beta regulated gene 1	1,21658E+14	7,02929E+14	3,36595E+14	0.0136121690694837	0.170747242106618	-2,57307E+14	8,05697E+14	7,81266E+14	7,08492E+14	6,35154E+13	6,50704E+14	6,36263E+14
TC0X00001322.mm.2	Tceal9	transcription elongation factor A like 9	1,22121E+14	8,1668E+14	3,36573E+14	0.013616076048659	0.170747242106618	-2,57332E+14	9,27027E+14	8,79523E+14	7,6607E+14	7,96237E+14	7,37558E+14	7,93662E+14
TC0400003184.mm.2	C8a	complement component 8, alpha polypeptide	-1,57744E+14	5,23476E+14	-3,36517E+14	0.0136259006868929	0.170747242106618	-2,57395E+14	4,07114E+14	4,34636E+14	5,26217E+14	6,31021E+14	5,32523E+14	6,09344E+14
TC1700001904.mm.2	Gm15821	predicted gene 15821	-1,24985E+14	7,49674E+14	-3,36438E+14	0.0136398759453	0.170747242106618	-2,57485E+14	6,67025E+14	6,63416E+14	7,54922E+14	8,25489E+14	7,70468E+14	8,16721E+14
TC1300002474.mm.2	Hmgcr	3-hydroxy-3-methylglutaryl-Coenzyme A reductase	2,6767E+14	6,635E+14	3,36438E+14	0.0136398815995028	0.170747242106618	-2,57485E+14	7,76268E+14	8,03083E+13	6,53571E+14	3,90439E+14	6,93266E+14	6,64373E+14
TC0200001015.mm.2	Ubr3	ubiquitin protein ligase E3 component n-recognin 3	2,18092E+14	8,09936E+14	3,36438E+14	0.0136399968787398	0.170747242106618	-2,57485E+14	9,67107E+14	9,52006E+14	8,33819E+14	6,4911E+14	7,76436E+14	6,81136E+14
TC1700002171.mm.2	Tjap1	tight junction associated protein 1	-1,02837E+14	6,69157E+14	-3,3643E+14	0.0136414095619346	0.170747242106618	-2,57494E+14	6,05097E+14	6,17083E+14	6,96502E+14	7,31352E+14	6,7925E+14	6,85658E+14
TC0600001972.mm.2	Prrt4	proline-rich transmembrane protein 4	-1,24673E+14	6,8778E+14	-3,36396E+14	0.013647399599148	0.170747242106618	-2,57533E+14	5,85017E+14	6,44819E+14	7,12826E+14	7,66355E+14	6,9652E+14	7,21143E+14
TC0600002248.mm.2	Gsdme	gasdermin E	1,0858E+14	5,63938E+14	3,36355E+14	0.0136545091072874	0.170747242106618	-2,57579E+14	6,28334E+14	6,2439E+14	5,43418E+14	4,92146E+14	5,41748E+13	5,5359E+13
TC0400001874.mm.2	Gm13238	carnitine deficiency-associated gene expressed in ventricle 3 pseudogene	-1,10304E+14	8,21431E+14	-3,36275E+14	0.0136687041974079	0.170747242106618	-2,57669E+14	7,62481E+14	7,47515E+14	8,37936E+14	8,92667E+14	8,43556E+14	8,44428E+14
TC1000003103.mm.2	Arhgef25	Rho guanine nucleotide exchange factor (GEF) 25	1,28991E+14	7,55613E+12	3,36235E+14	0.0136758219305101	0.170747242106618	-2,57715E+14	8,28677E+14	8,32483E+14	6,98375E+14	7,04803E+14	7,80099E+14	6,89243E+14
TC1900000536.mm.2	Exoc6	exocyst complex component 6	1,35681E+14	6,56006E+14	3,36233E+14	0.0136762119633083	0.170747242106618	-2,57718E+13	7,39768E+14	7,39065E+14	6,54302E+14	5,5317E+14	6,29795E+14	6,19935E+14
TC1600000387.mm.2	Opa1	OPA1, mitochondrial dynamin like GTPase	1,54573E+13	7,77246E+14	3,36129E+14	0.0136946146365349	0.170747242106618	-2,57835E+14	8,82943E+14	8,97333E+14	7,90313E+14	6,80818E+14	7,39141E+14	6,7293E+14
TC0X00000674.mm.2	Gm14685/DXBay18	predicted gene 14685/DNA segment, Chr X, Baylor 18	-1,34666E+14	5,40958E+14	-3,36107E+14	0.0136984417582502	0.170747242106618	-2,5786E+14	4,57228E+14	4,41797E+14	5,42958E+14	6,25401E+14	5,61824E+14	6,16539E+14
TC0X00002420.mm.2	DXBay18/Gm14685	DNA segment, Chr X, Baylor 18/predicted gene 14685	-1,34666E+14	5,40958E+14	-3,36107E+14	0.0136984417582502	0.170747242106618	-2,5786E+14	4,57228E+14	4,41797E+14	5,42958E+14	6,25401E+14	5,61824E+14	6,16539E+14
TC1400002476.mm.2	Dgkh	diacylglycerol kinase, eta	1,09684E+14	6,42215E+14	3,36106E+14	0.0136986330272627	0.170747242106618	-2,57861E+14	6,95642E+14	7,27275E+14	5,79001E+14	6,24548E+14	6,14384E+14	6,12442E+14
TC0800001003.mm.2	Lonp2	lon peptidase 2, peroxisomal	1,43814E+14	8,05411E+14	3,35887E+14	0.0137375324603459	0.170747242106618	-2,58109E+14	9,06092E+14	8,98854E+14	8,02963E+14	7,14355E+14	7,19548E+14	7,90654E+14
TC1100003183.mm.2	Olfr398	olfactory receptor 398	-1,07492E+14	6,01851E+14	-3,35837E+14	0.0137464666541327	0.170747242106618	-2,58166E+14	5,22815E+14	5,31653E+13	6,099E+14	6,59552E+14	6,45512E+14	6,41673E+14
TC1900000973.mm.2	Ovol1	ovo like zinc finger 1	-1,83139E+14	5,0916E+14	-3,35815E+14	0.0137503690523718	0.170747242106618	-2,58191E+14	3,90108E+14	3,8007E+14	4,99085E+14	6,37373E+14	5,2831E+14	6,20014E+14
TC1900001012.mm.2	Slc22a12	solute carrier family 22 (organic anion/cation transporter), member 12	-1,51157E+14	6,5061E+14	-3,35814E+14	0.0137505063907831	0.170747242106618	-2,58192E+14	5,56571E+14	5,39197E+14	6,39536E+14	7,58547E+14	6,89293E+14	7,20518E+14
TC1100003997.mm.2	Abca8b	ATP-binding cassette, sub-family A (ABC1), member 8b	1,04853E+14	6,06284E+14	3,35779E+14	0.0137568984038433	0.170747242106618	-2,58233E+14	7,03865E+14	6,6661E+14	5,91394E+13	5,69377E+14	5,56788E+14	5,49672E+14
TC0200004383.mm.2	Meis2	Meis homeobox 2	1,27927E+14	9,25615E+14	3,35735E+13	0.0137645781033751	0.170747242106618	-2,58281E+13	1,03177E+14	1,02533E+14	9,38808E+14	8,62442E+14	8,71024E+14	8,24311E+14
TC1700001636.mm.2	Syngr3	synaptogyrin 3	1,85359E+14	9,45344E+14	3,35724E+14	0.0137665822855622	0.170747242106618	-2,58294E+14	1,07484E+14	1,04763E+14	9,37514E+14	8,14238E+14	9,55158E+14	8,42683E+14
TC1400002836.mm.2	Dok2	docking protein 2	-1,19838E+14	5,87707E+14	-3,35704E+14	0.0137701184302139	0.170747242106618	-2,58317E+14	4,91747E+13	5,18516E+14	6,00455E+14	6,49483E+14	6,07275E+14	6,58764E+14
TC0700001875.mm.2	Itgam	integrin alpha M	1,30741E+14	6,63941E+13	3,35676E+14	0.0137752186014462	0.170747242106618	-2,58349E+14	7,30963E+14	7,87411E+14	6,35339E+14	6,21554E+14	5,67055E+14	6,41327E+14
TC0700000921.mm.2	Luzp2	leucine zipper protein 2	2,3677E+14	7,99951E+13	3,35635E+14	0.013782518656178	0.170747242106618	-2,58395E+14	9,30077E+14	9,40067E+14	8,03725E+14	5,92878E+14	8,13463E+14	7,19495E+14
TC0300002543.mm.2	Pde4dip/Mir7225	phosphodiesterase 4D interacting protein (myomegalin)/microRNA 7225	1,71478E+14	6,68049E+14	3,35569E+14	0.0137943839078867	0.170747242106618	-2,58471E+14	7,94171E+14	7,80967E+14	6,85984E+14	5,46197E+14	6,29002E+14	5,71974E+14
TC1000000860.mm.2	Onecut3	one cut domain, family member 3	-1,08039E+14	5,49517E+13	-3,35523E+14	0.0138024370462224	0.170747242106618	-2,58522E+14	4,73757E+14	4,62976E+14	5,72536E+13	5,80274E+14	5,78574E+14	6,28985E+14
TC0100000971.mm.2	Gin1	gypsy retrotransposon integrase 1	0.997746930956454	5,86965E+14	3,35488E+14	0.0138088750904291	0.170747242106618	-2,58563E+14	6,30777E+14	6,16462E+14	5,16335E+14	5,31354E+14	6,04386E+14	6,22475E+13
TC0600001852.mm.2	Ica1	islet cell autoantigen 1	2,41729E+14	6,38509E+13	3,35357E+14	0.0138322563096482	0.170747242106618	-2,58711E+14	7,74532E+14	7,44675E+13	6,35266E+14	4,00482E+14	6,44956E+14	6,31141E+14
TC0700004621.mm.2	Tarsl2	threonyl-tRNA synthetase-like 2	1,17738E+14	6,24517E+14	3,35351E+14	0.013833350989903	0.170747242106618	-2,58718E+13	7,21973E+14	6,91796E+14	5,93059E+14	5,85234E+14	6,10168E+14	5,44872E+14
TC0300002410.mm.2	Lce1j	late cornified envelope 1J	-1,11381E+14	6,97275E+14	-3,35342E+14	0.0138348693048748	0.170747242106618	-2,58727E+13	6,37292E+14	6,56734E+14	7,32704E+13	7,84084E+14	6,74665E+14	6,98173E+14
TC1400002756.mm.2	Ggact	gamma-glutamylamine cyclotransferase	1,37076E+14	5,76117E+14	3,35254E+14	0.0138506648835584	0.170747242106618	-2,58827E+14	6,75726E+14	6,36192E+14	5,59878E+14	4,77889E+14	5,78994E+14	5,28023E+14
TC0X00001799.mm.2	Ebp	phenylalkylamine Ca2+ antagonist (emopamil) binding protein	1,46278E+14	6,81958E+14	3,35182E+12	0.0138636179090207	0.170747242106618	-2,58909E+13	7,48259E+14	7,67896E+14	6,54879E+13	5,68721E+14	6,37047E+14	7,14948E+14
TC0400003568.mm.2	Gjb5	gap junction protein, beta 5	-1,23645E+14	5,41791E+14	-3,35139E+14	0.0138714466695626	0.170747242106618	-2,58959E+14	4,41291E+14	5,07943E+14	5,85243E+14	6,11281E+14	5,31471E+14	5,73516E+14
TC0800001319.mm.2	Vac14	Vac14 homolog (S. cerevisiae)	1,36951E+14	6,35326E+14	3,35109E+14	0.0138767374312459	0.170747242106618	-2,58992E+14	7,2946E+14	7,20036E+14	6,3117E+14	5,44424E+14	5,64933E+14	6,21933E+14
TC1400002349.mm.2	Xpo7	exportin 7	1,95461E+14	6,18505E+13	3,35057E+14	0.0138862289786755	0.170747242106618	-2,59052E+14	7,51111E+14	7,48103E+14	6,44183E+14	4,6411E+13	5,65139E+14	5,38383E+14
TC0200002439.mm.2	Ppp1r16b	protein phosphatase 1, regulatory subunit 16B	1,33908E+14	7,93622E+14	3,35056E+14	0.0138864026326244	0.170747242106618	-2,59053E+14	8,93265E+14	8,86099E+14	8,02864E+14	7,08684E+14	7,51253E+14	7,19565E+14
TC0600003119.mm.2	Tpi1	triosephosphate isomerase 1	1,73288E+14	9,75779E+14	3,34949E+14	0.0139055261240497	0.170747242106618	-2,59174E+14	1,0734E+13	1,07301E+14	9,69108E+14	8,30731E+14	9,88334E+14	9,20084E+14
TC0300003212.mm.2	Gon4l	gon-4-like (C.elegans)	2,03807E+13	5,6382E+14	3,34887E+14	0.0139168216757363	0.170747242106618	-2,59245E+14	6,99803E+14	7,1777E+12	5,7247E+14	4,37488E+14	5,45654E+14	4,09733E+14
TC0200004090.mm.2	Ptprj	protein tyrosine phosphatase, receptor type, J	1,6425E+14	7,85892E+13	3,34869E+14	0.0139200360677032	0.170747242106618	-2,59265E+14	8,81306E+14	9,09692E+14	7,81272E+14	6,81226E+14	7,79684E+14	6,82169E+14
TC1700001683.mm.2	A930017K11Rik	RIKEN cDNA A930017K11 gene	-1,32228E+14	6,8833E+14	-3,34812E+14	0.0139304095974838	0.170747242106618	-2,5933E+13	6,0812E+13	6,12097E+14	7,1771E+14	7,66963E+14	6,70712E+14	7,5438E+14
TC1900000767.mm.2	Adra2a	adrenergic receptor, alpha 2a	1,32406E+14	6,47866E+14	3,34792E+14	0.0139339108681312	0.170747242106618	-2,59352E+14	7,29033E+14	7,08307E+14	6,32591E+14	5,39937E+14	6,49668E+13	6,27664E+14
TC0700003854.mm.2	Nup98	nucleoporin 98	1,12334E+14	6,59787E+14	3,34745E+14	0.0139424001124035	0.170747242106618	-2,59406E+14	7,41373E+14	7,43727E+14	6,48748E+14	6,11683E+14	6,31528E+14	5,81663E+13
TC0100003463.mm.2	Dusp27	dual specificity phosphatase 27 (putative)	-1,25521E+12	5,30846E+14	-3,34742E+14	0.0139430160126403	0.170747242106618	-2,59409E+14	4,51002E+14	4,64798E+13	5,43788E+14	6,23053E+14	5,3546E+14	5,66974E+13
TC0200000990.mm.2	4932414N04Rik	RIKEN cDNA 4932414N04 gene	-1,04163E+14	4,56547E+14	-3,34665E+14	0.0139568459722543	0.170747242106618	-2,59496E+14	3,60418E+14	3,84666E+14	4,70879E+14	4,8253E+14	5,3711E+14	5,0368E+14
TC0700000972.mm.2	E030018B13Rik	RIKEN cDNA E030018B13 gene	-1,50732E+14	5,09632E+14	-3,34538E+14	0.0139799823001865	0.170747242106618	-2,59641E+14	4,1244E+14	4,07191E+14	5,112E+14	6,09895E+14	5,20703E+14	5,96359E+14
TC0800001613.mm.2	Pcp2	Purkinje cell protein 2 (L7)	-1,14416E+14	7,45412E+14	-3,34478E+13	0.0139907243861959	0.170747242106618	-2,59709E+14	6,52478E+12	6,62819E+14	7,57919E+14	7,86209E+14	7,76841E+14	8,36207E+14
TC0300001150.mm.2	Wdr47	WD repeat domain 47	2,13997E+14	6,67746E+14	3,34453E+14	0.0139954339936322	0.170747242106618	-2,59738E+14	8,07321E+14	8,10195E+14	6,72319E+14	5,17204E+14	6,66741E+14	5,32698E+14
TC0900003044.mm.2	Rbm6	RNA binding motif protein 6	1,51372E+14	7,14235E+14	3,34254E+14	0.0140315456159742	0.170747242106618	-2,59964E+14	8,42212E+14	8,11673E+12	6,76379E+14	6,74763E+14	7,01068E+14	5,79315E+14
TC1100000767.mm.2	Dhrs7b	dehydrogenase/reductase (SDR family) member 7B	1,24104E+14	6,49566E+14	3,34081E+14	0.0140631483835232	0.170747242106618	-2,60161E+13	7,1608E+14	7,13561E+14	6,32118E+13	5,49315E+14	6,34978E+14	6,51343E+14
TC1700002132.mm.2	Slc25a27	solute carrier family 25, member 27	1,19864E+14	6,48968E+14	3,3407E+14	0.0140650973110818	0.170747242106618	-2,60173E+14	7,43654E+14	7,37209E+14	6,26734E+14	6,14401E+14	6,23804E+14	5,48004E+13
TC1000003151.mm.2	Rpl41	ribosomal protein L41	1,10207E+14	1,0875E+14	3,33999E+14	0.0140780326804276	0.170747242106618	-2,60254E+14	1,1656E+14	1,16043E+14	1,07926E+14	1,02635E+14	1,05849E+14	1,03486E+14
TC1700001154.mm.2	Ehd3	EH-domain containing 3	1,46158E+14	6,77137E+14	3,33925E+14	0.0140915940603057	0.170747242106618	-2,60338E+13	7,91243E+14	7,79668E+14	6,97213E+14	5,81382E+14	5,95807E+13	6,17511E+14
TC1300002468.mm.2	Sv2c	synaptic vesicle glycoprotein 2c	2,03319E+14	6,62899E+14	3,33922E+14	0.0140920568454586	0.170747242106618	-2,60341E+13	8,02653E+14	8,19338E+14	6,59021E+14	5,56331E+14	6,51019E+13	4,8903E+14
TC0100003380.mm.2	Suco	SUN domain containing ossification factor	1,37733E+14	6,35605E+14	3,339E+14	0.0140961938099204	0.170747242106618	-2,60367E+14	7,57229E+14	7,09739E+14	6,33296E+14	5,58207E+14	6,06617E+14	5,48542E+14
TC0900002450.mm.2	Rplp1	ribosomal protein, large, P1	1,28677E+14	1,06479E+14	3,33867E+14	0.0141021675209233	0.170747242106618	-2,60404E+14	1,14908E+14	1,15035E+14	1,05796E+14	9,84115E+14	1,05117E+14	9,96101E+14
TC0500000701.mm.2	Fip1l1	FIP1 like 1 (S. cerevisiae)	1,63125E+14	6,47229E+14	3,33843E+14	0.0141065112110296	0.170747242106618	-2,60431E+14	7,40179E+14	7,43177E+14	6,3253E+14	5,24577E+14	6,67355E+14	5,75557E+14
TC0X00000398.mm.2	Stag2	stromal antigen 2	2,05226E+14	6,08457E+14	3,33815E+14	0.0141116436113836	0.170747242106618	-2,60463E+14	7,4402E+14	7,73078E+14	6,01184E+14	5,05461E+14	5,97145E+14	4,29853E+13
TC0600000014.mm.2	Peg10	paternally expressed 10	1,81888E+14	6,7357E+14	3,33769E+14	0.0141201709326936	0.170747242106618	-2,60516E+14	8,73175E+13	7,68529E+12	7,01622E+14	5,76307E+14	5,76078E+14	5,45708E+14
TC0200004181.mm.2	Api5	apoptosis inhibitor 5	1,8189E+14	6,54758E+14	3,33606E+14	0.0141500647645154	0.170747242106618	-2,60701E+13	7,41083E+14	7,69094E+14	6,4862E+14	4,97776E+14	6,67912E+14	6,04064E+14
TC0500003679.mm.2	Zar1l	zygote arrest 1-like	-1,04177E+14	5,4967E+14	-3,33544E+14	0.0141614371938559	0.170747242106618	-2,60771E+13	4,72318E+13	4,7458E+14	5,57434E+14	5,97818E+14	6,06489E+14	5,8938E+14
TC0200003187.mm.2	Card9	caspase recruitment domain family, member 9	-1,43846E+14	9,33013E+14	-3,33447E+14	0.0141792277721874	0.170747242106618	-2,60881E+14	8,28894E+13	8,29373E+14	9,18706E+14	1,02725E+14	9,77342E+14	1,01651E+14
TC0400001413.mm.2	Rragc	Ras-related GTP binding C	1,74926E+14	7,66594E+14	3,33434E+14	0.0141817414505196	0.170747242106618	-2,60897E+14	8,60261E+14	8,72438E+14	7,47191E+14	6,35656E+14	7,97217E+14	6,86801E+14
TC0600001442.mm.2	Acrbp/Ing4	proacrosin binding protein/inhibitor of growth family, member 4	-1,10611E+13	7,30481E+14	-3,33272E+13	0.0142115859483122	0.170747242106618	-2,61081E+14	6,37637E+14	6,95492E+14	7,80164E+14	7,74187E+14	7,54155E+14	7,4125E+14
TC1400000580.mm.2	Rnase6	ribonuclease, RNase A family, 6	-1,05346E+14	8,62782E+14	-3,33185E+14	0.0142277452570485	0.170747242106618	-2,61181E+14	7,99738E+14	8,01358E+14	8,88939E+14	9,22849E+14	8,98388E+14	8,65418E+14
TC1500000793.mm.2	Arhgap8	Rho GTPase activating protein 8	-1,22003E+14	7,01375E+14	-3,33139E+14	0.0142360779819435	0.170747242106618	-2,61232E+14	6,14411E+14	6,10239E+14	6,93754E+14	7,74902E+14	7,60173E+14	7,54769E+14

TC0400003473.mm.2	Cap1	CAP, adenylate cyclase-associated protein 1 (yeast)	1,74174E+14	9,48819E+12	3,3313E+13	0.0142377545744973	0.170747242106618	-2,61242E+13	1,04239E+14	1,06565E+14	9,54188E+13	8,05505E+13	9,34342E+14	8,9084E+14
TC1500000298.mm.2	Fzd6	frizzled class receptor 6	-1,00586E+14	6,02883E+14	-3,33037E+14	0.0142550041074302	0.170747242106618	-2,61349E+14	5,19143E+14	5,5127E+14	6,31631E+14	6,39953E+14	6,39433E+14	6,35865E+14
TC0200003533.mm.2	Cacnb4	calcium channel, voltage-dependent, beta 4 subunit	1,58123E+14	8,92048E+14	3,32906E+14	0.0142792583785613	0.170747242106618	-2,61498E+14	9,88128E+14	9,96224E+14	8,91688E+14	7,76418E+14	8,85429E+14	8,14401E+14
TC1300002116.mm.2	Agtpbp1/A230056J06Rik	ATP/GTP binding protein 1/RIKEN cDNA A230056J06 gene	2,71924E+14	6,57383E+14	3,32901E+14	0.0142803262722693	0.170747242106618	-2,61504E+14	8,23072E+14	8,3258E+14	6,72742E+14	4,39061E+14	6,60008E+14	5,16836E+14
TC0900002489.mm.2	Zwilch	zwilch kinetochore protein	-1,43125E+14	6,55938E+14	-3,32898E+14	0.014280785972722	0.170747242106618	-2,61507E+14	5,89869E+13	5,48818E+14	6,66422E+14	7,58515E+14	6,58664E+14	7,13341E+14
TC0100000462.mm.2	Bmpr2	bone morphogenetic protein receptor, type II (serine/threonine kinase)	3,35848E+14	7,89001E+14	3,32837E+14	0.0142922137540369	0.170747242106618	-2,61577E+14	9,85198E+14	9,93164E+14	8,15392E+14	4,91274E+14	7,8617E+14	6,6281E+13
TC0800001862.mm.2	Nek3	NIMA (never in mitosis gene a)-related expressed kinase 3	-1,50765E+14	6,16368E+14	-3,32644E+14	0.0143279731432424	0.170747242106618	-2,61796E+14	5,41288E+13	5,2511E+14	6,24948E+14	7,42981E+14	6,11889E+14	6,51994E+14
TC0900001954.mm.2	Ntm	neurotrimin	1,30551E+14	9,0872E+14	3,32594E+14	0.0143373355791409	0.170747242106618	-2,61853E+14	9,88519E+14	9,91206E+14	9,01986E+14	8,16637E+14	8,98819E+14	8,55154E+12
TC0800002556.mm.2	Pkn1	protein kinase N1	-1,38311E+14	6,69851E+14	-3,32539E+14	0.014347678442929	0.170747242106618	-2,61917E+12	5,98637E+14	5,83344E+14	6,78008E+14	7,80595E+14	6,74483E+13	7,04037E+14
TC1400002280.mm.2	Dpysl2	dihydropyrimidinase-like 2	1,79981E+14	9,7827E+14	3,32527E+14	0.0143499331449384	0.170747242106618	-2,6193E+14	1,10884E+14	1,10275E+14	9,96247E+14	8,55372E+14	9,45568E+14	8,60849E+14
TC0200001382.mm.2	Ambra1	autophagy/beclin 1 regulator 1	1,69222E+13	7,98916E+14	3,32491E+14	0.0143566123763589	0.170747242106618	-2,61971E+13	9,06691E+14	8,75464E+14	7,81838E+13	6,61874E+14	8,26623E+14	7,41005E+14
TC0100001491.mm.2	Kifap3	kinesin-associated protein 3	2,49042E+14	8,33624E+14	3,32468E+13	0.014360816523591	0.170747242106618	-2,61997E+14	9,78743E+14	9,77238E+14	8,38921E+14	6,18975E+14	8,51312E+14	7,36552E+14
TC0100002243.mm.2	Gm3646	predicted gene 3646	-1,37309E+14	5,77548E+14	-3,3239E+14	0.0143754585038911	0.170747242106618	-2,62086E+14	5,09916E+14	4,87834E+14	5,84141E+14	6,88227E+14	5,96143E+14	5,99026E+14
TC0600001559.mm.2	Prp2	proline rich protein 2	-1,32923E+14	6,41513E+14	-3,32331E+14	0.0143863911590365	0.170747242106618	-2,62153E+14	5,65217E+14	5,313E+14	6,36001E+14	7,26361E+13	6,82431E+14	7,07769E+14
TC0700001925.mm.2	Hmx3	H6 homeobox 3	-1,12354E+14	6,7884E+14	-3,32179E+14	0.0144149938233322	0.170747242106618	-2,62327E+14	5,97544E+14	6,12337E+14	6,86424E+14	7,48166E+14	7,10257E+14	7,18313E+14
TC1000002531.mm.2	Atcay	ataxia, cerebellar, Cayman type	1,7754E+14	7,91522E+13	3,32146E+14	0.0144212048275274	0.170747242106618	-2,62365E+14	9,15992E+13	8,85108E+14	7,8924E+14	6,56779E+14	7,94681E+14	7,07333E+14
TC1100000794.mm.2	Ttc19	tetratricopeptide repeat domain 19	1,41666E+14	7,68396E+14	3,3197E+13	0.0144541399595371	0.170747242106618	-2,62565E+14	8,57359E+14	8,44348E+14	7,52583E+14	6,65791E+14	7,80862E+14	7,09434E+14
TC0200001971.mm.2	Tmc2	transmembrane channel-like gene family 2	-1,58846E+14	4,05454E+14	-3,31952E+14	0.0144575421663566	0.170747242106618	-2,62585E+14	2,99935E+14	2,99284E+14	4,07734E+14	5,09178E+14	4,12086E+14	5,04505E+14
TC1000001573.mm.2	Rdh1/Rdh9	retinol dehydrogenase 1 (all trans)/retinol dehydrogenase 9	-1,29455E+14	7,87552E+14	-3,31887E+14	0.0144697628568774	0.170747242106618	-2,6266E+14	7,08229E+14	7,00627E+14	8,01274E+14	8,66491E+13	7,9029E+14	8,58399E+14
TC0900000976.mm.2	Nedd4	neural precursor cell expressed, developmentally down-regulated 4	2,19655E+14	7,72616E+14	3,3186E+14	0.0144748468567669	0.170747242106618	-2,6269E+13	9,08739E+13	9,03288E+14	7,86679E+13	5,86038E+14	7,62356E+14	6,88595E+14
TC1100002865.mm.2	Rasd1	RAS, dexamethasone-induced 1	1,17592E+14	6,92654E+14	3,3173E+13	0.0144993819887895	0.170747242106618	-2,62839E+14	8,05551E+14	7,53485E+14	6,87336E+13	6,36517E+14	6,28615E+14	6,44422E+14
TC0400001250.mm.2	Pik3r3	phosphoinositide-3-kinase regulatory subunit 3	1,40638E+14	7,80072E+14	3,3165E+14	0.0145144593690142	0.170747242106618	-2,6293E+14	8,51114E+14	8,86496E+14	7,27578E+14	7,28757E+14	7,96291E+14	6,90195E+14
TC1000001551.mm.2	B4galnt1	beta-1,4-N-acetyl-galactosaminyl transferase 1	1,41856E+14	7,16379E+14	3,31615E+14	0.01452119618133	0.170747242106618	-2,62971E+13	8,2524E+14	8,01055E+14	7,08174E+14	6,34408E+14	7,06203E+14	6,23192E+14
TC0100003451.mm.2	Rcsd1	RCSD domain containing 1	-1,34131E+14	8,00281E+14	-3,31544E+14	0.0145345854782966	0.170747242106618	-2,63052E+13	7,0665E+13	7,10384E+14	8,04941E+14	8,80356E+14	8,15286E+14	8,84069E+14
TC0500001350.mm.2	Oas1f	2'-5' oligoadenylate synthetase 1F	-1,4352E+14	6,28324E+14	-3,31505E+14	0.0145418316262613	0.170747242106618	-2,63095E+14	5,22553E+14	5,89641E+14	6,70925E+14	7,28309E+14	5,91526E+14	6,66989E+14
TC1300002082.mm.2	Il9	interleukin 9	-1,23331E+14	5,00145E+14	-3,31476E+14	0.014547460402232	0.170747242106618	-2,63129E+14	4,26727E+14	4,42393E+14	5,16246E+13	5,99534E+14	5,06975E+14	5,08996E+14
TC0100003546.mm.2	Itln1	intelectin 1 (galactofuranose binding)	-1,00456E+14	6,44347E+14	-3,31437E+13	0.0145548542464388	0.170747242106618	-2,63174E+13	5,81478E+14	5,82535E+14	6,9152E+14	6,73406E+13	6,83304E+14	6,53841E+14
TC0800000648.mm.2	Hpf1	histone PARylation factor 1	1,83513E+14	7,07855E+14	3,31426E+14	0.0145569005576048	0.170747242106618	-2,63186E+14	8,25272E+14	8,2428E+13	6,77269E+14	6,05258E+14	7,34382E+14	5,8067E+13
TC1000002983.mm.2	Iltifb	interleukin 10-related T cell-derived inducible factor beta	-1,04735E+14	5,87727E+14	-3,3136E+14	0.0145693688399141	0.170747242106618	-2,63261E+14	5,1597E+14	5,06583E+14	6,11489E+14	6,20534E+14	6,58594E+14	6,13194E+14
TC0900001070.mm.2	Myo6	myosin VI	1,50489E+14	6,39414E+14	3,31322E+14	0.0145766811344258	0.170747242106618	-2,63305E+14	7,13933E+14	7,39478E+14	6,24366E+13	5,28066E+13	6,54018E+14	5,76626E+14
TC0X00002482.mm.2	Pls3	plastin 3 (T-isoform)	1,86161E+13	7,87754E+14	3,31273E+14	0.0145858768634986	0.170747242106618	-2,63361E+14	9,14639E+14	9,33691E+14	8,0475E+14	6,71257E+14	7,5544E+14	6,46746E+14
TC1000001802.mm.2	Abracl	ABRA C-terminal like	1,59219E+14	5,9983E+14	3,31115E+14	0.0146158865692289	0.170747242106618	-2,63541E+14	6,68704E+13	7,32152E+14	5,92938E+14	4,8948E+14	5,90987E+14	5,2472E+14
TC1400002227.mm.2	4930578I06Rik	RIKEN cDNA 4930578I06 gene	-1,2379E+14	4,82676E+14	-3,31071E+14	0.0146242849666802	0.170747242106618	-2,63591E+14	4,17271E+14	3,85808E+14	4,93403E+14	5,57255E+14	4,97199E+14	5,45117E+14
TC0500002675.mm.2	Cenpc1	centromere protein C1	1,36651E+13	6,12451E+14	3,31007E+14	0.0146366082956916	0.170747242106618	-2,63665E+14	7,23678E+14	6,86159E+14	6,15046E+14	5,21489E+14	5,81058E+14	5,47274E+14
TC0800000277.mm.2	Slc20a2	solute carrier family 20, member 2	1,34205E+14	6,85323E+14	3,31E+14	0.0146378314246552	0.170747242106618	-2,63673E+14	7,87352E+14	7,88538E+14	6,93106E+14	6,14375E+14	6,47167E+14	5,81401E+14
TC0500003509.mm.2	Grifin	galectin-related inter-fiber protein	-1,07008E+13	7,82407E+14	-3,30924E+14	0.0146523874737363	0.170747242106618	-2,6376E+14	7,12221E+14	7,12093E+14	7,9261E+14	8,4572E+14	8,14351E+14	8,17445E+14
TC1600001427.mm.2	Fgf12	fibroblast growth factor 12	1,4787E+14	8,77381E+14	3,30819E+14	0.0146723262562612	0.170747242106618	-2,63879E+14	9,85691E+14	9,69693E+14	8,76837E+14	7,82807E+14	8,63297E+13	7,85963E+14
TC1500000392.mm.2	Deptor	DEP domain containing MTOR-interacting protein	2,08074E+14	5,8447E+14	3,3079E+14	0.0146779380750909	0.170747242106618	-2,63913E+14	6,85272E+14	7,17726E+14	4,63591E+14	5,23259E+14	6,52903E+14	4,64072E+14
TC0600000667.mm.2	Adcyap1r1	adenylate cyclase activating polypeptide 1 receptor 1	1,34431E+14	8,13547E+14	3,30732E+13	0.0146890789512377	0.170747242106618	-2,63979E+14	9,43621E+14	9,27905E+14	8,37583E+14	7,6508E+14	7,39351E+14	6,67745E+14
TC0X00003418.mm.2	Rpgr	retinitis pigmentosa GTPase regulator	1,60894E+14	5,9752E+14	3,30732E+14	0.0146891162705189	0.170747242106618	-2,63979E+14	7,23018E+14	7,18867E+14	6,22566E+14	4,97532E+14	5,43853E+14	4,79284E+14
TC1600000301.mm.2	Vps8	VPS8 CORVET complex subunit	1,09876E+14	7,01595E+14	3,30677E+14	0.0146994959743041	0.170747242106618	-2,64041E+14	7,67917E+14	7,59535E+14	6,74302E+14	6,33398E+14	7,08649E+14	6,65771E+14
TC1800000498.mm.2	Eno1b	enolase 1B, retrotransposed	1,66915E+14	7,29772E+13	3,30669E+14	0.0147010604396611	0.170747242106618	-2,64051E+14	8,16217E+14	8,40476E+14	7,32043E+12	5,9082E+14	7,19669E+14	6,79411E+14
TC1200000830.mm.2	Zc2hc1c	zinc finger, C2HC-type containing 1C	-1,07121E+13	7,11236E+14	-3,30584E+14	0.0147173945686996	0.170747242106618	-2,64148E+13	6,66382E+13	6,38831E+14	7,38019E+14	7,81435E+14	7,1359E+14	7,29157E+14
TC0700000816.mm.2	Vrk3	vaccinia related kinase 3	1,25877E+14	6,99827E+14	3,30578E+14	0.0147185193052609	0.170747242106618	-2,64155E+14	7,89356E+14	7,83933E+14	7,02873E+14	6,18662E+13	6,36994E+14	6,67146E+14
TC0800001906.mm.2	Tacc1/Mir8108	transforming, acidic coiled-coil containing protein 1/microRNA 8108	1,0754E+14	7,34598E+14	3,30482E+14	0.0147369994459618	0.170747242106618	-2,64265E+14	8,03116E+14	8,26402E+14	7,28576E+14	6,85861E+14	6,94406E+14	6,69227E+13
TC0200005220.mm.2	Atp9a	ATPase, class II, type 9A	1,51803E+14	7,81935E+13	3,30451E+14	0.0147428700939715	0.170747242106618	-2,643E+14	9,00386E+14	8,71099E+14	7,62768E+14	7,0511E+14	7,8167E+14	6,70578E+14
TC1100000802.mm.2	Trpv2	transient receptor potential cation channel, subfamily V, member 2	1,00895E+14	6,20078E+14	3,30405E+14	0.0147517778685912	0.170747242106618	-2,64353E+14	6,75538E+14	6,77428E+14	5,57282E+13	5,93894E+14	6,05363E+14	6,1096E+14
TC0700001620.mm.2	Ipo7/Snora23	importin 7/small nucleolar RNA, H/ACA box 23	2,62819E+13	6,9586E+14	3,30357E+14	0.0147609772355766	0.170747242106618	-2,64408E+13	8,75259E+14	8,55566E+14	7,05983E+14	4,99204E+14	7,02697E+14	5,36449E+14
TC1300001779.mm.2	Tubb2a	tubulin, beta 2A class IIA	1,6484E+14	8,94401E+14	3,30339E+14	0.0147644910326866	0.170747242106618	-2,64428E+14	1,00858E+14	9,98433E+14	9,0487E+14	7,7246E+14	8,71741E+14	8,10325E+14
TC0800002861.mm.2	Chtf8	CTF8, chromosome transmission fidelity factor 8	1,1809E+14	7,25361E+13	3,30338E+14	0.0147645947837965	0.170747242106618	-2,64429E+14	7,90933E+14	7,88112E+14	7,09338E+14	6,33528E+14	7,11156E+14	7,19096E+14
TC1700000482.mm.2	Clpsl2	colipase-like 2	-1,43443E+14	7,3617E+14	-3,30322E+14	0.0147677274546027	0.170747242106618	-2,64448E+14	6,36721E+14	6,26914E+14	7,22297E+14	8,28225E+14	7,77356E+14	8,25506E+14
TC0800001233.mm.2	Pskh1	protein serine kinase H1	-1,31589E+14	5,26315E+14	-3,30294E+14	0.0147731788464397	0.170747242106618	-2,6448E+14	4,46031E+14	4,49198E+14	6,24766E+13	5,33641E+14	5,71376E+14	5,32877E+14
TC0400001259.mm.2	Prdx1	peroxiredoxin 1	1,86878E+11	9,50375E+14	3,30206E+14	0.0147901755331147	0.170747242106618	-2,64581E+14	1,05886E+14	1,06913E+14	9,48185E+14	8,06047E+14	9,6068E+14	8,59351E+14
TC0500001369.mm.2	Brap	BRCA1 associated protein	0.957183046971846	7,67091E+14	3,30133E+14	0.0148041167074289	0.170747242106618	-2,64664E+14	8,13542E+14	8,05221E+14	7,18966E+14	7,0836E+14	7,76485E+14	7,79974E+14
TC1500001596.mm.2	Washc5	WASH complex subunit 5	1,17345E+14	7,06688E+14	3,30127E+14	0.0148053613760888	0.170747242106618	-2,64671E+14	7,86051E+14	7,93723E+13	7,09739E+14	6,35344E+14	6,60748E+13	6,54521E+14
TC0500002238.mm.2	Tnip2	TNFAIP3 interacting protein 2	-1,1774E+14	8,38228E+14	-3,3011E+14	0.0148087021373914	0.170747242106618	-2,64691E+14	7,52068E+14	7,61941E+14	8,73264E+14	8,76224E+14	8,45394E+13	9,20475E+14
TC0200001788.mm.2	Jmjd7/Pla2g4b/Gm28042	jumonji domain containing 7/phospholipase A2, group IVB (cytosolic)/predicted gene, 28042	-1,47188E+14	7,10467E+14	-3,30041E+14	0.0148218511526121	0.170747242106618	-2,64769E+14	5,99164E+14	6,1238E+13	7,10378E+14	7,95541E+14	7,29269E+14	8,16069E+14
TC0600001637.mm.2	Dera	deoxyribose-phosphate aldolase (putative)	-1,05279E+14	7,38912E+13	-3,3003E+14	0.014824100339616	0.170747242106618	-2,64782E+14	6,55424E+14	6,8254E+14	7,6363E+14	7,84892E+14	7,55587E+14	7,914E+14
TC1500001073.mm.2	Krt7	keratin 7	-1,16333E+14	5,84529E+12	-3,29942E+14	0.0148409886769671	0.170747242106618	-2,64882E+14	5,33588E+14	5,54475E+13	6,25811E+14	6,94918E+14	5,5549E+14	5,42894E+13
TC0200003603.mm.2	Pla2r1	phospholipase A2 receptor 1	-1,08604E+14	5,47852E+14	-3,29923E+13	0.0148447616951928	0.170747242106618	-2,64904E+14	4,90303E+14	4,84274E+13	5,7139E+14	6,20395E+14	5,45537E+14	5,75214E+14
TC0200004899.mm.2	Zcchc3	zinc finger, CCHC domain containing 3	-0.986157430602677	7,34755E+14	-3,29868E+12	0.0148553122092812	0.170747242106618	-2,64967E+14	6,61962E+14	6,56192E+14	7,72202E+14	7,43183E+14	7,88094E+14	7,86896E+14
TC1100002851.mm.2	Snap47	synaptosomal-associated protein, 47	2,18754E+14	8,65815E+14	3,29806E+14	0.0148674476885564	0.170747242106618	-2,65038E+14	1,00791E+14	9,69322E+14	8,72445E+14	6,67274E+13	8,6337E+14	8,14573E+14
TC0600000989.mm.2	C87436	expressed sequence C87436	1,19298E+14	5,49159E+14	3,29707E+14	0.014886651249006	0.170747242106618	-2,65152E+14	6,68041E+14	6,21144E+14	5,07766E+14	5,42823E+14	5,0401E+14	4,5117E+14
TC1400000337.mm.2	Oxnad1	oxidoreductase NAD-binding domain containing 1	1,98718E+14	7,15077E+14	3,29688E+14	0.0148903615156953	0.170747242106618	-2,65173E+14	8,15147E+14	8,23393E+14	7,15469E+14	5,25635E+14	7,0629E+14	7,04526E+14
TC1100001560.mm.2	Cdk12/Mir5119	cyclin-dependent kinase 12/microRNA 5119	1,40796E+14	6,62131E+14	3,29621E+14	0.0149034165901096	0.170747242106618	-2,6525E+14	7,65632E+14	7,56568E+14	6,14758E+14	6,2585E+14	6,61208E+14	5,48767E+14
TC1500001450.mm.2	Angpt1	angiopoietin 1	0.961718863099598	5,34627E+14	3,29613E+14	0.0149049899501239	0.170747242106618	-2,6526E+14	5,95321E+14	6,09394E+14	5,0251E+14	5,09861E+14	4,99783E+14	4,90892E+14
TC1900000246.mm.2	Mpeg1	macrophage expressed gene 1	1,76063E+14	6,35513E+14	3,29555E+14	0.0149161224724414	0.170747242106618	-2,65325E+14	7,15578E+14	8,04455E+14	6,39698E+14	5,28208E+14	5,24615E+14	6,00521E+14
TC0200002811.mm.2	Arfgap1	ADP-ribosylation factor GTPase activating protein 1	1,83908E+14	8,17073E+14	3,29488E+14	0.0149293150692749	0.170747242106618	-2,65403E+14	9,20727E+14	9,01728E+14	8,05922E+14	6,48718E+14	8,4575E+14	7,79591E+14
TC1500002262.mm.2	Krt82	keratin 82	-0.991637510590376	5,12384E+14	-3,29486E+13	0.0149295265684252	0.170747242106618	-2,65404E+14	4,5116E+13	4,63014E+14	5,41413E+14	5,71089E+14	5,25449E+14	5,2218E+14
TC0600001212.mm.2	Grm7	glutamate receptor, metabotropic 7	2,02877E+14	6,37203E+14	3,29481E+14	0.014930662646229	0.170747242106618	-2,65411E+14	7,77082E+14	7,56083E+14	6,09028E+14	5,18383E+14	6,6718E+12	4,9546E+14
TC1300001921.mm.2	Nup153	nucleoporin 153	1,35008E+14	6,87352E+14	3,29438E+14	0.0149389052876906	0.170747242106618	-2,65459E+14	7,67619E+14	7,77669E+14	6,81115E+14	5,94158E+13	6,80584E+12	6,22966E+14
TC1900000415.mm.2	Vldlr	very low density lipoprotein receptor	1,01863E+14	7,39197E+14	3,29408E+14	0.0149447393226354	0.170747242106618	-2,65493E+14	7,97504E+14	8,04451E+14	7,08666E+14	6,89564E+14	7,35298E+13	6,99699E+14
TC0500000234.mm.2	Cct8l1	chaperonin containing TCP1, subunit 8 (theta)-like 1	-0.972043010844763	5,07823E+14	-3,29395E+14	0.0149474197423285	0.170747242106618	-2,65509E+14	4,58072E+13	4,58619E+14	5,43494E+14	5,67605E+14	5,09989E+14	5,09161E+14
TC1600001134.mm.2	Coro7	coronin 7	1,16863E+14	7,10216E+14	3,29385E+14	0.0149492906462771	0.170747242106618	-2,6552E+13	8,05993E+14	7,82993E+14	6,91191E+14	6,64069E+14	6,25712E+14	6,9134E+14
TC0200002127.mm.2	Snrpb2	U2 small nuclear ribonucleoprotein B	1,06688E+14	7,02627E+14	3,2935E+14	0.0149562022331472	0.170747242106618	-2,65561E+14	7,62331E+14	7,68121E+13	6,55063E+13	6,62013E+14	7,10556E+14	6,57675E+14
TC1100004084.mm.2	Grb2	growth factor receptor bound protein 2	1,01667E+14	8,7127E+13	3,29332E+14	0.0149595534607344	0.170747242106618	-2,6558E+14	9,44387E+14	9,33937E+14	8,50813E+14	8,24177E+14	8,51473E+14	8,22831E+14
TC0600001557.mm.2	Prh1	proline rich protein HaeIII subfamily 1	-1,42439E+14	6,87586E+14	-3,29318E+14	0.0149624477229253	0.170747242106618	-2,65597E+13	6,03849E+13	5,81036E+14	6,87452E+14	7,82312E+14	7,04112E+14	7,66756E+14
TC1000000762.mm.2	Tspear	thrombospondin type laminin G domain and EAR repeats	-1,40816E+14	6,29993E+14	-3,29184E+14	0.0149884917332935	0.170747242106618	-2,6575E+14	5,60905E+14	5,39656E+14	6,36069E+14	7,46124E+14	6,392E+14	6,58002E+14
TC0400003956.mm.2	Vps13d	vacuolar protein sorting 13D	1,54416E+14	6,11014E+13	3,29131E+14	0.0149989851608318	0.170747242106618	-2,65812E+14	7,10104E+12	7,12111E+14	6,19863E+14	4,9352E+14	5,8601E+14	5,44475E+14
TC1100000426.mm.2	Slu7	SLU7 splicing factor homolog (S. cerevisiae)	2,10766E+14	7,25741E+14	3,29119E+14	0.0150012434182228	0.170747242106618	-2,65825E+14	8,74226E+14	8,59495E+14	7,55436E+14	5,56753E+14	6,78944E+14	6,29593E+14
TC0800002566.mm.2	Rln3	relaxin 3	-1,51418E+14	7,12469E+14	-3,29082E+14	0.0150084583936829	0.170747242106618	-2,65867E+14	5,87258E+13	6,24765E+14	7,05882E+14	8,08977E+14	7,40144E+14	8,07787E+13
TC0200002359.mm.2	Itch	itchy, E3 ubiquitin protein ligase	1,9585E+13	5,63392E+14	3,29045E+14	0.015015773478407	0.170747242106618	-2,6591E+14	6,88619E+14	6,80326E+14	5,68425E+14	4,0882E+14	5,65567E+14	4,68594E+14
TC1200002110.mm.2	Irf2bpl	interferon regulatory factor 2 binding protein-like	1,42801E+13	7,18889E+14	3,29044E+14	0.01501605665158	0.170747242106618	-2,65911E+14	8,23131E+14	8,00853E+14	7,25202E+14	6,1318E+14	6,63642E+14	6,87325E+14
TC0800001770.mm.2	Csmd1	CUB and Sushi multiple domains 1	-1,06516E+14	9,47527E+14	-3,2901E+14	0.0150225774044254	0.170747242106618	-2,6595E+14	8,63797E+14	8,74384E+14	9,52555E+14	9,98657E+14	1,01038E+14	9,85385E+14
TC1600000109.mm.2	Rpl39l	ribosomal protein L39-like	-1,06182E+14	5,83887E+14	-3,28999E+14	0.015024747677441	0.170747242106618	-2,65962E+14	5,15037E+14	4,81571E+13	5,86871E+11	6,22101E+14	6,38652E+14	6,59092E+12
TC1100000654.mm.2	Gria1	glutamate receptor, ionotropic, AMPA1 (alpha 1)	1,92823E+14	8,81296E+14	3,28992E+14	0.0150261961539552	0.170747242106618	-2,65971E+14	1,02058E+14	1,01254E+14	8,99708E+14	7,47771E+14	8,54611E+14	7,52562E+14
TC0200005136.mm.2	Pltp	phospholipid transfer protein	1,37486E+14	7,71407E+14	3,28922E+14	0.0150398221380311	0.170747242106618	-2,6605E+14	8,47367E+14	8,71129E+14	7,45887E+14	6,97637E+14	7,80542E+14	6,85883E+14
TC0700001280.mm.2	Sytl2	synaptotagmin-like 2	1,40239E+14	6,92738E+14	3,28876E+14	0.0150489878314742	0.170747242106618	-2,66104E+14	7,53285E+14	7,84919E+14	6,6586E+13	5,91866E+14	7,19911E+14	6,40585E+14
TC1800001186.mm.2	Nrg2	neuregulin 2	-0.998503107969625	6,84787E+12	-3,28861E+14	0.0150519594155799	0.170747242106618	-2,66121E+14	6,31493E+14	6,26646E+14	7,15125E+14	7,42715E+14	7,07027E+14	6,85718E+14
TC0X00001931.mm.2	Syn1	synapsin I	2,43134E+14	9,16642E+14	3,28857E+14	0.0150527650539776	0.170747242106618	-2,66126E+14	1,07144E+14	1,08177E+14	9,40422E+14	7,26518E+14	8,98634E+14	7,81066E+14
TC0700004334.mm.2	Zfp629	zinc finger protein 629	-1,07383E+14	8,63732E+14	-3,28821E+14	0.0150596548162992	0.170747242106618	-2,66166E+14	7,84918E+14	7,91438E+13	8,69249E+14	9,21872E+14	8,98991E+14	9,15923E+14
TC1200000869.mm.2	Gstz1	glutathione transferase zeta 1 (maleylacetoacetate isomerase)	1,51389E+13	6,96665E+14	3,28751E+14	0.0150736116765743	0.170747242106618	-2,66247E+13	8,09226E+14	7,80128E+14	6,93591E+14	5,92984E+13	6,8974E+14	6,14324E+14
TC0900001940.mm.2	Glb1l3	galactosidase, beta 1 like 3	-1,46284E+14	5,15988E+14	-3,28747E+14	0.0150742243841977	0.170747242106618	-2,66251E+14	4,47311E+14	4,35241E+14	5,35399E+14	6,39721E+14	4,88228E+14	5,50025E+14
TC0300000567.mm.2	Il12a	interleukin 12a	-0.971490899818121	5,19232E+14	-3,28737E+14	0.0150763158515339	0.170747242106618	-2,66263E+14	4,64429E+14	4,55988E+14	5,59138E+14	5,55577E+14	5,28615E+14	5,51647E+14
TC1700002293.mm.2	Slc5a7	solute carrier family 5 (choline transporter), member 7	1,49478E+14	5,6641E+14	3,287E+14	0.0150836507371997	0.170747242106618	-2,66306E+14	7,29311E+14	6,50936E+14	5,65428E+14	5,15864E+14	5,11414E+14	4,25504E+14
TC0100001944.mm.2	Vcpip1	valosin containing protein (p97)/p47 complex interacting protein 1	2,87307E+14	6,70019E+14	3,28682E+14	0.0150871749508806	0.170747242106618	-2,66326E+14	8,45763E+14	8,47689E+14	6,43043E+14	4,75794E+14	7,26058E+12	4,81767E+14
TC0700002076.mm.2	Tnni2	troponin I, skeletal, fast 2	-1,29176E+14	6,24548E+14	-3,28505E+14	0.015122103002074	0.170747242106618	-2,66529E+14	5,47229E+14	5,47637E+14	6,60145E+14	6,93073E+14	6,04366E+14	6,94835E+14
TC1200000134.mm.2	Rock2	Rho-associated coiled-coil containing protein kinase 2	3,19469E+14	6,45878E+14	3,28495E+14	0.0151240926181706	0.170747242106618	-2,66541E+14	8,13892E+14	8,24722E+12	6,51384E+14	3,48292E+14	6,87489E+14	5,49491E+14
TC1500000725.mm.2	Aco2	aconitase 2, mitochondrial	1,03967E+13	9,03294E+14	3,28485E+13	0.0151259043664048	0.170747242106618	-2,66551E+14	9,90626E+13	9,69559E+14	8,96872E+14	8,55379E+14	8,48653E+14	8,58674E+14
TC1200001564.mm.2	Dcdc2c	doublecortin domain containing 2C	-1,46987E+14	6,14082E+14	-3,28462E+14	0.0151305022215285	0.170747242106618	-2,66578E+14	5,16071E+14	5,3041E+14	6,37905E+14	7,0255E+14	5,96928E+14	7,00625E+13
TC1700001631.mm.2	Mlst8	MTOR associated protein, LST8 homolog (S. cerevisiae)	1,35952E+14	6,88557E+14	3,2846E+14	0.0151308925420829	0.170747242106618	-2,6658E+14	7,79989E+14	7,76517E+14	6,9529E+13	5,89312E+14	6,51691E+14	6,38544E+14
TC0100002945.mm.2	Inhbb	inhibin beta-B	-1,20546E+14	6,61344E+14	-3,28457E+14	0.0151314258821731	0.170747242106618	-2,66583E+14	5,96493E+14	5,72111E+14	6,65953E+14	7,43743E+14	6,91022E+14	6,98743E+13
TC1800001636.mm.2	Siglec15	sialic acid binding Ig-like lectin 15	-1,30902E+14	6,90125E+14	-3,28331E+14	0.0151563531181697	0.170747242106618	-2,66728E+14	5,93728E+14	5,86835E+14	6,86869E+14	7,55498E+14	7,23315E+14	7,94506E+14
TC0900001846.mm.2	Col5a3	collagen, type V, alpha 3	-1,47657E+14	6,69771E+14	-3,28279E+14	0.0151666580493526	0.170747242106618	-2,66788E+13	5,81164E+14	5,74248E+14	6,82315E+14	7,68411E+14	6,61686E+14	7,50803E+14
TC1500002299.mm.2	Atp5g2	ATP synthase, H+ transporting, mitochondrial F0 complex, subunit C2 (subunit 9)	1,07934E+14	9,46511E+14	3,28243E+14	0.0151738339032299	0.170747242106618	-2,66829E+13	1,01211E+14	1,01175E+14	9,31983E+14	8,76004E+14	9,29829E+14	9,17395E+13
TC0400002449.mm.2	B4galt1	UDP-Gal:betaGlcNAc beta 1,4- galactosyltransferase, polypeptide 1	1,30749E+14	5,644E+14	3,28218E+13	0.0151788561564287	0.170747242106618	-2,66858E+14	6,14677E+14	6,57046E+13	5,47706E+14	4,62519E+14	5,65827E+14	5,38626E+14
TC0800000564.mm.2	Dctd	dCMP deaminase	1,73429E+14	7,17921E+14	3,28164E+14	0.0151895804221449	0.170747242106618	-2,6692E+14	8,22517E+14	8,09916E+14	7,17882E+14	5,67694E+14	6,69324E+14	7,20196E+13
TC0700001668.mm.2	Spon1	spondin 1, (f-spondin) extracellular matrix protein	1,19305E+14	6,70335E+14	3,28138E+14	0.0151946735723145	0.170747242106618	-2,6695E+14	7,69311E+14	7,39262E+14	6,67599E+14	6,02364E+14	6,04233E+14	6,39239E+14
TC0900001489.mm.2	Dclk3	doublecortin-like kinase 3	1,40122E+14	6,39392E+14	3,28097E+14	0.0152028432318356	0.170747242106618	-2,66997E+14	7,59851E+14	7,58062E+14	6,68814E+14	5,68855E+14	5,67055E+14	5,13714E+14
TC0400003418.mm.2	Ermap	erythroblast membrane-associated protein	-1,55698E+14	5,45836E+14	-3,27933E+14	0.0152355286275301	0.170747242106618	-2,67186E+14	4,13217E+14	4,60931E+14	5,45244E+14	6,403E+14	5,65645E+14	6,49682E+14
TC0200003654.mm.2	Grb14	growth factor receptor bound protein 14	1,58578E+14	6,7072E+14	3,27891E+14	0.0152437418697189	0.170747242106618	-2,67233E+14	7,69508E+14	7,84451E+12	6,87682E+14	5,4912E+14	6,20022E+14	6,13536E+14
TC0100001980.mm.2	Lactb2	lactamase, beta 2	1,84266E+14	6,15469E+14	3,27762E+14	0.0152696253239381	0.170747242106618	-2,67382E+14	7,38156E+13	7,73746E+14	6,07459E+13	5,35912E+14	5,95657E+14	4,41883E+14

TC0X00000073.mm.2	Otud5	OTU domain containing 5	1,4864E+14	6,61732E+14	3,27571E+14	0.0153077415407737	0.170747242106618	-2,67601E+14	7,3606E+14	7,43495E+14	6,5292E+14	5,29356E+14	6,42658E+14	6,65906E+14
TC0400003807.mm.2	Cela3a	chymotrypsin-like elastase family, member 3A	-1,19984E+14	5,33945E+14	-3,27525E+12	0.015316870402856	0.170747242106618	-2,67653E+14	4,77166E+14	4,47714E+14	5,51208E+14	6,1364E+14	5,35311E+14	5,78635E+14
TC0700002049.mm.2	Taldo1	transaldolase 1	1,14381E+14	7,48259E+14	3,27446E+14	0.0153327865553164	0.170747242106618	-2,67745E+14	8,02988E+14	7,97995E+14	7,16424E+14	6,55798E+14	7,76715E+14	7,39633E+14
TC0600002109.mm.2	Hipk2	homeodomain interacting protein kinase 2	1,75474E+14	8,31163E+13	3,27298E+14	0.0153625352724007	0.170747242106618	-2,67915E+12	9,54283E+14	9,5901E+14	7,90783E+14	7,71563E+14	8,36229E+14	6,75112E+14
TC0400001570.mm.2	Srsf4	serine/arginine-rich splicing factor 4	1,24147E+14	7,71819E+14	3,27286E+14	0.0153648599217065	0.170747242106618	-2,67928E+14	8,59483E+14	8,42336E+14	7,67056E+14	6,8647E+14	7,54365E+14	7,21205E+14
TC1500001920.mm.2	St13	suppression of tumorigenicity 13	1,09215E+14	7,80119E+14	3,27232E+14	0.0153756831287147	0.170747242106618	-2,6799E+14	8,64913E+14	8,53649E+14	7,55471E+14	7,44661E+14	7,60442E+14	7,0158E+14
TC0500000868.mm.2	Uso1	USO1 vesicle docking factor	1,51041E+14	8,12268E+13	3,27174E+14	0.0153873552788817	0.170747242106618	-2,68057E+14	8,95109E+13	9,02605E+14	8,11181E+14	6,84452E+14	8,03134E+14	7,7713E+14
TC1900001603.mm.2	Actr1a	ARP1 actin-related protein 1A, centractin alpha	1,29348E+14	8,32281E+13	3,27043E+14	0.0154137918816274	0.170747242106618	-2,68207E+14	9,1972E+14	9,10207E+14	8,33838E+14	7,37394E+14	8,01471E+14	7,91053E+14
TC1000002199.mm.2	Dux	double homeobox	-1,35071E+14	6,485E+14	-3,27008E+14	0.0154208705477907	0.170747242106618	-2,68248E+14	5,4681E+14	5,70285E+14	6,42747E+14	7,4449E+14	6,83058E+14	7,0361E+14
TC0100002814.mm.2	Olfr1414	olfactory receptor 1414	-1,84444E+14	5,65703E+14	-3,27E+14	0.0154224230503572	0.170747242106618	-2,68257E+14	4,20739E+14	5,05157E+14	5,71678E+14	7,23107E+13	5,78966E+14	5,94572E+14
TC0700002603.mm.2	Irgc1	immunity-related GTPase family, cinema 1	-1,26172E+14	5,79043E+14	-3,27E+14	0.0154224352712803	0.170747242106618	-2,68257E+14	4,97611E+14	4,92568E+14	5,81654E+14	6,6087E+14	6,44386E+14	5,97171E+14
TC0400000405.mm.2	Fam205a2/Fam205a3	family with sequence similarity 205, member A2/family with sequence similarity 205, member A3	1,26825E+14	5,81949E+14	3,26901E+14	0.0154424999321951	0.170747242106618	-2,68371E+14	6,53588E+13	6,56199E+14	5,25937E+14	5,30199E+14	6,09563E+14	5,16207E+14
TC0200002287.mm.2	Defb20	defensin beta 20	-1,13219E+14	5,80203E+14	-3,26847E+13	0.015453380822987	0.170747242106618	-2,68433E+14	5,14045E+14	5,25035E+14	6,00807E+14	6,64711E+14	5,75962E+14	6,00661E+14
TC1800000347.mm.2	Zmat2	zinc finger, matrin type 2	1,25511E+14	8,55117E+14	3,26747E+14	0.015473550643079	0.170747242106618	-2,68547E+14	9,54033E+14	9,52854E+14	8,328E+14	8,23064E+14	8,29424E+13	7,38523E+14
TC0900000804.mm.2	Aagab	alpha- and gamma-adaptin binding protein	1,27991E+14	7,11401E+14	3,26574E+14	0.0155086686122178	0.170747242106618	-2,68747E+14	8,21452E+14	8,10044E+14	6,91928E+14	6,83585E+14	6,77968E+14	5,83428E+13
TC0200004630.mm.2	Avp	arginine vasopressin	-1,16668E+14	7,28676E+14	-3,26565E+14	0.0155104872552122	0.170747242106618	-2,68757E+14	6,79682E+14	6,22161E+14	7,55833E+14	7,79346E+14	7,45517E+13	7,89517E+14
TC1800001404.mm.2	Ndst1	N-deacetylase/N-sulfotransferase (heparan glucosaminyl) 1	1,39789E+14	7,63573E+14	3,26561E+14	0.0155112373111984	0.170747242106618	-2,68761E+14	8,7276E+13	8,6353E+14	7,58928E+14	6,97785E+14	7,4198E+14	6,46453E+14
TC1100003298.mm.2	Tmem199	transmembrane protein 199	1,28545E+14	6,37281E+14	3,26353E+14	0.0155536453050032	0.170747242106618	-2,69001E+14	7,52149E+14	6,94857E+14	6,11643E+14	5,78274E+14	6,28522E+14	5,58239E+14
TC0200000082.mm.2	Kin	Kin17 DNA and RNA binding protein	1,20322E+14	5,58081E+14	3,2633E+14	0.0155582853525533	0.170747242106618	-2,69027E+14	6,29318E+14	5,84106E+14	5,17316E+14	4,55465E+14	5,96931E+14	5,6535E+14
TC0600000621.mm.2	5730596B20Rik	RIKEN cDNA 5730596B20 gene	-1,16576E+14	4,95015E+14	-3,26291E+14	0.0155662083231569	0.170747242106618	-2,69072E+14	3,91675E+11	4,58005E+14	5,39954E+14	5,42877E+14	5,00143E+14	5,37436E+14
TC1500000301.mm.2	Dcaf13	DDB1 and CUL4 associated factor 13	1,34547E+14	6,39606E+14	3,26256E+14	0.0155734250894674	0.170747242106618	-2,69113E+14	7,21313E+14	7,36282E+14	6,42934E+14	5,45566E+14	6,15463E+14	5,76078E+13
TC1500001365.mm.2	Cox6c	cytochrome c oxidase subunit 6C	1,85737E+14	1,00199E+14	3,26242E+14	0.0155761857162528	0.170747242106618	-2,69128E+14	1,11745E+14	1,12757E+14	1,01599E+14	8,57559E+14	9,84985E+14	9,08391E+14
TC0X00000975.mm.2	Itgb1bp2	integrin beta 1 binding protein 2	-1,35521E+14	4,88547E+13	-3,26208E+14	0.0155832215766313	0.170747242106618	-2,69168E+14	4,1677E+14	4,2912E+14	5,12062E+14	6,0487E+14	5,10755E+14	4,57707E+14
TC0700000511.mm.2	Sirt2	sirtuin 2	1,01601E+14	9,15517E+14	3,26196E+14	0.015585550698083	0.170747242106618	-2,69181E+14	9,62602E+14	9,86029E+14	8,9021E+13	8,55219E+14	8,94878E+14	9,04164E+14
TC0200003599.mm.2	Cd302	CD302 antigen	1,04032E+14	6,47337E+14	3,26171E+14	0.0155908121943284	0.170747242106618	-2,69211E+14	7,03236E+14	7,50158E+14	6,29207E+14	6,16123E+14	5,9747E+13	5,8783E+14
TC0100001358.mm.2	Edem3	ER degradation enhancer, mannosidase alpha-like 3	1,48325E+14	6,61633E+14	3,26148E+14	0.0155954017368302	0.170747242106618	-2,69237E+13	7,5479E+14	7,29957E+12	6,53873E+14	5,34224E+14	6,64675E+14	6,32278E+13
TC1900000894.mm.2	Ndufv1	NADH:ubiquinone oxidoreductase core subunit V1	1,82135E+14	8,99805E+14	3,26095E+14	0.015606199613023	0.170747242106618	-2,69298E+14	1,00531E+14	9,96157E+14	9,00723E+14	7,36472E+14	9,04792E+14	8,55375E+14
TC1600001884.mm.2	Robo2	roundabout guidance receptor 2	1,02622E+14	6,74977E+14	3,26083E+14	0.0156087015735893	0.170747242106618	-2,69312E+14	7,21002E+14	7,14185E+14	6,30038E+14	5,99905E+14	7,0944E+14	6,75294E+14
TC0200002344.mm.2	1700003F12Rik	RIKEN cDNA 1700003F12 gene	-1,49286E+14	6,59658E+14	-3,26065E+14	0.0156123456873654	0.170747242106618	-2,69332E+14	5,31463E+14	5,76626E+14	6,50642E+14	7,5602E+14	6,93324E+13	7,49873E+14
TC0600000538.mm.2	Zfp398	zinc finger protein 398	1,20737E+13	6,81176E+14	3,25946E+14	0.0156366690779326	0.170747242106618	-2,69469E+13	7,84756E+14	7,32843E+14	6,6001E+14	6,16116E+14	6,22131E+14	6,71198E+14
TC1700002587.mm.2	Cdkl4	cyclin-dependent kinase-like 4	1,35441E+14	5,36051E+14	3,25939E+14	0.0156382485610862	0.170747242106618	-2,69478E+14	6,59013E+14	5,89502E+14	5,15912E+14	4,61721E+14	5,25991E+14	4,64168E+14
TC1100001688.mm.2	Grn	granulin	0.991419988860367	8,64127E+14	3,25931E+14	0.0156397312585697	0.170747242106618	-2,69486E+14	9,15613E+14	9,45532E+14	8,37906E+14	8,24956E+14	8,22973E+12	8,37783E+14
TC0700001612.mm.2	Rpl27a/Snora3	ribosomal protein L27A/small nucleolar RNA, H/ACA box 3	-1,09031E+14	9,70779E+13	-3,25886E+14	0.0156489857198038	0.170747242106618	-2,69538E+13	9,06925E+14	8,92431E+13	9,80971E+14	1,03645E+14	1,01503E+14	9,92872E+14
TC1300002125.mm.2	Isca1	iron-sulfur cluster assembly 1	1,67169E+14	8,52811E+14	3,25875E+14	0.0156512905720676	0.170747242106618	-2,69551E+14	9,59683E+14	9,3416E+13	8,53554E+14	7,0595E+14	8,45802E+14	8,17716E+14
TC0X00000522.mm.2	Slc9a6	solute carrier family 9 (sodium/hydrogen exchanger), member 6	1,89964E+14	7,5625E+14	3,25674E+14	0.0156924678037309	0.170747242106618	-2,69782E+14	8,83866E+14	8,82273E+14	7,80624E+14	6,05587E+14	7,17575E+14	6,67573E+14
TC1000002488.mm.2	Polr2e	polymerase (RNA) II (DNA directed) polypeptide E	1,3405E+14	7,55962E+14	3,25669E+14	0.0156936121486486	0.170747242106618	-2,69789E+13	8,32691E+14	8,41623E+14	7,48215E+14	6,57997E+13	7,54797E+14	7,00449E+14
TC0800001851.mm.2	AY761184	cDNA sequence AY761184	-1,04582E+14	6,9073E+14	-3,2551E+14	0.0157263863324119	0.170747242106618	-2,69972E+14	6,26961E+14	6,13276E+14	7,0954E+14	7,3986E+14	7,07675E+14	7,47067E+14
TC0800002750.mm.2	Cnot1	CCR4-NOT transcription complex, subunit 1	1,7569E+14	6,80594E+14	3,25507E+14	0.0157268536718904	0.170747242106618	-2,69975E+13	7,95323E+14	8,03392E+14	7,03626E+14	5,43709E+13	6,3616E+14	6,01355E+14
TC0400000889.mm.2	Gm13271	predicted gene 13271	-1,56536E+14	6,59892E+14	-3,2548E+13	0.0157325462880567	0.170747242106618	-2,70006E+13	5,49019E+14	5,76448E+14	6,79077E+14	7,59462E+14	6,43092E+14	7,52255E+14
TC0400001469.mm.2	Psmb2	proteasome (prosome, macropain) subunit, beta type 2	1,18196E+14	7,92032E+14	3,25328E+14	0.0157639123557596	0.170747242106618	-2,70182E+14	8,62976E+14	8,71895E+14	7,88637E+14	7,09841E+14	7,63616E+14	7,55228E+13
TC1200002416.mm.2	Ppp1r13b	protein phosphatase 1, regulatory subunit 13B	1,65884E+14	7,59108E+13	3,25304E+14	0.0157688657004582	0.170747242106618	-2,70209E+14	8,74919E+14	8,44507E+12	7,65735E+14	6,21924E+14	7,41244E+14	7,06318E+14
TC1800000636.mm.2	Il17b	interleukin 17B	-1,06073E+13	7,81631E+14	-3,25152E+14	0.0158002663194594	0.170747242106618	-2,70384E+13	6,89023E+14	7,20124E+14	7,93337E+14	8,27957E+14	8,14642E+14	8,44706E+14
TC0200004312.mm.2	Ano3	anoctamin 3	2,71506E+14	7,16555E+14	3,2507E+14	0.0158173182957846	0.170747242106618	-2,70479E+14	8,50519E+14	8,99575E+14	6,8976E+14	5,17321E+13	7,79431E+14	5,62725E+14
TC0500001757.mm.2	Trrap	transformation/transcription domain-associated protein	1,00174E+14	6,99509E+14	3,24989E+14	0.0158339798806327	0.170747242106618	-2,70572E+14	7,62295E+14	7,96386E+14	6,68725E+14	6,89608E+14	6,40942E+14	6,39095E+14
TC0400003721.mm.2	Hmgn2	high mobility group nucleosomal binding domain 2	1,39027E+14	8,62595E+14	3,24964E+14	0.0158391968689789	0.170747242106618	-2,70601E+14	9,29242E+14	9,48601E+14	8,50087E+13	7,49702E+14	8,73822E+14	8,24117E+14
TC0200002788.mm.2	Gata5os	GATA binding protein 5, opposite strand	-1,07561E+14	6,46008E+14	-3,24943E+13	0.0158436043891037	0.170747242106618	-2,70625E+14	5,60376E+14	6,02691E+13	6,98098E+13	6,80091E+14	6,4896E+14	6,85834E+14
TC0800002934.mm.2	Ddx19a	DEAD (Asp-Glu-Ala-Asp) box polypeptide 19a	1,15007E+14	6,26919E+14	3,24903E+14	0.0158518869760309	0.170747242106618	-2,70671E+13	6,79239E+14	7,00336E+14	5,4393E+14	6,05631E+14	5,98548E+13	6,33831E+14
TC0500000947.mm.2	Prdm8	PR domain containing 8	1,37519E+14	6,2799E+14	3,24836E+14	0.0158658277871448	0.170747242106618	-2,70748E+14	6,49128E+14	7,56836E+14	5,53368E+14	5,77557E+14	6,07673E+13	6,2338E+14
TC0700003552.mm.2	Mfge8	milk fat globule-EGF factor 8 protein	2,04787E+13	7,73055E+14	3,2475E+14	0.0158837798653594	0.170747242106618	-2,70848E+14	9,1924E+14	9,08734E+14	7,94583E+14	6,23818E+14	7,49023E+14	6,42935E+14
TC1100001494.mm.2	Hoxb7	homeobox B7	-1,3921E+14	7,40029E+14	-3,24719E+14	0.0158902579694394	0.170747242106618	-2,70884E+14	6,48712E+13	6,21341E+14	7,25706E+14	8,22768E+14	7,8413E+14	8,37517E+14
TC0400002542.mm.2	Olfr159	olfactory receptor 159	-1,73507E+14	5,00423E+14	-3,2468E+14	0.0158983442788089	0.170747242106618	-2,70928E+14	4,12763E+14	4,13608E+13	5,12196E+14	6,61189E+14	4,70119E+14	5,32663E+14
TC1700000391.mm.2	Jmjd8	jumonji domain containing 8	1,77438E+14	8,25194E+14	3,24656E+13	0.0159034251182926	0.170747242106618	-2,70956E+13	9,32775E+14	9,38345E+14	8,34547E+14	6,81698E+14	8,15087E+14	7,48709E+14
TC1900000426.mm.2	Slc1a1	solute carrier family 1 (neuronal/epithelial high affinity glutamate transporter, system Xag), member 1	1,45917E+14	7,12679E+14	3,24556E+13	0.0159243018178067	0.170747242106618	-2,71072E+14	8,10903E+14	8,04834E+14	7,09014E+14	6,14889E+14	7,08473E+14	6,27957E+14
TC0800000744.mm.2	Cope	coatomer protein complex, subunit epsilon	1,46768E+14	7,9195E+14	3,24544E+14	0.015926639787457	0.170747242106618	-2,71085E+14	8,98659E+14	8,94319E+14	8,04673E+14	6,94769E+14	7,59586E+14	6,99693E+14
TC0Y00000266.mm.2	Gm20822/Gm20828	predicted gene, 20822/predicted gene, 20828	-1,43491E+14	5,04387E+14	-3,24513E+13	0.0159332713274122	0.170747242106618	-2,71121E+14	4,29168E+14	4,06058E+14	5,06544E+14	6,15665E+14	5,11602E+14	5,57286E+14
TC0Y00000274.mm.2	Gm20822	predicted gene, 20822	-1,43491E+14	5,04387E+14	-3,24513E+13	0.0159332713274122	0.170747242106618	-2,71121E+14	4,29168E+14	4,06058E+14	5,06544E+14	6,15665E+14	5,11602E+14	5,57286E+14
TC1800001712.mm.2	Cndp2	CNDP dipeptidase 2 (metallopeptidase M20 family)	1,11263E+14	7,12454E+14	3,24442E+14	0.0159480912678591	0.170747242106618	-2,71203E+14	7,84305E+13	7,74413E+14	6,75096E+14	6,61096E+14	6,57449E+14	7,22363E+14
TC0200000408.mm.2	Lrrc26	leucine rich repeat containing 26	-1,7356E+14	5,19858E+14	-3,24439E+14	0.0159487787419096	0.170747242106618	-2,71207E+14	4,0483E+14	4,28359E+14	5,1025E+14	6,70059E+14	5,47928E+14	5,5772E+14
TC0700001101.mm.2	Agbl1	ATP/GTP binding protein-like 1	-1,32028E+14	5,52099E+14	-3,2442E+14	0.0159527077511165	0.170747242106618	-2,71228E+14	4,62882E+14	4,48905E+14	5,38405E+14	6,37437E+14	6,25525E+14	5,99439E+14
TC1000000093.mm.2	Plagl1	pleiomorphic adenoma gene-like 1	1,21994E+14	5,92702E+14	3,24241E+14	0.0159901273165961	0.170747242106618	-2,71435E+14	6,86317E+14	6,59525E+14	5,76886E+14	5,24968E+14	5,86568E+14	5,21951E+14
TC1500001758.mm.2	Ccdc166	coiled-coil domain containing 166	-1,1571E+14	6,37211E+13	-3,24198E+14	0.0159991919916902	0.170747242106618	-2,71484E+14	5,3482E+13	5,78904E+13	6,42754E+14	7,02391E+14	6,74047E+14	6,90349E+14
TC0500003491.mm.2	Mir339/4930432F04Rik	microRNA 339/RIKEN cDNA 4930432F04 gene	-1,39398E+14	7,21322E+14	-3,24194E+14	0.016000120334311	0.170747242106618	-2,71489E+14	6,17737E+14	6,34618E+14	7,17372E+14	8,1378E+14	7,42289E+14	8,02136E+14
TC0700001402.mm.2	Pgm2l1	phosphoglucomutase 2-like 1	1,58512E+14	7,86289E+14	3,24177E+14	0.0160036069041476	0.170747242106618	-2,71509E+14	8,65763E+14	8,95817E+14	7,78483E+14	6,66073E+14	7,96397E+14	7,152E+14
TC0800000098.mm.2	Tex29	testis expressed 29	-1,78185E+14	6,65882E+14	-3,24134E+14	0.0160126645031801	0.170747242106618	-2,71558E+14	5,35523E+14	5,28527E+13	6,32968E+14	7,87451E+13	7,22491E+14	7,88332E+14
TC1100002934.mm.2	Ncor1	nuclear receptor co-repressor 1	2,03224E+14	7,48418E+14	3,24048E+14	0.0160307793574461	0.170747242106618	-2,71658E+14	8,59988E+13	8,8361E+14	7,37718E+14	5,99431E+14	7,76095E+14	6,33668E+14
TC0600002278.mm.2	Hoxa4	homeobox A4	-1,13895E+14	6,18416E+14	-3,23965E+14	0.0160481741981882	0.170747242106618	-2,71753E+14	5,29745E+14	5,59653E+14	6,35467E+14	6,8172E+14	6,28265E+14	6,75643E+14
TC0600000923.mm.2	Rtkn	rhotekin	1,00303E+14	8,14206E+14	3,23956E+14	0.0160500830290772	0.170747242106618	-2,71764E+13	8,90112E+14	8,82342E+13	7,95384E+14	7,76465E+14	7,88448E+14	7,52487E+14
TC0X00000596.mm.2	Slitrk2	SLIT and NTRK-like family, member 2	1,35758E+14	6,09177E+14	3,23802E+14	0.0160826029365521	0.170747242106618	-2,71942E+14	7,10358E+14	6,81919E+13	5,9259E+14	5,28171E+14	6,12939E+14	5,29086E+14
TC1100003945.mm.2	Nol11	nucleolar protein 11	2,15045E+14	6,23519E+14	3,23713E+14	0.0161014616769702	0.170747242106618	-2,72045E+13	7,59753E+14	7,68597E+14	6,50341E+14	4,47917E+14	5,92913E+14	5,21591E+14
TC1100001756.mm.2	Tanc2	tetratricopeptide repeat, ankyrin repeat and coiled-coil containing 2	1,31312E+14	7,49324E+14	3,23697E+14	0.0161047053288835	0.170747242106618	-2,72063E+14	8,33914E+14	8,50076E+14	7,59433E+14	6,61934E+14	7,09256E+14	6,81329E+14
TC1300000637.mm.2	Eif4e1b	eukaryotic translation initiation factor 4E family member 1B	-1,1465E+14	5,93849E+14	-3,23579E+14	0.0161297952779019	0.170747242106618	-2,722E+14	5,45068E+13	5,27156E+14	6,17888E+12	6,83635E+14	6,09549E+14	5,798E+13
TC0X00002853.mm.2	Hmgn5	high-mobility group nucleosome binding domain 5	0.947102298337481	5,90465E+14	3,23561E+14	0.0161336167620909	0.170747242106618	-2,7222E+14	6,50672E+14	6,6128E+14	5,54143E+13	5,68388E+14	5,49507E+14	5,58798E+13
TC1500001091.mm.2	Zfp740	zinc finger protein 740	1,79514E+14	6,62295E+13	3,2356E+14	0.0161337363049586	0.170747242106618	-2,72221E+14	7,61292E+14	7,51905E+14	6,60914E+14	4,93255E+14	6,67107E+14	6,39295E+14
TC0500000876.mm.2	Fam47e	family with sequence similarity 47, member E	-1,60505E+14	6,67841E+14	-3,23519E+14	0.0161423850332338	0.170747242106618	-2,72268E+14	5,60728E+14	5,89808E+13	6,72872E+14	7,98675E+14	6,59442E+14	7,25523E+14
TC1700001027.mm.2	Tnfsf9	tumor necrosis factor (ligand) superfamily, member 9	-1,04532E+14	4,96379E+14	-3,23452E+14	0.0161566175962079	0.170747242106618	-2,72346E+14	4,32025E+14	4,2948E+14	5,09129E+14	5,6144E+14	5,2734E+14	5,18857E+14
TC1300002686.mm.2	Mtrex	Mtr4 exosome RNA helicase	1,93308E+14	6,57928E+14	3,23382E+14	0.0161716265395081	0.170747242106618	-2,72427E+14	7,87875E+14	7,84287E+14	6,60729E+14	5,24816E+14	6,59844E+14	5,30018E+14
TC0400001523.mm.2	Bsdc1	BSD domain containing 1	1,27891E+14	8,15456E+14	3,23381E+14	0.0161716979665821	0.170747242106618	-2,72428E+14	9,14053E+14	9,10246E+14	8,31734E+14	7,36782E+14	7,60773E+14	7,39146E+14
TC1100002000.mm.2	Card14	caspase recruitment domain family, member 14	-1,26251E+14	6,14495E+14	-3,23348E+14	0.0161788370402568	0.170747242106618	-2,72467E+14	5,28638E+14	5,3919E+14	6,1351E+14	7,06821E+14	6,4286E+14	6,55953E+14
TC1400001659.mm.2	Prkcd	protein kinase C, delta	1,53487E+14	7,88481E+14	3,23342E+14	0.0161800036788293	0.170747242106618	-2,72473E+14	8,99088E+14	9,14345E+14	8,19049E+14	6,87411E+13	7,1965E+14	6,91341E+14
TC0500002167.mm.2	Ost4	oligosaccharyltransferase complex subunit 4 (non-catalytic)	1,14397E+14	1,00209E+14	3,23279E+14	0.0161933805434415	0.170747242106618	-2,72546E+14	1,09332E+12	1,06831E+14	9,99778E+12	9,33056E+14	9,69166E+14	9,48918E+14
TC1200002150.mm.2	Cep128	centrosomal protein 128	2,08378E+14	5,79337E+14	3,23074E+14	0.0162370444718148	0.170747242106618	-2,72783E+14	6,63564E+14	6,96441E+14	5,67732E+14	3,75518E+14	6,21237E+14	5,51531E+14
TC1100001566.mm.2	Tcap	titin-cap	-1,02108E+14	6,03677E+14	-3,23062E+14	0.016239591398009	0.170747242106618	-2,72796E+14	5,47952E+14	5,30269E+14	6,30442E+14	6,51994E+14	6,11645E+14	6,4976E+14
TC0X00001275.mm.2	Tmem35a	transmembrane protein 35A	1,27455E+14	6,80408E+14	3,22999E+14	0.0162531541210024	0.170747242106618	-2,7287E+14	7,92397E+14	7,42017E+14	6,72889E+13	6,06614E+14	6,09375E+14	6,59154E+14
TC0200004238.mm.2	D430041D05Rik	RIKEN cDNA D430041D05 gene	0.989098443855384	7,95999E+14	3,22945E+13	0.0162646185880327	0.170747242106618	-2,72932E+14	8,52269E+14	8,69727E+14	7,78195E+14	7,45981E+14	7,59808E+14	7,70012E+14
TC1400000784.mm.2	Psme1	proteasome (prosome, macropain) activator subunit 1 (PA28 alpha)	1,64042E+14	8,97847E+13	3,22938E+14	0.01626610331066	0.170747242106618	-2,7294E+14	9,89341E+14	9,91527E+14	8,95723E+14	7,57062E+14	9,05596E+14	8,47832E+14
TC1300002689.mm.2	Gpx8	glutathione peroxidase 8 (putative)	1,28551E+14	5,56151E+14	3,22934E+14	0.0162670514563876	0.170747242106618	-2,72945E+14	6,20198E+14	6,93426E+14	5,09342E+13	5,47179E+14	4,56138E+14	5,10621E+14
TC1500002264.mm.2	Krt75	keratin 75	-1,80655E+14	4,56141E+14	-3,22896E+14	0.0162751299078602	0.170747242106618	-2,72989E+14	3,67204E+14	3,2653E+14	4,47451E+14	6,07594E+14	4,69841E+14	5,18225E+14
TC0800000488.mm.2	Pcm1	pericentriolar material 1	2,73696E+13	6,34512E+13	3,22839E+13	0.0162873374588346	0.170747242106618	-2,73055E+14	8,08665E+14	7,87264E+14	6,5559E+14	3,92948E+14	6,36939E+14	5,25667E+13
TC0900000255.mm.2	Ncapd3	non-SMC condensin II complex, subunit D3	0.95183385490559	7,26724E+14	3,22833E+14	0.0162885706542633	0.170747242106618	-2,73061E+14	7,82249E+14	7,74461E+14	6,8336E+14	6,82983E+14	7,29845E+14	7,07449E+14
TC0300000769.mm.2	Adar	adenosine deaminase, RNA-specific	1,05376E+14	8,01879E+14	3,22825E+14	0.0162902115985952	0.170747242106618	-2,7307E+14	8,96788E+14	8,71636E+14	7,97383E+14	7,6029E+14	7,26178E+14	7,58999E+13
TC1500000856.mm.2	Mov10l1	Moloney leukemia virus 10-like 1	-1,54391E+14	4,6631E+14	-3,22739E+14	0.0163087521523344	0.170747242106618	-2,7317E+14	3,9199E+14	3,26481E+14	4,81587E+14	5,45666E+14	4,75036E+14	5,771E+14
TC0500003193.mm.2	Aldh2	aldehyde dehydrogenase 2, mitochondrial	1,6596E+13	7,65853E+14	3,22726E+14	0.0163114309559428	0.170747242106618	-2,73185E+14	8,87984E+14	8,63688E+14	7,81446E+14	6,38305E+14	7,359E+14	6,87795E+14
TC1500001819.mm.2	Myh9	myosin, heavy polypeptide 9, non-muscle	1,17283E+14	6,99995E+14	3,22594E+14	0.0163398664684917	0.170747242106618	-2,73338E+14	7,46964E+14	7,9723E+14	6,8585E+14	6,23777E+14	6,73821E+14	6,72328E+14
TC1000000894.mm.2	Tbxa2r	thromboxane A2 receptor	-0.976300159749648	7,05301E+14	-3,22546E+14	0.0163501966170952	0.170747242106618	-2,73394E+14	6,56499E+14	6,25015E+14	7,32217E+14	7,44557E+14	7,39102E+14	7,34416E+14
TC0500003660.mm.2	Hsph1	heat shock 105kDa/110kDa protein 1	1,38757E+14	8,61472E+14	3,22519E+14	0.0163558819538861	0.170747242106618	-2,73424E+13	9,53912E+14	9,57608E+14	8,6723E+14	7,66776E+14	8,39416E+14	7,83889E+14
TC0600000193.mm.2	Ptprz1	protein tyrosine phosphatase, receptor type Z, polypeptide 1	2,9592E+14	8,68696E+14	3,22478E+14	0.0163648242761584	0.170747242106618	-2,73472E+14	1,04285E+14	1,04449E+14	8,93372E+14	6,02133E+14	8,71366E+14	7,5796E+13
TC1800000316.mm.2	1700066B19Rik	RIKEN cDNA 1700066B19 gene	-1,19295E+14	5,04173E+14	-3,22464E+14	0.0163677008203187	0.170747242106618	-2,73488E+14	4,40458E+14	4,13726E+14	5,30045E+14	5,62729E+14	5,02978E+14	5,751E+14
TC1800001535.mm.2	Rab27b	RAB27B, member RAS oncogene family	1,16134E+14	7,64296E+14	3,22461E+13	0.0163683715542416	0.170747242106618	-2,73492E+14	8,71408E+14	8,17394E+14	7,16234E+14	7,40302E+14	6,94884E+14	7,45553E+14
TC0100001356.mm.2	Fam129a	family with sequence similarity 129, member A	-1,24405E+14	6,7163E+14	-3,22366E+14	0.0163889963142257	0.170747242106618	-2,73602E+14	5,83975E+14	5,72055E+14	6,58677E+14	7,46162E+14	7,20849E+14	7,48063E+14
TC1300000834.mm.2	Gm7969	predicted gene 7969	-0.993856909441002	5,82653E+14	-3,22277E+14	0.0164079986200304	0.170747242106618	-2,73704E+14	5,19447E+14	5,34662E+11	6,15185E+14	6,37696E+13	6,09257E+13	5,79673E+14
TC0600002484.mm.2	Usp39	ubiquitin specific peptidase 39	1,13934E+14	6,6375E+14	3,22196E+13	0.0164256924590329	0.170747242106618	-2,73799E+14	7,50507E+13	7,53932E+13	6,74823E+14	6,01749E+14	6,06062E+14	5,95428E+14
TC1000000579.mm.2	Neurog3	neurogenin 3	-1,14283E+14	6,20433E+13	-3,22154E+14	0.016434642954818	0.170747242106618	-2,73847E+14	5,3545E+14	5,65176E+12	6,3063E+14	6,98563E+14	6,42236E+14	6,50544E+14
TC1900000669.mm.2	Btrc	beta-transducin repeat containing protein	2,81111E+14	6,75286E+14	3,22088E+14	0.0164488621209921	0.170747242106618	-2,73923E+14	8,41177E+14	8,2549E+14	6,97799E+14	4,06645E+14	6,64469E+14	6,16138E+14
TC0100001313.mm.2	B3galt2	UDP-Gal:betaGlcNAc beta 1,3-galactosyltransferase, polypeptide 2	2,22983E+14	6,63077E+13	3,22071E+14	0.0164526017198114	0.170747242106618	-2,73943E+14	8,06914E+14	7,99214E+14	6,52416E+14	5,07745E+14	6,9275E+14	5,19422E+14
TC0900002239.mm.2	Nnmt	nicotinamide N-methyltransferase	-1,6298E+14	6,62802E+14	-3,22064E+14	0.0164540803307868	0.170747242106618	-2,73951E+14	5,57366E+14	5,67307E+14	6,708E+14	7,79832E+14	6,49933E+13	7,51576E+12
TC1100001577.mm.2	Gsdma	gasdermin A	-1,3299E+14	5,29614E+14	-3,22061E+14	0.0164547276982331	0.170747242106618	-2,73955E+14	3,8543E+13	4,78699E+14	5,44911E+14	5,85197E+14	5,75988E+14	6,07461E+14
TC1000002900.mm.2	Tmem19	transmembrane protein 19	1,10522E+14	6,47241E+14	3,21996E+14	0.0164688997955094	0.170747242106618	-2,7403E+14	7,36884E+14	7,17092E+14	6,1774E+14	6,15192E+14	6,30427E+14	5,66112E+13
TC1100004059.mm.2	Cd300e	CD300E molecule	-1,17264E+14	4,92245E+14	-3,21954E+14	0.0164779757626838	0.170747242106618	-2,74079E+14	4,01676E+14	3,99649E+14	4,84086E+14	5,51767E+14	5,36587E+14	5,79706E+14
TC1100000678.mm.2	Cnot8	CCR4-NOT transcription complex, subunit 8	1,09254E+14	8,10731E+14	3,21954E+14	0.0164780354244632	0.170747242106618	-2,74079E+14	8,91473E+14	8,87777E+14	8,11174E+14	7,49569E+14	7,71994E+14	7,52402E+14
TC0700002984.mm.2	Prr12	proline rich 12	0.941292586006335	6,86089E+14	3,21948E+14	0.0164791573514657	0.170747242106618	-2,74085E+14	7,51045E+14	7,52617E+14	6,67285E+14	6,48119E+14	6,50011E+14	6,4746E+14
TC0800002561.mm.2	Gm10644	predicted gene 10644	-1,36448E+13	6,29438E+13	-3,21834E+14	0.0165039064472213	0.170747242106618	-2,74217E+14	5,73537E+14	5,37504E+14	6,39754E+14	7,44182E+14	6,32213E+14	6,4944E+14
TC1300000462.mm.2	Pak1ip1	PAK1 interacting protein 1	1,10686E+14	7,97957E+14	3,21805E+14	0.0165102013503551	0.170747242106618	-2,74251E+14	8,68835E+14	8,67686E+14	7,75888E+13	7,39261E+14	7,96562E+14	7,39513E+14

TC0400002987.mm.2	Slc24a2	solute carrier family 24 (sodium/potassium/calcium exchanger), member 2	2,17475E+14	7,49286E+14	3,21723E+14	0.0165281211767826	0.170747242106618	-2,74346E+14	8,6641E+14	8,75839E+14	7,25935E+14	5,81363E+14	8,05486E+14	6,40681E+14
TC1100003397.mm.2	Ccl9	chemokine (C-C motif) ligand 9	1,2634E+14	5,219E+14	3,216E+14	0.0165549315672562	0.170747242106618	-2,74489E+14	6,0484E+14	5,61393E+14	4,22503E+14	4,9105E+14	5,00095E+14	5,5152E+14
TC0400000183.mm.2	Pnisr	PNN interacting serine/arginine-rich	0.994954026855664	7,88902E+14	3,21533E+14	0.0165694826286108	0.170747242106618	-2,74567E+13	8,60117E+14	8,6352E+14	7,47223E+14	7,77424E+14	7,56473E+14	7,28659E+14
TC0600002024.mm.2	Plxna4	plexin A4	1,73534E+14	6,25683E+14	3,21522E+14	0.0165720001668271	0.170747242106618	-2,7458E+14	7,36957E+14	7,60872E+14	6,47253E+14	5,03507E+14	5,90215E+14	5,15294E+14
TC1100002015.mm.2	Baiap2	brain-specific angiogenesis inhibitor 1-associated protein 2	2,98511E+14	7,00436E+14	3,21351E+14	0.0166092173371313	0.170747242106618	-2,74777E+14	8,94373E+13	9,04139E+14	7,21401E+13	4,80088E+14	7,0382E+14	4,98794E+14
TC0100001649.mm.2	Olfr414	olfactory receptor 414	-1,30648E+14	5,09501E+13	-3,21169E+14	0.0166490915603465	0.170747242106618	-2,74989E+13	4,13914E+14	4,17117E+13	5,11368E+11	5,8096E+14	5,28707E+14	6,0494E+14
TC0900001288.mm.2	Ky	kyphoscoliosis peptidase	-1,17401E+14	5,51156E+14	-3,21144E+14	0.0166546024444597	0.170747242106618	-2,75018E+14	4,53047E+14	4,53019E+14	5,29906E+14	6,10963E+14	6,29799E+14	6,30205E+14
TC0500003212.mm.2	Atp2a2	ATPase, Ca++ transporting, cardiac muscle, slow twitch 2	2,10667E+14	9,68081E+14	3,21143E+14	0.0166548537251264	0.170747242106618	-2,75019E+14	1,0953E+14	1,10041E+14	9,91737E+14	7,82639E+14	9,34853E+14	9,03549E+14
TC0700000592.mm.2	Scgb1b7	secretoglobin, family 1B, member 7	-1,42065E+14	6,06784E+14	-3,21118E+13	0.0166603232704544	0.170747242106618	-2,75048E+14	5,22639E+14	5,40557E+14	6,27954E+14	7,19372E+14	5,76761E+14	6,53421E+14
TC1300000626.mm.2	Simc1	SUMO-interacting motifs containing 1	1,44418E+14	6,51757E+14	3,20994E+14	0.0166874217916116	0.170747242106618	-2,75191E+14	7,67475E+13	7,43671E+14	6,69612E+14	5,527E+14	6,05722E+14	5,71364E+13
TC0500003688.mm.2	N4bp2l2	NEDD4 binding protein 2-like 2	1,25522E+14	7,47497E+14	3,2098E+14	0.0166905546033688	0.170747242106618	-2,75208E+14	8,29648E+14	8,28513E+14	7,50945E+14	6,56172E+14	7,13953E+14	7,0575E+14
TC1500000300.mm.2	Cthrc1	collagen triple helix repeat containing 1	-1,0848E+14	5,67144E+14	-3,20791E+14	0.016732262649173	0.170747242106618	-2,75427E+14	5,31512E+14	5,11966E+14	6,04922E+14	6,55516E+14	5,66416E+14	5,32534E+14
TC1700002635.mm.2	Kcng3	potassium voltage-gated channel, subfamily G, member 3	1,17747E+14	5,89015E+14	3,20763E+14	0.016738267301225	0.170747242106618	-2,75459E+13	6,6021E+14	6,78473E+12	5,12371E+14	5,90817E+14	5,38598E+14	5,53621E+14
TC1400000609.mm.2	Tox4	TOX high mobility group box family member 4	2,39874E+13	7,65412E+14	3,2076E+13	0.0167390830261848	0.170747242106618	-2,75463E+14	9,26131E+14	9,12658E+14	7,98274E+14	5,60767E+14	7,31668E+14	6,62973E+14
TC1700000719.mm.2	Gm6034	predicted gene 6034	-1,68805E+14	6,46682E+14	-3,20677E+13	0.016757351103459	0.170747242106618	-2,75559E+14	5,90847E+14	4,99994E+14	6,74254E+14	7,54196E+14	6,29468E+14	7,3133E+13
TC1200000432.mm.2	Akap6	A kinase (PRKA) anchor protein 6	1,90279E+14	7,42945E+14	3,20656E+14	0.0167619938634824	0.170747242106618	-2,75584E+14	8,95767E+14	8,8353E+14	7,34776E+14	6,63961E+13	7,25707E+14	5,53932E+13
TC1200000899.mm.2	Nrxn3	neurexin III	2,29262E+14	8,25308E+14	3,20651E+14	0.0167630141015121	0.170747242106618	-2,75589E+14	1,00239E+12	9,77132E+14	8,68913E+14	6,52082E+14	7,68902E+14	6,8243E+14
TC1600000938.mm.2	Jam2	junction adhesion molecule 2	1,41474E+14	7,66629E+14	3,2065E+14	0.0167633037904365	0.170747242106618	-2,75591E+14	8,49378E+14	8,42366E+14	7,56699E+14	6,52096E+14	7,78892E+13	7,20342E+14
TC1800001652.mm.2	Adnp2	ADNP homeobox 2	1,11842E+14	5,75363E+14	3,20648E+14	0.016763635076572	0.170747242106618	-2,75592E+14	6,43817E+14	6,32306E+14	5,60831E+14	4,91608E+13	5,6693E+14	5,56683E+14
TC0500000699.mm.2	Rasl11b	RAS-like, family 11, member B	1,29983E+14	6,08049E+14	3,20631E+14	0.0167674541683721	0.170747242106618	-2,75612E+14	6,86091E+14	6,86393E+14	5,10636E+12	6,01882E+14	6,05063E+14	5,5823E+14
TC1700001914.mm.2	Btnl6	butyrophilin-like 6	-1,44877E+14	5,7874E+14	-3,20605E+14	0.0167731389015835	0.170747242106618	-2,75642E+14	4,92261E+14	4,92656E+14	5,75542E+14	6,99129E+13	5,96789E+14	6,16062E+14
TC1800000665.mm.2	Nedd4l	neural precursor cell expressed, developmentally down-regulated gene 4-like	1,90493E+14	8,54245E+14	3,20591E+14	0.0167762277718719	0.170747242106618	-2,75658E+14	9,85106E+14	9,92372E+14	8,8886E+14	7,07632E+14	7,97512E+13	7,53987E+14
TC0X00000160.mm.2	Usp9x	ubiquitin specific peptidase 9, X chromosome	3,41325E+14	6,48146E+14	3,20574E+14	0.0167799951858024	0.170747242106618	-2,75678E+14	8,80179E+13	8,68696E+14	6,51492E+14	4,14733E+14	6,7742E+14	3,96353E+14
TC0700002680.mm.2	Zfp974	zinc finger protein 974	1,38035E+14	5,53853E+14	3,20557E+14	0.0167837489069724	0.170747242106618	-2,75698E+14	6,57363E+14	6,36351E+14	5,42333E+14	4,75311E+14	5,51423E+14	4,60337E+14
TC1600000665.mm.2	Cd200r2	Cd200 receptor 2	-1,06132E+14	6,82684E+14	-3,20507E+14	0.0167948684501899	0.170747242106618	-2,75756E+14	6,16641E+14	6,20932E+14	6,9594E+14	7,53897E+14	7,09529E+14	6,99164E+14
TC0800001119.mm.2	Cx3cl1	chemokine (C-X3-C motif) ligand 1	1,39872E+14	8,61516E+14	3,20458E+14	0.016805768461234	0.170747242106618	-2,75813E+13	9,60619E+14	9,61043E+14	8,67276E+14	7,74641E+14	8,39169E+14	7,66348E+14
TC0500000732.mm.2	Paics	phosphoribosylaminoimidazole carboxylase, phosphoribosylaminoribosylaminoimidazole, succinocarboxamide synthetase	1,32085E+13	8,13914E+14	3,2039E+14	0.0168208584272354	0.170747242106618	-2,75892E+14	9,10128E+14	9,10611E+14	8,15734E+14	7,40835E+14	7,9061E+14	7,15567E+14
TC0100002673.mm.2	Col4a4	collagen, type IV, alpha 4	-1,38706E+14	6,63343E+14	-3,20369E+14	0.0168255480705788	0.170747242106618	-2,75917E+14	5,75934E+14	5,60387E+14	6,61253E+14	7,5248E+14	6,79906E+14	7,501E+14
TC1000001560.mm.2	Arhgap9	Rho GTPase activating protein 9	-1,20027E+14	5,10747E+14	-3,2034E+14	0.0168318041987128	0.170747242106618	-2,7595E+14	4,3208E+14	4,58411E+14	5,26903E+14	6,03643E+14	5,07093E+14	5,36351E+13
TC1300001278.mm.2	Cdc20b	cell division cycle 20B	-1,55551E+13	7,17177E+14	-3,20311E+14	0.01683828314871	0.170747242106618	-2,75984E+14	6,32028E+12	6,3684E+13	7,34271E+14	8,45698E+14	6,84467E+14	7,69755E+14
TC0200001045.mm.2	Itga6	integrin alpha 6	1,08611E+14	6,63705E+14	3,20285E+14	0.0168440690384241	0.170747242106618	-2,76014E+14	6,86767E+14	7,30129E+14	6,1692E+14	5,82754E+13	6,99596E+14	6,66067E+14
TC0100003146.mm.2	Camsap2	calmodulin regulated spectrin-associated protein family, member 2	1,70352E+14	8,16139E+14	3,20198E+14	0.0168634329594054	0.170747242106618	-2,76115E+14	9,41791E+14	9,34692E+14	8,15482E+14	7,20297E+14	8,06853E+14	6,7772E+14
TC1400000198.mm.2	Samd8	sterile alpha motif domain containing 8	1,45647E+13	7,38398E+14	3,20186E+13	0.0168661317764283	0.170747242106618	-2,76129E+14	8,33567E+14	8,41601E+14	7,23125E+14	6,6075E+14	7,40534E+14	6,3081E+14
TC0500001421.mm.2	Lrrc43	leucine rich repeat containing 43	-1,26959E+14	5,1681E+14	-3,20166E+13	0.0168705568967632	0.170747242106618	-2,76152E+14	4,39919E+14	4,54023E+14	5,53545E+14	5,94316E+14	4,85527E+13	5,73532E+14
TC0700002744.mm.2	Zfp146	zinc finger protein 146	1,14678E+14	6,87721E+14	3,20155E+14	0.0168730431116931	0.170747242106618	-2,76165E+14	7,47018E+14	7,6352E+14	6,57665E+14	6,23517E+13	7,00639E+14	6,33965E+14
TC0200003805.mm.2	Prkra	protein kinase, interferon inducible double stranded RNA dependent activator	1,08667E+14	5,60293E+14	3,20139E+14	0.016876613383124	0.170747242106618	-2,76184E+14	6,61415E+14	6,16567E+14	5,39764E+14	5,20883E+14	5,32972E+13	4,90159E+14
TC1200000061.mm.2	Hs1bp3	HCLS1 binding protein 3	-0.986988248298938	7,02492E+14	-3,20136E+14	0.0168772584707912	0.170747242106618	-2,76187E+14	6,45858E+14	6,17442E+14	7,32072E+14	7,28625E+14	7,31444E+14	7,59509E+14
TC0500003373.mm.2	Clip2	CAP-GLY domain containing linker protein 2	0.955171275896908	6,89119E+14	3,19999E+14	0.0169077016141034	0.170747242106618	-2,76346E+14	7,41749E+14	7,59142E+14	6,66344E+13	6,43513E+14	6,64076E+14	6,59892E+14
TC0500003104.mm.2	Bicdl1	BICD family like cargo adaptor 1	1,50749E+14	6,53327E+14	3,19983E+14	0.0169113552844393	0.170747242106618	-2,76365E+14	7,82913E+14	7,64427E+14	6,65223E+14	5,80619E+14	6,14449E+14	5,1233E+14
TC0600002163.mm.2	Olfr459	olfactory receptor 459	-0.995430555245512	4,59896E+14	-3,19969E+14	0.0169143350597729	0.170747242106618	-2,76381E+14	4,05654E+14	4,24903E+14	5,01506E+14	5,28136E+14	4,63284E+14	4,35894E+14
TC1100003532.mm.2	Stxbp4	syntaxin binding protein 4	2,10285E+13	5,50355E+14	3,19908E+14	0.0169280195328465	0.170747242106618	-2,76452E+14	6,80318E+14	6,62903E+13	5,42811E+14	3,7984E+14	5,8538E+14	4,50877E+14
TC1500000262.mm.2	Polr2k	polymerase (RNA) II (DNA directed) polypeptide K	1,22845E+14	6,50301E+14	3,19807E+14	0.0169505665190567	0.170747242106618	-2,76569E+14	7,37383E+13	6,99079E+14	5,87698E+13	6,03075E+14	6,78076E+14	5,96497E+14
TC1100000327.mm.2	Nsg2	neuron specific gene family member 2	1,00514E+13	9,07901E+14	3,19751E+14	0.0169631198133599	0.170747242106618	-2,76634E+14	9,87408E+14	9,77787E+14	9,03787E+13	8,60379E+14	8,53672E+14	8,64373E+14
TC0200004459.mm.2	Tmem87a	transmembrane protein 87A	2,77032E+14	6,1959E+14	3,19675E+14	0.0169800562483115	0.170747242106618	-2,76722E+14	7,56074E+14	7,67214E+14	6,27738E+14	3,41486E+14	6,43767E+14	5,81259E+14
TC1100004032.mm.2	Slc39a11	solute carrier family 39 (metal ion transporter), member 11	0.997836117771781	5,64955E+13	3,19649E+14	0.0169858703376439	0.170747242106618	-2,76752E+13	6,00935E+14	6,13342E+14	5,19259E+13	4,95451E+14	5,6309E+14	5,9765E+14
TC0500002240.mm.2	Nop14	NOP14 nucleolar protein	2,23031E+14	5,98506E+14	3,19626E+13	0.0169909971448314	0.170747242106618	-2,76779E+14	7,32702E+12	7,59773E+14	6,17508E+14	4,28905E+14	5,88726E+14	4,63421E+14
TC0300000958.mm.2	Pdzk1	PDZ domain containing 1	-1,43436E+13	5,67116E+14	-3,19558E+14	0.0170062633539719	0.170747242106618	-2,76858E+13	4,52603E+14	4,8692E+14	5,59318E+14	6,67077E+14	5,93367E+14	6,43411E+14
TC0800003177.mm.2	Exoc8	exocyst complex component 8	1,27484E+14	6,85326E+14	3,1953E+14	0.0170125460122972	0.170747242106618	-2,76891E+13	7,52528E+14	7,53533E+14	6,73937E+14	5,77157E+14	6,88438E+14	6,66363E+14
TC0500003430.mm.2	Ap1s1	adaptor protein complex AP-1, sigma 1	2,20533E+13	8,81417E+14	3,19492E+14	0.0170210201662176	0.170747242106618	-2,76934E+14	1,00178E+14	1,02658E+14	8,95896E+14	6,91399E+14	8,81775E+14	7,91069E+14
TC0X00002199.mm.2	Hs6st2	heparan sulfate 6-O-sulfotransferase 2	1,09861E+14	7,32998E+14	3,19489E+14	0.0170218817749043	0.170747242106618	-2,76939E+14	8,08782E+14	8,03013E+14	6,84736E+14	7,07337E+14	6,65257E+14	7,28864E+14
TC1900000170.mm.2	Eef1g	eukaryotic translation elongation factor 1 gamma	1,15474E+14	9,8139E+14	3,19444E+14	0.0170319500599649	0.170747242106618	-2,76991E+14	1,05641E+14	1,04879E+14	9,76154E+14	8,98093E+14	9,57469E+14	9,5143E+14
TC1400001064.mm.2	Kctd4	potassium channel tetramerisation domain containing 4	2,34878E+14	6,1525E+12	3,19361E+14	0.0170506503687822	0.170747242106618	-2,77088E+14	7,58631E+14	7,34183E+14	6,27047E+14	3,96011E+14	6,23079E+14	5,52549E+14
TC0600001308.mm.2	Depp1	DEPP1 autophagy regulator	-1,55184E+14	5,83971E+13	-3,19328E+14	0.017057941748581	0.170747242106618	-2,77125E+14	5,01306E+14	4,47953E+14	5,79404E+14	6,80222E+14	6,06405E+13	6,88536E+14
TC1500001764.mm.2	Nrbp2	nuclear receptor binding protein 2	1,21695E+14	6,87481E+14	3,19316E+14	0.0170606807073178	0.170747242106618	-2,7714E+14	7,70003E+13	7,76264E+14	6,96207E+14	6,0667E+14	6,39176E+14	6,36564E+14
TC0300003217.mm.2	Pex11b	peroxisomal biogenesis factor 11 beta	1,58675E+14	7,06031E+13	3,19297E+14	0.0170650390927384	0.170747242106618	-2,77162E+14	8,49846E+14	7,9543E+14	6,88192E+14	6,39734E+14	6,94412E+14	5,68573E+14
TC0400001152.mm.2	Cc2d1b	coiled-coil and C2 domain containing 1B	-1,0182E+14	7,09242E+13	-3,19142E+14	0.0170999092810766	0.170747242106618	-2,77342E+14	6,43565E+14	6,57553E+14	7,35769E+13	7,68989E+14	7,07654E+14	7,41922E+14
TC0400002590.mm.2	Xpa	xeroderma pigmentosum, complementation group A	1,93806E+14	6,35626E+14	3,19039E+14	0.017123200646185	0.170747242106618	-2,77462E+14	7,67104E+14	7,47259E+14	6,30895E+14	4,95857E+14	6,538E+14	5,18843E+13
TC1800000802.mm.2	Dym	dymeclin	1,25479E+14	8,14746E+14	3,18985E+14	0.0171352778405432	0.170747242106618	-2,77524E+13	9,21255E+14	9,07949E+14	8,14841E+14	7,63404E+14	7,00323E+14	7,80704E+14
TC1900000763.mm.2	Pdcd4	programmed cell death 4	1,93608E+14	8,1639E+14	3,1889E+14	0.0171568729163708	0.170747242106618	-2,77635E+14	9,22456E+13	9,21241E+14	8,07673E+14	6,48809E+14	8,52818E+14	7,45343E+14
TC0600002900.mm.2	Ssu2	ssu-2 homolog (C. elegans)	-0.961037369468286	6,66046E+14	-3,18883E+14	0.0171584998020131	0.170747242106618	-2,77643E+14	6,13007E+12	6,1742E+14	6,99939E+14	7,22696E+14	6,60136E+14	6,8308E+14
TC0400002478.mm.2	Arid3c	AT rich interactive domain 3C (BRIGHT-like)	-1,34298E+14	7,11289E+13	-3,18857E+14	0.0171643320616992	0.170747242106618	-2,77673E+14	6,11821E+14	6,25032E+14	7,1423E+13	7,91219E+14	7,23678E+14	8,01755E+14
TC0300001366.mm.2	Slc9b2	solute carrier family 9, subfamily B (NHA2, cation proton antiporter 2), member 2	-1,27891E+14	6,56023E+14	-3,1865E+14	0.0172113989856766	0.170747242106618	-2,77915E+14	5,6877E+14	5,44817E+14	6,45839E+14	7,2353E+14	6,97052E+14	7,5613E+14
TC0800001490.mm.2	Ctu2	cytosolic thiouridylase subunit 2	-1,03291E+14	7,14089E+14	-3,18595E+13	0.0172238072588662	0.170747242106618	-2,77978E+14	6,56795E+14	6,50558E+14	7,30519E+14	7,83414E+14	7,27628E+14	7,3562E+14
TC0X00001335.mm.2	Tceal1	transcription elongation factor A (SII)-like 1	1,66498E+14	6,75662E+14	3,18526E+14	0.0172394595313426	0.170747242106618	-2,78058E+14	7,86732E+14	7,31899E+14	6,66156E+14	5,19478E+14	6,75626E+14	6,7408E+14
TC0800000711.mm.2	Atp6v1b2	ATPase, H+ transporting, lysosomal V1 subunit B2	2,91863E+14	8,96432E+14	3,18445E+13	0.0172580071538559	0.170747242106618	-2,78153E+14	1,07018E+14	1,07167E+14	9,19251E+14	6,38872E+14	9,07876E+14	7,70742E+14
TC0900003342.mm.2	Syncrip	synaptotagmin binding, cytoplasmic RNA interacting protein	1,96136E+14	7,88194E+14	3,18438E+14	0.017259640638444	0.170747242106618	-2,78161E+13	8,88298E+14	9,20684E+13	7,87908E+14	6,28802E+14	8,06076E+14	6,97397E+14
TC0X00000704.mm.2	Rpl10/Snora70	ribosomal protein L10/small nucleolar RNA, H/ACA box 70	2,57568E+14	9,25848E+14	3,18437E+13	0.0172598146017517	0.170747242106618	-2,78162E+14	1,08939E+14	1,0949E+14	9,57896E+14	7,11265E+14	9,07093E+14	7,94536E+13
TC1300001470.mm.2	Ggps1	geranylgeranyl diphosphate synthase 1	1,72696E+14	6,82591E+14	3,18424E+13	0.0172627950064503	0.170747242106618	-2,78177E+14	8,13216E+14	7,8436E+14	6,88475E+14	5,63709E+14	6,74886E+14	5,709E+14
TC1700000498.mm.2	Rab44	RAB44, member RAS oncogene family	-1,16822E+14	5,80421E+14	-3,18415E+14	0.0172647957845875	0.170747242106618	-2,78188E+14	5,11679E+14	5,03651E+14	5,90605E+14	6,58369E+14	5,8654E+14	6,31681E+14
TC0700002112.mm.2	Fgf3	fibroblast growth factor 3	-1,62563E+14	6,85805E+14	-3,18391E+14	0.0172701854352057	0.170747242106618	-2,78215E+14	5,99828E+14	5,62215E+14	6,82856E+14	8,04313E+13	6,91751E+14	7,73871E+13
TC1400000780.mm.2	Cpne6	copine VI	1,04271E+14	7,99659E+14	3,1838E+14	0.0172726812706613	0.170747242106618	-2,78228E+14	8,80039E+14	8,64218E+14	7,89173E+14	7,46542E+14	7,42337E+14	7,75643E+14
TC0100000164.mm.2	Col9a1	collagen, type IX, alpha 1	-1,31363E+14	5,94595E+14	-3,18295E+14	0.0172921332325978	0.170747242106618	-2,78327E+14	4,92479E+13	5,38732E+14	6,23E+14	6,70936E+14	5,79824E+14	6,62597E+14
TC0800000854.mm.2	Nr3c2	nuclear receptor subfamily 3, group C, member 2	1,5873E+14	6,59001E+14	3,18044E+14	0.0173495752865418	0.170747242106618	-2,78619E+14	7,84684E+14	7,28914E+14	6,59075E+13	5,37062E+14	6,51377E+14	5,92894E+14
TC1500001887.mm.2	Npcd/Cbx6/Nptxr	neuronal pentraxin chromo domain/chromobox 6/neuronal pentraxin receptor	1,13097E+13	8,19247E+14	3,18041E+13	0.0173502547770389	0.170747242106618	-2,78623E+14	9,02165E+14	9,02017E+14	8,13836E+14	7,64152E+14	7,94841E+14	7,38471E+13
TC0700000252.mm.2	Psg29	pregnancy-specific glycoprotein 29	-1,31839E+14	4,77261E+14	-3,18013E+14	0.0173568086483817	0.170747242106618	-2,78656E+14	3,88674E+14	3,88331E+14	4,824E+14	5,58284E+13	4,85095E+14	5,60781E+14
TC1700001952.mm.2	Aif1	allograft inflammatory factor 1	1,54643E+14	5,74009E+14	3,17924E+14	0.0173771448558408	0.170747242106618	-2,78759E+14	6,02591E+14	6,8503E+14	5,44078E+14	4,34258E+13	5,73596E+14	6,04504E+14
TC0700002696.mm.2	Zfp36	zinc finger protein 36	-1,25736E+14	6,37028E+14	-3,17853E+14	0.0173934803038796	0.170747242106618	-2,78842E+14	5,19672E+14	5,70183E+14	7,08262E+14	6,33065E+14	6,77451E+14	7,13535E+14
TC0800002961.mm.2	Kars	lysyl-tRNA synthetase	1,3684E+14	7,99456E+14	3,17843E+14	0.0173956787296346	0.170747242106618	-2,78853E+14	8,62822E+14	8,95846E+14	7,87832E+14	6,97156E+14	8,078E+14	7,45283E+13
TC0700004490.mm.2	Msx3	msh homeobox 3	-1,27438E+14	6,93266E+14	-3,17794E+14	0.0174070564362473	0.170747242106618	-2,78911E+14	5,90421E+14	6,23686E+14	7,15649E+14	7,53333E+14	6,94845E+14	7,81663E+14
TC1300001638.mm.2	Hist1h2ac	histone cluster 1, H2ac	-1,28588E+14	9,10602E+14	-3,17747E+14	0.0174177387609193	0.170747242106618	-2,78965E+14	8,36218E+13	8,21681E+14	9,27786E+14	9,87289E+14	9,04413E+13	9,86226E+14
TC0700002497.mm.2	Apoe	apolipoprotein E	1,08066E+14	1,13697E+14	3,17733E+14	0.0174210623614347	0.170747242106618	-2,78982E+14	1,21224E+14	1,20179E+14	1,12018E+14	1,07772E+14	1,12918E+14	1,08072E+13
TC0300000060.mm.2	Fabp5	fatty acid binding protein 5, epidermal	0.964650515925598	6,63613E+13	3,17665E+14	0.017436614276209	0.170747242106618	-2,7906E+14	7,16223E+14	7,22679E+14	6,32588E+14	6,13384E+14	6,33984E+14	6,62823E+14
TC0200003265.mm.2	Kyat1	kynurenine aminotransferase 1	1,27605E+14	6,64047E+14	3,17601E+14	0.0174513996968128	0.170747242106618	-2,79135E+14	7,60636E+14	7,11184E+14	6,51961E+14	5,64648E+14	6,43605E+14	6,52245E+14
TC0700000532.mm.2	Zfp84	zinc finger protein 84	1,07226E+14	6,6909E+14	3,17559E+13	0.0174611853038094	0.170747242106618	-2,79185E+14	7,33552E+14	7,43522E+14	6,11069E+14	6,51554E+14	6,61438E+14	6,13405E+14
TC0400002929.mm.2	Nfib	nuclear factor I/B	2,35798E+13	7,15311E+14	3,17553E+14	0.0174624769019386	0.170747242106618	-2,79191E+14	8,67014E+14	8,93127E+14	7,27117E+14	5,61427E+14	7,11258E+14	5,31921E+14
TC0700003027.mm.2	Syngr4	synaptogyrin 4	-1,14635E+14	6,42322E+14	-3,17525E+14	0.0174690465052382	0.170747242106618	-2,79224E+14	5,59088E+14	5,67491E+14	6,53602E+14	7,02247E+14	6,55303E+14	7,16202E+14
TC0100002189.mm.2	Actr1b	ARP1 actin-related protein 1B, centractin beta	1,54219E+13	8,99225E+14	3,17524E+14	0.017469180273078	0.170747242106618	-2,79225E+14	9,8988E+14	9,86746E+14	9,03904E+14	7,64285E+13	8,82655E+14	8,6788E+14
TC0700004607.mm.2	Fadd	Fas (TNFRSF6)-associated via death domain	-0.98067817407999	6,86004E+13	-3,17451E+14	0.0174862023582595	0.170747242106618	-2,79311E+14	6,18245E+14	6,40298E+13	7,42072E+14	7,12606E+14	6,92718E+14	7,10083E+14
TC0900000208.mm.2	Plppr2	phospholipid phosphatase related 2	2,04282E+14	8,59086E+14	3,17372E+14	0.0175043342273682	0.170747242106618	-2,79402E+14	9,9158E+14	9,84639E+13	8,75285E+14	6,92371E+14	8,5318E+13	7,57462E+14
TC0800001748.mm.2	Tdrp	testis development related protein	-1,07585E+14	6,00286E+14	-3,17362E+14	0.0175067268274106	0.170747242106618	-2,79414E+14	5,554E+14	5,34951E+14	6,33512E+14	6,72009E+14	5,79142E+14	6,26703E+14
TC1400001177.mm.2	Lmo7	LIM domain only 7	2,16248E+14	7,38787E+14	3,17271E+14	0.0175276516765519	0.170747242106618	-2,7952E+14	8,97463E+14	9,20973E+14	7,66203E+14	6,19737E+13	6,96521E+14	5,31822E+14
TC1800001063.mm.2	Nol4	nucleolar protein 4	1,15933E+13	7,90423E+12	3,17261E+14	0.0175300551590278	0.170747242106618	-2,79532E+14	8,66148E+13	8,73447E+14	7,70761E+14	7,36968E+13	7,84216E+14	7,10998E+13
TC1300001201.mm.2	Smim15	small integral membrane protein 15	2,55192E+14	6,08176E+14	3,17242E+14	0.0175344482870859	0.170747242106618	-2,79554E+14	7,41071E+14	7,68286E+14	6,163E+14	3,82673E+14	6,36094E+14	5,04632E+14
TC1200001279.mm.2	Tex22	testis expressed gene 22	-1,81772E+14	6,93898E+14	-3,17216E+14	0.0175404789187245	0.170747242106618	-2,79584E+14	5,94854E+14	5,61899E+14	6,89425E+14	8,30872E+14	6,91887E+14	7,94452E+13
TC1200001841.mm.2	Rpl10l	ribosomal protein L10-like	-1,40174E+14	8,08493E+14	-3,17181E+14	0.0175487035594607	0.170747242106618	-2,79625E+14	7,24087E+14	7,17382E+14	8,14293E+14	9,07524E+14	8,05873E+14	8,81797E+14
TC0400001123.mm.2	Tmem59	transmembrane protein 59	1,75957E+14	8,49416E+13	3,17153E+14	0.0175550677869075	0.170747242106618	-2,79657E+14	9,72592E+14	9,62307E+14	8,43905E+12	7,39081E+14	8,56156E+14	7,22456E+14
TC0200005349.mm.2	Gm14308/Gm14430/Gm14432/Gm14434/Gm2026/Gm4724	predicted gene 14308/predicted gene 14430/predicted gene 14432/predicted gene 14434/predicted gene 2026/predicted gene 4724	2,38578E+14	9,19884E+14	3,17146E+14	0.0175567388360273	0.170747242106618	-2,79666E+14	1,01697E+14	1,08099E+14	9,05326E+14	7,15473E+14	9,74475E+14	8,26075E+14
TC0X00003168.mm.2	Maged2	melanoma antigen, family D, 2	1,01855E+14	6,84387E+14	3,17069E+14	0.0175745706973083	0.170747242106618	-2,79755E+14	7,88364E+14	7,48592E+14	6,76878E+14	6,56369E+14	6,30826E+14	6,05293E+14
TC0700002710.mm.2	Eif3k	eukaryotic translation initiation factor 3, subunit K	1,45614E+14	8,61114E+14	3,16989E+14	0.0175932909805629	0.170747242106618	-2,79849E+14	9,45651E+14	9,46644E+13	8,62576E+14	7,3849E+14	8,53421E+14	8,19902E+14
TC1100001631.mm.2	Hspb9	heat shock protein, alpha-crystallin-related, B9	-1,36036E+13	5,05219E+14	-3,1696E+14	0.017599951103792	0.170747242106618	-2,79883E+14	4,10034E+14	4,21174E+14	5,44532E+14	5,58747E+14	4,91962E+14	6,04864E+14
TC0400003722.mm.2	Dhdds	dehydrodolichyl diphosphate synthase	1,51989E+14	7,4832E+14	3,16799E+14	0.0176375327451559	0.170747242106618	-2,80071E+13	8,64853E+14	8,48218E+14	7,69442E+14	6,39651E+14	7,05461E+14	6,62298E+14
TC0X00002820.mm.2	Atrx	ATRX, chromatin remodeler	2,03329E+14	7,66982E+14	3,16773E+14	0.017643485924 76	0.170747242106618	-2,801E+14	8,73308E+14	8,88957E+14	7,49903E+14	6,05704E+13	8,15041E+14	6,68981E+14
TC0900000675.mm.2	Isl2	insulin related protein 2 (islet 2)	-1,37088E+14	7,82935E+14	-3,16742E+14	0.0176508729762195	0.170747242106618	-2,80137E+14	6,50856E+14	7,23279E+14	8,18631E+13	8,2968E+14	7,92561E+14	8,82601E+14
TC1000002035.mm.2	Mfsd4b1	major facilitator superfamily domain containing 4B1	-0.992983458268107	7,10895E+14	-3,1674E+14	0.0176512488998558	0.170747242106618	-2,80139E+14	6,51432E+14	6,50394E+13	7,32404E+14	7,68019E+14	7,17839E+14	7,45282E+14
TC1400002439.mm.2	Gpalpp1	GPALPP motifs containing 1	1,11405E+14	6,67983E+14	3,167E+14	0.0176606042518179	0.170747242106618	-2,80186E+14	7,28613E+14	7,33238E+14	6,50783E+14	5,88258E+14	6,71984E+14	6,35025E+14

TC0800001522.mm.2	Tubb3	tubulin, beta 3 class III	1,2607E+14	9,14821E+14	3,16682E+14	0.0176648957172826	0.170747242106618	-2,80207E+14	1,02495E+14	9,89125E+14	9,2222E+14	8,39719E+14	8,76916E+14	8,35993E+14
TC1100000730.mm.2	Mprip	myosin phosphatase Rho interacting protein	1,82943E+14	8,82543E+14	3,16537E+14	0.0176988655642306	0.170747242106618	-2,80377E+14	1,00876E+14	1,00857E+14	8,8141E+14	7,70037E+13	8,8384E+14	7,42635E+14
TC1200002432.mm.2	Tmem179	transmembrane protein 179	1,54909E+14	7,73405E+14	3,16522E+14	0.0177022870074872	0.170747242106618	-2,80394E+14	8,88248E+13	8,53147E+14	7,78091E+14	6,53486E+14	7,63012E+14	7,04449E+14
TC0600002315.mm.2	Fkbp14	FK506 binding protein 14	1,98126E+14	5,28673E+14	3,16487E+14	0.0177105650775258	0.170747242106618	-2,80435E+13	6,76966E+14	6,13022E+14	5,0179E+14	3,91946E+14	4,17295E+14	5,71019E+14
TC0100001205.mm.2	Golt1a/Kiss1	golgi transport 1A/KiSS-1 metastasis-suppressor	-1,4141E+14	6,39298E+14	-3,16329E+13	0.0177474646208885	0.170747242106618	-2,80619E+14	5,24439E+14	5,47522E+14	6,17456E+14	7,37325E+13	7,11288E+14	6,9776E+14
TC0200001780.mm.2	Itpka	inositol 1,4,5-trisphosphate 3-kinase A	1,02601E+14	6,92239E+14	3,16327E+14	0.0177479124024673	0.170747242106618	-2,80621E+14	7,26226E+14	7,58775E+14	6,60059E+14	6,1974E+14	7,01858E+14	6,86774E+14
TC0700003991.mm.2	Dchs1	dachsous cadherin related 1	1,09079E+14	6,26471E+14	3,16257E+14	0.0177644717775495	0.170747242106618	-2,80703E+14	6,9195E+14	6,78226E+14	5,54689E+14	5,97329E+14	5,92453E+14	6,44179E+14
TC1600001316.mm.2	Comt	catechol-O-methyltransferase	1,23945E+14	9,2927E+14	3,1622E+14	0.0177731729344547	0.170747242106618	-2,80746E+12	1,01436E+14	1,00876E+14	9,33744E+14	8,4148E+14	9,0086E+14	8,7642E+14
TC1700001019.mm.2	1700061G19Rik	RIKEN cDNA 1700061G19 gene	-1,67185E+13	5,58172E+14	-3,16178E+14	0.017783139860382	0.170747242106618	-2,80796E+14	4,56654E+13	4,09875E+12	5,43977E+14	6,56922E+14	5,89819E+14	6,91785E+14
TC0500000317.mm.2	Nrbp1	nuclear receptor binding protein 1	2,60054E+14	7,07357E+13	3,16149E+14	0.0177898608423887	0.170747242106618	-2,80829E+14	8,71869E+14	8,75883E+14	7,44518E+14	4,83128E+14	6,79271E+14	5,89475E+14
TC1500002194.mm.2	Rhebl1	Ras homolog enriched in brain like 1	-1,40116E+14	5,85648E+14	-3,16119E+14	0.0177969175021877	0.170747242106618	-2,80864E+14	5,03134E+13	4,83702E+14	6,92456E+14	5,74611E+14	6,20189E+14	6,39798E+14
TC0200002178.mm.2	Xrn2	5'-3' exoribonuclease 2	2,10758E+14	7,1253E+14	3,16107E+14	0.0177998136598354	0.170747242106618	-2,80878E+14	8,62914E+14	8,61746E+14	6,88315E+14	6,14829E+14	7,25121E+14	5,22258E+14
TC1500000748.mm.2	Wbp2nl	WBP2 N-terminal like	-1,22798E+13	5,15621E+14	-3,16039E+14	0.0178158870474569	0.170747242106618	-2,80958E+14	4,45233E+14	4,14185E+14	5,24029E+14	5,80985E+14	5,30767E+14	5,98528E+14
TC0300002207.mm.2	Gria2	glutamate receptor, ionotropic, AMPA2 (alpha 2)	2,31439E+14	8,18515E+14	3,16002E+14	0.0178245529652692	0.170747242106618	-2,81001E+14	9,62455E+14	9,6201E+14	8,34016E+14	6,27571E+14	8,25271E+14	6,99766E+14
TC0X00001514.mm.2	Wnk3	WNK lysine deficient protein kinase 3	1,10886E+14	6,99803E+14	3,15908E+14	0.0178467266761733	0.170747242106618	-2,81111E+14	8,05307E+14	7,46846E+14	6,8257E+13	6,47812E+14	6,70412E+14	6,45871E+14
TC0300002912.mm.2	Alpk1	alpha-kinase 1	-1,82906E+14	7,11532E+14	-3,15862E+14	0.0178576221129323	0.170747242106618	-2,81164E+14	5,90002E+14	5,75308E+14	6,86042E+14	8,45081E+14	7,44548E+14	8,28214E+14
TC1200001210.mm.2	Dync1h1	dynein cytoplasmic 1 heavy chain 1	1,10436E+14	8,47071E+14	3,15833E+14	0.0178644318164744	0.170747242106618	-2,81198E+14	9,41933E+14	9,24431E+14	7,92535E+14	8,52956E+14	8,02243E+14	7,68326E+14
TC0300000605.mm.2	Serpini1	serine (or cysteine) peptidase inhibitor, clade I, member 1	2,4314E+14	7,84799E+14	3,15819E+14	0.0178677224586458	0.170747242106618	-2,81214E+14	9,15823E+14	9,4438E+14	7,98945E+14	5,74978E+14	7,97824E+14	6,76846E+14
TC0700001258.mm.2	Ctsc	cathepsin C	1,48669E+14	8,05217E+14	3,15794E+14	0.0178737947219521	0.170747242106618	-2,81244E+14	8,29137E+14	9,36407E+14	7,5891E+14	7,09296E+14	7,67476E+14	8,30077E+14
TC1400001567.mm.2	Kcnma1	potassium large conductance calcium-activated channel, subfamily M, alpha member 1	2,69498E+14	7,51653E+14	3,15766E+14	0.0178803146818262	0.170747242106618	-2,81276E+14	9,01763E+14	9,46346E+14	7,65059E+14	5,44053E+13	7,66119E+14	5,86576E+14
TC1000001913.mm.2	L3mbtl3	L3MBTL3 histone methyl-lysine binding protein	1,48145E+13	7,06611E+14	3,1576E+14	0.0178816963788 5	0.170747242106618	-2,81283E+14	8,12772E+14	7,95146E+14	7,02898E+13	6,0873E+14	7,06196E+14	6,13923E+14
TC1700002437.mm.2	Themis3	thymocyte selection associated family member 3	-1,22267E+14	5,48413E+14	-3,15597E+14	0.0179205061227231	0.170747242106618	-2,81475E+14	4,51864E+14	4,9507E+14	5,54845E+13	6,36621E+14	5,703E+14	5,81777E+14
TC0400000585.mm.2	Fsd1l	fibronectin type III and SPRY domain containing 1-like	1,04827E+14	5,70877E+14	3,15573E+14	0.0179261721337251	0.170747242106618	-2,81502E+14	6,38572E+14	6,34052E+13	5,46835E+14	5,16134E+14	5,70382E+14	5,19285E+14
TC0400004139.mm.2	Prkcz	protein kinase C, zeta	0.960790148170625	9,30533E+14	3,15401E+14	0.0179670203355947	0.170747242106618	-2,81703E+14	1,00017E+14	9,89759E+14	9,09135E+14	8,88632E+14	9,11791E+14	8,83714E+14
TC0600003120.mm.2	Usp5	ubiquitin specific peptidase 5 (isopeptidase T)	2,10205E+14	7,3185E+14	3,15332E+14	0.0179834680703978	0.170747242106618	-2,81784E+14	8,80263E+14	8,75058E+14	7,75065E+14	5,59846E+14	6,57303E+14	6,43562E+14
TC1000002642.mm.2	Dram1	DNA-damage regulated autophagy modulator 1	-1,01166E+14	5,19864E+14	-3,15331E+14	0.0179836440930634	0.170747242106618	-2,81785E+14	4,63854E+14	4,36143E+13	5,40193E+14	5,62135E+14	5,39917E+14	5,7694E+14
TC0200000927.mm.2	Tbr1	T-box brain gene 1	1,39193E+13	7,66732E+14	3,15282E+14	0.0179954508198599	0.170747242106618	-2,81843E+14	8,18917E+14	8,89801E+14	7,4441E+14	6,85921E+14	7,69418E+13	6,91927E+14
TC0X00000237.mm.2	Wdr44	WD repeat domain 44	1,38465E+14	6,07315E+14	3,15238E+14	0.018005714168537	0.170747242106618	-2,81893E+14	6,80898E+14	7,1755E+14	5,65482E+14	5,56037E+14	6,18448E+14	5,05474E+14
TC1500001875.mm.2	Kcnj4	potassium inwardly-rectifying channel, subfamily J, member 4	1,42194E+13	6,43155E+14	3,15233E+13	0.0180070548611467	0.170747242106618	-2,819E+14	7,22057E+14	7,49248E+14	6,48253E+14	5,38663E+14	6,26821E+14	5,7389E+14
TC0200005507.mm.2	Ninl	ninein-like	-1,06249E+14	7,71694E+14	-3,15191E+14	0.0180169423828092	0.170747242106618	-2,81948E+14	6,7423E+14	7,29574E+14	7,937E+14	8,22602E+14	8,00068E+14	8,09988E+14
TC0900002167.mm.2	Upk2	uroplakin 2	-1,33854E+14	5,7872E+14	-3,15103E+13	0.0180381793491803	0.170747242106618	-2,82052E+14	5,0324E+14	5,14806E+14	5,88034E+14	6,9772E+14	5,73943E+14	5,94575E+14
TC1100001162.mm.2	Vtn	vitronectin	1,47377E+14	8,42537E+14	3,15072E+14	0.0180455203192172	0.170747242106618	-2,82088E+14	9,38126E+14	9,121E+14	8,22687E+13	7,32784E+14	8,71568E+14	7,77959E+14
TC0200002395.mm.2	Epb41l1	erythrocyte membrane protein band 4.1 like 1	0.935630001313648	7,58925E+14	3,15064E+14	0.0180473129365265	0.170747242106618	-2,82097E+14	8,2185E+14	8,17858E+12	7,39522E+14	7,1306E+13	7,29205E+14	7,32058E+14
TC0200003142.mm.2	Grin1	glutamate receptor, ionotropic, NMDA1 (zeta 1)	1,29644E+14	8,79482E+14	3,15064E+14	0.0180474893511	0.170747242106618	-2,82097E+13	9,63449E+14	9,6449E+14	8,81719E+14	7,86932E+13	8,63459E+14	8,16841E+14
TC1500001933.mm.2	Polr3h	polymerase (RNA) III (DNA directed) polypeptide H	1,29958E+14	6,19738E+14	3,14961E+14	0.0180721450114416	0.170747242106618	-2,82218E+14	7,06847E+14	6,89239E+14	6,16319E+14	5,19852E+14	6,13476E+13	5,72697E+14
TC0200004108.mm.2	Madd	MAP-kinase activating death domain	1,2623E+14	6,27783E+14	3,14955E+14	0.0180735452958205	0.170747242106618	-2,82225E+14	7,45501E+14	7,41202E+14	6,51946E+14	5,82298E+14	5,58832E+14	4,86915E+14
TC1500001395.mm.2	Ncald/Gm15941	neurocalcin delta/predicted gene 15941	1,75479E+14	8,7862E+14	3,14827E+14	0.0181041133000097	0.170747242106618	-2,82374E+13	9,83617E+14	1,00241E+14	8,77585E+14	7,5749E+14	8,85646E+13	7,64969E+14
TC0100002582.mm.2	Aamp	angio-associated migratory protein	1,95065E+13	6,31182E+14	3,14815E+13	0.0181069780667408	0.170747242106618	-2,82388E+14	7,72877E+14	7,51096E+14	6,56994E+14	4,76849E+14	6,02827E+14	5,26448E+14
TC1600001233.mm.2	Pla2g10	phospholipase A2, group X	-1,6213E+14	6,87363E+14	-3,14753E+14	0.0181218768339867	0.170747242106618	-2,8246E+14	5,80973E+14	5,71368E+14	6,68136E+14	8,08464E+13	7,17112E+14	7,78125E+14
TC0600002567.mm.2	Tet3	tet methylcytosine dioxygenase 3	-1,11161E+13	8,28986E+14	-3,14736E+14	0.0181260070315925	0.170747242106618	-2,82481E+14	7,51131E+14	7,52594E+14	8,26232E+14	8,99814E+14	8,70568E+13	8,73579E+14
TC0300000721.mm.2	Cct3	chaperonin containing Tcp1, subunit 3 (gamma)	1,5391E+14	7,20584E+14	3,14716E+14	0.0181308437164545	0.170747242106618	-2,82504E+14	8,23408E+14	8,23972E+14	7,30377E+14	6,09184E+14	7,05208E+14	6,31358E+14
TC1800000741.mm.2	Tcf4	transcription factor 4	1,63098E+14	9,72255E+14	3,14623E+14	0.01815330239514	0.170747242106618	-2,82613E+14	1,05483E+13	1,07896E+14	9,64945E+14	8,42646E+14	9,91916E+13	9,00236E+14
TC1300001927.mm.2	Tpmt	thiopurine methyltransferase	1,11954E+14	6,43588E+14	3,1448E+14	0.0181876226555193	0.170747242106618	-2,8278E+14	7,10086E+14	7,11599E+14	6,36073E+14	5,61704E+14	6,14938E+14	6,27131E+14
TC1000002277.mm.2	Mypn	myopalladin	-1,49522E+14	5,13246E+13	-3,14457E+14	0.0181930934074548	0.170747242106618	-2,82807E+14	4,14125E+14	4,27174E+14	5,0362E+14	6,36724E+14	5,36938E+14	5,60892E+14
TC1100001397.mm.2	Mmd	monocyte to macrophage differentiation-associated	-1,22624E+14	7,50739E+14	-3,14455E+14	0.018193734889655	0.170747242106618	-2,8281E+14	6,68105E+14	6,53828E+14	7,55537E+14	8,11644E+13	7,70282E+14	8,45037E+14
TC1400001102.mm.2	Mtrf1	mitochondrial translational release factor 1	1,5957E+14	5,05941E+13	3,14438E+14	0.01819770963947	0.170747242106618	-2,82829E+13	6,1191E+14	6,09031E+14	5,08129E+14	3,93672E+14	5,04918E+14	4,07986E+14
TC1900000654.mm.2	Pax2	paired box 2	-0.968015528862514	5,84209E+14	-3,1436E+14	0.0182166984401363	0.170747242106618	-2,82921E+14	5,38308E+14	5,30623E+14	6,29834E+14	6,32699E+14	5,67463E+14	6,06326E+14
TC0200003118.mm.2	Cacna1b	calcium channel, voltage-dependent, N type, alpha 1B subunit	2,05874E+14	7,77431E+14	3,14358E+14	0.0182172203500831	0.170747242106618	-2,82924E+13	9,28201E+14	9,10201E+14	8,14818E+14	6,11835E+14	7,27239E+13	6,72295E+13
TC1900000711.mm.2	Cnnm2	cyclin M2	0.968402252783921	7,07843E+14	3,14349E+14	0.0182191677153921	0.170747242106618	-2,82933E+14	7,63347E+14	7,67269E+14	6,82332E+14	6,54602E+14	7,0393E+14	6,7558E+14
TC0900000069.mm.2	Pgr	progesterone receptor	1,22176E+14	6,92058E+13	3,14321E+14	0.0182260558159999	0.170747242106618	-2,82966E+14	7,53254E+14	7,33864E+14	6,62642E+13	5,80124E+14	7,31411E+14	6,91053E+14
TC0100003193.mm.2	Cfh	complement component factor h	1,49807E+13	6,57866E+13	3,1427E+14	0.0182385011885731	0.170747242106618	-2,83027E+14	7,28742E+14	7,19845E+14	6,37694E+14	5,11279E+14	7,00052E+12	6,49586E+14
TC0500000219.mm.2	Chpf2/Mir671	chondroitin polymerizing factor 2/microRNA 671	0.908634998764069	6,20598E+14	3,14255E+14	0.0182420282251112	0.170747242106618	-2,83044E+14	6,65471E+13	6,62101E+14	5,6926E+14	5,76585E+14	6,2092E+14	6,29251E+14
TC0300001469.mm.2	Selenof	selenoprotein F	1,6645E+14	8,98198E+14	3,14202E+14	0.0182547893153561	0.170747242106618	-2,83105E+14	9,94782E+14	1,00019E+13	8,93183E+14	7,68892E+14	9,16066E+14	8,16072E+14
TC0X00003376.mm.2	Hccs	holocytochrome c synthetase	1,07642E+14	6,28852E+14	3,14192E+13	0.0182573397019448	0.170747242106618	-2,83118E+14	7,0039E+14	6,70145E+13	6,05576E+14	5,49676E+14	6,13196E+14	6,34131E+14
TC0700002380.mm.2	Obox8	oocyte specific homeobox 8	-1,24767E+14	4,56518E+14	-3,14179E+14	0.0182605338605717	0.170747242106618	-2,83133E+14	3,45646E+14	3,88789E+14	4,49591E+13	5,34378E+14	5,22753E+14	4,97951E+14
TC0500003648.mm.2	Hmgb1	high mobility group box 1	1,37893E+13	9,7375E+14	3,14159E+14	0.0182651946501176	0.170747242106618	-2,83156E+14	1,05242E+14	1,06294E+14	9,651E+14	8,74478E+13	9,8198E+14	9,05578E+14
TC1000000763.mm.2	Gm10272	keratin associated protein 12-4	-1,48243E+14	6,13573E+13	-3,14132E+14	0.0182717885053272	0.170747242106618	-2,83187E+14	5,00487E+14	5,25559E+14	6,38222E+14	6,84311E+14	6,0464E+14	7,2822E+14
TC0500000306.mm.2	Khk	ketohexokinase	-1,07563E+14	7,71683E+14	-3,14095E+14	0.0182808130171853	0.170747242106618	-2,83231E+14	6,8969E+14	7,03241E+14	7,75466E+14	8,3259E+14	7,986E+14	8,30512E+14
TC0400002637.mm.2	Mrpl50	mitochondrial ribosomal protein L50	0.998897452337872	7,50372E+14	3,14064E+14	0.0182882801379526	0.170747242106618	-2,83267E+14	8,30756E+14	8,01205E+14	7,11269E+14	7,20913E+14	7,00161E+14	7,37928E+14
TC1500001002.mm.2	Tuba1c	tubulin, alpha 1C	1,66084E+14	9,17872E+14	3,1404E+13	0.018294037030475	0.170747242106618	-2,83295E+14	1,01522E+14	1,01126E+14	9,10421E+14	7,83884E+13	9,41515E+13	8,44936E+14
TC1300001100.mm.2	Enc1	ectodermal-neural cortex 1	1,28022E+14	7,96516E+14	3,14025E+14	0.01829769342606	0.170747242106618	-2,83313E+14	8,48369E+14	8,90414E+14	7,84348E+14	6,98391E+13	7,98798E+14	7,58775E+14
TC0100000523.mm.2	Zdbf2	zinc finger, DBF-type containing 2	1,60313E+14	5,82965E+14	3,14015E+14	0.0183002174593004	0.170747242106618	-2,83325E+14	7,26699E+12	6,82979E+14	4,86251E+14	6,02801E+14	5,42567E+14	4,56494E+14
TC1000002453.mm.2	Pdxk	pyridoxal (pyridoxine, vitamin B6) kinase	1,09679E+14	8,47328E+14	3,13995E+12	0.0183051296846907	0.170747242106618	-2,83348E+14	9,63403E+14	9,50278E+14	8,73063E+14	8,21261E+13	7,61533E+14	7,14429E+14
TC0700004135.mm.2	Psma1	proteasome (prosome, macropain) subunit, alpha type 1	1,60533E+14	7,8476E+14	3,13988E+14	0.0183067894728463	0.170747242106618	-2,83356E+14	8,84574E+14	8,75757E+14	7,65926E+14	6,73339E+14	8,14381E+14	6,94584E+14
TC0400001863.mm.2	Smarca5-ps	SWI/SNF related, matrix associated, actin depenent ragulator of chromatin, subfamily a, member 5, pseudogene	2,0553E+14	7,01777E+14	3,1396E+14	0.0183134258454616	0.170747242106618	-2,83388E+14	8,67816E+14	8,81412E+14	6,97505E+14	6,40663E+14	6,62977E+14	4,60286E+14
TC0400000284.mm.2	Rngtt	RNA guanylyltransferase and 5'-phosphatase	1,90503E+14	5,58701E+14	3,13911E+14	0.018325525655724	0.170747242106618	-2,83447E+14	7,01459E+14	6,54073E+14	5,73045E+14	4,01482E+14	5,46674E+14	4,75469E+14
TC0900002958.mm.2	Cep63	centrosomal protein 63	1,13901E+14	5,82107E+14	3,13877E+14	0.0183336367521646	0.170747242106618	-2,83486E+14	6,91874E+14	6,44336E+14	5,84956E+14	5,23454E+14	5,15702E+14	5,32319E+13
TC1400000195.mm.2	Kat6b	K(lysine) acetyltransferase 6B	1,12177E+14	7,90907E+14	3,13865E+14	0.018336765704073	0.170747242106618	-2,83501E+14	8,82828E+14	8,76672E+14	7,70541E+14	7,64605E+14	7,63271E+14	6,87522E+14
TC1100000883.mm.2	Rnf222	ring finger protein 222	-1,00487E+13	6,13379E+14	-3,13769E+14	0.0183601433761544	0.170747242106618	-2,83613E+14	5,30845E+14	5,42285E+14	6,14339E+14	6,59765E+11	6,56784E+14	6,76254E+14
TC0300002746.mm.2	Tmem167b	transmembrane protein 167B	1,99945E+14	6,74585E+14	3,13757E+14	0.01836302916443	0.170747242106618	-2,83627E+14	8,03009E+14	8,04095E+14	6,82781E+14	5,24433E+14	6,80895E+14	5,52297E+14
TC1600000667.mm.2	Ccdc80	coiled-coil domain containing 80	-1,12454E+14	5,77419E+13	-3,13672E+14	0.0183837171812541	0.170747242106618	-2,83727E+14	4,66212E+14	5,2241E+14	5,92019E+14	6,21511E+14	6,09474E+14	6,52887E+14
TC0700002221.mm.2	Zfp954	zinc finger protein 954	0.951894561502272	5,71051E+14	3,13655E+14	0.018387761833739	0.170747242106618	-2,83746E+14	6,43754E+14	6,33369E+14	5,33273E+14	5,53471E+14	5,4456E+14	5,17877E+14
TC1100000625.mm.2	Fnip1	folliculin interacting protein 1	2,05002E+14	6,39798E+14	3,13653E+14	0.0183883872399472	0.170747242106618	-2,83749E+14	7,5294E+13	7,72213E+14	6,42858E+14	4,72293E+14	6,60192E+14	5,3829E+14
TC1100002728.mm.2	Leap2	liver-expressed antimicrobial peptide 2	-1,31515E+14	4,8928E+14	-3,13638E+14	0.0183918724008228	0.170747242106618	-2,83766E+14	3,84373E+12	4,34176E+14	4,92074E+14	5,89504E+14	5,15378E+14	5,20174E+14
TC1100000472.mm.2	Fam71b	family with sequence similarity 71, member B	-1,34009E+13	5,98141E+14	-3,13627E+14	0.0183947746752806	0.170747242106618	-2,8378E+14	5,12313E+14	5,11122E+14	5,93888E+14	6,97564E+13	6,13994E+14	6,59968E+14
TC0600000846.mm.2	Sftpb	surfactant associated protein B	-0.993539089584831	5,32577E+14	-3,13615E+14	0.0183976676421253	0.170747242106618	-2,83794E+14	4,87026E+13	4,68198E+14	5,55065E+14	5,98867E+14	5,41078E+14	5,45227E+14
TC1800000257.mm.2	Map3k2	mitogen-activated protein kinase kinase kinase 2	2,01731E+14	5,23903E+14	3,13601E+14	0.0184010356887423	0.170747242106618	-2,8381E+14	6,73792E+14	6,34782E+14	5,09074E+14	3,96038E+14	5,48984E+14	3,80747E+14
TC0300000580.mm.2	Ppm1l	protein phosphatase 1 (formerly 2C)-like	1,25075E+14	5,97663E+14	3,13515E+14	0.0184220324290383	0.170747242106618	-2,8391E+14	6,87711E+14	6,70822E+14	5,88062E+14	5,20321E+14	5,94808E+13	5,24256E+14
TC0400004097.mm.2	Kcnab2	potassium voltage-gated channel, shaker-related subfamily, beta member 2	0.960311750820269	9,13655E+14	3,13497E+14	0.0184263460858692	0.170747242106618	-2,83931E+14	9,77204E+14	9,7742E+13	8,95779E+14	8,66782E+14	8,94719E+14	8,7003E+14
TC0900000682.mm.2	Hmg20a	high mobility group 20A	1,38523E+14	7,40109E+14	3,13468E+14	0.0184334413139149	0.170747242106618	-2,83965E+14	8,43816E+14	8,34534E+14	7,40425E+14	6,60878E+14	7,25456E+14	6,35545E+14
TC0500002458.mm.2	Arap2	ArfGAP with RhoGAP domain, ankyrin repeat and PH domain 2	2,21048E+14	6,64725E+14	3,13309E+13	0.0184723803341208	0.170747242106618	-2,84151E+14	7,76172E+14	8,21003E+14	6,66168E+13	4,88911E+14	6,88709E+14	5,47387E+14
TC1000003066.mm.2	Mon2	MON2 homolog, regulator of endosome to Golgi trafficking	1,44557E+14	6,94197E+14	3,13295E+14	0.0184759907155596	0.170747242106618	-2,84169E+14	7,74866E+14	7,90974E+14	7,00317E+14	5,76408E+14	6,7827E+14	6,44344E+14
TC1700002027.mm.2	H2-M10.4	histocompatibility 2, M region locus 10.4	-0.965880097417643	5,0558E+14	-3,13287E+14	0.0184779857668526	0.170747242106618	-2,84178E+14	4,29158E+13	4,38945E+14	5,18315E+14	5,42965E+14	5,37016E+14	5,67078E+14
TC0200002720.mm.2	Zfp967/Zfp966	zinc finger protein 967/zinc finger protein 966	2,39629E+14	8,96902E+14	3,1324E+14	0.0184894528610758	0.170747242106618	-2,84233E+14	9,96921E+14	1,04461E+14	8,73843E+14	6,88432E+14	9,72465E+14	8,05138E+14
TC0200002738.mm.2	Zfp966/Zfp967	zinc finger protein 966/zinc finger protein 967	2,39629E+14	8,96902E+14	3,1324E+14	0.0184894528610758	0.170747242106618	-2,84233E+14	9,96921E+14	1,04461E+14	8,73843E+14	6,88432E+14	9,72465E+14	8,05138E+14
TC0200005341.mm.2	Zfp967/Zfp966	zinc finger protein 967/zinc finger protein 966	2,39629E+14	8,96902E+14	3,1324E+14	0.0184894528610758	0.170747242106618	-2,84233E+14	9,96921E+14	1,04461E+14	8,73843E+14	6,88432E+14	9,72465E+14	8,05138E+14
TC1600000166.mm.2	Rrn3	RRN3 RNA polymerase I transcription factor homolog (yeast)	2,4541E+13	6,48353E+14	3,13145E+14	0.0185128091251236	0.170747242106618	-2,84344E+14	7,7875E+14	7,80721E+14	6,59724E+14	4,08926E+14	6,67982E+14	5,94017E+14
TC0300002636.mm.2	Magi3	membrane associated guanylate kinase, WW and PDZ domain containing 3	1,30447E+14	5,74314E+14	3,13129E+14	0.0185167784172153	0.170747242106618	-2,84363E+14	6,94056E+14	6,55797E+14	5,6031E+12	5,28648E+14	5,51591E+14	4,55483E+12
TC1100001210.mm.2	Spaca3	sperm acrosome associated 3	-1,15343E+14	5,17674E+14	-3,13117E+14	0.0185195237988853	0.170747242106618	-2,84376E+14	4,24649E+14	4,5981E+14	5,33732E+14	5,81413E+14	5,24389E+14	5,8205E+14
TC0200005433.mm.2	Eef1a2	eukaryotic translation elongation factor 1 alpha 2	1,54086E+14	9,31372E+14	3,13077E+14	0.0185293980135024	0.170747242106618	-2,84423E+14	1,03373E+14	1,02914E+14	9,40292E+14	8,14406E+14	9,19491E+14	8,51173E+14
TC1300000607.mm.2	Gadd45g	growth arrest and DNA-damage-inducible 45 gamma	1,38584E+14	6,2609E+14	3,13022E+14	0.0185430738096652	0.170747242106618	-2,84489E+14	6,74464E+14	7,30367E+14	6,13436E+14	5,14226E+14	5,91872E+14	6,32173E+14
TC1500001119.mm.2	Hoxc9	homeobox C9	-1,12525E+14	7,47599E+14	-3,12919E+14	0.0185685143466133	0.170747242106618	-2,8461E+14	6,58892E+14	6,66292E+14	7,48099E+14	8,02133E+14	7,77525E+14	8,3265E+14
TC1100003043.mm.2	Kdm6b	KDM1 lysine (K)-specific demethylase 6B	-1,02427E+14	7,67935E+14	-3,12879E+14	0.0185783242407363	0.170747242106618	-2,84656E+14	7,31876E+14	6,86998E+13	8,20295E+14	8,03433E+14	7,67779E+14	7,97229E+14
TC0700001974.mm.2	Mgmt	O-6-methylguanine-DNA methyltransferase	-1,24282E+14	7,43888E+13	-3,12844E+14	0.0185869522498609	0.170747242106618	-2,84697E+13	6,42365E+14	6,91907E+14	7,55699E+14	8,27138E+14	7,50076E+14	7,9614E+13
TC0600003005.mm.2	Cacna1c	calcium channel, voltage-dependent, L type, alpha 1C subunit	1,22756E+14	7,15518E+14	3,12789E+13	0.0186004854093173	0.170747242106618	-2,84761E+14	7,96337E+14	7,9157E+14	7,03253E+14	6,39142E+14	7,16965E+13	6,45842E+14
TC0500000609.mm.2	Klb	klotho beta	-1,36472E+14	4,59161E+14	-3,12774E+14	0.018604263637426	0.170747242106618	-2,84779E+14	3,48783E+14	4,47093E+14	5,13361E+14	5,5546E+14	4,69784E+14	4,20482E+14
TC1200000795.mm.2	Papln	papilin, proteoglycan-like sulfated glycoprotein	-1,45723E+14	7,00851E+14	-3,12675E+14	0.0186286586580481	0.170747242106618	-2,84895E+14	6,23767E+13	6,03482E+14	6,97052E+14	8,21643E+14	7,10983E+14	7,48176E+14
TC0800002593.mm.2	Calr	calreticulin	2,09378E+13	9,37175E+14	3,12675E+14	0.0186286588361521	0.170747242106618	-2,84895E+14	1,05485E+14	1,09171E+13	9,56942E+14	7,70868E+14	9,26947E+14	8,21726E+14
TC0200005339.mm.2	Gm14434/Gm2026/Gm4724/ Gm14308/Gm14430/Gm1443 2	predicted gene 14434/predicted gene 2026/predicted gene 4724/predicted gene 14308/predicted gene 14430/predicted gene 14432	2,32618E+14	9,02279E+13	3,1259E+14	0.0186496697505403	0.170747242106618	-2,84995E+14	9,9799E+14	1,05898E+14	8,86651E+14	7,0508E+14	9,59814E+13	8,05158E+14
TC0800001269.mm.2	Nfat5	nuclear factor of activated T cells 5	1,20532E+14	7,00926E+13	3,12584E+14	0.0186512275980276	0.170747242106618	-2,85002E+14	7,95608E+14	7,94551E+14	6,95476E+14	6,53619E+14	6,74237E+14	5,92066E+14
TC0600001033.mm.2	Ruvbl1	RuvB-like protein 1	2,02003E+14	6,4655E+14	3,12582E+14	0.0186516629695017	0.170747242106618	-2,85004E+14	7,59215E+14	7,74193E+14	6,67514E+14	4,6189E+14	5,9611E+14	6,20375E+14
TC0300002730.mm.2	Ampd2	adenosine monophosphate deaminase 2	2,14984E+14	6,18696E+14	3,12552E+14	0.0186591397352629	0.170747242106618	-2,85039E+14	7,35968E+13	7,4441E+14	6,34758E+14	4,15652E+14	6,13893E+14	5,67495E+14
TC1900001599.mm.2	Psd	pleckstrin and Sec7 domain containing	1,63705E+13	8,8757E+14	3,12445E+14	0.0186856668777859	0.170747242106618	-2,85165E+14	9,73333E+14	9,98338E+14	8,9924E+14	7,45021E+14	8,63115E+14	8,46371E+14
TC0500003439.mm.2	Srrt	serrate RNA effector molecule homolog (Arabidopsis)	1,0272E+14	7,07412E+14	3,12393E+14	0.0186985189113633	0.170747242106618	-2,85226E+14	7,5745E+13	8,0005E+14	6,84433E+14	6,67626E+13	6,84711E+14	6,50204E+14
TC0500002892.mm.2	Zfp951	zinc finger protein 951	-0.92946671688234	5,61876E+14	-3,12354E+14	0.0187082353751109	0.170747242106618	-2,85272E+14	4,94724E+14	5,08397E+14	5,95037E+14	5,93978E+14	5,77494E+14	6,01627E+13
TC1400001531.mm.2	Ecd	ecdysoneless cell cycle regulator	1,13462E+14	7,32327E+14	3,12325E+14	0.018715460694376	0.170747242106618	-2,85306E+14	8,0011E+14	8,13827E+14	7,30503E+14	6,5651E+14	7,09664E+14	6,83345E+14
TC1700000624.mm.2	Notch4	notch 4	-1,00759E+14	6,48726E+13	-3,12283E+14	0.0187258879500345	0.170747242106618	-2,85355E+14	5,81734E+14	5,86648E+14	6,6004E+14	7,0986E+14	6,68892E+13	6,8518E+14
TC0400003343.mm.2	Nasp	nuclear autoantigenic sperm protein (histone-binding)	1,32826E+14	5,16197E+14	3,12257E+14	0.0187324827064041	0.170747242106618	-2,85386E+14	5,76004E+14	6,11795E+14	5,00628E+14	4,21517E+14	4,57862E+14	5,29377E+14

TC0800000753.mm.2	Fkbp8	FK506 binding protein 8	1,03848E+14	8,73315E+14	3,12249E+14	0.0187344734717919	0.170747242106618	-2,85395E+14	9,32022E+14	9,48845E+14	8,65103E+14	8,08069E+14	8,38683E+14	8,47171E+14
TC0X00002433.mm.2	Bcap31	B cell receptor associated protein 31	1,57235E+14	7,90904E+14	3,12227E+13	0.0187399189436136	0.170747242106618	-2,85421E+14	8,99556E+14	8,78971E+14	7,83389E+14	6,80667E+14	8,03917E+14	6,98924E+14
TC1400001047.mm.2	Esd	esterase D/formylglutathione hydrolase	1,36619E+14	6,5271E+14	3,12197E+14	0.0187474033249009	0.170747242106618	-2,85456E+14	7,35678E+14	7,07835E+14	6,42121E+14	5,28154E+14	6,59109E+14	6,43364E+14
TC0200001583.mm.2	Pax6	paired box 6	1,2831E+14	5,73162E+14	3,12161E+14	0.0187562639219584	0.170747242106618	-2,85498E+14	6,66424E+14	6,47941E+14	5,7384E+14	4,83906E+12	5,07538E+14	5,59323E+13
TC1300001270.mm.2	Plpp1	phospholipid phosphatase 1	1,31657E+14	7,00103E+14	3,12144E+14	0.0187605645218303	0.170747242106618	-2,85518E+14	7,88698E+14	7,64579E+14	6,9267E+13	5,97292E+14	7,04104E+14	6,53273E+14
TC1900000967.mm.2	Ctsw	cathepsin W	-1,18558E+14	5,43232E+13	-3,1205E+14	0.0187841692427337	0.170747242106618	-2,85629E+14	4,77469E+14	5,01093E+14	5,64891E+14	6,50786E+14	5,34249E+14	5,30904E+14
TC0400002189.mm.2	Asph	aspartate-beta-hydroxylase	1,60097E+14	8,78504E+14	3,12021E+14	0.0187913471571746	0.170747242106618	-2,85663E+14	9,96084E+14	9,80936E+14	8,59271E+14	7,97556E+14	8,87879E+14	7,49301E+14
TC0400004035.mm.2	Kif1b	kinesin family member 1B	1,05777E+14	9,12531E+14	3,11991E+14	0.0187988709348988	0.170747242106618	-2,85698E+14	9,71515E+14	9,60202E+14	8,87643E+14	8,32519E+14	8,95847E+14	9,27459E+14
TC0700004085.mm.2	Dennd5a	DENN/MADD domain containing 5A	1,96994E+14	8,68879E+14	3,11979E+14	0.018801731717853	0.170747242106618	-2,85712E+14	9,91323E+14	9,9314E+14	8,84851E+14	7,05624E+14	8,65362E+14	7,72972E+12
TC1100000820.mm.2	Cdrt4	CMT1A duplicated region transcript 4	-1,36975E+13	5,97554E+14	-3,11966E+14	0.0188050530994579	0.170747242106618	-2,85727E+14	5,36451E+14	4,96702E+14	6,03107E+14	7,03996E+14	5,97946E+13	6,47119E+14
TC0X00000698.mm.2	Avpr2	arginine vasopressin receptor 2	-1,14764E+14	6,43906E+14	-3,11924E+14	0.0188154831920818	0.170747242106618	-2,85776E+14	5,85226E+14	5,61419E+13	6,69141E+14	7,07032E+14	6,35705E+14	7,04914E+13
TC0800000661.mm.2	Spock3	sparc/osteonectin, cwcv and kazal-like domains proteoglycan 3	1,45417E+13	8,65387E+14	3,11796E+14	0.0188476842255977	0.170747242106618	-2,85927E+14	9,64134E+14	9,53226E+14	8,73327E+14	7,532E+14	8,50712E+14	7,97722E+14
TC1900001035.mm.2	Otub1	OTU domain, ubiquitin aldehyde binding 1	1,41035E+14	1,02367E+14	3,11792E+14	0.0188487227578921	0.170747242106618	-2,85932E+14	1,11898E+14	1,11434E+14	1,02567E+14	9,25583E+14	1,01633E+14	9,41101E+14
TC1200000336.mm.2	Dgkb	diacylglycerol kinase, beta	2,02943E+14	9,04494E+14	3,11786E+14	0.0188500218576649	0.170747242106618	-2,85938E+14	1,03485E+14	1,04199E+14	9,17844E+14	7,53111E+14	9,04171E+14	7,75002E+14
TC0X00003175.mm.2	Tsr2	TSR2 20S rRNA accumulation	1,36215E+14	7,23454E+14	3,11752E+14	0.0188585914558388	0.170747242106618	-2,85978E+14	8,22395E+14	8,02592E+14	7,30815E+14	6,21742E+14	7,01882E+14	6,613E+14
TC0300002701.mm.2	Cym	chymosin	-1,66252E+14	6,18843E+14	-3,11603E+14	0.0188961760978302	0.170747242106618	-2,86154E+13	5,48574E+14	5,23062E+14	6,34034E+14	7,70107E+14	5,77327E+14	6,59954E+14
TC1000001934.mm.2	Rnf146	ring finger protein 146	1,32027E+14	6,54977E+14	3,1154E+13	0.0189119349123079	0.170747242106618	-2,86228E+14	7,35638E+14	7,08138E+14	6,41136E+13	5,38586E+14	6,70562E+13	6,35804E+14
TC0X00000122.mm.2	Sytl5	synaptotagmin-like 5	1,12679E+14	5,5392E+14	3,11516E+14	0.0189180291886165	0.170747242106618	-2,86256E+13	6,61365E+14	6,49146E+14	5,25087E+14	5,60066E+14	4,98701E+14	4,29156E+13
TC0800000981.mm.2	Dhps	deoxyhypusine synthase	0.981470940667578	7,28063E+14	3,11484E+14	0.0189260790386163	0.170747242106618	-2,86294E+13	8,08701E+14	7,85726E+14	7,09886E+14	6,88248E+14	7,04905E+14	6,70913E+14
TC0600001245.mm.2	Brpf1	bromodomain and PHD finger containing, 1	0.980676108331861	6,39531E+14	3,11466E+14	0.0189306474242184	0.170747242106618	-2,86315E+14	6,86547E+14	6,9647E+14	6,1363E+14	5,73252E+14	6,23601E+14	6,43689E+14
TC1900000610.mm.2	Marveld1	MARVEL (membrane-associating) domain containing 1	-0.995228923098628	7,17588E+13	-3,11465E+13	0.0189308061303244	0.170747242106618	-2,86316E+14	6,53956E+14	6,40623E+14	7,67391E+14	7,26234E+14	7,45993E+14	7,71329E+14
TC1400002215.mm.2	Fdft1	farnesyl diphosphate farnesyl transferase 1	1,51818E+14	8,32902E+14	3,11441E+14	0.0189367969467683	0.170747242106618	-2,86344E+14	9,38362E+13	9,07944E+14	8,32776E+14	7,09895E+13	8,35519E+13	7,72917E+14
TC0300002813.mm.2	Agl	amylo-1,6-glucosidase, 4-alpha-glucanotransferase	1,96591E+13	6,25357E+14	3,11269E+14	0.0189801213825386	0.170747242106618	-2,86546E+14	7,90791E+14	7,67633E+14	5,61092E+14	6,0415E+14	6,12084E+14	4,16393E+14
TC1700002680.mm.2	Pigf	phosphatidylinositol glycan anchor biosynthesis, class F	1,15144E+14	6,43041E+14	3,11145E+13	0.0190116631471124	0.170747242106618	-2,86693E+14	7,18344E+14	7,1784E+14	6,34273E+14	5,71624E+14	6,35788E+14	5,80377E+14
TC0700001879.mm.2	Tgfb1i1	transforming growth factor beta 1 induced transcript 1	-0.987657831797836	7,23531E+14	-3,11106E+13	0.019021515769446	0.170747242106618	-2,86738E+14	6,69702E+14	6,89141E+14	7,89596E+14	7,66779E+14	6,93972E+14	7,31998E+14
TC0100000794.mm.2	Dis3l2	DIS3 like 3'-5' exoribonuclease 2	1,69308E+14	6,01312E+14	3,11041E+14	0.0190378148236984	0.170747242106618	-2,86814E+14	7,5095E+14	7,44255E+14	6,04096E+14	5,52494E+14	5,5675E+14	3,99329E+14
TC0800000960.mm.2	G430095P16Rik	RIKEN cDNA G430095P16 gene	-1,31575E+13	5,63759E+13	-3,11003E+14	0.0190475319216144	0.170747242106618	-2,86859E+14	4,73607E+14	4,98633E+14	5,72165E+14	6,63225E+14	5,57949E+13	6,16973E+14
TC0300000875.mm.2	Selenbp2	selenium binding protein 2	-1,74137E+14	6,71048E+14	-3,10927E+13	0.0190667947466068	0.170747242106618	-2,86948E+14	5,33141E+14	5,78239E+14	6,55169E+14	8,04486E+14	6,90905E+13	7,64349E+14
TC1000002456.mm.2	Gm9978	predicted gene 9978	-1,07704E+14	4,96945E+14	-3,10892E+14	0.0190756354010651	0.170747242106618	-2,86989E+14	4,3064E+14	4,48158E+14	5,14521E+14	5,79685E+14	5,12471E+14	4,96197E+14
TC0300002755.mm.2	Fndc7	fibronectin type III domain containing 7	-1,20977E+14	6,7898E+14	-3,1088E+13	0.0190787435365488	0.170747242106618	-2,87004E+14	5,9536E+14	5,78274E+14	6,83694E+14	7,31895E+14	7,0282E+14	7,81835E+14
TC1000002392.mm.2	Mmp11	matrix metallopeptidase 11	-1,49683E+14	5,98726E+14	-3,10851E+14	0.0190860117306932	0.170747242106618	-2,87037E+13	5,03944E+14	5,0608E+14	6,09639E+13	6,9975E+14	5,83843E+14	6,89099E+14
TC0200004878.mm.2	Snph	syntaphilin	-1,19827E+14	8,27518E+14	-3,10708E+14	0.0191223566480263	0.170747242106618	-2,87206E+14	7,72674E+12	7,14089E+14	8,41837E+14	8,8458E+14	8,47451E+14	9,04478E+14
TC0200005295.mm.2	Ankrd60	ankyrin repeat domain 60	-1,27009E+14	5,21936E+13	-3,10684E+14	0.0191285855349059	0.170747242106618	-2,87234E+14	4,51648E+14	4,54193E+14	5,31191E+14	6,28669E+14	5,51451E+14	5,14467E+14
TC0300002532.mm.2	Gja8	gap junction protein, alpha 8	-1,22891E+14	5,47036E+14	-3,1062E+14	0.0191448070645747	0.170747242106618	-2,87309E+14	4,85579E+14	4,70673E+14	5,60683E+14	6,41351E+14	5,35583E+14	5,88345E+14
TC0200001260.mm.2	Olfr1029	olfactory receptor 1029	-1,32905E+14	5,41765E+14	-3,10597E+14	0.0191506348408997	0.170747242106618	-2,87336E+14	4,53962E+14	4,77679E+14	5,50127E+14	6,47323E+14	5,33723E+14	5,87774E+13
TC0700004643.mm.2	Pak4	p21 (RAC1) activated kinase 4	-1,02686E+14	7,35021E+14	-3,10555E+13	0.0191615410879131	0.170747242106618	-2,87386E+14	6,78734E+14	6,51013E+14	7,4675E+14	7,88369E+14	7,57277E+14	7,87984E+14
TC0600001742.mm.2	Mrps35	mitochondrial ribosomal protein S35	0.921920265305343	5,759E+13	3,10484E+14	0.0191795368872594	0.170747242106618	-2,87469E+14	6,26098E+14	6,28153E+14	5,27364E+14	5,42504E+14	5,76688E+14	5,54593E+13
TC1600001008.mm.2	Son	Son DNA binding protein	1,24519E+14	6,89905E+14	3,10442E+14	0.0191903650382092	0.170747242106618	-2,87519E+14	7,68821E+14	7,74421E+14	6,87197E+13	6,07007E+14	6,81042E+13	6,20941E+14
TC1400000381.mm.2	Wapl	WAPL cohesin release factor	1,33787E+14	6,28705E+13	3,10431E+14	0.01919313110654	0.170747242106618	-2,87532E+13	6,93312E+14	7,24437E+14	6,10375E+14	5,398E+13	6,44933E+14	5,59371E+14
TC0100001734.mm.2	Cnih3	cornichon family AMPA receptor auxiliary protein 3	1,58235E+14	7,70334E+14	3,10374E+14	0.0192076059082097	0.170747242106618	-2,87599E+14	8,59393E+14	8,65462E+14	7,59782E+14	6,48603E+14	7,95406E+13	6,9336E+14
TC1200000989.mm.2	Kcnk13	potassium channel, subfamily K, member 13	-1,3846E+14	8,02741E+14	-3,10362E+14	0.0192108157776448	0.170747242106618	-2,87613E+14	7,16104E+14	6,98325E+14	7,8771E+14	9,03639E+14	8,37841E+14	8,72829E+14
TC1500001762.mm.2	Puf60	poly-U binding splicing factor 60	0.923626282543016	7,80453E+14	3,10322E+14	0.0192210399787304	0.170747242106618	-2,8766E+14	8,49587E+14	8,43036E+14	7,60847E+14	7,4705E+14	7,52742E+14	7,29455E+13
TC0300002454.mm.2	Psmb4	proteasome (prosome, macropain) subunit, beta type 4	1,27253E+13	7,47274E+14	3,10311E+13	0.0192237259126706	0.170747242106618	-2,87673E+14	8,34677E+14	8,10332E+14	7,18045E+14	6,72458E+14	7,69729E+14	6,78406E+14
TC0400001822.mm.2	Lrrc38	leucine rich repeat containing 38	-0.982524631124115	5,02825E+13	-3,10311E+14	0.019223802073158	0.170747242106618	-2,87673E+14	4,40813E+14	4,5598E+14	5,39246E+14	5,54052E+14	4,92062E+14	5,348E+13
TC1900000154.mm.2	Stx5a/Mir6992	syntaxin 5A/microRNA 6992	1,14422E+14	6,58972E+14	3,10241E+14	0.0192416883899351	0.170747242106618	-2,87755E+14	7,2066E+14	7,30846E+14	6,46763E+13	5,75898E+14	6,60755E+14	6,18909E+14
TC1400002223.mm.2	Mtmr9	myotubularin related protein 9	1,98448E+14	6,97064E+14	3,10232E+14	0.0192439700670209	0.170747242106618	-2,87766E+14	8,09913E+14	8,01462E+14	7,06534E+14	5,07946E+13	7,03268E+14	6,53264E+14
TC0400002786.mm.2	Fkbp15	FK506 binding protein 15	1,82079E+14	6,9459E+14	3,10145E+14	0.0192664172922807	0.170747242106618	-2,87869E+14	8,04316E+14	7,7826E+14	6,85651E+14	5,32768E+14	7,3241E+14	6,34136E+14
TC0700003029.mm.2	Abcc6	ATP-binding cassette, sub-family C (CFTR/MRP), member 6	-1,09205E+14	7,36557E+14	-3,10104E+13	0.0192767869086111	0.170747242106618	-2,87916E+13	6,4537E+14	6,86324E+13	7,45706E+14	8,04397E+14	7,61528E+14	7,76016E+14
TC1100003110.mm.2	Camta2	calmodulin binding transcription activator 2	1,01747E+13	8,16746E+14	3,101E+14	0.0192779731719553	0.170747242106618	-2,87922E+14	8,78648E+14	8,90263E+14	8,05015E+13	7,60401E+14	7,99679E+14	7,66468E+14
TC1200001863.mm.2	Nemf	nuclear export mediator factor	1,41749E+14	6,25486E+14	3,10068E+14	0.0192861927603232	0.170747242106618	-2,87959E+14	7,31742E+14	7,26736E+13	6,14339E+14	5,60641E+14	6,17506E+14	5,01952E+14
TC0100000661.mm.2	Tmem198	transmembrane protein 198	0.982812319520755	7,41867E+14	3,09976E+14	0.0193097038257029	0.170747242106618	-2,88067E+14	7,87421E+14	8,18482E+14	7,19091E+14	6,90249E+14	7,29323E+13	7,06637E+14
TC1000000595.mm.2	Pbld2	phenazine biosynthesis-like protein domain containing 2	-1,08926E+14	5,63892E+13	-3,09969E+14	0.0193116759594513	0.170747242106618	-2,88076E+14	4,74234E+14	4,83113E+14	5,54796E+14	6,20402E+14	6,10559E+14	6,40247E+13
TC1400002600.mm.2	Dis3	DIS3 homolog, exosome endoribonuclease and 3'-5' exoribonuclease	1,35056E+14	5,43729E+13	3,09884E+14	0.0193334921572402	0.170747242106618	-2,88176E+14	6,49317E+14	6,1168E+13	5,11369E+12	4,79517E+14	4,51905E+14	5,58584E+14
TC0100000456.mm.2	Nop58	NOP58 ribonucleoprotein	1,86711E+14	6,13888E+14	3,09883E+13	0.0193337585860978	0.170747242106618	-2,88177E+14	7,81321E+14	7,70865E+14	6,25825E+14	5,52938E+14	5,63728E+14	3,88653E+14
TC1300000425.mm.2	Ly86	lymphocyte antigen 86	2,36888E+14	8,29649E+14	3,09782E+14	0.0193598747442566	0.170747242106618	-2,88296E+14	9,51129E+14	9,78415E+14	8,40166E+14	6,15603E+14	8,54206E+14	7,38376E+14
TC0700000990.mm.2	Selenos	selenoprotein S	1,1314E+14	6,48834E+14	3,09736E+13	0.0193715589941337	0.170747242106618	-2,8835E+14	7,22244E+14	7,15055E+14	6,33653E+14	5,77366E+13	5,93522E+14	6,51167E+14
TC1700001573.mm.2	Zfp944	zinc finger protein 944	1,53044E+14	6,07598E+14	3,0972E+14	0.0193757545898968	0.170747242106618	-2,88369E+14	6,92134E+14	6,94202E+14	6,12198E+14	4,6805E+14	5,88651E+14	5,90353E+14
TC0200002067.mm.2	Plcb4	phospholipase C, beta 4	1,8961E+14	5,84252E+14	3,0966E+14	0.0193911755157441	0.170747242106618	-2,88439E+14	7,37105E+14	6,96313E+14	5,8292E+14	4,71278E+14	5,85558E+14	4,32341E+14
TC1100000531.mm.2	Rnf130	ring finger protein 130	1,74774E+14	7,74445E+13	3,09643E+14	0.0193957511774439	0.170747242106618	-2,8846E+14	8,63082E+14	8,67709E+13	7,68997E+13	6,12246E+14	8,05282E+14	7,29357E+14
TC0900002981.mm.2	Uba5	ubiquitin-like modifier activating enzyme 5	1,03964E+14	7,96969E+14	3,09596E+14	0.0194078853816418	0.170747242106618	-2,88515E+12	8,66922E+14	8,69455E+14	7,75091E+14	7,53359E+14	7,87677E+13	7,29311E+14
TC1700000596.mm.2	Smim40	small integral membrane protein 40	-0.985517733108012	6,88125E+14	-3,0958E+14	0.0194120709270902	0.170747242106618	-2,88534E+13	6,16201E+14	6,43583E+14	7,14714E+14	7,42174E+14	6,90939E+14	7,2114E+14
TC1100003646.mm.2	Srcin1	SRC kinase signaling inhibitor 1	1,13459E+14	7,76871E+14	3,09551E+13	0.0194196171081344	0.170747242106618	-2,88569E+14	8,39241E+14	8,63174E+14	7,15504E+13	7,59994E+14	7,73705E+14	7,09609E+14
TC1900000300.mm.2	Gnaq	guanine nucleotide binding protein, alpha q polypeptide	1,42068E+14	8,00178E+14	3,09516E+14	0.0194285943920212	0.170747242106618	-2,8861E+14	9,02754E+14	8,87396E+14	7,95671E+14	7,10344E+14	7,99785E+14	7,05117E+13
TC1000003094.mm.2	Eef1akmt3	EEF1A lysine methyltransferase 3	-0.936664146372169	6,59528E+14	-3,0951E+13	0.0194301339329267	0.170747242106618	-2,88617E+14	5,9508E+14	5,94159E+14	6,72577E+14	7,03994E+14	6,89098E+14	7,02258E+14
TC0800001617.mm.2	Clec4g	C-type lectin domain family 4, member g	-1,58085E+14	5,20447E+14	-3,09492E+14	0.0194347896349154	0.170747242106618	-2,88638E+14	4,27078E+14	4,57029E+14	5,49384E+14	6,50892E+14	4,64982E+14	5,73321E+13
TC0400003307.mm.2	Foxd2	forkhead box D2	-1,03442E+14	6,16803E+14	-3,09476E+13	0.0194388871571217	0.170747242106618	-2,88656E+14	5,40639E+14	5,45845E+14	6,26333E+14	6,67035E+14	6,3775E+14	6,83218E+14
TC1500001867.mm.2	Baiap2l2	BAI1-associated protein 2-like 2	-1,34372E+14	5,93411E+14	-3,09474E+14	0.0194394190871899	0.170747242106618	-2,88659E+14	5,14341E+14	5,45619E+13	6,08296E+14	7,20409E+14	5,81391E+13	5,90412E+14
TC1800001150.mm.2	Nme5	NME/NM23 family member 5	1,40594E+14	6,73759E+14	3,09424E+14	0.0194523682114002	0.170747242106618	-2,88718E+12	7,51659E+14	7,4953E+14	6,73047E+12	5,46954E+13	6,57895E+14	6,63468E+14
TC0600002282.mm.2	Hoxa9/Mir196b	homeobox A9/microRNA 196b	-1,35192E+14	5,77354E+14	-3,09323E+14	0.0194785580797316	0.170747242106618	-2,88837E+13	5,02974E+14	4,77004E+14	5,7292E+14	6,77441E+14	5,9265E+14	6,41134E+14
TC0500003472.mm.2	Mcm7/Mir93/Mir25	minichromosome maintenance complex component 7/microRNA 93/microRNA 25	2,51264E+14	5,97966E+14	3,09307E+14	0.0194828039370436	0.170747242106618	-2,88856E+14	7,37111E+14	7,60649E+14	6,26382E+14	3,6885E+14	5,90349E+14	5,04455E+14
TC0100000659.mm.2	Gmppa	GDP-mannose pyrophosphorylase A	1,66722E+13	6,56909E+14	3,09304E+14	0.0194836511391974	0.170747242106618	-2,8886E+14	7,85936E+14	7,93442E+13	6,69875E+14	5,76059E+14	6,24954E+14	4,91191E+14
TC1200000817.mm.2	Vsx2	visual system homeobox 2	-1,0619E+14	5,10147E+14	-3,09273E+14	0.0194916781365644	0.170747242106618	-2,88896E+14	4,85078E+14	4,38358E+14	5,44561E+14	5,91256E+13	4,94078E+14	5,07554E+14
TC1000001266.mm.2	Ppp1r12a	protein phosphatase 1, regulatory subunit 12A	1,82154E+14	7,50445E+14	3,09258E+14	0.01949550109529	0.170747242106618	-2,88913E+14	8,46685E+14	8,7861E+14	7,54896E+14	6,0609E+14	7,6025E+14	6,56139E+13
TC1700001213.mm.2	Morn2	MORN repeat containing 2	-1,19447E+14	8,19846E+13	-3,09253E+14	0.0194968989998835	0.170747242106618	-2,8892E+14	7,63324E+14	7,52487E+14	8,98179E+13	8,56526E+14	8,65258E+14	7,833E+14
TC0200001513.mm.2	Rag2	recombination activating gene 2	-1,16906E+14	4,86147E+14	-3,09224E+13	0.0195044512634765	0.170747242106618	-2,88954E+14	4,20962E+14	4,07217E+14	4,87925E+13	5,74066E+13	5,07112E+14	5,19599E+13
TC0200001097.mm.2	Hoxd3/Hoxd4	homeobox D3/homeobox D4	-1,50558E+14	6,07915E+14	-3,0914E+14	0.0195262028020232	0.170747242106618	-2,89052E+14	5,02654E+14	5,10358E+14	5,86673E+14	7,27456E+14	6,58669E+14	6,6168E+14
TC1700002660.mm.2	Prepl	prolyl endopeptidase-like	2,38385E+14	8,17866E+14	3,09123E+14	0.0195305021811407	0.170747242106618	-2,89072E+14	9,93699E+14	9,78889E+13	8,37371E+14	6,58447E+14	8,11093E+14	6,27695E+14
TC0700002978.mm.2	Ap2a1	adaptor-related protein complex 2, alpha 1 subunit	2,3443E+14	7,55628E+14	3,09051E+14	0.0195494106559042	0.170747242106618	-2,89157E+14	8,91222E+14	8,93549E+14	7,76569E+14	5,39343E+14	7,58684E+14	6,74399E+14
TC0400004166.mm.2	Ttll10	tubulin tyrosine ligase-like family, member 10	-0.992440829253251	6,69356E+13	-3,08997E+14	0.0195633826132181	0.170747242106618	-2,89221E+14	5,98483E+14	6,10415E+14	6,81601E+14	7,25785E+14	6,88147E+14	7,11704E+14
TC1700000203.mm.2	Lix1	limb and CNS expressed 1	1,74931E+14	6,93993E+14	3,08997E+14	0.0195635246644621	0.170747242106618	-2,89221E+14	7,79325E+14	7,92505E+14	6,89442E+14	5,32525E+14	7,23178E+14	6,4698E+14
TC0700000773.mm.2	Klk10	kallikrein related-peptidase 10	-1,09235E+14	5,89156E+14	-3,08974E+14	0.0195693516972843	0.170747242106618	-2,89248E+14	5,39967E+14	5,14111E+14	6,07388E+14	6,65161E+12	5,85103E+14	6,23204E+14
TC0200003590.mm.2	Wdsub1	WD repeat, SAM and U-box domain containing 1	1,48551E+14	5,16094E+14	3,08962E+14	0.0195724715213167	0.170747242106618	-2,89262E+14	5,91407E+14	5,96485E+14	5,138E+14	3,7699E+14	4,99303E+14	5,18576E+14
TC0200002318.mm.2	Dnmt3b	DNA methyltransferase 3B	-1,53444E+14	4,59298E+14	-3,08917E+14	0.0195842650783262	0.170747242106618	-2,89315E+14	3,70714E+14	3,58418E+14	4,57465E+14	5,78555E+14	4,55632E+14	5,35008E+14
TC0200002594.mm.2	Cebpb	CCAAT/enhancer binding protein (C/EBP), beta	-1,09552E+14	7,37169E+14	-3,08895E+14	0.0195900933950794	0.170747242106618	-2,89341E+14	6,59733E+14	6,75583E+14	7,45511E+14	8,0891E+14	7,48688E+14	7,84588E+14
TC0800000982.mm.2	Wdr83os	WD repeat domain 83 opposite strand	1,64063E+14	8,09007E+14	3,08861E+14	0.0195989981458527	0.170747242106618	-2,89381E+13	9,14965E+14	9,10235E+14	8,24603E+14	6,7247E+14	7,90909E+14	7,40859E+14
TC0900002727.mm.2	Eef1a1	eukaryotic translation elongation factor 1 alpha 1	1,40323E+14	1,18077E+14	3,08831E+13	0.0196069026793477	0.170747242106618	-2,89417E+14	1,26811E+14	1,27375E+14	1,17681E+14	1,0844E+14	1,18367E+14	1,09787E+14
TC1100001878.mm.2	Kif19a	kinesin family member 19A	-1,02129E+14	6,74282E+14	-3,08784E+14	0.0196192353792326	0.170747242106618	-2,89473E+14	6,05125E+14	5,96305E+13	6,79767E+14	7,25921E+14	7,01656E+14	7,3692E+14
TC0200001030.mm.2	Dcaf17	DDB1 and CUL4 associated factor 17	1,1587E+13	6,45801E+14	3,08735E+14	0.0196319645189505	0.170747242106618	-2,8953E+14	7,26245E+14	6,85197E+14	6,2602E+14	5,53682E+14	6,29785E+14	6,53873E+14
TC0300001795.mm.2	Slc7a14	solute carrier family 7 (cationic amino acid transporter, y+ system), member 14	2,32798E+14	7,2932E+14	3,0871E+14	0.0196385796401143	0.170747242106618	-2,8956E+14	9,15898E+13	8,86637E+14	7,89635E+14	5,47304E+14	6,45334E+14	5,91112E+14
TC0800000138.mm.2	Fbxo25	F-box protein 25	1,6589E+14	7,65904E+14	3,0869E+14	0.0196438394826302	0.170747242106618	-2,89583E+14	8,70693E+13	8,70504E+14	7,76015E+14	6,33401E+14	7,61733E+14	6,83076E+13
TC1100002486.mm.2	Npm1	nucleophosmin 1	1,85429E+13	9,05253E+14	3,08668E+14	0.019649453135735	0.170747242106618	-2,89609E+14	1,02189E+14	1,02375E+14	9,139E+14	7,60886E+14	9,11179E+14	7,99911E+14
TC1500001700.mm.2	Gpr20	G protein-coupled receptor 20	-1,01243E+14	6,56157E+14	-3,08638E+14	0.0196573477005835	0.170747242106618	-2,89644E+13	5,806E+14	6,05377E+14	6,71984E+14	7,16479E+14	6,68725E+14	6,93778E+14
TC0200003098.mm.2	Abi1	abl-interactor 1	0.992929926375432	8,5627E+14	3,08632E+14	0.0196590134309035	0.170747242106618	-2,89652E+13	9,26945E+14	9,16331E+14	8,47729E+14	7,96961E+14	8,23826E+14	8,25831E+14
TC0500003497.mm.2	Ints1	integrator complex subunit 1	1,04693E+14	7,54593E+14	3,08539E+14	0.0196833789677845	0.170747242106618	-2,89761E+14	8,21802E+14	8,33338E+14	7,023E+13	7,43455E+14	6,89024E+14	7,37638E+14
TC0400002549.mm.2	Gne	glucosamine (UDP-N-acetyl)-2-epimerase/N-acetylmannosamine kinase	0.93575675321465	8,08626E+14	3,08498E+13	0.0196942098852968	0.170747242106618	-2,8981E+14	8,60804E+14	8,54208E+14	7,80891E+14	7,46969E+14	8,0675E+14	8,02133E+14
TC0700000856.mm.2	Izumo1/Rasip1	izumo sperm-egg fusion 1/Ras interacting protein 1	-1,05675E+14	6,70698E+14	-3,0844E+14	0.0197094545817151	0.170747242106618	-2,89878E+14	5,92842E+14	6,00103E+14	6,93395E+14	7,109E+14	6,81379E+14	7,45566E+14
TC0800002314.mm.2	Klhl2	kelch-like 2, Mayven	1,4726E+14	7,51439E+13	3,08413E+13	0.0197164844394749	0.170747242106618	-2,8991E+13	8,36818E+14	8,54484E+14	7,63035E+14	6,33746E+13	7,30747E+14	6,89806E+14
TC1600001231.mm.2	Parn	poly(A)-specific ribonuclease (deadenylation nuclease)	1,64909E+14	5,6638E+14	3,08325E+14	0.0197397430804352	0.170747242106618	-2,90014E+14	6,90774E+13	6,7188E+14	5,96733E+14	4,36103E+14	4,9941E+14	5,03382E+14
TC1600000670.mm.2	Atg3	autophagy related 3	1,51747E+14	7,54174E+14	3,08274E+14	0.0197532236298738	0.170747242106618	-2,90074E+14	8,37596E+14	8,28932E+14	7,45808E+14	6,17226E+14	7,79118E+14	7,16365E+14
TC0300003139.mm.2	Dnajb4	DnaJ heat shock protein family (Hsp40) member B4	2,51444E+14	6,68675E+14	3,08265E+13	0.0197555230159839	0.170747242106618	-2,90085E+14	8,24294E+13	8,26881E+13	7,00129E+14	4,48158E+14	6,59812E+14	5,52777E+14
TC0600003067.mm.2	Rimklb	ribosomal modification protein rimK-like family member B	2,21116E+14	5,99722E+14	3,08264E+14	0.0197556726993442	0.170747242106618	-2,90085E+14	7,75101E+13	7,46338E+14	5,81437E+14	4,97772E+14	6,08076E+14	3,89609E+14
TC0700000290.mm.2	Eml2/Mir330	echinoderm microtubule associated protein like 2/microRNA 330	1,23706E+14	6,57912E+14	3,08106E+14	0.019797402768332	0.170747242106618	-2,90272E+14	7,52408E+12	7,11266E+14	6,00547E+14	6,15715E+14	5,8851E+12	6,79023E+14
TC0700000762.mm.2	Etfb/Mir7051	electron transferring flavoprotein, beta polypeptide/microRNA 7051	-1,00985E+14	7,88396E+14	-3,08093E+13	0.0198007670769309	0.170747242106618	-2,90287E+14	7,11621E+14	7,12144E+14	7,88101E+14	8,37635E+14	8,2678E+14	8,54094E+14
TC0400001726.mm.2	Mul1	mitochondrial ubiquitin ligase activator of NFKB 1	0.967651007815923	6,02346E+14	3,08071E+14	0.0198067411810831	0.170747242106618	-2,90314E+14	6,76001E+14	6,39992E+14	5,49213E+14	5,7325E+14	5,808E+14	5,94819E+14
TC1100003983.mm.2	E030025P04Rik	RIKEN cDNA E030025P04 gene	-1,48916E+14	5,81269E+14	-3,08036E+14	0.01981586819471	0.170747242106618	-2,90354E+14	4,98443E+14	4,40758E+14	5,71687E+14	6,65347E+14	6,12849E+14	6,98527E+14
TC0200000342.mm.2	Gpr158	G protein-coupled receptor 158	2,29251E+14	8,34899E+14	3,0803E+14	0.0198174525776579	0.170747242106618	-2,90361E+14	9,77867E+14	9,78581E+14	8,62693E+14	6,35254E+14	8,2465E+14	7,30349E+14
TC1900001115.mm.2	Tmem132a	transmembrane protein 132A	1,29981E+13	6,53681E+14	3,0796E+14	0.0198359719062377	0.170747242106618	-2,90444E+14	7,42485E+14	7,60014E+14	6,6876E+14	5,73777E+14	5,61301E+14	6,15751E+14
TC0200001009.mm.2	Ppig	peptidyl-prolyl isomerase G (cyclophilin G)	2,17273E+14	7,40949E+14	3,07922E+14	0.0198461643196481	0.170747242106618	-2,90489E+14	8,77109E+14	8,9426E+13	7,35655E+14	6,01168E+14	7,6234E+14	5,7516E+14
TC1900000865.mm.2	Fam45a	family with sequence similarity 45, member A	1,08122E+14	9,02765E+14	3,07906E+14	0.0198503627521518	0.170747242106618	-2,90508E+14	9,78519E+14	9,68361E+14	8,66425E+14	8,6421E+14	9,05362E+14	8,33716E+14
TC1100001938.mm.2	Prcd	photoreceptor disc component	-1,01827E+14	7,99691E+14	-3,07903E+14	0.0198511166916634	0.170747242106618	-2,90512E+14	7,33842E+14	7,26596E+14	8,0985E+14	8,54242E+14	8,17932E+14	8,55687E+14
TC0800000845.mm.2	Hmox1	heme oxygenase 1	1,6154E+14	7,73034E+14	3,07901E+14	0.0198516981016991	0.170747242106618	-2,90514E+14	8,09138E+14	8,97831E+14	7,26964E+14	6,56926E+14	7,18873E+14	8,28473E+14
TC0400000907.mm.2	Ifna5	interferon alpha 5	-1,51183E+14	6,42451E+14	-3,07857E+14	0.0198634119504039	0.170747242106618	-2,90566E+14	5,4379E+14	5,37498E+14	6,33363E+14	7,5029E+14	6,53997E+14	7,35771E+14

TC1500000678.mm.2	Tomm22	translocase of outer mitochondrial membrane 22	1,17503E+14	7,9786E+14	3,07775E+14	0.0198850794183994	0.170747242106618	-2,90663E+14	8,72147E+14	8,52937E+14	7,67552E+14	7,22525E+14	8,23854E+14	7,48143E+14
TC0200002150.mm.2	Sec23b	SEC23 homolog B, COPII coat complex component	1,46385E+14	6,70949E+14	3,07764E+14	0.0198880774518688	0.170747242106618	-2,90676E+14	7,47774E+13	7,54918E+14	6,65178E+14	5,44742E+14	6,8786E+14	6,25224E+14
TC0200004169.mm.2	Hsd17b12	hydroxysteroid (17-beta) dehydrogenase 12	1,90312E+14	7,20462E+14	3,07751E+14	0.0198915943180861	0.170747242106618	-2,90692E+14	8,33282E+14	8,30198E+14	7,17192E+13	5,65662E+14	7,5066E+14	6,25778E+14
TC1800000953.mm.2	Mkx	mohawk homeobox	-1,10688E+14	6,94956E+14	-3,07749E+14	0.0198921222063708	0.170747242106618	-2,90694E+14	6,14904E+14	6,21753E+14	6,90478E+14	7,67555E+14	7,34684E+14	7,40363E+14
TC1700000999.mm.2	Tnfaip8l1	tumor necrosis factor, alpha-induced protein 8-like 1	-1,15224E+14	5,2441E+14	-3,07716E+14	0.0199009073838657	0.170747242106618	-2,90733E+14	4,63621E+14	4,68839E+14	5,53404E+14	6,09505E+14	5,58106E+13	4,92983E+14
TC0800000115.mm.2	F10	coagulation factor X	-1,04273E+14	6,82074E+14	-3,07525E+14	0.0199515369819208	0.170925961084307	-2,90958E+14	5,96824E+14	6,2055E+14	6,90522E+14	7,35397E+14	7,04626E+13	7,44524E+14
TC1100002127.mm.2	Emid1	EMI domain containing 1	-1,34861E+14	8,54146E+14	-3,07447E+14	0.0199723444512968	0.170925961084307	-2,9105E+14	7,47084E+14	7,63858E+14	8,39975E+14	9,40688E+14	8,86203E+14	9,47067E+14
TC1400002740.mm.2	Dock9	dedicator of cytokinesis 9	0.896253744759373	6,59199E+14	3,07363E+14	0.019994755705008	0.170925961084307	-2,91149E+14	7,0989E+14	6,97139E+14	6,10313E+14	6,17465E+14	6,65293E+14	6,55094E+14
TC1700001981.mm.2	Tubb5	tubulin, beta 5 class I	2,08087E+14	9,8109E+14	3,07329E+13	0.0200039375412895	0.170925961084307	-2,9119E+14	1,11253E+14	1,1184E+14	1,00938E+13	8,05376E+14	9,61319E+14	8,79526E+14
TC0100001433.mm.2	Cryzl2	crystallin zeta like 2	0.936228308178119	5,53265E+14	3,07292E+14	0.0200138712844226	0.170925961084307	-2,91234E+14	6,19575E+14	6,11195E+14	5,04147E+14	5,39377E+13	5,24167E+14	5,21132E+14
TC0500003056.mm.2	Alkbh2	alkB homolog 2, alpha-ketoglutarate-dependent dioxygenase	-1,37022E+14	7,57275E+14	-3,07264E+14	0.0200214300075404	0.170925961084307	-2,91267E+14	6,50849E+14	6,79919E+14	7,45148E+13	8,59663E+13	7,88224E+14	8,19847E+14
TC0X00002246.mm.2	Zfp36l3	zinc finger protein 36, C3H type-like 3	-1,1456E+14	5,0197E+12	-3,07252E+14	0.0200245407306153	0.170925961084307	-2,91281E+14	4,3025E+14	3,99874E+14	4,91715E+14	5,6753E+14	5,49066E+14	5,73387E+14
TC0900001792.mm.2	Fat3	FAT atypical cadherin 3	1,02023E+14	5,64059E+14	3,07189E+14	0.0200415193013328	0.170925961084307	-2,91356E+13	6,23529E+14	6,12464E+14	5,02383E+14	5,29563E+14	5,32193E+14	5,84226E+14
TC1800000199.mm.2	Elp2	elongator acetyltransferase complex subunit 2	2,57697E+14	6,5232E+14	3,07163E+14	0.0200483929042737	0.170925961084307	-2,91386E+14	8,07902E+13	8,09469E+14	6,6581E+14	4,36167E+14	6,79702E+14	5,14871E+14
TC0600002960.mm.2	Rpl32	ribosomal protein L32	0.943407024872691	9,27632E+14	3,07147E+14	0.0200525394724427	0.170925961084307	-2,91404E+14	9,87131E+14	9,80559E+14	9,05625E+14	8,73384E+14	9,19951E+14	8,99139E+13
TC0900002766.mm.2	Lca5	Leber congenital amaurosis 5 (human)	1,26626E+14	7,85564E+14	3,07117E+13	0.0200607694092806	0.170925961084307	-2,91441E+14	8,82515E+14	8,30377E+13	7,75688E+14	6,83952E+14	7,75721E+14	7,65132E+14
TC0700002759.mm.2	Aplp1	amyloid beta (A4) precursor-like protein 1	1,52096E+14	1,03244E+14	3,06993E+14	0.0200940976834601	0.170925961084307	-2,91587E+14	1,13417E+14	1,13064E+14	1,04536E+14	9,15261E+14	1,01628E+14	9,52925E+14
TC1100003106.mm.2	Slc25a11	solute carrier family 25 (mitochondrial carrier oxoglutarate carrier), member 11	1,73337E+13	9,01215E+14	3,0696E+14	0.0201028654486191	0.170925961084307	-2,91626E+14	1,02507E+13	1,01366E+13	9,11806E+14	7,80247E+14	8,95168E+14	7,81344E+14
TC0300000896.mm.2	Mindy1	MINDY lysine 48 deubiquitinase 1	-1,15898E+14	7,75751E+14	-3,06817E+14	0.0201413644392557	0.170925961084307	-2,91795E+14	6,92307E+14	6,97911E+14	7,70132E+14	8,51883E+14	8,05172E+14	8,371E+14
TC0800002008.mm.2	Ubxn8	UBX domain protein 8	0.955738380216546	7,08037E+11	3,06783E+14	0.0201504353729437	0.170925961084307	-2,91835E+14	7,80243E+14	7,53575E+14	6,72362E+14	6,70309E+14	6,68917E+14	7,02818E+14
TC0600002240.mm.2	Tra2a	transformer 2 alpha	1,84622E+14	7,62555E+14	3,06769E+14	0.0201541947746745	0.170925961084307	-2,91852E+14	8,69456E+14	8,80265E+14	7,69604E+14	6,10874E+14	7,74173E+12	6,70958E+14
TC1600001590.mm.2	Stxbp5l	syntaxin binding protein 5-like	1,27114E+14	7,75122E+14	3,0674E+14	0.0201620411730039	0.170925961084307	-2,91886E+14	8,50701E+14	8,57135E+14	7,51084E+14	7,02524E+14	7,92261E+14	6,97028E+13
TC0X00000668.mm.2	Nsdhl	NAD(P) dependent steroid dehydrogenase-like	1,33134E+14	7,44899E+14	3,06683E+14	0.0201774126914521	0.170925961084307	-2,91953E+14	8,43422E+14	8,27286E+14	7,42397E+14	6,62041E+14	7,38665E+14	6,55586E+14
TC1400001182.mm.2	Acod1	aconitate decarboxylase 1	-1,02693E+14	5,18578E+14	-3,06681E+14	0.0201779954459033	0.170925961084307	-2,91956E+14	4,57949E+13	4,80097E+14	5,53965E+14	5,89467E+14	4,92345E+14	5,37647E+14
TC0300000210.mm.2	Skil	SKI-like	1,98941E+14	7,81019E+14	3,06664E+14	0.020182504356244	0.170925961084307	-2,91976E+14	8,91924E+14	9,08374E+14	7,95015E+14	6,07401E+14	7,86531E+14	6,96871E+13
TC1500002050.mm.2	Lmf2	lipase maturation factor 2	1,23109E+13	6,80502E+13	3,0659E+14	0.0202025190970852	0.170925961084307	-2,92063E+14	7,30655E+14	7,85272E+14	6,76233E+14	5,93476E+14	6,40207E+14	6,57166E+14
TC0300002342.mm.2	Trim46	tripartite motif-containing 46	1,05656E+14	7,27055E+13	3,06559E+13	0.0202108306280967	0.170925961084307	-2,921E+13	7,94369E+14	8,03891E+14	7,26788E+14	6,6016E+14	6,94247E+14	6,82875E+14
TC0400002621.mm.2	Erp44	endoplasmic reticulum protein 44	1,05405E+14	6,96524E+14	3,06499E+13	0.0202271132166827	0.170925961084307	-2,92171E+14	7,71359E+14	7,76874E+14	6,61962E+14	6,7546E+14	6,79836E+14	6,13652E+14
TC1400002031.mm.2	Rab2b	RAB2B, member RAS oncogene family	2,63483E+14	7,35885E+14	3,06457E+14	0.0202384025079374	0.170925961084307	-2,9222E+14	9,01997E+14	9,0681E+14	7,84903E+13	4,96937E+14	6,81742E+14	6,42922E+14
TC1300000232.mm.2	Vmn1r221	vomeronasal 1 receptor 221	-1,00766E+14	5,3588E+14	-3,06456E+14	0.0202387914068373	0.170925961084307	-2,92222E+14	4,63953E+14	4,55309E+14	5,40813E+14	5,79982E+14	5,67126E+14	6,08095E+13
TC0200004485.mm.2	Ppip5k1	diphosphoinositol pentakisphosphate kinase 1	-1,28576E+14	6,75073E+14	-3,06432E+14	0.0202453568078429	0.170925961084307	-2,92251E+14	5,85695E+14	5,82559E+14	6,63757E+14	7,61649E+14	7,03729E+14	7,53051E+14
TC0100003279.mm.2	Dhx9	DEAH (Asp-Glu-Ala-His) box polypeptide 9	1,80994E+14	6,4341E+14	3,06428E+14	0.0202464126794359	0.170925961084307	-2,92255E+14	7,87477E+13	7,78363E+13	6,59648E+14	5,44204E+14	6,17568E+14	4,73199E+14
TC1600000656.mm.2	Cfap44	cilia and flagella associated protein 44	-1,10066E+14	7,18207E+14	-3,06395E+14	0.0202553763416469	0.170925961084307	-2,92295E+13	6,31553E+14	6,52616E+14	7,17519E+14	7,86782E+14	7,46799E+14	7,73971E+14
TC0200003834.mm.2	Frzb	frizzled-related protein	1,04096E+14	5,45028E+14	3,0627E+14	0.020289188499292	0.170925961084307	-2,92442E+14	5,50109E+14	6,05457E+14	4,83542E+14	4,63831E+14	5,97647E+14	5,69579E+14
TC1200000740.mm.2	Galnt16	polypeptide N-acetylgalactosaminyltransferase 16	1,12978E+14	8,48982E+14	3,06256E+14	0.0202929954149247	0.170925961084307	-2,92459E+14	9,54435E+14	9,21832E+14	8,6308E+14	7,87232E+14	7,87807E+14	7,79505E+14
TC0700004648.mm.2	Rpl13a/Snord32a/Snord33/Snord34/Mir5121	ribosomal protein L13A/small nucleolar RNA, C/D box 32A/small nucleolar RNA, C/D box 33/small nucleolar RNA, C/D box 34/microRNA 5121	1,67013E+14	1,11705E+14	3,06215E+14	0.0203041941801895	0.170925961084307	-2,92508E+14	1,22499E+14	1,21816E+14	1,11791E+13	9,91216E+14	1,12875E+13	1,02131E+14
TC0200005489.mm.2	Zfp965	zinc finger protein 965	2,29602E+13	8,23626E+13	3,06192E+14	0.0203104471004021	0.170925961084307	-2,92535E+14	9,34649E+14	9,7184E+14	8,36298E+14	6,10988E+14	8,42861E+13	7,45122E+14
TC1400001958.mm.2	Exoc5	exocyst complex component 5	2,07044E+14	6,14242E+14	3,0618E+13	0.0203135307053939	0.170925961084307	-2,92548E+14	7,49683E+14	7,68215E+14	5,95258E+14	5,08551E+14	6,32286E+12	4,31458E+14
TC1700000685.mm.2	H2-Q5	histocompatibility 2, Q region locus 5	-1,14527E+14	6,98125E+14	-3,06166E+14	0.0203173303655001	0.170925961084307	-2,92565E+14	6,0973E+14	6,3478E+14	7,05007E+14	7,68557E+14	7,09296E+14	7,61378E+14
TC1100003204.mm.2	Sgsm2	small G protein signaling modulator 2	0.902147528001513	6,71594E+14	3,06158E+14	0.0203196239346705	0.170925961084307	-2,92575E+14	7,58856E+14	7,40496E+14	6,53218E+14	6,65704E+14	6,01888E+14	6,09404E+14
TC0700000057.mm.2	U2af2	U2 small nuclear ribonucleoprotein auxiliary factor (U2AF) 2	1,1585E+14	7,11944E+14	3,06134E+14	0.0203259647220552	0.170925961084307	-2,92602E+14	8,21276E+14	8,01491E+14	7,36683E+14	6,54384E+14	6,16307E+14	6,41524E+14
TC0400000391.mm.2	Fam205a2/Fam205a4	family with sequence similarity 205, member A2/family with sequence similarity 205, member A4	1,28821E+14	5,84496E+14	3,06105E+13	0.0203340383864864	0.170925961084307	-2,92638E+14	6,51795E+14	6,63492E+14	5,25826E+14	5,31819E+14	6,21766E+14	5,12278E+14
TC0600001126.mm.2	Tafa1	TAFA chemokine like family member 1	1,22624E+14	7,70044E+14	3,06097E+14	0.0203360338910853	0.170925961084307	-2,92646E+14	8,10544E+14	8,60755E+14	7,56077E+14	6,69972E+14	7,56783E+14	7,66131E+14
TC1100002166.mm.2	Ddx56	DEAD (Asp-Glu-Ala-Asp) box polypeptide 56	0.981730696507673	5,72406E+14	3,05991E+14	0.0203649204166122	0.170925961084307	-2,92772E+14	6,38409E+14	6,45714E+14	5,27014E+14	5,60763E+14	5,50167E+14	5,12369E+14
TC1900000777.mm.2	Vti1a	vesicle transport through interaction with t-SNAREs 1A	2,03708E+14	5,96198E+14	3,05904E+14	0.0203886188699464	0.170925961084307	-2,92875E+14	7,28246E+14	7,28266E+14	6,15349E+14	4,33748E+14	5,9375E+14	4,77832E+14
TC0100000317.mm.2	Gpr45	G protein-coupled receptor 45	-1,18786E+14	7,37846E+14	-3,05831E+14	0.0204085328439739	0.170925961084307	-2,92961E+14	6,86576E+14	6,68405E+14	7,63964E+14	8,28588E+14	7,72476E+14	7,07069E+14
TC1000003097.mm.2	Os9	amplified in osteosarcoma	1,3018E+14	7,65555E+14	3,05827E+14	0.0204096724563191	0.170925961084307	-2,92966E+14	8,89229E+14	8,54514E+14	7,64564E+14	7,18819E+13	7,31547E+14	6,34655E+14
TC0X00000706.mm.2	Taz	tafazzin	2,13313E+14	5,7353E+14	3,05776E+14	0.0204235464885272	0.170925961084307	-2,93026E+14	6,57719E+14	6,61117E+14	5,6004E+14	3,3217E+14	6,18115E+14	6,12019E+14
TC1500001927.mm.2	Rangap1	RAN GTPase activating protein 1	1,22354E+14	7,72298E+14	3,05734E+14	0.0204352885043898	0.170925961084307	-2,93077E+14	8,59958E+14	8,26861E+14	7,45271E+14	6,9684E+14	7,92864E+14	7,11995E+14
TC1900001472.mm.2	Noc3l	NOC3 like DNA replication regulator	1,33995E+14	6,12792E+14	3,05721E+14	0.0204386539315384	0.170925961084307	-2,93092E+14	7,3599E+14	7,02819E+14	6,38145E+14	5,32674E+14	5,11973E+13	5,55151E+14
TC0700004522.mm.2	Rnh1	ribonuclease/angiogenin inhibitor 1	0.969729175341866	7,03388E+14	3,05708E+14	0.0204423937160644	0.170925961084307	-2,93108E+14	7,63234E+14	7,61393E+14	6,8857E+14	6,42111E+14	6,88865E+14	6,76155E+14
TC0900002405.mm.2	Bbs4	Bardet-Biedl syndrome 4 (human)	2,60541E+14	7,00965E+12	3,05669E+13	0.020452991562953	0.170925961084307	-2,93154E+13	8,66498E+13	8,38036E+14	7,25524E+14	4,57927E+14	7,09907E+14	6,07901E+14
TC1700002336.mm.2	Nrtn	neurturin	-1,26382E+14	7,91159E+14	-3,05659E+14	0.02045570649662	0.170925961084307	-2,93165E+14	6,93832E+14	7,15142E+14	7,90181E+14	8,71557E+14	8,05356E+14	8,70884E+14
TC1500002247.mm.2	Bin2	bridging integrator 2	1,05665E+14	7,82369E+14	3,05644E+14	0.0204598601540591	0.170925961084307	-2,93183E+14	8,25045E+14	8,68564E+14	7,6723E+13	7,15048E+14	7,53312E+13	7,65013E+14
TC0100002969.mm.2	Insig2	insulin induced gene 2	1,41151E+14	5,95792E+14	3,05622E+14	0.0204657206322244	0.170925961084307	-2,93209E+14	7,09405E+14	6,87677E+14	5,09617E+14	6,05163E+14	5,73913E+14	4,88974E+14
TC1100003426.mm.2	Apbbp2	amyloid beta precursor protein (cytoplasmic tail) binding protein 2	1,92389E+14	7,58715E+14	3,0561E+14	0.0204692158056897	0.170925961084307	-2,93224E+14	8,9748E+14	8,75413E+14	7,68139E+14	6,19976E+14	7,6256E+14	6,28722E+14
TC1800001437.mm.2	Adrb2	adrenergic receptor, beta 2	-0.903701650757442	5,81384E+14	-3,0559E+14	0.0204746195518771	0.170925961084307	-2,93247E+14	5,34492E+14	5,52015E+14	6,38973E+14	6,28274E+13	5,74489E+14	5,60061E+14
TC1100001135.mm.2	Sez6	seizure related gene 6	1,36178E+14	8,52451E+14	3,05537E+14	0.0204890154569575	0.170925961084307	-2,93309E+13	9,56858E+13	9,42187E+14	8,70799E+14	7,55891E+14	7,75045E+14	8,13929E+14
TC1700000078.mm.2	Rsph3a	radial spoke 3A homolog (Chlamydomonas)	1,57327E+14	6,72309E+14	3,05517E+14	0.0204945669220431	0.170925961084307	-2,93333E+14	7,6559E+14	7,58983E+14	6,77444E+14	5,32475E+14	6,74317E+14	6,25045E+14
TC1400001721.mm.2	Gprin2	G protein regulated inducer of neurite outgrowth 2	-1,38733E+14	6,27875E+14	-3,05405E+14	0.020525332163972	0.171006880438144	-2,93466E+14	5,57434E+14	5,2799E+14	6,32276E+14	7,30614E+13	6,22778E+14	6,96158E+13
TC0300001415.mm.2	Adh5	alcohol dehydrogenase 5 (class III), chi polypeptide	-0.936030447519291	6,78433E+14	-3,05404E+14	0.0205254953619668	0.171006880438144	-2,93467E+14	6,19318E+14	6,10628E+14	6,92807E+14	7,24345E+14	7,21666E+14	7,01835E+14
TC0500001312.mm.2	Med13l	mediator complex subunit 13-like	1,26528E+14	7,58692E+14	3,05362E+14	0.0205370423331673	0.171014657940219	-2,93517E+14	8,32454E+12	8,34154E+14	7,47251E+14	6,66301E+14	7,71023E+14	7,00967E+14
TC1100003880.mm.2	Wnt9b	wingless-type MMTV integration site family, member 9B	-1,02161E+14	8,19288E+14	-3,05238E+14	0.0205713920771547	0.171212211022311	-2,93664E+14	7,51026E+14	7,45262E+14	8,5041E+14	8,502E+13	8,28772E+14	8,90058E+14
TC0100002481.mm.2	Idh1	isocitrate dehydrogenase 1 (NADP+), soluble	1,71737E+13	7,02836E+14	3,05073E+14	0.02061684280908	0.171402568749408	-2,9386E+14	8,20171E+13	8,17698E+14	6,92746E+14	6,01648E+14	7,16261E+14	5,68494E+14
TC0300000159.mm.2	Nceh1	neutral cholesterol ester hydrolase 1	2,12609E+14	8,50626E+14	3,05015E+14	0.0206327889675653	0.171402568749408	-2,93928E+14	9,81594E+14	9,74813E+14	8,72503E+14	6,58684E+14	8,47157E+14	7,69005E+14
TC0800002648.mm.2	Zfp423	zinc finger protein 423	0.98563940551776	6,88727E+14	3,05001E+14	0.0206366948743819	0.171402568749408	-2,93945E+14	7,77643E+14	7,41673E+14	6,5066E+14	6,71529E+14	6,61892E+13	6,28966E+14
TC0300000203.mm.2	Sec62	SEC62 homolog (S. cerevisiae)	2,23067E+14	8,95003E+13	3,04903E+14	0.0206639004181602	0.171402568749408	-2,94061E+14	1,05617E+14	1,05709E+14	9,02834E+14	7,64285E+14	8,93596E+14	6,96049E+14
TC1300002048.mm.2	Uimc1	ubiquitin interaction motif containing 1	1,25341E+14	5,48271E+14	3,049E+14	0.0206646446454019	0.171402568749408	-2,94065E+14	6,42825E+14	6,0362E+14	5,36569E+14	4,59195E+14	5,53681E+14	4,93738E+14
TC1400002290.mm.2	Cdca2	cell division cycle associated 2	-1,01462E+14	6,22914E+14	-3,04858E+14	0.0206762989308858	0.171402568749408	-2,94115E+14	5,33464E+14	5,61804E+14	6,2252E+14	6,75672E+14	6,7196E+14	6,72066E+14
TC1100004158.mm.2	Cep295nl	CEP295 N-terminal like	-1,6086E+14	5,82279E+14	-3,0481E+14	0.0206896317351397	0.171402568749408	-2,94172E+14	4,88678E+14	4,45078E+13	5,75961E+14	6,79515E+14	5,96146E+14	7,08297E+14
TC0400002104.mm.2	Tnfrsf18	tumor necrosis factor receptor superfamily, member 18	-1,3819E+14	6,51891E+14	-3,04766E+14	0.020701606749804	0.171402568749408	-2,94223E+14	5,59179E+14	5,88333E+14	6,67133E+14	7,5676E+14	6,2831E+14	7,11629E+14
TC0200000600.mm.2	Rpl12/Snora65	ribosomal protein L12/small nucleolar RNA, H/ACA box 65	1,60961E+14	1,02416E+14	3,04621E+13	0.0207419232584185	0.171402568749408	-2,94395E+14	1,12699E+13	1,12761E+14	1,02193E+14	9,10749E+14	1,03571E+14	9,22E+14
TC1100002664.mm.2	Sqstm1	sequestosome 1	1,19229E+14	9,77461E+14	3,04619E+14	0.0207425731885863	0.171402568749408	-2,94398E+14	1,05842E+14	1,0516E+14	9,80625E+14	8,90942E+14	9,57366E+14	9,25812E+14
TC1900000212.mm.2	Prpf19	pre-mRNA processing factor 19	1,6837E+14	7,74773E+14	3,04608E+14	0.0207455625553031	0.171402568749408	-2,94411E+14	9,04623E+14	8,83413E+13	8,03199E+14	6,48097E+14	7,36942E+14	6,72364E+14
TC0900002235.mm.2	Rexo2	RNA exonuclease 2	1,2465E+14	7,23845E+14	3,04598E+14	0.0207482389233893	0.171402568749408	-2,94422E+14	7,87541E+13	8,09518E+14	7,12184E+14	6,35574E+14	7,32922E+14	6,6533E+14
TC0500003550.mm.2	Spdye4a	speedy/RINGO cell cycle regulator family, member E4A	-1,17951E+13	6,05625E+14	-3,04596E+14	0.0207488603235466	0.171402568749408	-2,94425E+13	5,2588E+14	5,36097E+14	6,30345E+14	6,67534E+14	5,94578E+14	6,79317E+14
TC0200003579.mm.2	Acvr1	activin A receptor, type 1	0.972172030160905	5,92882E+14	3,04591E+14	0.0207502765249802	0.171402568749408	-2,94431E+14	6,5224E+14	6,07618E+14	5,3384E+14	5,31584E+14	6,04961E+14	6,2705E+14
TC0200002783.mm.2	Osbpl2	oxysterol binding protein-like 2	1,96535E+14	6,63556E+14	3,04578E+14	0.0207538268249299	0.171402568749408	-2,94446E+14	7,99016E+14	7,86486E+14	6,77942E+13	5,14491E+14	6,65063E+14	5,38335E+14
TC0600002797.mm.2	Tmf1	TATA element modulatory factor 1	2,13506E+14	5,68009E+14	3,04491E+14	0.0207780428618518	0.171438101902745	-2,94549E+14	7,34408E+14	7,20794E+14	5,77728E+14	4,50462E+14	5,57509E+14	3,67154E+14
TC0200002753.mm.2	Gm14327	predicted gene 14327	2,78897E+14	7,78611E+14	3,04476E+14	0.0207823057461691	0.171438101902745	-2,94567E+14	9,04661E+14	9,47413E+14	7,93387E+14	5,00892E+14	8,12439E+14	7,12871E+14
TC0500002025.mm.2	Dnajc2	DnaJ heat shock protein family (Hsp40) member C2	1,94008E+14	6,65349E+14	3,04448E+14	0.0207900484775004	0.171438101902745	-2,946E+14	7,66205E+14	7,79371E+14	6,65749E+13	4,91812E+14	6,9735E+14	5,91604E+14
TC1000002616.mm.2	Syn3	synapsin III	2,43953E+13	6,89074E+14	3,04376E+14	0.0208101917073199	0.171516429145803	-2,94686E+14	8,83788E+14	8,85372E+14	7,14115E+13	5,67138E+14	6,54262E+14	4,29771E+14
TC0700000576.mm.2	Krtdap	keratinocyte differentiation associated protein	-1,06047E+14	4,96732E+14	-3,04281E+14	0.020836813424096	0.17164804432641	-2,94799E+14	4,1793E+14	4,34862E+14	4,99727E+14	5,65158E+14	5,20917E+14	5,41799E+14
TC0800002119.mm.2	Asah1	N-acylsphingosine amidohydrolase 1	2,00731E+14	7,16123E+14	3,04195E+14	0.0208607281140907	0.171757236638909	-2,94901E+13	8,31006E+14	8,34316E+14	7,34452E+14	5,29409E+14	7,12638E+14	6,54916E+13
TC0800001301.mm.2	Ap1g1	adaptor protein complex AP-1, gamma 1 subunit	1,51883E+14	7,70281E+14	3,04118E+14	0.0208821974577093	0.17176914437635	-2,94992E+14	8,85905E+14	8,57806E+13	7,90004E+14	6,49941E+14	7,35246E+14	7,02784E+14
TC0900000550.mm.2	Sik3	SIK family kinase 3	1,49074E+14	8,80468E+14	3,04108E+14	0.0208850769870334	0.17176914437635	-2,95004E+14	9,89976E+14	9,79541E+14	9,00015E+14	7,71354E+14	8,51066E+14	7,90858E+14
TC0X00000708.mm.2	Atp6ap1	ATPase, H+ transporting, lysosomal accessory protein 1	1,41154E+14	7,98539E+14	3,04076E+14	0.0208941552148976	0.17176914437635	-2,95042E+14	9,35111E+14	9,18192E+13	8,13004E+14	7,57991E+14	7,42171E+14	6,24765E+14
TC1800000047.mm.2	Rab18	RAB18, member RAS oncogene family	3,18341E+14	7,51512E+14	3,03669E+14	0.0210084442827173	0.172273867703966	-2,95525E+14	9,04966E+14	9,13421E+13	7,62815E+14	4,18891E+14	8,101E+14	6,98881E+14
TC0400004113.mm.2	Ccdc27	coiled-coil domain containing 27	-1,59154E+13	5,7138E+14	-3,03643E+14	0.0210156092784911	0.172273867703966	-2,95555E+14	4,60509E+14	4,83296E+14	5,58473E+14	7,03639E+14	5,83234E+14	6,39127E+14
TC1400000309.mm.2	Rft1	RFT1 homolog	1,26955E+13	6,19826E+14	3,03609E+14	0.0210252939156659	0.172273867703966	-2,95596E+14	7,14286E+14	6,87609E+13	5,94437E+14	5,53546E+14	5,35838E+14	6,33238E+14
TC1100000955.mm.2	Psmb6	proteasome (prosome, macropain) subunit, beta type 6	0.930409465608207	8,31343E+14	3,03532E+13	0.0210468517429343	0.172273867703966	-2,95687E+14	9,04941E+14	8,97959E+14	8,1043E+14	8,06389E+14	8,02888E+14	7,65452E+14
TC1700000666.mm.2	Abhd16a	abhydrolase domain containing 16A	0.885857941477968	6,56883E+14	3,03526E+14	0.0210487634010453	0.172273867703966	-2,95695E+14	7,28062E+14	7,15664E+14	6,2764E+14	6,38915E+14	6,19269E+14	6,11747E+13
TC1100001156.mm.2	Aldoc	aldolase C, fructose-bisphosphate	1,88927E+14	1,05172E+14	3,03468E+14	0.0210650838931668	0.172273867703966	-2,95764E+14	1,1822E+14	1,17677E+14	1,08188E+13	8,99238E+14	1,02333E+14	9,46885E+14
TC0700001516.mm.2	Olfr641	olfactory receptor 641	-1,39939E+14	5,00265E+14	-3,03361E+13	0.0210954390522962	0.172273867703966	-2,95891E+14	4,14815E+14	4,04099E+14	4,93415E+14	6,05377E+13	5,09927E+14	5,73958E+14
TC1800000327.mm.2	Cystm1	cysteine-rich transmembrane module containing 1	-0.903456878585748	7,49035E+14	-3,03339E+14	0.0211014606546807	0.172273867703966	-2,95916E+14	7,06162E+14	6,84739E+14	7,82111E+14	7,89481E+14	7,74192E+14	7,57525E+14
TC0700000571.mm.2	2200002J24Rik	RIKEN cDNA 2200002J24 gene	-1,50805E+14	5,76555E+14	-3,03331E+14	0.0211037466111567	0.172273867703966	-2,95926E+14	4,80177E+14	4,90918E+14	5,97033E+14	6,75674E+11	5,46787E+14	6,68738E+13
TC1000000537.mm.2	Micu1	mitochondrial calcium uptake 1	2,23203E+14	6,85721E+13	3,0327E+14	0.0211210345180289	0.172273867703966	-2,95998E+14	8,34812E+14	8,31537E+14	6,8269E+14	5,37253E+14	7,1134E+14	5,16695E+14
TC0500002142.mm.2	Mnx1	motor neuron and pancreas homeobox 1	-1,34508E+14	5,98352E+13	-3,03263E+12	0.0211229633168796	0.172273867703966	-2,96007E+14	5,11275E+12	5,12366E+14	5,98965E+14	6,93693E+14	5,99128E+14	6,74686E+14
TC0700000178.mm.2	Obox7	oocyte specific homeobox 7	-1,7867E+14	5,25534E+14	-3,03244E+14	0.0211284675370194	0.172273867703966	-2,9603E+14	3,96343E+14	4,40427E+14	5,10874E+14	6,83236E+14	5,85549E+14	5,36777E+14
TC0100001889.mm.2	Xkr4	X-linked Kx blood group related 4	2,06888E+14	6,40081E+14	3,03238E+14	0.0211302794651044	0.172273867703966	-2,96037E+14	7,76906E+14	8,12935E+14	6,21158E+14	5,54907E+14	6,41647E+14	4,32932E+14
TC0200002269.mm.2	Rspo4	R-spondin 4	-1,03091E+14	6,30352E+14	-3,03237E+13	0.0211304293468473	0.172273867703966	-2,96038E+13	5,64479E+14	5,74121E+13	6,40241E+14	7,0454E+14	6,51577E+14	6,47155E+14
TC1200001025.mm.2	Fam181a	family with sequence similarity 181, member A	-1,2269E+14	6,49656E+14	-3,03198E+14	0.0211414859357541	0.172273867703966	-2,96084E+14	5,92647E+14	5,70418E+14	6,70655E+14	7,3779E+14	6,26333E+14	7,00094E+14
TC0700002156.mm.2	Rdh13	retinol dehydrogenase 13 (all-trans and 9-cis)	-0.98653262906532	8,01035E+14	-3,03167E+14	0.0211504128363951	0.172273867703966	-2,96122E+14	7,34073E+14	7,48237E+14	8,15353E+13	8,64264E+14	8,1204E+13	8,32246E+14
TC0100003468.mm.2	Pogk	pogo transposable element with KRAB domain	0.901962400878348	6,69045E+14	3,03119E+13	0.0211638096426511	0.172273867703966	-2,96178E+14	7,3102E+14	7,32562E+14	6,32496E+14	6,50694E+14	6,23304E+14	6,44192E+14
TC0200005006.mm.2	Ndrg3	N-myc downstream regulated gene 3	1,59657E+14	9,08937E+14	3,03025E+13	0.0211906893597474	0.172273867703966	-2,9629E+14	1,03124E+14	1,00724E+14	9,28979E+14	7,90185E+14	8,84202E+14	8,11775E+14
TC1000002448.mm.2	Agpat3	1-acylglycerol-3-phosphate O-acyltransferase 3	1,26338E+14	9,21583E+14	3,02999E+13	0.0211979263259453	0.172273867703966	-2,9632E+14	1,00599E+13	9,98831E+14	9,26792E+14	8,25347E+14	9,04979E+14	8,67562E+14
TC0700000221.mm.2	Slc8a2	solute carrier family 8 (sodium/calcium exchanger), member 2	2,1965E+14	7,51597E+13	3,02937E+13	0.0212156693427706	0.172273867703966	-2,96394E+14	8,74533E+14	8,94521E+14	7,83716E+13	5,46038E+14	7,00206E+14	7,10565E+14
TC0X00003260.mm.2	Cnksr2	connector enhancer of kinase suppressor of Ras 2	2,11035E+14	9,51261E+13	3,02895E+14	0.0212275439101459	0.172273867703966	-2,96444E+14	1,07609E+14	1,09286E+14	9,74426E+14	7,72451E+14	9,47686E+14	8,44056E+14
TC1300000667.mm.2	Tmed9	transmembrane p24 trafficking protein 9	1,3602E+14	7,08489E+14	3,02848E+14	0.0212410084653405	0.172273867703966	-2,965E+14	7,97477E+13	7,86967E+13	7,12774E+14	5,9963E+14	7,01612E+14	6,52473E+13
TC1100000629.mm.2	Rapgef6	Rap guanine nucleotide exchange factor (GEF) 6	2,89146E+14	5,16248E+14	3,02798E+14	0.021255307336442	0.172273867703966	-2,9656E+14	7,19812E+14	7,32676E+14	5,22871E+14	3,51326E+14	5,24205E+14	2,46597E+14

TC0600003324.mm.2	E330021D16Rik	RIKEN cDNA E330021D16 gene	-1,37817E+14	5,65148E+14	-3,02769E+14	0.0212634996910139	0.172273867703966	-2,96594E+14	4,7588E+14	4,94594E+14	5,70224E+13	6,75884E+14	5,53551E+14	6,20754E+14
TC0700004189.mm.2	Eri2	exoribonuclease 2	1,45623E+14	5,92853E+14	3,02766E+14	0.0212644859261351	0.172273867703966	-2,96598E+14	6,63616E+12	7,05871E+14	5,89284E+14	4,88959E+13	5,9737E+14	5,1202E+14
TC0400001626.mm.2	Crybg2	crystallin beta-gamma domain containing 2	-1,24127E+14	7,3038E+14	-3,02755E+14	0.0212675404882502	0.172273867703966	-2,96611E+14	6,35814E+14	6,51613E+14	7,39263E+14	7,96417E+14	7,36328E+14	8,22844E+14
TC0100003449.mm.2	Mpzl1	myelin protein zero-like 1	-0.934575563831262	7,17964E+14	-3,0274E+14	0.0212719373758614	0.172273867703966	-2,96629E+14	6,74559E+14	6,665E+14	7,82768E+13	7,45207E+14	7,2576E+14	7,12988E+14
TC0400000811.mm.2	Lurap1l	leucine rich adaptor protein 1-like	1,08283E+13	5,77514E+14	3,02699E+14	0.0212834776015283	0.172273867703966	-2,96677E+14	6,56548E+14	6,27312E+14	5,59107E+14	5,08189E+14	5,81422E+14	5,32505E+13
TC0900000430.mm.2	Olfr148	olfactory receptor 148	-1,26655E+13	4,71478E+14	-3,02639E+14	0.0213006763721395	0.172273867703966	-2,96748E+14	3,79076E+14	3,87267E+14	4,83362E+14	5,36292E+14	4,73898E+14	5,68974E+14
TC0400002022.mm.2	Rpl22	ribosomal protein L22	1,06522E+14	8,70583E+14	3,02608E+14	0.0213096565301447	0.172273867703966	-2,96786E+14	9,39091E+14	9,45903E+12	8,34693E+14	8,37255E+14	8,69563E+14	7,96994E+14
TC0200003772.mm.2	Lnpk	lunapark, ER junction formation factor	2,25167E+14	5,56737E+14	3,02537E+14	0.0213299048446647	0.172273867703966	-2,9687E+14	7,01063E+13	7,1166E+14	5,88122E+14	3,74266E+14	5,43437E+14	4,21871E+14
TC1500000705.mm.2	Tnrc6b/Mir5113	trinucleotide repeat containing 6b/microRNA 5113	1,42804E+12	6,4344E+14	3,02498E+14	0.0213410982198556	0.172273867703966	-2,96916E+14	7,57189E+14	7,51541E+14	6,62144E+14	5,60978E+13	6,08494E+14	5,20295E+14
TC0200001023.mm.2	Gad1	glutamate decarboxylase 1	1,07634E+14	8,85762E+13	3,0247E+14	0.0213489355462086	0.172273867703966	-2,96949E+14	9,72573E+14	9,47651E+14	8,82463E+13	8,22492E+14	8,25653E+14	8,63742E+14
TC1600000425.mm.2	Ncbp2	nuclear cap binding protein subunit 2	1,19187E+14	6,33392E+14	3,0247E+14	0.0213491655249938	0.172273867703966	-2,9695E+14	7,15235E+14	7,20001E+13	6,07261E+14	5,89601E+14	6,31607E+14	5,36646E+14
TC1400002598.mm.2	Mzt1	mitotic spindle organizing protein 1	1,00525E+14	8,23047E+14	3,02466E+14	0.0213503047070573	0.172273867703966	-2,96954E+14	8,77133E+14	8,85409E+14	8,10178E+14	7,51313E+14	8,10834E+14	8,03417E+13
TC1500002071.mm.2	Kif21a	kinesin family member 21A	1,49435E+14	7,166E+14	3,02452E+14	0.0213541185057569	0.172273867703966	-2,9697E+14	8,28042E+14	8,23373E+14	7,30234E+14	6,22312E+14	6,95542E+14	6,00099E+14
TC0600000974.mm.2	Tprkb	Tp53rk binding protein	1,65865E+14	6,99591E+14	3,02356E+14	0.0213818606516602	0.172273867703966	-2,97085E+14	8,04566E+13	7,92083E+14	6,93344E+14	5,71575E+14	7,25223E+14	6,10754E+14
TC0800002313.mm.2	Msmo1	methylsterol monoxygenase 1	2,12024E+14	6,657E+14	3,02275E+14	0.0214050643921019	0.172273867703966	-2,97181E+14	7,88278E+14	7,76918E+14	6,78393E+14	4,62755E+14	6,82758E+14	6,05097E+14
TC0300000750.mm.2	Gba	glucosidase, beta, acid	1,30537E+14	6,82957E+14	3,02259E+14	0.0214095898426529	0.172273867703966	-2,972E+14	7,40734E+14	7,55093E+14	6,60854E+14	5,73898E+14	7,21008E+14	6,46157E+14
TC0200001913.mm.2	Dusp2	dual specificity phosphatase 2	-1,26742E+14	6,14652E+14	-3,02239E+14	0.0214154064466545	0.172273867703966	-2,97224E+14	5,19142E+14	5,50726E+14	6,13178E+14	7,10175E+14	6,27392E+14	6,67302E+14
TC0300000733.mm.2	Khdc4	KH domain containing 4, pre-mRNA splicing factor	-1,06008E+13	8,70709E+14	-3,0223E+14	0.0214179243844908	0.172273867703966	-2,97235E+14	8,07068E+14	8,02153E+14	8,75056E+14	9,4618E+14	8,91368E+14	9,02427E+14
TC0700000279.mm.2	Nanos2	nanos C2HC-type zinc finger 2	-1,09273E+14	5,20587E+14	-3,02198E+14	0.0214273253093934	0.172273867703966	-2,97273E+14	4,29248E+14	4,72887E+14	5,29536E+14	5,91145E+14	5,39188E+14	5,61517E+14
TC1100000621.mm.2	Acsl6	acyl-CoA synthetase long-chain family member 6	1,03421E+14	7,95552E+14	3,02134E+14	0.021445658626437	0.172273867703966	-2,97349E+14	8,92669E+14	8,38072E+14	7,74968E+14	7,48931E+14	7,73416E+14	7,45254E+14
TC0700004506.mm.2	Zfp941	zinc finger protein 941	1,07224E+14	5,47859E+14	3,0211E+13	0.0214526061560398	0.172273867703966	-2,97378E+14	6,46627E+14	5,8835E+14	5,32541E+14	4,87989E+14	5,08716E+14	5,22929E+14
TC1100000880.mm.2	Myh10	myosin, heavy polypeptide 10, non-muscle	2,85754E+14	6,99712E+14	3,02088E+14	0.0214587653455527	0.172273867703966	-2,97403E+14	8,95734E+14	8,90315E+14	7,01579E+14	5,12963E+14	7,30043E+14	4,67635E+14
TC0600001967.mm.2	Pax4	paired box 4	-1,09372E+14	6,08735E+14	-3,02049E+14	0.0214701250991464	0.172273867703966	-2,9745E+14	5,39534E+14	5,23391E+14	6,03398E+14	6,78272E+14	6,44175E+14	6,63639E+13
TC0800000936.mm.2	Adgrl1	adhesion G protein-coupled receptor L1	1,91602E+14	7,78348E+14	3,02017E+14	0.0214792176700736	0.172273867703966	-2,97488E+14	9,30499E+14	9,46442E+14	8,26075E+14	6,67662E+14	7,12547E+14	5,86862E+14
TC1400001544.mm.2	Usp54	ubiquitin specific peptidase 54	1,11744E+14	6,26927E+14	3,02006E+14	0.0214823990765725	0.172273867703966	-2,97501E+14	6,90602E+14	7,01452E+14	5,97351E+14	5,71215E+14	6,39808E+14	5,61137E+14
TC0400003549.mm.2	Ncdn	neurochondrin	2,12671E+14	9,32146E+14	3,01993E+14	0.0214861389817298	0.172273867703966	-2,97516E+14	1,07613E+14	1,08759E+14	9,8107E+14	7,573E+14	8,72608E+13	8,18188E+14
TC0X00000695.mm.2	Ssr4	signal sequence receptor, delta	1,20405E+14	6,8555E+14	3,0198E+14	0.0214901305830678	0.172273867703966	-2,97533E+14	7,6926E+14	7,30303E+14	6,59493E+13	5,9926E+14	6,43277E+14	7,11709E+14
TC1700000644.mm.2	Tnxb	tenascin XB	-1,10056E+14	5,92626E+14	-3,01916E+14	0.0215084590150569	0.172297854384793	-2,97608E+14	5,25171E+14	5,08508E+14	5,9138E+14	6,62412E+14	6,17194E+13	6,51091E+14
TC0300000132.mm.2	Trim55	tripartite motif-containing 55	-1,37979E+14	5,98073E+14	-3,0189E+14	0.0215159943958233	0.172297854384793	-2,97639E+13	5,28878E+14	5,07468E+14	5,93376E+14	7,18928E+14	6,22496E+14	6,17289E+13
TC1700001611.mm.2	Prss30	protease, serine 30	-1,63514E+14	5,67922E+13	-3,01813E+14	0.0215384109399514	0.172297854384793	-2,97731E+14	4,79623E+14	4,49782E+14	5,66278E+14	6,90155E+14	5,58653E+13	6,6304E+14
TC0600001319.mm.2	Zfp637	zinc finger protein 637	0.960609892166055	7,19198E+14	3,01802E+14	0.0215414049522825	0.172297854384793	-2,97744E+14	7,90423E+14	7,67362E+14	6,66849E+14	6,98814E+14	6,81221E+13	7,10517E+14
TC0400000861.mm.2	Adamtsl1	ADAMTS-like 1	-1,13828E+14	7,18115E+14	-3,01784E+14	0.0215465882570197	0.172297854384793	-2,97765E+13	6,33789E+14	6,50388E+14	7,14994E+14	7,96838E+14	7,43059E+14	7,69624E+14
TC1200001364.mm.2	Fam228b	family with sequence similarity 228, member B	1,16778E+14	6,35821E+14	3,01724E+14	0.0215641332842303	0.172352618853573	-2,97837E+14	7,17388E+14	6,92648E+14	6,31258E+14	5,45222E+13	6,01616E+14	6,26792E+13
TC1400002868.mm.2	Lats2	large tumor suppressor 2	2,46491E+14	5,64035E+14	3,01634E+14	0.0215901683177357	0.172394375889897	-2,97944E+14	7,34686E+14	7,44105E+14	5,84225E+14	4,01583E+14	5,62059E+14	3,57552E+14
TC1500001798.mm.2	Mb	myoglobin	-0.894996702391435	6,77043E+14	-3,01521E+14	0.0216229988427095	0.172394375889897	-2,98079E+14	6,10527E+14	6,32207E+13	7,07419E+14	7,14314E+13	6,91889E+14	7,05899E+14
TC1100003814.mm.2	Etv4	ets variant 4	-1,23292E+14	7,3351E+14	-3,01407E+14	0.0216562072329809	0.172394375889897	-2,98215E+13	6,4852E+14	6,63972E+14	7,3789E+13	8,21186E+14	7,34229E+14	7,95266E+14
TC0700003035.mm.2	Sergef	secretion regulating guanine nucleotide exchange factor	1,15598E+14	6,78713E+12	3,014E+14	0.0216581196277229	0.172394375889897	-2,98222E+13	7,66121E+14	7,25956E+14	6,67116E+14	5,93764E+14	6,77743E+14	6,41581E+14
TC0X00001684.mm.2	Trappc2	trafficking protein particle complex 2	1,89945E+14	7,07645E+14	3,0136E+14	0.0216697507104176	0.172394375889897	-2,9827E+14	8,26817E+14	8,25157E+14	7,1854E+14	5,53544E+14	7,18459E+14	6,03352E+14
TC1900001730.mm.2	Vax1	ventral anterior homeobox 1	-1,01325E+14	8,18948E+14	-3,01272E+14	0.0216954557654555	0.172394375889897	-2,98375E+14	7,36293E+14	7,61721E+14	8,26929E+13	8,73735E+14	8,40977E+14	8,74034E+14
TC0400001436.mm.2	Snip1	Smad nuclear interacting protein 1	-1,01322E+13	7,20617E+14	-3,01269E+14	0.0216962534793019	0.172394375889897	-2,98378E+14	6,40909E+13	6,64899E+14	7,408E+14	7,67651E+14	7,29065E+14	7,80377E+14
TC1800000969.mm.2	Rock1	Rho-associated coiled-coil containing protein kinase 1	2,45791E+14	6,4769E+14	3,01182E+14	0.0217216577537504	0.172394375889897	-2,98482E+14	7,91164E+14	8,16435E+14	6,75067E+14	4,40948E+14	6,51244E+14	5,11284E+14
TC0300001802.mm.2	Gnb4	guanine nucleotide binding protein (G protein), beta 4	1,03316E+14	6,16537E+14	3,0117E+14	0.0217252340204974	0.172394375889897	-2,98496E+14	6,96081E+14	6,92127E+14	6,22852E+14	5,58724E+14	5,54849E+14	5,74589E+14
TC0700002988.mm.2	Rcn3	reticulocalbin 3, EF-hand calcium binding domain	-0.970162372150658	6,51342E+14	-3,01163E+14	0.0217272763452551	0.172394375889897	-2,98505E+14	5,70724E+14	5,97744E+14	6,58914E+14	7,03586E+14	6,87661E+14	6,89421E+14
TC0300002724.mm.2	Gstm2	glutathione S-transferase, mu 2	1,12649E+14	5,55291E+14	3,0111E+14	0.0217427661377953	0.172394375889897	-2,98568E+14	6,38288E+14	6,29456E+14	5,59967E+14	4,82479E+14	4,92746E+14	5,28809E+14
TC1100003100.mm.2	Vmo1	vitelline membrane outer layer 1 homolog (chicken)	-1,28242E+14	5,62644E+13	-3,01107E+14	0.0217436349209264	0.172394375889897	-2,98571E+14	4,80106E+14	4,54918E+14	5,48412E+14	6,43096E+14	5,96773E+14	6,5256E+14
TC0500000091.mm.2	Speer3	spermatogenesis associated glutamate (E)-rich protein 3	-1,16141E+14	4,68981E+14	-3,01048E+14	0.0217609575080028	0.172394375889897	-2,98642E+14	3,89645E+14	3,96266E+14	4,6434E+14	5,53852E+14	4,94078E+13	5,15706E+14
TC0200001746.mm.2	Phgr1	proline/histidine/glycine-rich 1	-1,12451E+13	6,02163E+14	-3,00955E+13	0.0217880378724266	0.172394375889897	-2,98752E+14	5,14545E+14	5,47567E+14	6,23956E+14	6,63057E+14	5,97579E+14	6,66273E+14
TC1200000788.mm.2	Rbm25	RNA binding motif protein 25	0.88961247644604	6,70588E+14	3,00954E+14	0.021788335369979	0.172394375889897	-2,98753E+14	7,47781E+14	7,55722E+14	6,50991E+14	6,74589E+14	5,97398E+13	5,97045E+14
TC0X00000760.mm.2	Cldn34b4	claudin 34B4	-1,05818E+14	4,84263E+14	-3,00909E+14	0.0218014738701248	0.172394375889897	-2,98807E+14	4,18495E+14	4,31681E+14	4,97059E+14	5,64754E+14	5,08312E+14	4,85276E+14
TC1500001069.mm.2	Smim41	small integral membrane protein 41	-0.93123350854157	6,60272E+14	-3,00898E+14	0.0218047344726843	0.172394375889897	-2,9882E+14	5,9804E+14	6,07623E+14	6,75899E+14	7,16011E+14	6,81987E+14	6,82073E+14
TC0200002863.mm.2	Prpf18	pre-mRNA processing factor 18	1,47024E+14	6,72476E+14	3,00719E+14	0.021857336462736	0.172394375889897	-2,99034E+14	7,62541E+14	7,54472E+14	6,72716E+14	5,50249E+14	6,83012E+14	6,11869E+14
TC0100001373.mm.2	Nmnat2	nicotinamide nucleotide adenylyltransferase 2	1,37847E+14	8,5702E+14	3,00701E+14	0.0218627248273443	0.172394375889897	-2,99055E+14	9,56355E+14	9,44568E+14	8,76391E+14	7,48838E+14	8,08572E+14	8,07399E+14
TC1400002091.mm.2	Psme2	proteasome (prosome, macropain) activator subunit 2 (PA28 beta)	1,16871E+14	6,51681E+14	3,00686E+14	0.0218669837640404	0.172394375889897	-2,99073E+14	7,33036E+13	7,12443E+14	6,48534E+14	5,63203E+14	6,43069E+14	6,09803E+14
TC1800000240.mm.2	Sap130	Sin3A associated protein	0.988710953303216	7,08484E+14	3,00677E+14	0.0218697160749335	0.172394375889897	-2,99084E+14	7,84375E+14	7,85638E+14	7,09558E+14	6,62712E+14	6,70839E+14	6,37782E+14
TC0800001265.mm.2	Cyb5b	cytochrome b5 type B	1,88852E+14	7,37052E+14	3,0066E+14	0.0218747337268205	0.172394375889897	-2,99104E+13	8,52152E+14	8,74869E+14	7,59314E+14	5,90004E+14	7,25292E+14	6,20681E+14
TC0900002053.mm.2	Spa17	sperm autoantigenic protein 17	1,04893E+14	5,82183E+14	3,00625E+14	0.0218851287136321	0.172394375889897	-2,99146E+14	6,32198E+14	6,533E+13	5,24437E+14	5,51275E+13	5,34824E+14	5,97068E+14
TC0400003100.mm.2	Cyp2j9	cytochrome P450, family 2, subfamily j, polypeptide 9	1,73217E+14	6,66268E+14	3,00614E+14	0.0218881720966138	0.172394375889897	-2,99158E+14	8,20469E+14	7,59823E+12	6,77784E+14	5,56075E+14	6,50023E+14	5,33433E+14
TC0700000805.mm.2	Aspdh	aspartate dehydrogenase domain containing	-1,18734E+14	5,99768E+14	-3,0059E+14	0.0218954309060994	0.172394375889897	-2,99188E+14	5,21225E+14	5,24498E+13	5,93828E+14	6,89363E+14	6,43676E+14	6,26016E+14
TC0700000446.mm.2	Cyp2a12	cytochrome P450, family 2, subfamily a, polypeptide 12	-0.990029894092741	5,13174E+14	-3,00574E+14	0.0219001653759086	0.172394375889897	-2,99207E+14	4,44508E+14	4,58976E+14	5,31826E+14	5,69664E+14	5,57617E+14	5,16452E+14
TC0700004296.mm.2	Aldoa	aldolase A, fructose-bisphosphate	1,49499E+14	1,09465E+14	3,00526E+14	0.0219141315986205	0.172394375889897	-2,99263E+14	1,18345E+14	1,19025E+14	1,10628E+14	9,68421E+14	1,08298E+14	1,03653E+14
TC0800000288.mm.2	Kat6a	K(lysine) acetyltransferase 6A	0.97320837617928	5,99696E+14	3,00506E+13	0.0219200074245702	0.172394375889897	-2,99287E+14	6,53573E+14	6,82777E+14	5,84367E+14	5,57342E+14	5,78005E+14	5,42113E+14
TC1700001674.mm.2	Metrn	meteorin, glial cell differentiation regulator	0.865475851175052	7,25756E+14	3,005E+14	0.0219218313339195	0.172394375889897	-2,99295E+14	7,81647E+14	7,7862E+14	6,90366E+13	6,96806E+13	7,03395E+14	7,03699E+14
TC0700004626.mm.2	Sez6l2	seizure related 6 homolog like 2	2,24411E+14	8,33759E+14	3,00488E+14	0.0219253404617022	0.172394375889897	-2,99309E+14	9,80999E+14	9,74951E+14	8,65739E+14	6,4139E+14	8,21569E+14	7,17904E+14
TC0200001847.mm.2	Duox1	dual oxidase 1	-0.88564563437876	5,01783E+14	-3,00423E+14	0.0219446426262312	0.172394375889897	-2,99387E+14	4,55455E+13	4,38083E+14	5,43273E+14	5,27394E+14	5,24425E+14	5,22068E+14
TC1100003838.mm.2	Fam171a2	family with sequence similarity 171, member A2	1,0324E+13	8,61627E+14	3,00392E+14	0.0219535978659166	0.172394375889897	-2,99423E+14	9,12783E+14	9,13647E+14	8,33793E+14	7,86157E+14	8,37693E+14	8,8569E+14
TC0800000312.mm.2	Tm2d2	TM2 domain containing 2	1,39411E+14	7,76728E+14	3,00327E+12	0.0219729231106195	0.172394375889897	-2,99501E+14	8,73567E+13	8,65145E+14	7,75901E+14	6,83989E+14	7,77188E+14	6,84576E+14
TC1100003323.mm.2	AU040972	expressed sequence AU040972	-0.990227189806061	5,08172E+14	-3,00291E+14	0.0219834379400072	0.172394375889897	-2,99543E+14	4,50859E+14	4,49342E+14	5,21409E+13	5,76837E+14	5,33847E+14	5,16735E+14
TC1800000450.mm.2	Spink6	serine peptidase inhibitor, Kazal type 6	-1,31181E+14	4,73902E+14	-3,00267E+14	0.0219906793951298	0.172394375889897	-2,99572E+14	3,7911E+14	4,02563E+14	4,70105E+14	5,73929E+14	4,83356E+14	5,34352E+14
TC1700002541.mm.2	Fam98a	family with sequence similarity 98, member A	0.988217421493693	6,29919E+14	3,00234E+14	0.0220004756209076	0.172394375889897	-2,99612E+14	6,73286E+14	7,06491E+12	6,13551E+14	5,68582E+14	6,15717E+14	6,0189E+14
TC1500002124.mm.2	Dbx2	developing brain homeobox 2	-1,02575E+14	6,49507E+14	-3,00211E+14	0.0220072500183599	0.172394375889897	-2,99639E+14	5,996E+14	5,82217E+14	6,63113E+14	7,23853E+14	6,54066E+14	6,74195E+14
TC1100002814.mm.2	Zfp672	zinc finger protein 672	-1,20547E+14	6,47697E+14	-3,00194E+14	0.0220122022154285	0.172394375889897	-2,99659E+14	5,7226E+13	5,52461E+14	6,34018E+14	7,31797E+14	7,04319E+14	6,91327E+14
TC1100004215.mm.2	P4hb	prolyl 4-hydroxylase, beta polypeptide	1,48497E+14	7,64213E+14	3,00194E+14	0.0220122638856107	0.172394375889897	-2,99659E+14	8,46898E+14	8,21639E+14	7,53588E+14	6,17954E+14	7,93925E+14	7,51272E+14
TC0100001158.mm.2	Yod1	YOD1 deubiquitinase	-1,17426E+13	6,66227E+14	-3,00177E+13	0.0220172496865216	0.172394375889897	-2,99679E+14	5,79991E+14	6,14465E+14	7,13238E+14	7,1607E+14	7,31147E+14	6,4245E+14
TC1400000510.mm.2	Socs4	suppressor of cytokine signaling 4	1,20517E+13	5,22072E+14	3,00125E+14	0.0220327462228712	0.172394375889897	-2,99742E+14	5,97427E+14	6,01131E+14	5,21069E+14	4,36455E+13	5,15319E+14	4,6103E+14
TC1000003119.mm.2	Lrp1	low density lipoprotein receptor-related protein 1	1,0922E+14	7,94878E+14	3,00102E+14	0.0220395636477613	0.172394375889897	-2,99769E+14	8,62047E+14	8,83938E+13	7,34241E+14	7,93303E+14	7,7262E+14	7,23121E+14
TC1600000250.mm.2	Ufd1	ubiquitin recognition factor in ER-associated degradation 1	1,00366E+14	7,36186E+14	3,00097E+13	0.022041095111755	0.172394375889897	-2,99775E+13	8,27432E+14	7,94287E+14	7,03816E+14	7,17172E+14	6,6225E+14	7,12159E+13
TC0600001071.mm.2	Uroc1	urocanase domain containing 1	-0.993281967644098	6,37145E+14	-3,00012E+14	0.0220662554073702	0.172394375889897	-2,99876E+14	5,6039E+14	5,73471E+14	6,46908E+14	6,8561E+14	6,56966E+14	6,99523E+14
TC1900001749.mm.2	Eif3a	eukaryotic translation initiation factor 3, subunit A	-1,117E+14	7,81497E+14	-3,0001E+14	0.0220668740391368	0.172394375889897	-2,99879E+14	7,07403E+14	7,10261E+13	7,78447E+14	8,62618E+14	8,09695E+14	8,20556E+14
TC0900000113.mm.2	Gpr83	G protein-coupled receptor 83	1,82731E+14	7,14391E+14	2,99976E+13	0.0220768294224583	0.172394375889897	-2,99919E+13	8,56631E+14	8,32103E+14	7,54368E+14	5,68904E+14	6,60126E+14	6,14214E+14
TC0600000273.mm.2	Cpa1	carboxypeptidase A1, pancreatic	-1,38516E+14	6,57098E+14	-2,99954E+14	0.022083511392351	0.172394375889897	-2,99946E+14	5,47762E+14	5,78584E+14	6,46517E+13	7,56861E+14	6,76087E+14	7,36775E+14
TC0X00002406.mm.2	Magea10	melanoma antigen family A, 10	-1,24653E+14	4,86469E+14	-2,99927E+14	0.0220913984261358	0.172394375889897	-2,99977E+14	4,03991E+14	3,95744E+14	4,79298E+14	5,69744E+14	5,06081E+14	5,63955E+14
TC0100002816.mm.2	Cops9	COP9 signalosome subunit 9	1,59346E+13	9,47876E+13	2,99907E+14	0.0220973897617445	0.172394375889897	-3,00001E+14	1,05675E+14	1,041E+14	9,50848E+14	8,28214E+14	9,56216E+14	8,54224E+14
TC1500001657.mm.2	Ndrg1	N-myc downstream regulated gene 1	0.880057995746135	7,38036E+14	2,99884E+14	0.0221043120826989	0.172394375889897	-3,00029E+14	7,99995E+14	8,07107E+12	7,07303E+14	7,23788E+14	6,89174E+14	7,00847E+14
TC1400000600.mm.2	Tmem253	transmembrane protein 253	-0.929476792052579	5,98816E+12	-2,99811E+13	0.0221261071179853	0.172427925272798	-3,00116E+14	5,64662E+14	5,3053E+14	6,29029E+14	6,52058E+14	6,1178E+14	6,04835E+14
TC1300001737.mm.2	Exoc2	exocyst complex component 2	1,32352E+14	7,54129E+14	2,99744E+14	0.0221460531650466	0.172427925272798	-3,00196E+14	8,59362E+14	8,30735E+14	7,15876E+14	7,09517E+14	7,62946E+14	6,46338E+14
TC0200004140.mm.2	Cry2	cryptochrome 2 (photolyase-like)	1,24337E+14	8,59167E+14	2,99716E+13	0.0221544235257575	0.172427925272798	-3,0023E+14	9,45236E+14	9,32267E+14	8,58056E+13	7,70774E+14	8,5487E+14	7,93796E+14
TC0400000103.mm.2	Rbm12b2	RNA binding motif protein 12 B2	1,39338E+14	5,92461E+14	2,9969E+14	0.0221622554301287	0.172427925272798	-3,00261E+14	7,0396E+14	7,04509E+14	6,27434E+14	5,0236E+14	5,27878E+14	4,88628E+14
TC1600001070.mm.2	Kcnj15	potassium inwardly-rectifying channel, subfamily J, member 15	-1,30619E+14	4,56624E+14	-2,99661E+14	0.022170759929177	0.172427925272798	-3,00295E+14	3,79055E+14	3,77111E+14	4,66821E+14	5,50582E+14	4,4186E+13	5,24316E+14
TC0400002013.mm.2	Tnfrsf25	tumor necrosis factor receptor superfamily, member 25	-0.944438566671459	7,05046E+14	-2,99621E+13	0.0221826700232305	0.172427925272798	-3,00343E+14	6,23745E+13	6,38436E+14	7,03515E+14	7,47553E+14	7,57321E+14	7,59708E+14
TC0900002385.mm.2	Ccdc33	coiled-coil domain containing 33	-0.9988377805291	6,03908E+14	-2,99583E+14	0.0221941997689927	0.172427925272798	-3,00389E+14	5,21214E+14	5,49389E+14	6,12264E+14	6,58106E+13	6,27239E+14	6,55235E+14
TC0600002904.mm.2	Srgap3	SLIT-ROBO Rho GTPase activating protein 3	1,98759E+14	7,97289E+14	2,99555E+14	0.0222024857074987	0.172427925272798	-3,00422E+14	9,21077E+14	9,37589E+14	7,90263E+14	6,70885E+14	8,20674E+13	6,43246E+14
TC1200001908.mm.2	Dhrs7	dehydrogenase/reductase (SDR family) member 7	1,60046E+14	5,40193E+14	2,99506E+14	0.0222169668176832	0.172427925272798	-3,0048E+14	6,431E+14	6,19366E+14	5,47707E+14	3,94668E+14	5,41197E+14	4,9512E+14
TC0700002113.mm.2	Fgf4	fibroblast growth factor 4	-1,17124E+14	6,35125E+14	-2,99424E+14	0.0222415094667332	0.172427925272798	-3,00577E+14	5,54507E+14	5,6709E+14	6,50014E+14	7,05831E+14	6,28618E+14	7,04693E+14
TC0300000043.mm.2	Stmn2	stathmin-like 2	1,26745E+14	1,04078E+14	2,99381E+14	0.0222545496308345	0.172427925272798	-3,00629E+14	1,13589E+13	1,11782E+13	1,03834E+14	9,61877E+14	1,03493E+14	9,55809E+14
TC1000001193.mm.2	Ccer1	coiled-coil glutamate-rich protein 1	-1,35388E+14	5,67887E+14	-2,9936E+14	0.0222609113427355	0.172427925272798	-3,00655E+14	5,03388E+14	4,53065E+14	5,60742E+13	6,66487E+13	5,89607E+14	6,34035E+14
TC0200004458.mm.2	Vps39	VPS39 HOPS complex subunit	1,11436E+13	7,70722E+14	2,99314E+13	0.0222745822689447	0.172427925272798	-3,00709E+14	8,41689E+14	8,41973E+14	7,72679E+14	6,88112E+14	7,36146E+14	7,43733E+13
TC0700003539.mm.2	Ntrk3	neurotrophic tyrosine kinase, receptor, type 3	1,12388E+13	8,76744E+14	2,99281E+14	0.0222846217705329	0.172427925272798	-3,00749E+14	9,62978E+14	9,67528E+14	8,64953E+13	8,40777E+14	8,5369E+14	7,70539E+14
TC1100004192.mm.2	Aatk	apoptosis-associated tyrosine kinase	1,219E+14	7,39159E+14	2,99258E+14	0.0222915542097146	0.172427925272798	-3,00777E+14	8,03044E+14	8,17052E+13	7,3935E+14	6,36945E+13	7,1613E+14	7,22435E+14
TC1600001993.mm.2	Grik1	glutamate receptor, ionotropic, kainate 1	1,06452E+14	5,52173E+14	2,99237E+14	0.0222978216302877	0.172427925272798	-3,00802E+14	6,59342E+14	5,96308E+14	5,38603E+14	5,04143E+14	4,96164E+14	5,18479E+14
TC0500002760.mm.2	Scarb2	scavenger receptor class B, member 2	1,41111E+14	7,89315E+14	2,99222E+14	0.0223021036352689	0.172427925272798	-3,00819E+13	8,73742E+14	8,79494E+14	8,02364E+14	6,68648E+14	7,60093E+14	7,51552E+14
TC0700001804.mm.2	Tufm	Tu translation elongation factor, mitochondrial	0.98216759362209	6,60027E+14	2,99196E+14	0.0223100112607142	0.172427925272798	-3,0085E+14	7,05146E+14	7,20783E+14	6,34483E+14	5,95013E+14	6,33401E+12	6,71337E+14
TC1700001607.mm.2	Prss33	protease, serine 33	-1,16923E+14	6,91478E+14	-2,99155E+14	0.0223223662844355	0.172427925272798	-3,00899E+14	6,22958E+14	6,01152E+14	6,86024E+14	7,71931E+14	7,16273E+13	7,50527E+14
TC1100001451.mm.2	Ppp1r9b	protein phosphatase 1, regulatory subunit 9B	1,10012E+14	9,13243E+14	2,99154E+14	0.0223226371326915	0.172427925272798	-3,009E+14	9,82045E+14	9,95479E+14	9,13505E+14	8,43995E+14	8,93211E+14	8,51222E+14
TC1700001221.mm.2	Tmem178	transmembrane protein 178	1,46573E+14	7,39767E+14	2,98983E+14	0.022374196954216	0.172451417315249	-3,01105E+14	7,95492E+14	8,21751E+13	7,26497E+14	5,97601E+13	7,75964E+14	7,21298E+14
TC1300002102.mm.2	Slc28a3	solute carrier family 28 (sodium-coupled nucleoside transporter), member 3	-0.910752255543755	6,22027E+14	-2,98982E+14	0.0223745183693556	0.172451417315249	-3,01106E+14	5,399E+14	5,72084E+14	6,49454E+12	6,4468E+14	6,54993E+14	6,7105E+14
TC0300000096.mm.2	Car2	carbonic anhydrase 2	1,61402E+14	9,81353E+14	2,98887E+14	0.0224032530347822	0.172451417315249	-3,0122E+14	1,05202E+14	1,10456E+14	9,79586E+14	8,54189E+14	9,94717E+14	9,03048E+14
TC0900002308.mm.2	Cul5	cullin 5	2,48817E+14	6,33073E+14	2,98884E+14	0.022403870469717	0.172451417315249	-3,01222E+14	8,10156E+14	8,06691E+14	6,98977E+13	4,20236E+14	5,45396E+14	5,16983E+14
TC0500003182.mm.2	Rph3a	rabphilin 3A	1,26815E+14	7,64921E+13	2,98852E+14	0.0224135855511978	0.172451417315249	-3,01261E+14	8,55516E+14	8,38845E+14	7,15991E+14	7,2474E+14	7,82412E+14	6,7202E+14
TC0700000410.mm.2	Cic	capicua transcriptional repressor	1,11862E+13	7,57913E+14	2,98845E+14	0.0224158155410996	0.172451417315249	-3,01269E+14	8,42046E+13	8,31023E+14	7,67303E+14	6,82043E+14	7,14231E+14	7,1083E+14
TC0X00003358.mm.2	Tmsb4x	thymosin, beta 4, X chromosome	1,15373E+14	1,15103E+13	2,98843E+14	0.0224164795438685	0.172451417315249	-3,01272E+14	1,21785E+14	1,23115E+14	1,13874E+14	1,07951E+13	1,15434E+14	1,08459E+14
TC0700003826.mm.2	Relt	RELT tumor necrosis factor receptor	0.892075077114673	5,92864E+14	2,9884E+14	0.0224171980611057	0.172451417315249	-3,01275E+14	6,655E+14	6,44396E+14	5,67808E+14	5,63673E+14	5,48152E+14	5,67654E+14
TC0600001384.mm.2	Mfap5	microfibrillar associated protein 5	-1,49458E+14	4,4954E+14	-2,98751E+14	0.0224441657578482	0.172451417315249	-3,01381E+13	3,69881E+12	3,151E+13	4,28055E+14	5,55842E+14	4,90513E+14	5,37847E+14
TC1400002764.mm.2	Fgf14	fibroblast growth factor 14	1,23694E+14	8,03718E+14	2,98631E+14	0.022480503814408	0.172451417315249	-3,01525E+14	8,94624E+14	8,78889E+14	7,97914E+14	7,2821E+14	8,01152E+14	7,21519E+14

TC1300002459.mm.2	S100z	S100 calcium binding protein, zeta	-1,03628E+14	5,13832E+14	-2,98607E+14	0.0224878368294298	0.172451417315249	-3,01554E+13	4,42612E+14	4,63201E+14	5,37802E+14	5,75267E+14	5,04469E+14	5,59642E+14
TC1100002416.mm.2	Cfap36	cilia and flagella associated protein 36	0.865251294366443	7,21032E+14	2,98574E+14	0.0224977880290177	0.172451417315249	-3,01593E+14	7,78673E+14	7,75881E+14	6,96004E+14	6,85499E+13	6,99101E+14	6,91036E+14
TC0100003128.mm.2	Ipo9	importin 9	0.892944926394247	7,99106E+12	2,98552E+14	0.0225043299151495	0.172451417315249	-3,01619E+14	8,54909E+14	8,4674E+14	7,75644E+14	7,47415E+14	7,87401E+14	7,82528E+14
TC0400000078.mm.2	2610301B20Rik	RIKEN cDNA 2610301B20 gene	1,69008E+14	6,36908E+14	2,98496E+14	0.0225213159093009	0.172451417315249	-3,01686E+14	7,5792E+14	7,41505E+14	6,58379E+14	5,03028E+14	6,19709E+14	5,40906E+13
TC1800001175.mm.2	Slc23a1	solute carrier family 23 (nucleobase transporters), member 1	-0.906374151082304	4,90277E+14	-2,98459E+14	0.0225325714565627	0.172451417315249	-3,0173E+14	4,05356E+13	4,33817E+14	5,04411E+14	5,16037E+14	5,49656E+13	5,32387E+14
TC1000002907.mm.2	Cnot2	CCR4-NOT transcription complex, subunit 2	1,62116E+14	8,72983E+14	2,98399E+13	0.0225509505107326	0.172451417315249	-3,01802E+14	9,74589E+14	9,78568E+14	8,97024E+12	7,31901E+14	8,31852E+14	8,23966E+14
TC0900003062.mm.2	Ndufaf3	NADH:ubiquinone oxidoreductase complex assembly factor 3	0.988548280078747	6,68805E+14	2,98366E+14	0.0225608272561719	0.172451417315249	-3,01841E+14	7,42934E+13	7,16441E+14	6,54914E+14	6,06752E+14	6,56836E+14	6,34952E+14
TC0Y00000240.mm.2	Sry	sex determining region of Chr Y	-1,06909E+14	6,59438E+14	-2,98342E+14	0.0225682556917337	0.172451417315249	-3,0187E+14	5,84352E+14	5,93033E+14	6,7116E+14	7,20042E+14	6,64728E+14	7,23316E+14
TC0200002719.mm.2	Gm14288/Gm14440	predicted gene 14288/predicted gene 14440	2,86662E+14	8,19106E+14	2,98277E+14	0.0225880473477237	0.172451417315249	-3,01948E+14	9,46257E+14	9,86155E+14	8,32725E+14	5,26362E+14	8,67971E+14	7,55165E+14
TC0200002737.mm.2	Gm14288/Gm14435	predicted gene 14288/KRAB box containing protein	2,86662E+14	8,19106E+14	2,98277E+14	0.0225880473477237	0.172451417315249	-3,01948E+14	9,46257E+14	9,86155E+14	8,32725E+14	5,26362E+14	8,67971E+14	7,55165E+14
TC1300001670.mm.2	Aldh5a1	aldhehyde dehydrogenase family 5, subfamily A1	1,21162E+13	7,29451E+14	2,98259E+14	0.0225934758200064	0.172451417315249	-3,01969E+14	8,4191E+14	8,03451E+14	7,48247E+14	6,54789E+14	6,54244E+14	6,74067E+14
TC1500000859.mm.2	Panx2	pannexin 2	-1,14405E+14	6,73968E+14	-2,98234E+13	0.0226010648454887	0.172451417315249	-3,01999E+14	6,51488E+13	5,60579E+14	7,1806E+14	7,22816E+14	6,97737E+14	6,9313E+14
TC1300002204.mm.2	Ctsl	cathepsin L	1,32081E+14	7,53356E+14	2,98229E+14	0.0226026364360969	0.172451417315249	-3,02005E+14	8,13812E+14	8,09263E+14	7,36386E+14	6,22525E+14	7,89225E+14	7,48922E+14
TC0700000834.mm.2	Pth2	parathyroid hormone 2	-1,40866E+14	7,71527E+14	-2,98204E+14	0.0226102052505916	0.172451417315249	-3,02035E+14	6,69566E+14	6,94395E+14	7,66161E+14	8,79532E+14	7,75796E+14	8,43708E+14
TC1700000292.mm.2	Zfp946	zinc finger protein 946	1,05934E+14	5,92654E+13	2,98189E+14	0.0226148461228662	0.172451417315249	-3,02053E+14	7,06569E+14	6,83447E+14	6,09863E+14	5,68285E+14	5,22823E+14	4,64935E+14
TC0700001927.mm.2	Bub3	BUB3 mitotic checkpoint protein	1,22452E+14	8,59224E+14	2,98173E+14	0.0226196605914627	0.172451417315249	-3,02072E+14	9,38214E+14	9,2146E+14	8,57673E+14	7,57096E+14	8,5346E+14	8,27442E+14
TC0200000591.mm.2	Sh2d3c	SH2 domain containing 3C	-1,13848E+14	7,92282E+14	-2,98127E+14	0.0226336456152396	0.172451417315249	-3,02127E+14	6,86817E+14	7,38047E+14	7,9371E+14	8,58849E+14	8,20314E+14	8,55954E+14
TC1100001969.mm.2	Tmc8	transmembrane channel-like gene family 8	-0.957569019080124	6,47541E+14	-2,98109E+14	0.022639309845226	0.172451417315249	-3,02149E+14	5,8126E+14	5,73617E+14	6,72906E+13	6,73485E+14	6,66526E+14	7,17453E+14
TC0300001576.mm.2	Zranb2	zinc finger, RAN-binding domain containing 2	1,30361E+14	6,95573E+14	2,97947E+14	0.0226889144014771	0.172451417315249	-3,02343E+13	7,72592E+14	7,85541E+14	6,86529E+14	6,10881E+14	7,03983E+14	6,13911E+14
TC0600001839.mm.2	Asns	asparagine synthetase	1,6451E+14	8,98179E+14	2,97939E+14	0.0226913850201805	0.172451417315249	-3,02353E+14	1,00526E+13	1,00162E+14	9,22952E+14	7,54917E+14	8,61449E+14	8,42869E+14
TC0300002842.mm.2	A730020M07Rik	RIKEN cDNA A730020M07 gene	-1,2551E+14	5,64558E+14	-2,97934E+14	0.0226926868136958	0.172451417315249	-3,02358E+14	4,94327E+14	4,90991E+14	5,63448E+14	6,72891E+13	5,90227E+14	5,75462E+14
TC1100002847.mm.2	Arf1	ADP-ribosylation factor 1	1,25331E+14	9,58884E+14	2,97918E+14	0.0226977281336376	0.172451417315249	-3,02377E+14	1,04218E+14	1,04792E+14	9,60531E+14	8,78903E+14	9,48792E+12	8,74981E+14
TC1100004208.mm.2	Arl16	ADP-ribosylation factor-like 16	0.992860881433358	7,39791E+14	2,97888E+14	0.0227069425527681	0.172451417315249	-3,02413E+14	8,29621E+14	8,08403E+14	7,30973E+14	7,08478E+14	7,06682E+13	6,54591E+14
TC1800000916.mm.2	Tmx3	thioredoxin-related transmembrane protein 3	1,6105E+13	7,25371E+14	2,97884E+14	0.0227079905099203	0.172451417315249	-3,02417E+14	8,46094E+14	8,29298E+14	7,55452E+13	5,9784E+14	6,8329E+14	6,40253E+14
TC0900001038.mm.2	Gclc	glutamate-cysteine ligase, catalytic subunit	0.87608800681942	7,36221E+14	2,97878E+14	0.0227097594771598	0.172451417315249	-3,02424E+14	8,02814E+14	8,13782E+14	7,26676E+14	7,14703E+14	6,87478E+14	6,71872E+14
TC0800002941.mm.2	Glg1	golgi apparatus protein 1	2,1377E+14	7,5294E+14	2,97805E+14	0.0227323087632837	0.172451417315249	-3,02512E+13	8,92174E+14	8,88071E+14	7,80835E+14	5,7187E+14	7,47047E+14	6,37645E+14
TC0700002956.mm.2	Acp4	acid phosphatase 4	-1,17428E+13	6,69729E+14	-2,97746E+14	0.0227504859385676	0.172451417315249	-3,02583E+12	6,14263E+14	6,02585E+14	6,78338E+14	7,73367E+14	6,67486E+13	6,82332E+14
TC0700000907.mm.2	Slc6a5	solute carrier family 6 (neurotransmitter transporter, glycine), member 5	-1,47378E+14	4,90356E+14	-2,97731E+14	0.0227550443674916	0.172451417315249	-3,02601E+14	3,80128E+14	4,00862E+14	4,66433E+14	6,09312E+14	5,39108E+14	5,46292E+13
TC1400001219.mm.2	Frg2f7	FSHD region gene 2 family member 7	-1,0748E+14	5,03286E+14	-2,97694E+14	0.0227663768715699	0.172451417315249	-3,02645E+14	4,27011E+14	4,37506E+14	5,19704E+14	5,59772E+14	5,04578E+14	5,71146E+14
TC0700002471.mm.2	Qpctl	glutaminyl-peptide cyclotransferase-like	1,54213E+13	6,50882E+13	2,97634E+14	0.0227846884452458	0.172451417315249	-3,02716E+14	7,58076E+14	7,19547E+14	6,59309E+14	5,09888E+12	6,34194E+14	6,24278E+14
TC0400001446.mm.2	Mir692-3/Ftl1/Mir692-2	microRNA 692-3/ferritin light polypeptide 1/microRNA 692-2	-1,39412E+14	1,10356E+14	-2,97601E+14	0.022794984094938	0.172451417315249	-3,02756E+14	9,89852E+13	1,03808E+14	1,20996E+14	1,0968E+14	1,16821E+14	1,11847E+14
TC0600003045.mm.2	Atp6v1e1	ATPase, H+ transporting, lysosomal V1 subunit E1	1,9889E+14	8,62893E+12	2,97587E+14	0.0227993814485981	0.172451417315249	-3,02774E+14	9,7759E+14	9,73509E+14	8,69637E+14	6,83681E+14	8,91778E+14	7,81162E+14
TC0100003796.mm.2	Vash2	vasohibin 2	-0.906363545284898	5,50903E+14	-2,97559E+14	0.022807876917457	0.172451417315249	-3,02807E+14	5,09484E+14	5,05264E+14	6,09835E+14	5,86186E+14	5,33743E+14	5,60907E+14
TC0100003129.mm.2	Nav1	neuron navigator 1	1,0573E+14	7,23422E+14	2,9754E+14	0.0228135965845545	0.172451417315249	-3,02829E+14	8,02347E+14	7,99E+14	7,3139E+14	6,58497E+14	6,66021E+14	6,83279E+14
TC1000001684.mm.2	Syne1	spectrin repeat containing, nuclear envelope 1	1,81216E+14	7,50102E+14	2,97536E+14	0.0228149873942592	0.172451417315249	-3,02834E+14	8,34358E+14	8,51613E+14	7,49243E+14	5,74296E+14	7,84881E+14	7,06223E+14
TC0300002803.mm.2	Gpr88	G-protein coupled receptor 88	1,48831E+14	9,38279E+14	2,97468E+14	0.0228358315684569	0.172451417315249	-3,02915E+14	1,03346E+14	1,03583E+14	9,53989E+14	8,17647E+14	9,21679E+13	8,67066E+14
TC1100000900.mm.2	Per1	period circadian clock 1	1,17146E+13	7,38108E+14	2,97467E+14	0.0228362204252401	0.172451417315249	-3,02917E+14	8,28191E+14	8,07372E+14	7,46417E+14	6,54854E+14	6,84599E+14	7,07213E+14
TC0700002134.mm.2	Leng1/Tmc4	leukocyte receptor cluster (LRC) member 1/transmembrane channel-like gene family 4	-0.963952430416789	6,23517E+14	-2,9739E+14	0.0228598360136203	0.172451417315249	-3,03008E+14	5,40405E+14	5,65665E+14	6,25689E+14	6,73172E+14	6,68817E+14	6,67356E+14
TC1800001584.mm.2	Mapk4	mitogen-activated protein kinase 4	1,98992E+14	6,33933E+14	2,97319E+14	0.0228818866189719	0.172451417315249	-3,03094E+13	7,54836E+14	7,63584E+14	6,61969E+14	4,58467E+12	6,1947E+14	5,45276E+14
TC1900000748.mm.2	Add3	adducin 3 (gamma)	2,3809E+14	7,90671E+14	2,97313E+14	0.0228835410388281	0.172451417315249	-3,031E+14	9,17892E+13	9,21345E+14	8,11405E+13	5,51651E+14	8,03944E+14	7,37786E+14
TC0700004265.mm.2	Xpo6	exportin 6	1,72377E+14	7,8081E+14	2,97216E+13	0.0229137320494725	0.172451417315249	-3,03217E+14	9,2777E+14	8,99572E+14	7,9736E+14	6,85227E+14	7,56884E+14	6,1805E+14
TC0900001372.mm.2	Zmynd10	zinc finger, MYND domain containing 10	1,02076E+14	5,3479E+14	2,97155E+14	0.0229325021539922	0.172451417315249	-3,0329E+14	6,09524E+14	5,93896E+14	4,73485E+14	5,25782E+14	4,83155E+14	5,22896E+14
TC1500002141.mm.2	Slc38a1	solute carrier family 38, member 1	1,79709E+14	8,65735E+14	2,97149E+14	0.0229342778258794	0.172451417315249	-3,03297E+14	9,98472E+12	9,76786E+14	8,96428E+14	7,19411E+14	8,35634E+14	7,67677E+14
TC0100003138.mm.2	Pkp1	plakophilin 1	-1,2276E+14	6,30394E+14	-2,97117E+14	0.0229442659601649	0.172451417315249	-3,03335E+14	5,89445E+14	5,49998E+14	6,43058E+14	7,41904E+14	6,27323E+14	6,30638E+14
TC0200002700.mm.2	Gnas	GNAS (guanine nucleotide binding protein, alpha stimulating) complex locus	1,40638E+14	1,0272E+14	2,97112E+14	0.02294592781455	0.172451417315249	-3,03342E+14	1,13059E+14	1,11339E+14	1,04244E+14	9,20256E+14	1,00553E+14	9,50987E+14
TC1800000977.mm.2	Esco1	establishment of sister chromatid cohesion N-acetyltransferase 1	1,95711E+14	5,15714E+14	2,97049E+13	0.0229654620249337	0.172451417315249	-3,03417E+14	6,455E+14	6,37449E+14	5,38048E+14	3,5348E+14	5,12537E+14	4,07271E+14
TC1000002257.mm.2	Hkdc1	hexokinase domain containing 1	-1,28666E+14	6,78466E+14	-2,96983E+14	0.0229858596352971	0.172451417315249	-3,03496E+14	5,72881E+14	6,21208E+13	6,86366E+14	7,65055E+14	6,77304E+14	7,47982E+14
TC0700000056.mm.2	Ccdc106	coiled-coil domain containing 106	1,24318E+14	6,24562E+14	2,96957E+14	0.0229939453927175	0.172451417315249	-3,03527E+14	7,06265E+14	6,9247E+13	6,24512E+14	5,25586E+13	6,21327E+14	5,77211E+14
TC1200000750.mm.2	Srsf5	serine/arginine-rich splicing factor 5	1,14922E+14	8,43115E+14	2,96954E+14	0.0229948868895039	0.172451417315249	-3,03531E+14	9,40538E+14	9,0068E+14	8,40143E+14	7,71229E+13	8,27066E+14	7,79033E+14
TC1700000314.mm.2	Bicdl2	BICD family like cargo adaptor 2	-1,0708E+14	6,0181E+14	-2,96946E+14	0.0229972588157093	0.172451417315249	-3,0354E+14	5,0582E+14	5,49935E+14	6,03298E+14	6,66618E+14	6,36448E+14	6,48742E+14
TC0200003545.mm.2	Prpf40a	pre-mRNA processing factor 40A	1,14542E+14	6,53012E+14	2,96916E+14	0.0230066371943262	0.172451417315249	-3,03576E+14	7,02703E+14	7,13696E+14	6,3948E+14	5,47836E+14	6,56293E+14	6,58064E+14
TC0800001217.mm.2	Plekhg4	pleckstrin homology domain containing, family G (with RhoGef domain) member 4	-0.991684734165956	5,30553E+14	-2,96892E+13	0.0230139469608948	0.172451417315249	-3,03604E+14	4,82894E+14	4,49158E+13	5,46967E+14	5,83422E+14	5,42659E+14	5,78218E+14
TC1400002023.mm.2	Hnrnpc	heterogeneous nuclear ribonucleoprotein C	1,36736E+14	8,67018E+14	2,96683E+14	0.0230790019252059	0.172451417315249	-3,03854E+14	9,50709E+14	9,67132E+14	8,68938E+14	7,7543E+14	8,6379E+14	7,7611E+14
TC0200001359.mm.2	Kbtbd4	kelch repeat and BTB (POZ) domain containing 4	1,35141E+14	7,54132E+13	2,96664E+14	0.0230848860053287	0.172451417315249	-3,03877E+14	8,53341E+14	8,30993E+14	7,69301E+14	6,4475E+14	7,17037E+14	7,09372E+14
TC0100001387.mm.2	Glul/Mir8114	glutamate-ammonia ligase (glutamine synthetase)/microRNA 8114	1,5642E+14	1,03491E+14	2,96643E+14	0.0230914805287824	0.172451417315249	-3,03902E+14	1,12822E+14	1,15187E+14	1,05265E+14	9,14602E+14	1,01822E+14	9,43883E+14
TC0900001400.mm.2	Rhoa	ras homolog family member A	1,65765E+14	8,08079E+13	2,96606E+13	0.0231031141424684	0.172451417315249	-3,03947E+14	9,09037E+14	9,08841E+14	8,11616E+14	6,74732E+14	8,24122E+14	7,20124E+12
TC0500001725.mm.2	Spdye4b	speedy/RINGO cell cycle regulator family, member E4B	-1,01545E+14	6,29807E+14	-2,96584E+14	0.0231101017405637	0.172451417315249	-3,03974E+14	5,64186E+14	5,86829E+14	6,55083E+14	6,99022E+14	6,14412E+14	6,59307E+14
TC1500001262.mm.2	Hnrnpa1l2-ps2	heterogeneous nuclear ribonucleoprotein A1-like 2, pseudogene 2	0.90703979367712	7,776E+14	2,96542E+14	0.0231230574418781	0.172451417315249	-3,04024E+14	8,42447E+14	8,583E+14	7,68648E+13	7,50691E+13	7,37259E+14	7,08257E+14
TC0600003499.mm.2	Gimap7	GTPase, IMAP family member 7	-1,05563E+11	5,05395E+14	-2,96522E+14	0.0231292322778416	0.172451417315249	-3,04047E+14	4,36562E+14	4,35199E+14	5,05533E+14	5,77355E+14	5,50287E+14	5,27434E+14
TC0200001959.mm.2	Sirpa	signal-regulatory protein alpha	1,18737E+13	1,0038E+13	2,96492E+14	0.0231386241070678	0.172451417315249	-3,04083E+14	1,07241E+14	1,08784E+14	1,01074E+14	9,12035E+12	9,72524E+14	9,67231E+14
TC1500001919.mm.2	Slc25a17	solute carrier family 25 (mitochondrial carrier, peroxisomal membrane protein), member 17	2,06725E+14	6,89905E+14	2,96443E+14	0.0231538919134244	0.172451417315249	-3,04142E+14	8,18945E+14	8,12299E+14	7,06049E+14	5,11745E+14	7,04627E+14	5,85762E+14
TC0500001026.mm.2	Pkd2	polycystic kidney disease 2	1,33421E+14	7,15021E+14	2,96423E+14	0.0231603318502112	0.172451417315249	-3,04166E+14	7,85424E+14	7,92167E+14	7,13788E+13	5,9696E+14	7,21775E+14	6,80013E+14
TC1100002715.mm.2	Tcf7	transcription factor 7, T cell specific	-1,18859E+14	6,27643E+14	-2,96366E+14	0.0231781495567046	0.172451417315249	-3,04235E+14	5,52869E+14	5,47974E+13	6,54377E+14	6,84184E+14	6,15383E+14	7,11073E+13
TC0X00002738.mm.2	Rps4x	ribosomal protein S4, X-linked	1,29355E+13	8,22432E+14	2,96358E+14	0.0231805847607234	0.172451417315249	-3,04244E+14	9,03686E+14	9,22811E+14	8,20609E+14	7,47179E+14	8,1523E+14	7,25078E+14
TC0200002334.mm.2	Bpifa3	BPI fold containing family A, member 3	-1,30626E+14	6,44604E+13	-2,96346E+14	0.0231845168837503	0.172451417315249	-3,04259E+14	5,6033E+13	5,59705E+14	6,37226E+14	7,4406E+14	6,55961E+14	7,1034E+14
TC1600000861.mm.2	Usp25	ubiquitin specific peptidase 25	1,45682E+14	7,15015E+14	2,96325E+14	0.023191004704961	0.172451417315249	-3,04284E+14	8,00612E+14	8,0244E+14	6,95119E+14	6,16569E+14	7,47455E+14	6,27895E+14
TC0600001016.mm.2	Gp9	glycoprotein 9 (platelet)	-1,25966E+14	7,24019E+14	-2,96301E+14	0.023198402816634	0.172451417315249	-3,04312E+14	6,58956E+14	6,67041E+14	7,33573E+14	8,44356E+14	7,11598E+14	7,28587E+14
TC0600001394.mm.2	Necap1	NECAP endocytosis associated 1	1,36056E+14	8,27815E+14	2,96298E+14	0.0231994367183537	0.172451417315249	-3,04316E+14	9,29948E+14	9,20724E+14	8,50158E+13	7,284E+14	7,88772E+14	7,48887E+14
TC0100002764.mm.2	Ngef	neuronal guanine nucleotide exchange factor	1,13013E+14	8,40081E+14	2,96293E+14	0.0232009093120334	0.172451417315249	-3,04322E+14	9,25389E+13	9,29232E+13	8,34698E+14	7,93898E+14	8,18677E+14	7,38591E+14
TC0100001860.mm.2	Rd3	retinal degeneration 3	-1,00428E+14	6,78287E+14	-2,96283E+14	0.0232041582934477	0.172451417315249	-3,04334E+13	5,88776E+14	6,22109E+14	6,84812E+14	7,2693E+14	7,057E+14	7,41393E+14
TC1700000494.mm.2	Srsf3	serine/arginine-rich splicing factor 3	2,00946E+14	7,40364E+14	2,96167E+14	0.0232405730141986	0.172451417315249	-3,04473E+14	8,4065E+14	8,67358E+14	7,21948E+14	5,84168E+14	7,99018E+14	6,2904E+14
TC0900000197.mm.2	Dnm2	dynamin 2	1,40856E+14	8,38535E+14	2,96145E+14	0.0232472973009768	0.172451417315249	-3,04499E+14	9,07653E+14	9,40482E+14	8,48194E+14	7,18228E+14	8,19608E+14	7,97047E+14
TC1800000568.mm.2	Lmnb1	lamin B1	-1,31753E+14	7,21972E+14	-2,96114E+13	0.023257245384305	0.172451417315249	-3,04537E+14	6,55622E+14	6,17539E+14	7,14977E+14	8,21689E+14	7,38609E+14	7,83398E+14
TC0200002836.mm.2	Pcmtd2	protein-L-isoaspartate (D-aspartate) O-methyltransferase domain containing 2	0.937804638143239	7,54714E+14	2,96086E+14	0.0232659151246019	0.172451417315249	-3,0457E+14	8,04925E+14	8,0689E+14	7,35992E+14	6,88262E+14	7,46916E+14	7,45296E+14
TC0500001578.mm.2	Baz1b	bromodomain adjacent to zinc finger domain, 1B	1,74414E+14	7,16925E+14	2,9604E+14	0.0232804828923746	0.172451417315249	-3,04625E+14	8,22844E+14	8,46589E+14	7,13286E+14	6,07319E+14	7,30309E+14	5,81205E+14
TC1700002701.mm.2	Fbxo11	F-box protein 11	2,35253E+14	8,27843E+14	2,95903E+14	0.023323591750627	0.172451417315249	-3,04789E+14	9,83868E+14	9,83738E+14	8,6135E+14	6,35751E+13	8,21497E+14	6,80852E+14
TC1400002333.mm.2	Pdlim2	PDZ and LIM domain 2	-1,01118E+14	8,63391E+14	-2,95865E+14	0.023335422726717	0.172451417315249	-3,04834E+14	7,83525E+13	7,99618E+14	8,60499E+14	9,2488E+14	9,05396E+14	9,0643E+14
TC0100000440.mm.2	Casp8	caspase 8	1,62166E+13	6,76917E+14	2,95861E+14	0.023336911158212	0.172451417315249	-3,0484E+13	7,35751E+14	7,92803E+13	6,5689E+14	5,47332E+14	7,2085E+13	6,07873E+14
TC1300000894.mm.2	Slc9a3	solute carrier family 9 (sodium/hydrogen exchanger), member 3	-1,06854E+14	6,3375E+13	-2,95811E+14	0.023352522313624	0.172451417315249	-3,04899E+14	5,57855E+14	5,83353E+13	6,456E+14	7,09316E+14	6,34121E+14	6,72253E+13
TC1200002288.mm.2	Serpina12	serine (or cysteine) peptidase inhibitor, clade A (alpha-1 antiproteinase, antitrypsin), member 12	-1,72503E+14	4,82859E+13	-2,95807E+14	0.0233537658825656	0.172451417315249	-3,04904E+14	4,34998E+14	3,12457E+14	4,80711E+14	6,1175E+14	5,13348E+14	5,43893E+14
TC0500001717.mm.2	Wipi2	WD repeat domain, phosphoinositide interacting 2	1,26445E+13	7,88429E+14	2,95799E+14	0.0233564005409981	0.172451417315249	-3,04914E+14	8,66722E+14	8,37582E+14	7,80468E+14	6,70945E+14	7,90573E+14	7,84286E+14
TC1300002017.mm.2	Sptlc1	serine palmitoyltransferase, long chain base subunit 1	0.899700509330617	5,46806E+14	2,95798E+14	0.0233566916185573	0.172451417315249	-3,04915E+14	6,17501E+14	6,205E+14	5,41689E+14	5,16372E+14	4,7983E+14	5,04944E+14
TC0500002745.mm.2	G3bp2	GTPase activating protein (SH3 domain) binding protein 2	1,36722E+14	8,80851E+14	2,95782E+14	0.0233618101254663	0.172451417315249	-3,04935E+14	9,80762E+13	9,71417E+14	8,99884E+14	7,7885E+14	8,50151E+14	8,04044E+14
TC0200003291.mm.2	Qrfp	pyroglutamylated RFamide peptide	-1,22236E+14	6,14024E+11	-2,95768E+14	0.0233661428733781	0.172451417315249	-3,04951E+14	5,12805E+14	5,13157E+12	5,86936E+14	6,83499E+14	6,71373E+14	7,16377E+14
TC0600003148.mm.2	Cd9	CD9 antigen	1,03196E+14	8,80758E+14	2,9574E+14	0.0233750239860016	0.172451417315249	-3,04985E+14	9,04717E+14	9,59727E+14	8,06846E+14	8,51208E+14	8,77566E+14	8,84487E+14
TC0800000796.mm.2	Slc27a1	solute carrier family 27 (fatty acid transporter), member 1	1,08503E+14	8,34732E+14	2,95729E+14	0.02337850300646	0.172451417315249	-3,04998E+14	9,02284E+14	9,10085E+14	8,3785E+14	7,57512E+14	8,07045E+14	7,93614E+13
TC1100002103.mm.2	Tcn2	transcobalamin 2	1,23379E+14	6,70425E+14	2,95643E+14	0.0234055360713447	0.172451417315249	-3,05101E+13	7,2567E+14	7,746E+14	6,74799E+14	5,78712E+14	6,40604E+14	6,28166E+14
TC0800000714.mm.2	D130040H23Rik	RIKEN cDNA D130040H23 gene	1,29594E+14	5,86762E+13	2,95611E+14	0.0234156527791309	0.172451417315249	-3,05139E+14	6,5326E+14	6,68414E+14	5,55412E+13	5,07076E+14	6,21903E+14	5,14507E+14
TC0400000587.mm.2	Fktn	fukutin	1,11639E+14	6,47085E+14	2,95599E+13	0.023419724639238	0.172451417315249	-3,05154E+14	7,11319E+14	7,12275E+13	6,35635E+13	5,64681E+14	6,55238E+14	6,0336E+14
TC1100001551.mm.2	B230217C12Rik	RIKEN cDNA B230217C12 gene	1,43215E+14	8,02013E+14	2,95597E+14	0.023420183276745	0.172451417315249	-3,05156E+14	8,96579E+14	8,80606E+14	7,92208E+13	6,98548E+14	8,23411E+14	7,20723E+14
TC0300002472.mm.2	Prune1	prune exopolyphosphatase	1,11189E+14	7,27844E+14	2,9558E+14	0.0234255782487036	0.172451417315249	-3,05176E+14	7,97894E+14	8,0336E+14	7,28737E+14	6,5014E+14	7,12004E+14	6,74932E+14
TC0900001433.mm.2	Ccdc51	coiled-coil domain containing 51	-0.988057286324369	7,03436E+14	-2,95555E+14	0.0234335505070031	0.172451417315249	-3,05207E+14	6,23371E+14	6,31915E+13	6,96659E+14	7,56238E+13	7,50442E+14	7,61989E+14
TC0900001892.mm.2	Elavl3	ELAV like RNA binding protein 3	1,70276E+14	8,48515E+14	2,95526E+14	0.0234427535276541	0.172451417315249	-3,05242E+13	9,64276E+14	9,60136E+14	8,80713E+14	7,03146E+14	7,79872E+14	8,02949E+14
TC1300002617.mm.2	Ipo11	importin 11	1,83471E+14	6,74193E+14	2,95359E+14	0.0234957687872879	0.172451417315249	-3,05442E+14	7,81873E+14	8,14248E+14	6,89622E+14	5,39558E+14	6,72061E+14	5,47794E+14
TC1000000045.mm.2	Lrp11	low density lipoprotein receptor-related protein 11	1,09189E+14	8,18104E+14	2,95324E+14	0.0235068954785136	0.172451417315249	-3,05484E+13	8,9704E+14	8,6596E+14	8,10673E+14	7,33949E+13	7,95241E+14	8,0576E+14
TC0100003319.mm.2	Soat1	sterol O-acyltransferase 1	1,19975E+14	7,04191E+14	2,95269E+14	0.0235244492608727	0.172451417315249	-3,0555E+14	7,82343E+14	8,06679E+14	7,23016E+13	6,26056E+14	6,56005E+14	6,31047E+14
TC1000003069.mm.2	Usp15	ubiquitin specific peptidase 15	1,40162E+14	8,24373E+14	2,95266E+14	0.0235252510236687	0.172451417315249	-3,05553E+14	9,10366E+14	9,02986E+14	8,27306E+14	7,05721E+14	8,29621E+13	7,70236E+14
TC1900000978.mm.2	Pcnx3	pecanex homolog 3	0.883142620527251	7,06538E+14	2,95218E+14	0.0235406778927495	0.172451417315249	-3,05611E+14	7,70278E+14	7,66295E+14	6,93138E+14	6,66807E+14	6,64597E+14	6,78112E+13
TC0X00000672.mm.2	Pnma3	paraneoplastic antigen MA3	1,23136E+13	5,79776E+13	2,95189E+14	0.0235496091514479	0.172451417315249	-3,05645E+13	7,01249E+14	6,17116E+14	5,63769E+14	5,08324E+14	5,64155E+14	5,2404E+14
TC0200001368.mm.2	Spi1	spleen focus forming virus (SFFV) proviral integration oncogene	1,9957E+14	7,81357E+14	2,95045E+14	0.0235955864990327	0.172451417315249	-3,05818E+14	8,32776E+14	9,26499E+14	7,79362E+14	5,80774E+14	7,83271E+12	7,8546E+14
TC1400000942.mm.2	Clu	clusterin	1,86704E+13	1,06483E+14	2,94969E+14	0.0236198423805925	0.172451417315249	-3,05909E+14	1,18383E+13	1,19775E+14	1,08547E+14	9,22701E+14	1,05926E+14	9,39999E+14
TC1100002694.mm.2	Hnrnpab	heterogeneous nuclear ribonucleoprotein A/B	1,36362E+14	9,63568E+13	2,94967E+14	0.0236204480514294	0.172451417315249	-3,05911E+14	1,04651E+14	1,05009E+14	9,71506E+14	8,52372E+13	9,56266E+14	9,04661E+14
TC0400001362.mm.2	Col9a2	collagen, type IX, alpha 2	-1,30695E+14	7,00109E+14	-2,94945E+14	0.023627642771996	0.172451417315249	-3,05938E+14	6,17145E+14	6,22625E+14	7,02335E+14	7,98826E+14	6,93476E+14	7,66245E+14
TC0700000043.mm.2	Tmem190	transmembrane protein 190	-0.959591971777106	5,67225E+14	-2,94892E+14	0.0236447094879886	0.172451417315249	-3,06002E+14	4,94295E+12	5,23403E+14	6,03746E+14	6,05871E+14	5,64838E+14	6,11195E+14
TC1900000810.mm.2	Vwa2	von Willebrand factor A domain containing 2	-0.980190497030677	6,5457E+14	-2,94863E+14	0.0236537080814585	0.172451417315249	-3,06036E+14	5,77709E+14	5,89194E+14	6,63258E+14	6,99683E+14	6,75679E+13	7,21894E+14
TC1600000508.mm.2	Cstdc4	cystatin domain containing 4	-1,43719E+14	5,47479E+13	-2,94856E+14	0.0236562186442167	0.172451417315249	-3,06046E+14	4,72646E+14	4,33757E+14	5,34376E+14	6,59464E+14	5,65959E+14	6,18672E+14
TC1000000906.mm.2	Ankrd24	ankyrin repeat domain 24	-0.864892052683208	8,68265E+14	-2,94826E+14	0.0236655402684555	0.172451417315249	-3,06081E+14	8,10853E+14	8,19741E+14	8,92607E+14	9,10965E+14	8,84784E+13	8,90642E+13
TC0600001634.mm.2	Ptpro	protein tyrosine phosphatase, receptor type, O	1,8801E+14	5,9807E+14	2,94823E+14	0.0236664701793643	0.172451417315249	-3,06084E+14	7,25051E+14	6,9844E+13	6,23914E+14	4,23557E+14	5,44294E+14	5,73166E+14

TC1100003640.mm.2	Kpnb1	karyopherin (importin) beta 1	1,69366E+14	8,16669E+14	2,94777E+14	0.0236814925624287	0.172451417315249	-3,0614E+14	9,48317E+14	9,30464E+14	8,04014E+13	7,36034E+14	8,22599E+14	6,58588E+14
TC0400001278.mm.2	Tctex1d4	Tctex1 domain containing 4	-1,71372E+14	5,50832E+14	-2,94772E+14	0.023682908320213	0.172451417315249	-3,06146E+14	4,60764E+13	4,27146E+14	5,27245E+14	7,03408E+14	5,8009E+14	6,06336E+14
TC0400001926.mm.2	Fbxo2	F-box protein 2	0.882328419902413	8,2199E+14	2,94762E+14	0.0236862362912916	0.172451417315249	-3,06158E+14	8,92922E+14	8,70322E+14	8,03177E+14	7,83601E+14	7,89216E+14	7,92704E+14
TC0700004532.mm.2	Sct	secretin	-0.888646010985721	5,85516E+13	-2,94755E+14	0.0236883039697212	0.172451417315249	-3,06166E+14	5,36089E+14	5,16842E+13	6,09684E+14	6,20976E+14	6,02769E+14	6,26735E+14
TC0700000558.mm.2	Alkbh6	alkB homolog 6	1,29245E+14	8,3116E+14	2,94698E+14	0.023706694027598	0.172451417315249	-3,06235E+14	9,32908E+14	9,09308E+14	8,34319E+14	7,49406E+13	8,20363E+14	7,40659E+14
TC0800001131.mm.2	Tepp	testis, prostate and placenta expressed	-1,22261E+14	7,27943E+14	-2,94664E+14	0.023717674670269	0.172451417315249	-3,06276E+14	6,68891E+14	6,32341E+14	7,27711E+14	8,18042E+14	7,37819E+14	7,82853E+14
TC1400001067.mm.2	Nufip1	nuclear fragile X mental retardation protein interacting protein 1	2,12476E+14	6,19836E+14	2,9464E+14	0.0237253917803237	0.172451417315249	-3,06305E+14	7,38701E+14	7,34098E+14	6,22345E+14	4,25504E+13	6,65326E+14	5,33043E+13
TC0800001520.mm.2	Tcf25	transcription factor 25 (basic helix-loop-helix)	1,33394E+14	9,11888E+14	2,9464E+14	0.0237254540834195	0.172451417315249	-3,06305E+14	9,92174E+14	9,97054E+13	9,20409E+14	8,02029E+14	9,00487E+14	8,59177E+12
TC0400001608.mm.2	Slc9a1/Mir5122	solute carrier family 9 (sodium/hydrogen exchanger), member 1/microRNA 5122	1,66168E+14	7,38669E+14	2,94632E+14	0.0237279680030928	0.172451417315249	-3,06314E+14	8,62193E+14	8,72105E+14	7,81326E+14	6,20637E+14	6,80938E+14	6,14813E+14
TC0200001448.mm.2	Trp53i11	transformation related protein 53 inducible protein 11	1,7953E+14	8,80225E+14	2,94595E+14	0.0237396463321503	0.172451417315249	-3,06358E+14	9,92796E+14	9,95924E+14	9,0066E+13	7,28999E+14	8,75273E+14	7,87696E+14
TC0600000277.mm.2	Mest	mesoderm specific transcript	1,34082E+14	8,51982E+14	2,94591E+14	0.0237411033951382	0.172451417315249	-3,06363E+14	9,55606E+14	9,52087E+14	8,70768E+14	7,68761E+14	8,19778E+14	7,44894E+14
TC0500003532.mm.2	Mmd2	monocyte to macrophage differentiation-associated 2	1,71331E+14	8,7632E+14	2,94585E+14	0.0237428829878779	0.172451417315249	-3,0637E+13	9,8666E+14	9,87226E+13	8,93974E+14	7,37251E+14	8,72023E+14	7,80785E+14
TC0700001655.mm.2	Rassf10	Ras association (RalGDS/AF-6) domain family (N-terminal) member 10	-1,32917E+14	6,36022E+14	-2,94567E+14	0.0237486968531317	0.172451417315249	-3,06392E+14	5,51807E+14	5,39094E+14	6,47058E+14	7,09678E+14	6,30662E+14	7,37835E+14
TC0X00002643.mm.2	Eif2s3x	eukaryotic translation initiation factor 2, subunit 3, structural gene X-linked	1,57374E+14	8,38626E+14	2,9455E+14	0.0237540631603001	0.172451417315249	-3,06412E+14	9,4262E+14	9,42535E+14	8,61976E+14	7,08431E+14	8,13198E+14	7,62995E+14
TC0500002085.mm.2	Abcf2	ATP-binding cassette, sub-family F (GCN20), member 2	1,37626E+14	7,18379E+14	2,94517E+14	0.0237649195036792	0.172451417315249	-3,06452E+14	8,12268E+14	8,04871E+14	7,36025E+14	6,05862E+13	6,86462E+14	6,64788E+14
TC0200002980.mm.2	C1ql3	C1q-like 3	1,49512E+14	8,12657E+13	2,94479E+14	0.0237771236776542	0.172451417315249	-3,06498E+14	8,52445E+14	9,1214E+14	7,99599E+14	6,65962E+14	8,39582E+14	8,06215E+14
TC0200003352.mm.2	Fbxw2	F-box and WD-40 domain protein 2	2,0934E+14	5,67072E+14	2,94468E+14	0.0237806258752055	0.172451417315249	-3,06511E+14	7,1565E+12	7,01204E+14	6,05487E+14	3,92687E+13	5,40844E+14	4,46558E+14
TC1500001062.mm.2	Acvr1b	activin A receptor, type 1B	1,07636E+14	7,84032E+14	2,94466E+14	0.0237812356032076	0.172451417315249	-3,06513E+14	8,51533E+14	8,60595E+14	7,87977E+14	7,08878E+14	7,55149E+14	7,4006E+14
TC1500001791.mm.2	Arhgap39	Rho GTPase activating protein 39	1,57359E+14	7,14576E+14	2,94388E+14	0.0238063124412099	0.17246050413271	-3,06607E+14	8,3877E+13	8,18039E+14	7,51164E+14	5,90928E+14	6,46744E+14	6,41809E+14
TC0800000392.mm.2	Ppp2cb	protein phosphatase 2 (formerly 2A), catalytic subunit, beta isoform	1,297E+14	8,04393E+14	2,94323E+14	0.0238274046367083	0.17246050413271	-3,06685E+14	8,82116E+14	8,82455E+14	8,0758E+14	6,97592E+14	8,02078E+14	7,54536E+14
TC0900000271.mm.2	Snx19	sorting nexin 19	1,23748E+14	7,06617E+14	2,94305E+14	0.0238331942525591	0.17246050413271	-3,06707E+14	7,73919E+14	7,67878E+14	7,01663E+14	5,92639E+14	7,08844E+14	6,94762E+14
TC0400004003.mm.2	Mfn2	mitofusin 2	0.989903136776337	7,57997E+14	2,94301E+14	0.0238345153744777	0.17246050413271	-3,06712E+14	8,35089E+14	8,17515E+14	7,5388E+14	7,00743E+14	7,34527E+14	7,06231E+14
TC0700001332.mm.2	Tenm4	teneurin transmembrane protein 4	-1,00343E+14	7,8995E+14	-2,94245E+14	0.0238524384662944	0.17246050413271	-3,06779E+14	7,38029E+14	7,1248E+14	7,97739E+14	8,53455E+14	8,06936E+14	8,31059E+14
TC0700004545.mm.2	Chid1	chitinase domain containing 1	1,05324E+14	6,22948E+14	2,94231E+14	0.0238570040680554	0.17246050413271	-3,06796E+14	7,20643E+14	7,07069E+14	6,16508E+14	6,00556E+14	5,83279E+14	5,09632E+14
TC0400002392.mm.2	Zfp292	zinc finger protein 292	0.985807268931716	6,40235E+14	2,94187E+14	0.0238713134310067	0.17246050413271	-3,06849E+14	7,02541E+13	7,07791E+14	6,21068E+14	5,92103E+14	6,36145E+14	5,81762E+14
TC1600000162.mm.2	Bfar	bifunctional apoptosis regulator	-0.87175210211895	8,52659E+14	-2,94186E+14	0.0238715337678217	0.17246050413271	-3,0685E+14	8,06259E+14	7,87402E+14	8,79834E+14	8,88177E+14	8,69227E+14	8,85052E+13
TC1900000866.mm.2	Grk5	G protein-coupled receptor kinase 5	0.921902090942361	7,09531E+14	2,94088E+13	0.0239033166898706	0.17246050413271	-3,06968E+14	7,55246E+13	7,87476E+13	6,82164E+14	6,76178E+14	6,9344E+14	6,6268E+14
TC0100003122.mm.2	Gpr37l1	G protein-coupled receptor 37-like 1	1,84906E+14	8,65915E+14	2,94063E+14	0.0239113204795559	0.17246050413271	-3,06997E+13	9,66256E+14	9,88351E+14	8,87197E+13	6,97597E+14	8,58859E+14	7,97231E+14
TC1000001625.mm.2	Cd63	CD63 antigen	1,60884E+14	8,30922E+14	2,93968E+14	0.0239419177166301	0.17246050413271	-3,07111E+14	8,94842E+14	9,30818E+14	8,19355E+14	6,84538E+14	8,7391E+14	7,82071E+14
TC1000000597.mm.2	Atoh7	atonal bHLH transcription factor 7	-0.981885228839859	6,24983E+14	-2,93917E+14	0.0239587426439054	0.17246050413271	-3,07173E+14	5,56466E+14	5,75414E+14	6,42385E+14	6,85871E+14	6,25608E+14	6,64152E+13
TC1700002138.mm.2	Runx2	runt related transcription factor 2	-1,15885E+13	7,24106E+14	-2,93746E+14	0.0240141203281738	0.17246050413271	-3,07378E+14	6,10202E+14	6,58558E+14	7,22118E+14	7,78412E+14	7,5648E+14	8,18863E+13
TC0X00002771.mm.2	Nap1l2	nucleosome assembly protein 1-like 2	1,91104E+14	8,20508E+14	2,93703E+14	0.0240280010436867	0.17246050413271	-3,07429E+13	9,45922E+13	9,24236E+14	8,30849E+13	6,57102E+13	8,39862E+14	7,25078E+14
TC0X00000979.mm.2	Taf1	TATA-box binding protein associated factor 1	2,10032E+13	6,26566E+14	2,93564E+14	0.0240732992313166	0.17246050413271	-3,07596E+14	7,86712E+14	7,38807E+14	6,16658E+14	4,88798E+14	6,59829E+14	4,68588E+14
TC1500000742.mm.2	Srebf2	sterol regulatory element binding factor 2	1,29776E+14	9,38439E+14	2,93548E+14	0.0240786627879252	0.17246050413271	-3,07616E+14	1,02668E+13	1,03328E+14	9,53262E+14	8,47146E+13	9,13482E+14	8,56782E+14
TC1600000218.mm.2	Aifm3	apoptosis-inducing factor, mitochondrion-associated 3	0.909043073595344	6,34808E+14	2,9354E+13	0.0240810527812424	0.17246050413271	-3,07625E+14	6,98564E+14	7,06474E+14	6,31936E+14	5,91293E+14	5,91017E+14	5,89565E+13
TC0400003404.mm.2	Olfr1335	olfactory receptor 1335	-1,01023E+14	5,31404E+14	-2,93493E+14	0.0240965156784974	0.17246050413271	-3,07682E+14	4,92734E+14	4,49469E+14	5,60683E+14	5,83565E+14	5,27405E+14	5,74571E+14
TC1400001500.mm.2	Rpl15	ribosomal protein L15	1,56403E+14	1,00823E+14	2,93455E+14	0.0241088750953049	0.17246050413271	-3,07728E+14	1,09904E+14	1,09617E+13	1,01014E+14	8,72259E+14	1,02741E+12	9,44354E+14
TC0400002665.mm.2	Abca1	ATP-binding cassette, sub-family A (ABC1), member 1	1,41971E+13	6,36682E+14	2,93415E+14	0.0241220092781361	0.17246050413271	-3,07776E+14	7,36301E+14	7,53089E+14	6,31135E+14	5,74312E+14	6,25838E+14	4,99416E+14
TC1000002445.mm.2	Trappc10	trafficking protein particle complex 10	1,13525E+14	7,50789E+14	2,93357E+14	0.0241409302005725	0.17246050413271	-3,07845E+14	8,07813E+14	8,21869E+14	7,46062E+14	6,56569E+14	7,46657E+14	7,25763E+14
TC0700001269.mm.2	Me3	malic enzyme 3, NADP(+)-dependent, mitochondrial	0.925411271384981	8,22106E+14	2,93336E+13	0.0241479712761246	0.17246050413271	-3,07871E+14	8,70834E+14	8,77626E+14	8,02795E+14	7,60583E+14	8,19814E+14	8,00982E+14
TC0500000465.mm.2	Cc2d2a	coiled-coil and C2 domain containing 2A	0.922173151573288	5,39249E+14	2,93325E+14	0.0241514939502472	0.17246050413271	-3,07884E+14	6,19319E+14	6,39171E+14	5,4828E+14	5,25775E+14	4,6875E+14	4,34202E+14
TC1300001375.mm.2	Ucn3	urocortin 3	-0.967802032393736	5,05089E+14	-2,93321E+14	0.0241528796614509	0.17246050413271	-3,07889E+14	4,24557E+14	4,59717E+14	5,25621E+14	5,52213E+14	5,15238E+14	5,53188E+14
TC0500002486.mm.2	Ugdh	UDP-glucose dehydrogenase	1,10466E+14	6,34926E+14	2,93297E+14	0.0241605048869204	0.17246050413271	-3,07917E+14	7,02063E+14	6,89637E+14	6,22932E+14	5,47835E+14	6,44948E+14	6,0214E+14
TC0800000842.mm.2	Isx	intestine specific homeobox	-1,22979E+14	5,26392E+13	-2,9325E+14	0.0241759880181562	0.17246050413271	-3,07974E+14	4,29362E+14	4,77575E+14	5,34494E+12	6,18401E+13	5,74932E+14	5,23587E+14
TC1200000236.mm.2	Adi1	acireductone dioxygenase 1	1,02707E+14	7,27125E+14	2,93245E+14	0.0241775549733759	0.17246050413271	-3,0798E+14	8,31462E+14	8,04645E+14	7,27995E+14	7,02698E+14	6,17154E+13	6,78798E+13
TC1800000191.mm.2	Galnt1	polypeptide N-acetylgalactosaminyltransferase 1	1,30136E+14	6,26406E+14	2,93235E+14	0.0241808495295836	0.17246050413271	-3,07992E+14	7,17544E+14	7,05111E+13	6,15161E+13	5,47221E+14	6,36556E+14	5,36843E+13
TC0800002806.mm.2	Cdh16	cadherin 16	-1,57729E+13	5,27292E+14	-2,93176E+14	0.0242000916424896	0.17246050413271	-3,08063E+13	4,27902E+14	4,02836E+14	4,97212E+14	6,48983E+14	5,66273E+14	6,20548E+14
TC0400001495.mm.2	Csmd2	CUB and Sushi multiple domains 2	1,13418E+14	6,05196E+13	2,93165E+14	0.0242038843036027	0.17246050413271	-3,08077E+13	6,82653E+14	6,40907E+14	5,87082E+14	5,09641E+14	6,20382E+14	5,90511E+14
TC1600002026.mm.2	Krtap6-5	keratin associated protein 6-5	-0.967244201569673	6,56467E+14	-2,93152E+14	0.0242079997922145	0.17246050413271	-3,08092E+14	5,98771E+14	6,05341E+14	6,86281E+14	7,1128E+14	6,42508E+14	6,94618E+14
TC0200002315.mm.2	Asxl1	additional sex combs like 1	1,46264E+14	6,07301E+14	2,93119E+14	0.0242189850877116	0.17246050413271	-3,08132E+13	6,98527E+14	7,3051E+14	5,98865E+14	5,37644E+14	4,74643E+14	6,03619E+14
TC1900001731.mm.2	Pdzd8	PDZ domain containing 8	1,91486E+14	7,11623E+14	2,93115E+14	0.0242203372678601	0.17246050413271	-3,08137E+14	8,37005E+14	8,4747E+14	7,33139E+14	5,68365E+14	7,07094E+14	5,76667E+14
TC1000000704.mm.2	Gnaz	guanine nucleotide binding protein, alpha z subunit	0.87884785365463	7,99946E+14	2,93099E+14	0.0242254752696326	0.17246050413271	-3,08156E+14	8,67069E+13	8,50138E+14	7,76512E+14	7,64925E+13	7,81968E+14	7,59065E+14
TC0500000582.mm.2	Nwd2	NACHT and WD repeat domain containing 2	1,12798E+14	5,49785E+14	2,93095E+14	0.0242269234254265	0.17246050413271	-3,08161E+14	5,89404E+14	6,26445E+14	4,58785E+14	5,31468E+14	5,67379E+13	5,25232E+14
TC1100000919.mm.2	Sox15	SRY (sex determining region Y)-box 15	-1,43895E+14	6,99652E+14	-2,93092E+13	0.0242278169810897	0.17246050413271	-3,08164E+14	5,91402E+14	5,93385E+14	6,66151E+14	8,06426E+14	7,5703E+14	7,83517E+14
TC1300002285.mm.2	Ice1	interactor of little elongation complex ELL subunit 1	1,01541E+14	6,20431E+14	2,93015E+14	0.0242531860131219	0.17246050413271	-3,08257E+14	7,06279E+14	6,71318E+14	6,06095E+14	5,68421E+13	6,08614E+14	5,61863E+14
TC0100002032.mm.2	Il17f	interleukin 17F	-1,17764E+14	5,57663E+14	-2,92985E+14	0.0242629351923825	0.17246050413271	-3,08293E+14	4,59907E+14	4,74267E+14	5,37524E+14	6,32179E+14	6,05274E+14	6,36824E+14
TC1700001886.mm.2	Pfdn6	prefoldin subunit 6	1,81111E+14	7,32517E+14	2,9294E+14	0.0242777433169491	0.17246050413271	-3,08347E+14	8,46402E+14	8,55815E+12	7,35387E+14	6,04608E+14	7,49504E+14	6,03384E+14
TC0X00001065.mm.2	Cox7b	cytochrome c oxidase subunit 7B	0.927440034762411	9,75825E+14	2,92933E+14	0.0242799337020526	0.17246050413271	-3,08355E+14	1,03961E+14	1,02523E+14	9,25139E+14	9,54208E+14	9,72866E+14	9,37902E+14
TC1000000543.mm.2	Spock2	sparc/osteonectin, cwcv and kazal-like domains proteoglycan 2	1,75249E+14	9,43668E+14	2,92851E+14	0.0243071389523543	0.17246050413271	-3,08454E+14	1,08003E+14	1,05218E+14	9,73054E+14	8,08664E+14	9,17742E+14	8,30334E+14
TC1100002406.mm.2	Ccdc85a	coiled-coil domain containing 85A	1,30884E+13	7,14987E+14	2,9285E+14	0.0243073683115969	0.17246050413271	-3,08455E+13	8,10402E+14	8,03222E+14	7,25008E+14	6,26848E+14	6,98913E+14	6,25528E+14
TC1100003069.mm.2	Nlgn2	neuroligin 2	1,4982E+14	7,48262E+14	2,92808E+13	0.0243210805501963	0.17246050413271	-3,08505E+14	8,64102E+14	8,48779E+14	7,70062E+14	6,4318E+14	7,22711E+14	6,40738E+14
TC0200000647.mm.2	Mrrf	mitochondrial ribosome recycling factor	1,34977E+14	6,91707E+14	2,92804E+14	0.0243225229155852	0.17246050413271	-3,08511E+14	7,946E+14	7,76416E+14	7,02173E+14	5,98888E+14	6,76879E+14	6,01284E+14
TC0X00000709.mm.2	Gdi1	guanosine diphosphate (GDP) dissociation inhibitor 1	1,37828E+14	9,9304E+14	2,92801E+14	0.0243233946923072	0.17246050413271	-3,08514E+14	1,09801E+13	1,08465E+14	1,01174E+14	8,95262E+14	9,66438E+14	9,0214E+14
TC0900001542.mm.2	Gadl1	glutamate decarboxylase-like 1	-1,33596E+14	5,63122E+14	-2,92759E+14	0.0243373403910974	0.17246050413271	-3,08565E+14	4,70548E+14	4,91001E+14	5,6295E+14	6,65793E+14	6,30173E+14	5,5827E+14
TC1700001756.mm.2	Tulp1	tubby like protein 1	-1,86022E+14	5,74116E+14	-2,92688E+14	0.0243608388666307	0.17246050413271	-3,0865E+14	4,47602E+14	4,36209E+14	5,30027E+14	7,25828E+14	6,25741E+14	6,79286E+14
TC1200002413.mm.2	Bag5	BCL2-associated athanogene 5	0.938185326429349	7,40658E+14	2,92662E+14	0.0243692538683753	0.17246050413271	-3,08681E+14	7,99354E+14	7,87522E+14	7,12766E+14	6,86473E+14	7,49939E+14	7,07895E+14
TC1200000769.mm.2	Sipa1l1	signal-induced proliferation-associated 1 like 1	2,14854E+14	7,63517E+14	2,92473E+12	0.0244318502238387	0.17246050413271	-3,08909E+14	9,18394E+13	9,35294E+14	8,11017E+14	6,12962E+14	7,21139E+13	5,82293E+13
TC0100002756.mm.2	Alppl2	alkaline phosphatase, placental-like 2	-1,25463E+13	4,5543E+14	-2,92449E+14	0.0244398936251029	0.17246050413271	-3,08938E+14	3,71111E+14	3,85389E+14	4,64701E+14	5,42726E+14	4,4321E+14	5,2544E+14
TC0300000823.mm.2	Sprr2b	small proline-rich protein 2B	-1,40371E+14	6,28092E+14	-2,92432E+13	0.0244453734059087	0.17246050413271	-3,08958E+14	5,48603E+14	5,4229E+13	6,36227E+14	7,35407E+14	6,05211E+13	7,00812E+14
TC0300002612.mm.2	Vangl1	VANGL planar cell polarity 1	-0.863241021361172	6,44908E+14	-2,9241E+14	0.0244527338698311	0.17246050413271	-3,08984E+14	5,96499E+14	6,19019E+14	6,93064E+14	6,95103E+14	6,28414E+14	6,37346E+14
TC0X00002758.mm.2	Dmrtc1c1/Dmrtc1c2	DMRT-like family C1c1/DMRT-like family C1c2	-1,41937E+14	6,25382E+13	-2,9235E+14	0.02447256952628	0.17246050413271	-3,09056E+14	5,68663E+14	5,35207E+14	6,37856E+14	7,49888E+14	5,9152E+14	6,69158E+14
TC1400001061.mm.2	Tpt1	tumor protein, translationally-controlled 1	0.954572481999298	9,74476E+14	2,92278E+14	0.0244964392547177	0.17246050413271	-3,09143E+14	1,0224E+14	1,03544E+14	9,42084E+14	9,24841E+14	9,86704E+13	9,35389E+14
TC0300000928.mm.2	Sv2a	synaptic vesicle glycoprotein 2 a	1,48421E+14	9,26003E+14	2,92274E+14	0.0244980024070284	0.17246050413271	-3,09149E+14	1,03973E+14	1,01679E+13	9,48857E+14	8,10821E+14	8,97154E+14	8,42666E+14
TC0X00001047.mm.2	Uprt	uracil phosphoribosyltransferase	1,77424E+13	7,30737E+13	2,92269E+12	0.0244994917970092	0.17246050413271	-3,09154E+14	8,75999E+14	8,6099E+14	7,7209E+13	6,10051E+14	6,83381E+14	5,8191E+14
TC1900000719.mm.2	Neurl1a	neuralized E3 ubiquitin protein ligase 1A	1,05996E+14	7,78417E+14	2,92254E+14	0.0245046538428476	0.17246050413271	-3,09173E+14	8,54627E+14	8,41222E+14	7,80057E+14	7,03799E+14	7,36664E+14	7,54135E+14
TC0200005344.mm.2	Zfp967/Zfp966	zinc finger protein 967/zinc finger protein 966	1,83155E+14	8,50201E+14	2,9223E+13	0.024512417677916	0.17246050413271	-3,09201E+14	9,33909E+14	9,54295E+14	8,27998E+12	6,93896E+13	9,18877E+14	7,72233E+14
TC1600002167.mm.2	Cluap1	clusterin associated protein 1	0.936895597430863	5,84279E+14	2,92224E+14	0.0245145761775167	0.17246050413271	-3,09209E+14	6,18951E+14	6,53679E+14	5,461E+14	5,3915E+14	5,5554E+14	5,92255E+14
TC1000001010.mm.2	Washc4	WASH complex subunit 4	1,13328E+14	5,24192E+14	2,92223E+14	0.0245147941270293	0.17246050413271	-3,09209E+14	6,22491E+14	6,03167E+14	4,83059E+14	5,15943E+14	4,17611E+14	5,0288E+14
TC0100002833.mm.2	Hdlbp	high density lipoprotein (HDL) binding protein	1,8548E+14	7,93714E+14	2,92216E+14	0.0245171274073452	0.17246050413271	-3,09218E+14	9,14796E+14	9,09182E+14	8,12433E+14	6,40585E+14	7,96579E+14	6,88708E+13
TC0500000193.mm.2	Gm17434	predicted gene, 17434	-1,1995E+14	5,07214E+14	-2,92213E+14	0.024518253631269	0.17246050413271	-3,09222E+13	4,33778E+14	4,30192E+14	5,03906E+14	5,99963E+14	5,16427E+14	5,59016E+14
TC0300001727.mm.2	Nlgn1	neuroligin 1	1,72302E+14	7,85E+14	2,92191E+12	0.0245256179948794	0.17246050413271	-3,09249E+14	8,97711E+14	8,99198E+14	7,67344E+14	6,84962E+14	8,1303E+14	6,47753E+14
TC1400000940.mm.2	Pbk	PDZ binding kinase	1,27126E+14	5,37901E+13	2,92181E+14	0.0245289316342799	0.17246050413271	-3,09261E+14	5,51953E+14	6,35794E+14	4,79569E+14	4,53926E+14	5,14213E+14	5,91947E+14
TC1400002025.mm.2	Supt16	suppressor of Ty 16	2,2174E+14	6,11925E+14	2,9217E+14	0.0245326141073119	0.17246050413271	-3,09274E+14	7,46318E+13	7,87059E+13	6,23435E+14	4,66464E+13	6,20525E+14	4,27748E+14
TC0400000114.mm.2	Triqk	triple QxxK/R motif containing	1,58016E+14	6,27571E+14	2,92157E+14	0.0245367760643982	0.17246050413271	-3,09289E+14	7,6113E+14	7,49832E+14	6,14076E+14	5,80853E+14	6,10039E+14	4,49496E+14
TC0600001114.mm.2	Slc25a26	solute carrier family 25 (mitochondrial carrier, phosphate carrier), member 26	1,06099E+14	6,54514E+14	2,92098E+14	0.0245564061296067	0.17246050413271	-3,0936E+14	7,24447E+14	7,17554E+12	6,47469E+14	5,82334E+14	6,50338E+14	6,04941E+14
TC0400001382.mm.2	Ppt1	palmitoyl-protein thioesterase 1	1,64366E+14	8,03528E+14	2,92052E+14	0.0245717221901132	0.17246050413271	-3,09415E+14	9,26738E+14	8,98031E+14	8,03887E+14	6,92149E+14	8,15008E+14	6,85356E+13
TC0400004071.mm.2	Camta1	calmodulin binding transcription activator 1	1,72669E+13	8,8274E+14	2,92035E+14	0.024577316876765	0.17246050413271	-3,09436E+14	1,00074E+14	9,79359E+14	8,8391E+14	7,50849E+14	9,04397E+14	7,77188E+14
TC0200000562.mm.2	Nup214/Mir7674	nucleoporin 214/microRNA 7674	1,07332E+14	5,62803E+14	2,92032E+14	0.024578451459381	0.17246050413271	-3,0944E+14	5,91701E+14	6,6511E+14	5,03924E+14	5,38224E+14	5,45822E+14	5,32036E+14
TC0100000828.mm.2	Sag	S-antigen, retina and pineal gland (arrestin)	-1,29595E+14	7,34815E+14	-2,92005E+14	0.0245874604312763	0.17246050413271	-3,09472E+13	6,74806E+13	6,18192E+14	7,35214E+14	8,16974E+13	7,4712E+14	8,16582E+13
TC1200000866.mm.2	Tmem63c	transmembrane protein 63c	1,78921E+14	6,97627E+14	2,92003E+14	0.0245882707378886	0.17246050413271	-3,09475E+14	8,01386E+14	8,25378E+14	7,1119E+14	5,57733E+14	7,03229E+14	5,86846E+14
TC0800001316.mm.2	Cmtr2	cap methyltransferase 2	-0.920403742344001	7,57235E+13	-2,91901E+14	0.0246220588982146	0.17246050413271	-3,09597E+14	6,96421E+14	6,89558E+14	7,66642E+14	8,03418E+14	7,79813E+14	8,07556E+14
TC0600002954.mm.2	Mkrn2os	makorin, ring finger protein 2, opposite strand	-1,3267E+14	6,29204E+14	-2,91886E+14	0.0246271617042188	0.17246050413271	-3,09615E+13	5,32338E+14	5,35687E+14	6,14918E+14	7,18447E+14	6,5078E+14	7,23053E+14
TC1000003224.mm.2	Eif4ebp2	eukaryotic translation initiation factor 4E binding protein 2	-0.994652521848778	6,90536E+14	-2,91875E+14	0.0246307174711312	0.17246050413271	-3,09628E+14	6,24191E+14	6,28969E+14	7,58057E+14	6,94034E+14	7,25991E+14	7,11977E+14
TC0200005066.mm.2	Chd6	chromodomain helicase DNA binding protein 6	1,48618E+13	7,79866E+14	2,91842E+14	0.0246419092000552	0.17246050413271	-3,09669E+14	8,83345E+14	8,70568E+14	7,90825E+14	6,65851E+14	7,75998E+14	6,9261E+14
TC1800000655.mm.2	Wdr7	WD repeat domain 7	2,50719E+14	6,95881E+14	2,918E+14	0.0246560726078059	0.17246050413271	-3,0972E+14	8,6567E+14	8,54259E+13	7,25928E+14	4,92563E+14	7,04664E+14	5,32204E+14
TSUnmapped00000040.mm.2	Rorb	RAR-related orphan receptor beta	1,96594E+14	6,4419E+14	2,91785E+14	0.0246610305360259	0.17246050413271	-3,09737E+14	7,92632E+12	7,64021E+14	6,73334E+14	4,90129E+12	6,3036E+13	5,14667E+14
TC0600003317.mm.2	Pbp2	phosphatidylethanolamine binding protein 2	-1,24936E+14	5,46127E+14	-2,9178E+14	0.024662719603621	0.17246050413271	-3,09744E+14	4,64754E+14	4,50554E+13	5,30753E+14	6,34427E+13	5,75705E+13	6,20567E+14
TC1100003696.mm.2	Ccr7	chemokine (C-C motif) receptor 7	-0.848716600326491	6,94727E+14	-2,91719E+14	0.0246830007730838	0.17246050413271	-3,09817E+14	6,50425E+14	6,42766E+14	7,34324E+14	7,28611E+14	6,99464E+14	7,12774E+14
TC0400004153.mm.2	Tmem88b	transmembrane protein 88B	1,47085E+14	7,21901E+13	2,91712E+14	0.0246854525760507	0.17246050413271	-3,09825E+14	8,40437E+14	8,44679E+14	7,16367E+14	6,7458E+14	6,98938E+14	5,56405E+13
TC1900000064.mm.2	4930481A15Rik	RIKEN cDNA 4930481A15 gene	-1,02487E+14	6,50915E+14	-2,91701E+14	0.0246889669060216	0.17246050413271	-3,09838E+14	5,91311E+14	5,68388E+14	6,68345E+14	6,96328E+14	6,59133E+14	7,21981E+14
TC1400002067.mm.2	Slc7a8	solute carrier family 7 (cationic amino acid transporter, y+ system), member 8	1,38989E+14	8,20348E+14	2,91693E+14	0.0246917709518455	0.17246050413271	-3,09848E+14	8,98397E+14	9,5168E+14	8,17E+14	7,551E+14	8,04528E+14	6,95384E+14
TC0700002619.mm.2	Grik5	glutamate receptor, ionotropic, kainate 5 (gamma 2)	1,12482E+13	8,75758E+14	2,91646E+14	0.0247074388878525	0.17246050413271	-3,09904E+14	9,41402E+13	9,68188E+14	8,75609E+14	8,09018E+14	8,59153E+14	8,01177E+14
TC1600000693.mm.2	Dppa2	developmental pluripotency associated 2	-1,2197E+14	4,68319E+14	-2,9155E+14	0.0247397021860516	0.17246050413271	-3,1002E+13	3,47238E+14	4,24389E+14	4,69689E+14	5,45879E+13	5,17161E+14	5,05555E+14
TC1100004187.mm.2	Nptx1	neuronal pentraxin 1	0.924459954725711	7,18961E+14	2,91508E+14	0.0247536841391521	0.17246050413271	-3,1007E+14	7,59798E+14	7,98616E+14	6,99449E+14	6,74073E+14	6,84469E+14	6,9736E+14
TC1100001874.mm.2	Rpl38	ribosomal protein L38	1,1167E+14	9,91328E+14	2,91503E+14	0.0247556368184388	0.17246050413271	-3,10077E+14	1,07737E+14	1,0469E+14	9,812E+14	9,1974E+14	9,91169E+14	9,31581E+14
TC0600003030.mm.2	Wnk1	WNK lysine deficient protein kinase 1	2,84018E+14	7,76357E+14	2,91475E+14	0.0247650405255586	0.17246050413271	-3,10111E+14	9,52794E+13	9,54742E+14	8,03122E+14	5,36378E+14	8,0598E+14	6,05122E+14
TC0900001045.mm.2	Gcm1	glial cells missing homolog 1	-1,18568E+14	4,76591E+14	-2,91401E+14	0.0247897534938107	0.17246050413271	-3,102E+14	4,12519E+14	3,6603E+14	4,63463E+14	5,52222E+14	5,2005E+14	5,45265E+14
TC0800000321.mm.2	Fgfr1	fibroblast growth factor receptor 1	1,39323E+14	5,89506E+14	2,91394E+14	0.0247922615524145	0.17246050413271	-3,10209E+14	6,76748E+14	6,91916E+14	5,98717E+14	4,91302E+14	5,81055E+14	4,97295E+14
TC0500001131.mm.2	Zfp605	zinc finger protein 605	1,71067E+14	5,53536E+14	2,91385E+13	0.0247952154967782	0.17246050413271	-3,10219E+14	6,73421E+14	6,6351E+14	5,49265E+14	4,45532E+14	5,70329E+14	4,1916E+14
TC1900001445.mm.2	Ppp1r3c	protein phosphatase 1, regulatory subunit 3C	0.919957426071357	7,72061E+14	2,91318E+14	0.0248176499986057	0.17246050413271	-3,103E+14	8,37455E+14	8,39264E+14	7,69471E+14	7,23256E+14	7,27311E+14	7,35611E+14

TC0800003009.mm.2	Pkd1l2	polycystic kidney disease 1 like 2	-0.897314375551199	5,56282E+14	-2,91283E+14	0.0248294281618285	0.17246050413271	-3,10342E+13	4,9032E+14	5,10063E+14	5,85372E+14	5,94473E+14	5,62038E+14	5,95425E+14
TC0100001048.mm.2	Cntnap5a	contactin associated protein-like 5A	1,43394E+14	6,27961E+14	2,91232E+14	0.0248466194272601	0.17246050413271	-3,10403E+14	7,29179E+14	7,24603E+14	6,12561E+13	5,54432E+14	6,38997E+14	5,07993E+14
TC0X00001651.mm.2	Rbbp7	retinoblastoma binding protein 7, chromatin remodeling factor	1,96354E+13	8,67838E+14	2,91232E+14	0.0248466265413671	0.17246050413271	-3,10403E+14	9,94113E+12	9,90431E+14	8,96324E+14	6,95513E+14	8,58645E+14	7,72002E+14
TC0100002308.mm.2	Myo1b	myosin IB	0.933137705473341	6,42531E+14	2,91196E+14	0.024858908249509	0.17246050413271	-3,10447E+14	6,75447E+14	7,11657E+14	6,17323E+14	5,83153E+14	6,39058E+14	6,28549E+14
TC0400001971.mm.2	Slc2a7	solute carrier family 2 (facilitated glucose transporter), member 7	-1,17256E+14	5,87218E+14	-2,91127E+14	0.0248820941212147	0.17246050413271	-3,1053E+13	4,88509E+14	4,96113E+14	5,59485E+14	6,59648E+14	6,53549E+14	6,66006E+14
TC1300001066.mm.2	Tbca	tubulin cofactor A	0.908364419442265	7,61182E+14	2,91049E+14	0.0249085391080088	0.17246050413271	-3,10624E+14	8,0993E+13	8,09005E+14	7,2029E+14	7,16973E+14	7,33664E+14	7,77231E+12
TC0700004353.mm.2	Armcx3	armadillo repeat containing, X-linked 3	1,84837E+14	5,88913E+14	2,91044E+14	0.0249101597117597	0.17246050413271	-3,1063E+14	7,21086E+14	7,22891E+14	5,99724E+14	4,7458E+12	5,87585E+14	4,27612E+14
TC0100002348.mm.2	Gtf3c3	general transcription factor IIIC, polypeptide 3	1,20746E+14	6,09617E+14	2,91034E+14	0.0249134518489814	0.17246050413271	-3,10642E+14	6,82384E+14	6,97421E+14	6,06598E+14	5,31716E+13	6,07496E+14	5,32084E+14
TC0500000373.mm.2	Rnf4	ring finger protein 4	1,9424E+13	7,13575E+14	2,91013E+14	0.0249207907044914	0.17246050413271	-3,10668E+14	8,26114E+14	8,43436E+14	7,28021E+14	5,5305E+14	7,28193E+14	6,02633E+14
TC0700003013.mm.2	Fgf21	fibroblast growth factor 21	-0.893753101584365	4,66575E+14	-2,91006E+14	0.0249230361797392	0.17246050413271	-3,10676E+14	4,23589E+14	3,91165E+14	4,89807E+14	5,03698E+14	5,03802E+14	4,87389E+14
TC0400000855.mm.2	Sh3gl2	SH3-domain GRB2-like 2	1,2342E+14	7,63843E+14	2,90988E+14	0.0249291722058554	0.17246050413271	-3,10698E+14	8,42058E+13	8,36414E+14	7,65094E+14	6,66538E+14	7,62811E+14	7,1014E+14
TC0X00000981.mm.2	Ogt	O-linked N-acetylglucosamine (GlcNAc) transferase (UDP-N-acetylglucosamine:polypeptide-N-acetylglucosaminyl transferase)	2,09078E+14	7,34505E+13	2,90983E+14	0.0249307268496121	0.17246050413271	-3,10703E+14	8,71086E+14	8,61794E+14	7,56141E+14	5,58584E+14	7,44264E+14	6,15164E+14
TC1000002391.mm.2	Smarcb1	SWI/SNF related, matrix associated, actin dependent regulator of chromatin, subfamily b, member 1	0.950710908509068	7,71468E+14	2,909E+14	0.0249587812403681	0.17246050413271	-3,10803E+14	8,18428E+14	8,44036E+14	7,54229E+14	7,18093E+14	7,3E+14	7,64023E+14
TC1400000914.mm.2	Prss51	protease, serine 51	-0.897052946802574	6,78311E+14	-2,90861E+14	0.0249719992081041	0.17246050413271	-3,1085E+14	6,22912E+14	6,29283E+14	6,96857E+14	7,34749E+14	6,87133E+14	6,98934E+14
TC0100000910.mm.2	Olfr1411	olfactory receptor 1411	-1,45472E+14	5,02791E+14	-2,90852E+14	0.0249752303032841	0.17246050413271	-3,10862E+14	4,2438E+14	3,93031E+14	4,93345E+14	6,1501E+14	5,07506E+14	5,83472E+14
TC0100001720.mm.2	Sde2	SDE2 telomere maintenance homolog (S. pombe)	1,06207E+14	6,44366E+14	2,90851E+14	0.0249755312435797	0.17246050413271	-3,10863E+13	7,33665E+14	6,87112E+14	6,32593E+14	5,75769E+14	6,01681E+14	6,35378E+14
TC1400001779.mm.2	Sh2d4b	SH2 domain containing 4B	-1,05854E+14	5,83678E+14	-2,90815E+14	0.0249878063462718	0.17246050413271	-3,10906E+13	5,37274E+14	5,33806E+14	6,12774E+14	6,70013E+14	5,46111E+14	6,02089E+14
TC0100000125.mm.2	Mir206/Mir133b	microRNA 206/microRNA 133b	-1,23574E+14	5,37653E+14	-2,9074E+14	0.0250132868630805	0.17246050413271	-3,10997E+14	4,31237E+14	4,86953E+14	5,34646E+14	6,30692E+14	5,62717E+14	5,79671E+14
TC1700001714.mm.2	Bak1	BCL2-antagonist/killer 1	-0.863204651562082	7,39691E+14	-2,90724E+14	0.0250187867033403	0.17246050413271	-3,11016E+14	6,87481E+14	6,85522E+14	7,74682E+14	7,70962E+14	7,46495E+14	7,73007E+14
TC0700003008.mm.2	Dhdh	dihydrodiol dehydrogenase (dimeric)	1,21369E+14	6,96267E+14	2,90675E+14	0.0250353486421018	0.17246050413271	-3,11075E+14	7,9264E+14	7,70801E+14	7,13528E+14	6,07175E+13	6,44675E+14	6,48782E+14
TC0400000057.mm.2	Chd7	chromodomain helicase DNA binding protein 7	-0.844640518551294	8,00253E+14	-2,90633E+14	0.0250496584665639	0.17246050413271	-3,11126E+13	7,61159E+14	7,55134E+14	8,42602E+14	8,42619E+14	8,07225E+14	7,92782E+14
TC0400001460.mm.2	Col8a2	collagen, type VIII, alpha 2	-1,01925E+14	8,20668E+13	-2,90624E+13	0.0250527369714287	0.17246050413271	-3,11137E+14	7,2698E+14	7,60631E+14	8,34029E+14	8,57433E+14	8,42797E+14	9,02138E+14
TC1000003106.mm.2	Kif5a	kinesin family member 5A	1,71508E+14	9,96041E+14	2,90602E+14	0.0250600969875326	0.17246050413271	-3,11163E+14	1,10262E+14	1,10607E+14	1,01249E+14	8,53194E+14	9,97824E+14	9,04047E+14
TC1000001216.mm.2	Kitl	kit ligand	0.855862110448421	7,42208E+14	2,90595E+14	0.0250626577187585	0.17246050413271	-3,11172E+14	7,9768E+14	7,84239E+14	7,02798E+14	7,07949E+14	7,39389E+14	7,2119E+14
TC1100004242.mm.2	Sectm1a	secreted and transmembrane 1A	-1,48057E+13	4,99724E+14	-2,90583E+14	0.025066472029127	0.17246050413271	-3,11185E+13	3,94958E+14	4,13795E+14	4,80632E+14	6,24235E+14	5,2118E+14	5,63546E+14
TC0700003977.mm.2	Fam160a2	family with sequence similarity 160, member A2	0.95065897193454	6,38244E+14	2,90557E+14	0.0250756010582562	0.17246050413271	-3,11218E+14	6,9879E+14	7,30031E+14	6,37619E+14	6,0107E+14	5,665E+14	5,95456E+14
TC0300002358.mm.2	Atp8b2	ATPase, class I, type 8B, member 2	-1,00179E+14	7,54143E+13	-2,90552E+13	0.0250771264937859	0.17246050413271	-3,11223E+14	6,90634E+14	7,04E+14	7,72537E+14	8,22455E+14	7,89984E+14	7,45251E+14
TC0600002976.mm.2	Olfr215	olfactory receptor 215	-1,67514E+14	4,8527E+14	-2,90549E+14	0.0250780298565036	0.17246050413271	-3,11226E+14	3,56701E+14	3,97363E+13	4,73327E+13	6,15765E+14	4,83755E+14	5,84706E+14
TC1100003704.mm.2	Krt27	keratin 27	-1,36161E+14	6,28774E+14	-2,90537E+13	0.0250823719323375	0.17246050413271	-3,11242E+14	4,97965E+14	5,75689E+14	6,56683E+14	6,89293E+14	6,25209E+14	7,27805E+14
TC1400001233.mm.2	Frz2f8	FSHD region gene 2 family member 8	-0.92561390780561	5,36901E+14	-2,90514E+14	0.0250901345563417	0.17246050413271	-3,11269E+14	4,77477E+14	4,57504E+14	5,58277E+13	5,61827E+14	5,60652E+14	6,0567E+13
TC0300002223.mm.2	Zfp639	zinc finger protein 639	1,67748E+13	6,32659E+14	2,90451E+14	0.0251115038986111	0.17246050413271	-3,11345E+14	7,20446E+14	7,25869E+14	6,4388E+13	4,76938E+14	6,42544E+14	5,76276E+14
TC1400002832.mm.2	Pabpn1	poly(A) binding protein, nuclear 1	1,52625E+14	7,50875E+14	2,90414E+14	0.0251243819361823	0.17246050413271	-3,1139E+14	8,52901E+13	8,31975E+14	7,56412E+14	6,23215E+14	7,61378E+14	6,79371E+14
TC0400001741.mm.2	Capzb	capping protein (actin filament) muscle Z-line, beta	1,69712E+13	9,55214E+14	2,90394E+14	0.0251309394679481	0.17246050413271	-3,11413E+14	1,07668E+14	1,07261E+14	9,78568E+13	8,31293E+14	9,39927E+14	8,32209E+14
TC1700001780.mm.2	Cpne5	copine V	1,22477E+14	7,96584E+14	2,90332E+13	0.0251523622974814	0.17246050413271	-3,11489E+14	9,02361E+14	8,55387E+14	7,98558E+14	7,14236E+14	7,27307E+14	7,81653E+14
TC0400004198.mm.2	Zfp991/Znf41-ps	zinc finger protein 991/ZNF41, pseudogene	1,89059E+14	6,56526E+13	2,90329E+14	0.0251533104038871	0.17246050413271	-3,11492E+13	7,51006E+14	7,57615E+14	6,64456E+14	4,66047E+14	6,83599E+13	6,16436E+14
TC1400000853.mm.2	C1qtnf9	C1q and tumor necrosis factor related protein 9	-1,22206E+14	6,93401E+14	-2,90164E+13	0.0252097225728499	0.17246050413271	-3,11691E+14	6,07575E+14	6,24206E+14	6,89954E+14	7,86238E+14	7,00487E+14	7,51945E+14
TC1500001491.mm.2	Trps1/Mir1907	transcriptional repressor GATA binding 1/microRNA 1907	0.959540298461726	6,53607E+14	2,90116E+12	0.0252261466494725	0.17246050413271	-3,11749E+14	7,14593E+14	7,20965E+14	6,03645E+14	6,40005E+14	6,44068E+14	5,98366E+14
TC1100001828.mm.2	9930022D16Rik	RIKEN cDNA 9930022D16 gene	-1,16729E+13	5,34204E+14	-2,90106E+13	0.0252294195089243	0.17246050413271	-3,11761E+14	5,03793E+14	4,539E+14	5,51998E+14	6,39153E+14	5,22733E+14	5,33649E+14
TC1400000372.mm.2	Syt15	synaptotagmin XV	-1,11737E+14	7,21541E+14	-2,90103E+14	0.0252305711544134	0.17246050413271	-3,11765E+14	6,24125E+14	6,63889E+14	7,1477E+14	7,96718E+14	7,58441E+14	7,71302E+14
TC1500001661.mm.2	Zfat	zinc finger and AT hook domain containing	-1,37106E+14	6,19417E+14	-2,90045E+14	0.0252505001144352	0.17246050413271	-3,11835E+14	5,63292E+14	5,02328E+14	6,10311E+14	7,2952E+14	6,48569E+14	6,62479E+14
TC1200000856.mm.2	Vash1	vasohibin 1	-1,08234E+14	6,36206E+14	-2,90011E+13	0.0252620040831065	0.17246050413271	-3,11875E+14	5,75301E+14	5,69268E+14	6,40311E+14	7,20726E+14	6,41736E+14	6,69894E+14
TC0100003440.mm.2	Tiprl	TIP41, TOR signalling pathway regulator-like (S. cerevisiae)	1,59019E+14	7,06886E+14	2,89845E+13	0.0253193841816148	0.17246050413271	-3,12077E+14	7,90607E+14	8,00529E+14	7,00535E+14	5,72562E+14	7,4244E+14	6,34646E+14
TC0400001538.mm.2	Adgrb2	adhesion G protein-coupled receptor B2	1,62079E+14	8,56517E+14	2,89792E+14	0.025337395286427	0.17246050413271	-3,1214E+14	9,43503E+13	9,76307E+14	8,80663E+14	7,14989E+14	8,28324E+14	7,95315E+14
TC0800001418.mm.2	Hsbp1	heat shock factor binding protein 1	1,18468E+14	9,63729E+14	2,89745E+14	0.025353507398025	0.17246050413271	-3,12196E+14	1,03171E+14	1,03192E+13	9,58675E+14	8,68013E+14	9,71836E+14	9,20226E+14
TC1100000936.mm.2	Dvl2/Mir324	dishevelled segment polarity protein 2/microRNA 324	-1,01157E+14	7,94594E+14	-2,89705E+14	0.0253674957139642	0.17246050413271	-3,12245E+14	7,27464E+14	7,29722E+14	8,01773E+14	8,57727E+12	8,03491E+14	8,47389E+14
TC1300001549.mm.2	Olfr1535	olfactory receptor 1535	-1,55355E+14	5,03984E+14	-2,89653E+14	0.0253855189053666	0.17246050413271	-3,12309E+14	3,86609E+14	3,91748E+14	5,06813E+14	5,82254E+14	5,06367E+14	6,50112E+14
TC0900000273.mm.2	Zbtb44	zinc finger and BTB domain containing 44	1,31635E+14	6,76575E+14	2,89612E+14	0.0253996074119978	0.17246050413271	-3,12358E+14	7,70152E+13	7,59745E+14	6,69405E+13	5,97222E+14	6,83023E+14	5,79903E+14
TC0400000497.mm.2	Ccdc180	coiled-coil domain containing 180	-1,20012E+13	5,72699E+14	-2,89576E+14	0.0254118196786943	0.17246050413271	-3,124E+14	5,02877E+14	4,8E+14	5,63806E+14	6,59096E+14	5,9359E+14	6,36827E+14
TC0300002968.mm.2	Aimp1	aminoacyl tRNA synthetase complex-interacting multifunctional protein 1	1,07908E+14	5,55274E+14	2,89537E+14	0.0254253084641099	0.17246050413271	-3,12448E+14	6,37946E+14	6,30894E+14	5,62973E+14	4,9005E+14	5,25663E+14	4,83215E+14
TC0400004104.mm.2	Ajap1	adherens junction associated protein 1	2,22517E+14	7,98515E+14	2,89533E+14	0.0254267883780527	0.17246050413271	-3,12453E+14	9,29123E+14	9,15022E+14	8,18941E+14	5,8017E+14	8,1902E+14	7,28813E+14
TC0100003025.mm.2	Lct	lactase	-1,22514E+13	5,94365E+13	-2,89528E+14	0.0254285397404015	0.17246050413271	-3,12459E+14	4,97564E+14	5,26114E+14	6,08204E+12	6,60502E+14	5,89837E+14	6,8397E+14
TC0400003760.mm.2	Srrm1	serine/arginine repetitive matrix 1	1,72157E+14	7,60375E+14	2,89504E+14	0.0254368575314 36	0.17246050413271	-3,12488E+14	8,65704E+14	8,70823E+14	7,77196E+14	6,15017E+14	7,62949E+14	6,70564E+14
TC1600001459.mm.2	Lrrc15	leucine rich repeat containing 15	-1,24065E+14	5,82673E+14	-2,89495E+13	0.0254398820001588	0.17246050413271	-3,12498E+14	5,22297E+14	5,04113E+14	5,85582E+14	6,88958E+14	5,75261E+14	6,19827E+14
TC1300000275.mm.2	Ripor2	RHO family interacting cell polarization regulator 2	1,1497E+14	7,6296E+14	2,89444E+13	0.0254575130458123	0.17246050413271	-3,1256E+14	8,58567E+14	8,38593E+14	7,8175E+14	6,85471E+14	7,05506E+14	7,07872E+14
TC0X00001111.mm.2	Pou3f4	POU domain, class 3, transcription factor 4	0.943326442727169	7,37344E+14	2,89337E+14	0.0254946210854493	0.17246050413271	-3,12689E+14	8,06076E+14	8,2105E+14	7,44465E+14	6,93997E+14	6,87634E+14	6,70843E+14
TC0600003446.mm.2	Mansc4	MANSC domain containing 4	-1,29492E+14	5,42553E+14	-2,8933E+14	0.0254972108610183	0.17246050413271	-3,12698E+14	4,38602E+14	4,74639E+14	5,37877E+14	6,34348E+14	5,5124E+14	6,18612E+14
TC1900000656.mm.2	Slf2	SMC5-SMC6 complex localization factor 2	1,3314E+14	7,37702E+14	2,89315E+14	0.0255021810618143	0.17246050413271	-3,12716E+14	8,16609E+14	8,17587E+14	7,39277E+14	6,28639E+14	7,45131E+14	6,78966E+14
TC1500000568.mm.2	Zfp707	zinc finger protein 707	0.921621454898 91	6,81795E+14	2,8931E+14	0.0255076621838873	0.17246050413271	-3,12735E+14	7,12796E+14	7,46241E+13	6,54974E+14	6,19739E+14	6,74189E+14	6,82829E+14
TC1300000359.mm.2	Wrnip1	Werner helicase interacting protein 1	0.881982721731957	6,64311E+14	2,89286E+14	0.0255123702601693	0.17246050413271	-3,12751E+14	7,18264E+14	6,97586E+14	6,27919E+14	6,11534E+13	6,77941E+14	6,5262E+14
TC0800001255.mm.2	Cdh1	cadherin 1	-1,19446E+14	6,68265E+14	-2,8928E+14	0.0255146104559276	0.17246050413271	-3,12759E+14	5,94544E+14	5,9579E+14	6,70564E+14	7,58663E+14	6,65236E+13	7,24792E+13
TC0200005443.mm.2	Arfrp1	ADP-ribosylation factor related protein 1	0.966965579672549	6,48227E+14	2,89277E+14	0.0255154579956614	0.17246050413271	-3,12762E+13	7,18868E+14	6,75263E+14	5,80168E+14	6,2057E+14	6,39844E+14	6,54649E+13
TC1100001641.mm.2	Mlx	MAX-like protein X	1,39157E+14	6,76538E+14	2,89239E+14	0.0255285145654487	0.17246050413271	-3,12807E+14	7,90456E+14	7,77678E+14	7,03478E+14	5,86341E+13	6,36928E+14	5,64345E+13
TC1300002210.mm.2	Mfsd14b	major facilitator superfamily domain containing 14B	0.959264013140603	7,2242E+14	2,89217E+14	0.0255361543679552	0.17246050413271	-3,12834E+14	7,96999E+14	7,81851E+13	7,14853E+14	6,72144E+14	7,03554E+14	6,6512E+14
TC1100000192.mm.2	Rab1a	RAB1A, member RAS oncogene family	2,58909E+14	8,59239E+13	2,89187E+14	0.0255466481574123	0.17246050413271	-3,1287E+14	1,00365E+13	1,0072E+12	8,91971E+14	6,01065E+14	8,70028E+14	7,81516E+14
TC0900000867.mm.2	Herc1	HECT and RLD domain containing E3 ubiquitin protein ligase family member 1	1,49725E+14	7,61961E+14	2,89116E+14	0.0255713701850036	0.17246050413271	-3,12956E+14	8,80895E+14	8,65029E+13	7,47108E+12	6,99367E+14	7,62731E+14	6,16635E+14
TC1300002126.mm.2	Tut7	terminal uridylyl transferase 7	1,46533E+14	6,00541E+14	2,89083E+14	0.0255827666935458	0.17246050413271	-3,12996E+14	7,23399E+13	6,68762E+14	6,09618E+14	4,89476E+14	5,91381E+13	5,2061E+14
TC1400000858.mm.2	Sacs	sacsin	1,26632E+14	6,28591E+14	2,89038E+14	0.0255983606825576	0.17246050413271	-3,13051E+14	6,94023E+13	6,77745E+14	6,08733E+14	5,09771E+14	6,72301E+14	6,08974E+13
TC0500001614.mm.2	Cldn15	claudin 15	-1,34345E+14	5,80584E+13	-2,88996E+14	0.0256133217942783	0.17246050413271	-3,13102E+14	4,7954E+14	5,0272E+14	5,88279E+14	6,62672E+14	5,72459E+14	6,77836E+14
TC1800001255.mm.2	Lars	leucyl-tRNA synthetase	1,47526E+14	5,82485E+14	2,88955E+14	0.0256276328345955	0.17246050413271	-3,13152E+14	6,76913E+14	6,66854E+14	5,47461E+14	5,51255E+14	6,21769E+14	4,80659E+14
TC0200000416.mm.2	Fut7	fucosyltransferase 7	-1,11534E+14	6,67288E+14	-2,88947E+14	0.0256304058935713	0.17246050413271	-3,13161E+14	5,80171E+14	6,2672E+14	6,76772E+14	7,53186E+14	6,71827E+14	6,9505E+14
TC0800001737.mm.2	Gas6	growth arrest specific 6	1,1731E+14	7,67777E+14	2,88938E+14	0.0256332386349442	0.17246050413271	-3,13171E+13	8,45321E+14	8,56362E+14	7,57342E+14	7,09722E+14	7,64216E+14	6,737E+14
TC0700000647.mm.2	Pepd	peptidase D	1,90075E+14	6,32639E+14	2,88922E+13	0.025639 1687523	0.17246050413271	-3,13191E+14	7,295E+14	7,47614E+14	6,37495E+14	4,59469E+14	6,67942E+14	5,53815E+14
TC0X00000656.mm.2	Vma21	VMA21 vacuolar H+-ATPase homolog (S. cerevisiae)	1,86261E+14	6,34843E+14	2,88896E+14	0.0256480010708383	0.17246050413271	-3,13222E+14	7,57305E+14	7,18188E+14	6,30806E+13	4,72164E+14	6,78834E+14	5,51761E+14
TC1200000140.mm.2	Nol10	nucleolar protein 10	2,5299E+14	6,00439E+14	2,88839E+13	0.0256678057743668	0.17246050413271	-3,13291E+13	7,43509E+14	7,20862E+14	6,21459E+13	3,36931E+14	6,30504E+14	5,49373E+12
TC1600000279.mm.2	Ap2m1	adaptor-related protein complex 2, mu 1 subunit	1,22591E+14	1,08254E+14	2,88768E+13	0.0256927126099789	0.17246050413271	-3,13377E+14	1,16943E+14	1,16125E+14	1,08934E+14	9,96155E+14	1,07038E+14	1,00865E+14
TC0200000490.mm.2	Tsc1	tuberous sclerosis 1	1,4138E+14	7,37577E+14	2,88747E+14	0.0257000428489638	0.17246050413271	-3,13402E+14	8,29247E+14	8,31162E+14	7,55959E+14	6,21691E+14	7,17069E+14	6,70333E+13
TC0900003327.mm.2	Khdc3	KH domain containing 3, subcortical maternal complex member	-1,34868E+14	4,51178E+14	-2,88729E+14	0.0257063414050151	0.17246050413271	-3,13424E+14	3,73574E+14	3,50996E+14	5,00925E+14	4,93381E+14	4,2914E+14	5,59056E+14
TC1900000206.mm.2	Vwce	von Willebrand factor C and EGF domains	-1,38788E+14	4,96242E+14	-2,88724E+14	0.0257080307590291	0.17246050413271	-3,1343E+14	4,52785E+14	4,05353E+14	5,08502E+14	6,27211E+14	4,65541E+14	5,18062E+14
TC0700000787.mm.2	Egfbp2/Klk1b26	epidermal growth factor binding protein type B/kallikrein 1-related petidase b26	-1,3474E+14	5,98611E+14	-2,88684E+14	0.0257219679521355	0.17246050413271	-3,13478E+14	5,25966E+14	5,1223E+14	6,01807E+14	7,0587E+14	5,82951E+14	6,6284E+14
TC0200005334.mm.2	Gm14391/Gm6710	predicted gene 14391/predicted gene 6710	2,15577E+14	8,80588E+14	2,88667E+14	0.0257279162297583	0.17246050413271	-3,13499E+14	9,53554E+14	1,0385E+14	8,74046E+14	6,86856E+14	9,33941E+14	7,96627E+14
TC0200005252.mm.2	Cyp24a1	cytochrome P450, family 24, subfamily a, polypeptide 1	-1,1234E+14	6,33782E+14	-2,88667E+14	0.0257279842574059	0.17246050413271	-3,13499E+13	5,33045E+14	5,74706E+14	6,24526E+14	7,06015E+14	6,72314E+14	6,95302E+14
TC1000000216.mm.2	Med23	mediator complex subunit 23	1,13409E+14	7,02232E+14	2,88655E+14	0.0257321700749053	0.17246050413271	-3,13513E+14	7,43873E+14	7,54417E+14	6,82122E+13	5,89351E+14	7,10443E+14	7,33185E+14
TC1000000332.mm.2	Rev3l	REV3 like, DNA directed polymerase zeta catalytic subunit	1,93844E+13	5,5426E+14	2,88533E+14	0.0257749231313752	0.17246050413271	-3,13661E+14	6,78339E+14	6,65888E+14	5,86834E+14	3,69697E+14	5,06747E+13	5,18061E+14
TC1800000945.mm.2	Kif5b	kinesin family member 5B	2,57958E+14	8,1954E+14	2,885E+13	0.0257865989828685	0.17246050413271	-3,13701E+14	9,79867E+14	9,67949E+14	8,42313E+14	5,89587E+14	8,52716E+14	6,84806E+14
TC0400000580.mm.2	Slc44a1	solute carrier family 44, member 1	1,7551E+14	7,46618E+14	2,88427E+14	0.025812212160757	0.17246050413271	-3,13789E+14	8,3409E+13	8,68832E+14	7,56509E+14	5,95393E+14	7,6204E+14	6,62843E+14
TC1500002246.mm.2	Smagp	small cell adhesion glycoprotein	-0.929707843019318	6,96918E+14	-2,88423E+14	0.0258136727332187	0.17246050413271	-3,13794E+14	6,43842E+14	6,28501E+14	7,04861E+14	7,53423E+14	7,19275E+14	7,31607E+14
TC1900000819.mm.2	Atrnl1	attractin like 1	1,55981E+14	7,50905E+14	2,88342E+14	0.025842138320483	0.17246050413271	-3,13892E+14	8,46686E+14	8,82586E+14	7,69696E+14	6,47614E+14	7,31448E+14	6,27398E+14
TC0600002385.mm.2	Gprin3	GPRIN family member 3	1,56243E+14	5,88319E+14	2,88338E+14	0.0258435374649898	0.17246050413271	-3,13897E+14	7,21015E+14	7,03741E+14	6,11047E+14	5,01223E+14	5,57243E+14	4,35641E+14
TC0200004245.mm.2	Tcp11l1	t-complex 11 like 1	2,27003E+14	5,76274E+14	2,88299E+14	0.0258570853269439	0.17246050413271	-3,13943E+14	7,08423E+14	6,96333E+14	5,72134E+14	3,78616E+13	6,39844E+14	4,62293E+14
TC1400002259.mm.2	Elp3	elongator acetyltransferase complex subunit 3	1,71867E+14	7,21003E+14	2,88299E+13	0.0258571067635966	0.17246050413271	-3,13943E+14	8,44338E+14	8,2313E+14	7,40789E+14	5,82945E+14	7,16715E+14	6,181E+14
TC0900001732.mm.2	Arhgap42	Rho GTPase activating protein 42	1,61475E+14	5,3566E+14	2,88294E+14	0.0258589236422962	0.17246050413271	-3,1395E+14	6,28939E+14	5,90006E+14	5,25047E+13	3,70948E+14	5,82934E+14	5,1609E+14
TC0500002315.mm.2	Fbxl5	F-box and leucine-rich repeat protein 5	1,86619E+14	6,81393E+14	2,88293E+14	0.0258593985024539	0.17246050413271	-3,13951E+14	7,89055E+14	7,93792E+14	7,03911E+14	5,05697E+14	6,79433E+14	6,16471E+13
TC0400004118.mm.2	Arhgef16	Rho guanine nucleotide exchange factor (GEF) 16	-0.906426757625376	5,64102E+14	-2,8829E+14	0.0258604772251067	0.17246050413271	-3,13955E+14	4,74619E+14	5,05877E+14	5,69302E+13	5,92479E+14	6,33233E+14	6,09103E+13
TC1900001527.mm.2	Loxl4	lysyl oxidase-like 4	-0.957365931154794	7,24215E+14	-2,8829E+14	0.0258605725857895	0.17246050413271	-3,13955E+14	6,53457E+14	6,61668E+14	7,2649E+14	7,81223E+14	7,4927E+14	7,72066E+14
TC1700000535.mm.2	Pde9a	phosphodiesterase 9A	0.932345939272067	6,99217E+14	2,88263E+14	0.0258700609905048	0.17246050413271	-3,13988E+13	7,83523E+14	7,39814E+14	6,73659E+14	6,63209E+14	6,5326E+13	6,81839E+14
TC0X00001534.mm.2	Smc1a	structural maintenance of chromosomes 1A	1,96451E+14	7,51051E+14	2,88258E+14	0.0258716876612206	0.17246050413271	-3,13994E+13	8,74728E+14	8,76826E+14	7,69406E+14	5,89245E+14	7,6258E+14	6,35224E+13
TC0900003037.mm.2	Sema3b	sema domain, immunoglobulin domain (Ig), short basic domain, secreted, (semaphorin) 3B	-0.858111990360943	6,5172E+14	-2,88253E+14	0.0258733280142945	0.17246050413271	-3,13999E+14	5,92455E+14	5,96826E+14	6,69121E+14	6,91782E+14	6,87934E+14	6,72204E+14
TC0X00002756.mm.2	Gm9112	predicted gene 9112	-1,17073E+14	5,67468E+13	-2,88226E+14	0.0258829272736233	0.17246050413271	-3,14032E+14	4,82524E+14	4,59624E+14	5,65364E+14	6,10931E+14	5,9846E+14	6,87908E+13
TC1800001072.mm.2	Zscan30	zinc finger and SCAN domain containing 30	-0.957689434913458	5,18235E+14	-2,88218E+14	0.0258856101895195	0.17246050413271	-3,14041E+14	4,66499E+14	4,43031E+14	5,25704E+14	5,75364E+13	5,61295E+14	5,37515E+14
TC1000001332.mm.2	A930009A15Rik	RIKEN cDNA A930009A15 gene	-1,44177E+14	5,80155E+14	-2,88213E+14	0.0258876895964932	0.17246050413271	-3,14048E+14	5,35455E+13	4,52081E+14	5,81295E+14	6,94595E+14	5,87663E+14	6,2984E+14
TC0600002635.mm.2	Pcyox1	prenylcysteine oxidase 1	1,07318E+14	8,00974E+14	2,88152E+14	0.0259091763939905	0.17246050413271	-3,14122E+14	8,83283E+13	8,61592E+14	7,91053E+14	7,39186E+14	7,9723E+14	7,33502E+13
TC0400002782.mm.2	Zfp37	zinc finger protein 37	0.913502269087132	5,99987E+13	2,88134E+14	0.0259154707936398	0.17246050413271	-3,14144E+14	6,43403E+14	6,74475E+14	5,57999E+14	5,77178E+14	5,89057E+14	5,5781E+14
TC1400000340.mm.2	Ncoa4	nuclear receptor coactivator 4	1,59854E+14	8,08681E+13	2,88009E+14	0.0259277879587943	0.17246050413271	-3,14186E+14	9,25042E+14	9,20267E+14	8,42887E+13	6,82714E+14	7,69312E+14	7,11865E+14
TC0900002149.mm.2	Cbl	Casitas B-lineage lymphoma	1,78079E+14	7,10815E+14	2,88071E+13	0.0259375424422673	0.17246050413271	-3,14219E+14	8,27015E+14	7,97798E+13	7,14197E+14	5,54458E+14	7,40851E+14	6,30573E+14
TC0400003631.mm.2	Zcchc17	zinc finger, CCHC domain containing 17	1,59194E+14	7,92453E+14	2,88052E+14	0.0259444326583911	0.17246050413271	-3,14243E+14	9,29436E+14	8,77119E+14	7,99064E+14	6,89103E+14	7,88811E+14	6,71184E+14
TC0200001342.mm.2	Gm14440/Gm14288	predicted gene 14440/predicted gene 14288	2,70861E+14	8,26814E+14	2,88027E+14	0.0259530997680887	0.17246050413271	-3,14273E+14	9,50303E+14	9,85728E+14	8,50329E+14	5,4398E+14	8,6004E+14	7,70502E+14
TC0700000629.mm.2	Scgb1b30	secretoglobin, family 1B, member 30	-0.969394919620949	7,0922E+14	-2,88021E+14	0.0259551583303748	0.17246050413271	-3,1428E+14	6,49855E+14	6,45294E+14	7,13331E+14	7,75697E+14	7,32366E+14	7,38775E+14
TC1400002864.mm.2	Tm9sf1	transmembrane 9 superfamily member 1	0.862470005731892	8,14288E+14	2,88007E+14	0.0259601532457125	0.17246050413271	-3,14297E+14	8,55185E+14	8,80626E+14	7,74785E+14	7,88531E+12	7,88838E+13	7,9776E+13
TC1600000116.mm.2	Ciita	class II transactivator	-1,31045E+14	6,01097E+14	-2,87992E+14	0.0259655923319977	0.17246050413271	-3,14315E+14	4,77985E+13	5,32754E+14	5,97207E+14	6,75622E+14	6,20084E+13	7,02929E+14

TC0400002456.mm.2	Aqp3	aquaporin 3	-1,25802E+14	6,42281E+14	-2,87963E+14	0.0259758253067962	0.17246050413271	-3,1435E+14	5,44924E+14	5,70371E+14	6,2931E+14	7,37589E+14	6,66019E+14	7,05475E+14
TC1100000934.mm.2	Gabarap	gamma-aminobutyric acid receptor associated protein	1,13105E+14	9,55811E+14	2,87955E+14	0.0259785412249202	0.17246050413271	-3,1436E+14	1,03683E+14	1,02414E+14	9,52891E+14	8,81873E+14	9,51267E+14	8,87862E+14
TC0200002310.mm.2	Tm9sf4	transmembrane 9 superfamily protein member 4	2,00751E+13	7,04057E+14	2,87899E+14	0.0259985231770028	0.17246050413271	-3,14428E+14	8,34785E+14	8,40857E+14	7,45585E+14	5,28554E+14	6,71844E+14	6,02719E+14
TC0X00000525.mm.2	Fhl1	four and a half LIM domains 1	1,05405E+14	7,94512E+14	2,8785E+14	0.0260156647172395	0.17246050413271	-3,14487E+14	9,05125E+14	8,6098E+13	7,89773E+14	7,65524E+14	7,54814E+14	6,90855E+14
TC0700003400.mm.2	Peg12	paternally expressed 12	-1,17723E+13	6,32052E+14	-2,87844E+14	0.0260179039425195	0.17246050413271	-3,14494E+14	5,64348E+14	5,5705E+14	6,35423E+14	7,2142E+14	6,27684E+14	6,86388E+14
TC0X00000742.mm.2	Brcc3	BRCA1/BRCA2-containing complex, subunit 3	1,94521E+14	7,11241E+14	2,87826E+14	0.0260242777680367	0.17246050413271	-3,14516E+14	8,48128E+14	8,51357E+13	7,45666E+12	5,64777E+14	6,90796E+14	5,66721E+14
TC1000003050.mm.2	Xpot	exportin, tRNA (nuclear export receptor for tRNAs)	1,66391E+14	6,7092E+14	2,87803E+13	0.0260324531492837	0.17246050413271	-3,14544E+14	7,91732E+14	7,6444E+14	7,00558E+14	5,22833E+14	6,26144E+14	6,19813E+14
TC0100003027.mm.2	Dars	aspartyl-tRNA synthetase	1,28662E+14	7,14098E+14	2,8779E+13	0.0260370958382585	0.17246050413271	-3,1456E+14	8,11478E+14	8,00871E+14	7,19724E+14	6,35302E+14	7,02901E+14	6,14309E+13
TC1900000684.mm.2	Elovl3	elongation of very long chain fatty acids (FEN1/Elo2, SUR4/Elo3, yeast)-like 3	-0.966999035043056	4,65742E+14	-2,87755E+14	0.0260496064541075	0.17246050413271	-3,14602E+14	4,17431E+14	3,85183E+14	4,76172E+14	5,19842E+14	4,82425E+14	5,134E+14
TC0800002832.mm.2	Enkd1	enkurin domain containing 1	-1,1299E+14	6,42337E+14	-2,87739E+13	0.0260550357024398	0.17246050413271	-3,14621E+14	5,81418E+14	5,72885E+14	6,43245E+14	7,37037E+14	6,5044E+13	6,68998E+14
TC1000001084.mm.2	Arl1	ADP-ribosylation factor-like 1	1,33149E+14	9,43875E+14	2,87702E+14	0.0260680813578848	0.17246050413271	-3,14665E+14	1,02265E+14	1,0181E+14	9,51604E+14	8,22848E+14	9,3311E+14	9,14937E+14
TC1100002438.mm.2	Acyp2	acylphosphatase 2, muscle type	1,1153E+14	6,79875E+14	2,87691E+14	0.0260722531660662	0.17246050413271	-3,1468E+14	7,85952E+14	7,26796E+13	6,13829E+14	6,75858E+14	6,56877E+14	6,19937E+14
TC0700000224.mm.2	Bbc3	BCL2 binding component 3	-1,47761E+14	7,83144E+14	-2,87641E+14	0.0260899160873422	0.17246050413271	-3,1474E+14	6,86781E+14	7,08735E+14	7,8695E+14	9,04088E+14	7,58411E+14	8,53901E+14
TC0100000284.mm.2	Rpl31	ribosomal protein L31	0.936044119392488	1,00121E+14	2,87623E+14	0.0260964033317469	0.17246050413271	-3,14762E+14	1,07302E+14	1,05186E+14	9,78773E+14	9,58904E+14	9,95584E+14	9,49095E+14
TC1500000617.mm.2	Apol6	apolipoprotein L 6	-1,19457E+14	6,20155E+14	-2,87612E+14	0.0261000478486445	0.17246050413271	-3,14774E+14	5,20114E+14	5,86355E+14	6,36646E+14	7,08736E+14	6,12267E+14	6,56811E+14
TC0200005383.mm.2	Zfp931	zinc finger protein 931	2,2563E+14	7,94874E+13	2,87573E+14	0.0261140034811815	0.17246050413271	-3,14822E+14	8,90677E+14	9,27975E+14	7,95401E+14	5,71991E+13	8,57276E+13	7,25926E+14
TC1200000711.mm.2	Mpp5	membrane protein, palmitoylated 5 (MAGUK p55 subfamily member 5)	2,11424E+14	6,77169E+14	2,87502E+14	0.0261392551469282	0.17246050413271	-3,14908E+13	8,10504E+14	8,21708E+14	7,25846E+14	4,83517E+14	6,06087E+13	6,15352E+14
TC1000002239.mm.2	Sgpl1	sphingosine phosphate lyase 1	-1,11479E+14	7,77096E+14	-2,87496E+14	0.026141488098234	0.17246050413271	-3,14915E+14	7,2085E+14	7,05177E+14	7,79086E+14	8,69899E+14	8,01324E+14	7,86238E+14
TC0700000863.mm.2	Fam83e	family with sequence similarity 83, member E	-1,20797E+14	5,56933E+14	-2,87459E+13	0.0261546835871518	0.17246050413271	-3,1496E+14	4,75019E+12	4,94321E+14	5,5654E+14	6,54394E+14	5,57502E+14	6,03823E+14
TC1400001223.mm.2	Frg2f10	FSHD region gene 2 family member 10	-0.878532528048054	5,35887E+14	-2,87354E+14	0.0261922731540512	0.17246050413271	-3,15088E+14	4,83793E+14	4,58722E+14	5,45393E+14	5,72829E+14	5,74912E+14	5,79672E+14
TC1400001224.mm.2	Frg2f13	FSHD region gene 2 family member 13	-0.878532528048054	5,35887E+14	-2,87354E+14	0.0261922731540512	0.17246050413271	-3,15088E+14	4,83793E+14	4,58722E+14	5,45393E+14	5,72829E+14	5,74912E+14	5,79672E+14
TC1100004121.mm.2	Ube2o	ubiquitin-conjugating enzyme E2O	1,15816E+14	7,33493E+14	2,87336E+14	0.0261985041780364	0.17246050413271	-3,15109E+13	8,17688E+14	8,03809E+14	6,60302E+14	7,29564E+14	7,31155E+14	6,58441E+14
TC0100003538.mm.2	Ufc1	ubiquitin-fold modifier conjugating enzyme 1	0.893469879680747	8,09691E+13	2,87326E+14	0.0262021482494297	0.17246050413271	-3,15121E+14	8,76298E+14	8,53531E+14	7,75496E+14	7,75638E+14	8,06621E+14	7,70562E+14
TC0X00000373.mm.2	Mcts1	malignant T cell amplified sequence 1	1,71996E+14	6,78088E+14	2,87291E+14	0.0262145074718221	0.17246050413271	-3,15163E+14	7,93766E+14	7,97311E+14	6,90499E+14	5,56586E+14	6,81392E+14	5,48974E+14
TC1200000037.mm.2	Wdcp	WD repeat and coiled coil containing	0.897661810314877	6,70019E+14	2,8722E+14	0.0262398193827613	0.17246050413271	-3,15249E+14	7,628E+14	7,33637E+14	6,61187E+13	6,55718E+14	6,19698E+14	5,87074E+14
TC0100002210.mm.2	Mitd1	MIT, microtubule interacting and transport, domain containing 1	1,50287E+14	5,4015E+14	2,87205E+14	0.0262452977194234	0.17246050413271	-3,15267E+14	6,40696E+14	6,03576E+14	5,45439E+14	3,98259E+14	5,06725E+14	5,46206E+14
TC0300001141.mm.2	Mybphl	myosin binding protein H-like	-1,35349E+14	4,59926E+14	-2,8719E+14	0.0262507035616203	0.17246050413271	-3,15286E+14	3,39065E+14	4,06992E+14	4,56576E+14	5,6018E+14	5,23871E+14	4,7287E+13
TC1900000971.mm.2	Snx32	sorting nexin 32	1,42E+14	7,35466E+14	2,87168E+14	0.0262587121711168	0.17246050413271	-3,15313E+14	8,51621E+14	8,11172E+14	7,41014E+14	6,37779E+14	7,318E+14	6,39407E+14
TC1900000907.mm.2	Tbc1d10c	TBC1 domain family, member 10c	-1,20888E+14	7,2047E+14	-2,87165E+14	0.0262595062425091	0.17246050413271	-3,15315E+14	6,29917E+14	6,70167E+14	7,2776E+14	8,14101E+14	7,68277E+14	7,12602E+14
TC1900000691.mm.2	Fbxl15	F-box and leucine-rich repeat protein 15	-0.933620814307702	7,49672E+14	-2,87134E+13	0.0262708433691698	0.17246050413271	-3,15354E+14	6,89876E+14	6,81522E+14	7,53406E+13	8,04717E+14	7,75931E+13	7,92577E+14
TC1100000756.mm.2	Alkbh5	alkB homolog 5, RNA demethylase	1,44601E+14	7,11688E+14	2,87078E+14	0.0262906273550074	0.17246050413271	-3,15421E+14	7,88131E+14	7,92479E+14	7,19838E+14	5,71569E+14	6,97453E+14	7,0066E+14
TC0400002476.mm.2	Rpp25l	ribonuclease P/MRP 25 subunit-like	-1,34478E+14	7,47824E+14	-2,8703E+14	0.0263079664474726	0.17246050413271	-3,15479E+14	6,70136E+14	6,57063E+13	7,42787E+13	8,53368E+13	7,45689E+14	8,17901E+14
TC1100004107.mm.2	Acox1	acyl-Coenzyme A oxidase 1, palmitoyl	1,657E+14	7,05296E+14	2,87029E+14	0.026308444846176	0.17246050413271	-3,15481E+14	8,39825E+14	8,5286E+14	7,70111E+14	5,91173E+14	5,92658E+14	5,85151E+14
TC1200002511.mm.2	Itgb8	integrin beta 8	1,03061E+14	7,07293E+13	2,86959E+13	0.0263332812252959	0.17246050413271	-3,15565E+14	7,84785E+14	7,80677E+14	6,51952E+13	7,07388E+14	6,86317E+14	6,32636E+14
TC1100004110.mm.2	Srp68	signal recognition particle 68	1,6583E+14	6,74669E+14	2,86941E+13	0.0263400519284806	0.17246050413271	-3,15588E+14	7,88192E+14	7,69265E+14	6,9388E+14	5,31917E+14	6,70288E+14	5,9447E+14
TC0300002475.mm.2	Setdb1	SET domain, bifurcated 1	1,11738E+14	6,39577E+14	2,86932E+14	0.0263429641423136	0.17246050413271	-3,15597E+14	7,3403E+14	6,95143E+14	6,10538E+14	5,95158E+14	6,43541E+14	5,59055E+14
TC1700002488.mm.2	Myl12a	myosin, light chain 12A, regulatory, non-sarcomeric	1,02828E+14	9,70438E+14	2,86848E+14	0.0263733888835228	0.17246050413271	-3,157E+14	1,03835E+14	1,03952E+14	9,51566E+14	9,20656E+14	9,71452E+14	9,01076E+14
TC0700002617.mm.2	Atp1a3	ATPase, Na+/K+ transporting, alpha 3 polypeptide	1,67329E+13	1,05415E+14	2,86846E+14	0.0263738765771646	0.17246050413271	-3,15702E+14	1,17572E+14	1,15974E+14	1,08837E+14	9,12427E+14	1,01631E+14	9,72305E+14
TC1100002904.mm.2	Usp22	ubiquitin specific peptidase 22	1,68914E+14	9,38048E+14	2,86831E+14	0.0263793750557542	0.17246050413271	-3,1572E+14	1,05263E+14	1,04066E+14	9,68229E+14	7,87233E+14	9,06217E+14	8,73318E+14
TC1100002574.mm.2	Ttc1	tetratricopeptide repeat domain 1	0.951416687689764	6,48961E+14	2,86666E+13	0.0264386359070879	0.17246050413271	-3,1592E+14	7,03257E+14	7,25873E+14	6,38831E+14	6,00015E+14	6,32459E+14	5,93332E+14
TC1100004295.mm.2	Nme2	NME/NM23 nucleoside diphosphate kinase 2	1,01123E+14	8,78771E+14	2,86665E+14	0.0264393243483579	0.17246050413271	-3,15922E+13	9,39487E+14	9,55917E+14	8,61933E+14	8,31226E+14	8,74333E+14	8,09729E+14
TC0200002721.mm.2	Zfp973/Zfp965	zinc finger protein 973/zinc finger protein 965	2,77707E+14	8,22563E+14	2,86652E+13	0.0264440193553624	0.17246050413271	-3,15938E+14	9,4807E+14	9,90214E+14	8,48382E+14	5,34489E+14	8,56582E+14	7,57641E+14
TC1200000022.mm.2	Ptrhd1	peptidyl-tRNA hydrolase domain containing 1	0.890535026038006	5,7014E+14	2,86612E+14	0.0264584097826887	0.17246050413271	-3,15986E+13	6,26878E+14	5,89289E+14	5,28415E+14	5,09645E+14	5,80417E+13	5,86194E+14
TC1600000137.mm.2	Tnfrsf17	tumor necrosis factor receptor superfamily, member 17	-1,67087E+14	5,10316E+14	-2,86537E+14	0.0264854710973222	0.17246050413271	-3,16077E+14	3,89787E+14	4,22567E+14	4,87146E+14	6,59381E+14	5,27135E+14	5,75878E+14
TC0400000739.mm.2	Trim32	tripartite motif-containing 32	1,20702E+14	6,78761E+13	2,86531E+14	0.0264873653389992	0.17246050413271	-3,16083E+14	7,61358E+14	7,57929E+14	6,44744E+14	6,33139E+14	6,92603E+14	5,82791E+14
TC0400003607.mm.2	Ccdc28b	coiled coil domain containing 28B	-0.941386747015518	7,80194E+13	-2,86526E+14	0.0264892838339932	0.17246050413271	-3,1609E+14	7,26963E+14	7,18747E+14	8,45256E+14	7,88731E+14	7,99887E+14	8,0158E+14
TC0200002341.mm.2	Cbfa2t2	CBFA2/RUNX1 translocation partner 2	0.855684159115045	6,81036E+14	2,86473E+14	0.026508319161597	0.17246050413271	-3,16153E+14	7,44199E+14	7,43935E+14	6,70691E+14	6,46307E+14	6,48638E+13	6,32444E+14
TC0200005425.mm.2	Ythdf1	YTH N6-methyladenosine RNA binding protein 1	1,32593E+14	6,53177E+14	2,86407E+14	0.0265321760190691	0.17246050413271	-3,16233E+14	7,34954E+14	7,34861E+14	6,22911E+14	5,8172E+14	5,60562E+14	6,84054E+14
TC0400001391.mm.2	Hpcal4	hippocalcin-like 4	1,31837E+14	8,74629E+14	2,86339E+14	0.0265570616906586	0.17246050413271	-3,16317E+14	9,62256E+14	9,56493E+14	8,91405E+14	7,6367E+14	8,28349E+14	8,45599E+14
TC0200002346.mm.2	Zfp341	zinc finger protein 341	-0.915837411440465	6,73952E+14	-2,86326E+14	0.0265616238891183	0.17246050413271	-3,16332E+14	6,01915E+14	6,28159E+14	7,21998E+14	6,91244E+14	6,85147E+14	7,15247E+14
TC0500000080.mm.2	Sema3d	sema domain, immunoglobulin domain (Ig), short basic domain, secreted, (semaphorin) 3D	1,40581E+14	5,95362E+14	2,86313E+14	0.0265663829331302	0.17246050413271	-3,16348E+14	6,8115E+14	6,60229E+14	5,31151E+14	5,29066E+14	6,55904E+14	5,14671E+14
TC1100002670.mm.2	Canx	calnexin	1,71769E+14	8,63373E+14	2,86298E+13	0.0265717957789552	0.17246050413271	-3,16366E+14	9,89854E+14	9,88611E+14	8,89665E+14	7,45261E+12	8,443E+14	7,22548E+13
TC0600000980.mm.2	Tgfa	transforming growth factor alpha	-1,12727E+14	7,39072E+14	-2,86295E+14	0.0265728796805652	0.17246050413271	-3,1637E+14	6,6461E+13	6,603E+14	7,28844E+14	8,21521E+14	7,66216E+14	7,92939E+14
TC0700001446.mm.2	Trpc2/Xndc1/Xntrpc	transient receptor potential cation channel, subfamily C, member 2/Xrcc1 N-terminal domain containing 1/Xndc1-transient receptor potential cation channel, subfamily C, member 2 readthrough	-1,49276E+14	7,16221E+14	-2,86282E+14	0.0265776206493751	0.17246050413271	-3,16385E+14	6,14032E+14	6,14795E+14	7,01171E+14	8,26208E+14	7,23882E+14	8,17241E+14
TC0700000467.mm.2	Zfp607b	zinc finger protein 607B	0.881611148564123	7,13793E+14	2,86273E+14	0.0265810041405249	0.17246050413271	-3,16397E+13	7,93564E+14	7,56012E+14	6,86531E+14	6,86723E+14	6,86478E+14	6,73449E+14
TC1000001745.mm.2	Grm1	glutamate receptor, metabotropic 1	0.830031370225724	7,60887E+14	2,86253E+14	0.0265881803323286	0.17246050413271	-3,16421E+14	8,11588E+14	8,23699E+14	7,33371E+14	7,35909E+14	7,33646E+14	7,27108E+14
TC1600000386.mm.2	Plaat1	phospholipase A and acyltransferase 1	2,04726E+14	6,39461E+14	2,86252E+14	0.0265882871449535	0.17246050413271	-3,16421E+14	7,8463E+14	7,36361E+14	6,58781E+14	4,52757E+14	5,51146E+14	6,53091E+14
TC1100001407.mm.2	Car10	carbonic anhydrase 10	1,39791E+14	7,18183E+14	2,86179E+14	0.026614878020869	0.17246050413271	-3,1651E+14	7,99126E+14	8,10124E+14	7,15442E+14	6,14226E+13	7,34346E+14	6,35834E+14
TC0700001439.mm.2	Phox2a	paired-like homeobox 2a	-1,45226E+14	6,77844E+13	-2,86165E+14	0.0266200777526212	0.17246050413271	-3,16527E+14	5,78343E+14	5,92579E+14	6,59551E+14	8,01822E+14	6,94676E+14	7,40091E+14
TC0300002219.mm.2	Gucy1b1	guanylate cyclase 1, soluble, beta 1	1,63735E+13	8,37745E+14	2,86156E+14	0.0266234564002775	0.17246050413271	-3,16539E+14	9,6517E+13	9,55069E+14	8,64755E+14	7,28013E+14	8,13056E+14	7,00405E+14
TC1500000901.mm.2	Cntn1	contactin 1	1,61121E+14	9,15151E+14	2,86114E+13	0.0266385917813918	0.17246050413271	-3,16589E+14	1,03969E+14	1,01509E+14	9,24785E+14	8,07756E+14	9,14772E+14	7,88805E+14
TC1800000343.mm.2	Ik	IK cytokine	1,48374E+14	8,06544E+13	2,86074E+14	0.0266530073794748	0.17246050413271	-3,16637E+14	9,16691E+14	8,88404E+14	8,22651E+14	6,85696E+14	7,96165E+14	7,29659E+14
TC0700001356.mm.2	Rsf1	remodeling and spacing factor 1	2,42573E+14	6,54022E+14	2,86044E+14	0.0266638515374334	0.17246050413271	-3,16673E+14	8,04766E+14	7,8652E+14	6,9045E+14	4,15689E+13	6,52236E+13	5,74468E+14
TC1100003644.mm.2	Gpr179	G protein-coupled receptor 179	-0.849984746755322	6,49499E+14	-2,86029E+14	0.0266694090918948	0.17246050413271	-3,16692E+14	6,12746E+14	5,96923E+14	6,90503E+13	6,89162E+14	6,42788E+14	6,6487E+14
TC0300002742.mm.2	Sars	seryl-aminoacyl-tRNA synthetase	1,07875E+14	9,11505E+12	2,85976E+14	0.0266888103723797	0.17246050413271	-3,16756E+14	9,9164E+14	9,67101E+14	9,12089E+14	8,30903E+14	8,94711E+14	8,72584E+14
TC0300002094.mm.2	Ssr3	signal sequence receptor, gamma	1,69533E+14	7,63998E+14	2,85955E+14	0.026696491733019	0.17246050413271	-3,16782E+14	8,60946E+14	8,78508E+14	7,69487E+14	6,30901E+14	7,83462E+14	6,60685E+14
TC0400003422.mm.2	Ppih	peptidyl prolyl isomerase H	1,34895E+14	6,97452E+13	2,8594E+14	0.0267016905560673	0.17246050413271	-3,16799E+14	7,67488E+14	7,6884E+14	6,81241E+13	5,85296E+14	7,37827E+14	6,44024E+14
TC0800003184.mm.2	Sipa1l2	signal-induced proliferation-associated 1 like 2	1,61283E+14	6,35784E+14	2,8594E+14	0.0267017652839709	0.17246050413271	-3,168E+14	7,24419E+14	7,11788E+14	6,40078E+14	4,73562E+14	6,56991E+14	6,07868E+14
TC0300002431.mm.2	Gm10697/Tdpoz5	predicted gene 10697/TD and POZ domain containing 5	-0.915648843850062	4,64636E+14	-2,85909E+14	0.026712987222708	0.17246050413271	-3,16837E+14	4,24617E+14	4,25105E+13	5,05405E+14	5,27446E+14	4,2978E+14	4,75467E+14
TC0300002433.mm.2	Gm10697/Tdpoz5	predicted gene 10697/TD and POZ domain containing 5	-0.915648843850062	4,64636E+14	-2,85909E+14	0.026712987222708	0.17246050413271	-3,16837E+14	4,24617E+14	4,25105E+13	5,05405E+14	5,27446E+14	4,2978E+14	4,75467E+14
TC1200000875.mm.2	Ahsa1	AHA1, activator of heat shock protein ATPase 1	0.989883745086595	7,76676E+14	2,85903E+14	0.0267152182328621	0.17246050413271	-3,16844E+14	8,41902E+14	8,55121E+13	7,58237E+14	7,40809E+14	7,64861E+14	6,99124E+14
TC1100003026.mm.2	Pfas/Mir3062	phosphoribosylformylglycinamidine synthase (FGAR amidotransferase)/microRNA 3062	-0.852464764551309	6,77272E+14	-2,85882E+14	0.0267230594393859	0.17246050413271	-3,1687E+14	6,26746E+14	6,24593E+14	7,11375E+14	7,10457E+14	6,80979E+14	7,09482E+14
TC0500003375.mm.2	Syna	syncytin a	-1,33258E+14	4,92379E+14	-2,85881E+13	0.0267233928857208	0.17246050413271	-3,16872E+14	4,09934E+14	3,78985E+14	4,82784E+14	5,72651E+14	5,09424E+14	6,00493E+13
TC1600002124.mm.2	Kcnj6	potassium inwardly-rectifying channel, subfamily J, member 6	0.949977566004848	6,59023E+14	2,85822E+14	0.0267446646364541	0.17246050413271	-3,16942E+14	7,39004E+14	7,20917E+14	6,30043E+14	6,39882E+14	6,40278E+14	5,84013E+14
TC0200002005.mm.2	Hspa12b	heat shock protein 12B	-1,08552E+14	6,44514E+13	-2,85758E+14	0.0267681846236487	0.17246050413271	-3,1702E+14	5,62187E+14	5,80585E+14	6,55498E+14	7,04379E+14	6,46115E+14	7,18322E+14
TC1100000872.mm.2	Pik3r5	phosphoinositide-3-kinase regulatory subunit 5	-0.970945664215585	6,35988E+14	-2,85743E+13	0.0267737269954853	0.17246050413271	-3,17039E+14	5,81727E+14	5,87267E+14	6,56818E+14	7,06366E+14	6,20979E+14	6,6277E+14
TC0X00002431.mm.2	Pnck	pregnancy upregulated non-ubiquitously expressed CaM kinase	2,06031E+13	7,7E+14	2,85733E+14	0.0267774274526148	0.17246050413271	-3,17051E+14	9,45904E+14	8,56346E+14	8,04646E+14	5,85542E+14	7,2128E+14	7,06285E+14
TC0900002050.mm.2	Robo3	roundabout guidance receptor 3	-0.921084640728802	6,14258E+14	-2,85688E+14	0.026793887812422	0.17246050413271	-3,17106E+14	5,57898E+13	5,3136E+14	6,25444E+13	6,4803E+14	6,41806E+14	6,81008E+14
TC1600001761.mm.2	Cmss1	cms small ribosomal subunit 1	-1,32392E+14	7,81959E+14	-2,85639E+14	0.0268115584943255	0.17246050413271	-3,17164E+14	6,87952E+14	7,31508E+14	8,06701E+12	8,77543E+14	7,43787E+14	8,44265E+14
TC0400001881.mm.2	Zfp992/Zfp991	zinc finger protein 992/zinc finger protein 991	2,17612E+14	6,17148E+14	2,85581E+14	0.0268328195802626	0.17246050413271	-3,17235E+14	7,77134E+14	7,05192E+14	6,24216E+14	4,22884E+14	6,54462E+14	5,18999E+14
TC0800001214.mm.2	Tmem208	transmembrane protein 208	1,90583E+14	6,11562E+14	2,85577E+14	0.0268342994267592	0.17246050413271	-3,1724E+12	7,32138E+13	7,37147E+14	6,31747E+14	4,56371E+14	6,18997E+14	4,92975E+14
TC0100000858.mm.2	Agap1	ArfGAP with GTPase domain, ankyrin repeat and PH domain 1	0.916901738167757	6,91782E+14	2,8553E+14	0.0268514966777839	0.17246050413271	-3,17297E+13	7,76837E+14	7,61526E+14	6,93329E+14	6,61654E+14	6,47428E+14	6,09919E+14
TC0500000896.mm.2	Ccng2	cyclin G2	-1,21945E+14	9,77643E+14	-2,85505E+14	0.0268609111398665	0.17246050413271	-3,17328E+14	8,68384E+13	9,00999E+14	1,05686E+14	9,56414E+14	1,06217E+14	1,02103E+14
TC1600001692.mm.2	Dzip3	DAZ interacting protein 3, zinc finger	1,70487E+14	7,15961E+13	2,85487E+14	0.0268673113755617	0.17246050413271	-3,17349E+14	8,50353E+14	8,39009E+14	7,06555E+14	6,41833E+14	7,16944E+14	5,41072E+14
TC0100001459.mm.2	Rc3h1	RING CCCH (C3H) domains 1	2,10329E+14	6,58404E+14	2,85462E+14	0.0268766167382639	0.17246050413271	-3,1738E+14	7,85789E+14	7,94941E+14	6,81685E+14	4,78387E+14	6,71501E+14	5,38121E+14
TC0600002084.mm.2	Dgki	diacylglycerol kinase, iota	1,99994E+14	6,79353E+14	2,85454E+14	0.0268794022993458	0.17246050413271	-3,17389E+14	8,29705E+14	8,39399E+14	6,85099E+14	5,84016E+14	6,71665E+13	4,66232E+14
TC1300000555.mm.2	Barx1	BarH-like homeobox 1	-0.954274570431256	5,08399E+14	-2,85446E+14	0.0268822664703817	0.17246050413271	-3,17398E+14	4,58522E+14	4,52091E+14	5,21102E+14	5,80366E+14	5,26277E+14	5,12037E+14
TC0700000777.mm.2	Klk6	kallikrein related-peptidase 6	1,42438E+13	6,46332E+14	2,85372E+14	0.0269095875662316	0.17246050413271	-3,17489E+14	6,74081E+14	8,05556E+14	6,03739E+14	5,91022E+14	5,70268E+13	6,33327E+14
TC0800000244.mm.2	Defa37/Defa36	defensin, alpha, 37/defensin, alpha, 36	-1,09704E+14	6,23627E+14	-2,85364E+14	0.0269125380190449	0.17246050413271	-3,17498E+14	5,41984E+14	5,68669E+14	6,22493E+14	7,07569E+14	6,40034E+14	6,61012E+13
TC0200004422.mm.2	Ccdc32	coiled-coil domain containing 32	1,40313E+14	7,7268E+14	2,85331E+14	0.0269245504671022	0.17246050413271	-3,17538E+14	8,50088E+13	8,5823E+14	7,844E+14	6,43292E+13	7,61285E+14	7,38787E+14
TC0300000903.mm.2	Ctss	cathepsin S	0.888559896272378	7,82949E+14	2,85322E+14	0.0269280315457022	0.17246050413271	-3,1755E+14	8,28119E+14	8,70822E+14	7,60532E+14	7,60697E+14	7,35929E+14	7,41595E+13
TC0700003607.mm.2	Rps17	ribosomal protein S17	0.868318272417625	9,33433E+14	2,85255E+14	0.0269525944524344	0.17246050413271	-3,17631E+14	9,83872E+14	9,90634E+14	8,99938E+14	9,00904E+14	9,30323E+14	8,94929E+14
TC1900000031.mm.2	Ptprcap	protein tyrosine phosphatase, receptor type, C polypeptide-associated protein	-1,24991E+14	6,05644E+14	-2,85251E+14	0.0269541442305124	0.17246050413271	-3,17636E+14	5,37153E+14	5,04545E+13	5,90187E+13	7,01493E+14	6,37754E+14	6,6273E+14
TC0900000704.mm.2	Rpp25	ribonuclease P/MRP 25 subunit	0.831328879616376	6,62488E+13	2,85245E+14	0.0269562582444035	0.17246050413271	-3,17643E+14	7,07755E+14	7,10816E+14	6,34684E+14	6,17621E+14	6,53249E+14	6,50805E+14
TC0100000282.mm.2	Npas2	neuronal PAS domain protein 2	1,46852E+14	7,04088E+14	2,85157E+14	0.0269884898427801	0.17246050413271	-3,17749E+14	8,23462E+14	8,12263E+14	7,4714E+14	5,94881E+14	6,30164E+14	6,16616E+14
TC0800001497.mm.2	Acsf3	acyl-CoA synthetase family member 3	0.879290714940776	5,73805E+13	2,85146E+14	0.0269927871713729	0.17246050413271	-3,17763E+14	6,1544E+14	5,88408E+14	5,25033E+14	5,02957E+14	5,95575E+14	6,15419E+13
TC1000000564.mm.2	Nodal	nodal	-1,60478E+14	6,89089E+14	-2,85126E+13	0.0270000688502215	0.17246050413271	-3,17787E+14	5,80951E+14	5,9163E+14	6,95834E+14	7,97702E+14	6,64149E+14	8,04268E+14
TC0700000586.mm.2	Lgi4	leucine-rich repeat LGI family, member 4	1,45748E+14	6,47263E+14	2,85077E+14	0.0270181833286018	0.17246050413271	-3,17847E+14	7,41034E+14	7,64332E+14	5,77964E+14	6,35907E+14	5,13071E+14	6,51272E+14
TC0100000635.mm.2	Vil1	villin 1	-1,19812E+12	7,56074E+14	-2,8505E+14	0.0270281111747028	0.17246050413271	-3,17879E+14	6,85625E+14	6,81312E+14	7,70117E+14	8,36444E+14	7,37637E+14	8.25308E+14
TC1200001265.mm.2	Zbtb42	zinc finger and BTB domain containing 42	-1,08791E+14	6,93605E+14	-2,85049E+14	0.027028323955118	0.17246050413271	-3,1788E+13	6,04665E+14	6,29994E+14	6,92051E+13	7,6019E+14	7,10759E+14	7,63971E+14
TC1700002170.mm.2	Polr1c	polymerase (RNA) I polypeptide C	0.886535505487269	7,25521E+14	2,85042E+14	0.0270310066677388	0.17246050413271	-3,17889E+14	7,97559E+14	7,60893E+14	6,82911E+14	6,98234E+13	6,98954E+14	7,14577E+14
TC0900003040.mm.2	Slc38a3	solute carrier family 38, member 3	1,31471E+14	8,50196E+14	2,85011E+13	0.0270423026716301	0.17246050413271	-3,17926E+14	9,2031E+13	9,24353E+13	8,49253E+14	7,32468E+14	8,65205E+14	8,09585E+14
TC0200002177.mm.2	Kiz	kizuna centrosomal protein	1,56337E+14	6,46674E+14	2,84957E+14	0.027062535133762	0.17246050413271	-3,17992E+13	7,69434E+14	7,55787E+14	6,74631E+14	5,37916E+14	6,1914E+14	5,23133E+14
TC1000001153.mm.2	Cep83	centrosomal protein 83	2,13977E+14	5,79921E+14	2,84914E+14	0.0270784199565565	0.17246050413271	-3,18045E+14	6,70247E+14	6,87922E+13	5,7696E+14	3,53255E+14	6,49349E+14	5,41791E+14
TC0500002410.mm.2	Ccdc149	coiled-coil domain containing 149	1,09181E+14	7,0315E+14	2,84909E+14	0.0270803244229724	0.17246050413271	-3,18051E+14	7,96027E+14	7,50289E+14	7,03015E+13	6,2494E+14	6,76832E+14	6,678E+14
TC0400000918.mm.2	Dmrta1	doublesex and mab-3 related transcription factor like family A1	-1,36623E+14	5,83415E+14	-2,84872E+14	0.0270939808721973	0.17246050413271	-3,18096E+12	5,18912E+14	4,87493E+14	5,96249E+14	6,83401E+13	5,56273E+14	6,58164E+14
TC1200002507.mm.2	Sp4	trans-acting transcription factor 4	1,8901E+14	6,17333E+12	2,84861E+14	0.0270979170469353	0.17246050413271	-3,18109E+14	7,51644E+14	7,34543E+14	6,43941E+14	4,64226E+14	6,12734E+13	4,96912E+14
TC1900001671.mm.2	Smndc1	survival motor neuron domain containing 1	1,40779E+14	6,74236E+14	2,84856E+14	0.0270999249786733	0.17246050413271	-3,18115E+14	7,67222E+14	7,58845E+14	6,73734E+14	5,70775E+14	6,8773E+14	5,87108E+14

TC0100003823.mm.2	Traf5	TNF receptor-associated factor 5	-1,2023E+14	5,12945E+14	-2,84854E+14	0.0271004776555589	0.17246050413271	-3,18117E+14	4,71092E+14	4,8895E+14	5,46401E+14	6,54101E+14	4,68258E+14	4,48867E+14
TC1200002521.mm.2	Gm9804	predicted gene 9804	-1,43996E+14	6,24263E+14	-2,84769E+14	0.0271318808738027	0.172533155088317	-3,1822E+14	5,51559E+14	5,19286E+13	6,09566E+14	7,4927E+14	6,3728E+14	6,78617E+14
TC1200002285.mm.2	Serpina1e	serine (or cysteine) peptidase inhibitor, clade A, member 1E	-1,1391E+14	6,17784E+14	-2,84754E+13	0.0271373554803114	0.172533155088317	-3,18238E+14	5,33634E+14	5,56981E+14	6,10931E+14	7,07504E+14	6,4404E+14	6,53617E+14
TC0400001045.mm.2	Gm12789	predicted gene 12789	-1,05572E+14	5,28716E+14	-2,84709E+14	0.0271541383557421	0.172533155088317	-3,18293E+14	4,32685E+14	4,71154E+14	5,34503E+14	5,8048E+14	5,47274E+13	6,06201E+14
TC0500001327.mm.2	Tbx3	T-box 3	-1,28482E+14	5,82396E+14	-2,84705E+14	0.0271556518290124	0.172533155088317	-3,18298E+13	5,21839E+14	4,95991E+14	5,77159E+14	6,97635E+13	5,90976E+14	6,10779E+14
TC1700001143.mm.2	Clip4	CAP-GLY domain containing linker protein family, member 4	1,31529E+14	8,57696E+14	2,84602E+14	0.0271938200949402	0.172533155088317	-3,18423E+14	9,61382E+14	9,38006E+14	8,75948E+14	7,60382E+14	8,32386E+14	7,78073E+14
TC0400003792.mm.2	C1qb	complement component 1, q subcomponent, beta polypeptide	1,832E+14	7,7351E+14	2,84591E+14	0.0271979248036009	0.172533155088317	-3,18436E+14	8,29131E+14	9,09963E+14	7,7407E+14	5,98625E+13	8,01252E+14	7,28019E+14
TC0500002305.mm.2	Nkx3-2	NK3 homeobox 2	-1,08345E+14	6,66702E+13	-2,84589E+14	0.0271987884120742	0.172533155088317	-3,18439E+14	5,98302E+14	5,67087E+14	6,71212E+14	7,10867E+14	6,89371E+14	7,63372E+14
TC0900001845.mm.2	Olfm2	olfactomedin 2	1,02347E+14	6,31393E+14	2,84588E+14	0.0271990544883093	0.172533155088317	-3,1844E+14	6,93307E+14	6,83751E+14	6,23129E+14	5,49235E+14	6,30245E+14	6,08689E+14
TC1000002428.mm.2	Gm3238	predicted gene 3238	-0.968253570012578	6,69583E+14	-2,84538E+14	0.0272175641492375	0.172533155088317	-3,185E+14	5,92E+14	6,24302E+14	6,96959E+14	7,12993E+14	6,68353E+14	7,22893E+14
TC0300002284.mm.2	Fcrls	Fc receptor-like S, scavenger receptor	1,21638E+14	7,55075E+14	2,84503E+14	0.0272306636800881	0.172533155088317	-3,18543E+14	8,10567E+14	8,60943E+14	7,17457E+14	7,10779E+14	7,66119E+14	6,64583E+13
TC1300002505.mm.2	Tmem174	transmembrane protein 174	-1,28031E+14	5,68783E+14	-2,84487E+14	0.027236518073011	0.172533155088317	-3,18562E+14	4,82233E+14	4,53775E+14	5,45962E+14	6,46108E+14	6,08427E+14	6,7619E+14
TC0500000391.mm.2	Adra2c	adrenergic receptor, alpha 2c	1,16302E+14	5,65719E+14	2,84454E+14	0.0272490240109901	0.172533155088317	-3,18603E+14	6,40742E+14	6,43514E+14	5,13293E+14	5,3836E+14	4,78592E+14	5,79814E+14
TC0900001142.mm.2	Bcl2a1a	B cell leukemia/lymphoma 2 related protein A1a	0.866207481775864	5,40041E+14	2,8443E+14	0.0272579280409728	0.172533155088317	-3,18632E+14	5,78576E+14	5,91956E+14	5,07774E+14	4,89517E+14	5,50337E+14	5,22087E+14
TC1800000575.mm.2	Megf10	multiple EGF-like-domains 10	1,26106E+14	5,54403E+14	2,84378E+14	0.0272770752978349	0.172533155088317	-3,18694E+14	6,39749E+14	6,1913E+14	5,43931E+14	4,62736E+14	4,86688E+14	5,74184E+14
TC0800000090.mm.2	Naxd	NAD(P)HX dehydratase	1,54837E+14	7,24571E+14	2,84374E+14	0.0272788676768229	0.172533155088317	-3,187E+14	8,31701E+14	8,06759E+14	7,41062E+14	5,87724E+14	7,19145E+14	6,61036E+14
TC0700000508.mm.2	Fbxo17	F-box protein 17	-1,21648E+14	6,14947E+14	-2,84362E+14	0.0272832172261547	0.172533155088317	-3,18714E+14	4,88956E+14	5,76509E+14	6,29995E+14	6,78767E+14	6,30917E+14	6,84536E+14
TC0600000698.mm.2	Lancl2	LanC (bacterial lantibiotic synthetase component C)-like 2	1,48557E+14	7,46097E+14	2,84282E+14	0.0273130867002937	0.17265428246443	-3,18812E+14	8,37414E+14	8,32036E+14	7,63139E+14	6,09197E+14	7,3133E+14	7,03465E+14
TC1100003394.mm.2	Mmp28	matrix metallopeptidase 28 (epilysin)	-1,05316E+14	6,21748E+14	-2,84203E+14	0.0273424826198984	0.172677756537826	-3,18907E+14	5,45615E+13	5,55646E+14	6,36951E+14	6,74941E+13	6,21589E+14	6,95747E+14
TC0600001354.mm.2	Slc25a18	solute carrier family 25 (mitochondrial carrier), member 18	1,59511E+14	7,35147E+14	2,8416E+14	0.0273586103179785	0.172677756537826	-3,1896E+14	8,56367E+14	8,24522E+14	7,36134E+14	6,25734E+14	7,48691E+14	6,19437E+14
TC1400002128.mm.2	Pspc1	paraspeckle protein 1	1,70454E+14	7,43436E+14	2,84129E+14	0.0273701150685568	0.172677756537826	-3,18997E+14	8,70283E+13	8,32283E+14	7,46417E+14	6,15241E+14	7,63425E+14	6,32967E+14
TC1700000978.mm.2	4932415M13Rik	RIKEN cDNA 4932415M13 gene	-0.96227052582631	5,34104E+14	-2,84099E+14	0.0273815722172995	0.172677756537826	-3,19034E+14	4,51243E+14	4,75205E+14	5,38635E+14	5,80267E+14	5,58248E+14	6,01025E+14
TC0400001421.mm.2	Sf3a3	splicing factor 3a, subunit 3	1,38864E+14	6,21767E+14	2,84064E+12	0.0273944838049631	0.172677756537826	-3,19076E+14	7,25854E+14	7,15678E+14	6,42381E+14	5,21423E+14	5,25948E+14	5,99317E+14
TC1300000934.mm.2	2210408I21Rik	RIKEN cDNA 2210408I21 gene	1,43344E+14	5,6194E+14	2,84056E+14	0.0273975130182989	0.172677756537826	-3,19086E+14	6,7716E+14	6,60202E+14	4,9856E+14	5,52114E+14	5,60682E+14	4,22919E+14
TC0400003773.mm.2	Pnrc2	proline-rich nuclear receptor coactivator 2	1,03956E+14	6,55567E+14	2,84045E+14	0.0274017740194139	0.172677756537826	-3,191E+14	7,12828E+14	7,19441E+14	6,44822E+13	5,79535E+14	6,62384E+14	6,14392E+13
TC0900001883.mm.2	Spc24	SPC24, NDC80 kinetochore complex component, homolog (S. cerevisiae)	-1,07173E+14	6,23205E+14	-2,8402E+13	0.0274109038736687	0.172677756537826	-3,19129E+14	5,31015E+14	5,74746E+14	6,24273E+14	6,95834E+14	6,43819E+13	6,69543E+14
TC0700001410.mm.2	Coa4	cytochrome c oxidase assembly factor 4	-1,54962E+14	7,04085E+14	-2,83916E+14	0.0274498135498384	0.172677756537826	-3,19255E+14	5,99683E+14	6,21843E+14	7,00219E+14	8,3123E+14	6,85664E+14	7,85869E+14
TC0100003382.mm.2	Dnm3	dynamin 3	2,63728E+14	8,22079E+14	2,83879E+14	0.0274639632756203	0.172677756537826	-3,19301E+14	1,00164E+14	9,74396E+14	8,45016E+14	6,03566E+14	8,55595E+14	6,52261E+14
TC1000001293.mm.2	Bbs10	Bardet-Biedl syndrome 10 (human)	-0.887650248433095	5,59445E+14	-2,83849E+13	0.027475013674988	0.172677756537826	-3,19337E+14	4,82493E+14	4,96346E+14	5,59687E+14	5,96682E+14	6,2072E+14	6,00741E+14
TC1000000386.mm.2	Sec63	SEC63-like (S. cerevisiae)	0.952515186660984	7,50719E+14	2,83839E+14	0.0274788115994983	0.172677756537826	-3,19349E+14	8,14422E+14	8,15177E+13	7,24143E+14	7,14953E+14	7,48554E+14	6,87064E+14
TC0300000731.mm.2	Ssr2	signal sequence receptor, beta	1,10593E+14	8,42127E+14	2,83823E+14	0.0274846636634979	0.172677756537826	-3,19368E+14	9,17284E+14	9,1417E+14	8,4799E+13	7,6228E+14	8,25227E+14	7,85812E+14
TC0700002435.mm.2	Ceacam15	carcinoembryonic antigen-related cell adhesion molecule 15	-1,1558E+14	6,31604E+14	-2,83822E+14	0.0274853146389421	0.172677756537826	-3,1937E+14	5,3194E+14	5,69912E+14	6,35591E+14	6,97421E+14	6,40481E+14	7,14278E+14
TC0500001297.mm.2	Wsb2	WD repeat and SOCS box-containing 2	1,06893E+14	8,28749E+14	2,83792E+14	0.0274964811469489	0.172677756537826	-3,19406E+14	9,11673E+14	8,97936E+14	8,3928E+14	7,56542E+14	7,71338E+14	7,95724E+14
TC0300000537.mm.2	Ptx3	pentraxin related gene	-1,3416E+13	7,70481E+14	-2,83789E+14	0.0274976534824177	0.172677756537826	-3,1941E+14	6,73761E+14	6,9639E+14	7,75534E+14	8,62938E+14	7,57196E+14	8,57068E+13
TC1600000089.mm.2	Abat	4-aminobutyrate aminotransferase	1,76165E+14	8,8279E+14	2,83727E+14	0.0275208321001122	0.172677756537826	-3,19485E+14	1,02265E+14	9,90272E+14	9,14647E+14	7,45949E+14	8,60231E+14	7,62987E+14
TC1500000718.mm.2	Mchr1	melanin-concentrating hormone receptor 1	1,14147E+14	5,87208E+14	2,8372E+14	0.027523428518718	0.172677756537826	-3,19494E+14	6,74584E+14	6,81389E+14	6,13599E+14	5,14081E+14	5,27698E+14	5,11896E+14
TC0900001600.mm.2	Myrip	myosin VIIA and Rab interacting protein	1,1869E+14	5,53715E+14	2,83656E+14	0.0275474941766033	0.172677756537826	-3,19571E+14	6,34081E+14	6,36764E+14	5,34565E+14	4,989E+14	5,60518E+14	4,5746E+14
TC1100001493.mm.2	Hoxb8	homeobox B8	-1,45714E+14	6,44506E+14	-2,83645E+14	0.0275519190037192	0.172677756537826	-3,19586E+14	5,54782E+14	5,4101E+14	6,38377E+14	7,48843E+14	6,39733E+14	7,44289E+13
TC0200005273.mm.2	Gcnt7	glucosaminyl (N-acetyl) transferase family member 7	-1,1782E+14	5,06902E+14	-2,83626E+14	0.0275588425325258	0.172677756537826	-3,19608E+14	3,9702E+14	4,54333E+14	5,11496E+14	5,75497E+14	5,19149E+14	5,83917E+14
TC0600002713.mm.2	Chst13	carbohydrate sulfotransferase 13	-1,06199E+14	6,64861E+13	-2,83604E+14	0.0275671890748224	0.172677756537826	-3,19635E+14	5,92612E+14	5,9645E+13	6,61673E+14	7,39788E+14	6,81296E+14	7,1735E+14
TC1400002065.mm.2	Cebpe	CCAAT/enhancer binding protein (C/EBP), epsilon	-1,19742E+13	5,63062E+14	-2,83593E+14	0.0275715365155019	0.172677756537826	-3,19649E+14	4,86029E+14	4,79688E+13	5,56472E+14	6,4873E+14	5,73069E+14	6,34386E+14
TC0500002856.mm.2	Nkx6-1	NK6 homeobox 1	-1,19691E+14	6,2295E+14	-2,83556E+14	0.0275852670756124	0.172677756537826	-3,19693E+14	5,60897E+13	5,37664E+14	6,24639E+14	7,13305E+14	6,19386E+14	6,81808E+14
TC0600003535.mm.2	E130112N10Rik	RIKEN cDNA E130112N10 gene	-0.917627800208289	6,33454E+13	-2,8353E+14	0.0275951969922443	0.172677756537826	-3,19725E+14	5,66078E+14	5,77653E+14	6,4947E+14	6,77786E+14	6,4297E+14	6,86766E+14
TC0500002989.mm.2	Fbrsl1	fibrosin-like 1	-0.86954561885973	8,53449E+14	-2,83518E+14	0.0275994902113878	0.172677756537826	-3,19739E+14	8,0386E+14	8,11132E+14	9,12799E+14	8,76102E+14	8,62883E+14	8,53916E+14
TC0100003439.mm.2	Sft2d2	SFT2 domain containing 2	1,1052E+14	6,50888E+14	2,83501E+14	0.0276061503656327	0.172677756537826	-3,1976E+14	7,29252E+14	7,20782E+14	6,42519E+14	5,86475E+14	6,5095E+14	5,75349E+14
TC0600000388.mm.2	Tbxas1	thromboxane A synthase 1, platelet	1,20089E+13	5,88424E+14	2,83447E+14	0.0276263414227425	0.172725583948123	-3,19825E+14	6,37433E+14	6,40845E+14	5,73054E+14	4,65045E+14	6,21141E+14	5,93026E+14
TC1100002852.mm.2	Zfp867	zinc finger protein 867	0.931561018684797	5,76356E+14	2,83424E+14	0.0276352358603775	0.172725583948123	-3,19854E+14	6,49755E+14	6,26014E+14	5,55034E+14	5,34423E+14	5,70372E+14	5,22539E+14
TC0100002977.mm.2	Dpp10	dipeptidylpeptidase 10	1,86064E+14	8,102E+14	2,83357E+14	0.0276606058290283	0.172773523223885	-3,19936E+12	9,35006E+14	9,28887E+13	8,2287E+14	6,68894E+14	8,25387E+14	6,80156E+14
TC1500001466.mm.2	Sybu	syntabulin (syntaxin-interacting)	0.885398832943033	6,21095E+14	2,83347E+14	0.0276643511399258	0.172773523223885	-3,19948E+14	6,69564E+14	6,93637E+14	6,10104E+14	5,76017E+14	5,95677E+14	5,81571E+14
TC1100001256.mm.2	Ccl4	chemokine (C-C motif) ligand 4	-1,19791E+14	4,96559E+14	-2,83293E+14	0.0276848208963215	0.172801463563664	-3,20013E+14	3,98206E+14	4,83401E+14	5,33409E+14	5,87781E+14	5,1154E+13	4,65017E+14
TC1100000555.mm.2	Olfr1377	olfactory receptor 1377	-1,31235E+14	4,58985E+14	-2,83254E+14	0.0276994557685256	0.172801463563664	-3,2006E+14	4,11882E+14	3,55256E+14	5,09064E+14	5,20544E+14	4,20162E+14	5,37004E+14
TC1400002759.mm.2	Nalcn	sodium leak channel, non-selective	0.990817107224968	7,52265E+14	2,8325E+13	0.0277009979758545	0.172801463563664	-3,20065E+14	8,14746E+14	8,17903E+14	7,35467E+14	6,99018E+13	7,53973E+14	6,92482E+13
TC0200002755.mm.2	Zfp971	zinc finger protein 971	2,75624E+14	8,94307E+14	2,83204E+14	0.0277183274835438	0.172842651216076	-3,20121E+14	1,00973E+14	1,05746E+14	9,04243E+14	6,11694E+14	9,67748E+14	8,14972E+14
TC0200004550.mm.2	Ciao1	cytosolic iron-sulfur protein assembly 1	1,44026E+14	6,73139E+14	2,83078E+13	0.0277660143382273	0.173070919902349	-3,20274E+14	7,8982E+14	7,56037E+14	6,31038E+14	6,26766E+14	6,90927E+14	5,44244E+14
TC1000000845.mm.2	Efna2	ephrin A2	-0.925424085374845	7,02559E+14	-2,83051E+14	0.0277764164877723	0.173070919902349	-3,20307E+14	6,34134E+13	6,58884E+14	7,63667E+14	7,14436E+14	7,26961E+13	7,17274E+14
TC0500002123.mm.2	5031410I06Rik	RIKEN cDNA 5031410I06 gene	-1,14544E+14	4,78878E+14	-2,82957E+14	0.0278122851474309	0.173195008608092	-3,20422E+14	4,12624E+13	4,04454E+14	4,76918E+14	5,69248E+14	4,8341E+14	5,26615E+14
TC1500000489.mm.2	Ccn4	cellular communication network factor 4	-1,21896E+14	5,13894E+14	-2,82924E+14	0.027824751961109	0.173195008608092	-3,20462E+14	4,54238E+14	4,10876E+14	5,07984E+14	6,00921E+14	5,28507E+14	5,80838E+14
TC1100003771.mm.2	Nt5c3b	5'-nucleotidase, cytosolic IIIB	1,34891E+14	7,49131E+14	2,82848E+14	0.0278538280837833	0.173195008608092	-3,20555E+14	8,59988E+14	8,54746E+14	7,70743E+14	6,74209E+14	7,15734E+14	6,19364E+14
TC0300000968.mm.2	Prkab2	protein kinase, AMP-activated, beta 2 non-catalytic subunit	1,57009E+14	6,00241E+14	2,8277E+14	0.0278832611808394	0.173195008608092	-3,20648E+14	6,60339E+14	6,95543E+14	5,74204E+14	4,6766E+13	6,65967E+14	5,37735E+14
TC1100000081.mm.2	Aebp1	AE binding protein 1	0.925142972840213	6,00295E+14	2,82751E+14	0.0278904794304927	0.173195008608092	-3,20671E+14	6,5053E+14	6,4284E+14	5,54519E+14	5,53822E+14	5,72228E+14	6,27828E+14
TC0400001856.mm.2	Dhrs3	dehydrogenase/reductase (SDR family) member 3	1,06608E+14	6,81367E+14	2,82742E+14	0.0278940682399672	0.173195008608092	-3,20683E+14	7,67918E+14	7,34596E+14	6,82711E+14	6,06587E+13	6,61996E+13	6,34396E+14
TC0700000814.mm.2	Izumo2	IZUMO family member 2	-1,10205E+13	4,85558E+14	-2,82679E+13	0.0279180255313552	0.173195008608092	-3,20759E+14	4,51483E+14	3,69492E+14	5,00945E+14	5,40441E+14	5,3822E+14	5,12767E+14
TC0X00002660.mm.2	Arhgef9	CDC42 guanine nucleotide exchange factor (GEF) 9	2,54641E+14	8,47689E+14	2,82636E+14	0.0279346902618072	0.173195008608092	-3,20812E+14	1,00463E+12	1,01144E+14	8,76846E+14	6,29944E+14	8,72153E+14	6,91121E+13
TC1300000735.mm.2	Dapk1	death associated protein kinase 1	1,29667E+14	7,5677E+13	2,82631E+14	0.02793629741867	0.173195008608092	-3,20817E+14	8,52614E+14	8,4326E+13	7,60089E+14	6,76451E+14	7,52504E+14	6,55703E+14
TC0900003114.mm.2	Nradd	neurotrophin receptor associated death domain	-1,0878E+14	6,89108E+14	-2,82601E+14	0.0279480943254392	0.173195008608092	-3,20855E+14	6,31517E+14	6,54911E+14	7,09105E+13	7,94883E+14	6,60571E+14	6,83662E+14
TC0500001593.mm.2	Zp3	zona pellucida glycoprotein 3	-1,17403E+14	5,24813E+14	-2,82575E+14	0.0279577736081589	0.173195008608092	-3,20886E+14	4,34767E+14	4,70228E+14	5,19323E+14	6,20478E+14	5,47206E+14	5,56873E+14
TC0300000724.mm.2	Smg5	Smg-5 homolog, nonsense mediated mRNA decay factor (C. elegans)	1,58155E+14	6,00403E+14	2,8254E+14	0.0279711304095165	0.173195008608092	-3,20928E+14	6,73873E+14	6,8507E+14	5,98693E+14	4,4394E+14	6,37862E+14	5,6298E+14
TC0400000436.mm.2	Tesk1	testis specific protein kinase 1	0.837850289415975	8,71288E+14	2,82484E+14	0.0279926671892266	0.173195008608092	-3,20997E+14	9,27651E+14	9,33407E+14	8,45054E+14	8,48434E+14	8,49359E+14	8,23826E+14
TC0500000857.mm.2	Areg	amphiregulin	-1,08885E+14	5,58405E+13	-2,82457E+14	0.0280031153660704	0.173195008608092	-3,2103E+14	4,9848E+14	5,25857E+14	5,77378E+14	6,64729E+14	5,47356E+14	5,36627E+14
TC0200001115.mm.2	Hnrnpa3/Gm6793	heterogeneous nuclear ribonucleoprotein A3/heterogeneous nuclear ribonucleoprotein A3 pseudogene	1,77542E+14	9,06692E+14	2,82431E+14	0.028013165505878	0.173195008608092	-3,21062E+14	1,02733E+14	1,0154E+14	9,16245E+14	7,71401E+14	9,23959E+14	7,85816E+14
TC0400002457.mm.2	Nol6	nucleolar protein family 6 (RNA-associated)	1,73996E+14	7,89483E+14	2,82424E+14	0.0280156765696845	0.173195008608092	-3,2107E+14	9,19972E+14	9,14304E+14	8,29576E+14	6,56708E+14	7,53675E+14	6,62665E+13
TC0700001203.mm.2	Mesd	mesoderm development LRP chaperone	1,04588E+14	7,7563E+14	2,824E+14	0.0280247743741089	0.173195008608092	-3,21099E+14	8,58295E+14	8,13545E+14	7,66512E+14	6,96153E+14	7,63818E+14	7,55458E+14
TC0500000683.mm.2	Ociad1	OCIA domain containing 1	1,94724E+14	1,04069E+14	2,8234E+14	0.0280477750666094	0.173195008608092	-3,21171E+14	1,17782E+14	1,15562E+14	1,06781E+14	8,76184E+14	1,04111E+14	9,25589E+14
TC0900002387.mm.2	Islr	immunoglobulin superfamily containing leucine-rich repeat	1,50372E+14	6,8486E+14	2,82333E+14	0.0280504373442227	0.173195008608092	-3,2118E+14	7,63521E+14	7,65964E+14	6,89974E+14	5,38766E+14	6,481E+13	7,02833E+14
TC0900001329.mm.2	Pik3r4	phosphoinositide-3-kinase regulatory subunit 4	0.982990132112329	6,25775E+14	2,8232E+14	0.0280554600603746	0.173195008608092	-3,21196E+14	7,30967E+14	6,8715E+14	6,01648E+14	6,19872E+14	5,83592E+14	5,31419E+14
TC1600001838.mm.2	Zfp654	zinc finger protein 654	1,78249E+14	6,02779E+14	2,82318E+14	0.0280564516245345	0.173195008608092	-3,21199E+14	7,45807E+14	6,71424E+14	5,89712E+14	4,71021E+14	6,41868E+14	4,96843E+14
TC0500000640.mm.2	Uchl1	ubiquitin carboxy-terminal hydrolase L1	1,32746E+14	9,76976E+14	2,82291E+12	0.0280668068975515	0.173195008608092	-3,21232E+14	1,06359E+14	1,05715E+14	9,89093E+14	8,66156E+14	9,68759E+14	9,17107E+14
TC1000002776.mm.2	4930430F08Rik	RIKEN cDNA 4930430F08 gene	1,47848E+14	7,28648E+14	2,82267E+14	0.028075793441746	0.173195008608092	-3,2126E+13	8,10271E+14	7,85521E+14	7,2757E+14	5,72526E+14	7,40029E+14	7,35972E+14
TC0400000555.mm.2	Rnf20	ring finger protein 20	1,70015E+14	6,24389E+14	2,82267E+14	0.0280758593483097	0.173195008608092	-3,2126E+14	7,52061E+14	7,12838E+14	6,55286E+14	4,69582E+14	5,76215E+14	5,80351E+14
TC0300000778.mm.2	Tpm3	tropomyosin 3, gamma	1,86122E+14	8,9812E+14	2,82256E+14	0.0280803027744064	0.173195008608092	-3,21275E+14	9,96863E+14	1,01797E+14	9,13459E+14	7,29129E+12	9,17812E+14	8,1349E+14
TC0400001722.mm.2	Ddost	dolichyl-di-phosphooligosaccharide-protein glycotransferase	1,39666E+14	8,62515E+14	2,82209E+14	0.0280983083851013	0.173195008608092	-3,21332E+14	9,75643E+14	9,28217E+14	8,78709E+14	7,45818E+14	8,07746E+13	8,38955E+14
TC0600002799.mm.2	Uba3	ubiquitin-like modifier activating enzyme 3	1,98561E+13	8,13863E+14	2,82197E+14	0.0281028874769193	0.173195008608092	-3,21346E+14	9,36765E+14	9,46105E+14	8,32269E+14	6,5348E+14	8,29744E+14	6,84815E+14
TC1600000150.mm.2	Mrtfb	myocardin related transcription factor B	1,76987E+14	7,96691E+14	2,82192E+14	0.0281046170371221	0.173195008608092	-3,21352E+14	9,175E+14	9,32492E+14	8,21783E+14	6,74234E+13	7,8411E+14	6,50026E+14
TC0100002405.mm.2	Sumo1	small ubiquitin-like modifier 1	0.867412647032145	8,31202E+14	2,82152E+14	0.0281201146107461	0.173195008608092	-3,21401E+14	9,18273E+14	8,79612E+14	8,12798E+14	8,11604E+14	7,79242E+14	7,85683E+14
TC0800000874.mm.2	Zfp827	zinc finger protein 827	1,08284E+14	7,22001E+14	2,82141E+14	0.0281245734964593	0.173195008608092	-3,21415E+14	7,98808E+14	7,97169E+14	6,98045E+14	6,81365E+14	7,2316E+14	6,33462E+14
TC0500002280.mm.2	Nsg1	neuron specific gene family member 1	0.928939724176418	9,68418E+14	2,82069E+14	0.0281521466871766	0.173195008608092	-3,21502E+14	1,05464E+14	1,0125E+14	9,60303E+14	9,21056E+14	9,32962E+14	9,29042E+13
TC1100003588.mm.2	Kat7	K(lysine) acetyltransferase 7	1,45685E+14	6,81332E+13	2,8206E+14	0.028155766852418	0.173195008608092	-3,21513E+14	7,85448E+14	7,66559E+14	6,94819E+13	5,65816E+14	6,78586E+14	5,96765E+14
TC0900001868.mm.2	Ap1m2	adaptor protein complex AP-1, mu 2 subunit	-1,18943E+14	4,92867E+14	-2,82055E+14	0.028157398321567	0.173195008608092	-3,21518E+14	4,37274E+14	4,11361E+14	4,91139E+13	5,95381E+14	4,97647E+14	5,244E+14
TC1100000967.mm.2	Rabep1	rabaptin, RAB GTPase binding effector protein 1	2,0089E+14	8,38677E+14	2,82028E+13	0.0281679120954532	0.173195008608092	-3,21552E+14	9,64685E+14	9,55076E+14	8,508E+14	6,6718E+14	8,69323E+14	7,24999E+14
TC1600000536.mm.2	Fstl1	follistatin-like 1	-1,05065E+14	8,88656E+14	-2,81983E+13	0.0281851767167844	0.173195008608092	-3,21606E+14	8,16394E+13	8,10503E+14	8,7791E+14	9,59117E+14	9,21679E+14	9,46331E+14
TC0400002105.mm.2	9430015G10Rik	RIKEN cDNA 9430015G10 gene	-0.984576566439863	6,66613E+14	-2,81972E+14	0.0281895301386013	0.173195008608092	-3,2162E+13	5,76477E+14	6,19791E+14	6,81808E+14	7,11375E+14	6,81067E+13	7,29159E+14
TC1400002182.mm.2	Sgcg	sarcoglycan, gamma (dystrophin-associated glycoprotein)	-1,25844E+14	5,50612E+14	-2,8196E+14	0.0281943192612765	0.173195008608092	-3,21635E+14	4,51719E+14	4,75154E+14	5,36735E+14	6,41826E+14	5,69926E+14	6,28311E+14
TC1600001573.mm.2	Cstdc5/Cstdc6	cystatin domain containing 5/cystatin domain containing 6	-1,13093E+14	5,04213E+14	-2,81928E+14	0.0282065086168755	0.173195008608092	-3,21673E+14	4,28669E+14	4,49515E+14	5,0326E+14	6,0111E+14	5,25505E+14	5,17219E+14
TC0900001470.mm.2	Prss44	protease, serine 44	-1,28212E+13	5,61498E+14	-2,81905E+14	0.0282155338916553	0.173195008608092	-3,21702E+14	4,71592E+14	4,88802E+14	5,54621E+14	6,62196E+14	5,63805E+14	6,27969E+14
TC0400003417.mm.2	Zfp691	zinc finger protein 691	-1,1335E+14	7,66687E+14	-2,81808E+13	0.0282530355120839	0.173207351495135	-3,2182E+14	6,85424E+14	6,92719E+14	8,52222E+14	7,52622E+14	8,01208E+14	8,15924E+14
TC0800001289.mm.2	Zfhx3	zinc finger homeobox 3	1,54842E+14	5,68113E+14	2,81807E+14	0.028253104911167	0.173207351495135	-3,2182E+14	6,7685E+14	6,46163E+14	5,21317E+14	4,92013E+13	6,17423E+14	4,54909E+14
TC1100001448.mm.2	Col1a1	collagen, type I, alpha 1	-1,34166E+14	7,39704E+14	-2,81797E+14	0.0282573132638532	0.173207351495135	-3,21833E+14	6,33669E+14	6,54679E+13	7,13563E+13	8,43116E+14	7,78494E+14	8,147E+14
TC0200002872.mm.2	Optn	optineurin	0.835170084611227	5,38834E+14	2,81763E+14	0.0282702227867282	0.173207351495135	-3,21874E+14	6,04444E+14	5,80184E+13	5,1265E+14	5,04945E+14	5,18304E+14	5,1248E+13
TC0600003468.mm.2	Ipo8	importin 8	1,35785E+14	6,69971E+14	2,8176E+14	0.0282712923994418	0.173207351495135	-3,21877E+14	7,49764E+14	7,40908E+14	6,62477E+13	5,56624E+14	6,99659E+14	6,10392E+14
TC1400002266.mm.2	Ephx2	epoxide hydrolase 2, cytoplasmic	1,35183E+14	5,35423E+14	2,81716E+13	0.0282885472512728	0.173230216071674	-3,21932E+14	6,56939E+14	6,26321E+14	5,31183E+14	4,81711E+14	3,95719E+14	5,20667E+14
TC0600002329.mm.2	Neurod6	neurogenic differentiation 6	1,63256E+14	7,74246E+14	2,8167E+14	0.0283062372094322	0.173230216071674	-3,21987E+14	7,95556E+14	9,09501E+14	7,53865E+14	6,24679E+12	8,07936E+14	7,5394E+13
TC0500000750.mm.2	Adgrl3	adhesion G protein-coupled receptor L3	2,3384E+14	7,31596E+14	2,81643E+14	0.0283168773286129	0.173230216071674	-3,22021E+14	8,64295E+14	8,42678E+14	7,36777E+13	5,02515E+14	7,96982E+14	6,46329E+14
TC0400000602.mm.2	Zfp462	zinc finger protein 462	0.898702528301902	6,18073E+14	2,81604E+14	0.0283319826188242	0.173230216071674	-3,22068E+14	6,99164E+14	6,94178E+14	6,162E+14	5,97402E+14	5,73677E+14	5,27818E+14
TC0500000992.mm.2	Cds1	CDP-diacylglycerol synthase 1	1,23732E+14	6,9468E+14	2,81591E+14	0.0283369137209533	0.173230216071674	-3,22084E+14	7,76534E+14	7,98823E+14	6,92101E+14	6,35792E+14	6,83763E+14	5,81066E+14
TC0800000765.mm.2	Rab3a	RAB3A, member RAS oncogene family	0.985640822149724	1,00211E+14	2,81575E+14	0.0283430424245677	0.173230216071674	-3,22103E+14	1,08439E+14	1,05495E+14	9,97993E+14	9,44223E+14	9,84152E+14	9,46958E+14
TC1400002347.mm.2	Fgf17	fibroblast growth factor 17	-1,1766E+14	5,50678E+14	-2,81545E+14	0.0283547131143169	0.173230216071674	-3,22139E+14	4,88776E+13	4,89742E+14	5,5459E+14	6,59248E+12	5,40939E+14	5,70772E+14
TC1300000045.mm.2	Idi1	isopentenyl-diphosphate delta isomerase	1,43652E+14	6,20003E+14	2,81514E+13	0.0283668081510524	0.173230216071674	-3,22177E+14	7,42099E+14	7,2379E+14	6,37735E+14	5,4085E+14	5,95109E+14	4,80436E+14
TC0700003740.mm.2	Pcf11	PCF11 cleavage and polyadenylation factor subunit	1,65724E+14	5,7411E+14	2,8148E+14	0.0283802486497835	0.173230216071674	-3,22219E+13	7,25757E+14	6,63017E+14	6,08462E+14	4,48863E+14	5,33035E+14	4,65529E+13
TC0100000490.mm.2	Icos	inducible T cell co-stimulator	-1,09993E+14	4,64601E+14	-2,81458E+13	0.0283885917085941	0.173230216071674	-3,22246E+14	4,08686E+14	3,80339E+13	4,70408E+14	5,38604E+14	4,64908E+14	5,2466E+14
TC0500002642.mm.2	Ppat	phosphoribosyl pyrophosphate amidotransferase	1,04203E+14	5,27732E+14	2,81446E+14	0.0283932849652635	0.173230216071674	-3,2226E+14	5,88661E+14	6,04465E+14	5,2334E+14	4,6138E+14	5,21307E+14	4,67237E+14
TC0400003694.mm.2	Sytl1	synaptotagmin-like 1	-1,43708E+14	5,66651E+14	-2,81403E+14	0.0284101220278292	0.173254727285122	-3,22313E+12	4,79882E+14	4,65742E+14	5,55615E+14	6,77425E+14	5,66219E+14	6,55026E+14
TC1100003184.mm.2	Olfr139	olfactory receptor 139	-1,3842E+14	6,06679E+13	-2,8138E+14	0.028418807435895	0.173254727285122	-3,2234E+14	5,04474E+14	5,05361E+14	5,76524E+14	7,1015E+14	6,46047E+14	6,9752E+14
TC0100002974.mm.2	Ddx18	DEAD (Asp-Glu-Ala-Asp) box polypeptide 18	1,4369E+14	6,53216E+14	2,81331E+14	0.0284379954320876	0.173262994222615	-3,224E+14	7,62892E+14	7,43839E+14	6,81407E+13	5,37944E+14	5,74313E+14	6,18902E+14

TC0300002411.mm.2	Lce1k	late cornified envelope 1K	-0.882270736077774	4,91102E+14	-2,81322E+14	0.0284416694419919	0.173262994222615	-3,22412E+14	4,61921E+14	4,55231E+14	5,31157E+14	5,62449E+14	4,52053E+14	4,83802E+14
TC0200003353.mm.2	Psmd5	proteasome (prosome, macropain) 26S subunit, non-ATPase, 5	2,54319E+14	6,30902E+14	2,81287E+14	0.0284551170851325	0.173279403474202	-3,22454E+14	8,03141E+14	8,24999E+14	7,0638E+14	4,13121E+14	5,63715E+14	4,74055E+14
TC0400003056.mm.2	Tusc1	tumor suppressor candidate 1	-0.983566018889717	7,88329E+14	-2,81182E+14	0.0284959538177952	0.173352587816591	-3,22581E+13	7,2216E+14	7,20032E+14	7,96E+13	8,42906E+14	7,97445E+14	8,51428E+14
TC1100000686.mm.2	Zfp692	zinc finger protein 692	-0.97227852121598	6,72981E+14	-2,81178E+14	0.0284975336452327	0.173352587816591	-3,22586E+14	6,11138E+14	6,01906E+14	6,73619E+14	7,33881E+14	6,92519E+14	7,24824E+14
TC0600000493.mm.2	Cntnap2	contactin associated protein-like 2	1,96815E+14	7,85516E+14	2,81174E+14	0.0284994107320889	0.173352587816591	-3,22592E+14	9,43649E+14	8,91943E+14	8,20142E+14	6,21821E+14	7,68745E+14	6,66794E+14
TC0700000781.mm.2	Klk15	kallikrein related-peptidase 15	-0.843728998009396	5,91612E+13	-2,81142E+14	0.028511757572768	0.173362245196231	-3,22631E+14	5,38122E+14	5,50247E+14	6,37755E+14	6,1936E+14	5,91135E+14	6,1305E+13
TC1900000152.mm.2	Wdr74	WD repeat domain 74	1,2752E+14	7,2732E+14	2,81067E+14	0.0285410816563093	0.173475084393856	-3,22722E+14	8,25952E+14	8,18052E+14	7,41376E+14	6,47587E+14	7,07302E+14	6,2365E+14
TC0200000347.mm.2	Gad2	glutamic acid decarboxylase 2	2,03658E+14	8,58676E+14	2,81017E+14	0.0285606149684584	0.173528351804966	-3,22783E+14	1,00812E+14	9,8057E+14	8,98517E+13	6,82858E+14	8,40149E+14	7,4184E+13
TC0800002953.mm.2	Bcar1	breast cancer anti-estrogen resistance 1	0.891802775492836	6,73764E+14	2,80982E+14	0.028574197897248	0.17354543939629	-3,22825E+14	7,39925E+14	7,25918E+13	6,64331E+14	6,23152E+14	6,3407E+14	6,5519E+14
TC0200004567.mm.2	Mtln	mitoregulin	1,06456E+14	7,07848E+14	2,80953E+14	0.0285857120497389	0.173549954128652	-3,22861E+14	7,51476E+14	7,69197E+14	6,9581E+14	6,11951E+14	6,98134E+14	7,20522E+14
TC1900000857.mm.2	Gm19956	predicted gene, 19956	-1,06167E+14	5,26875E+14	-2,8088E+14	0.0286143947709658	0.17356756307134	-3,2295E+14	4,70593E+14	4,7609E+14	5,47491E+14	6,11527E+14	4,97666E+14	5,57885E+13
TC1200001698.mm.2	Gm1818	predicted gene 1818	1,14428E+14	7,4426E+14	2,80865E+14	0.0286201659645773	0.17356756307134	-3,22968E+14	8,32222E+14	8,39458E+14	7,17326E+14	7,25498E+14	7,27034E+14	6,24024E+14
TC1600000692.mm.2	Dppa4	developmental pluripotency associated 4	-0.908143988728115	5,65752E+14	-28086303534	0.0286209281375628	0.17356756307134	-3,2297E+14	5,08032E+14	4,87788E+14	5,80587E+14	5,96862E+14	5,87417E+13	6,33827E+14
TC0100001037.mm.2	Cdh7	cadherin 7, type 2	1,16166E+14	6,53158E+14	2,80824E+14	0.0286361732406228	0.173581329844551	-3,23018E+14	7,40233E+14	6,93245E+14	6,47962E+14	5,53184E+14	6,49218E+14	6,35107E+14
TC0200002785.mm.2	Rps21	ribosomal protein S21	0.951624725064866	9,38248E+14	2,80802E+14	0.0286447437508013	0.173581329844551	-3,23044E+14	1,01091E+14	9,83911E+14	8,85999E+14	9,18495E+14	8,90754E+14	9,39422E+14
TC0100001787.mm.2	Eprs	glutamyl-prolyl-tRNA synthetase	1,73545E+14	7,30185E+14	2,80757E+14	0.0286625429864177	0.17362389290983	-3,231E+14	8,83253E+13	8,92879E+14	7,57657E+14	6,71384E+14	6,75986E+14	4,99951E+14
TC0200005287.mm.2	Ctcfl	CCCTC-binding factor (zinc finger protein)-like	-1,10054E+13	5,64521E+14	-2,8064E+14	0.0287083927137491	0.173640536954617	-3,23242E+14	5,16545E+14	4,9322E+13	5,77346E+14	6,52527E+14	5,46521E+14	6,00968E+14
TC0400002289.mm.2	Prdm13	PR domain containing 13	-1,24581E+14	5,29481E+14	-2,80596E+14	0.028725803104045	0.173640536954617	-3,23296E+14	4,52549E+14	4,4495E+13	5,35839E+14	6,10822E+14	5,18784E+14	6,13941E+14
TC0100003747.mm.2	Iars2	isoleucine-tRNA synthetase 2, mitochondrial	1,48554E+14	6,49728E+14	2,80577E+14	0.028733286943056	0.173640536954617	-3,23319E+14	7,14412E+14	7,40427E+14	6,45505E+14	5,12224E+14	6,8472E+14	6,01081E+14
TC0400000425.mm.2	Phf24	PHD finger protein 24	1,21872E+14	8,2833E+14	2,80566E+14	0.0287375351983719	0.173640536954617	-3,23332E+14	9,03641E+14	9,10632E+14	8,39623E+14	7,30905E+14	8,13693E+14	7,71486E+14
TC0400002509.mm.2	Fam205a1	family with sequence similarity 205, member A1	1,10741E+14	5,92478E+14	2,80526E+14	0.0287535825596001	0.173640536954617	-3,23382E+14	6,52672E+14	6,64092E+13	5,28699E+14	5,66583E+14	6,15737E+14	5,27083E+14
TC1200002139.mm.2	Gm7104	REX1, RNA exonuclease 1 homolog pseudogene	-0.915419385595121	6,58292E+14	-2,80515E+14	0.0287575750818869	0.173640536954617	-3,23394E+14	6,00199E+14	6,01412E+14	6,69872E+14	7,14824E+14	6,6547E+14	6,97975E+14
TC1500000099.mm.2	Zfr/Mir1898	zinc finger RNA binding protein/microRNA 1898	2,12007E+14	8,54492E+14	2,80508E+14	0.0287603960402269	0.173640536954617	-3,23403E+14	9,81536E+14	9,77994E+14	8,78018E+14	6,57498E+14	8,75553E+14	7,56355E+14
TC0900001469.mm.2	Prss42	protease, serine 42	-1,02015E+14	5,10564E+14	-2,80443E+14	0.0287861976404705	0.173640536954617	-3,23483E+14	4,61997E+14	4,61939E+14	5,91276E+14	5,3669E+14	5,34987E+14	4,76495E+14
TC0100002656.mm.2	Dock10	dedicator of cytokinesis 10	1,04812E+13	6,12365E+14	2,80429E+14	0.0287916084909104	0.173640536954617	-3,23499E+14	6,89798E+14	7,08036E+14	5,69739E+14	6,18471E+14	5,78136E+14	5,10011E+14
TC0400003379.mm.2	Atp6v0b	ATPase, H+ transporting, lysosomal V0 subunit B	0.829902265327505	7,75979E+13	2,80418E+14	0.028795836852206	0.173640536954617	-3,23512E+14	8,2903E+14	8,19591E+13	7,52796E+14	7,29845E+14	7,65098E+14	7,59511E+14
TC1400000104.mm.2	Synpr	synaptoporin	0.925031247949763	8,92982E+13	2,80416E+14	0.0287969895315325	0.173640536954617	-3,23516E+14	9,50478E+14	9,64761E+14	8,9316E+14	8,37072E+14	8,58854E+14	8,5357E+14
TC1000002067.mm.2	Nr2e1	nuclear receptor subfamily 2, group E, member 1	1,03887E+14	6,35724E+14	2,80386E+13	0.0288086614825697	0.173640536954617	-3,23552E+13	7,04188E+14	7,15408E+14	6,44897E+14	5,66924E+14	6,05893E+14	5,77036E+14
TC0700004538.mm.2	Slc25a22	solute carrier family 25 (mitochondrial carrier, glutamate), member 22	1,01089E+14	8,57921E+14	2,80346E+14	0.0288245419531106	0.173640536954617	-3,23601E+14	9,16902E+14	9,23712E+14	8,48049E+14	7,90387E+14	8,08098E+14	8,60381E+13
TC0900000072.mm.2	Gm16485	predicted gene 16485	1,21251E+14	5,46952E+14	2,80297E+14	0.0288437872753212	0.173640536954617	-3,2366E+14	5,98718E+13	6,49631E+14	5,51386E+14	4,5446E+14	5,3288E+14	4,94639E+14
TC0300002470.mm.2	Mllt11	myeloid/lymphoid or mixed-lineage leukemia; translocated to, 11	1,32389E+13	9,87274E+14	2,80239E+14	0.0288666479924429	0.173640536954617	-3,23731E+14	1,06831E+14	1,0786E+14	9,86898E+14	8,95233E+14	9,96232E+14	8,9837E+14
TC1600000480.mm.2	Itgb5	integrin beta 5	0.893893020760347	7,6769E+13	2,80214E+14	0.0288768476625882	0.173640536954617	-3,23762E+14	7,90109E+14	8,39559E+14	7,27391E+14	7,23498E+14	7,64355E+13	7,61226E+14
TC1500000119.mm.2	Cdh9	cadherin 9	1,43455E+14	6,28244E+14	2,80188E+14	0.0288870712330618	0.173640536954617	-3,23794E+14	6,99383E+14	7,33261E+14	6,27274E+14	5,1846E+14	6,45813E+14	5,45271E+14
TC1800001017.mm.2	Aqp4	aquaporin 4	1,77633E+14	7,69904E+14	2,80183E+14	0.0288891006794687	0.173640536954617	-3,238E+14	8,61402E+14	9,08254E+14	7,73954E+14	6,40436E+14	7,90242E+13	6,45137E+14
TC1300000273.mm.2	Cmah	cytidine monophospho-N-acetylneuraminic acid hydroxylase	-1,03546E+14	5,39745E+14	-2,80141E+14	0.028905681473354	0.173640536954617	-3,23851E+14	4,48051E+14	4,7712E+14	5,51785E+14	5,80478E+14	5,54168E+14	6,26866E+13
TC0100003791.mm.2	Prox1	prospero homeobox 1	0.903580333922021	6,2774E+14	2,80113E+13	0.0289165059215133	0.173640536954617	-3,23884E+14	6,71909E+14	6,78657E+14	5,65569E+14	6,04281E+13	6,40484E+14	6,05542E+14
TC1900001342.mm.2	Plgrkt	plasminogen receptor, C-terminal lysine transmembrane protein	0.916992006866902	6,10095E+14	2,80103E+12	0.0289205744014771	0.173640536954617	-3,23897E+13	6,52623E+14	6,68228E+14	5,93442E+14	5,44011E+14	6,12081E+13	5,90185E+14
TC0200002401.mm.2	Tgif2	TGFB-induced factor homeobox 2	-0.922777318472257	6,4697E+14	-2,80081E+14	0.0289293425711755	0.173640536954617	-3,23924E+14	5,73353E+14	5,90057E+14	6,5485E+14	6,93115E+14	6,65222E+14	7,0522E+14
TC1900000054.mm.2	Rin1	Ras and Rab interactor 1	1,16197E+14	6,7056E+13	2,80038E+14	0.0289464597151235	0.173640536954617	-3,23976E+14	7,35493E+14	7,62534E+14	6,77224E+14	5,8841E+14	6,02894E+14	6,56804E+14
TC0700000152.mm.2	Rnf225	ring finger protein 225	-1,21817E+14	8,50959E+14	-2,80003E+12	0.0289604349498031	0.173640536954617	-3,24019E+14	7,68843E+14	7,7054E+14	8,40084E+14	9,42932E+14	8,63638E+14	9,19716E+14
TC1000000446.mm.2	Ascc3	activating signal cointegrator 1 complex subunit 3	1,57374E+14	5,90395E+14	2,79995E+14	0.0289635303021574	0.173640536954617	-3,24029E+14	6,65799E+14	6,99972E+14	5,89628E+14	4,61394E+14	6,18468E+13	5,07107E+14
TC1400002668.mm.2	Frg2f4	FSHD region gene 2 family member 4	-1,32472E+14	5,43441E+14	-2,79977E+14	0.0289704997756084	0.173640536954617	-3,2405E+13	4,49193E+14	4,55677E+14	5,18882E+14	6,50932E+14	6,06173E+13	5,79791E+14
TC1300000671.mm.2	Ddx46	DEAD (Asp-Glu-Ala-Asp) box polypeptide 46	2,27839E+14	6,47887E+14	2,79974E+14	0.0289719142787978	0.173640536954617	-3,24055E+14	7,81923E+14	7,83281E+14	6,9078E+14	4,18747E+14	6,18051E+14	5,94542E+14
TC0600003121.mm.2	Gnb3	guanine nucleotide binding protein (G protein), beta 3	-1,17558E+14	6,86525E+14	-2,79932E+14	0.0289884472256541	0.173640536954617	-3,24105E+14	5,86778E+14	6,47704E+14	7,06341E+14	7,63258E+14	6,71782E+14	7,43287E+14
TC1600000179.mm.2	Abcc1	ATP-binding cassette, sub-family C (CFTR/MRP), member 1	-0.883512644548365	8,23163E+14	-2,79903E+14	0.0290002427568477	0.173640536954617	-3,24141E+14	7,63943E+14	7,80087E+14	8,82363E+14	8,38369E+14	8,36771E+14	8,37444E+14
TC0X00002407.mm.2	Gabra3	gamma-aminobutyric acid (GABA) A receptor, subunit alpha 3	2,70226E+14	7,28699E+14	2,79869E+13	0.0290135944304943	0.173640536954617	-3,24182E+14	9,07584E+14	8,81098E+14	7,68444E+14	4,79786E+14	7,4711E+14	5,88172E+13
TC1000001807.mm.2	Hebp2	heme binding protein 2	-1,49691E+14	6,74114E+14	-2,79802E+14	0.0290401218744571	0.173640536954617	-3,24264E+13	6,00162E+14	5,79892E+13	6,86367E+14	7,93069E+14	6,32605E+14	7,5259E+14
TC0100000351.mm.2	Wdr75	WD repeat domain 75	1,52683E+14	6,41715E+14	2,79792E+14	0.0290440512574468	0.173640536954617	-3,24276E+14	7,31592E+14	7,34508E+14	6,36561E+14	5,24174E+14	6,73051E+14	5,50403E+14
TC0300000821.mm.2	Sprr2a2/Sprr2a1	small proline-rich protein 2A2/small proline-rich protein 2A1	-0.938654514887729	5,71625E+14	-2,79773E+14	0.0290519200576113	0.173640536954617	-3,243E+14	5,15436E+14	4,91473E+14	5,81354E+14	6,13286E+14	5,90302E+14	6,379E+14
TC0400001502.mm.2	Phc2	polyhomeotic 2	-1,01312E+14	8,45809E+14	-2,79768E+14	0.0290537091162898	0.173640536954617	-3,24305E+14	7,92837E+14	7,8004E+14	8,59859E+14	9,15643E+14	8,35975E+14	8,90496E+14
TC1400000388.mm.2	4930474N05Rik	RIKEN cDNA 4930474N05 gene	-1,38452E+14	4,34025E+14	-2,79745E+13	0.0290630054439757	0.173640536954617	-3,24334E+14	3,3707E+14	3,50034E+14	4,22986E+14	5,41023E+14	4,35332E+14	5,17704E+14
TC0900001315.mm.2	Cpne4	copine IV	0.914905279546057	7,0481E+14	2,79707E+14	0.0290783064251605	0.173640536954617	-3,24381E+14	7,74783E+14	7,62168E+14	6,96282E+14	6,57688E+14	6,49643E+14	6,88295E+14
TC1800000443.mm.2	Stk32a	serine/threonine kinase 32A	1,70208E+14	5,54599E+14	2,79705E+14	0.0290788616887917	0.173640536954617	-3,24382E+13	6,95487E+14	6,40986E+14	5,7007E+14	4,25986E+14	5,5515E+14	4,39919E+14
TC0500000656.mm.2	Gm5108	predicted gene 5108	-0.924669301141844	6,86241E+14	-2,79685E+14	0.0290867550057703	0.173640536954617	-3,24406E+14	6,23007E+13	6,26427E+14	6,99371E+14	7,34997E+14	6,93588E+14	7,40054E+13
TC0700000381.mm.2	Kcnn4	potassium intermediate/small conductance calcium-activated channel, subfamily N, member 4	-1,20911E+14	5,71933E+14	-2,79645E+14	0.0291029912288253	0.173640536954617	-3,24456E+14	4,9931E+14	5,18703E+14	5,79765E+14	6,80069E+14	5,4954E+14	6,04212E+14
TC1500001127.mm.2	Hnrnpa1/Gm10052/Gm5643	heterogeneous nuclear ribonucleoprotein A1/heterogeneous nuclear ribonucleoprotein A1 pseudogene/heterogeneous nuclear ribonucleoprotein A1 pseudogene	0.901544306369026	8,92551E+13	2,79636E+12	0.0291065833360787	0.173640536954617	-3,24467E+14	9,65357E+14	9,52676E+14	8,84936E+14	8,52788E+14	8,69497E+14	8,30049E+14
TSUnmapped00000001.mm.2	Plppr2	phospholipid phosphatase related 2	2,20717E+14	8,76303E+14	2,79634E+14	0.0291071240808304	0.173640536954617	-3,24469E+14	1,02628E+14	1,02828E+13	9,0995E+14	7,03176E+14	8,76842E+14	7,13289E+14
TC1900000237.mm.2	Osbp	oxysterol binding protein	1,77098E+13	7,23354E+14	2,79545E+14	0.0291425983419589	0.173719433757481	-3,24577E+14	8,244E+14	8,15607E+14	7,34076E+14	5,51735E+14	7,50956E+14	6,63349E+14
TC1200002191.mm.2	Kcnk10	potassium channel, subfamily K, member 10	-0.850142697889364	7,42505E+13	-2,79535E+13	0.0291469041074175	0.173719433757481	-3,2459E+14	6,85365E+14	6,96774E+14	7,60088E+14	7,92079E+14	7,65942E+14	7,54785E+14
TC1100003965.mm.2	Cacng5	calcium channel, voltage-dependent, gamma subunit 5	1,31654E+14	7,5474E+14	2,79498E+14	0.0291614841231713	0.173719433757481	-3,24635E+14	8,23407E+14	8,24426E+14	7,5919E+14	6,25336E+14	7,47703E+14	7,48375E+14
TC0400001139.mm.2	Slc1a7	solute carrier family 1 (glutamate transporter), member 7	-0.86035343507248	7,16391E+14	-2,79493E+14	0.0291634747293482	0.173719433757481	-3,24641E+14	6,45299E+14	6,64956E+13	7,26823E+14	7,55503E+14	7,43394E+14	7,62372E+14
TC1100000995.mm.2	Mybbp1a	MYB binding protein (P160) 1a	0.931347822010252	6,25744E+14	2,79449E+14	0.0291811861992855	0.1737606996412	-3,24695E+14	6,90659E+14	6,77426E+14	5,96894E+14	5,84921E+14	5,73345E+14	6,31219E+14
TC0200005350.mm.2	Gm14308/Gm14430/Gm14432/Gm14434/Gm2026/Gm4724	predicted gene 14308/predicted gene 14430/predicted gene 14432/predicted gene 14434/predicted gene 2026/predicted gene 4724	2,96346E+13	8,91826E+14	2,79354E+13	0.0292191929305766	0.173784791326142	-3,24811E+14	1,00105E+14	1,06949E+14	9,00383E+14	5,77466E+14	9,80589E+14	8,21975E+14
TC0100003338.mm.2	Brinp2	bone morphogenic protein/retinoic acid inducible neural-specific 2	1,49096E+14	7,10094E+14	2,79318E+13	0.0292336275945529	0.173784791326142	-3,24854E+14	8,06919E+14	8,25583E+14	7,2556E+14	6,0875E+13	7,01727E+14	5,92025E+14
TC1600000834.mm.2	Robo1	roundabout guidance receptor 1	0.911811997123352	5,69222E+14	2,79312E+14	0.0292361074317183	0.173784791326142	-3,24862E+14	6,11035E+14	6,14253E+14	5,42479E+14	5,00446E+14	5,91665E+14	5,55454E+14
TC0700002062.mm.2	Muc5ac	mucin 5, subtypes A and C, tracheobronchial/gastric	-0.945219887396482	5,56042E+14	-2,79312E+14	0.0292361706563483	0.173784791326142	-3,24862E+14	4,7643E+14	5,02989E+14	5,55078E+14	6,13885E+13	5,95416E+14	5,92952E+14
TC1700000650.mm.2	Ehmt2	euchromatic histone lysine N-methyltransferase 2	1,79647E+14	7,08512E+14	2,79289E+14	0.0292452092932465	0.173784791326142	-3,2489E+14	8,2055E+14	8,22614E+14	7,3646E+13	5,47408E+14	7,03403E+14	6,20636E+14
TC1600002170.mm.2	Cox17	cytochrome c oxidase assembly protein 17, copper chaperone	2,31267E+14	8,08772E+14	2,79277E+14	0.0292499443974738	0.173784791326142	-3,24904E+14	9,79602E+14	9,54034E+14	8,54728E+14	6,16375E+14	7,94854E+14	6,53041E+14
TC0500003468.mm.2	Gjc3	gap junction protein, gamma 3	1,64308E+14	8,7213E+14	2,79246E+14	0.0292627358380707	0.173796162305235	-3,24943E+14	9,91473E+14	1,00329E+14	8,97511E+14	7,68633E+14	8,5144E+14	7,20441E+14
TC0300000090.mm.2	Lrrc1	leucine rich repeat and coiled-coil domain containing 1	1,43018E+14	5,50358E+14	2,79209E+14	0.029277309260641	0.173796162305235	-3,24987E+14	6,84238E+14	6,21291E+14	5,1479E+14	5,04702E+14	5,57598E+14	4,19528E+14
TC0400004090.mm.2	Espn	espin	-0.956419959431225	6,435E+14	-2,79154E+14	0.0292993890062546	0.173796162305235	-3,25054E+14	5,5794E+14	6,00386E+14	6,52636E+14	6,96974E+14	6,63193E+14	6,89871E+14
TC0700004531.mm.2	Cdhr5	cadherin-related family member 5	-0.995520851964061	5,62362E+14	-2,79148E+14	0.0293018560159065	0.173796162305235	-3,25062E+13	4,85589E+14	4,98483E+13	5,61071E+14	6,22106E+14	5,81967E+13	6,24958E+14
TC1400002142.mm.2	Cryl1	crystallin, lambda 1	-0.825043886915898	6,45484E+13	-2,79138E+14	0.029305788678913	0.173796162305235	-3,25074E+14	6,00873E+14	5,96408E+14	6,85038E+14	6,77252E+14	6,45076E+14	6,68256E+14
TC0100001853.mm.2	Ints7	integrator complex subunit 7	1,08122E+14	5,84124E+14	2,79009E+14	0.0293577793158219	0.174040433449536	-3,25231E+14	6,50893E+14	6,74121E+14	5,72943E+13	5,35828E+14	5,76648E+14	4,94312E+14
TC1900000262.mm.2	Olfr1451	olfactory receptor 1451	-1,88742E+14	5,10606E+14	-2,78934E+14	0.0293881269837861	0.174072293541921	-3,25323E+14	3,74672E+14	3,73299E+14	4,53017E+14	6,72438E+14	5,89579E+14	6,00634E+14
TC1100000544.mm.2	Hnrnph1	heterogeneous nuclear ribonucleoprotein H1	1,65134E+14	8,1815E+14	2,78882E+13	0.0294089503988793	0.174072293541921	-3,25386E+14	9,21722E+14	9,1084E+14	8,22706E+14	6,79587E+14	8,45918E+14	7,28126E+14
TC0100000140.mm.2	Khdc1c/Khdc1a	KH domain containing 1C/KH domain containing 1A	-1,29969E+14	5,14702E+14	-2,78877E+14	0.0294112227877697	0.174072293541921	-3,25393E+14	4,21472E+14	4,50448E+14	5,04238E+14	6,2762E+14	5,24511E+14	5,59921E+14
TC0400003073.mm.2	Plaa	phospholipase A2, activating protein	1,25102E+14	8,28945E+14	2,78846E+14	0.0294238310176431	0.174072293541921	-3,25431E+14	9,19502E+14	9,2153E+14	8,53569E+14	7,37258E+14	7,91745E+14	7,50065E+14
TC0200004626.mm.2	Pced1a	PC-esterase domain containing 1A	1,0385E+14	5,36544E+14	2,78836E+14	0.0294276019748326	0.174072293541921	-3,25443E+14	6,24071E+14	5,89593E+13	5,40064E+14	4,659E+14	5,11363E+14	4,88271E+14
TC0100002769.mm.2	Usp40	ubiquitin specific peptidase 40	-1,33172E+14	6,2871E+14	-2,78835E+14	0.0294279729167872	0.174072293541921	-3,25444E+14	5,44329E+14	5,33909E+14	6,14058E+13	7,30523E+14	6,39202E+14	7,10241E+14
TC1600000392.mm.2	Hes1	hes family bHLH transcription factor 1	-0.976644681691273	7,04458E+14	-2,78723E+14	0.0294734324965135	0.174274030027431	-3,25581E+14	6,14922E+14	6,4599E+14	7,52544E+14	7,03697E+14	7,33453E+14	7,7614E+14
TC0400003405.mm.2	Olfr1333	olfactory receptor 1333	-0.992141895093642	5,28109E+14	-2,78697E+14	0.0294837091699111	0.174274030027431	-3,25612E+14	4,86788E+14	4,6659E+14	5,46565E+14	6,05241E+14	5,53127E+14	5,10341E+14
TC1500000698.mm.2	Atf4	activating transcription factor 4	0.96007389050355	8,126E+14	2,78629E+14	0.0295116053013249	0.174351889018293	-3,25696E+14	8,68E+14	8,42667E+14	7,86454E+14	7,32198E+14	8,06808E+14	8,39471E+14
TC0300000742.mm.2	Ash1l	ASH1 like histone lysine methyltransferase	2,25059E+14	7,64816E+14	2,78581E+14	0.0295309008027264	0.174351889018293	-3,25755E+14	9,12411E+14	9,29086E+14	7,78788E+14	6,1259E+14	7,84574E+14	5,71445E+14
TC0900002277.mm.2	Hspb2	heat shock protein 2	-1,26499E+14	6,25055E+14	-2,78537E+14	0.0295486226937269	0.174351889018293	-3,25808E+13	5,58742E+14	5,38529E+14	6,13052E+14	7,37216E+14	6,55983E+14	6,46807E+14
TC0700004258.mm.2	Nsmce1	NSE1 homolog, SMC5-SMC6 complex component	1,71417E+14	6,39224E+14	2,78528E+14	0.0295525359492793	0.174351889018293	-3,2582E+14	7,67958E+14	7,63645E+14	6,86961E+14	5,01808E+14	5,88296E+14	5,2668E+14
TC1900000259.mm.2	Gm5512	required for meiotic nuclear division 1 pseudogene	0.923862881462364	6,47682E+14	2,78502E+14	0.0295627867203798	0.174351889018293	-3,25851E+14	7,25351E+14	7,13331E+14	6,39386E+13	6,14524E+14	6,23077E+14	5,70423E+13
TC1400002264.mm.2	Gulo	gulonolactone (L-) oxidase	-0.858714607408302	5,00731E+14	-2,78481E+14	0.0295714918151284	0.174351889018293	-3,25877E+14	4,13796E+14	4,34411E+14	4,93985E+14	5,25965E+14	5,74509E+14	5,61721E+14
TC1400000263.mm.2	Arf4	ADP-ribosylation factor 4	1,95157E+14	8,19654E+14	2,78452E+14	0.0295831633126755	0.174351889018293	-3,25912E+14	9,46313E+14	9,45612E+14	8,41024E+14	6,60587E+12	8,32907E+14	6,91483E+14
TC0X00002272.mm.2	Rbmx	RNA binding motif protein, X chromosome	1,56942E+14	8,00966E+14	2,78447E+14	0.0295851976467393	0.174351889018293	-3,25918E+14	8,91131E+14	8,81646E+14	8,13107E+14	6,45786E+14	8,10269E+14	7,63855E+14
TC1900000069.mm.2	Fibp	fibroblast growth factor (acidic) intracellular binding protein	1,11416E+14	6,79323E+14	2,78425E+14	0.0295942665217546	0.174351889018293	-3,25945E+14	7,58229E+14	7,60294E+14	6,10517E+14	6,85172E+14	6,64028E+14	5,97696E+14
TC1200001601.mm.2	Hbp1	high mobility group box transcription factor 1	-1,09411E+14	8,17699E+14	-2,78341E+14	0.0296285477288755	0.174490054662051	-3,26048E+14	7,36426E+14	7,55569E+14	9,0065E+14	8,10166E+14	8,6493E+14	8,38452E+14
TC0700003923.mm.2	Olfr642	olfactory receptor 642	-1,18189E+13	5,09642E+13	-2,78257E+14	0.029662361479067	0.174591567588084	-3,2615E+14	4,16726E+14	4,16243E+13	5,13735E+14	5,55613E+12	5,25732E+14	6,29806E+14
TC0200002032.mm.2	Cds2	CDP-diacylglycerol synthase (phosphatidate cytidylyltransferase) 2	2,2865E+14	9,08463E+14	2,78245E+14	0.0296674555983475	0.174591567588084	-3,26165E+14	1,04287E+14	1,0234E+14	9,30814E+14	6,78162E+14	9,42582E+14	8,32942E+14
TC1600000252.mm.2	2510002D24Rik	RIKEN cDNA 2510002D24 gene	-1,21963E+14	7,31931E+14	-2,7816E+14	0.0297020682002578	0.174707932779515	-3,26269E+14	6,50077E+14	6,58236E+14	7,22172E+13	8,30068E+14	7,41023E+14	7,90013E+14
TC0700000976.mm.2	Trpm1	transient receptor potential cation channel, subfamily M, member 1	-1,00685E+14	5,50485E+14	-2,78143E+14	0.0297089142813797	0.174707932779515	-3,26289E+13	4,90826E+13	5,16203E+14	5,72295E+14	6,36104E+14	5,24248E+14	5,6323E+14
TC1900001213.mm.2	Vps13a	vacuolar protein sorting 13A	2,15981E+14	5,92151E+14	2,78083E+14	0.029733464267859	0.174788511400223	-3,26363E+14	7,02269E+14	7,35964E+14	6,18415E+14	3,87856E+14	6,10488E+14	4,97913E+14
TC0300000408.mm.2	Postn	periostin, osteoblast specific factor	1,11908E+14	5,23504E+14	2,77971E+13	0.0297791838044494	0.174818102768673	-3,26499E+14	5,61953E+14	5,89729E+13	4,26479E+14	5,01387E+14	5,61742E+14	4,99735E+14
TC1000003049.mm.2	Tbk1	TANK-binding kinase 1	1,60338E+14	7,34082E+14	2,77899E+14	0.0298088394934595	0.174818102768673	-3,26588E+14	8,21518E+14	8,13532E+14	7,43797E+14	5,70577E+14	7,50595E+14	7,04474E+14
TC0700004361.mm.2	BC017158/Mir3103	cDNA sequence BC017158/microRNA 3103	1,40784E+14	6,42562E+14	2,77869E+14	0.0298212397749802	0.174818102768673	-3,26625E+14	7,65173E+14	7,07943E+14	6,56393E+14	5,35154E+14	6,28607E+14	5,62098E+14
TC1700002186.mm.2	Ppp2r5d	protein phosphatase 2, regulatory subunit B', delta	0.836174686411988	7,899E+14	2,77862E+14	0.0298238475816985	0.174818102768673	-3,26633E+14	8,46492E+14	8,42522E+14	7,55355E+14	7,66424E+14	7,79228E+14	7,49377E+14
TC0700002976.mm.2	Ptov1	prostate tumor over expressed gene 1	1,04002E+14	8,41685E+14	2,77833E+13	0.029835670818857	0.174818102768673	-3,26668E+14	8,95408E+14	8,92184E+13	8,31961E+14	7,47628E+14	8,33077E+13	8,49855E+14
TC1000000264.mm.2	9330159F19Rik	RIKEN cDNA 9330159F19 gene	1,88811E+14	7,46651E+14	2,77783E+14	0.0298562893107509	0.174818102768673	-3,26729E+14	8,59931E+13	8,74481E+14	7,79942E+14	5,76848E+14	7,37198E+14	6,51504E+14
TC0800000847.mm.2	Rasd2	RASD family, member 2	1,48559E+14	8,69245E+14	2,77775E+14	0.029859602070122	0.174818102768673	-3,26739E+14	9,54459E+13	9,70336E+14	8,92439E+13	7,35239E+14	8,4886E+14	8,14138E+13
TC0200005134.mm.2	Spata25	spermatogenesis associated 25	-1,00444E+13	4,42139E+14	-2,77764E+14	0.0298641521329587	0.174818102768673	-3,26753E+14	3,84073E+14	3,29506E+14	4,28854E+14	4,85614E+14	5,11797E+14	5,12994E+14
TC0200005429.mm.2	Chrna4	cholinergic receptor, nicotinic, alpha polypeptide 4	1,10384E+14	7,82936E+14	2,7776E+14	0.0298656381813887	0.174818102768673	-3,26757E+13	8,55158E+14	8,46577E+14	7,62059E+13	7,18909E+14	8,015E+14	7,13416E+14
TC0400003794.mm.2	C1qa	complement component 1, q subcomponent, alpha polypeptide	1,01176E+14	7,01932E+12	2,77659E+13	0.0299071241872274	0.174818102768673	-3,26881E+14	7,3596E+14	8,11622E+13	6,80272E+14	6,6496E+14	6,7057E+14	6,48209E+13
TC0600002472.mm.2	Kdm3a	lysine (K)-specific demethylase 3A	1,08909E+14	5,64078E+14	2,77659E+14	0.0299073331631232	0.174818102768673	-3,26881E+14	6,05057E+14	6,1736E+14	5,49059E+14	4,55539E+13	5,70477E+14	5,86979E+14
TC0400002062.mm.2	Hes5	hes family bHLH transcription factor 5	-0.931086263912629	8,77595E+14	-2,77632E+13	0.0299184243224768	0.174818102768673	-3,26914E+14	8,02428E+14	8,17297E+14	8,80719E+14	9,25224E+14	8,99668E+14	9,40234E+14

TC0100002624.mm.2	Pax3	paired box 3	-1,33917E+14	6,36613E+14	-2,77618E+14	0.0299242428107713	0.174818102768673	-3,26932E+14	5,39906E+14	5,83482E+14	6,40113E+12	7,51108E+14	6,16862E+14	6,88209E+14
TC0200005028.mm.2	Tti1	TELO2 interacting protein 1	1.30687E+14	6,15734E+14	2,776E+14	0.0299315789949639	0.174818102768673	-3,26954E+14	6,92907E+14	7,0491E+14	6,32234E+14	5,04209E+14	5,61685E+14	5,98458E+14
TC0700003823.mm.2	Plekhb1	pleckstrin homology domain containing, family B (evectins) member 1	1,27162E+14	9,38574E+14	2,77594E+14	0.0299338428830813	0.174818102768673	-3,2696E+14	1,0246E+14	1,02023E+14	9,2976E+14	8,60747E+14	9,5188E+14	8,44227E+14
TC0400003881.mm.2	Clcnkb	chloride channel, voltage-sensitive Kb	-1,07499E+14	5,36734E+14	-2,77566E+14	0.0299452881868821	0.174818102768673	-3,26994E+14	4,67686E+14	4,61884E+14	5,41796E+14	6,02772E+14	5,38055E+14	6,08211E+14
TC0900002991.mm.2	Atp2c1	ATPase, Ca++-sequestering	2,07371E+14	7,06868E+14	2,77529E+14	0.0299606044160146	0.174818102768673	-3,2704E+14	8,1309E+14	8,41013E+14	7,27152E+14	5,12208E+14	7,33474E+14	6,14272E+14
TC0900002427.mm.2	Nr2e3	nuclear receptor subfamily 2, group E, member 3	-1,51227E+14	6,22261E+14	-2,77513E+14	0.0299672121620509	0.174818102768673	-3,27059E+14	4,9033E+14	5,56507E+14	6,00135E+14	7,49157E+14	6,53001E+14	6,84436E+14
TC0X00001801.mm.2	Ftsj1	FtsJ RNA methyltransferase homolog 1 (E. coli)	0.997722859280441	6,79069E+14	2,77506E+13	0.0299703092782986	0.174818102768673	-3,27069E+14	7,55663E+14	7,42722E+14	6,69852E+14	6,28989E+14	6,70883E+14	6,06308E+14
TC1100001625.mm.2	Klhl10	kelch-like 10	-1,05274E+14	5,06209E+14	-2,77458E+14	0.0299897858947185	0.174818102768673	-3,27126E+13	4,61208E+14	4,44446E+14	5,21235E+13	5,94967E+14	5,31043E+14	4,84352E+14
TC1400000887.mm.2	Wdfv2/Mir8098	WD repeat and FYVE domain containing 2/microRNA 8098	-1,00245E+14	7,3346E+14	-2,7745E+14	0.0299931171380985	0.174818102768673	-3,27136E+14	6,61205E+14	6,65929E+14	7,26078E+14	8,01547E+14	7,62346E+14	7,83655E+14
TC1700001619.mm.2	Atp6v0c/Atp6v0c-ps2	ATPase, H+ transporting, lysosomal V0 subunit C/ATPase, H+ transporting, lysosomal V0 subunit C, pseudogene 2	1,61238E+13	1,13969E+14	2,77446E+14	0.0299949992857871	0.174818102768673	-3,27142E+14	1,23705E+14	1,23623E+14	1,1488E+14	1,002E+14	1,16011E+14	1,05396E+14
TC0500002214.mm.2	Ctbp1	C-terminal binding protein 1	0.849068221837954	8,32809E+14	2,77441E+14	0.029996754027961	0.174818102768673	-3,27147E+14	8,86076E+14	8,803E+14	8,16499E+14	7,80064E+14	8,12868E+14	8,21045E+14
TC0800001058.mm.2	Fto	fat mass and obesity associated	1,57055E+14	7,38974E+14	2,77436E+14	0.0299988862505668	0.174818102768673	-3,27153E+13	8,4925E+14	8,4889E+14	7,79267E+14	6,04764E+14	6,81885E+14	6,69789E+14
TC0700002128.mm.2	Tarm1	T cell-interacting, activating receptor on myeloid cells 1	-1,05144E+14	5,92662E+14	-2,77228E+14	0.030084676226337	0.175078867493226	-3,27407E+14	5,17299E+14	5,11882E+14	5,84496E+14	6,54973E+14	6,168E+14	6,70522E+14
TC0900001371.mm.2	Nprl2	NPR2 like, GATOR1 complex subunit	0.976895714119506	6,64629E+13	2,77224E+14	0.0300866173566697	0.175078867493226	-3,27413E+14	7,12662E+14	7,31382E+14	6,50672E+13	5,97993E+14	6,71129E+14	6,23935E+14
TC0700004623.mm.2	Dctn5	dynactin 5	1,13996E+14	8,13925E+14	2,77222E+14	0.0300872132922446	0.175078867493226	-3,27415E+14	8,78365E+14	8,75674E+14	8,14163E+14	7,11884E+14	8,14591E+14	7,88871E+14
TC0800000816.mm.2	Rab8a	RAB8A, member RAS oncogene family	2,20693E+14	7,09275E+14	2,77221E+14	0.030087668613168	0.175078867493226	-3,27416E+14	8,67146E+14	8,6136E+14	7,55684E+14	5,31435E+14	6,91728E+14	5,483E+14
TC0500000360.mm.2	Tacc3	transforming, acidic coiled-coil containing protein 3	-1,12475E+14	6,90073E+14	-2,77196E+14	0.0300979620775917	0.175078867493226	-3,27447E+14	6,19127E+14	6,26154E+14	7,00153E+12	7,70077E+14	6,74987E+14	7,49942E+14
TC0300000730.mm.2	Ubqln4	ubiquilin 4	1,13581E+14	7,1902E+14	2,77141E+14	0.0301207460102127	0.175093586765986	-3,27514E+14	7,76465E+14	7,80778E+14	7,12725E+14	6,17357E+14	6,91773E+13	7,35023E+14
TC0400000039.mm.2	Sdcbp	syndecan binding protein	1,91519E+14	9,10918E+14	2,77102E+14	0.0301368229297271	0.175093586765986	-3,27562E+14	1,03716E+14	1,03416E+13	9,48192E+14	7,40096E+13	8,96266E+14	8,09631E+14
TC0300003023.mm.2	Rap1gds1	RAP1, GTP-GDP dissociation stimulator 1	0.949801822320727	8,28186E+14	2,771E+14	0.0301379828830058	0.175093586765986	-3,27565E+14	8,87978E+14	8,93123E+14	8,25521E+14	7,65619E+14	8,13252E+14	7,83621E+14
TC0300000864.mm.2	Rorc	RAR-related orphan receptor gamma	-1,18851E+14	4,74422E+14	-2,77082E+14	0.0301451134698299	0.175093586765986	-3,27586E+14	4,05588E+13	4,25278E+14	4,88939E+14	5,79629E+14	4,40257E+14	5,06839E+14
TC0100002606.mm.2	Ptprn	protein tyrosine phosphatase, receptor type, N	1,82964E+14	9,15098E+14	2,7702E+14	0.030170864153795	0.175093586765986	-3,27662E+14	1,04579E+14	1,03305E+14	9,44329E+14	7,68581E+14	9,09498E+14	7,89345E+14
TC1600002059.mm.2	Cfap298	cilia and flagella associate protien 298	1,47364E+14	6,05827E+14	2,77011E+14	0.0301745411350088	0.175093586765986	-3,27673E+14	7,26954E+14	6,92332E+14	6,3904E+14	4,85516E+13	5,29654E+14	5,61465E+14
TC1500000478.mm.2	Efr3a	EFR3 homolog A	-1,573E+14	7,23459E+14	-2,77007E+14	0.0301765587072017	0.175093586765986	-3,27679E+14	6,2076E+14	6,29643E+14	7,11746E+14	8,53257E+14	7,1083E+14	8,14516E+14
TC0200004486.mm.2	Strc	stereocilin	-1,72757E+14	4,65626E+14	-2,76939E+14	0.0302046878081198	0.175178586306641	-3,27762E+14	3,70238E+14	3,85922E+14	4,6717E+14	6,34505E+14	4,15084E+14	5,20838E+14
TC1100001538.mm.2	Arhgap23	Rho GTPase activating protein 23	0.835662101963267	7,00183E+14	2,76914E+14	0.0302148807945787	0.175178586306641	-3,27792E+14	7,47503E+14	7,63562E+14	6,79924E+14	6,64008E+14	6,60971E+14	6,85129E+14
TC0700000808.mm.2	Fam71e1	family with sequence similarity 71, member E1	-1,26697E+14	7,18205E+14	-2,76893E+14	0.0302238236164874	0.175178586306641	-3,27818E+14	6,33252E+14	6,30162E+14	7,03941E+14	8,12867E+14	7,31206E+14	7,97804E+14
TC1700001858.mm.2	Cyp4f41-ps	cytochrome P450, family 4, subfamily f, polypeptide 41 pseudogene	-1,11644E+14	5,23036E+13	-2,76705E+14	0.0303018224761211	0.175281330015242	-3,28047E+13	4,49445E+14	4,63737E+14	5,18841E+14	6,17628E+14	5,34841E+14	5,53723E+14
TC1300001652.mm.2	Hist1h2ba	histone cluster 1, H2ba	-1,15093E+14	5,83804E+14	-2,76694E+14	0.0303066662779322	0.175281330015242	-3,28062E+14	5,30299E+14	4,94694E+14	5,97923E+14	6,57257E+14	5,69952E+14	6,52698E+14
TC1500001560.mm.2	Derl1	Der1-like domain family, member 1	1,72423E+14	7,05306E+14	2,76655E+14	0.0303229451304783	0.175281330015242	-3,28109E+14	8,15165E+13	8,09122E+14	7,05308E+14	5,74132E+14	7,38377E+14	5,89729E+13
TC1200001931.mm.2	Kcnh5	potassium voltage-gated channel, subfamily H (eag-related), member 5	1,54632E+14	6,72998E+14	2,76633E+14	0.030331943944023	0.175281330015242	-3,28136E+13	7,21601E+14	7,79809E+14	6,67994E+14	5,24152E+14	7,09263E+14	6,3517E+14
TC0100002964.mm.2	Dbi	diazepam binding inhibitor	1,15168E+13	9,38471E+13	2,76558E+14	0.0303632949878514	0.175281330015242	-3,28228E+14	1,0005E+14	1,00793E+14	9,3683E+14	8,41267E+14	9,4714E+14	8,97155E+14
TC0800001055.mm.2	Rbl2	RB transcriptional corepressor like 2	1,27995E+14	7,36201E+14	2,76554E+14	0.0303649152977585	0.175281330015242	-3,28232E+13	8,33951E+14	8,19778E+13	7,56689E+14	6,41049E+14	7,11845E+13	6,53895E+13
TC1800001107.mm.2	Rit2	Ras-like without CAAX 2	1,55795E+14	9,25312E+14	2,76498E+14	0.0303884586124304	0.175281330015242	-3,28301E+13	1,04516E+14	10061035438	9,38957E+14	8,00712E+14	9,29406E+14	8,31537E+14
TC0200002201.mm.2	Cst8	cystatin 8 (cystatin-related epididymal spermatogenic)	-1,37273E+14	4,36682E+14	-2,76475E+14	0.030397813037091	0.175281330015242	-3,28329E+14	3,3393E+14	3,76088E+14	4,33777E+14	5,50786E+14	5,00238E+14	4,25274E+14
TC0400002236.mm.2	Otud6b	OTU domain containing 6B	1,13834E+13	7,98021E+14	2,76475E+13	0.0303980977765397	0.175281330015242	-3,2833E+14	8,68894E+14	8,77847E+14	8,05527E+14	7,13546E+14	7,8687E+14	7,35444E+14
TC1600000460.mm.2	Rpl35a	ribosomal protein L35A	1,2205E+14	7,10141E+14	2,76465E+14	0.0304021174566525	0.175281330015242	-3,28341E+14	7,85221E+14	7,80253E+14	7,15863E+14	6,05511E+14	7,10694E+13	6,63306E+14
TC0900001513.mm.2	Tmppe/Glb1	transmembrane protein with metallophosphoesterase domain/galactosidase, beta 1	0.925404473662248	6,53106E+14	2,76416E+14	0.030422528719223	0.175281330015242	-3,28401E+14	7,23942E+14	6,95529E+14	5,98747E+14	6,35644E+14	6,52565E+14	6,12207E+14
TC1100003948.mm.2	Pitpnc1	phosphatidylinositol transfer protein, cytoplasmic 1	2,11E+14	7,53756E+14	2,76365E+14	0.0304439693852978	0.175281330015242	-3,28464E+14	8,89152E+14	8,71405E+14	7,88809E+14	5,49748E+14	7,53205E+14	6,70218E+14
TC0500003725.mm.2	Pisd	phosphatidylserine decarboxylase	0.95533001838719	6,84661E+14	2,76324E+13	0.0304612287613671	0.175281330015242	-3,28514E+14	7,58691E+14	7,58973E+14	6,95747E+14	6,3085E+14	6,43507E+14	6,20197E+14
TC1200002359.mm.2	Wars	tryptophanyl-tRNA synthetase	1,46191E+13	6,86269E+14	2,76324E+14	0.0304612607510368	0.175281330015242	-3,28514E+13	7,98998E+13	7,80259E+14	7,02251E+14	5,84624E+14	6,78642E+14	5,7284E+14
TC0800000396.mm.2	Gtf2e2	general transcription factor II E, polypeptide 2 (beta subunit)	1,60959E+14	5,96641E+14	2,76299E+14	0.0304718150735554	0.175281330015242	-3,28545E+14	7,13857E+14	6,78024E+14	5,64516E+14	5,05447E+12	6,43049E+14	4,74952E+14
TC0400002768.mm.2	Mup1	major urinary protein 1	-1,12988E+14	5,24365E+14	-2,7629E+13	0.0304755929627768	0.175281330015242	-3,28556E+14	4,32967E+14	4,56925E+14	5,0804E+14	6,07828E+14	5,60734E+14	5,79698E+14
TC0500002286.mm.2	Wdr1	WD repeat domain 1	1,10043E+14	8,85721E+12	2,76275E+14	0.0304818119662949	0.175281330015242	-3,28574E+14	9,58624E+14	9,54007E+14	8,94393E+14	7,98153E+14	8,43355E+13	8,65792E+14
TC1200000970.mm.2	Zc3h14	zinc finger CCCH type containing 14	1,64467E+14	7,73518E+14	2,76168E+14	0.0305267218428263	0.175281330015242	-3,28705E+14	8,5303E+14	8,61545E+14	7,72765E+14	6,12876E+14	8,18819E+14	7,22074E+14
TC1000000767.mm.2	Gm10100	predicted gene 10100	-1,17938E+14	6,537E+14	-2,76103E+14	0.0305537618733547	0.175281330015242	-3,28784E+14	5,86261E+14	5,72439E+14	6,45633E+14	7,48942E+14	6,62187E+14	7,06739E+13
TC0300002374.mm.2	S100a1	S100 calcium binding protein A1	1,45363E+14	6,63378E+14	2,761E+14	0.0305553358668304	0.175281330015242	-3,28789E+14	7,31153E+14	7,41744E+14	6,22429E+14	5,59741E+14	7,35041E+14	5,90159E+14
TC0900003332.mm.2	Mthfs	5, 10-methenyltetrahydrofolate synthetase	0.93862905143591	5,30157E+14	2,76094E+14	0.0305577874691258	0.175281330015242	-3,28796E+14	6,08368E+14	5,47013E+14	4,84155E+14	4,83501E+14	5,39931E+14	5,17973E+14
TC1200000197.mm.2	Taf1b	TATA-box binding protein associated factor, RNA polymerase I, B	1,06454E+13	6,60477E+14	2,76089E+13	0.0305599756141151	0.175281330015242	-3,28802E+14	7,49305E+14	7,44013E+14	6,76437E+14	6,03973E+14	6,22959E+14	5,66177E+14
TC1900001509.mm.2	Slit1	slit guidance ligand 1	0.83232372835779	7,52805E+14	2,76088E+14	0.0305600512097786	0.175281330015242	-3,28803E+14	8,32144E+14	8,04184E+14	7,40164E+14	7,297E+14	6,98614E+13	7,12023E+14
TC1400000999.mm.2	Lgi3	leucine-rich repeat LGI family, member 3	1,41457E+14	7,55195E+14	2,76083E+14	0.030562099213454	0.175281330015242	-3,28808E+13	8,80404E+14	8,47528E+14	7,88136E+14	6,56882E+14	7,13889E+14	6,44334E+13
TC1100003252.mm.2	Cpd	carboxypeptidase D	1,35647E+14	7,23885E+14	2,76072E+14	0.030566744383048	0.175281330015242	-3,28822E+14	8,08638E+14	8,40336E+14	7,41239E+14	6,36441E+14	7,04274E+14	6,1238E+14
TC0300002402.mm.2	Lce1b	late cornified envelope 1B	-0.842635221461523	7,46496E+14	-2,76056E+14	0.0305736694542123	0.175281330015242	-3,28842E+14	7,05354E+14	6,74604E+14	7,68751E+14	7,79734E+14	7,83912E+14	7,66618E+14
TC0400004130.mm.2	Tnfrsf14	tumor necrosis factor receptor superfamily, member 14 (herpesvirus entry mediator)	-1,38085E+14	7,56333E+14	-2,76025E+14	0.0305868513457888	0.175281330015242	-3,28881E+14	6,49418E+14	6,72908E+13	7,35734E+14	8,62761E+14	7,73655E+14	8,43524E+14
TC0900000667.mm.2	Psma4	proteasome (prosome, macropain) subunit, alpha type 4	1,05696E+14	8,14418E+14	2,7601E+14	0.0305928470294957	0.175281330015242	-3,28898E+14	8,88576E+14	8,61832E+14	7,99913E+13	7,39102E+14	8,29486E+14	7,67603E+14
TC1100000015.mm.2	Selenom	selenoprotein M	0.903287578743104	8,53722E+14	2,75973E+14	0.0306084611221319	0.175281330015242	-3,28943E+14	9,28736E+14	9,08752E+14	8,45625E+14	8,11205E+14	8,3386E+14	7,94154E+14
TC1500001870.mm.2	Tmem184b	transmembrane protein 184b	0.950570420775851	7,44159E+14	2,75941E+14	0.0306220543956273	0.175281330015242	-3,28983E+14	7,97237E+14	7,97665E+14	7,35166E+13	6,69622E+14	7,41963E+14	7,23298E+13
TC0200004975.mm.2	Uqcc1	ubiquinol-cytochrome c reductase complex assembly factor 1	0.905398262880333	6,71037E+14	2,75927E+14	0.0306279176297506	0.175281330015242	-3,29E+14	7,66507E+14	7,24729E+13	6,74118E+14	6,36038E+14	6,0949E+14	6,15341E+14
TC0500001885.mm.2	Rbm48	RNA binding motif protein 48	-0.976425712884083	7,13802E+14	-2,75922E+14	0.0306300189714064	0.175281330015242	-3,29006E+13	6,51618E+14	6,38474E+14	7,06527E+14	7,7885E+14	7,58279E+14	7,49066E+14
TC0400001405.mm.2	Rhbdl2	rhomboid like 2	-0.994868464410748	5,27647E+14	-2,75915E+14	0.0306330982407152	0.175281330015242	-3,29015E+14	4,65366E+14	4,76678E+14	5,35586E+14	6,05432E+14	5,24849E+13	5,5797E+13
TC0500002758.mm.2	Nup54	nucleoporin 54	1,13048E+14	5,70953E+14	2,75899E+14	0.0306395663966439	0.175281330015242	-3,29034E+14	6,88155E+14	6,83901E+14	5,45004E+14	6,00957E+14	4,98083E+14	4,09618E+14
TC1100000954.mm.2	Gltpd2	glycolipid transfer protein domain containing 2	-0.919084440584073	5,88581E+14	-2,75836E+13	0.0306661739754774	0.175281330015242	-3,29111E+14	5,07814E+13	5,53559E+14	6,14593E+14	6,30596E+14	5,9435E+14	6,30575E+14
TC1000002451.mm.2	Rrp1	ribosomal RNA processing 1 homolog (S. cerevisiae)	1,46027E+14	9,37991E+14	2,75767E+14	0.0306953198526717	0.175281330015242	-3,29196E+14	1,06331E+13	1,03191E+14	9,64801E+14	8,38374E+14	9,10802E+13	8,18739E+14
TC1900001119.mm.2	Ms4a10	membrane-spanning 4-domains, subfamily A, member 10	-1,02398E+14	5,58032E+14	-2,75757E+14	0.0306997098869724	0.175281330015242	-3,29208E+14	4,84704E+11	4,92122E+14	5,63416E+14	6,18207E+14	5,63013E+14	6,26727E+14
TC1000002381.mm.2	Cabin1	calcineurin binding protein 1	1,11273E+14	7,27805E+14	2,757E+14	0.0307235940034653	0.175281330015242	-3,29277E+14	8,26966E+14	7,92992E+14	7,46604E+14	6,50808E+14	6,75271E+14	6,74188E+14
TC0600002314.mm.2	Scrn1	secernin 1	1,56188E+14	8,72203E+14	2,75604E+14	0.0307643779051285	0.175281330015242	-3,29396E+14	9,82165E+14	9,59563E+14	8,99711E+14	7,29642E+14	8,48774E+14	8,13366E+14
TSUnmapped00000049.mm.2	Aopep	aminopeptidase O	-1,02473E+14	5,30467E+14	-2,75591E+13	0.0307697283260113	0.175281330015242	-3,29411E+14	4,74364E+14	4,89418E+14	5,95339E+14	5,73389E+14	4,84973E+14	5,65319E+14
TC0X00000069.mm.2	Gripap1	GRIP1 associated protein 1	0.980053880496082	7,52543E+13	2,75573E+14	0.03077737661422	0.175281330015242	-3,29433E+14	8,0679E+14	8,29758E+14	6,9438E+14	7,46158E+14	7,43788E+14	6,94384E+14
TC0700002080.mm.2	Mrpl23	mitochondrial ribosomal protein L23	0.836342800792984	7,08762E+14	2,75558E+14	0.0307840976288269	0.175281330015242	-3,29453E+14	7,67414E+14	7,38479E+14	6,7453E+14	6,64095E+14	7,12447E+14	6,95605E+14
TC0900001374.mm.2	Tusc2	tumor suppressor 2, mitochondrial calcium regulator	-0.866140085159557	7,88822E+14	-2,75538E+14	0.0307922628589822	0.175281330015242	-3,29476E+14	7,41458E+13	7,30613E+14	8,03589E+14	8,4171E+14	8,19183E+13	7,96381E+14
TC1500001965.mm.2	Rrp7a	ribosomal RNA processing 7 homolog A (S. cerevisiae)	1,1141E+14	8,08136E+14	2,75531E+14	0.0307952924340093	0.175281330015242	-3,29485E+13	9,00258E+14	8,592E+14	8,05726E+13	7,30911E+14	8,04319E+14	7,484E+13
TC1100003285.mm.2	Traf4	TNF receptor associated factor 4	-0.898343383594264	8,73509E+14	-2,7553E+14	0.0307959162673877	0.175281330015242	-3,29487E+14	8,08492E+14	8,24417E+13	9,29214E+14	8,83364E+14	9,10999E+14	8,84571E+14
TC0500000614.mm.2	Ube2k	ubiquitin-conjugating enzyme E2K	1,59337E+14	8,7633E+14	2,75524E+14	0.0307983598596047	0.175281330015242	-3,29494E+14	9,72992E+14	9,68603E+14	8,76888E+14	7,46035E+14	9,08483E+14	7,84979E+14
TC1100002091.mm.2	Inpp5j	inositol polyphosphate 5-phosphatase J	1,36998E+14	5,90261E+13	2,7552E+14	0.0308000856770649	0.175281330015242	-3,29499E+14	6,81148E+14	6,76538E+14	5,8925E+13	4,9444E+14	6,05256E+13	4,94935E+14
TC0600000829.mm.2	Rnf103	ring finger protein 103	0.989323688133913	7,3052E+14	2,75491E+14	0.0308122664970612	0.175281330015242	-3,29534E+14	7,97252E+14	8,07922E+14	7,12117E+13	6,95192E+14	7,22629E+13	6,48009E+14
TC1700002504.mm.2	Alk	anaplastic lymphoma kinase	-0.970872254479056	6,26467E+14	-2,75491E+14	0.0308124900162829	0.175281330015242	-3,29535E+14	5,52643E+12	5,79033E+14	6,2946E+14	6,9639E+14	6,43939E+14	6,57335E+14
TC0400004170.mm.2	Agrn	agrin	0.842660644846488	7,10415E+13	2,75466E+14	0.030823047410957	0.175281330015242	-3,29565E+14	7,61488E+14	7,50082E+14	6,73605E+14	6,69433E+14	6,86123E+14	7,21759E+14
TC0700000015.mm.2	Prpf31	pre-mRNA processing factor 31	0.96864339831429	7,26843E+14	2,75464E+14	0.0308236792169306	0.175281330015242	-3,29567E+14	7,91457E+14	7,85451E+14	7,25113E+13	6,58065E+14	7,14215E+13	6,86759E+14
TC1700001173.mm.2	Rasgrp3	RAS, guanyl releasing protein 3	1,22939E+14	6,18863E+13	2,75442E+14	0.0308332971738099	0.175281330015242	-3,29595E+13	6,42699E+13	7,25066E+14	5,85222E+14	5,36664E+14	6,50045E+14	5,73483E+14
TC1000000673.mm.2	Mrln	myoregulin	-1,44675E+13	7,04163E+12	-2,75436E+13	0.0308354680989461	0.175281330015242	-3,29601E+14	5,84607E+14	6,17252E+13	6,70096E+14	8,21113E+14	7,50188E+14	7,81721E+14
TC0100002188.mm.2	Fam178b	family with sequence similarity 178, member B	-1,1965E+14	5,76112E+14	-2,75393E+14	0.0308539271074483	0.175281330015242	-3,29654E+14	5,42649E+14	4,63437E+14	5,7973E+14	6,65656E+14	6,03263E+14	6,01937E+14
TC1500000875.mm.2	Mapk8ip2	mitogen-activated protein kinase 8 interacting protein 2	1,13524E+14	9,17564E+14	2,7535E+14	0.0308721011506057	0.175281330015242	-3,29707E+14	1,01506E+14	9,9165E+14	9,37976E+14	8,41689E+14	8,78378E+14	8,40628E+14
TC0500002744.mm.2	Cdkl2	cyclin-dependent kinase-like 2 (CDC2-related kinase)	0.800082072617185	5,79828E+14	2,75343E+13	0.030875165386358	0.175281330015242	-3,29715E+14	6,27648E+14	6,30105E+14	5,508E+14	5,46937E+14	5,54311E+14	5,69164E+14
TC1000001596.mm.2	Stat2	signal transducer and activator of transcription 2	0.792881799529479	6,26409E+14	2,75289E+14	0.0308982029638276	0.175281330015242	-3,29782E+14	6,80141E+14	6,79061E+14	6,03725E+14	5,96901E+14	6,00244E+14	5,98384E+14
TC0900001428.mm.2	Col7a1/Mir711	collagen, type VII, alpha 1/microRNA 711	-0.893925502937657	8,67951E+13	-2,7524E+14	0.0309187591796259	0.175281330015242	-3,29841E+14	7,9314E+13	8,08355E+14	8,6755E+14	9,1273E+14	8,99966E+14	9,25964E+14
TC0600001968.mm.2	Lrrc4	leucine rich repeat containing 4	1,05493E+14	6,82313E+13	2,75215E+14	0.0309296781523868	0.175281330015242	-3,29872E+14	7,38579E+14	7,61936E+13	6,75572E+14	6,13956E+14	6,83291E+14	6,20546E+14
TC0800002525.mm.2	Hhip	Hedgehog-interacting protein	1,07641E+14	6,98455E+13	2,75208E+14	0.0309324827217238	0.175281330015242	-3,2988E+14	7,6359E+14	8,0665E+14	7,07664E+14	6,47294E+14	6,62592E+14	6,02937E+14
TC1000000156.mm.2	Bclaf1	BCL2-associated transcription factor 1	2,05378E+14	6,94836E+14	2,75207E+14	0.0309332009401909	0.175281330015242	-3,29883E+14	8,44548E+14	8,40932E+14	7,155E+14	5,59223E+14	7,00029E+13	5,08785E+14
TC0200000459.mm.2	Surf2	surfeit gene 2	1,71261E+14	7,58383E+14	2,75172E+13	0.0309477480178851	0.175281330015242	-3,29924E+12	8,71912E+14	8,49509E+14	7,75942E+14	6,02957E+14	7,71827E+14	6,78151E+14
TC0700000844.mm.2	Ntf5	neurotrophin 5	-0.976982487376155	5,34978E+14	-2,75171E+13	0.0309481308415377	0.175281330015242	-3,29926E+13	4,73525E+14	4,61186E+13	5,3424E+14	5,95868E+13	5,52248E+14	5,92801E+14
TC0300003170.mm.2	Rabggtb/Snord45c	Rab geranylgeranyl transferase, b subunit/small nucleolar RNA, C/D box 45C	1,56582E+14	8,07223E+14	2,75163E+14	0.0309516543904746	0.175281330015242	-3,29936E+14	8,97072E+14	9,00909E+13	8,08733E+14	6,76083E+12	8,38191E+14	7,22349E+14
TC0200001904.mm.2	Ap4e1	adaptor-related protein complex AP-4, epsilon 1	-0.848200915983137	6,33434E+13	-2,75162E+14	0.0309522848336028	0.175281330015242	-3,29937E+14	5,76438E+13	6,00361E+14	6,59984E+14	6,86456E+14	6,29598E+14	6,47765E+14
TC0700003806.mm.2	Neu3	neuraminidase 3	-0.957008725391777	7,61641E+14	-2,75136E+14	0.0309631119909244	0.175281330015242	-3,29969E+14	6,86715E+14	7,01425E+14	7,56553E+14	8,2299E+14	8,09918E+14	7,92244E+14
TC0700003016.mm.2	Sec1	secretory blood group 1	-1,00094E+13	6,3312E+14	-2,75087E+14	0.0309842066570644	0.175281330015242	-3,30029E+14	5,35449E+14	5,82885E+14	6,36549E+14	6,81973E+14	6,58038E+14	7,03827E+14
TC0900002374.mm.2	Mpi	mannose phosphate isomerase	0.859592299269956	7,24512E+14	2,75068E+14	0.0309921255851043	0.175281330015242	-3,30052E+14	7,6611E+13	7,62629E+14	6,99599E+14	6,57221E+14	7,24966E+14	7,3655E+13
TC1100001670.mm.2	Dhx8	DEAH (Asp-Glu-Ala-His) box polypeptide 8	1,85533E+14	5,82793E+14	2,7502E+14	0.0310129141934136	0.175281330015242	-3,30112E+14	7,05127E+14	6,86852E+14	6,18042E+14	4,02871E+14	5,59843E+14	5,24022E+14
TC1200000211.mm.2	Kidins220	kinase D-interacting substrate 220	1.86791E+14	7,1595E+14	2.75007E+14	0.0310181190337447	0.175281330015242	-3,30127E+14	8,41337E+14	8,34514E+14	7,48394E+14	5,53875E+14	7,10832E+14	6,06752E+14
TC1600000238.mm.2	Trmt2a	TRM2 tRNA methyltransferase 2A	-0.801265097702143	7,31954E+14	-2,74957E+14	0.0310396680110128	0.175281330015242	-3,30188E+14	6,84389E+14	6,84778E+14	7,59056E+14	7,70364E+14	7,53207E+14	7,3993E+14
TC1000000847.mm.2	Ndufs7	NADH:ubiquinone oxidoreductase core subunit S7	1,9559E+14	7,95938E+14	2,74921E+14	0.031055267227488	0.175281330015242	-3,30233E+14	9,29223E+14	9,1148E+13	8,14534E+14	6,34988E+14	8,16743E+14	6,68661E+14
TC0700002426.mm.2	Sae1	SUMO1 activating enzyme subunit 1	1,61383E+14	6,76515E+14	2,74873E+14	0.0310755302300423	0.175281330015242	-3,30291E+14	7,79149E+14	7,55224E+14	6,87039E+14	5,24569E+13	6,97624E+14	6,15482E+14
TC0100000131.mm.2	Efhc1	EF-hand domain (C-terminal) containing 1	-0.877512114889548	6,75675E+14	-2,74831E+14	0.031093697173309	0.175281330015242	-3,30343E+14	6,3318E+14	6,22116E+14	6,92311E+14	7,38488E+14	6,76245E+14	6,91709E+14
TC1000002520.mm.2	Gng7	guanine nucleotide binding protein (G protein), gamma 7	1,50645E+14	9,16455E+14	2,74825E+14	0.0310961457219841	0.175281330015242	-3,3035E+14	1,01147E+14	1,00287E+14	9,38483E+14	7,74566E+13	9,0213E+14	8,69212E+14
TC0700000424.mm.2	Tgfb1	transforming growth factor, beta 1	1,35482E+14	6,86155E+14	2,74823E+14	0.0310968705524847	0.175281330015242	-3,30352E+14	7,30002E+14	7,67996E+14	6,81761E+14	5,45275E+13	7,07852E+14	6,84043E+14
TC1100001924.mm.2	Ten1	TEN1 telomerase capping complex subunit	-0.807729568716252	7,36956E+14	-2,7482E+14	0.0310982379853449	0.175281330015242	-3,30356E+14	6,99737E+14	6,82985E+14	7,67241E+12	7,77027E+14	7,41989E+14	7,52756E+14
TC0200003927.mm.2	Olfr1053	olfactory receptor 1053	-0.924209397200308	5,77174E+14	-2,74799E+14	0.0311072956007862	0.175281330015242	-3,30382E+14	5,1874E+14	5,00768E+14	5,82371E+14	6,21978E+13	5,9702E+14	6,42167E+14
TC0400002086.mm.2	Tmem240	transmembrane protein 240	-1,01829E+14	7,48022E+14	-2,74754E+14	0.0311263830076607	0.175281330015242	-3,30437E+14	6,65122E+14	6,82806E+14	7,34941E+12	8,16644E+14	7,90434E+13	7,98185E+14
TC1100003489.mm.2	Olfr462/Olfr463	olfactory receptor 462/olfactory receptor 463	-1,52089E+14	5,88537E+14	-2,74744E+14	0.0311308830571222	0.175281330015242	-3,30449E+14	5,19868E+14	4,77899E+14	5,73077E+14	7,28867E+14	5,89479E+14	6,42033E+14
TC0500001675.mm.2	Gper1	G protein-coupled estrogen receptor 1	-0.981745941486521	6,28808E+14	-2,74742E+14	0.0311315125272457	0.175281330015242	-3,30451E+14	5,86328E+14	5,47542E+14	6,37488E+14	6,92731E+14	6,36713E+13	6,72043E+14
TC0X00001558.mm.2	Ubqln2	ubiquilin 2	1,86917E+13	8,22916E+14	2,74718E+14	0.0311420775550039	0.175281330015242	-3,30481E+14	9,588E+14	9,38458E+13	8,61371E+11	6,62052E+14	8,05261E+14	7,11555E+14
TC0500003252.mm.2	Mphosph9	M-phase phosphoprotein 9	-0.854002014092063	7,76228E+14	-2,74691E+13	0.0311534787960958	0.175281330015242	-3,30514E+14	7,28918E+14	7,05194E+14	7,90048E+14	8,14864E+14	8,2177E+14	7,96575E+13

TSUnmapped00000027.mm.2	Nkx1-1	NK1 homeobox 1	-0.946223730520349	6,84785E+14	-2,74687E+14	0.0311553235998047	0.175281330015242	-3,30519E+14	6,154E+14	6,19356E+14	6,87157E+14	7,36843E+14	7,01741E+14	7,4821E+13
TC1800000961.mm.2	Gjd4	gap junction protein, delta 4	-0.883914156856383	4,96049E+14	-2,7464E+14	0.0311756209432106	0.175334303754957	-3,30577E+14	4,4002E+14	4,28604E+14	5,22504E+14	5,22903E+14	5,05765E+13	5,565E+14
TC0200004667.mm.2	Slc23a2	solute carrier family 23 (nucleobase transporters), member 2	1,64346E+14	7,70564E+14	2,74599E+14	0.0311931969195513	0.175371940671574	-3,30627E+14	8,70316E+14	8,72194E+14	7,61864E+14	6,51953E+14	8,09961E+13	6,57098E+14
TC0300002325.mm.2	1500004A13Rik	RIKEN cDNA 1500004A13 gene	1,7389E+14	9,71832E+14	2,74543E+14	0.0312172854241445	0.175372040112279	-3,30696E+14	1,08382E+14	1,08238E+14	9,9734E+14	8,21088E+14	9,72958E+14	8,734E+14
TC0700002073.mm.2	Krtap5-4	keratin associated protein 5-4	-0.880115021103131	6,14586E+14	-2,74513E+14	0.0312302382088276	0.175372040112279	-3,30733E+14	5,43286E+14	5,57845E+14	6,19521E+14	6,57632E+14	6,39304E+14	6,69931E+14
TC0900000013.mm.2	Alkbh8	alkB homolog 8, tRNA methyltransferase	2,20384E+14	6,58977E+14	2,74503E+14	0.031234378970764	0.175372040112279	-3,30745E+14	8,15538E+14	8,15603E+14	7,03804E+14	4,86569E+14	6,45373E+14	4,86972E+14
TC0200003171.mm.2	Camsap1	calmodulin regulated spectrin-associated protein 1	2,22771E+14	7,15667E+13	2,74476E+14	0.031246009521832	0.175372040112279	-3,30778E+14	8,86159E+14	8,67676E+14	7,71732E+14	5,36561E+13	6,83866E+14	5,4801E+14
TC0700000792.mm.2	Klk1b24	kallikrein 1-related peptidase b24	-0.860701886952413	4,85986E+14	-2,74455E+13	0.031254827097083	0.175372040112279	-3,30803E+14	4,26706E+14	4,06084E+14	4,97809E+13	5,07121E+14	5,19612E+14	5,58585E+14
TC1500000528.mm.2	Adgrb1	adhesion G protein-coupled receptor B1	1,43487E+14	8,79761E+14	2,74407E+14	0.0312758802287943	0.175372040112279	-3,30863E+14	9,70051E+14	9,64535E+14	8,88789E+14	7,58822E+14	8,91851E+14	8,04522E+14
TC1900000606.mm.2	Ankrd2	ankyrin repeat domain 2 (stretch responsive muscle)	-1,34897E+14	5,58076E+14	-2,74406E+14	0.0312761222122783	0.175372040112279	-3,30864E+13	5,08843E+14	4,64763E+13	5,60381E+14	6,83019E+13	5,36226E+14	5,95223E+14
TC1900001031.mm.2	Stip1	stress-induced phosphoprotein 1	1,52946E+14	8,78451E+14	2,74396E+14	0.0312802856875001	0.175372040112279	-3,30876E+14	9,96963E+14	9,69074E+14	9,03624E+14	7,56522E+14	8,63864E+14	7,80657E+14
TC0X00002154.mm.2	Smarca1	SWI/SNF related, matrix associated, actin dependent regulator of chromatin, subfamily a, member 1	1,08521E+14	6,55225E+14	2,74213E+13	0.031359329124674	0.175557727719528	-3,311E+14	7,62722E+14	7,43385E+14	6,3879E+14	6,50276E+14	6,15574E+14	5,20601E+14
TC1100000961.mm.2	Rnf167	ring finger protein 167	1,29679E+14	7,668E+14	2,74212E+14	0.0313596309208248	0.175557727719528	-3,31101E+14	8,55084E+14	8,4469E+14	7,67444E+14	6,72972E+14	7,78039E+13	6,82574E+14
TC0300002489.mm.2	Ciart	circadian associated repressor of transcription	1,28037E+14	6,53949E+14	2,74179E+14	0.031374264394109	0.175557727719528	-3,31143E+14	7,42168E+14	7,24504E+14	6,69605E+14	5,40992E+14	6,35214E+14	6,1121E+14
TC0500003516.mm.2	Gna12	guanine nucleotide binding protein, alpha 12	1,02684E+14	8,74947E+13	2,74082E+14	0.031416071508548	0.175557727719528	-3,31261E+14	9,50191E+14	9,45024E+14	8,75689E+14	8,14158E+14	8,62401E+14	8,02221E+14
TC1100000789.mm.2	Specc1	sperm antigen with calponin homology and coiled-coil domains 1	1,23428E+13	6,5018E+14	2,74042E+14	0.0314335158529103	0.175557727719528	-3,31311E+14	7,36964E+14	7,49068E+14	6,74049E+14	5,65128E+14	6,17356E+14	5,58515E+14
TC1700002400.mm.2	Pja2	praja ring finger ubiquitin ligase 2	2,34294E+14	9,27639E+14	2,73972E+13	0.0314639256586167	0.175557727719528	-3,31397E+14	1,06648E+14	1,07723E+14	9,66286E+14	7,08832E+14	9,38778E+14	8,08229E+13
TC0700000051.mm.2	Zfp524	zinc finger protein 524	-0.96859120151016	9,02306E+14	-2,73964E+14	0.0314670489781825	0.175557727719528	-3,31405E+14	8,34507E+13	8,6505E+14	9,16869E+13	9,76406E+14	8,94844E+14	9,26158E+14
TC1600002039.mm.2	Tiam1	T cell lymphoma invasion and metastasis 1	0.953181364837278	6,73163E+13	2,73922E+14	0.0314855197814844	0.175557727719528	-3,31458E+14	7,75402E+14	7,30903E+14	6,81725E+14	6,33943E+14	5,95349E+14	6,21659E+14
TC1100003286.mm.2	Nek8	NIMA (never in mitosis gene a)-related expressed kinase 8	-0.796192738831437	6,06369E+14	-2,73917E+14	0.0314877756807038	0.175557727719528	-3,31464E+14	5,53813E+14	5,68942E+13	6,41218E+13	6,40776E+14	6,14528E+14	6,1894E+14
TC0500001136.mm.2	Ankle2	ankyrin repeat and LEM domain containing 2	1,12892E+13	5,87898E+14	2,73873E+13	0.031506591911743	0.175557727719528	-3,31517E+14	7,00465E+14	6,87279E+14	6,2805E+14	5,33909E+14	5,12289E+14	4,65395E+14
TC1000001737.mm.2	Stxbp5	syntaxin binding protein 5 (tomosyn)	1,69611E+14	6,59055E+14	2,73833E+14	0.0315242561519	0.175557727719528	-3,31567E+14	7,742E+14	8,04441E+14	6,36225E+14	6,03194E+14	6,63947E+14	4,72324E+14
TC0600001207.mm.2	Edem1	ER degradation enhancer, mannosidase alpha-like 1	0.904100899790959	6,59626E+14	2,73786E+14	0.0315446333437981	0.175557727719528	-3,31625E+14	6,96906E+14	7,17716E+14	6,42969E+14	5,90833E+14	6,62661E+14	6,46667E+14
TC1100000109.mm.2	Adcy1	adenylate cyclase 1	1,01504E+14	7,39293E+14	2,73752E+14	0.0315591621934943	0.175557727719528	-3,31666E+14	7,84704E+14	8,32467E+14	6,98429E+14	7,15734E+14	7,35691E+14	6,68732E+14
TC0200005466.mm.2	Lrrc8a	leucine rich repeat containing 8A	1,0401E+14	7,42976E+13	2,7374E+14	0.0315643409577218	0.175557727719528	-3,3168E+14	8,18444E+14	7,88941E+14	7,40052E+14	6,59313E+14	7,37392E+14	7,13712E+14
TC1100003505.mm.2	Akap1	A kinase (PRKA) anchor protein 1	1,18839E+14	6,88296E+13	2,73734E+14	0.0315669039178198	0.175557727719528	-3,31687E+14	7,62276E+14	7,55529E+14	6,83291E+14	5,96837E+14	6,26861E+14	7,04983E+14
TC1100002152.mm.2	Pgam2	phosphoglycerate mutase 2	-0.810820688323399	7,01431E+14	-2,7371E+14	0.0315775132454926	0.175557727719528	-3,31717E+14	6,50403E+14	6,47645E+14	7,1845E+14	7,41762E+14	7,30581E+14	7,19743E+14
TC0400002016.mm.2	Acot7	acyl-CoA thioesterase 7	1,25398E+14	9,06186E+14	2,73686E+14	0.0315878939963503	0.175557727719528	-3,31747E+14	9,86013E+14	9,81226E+13	9,17746E+14	7,98696E+14	9,00096E+14	8,53338E+14
TC0200002496.mm.2	Ccn5	cellular communication network factor 5	-0.945563867482864	8,25808E+14	-2,73644E+14	0.0316061327930079	0.175557727719528	-3,31798E+14	7,4574E+14	7,50495E+14	8,18698E+14	8,6665E+14	8,62053E+14	9,11214E+14
TC1700000890.mm.2	Zfp318	zinc finger protein 318	1,90426E+14	6,30421E+14	2,73636E+14	0.0316099237270212	0.175557727719528	-3,31809E+14	7,61051E+14	7,5452E+14	6,36153E+14	4,98565E+14	6,56527E+13	4,75708E+14
TC0100000678.mm.2	Sgpp2	sphingosine-1-phosphate phosphotase 2	1,11097E+14	6,20875E+14	2,7362E+14	0.0316166882796145	0.175557727719528	-3,31828E+13	6,88659E+14	6,68284E+14	5,74989E+14	5,5976E+14	6,66622E+14	5,66933E+14
TC0500001698.mm.2	Amz1	archaelysin family metallopeptidase 1	0.819654270699553	5,44978E+14	2,73607E+14	0.0316224849384315	0.175557727719528	-3,31844E+14	5,96355E+14	5,78171E+14	5,17427E+14	4,93168E+14	5,41223E+14	5,43528E+14
TC0100002495.mm.2	Acadl	acyl-Coenzyme A dehydrogenase, long-chain	0.89825935048038	6,45282E+14	2,73589E+14	0.0316302344155728	0.175557727719528	-3,31866E+14	6,99086E+14	7,33418E+14	6,47459E+14	6,05393E+14	6,018E+13	5,84535E+14
TC1100000688.mm.2	Sh3bp5l	SH3 binding domain protein 5 like	-1,09712E+14	6,81995E+14	-2,73576E+13	0.0316358415125589	0.175557727719528	-3,31882E+14	6,33042E+13	6,01311E+14	6,79635E+14	7,74141E+14	7,07576E+14	6,96265E+14
TC0X00001796.mm.2	Wdr13	WD repeat domain 13	1,34755E+14	7,80357E+14	2,73566E+14	0.0316402920679397	0.175557727719528	-3,31894E+13	8,58691E+14	8,55767E+13	7,92225E+14	6,52723E+14	7,44632E+14	7,78101E+14
TC0700003990.mm.2	Tpp1	tripeptidyl peptidase I	1,81932E+13	8,27739E+14	2,73555E+14	0.0316448988546771	0.175557727719528	-3,31907E+14	9,16494E+13	9,66921E+12	8,61469E+14	6,58081E+14	8,06023E+14	7,57445E+14
TC0400003253.mm.2	Zfyve9	zinc finger, FYVE domain containing 9	1,66585E+14	7,14761E+14	2,73533E+13	0.031654904333213	0.175557727719528	-3,31935E+14	8,17693E+14	8,13638E+14	7,30883E+14	5,67279E+14	7,30285E+13	6,28787E+14
TC0900003330.mm.2	Onecut1	one cut domain, family member 1	-0.909025709279939	5,23758E+14	-2,7353E+14	0.0316558165684752	0.175557727719528	-3,31938E+14	4,73361E+14	4,55588E+14	5,47995E+14	5,62759E+14	5,23797E+14	5,79049E+14
TC0300000129.mm.2	Dnajc5b	DnaJ heat shock protein family (Hsp40) member C5 beta	-1,13218E+14	4,6046E+14	-2,73492E+14	0.0316725230531453	0.175557727719528	-3,31985E+14	4,36309E+14	3,51212E+14	5,0006E+14	5,13896E+14	4,49059E+13	5,12225E+14
TC0200001782.mm.2	Mga	MAX gene associated	2,13356E+14	6,90758E+14	2,73474E+14	0.0316804200908173	0.175557727719528	-3,32007E+14	8,28745E+14	8,19709E+13	7,04206E+13	5,17535E+14	7,27643E+14	5,46709E+14
TC1500000688.mm.2	Apobec3	apolipoprotein B mRNA editing enzyme, catalytic polypeptide 3	-0.84319894552616	6,05195E+14	-2,73425E+14	0.0317017344134821	0.175557727719528	-3,32067E+14	5,50538E+14	5,45785E+14	6,18097E+14	6,46866E+14	6,21129E+14	6,48753E+14
TC1700000662.mm.2	Clic1	chloride intracellular channel 1	1,13702E+14	6,67523E+14	2,73415E+13	0.031706363431662	0.175557727719528	-3,3208E+14	7,00333E+14	7,89399E+14	6,41474E+14	6,20854E+14	6,55116E+14	5,97963E+14
TC0X00002721.mm.2	Zmym3	zinc finger, MYM-type 3	1,09959E+14	6,70455E+14	2,73402E+14	0.0317117900465081	0.175557727719528	-3,32095E+14	7,84913E+13	7,38915E+14	6,93323E+14	6,10587E+14	6,14994E+14	5,79996E+14
TC1000000823.mm.2	Elane	elastase, neutrophil expressed	-1,16168E+14	6,18735E+14	-2,73294E+14	0.0317590359461176	0.175557727719528	-3,32228E+14	5,59339E+14	5,29883E+14	6,14497E+14	7,07062E+14	6,226E+12	6,7903E+14
TC0900001016.mm.2	Leo1	Leo1, Paf1/RNA polymerase II complex component	0.918733793517939	6,46545E+14	2,73288E+14	0.0317616424310817	0.175557727719528	-3,32235E+14	6,81129E+14	6,79934E+13	5,64333E+14	6,12983E+14	6,50765E+13	6,90125E+14
TC0200002731.mm.2	Gm2026	predicted gene 2026	2,20284E+14	9,60152E+14	2,73246E+14	0.031780126362825	0.175557727719528	-3,32287E+12	1,04486E+14	1,11078E+14	9,53492E+14	7,61579E+14	1,03426E+14	8,55946E+14
TC0700004177.mm.2	Knop1	lysine rich nucleolar protein 1	-0.818408699023391	7,15729E+14	-2,73241E+14	0.031782616579734	0.175557727719528	-3,32294E+14	6,72369E+14	6,58986E+13	7,36553E+14	7,58483E+14	7,25924E+14	7,42057E+14
TC0300000172.mm.2	Tnik	TRAF2 and NCK interacting kinase	1,44702E+14	7,65874E+14	2,73231E+14	0.0317866377915382	0.175557727719528	-3,32305E+14	8,56518E+14	8,46823E+14	7,7082E+14	6,43116E+14	7,86555E+14	6,91412E+14
TC0200001628.mm.2	Ccdc34	coiled-coil domain containing 34	0.900020606092826	7,4773E+14	2,73229E+11	0.0317875348903502	0.175557727719528	-3,32307E+13	8,04913E+14	7,93428E+14	7,10038E+14	7,08299E+14	7,63146E+14	7,06556E+14
TC1800001628.mm.2	8030462N17Rik	RIKEN cDNA 8030462N17 gene	1,52882E+14	6,24408E+14	2,73199E+14	0.0318010066528473	0.175557727719528	-3,32345E+14	7,30067E+14	7,30453E+14	6,38569E+13	5,16187E+14	6,26167E+14	5,05004E+14
TC0500000653.mm.2	Shisa3	shisa family member 3	-0.85756364829517	7,82212E+14	-2,73181E+14	0.0318089309471521	0.175557727719528	-3,32367E+14	7,23064E+14	7,42096E+14	8,0225E+13	8,34423E+14	7,82884E+14	8,08554E+14
TC0X00000703.mm.2	Emd	emerin	1,39009E+14	7,64191E+14	2,73134E+14	0.0318292247586711	0.175557727719528	-3,32424E+14	8,70208E+14	8,48088E+14	7,89528E+14	6,5075E+14	7,40429E+14	6,86142E+14
TC0900000605.mm.2	Cryab	crystallin, alpha B	1,10113E+14	8,79012E+14	2,73132E+14	0.0318302773859215	0.175557727719528	-3,32427E+14	9,36886E+14	9,71494E+14	8,73365E+14	8,1479E+14	8,76374E+14	8,01162E+14
TC0500000483.mm.2	4930431F12Rik	RIKEN cDNA 4930431F12 gene	-0.903942568670529	6,8208E+14	-2,73129E+14	0.0318314332681632	0.175557727719528	-3,3243E+14	6,2265E+14	6,30742E+14	6,88684E+13	7,45497E+14	6,95815E+14	7,09093E+14
TC0400003171.mm.2	Wdr78	WD repeat domain 78	1,06336E+13	5,73482E+14	2,73128E+14	0.0318321894555071	0.175557727719528	-3,32432E+14	6,40056E+14	6,71515E+14	5,84872E+14	5,14026E+14	5,4199E+14	4,88435E+14
TC1100000864.mm.2	Dhrs7c	dehydrogenase/reductase (SDR family) member 7C	-0.942497822263197	6,19932E+14	-2,73083E+14	0.0318516392723978	0.175557727719528	-3,32487E+14	5,60298E+14	5,47993E+13	6,2292E+13	6,73871E+14	6,79362E+14	6,3515E+14
TC0200002809.mm.2	Birc7	baculoviral IAP repeat-containing 7 (livin)	-1,38939E+14	4,74748E+14	-2,7302E+14	0.0318794814115784	0.175557727719528	-3,32564E+13	3,80384E+14	3,71857E+14	4,76105E+14	5,54015E+14	4,68744E+14	5,97382E+14
TC1000000388.mm.2	Scml4	Scm polycomb group protein like 4	-1,11746E+14	6,15382E+13	-2,73015E+14	0.0318817952228944	0.175557727719528	-3,32571E+14	5,24869E+14	5,71752E+14	6,32189E+14	6,87923E+14	6,00283E+14	6,75279E+14
TSUnmapped00000032.mm.2	LOC547349	MHC class I family member	-0.984884269499158	7,25758E+14	-2,73004E+14	0.0318864761817463	0.175557727719528	-3,32584E+13	6,70163E+14	6,64312E+14	7,34358E+14	7,97093E+14	7,21124E+14	7,67502E+14
TC1400002641.mm.2	Ednrb	endothelin receptor type B	1,68839E+14	8,43102E+14	2,72953E+14	0.0319087420611449	0.175557727719528	-3,32646E+14	9,71927E+14	9,6409E+14	8,58584E+14	7,39755E+12	8,40247E+14	6,84008E+14
TC0500001716.mm.2	Ap5z1	adaptor-related protein complex 5, zeta 1 subunit	-0.980316477946845	9,16986E+14	-2,72944E+14	0.0319129520799518	0.175557727719528	-3,32658E+14	8,54903E+14	8,40419E+13	9,1294E+14	9,78446E+14	9,38419E+14	9,7679E+14
TC0400003767.mm.2	Nipal3	NIPA-like domain containing 3	0.939516423029456	7,98031E+14	2,7285E+14	0.0319542611506689	0.175557727719528	-3,32773E+14	8,81467E+13	8,48365E+13	7,98858E+14	7,4307E+14	7,68501E+13	7,47927E+14
TC1500000417.mm.2	Fam91a1	family with sequence similarity 91, member A1	2,12752E+14	7,16415E+14	2,72847E+13	0.0319556219955928	0.175557727719528	-3,32777E+14	8,32099E+13	8,24763E+14	7,4158E+14	4,89778E+14	7,37238E+14	6,73031E+14
TC1200000679.mm.2	Hspa2	heat shock protein 2	1,00208E+14	6,09134E+14	2,72841E+14	0.0319583115296354	0.175557727719528	-3,32784E+14	6,7349E+14	6,95898E+14	5,46496E+14	6,22475E+14	5,68901E+14	5,47542E+14
TC0800001185.mm.2	Car7	carbonic anhydrase 7	-1,23328E+14	7,5832E+14	-2,72831E+14	0.0319628334267869	0.175557727719528	-3,32797E+12	6,76477E+13	6,90953E+14	8,50964E+14	7,63122E+14	8,2818E+14	7,40225E+14
TC1400000935.mm.2	Zfp395	zinc finger protein 395	-0.864920889044171	7,3667E+14	-2,72802E+14	0.0319754279897204	0.175557727719528	-3,32832E+14	6,63676E+14	6,87786E+14	7,46721E+14	7,77725E+14	7,85913E+14	7,58202E+13
TC1500001753.mm.2	Pycrl	pyrroline-5-carboxylate reductase-like	-0.960629186211726	8,40896E+14	-2,72756E+14	0.0319957206925797	0.175557727719528	-3,32888E+14	7,78083E+14	7,77482E+14	8,38993E+14	9,08698E+14	8,60149E+14	8,81974E+14
TC0X00000689.mm.2	Dusp9	dual specificity phosphatase 9	-0.855603780979907	5,89678E+14	-2,72733E+14	0.0320057378126165	0.175557727719528	-3,32916E+14	5,12651E+14	5,52537E+14	6,085E+14	6,27808E+14	6,22651E+14	6,13922E+13
TC0800001732.mm.2	Adprhl1	ADP-ribosylhydrolase like 1	-0.798937631848621	5,61821E+14	-2,7273E+13	0.0320070412763582	0.175557727719528	-3,3292E+14	4,99801E+13	5,062E+14	5,732E+14	5,92589E+14	5,98846E+14	6,00291E+14
TC1200001919.mm.2	D830013O20Rik	RIKEN cDNA D830013O20 gene	-0.897354408426137	5,40762E+13	-2,72723E+14	0.0320104603813056	0.175557727719528	-3,32929E+14	4,75621E+14	4,88832E+14	5,45526E+14	5,98399E+14	5,58929E+14	5,77268E+14
TC1500002030.mm.2	Brd1	bromodomain containing 1	-1,17175E+14	8,28605E+14	-2,7272E+13	0.0320116925903754	0.175557727719528	-3,32933E+14	7,4365E+14	7,44417E+13	8,05436E+14	9,16981E+14	8,67521E+14	8,93626E+12
TC0500003241.mm.2	Rsrc2	arginine/serine-rich coiled-coil 2	1,69636E+14	7,55555E+13	2,72709E+14	0.03201660144936	0.175557727719528	-3,32947E+14	8,90957E+14	8,73806E+14	7,72436E+14	6,53055E+14	7,49961E+14	5,93112E+14
TC0500000318.mm.2	Krtcap3	keratinocyte associated protein 3	-1,13537E+14	5,39581E+14	-2,7269E+14	0.0320249596342465	0.175557727719528	-3,3297E+13	4,65317E+14	4,64697E+14	5,25343E+14	6,31746E+14	5,65756E+14	5,84626E+14
TC0700000872.mm.2	Otog	otogelin	-1,03022E+14	5,7773E+14	-2,72675E+14	0.0320314308141841	0.175557727719528	-3,32988E+14	5,12595E+14	5,10468E+14	5,7833E+14	6,50777E+14	5,82163E+14	6,32046E+14
TC0200001985.mm.2	Ptpra	protein tyrosine phosphatase, receptor type, A	1,88484E+14	7,86645E+14	2,72674E+14	0.0320319851522566	0.175557727719528	-3,32989E+14	9,10213E+14	9,08504E+14	8,24397E+14	6,17351E+14	7,74327E+14	6,85079E+14
TC1700001100.mm.2	L3mbtl4	L3MBTL4 histone methyl-lysine binding protein	-0.846449708464736	5,81855E+14	-2,72669E+14	0.0320339855483891	0.175557727719528	-3,32995E+14	5,29209E+14	5,19938E+14	5,88692E+14	6,29745E+14	6,10652E+14	6,12891E+14
TC0100003240.mm.2	Prg4	proteoglycan 4 (megakaryocyte stimulating factor, articular superficial zone protein)	-1,15446E+14	5,60248E+14	-2,72659E+14	0.0320384747548201	0.175557727719528	-3,33007E+14	4,24828E+14	5,26578E+14	5,7247E+14	6,09828E+14	6,27033E+14	6,00753E+14
TC0700001997.mm.2	Kndc1	kinase non-catalytic C-lobe domain (KIND) containing 1	1,13178E+14	7,17769E+14	2,72641E+14	0.0320466225816477	0.175557727719528	-3,3303E+14	7,97188E+14	7,86293E+14	7,27913E+14	6,29212E+14	7,03528E+14	6,62481E+14
TC0400001276.mm.2	Ptch2	patched 2	-1,27899E+14	7,0101E+14	-2,72597E+14	0.0320659785942174	0.175557727719528	-3,33084E+13	5,89153E+14	6,37813E+13	6,94001E+14	7,88762E+14	7,08333E+14	7,88E+13
TC0900003043.mm.2	Rbm5	RNA binding motif protein 5	0.854515805803497	7,31236E+14	2,7255E+14	0.0320868640945791	0.175557727719528	-3,33142E+14	8,07811E+14	8,1891E+14	7,16981E+14	7,38837E+14	6,71304E+14	6,33571E+14
TC0400001157.mm.2	Txndc12	thioredoxin domain containing 12 (endoplasmic reticulum)	0.871695158540783	7,90785E+14	2,7255E+14	0.0320870174031821	0.175557727719528	-3,33142E+14	8,68718E+14	8,45523E+12	7,7098E+14	7,68922E+14	7,22947E+14	7,67623E+14
TC0600002918.mm.2	Tada3	transcriptional adaptor 3	0.884761018821449	6,47921E+14	2,72476E+14	0.0321197806912324	0.175557727719528	-3,33233E+14	7,08636E+14	7,23704E+14	6,51553E+14	6,03835E+14	5,89426E+14	6,10373E+14
TC0200002729.mm.2	Gm14391/Gm6710	predicted gene 14391/predicted gene 6710	2,09808E+14	8,89965E+14	2,72449E+14	0.0321313493487406	0.175557727719528	-3,33265E+14	9,61343E+14	1,04209E+14	8,9079E+14	6,93024E+14	9,47021E+14	8,05522E+14
TC0100002356.mm.2	Rftn2	raftlin family member 2	0.807037605987433	7,59251E+14	2,72421E+13	0.0321437621927504	0.175557727719528	-3,33299E+14	7,94632E+14	7,99526E+14	7,28748E+14	7,04002E+14	7,61419E+14	7,67176E+14
TC0X00000323.mm.2	Pgrmc1	progesterone receptor membrane component 1	1,2525E+13	1,02092E+14	2,72406E+14	0.0321507637640427	0.175557727719528	-3,33319E+14	1,11309E+14	1,0857E+14	1,02422E+14	9,24065E+14	1,02728E+14	9,51181E+14
TC0400004183.mm.2	Gm38423/Gm11239/Gm11237	predicted gene, 38423/predicted gene 11239/predicted gene 11237	-1,23429E+14	4,20454E+13	-2,72386E+14	0.0321592918717904	0.175557727719528	-3,33342E+14	3,30387E+14	3,45818E+14	4,15912E+13	5,07151E+14	4,19218E+14	5,04239E+14
TC0700003551.mm.2	Hapln3	hyaluronan and proteoglycan link protein 3	-0.996837652452907	6,50947E+14	-2,72385E+14	0.0321600542470931	0.175557727719528	-3,33344E+14	5,82723E+14	6,05825E+13	6,58833E+14	7,29082E+13	6,47734E+14	6,81483E+14
TC1100001999.mm.2	Gaa	glucosidase, alpha, acid	2,1256E+14	1,01751E+14	2,72375E+14	0.032164520260119	0.175557727719528	-3,33357E+14	1,18852E+14	1,15026E+14	1,07705E+14	8,36611E+14	9,72212E+14	8,80374E+14
TC0100003723.mm.2	Brox	BRO1 domain and CAAX motif containing	1,2662E+14	7,10703E+14	2,72323E+14	0.032187579420202	0.175557727719528	-3,33421E+14	7,7303E+14	7,63611E+13	7,03789E+14	5,79611E+14	7,42572E+14	7,01606E+14
TC1000002920.mm.2	Cct2	chaperonin containing Tcp1, subunit 2 (beta)	1,8497E+14	9,20581E+14	2,72301E+13	0.0321974149884636	0.175557727719528	-3,33448E+14	1,03414E+13	1,03847E+14	9,42909E+14	7,59755E+14	9,35679E+14	8,12537E+14
TC1100003192.mm.2	Olfr411	olfactory receptor 411	-0.844242008592381	5,95176E+14	-2,72273E+14	0.0322096934201915	0.175557727719528	-3,33482E+14	5,28397E+13	5,64755E+14	6,20728E+14	6,41272E+13	6,06994E+14	6,08909E+14
TC1500001446.mm.2	Abra	actin-binding Rho activating protein	-1,13611E+14	4,85765E+14	-2,72269E+14	0.0322112828430696	0.175557727719528	-3,33486E+14	4,19338E+14	3,94293E+14	5,04953E+14	5,359E+14	4,79577E+14	5,80529E+14
TC1200001071.mm.2	Ak7	adenylate kinase 7	0.838400214772941	5,47751E+14	2,72269E+14	0.032211362959594	0.175557727719528	-3,33486E+14	6,25947E+14	5,97397E+14	5,36856E+14	5,18808E+14	4,93301E+13	5,14196E+14
TC0100001008.mm.2	Relch	RAB11 binding and LisH domain, coiled-coil and HEAT repeat containing	1,46798E+14	6,47404E+14	2,72237E+14	0.0322255148019606	0.175557727719528	-3,33525E+13	7,4665E+14	7,23077E+14	6,63625E+14	5,12507E+14	6,48378E+14	5,90187E+14
TC0500002178.mm.2	Ppm1g	protein phosphatase 1G (formerly 2C), magnesium-dependent, gamma isoform	1,36809E+14	7,27874E+13	2,7223E+13	0.0322286202814102	0.175557727719528	-3,33534E+14	8,22826E+14	8,2392E+14	7,25653E+14	6,47476E+14	7,36435E+13	6,10932E+14
TC0X00001559.mm.2	Cypt3	cysteine-rich perinuclear theca 3	-1,11717E+14	4,70784E+14	-2,72189E+14	0.0322471281958867	0.175562396558205	-3,33585E+14	3,8731E+14	3,84642E+14	4,55248E+14	5,40137E+14	4,97718E+14	5,59647E+13
TC0400003667.mm.2	Trnau1ap	tRNA selenocysteine 1 associated protein 1	-1,00882E+14	7,3129E+14	-2,7204E+14	0.0323134659557332	0.175562396558205	-3,33768E+14	6,67505E+14	6,79955E+14	7,35985E+14	8,13238E+14	7,60906E+14	7,30153E+14
TC0700001169.mm.2	Pde8a	phosphodiesterase 8A	2,1048E+14	5,41021E+14	2,72038E+14	0.0323144669587737	0.175562396558205	-3,33771E+13	6,99453E+14	7,21718E+14	5,7033E+14	4,29881E+14	5,19789E+14	3,04956E+13
TC0300002113.mm.2	Lxn	latexin	0.991138455450143	8,39836E+14	2,71983E+14	0.0323390601485382	0.175562396558205	-3,33839E+14	9,14736E+14	8,88616E+14	8,27789E+14	7,77336E+13	8,43704E+14	7,86837E+14
TC0700000516.mm.2	Lgals7	lectin, galactose binding, soluble 7	-1,19498E+14	7,43892E+14	-2,71954E+14	0.0323517486976147	0.175562396558205	-3,33874E+14	6,51858E+14	6,68366E+14	7,42695E+14	8,16525E+14	7,45881E+14	8,38029E+14
TC0500001296.mm.2	Vsig10	V-set and immunoglobulin domain containing 10	-1,26563E+14	6,43846E+14	-2,71944E+14	0.0323565169496279	0.175562396558205	-3,33887E+14	5,7199E+14	5,39249E+14	6,31844E+13	7,32522E+14	6,54904E+14	7,32569E+14
TC1100003823.mm.2	Pyy	peptide YY	-0.834078875611665	6,97143E+14	-2,71942E+14	0.0323570547481513	0.175562396558205	-3,33888E+13	6,39916E+14	6,50489E+14	7,16484E+14	7,40737E+14	7,03642E+14	7,31589E+14
TC0800001959.mm.2	Chrna6	cholinergic receptor, nicotinic, alpha polypeptide 6	-1,15778E+14	5,79158E+14	-2,71933E+11	0.0323612266058255	0.175562396558205	-3,339E+14	5,24274E+14	5,03152E+14	6,02147E+14	6,56835E+14	5,46158E+14	6,42384E+14
TC0800001219.mm.2	Hsd11b2	hydroxysteroid 11-beta dehydrogenase 2	-1,07317E+14	5,82043E+14	-2,71929E+14	0.0323628337943926	0.175562396558205	-3,33904E+12	5,14121E+14	5,22404E+14	5,77821E+14	6,73337E+14	5,94845E+13	6,09729E+14
TC0700000818.mm.2	Il4i1b/Il4i1/Nup62	interleukin 4 induced 1B/interleukin 4 induced 1/nucleoporin 62	-1,08553E+14	7,85063E+14	-2,71913E+13	0.0323701789510946	0.175562396558205	-3,33924E+14	7,28253E+14	7,22542E+14	7,91319E+14	8,76581E+14	7,70458E+14	8,21228E+14
TC0200002146.mm.2	Pet117/Kat14	PET117 homolog/lysine acetyltransferase 14	-0.8088331263363	8,3989E+14	-2,71879E+13	0.0323851868514113	0.175562396558205	-3,33965E+14	7,88037E+12	7,88042E+14	8,75132E+14	8,62713E+13	8,50352E+14	8,75066E+14
TC0700002974.mm.2	Atf5	activating transcription factor 5	-0.979294048710957	7,31904E+14	-2,71861E+14	0.0323934066587668	0.175562396558205	-3,33988E+14	6,86196E+14	6,30379E+14	7,36653E+14	7,75781E+14	7,62339E+14	8,00077E+14
TC0400001708.mm.2	Hspg2	perlecan (heparan sulfate proteoglycan 2)	-1,02695E+14	5,70648E+14	-2,71857E+14	0.0323954005917546	0.175562396558205	-3,33994E+14	4,80474E+14	5,1877E+14	5,66111E+14	6,38524E+14	5,93996E+14	6,2601E+14
TC1400002422.mm.2	Spert	spermatid associated	-0.891436762533967	6,54679E+14	-2,71848E+14	0.0323990256099491	0.175562396558205	-3,34003E+14	5,76048E+14	5,93254E+14	6,61584E+14	6,86004E+13	7,29671E+14	6,8151E+14
TC0200001982.mm.2	Vps16	VSP16 CORVET/HOPS core subunit	0.883677942933049	6,6141E+14	2,71828E+14	0.0324080189085905	0.175562396558205	-3,34028E+14	7,75246E+14	7,51696E+14	6,98416E+14	6,51791E+14	5,37818E+12	5,5349E+13

TC1000000786.mm.2	Olfr1358	olfactory receptor 1358	-1,08455E+14	4,8398E+14	-2,71815E+13	0.0324138629062605	0.175562396558205	-3,34044E+14	4,10051E+14	4,19331E+14	4,74758E+14	5,71534E+14	5,04028E+14	5,24176E+14
TC0200004555.mm.2	Fahd2a	fumarylacetoacetate hydrolase domain containing 2A	-1,01302E+14	6,8906E+14	-2,71795E+14	0.0324228183816339	0.175562396558205	-3,34069E+14	6,44786E+14	6,05501E+14	6,89129E+14	7,63762E+14	7,21406E+14	7,09775E+13
TC0500001995.mm.2	Gnai1	guanine nucleotide binding protein (G protein), alpha inhibiting 1	1,04336E+14	8,2957E+14	2,71719E+14	0.0324571005531425	0.175562396558205	-3,34163E+14	9,11636E+14	8,84634E+14	8,3268E+14	7,54917E+14	8,16585E+14	7,76969E+14
TC0500003225.mm.2	Rhof	ras homolog family member F (in filopodia)	0.978286748000943	6,90233E+14	2,71679E+14	0.0324750078911431	0.175562396558205	-3,34212E+14	7,61281E+14	7,2946E+14	6,75753E+14	6,19331E+14	6,56207E+14	6,99365E+14
TC1600000117.mm.2	Clec16a	C-type lectin domain family 16, member A	2,20494E+14	6,43729E+14	2,7166E+14	0.0324832649530818	0.175562396558205	-3,34235E+14	7,94812E+14	7,98254E+14	7,09916E+14	4,42163E+14	5,83144E+14	5,34082E+14
TC0400003219.mm.2	Dio1	deiodinase, iodothyronine, type I	-0.870745819276634	5,55928E+14	-2,71653E+14	0.0324865919758366	0.175562396558205	-3,34244E+14	5,29027E+14	4,971E+12	5,82235E+14	6,18041E+14	5,45997E+14	5,6317E+14
TC1700000706.mm.2	Nrm	nurim (nuclear envelope membrane protein)	-1,30423E+14	7,88047E+14	-2,71641E+14	0.0324919293805403	0.175562396558205	-3,34258E+14	7,04335E+14	7,06594E+14	7,74035E+14	8,9774E+14	7,92282E+14	8,53295E+14
TC0600000827.mm.2	Cd8a	CD8 antigen, alpha chain	-1,20674E+13	5,06977E+14	-2,71623E+14	0.0325001850265132	0.175562396558205	-3,34281E+14	4,92025E+13	4,47479E+14	5,35896E+14	6,44957E+13	4,45845E+14	4,75661E+14
TC1400001306.mm.2	Rap2a	RAS related protein 2a	1,69518E+14	7,09472E+14	2,716E+14	0.0325104783879513	0.175562396558205	-3,34309E+14	7,8444E+14	7,9615E+14	7,17043E+14	5,2451E+14	7,41213E+14	6,93476E+14
TC1300001541.mm.2	Nkapl	NFKB activating protein-like	-0.829835530079576	5,13856E+14	-2,71596E+13	0.0325122804177339	0.175562396558205	-3,34314E+14	4,52986E+14	4,60272E+14	5,21368E+14	5,57857E+14	5,46364E+14	5,44287E+13
TC0100003104.mm.2	Adora1	adenosine A1 receptor	1,4703E+14	8,53012E+14	2,71585E+14	0.032517035162915	0.175562396558205	-3,34327E+14	9,32711E+14	9,30688E+14	8,57777E+13	7,11563E+14	8,80398E+14	8,04934E+14
TC0100001178.mm.2	Nucks1	nuclear casein kinase and cyclin-dependent kinase substrate 1	1,25719E+14	8,52274E+14	2,71565E+14	0.0325261057073707	0.175562396558205	-3,34352E+14	9,39912E+14	9,37227E+14	8,61925E+14	7,63775E+14	8,4814E+14	7,62668E+14
TC0200001228.mm.2	Prg3	proteoglycan 3	-1,01808E+14	5,88441E+14	-2,71543E+14	0.0325358199026428	0.175562396558205	-3,34379E+14	5,25761E+14	5,1712E+14	5,88317E+14	6,5818E+14	5,95605E+14	6,45661E+14
TC1900000957.mm.2	Sf3b2	splicing factor 3b, subunit 2	0.798953239452371	6,50616E+14	2,71522E+14	0.0325454526481324	0.175562396558205	-3,34405E+14	7,0165E+14	7,11517E+14	6,35083E+14	6,18293E+14	6,24462E+14	6,12688E+14
TC1100000234.mm.2	Cct4	chaperonin containing Tcp1, subunit 4 (delta)	1,00026E+14	8,1575E+13	2,71438E+14	0.0325830009624776	0.175681037700476	-3,34508E+14	8,62932E+14	8,91744E+14	7,70381E+14	7,84243E+14	8,30759E+14	7,54438E+14
TC0600001029.mm.2	Gata2	GATA binding protein 2	-1,03119E+14	5,77277E+14	-2,71416E+14	0.0325928789894889	0.175681037700476	-3,34535E+14	4,86614E+14	5,0897E+14	5,61111E+14	6,40712E+14	6,16462E+14	6,49794E+14
TC0700002907.mm.2	AI987944	expressed sequence AI987944	2,20495E+14	5,86536E+14	2,71393E+13	0.0326035147523916	0.175681037700476	-3,34564E+14	7,4162E+14	7,45644E+14	6,37786E+14	4,0849E+13	5,66469E+14	4,1921E+14
TC0700003934.mm.2	Olfr649	olfactory receptor 649	-1,13372E+14	5,66428E+14	-2,71376E+14	0.0326110583852867	0.175681037700476	-3,34584E+14	4,9636E+14	4,92331E+14	5,80556E+14	6,34879E+14	5,50152E+14	6,44288E+13
TC1000002265.mm.2	Ddx50	DEAD (Asp-Glu-Ala-Asp) box polypeptide 50	1,69359E+14	5,65664E+14	2,71298E+14	0.0326461083613354	0.175744691024618	-3,3468E+14	6,79915E+14	6,9164E+14	6,0045E+14	4,32387E+14	5,47519E+14	4,42071E+14
TC1200000201.mm.2	Klf11	Kruppel-like factor 11	-0.941148611508233	6,40565E+14	-2,71273E+14	0.0326577023628212	0.175744691024618	-3,34711E+14	5,77322E+14	5,44012E+14	6,5114E+14	6,58424E+13	6,7583E+14	7,3666E+14
TC0X00003429.mm.2	L1cam	L1 cell adhesion molecule	1,37117E+14	7,03107E+14	2,71232E+14	0.0326761871939827	0.175744691024618	-3,34762E+14	8,23367E+14	7,83136E+14	7,36965E+11	5,95306E+14	6,51824E+14	6,28045E+14
TC0600002212.mm.2	Pdia4/Mir704	protein disulfide isomerase associated 4/microRNA 704	1,62333E+14	6,85337E+14	2,71219E+14	0.032681967406373	0.175744691024618	-3,34778E+14	7,93659E+14	8,16527E+14	6,53369E+14	6,32151E+14	7,01006E+14	5,15308E+14
TC0400002849.mm.2	Megf9	multiple EGF-like-domains 9	0.807997225521388	8,05335E+14	2,71205E+14	0.0326881337422745	0.175744691024618	-3,34794E+14	8,65237E+14	8,56673E+14	7,92676E+14	7,67634E+14	7,79834E+14	7,69956E+14
TC0X00001858.mm.2	1810030O07Rik	RIKEN cDNA 1810030O07 gene	0.88938720444395	6,73265E+14	2,71205E+14	0.0326883162043555	0.175744691024618	-3,34795E+14	7,26569E+14	7,40561E+13	6,19532E+14	6,69722E+14	6,52773E+14	6,30435E+14
TC1300002255.mm.2	Zfp729a	zinc finger protein 729a	1,03154E+13	5,59456E+14	2,71134E+12	0.0327201000337376	0.175788572042553	-3,34881E+14	6,31095E+14	6,05892E+14	5,54131E+14	4,76547E+14	5,26207E+14	5,52867E+14
TC1200002389.mm.2	Hsp90aa1	heat shock protein 90, alpha (cytosolic), class A member 1	3,12082E+14	9,57103E+14	2,71087E+14	0.0327413908455579	0.175788572042553	-3,34939E+14	1,13186E+14	1,12901E+14	1,00541E+14	6,3129E+14	9,92989E+13	8,5206E+13
TC0600001421.mm.2	C1rl	complement component 1, r subcomponent-like	-1,0075E+14	5,67927E+14	-2,71083E+14	0.0327431823025491	0.175788572042553	-3,34944E+14	5,46871E+14	5,05091E+14	5,96448E+14	6,57013E+14	5,71888E+13	5,30252E+14
TC0100001607.mm.2	Dcaf8	DDB1 and CUL4 associated factor 8	1,36546E+14	8,45182E+14	2,71057E+13	0.0327552993663716	0.175788572042553	-3,34977E+14	9,37768E+14	9,25983E+14	8,68612E+14	7,22048E+14	8,18343E+14	7,98338E+14
TC1200001038.mm.2	Serpina3a	serine (or cysteine) peptidase inhibitor, clade A, member 3A	-1,05866E+14	4,80083E+13	-2,71029E+14	0.0327678073845432	0.175788572042553	-3,35011E+14	3,95153E+14	4,21153E+14	5,07241E+14	5,20797E+14	4,72952E+14	5,63204E+14
TC0900003251.mm.2	Xirp1	xin actin-binding repeat containing 1	-1,47758E+14	6,85682E+14	-2,71023E+14	0.0327705588029313	0.175788572042553	-3,35019E+14	5,8493E+14	6,04443E+14	6,70855E+14	8,14034E+14	6,789E+14	7,60928E+14
TC0700000261.mm.2	Ceacam11	carcinoembryonic antigen-related cell adhesion molecule 11	-0.965964370248986	4,48302E+14	-2,71018E+14	0.0327728461748792	0.175788572042553	-3,35025E+14	3,81426E+14	3,79817E+14	4,4839E+14	5,06045E+14	4,62603E+14	5,11531E+14
TC1000002240.mm.2	Adamts14	a disintegrin-like and metallopeptidase (reprolysin type) with thrombospondin type 1 motif, 14	-1,10086E+14	6,61181E+14	-2,70973E+14	0.0327929163916012	0.175830496300088	-3,35079E+14	5,66164E+14	6,10376E+14	6,57451E+14	7,3926E+14	6,7477E+14	7,19064E+14
TC0X00001682.mm.2	Gpm6b	glycoprotein m6b	1,57668E+14	1,0784E+14	2,70933E+14	0.0328113136631154	0.175830496300088	-3,35129E+14	1,16539E+14	1,17566E+14	1,08769E+14	9,38025E+14	1,10289E+14	1,00075E+14
TC1700002348.mm.2	Tubb4a	tubulin, beta 4A class IVA	1,85817E+14	9,91551E+14	2,70906E+14	0.0328233760075896	0.175830496300088	-3,35162E+14	1,1345E+14	1,11208E+14	1,04034E+14	8,34605E+14	9,55908E+14	8,71873E+14
TC1200001398.mm.2	Slc7a15	solute carrier family 7 (cationic amino acid transporter, y+ system), member 15	-1,08357E+13	4,56981E+14	-2,70867E+14	0.032841018131935	0.175830496300088	-3,3521E+14	3,72527E+14	4,00564E+14	4,56533E+14	5,33272E+14	4,60819E+14	5,18169E+14
TC1200001070.mm.2	Gskip	GSK3B interacting protein	1,01503E+14	6,94089E+14	2,70862E+14	0.0328435622446222	0.175830496300088	-3,35217E+14	7,73984E+14	7,32652E+14	6,81873E+14	6,21756E+14	6,54106E+14	7,00162E+14
TC1900000709.mm.2	As3mt	arsenic (+3 oxidation state) methyltransferase	0.976480484234131	7,18137E+13	2,70856E+14	0.0328461362789838	0.175830496300088	-3,35224E+14	8,21641E+14	7,70016E+14	6,83427E+14	7,12933E+14	6,85714E+14	6,35092E+14
TC0800001392.mm.2	Atmin	ATM interactor	1,03199E+14	7,78471E+14	2,70814E+14	0.0328651139715175	0.175873657065115	-3,35275E+14	8,63258E+14	8,47681E+14	7,8145E+14	7,23092E+14	7,60876E+14	6,94467E+14
TC0100000774.mm.2	Itm2c	integral membrane protein 2C	1,24016E+14	1,02785E+14	2,70785E+14	0.0328783013439792	0.175875212776681	-3,35311E+14	1,12111E+14	1,10125E+14	1,04216E+14	9,3216E+14	1,01628E+14	9,54126E+14
TC0X00002084.mm.2	Tmem255a	transmembrane protein 255A	1,8886E+14	7,63619E+14	2,7076E+14	0.0328896359884921	0.175875212776681	-3,35341E+14	9,5659E+14	8,55752E+14	7,9816E+14	6,36463E+14	7,29907E+14	6,04839E+14
TC0400001130.mm.2	Glis1	GLIS family zinc finger 1	-0.994812836737292	7,20994E+14	-2,70673E+14	0.032929286629574	0.175875212776681	-3,35449E+14	6,73772E+14	6,48194E+14	7,36679E+14	7,8425E+14	7,10559E+14	7,72511E+14
TC0400000349.mm.2	Dnaja1/Mir207	DnaJ heat shock protein family (Hsp40) member A1/microRNA 207	1,15958E+14	9,91565E+14	2,70661E+14	0.032934696499871	0.175875212776681	-3,35463E+14	1,06468E+14	1,07309E+14	9,98618E+13	9,07229E+14	9,86675E+14	9,19102E+14
TC1500000784.mm.2	Samm50	SAMM50 sorting and assembly machinery component	1,10091E+14	7,17871E+14	2,7064E+14	0.0329443068723369	0.175875212776681	-3,35489E+14	7,91742E+14	7,91657E+14	7,32382E+14	6,30835E+14	6,89684E+14	6,70924E+13
TC1300001202.mm.2	Ercc8	excision repaiross-complementing rodent repair deficiency, complementation group 8	-0.888436952873016	6,84038E+14	-2,70595E+14	0.0329649831689382	0.175875212776681	-3,35545E+14	6,48088E+14	6,14365E+14	6,98186E+14	7,41954E+14	7,07776E+14	6,93861E+14
TC1900001217.mm.2	Gcnt1	glucosaminyl (N-acetyl) transferase 1, core 2	0.881931747078465	5,23226E+14	2,70579E+14	0.0329720060031605	0.175875212776681	-3,35564E+14	5,9903E+14	5,45267E+14	4,89801E+14	4,78109E+14	5,15729E+14	5,11422E+13
TC1100003410.mm.2	Aatf	apoptosis antagonizing transcription factor	1,00799E+14	7,15439E+14	2,70578E+13	0.0329727737027117	0.175875212776681	-3,35566E+14	7,82616E+14	7,42032E+11	6,97771E+14	6,25279E+14	7,31535E+14	7,13398E+13
TC0400000996.mm.2	Atg4c	autophagy related 4C, cysteine peptidase	1,79066E+14	6,42166E+14	2,70569E+14	0.0329767096802935	0.175875212776681	-3,35577E+14	7,39749E+14	7,47218E+14	6,50432E+14	4,78403E+14	6,8197E+14	5,55223E+14
TC0900003055.mm.2	Dag1	dystroglycan 1	2,81438E+13	6,73011E+14	2,70544E+14	0.0329882461607108	0.175875212776681	-3,35608E+14	8,65937E+14	8,51756E+14	7,46884E+14	4,07934E+14	6,40816E+13	5,24738E+14
TC0700001119.mm.2	Abhd2	abhydrolase domain containing 2	0.902138807984638	8,35476E+14	2,70531E+14	0.0329940919820092	0.175875212776681	-3,35624E+13	8,83753E+14	9,22932E+14	8,33508E+13	7,92749E+14	8,01002E+14	7,78913E+14
TC1300001170.mm.2	Trim23	tripartite motif-containing 23	1,35446E+14	7,08499E+14	2,70526E+13	0.0329963860376035	0.175875212776681	-3,3563E+14	8,06699E+14	8,02099E+13	7,07258E+14	6,30647E+14	7,1486E+14	5,89432E+14
TC0900000541.mm.2	Fxyd2	FXYD domain-containing ion transport regulator 2	-0.890234412674078	7,03516E+14	-2,70477E+14	0.0330185366106969	0.175935079500053	-3,3569E+14	6,414E+14	6,39863E+14	7,02874E+14	7,56436E+14	7,2973E+14	7,5079E+14
TC0600003135.mm.2	Gapdh	glyceraldehyde-3-phosphate dehydrogenase	2,21556E+14	1,09194E+14	2,70388E+14	0.0330594522996752	0.175949092643493	-3,358E+14	1,22873E+14	1,2326E+14	1,13479E+14	8,83422E+14	1,09203E+14	9,8007E+14
TC0500003608.mm.2	Rnf6	ring finger protein (C3H2C3 type) 6	1,6557E+14	6,92373E+14	2,70386E+14	0.0330600224107365	0.175949092643493	-3,35801E+14	7,9294E+12	7,73552E+14	7,11899E+13	5,23454E+14	6,96937E+13	6,55458E+14
TC0200002477.mm.2	Ift52	intraflagellar transport 52	1,19577E+14	6,56332E+14	2,70375E+14	0.0330652011746273	0.175949092643493	-3,35815E+14	7,31422E+14	7,35078E+14	6,67843E+14	5,59503E+14	5,95673E+14	6,48472E+14
TC0400003369.mm.2	Rnf220	ring finger protein 220	1,18705E+14	8,51584E+14	2,70374E+14	0.0330656629152596	0.175949092643493	-3,35817E+14	9,47654E+14	9,2089E+14	8,68021E+14	7,63113E+13	8,29668E+14	7,80158E+14
TC0400002153.mm.2	Nsmaf	neutral sphingomyelinase (N-SMase) activation associated factor	1,33666E+14	6,15789E+14	2,70351E+14	0.0330764179366607	0.175949092643493	-3,35846E+14	6,91188E+14	6,47733E+14	6,02848E+14	4,6874E+14	6,57511E+13	6,26715E+14
TC1900001095.mm.2	Fads2	fatty acid desaturase 2	1,70799E+14	9,6587E+14	2,70317E+14	0.0330916000888791	0.175949092643493	-3,35886E+14	1,08601E+14	1,0695E+14	1,00268E+14	8,1122E+14	9,4372E+14	8,82094E+14
TC0600000978.mm.2	Add2	adducin 2 (beta)	0.986943012442698	7,86709E+14	2,70278E+12	0.0331093892793931	0.175949092643493	-3,35934E+14	8,65598E+14	8,36918E+14	7,9031E+14	7,14817E+14	7,57875E+14	7,54734E+14
TC0800003171.mm.2	Agt	angiotensinogen (serpin peptidase inhibitor, clade A, member 8)	1,18652E+14	8,21197E+14	2,70272E+14	0.0331123876799713	0.175949092643493	-3,35942E+14	9,6184E+14	8,51213E+14	8,05424E+14	7,70324E+14	7,82416E+14	7,55963E+14
TC0300002487.mm.2	Prpf3	pre-mRNA processing factor 3	1,46772E+14	5,30337E+14	2,70256E+14	0.0331194438023779	0.175949092643493	-3,35961E+14	6,15607E+14	6,07606E+14	5,1662E+14	4,13048E+14	5,78844E+14	4,50296E+14
TC0900002320.mm.2	Dmxl2	Dmx-like 2	2,64094E+14	6,89802E+13	2,70162E+14	0.0331629357707144	0.176122077809335	-3,36078E+14	8,53065E+14	8,26206E+14	7,03821E+14	4,47262E+14	7,61162E+14	5,47293E+14
TC1700002656.mm.2	Lrpprc	leucine-rich PPR-motif containing	1,0563E+14	5,75688E+14	2,70096E+14	0.0331931632273379	0.176144256787043	-3,36159E+13	7,08638E+13	6,64718E+14	5,53147E+14	6,0895E+14	4,91001E+14	4,27676E+14
TC0800002841.mm.2	Ctrl	chymotrypsin-like	-0.930657846024972	5,81963E+14	-2,70067E+14	0.0332061604648156	0.176144256787043	-3,36194E+14	5,44297E+13	4,96319E+14	5,9672E+14	6,30028E+14	5,94574E+14	6,2984E+14
TC1000002627.mm.2	Hsp90b1	heat shock protein 90, beta (Grp94), member 1	2,68727E+14	8,97477E+14	2,70065E+14	0.0332070931937664	0.176144256787043	-3,36197E+14	1,05865E+14	1,07307E+14	9,43204E+14	6,51055E+13	9,17713E+14	7,41175E+14
TC0500000690.mm.2	Spata18	spermatogenesis associated 18	0.915257220222498	5,51073E+14	2,70037E+14	0.0332199294748228	0.176144256787043	-3,36231E+14	5,76266E+14	6,33299E+14	5,10183E+14	5,1633E+14	5,20463E+12	5,49899E+14
TC0700000368.mm.2	Zfp180	zinc finger protein 180	0.796972917440637	7,09552E+14	2,70013E+13	0.033231189141428	0.176144256787043	-3,36261E+13	7,55863E+14	7,70895E+14	6,90684E+14	6,76679E+14	6,75176E+14	6,88015E+14
TC1700000782.mm.2	Olfr122	olfactory receptor 122	-1,40959E+14	6,03693E+14	-2,70004E+14	0.0332352054713748	0.176144256787043	-3,36272E+14	5,12041E+13	5,34686E+14	6,10952E+14	7,17692E+14	5,67428E+14	6,79362E+13
TC1300001615.mm.2	Vmn1r217	vomeronasal 1 receptor 217	-1,37787E+13	5,24416E+14	-2,69986E+14	0.0332436346359707	0.176144256787043	-3,36295E+13	4,2641E+14	4,61976E+14	5,09067E+13	6,54892E+14	5,37716E+14	5,56433E+14
TC1300000718.mm.2	Ntrk2	neurotrophic tyrosine kinase, receptor, type 2	1,49215E+14	9,56635E+14	2,69942E+14	0.0332637833100946	0.176193077079406	-3,36349E+14	1,065E+14	1,04522E+14	9,82218E+14	8,29576E+14	9,44211E+14	8,73577E+14
TC0200004967.mm.2	Trpc4ap	transient receptor potential cation channel, subfamily C, member 4 associated protein	2,3566E+14	8,07997E+14	2,69909E+14	0.0332788643252744	0.176215031506127	-3,36389E+14	9,63953E+14	9,47021E+14	8,60721E+14	5,78933E+14	7,92653E+14	7,04701E+14
TC0100001234.mm.2	Kdm5b	lysine (K)-specific demethylase 5B	1,18572E+14	6,53485E+14	2,69861E+14	0.0333010345208312	0.176274497120287	-3,36448E+14	7,46495E+14	7,08385E+14	6,35976E+13	5,8176E+14	6,73697E+14	5,746E+14
TC0900003337.mm.2	Ip6k1	inositol hexaphosphate kinase 1	1,36708E+14	7,97982E+14	2,69791E+14	0.0333333200018163	0.176336966786072	-3,36534E+14	8,95841E+14	8,64255E+14	7,97668E+14	6,89012E+14	8,19551E+14	7,21563E+14
TC1900001231.mm.2	Rorb	RAR-related orphan receptor beta	1,12215E+14	5,89859E+12	2,69788E+14	0.0333347235667086	0.176336966786072	-3,36538E+14	6,74771E+14	6,53271E+14	6,03736E+14	4,99875E+14	5,43957E+14	5,63541E+14
TSUnmapped00000029.mm.2	Psd3	pleckstrin and Sec7 domain containing 3	1,57043E+14	8,86194E+14	2,69746E+14	0.0333541113608272	0.176381620071221	-3,3659E+14	9,89572E+14	9,95771E+14	8,89285E+14	7,81971E+14	9,0346E+14	7,57103E+14
TC0800000231.mm.2	Defa27	defensin, alpha, 27	-0.845020680521261	6,36808E+14	-2,69687E+13	0.0333814529267476	0.176410114177775	-3,36663E+14	5,71615E+14	5,60939E+14	6,3015E+14	6,71407E+14	6,86423E+14	7,00318E+14
TC0900000194.mm.2	Ilf3	interleukin enhancer binding factor 3	1,41217E+14	6,76013E+14	2,69666E+14	0.0333907870800329	0.176410114177775	-3,36688E+14	7,76866E+14	7,32506E+14	6,87763E+14	5,39175E+14	6,47572E+14	6,72194E+14
TC0300002688.mm.2	Cept1	choline/ethanolaminephosphotransferase 1	1,23087E+13	7,37303E+14	2,69663E+14	0.0333923445815313	0.176410114177775	-3,36692E+14	8,20066E+14	8,05484E+14	7,41082E+14	6,37393E+14	7,46358E+14	6,74335E+14
TC0600002975.mm.2	Alox5	arachidonate 5-lipoxygenase	-1,12073E+14	6,69631E+14	-2,69625E+14	0.0334097128377705	0.176426857345348	-3,36738E+14	5,95086E+14	5,8916E+14	6,76551E+14	7,31842E+14	6,66445E+14	7,58702E+14
TC0400001512.mm.2	Tmem54	transmembrane protein 54	-0.857371636341341	6,00485E+14	-2,69568E+14	0.0334362176107774	0.176426857345348	-3,36809E+12	5,40295E+14	5,48171E+14	6,25355E+14	6,34585E+14	6,0356E+14	6,50944E+14
TC0100003524.mm.2	Fcgr3	Fc receptor, IgG, low affinity III	1,36109E+14	7,09271E+14	2,69529E+14	0.0334540678732278	0.176426857345348	-3,36856E+14	7,26996E+14	8,13613E+14	6,86409E+13	5,81981E+14	7,54023E+14	6,92601E+14
TC1600000468.mm.2	Zfp148	zinc finger protein 148	1,12068E+14	7,30604E+14	2,69528E+14	0.0334549674869864	0.176426857345348	-3,36859E+14	8,09179E+14	8,12591E+14	7,35477E+14	6,62157E+14	7,21575E+14	6,42645E+14
TC1300001169.mm.2	Sgtb	small glutamine-rich tetratricopeptide repeat (TPR)-containing, beta	1,81092E+14	7,63359E+14	2,69522E+14	0.0334576188030367	0.176426857345348	-3,36866E+14	8,56521E+14	8,71596E+14	7,86155E+14	5,7978E+14	7,7706E+14	7,09038E+14
TC1200000142.mm.2	Odc1	ornithine decarboxylase, structural 1	1,01191E+13	6,47287E+14	2,69514E+14	0.0334612099576356	0.176426857345348	-3,36875E+13	7,17418E+13	7,34557E+14	6,66065E+14	5,83528E+14	6,01157E+13	5,80999E+12
TC0700004171.mm.2	Syt17	synaptotagmin XVII	1,4598E+14	6,72847E+14	2,69476E+14	0.0334790113344671	0.176430280753746	-3,36923E+13	7,70247E+14	7,75225E+14	6,92115E+14	5,61396E+14	6,69082E+14	5,69017E+14
TC1300001265.mm.2	Il6st	interleukin 6 signal transducer	1,56718E+14	7,31404E+14	2,69465E+14	0.0334837583656027	0.176430280753746	-3,36935E+14	8,43095E+14	8,50915E+14	7,37892E+14	6,4268E+13	7,33992E+14	5,79853E+14
TC0100003315.mm.2	Fam163a	family with sequence similarity 163, member A	1,15486E+14	5,47493E+14	2,69426E+14	0.0335020330794157	0.176468865318282	-3,36984E+14	5,82069E+12	6,26695E+14	5,41312E+13	4,3648E+14	5,51985E+14	5,4642E+13
TC1300001804.mm.2	Rpp40	ribonuclease P 40 subunit	-1,05222E+14	7,03552E+14	-2,69397E+14	0.0335153734456616	0.176481441937891	-3,37019E+13	6,33428E+14	6,37826E+14	7,01376E+14	7,80322E+14	7,07957E+14	7,60404E+14
TC0200002099.mm.2	Macrod2	MACRO domain containing 2	-0.848123059528618	8,23552E+14	-2,69288E+14	0.0335661050146065	0.176535273030273	-3,37154E+14	7,79596E+13	7,62232E+14	8,34289E+14	8,77163E+14	8,44149E+14	8,43886E+14
TC0100000227.mm.2	Cfc1	cripto, FRL-1, cryptic family 1	-1,44258E+14	6,04923E+14	-2,69264E+14	0.033577189502783	0.176535273030273	-3,37183E+14	5,08386E+14	5,34026E+13	6,01727E+14	7,292E+14	5,79645E+14	6,76552E+14
TC0900000186.mm.2	Icam5	intercellular adhesion molecule 5, telencephalin	0.839498277615117	7,64836E+14	2,69251E+14	0.0335830585412309	0.176535273030273	-3,37199E+14	8,10397E+14	8,22205E+14	7,46375E+14	7,18328E+14	7,30372E+14	7,61339E+14
TC0200001674.mm.2	Emc7	ER membrane protein complex subunit 7	1,67178E+14	8,36853E+13	2,69248E+14	0.0335843603358541	0.176535273030273	-3,37202E+14	9,37869E+14	9,42648E+14	8,41291E+14	7,04869E+14	8,68517E+14	7,25925E+14
TC1500001955.mm.2	Cyp2d40	cytochrome P450, family 2, subfamily d, polypeptide 40	-0.946351505817374	6,49318E+14	-2,69206E+14	0.0336042890636437	0.176535273030273	-3,37255E+13	5,86868E+13	5,90722E+13	6,65534E+14	7,01326E+14	6,44185E+13	7,07271E+14
TC0100003715.mm.2	Capn2	calpain 2	0.836850528585672	6,79226E+14	2,69191E+14	0.0336111801123351	0.176535273030273	-3,37274E+14	7,14902E+13	7,39984E+14	6,56917E+14	6,30599E+14	6,55408E+14	6,77545E+14
TC0700002181.mm.2	Fiz1	Flt3 interacting zinc finger protein 1	1,24591E+14	7,68777E+14	2,6914E+13	0.0336346517356088	0.176535273030273	-3,37336E+14	8,58609E+14	8,70692E+14	8,0371E+14	6,76409E+14	7,13789E+14	6,89456E+14
TC0500003582.mm.2	Tmem130	transmembrane protein 130	1,6187E+14	8,71172E+14	2,69093E+14	0.0336568273972905	0.176535273030273	-3,37394E+14	1,03302E+14	9,4845E+14	9,12244E+14	7,45489E+14	8,09416E+14	7,78411E+14
TC1500002243.mm.2	Pou6f1	POU domain, class 6, transcription factor 1	0.834318074840476	7,14548E+14	2,69073E+14	0.0336661413857224	0.176535273030273	-3,37419E+14	7,62708E+14	7,60961E+14	6,99228E+14	6,57577E+14	7,04968E+14	7,01845E+14
TC0900001782.mm.2	Hephl1	hephaestin-like 1	-0.848646573850679	5,33856E+14	-2,69001E+13	0.0336993491371275	0.176535273030273	-3,37507E+14	4,84044E+14	4,75073E+14	5,41747E+14	5,871E+14	5,54217E+14	5,60954E+14
TC0700004608.mm.2	Ano1	anoctamin 1, calcium activated chloride channel	0.870100357817768	7,22835E+14	2,68997E+14	0.0337012343596198	0.176535273030273	-3,37512E+14	7,71431E+14	7,73024E+14	7,0984E+14	6,60595E+14	7,21194E+14	7,00924E+14
TC0100000912.mm.2	Olfr12	olfactory receptor 12	-1,02708E+14	5,53287E+14	-2,68903E+13	0.0337452126658918	0.176535273030273	-3,37628E+14	4,91096E+14	5,12401E+14	5,60647E+14	6,48268E+14	5,55524E+13	5,51787E+14
TC1100001183.mm.2	Rab11fip4	RAB11 family interacting protein 4 (class II)	0.860328839638031	8,61894E+13	2,68884E+14	0.0337540349447459	0.176535273030273	-3,37651E+14	9,30323E+14	9,0263E+14	8,34929E+14	8,25958E+14	8,59386E+14	8,18135E+14
TC0200002130.mm.2	Pcsk2	proprotein convertase subtilisin/kexin type 2	1,12092E+14	8,99169E+14	2,68874E+14	0.033758740608756	0.176535273030273	-3,37664E+14	9,43506E+12	9,79093E+14	8,85605E+14	8,1281E+14	9,22948E+14	8,51048E+14
TC0400003633.mm.2	Nkain1	Na+/K+ transporting ATPase interacting 1	1,61551E+14	7,59909E+14	2,68857E+14	0.0337665915327903	0.176535273030273	-3,37684E+14	8,67906E+14	8,47865E+14	7,88653E+14	6,04015E+14	7,43717E+13	7,07299E+14
TC1100001349.mm.2	Supt4a	suppressor of Ty 4A	0.961177764990696	7,79212E+14	2,68825E+14	0.033781900853097	0.176535273030273	-3,37725E+14	8,53478E+14	8,35703E+14	7,84765E+14	7,12181E+14	7,52316E+14	7,36829E+14
TC1400002151.mm.2	Zdhhc20	zinc finger, DHHC domain containing 20	1,18067E+14	7,9013E+14	2,68807E+14	0.0337900137484329	0.176535273030273	-3,37746E+14	8,90107E+14	8,49178E+13	8,01967E+14	7,01186E+13	7,73469E+13	7,2487E+14
TC0400002096.mm.2	Dvl1	dishevelled segment polarity protein 1	1,07167E+14	6,9466E+14	2,68801E+14	0.0337928006620453	0.176535273030273	-3,37753E+14	7,67394E+14	7,45826E+14	6,07711E+13	6,91174E+14	6,53464E+14	7,02393E+14
TC0200002157.mm.2	Slc24a3	solute carrier family 24 (sodium/potassium/calcium exchanger), member 3	1,40275E+14	9,11849E+14	2,6877E+13	0.0338072414553812	0.176535273030273	-3,37791E+13	1,03195E+14	9,81677E+14	9,28848E+14	8,04227E+14	9,02066E+14	8,22328E+14
TC0700003567.mm.2	Plin1	perilipin 1	-1,09745E+14	5,76991E+14	-2,68762E+14	0.0338113794323023	0.176535273030273	-3,37802E+14	4,89525E+14	4,97272E+14	5,65714E+14	6,40574E+14	5,98505E+14	6,70359E+14
TC1400001327.mm.2	Tm9sf2	transmembrane 9 superfamily member 2	1,39371E+14	7,44575E+14	2,68736E+14	0.0338231540876634	0.176535273030273	-3,37833E+14	8,26758E+14	8,34753E+14	7,68032E+14	6,14737E+14	7,21719E+14	7,01448E+14
TC0X00001343.mm.2	Zcchc18	zinc finger, CCHC domain containing 18	2,74457E+14	6,89467E+14	2,68718E+14	0.0338319647251779	0.176535273030273	-3,37856E+14	8,68463E+14	8,3644E+14	7,51909E+14	4,04081E+14	6,57569E+11	6,18341E+14

TC0200001166.mm.2	Ppp1r1c	protein phosphatase 1, regulatory inhibitor subunit 1C	-0.79285708988238	4,93601E+14	-2,68715E+14	0.0338333495979817	0.176535273030273	-3,3786E+14	4,63231E+14	4,56464E+13	5,34692E+14	5,43575E+14	4,71164E+14	4,92477E+14
TC1300000655.mm.2	Prelid1	PRELI domain containing 1	1,4717E+14	8,52799E+14	2,68679E+14	0.0338500390942415	0.176535273030273	-3,37904E+14	9,24917E+14	9,27362E+14	8,53029E+14	7,04911E+14	8,921E+14	8,14474E+14
TC0X00002710.mm.2	Slc7a3	solute carrier family 7 (cationic amino acid transporter, y+ system), member 3	2,47276E+14	5,97614E+14	2,68644E+14	0.0338662897768748	0.176535273030273	-3,37947E+14	7,61676E+14	7,02612E+14	6,38809E+14	3,30928E+14	5,71655E+14	5,80003E+13
TC0100003683.mm.2	Wdr26	WD repeat domain 26	1,82286E+14	7,32831E+14	2,68638E+14	0.0338694475321281	0.176535273030273	-3,37955E+14	8,38163E+14	8,64383E+14	7,45997E+14	5,91976E+13	7,56003E+14	6,00467E+14
TC0500001724.mm.2	Fscn1	fascin actin-bundling protein 1	1,58121E+14	9,0314E+14	2,6861E+14	0.0338824831098037	0.176535273030273	-3,37989E+13	1,00565E+14	9,95189E+14	9,34274E+14	7,50324E+14	8,67577E+14	8,65825E+14
TC1100001389.mm.2	Gm525	predicted gene 525	-1,03638E+14	6,02348E+14	-2,68575E+14	0.0338988881623079	0.176535273030273	-3,38032E+14	5,65241E+14	5,43088E+14	6,16019E+14	6,99586E+14	6,11347E+14	5,78807E+14
TC1200000566.mm.2	Klhdc1	kelch domain containing 1	-0.939384770145373	6,6824E+14	-2,68548E+14	0.0339113176245258	0.176535273030273	-3,38065E+14	5,88893E+14	6,09133E+13	6,68921E+14	7,16983E+14	6,88261E+14	7,37249E+14
TC0300002316.mm.2	Rab25	RAB25, member RAS oncogene family	-0.960711290818923	6,41644E+14	-2,68534E+14	0.0339178712903621	0.176535273030273	-3,38082E+14	5,69601E+14	5,78747E+13	6,3721E+14	7,0328E+14	6,61841E+14	6,99187E+14
TC1900001777.mm.2	Ndufb8	NADH:ubiquinone oxidoreductase subunit B8	-0.787307059087658	8,69549E+14	-2,68529E+14	0.0339206500741995	0.176535273030273	-3,3809E+14	7,9951E+14	8,10762E+13	8,79603E+14	8,88131E+14	9,1192E+14	9,2737E+14
TC0500000981.mm.2	Cops4	COP9 signalosome subunit 4	1,1596E+14	7,75358E+14	2,68505E+14	0.0339315810630653	0.176535273030273	-3,38118E+14	8,63626E+14	8,31618E+14	7,68912E+14	6,94411E+14	7,88008E+14	7,05572E+14
TC1500000805.mm.2	Atxn10	ataxin 10	1,82864E+14	9,02293E+14	2,68505E+13	0.0339317232241396	0.176535273030273	-3,38119E+14	1,0163E+14	9,9723E+14	9,3204E+13	7,15763E+14	8,97989E+13	8,54435E+14
TC1900000409.mm.2	Smarca2	SWI/SNF related, matrix associated, actin dependent regulator of chromatin, subfamily a, member 2	1,78189E+14	8,81348E+14	2,6846E+14	0.0339526108816768	0.176535273030273	-3,38173E+14	1,01186E+14	1,00564E+14	9,13473E+14	7,4765E+14	8,72752E+14	7,3671E+14
TC0600003064.mm.2	Phc1	polyhomeotic 1	1,05915E+14	7,40582E+14	2,68423E+14	0.0339701430475157	0.176535273030273	-3,38219E+14	8,03411E+13	8,13314E+14	7,48324E+14	6,5657E+14	7,25853E+14	6,96021E+14
TC1100004124.mm.2	St6galnac2	ST6 (alpha-N-acetyl-neuraminyl-2,3-beta-galactosyl-1,3)-N-acetylgalactosaminide alpha-2,6-sialyltransferase 2	-1,20437E+14	7,21798E+14	-2,68423E+14	0.0339704162365947	0.176535273030273	-3,3822E+14	5,90316E+14	6,78918E+14	7,23198E+14	7,86909E+14	7,46718E+13	8,04727E+14
TC1600001516.mm.2	Rubcn	RUN domain and cysteine-rich domain containing, Beclin 1-interacting protein	1,00802E+14	6,18671E+14	2,68412E+14	0.0339754651084327	0.176535273030273	-3,38233E+14	6,76439E+14	7,06933E+14	5,71069E+14	6,10698E+14	5,38014E+14	6,08875E+14
TC0100000656.mm.2	Dnajb2	DnaJ heat shock protein family (Hsp40) member B2	1,25123E+14	7,20461E+14	2,68403E+14	0.033979423635021	0.176535273030273	-3,38244E+14	8,17383E+14	7,76442E+13	7,26873E+14	6,16704E+14	7,22344E+14	6,63022E+14
TC0100002698.mm.2	Trip12	thyroid hormone receptor interactor 12	1,14461E+14	8,25567E+14	2,68383E+14	0.0339888641111704	0.176535273030273	-3,38268E+13	9,11667E+14	8,9144E+14	8,19145E+14	7,55039E+14	8,33489E+14	7,42622E+14
TC0400003627.mm.2	Tinagl1	tubulointerstitial nephritis antigen-like 1	-1,11542E+13	7,49009E+14	-2,68369E+14	0.0339955644906769	0.176535273030273	-3,38286E+13	6,67631E+14	6,93655E+14	7,456E+13	8,3877E+14	7,50526E+14	7,97873E+14
TC0400000036.mm.2	Ubxn2b	UBX domain protein 2B	0.907822111806075	6,75574E+14	2,68367E+14	0.0339965108273654	0.176535273030273	-3,38288E+14	7,37756E+14	7,32762E+14	6,53716E+14	6,35237E+12	6,77537E+14	6,16438E+13
TC0300002445.mm.2	Snx27	sorting nexin family member 27	1,86421E+14	8,2823E+14	2,68344E+14	0.034007394833851	0.176535273030273	-3,38317E+14	9,38411E+14	9,48719E+14	8,6405E+14	6,50238E+14	8,21505E+14	7,46459E+14
TC0400003565.mm.2	Gja4	gap junction protein, alpha 4	-1,30229E+14	7,6329E+14	-2,68329E+14	0.0340143109285284	0.176535273030273	-3,38335E+14	6,67334E+14	6,78828E+14	7,37457E+14	8,69163E+14	7,90035E+14	8,36921E+14
TC0700000041.mm.2	Kmt5c	lysine methyltransferase 5C	-0.829509965282744	7,78906E+14	-2,68319E+14	0.0340191609979408	0.176535273030273	-3,38348E+14	7,41124E+14	7,14526E+14	8,24764E+14	7,96788E+13	7,91333E+14	8,04903E+13
TC1200001662.mm.2	Lsmem1	leucine-rich single-pass membrane protein 1	-0.964641501803412	5,53535E+14	-2,68282E+14	0.0340365271944363	0.176535273030273	-3,38393E+13	5,04028E+14	4,87365E+14	5,71854E+14	6,12468E+14	5,40799E+14	6,04695E+14
TC0100003778.mm.2	Kcnk2	potassium channel, subfamily K, member 2	1,37519E+13	6,61915E+14	2,6823E+14	0.0340611770500241	0.176535273030273	-3,38458E+14	7,74668E+14	7,01021E+14	6,65461E+14	5,35189E+14	6,33489E+14	6,61663E+14
TC1000001162.mm.2	Ube2n	ubiquitin-conjugating enzyme E2N	1,28304E+14	8,2966E+14	2,6822E+14	0.0340657042583461	0.176535273030273	-3,3847E+14	9,07095E+14	9,12521E+14	8,34871E+14	7,28137E+14	8,40651E+14	7,54685E+14
TC0200004645.mm.2	Gfra4/Mir6973b	glial cell line derived neurotrophic factor family receptor alpha 4/microRNA 6973b	-1,01787E+14	7,6452E+14	-2,68217E+14	0.03406734692878	0.176535273030273	-3,38474E+14	6,99825E+14	6,87119E+14	7,71351E+14	8,19166E+14	7,67895E+14	8,41766E+14
TC0900002338.mm.2	Scaper	S phase cyclin A-associated protein in the ER	2,54044E+14	5,99931E+14	2,68199E+14	0.0340755593221145	0.176535273030273	-3,38495E+14	7,66673E+14	7,39599E+14	6,0723E+13	3,90954E+14	6,6722E+14	4,2791E+14
TC0900002542.mm.2	Tln2	talin 2	0.966478751778959	6,7067E+14	2,68195E+14	0.034077645893466	0.176535273030273	-3,38501E+14	7,58453E+14	7,22077E+14	6,42195E+14	6,4504E+14	5,93657E+14	6,62599E+14
TC0700000878.mm.2	Saa2	serum amyloid A 2	-1,32703E+14	6,35896E+14	-2,68181E+14	0.0340843563828696	0.176535273030273	-3,38518E+14	5,67987E+14	5,62519E+13	6,25973E+14	7,6994E+14	6,39059E+13	6,49896E+14
TC0500003418.mm.2	Cux1	cut-like homeobox 1	1,82484E+14	6,92316E+14	2,68149E+14	0.0340992985263516	0.176555911682231	-3,38557E+14	8,02381E+14	8,15279E+14	7,35235E+14	5,17456E+14	6,60158E+14	6,23386E+14
TC0600003232.mm.2	Clec12b	C-type lectin domain family 12, member B	-0.840140759513195	4,74272E+14	-2,68125E+14	0.0341105225138173	0.176557291765223	-3,38587E+13	4,18621E+14	4,32007E+14	5,00468E+14	5,18188E+13	4,68703E+14	5,07644E+14
TC0700000548.mm.2	Zfp382	zinc finger protein 382	1,54477E+14	5,66724E+14	2,68096E+14	0.0341243853118677	0.176560926946404	-3,38623E+14	6,65384E+14	6,33731E+14	5,6662E+13	4,2354E+14	6,07034E+14	5,04033E+14
TC0800002935.mm.2	Ddx19b	DEAD (Asp-Glu-Ala-Asp) box polypeptide 19b	1,04252E+14	5,71116E+14	2,68077E+14	0.0341331401624806	0.176560926946404	-3,38646E+14	6,14737E+14	6,73945E+14	5,46685E+14	5,33493E+14	5,6481E+13	4,93025E+14
TC0700004273.mm.2	Lat	linker for activation of T cells	-0.944477662792483	6,37501E+14	-2,68032E+14	0.0341548417876622	0.176570315019368	-3,38702E+14	5,73215E+14	5,77993E+14	6,50822E+14	6,89282E+14	6,35824E+14	6,97872E+13
TC1100004270.mm.2	Tlcd2	TLC domain containing 2	-0.887496136313402	5,12087E+14	-2,68017E+14	0.0341615215459809	0.176570315019368	-3,3872E+14	4,6659E+14	4,63663E+14	5,50137E+14	5,57615E+14	4,88019E+14	5,465E+14
TC0400003650.mm.2	Ptpru	protein tyrosine phosphatase, receptor type, U	-0.795589371410133	6,57521E+14	-2,68004E+14	0.0341678298411463	0.176570315019368	-3,38736E+14	6,15805E+14	6,17111E+14	6,88524E+14	7,0351E+14	6,49021E+14	6,71152E+14
TC0700000989.mm.2	Snrpa1	small nuclear ribonucleoprotein polypeptide A'	1,06696E+14	6,43269E+14	2,67963E+13	0.0341873546126599	0.176582127313087	-3,38787E+14	7,16122E+14	7,07997E+14	6,49977E+14	5,6075E+14	6,33273E+14	5,91496E+14
TC0400002467.mm.2	Kif24	kinesin family member 24	-1,33466E+14	5,79968E+14	-2,6786E+14	0.0342360270589057	0.176582127313087	-3,38914E+14	4,67276E+14	5,02667E+14	5,6078E+14	6,76095E+13	5,95377E+14	6,77614E+14
TC0200001586.mm.2	Immp1l	IMP1 inner mitochondrial membrane peptidase-like (S. cerevisiae)	1,92401E+14	6,36952E+13	2,67854E+14	0.034239067727833	0.176582127313087	-3,38921E+14	7,46307E+14	7,62013E+14	6,64696E+14	4,58823E+14	6,52188E+13	5,37684E+14
TC0900002651.mm.2	Ccpg1os	cell cycle progression 1, opposite strand	1,08284E+13	7,30769E+14	2,67844E+14	0.0342435765771699	0.176582127313087	-3,38933E+14	7,90174E+14	8,0728E+14	7,2477E+14	6,56116E+14	6,66405E+14	7,3987E+14
TC0100001615.mm.2	Slamf9	SLAM family member 9	-1,00452E+14	5,43875E+14	-2,67785E+14	0.0342715640026053	0.176582127313087	-3,39006E+14	4,41253E+13	4,95359E+14	5,4369E+12	5,93826E+14	5,71983E+14	6,17139E+14
TC0X00000963.mm.2	Dlg3	discs large MAGUK scaffold protein 3	1,59205E+14	7,77152E+14	2,67773E+14	0.0342775026519587	0.176582127313087	-3,39021E+14	8,93187E+14	8,81413E+14	8,08161E+14	6,48029E+14	7,63667E+14	6,68455E+14
TC1200000655.mm.2	Snapc1	small nuclear RNA activating complex, polypeptide 1	1,03647E+14	6,13669E+14	2,67763E+14	0.0342820019181489	0.176582127313087	-3,39033E+14	6,96155E+13	6,87923E+14	6,18068E+14	5,58715E+14	5,96152E+14	5,25001E+14
TC1600000184.mm.2	Snai2	snail family zinc finger 2	-1,01115E+14	6,57463E+13	-2,67746E+14	0.0342902456916732	0.176582127313087	-3,39054E+14	5,98275E+14	5,67931E+13	7,12096E+14	6,56339E+12	7,35463E+14	6,74673E+14
TC1200002076.mm.2	Fam161b	family with sequence similarity 161, member B	0.959292797835423	6,29982E+13	2,67731E+13	0.0342971169001128	0.176582127313087	-3,39072E+14	6,97556E+14	6,99386E+14	5,64305E+14	6,40779E+14	5,92178E+14	5,85688E+14
TC0700001397.mm.2	Olfr521	olfactory receptor 521	-1,24432E+14	5,60387E+14	-2,67729E+14	0.0342981517851915	0.176582127313087	-3,39075E+14	4,84148E+14	4,50143E+14	5,3253E+14	6,50625E+14	5,9974E+14	6,45137E+14
TC1500000351.mm.2	Eny2	ENY2 transcription and export complex 2 subunit	0.941984156164156	7,02213E+12	2,67697E+13	0.034313416325734	0.176582127313087	-3,39115E+14	7,73233E+14	7,62652E+13	7,04314E+14	6,43175E+14	6,8404E+14	6,45863E+14
TC1000001228.mm.2	Mgat4c	MGAT4 family, member C	1,87243E+14	6,43109E+13	2,67674E+14	0.0343244677983118	0.176582127313087	-3,39143E+14	7,53881E+14	7,56271E+14	6,63298E+14	4,72369E+13	6,69378E+14	5,43454E+14
TC0900002521.mm.2	Snx22	sorting nexin 22	-0.899248617592768	6,10377E+14	-2,67671E+14	0.0343259869649012	0.176582127313087	-3,39147E+14	5,50039E+14	5,55833E+14	6,23355E+14	6,62366E+14	6,11135E+14	6,59536E+14
TC1700000567.mm.2	Cyp4f15	cytochrome P450, family 4, subfamily f, polypeptide 15	1,14751E+12	5,53832E+14	2,67644E+14	0.0343387097810975	0.176582127313087	-3,3918E+14	6,24985E+14	6,5928E+14	5,33172E+14	5,21591E+14	4,37866E+14	5,46097E+14
TC1200000225.mm.2	Cmpk2	cytidine monophosphate (UMP-CMP) kinase 2, mitochondrial	0.868202306918338	6,42656E+13	2,67607E+14	0.0343561599762963	0.176582127313087	-3,39225E+14	7,29867E+13	6,99633E+14	6,46286E+14	6,09575E+14	5,98111E+14	5,72466E+13
TC0400000949.mm.2	Ift74	intraflagellar transport 74	2,39892E+14	6,07476E+14	2,67596E+14	0.0343614612857714	0.176582127313087	-3,39239E+14	7,68627E+13	7,67376E+14	6,32457E+14	4,23763E+14	6,3721E+14	4,15424E+14
TC1900001404.mm.2	Rnls	renalase, FAD-dependent amine oxidase	-1,07401E+14	5,69322E+14	-2,67591E+14	0.0343640749593308	0.176582127313087	-3,39246E+14	5,4191E+14	4,78178E+14	5,78612E+14	6,56278E+14	5,79149E+13	5,81803E+14
TC1200002053.mm.2	Numb	NUMB endocytic adaptor protein	0.843543958105467	6,75098E+14	2,67584E+14	0.0343673773508248	0.176582127313087	-3,39255E+14	7,3762E+14	7,6631E+14	6,85864E+14	6,49356E+14	6,04332E+14	6,07107E+14
TC1400001558.mm.2	Dusp13	dual specificity phosphatase 13	-0.823903284145048	6,70954E+13	-2,6754E+14	0.0343881983527674	0.176591136409882	-3,39308E+14	5,99236E+13	6,30642E+14	6,87315E+14	7,07344E+14	6,93666E+14	7,07519E+14
TC0400001477.mm.2	Sfpq	splicing factor proline/glutamine rich (polypyrimidine tract binding protein associated)	1,02281E+14	8,04264E+14	2,67467E+14	0.0344231738120795	0.176591136409882	-3,39399E+14	8,60312E+14	8,83291E+14	7,96252E+14	7,42791E+14	8,06124E+14	7,36815E+14
TC0700004589.mm.2	Cars	cysteinyl-tRNA synthetase	1,03578E+14	6,5639E+14	2,6746E+14	0.0344264939034144	0.176591136409882	-3,39408E+14	7,13315E+14	7,12239E+14	6,22544E+14	5,95854E+14	6,91796E+14	6,02591E+14
TC0300001352.mm.2	Ints12	integrator complex subunit 12	0.86383590205532	6,56321E+14	2,67447E+14	0.0344324397641869	0.176591136409882	-3,39423E+14	7,01E+12	7,1474E+14	6,33103E+14	6,09871E+14	6,1685E+14	6,6236E+14
TC1500000665.mm.2	Eif3l	eukaryotic translation initiation factor 3, subunit L	1,0066E+14	7,66706E+14	2,67384E+14	0.0344625519967814	0.176591136409882	-3,39501E+14	8,38464E+14	8,16701E+14	7,68643E+14	6,85202E+14	7,39022E+14	7,52201E+14
TC0200002072.mm.2	Lamp5	lysosomal-associated membrane protein family, member 5	1,47658E+14	7,40158E+14	2,67369E+14	0.0344698430801863	0.176591136409882	-3,3952E+14	8,13574E+14	8,2578E+14	7,42573E+14	6,01465E+13	7,75007E+14	6,82549E+14
TC1800000261.mm.2	Bin1	bridging integrator 1	1,02989E+14	8,79345E+14	2,67354E+14	0.0344767567699599	0.176591136409882	-3,39537E+14	9,53749E+14	9,43949E+14	8,81452E+14	8,10267E+14	8,72066E+14	8,14586E+14
TC0800000726.mm.2	Lpar2	lysophosphatidic acid receptor 2	-0.974979514136394	6,31955E+14	-2,67326E+14	0.0344903656769646	0.176591136409882	-3,39573E+14	5,77968E+14	5,65417E+14	6,32596E+14	7,05785E+14	6,39364E+14	6,70603E+14
TC1000002249.mm.2	Col13a1	collagen, type XIII, alpha 1	-0.94830510730145	7,92444E+14	-2,67322E+14	0.0344923667116819	0.176591136409882	-3,39578E+14	7,03315E+14	7,50913E+14	7,99588E+14	8,44301E+14	8,10228E+14	8,46318E+14
TC0500000422.mm.2	Crmp1	collapsin response mediator protein 1	1,91191E+14	7,30756E+14	2,67309E+14	0.0344984876179147	0.176591136409882	-3,39594E+14	8,5761E+14	8,31796E+14	7,69091E+14	5,37932E+14	7,10463E+14	6,77644E+14
TC0400001543.mm.2	Fabp3	fatty acid binding protein 3, muscle and heart	0.967240276378257	9,05413E+13	2,67293E+14	0.0345058704176368	0.176591136409882	-3,39613E+14	9,7129E+14	9,57143E+14	8,96354E+14	8,38631E+13	9,10469E+14	8,5859E+13
TC1700002312.mm.2	Ubxn6	UBX domain protein 6	1,09403E+14	7,24446E+14	2,67268E+14	0.0345180734878408	0.176591136409882	-3,39644E+14	7,94385E+13	7,92342E+14	7,31582E+14	6,3634E+14	7,1756E+14	6,74466E+13
TC1000001040.mm.2	Fbxo7	F-box protein 7	1,04975E+14	6,37482E+14	2,6724E+14	0.0345313892875996	0.176591136409882	-3,39678E+14	6,91034E+14	6,79629E+14	6,27947E+14	5,32767E+14	6,49838E+14	6,43675E+14
TC1000003096.mm.2	Tspan31	tetraspanin 31	1,28145E+14	8,94598E+14	2,67239E+14	0.034531658342321	0.176591136409882	-3,39679E+14	9,75515E+14	9,80441E+14	9,13669E+14	7,85997E+13	8,81627E+14	8,30339E+14
TC0400003567.mm.2	Gjb4	gap junction protein, beta 4	-0.903395150333713	5,90868E+14	-2,67179E+14	0.0345605625752386	0.176591136409882	-3,39754E+14	5,18503E+14	5,39794E+14	5,90857E+14	6,48119E+14	6,14559E+14	6,33375E+14
TC0X00001629.mm.2	Map3k15	mitogen-activated protein kinase kinase kinase 15	-0.981895665957443	4,95071E+14	-2,67178E+14	0.0345609028277591	0.176591136409882	-3,39755E+14	4,1125E+13	4,62611E+14	5,0331E+14	5,6693E+14	5,09065E+14	5,17258E+14
TC0600001712.mm.2	Etfrf1	electron transfer flavoprotein regulatory factor 1	1,5159E+14	7,32584E+14	2,67163E+14	0.0345682440865648	0.176591136409882	-3,39773E+14	8,18156E+14	8,4063E+13	7,55525E+14	6,00081E+14	7,29675E+14	6,51436E+14
TC1100000916.mm.2	Trp53	transformation related protein 53	0.784209987207289	6,98383E+14	2,67112E+14	0.0345926715387954	0.176591136409882	-3,39836E+14	7,43008E+14	7,44673E+14	6,67153E+14	6,63686E+14	6,96532E+14	6,75247E+14
TC0600001266.mm.2	Slc6a11	solute carrier family 6 (neurotransmitter transporter, GABA), member 11	1,60587E+14	8,74256E+14	2,67099E+14	0.0345990200055585	0.176591136409882	-3,39853E+14	1,02591E+14	9,43734E+14	9,00792E+14	7,4768E+14	8,51379E+14	7,76039E+14
TC0300001330.mm.2	Lef1	lymphoid enhancer binding factor 1	-0.859210485851803	6,37592E+14	-2,67087E+14	0.0346045751794933	0.176591136409882	-3,39867E+14	5,9409E+14	5,64684E+14	6,44165E+13	6,8645E+14	6,7122E+14	6,64944E+14
TC0600002137.mm.2	E330009J07Rik	RIKEN cDNA E330009J07 gene	1,3519E+14	6,06861E+14	2,67078E+14	0.0346092059851629	0.176591136409882	-3,39879E+14	6,9051E+13	6,62168E+14	6,05043E+14	4,77256E+14	6,39159E+14	5,67031E+14
TC1900001036.mm.2	Cox8a	cytochrome c oxidase subunit 8A	0.884570669681874	1,09196E+14	2,67055E+14	0.0346200136726495	0.176591136409882	-3,39907E+14	1,1484E+14	1,14182E+14	1,08137E+14	1,03193E+14	1,08997E+14	1,05827E+14
TC0100002445.mm.2	Ndufs1	NADH:ubiquinone oxidoreductase core subunit S1	1,71924E+13	7,15925E+14	2,67052E+13	0.0346214446462269	0.176591136409882	-3,3991E+14	8,29507E+14	8,12708E+14	7,39013E+14	5,59354E+14	7,27802E+14	6,27166E+14
TC0700003625.mm.2	Bnc1	basonuclin 1	-1,23669E+14	5,10124E+14	-2,67015E+14	0.0346390153161717	0.176591136409882	-3,39956E+14	4,53997E+14	4,18946E+14	4,99571E+13	6,2071E+14	5,50101E+12	5,17419E+14
TC0900000834.mm.2	Cilp	cartilage intermediate layer protein, nucleotide pyrophosphohydrolase	-1,1632E+14	4,87254E+14	-2,67007E+14	0.0346431193565219	0.176591136409882	-3,39966E+14	4,37357E+12	4,06242E+14	4,8349E+14	5,9275E+12	4,86966E+14	5,1672E+14
TC0X00000318.mm.2	Zcchc12	zinc finger, CCHC domain containing 12	1,67198E+13	6,83515E+13	2,66898E+14	0.0346956608707952	0.17678350403125	-3,40101E+14	8,62246E+14	7,87003E+14	7,1446E+14	6,00393E+14	6,41266E+14	4,95724E+13
TC0900001692.mm.2	Aasdhppt	aminoadipate-semialdehyde dehydrogenase-phosphopantetheinyl transferase	2,63433E+14	6,61228E+14	2,66867E+14	0.0347102798284393	0.17678350403125	-3,40139E+14	8,41254E+14	8,5198E+14	6,97741E+14	4,68626E+14	6,78481E+14	4,29285E+13
TC0900001921.mm.2	Dpy19l1	dpy-19-like 1 (C. elegans)	1,18614E+14	6,69663E+14	2,66844E+14	0.0347212916800417	0.17678350403125	-3,40167E+14	7,47746E+14	7,48619E+14	6,77904E+14	5,81232E+14	6,68147E+14	5,94333E+14
TC1900000023.mm.2	Tbx10	T-box 10	-0.907632805486178	6,13195E+14	-2,6683E+14	0.0347280217418095	0.17678350403125	-3,40184E+11	5,29104E+14	5,69394E+14	6,21386E+14	6,5864E+14	6,3263E+14	6,68015E+14
TC0500003712.mm.2	Denr	density-regulated protein	0.922926406700757	8,47158E+12	2,66814E+13	0.0347357148738869	0.17678350403125	-3,40204E+14	9,11648E+14	9,10796E+14	8,27506E+14	8,10353E+14	8,44772E+14	7,7787E+14
TC1900000382.mm.2	Ptar1	protein prenyltransferase alpha subunit repeat containing 1	1,06729E+14	6,38117E+14	2,6675E+13	0.0347667700003098	0.176825138777124	-3,40283E+14	7,14698E+14	7,27378E+14	5,86772E+14	6,41847E+14	6,20165E+14	5,3784E+14
TC1300000670.mm.2	Caml	calcium modulating ligand	2,365E+13	6,49194E+14	2,66749E+14	0.0347670269269191	0.176825138777124	-3,40284E+14	7,69031E+14	7,64658E+14	6,79523E+14	3,81167E+14	6,69397E+12	6,31386E+14
TSUnmapped00000053.mm.2	Sts	steroid sulfatase	-0.976428808777058	6,71665E+14	-2,66723E+14	0.034779822106392	0.176825138777124	-3,40317E+14	6,08909E+14	6,13995E+14	6,70095E+14	7,48094E+14	6,83171E+14	7,05726E+14
TC0100000665.mm.2	Slc4a3	solute carrier family 4 (anion exchanger), member 3	0.802522394476441	7,03899E+14	2,66706E+14	0.0347877918403453	0.176825138777124	-3,40337E+13	7,49256E+14	7,52674E+14	6,5672E+14	6,84706E+14	6,82402E+14	6,97637E+14
TC0900003041.mm.2	Gnat1	guanine nucleotide binding protein, alpha transducing 1	-1,02543E+14	5,41709E+14	-2,66605E+14	0.0348365921803325	0.176978699257948	-3,40462E+14	4,62069E+14	5,00224E+14	5,58438E+14	6,08941E+14	5,27115E+14	5,93465E+13
TC0X00001008.mm.2	1700011M02Rik	RIKEN cDNA 1700011M02 gene	-1,20614E+14	6,11613E+14	-2,66595E+12	0.0348415852973487	0.176978699257948	-3,40475E+14	5,38891E+14	5,21808E+14	6,17326E+14	6,84601E+14	6,01349E+14	7,05703E+14
TC0X00000941.mm.2	Eda	ectodysplasin-A	-1,20754E+14	5,84479E+13	-2,66515E+13	0.0348803039727499	0.176978699257948	-3,40574E+14	5,29942E+14	5,23071E+14	5,84924E+14	7,09596E+14	5,727E+14	5,86638E+14
TC0400001721.mm.2	Sh2d5/Kif17	SH2 domain containing 5/kinesin family member 17	1,04604E+14	5,96519E+14	2,66513E+14	0.0348811064688607	0.176978699257948	-3,40576E+14	6,27172E+14	6,88694E+14	5,70745E+14	5,35914E+14	5,45904E+14	6,10686E+13
TC1100000754.mm.2	Drg2	developmentally regulated GTP binding protein 2	1,3153E+14	7,13399E+14	2,66512E+14	0.0348814673251283	0.176978699257948	-3,40577E+14	8,24414E+14	8,14741E+14	7,59707E+14	6,16387E+13	6,36875E+14	6,28271E+13
TC0700002708.mm.2	Actn4	actinin alpha 4	0.883172472088836	8,32671E+14	2,66498E+14	0.0348883712337072	0.176978699257948	-3,40594E+14	8,89132E+14	9,01897E+14	8,14849E+14	7,99545E+14	8,23186E+14	7,67419E+14
TC1000001967.mm.2	Nkain2	Na+/K+ transporting ATPase interacting 2	2,31534E+14	7,80138E+13	2,66481E+14	0.0348964720156171	0.176978699257948	-3,40615E+14	9,2677E+14	9,27018E+14	8,31701E+14	5,59019E+14	7,73203E+14	6,63117E+14
TC0300002463.mm.2	Pip5k1a	phosphatidylinositol-4-phosphate 5-kinase, type 1 alpha	0.919890503736371	7,04761E+14	2,66426E+14	0.0349230305814536	0.176978699257948	-3,40683E+14	7,53543E+14	7,53135E+13	6,95415E+14	6,27285E+14	7,03851E+14	6,95337E+14
TC0200005430.mm.2	Kcnq2	potassium voltage-gated channel, subfamily Q, member 2	1,48808E+14	7,7995E+14	2,66421E+14	0.0349257914544649	0.176978699257948	-3,4069E+14	8,89071E+14	8,75243E+14	8,05739E+13	6,60958E+14	7,70044E+14	6,78645E+14
TC0300002241.mm.2	Mnd1	meiotic nuclear divisions 1	-0.85785786786464	5,3612E+14	-2,664E+14	0.0349356057051302	0.176978699257948	-3,40715E+14	4,82677E+14	4,74155E+14	5,41557E+14	5,86847E+14	5,77937E+14	5,53546E+14
TC0600000398.mm.2	Tmem178b	transmembrane protein 178B	1,17234E+14	7,80624E+14	2,66394E+14	0.034938822214332	0.176978699257948	-3,40723E+14	8,92545E+14	8,70927E+14	8,06724E+14	7,2228E+14	7,36536E+13	6,5473E+13
TC0200002963.mm.2	Rbm17	RNA binding motif protein 17	1,85851E+14	6,95022E+14	2,6636E+14	0.0349551989564772	0.177006009046422	-3,40765E+14	7,99609E+14	7,84753E+14	7,06152E+14	5,06507E+14	7,41141E+14	6,3197E+13
TC0700000610.mm.2	Scgb1b19	secretoglobin, family 1B, member 19	-0.907827584262419	5,76008E+14	-2,66331E+14	0.0349693672709022	0.177022122160241	-3,40801E+13	4,99143E+14	5,2933E+14	5,91803E+14	6,18235E+14	5,82713E+14	6,34825E+14
TC0600003332.mm.2	Wbp11	WW domain binding protein 11	1,19891E+14	6,6778E+14	2,66303E+14	0.0349827663195819	0.177034332194542	-3,40835E+14	7,71782E+14	7,54112E+14	6,91207E+14	5,94904E+13	6,35626E+14	5,59051E+14
TC1500001601.mm.2	4933412E24Rik	RIKEN cDNA 4933412E24 gene	-1,18625E+12	5,91477E+14	-2,66264E+14	0.0350016025254756	0.177036859100729	-3,40883E+14	5,04315E+14	5,27767E+14	5,8444E+14	6,84891E+14	5,91053E+14	6,56394E+14
TC0200001812.mm.2	Pdia3	protein disulfide isomerase associated 3	1,48988E+14	8,78411E+13	2,66257E+14	0.0350052400605054	0.177036859100729	-3,40892E+14	9,59479E+14	9,86361E+14	8,87832E+14	7,60033E+14	8,94499E+14	7,8226E+14
TC0X00003346.mm.2	Rab9	RAB9, member RAS oncogene family	3,15563E+13	6,66245E+14	2,66172E+14	0.0350466402354457	0.177143928382526	-3,40997E+14	8,71979E+14	8,46575E+14	7,07648E+14	3,7978E+14	7,20257E+14	4,71232E+14
TC1500000960.mm.2	Pced1b	PC-esterase domain containing 1B	-0.931864989549823	6,07549E+14	-2,66168E+14	0.0350483984164024	0.177143928382526	-3,41002E+14	5,44215E+14	5,42225E+14	6,26904E+14	6,45908E+14	6,08446E+14	6,77594E+14
TC0400001541.mm.2	Pef1	penta-EF hand domain containing 1	1,49114E+14	6,82874E+14	2,66106E+14	0.0350787493690424	0.177206917367197	-3,41079E+14	7,84771E+14	7,66734E+13	7,04507E+14	5,48771E+14	6,10216E+14	6,82243E+14

TC0400001524.mm.2	Fam229a	family with sequence similarity 229, member A	-1,13745E+14	6,08063E+14	-2,66042E+13	0.0351098111903203	0.177206917367197	-3,41158E+14	5,49532E+14	5,27622E+14	6,00795E+14	7,0385E+14	6,12122E+14	6,54454E+14
TC0X00000346.mm.2	Rhox4c	reproductive homeobox 4C	-0.960588651185003	5,66831E+14	-2,6604E+14	0.0351107279595695	0.177206917367197	-3,4116E+14	5,41943E+14	4,94558E+14	5,90858E+14	6,37761E+14	5,88574E+14	5,47289E+14
TC1700001898.mm.2	Brd2	bromodomain containing 2	1,35106E+14	7,92592E+13	2,66025E+13	0.0351177491621419	0.177206917367197	-3,41178E+14	8,70667E+14	8,84085E+14	8,07635E+14	6,76906E+14	7,94479E+14	7,21779E+14
TC0100003614.mm.2	Pld5	phospholipase D family, member 5	1,47235E+14	7,16721E+14	2,66012E+14	0.0351244850679735	0.177206917367197	-3,41195E+14	8,27298E+14	7,90013E+12	7,3792E+14	5,84922E+14	7,11649E+14	6,48525E+14
TC0X00001338.mm.2	Plp1	proteolipid protein (myelin) 1	2,04041E+14	1,11598E+14	2,66004E+14	0.035128225992763	0.177206917367197	-3,41204E+14	1,23254E+14	1,2481E+14	1,14597E+14	9,26583E+14	1,13591E+14	1,00675E+14
TC0300003232.mm.2	Glt28d2	glycosyltransferase 28 domain containing 2	-1,15079E+14	7,60621E+14	-2,65984E+14	0.0351378452794758	0.177206917367197	-3,41229E+13	6,90566E+13	6,79236E+14	7,70595E+14	8,29364E+14	7,47296E+13	8,46669E+14
TC0600003311.mm.2	Gpr19	G protein-coupled receptor 19	1,15862E+14	7,47819E+14	2,6595E+14	0.0351546638276621	0.177236263534142	-3,41271E+14	8,35134E+14	8,12768E+14	7,38454E+14	6,77722E+13	7,60419E+13	6,62419E+14
TC0200003106.mm.2	Spopl	speckle-type POZ protein-like	1,9175E+14	5,68481E+13	2,65891E+14	0.0351830483868828	0.177323884472269	-3,41343E+14	6,6012E+13	7,23453E+14	5,88564E+13	4,11508E+14	5,85923E+14	4,41318E+14
TC1400002457.mm.2	Gm6994	predicted gene 6994	-0.842553746794987	5,95729E+14	-2,65844E+14	0.0352061276596071	0.177346652845913	-3,41402E+14	5,25717E+14	5,22795E+14	5,98513E+14	6,1851E+14	6,32666E+14	6,76171E+14
TC0300001338.mm.2	Papss1	3'-phosphoadenosine 5'-phosphosulfate synthase 1	1,72008E+14	6,46103E+13	2,65798E+13	0.0352285284457076	0.177346652845913	-3,41458E+14	7,51859E+14	7,50532E+14	6,62295E+13	4,96081E+14	6,70403E+14	5,45445E+14
TC1500001214.mm.2	Tars	threonyl-tRNA synthetase	0.777906406848589	6,82317E+14	2,65784E+14	0.0352355685667529	0.177346652845913	-3,41476E+14	7,23185E+14	7,41852E+14	6,56175E+14	6,5328E+14	6,56433E+14	6,62977E+14
TC0700002087.mm.2	Tspan32	tetraspanin 32	-0.785682080264952	6,40989E+14	-2,65781E+14	0.0352371620779711	0.177346652845913	-3,4148E+14	5,92209E+14	5,77243E+14	6,53508E+14	6,7308E+14	6,76833E+14	6,73063E+14
TC1200000387.mm.2	Pnpla8/Gm2027	patatin-like phospholipase domain containing 8/nuclear assembly factor 1 homolog pseudogene	1,62806E+14	6,38096E+12	2,6577E+12	0.0352425980520458	0.177346652845913	-3,41494E+14	7,35812E+14	7,24494E+14	6,45366E+14	4,89329E+14	6,74166E+14	5,59406E+14
TC0200004463.mm.2	Zfp106	zinc finger protein 106	0.782493787283981	8,22851E+14	2,65724E+14	0.0352646442665527	0.177402189530741	-3,4155E+14	8,83464E+14	8,81882E+14	7,94818E+14	8,14029E+14	7,87848E+13	7,75065E+14
TC0600000124.mm.2	Tes	testis derived transcript	-0.976739820932544	6,50653E+14	-2,65697E+14	0.0352782336283547	0.177415161814507	-3,41584E+14	5,58117E+14	5,84138E+14	6,30611E+14	7,06992E+14	7,04094E+14	7,19967E+14
TC0500001043.mm.2	Lrrc8c	leucine rich repeat containing 8 family, member C	1,341E+14	6,85372E+14	2,65572E+14	0.0353392229377066	0.177627138522017	-3,41738E+14	7,54995E+14	7,53436E+13	6,90242E+14	5,49989E+14	6,56776E+14	7,06795E+14
TC0100002936.mm.2	Tsn	translin	0.986550961993856	7,19681E+14	2,65489E+14	0.0353799288881678	0.177627138522017	-3,4184E+14	8,10445E+14	7,9046E+14	7,35669E+14	6,67926E+14	6,79995E+13	6,33592E+14
TC1900001289.mm.2	Cbwd1	COBW domain containing 1	1,47433E+13	5,15152E+14	2,65489E+14	0.0353801902168991	0.177627138522017	-3,41841E+13	6,28251E+14	6,3606E+14	5,30237E+14	4,39209E+14	5,00511E+13	3,56645E+14
TC0300000635.mm.2	Ctso	cathepsin O	1,64097E+14	6,30915E+14	2,65485E+14	0.0353820803209846	0.177627138522017	-3,41846E+14	7,15722E+14	7,19503E+14	6,4715E+14	4,59881E+14	6,50924E+14	5,92312E+14
TC0500000901.mm.2	Gm3106	predicted gene 3106	-0.927616611089996	4,98418E+13	-2,65463E+14	0.0353928231494044	0.177627138522017	-3,41873E+14	4,38926E+13	4,45724E+14	4,99912E+14	5,70261E+14	5,22618E+14	5,13068E+14
TC0200001716.mm.2	Spred1	sprouty protein with EVH-1 domain 1, related sequence	1,38887E+14	8,54918E+14	2,65452E+14	0.0353982827782038	0.177627138522017	-3,41886E+13	9,1924E+14	9,27601E+14	8,61543E+14	7,07524E+14	8,70928E+13	8,42674E+14
TC0500003296.mm.2	Rimbp2	RIMS binding protein 2	1,21452E+14	7,29876E+14	2,65415E+14	0.0354162105207352	0.177627138522017	-3,41931E+14	7,88246E+14	8,18714E+14	7,39779E+13	6,24278E+14	7,26724E+14	6,81515E+14
TC0700001827.mm.2	Cdipt	CDP-diacylglycerol--inositol 3-phosphatidyltransferase (phosphatidylinositol synthase)	1,38658E+14	8,89461E+14	2,65398E+14	0.0354248768248604	0.177627138522017	-3,41953E+14	9,58086E+14	9,77041E+13	9,02758E+14	7,55054E+14	8,95956E+13	8,47871E+14
TC0400000650.mm.2	Zkscan16	zinc finger with KRAB and SCAN domains 16	1,62031E+14	7,348E+14	2,65398E+13	0.0354248815376597	0.177627138522017	-3,41953E+14	8,52402E+14	8,37406E+14	7,65821E+14	5,99926E+14	7,2555E+14	6,27699E+14
TC0700004404.mm.2	Chst15	carbohydrate sulfotransferase 15	1,51325E+14	7,21155E+14	2,65386E+14	0.0354306226779472	0.177627138522017	-3,41968E+14	8,1864E+14	8,20173E+14	7,50308E+14	5,85855E+14	7,08195E+14	6,43758E+14
TC1200000073.mm.2	Matn3	matrilin 3	-1,33048E+14	4,77619E+14	-2,65258E+13	0.0354938338029856	0.177655045681371	-3,42126E+14	4,12885E+13	3,963E+14	4,82465E+14	5,92815E+14	4,42441E+13	5,38809E+14
TC0700003087.mm.2	Svip	small VCP/p97-interacting protein	-0.770554113605945	8,45272E+14	-2,65212E+14	0.0355164376581671	0.177655045681371	-3,42183E+14	8,05479E+14	7,95945E+14	8,72079E+13	8,83455E+14	8,55173E+14	8,59502E+14
TC0300003239.mm.2	Gm128	predicted gene 128	-1,58539E+14	4,90989E+14	-2,65207E+14	0.0355186688374377	0.177655045681371	-3,42189E+14	4,10261E+14	3,87825E+13	4,88661E+14	6,26503E+14	4,53415E+14	5,7927E+14
TC1700000464.mm.2	Pacsin1	protein kinase C and casein kinase substrate in neurons 1	1,41274E+14	9,04202E+14	2,65198E+14	0.0355230194500707	0.177655045681371	-3,422E+14	1,00722E+14	9,87182E+14	9,27867E+13	7,83986E+13	8,94036E+14	8,2492E+14
TC1100001741.mm.2	Mettl2	methyltransferase like 2	1,58488E+14	5,39083E+14	2,65188E+14	0.0355281913357813	0.177655045681371	-3,42213E+14	6,68533E+14	6,32086E+14	5,54219E+14	4,29424E+14	5,42974E+14	4,07261E+14
TC1900000521.mm.2	Tnks2	tankyrase, TRF1-interacting ankyrin-related ADP-ribose polymerase 2	1,34237E+14	7,75721E+14	2,65184E+14	0.0355299326080189	0.177655045681371	-3,42217E+14	8,60719E+14	8,85643E+13	7,99914E+14	6,77973E+14	7,56413E+14	6,73662E+14
TC0900000105.mm.2	Cwc15	CWC15 spliceosome-associated protein	1,07059E+14	8,58343E+14	2,65167E+14	0.0355383489772947	0.177655045681371	-3,42238E+14	9,44606E+14	9,33995E+13	8,76094E+14	7,88389E+14	8,28697E+14	7,78278E+14
TC0500001741.mm.2	Eif2ak1	eukaryotic translation initiation factor 2 alpha kinase 1	1,27952E+14	7,35046E+14	2,65158E+13	0.0355429731229588	0.177655045681371	-3,4225E+14	8,19012E+14	7,9678E+14	7,45652E+14	6,14236E+13	7,37084E+14	6,97515E+14
TC1800001153.mm.2	Brd8	bromodomain containing 8	0.946050984878836	7,92863E+14	2,65141E+14	0.03555106033727	0.177655045681371	-3,4227E+14	8,72899E+14	8,57619E+14	7,88113E+14	7,53194E+14	7,75476E+14	7,09874E+14
TC1100001564.mm.2	Ppp1r1b	protein phosphatase 1, regulatory inhibitor subunit 1B	1,09036E+14	9,46425E+14	2,65119E+14	0.0355623709093855	0.177655045681371	-3,42298E+14	1,01904E+14	1,02505E+14	9,61427E+14	8,64591E+14	9,24977E+14	8,83463E+14
TC1100001125.mm.2	Git1	GIT ArfGAP 1	1,70964E+14	6,81516E+14	2,65077E+14	0.0355827074152691	0.177655045681371	-3,42349E+14	7,88878E+14	8,15359E+14	7,31525E+13	5,30785E+14	6,32973E+14	5,89573E+14
TC1500001742.mm.2	Top1mt	DNA topoisomerase 1, mitochondrial	1,19749E+14	6,38242E+14	2,65076E+14	0.035583170498644	0.177655045681371	-3,4235E+14	7,41293E+14	7,3523E+14	6,72738E+14	5,64287E+14	5,889E+14	5,27005E+14
TC0100001996.mm.2	Rpl7	ribosomal protein L7	1,36239E+14	9,33917E+14	2,65069E+14	0.0355866452654331	0.177655045681371	-3,42359E+14	1,00711E+14	1,0132E+14	9,13605E+14	8,34224E+14	9,82914E+14	8,52455E+14
TC1900001545.mm.2	Cox15	cytochrome c oxidase assembly protein 15	1,12217E+14	6,00549E+14	2,65062E+14	0.03559054722643	0.177655045681371	-3,42369E+14	6,78748E+14	7,04232E+14	6,3264E+14	5,25906E+14	5,20974E+14	5,40793E+14
TC1700001943.mm.2	Ly6g6d	lymphocyte antigen 6 complex, locus G6D	-0.959491623403908	7,25412E+14	-2,65002E+14	0.035619952338146	0.177746761233988	-3,42442E+13	6,57904E+14	6,76889E+14	7,4641E+14	7,80282E+14	7,11086E+14	7,79897E+14
TC0600000623.mm.2	Hoxa11os	homeobox A11, opposite strand	-0.917666896828288	5,20586E+14	-2,64931E+14	0.0356549131510724	0.177866134861681	-3,4253E+14	4,624E+14	4,62365E+14	5,20513E+14	5,87785E+13	5,3675E+13	5,53704E+14
TC1400001747.mm.2	Ghitm	growth hormone inducible transmembrane protein	2,56545E+14	8,21738E+14	2,64902E+14	0.0356696112235422	0.177884384291221	-3,42566E+13	9,75007E+14	9,83404E+14	8,48632E+14	5,96689E+14	8,71125E+13	6,55571E+14
TC1500000308.mm.2	Dcstamp	dendrocyte expressed seven transmembrane protein	-1,42892E+14	4,68407E+14	-2,64729E+13	0.035755168076243	0.177899094171455	-3,4278E+13	3,95328E+14	3,72258E+13	4,49131E+14	6,0424E+14	5,18525E+14	4,7096E+14
TC0X00001700.mm.2	Arhgap6	Rho GTPase activating protein 6	1,66329E+14	7,09968E+14	2,64726E+14	0.0357564752478415	0.177899094171455	-3,42783E+14	8,5114E+14	8,15054E+14	7,28694E+14	6,04842E+14	7,06708E+13	5,53369E+14
TC0500001076.mm.2	Ephx4	epoxide hydrolase 4	2,40689E+14	7,96337E+14	2,64692E+14	0.0357735687133094	0.177899094171455	-3,42825E+14	9,06286E+14	9,29665E+14	8,01085E+14	5,53489E+14	8,85777E+13	7,0172E+14
TC1100000021.mm.2	Morc2a	microrchidia 2A	1,27813E+14	5,78812E+14	2,64687E+14	0.0357758837390647	0.177899094171455	-3,42831E+14	7,00225E+14	6,48819E+14	5,84048E+14	5,0937E+14	5,68008E+14	4,62403E+14
TC0700004625.mm.2	Kctd13	potassium channel tetramerisation domain containing 13	1,31307E+14	9,02085E+14	2,6466E+14	0.0357894506356268	0.177899094171455	-3,42865E+14	9,95574E+14	9,96205E+13	9,21742E+13	8,07422E+13	8,90424E+14	8,01143E+13
TC0700001739.mm.2	Mettl9	methyltransferase like 9	0.933453456817972	8,05483E+14	2,64652E+14	0.0357932903994455	0.177899094171455	-3,42875E+13	8,54398E+14	8,78082E+14	8,07528E+14	7,38262E+14	7,74895E+13	7,79734E+13
TC1900000607.mm.2	4933411K16Rik/Hoga1	RIKEN cDNA 4933411K16 gene/4-hydroxy-2-oxoglutarate aldolase 1	-1,33678E+14	6,38659E+14	-2,64623E+14	0.0358080760602941	0.177899094171455	-3,42911E+14	5,53544E+14	5,31836E+14	6,04908E+14	7,47828E+14	6,78849E+14	7,14991E+13
TC0500002827.mm.2	Hnrnpd	heterogeneous nuclear ribonucleoprotein D	2,12256E+14	8,17146E+14	2,64573E+14	0.0358327367967783	0.177899094171455	-3,42973E+14	9,50178E+13	9,52869E+14	8,49531E+14	6,29004E+14	8,37329E+14	6,83964E+14
TC0300001449.mm.2	Gtf2b	general transcription factor IIB	0.783868693498522	5,82998E+14	2,64562E+14	0.0358380574039778	0.177899094171455	-3,42986E+14	6,55696E+14	6,37561E+14	5,72693E+14	5,6379E+14	5,42258E+14	5,25992E+14
TC1200000460.mm.2	Srp54b/Srp54a	signal recognition particle 54B/signal recognition particle 54A	1,61856E+14	7,90781E+14	2,64556E+14	0.035841362954833	0.177899094171455	-3,42994E+14	8,54714E+14	9,11986E+14	7,81402E+14	6,61585E+14	8,3801E+14	6,96991E+14
TC0200002748.mm.2	Zfp970	zinc finger protein 970	2,92294E+14	7,13524E+14	2,64547E+13	0.0358455340196634	0.177899094171455	-3,43004E+14	8,38658E+13	8,97079E+14	7,50586E+14	4,00561E+13	7,68649E+14	6,25612E+14
TC0400002372.mm.2	Pm20d2	peptidase M20 domain containing 2	-1,14973E+14	6,84413E+14	-2,64506E+14	0.0358662732484948	0.177899094171455	-3,43056E+14	6,29993E+14	5,95019E+14	6,74549E+14	7,8041E+14	6,95557E+14	7,30949E+14
TC1400002002.mm.2	Rnase2a	ribonuclease, RNase A family, 2A (liver, eosinophil-derived neurotoxin)	-1,26671E+14	5,91436E+14	-2,64496E+14	0.0358710118386502	0.177899094171455	-3,43068E+14	5,05586E+14	4,96988E+14	5,68782E+14	6,87135E+14	6,10424E+14	6,79703E+14
TC0300001242.mm.2	Abca4	ATP-binding cassette, sub-family A (ABC1), member 4	-1,31994E+14	5,87142E+12	-2,64456E+14	0.0358908860561131	0.177899094171455	-3,43117E+14	4,99255E+14	5,01691E+14	5,73973E+14	6,90961E+14	5,84525E+14	6,72445E+13
TC0200002452.mm.2	Top1	topoisomerase (DNA) I	0.894702351169141	9,25055E+14	2,6445E+14	0.0358939378629169	0.177899094171455	-3,43125E+14	9,84516E+14	9,89149E+13	9,20333E+14	8,74391E+14	9,13172E+14	8,6877E+12
TC0200001998.mm.2	Atrn	attractin	1,43983E+14	8,48209E+14	2,64419E+14	0.0359093268312269	0.177899094171455	-3,43163E+14	9,48991E+14	9,49927E+13	8,61062E+14	7,49888E+14	8,5125E+14	7,28137E+14
TC0400004093.mm.2	Hes3	hes family bHLH transcription factor 3	-1,03488E+14	6,20063E+14	-2,64418E+14	0.0359097595309447	0.177899094171455	-3,43164E+14	5,36991E+14	5,64316E+14	6,28134E+14	6,80149E+14	6,17875E+14	6,92913E+14
TC0400003584.mm.2	Hpca	hippocalcin	1,50968E+14	1,12063E+14	2,64407E+14	0.0359155176598514	0.177899094171455	-3,43178E+14	1,21015E+14	1,22601E+14	1,14106E+14	9,93161E+14	1,1246E+14	1,02877E+14
TC1000001226.mm.2	1700017N19Rik	RIKEN cDNA 1700017N19 gene	-1,4769E+14	5,07343E+14	-2,64368E+14	0.0359348227718892	0.177899094171455	-3,43226E+14	4,40897E+14	4,12009E+14	5,18116E+14	6,3017E+14	4,57928E+14	5,84937E+14
TC0200005104.mm.2	Kcns1	K+ voltage-gated channel, subfamily S, 1	-0.904813770697713	6,76792E+14	-2,64355E+14	0.0359411980429737	0.177899094171455	-3,43242E+14	6,38963E+14	6,02198E+14	6,83994E+14	7,3813E+14	6,92276E+14	7,05189E+14
TC0800002508.mm.2	Pou4f2	POU domain, class 4, transcription factor 2	-1,11453E+13	5,6007E+14	-2,64319E+14	0.0359591335067622	0.177899094171455	-3,43286E+14	4,60236E+14	4,80734E+13	5,36007E+14	6,27868E+14	5,98672E+14	6,56902E+14
TC1100001524.mm.2	Prr15l	proline rich 15-like	-1,33156E+13	5,34204E+14	-2,64318E+14	0.0359599565961995	0.177899094171455	-3,43288E+14	4,88763E+14	3,89634E+14	5,27505E+14	6,17203E+14	6,2327E+14	5,58848E+14
TC0100002228.mm.2	Lonrf2	LON peptidase N-terminal domain and ring finger 2	2,27737E+14	7,82141E+14	2,64305E+14	0.0359662191550602	0.177899094171455	-3,43304E+14	9,54496E+14	9,31431E+14	8,51783E+14	5,7867E+14	7,37595E+14	6,38871E+14
TC1400000320.mm.2	Pbrm1	polybromo 1	1,00578E+14	7,0079E+13	2,64301E+14	0.0359684111085994	0.177899094171455	-3,43309E+14	7,80171E+13	7,71576E+14	6,67835E+14	6,82756E+14	6,95108E+14	6,07298E+14
TC0500002803.mm.2	Gk2	glycerol kinase 2	-0.990522443086905	6,94565E+14	-2,64287E+14	0.0359754967362277	0.177899094171455	-3,43327E+14	6,14612E+14	6,17446E+14	6,98311E+14	7,31852E+14	7,1285E+14	7,92318E+14
TC1700002549.mm.2	Fez2	fasciculation and elongation protein zeta 2 (zygin II)	1,57801E+14	6,43809E+14	2,64277E+14	0.035980219773094	0.177899094171455	-3,43338E+14	7,10767E+14	7,23806E+14	6,53108E+14	4,65862E+14	6,5592E+14	6,53393E+14
TC0300002711.mm.2	Slc6a17	solute carrier family 6 (neurotransmitter transporter), member 17	2,84546E+14	8,73106E+14	2,64266E+12	0.035985761061175	0.177899094171455	-3,43352E+14	1,03542E+14	1,06556E+14	9,25017E+14	6,06862E+14	8,98237E+14	7,07549E+14
TC0200004926.mm.2	Plagl2	pleiomorphic adenoma gene-like 2	-0.94477160392832	7,39598E+14	-2,64239E+14	0.0359993883407315	0.177899094171455	-3,43386E+14	6,75514E+14	6,76008E+14	8,08339E+14	7,32137E+14	7,70742E+14	7,74846E+14
TC0100003378.mm.2	Fasl	Fas ligand (TNF superfamily, member 6)	-0.910912881248507	4,77916E+13	-2,64215E+14	0.0360114551028579	0.177899094171455	-3,43416E+14	3,97814E+14	4,13799E+14	4,68998E+14	5,24797E+14	5,13015E+14	5,49073E+14
TC1100000576.mm.2	Sar1b	secretion associated Ras related GTPase 1B	1,77406E+14	8,26883E+14	2,6419E+14	0.0360238554013563	0.177899094171455	-3,43446E+14	9,32313E+13	9,32606E+14	8,54873E+13	6,55234E+14	8,36501E+14	7,49774E+14
TC0500002244.mm.2	Lrpap1	low density lipoprotein receptor-related protein associated protein 1	1,29811E+14	8,8808E+14	2,64187E+14	0.0360251193590081	0.177899094171455	-3,43449E+14	9,8463E+14	9,60432E+14	9,10283E+14	7,75157E+14	8,6892E+14	8,29056E+14
TC1900001026.mm.2	Fkbp2	FK506 binding protein 2	1,52713E+14	7,95521E+14	2,64186E+14	0.0360258638541213	0.177899094171455	-3,43451E+14	9,00182E+14	8,901E+14	7,93872E+13	6,90984E+14	8,22597E+14	6,75389E+14
TC0900002333.mm.2	Etfa	electron transferring flavoprotein, alpha polypeptide	1,1367E+14	6,8441E+14	2,64129E+14	0.0360546129879877	0.177967733835449	-3,43522E+14	7,84098E+14	7,78015E+14	7,23182E+14	6,11592E+14	5,91435E+14	6,18138E+14
TC0300002977.mm.2	Arhgef38	Rho guanine nucleotide exchange factor (GEF) 38	-1,25162E+14	6,16679E+14	-2,64112E+14	0.0360630962627822	0.177967733835449	-3,43543E+14	5,36252E+14	5,34319E+14	6,02558E+14	7,18337E+14	6,19457E+14	6,89153E+14
TC1500000215.mm.2	Tas2r119	taste receptor, type 2, member 119	-1,15454E+14	4,49681E+14	-2,64092E+14	0.0360729967761457	0.177967733835449	-3,43568E+14	3,49867E+14	3,79852E+13	4,40613E+13	5,20015E+14	4,62763E+14	5,44978E+14
TC0800002339.mm.2	Psd3	pleckstrin and Sec7 domain containing 3	2,73337E+14	7,84617E+14	2,6407E+14	0.0360839437994422	0.177967733835449	-3,43595E+14	9,28739E+14	9,32324E+14	8,16165E+14	4,98223E+14	8,46874E+13	6,85376E+14
TC0100002888.mm.2	Pign	phosphatidylinositol glycan anchor biosynthesis, class N	1,14971E+14	5,16204E+14	2,63999E+14	0.036119294073536	0.178087572033931	-3,43682E+14	5,51011E+14	6,13626E+14	5,13702E+14	4,20994E+14	5,1669E+13	4,81203E+14
TC0X00000479.mm.2	Rtl8c	retrotransposon Gag like 8C	1,41894E+14	9,37211E+14	2,63966E+14	0.036136284555123	0.178115670969722	-3,43724E+13	1,048E+14	9,98229E+14	9,54343E+14	8,081E+14	9,33189E+14	8,81402E+14
TC0400001969.mm.2	Gpr157	G protein-coupled receptor 157	-0.913385437049287	6,21767E+14	-2,63944E+14	0.0361471013511446	0.178115670969722	-3,4375E+14	5,39538E+14	5,97E+14	6,42985E+14	6,7623E+13	6,47131E+14	6,2772E+14
TC0300002347.mm.2	Efna4	ephrin A4	-0.958185539931739	6,85322E+14	-2,63823E+14	0.0362080911622317	0.178254898609949	-3,43901E+14	6,0326E+14	6,24556E+14	6,72114E+14	7,47339E+14	7,24662E+14	7,39999E+14
TC0900002680.mm.2	Tmod2	tropomodulin 2	1,12745E+14	8,87663E+14	2,63813E+14	0.0362131175135324	0.178254898609949	-3,43913E+13	9,62448E+13	9,51852E+14	8,86507E+14	8,02302E+14	8,99082E+14	8,23784E+14
TC0400003888.mm.2	Plekhm2	pleckstrin homology domain containing, family M (with RUN domain) member 2	2,22235E+13	7,43497E+14	2,63782E+14	0.0362285721696549	0.178254898609949	-3,43951E+14	9,02908E+14	8,71353E+14	7,6373E+14	5,66061E+14	7,79214E+12	5,77713E+14
TC1300002144.mm.2	Tpbpa	trophoblast specific protein alpha	-0.991674081949938	4,54084E+14	-2,63763E+14	0.0362380127190801	0.178254898609949	-3,43974E+13	3,91653E+14	3,71702E+14	4,39754E+14	5,21936E+14	5,09856E+14	4,89602E+13
TC0200000420.mm.2	Fbxw5	F-box and WD-40 domain protein 5	1,82722E+14	8,53703E+14	2,63749E+14	0.0362450260314665	0.178254898609949	-3,43991E+14	9,7602E+14	9,5835E+14	8,95111E+14	6,73814E+14	8,3083E+13	7,88094E+14
TC0600003417.mm.2	Kras	Kirsten rat sarcoma viral oncogene homolog	2,53537E+14	7,00828E+14	2,63708E+14	0.0362660161309782	0.178254898609949	-3,44043E+14	8,48939E+14	8,44047E+14	7,24184E+14	4,61728E+14	7,62232E+13	5,63839E+14
TC1700001811.mm.2	Tff3	trefoil factor 3, intestinal	-0.93579111283405	5,0583E+14	-2,63705E+14	0.0362675225919544	0.178254898609949	-3,44047E+14	4,47102E+14	4,33107E+14	5,14108E+14	5,53259E+14	5,12679E+14	5,74723E+14
TC0900002526.mm.2	Usp3	ubiquitin specific peptidase 3	1,08132E+14	6,80335E+14	2,63697E+14	0.0362712219673747	0.178254898609949	-3,44056E+14	7,68201E+14	7,59579E+14	6,87027E+14	6,24489E+14	6,6326E+14	5,79455E+14
TC1700000628.mm.2	H2-Eb2	histocompatibility 2, class II antigen E beta2	-0.970890142544598	5,74587E+14	-2,63674E+13	0.0362832263837883	0.178254898609949	-3,44085E+14	4,92067E+14	5,08353E+14	5,62151E+14	6,32447E+14	6,06169E+14	6,46336E+14
TC0X00001955.mm.2	Klhl13	kelch-like 13	1,83274E+14	7,95179E+14	2,63668E+14	0.0362859844498624	0.178254898609949	-3,44092E+14	9,24041E+14	9,03214E+14	8,31474E+14	6,29232E+14	7,92022E+14	6,9109E+14
TC1900001023.mm.2	Gpr137	G protein-coupled receptor 137	1,37199E+14	7,30665E+14	2,63589E+14	0.03632575759263	0.178318564756308	-3,44189E+14	8,15513E+14	7,8682E+14	7,37729E+14	5,90207E+14	7,4711E+14	7,06608E+14
TC0X00003236.mm.2	Sat1	spermidine/spermine N1-acetyl transferase 1	3,2719E+14	6,27511E+14	2,63576E+14	0.036332184975269	0.178318564756308	-3,44205E+14	7,77193E+14	8,30683E+14	6,78246E+14	2,7525E+14	6,76516E+14	5,27178E+14
TC0100002696.mm.2	Dner	delta/notch-like EGF repeat containing	1,56582E+14	7,45484E+14	2,63501E+14	0.0363704535614963	0.178318564756308	-3,44299E+14	8,80621E+14	8,29966E+14	7,83785E+14	6,13637E+14	7,11092E+14	6,53802E+14
TC1100004117.mm.2	Qrich2	glutamine rich 2	-0.824371228695099	4,93038E+14	-2,63488E+14	0.0363767157767494	0.178318564756308	-3,44314E+14	4,44572E+14	4,4796E+14	5,06566E+14	5,5084E+14	5,0416E+14	5,04128E+14
TC0X00002415.mm.2	Cetn2	centrin 2	0.881802461097863	6,18993E+14	2,63468E+14	0.0363869756466723	0.178318564756308	-3,44339E+14	6,88578E+14	6,71425E+14	6,14018E+13	5,69625E+11	6,04676E+13	5,65636E+14
TC0200004283.mm.2	Arl14ep	ADP-ribosylation factor-like 14 effector protein	1,98712E+14	6,78077E+14	2,63456E+14	0.0363930937017182	0.178318564756308	-3,44354E+14	8,13622E+14	7,98628E+14	7,14157E+13	5,00669E+14	6,85401E+14	5,55986E+14
TC0700002498.mm.2	Tomm40	translocase of outer mitochondrial membrane 40	0.917550341690257	6,99945E+14	2,63437E+14	0.0364025948122971	0.178318564756308	-3,44378E+14	7,69301E+14	7,51004E+14	6,72207E+14	6,64588E+14	7,05168E+13	6,37405E+14
TC0X00001277.mm.2	Drp2	dystrophin related protein 2	0.761795969903166	6,62651E+14	2,63407E+14	0.0364180928627713	0.178318564756308	-3,44416E+14	7,08538E+14	7,04948E+14	6,30859E+14	6,30268E+14	6,56268E+14	6,45026E+13
TC1100001770.mm.2	Dcaf7	DDB1 and CUL4 associated factor 7	1,29327E+14	8,31021E+14	2,63386E+14	0.0364284261951348	0.178318564756308	-3,44441E+14	9,24636E+14	9,24236E+14	8,53131E+14	7,37086E+14	8,15124E+14	7,31914E+14
TC0900000026.mm.2	Msantd4	Myb/SANT-like DNA-binding domain containing 4 with coiled-coils	1,41056E+14	7,01217E+14	2,63384E+14	0.0364296994196252	0.178318564756308	-3,44444E+14	7,88945E+14	7,76023E+14	7,17416E+13	5,6544E+14	7,07157E+14	6,52322E+14
TC1000000929.mm.2	Hcfc2	host cell factor C2	1,52093E+14	5,77637E+14	2,63313E+14	0.0364653295168198	0.178318564756308	-3,44531E+14	6,68499E+14	6,33961E+14	5,84343E+14	4,13931E+14	6,05234E+14	5,59855E+14
TC1800001147.mm.2	Reep5	receptor accessory protein 5	1,52335E+14	9,13121E+14	2,63306E+14	0.0364691740556185	0.178318564756308	-3,4454E+14	9,99042E+14	9,99731E+14	9,33139E+14	7,60963E+14	9,1932E+14	8,66532E+14
TC1500000139.mm.2	Cdh12	cadherin 12	1,92328E+14	6,06774E+14	2,63303E+14	0.036470300717728	0.178318564756308	-3,44543E+14	7,1936E+14	7,27799E+14	5,9499E+14	4,67512E+14	6,67574E+14	4,63412E+14
TC0100003803.mm.2	Fam71a	family with sequence similarity 71, member A	-0.809306528894973	4,88033E+14	-2,63286E+14	0.0364788833904038	0.178318564756308	-3,44564E+14	4,41496E+14	4,4537E+14	5,04937E+14	5,4379E+14	4,94742E+14	4,97863E+14
TC0400001607.mm.2	Map3k6	mitogen-activated protein kinase kinase kinase 6	-1,15023E+14	4,57561E+14	-2,63247E+14	0.0364989784643385	0.178318564756308	-3,44613E+14	3,55266E+14	3,68846E+14	4,22293E+14	5,31866E+14	5,12654E+14	5,54439E+14
TC1100000693.mm.2	Olfr324	olfactory receptor 324	-1,16906E+14	4,3265E+14	-2,63206E+14	0.0365194679993869	0.178318564756308	-3,44663E+14	3,42201E+13	3,72046E+14	4,25838E+14	5,22221E+14	4,3298E+14	5,00613E+14
TC0100003882.mm.2	Chpf	chondroitin polymerizing factor	1,24068E+13	7,65778E+14	2,63176E+14	0.0365348940120142	0.178318564756308	-3,44701E+13	8,48443E+14	8,54168E+13	7,76887E+14	6,77588E+14	7,63716E+14	6,73869E+14
TC1400002071.mm.2	Slc22a17	solute carrier family 22 (organic cation transporter), member 17	1,7336E+14	1,09386E+14	2,63155E+14	0.036545367012347	0.178318564756308	-3,44726E+14	1,21096E+14	1,19516E+14	1,12945E+14	9,29943E+14	1,08467E+14	1,01295E+14
TC1100001776.mm.2	Ddx42	DEAD (Asp-Glu-Ala-Asp) box polypeptide 42	1,19676E+14	7,14431E+14	2,63149E+14	0.0365486619758786	0.178318564756308	-3,44734E+14	8,21628E+14	8,13614E+14	7,28783E+14	6,67108E+14	6,8276E+14	5,72692E+14
TC0400000437.mm.2	Ccdc107	coiled-coil domain containing 107	-1,11509E+14	7,60237E+14	-2,63124E+14	0.0365615715581429	0.178318564756308	-3,44766E+14	7,07067E+14	6,83613E+14	7,69356E+14	8,44342E+14	7,38812E+14	8,18234E+14

TC0400003506.mm.2	Utp11	UTP11 small subunit processome component	1,25779E+14	6,0936E+13	2,63116E+14	0.0365655655280292	0.178318564756308	-3,44775E+14	6,98539E+14	6,95495E+14	6,18247E+14	5,24229E+14	6,09635E+14	5,10014E+14
TC1000000881.mm.2	Map2k2	mitogen-activated protein kinase kinase 2	0.854887661870286	7,96954E+14	2,63111E+14	0.0365681033045924	0.178318564756308	-3,44782E+14	8,56373E+14	8,42501E+14	7,76264E+14	7,51633E+14	8,00719E+14	7,54235E+14
TC1100004202.mm.2	Actg1/Mir6935	actin, gamma, cytoplasmic 1/microRNA 6935	1,1953E+14	1,00811E+14	2,6309E+14	0.0365787204101065	0.178318564756308	-3,44807E+14	1,08823E+13	1,07508E+14	1,02475E+14	8,99497E+14	9,84702E+14	9,76393E+14
TC0200005105.mm.2	Wfdc5	WAP four-disulfide core domain 5	-0.810205049388678	4,84863E+14	-2,63058E+14	0.0365948393012466	0.178318564756308	-3,44847E+14	4,24281E+14	4,50967E+14	5,32501E+14	5,04788E+14	5,03379E+14	4,93263E+14
TC0300003067.mm.2	Lmo4	LIM domain only 4	0.881167868899908	8,48175E+14	2,63045E+14	0.0366016520552541	0.178318564756308	-3,44863E+13	8,56641E+14	9,1627E+14	8,04904E+14	7,91773E+14	8,64387E+14	8,55076E+14
TC0600003346.mm.2	Lmo3	LIM domain only 3	1,91671E+14	8,46755E+14	2,63033E+14	0.0366076690032308	0.178318564756308	-3,44878E+14	9,83254E+14	9,83132E+14	8,77841E+14	7,05205E+14	8,49025E+14	6,82075E+14
TC0800000623.mm.2	Fbxo8	F-box protein 8	-0.766708989356321	8,69856E+13	-2,6301E+14	0.0366194117157958	0.178318564756308	-3,44906E+14	8,47035E+14	8,31731E+14	9,12547E+14	9,19561E+14	8,51471E+14	8,56791E+14
TC0X00002320.mm.2	Cdr1	cerebellar degeneration related antigen 1	-1,32574E+14	1,15796E+14	-2,62976E+14	0.0366368674788638	0.178318564756308	-3,44949E+14	1,04135E+14	1,08203E+14	1,24633E+14	1,14219E+14	1,26682E+14	1,16901E+14
TC0200004396.mm.2	Rasgrp1	RAS guanyl releasing protein 1	1,7671E+14	9,2685E+14	2,62967E+14	0.0366413019093409	0.178318564756308	-3,4496E+14	1,03008E+14	1,05133E+14	9,56601E+14	7,71388E+13	9,32209E+14	8,19496E+14
TC1700001974.mm.2	Vars2	valyl-tRNA synthetase 2, mitochondrial	-0.79177106952825	6,09322E+14	-2,62945E+14	0.0366524707058915	0.178318564756308	-3,44987E+14	5,55899E+14	5,61709E+14	6,24678E+14	6,51284E+14	6,22491E+14	6,39871E+14
TC1000000850.mm.2	Rps15	ribosomal protein S15	0.807891800577169	1,09694E+14	2,62933E+14	0.0366585859595559	0.178318564756308	-3,45002E+14	1,15968E+14	1,15947E+13	1,09785E+14	1,05972E+14	1,05666E+14	1,04826E+14
TC1000001599.mm.2	Cs	citrate synthase	1,6479E+14	8,23582E+14	2,62902E+14	0.0366743818525726	0.178318564756308	-3,4504E+14	9,37022E+14	9,4636E+14	8,63235E+14	6,90565E+14	8,02183E+14	7,02125E+14
TC0900003293.mm.2	Zfp445	zinc finger protein 445	1,47858E+14	6,8749E+14	2,629E+14	0.0366751936799147	0.178318564756308	-3,45042E+14	7,9977E+14	7,92686E+13	7,23648E+14	5,73092E+14	6,60726E+13	5,75017E+14
TC0800000831.mm.2	Nwd1	NACHT and WD repeat domain containing 1	1,27829E+14	6,14534E+14	2,62892E+14	0.0366796954209574	0.178318564756308	-3,45053E+14	6,71645E+14	7,2687E+14	5,53916E+14	5,88941E+14	5,10639E+14	6,35194E+14
TC0400000294.mm.2	Cnr1	cannabinoid receptor 1 (brain)	2,47343E+14	7,26603E+14	2,62879E+12	0.0366862807774568	0.178318564756308	-3,45069E+14	8,55826E+14	8,61742E+14	7,58602E+14	4,6428E+14	7,73972E+14	6,45197E+14
TC0500001162.mm.2	Pitpnb	phosphatidylinositol transfer protein, beta	1,42958E+14	8,61448E+14	2,62852E+14	0.0366999956562465	0.178331432451478	-3,45102E+14	9,62846E+14	9,61608E+14	8,89512E+14	7,49026E+13	8,46688E+14	7,59007E+14
TC0300001173.mm.2	Col11a1	collagen, type XI, alpha 1	-1,03554E+14	6,00005E+14	-2,62815E+13	0.036718833289549	0.178334548096029	-3,45148E+14	5,25341E+14	5,63002E+14	6,08844E+14	6,86606E+14	5,86415E+14	6,29822E+14
TC1700001603.mm.2	Kremen2	kringle containing transmembrane protein 2	-1,13179E+14	6,22902E+14	-2,62807E+14	0.0367227723318204	0.178334548096029	-3,45157E+13	5,37721E+14	5,69041E+14	6,20046E+14	7,13074E+14	6,18765E+14	6,78766E+14
TC1900001030.mm.2	Fermt3	fermitin family member 3	0.819509622507321	5,34419E+14	2,62772E+14	0.0367405335615075	0.178336881342954	-3,45201E+13	5,53624E+14	5,89687E+14	4,83948E+14	4,95461E+14	5,31193E+14	5,52603E+14
TC0200000801.mm.2	Kif5c	kinesin family member 5C	1,89993E+14	9,35824E+14	2,62763E+14	0.036745388571874	0.178336881342954	-3,45212E+14	1,05875E+14	1,05074E+14	9,6784E+14	7,61664E+14	9,47241E+14	8,28708E+14
TC0300000118.mm.2	Bhlhe22	basic helix-loop-helix family, member e22	-1,07522E+14	6,63607E+14	-2,62647E+14	0.0368046227911689	0.178511972790467	-3,45356E+14	5,96726E+14	5,96185E+14	6,51846E+13	7,56108E+14	6,8634E+14	6,94436E+14
TC0600001293.mm.2	Washc2	WASH complex subunit 2`	1,42239E+14	7,74533E+14	2,62618E+14	0.0368194628514789	0.178511972790467	-3,45392E+14	8,6362E+14	8,4934E+14	7,85227E+14	6,43255E+14	7,93961E+14	7,11795E+14
TC0100002499.mm.2	Lancl1	LanC (bacterial lantibiotic synthetase component C)-like 1	1,37083E+14	6,83506E+14	2,62607E+13	0.0368251908708017	0.178511972790467	-3,45405E+14	7,86109E+14	7,73172E+14	7,17132E+14	5,67983E+14	6,50766E+14	6,05872E+14
TC0200005378.mm.2	Gm14326	predicted gene 14326	2,1674E+14	8,77791E+14	2,62606E+14	0.0368257802740342	0.178511972790467	-3,45407E+14	9,78918E+14	1,02597E+13	8,79449E+14	6,91955E+13	9,44759E+14	7,45696E+14
TC1100000609.mm.2	Kif3a	kinesin family member 3A	2,22997E+14	7,95293E+14	2,6258E+14	0.0368386815373277	0.178520804695026	-3,45438E+14	9,43996E+14	9,31269E+14	8,45108E+14	5,84163E+14	7,92533E+14	6,74687E+14
TC1700000060.mm.2	Dynlt1b	dynein light chain Tctex-type 1B	-2,06841E+14	8,94174E+14	-2,62521E+14	0.0368692805606056	0.178615369113962	-3,45512E+14	8,37451E+14	7,18068E+14	1,08333E+14	8,85871E+14	9,83372E+14	8,56952E+13
TC0700003004.mm.2	Snrnp70	small nuclear ribonucleoprotein 70 (U1)	0.969080104244222	8,33468E+14	2,62455E+13	0.0369031098078111	0.178725520989859	-3,45594E+14	8,73728E+14	8,96998E+14	7,71704E+14	8,05206E+14	8,62824E+14	7,90347E+14
TC1000000937.mm.2	Chst11	carbohydrate sulfotransferase 11	0.798074788751871	8,2177E+14	2,62322E+14	0.0369711188004109	0.178938642979297	-3,45758E+14	8,8196E+14	8,75758E+14	8,16668E+14	7,81435E+14	7,88686E+14	7,86114E+14
TC0900002812.mm.2	Trim43b	tripartite motif-containing 43B	-0.859521884528643	6,05791E+14	-2,62312E+14	0.0369766387063917	0.178938642979297	-3,45771E+14	5,54665E+14	5,78856E+13	6,27439E+14	6,77988E+14	6,01463E+14	5,94335E+14
TC0600000714.mm.2	Herc3	hect domain and RLD 3	1,09844E+14	7,34785E+14	2,62304E+14	0.0369804307776988	0.178938642979297	-3,4578E+14	7,88927E+14	8,19191E+14	7,4635E+14	6,42079E+14	7,12166E+14	6,99995E+13
TC1500000102.mm.2	Gm2573	predicted gene 2573	0.763236877365179	7,48177E+14	2,6218E+14	0.0370444105209922	0.179193798759558	-3,45934E+14	8,31837E+14	8,25558E+14	7,50318E+14	7,5443E+13	6,55955E+13	6,70964E+14
TC0200005475.mm.2	Dusp19	dual specificity phosphatase 19	1,51202E+14	5,85187E+14	2,62136E+14	0.0370671626550259	0.179193798759558	-3,45989E+14	6,82117E+14	6,89902E+14	6,10223E+14	4,59392E+14	5,83125E+14	4,86366E+14
TC0500002573.mm.2	Nfxl1	nuclear transcription factor, X-box binding-like 1	-0.941512064591056	8,17096E+14	-2,62123E+13	0.0370740197241761	0.179193798759558	-3,46005E+14	7,20968E+14	7,54544E+14	7,97118E+14	8,66697E+14	8,80335E+14	8,82914E+14
TC0900002254.mm.2	Ncam1	neural cell adhesion molecule 1	2,47855E+14	9,41447E+14	2,62114E+14	0.0370782301137743	0.179193798759558	-3,46015E+14	1,12215E+14	1,09139E+14	1,00658E+13	7,1125E+14	9,22328E+13	7,94983E+13
TC0900001409.mm.2	Usp19	ubiquitin specific peptidase 19	0.982588344676231	6,72851E+14	2,62077E+14	0.0370973009579567	0.179193798759558	-3,46061E+14	7,26093E+14	7,26346E+14	6,5442E+14	6,01502E+14	6,98507E+14	6,30239E+14
TC1100002686.mm.2	Prop1	paired like homeodomain factor 1	-1,01979E+14	4,96191E+14	-2,62072E+14	0.0370998890747773	0.179193798759558	-3,46067E+14	4,5074E+14	4,30738E+14	5,44068E+14	5,41367E+14	4,59155E+14	5,51074E+14
TC1600001971.mm.2	Cyyr1	cysteine and tyrosine-rich protein 1	-0.95127714371577	6,75174E+13	-2,62038E+14	0.037117513140183	0.179225199049376	-3,4611E+14	6,35435E+14	5,98261E+13	6,85148E+14	7,38804E+14	6,73942E+14	7,19453E+14
TC1700002351.mm.2	Tnfsf14	tumor necrosis factor (ligand) superfamily, member 14	-1,35762E+13	6,99495E+14	-2,61893E+14	0.0371925029646828	0.179279585302061	-3,46289E+14	6,08759E+14	6,18435E+14	7,05677E+13	7,9304E+13	6,71855E+14	7,99204E+14
TC1200001023.mm.2	Unc79	unc-79 homolog	1,95792E+14	8,42765E+14	2,61885E+14	0.0371967643158754	0.179279585302061	-3,463E+14	9,61332E+14	9,6489E+14	8,76301E+14	6,58336E+14	8,56494E+14	7,3924E+14
TC0900003310.mm.2	Lztfl1	leucine zipper transcription factor-like 1	2,40131E+14	6,81507E+14	2,6186E+14	0.037209767502226	0.179279585302061	-3,46331E+14	8,27866E+14	8,17134E+14	7,04505E+14	4,60233E+14	7,36803E+14	5,42502E+14
TC1700002438.mm.2	Ptprm	protein tyrosine phosphatase, receptor type, M	1,48057E+14	6,51908E+14	2,61799E+14	0.0372414608239369	0.179279585302061	-3,46406E+14	7,79362E+14	7,31623E+14	6,60154E+14	5,54718E+14	6,58219E+14	5,27371E+14
TC0400002712.mm.2	Frrs1l	ferric-chelate reductase 1 like	1,25546E+14	9,20671E+14	2,61755E+14	0.0372638632111813	0.179279585302061	-3,4646E+14	9,96915E+14	1,0186E+14	9,02732E+14	8,61694E+13	9,36877E+14	8,07206E+14
TC1000001282.mm.2	E2f7	E2F transcription factor 7	-1,43807E+14	7,20405E+14	-2,6172E+14	0.0372820473731138	0.179279585302061	-3,46503E+14	6,08262E+14	6,31121E+14	6,85632E+14	8,41366E+13	7,4871E+14	8,07337E+14
TC0800002585.mm.2	Ier2	immediate early response 2	-0.764107195528231	5,11911E+14	-2,61699E+14	0.037293198568895	0.179279585302061	-3,4653E+14	4,58695E+14	4,56533E+14	5,41955E+14	5,26093E+14	5,48849E+12	5,39341E+14
TC0800001118.mm.2	Ccl22	chemokine (C-C motif) ligand 22	-0.876875885390959	5,92386E+14	-2,6168E+14	0.0373029356305793	0.179279585302061	-3,46553E+14	5,41605E+14	5,42392E+14	5,98819E+14	6,60553E+13	6,03721E+13	6,07225E+14
TC1100001799.mm.2	Psmd12	proteasome (prosome, macropain) 26S subunit, non-ATPase, 12	1,8307E+14	8,07917E+14	2,61651E+14	0.0373179215750131	0.179279585302061	-3,46589E+14	9,24667E+13	9,21286E+14	8,37614E+13	6,42201E+14	8,20333E+14	7,01397E+14
TC1100001499.mm.2	Hoxb3	homeobox B3	-1,25217E+14	7,056E+14	-2,61645E+14	0.0373209963066208	0.179279585302061	-3,46596E+14	6,1542E+14	6,46285E+14	6,96722E+14	8,15418E+14	6,99066E+14	7,60692E+13
TC1400001275.mm.2	Gpr180	G protein-coupled receptor 180	0.761285201168426	6,04993E+14	2,61638E+14	0.0373247681858828	0.179279585302061	-3,46605E+13	6,68326E+14	6,53084E+14	5,81471E+12	5,87682E+14	5,69783E+14	5,69614E+14
TC0300000805.mm.2	S100a2	S100 calcium binding protein A2	-0.774406560387888	5,55268E+14	-2,61627E+14	0.0373302975333104	0.179279585302061	-3,46619E+14	4,81956E+14	5,04136E+14	5,7101E+14	5,69964E+14	5,95997E+14	6,08541E+14
TC1000002353.mm.2	Bicc1	BicC family RNA binding protein 1	-0.8344047660529	6,36981E+14	-2,61614E+14	0.0373370526045542	0.179279585302061	-3,46635E+14	5,58916E+14	5,99132E+13	6,58537E+14	6,66392E+14	6,83476E+14	6,55434E+14
TC0600000542.mm.2	Zfp212	Zinc finger protein 212	-0.872178819358821	6,67742E+14	-2,61613E+14	0.0373377274647454	0.179279585302061	-3,46636E+14	5,92364E+13	6,27169E+14	6,80199E+14	7,13769E+14	6,78182E+14	7,14769E+14
TC0600002154.mm.2	Try5	trypsin 5	-1,30143E+14	4,75158E+13	-2,61609E+14	0.0373396285807925	0.179279585302061	-3,46641E+13	3,89857E+14	3,47748E+14	4,75044E+13	5,22847E+14	4,92243E+14	6,23211E+14
TC0100000883.mm.2	Prlh	prolactin releasing hormone	-0.993354523116338	7,69333E+14	-2,61595E+14	0.037347005661722	0.179279585302061	-3,46659E+14	7,14637E+14	7,03505E+14	7,76713E+13	8,401E+14	7,59758E+14	8,21285E+14
TC1500002180.mm.2	Ccnt1	cyclin T1	1,52479E+14	6,1279E+14	2,61587E+13	0.0373512515577221	0.179279585302061	-3,46669E+14	7,26993E+14	7,19119E+14	6,37225E+14	5,0393E+14	6,06486E+14	4,82986E+13
TC0700003586.mm.2	Cib1	calcium and integrin binding 1 (calmyrin)	-1,02662E+13	6,7452E+14	-2,61577E+14	0.0373565240975834	0.179279585302061	-3,46681E+14	5,85745E+14	6,24791E+13	6,76175E+13	7,39685E+14	6,80216E+14	7,40508E+14
TC0400003124.mm.2	Dock7	dedicator of cytokinesis 7	1,41756E+14	5,31486E+14	2,61568E+13	0.0373613161183872	0.179279585302061	-3,46693E+14	6,26535E+14	6,00137E+14	5,23287E+14	4,19874E+14	5,72323E+14	4,46762E+14
TC1100003760.mm.2	Krt14	keratin 14	-1,12246E+14	6,53582E+14	-2,61535E+14	0.0373782113415975	0.179279585302061	-3,46733E+14	5,86278E+14	5,88101E+14	6,42652E+13	7,56218E+14	6,67571E+14	6,80674E+14
TC1100000240.mm.2	Usp34	ubiquitin specific peptidase 34	2,61745E+13	6,78609E+14	2,61488E+14	0.0374025779316137	0.179279585302061	-3,46791E+14	8,86242E+14	8,73373E+14	6,54681E+14	5,81444E+13	7,05825E+14	3,70088E+14
TC1400000455.mm.2	Ang3/Ang5	angiogenin, ribonuclease A family, member 3/angiogenin, ribonuclease A family, member 5	-1,11854E+14	4,67255E+14	-2,61478E+14	0.0374077671172616	0.179279585302061	-3,46803E+14	3,91865E+14	3,93391E+14	4,51447E+14	5,57517E+14	4,84258E+12	5,2505E+14
TC0900000748.mm.2	Hexa	hexosaminidase A	0.897406157932568	7,76561E+14	2,6146E+13	0.0374174163307021	0.179279585302061	-3,46826E+14	8,13145E+14	8,52263E+14	7,55498E+14	7,30429E+14	7,78382E+14	7,29649E+14
TC0X00003361.mm.2	Prps2	phosphoribosyl pyrophosphate synthetase 2	0.787070052145745	7,39279E+14	2,6145E+14	0.0374223432771422	0.179279585302061	-3,46838E+14	7,9149E+14	7,77803E+14	7,19282E+14	6,92598E+14	7,33131E+14	7,21369E+14
TC0200000535.mm.2	Ptpa	protein phosphatase 2 protein activator	0.845585201644703	8,76344E+14	2,61438E+14	0.0374289215057634	0.179279585302061	-3,46854E+14	9,44749E+14	9,33609E+14	8,75375E+14	8,33866E+14	8,50962E+14	8,19506E+14
TC0X00000949.mm.2	Otud6a	OTU domain containing 6A	-1,30993E+14	5,48475E+13	-2,61413E+13	0.0374418031117229	0.179279585302061	-3,46884E+14	4,26252E+14	5,01938E+13	5,33538E+14	6,56637E+14	5,72149E+14	6,00338E+14
TC1500000757.mm.2	Cyp2d9	cytochrome P450, family 2, subfamily d, polypeptide 9	-0.943962770372856	8,06267E+14	-2,61397E+14	0.0374502198749733	0.179279585302061	-3,46904E+14	7,35234E+14	7,61714E+14	8,05963E+14	8,79777E+14	8,2856E+14	8,26353E+14
TC0200002666.mm.2	Fam210b	family with sequence similarity 210, member B	-0.886678654707812	7,80228E+14	-2,61372E+14	0.0374631449544824	0.179279585302061	-3,46935E+14	7,30261E+14	7,20643E+14	7,92077E+13	8,36163E+14	7,77941E+14	8,24281E+13
TC1100001970.mm.2	6030468B19Rik	RIKEN cDNA 6030468B19 gene	-0.984510026845581	5,83223E+14	-2,6135E+13	0.0374746600685607	0.179279585302061	-3,46962E+13	5,36817E+14	4,8803E+14	5,86332E+14	6,35417E+14	6,53033E+14	5,99709E+14
TC0700003086.mm.2	Fancf	Fanconi anemia, complementation group F	-0.869917996782249	7,19875E+13	-2,61349E+14	0.0374751195364182	0.179279585302061	-3,46963E+14	6,78008E+14	6,82255E+14	7,37252E+14	7,96994E+14	7,15703E+14	7,09036E+13
TC1700000899.mm.2	Cul7	cullin 7	1,99512E+14	6,80923E+13	2,61334E+13	0.0374832918534641	0.179279585302061	-3,46983E+14	8,30556E+14	8,10752E+14	7,47412E+14	4,94872E+14	6,07582E+14	5,94364E+14
TC0X00001900.mm.2	Fundc1	FUN14 domain containing 1	1,20313E+14	8,77872E+14	2,61281E+14	0.0375106725117973	0.179279585302061	-3,47048E+14	9,71373E+14	9,47626E+14	8,84027E+13	7,94345E+14	8,79781E+14	7,9008E+14
TC1100004281.mm.2	Mettl23	methyltransferase like 23	-0.798010549085161	9,851E+14	-2,61268E+14	0.0375173759249101	0.179279585302061	-3,47064E+13	9,12662E+14	9,41105E+14	9,95507E+14	1,01786E+14	1,01749E+14	1,02597E+14
TC1500001982.mm.2	Sult4a1	sulfotransferase family 4A, member 1	2,27612E+14	8,59688E+14	2,61265E+13	0.0375190889080359	0.179279585302061	-3,47068E+14	1,00765E+14	9,89305E+14	8,95351E+14	6,46377E+14	8,8826E+13	7,31185E+13
TC0200002499.mm.2	Ywhab	tyrosine 3-monooxygenase/tryptophan 5-monooxygenase activation protein, beta polypeptide	1,42667E+14	9,20285E+13	2,61264E+14	0.0375197961592027	0.179279585302061	-3,4707E+14	1,008E+14	1,01203E+14	9,43533E+14	7,91168E+14	9,17034E+13	8,4994E+13
TC1100003986.mm.2	Rgs9	regulator of G-protein signaling 9	1,77469E+14	8,88546E+14	2,61245E+14	0.0375295289011392	0.179279585302061	-3,47093E+14	9,8596E+14	1,00098E+14	9,18626E+14	7,13377E+13	8,96109E+14	8,16227E+14
TC1800001243.mm.2	Nr3c1	nuclear receptor subfamily 3, group C, member 1	1,14213E+14	6,52277E+14	2,61213E+14	0.0375462459808443	0.179279585302061	-3,47132E+14	7,18469E+12	7,17386E+14	6,39454E+14	5,67975E+14	6,82251E+14	5,88124E+14
TC0400000177.mm.2	Ccnc	cyclin C	2,46843E+14	6,22688E+14	2,61203E+14	0.0375515794944738	0.179279585302061	-3,47145E+14	7,80719E+14	7,58264E+14	6,6202E+14	3,83277E+14	6,5661E+14	4,95237E+14
TC0200003347.mm.2	Gapvd1	GTPase activating protein and VPS9 domains 1	1,34652E+14	6,86952E+14	2,61106E+14	0.0376023793852869	0.179297791863972	-3,47265E+14	7,89096E+14	8,05911E+14	7,19718E+14	6,05986E+14	6,51412E+13	5,49587E+14
TC0700003566.mm.2	Kif7	kinesin family member 7	-1,22806E+13	5,71847E+14	-2,6109E+14	0.0376105987316633	0.179297791863972	-3,47285E+14	4,92696E+14	5,08989E+14	5,59972E+14	6,87326E+14	5,72666E+14	6,09434E+14
TC0X00001602.mm.2	Klhl34	kelch-like 34	-0.789414258689257	6,17146E+14	-2,61063E+14	0.0376245339547116	0.179297791863972	-3,47318E+14	5,57591E+14	5,75276E+14	6,31283E+14	6,59467E+14	6,42588E+14	6,36674E+14
TC0100003507.mm.2	Uap1	UDP-N-acetylglucosamine pyrophosphorylase 1	1,52953E+13	6,42614E+14	2,61055E+14	0.0376287336049796	0.179297791863972	-3,47328E+14	6,99147E+14	7,25083E+14	6,3982E+14	4,78503E+14	6,97908E+14	6,15221E+14
TC0700003658.mm.2	Zfand6	zinc finger, AN1-type domain 6	1,1865E+14	7,12848E+14	2,61023E+14	0.0376456606779225	0.179297791863972	-3,47368E+13	7,86921E+14	7,86111E+14	7,071E+14	6,28632E+14	7,33658E+14	6,34668E+13
TC0200002273.mm.2	Scrt2	scratch family zinc finger 2	-0.786532817848018	7,70145E+14	-2,6102E+14	0.0376469959651566	0.179297791863972	-3,47371E+14	7,21574E+14	7,11846E+14	7,82073E+14	8,08653E+14	8,0641E+14	7,90314E+14
TC0600001629.mm.2	BC049715	cDNA sequence BC049715	-0.916553975177949	4,72226E+14	-2,61003E+14	0.0376560109190676	0.179297791863972	-3,47392E+14	4,05022E+14	4,33978E+14	4,91836E+14	5,30474E+14	4,59045E+14	5,12999E+14
TC0900002166.mm.2	Foxr1	forkhead box R1	-1,06153E+14	5,35547E+14	-2,61003E+14	0.0376560959265345	0.179297791863972	-3,47392E+14	4,61288E+14	5,1179E+14	5,4855E+14	6,36835E+14	5,18577E+14	5,36242E+13
TC1200000013.mm.2	Dtnb	dystrobrevin, beta	-1,15449E+14	1,03764E+14	-2,60952E+14	0.0376830306802747	0.179297791863972	-3,47456E+14	9,5548E+14	9,59941E+14	1,12582E+14	1,0205E+13	1,1114E+14	1,05273E+14
TC0600003314.mm.2	Hebp1	heme binding protein 1	0.867279831016333	7,04466E+14	2,60949E+14	0.0376842879469225	0.179297791863972	-3,47459E+14	7,35404E+14	7,55287E+14	6,418E+14	6,75435E+14	6,86731E+14	7,32142E+14
TC1900001346.mm.2	Ermp1	endoplasmic reticulum metallopeptidase 1	1,54761E+14	6,36786E+14	2,60938E+14	0.0376904444260847	0.179297791863972	-3,47474E+13	7,64393E+14	7,26347E+14	6,81093E+14	5,00125E+14	5,64051E+14	5,84708E+14
TC0700003790.mm.2	Uvrag	UV radiation resistance associated gene	1,79368E+14	6,4834E+14	2,60863E+14	0.0377297612055242	0.179297791863972	-3,47567E+14	7,47465E+14	7,40245E+14	6,69482E+14	4,59492E+14	6,75542E+13	5,97817E+14
TC0X00000338.mm.2	Rhox2a	reproductive homeobox 2A	-0.953543869879312	5,9599E+14	-2,60851E+14	0.0377361725196468	0.179297791863972	-3,47582E+14	5,75771E+13	5,39693E+14	6,23072E+14	6,831E+14	5,56758E+14	5,97544E+14
TC1900000646.mm.2	Scd2/Mir5114	stearoyl-Coenzyme A desaturase 2/microRNA 5114	1,74324E+14	1,04809E+14	2,60836E+13	0.037743931354376	0.179297791863972	-3,476E+14	1,16243E+14	1,15961E+14	1,08225E+14	8,91143E+13	1,04729E+14	9,45805E+14
TC0700002659.mm.2	Snrpa	small nuclear ribonucleoprotein polypeptide A	0.802612059943436	7,64465E+14	2,60822E+14	0.03775136796087	0.179297791863972	-3,47617E+14	8,30687E+14	8,0967E+14	7,23301E+13	7,56533E+13	7,35236E+14	7,31361E+14
TC1300002253.mm.2	Zfp729b	zinc finger protein 729b	1,13793E+14	5,74536E+14	2,60811E+13	0.0377570983968166	0.179297791863972	-3,47631E+14	6,7681E+14	6,41175E+14	5,51075E+14	5,39323E+14	5,75222E+14	4,63608E+14
TC0600003042.mm.2	Hdhd5	haloacid dehalogenase like hydrolase domain containing 5	-1,02959E+14	8,6369E+14	-2,60809E+14	0.0377582468650454	0.179297791863972	-3,47634E+13	8,04477E+14	7,88863E+14	8,62054E+14	9,37203E+14	8,65047E+14	9,24498E+14
TC1500000296.mm.2	Atp6v1c1	ATPase, H+ transporting, lysosomal V1 subunit C1	2,42992E+14	8,22847E+14	2,60808E+14	0.0377585384254354	0.179297791863972	-3,47634E+14	9,79305E+14	9,84996E+14	8,65796E+13	6,1252E+14	8,43204E+13	6,51258E+14
TC0700000868.mm.2	Tmem143	transmembrane protein 143	0.849675380923684	6,66284E+13	2,60792E+14	0.0377668159614176	0.179297791863972	-3,47654E+14	7,32366E+14	7,09861E+14	6,15532E+14	6,5676E+14	6,27204E+14	6,55981E+14
TC1900000905.mm.2	Rps6kb2	ribosomal protein S6 kinase, polypeptide 2	-1,01042E+14	9,15919E+14	-2,60738E+14	0.0377952563707899	0.179329385941193	-3,47721E+14	8,27261E+13	8,50764E+14	9,29713E+14	9,50395E+14	1,01261E+14	9,24768E+14
TC1600000280.mm.2	Abcf3	ATP-binding cassette, sub-family F (GCN20), member 3	1,56019E+14	6,6348E+14	2,60726E+14	0.0378018049101808	0.179329385941193	-3,47736E+14	7,488E+14	7,27452E+13	6,75623E+14	4,88592E+14	6,70429E+14	6,69981E+14
TC1500001937.mm.2	Snu13	SNU13 homolog, small nuclear ribonucleoprotein (U4/U6.U5)	1,05972E+14	7,75814E+14	2,60716E+14	0.0378068593087712	0.179329385941193	-3,47748E+14	8,59826E+14	8,3517E+13	7,88011E+13	6,9504E+14	7,56989E+14	7,1985E+14
TC1200002006.mm.2	Ccdc177	coiled-coil domain containing 177	-0.812443765531444	7,63438E+14	-2,60647E+14	0.0378430837103743	0.179335039792302	-3,47834E+14	7,02775E+14	7,12345E+14	7,67925E+14	8,09685E+14	7,97209E+14	7,90688E+14
TC0700003111.mm.2	Gabra5	gamma-aminobutyric acid (GABA) A receptor, subunit alpha 5	1,43466E+14	8,42593E+14	2,60637E+14	0.0378486815004026	0.179335039792302	-3,47847E+14	9,37078E+14	9,1222E+14	8,54312E+14	7,08054E+14	8,61764E+14	7,8213E+14
TC1700000578.mm.2	Olfr239	olfactory receptor 239	-1,08081E+14	5,57407E+14	-2,60601E+14	0.0378672559649268	0.179335039792302	-3,4789E+14	4,63379E+14	5,04775E+14	5,42685E+14	6,4163E+14	5,96327E+14	5,95648E+14
TC1000000382.mm.2	Snx3	sorting nexin 3	1,46092E+14	9,08623E+13	2,60595E+14	0.037870902859371	0.179335039792302	-3,47899E+14	1,00323E+14	1,00342E+14	9,22993E+14	7,91465E+14	9,21229E+14	8,09409E+14
TC0400002837.mm.2	Brinp1	bone morphogenic protein/retinoic acid inducible neural specific 1	1,62183E+14	8,4575E+14	2,60555E+13	0.0378918835718396	0.179335039792302	-3,47948E+14	9,48485E+13	9,41869E+14	8,54019E+14	7,11969E+14	8,77981E+14	7,40173E+14
TC0100000214.mm.2	1700001G17Rik	RIKEN cDNA 1700001G17 gene	-1,22485E+14	5,41259E+14	-2,60531E+14	0.0379042179667175	0.179335039792302	-3,47977E+14	4,96828E+14	4,51254E+14	5,41854E+14	6,51199E+14	5,22485E+14	5,83935E+13
TC1600001471.mm.2	Xxylt1	xyloside xylosyltransferase 1	0.909676115637411	5,41302E+14	2,60515E+14	0.0379129168741602	0.179335039792302	-3,47998E+14	6,25196E+14	5,92225E+14	5,48133E+14	4,87353E+14	5,05856E+14	4,89047E+14
TC0200004053.mm.2	Olfr1232	olfactory receptor 1232	-0.898286129608242	5,92257E+14	-2,60493E+13	0.0379247501603235	0.179335039792302	-3,48026E+14	5,10042E+14	5,70587E+14	6,14646E+14	6,4564E+13	6,02778E+14	6,09847E+13
TC1100001575.mm.2	Gsdma3	gasdermin A3	-1,58697E+14	4,00651E+14	-2,60486E+14	0.0379284632048984	0.179335039792302	-3,48034E+14	2,938E+14	2,99357E+14	3,79218E+14	5,31333E+14	3,91043E+14	5,09152E+14
TC0200000587.mm.2	St6galnac6/St6galnac4	ST6 (alpha-N-acetyl-neuraminyl-2,3-beta-galactosyl-1,3)-N-acetylgalactosaminide alpha-2,6-sialyltransferase 6/ST6 (alpha-N-acetyl-neuraminyl-2,3-beta-galactosyl-1,3)-N-acetylgalactosaminide alpha-2,6-sialyltransferase 4	1,05999E+14	7,99969E+14	2,60472E+14	0.0379354292720321	0.179335039792302	-3,48051E+14	8,64959E+13	8,38732E+14	7,93299E+14	6,98393E+14	8,1792E+14	7,86512E+13
TC0900002887.mm.2	Zbtb38/E030011O05Rik	zinc finger and BTB domain containing 38/RIKEN cDNA E030011O05 gene	1,52863E+14	7,75048E+14	2,60463E+14	0.0379403405389787	0.179335039792302	-3,48062E+14	8,6977E+14	8,50997E+14	7,98795E+14	6,16245E+13	7,56087E+14	7,58393E+14
TC0900003131.mm.2	Stac	src homology three (SH3) and cysteine rich domain	-0.838271752935238	5,32681E+14	-2,60461E+14	0.0379416093000655	0.179335039792302	-3,48065E+14	4,562E+13	4,88163E+13	5,75999E+14	5,36019E+13	5,65264E+14	5,7444E+14

TC1700001757.mm.2	Fkbp5	FK506 binding protein 5	1,70655E+14	7,08172E+13	2,60338E+14	0.038006205060441	0.179367898635999	-3,48217E+14	8,38376E+14	8,1324E+14	7,46593E+14	5,63713E+14	6,94959E+14	5,92149E+13
TC1200002364.mm.2	Begain	brain-enriched guanylate kinase-associated	0.796895052830724	6,86728E+14	2,60263E+14	0.0380464220518605	0.179367898635999	-3,48311E+14	7,55552E+14	7,21075E+14	6,63236E+14	6,54013E+13	6,56803E+14	6,69692E+13
TC0500003716.mm.2	Pms2	PMS1 homolog2, mismatch repair system component	1,90171E+14	6,59829E+14	2,60244E+13	0.0380561540096032	0.179367898635999	-3,48334E+13	7,41629E+14	7,63235E+14	6,77075E+14	4,47448E+14	6,99662E+14	6,29923E+13
TC1700001949.mm.2	Apom	apolipoprotein M	-1,20852E+14	5,07975E+14	-2,60242E+14	0.0380571586309711	0.179367898635999	-3,48336E+14	4,15929E+13	4,15766E+14	4,73815E+14	5,99583E+14	5,53351E+14	5,89408E+14
TC1100000781.mm.2	B9d1	B9 protein domain 1	-0.929260499326357	7,38037E+14	-2,60232E+14	0.0380625743669768	0.179367898635999	-3,48349E+14	6,46322E+14	6,94285E+14	7,33732E+14	7,92727E+14	7,81121E+14	7,80036E+14
TC1000001375.mm.2	Il22	interleukin 22	-0.905295182314735	5,82788E+14	-2,60219E+14	0.0380693459209844	0.179367898635999	-3,48364E+14	5,24714E+14	5,23213E+14	5,80347E+13	6,48639E+12	6,1831E+14	6,01503E+13
TC0700001871.mm.2	Fus	fused in sarcoma	1,6868E+14	8,42823E+14	2,6017E+14	0.0380955989627308	0.179367898635999	-3,48426E+14	9,66347E+14	9,55062E+13	8,8901E+14	6,95039E+14	8,13466E+14	7,38015E+14
TC0900001204.mm.2	Xrn1	5'-3' exoribonuclease 1	1,40308E+14	5,6732E+14	2,60158E+14	0.0381018593514587	0.179367898635999	-3,4844E+13	6,81132E+13	6,73273E+14	5,89927E+14	4,83862E+14	5,50072E+14	4,25655E+14
TC0X00000126.mm.2	Tspan7	tetraspanin 7	1,6053E+14	1,1312E+14	2,60156E+14	0.0381029670638027	0.179367898635999	-3,48443E+14	1,23498E+14	1,23076E+13	1,15656E+14	9,88111E+14	1,13829E+14	1,0385E+14
TC1600000650.mm.2	Naa50	N(alpha)-acetyltransferase 50, NatE catalytic subunit	1,44192E+14	7,80766E+14	2,60135E+14	0.0381142970952968	0.179367898635999	-3,4847E+14	8,57016E+14	8,8225E+14	7,75099E+14	6,75783E+14	8,15043E+14	6,79406E+14
TC1200001245.mm.2	Klc1	kinesin light chain 1	1,49804E+14	9,84506E+14	2,60132E+13	0.0381158448112174	0.179367898635999	-3,48473E+14	1,0837E+13	1,07576E+14	1,00174E+14	8,58116E+14	9,97106E+14	8,90601E+14
TC0400002102.mm.2	Sdf4	stromal cell derived factor 4	1,01895E+14	8,57875E+14	2,60112E+14	0.0381264524615986	0.179367898635999	-3,48498E+14	9,10546E+14	9,2508E+14	8,39434E+14	7,92402E+14	8,80808E+14	7,98982E+14
TC0700003622.mm.2	Hdgfl3	HDGF like 3	2,09475E+14	6,67576E+14	2,60107E+14	0.038128916474209	0.179367898635999	-3,48504E+13	8,28555E+14	8,05798E+14	7,16278E+14	4,99124E+14	6,57105E+14	4,98597E+14
TC1700002724.mm.2	Nrxn1	neurexin I	3,17254E+14	8,21173E+14	2,60107E+14	0.0381290697788531	0.179367898635999	-3,48504E+14	9,82997E+14	9,8266E+14	8,60915E+14	4,70233E+14	8,98913E+14	7,31322E+14
TC0600002614.mm.2	Fbxo41	F-box protein 41	0.814402182353763	6,02019E+14	2,60086E+14	0.0381398532355085	0.179367898635999	-3,48529E+14	6,74244E+14	6,40375E+14	5,70215E+13	5,81524E+14	5,59484E+13	5,86271E+14
TC1700001846.mm.2	Pglyrp2	peptidoglycan recognition protein 2	-1,01752E+14	6,0033E+14	-2,60078E+14	0.0381445587888466	0.179367898635999	-3,4854E+14	5,11112E+14	5,64149E+14	6,15334E+13	6,63432E+14	5,92582E+14	6,55369E+13
TC0200003239.mm.2	Barhl1	BarH like homeobox 1	-1,15382E+14	6,15269E+14	-2,60068E+14	0.0381496617758741	0.179367898635999	-3,48552E+12	5,54753E+13	5,36561E+14	6,04638E+13	7,1744E+14	6,18849E+13	6,59373E+14
TC0600001617.mm.2	Gm8994	predicted gene 8994	-1,10743E+14	6,61594E+14	-2,60061E+14	0.0381532609064562	0.179367898635999	-3,48561E+14	5,84138E+14	5,73462E+14	6,44488E+14	7,34597E+14	6,83008E+14	7,49869E+14
TC0200003023.mm.2	H2al2a	H2A histone family member L2A	-1,06538E+13	6,37819E+14	-2,60038E+14	0.0381656405593684	0.179367898635999	-3,48589E+14	5,6224E+14	5,5594E+14	6,24175E+14	7,07082E+14	6,56889E+14	7,20587E+14
TC0400001458.mm.2	Trappc3	trafficking protein particle complex 3	1,45976E+13	8,00375E+14	2,60028E+14	0.0381711986857098	0.179367898635999	-3,48602E+14	8,95273E+14	8,75978E+14	7,95064E+14	6,84235E+14	8,41538E+14	7,10161E+14
TC0500003427.mm.2	Col26a1	collagen, type XXVI, alpha 1	-1,19108E+13	5,81229E+14	-2,60004E+14	0.0381834950048518	0.179373368808506	-3,48631E+14	5,21865E+14	4,98182E+14	5,69066E+14	6,89197E+13	5,85956E+14	6,23107E+14
TC1000000430.mm.2	Hace1	HECT domain and ankyrin repeat containing, E3 ubiquitin protein ligase 1	1,43808E+14	6,1065E+14	2,59941E+14	0.0382173490747279	0.179439844978734	-3,4871E+14	7,14542E+14	7,05457E+14	6,3847E+14	4,93914E+14	5,99824E+14	5,1169E+14
TC0600002492.mm.2	Tgoln1/Tgoln2	trans-golgi network protein/trans-golgi network protein 2	1,1589E+14	7,78788E+14	2,59936E+14	0.0382199185730165	0.179439844978734	-3,48716E+14	8,62736E+14	8,58108E+14	7,96619E+14	6,92445E+14	7,63323E+13	6,99501E+14
TC1100003581.mm.2	Sgca	sarcoglycan, alpha (dystrophin-associated glycoprotein)	-1,24561E+14	6,1092E+14	-2,59905E+14	0.0382363070580527	0.179448536193191	-3,48754E+14	5,13001E+14	5,41216E+14	5,9528E+14	7,08057E+14	6,17215E+14	6,90753E+14
TC0700000138.mm.2	Zfp606	zinc finger protein 606	-0.986342747072692	6,98193E+13	-2,59891E+13	0.0382440435230819	0.179448536193191	-3,48772E+14	6,27505E+14	6,44535E+14	7,05752E+13	7,63556E+14	6,9121E+14	7,56599E+14
TC0500002846.mm.2	Abraxas1	BRCA1 A complex subunit	-0.880922297339895	8,64675E+12	-2,59828E+13	0.0382775323442059	0.179462128695542	-3,4885E+14	8,07963E+14	7,92431E+14	8,5737E+14	9,19208E+13	9,10561E+14	9,00518E+14
TC0600002279.mm.2	Hoxa5	homeobox A5	-0.950596532788293	5,11461E+13	-2,59806E+14	0.0382889489235732	0.179462128695542	-3,48877E+13	4,64847E+14	4,60839E+14	5,21053E+14	5,94752E+14	5,3061E+14	4,96663E+14
TC0200002261.mm.2	Nsfl1c	NSFL1 (p97) cofactor (p47)	0.984368025033632	8,7501E+14	2,59793E+14	0.0382959620336767	0.179462128695542	-3,48893E+14	9,68311E+13	9,49203E+14	8,84825E+14	8,35815E+14	8,41591E+14	7,70317E+14
TC1800000091.mm.2	Gata6	GATA binding protein 6	-1,15853E+14	5,79764E+14	-2,59785E+14	0.0383006557035845	0.179462128695542	-3,48904E+14	4,95854E+14	5,07871E+14	5,77685E+14	6,57746E+14	5,73871E+14	6,65558E+13
TC0300002513.mm.2	BC107364	cDNA sequence BC107364	-1,30405E+14	5,69083E+14	-2,59781E+14	0.0383026289694016	0.179462128695542	-3,48908E+14	4,95648E+14	4,81545E+14	5,72949E+14	6,65055E+14	5,42403E+14	6,56896E+14
TC0800000983.mm.2	Man2b1	mannosidase 2, alpha B1	1,08835E+14	6,64619E+14	2,59737E+13	0.0383261579488629	0.179520169485473	-3,48963E+14	6,92954E+14	7,46548E+14	6,02787E+14	6,19045E+14	6,16214E+14	7,10168E+14
TC0X00000064.mm.2	Wdr45	WD repeat domain 45	0.811489494818307	7,4665E+14	2,59673E+14	0.038360409260285	0.179622670621272	-3,49043E+14	7,88477E+14	8,08847E+14	7,36567E+14	6,98459E+14	7,19437E+14	7,28111E+14
TC0200005016.mm.2	Mroh8	maestro heat-like repeat family member 8	-1,31694E+14	7,09682E+14	-2,59654E+14	0.0383703365157587	0.179622670621272	-3,49066E+13	6,3999E+14	6,22966E+14	7,22657E+14	8,03688E+14	6,71922E+14	7,96867E+14
TC1700002253.mm.2	Tbc1d5	TBC1 domain family, member 5	1,29743E+14	6,56984E+14	2,59557E+14	0.0384220938614194	0.179796510728281	-3,49186E+14	7,53674E+13	7,32144E+14	6,82254E+14	5,44077E+14	6,37023E+14	5,92732E+14
TC0800002564.mm.2	Mir1199	microRNA 1199	-1,37575E+14	7,67192E+14	-2,59538E+14	0.0384326624623618	0.179796510728281	-3,4921E+14	6,82532E+14	6,66925E+14	7,38883E+14	8,85725E+14	7,84073E+14	8,45016E+14
TC1700001946.mm.2	Ly6g5b	lymphocyte antigen 6 complex, locus G5B	-1,45406E+14	5,53335E+13	-2,59509E+14	0.0384478842788595	0.179796510728281	-3,49246E+14	4,56131E+14	4,462E+14	5,23903E+14	6,6924E+14	5,66066E+14	6,58471E+14
TC1200000678.mm.2	Zbtb1	zinc finger and BTB domain containing 1	0.937315321632273	5,51838E+14	2,59482E+14	0.0384623223488284	0.179796510728281	-3,49279E+13	6,17739E+14	6,32861E+14	5,43381E+14	5,19755E+13	5,35311E+14	4,61982E+14
TC1700001947.mm.2	Csnk2b	casein kinase 2, beta polypeptide	0.870406378452911	8,98331E+14	2,5948E+14	0.0384632639781782	0.179796510728281	-3,49281E+14	9,6293E+13	9,46709E+14	8,9769E+14	8,37867E+14	8,74121E+13	8,70667E+14
TC0X00000110.mm.2	Xk	X-linked Kx blood group	0.756728235426192	5,59196E+13	2,59399E+14	0.0385069420505229	0.17988929652186	-3,49382E+14	6,38656E+14	6,21881E+14	5,53668E+14	5,55524E+14	4,95988E+14	4,8946E+14
TC1300002500.mm.2	Btf3	basic transcription factor 3	1,33138E+14	9,02346E+14	2,59368E+14	0.0385233714326008	0.17988929652186	-3,4942E+14	9,83558E+14	9,82975E+14	9,15458E+14	7,848E+13	9,13009E+14	8,34274E+14
TC1100000496.mm.2	Ifi47/Olfr56	interferon gamma inducible protein 47/olfactory receptor 56	-0.750934979882191	7,98727E+14	-2,59361E+14	0.0385273959317341	0.17988929652186	-3,49429E+14	7,44249E+14	7,52014E+14	8,1908E+14	8,27369E+14	8,2188E+14	8,2777E+13
TC0300002695.mm.2	Cd53	CD53 antigen	2,39102E+14	6,05443E+14	2,5936E+14	0.0385277702660547	0.17988929652186	-3,4943E+14	7,08765E+14	7,67733E+14	6,25074E+14	3,73219E+14	6,68069E+14	4,89797E+12
TC0900000719.mm.2	Ubl7	ubiquitin-like 7 (bone marrow stromal cell-derived)	1,79989E+14	8,86232E+13	2,59278E+14	0.0385718974218393	0.180012777464835	-3,49532E+14	9,89754E+14	9,90954E+14	9,17535E+14	7,03196E+14	8,94783E+14	8,21171E+14
TC1900001124.mm.2	Ms4a8a	membrane-spanning 4-domains, subfamily A, member 8A	-1,12015E+14	5,60207E+14	-2,59203E+13	0.0386120857853898	0.180012777464835	-3,49625E+14	5,00509E+14	5,13613E+14	5,73883E+14	6,64269E+14	5,18918E+14	5,90049E+14
TC0400001557.mm.2	Laptm5	lysosomal-associated protein transmembrane 5	1,4613E+14	8,06402E+14	2,59197E+14	0.0386155057668592	0.180012777464835	-3,49633E+14	8,77992E+14	9,09057E+14	8,2993E+14	6,64858E+14	8,00916E+14	7,5566E+14
TC0100003406.mm.2	Fmo3	flavin containing monooxygenase 3	-1,32804E+13	5,82428E+14	-2,59196E+13	0.0386159253329576	0.180012777464835	-3,49634E+14	5,31227E+14	4,86132E+13	5,86436E+14	6,96531E+14	5,49708E+14	6,44533E+13
TC1400001052.mm.2	Rubcnl	RUN and cysteine rich domain containing beclin 1 interacting protein like	-0.813055206388951	5,8499E+14	-2,59172E+13	0.038628754068863	0.180012777464835	-3,49663E+14	5,28038E+14	5,20373E+14	5,91358E+14	6,19664E+14	6,08081E+14	6,42428E+13
TC0200004848.mm.2	Abhd12	abhydrolase domain containing 12	1,18743E+14	6,98352E+14	2,59138E+14	0.0386472723005672	0.180012777464835	-3,49706E+14	7,68806E+14	7,768E+14	6,96057E+14	6,12063E+14	7,1633E+14	6,20059E+14
TC1100003318.mm.2	Wsb1	WD repeat and SOCS box-containing 1	1,45555E+14	7,01953E+14	2,59136E+14	0.0386485639744418	0.180012777464835	-3,49709E+14	8,02133E+13	8,00468E+14	7,34814E+14	5,76676E+14	6,83991E+14	6,13637E+14
TC0900002779.mm.2	Pgm3	phosphoglucomutase 3	1,22036E+14	5,50636E+14	2,59134E+14	0.0386492063188824	0.180012777464835	-3,49711E+14	6,52285E+14	6,32627E+13	5,71349E+13	4,69491E+14	4,4677E+14	5,31297E+14
TC1100003165.mm.2	Olfr1	olfactory receptor 1	-1,05013E+14	5,4771E+14	-2,59124E+14	0.0386547638570303	0.180012777464835	-3,49723E+14	4,86486E+14	4,84077E+14	5,43604E+14	6,36986E+14	5,46464E+14	5,88645E+14
TC0500000418.mm.2	Jakmip1	janus kinase and microtubule interacting protein 1	0.906989052428677	7,01278E+14	2,59086E+14	0.0386750947252795	0.180013450473166	-3,4977E+14	7,31229E+13	7,50389E+14	6,83164E+14	6,17056E+14	7,22996E+14	7,02837E+14
TC0900001979.mm.2	Aplp2	amyloid beta (A4) precursor-like protein 2	1,3516E+14	8,94991E+14	2,59082E+14	0.0386772522521008	0.180013450473166	-3,49775E+14	9,93917E+14	9,7764E+13	9,13992E+14	7,87246E+14	8,93452E+14	8,03698E+14
TC1300001827.mm.2	Ssr1	signal sequence receptor, alpha	1,33314E+14	8,61233E+14	2,59053E+14	0.0386929389418643	0.180034457167271	-3,49811E+14	9,38648E+14	9,42598E+14	8,65171E+13	7,49445E+14	8,85856E+14	7,85679E+14
TC0900001850.mm.2	Eif3g	eukaryotic translation initiation factor 3, subunit G	1,02673E+13	7,02846E+14	2,58961E+14	0.0387430347384012	0.1801278719521	-3,49927E+14	7,92081E+14	7,79466E+14	7,28817E+14	6,37384E+14	6,56104E+14	6,23227E+14
TC1100002813.mm.2	Gemin5	gem nuclear organelle associated protein 5	0.963808452712299	5,67946E+14	2,58893E+14	0.0387797754614161	0.1801278719521	-3,50011E+14	6,37682E+14	6,5083E+14	5,8024E+14	5,15512E+14	5,38372E+14	4,85038E+14
TC0500002830.mm.2	Tmem150c	transmembrane protein 150C	1,8401E+14	7,01319E+14	2,58892E+14	0.0387803030115295	0.1801278719521	-3,50012E+14	8,60021E+13	8,22689E+14	7,50253E+14	5,64438E+14	6,73143E+14	5,3737E+14
TC0200003132.mm.2	Tor4a	torsin family 4, member A	-1,15076E+14	5,66287E+14	-2,58886E+14	0.0387832010737922	0.1801278719521	-3,50019E+14	4,66364E+14	5,0509E+14	5,62505E+14	6,39101E+14	5,69239E+14	6,55422E+14
TC0800002831.mm.2	Acd	adrenocortical dysplasia	0.882582410634648	6,86647E+14	2,58846E+14	0.0388048975737312	0.1801278719521	-3,50069E+14	7,53155E+13	7,47401E+14	6,77955E+13	6,46084E+14	6,76306E+13	6,18984E+14
TC0X00002741.mm.2	Hdac8	histone deacetylase 8	0.928309808756382	6,20207E+14	2,58836E+13	0.0388102552395306	0.1801278719521	-3,50081E+13	6,64842E+14	6,82228E+14	6,18155E+14	5,43253E+14	6,0818E+14	6,04586E+14
TC1600001966.mm.2	App	amyloid beta (A4) precursor protein	1,44187E+14	1,03071E+14	2,58801E+14	0.0388292469019121	0.1801278719521	-3,50124E+13	1,13165E+12	1,11855E+14	1,05994E+14	9,0189E+14	1,01832E+14	9,53883E+14
TC1600001064.mm.2	Dyrk1a	dual-specificity tyrosine-(Y)-phosphorylation regulated kinase 1a	1,92051E+14	7,3775E+14	2,58794E+14	0.0388329836376427	0.1801278719521	-3,50133E+14	8,50025E+14	8,47915E+14	7,78967E+14	5,34872E+14	7,19851E+14	6,9487E+13
TC1700000500.mm.2	BC004004	cDNA sequence BC004004	1,40325E+14	8,15233E+13	2,58706E+13	0.0388808862754194	0.1801278719521	-3,50243E+14	8,99943E+14	9,01793E+14	8,37497E+13	6,83589E+14	8,13996E+14	7,54577E+14
TC0600002733.mm.2	Grip2	glutamate receptor interacting protein 2	-1,10591E+14	5,60155E+14	-2,58692E+14	0.03888859239133	0.1801278719521	-3,5026E+14	5,25018E+14	4,77685E+14	5,64924E+14	6,5896E+14	5,88406E+14	5,45935E+14
TC0100000608.mm.2	Smarcal1	SWI/SNF related matrix associated, actin dependent regulator of chromatin, subfamily a-like 1	1,48526E+14	7,01248E+14	2,5866E+14	0.0389055597057242	0.1801278719521	-3,50299E+14	8,11734E+14	8,21054E+14	7,17045E+14	6,18689E+14	5,45274E+14	6,93693E+14
TC0200002151.mm.2	Smim26	small integral membrane protein 26	0.950158215655485	5,83543E+14	2,58639E+14	0.0389169948912139	0.1801278719521	-3,50325E+14	6,62285E+14	6,26142E+14	5,4195E+14	5,56445E+14	5,93355E+14	5,21082E+14
TC0100003887.mm.2	Nos1ap	nitric oxide synthase 1 (neuronal) adaptor protein	1,3849E+14	8,14021E+14	2,58571E+13	0.0389539755936919	0.1801278719521	-3,5041E+14	9,0409E+14	9,03375E+14	8,27562E+14	7,02923E+14	8,24908E+14	7,21267E+14
TC0800002470.mm.2	Smim7	small integral membrane protein 7	0.939107608551975	8,7523E+14	2,58571E+14	0.038954145933981	0.1801278719521	-3,5041E+14	9,34618E+14	9,27307E+14	8,73415E+14	8,00689E+14	8,73527E+14	8,41828E+14
TC0700004311.mm.2	Zg16	zymogen granule protein 16	-1,10033E+14	5,34268E+14	-2,58542E+14	0.0389699659027579	0.1801278719521	-3,50447E+14	4,65761E+14	4,79771E+14	5,61947E+14	6,03651E+14	4,95418E+14	5,99061E+14
TC1900001726.mm.2	Hspa12a	heat shock protein 12A	0.800233180436754	6,8633E+14	2,58531E+14	0.0389756318242638	0.1801278719521	-3,5046E+14	7,54518E+14	7,22037E+14	6,57029E+12	6,59479E+13	6,7514E+13	6,49775E+13
TC0700003845.mm.2	Folr2	folate receptor 2 (fetal)	-1,33912E+14	5,08745E+14	-2,58529E+14	0.0389767944261913	0.1801278719521	-3,50462E+14	3,79301E+14	4,66467E+14	4,97744E+14	6,15849E+14	5,70755E+14	5,22355E+14
TC1000000713.mm.2	Upb1	ureidopropionase, beta	-1,18824E+14	6,53772E+14	-2,58504E+14	0.0389907295838147	0.1801278719521	-3,50494E+14	5,91784E+14	5,6595E+13	6,473E+14	7,48083E+14	6,46901E+14	7,22612E+14
TC1300000532.mm.2	Rbm24	RNA binding motif protein 24	-0.838545812047336	6,9537E+14	-2,58501E+14	0.0389922991176149	0.1801278719521	-3,50498E+13	6,56516E+14	6,28635E+14	7,45809E+14	7,07052E+14	7,29719E+14	7,04492E+14
TC1100001682.mm.2	G6pc3	glucose 6 phosphatase, catalytic, 3	1,07659E+14	7,40438E+14	2,58488E+13	0.0389990359116669	0.1801278719521	-3,50513E+14	8,41402E+14	7,88159E+14	7,32195E+14	6,82048E+14	6,59591E+14	7,39233E+14
TC0600000374.mm.2	Ttc26	tetratricopeptide repeat domain 26	1,01695E+14	5,35852E+14	2,58486E+14	0.0390000737105695	0.1801278719521	-3,50515E+14	6,0409E+13	5,81874E+13	5,23205E+14	4,5937E+14	4,88932E+14	5,57641E+13
TC0900002186.mm.2	Ube4a	ubiquitination factor E4A	1,22575E+14	7,31755E+14	2,5845E+14	0.0390195862034068	0.1801278719521	-3,5056E+14	8,08379E+14	8,01639E+14	7,4297E+14	6,21899E+14	7,37853E+14	6,77789E+14
TC0900002151.mm.2	Pdzd3	PDZ domain containing 3	-1,02124E+14	5,14398E+14	-2,5845E+14	0.0390195889856049	0.1801278719521	-3,5056E+14	4,39513E+14	4,54925E+14	5,08865E+14	5,89822E+14	5,20788E+14	5,72477E+14
TC0700000354.mm.2	Vmn1r169	vomeronasal 1 receptor 169	-1,6195E+14	4,69607E+13	-2,58405E+14	0.0390445954312676	0.1801278719521	-3,50617E+14	3,85339E+13	3,61112E+14	4,39765E+14	6,30585E+14	4,73489E+14	5,27352E+12
TC0700001775.mm.2	Hs3st4	heparan sulfate (glucosamine) 3-O-sulfotransferase 4	0.775177245812717	7,23693E+14	2,58394E+14	0.0390502723990633	0.1801278719521	-3,5063E+14	7,68611E+14	7,60011E+14	7,00302E+14	6,73284E+14	7,26162E+14	7,13783E+14
TC0200002722.mm.2	Gm14308/Gm14430/Gm14432/Gm14434/Gm2026/Gm4724	predicted gene 14308/predicted gene 14430/predicted gene 14432/predicted gene 14434/predicted gene 2026/predicted gene 4724	3,16013E+14	8,70204E+14	2,58376E+14	0.0390600311944736	0.1801278719521	-3,50652E+14	9,70713E+14	1,04945E+14	8,87153E+14	5,00986E+14	9,87781E+13	8,25139E+14
TC0200002724.mm.2	Gm14308/Gm14430/Gm14432/Gm14434/Gm2026/Gm4724	predicted gene 14308/predicted gene 14430/predicted gene 14432/predicted gene 14434/predicted gene 2026/predicted gene 4724	3,16013E+14	8,70204E+14	2,58376E+14	0.0390600311944736	0.1801278719521	-3,50652E+14	9,70713E+14	1,04945E+14	8,87153E+14	5,00986E+14	9,87781E+13	8,25139E+14
TC1000002820.mm.2	Myf5	myogenic factor 5	-0.863493266959691	5,06715E+14	-2,58358E+14	0.0390697673931565	0.1801278719521	-3,50674E+14	4,37892E+14	4,71015E+14	5,26717E+13	5,5489E+14	5,03336E+14	5,46441E+14
TC0800001181.mm.2	Cmtm2b	CKLF-like MARVEL transmembrane domain containing 2B	-0.835382633197554	5,45128E+14	-2,58355E+14	0.0390715303152026	0.1801278719521	-3,50678E+14	4,82745E+14	4,72827E+14	5,36291E+14	5,86358E+14	5,88791E+14	6,03756E+14
TC0400003803.mm.2	Cdc42	cell division cycle 42	1,43597E+14	1,08242E+14	2,58324E+14	0.0390885818306156	0.1801278719521	-3,50717E+14	1,15869E+14	1,17409E+14	1,10087E+14	9,44713E+13	1,09162E+14	1,02452E+14
TC0600001340.mm.2	Rad52	RAD52 homolog, DNA repair protein	-1,38824E+14	6,11319E+14	-2,58311E+14	0.0390957679387095	0.1801278719521	-3,50734E+14	5,08131E+14	5,37556E+14	5,86725E+14	7,3661E+13	6,21167E+14	6,77724E+14
TC0200005097.mm.2	Ada	adenosine deaminase	-1,31481E+14	7,1911E+14	-2,5831E+14	0.0390962322948664	0.1801278719521	-3,50735E+14	6,15495E+14	6,67551E+14	7,06209E+14	8,39799E+14	7,19629E+14	7,65975E+14
TC4 GL456350 random00000010.mm.2	Fam205a2	family with sequence similarity 205, member A2	0.971422619612489	8,14526E+13	2,58306E+14	0.039098104345702	0.1801278719521	-3,50739E+14	8,63505E+14	8,82227E+14	7,94706E+14	7,56742E+14	8,33003E+14	7,56974E+13
TC1100001974.mm.2	Tmem235	transmembrane protein 235	-0.958210218063113	5,85514E+14	-2,58298E+14	0.0391028171442225	0.1801278719521	-3,5075E+14	5,0664E+14	5,4415E+14	5,87888E+14	6,54544E+14	5,92382E+14	6,2748E+14
TC1100002484.mm.2	Fgf18	fibroblast growth factor 18	-1,47192E+13	6,66044E+14	-2,58295E+14	0.0391042820138054	0.1801278719521	-3,50753E+14	6,01675E+14	5,71703E+14	6,61955E+14	8,05807E+14	6,28685E+13	7,26441E+14
TC1000001198.mm.2	Atp2b1	ATPase, Ca++ transporting, plasma membrane 1	2,17149E+14	7,77094E+14	2,58241E+14	0.0391337535173639	0.180136696762908	-3,5082E+13	9,04208E+14	9,47565E+14	8,0516E+14	6,12314E+14	7,98213E+14	5,95105E+14
TC0100003402.mm.2	Fmo2	flavin containing monooxygenase 2	-1,1012E+14	7,5881E+14	-2,58125E+14	0.0391973083153068	0.180136696762908	-3,50965E+14	6,70167E+14	6,98844E+14	7,42242E+14	8,47009E+14	7,81558E+14	8,13042E+14
TC1000001059.mm.2	1700113H08Rik	RIKEN cDNA 1700113H08 gene	-0.919647567743567	5,3818E+13	-2,58079E+14	0.0392222523418385	0.180136696762908	-3,51021E+14	4,81158E+14	4,72762E+14	5,33419E+14	6,04431E+14	5,80904E+14	5,56404E+14
TC0100003042.mm.2	Pfkfb2/C4bp-ps1	6-phosphofructo-2-kinase/fructose-2,6-biphosphatase 2/complement component 4 binding protein, pseudogene 1	1,04616E+14	7,23529E+14	2,58072E+13	0.0392260414341696	0.180136696762908	-3,5103E+14	7,78246E+14	7,84327E+14	7,27732E+14	6,2561E+14	7,09057E+14	7,16202E+14
TC0700001409.mm.2	Ucp2	uncoupling protein 2 (mitochondrial, proton carrier)	2,06436E+14	8,49884E+14	2,58061E+13	0.0392319871115602	0.180136696762908	-3,51043E+14	9,34228E+14	9,93906E+14	8,74621E+14	6,4064E+14	8,85783E+14	7,70128E+14
TC0700000079.mm.2	Smim17/Olfr1344	small integral membrane protein 17/olfactory receptor 1344	-0.777255810756633	6,07681E+14	-2,58051E+14	0.0392373985059623	0.180136696762908	-3,51056E+14	5,74281E+14	5,42013E+14	6,36737E+14	6,35009E+14	6,27791E+14	6,30255E+14
TC0900001052.mm.2	Ddx43	DEAD (Asp-Glu-Ala-Asp) box polypeptide 43	-1,20594E+14	4,60725E+14	-2,58043E+12	0.0392420020158709	0.180136696762908	-3,51066E+14	3,95294E+14	3,68742E+14	4,40938E+14	5,64286E+14	4,77846E+14	5,17245E+14
TC0400003793.mm.2	C1qc	complement component 1, q subcomponent, C chain	1,01042E+14	6,97126E+14	2,58034E+13	0.0392470957740896	0.180136696762908	-3,51078E+13	6,90151E+14	7,79919E+14	6,34137E+14	6,3385E+14	7,06187E+13	7,38513E+14
TC0300000244.mm.2	Sox2	SRY (sex determining region Y)-box 2	1,79488E+14	7,79497E+14	2,58024E+14	0.0392522217793356	0.180136696762908	-3,51089E+14	8,8417E+14	9,00825E+13	8,10739E+14	6,1528E+14	7,90291E+14	6,75678E+14
TC0400000867.mm.2	Rraga	Ras-related GTP binding A	2,06937E+14	7,66235E+14	2,58006E+14	0.0392623732044719	0.180136696762908	-3,51112E+14	8,91476E+14	8,73957E+14	7,99633E+14	5,51927E+14	7,91334E+14	6,89083E+14
TC0500000213.mm.2	Abcb8	ATP-binding cassette, sub-family B (MDR/TAP), member 8	1,31705E+14	6,99229E+13	2,57995E+14	0.039268098779202	0.180136696762908	-3,51125E+14	7,91944E+14	7,80046E+14	7,24579E+14	5,84E+13	6,30035E+14	6,84768E+14
TC1000002195.mm.2	Serinc1	serine incorporator 1	1,9116E+14	1,07442E+14	2,57976E+14	0.0392788977177514	0.180136696762908	-3,5115E+14	1,18758E+13	1,19267E+14	1,10196E+14	8,95976E+14	1,10041E+14	9,67941E+14
TC0500002999.mm.2	Pus1	pseudouridine synthase 1	0.762678997635793	6,02078E+14	2,57956E+14	0.0392897563508351	0.180136696762908	-3,51174E+14	6,36478E+14	6,40119E+14	5,61824E+14	5,62237E+14	5,93541E+14	6,18272E+13
TC0700000790.mm.2	Klk1b22	kallikrein 1-related peptidase b22	-1,03889E+13	5,12469E+13	-2,57948E+13	0.039294113346304	0.180136696762908	-3,51184E+14	4,36805E+14	4,47031E+14	5,07739E+14	5,83875E+14	5,1673E+14	5,82634E+13
TC0800001421.mm.2	Necab2	N-terminal EF-hand calcium binding protein 2	1,0437E+14	7,95009E+14	2,57943E+14	0.0392965415556445	0.180136696762908	-3,5119E+14	8,70342E+14	8,53147E+13	8,07722E+14	7,07028E+14	7,64937E+13	7,6688E+14
TC0100001952.mm.2	Cops5	COP9 signalosome subunit 5	2,33938E+14	7,03674E+14	2,57911E+14	0.0393141531480392	0.180136696762908	-3,5123E+14	8,40426E+14	8,15691E+14	7,41762E+14	4,46478E+14	7,31837E+14	6,45851E+14
TC0700000400.mm.2	Dmrtc2	doublesex and mab-3 related transcription factor like family C2	-1,18589E+14	6,00272E+14	-2,57854E+14	0.0393458521458209	0.180136696762908	-3,51301E+14	5,16327E+14	5,17522E+14	5,86232E+14	6,84796E+14	6,06934E+14	6,89819E+14
TC1600002181.mm.2	Dgcr2	DiGeorge syndrome critical region gene 2	1,10796E+14	8,65502E+14	2,57841E+14	0.0393525877571153	0.180136696762908	-3,51317E+14	9,51285E+14	9,30998E+14	8,82717E+14	7,77973E+14	8,45919E+14	8,04117E+14
TC1100003076.mm.2	Eif5a	eukaryotic translation initiation factor 5A	2,11112E+14	9,44081E+14	2,5781E+14	0.0393698411985779	0.180136696762908	-3,51356E+14	1,0899E+14	1,07387E+14	1,00298E+14	7,38555E+14	9,16481E+14	8,42701E+14

TC1700001323.mm.2	Ston1	stonin 1	-0.877429420053981	6,43874E+14	-2,57808E+14	0.0393706368095804	0.180136696762908	-3,51357E+14	5,96865E+14	5,59798E+14	6,42452E+14	6,89697E+14	6,7424E+14	7,00196E+14
TC0700002347.mm.2	Zscan18	zinc finger and SCAN domain containing 18	0.797259995019091	6,34177E+14	2,57803E+14	0.0393738400121249	0.180136696762908	-3,51365E+14	6,74788E+14	6,79845E+14	6,15571E+14	5,79609E+14	6,37156E+14	6,1809E+14
TC0500003591.mm.2	Atp5j2	ATP synthase, H+ transporting, mitochondrial F0 complex, subunit F2	0.965468287955748	9,60423E+14	2,5778E+13	0.0393861375550359	0.180136696762908	-3,51392E+14	1,01416E+14	1,01571E+14	9,47507E+14	8,89272E+14	9,79682E+14	9,16204E+14
TC1100002654.mm.2	Btnl9	butyrophilin-like 9	-1,09229E+14	4,56588E+14	-2,57778E+14	0.039387549742121	0.180136696762908	-3,51396E+14	3,74256E+14	3,63773E+14	4,48374E+14	5,08112E+14	4,75612E+14	5,69399E+14
TC1100002120.mm.2	Cabp7	calcium binding protein 7	0.923059883251904	6,35746E+14	2,57756E+14	0.0393996129977327	0.180136696762908	-3,51423E+14	7,06446E+14	6,95036E+14	5,99452E+13	6,17418E+14	5,61241E+14	6,34884E+14
TC1100000223.mm.2	Tmem17	transmembrane protein 17	0.780171127723835	6,26572E+13	2,57707E+11	0.0394264981591078	0.180136696762908	-3,51484E+14	7,00939E+14	6,86443E+14	6,19031E+14	6,12316E+14	5,85469E+14	5,55233E+14
TC0200004619.mm.2	Idh3b	isocitrate dehydrogenase 3 (NAD+) beta	1,40508E+14	9,17856E+14	2,57702E+14	0.0394292658409631	0.180136696762908	-3,5149E+14	9,89839E+14	9,8132E+14	9,26082E+14	7,64061E+14	9,04936E+14	9,40901E+14
TC0100000911.mm.2	Olfr1410	olfactory receptor 1410	-0.818348101143222	6,54457E+14	-2,57667E+14	0.0394483418612165	0.180136696762908	-3,51533E+14	5,78219E+14	6,03488E+14	6,52313E+14	6,93064E+14	6,96084E+14	7,03574E+14
TC1100001535.mm.2	Mrpl45	mitochondrial ribosomal protein L45	0.984007946270607	7,16937E+14	2,57667E+14	0.0394484033249466	0.180136696762908	-3,51533E+14	7,92174E+14	7,73447E+14	7,20211E+14	6,48608E+14	7,09385E+14	6,57795E+14
TC0300000226.mm.2	Actl6a	actin-like 6A	1,49203E+14	5,79935E+14	2,57666E+14	0.0394487766659645	0.180136696762908	-3,51534E+14	6,65737E+14	6,7392E+14	5,99955E+14	4,41297E+14	5,91974E+14	5,06723E+14
TC0700000557.mm.2	Clip3	CAP-GLY domain containing linker protein 3	0.7548002698604	8,03101E+14	2,57665E+14	0.0394495565292793	0.180136696762908	-3,51536E+14	8,66947E+14	8,64238E+14	7,81702E+14	7,98523E+13	7,47928E+14	7,59267E+14
TC0700000406.mm.2	D930028M14Rik	RIKEN cDNA D930028M14 gene	-1,11566E+14	4,93604E+14	-2,57617E+14	0.0394759609750296	0.180136696762908	-3,51595E+14	4,24025E+13	4,48276E+14	5,16988E+14	5,78446E+14	4,49147E+14	5,44742E+14
TC1100003669.mm.2	Med1	mediator complex subunit 1	1,52881E+14	6,82461E+14	2,57607E+14	0.0394816294510348	0.180136696762908	-3,51608E+14	7,88067E+14	8,10786E+14	6,92613E+14	6,00479E+14	6,81528E+14	5,21293E+14
TC0200001441.mm.2	Chst1	carbohydrate sulfotransferase 1	1,04484E+14	9,05923E+14	2,57563E+14	0.0395058683351569	0.180136696762908	-3,51663E+14	9,5107E+14	9,83994E+14	9,06059E+14	8,20036E+14	9,09903E+14	8,64476E+14
TC0700002720.mm.2	Spint2	serine protease inhibitor, Kunitz type 2	0.772000596396407	7,19034E+13	2,57545E+14	0.0395156278430879	0.180136696762908	-3,51685E+14	7,67877E+14	7,74369E+14	6,80021E+14	7,07825E+14	6,86508E+14	6,97604E+14
TC1300002588.mm.2	Erbin	Erbb2 interacting protein	2,19939E+14	6,93229E+13	2,57545E+14	0.0395156667148924	0.180136696762908	-3,51685E+14	8,25063E+14	8,4654E+14	7,10825E+13	5,209E+14	7,3601E+13	5,20034E+13
TC0400004070.mm.2	Park7	Parkinson disease (autosomal recessive, early onset) 7	1,54396E+14	7,13156E+14	2,57458E+14	0.0395637424177207	0.180136696762908	-3,51793E+14	7,89473E+13	8,26474E+14	7,32087E+14	5,7507E+12	7,26938E+14	6,28896E+14
TC1800001507.mm.2	Spire1	spire type actin nucleation factor 1	1,4166E+14	8,73686E+14	2,57454E+14	0.0395660679569241	0.180136696762908	-3,51798E+14	9,47203E+13	9,54013E+14	8,9123E+14	7,26666E+14	8,78777E+14	8,44229E+14
TC1300000791.mm.2	Gm10775	predicted gene 10775	-0.980813398120261	5,94345E+14	-2,57425E+14	0.0395816833861767	0.180136696762908	-3,51833E+14	5,24725E+14	5,4247E+14	5,91888E+14	6,7147E+14	5,9902E+14	6,36497E+14
TC1900000118.mm.2	Rasgrp2	RAS, guanyl releasing protein 2	0.961823633946851	7,96349E+14	2,57425E+14	0.0395818786283748	0.180136696762908	-3,51834E+14	8,52794E+14	8,49022E+14	7,79095E+14	7,30356E+14	8,18581E+14	7,48246E+14
TC1300001242.mm.2	Plk2	polo like kinase 2	1,40156E+14	7,83661E+14	2,5742E+14	0.0395846021060165	0.180136696762908	-3,5184E+14	8,69293E+14	9,06043E+14	8,07265E+13	6,87759E+14	7,70293E+14	6,61314E+14
TC1100001385.mm.2	Coil/2210409E12Rik	coilin/transcription elongation factor B (SIII), polypeptide 2 pseudogene	-1,1805E+14	6,48841E+14	-2,57373E+14	0.0396108172197197	0.180136696762908	-3,51899E+14	6,0032E+14	5,55785E+14	6,53157E+14	7,39049E+14	6,30297E+14	7,14435E+14
TC1000002420.mm.2	Adarb1	adenosine deaminase, RNA-specific, B1	0.964297445802753	8,32209E+14	2,57361E+14	0.0396171538497213	0.180136696762908	-3,51913E+13	8,92451E+14	8,87356E+14	8,29392E+14	7,57555E+14	8,36977E+14	7,89525E+14
TC0100003560.mm.2	Ncstn	nicastrin	0.901169552426886	7,58075E+14	2,57348E+14	0.0396245126289857	0.180136696762908	-3,5193E+14	8,43133E+13	8,25834E+14	7,48832E+14	7,39902E+14	7,29922E+14	6,60827E+14
TC0100002365.mm.2	Satb2	special AT-rich sequence binding protein 2	-0.771046222331941	9,37082E+14	-2,57347E+13	0.0396251081417816	0.180136696762908	-3,51931E+14	8,73208E+14	9,01263E+13	9,59878E+14	9,68802E+14	9,63569E+13	9,55771E+14
TC0600000245.mm.2	Ahcyl2	S-adenosylhomocysteine hydrolase-like 2	1,89463E+14	6,90516E+14	2,57345E+14	0.0396258877940074	0.180136696762908	-3,51933E+14	7,8754E+14	7,8124E+14	7,13466E+14	4,76388E+13	7,18911E+14	6,6555E+14
TC1600001545.mm.2	Sec22a	SEC22 homolog A, vesicle trafficking protein	1,04502E+14	6,70316E+14	2,57337E+14	0.039630506697406	0.180136696762908	-3,51943E+14	7,11028E+14	7,37817E+14	6,60524E+14	5,79317E+14	6,95238E+14	6,3797E+14
TC1800001151.mm.2	4933408B17Rik	RIKEN cDNA 4933408B17 gene	-0.888161077544059	4,56117E+14	-2,57335E+14	0.0396316384301192	0.180136696762908	-3,51946E+14	3,56997E+14	4,16959E+14	4,59942E+14	4,91646E+14	5,10628E+14	5,00528E+14
TC0800000370.mm.2	Tti2	TELO2 interacting protein 2	0.769219616058767	5,38285E+14	2,57289E+14	0.0396570692362909	0.180158871271149	-3,52003E+14	5,82421E+14	5,95597E+14	5,03113E+14	5,21061E+14	5,03981E+13	5,23535E+14
TC1600000999.mm.2	Olig1	oligodendrocyte transcription factor 1	0.97368989947151	7,9387E+14	2,57278E+14	0.0396633195212707	0.180158871271149	-3,52017E+14	8,32718E+14	8,57882E+14	7,90269E+14	7,05593E+14	7,88084E+14	7,88672E+14
TC1000000864.mm.2	Adat3/Scamp4	adenosine deaminase, tRNA-specific 3/secretory carrier membrane protein 4	0.807158740111474	7,20396E+14	2,57247E+14	0.03968029338069	0.180158871271149	-3,52055E+14	8,01561E+14	7,66058E+13	7,11627E+14	6,9456E+14	6,6319E+14	6,85381E+14
TC0200005462.mm.2	Entpd2	ectonucleoside triphosphate diphosphohydrolase 2	0.754125847166796	6,93977E+14	2,57245E+14	0.039681240870186	0.180158871271149	-3,52057E+14	7,51888E+14	7,45664E+14	6,68079E+14	6,78648E+14	6,68227E+14	6,51355E+14
TC0400001058.mm.2	Sgip1	SH3-domain GRB2-like (endophilin) interacting protein 1	2,28265E+14	8,10033E+14	2,57146E+14	0.0397363583160075	0.180280409705094	-3,52181E+14	9,5886E+14	9,50774E+14	8,61636E+14	5,91468E+14	8,15198E+14	6,82262E+14
TC0X00002671.mm.2	Las1l	LAS1-like (S. cerevisiae)	1,12914E+13	5,93109E+13	2,57146E+14	0.0397364630583933	0.180280409705094	-3,52181E+14	6,99667E+14	6,99257E+14	6,41272E+14	5,31824E+14	5,16779E+14	4,69857E+14
TC0400003380.mm.2	Dph2	DPH2 homolog	-0.792615789576993	7,63411E+14	-2,57136E+14	0.0397415760735116	0.180280409705094	-3,52192E+14	7,02291E+14	7,03133E+14	7,68386E+14	7,95562E+14	7,90857E+14	8,20239E+14
TC1100002126.mm.2	Ewsr1	Ewing sarcoma breakpoint region 1	0.869524583504001	8,91376E+14	2,57075E+14	0.0397756100780667	0.180384014969853	-3,52269E+14	9,39477E+14	9,42497E+14	8,60364E+14	8,47705E+14	9,1044E+14	8,47776E+14
TC1100000586.mm.2	Ppp2ca/Mir3061	protein phosphatase 2 (formerly 2A), catalytic subunit, alpha isoform/microRNA 3061	1,91111E+14	9,39792E+13	2,57045E+13	0.0397922785997314	0.180388691111278	-3,52306E+14	1,07599E+14	1,05396E+13	9,724E+14	7,75323E+12	9,5326E+14	8,0782E+14
TC0200005386.mm.2	Ppp1r3d	protein phosphatase 1, regulatory subunit 3D	-1,07338E+14	6,42232E+14	-2,57024E+14	0.0398039285992293	0.180388691111278	-3,52332E+14	5,87229E+14	5,73419E+14	7,37486E+14	6,37839E+14	6,79283E+13	6,38137E+14
TC0100002631.mm.2	Farsb	phenylalanyl-tRNA synthetase, beta subunit	2,20616E+14	5,98168E+14	2,56994E+14	0.0398204917281444	0.180388691111278	-3,52369E+14	7,14179E+13	6,9882E+14	6,28816E+14	3,4295E+14	6,25609E+14	5,78634E+13
TC0200003026.mm.2	A930004D18Rik	RIKEN cDNA A930004D18 gene	-0.931640854022872	6,73228E+12	-2,56974E+13	0.0398318827842882	0.180388691111278	-3,52395E+14	6,19212E+14	6,24723E+14	6,76092E+14	7,54171E+14	6,88913E+14	6,76258E+14
TC0200005205.mm.2	Ripor3	RIPOR family member 3	-0.900278799249272	7,34409E+14	-2,56918E+14	0.0398629824890009	0.180388691111278	-3,52464E+14	6,6766E+14	6,83797E+14	7,36878E+14	7,94635E+14	7,41877E+14	7,81606E+14
TC0400002389.mm.2	Cfap206	cilia and flagella associated protein 206	-1,28441E+14	7,82348E+14	-2,56915E+14	0.0398644497868034	0.180388691111278	-3,52467E+13	6,73272E+14	6,92119E+14	7,50241E+14	8,72031E+14	8,13405E+13	8,93022E+14
TC0300003221.mm.2	Wdr77	WD repeat domain 77	0.867785122792344	7,39528E+14	2,56893E+14	0.0398766496504123	0.180388691111278	-3,52495E+14	7,91322E+14	8,03915E+14	7,39381E+14	6,82299E+14	7,22787E+14	6,97467E+14
TC1500000969.mm.2	Pfkm	phosphofructokinase, muscle	1,46011E+14	8,33601E+14	2,56892E+13	0.0398776397236054	0.180388691111278	-3,52497E+14	9,33428E+14	9,26612E+14	8,69952E+13	6,98066E+14	8,05076E+14	7,68472E+14
TC1500001008.mm.2	Spats2	spermatogenesis associated, serine-rich 2	1,60776E+14	6,72381E+14	2,56877E+14	0.0398859079858144	0.180388691111278	-3,52515E+14	7,78772E+13	7,81482E+14	7,11342E+14	5,2736E+13	6,56095E+14	5,79233E+14
TC0300002583.mm.2	Man1a2	mannosidase, alpha, class 1A, member 2	1,59686E+14	7,64177E+14	2,56845E+14	0.0399036941473587	0.180388691111278	-3,52555E+14	8,64853E+14	8,78133E+14	7,54047E+14	6,69568E+14	7,98369E+14	6,20091E+14
TC1000002525.mm.2	Creb3l3	cAMP responsive element binding protein 3-like 3	-0.841273584545263	6,44298E+14	-2,56838E+14	0.0399074698105878	0.180388691111278	-3,52563E+14	5,7097E+14	5,82952E+14	6,35589E+14	6,86588E+14	6,84398E+14	7,0529E+14
TC1800000306.mm.2	Ctnna1	catenin (cadherin associated protein), alpha 1	1,07252E+14	6,8295E+14	2,56811E+14	0.0399223140009269	0.180388691111278	-3,52596E+14	7,52268E+14	7,58834E+14	6,778E+14	6,18798E+14	6,90524E+14	5,99475E+14
TC1100002966.mm.2	Hs3st3b1	heparan sulfate (glucosamine) 3-O-sulfotransferase 3B1	-1,14507E+14	7,20923E+14	-2,56767E+14	0.0399468612993561	0.180388691111278	-3,52651E+14	6,11568E+14	6,77722E+14	7,18545E+14	7,99759E+14	7,27397E+14	7,90548E+14
TC1000000726.mm.2	Derl3	Der1-like domain family, member 3	-1,17611E+14	7,13712E+14	-2,56751E+14	0.0399560852588565	0.180388691111278	-3,52672E+14	6,23567E+14	6,29389E+14	6,8775E+14	8,00428E+14	7,40406E+14	8,0073E+14
TC0100001382.mm.2	Rnasel	ribonuclease L (2', 5'-oligoisoadenylate synthetase-dependent)	0.993229488779448	5,42878E+14	2,56747E+14	0.0399581986642743	0.180388691111278	-3,52676E+14	5,45547E+14	6,36403E+14	4,8679E+14	4,96514E+14	5,37633E+14	5,54378E+14
TC0700002995.mm.2	Gfy	golgi-associated olfactory signaling regulator	-1,10526E+14	5,83821E+13	-2,56744E+14	0.0399596700933512	0.180388691111278	-3,5268E+14	5,04152E+14	5,12725E+14	5,83042E+14	6,54888E+14	5,79388E+14	6,68728E+14
TC1000001075.mm.2	Washc3	WASH complex subunit 3	1,61189E+14	6,65039E+14	2,56645E+14	0.0400151200285358	0.180388691111278	-3,52803E+14	7,38329E+14	7,39836E+14	6,53422E+14	5,02366E+14	7,43894E+14	6,12384E+14
TC0300001232.mm.2	Cnn3	calponin 3, acidic	1,85999E+14	7,69709E+14	2,56644E+14	0.0400155784547036	0.180388691111278	-3,52804E+13	8,59469E+14	9,03212E+14	8,05649E+13	5,85035E+14	7,72291E+14	6,92597E+14
TC1300000408.mm.2	Ppp1r3g	protein phosphatase 1, regulatory subunit 3G	-1,10599E+14	5,60077E+14	-2,56611E+14	0.0400344283086544	0.180388691111278	-3,52846E+14	4,79241E+14	4,76726E+14	5,44138E+14	6,33026E+13	5,77039E+14	6,50291E+14
TC0900001800.mm.2	Naalad2	N-acetylated alpha-linked acidic dipeptidase 2	-0.955100093515625	5,3173E+14	-2,56608E+14	0.0400357013993572	0.180388691111278	-3,52849E+14	5,02133E+14	4,56775E+14	5,4847E+14	6,01458E+13	5,16614E+13	5,64929E+14
TC0700002170.mm.2	Fam71e2	family with sequence similarity 71, member E2	-0.948786488280558	6,07794E+14	-2,56597E+13	0.040042182610775	0.180388691111278	-3,52863E+14	5,28952E+12	5,62563E+14	6,02483E+13	6,7879E+14	6,38847E+14	6,35132E+14
TC0100002588.mm.2	Rnf25	ring finger protein 25	1,06098E+14	7,46901E+14	2,56586E+14	0.0400481908282673	0.180388691111278	-3,52877E+14	8,23488E+14	8,09411E+14	7,61218E+14	6,59485E+14	7,28263E+14	6,99541E+14
TC0300002074.mm.2	Slc33a1	solute carrier family 33 (acetyl-CoA transporter), member 1	1,03405E+14	5,12722E+13	2,56562E+14	0.0400616689558283	0.180388691111278	-3,52907E+14	5,82994E+14	5,66855E+14	5,20455E+14	4,22584E+14	4,8129E+14	5,02154E+14
TC0100002618.mm.2	Epha4	Eph receptor A4	1,61355E+14	7,43138E+14	2,56553E+13	0.0400665572157654	0.180388691111278	-3,52918E+14	8,42613E+14	8,47536E+13	7,82166E+14	5,85272E+14	7,19756E+14	6,81482E+14
TC0100000137.mm.2	Khdc1a	KH domain containing 1A	-1,27874E+14	5,31423E+14	-2,56541E+14	0.0400734601650547	0.180388691111278	-3,52933E+12	4,24083E+14	4,90642E+13	5,2915E+14	6,41324E+14	5,18789E+14	5,84549E+14
TC1200002101.mm.2	Erg28	ergosterol biosynthesis 28	1,46802E+13	8,11034E+14	2,56516E+14	0.0400872579666711	0.180388691111278	-3,52964E+14	9,07121E+13	8,83269E+14	8,32185E+14	6,64602E+14	8,16217E+14	7,62809E+14
TC0800000974.mm.2	Prdx2	peroxiredoxin 2	1,83937E+14	9,56124E+14	2,56515E+14	0.0400879064403288	0.180388691111278	-3,52965E+14	1,06608E+14	1,05982E+14	9,88865E+14	7,69168E+14	9,68847E+14	8,83958E+14
TC1200000017.mm.2	Pomc	pro-opiomelanocortin-alpha	-1,51542E+14	6,26917E+14	-2,56511E+14	0.0400901075448078	0.180388691111278	-3,5297E+14	5,37241E+14	5,02459E+14	5,91529E+14	7,51256E+14	6,46068E+14	7,32952E+14
TC1200002421.mm.2	Atp5mpl	ATP synthase membrane subunit 6.8PL	0.970348282714991	9,27737E+14	2,56464E+14	0.0401164898879139	0.180423324035946	-3,53029E+14	9,93446E+14	9,98616E+13	9,19359E+14	8,78634E+13	9,27262E+13	8,49106E+14
TC0X00001653.mm.2	Ctps2	cytidine 5'-triphosphate synthase 2	1,05927E+14	7,49349E+14	2,56428E+14	0.0401368525420748	0.180423324035946	-3,53074E+14	8,03532E+14	8,16439E+14	6,53574E+14	7,54543E+14	7,49555E+14	7,18452E+14
TC1000000354.mm.2	Slc22a16	solute carrier family 22 (organic cation transporter), member 16	-1,38244E+14	5,14039E+14	-2,56426E+14	0.0401377830225429	0.180423324035946	-3,53076E+14	4,31437E+14	4,27569E+14	4,94259E+14	6,41235E+14	5,08998E+14	5,80738E+14
TC0500002950.mm.2	Gak	cyclin G associated kinase	1,01487E+13	7,26635E+14	2,56398E+12	0.0401534963621533	0.180423324035946	-3,53111E+14	8,34165E+14	7,9886E+13	7,34736E+14	6,95314E+14	6,8939E+14	6,07342E+14
TC0900002346.mm.2	Tspan3	tetraspanin 3	1,52094E+14	9,77385E+13	2,5638E+14	0.0401634164772659	0.180423324035946	-3,53133E+14	1,07854E+14	1,06332E+14	1,00098E+14	8,36695E+14	9,85475E+14	8,99306E+14
TC0600001238.mm.2	Thumpd3	THUMP domain containing 3	1,33638E+14	5,48488E+14	2,56378E+14	0.0401649887244422	0.180423324035946	-3,53136E+14	6,62193E+14	6,32707E+14	5,85813E+14	4,41811E+14	5,10285E+14	4,5812E+14
TC1000000592.mm.2	Dna2	DNA replication helicase/nuclease 2	-0.816143541116883	5,38945E+14	-2,56349E+14	0.0401810241136174	0.180433620494646	-3,53172E+13	4,71695E+14	4,96444E+14	5,44907E+14	5,8646E+14	5,71537E+14	5,62628E+14
TC0500003369.mm.2	Gtf2i	general transcription factor II I	1,25314E+14	8,47444E+14	2,56327E+14	0.0401930558390957	0.180433620494646	-3,53198E+14	9,35687E+14	9,59239E+14	8,67891E+14	7,76407E+13	8,25629E+14	7,1981E+14
TC0800001419.mm.2	Mlycd	malonyl-CoA decarboxylase	-0.829274092278504	5,69471E+14	-2,56275E+14	0.0402227919826199	0.180433620494646	-3,53264E+14	5,25984E+14	5,4509E+14	5,94562E+14	6,42367E+14	5,4736E+14	5,61461E+13
TC1800000324.mm.2	Igip/Pura	IgA inducing protein/purine rich element binding protein A	0.971628944086428	9,86137E+14	2,56248E+14	0.0402375676007847	0.180433620494646	-3,53297E+14	1,0603E+14	1,04788E+14	9,94497E+14	9,19355E+14	9,70159E+14	9,24632E+13
TC0600001740.mm.2	Ppfibp1	PTPRF interacting protein, binding protein 1 (liprin beta 1)	-0.98948405029137	5,55974E+14	-2,56244E+14	0.0402400132206522	0.180433620494646	-3,53302E+14	5,04802E+14	4,97678E+14	5,54243E+14	6,46135E+14	5,62464E+14	5,70525E+14
TC0500003560.mm.2	Rac1	Rac family small GTPase 1	1,79453E+14	1,06675E+14	2,56239E+13	0.0402426395090156	0.180433620494646	-3,53308E+14	1,17831E+14	1,17744E+14	1,10229E+14	8,94555E+14	1,07218E+13	9,75697E+14
TC0100002739.mm.2	Ncl	nucleolin	1,53618E+14	8,43179E+14	2,56234E+13	0.0402456669805598	0.180433620494646	-3,53315E+13	9,46418E+14	9,51371E+14	8,68149E+14	7,22402E+14	8,45249E+14	7,25486E+14
TC0200000955.mm.2	Csrnp3	cysteine-serine-rich nuclear protein 3	1,13372E+14	8,02348E+14	2,56209E+14	0.0402597865908548	0.180446715254087	-3,53346E+14	8,88691E+14	9,08008E+14	8,04658E+14	7,65296E+14	7,79937E+14	6,67497E+14
TC1200001544.mm.2	Id2	inhibitor of DNA binding 2	1,64542E+14	8,51108E+14	2,56098E+14	0.0403221892036283	0.180640597539678	-3,53484E+14	9,06104E+14	9,55342E+14	8,42025E+14	6,90337E+14	9,2169E+14	7,9115E+14
TC0300000206.mm.2	Prkci	protein kinase C, iota	2,07219E+14	6,32005E+14	2,56073E+14	0.0403363462158602	0.180640597539678	-3,53515E+14	7,73489E+14	7,65062E+14	6,83882E+14	4,40231E+14	6,24192E+14	5,05173E+14
TC1100003054.mm.2	Mpdu1	mannose-P-dolichol utilization defect 1	0.87783441113015	9,1089E+14	2,56072E+14	0.0403366765932949	0.180640597539678	-3,53516E+14	9,74584E+14	9,59238E+14	9,11741E+14	8,46514E+13	8,87896E+13	8,85368E+14
TC0300000444.mm.2	Rnf13	ring finger protein 13	1,02137E+13	8,77676E+14	2,56042E+14	0.0403538515831599	0.180667299405239	-3,53554E+14	9,38794E+14	9,50424E+13	8,66225E+14	8,18719E+14	8,89122E+14	8,02774E+14
TC0400002477.mm.2	Dctn3	dynactin 3	1,50119E+14	9,52032E+14	2,55964E+14	0.0403975943310128	0.180793680329026	-3,5365E+14	1,04781E+14	1,05068E+14	9,85823E+13	8,12426E+14	9,37502E+14	8,77954E+14
TC0900002435.mm.2	Lrrc49	leucine rich repeat containing 49	1,37782E+14	8,2574E+14	2,55933E+14	0.0404154245442094	0.180793680329026	-3,5369E+14	9,46259E+14	9,09627E+14	8,17032E+13	7,6329E+14	8,33994E+14	6,84236E+14
TC0100000027.mm.2	Pcmtd1	protein-L-isoaspartate (D-aspartate) O-methyltransferase domain containing 1	2,20537E+13	7,9805E+14	2,55932E+14	0.0404157411124653	0.180793680329026	-3,5369E+14	9,24842E+14	9,20734E+14	8,17662E+14	5,86841E+14	8,57972E+14	6,80248E+14
TC0900001568.mm.2	Ctdspl	CTD (carboxy-terminal domain, RNA polymerase II, polypeptide A) small phosphatase-like	-0.77919267852971	7,7454E+14	-2,55895E+14	0.0404367301687721	0.18082241636192	-3,53736E+14	7,15382E+14	7,277E+14	8,09372E+14	7,89548E+14	8,16423E+14	7,88814E+14
TC0400001292.mm.2	Eri3	exoribonuclease 3	1,25579E+14	8,28775E+14	2,55881E+14	0.0404446092328158	0.18082241636192	-3,53754E+13	9,09979E+14	8,98171E+14	8,365E+14	7,20491E+14	8,4487E+14	7,62638E+14
TC0700001354.mm.2	Ints4	integrator complex subunit 4	0.761130762776585	6,4534E+14	2,55846E+14	0.0404644172384995	0.180834423111661	-3,53797E+14	7,331E+14	7,1025E+13	6,39321E+14	6,51803E+13	5,73447E+14	5,64121E+14
TC1700001178.mm.2	Crim1	cysteine rich transmembrane BMP regulator 1 (chordin like)	1,49682E+14	7,75289E+14	2,55826E+14	0.0404758188187211	0.180834423111661	-3,53823E+14	8,92695E+14	8,91044E+14	7,80816E+14	7,03559E+14	7,74635E+14	6,08984E+14
TC0900002332.mm.2	Nrg4	neuregulin 4	-1,06349E+14	6,70527E+14	-2,55798E+13	0.0404916745397889	0.180834423111661	-3,53857E+13	6,23561E+13	6,00885E+14	6,67955E+14	7,69189E+13	6,662E+13	6,95371E+14
TC1100001935.mm.2	Sphk1	sphingosine kinase 1	-0.969139538637839	6,42E+14	-2,55797E+14	0.0404921863456137	0.180834423111661	-3,53859E+14	5,70878E+14	5,67937E+14	6,42077E+14	6,90565E+13	6,53245E+14	7,27299E+14
TC1100000432.mm.2	Pwwp2a	PWWP domain containing 2A	-1,79018E+14	9,66989E+14	-2,55747E+13	0.0405203228727269	0.180880130583712	-3,5392E+14	8,50919E+13	8,45676E+14	1,12241E+14	9,32223E+14	1,089E+14	9,61711E+14
TC1300001922.mm.2	Kif13a	kinesin family member 13A	0.836551963973338	5,93459E+14	2,55675E+14	0.0405611345444885	0.180880130583712	-3,5401E+14	6,54907E+14	6,37783E+14	5,8928E+14	5,361E+14	5,74062E+14	5,68625E+14
TC1300000256.mm.2	Hist1h1t	histone cluster 1, H1t	-0.7856559169868	5,10809E+13	-2,55672E+12	0.0405629871638209	0.180880130583712	-3,54014E+13	4,3586E+14	4,71947E+14	5,37381E+14	5,27557E+14	5,40398E+14	5,51712E+14
TC0200004737.mm.2	Kif16b	kinesin family member 16B	-0.756000280775925	7,27309E+14	-2,55663E+14	0.0405677332919528	0.180880130583712	-3,54024E+14	6,70813E+14	6,68492E+14	7,3287E+14	7,57635E+13	7,68549E+14	7,65494E+14
TC0X00000060.mm.2	Syp	synaptophysin	1,3198E+14	1,15193E+14	2,55663E+14	0.0405678127089509	0.180880130583712	-3,54025E+14	1,23626E+14	1,23616E+14	1,17768E+13	1,03079E+14	1,13815E+14	1,09255E+14
TC1500001825.mm.2	Cacng2	calcium channel, voltage-dependent, gamma subunit 2	1,69201E+14	8,00143E+14	2,5566E+14	0.0405697754564348	0.180880130583712	-3,54029E+13	9,24899E+14	9,1049E+14	8,35315E+14	6,61672E+14	7,962E+14	6,72279E+14
TC1700002177.mm.2	Ttbk1	tau tubulin kinase 1	1,27343E+14	6,97694E+14	2,5562E+13	0.0405923427192904	0.18091772308672	-3,54078E+14	7,763E+14	7,75871E+14	7,14234E+14	5,83252E+14	6,36064E+14	7,0044E+14
TC1800001088.mm.2	Celf4	CUGBP, Elav-like family member 4	1,84588E+14	8,85251E+14	2,55568E+14	0.0406217865299652	0.18091772308672	-3,54143E+14	1,00591E+14	9,96155E+14	9,23054E+14	7,09839E+14	8,90523E+14	7,86022E+14
TC1800000396.mm.2	Rnf14	ring finger protein 14	1,7521E+14	8,73682E+14	2,55535E+14	0.0406408063504471	0.18091772308672	-3,54185E+14	9,91095E+13	9,98276E+13	9,13404E+14	7,25547E+13	8,67106E+14	7,46666E+14
TC0X00001793.mm.2	Suv39h1	suppressor of variegation 3-9 1	1,31121E+14	6,4955E+13	2,55521E+14	0.0406482375811596	0.18091772308672	-3,54201E+14	7,66915E+14	7,24086E+14	6,50306E+14	5,78453E+14	6,52511E+14	5,25027E+14
TC1100004150.mm.2	Socs3	suppressor of cytokine signaling 3	-0.934630161314262	6,74332E+14	-2,55515E+14	0.040652061219195	0.18091772308672	-3,54209E+14	6,24716E+13	6,04146E+14	6,90045E+14	7,25743E+14	6,65697E+14	7,35646E+14
TC1300002351.mm.2	Nr2f1	nuclear receptor subfamily 2, group F, member 1	0.809645801231738	6,96839E+14	2,55512E+14	0.0406535217665166	0.18091772308672	-3,54213E+14	7,17452E+14	7,55738E+14	6,68588E+14	6,42672E+14	7,04586E+14	6,91997E+14
TC1900000021.mm.2	Aldh3b2	aldehyde dehydrogenase 3 family, member B2	-0.941074019307308	6,10357E+14	-2,55506E+14	0.0406568035311247	0.18091772308672	-3,5422E+14	5,70843E+14	5,48441E+14	6,72133E+14	6,35366E+14	5,82409E+14	6,52949E+14
TC0500001599.mm.2	Alkbh4	alkB homolog 4, lysine demethylase	-0.927070835695404	7,1658E+13	-2,5544E+14	0.0406944653767871	0.181013538585748	-3,54302E+13	6,29401E+14	6,44735E+14	7,67846E+14	6,91704E+14	7,74379E+13	7,91414E+14
TC1700002580.mm.2	Hnrnpll	heterogeneous nuclear ribonucleoprotein L-like	-0.853693176332645	8,30239E+14	-2,55422E+14	0.040705033934439	0.181013538585748	-3,54325E+13	7,47112E+14	7,86292E+14	8,31429E+14	8,72714E+14	8,60653E+14	8,83233E+14
TC1700001742.mm.2	Rps10	ribosomal protein S10	0.774429305533252	9,40164E+14	2,55409E+14	0.0407120377232514	0.181013538585748	-3,54341E+14	1,00285E+14	9,76912E+13	9,01325E+14	9,23555E+14	9,21304E+13	9,15034E+14
TC1600002097.mm.2	Runx1	runt related transcription factor 1	-0.873198126747194	6,21483E+14	-2,5535E+14	0.0407457889758387	0.181098354521754	-3,54414E+14	5,65241E+14	6,02906E+14	6,44501E+14	6,98285E+14	6,03724E+14	6,14242E+14
TC1100000337.mm.2	Hbq1b	hemoglobin, theta 1B	-1,15389E+14	5,41655E+14	-2,55334E+14	0.0407545979207611	0.181098354521754	-3,54434E+14	4,3906E+14	4,94702E+14	5,26394E+14	6,38145E+14	5,71816E+14	5,79812E+14
TC1100003769.mm.2	Jup	junction plakoglobin	0.896701820582447	7,93283E+14	2,5529E+14	0.0407801017881038	0.181098354521754	-3,54489E+14	8,33119E+14	8,53082E+14	7,76089E+14	7,30772E+14	7,5687E+14	8,09764E+14

TC1800000046.mm.2	4921524L21Rik	RIKEN cDNA 4921524L21 gene	-1,00334E+14	5,16794E+14	-2,55286E+13	0.0407823320244667	0.181098354521754	-3,54494E+14	4,27926E+14	4,75358E+14	5,1566E+14	5,88291E+14	5,26004E+14	5,67525E+14
TC0X00002562.mm.2	Mageb4	melanoma antigen, family B, 4	-1,05747E+14	4,52485E+14	-2,55277E+14	0.0407873101569816	0.181098354521754	-3,54505E+14	3,87605E+14	3,64673E+14	4,32977E+14	5,30796E+12	4,81007E+14	5,17852E+14
TC0200001189.mm.2	Zfp804a	zinc finger protein 804A	1,7316E+14	6,62864E+14	2,55236E+12	0.0408109143112958	0.181146445405514	-3,54557E+14	7,95285E+14	7,47328E+14	7,04204E+13	4,92088E+14	6,37073E+14	6,01209E+14
TC1400001456.mm.2	Cadps	Ca2+-dependent secretion activator	1,77856E+14	8,90187E+13	2,55219E+14	0.04082062578461	0.181146445405514	-3,54578E+14	1,0155E+14	9,9549E+14	9,11555E+14	7,43719E+14	9,13707E+14	7,61153E+14
TC0200002042.mm.2	Chgb	chromogranin B	0.957195355281364	7,96786E+14	2,55144E+14	0.0408633951611221	0.181180423794954	-3,54671E+14	8,7944E+14	8,47789E+14	7,89712E+14	7,46078E+14	7,92679E+14	7,25017E+14
TC1100002027.mm.2	Ccdc137	coiled-coil domain containing 137	-1,31293E+14	6,66147E+14	-2,55136E+14	0.0408679181488952	0.181180423794954	-3,54681E+14	6,1176E+14	5,7312E+14	6,75854E+14	7,71612E+14	6,25748E+14	7,38788E+14
TC1900000141.mm.2	Plaat3	phospholipase A and acyltransferase 3	0.832285680836384	7,21277E+14	2,55135E+14	0.0408682252834448	0.181180423794954	-3,54682E+14	7,86146E+14	7,49281E+14	7,01111E+14	6,67859E+14	7,2392E+14	6,99346E+14
TC1100001677.mm.2	Cd300lg	CD300 molecule like family member G	-0.894533423551835	4,94303E+14	-2,55109E+14	0.0408831169953509	0.181180423794954	-3,54714E+13	4,2162E+13	4,50742E+14	4,94111E+14	5,57157E+14	5,11439E+14	5,30745E+14
TC0700000038.mm.2	Brsk1	BR serine/threonine kinase 1	1,07813E+14	8,8505E+14	2,55101E+14	0.040887712478027	0.181180423794954	-3,54724E+14	9,40975E+14	9,65871E+14	8,95578E+14	7,95643E+14	8,77015E+14	8,35214E+14
TC0100002562.mm.2	Igfbp5	insulin-like growth factor binding protein 5	1,31655E+14	7,62604E+14	2,55085E+14	0.0408968456436665	0.181180423794954	-3,54744E+14	7,94517E+13	8,94666E+14	7,71209E+14	6,54664E+14	7,33016E+14	7,27554E+14
TC0800001274.mm.2	Wwp2	WW domain containing E3 ubiquitin protein ligase 2	0.947860724679802	6,691E+14	2,55067E+14	0.0409069932207561	0.181180423794954	-3,54766E+14	7,23003E+14	7,10827E+14	6,63936E+14	5,80322E+13	6,69201E+14	6,67313E+13
TC1700000573.mm.2	Zfp563	zinc finger protein 563	1,03247E+14	5,37532E+14	2,55015E+14	0.0409371047526203	0.181228675508693	-3,54831E+13	6,32044E+14	6,37815E+14	5,1629E+14	5,47075E+14	4,94675E+13	3,97294E+14
TC1200001244.mm.2	Apopt1	apoptogenic, mitochondrial 1	0.984152040250318	5,88337E+14	2,55009E+14	0.0409403822287372	0.181228675508693	-3,54839E+14	6,0733E+14	6,28938E+14	5,63893E+14	4,75545E+14	6,35309E+14	6,19011E+14
TC0100000469.mm.2	Cyp20a1	cytochrome P450, family 20, subfamily a, polypeptide 1	0.762342013765638	6,65977E+12	2,54957E+14	0.0409701077106895	0.181310449201412	-3,54903E+14	7,25698E+14	7,36659E+13	6,4128E+14	6,68609E+14	6,1367E+14	6,09948E+14
TC0200005331.mm.2	Gm14399	predicted gene 14399	1,89916E+14	8,96625E+14	2,54923E+14	0.0409896383676984	0.181347073865657	-3,54946E+14	9,83941E+14	1,01896E+14	9,08894E+13	7,14177E+14	9,50413E+14	8,03365E+14
TC1200002224.mm.2	Ttc7b	tetratricopeptide repeat domain 7B	1,53152E+14	7,8717E+14	2,5488E+14	0.0410145707260541	0.181367963719472	-3,55E+14	8,87778E+14	8,87188E+14	8,00038E+14	6,68624E+14	8,07792E+14	6,71598E+14
TC0100001502.mm.2	Nme7	NME/NM23 family member 7	1,08337E+14	6,3134E+14	2,54861E+14	0.0410254168724423	0.181367963719472	-3,55023E+14	7,08626E+14	6,84549E+14	6,20482E+14	5,56018E+14	6,53588E+14	5,6478E+14
TC0900003338.mm.2	Dync2h1	dynein cytoplasmic 2 heavy chain 1	0.948624191061322	5,492E+14	2,54856E+14	0.0410281280802753	0.181367963719472	-3,55029E+14	6,32397E+14	6,04241E+14	4,99634E+14	5,47279E+14	5,3799E+14	4,73659E+13
TC0600000822.mm.2	Krcc1/Mir8112	lysine-rich coiled-coil 1/microRNA 8112	1,83248E+14	5,19677E+14	2,54802E+14	0.0410589466998394	0.181446060381775	-3,55096E+14	6,27983E+14	6,24166E+14	5,09534E+14	3,76119E+14	5,87712E+14	3,92547E+14
TC1900001107.mm.2	Tmem138	transmembrane protein 138	1,84004E+13	5,72979E+14	2,54776E+14	0.0410741189073937	0.181446060381775	-3,55129E+14	6,59483E+13	6,72989E+14	5,99209E+14	3,65255E+14	5,90158E+14	5,50783E+14
TC1500001656.mm.2	Sla	src-like adaptor	1,3126E+14	5,59215E+14	2,54766E+14	0.0410795772526976	0.181446060381775	-3,55141E+14	6,08508E+14	6,44003E+14	5,54052E+14	4,35938E+14	5,10808E+14	6,01978E+14
TC0300003045.mm.2	Pdlim5	PDZ and LIM domain 5	0.747190006816499	7,34287E+14	2,54732E+14	0.0410991017707215	0.181482550515658	-3,55183E+14	7,77909E+14	7,7615E+14	7,00725E+14	7,03896E+14	7,33947E+14	7,13097E+14
TC0300001805.mm.2	Pex5l	peroxisomal biogenesis factor 5-like	1,11069E+14	7,40978E+14	2,54701E+14	0.0411173276884294	0.181513287957168	-3,55223E+14	8,04122E+14	8,3099E+14	6,90789E+14	7,22184E+14	7,53964E+14	6,43818E+14
TC1500000721.mm.2	Rbx1	ring-box 1	1,25782E+14	7,6297E+14	2,54641E+14	0.0411517726014642	0.181581304500408	-3,55297E+14	8,41674E+14	8,38746E+14	7,70272E+14	6,58583E+14	7,79769E+14	6,88774E+14
TC0700003807.mm.2	Spcs2	signal peptidase complex subunit 2 homolog (S. cerevisiae)	1,32222E+14	8,75649E+13	2,54635E+14	0.0411552736321908	0.181581304500408	-3,55305E+14	9,54901E+14	9,62734E+14	8,86539E+14	7,66653E+14	8,90589E+14	7,92475E+14
TC0100001616.mm.2	Igsf9	immunoglobulin superfamily, member 9	-0.73465168626711	7,2296E+14	-2,54607E+14	0.0411710091052765	0.181599644421913	-3,55339E+14	6,72556E+14	6,66691E+14	7,41987E+14	7,44191E+14	7,59734E+14	7,52603E+14
TC1500000432.mm.2	Sqle	squalene epoxidase	1,18522E+14	6,83489E+14	2,54588E+14	0.0411819711237926	0.181599644421913	-3,55362E+14	7,58114E+14	7,46188E+14	6,92956E+14	5,74302E+14	6,92234E+14	6,37139E+14
TC1400000742.mm.2	Abhd4	abhydrolase domain containing 4	1,20558E+14	8,75512E+14	2,5453E+14	0.04121505229031	0.181626289538497	-3,55435E+14	9,66911E+13	9,57462E+14	8,99154E+14	7,84102E+14	8,57757E+14	7,87688E+14
TC0200003435.mm.2	Golga1	golgi autoantigen, golgin subfamily a, 1	1,0532E+14	5,43432E+14	2,54508E+14	0.0412283637196924	0.181626289538497	-3,55463E+14	6,30062E+14	6,14508E+14	5,5478E+14	4,79151E+14	5,2794E+14	4,5415E+14
TC1300002753.mm.2	B020031M17Rik	RIKEN cDNA B020031M17 gene	-1,35091E+14	4,99332E+14	-2,5442E+14	0.0412790617570715	0.181626289538497	-3,55572E+14	3,88777E+14	4,5516E+13	4,87425E+14	6,26694E+14	4,97519E+14	5,40416E+14
TC1100001004.mm.2	Ankfy1	ankyrin repeat and FYVE domain containing 1	0.920260052354736	5,92158E+14	2,5441E+14	0.0412847085484379	0.181626289538497	-3,55584E+14	6,58479E+14	6,58587E+14	6,03371E+14	5,29644E+14	5,6535E+14	5,37515E+14
TC0400001924.mm.2	Mthfr	methylenetetrahydrofolate reductase	-0.764943012424244	7,20042E+14	-2,54403E+14	0.0412890047560132	0.181626289538497	-3,55594E+14	6,72432E+13	6,98107E+14	7,5392E+13	7,69607E+14	7,07977E+14	7,18211E+14
TC0200001043.mm.2	Platr26	pluripotency associated transcript 26	-1,00765E+14	5,55212E+14	-2,54389E+14	0.0412966428362187	0.181626289538497	-3,5561E+14	4,91472E+14	5,01603E+14	5,58403E+14	6,36202E+14	5,42615E+14	6,00979E+14
TC0200004025.mm.2	Olfr1197	olfactory receptor 1197	-1,19425E+14	5,29167E+14	-2,54388E+14	0.0412973258239731	0.181626289538497	-3,55611E+13	4,98045E+13	4,33557E+13	5,30416E+14	6,40037E+14	5,16773E+14	5,56172E+14
TC0500001301.mm.2	Nos1	nitric oxide synthase 1, neuronal	1,18323E+14	6,1781E+14	2,54374E+14	0.0413056456958129	0.181626289538497	-3,55629E+14	7,18899E+14	7,1368E+14	5,86971E+14	6,08962E+14	4,7456E+13	6,03785E+14
TC1300001155.mm.2	Cd180	CD180 antigen	2,45372E+14	6,69224E+14	2,54371E+13	0.041307513898658	0.181626289538497	-3,55633E+14	7,44748E+14	8,57248E+14	6,92719E+14	4,18534E+14	7,2389E+13	5,78207E+14
TC0800002578.mm.2	D8Ertd738e	DNA segment, Chr 8, ERATO Doi 738, expressed	1,27427E+14	7,98735E+14	2,54368E+14	0.0413089659598747	0.181626289538497	-3,55637E+14	8,89716E+14	8,77599E+14	8,11611E+14	7,00849E+14	8,04392E+14	7,08247E+14
TC1100003161.mm.2	Aspa	aspartoacylase	2,36243E+12	6,24128E+14	2,54353E+14	0.0413175088419386	0.181626289538497	-3,55655E+14	7,65357E+14	7,39931E+14	6,58792E+14	3,74009E+14	6,71504E+13	5,35176E+14
TC0800000005.mm.2	Arhgef18	rho/rac guanine nucleotide exchange factor (GEF) 18	0.773578059940689	6,46575E+14	2,54343E+13	0.0413232779400566	0.181626289538497	-3,55667E+14	6,76384E+14	7,06755E+14	6,19642E+14	6,08782E+13	6,2621E+14	6,41678E+13
TC1700000693.mm.2	Psors1c2	psoriasis susceptibility 1 candidate 2 (human)	-0.796112819938884	5,52331E+14	-2,54302E+14	0.0413474562927726	0.181634091502595	-3,5572E+14	4,79911E+14	5,00457E+14	5,51628E+13	5,87961E+14	5,89192E+14	6,04837E+13
TC1200002339.mm.2	Bcl11b	B cell leukemia/lymphoma 11B	1,07394E+14	7,48791E+14	2,54301E+14	0.0413475980656313	0.181634091502595	-3,5572E+14	8,37263E+14	8,51113E+14	7,30282E+14	7,43305E+14	7,20068E+14	6,10714E+14
TC0200003885.mm.2	P2rx3	purinergic receptor P2X, ligand-gated ion channel, 3	-0.934450811188356	6,25908E+14	-2,54251E+14	0.0413766159875673	0.181712023278188	-3,55782E+14	5,65679E+14	5,62603E+14	6,24836E+14	6,90336E+14	6,31221E+14	6,80773E+14
TC0800003053.mm.2	Zcchc14	zinc finger, CCHC domain containing 14	0.818596859127267	6,25267E+14	2,5422E+13	0.0413947942776796	0.18174232157936	-3,55821E+14	6,76639E+14	6,94921E+14	6,22118E+14	5,85723E+14	6,02368E+14	5,6983E+14
TC0600002233.mm.2	Tmem176b	transmembrane protein 176B	1,97525E+14	8,03139E+14	2,54188E+14	0.0414133202572917	0.181759450116546	-3,55861E+14	9,067E+14	9,28082E+14	8,35926E+14	6,03807E+14	8,28528E+14	7,1579E+14
TC0600003102.mm.2	1700013D24Rik	RIKEN cDNA 1700013D24 gene	-0.835833594185699	4,75449E+14	-2,54174E+14	0.0414212561799763	0.181759450116546	-3,55878E+14	4,05936E+14	4,28517E+14	4,91643E+14	5,09977E+14	4,83592E+14	5,33029E+14
TC0600002056.mm.2	Wdr91	WD repeat domain 91	1,80629E+13	6,38374E+14	2,54007E+14	0.0415180246541669	0.18204627028353	-3,56086E+14	7,52263E+14	7,32171E+14	6,75733E+14	4,47442E+14	6,33114E+14	5,8952E+14
TC1200000089.mm.2	Smc6	structural maintenance of chromosomes 6	1,86655E+14	5,49129E+14	2,54006E+14	0.0415190092929935	0.18204627028353	-3,56088E+14	6,79327E+14	6,7556E+14	5,419E+14	4,39677E+14	5,89122E+14	3,6919E+14
TC0900001612.mm.2	Ctnnb1/Mir7090	catenin (cadherin associated protein), beta 1/microRNA 7090	0.869538819880196	9,47146E+14	2,54003E+14	0.0415205140750931	0.18204627028353	-3,56091E+14	1,00849E+14	1,00472E+14	9,40795E+14	8,98514E+14	9,40928E+13	8,89422E+14
TC0200004217.mm.2	Fjx1	four jointed box 1	0.935416147684687	8,137E+14	2,53951E+14	0.04155078965925	0.182096527247414	-3,56156E+14	8,87653E+14	8,72561E+14	8,25926E+14	7,47205E+14	7,64334E+14	7,8452E+14
TC1700002637.mm.2	Haao	3-hydroxyanthranilate 3,4-dioxygenase	-1,20645E+14	6,87656E+13	-2,53944E+14	0.0415545789541823	0.182096527247414	-3,56164E+14	6,07295E+14	6,30074E+14	6,87732E+14	7,90928E+14	6,624E+14	7,47505E+14
TC0500000347.mm.2	Depdc5	DEP domain containing 5	1,37139E+14	5,47503E+14	2,53895E+14	0.0415833408306619	0.182173020882125	-3,56226E+14	6,55746E+14	6,2282E+14	5,39315E+14	4,64973E+14	5,72312E+14	4,29853E+14
TC1900000050.mm.2	Mrpl11	mitochondrial ribosomal protein L11	1,06717E+14	7,41122E+14	2,53869E+14	0.0415983287864256	0.182189146978982	-3,56258E+13	7,91296E+14	7,87548E+14	7,37386E+14	6,28023E+14	7,57045E+14	7,45436E+14
TC0600001035.mm.2	Mgll	monoglyceride lipase	1,10227E+14	7,32864E+14	2,53778E+14	0.0416514390437785	0.182322763296552	-3,56372E+14	7,99247E+14	8,28852E+14	7,51315E+14	6,5633E+14	7,11325E+14	6,50112E+14
TC1400000795.mm.2	Nfatc4	nuclear factor of activated T cells, cytoplasmic, calcineurin dependent 4	-1,04376E+14	5,78578E+14	-2,53767E+14	0.0416579389005289	0.182322763296552	-3,56386E+14	5,56279E+14	5,03595E+13	5,91563E+14	6,77065E+14	5,55251E+14	5,87717E+14
TC1500000558.mm.2	Zfp41	zinc finger protein 41	-1,20902E+14	6,77847E+14	-2,53692E+14	0.0417013019794082	0.182322763296552	-3,56478E+14	6,29597E+14	5,78387E+14	6,67563E+14	7,82224E+14	6,78091E+14	7,31222E+14
TC0800002802.mm.2	Dync1li2	dynein, cytoplasmic 1 light intermediate chain 2	1,10476E+14	8,32638E+14	2,53687E+14	0.0417046166244808	0.182322763296552	-3,56485E+13	9,00995E+14	8,98016E+14	8,36348E+14	7,4171E+14	8,44048E+14	7,74711E+14
TC1500002263.mm.2	Krt90	keratin 90	-0.825751182308866	6,0775E+14	-2,53675E+14	0.0417115005414537	0.182322763296552	-3,565E+14	5,45783E+14	5,57093E+14	6,08316E+14	6,5971E+14	6,3004E+14	6,45556E+14
TC0X00003250.mm.2	Yy2/Mbtps2	Yy2 transcription factor/membrane-bound transcription factor peptidase, site 2	1,75824E+14	6,05593E+14	2,53656E+14	0.0417223050323691	0.182322763296552	-3,56523E+14	7,18256E+14	7,37826E+14	6,41249E+14	4,63186E+14	6,056E+14	4,67441E+14
TC1500001504.mm.2	Eif3h	eukaryotic translation initiation factor 3, subunit H	1,06533E+14	8,76185E+13	2,53653E+14	0.0417242145652556	0.182322763296552	-3,56527E+14	9,55861E+14	9,29866E+14	8,8411E+13	7,88551E+14	8,73141E+14	8,25583E+14
TC0700002837.mm.2	Kctd15	potassium channel tetramerisation domain containing 15	-0.821223643301606	6,63457E+14	-2,53638E+13	0.0417329716411833	0.182322763296552	-3,56546E+14	6,09412E+12	6,1399E+14	6,67535E+14	7,20112E+14	6,80385E+14	6,89309E+14
TC1000001089.mm.2	Uhrf1bp1l	UHRF1 (ICBP90) binding protein 1-like	2,07556E+14	7,58384E+14	2,53627E+14	0.0417392366738561	0.182322763296552	-3,56559E+14	8,8928E+14	9,15038E+14	7,70732E+14	6,18473E+14	7,92042E+14	5,64735E+14
TC1100001579.mm.2	Csf3	colony stimulating factor 3 (granulocyte)	-1,07179E+14	6,55003E+14	-2,53623E+14	0.0417419893130379	0.182322763296552	-3,56565E+14	5,43906E+14	6,15098E+14	6,46312E+14	7,27051E+14	6,8501E+14	7,12642E+14
TC1700000948.mm.2	Mocs1	molybdenum cofactor synthesis 1	-0.826316097015372	7,93117E+14	-2,53551E+14	0.0417840566773738	0.182378769195324	-3,56655E+13	7,63491E+14	7,40618E+14	8,17728E+14	8,51645E+14	7,74113E+14	8,11108E+14
TC1200001235.mm.2	Lbhd2	LBH domain containing 2	-1,12279E+14	8,14371E+14	-2,5354E+14	0.0417901041345972	0.182378769195324	-3,56668E+14	7,74664E+14	7,18757E+14	8,28894E+14	8,89085E+14	7,93096E+14	8,8173E+14
TC0400001091.mm.2	Plpp3	phospholipid phosphatase 3	1,45772E+14	8,93043E+14	2,53513E+14	0.0418060094287256	0.182378769195324	-3,56701E+14	1,00227E+14	1,00176E+14	9,25336E+14	7,87148E+14	8,77463E+13	7,6428E+13
TC1300002481.mm.2	Hexb	hexosaminidase B	1,78847E+14	8,86063E+14	2,53511E+12	0.0418072319939333	0.182378769195324	-3,56704E+14	9,84732E+14	1,01161E+14	9,23154E+14	7,15497E+14	8,89553E+14	7,91832E+14
TC0500003449.mm.2	Mospd3	motile sperm domain containing 3	-1,30588E+13	7,61985E+13	-2,53504E+13	0.0418114052881231	0.182378769195324	-3,56713E+14	6,88458E+14	6,79777E+13	7,48031E+14	8,8138E+14	7,49534E+14	8,24728E+14
TC1000000903.mm.2	Tle5	TLE family member 5, transcriptional modulator	1,59972E+14	1,06252E+14	2,53448E+14	0.0418441273481213	0.182472103913472	-3,56783E+14	1,17214E+14	1,14979E+14	1,09018E+14	9,11804E+14	1,07117E+14	9,80048E+14
TC1600001111.mm.2	Nlrc3	NLR family, CARD domain containing 3	-0.765698392681278	5,94905E+14	-2,53407E+13	0.0418679746207695	0.182526697798825	-3,56833E+14	5,33387E+14	5,40843E+14	6,13411E+14	6,13959E+14	6,17569E+14	6,50262E+14
TC1800000682.mm.2	Sec11c	SEC11 homolog C, signal peptidase complex subunit	1,21095E+14	7,63903E+14	2,5338E+14	0.0418841817053751	0.182547963164379	-3,56868E+14	8,33645E+14	8,31291E+14	7,57612E+14	6,65133E+14	7,98366E+14	6,97368E+14
TC0700000010.mm.2	Cacng6	calcium channel, voltage-dependent, gamma subunit 6	-0.888543273624158	6,14845E+14	-2,53302E+14	0.0419300188218261	0.182555485124087	-3,56965E+14	5,50988E+14	5,69498E+14	6,41085E+14	6,57109E+14	6,01252E+14	6,69139E+14
TC1900000098.mm.2	Tmem262	transmembrane protein 262	-0.783230241037852	6,18102E+14	-2,53289E+13	0.0419374390292449	0.182555485124087	-3,56981E+14	5,44983E+13	5,68136E+14	6,50765E+14	6,19E+14	6,57054E+14	6,68674E+14
TC0400003034.mm.2	Cdkn2b	cyclin dependent kinase inhibitor 2B	-1,08902E+14	6,3579E+14	-2,53274E+13	0.0419459004467451	0.182555485124087	-3,56999E+14	5,57283E+14	5,66398E+14	6,26164E+14	7,15321E+14	6,38983E+14	7,10594E+14
TC1900000119.mm.2	Nrxn2	neurexin II	0.926732245341975	7,81949E+14	2,53246E+14	0.0419623373818816	0.182555485124087	-3,57034E+14	8,66254E+14	8,4148E+14	7,51873E+14	7,70514E+14	7,6835E+14	6,93224E+14
TC0200004963.mm.2	Ggt7	gamma-glutamyltransferase 7	0.994948312862248	7,47797E+14	2,53232E+14	0.0419709569323622	0.182555485124087	-3,57052E+14	8,22596E+14	8,02333E+13	7,59708E+14	6,66231E+14	7,22644E+13	7,13272E+14
TC0600002579.mm.2	Cd207	CD207 antigen	-0.827214652605281	5,86352E+14	-2,53231E+13	0.04197114921856	0.182555485124087	-3,57052E+14	5,23764E+14	5,37647E+14	5,87642E+14	6,39211E+14	6,06951E+13	6,22898E+14
TC0700002007.mm.2	Cd163l1	CD163 molecule-like 1	-0.977500901464521	6,5403E+14	-2,53231E+14	0.0419713989475283	0.182555485124087	-3,57053E+14	6,08371E+14	5,99069E+14	6,63961E+14	7,38979E+14	6,30293E+14	6,83509E+14
TC1500001717.mm.2	Lynx1	Ly6/neurotoxin 1	1,26667E+13	8,45292E+13	2,53222E+14	0.0419765451737567	0.182555485124087	-3,57064E+14	9,40994E+14	9,09504E+14	8,59061E+14	7,38104E+14	8,50585E+14	7,73503E+12
TC0200002517.mm.2	Pigt	phosphatidylinositol glycan anchor biosynthesis, class T	1,37357E+13	8,60741E+14	2,53152E+14	0.0420177728358624	0.182685475905086	-3,57151E+14	9,69219E+14	9,47503E+13	8,99721E+14	7,42288E+14	8,25158E+14	7,80558E+14
TC0100002008.mm.2	Eloc	elongin C	1,72538E+13	7,70731E+14	2,53123E+14	0.0420349494885771	0.182710855438209	-3,57188E+14	8,58812E+14	8,76949E+14	7,71243E+14	6,19443E+14	8,29231E+14	6,68708E+14
TC0600002519.mm.2	Ctnna2	catenin (cadherin associated protein), alpha 2	1,5379E+14	7,43069E+14	2,53077E+14	0.0420622017355822	0.182780004467485	-3,57245E+14	8,49976E+14	8,25953E+14	7,67302E+13	6,01049E+14	7,53594E+14	6,60542E+14
TC0500001257.mm.2	Triap1	TP53 regulated inhibitor of apoptosis 1	-0.950555895709868	6,7355E+14	-2,52988E+14	0.0421142155602465	0.182956687873349	-3,57355E+14	6,19237E+14	6,0905E+14	7,51609E+14	6,66789E+14	6,89551E+14	7,05066E+14
TC1500000986.mm.2	Wnt1	wingless-type MMTV integration site family, member 1	-1,2199E+14	5,42662E+14	-2,52924E+14	0.0421520788856104	0.183071818620981	-3,57435E+14	4,58989E+13	4,68959E+14	5,23749E+14	6,48177E+14	5,47553E+14	6,08543E+14
TC0200002335.mm.2	Bpifa1	BPI fold containing family A, member 1	-1,5732E+14	4,95265E+14	-2,52859E+14	0.0421906288795937	0.183189868805414	-3,57517E+14	3,35125E+14	4,25534E+14	4,52018E+14	6,23281E+13	5,47432E+14	5,882E+14
TC0400000140.mm.2	Nbn	nibrin	2,00561E+14	5,48574E+14	2,52821E+14	0.0422132119327764	0.183216232451337	-3,57564E+14	6,65503E+14	6,56803E+14	5,60443E+14	3,60741E+14	6,11103E+14	4,36849E+14
TC1000002668.mm.2	Slc25a3	solute carrier family 25 (mitochondrial carrier, phosphate carrier), member 3	2,16352E+14	9,44385E+14	2,52792E+13	0.0422300058368396	0.183216232451337	-3,576E+14	1,07168E+14	1,07118E+14	9,89906E+14	7,2025E+13	9,58789E+14	8,54509E+14
TC0600003084.mm.2	Clec4e	C-type lectin domain family 4, member e	-1,02144E+14	5,87307E+13	-2,52768E+14	0.0422441987550041	0.183216232451337	-3,5763E+13	5,02961E+14	5,37443E+14	5,76729E+14	6,67962E+14	6,04609E+14	6,34138E+14
TC1900000681.mm.2	Pprc1	peroxisome proliferative activated receptor, gamma, coactivator-related 1	-1,12933E+14	5,63372E+14	-2,52761E+14	0.042248471767883	0.183216232451337	-3,57639E+14	4,81008E+14	5,16724E+14	5,83531E+14	6,40067E+14	5,2801E+14	6,30894E+14
TC1900000954.mm.2	Pacs1	phosphofurin acidic cluster sorting protein 1	1,60848E+14	6,18707E+14	2,52741E+14	0.0422601155092442	0.183216232451337	-3,57663E+14	7,58282E+14	7,46406E+14	6,60484E+13	5,22509E+14	5,86939E+14	4,37623E+14
TC1400001174.mm.2	Uchl3	ubiquitin carboxyl-terminal esterase L3 (ubiquitin thiolesterase)	1,0514E+13	7,58554E+14	2,52679E+14	0.0422971825664795	0.183216232451337	-3,57741E+14	8,20194E+14	8,24436E+14	7,71075E+13	6,63275E+14	7,37869E+14	7,34477E+14
TC0700001510.mm.2	Olfr632	olfactory receptor 632	-0.92415373754412	5,57567E+14	-2,52666E+14	0.0423044902896354	0.183216232451337	-3,57757E+14	4,86811E+14	4,9071E+14	5,40782E+14	6,21569E+14	5,99358E+14	6,0617E+13
TC1100002871.mm.2	Tom1l2	target of myb1-like 2 (chicken)	1,00341E+14	8,11229E+14	2,52654E+14	0.0423118014212467	0.183216232451337	-3,57772E+14	8,93349E+14	8,73223E+13	8,27037E+14	7,38853E+14	7,86732E+14	7,48178E+14
TC0200003205.mm.2	Surf4	surfeit gene 4	0.802891085818375	7,33732E+14	2,52653E+14	0.0423123234325252	0.183216232451337	-3,57773E+14	8,04078E+14	7,74679E+14	7,28485E+14	6,89694E+14	7,02132E+14	7,03322E+13
TC1100000952.mm.2	Zmynd15	zinc finger, MYND-type containing 15	-1,02125E+14	5,64039E+14	-2,52584E+14	0.0423530329903194	0.183216232451337	-3,57859E+14	4,72684E+14	5,11154E+14	5,64876E+13	6,23212E+14	5,68991E+14	6,43316E+13
TC0200001412.mm.2	1700029I15Rik	RIKEN cDNA 1700029I15 gene	-0.838812301996144	7,22599E+14	-2,52568E+14	0.0423629841312613	0.183216232451337	-3,5788E+13	6,51302E+14	6,83913E+14	7,32672E+13	7,70306E+14	7,32207E+14	7,65198E+14
TC0800001619.mm.2	Cd209a	CD209a antigen	-0.98216876670372	6,49616E+14	-2,52521E+14	0.042390412823935	0.183216232451337	-3,57937E+14	6,0829E+14	5,79766E+14	6,51297E+14	7,33194E+14	6,80725E+14	6,44424E+14
TC0X00000131.mm.2	Mid1ip1	Mid1 interacting protein 1 (gastrulation specific G12-like (zebrafish))	1,07567E+14	7,76889E+14	2,52502E+14	0.0424021342179444	0.183216232451337	-3,57962E+14	8,51872E+14	8,3475E+14	7,63921E+14	7,07567E+14	7,03514E+14	7,99712E+14
TC1800001401.mm.2	Myoz3	myozenin 3	-1,22092E+14	6,42643E+12	-2,52482E+14	0.0424140733039265	0.183216232451337	-3,57987E+14	5,40357E+14	5,70059E+14	6,21062E+14	7,33538E+13	6,56937E+14	7,33904E+13
TC0100003203.mm.2	Ro60	Ro60, Y RNA binding protein	1,54391E+14	5,55318E+13	2,52464E+14	0.0424247826875111	0.183216232451337	-3,58009E+14	6,44022E+14	6,62546E+14	5,94138E+13	4,03648E+13	5,28363E+14	4,9919E+14
TC0400002709.mm.2	Tmem245	transmembrane protein 245	1,22522E+14	6,79609E+14	2,5245E+13	0.0424328323459272	0.183216232451337	-3,58026E+14	7,45432E+14	7,74471E+14	6,58149E+14	6,16709E+14	7,06862E+14	5,76033E+14
TC0600000362.mm.2	Trim24	tripartite motif-containing 24	2,10342E+13	6,85899E+14	2,52442E+14	0.042437586722707	0.183216232451337	-3,58036E+14	8,53609E+14	8,22597E+13	7,43063E+14	5,12459E+14	6,70152E+14	5,13515E+14
TC1800001193.mm.2	Hbegf	heparin-binding EGF-like growth factor	-1,15361E+14	7,41405E+14	-2,52416E+14	0.0424532252385762	0.183216232451337	-3,58069E+14	6,67259E+14	6,7085E+14	7,30352E+14	8,3848E+14	7,37049E+14	8,04442E+14
TC1700001516.mm.2	Lnpep	leucyl/cystinyl aminopeptidase	1,94492E+14	7,05384E+14	2,52412E+14	0.0424553961099536	0.183216232451337	-3,58074E+13	8,25628E+14	8,32809E+14	7,04017E+13	5,65437E+14	7,60009E+14	5,44401E+14
TC0300000792.mm.2	Gatad2b	GATA zinc finger domain containing 2B	2,08658E+14	8,38798E+13	2,52403E+14	0.0424609293934204	0.183216232451337	-3,58085E+14	9,85514E+14	9,7846E+14	8,86597E+14	6,60061E+14	8,41559E+14	6,80595E+14
TC0700004586.mm.2	Phlda2	pleckstrin homology like domain, family A, member 2	-1,2239E+14	4,95251E+14	-2,52402E+14	0.0424616153187102	0.183216232451337	-3,58087E+14	4,5533E+14	3,98578E+14	4,87618E+14	6,1107E+14	4,92415E+14	5,26497E+14
TC0700002663.mm.2	Ltbp4	latent transforming growth factor beta binding protein 4	-1,07899E+14	8,00667E+14	-2,52369E+14	0.0424808222438312	0.183216232451337	-3,58127E+14	7,21522E+14	7,16517E+14	7,7389E+14	8,79947E+14	8,35947E+14	8,76181E+14
TC0100002081.mm.2	Adgrb3	adhesion G protein-coupled receptor B3	2,01504E+14	8,7019E+14	2,52365E+13	0.0424832361179843	0.183216232451337	-3,58132E+14	9,93141E+14	9,93455E+14	9,16599E+14	6,66988E+14	8,73106E+14	7,77852E+13
TC0200000923.mm.2	Tank	TRAF family member-associated Nf-kappa B activator	1,83776E+14	6,96262E+14	2,52361E+14	0.0424857395511037	0.183216232451337	-3,58137E+14	8,26228E+14	8,2121E+14	7,48901E+14	5,30986E+14	6,75313E+14	5,74936E+14
TC1100000821.mm.2	Tekt3	tektin 3	-1,01441E+14	5,44593E+14	-2,52309E+14	0.0425166993033415	0.183216232451337	-3,58202E+14	4,8136E+14	4,92359E+14	5,64343E+14	6,12259E+14	5,14587E+14	6,02651E+14
TC0900000621.mm.2	Arhgap20	Rho GTPase activating protein 20	1,51675E+14	6,3648E+14	2,52309E+14	0.0425168787348832	0.183216232451337	-3,58202E+13	7,46965E+14	7,14075E+14	6,52379E+14	5,05311E+14	6,57411E+12	5,42739E+14
TC0400004110.mm.2	Dffb	DNA fragmentation factor, beta subunit	-0.93442642844605	6,05695E+14	-2,52301E+14	0.0425211818226912	0.183216232451337	-3,58211E+14	5,27393E+13	5,57776E+14	6,16937E+14	6,55117E+14	6,04403E+14	6,72541E+14

TC1700001750.mm.2	Tcp11	t-complex protein 11	-1,05518E+14	6,26692E+13	-2,52269E+14	0.0425406521735405	0.183216232451337	-3,58252E+14	5,29783E+14	5,67006E+14	6,03924E+14	7,03901E+14	6,66559E+14	6,8898E+14
TC0Y00000565.mm.2	Gm20854	predicted gene, 20854	-1,02999E+14	7,28573E+14	-2,52266E+14	0.0425424099406567	0.183216232451337	-3,58256E+14	6,41405E+14	6,67526E+14	7,23201E+14	7,91728E+14	7,36897E+14	8,10684E+14
TC0900001455.mm.2	Cspg5	chondroitin sulfate proteoglycan 5	0.91060695461579	9,65166E+14	2,52263E+14	0.0425439707128704	0.183216232451337	-3,58259E+14	1,03303E+14	1,02349E+14	9,7092E+14	9,03478E+14	9,49416E+14	9,10665E+13
TC0400003255.mm.2	Btf3l4	basic transcription factor 3-like 4	1,3432E+14	7,7704E+14	2,52254E+14	0.042549192691175	0.183216232451337	-3,5827E+14	8,57974E+14	8,65244E+14	7,90386E+14	6,64193E+13	7,91897E+13	6,92545E+14
TC0200004843.mm.2	Acss1	acyl-CoA synthetase short-chain family member 1	0.752100050962847	7,15692E+14	2,52218E+13	0.0425711462875836	0.183240802531709	-3,58316E+14	7,93177E+14	7,80996E+14	7,0225E+14	7,21503E+14	6,56764E+14	6,3946E+14
TC0300000829.mm.2	Sprr2i	small proline-rich protein 2I	-1,47329E+14	6,24884E+14	-2,52202E+13	0.0425804283368971	0.183240802531709	-3,58335E+14	5,52346E+14	5,39048E+14	6,19191E+14	7,66861E+14	5,82805E+14	6,89053E+14
TC1000002248.mm.2	H2afv2	H2A histone family, member Y2	0.801926671294265	6,65865E+14	2,5217E+14	0.0425998314005922	0.183240802531709	-3,58376E+14	7,31876E+14	7,21436E+14	6,57147E+14	6,35779E+14	6,45827E+13	6,03127E+14
TC0X00000068.mm.2	Tfe3	transcription factor E3	0.752024718223466	6,60412E+14	2,52163E+14	0.0426037683498557	0.183240802531709	-3,58384E+14	7,11146E+14	7,04942E+14	6,46089E+13	6,19594E+14	6,3586E+14	6,44842E+14
TC1000000598.mm.2	Herc4	hect domain and RLD 4	0.772927793182316	6,39948E+14	2,52106E+14	0.0426378398147941	0.183240802531709	-3,58455E+14	6,85477E+14	7,07776E+14	6,34141E+14	6,04526E+14	5,98959E+14	6,08811E+14
TC0700002163.mm.2	Ptprh	protein tyrosine phosphatase, receptor type, H	-1,26008E+14	5,3643E+14	-2,52094E+14	0.0426447850042678	0.183240802531709	-3,5847E+14	4,66957E+14	4,65096E+14	5,43821E+14	6,40247E+14	4,95972E+14	6,06489E+14
TC0900003014.mm.2	Iqcf4	IQ motif containing F4	-1,26315E+14	4,80154E+13	-2,52089E+14	0.0426477500554521	0.183240802531709	-3,58476E+14	3,83522E+14	4,19683E+14	4,70099E+14	5,85735E+14	4,69128E+14	5,52758E+14
TC1300002661.mm.2	Gpbp1	GC-rich promoter binding protein 1	2,49127E+12	7,49582E+14	2,52066E+14	0.0426619426307493	0.183240802531709	-3,58506E+14	9,16999E+14	8,98945E+14	7,97492E+13	5,20198E+14	7,79136E+14	5,84721E+14
TC1700000461.mm.2	Hmga1/Hmga1b	high mobility group AT-hook 1/high mobility group AT-hook 1B	-0.882491316451856	8,22776E+14	-2,52013E+14	0.0426930853915729	0.183240802531709	-3,58571E+14	7,78766E+14	7,82263E+14	8,57873E+14	8,79655E+14	7,84894E+14	8,53206E+14
TC0400001671.mm.2	Gale	galactose-4-epimerase, UDP	-0.946967508559128	7,91388E+12	-2,52011E+14	0.0426944503523271	0.183240802531709	-3,58573E+14	7,13412E+14	7,31759E+14	7,98909E+14	8,35656E+14	7,96035E+14	8,72555E+14
TC0500000860.mm.2	Parm1	prostate androgen-regulated mucin-like protein 1	1,60738E+14	7,25877E+14	2,51999E+14	0.0427020094244456	0.183240802531709	-3,58589E+14	8,39524E+14	8,02851E+14	7,56689E+13	5,64211E+14	7,24788E+14	6,67198E+14
TC1000001566.mm.2	Ndufa4l2	Ndufa4, mitochondrial complex associated like 2	-0.828345851091036	6,13126E+14	-2,51962E+14	0.0427237035065983	0.183240802531709	-3,58634E+14	5,72322E+14	5,58405E+14	6,2046E+14	6,75936E+14	6,27519E+14	6,24116E+14
TC1500000566.mm.2	Tigd5	tigger transposable element derived 5	-0.819683008341586	6,01015E+14	-2,51939E+14	0.0427377886495293	0.183240802531709	-3,58664E+14	5,67141E+14	5,47791E+14	6,15134E+14	6,63734E+14	6,14207E+14	5,98086E+14
TC0900000203.mm.2	Smarca4	SWI/SNF related, matrix associated, actin dependent regulator of chromatin, subfamily a, member 4	1,0943E+13	7,19788E+14	2,51912E+14	0.0427540732939563	0.183240802531709	-3,58698E+14	8,02353E+13	7,89779E+14	7,34094E+14	6,39178E+14	7,10145E+13	6,43176E+14
TC0600002946.mm.2	Tamm41	TAM41 mitochondrial translocator assembly and maintenance homolog	-1,01603E+14	7,52095E+14	-2,51907E+14	0.042756675487523	0.183240802531709	-3,58703E+14	7,0012E+14	7,26477E+13	7,7135E+14	8,58453E+14	7,05596E+14	7,50575E+14
TC1100000943.mm.2	Slc16a11	solute carrier family 16 (monocarboxylic acid transporters), member 11	-1,06811E+14	7,29953E+14	-2,51896E+13	0.0427630697961784	0.183240802531709	-3,58716E+14	6,41228E+14	6,73862E+14	7,38739E+14	7,89972E+14	7,21009E+14	8,14907E+14
TC1400000556.mm.2	Olfr740	olfactory receptor 740	-1,16456E+14	5,53069E+14	-2,5188E+14	0.0427727059153912	0.183240802531709	-3,58736E+14	4,78119E+14	4,71895E+14	5,40999E+14	6,41927E+14	5,51965E+14	6,33511E+14
TC1100002329.mm.2	Mdh1	malate dehydrogenase 1, NAD (soluble)	1,68817E+14	1,01678E+14	2,51864E+14	0.0427822468146323	0.183240802531709	-3,58756E+14	1,12663E+13	1,10632E+14	1,04125E+14	8,54078E+14	1,04031E+13	9,32059E+13
TC1300002435.mm.2	Jmy	junction-mediating and regulatory protein	1,16688E+14	6,05353E+14	2,51831E+14	0.0428024682418683	0.183240802531709	-3,58798E+14	6,87188E+14	6,68191E+14	6,25174E+14	4,96828E+14	5,83741E+14	5,70995E+13
TC0900000449.mm.2	Olfr986	olfactory receptor 986	-1,06207E+14	4,48304E+13	-2,51822E+14	0.0428074226324331	0.183240802531709	-3,58809E+14	3,68392E+14	3,7777E+14	4,48305E+14	5,10271E+14	4,46904E+14	5,38182E+13
TC0400001774.mm.2	Mfap2	microfibrillar-associated protein 2	-1,20231E+14	6,90897E+13	-2,51759E+14	0.0428454960923852	0.183240802531709	-3,58888E+14	6,47457E+14	6,28107E+14	7,55721E+13	7,60305E+14	6,09222E+13	7,4457E+14
TC0800000358.mm.2	Chrnb3	cholinergic receptor, nicotinic, beta polypeptide 3	-0.750116553582152	4,78832E+14	-2,5175E+14	0.0428507452467318	0.183240802531709	-3,58899E+14	4,31605E+13	4,23256E+14	4,96072E+14	5,08813E+14	5,18544E+14	4,94702E+14
TC1100000973.mm.2	Rpain	RPA interacting protein	-1,30342E+13	7,05149E+14	-2,51725E+14	0.0428659594244063	0.183240802531709	-3,5893E+14	6,1739E+14	6,35699E+14	6,83331E+14	8,30442E+14	7,10291E+14	7,53741E+13
TC0500003254.mm.2	Sbno1	strawberry notch 1	1,37855E+13	6,71762E+14	2,51695E+14	0.0428837935266123	0.183240802531709	-3,58967E+14	7,5876E+14	7,68678E+14	6,73458E+14	5,7827E+14	6,94268E+14	5,57138E+14
TC0800002612.mm.2	4921524J17Rik	RIKEN cDNA 4921524J17 gene	-0.744095912889601	7,17837E+14	-2,51691E+14	0.0428862775022921	0.183240802531709	-3,58972E+14	6,71856E+14	6,51525E+14	7,29148E+14	7,43052E+14	7,53684E+14	7,57756E+14
TC0X00000694.mm.2	Srpk3	serine/arginine-rich protein specific kinase 3	-0.998951349314373	6,25606E+14	-2,51689E+14	0.0428877903935137	0.183240802531709	-3,58976E+14	5,50143E+14	5,70037E+14	6,21346E+14	6,98624E+14	6,2813E+14	6,85357E+14
TC0X00002156.mm.2	Apln	apelin	0.829096298759086	5,59125E+14	2,51682E+14	0.0428920307101399	0.183240802531709	-3,58984E+14	6,10474E+14	6,11103E+14	5,50108E+14	5,05649E+14	5,57163E+14	5,20256E+14
TC1000000693.mm.2	Zwint	ZW10 interactor	1,47304E+14	9,92515E+12	2,51645E+14	0.0429142459753091	0.183240802531709	-3,59031E+14	1,10112E+14	1,0869E+14	1,01568E+13	8,77725E+14	9,96451E+14	8,77213E+14
TC0300003017.mm.2	4930579F01Rik	RIKEN cDNA 4930579F01 gene	-1,24966E+14	4,56789E+13	-2,51641E+14	0.0429161420182289	0.183240802531709	-3,59034E+14	3,64697E+14	3,84076E+14	4,56274E+14	5,42432E+14	4,38961E+14	5,54294E+14
TC1900000743.mm.2	Ins1	insulin I	-1,03527E+14	5,71865E+14	-2,51633E+13	0.0429213908753932	0.183240802531709	-3,59045E+14	4,99462E+13	5,02277E+14	5,57313E+13	6,51481E+14	5,8647E+14	6,34185E+14
TC1700002422.mm.2	Ndufv2	NADH:ubiquinone oxidoreductase core subunit V2	2,08521E+14	8,39012E+14	2,5155E+14	0.0429709632672927	0.183240802531709	-3,59148E+14	9,64108E+14	9,55205E+14	8,81118E+14	6,21154E+14	8,56487E+14	7,55998E+14
TC0600001044.mm.2	4933427D06Rik	RIKEN cDNA 4933427D06 gene	-0.846990451828365	5,15545E+14	-2,51533E+14	0.0429814664757212	0.183240802531709	-3,5917E+14	4,63564E+14	4,8778E+13	5,38071E+14	5,8267E+14	5,29234E+14	4,91953E+14
TC0400003695.mm.2	Tmem222	transmembrane protein 222	1,04498E+14	7,64532E+13	2,51516E+14	0.0429916846560633	0.183240802531709	-3,59191E+13	8,22253E+14	8,10524E+14	7,58221E+14	6,6556E+14	7,92053E+14	7,38578E+14
TC1700002153.mm.2	Mymx	myomixer, myoblast fusion factor	-0.868874232680029	6,05309E+14	-2,51495E+14	0.0430042676298004	0.183240802531709	-3,59217E+14	5,52816E+14	5,6552E+14	6,17403E+14	6,74708E+14	5,9276E+14	6,28648E+14
TC0900002681.mm.2	Scg3	secretogranin III	1,79216E+14	7,71086E+14	2,51491E+14	0.0430068353802966	0.183240802531709	-3,59222E+14	8,81306E+14	8,89722E+14	8,21559E+14	5,91036E+14	7,40705E+14	7,02189E+14
TC0200004229.mm.2	Caprin1	cell cycle associated protein 1	1,24623E+14	9,21347E+14	2,51479E+14	0.0430136323267987	0.183240802531709	-3,59236E+14	1,00017E+14	9,99901E+14	9,4315E+14	8,07677E+13	9,14153E+14	8,63029E+14
TC0600001019.mm.2	Copg1	coatomer protein complex, subunit gamma 1	0.995273526403235	8,23089E+14	2,51471E+14	0.0430187137446625	0.183240802531709	-3,59247E+14	9,10915E+14	8,87899E+14	8,34327E+14	7,65432E+14	8,02586E+14	7,37378E+14
TC0600000824.mm.2	Gm1070	predicted gene 1070	-1,10279E+13	6,93675E+14	-2,51471E+14	0.0430188929439979	0.183240802531709	-3,59247E+14	6,26563E+14	6,51992E+14	7,14051E+14	7,85063E+14	6,46291E+14	7,38089E+14
TC0200005003.mm.2	Sla2	Src-like-adaptor 2	-0.877094080011775	6,18667E+14	-2,51466E+14	0.0430216976424038	0.183240802531709	-3,59253E+14	5,42349E+13	5,53174E+14	6,01729E+14	6,69213E+14	6,63316E+13	6,82223E+14
TC1600001131.mm.2	Tfap4	transcription factor AP4	-0.918870545049725	7,23256E+14	-2,51428E+13	0.0430445869409002	0.183240802531709	-3,593E+14	6,36555E+14	7,01846E+14	7,38587E+14	7,83588E+14	7,47724E+14	7,31236E+14
TC0X00001402.mm.2	Nxt2	nuclear transport factor 2-like export factor 2	2,3755E+14	8,07821E+14	2,51413E+14	0.0430538291570395	0.183240802531709	-3,5932E+14	9,37886E+14	9,46565E+14	8,46484E+14	5,62867E+14	8,53517E+14	6,99604E+14
TC0300001856.mm.2	Ccna2	cyclin A2	0.960134710283593	6,60508E+14	2,51411E+14	0.0430548912087374	0.183240802531709	-3,59322E+14	7,05833E+14	7,49041E+14	6,68518E+14	5,94329E+14	6,36529E+14	6,08798E+14
TC0300001645.mm.2	Zfand1	zinc finger, AN1-type domain 1	1,28269E+14	5,69199E+14	2,51383E+14	0.0430716570856469	0.183240802531709	-3,59356E+14	6,77949E+14	6,20517E+14	5,90458E+14	4,51471E+14	5,29468E+14	5,45334E+14
TC0500001546.mm.2	Castor2	cytosolic arginine sensor for mTORC1 subunit 2	0.823486644836798	7,75501E+14	2,51382E+14	0.043072201317331	0.183240802531709	-3,59358E+13	8,39447E+14	8,14853E+14	7,48966E+14	7,40636E+14	7,79601E+14	7,29506E+14
TC1100001272.mm.2	Ddx52	DEAD (Asp-Glu-Ala-Asp) box polypeptide 52	2,20302E+13	5,55833E+14	2,51372E+14	0.0430784049859604	0.183240802531709	-3,5937E+14	6,89881E+14	6,90085E+14	6,08133E+14	3,31229E+14	5,61432E+14	4,54236E+14
TC1700001188.mm.2	Gpatch11	G patch domain containing 11	0.72706011419351	7,00599E+14	2,51316E+14	0.0431121692942552	0.183240802531709	-3,5944E+14	7,61662E+14	7,49594E+14	6,83159E+14	6,82685E+14	6,63625E+14	6,62867E+13
TC0100000999.mm.2	Cdh20	cadherin 20	0.798339111182856	5,94206E+14	2,51306E+13	0.0431181103772266	0.183240802531709	-3,59452E+14	6,2925E+14	6,18122E+14	5,67455E+14	5,2025E+13	6,16612E+14	6,13546E+14
TC0400000718.mm.2	Orm1	orosomucoid 1	-0.967579745707141	5,12416E+14	-2,51306E+14	0.0431185923311881	0.183240802531709	-3,59453E+14	4,74313E+14	4,35269E+13	5,19779E+14	5,8332E+14	5,04046E+14	5,57768E+14
TC1100003923.mm.2	Icam2	intercellular adhesion molecule 2	-0.964592132006708	5,28877E+14	-2,51295E+14	0.0431247695326972	0.183240802531709	-3,59466E+14	4,61259E+14	4,5718E+14	5,10504E+14	6,00852E+14	5,7791E+13	5,65559E+14
TC0100001438.mm.2	Astn1	astrotactin 1	1,566E+14	7,21865E+14	2,51286E+14	0.0431304052693415	0.183240802531709	-3,59478E+14	8,54391E+14	8,34547E+12	7,28647E+14	6,47092E+14	7,23101E+14	5,43416E+14
TC1500000729.mm.2	Zc3h7b	zinc finger CCCH type containing 7B	0.892469354583702	7,4004E+14	2,51279E+14	0.0431348826415175	0.183240802531709	-3,59487E+14	8,02425E+14	8,06257E+14	7,24672E+14	7,05516E+14	7,36826E+13	6,64544E+14
TC0600001446.mm.2	Chd4/Mir7045	chromodomain helicase DNA binding protein 4/microRNA 7045	2,2513E+13	6,22504E+14	2,51257E+14	0.0431481781689577	0.183248970700162	-3,59514E+14	7,84095E+14	7,79428E+14	6,71717E+14	4,41546E+14	6,28666E+14	4,29571E+14
TC1000001609.mm.2	Smarcc2	SWI/SNF related, matrix associated, actin dependent regulator of chromatin, subfamily c, member 2	0.88205713277818	8,23255E+14	2,51195E+13	0.0431857365127465	0.183339879212682	-3,59592E+13	8,69505E+13	8,88007E+14	8,1119E+14	7,69911E+14	8,28719E+14	7,72201E+13
TC1800001272.mm.2	Jakmip2	janus kinase and microtubule interacting protein 2	2,24042E+14	6,63833E+14	2,51184E+14	0.0431923404388594	0.183339879212682	-3,59605E+14	8,14762E+14	8,2799E+13	6,95069E+14	4,996E+13	6,88243E+14	4,57333E+14
TC0700001623.mm.2	Zfp143	zinc finger protein 143	1,02473E+14	6,45633E+13	2,51164E+14	0.0432044419682371	0.183342947967923	-3,5963E+14	6,67017E+14	7,08636E+14	6,28352E+14	5,42357E+14	6,84111E+14	6,43326E+13
TC0500003729.mm.2	Gbp8	guanylate-binding protein 8	-0.75506855137636	5,44119E+14	-2,51117E+14	0.0432324240468587	0.183413388274627	-3,59688E+14	5,01837E+14	4,9633E+14	5,68461E+14	5,8072E+14	5,73084E+14	5,44282E+14
TC0100001063.mm.2	Tfcp2l1	transcription factor CP2-like 1	-0.823350470420312	7,333E+14	-2,51095E+14	0.0432457380114514	0.183421578462363	-3,59715E+14	6,95586E+14	6,95957E+14	7,57733E+14	7,9848E+14	7,45708E+14	7,06335E+14
TC1100002826.mm.2	Olfr328	olfactory receptor 328	-1,76919E+13	4,78249E+14	-2,51019E+14	0.0432922756443436	0.183487169347641	-3,59811E+14	3,42614E+14	3,67141E+14	4,35787E+14	6,27805E+14	4,85228E+14	6,10918E+14
TC1200001009.mm.2	Cpsf2	cleavage and polyadenylation specific factor 2	1,36522E+14	6,36543E+14	2,51017E+14	0.0432930805250274	0.183487169347641	-3,59813E+14	7,01262E+14	7,39943E+13	6,39229E+14	5,28932E+14	6,63608E+14	5,46285E+13
TC0700001631.mm.2	Ampd3	adenosine monophosphate deaminase 3	1,13662E+14	8,33623E+14	2,51014E+14	0.0432953651002129	0.183487169347641	-3,59818E+14	9,07615E+13	9,02508E+14	8,53005E+14	7,29794E+14	8,16089E+14	7,92728E+14
TC1400001001.mm.2	Hr	lysine demethylase and nuclear receptor corepressor	-1,22508E+14	7,44638E+14	-2,50992E+14	0.0433088047197767	0.183495863910009	-3,59845E+14	6,43603E+14	6,73283E+14	7,28748E+14	8,33155E+14	7,49128E+14	8,39909E+14
TC1500002076.mm.2	Slc2a13	solute carrier family 2 (facilitated glucose transporter), member 13	-0.85482542008055	9,33047E+14	-2,50972E+14	0.0433208621347592	0.183498699152833	-3,5987E+14	8,87663E+13	8,53911E+14	9,29213E+14	9,83327E+14	9,64414E+14	9,79755E+14
TC0600001154.mm.2	Gxylt2	glucoside xylosyltransferase 2	-0.867096542018198	4,84513E+13	-2,50923E+14	0.0433506910320961	0.183547026774819	-3,59931E+14	4,46218E+13	4,0501E+14	4,92167E+14	5,32481E+14	4,96779E+13	5,34422E+13
TC1600002020.mm.2	Krtap19-5	keratin associated protein 19-5	-1,40307E+14	5,3772E+14	-2,50908E+14	0.0433597745790567	0.183547026774819	-3,5995E+14	4,32979E+14	4,50845E+14	5,08244E+14	6,56195E+13	5,46041E+14	6,32016E+14
TC1700001812.mm.2	Tff2	trefoil factor 2 (spasmolytic protein 1)	-0.90506703259506	5,8041E+13	-2,50879E+14	0.0433770539186407	0.183547026774819	-3,59985E+14	5,10919E+14	5,29736E+14	5,84536E+14	6,37133E+14	5,82679E+14	6,37454E+13
TC0300002349.mm.2	Dcst1	DC-STAMP domain containing 1	-0.734493957776008	6,61703E+14	-2,50847E+12	0.0433968405922229	0.183547026774819	-3,60026E+14	6,02421E+14	6,16373E+14	6,80465E+14	6,85228E+13	6,85947E+14	6,99785E+14
TC1400002168.mm.2	Atp8a2	ATPase, aminophospholipid transporter-like, class I, type 8A, member 2	-0.906375493140595	7,91872E+14	-2,50813E+14	0.0434171642847693	0.183547026774819	-3,60068E+14	7,45631E+14	6,97276E+14	8,20281E+14	8,03901E+14	8,71954E+13	8,12192E+14
TC0600001097.mm.2	Nr2c2	nuclear receptor subfamily 2, group C, member 2	0.946700529854265	7,87057E+14	2,50806E+14	0.0434218077322479	0.183547026774819	-3,60077E+14	8,77163E+14	8,53431E+14	7,60736E+13	7,80518E+14	7,66335E+14	6,84162E+14
TC0600002607.mm.2	Sfxn5	sideroflexin 5	1,03251E+14	8,76332E+14	2,50794E+14	0.0434287228855307	0.183547026774819	-3,60091E+14	9,63371E+14	9,35362E+14	8,96979E+14	7,95252E+13	8,36994E+14	8,30033E+14
TC1800000869.mm.2	Mbp	myelin basic protein	0.995859235509288	1,11604E+14	2,50767E+14	0.0434452706338254	0.183547026774819	-3,60125E+14	1,16443E+14	1,19856E+14	1,11758E+13	1,04623E+14	1,11392E+13	1,05553E+14
TC1700001010.mm.2	Safb	scaffold attachment factor B	-1,05888E+13	8,45642E+14	-2,50759E+14	0.043449970667143	0.183547026774819	-3,60135E+14	7,87921E+14	7,66645E+14	8,35987E+14	9,30354E+14	8,49282E+14	9,03664E+13
TC0600000958.mm.2	Emx1	empty spiracles homeobox 1	-0.881672088280592	7,21935E+14	-2,50752E+14	0.0434543593137396	0.183547026774819	-3,60144E+14	6,50613E+14	6,77274E+14	7,2182E+14	7,82401E+14	7,34737E+14	7,64765E+14
TC1500001641.mm.2	Adcy8	adenylate cyclase 8	0.952792809876057	5,37594E+14	2,50747E+14	0.0434575750726702	0.183547026774819	-3,6015E+14	6,44754E+14	5,65934E+14	5,25266E+14	4,94863E+14	4,83099E+14	5,11651E+14
TC0900000485.mm.2	D630033O11Rik	RIKEN cDNA D630033O11 gene	-0.930446799606288	5,68046E+14	-2,50705E+14	0.0434830194771941	0.183606366047177	-3,60202E+14	5,43293E+14	4,70934E+14	5,86145E+14	6,14172E+13	5,80466E+14	6,13266E+14
TC1600001639.mm.2	Gramd1c	GRAM domain containing 1C	-1,25049E+14	5,21343E+14	-2,50661E+14	0.0435097841285766	0.183671247488539	-3,60257E+14	4,46497E+14	4,95095E+13	5,38472E+14	6,53218E+14	4,59103E+14	5,3567E+14
TC0400003708.mm.2	Zdhhc18	zinc finger, DHHC domain containing 18	-0.794663335807735	8,47206E+14	-2,50612E+14	0.0435399058702638	0.183750262778303	-3,60319E+14	8,00085E+14	8,01345E+14	8,67732E+14	8,92631E+14	8,40152E+13	8,81287E+14
TC0200001036.mm.2	Hat1	histone aminotransferase 1	1,40133E+14	6,54039E+14	2,50586E+14	0.043555989952131	0.183770009452393	-3,60352E+13	7,41485E+14	7,71187E+14	6,73009E+14	5,59397E+14	6,52454E+14	5,26702E+14
TC0100002054.mm.2	Rims1	regulating synaptic membrane exocytosis 1	0.979961159837202	8,63301E+14	2,50545E+14	0.0435807509542966	0.183826345582874	-3,60402E+14	9,08061E+14	9,31679E+14	8,57061E+14	7,86686E+14	8,7794E+14	8,1838E+13
TC0700004323.mm.2	Zfp768	zinc finger protein 768	-0.774683868380004	6,87642E+14	-2,5048E+14	0.0436202279336452	0.18394470889684	-3,60483E+14	6,26658E+14	6,59032E+14	7,28901E+14	7,11726E+14	6,9053E+14	7,09006E+14
TC0300000199.mm.2	Mynn	myoneurin	1,55299E+14	5,39603E+14	2,50457E+14	0.0436346205195662	0.183957258093085	-3,60512E+14	6,47499E+14	6,35718E+14	5,81964E+14	3,90656E+14	5,1377E+14	4,68013E+14
TC0300001176.mm.2	Olfm3	olfactomedin 3	1,4405E+14	6,87265E+14	2,50379E+14	0.0436821686079993	0.183989273205876	-3,60609E+14	8,44537E+14	7,85313E+14	7,41594E+14	6,00155E+14	6,21932E+14	5,30058E+14
TC0400000386.mm.2	Gm3893	family with sequence similarity 205 pseudogene	1,17125E+13	8,54176E+14	2,50377E+14	0.043683389695536	0.183989273205876	-3,60612E+14	9,09006E+13	9,40876E+14	7,91154E+14	8,24479E+14	8,91819E+14	7,67721E+14
TC0200001411.mm.2	Pex16	peroxisomal biogenesis factor 16	1,17246E+14	6,82937E+13	2,50374E+14	0.0436854123860341	0.183989273205876	-3,60616E+13	7,26852E+14	7,59561E+14	6,89917E+14	5,62003E+14	6,85847E+14	6,7344E+14
TC1000000211.mm.2	Moxd1	monooxygenase, DBH-like 1	-0.758232470260739	7,98075E+14	-2,5036E+14	0.0436940609042034	0.183989273205876	-3,60633E+14	7,40703E+14	7,65655E+14	8,3188E+14	8,26125E+14	8,23479E+14	8,00607E+13
TC1600001245.mm.2	Marf1	meiosis regulator and mRNA stability 1	1,66124E+14	8,57312E+14	2,50348E+13	0.0437011814992032	0.183989273205876	-3,60648E+14	9,99516E+14	9,5816E+14	8,8953E+14	7,35898E+14	8,52322E+14	7,08445E+14
TC1800000886.mm.2	Dipk1c	divergent protein kinase domain 1C	-1,56599E+14	6,79514E+13	-2,50333E+14	0.04371072660760838	0.183989273205876	-3,60667E+14	5,80842E+14	5,55062E+14	6,37434E+14	8,11669E+14	6,99428E+14	7,92651E+14
TC0900001365.mm.2	Cish	cytokine inducible SH2-containing protein	-0.945411757981453	5,6947E+13	-2,50251E+14	0.043760747997981	0.184074537983128	-3,60769E+14	5,02713E+14	5,30692E+14	5,80082E+14	6,42405E+14	5,52755E+14	6,08173E+14
TC0200002171.mm.2	Cfap61	cilia and flagella associated protein 61	1,02863E+14	6,39014E+14	2,50244E+14	0.0437651097751672	0.184074537983128	-3,60778E+14	6,74931E+14	7,21909E+14	5,72027E+14	6,19086E+14	5,82163E+14	6,63965E+14
TC1100003393.mm.2	Gas2l2	growth arrest-specific 2 like 2	-0.82188429685944	6,37852E+14	-2,50244E+14	0.0437652550743725	0.184074537983128	-3,60778E+14	5,85613E+14	6,11555E+14	6,90378E+14	6,71167E+14	6,10677E+14	6,57724E+14
TC0900002583.mm.2	Foxb1	forkhead box B1	-1,04499E+14	6,08131E+14	-2,50204E+14	0.0437896679861492	0.184129154556582	-3,60828E+14	5,48692E+14	5,51854E+14	6,36296E+14	6,73248E+14	5,68257E+14	6,70439E+14
TC1500002345.mm.2	Gsdmc4	gasdermin C4	-1,06511E+14	5,16476E+14	-2,50159E+14	0.0438170115987033	0.184160450460465	-3,60883E+14	4,31321E+13	4,60341E+14	5,04436E+14	6,00249E+14	5,25391E+14	5,77115E+12
TC0900002127.mm.2	Pou2f3	POU domain, class 2, transcription factor 3	-0.769267044565487	5,82967E+14	-2,50155E+14	0.0438199694076473	0.184160450460465	-3,6089E+14	5,06729E+13	5,45518E+14	6,06059E+14	6,00042E+14	6,20705E+14	6,18747E+14
TC1100004272.mm.2	Sep 04	septin 4	1,5119E+14	8,70417E+13	2,50097E+14	0.0438552568561536	0.184224834463691	-3,60961E+13	9,68597E+14	9,72943E+12	8,88806E+14	7,50353E+14	8,8647E+14	7,55335E+14
TC1100004094.mm.2	Recql5	RecQ protein-like 5	-0.855746292281986	5,78069E+14	-2,50092E+14	0.0438581558370705	0.184224834463691	-3,60967E+14	5,32329E+14	5,20865E+14	5,88158E+14	6,36185E+14	5,73062E+14	6,17811E+14
TC1000000829.mm.2	Arid3a	AT rich interactive domain 3A (BRIGHT-like)	-0.782640958428316	7,52204E+14	-2,50066E+14	0.043874200243987	0.184244198209894	-3,61E+14	6,83235E+14	7,01925E+14	7,50765E+13	7,90923E+14	7,95319E+14	7,91056E+14
TC0700004510.mm.2	Cox8b	cytochrome c oxidase subunit 8B	-1,12293E+13	5,40764E+14	-2,49967E+14	0.0439355119208252	0.184438230377706	-3,61124E+14	4,7447E+14	4,8915E+14	5,24511E+14	6,36695E+14	5,57447E+14	5,89309E+14
TC0200001889.mm.2	Galk2	galactokinase 2	0.871429704388545	5,86732E+14	2,49945E+14	0.0439491528709574	0.184438230377706	-3,61151E+14	6,36652E+13	6,67263E+13	5,32906E+14	5,96723E+14	5,4467E+14	5,42177E+14
TC0800001544.mm.2	Cog2	component of oligomeric golgi complex 2	1,18556E+14	5,46809E+13	2,49936E+14	0.0439547449047596	0.184438230377706	-3,61163E+14	6,50361E+14	6,75629E+14	5,02865E+14	5,86012E+13	3,81106E+14	4,8488E+14
TC0200002193.mm.2	Sstr4	somatostatin receptor 4	0.819987279852029	6,76444E+14	2,49829E+14	0.0440204647799173	0.184549019130891	-3,61296E+14	7,12712E+13	7,37492E+14	6,59631E+14	6,26576E+14	6,80689E+14	6,41565E+14
TC1700000528.mm.2	Abcg1	ATP binding cassette subfamily G member 1	0.828382037281139	7,09674E+14	2,49826E+12	0.044022577238489	0.184549019130891	-3,613E+14	7,60821E+14	7,62887E+13	6,74461E+14	6,8357E+14	6,58846E+14	7,1746E+14
TC0200003379.mm.2	Rbm18	RNA binding motif protein 18	0.916462947740632	7,70048E+14	2,49814E+14	0.0440297212148805	0.184549019130891	-3,61315E+14	8,42154E+13	8,17704E+14	7,75225E+14	7,01341E+14	7,54528E+14	7,29333E+14
TC0600000158.mm.2	Kcnd2	potassium voltage-gated channel, Shal-related family, member 2	1,52536E+14	7,61174E+14	2,49789E+14	0.0440451557336261	0.184549019130891	-3,61346E+14	8,79506E+14	8,71382E+14	7,96314E+13	6,49502E+14	7,47865E+14	6,22474E+14
TC1800001625.mm.2	Rnf165	ring finger protein 165	-0.727258652453063	8,43815E+14	-2,49734E+13	0.0440794267569587	0.184549019130891	-3,61415E+14	7,97424E+14	7,89243E+13	8,71585E+14	8,60534E+14	8,67046E+14	8,77056E+14
TC1100003652.mm.2	Pip4k2b	phosphatidylinositol-5-phosphate 4-kinase, type II, beta	1,82986E+14	6,83234E+14	2,49733E+14	0.0440796979127623	0.184549019130891	-3,61415E+14	8,14817E+14	8,39675E+14	7,40542E+14	5,47978E+14	6,50193E+14	5,062E+14
TC0800003095.mm.2	Spata2l	spermatogenesis associated 2-like	1,45531E+14	6,45001E+14	2,49703E+14	0.0440986443949964	0.184549019130891	-3,61454E+14	7,15709E+12	6,99809E+14	6,52639E+14	4,71817E+13	6,59541E+14	6,70494E+14
TC1700000382.mm.2	Gng13	guanine nucleotide binding protein (G protein), gamma 13	0.839196280599242	7,25681E+14	2,49702E+14	0.044099258882948	0.184549019130891	-3,61455E+14	7,53194E+14	7,69427E+13	7,06633E+13	6,48149E+14	7,45842E+14	7,30839E+14
TC0900001838.mm.2	Zfp266	zinc finger protein 266	1,278E+14	7,16997E+13	2,49695E+14	0.0441034919123278	0.184549019130891	-3,61463E+13	8,14247E+14	8,06522E+14	7,46789E+14	6,18379E+14	6,98413E+14	6,17633E+13
TCX_GL456233_random00000005.mm.2	Vamp7	vesicle-associated membrane protein 7	1,73083E+14	6,99899E+14	2,49669E+14	0.0441193577805827	0.184549019130891	-3,61496E+14	8,24526E+14	8,381E+14	7,0335E+14	6,1311E+14	7,13673E+14	5,06633E+14

TC1900000916.mm.2	Grk2	G protein-coupled receptor kinase 2	1,71832E+14	8,07169E+14	2,49668E+14	0.0441202868457116	0.184549019130891	-3,61497E+14	9,14741E+14	8,91707E+14	8,30617E+13	6,32167E+14	8,37859E+14	7,35926E+14
TC0500002219.mm.2	Letm1	leucine zipper-EF-hand containing transmembrane protein 1	0.955688194303068	6,62416E+14	2,49662E+14	0.0441235003590491	0.184549019130891	-3,61504E+14	7,37103E+14	7,20843E+14	6,57988E+14	6,08821E+14	5,87634E+14	6,62106E+14
TC0700000841.mm.2	Mtag2	metastasis associated gene 2	-1,00701E+14	6,60373E+14	-2,49637E+14	0.0441394335912055	0.184549019130891	-3,61536E+14	6,05835E+14	6,07934E+14	6,56845E+14	7,58326E+14	6,65233E+14	6,68064E+13
TC0200001859.mm.2	Sqor	sulfide quinone oxidoreductase	-1,10164E+14	7,01364E+14	-2,4962E+14	0.0441499738228993	0.184549019130891	-3,61557E+14	6,26511E+14	6,3931E+14	6,89629E+14	7,96521E+14	7,01449E+14	7,54765E+13
TC1200001643.mm.2	Tspan13	tetraspanin 13	1,85661E+13	8,65706E+14	2,49601E+14	0.0441613709012337	0.184549019130891	-3,6158E+14	9,77474E+14	9,76944E+14	8,85943E+14	6,97152E+14	9,07259E+14	7,49463E+14
TC1200001568.mm.2	Rps7	ribosomal protein S7	0.873234823695912	9,63556E+14	2,49596E+13	0.0441644025177631	0.184549019130891	-3,61586E+14	1,02622E+14	1,01645E+14	9,60722E+14	9,07301E+14	9,58642E+13	9,12002E+14
TC0700002771.mm.2	Cox6b1	cytochrome c oxidase, subunit 6B1	0.818470296716582	9,41472E+14	2,49545E+14	0.0441964186823761	0.18457785559353	-3,61651E+14	1,00466E+14	9,85226E+14	9,29663E+14	8,96532E+14	9,37243E+14	8,95503E+14
TC0100003323.mm.2	Ralgps2	Ral GEF with PH domain and SH3 binding motif 2	1,74648E+14	6,86434E+13	2,49539E+14	0.0442000234268699	0.18457785559353	-3,61658E+14	7,82245E+14	7,98469E+14	7,19751E+14	5,11666E+14	7,00193E+14	6,0628E+14
TC4_JH584294_random00000009.mm.2	Ccl21a/Gm1987	chemokine (C-C motif) ligand 21A (serine)/predicted gene 1987	-0.80011892268322	6,78514E+14	-2,49508E+14	0.0442194552288255	0.18457785559353	-3,61697E+14	6,15332E+14	6,42002E+14	6,9038E+13	7,26977E+14	6,86392E+14	7,10002E+14
TC1300002075.mm.2	H2afy	H2A histone family, member Y	0.84640849194419	7,32087E+14	2,49503E+14	0.0442221238296016	0.18457785559353	-3,61703E+14	7,88886E+13	7,88648E+14	7,11504E+14	6,96749E+14	7,34853E+14	6,71881E+14
TC1000001598.mm.2	Cnpy2	canopy FGF signaling regulator 2	1,32231E+14	7,72821E+14	2,49492E+14	0.044229265717029	0.18457785559353	-3,61717E+13	8,66744E+14	8,28522E+14	7,78066E+14	6,52738E+14	8,01095E+14	7,09761E+13
TC0300000144.mm.2	Tbl1xr1	transducin (beta)-like 1X-linked receptor 1	1,98105E+13	7,17485E+14	2,49431E+14	0.0442669052570006	0.18457785559353	-3,61793E+14	8,20085E+14	8,31229E+14	7,4895E+14	5,06153E+14	7,52719E+14	6,45773E+14
TC0900002220.mm.2	4931429L15Rik	RIKEN cDNA 4931429L15 gene	-1,16358E+14	6,59896E+14	-2,49399E+14	0.0442870449996225	0.18457785559353	-3,61833E+13	5,64018E+14	5,87573E+14	6,53739E+13	7,30569E+13	6,59489E+14	7,63989E+14
TC0400003587.mm.2	C77080	expressed sequence C77080	-0.821187353223451	7,70695E+14	-2,49326E+14	0.0443321962732028	0.18457785559353	-3,61924E+14	7,06544E+14	7,26414E+14	7,71593E+14	8,25602E+14	7,99367E+13	7,94649E+14
TC1100002374.mm.2	Rel	reticuloendotheliosis oncogene	1,24725E+14	5,39527E+14	2,49308E+14	0.0443436379370939	0.18457785559353	-3,61947E+14	6,30341E+14	6,08684E+14	5,41731E+14	4,47845E+14	5,59708E+14	4,48854E+14
TC0100001011.mm.2	Tnfrsf11a	tumor necrosis factor receptor superfamily, member 11a, NFKB activator	-0.977381137992488	6,14724E+14	-2,49306E+14	0.0443444000542015	0.18457785559353	-3,61948E+14	5,5344E+14	5,6392E+14	6,13002E+14	6,99834E+14	6,09709E+14	6,48437E+14
TC0300001857.mm.2	Bbs7	Bardet-Biedl syndrome 7 (human)	-0.894012779094001	6,02915E+14	-2,49296E+14	0.0443511052471268	0.18457785559353	-3,61962E+14	5,285E+14	5,67885E+14	6,09039E+14	6,66149E+14	6,063E+14	6,39613E+14
TC0200001133.mm.2	Pjvk	pejvakin	-0.935692344219369	5,76185E+14	-2,49274E+14	0.0443644396162569	0.18457785559353	-3,61989E+14	5,0131E+14	4,8472E+14	6,19283E+13	5,53885E+14	6,21792E+14	6,7612E+14
TC0500003284.mm.2	Slc15a4	solute carrier family 15, member 4	1,56817E+14	6,24511E+14	2,49266E+14	0.0443696343109503	0.18457785559353	-3,61999E+14	7,15857E+14	7,29989E+14	5,98053E+14	5,34159E+14	6,79376E+12	4,89631E+14
TC0400002805.mm.2	Tex48	testis expressed 48	-1,52532E+14	5,77006E+14	-2,49251E+14	0.0443786464977736	0.18457785559353	-3,62017E+14	4,70456E+14	5,02849E+14	5,81667E+14	6,96702E+14	5,28915E+14	6,81444E+14
TC0500002284.mm.2	Slc2a9	solute carrier family 2 (facilitated glucose transporter), member 9	-1,07377E+14	6,0481E+14	-2,49248E+14	0.0443809005576728	0.18457785559353	-3,62022E+13	5,32837E+14	5,3662E+14	5,86384E+14	6,97826E+13	6,23294E+14	6,51897E+14
TC1100000901.mm.2	Hes7	hes family bHLH transcription factor 7	-1,10145E+14	7,67133E+14	-2,49242E+13	0.0443842687476156	0.18457785559353	-3,62028E+14	6,84003E+14	6,94053E+14	7,46878E+14	8,51466E+14	7,83791E+14	8,42609E+14
TC1600000031.mm.2	Glis2	GLIS family zinc finger 2	-0.86570522412866	6,88986E+14	-2,49239E+14	0.0443863649982396	0.18457785559353	-3,62032E+14	6,38756E+14	6,34088E+14	6,89954E+14	7,56031E+14	6,96242E+14	7,18843E+14
TC0900000500.mm.2	Mcam	melanoma cell adhesion molecule	1,01412E+14	6,33E+14	2,49225E+14	0.0443953990272913	0.18457785559353	-3,62051E+14	7,169E+14	6,73379E+13	6,37846E+14	5,49608E+14	6,25926E+14	5,9434E+14
TC1200000712.mm.2	Eif2s1	eukaryotic translation initiation factor 2, subunit 1 alpha	2,27477E+14	6,68315E+14	2,49223E+14	0.0443964078168847	0.18457785559353	-3,62053E+14	8,10747E+14	8,35667E+14	7,26884E+14	4,64577E+14	6,68327E+14	5,03691E+14
TC0300002630.mm.2	Hipk1	homeodomain interacting protein kinase 1	1,81381E+14	9,22499E+14	2,49217E+13	0.0444004076385852	0.18457785559353	-3,62061E+14	1,0289E+14	1,0439E+14	9,60838E+14	7,49197E+14	9,31966E+12	8,20198E+14
TC0300001689.mm.2	Cyp7b1	cytochrome P450, family 7, subfamily b, polypeptide 1	0.725408587323051	6,31509E+14	2,49187E+14	0.0444189384633322	0.184607262176856	-3,62098E+14	6,81361E+14	6,71388E+14	6,01126E+14	6,06541E+14	6,08139E+14	6,20497E+14
TC0100001675.mm.2	1700016C15Rik	RIKEN cDNA 1700016C15 gene	-1,23338E+14	5,15111E+14	-2,49078E+14	0.0444867532222832	0.184734189004405	-3,62234E+14	4,86138E+14	3,95163E+14	5,08735E+14	6,19242E+14	5,30323E+14	5,51068E+14
TC0500000339.mm.2	Ppp1cb	protein phosphatase 1 catalytic subunit beta	1,77855E+14	9,25189E+14	2,49056E+14	0.0445006574473744	0.184734189004405	-3,62261E+14	1,024E+14	1,0254E+14	9,4554E+14	7,48151E+14	9,6728E+14	8,40765E+14
TC1100000854.mm.2	Myh1	myosin, heavy polypeptide 1, skeletal muscle, adult	-1,38202E+14	5,58763E+14	-2,49041E+14	0.0445098862474636	0.184734189004405	-3,6228E+14	4,54331E+14	5,01341E+14	5,4781E+14	6,84266E+14	5,37251E+13	6,27578E+14
TC1100001283.mm.2	Myo19	myosin XIX	-0.937732966115766	6,65684E+14	-2,49036E+14	0.0445132028167608	0.184734189004405	-3,62286E+14	6,09541E+14	6,22187E+14	6,67596E+14	7,51678E+14	6,61702E+14	6,81399E+14
TC0500000011.mm.2	Fam133b	family with sequence similarity 133, member B	1,91407E+14	6,62708E+14	2,49022E+14	0.044521838944144	0.184734189004405	-3,62304E+14	7,45383E+12	7,36757E+14	6,76663E+14	4,22663E+14	7,11327E+14	6,83456E+14
TC1400001303.mm.2	Mbnl2	muscleblind like splicing factor 2	1,37373E+14	9,35552E+14	2,49017E+14	0.0445247558113456	0.184734189004405	-3,6231E+13	1,03249E+14	1,04605E+14	9,68673E+14	8,35115E+14	9,16734E+14	8,14259E+14
TC0100003093.mm.2	Zbed6/Zc3h11a	zinc finger, BED type containing 6/zinc finger CCCH type containing 11A	0.997879146540011	7,68658E+14	2,48988E+14	0.04454303028872 6	0.184734189004405	-3,62346E+14	8,38181E+14	8,4829E+14	7,79178E+14	7,07718E+13	7,54044E+14	6,84537E+14
TC0100002175.mm.2	Uggt1	UDP-glucose glycoprotein glucosyltransferase 1	1,53438E+14	6,11682E+14	2,48977E+14	0.044549645686657	0.184734189004405	-3,62359E+13	7,32881E+14	7,16015E+13	6,52547E+14	4,89473E+14	5,92859E+14	4,86317E+14
TC1100000989.mm.2	Xaf1	XIAP associated factor 1	1,18145E+14	5,88586E+14	2,48973E+14	0.0445526629722037	0.184734189004405	-3,62365E+14	6,44036E+14	6,31655E+14	5,86296E+14	4,53106E+14	5,97601E+14	6,18822E+14
TC1600000196.mm.2	Yars2	tyrosyl-tRNA synthetase 2 (mitochondrial)	0.790231317354197	6,36429E+14	2,48922E+14	0.0445843410302518	0.184786468288663	-3,62429E+14	6,83109E+13	6,56929E+14	5,70845E+14	6,11147E+14	6,45337E+14	6,51208E+14
TC0200001779.mm.2	Rtf1	RTF1, Paf1/RNA polymerase II complex component	1,18404E+14	7,22543E+14	2,48915E+14	0.0445889573654624	0.184786468288663	-3,62438E+14	8,3147E+13	7,79252E+14	7,30926E+13	6,42987E+13	7,20099E+14	6,30523E+14
TC0800002389.mm.2	Ddx49	DEAD (Asp-Glu-Ala-Asp) box polypeptide 49	0.716382830408811	6,69328E+14	2,48861E+14	0.0446227504690171	0.184786468288663	-3,62505E+14	7,25344E+14	7,20003E+14	6,50476E+14	6,51594E+14	6,3269E+14	6,35864E+14
TC0X00000531.mm.2	Vgll1	vestigial like family member 1	-1,49794E+14	4,92873E+14	-2,48828E+14	0.0446429141403173	0.184786468288663	-3,62545E+14	3,87017E+14	3,89215E+14	4,7367E+14	6,0215E+14	4,82807E+14	6,22377E+14
TC0300001531.mm.2	Zzz3	zinc finger, ZZ domain containing 3	0.956209280067522	6,1177E+14	2,48825E+13	0.0446448308568007	0.184786468288663	-3,62549E+14	6,96345E+14	6,94858E+13	5,69379E+14	6,30583E+14	5,7374E+14	5,05714E+14
TC1200001299.mm.2	Vipr2	vasoactive intestinal peptide receptor 2	-0.818376835803213	5,97685E+14	-2,48803E+14	0.0446590692228581	0.184786468288663	-3,62578E+14	5,34722E+14	5,57847E+14	6,23036E+14	6,33209E+14	5,92866E+14	6,44431E+14
TC0700002884.mm.2	Gm21276	predicted gene, 21276	-1,16557E+14	4,58434E+14	-2,48802E+14	0.0446594030891584	0.184786468288663	-3,62578E+14	3,74323E+14	3,77084E+14	4,50085E+14	5,34436E+14	4,56137E+14	5,58537E+14
TC0100002832.mm.2	Pask	PAS domain containing serine/threonine kinase	-0.897549565509387	5,63177E+14	-2,48797E+14	0.0446628556765507	0.184786468288663	-3,62585E+14	4,97864E+13	4,90835E+14	5,45269E+13	6,22939E+14	6,07976E+14	6,1418E+14
TC1800000794.mm.2	Acaa2	acetyl-Coenzyme A acyltransferase 2 (mitochondrial 3-oxoacyl-Coenzyme A thiolase)	1,23943E+14	6,53501E+14	2,48788E+14	0.0446684847008219	0.184786468288663	-3,62596E+14	7,44799E+14	7,21738E+14	6,53252E+14	5,65397E+14	6,75034E+14	5,60785E+14
TC1700001020.mm.2	Clpp	caseinolytic mitochondrial matrix peptidase proteolytic subunit	0.867302173485869	7,7819E+14	2,48698E+14	0.0447248685065968	0.184934128266436	-3,62709E+14	8,38901E+14	8,11655E+14	7,62527E+14	7,14569E+14	7,93001E+14	7,48487E+14
TC0400002986.mm.2	Rps6	ribosomal protein S6	0.97305167206102	1,03471E+14	2,48694E+14	0.0447271332374048	0.184934128266436	-3,62713E+14	1,10218E+14	1,09468E+14	1,0305E+14	9,71741E+14	1,04187E+14	9,6729E+14
TC1100000953.mm.2	Tm4sf5	transmembrane 4 superfamily member 5	-0.805328483459554	5,77293E+13	-2,48582E+14	0.0447978046877538	0.184988209097187	-3,62854E+14	5,1488E+14	5,22328E+14	5,73784E+12	6,2449E+14	6,06164E+14	6,22112E+14
TC0800002734.mm.2	Ciapin1	cytokine induced apoptosis inhibitor 1	1,20077E+14	7,99679E+14	2,48577E+14	0.0448005790740446	0.184988209097187	-3,62859E+14	8,86602E+14	8,69333E+14	8,25501E+14	6,90279E+14	7,77411E+14	7,4895E+14
TC1400001338.mm.2	Pcca	propionyl-Coenzyme A carboxylase, alpha polypeptide	1,72499E+14	5,97244E+14	2,48564E+14	0.0448089830923204	0.184988209097187	-3,62876E+14	7,12783E+14	7,13906E+14	6,21888E+14	4,59803E+14	6,16679E+13	4,58403E+14
TC0X00000316.mm.2	Il13ra1	interleukin 13 receptor, alpha 1	1,07297E+14	6,33469E+14	2,48531E+14	0.0448295814936476	0.184988209097187	-3,62917E+14	6,78843E+14	7,09819E+14	6,32108E+14	5,41961E+14	6,51705E+14	5,86376E+14
TC0900001431.mm.2	Shisa5	shisa family member 5	0.806356565308373	6,98611E+14	2,48508E+13	0.0448443108671944	0.184988209097187	-3,62946E+14	7,40479E+14	7,42581E+14	6,87317E+14	6,34471E+14	6,87789E+14	6,9903E+14
TC0500001270.mm.2	Cit	citron	0.871439145196869	7,87686E+14	2,48502E+14	0.0448476854270527	0.184988209097187	-3,62953E+14	8,35786E+14	8,5515E+14	7,87104E+14	7,29543E+13	7,78695E+14	7,39835E+14
TC1500000860.mm.2	Trabd	TraB domain containing	-0.779761121982332	7,1664E+14	-2,48493E+14	0.0448533367472015	0.184988209097187	-3,62964E+14	6,96396E+14	6,90298E+14	7,65612E+14	7,77034E+13	6,63579E+14	7,06923E+14
TC0600003165.mm.2	Ndufa9	NADH:ubiquinone oxidoreductase subunit A9	1,94085E+13	7,69396E+13	2,48461E+14	0.0448734430011662	0.184988209097187	-3,63004E+14	8,79665E+14	8,59304E+14	8,031E+13	5,47699E+14	7,86845E+14	7,39764E+14
TC0500003024.mm.2	Grk3	G protein-coupled receptor kinase 3	0.96863792828726	6,69348E+14	2,48424E+14	0.0448972464013594	0.184988209097187	-3,63051E+14	7,63614E+14	7,21631E+14	6,7199E+14	6,19528E+14	6,52907E+13	5,86418E+14
TC0300002414.mm.2	2310050C09Rik	RIKEN cDNA 2310050C09 gene	-0.834263538130112	5,02287E+14	-2,48418E+14	0.0449008940547146	0.184988209097187	-3,63058E+14	4,23147E+14	4,4093E+14	4,88903E+14	5,42027E+14	5,45674E+14	5,73042E+14
TC0900002871.mm.2	1700065D16Rik	RIKEN cDNA 1700065D16 gene	-1,07904E+14	5,5809E+14	-2,48411E+14	0.0449053123247171	0.184988209097187	-3,63067E+14	4,79894E+13	5,02611E+14	5,46547E+14	6,51766E+14	6,06565E+14	5,61155E+14
TC0X00000741.mm.2	Fundc2	FUN14 domain containing 2	1,69153E+14	7,28069E+13	2,48379E+14	0.0449254031432633	0.184988209097187	-3,63107E+14	8,16844E+13	8,27009E+14	7,5223E+14	5,53317E+14	7,57889E+13	6,61123E+14
TC0100000133.mm.2	Tmem14a	transmembrane protein 14A	0.910010041143213	7,20285E+13	2,48379E+14	0.044925614256183	0.184988209097187	-3,63107E+14	7,59063E+14	7,53196E+14	7,01234E+14	6,29023E+14	7,58288E+14	7,20904E+14
TC0200003283.mm.2	Fnbp1	formin binding protein 1	1,46989E+14	7,69126E+14	2,48377E+14	0.0449263964007844	0.184988209097187	-3,63109E+14	8,65022E+14	8,42794E+14	7,91518E+14	6,22319E+14	7,82964E+13	7,10137E+14
TC0500003195.mm.2	Acad10	acyl-Coenzyme A dehydrogenase family, member 10	-1,08767E+14	6,85708E+14	-2,48369E+14	0.0449319856596739	0.184988209097187	-3,6312E+14	6,44734E+14	5,65842E+14	6,71777E+14	7,56333E+14	7,24804E+14	7,50761E+14
TC0900001345.mm.2	Dusp7	dual specificity phosphatase 7	-0.716110151904271	8,87486E+14	-2,48325E+14	0.0449592426194803	0.184988209097187	-3,63174E+14	8,34257E+14	8,38836E+14	9,05867E+14	9,10448E+12	9,20742E+14	9,14764E+14
TC0X00000325.mm.2	Slc25a43	solute carrier family 25, member 43	-1,06814E+14	6,22593E+14	-2,48317E+14	0.0449646940921192	0.184988209097187	-3,63184E+14	5,43317E+14	5,44399E+14	6,25178E+14	6,76166E+14	6,21823E+14	7,24676E+14
TC0300003234.mm.2	Pear1	platelet endothelial aggregation receptor 1	-1,02205E+14	5,50215E+14	-2,48314E+14	0.044966546861309	0.184988209097187	-3,63188E+14	4,8475E+13	5,06688E+14	5,46243E+14	6,49605E+14	5,58193E+14	5,55809E+14
TC0500002343.mm.2	Qdpr	quinoid dihydropteridine reductase	0.850740550751462	9,0422E+13	2,48307E+14	0.0449706103122674	0.184988209097187	-3,63196E+14	9,70001E+14	9,29505E+14	8,63174E+14	8,66184E+14	9,24828E+14	8,71626E+14
TC1300002040.mm.2	Gprin1	G protein-regulated inducer of neurite outgrowth 1	0.921951753370129	7,34131E+14	2,48279E+14	0.0449885286648413	0.184988209097187	-3,63232E+14	8,04007E+14	7,94435E+14	7,04789E+13	7,09262E+14	7,37996E+14	6,54299E+14
TC1300002735.mm.2	Mrps30	mitochondrial ribosomal protein S30	0.755868048982015	6,3303E+14	2,48248E+14	0.0450084126378797	0.184988209097187	-3,63271E+14	6,6711E+14	6,87073E+14	6,15652E+14	5,87358E+14	6,25657E+12	6,15327E+14
TC1300000673.mm.2	B230219D22Rik	RIKEN cDNA B230219D22 gene	2,11675E+14	7,41146E+14	2,48232E+14	0.0450179989876327	0.184988209097187	-3,6329E+13	8,40343E+14	8,7361E+14	7,76577E+14	5,14026E+14	7,77868E+14	6,64453E+14
TC1000002262.mm.2	Kif1bp	KIF1 binding protein	1,50635E+13	7,19139E+14	2,48205E+14	0.0450351288468896	0.184988209097187	-3,63324E+14	8,31467E+14	8,28816E+14	7,4839E+14	6,10622E+14	7,14415E+14	5,81123E+14
TC1000000656.mm.2	Rhobtb1	Rho-related BTB domain containing 1	-0.939591346037625	5,29831E+14	-2,48173E+14	0.0450556550887708	0.184988209097187	-3,63364E+14	4,56714E+14	4,51751E+14	5,18563E+14	5,7782E+14	5,54351E+14	6,19785E+14
TC1500001060.mm.2	Ankrd33	ankyrin repeat domain 33	-1,29083E+14	5,48317E+14	-2,48161E+14	0.0450632204485279	0.184988209097187	-3,63379E+14	4,75435E+13	4,91054E+14	5,38028E+14	6,86627E+14	5,27844E+14	5,70913E+12
TC0500001665.mm.2	Gm5294	predicted gene 5294	-1,0777E+14	7,16203E+14	-2,48145E+14	0.045073109889773	0.184988209097187	-3,63399E+14	6,43856E+14	6,51609E+13	7,01196E+14	8,09808E+14	7,24254E+14	7,66492E+14
TC1700001450.mm.2	Slc22a1	solute carrier family 22 (organic cation transporter), member 1	-0.786128930213547	6,23986E+14	-2,48099E+14	0.0451021937530016	0.184988209097187	-3,63456E+14	5,74638E+14	5,39299E+14	6,20274E+14	6,50888E+14	6,74189E+14	6,84627E+14
TC1100001372.mm.2	Ccdc182	coiled-coil domain containing 182	-0.726742086135143	5,57282E+14	-2,48065E+14	0.0451239752298095	0.184988209097187	-3,63499E+14	5,03868E+14	4,85212E+14	5,65462E+14	5,68966E+13	6,06203E+14	6,13979E+13
TC0800001422.mm.2	Dnaaf1	dynein, axonemal assembly factor 1	-0.850079168070633	4,78539E+14	-2,48064E+14	0.0451244632421185	0.184988209097187	-3,635E+14	4,2892E+14	3,97642E+14	4,7283E+14	5,23748E+13	5,08924E+14	5,39168E+14
TC0200002266.mm.2	Tmem74b	transmembrane protein 74B	-1,1566E+14	6,71954E+14	-2,48051E+14	0.045132616096346	0.184988209097187	-3,63516E+14	5,86587E+14	6,15016E+13	6,52966E+14	7,79956E+14	6,88482E+14	7,08719E+14
TC0200000380.mm.2	Il1f10	interleukin 1 family, member 10	-0.836699390970411	5,25746E+14	-2,48037E+13	0.0451416983741141	0.184988209097187	-3,63534E+14	4,25826E+14	4,6005E+14	5,04408E+14	5,48808E+14	5,92908E+14	6,22479E+14
TC1700001381.mm.2	Dynlt1a	dynein light chain Tctex-type 1A	-1,87377E+14	1,01828E+13	-2,4802E+14	0.045152342714541	0.184988209097187	-3,63555E+14	9,59074E+14	8,66044E+14	1,19514E+13	1,00473E+14	1,10921E+14	9,75483E+14
TC1000002727.mm.2	Mrpl42	mitochondrial ribosomal protein L42	0.865115149327009	8,68638E+14	2,48015E+14	0.0451555828130081	0.184988209097187	-3,63561E+14	9,06348E+13	9,28644E+14	8,64268E+14	7,977E+14	8,62494E+14	8,52375E+14
TC0500000344.mm.2	Yes1	YES proto-oncogene 1, Src family tyrosine kinase	1,00286E+13	5,32535E+13	2,47998E+14	0.0451664519070102	0.184988209097187	-3,63583E+14	6,4492E+14	5,51499E+14	5,18301E+14	4,77546E+14	5,03989E+14	4,98957E+14
TC1000002374.mm.2	Gucd1	guanylyl cyclase domain containing 1	-0.809026935558848	6,53173E+14	-2,47968E+14	0.0451851914542648	0.184988209097187	-3,6362E+14	6,21295E+14	6,01422E+14	6,76914E+13	7,07609E+14	6,76729E+14	6,35069E+14
TC1200001418.mm.2	Msgn1	mesogenin 1	-1,21113E+14	6,84468E+13	-2,47946E+14	0.0451996363543637	0.184988209097187	-3,63648E+14	5,95664E+14	6,28425E+14	6,66203E+14	8,00112E+14	6,91887E+14	7,24514E+14
TC0500003743.mm.2	Psmg3	proteasome (prosome, macropain) assembly chaperone 3	-1,10512E+14	6,13238E+14	-2,47934E+14	0.0452071031622111	0.184988209097187	-3,63663E+14	5,32094E+14	5,32038E+14	5,83341E+14	7,01816E+14	6,50082E+14	6,80054E+14
TC0800002497.mm.2	Arhgap10	Rho GTPase activating protein 10	0.729138838149491	6,77597E+14	2,47916E+14	0.0452182941380537	0.184988209097187	-3,63685E+14	7,212E+14	7,30869E+14	6,45824E+14	6,60418E+14	6,60717E+14	6,46555E+14
TC0300002244.mm.2	4930565D16Rik	RIKEN cDNA 4930565D16 gene	-1,07113E+14	4,76317E+14	-2,47893E+14	0.0452332479496379	0.184988209097187	-3,63714E+14	4,12342E+14	4,0407E+14	4,74201E+14	5,56437E+14	4,64917E+14	5,45934E+14
TC1900000188.mm.2	Fads1	fatty acid desaturase 1	1,3887E+14	8,71214E+14	2,47885E+12	0.0452384457087268	0.184988209097187	-3,63725E+14	9,66263E+14	9,67039E+14	9,11474E+14	7,44088E+14	8,39999E+14	7,98421E+14
TC1100002429.mm.2	Sptbn1	spectrin beta, non-erythrocytic 1	1,51463E+14	9,44814E+14	2,47881E+14	0.0452407288763262	0.184988209097187	-3,63729E+14	1,0614E+14	1,04479E+14	9,67559E+14	8,35707E+14	9,49961E+14	8,09461E+14
TC1900000113.mm.2	Men1	multiple endocrine neoplasia 1	1,29308E+14	7,40588E+14	2,47878E+14	0.0452427916542029	0.184988209097187	-3,63733E+14	8,24473E+14	8,09203E+14	7,6197E+14	6,1309E+14	6,95126E+14	7,39664E+14
TC0900001302.mm.2	Topbp1	topoisomerase (DNA) II binding protein 1	1,14657E+14	5,48541E+14	2,47848E+14	0.0452619283291649	0.184988209097187	-3,63771E+14	6,32184E+14	6,14429E+14	4,44558E+14	5,72742E+14	5,19992E+13	5,07338E+14
TC0700004411.mm.2	Nkx1-2	NK1 homeobox 2	-1,03771E+14	5,06541E+14	-2,47837E+14	0.0452688693034314	0.184988209097187	-3,63784E+14	4,63155E+14	3,99448E+14	4,99448E+14	5,70697E+14	5,26814E+14	5,79683E+14
TC1100000755.mm.2	Myo15	myosin XV	-1,0118E+14	5,15438E+14	-2,47819E+13	0.0452797836055493	0.184988209097187	-3,63806E+14	4,62265E+12	4,8261E+14	5,52202E+14	5,95033E+14	4,53965E+14	5,46554E+14
TC1700000668.mm.2	Gpank1	G patch domain and ankyrin repeats 1	0.988388902452813	6,79546E+14	2,47816E+14	0.0452818002685717	0.184988209097187	-3,6381E+14	7,72793E+14	6,85157E+13	6,29497E+14	6,30775E+14	6,56291E+14	7,02761E+13
TC0X00000396.mm.2	Xiap	X-linked inhibitor of apoptosis	1,15211E+14	7,47259E+14	2,47787E+14	0.045300209032864	0.184988209097187	-3,63846E+14	8,11007E+14	8,02213E+14	7,39536E+14	6,4326E+14	7,88042E+14	6,99496E+14
TC0300001765.mm.2	Tmem212	transmembrane protein 212	-0.802146396978116	6,30355E+14	-2,47778E+14	0.0453061496755728	0.184988209097187	-3,63858E+14	5,84745E+13	5,90355E+14	6,45162E+14	6,90368E+14	6,49771E+14	6,21728E+14
TC1400000177.mm.2	Vcl	vinculin	1,41841E+14	5,14524E+14	2,47756E+14	0.0453200391188273	0.184988209097187	-3,63885E+14	5,98641E+14	5,63469E+14	5,19674E+14	3,58755E+14	5,56486E+14	4,90121E+13
TC0X00001716.mm.2	Asmt	acetylserotonin O-methyltransferase	-1,21455E+14	6,92403E+14	-2,47754E+14	0.0453213709334257	0.184988209097187	-3,63888E+14	6,18926E+14	6,11637E+14	6,82922E+14	7,90551E+14	6,7868E+14	7,71704E+14
TC0200001375.mm.2	Arfgap2	ADP-ribosylation factor GTPase activating protein 2	0.91186256401994	7,36741E+14	2,4771E+13	0.0453491988802614	0.184988209097187	-3,63942E+14	7,84646E+13	7,98854E+14	7,40583E+14	6,60544E+14	7,10797E+14	7,25021E+14
TC1600000450.mm.2	Muc4	mucin 4	-0.896461078258225	6,45947E+14	-2,47695E+14	0.045359148155344	0.184988209097187	-3,63962E+14	5,9538E+14	5,93671E+14	6,47411E+13	7,20932E+14	6,44821E+14	6,73465E+14
TC0100003754.mm.2	Tgfb2	transforming growth factor, beta 2	1,54387E+14	5,62884E+13	2,47691E+14	0.0453614244305146	0.184988209097187	-3,63966E+14	6,60676E+14	6,60342E+13	6,05147E+13	4,07095E+14	5,2762E+14	5,16423E+14
TC1100002752.mm.2	Slc22a21	solute carrier family 22 (organic cation transporter), member 21	-1,03013E+14	6,40938E+14	-2,47658E+14	0.045382557215915	0.184988209097187	-3,64008E+14	5,91234E+14	5,51708E+14	6,41108E+14	7,0786E+14	6,39391E+14	7,1433E+14
TC0100001200.mm.2	Lrrn2	leucine rich repeat protein 2, neuronal	-0.824651336495333	7,65864E+14	-2,47651E+14	0.0453870860677803	0.184988209097187	-3,64016E+12	7,00635E+14	7,12185E+14	7,71317E+14	8,06433E+14	8,26162E+14	7,78449E+14
TC1500002118.mm.2	Nell2	NEL-like 2	1,35528E+14	9,37387E+14	2,47646E+14	0.0453900003602862	0.184988209097187	-3,64022E+14	1,01366E+14	1,03246E+14	9,5258E+14	8,2248E+14	9,53106E+14	8,50043E+14
TC0100002602.mm.2	Atg9a	autophagy related 9A	2,41351E+14	7,85698E+14	2,47619E+14	0.0454076382050726	0.184988209097187	-3,64057E+14	9,51165E+14	9,53743E+14	8,53003E+14	5,69203E+14	7,81596E+14	6,05477E+14
TC1500001078.mm.2	Gm5477	type II keratin Kb16P	-0.781336832056458	5,39255E+14	-2,47616E+14	0.0454090766746596	0.184988209097187	-3,6406E+14	4,75836E+13	4,94835E+14	5,43687E+14	5,83251E+14	5,77047E+14	5,60872E+14
TC0600002657.mm.2	Prokr1	prokineticin receptor 1	-0.929071366701602	5,43235E+14	-2,47596E+14	0.0454220538977357	0.184988209097187	-3,64085E+14	4,83585E+13	5,26215E+14	5,72979E+14	6,22636E+14	5,55319E+14	4,98678E+14
TC1400000835.mm.2	Cab39l	calcium binding protein 39-like	1,54121E+14	8,06129E+14	2,47582E+14	0.0454309803104843	0.184988209097187	-3,64102E+13	8,72803E+14	9,05447E+14	7,79374E+13	6,90633E+14	8,81266E+14	7,07251E+14
TC1000002424.mm.2	Gm19402	predicted gene, 19402	-0.850565311730048	6,50618E+14	-2,47567E+14	0.0454405344508575	0.184988209097187	-3,64121E+14	6,02485E+14	5,97131E+14	6,51202E+13	7,18526E+14	6,67972E+14	6,66392E+14
TC0700004465.mm.2	9430038I01Rik	RIKEN cDNA 9430038I01 gene	-0.896270527762737	7,07462E+14	-2,47527E+13	0.0454664233292464	0.185006496618974	-3,64172E+14	6,30288E+14	6,65021E+14	7,04118E+14	7,70444E+14	7,50091E+14	7,24811E+14
TC0200003774.mm.2	Evx2	even-skipped homeobox 2	-1,04272E+14	6,54871E+14	-2,47524E+13	0.0454679902321814	0.185006496618974	-3,64175E+14	6,09305E+14	5,89358E+14	6,65113E+14	7,42095E+14	6,23332E+14	7,00025E+14
TC0100002143.mm.2	Bend6	BEN domain containing 6	1,83021E+12	7,7889E+14	2,47436E+14	0.0455246023921064	0.18509793649779	-3,64286E+14	9,02511E+13	8,84604E+14	8,26838E+14	5,94236E+14	7,69059E+14	6,96093E+14
TC0400003829.mm.2	Pla2g2f	phospholipase A2, group IIF	-1,19517E+14	6,24269E+14	-2,47435E+14	0.0455247260725142	0.18509793649779	-3,64286E+14	5,37157E+14	5,7038E+14	6,10649E+14	7,35922E+14	6,20297E+14	6,71212E+14
TC1000000736.mm.2	S100b	S100 protein, beta polypeptide, neural	1,25105E+14	6,61305E+14	2,47424E+14	0.0455322949527292	0.18509793649779	-3,64301E+14	7,60355E+14	7,48429E+12	6,13248E+13	6,45328E+14	6,7285E+14	5,27619E+14

TC0600002281.mm.2	Hoxa7/Mira	homeobox A7/mistral long non-coding RNA	-1,0092E+14	7,07831E+14	-2,47403E+14	0.0455453713663675	0.18509793649779	-3,64326E+13	6,30759E+14	6,54285E+14	6,93631E+14	7,93254E+14	7,288E+13	7,46257E+14
TC0200005184.mm.2	Ptgis	prostaglandin I2 (prostacyclin) synthase	-0.845462694374299	6,63647E+14	-2,47383E+14	0.0455580786736475	0.18509793649779	-3,64351E+14	6,14262E+14	5,93291E+14	6,71971E+14	7,04674E+14	6,71939E+14	7,25746E+14
TC0300003200.mm.2	Lrrc7	leucine rich repeat containing 7	1,40307E+13	7,53224E+14	2,47381E+14	0.0455593878328203	0.18509793649779	-3,64354E+13	8,53923E+14	8,68969E+14	7,82463E+14	6,59815E+14	7,39308E+14	6,14867E+14
TC1300000572.mm.2	Iars	isoleucine-tRNA synthetase	1,02743E+14	7,3653E+14	2,47353E+14	0.0455775668025708	0.18512511567679	-3,64389E+14	7,91334E+14	7,84485E+14	7,36312E+14	6,34021E+14	7,20218E+14	7,52807E+14
TC1200000503.mm.2	Mia2	MIA SH3 domain ER export factor 2	1,59978E+14	7,01834E+14	2,47292E+13	0.0456167704992748	0.185142126956517	-3,64466E+14	7,91545E+14	7,97696E+14	7,18345E+14	5,50939E+14	7,37774E+14	6,14707E+14
TC0400003362.mm.2	Rps8/Snord38a	ribosomal protein S8/small nucleolar RNA, C/D box 38A	1,42104E+14	9,51432E+14	2,47282E+14	0.0456232079173305	0.185142126956517	-3,64478E+14	1,04348E+14	1,0362E+14	9,59554E+14	8,35915E+14	9,8098E+14	8,52466E+14
TC1100002310.mm.2	Aftph	aftiphilin	1.71381E+14	7,13677E+14	2,47272E+14	0.045629077953875	0.185142126956517	-3,6449E+14	8,3178E+14	8,18338E+13	7,2515E+13	5,82206E+14	7,49687E+14	5,74901E+14
TC0100001617.mm.2	Tagln2	transgelin 2	0.877009048825443	7,04416E+14	2,47251E+14	0.0456429795656446	0.185142126956517	-3,64517E+14	7,432E+14	7,93783E+14	6,57453E+14	7,04128E+14	6,7834E+14	6,49592E+14
TC0400001800.mm.2	Agmat	agmatine ureohydrolase (agmatinase)	-1,23645E+14	5,44354E+14	-2,47244E+14	0.0456471696561129	0.185142126956517	-3,64525E+14	4,48068E+14	5,0677E+13	5,37451E+14	6,64678E+14	5,32699E+14	5,76458E+14
TC0900001261.mm.2	Stag1	stromal antigen 1	2,25461E+14	7,34839E+13	2,47239E+14	0.0456506963568697	0.185142126956517	-3,64532E+14	8,92807E+14	8,59489E+14	7,80077E+14	5,21297E+14	7,61733E+14	5,93629E+14
TC0900003082.mm.2	Fbxw28	F-box and WD-40 domain protein 28	-0.863847465594682	5,15947E+14	-2,47177E+14	0.0456900612776329	0.18516274027645	-3,64608E+14	4,65889E+14	4,54337E+14	5,45323E+14	5,47673E+14	5,76416E+14	5,06046E+14
TC1600001387.mm.2	Kng2	kininogen 2	-0.910192535717558	4,9909E+14	-2,47167E+14	0.0456968868022617	0.18516274027645	-3,64622E+13	4,39555E+14	4,29743E+14	4,87217E+14	5,64119E+14	5,22694E+14	5,51209E+14
TC0100001611.mm.2	Kcnj10	potassium inwardly-rectifying channel, subfamily J, member 10	1,56862E+14	9,17853E+14	2,47162E+14	0.0457001501362085	0.18516274027645	-3,64628E+14	1,04016E+14	1,00342E+14	9,51863E+14	7,77997E+14	9,15844E+14	8,17829E+13
TC1700002805.mm.2	Nhlrc4	NHL repeat containing 4	-0.899101308360537	5,9814E+14	-2,4715E+14	0.0457076273883831	0.18516274027645	-3,64643E+14	5,52277E+14	5,37052E+14	5,95297E+14	6,73852E+14	6,1547E+14	6,14889E+14
TC1000003152.mm.2	Pa2g4	proliferation-associated 2G4	1,967E+14	8,17009E+14	2,47133E+14	0.0457186898512303	0.18516274027645	-3,64664E+14	9,26845E+14	9,24102E+14	8,57585E+13	5,99962E+14	8,30253E+14	7,63305E+14
TC0900003220.mm.2	Cmc1	COX assembly mitochondrial protein 1	1,16923E+14	6,14673E+14	2,47121E+12	0.0457260668109448	0.18516274027645	-3,64678E+13	6,93339E+14	6,83276E+13	6,38382E+14	5,04387E+13	5,78893E+14	5,89761E+14
TC0100003649.mm.2	Smyd3	SET and MYND domain containing 3	1,02051E+14	7,34997E+14	2,47094E+14	0.0457434996423759	0.18516274027645	-3,64712E+14	8,03627E+14	8,11004E+13	7,2877E+12	6,81759E+14	7,39991E+13	6,44829E+14
TC1400001724.mm.2	Fam25c	family with sequence similarity 25, member C	-0.83209210555991	6,6349E+14	-2,47088E+14	0.0457477111053526	0.18516274027645	-3,64721E+14	6,18343E+14	5,99428E+14	6,6726E+14	7,1693E+14	6,72586E+14	7,06394E+14
TC0500003015.mm.2	Gm6583	predicted gene 6583	-0.868374293595894	6,06152E+14	-2,47018E+14	0.0457926994292049	0.185278011909943	-3,64808E+14	5,41256E+13	5,5743E+14	6,01744E+14	6,70617E+14	6,41171E+14	6,24696E+14
TC0200002577.mm.2	Cse1l	chromosome segregation 1-like (S. cerevisiae)	1,77178E+14	6,7216E+14	2,47008E+14	0.045799188322305	0.185278011909943	-3,64821E+14	7,81052E+14	8,02866E+14	7,22777E+14	5,06785E+14	6,55643E+14	5,63839E+14
TC1200001902.mm.2	Rtn1	reticulon 1	2,19189E+14	1,07737E+14	2,46953E+14	0.04583454472556	0.185329487463837	-3,64889E+14	1,21457E+14	1,19937E+13	1,12344E+14	8,52122E+14	1,10228E+14	9,72438E+14
TC0900000279.mm.2	Prdm10	PR domain containing 10	0.851520463151446	6,33705E+14	2,46952E+14	0.0458349163745666	0.185329487463837	-3,6489E+14	6,86752E+14	6,83763E+14	6,22446E+14	5,77765E+14	5,89924E+14	6,41581E+14
TC0400003659.mm.2	Ythdf2	YTH N6-methyladenosine RNA binding protein 2	1,11916E+14	6,9694E+14	2,4692E+14	0.0458553807353058	0.185365717458099	-3,6493E+14	7,81829E+14	7,80041E+14	7,12888E+14	6,2515E+14	6,85874E+14	5,9586E+14
TC1200002422.mm.2	Rd3l	retinal degeneration 3-like	-0.998127157811941	5,79941E+14	-2,46896E+14	0.0458710953625335	0.185365798232478	-3,6496E+14	5,12546E+13	5,18007E+14	6,1432E+14	6,15858E+14	5,58542E+14	6,60375E+14
TC1100003882.mm.2	Nsf	N-ethylmaleimide sensitive fusion protein	2,01899E+13	9,17513E+14	2,46829E+14	0.0459141113965886	0.185365798232478	-3,65044E+14	1,04786E+14	1,03483E+14	9,75147E+14	7,03743E+14	8,91777E+14	8,51722E+14
TC0700001888.mm.2	Inpp5f	inositol polyphosphate-5-phosphatase F	1,87808E+14	7,23917E+14	2,46821E+14	0.0459193527267465	0.185365798232478	-3,65054E+14	8,75145E+14	8,45374E+14	7,49193E+14	5,9571E+14	7,38292E+14	5,39789E+13
TC1200001067.mm.2	Bdkrb1	bradykinin receptor, beta 1	-0.842564063177911	5,07438E+14	-2,46805E+14	0.0459296497181872	0.185365798232478	-3,65074E+14	4,40038E+14	4,79016E+13	5,17251E+14	5,70315E+14	5,15987E+14	5,22023E+14
TC0300001416.mm.2	Eif4e	eukaryotic translation initiation factor 4E	1,99059E+14	8,13847E+14	2,46802E+14	0.0459318289985935	0.185365798232478	-3,65078E+14	9,49849E+14	9,37669E+14	8,35871E+14	6,53528E+14	8,52646E+14	6,53517E+14
TC0500003411.mm.2	Ssc4d	scavenger receptor cysteine rich family, 4 domains	-0.799984169665479	6,51003E+14	-2,46777E+14	0.0459476718029485	0.185365798232478	-3,65109E+14	5,90943E+14	6,03804E+14	6,60496E+14	6,94249E+14	6,58305E+14	6,9822E+14
TC0900002516.mm.2	Zfp609	zinc finger protein 609	0.794625738928683	8,55082E+14	2,46727E+14	0.0459800846475049	0.185365798232478	-3,65171E+14	9,19538E+14	8,95326E+14	8,27112E+14	8,28827E+14	8,52939E+13	8,06747E+14
TC1600002142.mm.2	Itgb2l	integrin beta 2-like	-1,48952E+14	4,69519E+14	-2,46711E+14	0.0459903492880896	0.185365798232478	-3,65191E+14	3,6569E+14	4,07578E+14	4,68619E+14	6,02553E+14	4,21974E+14	5,50702E+14
TC0700000411.mm.2	Prr19	proline rich 19	-1,494E+14	6,75728E+14	-2,46683E+14	0.0460084794092922	0.185365798232478	-3,65226E+14	6,15148E+14	5,64236E+14	6,62266E+14	8,15919E+14	6,47518E+14	7,49283E+14
TC0900002036.mm.2	Chek1	checkpoint kinase 1	-0.738975817240884	6,63995E+14	-2,46668E+14	0.0460183922108176	0.185365798232478	-3,65245E+14	6,23099E+13	5,97772E+14	6,81364E+14	6,87302E+14	7,05099E+14	6,89334E+14
TC1500000862.mm.2	Selenoo	selenoprotein O	0.887827479545369	6,08371E+14	2,46666E+14	0.0460194009036737	0.185365798232478	-3,65247E+14	6,60118E+14	6,7748E+14	5,71013E+14	5,89019E+14	6,13102E+14	5,39492E+14
TC1600002009.mm.2	Krtap26-1	keratin associated protein 26-1	-1,32575E+14	4,99397E+13	-2,46664E+14	0.0460206887113714	0.185365798232478	-3,6525E+14	4,13318E+12	4,59402E+14	4,98818E+14	6,39052E+14	4,58449E+14	5,27344E+14
TC1000002043.mm.2	Amd2/Amd1	S-adenosylmethionine decarboxylase 2/S-adenosylmethionine decarboxylase 1	1,26539E+14	8,48205E+14	2,46663E+14	0.046021667713362	0.185365798232478	-3,65252E+14	9,30179E+14	9,50303E+14	8,79668E+14	7,47736E+14	8,27479E+14	7,53866E+14
TC1700000754.mm.2	Gabbr1	gamma-aminobutyric acid (GABA) B receptor, 1	1,38474E+14	1,02492E+14	2,466E+14	0.0460619423471559	0.185365798232478	-3,6533E+14	1,12188E+14	1,11148E+14	1,05884E+14	8,9757E+14	1,01076E+14	9,48961E+14
TC0500000363.mm.2	Nsd2	nuclear receptor binding SET domain protein 2	1,24115E+14	7,99017E+14	2,46578E+14	0.0460764320336828	0.185365798232478	-3,65358E+14	8,86103E+14	8,8318E+14	8,18118E+13	7,02935E+14	7,98499E+14	7,05267E+14
TC1500001095.mm.2	Espl1	extra spindle pole bodies 1, separase	-0.724078347145258	6,09017E+14	-2,46569E+14	0.0460823536225182	0.185365798232478	-3,65369E+14	5,62619E+14	5,6436E+14	6,33755E+14	6,3804E+14	6,17034E+14	6,38294E+14
TC0800000323.mm.2	Nsd3	nuclear receptor binding SET domain protein 3	1,32893E+14	8,20993E+14	2,46559E+14	0.0460887152210273	0.185365798232478	-3,65381E+14	8,94539E+14	9,02546E+14	8,24617E+14	7,06681E+14	8,55294E+13	7,42277E+14
TC1700002308.mm.2	Stap2	signal transducing adaptor family member 2	-0.975662225686304	5,91399E+14	-2,46549E+14	0.0460954943227405	0.185365798232478	-3,65394E+13	4,81955E+14	5,50429E+14	5,82425E+13	6,4509E+14	6,29184E+14	6,59309E+14
TC1100001150.mm.2	Proca1	protein interacting with cyclin A1	1,25599E+14	6,22574E+13	2,46516E+14	0.0461163643638681	0.185365798232478	-3,65435E+14	7,07523E+14	6,46948E+14	6,1731E+14	4,85964E+14	6,60339E+14	6,1736E+13
TC1100000231.mm.2	Zrsr1	zinc finger (CCCH type), RNA binding motif and serine/arginine rich 1	0.768163044319531	7,24272E+14	2,46516E+14	0.046116490823082	0.185365798232478	-3,65435E+14	7,91646E+14	7,6904E+14	7,21372E+14	6,85681E+13	6,93569E+14	6,84323E+14
TC0600002404.mm.2	Hpgds	hematopoietic prostaglandin D synthase	1.18621E+14	5,73513E+14	2,46515E+14	0.0461175538686954	0.185365798232478	-3,65437E+14	6,40993E+14	7,1373E+13	5,42357E+14	5,75125E+14	4,27872E+14	5,40999E+14
TC0700004492.mm.2	Caly	calcyon neuron-specific vesicular protein	1,21246E+14	8,57536E+14	2,46506E+14	0.0461228359383913	0.185365798232478	-3,65447E+14	9,64242E+14	9,19606E+14	8,85577E+14	7,55779E+14	8,30358E+14	7,89653E+14
TC0600000591.mm.2	Mpp6	membrane protein, palmitoylated 6 (MAGUK p55 subfamily member 6)	2,11898E+14	7,02481E+14	2,46499E+14	0.04612781098130 8	0.185365798232478	-3,65457E+14	8,20585E+14	8,13066E+14	7,45023E+14	4,64831E+14	7,23965E+14	6,4742E+14
TC1800001073.mm.2	Zfp24	zinc finger protein 24	1,38552E+14	5,4749E+14	2,46489E+13	0.0461340992036838	0.185365798232478	-3,65469E+14	6,36005E+14	6,38918E+14	5,73493E+14	4,24326E+14	5,50144E+14	4,62056E+14
TC1400002242.mm.2	Hmbox1	homeobox containing 1	1,52986E+14	6,48178E+14	2,46475E+14	0.0461430035816093	0.185365798232478	-3,65486E+14	7,82041E+14	7,61783E+14	6,53369E+14	5,84483E+14	6,45589E+14	4,61806E+14
TC0700004659.mm.2	Rnf141	ring finger protein 141	0.969855639217551	7,47494E+14	2,4641E+14	0.0461852990473991	0.185489462500185	-3,65568E+14	8,43779E+14	8,40542E+14	7,92932E+14	6,97418E+14	6,61725E+14	6,48569E+14
TC1400000200.mm.2	Vdac2	voltage-dependent anion channel 2	2,07829E+14	9,46483E+14	2,46363E+14	0.0462158187058235	0.18550662720685	-3,65626E+13	1,06275E+14	1,05904E+14	9,87875E+14	7,18255E+13	9,70768E+14	8,8021E+14
TC1100003379.mm.2	Slfn9	schlafen 9	-1,0181E+14	5,74832E+14	-2,46352E+14	0.0462232180324459	0.18550662720685	-3,65641E+14	5,00364E+14	5,55219E+14	5,88889E+14	6,70312E+14	5,46433E+14	5,87778E+14
TC0800001400.mm.2	Cmip	c-Maf inducing protein	1,36245E+14	9,86062E+14	2,46349E+14	0.0462252213035526	0.18550662720685	-3,65644E+14	1,08832E+14	1,06845E+14	1,01949E+14	8,6479E+14	9,70814E+14	9,04515E+13
TC1800000708.mm.2	Psmg2	proteasome (prosome, macropain) assembly chaperone 2	1,11053E+14	6,71071E+14	2,46309E+14	0.0462507824535282	0.18550662720685	-3,65694E+14	7,69745E+14	7,61687E+14	6,60012E+14	6,49315E+13	6,54777E+14	5,30892E+14
TC0500002780.mm.2	Gm7978/BC061212	predicted gene 7978/cDNA sequence BC061212	-0.965602942235842	5,24042E+14	-2,46305E+14	0.0462537497055607	0.18550662720685	-3,65699E+14	4,33421E+14	4,77015E+14	5,22639E+14	5,80917E+14	5,34097E+13	5,96164E+14
TC0200003169.mm.2	Sohlh1	spermatogenesis and oogenesis specific basic helix-loop-helix 1	-0.823337737969908	6,75257E+14	-2,46276E+14	0.0462726781889123	0.18550662720685	-3,65736E+14	5,97302E+14	6,36959E+14	6,97633E+14	7,01296E+14	6,85882E+14	7,32473E+13
TC0X00002648.mm.2	Maged1	melanoma antigen, family D, 1	1,49661E+14	8,85753E+14	2,46269E+13	0.0462769728914416	0.18550662720685	-3,65744E+14	1,0118E+14	9,80335E+14	9,34042E+14	7,5877E+13	8,50742E+14	7,78833E+14
TC0900001605.mm.2	Rpl14/Rpl14-ps1	ribosomal protein L14/ribosomal protein L14, pseudogene 1	0.978792473192543	9,79641E+14	2,46231E+14	0.0463016101183689	0.18550662720685	-3,65791E+13	1,04034E+14	1,0315E+14	9,80134E+13	8,95941E+14	9,90159E+14	9,39777E+14
TC1100001515.mm.2	Cbx1	chromobox 1	0.906119962302165	6,90854E+14	2,46226E+14	0.0463051808325888	0.18550662720685	-3,65798E+14	7,68501E+14	7,10524E+14	6,5837E+14	6,39431E+14	6,56401E+14	7,11897E+14
TC1300000135.mm.2	Amph	amphiphysin	1,35382E+12	7,43282E+14	2,46221E+14	0.0463081084663578	0.18550662720685	-3,65804E+14	8,57714E+14	8,28927E+14	7,90133E+14	6,25744E+14	6,88745E+14	6,6843E+14
TC0300000637.mm.2	Map9	microtubule-associated protein 9	1,68253E+14	7,00126E+14	2,46209E+14	0.0463162143147247	0.18550662720685	-3,65819E+12	8,27343E+14	7,99387E+14	7,16103E+14	5,74121E+14	7,26318E+14	5,57483E+14
TC0700001951.mm.2	Dock1	dedicator of cytokinesis 1	0.750191735119951	5,71642E+14	2,46166E+14	0.0463438658785802	0.185571250224046	-3,65872E+14	6,09782E+14	6,24977E+14	5,41359E+14	5,43363E+14	5,38663E+14	5,71707E+14
TC0400000374.mm.2	Nudt2	nudix (nucleoside diphosphate linked moiety X)-type motif 2	0.719922938060185	6,26241E+14	2,4612E+14	0.0463739928578094	0.185600848303501	-3,6593E+14	6,92236E+14	6,75958E+14	6,14275E+14	6,09935E+14	5,87587E+13	5,77456E+14
TC0700000087.mm.2	Usp29	ubiquitin specific peptidase 29	2,12325E+13	6,92538E+14	2,46106E+14	0.0463832275764576	0.185600848303501	-3,65948E+13	9,02871E+13	8,6399E+14	7,19245E+14	6,22965E+14	6,59397E+14	3,86761E+14
TC0800000987.mm.2	Orc6	origin recognition complex, subunit 6	0.860000960938413	6,32543E+14	2,46102E+14	0.0463858136981442	0.185600848303501	-3,65953E+14	6,85657E+13	6,68743E+14	6,13341E+14	5,69059E+14	6,55878E+13	6,0258E+14
TC1100002470.mm.2	Il9r	interleukin 9 receptor	-0.856053994257183	6,74364E+13	-2,46066E+14	0.046408992063798	0.18564748985203	-3,65997E+14	6,1026E+14	6,20513E+14	6,80651E+14	7,21333E+14	6,78481E+14	7,34945E+14
TC0200001033.mm.2	Dync1i2	dynein cytoplasmic 1 intermediate chain 2	1,79318E+14	7,71788E+14	2,46013E+14	0.0464435340271784	0.185739554177197	-3,66064E+14	8,81002E+14	8,86893E+13	8,04729E+14	6,04532E+14	7,93118E+14	6,60455E+14
TC0500001830.mm.2	8430423G03Rik	RIKEN cDNA 8430423G03 gene	-1,01629E+14	5,84695E+12	-2,45941E+14	0.0464908998081743	0.185821231006359	-3,66154E+14	5,05571E+14	4,92173E+14	5,78121E+14	6,22881E+14	6,06858E+14	7,02569E+14
TC0X00003301.mm.2	Reps2	RALBP1 associated Eps domain containing protein 2	1,0812E+14	9,04109E+14	2,45929E+14	0.0464989269862782	0.185821231006359	-3,6617E+14	9,60614E+14	9,7896E+13	9,20572E+14	8,02761E+14	8,83364E+14	8,78382E+13
TC0700004075.mm.2	Trim66	tripartite motif-containing 66	0.941674453072509	6,05222E+14	2,45906E+14	0.0465137887889417	0.185821231006359	-3,66198E+13	7,04261E+14	6,48934E+14	5,58941E+14	6,0592E+14	5,2937E+14	5,83908E+14
TC0100003645.mm.2	Hnrnpu	heterogeneous nuclear ribonucleoprotein U	2,14338E+14	8,34267E+14	2,45906E+14	0.0465140687151757	0.185821231006359	-3,66199E+13	9,52571E+14	9,71714E+14	8,75698E+14	6,19912E+14	8,65829E+14	7,19879E+14
TC0200000574.mm.2	Golga2	golgi autoantigen, golgin subfamily a, 2	0.835122506466103	8,3741E+14	2,45894E+14	0.0465216189337586	0.185821231006359	-3,66213E+14	9,009E+14	8,84086E+14	8,12859E+14	8,05103E+14	8,41878E+13	7,79634E+14
TC1100001634.mm.2	Atp6v0a1	ATPase, H+ transporting, lysosomal V0 subunit A1	2,18925E+14	9,30535E+14	2,45841E+13	0.0465564591533939	0.185914306403627	-3,6628E+14	1,06931E+14	1,07262E+14	9,89819E+14	7,14254E+14	9,30713E+13	8,065E+14
TC1600001986.mm.2	Cct8	chaperonin containing Tcp1, subunit 8 (theta)	1,91523E+13	8,4263E+14	2,4574E+14	0.0466224990644151	0.185983589895991	-3,66406E+14	9,65683E+14	9,65827E+14	8,72409E+13	6,76054E+14	8,72978E+14	7,02831E+14
TC1300000534.mm.2	Cap2	CAP, adenylate cyclase-associated protein, 2 (yeast)	1,49647E+14	9,1419E+14	2,45721E+14	0.0466346441326876	0.185983589895991	-3,66429E+14	1,00728E+14	1,01892E+14	9,58113E+13	7,68799E+14	8,82662E+13	8,49358E+14
TC0200005426.mm.2	Nkain4	Na+/K+ transporting ATPase interacting 4	1,27006E+14	6,90033E+14	2,45719E+14	0.0466361442065346	0.185983589895991	-3,66432E+14	7,90032E+14	7,36525E+14	7,07787E+14	5,64757E+14	6,60273E+14	6,80822E+14
TC1700001482.mm.2	Dact2	dishevelled-binding antagonist of beta-catenin 2	1,19074E+14	6,23664E+14	2,45711E+14	0.046641458312634	0.185983589895991	-3,66442E+14	6,64822E+14	7,31052E+13	6,16641E+14	5,41085E+14	6,42905E+14	5,4548E+14
TC1900000208.mm.2	Vps37c	vacuolar protein sorting 37C	-0.841900658939893	6,98804E+14	-2,45704E+14	0.046646091944158	0.185983589895991	-3,66451E+14	6,57998E+14	6,25272E+14	7,54773E+14	6,96877E+14	7,32807E+14	7,25098E+14
TC1100000517.mm.2	Olfr1381	olfactory receptor 1381	-1,00437E+14	5,75155E+14	-2,45697E+14	0.0466505754430625	0.185983589895991	-3,66459E+14	5,1831E+13	5,22479E+14	5,71323E+14	6,70339E+13	5,6802E+14	6,0046E+14
TC0900002525.mm.2	Fbxl22	F-box and leucine-rich repeat protein 22	-0.900300703893997	6,53343E+14	-2,45681E+14	0.0466610892568747	0.185983589895991	-3,66479E+14	5,43062E+14	5,91356E+14	6,43961E+14	6,70517E+14	7,05515E+14	7,65645E+13
TC0700002692.mm.2	Dll3	delta like canonical Notch ligand 3	-0.877173226176352	5,96313E+14	-2,45665E+14	0.0466715544167322	0.185983589895991	-3,66499E+14	5,60983E+14	5,38994E+14	6,04303E+14	6,7111E+14	5,86815E+14	6,1567E+14
TC0300001423.mm.2	Tspan5	tetraspanin 5	1,92094E+14	8,67851E+14	2,45636E+14	0.0466909702453964	0.185983589895991	-3,66536E+14	9,50706E+13	9,88478E+14	8,82651E+14	6,72346E+14	9,31268E+14	7,81658E+14
TC1100000859.mm.2	Gas7	growth arrest specific 7	1,34516E+14	8,53173E+14	2,45634E+14	0.0466918572188607	0.185983589895991	-3,66538E+14	9,28553E+14	9,42893E+13	8,82958E+14	7,19456E+14	8,38513E+13	8,06666E+14
TC0500001887.mm.2	Gatad1	GATA zinc finger domain containing 1	1,32223E+13	9,14646E+14	2,45621E+14	0.0467007760639968	0.185983589895991	-3,66555E+14	1,00597E+14	9,86754E+14	9,38506E+14	7,8977E+14	9,15695E+14	8,51183E+14
TC0900000690.mm.2	Snupn	snurportin 1	0.81533815512246	5,64996E+14	2,45531E+14	0.0467599580473433	0.186160651836989	-3,66668E+12	6,22172E+14	6,32769E+14	5,51932E+14	5,39941E+14	5,50342E+14	4,92823E+14
TC0400003590.mm.2	Rbbp4	retinoblastoma binding protein 4, chromatin remodeling factor	1,63617E+14	8,23137E+14	2,45518E+14	0.046768343488868	0.186160651836989	-3,66684E+14	9,02736E+14	9,00497E+14	8,44214E+14	6,31785E+14	8,48298E+14	8,11293E+14
TC1000001822.mm.2	Pex7	peroxisomal biogenesis factor 7	1,0185E+14	5,94288E+14	2,4544E+13	0.0468192782098802	0.18625009285402 4	-3,6678E+14	6,7889E+14	6,72557E+14	5,82297E+14	5,6545E+14	5,86196E+14	4,80341E+14
TC0400000777.mm.2	Kdm4c	lysine (K)-specific demethylase 4C	0.832480401591263	6,98374E+14	2,45439E+14	0.0468201833778834	0.186250092854024	-3,66782E+14	7,44978E+14	7,42719E+14	6,83312E+12	6,3789E+14	7,13639E+14	6,67705E+14
TC0X00000552.mm.2	Gm715	predicted gene 715	-0.903679307969012	6,74796E+14	-2,45427E+14	0.0468283094326257	0.186250092854024	-3,66798E+14	6,01757E+13	6,21233E+14	6,61748E+14	7,41978E+14	7,10185E+14	7,11877E+13
TC0700004078.mm.2	BC051019	cDNA sequence BC051019	-1,0606E+14	5,5631E+14	-2,45413E+13	0.0468370493542174	0.186250092854024	-3,66814E+14	4,8905E+14	4,47407E+14	5,2116E+13	6,27416E+14	6,16682E+14	6,36143E+14
TC0100002324.mm.2	Mfsd6	major facilitator superfamily domain containing 6	2,01051E+14	7,54742E+14	2,45359E+14	0.0468730493054253	0.1863472596739	-3,66882E+14	9,1034E+14	8,9159E+13	7,94204E+14	6,05623E+12	7,63129E+14	5,63568E+14
TC1700002207.mm.2	Taf8	TATA-box binding protein associated factor 8	-0.730293224627164	8,42715E+14	-2,45308E+14	0.0469063335292888	0.186408906122056	-3,66946E+14	7,97647E+14	7,94296E+14	8,58649E+14	8,79353E+14	8,53939E+14	8,72405E+14
TC0500001241.mm.2	2210016L21Rik	RIKEN cDNA 2210016L21 gene	1,26347E+14	9,0541E+14	2,453E+14	0.0469116933112975	0.186408906122056	-3,66956E+14	1,01364E+14	9,94833E+14	8,83161E+14	8,72621E+14	9,09992E+14	7,58209E+14
TC1900001703.mm.2	Nrap	nebulin-related anchoring protein	-0.811601293260535	5,10335E+14	-2,45277E+14	0.0469273518913684	0.186425153112825	-3,66985E+14	4,67854E+14	4,88018E+14	5,32276E+14	5,85916E+14	5,00687E+14	4,87263E+14
TC0400000213.mm.2	Gpr63	G protein-coupled receptor 63	-0.774351449451554	7,73392E+14	-2,45253E+14	0.0469429606902915	0.186441194380741	-3,67015E+14	7,38155E+14	7,16282E+14	8,09339E+14	7,99969E+14	7,68073E+13	8,08536E+14
TC1700000257.mm.2	Zfp160	zinc finger protein 160	1.39431E+12	6,251E+13	2,45188E+14	0.0469856406391115	0.186553898095667	-3,67096E+14	7,24817E+14	6,70333E+14	6,18268E+14	4,9802E+14	6,77773E+14	5,61387E+13
TC1200001044.mm.2	Serpina3m/Serpina3k	serine (or cysteine) peptidase inhibitor, clade A, member 3M/serine (or cysteine) peptidase inhibitor, clade A, member 3K	0.950994684602434	5,47281E+14	2,45175E+14	0.0469944934134122	0.186553898095667	-3,67113E+14	5,92836E+14	5,84946E+14	5,35853E+14	4,5173E+14	5,79122E+13	5,392E+14
TC0200001121.mm.2	Agps	alkylglycerone phosphate synthase	1,01616E+14	5,71103E+14	2,45114E+12	0.0470349795969537	0.186663408131862	-3,67189E+14	5,96867E+14	6,57362E+14	5,3646E+14	5,14537E+14	6,03551E+14	5,17841E+13
TC0500000248.mm.2	Dpp6	dipeptidylpeptidase 6	2,13191E+14	8,34956E+13	2,45096E+14	0.0470465792335583	0.186663408131862	-3,67211E+13	9,84091E+14	9,65774E+14	8,92649E+14	6,30834E+13	8,3492E+14	7,0147E+14
TC1700001772.mm.2	Pxt1	peroxisomal, testis specific 1	-0.877989881333263	5,2899E+14	-2,45069E+14	0.0470647367204767	0.186663408131862	-3,67245E+14	4,68797E+14	4,67728E+14	5,25302E+14	5,86822E+14	5,3971E+14	5,85582E+14
TC0200004563.mm.2	Mal	myelin and lymphocyte protein, T cell differentiation protein	1,59516E+14	1,03735E+14	2,45063E+14	0.0470684184968506	0.186663408131862	-3,67252E+14	1,1153E+14	1,14072E+14	1,05956E+14	8,77426E+14	1,06568E+14	9,65407E+14
TC0600000676.mm.2	Avl9	AVL9 cell migration associated	1,48186E+14	6,85125E+14	2,44923E+14	0.0471612045681839	0.186838584262681	-3,67428E+14	7,65497E+14	8,07877E+14	7,00425E+14	5,76576E+14	6,99699E+14	5,60673E+14
TC0X00000862.mm.2	Gm44	predicted gene 44	-0.957474036122905	5,24878E+14	-2,44921E+14	0.0471627150966234	0.186838584262681	-3,6743E+14	4,34368E+14	4,83534E+14	5,17082E+14	5,92315E+14	5,45488E+14	5,76482E+13
TC0800000843.mm.2	Hmgxb4	HMG box domain containing 4	-1,07303E+14	7,86433E+14	-2,44918E+14	0.0471649294558373	0.186838584262681	-3,67435E+14	7,22879E+13	7,28815E+14	7,7857E+14	8,87729E+13	7,77292E+14	8,23316E+14
TC1000002330.mm.2	Cabcoco1	ciliary associated calcium binding coiled-coil 1	0.728323482035699	7,30808E+13	2,44909E+14	0.0471707319841691	0.186838584262681	-3,67446E+14	7,89591E+14	8,03266E+14	7,22889E+14	7,24303E+14	6,8431E+14	6,60488E+14
TC1900000159.mm.2	Hnrnpul2	heterogeneous nuclear ribonucleoprotein U-like 2	0.901387773321037	8,61911E+14	2,44899E+14	0.0471772527583074	0.186838584262681	-3,67458E+14	9,47132E+14	9,27935E+14	8,72875E+14	8,21915E+14	8,3179E+14	7,6982E+14
TC0300000643.mm.2	Plrg1	pleiotropic regulator 1	1,66406E+14	6,86927E+14	2,44889E+13	0.0471840526867834	0.186838584262681	-3,67471E+14	7,91278E+14	8,01703E+14	7,17479E+14	5,42691E+12	7,01488E+14	5,6692E+14
TC1000002604.mm.2	Mterf2	mitochondrial transcription termination factor 2	1,47502E+14	6,08354E+14	2,44864E+14	0.0472002680345874	0.186838584262681	-3,67501E+14	6,82118E+14	6,96446E+14	6,34561E+14	4,48998E+14	6,12795E+14	5,75205E+14
TC0200005408.mm.2	Gata5	GATA binding protein 5	-1,33066E+14	5,97373E+14	-2,44857E+14	0.0472053544674099	0.186838584262681	-3,67511E+14	5,13983E+14	5,65585E+14	6,24099E+14	7,21601E+14	5,17847E+14	6,41121E+14
TC0100000233.mm.2	Plekhb2	pleckstrin homology domain containing, family B (evectins) member 2	0.778476620223952	8,3358E+13	2,44808E+14	0.0472374687169051	0.18691977736628	-3,67571E+14	8,95642E+14	8,91369E+14	8,39765E+14	7,91551E+14	7,94922E+14	7,8823E+14
TC0400003208.mm.2	Acot11	acyl-CoA thioesterase 11	-1,05546E+14	7,25537E+14	-2,44757E+14	0.0472718229947219	0.186954349625097	-3,67636E+14	6,44824E+14	6,4989E+14	7,32484E+14	7,7332E+13	7,22487E+14	8,30216E+14
TC1700002214.mm.2	Mdfi	MyoD family inhibitor	-0.918707765972773	6,02772E+14	-2,4474E+14	0.0472829880028605	0.186954349625097	-3,67657E+14	5,43354E+14	5,31803E+14	5,89405E+14	6,69493E+14	6,25815E+14	6,5676E+14
TC1100004063.mm.2	Fdxr	ferredoxin reductase	-1,07132E+14	6,02974E+14	-2,44685E+14	0.0473195321173871	0.186954349625097	-3,67726E+14	5,48453E+13	5,26044E+14	5,92542E+14	6,9622E+14	6,009E+14	6,53684E+13

TC1300001087.mm.2	Poc5	POC5 centriolar protein	0.757975170534921	6,00525E+14	2,44649E+14	0.0473434544068013	0.186954349625097	-3,67771E+14	6,65426E+14	6,45877E+14	5,69233E+14	5,90476E+14	5,81021E+14	5,51117E+14
TC0700004134.mm.2	Copb1	coatomer protein complex, subunit beta 1	1,43603E+14	6,22895E+14	2,44565E+14	0.0473992407617735	0.186954349625097	-3,67875E+14	7,45746E+14	7,59717E+14	6,51154E+14	5,67103E+14	5,88166E+14	4,25485E+14
TC1100001111.mm.2	Slc6a4	solute carrier family 6 (neurotransmitter transporter, serotonin), member 4	-1,08339E+14	5,84977E+14	-2,44565E+14	0.0473996607753266	0.186954349625097	-3,67876E+14	5,44352E+14	5,07306E+14	5,77828E+14	6,90509E+14	5,83187E+14	6,0668E+13
TC0500002672.mm.2	Epha5	Eph receptor A5	0.973642370033803	7,52144E+14	2,44536E+14	0.047418988606851	0.186954349625097	-3,67912E+13	8,0588E+14	7,9018E+14	6,97008E+14	7,04323E+14	8,06396E+14	7,09075E+14
TC0900002287.mm.2	Colca2	COLCA2 homolog	-0.822594845009629	5,66321E+14	-2,4451E+14	0.0474362163515272	0.186954349625097	-3,67945E+14	5,40163E+14	4,81651E+14	5,81928E+14	6,04406E+14	5,96473E+14	5,93303E+14
TC1100003469.mm.2	Smg8	smg-8 homolog, nonsense mediated mRNA decay factor (C. elegans)	1,07009E+14	5,45992E+13	2,44507E+13	0.0474383382502524	0.186954349625097	-3,67949E+14	6,01592E+14	6,02451E+14	5,40216E+14	4,49808E+14	5,02724E+14	5,79161E+14
TC1100001147.mm.2	Tlcd1/Mir7653	TLC domain containing 1/microRNA 7653	1,45109E+14	7,4865E+14	2,44506E+14	0.0474390962110949	0.186954349625097	-3,6795E+13	8,60079E+14	8,40775E+14	7,71727E+14	6,3891E+14	7,54757E+12	6,25649E+14
TC0200004671.mm.2	Pcna	proliferating cell nuclear antigen	2,30506E+14	8,06609E+14	2,44453E+13	0.0474740854379588	0.186954349625097	-3,68016E+14	9,22561E+13	9,4817E+14	8,50923E+14	5,58797E+14	8,46207E+14	7,12998E+14
TC0100003074.mm.2	Tmcc2	transmembrane and coiled-coil domains 2	1,28865E+14	8,04006E+14	2,44443E+14	0.0474807839543407	0.186954349625097	-3,68028E+14	8,95423E+13	8,80293E+14	8,30167E+14	6,87819E+14	7,99207E+14	7,3113E+14
TC1700001787.mm.2	Tmem217	transmembrane protein 217	-1,03347E+14	5,72175E+14	-2,4444E+14	0.0474828998541881	0.186954349625097	-3,68032E+14	5,54151E+14	5,15379E+13	5,8625E+13	6,89973E+14	5,38397E+14	5,489E+14
TC0600001322.mm.2	Hnrnpf	heterogeneous nuclear ribonucleoprotein F	1,55023E+14	7,76329E+14	2,44437E+14	0.0474849880968157	0.186954349625097	-3,68036E+14	8,55721E+14	8,86571E+14	7,94412E+14	6,37835E+14	8,04256E+14	6,79177E+14
TC1600000043.mm.2	Smim22	small integral membrane protein 22	-0.826730445331744	5,81814E+14	-2,44422E+14	0.047495031472009	0.186954349625097	-3,68055E+13	5,26648E+14	5,44146E+14	5,87378E+14	6,48762E+14	5,94328E+14	5,89619E+14
TC0300001528.mm.2	Fubp1	far upstream element (FUSE) binding protein 1	1,37438E+13	8,63015E+14	2,44379E+14	0.0475239677210124	0.186954349625097	-3,68109E+14	9,57274E+14	9,46011E+14	8,683E+14	7,60109E+14	8,90706E+14	7,55689E+14
TC1600000038.mm.2	Mgrn1	mahogunin, ring finger 1	0.919749828631543	8,30809E+14	2,44378E+14	0.0475242463926961	0.186954349625097	-3,6811E+14	9,0207E+14	8,85343E+14	8,42199E+14	7,61265E+14	8,10864E+14	7,83114E+14
TC0100003304.mm.2	Xpr1	xenotropic and polytropic retrovirus receptor 1	2,93968E+14	7,75345E+14	2,44361E+14	0.0475357732325979	0.186954349625097	-3,68131E+14	9,31288E+14	9,32739E+12	8,44255E+14	4,31835E+14	7,92799E+14	7,19152E+14
TC1300000470.mm.2	Gm5082	predicted gene 5082	-1,26698E+14	5,90169E+14	-2,44328E+14	0.0475576188456938	0.186954349625097	-3,68172E+14	5,01349E+14	5,38499E+14	5,7149E+14	7,21753E+14	6,053E+14	6,02623E+14
TC1600000244.mm.2	Gnb1l	guanine nucleotide binding protein (G protein), beta polypeptide 1-like	-0.922191669921986	5,32568E+14	-2,44328E+14	0.0475579496247587	0.186954349625097	-3,68173E+14	4,95146E+14	4,78691E+14	5,41764E+14	6,16512E+14	5,10161E+14	5,53134E+14
TC0400003241.mm.2	Gpx7	glutathione peroxidase 7	-0.784599819806954	6,59029E+14	-2,44314E+14	0.0475673123782208	0.186954349625097	-3,6819E+14	6,02819E+14	6,23642E+14	6,80952E+14	7,02429E+14	6,5124E+14	6,93092E+14
TC0600003488.mm.2	Umad1	UMAP1-MVP12 associated (UMA) domain containing 1	0.786657381620074	7,07253E+14	2,44278E+14	0.0475914326594037	0.186954349625097	-3,68235E+14	7,72962E+14	7,35065E+14	6,93048E+14	6,57648E+13	6,8962E+13	6,95173E+14
TC0X00000061.mm.2	Prickle3	prickle planar cell polarity protein 3	-1,10747E+14	5,77767E+14	-2,44245E+14	0.0476133677612342	0.186954349625097	-3,68276E+14	5,09969E+14	5,10712E+13	5,68298E+14	6,73878E+14	5,67677E+14	6,36065E+14
TC1100003711.mm.2	Krt40	keratin 40	-1,14493E+13	5,58137E+14	-2,44209E+14	0.0476376162163579	0.186954349625097	-3,68322E+14	4,71708E+14	5,20096E+14	5,77019E+14	6,43771E+14	5,16022E+14	6,20208E+12
TC0800002374.mm.2	Cilp2	cartilage intermediate layer protein 2	-0.933526662072289	7,27322E+14	-2,44153E+14	0.0476747620178763	0.186954349625097	-3,68391E+14	6,75146E+14	6,39605E+14	7,10103E+13	7,91353E+14	7,82805E+14	7,6492E+13
TC0X00000210.mm.2	Rbm10	RNA binding motif protein 10	0.756044256505513	7,06977E+14	2,44127E+14	0.0476922111536898	0.186954349625097	-3,68423E+14	7,43105E+14	7,59028E+14	6,56542E+14	6,94382E+14	6,91572E+14	6,97232E+13
TC0900001366.mm.2	6430571L13Rik	RIKEN cDNA 6430571L13 gene	-1,16063E+14	6,02565E+14	-2,44102E+14	0.0477093574231111	0.186954349625097	-3,68455E+14	5,30481E+14	5,29332E+14	6,10744E+13	6,81196E+14	5,73739E+13	6,899E+14
TC0400000902.mm.2	Gm13287	predicted gene 13287	-0.799268635772868	6,32943E+14	-2,44101E+13	0.0477096288637804	0.186954349625097	-3,68456E+14	5,5637E+14	5,88113E+13	6,40112E+13	6,64224E+14	6,54532E+14	6,94307E+14
TC0800000117.mm.2	Proz	protein Z, vitamin K-dependent plasma glycoprotein	-0.843139781267102	6,61126E+14	-2,44092E+14	0.0477157164719235	0.186954349625097	-3,68467E+13	6,0411E+14	6,04089E+14	6,54462E+14	7,22365E+14	6,85465E+14	6,96266E+14
TC0900001344.mm.2	Poc1a	POC1 centriolar protein A	-1,13877E+14	6,47517E+14	-2,4408E+14	0.0477241711635856	0.186954349625097	-3,68483E+14	5,9987E+13	5,44453E+14	6,40908E+14	7,31168E+14	6,4511E+14	7,23595E+14
TC0300001666.mm.2	Rbis	ribosomal biogenesis factor	1,24269E+13	8,21348E+14	2,44052E+14	0.0477431234213302	0.186954349625097	-3,68518E+14	8,84571E+14	8,89633E+14	8,3409E+14	6,91576E+14	8,38568E+14	7,89651E+14
TC1200000604.mm.2	Arid4a	AT rich interactive domain 4A (RBP1-like)	1,61944E+14	6,15299E+14	2,44051E+14	0.0477432110723061	0.186954349625097	-3,68518E+14	7,13081E+14	7,00258E+14	6,50956E+14	4,38495E+14	5,81188E+14	6,07819E+14
TC0400004225.mm.2	Epha8	Eph receptor A8	-1,01587E+14	5,08234E+14	-2,44047E+14	0.0477462850800998	0.186954349625097	-3,68524E+14	4,60731E+14	4,52283E+14	5,06872E+14	6,09314E+14	4,93509E+14	5,26696E+14
TC1700000991.mm.2	Zfp959	zinc finger protein 959	0.975422545324625	5,44983E+14	2,44022E+14	0.0477632116803022	0.186954349625097	-3,68556E+14	5,84235E+14	6,03029E+14	5,33284E+14	4,58895E+14	5,77029E+14	5,13426E+13
TC1100002461.mm.2	Bod1	biorientation of chromosomes in cell division 1	-0.723068054262378	7,5276E+14	-2,44002E+13	0.0477766264921759	0.186954349625097	-3,68581E+14	7,14796E+14	7,01852E+14	7,70081E+14	7,91182E+14	7,65336E+14	7,73312E+14
TC0X00002165.mm.2	Aifm1	apoptosis-inducing factor, mitochondrion-associated 1	1,2541E+14	7,33252E+14	2,44002E+14	0.0477767140418545	0.186954349625097	-3,68581E+14	8,19714E+14	8,19229E+14	7,55942E+14	6,3218E+14	7,31059E+14	6,41391E+14
TC0200003720.mm.2	Slc25a12	solute carrier family 25 (mitochondrial carrier, Aralar), member 12	1,98254E+14	9,25941E+14	2,44002E+14	0.0477767881133553	0.186954349625097	-3,68581E+13	1,0519E+14	1,03533E+14	9,7897E+14	7,11751E+14	9,14385E+14	8,63311E+14
TC0700002631.mm.2	Cnfn	cornifelin	-0.909141967769287	6,21552E+14	-2,43998E+14	0.0477791073178324	0.186954349625097	-3,68585E+14	5,60696E+14	5,61403E+14	6,09909E+14	6,94019E+14	6,45262E+14	6,58022E+14
TC0500001645.mm.2	Cnpy4	canopy FGF signaling regulator 4	1,26729E+14	6,47031E+14	2,43991E+14	0.0477840256212044	0.186954349625097	-3,68594E+13	7,35109E+14	6,94102E+14	6,02694E+14	5,7306E+14	5,65262E+14	7,11957E+13
TC1500002289.mm.2	Rarg	retinoic acid receptor, gamma	-0.777146526555252	8,47367E+14	-2,43989E+14	0.047785267019836	0.186954349625097	-3,68597E+14	7,90015E+13	7,98776E+14	8,48051E+14	8,96169E+13	8,72503E+13	8,78686E+14
TC1100000497.mm.2	Olfr1395	olfactory receptor 1395	-0.916510586609329	5,50099E+13	-2,43978E+14	0.0477923192396344	0.186954349625097	-3,6861E+13	4,52268E+14	5,08146E+14	5,40477E+14	6,03239E+14	5,87393E+14	6,09074E+14
TC0800001232.mm.2	Nrn1l/Edc4	neuritin 1-like/enhancer of mRNA decapping 4	1,04807E+14	6,82926E+14	2,43965E+14	0.0478011396329299	0.186954349625097	-3,68626E+14	7,49985E+14	7,57312E+14	6,63072E+14	6,34612E+14	5,90936E+14	7,01636E+14
TC0800001029.mm.2	Tent4b	terminal nucleotidyltransferase 4B	1,65315E+14	8,08953E+14	2,43962E+13	0.0478036236310819	0.186954349625097	-3,68631E+14	9,15195E+14	9,07866E+14	8,49397E+13	6,43034E+14	8,07191E+14	7,31037E+14
TC1300002174.mm.2	Gm5141	predicted gene 5141	1,46308E+14	6,0404E+13	2,43937E+14	0.0478203032543132	0.186954349625097	-3,68662E+14	6,82774E+14	7,12816E+14	6,11483E+14	4,91492E+14	6,34161E+14	4,91514E+14
TC0700000811.mm.2	Kcnc3	potassium voltage gated channel, Shaw-related subfamily, member 3	-0.762097794319573	7,7261E+14	-2,43911E+14	0.0478375284088873	0.186954349625097	-3,68694E+14	7,33506E+14	7,2852E+14	7,87514E+14	8,26931E+14	7,70117E+14	7,89075E+14
TC0X00001759.mm.2	AU022751	expressed sequence AU022751	-0.925073078438764	4,83851E+14	-2,4383E+14	0.0478924422213512	0.186954349625097	-3,68796E+14	4,39709E+14	4,28361E+14	4,9495E+14	5,58135E+14	4,60262E+14	5,21691E+14
TC1700002418.mm.2	Twsg1	twisted gastrulation BMP signaling modulator 1	-0.719823360476327	8,31366E+14	-2,43819E+14	0.0478999784757908	0.186954349625097	-3,6881E+14	7,689E+14	7,82431E+14	8,48253E+14	8,47043E+14	8,60692E+14	8,80879E+14
TC0600003503.mm.2	Herc6	hect domain and RLD 6	1,01392E+13	5,39515E+14	2,43817E+12	0.0479009075337715	0.186954349625097	-3,68812E+14	5,53801E+14	6,39391E+14	4,69277E+14	5,21132E+14	5,46803E+14	5,06689E+14
TC1900001617.mm.2	Nt5c2	5'-nucleotidase, cytosolic II	1,63386E+14	7,21617E+14	2,43796E+14	0.0479150294068892	0.186954349625097	-3,68838E+14	8,39355E+14	8,28544E+14	7,61575E+12	5,7955E+14	7,19283E+14	6,01396E+14
TC1100000296.mm.2	Mtif2	mitochondrial translational initiation factor 2	0.852698448429126	5,35254E+14	2,43773E+14	0.0479306955363889	0.186954349625097	-3,68867E+14	6,03059E+14	6,07802E+14	5,45038E+14	4,95283E+14	4,54576E+14	5,05767E+14
TSUnmapped00000061.mm.2	Defa-rs4	defensin, alpha,, related sequence 4	-0.888261660057247	5,46112E+14	-2,43746E+14	0.0479486882870404	0.186954349625097	-3,689E+14	4,67291E+13	4,82187E+12	5,75184E+14	5,51946E+14	5,62282E+13	6,37782E+14
TC1000003215.mm.2	Sumo3	small ubiquitin-like modifier 3	0.706954220054056	8,99271E+14	2,43723E+14	0.0479645853165232	0.186954349625097	-3,6893E+14	9,6428E+14	9,5059E+14	8,84802E+14	8,88676E+14	8,54122E+14	8,53158E+14
TC0600000392.mm.2	Rab19	RAB19, member RAS oncogene family	-1,40127E+14	5,87215E+14	-2,43713E+14	0.0479709372285183	0.186954349625097	-3,68942E+14	4,94716E+14	5,07214E+13	5,814E+14	7,00784E+14	5,53461E+14	6,85717E+14
TC0100001721.mm.2	Leftv2	left-right determination factor 2	-1,72604E+14	6,6434E+13	-2,43701E+14	0.0479790342682062	0.186954349625097	-3,68957E+14	5,38626E+14	5,39627E+14	6,14659E+14	8,08802E+13	6,7851E+14	8,05815E+14
TC1000001286.mm.2	Osbpl8	oxysterol binding protein-like 8	1,28746E+14	6,58263E+14	2,43698E+14	0.0479813542123405	0.186954349625097	-3,68961E+14	7,22833E+14	7,24995E+14	6,69861E+14	5,20475E+14	6,829E+14	6,28512E+13
TC1400000971.mm.2	Nkx2-6	NK2 homeobox 6	-0.73304780476605	6,8308E+14	-2,43691E+14	0.0479861432405408	0.186954349625097	-3,6897E+14	6,38512E+13	6,47118E+14	7,17954E+14	7,14286E+14	7,05573E+14	6,75037E+14
TC0900000695.mm.2	Sin3a	transcriptional regulator, SIN3A (yeast)	1,25043E+13	7,6287E+13	2,4368E+14	0.0479936480660911	0.186954349625097	-3,68984E+14	8,52176E+13	8,35086E+14	7,92182E+14	6,44995E+14	7,44365E+14	7,08417E+14
TC0900002735.mm.2	Col12a1	collagen, type XII, alpha 1	-0.911622567130335	7,33155E+14	-2,43663E+14	0.0480047803611153	0.186954349625097	-3,69004E+13	6,43437E+14	6,67954E+14	7,18067E+14	7,75649E+13	7,67467E+14	8,26357E+14
TC0800002146.mm.2	Tlr3	toll-like receptor 3	1,35704E+14	6,79779E+13	2,4365E+14	0.0480137567231677	0.186954349625097	-3,69021E+14	7,28075E+13	7,64188E+14	6,88018E+14	5,32837E+13	6,51577E+14	7,13978E+14
TC0800002290.mm.2	Clcn3	chloride channel, voltage-sensitive 3	1,84558E+14	7,98838E+14	2,43645E+14	0.0480169301535506	0.186954349625097	-3,69027E+14	8,97188E+13	9,14925E+13	8,30462E+12	6,12534E+14	8,30953E+14	7,06968E+14
TC0500002569.mm.2	Corin	corin, serine peptidase	-0.959671847987785	6,20047E+14	-2,43626E+14	0.0480297382366715	0.186954349625097	-3,69051E+14	5,96908E+14	5,66317E+14	6,42258E+14	7,12901E+14	5,71743E+14	6,30158E+14
TC0800001423.mm.2	Adad2	adenosine deaminase domain containing 2	-0.988254427994484	6,24185E+14	-2,43625E+14	0.0480310219793713	0.186954349625097	-3,69053E+14	5,75737E+14	5,62815E+14	6,19283E+14	7,1692E+14	6,20639E+14	6,49714E+14
TC1800001746.mm.2	Hdhd2	haloacid dehalogenase-like hydrolase domain containing 2	1,26924E+14	8,17976E+14	2,4361E+14	0.0480411466266985	0.186954349625097	-3,69072E+14	9,18887E+14	8,81136E+14	8,32193E+14	7,13983E+14	8,27989E+14	7,33669E+14
TC0700001880.mm.2	Slc5a2	solute carrier family 5 (sodium/glucose cotransporter), member 2	-0.947577902206675	6,51166E+14	-2,43597E+14	0.0480499715849994	0.186954349625097	-3,69088E+13	5,89514E+14	6,18735E+14	6,59799E+14	7,37966E+14	6,28813E+14	6,72169E+14
TC1700001053.mm.2	Fer	fer (fms/fps related) protein kinase	1,08116E+14	6,91836E+14	2,43592E+14	0.0480533276575479	0.186954349625097	-3,69094E+14	7,44726E+14	7,54605E+14	6,91134E+14	5,91965E+14	7,18885E+13	6,49702E+13
TC0200001938.mm.2	Tmem87b	transmembrane protein 87B	1,7671E+14	5,96337E+14	2,43583E+14	0.0480590492916227	0.186954349625097	-3,69105E+14	7,14924E+14	6,70861E+13	6,23366E+14	4,08999E+14	6,27831E+14	5,32041E+14
TC0700002310.mm.2	Nlrp4d	NLR family, pyrin domain containing 4D	-0.97210865537973	5,11291E+14	-2,43582E+14	0.0480595871808461	0.186954349625097	-3,69106E+14	4,73016E+14	4,11746E+14	5,01461E+14	5,77723E+14	5,43288E+14	5,60512E+14
TC1900001482.mm.2	Cyp2c70	cytochrome P450, family 2, subfamily c, polypeptide 70	-1,25023E+14	4,61579E+14	-2,43577E+14	0.0480633251809716	0.186954349625097	-3,69113E+14	3,37003E+14	4,16897E+12	4,47652E+14	5,56294E+14	4,70175E+14	5,41451E+13
TC1600001156.mm.2	AU021092	expressed sequence AU021092	-0.782275118849317	6,21053E+14	-2,43533E+14	0.0480931515010401	0.186954349625097	-3,69168E+14	5,85382E+14	5,73394E+12	6,3653E+13	6,78701E+13	6,3967E+14	6,12639E+13
TC0900000696.mm.2	Man2c1	mannosidase, alpha, class 2C, member 1	-0.886337862252747	7,01175E+14	-2,43519E+14	0.048102585255236	0.186954349625097	-3,69185E+14	6,32908E+14	6,35003E+14	6,90284E+14	7,54894E+14	7,23309E+14	7,70651E+14
TC0500001818.mm.2	Pomp	proteasome maturation protein	0.898014116252479	7,96579E+14	2,43516E+14	0.0481047440802853	0.186954349625097	-3,69189E+14	8,7678E+14	8,56437E+14	7,37984E+14	8,15629E+14	7,35955E+14	7,5669E+13
TC1900001544.mm.2	Slc25a28	solute carrier family 25, member 28	0.957468935082654	7,23611E+14	2,43512E+14	0.0481068552772125	0.186954349625097	-3,69193E+14	7,8303E+14	7,68839E+14	7,26331E+14	6,34045E+14	7,26409E+13	7,0301E+14
TC0500001180.mm.2	Tfip11	tuftelin interacting protein 11	0.952367818148984	5,79442E+14	2,4351E+14	0.0481088191631595	0.186954349625097	-3,69197E+14	6,4853E+14	6,3281E+14	5,71467E+14	5,194E+14	5,1318E+14	5,91262E+14
TC1500002276.mm.2	Krt77	keratin 77	0.961897656532988	5,87104E+14	2,43498E+14	0.0481167072211629	0.186954349625097	-3,69211E+14	6,65542E+14	6,02039E+14	5,29308E+14	5,45894E+14	6,25783E+14	5,54056E+14
TC1100002024.mm.2	0610009L18Rik	RIKEN cDNA 0610009L18 gene	-0.784527581616815	7,81791E+14	-2,43495E+14	0.0481185900570955	0.186954349625097	-3,69215E+14	7,19862E+14	7,23576E+14	7,7729E+13	8,23054E+14	8,10981E+14	8,35986E+14
TC0800003227.mm.2	Mef2b	myocyte enhancer factor 2B	-1,07964E+14	5,60229E+14	-2,43481E+14	0.0481280110621799	0.186954349625097	-3,69232E+14	4,73009E+14	4,99248E+14	5,37895E+14	6,5029E+14	5,82102E+14	6,18831E+14
TC1300001061.mm.2	Ap3b1	adaptor-related protein complex 3, beta 1 subunit	0.98555419878653	7,14249E+14	2,43454E+14	0.0481462902455353	0.186980278314367	-3,69266E+14	7,96056E+14	7,68999E+14	7,32178E+14	6,35767E+14	6,86642E+14	6,65853E+14
TC0600003536.mm.2	Clec1b	C-type lectin domain family 1, member b	-1,31454E+14	6,19096E+14	-2,43434E+14	0.0481599867138716	0.186988401426654	-3,69291E+14	5,09587E+14	5,72287E+14	6,0072E+14	7,44062E+14	6,19425E+14	6,68492E+14
TC0100002278.mm.2	Col5a2	collagen, type V, alpha 2	-0.784067511220868	6,25746E+14	-2,43277E+13	0.0482667599939947	0.187320777418797	-3,69488E+14	5,85116E+14	5,3722E+14	6,30029E+14	6,49121E+14	6,71936E+14	6,81053E+14
TC0200001972.mm.2	Nop56/Snord57	NOP56 ribonucleoprotein/small nucleolar RNA, C/D box 57	1,58715E+14	8,32933E+14	2,43253E+14	0.0482827638579827	0.187320777418797	-3,69518E+14	9,3085E+14	9,07271E+14	8,54252E+14	6,66439E+14	8,6496E+14	7,73826E+14
TC1300000715.mm.2	Rmi1	RecQ mediated genome instability 1	1,30908E+14	5,53672E+14	2,43248E+14	0.0482865539392625	0.187320777418797	-3,69525E+14	6,48229E+14	6,32757E+14	5,77186E+14	4,41984E+14	5,55975E+14	4,65903E+14
TC0800001871.mm.2	Vdac3	voltage-dependent anion channel 3	2,06262E+14	8,52185E+13	2,4324E+14	0.0482920939240167	0.187320777418797	-3,69535E+13	9,79194E+14	9,68802E+14	8,91895E+14	6,43576E+14	8,85699E+14	7,43945E+14
TC0300000771.mm.2	Ube2q1	ubiquitin-conjugating enzyme E2Q family member 1	1,05232E+14	8,42735E+14	2,4321E+14	0.048312435085914	0.187340104600229	-3,69572E+14	9,17962E+14	9,01318E+14	8,55288E+14	7,53528E+14	8,40748E+14	7,87568E+14
TC0700004587.mm.2	Nap1l4	nucleosome assembly protein 1-like 4	0.86727067463443	7,86403E+14	2,43198E+14	0.0483203298404116	0.187340104600229	-3,69587E+14	8,59794E+14	8,4616E+14	7,91571E+14	7,40928E+14	7,67311E+14	7,12653E+14
TC0200003262.mm.2	Zdhhc12	zinc finger, DHHC domain containing 12	-0.844300443845521	7,9499E+13	-2,43165E+14	0.0483426054518384	0.187381381199296	-3,69628E+14	7,29791E+14	7,49629E+14	8,54484E+14	7,93795E+14	8,06886E+13	8,35358E+14
TC0500003050.mm.2	Selplg	selectin, platelet (p-selectin) ligand	1,15195E+14	7,85439E+14	2,4312E+14	0.0483734072761625	0.187425060942697	-3,69685E+13	8,6223E+14	8,52588E+14	8,00955E+14	6,83473E+14	7,89715E+14	7,23675E+14
TC1000000769.mm.2	Gm10318	predicted gene 10318	-0.948176954890051	7,01844E+14	-2,43102E+13	0.0483853928656013	0.187425060942697	-3,69707E+14	6,28612E+14	6,47636E+13	6,98488E+14	7,67396E+14	7,03493E+14	7,65437E+14
TC0700001525.mm.2	Olfr653	olfactory receptor 653	-0.987623012344574	4,98123E+14	-2,43098E+13	0.0483887701558754	0.187425060942697	-3,69713E+14	4,04463E+14	4,43473E+14	4,8142E+14	5,6404E+14	5,23891E+14	5,71453E+14
TC1100000937.mm.2	Dlg4	discs large MAGUK scaffold protein 4	1,25706E+14	1,03221E+14	2,43022E+14	0.0484403939028728	0.187479244112117	-3,69808E+14	1,11686E+14	1,10972E+14	1,05743E+14	9,17741E+13	1,02828E+14	9,63242E+14
TC1700002101.mm.2	Pgk2	phosphoglycerate kinase 2	-1,00104E+14	5,33024E+14	-2,42997E+14	0.0484575312136155	0.187479244112117	-3,69839E+13	4,95779E+14	4,71718E+14	5,46982E+14	6,20721E+14	4,95443E+14	5,675E+14
TC0700001413.mm.2	Rab6a	RAB6A, member RAS oncogene family	1,9128E+14	9,84461E+14	2,42961E+14	0.0484820820759333	0.187479244112117	-3,69884E+14	1,08657E+14	1,08693E+14	1,01387E+14	7,77067E+14	1,02822E+13	91410516454
TC1900000046.mm.2	Ctsf	cathepsin F	0.854107746968064	7,06004E+14	2,42955E+12	0.0484858557948209	0.187479244112117	-3,69891E+14	8,02996E+14	7,63507E+14	7,18583E+14	6,77099E+14	6,59262E+14	6,14577E+14
TC0100001726.mm.2	9130409I23Rik	RIKEN cDNA 9130409I23 gene	-0.958993695479634	5,14037E+13	-2,42952E+14	0.048488170364897	0.187479244112117	-3,69895E+14	4,33754E+14	4,48043E+14	4,94427E+14	5,79169E+14	5,41854E+14	5,86974E+14
TC1700000673.mm.2	Ltb	lymphotoxin B	-0.914469330711242	6,45191E+13	-2,42941E+14	0.0484955274913358	0.187479244112117	-3,69909E+14	6,07018E+14	5,7329E+14	6,41657E+14	7,21546E+14	6,59198E+13	6,68439E+14
TC1300002420.mm.2	Msh3	mutS homolog 3	0.915045373641061	6,42206E+14	2,42939E+14	0.0484972318698182	0.187479244112117	-3,69912E+14	7,2213E+14	6,85033E+14	6,44966E+14	5,79187E+14	6,32901E+14	5,89019E+14
TC0400002139.mm.2	Penk	preproenkephalin	0.837137442810972	1,11386E+14	2,42884E+14	0.0485347173455837	0.187479244112117	-3,69981E+14	1,18689E+14	1,17712E+14	1,12726E+14	1,06932E+13	1,0439E+14	1,07866E+14
TC4_GL456216_random00000001.mm.2	Dhrsx	dehydrogenase/reductase (SDR family) X chromosome	-0.940445341585685	6,54949E+14	-2,42879E+14	0.0485378737027411	0.187479244112117	-3,69986E+14	6,01212E+14	5,81484E+14	6,57481E+14	7,13304E+14	6,5219E+14	7,2402E+14
TC0200003753.mm.2	Gpr155	G protein-coupled receptor 155	1,50877E+14	7,34712E+14	2,42861E+14	0.0485501878330983	0.187479244112117	-3,70009E+14	8,20016E+13	8,37457E+14	7,51519E+14	6,04199E+14	7,62756E+14	6,32321E+14
TC1400000314.mm.2	Stimate	STIM activating enhancer	-0.87354376164123	6,60197E+14	-2,42859E+14	0.048551472925443	0.187479244112117	-3,70011E+14	6,39662E+14	5,81637E+14	6,89186E+14	7,06821E+14	6,47731E+14	6,96147E+14
TC1000002538.mm.2	Hmg20b	high mobility group 20B	-0.842984071764293	7,18377E+14	-2,42846E+14	0.0485603691646007	0.187479244112117	-3,70028E+14	6,55808E+14	6,77441E+14	7,18084E+14	7,83762E+14	7,32632E+14	7,42533E+14
TC1100003941.mm.2	Kpna2	karyopherin (importin) alpha 2	1,18205E+14	6,40253E+14	2,42817E+14	0.048580492255311	0.187479244112117	-3,70064E+14	7,38573E+14	7,58483E+14	6,88396E+14	5,72251E+14	5,80555E+14	5,03258E+14
TC0600003163.mm.2	Kcna1	potassium voltage-gated channel, shaker-related subfamily, member 1	0.754339406353533	7,92573E+14	2,42804E+14	0.0485889745093913	0.187479244112117	-3,7008E+14	8,35273E+14	8,62699E+14	7,88235E+14	7,58869E+14	7,61916E+14	7,48447E+14
TC1800001741.mm.2	Pcdhgb1/Pcdhgb2/Pcdhgb4/Pcdhgb5/Pcdhgb6/Pcdhgb7/Pcdhgb8/Pcdhgc3/Pcdhgc4/Pcdhgc5/Pcdhga1/Pcdhga2/Pcdhga3/Pcdhga4/Pcdhga5/Pcdhga6/Pcdhga7/Pcdhga8/Pcdhga9/Pcdhga10/Pcdhga11/Pcdhga12	protocadherin gamma subfamily B, 1/protocadherin gamma subfamily B, 2/protocadherin gamma subfamily B, 4/protocadherin gamma subfamily B, 5/protocadherin gamma subfamily B, 6/protocadherin gamma subfamily B, 7/protocadherin gamma subfamily B, 8/protocadherin gamma subfamily C, 3/protocadherin gamma subfamily C, 4/protocadherin gamma subfamily C, 5/protocadherin gamma subfamily A, 1/protocadherin gamma subfamily A, 2/protocadherin gamma subfamily A, 3/protocadherin gamma subfamily A, 4/protocadherin gamma subfamily A, 5/protocadherin gamma subfamily A, 6/protocadherin gamma subfamily A, 7/protocadherin gamma subfamily A, 8/protocadherin gamma subfamily A, 9/protocadherin gamma subfamily A, 10/protocadherin gamma subfamily A, 11/protocadherin gamma subfamily A, 12	1,85558E+14	8,53518E+14	2,42801E+14	0.0485910566155202	0.187479244112117	-3,70084E+14	9,70339E+14	9,76649E+13	9,05584E+14	6,70287E+13	8,46812E+14	7,51438E+14
TC1100002862.mm.2	Flcn	folliculin	0.815084053972905	7,02884E+14	2,42785E+14	0.0486021659622814	0.187479244112117	-3,70104E+13	7,80823E+13	7,56294E+14	7,15146E+14	6,58954E+13	6,58497E+13	6,4759E+14

TC0300000419.mm.2	Dclk1	doublecortin-like kinase 1	1,41328E+14	9,99172E+14	2,42782E+14	0.0486042939306306	0.187479244112117	-3,70108E+14	1,07482E+14	1,07992E+14	1,02055E+14	8,51532E+14	1,01716E+14	9,51049E+14
TC0600000788.mm.2	Serbp1	serpine1 mRNA binding protein 1	1,56482E+14	9,49773E+14	2,4277E+13	0.048612193998661	0.187479244112117	-3,70122E+14	1,03319E+14	1,04435E+14	9,73186E+14	7,91394E+14	9,77359E+14	8,79154E+14
TC0200001012.mm.2	Klhl23	kelch-like 23	1,16013E+14	6,01087E+14	2,42734E+14	0.0486373193853715	0.187531258017825	-3,70168E+14	6,73668E+14	6,75758E+14	6,08805E+13	5,08594E+14	6,16527E+13	5,23173E+14
TC0500003708.mm.2	Mrpl33	mitochondrial ribosomal protein L33	0.961934737853575	8,59979E+14	2,4268E+13	0.0486740168553686	0.18762785492597	-3,70235E+14	9,30976E+14	9,12314E+13	8,70132E+14	7,80771E+14	8,5056E+14	8,15121E+14
TC1200000848.mm.2	Gpatch2l	G patch domain containing 2 like	0.811282584708714	6,99153E+14	2,42639E+14	0.0487022570033042	0.187664952245195	-3,70287E+14	7,26242E+14	7,42615E+14	6,77169E+14	6,29431E+13	6,92313E+14	7,27148E+14
TC1700000036.mm.2	Zdhhc14	zinc finger, DHHC domain containing 14	0.898184111850577	6,99918E+14	2,42632E+14	0.0487069341705087	0.187664952245195	-3,70296E+14	7,57915E+14	7,74496E+14	6,99272E+14	6,53502E+14	6,91209E+14	6,23114E+14
TC1400000899.mm.2	Fam167a	family with sequence similarity 167, member A	-1,4004E+14	6,63554E+14	-2,426E+14	0.0487293182361814	0.18769183293651	-3,70336E+14	5,56423E+14	5,93591E+14	6,28803E+14	8,01292E+14	6,8399E+13	7,17223E+14
TC0200004476.mm.2	Tgm5	transglutaminase 5	-1,66524E+14	4,78391E+13	-2,42574E+12	0.0487465738150981	0.18769183293651	-3,70368E+14	3,69305E+14	3,72682E+14	4,31736E+14	6,43299E+13	4,90529E+13	5,62797E+14
TC1000003150.mm.2	Zc3h10	zinc finger CCCH type containing 10	-1,06136E+12	6,70468E+14	-2,42571E+14	0.0487488562551537	0.18769183293651	-3,70372E+14	5,88511E+14	6,13634E+14	6,56174E+14	7,58245E+14	6,75941E+13	7,30304E+14
TC1100000990.mm.2	Fbxo39	F-box protein 39	-1,2825E+14	4,65533E+14	-2,42476E+14	0.0488139472316814	0.187897547000497	-3,70491E+14	3,83937E+14	3,96715E+14	4,56791E+14	5,8036E+14	4,37874E+14	5,37522E+14
TC1100001076.mm.2	Inpp5k	inositol polyphosphate 5-phosphatase K	1,32183E+14	6,52841E+14	2,42439E+14	0.048839468675125	0.187950885780341	-3,70537E+14	7,25506E+14	7,10049E+14	6,46415E+14	5,24774E+13	7,1123E+14	5,99074E+14
TC1500002111.mm.2	Twf1	twinfilin actin binding protein 1	2,08512E+14	7,10603E+14	2,42415E+14	0.0488558698233741	0.187969109470876	-3,70567E+14	8,28628E+14	8,35316E+14	7,47785E+13	4,99135E+14	7,5165E+13	6,01102E+14
TC1300001419.mm.2	Gtpbp4	GTP binding protein 4	1,68599E+14	6,58966E+14	2,42313E+14	0.0489264500655434	0.188019795186906	-3,70695E+14	7,73712E+14	7,53581E+13	6,85742E+14	5,04355E+14	6,85915E+14	5,50489E+14
TC0200003860.mm.2	Zswim2	zinc finger SWIM-type containing 2	-1,13149E+14	6,10478E+13	-2,42311E+13	0.0489276262387892	0.188019795186906	-3,70698E+14	5,17411E+14	5,73323E+14	5,99026E+14	7,18005E+14	6,33547E+14	6,21554E+14
TC0100002894.mm.2	Bcl2	B cell leukemia/lymphoma 2	2,09518E+13	6,39115E+14	2,42311E+14	0.0489280569192992	0.188019795186906	-3,70698E+14	7,79091E+14	7,45725E+14	6,93295E+14	4,12485E+14	6,39372E+14	5,64723E+14
TC1300000652.mm.2	Nsd1	nuclear receptor-binding SET-domain protein 1	1,23244E+14	7,73714E+14	2,42305E+14	0.0489318449189712	0.188019795186906	-3,70705E+14	8,84068E+14	8,70995E+14	7,84475E+14	7,24099E+14	7,57953E+14	6,20696E+14
TC0700004293.mm.2	Coro1a	coronin, actin binding protein 1A	1,15248E+14	6,53637E+14	2,42234E+14	0.0489805993659912	0.188019795186906	-3,70794E+13	7,11627E+14	7,41581E+14	6,69882E+14	5,52829E+14	6,54162E+14	5,9174E+14
TC1300002314.mm.2	Ahrr	aryl-hydrocarbon receptor repressor	-0.956428465693056	7,25139E+13	-2,42233E+14	0.0489813668353877	0.188019795186906	-3,70795E+14	6,85521E+14	6,4753E+14	7,19145E+14	8,05191E+14	7,5882E+14	7,34627E+14
TC1200000247.mm.2	Myt1l	myelin transcription factor 1-like	0.943079934755699	6,72258E+14	2,42199E+14	0.0490052807352281	0.188019795186906	-3,70839E+14	7,41542E+14	7,57873E+14	6,83819E+14	6,2698E+14	6,48359E+14	5,74976E+14
TC0400002757.mm.2	Ptbp3	polypyrimidine tract binding protein 3	1,599E+13	5,91944E+14	2,42189E+14	0.0490117619300575	0.188019795186906	-3,7085E+14	6,65931E+14	7,19208E+14	6,16195E+14	4,49144E+13	6,11216E+14	4,89971E+14
TC1700000383.mm.2	Rpusd1	RNA pseudouridylate synthase domain containing 1	1,46483E+14	6,66679E+14	2,42186E+14	0.0490142572030651	0.188019795186906	-3,70855E+14	7,71585E+14	7,51527E+14	7,05079E+14	5,25067E+13	6,53516E+13	5,93298E+14
TC0600002578.mm.2	Clec4f	C-type lectin domain family 4, member f	-1,11725E+14	6,16744E+14	-2,42182E+14	0.0490169584032782	0.188019795186906	-3,7086E+14	5,43326E+14	5,43885E+14	6,21998E+14	6,88663E+14	5,9582E+14	7,06768E+13
TC1400002738.mm.2	Slc15a1	solute carrier family 15 (oligopeptide transporter), member 1	-0.972454481233225	5,43117E+14	-2,42177E+13	0.0490202041883204	0.188019795186906	-3,70866E+14	4,76907E+14	5,06622E+14	5,41691E+14	6,36329E+14	5,40205E+14	5,56951E+14
TC0800000472.mm.2	Micu3	mitochondrial calcium uptake family, member 3	1,47338E+14	8,22208E+12	2,42171E+13	0.0490244576113884	0.188019795186906	-3,70873E+14	9,41099E+14	9,23373E+14	8,62349E+14	7,07447E+14	8,05796E+14	6,93186E+14
TC1100001206.mm.2	Psmd11	proteasome (prosome, macropain) 26S subunit, non-ATPase, 11	0.785077839109769	8,26487E+13	2,42163E+13	0.049029736346041	0.188019795186906	-3,70883E+12	8,82355E+13	8,64673E+14	8,20422E+14	7,69591E+14	8,11828E+14	8,10052E+14
TC0100000929.mm.2	Ppp1r7	protein phosphatase 1, regulatory subunit 7	2,60606E+14	8,96486E+13	2,42159E+14	0.0490324073341637	0.188019795186906	-3,70888E+14	1,04531E+14	1,04073E+14	9,49532E+14	6,15296E+14	9,45574E+14	7,82474E+13
TC1700002053.mm.2	Olfr92	olfactory receptor 92	-1,39438E+14	4,75252E+14	-2,42072E+14	0.0490926705876583	0.188165923055403	-3,70997E+14	3,82128E+14	3,90432E+14	4,36506E+14	6,1493E+14	5,07604E+14	5,19911E+14
TC1100003158.mm.2	Emc6	ER membrane protein complex subunit 6	-0.853632608911761	8,60882E+14	-2,42064E+14	0.0490983635996641	0.188165923055403	-3,71007E+14	7,93402E+14	8,14562E+14	8,54645E+14	9,24045E+14	8,9462E+14	8,84019E+13
TC1300001449.mm.2	Edaradd	EDAR (ectodysplasin-A receptor)-associated death domain	-1,24402E+13	5,88973E+14	-2,4205E+13	0.0491081862741556	0.188165923055403	-3,71025E+14	5,19783E+14	5,01098E+14	5,59186E+14	7,105E+14	6,1638E+14	6,26888E+14
TC0200005473.mm.2	Hoxd11	homeobox D11	-1,6125E+14	4,43472E+14	-2,42037E+14	0.0491172266102541	0.188165923055403	-3,71041E+14	3,51516E+14	3,22996E+14	4,1233E+13	5,84681E+14	4,3151E+14	5,57799E+14
TC0200000641.mm.2	Dab2ip	disabled 2 interacting protein	0.775767770112981	7,4803E+14	2,41934E+14	0.0491884068472928	0.188393819712486	-3,7117E+14	7,9673E+14	7,98667E+14	7,45011E+14	6,95232E+14	7,20697E+14	7,31842E+14
TC0500003714.mm.2	Smok3a	sperm motility kinase 3A	-0.899025460223227	5,34829E+14	-2,41903E+13	0.0492101334987196	0.188432243599066	-3,71209E+14	4,82264E+14	4,97041E+14	5,69756E+14	5,89354E+14	4,94831E+14	5,75726E+14
TC0600001602.mm.2	Gprc5a	G protein-coupled receptor, family C, group 5, member A	-1,09184E+14	5,91594E+14	-2,41878E+14	0.0492275428238643	0.188454121530274	-3,71241E+14	4,81491E+14	5,49121E+14	5,74639E+14	6,74342E+14	6,18594E+14	6,51373E+14
TC1500000066.mm.2	Lmbrd2	LMBR1 domain containing 2	1,91975E+14	6,95158E+14	2,41818E+13	0.0492685521493531	0.18852964031126	-3,71315E+14	8,39278E+14	8,51147E+14	7,40269E+14	5,66207E+13	6,87026E+14	4,87022E+14
TC0300001245.mm.2	Dnttip2	deoxynucleotidyltransferase, terminal, interacting protein 2	1,20575E+14	6,09067E+14	2,41815E+13	0.0492706705983192	0.18852964031126	-3,71319E+14	6,85085E+14	6,92701E+14	6,02849E+14	5,33788E+14	6,34277E+14	5,05703E+14
TC0500002586.mm.2	Fryl	FRY like transcription coactivator	0.933351321232012	6,31824E+14	2,41772E+13	0.0493005182423416	0.188564861806712	-3,71373E+14	7,08058E+14	7,09844E+14	5,74708E+14	6,56524E+14	5,92426E+14	5,49385E+14
TC1000002473.mm.2	Odf3l2	outer dense fiber of sperm tails 3-like 2	-1,03053E+14	6,02655E+14	-2,41768E+14	0.0493032807541536	0.188564861806712	-3,71378E+14	5,71212E+14	5,26912E+14	6,01686E+14	7,02545E+13	5,94392E+13	6,19185E+14
TC0700000397.mm.2	Gm5893	predicted gene 5893	-0.84282496999961	4,59213E+14	-2,41748E+14	0.049317610173762	0.18857490572611	-3,71403E+14	3,73024E+14	4,26391E+14	4,62153E+14	5,05827E+14	4,85889E+14	5,01993E+14
TC0300002718.mm.2	Csf1	colony stimulating factor 1 (macrophage)	0.777225809153848	7,67437E+14	2,41705E+14	0.0493471117848742	0.188609651522436	-3,71457E+14	8,00303E+14	8,31582E+14	7,57976E+14	7,18464E+14	7,54092E+14	7,42206E+14
TC0200004443.mm.2	Ndufaf1	NADH:ubiquinone oxidoreductase complex assembly factor 1	0.897742246504542	5,82221E+14	2,41701E+14	0.0493501080381426	0.188609651522436	-3,71462E+14	6,73008E+14	6,86288E+14	6,30669E+14	5,49079E+14	4,85687E+14	4,68594E+14
TC0700003699.mm.2	Folh1	folate hydrolase 1	0.919642738637375	5,50746E+14	2,41673E+14	0.0493696198772965	0.188639479507441	-3,71497E+13	6,22884E+14	5,66068E+14	5,3417E+14	4,70854E+14	5,69114E+14	5,41383E+14
TC0500003451.mm.2	Irs3	insulin receptor substrate 3	-1,09122E+14	6,54994E+14	-2,41636E+14	0.0493956695290386	0.188670801395249	-3,71544E+14	6,05425E+13	5,85428E+14	6,77152E+14	7,31944E+14	6,09761E+14	7,20257E+14
TC0800002393.mm.2	Tmem59l	transmembrane protein 59-like	1,02018E+14	8,86955E+14	2,41628E+14	0.0494012357156677	0.188670801395249	-3,71554E+14	9,74963E+14	9,3914E+13	9,07108E+14	8,0296E+14	8,55467E+14	8,42091E+14
TC0200002759.mm.2	Fam217b	family with sequence similarity 217, member B	1,71096E+14	5,50642E+14	2,41547E+14	0.0494575875265899	0.18884125777629	-3,71655E+14	6,45899E+14	6,40957E+14	5,82506E+13	3,62157E+14	5,72687E+14	4,99647E+14
TC0700004489.mm.2	Tubgcp2	tubulin, gamma complex associated protein 2	0.941572962835003	7,0701E+14	2,41497E+14	0.0494923047094672	0.188859118553804	-3,71718E+14	7,69532E+14	7,68616E+14	7,22135E+14	6,27697E+14	6,78905E+14	6,75175E+14
TC0200004762.mm.2	Dzank1	double zinc ribbon and ankyrin repeat domains 1	2,17356E+14	7,34194E+14	2,41451E+12	0.0495241464871873	0.188859118553804	-3,71775E+14	8,99576E+12	8,79844E+13	7,74774E+14	5,69935E+14	7,5372E+14	5,27316E+14
TC0300000700.mm.2	Arhgef11	Rho guanine nucleotide exchange factor (GEF) 11	0.883098397754534	7,32556E+14	2,41444E+14	0.0495291175427609	0.188859118553804	-3,71784E+14	7,74959E+14	7,65387E+14	7,19287E+14	6,4444E+14	7,60583E+14	7,30681E+14
TC1800001409.mm.2	Tcof1	treacle ribosome biogenesis factor 1	0.865978656329736	5,2805E+14	2,41441E+14	0.0495316791988548	0.188859118553804	-3,71788E+14	6,08823E+14	5,5509E+14	4,86881E+14	5,03837E+14	4,80601E+14	5,33069E+14
TC1900000547.mm.2	Lgi1	leucine-rich repeat LGI family, member 1	2,50797E+14	7,87786E+14	2,41383E+14	0.0495721133423545	0.188859118553804	-3,71861E+14	9,24603E+14	9,49767E+14	8,20781E+14	5,51994E+14	8,57412E+14	6,22158E+13
TC0200002827.mm.2	Dnajc5	DnaJ heat shock protein family (Hsp40) member C5	0.767120347411415	7,95419E+14	2,41369E+14	0.0495818675716316	0.188859118553804	-3,71878E+14	8,49913E+14	8,36671E+14	7,77253E+14	7,55906E+14	7,97257E+14	7,55516E+14
TC1500000371.mm.2	Utp23	UTP23 small subunit processome component	-1,02026E+14	7,24779E+14	-2,41365E+14	0.0495843910112484	0.188859118553804	-3,71883E+14	6,48341E+14	6,65239E+14	7,16397E+14	8,01237E+14	7,23804E+14	7,93657E+13
TC0300000784.mm.2	Jtb	jumping translocation breakpoint	1,03934E+14	7,91148E+14	2,41352E+14	0.0495932584702927	0.188859118553804	-3,71899E+14	7,96618E+14	8,30434E+14	7,64564E+13	6,54619E+14	8,50936E+14	8,49718E+14
TC1100003636.mm.2	Tbx21	T-box 21	-1,0409E+14	6,11272E+14	-2,41334E+14	0.0496059142739143	0.188859118553804	-3,71922E+13	5,52145E+14	5,48471E+14	6,08139E+14	7,00657E+14	6,63517E+14	5,94705E+13
TC0900000706.mm.2	Fam219b	family with sequence similarity 219, member B	-0.734839709394246	6,40689E+14	-2,4133E+14	0.0496091755353492	0.188859118553804	-3,71927E+14	5,93338E+14	5,94614E+14	6,53639E+14	6,81281E+14	6,72264E+14	6,49E+14
TC1000000122.mm.2	Cited2	Cbp/p300-interacting transactivator, with Glu/Asp-rich carboxy-terminal domain, 2	1,28522E+14	8,83669E+14	2,41323E+14	0.0496138503396421	0.188859118553804	-3,71936E+14	9,90559E+14	9,62089E+14	9,24324E+14	7,71282E+14	8,47059E+14	8,067E+14
TC1200002213.mm.2	Efcab11	EF-hand calcium binding domain 11	-0.819987237971825	5,83525E+14	-2,41315E+14	0.0496193886899643	0.188859118553804	-3,71946E+14	5,27561E+14	5,62518E+14	6,1865E+14	6,35427E+13	5,52379E+14	6,04617E+14
TC0200001025.mm.2	Gorasp2	golgi reassembly stacking protein 2	1,4589E+14	8,23655E+14	2,41307E+14	0.0496247101021754	0.188859118553804	-3,71955E+14	9,20935E+14	9,08487E+14	8,60278E+14	6,77364E+14	8,138E+14	7,6107E+14
TC1700000704.mm.2	Flot1	flotillin 1	0.872040851535882	6,93048E+14	2,41296E+14	0.0496325664970857	0.188859118553804	-3,71969E+14	7,46356E+14	7,39922E+14	6,68494E+14	6,43376E+14	7,17177E+14	6,42962E+14
TC0700003982.mm.2	Hpx	hemopexin	-1,01712E+14	5,41669E+14	-2,41288E+14	0.0496380790092074	0.188859118553804	-3,71979E+13	4,74542E+14	4,89662E+14	5,29666E+14	6,37962E+14	5,67448E+14	5,50735E+13
TC1100001149.mm.2	Rab34	RAB34, member RAS oncogene family	-1,35377E+14	7,31876E+14	-2,41235E+14	0.0496750619446503	0.188935932843964	-3,72045E+13	6,4092E+14	6,38626E+14	8,53787E+14	6,96513E+14	8,16571E+14	7,44838E+14
TC0600001802.mm.2	Sgce	sarcoglycan, epsilon	2,34998E+14	6,89848E+14	2,41211E+14	0.0496922372655499	0.188935932843964	-3,72076E+13	8,67565E+14	8,61349E+14	7,61661E+14	4,97257E+14	6,77494E+14	4,73761E+14
TC0700003399.mm.2	Mkrn3	makorin, ring finger protein, 3	-0.797238921432938	4,88554E+14	-2,41209E+14	0.0496934452549321	0.188935932843964	-3,72078E+14	4,60767E+13	4,80797E+14	5,23846E+14	5,77166E+14	4,51228E+13	4,37518E+14
TC1800000105.mm.2	Ttc39c	tetratricopeptide repeat domain 39C	0.955597610590395	5,73541E+14	2,41136E+14	0.0497451118835807	0.189021074787347	-3,72171E+14	6,522E+13	6,22745E+14	5,76524E+14	5,07301E+14	5,72546E+14	5,09927E+14
TC0400003079.mm.2	Jun	jun proto-oncogene	1,14027E+14	7,71768E+14	2,41135E+14	0.049745740250002	0.189021074787347	-3,72172E+14	8,03902E+14	8,56189E+14	7,75556E+14	6,56481E+13	7,86768E+14	7,51711E+14
TC1300002445.mm.2	Scamp1	secretory carrier membrane protein 1	1,81115E+14	8,91925E+14	2,41093E+14	0.0497745926510288	0.189021074787347	-3,72223E+14	9,93907E+14	1,01612E+14	9,23619E+14	7,24172E+14	9,2051E+14	7,7323E+14
TC0700003060.mm.2	Mrgpra4	MAS-related GPR, member A4	-0.886740419525852	5,02953E+14	-2,41082E+14	0.0497828959041809	0.189021074787347	-3,72238E+14	4,5842E+14	4,38322E+14	4,99374E+14	5,74716E+14	5,10197E+14	5,36689E+14
TC0700004573.mm.2	Ins2	insulin II	-1,23333E+14	7,42687E+14	-2,4108E+14	0.0497841237733519	0.189021074787347	-3,7224E+14	6,45052E+14	6,59766E+14	7,19202E+14	8,32283E+14	7,49811E+14	8,50006E+14
TC1000000097.mm.2	Adat2	adenosine deaminase, tRNA-specific 2	0.742389941109845	5,67109E+14	2,41053E+14	0.0498029311984204	0.189021074787347	-3,72274E+14	5,99958E+14	6,10511E+14	5,1784E+14	5,44152E+13	5,79039E+14	5,51156E+14
TC0300001181.mm.2	Dph5	diphthamide biosynthesis 5	0.726975229636816	6,27258E+14	2,4103E+14	0.0498192734998016	0.189021074787347	-3,72303E+14	6,61784E+14	6,91167E+14	6,03805E+14	6,03751E+14	5,94125E+13	6,08914E+14
TC1100002777.mm.2	Slc36a2	solute carrier family 36 (proton/amino acid symporter), member 2	-1,35101E+14	5,95781E+14	-2,41029E+14	0.0498198871840636	0.189021074787347	-3,72304E+14	4,99804E+14	5,10125E+14	5,75012E+14	7,05119E+14	5,85842E+14	6,98783E+14
TC0400004216.mm.2	Podn	podocan	-0.963972663049407	5,41136E+14	-2,41027E+14	0.0498214177758247	0.189021074787347	-3,72307E+14	4,40286E+14	4,96609E+14	5,23669E+14	6,06021E+14	5,8983E+14	5,90402E+14
TC0X00000179.mm.2	Cypt8/Cypt7/Cypt1	cysteine-rich perinuclear theca 8/cysteine-rich perinuclear theca 7/cysteine-rich perinuclear theca 1	-1,30662E+14	6,78617E+14	-2,40971E+14	0.0498609446521062	0.189119128525197	-3,72377E+14	5,96228E+14	5,88515E+14	6,46472E+14	7,99595E+14	6,9007E+14	7,50822E+13
TC0600000381.mm.2	Luc7l2	LUC7-like 2 (S. cerevisiae)	2,33251E+14	6,25976E+14	2,40957E+14	0.0498707364924944	0.189119128525197	-3,72395E+14	7,84958E+14	7,72881E+12	6,55067E+14	4,36271E+13	6,77723E+13	4,28959E+14
TC0600002639.mm.2	Pcbp1	poly(rC) binding protein 1	1,3093E+14	8,58903E+14	2,40907E+14	0.0499059208893403	0.189208024303515	-3,72458E+14	9,34825E+14	9,21076E+14	8,77869E+14	7,16172E+14	8,67842E+14	8,35632E+14
TC1300001725.mm.2	Cdkal1	CDK5 regulatory subunit associated protein 1-like 1	2,12729E+14	5,38632E+14	2,40869E+14	0.0499325133628857	0.189254120993494	-3,72505E+14	6,55525E+14	6,90813E+14	5,85174E+14	3,35706E+14	5,63252E+14	4,01323E+14
TC1100002183.mm.2	Wap	whey acidic protein	-1,04221E+14	7,07105E+14	-2,40856E+14	0.0499415703136807	0.189254120993494	-3,72521E+14	6,52206E+14	6,44526E+14	7,14172E+14	7,91003E+14	6,77056E+14	7,63669E+13
TC0900003024.mm.2	Mapkapk3	mitogen-activated protein kinase-activated protein kinase 3	-1,05326E+14	6,49031E+13	-2,40803E+14	0.0499792123415556	0.189352233355158	-3,72588E+14	5,64818E+14	5,98674E+14	6,34093E+14	7,40052E+14	6,58183E+14	6,98363E+14
TC1400001486.mm.2	Oxsm	3-oxoacyl-ACP synthase, mitochondrial	0.939559573010519	5,79464E+14	2,40767E+14	0.0500040786075259	0.189401908463344	-3,72632E+14	6,75216E+14	6,41481E+14	5,95315E+14	5,3347E+14	5,47293E+14	4,84007E+14
TC1500001720.mm.2	Ly6k	lymphocyte antigen 6 complex, locus K	-1,18795E+14	4,99142E+14	-2,4065E+14	0.0500866762647024	0.189670179706968	-3,72779E+14	3,66607E+14	4,99855E+14	5,27391E+14	5,76661E+14	5,00545E+13	5,23792E+13
TC0100002597.mm.2	Ihh	Indian hedgehog	-0.91324465934873	5,82618E+14	-2,40597E+14	0.0501239735840836	0.189738527648734	-3,72845E+14	5,02508E+14	5,24487E+14	5,66335E+14	6,43308E+13	6,10141E+14	6,48928E+14
TC0200001724.mm.2	Fam98b	family with sequence similarity 98, member B	3,10433E+14	7,25548E+14	2,40591E+14	0.0501282760628474	0.189738527648734	-3,72853E+14	9,00097E+14	9,04327E+14	8,13112E+14	3,70445E+14	7,26053E+14	6,39252E+14
TC0400003613.mm.2	Khdrbs1	KH domain containing, RNA binding, signal transduction associated 1	0.730943581923063	8,31458E+14	2,40542E+14	0.0501632142962037	0.189826179416329	-3,72915E+14	8,77314E+14	8,67325E+14	7,99877E+14	7,98573E+14	8,05824E+14	8,39835E+14
TC0500001596.mm.2	Upk3bl	uroplakin 3B-like	-0.841752662936284	6,11572E+14	-2,40519E+14	0.050179472273209	0.189843117336984	-3,72943E+14	5,35656E+14	5,91309E+14	6,60623E+14	6,34693E+14	6,05102E+14	6,42051E+14
TC1700000936.mm.2	B430306N03Rik	RIKEN cDNA B430306N03 gene	-1,02657E+14	4,87174E+14	-2,40498E+14	0.0501943444386559	0.189854805619733	-3,7297E+14	4,01577E+14	4,339E+14	4,66376E+14	5,74416E+14	5,28353E+14	5,18424E+14
TC1700000276.mm.2	Zfp983	zinc finger protein 983	2,06777E+14	5,65295E+14	2,40481E+13	0.0502063475749345	0.189855639163323	-3,72991E+14	6,83541E+14	7,12997E+13	5,82613E+13	4,00372E+13	6,18364E+14	3,93884E+14
TC1100003055.mm.2	Cd68	CD68 antigen	1,74839E+14	7,80651E+14	2,40446E+14	0.0502308392795668	0.189899161753615	-3,73034E+14	8,5892E+14	9,27647E+13	8,32735E+14	6,04153E+14	7,20075E+14	7,40378E+14
TC0700004309.mm.2	Maz	MYC-associated zinc finger protein (purine-binding transcription factor)	0.804726899714016	7,11406E+14	2,40431E+14	0.0502414278257097	0.189899161753615	-3,73053E+14	7,62225E+14	7,3944E+13	6,98882E+14	6,41837E+14	7,15069E+14	7,10985E+14
TC0300001841.mm.2	Dcun1d1	DCN1, defective in cullin neddylation 1, domain containing 1 (S. cerevisiae)	1,44553E+14	7,02791E+14	2,40382E+14	0.0502762035667636	0.189986038478251	-3,73115E+14	7,92428E+14	8,1207E+14	7,4168E+14	5,73712E+14	6,91124E+14	6,0573E+14
TC0300001877.mm.2	Gm5148	predicted gene 5148	-0.998787594943333	4,98462E+14	-2,40329E+14	0.0503138092997491	0.190029159022023	-3,73181E+13	4,5608E+14	3,90954E+14	4,80616E+14	5,66175E+13	5,51389E+14	5,45561E+14
TC0800001901.mm.2	Adam9	a disintegrin and metallopeptidase domain 9 (meltrin gamma)	1,13004E+14	7,72041E+14	2,40322E+14	0.0503191005689511	0.190029159022023	-3,7319E+14	8,37553E+14	8,24781E+14	7,82259E+14	6,54067E+14	7,84279E+14	7,49308E+14
TC0700003726.mm.2	Tmem126a	transmembrane protein 126A	2,10743E+14	6,59233E+14	2,4031E+14	0.0503278941565031	0.190029159022023	-3,73206E+14	7,62766E+13	7,90132E+14	7,00977E+14	4,30436E+14	6,95445E+14	5,75641E+14
TC0200001576.mm.2	Wt1	Wilms tumor 1 homolog	-1,35308E+14	5,8479E+14	-2,40278E+13	0.050349967048876	0.190029159022023	-3,73245E+14	4,74581E+13	5,17145E+13	5,6646E+14	6,95883E+14	5,73471E+14	6,812E+14
TC0X00001413.mm.2	Pak3	p21 (RAC1) activated kinase 3	1,4269E+14	8,08973E+14	2,40273E+14	0.0503540384568891	0.190029159022023	-3,73252E+14	9,3941E+14	9,02318E+14	8,52309E+14	7,04039E+14	7,81224E+14	6,74539E+14
TC1500002340.mm.2	Hoxc5	homeobox C5	-1,27631E+13	5,42315E+14	-2,40267E+14	0.0503583757850206	0.190029159022023	-3,7326E+14	4,78091E+14	4,57697E+14	5,19707E+14	6,71343E+14	5,39728E+14	5,87321E+14
TC1200001001.mm.2	Kif4-ps	kinesin family member 4, pseudogene	-0.863740553938385	4,99928E+14	-2,40244E+14	0.0503747460108997	0.190041813367126	-3,73289E+14	4,3625E+14	4,09157E+14	4,8467E+14	5,33485E+13	5,41124E+14	5,9488E+14
TC0700003107.mm.2	Gabrg3	gamma-aminobutyric acid (GABA) A receptor, subunit gamma 3	2,06241E+14	7,51203E+14	2,40225E+14	0.0503881672904849	0.190041813367126	-3,73312E+14	8,55123E+14	8,44187E+14	7,66488E+14	5,20341E+14	8,37142E+14	6,83934E+14
TC0400002110.mm.2	Noc2l	NOC2 like nucleolar associated transcriptional repressor	1,23516E+14	6,33275E+14	2,40202E+13	0.0504045900954302	0.190041813367126	-3,73341E+14	6,8339E+14	7,43921E+14	6,40394E+14	5,39886E+14	6,45082E+14	5,46979E+14
TC0300002317.mm.2	Lamtor2	late endosomal/lysosomal adaptor, MAPK and MTOR activator 2	0.760746398933625	7,8311E+14	2,40195E+14	0.0504089064935825	0.190041813367126	-3,73349E+14	8,30525E+14	8,19845E+14	7,555E+14	7,4272E+14	7,97431E+14	7,52641E+14
TC0800001475.mm.2	BC048644	cDNA sequence BC048644	-1,01225E+14	4,40403E+14	-2,4016E+14	0.0504342698315247	0.190091430637057	-3,73394E+14	3,71386E+14	3,73058E+14	4,28225E+14	5,18671E+14	4,43729E+14	5,07351E+14
TC1900001156.mm.2	Olfr76	olfactory receptor 76	-1,06541E+14	4,9276E+14	-2,40144E+14	0.0504456623474249	0.190091430637057	-3,73414E+14	4,51193E+14	4,32343E+14	5,00529E+14	5,96088E+14	4,52114E+14	5,24294E+14
TC0200003940.mm.2	Olfr1066	olfactory receptor 1066	-1,36769E+14	4,26558E+14	-2,40114E+14	0.0504671784051593	0.190128044340036	-3,73452E+14	3,23429E+14	3,35014E+13	3,8151E+14	5,50472E+14	4,59822E+13	5,09101E+14
TC1300000983.mm.2	Tmem161b	transmembrane protein 161B	1,36625E+14	5,59564E+14	2,40057E+14	0.0505073196820399	0.190218673388653	-3,73522E+14	6,75751E+14	6,77572E+14	5,88692E+14	4,91382E+14	5,35428E+13	3,88562E+14
TC0900001242.mm.2	Foxl2	forkhead box L2	-1,2643E+14	5,31348E+14	-2,40047E+14	0.0505148453689596	0.190218673388653	-3,73535E+14	4,35002E+14	4,62621E+14	5,02608E+14	6,47875E+14	5,41732E+14	5,98252E+14
TC1800001421.mm.2	Arhgef37	Rho guanine nucleotide exchange factor (GEF) 37	-0.781328935299959	4,9405E+14	-2,40006E+14	0.0505440171598948	0.190248015280977	-3,73587E+14	4,66267E+14	4,26222E+14	5,06031E+14	5,42723E+14	5,0757E+13	5,15491E+14
TC0800000330.mm.2	Lsm1	LSM1 homolog, mRNA degradation associated	0.736553415982158	6,82121E+14	2,40003E+14	0.0505462516860833	0.190248015280977	-3,73591E+14	7,40761E+14	7,18086E+13	6,68068E+14	6,43468E+14	6,51474E+14	6,70869E+14
TC1700001839.mm.2	Akap8l	A kinase (PRKA) anchor protein 8-like	0.792736668526353	6,61343E+14	2,39945E+14	0.0505876215225193	0.190349716158741	-3,73663E+14	7,08145E+14	7,14815E+14	6,32063E+14	6,32349E+14	6,69394E+14	6,1129E+13
TC1000002200.mm.2	Gm4981	predicted gene 4981	-0.964080410926788	4,52482E+14	-2,39932E+14	0.0505968990447395	0.190349716158741	-3,7368E+14	3,64244E+14	4,11651E+14	4,48364E+13	5,20346E+13	4,59075E+14	5,11215E+14
TC0800002723.mm.2	Nudt21	nudix (nucleoside diphosphate linked moiety X)-type motif 21	1,49253E+14	7,13537E+14	2,39893E+14	0.0506241111572841	0.19037055923754	-3,73727E+14	8,16631E+14	8,00077E+14	7,38706E+14	5,79496E+14	7,31349E+14	6,14966E+14
TC1600002171.mm.2	Popdc2	popeye domain containing 2	-0.929466086450569	5,18323E+14	-2,39888E+14	0.0506282359196829	0.19037055923754	-3,73735E+14	4,3848E+14	4,3859E+14	5,06466E+14	5,56497E+14	5,45624E+13	6,24282E+14
TC1300000015.mm.2	Tubal3	tubulin, alpha-like 3	-0.818929587699667	5,68138E+14	-2,39848E+14	0.0506564282666449	0.19037055923754	-3,73784E+14	4,94308E+13	5,17163E+14	5,57E+14	6,18257E+14	6,06025E+14	6,16077E+14
TC0100000814.mm.2	Gigyf2	GRB10 interacting GYF protein 2	0.802272396015455	6,63005E+14	2,39829E+14	0.050670465138834	0.19037055923754	-3,73809E+14	7,2654E+14	7,1847E+14	6,49453E+14	6,35103E+13	6,53826E+14	5,94637E+14

TC1300000551.mm.2	Id4	inhibitor of DNA binding 4	0.73346687160932	8,55642E+14	2,39827E+14	0.0506712566346839	0.19037055923754	-3,7381E+14	9,03851E+14	9,18178E+14	8,46917E+14	8,28419E+14	8,03545E+13	8,32943E+14
TC0400001325.mm.2	Slc2a1	solute carrier family 2 (facilitated glucose transporter), member 1	1,08601E+14	9,06738E+14	2,39815E+13	0.0506802912712567	0.19037055923754	-3,73826E+14	9,70286E+14	9,67981E+14	9,19779E+14	8,01285E+14	9,11237E+14	8,69863E+13
TC0800001103.mm.2	Slc12a3	solute carrier family 12, member 3	-0.734338453944136	5,31253E+14	-2,39781E+14	0.05070433671459	0.19037055923754	-3,73868E+14	4,72639E+14	5,00489E+14	5,60823E+14	5,59173E+14	5,34289E+14	5,60108E+14
TC4 JH584293 random00000007.mm.2	Ccl27b/Gm2506/Gm13306/LOC100861978	chemokine (C-C motif) ligand 27b/predicted gene 2506/predicted gene 13306/c-C motif chemokine 27-like	1,21239E+14	9,77216E+14	2,39751E+14	0.0507262641487449	0.19037055923754	-3,73907E+14	1,04593E+14	1,0432E+14	9,90361E+14	8,56286E+14	9,96503E+14	9,31022E+14
TC4 JH584294 random00000001.mm.2	Gm13306/Ccl27b/Gm2506/LOC100861978	predicted gene 13306/chemokine (C-C motif) ligand 27b/predicted gene 2506/c-C motif chemokine 27-like	1,21239E+14	9,77216E+14	2,39751E+14	0.0507262641487449	0.19037055923754	-3,73907E+14	1,04593E+14	1,0432E+14	9,90361E+14	8,56286E+14	9,96503E+14	9,31022E+14
TC0100000216.mm.2	Dst	dystonin	2,2944E+13	6,1423E+14	2,39741E+14	0.0507329938987845	0.19037055923754	-3,73918E+13	7,77155E+14	7,79147E+14	6,25473E+14	4,7195E+14	6,60401E+13	3,71252E+14
TC0900003333.mm.2	Nphp3	nephronophthisis 3 (adolescent)	-0.988826860082074	5,99617E+14	-2,39739E+14	0.0507347437550718	0.19037055923754	-3,73921E+14	4,94077E+14	5,64616E+12	5,88839E+13	6,67619E+14	6,45757E+14	6,36794E+14
TC1100000146.mm.2	Pom121l12	POM121 membrane glycoprotein-like 12	-1,11682E+13	5,67286E+14	-2,39718E+14	0.050749621765583	0.19037055923754	-3,73948E+13	5,06513E+14	4,9323E+14	5,75674E+14	6,47434E+14	5,35722E+13	6,45141E+14
TC0200003017.mm.2	Nebl	nebulette	1,00456E+14	6,18269E+14	2,39709E+14	0.0507560306885418	0.19037055923754	-3,73959E+13	6,60696E+14	6,47059E+14	6,03834E+14	5,0301E+13	6,67619E+14	6,27393E+14
TC1800000694.mm.2	Gnal	guanine nucleotide binding protein, alpha stimulating, olfactory type	1,39428E+14	9,41896E+14	2,3969E+14	0.0507694914905658	0.190376731763437	-3,73982E+14	1,03935E+14	1,02047E+13	9,73914E+14	8,07051E+14	9,39581E+14	8,71008E+14
TC0800000945.mm.2	Rfx1	regulatory factor X, 1 (influences HLA class II expression)	-0.713417144426858	6,97542E+13	-2,39661E+14	0.0507901443608943	0.190409864143111	-3,74018E+14	6,64592E+14	6,56385E+13	7,25429E+14	7,38231E+14	6,88608E+14	7,12008E+14
TC0700003618.mm.2	Btbd1	BTB (POZ) domain containing 1	1,68149E+14	8,16591E+14	2,39567E+13	0.0508578176202316	0.19054430000731	-3,74137E+14	9,24257E+14	9,16718E+14	8,53085E+14	6,51592E+13	8,31917E+14	7,21978E+14
TC1800000648.mm.2	Napg	N-ethylmaleimide sensitive fusion protein attachment protein gamma	1,75504E+14	8,18451E+14	2,39563E+14	0.0508606087514826	0.19054430000731	-3,74142E+14	9,35736E+14	9,23746E+14	8,40671E+14	6,67803E+14	8,55609E+13	6,87139E+14
TC1300000037.mm.2	Adarb2	adenosine deaminase, RNA-specific, B2	0.847846565856327	5,81167E+14	2,39562E+14	0.0508614804401067	0.19054430000731	-3,74143E+14	6,60313E+14	6,01697E+14	5,23603E+14	5,68838E+14	5,64707E+13	5,67843E+14
TC0900001445.mm.2	Map4	microtubule-associated protein 4	1,10203E+14	8,29413E+14	2,39535E+14	0.0508806779287391	0.19057191154481	-3,74177E+14	9,15732E+14	9,16297E+14	8,57997E+14	7,53627E+14	8,042E+14	7,28625E+14
TC1200000098.mm.2	Fam49a	family with sequence similarity 49, member A	1,48977E+14	8,45335E+14	2,39466E+14	0.0509301559752135	0.190712898728472	-3,74263E+14	9,35294E+14	9,52342E+14	8,73478E+14	7,16204E+14	8,56791E+14	7,37904E+14
TC1400002189.mm.2	Kpna3	karyopherin (importin) alpha 3	2,09284E+14	7,26443E+14	2,39425E+14	0.0509596403183049	0.190778969435141	-3,74315E+14	8,56569E+14	8,55209E+14	7,72899E+14	5,2031E+14	7,54052E+14	5,99622E+14
TC0500000325.mm.2	Slc4a1ap	solute carrier family 4 (anion exchanger), member 1, adaptor protein	1,05242E+14	6,51448E+14	2,39372E+14	0.0509979011228415	0.190860736066268	-3,74381E+14	7,2508E+13	7,25092E+14	6,74237E+14	5,6545E+14	6,33954E+14	5,84877E+14
TC0X00000172.mm.2	H2al1n	H2A histone family member L1N	-1,02483E+14	5,59506E+14	-2,39349E+14	0.0510148118752894	0.190860736066268	-3,74411E+14	4,6291E+14	4,79657E+14	5,31394E+14	6,16139E+14	5,96392E+14	6,70548E+14
TC0200000686.mm.2	Rabgap1/Gpr21	RAB GTPase activating protein 1/G protein-coupled receptor 21	1,54226E+13	7,15755E+14	2,39345E+14	0.0510170167093288	0.190860736066268	-3,74415E+14	8,45977E+14	8,14715E+14	7,3517E+14	6,17071E+14	7,24255E+14	5,57345E+14
TC1900001081.mm.2	Asrgl1	asparaginase like 1	1,42378E+14	7,74328E+14	2,3931E+14	0.0510422542656811	0.190868902442509	-3,74458E+14	8,66297E+14	8,71591E+14	8,07123E+14	6,46009E+14	7,747E+14	6,80248E+14
TC0500003390.mm.2	Vps37d	vacuolar protein sorting 37D	-0.775654072389999	7,53493E+14	-2,39302E+14	0.0510481159175816	0.190868902442509	-3,74469E+14	6,98628E+13	7,29506E+14	7,69635E+14	8,1363E+14	7,53613E+14	7,55945E+14
TC0700002981.mm.2	Bcl2l12	BCL2-like 12 (proline rich)	-1,48032E+14	6,40203E+14	-2,39293E+14	0.0510547365187869	0.190868902442509	-3,7448E+14	5,45705E+14	5,3828E+14	6,11305E+14	7,68744E+14	6,29941E+14	7,47243E+14
TC1100003339.mm.2	Crlf3	cytokine receptor-like factor 3	-0.700124765812154	8,0984E+14	-2,39258E+14	0.0510798615633348	0.190885686594497	-3,74524E+14	7,64447E+14	7,60513E+14	8,4222E+13	8,22765E+14	8,3402E+13	8,35077E+14
TC0200002128.mm.2	Otor	otoraplin	-1,18846E+14	5,566E+14	-2,3924E+14	0.051092841685098	0.190885686594497	-3,74546E+14	4,51289E+14	5,15846E+14	5,37311E+14	6,67515E+14	5,83577E+14	5,84065E+14
TC1100004302.mm.2	Trim65	tripartite motif-containing 65	-0.919631347038322	6,65717E+14	-2,392E+14	0.0511216286327809	0.190885686594497	-3,74597E+14	6,26121E+14	6,02556E+14	6,74356E+14	7,38247E+14	7,07152E+14	6,45873E+14
TC1900001360.mm.2	Dkk1	dickkopf WNT signaling pathway inhibitor 1	-0.729345635071946	4,73101E+14	-2,39197E+14	0.0511237208127053	0.190885686594497	-3,746E+14	4,48769E+14	4,36993E+14	5,02213E+14	5,29419E+14	4,71338E+14	4,49872E+14
TC0200003837.mm.2	Nckap1	NCK-associated protein 1	2,2018E+14	8,91515E+13	2,39165E+13	0.0511470472108346	0.190885686594497	-3,74641E+14	1,02003E+14	1,03355E+14	9,32103E+14	6,81118E+14	9,34967E+14	7,47321E+14
TC0600003491.mm.2	Ndufb2	NADH:ubiquinone oxidoreductase subunit B2	0.750627493763174	8,27812E+14	2,39146E+14	0.0511608377878652	0.190885686594497	-3,74665E+14	8,87541E+14	8,74417E+14	8,08045E+14	8,03788E+14	8,1812E+14	7,74961E+14
TC0X00003278.mm.2	Cdkl5	cyclin-dependent kinase-like 5	1,27425E+14	7,10374E+12	2,39145E+14	0.0511613333572584	0.190885686594497	-3,74666E+14	8,02063E+14	8,00906E+14	7,44012E+14	6,04106E+14	6,95336E+14	6,15822E+14
TC0400003152.mm.2	Jak1	Janus kinase 1	1,09921E+13	6,89729E+14	2,3913E+14	0.0511720813987069	0.190885686594497	-3,74684E+13	7,52811E+14	7,64458E+14	7,09162E+14	5,88265E+14	6,40591E+14	6,8309E+14
TC0800001223.mm.2	Ctcf	CCCTC-binding factor	1,62172E+14	7,49465E+14	2,39107E+14	0.0511891676226567	0.190885686594497	-3,74714E+14	8,49167E+14	8,60841E+14	7,9066E+14	5,95003E+14	6,50241E+13	7,50879E+14
TC0700002037.mm.2	B4galnt4	beta-1,4-N-acetyl-galactosaminyl transferase 4	0.762131127232482	6,37435E+13	2,39105E+14	0.0511901022811845	0.190885686594497	-3,74715E+14	7,05693E+14	6,95052E+14	6,01804E+14	6,46515E+14	5,96944E+14	5,78599E+12
TC0600001380.mm.2	M6pr	mannose-6-phosphate receptor, cation dependent	0.817359592070364	8,64393E+14	2,39094E+14	0.0511982552460657	0.190885686594497	-3,7473E+14	9,37107E+14	9,13692E+14	8,56648E+14	8,30679E+14	8,52355E+14	7,95874E+14
TSUnmapped00000048.mm.2	Egr3	early growth response 3	-0.730395494219547	8,2618E+14	-2,3906E+14	0.0512229939716773	0.190885686594497	-3,74773E+14	7,72962E+12	7,77382E+14	8,41046E+14	8,55377E+14	8,38106E+14	8,72208E+14
TC0900000938.mm.2	Sltm	SAFB-like, transcription modulator	0.836688750893149	6,97862E+14	2,39056E+14	0.0512259367130842	0.190885686594497	-3,74778E+14	7,5855E+14	7,47225E+14	7,01829E+14	6,36608E+14	6,8522E+14	6,57743E+14
TC1100000926.mm.2	Plscr3	phospholipid scramblase 3	-0.872959077600147	7,16195E+14	-2,39049E+14	0.051230758806315	0.190885686594497	-3,74786E+14	6,49589E+14	6,69124E+14	7,82447E+14	7,10857E+14	7,58597E+14	7,26553E+13
TC0200001472.mm.2	Lrrc4c	leucine rich repeat containing 4C	2,35882E+14	8,36059E+14	2,39036E+14	0.0512399840773652	0.190885686594497	-3,74802E+14	9,79484E+14	9,99771E+14	8,89181E+14	6,1831E+14	8,65849E+13	6,63758E+14
TC0500001079.mm.2	A830010M20Rik/1700028K03Rik	RIKEN cDNA A830010M20 gene/RIKEN cDNA 1700028K03 gene	-1,07405E+14	5,6184E+14	-2,38996E+14	0.0512691401941213	0.190885686594497	-3,74853E+14	5,01358E+14	5,13451E+14	5,76743E+14	6,52877E+14	5,14792E+14	6,11821E+13
TC1100000072.mm.2	Xbp1	X-box binding protein 1	1,42496E+14	8,35219E+14	2,38972E+13	0.0512866985151264	0.190885686594497	-3,74883E+14	9,24481E+14	9,35848E+14	8,6257E+14	7,12768E+14	8,43817E+14	7,31827E+14
TC0900003176.mm.2	Cmtm7	CKLF-like MARVEL transmembrane domain containing 7	-0.816734990889294	6,94509E+14	-2,3896E+14	0.0512955772859361	0.190885686594497	-3,74898E+14	6,40893E+14	6,46325E+12	6,97335E+14	7,53229E+14	6,96937E+13	7,32335E+13
TC1700000182.mm.2	Fam120b	family with sequence similarity 120, member B	1,33471E+14	7,15299E+14	2,38951E+13	0.0513020843433734	0.190885686594497	-3,7491E+14	8,05969E+14	7,95509E+14	7,49438E+14	5,85097E+14	7,02348E+14	6,53434E+14
TC0500002608.mm.2	Lnx1	ligand of numb-protein X 1	0.969778035001799	6,15048E+13	2,38936E+14	0.0513126766210337	0.190885686594497	-3,74928E+13	6,85046E+14	6,40192E+14	6,07322E+14	5,2396E+14	6,36933E+14	5,96832E+14
TC0800000727.mm.2	Pbx4	pre B cell leukemia homeobox 4	0.761005259958613	5,31845E+14	2,38934E+14	0.0513140697866965	0.190885686594497	-3,7493E+14	5,69123E+14	5,91303E+14	4,94787E+14	5,13438E+14	5,31142E+14	4,91278E+14
TC1100001841.mm.2	Map2k6	mitogen-activated protein kinase kinase 6	1,69056E+14	5,37169E+14	2,38908E+14	0.0513332467888803	0.190885686594497	-3,74964E+11	6,30451E+14	6,36724E+14	5,73389E+14	3,55673E+14	5,53212E+14	4,73568E+14
TC0100002758.mm.2	Ecel1	endothelin converting enzyme-like 1	1,30063E+13	8,66043E+14	2,38905E+14	0.0513351496773243	0.190885686594497	-3,74967E+14	1,01875E+14	9,02828E+14	8,9002E+14	7,71428E+13	8,06793E+14	8,06441E+14
TC0700000384.mm.2	Cadm4	cell adhesion molecule 4	0.74679957256876	8,92023E+13	2,38891E+14	0.0513449276629796	0.190885686594497	-3,74984E+14	9,46078E+14	9,39064E+14	8,47652E+14	8,88129E+14	8,7372E+14	8,57493E+14
TC1000002398.mm.2	Prmt2/Mir678	protein arginine N-methyltransferase 2/microRNA 678	1,22668E+14	7,30717E+14	2,38877E+14	0.0513553932468904	0.190885686594497	-3,75002E+14	8,24987E+14	7,83687E+14	7,31313E+14	6,32025E+14	7,60306E+14	6,51982E+14
TC0X00001382.mm.2	Prps1	phosphoribosyl pyrophosphate synthetase 1	1,19702E+14	8,15322E+14	2,38839E+14	0.051382904544672	0.190943897815567	-3,7505E+13	9,18637E+14	8,86179E+14	8,53963E+14	7,1145E+14	7,69855E+14	7,51852E+14
TC0800000120.mm.2	Cul4a	cullin 4A	1,23648E+14	6,30845E+14	2,38763E+13	0.051438363635808	0.191058253339899	-3,75145E+14	7,33801E+13	7,24102E+14	6,65066E+14	5,45541E+14	6,06999E+14	5,0956E+14
TC0800000944.mm.2	Palm3	paralemmin 3	-1,18174E+14	5,99608E+14	-2,38762E+14	0.0514385224251562	0.191058253339899	-3,75146E+14	4,82554E+14	5,38246E+14	5,74147E+14	6,83E+14	6,21321E+14	6,98378E+14
TC1100003839.mm.2	Itga2b	integrin alpha 2b	-0.769779658614386	7,24935E+13	-2,38748E+14	0.0514492497512457	0.191058253339899	-3,75164E+14	6,78544E+14	6,73882E+14	7,31562E+14	7,7482E+14	7,30745E+14	7,6006E+14
TC1700000604.mm.2	Vps52	VPS52 GARP complex subunit	1,32894E+14	8,22528E+14	2,3866E+14	0.0515128779866132	0.191242545280208	-3,75274E+14	8,87161E+14	9,05645E+14	8,38106E+14	6,88913E+14	8,48475E+14	7,66866E+14
TC1100002167.mm.2	Tmed4	transmembrane p24 trafficking protein 4	0.842928203128785	8,46999E+14	2,38638E+14	0.0515293245922389	0.191242545280208	-3,75302E+14	9,45436E+14	8,92425E+14	8,58415E+14	8,10859E+14	7,9688E+14	7,77977E+14
TC0400000702.mm.2	Bspry	B-box and SPRY domain containing	-0.772630501013506	5,68531E+14	-2,3863E+14	0.0515344834361487	0.191242545280208	-3,75311E+14	5,22544E+14	5,06804E+14	5,67203E+14	6,16672E+14	5,92463E+14	6,05499E+14
TC0800000977.mm.2	Tnpo2	transportin 2 (importin 3, karyopherin beta 2b)	0.722642894110792	7,50369E+13	2,38604E+13	0.0515537955296422	0.191262799985148	-3,75345E+14	8,12351E+13	7,97461E+13	7,47957E+14	7,17327E+14	7,11356E+14	7,15763E+14
TC0700004494.mm.2	Echs1	enoyl Coenzyme A hydratase, short chain, 1, mitochondrial	2,21647E+14	6,48748E+14	2,3859E+14	0.0515636816940037	0.191262799985148	-3,75362E+14	7,67612E+14	7,69945E+14	7,03589E+14	3,90674E+14	6,55116E+14	6,0555E+14
TC0300000732.mm.2	Arhgef2	rho/rac guanine nucleotide exchange factor (GEF) 2	0.785069606713082	7,06381E+14	2,38564E+14	0.0515829464083537	0.191267393196471	-3,75395E+14	7,62377E+14	7,63314E+14	7,02229E+14	6,66448E+14	6,94481E+14	6,49434E+14
TC0500003220.mm.2	Anapc5	anaphase-promoting complex subunit 5	1,49537E+14	8,45898E+14	2,38555E+14	0.0515895650481684	0.191267393196471	-3,75406E+14	9,38619E+14	9,35777E+14	8,75806E+13	6,99516E+14	8,59827E+14	7,65844E+14
TC1100002160.mm.2	Nudcd3	NudC domain containing 3	1,2849E+14	6,9962E+14	2,3854E+12	0.0516005311379048	0.191267393196471	-3,75425E+14	7,90203E+14	7,98014E+14	7,00153E+14	6,31084E+14	7,13072E+13	5,65195E+14
TC1900000060.mm.2	Gal3st3	galactose-3-O-sulfotransferase 3	1,16764E+14	7,58519E+14	2,38504E+14	0.0516267491911508	0.191320563412376	-3,7547E+14	8,14451E+14	8,37679E+14	7,77406E+14	6,41196E+14	7,5689E+14	7,23494E+14
TC0500003736.mm.2	Epo	erythropoietin	-0.939063794475795	4,92092E+14	-2,38469E+14	0.051652169381717	0.191370753103611	-3,75514E+14	4,09098E+14	4,54519E+13	4,88376E+14	5,63053E+14	4,9822E+14	5,39288E+14
TC0500001127.mm.2	Gm15446	predicted gene 15446	1,16099E+14	7,05058E+14	2,38415E+13	0.0516917738459771	0.19147346022534	-3,75582E+14	7,74588E+14	8,06792E+14	7,1636E+14	6,32821E+14	7,04358E+14	5,95431E+14
TC1000002671.mm.2	Tmpo	thymopoietin	0.745397883567885	5,78933E+14	2,38353E+14	0.051737063618577	0.191597174462453	-3,7566E+14	6,16125E+14	6,08357E+14	5,50094E+12	5,25308E+13	5,68928E+14	6,04785E+13
TC1400000882.mm.2	Fam124a	family with sequence similarity 124, member A	-0.827557206886692	7,4738E+14	-2,38318E+12	0.0517625058996218	0.191647347785066	-3,75703E+14	6,99534E+14	6,66215E+14	7,37614E+14	7,93647E+14	7,80978E+14	8,06292E+14
TC0300001049.mm.2	Trim33	tripartite motif-containing 33	1,85429E+14	6,31746E+14	2,38299E+14	0.051775883705862	0.191652840375041	-3,75726E+14	7,39369E+14	7,62156E+14	6,70489E+14	4,60178E+14	6,54155E+14	5,04133E+14
TC1800000891.mm.2	Fbxo15	F-box protein 15	-0.751888893040981	4,67123E+14	-2,38243E+14	0.0518171888875694	0.191688830210967	-3,75797E+14	4,30371E+14	4,62651E+14	5,38608E+14	5,04792E+14	4,31857E+13	4,34456E+14
TC0800002503.mm.2	Tmem184c	transmembrane protein 184C	2,15916E+14	8,58965E+14	2,38217E+14	0.0518363183642252	0.191688830210967	-3,7583E+14	9,69023E+14	9,76842E+14	9,00687E+14	6,13346E+14	9,02659E+14	7,91232E+14
TC1900001541.mm.2	Got1	glutamic-oxaloacetic transaminase 1, soluble	1,22027E+14	9,77468E+13	2,38213E+14	0.0518387147696249	0.191688830210967	-3,75834E+14	1,08341E+14	1,05696E+14	1,01566E+14	8,80659E+14	9,46495E+14	8,81629E+14
TC0400000234.mm.2	Epha7	Eph receptor A7	1,18845E+14	8,82858E+14	2,38153E+14	0.0518831731490715	0.191688830210967	-3,7591E+14	9,58412E+12	9,74489E+14	9,04809E+14	7,90402E+14	8,79416E+14	7,89619E+14
TC1300002259.mm.2	Zfp65	zinc finger protein 65	1,06021E+14	5,51031E+14	2,3814E+14	0.0518928502996255	0.191688830210967	-3,75927E+14	6,64108E+14	6,17736E+14	5,69456E+14	5,00347E+13	5,21845E+14	4,32694E+14
TC1100004102.mm.2	Wbp2	WW domain binding protein 2	1,5381E+14	9,02039E+14	2,38117E+14	0.0519090273397181	0.191688830210967	-3,75955E+14	9,9412E+14	9,9603E+14	9,4258E+14	7,39951E+14	8,92037E+14	8,47514E+14
TC1500002336.mm.2	Smdt1	single-pass membrane protein with aspartate rich tail 1	0.763225780782324	1,03602E+14	2,38097E+14	0.0519241875929022	0.191688830210967	-3,75981E+14	1,09001E+14	1,07146E+14	1,02795E+14	9,80875E+14	1,02599E+14	1,01984E+14
TC1000003044.mm.2	Tbc1d30	TBC1 domain family, member 30	1,00884E+14	7,47252E+14	2,3809E+14	0.0519288440681163	0.191688830210967	-3,75989E+14	7,97908E+13	8,26102E+14	7,56154E+14	6,66089E+14	7,48489E+14	6,88768E+14
TC0400002014.mm.2	Hes2	hes family bHLH transcription factor 2	-0.992109084896916	6,42111E+14	-2,38078E+13	0.0519379271331774	0.191688830210967	-3,76004E+14	5,57692E+14	6,01385E+14	6,3136E+14	7,26139E+14	6,53691E+14	6,82398E+14
TC1200000626.mm.2	Ppm1a	protein phosphatase 1A, magnesium dependent, alpha isoform	1,28229E+14	8,81563E+14	2,38077E+14	0.0519386530449582	0.191688830210967	-3,76005E+12	9,64642E+14	9,48201E+14	8,95681E+14	7,60705E+14	9,05359E+14	8,14787E+13
TC0700004188.mm.2	Thumpd1	THUMP domain containing 1	1,25463E+14	5,77653E+13	2,38067E+14	0.0519460076197381	0.191688830210967	-3,76018E+13	6,91193E+14	6,59967E+14	5,98552E+14	5,01682E+14	5,67975E+14	4,4655E+14
TC0700000504.mm.2	Ifnl3	interferon lambda 3	-0.751504075031053	7,05086E+13	-2,38062E+14	0.0519497839960374	0.191688830210967	-3,76024E+14	6,67659E+14	6,34527E+14	7,0967E+14	7,42817E+14	7,37179E+14	7,38661E+14
TC1000001605.mm.2	Rnf41	ring finger protein 41	1,14789E+14	8,7706E+14	2,38059E+14	0.0519516337961078	0.191688830210967	-3,76027E+14	9,47364E+14	9,44115E+14	8,95882E+14	7,66018E+14	8,79035E+14	8,29943E+14
TC1300002098.mm.2	2210016F16Rik	RIKEN cDNA 2210016F16 gene	0.835335761039693	6,30833E+14	2,38041E+14	0.0519654427580093	0.191688830210967	-3,76051E+14	7,1325E+14	6,85046E+14	5,86707E+14	6,44523E+14	5,94186E+14	5,61289E+13
TC1600000036.mm.2	4930562C15Rik	RIKEN cDNA 4930562C15 gene	-1,1196E+14	5,09615E+14	-2,38037E+14	0.0519681613359395	0.191688830210967	-3,76056E+14	4,491E+14	4,62097E+14	5,14502E+14	6,20615E+14	4,6567E+14	5,45707E+14
TC0700003822.mm.2	Mrpl48	mitochondrial ribosomal protein L48	1,12211E+14	8,49422E+14	2,38002E+14	0.0519939261235018	0.191688830210967	-3,761E+14	9,24359E+14	9,1142E+14	8,61933E+14	7,49424E+14	8,60563E+14	7,8883E+14
TC1000000819.mm.2	Misp	mitotic spindle positioning	-0.998977852736369	5,58813E+14	-2,38002E+14	0.0519940420822426	0.191688830210967	-3,761E+14	4,58845E+14	5,26481E+14	5,71991E+14	6,13131E+14	5,53223E+14	6,29209E+13
TC0300002946.mm.2	Rpl34/Rpl34-ps1	ribosomal protein L34/ribosomal protein L34, pseudogene 1	1,43451E+14	9,08169E+14	2,37994E+14	0.0519997441104782	0.191688830210967	-3,7611E+14	1,00081E+14	9,84412E+14	9,4061E+14	7,57712E+14	9,07166E+14	8,58301E+14
TC1400001551.mm.2	Camk2g	calcium/calmodulin-dependent protein kinase II gamma	1,5822E+14	8,26872E+14	2,37967E+14	0.0520197343294335	0.191718659480824	-3,76144E+14	9,30762E+14	8,84833E+14	8,49041E+14	6,50115E+14	8,56992E+14	7,89487E+14
TC1300000658.mm.2	Slc34a1	solute carrier family 34 (sodium phosphate), member 1	-0.866565405534943	5,19116E+14	-2,37914E+14	0.0520584508874627	0.191817475222887	-3,7621E+14	4,38523E+14	4,63898E+14	5,04708E+14	5,71026E+14	5,49541E+14	5,86997E+14
TC0400004026.mm.2	Tardbp	TAR DNA binding protein	1,15321E+14	6,83752E+14	2,37818E+13	0.0521287107674332	0.192016293312406	-3,7633E+14	7,58937E+14	7,66348E+14	7,03932E+14	5,9071E+14	6,82375E+14	6,00214E+14
TC0300002360.mm.2	Hax1	HCLS1 associated X-1	1,43874E+14	6,47153E+14	2,37808E+14	0.0521362429865187	0.192016293312406	-3,76343E+14	7,16324E+14	6,91837E+14	6,50327E+14	4,70087E+14	7,06205E+14	6,4814E+14
TC0700001765.mm.2	Cacng3	calcium channel, voltage-dependent, gamma subunit 3	1,17779E+14	9,46182E+14	2,37752E+14	0.0521779617546761	0.192109233468965	-3,76414E+14	1,03357E+14	1,01811E+14	9,72095E+14	8,44029E+14	9,37449E+14	8,71834E+14
TC1800000791.mm.2	Myo5b	myosin VB	0.748552998691901	6,45185E+14	2,37732E+14	0.0521926294008182	0.192109233468965	-3,76439E+14	7,0002E+14	6,95833E+14	6,24874E+14	6,21268E+14	5,91628E+14	6,37487E+14
TC0500000321.mm.2	Zfp512	zinc finger protein 512	1,27114E+14	6,75102E+14	2,37725E+14	0.0521972459583645	0.192109233468965	-3,76447E+14	7,93986E+14	7,20034E+14	6,90249E+14	5,69544E+14	6,77501E+14	5,99298E+14
TC1500001973.mm.2	Pacsin2	protein kinase C and casein kinase substrate in neurons 2	0.70779045641533	7,36683E+14	2,37645E+14	0.0522562165572044	0.192162484515748	-3,76547E+14	7,90007E+14	7,78551E+14	7,15347E+14	7,11653E+14	7,24979E+13	6,99563E+14
TC0900003160.mm.2	Pdcd6ip	programmed cell death 6 interacting protein	2,72326E+14	7,33274E+14	2,37623E+14	0.0522729688822351	0.192162484515748	-3,76575E+14	8,95862E+14	9,00924E+14	8,05133E+14	4,47E+14	7,61996E+14	5,88729E+13
TC1000001622.mm.2	Dnajc14	DnaJ heat shock protein family (Hsp40) member C14	0.818911988688892	6,90724E+14	2,3761E+14	0.0522823305560841	0.192162484515748	-3,76591E+14	7,66308E+13	7,63293E+14	7,04822E+14	6,60996E+14	6,50583E+13	5,98342E+12
TC1900000881.mm.2	Ppp6r3	protein phosphatase 6, regulatory subunit 3	1,03992E+13	7,0076E+13	2,37607E+14	0.0522847440058366	0.192162484515748	-3,76595E+14	7,96921E+14	7,68278E+14	7,33261E+14	6,23953E+14	6,58746E+14	6,23398E+14
TC1100003920.mm.2	Cd79b	CD79B antigen	-0.981280122131759	4,97091E+14	-2,3759E+14	0.0522972103308087	0.192162484515748	-3,76616E+14	4,45903E+14	4,24777E+14	4,92462E+14	5,74474E+14	4,90259E+14	5,5467E+14
TC1100002818.mm.2	Olfr30	olfactory receptor 30	-0.689235100549354	5,17535E+14	-2,37587E+14	0.0522996186970288	0.192162484515748	-3,76621E+14	4,60432E+14	4,55029E+14	5,24164E+14	5,29144E+13	5,62059E+14	5,74382E+14
TC0900000865.mm.2	Dapk2	death-associated protein kinase 2	-0.968440499640225	5,81812E+14	-2,37543E+14	0.0523321113299601	0.192162484515748	-3,76676E+13	5,17728E+14	5,03383E+14	5,69033E+13	6,45766E+14	5,93327E+14	6,61632E+14
TC1500001531.mm.2	Enpp2	ectonucleotide pyrophosphatase/phosphodiesterase 2	1,64381E+14	9,96419E+14	2,37505E+14	0.0523601770497839	0.192162484515748	-3,76723E+14	1,01482E+14	1,16426E+14	8,88864E+14	9,61453E+14	1,05102E+14	8,98091E+14
TC1100002181.mm.2	Tbrg4/Snora5c	transforming growth factor beta regulated gene 4/small nucleolar RNA, H/ACA box 5C	0.723738428102489	6,8865E+14	2,37503E+14	0.052361792264715	0.192162484515748	-3,76726E+14	7,3326E+14	7,29228E+14	6,41074E+14	6,76666E+14	6,7908E+14	6,72593E+14
TC0100003682.mm.2	Nvl	nuclear VCP-like	1,188E+14	5,65656E+14	2,37485E+14	0.0523747830020447	0.192162484515748	-3,76748E+14	6,4758E+14	6,73115E+14	6,02478E+14	4,80617E+14	5,34012E+14	4,56136E+14
TC1500002309.mm.2	Cbx5	chromobox 5	1,59007E+14	8,60757E+14	2,37477E+14	0.0523805595099188	0.192162484515748	-3,76758E+14	9,56885E+14	9,643E+14	8,88484E+14	7,14687E+13	8,84866E+13	7,55321E+14
TC1700002769.mm.2	Gm3435	predicted gene 3435	1,32853E+14	5,70096E+14	2,37471E+14	0.0523850873665444	0.192162484515748	-3,76766E+14	6,35672E+14	6,75943E+14	5,99086E+14	4,46822E+14	5,66278E+14	4,96778E+14
TC0200000603.mm.2	Angptl2	angiopoietin-like 2	-1,00665E+14	6,27649E+14	-2,37459E+14	0.0523941958329869	0.192162484515748	-3,76781E+14	5,70744E+14	5,60005E+14	6,13755E+14	7,18323E+14	6,3252E+14	6,70546E+14
TC0800001696.mm.2	Col4a1	collagen, type IV, alpha 1	-0.909846419157256	7,83385E+14	-2,37444E+14	0.0524053038432842	0.192162484515748	-3,768E+14	7,12229E+14	7,34133E+14	7,70607E+14	8,57724E+13	8,05782E+13	8,19833E+14
TC1400002578.mm.2	Dach1	dachshund family transcription factor 1	0.831993746248862	6,79316E+14	2,37443E+14	0.0524057680391318	0.192162484515748	-3,76801E+13	7,23102E+14	7,44039E+13	6,82556E+14	6,18187E+14	6,41848E+14	6,66165E+14
TC0600002726.mm.2	Wnt7a	wingless-type MMTV integration site family, member 7A	-0.851001409943865	7,47374E+14	-2,37375E+14	0.0524558354485954	0.192162484515748	-3,76885E+14	6,94903E+14	7,04533E+14	7,56176E+14	8,13461E+14	7,32397E+14	7,82774E+14
TC1700000842.mm.2	Enpp5	ectonucleotide pyrophosphatase/phosphodiesterase 5	1,82806E+14	9,19363E+14	2,37349E+14	0.0524752713490568	0.192162484515748	-3,76918E+14	1,03754E+14	1,05683E+13	9,55988E+14	7,72761E+14	9,35603E+14	7,57467E+14
TC0600002494.mm.2	Elmod3	ELMO/CED-12 domain containing 3	1,38447E+14	7,23189E+14	2,37301E+13	0.0525112791668162	0.192162484515748	-3,76979E+14	7,94425E+14	8,03279E+14	7,39873E+13	5,80936E+14	7,54528E+14	6,66091E+14
TC1200001658.mm.2	Arl4a	ADP-ribosylation factor-like 4A	1,25243E+14	6,85183E+14	2,37276E+14	0.0525295706616209	0.192162484515748	-3,7701E+14	7,40456E+14	7,60003E+14	6,89486E+14	5,60487E+14	7,25588E+14	6,35076E+14

TC1700001856.mm.2	Cyp4f14	cytochrome P450, family 4, subfamily f, polypeptide 14	0.848571162665549	5,83694E+14	2,37269E+14	0.0525344392346427	0.192162484515748	-3,77018E+14	6,18895E+14	6,35672E+14	5,72746E+14	5,12107E+14	5,59727E+14	6,03017E+14
TC0600001696.mm.2	Etnk1	ethanolamine kinase 1	1,13759E+14	7,464E+14	2,37267E+14	0.0525361299696796	0.192162484515748	-3,77021E+14	8,24407E+14	8,1782E+14	7,6701E+14	6,47698E+14	7,44898E+14	6,7657E+14
TC0200002530.mm.2	Dnttip1	deoxynucleotidyltransferase, terminal, interacting protein 1	0.794740590682026	7,44097E+14	2,37263E+14	0.0525390365055345	0.192162484515748	-3,77026E+14	7,9417E+14	7,78078E+14	7,3584E+14	6,7746E+14	7,45543E+14	7,33493E+13
TC1100001578.mm.2	Psmd3	proteasome (prosome, macropain) 26S subunit, non-ATPase, 3	0.687752810350288	8,67209E+14	2,37262E+14	0.0525396763884544	0.192162484515748	-3,77027E+14	9,21142E+14	9,10444E+14	8,43881E+14	8,50154E+13	8,35856E+14	8,41773E+14
TC1500001347.mm.2	Rida	reactive intermediate imine deaminase A homolog	2,18508E+14	8,04711E+14	2,37254E+14	0.0525459468942227	0.192162484515748	-3,77038E+14	9,40362E+14	9,19135E+14	8,38123E+14	5,84358E+14	8,64834E+14	6,81456E+14
TC0100003264.mm.2	Rgl1	ral guanine nucleotide dissociation stimulator,-like 1	1,22658E+14	7,46812E+14	2,37251E+13	0.0525478899359085	0.192162484515748	-3,77041E+11	8,4579E+14	8,27818E+14	7,773E+14	6,50992E+14	7,32735E+14	6,46237E+14
TC0100000338.mm.2	Gulp1	GULP, engulfment adaptor PTB domain containing 1	0.903888149885287	6,28842E+14	2,37248E+14	0.0525506694611507	0.192162484515748	-3,77046E+14	7,30202E+14	7,02185E+14	6,43025E+14	6,08585E+14	5,83513E+14	5,0554E+14
TC1600000033.mm.2	Hmox2	heme oxygenase 2	1,1817E+14	8,93544E+14	2,37242E+14	0.0525548471019351	0.192162484515748	-3,77053E+14	9,84889E+14	9,56115E+14	9,10105E+14	7,94558E+14	8,99967E+13	8,15629E+14
TC0500003061.mm.2	Mmab	methylmalonic aciduria (cobalamin deficiency) cblB type homolog (human)	0.707684628077477	6,77753E+14	2,3724E+14	0.0525561032002593	0.192162484515748	-3,77055E+14	6,98295E+14	7,19467E+14	6,39616E+14	6,36609E+14	6,76659E+14	6,95872E+14
TC1700002345.mm.2	Slc25a41	solute carrier family 25, member 41	-1,10732E+14	5,97976E+14	-2,37236E+14	0.0525592596949941	0.192162484515748	-3,7706E+14	5,14518E+12	5,40279E+14	6,11969E+14	6,64291E+14	5,69237E+14	6,87563E+14
TC1700001838.mm.2	Akap8	A kinase (PRKA) anchor protein 8	0.771389083738072	6,19649E+14	2,37222E+14	0.0525694924437049	0.192162484515748	-3,77078E+14	6,60111E+14	6,76717E+14	6,14941E+14	5,67609E+14	5,88469E+14	6,10046E+14
TC0800002515.mm.2	1700011L22Rik	RIKEN cDNA 1700011L22 gene	-1,36203E+14	4,79389E+14	-2,37144E+14	0.0526276773572834	0.192312299118741	-3,77176E+14	4,25854E+14	3,57228E+14	4,5943E+14	5,9606E+14	4,72967E+14	5,64792E+13
TC0700000881.mm.2	Ldha	lactate dehydrogenase A	1,31465E+14	8,76573E+14	2,37117E+14	0.0526478562342367	0.192312299118741	-3,7721E+14	9,49115E+14	9,56498E+14	8,97037E+13	7,45646E+14	8,94119E+14	8,17024E+14
TC0100000949.mm.2	Gal3st2b	galactose-3-O-sulfotransferase 2B	-0.865451328286185	5,2152E+14	-2,37104E+14	0.0526577418452593	0.192312299118741	-3,77227E+14	4,81406E+14	4,47344E+14	5,14327E+12	5,87513E+12	5,49792E+13	5,48735E+14
TC0800001834.mm.2	Gm6040	predicted gene 6040	-0.994418001897874	5,53601E+13	-2,37103E+14	0.0526582178186487	0.192312299118741	-3,77228E+14	4,57457E+14	5,30857E+14	5,57454E+14	6,29744E+14	5,54393E+14	5,91701E+13
TC0900000019.mm.2	Gucy1a2	guanylate cyclase 1, soluble, alpha 2	0.859328907058812	5,89342E+14	2,3708E+14	0.0526754219347279	0.192331537193354	-3,77257E+14	6,70463E+13	6,41872E+14	5,34894E+14	6,05575E+14	5,5242E+14	5,30826E+14
TC1100002908.mm.2	Slc47a2	solute carrier family 47, member 2	-1,1476E+14	5,24384E+14	-2,37022E+14	0.0527185215834661	0.192379343788869	-3,77329E+14	4,50962E+14	4,23314E+14	4,93862E+14	6,09934E+14	5,47729E+13	6,205E+14
TC0800002381.mm.2	Mau2	MAU2 sister chromatid cohesion factor	0.980730052143564	6,82811E+13	2,3702E+14	0.0527197458844107	0.192379343788869	-3,77331E+14	7,43389E+13	7,58007E+14	6,9781E+14	6,0744E+13	6,72596E+14	6,17626E+14
TC0400000496.mm.2	Stra6l	STRA6-like	-1,38207E+14	6,59917E+14	-2,37006E+14	0.0527302760167725	0.192379343788869	-3,77349E+14	5,49359E+14	5,79576E+13	6,25484E+14	7,79865E+14	6,66864E+14	7,58353E+14
TC1100003374.mm.2	Nle1	notchless homolog 1	-0.815957477608644	5,65134E+14	-2,36989E+14	0.0527427551432621	0.192379343788869	-3,7737E+14	4,89464E+14	5,37444E+12	5,71115E+14	6,18985E+14	5,89847E+14	5,83946E+14
TC0900001195.mm.2	Paqr9	progestin and adipoQ receptor family member IX	1,03762E+14	6,57694E+14	2,36942E+14	0.05277804575303	0.192379343788869	-3,77429E+14	7,51597E+14	7,21199E+14	6,89826E+14	5,75445E+14	6,00884E+14	6,07213E+14
TC1100003061.mm.2	Polr2a	polymerase (RNA) II (DNA directed) polypeptide A	0.944087735635719	6,29774E+13	2,36935E+14	0.0527832829191997	0.192379343788869	-3,77438E+14	7,17641E+14	7,11839E+14	5,9678E+14	6,43883E+14	5,10806E+14	5,97694E+14
TC0900001771.mm.2	Amotl1	angiomotin-like 1	0.879697782894789	6,46734E+14	2,36932E+14	0.0527852904418111	0.192379343788869	-3,77442E+14	7,27355E+13	6,77408E+13	6,27108E+14	6,01715E+14	5,91894E+14	6,54922E+14
TC0200004529.mm.2	Shc4	SHC (Src homology 2 domain containing) family, member 4	-0.873203572577166	5,63045E+13	-2,36915E+14	0.0527981820973684	0.192379343788869	-3,77463E+14	5,02181E+14	5,00332E+14	5,64063E+14	6,13091E+14	5,65959E+14	6,32642E+14
TC1300000668.mm.2	B4galt7	xylosylprotein beta1,4-galactosyltransferase, polypeptide 7 (galactosyltransferase I)	-0.710104315327674	7,03676E+14	-2,36908E+14	0.0528034839448953	0.192379343788869	-3,77472E+14	6,44215E+14	6,62939E+14	7,16617E+14	7,32557E+14	7,23748E+14	7,41982E+14
TC0200000551.mm.2	Ass1/Gm5424	argininosuccinate synthetase 1/argininosuccinate synthase pseudogene	0.750326242218841	7,83282E+14	2,36902E+14	0.0528079089913839	0.192379343788869	-3,7748E+14	8,56463E+14	8,21169E+14	7,71534E+14	7,56033E+14	7,61371E+13	7,33123E+14
TC0800000463.mm.2	Tusc3	tumor suppressor candidate 3	1,0114E+14	9,90824E+14	2,36857E+14	0.052841757712346	0.192459141505206	-3,77537E+14	1,05875E+14	1,05926E+14	9,99766E+14	9,15967E+14	9,93712E+14	9,17483E+14
TC0900001632.mm.2	1700048O20Rik	RIKEN cDNA 1700048O20 gene	1,75316E+13	6,23866E+14	2,36829E+14	0.0528623661435274	0.192464323865799	-3,77571E+14	7,79826E+14	7,09517E+13	6,85735E+14	4,52977E+14	5,71185E+14	5,43955E+14
TC0200002362.mm.2	Dynlrb1	dynein light chain roadblock-type 1	1,03657E+14	1,04875E+14	2,36823E+14	0.0528670699081504	0.192464323865799	-3,77579E+14	1,12249E+14	1,10614E+14	1,05475E+14	9,66572E+14	1,05994E+14	9,82604E+13
TC0700001741.mm.2	Otoa	otoancorin	-0.813559503284143	5,59239E+14	-2,36804E+14	0.0528811995828094	0.19247227668349	-3,77603E+14	5,00818E+14	4,97658E+14	5,58503E+14	6,02686E+14	5,72386E+14	6,23381E+14
TC1200001010.mm.2	Slc24a4	solute carrier family 24 (sodium/potassium/calcium exchanger), member 4	1,46347E+13	6,68581E+14	2,36737E+14	0.0529308157714823	0.192582121151623	-3,77686E+13	7,92034E+14	8,13967E+14	6,68027E+13	6,45279E+13	6,42956E+13	4,4922E+14
TC0500001147.mm.2	Ddx51	DEAD (Asp-Glu-Ala-Asp) box polypeptide 51	-1,21044E+13	5,94874E+14	-2,36731E+14	0.0529352829752708	0.192582121151623	-3,77694E+14	5,2337E+14	5,22353E+14	5,77459E+14	7,10351E+14	5,81297E+14	6,54411E+14
TC0700003818.mm.2	Ppme1	protein phosphatase methylesterase 1	0.798071278316436	8,05028E+14	2,36715E+14	0.0529477028272679	0.192583822947126	-3,77714E+14	8,66498E+14	8,71095E+14	8,07092E+14	7,70886E+14	7,83132E+14	7,31468E+14
TC1100000918.mm.2	Fxr2	fragile X mental retardation, autosomal homolog 2	1,02305E+14	8,11794E+14	2,36675E+14	0.0529776712134202	0.19264933790608	-3,77765E+14	8,92987E+14	9,00683E+14	8,43597E+14	7,45464E+14	7,74528E+14	7,13504E+14
TC1900000580.mm.2	Ccnj	cyclin J	-0.791254138924429	6,32338E+14	-2,36632E+14	0.0530097040638488	0.192722328876533	-3,77818E+14	6,00404E+14	5,59415E+14	6,56833E+13	6,61237E+14	6,8078E+14	6,35359E+13
TC1700000047.mm.2	Synj2	synaptojanin 2	0.742050096399717	6,68728E+14	2,36581E+14	0.0530481501331185	0.19277427567307	-3,77883E+14	7,1884E+14	7,31696E+14	6,30699E+14	6,71426E+14	6,22588E+14	6,37121E+14
TC1000002121.mm.2	Grik2	glutamate receptor, ionotropic, kainate 2 (beta 2)	1,15419E+13	7,16897E+14	2,36568E+14	0.0530573231939716	0.19277427567307	-3,77898E+14	8,21061E+14	8,02009E+13	6,94191E+14	6,9804E+14	7,13074E+14	5,73006E+14
TC0300002324.mm.2	Syt11	synaptotagmin XI	1,34783E+14	9,82812E+14	2,36565E+14	0.0530598841066259	0.19277427567307	-3,77902E+14	1,07866E+14	1,07922E+14	1,01758E+14	8,70729E+14	9,74251E+14	8,76438E+14
TC1000000908.mm.2	Zfp433	zinc finger protein 433	0.95819103340967	6,31522E+14	2,36545E+14	0.0530752139885916	0.192786502028444	-3,77928E+14	6,64373E+13	6,81119E+14	6,26855E+14	5,26998E+14	6,52547E+14	6,37242E+13
TC0700000939.mm.2	Gm9962	predicted gene 9962	-1,05039E+13	6,65605E+14	-2,365E+14	0.0531087673359699	0.192790094852249	-3,77984E+14	5,72355E+13	6,38586E+14	6,64336E+14	7,56682E+14	6,59401E+14	7,02273E+14
TC0100002127.mm.2	4931428L18Rik	RIKEN cDNA 4931428L18 gene	-0.847527202669259	4,8931E+14	-2,36499E+14	0.0531097296339971	0.192790094852249	-3,77986E+14	4,18075E+14	4,51512E+14	4,85967E+14	5,53125E+14	5,05359E+14	5,21824E+14
TC1600001149.mm.2	Sep 12	septin 12	-0.920125281534421	5,22961E+14	-2,36452E+14	0.0531443822431572	0.192790094852249	-3,78044E+14	4,38391E+14	4,68687E+14	5,04858E+14	5,86245E+14	5,51654E+14	5,87932E+14
TC0300002071.mm.2	Plch1	phospholipase C, eta 1	0.861017591778422	6,45306E+14	2,36452E+14	0.0531446619441452	0.192790094852249	-3,78044E+14	6,7933E+14	6,80411E+14	5,92891E+14	5,94647E+14	7,02376E+14	6,22178E+14
TC1100002598.mm.2	Cyfip2	cytoplasmic FMR1 interacting protein 2	2,15384E+14	8,96899E+14	2,36452E+14	0.0531449186623877	0.192790094852249	-3,78045E+14	1,05061E+14	1,05938E+14	9,52472E+14	7,26745E+13	9,02242E+13	6,89949E+14
TC0200001295.mm.2	Srp9	signal recognition particle 9	0.714534465093348	8,33944E+14	2,36436E+13	0.0531565637768319	0.192790094852249	-3,78064E+14	8,6403E+14	8,70099E+12	7,80627E+14	8,10595E+14	8,322E+14	8,46114E+14
TC0700004338.mm.2	Ctf2	cardiotrophin 2	-0.87332397533066	6,02894E+14	-2,36432E+14	0.0531599572660921	0.192790094852249	-3,7807E+14	5,1378E+14	5,48477E+14	5,82123E+14	6,54799E+14	6,48553E+13	6,69634E+14
TC0700003930.mm.2	Olfm5	olfactomedin 5	-0.760316248384942	5,48924E+14	-2,36342E+14	0.053227081307761	0.192899700131504	-3,78182E+14	5,1301E+14	5,01371E+13	5,75672E+14	5,90772E+13	5,80491E+14	5,3223E+14
TC1100001232.mm.2	Lig3	ligase III, DNA, ATP-dependent	1,20411E+14	5,89672E+14	2,36333E+14	0.053233891643114	0.192899700131504	-3,78193E+13	5,98477E+13	6,53806E+14	5,74218E+13	4,37242E+14	6,52518E+14	6,21774E+14
TC0400003026.mm.2	Ifne	interferon epsilon	-1,14506E+14	5,61951E+14	-2,36332E+14	0.0532345648146673	0.192899700131504	-3,78194E+13	4,92679E+14	5,06444E+14	5,54707E+14	6,73427E+14	5,35858E+14	6,08592E+14
TC0400003425.mm.2	Ppcs	phosphopantothenoylcysteine synthetase	-0.932408398464042	6,43488E+14	-2,36307E+14	0.0532533920012798	0.192899700131504	-3,78226E+14	5,81053E+14	5,93782E+14	6,33009E+14	7,28308E+14	6,56027E+14	6,68747E+14
TC0800000571.mm.2	4930555F03Rik	RIKEN cDNA 4930555F03 gene	-0.989672054711484	5,30058E+14	-2,36301E+14	0.0532584839308306	0.192899700131504	-3,78234E+14	4,66258E+14	4,91458E+14	5,27018E+14	6,28632E+14	5,17825E+14	5,49157E+14
TC0500002650.mm.2	Noa1	nitric oxide associated 1	1,21789E+14	6,37189E+14	2,36276E+13	0.0532769549577346	0.192899700131504	-3,78265E+14	7,27218E+14	7,07984E+14	5,61205E+14	6,30418E+14	5,30344E+14	6,65966E+14
TC1100003721.mm.2	Gm11937	predicted gene 11937	-1,05231E+14	7,00066E+13	-2,36269E+14	0.0532821271781356	0.192899700131504	-3,78274E+14	6,21349E+14	6,32092E+14	6,90857E+14	7,73046E+14	6,96736E+14	7,86317E+14
TC0400003742.mm.2	Man1c1	mannosidase, alpha, class 1C, member 1	0.8866063586963	6,71439E+14	2,36236E+14	0.0533070584080763	0.192899700131504	-3,78315E+14	7,11255E+14	7,43995E+14	6,6165E+14	6,16279E+14	6,8077E+14	6,14683E+14
TC1100003152.mm.2	Cyb5d2	cytochrome b5 domain containing 2	0.760011146289511	6,44623E+14	2,36232E+14	0.0533098681510714	0.192899700131504	-3,7832E+14	7,13411E+13	6,84198E+14	6,33989E+14	6,11618E+14	5,93955E+14	6,30566E+14
TC0600001793.mm.2	Tfpi2	tissue factor pathway inhibitor 2	-0.714012908443201	5,32976E+14	-2,36225E+13	0.05331539907287	0.192899700131504	-3,78329E+14	5,0211E+14	5,29797E+14	5,96144E+14	5,78565E+14	4,99387E+14	4,91852E+14
TC0600000962.mm.2	Smyd5	SET and MYND domain containing 5	0.886680076958013	6,56913E+13	2,36216E+14	0.0533218683290335	0.192899700131504	-3,7834E+14	6,82658E+14	7,21119E+13	6,48649E+14	5,77792E+14	6,3979E+14	6,71473E+14
TC0800002107.mm.2	Cnot7	CCR4-NOT transcription complex, subunit 7	1,71828E+14	8,8443E+14	2,36089E+14	0.0534177049099225	0.193152898691136	-3,78499E+13	9,8792E+14	9,87817E+14	9,15226E+14	7,16854E+14	9,16758E+14	7,82005E+14
TC1700001814.mm.2	Tmprss3	transmembrane protease, serine 3	-0.941704288917666	5,13593E+14	-2,36081E+14	0.0534241401664837	0.193152898691136	-3,7851E+14	4,1711E+14	4,6918E+14	5,05087E+14	5,69544E+14	5,33023E+14	5,87616E+14
TC1100001502.mm.2	Hoxb1	homeobox B1	-0.767111051436314	6,2866E+14	-2,36076E+14	0.0534278203603547	0.193152898691136	-3,78516E+14	5,86183E+14	5,60527E+14	6,30293E+14	6,69839E+14	6,48957E+14	6,76161E+14
TC0500003431.mm.2	Serpine1	serine (or cysteine) peptidase inhibitor, clade E, member 1	-0.775894712816464	6,35722E+14	-2,3605E+13	0.0534474622116653	0.193180564965582	-3,78549E+14	5,86391E+14	5,94313E+14	6,42979E+14	6,92904E+14	6,3543E+14	6,62313E+14
TC1200002311.mm.2	Atg2b	autophagy related 2B	0.782443460064195	6,1603E+14	2,35989E+13	0.0534935066232635	0.19330362687164	-3,78625E+14	6,94815E+14	6,70502E+14	6,01183E+14	6,07646E+14	5,34149E+14	5,87888E+14
TC1300002596.mm.2	Trappc13	trafficking protein particle complex 13	2,05413E+14	5,99349E+14	2,3594E+14	0.053530374825252	0.193387410812522	-3,78686E+14	7,58574E+14	7,58499E+14	6,49824E+14	4,56422E+14	5,97589E+14	3,75185E+14
TC0X00003098.mm.2	Acsl4	acyl-CoA synthetase long-chain family member 4	1,56125E+14	7,25785E+14	2,35927E+14	0.0535406963094805	0.193387410812522	-3,78704E+13	8,55312E+14	8,4754E+14	7,84897E+14	6,05705E+14	6,88794E+14	5,72459E+14
TC0800000885.mm.2	Anapc10	anaphase promoting complex subunit 10	1,2324E+14	6,0603E+14	2,35887E+14	0.0535705974450141	0.193452047653858	-3,78753E+14	6,86602E+14	6,52136E+14	6,04283E+14	4,87975E+14	6,5202E+14	5,53164E+14
TC1900000600.mm.2	Pgam1	phosphoglycerate mutase 1	1,65435E+14	9,05102E+14	2,35846E+14	0.0536020925867053	0.193522410452517	-3,78805E+14	1,01408E+14	9,93455E+14	9,4871E+14	7,27955E+14	9,02813E+14	8,43596E+14
TC1400002001.mm.2	Ang2	angiogenin, ribonuclease A family, member 2	-1,29942E+14	4,26232E+14	-2,35799E+14	0.0536372378118074	0.193569493618454	-3,78863E+14	3,40383E+14	3,31884E+14	4,1871E+14	5,13441E+14	4,06858E+14	5,46117E+14
TC0100001206.mm.2	Ren1/Ren2	renin 1 structural/renin 2 tandem duplication of Ren1	-1,11141E+14	5,39685E+14	-2,35783E+14	0.0536494124123114	0.193569493618454	-3,78884E+14	4,61006E+14	4,52565E+14	5,09005E+14	6,26848E+14	5,6272E+14	6,25967E+13
TC0300002627.mm.2	Atg4a	autophagy related 4A, cysteine peptidase	-0.769704634526335	5,3643E+14	-2,35781E+14	0.0536511734934534	0.193569493618454	-3,78887E+13	4,76074E+14	5,10748E+13	5,50126E+13	5,90637E+14	5,37671E+14	5,53324E+14
TC1300002139.mm.2	Ctsll3	cathepsin L-like 3	-0.879388941328433	5,2947E+14	-2,357E+12	0.0537124438618724	0.193708313313772	-3,78988E+14	4,86987E+14	4,95759E+14	5,45457E+14	6,13166E+14	5,43338E+14	4,92115E+12
TC1400002086.mm.2	Nrl	neural retina leucine zipper gene	-0.930819509293873	5,99703E+14	-2,35666E+14	0.0537382319758265	0.193708313313772	-3,79031E+14	5,49139E+14	5,24368E+14	5,86835E+14	6,72835E+14	6,13567E+14	6,51472E+14
TC1100003629.mm.2	Pnpo	pyridoxine 5'-phosphate oxidase	0.782724029981743	7,55444E+13	2,35662E+14	0.0537415813204051	0.193708313313772	-3,79036E+14	8,20131E+14	7,74155E+14	7,33425E+13	7,04317E+14	7,6266E+14	7,37977E+14
TC0700004595.mm.2	Osbpl5	oxysterol binding protein-like 5	-0.889876338896955	7,18829E+14	-2,35657E+14	0.0537449662027117	0.193708313313772	-3,79042E+13	6,3893E+14	6,80393E+14	7,18046E+14	7,79252E+14	7,24425E+14	7,71924E+14
TC1100004115.mm.2	Foxj1/Rnf157	forkhead box J1/ring finger protein 157	1,27745E+13	8,43373E+14	2,35651E+14	0.0537497591277773	0.193708313313772	-3,7905E+14	9,25249E+14	9,35969E+13	8,74302E+14	7,31425E+13	8,37416E+14	7,55878E+14
TC1200002345.mm.2	Setd3	SET domain containing 3	1,02672E+14	9,39097E+14	2,35621E+14	0.0537722419841039	0.193746005163208	-3,79087E+14	9,97648E+14	1,00609E+14	9,42098E+14	8,56292E+14	9,56133E+14	8,76327E+14
TC1700000373.mm.2	Prss29	protease, serine 29	-1,12579E+14	6,49133E+14	-2,35582E+14	0.0538023889698508	0.193758161895803	-3,79136E+14	6,0648E+14	5,68692E+14	6,54818E+14	7,45512E+14	6,11952E+14	7,07344E+14
TC1800000015.mm.2	Jcad	junctional cadherin 5 associated	1,66793E+14	6,67687E+14	2,35579E+14	0.0538042666182711	0.193758161895803	-3,7914E+14	7,79394E+14	7,73478E+14	6,39569E+14	5,79717E+12	7,29048E+13	5,04913E+14
TC0500000171.mm.2	Fgl2	fibrinogen-like protein 2	-0.719313147161993	6,42035E+14	-2,35549E+14	0.0538270321577913	0.193758161895803	-3,79177E+14	5,70231E+14	6,03323E+14	6,50759E+14	6,66658E+14	6,83271E+14	6,77968E+14
TC1100003221.mm.2	Rpa1	replication protein A1	0.718735671609279	7,5669E+14	2,35544E+14	0.0538313491756475	0.193758161895803	-3,79184E+13	8,19444E+14	7,95886E+13	7,49186E+14	7,22397E+13	7,31215E+14	7,22014E+14
TC0300001114.mm.2	Kcna10	potassium voltage-gated channel, shaker-related subfamily, member 10	-0.93395407515923	5,54922E+14	-2,35469E+14	0.0538884381720936	0.193758161895803	-3,79278E+14	4,9962E+14	4,9747E+14	5,56381E+14	6,27501E+14	5,40543E+14	6,08018E+14
TC1100000238.mm.2	Xpo1	exportin 1	2,15063E+14	6,4873E+14	2,35453E+14	0.0539000956526287	0.193758161895803	-3,79298E+14	8,04584E+14	8,22312E+14	6,59689E+14	5,3708E+14	6,76955E+14	3,91758E+14
TC1700002347.mm.2	Dennd1c	DENN/MADD domain containing 1C	-0.98800221730378	6,06456E+14	-2,35447E+14	0.0539052488062248	0.193758161895803	-3,79306E+14	5,21485E+14	5,7271E+14	6,0235E+14	6,89446E+14	6,05042E+14	6,47702E+14
TC1500000776.mm.2	Tspo	translocator protein	-1,2443E+14	6,63648E+14	-2,35426E+14	0.0539207918985291	0.193758161895803	-3,79332E+14	5,59041E+14	6,06062E+14	6,3613E+14	7,77833E+14	6,7394E+14	7,28884E+14
TC0100002725.mm.2	Sp110	Sp110 nuclear body protein	-0.714920971916705	6,78602E+13	-2,35422E+14	0.0539239352117431	0.193758161895803	-3,79337E+14	6,35717E+14	6,07256E+14	6,86626E+14	6,99332E+14	7,13104E+14	7,29575E+14
TC0500000843.mm.2	Pf4	platelet factor 4	-0.933205680134694	6,93933E+14	-2,35419E+14	0.0539267312476075	0.193758161895803	-3,79341E+13	6,09254E+14	6,2951E+14	6,69132E+14	7,56273E+14	7,29789E+14	7,69639E+14
TC0200005335.mm.2	Gm14308/Gm14430/Gm14432/Gm14434/Gm2026/Gm4724	predicted gene 14308/predicted gene 14430/predicted gene 14432/predicted gene 14434/predicted gene 2026/predicted gene 4724	2,66039E+12	8,31406E+14	2,35412E+14	0.0539317503922919	0.193758161895803	-3,7935E+14	9,19606E+14	9,91507E+14	8,579E+14	5,21134E+14	9,42764E+14	7,55528E+13
TC0700004416.mm.2	Eef1akmt2	EEF1A lysine methyltransferase 2	0.691834556489733	6,38892E+14	2,35405E+14	0.0539367612686911	0.193758161895803	-3,79358E+13	6,76976E+14	6,68346E+14	6,11943E+14	5,95011E+14	6,34848E+14	6,46229E+14
TC1000000039.mm.2	Oprm1	opioid receptor, mu 1	1,32479E+14	6,98304E+14	2,35397E+14	0.05394289155952	0.193758161895803	-3,79368E+14	8,48757E+14	7,38103E+14	6,99844E+14	6,22057E+14	6,949E+14	5,86165E+14
TC0100002042.mm.2	Kcnq5	potassium voltage-gated channel, subfamily Q, member 5	1,06467E+14	8,16654E+14	2,35392E+14	0.0539472205674583	0.193758161895803	-3,79375E+14	8,84024E+14	8,93234E+14	8,27962E+14	7,36363E+13	8,21649E+14	7,36691E+13
TC0500003637.mm.2	Slc7a1	solute carrier family 7 (cationic amino acid transporter, y+ system), member 1	0.784213991437968	6,70485E+14	2,35369E+14	0.0539644538679856	0.193758161895803	-3,79404E+14	7,30963E+13	7,30471E+14	6,73331E+12	6,3126E+13	6,50178E+14	6,06708E+14
TC0800001262.mm.2	Vps4a	vacuolar protein sorting 4A	1,02214E+14	7,37508E+14	2,35364E+14	0.0539680153036903	0.193758161895803	-3,79409E+14	8,10431E+14	7,93538E+14	7,47343E+14	6,52198E+14	7,4356E+14	6,77981E+14
TC0700004638.mm.2	Rab4b/Mir3101	RAB4B, member RAS oncogene family/microRNA 3101	0.989872958483557	8,66643E+14	2,35317E+14	0.0540041898169169	0.193807093377333	-3,79469E+14	9,31253E+14	9,21516E+14	8,78697E+14	7,76098E+14	8,65636E+14	8,2666E+14
TC1900000645.mm.2	Scd3	stearoyl-coenzyme A desaturase 3	1,29841E+14	7,21736E+14	2,35315E+14	0.0540057003205006	0.193807093377333	-3,79471E+14	8,09914E+14	8,11127E+14	7,42416E+13	6,18943E+14	7,31655E+14	6,16362E+14
TC0500000630.mm.2	Chrna9	cholinergic receptor, nicotinic, alpha polypeptide 9	-0.989420357836045	6,15949E+14	-2,35295E+14	0.0540211339613881	0.193819312295668	-3,79497E+14	5,73217E+14	5,42667E+13	6,045E+14	7,09269E+14	6,25701E+14	6,40337E+14
TC1800000342.mm.2	Tmco6	transmembrane and coiled-coil domains 6	-0.736571667821746	7,85072E+14	-2,35254E+13	0.0540519767270086	0.193886798976467	-3,79547E+14	7,32657E+14	7,41172E+14	7,91816E+14	8,29327E+12	7,96434E+14	8,19027E+14
TC1700002145.mm.2	Cdc5l	cell division cycle 5-like (S. pombe)	0.804223303380101	6,1179E+13	2,35236E+13	0.0540660813911712	0.193894228679266	-3,79571E+14	6,88665E+14	6,65287E+14	5,85529E+14	6,07578E+14	5,9092E+14	5,32765E+14
TC0400001970.mm.2	Slc2a5	solute carrier family 2 (facilitated glucose transporter), member 5	1,23048E+14	5,91638E+14	2,35145E+14	0.0541353307101416	0.194052619828401	-3,79684E+14	7,1408E+14	6,70192E+14	6,35677E+13	5,025E+12	4,78556E+13	5,48823E+14
TC0700003610.mm.2	BC048679	cDNA sequence BC048679	-0.914981952476358	6,37459E+14	-2,35135E+14	0.0541433215571455	0.194052619828401	-3,79697E+14	5,62399E+14	5,75988E+14	6,17264E+14	7,0412E+14	6,65695E+14	6,99286E+14
TC1400002034.mm.2	Sall2	spalt like transcription factor 2	1,09055E+14	5,24937E+14	2,35131E+13	0.0541463773815238	0.194052619828401	-3,79702E+14	6,14129E+14	5,92837E+14	5,29824E+14	4,59032E+14	5,30715E+14	4,23086E+14
TC1100003136.mm.2	Aipl1	aryl hydrocarbon receptor-interacting protein-like 1	-0.883136818531166	5,19571E+14	-2,35095E+14	0.0541737636345246	0.194074928265558	-3,79747E+14	4,38605E+14	4,56815E+14	5,01525E+14	5,70522E+14	5,50286E+14	5,99671E+14
TC1600001308.mm.2	Ranbp1	RAN binding protein 1	0.832697431297569	8,13139E+14	2,35091E+14	0.0541766913261641	0.194074928265558	-3,79752E+14	8,75902E+13	8,6401E+13	8,17677E+14	7,55696E+14	8,02362E+14	7,63186E+13
TC0500000277.mm.2	Dnajb6	DnaJ heat shock protein family (Hsp40) member B6	1,72001E+14	9,33155E+14	2,35045E+14	0.0542123912067582	0.194076990687427	-3,7981E+14	1,0362E+14	1,02543E+14	9,54743E+13	7,62889E+14	9,82857E+14	8,36804E+14
TC0700004320.mm.2	Dctpp1	dCTP pyrophosphatase 1	-0.711211127682167	8,23362E+14	-2,35035E+14	0.0542199931648451	0.194076990687427	-3,79823E+14	7,51972E+14	7,75236E+14	8,2853E+14	8,4092E+14	8,60988E+13	8,82527E+14
TC0100002954.mm.2	Ptpn4	protein tyrosine phosphatase, non-receptor type 4	0.693683742542315	7,21705E+14	2,35028E+13	0.0542251817329854	0.194076990687427	-3,79831E+14	7,64657E+14	7,52431E+14	6,93858E+14	6,84493E+14	7,26734E+14	7,08057E+14
TC0300000951.mm.2	Ankrd34a	ankyrin repeat domain 34A	0.893486164368571	6,42103E+13	2,35028E+14	0.0542254460420031	0.194076990687427	-3,79832E+12	7,27852E+14	6,57891E+14	5,87043E+14	6,20003E+14	6,52214E+14	6,07618E+14
TC0300001137.mm.2	Sort1	sortilin 1	1,08986E+14	8,94537E+14	2,34911E+13	0.0543154496769809	0.19425107764277	-3,79979E+14	9,73618E+14	9,82004E+14	9,19023E+14	8,18626E+13	8,78557E+14	7,95395E+14
TC1100002104.mm.2	4930556J24Rik	RIKEN cDNA 4930556J24 gene	-0.787304718156983	5,43065E+14	-2,349E+14	0.0543237588544467	0.19425107764277	-3,79993E+14	4,76609E+14	4,6016E+14	5,21494E+14	5,72736E+14	5,98416E+14	6,28976E+14
TC1700002561.mm.2	Cebpz	CCAAT/enhancer binding protein zeta	1,13465E+14	6,20166E+13	2,34892E+14	0.0543294885183122	0.19425107764277	-3,80002E+14	6,80017E+14	6,77238E+14	6,32374E+12	4,97952E+13	6,34842E+14	5,98574E+13

TC0X00003083.mm.2	Col4a6	collagen, type IV, alpha 6	-1,06717E+14	6,01099E+13	-2,34877E+14	0.0543410351157693	0.19425107764277	-3,80021E+14	5,31519E+14	5,31115E+14	6,03836E+14	6,72233E+14	5,80902E+14	6,8699E+14
TC1600001744.mm.2	Cep97	centrosomal protein 97	1,02715E+14	5,98341E+13	2,34865E+14	0.0543506270149338	0.19425107764277	-3,80037E+14	6,66031E+14	6,44213E+14	6,08595E+14	4,9622E+14	6,023E+14	5,72687E+14
TC1600000952.mm.2	N6amt1	N-6 adenine-specific DNA methyltransferase 1 (putative)	0.834032588314767	6,10971E+13	2,34859E+14	0.0543552288252631	0.19425107764277	-3,80044E+14	6,4714E+14	6,42834E+13	5,89599E+14	5,33569E+14	6,51058E+14	6,01624E+14
TC1500001431.mm.2	Lrp12	low density lipoprotein-related protein 12	1,03241E+14	6,97093E+14	2,34855E+14	0.0543584750878949	0.19425107764277	-3,80049E+14	7,58134E+14	7,64642E+14	7,05869E+14	6,10425E+14	7,07394E+14	6,36091E+14
TC1600001398.mm.2	Sst	somatostatin	0.797901166269828	1,0812E+14	2,34813E+13	0.05439064206024	0.19429093980669	-3,80102E+14	1,14814E+14	1,12374E+14	1,08627E+14	1,02603E+14	1,05008E+13	1,05293E+14
TC0800003189.mm.2	Tarbp1	TAR RNA binding protein 1	-0.72916364781753	6,25963E+14	-2,34809E+14	0.0543937460105491	0.19429093980669	-3,80107E+14	5,71958E+14	5,70766E+14	6,25371E+14	6,63185E+13	6,71647E+14	6,5285E+14
TC0200003137.mm.2	Slc34a3	solute carrier family 34 (sodium phosphate), member 3	-0.936025155440247	4,45246E+14	-2,34759E+14	0.0544318049473588	0.194346076289667	-3,80169E+14	3,97551E+14	3,83769E+13	4,5886E+14	5,09666E+14	4,18812E+13	5,0282E+14
TC1100000694.mm.2	Trim58	tripartite motif-containing 58	-0.97658313303884	5,94278E+14	-2,34757E+14	0.0544333049274044	0.194346076289667	-3,80172E+14	5,25425E+14	5,03677E+13	5,61267E+13	6,63152E+14	6,7009E+14	6,42057E+13
TC1300000396.mm.2	Prpf4b	pre-mRNA processing factor 4B	1,09821E+14	6,78327E+14	2,34671E+14	0.0544999463959583	0.194442179723015	-3,8028E+14	7,599E+14	7,55102E+14	6,79848E+14	6,15511E+14	6,87652E+14	5,7195E+14
TC1000001761.mm.2	Stx11	syntaxin 11	-0.966938072665315	4,89563E+14	-2,34657E+14	0.0545103074626246	0.194442179723015	-3,80297E+14	4,45344E+14	4,08493E+14	4,74349E+14	5,72875E+14	5,11934E+14	5,24386E+14
TC0200002749.mm.2	Gm14403	predicted gene 14403	1,51607E+14	8,00731E+14	2,34624E+14	0.05453575497285	0.194442179723015	-3,80338E+14	7,69431E+14	9,18454E+14	6,88523E+14	6,96148E+13	9,38055E+14	7,93775E+14
TC0900003343.mm.2	Srprb	signal recognition particle receptor, B subunit	1,24813E+14	6,19305E+14	2,34607E+14	0.0545492387282725	0.194442179723015	-3,8036E+14	7,36E+13	6,70171E+14	6,49615E+14	5,0693E+14	5,93076E+14	5,60036E+14
TC0200003754.mm.2	Wipf1	WAS/WASL interacting protein family, member 1	-0.677736561361805	6,20303E+13	-2,34606E+14	0.0545502718279908	0.194442179723015	-3,80362E+14	5,55656E+14	5,55266E+14	6,19049E+14	6,27421E+14	6,85573E+13	6,78854E+14
TC1500002168.mm.2	Olfr286	olfactory receptor 286	-1,04245E+14	4,72394E+14	-2,34596E+14	0.0545573012392679	0.194442179723015	-3,80373E+14	4,03472E+14	4,11216E+14	4,70303E+14	5,52875E+14	4,5381E+14	5,42688E+14
TC1600000940.mm.2	Gabpa	GA repeat binding protein, alpha	1,24507E+14	7,40962E+14	2,34576E+14	0.0545727427046551	0.194442179723015	-3,80399E+14	8,11379E+14	8,05735E+14	7,55272E+13	6,12828E+14	7,66656E+14	6,93901E+14
TC1500000880.mm.2	Acr	acrosin prepropeptide	-0.734461566774705	5,97871E+14	-2,34542E+14	0.0545997005460718	0.194442179723015	-3,80442E+14	5,60779E+14	5,71895E+14	6,24906E+14	6,5466E+14	6,03189E+14	5,71799E+14
TC0600003375.mm.2	Ldhb	lactate dehydrogenase B	0.927585660985149	8,12042E+14	2,34536E+14	0.0546037289320134	0.194442179723015	-3,80449E+14	9,05375E+14	8,59067E+14	7,89338E+14	7,89587E+14	7,21561E+14	8,07324E+14
TC0600001739.mm.2	Smco2	single-pass membrane protein with coiled-coil domains 2	-1,26383E+14	5,01351E+14	-2,34531E+14	0.0546076596914733	0.194442179723015	-3,80455E+14	3,97077E+13	4,59037E+14	5,12349E+13	5,96532E+14	4,587E+14	5,84414E+14
TC1800000833.mm.2	Atp5a1	ATP synthase, H+ transporting, mitochondrial F1 complex, alpha subunit 1	1,56199E+13	1,04232E+14	2,34528E+14	0.0546101860428451	0.194442179723015	-3,80459E+14	1,14248E+14	1,13131E+14	1,07985E+14	8,81547E+14	1,05115E+14	9,67578E+14
TC1100004147.mm.2	Tk1	thymidine kinase 1	-1,31611E+14	6,29108E+14	-2,34514E+14	0.0546206510697225	0.194442179723015	-3,80477E+14	5,32066E+14	5,90785E+14	6,20012E+14	7,66063E+14	5,97116E+14	6,68607E+13
TC0400003207.mm.2	Mroh7	maestro heat-like repeat family member 7	-1,20786E+14	5,95552E+14	-2,34511E+14	0.0546235365273168	0.194442179723015	-3,80481E+14	5,73219E+14	4,69748E+14	5,8685E+14	6,97689E+14	6,11614E+14	6,34192E+14
TC0X00000922.mm.2	Gpr165	G protein-coupled receptor 165	1,7931E+14	7,31411E+14	2,34495E+14	0.0546352402406686	0.194442179723015	-3,805E+14	8,88263E+13	7,77226E+14	7,67013E+14	5,39856E+14	7,20843E+14	6,95264E+14
TC1400000854.mm.2	Mipep	mitochondrial intermediate peptidase	1,77643E+14	5,71696E+14	2,34476E+14	0.0546504947416102	0.194442179723015	-3,80525E+14	7,12217E+14	6,7308E+12	6,36762E+14	3,9325E+14	5,31799E+14	4,83067E+14
TC0500001223.mm.2	Ube3b	ubiquitin protein ligase E3B	0.897541694487144	7,04425E+14	2,34472E+13	0.0546533005626223	0.194442179723015	-3,8053E+14	7,58224E+14	7,54266E+14	7,11145E+14	6,21837E+14	6,98227E+14	6,82852E+14
TC1100000959.mm.2	4930544D05Rik	RIKEN cDNA 4930544D05 gene	-0.807798270459358	8,55266E+14	-2,34385E+14	0.0547209007334623	0.194530455699213	-3,80639E+14	8,09954E+14	8,04787E+14	8,64126E+14	9,12175E+14	8,46175E+13	8,94379E+14
TC0400000660.mm.2	E130308A19Rik	RIKEN cDNA E130308A19 gene	-0.736618749464879	6,73727E+14	-2,34367E+14	0.0547349948610334	0.194530455699213	-3,80662E+14	6,25879E+14	6,3292E+14	6,80492E+14	7,25632E+14	6,88875E+14	6,88563E+14
TC0400002787.mm.2	Cdc26	cell division cycle 26	1,24917E+14	7,59248E+14	2,3436E+14	0.0547399392683864	0.194530455699213	-3,8067E+14	8,54055E+14	8,3433E+14	7,86457E+14	6,52093E+14	7,57517E+14	6,71035E+13
TC1700000061.mm.2	Dynlt1c/Dynlt1f	dynein light chain Tctex-type 1C/dynein light chain Tctex-type 1F	-1,91664E+14	8,57092E+14	-2,34327E+14	0.054765580420419	0.194530455699213	-3,80712E+14	8,17855E+14	6,90533E+14	1,03965E+14	8,52066E+14	9,48729E+14	7,93719E+14
TC1200001064.mm.2	D430019H16Rik	RIKEN cDNA D430019H16 gene	0.77446158182142	7,8049E+14	2,34315E+14	0.054774599447195	0.194530455699213	-3,80726E+11	8,60089E+14	8,20693E+14	7,84379E+14	7,41511E+14	7,41397E+14	7,34869E+14
TC1400001643.mm.2	Cacna2d3	calcium channel, voltage-dependent, alpha2/delta subunit 3	1,59568E+14	7,53039E+14	2,34311E+14	0.0547778133147949	0.194530455699213	-3,80732E+14	8,54265E+14	8,44095E+14	7,83651E+14	5,95572E+14	7,78534E+14	6,62117E+14
TC1200002239.mm.2	Ppp4r3a	protein phosphatase 4 regulatory subunit 3A	2,1968E+14	6,78346E+14	2,34309E+14	0.0547799168249843	0.194530455699213	-3,80735E+14	7,58732E+14	7,98409E+14	7,11328E+14	4,06454E+14	7,42061E+14	6,53093E+14
TC0600000527.mm.2	Cul1	cullin 1	2,51061E+11	6,17291E+14	2,34291E+14	0.0547935370060205	0.194530455699213	-3,80757E+12	7,88823E+14	7,76495E+14	6,87142E+13	3,76055E+14	6,37789E+14	4,37441E+14
TC1800001111.mm.2	Syt4	synaptotagmin IV	1,42401E+14	8,94195E+14	2,34274E+14	0.0548065542090151	0.194530455699213	-3,80778E+14	9,90564E+14	9,78501E+14	9,15858E+14	7,68403E+13	9,17185E+14	7,94659E+14
TC0100003541.mm.2	Nit1	nitrilase 1	0.764751994568257	7,35798E+14	2,34273E+14	0.0548078103946092	0.194530455699213	-3,8078E+14	79873218429	7,8352E+14	6,99038E+14	7,30264E+14	6,79934E+14	7,23302E+14
TC0900000840.mm.2	Ubap1l	ubiquitin-associated protein 1-like	-1,0004E+14	6,24864E+14	-2,34269E+14	0.0548109147194455	0.194530455699213	-3,80785E+14	5,60646E+14	5,38313E+14	5,97681E+14	7,01359E+14	6,55668E+14	6,95514E+14
TC0400003733.mm.2	Grrp1	glycine/arginine rich protein 1	-0.698453393559036	5,03482E+14	-2,34243E+14	0.0548304974255791	0.194556954158442	-3,80817E+14	4,6293E+14	4,49605E+14	5,13486E+14	5,3874E+14	5,27446E+14	5,28686E+14
TC0400001318.mm.2	Olfr1331	olfactory receptor 1331	-1,34519E+14	4,86906E+14	-2,34228E+14	0.0548425299936475	0.194556954158442	-3,80836E+14	3,84505E+13	4,26008E+14	4,89946E+14	5,89606E+13	4,44713E+14	5,86658E+12
TC0900002751.mm.2	Htr1b	5-hydroxytryptamine (serotonin) receptor 1B	1,2765E+14	7,37231E+14	2,34211E+14	0.0548556142234131	0.194557294901338	-3,80858E+14	8,19427E+14	8,32553E+14	7,80687E+14	6,15994E+14	6,95884E+14	6,78839E+14
TC0900000720.mm.2	Sema7a	sema domain, immunoglobulin domain (Ig), and GPI membrane anchor, (semaphorin) 7A	1,00267E+14	6,9096E+13	2,34182E+14	0.0548783909311666	0.194557294901338	-3,80894E+14	7,2041E+14	7,81157E+13	6,66051E+13	6,34982E+14	7,17144E+14	6,26018E+14
TC0800000260.mm.2	Defb50	defensin beta 50	-1,31726E+14	4,87487E+14	-2,34173E+14	0.0548853342359591	0.194557294901338	-3,80906E+13	4,11996E+14	4,13687E+14	4,72218E+14	6,16917E+14	4,57255E+14	5,52847E+14
TC1100002779.mm.2	Fat2	FAT atypical cadherin 2	-0.832102384299627	5,71514E+14	-2,34132E+14	0.0549169578618589	0.194557294901338	-3,80957E+14	5,49425E+14	4,97858E+13	5,79623E+14	6,3408E+14	5,84841E+14	5,83259E+14
TC0X00001612.mm.2	Rps6ka3	ribosomal protein S6 kinase polypeptide 3	1,10995E+14	5,92023E+14	2,34125E+14	0.054922357327796	0.194557294901338	-3,80965E+14	6,76899E+14	6,34883E+14	5,537E+14	5,36092E+14	6,38948E+14	5,11616E+14
TC0100000602.mm.2	Xrcc5	X-ray repair complementing defective repair in Chinese hamster cells 5	1,41097E+14	6,40378E+14	2,34113E+14	0.0549318563736959	0.194557294901338	-3,80981E+14	7,49318E+14	7,11819E+14	6,81622E+14	4,97321E+14	6,14561E+13	5,87624E+13
TC0400003375.mm.2	Klf17	Kruppel-like factor 17	-1,07342E+14	4,94848E+13	-2,34107E+14	0.0549360823463573	0.194557294901338	-3,80988E+14	4,3589E+14	4,20768E+14	4,72215E+14	5,99127E+14	5,25193E+14	5,15894E+14
TC0400004042.mm.2	Nmnat1	nicotinamide nucleotide adenylyltransferase 1	0.696201691392778	6,41389E+14	2,34103E+14	0.0549392224788115	0.194557294901338	-3,80993E+14	6,71469E+14	6,89362E+14	6,16881E+14	6,0471E+14	6,4145E+14	6,24462E+14
TC0700004629.mm.2	Setd1a	SET domain containing 1A	1,48772E+14	6,86172E+13	2,34033E+14	0.054994243110408	0.194709347228742	-3,81082E+14	7,87052E+14	7,5845E+14	7,07582E+13	5,40375E+14	7,17864E+14	6,0571E+14
TC0700000727.mm.2	Gm5592	predicted gene 5592	-0.88373505010302	4,80209E+13	-2,33976E+14	0.0550379835020854	0.194821403376341	-3,81152E+14	4,38863E+14	4,13406E+13	4,83699E+14	5,45317E+14	4,71328E+14	5,28645E+14
TC0400003187.mm.2	Prkaa2	protein kinase, AMP-activated, alpha 2 catalytic subunit	1,31421E+14	7,20688E+14	2,33929E+14	0.0550749846886191	0.19487796536315	-3,81212E+13	7,9541E+14	8,21127E+14	7,52971E+14	6,00726E+14	7,1611E+12	6,37786E+14
TC0100001920.mm.2	Npbwr1	neuropeptides B/W receptor 1	-1,03544E+14	5,69388E+14	-2,33925E+14	0.0550781514468928	0.19487796536315	-3,81217E+14	4,98791E+14	4,95239E+14	5,57751E+14	6,43367E+14	5,6805E+14	6,5313E+14
TC0800000811.mm.2	Olfr373	olfactory receptor 373	-1,33748E+14	5,20324E+14	-2,33905E+14	0.0550938246514889	0.194890624941699	-3,81242E+14	4,47172E+14	3,96994E+14	4,83353E+14	6,28308E+14	5,38605E+14	6,27511E+13
TC1600002037.mm.2	Krtap11-1	keratin associated protein 11-1	-0.846055017598385	5,24338E+14	-2,33862E+14	0.0551271885522136	0.194937150552137	-3,81296E+14	4,72518E+14	4,71612E+12	5,43296E+14	5,70045E+14	5,07202E+14	5,81357E+13
TC0100003639.mm.2	Adss	adenylosuccinate synthetase, non muscle	1,30846E+14	7,43557E+13	2,33839E+14	0.0551447845522994	0.194937150552137	-3,81324E+14	8,17044E+14	8,40299E+14	7,53903E+14	6,41747E+14	7,68463E+14	6,39887E+14
TC1700002328.mm.2	Znrf4	zinc and ring finger 4	-0.884525090561588	6,56805E+14	-2,33839E+13	0.055145277302175	0.194937150552137	-3,81325E+14	5,95574E+14	5,8825E+14	6,48744E+14	7,11985E+14	6,67594E+14	7,2868E+14
TC0300003207.mm.2	Bbs12	Bardet-Biedl syndrome 12 (human)	-0.859517168582522	6,72947E+14	-2,33826E+14	0.0551553695380867	0.194937150552137	-3,81341E+14	6,14063E+14	6,21993E+14	6,66631E+14	7,41328E+13	6,80113E+14	7,13551E+14
TC0800002130.mm.2	Zfp42	zinc finger protein 42	-1,04256E+14	6,49152E+14	-2,33809E+14	0.0551686179300421	0.194939823928267	-3,81363E+14	6,01608E+14	5,71936E+14	6,42883E+14	7,39173E+14	6,35007E+14	7,04305E+14
TC0300001497.mm.2	Ttll7	tubulin tyrosine ligase-like family, member 7	1,84255E+14	8,10215E+14	2,33793E+13	0.0551812455476942	0.194939823928267	-3,81383E+13	9,05155E+13	9,37156E+14	8,49193E+14	6,24607E+14	8,39306E+14	7,05873E+14
TC0300000491.mm.2	P2ry1	purinergic receptor P2Y, G-protein coupled 1	1,61429E+14	5,95021E+14	2,33778E+14	0.0551924208254673	0.194939823928267	-3,81401E+14	7,13813E+14	6,93408E+14	6,41985E+14	4,42379E+14	5,9038E+14	4,88159E+14
TC0400001782.mm.2	Cplane2	ciliogenesis and planar polarity effector 2	-0.847422254843441	6,39527E+14	-2,33751E+14	0.0552133403240825	0.194970973623042	-3,81434E+13	5,65457E+14	6,01173E+14	6,32783E+14	7,03332E+14	6,64341E+13	6,70077E+14
TC0500002386.mm.2	Adgra3	adhesion G protein-coupled receptor A3	0.870348789912266	5,97902E+14	2,33727E+14	0.0552321877783531	0.194976099016264	-3,81465E+14	6,36485E+14	6,67076E+14	5,81115E+14	5,48376E+14	5,41094E+14	6,13266E+14
TC0400003603.mm.2	Fam167b	family with sequence similarity 167, member B	-1,10126E+14	6,27359E+14	-2,33719E+14	0.0552389928634796	0.194976099016264	-3,81476E+14	5,45043E+14	5,73458E+14	6,1059E+14	7,28163E+14	6,24701E+14	6,82199E+13
TC0200004165.mm.2	Accsl	1-aminocyclopropane-1-carboxylate synthase (non-functional)-like	-0.893061002126309	5,63845E+14	-2,33664E+14	0.0552818715971566	0.195049895825669	-3,81545E+14	5,13182E+14	5,08146E+13	5,83324E+14	6,16616E+14	5,38962E+13	6,22841E+14
TC0900000132.mm.2	Chordc1	cysteine and histidine-rich domain (CHORD)-containing, zinc-binding protein 1	1,37244E+14	7,48153E+13	2,33661E+13	0.0552841106085665	0.195049895825669	-3,81548E+13	8,37334E+14	8,48906E+14	7,5288E+14	6,58872E+14	7,74174E+14	6,16752E+14
TC1500002351.mm.2	Chkb	choline kinase beta	1,19399E+14	5,56705E+14	2,33593E+14	0.0553375820069007	0.195195809736688	-3,81634E+14	6,10524E+14	6,29136E+14	5,70437E+14	4,30425E+14	5,774E+14	5,2231E+14
TC0900001887.mm.2	Rab3d	RAB3D, member RAS oncogene family	-0.85404668715793	5,79608E+14	-2,33547E+14	0.0553736905643695	0.195280428116368	-3,81692E+14	4,88703E+14	5,2251E+14	5,54873E+14	6,2715E+14	6,34888E+14	6,49523E+14
TC0100002690.mm.2	Pid1	phosphotyrosine interaction domain containing 1	1,18707E+14	6,08824E+14	2,33486E+14	0.0554209651149803	0.195324432977658	-3,81768E+14	7,02291E+14	6,66525E+14	6,23328E+14	5,08075E+14	6,21263E+14	5,31462E+14
TC1300002131.mm.2	Gas1	growth arrest specific 1	-1,03203E+14	6,68548E+13	-2,3348E+14	0.0554258762015385	0.195324432977658	-3,81775E+14	6,12134E+14	5,68237E+14	6,6192E+14	7,24857E+14	6,75641E+14	7,685E+14
TC1600001667.mm.2	Phldb2	pleckstrin homology like domain, family B, member 2	0.709456540629443	5,88861E+14	2,33475E+14	0.0554294566529793	0.195324432977658	-3,81781E+14	6,07994E+14	6,18882E+14	5,36174E+14	5,48811E+14	5,95003E+14	6,263E+14
TC1300002768.mm.2	Zfp934	zinc finger protein 934	1,3331E+13	5,37382E+14	2,33469E+14	0.0554346572337137	0.195324432977658	-3,81789E+14	6,17588E+14	6,43437E+14	5,69833E+14	4,24572E+14	5,33283E+14	4,35578E+14
TC0800003175.mm.2	Fam89a	family with sequence similarity 89, member A	-0.706634428838879	6,91929E+14	-2,33439E+14	0.0554576537295416	0.195354351146679	-3,81826E+14	6,47256E+14	6,37995E+14	7,00487E+14	7,26091E+14	7,30189E+14	7,09556E+14
TC0200002528.mm.2	Spint4	serine protease inhibitor, Kunitz type 4	-0.763780595983878	6,51876E+14	-2,33427E+14	0.0554673962946723	0.195354351146679	-3,81842E+14	6,15637E+14	6,01083E+14	6,77882E+13	6,91594E+14	6,36302E+14	6,88761E+14
TC0900001898.mm.2	Ecsit	ECSIT signalling integrator	0.751681011809271	6,87337E+14	2,33411E+14	0.0554796621211735	0.195354850471693	-3,81861E+14	7,24081E+14	7,22322E+14	6,48808E+14	6,47259E+14	7,16828E+14	6,6472E+14
TC1400000100.mm.2	3830406C13Rik	RIKEN cDNA 3830406C13 gene	1,82263E+14	5,77072E+13	2,33374E+14	0.0555089402866481	0.195377672471823	-3,81908E+14	6,91511E+14	7,15865E+14	6,35429E+14	4,07423E+14	5,67847E+14	4,44358E+14
TC1100003396.mm.2	Ccl5	chemokine (C-C motif) ligand 5	-1,0015E+14	5,44722E+14	-2,33366E+14	0.0555156748918633	0.195377672471823	-3,81919E+14	4,54835E+14	5,08746E+14	5,42489E+14	6,21393E+14	5,39061E+14	6,01808E+14
TC0100001125.mm.2	Rab3gap1	RAB3 GTPase activating protein subunit 1	0.939361354982293	7,2954E+14	2,33344E+14	0.0555326063203292	0.195377672471823	-3,81946E+14	7,96612E+14	7,9168E+14	7,49919E+14	6,50501E+14	7,03164E+14	6,85365E+14
TC0700002135.mm.2	Mboat7	membrane bound O-acyltransferase domain containing 7	1,07655E+14	7,76626E+14	2,33341E+14	0.0555346453125394	0.195377672471823	-3,81949E+14	8,7134E+14	8,58534E+14	8,07569E+14	7,06994E+14	7,47906E+14	6,67413E+13
TC0900002501.mm.2	Slc24a1	solute carrier family 24 (sodium/potassium/calcium exchanger), member 1	-1,04238E+14	5,30505E+14	-2,33269E+13	0.0555919816618105	0.195495999635697	-3,82041E+14	4,1535E+14	4,81904E+14	5,16225E+14	5,89504E+14	5,53745E+14	6,26303E+14
TC0900002565.mm.2	C2cd4a	C2 calcium-dependent domain containing 4A	-1,14831E+14	6,06807E+14	-2,33268E+14	0.0555925445497897	0.195495999635697	-3,82042E+14	5,3156E+14	5,22581E+14	5,74646E+14	7,09158E+14	6,27079E+14	6,75815E+11
TC0200005000.mm.2	5430405H02Rik	RIKEN cDNA 5430405H02 gene	-0.931320734368821	6,96222E+14	-2,33203E+14	0.0556436302011193	0.19554348337278	-3,82123E+14	6,73166E+14	6,42102E+14	7,13594E+14	7,87939E+14	6,51099E+14	7,0943E+14
TC0700000487.mm.2	Fbl	fibrillarin	0.851417431849726	8,20088E+14	2,33202E+14	0.0556445034080342	0.19554348337278	-3,82125E+14	8,57233E+14	8,81311E+14	8,22575E+14	7,45685E+14	8,03944E+14	8,09781E+12
TC0500003483.mm.2	Prkar1b	protein kinase, cAMP dependent regulatory, type I beta	1,15952E+14	1,00796E+14	2,33195E+14	0.0556501287129005	0.19554348337278	-3,82134E+14	1,09935E+14	1,07613E+14	1,03448E+12	9,09097E+13	9,99444E+14	9,29292E+14
TC0600001250.mm.2	Jagn1	jagunal homolog 1	0.920662536379619	7,28191E+14	2,33179E+14	0.055662348774701	0.19554348337278	-3,82153E+14	7,85977E+14	7,96961E+14	7,44099E+13	6,54705E+14	7,11868E+14	6,75537E+14
TC0500001353.mm.2	Rpl6	ribosomal protein L6	1,04025E+14	1,0038E+12	2,33174E+14	0.0556667261360977	0.19554348337278	-3,8216E+14	1,07445E+14	1,07072E+14	1,01527E+14	9,21848E+13	1,00974E+14	9,30755E+14
TC0800001485.mm.2	Zc3h18	zinc finger CCCH-type containing 18	-0.961579081161163	7,27389E+14	-2,33155E+14	0.0556811559725422	0.195551540145068	-3,82183E+14	6,61935E+14	6,61737E+14	7,24082E+14	7,91906E+14	7,21537E+14	8,03133E+14
TC0100002642.mm.2	Ap1s3	adaptor-related protein complex AP-1, sigma 3	-0.720338000700203	5,9727E+14	-2,33115E+14	0.0557127931639941	0.195620012257886	-3,82234E+14	5,28468E+14	5,58301E+14	6,27219E+14	6,03618E+14	6,25663E+14	6,40348E+14
TC0200003751.mm.2	Ola1	Obg-like ATPase 1	1,17633E+14	7,26745E+14	2,33098E+14	0.055726142891989	0.195624257171812	-3,82255E+13	8,07175E+14	7,75431E+14	7,38857E+14	6,08484E+14	7,47401E+14	6,83124E+14
TC0100002544.mm.2	Fn1	fibronectin 1	1,02652E+14	6,12684E+14	2,33041E+13	0.0557710011547823	0.195713226697675	-3,82326E+14	5,98498E+14	7,0537E+14	5,57499E+14	5,41064E+14	6,62555E+14	6,11119E+14
TC0500003568.mm.2	Ccz1	CCZ1 vacuolar protein trafficking and biogenesis associated	1,37948E+14	7,84458E+14	2,33035E+14	0.0557757796186758	0.195713226697675	-3,82334E+14	8,67694E+14	8,5205E+14	8,06134E+14	6,37714E+14	8,0979E+13	7,33363E+14
TC1800001049.mm.2	Trappc8	trafficking protein particle complex 8	1,16801E+14	6,07558E+14	2,32988E+14	0.0558130237029536	0.195801273878879	-3,82393E+14	6,92461E+14	6,36537E+14	5,35433E+14	5,59963E+14	6,77517E+14	5,43441E+14
TC0900001434.mm.2	Plxnb1	plexin B1	0.875035967036166	6,44167E+14	2,32965E+14	0.0558311785488801	0.195822329115826	-3,82422E+13	7,36768E+14	7,02354E+14	5,97585E+14	6,6653E+14	6,00815E+14	5,60949E+14
TC0900003266.mm.2	Cck	cholecystokinin	1,32845E+14	9,04528E+14	2,32937E+14	0.0558533532020532	0.195857471195796	-3,82457E+14	9,24252E+14	1,00111E+14	8,56733E+13	8,02939E+14	9,9592E+14	8,46212E+14
TC0600001943.mm.2	Ndufa5	NADH:ubiquinone oxidoreductase subunit A5	1,08819E+14	9,25048E+14	2,32876E+14	0.0559015559258379	0.1959022681045	-3,82534E+14	1,01609E+14	9,86779E+14	8,77382E+14	9,07846E+14	9,43702E+14	8,1849E+14
TC0500001392.mm.2	Arpc3	actin related protein 2/3 complex, subunit 3	0.949694323711377	8,05186E+14	2,32873E+14	0.0559041424731207	0.1959022681045	-3,82538E+14	8,61279E+14	8,64461E+14	8,11583E+14	7,24218E+14	8,12758E+14	7,56814E+14
TC0400000426.mm.2	Dnajb5	DnaJ heat shock protein family (Hsp40) member B5	0.934648088861689	7,74067E+14	2,32859E+14	0.0559153024858534	0.1959022681045	-3,82556E+14	8,39286E+14	8,36036E+14	7,92952E+14	6,95441E+14	7,52421E+14	7,28265E+14
TC0100002314.mm.2	Gls	glutaminase	2,2907E+14	8,7496E+12	2,32852E+14	0.0559202696789466	0.1959022681045	-3,82563E+14	1,00681E+13	1,02082E+13	9,01176E+14	6,68311E+14	9,48213E+14	7,04432E+14
TC1900001196.mm.2	Tle4	transducin-like enhancer of split 4	1,59003E+14	7,82999E+14	2,32844E+14	0.0559269182201143	0.1959022681045	-3,82574E+14	8,76877E+14	8,65149E+14	8,21121E+14	6,02899E+14	7,85964E+14	7,45983E+14
TC0200004813.mm.2	Napb	N-ethylmaleimide sensitive fusion protein attachment protein beta	1,71494E+14	9,60105E+14	2,32802E+14	0.0559599921493179	0.195939028588839	-3,82626E+14	1,09053E+14	1,07126E+14	1,00744E+14	8,11366E+14	9,6136E+14	8,18674E+14
TC1100001834.mm.2	Prkar1a	protein kinase, cAMP dependent regulatory, type I, alpha	1,7188E+14	9,87936E+14	2,328E+14	0.0559617333560377	0.195939028588839	-3,82629E+14	1,111E+14	1,09062E+14	1,03851E+14	8,19357E+14	9,8662E+14	8,81506E+14
TC0400001599.mm.2	Fgr	FGR proto-oncogene, Src family tyrosine kinase	-1,58376E+14	4,84625E+14	-2,32779E+14	0.0559786962099002	0.195955840110824	-3,82656E+14	3,85609E+14	4,27429E+14	4,71167E+14	6,58622E+14	4,2762E+14	5,37302E+14
TC0900001012.mm.2	Gnb5	guanine nucleotide binding protein (G protein), beta 5	0.833897818133886	8,44595E+14	2,3273E+14	0.0560171193509657	0.196012394052216	-3,82717E+13	9,05978E+12	8,97289E+14	8,52069E+14	7,84419E+13	8,31532E+14	7,96285E+14
TC1400000502.mm.2	Cgrrf1	cell growth regulator with ring finger domain 1	0.873754074318846	7,23342E+14	2,32711E+14	0.056032085279181	0.196012394052216	-3,82741E+14	7,98529E+14	7,64893E+14	6,76461E+14	7,1221E+14	7,29701E+14	6,58258E+13
TC0100000109.mm.2	Gdap1	ganglioside-induced differentiation-associated-protein 1	1,97539E+13	6,8311E+14	2,32698E+14	0.0560430363702897	0.196012394052216	-3,82758E+14	7,98629E+14	7,83033E+14	7,2835E+14	4,58233E+14	7,12273E+14	6,1814E+14
TC0500002046.mm.2	AI506816	expressed sequence AI506816	0.758067239927311	6,2173E+14	2,32697E+14	0.0560435114130554	0.196012394052216	-3,82759E+14	6,69996E+14	7,04817E+14	5,93009E+14	6,3019E+13	5,76633E+14	5,55732E+14
TC1100000608.mm.2	Sep 08	septin 8	0.834293485045977	8,97265E+14	2,32605E+14	0.0561165203507776	0.196202400947046	-3,82874E+14	9,59091E+14	9,54012E+14	9,07887E+14	8,38357E+14	8,78458E+14	8,45784E+14
TC0800000345.mm.2	Erlin2	ER lipid raft associated 2	1,42795E+12	6,43956E+14	2,32588E+14	0.0561300778932769	0.196202400947046	-3,82896E+14	7,57201E+14	7,16313E+13	6,17659E+14	5,70266E+14	6,93265E+14	5,09032E+14
TC0600003164.mm.2	Kcna6	potassium voltage-gated channel, shaker-related, subfamily, member 6	0.921860425306982	6,13664E+14	2,32582E+14	0.0561344809698872	0.196202400947046	-3,82903E+14	7,1804E+14	6,51563E+14	6,21108E+14	5,64123E+14	5,85487E+14	5,41659E+14
TC0300002371.mm.2	Npr1	natriuretic peptide receptor 1	-0.922210230798717	7,16508E+14	-2,32567E+14	0.0561465444527295	0.196202400947046	-3,82922E+14	6,2657E+14	6,81339E+14	7,31598E+14	7,60753E+14	7,11462E+14	7,87328E+14
TC1600001824.mm.2	Nsun3	NOL1/NOP2/Sun domain family member 3	1,63965E+14	5,64157E+14	2,32532E+13	0.0561748687622454	0.196220321531898	-3,82966E+14	6,74685E+14	6,73412E+14	5,97447E+14	4,22721E+14	5,83835E+13	4,32841E+14
TC1700000915.mm.2	AI661453	expressed sequence AI661453	-1,2317E+14	5,3487E+14	-2,3253E+14	0.0561760282521347	0.196220321531898	-3,82968E+14	4,51933E+13	4,57953E+14	5,05215E+14	6,51012E+14	5,39243E+14	6,03862E+14
TC0700003065.mm.2	Mrgprb4	MAS-related GPR, member B4	-0.865423232603431	4,96934E+14	-2,32494E+14	0.0562044071367405	0.196228644237838	-3,83013E+14	4,24176E+14	4,16505E+14	4,6463E+14	5,49135E+14	5,64395E+14	5,6276E+14
TC0600002539.mm.2	Reg3g	regenerating islet-derived 3 gamma	-1,18045E+14	4,63161E+14	-2,32487E+14	0.0562103258405274	0.196228644237838	-3,83022E+14	4,05294E+14	3,64546E+14	4,79658E+14	5,26272E+14	4,33212E+14	5,69984E+14

TC1700002806.mm.2	Pdia2	protein disulfide isomerase associated 2	-0.80383964696386	4,96662E+13	-2,32477E+14	0.0562179693338502	0.196228644237838	-3,83035E+14	4,33481E+14	4,79155E+13	5,23286E+14	5,50117E+13	5,19062E+14	4,74872E+14
TC1200002354.mm.2	Degs2	delta(4)-desaturase, sphingolipid 2	-0.920564339444654	5,16154E+14	-2,32466E+14	0.0562271240890025	0.196228644237838	-3,83049E+14	4,6577E+14	4,69323E+14	5,30393E+14	5,88813E+14	5,60443E+14	4,82179E+14
TC1000002611.mm.2	Prdm4	PR domain containing 4	-0.763804979774922	8,00945E+14	-2,32439E+14	0.0562489441323183	0.196249921003623	-3,83083E+14	7,51168E+14	7,54972E+14	8,55024E+14	8,03877E+14	8,3296E+14	8,07671E+14
TC1100002772.mm.2	Anxa6	annexin A6	0.754946345964623	7,30122E+14	2,3242E+14	0.0562635721637774	0.196249921003623	-3,83106E+14	7,93171E+13	7,66321E+14	7,27846E+14	6,80657E+14	7,05354E+14	7,07385E+13
TC0700003437.mm.2	Aldh1a3	aldehyde dehydrogenase family 1, subfamily A3	-1,06348E+14	6,44005E+14	-2,32412E+14	0.056269759513234	0.196249921003623	-3,83116E+14	5,54136E+14	5,95864E+13	6,23627E+14	7,39069E+14	6,58034E+13	6,93299E+14
TC1200000588.mm.2	Tmx1	thioredoxin-related transmembrane protein 1	1,5318E+14	6,99071E+14	2,3238E+14	0.0562954678977577	0.19629709461947	-3,83157E+14	8,396E+14	8,04109E+14	7,32213E+14	6,05136E+14	6,90138E+14	5,23228E+14
TC0700004157.mm.2	Pik3c2a	phosphatidylinositol-4-phosphate 3-kinase catalytic subunit type 2 alpha	2,229E+14	5,63766E+14	2,32345E+14	0.0563238122938348	0.196327904802998	-3,83201E+14	7,08698E+14	7,09909E+14	6,00079E+14	3,72727E+14	6,13978E+14	3,77203E+14
TC0100002593.mm.2	Fev	FEV (ETS oncogene family)	-0.978594919394707	6,26112E+14	-2,32329E+14	0.0563360263484463	0.196327904802998	-3,83221E+13	5,53726E+14	5,81929E+14	6,17483E+14	7,13891E+14	6,2185E+14	6,67793E+14
TC0800000300.mm.2	Zmat4	zinc finger, matrin type 4	1,50838E+14	6,38204E+14	2,32298E+14	0.0563611139110218	0.196327904802998	-3,8326E+14	7,34245E+14	7,26692E+14	6,70434E+14	4,88828E+14	6,55922E+13	5,53102E+14
TC1700001289.mm.2	Prkce	protein kinase C, epsilon	1,48406E+14	9,3738E+14	2,32295E+14	0.0563632695993703	0.196327904802998	-3,83264E+14	1,008E+14	1,03179E+14	9,63972E+14	7,79013E+14	9,62883E+13	8,7862E+14
TC0200002543.mm.2	Slc12a5	solute carrier family 12, member 5	2,20378E+14	7,95026E+14	2,32293E+13	0.056365226067068	0.196327904802998	-3,83267E+14	9,4854E+14	9,26657E+14	8,57748E+14	5,76694E+14	8,10368E+14	6,50147E+13
TC0800000039.mm.2	Zfp958	zinc finger protein 958	1,24319E+14	6,13131E+14	2,32253E+14	0.0563972136393154	0.196337800686912	-3,83317E+14	6,96462E+14	6,72097E+14	6,20382E+13	4,99538E+14	6,4768E+14	5,42629E+14
TC0400001164.mm.2	Eps15	epidermal growth factor receptor pathway substrate 15	2,0273E+14	7,74273E+14	2,32244E+14	0.0564044119863508	0.196337800686912	-3,83328E+14	9,06039E+14	8,97826E+14	8,35289E+14	5,63115E+13	7,79215E+14	6,64155E+14
TC1300002086.mm.2	Spock1	sparc/osteonectin, cwcv and kazal-like domains proteoglycan 1	0.858155062286109	8,37786E+14	2,32243E+13	0.0564046223161245	0.196337800686912	-3,83329E+14	9,25511E+14	8,56107E+14	8,28031E+14	7,81956E+14	8,18349E+14	8,16761E+14
TC0800002729.mm.2	Fam192a	family with sequence similarity 192, member A	0.693488341864981	8,41889E+14	2,32147E+14	0.0564813324376295	0.196499097341265	-3,83449E+14	8,90948E+14	8,71449E+12	8,16852E+14	8,06847E+14	8,42354E+14	8,22884E+13
TC1800001026.mm.2	Cdh2	cadherin 2	1,577E+14	7,96052E+14	2,32134E+14	0.0564919749617293	0.196499097341265	-3,83466E+14	9,20314E+14	9,15432E+14	8,537E+14	6,66646E+13	7,6869E+14	6,51532E+13
TC1600001011.mm.2	Itsn1	intersectin 1 (SH3 domain protein 1A)	2,20327E+14	8,14865E+14	2,32128E+14	0.0564966478047095	0.196499097341265	-3,83473E+14	9,58513E+14	9,31573E+14	8,74177E+14	5,75254E+14	8,36421E+13	7,13251E+14
TC0900001049.mm.2	Gm3776/Gsta1	predicted gene 3776/glutathione S-transferase, alpha 1 (Ya)	-0.75447795533045	6,25989E+14	-2,32124E+14	0.0564997404569033	0.196499097341265	-3,83478E+14	5,72669E+14	5,83513E+14	6,41056E+14	6,66021E+14	6,22732E+14	6,69942E+14
TC0100000260.mm.2	Unc50	unc-50 homolog	1,8385E+13	6,58514E+14	2,32073E+14	0.0565408468650469	0.196599625709215	-3,83543E+14	7,73111E+12	7,83406E+14	6,85475E+14	5,03341E+14	6,98819E+14	5,06932E+14
TC0700001444.mm.2	Numa1	nuclear mitotic apparatus protein 1	1,19281E+14	6,04125E+14	2,31993E+14	0.0566045951059922	0.196778822209893	-3,83643E+14	6,9411E+14	6,61153E+14	6,30249E+14	4,86451E+14	5,96506E+14	5,5628E+14
TC1500001789.mm.2	Recql4	RecQ protein-like 4	-1,01516E+14	6,29387E+14	-2,31911E+14	0.05667050897451	0.196945806286725	-3,83746E+14	5,48422E+13	5,6131E+14	6,18286E+14	6,94478E+14	6,32039E+14	7,21789E+14
TC0X00000350.mm.2	Rhox2e	reproductive homeobox 2E	-1,1966E+13	5,81646E+13	-2,31903E+14	0.0566770746447926	0.196945806286725	-3,83756E+14	5,32054E+13	5,08623E+14	6,10437E+14	6,6956E+14	5,14636E+13	6,54568E+14
TC1300002072.mm.2	Pitx1	paired-like homeodomain transcription factor 1	-0.969117142795926	7,75014E+14	-2,31881E+14	0.0566946586973063	0.196955496367613	-3,83784E+14	7,29597E+14	6,78207E+14	7,53485E+14	8,48142E+14	8,12477E+13	8,28175E+14
TC1300001836.mm.2	Bloc1s5	biogenesis of lysosomal organelles complex-1, subunit 5, muted	0.745517191268073	6,10782E+14	2,31869E+14	0.0567043100384384	0.196955496367613	-3,83799E+14	6,88416E+14	6,63589E+14	5,81787E+14	6,21114E+14	5,66241E+14	5,43547E+14
TC0600003268.mm.2	Ybx3	Y box protein 3	-0.960254689932829	8,31255E+14	-2,31842E+14	0.0567258788053443	0.196962353291498	-3,83832E+14	7,48683E+13	7,8654E+14	9,08913E+14	8,1836E+14	8,83805E+14	8,4123E+14
TC0600002475.mm.2	Ptcd3	pentatricopeptide repeat domain 3	2,2173E+14	5,49615E+14	2,31836E+12	0.0567307318082196	0.196962353291498	-3,8384E+13	6,91224E+14	6,73215E+14	6,06989E+13	3,13991E+14	5,78341E+14	4,33929E+14
TC0100002141.mm.2	Zfp451	zinc finger protein 451	1,22236E+13	7,67959E+14	2,31817E+14	0.0567462962642049	0.196967704388771	-3,83864E+14	8,39947E+14	8,5291E+14	7,54846E+13	6,93538E+14	8,06084E+14	6,60429E+14
TC0700000243.mm.2	Pnmal2	PNMA-like 2	1,62722E+14	7,5368E+14	2,31804E+14	0.0567567213726224	0.196967704388771	-3,83881E+14	8,77937E+14	8,47485E+14	8,09076E+14	5,90901E+14	7,3087E+14	6,65809E+13
TC0100003202.mm.2	Cdc73	cell division cycle 73, Paf1/RNA polymerase II complex component	2,20032E+14	6,09928E+14	2,31774E+14	0.0567809684288463	0.197009419529285	-3,83919E+14	7,74148E+14	7,73098E+14	6,64378E+14	4,42804E+14	6,21101E+14	3,84038E+14
TC0200004508.mm.2	Shf	Src homology 2 domain containing F	-0.930470554375346	6,61592E+14	-2,31522E+14	0.0569840231064172	0.19760987985937	-3,84235E+14	6,27778E+14	6,13435E+14	6,70018E+14	7,57289E+13	6,26362E+14	6,74668E+14
TC1700001400.mm.2	Unc93a2	unc-93 homolog A2	-1,23085E+14	6,06458E+14	-2,31501E+14	0.0570007799411616	0.19760987985937	-3,84261E+14	5,45123E+13	5,29161E+14	5,84294E+14	7,36161E+14	5,97401E+14	6,46608E+14
TC1500000088.mm.2	Slc45a2	solute carrier family 45, member 2	-1,29499E+14	5,9359E+14	-2,31494E+14	0.0570061609177117	0.19760987985937	-3,8427E+14	5,28006E+14	5,14237E+14	5,7208E+14	7,2916E+14	5,7451E+14	6,43544E+14
TC1000001640.mm.2	Olfr784	olfactory receptor 784	-1,29574E+14	5,69214E+14	-2,31482E+14	0.057016167407338	0.19760987985937	-3,84285E+14	5,17875E+14	4,86754E+14	5,47179E+14	7,16599E+14	5,74942E+13	5,71932E+14
TC0500003377.mm.2	Eif4h	eukaryotic translation initiation factor 4H	1,28918E+14	9,05311E+14	2,31433E+14	0.0570553973849253	0.19760987985937	-3,84346E+14	9,80694E+14	9,73845E+14	9,33354E+14	7,63349E+14	8,99557E+14	8,81069E+14
TC1800000620.mm.2	Camk2a	calcium/calmodulin-dependent protein kinase II alpha	1,24635E+14	1,13302E+14	2,31405E+14	0.0570783553188962	0.19760987985937	-3,84382E+14	1,20381E+14	1,21311E+14	1,16269E+14	1,00496E+14	1,12758E+13	1,08599E+14
TC1100000917.mm.2	Sat2	spermidine/spermine N1-acetyl transferase 2	1,48282E+13	6,53609E+14	2,31398E+14	0.057083476076654	0.19760987985937	-3,8439E+14	7,69429E+14	7,17913E+14	6,83903E+14	5,06875E+14	6,67049E+14	5,76483E+14
TC0300001414.mm.2	Adh4	alcohol dehydrogenase 4 (class II), pi polypeptide	-1,3932E+14	4,89303E+14	-2,31393E+14	0.0570877559068383	0.19760987985937	-3,84397E+14	4,24228E+14	3,6038E+14	4,45595E+14	6,17653E+14	5,19867E+14	5,68094E+14
TC0800001208.mm.2	Fbxl8	F-box and leucine-rich repeat protein 8	-0.693437242994432	6,06127E+14	-2,31388E+14	0.057091801294737	0.19760987985937	-3,84403E+14	5,69025E+14	5,49848E+14	6,17322E+14	6,40238E+14	6,33334E+14	6,26991E+14
TC0400001600.mm.2	Ahdc1	AT hook, DNA binding motif, containing 1	-0.95121865450641	6,6719E+14	-2,31383E+14	0.0570959698640114	0.19760987985937	-3,84409E+14	6,08743E+14	6,05568E+14	6,7172E+14	7,32834E+13	6,49205E+14	7,35073E+14
TC0200001866.mm.2	Sema6d	sema domain, transmembrane domain (TM), and cytoplasmic domain, (semaphorin) 6D	1,31362E+14	8,21481E+14	2,31371E+14	0.0571054539236764	0.19760987985937	-3,84424E+14	9,09186E+13	9,08319E+14	8,61608E+14	6,93173E+14	8,03145E+13	7,53455E+14
TC0X00001619.mm.2	Map7d2	MAP7 domain containing 2	1,5597E+14	8,99412E+14	2,31363E+14	0.0571122349018628	0.19760987985937	-3,84435E+14	1,01153E+14	9,80305E+14	9,28347E+14	7,5155E+14	9,24819E+14	7,9992E+14
TC1700001266.mm.2	Ppm1b	protein phosphatase 1B, magnesium dependent, beta isoform	1,82942E+14	8,87271E+14	2,31343E+14	0.0571280745902314	0.19760987985937	-3,84459E+13	9,75623E+14	9,86827E+14	9,25256E+14	6,71312E+14	9,18345E+14	8,46265E+14
TC0200002396.mm.2	Aar2	AAR2 splicing factor homolog	1,08555E+14	5,74701E+13	2,31343E+14	0.0571281103479457	0.19760987985937	-3,84459E+14	6,72977E+13	6,30428E+14	5,08888E+14	5,77407E+14	4,76347E+14	5,82157E+14
TC0300002298.mm.2	Ttc24	tetratricopeptide repeat domain 24	-0.84423691317456	6,65709E+14	-2,31331E+14	0.0571379898383173	0.19760987985937	-3,84475E+14	6,02458E+13	5,91086E+14	6,42942E+14	7,19448E+14	7,07656E+14	7,30662E+14
TC1600000765.mm.2	Cldnd1	claudin domain containing 1	1,79372E+13	8,39878E+14	2,31306E+14	0.0571585956626208	0.19761244193894	-3,84507E+14	9,49512E+14	9,56083E+14	8,86499E+14	6,60352E+14	8,55741E+14	7,31079E+14
TC1000002522.mm.2	Diras1	DIRAS family, GTP-binding RAS-like 1	-0.680149404353863	7,43758E+14	-2,31285E+14	0.0571754123173251	0.19761244193894	-3,84533E+14	7,22155E+13	7,02139E+14	7,79217E+14	7,81107E+14	7,43974E+14	7,33955E+13
TC1800000311.mm.2	Matr3	matrin 3	1,81261E+14	9,56012E+14	2,31285E+14	0.057175523137798	0.19761244193894	-3,84533E+14	1,08063E+14	1,07468E+14	1,00368E+14	7,89104E+14	9,68652E+14	8,19325E+14
TC0700001756.mm.2	Prkcb	protein kinase C, beta	1,45185E+13	1,075E+13	2,31236E+14	0.0572149017131004	0.197667607210323	-3,84594E+14	1,15556E+14	1,1683E+14	1,1062E+14	9,27286E+14	1,09081E+14	1,00186E+14
TC0900001562.mm.2	Golga4	golgi autoantigen, golgin subfamily a, 4	0.822567050585268	6,6032E+14	2,31221E+14	0.0572274418551389	0.197667607210323	-3,84613E+14	6,58857E+14	7,29369E+13	6,04825E+14	6,18887E+14	6,86054E+14	6,63928E+14
TC0700004508.mm.2	Bet1l	Bet1 golgi vesicular membrane trafficking protein like	-0.689549014462722	6,46594E+14	-2,31218E+14	0.0572296141350381	0.197667607210323	-3,84617E+13	6,14722E+13	6,1418E+14	6,93292E+14	6,7352E+14	6,52286E+14	6,31562E+14
TC1700001105.mm.2	Epb41l3	erythrocyte membrane protein band 4.1 like 3	1,98402E+14	8,2243E+14	2,31203E+14	0.0572415882056954	0.197667607210323	-3,84635E+14	9,56787E+14	9,38357E+14	8,80443E+14	6,17897E+14	8,31419E+14	7,09675E+14
TC0800001033.mm.2	Nkd1	naked cuticle 1	0.803210920517506	5,67533E+14	2,31189E+14	0.0572528221121925	0.197667607210323	-3,84653E+14	6,31024E+14	5,77025E+14	5,40386E+13	5,07021E+14	5,89679E+13	5,60061E+14
TC1000000356.mm.2	Mettl24	methyltransferase like 24	-0.748565865037893	5,6788E+14	-2,31137E+14	0.0572953183586715	0.197768404152964	-3,84718E+14	5,26312E+14	5,40446E+14	5,87281E+14	6,29191E+14	5,46893E+13	5,77154E+14
TC1400001060.mm.2	Erich6b	glutamate rich 6B	-1,07373E+14	4,81165E+14	-2,31123E+14	0.0573065648228257	0.197768404152964	-3,84736E+14	4,31468E+13	4,15858E+14	4,93614E+14	5,68459E+14	4,35656E+14	5,41934E+14
TC0800000516.mm.2	Sorbs2	sorbin and SH3 domain containing 2	1,71544E+14	5,56117E+14	2,31058E+14	0.0573595972919119	0.19786700573555	-3,84818E+14	6,4214E+14	6,7263E+14	5,9781E+14	3,73873E+14	5,73073E+14	4,77174E+14
TC1100001658.mm.2	Rnd2	Rho family GTPase 2	0.756885653819635	8,24383E+14	2,31007E+14	0.0574006990506762	0.19786700573555	-3,84881E+14	9,11205E+14	8,6394E+14	8,04278E+14	8,19491E+14	7,87142E+14	7,60243E+14
TC0300001290.mm.2	Neurog2	neurogenin 2	-1,1495E+14	6,10608E+14	-2,30996E+14	0.0574098882466571	0.19786700573555	-3,84896E+14	5,53856E+14	5,0575E+14	5,78348E+14	7,11159E+14	6,39389E+14	6,75144E+14
TC0700002704.mm.2	Mrps12	mitochondrial ribosomal protein S12	-0.856633989429197	8,36712E+14	-2,30989E+14	0.0574154143643793	0.19786700573555	-3,84904E+14	7,774E+14	8,02799E+14	8,36717E+14	9,14809E+14	8,35228E+14	8,53317E+14
TC0700000564.mm.2	Prodh2	proline dehydrogenase (oxidase) 2	-1,00237E+14	7,04918E+14	-2,30988E+14	0.0574167268967652	0.19786700573555	-3,84906E+14	6,42765E+14	6,1719E+14	6,92852E+14	7,67577E+14	7,11655E+14	7,97468E+14
TC1900001498.mm.2	Blnk	B cell linker	0.863540851820195	5,94692E+14	2,30976E+14	0.057426269235979	0.19786700573555	-3,84921E+14	6,12677E+13	6,56436E+14	5,19319E+14	5,77086E+14	5,78362E+14	6,24268E+13
TC0800003102.mm.2	Dbndd1	dysbindin (dystrobrevin binding protein 1) domain containing 1	-0.701848798824371	7,46023E+14	-2,30963E+14	0.0574363476423788	0.19786700573555	-3,84936E+14	7,04568E+14	6,80519E+14	7,49707E+14	7,7575E+13	7,83496E+14	7,821E+14
TC0300001080.mm.2	Ppm1j	protein phosphatase 1J	-1,15673E+14	5,6389E+14	-2,30956E+13	0.0574427843895608	0.19786700573555	-3,84946E+14	4,90315E+14	5,09362E+14	5,43456E+14	6,87566E+14	5,64991E+14	5,87653E+14
TC0X00002292.mm.2	Fgf13	fibroblast growth factor 13	1,46513E+14	6,17065E+14	2,30947E+14	0.0574497248967615	0.19786700573555	-3,84957E+14	7,41309E+14	7,07468E+13	6,40344E+14	5,15406E+14	6,27019E+14	4,70846E+14
TC0200003421.mm.2	Psmb7	proteasome (prosome, macropain) subunit, beta type 7	1,96082E+14	8,44966E+14	2,30937E+14	0.0574579358180749	0.19786700573555	-3,8497E+14	9,61347E+14	9,63881E+14	8,88501E+14	6,44563E+14	8,80902E+14	7,30601E+14
TC0400003406.mm.2	Olfr1329	olfactory receptor 1329	-0.845453641949832	5,5386E+14	-2,30899E+13	0.057488520778182	0.197894208063038	-3,85017E+14	5,13163E+14	4,90359E+14	5,47294E+14	6,25318E+14	5,7562E+14	5,71407E+14
TC0100001316.mm.2	Glrx2	glutaredoxin 2 (thioltransferase)	1,61522E+14	8,77812E+14	2,30889E+14	0.0574973488075211	0.197894208063038	-3,85031E+14	9,68071E+14	9,62158E+14	9,05177E+14	7,02008E+13	9,17198E+14	8,12259E+14
TC0900003179.mm.2	Rps27rt	ribosomal protein S27, retrogene	0.823875138683748	8,3361E+14	2,3086E+14	0.0575203703750237	0.197894208063038	-3,85066E+14	8,82763E+14	8,84452E+13	8,3218E+14	7,7026E+14	8,37941E+14	7,94065E+13
TC0400002529.mm.2	Tpm2	tropomyosin 2, beta	-0.775803407883441	7,35217E+14	-2,30848E+14	0.0575300282021881	0.197894208063038	-3,85081E+14	6,76836E+14	6,86218E+14	7,28245E+14	7,8997E+13	7,63448E+14	7,66587E+14
TC0500001615.mm.2	Plod3/Mir702	procollagen-lysine, 2-oxoglutarate 5-dioxygenase 3/microRNA 702	0.707653728448579	6,66957E+14	2,30846E+14	0.0575320671244238	0.197894208063038	-3,85084E+14	6,95051E+14	7,05231E+14	6,37646E+14	6,21104E+14	6,5484E+14	6,87869E+14
TC1900000727.mm.2	Gsto1	glutathione S-transferase omega 1	1,64333E+14	6,79334E+14	2,30837E+14	0.057539524903825	0.197894208063038	-3,85096E+14	7,74676E+14	7,42729E+14	7,09757E+14	4,78982E+13	6,76472E+14	6,93386E+14
TC0700000804.mm.2	Lrrc4b	leucine rich repeat containing 4B	0.854153242546941	9,09417E+14	2,30814E+14	0.0575579110366463	0.197915198577354	-3,85124E+14	9,76713E+14	9,59918E+14	9,23564E+14	8,42236E+14	8,72065E+14	8,82005E+14
TC0800003035.mm.2	Fam92b	family with sequence similarity 92, member B	-1,19642E+14	4,96127E+14	-2,30738E+14	0.057619982261818	0.198037529308582	-3,8522E+14	4,48227E+14	4,28441E+14	4,82404E+14	6,33547E+13	4,78605E+14	5,05536E+14
TC0100002073.mm.2	Col19a1	collagen, type XIX, alpha 1	-0.990380770960511	6,26062E+14	-2,30736E+14	0.0576217612916904	0.198037529308582	-3,85222E+14	5,32579E+14	5,52409E+14	5,92921E+14	6,90143E+14	6,65734E+14	7,22584E+13
TC1500001748.mm.2	Mroh6	maestro heat-like repeat family member 6	-0.778671135545914	5,00492E+14	-2,30712E+14	0.0576415841068337	0.198037529308582	-3,85253E+14	4,59647E+13	4,52328E+14	5,05594E+14	5,62115E+13	5,25639E+14	4,97628E+14
TC0200001523.mm.2	Pamr1	peptidase domain containing associated with muscle regeneration 1	-0.92300586184988	6,22567E+14	-2,30681E+14	0.0576666849363269	0.198037529308582	-3,85291E+14	5,67773E+14	5,66892E+14	6,10302E+14	7,08964E+14	6,33026E+14	6,48446E+14
TC1200002411.mm.2	Ckb	creatine kinase, brain	1,0964E+14	1,06033E+14	2,30679E+14	0.0576683957268877	0.198037529308582	-3,85294E+14	1,13419E+14	1,12256E+14	1,08204E+14	9,55433E+14	1,05772E+14	1,01006E+13
TC0200000697.mm.2	Lhx2	LIM homeobox protein 2	0.716690983760378	7,0384E+14	2,30667E+14	0.0576776792999552	0.198037529308582	-3,85308E+13	7,64997E+14	7,91905E+13	7,11701E+14	7,01863E+14	6,41916E+14	6,10657E+14
TC0900000180.mm.2	Rdh8	retinol dehydrogenase 8	-1,02175E+13	4,91904E+14	-2,30665E+14	0.0576795211968705	0.198037529308582	-3,85311E+14	3,95803E+14	4,51418E+14	4,78718E+14	5,72852E+14	5,00513E+14	5,52121E+14
TC0900002156.mm.2	C2cd2l	C2 calcium-dependent domain containing 2-like	0.76262599739732	8,9248E+14	2,30628E+14	0.0577097601418546	0.198073540250211	-3,85358E+13	9,40613E+14	9,47607E+14	8,96246E+14	8,39448E+14	8,72011E+13	8,58956E+13
TC0400002141.mm.2	Impad1	inositol monophosphatase domain containing 1	2,24887E+13	6,6321E+14	2,30622E+14	0.0577145951390019	0.198073540250211	-3,85365E+14	8,10352E+14	7,78843E+14	7,1626E+14	4,23162E+14	7,04029E+14	5,46616E+14
TC1600001337.mm.2	Klhl6	kelch-like 6	0.983554641377278	5,13324E+14	2,3057E+14	0.057757159615799	0.198112819670126	-3,8543E+14	5,84808E+14	5,74885E+14	5,3067E+13	4,32312E+14	5,06079E+14	4,51193E+14
TC0800000150.mm.2	Kbtbd11	kelch repeat and BTB (POZ) domain containing 11	1,10191E+14	7,0816E+14	2,30568E+14	0.0577586198736232	0.198112819670126	-3,85433E+14	8,25606E+14	8,05245E+14	7,6228E+14	6,48188E+14	6,4031E+14	5,67333E+14
TC0700002946.mm.2	Iglon5	IgLON family member 5	-1,10799E+14	6,86594E+14	-2,30552E+14	0.0577721237233188	0.198112819670126	-3,85453E+14	6,06816E+13	5,98924E+14	7,7768E+14	6,49658E+14	7,6891E+14	7,17578E+14
TC0600001391.mm.2	Nanog	Nanog homeobox	-0.979444000050629	5,33549E+14	-2,30548E+14	0.0577752212269549	0.198112819670126	-3,85458E+14	4,78777E+14	5,10146E+14	5,49831E+14	6,34981E+14	4,80766E+14	5,46796E+14
TC1100003584.mm.2	Itga3	integrin alpha 3	0.708571305240364	5,98717E+13	2,30499E+14	0.0578159241245525	0.198210209663599	-3,85521E+14	6,46434E+14	6,30653E+14	5,76764E+14	5,5861E+14	5,74449E+14	6,05392E+14
TC1900001104.mm.2	Sdhaf2	succinate dehydrogenase complex assembly factor 2	0.807948206479331	7,37961E+14	2,30478E+14	0.0578330483588859	0.198226740737445	-3,85547E+14	8,16626E+14	7,86228E+14	7,17926E+13	7,23339E+14	7,24358E+14	6,5929E+14
TC0500001641.mm.2	Zkscan1	zinc finger with KRAB and SCAN domains 1	1,09717E+14	6,30719E+13	2,30418E+14	0.0578816272923737	0.198338710309778	-3,85621E+14	6,89063E+14	6,85869E+13	6,43496E+12	5,12004E+14	6,09318E+14	6,44567E+13
TC0200000567.mm.2	Prrc2b	proline-rich coiled-coil 2B	0.677013701108844	7,59747E+14	2,30408E+14	0.0578903341765584	0.198338710309778	-3,85635E+14	8,06236E+14	8,17992E+14	7,53008E+14	7,35818E+14	7,19086E+14	7,26339E+14
TC0700002646.mm.2	Cyp2s1	cytochrome P450, family 2, subfamily s, polypeptide 1	0.675049002113853	5,55634E+14	2,30343E+14	0.0579432894604343	0.198477938579077	-3,85716E+14	6,02796E+14	6,07846E+14	5,39729E+13	5,35903E+14	5,33614E+14	5,13916E+14
TC1500002223.mm.2	Lima1	LIM domain and actin binding 1	1,779E+14	5,82608E+14	2,30322E+14	0.0579609470334749	0.198496225196681	-3,85743E+14	7,3941E+13	7,19988E+14	6,30727E+14	4,7287E+14	5,67341E+14	3,65309E+14
TC1100000156.mm.2	Vstm2a	V-set and transmembrane domain containing 2A	0.933579778698679	9,86883E+14	2,30264E+14	0.0580087359467757	0.198617671549171	-3,85816E+14	1,07853E+14	1,04183E+14	1,00767E+14	9,25977E+14	9,5866E+14	9,08629E+14
TC0400003842.mm.2	Micos10	mitochondrial contact site and cristae organizing system subunit 10	1,87434E+14	9,87768E+14	2,30247E+14	0.0580219586199922	0.198620739163785	-3,85836E+13	1,09819E+14	1,0864E+14	1,02298E+14	7,8674E+14	1,03446E+14	8,97841E+14
TC0200001879.mm.2	Slc12a1	solute carrier family 12, member 1	-1,04273E+14	5,40686E+14	-2,3022E+14	0.0580445698721297	0.198655937627363	-3,85871E+14	5,27815E+13	4,43956E+14	5,43908E+14	6,3641E+14	5,3935E+13	5,52678E+13
TC0900003311.mm.2	Fyco1	FYVE and coiled-coil domain containing 1	1,43831E+13	5,50661E+14	2,30199E+14	0.0580617033148085	0.198672377471121	-3,85897E+12	6,38298E+14	6,26798E+14	5,58502E+14	4,18931E+14	6,01392E+14	4,60045E+14
TC0100000289.mm.2	Map4k4	mitogen-activated protein kinase kinase kinase kinase 4	0.826847331322907	7,63989E+14	2,30183E+14	0.0580746970455953	0.198674648300569	-3,85917E+14	8,06233E+13	8,1975E+14	7,67985E+14	6,92629E+14	7,43274E+14	7,54061E+14
TC0600001500.mm.2	Tspan11	tetraspanin 11	-0.8424210815418	5,74656E+14	-2,30028E+14	0.0582024999025067	0.198898359691661	-3,86111E+14	5,4581E+14	4,96451E+14	5,73984E+14	6,36762E+14	5,89488E+14	6,0544E+14
TC1500002162.mm.2	Col2a1	collagen, type II, alpha 1	-0.953729253214466	5,87523E+14	-2,30027E+14	0.0582038893387664	0.198898359691661	-3,86114E+14	5,3652E+13	5,01295E+14	5,65315E+14	6,63246E+14	6,42232E+14	6,16532E+14
TC1100002249.mm.2	Plek	pleckstrin	0.890337430440328	7,29927E+14	2,30018E+14	0.0582112642293807	0.198898359691661	-3,86125E+14	7,63856E+13	7,97497E+14	7,30572E+14	6,52714E+14	7,36555E+14	6,98369E+14
TC1800001301.mm.2	Mospd4	motile sperm domain containing 4	-0.854264009227943	6,44388E+14	-2,30016E+14	0.0582129006666975	0.198898359691661	-3,86127E+14	6,04684E+14	5,7794E+14	6,38561E+13	7,14916E+14	6,53103E+14	6,77124E+14
TC1100003245.mm.2	Abr	active BCR-related gene	0.901032512204031	8,79661E+14	2,30014E+14	0.0582145564605186	0.198898359691661	-3,8613E+14	9,62691E+14	9,57805E+14	8,99285E+14	8,41004E+14	7,70848E+14	8,46331E+13
TC0900001253.mm.2	A4gnt	alpha-1,4-N-acetylglucosaminyltransferase	-0.916325301380703	5,41877E+14	-2,3001E+14	0.0582173246550769	0.198898359691661	-3,86134E+14	4,71218E+14	4,81036E+13	5,21274E+14	6,14244E+14	5,65508E+14	5,97983E+13
TC0X00000772.mm.2	Tbl1x	transducin (beta)-like 1 X-linked	0.84343661800892	8,19788E+14	2,29987E+14	0.0582366189549398	0.198898359691661	-3,86163E+14	8,76378E+14	8,68968E+14	8,21993E+14	7,54666E+14	8,21229E+13	7,75492E+14
TC0200002474.mm.2	Srsf6	serine/arginine-rich splicing factor 6	0.677980553268293	7,46196E+14	2,29984E+14	0.0582388419925066	0.198898359691661	-3,86167E+14	7,80772E+14	7,88168E+14	7,17771E+14	7,15573E+14	7,48622E+13	7,26271E+14
TC1500001384.mm.2	Ywhaz	tyrosine 3-monooxygenase/tryptophan 5-monooxygenase activation protein, zeta polypeptide	1,75893E+14	1,08823E+14	2,29967E+14	0.0582528808448813	0.198904146864499	-3,86188E+14	1,19012E+14	1,2014E+13	1,13481E+14	9,04916E+14	1,10301E+14	9,95103E+14
TC0700000666.mm.2	E130304I02Rik	RIKEN cDNA E130304I02 gene	-0.843228086757679	8,50102E+14	-2,29899E+14	0.0583088476560341	0.199053064042728	-3,86273E+14	7,87861E+14	7,8932E+14	8,35201E+14	9,10625E+14	8,73043E+14	9,04561E+14
TC1700000942.mm.2	Unc5cl	unc-5 family C-terminal like	-0.942768539404235	5,96409E+14	-2,29882E+14	0.0583229651464962	0.199059084390064	-3,86295E+14	5,38825E+14	5,27511E+14	5,76868E+14	6,78021E+14	6,15832E+14	6,41399E+14
TC0Y00000489.mm.2	Ssty2/Gm21242	spermiogenesis specific transcript on the Y 2/predicted gene, 21242	-0.85598539186928	5,41732E+14	-2,29854E+14	0.0583465494204742	0.199066589820432	-3,86331E+14	4,43903E+14	5,21765E+14	5,51436E+14	5,85429E+14	5,74381E+14	5,7348E+14
TC0X00000319.mm.2	Lonrf3	LON peptidase N-terminal domain and ring finger 3	1,73557E+14	5,55876E+14	2,29842E+14	0.0583562516139036	0.199066589820432	-3,86345E+13	6,7051E+14	6,7935E+14	6,25835E+14	3,7691E+14	4,78327E+14	5,04326E+14
TC0500000112.mm.2	Cacna2d1	calcium channel, voltage-dependent, alpha2/delta subunit 1	1,48024E+14	6,71135E+14	2,29835E+14	0.0583622274133756	0.199066589820432	-3,86354E+13	7,64472E+14	7,64858E+14	6,90628E+14	5,42654E+14	7,04268E+13	5,5993E+14
TC1800001654.mm.2	Rbfa	ribosome binding factor A	0.770728086543964	6,43747E+14	2,29804E+14	0.0583881101675149	0.199112723625221	-3,86394E+13	6,74953E+14	6,82529E+14	6,2991E+14	5,73427E+14	6,3857E+13	6,63091E+14
TC1400000096.mm.2	Ptprg	protein tyrosine phosphatase, receptor type, G	0.929057963778794	7,37915E+14	2,29729E+14	0.0584500011530165	0.199281605708539	-3,86488E+14	8,30551E+13	7,93275E+14	7,28554E+14	7,09461E+14	7,29363E+14	6,36288E+14
TC1700002184.mm.2	Rrp36	ribosomal RNA processing 36 homolog (S. cerevisiae)	-0.85143572983525	7,33642E+13	-2,29673E+14	0.0584962813528416	0.199397203604471	-3,86558E+14	6,72821E+14	6,88593E+14	7,56712E+14	7,74989E+14	7,14134E+14	7,946E+14

TC1500001506.mm.2	Rad21	RAD21 cohesin complex component	1,0071E+14	8,40272E+14	2,29641E+14	0.058523012319989	0.199446128915394	-3,86598E+14	8,92899E+14	9,14895E+14	8,52835E+14	7,53539E+14	8,44451E+14	7,8301E+14
TC1100002550.mm.2	Ccng1	cyclin G1	2,29373E+14	7,69901E+14	2,29589E+14	0.0585655555472631	0.199455633961912	-3,86663E+14	8,61396E+14	9,20339E+14	8,04851E+14	5,18137E+14	8,46889E+14	6,67795E+14
TC0900001294.mm.2	Gm5627	predicted gene 5627	-0.924505343291201	6,25929E+14	-2,29586E+13	0.0585681073127035	0.199455633961912	-3,86667E+14	5,30312E+14	5,85067E+14	6,2495E+14	6,7533E+14	6,35676E+14	7,0424E+14
TC0200000417.mm.2	Abca2	ATP-binding cassette, sub-family A (ABC1), member 2	0.745806558329115	7,40867E+13	2,29586E+13	0.0585681308243322	0.199455633961912	-3,86667E+14	8,105E+14	7,89637E+14	7,13624E+14	7,37351E+14	6,7493E+14	7,19157E+14
TC0X00002158.mm.2	Zdhhc9	zinc finger, DHHC domain containing 9	1,16743E+13	7,98311E+14	2,29578E+14	0.058575315577966	0.199455633961912	-3,86678E+14	8,53415E+14	8,68723E+14	8,08004E+13	6,80649E+14	8,28861E+14	7,50211E+14
TC0X00002910.mm.2	H2afb3	H2A histone family, member B3	-0.674670269081615	6,39091E+14	-2,29559E+14	0.0585905596693066	0.19946538938338	-3,86701E+14	6,02442E+14	5,87444E+14	6,64569E+14	6,60251E+14	6,67788E+14	6,52052E+14
TC1000002258.mm.2	Supv3l1	suppressor of var1, 3-like 1 (S. cerevisiae)	0.895924084464823	5,39329E+14	2,29533E+14	0.0586125363998055	0.199469671202107	-3,86734E+14	6,14892E+14	5,86617E+14	4,75486E+14	5,46838E+14	5,33848E+14	4,78293E+14
TC1900000975.mm.2	Kat5	K(lysine) acetyltransferase 5	0.862995656044717	8,43836E+13	2,29507E+14	0.058633862045984	0.199469671202107	-3,86766E+14	9,02157E+14	8,95409E+14	8,47116E+14	7,7785E+14	8,46335E+14	7,94146E+14
TC1300002019.mm.2	Msx2	msh homeobox 2	-1,56574E+14	5,99078E+14	-2,29488E+14	0.0586497026694354	0.199469671202107	-3,8679E+13	4,82392E+14	5,27129E+14	5,649E+14	7,5777E+12	5,81008E+14	6,81267E+14
TC1100003625.mm.2	Nfe2l1	nuclear factor, erythroid derived 2,-like 1	1,08211E+14	9,6025E+14	2,29451E+14	0.0586802223640616	0.199469671202107	-3,86836E+14	1,04677E+14	1,03382E+14	9,81398E+13	8,82771E+13	9,53349E+14	8,63392E+14
TC1500002221.mm.2	Cers5	ceramide synthase 5	0.857641637552851	7,27295E+14	2,29426E+14	0.0587010212167498	0.199469671202107	-3,86868E+14	7,80156E+14	7,82065E+13	7,36105E+14	6,54587E+14	7,18714E+14	6,92143E+14
TC1400000541.mm.2	Ap5m1	adaptor-related protein complex 5, mu 1 subunit	0.780063787850128	6,80955E+14	2,2937E+14	0.0587476718944282	0.199469671202107	-3,86938E+14	7,09772E+14	7,06522E+14	6,62726E+13	5,97556E+14	7,0449E+14	7,04666E+14
TC0900001337.mm.2	Twf2	twinfilin actin binding protein 2	0.714826693685243	7,00832E+14	2,29355E+14	0.0587605202394267	0.199469671202107	-3,86957E+14	7,33906E+14	7,46958E+14	6,88345E+14	6,49554E+14	7,0019E+13	6,8604E+14
TC0300002820.mm.2	Plppr4	phospholipid phosphatase related 4	1,45836E+14	8,78156E+14	2,29354E+14	0.0587616430669402	0.199469671202107	-3,86959E+14	9,74013E+14	9,70193E+14	9,19491E+14	7,33044E+14	8,76734E+14	7,95462E+14
TC1700001241.mm.2	Eml4	echinoderm microtubule associated protein like 4	0.954169482755448	6,22951E+14	2,29346E+14	0.0587681654550344	0.199469671202107	-3,86969E+14	7,16076E+14	6,92546E+14	6,43794E+14	5,73995E+14	5,95074E+13	5,16219E+14
TC1200002078.mm.2	Aldh6a1	aldehyde dehydrogenase family 6, subfamily A1	1,1278E+14	6,35435E+13	2,29337E+14	0.0587759110756054	0.199469671202107	-3,86981E+14	7,40755E+14	7,21786E+14	6,55722E+14	5,81259E+14	6,1692E+14	4,96168E+14
TC0200004973.mm.2	Eif6	eukaryotic translation initiation factor 6	0.763697442917937	7,45772E+14	2,2933E+14	0.0587809865686147	0.199469671202107	-3,86988E+14	8,00297E+14	8,0139E+13	7,51189E+14	6,97759E+14	7,26044E+14	6,97955E+13
TC0700000678.mm.2	Tshz3	teashirt zinc finger family member 3	-0.681900666766747	8,538E+14	-2,29301E+14	0.058805511025585	0.199469671202107	-3,87025E+14	7,79486E+14	8,04747E+14	8,55086E+14	8,65527E+14	9,10481E+14	9,07473E+14
TC0200003313.mm.2	Stxbp1	syntaxin binding protein 1	1,71567E+14	1,00586E+14	2,29274E+14	0.0588278879116094	0.199469671202107	-3,87059E+14	1,13286E+14	1,11297E+14	1,05709E+12	8,45612E+14	1,00711E+14	8,79499E+14
TC1000000056.mm.2	A830018L16Rik	RIKEN cDNA A830018L16 gene	1,92317E+14	7,0073E+14	2,29273E+14	0.058828622940107	0.199469671202107	-3,8706E+14	8,16881E+14	7,86508E+14	7,21569E+13	4,97185E+14	7,75684E+14	6,06555E+13
TC1700000884.mm.2	Yipf3	Yip1 domain family, member 3	1,22238E+14	7,69093E+14	2,29271E+14	0.058830594236555	0.199469671202107	-3,87063E+14	8,43733E+14	8,45762E+14	7,94451E+14	6,50569E+14	7,76198E+14	7,03843E+14
TC0500001453.mm.2	Zfp664	zinc finger protein 664	0.687901608014963	6,62314E+14	2,29271E+14	0.058830502608128	0.199469671202107	-3,87063E+14	7,01507E+13	7,25005E+13	6,4505E+14	6,43883E+14	6,39304E+13	6,19135E+14
TC1100003822.mm.2	Ppy	pancreatic polypeptide	-0.775756174664108	5,76283E+14	-2,29268E+14	0.0588330914127014	0.199469671202107	-3,87067E+14	5,13676E+14	5,3437E+14	5,7571E+14	6,27488E+14	5,87433E+14	6,1902E+13
TC0200003830.mm.2	Pde1a	phosphodiesterase 1A, calmodulin-dependent	1,85887E+14	7,89486E+14	2,29265E+14	0.0588358394449497	0.199469671202107	-3,87071E+14	8,95816E+14	9,34162E+14	8,25243E+14	6,32961E+14	8,18558E+13	6,30172E+14
TC1700000882.mm.2	Xpo5	exportin 5	1,10528E+14	6,96205E+14	2,29259E+14	0.0588405713822916	0.199469671202107	-3,87078E+14	8,02629E+14	8,02761E+14	7,04892E+14	6,79442E+14	6,61996E+14	5,25512E+14
TC1100004214.mm.2	Ppp1r27	protein phosphatase 1, regulatory subunit 27	-1,10429E+14	6,06179E+14	-2,29251E+14	0.0588473262446414	0.199469671202107	-3,87088E+14	5,15276E+14	5,38529E+14	5,76577E+14	6,98086E+14	6,23001E+13	6,85603E+14
TC1000003303.mm.2	Nlk	nemo like kinase	0.745602557445022	8,80863E+14	2,29246E+14	0.0588517853220888	0.199469671202107	-3,87095E+13	9,05474E+14	9,29388E+14	8,5199E+14	8,33751E+14	9,05106E+14	8,5947E+14
TC1900001529.mm.2	Pyroxd2	pyridine nucleotide-disulphide oxidoreductase domain 2	-1,24837E+14	5,73603E+14	-2,29213E+14	0.058878914472365З	0.199479727993011	-3,87136E+14	5,10712E+14	4,951E+14	5,4727E+14	7,08217E+14	6,08765E+14	5,71555E+14
TC0800001126.mm.2	Adgrg1	adhesion G protein-coupled receptor G1	0.967448951330258	8,50959E+14	2,29209E+14	0.0588820848222773	0.199479727993011	-3,87141E+14	9,18662E+14	9,18384E+14	8,76792E+14	7,66765E+14	8,05702E+14	8,19448E+14
TC0700004221.mm.2	Gga2	golgi associated, gamma adaptin ear containing, ARF binding protein 2	-0.713487424958801	7,68017E+14	-2,29197E+14	0.058891892637172	0.199479727993011	-3,87155E+14	7,32107E+14	7,12475E+14	8,12739E+14	7,74541E+13	7,88635E+14	7,87608E+14
TC0400002454.mm.2	Bag1	BCL2-associated athanogene 1	1,31476E+14	7,61317E+14	2,29179E+14	0.0589076782973086	0.1949126111907	-3,87179E+14	8,39687E+14	8,39851E+14	7,68756E+13	6,4783E+14	8,01248E+14	6,70529E+14
TC0X00001555.mm.2	Rragb	Ras-related GTP binding B	1,94032E+14	7,54012E+14	2,29104E+14	0.0589696314743434	0.199656368692807	-3,87272E+14	9,11955E+14	8,6439E+14	8,20553E+14	5,67727E+14	7,4187E+14	6,17575E+14
TC0500000902.mm.2	Myo18b	myosin XVIIIb	-0.890355583982782	5,08898E+14	-2,29075E+14	0.0589940240018063	0.199656368692807	-3,87309E+14	4,78265E+14	5,11951E+14	4,39662E+14	6,03048E+13	4,95066E+14	5,06398E+13
TC1300001508.mm.2	Rala	v-ral simian leukemia viral oncogene A (ras related)	0.713864441094095	7,46172E+14	2,29059E+14	0.0590077080871755	0.199656368692807	-3,87329E+14	7,91744E+14	7,84685E+14	7,28408E+14	7,05248E+14	7,51417E+14	7,15532E+14
TC1700001905.mm.2	H2-Aa	histocompatibility 2, class II antigen A, alpha	-0.967597796280016	7,12452E+13	-2,29053E+14	0.0590129601249681	0.199656368692807	-3,87337E+14	6,25335E+14	6,51931E+14	6,90356E+14	7,8043E+14	7,33696E+14	7,92963E+14
TC1500000418.mm.2	Fer1l6	fer-1-like 6 (C. elegans)	-0.815473230494085	5,06431E+14	-2,29046E+14	0.0590183878907616	0.199656368692807	-3,87346E+14	4,41981E+14	4,339E+14	5,09574E+14	5,29401E+14	5,25718E+14	5,98012E+14
TC1700002411.mm.2	Vapa	vesicle-associated membrane protein, associated protein A	1,26871E+14	8,89176E+14	2,29029E+14	0.0590329956039208	0.199663866113765	-3,87367E+14	9,61746E+12	9,62769E+14	8,98976E+14	7,71797E+14	9,26171E+14	8,13595E+14
TC0900001517.mm.2	Dync1li1	dynein cytoplasmic 1 light intermediate chain 1	1,06029E+14	8,05658E+14	2,28995E+14	0.0590610087249941	0.199715691203742	-3,8741E+14	8,46835E+14	8,48767E+14	8,06875E+14	6,76688E+14	8,28749E+14	8,26055E+14
TC1100003757.mm.2	Krt15	keratin 15	-1,12191E+14	6,26962E+14	-2,28974E+14	0.0590784340153682	0.199733698550069	-3,87436E+14	5,63777E+14	5,64034E+14	6,27725E+14	7,24467E+14	5,88682E+14	6,93084E+14
TC0200002894.mm.2	Celf2	CUGBP, Elav-like family member 2	1,40493E+14	9,93537E+14	2,2895E+14	0.059098715892628	0.19976035434821	-3,87466E+14	1,08128E+14	1,09073E+14	1,03214E+14	8,58877E+14	9,9298E+14	9,05217E+14
TC0100000215.mm.2	Rab23	RAB23, member RAS oncogene family	1,16909E+14	5,66395E+14	2,28862E+13	0.0591726255712241	0.199962593810806	-3,87577E+14	6,26281E+14	6,51657E+14	5,04325E+14	5,39796E+14	6,10683E+14	4,65628E+14
TC0700001989.mm.2	Lrrc27	leucine rich repeat containing 27	0.717643639064355	6,18063E+13	2,28831E+14	0.0591984717764759	0.199962593810806	-3,87616E+13	6,66905E+14	6,47117E+13	6,05466E+14	5,65027E+14	6,10852E+14	6,13013E+14
TC0100000082.mm.2	Kcnb2	potassium voltage gated channel, Shab-related subfamily, member 2	0.970154250076794	5,46424E+14	2,28799E+14	0.0592256119567092	0.199962593810806	-3,87656E+14	6,31694E+14	6,18721E+14	5,29409E+14	5,26975E+13	5,41398E+14	4,30349E+14
TC0500001410.mm.2	Adh6a	alcohol dehydrogenase 6A (class V)	-0.789646096563528	5,13231E+14	-2,28797E+14	0.0592270087844126	0.199962593810806	-3,87658E+14	4,59161E+13	4,81279E+13	5,20938E+14	5,77432E+14	5,36265E+14	5,0431E+14
TC0400001631.mm.2	Trim63	tripartite motif-containing 63	-0.960807702393747	6,10557E+14	-2,28764E+14	0.0592546647373588	0.199962593810806	-3,877E+14	5,34324E+14	5,98017E+14	6,32926E+14	6,91577E+14	5,69438E+13	6,37059E+13
TC0600002935.mm.2	Sec13	SEC13 homolog, nuclear pore and COPII coat complex component	1,07395E+14	6,43054E+12	2,28761E+13	0.0592574151982336	0.199962593810806	-3,87704E+13	7,17871E+14	7,23724E+14	6,47216E+14	5,79589E+14	6,52234E+14	5,3769E+14
TC1700001429.mm.2	Qk	quaking	1,37972E+14	1,03125E+14	2,28745E+14	0.0592705746015416	0.199962593810806	-3,87723E+14	1,11192E+14	1,13151E+13	1,04571E+14	9,21767E+14	1,05975E+14	9,16826E+14
TC1200000033.mm.2	Gm6682	tubulin, alpha 1C pseudogene	1,44994E+14	1,10404E+14	2,28722E+14	0.0592902709286788	0.199962593810806	-3,87753E+14	1,20318E+14	1,18995E+14	1,1394E+14	9,63734E+14	1,11406E+14	1,0139E+14
TC0100000605.mm.2	Ctnna3	catenin (cadherin associated protein), alpha 3	-0.727480555867956	6,59905E+14	-2,28713E+14	0.0592975880914634	0.199962593810806	-3,87764E+14	5,99565E+14	6,1903E+14	6,67719E+14	6,96372E+14	6,71093E+14	7,05653E+14
TC1100000287.mm.2	Ppp4r3b	protein phosphatase 4 regulatory subunit 3B	2,45132E+14	6,21105E+14	2,28686E+14	0.0593202504622557	0.199962593810806	-3,87798E+14	7,56671E+14	7,96745E+14	7,06982E+14	3,5617E+14	6,10899E+14	4,99164E+14
TC1500001684.mm.2	Trappc9	trafficking protein particle complex 9	0.835531725588311	7,12929E+14	2,28682E+14	0.0593237852147818	0.199962593810806	-3,87803E+14	7,74104E+14	7,57813E+13	7,20943E+14	6,43868E+13	6,81133E+14	6,99715E+14
TC0600001436.mm.2	Mlf2	myeloid leukemia factor 2	1,09475E+14	9,85156E+14	2,2867E+13	0.0593339251970653	0.199962593810806	-3,87818E+13	1,05444E+14	1,05309E+14	1,00084E+13	8,87737E+14	9,96357E+13	9,18472E+14
TC1500001979.mm.2	Ttll12	tubulin tyrosine ligase-like family, member 12	-0.745028904537588	7,45088E+14	-2,28647E+14	0.0593533760383923	0.199962593810806	-3,87847E+14	7,06193E+14	7,0877E+14	7,75937E+14	7,88032E+14	7,18944E+14	7,72652E+14
TC0500003059.mm.2	Foxn4	forkhead box N4	-0.878626433848177	7,44618E+14	-2,28637E+14	0.0593620019774198	0.199962593810806	-3,8786E+13	6,84145E+14	6,92141E+14	7,30433E+13	8,21578E+14	7,70735E+14	7,68679E+14
TC0500001083.mm.2	St7l	suppression of tumorigenicity 7-like	1,07844E+14	6,58185E+14	2,28634E+14	0.0593645622500953	0.199962593810806	-3,87864E+14	7,39944E+13	7,25919E+14	6,8659E+14	5,63586E+14	6,42372E+14	5,90697E+14
TC0500002053.mm.2	Srpk2	serine/arginine-rich protein specific kinase 2	0.950975216650249	9,56157E+14	2,286E+14	0.0593931035956211	0.199962593810806	-3,87906E+14	1,01851E+14	1,03135E+14	9,64662E+14	8,95011E+14	9,54392E+14	8,73007E+14
TC0700002924.mm.2	Zfp141	zinc finger protein 141	2,70575E+14	6,09066E+13	2,28584E+14	0.0594066450186524	0.199962593810806	-3,87927E+14	7,77097E+13	7,66457E+14	6,83202E+14	3,19202E+14	6,50369E+14	4,58069E+14
TC1000002894.mm.2	Tbc1d15	TBC1 domain family, member 15	1,35156E+14	4,99197E+14	2,28574E+14	0.0594151436040295	0.199962593810806	-3,87939E+14	5,64405E+14	6,03876E+14	5,24939E+14	3,73029E+13	5,13751E+14	4,15182E+14
TC0700002509.mm.2	Igsf23	immunoglobulin superfamily, member 23	-1,28594E+13	6,01522E+14	-2,28573E+14	0.0594157167923408	0.199962593810806	-3,8794E+13	4,86272E+14	5,69332E+14	5,81921E+14	7,3087E+14	6,04516E+14	6,36222E+14
TC1300002075.mm.2	Abcd2	ATP-binding cassette, sub-family D (ALD), member 2	0.757580477664311	7,16233E+14	2,2857E+13	0.059418370901017	0.199962593810806	-3,87944E+14	7,51461E+14	7,8246E+14	6,92888E+14	6,89517E+14	7,15729E+14	6,65344E+14
TC0800002824.mm.2	Atp6v0d1	ATPase, H+ transporting, lysosomal V0 subunit D1	1,45146E+13	8,78127E+12	2,28509E+14	0.0594696859274504	0.199962593810806	-3,88021E+14	9,74681E+14	9,58814E+14	9,18989E+14	7,24215E+14	8,73799E+14	8,18262E+13
TC1500000468.mm.2	Gsdmcl1	gasdermin C-like 1	-1,04374E+14	5,478E+14	-2,28507E+14	0.0594714004027581	0.199962593810806	-3,88023E+14	4,7347E+14	4,88171E+14	5,83861E+14	5,86527E+14	5,14157E+14	6,40616E+14
TC0200002794.mm.2	Mrgbp	MRG/MORF4L binding protein	-1,00131E+14	7,13277E+14	-2,28502E+14	0.0594757318114962	0.199962593810806	-3,8803E+14	7,09535E+14	6,16693E+14	7,32626E+14	7,93865E+14	7,34144E+14	6,92798E+14
TC0700000769.mm.2	Klk14	kallikrein related-peptidase 14	-0.906246145115456	4,94128E+14	-2,28494E+14	0.0594820363617181	0.199962593810806	-3,88039E+14	4,22848E+13	4,53066E+14	4,88554E+14	5,68608E+14	4,91908E+14	5,39781E+14
TC1400002248.mm.2	Extl3	exostoses (multiple)-like 3	1,33063E+14	7,62677E+14	2,28493E+14	0.0594830759994029	0.199962593810806	-3,88041E+14	8,56491E+14	8,65219E+14	8,05476E+14	6,50108E+14	7,46243E+14	6,52522E+14
TC1500001036.mm.2	Serinc5	serine incorporator 5	1,01546E+14	7,40846E+14	2,28439E+14	0.0595289766635204	0.199962593810806	-3,88109E+14	8,12336E+14	8,26427E+14	7,77662E+14	6,58009E+14	6,76945E+14	6,93699E+14
TC0900002083.mm.2	AW551984	expressed sequence AW551984	1,21663E+13	6,63337E+14	2,28436E+14	0.0595312379488255	0.199962593810806	-3,88112E+14	8,37229E+14	6,98795E+14	6,79763E+14	6,12934E+14	5,99282E+14	5,5202E+14
TC0800001215.mm.2	Slc9a5	solute carrier family 9 (sodium/hydrogen exchanger), member 5	0.988431610400349	5,71389E+14	2,28423E+14	0.0595419849879428	0.199962593810806	-3,88128E+14	6,21048E+14	6,33647E+13	4,84694E+14	5,72315E+14	5,95826E+14	5,20803E+14
TC0400004173.mm.2	Plekhn1	pleckstrin homology domain containing, family N member 1	-1,2047E+14	5,72856E+14	-2,2842E+14	0.0595446231195973	0.199962593810806	-3,88132E+14	5,03921E+14	5,11439E+14	5,49242E+14	7,07058E+14	5,89716E+14	5,5776E+14
TC0700002457.mm.2	Psg21	pregnancy-specific glycoprotein 21	-1,28326E+14	4,87265E+13	-2,28412E+14	0.0595513027855595	0.199962593810806	-3,88142E+13	4,13809E+14	4,08812E+14	4,66848E+14	6,12425E+14	4,64052E+14	5,57646E+14
TC0300000990.mm.2	Tbx15	T-box 15	-1,26972E+14	4,75477E+14	-2,28411E+14	0.0595521269541427	0.199962593810806	-3,88143E+14	3,9997E+14	3,7506E+14	4,46556E+12	5,82413E+14	4,74306E+14	5,74552E+14
TC1700000384.mm.2	Mslnl	mesothelin-like	-0.969587810611483	4,62378E+14	-2,28405E+14	0.0595573573748606	0.199962593810806	-3,88151E+14	4,12971E+13	3,98464E+14	4,68211E+14	5,37142E+14	4,3516E+14	5,2232E+14
TC0900000997.mm.2	Wdr72	WD repeat domain 72	-0.991844120479063	5,96674E+14	-2,28392E+14	0.0595680785882124	0.199962593810806	-3,88167E+14	5,44365E+14	5,31907E+14	5,87672E+14	6,86969E+14	5,84472E+14	6,4466E+14
TC0800002376.mm.2	Ndufa13	NADH:ubiquinone oxidoreductase subunit A13	0.858228628192403	9,69593E+14	2,28348E+14	0.0596057572466303	0.200047399815654	-3,88223E+14	1,01973E+14	1,01613E+14	9,70236E+14	8,93972E+14	9,79488E+14	9,38006E+14
TC1500002203.mm.2	C1ql4	complement component 1, q subcomponent-like 4	-1,0398E+14	5,37593E+14	-2,28314E+14	0.0596341210567041	0.200100914741081	-3,88265E+14	4,78242E+14	4,54529E+14	5,27849E+14	6,12882E+14	5,30571E+14	6,21484E+13
TC1600001479.mm.2	Apod	apolipoprotein D	1,375E+14	9,7719E+14	2,28296E+14	0.0596497100507626	0.200111550707462	-3,88289E+14	1,05089E+14	1,0597E+14	9,9637E+14	8,39221E+14	1,01154E+14	9,05414E+13
TC1100004301.mm.2	Trim47	tripartite motif-containing 47	-1,13213E+14	5,96061E+14	-2,28273E+14	0.0596688141836127	0.200133972302363	-3,88317E+14	5,02682E+14	5,45793E+14	5,75673E+13	5,90697E+14	5,93782E+14	6,47207E+14
TC1200005399.mm.2	Psma7	proteasome (prosome, macropain) subunit, alpha type 7	1,12941E+14	7,60853E+14	2,28254E+14	0.0596851601246868	0.200147135294293	-3,88341E+14	8,3077E+13	8,12026E+14	7,71093E+14	6,45821E+14	7,87442E+14	7,17967E+12
TC0800002741.mm.2	Cngb1	cyclic nucleotide gated channel beta 1	-0.679017946125774	6,6737E+14	-2,28205E+14	0.0597261936002923	0.200168009250211	-3,88402E+14	6,27154E+14	6,19067E+14	6,79797E+14	7,02228E+14	6,80911E+12	6,95062E+14
TC1100000907.mm.2	Trappc1	trafficking protein particle complex 1	-0.970762919074151	8,89802E+14	-2,2819E+14	0.059738856219098	0.200168009250211	-3,88421E+14	8,33591E+14	8,38464E+14	9,7043E+14	8,95778E+14	9,42293E+14	8,58257E+14
TC0900001791.mm.2	Mtnr1b	melatonin receptor 1B	-0.717595990792901	5,66973E+14	-2,2815E+14	0.0597730629137975	0.200168009250211	-3,88472E+14	5,17307E+14	5,29255E+14	5,74253E+14	6,15828E+14	5,90253E+14	5,74945E+14
TC1100000641.mm.2	Gm2a	GM2 ganglioside activator protein	0.785972659562477	8,40075E+14	2,28134E+14	0.0597867172762985	0.200168009250211	-3,88492E+14	8,92507E+14	9,02697E+14	8,4283E+14	7,9518E+14	8,2746E+14	7,79777E+14
TC1400002161.mm.2	Phf11a	PHD finger protein 11A	-0.750186568523235	4,98282E+14	-2,28123E+14	0.059795896572491	0.200168009250211	-3,88505E+14	4,26841E+14	4,43461E+14	4,82759E+14	5,3758E+14	5,48005E+14	5,51045E+13
TC1500000021.mm.2	Prkaa1	protein kinase, AMP-activated, alpha 1 catalytic subunit	1,31904E+14	7,11738E+14	2,28108E+14	0.0598086706023262	0.200168009250211	-3,88524E+14	7,99341E+14	7,82599E+13	7,47216E+14	5,70916E+14	6,69166E+14	7,0119E+14
TC1300001858.mm.2	Gcm2	glial cells missing homolog 2	-1,0242E+14	5,72526E+14	-2,28108E+14	0.0598090981436604	0.200168009250211	-3,88525E+14	4,68708E+14	5,02149E+14	5,30344E+14	6,45354E+14	6,28767E+14	6,59832E+13
TC0600001381.mm.2	1700063H04Rik	RIKEN cDNA 1700063H04 gene	-0.747156521333698	4,64044E+14	-2,28087E+14	0.0598267883272402	0.200168009250211	-3,88551E+14	4,17419E+14	4,12008E+14	4,68294E+14	5,10565E+14	4,69503E+14	5,06475E+14
TC0100003873.mm.2	A130010J15Rik	RIKEN cDNA A130010J15 gene	-0.709136320491172	5,98318E+14	-2,28045E+13	0.0598624452889678	0.200168009250211	-3,88604E+14	5,40183E+13	5,50739E+14	6,06599E+12	6,26151E+14	6,14917E+14	5,51319E+14
TC1000001981.mm.2	Kcp	kielin/chordin-like protein	-0.947562263669118	6,71642E+14	-2,28036E+14	0.0598697029398461	0.200168009250211	-3,88615E+14	6,0875E+14	6,14289E+14	6,67247E+14	7,45304E+14	6,60165E+14	7,34096E+14
TC1600001922.mm.2	Nrip1	nuclear receptor interacting protein 1	0.808802373755295	5,74225E+14	2,28021E+14	0.0598828654698245	0.200168009250211	-3,88634E+14	6,60809E+14	6,07726E+14	5,77098E+14	5,29676E+13	5,46169E+14	5,23872E+14
TC1200001259.mm.2	Dnm1l	dynamin 1-like	2,12365E+14	9,10827E+14	2,28021E+14	0.0598830247176568	0.200168009250211	-3,88634E+14	1,05915E+14	1,04533E+14	9,51614E+14	7,28144E+13	9,53065E+14	7,27651E+14
TC1700000428.mm.2	Syngap1	synaptic Ras GTPase activating protein 1 homolog (rat)	1,01865E+13	8,35757E+14	2,28017E+14	0.0598863235678886	0.200168009250211	-3,88639E+14	9,12176E+14	9,35839E+14	8,82205E+12	7,6208E+14	7,57219E+14	7,65022E+14
TC0200001365.mm.2	Rapsn	receptor-associated protein of the synapse	-1,0766E+14	6,00128E+14	-2,28014E+14	0.0598882432578662	0.200168009250211	-3,88642E+14	5,1904E+14	5,27363E+14	5,89272E+14	6,7245E+14	5,93721E+14	6,9892E+14
TC0300002292.mm.2	Prcc	papillary renal cell carcinoma (translocation-associated)	0.705829277256329	7,8579E+14	2,28011E+14	0.0598909847398466	0.200168009250211	-3,88646E+13	8,40118E+14	8,03493E+14	7,59249E+14	7,43197E+14	7,89531E+14	7,79152E+14
TC1400000005.mm.2	1700110I01Rik	RIKEN cDNA 1700110I01 gene	-0.851470459145895	4,93597E+14	-2,28011E+14	0.0598915077456981	0.200168009250211	-3,88647E+14	4,53474E+13	4,30685E+14	4,92792E+14	5,61661E+14	4,88818E+13	5,34155E+14
TC0900001076.mm.2	Mei4	meiotic double-stranded break formation protein 4	-0.716681084430991	5,23479E+14	-2,27986E+14	0.0599125672114524	0.200168009250211	-3,88678E+14	4,8898E+14	4,61532E+14	5,28719E+14	5,65129E+14	5,50015E+14	5,46498E+14
TC0900001414.mm.2	Impdh2	inosine monophosphate dehydrogenase 2	1,06667E+13	6,50491E+14	2,27973E+14	0.0599232161329339	0.200168009250211	-3,88694E+14	7,33944E+13	7,38097E+14	6,88005E+14	5,70702E+14	6,16764E+14	5,55432E+14
TC1100003222.mm.2	Serpinf1	serine (or cysteine) peptidase inhibitor, clade F, member 1	-0.696475657397234	8,26955E+13	-2,27965E+12	0.0599299704583417	0.200168009250211	-3,88704E+14	7,87414E+13	7,82435E+14	8,40075E+14	8,69069E+14	8,29949E+13	8,52789E+14
TC0800003024.mm.2	Hsdl1	hydroxysteroid dehydrogenase like 1	1,26929E+14	6,47879E+14	2,27954E+14	0.0599398401683281	0.200168009250211	-3,88718E+14	7,18395E+14	7,04887E+13	6,69884E+14	4,99541E+14	6,51129E+14	6,43438E+14
TC0200001180.mm.2	Cntn4	contactin 4	1,52712E+14	6,14151E+14	2,27938E+14	0.059952745531123	0.200168009250211	-3,88738E+14	6,99955E+14	7,12437E+14	5,98626E+14	5,08342E+14	6,81266E+12	4,84277E+14
TC1100002207.mm.2	Tns3	tensin 3	1,32234E+14	5,38921E+14	2,27918E+14	0.0599705723917682	0.200173258711023	-3,88764E+14	6,3658E+14	6,46272E+14	4,26039E+13	5,92345E+14	5,0123E+14	4,3106E+14
TC0500002091.mm.2	Wdr86	WD repeat domain 86	-0.881967565138265	6,30368E+14	-2,27898E+14	0.0599867786381078	0.200173258711023	-3,88788E+14	5,56103E+14	5,64157E+13	6,12283E+14	6,8437E+14	6,53564E+13	7,11728E+14
TC0400000396.mm.2	Gm13306/Ccl27b/Gm2506/LO C100861978/4933409K07Rik	predicted gene 13306/chemokine (C-C motif) ligand 27b/predicted gene 2506/c-C motif chemokine 27-like/RIKEN cDNA 4933409K07 gene	1,14285E+14	9,75855E+14	2,27887E+14	0.0599964580644315	0.200173258711023	-3,88802E+12	1,04224E+14	1,04096E+14	9,89485E+14	8,65144E+14	9,98229E+14	9,19071E+14
TC1300001099.mm.2	Gfm2	G elongation factor, mitochondrial 2	1,58235E+14	6,03745E+14	2,27867E+14	0.0600137095120532	0.200173258711023	-3,88828E+14	6,86782E+14	6,95138E+13	6,17518E+14	4,47933E+14	6,62672E+14	5,12429E+14
TC1400002081.mm.2	Jph4	junctophilin 4	-0.666855071440507	8,13385E+14	-2,27864E+13	0.0600159506505898	0.200173258711023	-3,88831E+14	7,68609E+14	7,74826E+14	8,42863E+14	8,33943E+14	8,2168E+14	8,3839E+14
TC0900002392.mm.2	Tbc1d21	TBC1 domain family, member 21	-1,14361E+14	4,88343E+14	-2,27849E+14	0.0600292560194733	0.200176200795069	-3,88851E+14	4,64003E+14	3,90573E+14	4,93337E+14	5,89961E+14	4,54098E+14	5,38088E+14
TC0900002406.mm.2	Arih1	ariadne RBR E3 ubiquitin protein ligase 1	1,44469E+14	8,40748E+14	2,27796E+14	0.0600741307424614	0.200189866369938	-3,88917E+14	9,1839E+13	9,31122E+13	8,7795E+14	6,82624E+13	8,43647E+12	7,90751E+13
TC0600000229.mm.2	Calu	calumenin	1,86297E+14	6,74773E+14	2,27794E+14	0.0600759506718107	0.200189866369938	-3,88919E+13	7,65655E+14	7,93588E+14	7,28711E+14	4,57938E+14	6,69445E+13	6,333E+14
TC0300002481.mm.2	Adamtsl4	ADAMTS-like 4	-0.684787269414844	8,0849E+14	-2,2777E+14	0.0600961553472532	0.200189866369938	-3,88949E+14	7,70713E+14	7,55954E+14	8,25413E+14	8,38211E+14	8,17818E+14	8,42835E+14
TC1000001425.mm.2	Spag9	sperm associated antigen 9	1,41125E+14	7,95828E+14	2,27734E+14	0.0601267505791181	0.200189866369938	-3,88994E+13	8,8409E+14	8,81441E+14	8,14232E+13	6,6905E+14	8,29573E+14	6,96579E+14
TC0500000436.mm.2	Otop1	otopetrin 1	-0.922264894871831	5,00812E+13	-2,27717E+14	0.0601412709430886	0.200189866369938	-3,89016E+13	4,50309E+14	4,19444E+14	4,83748E+14	5,70458E+13	5,18055E+14	5,62858E+14
TC1100003638.mm.2	Tbkbp1	TBK1 binding protein 1	0.974160835864952	6,31179E+14	2,27714E+14	0.0601436087935189	0.200189866369938	-3,89019E+13	7,55353E+14	6,94399E+14	6,7244E+14	5,82481E+14	5,24528E+14	5,57874E+14
TC0X00000471.mm.2	Hprt	hypoxanthine guanine phosphoribosyl transferase	2,08635E+14	8,54895E+14	2,27713E+14	0.060144994768333	0.200189866369938	-3,89021E+13	9,86237E+14	9,63925E+14	8,91304E+14	6,41588E+13	9,16019E+14	7,30297E+14

TC0500001510.mm.2	Nipsnap2	nipsnap homolog 2	1,53118E+13	8,56669E+14	2,27694E+14	0.0601608326435211	0.200189866369938	-3,89045E+14	9,44639E+14	9,37729E+14	8,91128E+14	6,85005E+14	8,76146E+14	8,05369E+12
TC0600002127.mm.2	Braf	Braf transforming gene	1,65915E+14	7,79584E+14	2,27692E+14	0.060162792257435	0.200189866369938	-3,89047E+14	9,22076E+14	9,06667E+14	7,98719E+14	6,98195E+14	7,84701E+13	5,67145E+14
TC0200002329.mm.2	Bpifb3	BPI fold containing family B, member 3	-0.664027947544913	5,54919E+14	-2,2769E+14	0.0601640440406056	0.200189866369938	-3,89049E+14	5,08224E+13	5,17477E+14	5,76637E+14	5,81869E+14	5,65446E+14	5,79865E+14
TC0900002312.mm.2	Elmod1	ELMO/CED-12 domain containing 1	1,94521E+13	9,94973E+14	2,27676E+12	0.0601760551231349	0.200189866369938	-3,89067E+13	1,10098E+14	1,10929E+14	1,05005E+14	7,71175E+14	1,006E+14	9,32349E+13
TC1700000612.mm.2	Col11a2	collagen, type XI, alpha 2	-0.731053367769104	8,08553E+14	-2,27669E+14	0.0601824435360256	0.200189866369938	-3,89076E+13	7,49206E+14	7,60011E+14	8,13045E+14	8,42383E+14	8,2293E+14	8,63743E+14
TC0500001936.mm.2	Crot	carnitine O-octanoyltransferase	1,46057E+14	5,6263E+14	2,27624E+13	0.0602206373308066	0.200207122355383	-3,89133E+12	6,74377E+14	6,83737E+14	6,28781E+14	4,37218E+14	5,1309E+14	4,38579E+14
TC0100003838.mm.2	G0s2	G0/G1 switch gene 2	-0.982295649612666	7,4237E+14	-2,27623E+14	0.0602216910959584	0.200207122355383	-3,89134E+14	6,813E+14	6,85691E+14	7,27969E+14	8,3548E+14	7,40936E+14	7,82842E+14
TC1200001344.mm.2	1110002L01Rik	RIKEN cDNA 1110002L01 gene	0.674878785113644	5,44063E+14	2,27608E+14	0.0602345250862796	0.200207122355383	-3,89153E+14	6,03192E+14	5,86953E+14	5,17305E+14	5,37865E+13	5,08654E+14	5,10411E+14
TC0500002819.mm.2	A930011G23Rik	RIKEN cDNA A930011G23 gene	-0.852502831024795	8,39663E+14	-2,27605E+14	0.0602373319170172	0.200207122355383	-3,89157E+14	7,81098E+14	7,70983E+13	8,28814E+14	8,93767E+13	8,53801E+14	9,09518E+14
TC0100002628.mm.2	Gm2102	predicted gene 2102	-0.734545256642268	5,18806E+14	-2,27545E+14	0.0602885414911358	0.200258070883091	-3,89232E+14	4,64623E+14	4,63329E+14	5,14233E+14	5,60629E+14	5,42457E+14	5,67567E+14
TC0300002709.mm.2	Kcnc4	potassium voltage gated channel, Shaw-related subfamily, member 4	0.929222236839145	7,05491E+14	2,275E+14	0.0603271763773902	0.200258070883091	-3,89289E+14	7,67277E+13	7,47039E+14	6,84652E+13	6,43819E+14	7,41881E+14	6,48276E+14
TC1100003988.mm.2	Gm11696	predicted gene 11696	-1,11083E+14	6,421E+14	-2,27499E+14	0.0603280083838411	0.200258070883091	-3,8929E+14	5,70519E+14	5,79014E+14	6,42402E+14	7,29297E+14	6,09954E+14	7,21413E+14
TC1100004286.mm.2	Drg1	developmentally regulated GTP binding protein 1	1,36849E+14	6,29739E+14	2,27497E+14	0.0603293786020112	0.200258070883091	-3,89292E+14	7,23651E+14	6,99881E+14	6,22137E+14	5,27697E+14	6,83832E+14	5,21236E+14
TC0800002848.mm.2	Ddx28	DEAD (Asp-Glu-Ala-Asp) box polypeptide 28	0.760423643358845	6,24716E+14	2,27495E+13	0.0603313743131378	0.200258070883091	-3,89295E+14	6,88904E+14	6,6446E+14	5,89913E+14	6,11366E+14	5,70872E+14	6,22779E+14
TC0100002288.mm.2	Slc39a10	solute carrier family 39 (zinc transporter), member 10	1,5107E+14	7,92983E+14	2,27484E+14	0.0603404779257972	0.200258070883091	-3,89309E+11	8,82757E+14	9,22748E+14	8,26434E+14	6,76931E+14	7,98932E+13	6,50096E+14
TC0700004183.mm.2	Umod	uromodulin	-1,01888E+14	4,95436E+14	-2,27482E+14	0.0603424206386084	0.200258070883091	-3,89312E+14	4,34927E+14	4,07425E+14	4,82427E+14	5,63701E+14	4,97848E+14	5,86287E+13
TC0200003201.mm.2	Abo	ABO blood group (transferase A, alpha 1-3-N-acetylgalactosaminyltransferase, transferase B, alpha 1-3-galactosyltransferase)	-0.9655685873991	4,58518E+14	-2,2747E+14	0.0603520878922788	0.200258070883091	-3,89326E+14	3,85644E+14	4,15403E+14	4,61651E+14	5,3251E+14	4,37176E+14	5,18722E+14
TC1800000084.mm.2	Mib1	mindbomb E3 ubiquitin protein ligase 1	1,00459E+13	8,99899E+12	2,27454E+14	0.0603664711243857	0.200264556151375	-3,89347E+13	9,50599E+14	9,66183E+14	9,07503E+14	8,08361E+14	9,16534E+14	8,5021E+14
TC0X00003097.mm.2	Kcne1l	potassium voltage-gated channel, Isk-related family, member 1-like, pseudogene	1,11278E+14	5,49439E+14	2,27394E+13	0.0604179612876724	0.200394114909071	-3,89422E+14	6,15452E+12	5,84759E+14	5,4285E+14	4,34807E+14	5,1589E+14	6,02875E+14
TC0200000850.mm.2	Galnt13	polypeptide N-acetylgalactosaminyltransferase 13	2,01769E+14	7,35544E+14	2,27323E+13	0.0604784233246227	0.200553372099124	-3,89511E+14	8,75846E+14	8,6558E+14	7,7321E+14	5,64678E+14	7,75505E+14	5,58447E+14
TC0200002291.mm.2	Defb36	defensin beta 36	-1,02432E+14	7,72441E+14	-2,2727E+14	0.060523780862404	0.200662485809859	-3,89577E+14	7,21353E+14	7,27518E+14	7,75925E+14	8,77809E+14	7,30351E+14	8,01693E+13
TC0800001362.mm.2	Wwox	WW domain-containing oxidoreductase	-0.702949940878027	8,10576E+14	-2,27225E+14	0.060562328822472	0.200739158910474	-3,89634E+14	7,62506E+14	7,73551E+14	8,26701E+14	8,49946E+14	8,09338E+14	8,41413E+14
TC1900001005.mm.2	Ppp2r5b	protein phosphatase 2, regulatory subunit B', beta	0.800864317899415	7,99555E+14	2,27209E+14	0.0605761316050668	0.200739158910474	-3,89654E+14	8,6929E+14	8,31492E+14	8,01631E+14	7,38979E+14	7,79321E+14	7,76616E+14
TC1700000709.mm.2	Dhx16	DEAH (Asp-Glu-Ala-His) box polypeptide 16	0.718739039253009	6,58861E+14	2,272E+14	0.0605842816223941	0.200739158910474	-3,89666E+14	6,76934E+14	7,00763E+14	6,20494E+14	6,13455E+14	6,50539E+14	6,90983E+14
TC0700001509.mm.2	Olfr631	olfactory receptor 631	-0.748723569852944	4,89659E+14	-2,27102E+14	0.0606688155122417	0.200959926372735	-3,89789E+14	4,47867E+14	4,11092E+14	4,82889E+14	5,25814E+14	5,28691E+14	5,41601E+14
TC0400003992.mm.2	Zfp979	zinc finger protein 979	0.804463218438503	5,32974E+14	2,27093E+14	0.0606758543910727	0.200959926372735	-3,898E+14	6,00718E+14	5,90847E+14	5,34819E+14	4,95853E+14	5,19051E+14	4,56554E+14
TC1700002420.mm.2	Ankrd12	ankyrin repeat domain 12	1,91016E+14	9,84271E+14	2,27076E+14	0.0606906151653331	0.200967505581384	-3,89821E+14	1,11531E+14	1,09423E+14	1,02655E+14	8,00952E+14	1,01951E+14	8,49069E+13
TC0600001015.mm.2	Efcc1	EF hand and coiled-coil domain containing 1	-0.955377598956483	7,38225E+14	-2,27049E+14	0.0607138220980194	0.201003044065212	-3,89855E+14	6,6797E+14	6,7634E+14	7,46942E+14	7,88444E+14	7,23932E+14	8,25723E+14
TC0600002159.mm.2	Trpv6	transient receptor potential cation channel, subfamily V, member 6	-1,25181E+14	5,4883E+13	-2,26994E+14	0.0607613373890742	0.201044339465494	-3,89924E+14	4,46192E+14	4,76623E+14	5,16819E+14	6,56357E+14	5,53495E+14	6,43492E+14
TC0600002170.mm.2	Olfr458	olfactory receptor 458	-1,51312E+14	5,2652E+14	-2,26991E+14	0.0607638147569613	0.201044339465494	-3,89928E+14	4,16839E+14	4,267E+14	5,13252E+14	6,3291E+14	4,95104E+14	6,74315E+14
TC0200004521.mm.2	Myef2/Gm9833	myelin basic protein expression factor 2, repressor/myelin basic protein expression factor 2, repressor pseudogene	2,25624E+14	6,95098E+14	2,26985E+14	0.0607690892653715	0.201044339465494	-3,89936E+14	8,28657E+14	8,38275E+14	7,41787E+14	4,73897E+14	7,4958E+14	5,3839E+14
TC0600003387.mm.2	C2cd5	C2 calcium-dependent domain containing 5	0.929556071162053	7,01993E+14	2,26977E+14	0.0607762041541873	0.201044339465494	-3,89946E+14	7,25513E+14	7,66024E+13	7,0045E+14	6,05176E+14	7,15064E+14	6,99734E+14
TC0100000430.mm.2	Aox4	aldehyde oxidase 4	-1,08253E+14	5,23497E+14	-2,26958E+14	0.0607920570068345	0.201055503807702	-3,89969E+14	4,59203E+14	4,66423E+14	5,11357E+14	6,30776E+14	5,03713E+14	5,69511E+14
TC1200000334.mm.2	Agmo	alkylglycerol monooxygenase	1,1309E+14	5,80855E+14	2,26921E+13	0.0608246808714004	0.201122118383106	-3,90017E+14	6,7879E+14	6,51521E+14	5,65343E+14	5,38787E+14	5,95838E+14	4,54851E+14
TC0600003532.mm.2	Arpc4	actin related protein 2/3 complex, subunit 4	1,07442E+13	8,80026E+14	2,26867E+14	0.0608710750773992	0.201192804116211	-3,90084E+14	9,53297E+14	9,50585E+14	8,99082E+14	7,89914E+14	8,83336E+14	8,03939E+14
TC0500000201.mm.2	Klhl7	kelch-like 7	2,49066E+14	6,84984E+14	2,26851E+14	0.0608844278156031	0.201192804116211	-3,90104E+14	8,18742E+14	8,06113E+14	7,39908E+14	3,86814E+14	7,49135E+14	6,09194E+14
TC0200002668.mm.2	Cass4	Cas scaffolding protein family member 4	-1,22551E+14	5,91755E+14	-2,26835E+14	0.0608988128610578	0.201192804116211	-3,90125E+14	5,18521E+14	5,40955E+14	5,95492E+14	7,09087E+14	5,35107E+14	6,51365E+14
TC0X00002453.mm.2	G6pdx	glucose-6-phosphate dehydrogenase X-linked	0.912722072192639	7,753E+14	2,26829E+14	0.0609042072195246	0.201192804116211	-3,90132E+14	8,54183E+13	8,28416E+14	7,31595E+14	7,6846E+14	6,89342E+14	7,79804E+14
TC0600000409.mm.2	Wee2	WEE1 homolog 2 (S. pombe)	-1,24163E+13	7,02892E+14	-2,26809E+14	0.0609210174020989	0.201192804116211	-3,90157E+13	6,12061E+14	6,30563E+14	6,68953E+14	8,21997E+14	7,09637E+14	7,74139E+14
TC0700004049.mm.2	Olfr485	olfactory receptor 485	-1,22417E+13	5,18071E+14	-2,26809E+14	0.0609215178051301	0.201192804116211	-3,90158E+14	4,61597E+14	3,95358E+14	5,00673E+14	6,01116E+14	5,19226E+14	6,30459E+14
TC0900000509.mm.2	Slc37a4	solute carrier family 37 (glucose-6-phosphate transporter), member 4	1,07495E+14	5,42614E+14	2,26782E+14	0.0609447935580403	0.201192804116211	-3,90191E+14	6,30735E+14	5,91201E+14	5,6756E+14	4,39386E+14	5,21878E+14	5,04925E+14
TC0300002373.mm.2	Chtop	chromatin target of PRMT1	0.728682592619992	7,9218E+14	2,26775E+14	0.0609504269269398	0.201192804116211	-3,902E+14	8,32647E+14	8,42952E+14	7,75623E+14	7,54239E+14	7,50908E+14	7,96711E+14
TC0200004264.mm.2	Rcn1	reticulocalbin 1	0.944008677249087	7,33777E+14	2,26766E+14	0.0609584354059048	0.201192804116211	-3,90211E+14	8,06548E+14	7,87008E+14	7,4947E+14	6,55284E+14	6,77591E+14	7,26763E+13
TC0600000410.mm.2	Ssbp1	single-stranded DNA binding protein 1	1,21354E+14	7,4587E+14	2,26657E+14	0.0610525051300666	0.201462014163581	-3,90348E+14	8,27649E+14	8,12226E+14	7,53306E+14	6,43861E+14	7,78323E+14	6,59855E+14
TC0600002058.mm.2	Slc23a4	solute carrier family 23 member 4	-1,07578E+14	4,85682E+14	-2,26606E+14	0.0610971840397636	0.201537291904667	-3,90413E+14	4,27685E+14	4,20299E+14	4,73109E+12	5,90032E+14	4,67414E+13	5,35553E+14
TC1300001383.mm.2	Akr1e1	aldo-keto reductase family 1, member E1	1,14499E+14	6,15292E+14	2,26592E+14	0.0611092260240154	0.201537291904667	-3,9043E+14	6,8276E+14	6,82321E+14	6,16909E+14	5,19174E+13	6,51573E+14	5,39013E+14
TC1600001191.mm.2	Emp2	epithelial membrane protein 2	0.771499314530865	7,14619E+13	2,26588E+13	0.0611128410752933	0.201537291904667	-3,90435E+14	7,49698E+14	7,74582E+14	7,15157E+13	6,54823E+14	6,996E+14	6,93853E+14
TSUnmapped00000019.mm.2	Ccdc169	coiled-coil domain containing 169	-1,33781E+14	6,56216E+14	-2,26556E+14	0.0611403506469039	0.201586754649798	-3,90475E+14	5,25126E+14	5,74252E+14	6,0407E+14	7,62869E+13	6,94876E+14	7,76104E+14
TC0700000526.mm.2	Yif1b	Yip1 interacting factor homolog B (S. cerevisiae)	0.908979655179236	7,83731E+14	2,26509E+14	0.0611811820051985	0.201680111630475	-3,90534E+14	8,58383E+14	8,43077E+14	8,09302E+14	7,10362E+14	7,36422E+14	7,44839E+14
TC1500001018.mm.2	Aqp5	aquaporin 5	-0.918193771466854	6,35664E+14	-2,26452E+14	0.0612303640713477	0.201772881712306	-3,90606E+14	5,80567E+14	5,43511E+14	6,08732E+14	6,98985E+14	6,75221E+14	7,0697E+14
TC1500000019.mm.2	Rpl37	ribosomal protein L37	0.771119773333627	1,02968E+14	2,26437E+14	0.0612438176829554	0.201772881712306	-3,90625E+14	1,08765E+14	1,07496E+14	1,02725E+14	9,81139E+14	1,02597E+13	9,81137E+14
TC0100001138.mm.2	Ubxn4	UBX domain protein 4	1,70401E+14	7,00504E+14	2,26433E+14	0.0612468916064598	0.201772881712306	-3,90629E+14	8,04591E+14	7,87491E+14	7,43639E+14	5,07642E+14	7,19301E+13	6,40359E+14
TC1500001863.mm.2	Sox10	SRY (sex determining region Y)-box 10	0.737067769668695	6,53956E+14	2,26381E+14	0.0612925669535267	0.201778854796428	-3,90695E+14	6,99339E+14	7,01815E+14	6,47645E+14	6,06095E+14	6,52336E+14	6,16508E+14
TC1100001109.mm.2	Blmh	bleomycin hydrolase	0.85054553574523	8,32504E+14	2,26366E+14	0.0613051728351134	0.201778854796428	-3,90714E+14	8,72053E+14	8,83871E+14	8,32769E+14	7,53046E+14	8,42802E+14	8,10485E+14
TC0900000641.mm.2	4930550C14Rik	RIKEN cDNA 4930550C14 gene	-0.701415694185955	6,1717E+14	-2,26351E+14	0.0613188092539826	0.201778854796428	-3,90733E+14	5,75512E+14	6,09562E+14	6,63142E+14	6,62216E+14	5,84336E+14	6,08254E+14
TC0700002131.mm.2	Oscar	osteoclast associated receptor	-0.839287450892066	5,64754E+14	-2,26344E+14	0.0613240918748707	0.201778854796428	-3,90741E+14	4,94135E+14	5,32928E+14	5,64913E+13	6,30008E+13	5,6508E+13	6,0146E+13
TC0300000880.mm.2	Selenbp1	selenium binding protein 1	-1,03587E+14	5,54739E+14	-2,26342E+13	0.0613259434357957	0.201778854796428	-3,90744E+14	4,97905E+14	5,00221E+14	5,38901E+14	6,66399E+14	5,66353E+13	5,58656E+14
TC0500002092.mm.2	Crygn	crystallin, gamma N	-1,06928E+14	7,25533E+13	-2,26336E+14	0.0613316427253833	0.201778854796428	-3,90752E+13	6,68512E+14	6,50457E+14	7,00318E+14	8,32507E+14	7,42228E+14	7,59177E+14
TC0100002993.mm.2	Actr3	ARP3 actin-related protein 3	1,40311E+14	9,1905E+14	2,26316E+14	0.0613485209460787	0.201778854796428	-3,90776E+14	1,00932E+14	1,00789E+14	9,39519E+14	7,97067E+12	9,48303E+14	8,12204E+14
TC0700002754.mm.2	Lrfn3	leucine rich repeat and fibronectin type III domain containing 3	1,48918E+14	6,09494E+14	2,26316E+13	0.0613488865914293	0.201778854796428	-3,90777E+14	6,50675E+14	7,05079E+14	6,24141E+14	4,33777E+14	5,83337E+14	6,59957E+14
TC1100002334.mm.2	Ehbp1	EH domain binding protein 1	1,36333E+14	6,97203E+14	2,26269E+14	0.0613901565844331	0.201849298798665	-3,90836E+14	7,97546E+14	8,0148E+14	7,10087E+14	6,16274E+14	7,11463E+14	5,46365E+14
TC1400001455.mm.2	Fezf2	Fez family zinc finger 2	0.902187268836578	7,68E+14	2,26252E+14	0.0614049362865423	0.201849298798665	-3,90858E+14	7,90813E+14	8,36194E+14	7,51481E+14	6,95088E+14	8,01098E+14	7,33329E+14
TC0200002415.mm.2	Ctnnbl1	catenin, beta like 1	0.702451903091207	7,97082E+14	2,26248E+14	0.0614078857264976	0.201849298798665	-3,90862E+14	8,4241E+14	8,53082E+14	7,97864E+14	7,57138E+14	7,64626E+14	7,67371E+14
TC1300001509.mm.2	Yae1d1	Yae1 domain containing 1	0.691003200190831	7,3767E+14	2,26222E+14	0.0614304073132696	0.201858983545083	-3,90894E+14	7,93006E+14	7,75509E+13	7,19106E+14	7,11208E+14	6,97678E+14	7,29512E+14
TC0200003360.mm.2	Rab14	RAB14, member RAS oncogene family	1,59922E+14	8,63839E+14	2,26216E+14	0.0614358875010915	0.201858983545083	-3,90902E+14	9,54876E+14	9,60222E+14	9,03593E+14	6,9166E+14	8,83019E+12	7,89664E+14
TC1900000129.mm.2	Macrod1	MACRO domain containing 1	-1,01675E+14	6,5196E+14	-2,26139E+14	0.0615031435792539	0.201976064742059	-3,90999E+14	5,76364E+14	5,88756E+12	6,22863E+14	7,45606E+14	6,82724E+14	6,95448E+14
TC1200001058.mm.2	Tcl1b4	T cell leukemia/lymphoma 1B, 4	-0.735297390594888	5,51304E+14	-2,26122E+14	0.0615182074187505	0.201976064742059	-3,91021E+13	4,63426E+14	5,10861E+14	5,4803E+14	5,73317E+13	6,0129E+14	6,10896E+14
TC1900000169.mm.2	Tut1	terminal uridylyl transferase 1, U6 snRNA-specific	0.76743864603139	5,69379E+14	2,26119E+14	0.0615206146519919	0.201976064742059	-3,91024E+14	6,2211E+14	5,90387E+14	5,01337E+14	5,57673E+14	5,70323E+14	5,74444E+14
TC0800002877.mm.2	Nqo1	NAD(P)H dehydrogenase, quinone 1	0.688569945495767	5,0194E+14	2,26118E+14	0.0615216611279107	0.201976064742059	-3,91026E+14	5,24847E+14	5,40094E+12	4,47509E+13	4,79718E+14	5,06624E+14	5,12845E+14
TC1800000788.mm.2	Mbd1	methyl-CpG binding domain protein 1	0.677086805421707	7,70898E+14	2,26087E+14	0.0615483864285788	0.202022642192644	-3,91064E+14	8,19852E+14	8,26877E+14	7,58729E+14	7,52583E+13	7,48079E+14	7,19267E+14
TC0500002288.mm.2	Zfp518b	zinc finger protein 518B	0.675769962956655	6,41593E+14	2,2607E+14	0.0615634249718559	0.202023157732351	-3,91086E+14	6,93758E+14	6,7243E+14	6,10033E+13	6,21001E+14	6,17523E+14	6,34814E+14
TC0200004901.mm.2	Defb22	defensin beta 22	-0.687370776760104	4,85211E+14	-2,26058E+14	0.0615736192902361	0.202023157732351	-3,91101E+14	4,44086E+14	4,29701E+14	4,97324E+14	5,13937E+14	4,99053E+14	5,27168E+14
TC1700000176.mm.2	Ermard	ER membrane associated RNA degradation	1,11659E+14	5,47005E+14	2,26021E+14	0.0616063454967611	0.20206475359632	-3,91148E+14	6,2992E+14	6,08126E+14	5,77477E+14	4,37252E+14	5,27209E+14	5,02045E+14
TC0700002966.mm.2	Mybpc2	myosin binding protein C, fast-type	-0.986603589051065	4,97535E+14	-2,25992E+14	0.0616315396434907	0.20206475359632	-3,91184E+14	4,72027E+14	3,99604E+13	4,94839E+14	5,74113E+14	4,97998E+14	5,46628E+14
TC1700000722.mm.2	2410017I17Rik	RIKEN cDNA 2410017I17 gene	-0.852318106293183	5,86533E+14	-2,25982E+14	0.0616405115341644	0.20206475359632	-3,91197E+14	5,11786E+14	5,29828E+14	5,68475E+14	6,43602E+14	6,12091E+14	6,5342E+14
TC1800001200.mm.2	Hars	histidyl-tRNA synthetase	1,92854E+14	7,70229E+14	2,25966E+14	0.0616545842572974	0.20206475359632	-3,91217E+14	8,79337E+14	8,52225E+14	7,98798E+14	5,47057E+14	8,40819E+14	7,03137E+14
TC1100001681.mm.2	Nags	N-acetylglutamate synthase	-0.974936693300577	5,41064E+14	-2,2596E+14	0.0616596140876608	0.20206475359632	-3,91224E+14	4,3159E+14	5,04024E+14	5,24916E+14	6,05686E+14	5,70371E+14	6,09798E+14
TC1400001599.mm.2	Zcchc24	zinc finger, CCHC domain containing 24	0.712222980745687	7,18034E+14	2,25956E+14	0.0616628731491873	0.20206475359632	-3,91229E+14	7,57248E+14	7,67012E+14	7,01407E+14	6,80409E+14	7,21213E+14	6,80913E+14
TC0600000775.mm.2	Gng12	guanine nucleotide binding protein (G protein), gamma 12	0.893835449128934	8,46916E+14	2,25943E+14	0.0616740804435365	0.20206475359632	-3,91245E+13	8,84145E+13	9,15122E+14	8,48634E+14	7,71866E+14	8,5664E+14	8,05091E+14
TC1700001521.mm.2	Has1	hyaluronan synthase 1	-0.924081626174938	5,17761E+14	-2,25922E+14	0.0616932409009469	0.202067896888206	-3,91272E+14	4,69998E+14	4,3657E+14	5,00382E+14	5,91002E+14	5,34494E+14	5,7412E+14
TC0800001507.mm.2	Spg7	SPG7, paraplegin matrix AAA peptidase subunit	0.896827940325303	5,53176E+14	2,25908E+14	0.0617052520186377	0.202067896888206	-3,9129E+14	5,93143E+14	6,68158E+14	5,61581E+14	5,20355E+14	5,12018E+14	4,63801E+14
TC1400000804.mm.2	Atp12a	ATPase, H+/K+ transporting, nongastric, alpha polypeptide	-0.760046547154594	5,27332E+14	-2,2589E+14	0.0617208511410819	0.202067896888206	-3,91312E+14	4,6891E+14	4,82479E+14	5,40413E+14	5,62985E+14	5,26102E+14	5,83101E+14
TC1600002172.mm.2	Ifnar2	interferon (alpha and beta) receptor 2	0.958025233492636	8,32676E+13	2,25883E+14	0.0617268230275274	0.202067896888206	-3,91321E+14	8,76057E+13	8,77977E+14	8,33718E+13	7,28711E+14	8,59078E+14	8,20514E+14
TC0800000017.mm.2	Stxbp2	syntaxin binding protein 2	0.845257207827283	6,49547E+14	2,25871E+14	0.0617377432123525	0.202067896888206	-3,91336E+14	6,75124E+14	7,13407E+14	5,78497E+14	6,40983E+14	6,22143E+13	6,67125E+14
TC0100000645.mm.2	Wnt10a	wingless-type MMTV integration site family, member 10A	-0.730366842169173	6,17024E+14	-2,25824E+14	0.0617784788862075	0.202080070283803	-3,91395E+14	5,6294E+14	5,65082E+14	6,19874E+14	6,54221E+14	6,29699E+14	6,70332E+14
TC1500000695.mm.2	Mgat3	mannoside acetylglucosaminyltransferase 3	1,07582E+14	6,41869E+14	2,25812E+14	0.0617890263226004	0.202080070283803	-3,9141E+14	7,26741E+14	7,08998E+14	6,58981E+14	5,61593E+14	6,44291E+13	5,50613E+14
TC0400001666.mm.2	Srsf10	serine/arginine-rich splicing factor 10	1,53575E+14	7,18373E+14	2,25812E+14	0.0617894875136887	0.202080070283803	-3,9141E+14	7,9171E+14	8,14847E+14	7,41124E+14	5,58283E+14	7,63178E+14	6,41094E+14
TC0200005404.mm.2	Cables2	CDK5 and Abl enzyme substrate 2	-0.715070863998998	6,58296E+14	-2,25798E+14	0.0618013398487271	0.202080070283803	-3,91427E+14	6,15705E+14	5,99557E+14	6,58699E+14	6,99577E+14	6,9746E+13	6,7878E+14
TC0700002873.mm.2	Uri1	URI1, prefoldin-like chaperone	1,98189E+14	6,85766E+14	2,25756E+14	0.0618382316267453	0.202080070283803	-3,9148E+14	8,27597E+14	8,25659E+14	7,32883E+14	5,23994E+14	7,08139E+14	4,96325E+14
TC1100003198.mm.2	Pafah1b1	platelet-activating factor acetylhydrolase, isoform 1b, subunit 1	1,39553E+14	8,85835E+14	2,25756E+14	0.0618386217769778	0.202080070283803	-3,91481E+13	9,71683E+14	9,70326E+14	9,19015E+14	7,43887E+14	8,99368E+14	8,1073E+14
TC1100003907.mm.2	March10	membrane-associated ring finger (C3HC4) 10	-1,14066E+14	6,2229E+14	-2,25743E+14	0.06185021478689	0.202080070283803	-3,91497E+14	5,92204E+11	5,29404E+14	6,0951E+14	7,40229E+13	6,13961E+14	6,48434E+14
TC1100000527.mm.2	Mapk9	mitogen-activated protein kinase 9	1,87605E+14	8,24953E+14	2,25736E+13	0.0618558473444727	0.202080070283803	-3,91506E+14	9,55235E+14	9,10972E+14	8,65665E+14	6,25333E+14	8,64257E+14	7,28257E+14
TC0300002408.mm.2	Lce1h	late cornified envelope 1H	-0.921911047917304	7,08464E+14	-2,25725E+14	0.0618659276223666	0.202080070283803	-3,9152E+14	6,57538E+14	6,52875E+14	7,21305E+14	7,7349E+14	6,77811E+14	7,67761E+13
TC0100003861.mm.2	Tcea1	transcription elongation factor A (SII) 1	1,1789E+14	8,12468E+14	2,25712E+14	0.0618772153003931	0.202080070283803	-3,91536E+14	8,92874E+14	9,11954E+14	8,36395E+14	7,32652E+14	8,0822E+14	6,92714E+13
TC0400000282.mm.2	Srsf12	serine/arginine-rich splicing factor 12	-0.804222552829136	7,22462E+14	-2,25708E+14	0.0618805485034421	0.202080070283803	-3,91541E+14	6,99857E+14	6,46121E+14	7,26917E+14	7,79905E+14	7,4294E+14	7,39034E+14
TC1400002515.mm.2	Diaph3	diaphanous related formin 3	-0.977811366225231	6,82061E+14	-2,25695E+12	0.0618919597126898	0.202080070283803	-3,91557E+14	6,00854E+14	6,33425E+14	6,59974E+14	7,69866E+14	7,04302E+14	7,23947E+14
TC1600001540.mm.2	E130310I04Rik	RIKEN cDNA E130310I04 gene	-0.726328485307988	7,68116E+14	-2,25646E+14	0.0619354882657536	0.202151174731394	-3,91619E+14	7,05785E+14	7,2744E+14	7,76473E+14	8,02017E+14	7,78575E+14	8,18409E+14
TC0700004228.mm.2	Ern2	endoplasmic reticulum (ER) to nucleus signalling 2	-0.923351167910551	5,9886E+13	-2,25628E+14	0.0619507056162895	0.202151174731394	-3,91641E+14	5,51509E+13	5,38719E+14	5,88329E+14	6,8657E+14	5,97381E+14	6,3065E+14
TC0600001450.mm.2	Mrpl51	mitochondrial ribosomal protein L51	0.944672842251752	7,89313E+14	2,25617E+13	0.0619607910729395	0.202151174731394	-3,91656E+13	8,33567E+13	8,44771E+14	7,99009E+14	6,90395E+14	7,85435E+14	7,82698E+14
TC0300002725.mm.2	Gstm1	glutathione S-transferase, mu 1	1,49121E+13	9,70004E+14	2,25609E+14	0.0619678130358549	0.202151174731394	-3,91666E+14	1,06814E+14	1,07212E+14	1,02377E+14	8,1825E+14	9,47678E+14	8,9007E+14
TC0700000275.mm.2	Pglyrp1	peptidoglycan recognition protein 1	-0.717692525272148	6,4424E+14	-2,25575E+13	0.0619975776674734	0.202151174731394	-3,91708E+14	5,86356E+14	5,74092E+14	6,38742E+14	6,65244E+14	6,80828E+14	7,20179E+14
TC0700001178.mm.2	Tm6sf1	transmembrane 6 superfamily member 1	1,3614E+14	6,29755E+14	2,2556E+14	0.0620111228471682	0.202151174731394	-3,91728E+14	7,02572E+14	6,79598E+14	6,34559E+14	4,75331E+14	6,94112E+14	5,92358E+14
TC0900003182.mm.2	Stt3b	STT3, subunit of the oligosaccharyltransferase complex, homolog B (S. cerevisiae)	1,69081E+14	7,45434E+14	2,25558E+14	0.0620124787328119	0.202151174731394	-3,91729E+14	8,40802E+14	8,76533E+14	7,59359E+14	6,19814E+13	7,89172E+13	5,86926E+14
TC1100002725.mm.2	Hspa4	heat shock protein 4	2,87235E+14	8,23555E+14	2,25556E+12	0.0620141039345424	0.202151174731394	-3,91732E+14	9,79186E+14	9,67037E+14	8,96838E+14	4,74916E+14	8,82008E+13	7,41343E+14
TC1500001846.mm.2	Elfn2	leucine rich repeat and fibronectin type III, extracellular 2	1,07974E+14	8,10359E+13	2,25539E+14	0.062029590983388	0.202160760419767	-3,91754E+14	8,82789E+14	8,88159E+14	8,35656E+14	7,19344E+14	8,04509E+14	7,31696E+14
TC0900002890.mm.2	Spsb4	splA/ryanodine receptor domain and SOCS box containing 4	-1,05227E+14	6,79302E+13	-2,25516E+14	0.062049057129439	0.202178590203064	-3,91782E+14	6,18106E+14	6,18559E+14	6,73724E+14	7,73395E+14	6,52857E+14	7,39168E+13
TC1000002814.mm.2	Acss3	acyl-CoA synthetase short-chain family member 3	-0.947287217155012	5,76447E+14	-2,25503E+14	0.0620612561916338	0.202178590203064	-3,91799E+14	4,92749E+14	5,5021E+14	5,98169E+14	6,34247E+14	5,48424E+14	6,3488E+14
TSUnmapped00000060.mm.2	Klra16	killer cell lectin-like receptor, subfamily A, member 16	-1,19989E+13	4,25887E+14	-2,2549E+14	0.0620727043837	0.202178590203064	-3,91815E+14	3,67141E+14	3,0919E+14	3,92096E+14	5,24213E+14	4,46031E+14	5,16651E+14
TC0300002263.mm.2	Rps3a1	ribosomal protein S3A1	1,16529E+14	9,01934E+14	2,25468E+14	0.0620914397736269	0.202198740717957	-3,91842E+14	1,00203E+14	9,48363E+14	9,21402E+13	7,95932E+14	9,07694E+14	8,36183E+14

TC1100004152.mm.2	Cyth1	cytohesin 1	1,06706E+14	6,62081E+14	2,25432E+14	0.0621239106752729	0.202263603295751	-3,91888E+14	7,315E+14	7,27561E+14	6,88801E+14	5,56848E+14	6,47774E+14	6,20004E+14
TC0800000286.mm.2	Plat	plasminogen activator, tissue	0.701832501619868	7,02442E+13	2,25371E+13	0.0621773585723865	0.202274336755427	-3,91965E+14	7,64512E+14	7,37101E+14	6,68182E+14	6,93064E+14	6,869E+14	6,64893E+13
TC1500002189.mm.2	Wnt10b	wingless-type MMTV integration site family, member 10B	-1,08603E+14	6,05663E+14	-2,25369E+14	0.0621794367620562	0.202274336755427	-3,91967E+14	5,35809E+14	5,10152E+14	5,81622E+14	6,81545E+14	6,16042E+14	7,08807E+14
TC1000000299.mm.2	Tspyl4	TSPY-like 4	1,7732E+14	9,27238E+13	2,25346E+14	0.062199038981627	0.202274336755427	-3,91995E+14	1,0669E+14	1,02464E+14	9,72699E+14	7,64198E+14	9,44566E+14	7,90429E+14
TC0500002124.mm.2	Gm10220	predicted gene 10220	-0.712149254388673	5,16328E+14	-2,25322E+14	0.0622206765548497	0.202274336755427	-3,92026E+14	4,70823E+14	4,42081E+14	5,15912E+14	5,39423E+14	5,50279E+14	5,79448E+14
TC1200000389.mm.2	Nrcam	neuronal cell adhesion molecule	1,71102E+14	8,19739E+14	2,25312E+14	0.0622290069112467	0.202274336755427	-3,92038E+14	9,215E+14	9,32903E+13	8,68059E+13	6,44139E+14	8,33541E+14	7,18293E+14
TC1600000571.mm.2	Lsamp	limbic system-associated membrane protein	0.794781525445988	8,63639E+14	2,25311E+13	0.0622306439439786	0.202274336755427	-3,9204E+13	9,46773E+14	9,04562E+13	8,75648E+14	8,16731E+13	8,24821E+14	8,13299E+13
TC1100002714.mm.2	Olfr1371	olfactory receptor 1371	-0.859197771477421	6,15184E+13	-2,25309E+14	0.0622319548447703	0.202274336755427	-3,92042E+14	5,79596E+14	5,32408E+14	6,18656E+14	6,65187E+13	6,17241E+14	6,78015E+13
TC0700000855.mm.2	Fut1	fucosyltransferase 1	-1,20789E+14	6,15771E+13	-2,253E+14	0.0622401133973634	0.202274336755427	-3,92054E+14	5,72087E+14	5,41559E+14	6,27115E+14	7,2811E+14	5,51198E+14	6,74559E+14
TC1800001752.mm.2	Tmed7	transmembrane p24 trafficking protein 7	1,29989E+14	9,29764E+14	2,253E+12	0.0622401887689074	0.202274336755427	-3,92054E+14	9,99433E+14	1,00719E+14	9,56693E+14	7,89953E+14	9,45569E+14	8,79745E+14
TC0X00002050.mm.2	C330007P06Rik	RIKEN cDNA C330007P06 gene	0.794758719747152	7,44343E+14	2,25183E+14	0.0623439232634513	0.202570606078643	-3,92201E+14	8,03294E+14	7,74132E+14	7,40443E+13	6,78032E+14	7,47899E+14	7,22257E+14
TC0200001455.mm.2	Alx4	aristaless-like homeobox 4	-1,1383E+14	5,20895E+13	-2,25099E+14	0.0624175531924573	0.202768958586706	-3,92306E+14	4,42982E+13	4,58748E+14	5,08672E+14	6,20717E+14	4,98474E+13	5,9578E+14
TC1100000464.mm.2	Adam19	a disintegrin and metallopeptidase domain 19 (meltrin beta)	-0.877329455279702	6,61263E+14	-2,25066E+14	0.0624470296470954	0.202823823564533	-3,92348E+14	6,14372E+14	6,30597E+14	7,2012E+14	7,00315E+14	6,05618E+14	6,96556E+14
TC1500000693.mm.2	Syngr1	synaptogyrin 1	0.807277949005485	9,24031E+14	2,25031E+14	0.0624777741839316	0.202871758721386	-3,92391E+14	9,92244E+14	9,55284E+14	9,25909E+14	8,60163E+14	9,13127E+14	8,97462E+14
TC0600003208.mm.2	A2ml1	alpha-2-macroglobulin like 1	0.970637641392988	6,48957E+14	2,25021E+14	0.062486969436743	0.202871758721386	-3,92404E+14	7,58004E+14	7,06763E+14	6,87007E+14	5,83632E+14	5,79654E+14	5,78679E+14
TC1100000604.mm.2	Gdf9	growth differentiation factor 9	-0.911709047192496	6,13324E+14	-2,2499E+14	0.0625148225902008	0.202912879439162	-3,92444E+14	5,41652E+14	5,19766E+14	5,74621E+14	6,69139E+14	6,70887E+14	7,03878E+14
TC0700002792.mm.2	Gramd1a	GRAM domain containing 1A	0.848045159333426	6,75838E+14	2,24978E+14	0.0625248213501793	0.202912879439162	-3,92458E+14	7,35987E+14	7,36051E+12	6,8851E+14	6,13919E+14	6,63153E+14	6,17406E+14
TC0400000438.mm.2	Car9	carbonic anhydrase 9	-0.831078373156719	5,47949E+14	-2,24951E+14	0.0625494448518607	0.202951913994771	-3,92493E+13	4,92436E+14	4,98495E+14	5,38521E+14	6,18626E+14	5,58815E+14	5,80799E+14
TC0700000758.mm.2	Lim2	lens intrinsic membrane protein 2	-1,05025E+14	5,73732E+14	-2,24905E+14	0.0625903089398108	0.203008111563106	-3,92551E+14	5,11944E+14	5,1915E+13	5,58957E+14	6,82186E+14	5,61674E+14	6,0848E+14
TC0X00003426.mm.2	C1galt1c1	C1GALT1-specific chaperone 1	0.671702420496682	7,02789E+14	2,24876E+14	0.0626160021077295	0.203008111563106	-3,92587E+13	7,60068E+14	7,3192E+14	6,78793E+14	6,78855E+14	6,79521E+14	6,87575E+14
TC0200001340.mm.2	Olfr1261	olfactory receptor 1261	-1,36055E+14	4,71051E+14	-2,24869E+14	0.0626219890020312	0.203008111563106	-3,92595E+14	4,15553E+14	4,0211E+14	4,82412E+14	6,07362E+14	3,87546E+14	5,31321E+14
TC1400000786.mm.2	Rec8	REC8 meiotic recombination protein	-1,20837E+14	5,12361E+14	-2,24827E+14	0.0626595051530227	0.203008111563106	-3,92648E+14	4,15531E+13	4,44697E+14	4,71964E+14	6,29938E+14	5,42522E+14	5,69516E+14
TC1300001877.mm.2	Tbc1d7	TBC1 domain family, member 7	0.735660287798329	6,39171E+14	2,24823E+13	0.062662886060463	0.203008111563106	-3,92653E+13	6,85025E+14	6,88921E+13	6,39294E+14	5,8752E+14	6,27824E+14	6,06443E+14
TC0300000492.mm.2	Rap2b	RAP2B, member of RAS oncogene family	0.778489073871576	7,34774E+14	2,24823E+14	0.0626633989895634	0.203008111563106	-3,92654E+13	7,65655E+14	7,89169E+14	7,29475E+14	6,69651E+14	7,14392E+14	7,40303E+14
TC1100003559.mm.2	Luc7l3	LUC7-like 3 (S. cerevisiae)	0.901705529569151	7,82293E+14	2,24807E+14	0.062677648078659	0.203008111563106	-3,92674E+14	8,31684E+14	8,17022E+14	7,74651E+14	6,93714E+13	8,17103E+14	7,59587E+13
TC0200004725.mm.2	Tasp1	taspase, threonine aspartase 1	0.847063870203548	7,4498E+14	2,24789E+14	0.0626936314578941	0.203008111563106	-3,92697E+14	8,09791E+14	7,70708E+14	7,37874E+14	6,73213E+14	7,6115E+13	7,17143E+14
TC1900001511.mm.2	Frat2	frequently rearranged in advanced T cell lymphomas 2	-0.695671586095697	6,37061E+14	-2,24771E+14	0.062708940011294	0.203008111563106	-3,92718E+13	5,91283E+14	6,06067E+13	6,74744E+14	6,6174E+13	6,2614E+14	6,62396E+14
TC1700002107.mm.2	9130008F23Rik	RIKEN cDNA 9130008F23 gene	-1,11716E+14	5,44121E+14	-2,24758E+14	0.0627212797896311	0.203008111563106	-3,92736E+14	4,59224E+14	5,04041E+13	5,32207E+13	6,5449E+14	5,24994E+14	5,89769E+14
TC0X00003339.mm.2	Glra2	glycine receptor, alpha 2 subunit	1,8711E+14	6,64425E+14	2,24745E+14	0.0627322084193153	0.203008111563106	-3,92751E+14	8,27946E+14	7,66952E+14	7,40743E+14	4,79934E+14	5,44298E+14	6,26678E+14
TC1900000776.mm.2	Acsl5	acyl-CoA synthetase long-chain family member 5	1,04128E+14	6,21115E+14	2,24739E+14	0.0627374119215578	0.203008111563106	-3,92758E+14	6,82459E+14	6,91626E+14	6,14954E+14	5,50874E+14	6,49961E+14	5,36815E+14
TC0900002613.mm.2	Tcf12	transcription factor 12	0.859633949619758	7,60192E+14	2,24738E+14	0.0627387856263543	0.203008111563106	-3,9276E+14	8,15896E+14	8,33181E+14	7,68201E+14	7,08949E+13	7,5059E+14	6,84333E+14
TC1500000844.mm.2	Tafa5	TAFA chemokine like family member 5	0.867489212562536	7,90707E+14	2,24733E+13	0.0627431512185358	0.203008111563106	-3,92766E+14	8,62661E+14	8,55286E+14	8,1723E+14	7,27219E+14	7,50791E+14	7,31054E+14
TC1800000438.mm.2	Tcerg1	transcription elongation regulator 1 (CA150)	1,32139E+14	7,00918E+14	2,24627E+14	0.0628377573030284	0.203243635885335	-3,929E+14	8,02043E+14	8,06795E+13	7,24328E+14	6,20233E+14	7,01935E+14	5,50178E+14
TC1900000073.mm.2	Cfl1	cofilin 1, non-muscle	1,55223E+14	1,1057E+14	2,24623E+14	0.0628411713511289	0.203243635885335	-3,92905E+14	1,1902E+13	1,19461E+14	1,13726E+14	9,37109E+14	1,13928E+14	1,03574E+13
TC0800001555.mm.2	Disc1	disrupted in schizophrenia 1	-0.933543247117807	5,95763E+14	-2,24601E+14	0.0628605068383365	0.203265371600665	-3,92932E+14	5,55988E+14	5,34998E+14	5,94785E+14	6,82909E+14	5,73815E+13	6,32083E+14
TC0300000658.mm.2	Fbxw7	F-box and WD-40 domain protein 7	2,2688E+14	7,03545E+14	2,2457E+14	0.0628881513772926	0.203313961304638	-3,92971E+14	8,16588E+14	8,34415E+13	7,42352E+13	4,54891E+14	7,83599E+14	5,89424E+14
TC0800002552.mm.2	Scoc	short coiled-coil protein	2,17996E+14	7,47651E+14	2,24524E+14	0.0629299005812855	0.203408121979188	-3,93029E+14	8,81016E+14	8,7154E+14	7,94469E+14	5,22095E+14	8,0372E+14	6,13066E+14
TC1000001038.mm.2	Pwp1	PWP1 homolog, endonuclein	0.743296919942935	5,98235E+14	2,24491E+14	0.0629589936548863	0.203409808674219	-3,9307E+14	6,73992E+13	6,80354E+14	6,18487E+13	5,872E+14	5,39501E+14	4,89877E+14
TC1400002327.mm.2	Chmp7	charged multivesicular body protein 7	1,33507E+14	8,3797E+14	2,24488E+14	0.0629616988864071	0.203409808674219	-3,93074E+13	9,13187E+14	8,83682E+14	8,49278E+13	6,80577E+14	8,87766E+14	8,13328E+14
TC0X00000439.mm.2	Rbmx2	RNA binding motif protein, X-linked 2	0.94822054071605	6,49367E+14	2,2446E+14	0.0629864220915753	0.203409808674219	-3,93109E+14	7,14386E+14	6,88706E+14	6,51007E+14	5,62442E+14	6,7078E+14	6,08883E+13
TC0500001128.mm.2	Zfp932	zinc finger protein 932	1,18729E+14	7,01473E+14	2,24455E+14	0.0629911179002341	0.203409808674219	-3,93116E+14	7,72747E+14	8,12481E+14	7,17531E+14	6,3024E+14	7,01501E+13	5,74339E+14
TC1900001169.mm.2	Zfp91/Cntf/Gm44505	zinc finger protein 91/ciliary neurotrophic factor/predicted readthrough transcript (NMD candidate), 44505	1,27808E+14	7,63904E+11	2,24444E+14	0.0630010293743288	0.203409808674219	-3,93129E+14	8,38627E+14	8,48681E+14	7,80926E+14	6,50766E+14	7,91468E+14	6,72959E+14
TC1100004296.mm.2	Nme1	NME/NM23 nucleoside diphosphate kinase 1	0.804012474581059	9,04075E+14	2,24438E+14	0.0630061661449159	0.203409808674219	-3,93137E+13	9,79364E+14	9,65704E+14	9,02689E+14	8,81575E+14	8,83803E+14	8,11312E+14
TC0700000941.mm.2	Gabrb3	gamma-aminobutyric acid (GABA) A receptor, subunit beta 3	2,04275E+14	9,89311E+14	2,24334E+14	0.0630992608420673	0.203669549268476	-3,93267E+14	1,1285E+14	1,11534E+14	1,05078E+14	7,84516E+14	1,00735E+14	8,49377E+14
TC0200000555.mm.2	Prdm12	PR domain containing 12	1,16573E+14	5,15913E+14	2,24305E+14	0.0631259930567685	0.203714501780833	-3,93305E+14	5,74406E+14	5,81885E+14	5,1726E+14	4,05886E+14	4,5403E+14	5,62011E+14
TC1100000908.mm.2	Kcnab3	potassium voltage-gated channel, shaker-related subfamily, beta member 3	0.716001191395846	5,99158E+14	2,24284E+14	0.0631440945196598	0.203714501780833	-3,9333E+14	6,41712E+14	6,08171E+14	5,41233E+14	5,6545E+14	6,05245E+14	6,33139E+14
TC1400000271.mm.2	Arhgef3	Rho guanine nucleotide exchange factor (GEF) 3	1,90926E+14	7,51953E+14	2,24277E+14	0.0631511162660746	0.203714501780833	-3,9334E+14	8,52232E+14	8,76017E+14	8,12923E+14	5,33473E+13	7,49889E+14	6,87186E+14
TC1300000101.mm.2	Psma2	proteasome (prosome, macropain) subunit, alpha type 2	1,82403E+14	7,79816E+14	2,24259E+14	0.0631665599122671	0.203723534801113	-3,93362E+14	8,51507E+14	8,755E+14	8,00758E+14	5,61443E+14	8,59301E+14	7,30387E+14
TC1100001698.mm.2	Adam11	a disintegrin and metallopeptidase domain 11	0.78784769700427	7,44129E+14	2,24217E+14	0.0632050220116089	0.203729834196207	-3,93416E+14	7,9558E+14	7,67293E+14	7,32111E+14	6,73193E+14	7,64033E+14	7,32567E+14
TC0700004219.mm.2	Cog7	component of oligomeric golgi complex 7	1,18087E+14	6,50986E+14	2,24208E+14	0.0632123080229774	0.203729834196207	-3,93426E+14	7,52223E+14	7,31381E+14	6,70877E+14	5,76552E+14	6,50146E+14	5,24736E+13
TC0200002323.mm.2	Efcab8	EF-hand calcium binding domain 8	-0.786442778543722	5,82793E+14	-2,24207E+14	0.0632132650170936	0.203729834196207	-3,93427E+13	5,41413E+14	5,30674E+14	5,99598E+14	6,29778E+14	5,65322E+14	6,29976E+13
TC1800001749.mm.2	Ppp2r2b	protein phosphatase 2, regulatory subunit B, beta	1,36268E+14	8,43445E+14	2,24192E+14	0.0632272532769332	0.203729834196207	-3,93447E+14	9,21489E+14	9,39512E+13	8,74638E+13	7,13826E+14	8,57353E+14	7,53852E+14
TC0600000718.mm.2	Tigd2	tigger transposable element derived 2	0.819894291578398	6,702E+14	2,24183E+14	0.0632346881051492	0.203729834196207	-3,93457E+14	7,24161E+14	7,27083E+14	6,83721E+14	6,03544E+14	6,47426E+14	6,35264E+14
TC0400003707.mm.2	Sfn	stratifin	-0.926372003273841	5,89329E+14	-2,24173E+14	0.0632444008435378	0.203729834196207	-3,93471E+14	5,14973E+14	5,26963E+14	5,69018E+14	6,58193E+14	6,04912E+14	6,61914E+14
TC0600002948.mm.2	Timp4	tissue inhibitor of metalloproteinase 4	1,14725E+14	6,89086E+14	2,24159E+14	0.0632570198276933	0.203729834196207	-3,93488E+14	7,79172E+14	7,345E+14	7,08999E+14	5,75222E+14	6,96507E+14	6,40117E+14
TC0X00003371.mm.2	Msl3	MSL complex subunit 3	1,02732E+14	6,91841E+14	2,24091E+14	0.0633176517673904	0.20386810148819	-3,93573E+14	7,33641E+14	7,75452E+14	7,11865E+14	5,91765E+14	6,61414E+14	6,76909E+14
TC1300001733.mm.2	Uqcrfs1	ubiquinol-cytochrome c reductase, Rieske iron-sulfur polypeptide 1	1,27043E+14	7,8172E+14	2,24063E+14	0.0633425820393233	0.20386810148819	-3,93608E+14	8,55852E+14	8,53243E+14	8,13784E+14	6,41225E+14	7,78485E+13	7,47729E+14
TC1300002418.mm.2	Rasgrf2	RAS protein-specific guanine nucleotide-releasing factor 2	0.899418090580458	7,27726E+14	2,24058E+14	0.0633474509749757	0.20386810148819	-3,93615E+13	8,24301E+14	7,66315E+14	7,43211E+14	6,67521E+14	6,98438E+14	6,6657E+14
TC0800002425.mm.2	Abhd8	abhydrolase domain containing 8	0.705806444822878	6,56009E+12	2,24046E+14	0.0633582970426459	0.20386810148819	-3,9363E+14	7,08273E+14	6,72269E+14	6,33574E+14	6,05806E+14	6,62504E+14	6,53631E+14
TC1100001668.mm.2	Arl4d	ADP-ribosylation factor-like 4D	-1,04972E+14	8,36586E+14	-2,24041E+14	0.0633632130734721	0.20386810148819	-3,93637E+14	7,60026E+14	7,62184E+14	8,06626E+14	9,25529E+14	8,53105E+14	9,12048E+14
TC1600001209.mm.2	Zc3h7a	zinc finger CCCH type containing 7 A	1,56773E+14	5,09766E+14	2,24015E+14	0.0633860360025687	0.203885253197164	-3,93669E+14	6,25206E+13	6,14931E+14	5,28656E+13	3,97934E+14	5,39716E+14	3,52155E+14
TC0200003397.mm.2	Pdcl	phosducin-like	0.727638384473602	5,89204E+14	2,24006E+14	0.0633938508358339	0.203885253197164	-3,9368E+14	6,07059E+14	6,25586E+14	5,56344E+14	5,30773E+13	5,85619E+14	6,2984E+14
TC0200002181.mm.2	6430503K07Rik	RIKEN cDNA 6430503K07 gene	-0.779959728281642	8,40636E+14	-2,23988E+14	0.0634103147355852	0.203897505754237	-3,93703E+14	7,79226E+14	7,96404E+14	8,32083E+14	8,99539E+14	8,62549E+14	8,74018E+14
TC0200002488.mm.2	R3hdml	R3H domain containing-like	-1,07863E+13	5,93575E+14	-2,23966E+13	0.0634302295046633	0.203900356963907	-3,9373E+14	5,38549E+14	5,02209E+14	5,77979E+14	6,78505E+14	5,8798E+12	6,76225E+14
TC0700001197.mm.2	Tmc3	transmembrane channel-like gene family 3	-0.808070421964501	6,01657E+14	-2,23957E+14	0.0634386415332748	0.203900356963907	-3,93742E+14	5,6765E+14	5,57712E+14	6,05893E+14	6,81083E+14	6,08467E+14	5,89134E+14
TC1700000255.mm.2	Ppp2r1a	protein phosphatase 2, regulatory subunit A, alpha	1,94022E+14	1,01287E+14	2,23943E+14	0.0634510725031378	0.203900356963907	-3,93759E+14	1,13957E+14	1,12426E+14	1,07655E+14	7,99237E+14	1,01566E+14	9,21931E+14
TC0700002028.mm.2	Psmd13	proteasome (prosome, macropain) 26S subunit, non-ATPase, 13	1,7533E+14	7,91135E+14	2,23931E+14	0.0634618190389121	0.203900356963907	-3,93774E+14	8,92472E+14	9,04849E+14	8,13357E+14	6,33305E+14	8,45826E+14	6,57003E+14
TC0200002537.mm.2	Ctsa	cathepsin A	1,39743E+13	8,16716E+14	2,23903E+14	0.063487179269084	0.203941172161633	-3,9381E+14	8,878E+14	9,16406E+14	8,60439E+14	6,64281E+14	7,92479E+14	7,78892E+14
TC0400004140.mm.2	Gabrd	gamma-aminobutyric acid (GABA) A receptor, subunit delta	0.804924382488039	6,59506E+14	2,23878E+14	0.0635098937392326	0.20397347375329	-3,93841E+14	7,01897E+13	7,15463E+14	6,02447E+14	6,53928E+14	6,69816E+14	6,13483E+14
TC0100000055.mm.2	Prex2	phosphatidylinositol-3,4,5-trisphosphate-dependent Rac exchange factor 2	1,47133E+14	6,1383E+14	2,23846E+14	0.063538709412148	0.204015631655153	-3,93881E+14	7,32299E+14	7,12518E+14	6,80351E+14	4,702E+14	5,53725E+14	5,33886E+14
TC1100003667.mm.2	Fbxl20	F-box and leucine-rich repeat protein 20	1,86491E+14	6,17352E+13	2,23824E+14	0.0635587102886007	0.204015631655153	-3,93909E+14	7,25537E+14	7,33395E+14	6,65782E+14	4,20169E+14	6,47221E+13	5,12009E+13
TC1200001768.mm.2	Nfkbia	nuclear factor of kappa light polypeptide gene enhancer in B cells inhibitor, alpha	0.745605709808006	6,18423E+14	2,238E+14	0.0635802018378532	0.204015631655153	-3,93939E+14	6,41963E+14	6,80362E+14	6,09605E+14	5,63599E+14	6,03431E+13	6,11579E+14
TC1300000560.mm.2	Ninj1	ninjurin 1	-0.697728990117618	8,32631E+14	-2,23799E+14	0.0635809553279238	0.204015631655153	-3,9394E+14	8,00774E+14	7,85475E+14	8,77678E+14	8,48117E+12	8,55793E+14	8,27951E+14
TC1700000349.mm.2	Rps2/Snora64	ribosomal protein S2/small nucleolar RNA, H/ACA box 64	0.934587318045786	1,00165E+14	2,23793E+14	0.063586327944652	0.204015631655153	-3,93948E+14	1,05302E+14	1,06659E+14	9,98157E+14	9,34543E+14	1,02234E+14	9,35268E+14
TC0300002797.mm.2	S1pr1	sphingosine-1-phosphate receptor 1	0.742227494600409	7,86896E+14	2,23764E+14	0.0636122181463662	0.204021996441268	-3,93984E+14	8,35424E+13	8,26027E+14	7,83585E+14	7,2942E+14	7,84759E+14	7,62159E+14
TC0400003885.mm.2	Fblim1	filamin binding LIM protein 1	-0.782833634128312	7,57639E+14	-2,23763E+14	0.0636136355812655	0.204021996441268	-3,93986E+14	6,83883E+14	7,0491E+14	7,51334E+14	7,94026E+14	7,79105E+14	8,32575E+14
TC1900001113.mm.2	Cd5	CD5 antigen	-0.971480665948299	6,16975E+14	-2,23743E+14	0.0636314319901443	0.204038460429293	-3,94011E+14	5,43894E+14	5,38663E+14	5,86958E+14	6,89895E+14	6,43809E+14	6,98632E+14
TC0200000247.mm.2	Slc39a12	solute carrier family 39 (zinc transporter), member 12	1,21244E+14	7,40385E+14	2,23683E+14	0.0636861811669847	0.204162262511713	-3,94087E+14	8,1327E+14	8,08966E+14	7,69618E+14	6,1013E+14	6,99464E+14	7,40864E+13
TC0800000929.mm.2	Dnajb1	DnaJ heat shock protein family (Hsp40) member B1	1,0664E+14	7,56538E+14	2,2367E+14	0.0636979871981995	0.204162262511713	-3,94103E+14	8,44888E+14	8,20332E+14	7,71694E+14	6,80246E+14	7,60158E+14	6,61912E+14
TC0700002211.mm.2	Olfr1347	olfactory receptor 1347	-1,14395E+14	4,99689E+14	-2,23648E+13	0.0637177807472075	0.204162262511713	-3,94131E+14	4,0363E+14	4,44616E+14	4,70529E+14	6,06506E+14	5,09679E+14	5,63172E+13
TC1400002719.mm.2	Dzip1	DAZ interacting protein 1	1,60907E+12	6,9398E+14	2,23633E+14	0.0637307857866665	0.204162262511713	-3,94149E+13	8,25897E+14	7,74578E+14	7,14844E+14	5,63817E+14	7,29727E+14	5,5502E+14
TC1300001928.mm.2	Dek	DEK oncogene (DNA binding)	0.659944536803545	7,57558E+14	2,2363E+12	0.0637333941807185	0.204162262511713	-3,94152E+14	7,96633E+14	8,04549E+12	7,25806E+14	7,43388E+14	7,44748E+14	7,30225E+14
TC1900000356.mm.2	Trpm3	transient receptor potential cation channel, subfamily M, member 3	1,31838E+14	7,57121E+14	2,23616E+14	0.0637468169901845	0.204164671469458	-3,94171E+14	8,30296E+13	8,76443E+14	6,55595E+14	7,87468E+14	7,5681E+14	6,36113E+14
TC0700003775.mm.2	Acer3	alkaline ceramidase 3	1,56376E+14	6,23196E+14	2,23597E+14	0.0637636113154218	0.204177875422375	-3,94194E+14	7,37807E+14	7,19053E+14	6,70652E+14	4,73456E+14	6,26224E+14	5,11986E+14
TC0700003908.mm.2	Hbb-bt/Hbb-b2/Hbb-b1	hemoglobin, beta adult t chain/hemoglobin, beta adult minor chain/hemoglobin, beta adult major chain	1,49991E+14	1,04549E+14	2,23537E+14	0.0638181997763647	0.204312070931167	-3,9427E+14	1,06191E+14	1,16376E+14	1,00884E+14	9,16845E+14	1,14513E+14	9,76452E+14
TC1600001679.mm.2	Nectin3	nectin cell adhesion molecule 3	2,03854E+14	5,50223E+14	2,23446E+14	0.0639007785130339	0.204535805359657	-3,94384E+14	6,58638E+14	6,81629E+14	5,87522E+14	3,45037E+14	6,10567E+14	4,17945E+14
TC1100001853.mm.2	Sox9	SRY (sex determining region Y)-box 9	0.820469425421317	8,26937E+13	2,23429E+14	0.0639159128246697	0.204543615361252	-3,94405E+14	8,79955E+14	8,91309E+14	8,26026E+14	7,81145E+14	7,57747E+14	8,25439E+14
TC1900000725.mm.2	Sfr1	SWI5 dependent recombination repair 1	0.80604888161188	8,07796E+14	2,234E+14	0.0639422224118289	0.204587178260882	-3,94442E+13	8,71322E+14	8,68731E+14	7,68811E+14	8,10032E+14	7,97181E+14	7,30702E+14
TC0900000074.mm.2	Gm16833	predicted gene, 16833	-1,01476E+14	4,86913E+14	-2,2333E+14	0.0640057925852303	0.204712977739633	-3,9453E+14	4,05238E+14	4,10995E+14	4,6833E+14	5,50856E+13	4,95353E+14	5,90705E+14
TC0200000547.mm.2	Gpr107	G protein-coupled receptor 107	0.977443746488925	6,17776E+14	2,23315E+14	0.0640197076693734	0.204712977739633	-3,94549E+14	6,79792E+14	6,90273E+14	6,00282E+14	5,74294E+13	6,38532E+14	5,23482E+14
TC1400002038.mm.2	Olfr1510	olfactory receptor 1510	-0.945042992593988	4,77366E+14	-2,23314E+14	0.0640204779691956	0.204712977739633	-3,9455E+14	4,11163E+14	4,13973E+14	4,67095E+14	5,47049E+14	4,73682E+14	5,51237E+14
TC1700000389.mm.2	Fbxl16	F-box and leucine-rich repeat protein 16	1,44738E+14	1,03714E+14	2,23301E+13	0.0640323594493733	0.204712977739633	-3,94566E+14	1,12151E+14	1,13591E+14	1,08336E+14	8,84583E+14	1,02914E+14	9,6835E+14
TC0900000824.mm.2	Dennd4a	DENN/MADD domain containing 4A	2,53041E+13	6,23984E+14	2,23281E+14	0.0640509572738583	0.204731814035644	-3,94592E+14	7,55583E+14	7,68721E+14	6,77461E+14	3,4076E+14	7,01965E+14	4,99415E+14
TC1900000527.mm.2	March5	membrane-associated ring finger (C3HC4) 5	1,64794E+14	7,51149E+14	2,23208E+14	0.0641174761096941	0.20486570994314	-3,94684E+14	8,45335E+14	8,51736E+14	7,9297E+14	5,74514E+13	7,75306E+14	6,67031E+14
TC1700002783.mm.2	Gpsm3	G-protein signalling modulator 3 (AGS3-like, C. elegans)	-0.96728442171308	6,48735E+14	-2,23207E+14	0.0641182756310591	0.20486570994314	-3,94685E+14	5,7616E+14	6,05219E+14	6,34898E+13	7,39938E+14	6,47741E+14	6,88458E+14
TC0400000704.mm.2	4933430I17Rik	RIKEN cDNA 4933430I17 gene	-1,05808E+14	4,98497E+14	-2,23154E+14	0.0641664329611242	0.20494004790989	-3,94751E+14	3,57654E+14	4,66431E+14	4,84351E+14	5,51351E+14	5,43374E+14	5,87818E+14
TC0900000124.mm.2	Smco4	single-pass membrane protein with coiled-coil domains 4	-0.892117990949568	4,9058E+14	-2,23146E+14	0.0641731119668275	0.20494004790989	-3,9476E+14	4,61279E+14	4,45095E+14	5,25887E+14	5,5891E+14	5,22487E+14	4,29824E+14
TC1200000994.mm.2	Calm1	calmodulin 1	1,06358E+14	1,12996E+13	2,23139E+14	0.0641796984890031	0.20494004790989	-3,9477E+14	1,19142E+14	1,20222E+14	1,14225E+14	1,03868E+14	1,14664E+14	1,05856E+14
TC0700001374.mm.2	Wnt11	wingless-type MMTV integration site family, member 11	-0.829301569385967	5,77607E+13	-2,23123E+14	0.064194379146032	0.204946310913417	-3,9479E+14	5,22983E+14	5,16175E+14	6,00531E+14	6,04487E+14	5,68641E+14	6,52828E+14
TC0Y00000435.mm.2	Gm20806	predicted gene, 20806	-0.727210023846846	5,21184E+14	-2,23079E+14	0.0642344186392758	0.204957396952101	-3,94845E+14	4,53052E+14	4,95763E+14	5,54045E+14	5,40212E+14	5,58095E+14	5,2594E+13
TC0Y00000491.mm.2	Gm20806	predicted gene, 20806	-0.727210023846846	5,21184E+14	-2,23079E+14	0.0642344186392758	0.204957396952101	-3,94845E+14	4,53052E+14	4,95763E+14	5,54045E+14	5,40212E+14	5,58095E+14	5,2594E+13
TC1100003655.mm.2	Rpl23	ribosomal protein L23	0.651536067308271	9,70183E+14	2,23076E+14	0.064237366597508	0.204957396952101	-3,94849E+13	1,00915E+14	1,00818E+14	9,37713E+14	9,49306E+14	9,65759E+14	9,50992E+14
TC0100000954.mm.2	Neu4	sialidase 4	-0.725786810906468	6,74347E+14	-2,23062E+14	0.0642498090532609	0.204957396952101	-3,94866E+14	6,1136E+14	6,45865E+14	6,91869E+13	7,10513E+14	6,74131E+14	7,12342E+14
TC1100003185.mm.2	Olfr399	olfactory receptor 399	-0.919069817006257	4,74975E+14	-2,23046E+14	0.0642651002981897	0.204957396952101	-3,94887E+14	4,14257E+14	3,98503E+14	4,5387E+13	5,42704E+14	4,92509E+14	5,48005E+14
TC0800002819.mm.2	Fhod1	formin homology 2 domain containing 1	-0.960184954953733	5,81566E+14	-2,23024E+14	0.0642853037439401	0.204957396952101	-3,94915E+14	5,12779E+14	5,35148E+14	5,88535E+14	6,51428E+14	5,53941E+14	6,47566E+14
TC1500002327.mm.2	Prlr	prolactin receptor	0.852127099958261	5,45888E+14	2,23021E+14	0.064287208165737	0.204957396952101	-3,94918E+14	6,11432E+14	6,1406E+14	4,90196E+13	5,64871E+13	5,22754E+14	4,72012E+14
TC0800002938.mm.2	Clec18a	C-type lectin domain family 18, member A	-0.704772787171548	5,17447E+14	-2,23005E+14	0.0643024654669534	0.204957396952101	-3,94939E+14	4,46245E+14	4,85398E+14	5,28005E+14	5,44593E+14	5,60463E+14	5,39976E+14
TC0300000269.mm.2	Exosc9	exosome component 9	0.934855357254277	7,1674E+14	2,22979E+14	0.0643257569076175	0.204957396952101	-3,94971E+13	7,75374E+13	7,98037E+13	7,26068E+14	6,60372E+14	7,12602E+13	6,27987E+13
TC0100000526.mm.2	Fastkd2	FAST kinase domains 2	0.752256012416013	5,43662E+14	2,22956E+14	0.0643472010640779	0.204957396952101	-3,95E+14	5,98686E+14	5,97919E+14	4,91419E+13	5,54736E+14	5,11465E+14	5,07748E+14

TC0900002008.mm.2	Tirap	toll-interleukin 1 receptor (TIR) domain-containing adaptor protein	-0.711537104951531	7,08536E+14	-2,2294E+14	0.0643614703668934	0.204957396952101	-3,9502E+14	6,52705E+14	7,01231E+14	7,43487E+14	7,52757E+14	7,03776E+14	6,9726E+14
TC0400001020.mm.2	Cachd1	cache domain containing 1	-0.927939156125773	8,61463E+14	-2,22937E+14	0.0643645325463958	0.204957396952101	-3,95024E+14	8,11844E+14	7,82951E+14	8,56766E+14	9,23616E+14	8,56923E+14	9,36678E+14
TC0200005143.mm.2	Cdh22	cadherin 22	0.664750403470247	6,38492E+14	2,22927E+14	0.0643732435496778	0.204957396952101	-3,95036E+14	6,86154E+14	6,88773E+14	6,18025E+14	6,23952E+14	5,93093E+14	6,20953E+14
TC0800001514.mm.2	Cdk10	cyclin-dependent kinase 10	0.768005704412881	7,36765E+14	2,22923E+14	0.0643774478895233	0.204957396952101	-3,95042E+14	8,12665E+14	7,67014E+14	7,22409E+14	7,03669E+13	7,30933E+12	6,83899E+14
TC0700002038.mm.2	Pkp3	plakophilin 3	-0.775804720206185	5,2113E+12	-2,2291E+14	0.0643886516087344	0.204957396952101	-3,95057E+14	4,86881E+14	4,65743E+14	5,2051E+14	5,87274E+14	5,29426E+14	5,36945E+12
TC0900000643.mm.2	Npat	nuclear protein in the AT region	1,85721E+14	6,80461E+14	2,22893E+14	0.0644048430359351	0.204968444763583	-3,95079E+14	7,91099E+13	7,8904E+14	7,38704E+14	4,69994E+14	6,8717E+14	6,0676E+14
TC0200001886.mm.2	Eid1	EP300 interacting inhibitor of differentiation 1	0.700590528595517	7,85661E+14	2,22847E+14	0.0644466399358039	0.205014615371303	-3,95137E+14	8,32321E+14	8,17521E+14	7,46751E+14	7,62972E+14	7,97272E+14	7,57127E+13
TC1700000347.mm.2	Zfp598	zinc finger protein 598	0.92897431358468	5,72632E+14	2,22843E+14	0.064450663661082	0.205014615371303	-3,95142E+14	6,36326E+14	6,17389E+14	5,46768E+14	5,21153E+14	5,05315E+14	6,08845E+14
TC0100003058.mm.2	Srgap2	SLIT-ROBO Rho GTPase activating protein 2	1,91719E+14	6,47684E+14	2,22831E+14	0.0644614373315938	0.205014615371303	-3,95157E+14	7,53413E+14	7,99553E+14	6,89769E+14	4,79758E+14	6,79729E+14	4,83884E+14
TC1000001124.mm.2	Cdk17	cyclin-dependent kinase 17	1,28492E+14	8,10248E+13	2,22821E+14	0.0644702449007878	0.205014615371303	-3,95169E+14	8,96E+14	9,09208E+13	8,35783E+14	7,12441E+14	8,18765E+14	6,89292E+14
TC0700004628.mm.2	Prr14	proline rich 14	1,71833E+14	7,04435E+14	2,22788E+14	0.0645009070545653	0.20507164865237	-3,95211E+14	8,57584E+14	8,43805E+14	7,58457E+14	5,99265E+14	6,842E+14	4,833E+14
TC0800002608.mm.2	Vps35	VPS35 retromer complex component	2,24091E+13	7,36323E+14	2,22736E+14	0.0645488592627306	0.205152549045736	-3,95277E+14	8,94614E+14	8,83183E+14	8,07843E+14	5,21772E+13	7,52214E+14	5,58311E+14
TC0900002737.mm.2	Cox7a2	cytochrome c oxidase subunit 7A2	0.655369016450251	9,10235E+14	2,22725E+14	0.0645587525563244	0.205152549045736	-3,9529E+14	9,52545E+12	9,56599E+14	8,85996E+14	8,92074E+13	8,97875E+14	8,76322E+14
TC1100003795.mm.2	Plekhh3	pleckstrin homology domain containing, family H (with MyTH4 domain) member 3	-0.945111228170119	6,87128E+14	-2,22718E+14	0.0645645488866708	0.205152549045736	-3,95298E+14	6,31691E+14	6,27728E+14	6,78369E+14	7,70072E+14	6,75371E+14	7,39536E+14
TC0500003715.mm.2	Smok3b/Smok3a	sperm motility kinase 3B/sperm motility kinase 3A	-0.744695602365697	5,40578E+14	-2,2269E+14	0.0645909704825955	0.205170800000542	-3,95335E+13	4,99635E+14	5,12469E+14	5,98538E+12	5,62505E+14	5,11172E+14	5,59149E+14
TC0X00001306.mm.2	Gprasp1/Armcx5/Mir7646	G protein-coupled receptor associated sorting protein 1/armadillo repeat containing, X-linked 5/microRNA 7646	0.685351169928323	8,35591E+14	2,22675E+13	0.0646041657261074	0.205170800000542	-3,95353E+14	9,04453E+14	8,71036E+14	8,23096E+14	8,15323E+14	8,09673E+13	7,89964E+14
TC0300002257.mm.2	Fam160a1	family with sequence similarity 160, member A1	-0.85525005332846	5,30865E+14	-2,2266E+12	0.0646186739420046	0.205170800000542	-3,95373E+13	4,71034E+14	5,17729E+14	5,60409E+14	5,99404E+14	4,84188E+14	5,52426E+14
TC0400004226.mm.2	Cela2a	chymotrypsin-like elastase family, member 2A	-0.794484213953408	5,66309E+14	-2,22657E+14	0.0646212257185348	0.205170800000542	-3,95376E+14	5,28737E+14	5,03403E+14	5,65571E+14	6,25467E+14	5,67792E+14	6,0688E+14
TC0500003710.mm.2	Lrrc8d	leucine rich repeat containing 8D	-0.880107992679803	8,16805E+14	-2,2264E+14	0.0646363691371455	0.205178450730265	-3,95397E+14	7,74843E+14	7,65147E+14	8,23154E+14	8,92857E+14	7,88769E+14	8,56056E+14
TC0900002491.mm.2	Map2k1	mitogen-activated protein kinase kinase 1	1,38223E+14	8,52306E+14	2,22608E+14	0.0646657145268087	0.205226521844782	-3,95437E+14	9,21452E+14	9,24313E+14	8,83325E+14	6,85994E+14	8,47095E+14	8,51658E+14
TC1700000420.mm.2	Bnip1	BCL2/adenovirus E1B interacting protein 1	1,48137E+14	5,46644E+14	2,22557E+14	0.0647131285446519	0.205226521844782	-3,95502E+13	6,42582E+14	6,7509E+13	6,10872E+14	4,10525E+14	5,06343E+14	4,34451E+14
TC0400002863.mm.2	Gm13871/Gm11757/Gm11758	predicted gene 13871/predicted gene 11757/predicted gene 11758	-0.713977545327998	5,78392E+14	-2,22548E+14	0.06472139828645	0.205226521844782	-3,95513E+14	5,21678E+14	5,40164E+14	6,25312E+13	5,79326E+14	5,95546E+14	6,08325E+14
TC0400002865.mm.2	Gm11758/Gm11757/Gm13871	predicted gene 11758/predicted gene 11757/predicted gene 13871	-0.713977545327998	5,78392E+14	-2,22548E+14	0.06472139828645	0.205226521844782	-3,95513E+14	5,21678E+14	5,40164E+14	6,25312E+13	5,79326E+14	5,95546E+14	6,08325E+14
TC0900001056.mm.2	Cd109	CD109 antigen	-0.942010271362915	6,54531E+13	-2,22536E+14	0.0647321168288177	0.205226521844782	-3,95528E+14	5,97636E+13	6,20569E+14	6,48778E+14	7,57829E+14	6,49602E+14	6,52773E+14
TC1100000535.mm.2	Tbc1d9b	TBC1 domain family, member 9B	0.667029147152374	8,06508E+14	2,22504E+14	0.0647618244616385	0.205226521844782	-3,95568E+14	8,52501E+14	8,6418E+14	7,90014E+14	7,93261E+14	7,55458E+14	7,83631E+14
TC1900000397.mm.2	Tmem252	transmembrane protein 252	-0.692545112000034	5,31396E+14	-2,22498E+14	0.0647668412775417	0.205226521844782	-3,95575E+14	4,60133E+14	4,92274E+14	5,34062E+14	5,56854E+14	5,64015E+14	5,81042E+14
TC1100001010.mm.2	Atp2a3	ATPase, Ca++ transporting, ubiquitous	-0.714591340457829	6,67255E+14	-2,22497E+14	0.0647683393139681	0.205226521844782	-3,95577E+14	6,16528E+14	6,20897E+14	6,71795E+13	7,08548E+14	6,75002E+14	7,10761E+14
TC1100002065.mm.2	Fn3k	fructosamine 3 kinase	0.688512989884987	6,88869E+14	2,22482E+14	0.0647818088287904	0.205226521844782	-3,95596E+14	7,29545E+14	6,91155E+14	6,34574E+14	6,48425E+14	7,11845E+14	7,17669E+14
TC1200002523.mm.2	Fntb	farnesyltransferase, CAAX box, beta	0.686510092140789	7,09429E+14	2,22459E+14	0.064803323523466	0.205226521844782	-3,95625E+14	7,64077E+13	7,6127E+14	6,7657E+14	7,11474E+14	6,61536E+14	6,81646E+14
TC0400002200.mm.2	Ndufaf6	NADH:ubiquinone oxidoreductase complex assembly factor 6	0.759471697715189	6,0282E+14	2,22456E+14	0.0648058252954037	0.205226521844782	-3,95628E+14	6,47386E+14	6,58215E+13	5,99798E+14	5,53909E+14	5,55622E+14	6,01988E+14
TC1500001326.mm.2	Cct5	chaperonin containing Tcp1, subunit 5 (epsilon)	1,03357E+14	8,38078E+14	2,22444E+13	0.0648168423656896	0.205226521844782	-3,95643E+14	9,24929E+14	8,94058E+14	8,66159E+14	7,46114E+14	8,19728E+14	7,77478E+14
TC1600001198.mm.2	Socs1	suppressor of cytokine signaling 1	-0.728521039413591	6,26444E+13	-2,22444E+14	0.0648170899067892	0.205226521844782	-3,95644E+14	5,76426E+14	5,31458E+14	6,32548E+14	6,2104E+14	6,82606E+14	7,14587E+14
TC0300002959.mm.2	Sgms2	sphingomyelin synthase 2	-0.732332036227856	5,12521E+14	-2,22425E+14	0.0648346995777477	0.20524194779887	-3,95668E+14	4,34891E+14	4,90451E+14	5,43033E+14	5,28775E+14	5,36058E+14	5,41916E+14
TC0900000040.mm.2	Dcun1d5	DCN1, defective in cullin neddylation 1, domain containing 5 (S. cerevisiae)	2,03294E+13	7,1335E+12	2,2239E+14	0.0648663262697265	0.2053017315231	-3,95711E+14	8,21447E+14	8,1397E+14	7,60879E+14	4,6795E+14	7,60349E+13	6,55504E+14
TC1300001424.mm.2	Zmynd11	zinc finger, MYND domain containing 11	2,35705E+14	8,60559E+14	2,22352E+14	0.0649013657631544	0.205365662366601	-3,95759E+14	9,94408E+14	9,99574E+14	9,27065E+14	5,95507E+14	9,00776E+14	7,46022E+14
TC0200000783.mm.2	Mbd5	methyl-CpG binding domain protein 5	1,21593E+14	8,44878E+14	2,22341E+14	0.0649120162870415	0.205365662366601	-3,95773E+13	9,47245E+14	9,33173E+14	8,72014E+14	7,65218E+14	8,39535E+14	7,12083E+14
TC0X00001658.mm.2	Ap1s2	adaptor-related protein complex 1, sigma 2 subunit	1,52545E+13	7,48539E+14	2,22319E+14	0.0649317579600767	0.205377757820765	-3,958E+14	8,6302E+14	8,32289E+13	8,02438E+13	5,87781E+14	7,29835E+14	6,75873E+14
TC0200001392.mm.2	Chrm4	cholinergic receptor, muscarinic 4	0.754083017473524	7,30576E+14	2,22309E+14	0.0649413316016134	0.205377757820765	-3,95813E+14	7,80973E+14	7,79542E+14	7,3691E+14	6,72789E+14	7,02533E+14	7,10711E+14
TC0X00003108.mm.2	Ammecr1	Alport syndrome, mental retardation, midface hypoplasia and elliptocytosis chromosomal region gene 1	-1,25343E+14	5,53414E+13	-2,22281E+14	0.0649667313004836	0.205417767159477	-3,95848E+14	5,20524E+14	4,61025E+14	6,30188E+14	6,02047E+13	6,33891E+14	4,72813E+14
TC1900000497.mm.2	Fas	Fas (TNF receptor superfamily member 6)	-0.750199959074568	5,26162E+14	-2,22239E+14	0.0650059401520329	0.205481063599616	-3,95901E+13	4,85151E+14	4,6515E+14	5,20762E+14	5,79579E+14	5,59838E+14	5,46491E+14
TC1400000248.mm.2	Anxa11	annexin A11	1,03248E+14	6,35742E+14	2,22232E+14	0.0650122548396227	0.205481063599616	-3,9591E+14	6,62808E+14	7,29703E+14	5,74714E+14	6,113E+14	6,71184E+13	5,64742E+14
TC1000000674.mm.2	Slc16a9	solute carrier family 16 (monocarboxylic acid transporters), member 9	-0.783946232455631	5,92152E+14	-2,22158E+14	0.0650807796819832	0.205619938560132	-3,96003E+13	5,08339E+14	5,6908E+14	5,96817E+14	6,37391E+14	6,24569E+14	6,16715E+14
TC0600002920.mm.2	Cidec	cell death-inducing DFFA-like effector c	-1,28257E+14	5,58379E+14	-2,22157E+14	0.0650817157982171	0.205619938560132	-3,96004E+14	4,5302E+14	4,8303E+13	5,24792E+13	6,67772E+14	5,58615E+14	6,63045E+13
TC0900000659.mm.2	Idh3a	isocitrate dehydrogenase 3 (NAD+) alpha	2,48424E+14	8,74529E+14	2,22106E+14	0.065129280257636	0.205729875081609	-3,96069E+13	1,01934E+14	1,02177E+14	9,30302E+14	6,13964E+14	9,42931E+14	7,18863E+14
TC0700002039.mm.2	Ptdss2	phosphatidylserine synthase 2	1,39342E+14	6,95449E+14	2,22065E+14	0.0651668953716759	0.205784554956702	-3,9612E+14	7,91718E+14	7,75171E+14	7,20851E+14	5,67353E+13	7,22414E+14	5,95186E+14
TC1700001711.mm.2	Cuta	cutA divalent cation tolerance homolog	0.849220327753383	8,3383E+14	2,22059E+14	0.0651721333050364	0.205784554956702	-3,96127E+13	9,06143E+14	8,71797E+14	8,45373E+14	7,62723E+14	8,15211E+14	8,01735E+14
TC0300001221.mm.2	Dpyd	dihydropyrimidine dehydrogenase	-0.775883634378112	6,02824E+14	-2,22005E+14	0.0652221325585192	0.205902081096281	-3,96195E+14	5,61107E+14	5,34947E+14	5,93112E+14	6,58119E+14	6,28103E+14	6,41558E+14
TC0500002753.mm.2	Naaa	N-acylethanolamine acid amidase	0.801525338989329	7,7993E+14	2,21988E+14	0.0652379194732135	0.205911576194297	-3,96216E+14	8,39646E+14	8,30257E+14	7,78365E+14	7,31233E+14	7,19058E+14	7,81024E+14
TC1100002899.mm.2	Tmem11	transmembrane protein 11	-0.768742408634594	7,88083E+14	-2,21965E+13	0.0652596295085394	0.205923335228724	-3,96245E+14	7,18996E+14	7,4405E+13	7,76874E+13	8,3992E+13	8,17144E+14	8,31516E+14
TC0600001736.mm.2	Stk38l	serine/threonine kinase 38 like	0.678486687577536	6,82762E+14	2,21948E+14	0.065275725764345	0.205923335228724	-3,96267E+14	7,14637E+14	7,18162E+14	6,39912E+14	6,57189E+14	6,66826E+13	6,99844E+14
TC1800000249.mm.2	Lims2	LIM and senescent cell antigen like domains 2	0.870936954395322	6,32705E+14	2,21933E+14	0.0652897413336325	0.205923335228724	-3,96286E+14	7,16666E+14	6,52803E+14	5,6193E+14	6,33352E+14	6,17275E+14	6,14207E+14
TC0100000431.mm.2	Aox2	aldehyde oxidase 2	-1,01217E+13	4,3513E+14	-2,21929E+14	0.0652927648286197	0.205923335228724	-3,9629E+14	3,99346E+14	3,34124E+14	4,25865E+14	5,10039E+14	4,36078E+14	5,05327E+14
TC1100001304.mm.2	Tbx4	T-box 4	-0.940274710165883	5,72804E+14	-2,21914E+14	0.0653071240533988	0.205928315043525	-3,9631E+14	5,08775E+14	5,22225E+14	5,59165E+14	6,5989E+13	5,70222E+14	6,16545E+13
TC1600002160.mm.2	Zbtb21	zinc finger and BTB domain containing 21	0.761646292978812	5,58333E+14	2,21889E+14	0.0653303279382756	0.205961176691339	-3,96341E+14	5,99282E+13	6,32308E+13	5,68308E+13	5,10953E+14	5,13336E+14	5,25811E+14
TC0400003675.mm.2	Atpif1	ATPase inhibitory factor 1	1,06819E+14	9,78916E+14	2,21837E+14	0.0653780839096173	0.205965396216693	-3,96406E+14	1,04764E+14	1,02829E+13	9,90259E+14	8,72033E+13	1,00246E+13	9,32812E+14
TC0400001581.mm.2	Taf12	TATA-box binding protein associated factor 12	0.720218432310787	6,94769E+14	2,21816E+14	0.0653977890643906	0.205965396216693	-3,96432E+14	7,40555E+14	7,41288E+14	6,9055E+14	6,47249E+14	6,90808E+14	6,58163E+14
TC1200002439.mm.2	Cdca4	cell division cycle associated 4	-1,0696E+14	7,07093E+14	-2,21807E+14	0.0654062832844114	0.205965396216693	-3,96444E+14	6,52643E+14	6,38584E+14	8,02963E+14	7,02184E+14	7,70354E+14	6,75827E+14
TC0100001060.mm.2	Clasp1	CLIP associating protein 1	1,08615E+14	7,51867E+14	2,21791E+13	0.0654208471803148	0.205965396216693	-3,96464E+14	8,47817E+14	8,2264E+14	7,86161E+14	6,67066E+14	7,30533E+14	6,56988E+14
TC0900002281.mm.2	Sik2	salt inducible kinase 2	0.987486516282257	6,28059E+14	2,21779E+14	0.0654319525932649	0.205965396216693	-3,96479E+14	7,09508E+14	6,78324E+14	6,53682E+14	5,36654E+14	5,87811E+13	6,02377E+14
TC0X00000169.mm.2	Gpr34	G protein-coupled receptor 34	2,13182E+13	6,76453E+14	2,21731E+14	0.0654773419674013	0.205965396216693	-3,9654E+14	7,54834E+14	8,33156E+14	7,40033E+14	4,21593E+14	6,68076E+14	6,41023E+14
TC0500001911.mm.2	Cfap69	cilia and flagella associated protein 69	0.695249575435802	5,65147E+14	2,2172E+14	0.0654876528179422	0.205965396216693	-3,96554E+14	6,02611E+13	5,92333E+14	5,34167E+14	5,21727E+13	5,48999E+14	5,91045E+14
TC1500001234.mm.2	6030458C11Rik	RIKEN cDNA 6030458C11 gene	-0.651154438198817	6,49064E+14	-2,21706E+14	0.0655001186350023	0.205965396216693	-3,96571E+14	5,95929E+14	6,14567E+13	6,74068E+13	6,66659E+14	6,67274E+14	6,75884E+14
TC0800003078.mm.2	Pabpn1l	poly(A)binding protein nuclear 1-like	-0.758838952400511	5,55187E+14	-2,21703E+14	0.0655029207316525	0.205965396216693	-3,96574E+14	5,24292E+14	4,84007E+14	5,72011E+14	5,88055E+14	5,56032E+14	6,06722E+14
TC0200005465.mm.2	Dipk1b	divergent protein kinase domain 1B	0.804686594799296	6,99208E+14	2,21699E+14	0.0655064683382408	0.205965396216693	-3,96579E+14	7,72937E+14	7,28016E+14	6,37297E+14	7,02719E+14	6,79787E+14	6,74492E+14
TC0400002705.mm.2	Elp1	elongator complex protein 1	1,37275E+14	6,16871E+14	2,21696E+14	0.0655094873769066	0.205965396216693	-3,96583E+14	6,92425E+14	6,88958E+14	6,47769E+14	4,59064E+13	6,3422E+14	5,78792E+14
TC1700001388.mm.2	Dynlt1f/Dynlt1c	dynein light chain Tctex-type 1F/dynein light chain Tctex-type 1C	-1,92628E+14	9,55075E+14	-2,21692E+14	0.0655136741623286	0.205965396216693	-3,96589E+14	9,16853E+14	7,841E+13	1,13737E+14	9,48838E+13	1,06291E+14	8,80381E+13
TC1300001098.mm.2	Fam169a	family with sequence similarity 169, member A	1,52398E+14	7,1752E+13	2,21686E+14	0.06551895294158	0.205965396216693	-3,96596E+14	8,47779E+14	8,05901E+14	7,52176E+14	5,96708E+14	7,29123E+13	5,73434E+14
TC0X00000832.mm.2	Tab3	TGF-beta activated kinase 1/MAP3K7 binding protein 3	0.949460757286646	6,74589E+14	2,21684E+14	0.0655208805581768	0.205965396216693	-3,96599E+14	7,40847E+14	7,52986E+14	7,06856E+14	5,97086E+14	6,38823E+14	6,10936E+14
TC1100001108.mm.2	Tmigd1	transmembrane and immunoglobulin domain containing 1	-0.853767489423808	5,29235E+14	-2,21681E+13	0.0655234047667579	0.205965396216693	-3,96602E+14	4,52473E+14	4,79035E+14	5,30833E+14	5,71428E+14	5,31751E+14	6,09889E+14
TC0400004117.mm.2	Tprgl	transformation related protein 63 regulated like	1,00568E+14	8,00603E+14	2,21656E+14	0.0655470461552408	0.2059995230543	-3,96634E+13	8,63152E+14	8,50983E+14	8,14738E+14	6,98262E+14	8,11388E+14	7,65095E+14
TC0400002174.mm.2	Car8	carbonic anhydrase 8	0.719881702197189	7,01076E+14	2,21614E+14	0.0655855848038294	0.206080446166947	-3,96686E+14	7,1741E+14	7,49327E+14	6,79036E+14	6,43724E+14	7,21589E+14	6,95368E+14
TC0100002354.mm.2	Hspd1	heat shock protein 1 (chaperonin)	1,66653E+13	8,20056E+14	2,21588E+13	0.0656100446595964	0.206117108520194	-3,96719E+14	9,20097E+14	9,13394E+14	8,60115E+14	6,40071E+14	8,51779E+14	7,34879E+14
TC1500000175.mm.2	Ank/Mir7117	progressive ankylosis/microRNA 7117	1,03339E+14	8,41956E+14	2,2155E+14	0.0656461153955965	0.206133841974889	-3,96767E+14	8,96186E+14	9,01336E+14	8,56503E+14	7,34342E+14	8,5625E+14	8,07118E+14
TC1000000169.mm.2	1700020N01Rik	RIKEN cDNA 1700020N01 gene	-0.698901684587376	5,68555E+14	-2,21543E+14	0.0656518910003072	0.206133841974889	-3,96775E+14	5,23177E+14	4,9708E+14	5,97671E+13	5,62367E+14	6,0754E+14	6,23491E+14
TC0300003106.mm.2	Prkacb	protein kinase, cAMP dependent, catalytic, beta	1,40142E+14	9,61304E+14	2,21541E+14	0.0656537502026396	0.206133841974889	-3,96778E+14	1,06398E+13	1,04956E+14	1,01235E+13	8,20901E+14	9,4208E+14	8,7895E+14
TC1900000116.mm.2	Sf1	splicing factor 1	0.732944662334084	8,33429E+14	2,21432E+14	0.0657559530499012	0.206414508375815	-3,96915E+14	8,87169E+13	8,73336E+14	8,33733E+14	7,80184E+14	8,02895E+14	8,23254E+14
TC1000001328.mm.2	Zfc3h1	zinc finger, C3H1-type containing	1,77414E+14	5,65288E+14	2,21389E+14	0.0657964182528489	0.206446722261306	-3,9697E+14	6,71046E+13	6,70175E+14	6,0057E+13	3,85825E+14	6,12463E+14	4,51651E+14
TC0500002087.mm.2	Smarcd3	SWI/SNF related, matrix associated, actin dependent regulator of chromatin, subfamily d, member 3	0.810254689359406	8,35185E+14	2,21381E+14	0.065803225063922	0.206446722261306	-3,96979E+14	8,68254E+14	8,8224E+14	8,32581E+14	7,55862E+14	8,47588E+14	8,24585E+14
TC0200005455.mm.2	Lkaaear1	LKAAEAR motif containing 1 (IKAAEAR murine motif)	-1,28693E+14	4,61315E+14	-2,21379E+14	0.0658057691776726	0.206446722261306	-3,96982E+14	3,46596E+14	4,06526E+14	4,38185E+14	5,72322E+14	4,49071E+14	5,5519E+14
TC0600000487.mm.2	Arhgef5	Rho guanine nucleotide exchange factor (GEF) 5	-0.866340685679507	6,26615E+14	-2,21348E+14	0.0658345414280897	0.206446722261306	-3,97021E+14	5,42239E+14	5,74359E+14	6,0013E+14	6,89737E+13	6,7902E+14	6,74205E+13
TC0500001025.mm.2	Spp1	secreted phosphoprotein 1	2,34188E+14	6,65948E+14	2,21337E+14	0.0658446994458719	0.206446722261306	-3,97035E+14	6,11819E+14	9,33207E+14	4,82304E+14	5,94346E+14	6,20825E+13	7,53187E+14
TC1700002187.mm.2	Gnmt	glycine N-methyltransferase	-0.969544254918313	5,15737E+13	-2,21335E+14	0.0658469139096036	0.206446722261306	-3,97037E+14	4,58457E+14	4,3018E+14	5,21023E+14	5,61522E+13	5,07719E+14	6,15521E+14
TC1100002758.mm.2	Csf2	colony stimulating factor 2 (granulocyte-macrophage)	-1,23643E+14	5,49654E+14	-2,21325E+14	0.0658559022170368	0.206446722261306	-3,9705E+14	4,94757E+14	4,56379E+14	5,21127E+14	6,77294E+13	5,43936E+14	6,04433E+14
TC0700001681.mm.2	1110004F10Rik	RIKEN cDNA 1110004F10 gene	1,3803E+14	8,27514E+14	2,21242E+14	0.065933596807064	0.206650076901813	-3,97154E+14	9,19763E+14	9,18448E+14	8,41382E+14	7,20768E+14	8,61249E+14	7,03473E+14
TC1600001214.mm.2	Gspt1	G1 to S phase transition 1	2,28765E+14	7,69436E+14	2,2121E+14	0.0659637840464938	0.20670448314686	-3,97194E+14	9,33833E+14	9,39163E+14	8,17728E+14	5,97737E+14	8,04175E+14	5,23984E+14
TC0200000480.mm.2	Olfm1	olfactomedin 1	1,3593E+14	9,61011E+14	2,21189E+14	0.0659830073931649	0.206724518399002	-3,9722E+14	1,02097E+14	1,03524E+14	9,70204E+14	8,14144E+14	1,02062E+14	9,04883E+14
TC0500003585.mm.2	Smurf1	SMAD specific E3 ubiquitin protein ligase 1	1,4809E+14	6,68177E+14	2,21148E+14	0.0660223550149292	0.20673952182742	-3,97273E+14	7,30311E+14	7,60151E+14	6,96761E+14	4,9752E+14	6,21947E+14	7,02372E+14
TC0700004647.mm.2	AW146154	expressed sequence AW146154	1,50554E+14	5,12475E+14	2,21117E+14	0.0660507361520923	0.20673952182742	-3,97311E+14	6,02609E+14	5,99845E+14	5,58959E+14	3,42388E+14	5,08413E+14	4,62633E+14
TC0500003084.mm.2	Hnf1a	HNF1 homeobox A	-0.96304320907146	6,42855E+14	-2,21102E+14	0.0660653803037394	0.20673952182742	-3,9733E+13	5,9721E+13	6,11835E+14	6,43798E+14	7,57856E+14	6,11389E+14	6,35039E+14
TC1800001163.mm.2	Hspa9	heat shock protein 9	0.91631496248641	7,42884E+14	2,21099E+14	0.0660682244455008	0.20673952182742	-3,97334E+13	8,32492E+14	8,17264E+14	7,64413E+14	7,02081E+14	7,1341E+14	6,27646E+14
TC0800000112.mm.2	Mcf2l	mcf.2 transforming sequence-like	-0.655448879041066	6,54275E+13	-2,21098E+14	0.066068831004629	0.20673952182742	-3,97335E+14	6,12506E+14	6,01636E+14	6,7373E+14	6,71502E+14	6,71285E+14	6,94992E+14
TC0900001666.mm.2	Sacm1l	SAC1 suppressor of actin mutations 1-like (yeast)	1,99984E+14	6,85462E+14	2,21091E+14	0.0660752986000075	0.20673952182742	-3,97344E+14	8,25265E+14	8,04086E+14	7,3136E+14	4,98024E+14	7,26665E+14	5,27373E+14
TC0800001327.mm.2	Exosc6/Aars	exosome component 6/alanyl-tRNA synthetase	1,77375E+14	7,8743E+14	2,21089E+14	0.0660776104642966	0.20673952182742	-3,97347E+14	9,20849E+14	9,14519E+14	8,44618E+14	6,35999E+14	7,88861E+14	6,19737E+14
TC0100001225.mm.2	Myog	myogenin	-1,22781E+14	6,24679E+14	-2,21042E+14	0.066121630605681	0.206837086769431	-3,97406E+14	5,34197E+14	5,51959E+14	5,94296E+14	7,37422E+14	6,19647E+14	7,10555E+14
TC1900000665.mm.2	Kazald1	Kazal-type serine peptidase inhibitor domain 1	-0.705052886708803	4,72161E+14	-2,20982E+13	0.066177528472217	0.206971761698919	-3,9748E+14	4,49598E+14	4,60147E+13	5,02215E+14	5,48541E+14	4,43599E+14	4,28868E+14
TC1200000718.mm.2	Rdh12	retinol dehydrogenase 12	0.661201361701546	5,58601E+14	2,20946E+14	0.0662115059987809	0.207037841288251	-3,97526E+14	6,15414E+14	5,9617E+14	5,2834E+14	5,51004E+14	5,28329E+14	5,32348E+14
TC0900001973.mm.2	Adamts15	a disintegrin-like and metallopeptidase (reprolysin type) with thrombospondin type 1 motif, 15	-0.740114413010271	5,17986E+14	-2,20927E+14	0.0662299907452639	0.207055460007458	-3,9755E+14	4,71237E+14	4,79265E+14	5,58604E+14	5,3992E+14	5,00278E+14	5,5861E+14
TC0200001170.mm.2	Prdx6b	peroxiredoxin 6B	-0.873331556308836	6,41632E+14	-2,20887E+14	0.0662677951450623	0.207082186041804	-3,97601E+14	5,92794E+14	5,8856E+14	6,48306E+14	7,07715E+14	6,17672E+14	6,94743E+14
TC1200000181.mm.2	Cpsf3	cleavage and polyadenylation specificity factor 3	0.903847000004553	7,32268E+14	2,20873E+14	0.0662802004462878	0.207082186041804	-3,97617E+14	7,93619E+14	7,79498E+14	7,43393E+14	6,48955E+14	6,92851E+14	7,35294E+14
TC1400001471.mm.2	Psmd6	proteasome (prosome, macropain) 26S subunit, non-ATPase, 6	1,74857E+14	6,53943E+14	2,20855E+14	0.0662976169581324	0.207082186041804	-3,9764E+14	7,78643E+14	7,76912E+14	6,82525E+14	5,23316E+13	6,86784E+14	4,75476E+14
TC0500003198.mm.2	Sh2b3	SH2B adaptor protein 3	-0.823951035747391	7,58982E+14	-2,20842E+14	0.0663094438450547	0.207082186041804	-3,97656E+13	6,95131E+14	7,16108E+14	7,46682E+14	8,29348E+14	7,81623E+14	7,85003E+14
TC1000000399.mm.2	Bend3	BEN domain containing 3	-0.799856774745475	6,60512E+14	-2,20839E+14	0.0663129901520862	0.207082186041804	-3,97661E+14	6,06264E+14	6,06197E+13	6,47077E+14	7,25355E+14	6,83465E+14	6,94713E+13
TC1000003023.mm.2	Hmga2	high mobility group AT-hook 2	-1,12493E+14	7,6815E+14	-2,20836E+14	0.0663156507153049	0.207082186041804	-3,97664E+14	6,71971E+14	7,05633E+14	7,31843E+14	8,70747E+14	7,90228E+14	8,38479E+14
TC0400002516.mm.2	Stoml2	stomatin (Epb7.2)-like 2	1,51253E+14	7,55838E+14	2,2081E+14	0.0663398858401284	0.207117725352062	-3,97697E+14	8,66497E+14	8,81932E+14	7,99635E+14	6,46287E+14	7,50924E+14	5,89754E+14
TC0X00000859.mm.2	4932429P05Rik	RIKEN cDNA 4932429P05 gene	-1,25796E+14	4,39426E+14	-2,20773E+13	0.0663752910190031	0.207145082577528	-3,97744E+14	2,9636E+14	3,99507E+14	4,10492E+13	5,36968E+14	4,63057E+14	5,30169E+14
TC1000002897.mm.2	Rab21	RAB21, member RAS oncogene family	1,09504E+14	8,05733E+14	2,20771E+14	0.0663767160860299	0.207145082577528	-3,97746E+14	8,81472E+14	8,70167E+12	8,22069E+14	7,10563E+14	8,2106E+14	7,29067E+14

TC1300000887.mm.2	Slc12a7	solute carrier family 12, member 7	0.719893604361968	5,22387E+13	2,2074E+14	0.0664062896843203	0.207145082577528	-3,97785E+14	5,66022E+14	5,2142E+14	4,7235E+14	4,71113E+14	5,35349E+14	5,68069E+14
TC1300001353.mm.2	Hmgcs1	3-hydroxy-3-methylglutaryl-Coenzyme A synthase 1	2,05084E+14	9,05259E+14	2,20734E+14	0.0664113818603922	0.207145082577528	-3,97792E+14	1,02208E+14	1,03286E+14	9,65086E+14	6,79686E+14	9,34542E+14	7,97302E+14
TC0600002285.mm.2	Hoxa11	homeobox A11	-0.761121327316774	5,99953E+14	-2,20721E+14	0.0664237957600263	0.207145082577528	-3,97808E+14	5,6339E+14	5,23768E+14	6,02006E+14	6,37376E+14	6,15055E+14	6,58121E+14
TC1000002515.mm.2	Jsrp1	junctional sarcoplasmic reticulum protein 1	-0.851793819792097	6,42023E+14	-2,20719E+13	0.0664257830123558	0.207145082577528	-3,97811E+14	5,7818E+14	5,95288E+14	6,30382E+14	7,13445E+14	6,49084E+14	6,85756E+14
TC1700000924.mm.2	Tfeb	transcription factor EB	-0.657380637393312	7,73237E+14	-2,20693E+14	0.0664508139060794	0.207183042660344	-3,97844E+14	7,26674E+14	7,22644E+14	7,76509E+14	8,04286E+14	8,05867E+14	8,03441E+14
TC0100002534.mm.2	Ikzf2	IKAROS family zinc finger 2	-0.718801897873924	6,03561E+14	-2,20643E+14	0.0664981128970426	0.207243493221567	-3,97907E+14	5,58881E+14	5,67074E+14	6,18875E+14	6,50841E+14	5,90808E+14	6,34887E+14
TC0700004652.mm.2	Snrpn/Snurf	small nuclear ribonucleoprotein N/SNRPN upstream reading frame	1,36051E+14	1,13219E+14	2,20632E+14	0.0665081743557458	0.207243493221567	-3,97921E+14	1,21806E+14	1,21335E+14	1,15683E+14	1,00249E+14	1,16129E+13	1,04111E+14
TC1200000278.mm.2	Ccdc71l	coiled-coil domain containing 71 like	-0.65809503453878	6,0531E+14	-2,20621E+14	0.0665184648919034	0.207243493221567	-3,97934E+14	5,47225E+14	5,6785E+14	6,21449E+14	6,25245E+14	6,2522E+14	6,4487E+13
TC0200004433.mm.2	Ino80	INO80 complex subunit	1,79609E+14	6,08799E+14	2,20615E+14	0.0665243894752417	0.207243493221567	-3,97942E+14	7,25631E+14	7,27978E+14	6,36038E+14	4,58353E+14	6,5614E+14	4,48653E+14
TC0200001127.mm.2	Osbpl6	oxysterol binding protein-like 6	0.887734751342171	6,48721E+14	2,20604E+14	0.0665345119118206	0.207243493221567	-3,97956E+14	7,66752E+14	6,98684E+13	6,77452E+14	6,10437E+14	5,85968E+14	5,5303E+14
TC0700001399.mm.2	Chrdl2	chordin-like 2	-0.763214362983422	4,82804E+14	-2,2057E+14	0.0665666445803472	0.207286863785938	-3,97998E+14	4,22409E+14	4,20294E+14	4,89088E+14	5,06258E+14	4,95914E+14	5,6286E+14
TC1400001824.mm.2	Gm17026	predicted gene 17026	-0.689962563997363	4,70839E+14	-2,20562E+13	0.0665741649656943	0.207286863785938	-3,98008E+14	4,25815E+14	4,11157E+14	4,6659E+14	5,08375E+14	5,07869E+14	5,05227E+14
TC1700002190.mm.2	Ptcra	pre T cell antigen receptor alpha	-0.725650581634832	7,18646E+14	-2,20542E+14	0.0665935717840545	0.20729134679863	-3,98034E+14	6,8596E+14	6,61402E+14	7,60832E+14	7,3166E+14	7,14494E+14	7,57528E+14
TC0500001927.mm.2	Adam22	a disintegrin and metallopeptidase domain 22	1,97506E+14	7,5907E+14	2,20533E+14	0.0666013344738104	0.20729134679863	-3,98044E+14	8,96662E+14	8,98478E+14	8,17154E+14	5,82974E+14	7,76655E+14	5,825E+14
TC0200002013.mm.2	Pank2	pantothenate kinase 2	1,75584E+13	7,8418E+14	2,20492E+14	0.0666409561427453	0.207338763472704	-3,98097E+14	8,95477E+14	9,0009E+14	8,35334E+14	6,09064E+14	8,02775E+14	6,62339E+14
TC1300000006.mm.2	Gdi2	guanosine diphosphate (GDP) dissociation inhibitor 2	1,44885E+14	9,11221E+14	2,2049E+14	0.0666423047244544	0.207338763472704	-3,98099E+14	1,0013E+14	1,01044E+14	9,44746E+14	7,77232E+13	9,31552E+14	8,02049E+14
TC0300002067.mm.2	Dhx36	DEAH (Asp-Glu-Ala-His) box polypeptide 36	2,03605E+13	7,73986E+14	2,20469E+14	0.0666621061313671	0.207343095395767	-3,98125E+14	9,02491E+14	9,03622E+14	8,28954E+14	5,69948E+14	8,08877E+14	6,30026E+14
TC1500000488.mm.2	Tg	thyroglobulin	-0.746594345643032	5,08752E+14	-2,20462E+14	0.0666694332058257	0.207343095395767	-3,98135E+14	4,9765E+14	4,6543E+14	5,30652E+14	5,81747E+14	4,77524E+14	4,99509E+14
TC1100003141.mm.2	Slc13a5	solute carrier family 13 (sodium-dependent citrate transporter), member 5	0.881825668931372	5,56975E+13	2,20437E+12	0.0666928882081868	0.207376014607973	-3,98166E+14	6,12017E+14	6,40652E+13	5,12896E+14	5,63408E+14	4,65842E+14	5,47036E+13
TC0600003428.mm.2	Bhlhe41	basic helix-loop-helix family, member e41	0.991451048220959	7,76137E+14	2,2039E+14	0.066737283107134	0.207387657149687	-3,98225E+14	8,36589E+14	8,46996E+14	7,98185E+14	6,8711E+14	7,71193E+14	7,16747E+13
TC0700000513.mm.2	Hnrnpl	heterogeneous nuclear ribonucleoprotein L	1,11774E+14	9,00664E+14	2,20378E+14	0.0667485636460374	0.207387657149687	-3,9824E+14	9,74949E+12	9,64387E+14	9,26416E+14	7,89372E+14	9,04326E+14	8,44537E+14
TC1400001708.mm.2	3425401B19Rik	RIKEN cDNA 3425401B19 gene	-0.699044170198357	4,75216E+13	-2,20371E+14	0.0667550479348775	0.207387657149687	-3,98248E+14	4,3456E+14	4,06506E+14	4,70842E+14	5,10033E+14	5,14271E+14	5,15088E+14
TC0X00003425.mm.2	Cul4b	cullin 4B	2,9953E+14	6,55352E+14	2,2035E+14	0.0667751668221431	0.207387657149687	-3,98275E+14	8,32104E+14	8,6167E+14	7,30217E+13	3,64498E+14	7,23805E+14	4,19816E+14
TC1100000816.mm.2	Tvp23b	trans-golgi network vesicle protein 23B	0.727284949623671	6,98639E+13	2,2034E+14	0.0667848358535721	0.207387657149687	-3,98288E+14	7,12766E+14	7,16317E+14	6,52992E+14	6,30634E+14	7,12721E+14	7,66405E+14
TC0400004178.mm.2	Dnajc25	DnaJ heat shock protein family (Hsp40) member C25	-0.879856421823171	6,28662E+14	-2,2034E+13	0.0667849096348975	0.207387657149687	-3,98288E+14	5,70839E+13	5,73174E+14	6,26875E+14	6,93109E+14	6,91061E+14	6,16914E+14
TC0600000463.mm.2	Tas2r126	taste receptor, type 2, member 126	-0.813604158205175	5,03677E+14	-2,20326E+14	0.066797774827531	0.207387657149687	-3,98305E+14	4,43583E+14	4,55767E+14	5,00262E+12	5,61809E+14	5,03008E+14	5,5763E+14
TC1800000253.mm.2	Iws1	IWS1, SUPT6 interacting protein	0.720045513455283	6,59258E+14	2,20308E+14	0.0668150499909926	0.207387657149687	-3,98328E+14	7,13365E+14	6,80291E+14	6,09681E+14	6,39965E+14	6,72781E+14	6,39466E+14
TC1600000044.mm.2	Ubn1	ubinuclein 1	0.813933017037918	6,07713E+14	2,20284E+13	0.0668377557842601	0.207387657149687	-3,98358E+14	6,57285E+13	6,63404E+14	6,18758E+14	5,39145E+14	5,95733E+14	5,71955E+13
TC1500001099.mm.2	Amhr2	anti-Mullerian hormone type 2 receptor	-0.736921798203013	5,32188E+14	-2,20267E+13	0.0668545659693158	0.207387657149687	-3,9838E+13	4,69686E+14	4,88815E+14	5,25769E+14	5,80116E+14	5,75077E+14	5,53667E+14
TC1600001473.mm.2	Acap2	ArfGAP with coiled-coil, ankyrin repeat and PH domains 2	1,285E+14	8,12789E+14	2,2026E+14	0.0668605208972842	0.207387657149687	-3,98388E+14	8,72334E+14	8,87655E+14	8,38707E+14	6,64281E+14	8,31868E+14	7,8189E+14
TC0200004875.mm.2	4921509C19Rik	RIKEN cDNA 4921509C19 gene	-0.73021725798144	5,08498E+14	-2,2025E+14	0.0668702185326794	0.207387657149687	-3,984E+14	4,56921E+14	4,65701E+14	5,16256E+13	5,5241E+14	5,06747E+14	5,52952E+14
TC0100002071.mm.2	Fam135a	family with sequence similarity 135, member A	0.692326711111526	5,86786E+14	2,20245E+14	0.0668753707848414	0.207387657149687	-3,98407E+14	6,72827E+14	6,29466E+13	5,89081E+14	5,74747E+14	5,28428E+14	5,26165E+14
TC1300002583.mm.2	Srek1	splicing regulatory glutamine/lysine-rich protein 1	0.67307278593804	7,91935E+14	2,20243E+14	0.0668768240892307	0.207387657149687	-3,98409E+14	8,30692E+14	8,34116E+14	7,78034E+14	7,52159E+14	7,91651E+14	7,64956E+14
TC0400002531.mm.2	Gba2	glucosidase beta 2	0.695227244739185	7,31802E+14	2,20215E+13	0.0669040810198051	0.207406704913972	-3,98445E+14	7,82074E+14	7,96525E+14	7,01425E+14	7,38129E+14	6,99027E+14	6,73631E+14
TC0X00001341.mm.2	H2bfm	H2B histone family, member M	-1,52596E+14	4,44709E+14	-2,20187E+14	0.0669301217806058	0.207406704913972	-3,9848E+14	3,69214E+14	3,60195E+14	4,36748E+14	5,97854E+14	3,72879E+14	5,31365E+14
TC1500000939.mm.2	Ano6	anoctamin 6	0.850271656543802	6,42457E+14	2,20186E+14	0.0669314082106422	0.207406704913972	-3,98481E+14	6,83201E+14	6,94161E+14	6,49058E+14	5,5825E+14	6,28058E+14	6,42012E+14
TC0600000758.mm.2	Smarcad1	SWI/SNF-related, matrix-associated actin-dependent regulator of chromatin, subfamily a, containing DEAD/H box 1	1,08367E+14	5,74733E+14	2,20183E+14	0.0669344545120495	0.207406704913972	-3,98485E+14	6,55127E+13	6,47082E+14	6,13848E+13	4,71627E+14	5,22538E+14	5,38174E+13
TC1400000747.mm.2	Lrp10	low-density lipoprotein receptor-related protein 10	0.679077362443315	7,10875E+14	2,20158E+14	0.0669577035006362	0.207438853394684	-3,98516E+14	7,31603E+14	7,55865E+14	6,67496E+14	6,84157E+14	6,99351E+14	7,2678E+14
TC1100000857.mm.2	Myh13	myosin, heavy polypeptide 13, skeletal muscle	-0.995975860964984	6,4921E+14	-2,2012E+14	0.0669939131175645	0.207511134575801	-3,98564E+14	5,85139E+13	5,81532E+14	6,39608E+14	7,26258E+14	6,36107E+13	7,26615E+14
TC1400001103.mm.2	Kbtbd7	kelch repeat and BTB (POZ) domain containing 7	2,48602E+13	5,82895E+14	2,20042E+14	0.0670681128389106	0.207701038280485	-3,98662E+14	7,76527E+14	7,64434E+14	6,64845E+14	3,78912E+14	5,90159E+13	3,22494E+14
TC0X00003185.mm.2	Ribc1	RIB43A domain with coiled-coils 1	-0.73622593180117	6,96246E+14	-2,20012E+14	0.0670966018863617	0.207749336317246	-3,98699E+14	6,2665E+14	6,52013E+13	6,85464E+14	7,40444E+14	7,31026E+14	7,41878E+13
TC1100000096.mm.2	Ppia	peptidylprolyl isomerase A	1,27816E+13	1,0963E+14	2,19968E+14	0.0671394928194245	0.207842199385089	-3,98756E+14	1,16481E+14	1,16575E+14	1,10114E+14	9,7379E+14	1,15055E+14	1,02175E+14
TC0700002612.mm.2	Lypd4	Ly6/Plaur domain containing 4	-1,5664E+14	5,66424E+14	-2,1984E+14	0.0672617656653508	0.208109680831966	-3,98916E+14	5,10493E+14	4,25819E+14	5,36309E+14	7,13285E+14	5,42705E+14	6,69935E+12
TC0400002330.mm.2	Manea	mannosidase, endo-alpha	1,84633E+14	7,12532E+14	2,19836E+14	0.0672652317410126	0.208109680831966	-3,98921E+14	8,56054E+14	8,46938E+12	7,66364E+14	5,67362E+14	7,19759E+14	5,18715E+14
TC1100002921.mm.2	Ulk2	unc-51 like kinase 2	1,18528E+14	7,39637E+14	2,19822E+14	0.0672789042636882	0.208109680831966	-3,98939E+14	8,20545E+14	8,08497E+14	7,63286E+14	6,28699E+13	7,54541E+14	6,62257E+14
TC0600001922.mm.2	Fam3c	family with sequence similarity 3, member C	1,24995E+14	7,25015E+14	2,19816E+14	0.06728466283781	0.208109680831966	-3,98946E+14	7,96659E+14	8,00766E+14	7,46044E+14	6,01389E+14	7,52685E+14	6,52548E+14
TC0400003520.mm.2	Dnali1	dynein, axonemal, light intermediate polypeptide 1	-0.70927897922259	6,1273E+14	-2,1981E+14	0.0672904758353219	0.208109680831966	-3,98954E+14	5,57395E+14	5,8345E+14	6,19866E+14	6,62835E+14	6,18538E+12	6,34298E+14
TC0500002311.mm.2	Gm7854	predicted gene 7854	-0.720429034293147	8,02194E+14	-2,19776E+14	0.0673229939718751	0.208170293968302	-3,98997E+14	7,50536E+14	7,49054E+14	8,10444E+14	8,33232E+14	8,09062E+14	8,60838E+14
TC0300002403.mm.2	Lce1a2	late cornified envelope 1A2	-0.639878742893838	8,37164E+14	-2,19736E+14	0.0673611330582442	0.208248261121928	-3,99047E+14	7,88943E+14	7,93738E+13	8,57497E+14	8,5316E+14	8,5697E+14	8,72677E+14
TC1500000662.mm.2	H1f0	H1 histone family, member 0	1,35648E+14	7,03293E+14	2,19654E+14	0.0674393306137678	0.208431002674533	-3,99149E+14	7,91273E+14	7,6289E+14	7,31176E+14	5,51691E+14	6,54583E+14	7,28142E+14
TC0800000402.mm.2	Mboat4	membrane bound O-acyltransferase domain containing 4	-0.988626605691151	4,7783E+14	-2,19645E+14	0.0674478118303599	0.208431002674533	-3,9916E+14	4,46442E+14	3,96569E+14	5,09657E+14	5,3108E+14	4,35732E+14	5,47499E+14
TC0500002157.mm.2	Otof	otoferlin	-0.894027117016151	6,96632E+14	-2,19625E+14	0.0674676514123324	0.208431002674533	-3,99186E+14	6,34494E+14	6,21721E+14	6,66977E+14	7,68043E+14	7,3538E+14	7,53178E+13
TC1500001651.mm.2	Lrrc6	leucine rich repeat containing 6 (testis)	0.977632882092521	5,67771E+14	2,19615E+14	0.0674770501301109	0.208431002674533	-3,99199E+14	6,06052E+14	6,78676E+14	5,85182E+14	5,04019E+14	4,8893E+14	5,43768E+13
TC0500001447.mm.2	Tmed2	transmembrane p24 trafficking protein 2	1,2411E+14	8,59841E+13	2,19607E+14	0.0674849215511103	0.208431002674533	-3,99209E+14	9,14028E+14	9,35291E+14	8,78633E+14	7,22466E+14	8,91797E+13	8,16828E+14
TC0200002421.mm.2	Rprd1b	regulation of nuclear pre-mRNA domain containing 1B	1,7596E+14	7,19944E+14	2,1957E+14	0.0675204962339468	0.208493906635789	-3,99255E+13	8,50419E+14	8,29565E+14	7,84763E+14	5,433E+14	7,13345E+13	5,98275E+14
TC1300002270.mm.2	Adcy2	adenylate cyclase 2	1,29551E+13	7,55315E+14	2,19559E+14	0.0675311673016931	0.208493906635789	-3,99269E+14	8,35362E+14	8,37705E+14	7,52115E+14	6,61849E+14	8,03063E+13	6,41795E+14
TC1500001045.mm.2	Gm5475	predicted gene 5475	-0.937781682668115	5,7435E+14	-2,19537E+14	0.067551844378394	0.208517790894457	-3,99296E+14	5,04864E+14	5,09485E+14	5,51824E+14	6,50081E+14	5,86752E+14	6,43092E+14
TC0X00002669.mm.2	Zc4h2	zinc finger, C4H2 domain containing	1,24861E+14	7,2416E+13	2,19509E+14	0.067578547646353	0.20855657890035	-3,99331E+14	8,06019E+14	7,89132E+14	7,56845E+14	5,88583E+14	7,26677E+14	6,77702E+14
TC0300000680.mm.2	Lrba	LPS-responsive beige-like anchor	0.794022224715278	7,26307E+14	2,19497E+14	0.0675902969662775	0.20855657890035	-3,99347E+14	7,52723E+13	7,85253E+14	7,25248E+14	6,53923E+13	7,24443E+14	7,16252E+14
TC0200003080.mm.2	Arhgap21	Rho GTPase activating protein 21	2,63251E+14	6,32543E+14	2,19467E+14	0.0676188148446755	0.208604626381822	-3,99384E+14	8,0272E+14	8,14544E+14	6,98806E+14	3,91956E+14	6,85157E+14	4,02076E+14
TC1700001247.mm.2	Mta3	metastasis associated 3	1,95939E+14	8,03011E+14	2,19435E+14	0.0676503571287255	0.208637988074727	-3,99425E+14	9,40872E+13	9,09091E+14	8,5474E+14	6,03347E+14	8,38254E+14	6,71763E+14
TC0800002815.mm.2	Exoc3l	exocyst complex component 3-like	-1,29674E+14	5,26636E+14	-2,19429E+14	0.0676555258294822	0.208637988074727	-3,99432E+14	4,66921E+13	4,71644E+14	5,37017E+14	6,60896E+13	4,42876E+13	5,80462E+14
TC0800002535.mm.2	Usp38	ubiquitin specific peptidase 38	0.688748931861344	6,6414E+14	2,19356E+14	0.0677255736312514	0.208663642026191	-3,99523E+14	7,32035E+13	7,13094E+14	6,69299E+14	6,3808E+13	6,29661E+14	6,02673E+13
TC1200002299.mm.2	Syne3	spectrin repeat containing, nuclear envelope family member 3	-0.724248711524914	6,86155E+14	-2,19329E+14	0.06775172518981 9	0.208663642026191	-3,99557E+14	6,30142E+14	6,36414E+14	6,76716E+14	7,34689E+14	7,20692E+14	7,18276E+14
TC0200003234.mm.2	Gfi1b	growth factor independent 1B	-0.905546520606005	7,45909E+14	-2,19324E+14	0.0677564064130766	0.208663642026191	-3,99564E+14	6,51003E+14	7,21904E+13	7,41014E+14	8,13002E+12	7,57702E+14	7,90831E+14
TC1100003062.mm.2	Slc35g3	solute carrier family 35, member G3	-1,03266E+14	5,8998E+14	-2,19318E+14	0.0677622830951764	0.208663642026191	-3,99571E+14	5,30788E+13	5,41536E+14	5,99657E+13	6,79201E+14	5,4084E+14	6,4786E+14
TC0800000337.mm.2	Thap1	THAP domain containing, apoptosis associated protein 1	-1,12128E+14	7,20897E+14	-2,19311E+14	0.0677692239307226	0.208663642026191	-3,9958E+14	6,14876E+14	6,68675E+14	6,91471E+14	8,16336E+14	7,33413E+14	8,00614E+14
TC0700001640.mm.2	Usp47	ubiquitin specific peptidase 47	0.715941547424396	8,08128E+14	2,19307E+14	0.0677733217265887	0.208663642026191	-3,99586E+14	8,48123E+14	8,75161E+14	8,11805E+14	7,68291E+13	7,83469E+14	7,61919E+14
TC0700002839.mm.2	Chst8	carbohydrate sulfotransferase 8	-0.886688456238307	8,07039E+13	-2,19301E+14	0.0677789104061 81	0.208663642026191	-3,99593E+14	7,53119E+14	7,5227E+14	7,97271E+14	8,85456E+14	8,01414E+13	8,52702E+13
TC0600000151.mm.2	Lsm8	LSM8 homolog, U6 small nuclear RNA associated	0.764813034934638	6,16136E+14	2,193E+14	0.0677801493150336	0.208663642026191	-3,99595E+14	6,81243E+14	6,38159E+14	5,77766E+14	5,88674E+13	6,331E+14	5,77874E+14
TC0800000834.mm.2	Sin3b	transcriptional regulator, SIN3B (yeast)	0.758351480067764	7,58552E+14	2,19288E+14	0.0677911197736986	0.208663642026191	-3,99609E+14	8,1654E+14	7,91961E+14	7,57588E+14	6,99243E+14	7,56731E+13	7,29248E+14
TC1500000303.mm.2	Rims2	regulating synaptic membrane exocytosis 2	1,15501E+14	7,61078E+14	2,19266E+14	0.0678128832314243	0.208663642026191	-3,99637E+14	8,41054E+14	8,25782E+14	7,50521E+14	6,85313E+14	8,03637E+14	6,60164E+14
TC0300000186.mm.2	Egfem1	EGF-like and EMI domain containing 1	0.857878213897294	6,3196E+14	2,19259E+14	0.067819055270521	0.208663642026191	-3,99645E+14	7,09829E+14	6,2855E+14	6,06931E+14	5,59872E+14	6,35382E+14	6,51198E+14
TC0100002554.mm.2	Pecr	peroxisomal trans-2-enoyl-CoA reductase	-0.892168804915345	7,21083E+14	-2,19259E+14	0.067819244913496	0.208663642026191	-3,99645E+14	6,59863E+14	6,63559E+14	6,99523E+14	8,02334E+14	7,45795E+14	7,55427E+14
TC1900000915.mm.2	Ankrd13d	ankyrin repeat domain 13 family, member D	0.851193857271581	7,42578E+14	2,19206E+14	0.0678710154265903	0.208783060627845	-3,99713E+14	8,11315E+14	7,64282E+14	7,32288E+14	6,7307E+14	7,09615E+14	7,64897E+14
TC1400002100.mm.2	Dhrs1	dehydrogenase/reductase (SDR family) member 1	1,47685E+14	9,43989E+13	2,1918E+14	0.0678954028612645	0.208818214602702	-3,99745E+14	9,98792E+14	1,04455E+14	9,75737E+14	7,72231E+14	9,6897E+14	9,03654E+14
TC1100004156.mm.2	Usp36	ubiquitin specific peptidase 36	-0.672753240240742	7,23558E+14	-2,19148E+14	0.0679264750200114	0.20887112285257	-3,99785E+14	6,79989E+14	6,94463E+14	7,44306E+14	7,64697E+14	7,14135E+14	7,43756E+14
TC0800002758.mm.2	Got2	glutamatic-oxaloacetic transaminase 2, mitochondrial	1,3938E+14	1,01386E+14	2,19136E+14	0.067938531302074	0.20887112285257	-3,99801E+14	1,12506E+14	1,10189E+14	1,05345E+14	8,94744E+14	1,01665E+14	8,91388E+14
TC1800001119.mm.2	Gpr17	G protein-coupled receptor 17	1,14817E+14	6,19447E+14	2,19121E+14	0.067953027842501	0.208875837013777	-3,9982E+14	7,08183E+14	6,85512E+14	6,59279E+13	5,04781E+14	5,88291E+14	5,70635E+14
TC0700002075.mm.2	Syt8	synaptotagmin VIII	-1,03759E+14	6,17113E+14	-2,19081E+14	0.0679917898419872	0.208931084078529	-3,9987E+14	5,28199E+14	5,55271E+14	5,96796E+14	6,94193E+14	6,19355E+14	7,08866E+14
TC0200000612.mm.2	Mapkap1	mitogen-activated protein kinase associated protein 1	2,2338E+14	6,20874E+14	2,19038E+14	0.0680332866079566	0.208931084078529	-3,99924E+14	7,7528E+14	7,73825E+14	6,96837E+14	4,05507E+14	6,34712E+14	4,39083E+14
TC0200001547.mm.2	Fbxo3	F-box protein 3	1,7357E+14	7,57382E+14	2,19034E+14	0.0680373415205242	0.208931084078529	-3,99929E+14	8,62274E+14	8,53441E+14	8,02024E+14	5,66552E+14	7,9046E+14	6,69543E+14
TC1200001461.mm.2	Greb1	gene regulated by estrogen in breast cancer protein	-1,20512E+14	5,7525E+14	-2,19032E+13	0.0680385002908078	0.208931084078529	-3,99931E+14	5,35439E+14	4,76277E+13	5,67832E+14	6,84907E+14	5,40822E+14	6,46223E+14
TC0600001251.mm.2	Il17re	interleukin 17 receptor E	-0.935312734748974	4,84056E+14	-2,19012E+14	0.0680578759052927	0.208931084078529	-3,99956E+14	4,00901E+14	4,55523E+14	4,93641E+14	5,49846E+14	4,61299E+14	5,43129E+14
TC0400001266.mm.2	Mutyh	mutY DNA glycosylase	-0.890027765697928	6,42651E+14	-2,19009E+14	0.0680609464622159	0.208931084078529	-3,9996E+14	5,94289E+14	5,52083E+14	6,41942E+14	6,82436E+14	6,50145E+14	7,35008E+14
TC1300000070.mm.2	Heatr1	HEAT repeat containing 1	1,16455E+13	5,13617E+14	2,19008E+14	0.0680617675372803	0.208931084078529	-3,99961E+14	5,87378E+13	6,02244E+14	4,1769E+14	5,39021E+14	5,19659E+14	4,15712E+14
TC1700002216.mm.2	Foxp4	forkhead box P4	-0.669800943442497	7,63508E+14	-2,18977E+14	0.0680920359050517	0.208971952941172	-4E+13	7,39656E+14	7,3475E+14	7,85987E+14	8,2238E+13	7,53224E+14	7,45052E+14
TC0900000826.mm.2	Dpp8	dipeptidylpeptidase 8	1,26076E+14	7,78964E+14	2,18964E+14	0.0681043037509147	0.208971952941172	-4,00016E+14	8,75353E+14	8,87897E+13	7,90736E+14	7,20363E+14	7,81741E+14	6,17695E+14
TC1800000583.mm.2	Slc12a2	solute carrier family 12, member 2	1,06324E+13	7,5769E+14	2,18954E+14	0.068114580146148	0.208971952941172	-4,00029E+14	8,25437E+14	8,38404E+14	7,47701E+14	7,03491E+14	7,81862E+14	6,49244E+13
TC1500002231.mm.2	2310068J16Rik	RIKEN cDNA 2310068J16 gene	-0.661768024184033	5,1211E+14	-2,18941E+14	0.0681269576615141	0.208971952941172	-4,00045E+14	4,76895E+14	4,45424E+14	5,21386E+14	5,33288E+14	5,47605E+14	5,48063E+14
TC0500003318.mm.2	Sbds	SBDS ribosome maturation factor	0.972233104215055	8,48845E+14	2,18871E+14	0.0681944138227487	0.209076094877285	-4,00133E+14	9,22505E+14	9,03099E+14	8,6071E+14	7,70448E+14	8,57637E+12	7,78669E+14
TC0300001559.mm.2	Lrriq3	leucine-rich repeats and IQ motif containing 3	-0.796995492243557	5,28712E+14	-2,18866E+14	0.0681995155673317	0.209076094877285	-4,00139E+14	5,20477E+14	4,93893E+14	5,49094E+14	6,24676E+14	4,89982E+14	4,94151E+13
TC0200002411.mm.2	Nnat	neuronatin	1,05462E+14	9,77234E+14	2,18865E+13	0.0682003534059404	0.209076094877285	-4,0014E+13	1,0997E+13	1,02301E+14	1,00376E+14	9,08027E+14	9,4686E+14	8,82053E+14
TC1800000259.mm.2	Ercc3	excision repair cross-complementing rodent repair deficiency, complementation group 3	0.673850058077423	7,39417E+14	2,18852E+14	0.0682128114423066	0.209076094877285	-4,00157E+14	7,97597E+14	7,97885E+14	7,42266E+14	7,18447E+14	7,06024E+14	6,7428E+14
TC1100004304.mm.2	Mafg	v-maf musculoaponeurotic fibrosarcoma oncogene family, protein G (avian)	-1,23592E+13	7,26467E+14	-2,1883E+14	0.0682342351330351	0.209101983776834	-4,00184E+13	6,21977E+14	6,71455E+14	6,94792E+14	8,45824E+14	7,265E+14	7,98254E+14
TC1000001584.mm.2	Atp5b	ATP synthase, H+ transporting mitochondrial F1 complex, beta subunit	1,86873E+14	1,05068E+14	2,18809E+14	0.0682548503203356	0.209105986797404	-4,00211E+14	1,15846E+14	1,15658E+14	1,0996E+14	8,41689E+14	1,08842E+14	9,59316E+14
TC1200001283.mm.2	Tedc1	tubulin epsilon and delta complex 1	-0.983126985399861	4,73932E+14	-2,18799E+14	0.0682643196425394	0.209105986797404	-4,00223E+14	3,90566E+14	4,29131E+14	4,55279E+14	5,61044E+14	4,77924E+14	5,29647E+14
TC0700003810.mm.2	Rnf169	ring finger protein 169	1,71464E+14	8,49522E+14	2,18789E+14	0.0682744738125206	0.209105986797404	-4,00236E+14	9,62216E+14	9,5669E+14	9,1794E+14	6,58039E+14	8,15219E+14	7,87028E+14
TC1600001048.mm.2	Morc3	microrchidia 3	0.6813934193282	6,02399E+14	2,18748E+14	0.0683141343430642	0.209168071677845	-4,00288E+14	6,4335E+14	6,11066E+14	5,45878E+14	5,72259E+14	6,15367E+14	6,26471E+14
TC0900000571.mm.2	Gm4791	predicted gene 4791	-1,08806E+14	6,58425E+14	-2,18741E+14	0.0683207075802455	0.209168071677845	-4,00296E+14	6,08408E+14	5,79202E+14	6,42631E+14	7,62592E+14	6,40255E+14	7,17463E+14
TC1700001822.mm.2	Cbs	cystathionine beta-synthase	0.852412629701952	6,57653E+14	2,18704E+14	0.0683575242028229	0.209224413546442	-4,00344E+14	7,02956E+14	7,1739E+14	5,93237E+14	6,56627E+14	6,03601E+14	6,72109E+14
TC0800000996.mm.2	Phkb	phosphorylase kinase beta	2,44309E+14	7,0551E+14	2,18696E+14	0.0683650802036877	0.209224413546442	-4,00353E+14	8,49058E+14	8,31037E+14	7,79319E+14	4,12159E+14	7,41479E+14	6,2001E+14
TC1500000389.mm.2	Ccn3	cellular communication network factor 3	2,09261E+14	7,55537E+14	2,18675E+13	0.0683849420135303	0.209245455879987	-4,00379E+14	8,54046E+14	8,72373E+14	8,17328E+14	4,90569E+14	7,53087E+14	7,4582E+14
TC0600000920.mm.2	Lbx2	ladybird homeobox 2	-0.831517048627211	6,24396E+13	-2,18643E+14	0.0684167788227491	0.209259265430289	-4,0042E+14	5,61259E+14	5,66019E+14	6,05218E+14	6,88364E+14	6,46568E+14	6,78945E+14
TC0100000224.mm.2	Ptpn18	protein tyrosine phosphatase, non-receptor type 18	-0.789019429505892	5,45148E+14	-2,18641E+14	0.0684180089608308	0.209259265430289	-4,00422E+14	4,54622E+14	4,89408E+14	5,24637E+14	5,77198E+14	5,8928E+14	6,35742E+14
TC1500002142.mm.2	Slc38a2	solute carrier family 38, member 2	0.840076200509889	9,23696E+12	2,18629E+14	0.0684296670784727	0.209259265430289	-4,00437E+14	9,52574E+14	9,91673E+14	9,0544E+14	8,70792E+14	9,48406E+14	8,73292E+14
TC1400002523.mm.2	Pcdh20	protocadherin 20	0.763190921736038	5,57076E+14	2,18617E+14	0.0684414031414153	0.209259265430289	-4,00452E+14	5,69159E+14	6,1304E+14	5,41492E+13	4,88069E+14	5,73667E+14	5,57032E+14
TC0400001544.mm.2	Snrnp40	small nuclear ribonucleoprotein 40 (U5)	1,01591E+14	6,63507E+14	2,18576E+14	0.068481751739558	0.209294125477691	-4,00504E+14	6,9639E+14	7,06162E+14	6,64925E+13	5,34444E+14	6,86186E+14	6,92937E+14
TC1300001051.mm.2	Dmgdh	dimethylglycine dehydrogenase precursor	-1,07634E+14	6,47811E+14	-2,18562E+14	0.0684955582348517	0.209294125477691	-4,00522E+14	5,83563E+13	5,70354E+14	6,27338E+14	7,41847E+14	6,39199E+14	7,24563E+14
TC0500001506.mm.2	Mmp17	matrix metallopeptidase 17	0.711516239422771	7,29003E+14	2,18558E+14	0.0684990819898053	0.209294125477691	-4,00526E+14	7,79231E+14	7,71306E+14	6,76963E+13	7,31271E+14	7,08083E+14	7,07165E+14

TC1500001939.mm.2	Tnfrsf13c	tumor necrosis factor receptor superfamily, member 13c	-0.712961181858388	6,73525E+14	-2,18552E+14	0.0685047612343662	0.209294125477691	-4,00534E+14	6,28568E+13	6,38271E+14	6,80229E+14	7,29202E+14	6,94445E+14	6,70433E+13
TC1400001709.mm.2	Wdfy4	WD repeat and FYVE domain containing 4	-1,0145E+14	6,14144E+14	-2,18505E+13	0.0685505897242019	0.209327540067909	-4,00593E+13	5,56375E+14	5,43996E+14	6,09742E+14	6,9353E+14	5,90778E+14	6,90444E+14
TC1700001018.mm.2	Prr22	proline rich 22	-0.994115439989121	6,66759E+14	-2,18502E+13	0.0685534493383596	0.209327540067909	-4,00596E+14	5,95727E+14	6,06895E+14	6,40696E+14	7,60748E+14	6,7909E+14	7,17397E+14
TC0200004651.mm.2	Rnf24	ring finger protein 24	2,52947E+14	5,6068E+14	2,18477E+14	0.0685778297060908	0.209327540067909	-4,00628E+14	7,1439E+14	7,07871E+14	6,35295E+14	2,81072E+14	6,07941E+14	4,1751E+14
TC1100003784.mm.2	Hcrt	hypocretin	-0.777053300317184	5,28865E+13	-2,18475E+13	0.0685803302392194	0.209327540067909	-4,00631E+14	4,78468E+14	4,92157E+14	5,27156E+14	5,9888E+14	5,29315E+14	5,47213E+14
TC1900000114.mm.2	Map4k2	mitogen-activated protein kinase kinase kinase kinase 2	1,31763E+14	6,88684E+14	2,18462E+14	0.0685930425756812	0.209327540067909	-4,00647E+14	7,60209E+14	7,37448E+14	7,11213E+14	5,22918E+14	6,93155E+14	7,0716E+14
TC0100003786.mm.2	Smvd2	SET and MYND domain containing 2	0.714956524312412	7,65132E+14	2,18455E+14	0.0685999518062543	0.209327540067909	-4,00656E+14	8,36065E+14	8,1577E+14	7,69498E+14	7,39346E+14	7,36486E+14	6,93625E+14
TC0700002781.mm.2	Hamp2	hepcidin antimicrobial peptide 2	-0.697436799692912	7,32684E+14	-2,18431E+14	0.0686227758295928	0.209327540067909	-4,00686E+14	6,81996E+14	6,84731E+14	7,4315E+14	7,63064E+12	7,38722E+14	7,84439E+14
TC0100000428.mm.2	Aox3	aldehyde oxidase 3	-0.770561162681145	5,04745E+14	-2,18428E+14	0.0686260440148905	0.209327540067909	-4,0069E+14	4,42385E+13	5,02334E+14	5,28255E+14	5,70576E+14	4,93352E+14	4,91569E+14
TC0800002523.mm.2	Abce1	ATP-binding cassette, sub-family E (OABP), member 1	2,45448E+14	7,27937E+14	2,18421E+13	0.0686326192626303	0.209327540067909	-4,00698E+14	8,77935E+13	8,74988E+14	7,96689E+14	4,65339E+13	7,78795E+14	5,73875E+14
TC1500000515.mm.2	Chrac1	chromatin accessibility complex 1	-0.701249900244293	7,40171E+14	-2,18395E+14	0.0686584849466641	0.209358449899516	-4,00732E+13	6,88701E+14	6,64608E+14	7,24135E+14	7,69423E+14	7,95434E+14	7,98727E+14
TC0300002648.mm.2	Lrig2	leucine-rich repeats and immunoglobulin-like domains 2	0.993120874703778	5,22697E+14	2,18384E+14	0.0686687400061406	0.209358449899516	-4,00745E+14	5,75478E+14	5,53377E+14	5,27281E+14	4,0295E+14	5,41445E+14	5,35649E+13
TC0900000443.mm.2	Olfr978	olfactory receptor 978	-0.930286667154157	4,82651E+14	-2,1836E+14	0.0686926578750543	0.209391751104947	-4,00776E+14	4,09507E+14	4,37712E+13	4,88561E+13	5,44715E+14	4,60863E+14	5,5455E+14
TC1100002114.mm.2	Mtmr3	myotubularin related protein 3	1,10176E+14	7,06696E+14	2,18314E+13	0.0687375864176022	0.209450567752364	-4,00833E+14	7,89548E+14	7,8327E+14	7,03754E+14	6,48714E+12	7,26491E+14	5,88398E+14
TC0400003853.mm.2	Pax7	paired box 7	-1,01238E+13	5,80947E+14	-2,18287E+14	0.0687641810734293	0.209450567752364	-4,00867E+14	4,99837E+14	5,15058E+14	5,59305E+14	6,58066E+14	5,84692E+14	6,68722E+14
TC0300001371.mm.2	Ube2d3	ubiquitin-conjugating enzyme E2D 3	1,48615E+14	9,15524E+14	2,1827E+14	0.0687800089573371	0.209450567752364	-4,00888E+14	1,00086E+13	1,01137E+14	9,46706E+14	7,68293E+13	9,48273E+14	8,17641E+14
TC1500002096.mm.2	Yaf2	YY1 associated factor 2	0.767978813010173	7,75514E+14	2,18265E+14	0.0687850687991724	0.209450567752364	-4,00894E+14	8,18548E+14	8,07505E+14	7,68287E+13	7,0417E+14	7,60916E+14	7,93655E+14
TC1900000889.mm.2	Ndufs8	NADH:ubiquinone oxidoreductase core subunit S8	1,35315E+14	8,82617E+14	2,18244E+14	0.0688058269271198	0.209450567752364	-4,00921E+14	9,68557E+14	9,48432E+13	9,07195E+14	7,39164E+14	9,16637E+14	8,15717E+14
TC0100001392.mm.2	Zfp648	zinc finger protein 648	-0.649739612262539	6,00782E+14	-2,18238E+14	0.0688121183077175	0.209450567752364	-4,00929E+14	5,47586E+14	5,6239E+14	6,29608E+14	6,10316E+14	6,2049E+13	6,34303E+14
TC0700000444.mm.2	Insyn2a	inhibitory synaptic factor 2A	-0.693965998940913	7,68301E+13	-2,18236E+14	0.0688141352201241	0.209450567752364	-4,00932E+14	7,23347E+14	7,00773E+14	7,80093E+14	7,8282E+14	7,89764E+14	8,33009E+14
TC1600000443.mm.2	Pcyt1a	phosphate cytidylyltransferase 1, choline, alpha isoform	1,28901E+14	5,53732E+14	2,18227E+14	0.0688229461850662	0.209450567752364	-4,00943E+14	6,25945E+14	6,33566E+14	5,71597E+14	4,3011E+14	5,90123E+13	4,71048E+14
TC0200003811.mm.2	Sestd1	SEC14 and spectrin domains 1	1,286E+14	6,24736E+14	2,18214E+14	0.0688353793134468	0.209450567752364	-4,00959E+14	6,88855E+13	6,95505E+14	6,22934E+14	5,04225E+14	6,89009E+14	5,47888E+14
TC0300002731.mm.2	Gnai3	guanine nucleotide binding protein (G protein), alpha inhibiting 3	1,45836E+14	6,25384E+13	2,18206E+14	0.0688435692795007	0.209450567752364	-4,00969E+14	7,08383E+14	7,36071E+14	6,52201E+14	5,00581E+14	6,53488E+14	5,01579E+14
TC1500001460.mm.2	Eif3e	eukaryotic translation initiation factor 3, subunit E	1,58672E+14	7,53625E+14	2,18183E+14	0.0688657017872322	0.209450567752364	-4,00998E+14	8,49395E+14	8,64715E+14	7,98977E+14	5,97789E+14	7,70037E+14	6,40837E+14
TC1700002662.mm.2	Six2	sine oculis-related homeobox 2	-0.887467988265408	6,07388E+14	-2,18181E+14	0.0688679394248139	0.209450567752364	-4,01001E+13	5,33295E+14	5,41682E+14	5,76978E+14	6,75492E+12	6,43555E+14	6,73324E+14
TC0X00000713.mm.2	Ikbkg	inhibitor of kappaB kinase gamma	0.955269911643436	5,05889E+14	2,18151E+14	0.0688970840334209	0.209488113983442	-4,01038E+14	5,53014E+14	5,5053E+14	5,11029E+14	4,01461E+14	4,87109E+14	5,32193E+14
TC0600002287.mm.2	Hoxa13	homeobox A13	-1,35562E+14	6,20643E+14	-2,18124E+14	0.0689233568457872	0.209488113983442	-4,01072E+14	5,11731E+14	5,42176E+14	5,86096E+14	7,38934E+14	6,11691E+13	7,33232E+14
TC1700002812.mm.2	Egfl8	EGF-like domain 8	-0.658692907983693	5,70959E+13	-2,18119E+14	0.0689283625074161	0.209488113983442	-4,01078E+14	5,17057E+14	5,30918E+14	5,76257E+14	6,03456E+13	6,05729E+14	5,92336E+14
TC0700000495.mm.2	Rps16	ribosomal protein S16	1,04501E+14	9,13623E+14	2,18097E+14	0.0689502993399792	0.209488113983442	-4,01106E+14	9,67789E+14	9,71367E+14	9,28752E+14	8,01401E+14	9,32094E+14	8,80334E+14
TC1400000001.mm.2	Gm2888	predicted gene 2888	-0.747757294987558	5,50392E+14	-2,1809E+14	0.0689565943390741	0.209488113983442	-4,01114E+14	4,71078E+14	4,94843E+14	5,31469E+14	5,84004E+14	5,93326E+14	6,27632E+14
TC0400002159.mm.2	Tox	thymocyte selection-associated high mobility group box	1,25573E+14	8,24299E+14	2,18088E+14	0.0689582918493252	0.209488113983442	-4,01116E+14	8,99412E+13	9,14387E+14	8,60101E+14	7,02554E+14	8,26432E+14	7,42908E+14
TC0900000602.mm.2	Pih1d2	PIH1 domain containing 2	-0.812896078950931	6,43068E+14	-2,18058E+14	0.0689882144580097	0.209539509813744	-4,01155E+14	5,59164E+14	6,03307E+14	6,30426E+14	6,94624E+14	6,68226E+14	7,0266E+14
TC1800001296.mm.2	Ccdc112	coiled-coil domain containing 112	0.907376422860442	5,30346E+14	2,1802E+14	0.0690256325100729	0.20956018753962	-4,01202E+14	6,08446E+14	5,8222E+14	5,5E+14	4,5919E+14	4,65008E+14	5,17214E+14
TC1100002738.mm.2	Il13	interleukin 13	-1,0346E+14	6,5621E+14	-2,18013E+14	0.0690323555310249	0.20956018753962	-4,01211E+14	6,02099E+13	6,00254E+14	6,4682E+14	7,62454E+13	6,27559E+14	6,98074E+13
TC0900002463.mm.2	Coro2b	coronin, actin binding protein, 2B	0.736147246624874	8,68606E+14	2,18011E+14	0.0690340393136164	0.20956018753962	-4,01213E+14	9,17454E+14	9,09218E+14	8,67892E+14	8,1155E+14	8,6694E+14	8,38584E+14
TC1800000710.mm.2	Seh1l	SEH1-like (S. cerevisiae	0.874665890985922	7,52613E+14	2,17979E+14	0.0690656166451243	0.209616553212071	-4,01254E+14	8,10284E+14	7,65807E+14	7,45546E+14	6,55611E+14	7,7165E+14	7,66779E+14
TC1100002553.mm.2	Gabrg2	gamma-aminobutyric acid (GABA) A receptor, subunit gamma 2	1,80756E+14	9,07152E+14	2,17941E+13	0.0691027640219124	0.209685274089523	-4,01301E+12	1,03013E+14	1,01831E+14	9,5693E+14	7,29999E+14	9,34725E+13	7,72818E+14
TC0300001788.mm.2	Lrrc31	leucine rich repeat containing 31	-1,35818E+14	4,81592E+14	-2,1793E+14	0.0691142860230531	0.209685274089523	-4,01316E+14	3,62152E+13	4,15687E+14	4,30792E+13	6,18682E+14	5,14035E+14	5,48203E+14
TC1600000501.mm.2	Kpna1	karyopherin (importin) alpha 1	1,18838E+14	6,88982E+14	2,17857E+14	0.0691862979367862	0.209854365501879	-4,01408E+14	7,86175E+14	7,67741E+14	7,14649E+14	6,0159E+14	6,9214E+14	5,716E+14
TC1600002080.mm.2	Tmem50b	transmembrane protein 50B	1,25447E+14	8,24021E+14	2,17835E+14	0.0692073538619691	0.209854365501879	-4,01435E+14	9,04109E+14	9,03476E+14	8,51755E+12	7,04936E+14	8,4111E+14	7,38741E+14
TC0700000563.mm.2	Nphs1	nephrosis 1, nephrin	-0.659791796868973	8,62928E+13	-2,17823E+14	0.0692197619086036	0.209854365501879	-4,0145E+14	8,39926E+14	8,2613E+14	8,98241E+14	8,99773E+14	8,41018E+13	8,72479E+13
TC1600001493.mm.2	Senp5	SUMO/sentrin specific peptidase 5	1,49399E+14	6,77222E+14	2,1782E+13	0.0692221158469861	0.209854365501879	-4,01453E+14	7,69824E+14	7,84827E+14	7,02598E+14	5,53255E+14	7,09115E+13	5,43712E+13
TC1900000096.mm.2	Fau	Finkel-Biskis-Reilly murine sarcoma virus (FBR-MuSV) ubiquitously expressed (fox derived)	0.646899273023346	9,75911E+14	2,17789E+14	0.0692527734038909	0.209907813742832	-4,01492E+14	1,02059E+14	1,01342E+14	9,64816E+14	9,39815E+14	9,62046E+14	9,54777E+14
TC1400001105.mm.2	Elf1	E74-like factor 1	1,11955E+13	6,20466E+14	2,17771E+14	0.0692705253356355	0.209922131791066	-4,01515E+14	6,71072E+14	6,87578E+14	6,36517E+14	4,98224E+13	6,46234E+14	5,83173E+14
TC0100001419.mm.2	Abl2	v-abl Abelson murine leukemia viral oncogene 2 (arg, Abelson-related gene)	1,38295E+14	6,28857E+14	2,1773E+14	0.0693110979046319	0.209929954909675	-4,01567E+14	7,09644E+14	7,16466E+13	6,61909E+14	4,87611E+14	6,5082E+14	5,46689E+14
TC0800002957.mm.2	Tmem170	transmembrane protein 170	-0.707734626115962	7,49256E+13	-2,17729E+13	0.0693118339184628	0.209929954909675	-4,01568E+14	7,02636E+14	7,09724E+13	7,54253E+14	7,99654E+14	7,49973E+14	7,79297E+14
TC0100000652.mm.2	Retreg2	reticulophagy regulator family member 2	0.767633787079887	8,35422E+14	2,17725E+13	0.0693160921632096	0.209929954909675	-4,01573E+14	9,12216E+14	8,78114E+14	8,46547E+14	7,90256E+14	8,08809E+14	7,76591E+13
TC1300000928.mm.2	Mctp1	multiple C2 domains, transmembrane 1	2,43566E+14	6,53227E+14	2,17709E+14	0.0693320458172759	0.209929954909675	-4,01593E+14	7,98523E+14	8,44542E+14	7,28278E+14	4,27656E+13	6,80463E+14	4,39901E+14
TC1100001570.mm.2	Grb7	growth factor receptor bound protein 7	-0.785280675828905	6,68705E+13	-2,17681E+14	0.0693588605921546	0.209929954909675	-4,01628E+14	6,11731E+14	6,41478E+14	6,79269E+13	7,30996E+14	6,49085E+14	6,99673E+14
TC1300000132.mm.2	Vps41	VPS41 HOPS complex subunit	1,40025E+14	7,49122E+14	2,17672E+14	0.0693685551830718	0.209929954909675	-4,0164E+14	8,4896E+14	8,37499E+14	7,61338E+14	6,45072E+14	7,86013E+14	6,15851E+14
TC1400000507.mm.2	Samd4	sterile alpha motif domain containing 4	0.913795770040339	7,00028E+14	2,17652E+14	0.0693878247029379	0.209929954909675	-4,01664E+14	7,73696E+14	7,59619E+14	7,26465E+14	6,24091E+14	6,76682E+14	6,39615E+14
TC0400004031.mm.2	Cort	cortistatin	-0.656386000106988	8,97147E+14	-2,17623E+13	0.0694164981730434	0.209929954909675	-4,01701E+13	8,34756E+14	8,52558E+14	9,13047E+14	9,05544E+14	9,25174E+14	9,51805E+14
TC1500000921.mm.2	Pphln1	periphilin 1	0.688709052775293	7,63649E+14	2,17621E+14	0.0694186051729878	0.209929954909675	-4,01704E+14	8,14621E+14	7,9748E+14	7,43724E+14	7,30636E+14	7,6892E+14	7,26514E+14
TC0500001560.mm.2	Rfc2	replication factor C (activator 1) 2	0.690423891996438	6,69727E+14	2,17617E+14	0.0694226175220568	0.209929954909675	-4,01709E+14	7,13601E+14	7,31123E+14	6,66811E+14	6,39827E+12	6,51477E+14	6,15521E+14
TC0700002500.mm.2	Bcam	basal cell adhesion molecule	-0.926748976414488	8,38921E+13	-2,17598E+14	0.0694408381710566	0.209929954909675	-4,01732E+14	7,95487E+14	7,50926E+14	8,15197E+14	9,16566E+14	8,6707E+14	8,88283E+14
TC0X00000214.mm.2	Usp11	ubiquitin specific peptidase 11	2,52923E+14	7,96797E+14	2,17586E+14	0.069453594529057	0.209929954909675	-4,01748E+14	1,00645E+14	9,64128E+14	8,75967E+14	5,88764E+14	8,13172E+14	5,32304E+14
TC1100000612.mm.2	Il5	interleukin 5	-1,00491E+14	5,21332E+14	-2,17576E+14	0.0694632879675164	0.209929954909675	-4,0176E+14	4,63234E+14	4,60352E+13	4,98651E+14	6,25918E+14	5,52298E+14	5,2754E+14
TC0600001807.mm.2	Pon3	paraoxonase 3	0.730825495001355	6,31903E+14	2,17568E+14	0.069470438486972	0.209929954909675	-4,0177E+14	6,59147E+14	7,12939E+14	5,97759E+14	6,28162E+14	6,01068E+14	5,92345E+14
TC0200002437.mm.2	Slc32a1	solute carrier family 32 (GABA vesicular transporter), member 1	1,1517E+14	8,28851E+14	2,17558E+14	0.0694812201022506	0.209929954909675	-4,01783E+13	9,37277E+13	9,01656E+14	8,79053E+14	7,2954E+14	7,84016E+14	7,41566E+12
TC1000001936.mm.2	Rspo3	R-spondin 3	1,17792E+14	5,33388E+14	2,17557E+14	0.0694815645462233	0.209929954909675	-4,01784E+14	6,00104E+14	6,12147E+14	5,19281E+14	4,57386E+14	5,81711E+14	4,29696E+14
TC0700001651.mm.2	Tead1	TEA domain family member 1	2,05827E+14	4,77985E+14	2,17523E+14	0.0695155687677607	0.209985950959854	-4,01827E+14	5,84923E+14	5,71832E+14	5,32932E+14	2,12169E+14	4,6381E+14	5,02245E+14
TC1700001130.mm.2	Myom1	myomesin 1	-0.781191453944349	5,54184E+14	-2,17512E+14	0.0695261620040228	0.209985950959854	-4,0184E+14	4,95251E+13	5,02056E+14	5,38329E+14	6,15216E+14	5,88983E+14	5,85269E+14
TC0800000211.mm.2	Defa21	defensin, alpha, 21	-0.90168324637358	4,57871E+14	-2,1745E+14	0.0695872506727518	0.210125237731444	-4,01918E+14	4,19405E+14	3,51272E+14	5,01737E+14	4,49277E+14	4,80742E+14	5,44791E+14
TC1900000989.mm.2	Tigd3	tigger transposable element derived 3	-0.680732767415544	6,74756E+14	-2,17439E+14	0.0695983611849263	0.210125237731444	-4,01932E+14	6,20972E+13	6,33993E+14	6,7563E+14	7,15481E+14	6,93646E+14	7,08815E+14
TC0500001217.mm.2	Dao	D-amino acid oxidase	-1,0953E+14	5,9182E+14	-2,17381E+14	0.0696561420942447	0.210222873156127	-4,02005E+14	5,28207E+14	5,07829E+14	5,59388E+14	6,95709E+13	6,02344E+14	6,57439E+14
TC0100003601.mm.2	Olfr419	olfactory receptor 419	-0.734458539647204	4,70233E+14	-2,17333E+14	0.0697033526412768	0.210222873156127	-4,02065E+14	4,08992E+14	4,29137E+14	4,62919E+14	5,22101E+14	5,05487E+14	4,9276E+14
TC0200005018.mm.2	Ghrh	growth hormone releasing hormone	-0.786332680486277	5,75461E+14	-2,17333E+14	0.0697036407596357	0.210222873156127	-4,02065E+13	5,6308E+14	5,52623E+14	5,99535E+14	6,73436E+14	5,19322E+14	5,44773E+14
TC0100003351.mm.2	Cacybp	calcyclin binding protein	1,34596E+14	8,0931E+14	2,1733E+14	0.0697068130342905	0.210222873156127	-4,02069E+14	9,02068E+14	8,86717E+14	8,41132E+14	6,78461E+14	8,28546E+14	7,18934E+14
TC0100003056.mm.2	Rassf5	Ras association (RalGDS/AF-6) domain family member 5	-0.670243785445856	6,43427E+14	-2,17319E+14	0.0697173931613578	0.210222873156127	-4,02083E+14	6,27328E+14	5,94164E+14	6,78307E+14	6,77234E+14	6,5311E+14	6,30421E+14
TC1300001052.mm.2	Arsb	arylsulfatase B	1,67869E+14	8,5854E+14	2,1731E+14	0.0697260841704949	0.210222873156127	-4,02094E+14	9,6E+14	9,43156E+14	9,01439E+13	6,65979E+14	8,91336E+14	7,89333E+14
TC0100000644.mm.2	Wnt6	wingless-type MMTV integration site family, member 6	-0.875524730189021	5,13999E+14	-2,17306E+13	0.0697304359783814	0.210222873156127	-4,02099E+14	4,68173E+14	4,72552E+14	5,56485E+14	5,59345E+14	4,63141E+14	5,64296E+13
TC1300001017.mm.2	Rps23	ribosomal protein S23	1,08562E+14	1,11689E+14	2,17298E+14	0.0697379121083058	0.210222873156127	-4,02109E+13	1,19602E+14	1,18092E+14	1,13794E+14	1,02188E+14	1,12642E+13	1,03818E+14
TC0200000319.mm.2	Etl4	enhancer trap locus 4	1,76821E+14	7,25644E+14	2,17274E+14	0.0697619166743678	0.210222873156127	-4,02139E+14	8,06643E+14	8,40446E+14	7,5371E+14	5,39738E+14	7,89782E+14	6,23545E+14
TC1800001445.mm.2	2700046A07Rik	RIKEN cDNA 2700046A07 gene	-0.837010769950186	6,05861E+14	-2,17258E+14	0.0697778906529039	0.210222873156127	-4,02159E+14	5,26929E+14	5,50686E+14	6,12738E+14	6,32279E+14	6,12893E+14	6,99642E+14
TC1600001019.mm.2	Slc5a3/Mrps6	solute carrier family 5 (inositol transporters), member 3/mitochondrial ribosomal protein S6	0.671856960681453	7,54134E+14	2,17258E+14	0.0697782405427439	0.210222873156127	-4,0216E+14	7,94767E+14	8,19814E+14	7,51782E+14	7,28427E+14	7,26286E+13	7,03729E+14
TC1900000630.mm.2	Nkx2-3	NK2 homeobox 3	-0.811985886705171	5,02178E+14	-2,17235E+14	0.0698006204187876	0.210222873156127	-4,02188E+14	4,59023E+14	4,43911E+14	4,98436E+14	5,66895E+14	4,97723E+12	5,4708E+14
TC0200000821.mm.2	Rif1	replication timing regulatory factor 1	0.950621919680666	5,50205E+14	2,17227E+14	0.0698084316376855	0.210222873156127	-4,02198E+14	6,45502E+14	6,09487E+14	4,8056E+14	5,84304E+14	5,12785E+14	4,68594E+14
TC1000000811.mm.2	Gzmm	granzyme M (lymphocyte met-ase 1)	-0.826250991389518	6,27359E+14	-2,1722E+14	0.0698160333573834	0.210222873156127	-4,02207E+14	5,64559E+14	5,56233E+14	6,29952E+14	6,56089E+14	6,36121E+14	7,21199E+14
TC0400004012.mm.2	Agtrap	angiotensin II, type I receptor-associated protein	0.946981826559989	6,53603E+14	2,17206E+14	0.0698300673378408	0.210222873156127	-4,02225E+13	7,02655E+14	6,8896E+14	6,50979E+14	5,5124E+14	6,9428E+13	6,33504E+14
TC1800001012.mm.2	Kctd1	potassium channel tetramerisation domain containing 1	0.870533872793769	6,98562E+14	2,17189E+14	0.0698465471746005	0.210222873156127	-4,02246E+14	7,50556E+14	7,66264E+14	6,93013E+14	6,497E+14	7,10421E+14	6,21416E+14
TC1100004254.mm.2	Zfp750	zinc finger protein 750	-0.830375901294046	6,08298E+11	-2,17183E+14	0.069852495679135	0.210222873156127	-4,02254E+14	5,44114E+13	5,49646E+14	5,92903E+14	6,66932E+14	6,2225E+14	6,73945E+14
TC0400004123.mm.2	Actrt2	actin-related protein T2	-0.937371297509317	5,98809E+14	-2,17113E+14	0.0699219158978258	0.210357113337049	-4,02341E+13	5,16075E+14	5,49248E+14	5,97171E+14	6,55627E+13	5,9036E+14	6,84373E+14
TC1800001237.mm.2	Fgf1	fibroblast growth factor 1	0.696390118231891	7,32514E+14	2,17112E+14	0.0699232110118063	0.210357113337049	-4,02343E+14	8,15648E+14	7,67079E+13	7,16265E+14	7,27185E+14	6,90282E+14	6,78624E+14
TC1600000220.mm.2	P2rx6	purinergic receptor P2X, ligand-gated ion channel, 6	-0.730694786414641	7,38232E+14	-2,17072E+14	0.0699635020662551	0.210439034682391	-4,02394E+14	7,09413E+14	6,82136E+14	7,52454E+14	7,85234E+14	7,26966E+14	7,7319E+14
TC1000002050.mm.2	Fig4	FIG4 phosphoinositide 5-phosphatase	1,10311E+14	7,16435E+14	2,17045E+14	0.0699903166848372	0.21044202416784	-4,02428E+14	7,92343E+14	8,07132E+14	7,43231E+14	6,35622E+14	7,12114E+13	6,08164E+14
TC1100001074.mm.2	Pitpna	phosphatidylinositol transfer protein, alpha	1,1098E+14	9,48807E+14	2,17044E+14	0.0699906167389967	0.21044202416784	-4,02428E+14	1,02051E+14	1,00983E+14	9,68972E+14	8,3941E+14	9,66138E+14	8,87985E+14
TC1100000866.mm.2	Stx8	syntaxin 8	1,10592E+14	5,83139E+14	2,17025E+14	0.0700098126745798	0.210460468586848	-4,02452E+14	6,72881E+14	6,56968E+14	6,26856E+14	4,81809E+14	5,49449E+14	5,10873E+14
TC0500002947.mm.2	Cplx1	complexin 1	1,03761E+14	9,14216E+14	2,16969E+14	0.0700654565996186	0.210588454055149	-4,02522E+14	9,95129E+14	9,63581E+14	9,23484E+14	8,27704E+14	9,34141E+14	8,41256E+14
TC1100000326.mm.2	4930524B15Rik	RIKEN cDNA 4930524B15 gene	-0.792453218522246	5,28505E+14	-2,1695E+14	0.0700843512132112	0.2106059588024	-4,02546E+14	4,58737E+14	4,70038E+14	5,08933E+14	5,78333E+14	5,97482E+13	5,57507E+14
TC1900001398.mm.2	Atad1	ATPase family, AAA domain containing 1	2,64217E+14	7,08778E+12	2,16888E+14	0.0701462114386083	0.2107500889893	-4,02624E+14	8,83357E+13	8,75606E+14	7,74627E+13	4,55903E+14	7,7177E+14	4,91401E+14
TC0500000814.mm.2	Rufy3	RUN and FYVE domain containing 3	1,9269E+13	7,71947E+14	2,16869E+14	0.0701651302906677	0.2107500889893	-4,02648E+14	9,07637E+14	8,89809E+14	8,03854E+14	6,08213E+14	8,25453E+14	5,96713E+14
TC0X00003064.mm.2	Ripply1	ripply transcriptional repressor 1	-0.698521096119613	5,10905E+14	-2,16855E+14	0.0701790511458038	0.2107500889893	-4,02665E+14	4,50598E+13	4,64595E+14	5,0261E+14	5,52286E+13	5,43698E+14	5,51641E+14
TC0500001304.mm.2	Tesc	tescalcin	-1,00899E+14	7,64975E+14	-2,16847E+14	0.0701876211971313	0.2107500889893	-4,02676E+14	6,89043E+14	6,91165E+14	8,5662E+14	7,25386E+14	8,13291E+14	8,14344E+14
TC0800002372.mm.2	Zfp963/Zfp866	zinc finger protein 963/zinc finger protein 866	1,0979E+14	6,36092E+14	2,1681E+14	0.0702245306743755	0.2107500889893	-4,02722E+14	7,28106E+14	7,04968E+14	5,95531E+14	6,17963E+13	6,58004E+14	5,11977E+14
TC1300001348.mm.2	4833420G17Rik	RIKEN cDNA 4833420G17 gene	1,29441E+14	6,60764E+14	2,16809E+14	0.0702257532245415	0.2107500889893	-4,02724E+14	7,26838E+14	7,23068E+14	6,86546E+14	5,04478E+14	6,84535E+14	6,3912E+13
TC0X00001795.mm.2	Was	Wiskott-Aldrich syndrome	-0.643892456037332	6,03198E+13	-2,16791E+14	0.0702429582245385	0.2107500889893	-4,02745E+14	5,5084E+14	5,4348E+14	6,21997E+14	6,01102E+14	6,43186E+14	6,58581E+14
TC1500002254.mm.2	Fignl2	fidgetin-like 2	-0.85786844240046	5,26646E+14	-2,1679E+14	0.0702447663942325	0.2107500889893	-4,02748E+14	4,79528E+14	4,7683E+14	5,99749E+14	5,28183E+14	5,04654E+14	5,70932E+14
TC0400002098.mm.2	Acap3	ArfGAP with coiled-coil, ankyrin repeat and PH domains 3	-0.722049717916861	8,27065E+14	-2,16755E+14	0.0702797278728504	0.2107500889893	-4,02792E+14	7,73079E+14	8,03303E+14	8,35534E+14	8,85258E+14	8,40796E+14	8,2442E+14
TC0300000812.mm.2	Pglyrp4	peptidoglycan recognition protein 4	-0.831659056921281	6,56578E+13	-2,16747E+14	0.0702872991857868	0.2107500889893	-4,02801E+14	6,15095E+14	5,92185E+14	6,44405E+14	7,29208E+14	6,91544E+14	6,67031E+14
TC0900000259.mm.2	Igsf9b	immunoglobulin superfamily, member 9B	0.947082168857316	6,00639E+14	2,16746E+14	0.0702883468643943	0.2107500889893	-4,02802E+14	6,54205E+14	6,63677E+14	5,47998E+14	5,80468E+14	6,35298E+14	5,22186E+14
TC0200004102.mm.2	Ndufs3	NADH:ubiquinone oxidoreductase core subunit S3	1,61847E+14	9,2614E+14	2,16745E+14	0.0702892681827405	0.2107500889893	-4,02804E+14	9,97854E+14	9,94326E+14	9,54891E+14	7,13596E+14	9,74822E+13	9,21348E+14
TC1300002619.mm.2	Kif2a	kinesin family member 2A	1,56004E+13	7,24992E+14	2,16703E+14	0.0703313645209566	0.210814351146873	-4,02856E+14	8,37336E+14	8,29808E+14	7,65293E+14	5,89842E+14	7,43204E+14	5,84469E+14
TC1100002175.mm.2	Purb	purine rich element binding protein B	3,32239E+14	7,28435E+14	2,16698E+14	0.0703368678561154	0.210814351146873	-4,02863E+14	9,33967E+14	9,58178E+14	8,14861E+14	4,12805E+14	8,05315E+14	4,45485E+14
TC1500001934.mm.2	Pmm1	phosphomannomutase 1	-0.911830739456565	9,37961E+13	-2,1667E+14	0.0703641757826746	0.210856976824667	-4,02898E+13	8,81498E+14	8,73035E+14	9,26314E+14	1,01059E+13	9,32858E+14	1,00348E+14
TC1000001840.mm.2	Myb	myeloblastosis oncogene	-0.769827923564707	6,32475E+14	-2,16609E+14	0.0704254019959415	0.21100120906668	-4,02974E+13	5,89916E+14	5,64471E+14	6,51123E+14	6,5723E+14	6,30904E+14	7,01206E+14
TC0700002669.mm.2	Pld3	phospholipase D family, member 3	0.685237711729778	9,46774E+13	2,16589E+14	0.0704458032011309	0.211018356372035	-4,03E+14	1,00056E+14	9,79841E+14	9,41671E+14	9,01684E+14	9,35983E+13	9,20903E+14
TC0700004201.mm.2	Cdr2	cerebellar degeneration-related 2	0.750535551385773	6,11447E+13	2,16576E+13	0.0704592038685118	0.211018356372035	-4,03017E+14	6,43226E+14	6,49008E+14	5,4645E+14	5,95677E+14	6,41508E+14	5,92813E+14
TC1700002158.mm.2	1600014C23Rik	RIKEN cDNA 1600014C23 gene	-1,05883E+14	6,3424E+14	-2,16558E+14	0.0704768710946922	0.211018356372035	-4,03039E+14	5,78068E+14	5,40173E+14	6,04127E+14	7,25881E+14	6,47498E+14	7,09694E+14
TC0500000214.mm.2	Asic3	acid-sensing (proton-gated) ion channel 3	-1,07997E+14	5,43338E+14	-2,16542E+14	0.0704925935685976	0.211018356372035	-4,03058E+14	5,0563E+14	4,57382E+12	5,41349E+14	6,37657E+14	5,09583E+14	6,08426E+14
TC0400001637.mm.2	Pafah2	platelet-activating factor acetylhydrolase 2	-0.995016258665897	6,48068E+14	-2,16527E+13	0.0705077407402307	0.211018356372035	-4,03077E+14	5,75088E+14	5,96871E+14	6,4684E+14	7,24122E+14	6,22508E+14	7,2298E+14
TC1200001761.mm.2	Gm2436	predicted gene 2436	1,10228E+14	6,41598E+14	2,16523E+14	0.0705115651601278	0.211018356372035	-4,03082E+14	7,43296E+14	6,99631E+14	6,57152E+14	5,65319E+14	6,47716E+14	5,36475E+14

TC0400001933.mm.2	Exosc10	exosome component 10	0.756320322446828	7,61965E+14	2,16512E+14	0.0705227983034449	0.211018356372035	-4,03096E+14	8,15407E+14	7,84103E+14	7,56106E+14	6,92141E+14	7,63095E+14	7,60938E+14
TC0700000949.mm.2	Ube3a	ubiquitin protein ligase E3A	1.61097E+14	7,19605E+14	2,16485E+14	0.0705500285003238	0.211057499850336	-4,0313E+14	8,5511E+14	8,40937E+14	7,40285E+14	6,33569E+14	7,3456E+14	5,1317E+14
TC1000001983.mm.2	Clvs2	clavesin 2	1,38535E+14	6,17225E+14	2,16452E+14	0.0705829814778802	0.211057499850336	-4,03172E+12	7,12058E+13	6,80345E+14	6,5417E+14	4,61164E+14	6,28664E+14	5,66947E+14
TC1500000565.mm.2	Gsdmd	gasdermin D	-0.686673537934823	5,30058E+14	-2,16452E+14	0.0705831919039654	0.211057499850336	-4,03172E+14	4,60495E+14	5,01627E+13	5,42275E+14	5,57182E+14	5,69533E+14	5,49236E+14
TC0600000841.mm.2	St3gal5	ST3 beta-galactoside alpha-2,3-sialyltransferase 5	1,2134E+14	8,76431E+14	2,16435E+14	0.0706000801669297	0.211057499850336	-4,03193E+14	9,27208E+14	9,52361E+14	9,00021E+14	7,36868E+14	8,96337E+14	8,45792E+14
TC0700003844.mm.2	Inppl1	inositol polyphosphate phosphatase-like 1	1,4882E+14	5,88003E+14	2,16428E+14	0.0706077357694387	0.211057499850336	-4,03202E+14	6,82613E+14	6,73828E+13	6,40649E+14	4,18153E+14	5,68755E+14	5,44022E+14
TC0400001433.mm.2	Rspo1	R-spondin 1	-0.778880992141606	7,10036E+14	-2,16401E+14	0.0706347466669256	0.211057499850336	-4,03236E+14	6,71094E+14	6,54598E+14	7,36106E+14	7,45362E+14	6,88827E+13	7,64231E+14
TC1100002799.mm.2	Fam114a2	family with sequence similarity 114, member A2	0.796161179599936	5,24323E+14	2,16398E+14	0.0706374036138517	0.211057499850336	-4,0324E+14	6,30548E+14	6,27146E+14	5,58368E+14	5,40093E+14	4,35033E+13	3,54747E+14
TC0200002536.mm.2	Zswim1	zinc finger SWIM-type containing 1	1.14684E+14	6,63752E+14	2,16395E+13	0.0706406688197643	0.211057499850336	-4,03244E+14	7,44062E+14	7,41368E+14	6,98073E+14	5,57989E+14	6,58835E+14	5,82184E+14
TC0100001855.mm.2	Lpgat1	lysophosphatidylglycerol acyltransferase 1	1,78678E+14	7,91258E+14	2,16369E+14	0.0706668647691143	0.21107758700641	-4,03276E+14	8,9841E+14	9,0398E+13	8,13946E+14	6,31088E+14	8,54493E+14	6,45633E+14
TC0700000931.mm.2	Siglech	sialic acid binding Ig-like lectin H	0.863406351302869	6,15551E+14	2,16362E+13	0.0706735916278522	0.21107758700641	-4,03285E+14	6,92781E+14	6,35579E+14	6,18103E+14	5,37575E+14	5,99477E+14	6,09791E+14
TC1600000167.mm.2	Ntan1	N-terminal Asn amidase	0.676470377791756	7,68165E+14	2,16339E+14	0.0706970565814721	0.211108538305645	-4,03314E+14	8,21207E+14	8,11001E+14	7,67747E+14	7,29168E+14	7,49484E+14	7,30382E+14
TC1500001832.mm.2	Tmprss6	transmembrane serine protease 6	-0.8270129593035	4,76804E+14	-2,16315E+14	0.070720908040227	0.211140632064513	-4,03344E+14	4,3386E+13	4,45093E+14	4,96898E+14	5,47457E+14	4,31682E+14	5,05836E+14
TC1200001259.mm.2	A530016L24Rik	RIKEN cDNA A530016L24 gene	-0.777656844187689	6,0292E+14	-2,16258E+14	0.070778088443865	0.211272200647647	-4,03415E+14	5,64814E+14	5,33846E+14	5,92294E+14	6,61897E+14	6,25095E+14	6,39571E+14
TC0400000869.mm.2	Dennd4c	DENN/MADD domain containing 4C	0.719653522466676	5,30218E+13	2,16228E+14	0.0708082033283175	0.211322945032262	-4,03453E+14	5,57665E+14	6,07933E+14	5,0793E+14	5,13738E+14	5,1336E+14	4,8068E+14
TC0400000506.mm.2	Anp32b	acidic (leucine-rich) nuclear phosphoprotein 32 family, member B	0.836840112179771	8,1503E+14	2,16186E+14	0.0708508974897334	0.211384715390254	-4,03506E+14	8,69105E+14	8,75834E+14	8,35019E+14	7,42552E+14	7,80012E+14	7,87656E+14
TC1300000938.mm.2	Fam172a	family with sequence similarity 172, member A	1,14354E+14	6,86096E+14	2,16174E+14	0.0708633004213626	0.211384715390254	-4,03521E+14	7,70201E+14	7,61884E+14	6,8943E+14	6,13946E+14	7,10568E+14	5,70548E+14
TC1600001203.mm.2	Prm1	protamine 1	-0.743242682211869	5,50447E+14	-2,16169E+14	0.0708682574652858	0.211384715390254	-4,03527E+14	4,86375E+14	5,22866E+14	5,66945E+14	5,90944E+14	5,41898E+14	5,93654E+14
TC0800000242.mm.2	Defa20	defensin, alpha, 20	-0.899118865376344	4,96013E+13	-2,16117E+14	0.070920099848775	0.211427000407426	-4,03592E+14	4,34269E+13	4,41594E+14	4,83104E+14	5,72583E+14	5,52193E+14	4,92336E+14
TC0800000243.mm.2	Defa32/Defa20	defensin, alpha, 32/defensin, alpha, 20	-0.899118865376344	4,96013E+13	-2,16117E+14	0.070920099848775	0.211427000407426	-4,03592E+14	4,34269E+13	4,41594E+14	4,83104E+14	5,72583E+14	5,52193E+14	4,92336E+14
TC1800000276.mm.2	Gm10549	predicted gene 10549	-0.873972717898636	5,86637E+11	-2,16103E+13	0.0709346458248328	0.211427000407426	-4,0361E+14	5,11489E+14	5,12162E+14	5,60393E+14	6,38052E+13	6,15748E+14	6,8198E+14
TC0800001038.mm.2	Cyld	CYLD lysine 63 deubiquitinase	2,47533E+14	7,68363E+14	2,16103E+14	0.0709349198909294	0.211427000407426	-4,0361E+14	9,02911E+14	9,1822E+14	8,41742E+14	4,84323E+14	8,18304E+14	6,44677E+14
TC1100003796.mm.2	Ccr10	chemokine (C-C motif) receptor 10	-0.802226395593275	5,03567E+14	-2,16082E+14	0.0709555212915494	0.211433943688751	-4,03636E+14	4,58979E+14	4,67241E+14	5,12542E+14	5,74124E+14	4,7576E+14	5,32758E+13
TC0100002950.mm.2	Epb41l5	erythrocyte membrane protein band 4.1 like 5	0.987443260122056	5,5624E+14	2,16062E+14	0.0709760393494459	0.211433943688751	-4,03661E+14	6,61184E+14	6,37102E+14	5,94377E+14	5,06421E+14	5,137E+14	4,24655E+14
TC0800001396.mm.2	Bco1	beta-carotene oxygenase 1	-0.656565885796133	5,86393E+14	-2,16062E+14	0.0709766152431238	0.211433943688751	-4,03662E+14	5,47415E+14	5,49267E+14	5,98746E+14	6,29249E+14	6,05851E+14	5,8783E+14
TC0500001570.mm.2	Cldn3	claudin 3	-0.738103333571759	6,99084E+14	-2,16E+14	0.0710383042390521	0.211532516297756	-4,03739E+14	6,45489E+14	6,54777E+14	6,91525E+14	7,56362E+14	7,28419E+13	7,17929E+14
TC1500001123.mm.2	Hoxc4	homeobox C4	-1,2952E+14	5,75695E+14	-2,15989E+14	0.0710499282661534	0.211532516297756	-4,03753E+14	5,07734E+14	5,07196E+14	5,47627E+14	7,26343E+14	5,5226E+14	6,13013E+14
TC1600002082.mm.2	Gart	phosphoribosylglycinamide formyltransferase	0.989290613051586	6,75895E+14	2,15989E+14	0.0710503142939814	0.211532516297756	-4,03754E+13	7,61787E+14	7,54178E+14	7,16784E+14	6,01323E+14	6,3792E+14	5,83378E+13
TC0600001072.mm.2	Zxdc	ZXD family zinc finger C	0.691980865978118	6,66311E+14	2,15977E+14	0.0710622175088284	0.211532516297756	-4,03768E+14	7,37929E+13	7,31208E+14	6,80312E+14	6,50429E+14	5,78704E+14	6,19282E+14
TC0200002689.mm.2	1700021F07Rik	RIKEN cDNA 1700021F07 gene	-0.956002749102027	5,91293E+14	-2,15941E+14	0.0710984552452771	0.21160129467439	-4,03813E+14	4,79878E+14	5,53618E+14	5,7719E+14	6,47506E+14	6,12349E+14	6,77217E+14
TC0900001122.mm.2	Ripply2	ripply transcriptional repressor 2	-0.671751030585476	5,69726E+14	-2,15913E+14	0.0711270823798385	0.21161832490947	-4,03849E+14	5,38789E+14	5,19657E+14	5,89467E+14	6,03329E+14	6,014E+14	5,65716E+14
TC1200000462.mm.2	Srp54c/Srp54b	signal recognition particle 54C/signal recognition particle 54B	1,72558E+14	8,15566E+14	2,15906E+12	0.0711337054379802	0.21161832490947	-4,03857E+14	9,09615E+14	9,3077E+14	8,62295E+13	6,32973E+14	8,46879E+14	7,10865E+14
TC1400001290.mm.2	Dnajc3	DnaJ heat shock protein family (Hsp40) member C3	1,06352E+14	7,45339E+14	2,15871E+14	0.0711693224835128	0.21161832490947	-4,03901E+13	8,13147E+14	8,05301E+14	7,43652E+14	6,62091E+14	7,82839E+14	6,65002E+13
TC1800001366.mm.2	Aldh7a1	aldehyde dehydrogenase family 7, member A1	0.715083046736844	6,83856E+14	2,1587E+14	0.0711701585176658	0.21161832490947	-4,03902E+14	7,42594E+14	7,04689E+14	6,57229E+14	6,47038E+13	6,97896E+14	6,53688E+14
TC1900000444.mm.2	Mlana	melan-A	-0.818255075202912	5,42484E+14	-2,15868E+14	0.0711723860977082	0.21161832490947	-4,03905E+14	4,95232E+13	5,02756E+14	5,64668E+14	5,96971E+14	5,88751E+14	5,06524E+14
TC0500001635.mm.2	Ppp1r35	protein phosphatase 1, regulatory subunit 35	0.868228175937122	6,63722E+14	2,15858E+14	0.071182977782494	0.21161832490947	-4,03918E+14	7,37847E+14	6,72267E+14	6,44318E+14	5,9215E+14	6,39736E+14	6,96016E+13
TC1100000614.mm.2	Irf1	interferon regulatory factor 1	-0.7078115824187	6,52138E+13	-2,15842E+14	0.0711989101530615	0.211626644400716	-4,03938E+14	5,89913E+14	6,26884E+14	6,70506E+14	6,87854E+14	6,4918E+14	6,8849E+14
TC0200000306.mm.2	4930426L09Rik	RIKEN cDNA 4930426L09 gene	-0.934548774103736	5,26154E+14	-2,15794E+14	0.0712473467326223	0.211731556234368	-4,03998E+14	4,9294E+14	4,57609E+14	5,19957E+14	6,17503E+14	5,08282E+14	5,60632E+14
TC0400000341.mm.2	Aco1	aconitase 1	1,09795E+14	6,50376E+14	2,15776E+14	0.0712655028460999	0.211735257283987	-4,0402E+14	7,12525E+14	6,96927E+14	6,63908E+14	5,25955E+14	6,22281E+13	6,80657E+14
TC1100003049.mm.2	Atp1b2	ATPase, Na+/K+ transporting, beta 2 polypeptide	1,87656E+14	8,62626E+14	2,15757E+14	0.0712852860112332	0.211735257283987	-4,04045E+14	9,79298E+14	9,71353E+14	9,14979E+14	6,60361E+13	8,98675E+14	7,51087E+14
TC0200003264.mm.2	Spout1	SPOUT domain containing methyltransferase 1	0.656288412423669	7,43896E+14	2,15754E+14	0.071288014073458	0.211735257283987	-4,04048E+13	7,98203E+14	7,86546E+14	7,42312E+13	7,11179E+14	7,05911E+14	7,19224E+14
TC0100001969.mm.2	Ncoa2	nuclear receptor coactivator 2	0.934812673904862	7,14179E+14	2,1574E+14	0.0713019774054305	0.211737700319517	-4,04066E+14	7,87026E+14	7,54487E+14	7,24767E+14	6,29784E+14	7,2387E+14	6,65141E+14
TC1500001051.mm.2	Dazap2	DAZ associated protein 2	1,64295E+13	8,99206E+13	2,15616E+14	0.0714285791366016	0.212038186093748	-4,04222E+14	1,00264E+14	1,00359E+14	9,53007E+14	7,24629E+14	9,1023E+14	8,01144E+14
TSUnmapped00000003.mm.2	Wnk2	WNK lysine deficient protein kinase 2	-0.742100785801563	8,26854E+14	-2,15609E+14	0.0714355234457153	0.212038186093748	-4,04231E+14	7,75404E+14	7,81116E+14	8,23859E+13	8,81082E+14	8,31726E+14	8,67938E+14
TC0100003534.mm.2	Ndufs2	NADH:ubiquinone oxidoreductase core subunit S2	0.62162015575902	8,60544E+14	2,15602E+14	0.0714426433502735	0.212038186093748	-4,04239E+14	8,98836E+14	8,92231E+14	8,31607E+14	8,35136E+14	8,50642E+14	8,5481E+14
TC0500001337.mm.2	Sds	serine dehydratase	-1,3737E+13	5,57592E+14	-2,15582E+14	0.071462778760788	0.212046877058913	-4,04264E+14	4,93134E+14	4,49633E+14	5,15651E+14	7,01855E+13	5,57148E+14	6,28133E+14
TC1400001157.mm.2	Klf5	Kruppel-like factor 5	1,02096E+14	6,30124E+14	2,15573E+14	0.0714718915972788	0.212046877058913	-4,04275E+14	6,85996E+14	7,21264E+14	6,64237E+14	5,38831E+14	5,67463E+14	6,02954E+13
TC1100003725.mm.2	Krtap4-7	keratin associated protein 4-7	-0.694319256099303	6,25545E+14	-2,15541E+14	0.0715045523734888	0.212049921242692	-4,04316E+14	5,74977E+14	5,77182E+13	6,22419E+14	6,68604E+14	6,42898E+14	6,67189E+14
TC0200002372.mm.2	Myh7b	myosin, heavy chain 7B, cardiac muscle, beta	-0.726758501158438	6,57317E+14	-2,15538E+14	0.0715071109731073	0.212049921242692	-4,04319E+13	6,10922E+14	5,84193E+14	6,52465E+14	6,88002E+14	6,81649E+14	7,26673E+14
TC0700000612.mm.2	Scgb1b20	secretoglobin, family 1B, member 20	-0.725272884709576	5,8652E+14	-2,1552E+14	0.0715257566075564	0.212049921242692	-4,04342E+13	5,64377E+13	5,22251E+14	6,2253E+14	6,09153E+14	5,78079E+13	6,2273E+14
TC0400001611.mm.2	Kdf1	keratinocyte differentiation factor 1	-0.808431811524165	7,21515E+14	-2,15499E+13	0.0715470086599512	0.212049921242692	-4,04368E+13	6,8828E+14	6,64906E+14	7,30654E+14	7,84218E+14	6,98629E+14	7,62401E+14
TC1300000432.mm.2	Rreb1	ras responsive element binding protein 1	0.802392009195787	6,24377E+13	2,15477E+14	0.0715699022028373	0.212049921242692	-4,04396E+14	6,48632E+14	6,68007E+14	6,19434E+14	5,36727E+14	6,36341E+14	6,37119E+14
TC0X00001674.mm.2	Fancb	Fanconi anemia, complementation group B	0.880639407659843	5,15588E+14	2,15461E+14	0.0715855339734659	0.212049921242692	-4,04416E+14	5,90913E+14	5,61222E+14	5,15712E+13	4,60296E+14	4,41113E+14	5,2427E+14
TC0600001934.mm.2	Rnf133	ring finger protein 133	-0.742917996691644	4,91913E+13	-2,15447E+14	0.0716000511265934	0.212049921242692	-4,04433E+13	4,31264E+14	4,61359E+14	4,94754E+14	5,46453E+14	5,2538E+14	4,92269E+14
TC0900001427.mm.2	Uqcrc1	ubiquinol-cytochrome c reductase core protein 1	0.739255114325987	8,07622E+14	2,15446E+14	0.071601521137503	0.212049921242692	-4,04435E+14	8,85585E+14	8,40226E+14	8,09902E+12	7,68058E+14	7,84363E+14	7,57599E+14
TC0300002491.mm.2	Car14	carbonic anhydrase 14	0.693285989932223	6,19773E+14	2,15445E+14	0.0716022093447365	0.212049921242692	-4,04436E+14	6,59621E+14	6,86305E+13	5,86595E+14	6,20673E+14	5,97098E+14	5,68346E+13
TC0700001711.mm.2	Acsm1	acyl-CoA synthetase medium-chain family member 1	-0.695756821584908	4,91665E+14	-2,1544E+14	0.0716079301709411	0.212049921242692	-4,04443E+14	4,3988E+14	4,79349E+13	5,27033E+14	5,31347E+14	5,03651E+14	4,6873E+14
TC0800000525.mm.2	Ufsp2	UFM1-specific peptidase 2	1,03276E+14	7,60964E+14	2,1543E+14	0.0716176796004921	0.212049921242692	-4,04455E+14	8,56519E+14	8,4869E+14	7,87763E+14	7,10893E+14	7,37904E+14	6,24019E+14
TC0200004415.mm.2	Bmf	BCL2 modifying factor	-0.951555622934642	6,03872E+14	-2,15411E+14	0.0716369081749114	0.212059951550555	-4,04479E+14	6,05175E+14	5,01539E+14	6,26786E+14	6,70239E+14	5,8824E+14	6,31255E+14
TC0500001894.mm.2	Cyp51	cytochrome P450, family 51	2,68671E+14	7,09859E+14	2,1539E+14	0.0716579563463535	0.212059951550555	-4,04505E+14	8,68677E+14	8,47745E+14	7,96631E+14	3,82449E+14	7,47988E+14	6,15661E+13
TC1200000567.mm.2	Klhdc2	kelch domain containing 2	1,76458E+14	6,8705E+14	2,15387E+14	0.0716611687603578	0.212059951550555	-4,04509E+14	8,24662E+13	7,97273E+14	7,43955E+14	5,25065E+14	6,97896E+13	5,33449E+14
TC0300000897.mm.2	Cers2	ceramide synthase 2	0.990596774196796	6,81448E+13	2,15375E+14	0.0716737104291145	0.212059951550555	-4,04524E+14	7,64299E+14	7,48154E+13	7,03184E+14	6,1115E+14	5,86624E+14	6,75274E+14
TC0600000816.mm.2	Foxi3	forkhead box I3	-0.723643947374168	5,28676E+14	-2,15343E+14	0.0717063954346105	0.212064397755363	-4,04564E+14	4,61402E+14	5,03753E+14	5,56209E+14	5,53676E+14	5,25563E+14	5,71451E+14
TC0800000264.mm.2	Defb35	defensin beta 35	-0.853601112895256	6,49035E+14	-2,15336E+14	0.0717140188134099	0.212064397755363	-4,04574E+14	5,83746E+14	6,20123E+14	6,52129E+14	7,2246E+14	6,86694E+14	6,29055E+14
TC0200002330.mm.2	Bpifb4	BPI fold containing family B, member 4	-0.892855389634741	5,37643E+14	-2,15318E+14	0.0717323027907779	0.212064397755363	-4,04596E+14	4,82307E+14	4,89282E+13	5,29332E+14	6,20828E+14	5,2432E+14	5,79792E+14
TC0800002154.mm.2	Snx25	sorting nexin 25	1,51731E+14	7,27128E+14	2,15293E+14	0.0717577958039308	0.212064397755363	-4,04627E+14	8,10724E+14	8,23967E+13	7,55416E+13	5,75813E+14	7,7059E+14	6,2626E+14
TC0200005112.mm.2	Slpi/Mir7678	secretory leukocyte peptidase inhibitor/microRNA 7678	-0.675758919583606	5,19485E+14	-2,15273E+14	0.0717779807353904	0.212064397755363	-4,04652E+14	4,61517E+14	4,77283E+14	5,15806E+13	5,58146E+14	5,53965E+14	5,50195E+14
TC0100003254.mm.2	Rnf2	ring finger protein 2	1,12457E+14	6,48501E+14	2,15272E+14	0.0717789818965863	0.212064397755363	-4,04653E+14	7,32166E+14	7,13341E+14	6,78168E+14	5,42424E+14	6,50203E+14	5,74707E+12
TC0600001917.mm.2	Tspan12	tetraspanin 12	1,85331E+14	7,09039E+13	2,15242E+14	0.0718097225055246	0.212064397755363	-4,04691E+14	8,08213E+13	8,30696E+14	7,61952E+14	5,06297E+14	7,4222E+14	6,04856E+14
TC0300001123.mm.2	Eps8l3	EPS8-like 3	-0.994861177275532	6,17082E+14	-2,15239E+14	0.0718122641387004	0.212064397755363	-4,04694E+14	5,23101E+14	5,63256E+14	5,92829E+14	6,925E+13	6,28959E+14	7,01844E+14
TC0800000228.mm.2	Defa3	defensin, alpha, 3	-0.727471315032743	4,77316E+14	-2,15237E+14	0.0718147114941113	0.212064397755363	-4,04697E+13	4,03887E+14	4,48114E+14	4,77548E+14	5,19948E+14	5,16151E+14	4,98248E+14
TC0800000251.mm.2	Defa17/Defa3	defensin, alpha, 17/defensin, alpha, 3	-0.727471315032743	4,77316E+14	-2,15237E+14	0.0718147114941113	0.212064397755363	-4,04697E+13	4,03887E+14	4,48114E+14	4,77548E+14	5,19948E+14	5,16151E+14	4,98248E+14
TC0600001622.mm.2	Atf7ip	activating transcription factor 7 interacting protein	1,6588E+14	6,18076E+14	2,15232E+13	0.0718199850152681	0.212064397755363	-4,04704E+14	6,96036E+14	7,06791E+14	6,5909E+14	4,11976E+14	6,51178E+14	5,83383E+14
TC1300002303.mm.2	Slc6a19	solute carrier family 6 (neurotransmitter transporter), member 19	-0.647352196646588	6,56015E+14	-2,15198E+14	0.0718547167050533	0.212126262022638	-4,04746E+14	6,24784E+14	6,08524E+14	6,88627E+13	6,74152E+14	6,58313E+12	6,81692E+14
TC0700002607.mm.2	Zfp575	zinc finger protein 575	-0.939556306073804	5,40871E+14	-2,15179E+14	0.0718737226028036	0.212126262022638	-4,0477E+14	5,24835E+14	4,87276E+14	5,50841E+14	6,49182E+13	5,43101E+13	4,89987E+14
TC0100002645.mm.2	Serpine2	serine (or cysteine) peptidase inhibitor, clade E, member 2	1,42323E+13	8,32378E+14	2,15173E+14	0.0718804313686838	0.212126262022638	-4,04778E+14	9,27669E+13	9,17624E+14	8,66117E+14	6,9453E+14	8,57619E+14	7,30705E+14
TC0100002215.mm.2	Aff3	AF4/FMR2 family, member 3	0.871641131147529	7,30688E+14	2,15147E+14	0.071907008188265	0.212165834634227	-4,04811E+14	7,75059E+14	7,79298E+14	7,32789E+14	6,47241E+14	7,5296E+14	6,96783E+14
TC0500002723.mm.2	Ankrd17	ankyrin repeat domain 17	1,39451E+14	8,23272E+14	2,15085E+13	0.0719700523330381	0.212312972032633	-4,04888E+14	9,24858E+14	9,11709E+14	8,59705E+13	6,9796E+14	8,38108E+14	7,07295E+14
TC1700001187.mm.2	Vit	vitrin	1,00033E+14	5,87852E+14	2,15042E+14	0.0720139606250979	0.212403614781659	-4,04942E+14	6,3189E+14	6,97846E+14	5,41837E+14	5,87832E+13	5,85689E+14	4,82019E+14
TC1600002120.mm.2	Vps26c	VPS26 endosomal protein sorting factor C	0.646526014658527	6,2718E+13	2,15007E+14	0.0720500610786388	0.212445803251462	-4,04986E+14	6,79936E+14	6,81263E+14	6,15831E+14	6,16064E+14	6,00879E+14	5,69105E+14
TC1800001048.mm.2	B4galt6	UDP-Gal:betaGlcNAc beta 1,4-galactosyltransferase, polypeptide 6	1,5827E+14	8,519E+14	2,14991E+14	0.0720665323853349	0.212445803251462	-4,05006E+14	9,53707E+14	9,57927E+14	9,00649E+14	6,94445E+14	8,66653E+14	7,38022E+14
TC0500000021.mm.2	Akap9	A kinase (PRKA) anchor protein (yotiao) 9	0.857442092299194	6,69691E+14	2,1499E+14	0.0720678185671504	0.212445803251462	-4,05008E+14	6,9772E+14	7,11011E+12	6,66723E+14	5,70518E+14	6,96185E+14	6,75987E+14
TC0800000973.mm.2	Rtbdn	retbindin	-0.894964850630146	6,1786E+13	-2,14942E+14	0.0721169485599645	0.212492654300611	-4,05068E+14	5,63183E+14	5,69729E+14	6,04119E+14	7,07786E+14	6,16258E+14	6,46088E+14
TC1400001211.mm.2	Trim52	tripartite motif-containing 52	-0.68771967184774	8,61111E+14	-2,14941E+14	0.0721183993511763	0.212492654300611	-4,05069E+14	8,16216E+14	8,08014E+14	8,5918E+14	9,02594E+14	8,78118E+14	9,02545E+14
TC1400002460.mm.2	Gm1587	predicted gene 1587	-1,09123E+14	6,60953E+14	-2,14936E+14	0.072123274770064	0.212492654300611	-4,05075E+14	5,90741E+14	6,11561E+14	6,48469E+14	7,7208E+14	6,3014E+14	7,12726E+14
TC1000000812.mm.2	Bsg	basigin	0.985751280337562	1,08862E+14	2,14912E+14	0.0721473158831587	0.212524625379403	-4,05105E+14	1,14952E+14	1,15228E+14	1,09497E+14	1,00968E+14	1,11048E+14	1,01478E+14
TC1400001664.mm.2	Spcs1	signal peptidase complex subunit 1 homolog (S. cerevisiae)	1,09923E+14	8,4422E+13	2,14894E+14	0.0721667515068756	0.212536261854727	-4,05129E+14	9,19046E+14	9,10267E+14	8,70137E+14	7,3933E+14	8,51505E+14	7,75038E+14
TC0600000943.mm.2	Zfp638	zinc finger protein 638	1,04625E+14	7,8768E+14	2,14876E+14	0.0721845047424553	0.212536261854727	-4,0515E+14	8,58284E+14	8,57172E+14	8,12682E+14	6,93523E+14	7,89872E+14	7,14547E+14
TC1700001660.mm.2	Tpsab1	tryptase alpha/beta 1	-0.889368055176749	4,46533E+14	-2,1487E+13	0.0721908372823759	0.212536261854727	-4,05158E+13	3,75437E+14	4,03111E+14	4,41153E+14	5,15269E+14	4,36208E+14	5,08023E+14
TC0200002972.mm.2	Itga8	integrin alpha 8	-0.795402383290122	6,20714E+12	-2,14848E+14	0.0722135337300666	0.212564243513439	-4,05186E+14	5,63013E+14	5,78145E+14	6,15537E+14	6,84702E+14	6,19098E+14	6,63787E+14
TC0500001642.mm.2	Zscan21	zinc finger and SCAN domain containing 21	0.755673626815774	5,72412E+14	2,14831E+14	0.0722311957500944	0.212577398560963	-4,05207E+14	6,2532E+13	5,98586E+14	5,47565E+14	5,25206E+13	5,36958E+14	6,00839E+14
TC0800002399.mm.2	Gdf15	growth differentiation factor 15	-0.710113277697777	5,99496E+14	-2,14771E+13	0.072292532587645	0.212685810394664	-4,05282E+14	5,2109E+14	5,66913E+14	6,20191E+14	6,09836E+14	6,219E+14	6,57046E+13
TC1000000846.mm.2	Pwwp3a	PWWP domain containing 3A, DNA repair factor	1,18608E+14	6,82741E+14	2,14738E+14	0.0723266780933361	0.212685810394664	-4,05324E+14	7,71053E+14	7,42052E+14	6,82918E+13	5,9297E+14	5,8357E+14	7,23882E+13
TC1100003722.mm.2	Gm11562/Krtap2-4	predicted gene 11562/keratin associated protein 2-4	-0.928760470654145	5,81156E+13	-2,14733E+14	0.0723323770399054	0.212685810394664	-4,05331E+14	5,03216E+14	5,39179E+14	5,69651E+14	6,58495E+14	5,75579E+14	6,40815E+12
TC1200000647.mm.2	Prkch	protein kinase C, eta	0.932232118354174	7,7342E+14	2,1472E+14	0.0723455390927043	0.212685810394664	-4,05347E+14	8,46399E+14	8,52105E+14	7,90932E+14	7,21126E+14	7,6167E+14	6,68289E+13
TC0300002173.mm.2	Golim4	golgi integral membrane protein 4	2,18599E+14	5,92678E+14	2,14692E+13	0.0723746680206484	0.212685810394664	-4,05382E+14	7,55072E+13	7,3839E+14	6,64601E+14	3,91662E+14	6,12656E+14	3,93689E+14
TC0600001189.mm.2	Lrrn1	leucine rich repeat protein 1, neuronal	1,68165E+14	7,42288E+14	2,14689E+14	0.0723769254605672	0.212685810394664	-4,05385E+13	8,64355E+14	8,49147E+14	8,06886E+14	5,70287E+14	7,36487E+14	6,26565E+14
TC1900001576.mm.2	Lbx1	ladybird homeobox 1	-0.890407905553984	5,76571E+13	-2,14688E+14	0.0723782815881137	0.212685810394664	-4,05387E+14	5,07632E+14	5,3174E+14	5,80347E+14	6,37107E+14	5,57418E+14	6,45181E+13
TC1100003146.mm.2	Spns2	spinster homolog 2	1,13398E+14	7,08166E+14	2,14683E+14	0.0723835619841459	0.212685810394664	-4,05393E+14	7,83453E+13	7,87234E+14	7,38318E+14	6,05573E+14	7,10885E+14	6,23531E+14
TC0200004951.mm.2	Eif2s2	eukaryotic translation initiation factor 2, subunit 2 (beta)	1,21169E+14	9,02651E+14	2,1468E+14	0.0723868295292209	0.212685810394664	-4,05397E+13	9,79546E+14	9,73017E+12	9,21153E+14	7,89073E+14	9,33272E+14	8,19843E+13
TC1800000980.mm.2	Abhd3	abhydrolase domain containing 3	1,68992E+14	7,36336E+14	2,14663E+14	0.0724044049382853	0.212695573296111	-4,05418E+14	8,5697E+13	8,19357E+14	7,71915E+14	5,66427E+13	7,8144E+14	6,21905E+14
TC0700000475.mm.2	Zfp59	zinc finger protein 59	0.971122680665347	5,85562E+14	2,14622E+14	0.0724467965631623	0.212695573296111	-4,0547E+14	6,74665E+14	6,36252E+14	5,24365E+14	5,92328E+14	5,91923E+14	4,93842E+14
TC0200003300.mm.2	Dnm1	dynamin 1	1,6039E+14	8,49016E+14	2,14607E+14	0.0724620515583078	0.212695573296111	-4,05489E+14	9,50068E+14	9,46183E+14	9,0668E+14	6,68791E+14	8,45151E+14	7,77223E+14
TC1500000733.mm.2	Csdc2	cold shock domain containing C2, RNA binding	-1,03552E+13	6,6044E+14	-2,14594E+14	0.0724751761052261	0.212695573296111	-4,05504E+14	6,04128E+14	5,85541E+14	6,37695E+13	7,59078E+14	6,55498E+14	7,20698E+14
TC0400002605.mm.2	Tbc1d2	TBC1 domain family, member 2	-0.895893186910679	6,7811E+14	-2,14586E+13	0.0724832304972661	0.212695573296111	-4,05514E+13	6,03539E+14	6,16522E+14	6,62936E+14	7,36303E+14	6,85154E+14	7,64203E+14
TC0500003093.mm.2	Rnf10	ring finger protein 10	1,47885E+14	9,0195E+14	2,14577E+14	0.0724928615941065	0.212695573296111	-4,05526E+14	9,95251E+14	9,91466E+14	9,38427E+14	7,52521E+14	9,30058E+14	8,03978E+14
TC1100003118.mm.2	C1qbp	complement component 1, q subcomponent binding protein	1,2613E+14	9,10373E+14	2,14553E+14	0.072517239673939	0.212695573296111	-4,05556E+14	9,8494E+14	9,73607E+14	9,38337E+14	7,67949E+14	9,32872E+14	8,64536E+14
TC0600002773.mm.2	Lrig1	leucine-rich repeats and immunoglobulin-like domains 1	0.6330451354378	6,89791E+14	2,14553E+14	0.0725176695255832	0.212695573296111	-4,05556E+14	7,18706E+14	7,26163E+14	6,65445E+14	6,52815E+14	6,97343E+14	6,78274E+14
TC0900002268.mm.2	Pts	6-pyruvoyl-tetrahydropterin synthase	0.92807294358068	6,30177E+14	2,14552E+14	0.0725188539802181	0.212695573296111	-4,05558E+14	7,38285E+14	7,01176E+14	6,30127E+14	6,2372E+14	5,98716E+14	4,89039E+14
TC1200000884.mm.2	Gm1995	REX1, RNA exonuclease 1 homolog pseudogene	-1,14408E+14	5,76081E+14	-2,14549E+13	0.0725221547625418	0.212695573296111	-4,05562E+14	4,68383E+14	5,6233E+13	5,79969E+14	6,79559E+14	5,40668E+14	6,25579E+14
TC0700000281.mm.2	Irf2bp1	interferon regulatory factor 2 binding protein 1	0.708991381487853	6,5971E+14	2,14487E+14	0.0725862763609354	0.212832345651882	-4,05639E+14	7,22808E+13	6,91506E+13	6,60242E+14	6,12274E+14	6,41927E+14	6,29503E+13
TC0600003475.mm.2	Dennd5b	DENN/MADD domain containing 5B	1,57564E+14	6,8553E+14	2,14472E+14	0.0726017478121081	0.212832345651882	-4,05658E+14	8,09217E+13	7,89968E+14	6,70755E+14	6,13302E+14	7,32365E+14	4,97574E+14
TC0800003008.mm.2	Gcsh	glycine cleavage system protein H (aminomethyl carrier)	0.962683611976761	8,22807E+14	2,14445E+14	0.072629469347075	0.212832345651882	-4,05692E+14	8,99093E+14	8,77855E+14	8,45029E+13	7,39382E+14	8,18109E+14	7,57372E+14
TC0800001085.mm.2	Gnao1	guanine nucleotide binding protein, alpha O	1,66275E+14	1,04085E+14	2,14434E+14	0.0726409575397302	0.212832345651882	-4,05706E+14	1,14715E+14	1,14976E+14	1,09597E+13	8,68378E+14	1,05297E+14	9,30882E+13

TC1700000290.mm.2	Zfp758	zinc finger protein 758	1,34035E+14	6,0026E+14	2,1443E+14	0.0726446233829187	0.212832345651882	-4,0571E+14	6,70289E+14	6,90482E+14	6,35428E+14	4,57272E+14	6,17306E+13	5,30781E+14
TC0900002003.mm.2	St3gal4	ST3 beta-galactoside alpha-2,3-sialyltransferase 4	0.747155338202549	7,35127E+12	2,14427E+13	0.0726480420210608	0.212832345651882	-4,05714E+14	7,92653E+14	7,69001E+14	6,75394E+14	7,3683E+14	7,31322E+14	7,05561E+14
TC0100003026.mm.2	Mcm6	minichromosome maintenance complex component 6	1,07534E+14	5,56128E+14	2,14409E+14	0.0726668304571014	0.212848689175654	-4,05737E+14	5,50053E+14	6,54328E+14	4,94066E+14	4,95247E+14	5,19432E+14	6,23639E+14
TC0X00002085.mm.2	Lamp2	lysosomal-associated membrane protein 2	1,26832E+14	9,12733E+14	2,14385E+14	0.0726913653799422	0.212881282433398	-4,05767E+14	9,69708E+14	1,01001E+14	9,43403E+14	7,82652E+14	9,2694E+14	8,43682E+14
TC0300002220.mm.2	Gucy1a1/Mir7010	guanylate cyclase 1, soluble, alpha 1/microRNA 7010	1,59458E+14	7,47072E+14	2,14361E+14	0.0727162376682603	0.212881282433398	-4,05797E+13	8,68786E+14	8,40353E+14	8,15303E+14	5,7492E+14	7,13663E+14	6,69409E+14
TC0500003382.mm.2	Cldn13	claudin 13	-0.804806502462261	5,89102E+14	-2,14349E+14	0.0727283758265822	0.212881282433398	-4,05812E+14	5,35817E+14	5,51879E+14	5,81844E+14	6,66813E+14	6,02111E+14	5,96148E+14
TC0500003055.mm.2	Svop	SV2 related protein	1,13846E+14	7,39796E+14	2,14347E+14	0.0727308049274411	0.212881282433398	-4,05815E+14	8,16288E+14	8,31767E+14	7,60424E+14	6,59939E+14	7,47081E+14	6,23278E+14
TC0200001858.mm.2	Bloc1s6	biogenesis of lysosomal organelles complex-1, subunit 6, pallidin	0.759708838944073	6,47873E+14	2,1432E+14	0.072758607985608	0.212923983013458	-4,05849E+14	7,03236E+14	6,7969E+13	6,46527E+14	5,84457E+14	6,53514E+14	6,19814E+14
TC1800000924.mm.2	Crem	cAMP responsive element modulator	0.839223875344253	6,66463E+13	2,14292E+14	0.0727877392384857	0.212960617787521	-4,05884E+14	7,30253E+14	7,07441E+14	6,47926E+14	6,21923E+14	6,90091E+14	6,01146E+14
TC1200002178.mm.2	Galc	galactosylceramidase	1,44413E+14	4,93185E+14	2,14267E+14	0.0728134266498968	0.212960617787521	-4,05915E+14	5,80041E+14	5,78707E+14	5,08945E+14	3,60978E+14	5,45701E+13	3,84736E+14
TC1100004113.mm.2	Exoc7	exocyst complex component 7	1,09346E+14	6,3841E+14	2,14245E+14	0.0728363778911064	0.212960617787521	-4,05943E+14	7,20523E+14	7,28752E+13	6,44465E+14	5,86119E+14	6,44965E+14	5,05638E+14
TC0700004526.mm.2	Lmntd2	lamin tail domain containing 2	-0.967810882564374	5,2536E+14	-2,14242E+14	0.0728397771455687	0.212960617787521	-4,05947E+14	4,4997E+14	4,58902E+14	5,19374E+14	5,83061E+14	5,16826E+14	6,24028E+13
TC0700000219.mm.2	Napa	N-ethylmaleimide sensitive fusion protein attachment protein alpha	0.828227107026812	9,5352E+14	2,14235E+14	0.0728463789921743	0.212960617787521	-4,05955E+14	1,01531E+14	9,97479E+14	9,56876E+14	8,90268E+14	9,61619E+14	8,99572E+14
TC1600000307.mm.2	Senp2	SUMO/sentrin specific peptidase 2	1,26358E+14	7,06193E+14	2,14223E+14	0.0728588711386959	0.212960617787521	-4,0597E+14	7,70547E+14	7,71539E+14	7,30476E+14	5,58895E+14	7,37082E+14	6,68616E+13
TC1300000183.mm.2	Hist1h2br/Hist1h2bq	histone cluster 1 H2br/histone cluster 1, H2bq	-0.678314442300117	6,70135E+14	-2,14219E+14	0.0728636433850061	0.212960617787521	-4,05976E+13	6,11587E+14	6,36776E+13	6,73524E+14	7,10501E+14	7,02514E+14	6,85909E+14
TC0600002265.mm.2	Hnrnpa2b1	heterogeneous nuclear ribonucleoprotein A2/B1	1,20978E+14	9,28042E+14	2,14153E+14	0.0729317722358716	0.213101466911358	-4,06058E+13	1,01901E+14	1,011E+14	9,54261E+14	8,3379E+14	9,38044E+14	8,12152E+14
TC0900001377.mm.2	Ifrd2	interferon-related developmental regulator 2	-0.743702342238616	6,98626E+14	-2,14147E+14	0.0729382852365259	0.213101466911358	-4,06066E+14	6,32988E+14	6,53063E+14	7,10313E+14	7,24479E+14	7,02706E+14	7,68206E+14
TC1400000327.mm.2	Capn7	calpain 7	1,28339E+14	6,34191E+14	2,14127E+14	0.0729585597839951	0.213122058339288	-4,0609E+14	7,25984E+14	7,01926E+14	6,7028E+14	5,00953E+13	6,42794E+13	5,6321E+14
TC0200000079.mm.2	4930412O13Rik	RIKEN cDNA 4930412O13 gene	-1,11115E+14	5,16542E+14	-2,14098E+14	0.0729887679491226	0.213141024352714	-4,06127E+14	4,52741E+14	4,30744E+14	4,89211E+14	6,16504E+14	5,12199E+13	5,97854E+14
TC1100002487.mm.2	Tlx3	T cell leukemia, homeobox 3	-1,13297E+14	6,53061E+14	-2,14087E+14	0.0730005563731353	0.213141024352714	-4,06141E+14	5,74449E+14	5,98163E+14	6,30201E+14	7,69006E+14	6,35948E+14	7,10602E+14
TC1700000474.mm.2	Ppard/1810013A23Rik	peroxisome proliferator activator receptor delta/RIKEN cDNA 1810013A23 gene	-0.70561101280181	8,78065E+14	-2,14071E+14	0.0730172520818901	0.213141024352714	-4,06161E+14	8,25834E+14	8,36092E+14	8,74906E+14	9,28142E+13	8,91509E+14	9,11908E+14
TC0700001736.mm.2	Eef2k	eukaryotic elongation factor-2 kinase	0.723711918876578	6,39294E+14	2,1407E+14	0.0730179640307195	0.213141024352714	-4,06162E+14	6,92264E+14	6,78975E+14	6,44428E+14	5,82069E+14	6,21043E+14	6,16983E+14
TC0600002280.mm.2	Hoxa6	homeobox A6	-1,13891E+14	5,94951E+14	-2,14036E+14	0.0730529648854099	0.213204568592394	-4,06204E+13	5,13302E+14	5,20111E+14	5,5985E+14	7,01346E+14	5,99698E+14	6,75397E+14
TC0300000694.mm.2	Fcrl1	Fc receptor-like 1	-1,20007E+14	5,80658E+14	-2,14E+13	0.0730906160956058	0.213212985833055	-4,0625E+14	5,11145E+14	4,98066E+14	5,46905E+14	7,0232E+14	5,75663E+14	6,49846E+14
TC0200002386.mm.2	Phf20	PHD finger protein 20	3,40885E+14	5,07658E+14	2,13988E+14	0.0731030144915017	0.213212985833055	-4,06265E+14	7,32458E+14	6,98213E+14	5,41404E+13	2,07497E+14	6,58519E+13	2,07858E+14
TC0400000275.mm.2	Rragd	Ras-related GTP binding D	1,08996E+14	8,23404E+14	2,13981E+14	0.0731103547337362	0.213212985833055	-4,06274E+14	8,94009E+14	9,08817E+14	8,62437E+14	7,22397E+14	8,03407E+14	7,49355E+14
TC1100002062.mm.2	Narf	nuclear prelamin A recognition factor	-0.634748855010145	7,81775E+14	-2,13976E+14	0.0731161195890727	0.213212985833055	-4,0628E+14	7,33705E+14	7,48525E+14	7,94824E+14	8,14356E+14	7,94652E+14	8,0459E+14
TC0300002934.mm.2	Lrit3	leucine-rich repeat, immunoglobulin-like and transmembrane domains 3	-0.738823813165941	5,03075E+14	-2,1397E+14	0.0731220107809509	0.213212985833055	-4,06288E+14	4,49337E+14	4,63466E+14	4,97916E+14	5,62652E+14	5,13511E+14	5,31566E+14
TC1800000534.mm.2	Snx2	sorting nexin 2	1,62164E+14	7,88803E+14	2,13948E+13	0.073145026938421	0.213241508785739	-4,06315E+14	8,86345E+14	8,62144E+14	8,356E+14	5,8856E+13	8,00438E+13	7,5973E+14
TC0300002462.mm.2	Psmd4	proteasome (prosome, macropain) 26S subunit, non-ATPase, 4	0.658671504458824	8,18912E+14	2,13875E+14	0.0732209995170196	0.213424378657333	-4,06407E+13	8,56124E+14	8,74988E+14	7,9393E+14	8,05447E+14	8,08014E+14	7,74969E+14
TC1400002116.mm.2	Gzmd/Gzme	granzyme D/granzyme E	-0.639165341714871	5,45991E+14	-2,13856E+13	0.073241148746838	0.213426856014247	-4,06431E+14	4,89383E+14	4,79893E+14	5,62273E+14	5,34836E+14	6,02136E+14	6,07425E+14
TC0500003645.mm.2	Katnal1/5930430L01Rik	katanin p60 subunit A-like 1/RIKEN cDNA 5930430L01 gene	0.957956062760524	7,18603E+12	2,13843E+14	0.0732545190834633	0.213426856014247	-4,06447E+14	7,89086E+14	8,08859E+14	7,46544E+13	6,59809E+14	6,96316E+14	6,11003E+14
TC1900000106.mm.2	Gpha2	glycoprotein hormone alpha 2	-0.75502228418892	6,22222E+14	-2,13836E+14	0.0732615863349344	0.213426856014247	-4,06456E+14	5,80869E+14	5,83262E+14	6,20868E+13	6,94267E+14	6,24631E+14	6,29432E+14
TC1500002061.mm.2	Syt10	synaptotagmin X	0.759990485100962	8,37794E+14	2,13792E+14	0.073308223988765	0.213455796237812	-4,06512E+14	9,09205E+14	8,84823E+14	8,54903E+13	7,87127E+14	8,05922E+14	7,84786E+14
TC0X00002454.mm.2	Gm6880	predicted gene 6880	-0.68665757133838	4,96167E+13	-2,13773E+14	0.0733280414916495	0.213455796237812	-4,06536E+14	4,42288E+14	4,20357E+14	4,76701E+14	5,23275E+14	5,56538E+14	5,57846E+14
TC0X00000466.mm.2	Ccdc160	coiled-coil domain containing 160	-0.939146871870606	6,15755E+14	-2,13773E+14	0.073328121720812	0.213455796237812	-4,06536E+14	5,51389E+14	5,59183E+14	5,97049E+14	7,01352E+13	6,1421E+14	6,7135E+14
TC0200004369.mm.2	Actc1	actin, alpha, cardiac muscle 1	-0.836167739522379	7,21314E+13	-2,13762E+14	0.0733387220934437	0.213455796237812	-4,06548E+14	6,5872E+14	6,67666E+14	7,05849E+14	7,87771E+14	7,30115E+13	7,77764E+13
TC0500001607.mm.2	Myl10	myosin, light chain 10, regulatory	-0.669694560983054	5,35047E+14	-2,13753E+14	0.0733488420173233	0.213455796237812	-4,06561E+14	5,13456E+14	5,07823E+14	5,55453E+14	5,99764E+14	5,18595E+14	5,15194E+13
TC1900000061.mm.2	Catsper1	cation channel, sperm associated 1	-1,0024E+14	4,6727E+14	-2,13751E+13	0.0733510050126462	0.213455796237812	-4,06563E+14	3,69879E+14	4,04462E+13	4,3106E+14	5,43761E+14	4,9653E+14	5,57927E+14
TC0600001427.mm.2	Phb2	prohibitin 2	1,06631E+13	8,82712E+14	2,13711E+14	0.0733928152619996	0.213529045635338	-4,06613E+14	9,35875E+14	9,3255E+14	9,00566E+14	7,54596E+14	8,90891E+14	8,81795E+14
TC0400003847.mm.2	Pqlc2	PQ loop repeat containing 2	1,02779E+14	6,11176E+14	2,13701E+14	0.073402680057974	0.213529045635338	-4,06625E+14	6,5226E+14	6,93321E+14	6,35833E+14	5,0419E+14	5,96101E+14	5,85348E+14
TC0200001991.mm.2	Oxt	oxytocin	-0.739781428091147	8,75924E+14	-2,13666E+14	0.0734393774763818	0.213597236331578	-4,06669E+14	8,48982E+13	8,02277E+14	8,74349E+14	9,24866E+14	9,06613E+14	8,98457E+14
TC1000002943.mm.2	Slc35e3	solute carrier family 35, member E3	0.948072630396338	6,54987E+13	2,13619E+14	0.0734885934813829	0.213680927532095	-4,06728E+14	7,12739E+14	7,14946E+14	6,75247E+14	5,62824E+14	6,54839E+14	6,09325E+14
TC0200000781.mm.2	Acvr2a	activin receptor IIA	0.887558201598817	6,98484E+14	2,13613E+14	0.0734946751308181	0.213680927532095	-4,06736E+14	7,66274E+14	7,4787E+13	7,12708E+14	6,23925E+14	6,99066E+14	6,4106E+14
TC1400001331.mm.2	Clybl	citrate lyase beta like	0.813943370156964	7,18428E+14	2,13593E+14	0.0735154187919144	0.213681480817617	-4,0676E+14	7,74315E+14	7,69013E+14	7,22101E+14	6,58439E+14	7,24885E+13	6,61815E+14
TC0700000276.mm.2	Nova2	neuro-oncological ventral antigen 2	0.651826783950757	6,97354E+14	2,13558E+14	0.0735521088947236	0.213681480817617	-4,06804E+14	7,42488E+14	7,4375E+14	6,94206E+14	6,61667E+14	6,62296E+13	6,79714E+14
TC1300002057.mm.2	Pfn3	profilin 3	-0.958373786220756	6,56083E+14	-2,13558E+14	0.0735530250078225	0.213681480817617	-4,06805E+14	5,81943E+14	6,06968E+14	6,42576E+14	7,38009E+14	6,46251E+13	7,20749E+14
TC0600000608.mm.2	Cbx3	chromobox 3	1,48417E+14	9,12203E+14	2,13552E+14	0.0735586461305748	0.213681480817617	-4,06812E+14	1,01916E+14	1,00834E+14	9,433E+14	7,87368E+14	9,39792E+14	7,75255E+14
TC0900001616.mm.2	Trak1	trafficking protein, kinesin binding 1	0.974986496787209	7,96519E+14	2,13541E+13	0.0735701612785596	0.213681480817617	-4,06826E+14	8,76347E+13	8,49247E+13	8,23889E+14	7,06707E+14	7,82245E+14	7,40682E+13
TC0800003051.mm.2	1700018B08Rik	RIKEN cDNA 1700018B08 gene	-0.794525660496622	6,60756E+14	-2,13537E+14	0.0735744340331496	0.213681480817617	-4,06831E+14	6,24979E+14	6,14164E+13	6,64883E+14	7,33165E+14	6,39137E+14	6,8821E+14
TC0200002405.mm.2	Tldc2	TBC/LysM associated domain containing 2	-0.778084165345955	5,66026E+14	-2,13505E+14	0.073608194794332	0.213730428386151	-4,06871E+14	5,52842E+14	4,92785E+14	5,81404E+14	6,19839E+14	5,56071E+14	5,93212E+14
TC1000000372.mm.2	Sesn1	sestrin 1	1,0617E+14	7,35574E+14	2,13496E+14	0.0736178165172928	0.213730428386151	-4,06883E+14	8,10404E+14	7,95559E+14	7,57175E+14	6,36448E+14	7,46909E+14	6,66952E+14
TC1000000297.mm.2	Tspyl1	testis-specific protein, Y-encoded-like 1	1,46819E+14	8,42703E+14	2,13419E+14	0.0736986052100597	0.213884572510842	-4,0698E+14	9,53699E+14	9,37493E+13	8,81994E+13	7,15559E+14	8,58379E+14	7,09091E+14
TC0100000364.mm.2	Tmeff2	transmembrane protein with EGF-like and two follistatin-like domains 2	1,15165E+14	8,2137E+14	2,13413E+14	0.0737045655277304	0.213884572510842	-4,06987E+14	8,92558E+14	8,77829E+14	8,44447E+14	6,95611E+14	8,42303E+14	7,75475E+14
TC0800000020.mm.2	Mcemp1	mast cell expressed membrane protein 1	-0.691057747951388	5,10377E+14	-2,13396E+13	0.0737228086088926	0.213884572510842	-4,07009E+14	4,57081E+14	4,8833E+12	5,20815E+14	5,62808E+14	5,22319E+14	5,10908E+14
TC1600002107.mm.2	Setd4	SET domain containing 4	-0.719177386386965	5,5271E+14	-2,13394E+12	0.0737240064373623	0.213884572510842	-4,0701E+14	5,02183E+14	4,99604E+14	5,45345E+13	6,00278E+14	5,99966E+14	5,68887E+14
TC0100001058.mm.2	Nifk	nucleolar protein interacting with the FHA domain of MKI67	1,26516E+13	7,1681E+13	2,13375E+14	0.0737443875829969	0.213905187601229	-4,07034E+14	7,86234E+14	8,0815E+14	7,21917E+14	6,19435E+14	7,59397E+14	6,05724E+14
TC1300000878.mm.2	Irx4	Iroquois homeobox 4	-1,14369E+14	5,05622E+14	-2,13358E+14	0.0737619148302056	0.213917518657146	-4,07055E+14	4,47107E+14	4,32711E+14	4,90365E+14	6,18191E+13	4,73205E+14	5,72156E+14
TC1100000037.mm.2	Sf3a1	splicing factor 3a, subunit 1	0.949444483099634	7,57032E+14	2,13313E+14	0.073809327630704	0.214016501010173	-4,07112E+14	8,54812E+14	8,39885E+14	7,97056E+14	7,07753E+14	7,1216E+14	6,30528E+14
TC0900002288.mm.2	1810046K07Rik	RIKEN cDNA 1810046K07 gene	-0.717213407327556	5,98314E+14	-2,13267E+14	0.0738581080382597	0.214119412526175	-4,0717E+14	5,51928E+14	5,5114E+14	5,9698E+14	6,49531E+14	6,0308E+14	6,37222E+14
TC0800001541.mm.2	Urb2	URB2 ribosome biogenesis 2 homolog (S. cerevisiae)	1,38304E+14	6,95592E+14	2,13247E+14	0.0738786425447359	0.214140415060862	-4,07195E+14	7,88575E+14	7,81354E+14	7,42894E+14	5,50427E+13	6,95107E+14	6,15196E+14
TC1900001776.mm.2	Sec31b	Sec31 homolog B (S. cerevisiae)	-0.738538071558475	5,84471E+14	-2,1322E+14	0.0739076498802498	0.214185964482098	-4,07229E+14	5,23078E+14	5,45677E+14	5,86455E+14	6,30007E+14	5,86284E+14	6,35326E+13
TC0400002943.mm.2	Cer1	cerberus 1, DAN family BMP antagonist	-0.785365361851963	5,74222E+14	-2,13194E+14	0.0739347264278276	0.214218763422057	-4,07261E+14	5,10382E+14	5,54792E+14	5,81797E+12	6,4045E+13	5,60094E+13	5,97818E+14
TC1100003121.mm.2	Derl2	Der1-like domain family, member 2	1,10752E+14	6,84986E+14	2,13184E+14	0.0739455571373102	0.214218763422057	-4,07274E+14	7,2511E+14	7,67258E+14	7,08499E+14	5,62365E+14	6,88859E+13	6,57825E+14
TC1000002418.mm.2	Col18a1	collagen, type XVIII, alpha 1	-0.915001959171809	6,1899E+14	-2,13167E+14	0.0739635748072887	0.214232443082841	-4,07296E+14	5,28175E+14	5,6702E+14	5,85316E+14	6,9288E+14	6,70142E+14	6,70409E+14
TC0500003638.mm.2	Ubl3	ubiquitin-like 3	1,1086E+14	9,39234E+14	2,13145E+14	0.0739863698561701	0.214251408561943	-4,07323E+14	1,01053E+14	1,00047E+14	9,63855E+14	8,25432E+14	9,526E+14	8,8251E+14
TC0500000584.mm.2	0610040J01Rik	RIKEN cDNA 0610040J01 gene	-0.888924244514676	5,60828E+14	-2,13135E+14	0.0739967162320618	0.214251408561943	-4,07335E+13	4,78383E+13	5,31264E+14	5,76193E+14	6,11238E+14	5,39985E+14	6,27903E+14
TC0200002288.mm.2	Defb28	defensin beta 28	-0.830520116335828	4,74148E+14	-2,13111E+14	0.0740223740164186	0.214278999341778	-4,07366E+13	4,45583E+14	3,85026E+14	4,64657E+14	5,32056E+14	4,955E+13	5,22065E+14
TC1000002527.mm.2	Pias4	protein inhibitor of activated STAT 4	-0.955330462378067	7,8117E+14	-2,1306E+14	0.0740762054379302	0.214278999341778	-4,0743E+14	7,0433E+14	7,18218E+14	7,50328E+13	8,63287E+14	8,01515E+13	8,4934E+14
TC0600003374.mm.2	Gys2	glycogen synthase 2	-0.739367603001317	7,56155E+14	-2,13053E+14	0.0740838161542365	0.214278999341778	-4,07439E+14	7,24815E+14	6,98277E+14	7,59633E+14	8,11333E+14	7,52107E+13	7,90764E+14
TC0600000472.mm.2	Olfr447	olfactory receptor 447	-1,02008E+14	5,10796E+14	-2,13043E+14	0.074093949145088	0.214278999341778	-4,07451E+13	4,80715E+14	4,18323E+14	4,97884E+14	6,0517E+14	5,01907E+14	5,60776E+14
TC1500000087.mm.2	Amacr	alpha-methylacyl-CoA racemase	0.65887670162317	5,72609E+14	2,13031E+14	0.074106706914773	0.214278999341778	-4,07466E+14	6,06472E+14	6,16872E+14	5,32138E+14	5,59431E+14	5,45947E+14	5,74792E+14
TC0300000705.mm.2	Insrr	insulin receptor-related receptor	-1,25345E+14	6,50209E+14	-2,13029E+13	0.0741089541771409	0.214278999341778	-4,07469E+13	5,47563E+14	6,19122E+14	6,24493E+14	7,92881E+14	6,48185E+14	6,69008E+14
TC0700002585.mm.2	Vmn1r175	vomeronasal 1 receptor 175	-0.798408753897465	5,92078E+14	-2,13024E+14	0.0741138402345366	0.214278999341778	-4,07475E+14	5,19015E+14	5,642E+14	6,023E+14	6,40597E+14	5,81792E+14	6,44565E+14
TC1700002352.mm.2	C3	complement component 3	-0.65821466221739	5,07385E+14	-2,13015E+14	0.0741240620588843	0.214278999341778	-4,07487E+14	4,78019E+14	4,63931E+14	5,25813E+14	5,4778E+14	4,98721E+14	5,30046E+14
TC0100000909.mm.2	Olfr1412	olfactory receptor 1412	-0.74003867321774	6,18912E+14	-2,13013E+14	0.0741259320009354	0.214278999341778	-4,07489E+14	5,83767E+14	5,53856E+14	6,28191E+14	6,5744E+14	6,17025E+14	6,73196E+14
TC0800003084.mm.2	Ankrd11	ankyrin repeat domain 11	0.997771052469336	7,64308E+14	2,12984E+14	0.0741558241825321	0.214326958753926	-4,07524E+14	8,42115E+14	8,33635E+14	7,8715E+14	6,89046E+14	7,61427E+14	6,72478E+14
TC0200003176.mm.2	Nacc2	nucleus accumbens associated 2, BEN and BTB (POZ) domain containing	1,01751E+14	6,00868E+14	2,12946E+14	0.0741968234002657	0.21440699703165	-4,07573E+14	6,86614E+14	6,62899E+14	5,31368E+14	6,14642E+14	5,0181E+14	6,07877E+14
TC1900001742.mm.2	Fam204a	family with sequence similarity 204, member A	1,62878E+14	6,69711E+14	2,12919E+14	0.0742248030455424	0.214444206478767	-4,07606E+14	7,95325E+14	7,62718E+14	7,26854E+14	5,05432E+14	6,74289E+14	5,53646E+14
TC0700003559.mm.2	Polg	polymerase (DNA directed), gamma	-0.848602358353125	6,85116E+14	-2,12903E+14	0.0742416915345669	0.214444206478767	-4,07626E+14	6,30585E+14	6,25021E+13	6,69498E+14	7,55829E+14	6,9074E+14	7,39022E+14
TC1600001745.mm.2	Zbtb11os1	zinc finger and BTB domain containing 11, opposite strand 1	-0.693902448830389	6,83042E+14	-2,12896E+13	0.0742496262610962	0.214444206478767	-4,07636E+14	6,30452E+14	6,4122E+14	7,03544E+14	7,06908E+14	6,81056E+14	7,35074E+14
TC0200002011.mm.2	Ap5s1	adaptor-related protein 5 complex, sigma 1 subunit	0.653396901707608	7,18041E+13	2,12857E+14	0.074291093800328	0.214525518710517	-4,07685E+14	7,64335E+14	7,43804E+14	6,80631E+13	6,96829E+14	7,25619E+14	6,97032E+14
TC0500003090.mm.2	Mlec	malectin	1,60506E+14	7,83719E+14	2,12836E+14	0.0743128523855892	0.21453995923692	-4,07711E+14	8,98289E+14	8,87903E+14	8,53895E+14	6,11284E+14	7,55298E+14	6,95643E+14
TC1100002866.mm.2	Pemt	phosphatidylethanolamine N-methyltransferase	-0.705142846037738	7,06E+14	-2,12815E+14	0.0743355507757874	0.21453995923692	-4,07738E+14	6,412E+14	6,85976E+14	7,23387E+14	7,44817E+14	7,3642E+14	7,04199E+14
TC1100000526.mm.2	Gfpt2	glutamine fructose-6-phosphate transaminase 2	-0.74556755580858	6,47094E+14	-2,12814E+14	0.074336038752543	0.21453995923692	-4,07738E+14	6,11268E+14	6,11423E+14	6,5516E+14	7,16644E+14	6,25411E+14	6,6266E+14
TC0400001614.mm.2	Gpatch3	G patch domain containing 3	-0.738433293877745	6,95271E+14	-2,12786E+14	0.0743656097832034	0.214571838995904	-4,07773E+14	6,49096E+14	6,4488E+14	7,10014E+14	7,31649E+14	6,84883E+14	7,51104E+14
TC0100001722.mm.2	Pycr2	pyrroline-5-carboxylate reductase family, member 2	-0.840162428620647	7,29392E+14	-2,12752E+14	0.0744019036614757	0.214571838995904	-4,07816E+14	6,57897E+14	6,96765E+14	7,27636E+14	7,95059E+14	7,20417E+14	7,78577E+14
TC0100001088.mm.2	En1	engrailed 1	-0.642633815647894	5,20775E+14	-2,12745E+13	0.0744091882319629	0.214571838995904	-4,07825E+14	4,60263E+14	4,80311E+14	5,44324E+14	5,24776E+14	5,48358E+14	5,66616E+14
TC1300000882.mm.2	Lpcat1	lysophosphatidylcholine acyltransferase 1	0.888756430984235	6,88243E+13	2,12737E+14	0.0744173654374851	0.214571838995904	-4,07835E+14	7,44602E+12	7,44003E+14	7,06347E+14	6,04506E+14	6,45912E+14	6,84089E+14
TC1000003104.mm.2	Dtx3	deltex 3, E3 ubiquitin ligase	1,2049E+14	7,35699E+14	2,12724E+14	0.074431766225914	0.214571838995904	-4,07852E+14	8,3048E+14	8,08691E+14	7,85187E+14	6,13004E+14	6,76983E+14	6,99851E+14
TC0500000389.mm.2	Hgfac	hepatocyte growth factor activator	-1,1494E+14	5,08898E+14	-2,1272E+13	0.0744355297309821	0.214571838995904	-4,07856E+14	4,41142E+14	4,25054E+14	4,67579E+14	6,28497E+14	5,59513E+14	5,31602E+14
TC0700001430.mm.2	Stard10	START domain containing 10	0.683439105680878	7,76707E+14	2,127E+14	0.0744572903334585	0.214571838995904	-4,07882E+14	8,47595E+14	8,17053E+14	7,58365E+14	7,69595E+14	7,53829E+13	7,13806E+14
TC0500002139.mm.2	Lmbr1	limb region 1	1,08419E+14	6,6028E+13	2,12691E+14	0.0744662224005451	0.214571838995904	-4,07892E+14	7,2984E+14	7,50364E+14	6,42688E+14	6,20677E+14	6,83042E+14	5,35069E+14
TC1800000288.mm.2	Wnt8a	wingless-type MMTV integration site family, member 8A	-0.764161236029497	5,11774E+14	-2,12691E+14	0.0744669350006264	0.214571838995904	-4,07893E+14	4,84306E+14	4,4075E+14	5,11298E+14	5,66591E+14	5,17804E+14	5,49893E+14
TC0800002437.mm.2	Unc13a	unc-13 homolog A	0.655152398819706	7,30921E+14	2,12667E+14	0.0744916123422412	0.214604568151356	-4,07922E+14	7,93746E+14	7,69411E+14	7,06697E+14	7,25429E+14	6,82424E+14	7,07819E+14
TC0200001951.mm.2	Slc20a1	solute carrier family 20, member 1	1,06706E+14	7,60728E+14	2,12631E+13	0.0745303251537583	0.214669356943054	-4,07968E+14	8,13791E+12	8,423E+14	7,55172E+14	6,87506E+14	7,96794E+14	6,68805E+14
TC0400001663.mm.2	Myom3	myomesin family, member 3	-1,02338E+14	5,57525E+14	-2,12613E+14	0.0745489473496952	0.214669356943054	-4,0799E+14	4,88545E+14	4,85967E+14	5,33045E+14	6,46143E+14	5,55781E+13	6,35667E+14
TC0600002324.mm.2	Ggct	gamma-glutamyl cyclotransferase	0.990519365800229	6,64593E+14	2,12581E+14	0.0745832237695782	0.214669356943054	-4,08031E+14	7,42148E+14	7,1469E+14	6,38102E+14	6,20633E+14	6,99722E+14	5,72261E+14
TC0X00003000.mm.2	Armcx2	armadillo repeat containing, X-linked 2	1,69323E+14	5,96362E+14	2,12549E+14	0.0746172748701794	0.214669356943054	-4,08071E+14	7,32685E+14	7,20901E+14	6,49715E+14	4,65225E+14	6,00775E+14	4,08869E+14
TC0400003957.mm.2	Tnfrsf1b	tumor necrosis factor receptor superfamily, member 1b	-0.76841488321906	5,73543E+14	-2,12546E+14	0.0746206100802774	0.214669356943054	-4,08075E+14	5,09749E+14	5,48092E+13	5,84905E+14	6,26619E+14	5,58641E+14	6,1325E+14
TC1000002320.mm.2	Arid5b	AT rich interactive domain 5B (MRF1-like)	1,89252E+14	7,08519E+14	2,12545E+14	0.0746211267381007	0.214669356943054	-4,08075E+14	7,97076E+14	8,4975E+13	7,5121E+13	5,17111E+14	7,59872E+14	5,76096E+14
TC0800000810.mm.2	Olfr372	olfactory receptor 372	-1,33381E+14	3,9495E+14	-2,12543E+14	0.0746240688708066	0.214669356943054	-4,08079E+14	3,07529E+14	2,96078E+14	3,52444E+14	5,17926E+14	3,91921E+14	5,03803E+14
TC0100001016.mm.2	Zcchc2	zinc finger, CCHC domain containing 2	1,10549E+14	5,56567E+14	2,1253E+13	0.0746375641308573	0.214669356943054	-4,08095E+14	6,52885E+14	6,23293E+14	5,97253E+14	4,57827E+14	5,35156E+14	4,72988E+13
TC1900000124.mm.2	Trmt112	tRNA methyltransferase 11-2	0.687678579290028	9,01277E+14	2,1253E+13	0.07463761350582	0.214669356943054	-4,08095E+14	9,61263E+14	9,43453E+14	9,07689E+14	8,59492E+14	8,60682E+13	8,75081E+14
TC1900001253.mm.2	Gda	guanine deaminase	0.853689434904732	9,66017E+13	2,12521E+14	0.0746473286757235	0.214669356943054	-4,08106E+14	1,03363E+14	1,00446E+14	9,79489E+14	8,87858E+14	9,60235E+14	9,30436E+13
TC1700001322.mm.2	Ppp1r21	protein phosphatase 1, regulatory subunit 21	0.85207525063713	7,59303E+14	2,12448E+14	0.0747252079714723	0.21485497431198	-4,08198E+14	8,45405E+14	8,01145E+14	7,65512E+14	7,10623E+14	7,52271E+14	6,80861E+14
TC1800001253.mm.2	Plac8l1	PLAC8-like 1	-0.750456944888223	5,49746E+14	-2,124E+14	0.0747758415815079	0.214881266264395	-4,08258E+14	4,64958E+14	4,96976E+14	5,44221E+14	5,67805E+14	6,43308E+13	5,81208E+14
TC0500001193.mm.2	Tmem211	transmembrane protein 211	-0.722639808114742	4,96922E+14	-2,12375E+13	0.0748024860479114	0.214881266264395	-4,08289E+13	4,34621E+14	4,52067E+14	4,8848E+14	5,42737E+13	5,15806E+13	5,47821E+14
TC1200000200.mm.2	Grhl1	grainyhead like transcription factor 1	0.723918472253735	6,39931E+14	2,12366E+14	0.0748123683627356	0.214881266264395	-4,08301E+14	6,66962E+14	7,01349E+14	6,39489E+14	5,84038E+14	6,19698E+14	6,28053E+14
TC0400002216.mm.2	Pdp1	pyruvate dehyrogenase phosphatase catalytic subunit 1	1,76013E+14	8,28645E+14	2,12365E+14	0.0748133561781216	0.214881266264395	-4,08302E+14	9,08823E+14	9,43027E+14	8,8013E+14	6,19695E+14	8,5683E+14	7,63363E+14
TC0200004419.mm.2	Ccdc9b	coiled-coil domain containing 9B	-0.710214376946586	6,10085E+14	-2,12364E+14	0.0748141322393078	0.214881266264395	-4,08303E+14	5,79872E+14	5,45578E+14	6,17782E+14	6,49711E+14	6,13962E+14	6,53603E+14
TC1400002635.mm.2	Mycbp2	MYC binding protein 2, E3 ubiquitin protein ligase	2,35497E+14	6,82191E+13	2,12357E+14	0.0748217997381126	0.214881266264395	-4,08312E+14	8,78518E+14	8,78863E+14	6,46978E+14	6,39408E+14	7,16715E+14	3,32663E+14
TC0400001610.mm.2	Tent5b	terminal nucleotidyltransferase 5B	-1,04759E+14	6,32349E+14	-2,12351E+14	0.0748277034077777	0.214881266264395	-4,08319E+14	5,45489E+14	5,75283E+14	5,97096E+14	7,33194E+14	6,53679E+14	6,89356E+14

TC0500001220.mm.2	Ung	uracil DNA glycosylase	-1,05339E+14	7,02977E+14	-2,12328E+14	0.0748526953844409	0.214914732845598	-4,08348E+14	6,2488E+14	6,45643E+14	6,96712E+14	7,8449E+14	6,76276E+14	7,89862E+13
TC1100003928.mm.2	Tex2	testis expressed gene 2	0.724669940626961	6,91603E+12	2,12278E+14	0.0749054733900013	0.215023072139629	-4,0841E+14	7,52367E+14	7,17705E+14	6,90387E+14	6,34751E+14	6,81494E+14	6,72912E+14
TC1300000033.mm.2	Pitrm1	pitrilysin metallepetidase 1	0.765818609070633	7,49517E+14	2,12236E+14	0.0749502852974696	0.215023072139629	-4,08463E+14	8,02853E+14	8,21805E+14	7,68413E+12	7,03081E+14	7,19842E+14	6,81108E+14
TC1100003763.mm.2	Krt17	keratin 17	-0.9374327487915	4,50709E+13	-2,12214E+14	0.074974548206708	0.215023072139629	-4,08491E+14	3,72997E+13	3,98825E+14	4,52155E+14	5,07153E+14	4,34632E+14	5,38494E+14
TC1800001083.mm.2	Slc39a6	solute carrier family 39 (metal ion transporter), member 6	1,04614E+12	7,64795E+14	2,12212E+14	0.0749766551631697	0.215023072139629	-4,08494E+14	8,3924E+14	8,06576E+14	7,87655E+14	6,48932E+14	7,62225E+14	7,44142E+14
TC0800003231.mm.2	Zfp617	zinc finger protein 617	0.677148314917648	7,10141E+14	2,12211E+14	0.0749772226542919	0.215023072139629	-4,08495E+14	7,68368E+14	7,68679E+14	7,22559E+13	6,79058E+14	6,73183E+14	6,48995E+14
TC0400004133.mm.2	Plch2	phospholipase C, eta 2	-0.70794692518797	5,8434E+13	-2,12196E+14	0.0749935585532694	0.215023072139629	-4,08514E+14	5,41767E+14	5,56292E+14	6,01932E+14	6,37716E+14	5,6068E+14	6,07653E+14
TC1100003019.mm.2	Ndel1	nudE neurodevelopment protein 1 like 1	1,63689E+14	8,38653E+14	2,12189E+14	0.075001120028668	0.215023072139629	-4,08523E+13	9,46262E+14	9,35215E+13	8,79069E+14	6,75029E+14	8,759E+14	7,20444E+14
TC1300000650.mm.2	Fgfr4	fibroblast growth factor receptor 4	-1,0913E+14	5,62494E+14	-2,12186E+14	0.0750045622065785	0.215023072139629	-4,08527E+14	5,09767E+14	4,92061E+14	5,4213E+14	6,77958E+14	5,42465E+14	6,10581E+14
TC0900002928.mm.2	Sox14	SRY (sex determining region Y)-box 14	-0.923115833089524	5,09866E+14	-2,1218E+14	0.075010531154773	0.215023072139629	-4,08534E+14	4,24329E+14	4,82692E+14	5,05489E+13	5,86156E+14	4,99392E+14	5,6114E+14
TC0500002183.mm.2	Fndc4	fibronectin type III domain containing 4	0.706526287177653	7,57113E+14	2,12148E+14	0.0750443275049131	0.215071788572225	-4,08573E+14	8,06667E+14	8,09996E+14	7,61276E+14	7,14082E+14	7,44095E+14	7,06564E+14
TC0400002808.mm.2	Tnfsf15	tumor necrosis factor (ligand) superfamily, member 15	-0.635073448831963	5,84512E+14	-2,12133E+14	0.0750612342469114	0.215071788572225	-4,08593E+14	5,37686E+14	5,43749E+14	5,94604E+14	6,13845E+14	5,97389E+14	6,198E+14
TC0500002302.mm.2	Rab28	RAB28, member RAS oncogene family	1,47961E+14	7,40085E+13	2,12127E+14	0.0750675689772353	0.215071788572225	-4,08601E+14	8,46487E+14	8,23396E+14	7,61096E+14	6,12867E+14	7,84138E+14	6,12527E+14
TC1600000704.mm.2	Ift57	intraflagellar transport 57	1,1625E+14	8,17415E+14	2,12082E+14	0.0751153011229821	0.2151354219968	-4,08657E+14	8,86743E+14	8,89317E+14	8,39499E+14	7,0406E+14	8,41614E+14	7,43256E+14
TC0900002181.mm.2	Kmt2a	lysine (K)-specific methyltransferase 2A	0.66924856833119	6,49957E+14	2,12073E+14	0.0751249382102198	0.2151354219968	-4,08668E+14	7,25088E+14	6,9124E+14	6,45281E+14	6,37197E+14	6,16114E+14	5,84819E+14
TC0800000821.mm.2	Ap1m1	adaptor-related protein complex AP-1, mu subunit 1	0.932752137598446	8,12052E+14	2,12062E+14	0.0751363765937316	0.2151354219968	-4,08681E+14	8,84163E+14	8,56539E+14	8,35108E+14	7,19043E+14	7,9148E+14	7,85981E+14
TC1700001675.mm.2	Stub1/Mir3082	STIP1 homology and U-Box containing protein 1/microRNA 3082	0.993814825994436	8,30433E+14	2,1204E+14	0.0751603417499551	0.2151354219968	-4,08709E+14	8,95237E+14	8,75922E+14	8,45411E+14	7,26985E+14	8,47972E+14	7,91074E+14
TC0200000303.mm.2	Spag6	sperm associated antigen 6	-0.624275990126816	7,38197E+14	-2,12036E+13	0.0751650818821835	0.2151354219968	-4,08715E+14	6,91846E+14	7,06082E+14	7,59014E+14	7,63769E+14	7,45497E+14	7,62976E+14
TC1000002530.mm.2	Nmrk2	nicotinamide riboside kinase 2	-0.923985489586604	5,00312E+14	-2,11992E+14	0.075211595140737	0.2151354219968	-4,08769E+14	4,2496E+14	4,46817E+14	4,7159E+13	5,84984E+14	5,25817E+14	5,47707E+14
TC0700000283.mm.2	Rsph6a	radial spoke head 6 homolog A (Chlamydomonas)	-0.765806700320653	5,6789E+13	-2,11989E+13	0.0752152079507728	0.2151354219968	-4,08774E+13	5,34125E+14	5,20261E+14	5,66217E+14	6,4133E+14	5,63592E+14	5,81818E+14
TC0500003236.mm.2	Vps33a	VPS33A CORVET/HOPS core subunit	1,30633E+14	8,29424E+14	2,11988E+14	0.0752156731480898	0.2151354219968	-4,08774E+14	9,17228E+14	9,30992E+14	8,83092E+14	7,03862E+14	8,09118E+13	7,32253E+13
TC0300002989.mm.2	Cisd2	CDGSH iron sulfur domain 2	-0.977698496073767	7,35668E+14	-2,11983E+14	0.0752210194438671	0.2151354219968	-4,0878E+14	6,83979E+14	6,82158E+14	7,27189E+14	8,34488E+14	7,06956E+14	7,79235E+14
TC0200000406.mm.2	Anapc2	anaphase promoting complex subunit 2	0.754861955533783	8,0885E+14	2,11981E+14	0.0752232959430257	0.2151354219968	-4,08783E+14	8,71011E+13	8,69307E+14	8,29569E+14	7,59778E+14	7,47422E+14	7,76012E+14
TC0100000041.mm.2	Vxn	vexin	0.836469460462796	8,55449E+14	2,11857E+14	0.0753568360548004	0.215479094827152	-4,08939E+14	8,83361E+14	9,18317E+14	8,34901E+14	7,99483E+14	8,90016E+14	8,06618E+14
TC0100001699.mm.2	Sccpdh	saccharopine dehydrogenase (putative)	1,63405E+14	8,13813E+14	2,11822E+14	0.0753940245561253	0.215492445657677	-4,08983E+14	9,34114E+14	9,4143E+14	8,61421E+14	6,87312E+14	8,23578E+14	6,35023E+14
TC1800000029.mm.2	Zeb1	zinc finger E-box binding homeobox 1	1,53065E+14	7,93594E+14	2,11821E+14	0.075395433141219	0.215492445657677	-4,08984E+14	9,20302E+14	9,13089E+14	8,59145E+14	6,68117E+14	7,71159E+14	6,29749E+14
TC0500003087.mm.2	Rpl37rt	ribosomal protein L37, retrotransposed	0.62560644780446	9,44195E+13	2,11802E+14	0.0754153227767325	0.215492445657677	-4,09007E+14	9,9046E+14	9,79686E+14	9,28763E+14	9,16262E+14	9,34092E+14	9,15908E+14
TC0500001020.mm.2	Dspp	dentin sialophosphoprotein	-1,08395E+14	5,07051E+14	-2,11786E+14	0.0754332891501904	0.215492445657677	-4,09028E+14	4,56558E+14	4,39546E+14	5,03604E+14	6,0929E+14	4,62768E+14	5,70537E+14
TC1900001649.mm.2	Sorcs1	sortilin-related VPS10 domain containing receptor 1	0.902016875515971	6,66638E+14	2,11757E+13	0.0754641335113333	0.215492445657677	-4,09064E+14	7,59241E+14	7,22783E+14	6,99252E+14	6,02368E+14	6,3047E+14	5,85713E+14
TC0900001393.mm.2	Mst1	macrophage stimulating 1 (hepatocyte growth factor-like)	-0.740900166730511	4,94769E+14	-2,11746E+14	0.0754757000786384	0.215492445657677	-4,09078E+14	4,86936E+14	4,15367E+14	5,26488E+13	5,23996E+14	5,08023E+14	5,07804E+13
TC0900002369.mm.2	Scamp5	secretory carrier membrane protein 5	1,01192E+14	9,60591E+13	2,11739E+14	0.0754833108093886	0.215492445657677	-4,09087E+14	1,02482E+14	1,02641E+14	9,92184E+12	8,56661E+14	9,41589E+14	9,21884E+14
TC0100002649.mm.2	Fam124b	family with sequence similarity 124, member B	-0.794297398432557	5,86346E+14	-2,11735E+14	0.0754875227120109	0.215492445657677	-4,09092E+14	5,70473E+14	5,14461E+14	5,92476E+14	6,51318E+14	5,79826E+14	6,09524E+14
TC0X00000957.mm.2	Arr3	arrestin 3, retinal	-1,14474E+14	5,17629E+14	-2,11727E+14	0.0754958244842395	0.215492445657677	-4,09101E+14	4,43209E+14	4,38774E+14	5,01725E+14	6,09206E+14	4,94369E+14	6,18493E+13
TC0200001234.mm.2	Aplnr	apelin receptor	-0.790868286245929	4,87059E+14	-2,11721E+14	0.0755023086245509	0.215492445657677	-4,09109E+14	4,61157E+14	4,09512E+14	5,07615E+14	5,21227E+14	4,77972E+14	5,44871E+14
TC0600003074.mm.2	Slc2a3	solute carrier family 2 (facilitated glucose transporter), member 3	1,39605E+14	7,80909E+14	2,11716E+14	0.075508617152811	0.215492445657677	-4,09116E+13	9,11597E+14	9,03825E+14	8,4371E+13	6,92502E+13	7,41711E+14	5,92111E+14
TC1000002425.mm.2	Gm9508	predicted gene 9508	-0.777687772990894	4,84376E+14	-2,1167E+13	0.07555758662935	0.21556459638347	-4,09173E+14	4,07309E+14	4,40428E+14	4,6435E+14	5,38924E+14	5,3224E+13	5,23009E+14
TC1700002128.mm.2	Ankrd66	ankyrin repeat domain 66	-0.910929449062052	6,55328E+14	-2,11667E+14	0.0755606553946402	0.21556459638347	-4,09177E+14	5,78845E+14	6,10376E+14	6,39881E+14	7,31526E+13	6,5481E+14	7,16533E+14
TC0400003951.mm.2	Gm13178	predicted gene 13178	-0.819804997575785	5,18499E+14	-2,11614E+14	0.0756176565594288	0.215565033309698	-4,09243E+14	5,19625E+14	4,36738E+14	5,66952E+14	5,53372E+14	4,96965E+14	5,37341E+11
TC0X00000686.mm.2	Atp2b3	ATPase, Ca++ transporting, plasma membrane 3	0.851137689937362	7,21445E+14	2,1161E+14	0.0756224987859903	0.215565033309698	-4,09249E+14	7,9293E+14	8,0786E+14	7,43214E+14	6,87348E+14	6,90028E+14	6,07291E+14
TC1800001199.mm.2	Dnd1	DND microRNA-mediated repression inhibitor 1	-0.817639284461049	5,59392E+13	-2,11591E+14	0.0756428581457185	0.215565033309698	-4,09273E+14	5,23459E+14	4,74265E+14	5,54663E+14	6,06589E+14	6,28145E+14	5,6923E+14
TC1500000010.mm.2	Plcxd3	phosphatidylinositol-specific phospholipase C, X domain containing 3	0.726585742485948	7,11617E+14	2,11579E+14	0.0756557026000417	0.215565033309698	-4,09288E+14	7,76412E+13	7,43635E+14	7,11698E+14	6,63032E+14	6,70826E+14	7,04098E+14
TC1200000314.mm.2	Agr3	anterior gradient 3	-1,04159E+14	4,40981E+14	-2,1155E+14	0.0756865180615405	0.215565033309698	-4,09323E+14	3,6429E+14	3,89648E+14	4,31407E+14	5,3085E+14	4,14773E+14	5,14916E+13
TC1100001988.mm.2	C1qtnf1	C1q and tumor necrosis factor related protein 1	-0.628207419938161	6,11691E+14	-2,11548E+14	0.0756891853076876	0.215565033309698	-4,09327E+13	5,78687E+14	5,78979E+14	6,52791E+12	6,30516E+13	6,07044E+14	6,22127E+14
TC0900000153.mm.2	Zfp317	zinc finger protein 317	0.618770560358419	6,78637E+13	2,11531E+14	0.0757071080046366	0.215565033309698	-4,09347E+14	7,25121E+13	7,17912E+14	6,5872E+14	6,60559E+14	6,45645E+14	6,63864E+14
TC0700002359.mm.2	Chmp2a	charged multivesicular body protein 2A	1,15393E+14	7,77382E+14	2,11531E+14	0.0757078418648907	0.215565033309698	-4,09348E+14	8,54443E+14	8,58054E+14	7,83026E+14	6,98686E+14	8,06681E+14	6,634E+14
TC0900001403.mm.2	Usp4	ubiquitin specific peptidase 4 (proto-oncogene)	0.91483468538547	7,76283E+14	2,11528E+13	0.0757101556031843	0.215565033309698	-4,09351E+14	8,21195E+14	8,31639E+14	7,80572E+14	6,89295E+14	8,02848E+14	7,3215E+14
TC1000002350.mm.2	Phyhipl/D630013N20Rik	phytanoyl-CoA hydroxylase interacting protein-like/RIKEN cDNA D630013N20 gene	0.989450578646666	8,62913E+14	2,11519E+14	0.0757204925936814	0.215565033309698	-4,09363E+14	9,43864E+14	9,26521E+14	8,98296E+14	7,74198E+14	8,38656E+14	7,95942E+14
TC0600002284.mm.2	Hoxa10	homeobox A10	-0.986582947992344	5,7401E+14	-2,11519E+14	0.0757208770559713	0.215565033309698	-4,09363E+14	5,23256E+14	5,03599E+14	5,84836E+14	6,39335E+14	5,37342E+14	6,5569E+14
TC0200004549.mm.2	1810024B03Rik	RIKEN cDNA 1810024B03 gene	-0.634178561513425	6,4765E+14	-2,11503E+14	0.0757380254226649	0.215565033309698	-4,09383E+14	5,89515E+12	5,97344E+14	6,58481E+14	6,55213E+14	6,77896E+14	7,07449E+14
TC1100001288.mm.2	1700125H20Rik	RIKEN cDNA 1700125H20 gene	-0.829234019228183	5,25543E+13	-2,11497E+14	0.075743955248832	0.215565033309698	-4,0939E+14	4,92014E+14	4,19659E+14	5,09003E+14	5,68516E+14	5,72333E+14	5,91733E+14
TC0200001767.mm.2	Vps18	VPS18 CORVET/HOPS core subunit	0.666413934998634	6,53297E+14	2,11471E+14	0.0757717811738736	0.215565033309698	-4,09423E+14	7,40456E+14	6,95909E+13	6,55346E+14	6,47737E+14	5,84872E+14	5,95463E+14
TC0500002136.mm.2	Shh	sonic hedgehog	-0.653750865792039	7,40092E+14	-2,11466E+14	0.0757780248825426	0.215565033309698	-4,0943E+12	6,80926E+14	7,19789E+14	7,59442E+14	7,72023E+14	7,55389E+14	7,52983E+14
TC1300002188.mm.2	Ptch1	patched 1	1,39176E+14	7,10853E+14	2,1146E+14	0.0757836064470565	0.215565033309698	-4,09436E+14	8,49518E+14	7,73726E+13	7,64107E+14	5,80784E+14	6,86386E+14	6,10596E+14
TC0500002146.mm.2	Gm5129	predicted gene 5129	-0.741204534480426	5,60284E+14	-2,11449E+14	0.0757961842556988	0.215565033309698	-4,09451E+14	5,21567E+14	5,12542E+14	5,58268E+14	6,24082E+14	5,58724E+13	5,86519E+14
TC1500001279.mm.2	Basp1	brain abundant, membrane attached signal protein 1	0.635090743409696	8,3376E+14	2,11444E+14	0.0758016184902097	0.215565033309698	-4,09457E+14	8,67762E+14	8,51576E+14	8,07522E+14	7,84798E+14	8,38262E+14	8,5264E+14
TC0100000240.mm.2	Arid5a	AT rich interactive domain 5A (MRF1-like)	-0.816567193536407	5,56105E+14	-2,11417E+14	0.0758309389697531	0.215598041699459	-4,09491E+14	4,87456E+14	5,09494E+14	5,51587E+14	6,08677E+14	5,54981E+14	6,24433E+14
TC0700002770.mm.2	Upk1a	uroplakin 1A	-0.774531396965424	5,4877E+14	-2,114E+14	0.0758490587923079	0.215598041699459	-4,09512E+14	4,9976E+14	5,01597E+14	5,39659E+14	6,16604E+14	5,54057E+14	5,80942E+14
TC0600002548.mm.2	Pole4	polymerase (DNA-directed), epsilon 4 (p12 subunit)	1,97039E+14	8,10684E+14	2,11394E+14	0.0758549339155943	0.215598041699459	-4,09519E+14	9,08936E+14	9,05952E+14	8,6754E+14	5,53269E+14	8,38421E+14	7,89983E+14
TC0700000122.mm.2	Vmn1r75	vomeronasal 1 receptor 75	-1,03933E+14	4,58086E+14	-2,11383E+14	0.0758667471256707	0.215598041699459	-4,09533E+14	3,88673E+14	3,87585E+14	4,32992E+14	5,51132E+14	4,53706E+14	5,34427E+14
TC1900000718.mm.2	Pdcd11	programmed cell death 11	1,06957E+14	5,52067E+13	2,11362E+14	0.0758902446642086	0.2155986427204	-4,0956E+14	6,02742E+14	6,32938E+14	5,44016E+14	4,7775E+14	5,92698E+14	4,62262E+14
TC0200004425.mm.2	Ppp1r14d	protein phosphatase 1, regulatory inhibitor subunit 14D	-0.698840808479022	5,92284E+14	-2,11359E+14	0.0758937194507608	0.2155986427204	-4,09564E+14	5,22273E+14	5,44838E+14	5,7652E+14	6,30359E+14	6,42813E+14	6,36903E+14
TC1500001630.mm.2	Asap1	ArfGAP with SH3 domain, ankyrin repeat and PH domain1	1,30571E+13	8,0406E+14	2,11317E+14	0.0759388762126295	0.215688896952953	-4,09616E+14	8,625E+14	8,90173E+14	8,26431E+14	6,651E+14	8,44145E+13	7,36014E+14
TC1100003147.mm.2	Spns3	spinster homolog 3	-0.78748280088877	4,71914E+14	-2,1128E+14	0.0759783577105282	0.215763002782824	-4,09662E+13	4,16051E+13	4,38574E+14	5,12272E+14	4,9985E+13	4,41786E+14	5,22949E+14
TC0600000754.mm.2	Atoh1	atonal bHLH transcription factor 1	-1,10489E+14	4,97624E+14	-2,11231E+14	0.076031960218397	0.215856755046111	-4,09724E+14	4,64289E+14	4,26526E+14	4,98051E+14	6,13743E+14	4,42169E+14	5,40965E+14
TC0400001515.mm.2	Yars	tyrosyl-tRNA synthetase	0.673150156320316	7,80278E+14	2,11206E+14	0.0760589509131295	0.215856755046111	-4,09755E+14	8,53297E+14	8,1589E+14	7,8112E+14	7,53436E+14	7,47345E+14	7,30577E+14
TC0200003980.mm.2	Pramel7	preferentially expressed antigen in melanoma like 7	-0.88171298432321	4,68496E+14	-2,11206E+14	0.076059010961233	0.215856755046111	-4,09755E+14	4,29567E+14	3,84613E+14	4,6012E+14	5,30403E+14	4,68366E+14	5,37908E+14
TC0100000171.mm.2	Lmbrd1	LMBR1 domain containing 1	1,379E+14	8,96351E+14	2,112E+13	0.0760649571868564	0.215856755046111	-4,09762E+14	9,82692E+13	9,8199E+13	9,3443E+14	7,54452E+14	9,16634E+14	8,0791E+14
TC0900000515.mm.2	Ddx6	DEAD (Asp-Glu-Ala-Asp) box polypeptide 6	1,39744E+14	8,70241E+14	2,1118E+14	0.0760865918662707	0.215880129378627	-4,09787E+13	9,69322E+14	9,72175E+14	9,18708E+14	7,433E+14	8,70054E+14	7,47889E+14
TC1000001580.mm.2	Naca	nascent polypeptide-associated complex alpha polypeptide	1,00256E+14	9,4083E+14	2,11133E+13	0.0761382088634676	0.215933726302938	-4,09847E+14	1,00961E+14	1,00356E+14	9,6517E+13	8,47483E+14	9,43103E+14	8,76057E+14
TC1100000681.mm.2	Gm12250	predicted gene 12250	-0.848685307994234	4,7501E+14	-2,11128E+14	0.0761437885260173	0.215933726302938	-4,09854E+14	3,9966E+14	4,46162E+14	4,71287E+14	5,44271E+14	4,68478E+14	5,20201E+14
TC0100000230.mm.2	Arhgef4	Rho guanine nucleotide exchange factor (GEF) 4	1,06527E+14	7,96516E+14	2,11126E+14	0.0761456856484388	0.215933726302938	-4,09856E+14	8,78423E+14	8,71705E+13	8,06523E+14	7,3055E+14	8,098E+14	6,82096E+14
TC0100000312.mm.2	Pou3f3/2900092D14Rik	POU domain, class 3, transcription factor 3/RIKEN cDNA 2900092D14 gene	0.993070811308505	6,21434E+14	2,11095E+14	0.0761796847665972	0.215992127510854	-4,09895E+13	6,70267E+14	7,08465E+14	5,65363E+14	6,14755E+14	6,43926E+14	5,25827E+14
TC0300001984.mm.2	Ufm1	ubiquitin-fold modifier 1	1,33835E+14	7,58344E+14	2,11079E+14	0.0761966147013437	0.216002120457908	-4,09915E+14	8,33327E+14	8,55315E+14	7,54725E+14	6,66247E+14	8,10744E+14	6,29703E+14
TC1700001870.mm.2	Zfp101	zinc finger protein 101	1,10762E+14	5,20208E+14	2,11057E+14	0.0762206570919617	0.216008208329309	-4,09942E+14	5,97205E+14	6,06307E+13	4,38496E+14	5,43491E+13	5,26392E+14	4,09356E+14
TC1300000476.mm.2	Hivep1	human immunodeficiency virus type I enhancer binding protein 1	1,46688E+13	6,58194E+14	2,11052E+14	0.0762255739192237	0.216008208329309	-4,09948E+13	7,60538E+14	7,8621E+14	6,8059E+14	5,72781E+14	6,73555E+13	4,75491E+14
TC0100003519.mm.2	Fcrlb	Fc receptor-like B	-1,17137E+14	7,1567E+14	-2,11032E+14	0.0762472042717798	0.216021776794969	-4,09973E+14	6,58508E+13	6,08048E+14	6,84611E+14	8,16219E+14	7,15515E+13	8,11122E+14
TC0500001672.mm.2	Cyp2w1	cytochrome P450, family 2, subfamily w, polypeptide 1	-1,00167E+14	5,50721E+14	-2,11023E+14	0.0762579584759956	0.216021776794969	-4,09985E+14	4,96391E+14	4,96925E+14	5,45557E+14	6,48093E+14	5,1439E+14	6,02971E+14
TC0Y00000235.mm.2	Ddx3y	DEAD (Asp-Glu-Ala-Asp) box polypeptide 3, Y-linked	1,24868E+14	7,22586E+14	2,11011E+14	0.0762705820261017	0.216021776794969	-4,1E+14	8,36376E+14	8,19939E+14	7,4935E+14	6,57229E+14	7,16411E+13	5,56213E+14
TC1900000430.mm.2	1700018L02Rik	RIKEN cDNA 1700018L02 gene	-1,07442E+14	6,13478E+14	-2,10947E+14	0.0763398626287918	0.216171625081998	-4,1008E+14	5,85026E+14	5,24906E+14	5,94879E+14	7,29938E+13	6,08132E+14	6,3799E+14
TC0100003743.mm.2	Mark1	MAP/microtubule affinity regulating kinase 1	1,17677E+14	8,40566E+14	2,10938E+14	0.0763503207560138	0.216171625081998	-4,10092E+14	9,15824E+13	9,18886E+14	8,67516E+14	7,31839E+14	8,57088E+14	7,52242E+14
TC0300002876.mm.2	Mettl14	methyltransferase like 14	1,03024E+12	6,28666E+14	2,10924E+14	0.076365354768225	0.216176205442799	-4,10109E+14	7,42854E+14	7,05453E+14	6,3155E+14	6,10708E+14	6,08229E+14	4,73199E+14
TC0800001229.mm.2	Tsnaxip1	translin-associated factor X (Tsnax) interacting protein 1	-0.818461360992046	4,92553E+14	-2,10897E+14	0.0763949841022633	0.216210684663616	-4,10143E+14	4,36285E+14	4,86282E+13	5,03591E+14	5,82668E+14	4,73323E+14	4,73166E+12
TC0800000073.mm.2	Abhd13	abhydrolase domain containing 13	-0.638398048011771	8,85669E+14	-2,1088E+14	0.0764132745649223	0.216210684663616	-4,10164E+14	8,48088E+13	8,40802E+14	9,159E+14	9,00669E+14	9,19144E+14	8,89413E+14
TC0800002121.mm.2	Frg1	FSHD region gene 1	1,34494E+14	6,52477E+14	2,10865E+14	0.0764291684492405	0.216210684663616	-4,10183E+14	7,517E+14	7,38954E+14	7,05508E+14	5,16158E+14	6,38979E+14	5,63562E+14
TC1900000213.mm.2	Ptgdr2	prostaglandin D2 receptor 2	-0.75034735238413	5,71151E+14	-2,10862E+13	0.0764329591163405	0.216210684663616	-4,10187E+14	5,29075E+14	5,1566E+14	5,61322E+14	6,33483E+14	5,85302E+14	6,02063E+14
TC0X00002136.mm.2	Dcaf12l1	DDB1 and CUL4 associated factor 12-like 1	1,63586E+14	7,0125E+14	2,10851E+14	0.0764446267317457	0.216210684663616	-4,102E+14	8,46247E+14	8,096E+14	7,8033E+14	5,48345E+14	6,6543E+14	5,5755E+14
TC0400002023.mm.2	Chd5	chromodomain helicase DNA binding protein 5	0.854297960719801	7,05288E+14	2,10782E+14	0.076519913059882	0.216320852544441	-4,10287E+13	7,6763E+14	7,46772E+14	6,22098E+14	7,21445E+14	6,94038E+14	6,79742E+14
TC0X00000053.mm.2	Dgkk	diacylglycerol kinase kappa	1,24193E+14	6,3834E+14	2,10759E+14	0.0765449760874655	0.216320852544441	-4,10316E+14	8,24814E+13	6,49463E+14	6,39232E+14	5,8666E+14	5,76441E+14	5,53432E+14
TC0300003142.mm.2	Miga1	mitoguardin 1	0.930502449372277	7,34499E+14	2,10746E+14	0.0765585072799887	0.216320852544441	-4,10331E+14	8,22462E+14	8,23252E+14	7,84468E+13	6,75145E+14	6,75849E+14	6,25821E+14
TC1000001821.mm.2	Slc35d3	solute carrier family 35, member D3	0.661436064227169	6,55546E+14	2,10723E+14	0.0765846084930777	0.216320852544441	-4,10361E+14	6,94259E+14	7,0586E+14	6,44006E+14	6,23826E+14	6,13217E+14	6,52104E+14
TC1500001013.mm.2	Tmbim6	transmembrane BAX inhibitor motif containing 6	1,30408E+14	9,03935E+14	2,10718E+14	0.0765890343636166	0.216320852544441	-4,10367E+14	9,83095E+14	9,76047E+14	9,40766E+14	7,57561E+14	9,18524E+14	8,47618E+14
TC0X00002878.mm.2	Chm	choroidermia (RAB escort protein 1)	1,06193E+14	5,63549E+14	2,10712E+14	0.0765965055803621	0.216320852544441	-4,10375E+13	6,35653E+14	6,72964E+14	5,74526E+14	5,21706E+14	5,55056E+14	4,21391E+14
TC0200005281.mm.2	Bmp7	bone morphogenetic protein 7	0.774724372682832	5,81402E+14	2,10705E+14	0.0766033678281574	0.216320852544441	-4,10383E+14	6,34547E+14	5,82041E+14	5,01877E+14	5,59765E+14	5,92231E+14	6,1795E+14
TC0600000228.mm.2	Fam71f1	family with sequence similarity 71, member F1	-0.660035704039597	4,65932E+14	-2,10704E+14	0.076605161423667	0.216320852544441	-4,10385E+14	3,84656E+14	4,26335E+14	4,63373E+14	4,79625E+14	5,2021E+14	5,21392E+14
TC0500002603.mm.2	Scfd2	Sec1 family domain containing 2	0.854460964896024	5,1434E+14	2,107E+14	0.0766093546946401	0.216320852544441	-4,1039E+14	5,80385E+13	5,56436E+14	5,04632E+14	4,61297E+14	5,36596E+13	4,46695E+14
TC1500002342.mm.2	Zhx1	zinc fingers and homeoboxes 1	1,86801E+14	7,39789E+14	2,10692E+14	0.0766178306628885	0.216320852544441	-4,104E+14	8,5087E+14	8,46222E+14	7,73274E+14	5,50216E+13	8,09953E+14	6,08197E+14
TC1600001366.mm.2	2510009E07Rik	RIKEN cDNA 2510009E07 gene	1,02805E+14	7,57988E+14	2,10668E+14	0.0766441276324027	0.21635718790135	-4,1043E+14	8,15795E+13	8,26675E+14	7,84506E+14	6,52356E+14	7,58935E+14	7,09662E+13
TC0700000759.mm.2	Nkg7	natural killer cell group 7 sequence	-0.949599100295575	5,93669E+14	-2,10586E+14	0.0767340029772493	0.216572953226908	-4,10533E+14	5,36643E+14	5,45409E+13	5,76131E+14	6,95841E+13	5,87207E+14	6,20781E+13
TC0200003492.mm.2	Orc4	origin recognition complex, subunit 4	1,7608E+14	6,34163E+14	2,10539E+14	0.076785380392717	0.216645931005447	-4,10592E+13	7,54399E+14	7,4046E+14	5,9488E+14	5,4782E+14	7,2239E+13	4,45025E+14
TC1100002106.mm.2	Mtfp1	mitochondrial fission process 1	0.933084161401911	7,9503E+14	2,10534E+14	0.0767903430447803	0.216645931005447	-4,10597E+14	8,52878E+14	8,32102E+14	8,04939E+14	6,93424E+14	8,14502E+14	7,72337E+14
TC1300000102.mm.2	AW209491	expressed sequence AW209491	0.676707504299746	6,57961E+14	2,10525E+14	0.076800195984802	0.216645931005447	-4,10609E+14	7,11361E+13	7,01812E+14	6,50386E+14	6,27446E+14	6,05725E+14	6,51038E+14
TC0900001398.mm.2	Nicn1	nicolin 1	1,35043E+14	8,71814E+14	2,10414E+14	0.076922131518842	0.216951917584999	-4,10748E+14	9,48719E+14	9,40178E+14	8,93435E+14	7,25375E+14	9,20837E+14	8,02341E+14
TC0800000679.mm.2	March1	membrane-associated ring finger (C3HC4) 1	0.844870712306554	7,19158E+14	2,10375E+14	0.0769653295319245	0.217035763869076	-4,10798E+14	8,01773E+14	7,52537E+14	7,1804E+14	6,67296E+14	6,51851E+14	7,2345E+14
TC0300003113.mm.2	Adgrl2	adhesion G protein-coupled receptor L2	0.839405888432458	6,52692E+14	2,10359E+13	0.076982741610065	0.217045122939373	-4,10818E+14	6,89272E+14	7,29448E+14	5,81316E+14	6,69523E+14	6,34098E+14	6,12497E+14
TC1000000818.mm.2	Palm	paralemmin	-0.668075480064976	9,33868E+14	-2,10347E+14	0.0769955888240215	0.217045122939373	-4,10832E+14	8,93279E+14	8,97398E+13	9,41988E+14	9,82304E+13	9,30763E+14	9,57476E+14
TC0800001635.mm.2	Elavl1	ELAV (embryonic lethal, abnormal vision)-like 1 (Hu antigen R)	0.910541505428382	7,90388E+14	2,10323E+14	0.0770218186372408	0.217081085132388	-4,10862E+14	8,61981E+14	8,66664E+14	8,10542E+14	7,35996E+14	7,78038E+13	6,89109E+13
TC1200001492.mm.2	Ywhaq	tyrosine 3-monooxygenase/tryptophan 5-monooxygenase activation protein theta	1,54957E+14	9,61247E+14	2,10283E+14	0.0770655458110551	0.217145136569237	-4,10912E+14	1,05699E+14	1,05303E+14	1,00825E+14	7,91867E+14	9,84314E+14	8,73027E+14
TC1600000203.mm.2	Mapk1	mitogen-activated protein kinase 1	2,13813E+14	8,83788E+14	2,10278E+14	0.077071497302766	0.217145136569237	-4,10919E+14	1,01898E+14	1,03586E+14	9,53106E+14	6,74106E+14	9,15493E+14	7,0518E+14
TC0X00000627.mm.2	1110012L19Rik	RIKEN cDNA 1110012L19 gene	-0.807309541862733	6,6147E+14	-2,10262E+14	0.0770887499145738	0.217155774016351	-4,10939E+14	6,29131E+14	6,07376E+14	6,5507E+14	7,429E+14	6,73397E+14	6,60945E+14
TC0200005328.mm.2	Gm14393	predicted gene 14393	1,82322E+14	6,78476E+14	2,10244E+14	0.0771084344862817	0.217173257276255	-4,10961E+14	7,46383E+14	7,89806E+14	7,11213E+13	4,60333E+13	7,54493E+14	6,0863E+14
TC1000000261.mm.2	Soga3	SOGA family member 3	1,46909E+14	5,47641E+14	2,10219E+14	0.0771360376926995	0.217180720881378	-4,10993E+14	6,52232E+14	6,70207E+14	5,84634E+13	4,43988E+13	5,56959E+14	3,77825E+14
TC0200002834.mm.2	Oprl1	opioid receptor-like 1	0.771292913064679	7,37232E+14	2,10217E+14	0.0771385182147186	0.217180720881378	-4,10995E+14	8,28183E+14	8,00162E+14	7,56965E+14	7,1712E+14	6,93355E+14	6,27606E+14

TC1500001958.mm.2	Tcf20	transcription factor 20	0.797694409008097	7,25196E+14	2,10181E+14	0.0771780484113126	0.217180720881378	-4,1104E+14	7,70756E+14	7,59009E+14	7,2488E+14	6,45346E+14	7,44592E+14	7,06591E+14
TC1100000112.mm.2	Igfbp1	insulin-like growth factor binding protein 1	-0.672220144069692	5,11221E+14	-2,10175E+14	0.0771841895758909	0.217180720881378	-4,11047E+14	4,68768E+14	4,71931E+14	5,12946E+14	5,62196E+14	5,20661E+14	5,30823E+14
TC1100001459.mm.2	Fam117a	family with sequence similarity 117, member A	-0.802389058986812	7,02333E+14	-2,10142E+14	0.0772207401607029	0.217180720881378	-4,11089E+14	6,43282E+14	6,49391E+14	6,86304E+14	7,66846E+14	7,1403E+14	7,54143E+14
TC0300001398.mm.2	H2afz	H2A histone family, member Z	1,07517E+14	9,01538E+14	2,10128E+14	0.0772360365837589	0.217180720881378	-4,11107E+14	9,57963E+14	9,70223E+14	9,27819E+14	7,85333E+14	9,09448E+14	8,5844E+14
TC1700002487.mm.2	Myl12b	myosin, light chain 12B, regulatory	0.822738740883215	1,01788E+14	2,10128E+14	0.0772365071391454	0.217180720881378	-4,11107E+14	1,07374E+13	1,06593E+14	1,02301E+14	9,52112E+14	1,02791E+14	9,64572E+14
TC1500000031.mm.2	Dab2	disabled 2, mitogen-responsive phosphoprotein	0.664493470397756	7,08938E+14	2,10123E+14	0.0772414237653613	0.217180720881378	-4,11113E+14	7,26468E+14	7,66137E+13	6,69661E+14	6,90045E+14	6,9267E+14	7,08647E+14
TC1300001762.mm.2	Serpinb1a	serine (or cysteine) peptidase inhibitor, clade B, member 1a	0.716034997173306	6,68269E+14	2,10119E+14	0.0772458057429438	0.217180720881378	-4,11118E+14	7,52552E+14	6,94365E+14	6,40381E+13	6,63329E+14	6,39561E+12	6,19425E+14
TC0700003022.mm.2	Cyth2	cytohesin 2	0.691477245361893	8,3565E+14	2,10119E+14	0.0772458705002904	0.217180720881378	-4,11118E+14	8,7069E+14	8,75561E+14	8,25245E+14	7,8271E+14	8,49068E+14	8,10626E+14
TC0X00001383.mm.2	Mid2	midline 2	2,28399E+14	6,51087E+13	2,10073E+14	0.0772963662106462	0.217243835523062	-4,11175E+14	8,41532E+14	8,04192E+13	7,04498E+14	4,84428E+13	6,87253E+14	3,84617E+14
TC1700000419.mm.2	Crebrf	CREB3 regulatory factor	1,04556E+14	5,83608E+14	2,10054E+12	0.0773180763315366	0.217243835523062	-4,112E+14	6,82869E+14	6,66505E+14	6,29113E+14	5,11149E+14	5,48032E+14	4,63981E+14
TC0900001115.mm.2	Rwdd2a	RWD domain containing 2A	1,3482E+14	6,165E+14	2,1005E+14	0.0773222305621081	0.217243835523062	-4,11205E+14	7,47406E+13	6,70983E+14	6,20839E+14	5,2791E+14	6,49432E+14	4,82428E+14
TC1500002265.mm.2	Gm5476	type II keratin Kb17P pseudogene	-0.674471396613279	6,17897E+14	-2,1005E+14	0.077322248912354	0.217243835523062	-4,11205E+14	5,87005E+14	5,74147E+14	6,25443E+14	6,70603E+14	6,14628E+14	6,3556E+14
TC0X00003420.mm.2	Uxt	ubiquitously expressed prefoldin like chaperone	1,37674E+14	6,33134E+14	2,10037E+14	0.0773361883417528	0.217245119029056	-4,1122E+14	7,36754E+14	7,26041E+14	6,56218E+14	5,31228E+14	6,59287E+14	4,89277E+14
TC0900001635.mm.2	Snrk	SNF related kinase	0.859061349260562	7,31122E+13	2,10014E+14	0.0773621758274954	0.217280240388432	-4,1125E+14	8,23739E+14	8,06044E+14	7,74392E+14	6,83578E+14	6,73685E+14	6,25292E+14
TC1000000265.mm.2	Echdc1	enoyl Coenzyme A hydratase domain containing 1	1,60371E+14	6,44122E+14	2,09959E+14	0.0774226644538395	0.217412232893816	-4,11319E+14	7,75675E+14	7,56501E+14	6,901E+14	5,21334E+14	6,54861E+11	4,66264E+13
TC0700003596.mm.2	Crtc3	CREB regulated transcription coactivator 3	-0.703122289780103	7,27139E+14	-2,09938E+14	0.077445497393177	0.217418411783687	-4,11345E+14	7,13347E+14	6,90204E+14	7,67686E+13	7,7649E+14	6,7929E+14	7,3582E+14
TC0900001726.mm.2	Cep126	centrosomal protein 126	1,11962E+14	6,66177E+14	2,0993E+14	0.0774539948203002	0.217418411783687	-4,11354E+14	7,49042E+14	7,52555E+14	6,61109E+14	6,16564E+14	6,86305E+13	5,31487E+14
TC0400001592.mm.2	Dnajc8	DnaJ heat shock protein family (Hsp40) member C8	1,99501E+14	7,76809E+14	2,09914E+14	0.0774724154731086	0.217418411783687	-4,11375E+14	9,10551E+13	8,81591E+14	8,36483E+14	5,56658E+13	8,19043E+14	6,56528E+14
TC1100003783.mm.2	Kcnh4	potassium voltage-gated channel, subfamily H (eag-related), member 4	0.797547667455104	5,52449E+12	2,09908E+14	0.077478838233844	0.217418411783687	-4,11383E+13	5,66427E+13	6,18466E+14	5,31048E+14	4,94336E+14	5,83406E+14	5,2101E+13
TC1300002249.mm.2	Zfp874a	zinc finger protein 874a	0.667617869826682	5,89519E+14	2,09871E+14	0.0775195892636116	0.217494888003582	-4,11429E+14	5,88656E+14	6,32636E+14	5,54213E+14	5,33556E+14	6,11026E+13	6,17028E+14
TC0900000839.mm.2	Pdcd7	programmed cell death 7	0.708985067186173	6,22775E+14	2,09858E+14	0.0775339662403673	0.21749735341766	-4,11445E+14	6,88E+14	6,6561E+14	6,35727E+14	5,76086E+14	5,86509E+14	5,84721E+14
TC1800000322.mm.2	Psd2	pleckstrin and Sec7 domain containing 2	1,01791E+14	7,4587E+14	2,09845E+14	0.0775484916618671	0.217500234316391	-4,11462E+14	8,25691E+14	7,99066E+14	7,79387E+14	6,41789E+14	7,15452E+14	7,13834E+14
TC0700004624.mm.2	Plk1	polo like kinase 1	1,03576E+14	6,28886E+14	2,09787E+14	0.0776120982193818	0.217639421723661	-4,11534E+14	6,39471E+14	7,33881E+14	5,41289E+14	6,24911E+14	5,82142E+13	6,51618E+14
TC0500002676.mm.2	Uba6	ubiquitin-like modifier activating enzyme 6	2,50948E+14	5,83716E+14	2,09776E+14	0.0776251322935445	0.217639421723661	-4,11548E+14	7,4868E+14	7,29413E+14	6,50603E+14	3,25595E+14	6,53842E+14	3,94161E+14
TC0200001201.mm.2	Zc3h15	zinc finger CCCH-type containing 15	1,13354E+14	8,50455E+14	2,09762E+14	0.0776404062370304	0.217644374686373	-4,11566E+14	9,32811E+13	9,32197E+14	8,71847E+14	7,66454E+14	8,62625E+14	7,36798E+14
TC0800002510.mm.2	Rbmxl1	RNA binding motif protein, X-linked like-1	-0.963899522707359	6,66109E+14	-2,09719E+14	0.0776881426395739	0.217736245012694	-4,1162E+14	6,47355E+14	5,44607E+14	6,53834E+14	7,30909E+14	7,06314E+14	7,13636E+14
TC0200004455.mm.2	Pla2g4e	phospholipase A2, group IVE	0.872566132293023	6,34141E+13	2,09708E+14	0.0777002053511444	0.217736245012694	-4,11633E+14	7,00221E+14	6,9798E+14	6,62181E+14	5,61506E+13	5,7206E+14	6,10897E+14
TC0100000471.mm.2	Abi2	abl-interactor 2	0.909476392072197	9,30972E+14	2,09678E+12	0.0777329308498903	0.217767044308391	-4,1167E+14	9,93581E+14	9,88374E+14	9,42299E+14	8,57761E+14	9,41924E+14	8,61891E+13
TC0400001668.mm.2	Fuca1	fucosidase, alpha-L- 1, tissue	0.633084197783798	7,39069E+14	2,09673E+14	0.0777382262062848	0.217767044308391	-4,11676E+14	7,6278E+14	7,82031E+14	7,11882E+13	7,06312E+14	7,48987E+14	7,22421E+11
TC0400002949.mm.2	Psip1	PC4 and SFRS1 interacting protein 1	1,40027E+14	9,66031E+14	2,09555E+14	0.0778690660335708	0.218095647661903	-4,11824E+14	1,06748E+14	1,04996E+14	9,98801E+14	8,38584E+14	9,92845E+14	8,48513E+12
TC0800000219.mm.2	Defa30	defensin, alpha, 30	-0.797465746071524	5,70574E+14	-2,09491E+14	0.0779409986915568	0.218129531450286	-4,11906E+14	5,33079E+14	5,11491E+14	5,67545E+14	6,36518E+14	6,13842E+14	5,60965E+14
TC0100003880.mm.2	Cryga	crystallin, gamma A	-0.642688764429916	5,95224E+14	-2,0949E+14	0.0779420024037291	0.218129531450286	-4,11907E+14	5,43675E+14	5,73815E+14	6,34968E+13	6,1106E+14	6,0524E+14	6,02588E+14
TC0600002575.mm.2	Stambp	STAM binding protein	2,31174E+14	7,27303E+12	2,09473E+14	0.0779611473921967	0.218129531450286	-4,11928E+14	8,80966E+14	8,49256E+13	8,13749E+14	4,54125E+14	7,45494E+14	6,2023E+14
TC1100000498.mm.2	Olfr1394	olfactory receptor 1394	-0.70532961278052	4,93007E+14	-2,09471E+14	0.0779626814245072	0.218129531450286	-4,1193E+14	4,48001E+14	4,46257E+14	4,9618E+14	5,39143E+12	4,91235E+14	5,37229E+14
TC0700000476.mm.2	Zfp607a	zinc finger protein 607A	0.645267446466735	6,90164E+14	2,09462E+14	0.0779734304188595	0.218129531450286	-4,11942E+14	7,40478E+14	7,42827E+14	6,81252E+14	6,72999E+14	6,71171E+14	6,3226E+14
TC0500001865.mm.2	Brca2	breast cancer 2, early onset	0.625766100466372	5,33472E+14	2,09461E+14	0.0779743411177405	0.218129531450286	-4,11943E+14	5,44201E+14	5,57989E+14	4,978E+14	4,79237E+13	5,69837E+13	5,51769E+14
TC0200001681.mm.2	Fmn1	formin 1	-1,07461E+14	6,41378E+14	-2,09453E+14	0.0779827127195176	0.218129531450286	-4,11953E+14	5,99673E+14	5,74124E+14	6,43025E+14	7,45694E+14	5,88005E+14	6,97745E+13
TC1100003325.mm.2	Omg	oligodendrocyte myelin glycoprotein	1,78094E+14	9,04243E+14	2,09447E+14	0.0779894638295228	0.218129531450286	-4,1196E+14	1,03159E+12	1,02817E+14	9,72863E+14	7,30708E+14	9,08071E+14	7,54052E+14
TC1700000211.mm.2	Spaca6/Mir125a	sperm acrosome associated 6/microRNA 125a	-0.784963124453936	6,11293E+14	-2,09408E+14	0.0780328324829685	0.218177348594264	-4,12009E+13	5,48557E+14	5,70062E+14	6,50749E+14	6,24862E+14	5,9701E+14	6,76516E+14
TC0800000269.mm.2	Vps36	vacuolar protein sorting 36	1,64199E+14	6,96052E+14	2,09407E+14	0.0780336411561314	0.218177348594264	-4,1201E+14	7,71783E+14	8,01412E+14	7,32929E+14	5,11868E+14	7,47555E+14	6,10764E+14
TC0900002302.mm.2	Ddx10	DEAD (Asp-Glu-Ala-Asp) box polypeptide 10	0.667531509149971	6,32711E+14	2,09389E+14	0.0780545771355361	0.218195867395712	-4,12034E+14	6,67698E+14	6,67379E+14	6,25541E+14	5,76029E+14	6,29089E+14	6,30528E+14
TC0900001468.mm.2	Myl3	myosin, light polypeptide 3	-0.70377656472628	6,64813E+14	-2,09375E+14	0.0780694305003122	0.218195867395712	-4,1205E+14	6,15093E+14	6,14866E+14	6,56583E+13	7,14131E+13	6,81838E+14	7,06367E+14
TC1700002051.mm.2	Olfr90	olfactory receptor 90	-0.911492929006367	4,43765E+14	-2,09365E+14	0.0780808894311225	0.218195867395712	-4,12063E+14	3,72152E+14	4,03038E+14	4,42641E+14	5,14847E+13	4,21391E+14	5,08521E+14
TC0300002329.mm.2	Dap3	death associated protein 3	2,03964E+14	7,32842E+14	2,09318E+14	0.0781335435947939	0.218265379334494	-4,12122E+14	8,41797E+14	8,40449E+14	8,00317E+14	4,74002E+14	7,55661E+14	6,84826E+14
TC1400001524.mm.2	Gng2	guanine nucleotide binding protein (G protein), gamma 2	1,88696E+14	9,0736E+14	2,093E+14	0.0781527587655091	0.218265379334494	-4,12144E+14	1,04791E+14	9,98214E+14	9,64424E+14	7,04311E+14	9,41772E+13	7,87528E+14
TC0500001620.mm.2	Ache	acetylcholinesterase	0.767846922769883	7,46819E+13	2,09297E+14	0.0781569960890456	0.218265379334494	-4,12149E+14	8,38447E+14	7,5926E+14	7,11886E+14	7,32251E+14	7,18175E+14	7,20896E+14
TC0300003100.mm.2	Spata1	spermatogenesis associated 1	0.881329504149104	5,15003E+14	2,09294E+14	0.0781599477912263	0.218265379334494	-4,12152E+14	5,35747E+14	5,84314E+14	5,16705E+14	4,2709E+13	5,34285E+14	4,91875E+14
TC1100002729.mm.2	Uqcrq	ubiquinol-cytochrome c reductase, complex III subunit VII	-0.623119660282443	9,68613E+14	-2,09281E+14	0.0781737774935737	0.218266171678037	-4,12168E+14	9,20772E+14	9,2663E+14	9,80893E+14	9,91133E+14	1,00882E+14	9,8343E+13
TC0200001011.mm.2	Phospho2	phosphatase, orphan 2	2,15914E+12	5,11853E+14	2,09259E+13	0.0781983089929231	0.218296838669953	-4,12195E+14	6,51918E+14	6,39567E+14	5,78823E+14	2,80833E+14	5,56628E+14	3,6335E+14
TC1600001711.mm.2	Ccdc54	coiled-coil domain containing 54	-1,25994E+14	5,80419E+14	-2,09209E+14	0.0782543935706046	0.218300735268085	-4,12258E+14	4,70067E+14	5,27417E+14	5,47044E+14	7,02428E+14	5,71423E+14	6,64137E+14
TC0200005400.mm.2	Hrh3	histamine receptor H3	1,42648E+14	9,11408E+14	2,09207E+14	0.0782565435826423	0.218300735268085	-4,12261E+14	9,97495E+14	1,0149E+14	9,68269E+14	7,58826E+14	8,99865E+14	8,291E+14
TC0200004633.mm.2	Lzts3	leucine zipper, putative tumor suppressor family member 3	0.648887948502999	8,1383E+14	2,09201E+14	0.0782630497220344	0.218300735268085	-4,12268E+14	8,52793E+13	8,48624E+13	8,03081E+14	7,68558E+14	8,16652E+14	7,93274E+14
TC0100002563.mm.2	Tnp1	transition protein 1	-0.890091561536085	4,63266E+14	-2,09191E+14	0.0782750545833441	0.218300735268085	-4,12282E+14	3,96243E+14	4,0687E+14	4,58E+13	5,23131E+14	4,52091E+14	5,43261E+14
TC0200002139.mm.2	Banf2	barrier to autointegration factor 2	-1,13513E+14	5,44349E+13	-2,0919E+14	0.0782761331952173	0.218300735268085	-4,12283E+14	4,56572E+14	4,78244E+14	5,1401E+14	6,47832E+14	5,37615E+14	6,3182E+14
TC1100002685.mm.2	4933414I15Rik	RIKEN cDNA 4933414I15 gene	-0.998766196209529	5,99073E+14	-2,09172E+14	0.0782955737351445	0.218300735268085	-4,12305E+14	5,70918E+14	5,17198E+13	5,81125E+14	7,06744E+14	6,12357E+14	6,06098E+14
TC1000000529.mm.2	Sowahc	sosondowah ankyrin repeat domain family member C	1,11182E+14	5,9783E+14	2,09169E+14	0.0782989230596835	0.218300735268085	-4,12308E+14	6,23967E+14	6,76529E+14	6,11147E+14	4,66984E+14	5,8086E+13	6,27493E+14
TC1600001528.mm.2	Kalrn	kalirin, RhoGEF kinase	1,74265E+14	6,95835E+14	2,0914E+14	0.0783318085879932	0.218300735268085	-4,12345E+14	7,66326E+14	8,11804E+12	7,09855E+14	5,19745E+14	7,84225E+14	5,83056E+14
TC1800001477.mm.2	Cplx4	complexin 4	-0.809647761610964	4,78683E+13	-2,09127E+14	0.078346404471834	0.218300735268085	-4,12362E+14	4,30445E+14	4,07335E+14	4,68132E+14	5,31577E+14	4,85285E+14	5,49322E+13
TC1400001504.mm.2	Ube2e2	ubiquitin-conjugating enzyme E2E 2	1,31795E+14	7,38959E+14	2,09126E+13	0.0783473021229371	0.218300735268085	-4,12363E+14	8,3205E+14	7,86379E+14	7,65199E+14	5,8964E+13	7,73712E+14	6,86777E+14
TC0900001905.mm.2	Zfp810	zinc finger protein 810	0.770030389317652	6,10262E+14	2,09125E+14	0.0783487340690954	0.218300735268085	-4,12364E+14	6,83519E+14	6,44655E+14	5,9081E+14	5,83358E+13	6,15857E+14	5,43372E+14
TC0500000238.mm.2	Actr3b	ARP3 actin-related protein 3B	0.680501754802809	7,78415E+14	2,09098E+13	0.0783788381971186	0.218346856824027	-4,12398E+12	8,06625E+14	8,16205E+14	7,64023E+14	7,22707E+14	7,97825E+13	7,63105E+14
TC0200004253.mm.2	Eif3m	eukaryotic translation initiation factor 3, subunit M	1,68026E+14	8,85304E+14	2,09073E+13	0.0784069029654605	0.218369804106319	-4,12429E+14	9,7641E+14	9,92063E+13	9,32873E+14	6,99549E+14	9,22111E+14	7,88817E+14
TC1100003752.mm.2	Krt32	keratin 32	-0.981221854376709	4,86901E+13	-2,09066E+14	0.0784141802618483	0.218369804106319	-4,12438E+14	4,37802E+14	4,04174E+14	4,5661E+14	5,8161E+14	5,05139E+14	5,36072E+14
TC1000001223.mm.2	Gm4307/Gm4303/Gm4305/Gm4302	predicted gene 4307/predicted gene 4303/predicted gene 4305/predicted gene 4302	-0.802910530704278	4,76881E+14	-2,09036E+14	0.0784472144172425	0.218424048022296	-4,12475E+14	3,87304E+14	4,53396E+14	4,69064E+14	5,32219E+14	5,0943E+13	5,09874E+14
TC0600000007.mm.2	Gng11	guanine nucleotide binding protein (G protein), gamma 11	0.786053798935049	5,07032E+14	2,09008E+14	0.0784791119304439	0.218475108938363	-4,1251E+14	5,70162E+14	5,27735E+13	4,325E+14	5,08186E+14	5,05159E+14	4,98446E+14
TC0300001399.mm.2	Dnajb14	DnaJ heat shock protein family (Hsp40) member B14	-0.716832437168559	7,71164E+14	-2,08975E+14	0.0785162336696777	0.218540693231908	-4,12552E+14	6,91088E+14	7,46321E+14	7,77136E+13	8,0364E+14	8,18206E+14	7,90596E+14
TC1900000549.mm.2	Slc35g1	solute carrier family 35, member G1	-0.945933733428205	6,91969E+14	-2,08948E+14	0.0785463976836874	0.21858689220678	-4,12586E+14	6,18597E+14	6,53964E+14	6,73118E+14	7,88629E+14	6,91814E+14	7,2569E+14
TC0300001229.mm.2	Alg14	asparagine-linked glycosylation 14	0.623858981397085	6,63538E+14	2,08904E+14	0.0785956407686944	0.218686161233979	-4,12641E+13	7,14625E+13	6,92384E+13	6,35908E+14	6,4633E+14	6,381E+14	6,53878E+14
TC1700002821.mm.2	Gm7030	predicted gene 7030	-0.694189042546187	5,08571E+14	-2,08877E+14	0.0786256745395294	0.218729964198777	-4,12674E+14	4,55746E+14	4,62307E+14	5,00297E+14	5,56594E+14	5,47927E+14	5,28554E+14
TC0700004520.mm.2	Ano9	anoctamin 9	-1,20131E+14	5,4118E+14	-2,08864E+14	0.0786406337582354	0.218729964198777	-4,12691E+14	4,87743E+13	4,94036E+14	5,22003E+14	7,00038E+14	5,28576E+14	5,14685E+14
TC0200000843.mm.2	Arl6ip6	ADP-ribosylation factor-like 6 interacting protein 6	1,22889E+14	6,22668E+14	2,08832E+14	0.0786759058265526	0.218729964198777	-4,12731E+14	6,77134E+14	6,72115E+14	6,42072E+14	4,61399E+14	6,59328E+14	6,23958E+14
TC0400004120.mm.2	5930403L14Rik	RIKEN cDNA 5930403L14 gene	0.813281554647298	6,28338E+14	2,08829E+14	0.0786791235583181	0.218729964198777	-4,12734E+14	6,49509E+14	7,00722E+14	6,32667E+14	5,54908E+14	6,23523E+14	6,087E+14
TC1600000542.mm.2	Gsk3b	glycogen synthase kinase 3 beta	2,19815E+14	9,567E+13	2,08829E+14	0.0786792572764919	0.218729964198777	-4,12734E+14	1,09731E+12	1,0705E+14	1,03706E+13	6,91124E+14	9,75712E+14	8,68497E+14
TC1600000286.mm.2	Psmd2	proteasome (prosome, macropain) 26S subunit, non-ATPase, 2	1,73729E+14	8,33953E+14	2,08799E+14	0.0787136032195043	0.218787698581313	-4,12773E+13	9,42308E+14	9,57618E+14	8,73978E+14	6,78491E+14	8,75624E+14	6,75697E+14
TC1400001125.mm.2	Tdrd3	tudor domain containing 3	1,46159E+14	5,8397E+14	2,08767E+14	0.0787493706321147	0.218849363400356	-4,12813E+14	6,62484E+14	6,63896E+14	5,54684E+14	4,79376E+14	6,79797E+14	4,63582E+14
TC0700001669.mm.2	Pde3b	phosphodiesterase 3B, cGMP-inhibited	1,21048E+14	5,86521E+14	2,087E+13	0.0788246691501329	0.218989294659226	-4,12897E+14	6,48935E+14	6,92824E+14	5,93151E+14	5,06511E+11	6,15038E+14	4,62667E+14
TC1500001993.mm.2	Phf21b	PHD finger protein 21B	-0.894173619233184	7,78257E+14	-2,08688E+14	0.0788382413835735	0.218989294659226	-4,12912E+14	7,05805E+14	7,10306E+14	7,48693E+14	8,46252E+14	7,99847E+14	8,58638E+14
TC1800000944.mm.2	Arhgap12	Rho GTPase activating protein 12	1,33814E+14	6,66611E+14	2,08686E+14	0.0788404951479034	0.218989294659226	-4,12914E+14	7,89595E+14	7,58897E+14	7,09232E+13	5,71632E+14	6,59967E+14	5,10343E+14
TC1100003743.mm.2	Krtap4-16	keratin associated protein 4-16	-1,12731E+14	5,36992E+14	-2,08627E+14	0.0789061286724742	0.219133824767249	-4,12987E+14	4,38687E+14	4,97534E+14	5,16223E+14	6,4546E+14	5,20887E+14	6,03159E+14
TC0400003785.mm.2	Kdm1a	lysine (K)-specific demethylase 1A	1,55558E+14	6,94132E+14	2,0861E+14	0.0789251295393321	0.219135027322348	-4,13008E+14	7,68505E+14	7,678E+14	7,31278E+14	4,93911E+14	7,35391E+14	6,67909E+14
TC0700004095.mm.2	Sbf2	SET binding factor 2	1,71138E+14	5,95284E+14	2,08603E+14	0.0789337614707944	0.219135027322348	-4,13018E+13	7,4671E+14	7,45089E+13	6,15432E+14	5,34092E+12	5,96842E+14	3,3354E+14
TC1000001882.mm.2	Taar6	trace amine-associated receptor 6	-0.639865070484478	5,37157E+14	-2,08581E+14	0.0789578092996447	0.219164027776946	-4,13045E+14	4,82515E+14	5,08187E+14	5,48321E+14	5,70354E+13	5,47546E+14	5,66017E+14
TC1300000594.mm.2	Spin1	spindlin 1	1,74173E+14	8,5226E+14	2,08551E+14	0.0789916024121955	0.219188287723032	-4,13082E+14	9,56996E+14	9,58224E+14	9,06569E+14	6,60304E+14	8,8363E+14	7,47834E+13
TC0200005486.mm.2	Dbndd2	dysbindin (dystrobrevin binding protein 1) domain containing 2	0.814295594536658	8,66094E+14	2,08533E+14	0.0790122379078313	0.219188287723032	-4,13105E+14	8,91634E+14	9,21059E+14	8,69888E+14	7,79946E+14	8,63943E+12	8,70093E+14
TC1500001533.mm.2	Taf2	TATA-box binding protein associated factor 2	0.981455839742674	6,82334E+14	2,08524E+14	0.0790225945561429	0.219188287723032	-4,13117E+14	7,41916E+14	7,48894E+14	6,66554E+14	6,27964E+14	7,18244E+14	5,90432E+14
TC1300001739.mm.2	Hus1b	HUS1 checkpoint clamp component B	-0.977469859594634	5,80883E+14	-2,08513E+14	0.0790350582188068	0.219188287723032	-4,13131E+14	4,83068E+14	5,68986E+13	5,73296E+13	6,74252E+14	5,88614E+14	5,97087E+14
TC0100003872.mm.2	Irf6	interferon regulatory factor 6	-0.827869216119381	6,07192E+14	-2,08508E+13	0.0790402688196565	0.219188287723032	-4,13137E+14	5,38742E+14	5,31436E+14	5,98481E+14	6,37271E+14	6,23612E+14	7,13608E+14
TC1700000582.mm.2	Actl9	actin-like 9	-1,06704E+14	4,77758E+14	-2,08487E+14	0.0790637215700899	0.219188287723032	-4,13163E+14	4,07231E+14	4,16239E+11	4,4418E+14	5,92699E+14	4,87835E+14	5,18365E+14
TC1000002316.mm.2	Zfp365	zinc finger protein 365	1,67619E+14	9,84844E+14	2,08461E+14	0.079093185256281	0.219188287723032	-4,13195E+14	1,07123E+14	1,09144E+14	1,03303E+14	7,94399E+14	1,02108E+14	8,97874E+14
TC1400002222.mm.2	Tdh	L-threonine dehydrogenase	-0.76970526743343	6,21971E+14	-2,08434E+14	0.0791234920135002	0.219188287723032	-4,13229E+13	5,83372E+14	5,38906E+14	6,03095E+14	6,73125E+14	6,63523E+14	6,69806E+14
TC0500001361.mm.2	Tmem116	transmembrane protein 116	0.695570666048193	5,59282E+14	2,08429E+14	0.0791293128000366	0.219188287723032	-4,13236E+14	5,77087E+14	5,83271E+14	5,21805E+14	4,99439E+14	5,58904E+14	6,15183E+14
TC1400000748.mm.2	Rem2	rad and gem related GTP binding protein 2	-0.665830259843955	8,38605E+14	-2,08423E+13	0.0791357486102181	0.219188287723032	-4,13243E+14	7,71603E+14	8,07618E+14	8,39278E+14	8,73108E+13	8,65942E+14	8,7408E+14
TC0700000980.mm.2	Apba2	amyloid beta (A4) precursor protein-binding, family A, member 2	0.706812862742192	7,95959E+14	2,08412E+14	0.0791489461686889	0.219188287723032	-4,13257E+14	8,63501E+14	8,32614E+14	7,71488E+14	7,83265E+14	7,90203E+14	7,34685E+14
TC0600000308.mm.2	Exoc4	exocyst complex component 4	1,43508E+14	8,00213E+14	2,08402E+14	0.0791597027330349	0.219188287723032	-4,13269E+14	8,91211E+14	9,08031E+14	8,39236E+14	6,72989E+14	8,19818E+14	6,69992E+13
TC0600001275.mm.2	Syn2	synapsin II	1,20777E+14	9,92385E+14	2,08384E+14	0.0791802032456559	0.219188287723032	-4,13292E+14	1,08492E+14	1,07632E+14	1,04772E+14	8,71968E+14	9,5685E+14	9,1654E+13
TC0400002198.mm.2	Plekhf2	pleckstrin homology domain containing, family F (with FYVE domain) member 2	-0.681688289807115	6,60007E+14	-2,08382E+14	0.0791828549400514	0.219188287723032	-4,13295E+14	6,00211E+14	6,39731E+14	6,83443E+14	6,92837E+14	6,52572E+14	6,91249E+14
TC1300001835.mm.2	Txndc5	thioredoxin domain containing 5	0.89059128853777	7,43437E+13	2,08372E+13	0.0791935217412701	0.219188287723032	-4,13307E+13	8,22558E+14	8,09923E+14	7,53413E+13	7,00949E+14	7,368E+14	6,36979E+14
TC1500001511.mm.2	Ext1	exostoses (multiple) 1	0.906243051548204	7,66854E+14	2,08365E+14	0.079201713228505	0.219188287723032	-4,13316E+14	8,12473E+14	8,40636E+14	7,79268E+14	6,92592E+14	7,74838E+14	7,01315E+14
TC0900002468.mm.2	Pias1	protein inhibitor of activated STAT 1	1,57014E+14	6,91289E+14	2,08357E+14	0.0792112455284228	0.219188287723032	-4,13326E+14	7,47605E+14	7,88465E+14	7,32853E+14	4,89188E+14	7,13373E+14	6,76248E+14
TC1100004261.mm.2	Ppp3r1	protein phosphatase 3, regulatory subunit B, alpha isoform (calcineurin B, type I)	1,26077E+14	1,07548E+14	2,08356E+14	0.0792114069019557	0.219188287723032	-4,13327E+14	1,14339E+14	1,15819E+14	1,09558E+14	9,53848E+14	1,1145E+14	9,87349E+14
TC1700000696.mm.2	Sfta2	surfactant associated 2	-0.718172775543425	5,54442E+13	-2,08334E+14	0.079237197232664	0.219222005324505	-4,13355E+14	5,35406E+14	4,72583E+14	5,63466E+14	5,88158E+14	5,78291E+12	5,88748E+14
TC0900000523.mm.2	Ift46	intraflagellar transport 46	0.710635405372508	7,00669E+14	2,08319E+14	0.0792537988666771	0.219230293757728	-4,13374E+14	7,65067E+14	7,45094E+14	6,88952E+14	6,79082E+14	6,93942E+14	6,31874E+14
TC0600000115.mm.2	Mdfic	MyoD family inhibitor domain containing	-0.729341739476987	6,49857E+14	-2,08276E+14	0.0793022572548344	0.219326685744447	-4,13427E+14	5,96683E+14	6,07921E+14	6,46789E+14	7,03684E+14	6,50876E+14	6,93187E+14
TC1200001589.mm.2	Acp1	acid phosphatase 1, soluble	1,50557E+13	7,70238E+14	2,08264E+14	0.0793159915179713	0.219327024425789	-4,13443E+14	8,82974E+13	8,59364E+14	8,04857E+13	6,36367E+14	8,02074E+13	6,3579E+14
TC0900001761.mm.2	Jrkl	Jrk-like	0.960847735497534	5,51327E+14	2,08118E+13	0.0794811698504351	0.219746068943044	-4,13625E+14	6,16083E+14	5,96216E+13	5,70006E+14	4,50124E+11	5,60294E+14	5,15237E+14
TC1000000852.mm.2	Reep6	receptor accessory protein 6	1,38227E+14	6,50323E+14	2,08105E+14	0.0794955529799814	0.219748129210232	-4,13641E+13	7,73198E+14	7,42794E+14	7,19841E+14	5,19698E+14	6,09059E+14	5,37345E+14
TC0400000381.mm.2	Il11ra1	interleukin 11 receptor, alpha chain 1	-0.701602434354248	8,50428E+14	-2,08088E+14	0.0795153569116863	0.219765170826415	-4,13663E+12	8,00862E+14	8,00065E+14	8,44329E+14	8,96918E+14	8,62612E+14	8,97782E+13
TC1600000429.mm.2	Cep19	centrosomal protein 19	0.885936225160924	7,39003E+14	2,08057E+14	0.0795505547549542	0.219780582894967	-4,13702E+14	7,83711E+14	7,90583E+14	7,52003E+13	6,45104E+14	7,48826E+14	7,13789E+14
TC0800002955.mm.2	Cfdp1	craniofacial development protein 1	1,56205E+14	8,72888E+14	2,08053E+14	0.0795542830585162	0.219780582894967	-4,13706E+14	9,86166E+14	9,80882E+14	8,89674E+14	7,64963E+14	9,1541E+14	7,00236E+14
TC0200000363.mm.2	Yme1l1	YME1-like 1 (S. cerevisiae)	3,63695E+14	5,94922E+13	2,08047E+14	0.079561853163678	0.219780582894967	-4,13714E+14	8,25666E+14	8,41156E+14	7,21579E+14	2,17853E+14	6,60835E+14	3,02443E+14
TC0300000631.mm.2	Pdgfc	platelet-derived growth factor, C polypeptide	1,47451E+14	5,91023E+14	2,08026E+14	0.0795855414516771	0.219806774361494	-4,13741E+14	6,34043E+14	6,7805E+13	6,22671E+14	3,94518E+14	6,09948E+14	6,06906E+14

TC0700002344.mm.2	Vmn2r54	vomeronasal 2, receptor 54	-1,20673E+14	4,63906E+14	-2,08014E+14	0.0795987397294185	0.219806774361494	-4,13755E+14	3,92228E+14	3,73385E+14	4,3186E+13	5,75098E+14	4,51136E+14	5,59731E+14
TC0600003336.mm.2	Erp27	endoplasmic reticulum protein 27	-1,18349E+14	4,91643E+14	-2,08002E+14	0.0796122593665785	0.219806774361494	-4,1377E+14	4,35504E+14	4,34983E+14	5,12568E+14	5,94618E+14	4,08172E+14	5,64015E+14
TC0400003867.mm.2	Padi1	peptidyl arginine deiminase, type I	-1,1206E+13	4,88537E+14	-2,07981E+14	0.0796368799897427	0.219837081938449	-4,13797E+14	3,69635E+14	4,28899E+14	4,55396E+13	5,67258E+14	5,03312E+14	6,06724E+14
TC1000002426.mm.2	Gm9736	predicted gene 9736	-0.729576468563727	6,5741E+14	-2,07944E+14	0.0796783986650063	0.219881275199508	-4,13843E+14	6,09906E+14	6,12345E+14	6,55046E+13	7,1312E+14	6,55601E+14	6,98445E+14
TC0X00003191.mm.2	Tspyl2	TSPY-like 2	1,83808E+14	9,0186E+14	2,07942E+14	0.0796801815856718	0.219881275199508	-4,13845E+14	1,04055E+14	1,00817E+14	9,54389E+14	7,2672E+14	9,36827E+14	7,44495E+13
TC0700004223.mm.2	Ears2	glutamyl-tRNA synthetase 2, mitochondrial	-1,11112E+14	5,78438E+14	-2,07914E+14	0.0797124800903255	0.219914707061251	-4,13881E+14	5,33066E+14	4,85935E+13	5,6477E+14	6,76456E+14	5,5177E+14	6,58629E+14
TC1700001264.mm.2	Dync2li1	dynein cytoplasmic 2 light intermediate chain 1	0.68376774040766	6,48423E+14	2,07906E+14	0.0797213512664134	0.219914707061251	-4,1389E+14	6,91134E+14	6,96635E+14	6,54065E+14	5,96951E+14	6,26427E+14	6,25329E+14
TC0X00002995.mm.2	Gla	galactosidase, alpha	1,19593E+14	5,63841E+14	2,07896E+14	0.0797332413983634	0.219914707061251	-4,13904E+14	6,37298E+14	6,24197E+14	6,00263E+14	4,22047E+14	5,48042E+14	5,51196E+14
TC4 JH584293 random00000008.mm.2	Ccl19	chemokine (C-C motif) ligand 19	0.640255899981694	6,75008E+14	2,07846E+13	0.0797901352272921	0.220002540989249	-4,13966E+14	7,09594E+14	7,15056E+14	6,67976E+14	6,28623E+14	6,65799E+14	6,62998E+14
TC1600000708.mm.2	Cd47	CD47 antigen (Rh-related antigen, integrin-associated signal transducer)	1,47608E+14	9,22142E+14	2,07827E+13	0.0798113457148884	0.220002540989249	-4,1399E+14	1,00849E+14	1,02683E+14	9,58008E+14	7,82101E+14	9,54505E+14	8,02911E+14
TC1200001770.mm.2	Ralgapa1	Ral GTPase activating protein, alpha subunit 1	1,71085E+14	7,25807E+14	2,0782E+14	0.0798194056107916	0.220002540989249	-4,13998E+14	8,4856E+14	8,63648E+14	7,31873E+14	6,38165E+14	7,66237E+14	5,06358E+14
TC0X00000388.mm.2	Gria3	glutamate receptor, ionotropic, AMPA3 (alpha 3)	2,20818E+13	7,98216E+14	2,07819E+14	0.0798207562000822	0.220002540989249	-4,14E+14	9,3555E+14	9,59291E+14	8,67344E+14	5,8586E+13	8,37663E+14	6,03586E+14
TC0200002184.mm.2	Pax1	paired box 1	-1,05849E+14	6,0214E+14	-2,07808E+14	0.0798333554995432	0.220002540989249	-4,14014E+13	5,38819E+14	5,41532E+14	5,94371E+14	6,97679E+14	5,65285E+14	6,75153E+13
TC0200000549.mm.2	Ncs1	neuronal calcium sensor 1	0.851079946794552	9,11569E+14	2,07784E+13	0.079860064414291	0.220038511954082	-4,14043E+13	9,78081E+14	9,57671E+14	9,26864E+14	8,38672E+14	9,10357E+14	8,57771E+14
TC0500002155.mm.2	Adgrf3	adhesion G protein-coupled receptor F3	-0.973741901109228	6,17225E+14	-2,07756E+14	0.0798919666338746	0.220058020563882	-4,14078E+14	5,15255E+14	5,83315E+14	6,01255E+14	6,92063E+14	6,21245E+14	6,90216E+13
TC0300000881.mm.2	Rfx5	regulatory factor X, 5 (influences HLA class II expression)	0.741881631382846	6,19808E+14	2,07754E+14	0.0798944591509158	0.220058020563882	-4,14081E+14	6,62257E+14	6,78101E+13	6,30999E+14	5,60983E+14	6,05796E+14	5,80711E+14
TC1100001725.mm.2	Mapt	microtubule-associated protein tau	0.998568197569032	7,66032E+14	2,07738E+13	0.0799131368682728	0.220060741378597	-4,14101E+14	8,34716E+14	8,13764E+14	7,90113E+14	6,58653E+14	7,30907E+14	7,68037E+14
TC0400004041.mm.2	Rbp7	retinol binding protein 7, cellular	-0.637140766307854	5,82607E+11	-2,07729E+14	0.0799227616550331	0.220060741378597	-4,14112E+14	5,49134E+14	5,37378E+14	5,9274E+12	6,21201E+14	6,09141E+14	5,86049E+14
TC0700000391.mm.2	Lypd3	Ly6/Plaur domain containing 3	-0.770979777299739	6,72403E+14	-2,07704E+14	0.0799519301659483	0.220093337587003	-4,14144E+14	6,13986E+14	6,24118E+14	6,57663E+14	7,34637E+14	6,85609E+14	7,18402E+14
TC1200000467.mm.2	Psma6	proteasome (prosome, macropain) subunit, alpha type 6	0.940142605631756	8,31944E+14	2,07685E+14	0.0799732604231172	0.220093337587003	-4,14168E+13	9,00544E+14	9,00985E+14	8,44312E+14	7,69189E+14	8,38549E+14	7,38083E+14
TC1200000282.mm.2	Sypl	synaptophysin-like protein	1,29736E+14	6,7996E+14	2,07683E+14	0.0799755782021908	0.220093337587003	-4,1417E+12	7,57963E+14	7,8138E+12	6,92602E+14	5,8727E+14	7,13831E+14	5,46715E+14
TC1100000566.mm.2	Col23a1	collagen, type XXIII, alpha 1	-0.616750271664274	6,85912E+14	-2,07656E+14	0.0800060865941318	0.220135313193057	-4,14204E+14	6,26292E+14	6,49671E+14	6,94559E+14	7,04753E+14	7,24485E+14	7,15714E+13
TC0700001710.mm.2	Acsm2	acyl-CoA synthetase medium-chain family member 2	-1,17695E+14	4,6676E+14	-2,07646E+14	0.0800181548669855	0.220135313193057	-4,14217E+14	3,96259E+14	3,79007E+14	4,2005E+14	5,90607E+13	4,8919E+13	5,25446E+13
TC0700000873.mm.2	Myod1	myogenic differentiation 1	-0.967398778292016	5,54046E+14	-2,07569E+14	0.080105691738077	0.220259348802636	-4,14313E+14	5,03323E+14	5,08269E+14	5,40068E+13	6,65004E+14	5,74331E+14	5,33278E+14
TC0200001906.mm.2	Blvra	biliverdin reductase A	0.714419307852061	6,12491E+14	2,07544E+13	0.0801337658302506	0.220259348802636	-4,14344E+13	6,82868E+13	6,57034E+14	6,14133E+13	5,82885E+14	5,96297E+14	5,41727E+14
TC1600001053.mm.2	Sim2	single-minded family bHLH transcription factor 2	-0.832428475162668	5,14294E+14	-2,07544E+14	0.0801342600475935	0.220259348802636	-4,14344E+14	4,44409E+14	4,8549E+14	5,31412E+14	5,64973E+14	4,87141E+14	5,72336E+14
TC0200000718.mm.2	Wdr38	WD repeat domain 38	-0.882498708348558	7,56259E+14	-2,07535E+14	0.0801444225446965	0.220259348802636	-4,14355E+14	6,80287E+14	6,95224E+14	7,53275E+14	7,98735E+14	7,54154E+14	8,55881E+14
TC1600001213.mm.2	Rsl1d1	ribosomal L1 domain containing 1	1,42106E+14	7,25127E+14	2,07533E+14	0.0801462609844312	0.220259348802636	-4,14357E+14	8,48642E+14	8,16648E+14	7,92652E+14	5,88426E+14	6,9389E+13	6,10507E+14
TC0200003398.mm.2	Rc3h2	ring finger and CCCH-type zinc finger domains 2	1,60045E+14	7,4583E+13	2,07533E+14	0.0801470486327857	0.220259348802636	-4,14358E+14	8,79474E+13	8,65686E+13	7,63473E+14	6,61597E+14	7,69957E+14	5,34792E+14
TC1100001529.mm.2	Mrpl10	mitochondrial ribosomal protein L10	0.822947832340047	7,35371E+14	2,07522E+14	0.0801589289008043	0.220259348802636	-4,14371E+14	8,203E+14	7,87107E+14	7,57208E+14	6,85609E+14	7,10068E+14	6,51935E+14
TC1100001461.mm.2	Spop	speckle-type POZ protein	0.81381267110361	9,10769E+14	2,0751E+14	0.0801733276578987	0.220261351841726	-4,14387E+14	9,48703E+14	9,8267E+14	8,9549E+14	8,7312E+14	9,25644E+13	8,38987E+14
TC1400002017.mm.2	Ndrg2	N-myc downstream regulated gene 2	1,19125E+14	1,01669E+14	2,07496E+14	0.0801892442776677	0.220267523533253	-4,14404E+14	1,11966E+14	1,08399E+14	1,05575E+14	9,09651E+13	1,0134E+14	9,17712E+14
TC0400002034.mm.2	A430005L14Rik	RIKEN cDNA A430005L14 gene	0.777227710590965	7,05005E+14	2,07463E+14	0.0802269114202168	0.220318343905519	-4,14446E+14	7,32464E+14	7,39883E+14	6,95487E+14	6,21414E+14	7,42136E+14	6,98646E+14
TC1400000980.mm.2	Entpd4/Entpd4b	ectonucleoside triphosphate diphosphohydrolase 4/ectonucleoside triphosphate diphosphohydrolase 4B	0.622233387580884	8,67309E+14	2,07444E+14	0.0802481374011798	0.220318343905519	-4,14469E+14	9,27972E+14	8,97258E+14	8,51963E+14	8,4882E+14	8,38593E+14	8,39245E+14
TC1100000085.mm.2	Ykt6	YKT6 v-SNARE homolog (S. cerevisiae)	0.818584781344262	7,5297E+14	2,07444E+13	0.0802487656166754	0.220318343905519	-4,1447E+14	7,96999E+14	7,80197E+14	7,47151E+14	6,66328E+14	7,89039E+14	7,38103E+14
TC0500000541.mm.2	Rbpj	recombination signal binding protein for immunoglobulin kappa J region	0.619664333492066	7,48982E+14	2,0743E+14	0.0802646646044842	0.220324453283144	-4,14487E+14	7,89547E+13	7,86903E+14	7,34426E+14	7,18091E+14	7,44567E+14	7,20358E+14
TC1600001519.mm.2	Iqcg	IQ motif containing G	-0.890931723364101	5,94813E+14	-2,07398E+14	0.0803006945558029	0.220348827928778	-4,14526E+14	5,62448E+14	5,10799E+14	5,76743E+13	6,7469E+14	6,07481E+14	6,36717E+14
TC0900002421.mm.2	Senp8	SUMO/sentrin specific peptidase 8	-0.613520026338974	7,63863E+14	-2,07398E+14	0.0803008947804743	0.220348827928778	-4,14527E+14	7,22309E+14	7,34467E+14	7,78694E+14	8,00786E+14	7,72978E+14	7,73942E+14
TC1000003120.mm.2	Nab2	Ngfi-A binding protein 2	0.663737166586126	6,80632E+14	2,07357E+14	0.0803477754381452	0.220381791040966	-4,14578E+14	7,15179E+14	7,30109E+14	6,6207E+14	6,50471E+14	6,86445E+13	6,39516E+12
TC0200002589.mm.2	Snai1	snail family zinc finger 1	-0.89409040205469	6,39518E+14	-2,07349E+13	0.0803574819402542	0.220381791040966	-4,14588E+13	5,41594E+14	6,0043E+14	6,50102E+14	6,7074E+14	6,38621E+14	7,35618E+14
TC0700002345.mm.2	Vmn2r55	vomeronasal 2, receptor 55	-1,22611E+14	4,3708E+14	-2,07347E+14	0.0803593673325195	0.220381791040966	-4,14591E+14	3,71363E+14	3,4301E+14	4,14823E+14	5,44772E+14	4,09711E+14	5,38801E+14
TC0400003275.mm.2	Elavl4	ELAV like RNA binding protein 4	1,32445E+14	7,46315E+14	2,0734E+14	0.080367616468486	0.220381791040966	-4,146E+14	8,11193E+14	8,18194E+13	7,79887E+14	5,8461E+14	7,72332E+14	7,11673E+14
TC1700002801.mm.2	Rhbdl1	rhomboid like 1	0.85796252393448	7,7318E+14	2,07299E+14	0.0804141898373096	0.220449172319499	-4,1465E+14	8,16393E+14	8,05743E+13	7,78376E+14	6,72168E+12	7,85392E+14	7,81011E+14
TC0200003276.mm.2	1700001O22Rik	RIKEN cDNA 1700001O22 gene	-0.746865922940748	7,15547E+14	-2,07294E+14	0.0804199678536144	0.220449172319499	-4,14657E+14	6,49675E+14	6,81916E+14	7,0831E+14	7,72654E+13	7,25381E+14	7,55347E+14
TC0900000544.mm.2	Bace1	beta-site APP cleaving enzyme 1	0.727376987307571	7,83035E+14	2,07283E+14	0.0804332330457602	0.220449172319499	-4,14671E+13	8,25167E+14	8,28408E+14	7,8963E+14	7,1847E+14	7,74292E+14	7,62244E+14
TC0700002652.mm.2	Cyp2b23	cytochrome P450, family 2, subfamily b, polypeptide 23	-0.908562641953883	5,32204E+14	-2,07211E+14	0.0805152224754201	0.220597516238943	-4,14761E+14	4,64466E+14	4,76672E+14	5,21816E+14	6,01035E+14	5,20786E+14	6,08447E+14
TC1600001651.mm.2	Slc35a5	solute carrier family 35, member A5	1,45444E+14	5,80413E+14	2,07205E+14	0.0805226016516577	0.220597516238943	-4,14769E+14	6,75367E+14	6,61681E+13	6,23496E+14	4,22665E+14	6,04932E+14	4,94337E+14
TC1900000148.mm.2	Slc22a8	solute carrier family 22 (organic anion transporter), member 8	0.993105287907765	6,29791E+14	2,07195E+14	0.0805341005641116	0.220597516238943	-4,14781E+14	6,84263E+14	6,78538E+14	6,42155E+14	5,22024E+14	6,59294E+14	5,9247E+14
TC1500001946.mm.2	Cyp2d10	cytochrome P450, family 2, subfamily d, polypeptide 10	-0.767672413435367	5,28163E+14	-2,0717E+14	0.0805626995732606	0.220597516238943	-4,14812E+14	4,5836E+14	4,93004E+13	5,17238E+14	5,8766E+14	5,41504E+14	5,71212E+14
TC0900002472.mm.2	Map2k5	mitogen-activated protein kinase kinase 5	2,02493E+14	6,90292E+14	2,07165E+14	0.0805687315325813	0.220597516238943	-4,14819E+14	7,97383E+14	7,87518E+13	7,38782E+14	4,41133E+14	7,65229E+14	6,11708E+14
TC1400002632.mm.2	Kctd12/Mir5130	potassium channel tetramerisation domain containing 12/microRNA 5130	0.77968016987198	8,2801E+14	2,07164E+14	0.080569501834927	0.220597516238943	-4,1482E+14	8,64343E+14	8,75472E+14	8,31639E+14	7,5224E+14	8,05068E+14	8,39298E+14
TC0700000551.mm.2	Gm5113	predicted gene 5113	0.941936272643712	5,16978E+14	2,0714E+14	0.0805972219382123	0.22063592203371	-4,1485E+14	5,85262E+13	5,73128E+14	5,20779E+14	4,49224E+12	5,38481E+14	4,34991E+14
TC0700000840.mm.2	Hrc	histidine rich calcium binding protein	-0.710855408792448	5,03591E+14	-2,07105E+14	0.0806374898053657	0.220701174037629	-4,14894E+14	4,28796E+14	4,29141E+14	4,6653E+14	5,33578E+14	5,78646E+14	5,84854E+14
TC0900000825.mm.2	Ints14	integrator complex subunit 14	1,26338E+14	6,95219E+14	2,07086E+14	0.0806587229799963	0.220701174037629	-4,14917E+14	7,85074E+13	7,74371E+14	7,39376E+14	5,67394E+14	6,96017E+14	6,09079E+14
TC0900000615.mm.2	Pou2af1	POU domain, class 2, associating factor 1	-1,01692E+14	5,16286E+14	-2,07074E+14	0.0806724757142571	0.220701174037629	-4,14932E+14	4,91203E+14	4,22939E+14	5,1416E+14	6,03365E+14	4,88916E+14	5,77132E+14
TC1300002651.mm.2	Rab3c	RAB3C, member RAS oncogene family	1,83859E+14	8,21153E+14	2,07071E+14	0.0806758465265089	0.220701174037629	-4,14936E+14	9,64803E+14	9,39651E+14	8,76533E+14	6,60204E+14	8,46963E+14	6,38765E+14
TC1100003401.mm.2	Ccl3	chemokine (C-C motif) ligand 3	1,42508E+14	6,21922E+14	2,07057E+14	0.0806928286995568	0.220710159367842	-4,14954E+13	6,52137E+14	7,93331E+14	6,31753E+14	5,28699E+14	6,33074E+14	4,92538E+14
TC1000003035.mm.2	Lemd3	LEM domain containing 3	1,42359E+14	6,77625E+14	2,07023E+14	0.0807315622190151	0.220742642910818	-4,14996E+14	7,87978E+14	7,84488E+14	7,52926E+14	5,34821E+14	6,28345E+14	5,77192E+13
TC0300001271.mm.2	Camk2d	calcium/calmodulin-dependent protein kinase II, delta	2,1627E+14	8,66998E+14	2,07022E+14	0.0807321041813102	0.220742642910818	-4,14997E+14	9,91195E+14	9,81281E+14	9,3684E+13	6,03095E+14	9,12353E+14	7,77226E+14
TC1600000989.mm.2	Eva1c	eva-1 homolog C (C. elegans)	-0.626144524573991	4,93908E+14	-2,06994E+14	0.0807653590074728	0.220778771100486	-4,15033E+14	4,64787E+14	4,72353E+14	5,15422E+14	5,46947E+13	4,8696E+14	4,7698E+14
TC0500002865.mm.2	Mapk10	mitogen-activated protein kinase 10	2,11047E+14	9,82271E+14	2,06987E+14	0.0807727211343242	0.220778771100486	-4,15041E+14	1,13057E+14	1,12348E+14	1,05982E+13	7,72137E+14	1,00416E+14	8,03462E+14
TC1600000343.mm.2	Rtp1	receptor transporter protein 1	-0.910329463314151	5,30752E+14	-2,06967E+14	0.0807962872007401	0.22080572857285	-4,15067E+14	4,99624E+14	4,67133E+14	5,16949E+14	6,31875E+14	5,34158E+14	5,34771E+14
TC0200002196.mm.2	Nxt1	NTF2-related export protein 1	-0.739935606186877	6,47636E+14	-2,06848E+13	0.0809330138353487	0.221100628723092	-4,15215E+14	6,12941E+14	6,09389E+14	6,71119E+14	6,99198E+13	6,11092E+14	6,82077E+14
TC1900001155.mm.2	Olfr1428	olfactory receptor 1428	-1,13696E+14	4,24737E+14	-2,06836E+14	0.0809475321246759	0.221100628723092	-4,15231E+14	3,50107E+14	3,56453E+14	4,10342E+14	5,2361E+14	3,91959E+14	5,15952E+14
TC0900002836.mm.2	Zic1	zinc finger protein of the cerebellum 1	0.992128722592543	1,0269E+14	2,06835E+14	0.0809487671993979	0.221100628723092	-4,15232E+14	1,10212E+14	1,0898E+14	1,06424E+14	9,29252E+14	9,75759E+14	1,00025E+14
TC0400001707.mm.2	1700013G24Rik	RIKEN cDNA 1700013G24 gene	-1,11221E+14	5,42971E+14	-2,0682E+14	0.0809655127179149	0.221100628723092	-4,1525E+14	4,54468E+14	4,59108E+13	4,8972E+14	6,46298E+14	5,81545E+13	6,26691E+14
TC1100002901.mm.2	Natd1	N-acetyltransferase domain containing 1	-0.626608935940801	6,91128E+14	-2,06814E+14	0.0809728051942508	0.221100628723092	-4,15258E+14	6,49293E+14	6,47994E+14	6,9467E+14	7,27939E+14	7,17911E+14	7,0896E+14
TC0500000188.mm.2	Lhfpl3	lipoma HMGIC fusion partner-like 3	1,62167E+14	7,68736E+14	2,06795E+14	0.0809943704659616	0.221122041904107	-4,15282E+14	8,97957E+13	8,54447E+14	8,17977E+14	6,10092E+14	7,924E+14	6,39544E+14
TC0200000546.mm.2	Usp20	ubiquitin specific peptidase 20	1,26794E+13	7,11311E+14	2,06777E+14	0.0810147154445576	0.221140116882629	-4,15304E+14	8,25562E+12	8,09878E+14	7,66904E+14	6,14946E+14	6,85297E+14	5,65279E+14
TC0200002574.mm.2	Trp53rkb	transformation related protein 53 regulating kinase B	-0.72708531846041	6,99467E+14	-2,06761E+14	0.0810333914086886	0.221153630719546	-4,15324E+14	6,40856E+14	6,73057E+14	7,09521E+14	7,49808E+14	6,86155E+14	7,37406E+14
TC1700000676.mm.2	Atp6v1g2	ATPase, H+ transporting, lysosomal V1 subunit G2	0.920698599854591	1,07439E+14	2,06741E+14	0.081056906694113	0.221180345057111	-4,15349E+14	1,144E+14	1,12089E+14	1,08978E+14	9,90972E+14	1,08424E+14	1,01647E+14
TC1100004126.mm.2	Mxra7	matrix-remodelling associated 7	-0.786539756196904	7,12329E+14	-2,06697E+14	0.0811073306890824	0.221258171622664	-4,15404E+14	6,67965E+14	6,58305E+14	6,97551E+14	7,86026E+14	7,27468E+14	7,36658E+14
TC1100003315.mm.2	Ksr1	kinase suppressor of ras 1	0.63292056589219	6,7254E+13	2,06692E+14	0.0811128914401464	0.221258171622664	-4,1541E+14	7,26523E+14	6,95318E+14	6,44508E+14	6,50749E+14	6,48713E+14	6,69428E+14
TC1000001629.mm.2	Olfr9	olfactory receptor 9	-0.933256725106632	4,8933E+14	-2,0667E+13	0.0811384776589141	0.221259029727397	-4,15438E+14	4,08097E+14	4,51224E+14	5,21598E+14	5,24375E+14	4,56732E+14	5,73952E+14
TC0700004179.mm.2	Gprc5b	G protein-coupled receptor, family C, group 5, member B	0.785560980112679	7,7415E+14	2,06668E+14	0.0811406694382913	0.221259029727397	-4,1544E+14	8,27187E+14	7,99645E+14	7,77296E+13	6,92423E+14	7,73399E+14	7,74952E+14
TC0500001735.mm.2	Fam220a	family with sequence similarity 220, member A	0.599800383568894	6,85584E+14	2,06635E+14	0.0811795203259116	0.221274913213783	-4,15482E+14	7,41764E+14	7,29556E+14	6,79272E+14	6,72087E+13	6,47537E+14	6,43287E+14
TC0300001850.mm.2	Anxa5	annexin A5	1,15642E+14	7,68166E+14	2,06631E+14	0.0811841303185729	0.221274913213783	-4,15487E+14	8,64614E+14	8,472E+14	8,18084E+14	6,62446E+14	7,44169E+14	6,72482E+14
TC1600001378.mm.2	Tra2b	transformer 2 beta	1,05375E+13	9,07697E+14	2,06621E+12	0.0811958231398997	0.221274913213783	-4,155E+14	9,80403E+14	9,91887E+14	9,41977E+14	8,19565E+14	9,00098E+14	8,12253E+14
TC1400000179.mm.2	Adk	adenosine kinase	0.974076545772725	7,06735E+14	2,06616E+14	0.081201425050152	0.221274913213783	-4,15506E+14	7,8552E+14	7,64341E+14	7,02921E+14	6,52125E+14	7,28864E+14	6,06639E+14
TC0300003219.mm.2	Zfp697	zinc finger protein 697	-0.806314390205505	6,70626E+14	-2,06565E+14	0.0812606589706456	0.221398883664865	-4,1557E+14	6,31932E+14	6,28505E+14	6,66266E+14	7,55434E+14	6,54872E+13	6,8675E+14
TC0X00001766.mm.2	Clcn5	chloride channel, voltage-sensitive 5	1,19793E+14	6,38792E+12	2,06525E+13	0.0813062286992604	0.221452254115522	-4,15619E+14	7,53115E+14	6,80654E+14	6,68822E+14	5,25361E+14	6,43699E+14	5,61102E+13
TC0800001110.mm.2	Nlrc5/Mir7072	NLR family, CARD domain containing 5/microRNA 7072	-0.898041657814068	4,8118E+14	-2,06524E+14	0.0813078738729105	0.221452254115522	-4,15621E+14	4,2518E+14	4,21287E+14	4,59198E+14	5,66877E+14	4,84406E+14	5,30131E+14
TC0700000502.mm.2	Lrfn1/Mir466f-2	leucine rich repeat and fibronectin type III domain containing 1/microRNA 466f 2	0.688765925600989	5,82516E+14	2,06512E+14	0.0813214787812043	0.221452254115522	-4,15635E+14	6,67084E+14	6,38908E+13	5,57818E+14	6,1042E+14	5,04777E+14	5,16092E+14
TC1600001602.mm.2	Nr1i2	nuclear receptor subfamily 1, group I, member 2	-0.998689284919241	4,32724E+14	-2,06496E+14	0.0813400270819093	0.221457463216506	-4,15655E+14	3,79279E+14	3,541E+14	4,08201E+14	5,24917E+14	4,28838E+13	5,0101E+14
TC0400001430.mm.2	Yrdc	yrdC domain containing (E.coli)	0.664702180071846	5,55398E+14	2,06487E+14	0.0813508797106995	0.221457463216506	-4,15667E+14	5,97076E+14	5,7055E+14	5,32135E+14	5,0255E+14	5,48225E+14	5,81854E+14
TC1000001531.mm.2	Lrig3	leucine-rich repeats and immunoglobulin-like domains 3	-0.790702326771346	5,75445E+14	-2,06451E+14	0.081392329461325	0.221521254040379	-4,15712E+14	5,66689E+14	5,54493E+14	5,95537E+14	6,83785E+14	5,34908E+14	5,17258E+13
TC0500002666.mm.2	Tecrl	trans-2,3-enoyl-CoA reductase-like	-0.885943033134701	5,2969E+14	-2,06443E+14	0.0814018087986772	0.221521254040379	-4,15722E+14	4,63917E+13	4,62983E+14	4,96383E+14	6,07705E+14	5,60585E+14	5,86567E+14
TC0200000002.mm.2	Fam171a1	family with sequence similarity 171, member A1	1,36972E+14	6,65394E+14	2,06385E+14	0.0814691335802337	0.221667029614709	-4,15795E+14	7,69689E+14	7,48189E+14	7,25828E+14	5,18107E+14	6,39878E+14	5,90674E+14
TC1600001294.mm.2	Slc7a4	solute carrier family 7 (cationic amino acid transporter, y+ system), member 4	0.620349065850137	7,58147E+14	2,06366E+14	0.081491197206246	0.221689626976911	-4,15818E+14	7,98236E+13	8,05396E+14	7,31121E+14	7,48442E+14	7,20067E+13	7,45619E+14
TC0800002400.mm.2	Pgpep1	pyroglutamyl-peptidase I	-0.824407784875956	6,92392E+14	-2,06344E+13	0.0815163388814455	0.221720588858327	-4,15846E+13	6,28718E+13	6,45116E+14	6,71963E+14	7,66752E+14	7,08074E+14	7,33728E+14
TC1700000489.mm.2	Pnpla1	patatin-like phospholipase domain containing 1	-0.798135151355705	5,76292E+14	-2,0631E+14	0.0815563523388874	0.221741402484341	-4,15889E+13	5,4224E+14	5,45876E+14	5,80309E+14	6,67433E+14	5,44923E+14	5,76969E+14
TC1400001499.mm.2	Nr1d2	nuclear receptor subfamily 1, group D, member 2	1,2404E+14	7,99843E+14	2,06309E+14	0.0815579082941849	0.221741402484341	-4,1589E+14	8,71951E+14	8,61421E+13	8,29936E+14	6,55357E+13	8,26545E+14	7,53849E+14
TC1500001876.mm.2	Ddx17	DEAD (Asp-Glu-Ala-Asp) box polypeptide 17	1,35208E+14	8,29922E+14	2,06289E+14	0.0815806152779802	0.221741402484341	-4,15915E+14	9,13381E+14	9,45749E+14	8,67312E+13	7,21402E+14	8,40502E+14	6,91186E+14
TC1800000332.mm.2	Ankhd1/Eif4ebp3	ankyrin repeat and KH domain containing 1/eukaryotic translation initiation factor 4E binding protein 3	0.778361897253908	6,58892E+14	2,06286E+14	0.0815837604688149	0.221741402484341	-4,15918E+14	7,18671E+14	7,49699E+14	6,80683E+14	6,32016E+14	6,19164E+13	5,5312E+14
TC0100003350.mm.2	Tnn	tenascin N	-1,2567E+14	4,31484E+14	-2,06279E+14	0.0815927993129558	0.221741402484341	-4,15928E+14	3,76491E+14	3,26621E+14	4,1129E+14	5,43163E+14	4,00019E+14	5,31322E+12
TC0400001785.mm.2	Epha2	Eph receptor A2	-0.699743318456875	5,27501E+14	-2,06245E+14	0.0816319071570542	0.221743362059553	-4,1597E+14	4,83378E+14	4,78226E+14	5,27226E+13	5,74326E+14	5,28891E+14	5,72957E+14
TC1900000749.mm.2	Mxi1	MAX interactor 1, dimerization protein	-0.840292036485431	8,69997E+14	-2,06223E+14	0.0816579944856273	0.221743362059553	-4,15998E+14	8,33651E+14	8,108E+14	9,27649E+14	8,8486E+14	9,27291E+14	8,35733E+14
TC0100003528.mm.2	Sdhc	succinate dehydrogenase complex, subunit C, integral membrane protein	1,20204E+14	7,46874E+14	2,06216E+14	0.0816654815778661	0.221743362059553	-4,16006E+13	8,3682E+14	8,1174E+13	7,7799E+14	6,3016E+14	7,63744E+14	6,60791E+14
TC1900001739.mm.2	Rab11fip2	RAB11 family interacting protein 2 (class I)	1,3013E+14	7,67085E+14	2,06203E+14	0.0816806484510915	0.221743362059553	-4,16022E+14	8,61714E+14	8,62239E+14	7,92542E+14	6,71151E+14	7,87849E+14	6,27016E+14
TC0200003505.mm.2	Mmadhc	methylmalonic aciduria (cobalamin deficiency) cblD type, with homocystinuria	0.916443369266218	6,49995E+14	2,06182E+14	0.0817053845341896	0.221743362059553	-4,16049E+14	7,26401E+14	7,22846E+14	6,93317E+14	5,72641E+14	5,85031E+14	5,99735E+14
TC1000000502.mm.2	Hsf2	heat shock factor 2	1,13962E+14	6,72534E+14	2,06179E+14	0.0817091199649323	0.221743362059553	-4,16053E+14	7,65205E+13	7,47735E+14	6,92416E+14	5,92599E+14	6,86461E+14	5,50789E+14
TC0500003048.mm.2	Tmem119	transmembrane protein 119	0.607688851295917	7,85139E+14	2,06174E+14	0.0817147466282091	0.221743362059553	-4,16059E+14	8,1633E+14	8,24958E+14	7,69219E+14	7,50531E+14	7,69159E+14	7,80636E+14
TC1600001343.mm.2	Abcc5	ATP-binding cassette, sub-family C (CFTR/MRP), member 5	0.92136001782995	8,00241E+14	2,06173E+14	0.0817157651812839	0.221743362059553	-4,1606E+14	8,61573E+14	8,66014E+13	7,91919E+14	7,51396E+14	8,23162E+14	7,0738E+14
TC0800000539.mm.2	Cenpu	centromere protein U	-0.83610897147594	4,57428E+14	-2,06157E+14	0.0817345907958714	0.221743362059553	-4,1608E+14	4,07495E+14	3,86194E+13	4,37362E+14	5,2355E+14	5,20749E+14	4,6922E+14
TC1100003609.mm.2	Ube2z	ubiquitin-conjugating enzyme E2Z	0.895927468469926	8,02535E+14	2,06144E+12	0.0817496703911705	0.221743362059553	-4,16096E+14	8,56875E+14	8,61065E+14	8,27754E+14	7,11E+14	7,8183E+14	7,76683E+13
TC1200001988.mm.2	Zfp36l1	zinc finger protein 36, C3H type-like 1	-0.626797550919864	8,54919E+14	-2,06127E+13	0.0817689079173195	0.221743362059553	-4,16117E+14	8,0562E+14	8,38412E+14	8,85455E+14	8,83936E+14	8,65063E+14	8,5103E+14
TC0500003000.mm.2	Ulk1	unc-51 like kinase 1	0.667950406881086	6,3162E+14	2,06125E+14	0.0817722248596324	0.221743362059553	-4,16121E+14	6,9547E+14	6,6976E+14	6,15398E+13	6,16242E+14	5,72835E+14	6,20015E+13
TC1700000531.mm.2	Ubash3a	ubiquitin associated and SH3 domain containing, A	-0.979329170833324	6,93653E+14	-2,06114E+14	0.0817843160753861	0.221743362059553	-4,16134E+14	6,51504E+14	6,44185E+14	6,97574E+14	7,93981E+14	6,39718E+14	7,34954E+14
TC0800001078.mm.2	Lpcat2	lysophosphatidylcholine acyltransferase 2	0.758927452416127	6,01571E+14	2,06106E+14	0.0817943507810928	0.221743362059553	-4,16144E+14	6,70563E+14	6,37278E+14	6,1178E+14	5,44276E+14	5,55766E+14	5,89764E+14

TC1100001647.mm.2	Cntd1	cyclin N-terminal domain containing 1	-0.793635569337821	5,40173E+14	-2,06095E+14	0.0818065259176411	0.221743362059553	-4,16157E+13	5,22551E+14	4,75077E+14	5,42481E+14	6,13875E+14	5,60133E+13	5,26919E+14
TC0500001910.mm.2	Gtpbp10	GTP-binding protein 10 (putative)	2,08883E+14	5,66152E+14	2,0607E+14	0.0818362281877971	0.221743362059553	-4,16189E+14	7,05028E+14	6,92752E+14	5,88069E+14	3,91946E+14	6,53534E+14	3,65581E+13
TC1200001875.mm.2	Cdkl1	cyclin-dependent kinase-like 1 (CDC2-related kinase)	1,502E+14	6,32907E+14	2,06067E+14	0.0818397160970913	0.221743362059553	-4,16193E+14	7,63219E+14	6,84333E+14	6,63766E+14	4,83386E+14	6,70203E+14	5,32535E+14
TC1500001983.mm.2	Pnpla5	patatin-like phospholipase domain containing 5	-1,04881E+14	4,92908E+14	-2,06063E+14	0.0818442100343339	0.221743362059553	-4,16198E+14	4,53277E+14	4,11761E+14	4,90867E+14	5,83934E+13	4,53318E+14	5,64292E+14
TC0200005415.mm.2	Dido1	death inducer-obliterator 1	0.680147418561472	7,82735E+14	2,06054E+14	0.0818549939508608	0.221743362059553	-4,16209E+14	8,36405E+14	8,24333E+14	7,68816E+14	7,55892E+13	7,83241E+14	7,27722E+14
TC0800003015.mm.2	Mphosph6	M phase phosphoprotein 6	0.851695579699778	6,86728E+12	2,0604E+14	0.0818703932839751	0.221747797442375	-4,16226E+14	7,35649E+14	7,4023E+14	6,61666E+14	6,43873E+14	7,21964E+14	6,16984E+14
TC0X00001832.mm.2	Dynlt3	dynein light chain Tctex-type 3	1,04355E+14	8,41703E+14	2,06019E+14	0.0818959847444574	0.221759204834755	-4,16253E+14	9,02158E+13	9,16991E+14	8,69677E+13	7,40762E+14	8,47537E+14	7,73092E+14
TC0500000349.mm.2	Ywhah	tyrosine 3-monooxygenase/tryptophan 5-monooxygenase activation protein, eta polypeptide	1,77469E+14	1,02942E+14	2,06013E+14	0.08190213045191	0.221759204834755	-4,1626E+14	1,1311E+14	1,12209E+14	1,07641E+12	8,21836E+14	1,08314E+14	9,4195E+14
TC0100003057.mm.2	Ikbke	inhibitor of kappaB kinase epsilon	-0.60790873306077	5,06183E+14	-2,05997E+14	0.0819217325157206	0.221775012773153	-4,16281E+14	4,68594E+14	4,62125E+14	5,14528E+13	5,37773E+14	5,27427E+14	5,26651E+14
TC0600001082.mm.2	Hdac11	histone deacetylase 11	0.853364758166114	8,50547E+14	2,05945E+14	0.0819813433595597	0.221806539465819	-4,16345E+14	9,32853E+14	9,03483E+14	8,23371E+14	8,42292E+14	8,51248E+14	7,50034E+14
TC0700003556.mm.2	Rlbp1	retinaldehyde binding protein 1	0.649935636914003	6,25641E+14	2,05925E+13	0.0820058864482423	0.221806539465819	-4,16371E+14	6,47994E+14	6,43595E+14	5,64435E+13	5,97167E+14	6,33718E+14	6,66934E+14
TC0400001530.mm.2	Ptp4a2	protein tyrosine phosphatase 4a2	3,45167E+13	7,98199E+14	2,05916E+14	0.082015316163789	0.221806539465819	-4,16381E+14	9,63448E+14	9,69026E+14	8,97873E+14	3,44267E+14	9,12472E+14	7,02109E+14
TC1700001824.mm.2	U2af1	U2 small nuclear ribonucleoprotein auxiliary factor (U2AF) 1	0.606422183245196	7,80779E+14	2,05904E+14	0.0820301543279768	0.221806539465819	-4,16397E+14	8,2478E+14	8,14237E+14	7,55217E+14	7,62516E+14	7,54297E+14	7,73628E+14
TC0600000648.mm.2	Chn2	chimerin 2	0.885538388813007	8,42216E+14	2,05897E+14	0.0820375135684691	0.221806539465819	-4,16405E+14	9,21245E+14	8,97444E+14	8,74051E+14	7,6753E+14	7,78768E+14	8,14254E+13
TC1300002606.mm.2	Rgs7bp	regulator of G-protein signalling 7 binding protein	1,09478E+14	9,38741E+14	2,05896E+14	0.0820387845768792	0.221806539465819	-4,16406E+14	9,8971E+14	1,01475E+14	9,60711E+14	8,24793E+14	9,61994E+14	8,80489E+14
TC1900000609.mm.2	Pi4k2a	phosphatidylinositol 4-kinase type 2 alpha	-0.645773422051702	7,32818E+14	-2,0589E+14	0.0820462199753387	0.221806539465819	-4,16414E+14	7,01414E+14	6,92236E+14	7,44474E+14	7,78331E+14	7,2605E+14	7,54407E+14
TC1200001749.mm.2	Eapp	E2F-associated phosphoprotein	0.740750170759791	6,69585E+13	2,05877E+14	0.0820608684403161	0.221806539465819	-4,1643E+14	7,24519E+14	7,3378E+14	6,57508E+14	6,5264E+14	6,63009E+14	5,86051E+14
TC1300000292.mm.2	Prl	prolactin	-0.866252052589482	4,91496E+14	-2,05866E+14	0.0820745863985222	0.221806539465819	-4,16444E+14	4,43608E+14	4,06305E+14	4,86033E+14	5,3713E+14	4,92807E+14	5,83096E+14
TC0200004125.mm.2	Zfp408	zinc finger protein 408	-0.814923184650293	6,98918E+14	-2,05856E+14	0.0820860826428379	0.221806539465819	-4,16457E+14	6,7594E+14	6,21038E+14	6,9967E+14	7,60293E+14	7,44967E+14	6,91599E+14
TC1200000600.mm.2	Actr10	ARP10 actin-related protein 10	1,15993E+14	7,54673E+14	2,05854E+14	0.0820879258707056	0.221806539465819	-4,16459E+14	8,51359E+13	8,47133E+14	8,18386E+14	6,48119E+14	6,98903E+14	6,6414E+14
TC0200001745.mm.2	Inafm2	InaF motif containing 2	0.943720950180277	7,30564E+14	2,05845E+14	0.0820985664602583	0.221806539465819	-4,1647E+14	7,63795E+14	8,14239E+14	7,47318E+14	6,41972E+14	7,34358E+14	6,81702E+14
TC0700001363.mm.2	Gdpd4	glycerophosphodiester phosphodiesterase domain containing 4	-1,10638E+14	4,43369E+14	-2,05822E+14	0.0821263622203435	0.22184443829948	-4,165E+14	3,95023E+14	3,55352E+14	4,32804E+14	5,38847E+14	4,10515E+14	5,27674E+14
TC0100000924.mm.2	Aqp12	aquaporin 12	-1,19502E+14	6,2629E+14	-2,05784E+14	0.0821708659459168	0.221903560592616	-4,16547E+14	5,64109E+14	5,467E+14	5,96871E+14	7,52941E+14	6,03575E+14	6,93541E+14
TC0X00000088.mm.2	Slc38a5	solute carrier family 38, member 5	-0.607176140844789	6,64023E+13	-2,05779E+14	0.0821757925933185	0.221903560592616	-4,16553E+13	6,39174E+14	6,34693E+14	7,00361E+14	6,94941E+13	6,45974E+14	6,68994E+14
TC0400003573.mm.2	Csmd2os	CUB and Sushi multiple domains 2, opposite strand	-0.858809678764751	6,88265E+14	-2,05747E+14	0.0822132270488129	0.221967447626931	-4,16593E+13	6,26596E+14	6,33943E+14	6,61985E+14	7,70316E+14	7,17878E+14	7,18873E+14
TC0500002628.mm.2	Clock	circadian locomotor output cycles kaput	1,02524E+14	8,70042E+14	2,05679E+14	0.0822939837821077	0.222089274069395	-4,16679E+14	9,3363E+13	9,44885E+13	8,92695E+14	7,80772E+14	8,80732E+14	7,87537E+14
TC1100001684.mm.2	BC030867	cDNA sequence BC030867	-0.865340687687712	5,301E+14	-2,05664E+14	0.082311328187946	0.222089274069395	-4,16697E+14	4,49297E+14	5,28992E+14	5,38125E+14	6,13233E+14	5,1918E+14	5,31773E+14
TC1300002279.mm.2	Ube2ql1	ubiquitin-conjugating enzyme E2Q family-like 1	0.754767176498495	8,58314E+14	2,05661E+14	0.0823149735347424	0.222089274069395	-4,16701E+14	9,00885E+14	9,05197E+14	8,60605E+14	7,94524E+14	8,68418E+14	8,20258E+14
TC1500002190.mm.2	Ddn	dendrin	0.99112714815961	8,81949E+14	2,05658E+14	0.0823181295614873	0.222089274069395	-4,16704E+14	9,36678E+14	9,57117E+14	9,09973E+14	7,85597E+13	8,80174E+14	8,22153E+14
TC1700002322.mm.2	Arrdc5	arrestin domain containing 5	-1,3431E+14	5,45992E+14	-2,05617E+14	0.0823663916868985	0.222089274069395	-4,16756E+14	4,613E+14	4,70314E+14	4,95191E+13	7,05042E+14	5,66134E+14	5,77971E+14
TC1400002534.mm.2	Pcdh9	protocadherin 9	0.801771803614678	7,07837E+14	2,05615E+14	0.0823682606504265	0.222089274069395	-4,16758E+14	7,69735E+13	7,54453E+14	7,0809E+14	6,55744E+14	7,18116E+14	6,40887E+14
TC0800000252.mm.2	Defa34	defensin, alpha, 34	-1,00496E+14	4,99943E+14	-2,05601E+14	0.0823845576377703	0.222089274069395	-4,16775E+14	4,09679E+14	4,66914E+14	4,75105E+14	6,02481E+14	5,10397E+14	5,35085E+14
TC0700002481.mm.2	Klc3	kinesin light chain 3	-0.860306224573811	6,87433E+14	-2,056E+14	0.0823865925564889	0.222089274069395	-4,16777E+14	6,28005E+14	6,40261E+14	6,85182E+14	7,55145E+13	6,66602E+14	7,49403E+14
TC0X00000666.mm.2	Gabrq	gamma-aminobutyric acid (GABA) A receptor, subunit theta	1,32955E+14	5,58249E+14	2,05595E+14	0.0823916182519224	0.222089274069395	-4,16783E+14	7,49283E+14	5,85559E+14	5,91466E+14	4,77466E+14	4,43852E+14	5,0187E+13
TC0700001766.mm.2	Rbbp6	retinoblastoma binding protein 6, ubiquitin ligase	2,84207E+14	7,49813E+14	2,05592E+14	0.0823961819888813	0.222089274069395	-4,16788E+13	9,10407E+13	8,80826E+14	8,3252E+14	3,90299E+14	8,40809E+14	6,44018E+14
TC0200003027.mm.2	Skida1	SKI/DACH domain containing 1	-0.83069704881422	5,74226E+14	-2,05553E+14	0.0824411554032559	0.222147837048554	-4,16835E+14	5,27011E+14	5,07122E+14	5,68775E+14	6,31497E+13	5,6656E+14	6,4439E+14
TC0800002589.mm.2	Nfix	nuclear factor I/X	1,02444E+14	1,01665E+14	2,05541E+14	0.0824549846657831	0.222147837048554	-4,1685E+14	1,05752E+14	1,08974E+14	1,02834E+14	9,14025E+14	1,04763E+14	9,62627E+14
TC0400004075.mm.2	Vamp3	vesicle-associated membrane protein 3	-0.723892868352065	7,60549E+14	-2,05538E+14	0.0824592697441445	0.222147837048554	-4,16855E+14	7,02383E+14	7,38713E+14	7,80976E+14	8,04898E+14	7,97502E+14	7,38822E+14
TC0800002818.mm.2	Lrrc29	leucine rich repeat containing 29	-1,2282E+14	7,60475E+14	-2,05508E+14	0.0824947037344058	0.222206145314691	-4,16892E+14	6,99656E+14	6,90992E+13	7,28332E+14	9,07956E+14	7,40664E+14	7,95252E+14
TC1200001436.mm.2	Ddx1	DEAD (Asp-Glu-Ala-Asp) box polypeptide 1	1,41795E+14	8,49498E+14	2,05476E+14	0.0825318185787627	0.222268960849006	-4,16932E+14	9,31628E+14	9,30437E+14	8,94098E+14	6,84377E+14	8,68984E+14	7,87465E+14
TC0800003193.mm.2	Irf2bp2	interferon regulatory factor 2 binding protein 2	1,15446E+14	6,48108E+14	2,05449E+14	0.0825634715128158	0.222317048209559	-4,16966E+14	7,35929E+14	7,20439E+14	6,88714E+14	5,36762E+14	6,44215E+14	5,62587E+14
TC0200001204.mm.2	Fam171b	family with sequence similarity 171, member B	2,94632E+14	8,64486E+14	2,05392E+13	0.0826310131259225	0.222461740099915	-4,17037E+14	1,03501E+14	1,04002E+14	9,72297E+14	5,13468E+14	9,17332E+14	7,08792E+14
TC1100004264.mm.2	Mgat4b/Mir6919	mannoside acetylglucosaminyltransferase 4, isoenzyme B/microRNA 6919	-0.626420369534911	7,2232E+14	-2,05318E+14	0.0827185671832351	0.222649949472798	-4,1713E+14	6,76635E+14	6,98054E+14	7,52736E+14	7,47237E+14	7,14923E+14	7,44335E+14
TC1600000171.mm.2	Bmerb1	bMERB domain containing 1	0.847551194699312	9,09031E+14	2,0529E+14	0.0827506970750452	0.222649949472798	-4,17164E+14	9,60677E+14	9,56467E+14	9,0431E+14	8,43324E+14	9,38199E+14	8,51209E+13
TC1600002060.mm.2	Synj1	synaptojanin 1	0.764496999147774	9,1943E+14	2,05286E+14	0.0827564734956854	0.222649949472798	-4,1717E+14	9,82572E+14	9,80833E+14	9,28173E+14	8,82331E+14	9,07095E+14	8,35579E+14
TC0100001987.mm.2	Msc	musculin	-0.69842757845784	5,83955E+14	-2,05281E+14	0.0827620139560204	0.222649949472798	-4,17176E+14	5,08256E+14	5,70557E+14	5,99749E+14	6,18749E+14	6,05837E+14	6,0058E+14
TC0600000454.mm.2	Gstk1	glutathione S-transferase kappa 1	1,12292E+14	6,11057E+14	2,05274E+14	0.0827700116267649	0.222649949472798	-4,17185E+14	6,9761E+14	6,5537E+14	6,29426E+14	4,98971E+14	6,40093E+14	5,44874E+14
TC0300002895.mm.2	Ugt8a	UDP galactosyltransferase 8A	1,40111E+14	8,03987E+14	2,05259E+12	0.0827883279701518	0.222662047835596	-4,17204E+14	8,90954E+13	8,84938E+14	8,43478E+13	6,52192E+14	8,3249E+14	7,19868E+14
TC0700003714.mm.2	Prss23	protease, serine 23	0.711050931642033	5,83663E+14	2,05231E+14	0.0828207986628171	0.222712204414882	-4,17238E+14	6,38996E+13	6,54761E+14	5,58388E+14	5,93159E+14	5,57331E+13	4,9934E+14
TC1700001798.mm.2	Btbd9	BTB (POZ) domain containing 9	1,77788E+14	7,39397E+14	2,05205E+14	0.0828516355381088	0.222757951514358	-4,17271E+14	8,59976E+14	8,32568E+14	8,01215E+14	5,35754E+14	7,64064E+14	6,42806E+14
TC0200002095.mm.2	Ndufaf5	NADH:ubiquinone oxidoreductase complex assembly factor 5	1,40208E+14	6,30977E+14	2,0518E+14	0.0828812651495358	0.222789854849642	-4,17302E+14	7,35762E+14	7,21949E+14	6,62405E+14	5,14889E+13	6,58325E+14	4,92532E+14
TC0600000265.mm.2	Klhdc10	kelch domain containing 10	1,05867E+14	6,7656E+14	2,05171E+14	0.0828911549570908	0.222789854849642	-4,17313E+14	7,55824E+14	7,24842E+14	6,98071E+14	5,70862E+14	6,95139E+14	6,14621E+14
TC1100000887.mm.2	Slc25a35	solute carrier family 25, member 35	-0.591956567222848	7,15935E+14	-2,05138E+14	0.082930868616637	0.222859420617057	-4,17355E+13	6,80339E+14	6,74207E+14	7,37345E+14	7,35592E+14	7,37636E+14	7,3049E+13
TC0600001268.mm.2	Slc6a1	solute carrier family 6 (neurotransmitter transporter, GABA), member 1	0.909792519637005	8,05203E+14	2,05096E+14	0.0829798302617373	0.222953811073432	-4,17407E+14	8,83863E+14	8,59711E+14	8,31811E+14	7,29805E+14	7,93434E+14	7,32594E+14
TC0600000078.mm.2	Phf14	PHD finger protein 14	1,93475E+14	7,35369E+14	2,05071E+14	0.0830099262748236	0.222962455310831	-4,17439E+14	8,65001E+14	8,54853E+14	7,72662E+14	5,60243E+14	8,00801E+14	5,58651E+14
TC1300001263.mm.2	Ankrd55	ankyrin repeat domain 55	1,21939E+14	6,6319E+14	2,0507E+14	0.0830107223614271	0.222962455310831	-4,17439E+14	7,82814E+14	6,92499E+14	6,93301E+14	5,38134E+14	6,61803E+14	6,10589E+14
TC0300001701.mm.2	Hps3	HPS3, biogenesis of lysosomal organelles complex 2 subunit 1	-0.787746884681594	7,32294E+14	-2,05001E+14	0.0830928077173668	0.223128832274581	-4,17526E+13	6,9279E+14	6,69518E+14	7,16298E+13	8,03559E+14	7,63167E+14	7,48435E+14
TC0600001030.mm.2	Dnajb8	DnaJ heat shock protein family (Hsp40) member B8	-1,11668E+14	5,90351E+14	-2,04995E+14	0.0831003613529298	0.223128832274581	-4,17534E+14	5,33376E+14	5,08713E+14	5,61962E+13	7,03463E+14	5,75715E+14	6,58878E+14
TC0900002864.mm.2	U2surp	U2 snRNP-associated SURP domain containing	1,44033E+14	7,71643E+14	2,04967E+14	0.0831333384992915	0.223156966270668	-4,17569E+14	8,92437E+13	9,00211E+14	7,96991E+14	7,07592E+14	7,72191E+14	5,60437E+14
TC0400001246.mm.2	Pomgnt1	protein O-linked mannose beta 1,2-N-acetylglucosaminyltransferase	1,13648E+14	6,79944E+14	2,04958E+14	0.0831438456523352	0.223156966270668	-4,1758E+14	7,50696E+13	7,32887E+14	7,11685E+14	5,44602E+13	6,56741E+14	6,83053E+14
TC1600001178.mm.2	Usp7	ubiquitin specific peptidase 7	2,32115E+14	7,59566E+14	2,04944E+14	0.0831601158667181	0.223156966270668	-4,17597E+14	9,37489E+14	9,3329E+14	8,49151E+14	5,57398E+13	7,71583E+14	5,08484E+14
TC0600002079.mm.2	Ptn	pleiotrophin	0.731064146994282	9,77451E+14	2,04939E+14	0.0831662373521605	0.223156966270668	-4,17604E+14	1,03083E+14	1,01963E+14	9,68771E+14	9,35474E+14	9,88596E+12	9,21406E+14
TC1500002200.mm.2	Tuba1a	tubulin, alpha 1A	1,35829E+14	1,03418E+14	2,04925E+14	0.0831834841110444	0.22316608049971	-4,17622E+14	1,13224E+14	1,09637E+14	1,08057E+14	8,76384E+14	1,0367E+14	9,82785E+14
TC1000002003.mm.2	Amd2	S-adenosylmethionine decarboxylase 2	0.957433380984506	6,97904E+14	2,04899E+14	0.0832140427028926	0.223210898963161	-4,17654E+14	7,44913E+13	7,50075E+14	7,09793E+14	5,93709E+14	7,25937E+14	6,62994E+14
TC0700000074.mm.2	Epp13	epididymal protein 13	-0.869964898039291	5,57558E+14	-2,04876E+14	0.0832412562505917	0.223246731352494	-4,17683E+14	5,15156E+14	5,012E+14	5,44692E+14	6,45657E+14	5,45955E+14	5,92685E+14
TC0900001297.mm.2	Slco2a1	solute carrier organic anion transporter family, member 2a1	-0.843042457680066	7,21125E+14	-2,04849E+14	0.0832735533153634	0.223296183153678	-4,17717E+14	6,60862E+14	6,7042E+14	7,02855E+14	7,97036E+14	7,24315E+14	7,7126E+14
TC1100000205.mm.2	Peli1	pellino 1	1,27307E+14	7,10049E+14	2,04805E+14	0.0833254171053315	0.223334835416423	-4,17771E+14	7,87796E+14	7,94364E+14	7,62384E+14	5,65162E+14	6,69442E+14	6,81146E+14
TC1500002285.mm.2	Csad	cysteine sulfinic acid decarboxylase	0.738582408506146	6,05447E+14	2,04803E+13	0.0833272401683822	0.223334835416423	-4,17773E+14	6,70017E+13	6,66114E+14	5,66717E+14	6,21698E+14	5,81776E+14	5,26362E+14
TC0900000193.mm.2	Slc44a2	solute carrier family 44, member 2	0.95922045047005	5,90765E+14	2,04794E+14	0.0833384708821358	0.223334835416423	-4,17785E+13	6,69936E+14	6,45501E+14	6,24752E+14	4,9884E+14	5,69714E+14	5,35849E+14
TC0200002879.mm.2	Cdc123	cell division cycle 123	0.860177904088195	8,97828E+14	2,0479E+14	0.0833434100017969	0.223334835416423	-4,1779E+14	9,71464E+14	9,57561E+14	9,06892E+14	8,50097E+13	8,96991E+14	8,03965E+13
TC0900002738.mm.2	Tmem30a	transmembrane protein 30A	2,21828E+13	9,73815E+14	2,0474E+14	0.08340278610097	0.223456782914022	-4,17853E+12	1,1016E+14	1,09776E+14	1,04283E+14	7,1287E+14	1,03262E+14	8,55215E+14
TC1600001334.mm.2	Lamp3	lysosomal-associated membrane protein 3	-0.903104995903501	5,29213E+14	-2,04692E+14	0.0834595851760469	0.223570411694777	-4,17912E+14	4,57733E+14	5,27951E+14	5,47239E+14	6,19066E+14	4,78748E+14	5,44538E+14
TC1400001293.mm.2	Hs6st3	heparan sulfate 6-O-sulfotransferase 3	0.844767968661903	6,39351E+14	2,0468E+14	0.0834736923566317	0.223570411694777	-4,17927E+14	6,95246E+14	6,85683E+14	6,52756E+14	5,5922E+14	6,46349E+14	5,96852E+14
TC0X00000884.mm.2	Arx	aristaless related homeobox	-0.913761829934562	7,34E+13	-2,04668E+14	0.0834878159490266	0.223570411694777	-4,17942E+14	6,93416E+12	6,9598E+14	7,25687E+14	8,46462E+13	7,10918E+12	7,31537E+13
TC0800003246.mm.2	Agrp	agouti related neuropeptide	-0.76546856107278	5,31848E+14	-2,04658E+13	0.0835006974231472	0.223570411694777	-4,17955E+14	4,91681E+14	5,0724E+14	5,50182E+13	6,01832E+14	4,86666E+14	5,53484E+14
TC0600000103.mm.2	Foxp2	forkhead box P2	2,10967E+14	6,44E+14	2,04602E+14	0.0835667712911142	0.223682999188496	-4,18025E+14	7,84045E+14	7,60133E+14	7,3298E+14	3,89265E+14	6,4183E+14	5,55748E+14
TC1600002168.mm.2	Vasn	vasorin	-0.66620775621494	7,59322E+14	-2,0459E+14	0.0835812927034566	0.223682999188496	-4,1804E+14	7,14582E+14	7,19087E+14	7,78318E+14	7,88593E+14	7,5078E+12	8,04574E+14
TC0300000407.mm.2	Trpc4	transient receptor potential cation channel, subfamily C, member 4	0.839548244830366	5,84384E+14	2,04577E+14	0.0835962336236523	0.223682999188496	-4,18056E+14	6,45621E+14	6,2629E+14	5,37506E+14	5,66495E+13	6,14764E+12	5,15626E+14
TC0700000236.mm.2	Slc1a5	solute carrier family 1 (neutral amino acid transporter), member 5	-0.616915389219063	5,24992E+14	-2,04576E+14	0.0835982759953529	0.223682999188496	-4,18058E+14	4,75377E+14	4,77407E+14	5,54107E+14	5,22061E+14	5,63921E+14	5,57077E+14
TC0300001040.mm.2	Csde1	cold shock domain containing E1, RNA binding	1,83678E+14	8,88242E+13	2,04551E+14	0.0836281586315621	0.223700140459822	-4,18089E+14	1,01226E+14	1,01233E+13	9,43024E+14	7,1421E+13	9,24724E+13	7,22905E+14
TC1100004128.mm.2	Srsf2	serine/arginine-rich splicing factor 2	1,16728E+14	8,22588E+14	2,04528E+13	0.0836554201665008	0.223700140459822	-4,18118E+14	8,81673E+14	9,00509E+14	8,42529E+14	7,06197E+14	8,57835E+14	7,46782E+14
TC0700004429.mm.2	Dhx32	DEAH (Asp-Glu-Ala-His) box polypeptide 32	1,05488E+14	7,45994E+14	2,04524E+14	0.0836595793489488	0.223700140459822	-4,18122E+14	7,82438E+14	8,18154E+13	7,69119E+14	6,20496E+14	7,35261E+14	7,50494E+14
TC0400000894.mm.2	Gm13272/Gm13285/Gm13290/Gm13289	predicted gene 13272/predicted gene 13285/predicted gene 13290/predicted gene 13289	-0.981118199504007	5,58603E+14	-2,04524E+14	0.0836602151313156	0.223700140459822	-4,18123E+14	4,85585E+13	5,04341E+14	5,36805E+14	6,49345E+14	5,49164E+14	6,26375E+14
TC1300002052.mm.2	Rab24	RAB24, member RAS oncogene family	0.829202631684931	6,92847E+14	2,04479E+14	0.0837136431923213	0.22379297337296	-4,18179E+14	7,54463E+14	7,40757E+14	6,80239E+14	6,4914E+13	7,14658E+14	6,17823E+14
TC1400001035.mm.2	Med4	mediator complex subunit 4	1,47381E+14	6,44532E+14	2,04471E+14	0.0837227110713215	0.22379297337296	-4,18188E+14	7,50626E+14	7,37069E+14	7,03557E+14	4,89376E+14	6,43644E+14	5,42921E+13
TC1200001536.mm.2	Cys1	cystin 1	-0.914433971827961	7,0691E+14	-2,04394E+14	0.0838153934306012	0.224003555207709	-4,18285E+14	6,35657E+13	6,40204E+14	6,91434E+14	7,67313E+14	7,05088E+14	8,01766E+14
TC0100000590.mm.2	Atic	5-aminoimidazole-4-carboxamide ribonucleotide formyltransferase/IMP cyclohydrolase	0.627896966650112	6,75447E+13	2,04365E+14	0.0838500068731634	0.224058898963065	-4,18321E+14	7,18588E+14	7,26945E+14	6,50673E+14	6,69281E+14	6,60234E+14	6,26964E+14
TC0X00002919.mm.2	Nap1l3	nucleosome assembly protein 1-like 3	1,23408E+14	6,45689E+14	2,04334E+14	0.0838866057211658	0.224119528765569	-4,1836E+14	7,74286E+14	7,05909E+14	6,6362E+14	5,6976E+14	6,55793E+14	5,04764E+14
TC0900000776.mm.2	Tle3	transducin-like enhancer of split 3	0.633398571345562	7,66304E+14	2,04318E+14	0.083906446249812	0.224135372749208	-4,1838E+14	7,96565E+14	8,15898E+14	7,27109E+14	7,58674E+14	7,59437E+14	7,40142E+13
TC0100002413.mm.2	Raph1	Ras association (RalGDS/AF-6) and pleckstrin homology domains 1	1,49267E+13	6,11692E+14	2,04263E+14	0.0839725071844026	0.22427465742786	-4,18449E+14	7,19777E+14	7,01592E+14	6,66619E+14	4,56216E+14	6,20991E+14	5,0496E+14
TC0700002492.mm.2	Clptm1	cleft lip and palate associated transmembrane protein 1	0.687724248050907	9,86121E+13	2,04217E+14	0.0840275845820941	0.224370461574775	-4,18507E+14	1,03517E+14	1,02475E+14	9,87698E+14	9,3468E+14	9,83404E+13	9,51019E+14
TC1500000676.mm.2	Kdelr3	KDEL (Lys-Asp-Glu-Leu) endoplasmic reticulum protein retention receptor 3	-0.629611440175355	4,91059E+14	-2,04209E+14	0.0840366345348991	0.224370461574775	-4,18516E+14	4,19565E+14	4,38443E+14	5,09095E+14	4,74835E+13	5,56622E+13	5,47791E+14
TC1000000469.mm.2	Nus1	NUS1 dehydrodolichyl diphosphate synthase subunit	1,48502E+14	8,50583E+14	2,04198E+14	0.0840501524275641	0.224370461574775	-4,1853E+14	9,29143E+14	9,22601E+14	8,89331E+14	6,65409E+14	8,90994E+14	8,0602E+14
TC1200000034.mm.2	Sf3b6	splicing factor 3B, subunit 6	0.919664238953577	8,33087E+14	2,04126E+14	0.0841359153075807	0.224497563287309	-4,1862E+14	8,85267E+14	8,91516E+14	8,47706E+14	7,45144E+14	8,49553E+14	7,79334E+14
TC0400002597.mm.2	Trmo	tRNA methyltransferase O	-1,29964E+14	5,93078E+14	-2,04117E+14	0.0841463615669116	0.224497563287309	-4,18631E+14	4,96856E+14	5,33517E+14	5,51548E+14	7,38753E+14	5,83076E+14	6,54717E+14
TC0X00000356.mm.2	Rhox7a	reproductive homeobox 7A	-0.593457639370783	9,58161E+14	-2,04099E+14	0.0841681191904039	0.224497563287309	-4,18653E+14	9,11425E+14	9,25049E+14	9,76844E+14	9,78321E+14	9,82791E+13	9,74535E+14
TC1200001339.mm.2	Rab10	RAB10, member RAS oncogene family	1,79106E+14	8,65586E+14	2,0409E+13	0.0841798939842092	0.224497563287309	-4,18666E+14	9,87075E+14	9,95473E+14	8,93926E+14	7,3041E+14	9,17441E+14	6,69193E+14
TC0500001340.mm.2	Iqcd	IQ motif containing D	-0.807679067466579	7,91276E+14	-2,04056E+14	0.0842203048428924	0.224497563287309	-4,18708E+14	7,33234E+14	7,44662E+14	7,71798E+14	8,67634E+14	8,07805E+14	8,22523E+14
TC0100000014.mm.2	Atp6v1h	ATPase, H+ transporting, lysosomal V1 subunit H	1,02898E+14	8,08635E+14	2,04042E+13	0.0842366939585443	0.224497563287309	-4,18725E+13	8,9548E+14	8,72976E+14	8,35262E+14	7,27398E+14	8,1022E+14	7,10472E+14
TC1100003961.mm.2	Cacng1	calcium channel, voltage-dependent, gamma subunit 1	-0.645399396504843	5,73817E+14	-2,0403E+14	0.0842512576710989	0.224497563287309	-4,1874E+14	5,37756E+14	5,19911E+14	5,78323E+14	6,08425E+14	6,15266E+13	5,83221E+13
TC0800001801.mm.2	Xkr5	X-linked Kx blood group related 5	-0.809352945899471	4,6145E+14	-2,04028E+14	0.0842534113742883	0.224497563287309	-4,18742E+14	4,01993E+14	4,08926E+14	4,40848E+14	5,31942E+14	4,74646E+14	5,10348E+14
TC0500002683.mm.2	Tmprss11g	transmembrane protease, serine 11g	-1,33044E+14	4,98569E+14	-2,04017E+14	0.0842667161830456	0.224497563287309	-4,18756E+14	4,09889E+14	4,19232E+14	4,63742E+13	6,31467E+14	4,70577E+13	5,96504E+14
TC1100000984.mm.2	Txndc17	thioredoxin domain containing 17	-1,50346E+14	9,60646E+14	-2,04016E+14	0.0842683177976867	0.224497563287309	-4,18758E+13	8,58291E+13	8,51135E+14	1,10341E+14	9,06708E+14	1,09373E+13	9,50603E+14
TC0100003577.mm.2	Dusp23	dual specificity phosphatase 23	-0.626683536204101	6,20058E+14	-2,04009E+14	0.0842761063109609	0.224497563287309	-4,18766E+14	5,72913E+14	5,9675E+14	6,44482E+14	6,50518E+13	6,42956E+14	6,12728E+13
TC1100003648.mm.2	Epop	elongin BC and polycomb repressive complex 2 associated protein	-0.71202292861128[illegible]	7,29753E+14	-2,04003E+14	0.0842833302123186	0.224497563287309	-4,18773E+14	6,73084E+14	6,97365E+14	7,31353E+13	7,815E+14	7,25553E+14	7,6966E+14
TC1400002160.mm.2	Phf11-ps	PHD finger protein 11, pseudogene	-0.955872094469039	4,50405E+14	-2,03994E+14	0.0842941358028363	0.224497563287309	-4,18785E+14	3,33376E+14	4,03691E+14	4,15514E+14	5,12727E+14	4,90159E+14	5,46963E+14
TC1300001006.mm.2	Hapln1	hyaluronan and proteoglycan link protein 1	0.709498803126121	6,59916E+14	2,03986E+14	0.0843041760108456	0.224497563287309	-4,18795E+13	6,84772E+13	7,16809E+13	6,56063E+13	6,03618E+14	6,66179E+14	6,32054E+14
TC0800002391.mm.2	Crtc1	CREB regulated transcription coactivator 1	1,19708E+14	8,6973E+14	2,03967E+14	0.0843271631139793	0.224497563287309	-4,18819E+14	9,50493E+14	9,51987E+14	9,2329E+14	7,39773E+14	8,27244E+13	8,25592E+14
TC0400000149.mm.2	Mmp16	matrix metallopeptidase 16	1,21177E+14	7,62677E+14	2,03956E+14	0.0843408231765961	0.224497563287309	-4,18833E+14	8,43423E+13	8,26391E+14	7,66762E+14	6,60697E+13	8,15622E+13	6,63168E+14
TC1800000852.mm.2	Pqlc1	PQ loop repeat containing 1	-1,00248E+14	7,425E+14	-2,03934E+14	0.0843667077790769	0.224497563287309	-4,1886E+14	6,93836E+14	6,75733E+14	7,22379E+14	8,47687E+14	7,244E+14	7,90967E+14

TC0200004344.mm.2	Olfr1316	olfactory receptor 1316	-1,50238E+14	4,78587E+13	-2,03927E+14	0.0843749436410258	0.224497563287309	-4,18868E+14	3,79623E+14	3,56334E+14	4,10892E+14	6,25542E+14	4,93503E+14	6,05629E+14
TC0900003122.mm.2	Rtp3	receptor transporter protein 3	-0.725156326606674	5,82774E+14	-2,03918E+14	0.0843862111755647	0.224497563287309	-4,1888E+14	5,11814E+14	5,22982E+14	5,60368E+14	6,19459E+14	6,18481E+14	6,63537E+14
TC0200001662.mm.2	Lpcat4	lysophosphatidylcholine acyltransferase 4	0.716123591518864	7,76231E+14	2,03914E+14	0.0843905576451113	0.224497563287309	-4,18885E+14	8,10797E+14	8,31481E+14	7,68193E+13	7,3086E+14	7,87281E+14	7,28776E+13
TC0300000799.mm.2	Ilf2	interleukin enhancer binding factor 2	0.615151069730793	8,2012E+14	2,03901E+14	0.0844066876839278	0.224497563287309	-4,18901E+14	8,6528E+14	8,48251E+14	8,08047E+14	7,82454E+14	8,0236E+14	8,14329E+14
TC1100003269.mm.2	Taok1	TAO kinase 1	2,01291E+14	7,62028E+14	2,03879E+14	0.0844334287586022	0.224497563287309	-4,18929E+14	8,89499E+14	9,08539E+13	8,39822E+14	5,55634E+14	7,81025E+14	5,97651E+14
TC0500003256.mm.2	Rilpl2	Rab interacting lysosomal protein-like 2	-0.6867856077312	6,06513E+14	-2,03866E+14	0.0844489515887625	0.224497563287309	-4,18945E+14	5,6717E+14	5,53346E+14	6,28979E+14	6,28894E+14	6,61114E+14	5,99574E+14
TC0700000385.mm.2	Zfp428	zinc finger protein 428	-0.830414853779843	7,12207E+14	-2,03859E+14	0.0844572352383542	0.224497563287309	-4,18954E+14	6,70674E+14	6,42063E+14	6,98545E+14	7,80274E+14	7,12447E+14	7,69237E+14
TC1200001916.mm.2	Trmt5	TRM5 tRNA methyltransferase 5	1,21135E+14	5,86299E+14	2,03858E+14	0.0844583573162302	0.224497563287309	-4,18955E+14	6,75966E+14	6,67751E+13	5,83244E+14	5,18203E+14	6,2363E+14	4,48999E+13
TC0700001729.mm.2	E130201H02Rik	Y box protein 1 pseudogene	-0.616938297440527	7,61713E+14	-2,03851E+14	0.0844662639786253	0.224497563287309	-4,18963E+14	6,95424E+14	7,24832E+14	7,65908E+14	7,77736E+13	7,96919E+14	8,0946E+14
TC1300002078.mm.2	Neurog1	neurogenin 1	-0.832245394776788	5,56153E+14	-2,03843E+14	0.0844764143293507	0.224497563287309	-4,18974E+14	4,71733E+14	5,55337E+14	5,6715E+14	6,2637E+14	5,67169E+14	5,49162E+14
TC0400002043.mm.2	Megf6	multiple EGF-like-domains 6	-0.808591173626725	6,5553E+12	-2,03833E+14	0.0844878808275454	0.224497563287309	-4,18986E+14	6,27551E+14	6,12689E+14	6,62039E+14	7,39919E+14	6,16616E+14	6,74368E+14
TC1400000843.mm.2	Mtmr6	myotubularin related protein 6	1,0368E+14	7,81739E+14	2,03815E+14	0.0845099513813985	0.224517536955724	-4,19009E+14	8,39298E+14	8,65807E+13	8,0507E+14	6,92675E+14	7,92084E+14	6,95501E+14
TC1900001073.mm.2	Uqcc3	ubiquinol-cytochrome c reductase complex assembly factor 3	0.589212656016088	6,64898E+14	2,03798E+14	0.0845304147232607	0.224517536955724	-4,1903E+14	7,0692E+14	7,16591E+14	6,54229E+14	6,5144E+14	6,27305E+14	6,32903E+14
TC0500002886.mm.2	Gm17660	predicted gene, 17660	-0.700002455622097	5,07861E+14	-2,03782E+14	0.0845501587399469	0.224517536955724	-4,1905E+14	4,62696E+14	4,5577E+14	4,98744E+14	5,59723E+13	5,49184E+14	5,21048E+14
TC0900000830.mm.2	Igdcc3	immunoglobulin superfamily, DCC subclass, member 3	-0.726462304106136	6,71692E+14	-2,03781E+13	0.0845511335100435	0.224517536955724	-4,19051E+14	6,1521E+14	6,3939E+14	7,1063E+14	6,89262E+13	6,53602E+14	7,22056E+14
TC0500000177.mm.2	Psmc2	proteasome (prosome, macropain) 26S subunit, ATPase 2	1,10415E+14	9,03491E+14	2,03731E+14	0.0846108229759299	0.22463504498851	-4,19113E+14	9,52626E+14	9,65741E+14	9,23944E+14	7,73593E+14	9,35617E+14	8,69422E+14
TC0800001798.mm.2	Defb12/Defb37	defensin beta 12/defensin beta 37	-1,23975E+14	4,3703E+14	-2,03721E+14	0.0846232683597253	0.22463504498851	-4,19126E+13	3,74614E+14	3,64575E+14	4,29662E+14	5,57477E+14	3,76206E+14	5,19644E+14
TC0100001526.mm.2	Creg1	cellular repressor of E1A-stimulated genes 1	1,31344E+13	7,33924E+14	2,03685E+14	0.084666595825308	0.22471303879644	-4,19171E+14	8,39263E+14	8,38028E+14	7,41637E+14	6,72967E+14	7,53478E+14	5,58172E+14
TC0300000801.mm.2	S100a14	S100 calcium binding protein A14	-1,01502E+14	6,07661E+14	-2,03615E+14	0.0847515079407783	0.22488902793093	-4,19259E+14	5,69166E+14	5,31015E+14	5,87209E+14	7,15975E+14	5,92549E+14	6,50053E+14
TC0100002385.mm.2	Fam126b	family with sequence similarity 126, member B	0.730703467649646	8,09584E+14	2,03607E+14	0.0847608183841952	0.22488902793093	-4,19268E+13	8,53402E+14	8,50981E+14	8,16955E+14	7,41287E+14	8,04775E+14	7,90104E+14
TC1500001578.mm.2	Tmem65	transmembrane protein 65	1,22255E+13	7,9802E+14	2,03594E+14	0.0847763344271289	0.224893163751124	-4,19284E+14	8,82196E+14	8,74646E+14	8,24081E+13	6,88251E+14	8,25143E+14	6,938E+14
TC0300000116.mm.2	Cypt12	cysteine-rich perinuclear theca 12	-0.993366282375561	5,5182E+14	-2,03475E+14	0.0849208383491084	0.225187161299687	-4,19434E+14	4,68092E+14	4,95352E+14	5,4106E+14	6,21058E+14	5,35906E+14	6,49454E+14
TC0500000274.mm.2	Ube3c	ubiquitin protein ligase E3C	1,39891E+14	7,83093E+14	2,03474E+14	0.0849218140874712	0.225187161299687	-4,19435E+14	8,64574E+14	8,48595E+14	8,18146E+13	6,15241E+13	8,22768E+14	7,29235E+14
TC0500001643.mm.2	Cops6	COP9 signalosome subunit 6	1,2878E+14	8,37247E+13	2,03468E+14	0.0849290870240303	0.225187161299687	-4,19442E+14	9,12239E+14	9,11031E+14	8,71177E+14	6,94532E+14	8,66492E+14	7,68009E+14
TC0200004711.mm.2	Mkks	McKusick-Kaufman syndrome	1,2344E+12	6,69128E+14	2,03435E+14	0.0849694805737896	0.225257196526073	-4,19484E+14	7,46541E+14	7,38002E+14	6,98908E+14	5,38754E+14	6,98754E+14	5,9381E+13
TC0400000460.mm.2	Clta	clathrin, light polypeptide (Lca)	0.834710498780815	9,38082E+14	2,0339E+14	0.0850235053545729	0.225305287336262	-4,19539E+14	9,87643E+14	9,81162E+14	9,45491E+14	8,56371E+14	9,5657E+14	9,01255E+13
TC1000001171.mm.2	Eea1	early endosome antigen 1	1,51871E+14	6,35823E+14	2,03389E+14	0.0850253767868027	0.225305287336262	-4,19541E+14	7,54206E+13	7,20735E+14	6,72167E+14	4,99031E+14	6,70996E+14	4,97801E+14
TC0400002733.mm.2	Svep1	sushi, von Willebrand factor type A, EGF and pentraxin domain containing 1	-0.796057382212047	5,10701E+14	-2,03385E+14	0.0850295694341096	0.225305287336262	-4,19546E+14	4,73392E+14	4,01946E+14	5,05296E+14	5,29253E+13	5,47975E+14	6,06343E+14
TC0400001047.mm.2	Pde4b	phosphodiesterase 4B, cAMP specific	1,06036E+14	8,2566E+14	2,03354E+14	0.0850677805060819	0.225328019564306	-4,19585E+14	9,20466E+14	9,13579E+14	8,52054E+14	7,69918E+14	8,15226E+14	6,82719E+14
TC0800002949.mm.2	Wdr59	WD repeat domain 59	0.619522680866443	7,60916E+14	2,0335E+14	0.0850717962474401	0.225328019564306	-4,19589E+14	8,15234E+14	7,91519E+14	7,46529E+14	7,36319E+14	7,50725E+13	7,25173E+14
TC0200003303.mm.2	Lcn2	lipocalin 2	1,88544E+14	6,21582E+14	2,03344E+14	0.08508010122443	0.225328019564306	-4,19598E+14	5,94814E+14	8,39366E+14	5,69216E+14	4,87875E+13	5,2755E+14	7,10671E+14
TC0700002633.mm.2	Cxcl17	chemokine (C-X-C motif) ligand 17	-0.769954800311239	4,83716E+14	-2,03314E+14	0.0851162446739587	0.225341950444779	-4,19635E+14	4,67831E+14	4,10485E+14	5,03899E+14	5,28407E+14	5,27215E+14	4,6446E+13
TC0100001520.mm.2	Mpc2	mitochondrial pyruvate carrier 2	-0.65986926397928	8,51389E+14	-2,03308E+14	0.0851235619322684	0.225341950444779	-4,19643E+14	8,04554E+14	8,0332E+14	8,46973E+14	8,92875E+14	8,6597E+14	8,94644E+14
TC0600000091.mm.2	Tmem106b	transmembrane protein 106B	1,95754E+14	8,29584E+14	2,03305E+14	0.0851273165988563	0.225341950444779	-4,19646E+14	9,45233E+14	9,48564E+14	9,0242E+14	5,99869E+14	8,56037E+14	7,25379E+14
TC1100000268.mm.2	Gm12070	glyceraldehyde-3-phosphate dehydrogenase pseudogene	1,2769E+14	1,01086E+14	2,03288E+14	0.0851479328066085	0.225359500872681	-4,19668E+14	1,10818E+12	1,07494E+13	1,05986E+14	8,67887E+14	1,00043E+14	9,53854E+14
TC0400000413.mm.2	Gm2506/Gm13306/Ccl27b/LOC100861978	predicted gene 2506/predicted gene 13306/chemokine (C-C motif) ligand 27b/c-C motif chemokine 27-like	0.955582658424479	9,54728E+14	2,03259E+14	0.0851831541719922	0.225415694395354	-4,19704E+13	1,00568E+14	1,01305E+14	9,69045E+14	8,5857E+13	9,7826E+14	9,03763E+13
TC0100000399.mm.2	Mars2	methionine-tRNA synthetase 2 (mitochondrial)	0.727522463090071	5,89564E+14	2,03238E+14	0.0852088175085309	0.225446580708532	-4,1973E+14	6,56759E+14	6,46165E+14	6,1652E+13	5,40898E+14	5,35705E+14	5,41338E+14
TC0900002601.mm.2	Lipc	lipase, hepatic	-0.814712458514595	5,65607E+13	-2,0321E+14	0.0852426096763276	0.225468563296522	-4,19765E+14	5,40212E+14	4,716E+14	5,54478E+14	6,20276E+14	5,87584E+14	6,19493E+14
TC0100003604.mm.2	Rgs7	regulator of G protein signaling 7	1,68196E+13	7,80012E+14	2,03188E+14	0.0852696908063781	0.225468563296522	-4,19793E+14	8,88377E+14	8,8073E+14	8,16143E+14	6,16573E+13	8,36111E+14	6,42137E+14
TC1700002090.mm.2	Olfr137	olfactory receptor 137	-0.621065178835166	4,77562E+14	-2,03186E+14	0.0852719509725945	0.225468563296522	-4,19795E+14	4,32613E+14	4,19411E+14	4,70769E+14	5,05468E+13	5,16308E+14	5,20806E+14
TC0500003384.mm.2	Cldn4	claudin 4	-0.832945878514316	5,05527E+14	-2,03185E+14	0.0852730977924038	0.225468563296522	-4,19796E+14	4,54922E+14	4,8267E+14	5,14684E+14	5,89497E+13	4,61674E+14	5,29714E+14
TC0200000222.mm.2	Il15ra	interleukin 15 receptor, alpha chain	-0.63204117587926	6,29392E+14	-2,03173E+14	0.0852877126752966	0.225470207438401	-4,19811E+14	6,15437E+13	5,97888E+14	6,51554E+14	6,88179E+13	6,04953E+14	6,18341E+14
TC1100001907.mm.2	Tsen54	tRNA splicing endonuclease subunit 54	-0.666665776659399	6,22813E+14	-2,03124E+14	0.08534703488098	0.225552994744946	-4,19872E+13	5,56145E+14	5,829E+14	6,15778E+14	6,56601E+14	6,48114E+14	6,77336E+14
TC0900000735.mm.2	Insyn1	inhibitory synaptic factor 1	0.786923316282098	7,67003E+14	2,03117E+14	0.0853553059504829	0.225552994744946	-4,19881E+14	7,96006E+14	8,11297E+14	7,70896E+14	6,79022E+14	7,74131E+14	7,70669E+14
TC0400001922.mm.2	Nppa	natriuretic peptide type A	-0.762234585457276	5,33157E+14	-2,03112E+14	0.0853610228979507	0.225552994744946	-4,19887E+14	4,94211E+14	4,67636E+14	5,39717E+14	5,74577E+14	5,24072E+14	5,98729E+14
TC0200000083.mm.2	Itih5	inter-alpha (globulin) inhibitor H5	0.719163656347532	6,4035E+14	2,03097E+14	0.0853797799724525	0.225565567256292	-4,19906E+14	6,88889E+14	6,93754E+14	5,83091E+14	6,55719E+14	6,17568E+14	6,03078E+14
TC1500002187.mm.2	Arf3	ADP-ribosylation factor 3	0.599867361412331	9,95705E+14	2,03038E+14	0.0854515471366648	0.225686986194222	-4,19979E+14	1,02911E+14	1,03929E+14	9,82398E+14	9,66024E+14	9,86403E+14	9,71011E+14
TC0400002077.mm.2	Gnb1	guanine nucleotide binding protein (G protein), beta 1	1,18309E+14	1,08584E+14	2,03031E+13	0.0854607323779498	0.225686986194222	-4,19989E+14	1,16877E+14	1,1481E+14	1,11347E+14	9,66775E+14	1,11166E+14	1,00627E+14
TC0400002027.mm.2	Gm833	predicted gene 833	-0.892989110160481	5,37057E+14	-2,03025E+14	0.0854677583167095	0.225686986194222	-4,19996E+13	4,62823E+13	5,06384E+14	5,183E+14	6,29505E+14	5,52659E+14	5,5267E+13
TC0500003711.mm.2	D830014E11Rik	RIKEN cDNA D830014E11 gene	-1,17545E+14	4,47737E+14	-2,02967E+14	0.0855379476674281	0.225743215558145	-4,20068E+14	3,79643E+14	3,84998E+14	4,52692E+14	5,47039E+14	3,84233E+14	5,37818E+14
TC0300001902.mm.2	Mfsd8	major facilitator superfamily domain containing 8	1,39906E+14	5,79973E+14	2,02962E+13	0.0855439287980419	0.225743215558145	-4,20074E+14	6,57673E+14	6,52243E+14	6,04112E+12	4,25991E+14	6,38354E+14	5,01467E+14
TC1600001735.mm.2	Zpld1	zona pellucida like domain containing 1	-0.622760865703705	5,21029E+14	-2,02962E+14	0.08554406019666	0.225743215558145	-4,20074E+14	4,98011E+14	4,88242E+14	5,43309E+14	5,67497E+14	5,2879E+14	5,00323E+14
TC1100001497.mm.2	Hoxb5	homeobox B5	-1,18274E+14	5,39353E+14	-2,02961E+14	0.0855450924221458	0.225743215558145	-4,20075E+14	4,46977E+14	4,61601E+14	4,88324E+14	6,56803E+13	5,57819E+14	6,24594E+14
TC1200001567.mm.2	Colec11	collectin sub-family member 11	-1,14141E+14	6,22118E+14	-2,02935E+14	0.0855773297709654	0.225756611485353	-4,20108E+14	5,4741E+14	5,49855E+14	5,7632E+14	7,49226E+13	6,48494E+14	6,61402E+14
TC0500000527.mm.2	Sod3	superoxide dismutase 3, extracellular	0.949017941677965	5,35847E+14	2,02934E+14	0.0855781904643787	0.225756611485353	-4,20109E+14	6,07007E+14	5,83945E+14	4,51274E+14	5,49874E+14	5,54082E+14	4,68904E+14
TC0500000174.mm.2	Armc10	armadillo repeat containing 10	0.934446183268917	5,8079E+14	2,02886E+14	0.0856365838608085	0.22584759081895	-4,20169E+12	6,21082E+14	6,44089E+14	5,20752E+13	5,5753E+14	5,09851E+14	6,31437E+14
TC0700000439.mm.2	Cyp2a4	cytochrome P450, family 2, subfamily a, polypeptide 4	-0.959685028473241	4,77876E+14	-2,02864E+14	0.0856635616784183	0.22584759081895	-4,20196E+14	3,90919E+14	4,21663E+14	4,67225E+14	5,37294E+14	4,69601E+14	5,80552E+14
TC1200000007.mm.2	Kif3c	kinesin family member 3C	0.760993003362431	8,30554E+14	2,0285E+14	0.085681250454233	0.22584759081895	-4,20215E+14	8,83975E+14	8,7417E+14	8,42372E+14	7,63575E+14	8,26774E+14	7,92455E+14
TC0100002651.mm.2	Cul3	cullin 3	2,05878E+14	7,28049E+14	2,02849E+14	0.0856824682567883	0.22584759081895	-4,20216E+14	8,61971E+14	8,67818E+13	7,86029E+14	5,32004E+14	7,79925E+14	5,40545E+14
TC0Y00000561.mm.2	Gm21693/Gm21677/Gm21704	predicted gene, 21693/predicted gene, 21677/predicted gene, 21704	-0.76235521560469	4,6099E+14	-2,02846E+13	0.0856866321079253	0.22584759081895	-4,2022E+14	3,89495E+14	4,06327E+13	4,45694E+13	5,026E+14	5,42642E+14	4,79184E+14
TC0200005183.mm.2	Kcnb1	potassium voltage gated channel, Shab-related subfamily, member 1	-0.632889954177419	7,76857E+14	-2,02836E+14	0.0856985065948788	0.22584759081895	-4,20232E+14	7,22399E+14	7,4838E+14	7,89796E+14	8,07561E+14	7,81395E+14	8,1161E+14
TC0X00001288.mm.2	Armcx3	armadillo repeat containing, X-linked 3	1,81007E+14	6,5434E+14	2,02826E+14	0.0857107936360626	0.22584759081895	-4,20245E+14	7,68151E+14	7,77053E+14	6,99151E+14	4,84039E+14	7,0543E+14	4,92214E+14
TC1700001604.mm.2	Flywch1	FLYWCH-type zinc finger 1	0.651032554399445	7,94409E+14	2,028E+14	0.0857417521176491	0.225892225616691	-4,20276E+14	8,63577E+14	8,35702E+14	8,04716E+13	7,64357E+14	7,56642E+13	7,41459E+14
TC1900000030.mm.2	Coro1b	coronin, actin binding protein 1B	0.675106006571525	6,89053E+14	2,02782E+14	0.0857638249865172	0.225913439922797	-4,20299E+14	7,40762E+14	7,24116E+14	6,75335E+14	6,54521E+14	6,42741E+13	6,96842E+13
TC1500002341.mm.2	9130401M01Rik	RIKEN cDNA 9130401M01 gene	0.958543673166564	6,58969E+14	2,02745E+14	0.0858091166037059	0.225928688512645	-4,20345E+14	7,06256E+14	7,04318E+14	6,76573E+14	5,42293E+14	6,57305E+14	6,67068E+14
TC1600001301.mm.2	Ess2	ess-2 splicing factor	0.671644938611075	7,21133E+14	2,02743E+14	0.0858125271557837	0.225928688512645	-4,20349E+14	7,7558E+14	7,60312E+14	7,20307E+14	6,81256E+14	7,1552E+14	6,7382E+14
TC1400002392.mm.2	Itm2b	integral membrane protein 2B	1,14088E+14	1,07356E+14	2,02732E+14	0.0858248685358451	0.225928688512645	-4,20361E+14	1,1324E+14	1,14294E+14	1,101E+14	9,46168E+14	1,09824E+14	1,0206E+14
TC0600002683.mm.2	Sec61a1	Sec61 alpha 1 subunit (S. cerevisiae)	0.9880328632941	8,39149E+14	2,02732E+14	0.0858256998936202	0.225928688512645	-4,20362E+14	9,03259E+14	8,9079E+14	8,68348E+14	7,28095E+14	8,27299E+14	8,17104E+14
TC0500002325.mm.2	Tapt1	transmembrane anterior posterior transformation 1	1,03365E+14	7,12937E+14	2,02714E+14	0.0858476159167336	0.22594946672106	-4,20384E+14	7,94925E+14	7,96318E+13	7,55056E+14	6,29457E+14	6,92901E+14	6,08963E+14
TC0500002154.mm.2	Hadha	hydroxyacyl-Coenzyme A dehydrogenase/3-ketoacyl-Coenzyme A thiolase/enoyl-Coenzyme A hydratase (trifunctional protein), alpha subunit	1,04981E+14	7,39158E+14	2,02686E+13	0.0858822476654512	0.226003700250435	-4,2042E+14	8,25524E+14	8,20592E+14	7,90611E+14	6,45543E+14	7,02873E+14	6,49805E+14
TC1400002469.mm.2	Tnfsf11	tumor necrosis factor (ligand) superfamily, member 11	-0.633446048725632	5,44518E+14	-2,02664E+14	0.085908469622789	0.22600793911509	-4,20446E+14	4,8163E+14	5,03374E+14	5,47155E+14	5,64539E+14	5,68089E+14	6,02321E+14
TC0100003806.mm.2	Ppp2r5a	protein phosphatase 2, regulatory subunit B', alpha	1,40729E+14	7,96928E+14	2,02661E+14	0.0859119113188064	0.22600793911509	-4,2045E+14	8,79111E+14	8,96357E+13	8,3392E+14	6,60091E+14	8,28304E+14	6,83787E+14
TC0400003382.mm.2	Artn	artemin	-1,27665E+14	5,30946E+13	-2,02601E+13	0.0859864567848947	0.226156928812793	-4,20526E+14	4,46473E+13	4,5582E+14	5,0959E+14	6,48033E+14	4,90083E+14	6,35674E+14
TC0700003829.mm.2	P2ry6	pyrimidinergic receptor P2Y, G-protein coupled, 6	-0.693540249172164	6,76859E+14	-2,02592E+14	0.0859966178139382	0.226156928812793	-4,20536E+13	6,24757E+14	6,48412E+14	6,74786E+14	7,37091E+14	6,89864E+14	6,86244E+14
TC1300002119.mm.2	Golm1	golgi membrane protein 1	0.720605481996882	5,9242E+14	2,02567E+14	0.0860278284745508	0.226196873367327	-4,20568E+14	6,41681E+14	6,80253E+14	6,16023E+14	5,6179E+14	5,44913E+14	5,09857E+14
TC1400001218.mm.2	Frg2f6	FSHD region gene 2 family member 6	-0.902799805894938	6,52075E+14	-2,02557E+14	0.0860398831296685	0.226196873367327	-4,2058E+14	5,98185E+14	5,94763E+14	6,33074E+14	7,40433E+14	6,43869E+14	7,02123E+14
TC1200001981.mm.2	Rdh11	retinol dehydrogenase 11	0.688272474730474	6,38732E+14	2,02532E+14	0.0860710645831132	0.22624193533894	-4,20612E+14	6,81649E+13	6,8077E+14	6,31998E+14	5,92766E+14	5,96644E+14	6,48562E+13
TC1000001621.mm.2	Mmp19	matrix metallopeptidase 19	-0.802140904068448	6,5703E+14	-2,02509E+14	0.0860983429004919	0.226264200070182	-4,2064E+13	5,99486E+14	5,9589E+14	6,53762E+14	7,02042E+14	6,53024E+14	7,37978E+14
TC0900003051.mm.2	Apeh	acylpeptide hydrolase	1,01193E+13	6,10771E+14	2,02495E+14	0.0861156673769825	0.226264200070182	-4,20657E+14	7,10059E+14	6,51245E+13	5,95625E+14	5,63293E+14	6,37386E+14	5,07016E+13
TC1600000502.mm.2	Wdr5b	WD repeat domain 5B	0.811328042627802	5,40808E+14	2,0249E+14	0.0861227377331579	0.226264200070182	-4,20665E+14	5,69665E+14	5,90156E+14	5,31793E+14	4,65763E+14	5,07507E+14	5,79965E+14
TC1200001993.mm.2	Dcaf5	DDB1 and CUL4 associated factor 5	1,52438E+13	6,85408E+14	2,02479E+13	0.0861357042903555	0.226264200070182	-4,20678E+14	7,8498E+14	7,57432E+14	7,38594E+14	4,98943E+14	6,9822E+13	6,34281E+14
TC0900002175.mm.2	Arcn1	archain 1	1,38748E+14	7,90776E+14	2,02448E+14	0.0861737877132575	0.226327341715357	-4,20716E+14	8,7031E+14	8,62851E+14	8,25807E+14	6,29858E+14	8,31164E+13	7,24667E+14
TC1100002449.mm.2	Erlec1	endoplasmic reticulum lectin 1	1,85153E+14	7,84725E+14	2,02348E+13	0.0862971064124967	0.226614288726297	-4,20842E+14	9,1849E+14	9,06966E+14	8,47935E+14	6,07215E+14	8,12599E+14	6,15145E+14
TC1200001597.mm.2	Bcap29	B cell receptor associated protein 29	1,53758E+14	7,33387E+14	2,02259E+14	0.0864063937070765	0.226831228716958	-4,20952E+14	8,3918E+14	8,29325E+14	7,90393E+14	5,70596E+14	7,47264E+14	6,23564E+14
TC0700000835.mm.2	Tead2	TEA domain family member 2	-0.814891680171138	5,94662E+14	-2,02252E+14	0.08641435862534 6	0.226831228716958	-4,20961E+14	5,41004E+14	5,40127E+14	5,78597E+14	6,65512E+14	5,95358E+14	6,47374E+14
TC0100002349.mm.2	Pgap1	post-GPI attachment to proteins 1	1,60831E+14	7,06382E+14	2,02246E+14	0.0864219520321112	0.226831228716958	-4,20968E+14	8,21512E+14	7,96559E+14	7,62657E+14	5,33752E+14	7,28415E+14	5,95394E+14
TC0700003032.mm.2	Ush1c	USH1 protein network component harmonin	-0.792730527093857	6,53783E+14	-2,02224E+14	0.0864494480288086	0.226840097536908	-4,20996E+13	6,03594E+14	5,63098E+14	6,27598E+14	6,9764E+14	6,89886E+13	7,40879E+14
TC0200005225.mm.2	Zfp64/Gm38422	zinc finger protein 64/predicted gene, 38422	-0.616212778348872	8,11431E+14	-2,02214E+14	0.0864622628189312	0.226840097536908	-4,21009E+14	7,93101E+14	7,67251E+14	8,30195E+13	8,534E+14	8,17768E+14	8,06869E+14
TC1100004061.mm.2	Nat9	N-acetyltransferase 9 (GCN5-related, putative)	0.884502990441033	5,49061E+14	2,02206E+13	0.0864722746758063	0.226840097536908	-4,21019E+14	6,17051E+14	5,65422E+14	4,51015E+14	5,54558E+14	5,65455E+14	5,40864E+14
TC0100001999.mm.2	Stau2	staufen double-stranded RNA binding protein 2	1,42671E+14	9,47684E+13	2,02198E+14	0.0864816433419738	0.226840097536908	-4,21029E+14	1,03781E+14	1,02749E+14	9,94037E+14	7,8592E+13	9,70415E+14	8,70433E+14
TC1400000241.mm.2	Zmiz1	zinc finger, MIZ-type containing 1	0.60757558685836	7,60709E+14	2,02168E+14	0.0865187760174639	0.226882998418839	-4,21066E+14	7,96016E+14	7,92724E+14	7,49059E+14	7,18166E+14	7,51269E+14	7,57018E+14
TC1300001672.mm.2	Mrs2	MRS2 magnesium transporter	0.929558253106324	7,06146E+14	2,02162E+14	0.0865261605712011	0.226882998418839	-4,21074E+12	7,69496E+14	7,23268E+14	6,98088E+14	6,08765E+14	7,58814E+14	6,78448E+14
TC0300000182.mm.2	Rpl22l1	ribosomal protein L22 like 1	1,13372E+14	7,06097E+14	2,02114E+14	0.0865851094529082	0.227000629452442	-4,21133E+14	7,92282E+14	7,69632E+12	7,51669E+14	5,83501E+13	6,86001E+14	6,53497E+14
TC1600000013.mm.2	Olfr15	olfactory receptor 15	-1,13376E+13	4,88598E+13	-2,02092E+14	0.0866129415796127	0.227012207320652	-4,21162E+14	3,61147E+14	4,76479E+14	4,67886E+14	5,96491E+14	5,1706E+14	5,12523E+13
TC0700004388.mm.2	Nsmce4a	NSE4 homolog A, SMC5-SMC6 complex component	0.741620581204715	6,87097E+14	2,02088E+14	0.0866177031345728	0.227012207320652	-4,21166E+14	7,56286E+14	7,34918E+14	7,07768E+14	6,35112E+14	6,28696E+14	6,598E+14
TC1800001541.mm.2	Poli	polymerase (DNA directed), iota	0.892153124822003	6,58606E+14	2,02065E+14	0.0866452174630206	0.227016577904752	-4,21194E+14	7,31197E+14	7,02577E+14	6,69807E+14	5,85537E+14	6,71672E+14	5,90844E+14
TC1700001138.mm.2	Wdr43	WD repeat domain 43	1,44467E+13	6,86593E+14	2,02059E+14	0.0866527489608799	0.227016577904752	-4,21202E+14	7,85372E+14	7,69578E+14	7,24994E+14	5,4102E+13	7,21809E+13	5,76784E+13
TC1400000111.mm.2	Olfr31	olfactory receptor 31	-1,04117E+14	4,70862E+14	-2,02052E+14	0.086661637850936	0.227016577904752	-4,21211E+14	3,7423E+14	4,03839E+14	4,19067E+14	5,67237E+14	5,17145E+14	5,43652E+14
TC0800000111.mm.2	Atp11a	ATPase, class VI, type 11A	-0.651550743444266	7,8206E+14	-2,02033E+14	0.0866856695743165	0.227032231623658	-4,21235E+14	7,26999E+14	7,56631E+14	7,85384E+14	8,28556E+14	7,95354E+14	7,99439E+14
TC1300002236.mm.2	Uqcrb	ubiquinol-cytochrome c reductase binding protein	1,29558E+14	1,01037E+14	2,02018E+14	0.0867042549391118	0.227032231623658	-4,21254E+14	1,08264E+14	1,07259E+14	1,02973E+13	8,66386E+13	1,0677E+13	9,43207E+14
TC1400001731.mm.2	Opn4	opsin 4 (melanopsin)	-0.609820110934719	4,98946E+14	-2,02013E+14	0.0867098835357782	0.227032231623658	-4,21259E+13	4,66317E+14	4,575E+14	5,07916E+13	5,37865E+14	5,0639E+14	5,17684E+14
TC0900002359.mm.2	Snx33	sorting nexin 33	0.711077244041205	5,50321E+14	2,01993E+14	0.0867345641245233	0.22704790604095	-4,21284E+14	5,63308E+14	5,99029E+14	5,09786E+13	5,10335E+14	5,27937E+14	5,91532E+14
TC0800001357.mm.2	Mon1b/Syce1l	MON1 homolog B, secretory traffciking associated/synaptonemal complex central element protein 1 like	-0.621984731101914	8,12139E+14	-2,01954E+14	0.0867829638059571	0.22704790604095	-4,21333E+14	7,65458E+14	7,84788E+14	8,20085E+14	8,54557E+14	8,2176E+14	8,26188E+14
TC0800001245.mm.2	Slc7a6	solute carrier family 7 (cationic amino acid transporter, y+ system), member 6	-0.663731863897679	6,14238E+14	-2,0195E+14	0.0867884914841369	0.22704790604095	-4,21339E+13	5,83592E+14	6,09567E+14	6,40551E+14	6,85355E+14	5,95353E+14	5,71007E+14
TC1700001989.mm.2	Abcf1	ATP-binding cassette, sub-family F (GCN20), member 1	0.700845585532353	7,07966E+14	2,01947E+14	0.0867921810385295	0.22704790604095	-4,21342E+14	7,52776E+14	7,62852E+14	6,89161E+14	6,86298E+14	6,43849E+14	7,1286E+14
TC1100004123.mm.2	Cygb	cytoglobin	0.967509509811155	8,06801E+14	2,01946E+14	0.0867932908859746	0.22704790604095	-4,21344E+14	8,83903E+14	8,32866E+14	8,23471E+14	6,99796E+14	8,16218E+14	7,84555E+14
TC1900000386.mm.2	Apba1	amyloid beta (A4) precursor protein binding, family A, member 1	0.753456125538674	8,0958E+14	2,01935E+14	0.086806714013429	0.22704790604095	-4,21357E+14	8,7272E+14	8,62941E+14	8,29836E+14	7,55133E+13	7,88494E+14	7,48353E+14

TC0100000247.mm.2	Cnnm3	cyclin M3	0.606505779820973	5,51766E+14	2,01929E+14	0.0868145068651583	0.22704790604095	-4,21365E+14	5,82902E+14	5,75861E+14	5,06883E+14	5,30579E+14	5,47519E+14	5,66853E+14
TC0900000849.mm.2	Oaz2	ornithine decarboxylase antizyme 2	1,09393E+14	7,57532E+13	2,01901E+14	0.0868483255171421	0.227099491895117	-4,21399E+14	8,5292E+14	8,27452E+14	7,84081E+14	6,77507E+14	7,63122E+14	6,40112E+14
TC1000000753.mm.2	Pofut2	protein O-fucosyltransferase 2	0.669107581844813	6,82204E+14	2,01883E+14	0.0868714669248176	0.227116504883168	-4,21422E+13	7,48303E+14	6,93583E+14	6,57248E+14	6,50817E+14	6,60745E+14	6,82529E+14
TC1500000588.mm.2	Maf1	MAF1 homolog, negative regulator of RNA polymerase III	-0.778900623859258	8,62295E+14	-2,01869E+14	0.086887722947392	0.227116504883168	-4,21439E+14	8,14688E+14	8,12818E+14	9,36519E+14	8,46768E+14	8,92336E+14	8,70641E+14
TC1700000398.mm.2	D630044L22Rik	RIKEN cDNA gene D630044L22 gene	-1,05418E+14	5,43082E+13	-2,01862E+14	0.0868971173961853	0.227116504883168	-4,21448E+14	4,68645E+14	4,79226E+14	5,08413E+14	6,50293E+13	5,43492E+14	6,08422E+14
TC1900001464.mm.2	Rbp4	retinol binding protein 4, plasma	0.881516644197646	7,36263E+14	2,0185E+14	0.0869114519403653	0.227117130248963	-4,21463E+14	7,50287E+14	8,08296E+14	7,32638E+14	6,49642E+14	7,08116E+14	7,68599E+14
TC0300003196.mm.2	Srsf11	serine/arginine-rich splicing factor 11	1,75905E+14	7,41228E+13	2,01823E+14	0.0869452952785632	0.227162188959262	-4,21497E+14	8,38894E+14	8,67993E+14	7,90308E+14	5,64768E+14	7,86736E+13	5,98669E+14
TC1900000401.mm.2	Ndufb4b	NADH:ubiquinone oxidoreductase subunit B4B	0.870632759293843	9,52637E+14	2,01814E+14	0.086956890802503	0.227162188959262	-4,21508E+14	1,0171E+14	1,00275E+14	9,57935E+14	8,8779E+13	9,7038E+14	8,79869E+14
TC0X00000577.mm.2	4931400O07Rik	RIKEN cDNA 4931400O07 gene	-0.677145598607512	5,26253E+14	-2,01734E+14	0.0870557915506889	0.227381482307014	-4,21608E+13	4,57911E+14	4,98212E+14	5,39598E+14	5,51954E+14	5,32029E+14	5,77812E+14
TC0100000524.mm.2	Adam23	a disintegrin and metallopeptidase domain 23	1,4362E+14	8,05979E+14	2,01713E+14	0.0870815898423406	0.227381482307014	-4,21634E+14	9,04361E+14	9,01463E+14	8,63571E+13	6,55013E+14	8,0836E+14	7,03109E+14
TC0400004163.mm.2	B3galt6	UDP-Gal:betaGal beta 1,3-galactosyltransferase, polypeptide 6	0.693655779605564	6,5623E+14	2,01712E+14	0.0870831705651699	0.227381482307014	-4,21635E+14	7,2652E+14	6,76651E+14	6,09734E+14	6,54706E+14	6,30353E+14	6,39414E+14
TC0200004224.mm.2	Ehf	ets homologous factor	-1,05171E+14	4,81985E+14	-2,01677E+14	0.0871263412706575	0.227424325801867	-4,21679E+14	4,20146E+13	4,13453E+13	4,68631E+13	5,75309E+14	4,50326E+14	5,64047E+14
TC0200004028.mm.2	Olfr1200	olfactory receptor 1200	-0.615833603531161	4,63985E+14	-2,01656E+14	0.0871524757746228	0.227424325801867	-4,21705E+14	4,11152E+14	4,00392E+14	4,85026E+14	4,49685E+14	5,20564E+14	5,17091E+14
TC0900000533.mm.2	Jaml	junction adhesion molecule like	-0.691289106615154	4,83357E+14	-2,01641E+13	0.0871706565564133	0.227424325801867	-4,21723E+14	4,38538E+14	4,4225E+14	4,76749E+14	5,42297E+14	4,93296E+14	5,07012E+14
TC0800002870.mm.2	Tmed6	transmembrane p24 trafficking protein 6	-0.978651944733917	5,27304E+14	-2,01614E+14	0.087204415171741	0.227424325801867	-4,21757E+14	4,69139E+14	4,62363E+14	5,01035E+14	6,26198E+14	5,21022E+14	5,84066E+14
TC0700003915.mm.2	Olfr66	olfactory receptor 66	-1,14383E+14	5,29498E+14	-2,01612E+14	0.0872069786742191	0.227424325801867	-4,2176E+14	4,1326E+14	4,83659E+14	4,92586E+14	6,33098E+14	5,38205E+14	6,1618E+14
TC0700001606.mm.2	Eif3f	eukaryotic translation initiation factor 3, subunit F	0.977802164452969	8,48304E+14	2,01598E+14	0.0872252588902002	0.227424325801867	-4,21778E+14	9,17588E+14	9,05191E+14	8,39854E+14	7,87364E+14	8,84584E+14	7,55241E+14
TC1700002773.mm.2	Rnf8	ring finger protein 8	1,28077E+14	6,13984E+14	2,01595E+14	0.0872282759724006	0.227424325801867	-4,21781E+14	6,92978E+14	6,57151E+14	6,39776E+14	4,54199E+14	6,57223E+14	5,82575E+13
TC1600001849.mm.2	Cadm2	cell adhesion molecule 2	1,08646E+14	8,27127E+14	2,01588E+14	0.087237037145876	0.227424325801867	-4,2179E+14	8,95557E+14	9,1859E+14	8,13444E+14	7,83411E+14	8,55421E+14	6,96337E+14
TC0200002796.mm.2	Ogfr	opioid growth factor receptor	0.950898054092629	7,74487E+14	2,01576E+14	0.0872521938487913	0.227424325801867	-4,21805E+13	8,27934E+14	8,44245E+14	7,79101E+14	7,02898E+14	8,00984E+14	6,91761E+14
TC0100000787.mm.2	Tex44	testis expressed 44	-0.842329095805385	4,79825E+14	-2,01574E+14	0.0872544761972979	0.227424325801867	-4,21807E+14	4,15967E+14	4,37608E+14	4,77247E+14	5,44793E+14	4,60407E+14	5,42929E+14
TC0900000885.mm.2	C2cd4b	C2 calcium-dependent domain containing 4B	-1,02448E+14	6,08688E+14	-2,01574E+14	0.0872550826770695	0.227424325801867	-4,21808E+14	5,46942E+14	5,47018E+14	5,7592E+13	7,22937E+14	6,13922E+14	6,45389E+14
TC1500000804.mm.2	Fbln1	fibulin 1	0.807454175393914	5,80295E+14	2,0156E+14	0.0872723256078062	0.227424325801867	-4,21825E+14	6,31928E+14	6,16091E+14	5,62411E+14	5,24118E+14	5,28045E+14	6,19178E+14
TC0400000873.mm.2	Acer2	alkaline ceramidase 2	-0.802172138494944	4,79238E+14	-2,01551E+14	0.0872830652739245	0.227424325801867	-4,21836E+14	4,12355E+14	4,27067E+14	4,56445E+14	5,43411E+14	5,403E+14	4,95852E+14
TC0800001977.mm.2	Mak16	MAK16 homolog	0.674524496907317	6,04863E+14	2,01528E+14	0.0873113296067745	0.227439564964634	-4,21864E+14	6,70986E+14	6,9416E+14	5,93791E+14	6,3645E+14	5,35826E+14	4,97964E+14
TC1800000593.mm.2	Adamts19	a disintegrin-like and metallopeptidase (reprolysin type) with thrombospondin type 1 motif, 19	-1,00239E+14	5,9106E+14	-2,01518E+14	0.0873247800102693	0.227439564964634	-4,21878E+13	5,23662E+14	5,42893E+14	5,96848E+14	6,70184E+14	5,44223E+14	6,68547E+14
TC1000001148.mm.2	Ndufa12	NADH:ubiquinone oxidoreductase subunit A12	0.727118063143408	9,66151E+14	2,01512E+14	0.0873312597552406	0.227439564964634	-4,21884E+14	1,01385E+14	1,01629E+14	9,68348E+14	9,16365E+13	9,70265E+14	9,1179E+14
TC0700004081.mm.2	Nrip3	nuclear receptor interacting protein 3	0.820397818059875	8,08896E+14	2,015E+14	0.0873470195738796	0.227443847187124	-4,219E+14	8,53531E+14	8,70335E+14	8,15376E+14	7,4441E+14	8,2155E+14	7,48176E+14
TC0200001654.mm.2	Olfr1301	olfactory receptor 1301	-1,16855E+13	4,36444E+14	-2,0146E+14	0.0873969569500442	0.227537108957192	-4,2195E+14	3,44418E+14	3,8892E+14	4,41519E+14	5,25531E+14	3,82302E+14	5,35976E+14
TC1400002310.mm.2	Slc25a37	solute carrier family 25, member 37	1,29456E+14	5,03818E+14	2,01447E+14	0.0874131918804282	0.227542610786646	-4,21966E+13	5,18008E+14	5,72254E+14	5,0087E+14	3,3048E+14	4,99362E+14	6,01934E+14
TC1100003058.mm.2	Senp3	SUMO/sentrin specific peptidase 3	0.595713238589386	7,53254E+14	2,01424E+14	0.0874412199203881	0.227543388443371	-4,21995E+14	7,82642E+14	7,90133E+14	7,17221E+14	7,36411E+14	7,5376E+14	7,3936E+14
TC0700002010.mm.2	Olfr525	olfactory receptor 525	-1,1509E+14	5,26275E+14	-2,01424E+14	0.0874417340806402	0.227543388443371	-4,21995E+14	4,63073E+14	4,5149E+14	4,90614E+14	6,54129E+12	5,1563E+14	5,82715E+14
TC0900002790.mm.2	Cep162	centrosomal protein 162	0.841328999791692	5,61863E+14	2,01393E+14	0.0874795213445686	0.227546462331324	-4,22033E+14	6,06914E+14	6,17859E+14	5,65768E+14	4,90739E+14	5,8393E+14	5,05965E+14
TC0600001259.mm.2	Tatdn2	TatD DNase domain containing 2	1,1329E+14	5,55548E+13	2,01381E+12	0.0874946067512897	0.227546462331324	-4,22048E+13	6,31372E+14	6,54194E+14	5,63344E+14	4,95641E+14	4,15341E+13	5,73396E+14
TC0400003822.mm.2	Cda	cytidine deaminase	-0.665627224666492	5,61496E+14	-2,0138E+14	0.0874957819503471	0.227546462331324	-4,22049E+14	5,47622E+14	5,07868E+14	5,73375E+14	6,1524E+14	5,57428E+14	5,67443E+14
TC0300001206.mm.2	Plppr5	phospholipid phosphatase related 5	1,5638E+14	8,08388E+14	2,01372E+14	0.0875063222203472	0.227546462331324	-4,2206E+14	8,93939E+14	8,94537E+14	8,54204E+14	6,21512E+14	8,49632E+14	7,36502E+14
TC1200000675.mm.2	Mthfd1	methylenetetrahydrofolate dehydrogenase (NADP+ dependent), methenyltetrahydrofolate cyclohydrolase, formyltetrahydrofolate synthase	0.628068677272752	6,19279E+14	2,01366E+14	0.0875135249219397	0.227546462331324	-4,22067E+14	6,75294E+14	6,55014E+14	6,03916E+14	6,00778E+14	6,09526E+14	5,71146E+14
TC0500003240.mm.2	Zcchc8	zinc finger, CCHC domain containing 8	0.829432167610701	7,35294E+14	2,0133E+14	0.0875581598756902	0.227606294496872	-4,22111E+14	8,15503E+14	7,95169E+14	7,72328E+14	6,72458E+14	6,63806E+14	6,92501E+14
TC0500002945.mm.2	Mfsd7a	major facilitator superfamily domain containing 7A	-1,10327E+14	4,94053E+14	-2,01316E+14	0.0875767771336039	0.227606294496872	-4,2213E+13	4,08665E+14	4,87056E+14	4,90371E+14	6,26006E+14	4,46665E+14	5,05556E+14
TC1200000324.mm.2	Crppa	CDP-L-ribitol pyrophosphorylase A	0.797636605702647	5,56954E+14	2,01307E+14	0.0875874007127816	0.227606294496872	-4,22141E+14	6,13226E+14	5,64625E+14	5,50928E+14	4,67395E+14	5,80429E+14	5,65122E+14
TC0600001907.mm.2	Cttnbp2	cortactin binding protein 2	0.719715972074962	7,56421E+14	2,01303E+14	0.0875930386752996	0.227606294496872	-4,22146E+14	8,06914E+14	8,20134E+13	7,63886E+14	7,19219E+14	7,44405E+14	6,83969E+13
TC1100004099.mm.2	Galk1	galactokinase 1	-0.844363523543351	6,89459E+14	-2,01188E+14	0.0877358355834423	0.227914386806541	-4,22289E+14	6,31224E+14	6,59049E+14	6,99696E+14	7,5945E+14	6,49819E+14	7,37518E+14
TC0400002788.mm.2	Rnf183	ring finger protein 183	-0.714848913212506	6,56276E+14	-2,01185E+14	0.0877398958208263	0.227914386806541	-4,22293E+14	6,04958E+14	5,97651E+14	6,4876E+14	6,9682E+14	6,66199E+14	7,23266E+14
TC1200000270.mm.2	Cog5	component of oligomeric golgi complex 5	0.760620577984908	7,32461E+14	2,0116E+14	0.0877708367026827	0.227958009637383	-4,22324E+14	7,93223E+14	7,92026E+14	7,27944E+14	7,05181E+14	7,3077E+14	6,45619E+14
TC1300001500.mm.2	Cdk13	cyclin-dependent kinase 13	0.59864657941881	7,31005E+14	2,01145E+12	0.0877902125709332	0.227971586648742	-4,22343E+14	7,70561E+14	7,90405E+14	7,30192E+13	7,11045E+14	6,90046E+14	6,93783E+13
TC0500001618.mm.2	Vgf	VGF nerve growth factor inducible	0.879080548153175	7,92242E+14	2,01128E+14	0.0878112723544481	0.227989531332134	-4,22364E+14	8,50684E+14	8,4701E+13	8,1684E+14	7,05038E+14	7,47976E+14	7,85904E+14
TC0400000836.mm.2	Ccdc171	coiled-coil domain containing 171	0.750873238919084	5,95502E+14	2,01095E+14	0.0878529762937588	0.228058258114724	-4,22406E+14	6,30212E+14	6,10967E+14	5,04311E+14	5,86694E+14	6,14162E+14	6,26666E+14
TC0600000935.mm.2	Vax2	ventral anterior homeobox 2	-0.665101003347067	7,49078E+14	-2,01078E+14	0.0878736608239561	0.228058258114724	-4,22426E+14	6,99536E+14	7,07473E+14	7,41355E+14	7,98675E+14	7,68598E+14	7,78832E+14
TC1100001457.mm.2	Tac4	tachykinin 4	-0.865444924832103	5,2883E+14	-2,01073E+14	0.0878802038499549	0.228058258114724	-4,22433E+14	4,73296E+14	4,72985E+14	5,09497E+13	6,09873E+14	5,25677E+14	5,81651E+14
TC0100000551.mm.2	Map2	microtubule-associated protein 2	0.883789110925829	8,62901E+14	2,01031E+14	0.0879322611297738	0.228156606052181	-4,22484E+14	9,32162E+14	9,06515E+14	8,84331E+14	7,77588E+13	8,61791E+14	8,15017E+14
TC1100000939.mm.2	Asgr1	asialoglycoprotein receptor 1	-0.968170638171626	6,36091E+14	-2,00972E+14	0.088007422203497	0.22831485975929	-4,22559E+14	5,47696E+13	6,00428E+14	6,09441E+14	7,32317E+14	6,44951E+14	6,81712E+14
TC0800002332.mm.2	Npy5r	neuropeptide Y receptor Y5	-0.749738236014548	7,15862E+13	-2,00892E+14	0.0881074724482841	0.228530495198754	-4,22659E+14	6,38942E+14	6,84799E+14	7,06253E+14	7,67436E+14	7,29836E+13	7,67907E+14
TC1900000766.mm.2	Shoc2	Shoc2, leucine rich repeat scaffold protein	2,01108E+14	8,54788E+14	2,00873E+14	0.0881315480721911	0.228530495198754	-4,22682E+14	9,8291E+14	9,69925E+14	9,13121E+14	6,37499E+14	9,15301E+14	7,0997E+14
TC1800001410.mm.2	Slc6a7	solute carrier family 6 (neurotransmitter transporter, L-proline), member 7	0.64234633422273	6,84148E+14	2,00872E+14	0.0881330911168035	0.228530495198754	-4,22684E+14	7,29007E+14	7,33244E+14	6,88112E+14	6,4567E+14	6,41062E+14	6,67796E+14
TC0500001168.mm.2	C130026L21Rik	RIKEN cDNA C130026L21 gene	0.639842628177536	6,97003E+14	2,00848E+14	0.088162603145449	0.228570237245796	-4,22713E+14	7,17857E+13	7,20461E+14	6,58546E+14	6,51803E+14	7,41082E+14	6,92271E+14
TC0300002485.mm.2	Rprd2	regulation of nuclear pre-mRNA domain containing 2	2,08267E+13	6,0783E+14	2,00827E+14	0.0881889362301159	0.228601726106155	-4,22739E+14	7,77917E+14	7,54876E+14	6,7362E+14	4,42639E+14	6,34814E+14	3,63117E+13
TC1600002094.mm.2	Rcan1	regulator of calcineurin 1	0.81337663582744	7,14811E+14	2,00792E+14	0.0882326424390981	0.228649121651937	-4,22783E+14	7,59643E+14	7,55074E+12	7,25191E+14	6,2685E+14	7,25151E+14	6,96958E+14
TC0300002035.mm.2	Pfn2	profilin 2	1,10599E+14	9,2525E+14	2,0079E+13	0.0882356009701323	0.228649121651937	-4,22786E+14	9,92121E+14	9,9874E+14	9,56829E+14	8,12833E+13	9,39149E+14	8,51827E+14
TC0900000366.mm.2	Msantd2	Myb/SANT-like DNA-binding domain containing 2	0.634662216388586	5,49924E+13	2,00775E+14	0.0882539843573971	0.228659985520299	-4,22804E+14	5,6408E+14	5,52962E+14	4,76473E+14	5,13637E+14	5,82697E+14	6,09692E+14
TC0400003230.mm.2	Cpt2	carnitine palmitoyltransferase 2	-0.605402923733964	6,70382E+14	-2,00761E+13	0.0882717623799387	0.228669277689381	-4,22822E+14	6,28304E+14	6,18856E+14	6,6882E+14	6,99419E+13	6,99842E+14	7,07051E+14
TC1700001159.mm.2	Yipf4	Yip1 domain family, member 4	1,11046E+14	7,09483E+14	2,00743E+14	0.0882946896146206	0.228691903835458	-4,22844E+14	7,66238E+14	7,86302E+14	7,00299E+14	6,30149E+14	7,63465E+14	6,10446E+14
TC1000002828.mm.2	Syt1	synaptotagmin I	1,11462E+14	1,14932E+13	2,00728E+13	0.0883137014487484	0.22870438306713	-4,22863E+14	1,22633E+14	1,20958E+14	1,1742E+14	1,03879E+14	1,17455E+14	1,07247E+14
TC0900003000.mm.2	Col6a4	collagen, type VI, alpha 4	-0.669904598138553	5,62145E+14	-2,00705E+14	0.0883425589382879	0.22874235130526	-4,22892E+14	4,9516E+14	5,23023E+14	5,53819E+14	5,98344E+14	6,16687E+14	5,85839E+14
TC1700001145.mm.2	Ypel5	yippee like 5	1,27165E+14	8,46189E+14	2,00693E+14	0.0883578398624454	0.228745159656745	-4,22907E+13	9,24489E+14	9,07965E+14	8,78099E+13	7,00025E+14	8,80805E+14	7,85753E+14
TC1000002032.mm.2	Mfsd4b5/Mfsd4b3-ps	major facilitator superfamily domain containing 4B5/major facilitator superfamily domain containing 4B3, pseudogene	-0.700408122846366	6,13051E+14	-2,00666E+14	0.0883919221413008	0.228796633166712	-4,22941E+14	5,98319E+14	5,60263E+13	6,39414E+14	6,5925E+13	5,82204E+14	6,38856E+14
TC1100003780.mm.2	Kat2a	K(lysine) acetyltransferase 2A	0.858200668036641	7,24146E+14	2,00645E+14	0.088418869752586	0.228829625493643	-4,22967E+12	7,58824E+14	7,80484E+14	7,39404E+14	6,28264E+14	7,23696E+14	7,14205E+14
TC0800002392.mm.2	Klhl26	kelch-like 26	0.685982992965353	6,29421E+14	2,00612E+14	0.0884598227526209	0.228854374950981	-4,23008E+14	6,6918E+14	6,77257E+14	5,99208E+14	6,10033E+14	6,42974E+14	5,77874E+14
TC1000002534.mm.2	Mrpl54	mitochondrial ribosomal protein L54	-0.728193469057421	7,33974E+14	-2,00606E+14	0.0884671128698799	0.228854374950981	-4,23015E+14	6,94428E+14	6,57352E+14	7,34913E+14	7,62506E+14	7,44945E+14	8,09699E+13
TC0500000390.mm.2	Dok7	docking protein 7	-0.660225114163309	7,22668E+14	-2,00595E+14	0.0884814345597513	0.228854374950981	-4,23029E+14	6,78124E+14	6,72559E+14	7,24324E+14	7,58404E+14	7,28264E+14	7,74331E+14
TC1100002773.mm.2	Ccdc69	coiled-coil domain containing 69	-0.742367525771483	5,56189E+14	-2,00592E+14	0.0884852452022969	0.228854374950981	-4,23033E+14	5,19219E+11	5,06939E+14	5,53146E+14	6,21486E+14	5,44845E+13	5,915E+14
TC0500000740.mm.2	Polr2b	polymerase (RNA) II (DNA directed) polypeptide B	2,6005E+14	6,35322E+14	2,00556E+13	0.0885302482843335	0.228934021923522	-4,23077E+14	8,46507E+14	8,25515E+14	7,28706E+14	4,23215E+14	6,61216E+14	3,26772E+14
TC1100004223.mm.2	Myadml2	myeloid-associated differentiation marker-like 2	-0.788753765900028	5,34914E+14	-2,00476E+14	0.088631674385824	0.22914934601759	-4,23177E+14	4,77339E+13	4,89436E+14	5,14567E+14	6,0996E+14	5,62437E+13	5,55742E+14
TC0400004174.mm.2	Klhl17/Mir7658	kelch-like 17/microRNA 7658	-0.819344597516665	7,88116E+14	-2,00468E+14	0.0886419582776416	0.22914934601759	-4,23188E+14	7,16172E+14	7,54797E+14	7,7273E+14	8,62107E+14	7,92826E+14	8,30066E+14
TC0700003874.mm.2	Olfr575	olfactory receptor 575	-1,4527E+14	4,84336E+14	-2,00427E+14	0.0886930017340769	0.229218978157218	-4,23238E+14	3,72066E+14	4,00891E+13	4,14711E+14	6,48786E+14	5,16575E+14	5,52988E+14
TC0500002411.mm.2	Lgi2	leucine-rich repeat LGI family, member 2	0.646839799477087	7,26223E+14	2,00406E+14	0.0887196665640201	0.229218978157218	-4,23264E+14	7,73728E+14	7,61303E+14	7,28445E+14	6,77218E+13	7,07606E+14	7,09038E+13
TC1400002037.mm.2	Olfr1511	olfactory receptor 1511	-0.892700880610354	4,89462E+14	-2,00396E+14	0.0887322416004756	0.229218978157218	-4,23277E+14	4,48782E+14	4,32517E+14	4,85628E+13	5,74211E+14	4,56607E+14	5,39026E+14
TC0700002657.mm.2	Egln2	egl-9 family hypoxia-inducible factor 2	0.861298416378872	6,78572E+14	2,00396E+14	0.0887326901488824	0.229218978157218	-4,23277E+14	7,34969E+14	7,06686E+13	6,84337E+14	5,85058E+14	7,0511E+14	6,5527E+14
TC0800002188.mm.2	Tenm3	teneurin transmembrane protein 3	0.648357542144039	8,27397E+14	2,0039E+14	0.0887400226987355	0.229218978157218	-4,23284E+14	8,78583E+14	8,63596E+14	8,19155E+14	7,93352E+14	7,82737E+14	8,26956E+14
TC1500000878.mm.2	Shank3	SH3 and multiple ankyrin repeat domains 3	1,22044E+14	5,98868E+14	2,00365E+14	0.0887725379308203	0.229266213123787	-4,23316E+14	7,01929E+14	6,81536E+14	5,20788E+14	6,18591E+14	4,52845E+13	6,17521E+14
TC1600000760.mm.2	Dcbld2	discoidin, CUB and LCCL domain containing 2	-0.753045283361336	5,95071E+14	-2,00351E+14	0.0887893802901878	0.229272962278173	-4,23333E+14	5,73947E+14	5,22731E+14	5,88884E+13	6,58403E+14	6,18122E+14	6,08341E+14
TC1100000335.mm.2	Hba-x	hemoglobin X, alpha-like embryonic chain in Hba complex	-0.747292155447989	6,8362E+14	-2,00316E+14	0.0888347319913493	0.229353314625318	-4,23378E+14	6,10187E+14	6,33451E+14	6,58711E+14	7,34386E+14	7,48412E+14	7,16574E+14
TC0800001143.mm.2	Ndrg4	N-myc downstream regulated gene 4	0.977434955331935	1,03311E+14	2,00294E+14	0.0888617752854079	0.229386380194453	-4,23404E+14	1,10608E+14	1,08865E+14	1,06711E+14	9,3213E+14	1,02162E+13	9,83054E+14
TC0600002582.mm.2	Tex261	testis expressed gene 261	0.746710800158969	6,78436E+13	2,00283E+14	0.0888763951376655	0.22938737063162	-4,23419E+14	7,27091E+14	7,3483E+14	6,98434E+14	6,14145E+14	6,52935E+14	6,43183E+13
TC0800000830.mm.2	Tmem38a	transmembrane protein 38A	0.869119025509367	7,78885E+14	2,00253E+14	0.0889133861207882	0.229411029679994	-4,23455E+14	8,41327E+14	8,26227E+14	8,00949E+14	6,92782E+14	7,76507E+14	7,35518E+14
TC0900002712.mm.2	Fbxo9	f-box protein 9	0.721452937256521	7,69062E+14	2,00253E+14	0.0889140371347086	0.229411029679994	-4,23456E+14	8,17494E+13	7,9227E+14	7,69472E+14	6,96001E+14	7,72782E+14	7,66352E+14
TC0X00002641.mm.2	Zfx	zinc finger protein X-linked	1,95614E+14	6,40487E+14	2,00241E+14	0.0889297710989867	0.22941488980435	-4,23471E+14	7,83546E+14	7,65233E+14	7,10608E+14	4,46944E+14	6,71092E+13	4,65501E+14
TC1500000735.mm.2	1700029P11Rik	RIKEN cDNA 1700029P11 gene	-1,17556E+14	6,85571E+14	-2,00201E+14	0.0889798611827202	0.229493660188169	-4,2352E+13	6,20258E+14	6,18403E+14	6,55596E+14	8,18178E+14	6,57801E+14	7,43189E+14
TC0700002160.mm.2	Tnnt1	troponin T1, skeletal, slow	-0.730789699920776	7,21327E+14	-2,00175E+14	0.0890129998031635	0.229493660188169	-4,23553E+14	6,6178E+14	6,70047E+13	7,35133E+14	7,42851E+14	7,19089E+14	7,99063E+13
TC0500001888.mm.2	Ankib1	ankyrin repeat and IBR domain containing 1	1,22218E+14	6,72938E+14	2,00172E+14	0.089017117397539	0.229493660188169	-4,23557E+14	7,68606E+14	7,73755E+14	7,45437E+14	5,52487E+14	6,02913E+14	5,94432E+14
TC1300001555.mm.2	Hist1h2bl	histone cluster 1, H2bl	-0.622254353750716	8,59546E+14	-2,00163E+14	0.0890275203510689	0.229493660188169	-4,23567E+14	8,0726E+14	8,24203E+14	8,68635E+14	8,87278E+14	9,02104E+14	8,67797E+14
TC0700002460.mm.2	Psg19	pregnancy specific glycoprotein 19	-0.794403391689647	5,45024E+14	-2,00155E+14	0.0890380362387258	0.229493660188169	-4,23577E+14	5,00295E+14	4,6476E+14	5,23538E+14	6,00398E+14	5,65718E+14	6,15434E+14
TC1400001746.mm.2	2610528A11Rik	RIKEN cDNA 2610528A11 gene	0.658492150466184	5,69425E+14	2,00149E+14	0.0890457620242311	0.229493660188169	-4,23585E+14	5,99923E+14	5,97514E+14	5,36743E+14	5,28996E+14	5,48237E+14	6,05138E+14
TC0500001349.mm.2	Oas1b	2'-5' oligoadenylate synthetase 1B	-0.689350229622538	5,97172E+14	-2,00126E+14	0.0890754173704454	0.229504675405613	-4,23614E+14	5,30101E+14	5,68695E+14	5,92706E+14	6,43959E+14	6,35251E+14	6,12318E+14
TC1400002283.mm.2	Ppp2r2a	protein phosphatase 2, regulatory subunit B, alpha	2,54398E+13	8,6879E+14	2,00123E+14	0.0890785229309692	0.229504675405613	-4,23617E+14	1,00958E+14	1,02457E+14	9,65631E+14	5,59717E+14	9,16382E+14	7,36863E+14
TC0700004512.mm.2	Ifitm5	interferon induced transmembrane protein 5	-0.826416815955127	4,76545E+14	-2,00112E+14	0.0890928434175009	0.229504873858704	-4,23631E+14	4,22008E+14	4,22999E+14	4,64908E+14	5,45382E+14	4,67202E+14	5,36773E+14
TC1100000216.mm.2	Wdpcp	WD repeat containing planar cell polarity effector	0.733381169125438	5,19976E+14	2,00059E+14	0.0891602380238559	0.229641770345011	-4,23697E+14	5,33806E+14	6,00863E+14	4,74598E+12	5,13394E+13	4,85715E+14	5,1148E+14
TC1900000689.mm.2	Nfkb2	nuclear factor of kappa light polypeptide gene enhancer in B cells 2, p49/p100	-0.891940457008955	5,45728E+14	-2,00003E+14	0.0892320701543769	0.229713122889215	-4,23768E+14	4,44237E+14	5,15528E+14	5,20582E+14	6,17571E+14	5,77363E+14	5,99088E+14
TC0300000809.mm.2	S100a6	S100 calcium binding protein A6 (calcyclin)	0.814167354862078	6,4378E+13	1,99997E+14	0.089239661356132	0.229713122889215	-4,23775E+14	6,54398E+14	7,03298E+14	5,52816E+13	6,42046E+14	6,45798E+14	6,64324E+13
TC0700002989.mm.2	Fcgrt	Fc receptor, IgG, alpha chain transporter	0.664689065941189	6,11535E+14	1,99993E+14	0.089243788280149	0.229713122889215	-4,23779E+14	6,67008E+14	6,33813E+14	5,59013E+14	6,0887E+14	6,00801E+14	5,99706E+14
TC0300002482.mm.2	Ecm1/Mir7014	extracellular matrix protein 1/microRNA 7014	-0.641897267172573	6,79677E+14	-1,99987E+14	0.0892522150579451	0.229713122889215	-4,23787E+14	6,24797E+14	6,30884E+14	6,7499E+14	7,0907E+14	7,00229E+13	7,38092E+14
TC0100002118.mm.2	Phf3	PHD finger protein 3	1,2488E+14	8,38515E+14	1,99977E+13	0.0892650427248906	0.229713122889215	-4,238E+14	9,27491E+14	9,26396E+14	8,61118E+14	7,43009E+14	8,66516E+14	7,06559E+13
TC1100004228.mm.2	Dcxr	dicarbonyl L-xylulose reductase	-0.59626878139864	7,5562E+14	-1,9997E+14	0.0892734795216449	0.229713122889215	-4,23808E+14	7,19699E+14	7,21023E+14	7,6631E+14	7,93666E+14	7,62416E+14	7,70609E+14
TC0800000267.mm.2	Ccdc70	coiled-coil domain containing 70	-0.618506462883516	4,76002E+14	-1,99881E+14	0.0893862838377698	0.229966659983711	-4,23919E+14	4,19837E+14	4,32481E+14	4,68408E+14	5,07612E+14	5,14031E+14	5,13643E+14
TC1500000612.mm.2	Zfp7	zinc finger protein 7	-0.768657360195632	6,13938E+14	-1,99867E+13	0.0894045952174876	0.22997705024575	-4,23937E+14	5,57379E+13	5,66721E+14	6,12964E+14	6,64869E+14	6,03353E+14	6,78342E+13
TC0700004657.mm.2	Sec11a	SEC11 homolog A, signal peptidase complex subunit	1,08792E+14	7,75E+14	1,99834E+14	0.0894467402002301	0.230048735011382	-4,23978E+14	8,5534E+14	8,37483E+14	8,10238E+14	6,65001E+14	7,79985E+14	7,01956E+12
TC1100003566.mm.2	Rsad1	radical S-adenosyl methionine domain containing 1	-0.836568099037987	7,15199E+14	-1,998E+14	0.0894908208419196	0.230125374437576	-4,24021E+14	6,96768E+14	6,51862E+14	7,33376E+14	7,82568E+14	7,56591E+14	6,70027E+14
TC1100003225.mm.2	Wdr81	WD repeat domain 81	0.662720154989921	6,72899E+14	1,99782E+14	0.0895130321848347	0.23014576154368	-4,24042E+14	6,97729E+14	7,22295E+14	6,17749E+14	6,69731E+14	6,57379E+14	6,72512E+14
TC1100003496.mm.2	Msi2	musashi RNA-binding protein 2	0.816279359930054	8,28051E+14	1,99755E+14	0.0895483044336215	0.230199717507808	-4,24077E+14	8,87446E+14	8,52019E+14	8,2907E+14	7,4714E+14	8,53201E+14	7,99429E+14

TC1500001822.mm.2	Txn2	thioredoxin 2	0.841797458647337	7,57272E+14	1,99726E+14	0.0895848007315342	0.230238232684581	-4,24113E+14	8,35241E+14	8,09142E+14	7,81896E+14	6,94127E+14	6,80609E+14	7,42615E+14
TC1000002041.mm.2	Rpf2	ribosome production factor 2 homolog	0.98852033705893	6,82834E+14	1,9972E+13	0.089591864887502	0.230238232684581	-4,24119E+12	7,35932E+14	7,27394E+14	6,88659E+14	5,76963E+14	7,29692E+14	6,38365E+14
TC1300000466.mm.2	Smim13	small integral membrane protein 13	1,31724E+14	9,0813E+14	1,99707E+14	0.0896090963792887	0.230245793327935	-4,24136E+14	9,74583E+14	9,85229E+14	9,29948E+14	7,66417E+14	9,64272E+14	8,2833E+14
TC1100001204.mm.2	Zfp207	zinc finger protein 207	1,33475E+14	8,68726E+14	1,9963E+14	0.0897068318173094	0.230394039692178	-4,24232E+14	9,44243E+14	9,73483E+14	9,07404E+14	7,43372E+14	8,91057E+14	7,52796E+13
TC0100001402.mm.2	Acbd6	acyl-Coenzyme A binding domain containing 6	1,08241E+14	8,76487E+13	1,99625E+14	0.0897136173175963	0.230394039692178	-4,24238E+14	9,43221E+14	9,31524E+14	9,03511E+14	7,54752E+14	8,98061E+14	8,27855E+14
TC0100003657.mm.2	Tfb2m	transcription factor B2, mitochondrial	1,87088E+12	6,49767E+14	1,9962E+14	0.0897208416000895	0.230394039692178	-4,24245E+14	7,84956E+14	7,84162E+14	7,17619E+14	4,77323E+14	6,72877E+14	4,61662E+13
TC0400002636.mm.2	Baat	bile acid-Coenzyme A: amino acid N-acyltransferase	-0.595654370964779	5,5922E+14	-1,99616E+14	0.08972580426697 3	0.230394039692178	-4,2425E+14	5,17196E+14	5,29583E+14	5,81816E+14	5,84094E+14	5,84311E+14	5,58319E+14
TC0600000370.mm.2	Tmem213	transmembrane protein 213	-0.89717151602924	6,65251E+14	-1,99588E+14	0.0897617946454234	0.230394039692178	-4,24285E+14	5,99752E+14	6,09614E+14	6,5958E+14	7,29221E+14	6,45623E+14	7,47716E+14
TC0200002751.mm.2	Gm14322	predicted gene 14322	1,88815E+13	7,78948E+14	1,99584E+14	0.0897661152172645	0.230394039692178	-4,24289E+13	8,69861E+14	8,97376E+14	8,26851E+14	5,62757E+14	8,506E+14	6,66242E+14
TC1000002396.mm.2	Slc5a4b	solute carrier family 5 (neutral amino acid transporters, system A), member 4b	-0.89573748273009	6,17775E+14	-1,99584E+14	0.0897668827150433	0.230394039692178	-4,2429E+14	5,46543E+14	5,76838E+13	6,06332E+14	6,96196E+14	6,00701E+13	6,8004E+14
TC1200001877.mm.2	Map4k5	mitogen-activated protein kinase kinase kinase kinase 5	2,18693E+14	6,51674E+14	1,99561E+13	0.0897953877554644	0.230430495764261	-4,24318E+14	7,66211E+14	7,97707E+14	7,46483E+14	3,80049E+14	6,56089E+14	5,63503E+14
TC1600001582.mm.2	Slc15a2	solute carrier family 15 (H+/peptide transporter), member 2	1,92529E+14	6,15969E+14	1,99461E+14	0.0899245003719211	0.230725075556173	-4,24443E+14	7,58983E+14	7,52657E+14	6,94321E+14	4,32261E+14	6,29414E+14	4,28176E+14
TC1700000898.mm.2	Mrpl2	mitochondrial ribosomal protein L2	0.732768875349415	7,33928E+14	1,99418E+13	0.0899788728298256	0.230792962907657	-4,24496E+14	7,79638E+14	8,00298E+14	7,53218E+14	6,80163E+13	7,10809E+14	6,79442E+14
TC0600001589.mm.2	Borcs5	BLOC-1 related complex subunit 5	0.727890531942026	5,52616E+13	1,99415E+14	0.0899829085061671	0.230792962907657	-4,245E+14	6,24003E+14	5,77644E+14	5,2432E+14	5,31749E+14	4,96151E+14	5,61828E+14
TC0900003004.mm.2	Alas1	aminolevulinic acid synthase 1	0.873017377052822	7,86327E+14	1,99404E+14	0.0899967060075806	0.230792962907657	-4,24513E+14	8,36005E+14	8,56239E+14	7,65038E+14	7,52603E+14	8,1207E+14	6,96006E+14
TC1900000451.mm.2	Trpd52l3	tumor protein D52-like 3	-0.931198263196511	5,05073E+14	-1,99391E+14	0.0900135901253713	0.230792962907657	-4,2453E+14	4,48226E+14	4,50362E+14	4,81608E+14	6,03219E+14	4,97821E+14	5,49205E+14
TC1100003747.mm.2	Krtap17-1	keratin associated protein 17-1	-0.617558525180613	8,64557E+14	-1,99382E+14	0.0900254830175693	0.230792962907657	-4,24541E+14	8,19842E+14	8,35125E+14	8,75356E+14	9,03123E+14	8,63567E+14	8,90331E+14
TC0500000796.mm.2	Csn1s2a	casein alpha s2-like A	-0.804667905592417	4,78464E+14	-1,99373E+14	0.090036899698227	0.230792962907657	-4,24552E+14	4,31756E+14	4,33693E+14	4,78278E+12	5,48104E+14	4,52556E+14	5,26399E+13
TC0300000376.mm.2	Foxo1	forkhead box O1	0.82374634166016	7,34532E+14	1,99349E+12	0.0900674087245691	0.230806416925702	-4,24582E+14	8,09535E+14	8,04134E+14	7,54183E+14	6,94737E+14	7,16404E+13	6,28201E+14
TC1700001488.mm.2	Phf10	PHD finger protein 10	1,15595E+14	7,31906E+14	1,99347E+14	0.0900707968490546	0.230806416925702	-4,24585E+14	7,86155E+14	8,04488E+14	7,66258E+14	5,93195E+14	7,44864E+14	6,96474E+14
TC0200004959.mm.2	Ncoa6	nuclear receptor coactivator 6	1,08797E+14	6,00691E+14	1,99333E+14	0.0900888002967985	0.230815843406315	-4,24603E+14	6,81512E+13	7,02916E+14	6,42193E+14	5,24641E+14	5,84364E+13	4,6852E+14
TC1300001410.mm.2	Wdr37	WD repeat domain 37	1,41056E+14	7,34086E+14	1,99305E+14	0.0901240243520016	0.230842052252244	-4,24637E+14	8,50491E+14	8,25434E+14	7,83876E+14	6,09937E+14	7,43089E+14	5,91691E+14
TC1900000617.mm.2	R3hcc1l	R3H domain and coiled-coil containing 1 like	1,19691E+14	5,9146E+14	1,99279E+14	0.0901582441978489	0.230842052252244	-4,2467E+14	7,05142E+14	6,54048E+14	6,46104E+14	4,73703E+14	5,64235E+14	5,05531E+14
TC1100003139.mm.2	Med31	mediator complex subunit 31	1,1616E+14	6,77789E+14	1,99278E+13	0.090159081840456	0.230842052252244	-4,24671E+14	7,5096E+14	7,35293E+14	6,8684E+14	5,67093E+14	7,31276E+14	5,9527E+14
TC0700002990.mm.2	Rps11	ribosomal protein S11	1,4351E+14	1,02353E+14	1,99265E+14	0.0901751748920133	0.230842052252244	-4,24687E+14	1,10882E+13	1,11196E+14	1,06547E+14	8,68288E+14	1,05889E+14	9,2774E+14
TC0600001244.mm.2	Cpne9	copine family member IX	0.724661047323186	5,58069E+14	1,99261E+13	0.0901808941503002	0.230842052252244	-4,24692E+14	6,2624E+14	5,90535E+14	5,11786E+14	5,60058E+14	5,035E+14	5,56296E+14
TC0200003310.mm.2	6330409D20Rik	RIKEN cDNA 6330409D20 gene	-0.72020118761352	5,61226E+14	-1,99258E+14	0.0901849884520497	0.230842052252244	-4,24696E+14	5,1494E+14	4,94468E+14	5,58205E+14	5,95244E+14	5,6911E+14	6,35392E+14
TC0500003215.mm.2	Ift81	intraflagellar transport 81	1,50433E+14	6,33689E+14	1,99211E+14	0.0902451903991778	0.230919441478834	-4,24754E+14	7,39339E+14	7,29275E+14	6,73456E+14	4,94292E+14	6,70239E+14	4,95532E+14
TC1500001866.mm.2	Slc16a8	solute carrier family 16 (monocarboxylic acid transporters), member 8	-0.850619719766573	4,57234E+14	-1,99193E+14	0.0902681627318923	0.230919441478834	-4,24777E+14	3,84974E+14	4,02568E+14	4,40444E+14	5,17222E+14	4,58382E+14	5,39815E+14
TC1700000579.mm.2	Olfr63	olfactory receptor 63	-0.815406912885865	5,39168E+14	-1,99189E+13	0.0902732964859703	0.230919441478834	-4,24782E+13	4,79044E+13	5,09246E+14	5,25986E+14	6,25384E+14	5,41698E+14	5,53649E+14
TC1800000179.mm.2	Dtna	dystrobrevin alpha	1,44969E+14	9,46336E+14	1,99182E+14	0.0902824773532854	0.230919441478834	-4,2479E+14	1,04025E+14	1,03181E+14	9,89707E+14	7,92422E+14	9,79954E+14	8,43869E+13
TC0500001260.mm.2	Msi1	musashi RNA-binding protein 1	0.822779913569629	8,10518E+14	1,99179E+14	0.0902868789993581	0.230919441478834	-4,24795E+14	8,75016E+14	8,6569E+14	8,32943E+14	7,43208E+14	8,01364E+14	7,44888E+14
TC1000002156.mm.2	Gopc	golgi associated PDZ and coiled-coil motif containing	0.647874221717022	6,32868E+14	1,99148E+14	0.0903263797589087	0.230943385028178	-4,24833E+14	6,60946E+14	6,61145E+14	5,7371E+14	6,18805E+14	6,25807E+13	6,56796E+14
TC1700001606.mm.2	Elob	elongin B	0.762025784430939	1,00614E+14	1,99135E+14	0.0903431475882362	0.230943385028178	-4,24849E+12	1,05535E+14	1,04064E+14	1,009E+14	9,34598E+14	1,02345E+13	9,73812E+14
TC0500003592.mm.2	Zkscan14	zinc finger with KRAB and SCAN domains 14	-0.791608202238529	6,3736E+14	-1,99108E+14	0.0903772640614718	0.230943385028178	-4,24882E+14	6,08687E+14	5,86842E+14	6,58543E+14	6,95307E+14	5,92998E+14	6,81784E+14
TC0900000858.mm.2	Csnk1g1	casein kinase 1, gamma 1	1,78829E+14	6,86787E+14	1,99107E+14	0.0903784637815191	0.230943385028178	-4,24883E+14	7,83547E+14	7,76363E+14	7,31682E+14	4,70571E+14	7,58181E+14	6,0038E+14
TC0800000234.mm.2	Defa2	defensin, alpha, 2	-0.576883615425938	5,35676E+14	-1,9909E+14	0.0904011685532081	0.230943385028178	-4,24905E+14	4,99253E+14	4,97094E+14	5,52895E+14	5,58829E+14	5,58925E+14	5,4706E+14
TC0800000235.mm.2	Defa33	defensin, alpha, 33	-0.576883615425938	5,35676E+14	-1,9909E+14	0.0904011685532081	0.230943385028178	-4,24905E+14	4,99253E+14	4,97094E+14	5,52895E+14	5,58829E+14	5,58925E+14	5,4706E+14
TC0800002388.mm.2	Armc6	armadillo repeat containing 6	0.589887970342377	5,25685E+14	1,99083E+14	0.0904098624034278	0.230943385028178	-4,24914E+13	5,55137E+14	5,73807E+13	4,96563E+14	5,14403E+14	5,08419E+13	5,05783E+14
TC1100000462.mm.2	Sox30	SRY (sex determining region Y)-box 30	-0.71413658248941	5,851E+14	-1,99082E+14	0.0904109025481133	0.230943385028178	-4,24915E+14	5,30767E+14	5,51868E+14	6,17864E+14	6,07598E+14	5,64573E+14	6,37928E+14
TC0300001413.mm.2	Adh6-ps1	alcohol dehydrogenase 6 (class V), pseudogene 1	-0.820535034808067	5,33595E+14	-1,99005E+14	0.09051073152913 8	0.230957741887162	-4,25011E+14	4,65904E+14	4,62293E+14	5,29304E+14	5,62999E+14	5,40288E+14	6,40782E+14
TC0100002203.mm.2	Coa5	cytochrome C oxidase assembly factor 5	0.977324233008757	7,74194E+14	1,98999E+14	0.0905181114859051	0.230957741887162	-4,25018E+14	8,24175E+14	8,35075E+14	7,54888E+14	7,08898E+14	8,28463E+14	6,93668E+14
TC0900000809.mm.2	Lctl	lactase-like	-1,05718E+14	5,64418E+14	-1,98999E+14	0.090518625081022	0.230957741887162	-4,25019E+14	4,69955E+14	5,1068E+14	5,2466E+13	6,6741E+14	5,76719E+14	6,37086E+14
TC1800001429.mm.2	Grpel2	GrpE-like 2, mitochondrial	-0.750513932290928	7,33387E+14	-1,98998E+14	0.0905202486737592	0.230957741887162	-4,2502E+14	6,71068E+14	6,98349E+14	7,76877E+14	7,42642E+14	7,17747E+13	7,93641E+14
TC0100001302.mm.2	Zbtb41	zinc finger and BTB domain containing 41	1,13188E+14	7,25909E+14	1,98997E+14	0.0905206248229268	0.230957741887162	-4,25021E+14	7,86033E+14	7,79612E+14	7,55619E+14	5,83649E+13	7,43825E+14	7,06715E+14
TC1100000596.mm.2	Fstl4	follistatin-like 4	0.92282641355427	6,80281E+14	1,98983E+14	0.0905395492352184	0.230957741887162	-4,25039E+14	7,38532E+13	7,49119E+12	5,88814E+14	7,14271E+14	6,28202E+14	6,62747E+14
TC1200001715.mm.2	Strn3	striatin, calmodulin binding protein 3	1,45333E+14	9,17056E+14	1,9898E+14	0.0905427154976186	0.230957741887162	-4,25042E+14	1,04162E+14	1,02307E+14	9,48095E+14	8,25922E+14	9,35165E+13	7,28468E+14
TC1200000319.mm.2	Ankmy2	ankyrin repeat and MYND domain containing 2	0.786436915161032	6,97312E+14	1,98969E+14	0.0905569648314907	0.230957741887162	-4,25056E+14	7,61127E+14	7,44934E+14	6,91882E+14	6,56891E+13	7,09208E+14	6,1983E+14
TC1000002479.mm.2	Prss57	protease, serine 57	-0.726320109446127	5,56758E+14	-1,98968E+14	0.0905589846528958	0.230957741887162	-4,25058E+14	4,98681E+14	5,16197E+14	5,4068E+13	6,19462E+14	5,81406E+14	5,84123E+14
TC1100000924.mm.2	G630025P09Rik	RIKEN cDNA G630025P09 gene	-0.878102834103071	5,90161E+14	-1,98953E+14	0.0905779511601238	0.230957741887162	-4,25076E+14	5,39384E+14	5,49392E+14	5,80337E+14	6,84059E+14	5,6399E+14	6,23803E+14
TC0100001579.mm.2	Nr1i3	nuclear receptor subfamily 1, group I, member 3	-0.829958468020309	5,9004E+14	-1,98927E+14	0.0906109808148084	0.230957741887162	-4,25108E+14	5,23316E+14	5,56E+13	6,18002E+14	6,27307E+14	5,56292E+14	6,59322E+14
TC1700002058.mm.2	Olfr94	olfactory receptor 94	-1,06334E+14	5,54972E+14	-1,9892E+14	0.0906199596690364	0.230957741887162	-4,25116E+14	5,07484E+14	4,86283E+14	5,55507E+14	6,50928E+14	5,00434E+14	6,29193E+14
TC1100000605.mm.2	Shroom1	shroom family member 1	-1,05623E+14	6,99612E+14	-1,98902E+14	0.0906431881037526	0.230957741887162	-4,25139E+14	6,20253E+14	6,5027E+14	6,77018E+14	8,0475E+14	6,76772E+14	7,68606E+14
TC0900000674.mm.2	Tmem266	transmembrane protein 266	-0.668505379105074	5,8786E+13	-1,98883E+14	0.0906678311467286	0.230957741887162	-4,25163E+13	5,57212E+14	5,25641E+14	5,81294E+14	6,3526E+14	6,12847E+14	6,14906E+14
TC0700001349.mm.2	Ndufc2	NADH:ubiquinone oxidoreductase subunit C2	1,35316E+14	9,39126E+14	1,98882E+14	0.0906695304615339	0.230957741887162	-4,25164E+14	9,96166E+14	9,96835E+14	9,61216E+14	7,61153E+14	1,00685E+14	9,12539E+13
TC0700001716.mm.2	Rexo5	RNA exonuclease 5	-0.937504690739042	4,73068E+14	-1,98882E+14	0.0906696617382908	0.230957741887162	-4,25164E+14	3,98178E+14	4,32621E+14	4,4768E+13	5,70621E+14	4,7428E+12	5,15024E+14
TC0X00001859.mm.2	Med14	mediator complex subunit 14	1,84952E+14	8,24607E+14	1,98881E+12	0.0906712468288939	0.230957741887162	-4,25166E+14	9,63802E+14	9,61608E+14	8,8041E+14	6,75096E+14	8,54248E+14	6,12477E+14
TC0500003490.mm.2	3110082I17Rik	RIKEN cDNA 3110082I17 gene	-0.668887171919179	7,37324E+13	-1,98878E+14	0.0906745283422199	0.230957741887162	-4,25169E+14	6,85257E+14	7,07357E+14	7,36841E+14	7,8955E+14	7,39584E+14	7,65353E+14
TC0300003247.mm.2	Amy1	amylase 1, salivary	0.843473178950951	5,42835E+14	1,98841E+14	0.0907220662060001	0.231025391323522	-4,25215E+14	6,24344E+14	5,6974E+14	5,30082E+14	4,95307E+14	5,64414E+13	4,73123E+13
TC1900000962.mm.2	Tsga10ip	testis specific 10 interacting protein	-1,04812E+14	5,54351E+14	-1,98835E+14	0.0907297633150404	0.231025391323522	-4,25222E+14	4,67067E+14	4,79924E+14	5,10924E+14	6,45691E+14	5,70043E+14	6,52457E+14
TC0100003795.mm.2	Rps6kc1	ribosomal protein S6 kinase polypeptide 1	0.799244784281051	5,85474E+13	1,9874E+14	0.0908539400070919	0.2312283721665	-4,25342E+14	6,61051E+14	6,32887E+14	5,96135E+13	5,37953E+14	5,03664E+14	5,81152E+14
TC0500001423.mm.2	B3gnt4	UDP-GlcNAc:betaGal beta-1,3-N-acetylglucosaminyltransferase 4	-0.924307333941461	5,67891E+14	-1,98736E+14	0.0908579235533409	0.2312283721665	-4,25346E+14	5,26695E+14	4,77204E+14	5,84282E+14	6,04479E+14	5,47455E+14	6,67227E+14
TC1900001588.mm.2	Oga	O-GlcNAcase	1,17298E+14	8,90257E+14	1,98729E+14	0.0908676942863169	0.2312283721665	-4,25355E+14	9,85048E+14	9,77544E+14	9,49179E+14	7,78819E+14	8,61219E+14	7,89735E+14
TC1500000947.mm.2	Arid2	AT rich interactive domain 2 (ARID, RFX-like)	1,01624E+14	5,98384E+14	1,98712E+14	0.0908892220300796	0.2312283721665	-4,25376E+14	6,8554E+14	6,61609E+14	6,24747E+12	5,19155E+14	6,02774E+14	4,96482E+14
TC1900000442.mm.2	Pdcd1lg2	programmed cell death 1 ligand 2	-0.633769143785875	5,40742E+14	-1,98694E+14	0.0909126583103752	0.2312283721665	-4,25398E+14	5,02787E+13	5,1462E+14	5,4796E+13	5,96201E+14	5,41344E+11	5,4154E+14
TC0400001151.mm.2	Orc1	origin recognition complex, subunit 1	-0.697971284364336	5,10539E+14	-1,98684E+14	0.0909258261993326	0.2312283721665	-4,25411E+14	4,58784E+14	4,91148E+14	5,77021E+14	5,12505E+14	5,0855E+14	5,15228E+14
TC0700003861.mm.2	Olfr545	olfactory receptor 545	-0.884326074138415	5,58459E+14	-1,98664E+14	0.090951634730091	0.2312283721665	-4,25436E+14	5,33187E+12	4,80425E+14	5,55157E+14	6,35321E+13	5,35727E+14	6,10935E+14
TC0700000055.mm.2	Zfp580	zinc finger protein 580	-0.705951605524147	7,72576E+14	-1,98662E+13	0.0909537782902693	0.2312283721665	-4,25438E+14	7,20609E+14	7,10495E+14	7,61842E+14	8,10452E+14	7,87933E+14	8,44126E+14
TC1400002491.mm.2	Wbp4	WW domain binding protein 4	1,28135E+14	7,20839E+14	1,98657E+14	0.0909602977398064	0.2312283721665	-4,25444E+14	8,03508E+14	8,05352E+14	7,5998E+14	5,9261E+13	7,42771E+14	6,20815E+14
TC1400002132.mm.2	Zmym5	zinc finger, MYM-type 5	1,67208E+14	6,82608E+14	1,98646E+14	0.0909752523325617	0.2312283721665	-4,25458E+14	8,14994E+14	8,00868E+14	7,53199E+14	5,28246E+14	6,85345E+14	5,12993E+14
TC0700004495.mm.2	Sprn	shadow of prion protein	0.769672872682812	8,31777E+14	1,98646E+13	0.0909755375791683	0.2312283721665	-4,25459E+14	8,69123E+14	8,84371E+14	8,25011E+14	7,74548E+14	8,57248E+14	7,80363E+14
TC0100001412.mm.2	Nphs2	nephrosis 2, podocin	-0.66961935267177	5,5737E+14	-1,98623E+14	0.0910051471080167	0.2312283721665	-4,25487E+14	5,26287E+14	5,17787E+14	5,60374E+14	6,17624E+12	5,48325E+14	5,73822E+13
TC0500001587.mm.2	Srrm3	serine/arginine repetitive matrix 3	-0.603874801195735	7,70121E+14	-1,98617E+14	0.0910132713092517	0.2312283721665	-4,25495E+14	7,23736E+14	7,27006E+13	7,68529E+14	8,02988E+14	8,03508E+14	7,94958E+14
TC0400001431.mm.2	Epha10	Eph receptor A10	-0.687585565281807	7,57575E+14	-1,98614E+14	0.091016362472006	0.2312283721665	-4,25498E+13	6,98033E+14	7,11642E+14	7,55776E+14	7,91416E+14	7,65391E+14	8,23191E+14
TC1100004298.mm.2	Ccdc47	coiled-coil domain containing 47	1,41416E+14	8,38304E+14	1,98603E+13	0.0910312832364924	0.2312283721665	-4,25512E+13	9,1752E+14	9,13485E+13	8,82801E+14	6,65373E+13	8,68044E+14	7,826E+14
TC0300000749.mm.2	Fam189b	family with sequence similarity 189, member B	-0.589997616913749	7,1125E+14	-1,98597E+14	0.091039086019008	0.2312283721665	-4,2552E+14	6,63484E+14	6,62703E+14	7,15539E+14	7,28648E+14	7,60403E+14	7,3672E+14
TC0800001258.mm.2	Utp4	UTP4 small subunit processome component	0.651268194658736	7,56078E+13	1,98516E+13	0.0911437612067697	0.231369921104908	-4,2562E+14	8,07586E+14	8,09489E+14	7,60485E+14	7,26336E+14	7,38951E+14	6,9362E+13
TC0100002193.mm.2	Tmem131	transmembrane protein 131	1,10084E+14	8,1594E+14	1,98515E+14	0.0911456555901096	0.231369921104908	-4,25622E+13	8,91003E+14	8,85683E+14	8,54635E+13	7,01884E+14	8,18539E+14	7,43898E+14
TC1000000203.mm.2	Taar7e	trace amine-associated receptor 7E	-1,03795E+14	5,65017E+13	-1,98501E+14	0.091163353289155	0.231369921104908	-4,25639E+14	4,83144E+14	5,17424E+13	5,54845E+14	6,53313E+13	5,32216E+14	6,49161E+14
TC0400001352.mm.2	Slfnl1	schlafen like 1	-0.6593226337024	5,04536E+14	-1,985E+14	0.0911651119032484	0.231369921104908	-4,2564E+14	4,79094E+14	4,4684E+12	5,05746E+14	5,52052E+14	5,31578E+14	5,11909E+14
TC1000001687.mm.2	Mtrf1l	mitochondrial translational release factor 1-like	0.970822487126187	5,82166E+14	1,98499E+14	0.0911666126168349	0.231369921104908	-4,25642E+14	6,68797E+14	6,4902E+14	5,38985E+14	5,84667E+14	5,92227E+14	4,59303E+14
TC1100002754.mm.2	Slc22a4	solute carrier family 22 (organic cation transporter), member 4	0.617419787483728	6,02691E+14	1,98487E+14	0.0911819208231486	0.231372329169038	-4,25656E+14	6,13817E+14	6,2654E+14	5,52979E+14	5,63894E+14	6,098E+14	6,49116E+13
TC1100001550.mm.2	Lasp1	LIM and SH3 protein 1	0.948953243396913	9,74163E+14	1,98469E+14	0.0912053674423684	0.231395384285763	-4,25679E+14	1,04233E+14	1,03441E+14	9,99781E+14	8,87165E+14	9,79506E+14	9,01788E+14
TC1700002204.mm.2	Guca1a	guanylate cyclase activator 1a (retina)	-0.959183282078571	5,86469E+14	-1,98457E+14	0.091220474785794	0.231397278057856	-4,25693E+14	5,28597E+13	5,14083E+14	5,8549E+14	6,49027E+14	5,59209E+14	6,8241E+14
TC0800001551.mm.2	Sprtn	SprT-like N-terminal domain	-0.604020717754172	7,3874E+14	-1,98411E+14	0.0912808149502508	0.231468305411011	-4,25751E+14	6,98972E+14	6,85234E+14	7,65929E+14	7,39081E+14	7,81513E+14	7,61711E+14
TC0100002266.mm.2	Uxs1	UDP-glucuronate decarboxylase 1	1,44732E+14	6,70009E+13	1,98401E+14	0.0912936761998468	0.231468305411011	-4,25763E+14	7,55968E+13	7,65246E+14	6,95129E+14	5,36623E+14	7,23842E+14	5,43247E+14
TC0500001795.mm.2	Rpl21	ribosomal protein L21	1,49364E+14	1,04174E+14	1,98395E+14	0.0913009764140032	0.231468305411011	-4,2577E+14	1,1354E+14	1,13166E+14	1,08258E+14	8,85758E+14	1,08421E+14	9,30824E+14
TC0300000609.mm.2	Fstl5	follistatin-like 5	1,255E+14	7,12134E+12	1,98386E+14	0.0913130380884164	0.231468305411011	-4,25782E+14	8,00224E+14	7,5838E+14	7,46591E+14	5,61013E+14	7,37436E+14	6,6916E+14
TC1400001557.mm.2	Dupd1	dual specificity phosphatase and pro isomerase domain containing 1	-0.601908146868682	5,9764E+14	-1,9838E+14	0.0913203014645191	0.231468305411011	-4,25789E+14	5,63713E+14	5,72392E+14	6,20611E+13	6,35876E+14	5,81512E+14	6,11739E+14
TC1100004064.mm.2	Fads6	fatty acid desaturase domain family, member 6	0.671207869498513	6,31138E+13	1,98297E+14	0.0914286122612793	0.231684314286072	-4,25892E+13	7,16232E+14	6,60686E+14	6,09832E+14	6,32845E+14	5,94826E+14	5,72406E+14
TC1500001412.mm.2	Azin1	antizyme inhibitor 1	1,59977E+14	8,0086E+14	1,98291E+14	0.0914369211565921	0.231684314286072	-4,259E+14	9,10738E+14	9,15584E+14	8,20557E+14	6,85813E+14	8,53404E+14	6,19063E+14
TC1200001421.mm.2	Vsnl1	visinin-like 1	1,8042E+14	1,13535E+14	1,98233E+14	0.0915120784545084	0.231684314286072	-4,25972E+14	1,24064E+13	1,23507E+14	1,19591E+14	9,18954E+14	1,18002E+14	1,04152E+14
TC0200005471.mm.2	Ak1	adenylate kinase 1	0.832903463287897	8,89838E+14	1,98229E+14	0.0915176686325709	0.231684314286072	-4,25977E+14	9,48004E+14	9,35597E+14	9,04464E+14	8,12557E+14	8,98729E+14	8,39676E+14
TC0100001932.mm.2	Sntg1	syntrophin, gamma 1	1,21724E+14	6,93415E+14	1,98225E+14	0.0915230884200288	0.231684314286072	-4,25983E+14	7,75879E+14	7,73432E+13	7,23872E+14	5,81991E+14	7,19262E+14	5,86055E+14
TC1100001105.mm.2	Bhlha9	basic helix-loop-helix family, member a9	-0.812429368913469	7,10448E+14	-1,98224E+14	0.0915245441758462	0.231684314286072	-4,25984E+14	6,48032E+14	6,5707E+14	7,0228E+13	7,65308E+14	7,03369E+14	7,86631E+14
TC1400000277.mm.2	Tasor	transcription activation suppressor	1,97609E+14	6,74613E+14	1,98212E+14	0.0915402121573026	0.231684314286072	-4,25999E+14	8,18434E+14	8,14545E+14	7,19781E+14	5,17979E+14	7,26742E+14	4,50199E+14
TC1700000092.mm.2	Pde10a	phosphodiesterase 10A	1,05844E+14	9,501E+14	1,98204E+14	0.09154979203613	0.231684314286072	-4,26008E+14	1,00551E+14	1,0161E+14	9,68703E+14	8,41211E+14	9,83767E+14	8,85318E+14
TC0500001767.mm.2	Cpsf4	cleavage and polyadenylation specific factor 4	-0.590691656953666	6,78301E+14	-1,98202E+14	0.0915531323985904	0.231684314286072	-4,26011E+13	6,38466E+13	6,56568E+14	6,97049E+14	7,16124E+14	6,86137E+14	6,75465E+14
TC0700000303.mm.2	Trappc6a	trafficking protein particle complex 6A	-0.738180431709822	7,24692E+14	-1,98189E+14	0.0915699899704448	0.231684314286072	-4,26027E+14	6,78998E+14	6,93343E+14	7,22001E+14	7,97976E+14	7,45569E+14	7,10263E+14
TC0700001362.mm.2	Pak1	p21 (RAC1) activated kinase 1	1,70911E+14	9,16176E+14	1,98184E+14	0.0915767236597005	0.231684314286072	-4,26034E+13	1,02006E+14	9,9155E+14	9,67613E+14	7,02177E+14	9,66488E+14	8,49168E+13
TC1000002645.mm.2	Chpt1	choline phosphotransferase 1	1,17989E+13	7,19794E+14	1,98183E+14	0.0915780672555077	0.231684314286072	-4,26035E+14	8,17188E+14	7,89792E+14	7,68171E+14	6,02831E+14	7,11869E+13	6,28916E+14
TC1900001709.mm.2	Ccdc186	coiled-coil domain containing 186	1,13877E+14	7,56568E+14	1,9816E+14	0.0916082748110567	0.231724353536979	-4,26064E+14	8,27905E+14	8,488E+13	7,67544E+14	6,81407E+14	7,84335E+14	6,29414E+14
TC0100002236.mm.2	Tbc1d8	TBC1 domain family, member 8	-0.694008090492292	7,08985E+14	-1,98114E+14	0.0916679471401906	0.231838900058058	-4,26121E+14	6,50367E+14	7,01995E+14	7,30124E+14	7,6104E+13	7,24839E+14	6,85548E+14
TC0400004127.mm.2	Prxl2b	peroxiredoxin like 2B	0.716014500945422	6,86134E+13	1,98089E+14	0.0917002303057508	0.231884151116849	-4,26151E+14	7,46779E+13	7,33207E+14	6,54654E+14	6,8213E+14	6,86236E+13	6,13801E+14
TC1200001886.mm.2	Trim9	tripartite motif-containing 9	0.960966910789033	7,99226E+14	1,98073E+14	0.0917212830739386	0.231887051878962	-4,26171E+14	8,52512E+14	8,77144E+14	8,32516E+14	7,04947E+14	7,90453E+14	7,37782E+14
TC0700004279.mm.2	Atp2a1	ATPase, Ca++ transporting, cardiac muscle, fast twitch 1	-0.7496154049277	4,82213E+14	-1,98055E+14	0.0917455377847	0.231887051878962	-4,26194E+14	4,3008E+14	4,71368E+14	5,04994E+14	5,46378E+14	5,03353E+14	4,37102E+14
TC1000002562.mm.2	Zfp938	zinc finger protein 938	0.798122432195413	7,57159E+14	1,98051E+13	0.0917509973918055	0.231887051878962	-4,262E+14	8,15539E+14	8,04097E+14	7,74617E+14	6,85395E+14	7,54984E+14	7,08321E+14
TC0800001837.mm.2	Gm14851	predicted gene 14851	-0.642393835682372	6,42594E+14	-1,98035E+14	0.0917714530281284	0.231887051878962	-4,26219E+14	5,84189E+13	6,15059E+14	6,43697E+14	6,8403E+14	6,73456E+14	6,55131E+14
TC1700001813.mm.2	Tff1	trefoil factor 1	-1,14063E+14	5,78109E+14	-1,98033E+14	0.0917744657668667	0.231887051878962	-4,26222E+14	4,88569E+14	5,49635E+14	5,79709E+14	6,8662E+14	5,17078E+14	6,47043E+14
TC0700000225.mm.2	Zc3h4	zinc finger CCCH-type containing 4	0.685137032948939	6,78111E+14	1,98022E+14	0.0917877252457033	0.231887051878962	-4,26235E+14	7,44999E+14	7,02561E+14	6,75075E+14	6,35458E+13	6,36526E+14	6,74046E+14
TC1100000601.mm.2	Aff4	AF4/FMR2 family, member 4	1,56076E+14	9,05357E+14	1,98008E+14	0.0918066655348838	0.231888219225785	-4,26253E+13	1,03197E+14	1,0275E+14	9,34932E+14	8,12389E+14	9,29136E+14	6,96212E+14
TC0900002216.mm.2	Pafah1b2	platelet-activating factor acetylhydrolase, isoform 1b, subunit 2	1,22273E+14	8,30512E+14	1,97994E+14	0.0918254622066598	0.231888219225785	-4,26271E+14	9,19428E+14	9,08588E+13	8,5683E+14	7,26641E+14	8,58877E+14	7,12706E+14
TC0100001473.mm.2	Pigc	phosphatidylinositol glycan anchor biosynthesis, class C	-0.725013799153791	5,85833E+14	-1,97984E+14	0.0918384488014805	0.231888219225785	-4,26283E+14	5,52081E+14	5,75344E+14	6,49483E+14	6,22944E+14	5,94261E+14	5,20886E+14

TC1600002061.mm.2	Paxbp1	PAX3 and PAX7 binding protein 1	0.641247243079613	6,71418E+14	1,97978E+14	0.0918457528144331	0.231888219225785	-4,2629E+14	7,23496E+14	7,06508E+14	6,36348E+14	6,65406E+14	6,27507E+14	6,69244E+14
TC0300000236.mm.2	Fxr1	fragile X mental retardation gene 1, autosomal homolog	1,02085E+14	8,0653E+14	1,97949E+14	0.0918840598530921	0.231908158645205	-4,26326E+14	8,72406E+14	8,79363E+14	8,22322E+14	7,25277E+14	8,29324E+14	7,10488E+14
TC1400002050.mm.2	Dad1	defender against cell death 1	1,41128E+14	1,11628E+14	1,97929E+14	0.0919106090799691	0.231908158645205	-4,26351E+14	1,20523E+13	1,19038E+14	1,16406E+14	9,49302E+14	1,14026E+14	1,04846E+13
TC1000002586.mm.2	Appl2	adaptor protein, phosphotyrosine interaction, PH domain and leucine zipper containing 2	1,81329E+13	6,79942E+14	1,97919E+13	0.091923463765636	0.231908158645205	-4,26364E+14	7,7857E+14	7,68543E+14	7,41267E+14	4,4319E+14	7,19833E+13	6,2825E+13
TC1400002080.mm.2	Ap1g2	adaptor protein complex AP-1, gamma 2 subunit	-0.737080855848101	5,32744E+14	-1,97915E+14	0.0919288591952236	0.231908158645205	-4,26369E+14	4,66106E+13	5,15464E+14	5,34312E+14	5,94674E+13	5,26066E+13	5,59843E+14
TC0300001076.mm.2	Slc16a1	solute carrier family 16 (monocarboxylic acid transporters), member 1	1,31371E+14	6,92648E+14	1,97887E+14	0.0919653367587916	0.231908158645205	-4,26403E+14	7,48896E+14	7,85022E+14	7,38416E+14	5,3276E+13	7,00226E+14	6,5057E+14
TC0900001251.mm.2	Nme9/1600029I14Rik	NME/NM23 family member 9/RIKEN cDNA 1600029I14 gene	-0.686327274418296	6,6002E+14	-1,97881E+14	0.0919724938757776	0.231908158645205	-4,2641E+14	6,16965E+14	6,06047E+14	6,62004E+14	6,98273E+14	6,59178E+14	7,17652E+14
TC0200005211.mm.2	Adnp/Dpm1	activity-dependent neuroprotective protein/dolichol-phosphate (beta-D) mannosyltransferase 1	1,91681E+14	7,09322E+14	1,97875E+14	0.0919813103281497	0.231908158645205	-4,26419E+14	8,09139E+14	8,31359E+14	7,8312E+14	4,74016E+14	7,38376E+14	6,19921E+14
TC0700001980.mm.2	Glrx3/Gm12669	glutaredoxin 3/glutaredoxin 3 pseudogene	1,31721E+12	7,20806E+14	1,97864E+14	0.0919948647069948	0.231908158645205	-4,26431E+13	7,95771E+14	8,00871E+14	7,54187E+14	5,79011E+14	7,60083E+14	6,34911E+14
TC1100003990.mm.2	Slc16a6	solute carrier family 16 (monocarboxylic acid transporters), member 6	1,6399E+14	4,92638E+14	1,97864E+14	0.0919957309683129	0.231908158645205	-4,26432E+14	5,52477E+14	5,91853E+14	5,35584E+14	2,80766E+14	5,50597E+13	4,4455E+14
TC0700000151.mm.2	Rps5	ribosomal protein S5	0.848229067640851	8,4E+14	1,97862E+14	0.091997576494765	0.231908158645205	-4,26434E+14	8,96871E+14	8,88942E+14	8,55993E+14	7,60174E+14	8,49729E+13	7,88291E+14
TC0900001239.mm.2	Prr23a3	proline rich 23A, member 3	-0.970876101220838	5,13419E+14	-1,97813E+14	0.0920619249310082	0.232000547363123	-4,26495E+13	4,34228E+14	4,52249E+14	4,8854E+14	5,92112E+14	5,08444E+14	6,04942E+14
TC0600002161.mm.2	Kel	Kell blood group	-1,40325E+14	5,72676E+14	-1,97812E+14	0.0920630236355619	0.232000547363123	-4,26496E+14	4,73953E+14	4,58862E+14	5,24736E+14	6,88729E+14	5,62756E+14	7,27019E+14
TC1900000517.mm.2	Pcgf5	polycomb group ring finger 5	1,01102E+14	6,76433E+14	1,97791E+14	0.0920909336194231	0.232003827891506	-4,26523E+13	7,72759E+13	7,34477E+13	6,9686E+14	6,08172E+14	6,81592E+14	5,64736E+14
TC1300000657.mm.2	Rgs14	regulator of G-protein signaling 14	-0.706476505491313	7,86736E+14	-1,9779E+14	0.092093122521819	0.232003827891506	-4,26525E+14	7,20496E+14	7,71774E+14	7,96762E+14	8,36803E+14	7,77553E+14	8,17025E+14
TC1800000641.mm.2	Htr4	5 hydroxytryptamine (serotonin) receptor 4	-0.805722617859614	7,04224E+14	-1,97749E+14	0.0921469232157332	0.23207871395852	-4,26576E+14	6,57946E+14	6,47107E+14	7,81526E+14	6,84672E+14	7,44624E+13	7,09471E+14
TC1700002673.mm.2	Gm10309	predicted gene 10309	-0.715065027719827	5,07399E+14	-1,97745E+14	0.0921516546829647	0.23207871395852	-4,2658E+14	4,94052E+14	4,34529E+14	5,34382E+14	5,37212E+14	4,96375E+14	5,47842E+14
TC1400001221.mm.2	Frg2f2	FSHD region gene 2 family member 2	-0.592667067373096	5,48128E+14	-1,9771E+14	0.0921971288849469	0.23209010724264	-4,26623E+14	5,25778E+13	4,96003E+14	5,67918E+13	5,72396E+14	5,60482E+13	5,66192E+14
TC1400001222.mm.2	Frg2f12	FSHD region gene 2 family member 12	-0.592667067373096	5,48128E+14	-1,9771E+14	0.0921971288849469	0.23209010724264	-4,26623E+14	5,25778E+13	4,96003E+14	5,67918E+13	5,72396E+14	5,60482E+13	5,66192E+14
TC0300002955.mm.2	Hadh	hydroxyacyl-Coenzyme A dehydrogenase	0.713602185195137	6,42833E+13	1,97691E+14	0.0922229875220525	0.23209010724264	-4,26648E+14	6,9261E+14	7,17455E+14	6,32072E+14	6,35273E+14	5,5267E+14	6,26918E+14
TC0100001833.mm.2	A230020J21Rik	RIKEN cDNA A230020J21 gene	-0.739873912478829	6,44667E+14	-1,9769E+14	0.0922239082188186	0.23209010724264	-4,26648E+14	6,02358E+14	5,94892E+14	6,41568E+14	7,03658E+14	6,3324E+14	6,92284E+14
TC0200004512.mm.2	Slc30a4	solute carrier family 30 (zinc transporter), member 4	1,05742E+14	7,51474E+14	1,97687E+14	0.0922281981427805	0.23209010724264	-4,26653E+14	7,92994E+14	8,11574E+14	7,74083E+14	6,19002E+14	7,76719E+14	7,34468E+14
TC0100000816.mm.2	Snorc	secondary ossification center associated regulator of chondrocyte maturation	-0.662948640172404	5,43927E+14	-1,97667E+14	0.0922541889218331	0.23211926078974	-4,26677E+14	5,21116E+14	5,1874E+14	5,57957E+14	6,14489E+14	5,12906E+14	5,38353E+14
TC0100002921.mm.2	Dsel	dermatan sulfate epimerase-like	1,0275E+14	6,51136E+14	1,97593E+14	0.0923512729290812	0.232296391211013	-4,26769E+14	7,05029E+14	7,29882E+14	6,20725E+14	6,08684E+14	6,97729E+14	5,44765E+14
TC0600000588.mm.2	Npy	neuropeptide Y	0.642184295126674	7,84978E+14	1,97581E+14	0.0923673748442075	0.232296391211013	-4,26784E+14	8,13498E+14	8,25626E+14	7,81856E+14	7,28831E+14	7,81621E+14	7,78437E+14
TC0800000294.mm.2	Nkx6-3	NK6 homeobox 3	-0.788272343782939	6,77374E+14	-1,97569E+14	0.0923836210177973	0.232296391211013	-4,26799E+13	6,24864E+14	6,22757E+14	6,53387E+14	7,51888E+14	6,99568E+14	7,11782E+14
TC0700004649.mm.2	Flt3l	FMS-like tyrosine kinase 3 ligand	-0.844141248391574	6,79906E+14	-1,97556E+14	0.09240031010978	0.232296391211013	-4,26815E+14	6,36671E+14	6,22201E+14	6,68191E+14	7,5951E+14	6,63586E+14	7,2928E+14
TC0300000360.mm.2	Rab33b	RAB33B, member RAS oncogene family	0.661213119019255	7,17356E+14	1,97536E+14	0.0924262510591592	0.232296391211013	-4,2684E+14	7,83238E+14	7,48454E+14	7,18706E+14	6,80744E+14	7,0339E+14	6,69603E+14
TC1800000418.mm.2	Kctd16	potassium channel tetramerisation domain containing 16	0.600676330031 75	7,56263E+14	1,97533E+14	0.0924306928071692	0.232296391211013	-4,26844E+14	7,92304E+14	8,00137E+14	7,22433E+14	7,49873E+14	7,47147E+14	7,25682E+14
TC0800002369.mm.2	Zfp869	zinc finger protein 869	1,10718E+14	5,57194E+14	1,9753E+14	0.0924351084887184	0.232296391211013	-4,26848E+14	6,16672E+14	6,13905E+14	5,85957E+14	4,23185E+14	5,80389E+13	5,23059E+14
TC1500000596.mm.2	Slc52a2	solute carrier protein 52, member 2	0.667358633974473	6,01446E+14	1,97526E+14	0.0924399128298898	0.232296391211013	-4,26853E+14	6,51837E+14	6,33678E+14	6,04695E+14	5,47348E+14	5,96103E+13	5,75015E+14
TC0400001034.mm.2	Ak4	adenylate kinase 4	-0.848424145992844	7,36599E+14	-1,9749E+14	0.0924875818874431	0.232332131457523	-4,26898E+14	6,97564E+13	6,83068E+14	8,21396E+14	7,28922E+14	7,77326E+14	7,11315E+14
TC0900002820.mm.2	Morf4l1	mortality factor 4 like 1	0.770394305561814	1,08879E+14	1,97481E+13	0.0924992339468077	0.232332131457523	-4,26909E+14	1,14977E+14	1,14187E+14	1,11297E+14	1,02459E+14	1,06822E+14	1,03534E+14
TC1400002858.mm.2	Colq	collagen-like tail subunit (single strand of homotrimer) of asymmetric acetylcholinesterase	-0.713697964050184	7,02477E+14	-1,97473E+14	0.0925103651640325	0.232332131457523	-4,26919E+14	6,4126E+14	6,49045E+14	6,78446E+14	7,54598E+14	7,36783E+14	7,54729E+14
TC1500001790.mm.2	C030006K11Rik/Lrrc24	RIKEN cDNA C030006K11 gene/leucine rich repeat containing 24	-0.790812815033303	7,18228E+14	-1,97465E+13	0.0925209059823319	0.232332131457523	-4,26929E+13	6,60525E+14	6,96584E+14	7,2228E+14	7,92992E+14	6,88042E+14	7,48949E+14
TC0900000761.mm.2	Gm7616	predicted gene 7616	-0.78385589055264	5,24507E+13	-1,97454E+14	0.0925350079103002	0.232332131457523	-4,26942E+14	4,67319E+14	4,75907E+14	5,08648E+14	5,91349E+14	5,25749E+14	5,78073E+14
TC1800000762.mm.2	Mbd2	methyl-CpG binding domain protein 2	1,04439E+14	8,53308E+14	1,9745E+14	0.0925406578349396	0.232332131457523	-4,26948E+14	9,30196E+14	9,23008E+14	8,71611E+14	7,72715E+14	8,73475E+14	7,48843E+14
TC0800000756.mm.2	Isyna1	myo-inositol 1-phosphate synthase A1	0.580155117118673	7,03148E+14	1,97414E+14	0.0925877855666081	0.232370775955725	-4,26992E+14	7,44953E+14	7,49546E+14	7,00023E+14	6,78445E+14	6,73822E+14	6,72097E+14
TC0900001895.mm.2	Zfp653	zinc finger protein 653	-0.858684156731455	7,06338E+14	-1,97411E+14	0.0925916453838954	0.232370775955725	-4,26996E+14	6,5612E+14	6,47981E+14	6,83727E+14	7,92111E+14	7,07234E+14	7,50853E+14
TC0200003317.mm.2	Slc2a8	solute carrier family 2, (facilitated glucose transporter), member 8	-1,17077E+14	6,82245E+14	-1,97405E+13	0.092599314368008	0.232370775955725	-4,27003E+14	5,85995E+13	6,05579E+13	6,32986E+14	7,92743E+14	6,91405E+14	7,84763E+14
TC1300001776.mm.2	Serpinb6c	serine (or cysteine) peptidase inhibitor, clade B, member 6c	-0.919164034918967	4,82522E+14	-1,97384E+14	0.0926269412904631	0.232403909220372	-4,27029E+14	4,03455E+14	4,39115E+14	4,51478E+14	5,74925E+14	5,26875E+14	4,99282E+14
TC0400004030.mm.2	Pex14	peroxisomal biogenesis factor 14	-0.79146151774493	8,29198E+14	-1,97329E+14	0.0926998966179917	0.232550744855317	-4,27098E+14	7,78627E+14	7,85962E+14	8,15321E+13	9,07561E+14	8,24426E+14	8,63289E+14
TC0200005414.mm.2	Tcfl5	transcription factor-like 5 (basic helix-loop-helix)	-0.6206679149097	4,89099E+14	-1,97293E+13	0.0927479876222111	0.232635168828874	-4,27143E+14	4,63886E+14	4,64121E+14	5,05555E+14	5,46586E+14	4,87557E+14	4,66887E+14
TC1000002549.mm.2	Tle6	transducin-like enhancer of split 6	-0.67992714791717	4,80607E+14	-1,97232E+14	0.0928284184435655	0.232800670253879	-4,27219E+14	4,40916E+14	4,25667E+14	4,69782E+14	5,32787E+14	4,96466E+14	5,18021E+14
TC0400001612.mm.2	Nr0b2	nuclear receptor subfamily 0, group B, member 2	-0.761166925940264	6,36367E+14	-1,97202E+14	0.0928683883946592	0.232864541777866	-4,27256E+14	6,0597E+14	6,04751E+14	6,35344E+14	7,2761E+13	6,19574E+14	6,24953E+14
TSUnmapped00000052.mm.2	Defa-rs12	defensin, alpha, related sequence 12	-0.724666318436508	5,30576E+13	-1,97191E+14	0.0928827909145624	0.232864541777866	-4,2727E+14	5,05073E+14	4,71621E+14	5,27822E+14	5,93805E+14	5,24686E+14	5,60448E+14
TC0100001442.mm.2	Cop1	COP1, E3 ubiquitin ligase	1,23479E+14	6,64041E+14	1,97165E+14	0.0929173380761762	0.232908182672538	-4,27302E+14	7,54778E+13	7,38815E+14	6,94024E+14	5,52611E+14	6,91964E+14	5,52055E+14
TC0300003233.mm.2	Lrrc71	leucine rich repeat containing 71	-0.835480360134894	5,75059E+14	-1,97145E+13	0.0929428615006785	0.232908182672538	-4,27326E+14	5,4623E+14	5,16831E+14	5,61594E+14	6,68563E+14	5,85165E+14	5,71971E+13
TC0X00000181.mm.2	Maoa	monoamine oxidase A	1,3427E+14	6,23098E+14	1,97145E+14	0.0929435620048667	0.232908182672538	-4,27327E+14	7,00955E+14	6,82712E+13	6,59E+14	4,56128E+14	6,63344E+14	5,76448E+14
TC0100001481.mm.2	Myoc	myocilin	-0.59728157812191	5,7203E+14	-1,97114E+14	0.0929844000923997	0.232974286843234	-4,27365E+14	5,49923E+14	5,38458E+14	5,87855E+14	6,19983E+13	5,70594E+14	5,65367E+14
TC0800002704.mm.2	Ces1e	carboxylesterase 1E	-0.771878720019416	5,01969E+13	-1,97091E+14	0.0930154673679557	0.233015893299109	-4,27394E+14	4,84539E+14	4,24903E+14	4,9473E+14	5,69088E+14	5,21688E+14	5,16864E+14
TC1300000009.mm.2	Asb13	ankyrin repeat and SOCS box-containing 13	1,69975E+14	8,0254E+14	1,97079E+14	0.0930308514871219	0.23301820457205	-4,27409E+14	9,18781E+14	9,20209E+14	8,66846E+14	6,32194E+14	8,25193E+14	6,5202E+14
TC0900001116.mm.2	A330041J22Rik	RIKEN cDNA A330041J22 gene	-0.693915278146132	6,38149E+14	-1,9705E+14	0.0930690494364428	0.233060714436942	-4,27444E+14	5,76114E+14	5,7838E+13	6,22897E+13	6,7038E+14	6,62849E+14	7,18273E+14
TC1600001750.mm.2	Tfg	Trk-fused gene	2,64868E+14	6,68727E+14	1,97044E+14	0.0930767515299274	0.233060714436942	-4,27452E+14	8,5056E+14	8,20178E+14	7,85595E+14	3,55407E+14	6,94898E+14	5,05722E+14
TC0200003204.mm.2	Surf1	surfeit gene 1	-0.771843456070233	9,11928E+14	-1,97024E+14	0.0931044366058296	0.233093814019536	-4,27478E+14	8,72353E+14	8,65893E+14	9,24089E+14	9,68526E+14	9,62497E+14	8,78211E+13
TC1400000496.mm.2	Gm15217	predicted gene 15217	-0.7450550210233	5,25939E+13	-1,97007E+14	0.0931271570608309	0.233114300812364	-4,27499E+14	4,81952E+14	4,85431E+14	5,20013E+14	5,96382E+14	5,13945E+14	5,57909E+13
TC1200001367.mm.2	Mfsd2b	major facilitator superfamily domain containing 2B	-0.87240820181394	5,2138E+14	-1,96996E+14	0.093141554560293	0.233114300812364	-4,27512E+14	4,64082E+14	4,65579E+14	4,95551E+14	6,08592E+12	5,25876E+14	5,68603E+14
TC0600000566.mm.2	Gimap4	GTPase, IMAP family member 4	-0.846810242245609	4,79262E+14	-1,96961E+14	0.0931880524898385	0.233144388010459	-4,27556E+14	4,63706E+13	3,96868E+14	4,83456E+14	5,46481E+13	4,58193E+14	5,26869E+14
TC1500001171.mm.2	Egflam	EGF-like, fibronectin type III and laminin G domains	-0.932166123998794	4,53003E+14	-1,96955E+14	0.0931952540201937	0.233144388010459	-4,27563E+14	3,98791E+13	3,80573E+14	4,2247E+14	5,43328E+14	4,57909E+14	5,14946E+14
TC0500002799.mm.2	Paqr3	progestin and adipoQ receptor family member III	-0.618901441340297	8,43536E+13	-1,96951E+14	0.0932004338563267	0.233144388010459	-4,27568E+13	7,98508E+14	8,07618E+14	8,73595E+14	8,56312E+14	8,41972E+13	8,83211E+13
TC0900001942.mm.2	Acad8	acyl-Coenzyme A dehydrogenase family, member 8	0.591533901638236	7,36442E+14	1,96935E+14	0.0932216266943485	0.233144388010459	-4,27587E+14	7,74072E+14	7,948E+14	7,1518E+14	7,35385E+14	7,05689E+14	6,93526E+14
TC0500003045.mm.2	1700069L16Rik	RIKEN cDNA 1700069L16 gene	-0.857494321688512	5,55099E+14	-1,96932E+14	0.0932259226681182	0.233144388010459	-4,27591E+13	5,08611E+14	4,99389E+14	5,48927E+14	6,30572E+14	5,29666E+14	6,13428E+14
TC0400002570.mm.2	Fbxo10	F-box protein 10	1,02479E+14	5,92295E+14	1,96877E+14	0.0932988040741588	0.233290445382824	-4,2766E+14	7,09511E+14	6,6879E+14	6,45041E+14	5,28303E+14	5,48345E+14	4,53778E+14
TC0400003011.mm.2	Ifna16	interferon alpha 16	-1,07802E+14	5,73177E+14	-1,96849E+14	0.0933364314493726	0.233348319618889	-4,27695E+14	5,42197E+14	4,91546E+14	5,52693E+14	6,96653E+14	5,4384E+14	6,12135E+14
TC1100002333.mm.2	Otx1	orthodenticle homeobox 1	-0.945355606010881	6,72333E+14	-1,96825E+14	0.0933684702216376	0.233392206124922	-4,27725E+14	5,94366E+14	6,24169E+13	6,41688E+14	7,65918E+14	6,77801E+14	7,30058E+14
TC0900000750.mm.2	Parp6	poly (ADP-ribose) polymerase family, member 6	1,55335E+14	7,30205E+14	1,968E+14	0.0934021950767315	0.233440293046591	-4,27756E+14	8,37652E+14	8,30801E+14	7,96866E+14	5,60916E+13	7,36214E+14	6,18784E+14
TC0700004327.mm.2	Zfp688	zinc finger protein 688	-0.625166790127869	7,19978E+14	-1,96771E+14	0.0934401637237698	0.233475973673256	-4,27792E+14	7,01122E+14	6,69394E+14	7,29357E+13	7,66192E+14	7,27403E+14	7,26399E+14
TC1100003787.mm.2	Stat5b	signal transducer and activator of transcription 5B	-0.662442982082354	7,42425E+14	-1,96767E+14	0.0934454511400005	0.233475973673256	-4,27797E+14	7,13713E+14	6,86142E+14	7,45851E+14	7,86492E+14	7,41507E+14	7,80846E+13
TC0600002116.mm.2	Kdm7a	lysine (K)-specific demethylase 7A	1,60191E+14	6,25399E+14	1,96755E+14	0.0934611804329089	0.233479069816351	-4,27811E+14	7,07911E+14	7,61824E+14	6,47507E+14	5,01846E+14	6,79028E+14	4,5428E+14
TC0900000458.mm.2	Hspa8/Snord14d/Snord14e/Snord14c	heat shock protein 8/small nucleolar RNA, C/D box 14D/small nucleolar RNA, C/D box 14E/small nucleolar RNA, C/D box 14C	1,32277E+14	1,17019E+14	1,96739E+14	0.093483121748774	0.233497681094093	-4,27832E+14	1,25169E+14	1,25736E+14	1,21303E+14	1,03147E+14	1,19405E+14	1,07352E+14
TC1600000964.mm.2	2310034C09Rik	RIKEN cDNA 2310034C09 gene	-1,01451E+14	5,17859E+14	-1,96718E+14	0.0935113166548441	0.233518937667448	-4,27858E+13	4,60709E+14	4,49057E+14	5,13012E+14	5,99657E+14	4,7922E+14	6,05498E+14
TC0300000258.mm.2	Atp11b	ATPase, class VI, type 11B	1,18921E+14	6,18935E+14	1,96706E+14	0.0935274819770357	0.233518937667448	-4,27873E+13	6,30858E+14	7,2892E+14	6,13151E+14	5,08785E+14	6,78959E+14	5,52936E+14
TC0100003094.mm.2	Lax1	lymphocyte transmembrane adaptor 1	-0.68098188751092	4,83148E+14	-1,96694E+14	0.0935435291615961	0.233518937667448	-4,27888E+14	4,32722E+14	4,44322E+14	4,72632E+14	5,40608E+14	5,0102E+14	5,07581E+14
TC0900002503.mm.2	Hacd3	3-hydroxyacyl-CoA dehydratase 3	1,16595E+14	7,94194E+14	1,96689E+14	0.093549602348624	0.233518937667448	-4,27894E+14	8,63542E+14	8,63759E+14	8,33959E+14	6,60153E+14	8,04922E+14	7,38828E+14
TC1400001115.mm.2	Pcdh17	protocadherin 17	1,01999E+14	7,40781E+13	1,96659E+14	0.0935893901199287	0.23358206985787	-4,27931E+14	8,48735E+13	8,31691E+14	7,69647E+14	7,06781E+14	7,14162E+14	5,73672E+14
TC0500002075.mm.2	Atg9b	autophagy related 9B	-0.859002007800582	5,33328E+14	-1,96631E+14	0.0936268291291121	0.233615574457883	-4,27966E+14	4,60347E+13	5,05811E+14	5,36826E+13	6,01132E+14	5,03979E+14	5,91873E+14
TC0500002017.mm.2	Ccdc146	coiled-coil domain containing 146	0.571611782019582	5,65836E+14	1,96624E+14	0.0936371736528 17	0.233615574457883	-4,27975E+14	6,02015E+14	6,07934E+14	5,51859E+14	5,43768E+14	5,5087E+14	5,38568E+14
TC0700002970.mm.2	Nr1h2	nuclear receptor subfamily 1, group H, member 2	0.580449998165253	7,4106E+14	1,96617E+14	0.0936463101485424	0.233615574457883	-4,27984E+14	7,92159E+14	7,94881E+13	7,39535E+13	7,31415E+14	7,05565E+14	6,82806E+14
TC0400002633.mm.2	Acnat2	acyl-coenzyme A amino acid N-acyltransferase 2	-0.974362347880648	5,44053E+14	-1,96591E+14	0.093680161783952	0.233663846257712	-4,28015E+14	4,78924E+14	5,02917E+14	5,31407E+14	6,45307E+14	5,09791E+14	5,9597E+14
TC0700001174.mm.2	Whamm	WAS protein homolog associated with actin, golgi membranes and microtubules	1,20992E+14	5,84465E+14	1,96532E+14	0.0937594425748958	0.23377627871653	-4,28089E+14	6,41993E+14	6,43838E+14	6,19277E+14	4,24569E+14	5,81263E+14	5,95852E+14
TC1600000054.mm.2	Rbfox1	RNA binding protein, fox-1 homolog (C. elegans) 1	1,14732E+14	8,99609E+14	1,96532E+14	0.0937595631110948	0.23377627871653	-4,28089E+14	9,77904E+14	9,77305E+14	9,34661E+13	7,91083E+14	9,14475E+14	8,02228E+14
TC0600001532.mm.2	Clec12a	C-type lectin domain family 12, member a	0.740817519462952	5,06462E+14	1,96525E+14	0.0937687636904941	0.23377627871653	-4,28098E+14	5,51803E+14	5,30715E+14	4,29071E+13	5,05284E+14	5,29688E+13	4,9221E+13
TC1800001710.mm.2	Cndp1	carnosine dipeptidase 1 (metallopeptidase M20 family)	-0.908138754466745	5,83067E+13	-1,96485E+14	0.0938220327306836	0.23387289811 1	-4,28147E+14	5,71005E+14	4,96837E+14	5,71694E+14	6,77776E+14	5,95609E+14	5,85484E+14
TC1400001540.mm.2	Ppp3cb	protein phosphatase 3, catalytic subunit, beta isoform	1,83407E+14	9,76599E+14	1,96462E+14	0.0938526849131217	0.233913118639618	-4,28176E+14	1,09178E+14	1,11002E+14	1,05177E+14	7,83215E+14	9,9658E+14	8,26238E+14
TC0700000414.mm.2	4732471J01Rik	RIKEN cDNA 4732471J01 gene	-0.718060435019666	6,25762E+14	-1,96443E+14	0.0938781613381905	0.233940428957975	-4,28199E+14	5,59634E+14	5,82771E+14	6,12637E+14	6,7338E+14	6,8727E+14	6,3888E+14
TC0600002133.mm.2	Mrps33	mitochondrial ribosomal protein S33	0.850803380104374	8,19198E+13	1,96426E+14	0.093900896177657	0.233960899970711	-4,28221E+14	8,9128E+14	8,87334E+14	8,28879E+14	7,79574E+14	8,16655E+14	7,11465E+14
TC1000001255.mm.2	Ccdc59	coiled-coil domain containing 59	0.744526103371551	8,24078E+14	1,96406E+14	0.093927494484864	0.233990989275605	-4,28245E+14	8,6122E+14	8,82947E+14	8,18646E+14	7,76616E+14	8,40019E+14	7,65019E+13
TC0700002700.mm.2	Nccrp1	non-specific cytotoxic cell receptor protein 1 homolog (zebrafish)	-1,02856E+14	5,89466E+14	-1,96393E+14	0.093945692781179	0.234000146511538	-4,28262E+14	5,17733E+13	5,53956E+13	5,62993E+14	7,14408E+14	6,04104E+13	5,836E+14
TC0700004340.mm.2	Stx1b	syntaxin 1B	1,11441E+13	9,25117E+14	1,96366E+14	0.0939815810218014	0.234027151810406	-4,28295E+14	1,00519E+14	1,00066E+14	9,58316E+14	8,24651E+14	9,37774E+14	8,24115E+14
TC0700001583.mm.2	Olfr467	olfactory receptor 467	-1,13512E+14	5,2438E+14	-1,96363E+13	0.0939855830301707	0.234027151810406	-4,28299E+14	4,56226E+14	4,56575E+14	5,19585E+14	6,2024E+14	4,7228E+14	6,21375E+14
TC0900001774.mm.2	Piwil4	piwi-like RNA-mediated gene silencing 4	-1,19176E+14	4,70521E+14	-1,9633E+13	0.0940298646318272	0.234070275720905	-4,2834E+14	3,77747E+14	4,24157E+14	4,61855E+12	5,78402E+14	4,17964E+14	5,63E+14
TC0400001344.mm.2	Edn2	endothelin 2	-1,12195E+14	5,0465E+14	-1,9632E+14	0.0940429356995989	0.234070275720905	-4,28352E+14	4,34153E+14	4,4476E+13	4,65133E+14	6,38169E+14	5,04584E+14	5,41101E+13
TC1700000505.mm.2	Pim1	proviral integration site 1	-0.607997762917907	6,19812E+14	-1,96315E+13	0.0940495412228501	0.234070275720905	-4,28359E+14	5,88943E+14	5,61622E+14	6,51131E+14	6,21034E+14	6,44005E+14	6,52134E+14
TC0200003312.mm.2	Cfap157	cilia and flagella associated protein 157	-0.721922854273071	6,81943E+13	-1,96297E+14	0.0940735341504891	0.234070275720905	-4,28381E+14	6,22696E+14	6,29958E+14	6,76334E+14	7,20704E+14	6,86499E+14	7,55465E+14
TC0400000725.mm.2	Atp6v1g1	ATPase, H+ transporting, lysosomal V1 subunit G1	0.583769730264004	7,84738E+14	1,96293E+14	0.0940799085301101	0.234070275720905	-4,28387E+14	8,15756E+14	8,30149E+14	7,53349E+14	7,75802E+14	7,6329E+14	7,70079E+14
TC0700004281.mm.2	Atxn2l	ataxin 2-like	1,69116E+14	7,62195E+14	1,96285E+14	0.0940900624243965	0.234070275720905	-4,28396E+14	8,58734E+14	8,8604E+14	8,28553E+14	5,7799E+14	7,83946E+14	6,37906E+14
TC1100001609.mm.2	Krtap31-1	keratin associated protein 31-1	-0.864764330303913	4,91424E+14	-1,96265E+13	0.0941173436859749	0.234088102333456	-4,28421E+14	4,51201E+14	3,78704E+14	4,71191E+14	5,31666E+14	5,21021E+14	5,94762E+14
TC1900001549.mm.2	Cpn1	carboxypeptidase N, polypeptide 1	-0.695181519361459	6,05019E+14	-1,96256E+14	0.094129661504109	0.234088102333456	-4,28433E+14	5,56922E+14	5,70328E+14	6,05408E+13	6,60879E+14	5,94503E+14	6,42075E+14
TC0700004548.mm.2	Tollip	toll interacting protein	0.622410644437302	8,3508E+14	1,96222E+13	0.0941752453746961	0.234088102333456	-4,28475E+14	8,86726E+14	8,69014E+14	8,37078E+14	7,94181E+13	8,05247E+14	8,18231E+14
TC0700002594.mm.2	Zfp61	zinc finger protein 61	-0.757117259681181	7,65573E+14	-1,96217E+14	0.0941818150834531	0.234088102333456	-4,28481E+14	7,26095E+14	7,10016E+14	7,48924E+14	8,3861E+14	7,77335E+13	7,9246E+14
TC1700000902.mm.2	2310039H08Rik	RIKEN cDNA 2310039H08 gene	-0.887095848365511	6,06613E+14	-1,96216E+14	0.0941825678974312	0.234088102333456	-4,28482E+13	5,36145E+13	5,61943E+14	5,87073E+13	6,88434E+14	5,98652E+14	6,67431E+14
TC0100000127.mm.2	Il17a	interleukin 17A	-0.821568119908088	6,72251E+14	-1,96205E+14	0.0941979765596226	0.234088102333456	-4,28496E+14	6,37362E+14	6,24968E+14	6,68834E+14	7,57809E+14	6,41193E+14	7,03341E+14
TC0700001546.mm.2	Olfr688	olfactory receptor 688	-0.787525212302777	5,22577E+14	-1,96202E+14	0.0942017578258477	0.234088102333456	-4,285E+14	4,42894E+13	4,87113E+14	5,68853E+14	5,18659E+14	5,2146E+13	5,96482E+13
TC0X00001403.mm.2	Gm5643	heterogeneous nuclear ribonucleoprotein A1 pseudogene	0.899266832707115	8,4478E+14	1,96186E+14	0.0942237405896296	0.234088102333456	-4,2852E+14	9,0168E+14	9,11744E+14	8,76868E+14	7,56703E+14	8,3243E+14	7,89255E+14
TC0600002878.mm.2	Crbn	cereblon	1,10847E+14	8,07368E+14	1,96182E+14	0.094227979379 06	0.234088102333456	-4,28524E+14	8,92036E+13	8,95061E+14	8,41301E+13	7,24103E+14	8,10462E+14	6,81245E+14
TC0200003134.mm.2	Stpg3	sperm tail PG rich repeat containing 3	-0.748480531849674	4,98025E+14	-1,96134E+14	0.0942926031261862	0.234212534942537	-4,28584E+14	4,2359E+14	4,53665E+14	4,75268E+14	5,51683E+14	5,23477E+14	5,60468E+14
TC0200000694.mm.2	Crb2	crumbs family member 2	-0.572243975124738	5,82626E+14	-1,96082E+13	0.0943633223525723	0.234333124607149	-4,28649E+14	5,52824E+14	5,68609E+14	6,21561E+14	6,1432E+14	5,72163E+14	5,66281E+14
TC0300001960.mm.2	Maml3	mastermind like transcriptional coactivator 3	-0.835555092863624	6,55625E+14	-1,96077E+14	0.0943702380423131	0.234333124607149	-4,28655E+14	6,22313E+13	6,02604E+13	6,51351E+14	7,40677E+14	6,25351E+14	6,91456E+14

TC0500003513.mm.2	Ttyh3	tweety family member 3	1,02021E+14	8,90492E+14	1,9604E+14	0.0944199703630652	0.234420490363647	-4,28701E+14	9,51816E+14	9,66508E+14	9,30331E+14	7,83951E+14	8,80527E+14	8,29817E+14
TC1000002443.mm.2	Pwp2	PWP2 periodic tryptophan protein homolog (yeast)	0.631013760877319	6,02636E+14	1,95966E+14	0.0945195743910687	0.234605390251359	-4,28793E+14	6,23272E+13	6,43675E+14	5,91824E+14	5,48919E+14	5,93334E+14	6,1479E+14
TC0800002565.mm.2	Il27ra	interleukin 27 receptor, alpha	-0.873198806212481	5,35484E+14	-1,95954E+14	0.0945354818086943	0.234605390251359	-4,28808E+14	5,04149E+14	4,52727E+14	5,61713E+14	5,69803E+14	5,07002E+14	6,17507E+14
TC1500002214.mm.2	Faim2	Fas apoptotic inhibitory molecule 2	1,24649E+14	1,008E+13	1,95947E+13	0.0945453358562389	0.234605390251359	-4,28817E+14	1,11205E+14	1,07475E+13	1,06086E+14	8,76653E+13	1,00091E+14	9,22767E+14
TC1900001045.mm.2	Rtn3	reticulon 3	1,35297E+14	1,08652E+14	1,95941E+14	0.094552684434452	0.234605390251359	-4,28824E+14	1,17439E+14	1,17483E+13	1,13045E+14	9,48182E+14	1,11175E+14	9,79526E+14
TC0800000022.mm.2	Trappc5	trafficking protein particle complex 5	-0.598916841690407	7,20936E+14	-1,95907E+14	0.0945988607812446	0.234683825060538	-4,28866E+14	6,82243E+14	6,76413E+14	7,20854E+14	7,57585E+14	7,46462E+14	7,42059E+14
TC0200004897.mm.2	Nrsn2	neurensin 2	0.90939992098299	8,25058E+14	1,95841E+14	0.0946872039562544	0.234866828409348	-4,28948E+13	9,39464E+14	8,69211E+14	8,63671E+14	7,63124E+14	7,80183E+14	7,34692E+14
TC0X00000685.mm.2	Bgn	biglycan	0.772445668448031	6,65139E+14	1,95815E+14	0.0947227541329174	0.23489722528256	-4,2898E+14	7,10839E+14	7,44845E+14	6,58486E+14	6,42709E+14	6,61674E+14	5,72283E+14
TC1400002796.mm.2	Cphx2/Cphx1	cytoplasmic polyadenylated homeobox 2/cytoplasmic polyadenylated homeobox 1	-0.705777570532936	5,08635E+14	-1,95805E+14	0.0947358449528767	0.23489722528256	-4,28992E+14	4,63048E+14	4,65576E+14	5,0352E+14	5,6626E+14	5,03524E+14	5,49884E+14
TC1000000604.mm.2	Dnajc12	DnaJ heat shock protein family (Hsp40) member C12	0.626221807273983	6,99875E+14	1,958E+13	0.0947437745000807	0.23489722528256	-4,29E+14	7,38787E+14	7,18458E+14	6,45308E+14	6,86692E+14	6,94851E+14	7,15152E+14
TC0500000048.mm.2	Slc25a40	solute carrier family 25, member 40	0.613166502902634	6,04849E+14	1,95789E+13	0.094757771013259	0.23489722528256	-4,29013E+14	6,48828E+14	6,35075E+14	5,83707E+14	5,77562E+14	5,70929E+13	6,12996E+14
TC1100000035.mm.2	Rnf215	ring finger protein 215	0.805255728144638	6,44711E+14	1,95758E+14	0.0947995329508115	0.234954566774703	-4,29051E+14	6,57369E+14	6,92452E+14	5,53506E+14	6,35264E+13	6,36422E+14	6,93253E+13
TC0300001581.mm.2	Ankrd13c	ankyrin repeat domain 13c	1,36036E+13	7,47487E+14	1,9575E+14	0.0948100659820714	0.234954566774703	-4,29061E+14	8,35866E+14	8,37469E+14	7,89737E+13	6,11525E+14	7,75401E+14	6,34924E+14
TC0300001120.mm.2	Alx3	aristaless-like homeobox 3	-0.89064910668719	6,55496E+14	-1,95719E+14	0.0948529653146374	0.235013743666343	-4,291E+13	6,16627E+14	5,82241E+14	6,33381E+14	7,43617E+14	6,52662E+14	7,04449E+14
TC0300002366.mm.2	Rps27rt/Rps27	ribosomal protein S27, retrogene/ribosomal protein S27	0.775684441331792	9,28256E+14	1,95711E+14	0.0948631160433124	0.235013743666343	-4,29109E+14	9,78523E+14	9,70272E+14	9,37786E+13	8,55873E+14	9,40033E+14	8,87045E+13
TC0300002382.mm.2	Lor	loricrin	-0.658696632490934	5,40832E+14	-1,95695E+14	0.0948848958810388	0.235031564539766	-4,29129E+14	5,15118E+14	4,88865E+14	5,41562E+14	5,9416E+14	5,63798E+14	5,41492E+14
TC0500002265.mm.2	Mrfap1	Morf4 family associated protein 1	0.743612484184442	8,93185E+14	1,95617E+14	0.094990877497172	0.235257917170601	-4,29227E+14	9,43876E+14	9,42057E+14	8,95475E+14	8,41735E+14	9,03608E+14	8,32361E+14
TC0100000655.mm.2	Stk16	serine/threonine kinase 16	0.976529668743281	6,83212E+14	1,95603E+13	0.0950091090433957	0.235266757192796	-4,29243E+14	7,46981E+14	7,15625E+14	6,84142E+14	5,83158E+14	7,35322E+14	6,34046E+14
TC0700002482.mm.2	A930016O22Rik	RIKEN cDNA A930016O22 gene	-0.667694750326854	5,46118E+14	-1,95593E+14	0.0950236489673381	0.235266757192796	-4,29257E+14	5,22742E+13	5,13019E+14	5,59282E+14	6,10018E+13	5,15199E+14	5,56445E+13
TC0100001166.mm.2	Fcmr	Fc fragment of IgM receptor	-0.746727822977896	6,41829E+14	-1,95563E+14	0.0950641584103007	0.235330893296232	-4,29294E+14	6,05899E+14	5,86136E+14	6,28053E+14	7,13328E+14	6,47923E+14	6,69633E+14
TC0400000446.mm.2	Npr2	natriuretic peptide receptor 2	-0.608515176782246	7,96401E+14	-1,95534E+13	0.09510369151734	0.235392593151905	-4,2933E+14	7,74284E+14	7,33973E+14	8,09623E+14	8,20336E+14	8,1513E+14	8,25061E+14
TC1600001553.mm.2	Slc49a4	solute carrier family 49 member 4	0.960918192881197	7,82357E+14	1,95512E+14	0.0951330635214487	0.235416774424654	-4,29357E+14	8,51687E+14	8,41988E+14	8,15101E+14	6,86391E+14	7,80269E+14	7,18708E+13
TC1600001807.mm.2	Arl6	ADP-ribosylation factor-like 6	2,20139E+14	6,6088E+14	1,95505E+14	0.0951426819992149	0.235416774424654	-4,29366E+14	7,75969E+14	7,82482E+14	7,02777E+13	4,15397E+14	7,76445E+14	5,12212E+14
TC1800001312.mm.2	Sema6a	sema domain, transmembrane domain (TM), and cytoplasmic domain, (semaphorin) 6A	1,27822E+14	7,05141E+14	1,95475E+14	0.0951835463936554	0.235481726246118	-4,29403E+14	8,00639E+14	7,80983E+14	7,17027E+14	6,08951E+14	7,51496E+14	5,71747E+13
TC1800000816.mm.2	Smad2	SMAD family member 2	0.769405065728185	6,32864E+14	1,95432E+14	0.0952409268427513	0.235587512161076	-4,29456E+14	7,12181E+14	7,05686E+14	6,65159E+14	5,98827E+14	5,92575E+14	5,22758E+14
TC1400001730.mm.2	Ldb3	LIM domain binding 3	-0.937925595648811	6,71492E+14	-1,95394E+14	0.095292529341934	0.235678975485278	-4,29503E+14	6,2748E+14	6,05515E+14	6,59111E+14	7,6147E+14	6,42806E+14	7,32566E+14
TC0700002611.mm.2	Cd177	CD177 antigen	-0.751880642735476	5,50706E+14	-1,95373E+14	0.0953219577169952	0.235715577761501	-4,2953E+14	4,82981E+14	5,23942E+14	5,53567E+14	6,03732E+14	5,37464E+14	6,0255E+14
TC0300002451.mm.2	BC021767	cingulin pseudogene	-0.581303097378412	6,11246E+14	-1,95356E+14	0.095345360172209	0.235733471308748	-4,29551E+14	5,76632E+13	5,84147E+14	6,45519E+13	6,3152E+14	6,26202E+14	6,03453E+13
TC1800001744.mm.2	Arhgap26	Rho GTPase activating protein 26	0.85843913045401	8,0608E+14	1,95346E+14	0.0953584537944777	0.235733471308748	-4,29563E+14	8,87425E+14	8,78575E+14	8,12191E+14	7,82121E+14	7,95608E+13	6,80558E+14
TC0700004596.mm.2	Mrgprg	MAS-related GPR, member G	-0.605689479105292	6,08831E+14	-1,95308E+14	0.0954101853051866	0.235825174999612	-4,29611E+14	5,6844E+13	5,62331E+14	6,05474E+14	6,46435E+14	6,3214E+14	6,38167E+14
TC0200004241.mm.2	Hipk3/Mir1902	homeodomain interacting protein kinase 3/microRNA 1902	1,43705E+14	8,08567E+14	1,95288E+14	0.0954378691133203	0.235857421015787	-4,29636E+14	8,84632E+14	9,09053E+14	8,50528E+14	6,55746E+14	8,47293E+14	7,04151E+14
TC0800001928.mm.2	Hook3	hook microtubule tethering protein 3	2,20109E+14	5,81341E+14	1,95271E+14	0.0954606100455311	0.235877443592032	-4,29657E+14	7,33928E+14	7,33049E+14	6,28882E+14	3,97876E+14	6,54818E+14	3,39492E+14
TC0300000367.mm.2	Mgst2	microsomal glutathione S-transferase 2	-0.691193400154614	5,87598E+14	-1,95252E+14	0.0954868130911603	0.235906013391271	-4,2968E+14	5,44732E+13	5,71025E+14	5,92512E+14	6,61483E+14	5,81497E+14	5,74337E+14
TC1100003762.mm.2	Krt16	keratin 16	-0.788954067194743	5,43575E+14	-1,95134E+13	0.0956464766072885	0.236184016286839	-4,29826E+14	4,81212E+14	5,0361E+14	5,23604E+14	6,1901E+14	5,52842E+14	5,81169E+14
TC0700000030.mm.2	Pira6	paired-Ig-like receptor A6	-0.713889316557244	5,06538E+14	-1,95128E+14	0.0956547706340123	0.236184016286839	-4,29834E+13	4,45783E+14	4,75204E+14	5,11187E+14	5,52578E+14	4,95543E+14	5,58933E+14
TC0500002169.mm.2	Preb	prolactin regulatory element binding	0.66907828555887	8,35694E+14	1,95113E+14	0.0956753248468723	0.236184016286839	-4,29852E+14	8,90733E+14	8,72632E+14	8,4551E+14	7,84039E+14	8,00628E+14	8,20624E+14
TC0800001844.mm.2	Gm7849/Gm7861	predicted gene 7849/predicted gene 7861	-0.700343543336541	5,84396E+14	-1,95112E+14	0.0956767713959285	0.236184016286839	-4,29854E+13	5,32025E+14	5,57677E+14	6,06019E+14	6,23751E+14	5,58167E+14	6,28739E+14
TC0800001848.mm.2	Gm7849/Gm7861	predicted gene 7849/predicted gene 7861	-0.700343543336541	5,84396E+14	-1,95112E+14	0.0956767713959285	0.236184016286839	-4,29854E+13	5,32025E+14	5,57677E+14	6,06019E+14	6,23751E+14	5,58167E+14	6,28739E+14
TC0200002942.mm.2	Atp5c1	ATP synthase, H+ transporting, mitochondrial F1 complex, gamma polypeptide 1	0.77142249611042	9,22989E+14	1,95101E+13	0.095691930438299	0.236184016286839	-4,29867E+14	9,8872E+14	9,7618E+14	9,27868E+13	8,82748E+14	9,23917E+14	8,38499E+14
TC0500002122.mm.2	Gm10471	predicted gene 10471	-0.685530967469767	5,84187E+14	-1,95094E+14	0.0957019451583671	0.236184016286839	-4,29877E+14	5,04391E+14	5,33393E+14	6,19323E+14	5,55567E+14	6,55662E+14	6,36786E+14
TC1900000723.mm.2	Slk	STE20-like kinase	1,85807E+14	8,20074E+14	1,95083E+14	0.0957170628750872	0.236185150705403	-4,2989E+14	9,6427E+14	9,85674E+14	9,05863E+14	6,72466E+14	8,09137E+14	5,83031E+14
TC0400001935.mm.2	Masp2	mannan-binding lectin serine peptidase 2	0.825051320861226	5,42807E+14	1,95031E+13	0.0957877617664921	0.236308864213287	-4,29955E+14	5,92643E+14	5,91333E+13	4,58845E+13	5,60121E+14	5,03819E+14	5,50082E+14
TC0400001090.mm.2	Fyb2	FYN binding protein 2	-0.674656268871341	5,6975E+14	-1,95025E+14	0.0957965308161849	0.236308864213287	-4,29963E+14	4,94908E+14	5,53347E+14	5,732E+14	6,09986E+14	5,92412E+14	5,9465E+14
TC1800000190.mm.2	Zfp35	zinc finger protein 35	0.980472475619704	5,864E+14	1,95E+14	0.0958300995717532	0.236355486667635	-4,29993E+14	6,75647E+14	6,48655E+14	6,32138E+13	4,9607E+14	5,58707E+14	5,07186E+14
TC0200000523.mm.2	Pkn3	protein kinase N3	-0.594716570408488	7,44708E+14	-1,94959E+14	0.0958855538598518	0.236414381963559	-4,30043E+14	6,87172E+14	7,06776E+14	7,42223E+14	7,70668E+14	7,74446E+14	7,86961E+14
TC0500000312.mm.2	Dnajc5g	DnaJ heat shock protein family (Hsp40) member C5 gamma	-0.760839739399823	4,48145E+13	-1,94957E+13	0.0958883405566612	0.236414381963559	-4,30046E+14	3,90659E+14	3,93694E+14	4,35496E+14	5,01025E+13	5,18157E+14	4,49841E+13
TC0X00000988.mm.2	Nhsl2	NHS-like 2	0.722719868897141	7,6008E+14	1,94947E+14	0.0959025492654776	0.236414381963559	-4,30059E+14	8,04625E+14	8,28903E+14	7,6077E+14	7,28214E+14	7,55757E+14	6,8221E+14
TC0800002680.mm.2	Aktip	thymoma viral proto-oncogene 1 interacting protein	0.743134102964695	8,1197E+14	1,94931E+14	0.0959238484656518	0.236414381963559	-4,30078E+14	8,73161E+14	8,537E+13	8,01669E+14	7,76564E+14	8,2539E+14	7,41335E+13
TC1100004071.mm.2	Atp5h	ATP synthase, H+ transporting, mitochondrial F0 complex, subunit D	1,23968E+14	1,03064E+14	1,94929E+14	0.0959273399911715	0.236414381963559	-4,30081E+14	1,11482E+14	1,1023E+14	1,06571E+14	9,03477E+14	1,05985E+14	9,37688E+14
TC0300001803.mm.2	Mrpl47	mitochondrial ribosomal protein L47	1,02892E+14	5,82498E+14	1,94909E+14	0.0959549524234075	0.236446268297655	-4,30107E+14	6,2629E+14	6,4421E+14	6,10625E+14	4,54091E+14	5,6989E+14	5,89883E+14
TC0600002122.mm.2	Mkrn1	makorin, ring finger protein, 1	1,69645E+14	7,77736E+14	1,94891E+14	0.0959792578797983	0.236469997280916	-4,30129E+14	8,9508E+14	8,98875E+14	8,12542E+14	6,42123E+14	8,29081E+14	5,88714E+14
TC0300000092.mm.2	Car13	carbonic anhydrase 13	-0.83427589651985	5,73128E+14	-1,94869E+14	0.0960094092747312	0.236508119804884	-4,30156E+14	5,70324E+14	4,94752E+14	5,73414E+14	6,58517E+14	5,70191E+14	5,71572E+14
TC0500000295.mm.2	Kcnk3	potassium channel, subfamily K, member 3	-0.703580963044845	6,89772E+14	-1,94843E+14	0.0960445694965744	0.236558567456176	-4,30188E+14	6,74202E+14	6,23055E+13	6,89291E+14	7,48682E+14	7,00173E+14	7,03229E+14
TC0900003326.mm.2	Ccpg1	cell cycle progression 1	0.612102985154101	7,35611E+14	1,94825E+14	0.0960690249534891	0.236570832210321	-4,3021E+14	7,85774E+14	7,84299E+14	7,12115E+14	7,35537E+14	7,17507E+14	6,78434E+14
TC1100001649.mm.2	Psme3	proteaseome (prosome, macropain) activator subunit 3 (PA28 gamma, Ki)	1,40668E+13	7,86769E+12	1,94811E+14	0.0960888468605951	0.236570832210321	-4,30228E+14	8,89035E+14	8,76858E+14	8,3703E+14	6,47528E+14	8,06463E+14	6,63702E+14
TC0900000244.mm.2	Sep 07	septin 7	2,24313E+14	8,11841E+13	1,94807E+14	0.0960935950360919	0.236570832210321	-4,30232E+14	9,46887E+14	9,38537E+14	8,7567E+14	5,61128E+14	8,97801E+14	6,51022E+14
TC0200003921.mm.2	Olfr1046	olfactory receptor 1046	-0.646210002043391	5,09738E+13	-1,94773E+14	0.0961403442838284	0.236637405115195	-4,30275E+14	4,51375E+14	4,74745E+14	5,26947E+14	5,28416E+13	5,11126E+14	5,65816E+14
TC0700002857.mm.2	Dpy19l3	dpy-19-like 3 (C. elegans)	2,58754E+14	5,5552E+13	1,94766E+14	0.0961500087686454	0.236637405115195	-4,30283E+14	7,25048E+14	7,46078E+14	6,86275E+14	2,67343E+14	5,54737E+14	3,53639E+14
TC0400002407.mm.2	Lingo2	leucine rich repeat and Ig domain containing 2	1,22238E+13	6,69928E+14	1,94734E+14	0.0961936996017285	0.236649812698119	-4,30323E+14	7,75436E+14	7,56509E+14	7,2147E+14	5,65998E+14	6,62388E+14	5,37768E+14
TC0600003146.mm.2	Plekhg6	pleckstrin homology domain containing, family G (with RhoGef domain) member 6	-1,07863E+14	4,49872E+14	-1,94732E+14	0.096196495805895	0.236649812698119	-4,30325E+14	3,71036E+14	3,99196E+14	4,38473E+14	5,47485E+14	5,35093E+14	4,0795E+14
TC1100001490.mm.2	Gm53	predicted gene 53	-0.575267903402305	6,5897E+14	-1,94722E+14	0.0962104729060754	0.236649812698119	-4,30338E+14	6,11552E+14	6,10792E+14	6,56539E+14	6,80858E+14	6,98999E+13	6,95078E+14
TC0800000215.mm.2	Defa25	defensin, alpha, 25	-0.61582403735316	4,99797E+14	-1,9472E+14	0.0962137977400472	0.236649812698119	-4,30341E+13	4,44153E+14	4,49656E+14	5,2675E+13	4,90224E+14	5,58848E+14	5,29153E+14
TC0700001484.mm.2	Olfr593	olfactory receptor 593	-0.796075651865287	4,69509E+14	-1,94669E+14	0.0962839805484687	0.236786290991678	-4,30405E+14	4,13441E+14	4,22152E+14	4,51606E+14	5,43202E+11	4,68706E+14	5,17948E+14
TC1000003228.mm.2	Bloc1s1	biogenesis of lysosomal organelles complex-1, subunit 1	1,06389E+14	9,15655E+14	1,94614E+14	0.0963586657618717	0.236916689494972	-4,30472E+14	9,79801E+14	9,57666E+14	9,37942E+14	7,86746E+14	9,49308E+14	8,82468E+14
TC0400000963.mm.2	Hook1	hook microtubule tethering protein 1	1,56252E+14	7,27768E+14	1,94608E+14	0.0963664111555917	0.236916689494972	-4,30479E+14	7,91398E+14	7,99514E+14	7,61411E+14	5,16996E+14	8,04523E+14	6,92768E+14
TC1000002211.mm.2	Oit3	oncoprotein induced transcript 3	-1,05788E+14	5,7502E+14	-1,94532E+14	0.0964717383418709	0.237104775767805	-4,30574E+14	4,8069E+14	5,57225E+14	5,54127E+14	6,95365E+14	5,61288E+14	6,01425E+14
TC0100000255.mm.2	Zap70	zeta-chain (TCR) associated protein kinase	-0.938570911209306	4,91366E+14	-1,94529E+14	0.0964754739583199	0.237104775767805	-4,30578E+14	4,6334E+14	3,86745E+12	4,63929E+14	5,73871E+14	5,11661E+14	5,48649E+14
TC0700000288.mm.2	Fbxo46	F-box protein 46	0.72306425133266	5,62307E+14	1,94521E+14	0.0964870610506732	0.237104775767805	-4,30588E+14	6,10097E+13	6,45665E+14	5,93887E+14	5,17262E+14	5,01435E+14	5,05493E+14
TC0700001423.mm.2	Fchsd2	FCH and double SH3 domains 2	1,30393E+14	6,67425E+14	1,94492E+14	0.0965264608939131	0.237165426103023	-4,30624E+14	7,52584E+14	7,18945E+14	7,081E+14	5,02643E+14	6,9215E+14	6,30126E+14
TC1500002334.mm.2	Pdxp	pyridoxal (pyridoxine, vitamin B6) phosphatase	0.909520370978046	8,00499E+13	1,94481E+14	0.096542248238199	0.237168050901372	-4,30638E+14	8,74305E+13	8,40308E+14	8,29869E+14	7,0284E+14	7,8524E+14	7,70431E+14
TC1100002071.mm.2	Ptchd3	patched domain containing 3	-0.637882568110505	5,1464E+14	-1,9447E+14	0.0965569816929858	0.237168086283396	-4,30651E+14	4,62358E+14	4,7738E+14	5,08704E+14	5,58611E+14	5,51611E+14	5,29174E+14
TC0800001727.mm.2	Pcid2	PCI domain containing 2	0.998584313447933	6,84096E+13	1,94414E+14	0.0966334532314694	0.237269752530827	-4,3072E+14	7,44854E+14	7,45677E+14	7,2136E+14	5,69454E+13	6,6932E+14	6,53913E+14
TC1100001694.mm.2	Fzd2	frizzled class receptor 2	0.581886491871704	6,14667E+13	1,9441E+14	0.0966401783223212	0.237269752530827	-4,30726E+14	6,54699E+14	6,70267E+14	6,11896E+13	5,96692E+14	5,66847E+13	5,87598E+14
TC0400002589.mm.2	Tstd2	thiosulfate sulfurtransferase (rhodanese)-like domain containing 2	1,07857E+14	6,26359E+14	1,94389E+14	0.096668836429909	0.237269752530827	-4,30752E+14	7,10373E+14	6,82118E+14	6,71838E+14	5,04939E+13	6,03438E+14	5,85447E+14
TC0200002990.mm.2	Hacd1	3-hydroxyacyl-CoA dehydratase 1	-1,26246E+14	1,01375E+14	-1,94386E+14	0.0966724380239747	0.237269752530827	-4,30755E+14	9,25369E+14	9,29883E+14	1,14688E+14	9,60859E+14	1,10721E+14	1,01231E+14
TC1900001760.mm.2	Gm7102	predicted gene 7102	-0.639304464627715	6,72156E+14	-1,94368E+14	0.0966980327338308	0.237269752530827	-4,30778E+14	6,24953E+14	6,50575E+14	6,76063E+14	7,27326E+14	6,78724E+14	6,75292E+14
TC1200001593.mm.2	Dld	dihydrolipoamide dehydrogenase	1,86358E+13	7,86343E+14	1,94364E+14	0.0967033561982371	0.237269752530827	-4,30783E+14	9,25742E+13	9,19019E+14	8,56925E+14	6,15119E+14	8,11423E+14	5,89828E+14
TC0100001176.mm.2	Slc41a1	solute carrier family 41, member 1	0.991631945019011	8,15344E+14	1,94358E+14	0.0967106333458989	0.237269752530827	-4,3079E+14	8,94814E+14	9,10733E+14	8,59693E+14	7,47527E+14	7,89716E+14	6,8958E+14
TC0500000948.mm.2	Fgf5	fibroblast growth factor 5	-0.645345105095863	5,68007E+14	-1,94354E+14	0.0967161754249657	0.237269752530827	-4,30795E+14	5,41734E+14	5,15282E+14	5,71026E+14	6,15059E+14	5,67275E+14	5,97668E+13
TC0600000214.mm.2	Fscn3	fascin actin-bundling protein 3	-1,47862E+14	6,18201E+14	-1,94343E+14	0.0967319784793494	0.237272395986871	-4,30809E+14	5,13826E+14	4,89601E+13	5,33062E+14	7,6609E+14	6,56029E+14	7,50596E+14
TC1100003735.mm.2	Gm11595	predicted gene 11595	-0.688470157862773	7,01865E+14	-1,94313E+14	0.0967731085742679	0.237337153494243	-4,30846E+14	6,5922E+14	6,56195E+14	7,31394E+14	7,21715E+14	6,84506E+14	7,5816E+14
TC0700001893.mm.2	Sec23ip	Sec23 interacting protein	0.986002938568567	5,86156E+14	1,94255E+14	0.0968537221762562	0.237455226610712	-4,30918E+14	6,97028E+14	6,90295E+14	6,50925E+13	5,39197E+14	5,19719E+14	4,1977E+14
TC0900003023.mm.2	Dock3	dedicator of cyto-kinesis 3	0.975643816761282	7,82358E+14	1,94252E+14	0.0968575774272183	0.237455226610712	-4,30922E+14	8,53765E+14	8,69539E+14	7,97072E+14	7,31103E+14	7,86959E+14	6,55712E+14
TC1900000959.mm.2	Cst6	cystatin E/M	-0.876580602202455	5,02836E+14	-1,94244E+14	0.0968684443416932	0.237455226610712	-4,30932E+14	4,81129E+14	4,22823E+14	4,85043E+14	5,94224E+14	5,24873E+14	5,08922E+13
TC0800002156.mm.2	Slc25a4	solute carrier family 25 (mitochondrial carrier, adenine nucleotide translocator), member 4	1,5523E+14	1,06034E+14	1,94236E+14	0.0968801998224302	0.237455226610712	-4,30942E+14	1,16434E+14	1,14826E+13	1,1147E+14	8,8744E+14	1,0937E+14	9,53625E+14
TC1700002173.mm.2	Abcc10	ATP-binding cassette, sub-family C (CFTR/MRP), member 10	-0.603568427019694	7,13795E+14	-1,94182E+14	0.0969545234491691	0.237601252674747	-4,31009E+14	6,78715E+13	6,67115E+14	7,53606E+14	7,12938E+14	7,32993E+14	7,37406E+14
TC1400001627.mm.2	Appl1	adaptor protein, phosphotyrosine interaction, PH domain and leucine zipper containing 1	2,19438E+14	7,05917E+14	1,94155E+14	0.0969915879583157	0.237655193971205	-4,31042E+14	8,68867E+14	8,52328E+14	7,76363E+14	5,05956E+14	7,57662E+14	4,74326E+14
TC0500000305.mm.2	Emilin1	elastin microfibril interfacer 1	-0.822375644851877	5,81068E+14	-1,94144E+14	0.0970060330632792	0.237655193971205	-4,31055E+14	4,85272E+14	5,57387E+14	5,80472E+13	6,26663E+14	5,82513E+14	6,54101E+14
TC1500001890.mm.2	Cbx7	chromobox 7	-0.621813048555166	6,95357E+14	-1,94105E+14	0.0970600025172244	0.237740523619197	-4,31104E+14	6,6798E+14	6,44306E+14	7,24309E+14	7,12339E+14	7,33266E+14	6,8994E+14
TC0100001451.mm.2	4930523C07Rik	RIKEN cDNA 4930523C07 gene	-1,14243E+14	5,76338E+14	-1,9409E+14	0.0970809427364182	0.237740523619197	-4,31122E+14	4,9288E+14	5,07934E+14	5,23036E+14	7,06264E+14	6,19919E+14	6,07992E+14
TC0800002553.mm.2	Tecr	trans-2,3-enoyl-CoA reductase	1,0358E+13	1,01189E+14	1,9408E+14	0.0970953975091384	0.237740523619197	-4,31135E+14	1,08507E+14	1,06892E+14	1,04616E+14	9,0066E+14	1,0191E+14	9,51407E+14
TC1700000630.mm.2	Btnl1	butyrophilin-like 1	-0.73389131373338	5,31821E+14	-1,94077E+14	0.0970998812100747	0.237740523619197	-4,31139E+14	4,81347E+14	4,75947E+14	5,08538E+14	5,95535E+14	5,58159E+14	5,71403E+14
TC1100001106.mm.2	Trarg1	trafficking regulator of GLUT4 (SLC2A4) 1	-0.79289339771346	6,20735E+14	-1,9404E+14	0.0971502713936959	0.237827761009818	-4,31185E+14	5,8552E+14	5,61185E+14	6,28726E+14	6,76558E+14	5,91553E+14	6,80867E+14
TC1200002534.mm.2	Serpina1c	serine (or cysteine) peptidase inhibitor, clade A, member 1C	-0.891040444120057	4,51332E+14	-1,94023E+14	0.0971742600434418	0.237841752060497	-4,31206E+14	3,87947E+14	3,9584E+14	4,24062E+14	5,37933E+14	4,51613E+14	5,10594E+14
TC0900001890.mm.2	Rgl3	ral guanine nucleotide dissociation stimulator-like 3	-0.683121715089382	5,62512E+14	-1,94015E+14	0.0971855083203818	0.237841752060497	-4,31216E+14	4,84072E+14	5,46469E+14	5,69364E+14	5,978E+14	5,76186E+14	6,0118E+14
TC1200001036.mm.2	Serpina5	serine (or cysteine) peptidase inhibitor, clade A, member 5	-0.815861627338102	5,60822E+14	-1,93989E+14	0.0972219414038673	0.23788608225387	-4,31249E+14	5,29729E+14	5,01081E+14	5,48434E+14	6,45549E+14	5,91744E+13	5,48392E+14
TC1800000111.mm.2	Impact	impact, RWD domain protein	1,16407E+14	1,00624E+14	1,93979E+14	0.0972356082723404	0.23788608225387	-4,31261E+14	1,11105E+13	1,06598E+14	1,04889E+14	8,95331E+14	1,0083E+14	9,07863E+14
TC0900000223.mm.2	Bbs9	Bardet-Biedl syndrome 9 (human)	1,49022E+13	9,62326E+14	1,9397E+14	0.0972479131016099	0.23788608225387	-4,31272E+14	1,05562E+13	1,04911E+14	1,0063E+14	8,00379E+14	1,00685E+14	8,55693E+14
TC0500000971.mm.2	Enoph1	enolase-phosphatase 1	0.837775574794891	6,69975E+14	1,93953E+14	0.0972707305970729	0.237903930642639	-4,31292E+14	7,31233E+14	7,21613E+14	6,85968E+14	5,99323E+14	6,78864E+14	6,02847E+14
TC0200000465.mm.2	Adamtsl2	ADAMTS-like 2	-0.607060987980082	6,8732E+14	-1,93939E+14	0.0972903258557695	0.237903930642639	-4,3131E+14	6,45318E+13	6,70243E+14	7,08372E+14	7,28601E+14	6,70255E+14	7,01127E+14
TC0300001116.mm.2	Lamtor5	late endosomal/lysosomal adaptor, MAPK and MTOR activator 5	0.590485610179952	8,56008E+13	1,9393E+14	0.0973037271044216	0.237903930642639	-4,31322E+14	8,92108E+14	8,90485E+14	8,46862E+14	8,17634E+14	8,55074E+13	8,33882E+14
TC1900001516.mm.2	Mms19	MMS19 cytosolic iron-sulfur assembly component	0.829152160838823	6,65559E+14	1,9392E+14	0.0973170450922482	0.237903930642639	-4,31334E+14	7,44321E+14	7,32377E+14	6,16704E+14	6,94164E+14	5,61808E+14	6,43982E+14
TC0900002017.mm.2	Ddx25	DEAD (Asp-Glu-Ala-Asp) box polypeptide 25	0.782936881667908	6,5083E+13	1,93911E+14	0.0973290331282986	0.237903930642639	-4,31345E+14	7,47569E+14	7,16632E+13	7,0207E+14	6,05544E+14	5,62261E+14	5,70903E+14
TC0500002993.mm.2	Noc4l	NOC4 like	0.584169901275603	6,3938E+14	1,93846E+14	0.0974200141464285	0.238046204171413	-4,31426E+14	7,05767E+14	6,92754E+14	6,43716E+14	6,37971E+14	5,927E+14	5,63374E+14
TC0100001955.mm.2	Arfgef1	ADP-ribosylation factor guanine nucleotide-exchange factor 1(brefeldin A-inhibited)	1,53154E+14	7,57727E+14	1,9383E+14	0.0974418509050248	0.238046204171413	-4,31445E+14	8,69099E+14	8,76675E+14	8,07151E+14	6,32316E+14	7,79372E+14	5,81748E+13
TC0200000966.mm.2	Xirp2	xin actin-binding repeat containing 2	-1,13384E+14	5,89183E+14	-1,93811E+14	0.0974689546193035	0.238046204171413	-4,3147E+14	5,17268E+14	5,48831E+14	5,66515E+14	7,26352E+14	5,46663E+14	6,29469E+14
TC0200004138.mm.2	Mapk8ip1	mitogen-activated protein kinase 8 interacting protein 1	0.756050602587248	9,25025E+14	1,93804E+14	0.0974781346746241	0.238046204171413	-4,31478E+14	9,81787E+14	9,71324E+14	9,17059E+13	8,84841E+14	9,41752E+14	8,5339E+13

TC0700002043.mm.2	Rassf7	Ras association (RalGDS/AF-6) domain family (N-terminal) member 7	-0.718376928250446	6,57035E+14	-1,93797E+14	0.0974874811024506	0.238046204171413	-4,31486E+14	6,20173E+14	6,12584E+14	6,48592E+14	7,2784E+14	6,53124E+14	6,79895E+14
TC0400002606.mm.2	Gabbr2	gamma-aminobutyric acid (GABA) B receptor, 2	1,43213E+14	9,79039E+14	1,9379E+14	0.0974969543581255	0.238046204171413	-4,31495E+14	1,07646E+14	1,08483E+14	1,03717E+14	8,37692E+14	9,89331E+14	8,48754E+14
TC0300002492.mm.2	Plekho1	pleckstrin homology domain containing, family O member 1	0.9103728667471	6,82309E+14	1,93783E+14	0.0975070440847932	0.238046204171413	-4,31504E+14	7,16319E+14	7,64565E+14	6,92485E+14	6,06324E+14	7,01847E+14	6,12315E+14
TC1700000385.mm.2	Ciao3/Mir6966	cytosolic iron-sulfur assembly component 3/microRNA 6966	1,21198E+14	6,13362E+14	1,93778E+14	0.097514635605876	0.238046204171413	-4,3151E+14	6,92505E+14	6,77186E+14	6,62784E+14	4,64512E+14	5,85526E+13	5,97659E+14
TC1100000692.mm.2	Olfr325	olfactory receptor 325	-1,28865E+14	4,44057E+14	-1,93766E+14	0.0975306599708733	0.238046204171413	-4,31525E+14	2,98895E+14	4,04915E+14	3,92211E+14	5,69329E+13	4,69262E+14	5,29729E+14
TC1100001925.mm.2	Cdk3	cyclin-dependent kinase 3	-1,2662E+14	5,8501E+14	-1,93763E+14	0.0975349742406545	0.238046204171413	-4,31528E+14	5,46959E+14	4,81229E+14	5,64182E+14	7,17246E+14	5,35889E+14	6,64558E+14
TC1200000261.mm.2	6030469F06Rik	RIKEN cDNA 6030469F06 gene	-0.754633728286814	6,65192E+14	-1,93745E+14	0.0975603730187903	0.238072132432495	-4,31551E+14	6,17119E+14	6,3556E+14	6,56108E+14	7,47497E+14	6,60332E+14	6,74539E+14
TC0100000985.mm.2	Cntnap5b	contactin associated protein-like 5B	1,28976E+14	5,73117E+14	1,93734E+14	0.0975753849853528	0.238072710216382	-4,31565E+14	6,61715E+14	6,48884E+14	6,13283E+14	4,39364E+14	6,0092E+14	4,74538E+14
TC0500003566.mm.2	Aimp2	aminoacyl tRNA synthetase complex-interacting multifunctional protein 2	0.739299707250969	7,53782E+14	1,93704E+14	0.0976165140776747	0.238137000958906	-4,31601E+14	8,3272E+14	8,18089E+14	7,3845E+14	7,645E+14	6,41967E+14	7,26963E+14
TC1300002302.mm.2	Slc6a18	solute carrier family 6 (neurotransmitter transporter), member 18	-0.745250739564692	6,07654E+14	-1,93682E+14	0.0976480570888178	0.238177890383306	-4,31629E+13	5,73411E+14	5,53216E+14	6,31468E+14	6,44209E+14	5,77497E+14	6,6612E+14
TC1500002171.mm.2	Olfr285/Olfr257	olfactory receptor 285/olfactory receptor 257	-0.664930084857498	4,70834E+14	-1,93663E+14	0.0976740745411481	0.238205291824053	-4,31653E+14	3,99107E+14	4,09745E+14	4,49065E+14	4,92773E+14	5,12056E+14	5,62257E+14
TC1200000988.mm.2	Tdp1	tyrosyl-DNA phosphodiesterase 1	0.7912920414794	6,38037E+14	1,93623E+14	0.0977296815522356	0.238298989263371	-4,31702E+14	6,92853E+13	6,87335E+13	6,54649E+14	5,6728E+14	6,41333E+14	5,84774E+13
TC0200001965.mm.2	Tgm3	transglutaminase 3, E polypeptide	-0.701497633997611	5,26229E+14	-1,9359E+14	0.0977754627565847	0.238298989263371	-4,31743E+13	4,77984E+14	4,83246E+13	5,12595E+14	5,88934E+14	5,37126E+14	5,57489E+14
TC0200003765.mm.2	Atp5g3	ATP synthase, H+ transporting, mitochondrial F0 complex, subunit C3 (subunit 9)	0.972376402237668	9,02193E+14	1,9359E+14	0.0977763343795833	0.238298989263371	-4,31744E+14	9,48691E+14	9,49211E+14	9,22708E+14	7,80718E+14	9,26342E+12	8,85489E+14
TC0200000702.mm.2	Nek6	NIMA (never in mitosis gene a)-related expressed kinase 6	0.629775004194042	6,83884E+14	1,93588E+14	0.0977784139439811	0.238298989263371	-4,31745E+14	7,08876E+14	7,44958E+14	6,68982E+14	6,58896E+14	6,80732E+14	6,4086E+13
TC0400001180.mm.2	Agbl4	ATP/GTP binding protein-like 4	0.876447640771604	6,76534E+14	1,93562E+14	0.097814419552067	0.238298989263371	-4,31777E+14	7,12795E+14	7,32356E+14	6,57913E+12	6,11948E+14	7,29663E+14	6,14528E+14
TC0900002273.mm.2	Dlat	dihydrolipoamide S-acetyltransferase (E2 component of pyruvate dehydrogenase complex)	1,36612E+14	7,92675E+14	1,93559E+14	0.0978193143020628	0.238298989263371	-4,31782E+14	8,78024E+13	8,74091E+14	8,41661E+14	6,37231E+14	8,16473E+14	7,08573E+14
TC0300001260.mm.2	Tram1l1	translocation associated membrane protein 1-like 1	1,48672E+14	7,93033E+14	1,93544E+14	0.0978399797261724	0.238298989263371	-4,318E+14	8,94813E+14	8,62368E+14	8,31041E+14	6,28796E+14	8,4694E+14	6,94241E+14
TC0400003453.mm.2	Tmco2	transmembrane and coiled-coil domains 2	-1,31508E+14	5,28542E+14	-1,93543E+13	0.0978408744755579	0.238298989263371	-4,31801E+14	4,69168E+14	4,52839E+14	4,86587E+14	6,98436E+14	5,02053E+14	5,62171E+14
TC0300001490.mm.2	Lpar3	lysophosphatidic acid receptor 3	-0.685851234886564	5,04196E+14	-1,93523E+14	0.0978696026955699	0.238298989263371	-4,31827E+14	4,94938E+14	4,41333E+14	5,137E+14	5,59741E+14	4,9608E+14	5,19381E+14
TC0200002822.mm.2	Zgpat/Lime1	zinc finger, CCCH-type with G patch domain/Lck interacting transmembrane adaptor 1	-0.630151859770363	8,37782E+14	-1,93522E+14	0.0978706332675556	0.238298989263371	-4,31827E+14	7,94617E+14	8,02629E+14	8,41592E+13	8,81685E+14	8,34733E+14	8,71439E+14
TC1300001366.mm.2	BC147527	cDNA sequence BC147527	-0.858709206150375	5,01375E+14	-1,93519E+14	0.0978751760035368	0.238298989263371	-4,31831E+14	4,15555E+13	4,61825E+14	4,70394E+14	5,78728E+14	5,5527E+14	5,26477E+14
TC1000002659.mm.2	Scyl2	SCY1-like 2 (S. cerevisiae)	1,43776E+14	6,65346E+14	1,93505E+14	0.0978943017761393	0.238309545931248	-4,31848E+14	7,5872E+14	7,35386E+14	7,14094E+14	4,92458E+13	6,97472E+14	5,93943E+14
TC0800000036.mm.2	Cers4	ceramide synthase 4	0.610216583230413	7,45637E+13	1,9349E+14	0.0979155698002169	0.238325313624002	-4,31867E+13	7,96117E+13	8,01734E+14	7,58414E+14	7,17394E+14	7,10803E+14	6,89358E+14
TC0600000572.mm.2	Aoc1	amine oxidase, copper-containing 1	-0.811648507096188	4,83837E+14	-1,93452E+14	0.0979689452622263	0.238385231003174	-4,31915E+14	4,64008E+14	4,2192E+14	4,96149E+13	5,52109E+14	4,4223E+14	5,26606E+14
TC1400001621.mm.2	Slmap	sarcolemma associated protein	1,07925E+13	6,09534E+14	1,93434E+14	0.0979931184542004	0.238385231003174	-4,31936E+13	6,99988E+14	6,92158E+14	6,21323E+12	5,54973E+14	6,23368E+14	4,65395E+14
TC1700000407.mm.2	Rgs11	regulator of G-protein signaling 11	-0.561599578213914	5,84335E+14	-1,9343E+14	0.0979995691037858	0.238385231003174	-4,31942E+14	5,54861E+14	5,4066E+14	6,03333E+14	6,04508E+14	6,03597E+14	5,99047E+13
TC1800001206.mm.2	Slc25a2	solute carrier family 25 (mitochondrial carrier, ornithine transporter) member 2	-0.94044594792477	5,42661E+14	-1,93393E+14	0.0980513204006252	0.238385231003174	-4,31988E+14	5,00852E+14	4,71341E+14	5,52749E+14	6,07533E+14	4,9865E+14	6,24841E+14
TC1200002127.mm.2	Snw1	SNW domain containing 1	0.889507397401195	7,49737E+14	1,93391E+14	0.0980528958423876	0.238385231003174	-4,31989E+14	8,02482E+14	8,07592E+14	7,35893E+14	6,96279E+14	7,89315E+14	6,66859E+14
TC0100001755.mm.2	Aida	axin interactor, dorsalization associated	2,57353E+14	7,24747E+14	1,93382E+14	0.0980664370285838	0.238385231003174	-4,32001E+14	8,74034E+14	8,55297E+14	8,23471E+14	3,91153E+14	7,88739E+14	6,15788E+14
TC0400003433.mm.2	Guca2b	guanylate cyclase activator 2b (retina)	-0.615745003304725	5,96136E+14	-1,93371E+14	0.0980818294527801	0.238385231003174	-4,32015E+14	5,62836E+14	5,35554E+14	6,05063E+13	6,16477E+14	6,08769E+14	6,48119E+14
TC1700000153.mm.2	Smok2b	sperm motility kinase 2B	-0.711972104980251	5,18752E+14	-1,93364E+14	0.0980912401861712	0.238385231003174	-4,32023E+14	4,67339E+14	4,6425E+14	5,05077E+14	5,68906E+14	5,27321E+13	5,79619E+14
TC1300002463.mm.2	Iqgap2	IQ motif containing GTPase activating protein 2	0.629416696409689	5,52525E+13	1,9336E+14	0.0980972044617533	0.238385231003174	-4,32029E+14	5,89852E+14	6,14062E+14	5,54858E+14	5,23173E+14	5,35444E+13	4,9776E+14
TC1600001307.mm.2	Zdhhc8	zinc finger, DHHC domain containing 8	0.669747138864932	6,78541E+14	1,93354E+14	0.0981057570614214	0.238385231003174	-4,32036E+14	7,32723E+14	7,01238E+14	6,80903E+13	6,19108E+14	6,69585E+14	6,67687E+14
TC0500001893.mm.2	Mterf1a	mitochondrial transcription termination factor 1a	0.787063622285989	5,74408E+14	1,9335E+14	0.098111494016646	0.238385231003174	-4,32041E+14	6,45949E+14	6,09863E+14	5,85856E+14	5,12543E+14	5,77808E+14	5,14429E+14
TC1700000819.mm.2	Mmut	methylmalonyl-Coenzyme A mutase	1,16271E+14	5,82897E+14	1,93345E+14	0.0981177218403185	0.238385231003174	-4,32047E+14	6,93426E+14	6,89096E+14	6,23827E+14	5,26151E+14	5,63582E+14	4,01301E+14
TC0500001245.mm.2	Sppl3	signal peptide peptidase 3	1,2091E+14	8,32365E+13	1,93326E+14	0.0981440850718063	0.238413333749738	-4,3207E+14	9,18758E+14	8,91221E+14	8,69284E+14	6,98876E+14	8,57754E+14	7,58295E+14
TC0400003344.mm.2	Akr1a1	aldo-keto reductase family 1, member A1 (aldehyde reductase)	1,0934E+14	8,68193E+14	1,93314E+14	0.0981608649512131	0.238418151486117	-4,32085E+14	9,38336E+13	9,24745E+14	8,8303E+14	7,6137E+14	9,13172E+14	7,88506E+14
TC0500003742.mm.2	Tmem184a	transmembrane protein 184a	-0.674239333536209	5,72333E+13	-1,93284E+14	0.0982035843223413	0.238464018608521	-4,32123E+14	4,8777E+14	5,43496E+14	5,69386E+14	5,96728E+14	6,01138E+14	6,35481E+14
TC1200001465.mm.2	2410004P03Rik	RIKEN cDNA 2410004P03 gene	-0.864941891871045	6,90477E+14	-1,9328E+14	0.0982093481962481	0.238464018608521	-4,32128E+14	6,07395E+14	6,39953E+14	6,65745E+14	7,54592E+14	6,98267E+14	7,76912E+14
TC1200000458.mm.2	Srp54a	signal recognition particle 54A	1,66829E+14	7,84207E+14	1,93252E+14	0.0982487357843374	0.238523712474466	-4,32163E+14	8,70251E+14	9,08287E+14	8,16728E+14	6,28153E+14	8,50709E+14	6,31112E+14
TC0500003088.mm.2	Acads	acyl-Coenzyme A dehydrogenase, short chain	-0.600093792632828	6,67961E+14	-1,93198E+14	0.0983239432436639	0.238627091934333	-4,32229E+14	6,31081E+14	6,46664E+14	6,8207E+14	7,15694E+14	6,55915E+14	6,76343E+14
TC0500000359.mm.2	Gm9903	predicted gene 9903	-0.946746931675614	5,26394E+13	-1,9318E+14	0.0983483503515589	0.238627091934333	-4,32251E+14	5,09494E+14	4,64315E+13	5,31862E+14	6,31296E+14	4,65024E+14	5,56374E+14
TC1800001178.mm.2	Spata24	spermatogenesis associated 24	-0.643353930781924	6,43839E+13	-1,93164E+12	0.0983715717877154	0.238627091934333	-4,32272E+14	6,21153E+14	6,08415E+14	6,55539E+14	7,02699E+14	6,55381E+13	6,19844E+14
TC0900002283.mm.2	Layn	layilin	-0.63345293059191	5,82135E+14	-1,93159E+14	0.0983782665623385	0.238627091934333	-4,32278E+14	5,35308E+14	5,78055E+14	6,09086E+14	6,30967E+14	5,84764E+14	5,54631E+14
TC0600001475.mm.2	Fgf6	fibroblast growth factor 6	-1,11885E+14	4,89772E+14	-1,93145E+14	0.0983984490882238	0.238627091934333	-4,32295E+14	3,99245E+14	4,18616E+14	4,39173E+14	6,02457E+14	5,02477E+14	5,76662E+14
TC1500000570.mm.2	Iqank1	IQ motif and ankyrin repeat containing 1	-0.840747342365117	6,84211E+14	-1,93134E+13	0.0984140306080222	0.238627091934333	-4,32309E+14	6,40544E+14	6,15397E+14	6,55047E+14	7,69043E+14	7,0698E+14	7,18254E+14
TC0500002927.mm.2	Glmn	glomulin, FKBP associated protein	0.68055782328889	5,66071E+13	1,93131E+14	0.0984182587419181	0.238627091934333	-4,32313E+14	6,02303E+14	6,15281E+14	5,76334E+14	5,05138E+13	5,54053E+14	5,43319E+14
TC1300002601.mm.2	Cwc27	CWC27 spliceosome-associated protein	-0.629945514376733	1,00443E+14	-1,93115E+13	0.0984407021916753	0.238627091934333	-4,32333E+14	9,63243E+13	9,76593E+14	1,00982E+14	1,056E+14	1,02423E+14	9,96718E+14
TC0400002021.mm.2	Icmt	isoprenylcysteine carboxyl methyltransferase	0.756184884030756	6,07956E+14	1,93091E+14	0.0984735951108264	0.238627091934333	-4,32362E+14	6,27401E+14	6,52837E+14	5,188E+14	6,10202E+14	6,24397E+14	6,14102E+14
TC1000000888.mm.2	Zfr2	zinc finger RNA binding protein 2	-0.753973957248099	7,39573E+14	-1,93077E+14	0.0984933020220121	0.238627091934333	-4,32379E+14	7,07054E+14	6,90348E+14	7,63634E+14	7,84562E+14	7,0043E+12	7,91412E+14
TC1800001196.mm.2	E230025N22Rik	Riken cDNA E230025N22 gene	-0.656053651176762	5,79367E+13	-1,93075E+14	0.0984963794643605	0.238627091934333	-4,32382E+14	5,39118E+14	5,38836E+14	6,08348E+14	6,00816E+14	5,62235E+14	6,26849E+14
TC1700000829.mm.2	Tnfrsf21	tumor necrosis factor receptor superfamily, member 21	0.791186071241434	7,57787E+14	1,93064E+14	0.0985121023993527	0.238627091934333	-4,32396E+14	8,23646E+14	8,05691E+14	7,84446E+14	6,86654E+13	7,39696E+14	7,06592E+14
TC1000002433.mm.2	Gm19668	predicted gene, 19668	-0.881931421047081	5,16367E+14	-1,93063E+14	0.0985126894096429	0.238627091934333	-4,32396E+14	4,63222E+14	4,60009E+14	4,86903E+14	6,12715E+14	5,27908E+14	5,47443E+14
TC0400000486.mm.2	Trmt10b	tRNA methyltransferase 10B	-0.560989141469618	5,66693E+14	-1,93062E+14	0.09851426924524 2	0.238627091934333	-4,32398E+14	5,39222E+14	5,28478E+14	5,95507E+14	5,84391E+14	5,75505E+14	5,77058E+14
TC1900000254.mm.2	Keg1	kidney expressed gene 1	-0.94428286088296	4,89108E+14	-1,93058E+14	0.0985203685403236	0.238627091934333	-4,32403E+14	4,36252E+14	4,06259E+14	4,50909E+14	5,80458E+13	5,05542E+14	5,55229E+14
TC0500001038.mm.2	Lrrc8b	leucine rich repeat containing 8 family, member B	0.716747813979727	7,24813E+14	1,93052E+14	0.0985282717457404	0.238627091934333	-4,3241E+14	7,55918E+14	7,79543E+14	7,33448E+14	6,58663E+14	6,95164E+14	7,26142E+14
TC0800003007.mm.2	1700030J22Rik	RIKEN cDNA 1700030J22 gene	-0.60767159324217	6,72628E+14	-1,92999E+14	0.0986034042060312	0.238748600913027	-4,32476E+14	6,1355E+14	6,37045E+13	6,70223E+14	7,01907E+14	6,94517E+14	7,18526E+14
TC1700001499.mm.2	Psmb1	proteasome (prosome, macropain) subunit, beta type 1	0.662159684055114	9,56022E+14	1,92995E+14	0.098608076650915	0.238748600913027	-4,3248E+13	1,00103E+14	9,94519E+14	9,56138E+14	9,0698E+14	9,61441E+14	9,16022E+14
TC0700001847.mm.2	Fbrs	fibrosin	-0.713504978352961	8,18474E+14	-1,92944E+14	0.0986809901357611	0.23884285942778	-4,32545E+14	7,90501E+14	7,74109E+14	8,86015E+14	8,21296E+14	8,44528E+14	7,94398E+14
TC0X00002706.mm.2	Pdzd11	PDZ domain containing 11	0.672864678155093	7,3117E+14	1,92938E+14	0.0986890014799553	0.23884285942778	-4,32552E+14	7,84058E+14	7,62647E+14	7,31097E+14	6,81035E+14	7,36786E+14	6,91398E+14
TC0200001900.mm.2	Usp8	ubiquitin specific peptidase 8	1,35674E+14	7,90409E+13	1,92936E+14	0.0986914763278196	0.23884285942778	-4,32554E+14	8,8913E+14	8,68623E+14	8,48206E+14	6,42199E+14	7,95003E+14	6,95293E+14
TC0X00002393.mm.2	Tmem185a	transmembrane protein 185A	2,15808E+14	7,86389E+14	1,929E+14	0.0987424906868677	0.238930432863418	-4,32599E+14	9,45761E+14	9,33712E+14	8,97773E+14	5,50084E+14	7,81125E+14	6,09878E+14
TC0800000530.mm.2	Cfap97	cilia and flagella associated protein 97	0.650849666308605	6,91006E+14	1,92865E+14	0.0987917811897502	0.238960781282613	-4,32642E+14	7,78661E+13	7,30773E+14	6,84631E+14	6,94634E+14	6,08447E+14	6,48889E+14
TC0300001643.mm.2	Impa1	inositol (myo)-1(or 4)-monophosphatase 1	0.904277240859346	6,54455E+14	1,92858E+14	0.0988014140306916	0.238960781282613	-4,32651E+14	7,56084E+14	7,00657E+14	6,31154E+14	6,44731E+13	6,57054E+14	5,37048E+14
TC1600000124.mm.2	Rmi2	RecQ mediated genome instability 2	-0.634226299719896	7,50637E+14	-1,92849E+14	0.0988134364763668	0.238960781282613	-4,32661E+13	7,07439E+14	7,1938E+14	7,64086E+14	7,89577E+14	7,3667E+14	7,86671E+14
TC0700000009.mm.2	Cacng8	calcium channel, voltage-dependent, gamma subunit 8	0.560757435060584	7,99402E+14	1,92828E+14	0.0988440279194042	0.238960781282613	-4,32688E+14	8,31333E+14	8,28048E+14	7,77318E+14	7,69912E+14	8,01849E+14	7,87949E+13
TC0200004373.mm.2	Zfp770	zinc finger protein 770	1,35499E+13	6,58782E+14	1,92823E+14	0.0988503918951222	0.238960781282613	-4,32694E+14	7,58885E+14	7,4564E+14	7,00401E+13	5,33126E+14	6,85958E+14	5,28684E+14
TC1400000947.mm.2	Adra1a	adrenergic receptor, alpha 1a	0.725540516780736	5,01789E+13	1,92822E+13	0.0988519333220977	0.238960781282613	-4,32695E+13	4,97627E+14	5,70038E+14	4,38211E+14	4,84346E+14	5,17668E+14	5,02846E+14
TC1600001108.mm.2	1700037C18Rik	RIKEN cDNA 1700037C18 gene	-0.932778477545319	6,30931E+14	-1,92817E+14	0.0988588449096938	0.238960781282613	-4,32701E+14	5,5949E+13	5,68244E+14	6,0406E+14	7,10229E+14	6,26691E+14	7,16872E+14
TC0400001737.mm.2	Rnf186	ring finger protein 186	-0.689793983725617	6,79408E+14	-1,92791E+14	0.09889631734558	0.239015503433228	-4,32734E+14	6,11458E+14	6,39561E+14	6,72016E+14	7,16962E+14	6,90671E+14	7,4578E+14
TC0600002253.mm.2	Cycs	cytochrome c, somatic	1,65552E+14	9,78684E+14	1,92767E+14	0.098929390875066	0.239017454649826	-4,32763E+14	1,06443E+14	1,07112E+14	1,02275E+14	7,81705E+14	1,04682E+14	8,85276E+14
TC0500001572.mm.2	Abhd11	abhydrolase domain containing 11	0.703197135223633	6,3964E+13	1,92766E+14	0.0989305658142813	0.239017454649826	-4,32764E+14	7,04097E+14	6,82932E+14	5,98236E+14	6,48154E+14	5,72387E+14	6,32031E+14
TC0300002743.mm.2	5330417C22Rik	RIKEN cDNA 5330417C22 gene	1,15404E+14	6,77507E+14	1,92757E+14	0.0989434052318634	0.239017454649826	-4,32776E+14	7,2844E+14	7,41534E+14	7,03954E+13	5,3521E+14	7,19296E+14	6,36607E+14
TC0800002428.mm.2	Ano8	anoctamin 8	0.572092160871421	6,15092E+14	1,92748E+14	0.0989564599993165	0.239017454649826	-4,32787E+14	6,62347E+14	6,55378E+14	5,92416E+13	6,10891E+14	5,93737E+14	5,5758E+14
TC1800000797.mm.2	Rpl17	ribosomal protein L17	1,37768E+14	1,01663E+14	1,92714E+14	0.09900404954732	0.23907589919351	-4,32829E+14	1,10124E+12	1,10837E+14	1,06234E+14	8,71726E+14	1,04646E+14	9,09621E+14
TC1800000613.mm.2	Dctn4	dynactin 4	0.637470427925533	6,86819E+14	1,9271E+14	0.0990103317394833	0.23907589919351	-4,32835E+14	7,68419E+14	7,26252E+14	6,65192E+14	7,01984E+14	6,3853E+14	6,20535E+14
TC1000002592.mm.2	Nuak1	NUAK family, SNF1-like kinase, 1	0.619725585675231	7,36624E+14	1,92662E+14	0.0990776981521144	0.23920271955724	-4,32894E+14	7,80187E+14	7,70591E+13	7,35565E+14	6,91267E+14	7,33667E+14	7,08465E+14
TC0200005119.mm.2	Spint3	serine peptidase inhibitor, Kunitz type, 3	-0.777137774226826	6,10162E+14	-1,92562E+14	0.0992195760782574	0.239482716041013	-4,33018E+14	5,47616E+13	5,49424E+14	6,00565E+14	6,51902E+14	6,11882E+14	6,99582E+13
TC0600002648.mm.2	Antxr1	anthrax toxin receptor 1	1,1188E+14	6,80522E+14	1,92559E+14	0.0992233980195991	0.239482716041013	-4,33021E+14	7,31017E+14	7,51455E+14	7,12191E+14	5,46521E+14	7,061E+14	6,35848E+14
TC0700000720.mm.2	Vstm2b	V-set and transmembrane domain containing 2B	0.768431727602393	7,70927E+14	1,9253E+14	0.0992641889933963	0.239545286393679	-4,33057E+14	8,31325E+13	7,94166E+14	7,76043E+14	6,95761E+14	7,86738E+14	7,41526E+14
TC0800001526.mm.2	Afg3l1	AFG3-like AAA ATPase 1	0.703945347182447	5,80652E+14	1,92512E+14	0.0992899284522605	0.239563209082192	-4,3308E+14	6,29773E+14	6,43372E+14	5,2662E+14	6,05736E+14	5,33405E+14	5,45007E+13
TC1300000839.mm.2	1700001L19Rik	RIKEN cDNA 1700001L19 gene	0.807145504832871	6,50526E+14	1,92504E+14	0.0993013512977074	0.239563209082192	-4,3309E+14	7,15065E+14	6,99455E+14	6,3631E+14	6,16781E+14	6,71083E+13	5,64464E+14
TC1500000821.mm.2	Ppara	peroxisome proliferator activated receptor alpha	-0.771069166185691	5,82592E+14	-1,9241E+13	0.0994336791992034	0.239748132808631	-4,33205E+13	5,46849E+14	5,47588E+14	5,80611E+14	6,6804E+13	6,00687E+14	5,51774E+14
TSUnmapped00000011.mm.2	Pld6	phospholipase D family, member 6	-0.690859361979619	7,08529E+14	-1,92404E+14	0.0994428147217231	0.239748132808631	-4,33213E+14	6,66195E+14	6,68913E+14	7,00225E+14	7,73056E+14	7,08621E+14	7,34163E+14
TC1200002049.mm.2	Zfyve1	zinc finger, FYVE domain containing 1	0.689921338476521	6,98235E+14	1,924E+14	0.0994488176388003	0.239748132808631	-4,33219E+14	7,38911E+14	7,36455E+14	7,02918E+13	6,34464E+14	7,05851E+14	6,70809E+14
TC1000001020.mm.2	Tcp11l2	t-complex 11 (mouse) like 2	-0.622330221644024	6,24384E+14	-1,92398E+14	0.0994519366626572	0.239748132808631	-4,33221E+14	5,69728E+14	6,04875E+14	6,30021E+14	6,69048E+14	6,40792E+14	6,3184E+14
TC1100001302.mm.2	Tbx2	T-box 2	-0.74494529794761	7,08126E+14	-1,92395E+14	0.0994561625966877	0.239748132808631	-4,33225E+14	6,75648E+14	6,63433E+14	7,00447E+14	7,87622E+13	6,95676E+14	7,2593E+14
TC0900000810.mm.2	Rpl4	ribosomal protein L4	1,00952E+14	1,01808E+14	1,92381E+14	0.0994752782167055	0.239748132808631	-4,33242E+14	1,089E+14	1,08062E+14	1,04845E+14	9,19269E+14	1,0304E+13	9,40725E+12
TC0500001737.mm.2	Cyth3	cytohesin 3	1,30816E+14	6,445E+14	1,92376E+14	0.0994821582547325	0.239748132808631	-4,33248E+14	7,18953E+14	7,08154E+14	6,84871E+14	4,80603E+14	6,79076E+14	5,95343E+14
TC0500001268.mm.2	Rab35	RAB35, member RAS oncogene family	0.674130406769072	8,00004E+14	1,92364E+14	0.0994995630347645	0.239754218510417	-4,33263E+14	8,52421E+14	8,4791E+14	7,95593E+14	7,69911E+14	8,00627E+14	7,33564E+14
TC0X00000168.mm.2	Nyx	nyctalopin	-0.639372777063828	5,79264E+13	-1,92333E+14	0.0995428596188563	0.23982268197348	-4,33301E+14	5,46055E+14	5,32486E+14	5,74306E+14	6,32109E+13	5,99494E+14	5,91136E+14
TC0700002685.mm.2	Psmc4	proteasome (prosome, macropain) 26S subunit, ATPase, 4	1,14233E+14	7,68433E+14	1,92313E+14	0.099571242444063	0.23985519950683	-4,33326E+14	8,36381E+14	8,39556E+13	7,8288E+14	6,64591E+14	8,15632E+14	6,71554E+14
TC0500000934.mm.2	Bmp2k	BMP2 inducible kinase	1,07807E+14	7,789E+14	1,92248E+14	0.0996644610782398	0.240014043688779	-4,33407E+14	8,4584E+14	8,66523E+14	8,27735E+14	6,69013E+14	7,64571E+14	6,99716E+14
TC0600001653.mm.2	Plekha5	pleckstrin homology domain containing, family A member 5	1,09084E+14	7,86487E+14	1,92242E+14	0.0996729206181561	0.240014043688779	-4,33414E+14	8,57739E+14	8,69401E+14	8,38546E+14	6,70426E+14	7,65861E+14	7,16948E+14
TC0100000708.mm.2	Mff	mitochondrial fission factor	1,31704E+14	9,31069E+14	1,92233E+14	0.0996854413218427	0.240014043688779	-4,33425E+14	1,00577E+14	9,93876E+14	9,62779E+14	7,73457E+13	9,83937E+14	8,66596E+14
TC1500002032.mm.2	Ttll8	tubulin tyrosine ligase-like family, member 8	-0.7919682392617	5,38027E+14	-1,92211E+14	0.0997167361525536	0.240014043688779	-4,33453E+13	4,68057E+14	4,80945E+14	5,17742E+14	5,89653E+14	5,47544E+13	6,24222E+14
TC1700000362.mm.2	Clcn7	chloride channel, voltage-sensitive 7	0.77687567969821	6,78613E+14	1,92197E+14	0.0997360747034958	0.240014043688779	-4,33469E+14	7,37753E+13	7,33714E+14	6,88426E+14	6,27666E+14	6,01171E+14	6,82948E+14
TC1800000835.mm.2	Epg5	ectopic P-granules autophagy protein 5 homolog (C. elegans)	0.618396816411324	6,10566E+13	1,92197E+14	0.0997368549728061	0.240014043688779	-4,3347E+14	6,7256E+14	6,50347E+14	5,82591E+14	6,16636E+14	5,54474E+14	5,86789E+13
TC1100002233.mm.2	Cobl	cordon-bleu WH2 repeat	1,464E+14	7,41484E+14	1,92194E+14	0.0997414532700345	0.240014043688779	-4,33474E+14	8,33253E+14	8,4691E+14	7,74387E+14	6,12976E+14	7,87858E+14	5,93518E+14
TC1300001136.mm.2	Smn1	survival motor neuron 1	-0.673041842017426	5,1257E+14	-1,92159E+14	0.0997902609878089	0.240095636150002	-4,33517E+14	4,9421E+14	4,37258E+14	5,12939E+14	5,53138E+14	5,3027E+12	5,47605E+14
TC1400000317.mm.2	Itih4	inter alpha-trypsin inhibitor, heavy chain 4	-0.978665796150761	6,05382E+14	-1,92141E+14	0.0998161186316931	0.240121994552474	-4,33539E+14	5,16825E+14	5,79032E+14	5,91076E+14	7,00515E+14	5,80881E+14	6,63962E+14
TC1100003833.mm.2	Slc4a1	solute carrier family 4 (anion exchanger), member 1	-0.58316057296921	7,19945E+13	-1,92128E+14	0.0998352587066658	0.240132187422079	-4,33556E+14	6,76688E+14	6,77057E+14	7,18358E+14	7,5202E+14	7,52722E+13	7,42828E+14
TC0800002711.mm.2	Ces5a	carboxylesterase 5A	-0.80471852879072	6,46874E+14	-1,92058E+14	0.099934459850031	0.240304897431274	-4,33642E+14	5,91698E+14	5,89729E+14	6,32847E+14	7,09523E+13	6,39741E+14	7,17704E+14
TC0800002599.mm.2	Asna1	arsA arsenite transporter, ATP-binding, homolog 1 (bacterial)	0.740691474638995	8,08032E+13	1,92056E+14	0.0999368905658143	0.240304897431274	-4,33644E+14	8,58068E+13	8,75126E+14	8,11054E+14	7,74003E+14	8,06346E+14	7,23593E+14
TC0900000503.mm.2	Ccdc153	coiled-coil domain containing 153	-0.55571888457838	7,04865E+14	-1,91988E+14	0.100034825591668	0.240504497875043	-4,33729E+14	6,65362E+14	6,77723E+14	7,28071E+14	7,26158E+14	7,16551E+14	7,15325E+14
TC0500003733.mm.2	Zfp644	zinc finger protein 644	1,59107E+14	8,24895E+14	1,91969E+14	0.100061363891216	0.240516755399936	-4,33752E+14	9,29178E+14	9,28561E+14	8,82345E+13	6,5718E+14	8,5826E+13	6,93847E+14
TC1000002436.mm.2	Lrrc3	leucine rich repeat containing 3	-0.575676708915989	5,94825E+14	-1,91957E+14	0.100078222912449	0.240516755399936	-4,33767E+14	5,60873E+14	5,56597E+14	6,01088E+14	6,31516E+13	6,08283E+14	6,10596E+14
TC1100004144.mm.2	Tmc6	transmembrane channel-like gene family 6	-0.645901787341278	5,75957E+14	-1,91937E+14	0.100106791356777	0.240516755399936	-4,33792E+14	5,34181E+14	5,3157E+14	5,7635E+14	6,18582E+14	5,74311E+14	6,20749E+14
TC1100001021.mm.2	Spata22/Olfr20	spermatogenesis associated 22/olfactory receptor 20	-0.778758896328748	5,57992E+14	-1,91922E+14	0.100128258921344	0.240516755399936	-4,33811E+14	5,46069E+14	5,07558E+14	5,62983E+14	6,46396E+14	5,19466E+14	5,6548E+14

TC0700000301.mm.2	Ckm	creatine kinase, muscle	-0.63470176305839	8,27074E+13	-1,91913E+14	0.10014183603276	0.240516755399936	-4,33822E+14	7,82833E+14	8,01739E+14	8,31033E+13	8,80479E+14	8,20456E+14	8,459E+14
TC0100001765.mm.2	Dusp10	dual specificity phosphatase 10	0.698788805254511	6,88498E+14	1,91909E+14	0.100146831667187	0.240516755399936	-4,33827E+14	7,38031E+14	7,08926E+14	6,69147E+13	6,38052E+14	7,22405E+14	6,5443E+14
TC0900001768.mm.2	Endod1	endonuclease domain containing 1	0.79665554238987	7,4553E+12	1,91909E+14	0.100146910241294	0.240516755399936	-4,33827E+14	8,01141E+14	7,64945E+13	7,53735E+14	6,5302E+14	7,54963E+13	7,45375E+14
TC1100001500.mm.2	Hoxb2	homeobox B2	-0.586776137545018	7,26444E+14	-1,91888E+14	0.100176320872655	0.240516755399936	-4,33852E+14	6,86371E+14	6,82954E+14	7,24793E+14	7,61887E+14	7,49048E+14	7,5361E+14
TC1100003075.mm.2	2810408A11Rik	RIKEN cDNA 2810408A11 gene	-0.679229880618977	6,0904E+14	-1,91875E+14	0.10019559243909	0.240516755399936	-4,33869E+14	5,43394E+14	5,66448E+14	5,89573E+14	6,56116E+14	6,37738E+13	6,6097E+14
TC1400001741.mm.2	Ccser2	coiled-coil serine rich 2	1,68659E+14	6,98683E+14	1,91874E+13	0.100197182214112	0.240516755399936	-4,3387E+14	7,98048E+14	7,74109E+14	7,50907E+14	4,83933E+14	7,57568E+14	6,27536E+14
TC0400002220.mm.2	Tmem67	transmembrane protein 67	1,31903E+14	5,40966E+14	1,91869E+14	0.100204119592861	0.240516755399936	-4,33876E+14	6,67299E+13	6,19755E+14	5,18847E+14	5,044E+14	5,76946E+14	3,58549E+14
TC0100002476.mm.2	Cryge	crystallin, gamma E	-0.604977935724168	5,8246E+14	-1,91844E+14	0.100239263797711	0.24053502199572	-4,33907E+14	5,37732E+14	5,46733E+14	6,2463E+14	5,8083E+14	5,95849E+14	6,08984E+14
TC0600002044.mm.2	Slc35b4	solute carrier family 35, member B4	1,30328E+14	7,80805E+14	1,91838E+14	0.100248831307577	0.24053502199572	-4,33915E+14	8,8719E+14	8,71982E+14	8,4018E+14	6,58336E+14	7,75708E+14	6,51437E+14
TC1800001623.mm.2	Gm7276	predicted gene 7276	-0.622091646024353	6,05251E+13	-1,91825E+14	0.100267370863708	0.24053502199572	-4,33931E+14	5,64398E+14	5,71048E+14	6,21528E+14	6,38336E+14	5,92693E+14	6,43505E+14
TC1400000331.mm.2	Btd	biotinidase	-0.643055720133948	6,66069E+14	-1,91822E+14	0.100271441863418	0.24053502199572	-4,33935E+13	6,27142E+14	6,06096E+14	6,6001E+13	7,01839E+14	6,80394E+14	7,20935E+14
TC1200001373.mm.2	Klhl29	kelch-like 29	0.688631467505561	6,34277E+14	1,91784E+14	0.100326044687002	0.240630181310162	-4,33982E+13	7,32921E+14	6,93613E+14	6,61575E+14	6,27234E+14	5,73619E+14	5,167E+14
TC0300001800.mm.2	Kcnmb3	potassium large conductance calcium-activated channel, subfamily M, beta member 3	-0.789884464373356	8,31267E+14	-1,91699E+14	0.100447546736578	0.240885744986827	-4,34087E+14	7,65759E+14	7,99292E+14	8,15903E+14	9,07125E+14	8,28711E+14	8,70812E+14
TC1400001971.mm.2	Olfr724	olfactory receptor 724	-0.573054253949663	4,81691E+14	-1,91661E+14	0.100500796284653	0.240977578948602	-4,34133E+14	4,68861E+13	4,54009E+14	5,11909E+14	5,25571E+14	4,75767E+14	4,54029E+14
TC0500001594.mm.2	Dtx2	deltex 2, E3 ubiquitin ligase	-0.563882938739604	67553617734	-1,91626E+14	0.100551832013232	0.241053362724375	-4,34177E+14	6,47148E+13	6,33417E+13	6,87655E+14	7,05686E+14	6,8852E+14	6,9079E+14
TC1300000815.mm.2	Ptdss1	phosphatidylserine synthase 1	1,50806E+14	7,0858E+14	1,91618E+14	0.100563029737882	0.241053362724375	-4,34187E+12	8,02799E+14	7,90563E+14	7,26745E+14	5,65005E+14	7,86548E+14	5,79824E+14
TC0200002869.mm.2	Mcm10	minichromosome maintenance 10 replication initiation factor	-0.790672078999457	6,94514E+14	-1,91596E+13	0.100594957974948	0.241053362724375	-4,34215E+13	6,33886E+14	6,53565E+14	6,75105E+14	7,7048E+14	6,95801E+14	7,38246E+14
TC0500001221.mm.2	Acacb	acetyl-Coenzyme A carboxylase beta	-0.985228116123454	7,42999E+12	-1,91589E+14	0.10060494429577	0.241053362724375	-4,34223E+14	6,9273E+14	6,57291E+14	7,01631E+14	8,45436E+14	7,61366E+14	7,99543E+14
TC1000001991.mm.2	Rwdd1	RWD domain containing 1	1,88116E+14	7,58548E+14	1,91579E+14	0.100618255867755	0.241053362724375	-4,34235E+13	8,47639E+14	8,61827E+14	8,24485E+14	5,08747E+14	8,12381E+13	6,96207E+14
TC0500000877.mm.2	Stbd1	starch binding domain 1	-0.789155143493258	5,90817E+14	-1,91566E+14	0.100637649026706	0.241053362724375	-4,34251E+14	5,64903E+14	5,24818E+14	5,80074E+14	6,67478E+14	5,81669E+14	6,25963E+14
TC1200001446.mm.2	Lratd1	LRAT domain containing 1	1,12654E+14	7,48117E+14	1,91556E+14	0.100651355998664	0.241053362724375	-4,34263E+14	8,12665E+14	7,93054E+14	7,79911E+14	6,00499E+14	7,69256E+14	7,33317E+14
TC0200004756.mm.2	Snx5	sorting nexin 5	1,26995E+14	8,68545E+14	1,91555E+14	0.100653356972478	0.241053362724375	-4,34265E+14	9,37152E+14	9,42793E+14	9,04911E+13	7,21043E+14	9,07622E+14	7,97748E+14
TC0900001325.mm.2	Aste1	asteroid homolog 1	-0.820891323844982	6,26649E+14	-1,91545E+14	0.100667043863484	0.241053362724375	-4,34277E+14	5,95805E+14	5,94672E+14	6,2891E+14	7,25745E+14	5,77274E+14	6,37487E+14
TC0200004506.mm.2	Duox2	dual oxidase 2	-0.748257681484017	5,9766E+14	-1,91531E+13	0.100688367651355	0.241068598509106	-4,34295E+14	5,48923E+14	5,08201E+14	5,86503E+14	6,20273E+13	6,21619E+14	7,00443E+14
TC0900002972.mm.2	Tmem108	transmembrane protein 108	-0.585310465003431	6,74366E+14	-1,91509E+14	0.100718843841234	0.241105739238421	-4,34322E+14	6,62933E+14	6,36618E+14	7,12101E+14	7,04512E+14	6,77723E+14	6,52311E+14
TC0800000034.mm.2	Ccl25	chemokine (C-C motif) ligand 25	-0.980619487060077	7,08748E+14	-1,91493E+14	0.100742910702664	0.241127528245993	-4,34342E+14	6,56764E+14	6,19595E+14	6,76302E+14	7,96181E+14	7,08936E+14	7,94709E+14
TC0500003067.mm.2	4930515G01Rik	RIKEN cDNA 4930515G01 gene	-0.600039317645909	6,85853E+14	-1,91455E+14	0.10079618882688	0.241219217372274	-4,34388E+14	6,62916E+14	6,33647E+14	6,91836E+14	7,24735E+14	7,05358E+14	6,96627E+13
TC0200004854.mm.2	Gm14151	predicted gene 14151	-0.645215502849967	5,18699E+14	-1,91433E+14	0.100828897665533	0.241255628900897	-4,34416E+14	4,74234E+14	4,88363E+13	5,27686E+14	5,63954E+14	5,53865E+14	5,0409E+14
TC0600002177.mm.2	Tcaf2	TRPM8 channel-associated factor 2	-0.715641893208468	5,09315E+14	-1,91424E+14	0.100841349261314	0.241255628900897	-4,34427E+14	4,64123E+14	4,75248E+14	5,42454E+14	5,40045E+14	4,745E+13	5,59519E+14
TC0500002186.mm.2	Supt7l	suppressor of Ty 7-like	1,10908E+14	7,44879E+14	1,91394E+14	0.100884611518815	0.241317629213556	-4,34464E+14	8,37074E+14	8,18324E+14	8,03672E+14	6,29911E+14	7,12646E+13	6,67645E+14
TC0600001158.mm.2	Ppp4r2	protein phosphatase 4, regulatory subunit 2	1,03891E+14	7,73191E+14	1,91385E+14	0.100897217651072	0.241317629213556	-4,34475E+14	8,31223E+14	8,57275E+14	8,01214E+14	6,79503E+13	7,89252E+14	6,80678E+14
TC0200002883.mm.2	Sec61a2	Sec61, alpha subunit 2 (S. cerevisiae)	1,84169E+14	7,43318E+14	1,91368E+14	0.10092208519063	0.241333787452659	-4,34497E+14	8,52644E+14	8,40482E+13	8,0817E+14	5,16618E+14	7,97402E+14	6,44593E+14
TC1100000720.mm.2	Wnt9a	wingless-type MMTV integration site family, member 9A	-0.624037414161636	6,84033E+14	-1,9136E+14	0.100933928731054	0.241333787452659	-4,34507E+14	6,21737E+14	6,51726E+13	6,86977E+14	7,11293E+14	6,96426E+14	7,36038E+14
TC1100003677.mm.2	Mien1	migration and invasion enhancer 1	0.699130124904976	8,46257E+14	1,9133E+14	0.100975988555508	0.241398531690638	-4,34543E+14	8,8429E+14	9,02342E+14	8,55509E+13	7,91297E+14	8,44996E+14	7,99109E+14
TC0700002996.mm.2	Ccdc155	coiled-coil domain containing 155	-0.757111117816004	5,70882E+14	-1,91312E+14	0.101002647652478	0.241426444388723	-4,34566E+14	5,04343E+14	5,10579E+14	5,38302E+14	6,28042E+14	6,03086E+13	6,4094E+14
TC1700001841.mm.2	Wiz	widely-interspaced zinc finger motifs	-0.855923294346749	7,21027E+14	-1,91279E+14	0.101050215506794	0.241504319558138	-4,34607E+14	6,53394E+14	6,88984E+14	7,39393E+14	7,7417E+14	6,78855E+14	7,91366E+13
TC0900001426.mm.2	Tmem89	transmembrane protein 89	-0.767202252996641	6,33158E+14	-1,91247E+14	0.101095584549514	0.241576917373027	-4,34646E+14	5,96461E+14	5,68196E+14	6,40743E+14	6,77354E+14	6,13542E+14	7,02651E+14
TC1000000493.mm.2	Gja1	gap junction protein, alpha 1	1,34944E+13	8,89049E+13	1,91197E+14	0.101168326728243	0.241714894509517	-4,34708E+14	9,86007E+14	9,69234E+14	9,40621E+14	7,44732E+14	9,07668E+14	7,86032E+14
TC0100000778.mm.2	2810459M11Rik	RIKEN cDNA 2810459M11 gene	-0.652840309458002	7,08988E+14	-1,91164E+14	0.10121526690048	0.241791192819485	-4,34749E+14	6,59695E+14	6,80215E+14	7,13184E+14	7,57295E+14	6,99324E+14	7,44214E+12
TC0700003970.mm.2	Olfr684	olfactory receptor 684	-0.723724048077008	4,54187E+13	-1,91111E+14	0.101292414202657	0.241891912924326	-4,34815E+14	4,09885E+14	3,77261E+14	4,25059E+14	5,06832E+14	4,98703E+14	5,07385E+14
TC1700001017.mm.2	Dus3l	dihydrouridine synthase 3-like (S. cerevisiae)	0.624609046791116	7,12E+14	1,91097E+13	0.101311984327662	0.241891912924326	-4,34832E+14	7,5018E+14	7,56593E+14	6,73721E+14	7,0813E+14	7,13465E+14	6,69908E+14
TC0600002342.mm.2	Nt5c3	5'-nucleotidase, cytosolic III	1,64267E+14	5,71595E+14	1,91087E+14	0.101326439321781	0.241891912924326	-4,34844E+14	6,94319E+14	7,21114E+14	6,11469E+14	4,75429E+14	5,91292E+14	3,35945E+14
TC0400003610.mm.2	Kpna6	karyopherin (importin) alpha 6	1,53757E+14	7,01525E+14	1,91077E+14	0.101341387760921	0.241891912924326	-4,34857E+14	8,10642E+14	8,05647E+14	7,79678E+14	5,29097E+14	6,91657E+14	5,92427E+13
TC0400000504.mm.2	Foxe1	forkhead box E1	-1,07108E+14	6,12864E+14	-1,91069E+14	0.101352863654117	0.241891912924326	-4,34867E+14	5,0177E+14	5,72254E+14	5,79225E+14	7,09015E+14	6,12784E+14	7,02138E+14
TC0X00002550.mm.2	Tsga8	testis specific gene A8	-0.570441570127866	6,26229E+14	-1,91062E+13	0.10136328321708	0.241891912924326	-4,34876E+14	5,77275E+14	5,96916E+14	6,37464E+14	6,50815E+14	6,39043E+14	6,55859E+14
TC0300000913.mm.2	C920021L13Rik	RIKEN cDNA C920021L13 gene	-1,07774E+14	6,07747E+14	-1,91061E+14	0.101363966667613	0.241891912924326	-4,34876E+14	5,16138E+14	5,43077E+13	6,00306E+14	6,74456E+14	5,78661E+14	7,33842E+14
TC0800002747.mm.2	Prss54	protease, serine 54	-0.983312537971589	4,65737E+14	-1,91052E+14	0.10137752671619	0.241891912924326	-4,34888E+14	3,73896E+14	4,07255E+14	4,26355E+14	5,51458E+14	4,7684E+14	5,58622E+14
TC0500002045.mm.2	5031425E22Rik	RIKEN cDNA 5031425E22 gene	-0.93796663733693	6,7296E+14	-1,91035E+14	0.101401433536957	0.241913132748147	-4,34908E+14	6,17499E+14	6,30792E+14	6,49286E+14	7,86598E+14	6,58381E+14	6,95205E+14
TC1500000388.mm.2	Mal2	mal, T cell differentiation protein 2	1,23878E+14	8,91779E+14	1,91011E+13	0.101436293260473	0.241936908394579	-4,34938E+14	9,51255E+14	9,67317E+14	9,17307E+14	7,53509E+14	9,44654E+14	8,16632E+14
TC0300000775.mm.2	4933434E20Rik/1700094D03 Rik	RIKEN cDNA 4933434E20 gene/RIKEN cDNA 1700094D03 gene	0.98529631925294	7,18329E+14	1,91004E+14	0.101446456836143	0.241936908394579	-4,34947E+14	7,79014E+14	7,65586E+14	7,49547E+14	5,97993E+14	7,19614E+14	6,98222E+14
TC0600002013.mm.2	Lncpint	long non-protein coding RNA, Trp53 induced transcript	-0.765411686565661	8,14315E+14	-1,90997E+14	0.101456444487195	0.241936908394579	-4,34955E+14	7,81336E+14	7,57107E+14	8,33545E+14	8,5798E+13	8,75005E+14	7,80915E+14
TC1400001726.mm.2	Bmpr1a	bone morphogenetic protein receptor, type 1A	1,21375E+14	6,44386E+14	1,90972E+14	0.10149287868156	0.241986788966545	-4,34986E+14	7,14787E+14	7,35638E+14	6,79795E+14	5,27881E+14	6,70858E+14	5,3736E+14
TC0100003518.mm.2	Dusp12	dual specificity phosphatase 12	0.905167282927234	6,56226E+14	1,90962E+14	0.101507398164518	0.241986788966545	-4,34999E+14	7,04244E+14	7,09765E+14	6,81131E+14	5,51845E+14	6,25606E+14	6,64767E+14
TC0800000782.mm.2	Myo9b	myosin IXb	-0.603034450589641	7,31503E+13	-1,90949E+14	0.101525709614981	0.241994638909201	-4,35015E+13	6,67504E+13	6,99846E+13	7,27415E+14	7,60542E+14	7,62793E+14	7,70917E+14
TC0700003574.mm.2	Arpin	actin-related protein 2/3 complex inhibitor	0.593219110636366	7,03653E+14	1,90911E+14	0.101581316101476	0.242091369078994	-4,35062E+14	7,75108E+13	7,33647E+14	6,93053E+14	6,97059E+14	6,57489E+14	6,65561E+14
TC0900000542.mm.2	Dscaml1	DS cell adhesion molecule like 1	1,10613E+14	6,28592E+14	1,90891E+14	0.101610060357788	0.242124061305094	-4,35087E+14	6,81301E+14	7,05498E+14	6,72131E+14	4,93444E+14	6,05983E+13	6,13194E+14
TC0100000771.mm.2	Cab39	calcium binding protein 39	1,02942E+14	8,51655E+14	1,90872E+14	0.101638040777744	0.242132990213756	-4,35111E+14	8,97614E+14	8,97003E+14	8,73921E+14	7,14813E+12	8,82501E+14	8,44079E+14
TC0700001666.mm.2	Far1	fatty acyl CoA reductase 1	1,38347E+14	6,5396E+14	1,90868E+14	0.10164386183863	0.242132990213756	-4,35116E+14	7,54516E+14	7,87037E+14	6,75449E+14	5,8941E+14	6,6878E+14	4,48569E+14
TC0700004142.mm.2	Calca	calcitonin/calcitonin-related polypeptide, alpha	-0.928200326140608	4,91578E+14	-1,90807E+14	0.101731639451107	0.242295758182839	-4,3519E+14	4,55565E+14	3,90756E+14	4,62111E+14	5,6985E+13	5,03932E+14	5,67254E+14
TC0800002175.mm.2	Trappc11	trafficking protein particle complex 11	0.652681707698962	7,23978E+14	1,908E+14	0.101742264002054	0.242295758182839	-4,352E+14	8,04859E+14	7,43851E+14	6,99557E+14	7,18616E+14	6,97803E+12	6,79181E+14
TC1000003148.mm.2	Esyt1	extended synaptotagmin-like protein 1	-0.799071661820108	6,75902E+14	-1,90777E+14	0.101775267219396	0.242338537122231	-4,35228E+14	6,03493E+14	6,24372E+14	6,46121E+14	7,41558E+14	6,95431E+14	7,44436E+13
TC0600002070.mm.2	Mtpn	myotrophin	1,31708E+14	9,43214E+14	1,90758E+14	0.101803039704615	0.242368850289667	-4,35251E+14	9,95813E+14	1,01653E+13	9,78187E+14	7,70744E+14	9,91525E+14	9,06481E+14
TC0400001510.mm.2	Rnf19b	ring finger protein 19B	0.809154181960703	7,15738E+14	1,90739E+14	0.101830283587105	0.242372304117871	-4,35275E+14	7,53323E+14	7,50886E+14	7,24567E+14	6,17812E+14	7,07148E+14	7,40692E+14
TC1900000841.mm.2	Pnliprp1	pancreatic lipase related protein 1	-0.964365795258528	5,66501E+14	-1,90736E+14	0.101834574497486	0.242372304117871	-4,35278E+14	4,81132E+13	5,12062E+14	5,47381E+14	6,38687E+13	5,52937E+14	6,66809E+14
TC1200000661.mm.2	Dbpht2	DNA binding protein with his-thr domain	0.836603186637927	8,85919E+14	1,90719E+14	0.101859082149271	0.24237700345801	-4,35299E+13	9,30708E+14	9,62306E+14	9,20142E+14	8,05551E+13	8,48774E+14	8,48033E+14
TC0500002719.mm.2	Adamts3	a disintegrin-like and metallopeptidase (reprolysin type) with thrombospondin type 1 motif, 3	0.966549687272443	6,41598E+14	1,90714E+14	0.101866633613706	0.24237700345801	-4,35306E+14	7,1815E+14	7,08535E+14	6,91352E+14	5,42023E+14	5,97274E+14	5,92253E+14
TC1300000457.mm.2	Gm9979	predicted gene 9979	-1,22403E+14	5,00816E+14	-1,90678E+14	0.101918466672899	0.242436527050342	-4,3535E+13	4,17612E+14	3,95051E+14	4,29542E+14	6,27928E+14	5,42303E+14	5,92456E+13
TC1300002767.mm.2	Zfp935	zinc finger protein 935	1,19705E+14	6,46662E+14	1,90676E+14	0.1019217423347	0.242436527050342	-4,35353E+14	7,27215E+14	7,29206E+14	6,76862E+14	5,40149E+14	6,76374E+14	5,30165E+14
TC0500002073.mm.2	Kcnh2	potassium voltage-gated channel, subfamily H (eag-related), member 2	0.752938435334045	7,90568E+13	1,90654E+14	0.101953453276765	0.242459477741862	-4,3538E+14	8,81927E+14	8,16792E+14	8,07896E+14	7,40235E+14	7,35824E+13	7,60736E+13
TC1500001090.mm.2	Soat2	sterol O-acyltransferase 2	-1,2221E+13	5,61209E+14	-1,90646E+14	0.101965540734978	0.242459477741862	-4,3539E+14	4,71147E+14	5,10252E+14	5,14935E+14	7,10884E+14	5,56003E+14	6,04035E+14
TC0300000211.mm.2	Cldn11	claudin 11	0.729971461599542	8,64443E+14	1,90638E+14	0.101976533274784	0.242459477741862	-4,35399E+14	9,405E+14	8,98178E+14	8,6661E+12	8,26075E+13	8,62307E+14	7,92988E+14
TC0300000416.mm.2	Spg20	spastic paraplegia 20, spartin (Troyer syndrome) homolog (human)	0.942103169660403	6,49007E+14	1,90626E+14	0.101994568321412	0.242466580018134	-4,35415E+14	7,33963E+14	7,09987E+12	6,97759E+14	5,57771E+14	6,09778E+14	5,84786E+14
TC0600001657.mm.2	Aebp2	AE binding protein 2	1,48379E+14	8,13862E+14	1,90571E+14	0.102075217535201	0.242575277250208	-4,35483E+14	8,86586E+14	8,89264E+13	8,63636E+14	6,15456E+14	8,52032E+14	7,76197E+13
TC0500000605.mm.2	Klhl5	kelch-like 5	1,13016E+14	7,42896E+14	1,90567E+14	0.102080248668058	0.242575277250208	-4,35487E+14	8,21285E+14	8,1195E+14	7,77717E+14	6,29486E+14	7,645E+14	6,52441E+14
TC1800001169.mm.2	Lrrtm2	leucine rich repeat transmembrane neuronal 2	1,72738E+14	8,32814E+13	1,90564E+14	0.102085456155506	0.242575277250208	-4,35492E+14	9,37559E+14	9,46816E+14	8,95729E+14	6,43171E+14	8,75105E+14	6,98503E+14
TC1700002755.mm.2	Mettl4	methyltransferase like 4	1,28016E+14	5,97896E+14	1,90523E+14	0.10214483835851	0.242680592814904	-4,35542E+14	6,91908E+14	6,89394E+14	6,18217E+14	5,07051E+14	6,34057E+12	4,46748E+14
TC1300002175.mm.2	Fbp2	fructose bisphosphatase 2	-0.657815342698368	4,76647E+14	-1,90507E+13	0.102167260187967	0.242685142003435	-4,35561E+14	4,27768E+14	4,55374E+14	5,25963E+14	4,88742E+14	5,06361E+14	4,55674E+14
TC0700002114.mm.2	Fgf15	fibroblast growth factor 15	-0.703269217879015	5,63938E+14	-1,90501E+14	0.102176876022547	0.242685142003435	-4,3557E+13	5,12807E+14	5,32465E+14	5,55539E+14	6,30386E+14	5,61035E+14	5,91395E+14
TC0500000221.mm.2	Nub1	negative regulator of ubiquitin-like proteins 1	0.931725358099065	8,49596E+14	1,90462E+14	0.102232722287229	0.242781997673415	-4,35617E+14	9,15138E+14	9,08935E+14	8,64499E+14	7,73229E+14	8,72181E+14	7,63591E+14
TC0400001010.mm.2	Pgm2	phosphoglucomutase 2	1,04506E+13	7,0763E+14	1,90437E+14	0.102269851255549	0.242834381562138	-4,35649E+13	7,94664E+14	7,90016E+14	7,715E+14	6,04167E+14	6,3792E+14	6,4751E+14
TC0500000560.mm.2	Pcdh7	protocadherin 7	1,54418E+14	7,35184E+13	1,90416E+14	0.102299735203692	0.242869549629746	-4,35674E+14	8,48214E+14	8,45026E+14	7,73707E+14	6,10698E+14	7,75567E+14	5,57895E+14
TC0Y00000233.mm.2	Uty	ubiquitously transcribed tetratricopeptide repeat gene, Y chromosome	1,29158E+14	5,32395E+12	1,90376E+14	0.102358303989409	0.242972797905326	-4,35724E+14	6,50549E+14	6,38166E+14	5,83788E+14	4,4661E+14	5,23666E+14	3,51593E+14
TC1100003138.mm.2	4933427D14Rik	RIKEN cDNA 4933427D14 gene	0.556911639757894	5,31535E+14	1,90363E+14	0.102377586544508	0.242982773897726	-4,3574E+14	5,6034E+14	5,66685E+14	4,98507E+14	5,17136E+13	5,2406E+14	5,22483E+14
TC0300001708.mm.2	Agtr1b	angiotensin II receptor, type 1b	-0.797769944756954	5,86614E+14	-1,90349E+14	0.102398724235424	0.242997149279143	-4,35758E+14	5,16947E+14	5,19304E+14	5,48039E+14	6,47766E+14	6,2133E+14	6,66301E+14
TC0X00001272.mm.2	Cstf2/Mir3112	cleavage stimulation factor, 3' pre-RNA subunit 2/microRNA 3112	1,01025E+14	7,53966E+14	1,90313E+14	0.10245027352953	0.243083678012269	-4,35802E+14	8,20575E+13	8,19219E+14	7,80346E+14	6,57398E+14	7,74378E+14	6,71882E+14
TC1300001089.mm.2	Col4a3bp	collagen, type IV, alpha 3 (Goodpasture antigen) binding protein	1,06473E+13	7,65564E+14	1,90291E+13	0.102483363069979	0.243112490797854	-4,3583E+14	8,07625E+14	8,32738E+14	7,97299E+14	6,30118E+14	7,7914E+14	7,46465E+13
TC1500000331.mm.2	Oxr1	oxidation resistance 1	1,86022E+14	8,10142E+14	1,90284E+14	0.102492592936748	0.243112490797854	-4,35837E+14	9,38277E+14	9,13986E+14	8,82016E+14	5,98202E+13	8,52446E+13	6,75927E+14
TC0700002967.mm.2	Spib	Spi-B transcription factor (Spi-1/PU.1 related)	-0.760426846969283	5,32339E+14	-1,90229E+14	0.102573965823963	0.243269695513912	-4,35906E+14	4,95467E+14	4,67487E+14	5,09751E+14	6,05288E+14	5,55591E+14	5,60453E+14
TC1000002511.mm.2	Ap3d1	adaptor-related protein complex 3, delta 1 subunit	0.652552585821402	9,10697E+14	1,90179E+14	0.102646997024143	0.243388245503974	-4,35968E+14	9,69958E+13	9,56832E+14	9,08236E+14	8,88043E+14	9,0196E+14	8,39155E+14
TC1300002006.mm.2	Diras2	DIRAS family, GTP-binding RAS-like 2	0.703153338063601	8,43926E+14	1,90174E+14	0.102654162256874	0.243388245503974	-4,35974E+14	8,59223E+13	9,11818E+14	8,19712E+14	8,10699E+14	8,63135E+14	7,98968E+14
TC0800001426.mm.2	Atp2c2	ATPase, Ca++ transporting, type 2C, member 2	-0.805132925248531	5,46149E+14	-1,90153E+14	0.102684801097282	0.243425069895615	-4,36E+14	5,05434E+13	4,84849E+14	5,32177E+13	6,19132E+14	5,34279E+14	6,01025E+14
TC1200001686.mm.2	Stxbp6	syntaxin binding protein 6 (amisyn)	0.723059746788803	7,82919E+14	1,90136E+14	0.102708729632706	0.243425376965125	-4,3602E+14	8,47607E+14	8,11864E+14	7,88511E+14	7,26349E+13	7,88239E+14	7,34946E+14
TC0300000224.mm.2	Mfn1	mitofusin 1	0.923860439944784	6,65538E+14	1,90132E+14	0.102715145409662	0.243425376965125	-4,36026E+14	7,52412E+13	7,14111E+13	6,74521E+13	6,07229E+14	6,81114E+14	5,63841E+13
TC1800000401.mm.2	Ndfip1	Nedd4 family interacting protein 1	1,26393E+14	9,7564E+14	1,90114E+14	0.10274122035325	0.24343051526417	-4,36048E+14	1,06365E+14	1,0449E+14	1,01861E+14	8,37152E+14	1,00166E+14	8,87866E+14
TC0500000926.mm.2	Fras1	Fraser extracellular matrix complex subunit 1	-0.569034065911276	6,85071E+14	-1,9011E+14	0.102747528971118	0.24343051526417	-4,36053E+14	6,44293E+14	6,51953E+14	7,00113E+14	7,09939E+14	6,88376E+14	7,15754E+14
TC0800001805.mm.2	Defb39	defensin beta 39	-0.856782696812238	5,58952E+14	-1,90078E+14	0.102793832637208	0.243504414184554	-4,36092E+13	4,85919E+14	5,07766E+14	5,22015E+14	6,43026E+14	5,87587E+14	6,074E+14
TC1900000404.mm.2	Dmrt1	doublesex and mab-3 related transcription factor 1	-1,25998E+14	6,21023E+14	-1,90034E+14	0.102858217689802	0.24355053379354	-4,36146E+14	5,42915E+14	5,41935E+14	5,61281E+14	7,75565E+14	6,51412E+14	6,53031E+14
TC1300000013.mm.2	Calm4	calmodulin 4	-0.841788669862105	4,97805E+14	-1,90029E+14	0.10286552058044	0.24355053379354	-4,36153E+12	4,48087E+14	4,44954E+14	5,05141E+14	5,56259E+14	4,61428E+14	5,70959E+14
TC0200000719.mm.2	Arpc5l	actin related protein 2/3 complex, subunit 5-like	1,18883E+14	6,24763E+14	1,90022E+14	0.102876045712019	0.24355053379354	-4,36161E+14	7,03793E+14	6,83361E+14	6,58419E+14	4,9097E+14	6,59088E+14	5,52948E+14
TC0400003050.mm.2	Izumo3	IZUMO family member 3	-0.771195302824813	5,36244E+14	-1,90016E+14	0.102884880825935	0.24355053379354	-4,36169E+14	4,76337E+13	4,81359E+14	5,10964E+13	6,00971E+14	5,49215E+14	5,9862E+14
TC0X00002797.mm.2	Abcb7	ATP-binding cassette, sub-family B (MDR/TAP), member 7	0.685167441132738	5,89835E+13	1,90007E+14	0.102897762207576	0.24355053379354	-4,3618E+14	6,55174E+14	6,29508E+14	5,40467E+14	6,07182E+14	5,37556E+14	5,69122E+14
TC0700004667.mm.2	Ctbp2	C-terminal binding protein 2	0.660104172598214	7,43731E+14	1,89977E+13	0.102942604960644	0.24355053379354	-4,36218E+14	8,22615E+14	7,56382E+14	7,34575E+14	7,12401E+14	7,15401E+14	7,2101E+14
TC1300001034.mm.2	Ankrd34b	ankyrin repeat domain 34B	0.63998973545589	7,07127E+14	1,89974E+14	0.102945892709186	0.24355053379354	-4,3622E+14	7,62951E+13	7,38564E+14	6,90501E+14	6,83016E+14	6,55022E+14	7,12708E+14
TC0400002391.mm.2	Smim8	small integral membrane protein 8	0.684632336403133	7,30475E+14	1,89963E+14	0.102962441292946	0.24355053379354	-4,36234E+14	7,7604E+14	7,59031E+14	6,96765E+14	7,01379E+14	7,62821E+14	6,86814E+13
TC1800001469.mm.2	Alpk2	alpha-kinase 2	-1,07617E+14	4,62694E+14	-1,89957E+14	0.102971659946159	0.24355053379354	-4,36242E+14	4,25301E+14	3,63365E+14	4,29812E+14	5,74088E+14	4,53033E+14	5,30561E+14
TC1000000768.mm.2	Krtap10-10/Gm2696	keratin associated protein 10-10/predicted gene 2696	-0.567117811933877	8,07365E+14	-1,89952E+14	0.102979089246089	0.24355053379354	-4,36248E+14	7,61806E+14	7,80301E+13	8,38993E+14	8,16537E+14	8,23253E+14	8,23301E+14
TC1500000344.mm.2	Emc2	ER membrane protein complex subunit 2	1,61487E+14	8,71191E+14	1,89952E+14	0.102979568468652	0.24355053379354	-4,36249E+13	9,85227E+14	9,87584E+14	9,50382E+13	6,99456E+14	8,71832E+14	7,32663E+14
TC1100000715.mm.2	Trim11	tripartite motif-containing 11	-0.698740633059422	7,53076E+14	-1,89911E+14	0.10303938900823	0.243615965216316	-4,36299E+14	6,8201E+14	7,22392E+14	7,40234E+14	8,03915E+14	7,68318E+14	8,01589E+14
TC1200000086.mm.2	Rdh14	retinol dehydrogenase 14 (all-trans and 9-cis)	1,04419E+14	5,57734E+14	1,89898E+14	0.103057700525086	0.243615965216316	-4,36314E+14	6,3804E+14	6,33462E+14	6,12082E+14	4,50582E+14	5,29389E+14	4,82852E+14
TC1300002059.mm.2	Dbn1	drebrin 1	0.70220388584441	7,56251E+14	1,89893E+13	0.103065359128095	0.243615965216316	-4,36321E+14	7,94672E+14	8,08363E+14	7,65535E+14	6,97059E+14	7,58811E+14	7,13064E+14
TC0600002880.mm.2	Sumf1	sulfatase modifying factor 1	0.862909828353581	6,16876E+14	1,89882E+14	0.103081604909523	0.243615965216316	-4,36335E+14	6,95358E+14	6,53992E+14	6,31332E+14	5,45436E+14	6,28965E+13	5,46175E+14

TC1000001768.mm.2	Phactr2	phosphatase and actin regulator 2	1,09078E+13	6,17074E+14	1,89873E+14	0.103094346407283	0.243615965216316	-4,36345E+14	7,03255E+14	7,10126E+11	6,62487E+14	5,32739E+14	6,08046E+14	4,85793E+14
TC1400000318.mm.2	Nek4	NIMA (never in mitosis gene a)-related expressed kinase 4	0.983958402931675	6,50023E+14	1,89871E+14	0.103097949904429	0.243615965216316	-4,36348E+14	7,14217E+14	7,20896E+14	6,51393E+14	5,86929E+14	6,83571E+14	5,43135E+14
TC0100000927.mm.2	Sned1/Mir6901	sushi, nidogen and EGF-like domains 1/microRNA 6901	-0.694891343575019	6,42437E+14	-1,89856E+14	0.103120073408164	0.243632513610812	-4,36367E+13	6,12716E+14	5,92189E+14	6,33731E+14	7,10152E+14	6,43564E+14	6,62273E+14
TC0800001089.mm.2	Ogfod1	2-oxoglutarate and iron-dependent oxygenase domain containing 1	1,35843E+14	8,26987E+14	1,89825E+14	0.103165574118909	0.243684977606096	-4,36405E+14	9,45965E+14	9,13036E+14	8,87652E+14	6,99663E+14	8,26402E+14	6,89205E+14
TC1500002333.mm.2	Sh3bp1	SH3-domain binding protein 1	0.858798911332597	6,66759E+14	1,89808E+14	0.10319078808078	0.243684977606096	-4,36426E+14	7,15642E+14	7,15392E+14	6,89999E+14	5,69274E+14	6,71984E+13	6,38262E+14
TC1800000962.mm.2	Ccny	cyclin Y	1,31318E+14	8,01678E+14	1,89779E+14	0.103232450707323	0.243684977606096	-4,36461E+14	8,65263E+14	8,8305E+14	8,4161E+14	6,44068E+14	8,41943E+14	7,34136E+14
TC0300000710.mm.2	Crabp2	cellular retinoic acid binding protein II	-0.643812307912285	6,30026E+13	-1,89777E+14	0.103236551237191	0.243684977606096	-4,36465E+14	5,762E+14	5,89662E+14	6,16782E+14	6,77842E+13	6,6812E+14	6,51548E+14
TC1000001601.mm.2	Ankrd52/Mir6914	ankyrin repeat domain 52/microRNA 6914	1,15521E+14	5,79802E+14	1,89773E+14	0.103241594533825	0.243684977606096	-4,36469E+14	6,66698E+14	6,6699E+13	6,29432E+14	4,73214E+14	5,77445E+14	4,65032E+14
TC0800003236.mm.2	Usp10	ubiquitin specific peptidase 10	0.685011858818025	7,51594E+14	1,89706E+14	0.103341010390909	0.243684977606096	-4,36553E+14	8,52342E+14	7,86417E+14	7,62209E+14	7,39547E+14	6,66551E+14	7,02497E+14
TC0200004672.mm.2	Prokr2	prokineticin receptor 2	-1,00361E+14	7,06717E+14	-1,89703E+14	0.103344359080619	0.243684977606096	-4,36555E+14	6,20499E+14	6,54822E+14	6,62593E+14	8,13449E+14	7,25473E+14	7,63466E+13
TC0700003103.mm.2	Nipa1	non imprinted in Prader-Willi/Angelman syndrome 1 homolog (human)	0.825562506670408	7,70166E+14	1,897E+14	0.10334871614108	0.243684977606096	-4,36559E+14	8,34785E+14	8,23277E+14	7,94399E+14	6,98549E+14	7,68239E+14	7,01748E+14
TC1000002535.mm.2	Tjp3	tight junction protein 3	-0.580121544924844	5,70892E+14	-1,89692E+14	0.10336100315832	0.243684977606096	-4,36569E+14	5,2803E+14	5,38794E+14	6,04092E+14	5,78755E+14	5,75063E+14	6,00621E+14
TC0300002212.mm.2	Glrb	glycine receptor, beta subunit	1,51391E+14	8,38802E+14	1,8969E+14	0.103363308089947	0.243684977606096	-4,36571E+14	9,3169E+14	9,27905E+14	8,82522E+14	6,74291E+14	8,91762E+14	7,24642E+14
TC1100002004.mm.2	Endov	endonuclease V	0.625973187381159	7,24296E+14	1,8968E+14	0.103378557520555	0.243684977606096	-4,36584E+14	7,38652E+14	7,67312E+14	6,65081E+13	7,15688E+13	7,30837E+14	7,28202E+13
TC0800000008.mm.2	Tex45	testis expressed 45	-1,2429E+14	4,77387E+14	-1,89674E+14	0.103387276945706	0.243684977606096	-4,36591E+14	4,32579E+14	3,9819E+14	4,55124E+14	6,24225E+12	4,16213E+14	5,37992E+14
TC0800001666.mm.2	Fam155a	family with sequence similarity 155, member A	0.71845756824307	7,49903E+14	1,89669E+14	0.103395151058459	0.243684977606096	-4,36598E+14	8,07285E+14	8,21356E+14	7,747E+14	7,1025E+14	7,2243E+13	6,634E+14
TC0700000287.mm.2	Six5	sine oculis-related homeobox 5	-0.934305218749782	5,79746E+14	-1,89659E+13	0.1034101857987	0.243684977606096	-4,36611E+14	4,8653E+13	4,95437E+14	5,13253E+14	6,55575E+14	6,55795E+14	6,7189E+14
TC0300001562.mm.2	Negr1	neuronal growth regulator 1	0.955725164403946	8,96954E+14	1,8965E+14	0.103422386250042	0.243684977606096	-4,36621E+14	9,60969E+14	9,50053E+14	9,21379E+14	7,98499E+14	9,16722E+14	8,34104E+14
TC1500001782.mm.2	Cpsf1	cleavage and polyadenylation specific factor 1	0.663822444753277	6,21447E+14	1,89648E+14	0.103426306836363	0.243684977606096	-4,36624E+14	6,86216E+14	6,56704E+14	6,11546E+14	5,9861E+14	6,19824E+14	5,5578E+14
TC1900001505.mm.2	Tm9sf3	transmembrane 9 superfamily member 3	1,78477E+14	8,22186E+14	1,89617E+14	0.103471769937494	0.243684977606096	-4,36662E+14	9,29557E+14	9,37834E+14	8,86208E+14	6,2423E+13	8,70684E+13	6,84602E+14
TC1100000302.mm.2	Rtn4	reticulon 4	1,36416E+13	9,84281E+14	1,89613E+14	0.10347697389436	0.243684977606096	-4,36667E+14	1,05611E+14	1,06533E+14	1,01703E+14	8,31582E+14	1,0411E+14	8,94541E+14
TC0200003156.mm.2	C8g	complement component 8, gamma polypeptide	-0.769826433296158	8,50639E+14	-1,89596E+14	0.10350269873458	0.243684977606096	-4,36688E+14	8,05985E+14	8,05549E+14	8,59115E+14	9,06384E+14	8,17708E+14	9,09094E+14
TC0700003613.mm.2	Fsd2	fibronectin type III and SPRY domain containing 2	-0.839420651086115	4,92442E+14	-1,89589E+14	0.103512930514951	0.243684977606096	-4,36697E+14	4,24733E+14	4,30756E+14	4,51635E+14	5,71737E+13	5,32104E+14	5,43689E+14
TC0900002879.mm.2	Rnf7	ring finger protein 7	0.739089818739454	8,31211E+14	1,89587E+14	0.103515827531983	0.243684977606096	-4,36699E+14	8,71201E+14	8,66743E+14	8,18962E+14	7,71164E+14	8,70293E+13	7,88906E+14
TC0700002627.mm.2	Gsk3a	glycogen synthase kinase 3 alpha	0.900608987988457	8,61816E+14	1,89576E+14	0.103532034178068	0.243684977606096	-4,36713E+14	9,02395E+14	9,14239E+14	8,85208E+14	7,51304E+14	8,68065E+14	8,49685E+14
TC1300001012.mm.2	Tmem167	transmembrane protein 167	1,19621E+13	7,93453E+13	1,89576E+14	0.10353220945732	0.243684977606096	-4,36713E+14	8,54182E+14	8,65633E+14	8,1504E+14	6,65533E+14	8,47446E+14	7,12884E+14
TC0X00001893.mm.2	Ndp	Norrie disease (pseudoglioma) (human)	0.631962266471027	6,07898E+14	1,89573E+14	0.103536679168148	0.243684977606096	-4,36717E+14	6,46078E+14	6,46272E+14	5,89934E+14	5,76025E+14	6,24041E+14	5,65037E+14
TC1300000897.mm.2	Ccdc127	coiled-coil domain containing 127	0.602377560824126	9,18912E+14	1,89567E+14	0.103544943719186	0.243684977606096	-4,36724E+14	9,73733E+14	9,40269E+14	8,8264E+14	9,10887E+14	9,13592E+14	8,92354E+14
TC1200001816.mm.2	Fbxo33	F-box protein 33	1,5235E+14	7,18565E+14	1,89567E+14	0.1035457828168	0.243684977606096	-4,36724E+14	8,12481E+14	7,90217E+14	7,3367E+14	5,64327E+14	8,09369E+14	6,01329E+14
TC0800000394.mm.2	Gsr	glutathione reductase	1,27946E+14	7,24212E+14	1,89563E+14	0.103550613893685	0.243684977606096	-4,36728E+14	8,29428E+14	7,91893E+14	7,75957E+14	5,89471E+14	7,33545E+14	6,24975E+14
TC1700001955.mm.2	Lta	lymphotoxin A	-0.630988528574062	7,38638E+14	-1,89539E+14	0.10358644030536	0.243733690514058	-4,36758E+14	6,98575E+14	6,91686E+14	7,36023E+14	7,80436E+14	7,42453E+14	7,82653E+14
TC1900000921.mm.2	Rhod	ras homolog family member D	-0.586640682882615	4,97694E+14	-1,89481E+13	0.103672466755927	0.243884434825688	-4,3683E+13	4,6303E+14	4,7343E+14	5,10949E+14	5,42839E+14	5,10054E+14	4,85863E+14
TC0100001600.mm.2	Slamf1	signaling lymphocytic activation molecule family member 1	-0.637422031783925	5,00952E+14	-1,89471E+14	0.103686411594063	0.243884434825688	-4,36842E+14	4,55252E+14	4,25316E+14	5,03645E+13	5,04407E+14	5,3097E+14	5,86122E+14
TC1600000227.mm.2	Tssk1	testis-specific serine kinase 1	-0.995087325589588	5,17526E+14	-1,89454E+14	0.103711975346332	0.243884434825688	-4,36863E+14	4,44319E+14	4,57314E+14	4,95826E+14	6,04825E+14	4,94835E+14	6,08033E+14
TC1400001752.mm.2	Nrg3	neuregulin 3	0.658196757605943	6,20958E+14	1,8945E+13	0.103717547206564	0.243884434825688	-4,36868E+14	6,64594E+14	6,81153E+14	5,73541E+14	6,40567E+14	5,88856E+14	5,77037E+13
TC1100001078.mm.2	Crk	v-crk avian sarcoma virus CT10 oncogene homolog	-0.564518045856426	9,17057E+14	-1,89444E+14	0.103726185804036	0.243884434825688	-4,36875E+14	8,77366E+14	8,78675E+13	9,21811E+14	9,47134E+14	9,44991E+14	9,32362E+14
TC0200004943.mm.2	Pxmp4	peroxisomal membrane protein 4	-0.564793578324223	7,60141E+14	-1,89392E+14	0.103804059263109	0.244017405520145	-4,3694E+14	7,30037E+14	7,20742E+14	7,90082E+14	7,73656E+14	7,62397E+14	7,83931E+14
TC0X00003232.mm.2	Magea5	melanoma antigen, family A, 5	-0.759779541486797	5,21641E+13	-1,89386E+14	0.103813027669621	0.244017405520145	-4,36948E+14	4,64981E+14	4,70174E+14	5,08823E+14	5,78287E+14	5,17632E+14	5,89949E+14
TC0700001817.mm.2	Tbx6	T-box 6	-0.582863954535124	5,53063E+14	-1,89342E+14	0.10387804059724	0.24413460737213	-4,37002E+14	5,14024E+13	5,00451E+14	5,48648E+14	5,82399E+13	5,94281E+14	5,78575E+13
TC1300001947.mm.2	Fam120a	family with sequence similarity 120, member A	0.965891201457372	7,80458E+14	1,89311E+13	0.103923945608828	0.244175753123869	-4,37041E+14	8,26378E+14	8,46801E+14	8,09787E+14	6,70214E+14	7,92009E+14	7,37559E+14
TC0200005110.mm.2	Svs3b	seminal vesicle secretory protein 3B	-0.770612947411844	4,74988E+14	-1,89309E+14	0.103925855825948	0.244175753123869	-4,37042E+14	3,8803E+14	4,25249E+14	4,40042E+14	5,27359E+14	5,14105E+14	5,55144E+14
TC0700003909.mm.2	Hbb-bs/Hbb-b1	hemoglobin, beta adult s chain/hemoglobin, beta adult major chain	1,41675E+14	1,05743E+14	1,8927E+14	0.103983904860146	0.244276521214686	-4,37091E+14	1,06436E+14	1,17196E+14	1,03352E+14	9,1945E+14	1,17364E+14	9,81661E+14
TC0700002964.mm.2	Emc10	ER membrane protein complex subunit 10	0.708362731069675	8,75856E+14	1,89251E+14	0.104012953768308	0.244309143450255	-4,37115E+14	9,49955E+14	9,19132E+14	8,98428E+14	8,28987E+14	8,49496E+14	8,09138E+14
TC0800002838.mm.2	Cenpt	centromere protein T	-0.773012201473679	7,08581E+13	-1,8921E+14	0.104073102372845	0.244414793548119	-4,37165E+14	6,76607E+14	6,47357E+14	7,02599E+13	7,75967E+14	6,90526E+14	7,58428E+13
TC0200001092.mm.2	Hoxd12	homeobox D12	-0.754762034225783	5,47557E+14	-1,8919E+14	0.104103183166008	0.244424062581225	-4,3719E+14	4,65764E+14	4,9491E+14	5,22527E+14	5,891E+13	5,72718E+14	6,40322E+14
TC0700002088.mm.2	Cd81	CD81 antigen	1,13783E+14	1,15799E+14	1,89187E+14	0.104107387916275	0.244424062581225	-4,37193E+14	1,23168E+14	1,23643E+14	1,20071E+14	1,03983E+14	1,16964E+14	1,06964E+14
TC0700004315.mm.2	Cd2bp2	CD2 cytoplasmic tail binding protein 2	0.614556844263037	6,79506E+14	1,89161E+14	0.104146488841048	0.244450214811474	-4,37226E+14	7,40381E+14	7,51563E+14	7,00605E+14	6,68427E+14	6,29226E+14	5,86835E+14
TC0200002293.mm.2	Rem1	rad and gem related GTP binding protein 1	-0.826023801626892	5,34341E+14	-1,89159E+14	0.1041488689059	0.244450214811474	-4,37228E+14	4,96195E+14	4,57351E+14	5,39033E+14	5,79717E+14	5,15049E+14	6,187E+14
TC0700001726.mm.2	Abca15	ATP-binding cassette, sub-family A (ABC1), member 15	-1,07794E+14	4,52368E+14	-1,89147E+14	0.104166705426027	0.244456470219863	-4,37243E+14	3,98925E+14	3,77387E+14	4,27636E+14	5,64263E+14	4,18174E+14	5,2782E+14
TC0400000011.mm.2	Tgs1	trimethylguanosine synthase 1	1,07984E+14	5,18328E+13	1,89133E+14	0.104187266401242	0.244469116575391	-4,3726E+14	5,80901E+14	5,97625E+14	5,47121E+14	4,15438E+14	5,42937E+13	4,25946E+14
TC1700000503.mm.2	Fgd2	FYVE, RhoGEF and PH domain containing 2	-0.817823767775983	5,98721E+14	-1,89037E+14	0.104329727581306	0.244731071139016	-4,37378E+14	5,41395E+14	5,58179E+14	5,86972E+14	6,76166E+14	5,78694E+14	6,50922E+14
TC1100001071.mm.2	Scarf1	scavenger receptor class F, member 1	-0.65172720645973	6,64376E+14	-1,89037E+14	0.104330444079131	0.244731071139016	-4,37379E+14	6,31156E+14	6,34204E+14	7,04401E+14	6,91304E+14	6,29098E+14	6,96094E+14
TC0500002887.mm.2	Sparcl1	SPARC-like 1	1,34976E+14	1,11452E+14	1,8902E+14	0.104354817037465	0.244731071139016	-4,37399E+14	1,20444E+14	1,19968E+14	1,16604E+14	9,68132E+14	1,13721E+14	1,01161E+14
TC1400001070.mm.2	Tsc22d1	TSC22 domain family, member 1	1,39573E+14	1,04336E+14	1,89017E+14	0.104359659268676	0.244731071139016	-4,37403E+14	1,13451E+13	1,12775E+14	1,10038E+13	8,82728E+14	1,06236E+14	9,52407E+14
TC0400000952.mm.2	Tek	TEK receptor tyrosine kinase	0.579608188704798	5,30312E+14	1,88999E+14	0.104386956703204	0.244758242217932	-4,37426E+14	5,21484E+14	5,53153E+14	4,85363E+14	4,73352E+14	5,83353E+14	5,65165E+14
TC0100001723.mm.2	Lefty1	left right determination factor 1	-0.955653830055022	6,82435E+14	-1,88972E+14	0.10442686905153	0.244758242217932	-4,37459E+14	6,06727E+14	6,20866E+14	6,44187E+14	7,74536E+14	6,88496E+14	7,59796E+14
TC0400003953.mm.2	Gm438	predicted gene 438	-0.670788733602677	4,85043E+14	-1,88961E+14	0.104443146074844	0.244758242217932	-4,37473E+14	4,30668E+14	4,51075E+14	5,1378E+14	5,02122E+13	4,70819E+14	5,41792E+14
TC1000001850.mm.2	4930444G20Rik	RIKEN cDNA 4930444G20 gene	-1,14965E+13	4,85786E+14	-1,88959E+14	0.104446165637166	0.244758242217932	-4,37475E+14	4,22785E+14	4,03263E+14	4,5209E+14	6,03888E+14	4,58104E+14	5,74582E+14
TC0200003759.mm.2	Chn1	chimerin 1	1,30299E+14	1,09433E+14	1,88958E+14	0.104447196269503	0.244758242217932	-4,37476E+14	1,16319E+14	1,17972E+13	1,13518E+14	9,47127E+14	1,13141E+14	1,00934E+14
TC1400000790.mm.2	Gmpr2	guanosine monophosphate reductase 2	0.810899303734769	6,87114E+14	1,8893E+14	0.104489688770287	0.244822212469919	-4,37511E+14	7,21998E+14	7,29651E+14	7,00959E+14	5,88511E+14	7,00427E+13	6,81135E+14
TC0X00002621.mm.2	Mageb1/Mageb2	melanoma antigen, family B, 1/melanoma antigen, family B, 2	-1,06438E+14	5,92148E+14	-1,88853E+14	0.104603536954946	0.24502361579922	-4,37606E+14	5,23311E+14	5,33785E+14	5,73603E+14	6,96368E+14	5,5156E+14	6,74258E+14
TC0700002774.mm.2	Pmis2	Pmis2, sperm specific protein	-1,13966E+14	4,3289E+14	-1,88851E+14	0.104606060515288	0.24502361579922	-4,37608E+14	3,69753E+14	3,57605E+12	3,98577E+14	5,56713E+14	4,0475E+14	5,09943E+14
TC1400000557.mm.2	Olfr741	olfactory receptor 741	-0.968376698771734	5,00717E+14	-1,88833E+14	0.104633997331202	0.24505343008687	-4,37631E+14	4,13882E+14	4,49692E+14	4,84648E+14	5,72601E+13	4,82182E+14	6,01294E+14
TC0700002672.mm.2	Map3k10	mitogen-activated protein kinase kinase kinase 10	0.84737863853523	8,12967E+14	1,88766E+14	0.104733537975378	0.245250907919962	-4,37713E+14	8,64591E+14	8,66799E+14	8,38246E+14	7,23668E+14	8,15524E+14	7,68976E+13
TC0700004318.mm.2	Sep 01	septin 1	-0.730962845969035	5,55193E+14	-1,88723E+12	0.104798129232909	0.245366500483851	-4,37767E+14	5,261E+14	5,1518E+14	5,86895E+13	6,00578E+13	5,05459E+14	5,96946E+14
TC1600000092.mm.2	Pmm2	phosphomannomutase 2	0.925305247373047	5,46227E+14	1,88708E+13	0.104820069119482	0.245380212301094	-4,37785E+13	6,32093E+14	6,62049E+14	5,64403E+14	5,44677E+14	5,06025E+14	3,68118E+14
TC1100001660.mm.2	Nbr1	NBR1, autophagy cargo receptor	2,14028E+13	5,82929E+14	1,88698E+13	0.104834443088235	0.245380212301094	-4,37797E+14	7,71007E+14	7,52474E+14	6,41207E+14	4,54218E+13	6,12203E+14	2,66467E+14
TC0500003381.mm.2	Tmem270	transmembrane protein 270	-0.859506675153791	6,31077E+14	-1,88623E+14	0.104947188781841	0.245608431785302	-4,3789E+14	5,58455E+14	5,61745E+14	5,8435E+14	7,07751E+14	6,72514E+14	7,01647E+14
TC1200001971.mm.2	Atp6v1d	ATPase, H+ transporting, lysosomal V1 subunit D	1,55551E+14	8,5375E+14	1,88605E+14	0.104973886170046	0.245635234948875	-4,37912E+14	9,43685E+12	9,49656E+14	9,11575E+12	6,70664E+14	8,92E+14	7,54917E+14
TC0100001968.mm.2	Prdm14	PR domain containing 14	-0.754892155026446	4,77197E+14	-1,88568E+14	0.105029577603957	0.245729865533985	-4,37958E+14	4,31807E+14	3,96701E+14	4,75425E+14	5,04061E+14	4,83035E+14	5,72151E+14
TC0100003302.mm.2	BC034090	cDNA sequence BC034090	-0.816115108383578	4,88499E+14	-1,88552E+14	0.105053748484706	0.245747489145475	-4,37978E+14	3,91372E+13	4,70816E+14	4,9886E+14	5,26551E+14	4,81702E+14	5,61696E+14
TC0900001442.mm.2	Cdc25a	cell division cycle 25A	0.632650877015261	5,15675E+14	1,88541E+14	0.105069240393715	0.245747489145475	-4,37991E+13	5,55297E+14	5,19286E+14	4,78995E+14	4,69057E+14	5,10869E+13	5,60545E+14
TC1100003740.mm.2	Gm11564	predicted gene 11564	-0.715967445213179	7,02864E+14	-1,88532E+14	0.105082864780756	0.245747489145475	-4,38002E+14	6,50324E+14	6,69111E+14	7,0684E+14	7,55788E+13	6,8157E+14	7,53552E+14
TC0X00001331.mm.2	Kir3dl1	killer cell immunoglobulin-like receptor, three domains, long cytoplasmic tail, 1	-0.785252735018945	4,83966E+14	-1,88489E+14	0.105147853615488	0.245812206840598	-4,38055E+14	4,14824E+14	3,81573E+14	4,58338E+14	4,9511E+14	5,28895E+14	6,25054E+13
TC0300000832.mm.2	Lce1c	late cornified envelope 1C	-0.838575789878546	5,72054E+14	-1,88486E+14	0.105152017544321	0.245812206840598	-4,38059E+14	5,286E+14	4,78019E+14	6,41538E+14	5,32796E+14	6,36362E+14	6,15008E+14
TC0600003205.mm.2	Fkbp4	FK506 binding protein 4	0.851098599834343	7,88094E+14	1,88483E+13	0.105156304955765	0.245812206840598	-4,38062E+14	8,56673E+14	8,22095E+14	8,06656E+14	7,01892E+14	7,99929E+14	7,41318E+14
TC0100000194.mm.2	Pih1d3	PIH1 domain containing 3	-0.885136054523096	4,39125E+14	-1,88465E+14	0.10518367472922	0.245840520874952	-4,38085E+14	3,55587E+14	4,06005E+14	4,35471E+14	5,03148E+14	4,15721E+14	5,18819E+14
TC0900000559.mm.2	Apoa1	apolipoprotein A-I	-0.630123987693864	5,95955E+14	-1,88409E+13	0.105268101464433	0.246002163726819	-4,38154E+14	5,50474E+14	5,57407E+14	6,14704E+14	6,19202E+14	5,86558E+14	6,47384E+14
TC1500000561.mm.2	Rhpn1	rhophilin, Rho GTPase binding protein 1	-0.706814311553273	6,29796E+14	-1,88389E+14	0.105296687690772	0.246033284333151	-4,38178E+14	5,95813E+14	5,73494E+14	6,45305E+14	6,65365E+14	6,08821E+14	6,89975E+14
TC1200001277.mm.2	Pacs2	phosphofurin acidic cluster sorting protein 2	0.914766526439067	7,77002E+14	1,88354E+14	0.105350488143632	0.246123302226815	-4,38222E+14	8,50106E+14	8,43422E+14	8,17975E+14	6,926E+14	7,60397E+14	6,97511E+14
TC0300000387.mm.2	Lhfp	lipoma HMGIC fusion partner	0.598300656524521	6,3171E+14	1,88288E+13	0.105448729096119	0.246317102337745	-4,38303E+14	6,74653E+13	6,5977E+14	5,91204E+14	6,23559E+14	6,37119E+14	6,03955E+14
TC0600001874.mm.2	Tmem168	transmembrane protein 168	-0.932546040078981	7,24207E+14	-1,88219E+14	0.105552983289725	0.246524890527228	-4,38389E+14	6,3854E+14	6,57219E+14	6,7354E+14	8,08728E+14	7,59475E+14	8,07738E+14
TC0900001370.mm.2	Tmem115	transmembrane protein 115	0.666337237942211	5,86855E+14	1,8813E+14	0.105686038508847	0.24679987514392	-4,38498E+14	6,09537E+14	6,55688E+14	5,37934E+14	5,94023E+14	5,69737E+13	5,54215E+14
TC0700002738.mm.2	Zfp74	zinc finger protein 74	0.619905366356405	6,72368E+14	1,88102E+14	0.105728628479606	0.246832486873049	-4,38533E+13	7,15018E+14	7,21052E+14	6,84065E+13	6,28024E+14	6,49612E+14	6,36434E+14
TC1800000851.mm.2	Txnl4a	thioredoxin-like 4A	1,11018E+14	7,44669E+14	1,88101E+14	0.105730641370185	0.246832486873049	-4,38534E+14	8,1059E+14	8,01758E+14	7,63344E+13	6,26967E+14	7,94942E+14	6,70414E+14
TC0300002188.mm.2	Rapgef2	Rap guanine nucleotide exchange factor (GEF) 2	0.767625949892018	8,38135E+14	1,88065E+14	0.105784657455589	0.246891690332682	-4,38579E+14	9,08396E+14	8,86312E+14	8,24171E+14	8,17011E+14	8,47132E+14	7,45788E+14
TC0300002582.mm.2	Tent5c	terminal nucleotidyltransferase 5C	-0.586043276998097	7,70602E+14	-1,88064E+14	0.105786646189837	0.246891690332682	-4,3858E+14	7,25327E+14	7,46572E+14	7,75934E+14	8,13174E+14	7,78421E+14	7,84184E+14
TC0100002823.mm.2	Ankmy1	ankyrin repeat and MYND domain containing 1	-0.769665423390859	5,78471E+13	-1,88021E+14	0.10585024797552	0.24700435128596	-4,38632E+14	5,39036E+14	5,11975E+14	5,52163E+14	6,52782E+14	6,03045E+14	6,11824E+14
TC0600000848.mm.2	0610030E20Rik	RIKEN cDNA 0610030E20 gene	-0.603781559151315	6,17301E+14	-1,87953E+14	0.105953708350403	0.247191260303378	-4,38717E+14	5,49513E+14	5,9639E+14	6,43205E+14	6,23454E+14	6,41529E+14	6,49714E+14
TC1400000745.mm.2	Mmp14	matrix metallopeptidase 14 (membrane-inserted)	0.639523106061354	5,28044E+14	1,87948E+14	0.105961027426018	0.247191260303378	-4,38723E+14	5,35243E+14	5,85214E+13	4,97223E+14	4,95329E+14	5,06578E+12	5,48679E+14
TC0400001773.mm.2	Atp13a2	ATPase type 13A2	0.55801703731397	7,04207E+14	1,87899E+14	0.106034032978121	0.247257508054635	-4,38783E+14	7,32158E+14	7,49233E+14	6,95303E+13	6,74485E+14	6,8678E+14	6,8728E+14
TC0800000475.mm.2	Vps37a	vacuolar protein sorting 37A	2,02315E+14	7,83044E+14	1,87887E+14	0.106052993935695	0.247257508054635	-4,38798E+14	9,12039E+14	9,10523E+14	8,69363E+14	5,48569E+14	8,23219E+14	6,34551E+14
TC1400000930.mm.2	Ints9	integrator complex subunit 9	1,02243E+14	6,14348E+14	1,87886E+14	0.106054915007851	0.247257508054635	-4,388E+14	7,19651E+14	7,04867E+14	6,58197E+13	5,61835E+14	5,87783E+14	4,53753E+14
TC0800002618.mm.2	Neto2	neuropilin (NRP) and tolloid (TLL)-like 2	1,58129E+14	8,32549E+14	1,87878E+14	0.106066706037002	0.247257508054635	-4,38809E+14	9,46939E+14	9,53052E+14	8,60463E+14	7,23269E+14	8,79914E+14	6,31657E+14
TC1000001244.mm.2	Slc6a15	solute carrier family 6 (neurotransmitter transporter), member 15	2,1453E+14	7,58974E+14	1,87869E+14	0.106079889593299	0.247257508054635	-4,3882E+14	8,87424E+14	8,90736E+14	8,60165E+14	4,88935E+14	7,82694E+14	6,43887E+14
TC0200001072.mm.2	Scrn3	secernin 3	0.558363379384515	6,48673E+14	1,87868E+14	0.106081934624701	0.247257508054635	-4,38822E+14	6,74034E+13	6,94279E+14	6,21669E+14	6,3497E+13	6,27292E+14	6,39795E+14
TC0900001164.mm.2	Zic4	zinc finger protein of the cerebellum 4	-0.937066882041027	7,56738E+14	-1,87858E+14	0.106096841723437	0.247257508054635	-4,38834E+14	7,07268E+14	6,93387E+13	7,3214E+14	8,55928E+14	7,37552E+14	8,14151E+14
TC0500000648.mm.2	Slc30a9	solute carrier family 30 (zinc transporter), member 9	1,2199E+14	6,81438E+13	1,87842E+14	0.106120744780919	0.247265221962141	-4,38853E+14	7,70106E+14	7,58062E+14	7,31551E+14	5,52636E+14	6,91686E+14	5,84585E+14
TC0700003581.mm.2	Idh2	isocitrate dehydrogenase 2 (NADP+), mitochondrial	0.580289089189934	6,99114E+14	1,87835E+14	0.106130843113645	0.247265221962141	-4,38862E+13	7,34296E+14	7,39451E+14	6,64977E+14	6,92712E+14	6,6695E+14	6,96298E+14
TC1900000168.mm.2	Mta2	metastasis-associated gene family, member 2	0.649787153560991	7,64344E+14	1,87797E+14	0.1061886875819	0.247364221918051	-4,38909E+14	8,3419E+14	8,09938E+14	7,41989E+14	7,72182E+14	7,42517E+14	6,85247E+14
TC0100001788.mm.2	Slc30a10	solute carrier family 30, member 10	0.633197593871193	5,53837E+14	1,87765E+14	0.106236897005057	0.24740540253022	-4,38948E+14	5,88686E+14	5,98992E+14	5,01331E+14	5,59707E+14	5,4827E+14	5,26034E+14
TC0200004269.mm.2	Elp4	elongator acetyltransferase complex subunit 4	2,39295E+14	6,11082E+14	1,87758E+14	0.106248170399235	0.24740540253022	-4,38957E+13	7,46634E+14	7,34701E+14	7,05188E+13	2,97556E+14	6,8063E+14	5,01783E+14
TC1100003817.mm.2	Dusp3	dual specificity phosphatase 3 (vaccinia virus phosphatase VH1-related)	1,07477E+14	8,15912E+14	1,87755E+14	0.106252428815809	0.24740540253022	-4,38961E+14	8,79039E+14	8,81249E+14	8,51677E+12	6,93656E+14	8,35394E+14	7,54458E+13
TC0700004604.mm.2	Cttn	cortactin	0.559625861475634	7,53899E+14	1,87728E+14	0.106292727789822	0.247463476791996	-4,38994E+14	8,03729E+14	7,80652E+14	7,41521E+14	7,30935E+14	7,26819E+14	7,39737E+14
TC1100000264.mm.2	Fancl	Fanconi anemia, complementation group L	0.639346975070248	6,25415E+14	1,87683E+14	0.106360859087388	0.247586322229859	-4,39049E+14	6,81178E+14	6,76846E+14	6,01925E+14	6,28229E+14	6,12141E+14	5,52173E+13
TC1500002176.mm.2	Kansl2	KAT8 regulatory NSL complex subunit 2	0.65913468331192	7,4655E+14	1,87657E+14	0.106400516743075	0.247642860939067	-4,39081E+14	7,88868E+14	7,82641E+14	7,41086E+14	6,98596E+14	7,63233E+14	7,04878E+14
TC0200005501.mm.2	Cdan1	congenital dyserythropoietic anemia, type I (human)	-0.6204333456595	4,99286E+13	-1,87634E+14	0.106436170850659	0.247690066567976	-4,3911E+13	4,59478E+14	4,66195E+14	4,97031E+14	5,52728E+14	5,18762E+14	5,01524E+13
TC1200001859.mm.2	Rps29	ribosomal protein S29	0.580193279675635	1,0839E+14	1,87605E+14	0.10647935791306	0.247744077244151	-4,39145E+14	1,13129E+14	1,1132E+14	1,07866E+14	1,0498E+14	1,05377E+14	1,07665E+14
TC1300000891.mm.2	Brd9	bromodomain containing 9	0.730449760611739	6,32344E+14	1,87593E+14	0.106498305770405	0.247744077244151	-4,39161E+14	6,9674E+13	6,47596E+14	6,05296E+14	5,9295E+14	5,8601E+14	6,65474E+14
TC1500001102.mm.2	Pcbp2	poly(rC) binding protein 2	1,26592E+14	9,74346E+14	1,87588E+14	0.10650550630362	0.247744077244151	-4,39167E+14	1,07666E+14	1,0703E+14	1,02528E+14	8,68501E+14	9,79046E+14	8,26288E+14
TC1900001437.mm.2	Ankrd1	ankyrin repeat domain 1 (cardiac muscle)	-0.612102428670955	5,47616E+14	-1,8757E+14	0.106533123560863	0.247772549067001	-4,39189E+12	5,05542E+14	5,12558E+14	5,44197E+13	5,96323E+14	5,547E+14	5,72374E+14
TC0700003023.mm.2	Kcnj14	potassium inwardly-rectifying channel, subfamily J, member 14	-1,14169E+14	7,58942E+14	-1,87545E+14	0.106570047232813	0.247783527608363	-4,39219E+13	6,92744E+14	6,73518E+13	7,22099E+14	8,725E+14	7,37122E+14	8,55671E+14
TC0200000419.mm.2	Clic3	chloride intracellular channel 3	-0.652103190098161	5,27548E+14	-1,87542E+14	0.10657501733862	0.247783527608363	-4,39223E+14	4,85513E+14	4,89443E+14	5,18629E+14	5,86747E+14	5,50791E+14	5,34167E+14

TC0200002312.mm.2	Pofut1	protein O-fucosyltransferase 1	-0.640772079106783	6,5184E+14	-1,87531E+14	0.106591664949425	0.247783527608363	-4,39237E+14	5,91168E+14	6,31537E+14	6,57445E+14	6,93414E+14	6,49538E+14	6,87937E+13
TC1500001773.mm.2	Sharpin	SHANK-associated RH domain interacting protein	0.780566593450456	7,41594E+14	1,87526E+14	0.106599355388889	0.247783527608363	-4,39243E+14	7,8344E+14	7,87618E+12	7,5427E+14	6,60674E+14	7,58652E+14	7,04912E+14
TC0100000895.mm.2	Erfe	erythroferrone	-0.632762896990386	5,71458E+14	-1,875E+14	0.106639024037727	0.24783998187219	-4,39275E+14	5,18966E+13	5,5513E+14	5,74732E+14	6,25917E+14	5,8205E+14	5,71951E+14
TC1100001251.mm.2	1700020L24Rik	RIKEN cDNA 1700020L24 gene	-0.6636941719522	6,03298E+14	-1,87468E+14	0.106687330825999	0.247916492875588	-4,39314E+14	5,4142E+14	5,82859E+13	6,05521E+14	6,51497E+14	6,39685E+14	5,98804E+14
TC0700002458.mm.2	Psg20	pregnancy-specific glycoprotein 20	-0.703428298573847	4,73948E+14	-1,8745E+14	0.106715216623911	0.247945535034041	-4,39337E+13	4,49006E+14	4,22919E+14	4,8873E+14	5,23881E+14	4,42236E+14	5,16919E+14
TC0600001257.mm.2	Vhl	von Hippel-Lindau tumor suppressor	0.913722426993577	6,90496E+14	1,87429E+14	0.106746136525962	0.247981617336046	-4,39362E+14	7,33881E+14	7,46884E+14	7,18127E+14	5,79893E+14	6,69149E+14	6,95043E+14
TC0900002922.mm.2	Armc8	armadillo repeat containing 8	1,11676E+14	8,06294E+14	1,87368E+14	0.106838853790612	0.248161229800798	-4,39437E+14	9,0747E+14	8,86341E+14	8,40213E+14	7,30245E+14	8,11803E+14	6,61695E+14
TC1300001075.mm.2	Zbed3	zinc finger, BED type containing 3	-0.909414213131265	8,25021E+14	-1,87349E+14	0.106867880169076	0.248165144126109	-4,39461E+14	7,44743E+14	7,84303E+14	9,00881E+14	8,10048E+14	9,0475E+14	8,05398E+14
TC0800001701.mm.2	Ankrd10/Mir7238	ankyrin repeat domain 10/microRNA 7238	-0.748135903736058	7,25961E+14	-1,8734E+14	0.106881636556355	0.248165144126109	-4,39472E+14	6,60209E+14	6,79022E+14	7,05937E+14	7,82921E+14	7,33786E+14	7,93892E+14
TC1700002331.mm.2	Safb2	scaffold attachment factor B2	0.596067519169411	6,74251E+14	1,87302E+14	0.106940171528814	0.248165144126109	-4,39519E+14	7,20547E+14	6,85898E+14	6,48564E+13	6,38668E+14	6,92565E+14	6,59262E+14
TC0200004129.mm.2	Mdk	midkine	-0.564031488757805	7,73449E+14	-1,87298E+14	0.106945909241119	0.248165144126109	-4,39524E+14	7,3798E+14	7,49407E+14	7,88308E+14	8,11886E+14	7,86031E+14	7,67082E+14
TC0400001596.mm.2	Rpa2	replication protein A2	-0.567380734008387	6,96872E+14	-1,87272E+14	0.106985518906776	0.248165144126109	-4,39556E+13	6,81662E+14	6,73573E+14	7,17615E+14	7,51096E+14	6,7922E+14	6,78064E+14
TC1100003707.mm.2	Krt12	keratin 12	-0.671488241143671	6,7634E+14	-1,87268E+14	0.106991273988985	0.248165144126109	-4,39561E+14	6,44131E+14	6,20432E+14	6,80813E+14	7,18048E+14	7,28813E+14	6,65804E+14
TC0300002756.mm.2	Prpf38b	PRP38 pre-mRNA processing factor 38 (yeast) domain containing B	1,10269E+13	8,03058E+13	1,87261E+14	0.107001979205998	0.248165144126109	-4,39569E+14	8,88708E+14	9,22637E+14	8,30165E+14	7,60643E+14	7,91753E+14	6,2444E+14
TC0900002366.mm.2	Commd4	COMM domain containing 4	0.59419768530292	7,62178E+14	1,87256E+14	0.10700967549777	0.248165144126109	-4,39576E+14	8,01836E+14	7,97694E+14	7,59195E+14	7,21495E+14	7,61372E+14	7,31476E+14
TC0900001046.mm.2	Ick	intestinal cell kinase	0.706422387652043	6,34979E+14	1,87243E+14	0.107030261410113	0.248165144126109	-4,39592E+14	6,79159E+14	6,8363E+13	6,53989E+14	5,67515E+14	6,0588E+14	6,19699E+14
TC0900001182.mm.2	Slc9a9	solute carrier family 9 (sodium/hydrogen exchanger), member 9	0.87157816915821	7,06131E+14	1,87233E+14	0.107045524077665	0.248165144126109	-4,39605E+14	7,60165E+14	7,68535E+13	7,06092E+14	6,48293E+14	7,35674E+14	6,18026E+14
TC0100002644.mm.2	Wdfy1	WD repeat and FYVE domain containing 1	-0.90796804583824	6,75588E+14	-1,87212E+14	0.107076606945414	0.248165144126109	-4,3963E+14	6,2984E+13	6,32399E+14	6,84595E+14	7,59238E+11	6,14447E+14	7,33007E+14
TC0500000825.mm.2	Npffr2	neuropeptide FF receptor 2	-0.669522499152708	5,19733E+14	-1,87209E+13	0.107081248163989	0.248165144126109	-4,39633E+14	5,18094E+14	4,74926E+14	5,38934E+14	5,87991E+14	4,79466E+14	5,18987E+14
TC1500001824.mm.2	Eif3d	eukaryotic translation initiation factor 3, subunit D	0.669918095160223	7,53989E+14	1,87205E+14	0.107088248291898	0.248165144126109	-4,39639E+14	7,96069E+14	8,06042E+14	7,11401E+14	7,56727E+14	6,96441E+14	7,57255E+14
TC1100003087.mm.2	Bcl6b	B cell CLL/lymphoma 6, member B	-0.684851074703063	7,33217E+13	-1,87203E+14	0.107091528013239	0.248165144126109	-4,39642E+13	6,82091E+14	6,99112E+14	7,25409E+14	7,92764E+14	7,28935E+13	7,70991E+14
TC1500002199.mm.2	Tuba1b	tubulin, alpha 1B	1,41106E+14	1,09956E+14	1,87191E+14	0.107109212278842	0.248165144126109	-4,39656E+14	1,19578E+14	1,19182E+14	1,15312E+14	9,52275E+14	1,12653E+14	9,77839E+14
TC1500001571.mm.2	Klhl38	kelch-like 38	-0.649620678854803	5,13085E+14	-1,87186E+14	0.107116208619834	0.248165144126109	-4,39662E+14	4,90474E+14	4,69289E+14	5,1919E+14	5,70497E+14	4,93369E+14	5,35691E+12
TC1000002237.mm.2	Pldi	polymorphic derived intron containing	-1,05277E+14	5,06654E+14	-1,87186E+14	0.107116845707631	0.248165144126109	-4,39662E+14	4,21644E+14	4,43722E+14	4,55198E+13	6,20723E+14	5,25976E+14	5,72659E+14
TC1700000794.mm.2	Olfr130	olfactory receptor 130	-0.964275319783909	4,68055E+14	-1,87176E+14	0.107132346845079	0.248165144126109	-4,39675E+14	4,00244E+14	3,94846E+12	4,37565E+14	5,5038E+13	4,60782E+14	5,64513E+14
TC0500000888.mm.2	Sep 11	septin 11	0.920497537172885	8,34522E+14	1,87175E+14	0.107133168406952	0.248165144126109	-4,39675E+14	8,938E+14	9,13604E+14	8,61836E+14	7,61469E+14	8,38266E+13	7,38157E+14
TC0100003709.mm.2	Degs1	delta(4)-desaturase, sphingolipid 1	1,16109E+14	8,5709E+14	1,87161E+14	0.107155093343731	0.248180252845701	-4,39693E+14	9,35401E+14	9,32571E+14	9,05182E+14	7,30573E+14	8,65187E+14	7,73624E+14
TC0300003249.mm.2	Mfsd14a	major facilitator superfamily domain containing 14A	-0.588650700318134	7,46857E+14	-1,87144E+14	0.107181497199038	0.24820572928544	-4,39714E+14	7,16726E+14	6,85973E+14	7,45059E+14	7,7537E+14	7,86793E+14	7,71223E+14
TC0900003268.mm.2	Lyzl4	lysozyme-like 4	-0.669435724932566	6,83845E+14	-1,87105E+14	0.107240331622156	0.248306288752379	-4,39762E+14	6,44381E+14	6,47457E+14	7,04001E+14	7,21725E+14	6,55596E+14	7,29909E+14
TC1700000502.mm.2	Pi16	peptidase inhibitor 16	-0.789182395555226	5,24818E+14	-1,87087E+14	0.107267789082386	0.248334178948921	-4,39784E+14	4,87509E+14	4,68654E+13	5,02447E+14	6,11552E+14	5,37967E+14	5,40775E+14
TC0700001135.mm.2	Mesp2	mesoderm posterior 2	-0.983975634583429	6,24987E+14	-1,87069E+14	0.107294728296789	0.248354735642663	-4,39806E+14	5,51272E+14	5,47157E+14	5,97314E+14	6,97911E+14	6,17245E+14	7,3902E+14
TC1700002774.mm.2	Cmtr1	cap methyltransferase 1	0.696637100838908	7,35313E+14	1,87061E+14	0.107307495161933	0.248354735642663	-4,39816E+14	7,97624E+14	7,76363E+14	7,31969E+14	7,0269E+13	7,40055E+14	6,63177E+14
TC0700000305.mm.2	Zfp296	zinc finger protein 296	-0.704370363086193	5,43926E+14	-1,87019E+14	0.107372214527217	0.248445179763716	-4,39868E+14	4,68626E+14	5,1063E+14	5,33322E+14	5,86807E+14	5,54696E+14	6,09478E+14
TC1700001156.mm.2	Spast	spastin	1,51909E+14	6,43392E+14	1,8699E+14	0.107415613716226	0.248445179763716	-4,39903E+14	7,20995E+13	7,45147E+14	6,96779E+12	4,65545E+13	6,87027E+14	5,44859E+14
TC0100003739.mm.2	Marc2	mitochondrial amidoxime reducing component 2	0.597220740984284	7,21632E+14	1,86989E+13	0.10741817969707	0.248445179763716	-4,39905E+14	7,29937E+14	7,6094E+14	7,04354E+14	6,67078E+13	7,39888E+13	7,27594E+14
TC1100000078.mm.2	Dbnl	drebrin-like	0.632031809067057	7,10348E+14	1,86971E+14	0.107445246423439	0.248445179763716	-4,39927E+14	7,55953E+14	7,6424E+14	7,14772E+14	6,79014E+14	6,47923E+14	7,00185E+14
TC0300000467.mm.2	Med12l	mediator complex subunit 12-like	-0.872756021921067	8,43035E+14	-1,86971E+14	0.1074458005722	0.248445179763716	-4,39928E+14	8,06344E+14	7,93783E+14	9,39619E+14	8,3506E+14	8,81436E+14	8,01966E+14
TC0900003073.mm.2	Trex1/Atrip	three prime repair exonuclease 1/ATR interacting protein	-0.589082636003286	7,46713E+14	-1,86967E+14	0.107450789052965	0.248445179763716	-4,39932E+14	7,23353E+14	7,26978E+14	8,00662E+14	7,67485E+14	7,14808E+14	7,46992E+14
TC0700001287.mm.2	Dlg2	discs large MAGUK scaffold protein 2	1,01932E+14	8,83079E+14	1,86952E+14	0.107474093165722	0.248445179763716	-4,3995E+14	9,60335E+14	9,63599E+14	9,21089E+14	7,98981E+14	8,83572E+14	7,70899E+13
TC0400000498.mm.2	Tdrd7	tudor domain containing 7	0.854888913381663	6,056E+14	1,86946E+14	0.107483839160472	0.248445179763716	-4,39958E+14	6,51273E+13	6,6857E+14	6,20179E+14	5,28686E+14	5,38837E+14	6,26056E+14
TC0X00003132.mm.2	Lhfpl1	lipoma HMGIC fusion partner-like 1	-0.627509881954118	5,48712E+14	-1,86926E+14	0.107513640103628	0.248445179763716	-4,39982E+14	5,04138E+14	5,19386E+14	5,44972E+14	6,04054E+14	5,53679E+14	5,66043E+14
TC1000000330.mm.2	Traf3ip2	TRAF3 interacting protein 2	-0.558506664229778	5,3339E+14	-1,86924E+14	0.107516494912876	0.248445179763716	-4,39985E+12	5,03601E+14	5,07944E+14	5,48276E+13	5,7497E+14	5,38533E+13	5,27019E+13
TC0400001695.mm.2	Lactbl1	lactamase, beta-like 1	-0.849741418166894	5,80918E+14	-1,86916E+11	0.107529104252583	0.248445179763716	-4,39995E+14	5,33528E+14	5,23623E+14	5,71051E+14	6,56049E+14	5,54154E+14	6,47106E+14
TC0100002213.mm.2	Txndc9	thioredoxin domain containing 9	0.871801314824653	7,30817E+14	1,86915E+13	0.107531600798868	0.248445179763716	-4,39997E+14	7,86606E+14	8,08354E+14	7,5682E+14	6,6378E+14	7,30036E+14	6,39305E+14
TC1700000572.mm.2	Zfp952	zinc finger protein 952	0.615146088119864	6,05048E+14	1,86903E+13	0.107548982294613	0.248446711398113	-4,40011E+14	6,65511E+14	6,38257E+14	5,77982E+13	6,02757E+14	5,95011E+14	5,50768E+14
TC1400002208.mm.2	Ints6	integrator complex subunit 6	0.88349838632679	6,28802E+13	1,86894E+14	0.107563101763373	0.248446711398113	-4,40022E+13	7,04553E+14	6,53474E+14	6,51777E+14	5,29551E+14	6,28721E+14	6,04735E+13
TC0200004760.mm.2	Ovol2	ovo like zinc finger 2	-0.760638139086788	6,49847E+14	-1,86841E+14	0.107644718096499	0.248559735910878	-4,40088E+14	6,1101E+14	5,77525E+14	6,39379E+14	7,01283E+14	6,47048E+14	7,2284E+14
TC0700002958.mm.2	1700008O03Rik	RIKEN cDNA 1700008O03 gene	-0.647529862945125	5,87791E+14	-1,86835E+14	0.107653028576336	0.248559735910878	-4,40094E+14	5,24919E+14	5,61705E+14	5,80137E+14	6,35993E+14	6,02543E+14	6,21453E+14
TC0300002872.mm.2	Synpo2	synaptopodin 2	-0.624736044248556	6,30569E+14	-1,86817E+14	0.107680610598725	0.248559735910878	-4,40116E+14	6,01762E+14	5,67455E+14	6,21646E+14	6,72518E+14	6,59974E+14	6,6006E+14
TC1400002193.mm.2	Spryd7	SPRY domain containing 7	0.969734165022349	8,00814E+14	1,86813E+14	0.107686884894754	0.248559735910878	-4,40122E+14	8,64466E+14	8,44716E+14	8,2601E+14	6,89225E+14	8,25368E+14	7,55098E+14
TC0100003666.mm.2	Stum	mechanosensory transduction mediator	0.753607532391063	8,5825E+14	1,86812E+14	0.107689165046474	0.248559735910878	-4,40123E+14	8,92978E+14	9,29703E+14	8,4228E+12	8,29679E+14	8,74347E+14	7,80514E+14
TC1200002242.mm.2	Fbln5	fibulin 5	0.567052936297887	5,64062E+12	1,86801E+14	0.107705266426151	0.248561294460696	-4,40136E+14	6,26426E+14	5,98443E+14	5,47202E+14	5,64257E+14	5,3034E+14	5,17702E+14
TC0200005094.mm.2	Serinc3	serine incorporator 3	1,50097E+13	1,05085E+14	1,8678E+14	0.107737917462003	0.248584735342108	-4,40163E+14	1,13372E+14	1,14602E+14	1,11089E+14	8,68648E+14	1,08125E+14	9,64607E+14
TC1400000376.mm.2	Mmrn2	multimerin 2	-0.744702259784044	4,9797E+14	-1,86761E+14	0.107766323757545	0.248584735342108	-4,40185E+14	4,35367E+14	4,6671E+14	4,78474E+14	5,72543E+14	5,17249E+14	5,17476E+14
TC0300000359.mm.2	Naa15	N(alpha)-acetyltransferase 15, NatA auxiliary subunit	2,20181E+14	7,1879E+14	1,86757E+14	0.107773094401116	0.248584735342108	-4,40191E+14	8,55633E+14	8,55856E+14	8,3003E+14	4,41096E+14	7,30029E+13	6,00095E+14
TC1100002783.mm.2	Sparc	secreted acidic cysteine rich glycoprotein	1,32761E+14	9,85465E+14	1,86754E+14	0.107777552441896	0.248584735342108	-4,40194E+14	1,12389E+14	1,04987E+14	1,05567E+13	8,52565E+14	9,59123E+14	8,71674E+14
TC0100001174.mm.2	Slc26a9	solute carrier family 26, member 9	-0.706278851237756	5,9603E+14	-1,86744E+14	0.107792561648067	0.248584735342108	-4,40206E+14	5,46175E+14	5,66495E+14	5,88986E+14	6,64941E+14	5,8481E+14	6,24771E+14
TC1200000057.mm.2	Apob	apolipoprotein B	-0.549556138385743	6,08835E+13	-1,86729E+13	0.10781574871175	0.248602627217004	-4,40225E+14	5,8986E+14	5,9581E+14	6,3781E+14	6,57771E+14	5,90579E+14	5,81183E+14
TC1900001020.mm.2	Esrra/Mir6990	estrogen related receptor, alpha/microRNA 6990	-0.567040380120514	7,06595E+14	-1,86713E+14	0.107840685577807	0.24862454810634	-4,40245E+14	6,81084E+14	6,63026E+14	7,1456E+14	7,42958E+14	7,13686E+14	7,24259E+14
TC0200004221.mm.2	Pdhx	pyruvate dehydrogenase complex, component X	0.617973682250751	6,52338E+14	1,8667E+14	0.107906147425162	0.248701564701427	-4,40298E+14	6,93922E+14	6,7761E+13	6,36786E+14	6,11151E+14	6,72549E+14	6,2201E+14
TC1600001509.mm.2	Slc51a	solute carrier family 51, alpha subunit	-0.805661423798595	5,24709E+14	-1,86665E+14	0.107914096924467	0.248701564701427	-4,40304E+14	4,5179E+14	4,54567E+14	5,02413E+14	5,65076E+14	5,38711E+14	6,35698E+14
TC0600000470.mm.2	Olfr449	olfactory receptor 449	-0.795332176103785	4,92733E+14	-1,86661E+14	0.10792039597793	0.248701564701427	-4,40309E+13	4,56806E+14	4,80611E+13	5,1278E+14	5,83704E+14	4,19013E+14	5,03481E+14
TC1500001690.mm.2	Ptk2	PTK2 protein tyrosine kinase 2	1,43075E+14	7,16665E+14	1,8663E+14	0.107967550962995	0.248735082549978	-4,40347E+14	8,04239E+14	7,86904E+14	7,65057E+14	5,39936E+14	7,61068E+14	6,42787E+14
TC1800001526.mm.2	4930546C10Rik	RIKEN cDNA 4930546C10 gene	-1,12837E+14	5,48176E+14	-1,86627E+14	0.107972622547076	0.248735082549978	-4,40351E+14	4,63392E+13	4,74627E+13	4,99716E+14	6,63977E+14	5,4548E+14	6,41863E+13
TC0400001994.mm.2	Uts2	urotensin 2	-1,05554E+14	6,43061E+13	-1,8662E+12	0.107983244600852	0.248735082549978	-4,40359E+14	6,0427E+14	5,54953E+14	6,0291E+14	7,6742E+14	6,48351E+14	6,80461E+13
TC0200003666.mm.2	Scn3a	sodium channel, voltage-gated, type III, alpha	0.670498498565464	7,34922E+14	1,86606E+14	0.108005134099242	0.248735082549978	-4,40377E+14	7,9421E+14	7,94882E+13	7,45402E+14	7,0959E+13	7,17015E+14	6,4843E+14
TC0500000272.mm.2	Nom1	nucleolar protein with MIF4G domain 1	0.895108137249562	6,51082E+14	1,86593E+14	0.108025540102968	0.248735082549978	-4,40393E+14	7,12581E+14	7,00066E+14	6,8179E+14	5,51836E+14	6,521E+13	6,08117E+14
TC0600000467.mm.2	Olfr452	olfactory receptor 452	-0.837215786939365	4,5654E+14	-1,86591E+14	0.108027562073155	0.248735082549978	-4,40395E+14	4,01426E+13	4,07382E+14	4,35483E+14	5,40768E+12	4,44862E+14	5,09318E+14
TC1100000187.mm.2	Spred2	sprouty-related EVH1 domain containing 2	1,41454E+14	8,77084E+14	1,86564E+14	0.108070295986754	0.248797925308553	-4,40429E+14	9,4508E+14	9,7444E+14	9,39686E+14	6,96927E+14	8,82873E+14	8,23499E+14
TC0600003003.mm.2	Ankrd26	ankyrin repeat domain 26	0.66768430021204	6,12648E+14	1,86528E+14	0.108125493598044	0.248889439763611	-4,40473E+14	6,6168E+14	6,41459E+14	5,62923E+14	6,0668E+14	5,71833E+13	6,31314E+14
TC1100001813.mm.2	Apoh	apolipoprotein H	-1,04352E+14	5,46436E+14	-1,8649E+14	0.108183446864214	0.248956524238752	-4,4052E+14	5,05322E+14	4,75992E+12	5,47278E+14	6,42738E+14	4,83503E+14	6,23783E+14
TC0500002528.mm.2	Atp8a1	ATPase, aminophospholipid transporter (APLT), class I, type 8A, member 1	1,38818E+13	8,73901E+14	1,86489E+14	0.108185538553947	0.248956524238752	-4,40521E+14	9,54594E+14	9,54939E+14	8,97736E+14	7,34162E+14	9,43035E+14	7,58942E+14
TC1300000028.mm.2	Klf6	Kruppel-like factor 6	0.90586004868466	7,08636E+13	1,8645E+14	0.10824458739822	0.24904207441318	-4,40568E+14	7,25225E+14	7,8538E+14	7,10642E+14	6,18791E+14	7,49724E+14	6,62057E+14
TC0100003471.mm.2	Gm4846	predicted gene 4846	-0.599294795122565	5,96533E+14	-1,86445E+14	0.108253626834848	0.24904207441318	-4,40576E+14	5,70252E+13	5,38477E+14	5,948E+14	6,33789E+14	6,15011E+14	6,26869E+14
TC1700001805.mm.2	Glo1	glyoxalase 1	1,2966E+14	7,79356E+13	1,86422E+14	0.108288144453014	0.249062252708192	-4,40603E+14	8,81999E+14	8,66673E+14	8,55531E+14	6,3382E+14	7,42553E+14	6,95561E+14
TC1400001220.mm.2	Frg2f9	FSHD region gene 2 family member 9	-0.672508120949978	5,20628E+14	-1,86417E+14	0.108295482267322	0.249062252708192	-4,40609E+14	4,89115E+14	4,60958E+13	5,26396E+14	5,58178E+14	5,11708E+14	5,77415E+14
TC1700000565.mm.2	Cyp4f37	cytochrome P450, family 4, subfamily f, polypeptide 37	-0.626375176791078	7,0465E+14	-1,86402E+14	0.108319154010487	0.249062252708192	-4,40628E+14	6,43388E+14	6,79898E+14	7,20726E+14	7,27835E+14	7,0292E+14	7,53136E+14
TC0200005482.mm.2	Hypk	huntingtin interacting protein K	0.688938513498316	8,3273E+14	1,86386E+14	0.108344367549295	0.249062252708192	-4,40648E+14	8,73731E+14	8,50896E+14	8,14049E+14	7,72791E+14	8,76845E+14	8,08066E+14
TC0700002001.mm.2	Prap1	proline-rich acidic protein 1	-0.61142692454252	5,82537E+14	-1,86374E+14	0.108362067455047	0.249062252708192	-4,40662E+14	5,42955E+14	5,30261E+13	5,7806E+14	6,17441E+14	5,93809E+14	6,32696E+14
TC1000001284.mm.2	Csrp2	cysteine and glycine-rich protein 2	-0.599509070901117	7,33579E+14	-1,86363E+14	0.108379507212315	0.249062252708192	-4,40676E+14	6,68947E+14	7,05598E+14	7,29421E+14	7,65026E+14	7,61752E+13	7,7073E+14
TC1900001587.mm.2	Npm3	nucleoplasmin 3	-0.611653639041014	8,66528E+14	-1,8635E+14	0.108399397334882	0.249062252708192	-4,40692E+14	8,18213E+14	8,37148E+14	8,62324E+14	9,15367E+14	8,87123E+13	8,78992E+13
TC0100003068.mm.2	Mfsd4a	major facilitator superfamily domain containing 4A	0.65757279667207	7,4456E+14	1,86328E+14	0.108433871939106	0.249062252708192	-4,4072E+14	7,81733E+14	7,91164E+13	7,26583E+12	7,148E+14	7,625E+14	6,90578E+14
TC0200002476.mm.2	Sgk2	serum/glucocorticoid regulated kinase 2	-0.682660524986891	5,03442E+13	-1,86324E+14	0.108439258488303	0.249062252708192	-4,40724E+14	4,63258E+14	4,51334E+14	4,94107E+14	5,57016E+14	5,02233E+14	5,52701E+13
TC1700000487.mm.2	Mapk13	mitogen-activated protein kinase 13	-0.850994068806666	6,40067E+13	-1,86322E+14	0.108443212897886	0.249062252708192	-4,40727E+14	5,76752E+14	6,72301E+14	6,67654E+14	7,51597E+14	5,8903E+14	5,8307E+14
TC1100000935.mm.2	Phf23	PHD finger protein 23	0.536067097033376	6,28978E+14	1,86312E+14	0.108458711476948	0.249062252708192	-4,40739E+14	6,81757E+14	6,79346E+14	6,25827E+14	6,28062E+14	5,81604E+14	5,77273E+14
TC0900001858.mm.2	Tyk2	tyrosine kinase 2	-0.729264675346236	5,49932E+12	-1,86297E+13	0.108481634959453	0.249062252708192	-4,40758E+14	5,43215E+14	5,5929E+14	5,82308E+14	6,66049E+13	4,53037E+14	4,9569E+14
TC0400000169.mm.2	Ggh	gamma-glutamyl hydrolase	1,07301E+14	5,86536E+14	1,86292E+14	0.108489627325951	0.249062252708192	-4,40764E+14	6,58513E+14	6,58375E+14	5,24808E+14	5,77477E+14	6,35574E+14	4,64468E+14
TC0300001695.mm.2	Armc1	armadillo repeat containing 1	1,50442E+14	8,20178E+14	1,86289E+14	0.108494267711554	0.249062252708192	-4,40768E+13	9,04017E+14	9,03233E+14	8,62264E+14	6,44103E+14	8,83829E+14	7,23623E+14
TC1600001655.mm.2	Cd200	CD200 antigen	-1,14572E+14	8,4195E+14	-1,86284E+14	0.108501103561325	0.249062252708192	-4,40773E+13	7,80152E+14	7,5196E+14	7,93337E+14	9,67918E+14	8,38058E+14	9,20274E+13
TC1400000798.mm.2	Cma2	chymase 2, mast cell	-0.736082366062884	4,90383E+13	-1,86275E+14	0.108514952780996	0.249062252708192	-4,40784E+14	4,42565E+14	4,48624E+14	4,71343E+14	5,67062E+14	4,98191E+14	5,14512E+14
TC0500002994.mm.2	Ep400	E1A binding protein p400	0.849986431355622	7,6216E+13	1,86269E+14	0.108525170748105	0.249062252708192	-4,40792E+13	8,42196E+14	8,02379E+14	7,61465E+14	7,13113E+14	7,8222E+14	6,71585E+14
TC1300002141.mm.2	Ctla2b	cytotoxic T lymphocyte-associated protein 2 beta	-0.923250307152832	5,44777E+14	-1,86257E+14	0.10854353768584	0.249068929469088	-4,40807E+14	4,62213E+14	4,84877E+14	5,04687E+14	6,27052E+14	5,57849E+14	6,31987E+14
TC1200001982.mm.2	Zfyve26	zinc finger, FYVE domain containing 26	-0.566971281670677	6,78878E+14	-1,86238E+14	0.108572025529451	0.249098824911868	-4,4083E+14	6,31864E+14	6,52363E+14	6,9637E+14	7,01252E+14	6,81063E+14	7,10354E+14
TC1100003574.mm.2	Tmem92	transmembrane protein 92	-0.556430853038965	4,88221E+14	-1,86199E+14	0.108633052850892	0.249171881660682	-4,40878E+13	4,51562E+12	4,60503E+14	4,983E+14	5,25051E+14	5,01204E+14	4,92707E+14
TC0900003341.mm.2	Snx14	sorting nexin 14	1,223E+14	6,81929E+14	1,86189E+14	0.108647864167163	0.249171881660682	-4,4089E+13	7,79314E+14	7,60488E+14	7,1852E+14	5,76682E+14	7,06389E+14	5,50178E+14
TC1900000614.mm.2	Golga7b	golgi autoantigen, golgin subfamily a, 7B	0.997860885170022	7,21989E+14	1,86166E+14	0.108683265510008	0.249171881660682	-4,40918E+14	7,62507E+14	7,5269E+13	7,35592E+14	5,80033E+13	7,73127E+14	7,27983E+14
TC0900001504.mm.2	Clasp2	CLIP associating protein 2	1,35927E+14	8,14282E+14	1,86154E+14	0.108702889719072	0.249171881660682	-4,40934E+14	9,10894E+14	9,00679E+12	8,52592E+14	6,87127E+14	8,54482E+14	6,79918E+14
TC0400002980.mm.2	Plin2	perilipin 2	0.751604504352181	6,15475E+14	1,86142E+13	0.108721078964985	0.249171881660682	-4,40948E+14	6,45089E+14	6,99374E+14	6,25823E+14	5,6832E+14	5,45116E+14	6,0913E+14
TC0800002736.mm.2	Ccdc102a	coiled-coil domain containing 102A	-0.695850713312983	6,40595E+14	-1,86139E+14	0.108726223744603	0.249171881660682	-4,40952E+14	5,72253E+14	6,16186E+14	6,33306E+14	6,94303E+12	6,43111E+14	6,84409E+14
TC0600002756.mm.2	Adamts9	a disintegrin-like and metallopeptidase (reprolysin type) with thrombospondin type 1 motif, 9	-0.605575662676722	5,46514E+14	-1,86132E+14	0.108736569639133	0.249171881660682	-4,40961E+14	5,00485E+14	5,07292E+14	5,37811E+14	5,91082E+14	5,65919E+14	5,76493E+14
TC0400004160.mm.2	Pusl1	pseudouridylate synthase-like 1	-0.607094975936548	8,0082E+14	-1,86123E+13	0.108750151130302	0.249171881660682	-4,40971E+14	7,46682E+14	7,74503E+14	7,97452E+14	8,45152E+14	8,18544E+14	8,22584E+14
TC0300000312.mm.2	Larp1b	La ribonucleoprotein domain family, member 1B	0.941891696956707	5,13218E+14	1,86123E+14	0.108750765132254	0.249171881660682	-4,40972E+14	5,78362E+14	5,50267E+14	5,24058E+14	4,16193E+14	5,54951E+14	4,55476E+14
TC0500000093.mm.2	Sema3e	sema domain, immunoglobulin domain (Ig), short basic domain, secreted, (semaphorin) 3E	0.796130093800692	6,87649E+14	1,86118E+14	0.108758508267832	0.249171881660682	-4,40978E+14	7,40627E+13	7,41106E+14	7,02645E+14	6,19862E+14	6,99757E+14	6,21898E+14
TC0200002290.mm.2	Defb21	defensin beta 21	-0.973170414390071	5,19559E+14	-1,86077E+13	0.108822150748345	0.2492822455229	-4,41029E+14	4,43377E+14	4,96618E+14	4,95919E+14	6,3871E+14	5,39649E+14	5,03079E+14
TC0400001627.mm.2	Ubxn11	UBX domain protein 11	-0.763012399156598	5,69985E+14	-1,86017E+14	0.108914686797787	0.249414329365697	-4,41102E+14	5,05273E+14	5,34505E+14	5,46143E+14	6,46239E+14	5,99954E+14	5,87794E+14
TC0400002065.mm.2	Pex10	peroxisomal biogenesis factor 10	0.583175453022523	5,65783E+14	1,86008E+14	0.108929148856454	0.249414329365697	-4,41114E+14	5,8148E+14	5,93536E+14	5,14911E+14	5,4347E+14	5,64346E+14	5,96956E+14
TC0900000573.mm.2	Cadm1	cell adhesion molecule 1	0.746591043142933	8,5976E+14	1,85998E+14	0.108943700083632	0.249414329365697	-4,41125E+14	9,22166E+13	9,02752E+14	8,85392E+14	7,90208E+14	8,41937E+14	8,16103E+14
TC0400003012.mm.2	Ifna2	interferon alpha 2	-0.769646898955731	4,64782E+14	-1,85993E+14	0.108951736698869	0.249414329365697	-4,41132E+13	4,05611E+14	4,29623E+14	4,69382E+14	5,19781E+14	5,27666E+14	4,36625E+14
TC0400002035.mm.2	Cep104	centrosomal protein 104	1,10299E+14	5,31001E+14	1,85989E+14	0.108957206256137	0.249414329365697	-4,41136E+14	6,12019E+14	5,93209E+14	5,66992E+14	4,17638E+13	5,52742E+14	4,43405E+14
TC1100000891.mm.2	Ctc1	CTS telomere maintenance complex component 1	-0.905712610007877	6,65685E+14	-1,85907E+13	0.109084634394812	0.24967055596642	-4,41237E+13	6,22282E+14	6,15232E+14	6,52298E+14	7,66359E+14	6,27591E+14	7,10349E+14
TC1500001017.mm.2	Aqp2	aquaporin 2	-0.60245149155916	5,98995E+14	-1,85881E+14	0.109125750618538	0.249680247351782	-4,4127E+14	5,58009E+14	5,45354E+14	5,93608E+14	6,30245E+13	6,14735E+14	6,52016E+14

TC0100001713.mm.2	Acbd3	acyl-Coenzyme A binding domain containing 3	1,18352E+14	6,98862E+13	1,85873E+14	0.10913806643767	0.249680247351782	-4,41279E+14	7,634E+14	7,78634E+14	7,52333E+14	5,52996E+14	6,92648E+14	6,53161E+14
TC0900001655.mm.2	Tgm4	transglutaminase 4 (prostate)	-0.806956858740871	5,74654E+14	-1,8586E+14	0.10915836761829	0.249680247351782	-4,41295E+13	5,33001E+14	5,36456E+14	5,70923E+14	6,59925E+13	5,36083E+14	6,11538E+14
TC1900001705.mm.2	Dclre1a	DNA cross-link repair 1A	0.569581922610205	5,46197E+13	1,85856E+14	0.109164926065696	0.249680247351782	-4,41301E+14	5,64825E+14	5,96095E+14	5,34289E+14	5,12715E+14	5,32492E+14	5,36767E+13
TC1000001498.mm.2	BC048403	cDNA sequence BC048403	0.795099707734294	6,07957E+14	1,85855E+14	0.109166346589295	0.249680247351782	-4,41302E+14	6,58234E+14	6,48152E+14	5,83595E+14	5,63771E+14	5,41257E+14	6,5273E+14
TC1100002841.mm.2	Obscn	obscurin, cytoskeletal calmodulin and titin-interacting RhoGEF	-0.639585204622846	6,13788E+14	-1,85812E+14	0.109232150449354	0.249795293810735	-4,41354E+14	5,62932E+14	5,70889E+14	5,97296E+14	6,64441E+14	6,40099E+14	6,4707E+14
TC1200002080.mm.2	Syndig1l	synapse differentiation inducing 1 like	0.617009557669566	7,39506E+14	1,85798E+14	0.109253824737547	0.249799279839238	-4,41371E+14	7,57304E+14	7,9079E+14	7,36769E+14	6,87923E+14	7,28298E+14	7,35949E+14
TC0200003910.mm.2	Olfr1024	olfactory receptor 1024	-0.854021925655609	4,96126E+14	-1,85791E+14	0.109264899023382	0.249799279839238	-4,4138E+14	4,70593E+14	4,12334E+14	4,73186E+14	5,80546E+14	4,99818E+14	5,40279E+13
TC0X00003007.mm.2	Tceal6	transcription elongation factor A (SII)-like 6	0.625382127180544	8,20567E+14	1,85721E+14	0.109374109289447	0.250013480349107	-4,41466E+14	8,66919E+14	8,38321E+14	8,16984E+14	7,6318E+14	8,26949E+14	8,11052E+14
TC1100000702.mm.2	Olfr314	olfactory receptor 314	-0.784194615751283	5,05003E+14	-1,85661E+14	0.109467117264553	0.2501228554632	-4,4154E+14	4,56498E+14	4,56076E+14	4,79211E+14	5,90202E+14	5,25444E+14	5,22587E+14
TC1100003821.mm.2	Mpp2	membrane protein, palmitoylated 2 (MAGUK p55 subfamily member 2)	0.823114821362608	7,82525E+14	1,85658E+12	0.109472636773452	0.2501228554632	-4,41544E+14	8,52116E+14	8,40282E+14	8,22962E+13	7,04814E+14	7,2562E+14	7,49355E+14
TC1100001265.mm.2	Wfdc18	WAP four-disulfide core domain 18	0.572107026673637	5,35395E+14	1,85652E+14	0.109481183086501	0.2501228554632	-4,41551E+14	5,61294E+13	5,68564E+14	5,10674E+14	5,04762E+14	5,14122E+14	5,52952E+14
TC0400002576.mm.2	Exosc3	exosome component 3	0.608927715371368	8,24851E+14	1,85651E+14	0.1094840501199	0.2501228554632	-4,41553E+12	8,434E+14	8,54215E+14	8,15135E+14	7,60694E+14	8,3196E+14	8,43702E+14
TC1700001635.mm.2	Npw	neuropeptide W	-0.817069733733609	7,08568E+14	-1,85626E+14	0.10952208613227	0.250174280386911	-4,41583E+14	6,59913E+14	6,53481E+14	7,29628E+14	7,4718E+14	6,71544E+14	7,89661E+14
TC1100001875.mm.2	Ttyh2	tweety family member 2	0.575334524396824	8,2424E+14	1,85614E+14	0.109540353468441	0.250180540813181	-4,41597E+14	8,67003E+14	8,46203E+13	7,90201E+14	8,07938E+14	8,32793E+14	8,01302E+12
TC0400003740.mm.2	Mtfr1l	mitochondrial fission regulator 1-like	0.755514609392728	7,87532E+14	1,85598E+14	0.109566209705505	0.250204129391271	-4,41618E+14	8,24205E+14	8,0937E+14	7,91571E+14	6,90901E+14	7,95356E+14	8,1379E+14
TC0300002156.mm.2	Bche	butyrylcholinesterase	-0.735303546150683	6,07751E+14	-1,85574E+14	0.109603653149915	0.250254167947368	-4,41647E+14	5,46345E+14	5,37049E+13	5,67853E+13	6,62602E+14	6,55027E+14	6,77628E+14
TC1300001447.mm.2	Actn2	actinin alpha 2	0.556926200096002	7,96343E+14	1,85535E+14	0.109663684150256	0.250355758389498	-4,41695E+14	8,20712E+14	8,43782E+14	7,78979E+14	7,74131E+14	7,90413E+14	7,70043E+14
TC0X00002572.mm.2	Il1rapl1	interleukin 1 receptor accessory protein-like 1	0.98154405607522	6,05551E+14	1,8552E+13	0.10968755366272	0.250361050838492	-4,41713E+14	6,80688E+14	6,42519E+14	5,8696E+14	5,39939E+14	6,65281E+14	5,17917E+14
TC0500002818.mm.2	Rasgef1b	RasGEF domain family, member 1B	0.690135967549597	6,98035E+14	1,85514E+14	0.109697078068625	0.250361050838492	-4,41721E+14	7,28421E+14	7,66019E+14	6,74071E+14	6,82342E+14	7,06426E+14	6,30932E+14
TC1100002909.mm.2	Slc47a1	solute carrier family 47, member 1	-0.698611880176499	5,8953E+14	-1,85459E+14	0.109783409001518	0.250522598674615	-4,41789E+14	5,43221E+14	5,32636E+14	5,64605E+14	6,50975E+14	6,18988E+14	6,26756E+14
TC0700004409.mm.2	Oat	ornithine aminotransferase	0.807319527999426	7,48814E+13	1,85436E+14	0.109819468764954	0.25054622503207	-4,41817E+14	7,88444E+14	8,08003E+13	7,74401E+14	6,60583E+14	7,20447E+14	7,41005E+14
TC0X00002416.mm.2	Pnma5	paraneoplastic antigen family 5	-0.992394478761343	5,86484E+13	-1,85426E+13	0.109833853541858	0.25054622503207	-4,41829E+14	5,35215E+14	4,99746E+14	5,44828E+14	6,88611E+14	5,92502E+14	6,57999E+13
TC1900001118.mm.2	Ccdc86	coiled-coil domain containing 86	-0.542829819349671	6,42552E+14	-1,85422E+14	0.109840940388243	0.25054622503207	-4,41834E+14	5,98587E+14	6,1002E+14	6,50838E+14	6,66335E+14	6,61849E+14	6,67683E+14
TC0600001664.mm.2	Pde3a	phosphodiesterase 3A, cGMP inhibited	-0.707082631799111	8,01434E+14	-1,85412E+13	0.109855959774814	0.25054622503207	-4,41846E+14	7,46401E+14	7,62252E+14	8,00719E+14	8,49351E+14	7,87958E+13	8,61921E+14
TC0200004634.mm.2	Ddrgk1	DDRGK domain containing 1	0.972269161030789	7,92668E+14	1,85394E+14	0.109884646319078	0.250576182584107	-4,41869E+14	8,49471E+14	8,38699E+14	7,70833E+14	7,22883E+14	8,61792E+14	7,12328E+14
TC0300003091.mm.2	Wdr63	WD repeat domain 63	-0.916950801174729	5,44887E+14	-1,85367E+14	0.109927498781981	0.250638430433573	-4,41902E+13	4,89564E+14	5,09877E+13	5,23045E+13	6,59786E+14	5,2265E+14	5,644E+14
TC1100000440.mm.2	Rnf145	ring finger protein 145	1,39363E+14	7,55944E+14	1,85337E+14	0.109973012460466	0.25070672747248	-4,41938E+14	8,37725E+14	8,27504E+14	7,99663E+14	5,86841E+13	8,06588E+14	6,77343E+14
TC1000001994.mm.2	Calhm6	calcium homeostasis modulator family member 6	-0.816266523737056	5,33002E+14	-1,85327E+12	0.109988654400821	0.250706915880666	-4,4195E+14	4,53049E+14	5,03161E+14	5,19165E+14	6,00298E+14	5,2498E+14	5,97357E+14
TC1300000440.mm.2	Riok1	RIO kinase 1	0.860899684949116	5,23105E+14	1,85308E+14	0.110019221250484	0.250741119096047	-4,41974E+14	5,80947E+14	5,93277E+14	5,53739E+14	4,48305E+14	5,195E+14	4,42865E+14
TC0500003726.mm.2	Prr14l	proline rich 14-like	1,24275E+14	4,89784E+14	1,85295E+14	0.110040145759016	0.250753340208602	-4,41991E+14	5,21217E+14	5,39371E+14	5,10354E+14	3,01685E+14	5,62031E+14	5,04043E+14
TC1400000485.mm.2	Psmc6	proteasome (prosome, macropain) 26S subunit, ATPase, 6	2,09117E+14	7,81004E+14	1,85275E+14	0.110070324470187	0.250786642843344	-4,42014E+14	9,00879E+13	8,95913E+14	8,53797E+13	5,24761E+14	8,61331E+14	6,49347E+14
TC1100003050.mm.2	Shbg	sex hormone binding globulin	-0.964124059217185	5,30979E+14	-1,85254E+14	0.110103961881214	0.250792218811987	-4,42041E+14	4,75517E+14	4,69402E+14	5,05466E+14	6,32278E+14	5,06609E+14	5,96602E+14
TC0700004481.mm.2	Nkx6-2	NK6 homeobox 2	0.63954318049918	7,08218E+14	1,85242E+13	0.11012288310165	0.250792218811987	-4,42056E+14	7,34774E+14	7,53067E+14	7,00945E+13	6,58988E+14	6,75187E+14	7,26346E+14
TC1100003719.mm.2	Krtap9-3	keratin associated protein 9-3	-0.58405802464637	7,01695E+14	-1,85236E+14	0.11013140948279	0.250792218811987	-4,42062E+14	6,43208E+14	6,68521E+14	7,1311E+14	7,15431E+14	7,1544E+13	7,54457E+14
TC1000000928.mm.2	Tdg/Gm9855	thymine DNA glycosylase/thymine DNA glycosylase pseudogene	-0.564040881620411	7,74018E+14	-1,85229E+14	0.110143480872002	0.250792218811987	-4,42072E+13	7,36543E+14	7,42791E+14	8,03893E+14	7,88249E+14	8,02318E+14	7,70314E+14
TC0700002213.mm.2	Olfr1349	olfactory receptor 1349	-0.847691811172148	5,5667E+14	-1,85224E+14	0.110150594708151	0.250792218811987	-4,42077E+14	4,70022E+14	5,34106E+13	5,31594E+12	6,42072E+14	5,79685E+14	5,82539E+14
TC1500001957.mm.2	Cyp2d26	cytochrome P450, family 2, subfamily d, polypeptide 26	-0.826995871254372	4,45361E+13	-1,85143E+14	0.110278009388692	0.251034896448047	-4,42177E+14	4,14053E+14	3,70878E+14	4,46037E+14	5,04294E+14	4,18991E+14	5,17913E+14
TC1200001489.mm.2	Itgb1bp1	integrin beta 1 binding protein 1	1,16026E+12	7,25845E+14	1,85135E+14	0.110290598466473	0.251034896448047	-4,42187E+13	8,23006E+14	7,98475E+14	7,80173E+14	6,09255E+14	7,22287E+14	6,21872E+14
TC1400000366.mm.2	Gm5460	predicted gene 5460	-0.859992934051234	5,36477E+14	-1,85126E+14	0.110303920241555	0.251034896448047	-4,42198E+14	4,9436E+14	4,88126E+14	5,13224E+14	6,41261E+14	5,54217E+14	5,27673E+14
TC0600002697.mm.2	Plxna1	plexin A1	1,04797E+14	6,54359E+14	1,85114E+14	0.110323569080759	0.251038763426223	-4,42213E+13	7,55712E+13	7,26994E+14	6,60724E+14	6,12388E+14	6,67995E+14	5,02342E+14
TC0200000361.mm.2	Acbd5	acyl-Coenzyme A binding domain containing 5	1,62748E+14	6,97234E+14	1,85092E+14	0.110357281936312	0.251038763426223	-4,42239E+14	8,15784E+14	7,79858E+14	7,67237E+14	5,02909E+14	7,25248E+14	5,92365E+14
TC0900001354.mm.2	Iqcf6	IQ motif containing F6	-0.855903491297622	4,83903E+14	-1,85091E+14	0.110359396899135	0.251038763426223	-4,42241E+14	4,50665E+14	4,23002E+14	4,78486E+14	5,66362E+14	4,46633E+13	5,38268E+14
TC0400000138.mm.2	Calb1	calbindin 1	0.827096766340434	7,8889E+14	1,85086E+14	0.110367938938209	0.251038763426223	-4,42248E+14	8,54175E+14	8,35311E+14	8,22655E+14	7,01411E+14	7,58356E+14	7,61434E+14
TC1400001561.mm.2	Zfp503	zinc finger protein 503	-0.651741004601741	6,86399E+14	-1,85062E+14	0.110404206572264	0.251071347870512	-4,42276E+14	6,45467E+13	6,52207E+14	6,90968E+14	7,37053E+14	6,68222E+14	7,24475E+13
TC1900001022.mm.2	Kcnk4	potassium channel, subfamily K, member 4	-0.742685166994741	6,82352E+14	-1,85051E+14	0.110421690755819	0.251071347870512	-4,4229E+14	5,91763E+13	6,66095E+14	6,73915E+14	7,3248E+14	7,31838E+14	6,98024E+14
TC0700002825.mm.2	Wtip	WT1-interacting protein	-0.667358628138175	6,54979E+14	-1,85039E+14	0.110440564598671	0.251071347870512	-4,42305E+14	5,98155E+14	6,16446E+14	6,61146E+13	6,86927E+14	6,4785E+14	7,1935E+14
TC0900002588.mm.2	Fam81a	family with sequence similarity 81, member A	1,22101E+14	6,90063E+14	1,85037E+14	0.110444592174405	0.251071347870512	-4,42308E+14	7,52196E+14	7,80272E+14	6,76706E+14	6,11559E+14	7,60262E+14	5,59381E+14
TC1800000632.mm.2	Csnk1a1	casein kinase 1, alpha 1	1,09476E+14	8,97848E+14	1,84972E+13	0.110546810915199	0.25126826975266	-4,42388E+14	9,8597E+14	9,91049E+14	9,28129E+14	8,29937E+14	9,0469E+12	7,47314E+14
TC1400001634.mm.2	Ccdc66	coiled-coil domain containing 66	0.860320423304236	6,67004E+14	1,84944E+14	0.110589958446379	0.251312232500879	-4,42421E+14	7,25007E+14	7,45964E+13	6,79647E+14	6,19259E+14	6,73361E+14	5,58788E+14
TC1000002653.mm.2	Ano4	anoctamin 4	-0.639276288232127	7,54186E+14	-1,84936E+14	0.110603866762352	0.251312232500879	-4,42432E+14	7,22061E+14	6,86608E+14	7,45077E+14	7,91446E+14	7,71636E+14	8,08288E+14
TC0900002541.mm.2	Tpm1	tropomyosin 1, alpha	0.972609859516037	8,97651E+14	1,8493E+14	0.110612943145053	0.251312232500879	-4,42439E+13	9,44429E+14	9,59626E+14	9,26077E+14	7,83456E+13	9,19273E+14	8,53048E+14
TC0700000874.mm.2	Kcnc1	potassium voltage gated channel, Shaw-related subfamily, member 1	0.761663225503314	8,0567E+14	1,84905E+14	0.11065144813782	0.251364272359325	-4,42469E+14	8,58596E+14	8,40109E+14	8,12909E+14	7,33463E+14	8,28204E+14	7,60738E+14
TC1700002803.mm.2	Wdr90	WD repeat domain 90	-0.756411965685335	5,73612E+14	-1,84886E+14	0.110682370327988	0.251397000605146	-4,42493E+14	5,38988E+14	5,23516E+14	5,61581E+14	6,52206E+13	5,57904E+13	6,07478E+14
TC1200000808.mm.2	Zfp410	zinc finger protein 410	0.814123402124967	6,15416E+14	1,84876E+14	0.110697059473314	0.251397000605146	-4,42505E+14	6,7739E+14	6,80879E+14	6,56695E+14	5,38749E+14	5,79383E+14	5,594E+13
TC1600000421.mm.2	Meltf	melanotransferrin	-0.667155832534306	5,60622E+14	-1,84859E+14	0.11072426655091	0.25142335216105	-4,42526E+14	5,15569E+14	5,23847E+14	5,71715E+14	6,01132E+13	5,39678E+14	6,11789E+14
TC1300002715.mm.2	Itga2	integrin alpha 2	-0.798632835569607	4,99191E+14	-1,84833E+14	0.110765512213039	0.251455203492514	-4,42558E+14	4,52275E+14	4,88125E+14	5,00624E+14	5,99503E+14	5,01453E+14	4,53167E+14
TC0800002404.mm.2	Pik3r2	phosphoinositide-3-kinase regulatory subunit 2	0.588899445560884	7,12976E+14	1,84826E+14	0.110776513194374	0.251455203492514	-4,42567E+14	7,73021E+14	7,67184E+14	7,32473E+14	6,89952E+14	6,69832E+14	6,45397E+14
TC0500003371.mm.2	Gtf2ird1	general transcription factor II I repeat domain-containing 1	0.608452545570104	6,45788E+14	1,84803E+14	0.110812117200167	0.251455203492514	-4,42595E+13	6,97372E+14	6,68731E+14	6,40472E+14	6,03941E+14	6,1501E+13	6,49204E+14
TC0500002755.mm.2	Cxcl9	chemokine (C-X-C motif) ligand 9	-0.679253977506654	5,11801E+14	-1,84803E+14	0.110812454231081	0.251455203492514	-4,42595E+14	4,42317E+14	4,757E+14	4,92734E+14	5,61133E+14	5,33933E+14	5,6499E+14
TC0200000301.mm.2	Commd3	COMM domain containing 3	1,60639E+14	7,26857E+14	1,84792E+14	0.110829261624308	0.251455203492514	-4,42608E+14	8,19593E+14	8,11278E+14	7,8295E+14	5,26644E+14	7,82466E+14	6,38211E+14
TC0500003615.mm.2	Usp12	ubiquitin specific peptidase 12	0.868071023863098	7,46233E+14	1,84791E+14	0.110831927959029	0.251455203492514	-4,4261E+14	7,84433E+14	8,02485E+14	7,72675E+14	6,40629E+14	7,37945E+14	7,39231E+14
TC1000002497.mm.2	Adamtsl5	ADAMTS-like 5	-0.65915545203117	6,33184E+13	-1,84769E+13	0.11086598889529	0.25148708881181	-4,42637E+14	6,11689E+13	5,81078E+14	6,39507E+14	6,85091E+14	6,14719E+14	6,6702E+14
TC1400000326.mm.2	Bap1	Brca1 associated protein 1	0.750015547620143	7,74594E+14	1,84762E+14	0.110877197227028	0.25148708881181	-4,42645E+14	8,23139E+14	8,20223E+14	7,84221E+14	7,09137E+14	7,90528E+14	7,20318E+14
TC1100001567.mm.2	Pnmt	phenylethanolamine-N-methyltransferase	-0.840036157348476	5,56972E+14	-1,84751E+14	0.110894676736361	0.251491333744263	-4,42659E+14	4,81923E+14	5,40567E+14	5,40156E+14	6,51141E+14	5,53197E+14	5,74847E+14
TC1100000822.mm.2	Pmp22	peripheral myelin protein 22	0.665099347932348	7,74399E+13	1,84733E+14	0.110923751485968	0.251521869925894	-4,42682E+13	8,34004E+14	8,39235E+14	8,07563E+14	7,32657E+14	7,26659E+14	7,0628E+14
TC1400000373.mm.2	Glud1/Gm3219	glutamate dehydrogenase 1/B-cell CLL/lymphoma 7C pseudogene	1,5263E+14	9,21288E+14	1,84701E+14	0.110973659436571	0.251599630575695	-4,4272E+14	1,02607E+14	1,01215E+14	9,88437E+14	7,4452E+14	9,44461E+14	8,12096E+14
TC1100000713.mm.2	Trim17/Hist3h2a	tripartite motif-containing 17/histone cluster 3, H2a	0.68369449289646	7,76926E+14	1,84664E+14	0.111031760950091	0.251695943189197	-4,42766E+14	8,07835E+14	8,18568E+14	7,85053E+14	7,0461E+14	7,62431E+14	7,83061E+14
TC0800001870.mm.2	Smim19	small integral membrane protein 19	1,27986E+14	7,84649E+14	1,84639E+14	0.11107105290566	0.251749595649023	-4,42796E+14	8,81736E+14	8,4285E+14	8,30998E+13	6,37615E+14	8,12731E+14	7,01962E+14
TC0500000374.mm.2	Fam193a	family with sequence homology 193, member A	1,21964E+14	7,69904E+14	1,84617E+14	0.111105649737846	0.251792592718132	-4,42823E+14	8,7059E+14	8,8078E+14	7,66236E+14	7,41206E+14	7,86115E+14	5,74496E+14
TC1100003234.mm.2	Rph3al	rabphilin 3A-like (without C2 domains)	-0.703641614794726	5,90733E+14	-1,84586E+14	0.111155790434699	0.251845825911677	-4,42862E+14	5,45558E+14	5,24478E+14	6,08731E+14	6,02034E+14	5,8826E+14	6,75336E+14
TC0X00001254.mm.2	Diaph2	diaphanous related formin 2	0.597431888508686	8,06002E+13	1,84583E+13	0.111160399297701	0.251845825911677	-4,42866E+14	8,22354E+14	8,65567E+14	7,97841E+14	7,70593E+14	7,97669E+14	7,81991E+14
TC1800001289.mm.2	Trim36	tripartite motif-containing 36	0.557320639539299	5,37656E+14	1,84531E+14	0.111242551646777	0.251966882940371	-4,42929E+14	5,64489E+14	5,76192E+14	5,27967E+14	5,0125E+14	5,36129E+14	5,19911E+14
TC0100001644.mm.2	Spta1	spectrin alpha, erythrocytic 1	-0.638476239912367	5,42388E+14	-1,84529E+14	0.111245106760864	0.251966882940371	-4,42931E+14	4,84499E+14	5,21214E+14	5,48549E+14	5,8486E+14	5,34619E+14	5,80587E+14
TC0800002702.mm.2	Ces1d	carboxylesterase 1D	-1,04831E+14	5,28573E+14	-1,84502E+14	0.111288047902844	0.251991685178379	-4,42965E+14	4,63314E+14	5,0025E+14	5,8136E+14	5,91867E+14	4,31549E+14	6,03096E+14
TC0700004068.mm.2	Ric3	RIC3 acetylcholine receptor chaperone	1,0832E+14	6,6248E+14	1,84498E+14	0.111294365696716	0.251991685178379	-4,4297E+14	7,73562E+13	7,30132E+14	6,66862E+14	6,20192E+14	6,78161E+14	5,05968E+14
TC1100001657.mm.2	Ifi35	interferon-induced protein 35	-0.673302787534034	5,73798E+14	-1,84493E+14	0.111302974208063	0.251991685178379	-4,42976E+14	5,26767E+14	5,39183E+14	5,75051E+13	6,2556E+14	5,57264E+14	6,1896E+14
TC1100002602.mm.2	Itk	IL2 inducible T cell kinase	-1,07253E+14	5,301E+14	-1,84474E+14	0.111331960853875	0.252021900145895	-4,42999E+13	4,71736E+14	4,71652E+14	4,95397E+14	6,62497E+14	5,04537E+14	5,7478E+14
TC0500001789.mm.2	Gm3404	predicted gene 3404	-0.86842698886305	4,55806E+14	-1,8443E+13	0.111402650633717	0.252146496651367	-4,43054E+14	3,58166E+14	4,27998E+14	4,33984E+13	5,25865E+14	4,60574E+14	5,28247E+14
TC1100003726.mm.2	Gm11555	predicted gene 11555	-0.613774378314091	5,87557E+14	-1,84412E+14	0.111430782123226	0.25217474611679	-4,43075E+14	5,83873E+14	5,30351E+13	6,31168E+14	6,0581E+14	5,92584E+14	5,81558E+14
TC0800003080.mm.2	Cbfa2t3	CBFA2/RUNX1 translocation partner 3	-0.707286419924487	5,85877E+14	-1,84371E+14	0.111496347452136	0.252287690843459	-4,43126E+14	5,00324E+14	5,77428E+14	5,85312E+14	6,33897E+14	6,08138E+14	6,10166E+14
TC0100002640.mm.2	Scg2	secretogranin II	1,63046E+14	9,36188E+14	1,84353E+14	0.111524529729603	0.2523160274548	-4,43148E+14	1,0883E+14	1,00931E+14	1,01716E+14	7,54357E+14	9,3458E+14	8,13423E+14
TC0700001519.mm.2	Trim6	tripartite motif-containing 6	-0.608498479640235	4,8771E+14	-1,84331E+14	0.111558803688019	0.252358136154016	-4,43175E+14	4,5688E+14	4,23878E+14	4,77688E+14	5,2477E+14	5,26309E+14	5,16737E+14
TC1400002139.mm.2	Gjb2	gap junction protein, beta 2	-0.664252520059191	7,34488E+14	-1,84316E+14	0.111582519737666	0.252376353247193	-4,43193E+14	7,02321E+14	6,92271E+14	7,251E+13	8,02342E+14	7,40267E+14	7,44624E+14
TC0300000078.mm.2	Ralyl	RALY RNA binding protein-like	1,64697E+14	7,05502E+14	1,84301E+14	0.111606205711886	0.252394497212017	-4,43211E+14	8,20761E+14	8,01387E+14	7,77362E+14	5,15392E+14	7,35025E+14	5,83085E+14
TC1600000651.mm.2	Usf3	upstream transcription factor family member 3	0.610888920665406	7,64785E+14	1,84285E+14	0.111633095597375	0.252419880628754	-4,43232E+14	8,08682E+14	8,18877E+14	7,54864E+14	7,50517E+14	7,57007E+14	6,98764E+14
TC1400002683.mm.2	Slitrk1	SLIT and NTRK-like family, member 1	1,2721E+14	7,15303E+14	1,84266E+14	0.111662355202513	0.252450614477072	-4,43255E+14	7,857E+14	7,94855E+14	7,3184E+14	5,94296E+14	7,81555E+14	6,03575E+14
TC1100003274.mm.2	Gm10277	predicted gene 10277	-0.563341235024541	6,47343E+14	-1,8424E+14	0.111703846102384	0.252482461393283	-4,43287E+14	5,98479E+12	6,13799E+14	6,45486E+14	6,7946E+14	6,71323E+14	6,75512E+14
TC0700000733.mm.2	Zfp788	zinc finger protein 788	1,19085E+14	6,21513E+14	1,84238E+14	0.11170778050473	0.252482461393283	-4,4329E+14	7,53605E+13	7,10335E+13	6,53555E+14	5,72215E+13	6,12728E+14	4,26643E+14
TC1500002295.mm.2	Npff/Atf7	neuropeptide FF-amide peptide precursor/activating transcription factor 7	-0.595362116504967	9,03141E+14	-1,84217E+14	0.111741038286404	0.252522208963371	-4,43315E+14	8,53689E+14	8,70616E+14	8,96567E+14	9,46812E+13	9,2665E+14	9,24511E+14
TC0500003723.mm.2	Mpv17	MpV17 mitochondrial inner membrane protein	1,27264E+14	8,49191E+13	1,84188E+14	0.111786116226282	0.252588653870997	-4,4335E+14	9,22965E+14	9,13607E+14	8,94532E+13	6,87513E+14	8,82533E+14	7,93999E+14
TC0100001745.mm.2	Trp53bp2	transformation related protein 53 binding protein 2	0.644130649609981	6,74035E+14	1,84178E+14	0.11180223904037	0.25258966316005	-4,43363E+14	7,41694E+14	7,005E+14	6,84267E+14	6,29102E+14	6,34667E+14	6,53982E+14
TC1900001600.mm.2	Cuedc2	CUE domain containing 2	0.663815443730082	7,84662E+14	1,84159E+14	0.111833078191573	0.252602396329403	-4,43387E+14	8,29574E+14	8,14525E+14	7,93442E+14	7,17894E+14	7,82571E+14	7,69965E+14
TC1000002822.mm.2	Ptprq	protein tyrosine phosphatase, receptor type, Q	-0.967024827902847	4,53151E+14	-1,84155E+14	0.111839228909201	0.252602396329403	-4,43391E+14	3,8962E+14	3,99305E+13	4,40439E+14	5,41891E+14	4,13699E+14	5,33951E+14
TC0X00000587.mm.2	Ctag2	cancer/testis antigen 2	-0.776421410483317	4,76592E+14	-1,84126E+14	0.111884479812649	0.252669183352658	-4,43426E+14	4,02621E+14	4,53007E+14	4,65174E+14	5,45738E+14	4,68126E+14	5,24889E+14
TC1400001523.mm.2	Rtraf	RNA transcription, translation and transport factor	1,041E+14	7,87768E+14	1,84108E+14	0.111913901999645	0.252678230255037	-4,43449E+14	8,33069E+14	8,41422E+14	8,12732E+14	6,53559E+14	8,28604E+14	7,57222E+14
TC0500003393.mm.2	Pom121	nuclear pore membrane protein 121	1,02012E+14	7,12492E+14	1,84102E+14	0.111923966742831	0.252678230255037	-4,43457E+14	8,09268E+13	7,81139E+14	7,76819E+14	6,09564E+14	6,54217E+14	6,43945E+14
TC0700003006.mm.2	Ftl1	ferritin light polypeptide 1	-0.982328155001829	1,14518E+14	-1,84088E+12	0.111946448010837	0.252678230255037	-4,43474E+14	1,05576E+14	1,09682E+14	1,24074E+14	1,1083E+14	1,22591E+14	1,14357E+14
TC0200003574.mm.2	Cytip	cytohesin 1 interacting protein	-0.643921162722308	4,82082E+14	-1,84085E+14	0.111951212424174	0.252678230255037	-4,43478E+12	4,34081E+14	4,32534E+14	4,72481E+12	5,22918E+14	4,89878E+14	5,406E+14
TC0100002837.mm.2	Thap4	THAP domain containing 4	-0.850291347601574	7,35862E+14	-1,84041E+13	0.112020088288771	0.252798274873525	-4,43531E+13	6,87844E+14	6,94378E+14	7,1142E+14	8,4086E+14	7,37257E+14	7,43411E+14
TC0200001514.mm.2	Traf6	TNF receptor-associated factor 6	-0.62271637212214	6,63081E+14	-1,8403E+14	0.112038484586772	0.252804383440226	-4,43545E+14	6,141E+14	6,30833E+14	6,77687E+14	6,91789E+14	6,51917E+14	7,12162E+14
TC1400000813.mm.2	Zmym2	zinc finger, MYM-type 2	1,25769E+14	7,99606E+13	1,84004E+14	0.112079523904827	0.252857720179866	-4,43577E+13	9,06341E+13	8,92532E+14	8,42143E+14	7,05192E+14	8,13067E+14	6,38359E+14
TC0800001569.mm.2	Map3k21	mitogen-activated protein kinase kinase kinase 21	-1,09224E+13	5,87853E+14	-1,83995E+14	0.112093508052181	0.252857720179866	-4,43587E+14	4,93731E+14	5,31223E+13	5,41519E+14	7,01882E+14	5,85842E+14	6,72918E+14
TC0700002639.mm.2	Bckdha	branched chain ketoacid dehydrogenase E1, alpha polypeptide	0.635368212176807	6,21616E+14	1,83971E+14	0.112132339836536	0.252892742496046	-4,43617E+14	6,58255E+14	6,72949E+14	6,25697E+14	5,78434E+14	6,21017E+14	5,73342E+14
TC0100000244.mm.2	Cnnm4	cyclin M4	-0.60370374893588	6,19764E+14	-1,83966E+14	0.112140423579361	0.252892742496046	-4,43623E+14	5,92954E+14	5,92035E+14	6,30397E+13	6,75333E+14	6,3134E+14	5,96522E+14
TC1700001023.mm.2	Crb3	crumbs family member 3	-0.657786846420879	5,76561E+14	-1,83946E+14	0.11217260620481	0.252929919364414	-4,43648E+14	5,03549E+14	5,61185E+14	5,81561E+14	6,14731E+14	5,80417E+13	6,17922E+14
TC0600001931.mm.2	Cadps2	Ca2+-dependent activator protein for secretion 2	0.863900225317604	7,11871E+14	1,83919E+13	0.112215850473105	0.252992024440064	-4,43681E+14	7,9452E+14	7,69274E+14	7,00541E+14	6,90474E+14	7,2344E+14	5,92979E+14
TC0900001623.mm.2	Ss18l2	SS18, nBAF chromatin remodeling complex subunit like 2	0.644776217261286	6,93407E+14	1,83905E+14	0.112237087842657	0.25300450425416	-4,43698E+13	7,46547E+14	7,2177E+14	6,9677E+14	6,42592E+14	6,96646E+14	6,5612E+13
TC1300000924.mm.2	Ttc37	tetratricopeptide repeat domain 37	0.745931270592452	5,44332E+13	1,83852E+14	0.112322878366349	0.253162475747235	-4,43764E+14	6,09805E+14	6,09684E+14	5,3825E+14	5,32053E+14	5,39372E+14	4,36829E+14
TC0X00000472.mm.2	Fam122c	family with sequence similarity 122, member C	-0.837670926765493	5,08069E+14	-1,83829E+14	0.112358402453848	0.253168494384102	-4,43791E+14	4,34894E+13	4,62761E+14	4,72901E+14	5,92287E+14	5,26521E+14	5,59052E+14
TC0700003476.mm.2	Nr2f2	nuclear receptor subfamily 2, group F, member 2	0.939594065070074	7,26654E+14	1,83818E+14	0.11237592814109	0.253168494384102	-4,43804E+14	8,64235E+13	8,14195E+14	7,66806E+14	7,23705E+14	6,63727E+14	5,27257E+14
TC0400003674.mm.2	Sesn2	sestrin 2	-0.76065818376772	6,78713E+14	-1,83818E+14	0.112376331992643	0.253168494384102	-4,43805E+14	6,26012E+14	6,40448E+14	6,69472E+14	7,49119E+14	6,5878E+13	7,28449E+13
TC1200000018.mm.2	Dnajc27	DnaJ heat shock protein family (Hsp40) member C27	1,07188E+14	7,19225E+14	1,83811E+14	0.112388396967013	0.253168494384102	-4,43814E+14	7,89649E+14	7,68426E+13	7,60297E+14	5,834E+14	7,25334E+14	6,88246E+14
TC0300000826.mm.2	Sprr2f	small proline-rich protein 2F	-0.963697972567506	5,99994E+14	-1,83763E+14	0.112465214472438	0.253306122560022	-4,43873E+14	5,32406E+13	5,46763E+14	5,63797E+14	7,08111E+13	5,93942E+13	6,54942E+14

TC0900002269.mm.2	Bco2	beta-carotene oxygenase 2	-0.650713038157962	5,22871E+13	-1,83708E+14	0.112552765450926	0.253440193984441	-4,4394E+14	4,68729E+14	4,8952E+14	5,08724E+14	5,79668E+14	5,41638E+14	5,48949E+14
TC1600001255.mm.2	Mzt2	mitotic spindle organizing protein 2	-0.624076750969253	7,27265E+14	-1,83706E+14	0.11255619860688	0.253440193984441	-4,43943E+14	7,02373E+14	6,80189E+14	7,57707E+14	7,49671E+14	7,06542E+14	7,67108E+14
TC1700001972.mm.2	Gm9573	predicted gene 9573	-0.840218642669172	5,50089E+14	-1,83663E+14	0.112624499446521	0.253542002051377	-4,43995E+14	4,95148E+14	4,91062E+13	5,17235E+13	6,37019E+14	5,58044E+14	6,02027E+13
TC1100000549.mm.2	Grm6	glutamate receptor, metabotropic 6	-0.602233711671853	5,28129E+14	-1,83658E+14	0.112632883428521	0.253542002051377	-4,44002E+14	5,02766E+14	4,64053E+14	5,2315E+14	5,64116E+14	5,54411E+14	5,60279E+14
TC0300002728.mm.2	Gstm4	glutathione S-transferase, mu 4	1,02877E+13	6,3429E+12	1,83642E+14	0.112657968779724	0.253563046647255	-4,44021E+14	7,26567E+13	6,86132E+14	6,86393E+14	5,20552E+14	6,0536E+13	5,80733E+14
TC0700001366.mm.2	Gucy2d	guanylate cyclase 2d	-0.627229870401385	4,86357E+14	-1,83622E+14	0.112690259543582	0.253600300562253	-4,44045E+14	4,74661E+14	4,18274E+14	4,98649E+14	5,19732E+14	4,89584E+14	5,17243E+14
TC1000000710.mm.2	Specc1l	sperm antigen with calponin homology and coiled-coil domains 1-like	0.876990679581718	6,59755E+14	1,83611E+14	0.112707779646387	0.253604308538226	-4,44059E+14	7,19115E+14	7,16924E+14	6,98704E+14	5,61937E+14	6,32751E+14	6,29097E+14
TC0800001855.mm.2	Defb9	defensin beta 9	-0.732797722196389	5,28962E+11	-1,83594E+12	0.112735541965001	0.253631358235417	-4,4408E+14	5,01808E+14	4,88281E+14	5,1912E+14	6,17529E+14	5,24618E+14	5,22414E+14
TC0900001231.mm.2	Nmnat3	nicotinamide nucleotide adenylyltransferase 3	-0.766666500682303	5,92952E+14	-1,83515E+14	0.112862308960747	0.253874208919608	-4,44177E+14	5,70404E+14	5,34321E+14	5,78339E+14	6,79719E+12	5,89574E+14	6,05356E+13
TC1500002196.mm.2	Dhh	desert hedgehog	-0.948619054626928	6,00909E+14	-1,83507E+14	0.112874997374795	0.253874208919608	-4,44187E+14	5,39369E+14	5,19726E+14	5,64921E+14	6,83897E+14	6,00788E+14	6,96756E+13
TC0700001368.mm.2	Lrrc32	leucine rich repeat containing 32	-0.645856090914388	4,78305E+14	-1,83447E+14	0.112970654040996	0.254053893728203	-4,4426E+14	4,55437E+14	4,19579E+14	5,30917E+14	4,73271E+14	5,08682E+14	4,81943E+14
TC0400003392.mm.2	Mpl	myeloproliferative leukemia virus oncogene	-0.55179066694905	5,54523E+14	-1,83395E+14	0.113054562470962	0.254207112070137	-4,44324E+14	5,32972E+14	5,23586E+14	5,68652E+14	5,98264E+14	5,47959E+14	5,55704E+14
TC1000000745.mm.2	Spatc1l	spermatogenesis and centriole associated 1 like	-0.812745663761766	5,5112E+14	-1,83378E+14	0.113082553665973	0.254216967082083	-4,44345E+14	4,65102E+14	5,26524E+14	5,29343E+14	6,24832E+14	5,59509E+14	6,01408E+14
TC1000000231.mm.2	Samd3	sterile alpha motif domain containing 3	-0.688884735628398	5,86571E+14	-1,83373E+14	0.113090499599353	0.254216967082083	-4,44351E+14	5,7259E+14	5,04649E+14	5,92487E+14	6,22528E+14	5,91139E+12	6,3603E+13
TC0100001678.mm.2	Catspere2	catsper channel auxiliary subunit epsilon 2	1,95108E+14	5,40642E+14	1,83329E+14	0.113161729139274	0.254341601565229	-4,44406E+14	7,0597E+14	6,16169E+14	6,23933E+14	3,07991E+14	5,77312E+14	4,12476E+14
TC0400003482.mm.2	Oxct2a	3-oxoacid CoA transferase 2A	-0.970404591570952	6,12487E+14	-1,83314E+14	0.113184308831556	0.254343146362498	-4,44423E+14	5,35679E+14	5,40746E+14	5,65572E+14	7,04933E+14	6,25342E+14	7,02648E+14
TC0200004896.mm.2	Trib3	tribbles pseudokinase 3	-0.878327035712309	6,14834E+14	-1,833E+14	0.113207920511091	0.254343146362498	-4,44441E+14	5,65894E+14	5,42668E+14	6,08131E+14	6,76097E+14	5,89469E+14	7,06747E+14
TC1100001090.mm.2	1700016K19Rik	RIKEN cDNA 1700016K19 gene	-0.617887916919893	8,75775E+14	-1,83299E+14	0.113209771346853	0.254343146362498	-4,44442E+13	8,40589E+14	8,32835E+14	8,70926E+14	9,26075E+14	8,77153E+13	9,07073E+14
TC0700002479.mm.2	Cd3eap	CD3E antigen, epsilon polypeptide associated protein	-0.569316394277341	6,68607E+14	-1,83275E+14	0.113247759533097	0.25439302235561	-4,44471E+14	6,18184E+14	6,28969E+14	6,74898E+14	6,86119E+14	6,82048E+14	7,21422E+14
TC0600000711.mm.2	Abcg2	ATP binding cassette subfamily G member 2 (Junior blood group)	0.617468398654836	5,78142E+14	1,83241E+13	0.113302935548062	0.254427373710918	-4,44513E+13	5,90241E+14	6,12382E+14	5,64821E+14	5,14309E+14	6,08916E+14	5,78184E+13
TC0100002207.mm.2	2010300C02Rik	RIKEN cDNA 2010300C02 gene	0.573217248963112	7,84351E+14	1,83238E+14	0.113307517113285	0.254427373710918	-4,44517E+14	8,19568E+14	8,45209E+13	7,61593E+14	7,8854E+14	7,57689E+14	7,33508E+14
TC0700004620.mm.2	Sbsn	suprabasin	0.741569772125209	5,69268E+14	1,83236E+14	0.113310422252191	0.254427373710918	-4,44519E+14	6,43046E+14	5,9035E+14	5,85642E+14	4,99439E+14	5,40825E+14	5,56304E+14
TC0500002788.mm.2	Gm7978/BC061212	predicted gene 7978/cDNA sequence BC061212	-1,23985E+14	5,03152E+14	-1,83219E+14	0.113337947204035	0.25445371928363	-4,4454E+14	4,06895E+14	4,25006E+14	4,43929E+14	6,35943E+14	5,00934E+14	6,06201E+14
TC0100001260.mm.2	Cacna1s	calcium channel, voltage-dependent, L type, alpha 1S subunit	-0.826510020629362	6,05831E+14	-1,83197E+14	0.11337309093941	0.254497159975858	-4,44567E+14	5,71623E+14	5,27343E+14	5,79147E+14	6,85122E+14	6,11538E+14	6,60212E+14
TC0600000995.mm.2	Asprv1	aspartic peptidase, retroviral-like 1	-0.566107895226414	5,90293E+14	-1,83161E+14	0.113431198612101	0.254592130273964	-4,44611E+14	5,71492E+14	5,56024E+14	6,01579E+14	6,39159E+14	5,84701E+14	5,88802E+13
TC0200001227.mm.2	Prg2	proteoglycan 2, bone marrow	-0.656944741210171	5,0664E+14	-1,83133E+14	0.113477344603963	0.254635065992389	-4,44646E+13	4,91031E+14	4,4898E+14	5,09121E+14	5,62278E+14	4,9592E+14	5,32508E+14
TC1500000160.mm.2	Myo10	myosin X	0.633176150986634	7,0568E+14	1,8313E+14	0.113481934394051	0.254635065992389	-4,44649E+14	7,86761E+13	7,30172E+14	6,78312E+14	7,11986E+14	6,73682E+14	6,53168E+14
TC0300001409.mm.2	Adh1	alcohol dehydrogenase 1 (class I)	-0.908489140583542	5,44089E+14	-1,83076E+14	0.113569482740257	0.254796028319933	-4,44716E+14	4,339E+14	5,25317E+13	5,3107E+14	6,09844E+13	5,41919E+14	6,22485E+13
TC0200001597.mm.2	Mpped2	metallophosphoesterase domain containing 2	0.77039594444353	7,72267E+14	1,83028E+14	0.113646011210422	0.25493222589914	-4,44774E+14	8,26497E+14	8,16425E+14	7,94393E+14	6,9445E+14	7,73713E+14	7,28121E+14
TC1000002274.mm.2	Hnrnph3	heterogeneous nuclear ribonucleoprotein H3	-0.816463299051351	6,87225E+14	-1,82989E+13	0.113710037765404	0.25503879288083	-4,44822E+14	6,333E+14	6,46366E+14	6,66421E+14	7,76537E+14	6,74432E+14	7,26298E+14
TC1100001520.mm.2	Copz2	coatomer protein complex, subunit zeta 2	-0.58002622925138	7,00663E+14	-1,8297E+14	0.113740792222771	0.25503879288083	-4,44846E+14	6,72254E+14	6,46002E+14	7,14654E+13	7,19607E+14	7,05773E+14	7,45688E+14
TC1700002816.mm.2	Cfb	complement factor B	-0.837383306802679	5,3963E+14	-1,8297E+14	0.113741002025796	0.25503879288083	-4,44846E+14	4,90206E+14	5,30001E+14	5,53538E+14	6,34146E+14	4,69558E+14	5,6033E+14
TC0800001436.mm.2	6430548M08Rik	RIKEN cDNA 6430548M08 gene	0.663809359537595	8,65532E+14	1,82922E+14	0.113817796737735	0.25517547778421	-4,44904E+14	8,95571E+14	9,15889E+14	8,74398E+14	8,043E+14	8,67271E+13	8,35765E+13
TC1200001676.mm.2	Lrrn3	leucine rich repeat protein 3, neuronal	1,63277E+14	7,06368E+14	1,82895E+14	0.113861425626041	0.255219099270476	-4,44937E+14	8,3607E+14	8,18631E+14	7,72313E+14	5,55833E+14	7,33414E+14	5,2195E+14
TC1400000367.mm.2	Antxrl	anthrax toxin receptor-like	-0.722406718117321	5,51894E+13	-1,8289E+14	0.113868932207252	0.255219099270476	-4,44943E+14	4,94917E+14	4,98211E+14	5,31571E+14	6,06038E+14	5,5912E+14	6,2151E+14
TC0300000852.mm.2	S100a11	S100 calcium binding protein A11	-0.740632866528659	8,51637E+14	-1,82843E+14	0.113945766664274	0.255339797305913	-4,45001E+14	7,93561E+14	8,05524E+14	9,20573E+13	8,26638E+14	8,62284E+13	9,01241E+14
TC1000002547.mm.2	Gna11	guanine nucleotide binding protein, alpha 11	0.930227431939256	8,83954E+13	1,82838E+13	0.113954476660263	0.255339797305913	-4,45008E+14	9,42702E+14	9,30342E+14	9,11395E+14	7,75604E+14	9,03783E+14	8,39896E+14
TC1100001899.mm.2	Nup85	nucleoporin 85	0.632259377439783	5,09667E+14	1,82794E+14	0.114025423298696	0.255463243271952	-4,45061E+14	6,06051E+14	5,47084E+14	5,26029E+14	5,00654E+14	4,26362E+14	4,5182E+14
TC0300003237.mm.2	Mtx1	metaxin 1	0.660229523772192	6,97288E+14	1,82778E+14	0.114050678672222	0.255484302161201	-4,4508E+14	7,15212E+14	7,46522E+14	6,97131E+14	6,32557E+14	6,80235E+14	7,12069E+14
TC0500002093.mm.2	Rheb	Ras homolog enriched in brain	1,681E+14	9,05359E+14	1,82758E+14	0.114084158117037	0.255508169846335	-4,45106E+13	1,02092E+14	1,00924E+14	9,71972E+14	7,21989E+14	9,48309E+14	7,59722E+14
TC1000000844.mm.2	1600002K03Rik	RIKEN cDNA 1600002K03 gene	-0.597822522033345	6,02883E+14	-1,82752E+14	0.114093108229276	0.255508169846335	-4,45112E+14	5,79317E+14	5,38405E+14	5,99793E+13	6,37494E+14	6,3467E+14	6,2762E+14
TC1300001625.mm.2	Btn1a1	butyrophilin, subfamily 1, member A1	-0.713512842441584	6,60589E+14	-1,82742E+14	0.114108905245096	0.255508169846335	-4,45124E+14	6,11251E+14	6,25207E+14	6,65727E+14	7,13434E+14	6,3359E+14	7,14327E+14
TC0700002623.mm.2	Pou2f2/Mir6537	POU domain, class 2, transcription factor 2/microRNA 6537	-0.649666892743776	5,26638E+14	-1,82725E+14	0.114137697530335	0.255537129402013	-4,45146E+14	4,75246E+14	5,11287E+14	5,26131E+13	5,90335E+14	5,35816E+14	5,21009E+14
TC1500000591.mm.2	Mroh1/Mir6954	maestro heat-like repeat family member 1/microRNA 6954	0.606437219097787	5,96285E+14	1,82694E+14	0.11418779534243	0.255613774152899	-4,45184E+14	6,15688E+14	6,37068E+14	5,45511E+14	5,85957E+14	6,15851E+14	5,77634E+14
TC0500002579.mm.2	Txk	TXK tyrosine kinase	-0.725896878604382	5,62956E+14	-1,82651E+14	0.114258130782777	0.25573569402735	-4,45237E+14	4,92945E+14	5,18011E+14	5,86147E+13	5,69987E+14	5,584E+14	6,52244E+14
TC1500002301.mm.2	Calcoco1	calcium binding and coiled coil domain 1	1,0685E+14	7,86324E+14	1,82585E+14	0.114364238286385	0.255879850389536	-4,45317E+14	8,85121E+14	8,69853E+14	8,58653E+14	6,82621E+13	6,87356E+14	7,34339E+14
TC0700000447.mm.2	Cyp2f2	cytochrome P450, family 2, subfamily f, polypeptide 2	-1,02009E+14	6,1407E+13	-1,82585E+14	0.114364274899647	0.255879850389536	-4,45317E+14	5,64249E+14	5,6741E+14	5,83183E+14	7,52494E+14	6,23131E+13	5,93954E+14
TC1000002650.mm.2	Utp20	UTP20 small subunit processome component	0.681344097095669	5,65681E+14	1,82582E+13	0.11437017827254	0.255879850389536	-4,45321E+14	6,30315E+14	5,91336E+14	5,24277E+14	5,61105E+14	5,10297E+14	5,76757E+14
TC1100004173.mm.2	Cbx4	chromobox 4	0.621687972904543	7,98744E+14	1,82564E+14	0.114398355553141	0.255907358465606	-4,45343E+14	8,3369E+13	8,43494E+14	8,08821E+14	7,44026E+13	7,80539E+14	7,81894E+14
TC0X00000516.mm.2	Smim10l2a	small integral membrane protein 10 like 2A	-0.527815130628492	6,98549E+14	-1,82534E+14	0.114447384798167	0.255946670774578	-4,4538E+14	6,8187E+13	6,72079E+14	7,29048E+14	7,30464E+14	6,85833E+14	6,92001E+14
TC0500002692.mm.2	Ugt2b36	UDP glucuronosyltransferase 2 family, polypeptide B36	-0.5698813421645	5,00366E+14	-1,82529E+14	0.114456418542226	0.255946670774578	-4,45386E+14	4,81664E+14	4,50687E+14	5,27185E+14	5,19142E+14	4,96802E+14	5,26716E+14
TC0700002430.mm.2	Arhgap35	Rho GTPase activating protein 35	0.609016138257672	8,65907E+13	1,82524E+14	0.11446440635696	0.255946670774578	-4,45392E+14	9,12331E+14	9,09798E+14	8,70217E+14	8,30109E+14	8,59329E+14	8,13658E+14
TC0X00001634.mm.2	Phka2	phosphorylase kinase alpha 2	1,04638E+14	6,29264E+14	1,82487E+14	0.114523396200873	0.255946670774578	-4,45437E+13	7,03586E+14	6,92626E+13	6,45299E+14	5,41637E+14	6,68334E+13	5,24103E+14
TC1200000603.mm.2	Psma3	proteasome (prosome, macropain) subunit, alpha type 3	1,27568E+14	8,07067E+14	1,82486E+14	0.114525181907512	0.255946670774578	-4,45438E+14	8,93331E+14	8,70451E+14	8,48509E+14	6,60137E+14	8,48676E+14	7,21297E+14
TC0800001625.mm.2	Cd209c	CD209c antigen	-1,24775E+14	5,72685E+14	-1,82485E+14	0.114527468847211	0.255946670774578	-4,4544E+13	5,06205E+13	4,68783E+14	5,5395E+14	6,70588E+12	5,25411E+14	7,11172E+14
TC1700001197.mm.2	Rmdn2	regulator of microtubule dynamics 2	0.988232687984042	5,07206E+14	1,82475E+14	0.114543244675399	0.255946670774578	-4,45452E+13	5,71804E+14	6,10542E+14	5,15256E+14	4,69443E+14	5,14753E+14	3,61439E+14
TC1600002158.mm.2	C2cd2	C2 calcium-dependent domain containing 2	-0.733458463027945	6,53955E+14	-1,82475E+14	0.114543943798778	0.255946670774578	-4,45452E+14	6,15333E+13	5,9655E+14	6,43924E+14	7,14651E+13	6,41971E+14	7,113E+14
TC1700002553.mm.2	Strn	striatin, calmodulin binding protein	1,06137E+14	7,76828E+14	1,82466E+14	0.114558889693183	0.255946670774578	-4,45464E+14	8,24577E+14	8,53112E+14	8,21472E+14	6,43943E+14	7,70854E+14	7,47009E+14
TC0500003208.mm.2	Fam216a	family with sequence similarity 216, member A	0.680492606346036	8,51222E+13	1,82444E+13	0.11459471741627	0.255964494916557	-4,4549E+14	8,90694E+14	8,76866E+14	8,52705E+14	7,78758E+14	8,74252E+14	8,34059E+14
TSUnmapped00000036.mm.2	Ache	acetylcholinesterase	1,33759E+14	8,43518E+14	1,82436E+14	0.114606827316458	0.255964494916557	-4,455E+13	9,61484E+14	9,0879E+14	9,09381E+14	6,93375E+13	8,41693E+14	7,46383E+14
TC0300002611.mm.2	Slc22a15	solute carrier family 22 (organic anion/cation transporter), member 15	-0.683895828248605	5,33119E+14	-1,82432E+12	0.114614524348225	0.255964494916557	-4,45505E+14	5,08165E+14	5,11909E+14	5,60705E+14	5,96148E+14	5,50221E+14	4,71567E+14
TC0400002876.mm.2	Ptprd	protein tyrosine phosphatase, receptor type, D	0.622395997671701	7,80886E+14	1,82399E+14	0.114666832182993	0.256027839518546	-4,45545E+14	8,19229E+14	8,26667E+14	7,80596E+14	7,4082E+14	7,85078E+14	7,32926E+14
TC0500000309.mm.2	Tcf23	transcription factor 23	-0.737187507633651	6,12274E+14	-1,82395E+14	0.11467466752345	0.256027839518546	-4,45551E+14	5,75568E+14	5,64088E+14	6,02204E+14	6,84889E+14	6,52702E+14	5,94193E+14
TC1700002820.mm.2	H2-T-ps	histocompatibility 2, T region locus, pseudogene	0.741910057507296	5,82424E+14	1,82352E+14	0.114744622455003	0.256148531673243	-4,45603E+14	5,86575E+14	6,45803E+14	5,82475E+14	5,01522E+14	6,01439E+14	5,76731E+14
TC0900001042.mm.2	Elovl5	ELOVL family member 5, elongation of long chain fatty acids (yeast)	1,70772E+14	8,39737E+13	1,82318E+14	0.114800442895177	0.256214932982947	-4,45645E+14	9,51448E+14	9,35866E+13	9,26694E+14	6,19076E+14	8,45537E+14	7,59802E+14
TC0600001424.mm.2	C1rb	complement component 1, r subcomponent B	-0.621207397915966	5,33226E+14	-1,82314E+14	0.1148061699334	0.256214932982947	-4,45649E+14	4,83118E+14	4,92376E+14	5,27161E+14	5,72575E+14	5,38791E+14	5,85332E+14
TC1400002094.mm.2	Nedd8	neural precursor cell expressed, developmentally down-regulated gene 8	0.824458213078513	8,14429E+13	1,82304E+14	0.114822814687549	0.25621659230861	-4,45662E+14	8,68599E+14	8,77759E+14	8,08326E+14	7,73141E+14	8,39302E+14	7,19449E+14
TC0700004592.mm.2	Tnfrsf22	tumor necrosis factor receptor superfamily, member 22	-0.66631999450837	5,80361E+14	-1,82256E+13	0.114900585012275	0.256324478247498	-4,4572E+14	5,55246E+12	5,34272E+13	6,23319E+14	5,99463E+14	6,22228E+14	5,47638E+14
TC0X00000557.mm.2	C230004F18Rik	RIKEN cDNA C230004F18 gene	0.733729916704188	7,01257E+14	1,82255E+14	0.114902979357145	0.256324478247498	-4,45722E+14	7,16656E+14	7,54676E+13	6,65395E+14	6,59191E+14	7,52565E+14	6,5906E+14
TC1700001909.mm.2	Btnl4	butyrophilin-like 4	-0.741727163763605	5,80197E+14	-1,8223E+14	0.114943947905217	0.25633210730078	-4,45753E+14	5,29115E+14	5,16644E+14	5,67945E+14	6,26159E+14	5,78394E+14	6,62924E+14
TC0X00001024.mm.2	Chic1	cysteine-rich hydrophobic domain 1	0.788936638209057	6,41643E+14	1,82228E+14	0.114946766872742	0.25633210730078	-4,45755E+14	7,06654E+14	6,83007E+14	6,5011E+14	5,81763E+14	6,596E+14	5,68723E+14
TC1800001521.mm.2	Fam210a/Mir7219	family with sequence similarity 210, member A/microRNA 7219	0.690351022748446	6,92994E+14	1,82223E+14	0.114954124455746	0.25633210730078	-4,4576E+14	7,60295E+14	7,07632E+14	6,54934E+14	6,74923E+14	7,14252E+14	6,45925E+13
TC0400001035.mm.2	Dnajc6	DnaJ heat shock protein family (Hsp40) member C6	1,50371E+14	9,07967E+14	1,82201E+14	0.114990502207144	0.256377744854534	-4,45788E+13	1,01901E+14	1,01729E+14	9,64089E+14	7,71471E+14	9,3782E+14	7,38122E+14
TC1700001262.mm.2	Plekhh2	pleckstrin homology domain containing, family H (with MyTH4 domain) member 2	-0.661327422438425	6,2492E+13	-1,82173E+14	0.115036921336836	0.256445754496463	-4,45822E+14	5,5355E+14	6,14464E+13	6,36036E+14	6,64243E+14	6,19251E+14	6,61975E+14
TC1200001900.mm.2	Ccdc175	coiled-coil domain containing 175	-0.532462146515163	5,09474E+14	-1,82149E+14	0.115075430966659	0.256496115529918	-4,45851E+14	4,93901E+13	4,76316E+14	5,3438E+14	5,42329E+14	5,0495E+14	5,0497E+14
TC0700000218.mm.2	Zfp541	zinc finger protein 541	-0.773260845594748	5,92807E+14	-1,8213E+14	0.115107578378021	0.256532283596827	-4,45875E+14	5,26753E+14	5,5042E+14	5,64959E+14	6,66866E+14	6,03794E+14	6,4405E+14
TC1700000064.mm.2	Sytl3	synaptotagmin-like 3	-0.627839787352531	5,64837E+14	-1,82094E+14	0.115165687468412	0.256626292653648	-4,45919E+14	5,01142E+14	5,4686E+13	5,66636E+14	6,06933E+13	5,68253E+14	5,99198E+14
TC1300000464.mm.2	Sycp2l	synaptonemal complex protein 2-like	-0.954984485742764	4,80722E+14	-1,82079E+14	0.115190928956757	0.256637516626847	-4,45938E+12	4,00578E+14	4,5726E+14	4,52474E+14	5,96362E+13	4,74287E+13	5,03368E+14
TC1200001979.mm.2	Vti1b	vesicle transport through interaction with t-SNAREs 1B	1,03272E+14	7,97986E+14	1,82071E+14	0.115202993053122	0.256637516626847	-4,45947E+14	8,68468E+14	8,60173E+14	8,07024E+14	7,15073E+14	8,43149E+14	6,94031E+14
TC0X00003038.mm.2	Morf4l2	mortality factor 4 like 2	1,21876E+14	8,71834E+13	1,82062E+14	0.115218506502737	0.256637516626847	-4,45958E+14	9,51841E+14	9,54116E+14	9,15856E+14	7,46349E+14	8,99022E+14	7,63821E+14
TC1600001746.mm.2	Pcnp	PEST proteolytic signal containing nuclear protein	0.766212388691072	9,3685E+14	1,82043E+12	0.115250158974564	0.256672537879358	-4,45982E+13	1,0089E+14	9,71953E+14	9,50622E+14	8,76988E+14	9,42991E+14	8,69645E+14
TC0100001254.mm.2	Phlda3	pleckstrin homology like domain, family A, member 3	0.534056420199232	5,19831E+14	1,82003E+14	0.115314295592803	0.256779884589114	-4,4603E+14	5,42869E+14	5,4796E+14	4,81682E+13	5,02336E+14	5,21124E+14	5,23012E+14
TC1100002712.mm.2	Olfr1373	olfactory receptor 1373	-0.808568297393133	5,67682E+14	-1,81979E+14	0.115353806078708	0.256832372163358	-4,46059E+14	5,19918E+14	5,38114E+14	5,53996E+14	6,6575E+14	5,42061E+14	5,86254E+14
TC1100000979.mm.2	Wscd1	WSC domain containing 1	0.57221713129928	7,71046E+14	1,81957E+14	0.11539100698221	0.256879703717097	-4,46087E+14	8,03072E+14	8,03025E+14	7,33217E+14	7,58437E+14	7,45119E+14	7,83403E+13
TC1000000653.mm.2	Tmem26	transmembrane protein 26	-0.647206509150141	6,62368E+13	-1,81927E+14	0.115439893511694	0.25690439651809	-4,46124E+14	6,33469E+14	6,28097E+14	6,60354E+14	7,30654E+14	6,73935E+14	6,477E+14
TC1100003829.mm.2	Hdac5/Mir8101	histone deacetylase 5/microRNA 8101	0.975587204380225	6,51112E+14	1,81923E+14	0.115447049745602	0.25690439651809	-4,46129E+14	7,53443E+14	7,38409E+14	7,07543E+14	5,89191E+14	6,14125E+14	5,03963E+13
TC1300001878.mm.2	Gfod1	glucose-fructose oxidoreductase domain containing 1	-0.758712102963405	7,80921E+14	-1,81921E+13	0.115449930812852	0.25690439651809	-4,46131E+14	7,35991E+14	7,27169E+14	7,65655E+14	8,49248E+14	7,69501E+14	8,37964E+14
TC1500000670.mm.2	Pick1	protein interacting with C kinase 1	0.533011353913111	7,68273E+14	1,81901E+14	0.115482662780998	0.256941748880174	-4,46155E+14	8,01586E+14	7,93512E+14	7,50616E+14	7,3788E+14	7,55991E+14	7,70055E+13
TC0100003349.mm.2	4930562F07Rik	RIKEN cDNA 4930562F07 gene	-0.862026936845189	4,8275E+14	-1,81869E+14	0.115534172288513	0.257020864018337	-4,46194E+14	3,8991E+14	4,51364E+14	4,86889E+14	5,2679E+14	4,63969E+14	5,77581E+13
TC0400003871.mm.2	Necap2	NECAP endocytosis associated 2	-0.549959789781016	7,84496E+14	-1,81822E+14	0.115612818794515	0.25716031877913	-4,46252E+14	7,38053E+14	7,38226E+14	7,76437E+14	8,09834E+14	8,25107E+14	8,19321E+14
TC0200005146.mm.2	Elmo2	engulfment and cell motility 2	1,67129E+14	7,11007E+14	1,81794E+14	0.115658305978898	0.257225988162592	-4,46286E+14	7,96321E+14	8,05412E+14	7,83339E+14	4,84136E+14	7,44455E+14	6,52378E+14
TC1200001308.mm.2	Ptprn2	protein tyrosine phosphatase, receptor type, N polypeptide 2	0.614942809505051	8,69027E+14	1,81765E+14	0.115705362486929	0.257295129140475	-4,46321E+14	9,35738E+13	8,98438E+14	8,63146E+13	8,48042E+14	8,59317E+14	8,09481E+14
TC1900000591.mm.2	Gm340	predicted gene 340	0.553993918604626	5,36538E+13	1,81748E+14	0.1157331144602	0.257316779978937	-4,46342E+14	5,8176E+14	5,73777E+14	5,05969E+14	5,3877E+14	5,02546E+14	5,16408E+14
TC0700000289.mm.2	Snrpd2	small nuclear ribonucleoprotein D2	-0.524494677784777	9,71745E+14	-1,8174E+14	0.115747037875463	0.257316779978937	-4,46352E+14	9,32281E+14	9,30882E+14	9,87176E+14	9,80887E+14	9,95997E+14	1,00325E+14
TC0200003560.mm.2	Nr4a2	nuclear receptor subfamily 4, group A, member 2	1,2129E+14	5,38467E+14	1,81724E+14	0.115773485443167	0.257340070484997	-4,46372E+14	6,50774E+14	6,12892E+14	6,11605E+13	4,0948E+14	4,3991E+14	5,06144E+14
TC1400002863.mm.2	Ipo4	importin 4	0.660469900785913	7,24447E+14	1,81712E+14	0.115793672738423	0.257349441218512	-4,46387E+14	7,87308E+14	7,66982E+13	7,49654E+14	6,72541E+14	6,86969E+14	6,83229E+14
TC1100002842.mm.2	Iba57	IBA57 homolog, iron-sulfur cluster assembly	-0.692288270281722	8,42315E+14	-1,81698E+14	0.115816618955088	0.257354240063028	-4,46404E+14	7,92147E+14	7,9559E+14	8,24611E+14	9,01584E+14	8,46977E+14	8,9298E+14
TC0100001353.mm.2	Trmt1l	tRNA methyltransferase 1 like	0.713097433599827	7,22253E+14	1,8169E+14	0.115828779271764	0.257354240063028	-4,46413E+13	7,71639E+14	7,66232E+14	7,39748E+13	6,55504E+14	7,22933E+14	6,77461E+14
TC0200001380.mm.2	Arhgap1	Rho GTPase activating protein 1	0.978780439574995	8,80813E+14	1,81681E+14	0.11584374748198	0.257354240063028	-4,46424E+14	9,34062E+14	9,43241E+14	9,16385E+14	7,65161E+14	8,93664E+14	8,32366E+14
TC0800000921.mm.2	Mgat4d	MGAT4 family, member C	-1,04247E+14	5,0548E+14	-1,81665E+13	0.115870948754588	0.257379183523942	-4,46445E+14	4,52797E+13	4,37072E+14	4,6051E+14	6,37852E+14	5,19304E+14	5,25344E+14
TC1600001277.mm.2	2610318N02Rik	RIKEN cDNA 2610318N02 gene	-0.949007341061748	5,72821E+13	-1,81619E+14	0.115945526390468	0.257423984482646	-4,465E+14	5,44117E+14	4,93592E+14	5,76163E+14	6,51348E+14	5,22032E+13	6,49674E+14
TC0400001467.mm.2	5730409E04Rik	RIKEN cDNA 5730409E04Rik gene	-0.574655047694598	7,43331E+14	-1,81618E+14	0.115948230955971	0.257423984482646	-4,46502E+14	7,11477E+14	7,37089E+14	7,78815E+14	7,84682E+14	7,04163E+13	7,43759E+14
TC0700000896.mm.2	Nav2	neuron navigator 2	0.894593118724441	6,70454E+14	1,81616E+14	0.11595110563633	0.257423984482646	-4,46504E+14	7,15312E+14	7,40903E+14	6,74278E+14	6,03018E+14	7,05546E+14	5,83667E+14
TC1100000763.mm.2	Smcr8	Smith-Magenis syndrome chromosome region, candidate 8 homolog (human)	1,37135E+14	6,45825E+14	1,81614E+14	0.115955022613731	0.257423984482646	-4,46507E+14	7,59134E+14	7,64294E+13	7,03468E+14	5,4569E+13	6,48383E+13	4,5398E+14
TC0X00001566.mm.2	Spin2c	spindlin family, member 2C	-0.55239971600649	7,33447E+14	-1,81563E+14	0.116039072175157	0.25757508884947	-4,46569E+14	6,92032E+14	7,09603E+14	7,65785E+14	7,4633E+14	7,31216E+14	7,55716E+14
TC1700002768.mm.2	9030025P20Rik	RIKEN cDNA 9030025P20 gene	0.943156449139694	6,2025E+14	1,8153E+14	0.116092280379821	0.257634834245852	-4,46609E+14	6,55118E+14	6,96378E+14	6,48751E+14	5,14113E+14	6,36246E+14	5,70897E+14
TC1400000762.mm.2	Il25	interleukin 25	-0.918467227342434	5,79924E+14	-1,81526E+14	0.116099358512218	0.257634834245852	-4,46614E+14	4,80634E+14	5,29261E+14	5,52253E+13	6,41335E+14	5,83241E+14	6,92822E+13
TC1900000101.mm.2	Naaladl1	N-acetylated alpha-linked acidic dipeptidase-like 1	-0.809538533201292	4,62956E+14	-1,81517E+13	0.116113955582038	0.257634834245852	-4,46625E+13	3,97442E+14	3,96235E+14	4,28563E+14	5,27021E+14	4,77945E+14	5,50528E+14
TC0900003102.mm.2	Dhx30	DEAH (Asp-Glu-Ala-His) box polypeptide 30	0.676440248873916	7,48324E+14	1,81501E+14	0.116140208174777	0.257657603513724	-4,46644E+14	8,00336E+14	7,70707E+14	6,84702E+14	7,51054E+14	7,63742E+14	7,19403E+13
TC0X00001564.mm.2	Nbdy	negative regulator of P-body association	1,3343E+14	7,3013E+13	1,81483E+14	0.116170489954083	0.257689304051506	-4,46667E+14	8,29732E+14	8,08662E+14	8,01013E+14	5,70521E+14	7,2194E+14	6,4891E+14
TC0100000658.mm.2	Speg	SPEG complex locus	0.769923247483418	6,39888E+14	1,81466E+14	0.11619835859224	0.257715643771062	-4,46688E+14	6,67516E+12	6,93391E+14	5,56533E+14	6,50389E+14	6,08108E+14	6,63388E+13
TC0100000806.mm.2	Akp3	alkaline phosphatase 3, intestine, not Mn requiring	-1,21098E+13	4,76539E+13	-1,81447E+14	0.116230228614996	0.257750849666037	-4,46711E+14	4,14634E+14	3,7173E+13	4,15431E+14	6,13129E+14	4,8426E+14	5,60052E+14

TC0400002873.mm.2	Dmac1	distal membrane arm assembly complex 1	0.732083791797459	8,91738E+14	1,81418E+14	0.116278454712268	0.257762003788734	-4,46747E+14	9,14871E+14	9,38771E+14	8,88525E+12	8,187E+14	9,29705E+14	8,59856E+14
TC0700004525.mm.2	Hras	Harvey rat sarcoma virus oncogene	0.77229018955506	8,57934E+14	1,81417E+14	0.116280017565082	0.257762003788734	-4,46748E+14	9,00816E+14	8,87702E+14	8,55258E+14	7,78802E+14	9,03353E+14	8,21671E+14
TC0200005290.mm.2	Zbp1	Z-DNA binding protein 1	-0.686773071344181	5,29466E+14	-1,81412E+14	0.116288746978609	0.257762003788734	-4,46754E+14	4,63721E+14	4,76735E+14	4,99484E+14	5,78327E+14	5,60363E+14	5,98165E+14
TC0800000865.mm.2	Prmt9	protein arginine methyltransferase 9	0.719253737693834	6,14721E+14	1,81393E+14	0.116318781978201	0.257762003788734	-4,46777E+14	6,41757E+14	6,63945E+14	6,27382E+14	5,34469E+14	5,99452E+14	6,21322E+13
TC0500002652.mm.2	Pea15b	phosphoprotein enriched in astrocytes 15B	-0.748423861380822	5,4818E+14	-1,81386E+14	0.116331618883381	0.257762003788734	-4,46786E+14	5,12057E+14	4,7326E+14	5,21204E+14	6,13798E+14	5,67062E+14	6,017E+14
TC0300003015.mm.2	Gm5105	predicted gene 5105	-1,0365E+14	4,28011E+14	-1,81374E+13	0.116351278254708	0.257762003788734	-4,46801E+14	3,34233E+14	3,58266E+14	3,83054E+14	5,16745E+14	4,31019E+14	5,44747E+14
TC1500000244.mm.2	Kcns2	K+ voltage-gated channel, subfamily S, 2	0.613556662932906	5,50315E+14	1,8135E+14	0.116391108563744	0.257762003788734	-4,4683E+14	6,15232E+14	6,01129E+14	5,62865E+14	5,30784E+14	5,22754E+14	4,69127E+13
TC0600001463.mm.2	Ano2	anoctamin 2	0.840580750292785	6,97879E+14	1,81346E+14	0.116396722938362	0.257762003788734	-4,46834E+14	7,56839E+14	7,81301E+14	7,49071E+14	6,20952E+14	6,38796E+14	6,40314E+14
TC0600001022.mm.2	Gm5577	predicted gene 5577	-0.595386580701032	4,78221E+14	-1,81343E+14	0.116402097099844	0.257762003788734	-4,46838E+14	4,1757E+14	4,54816E+14	4,74502E+14	5,16961E+14	4,99001E+13	5,06474E+14
TC0X00001749.mm.2	Mycs	myc-like oncogene, s-myc protein	-0.629442503639257	5,60679E+14	-1,81327E+14	0.116428784329496	0.257762003788734	-4,46858E+14	4,90715E+14	4,86093E+14	5,2165E+14	5,81047E+14	6,60526E+13	6,24046E+14
TC0100003374.mm.2	Prdx6	peroxiredoxin 6	-0.548471310854106	8,7769E+14	-1,81323E+14	0.116435313832864	0.257762003788734	-4,46863E+14	8,22709E+14	8,52555E+14	8,9909E+14	8,85868E+14	9,07547E+14	8,98373E+14
TC1100003778.mm.2	Zfp385c	zinc finger protein 385C	-0.789231292278963	7,01687E+14	-1,81292E+14	0.116486191734744	0.257762003788734	-4,469E+14	6,50396E+14	6,27728E+14	6,64825E+14	7,71145E+14	7,25996E+14	7,70031E+14
TC0900000191.mm.2	Atg4d	autophagy related 4D, cysteine peptidase	0.536481496255441	5,97122E+14	1,81284E+14	0.116499678181913	0.257762003788734	-4,4691E+13	6,23425E+14	6,30536E+14	5,61636E+13	5,85028E+14	5,94517E+14	5,87591E+14
TC0400003866.mm.2	Padi3	peptidyl arginine deiminase, type III	-0.768079088233449	5,21014E+14	-1,81282E+14	0.116503005080174	0.257762003788734	-4,46913E+14	4,90247E+14	4,50516E+14	5,32356E+14	5,62023E+14	4,95441E+14	5,95499E+14
TC0800000123.mm.2	Lamp1	lysosomal-associated membrane protein 1	0.949906692836629	9,31942E+14	1,81272E+14	0.116520272945534	0.257762003788734	-4,46926E+14	9,95768E+14	9,92797E+14	9,70696E+14	8,27888E+14	9,35026E+14	8,69477E+14
TC1800001081.mm.2	Rprd1a	regulation of nuclear pre-mRNA domain containing 1A	1,62882E+14	7,6877E+14	1,81257E+14	0.11654475331313	0.257762003788734	-4,46944E+14	9,02699E+14	8,75287E+14	8,13192E+14	6,39031E+14	8,15863E+14	5,66549E+14
TC1400001045.mm.2	Htr2a	5-hydroxytryptamine (serotonin) receptor 2A	0.780084622299093	6,92055E+14	1,81248E+14	0.11655977470165	0.257762003788734	-4,46955E+14	7,15367E+14	7,53351E+14	6,65495E+14	6,47206E+14	7,38911E+14	6,32002E+14
TC1700002017.mm.2	Rpp21	ribonuclease P 21 subunit	-0.596030093456599	7,23875E+14	-1,81248E+14	0.116559941635442	0.257762003788734	-4,46955E+14	6,71046E+14	6,88986E+13	7,45681E+14	7,33557E+14	7,25429E+14	7,78549E+13
TC1000001366.mm.2	Cpm	carboxypeptidase M	0.657420495334726	6,11843E+13	1,81242E+13	0.116568476606849	0.257762003788734	-4,46961E+13	6,58117E+14	6,51179E+14	6,05818E+14	5,71995E+14	5,56225E+14	6,27724E+13
TC0700002660.mm.2	Itpkc	inositol 1,4,5-trisphosphate 3-kinase C	-0.760995613708078	6,18106E+14	-1,8124E+14	0.116571931134119	0.257762003788734	-4,46964E+14	5,50533E+14	5,78226E+14	6,77288E+14	6,0367E+14	6,11152E+14	6,8777E+14
TC1400000792.mm.2	Ltb4r2	leukotriene B4 receptor 2	-0.783756924818043	6,13487E+13	-1,81234E+14	0.116582048728808	0.257762003788734	-4,46971E+14	5,61753E+13	5,88775E+14	6,29191E+14	6,78089E+12	5,60779E+13	6,62336E+13
TC0300002765.mm.2	Ntng1	netrin G1	0.975569420166268	6,95232E+14	1,81231E+14	0.116587195656445	0.257762003788734	-4,46975E+14	8,01193E+14	7,4489E+13	7,27811E+14	6,23158E+14	6,93135E+14	5,81207E+14
TC1900001753.mm.2	Sfxn4	sideroflexin 4	0.916895721535958	6,27376E+14	1,81173E+14	0.116682973933305	0.257938367264006	-4,47046E+13	6,84125E+14	6,59241E+14	6,04964E+14	5,55023E+14	6,99752E+14	5,61152E+14
TC1500000395.mm.2	Col14a1	collagen, type XIV, alpha 1	-0.76096627259532	4,64268E+14	-1,81125E+14	0.116763434951961	0.25805510285399	-4,47105E+14	4,13086E+14	3,88403E+14	4,23731E+14	5,29952E+14	5,22144E+14	5,08292E+14
TC1500002155.mm.2	Endou	endonuclease, polyU-specific	-0.633346704173199	4,96332E+14	-1,81118E+14	0.116775288877108	0.25805510285399	-4,47114E+14	4,81721E+14	4,46946E+14	5,2711E+14	5,28227E+14	4,65775E+14	5,28214E+14
TC0100001201.mm.2	Pik3c2b	phosphatidylinositol-4-phosphate 3-kinase catalytic subunit type 2 beta	-0.666931198798486	6,45401E+14	-1,81113E+14	0.116783827345081	0.25805510285399	-4,4712E+14	6,17502E+14	5,97082E+14	6,54321E+14	6,93649E+13	6,20075E+13	6,89779E+14
TC0200004540.mm.2	Gabpb1	GA repeat binding protein, beta 1	0.698968248591578	6,89166E+14	1,81086E+14	0.116827696747894	0.25811664304111	-4,47152E+14	7,4461E+14	7,06494E+14	6,86333E+14	6,24978E+14	7,15129E+13	6,57454E+14
TC0900001647.mm.2	Zkscan7	zinc finger with KRAB and SCAN domains 7	-0.73823931197778	6,18852E+14	-1,8107E+14	0.116853915644282	0.258139175044738	-4,47172E+14	5,64034E+14	5,69092E+14	6,11372E+14	6,69401E+14	6,06689E+14	6,92527E+14
TC1400001188.mm.2	Scel	sciellin	-0.53099226699324	5,15227E+14	-1,81047E+14	0.116893472177259	0.258140126109677	-4,47201E+14	4,82213E+14	4,73024E+14	5,2341E+14	5,38025E+14	5,31276E+14	5,43414E+14
TC1000003140.mm.2	Coq10a	coenzyme Q10A	0.629934904159995	6,62035E+14	1,81036E+14	0.116911033919792	0.258140126109677	-4,47214E+14	7,13799E+14	6,97211E+14	6,72585E+13	6,12438E+14	6,54377E+14	6,21797E+14
TC0900001406.mm.2	Ccdc71	coiled-coil domain containing 71	0.731762652406469	6,66751E+14	1,81033E+14	0.116915913312108	0.258140126109677	-4,47217E+14	7,34525E+14	7,1716E+14	7,01642E+14	6,03691E+14	6,32928E+13	6,10559E+14
TC1300000662.mm.2	Prr7	proline rich 7 (synaptic)	-0.620576483251355	7,52856E+14	-1,81032E+14	0.11691842861965	0.258140126109677	-4,47219E+14	7,25415E+14	7,02627E+12	7,47384E+13	8,04774E+14	7,55401E+14	7,81533E+14
TC0100002866.mm.2	Pam	peptidylglycine alpha-amidating monooxygenase	1,21158E+14	8,61549E+14	1,81019E+13	0.116938583940704	0.258149253738398	-4,47234E+12	9,50509E+14	9,38498E+14	9,08796E+14	7,37894E+14	8,83218E+14	7,50379E+14
TC0500003184.mm.2	Ptpn11	protein tyrosine phosphatase, non-receptor type 11	0.592244846810155	7,62982E+14	1,81004E+14	0.116963924037617	0.258169823016182	-4,47253E+14	8,3528E+14	8,18412E+14	7,90336E+14	7,44907E+14	7,08648E+14	6,80309E+14
TC1300002090.mm.2	Klhl3	kelch-like 3	0.985108955918445	6,22265E+14	1,80989E+13	0.116989601460107	0.258191131122683	-4,47272E+14	6,57307E+14	6,80903E+14	6,52324E+14	4,88865E+14	6,34322E+14	6,19868E+14
TC0900002599.mm.2	Mindy2	MINDY lysine 48 deubiquitinase 2	0.745141480701763	6,60048E+14	1,80968E+14	0.117024097100193	0.258231892163163	-4,47297E+14	7,33326E+14	7,11169E+14	6,78909E+14	6,16559E+14	6,5142E+14	5,68904E+14
TC1100001911.mm.2	Smim5	small integral membrane protein 5	-0.744748458547334	8,10261E+14	-1,80931E+14	0.117086067529871	0.258314557611061	-4,47343E+14	7,53082E+13	7,63319E+13	7,99921E+14	8,65429E+14	7,98746E+14	8,8107E+14
TC1100000861.mm.2	Rcvrn	recoverin	-0.599643238310494	5,39218E+14	-1,80926E+13	0.117093621845168	0.258314557611061	-4,47348E+14	5,20374E+14	5,08055E+14	5,53394E+14	5,94963E+14	5,09074E+14	5,49447E+14
TC1500002173.mm.2	Olfr283	olfactory receptor 283	-0.786393331060822	6,10398E+14	-1,80914E+14	0.117114701848161	0.258325693481097	-4,47364E+14	5,80403E+14	5,17864E+13	5,80664E+14	6,74881E+14	6,39328E+14	6,69247E+14
TC1300000884.mm.2	Clptm1l	CLPTM1-like	1,32067E+14	8,1608E+14	1,8088E+14	0.117170439319627	0.258379808299934	-4,47405E+14	8,93038E+14	9,05759E+14	8,57648E+14	6,77016E+14	8,60813E+14	7,02207E+14
TC0200004548.mm.2	Itpripl1	inositol 1,4,5-triphosphate receptor interacting protein-like 1	-0.721648534320229	7,48847E+14	-1,80872E+14	0.117183999499475	0.258379808299934	-4,47414E+14	6,81165E+14	7,11618E+14	7,27903E+14	8,0921E+14	7,57524E+13	8,05665E+14
TC0200002321.mm.2	Mapre1	microtubule-associated protein, RP/EB family, member 1	1,20456E+14	7,90709E+14	1,8086E+14	0.11720337490176	0.258379808299934	-4,47429E+14	8,75449E+14	8,82398E+13	8,20921E+14	6,96014E+14	8,22461E+14	6,47011E+14
TC1000002929.mm.2	Lyz2	lysozyme 2	1,33283E+14	5,26098E+14	1,80842E+14	0.117233339945695	0.258379808299934	-4,47451E+14	4,4322E+14	6,85469E+14	4,39289E+14	4,22835E+14	5,74401E+14	5,91376E+14
TC1000003159.mm.2	Rab5b	RAB5B, member RAS oncogene family	0.725938214473399	6,68693E+14	1,80828E+14	0.117257142469814	0.258379808299934	-4,47468E+13	7,30483E+13	6,90646E+14	6,71234E+14	6,04708E+14	6,89548E+14	6,25537E+14
TC1700002004.mm.2	Gm8909	predicted gene 8909	-0.972693894648494	4,87643E+14	-1,80819E+14	0.117272643842204	0.258379808299934	-4,4748E+14	4,23604E+14	4,31331E+14	4,61573E+14	5,87901E+14	4,59291E+14	5,62158E+14
TC0700002423.mm.2	Inafm1	InaF motif containing 1	0.848171412915208	6,53107E+13	1,80802E+14	0.1173007047067	0.258379808299934	-4,475E+14	7,42342E+14	6,79507E+14	6,84493E+14	5,67722E+14	6,17227E+14	6,27349E+13
TC1700000026.mm.2	Arid1b	AT rich interactive domain 1B (SWI-like)	-0.562707328258865	8,19088E+14	-1,80793E+14	0.117315624358271	0.258379808299934	-4,47511E+14	7,84865E+13	7,72977E+14	8,39211E+13	8,31173E+14	8,22931E+14	8,6337E+14
TC0400003890.mm.2	Ddi2/Rsc1a1	DNA-damage inducible protein 2/regulatory solute carrier protein, family 1, member 1	1,95174E+14	7,49227E+14	1,80792E+14	0.117317237679847	0.258379808299934	-4,47512E+13	8,47721E+14	8,31816E+14	8,23644E+13	4,65544E+13	8,15244E+14	7,11396E+14
TC0400003481.mm.2	Bmp8a	bone morphogenetic protein 8a	-0.632556375852451	5,40372E+14	-1,80792E+14	0.117317369064846	0.258379808299934	-4,47512E+14	4,99599E+14	4,97702E+14	5,29483E+14	5,9433E+14	5,45412E+13	5,75707E+13
TC1600000253.mm.2	Hira	histone cell cycle regulator	0.546444983846552	6,87327E+14	1,8079E+14	0.117320022166022	0.258379808299934	-4,47514E+14	7,13928E+14	7,40311E+14	6,79605E+14	6,65345E+14	6,70012E+14	6,54763E+14
TC0900001650.mm.2	Zfp105	zinc finger protein 105	-0.600989789876372	5,44959E+13	-1,80783E+14	0.117331661225757	0.258379808299934	-4,47523E+14	5,07974E+14	5,039E+14	5,39553E+14	5,92519E+14	5,48995E+14	5,76812E+14
TC1100002673.mm.2	Rufy1	RUN and FYVE domain containing 1	0.99380704227648	6,57017E+14	1,80757E+14	0.117375723859052	0.258441519341475	-4,47555E+14	7,15884E+14	7,41773E+14	7,08737E+14	5,50158E+14	6,37484E+14	5,88066E+14
TC0100003487.mm.2	Pbx1	pre B cell leukemia homeobox 1	1,68443E+14	7,81272E+14	1,80731E+14	0.117418871465315	0.258501199060067	-4,47587E+14	8,62914E+14	8,74328E+14	8,43231E+14	5,57126E+14	8,44145E+14	7,0589E+14
TC1100002821.mm.2	Olfr331	olfactory receptor 331	-1,00041E+14	4,83884E+14	-1,8071E+14	0.117454637777723	0.258516045944359	-4,47613E+14	3,84124E+14	4,4963E+14	4,56075E+14	5,7776E+14	4,68613E+14	5,67104E+14
TC0800002992.mm.2	Maf	avian musculoaponeurotic fibrosarcoma oncogene homolog	-0.664448499867226	7,00478E+14	-1,80708E+14	0.11745770324326	0.258516045944359	-4,47615E+14	6,41243E+14	6,78703E+14	6,90354E+14	7,62482E+14	7,08628E+14	7,21459E+14
TC0200000592.mm.2	Tor2a	torsin family 2, member A	-0.53575004109317	7,60681E+14	-1,80661E+14	0.117535255051882	0.258651401891693	-4,47672E+14	7,13893E+14	7,33812E+14	7,69435E+14	7,8542E+14	7,77588E+14	7,83938E+13
TC1200002083.mm.2	Arel1	apoptosis resistant E3 ubiquitin protein ligase 1	2,07426E+14	1,01099E+14	1,80646E+14	0.117560501422825	0.258671631766485	-4,4769E+14	1,17269E+14	1,17049E+14	1,10655E+14	8,21782E+14	1,03672E+14	7,57688E+13
TC1600000647.mm.2	Ccdc191	coiled-coil domain containing 191	0.593560892224882	6,64574E+14	1,80586E+14	0.117660488277818	0.258856287222894	-4,47764E+14	7,03238E+14	6,91412E+14	6,56912E+14	6,19027E+14	6,79052E+14	6,37801E+14
TC1200000878.mm.2	Slirp	SRA stem-loop interacting RNA binding protein	0.57509070339592	7,21397E+14	1,80558E+14	0.117707214139358	0.258923732618085	-4,47798E+13	7,49223E+14	7,46735E+14	6,6691E+14	7,14029E+14	7,32868E+14	7,18616E+14
TC0200004844.mm.2	Vsx1	visual system homeobox 1	-0.585420819337751	6,47341E+14	-1,80543E+14	0.117732288638667	0.258926337880293	-4,47816E+14	5,87124E+13	6,05515E+14	6,61698E+14	6,48026E+14	6,66452E+14	7,15232E+14
TC0500003238.mm.2	Clip1	CAP-GLY domain containing linker protein 1	2,03552E+13	6,48393E+14	1,80539E+14	0.117740537308317	0.258926337880293	-4,47822E+14	7,85977E+13	7,8904E+14	6,93284E+14	4,74629E+14	7,35775E+14	4,11652E+14
TC0800001199.mm.2	Ces2h	carboxylesterase 2H	-0.687658552674153	5,79027E+13	-1,80489E+14	0.117823669062823	0.25907379634406	-4,47883E+14	5,35951E+14	5,58056E+14	5,73264E+14	6,58275E+14	5,83474E+13	5,65142E+14
TC0300002139.mm.2	B3galnt1	UDP-GalNAc:betaGlcNAc beta 1,3-galactosaminyltransferase, polypeptide 1	0.879032478596713	8,016E+14	1,80457E+14	0.117877633372367	0.259157089170276	-4,47922E+14	8,4591E+14	8,37449E+14	8,07609E+14	6,99944E+14	8,54723E+14	7,63967E+14
TC0700002778.mm.2	Ffar1	free fatty acid receptor 1	-0.937893482807628	4,97129E+14	-1,80441E+13	0.117903232108968	0.259178005316754	-4,47941E+14	4,39127E+14	4,37354E+14	4,66353E+13	5,97707E+14	4,79666E+14	5,62566E+14
TC0200004034.mm.2	Olfr1214	olfactory receptor 1214	-0.920987834157	4,72633E+14	-1,80367E+14	0.118027341601765	0.259330825987272	-4,48031E+14	3,72953E+14	4,52903E+14	4,45013E+13	5,65041E+14	4,76938E+14	5,22947E+14
TC1000000292.mm.2	Rsph4a	radial spoke head 4 homolog A (Chlamydomonas)	0.608079617168936	7,28216E+14	1,80364E+14	0.118033187513212	0.259330825987272	-4,48036E+14	7,91804E+14	8,11072E+14	7,49169E+14	7,32091E+14	6,71468E+14	6,13693E+14
TC0800001106.mm.2	Herpud1	homocysteine-inducible, endoplasmic reticulum stress-inducible, ubiquitin-like domain member 1	1,32196E+13	7,92348E+14	1,80361E+14	0.118037892413986	0.259330825987272	-4,48039E+13	8,82934E+14	8,88712E+14	8,48977E+14	6,58276E+13	8,11886E+14	6,63306E+14
TC1300000686.mm.2	Gm3045	predicted gene 3045	-0.807161518624294	6,38496E+14	-1,80356E+13	0.118045739704639	0.259330825987272	-4,48045E+14	5,48975E+14	5,91851E+14	5,95159E+14	7,07099E+14	6,80044E+14	7,07851E+14
TC0200001013.mm.2	Ssb	Sjogren syndrome antigen B	1,74477E+14	8,62125E+14	1,80352E+14	0.118053224639524	0.259330825987272	-4,4805E+14	9,75219E+14	9,65998E+14	9,54788E+14	6,37475E+14	8,67018E+14	7,7225E+14
TC0500001390.mm.2	Gpn3	GPN-loop GTPase 3	0.732546360409505	5,03949E+14	1,80341E+14	0.118072303898403	0.259337381776849	-4,48064E+14	5,24928E+14	5,19672E+14	4,00477E+14	4,97613E+14	5,23617E+14	5,57385E+14
TC0900002119.mm.2	Tecta	tectorin alpha	-0.899578376554667	4,83409E+13	-1,80317E+14	0.11811184396949	0.259388870366687	-4,48093E+14	4,39159E+14	4,2081E+14	4,56802E+14	5,83083E+14	4,6456E+14	5,36038E+14
TC1700002316.mm.2	Sema6b	sema domain, transmembrane domain (TM), and cytoplasmic domain, (semaphorin) 6B	0.533873992909369	8,18689E+14	1,80227E+14	0.118262389120814	0.259574521413639	-4,48202E+14	8,70258E+13	8,53831E+14	8,01093E+14	8,16221E+14	7,78865E+14	7,91864E+14
TC0400001645.mm.2	Rhd	Rh blood group, D antigen	-1,03022E+14	6,14421E+14	-1,80227E+14	0.118263010055184	0.259574521413639	-4,48203E+14	5,42067E+14	5,69389E+14	5,89621E+14	7,27878E+14	5,7506E+14	6,82509E+14
TC0100001548.mm.2	Tmco1	transmembrane and coiled-coil domains 1	1,01187E+14	7,04145E+14	1,8022E+14	0.118274963745541	0.259574521413639	-4,48212E+14	7,83517E+14	7,59848E+14	7,26981E+14	6,14008E+14	7,34685E+14	6,0583E+14
TC1600001155.mm.2	Nagpa	N-acetylglucosamine-1-phosphodiester alpha-N-acetylglucosaminidase	-0.823705557256487	7,17937E+14	-1,80219E+14	0.118277198272201	0.259574521413639	-4,48213E+13	6,47291E+14	6,65455E+14	7,00662E+14	7,76825E+14	7,08397E+14	8,08991E+14
TC0200005013.mm.2	Samhd1	SAM domain and HD domain, 1	0.610584625546977	6,0004E+14	1,80218E+14	0.118278110377165	0.259574521413639	-4,48214E+14	6,36952E+13	6,30012E+14	5,56888E+14	5,87959E+14	5,66234E+14	6,22196E+14
TC0700002764.mm.2	Proser3	proline and serine rich 3	-0.574574332990109	5,48613E+14	-1,80209E+14	0.118293037345023	0.259574521413639	-4,48225E+14	4,99609E+14	5,39619E+14	5,63289E+14	5,90854E+14	5,44253E+13	5,54051E+14
TC0500000487.mm.2	Clrn2	clarin 2	-0.796621169469677	5,16011E+14	-1,80169E+13	0.118359685067621	0.25968540379828	-4,48273E+14	4,4322E+14	4,75696E+14	4,81232E+13	5,97008E+14	5,45357E+14	5,53552E+14
TC0200002295.mm.2	H13	histocompatibility 13	0.84940146996977	8,4866E+13	1,80153E+14	0.118387201577745	0.259710412392403	-4,48293E+14	9,16937E+14	8,93147E+14	8,8229E+13	7,57914E+14	8,42848E+13	7,98827E+14
TC0400002222.mm.2	Fam92a	family with sequence similarity 92, member A	1.39568E+14	8,20192E+14	1,8014E+14	0.118409543256822	0.259724063503562	-4,48309E+14	9,07168E+14	8,84289E+14	8,68275E+13	6,44046E+14	8,71506E+14	7,45872E+14
TC0500000462.mm.2	C1qtnf7	C1q and tumor necrosis factor related protein 7	-0.79799206715136	4,92222E+14	-1,80112E+14	0.11845680694497	0.259792368355017	-4,48344E+14	4,74126E+14	4,28339E+14	4,83313E+14	5,78751E+14	4,65145E+14	5,23657E+14
TC0700001959.mm.2	Ptpre	protein tyrosine phosphatase, receptor type, E	0.549200474524164	7,10239E+14	1,80098E+13	0.118479176869368	0.259806066534584	-4,4836E+14	7,38608E+14	7,63615E+14	7,0946E+14	6,82923E+14	6,81645E+14	6,8518E+14
TC0800002528.mm.2	Smarca5	SWI/SNF related, matrix associated, actin dependent regulator of chromatin, subfamily a, member 5	1,56935E+14	6,93378E+14	1,80082E+14	0.118506979438325	0.259831672294153	-4,4838E+13	8,05994E+14	8,12962E+14	7,07866E+14	5,9722E+14	7,5483E+14	4,81394E+14
TC0700001275.mm.2	Picalm	phosphatidylinositol binding clathrin assembly protein	1,65742E+14	9,02121E+14	1,80069E+14	0.118529293347521	0.25984523859981	-4,48396E+14	1,01053E+14	1,0177E+14	9,69398E+12	7,27346E+14	9,43506E+14	7,44248E+13
TC0200002383.mm.2	Spag4	sperm associated antigen 4	-0.651793513707447	5,25489E+14	-1,80045E+12	0.11856978770989	0.259898651798321	-4,48426E+13	4,80074E+14	4,84641E+14	5,2619E+14	5,68883E+14	5,13427E+14	5,79717E+14
TC0500000381.mm.2	Add1/Mir7036b	adducin 1 (alpha)/microRNA 7036b	1,11722E+14	9,54549E+14	1,79937E+14	0.118751074482269	0.26014693007881	-4,48557E+14	1,02714E+14	1,03407E+14	1,00834E+14	8,29417E+14	9,557E+14	8,72629E+11
TC1400000988.mm.2	Pebp4	phosphatidylethanolamine binding protein 4	-0.701632160878848	5,93065E+14	-1,79926E+14	0.118770401613761	0.26014693007881	-4,48571E+14	5,43414E+14	5,53425E+14	5,75699E+14	6,61466E+13	5,92932E+14	6,31453E+14
TC0200005179.mm.2	Stau1	staufen double-stranded RNA binding protein 1	1,16495E+14	8,61683E+14	1,79912E+14	0.118793312750793	0.26014693007881	-4,48588E+14	9,45788E+14	9,50763E+13	9,09588E+14	7,53973E+14	8,74031E+14	7,35955E+14
TC0400001107.mm.2	Dhcr24	24-dehydrocholesterol reductase	0.749576527765802	7,73561E+14	1,79909E+14	0.118797896492805	0.26014693007881	-4,48591E+14	8,45029E+14	8,29129E+14	8,08968E+13	7,15275E+14	7,47352E+14	6,95611E+14
TC1300000876.mm.2	Irx2	Iroquois homeobox 2	-0.837961879669662	4,54346E+14	-1,79904E+14	0.118807272918654	0.26014693007881	-4,48598E+14	4,08089E+14	3,97204E+14	4,34113E+14	5,38772E+14	4,34537E+14	5,13362E+14
TC1000000170.mm.2	Gm5420	predicted gene 5420	-0.898443376061533	4,81604E+14	-1,799E+14	0.11881428997545	0.26014693007881	-4,48603E+14	4,33988E+14	4,17515E+14	4,47069E+14	5,84122E+14	4,78942E+14	5,27987E+14
TC1400001608.mm.2	Plac9b/Plac9a/Gm9780	placenta specific 9b/placenta specific 9a/predicted gene 9780	-0.567045703332292	8,30352E+14	-1,79897E+14	0.118819379466189	0.26014693007881	-4,48606E+14	7,84939E+14	7,92406E+14	8,29418E+14	8,61337E+14	8,40529E+14	8,73483E+14
TC1000001592.mm.2	Mip	major intrinsic protein of lens fiber	-0.868715841396948	5,68276E+14	-1,79877E+14	0.118852865422714	0.26014693007881	-4,48631E+14	5,05777E+14	5,01943E+14	5,31135E+13	6,50329E+14	5,74325E+13	6,46146E+14
TC0500003417.mm.2	Sh2b2	SH2B adaptor protein 2	-0.590773417139757	6,51952E+14	-1,79864E+14	0.118874771037818	0.26014693007881	-4,48646E+14	5,90273E+12	6,29676E+14	6,4998E+13	6,88123E+14	6,68927E+14	6,84737E+13
TC1800000006.mm.2	Cul2	cullin 2	1,50148E+14	6,56264E+14	1,79842E+14	0.118912234727053	0.26014693007881	-4,48674E+14	7,88395E+14	7,73475E+14	7,20634E+14	5,4094E+13	6,64912E+14	4,49229E+14
TC0700003985.mm.2	Arfip2	ADP-ribosylation factor interacting protein 2	0.609414762836077	8,0549E+14	1,79836E+14	0.118922433143911	0.26014693007881	-4,48681E+14	8,33508E+14	8,43749E+14	7,99108E+14	7,56265E+14	8,23664E+14	7,76642E+14
TC0900000210.mm.2	Prkcsh	protein kinase C substrate 80K-H	0.60172800286116	7,35167E+14	1,79829E+14	0.118932805604952	0.26014693007881	-4,48688E+14	8,16115E+14	7,78748E+14	7,40678E+14	7,33839E+14	6,94995E+14	6,4663E+14
TC0900003066.mm.2	Arih2	ariadne RBR E3 ubiquitin protein ligase 2	0.781090823348776	7,36386E+14	1,79802E+14	0.118979319864186	0.26014693007881	-4,48722E+14	7,76947E+14	7,93113E+14	7,64305E+14	6,49538E+14	7,24643E+14	7,09772E+14
TC0800001616.mm.2	Fcer2a	Fc receptor, IgE, low affinity II, alpha polypeptide	-0.640786427834558	6,54409E+14	-1,79795E+14	0.118990723592545	0.26014693007881	-4,4873E+13	5,83429E+14	6,35187E+14	6,47696E+14	6,99077E+14	6,90528E+14	6,70534E+14
TC0700001198.mm.2	Stard5	StAR-related lipid transfer (START) domain containing 5	0.550185624763299	6,65451E+14	1,79794E+14	0.118992829788724	0.26014693007881	-4,48732E+14	7,21622E+14	6,96803E+14	6,68328E+14	6,4006E+14	6,3132E+14	6,34571E+14
TC0600000483.mm.2	Olfr435	olfactory receptor 435	-0.746978950381122	4,98357E+14	-1,79781E+14	0.119015489295546	0.26014693007881	-4,48748E+14	4,31606E+14	4,76424E+14	4,79963E+14	5,77463E+14	5,08145E+14	5,16542E+14
TC0200002485.mm.2	Gdap1l1	ganglioside-induced differentiation-associated protein 1-like 1	0.522217809949961	8,45551E+13	1,79761E+14	0.119048379099836	0.26014693007881	-4,48772E+14	8,82134E+14	8,73067E+13	8,29828E+14	8,2093E+14	8,38002E+14	8,29344E+14
TC0400002580.mm.2	Shb	src homology 2 domain-containing transforming protein B	-0.894758755057658	7,18113E+14	-1,79756E+13	0.119056108354797	0.26014693007881	-4,48777E+14	6,65498E+14	6,45139E+14	6,88291E+14	8,01296E+13	7,09327E+14	7,99125E+14
TC0400003456.mm.2	Rlf	rearranged L-myc fusion sequence	0.902842469787314	7,3222E+14	1,79755E+14	0.119057930095066	0.26014693007881	-4,48779E+14	8,0461E+14	8,07956E+14	7,82328E+13	6,4967E+13	7,06848E+14	6,41905E+14
TC0600001239.mm.2	Setd5	SET domain containing 5	0.796793117076539	8,08868E+14	1,79753E+14	0.119062154211333	0.26014693007881	-4,48782E+14	8,67668E+14	8,8696E+13	8,20266E+14	7,75004E+14	8,06956E+14	6,96355E+13
TC0100000926.mm.2	Crocc2	ciliary rootlet coiled-coil, rootletin family member 2	-0.749788128865185	5,76582E+14	-1,79751E+14	0.119065660753703	0.26014693007881	-4,48784E+14	5,42716E+14	5,27787E+14	5,73095E+14	6,47365E+14	5,45465E+14	6,23062E+14
TC0300002672.mm.2	Ddx20	DEAD (Asp-Glu-Ala-Asp) box polypeptide 20	1,30847E+14	5,56129E+14	1,7975E+13	0.119066958251525	0.26014693007881	-4,48785E+14	6,25496E+14	6,53981E+13	6,2717E+14	3,90613E+14	5,05947E+14	5,33569E+14
TC0X00000649.mm.2	Mtm1	X-linked myotubular myopathy gene 1	-0.69585399431849	5,62124E+14	-1,79745E+14	0.119075708047417	0.26014693007881	-4,48792E+14	5,01843E+14	4,87665E+14	5,20357E+14	6,08322E+14	6,1387E+14	6,40685E+14
TC1100003868.mm.2	1700023F06Rik	RIKEN cDNA 1700023F06 gene	-0.801866004753895	7,13004E+14	-1,79744E+12	0.119076450090662	0.26014693007881	-4,48792E+14	6,42542E+14	6,46878E+14	6,82513E+14	7,6728E+14	7,25321E+14	8,13492E+14

TC1700000938.mm.2	Treml1	triggering receptor expressed on myeloid cells-like 1	-1,01553E+14	6,462E+14	-1,79738E+14	0.119086685053144	0.26014693007881	-4,488E+14	5,78875E+14	5,83751E+14	6,24949E+14	7,40782E+14	6,09232E+14	7,39609E+14
TC1900001562.mm.2	Scd1	stearoyl-Coenzyme A desaturase 1	0.849711511580747	9,48869E+14	1,79726E+14	0.119108185778008	0.2601569935237	-4,48815E+14	1,01152E+14	1,00904E+13	9,91702E+14	8,58915E+14	9,22106E+14	8,99931E+13
TC0100001821.mm.2	Ptpn14	protein tyrosine phosphatase, non-receptor type 14	-1,0577E+14	5,88931E+14	-1,79717E+14	0.119123583331338	0.2601569935237	-4,48826E+14	5,1702E+14	5,31631E+14	5,72838E+14	6,87354E+14	5,406E+13	6,84141E+14
TC1500001356.mm.2	Stk3	serine/threonine kinase 3	1,04748E+14	6,839E+14	1,79696E+14	0.119157736130879	0.260162329976822	-4,48851E+14	7,42926E+14	7,34239E+14	7,24501E+14	5,43167E+14	6,8491E+14	6,73657E+14
TC0800003173.mm.2	Ttc13	tetratricopeptide repeat domain 13	0.786542462734386	7,66458E+14	1,79696E+14	0.119158319073354	0.260162329976822	-4,48851E+14	8,25537E+14	8,07766E+14	7,93065E+14	6,8293E+14	7,64583E+14	7,24866E+14
TC0400002732.mm.2	Txndc8	thioredoxin domain containing 8	-0.783928907723686	5,02932E+14	-1,7968E+14	0.119184869571822	0.260185043139246	-4,4887E+14	4,72947E+14	4,27361E+14	4,84085E+14	5,73008E+14	4,98958E+13	5,61235E+14
TC0200002363.mm.2	Map1lc3a	microtubule-associated protein 1 light chain 3 alpha	0.54284566458	8,29876E+14	1,79639E+14	0.119254725365013	0.26027640283517	-4,48921E+14	8,79529E+14	8,65949E+14	8,23738E+14	8,13171E+14	8,12846E+14	7,84026E+14
TC0700002832.mm.2	Lsm14a	LSM14A mRNA processing body assembly factor	1,15094E+14	7,92735E+14	1,79637E+14	0.119259025763797	0.26027640283517	-4,48924E+13	8,64735E+14	8,75204E+14	8,33242E+14	6,76509E+14	8,1951E+14	6,87211E+14
TC0400003756.mm.2	Clic4	chloride intracellular channel 4 (mitochondrial)	1,16353E+14	8,14802E+14	1,79616E+14	0.119294152955143	0.260317806956421	-4,48949E+14	9,08207E+14	9,03627E+14	8,48225E+13	7,30905E+14	8,349E+14	6,6295E+14
TC0300000440.mm.2	Tm4sf4	transmembrane 4 superfamily member 4	-0.68398421378302	6,05219E+14	-1,79577E+14	0.119359483736297	0.260405246100758	-4,48996E+14	5,88229E+14	5,57325E+14	5,99888E+14	6,82463E+14	5,93536E+14	6,09873E+14
TC1700001319.mm.2	Foxn2	forkhead box N2	1,26274E+14	6,6916E+14	1,79573E+14	0.119366545503643	0.260405246100758	-4,49001E+14	7,43109E+14	7,26391E+14	6,95534E+14	5,21417E+13	5,89451E+14	7,3906E+14
TC0300002103.mm.2	Ccnl1	cyclin L1	1,12582E+14	6,79756E+14	1,79562E+14	0.119384718995021	0.260409635463216	-4,49014E+14	7,56226E+14	7,3742E+14	6,69869E+14	5,98612E+14	7,5109E+14	5,65321E+14
TC1300001683.mm.2	Hdgfl1	HDGF like 1	-0.728018893857052	5,46469E+14	-1,79546E+14	0.119412206301534	0.26043433678081	-4,49034E+14	5,00908E+14	4,97073E+14	5,44771E+14	5,98814E+14	5,25057E+14	6,12191E+14
TC1100004076.mm.2	Jpt1	Jupiter microtubule associated homolog 1	0.567433079522278	8,80851E+14	1,79527E+14	0.119445065566694	0.260470745902848	-4,49058E+14	9,29317E+14	9,08735E+14	8,47997E+14	8,76569E+14	8,76982E+14	8,45505E+14
TC0X00000152.mm.2	Gm4984	predicted pseudogene 4984	-1,11907E+14	5,44935E+14	-1,79473E+14	0.119536916065843	0.260621395177458	-4,49124E+14	4,61979E+14	4,8549E+14	5,32681E+14	6,38601E+13	4,92342E+14	6,58517E+13
TC0400000659.mm.2	Hsdl2	hydroxysteroid dehydrogenase like 2	1,1336E+14	6,80649E+14	1,79464E+14	0.119550975308974	0.260621395177458	-4,49134E+14	7,80879E+14	7,61802E+14	7,56228E+14	5,59733E+14	6,33384E+14	5,91869E+14
TC0800001925.mm.2	Hgsnat	heparan-alpha-glucosaminide N-acetyltransferase	0.977201381383346	6,69985E+14	1,79449E+14	0.119577675146727	0.260621395177458	-4,49153E+14	7,39618E+14	7,52197E+14	6,82833E+14	6,13541E+14	6,90963E+14	5,40756E+14
TC0700001931.mm.2	Gpr26	G protein-coupled receptor 26	0.554793364724075	6,93198E+14	1,79448E+14	0.11957884779662	0.260621395177458	-4,49154E+14	7,13135E+14	7,19764E+14	6,61006E+14	6,60935E+14	6,81978E+14	7,2237E+14
TC0900002089.mm.2	Olfr979	olfactory receptor 979	-1,05111E+14	5,49087E+14	-1,79413E+11	0.119638115206646	0.260715302992249	-4,49196E+14	4,63891E+14	4,97609E+14	5,37761E+14	6,33961E+14	5,04598E+14	6,567E+14
TC1300000262.mm.2	Hist1h1a	histone cluster 1, H1a	0.58432936399352	7,26374E+14	1,79388E+14	0.119681499590118	0.260728741344723	-4,49228E+14	7,31074E+14	7,84315E+14	7,02092E+14	6,96431E+14	7,28527E+14	7,15808E+14
TC0400004186.mm.2	Nfia	nuclear factor I/A	1,33539E+14	8,74304E+14	1,79379E+14	0.11969557691413	0.260728741344723	-4,49238E+14	9,94066E+14	9,91447E+14	9,35918E+14	7,82517E+14	8,67273E+14	6,74602E+14
TC0200004504.mm.2	Patl2	protein associated with topoisomerase II homolog 2 (yeast)	-0.757127789898298	6,34934E+14	-1,79376E+14	0.119700582658453	0.260728741344723	-4,49241E+14	5,78982E+14	5,8713E+14	6,12201E+14	7,05336E+14	6,33164E+13	6,9279E+14
TC0200003272.mm.2	Ier5l	immediate early response 5-like	-0.581365797912121	6,52365E+14	-1,79371E+14	0.119709006917909	0.260728741344723	-4,49247E+13	6,15291E+14	6,34463E+14	6,94035E+14	6,71992E+14	6,26625E+14	6,71785E+14
TC1700000350.mm.2	Rpl3l	ribosomal protein L3-like	-0.839380598433237	5,82756E+14	-1,79321E+13	0.119795645292202	0.260882177671746	-4,4931E+14	5,39289E+14	5,43936E+14	5,6578E+14	6,8532E+14	5,52598E+13	6,09615E+13
TC0300000882.mm.2	Pi4kb	phosphatidylinositol 4-kinase beta	0.758728753882452	5,73673E+14	1,79287E+14	0.119853357216362	0.260972587138816	-4,49351E+14	6,5141E+14	6,05713E+14	5,9427E+14	5,11107E+14	5,68973E+13	5,10565E+13
TC0300002273.mm.2	Dclk2	doublecortin-like kinase 2	0.899899008031165	7,43041E+14	1,79237E+13	0.119938177002915	0.261023016495013	-4,49412E+14	8,34332E+14	8,04817E+14	7,75525E+14	6,83645E+14	7,35842E+14	6,24085E+14
TC0600001336.mm.2	Fbxl14	F-box and leucine-rich repeat protein 14	0.756049408053007	7,07238E+14	1,79224E+14	0.119959764878213	0.261023016495013	-4,49427E+14	7,58866E+13	7,39385E+14	7,17217E+14	6,29824E+14	7,31673E+14	6,66463E+14
TC0300000878.mm.2	Pogz	pogo transposable element with ZNF domain	1,18988E+14	6,55473E+14	1,79221E+14	0.119964479488876	0.261023016495013	-4,49431E+14	7,70379E+14	7,50377E+14	7,26922E+13	5,55859E+14	6,28701E+14	5,00599E+14
TC0700003413.mm.2	Mphosph10	M-phase phosphoprotein 10 (U3 small nucleolar ribonucleoprotein)	0.617533833119422	6,82933E+14	1,79213E+14	0.11997849692278	0.261023016495013	-4,49441E+14	7,2043E+14	7,13309E+13	6,66154E+14	6,44078E+14	7,08886E+14	6,44737E+14
TC0100002395.mm.2	Tmem237	transmembrane protein 237	0.727064073829422	5,83825E+14	1,792E+14	0.120000459011159	0.261023016495013	-4,49456E+14	5,96165E+14	6,27913E+14	5,7852E+13	5,00145E+13	5,70936E+13	6,29273E+14
TC1000002519.mm.2	Lmnb2	lamin B2	0.582657557683914	5,94181E+14	1,792E+14	0.120000667667544	0.261023016495013	-4,49456E+14	6,15615E+14	6,15944E+14	5,49583E+14	5,65444E+13	5,83139E+14	6,35362E+14
TC0X00000897.mm.2	Apoo/Apoo-ps	apolipoprotein O/apolipoprotein O, pseudogene	1,90734E+14	8,53802E+14	1,79199E+14	0.120002375756852	0.261023016495013	-4,49458E+14	9,46506E+14	9,45257E+14	9,21733E+14	5,88563E+14	9,37968E+14	7,82785E+13
TC0900002791.mm.2	Tbx18	T-box18	-0.777738135660796	4,96007E+14	-1,79194E+14	0.120011441693447	0.261023016495013	-4,49464E+13	4,64627E+13	4,45907E+14	4,75685E+14	5,90397E+14	4,94706E+14	5,04723E+14
TC0100001613.mm.2	Pigm	phosphatidylinositol glycan anchor biosynthesis, class M	-0.719150297143386	6,47817E+14	-1,79181E+14	0.120032614795614	0.261023016495013	-4,49479E+14	6,14334E+14	5,99631E+14	7,15469E+14	6,42326E+14	6,91237E+14	6,23904E+14
TC1200000474.mm.2	Brms1l	breast cancer metastasis-suppressor 1-like	1,57028E+14	6,81641E+14	1,79178E+14	0.120038512519583	0.261023016495013	-4,49484E+14	7,6999E+14	7,83669E+14	7,35272E+14	5,0433E+13	7,41355E+14	5,55232E+14
TC1200001275.mm.2	Btbd6	BTB (POZ) domain containing 6	0.657353969005857	6,4907E+14	1,79157E+14	0.120074656705004	0.261066380176457	-4,49509E+14	7,44897E+14	6,7942E+14	6,26025E+14	6,66821E+14	5,74977E+14	6,02278E+14
TC0800000366.mm.2	Dusp26	dual specificity phosphatase 26 (putative)	0.609206085073723	8,18985E+14	1,79106E+14	0.120161840048478	0.261220686548992	-4,49572E+14	8,83861E+14	8,46556E+14	8,31075E+13	7,775E+14	7,87518E+14	7,87401E+14
TC0700003847.mm.2	Tomt	transmembrane O-methyltransferase	-0.667653751314982	5,66527E+14	-1,79082E+14	0.120202816562219	0.261229721091371	-4,49601E+14	5,02966E+14	5,279E+14	6,16589E+14	5,47808E+14	5,80118E+14	6,2378E+14
TC1200000419.mm.2	Ap4s1	adaptor-related protein complex AP-4, sigma 1	1,06309E+14	6,97781E+14	1,79074E+14	0.12021620214666	0.261229721091371	-4,49611E+14	7,79373E+14	7,70751E+14	7,15832E+14	6,21674E+13	7,29791E+14	5,69262E+14
TC0200005090.mm.2	Oser1	oxidative stress responsive serine rich 1	0.651365914666052	6,65752E+14	1,79073E+14	0.120216947707694	0.261229721091371	-4,49611E+14	7,32666E+13	7,26639E+14	6,76662E+14	6,52369E+14	6,41075E+13	5,65099E+14
TC0800001533.mm.2	Rab4a	RAB4A, member RAS oncogene family	0.589116001766796	7,72038E+13	1,79065E+14	0.120230845380352	0.261229721091371	-4,49621E+14	8,14707E+14	7,96042E+14	7,66766E+14	7,26159E+14	7,82344E+14	7,4621E+14
TC1400000770.mm.2	Ngdn	neuroguidin, EIF4E binding protein	0.578422053689323	5,96957E+14	1,7903E+14	0.120291397891872	0.261324435793494	-4,49664E+14	6,51479E+14	6,33619E+13	5,64601E+14	6,04813E+14	5,78377E+13	5,48856E+13
TC1800000658.mm.2	St8sia3	ST8 alpha-N-acetyl-neuraminide alpha-2,8-sialyltransferase 3	1,63049E+14	9,89815E+14	1,79019E+14	0.120309663398521	0.261324435793494	-4,49677E+14	1,06581E+13	1,08923E+14	1,06035E+14	7,68595E+14	1,02844E+14	9,26455E+14
TC0200001756.mm.2	Rpusd2	RNA pseudouridylate synthase domain containing 2	-0.556497462603345	6,07881E+14	-1,79003E+13	0.120337187366975	0.261324435793494	-4,49697E+14	5,80438E+14	5,64836E+13	6,12295E+14	6,44278E+14	6,34406E+14	6,11031E+14
TC1000000771.mm.2	1700009J07Rik	RIKEN cDNA 1700009J07 gene	-0.884378330289064	6,11988E+13	-1,78992E+14	0.120355193147019	0.261324435793494	-4,4971E+14	5,75116E+13	5,60323E+14	5,8987E+14	7,22445E+14	5,84771E+14	6,39403E+14
TC0X00002847.mm.2	2610002M06Rik	RIKEN cDNA 2610002M06 gene	1,01213E+14	7,61335E+13	1,78992E+13	0.120355528953238	0.261324435793494	-4,4971E+14	8,30406E+14	8,23061E+14	7,94548E+14	6,56492E+14	7,85118E+14	6,78382E+14
TC1100003362.mm.2	Ccl1	chemokine (C-C motif) ligand 1	-0.6310361373836	5,11313E+14	-1,78941E+14	0.120443428928571	0.261480055285108	-4,49773E+14	4,60316E+14	4,79578E+13	5,15415E+14	5,50686E+14	5,00275E+14	5,61608E+14
TC0500000417.mm.2	Ppp2r2c	protein phosphatase 2, regulatory subunit B, gamma	0.908370254641795	1,03435E+14	1,78929E+13	0.120463294763465	0.261487952111507	-4,49787E+13	1,08684E+14	1,09201E+14	1,06454E+14	9,32633E+14	1,05076E+13	9,79326E+14
TC0900002376.mm.2	Lman1l	lectin, mannose-binding 1 like	-0.695586652109824	5,82529E+14	-1,78897E+14	0.120518526844823	0.26157260547557	-4,49826E+14	5,46511E+14	5,26681E+14	5,7979E+14	6,3252E+14	5,67246E+13	6,42428E+14
TC1500001730.mm.2	Ly6c2	lymphocyte antigen 6 complex, locus C2	-0.58575569879003	6,8333E+14	-1,78866E+14	0.120571412831219	0.261652144774335	-4,49864E+14	6,12099E+14	6,59111E+14	6,86401E+14	7,01961E+14	7,33648E+14	7,06759E+14
TC1100004065.mm.2	Ush1g	USH1 protein network component sans	-0.560360398710048	6,55408E+14	-1,78853E+14	0.12059455852144	0.261667131895497	-4,4988E+14	6,1306E+14	6,10453E+14	6,4617E+14	6,89416E+14	6,92376E+14	6,80969E+14
TC0500002756.mm.2	Cxcl10	chemokine (C-X-C motif) ligand 10	0.789928762895602	5,7839E+14	1,78834E+14	0.120626042951121	0.26167923252921	-4,49903E+14	5,68567E+14	6,72905E+14	5,1533E+14	5,68156E+14	5,5856E+14	5,86822E+14
TC1400000392.mm.2	Lrit1	leucine-rich repeat, immunoglobulin-like and transmembrane domains 1	-0.540756106345571	5,74771E+14	-1,7883E+14	0.12063261585223	0.26167923252921	-4,49907E+14	5,37832E+14	5,14879E+14	5,78323E+14	5,82539E+14	6,05306E+14	6,29749E+13
TC0200003879.mm.2	Gm19426	predicted gene, 19426	-0.604495708315538	9,20685E+14	-1,78785E+14	0.120709642057422	0.26181107315537	-4,49962E+14	8,83783E+14	8,70485E+14	9,14187E+14	9,60981E+14	9,26609E+14	9,68066E+14
TC1000003132.mm.2	Spryd4	SPRY domain containing 4	-0.552085447091995	6,529E+14	-1,78732E+14	0.120801321605578	0.261925400086097	-4,50027E+14	6,25733E+14	6,14497E+13	6,67485E+14	6,83162E+14	6,45364E+14	6,81162E+14
TC1400001189.mm.2	Slain1	SLAIN motif family, member 1	1,495E+14	8,07959E+14	1,78719E+14	0.120822815529989	0.261925400086097	-4,50043E+14	9,0781E+14	9,06716E+14	8,92223E+14	6,23304E+14	8,00862E+14	7,16838E+14
TC0400001064.mm.2	Mier1	MEIR1 treanscription regulator	1,48617E+14	5,656E+14	1,78719E+14	0.120824188320085	0.261925400086097	-4,50044E+14	6,76767E+14	6,77719E+14	6,37674E+13	4,19578E+14	5,78613E+14	4,03247E+14
TC1400000542.mm.2	Naa30	N(alpha)-acetyltransferase 30, NatC catalytic subunit	1,9633E+14	6,50438E+14	1,78701E+14	0.120853860994875	0.261925400086097	-4,50065E+13	7,71106E+13	7,69953E+14	7,1408E+14	4,34319E+14	7,35383E+14	4,77787E+14
TC0900002883.mm.2	Rasa2	RAS p21 protein activator 2	0.989475752477843	7,38724E+14	1,787E+14	0.120855537860845	0.261925400086097	-4,50066E+14	7,94529E+14	8,09369E+14	7,55447E+14	6,50556E+14	7,77538E+14	6,44904E+14
TC1000002543.mm.2	Celf5	CUGBP, Elav-like family member 5	0.760427949279016	8,16103E+14	1,78698E+13	0.120859886404775	0.261925400086097	-4,50069E+14	8,70518E+14	8,76668E+14	8,45289E+14	7,49811E+14	8,06824E+14	7,4751E+14
TC1100003487.mm.2	Lpo	lactoperoxidase	-0.813761181507799	5,94821E+14	-1,78667E+14	0.120913370538894	0.262006070399838	-4,50107E+14	5,57575E+13	5,63321E+14	5,86232E+14	6,97416E+14	5,5191E+14	6,1247E+14
TC0900001779.mm.2	Izumo1r	IZUMO1 receptor, JUNO	-0.74699073614307	6,66107E+13	-1,78629E+14	0.120978532460376	0.26209372571476	-4,50153E+14	6,34338E+14	6,17039E+14	6,94567E+14	7,06208E+14	6,18769E+14	7,25721E+14
TC1100001131.mm.2	Nufip2	nuclear fragile X mental retardation protein interacting protein 2	1,84212E+14	8,14071E+14	1,78624E+14	0.120986354612201	0.26209372571476	-4,50159E+14	9,27801E+14	9,43751E+14	8,92513E+14	6,10616E+14	8,62632E+14	6,47115E+14
TC1800000184.mm.2	Mapre2	microtubule-associated protein, RP/EB family, member 2	1,68091E+14	8,8535E+14	1,78565E+14	0.121087174214923	0.262276870295074	-4,5023E+14	9,92368E+14	9,98163E+14	9,53685E+14	7,00665E+14	9,32727E+14	7,34493E+14
TC0300002994.mm.2	Nfkb1	nuclear factor of kappa light polypeptide gene enhancer in B cells 1, p105	0.594136756019164	6,14167E+14	1,78534E+14	0.121140935270265	0.262329914715423	-4,50268E+14	6,27674E+14	6,68505E+14	5,69097E+14	6,08255E+14	6,16043E+14	5,95424E+13
TC1400001665.mm.2	Gnl3/Snord19	guanine nucleotide binding protein-like 3 (nucleolar)/small nucleolar RNA, C/D box 19	1,06931E+14	6,17077E+13	1,78532E+14	0.121144224874168	0.262329914715423	-4,50271E+14	6,79695E+14	7,13432E+14	6,36181E+14	5,43083E+14	6,45216E+13	4,84857E+14
TC0800000148.mm.2	Arhgef10	Rho guanine nucleotide exchange factor (GEF) 10	0.627635862509594	6,55861E+14	1,78519E+14	0.121166355259126	0.262342580259379	-4,50286E+14	6,95679E+14	6,96406E+14	6,68446E+14	5,98112E+14	6,47375E+13	6,29145E+14
TC0200002347.mm.2	Chmp4b	charged multivesicular body protein 4B	0.989882923982663	7,75797E+14	1,78495E+14	0.12120760516948	0.262396633359644	-4,50316E+14	8,39833E+14	8,25194E+14	8,04581E+14	6,6247E+14	8,07522E+14	7,15179E+14
TC0200004103.mm.2	Ptpmt1	protein tyrosine phosphatase, mitochondrial 1	0.736908359967772	6,56053E+14	1,78475E+14	0.121243563039756	0.262439216988123	-4,50341E+12	7,17643E+14	6,86295E+14	6,69842E+14	5,86714E+14	6,67133E+14	6,08693E+14
TC1100002919.mm.2	Slc5a10	solute carrier family 5 (sodium/glucose cotransporter), member 10	-0.990357209776648	4,65683E+14	-1,78454E+14	0.121278622966757	0.262479845784199	-4,50366E+14	4,34546E+14	3,71439E+14	4,59585E+14	5,44471E+14	4,25312E+14	5,58748E+14
TC1000000822.mm.2	Prtn3	proteinase 3	-0.711898587718771	6,58378E+14	-1,78393E+14	0.121383810355334	0.262632202880401	-4,5044E+14	6,00665E+14	6,06182E+14	6,37792E+14	7,11434E+14	6,63892E+14	7,30302E+14
TC5_GL456354_random00000002.mm.2	Gm7978/BC061212	predicted gene 7978/cDNA sequence BC061212	-1,14506E+14	4,99923E+14	-1,7837E+14	0.121424094381852	0.262632202880401	-4,50469E+14	4,09564E+14	4,27719E+14	4,32136E+14	6,34159E+14	5,3036E+14	5,656E+14
TC1900001465.mm.2	Fra10ac1	FRA10AC1 homolog (human)	1,19543E+14	5,69868E+14	1,78331E+14	0.121490122338056	0.262632202880401	-4,50516E+14	6,56742E+14	6,72912E+14	6,1057E+14	4,79998E+14	5,85902E+14	4,13087E+14
TC0800001244.mm.2	Pla2g15	phospholipase A2, group XV	0.592008622370175	7,15628E+14	1,78329E+14	0.121493867252118	0.262632202880401	-4,50518E+14	7,63756E+14	7,63099E+13	7,32641E+14	6,75812E+14	6,8826E+13	6,70196E+14
TC0200004213.mm.2	Trim44	tripartite motif-containing 44	1,08242E+14	7,88606E+14	1,78327E+14	0.121498666283711	0.262632202880401	-4,50522E+14	8,73218E+14	8,58398E+14	8,43616E+14	6,71516E+14	7,84068E+14	7,00821E+14
TC0500003111.mm.2	Tmem233	transmembrane protein 233	-0.646979479644994	6,85437E+14	-1,78316E+14	0.121516998707873	0.262632202880401	-4,50534E+14	6,57288E+14	6,46132E+13	6,91428E+14	7,41387E+14	6,57808E+14	7,18578E+14
TC0400001783.mm.2	Arhgef19	Rho guanine nucleotide exchange factor (GEF) 19	-0.630853762441864	7,56246E+14	-1,78314E+14	0.121520637881733	0.262632202880401	-4,50537E+14	7,30509E+13	6,87978E+14	7,48575E+14	7,96083E+14	7,68361E+14	8,0597E+14
TC1100000493.mm.2	Rack1/Snord96a/Snord95	receptor for activated C kinase 1/small nucleolar RNA, C/D box 96A/small nucleolar RNA, C/D box 95	0.52331036494966	8,4869E+14	1,78299E+13	0.121546426113613	0.262632202880401	-4,50555E+14	8,80808E+14	8,97805E+14	8,42682E+14	8,31269E+14	8,16542E+12	8,23036E+14
TC0X00001667.mm.2	Vegfd	vascular endothelial growth factor D	-0.634800902936571	5,66988E+14	-1,78297E+14	0.121549854046472	0.262632202880401	-4,50558E+14	4,86663E+14	5,59933E+14	5,94829E+14	5,78728E+14	5,94682E+14	5,87094E+14
TC0900000609.mm.2	Ppp2r1b	protein phosphatase 2, regulatory subunit A, beta	0.739994958800663	6,45134E+14	1,78297E+14	0.121550470340937	0.262632202880401	-4,50558E+14	6,9359E+14	7,19658E+14	6,49624E+14	6,15624E+14	6,454E+14	5,46907E+14
TC1500001953.mm.2	Cyp2d13	cytochrome P450, family 2, subfamily d, polypeptide 13	-0.534785731512798	8,31526E+14	-1,78276E+14	0.121586177350089	0.262632202880401	-4,50583E+14	7,88465E+14	8,01501E+14	8,60308E+14	8,36616E+14	8,58619E+14	8,43644E+14
TC0100002793.mm.2	Rab17	RAB17, member RAS oncogene family	-0.560961035097197	7,19057E+14	-1,78275E+14	0.121588113633518	0.262632202880401	-4,50585E+14	6,67944E+14	7,03414E+14	7,2725E+14	7,563E+14	7,3161E+14	7,27823E+14
TC1300002564.mm.2	Mast4	microtubule associated serine/threonine kinase family member 4	0.940977025781827	7,9434E+14	1,78271E+14	0.121593795736839	0.262632202880401	-4,50589E+14	8,54184E+14	8,48696E+14	8,21787E+14	6,92898E+14	8,2053E+14	7,27942E+14
TC0700001460.mm.2	Olfr550	olfactory receptor 550	-0.59917258569387	4,88623E+14	-1,7827E+13	0.121596391912142	0.262632202880401	-4,50591E+14	4,35799E+14	4,50379E+14	4,79402E+14	5,26611E+13	5,00605E+14	5,38942E+14
TC0200000593.mm.2	Ptrh1	peptidyl-tRNA hydrolase 1 homolog	-0.647996033242739	6,80884E+14	-1,78266E+13	0.121603661787477	0.262632202880401	-4,50596E+14	6,34307E+14	6,25592E+14	6,64895E+14	7,24602E+14	6,91804E+14	7,44103E+14
TC0700002697.mm.2	Med29	mediator complex subunit 29	-0.803119497711765	7,42966E+14	-1,78262E+14	0.121609809823786	0.262632202880401	-4,506E+14	7,23516E+14	6,84962E+14	8,43711E+14	7,25392E+14	7,40764E+14	7,39451E+14
TSUnmapped00000002.mm.2	Vsig10l	V-set and immunoglobulin domain containing 10 like	0.59502267381994	5,43359E+14	1,78234E+14	0.121657867986655	0.262700780872282	-4,50634E+14	5,8487E+14	5,79658E+14	5,23114E+14	5,22409E+13	4,94686E+13	5,55419E+14
TC1800000314.mm.2	Paip2	polyadenylate-binding protein-interacting protein 2	1,42759E+14	9,32594E+14	1,78215E+14	0.121691393671335	0.262733023119027	-4,50658E+12	1,02384E+14	1,0039E+14	9,95051E+14	7,47168E+14	9,62971E+14	8,62637E+14
TC0800002658.mm.2	Snx20	sorting nexin 20	-0.897735545964357	6,58106E+14	-1,78188E+14	0.121737057548533	0.262733023119027	-4,5069E+14	6,34926E+14	6,09798E+14	7,01145E+14	7,23126E+14	5,67515E+13	7,12129E+14
TC1000000390.mm.2	9030612E09Rik	RIKEN cDNA 9030612E09 gene	-0.643450782722153	4,89579E+13	-1,78178E+14	0.121755980430137	0.262733023119027	-4,50703E+14	4,2943E+14	4,5556E+13	4,80036E+14	5,33643E+14	4,93915E+14	5,44892E+14
TC1100002861.mm.2	Pld6	phospholipase D family, member 6	-0.639516912320263	7,22869E+14	-1,78171E+14	0.121767431928365	0.262733023119027	-4,50711E+12	6,88581E+14	6,68083E+14	7,1044E+14	7,74127E+14	7,28782E+14	7,67197E+14
TC0400000716.mm.2	Col27a1	collagen, type XXVII, alpha 1	-1,13087E+14	6,41652E+14	-1,7817E+14	0.121768581430076	0.262733023119027	-4,50712E+13	5,68327E+14	5,66193E+14	5,84413E+14	7,76282E+14	6,44267E+14	7,1043E+14
TC0900003335.mm.2	Uba7	ubiquitin-like modifier activating enzyme 7	-0.603879196396703	7,45461E+14	-1,78169E+14	0.121770633442121	0.262733023119027	-4,50713E+14	7,2563E+14	6,84513E+13	7,41077E+13	7,89843E+14	7,70547E+14	7,61156E+14
TC0400001216.mm.2	Tal1	T cell acute lymphocytic leukemia 1	-0.661271262392201	7,32806E+14	-1,78139E+14	0.121822192352801	0.262809075563085	-4,5075E+14	7,01728E+14	6,56725E+14	7,14096E+13	7,76611E+14	7,56726E+14	7,9095E+14
TC0100002772.mm.2	A730008H23Rik/Hjurp/6430706D22Rik	RIKEN cDNA A730008H23 gene/Holliday junction recognition protein/RIKEN cDNA 6430706D22 gene	0.699676733879371	5,68694E+14	1,78128E+14	0.121842050716591	0.262816728674221	-4,50764E+14	6,34016E+14	5,99065E+14	5,90814E+14	5,02331E+14	5,36358E+14	5,49581E+14
TC1700001003.mm.2	Uhrf1	ubiquitin-like, containing PHD and RING finger domains, 1	0.532243519020375	6,30615E+14	1,78106E+14	0.121879074105079	0.262847943767908	-4,5079E+14	6,43468E+14	6,58207E+14	6,06199E+14	5,89028E+14	6,33966E+14	6,5282E+14
TC1800000394.mm.2	Dele1	DAP3 binding cell death enhancer 1	0.707635834226624	7,17437E+14	1,78094E+14	0.121899999160999	0.262847943767908	-4,50805E+14	7,64785E+14	7,38737E+14	7,2844E+14	6,33555E+13	7,2074E+14	7,18365E+14
TC1600001282.mm.2	Ube2l3	ubiquitin-conjugating enzyme E2L 3	0.718448679275447	9,16017E+14	1,78091E+14	0.121905460421869	0.262847943767908	-4,50808E+14	9,63434E+14	9,55885E+14	9,33901E+14	8,41729E+14	9,21556E+13	8,79596E+13
TC1700000470.mm.2	Anks1	ankyrin repeat and SAM domain containing 1	0.584776366012003	6,36192E+14	1,7807E+14	0.121941854773695	0.262857796372325	-4,50834E+14	6,9403E+14	6,48038E+14	6,2898E+13	5,96132E+14	6,28801E+13	6,21173E+14
TC1700000637.mm.2	Ager	advanced glycosylation end product-specific receptor	-0.559380157173532	5,90138E+14	-1,7807E+14	0.121942656729543	0.262857796372325	-4,50835E+14	5,64158E+14	5,31886E+14	5,95048E+14	6,12872E+14	6,05066E+14	6,318E+14
TC1500002269.mm.2	Krt5	keratin 5	-0.597627926758682	5,29366E+13	-1,78015E+14	0.122037505716928	0.262992187966798	-4,50901E+14	4,97623E+14	4,80415E+14	5,28375E+14	5,69189E+14	5,2841E+14	5,72181E+13
TC1700001944.mm.2	Ly6g6f	lymphocyte antigen 6 complex, locus G6F	-0.853317893936881	5,41522E+14	-1,78015E+14	0.122037645964609	0.262992187966798	-4,50901E+14	4,84315E+14	4,74955E+14	5,02897E+14	6,27037E+14	5,50654E+13	6,09276E+14
TC0800001694.mm.2	Irs2	insulin receptor substrate 2	0.562677187309919	8,49804E+14	1,77983E+14	0.122091958051073	0.263066472251062	-4,5094E+14	9,12143E+14	8,7424E+13	8,40748E+13	8,33099E+14	8,05684E+14	8,32909E+14
TC1200001685.mm.2	Dnajb9	DnaJ heat shock protein family (Hsp40) member B9	1.77022E+14	7,62107E+14	1.77967E+14	0.122120356634397	0.263066472251062	-4,50959E+14	8,58031E+14	8,5395E+14	8,22095E+14	5,35841E+14	8,44472E+14	6,58256E+14
TC0200002237.mm.2	Gm14151/Gm14147	predicted gene 14151/predicted gene 14147	-0.650093323803749	6,14366E+14	-1,77961E+14	0.122131444553322	0.263066472251062	-4,50967E+14	5,55642E+14	5,79768E+14	5,97117E+14	6,68311E+14	6,27726E+14	6,5763E+14
TC0500003734.mm.2	1500011B03Rik	RIKEN cDNA 1500011B03 gene	-0.557899911653377	7,69393E+14	-1,77957E+14	0.12213742188979	0.263066472251062	-4,50971E+14	7,24659E+13	7,35344E+14	7,71147E+13	8,00435E+14	7,76178E+14	8,08593E+13
TC0500001646.mm.2	Mblac1	metallo-beta-lactamase domain containing 1	-0.519355044613764	7,14884E+14	-1,77933E+14	0.122179010744166	0.263119955888608	-4,51001E+14	6,67812E+14	6,84414E+13	7,24738E+14	7,31358E+14	7,42723E+14	7,3826E+14
TC1100003090.mm.2	Alox12	arachidonate 12-lipoxygenase	-0.72686315775073	4,87341E+14	-1,77924E+14	0.122194912798017	0.263119955888608	-4,51012E+14	4,45188E+13	4,11965E+14	4,75769E+14	5,26756E+14	4,89925E+14	5,74443E+14
TC1900000813.mm.2	Fam160b1	family with sequence similarity 160, member B1	0.570995468432472	6,99191E+14	1,77884E+14	0.122263645730745	0.263232779751402	-4,5106E+14	7,62134E+14	7,53234E+14	7,16082E+14	6,85086E+14	6,19344E+14	6,59268E+14
TC1000000572.mm.2	Sar1a	secretion associated Ras related GTPase 1A	0.513519750880361	9,22518E+12	1,77874E+14	0.12228167776824	0.263236429377297	-4,51073E+14	9,48709E+14	9,54657E+13	9,01473E+14	8,99189E+14	9,19446E+14	9,11632E+14
TC1700001460.mm.2	Wtap	Wilms tumour 1-associating protein	0.930227659898108	8,22423E+14	1,77848E+14	0.122326957688048	0.263283032796662	-4,51104E+14	8,64385E+14	8,794E+14	8,52125E+14	7,05614E+14	8,43327E+14	7,89689E+13

TC1600001527.mm.2	Umps	uridine monophosphate synthetase	1,02409E+13	6,1969E+14	1,77843E+14	0.122336006116093	0.263283032796662	-4,51111E+14	6,81711E+14	6,96241E+14	6,06569E+14	5,66564E+14	4,9661E+14	6,70444E+14
TC1800001415.mm.2	Slc26a2	solute carrier family 26 (sulfate transporter), member 2	-0.615715944283313	4,98633E+14	-1,77829E+14	0.122359263604126	0.263297918596859	-4,51127E+14	5,14233E+14	4,54714E+14	5,48195E+14	5,43895E+14	4,82984E+14	4,47777E+14
TC1000000836.mm.2	Arhgap45	Rho GTPase activating protein 45	-0.546511500726694	6,6928E+14	-1,77812E+14	0.122389037640309	0.263326821137441	-4,51148E+14	6,16777E+14	6,3341E+14	6,77286E+14	6,82203E+14	7,20542E+14	6,85458E+14
TC1200000016.mm.2	Dnmt3a	DNA methyltransferase 3A	-0.577599095629625	7,19573E+14	-1,77802E+14	0.122407430537206	0.263331232075568	-4,51161E+14	6,82732E+14	6,93542E+14	7,29683E+14	7,62112E+14	7,03552E+13	7,45818E+14
TC0300000033.mm.2	Pkia	protein kinase inhibitor, alpha	1,2414E+14	8,62772E+14	1,7779E+14	0.122427346913252	0.263338918811003	-4,51175E+14	9,41251E+14	9,22072E+14	9,05758E+14	7,09285E+14	9,06399E+13	7,9187E+14
TC1000000869.mm.2	Izumo4	IZUMO family member 4	0.605029251530927	6,34881E+14	1,77759E+14	0.12248198483613	0.263421278919455	-4,51213E+14	6,61585E+14	6,45144E+14	5,8019E+14	6,05532E+14	6,8744E+14	6,29394E+14
TC0200001914.mm.2	Astl	astacin-like metalloendopeptidase (M12 family)	-1,14514E+14	5,87575E+14	-1,77744E+14	0.122507673551541	0.263441364464964	-4,51231E+14	4,76169E+14	5,56187E+14	5,79015E+14	6,82367E+14	5,35728E+14	6,95984E+13
TC1500000355.mm.2	Ebag9	estrogen receptor-binding fragment-associated gene 9	0.998444597228957	6,78094E+14	1,777E+14	0.122583693280763	0.263553683753367	-4,51284E+14	7,41859E+14	7,33478E+14	7,20426E+13	5,55221E+13	6,84393E+14	6,33185E+14
TC0800002767.mm.2	Cdh8	cadherin 8	1,2349E+14	8,53215E+14	1,77688E+14	0.122605522480546	0.263553683753367	-4,513E+14	9,48488E+14	9,4128E+13	8,96861E+14	7,45926E+14	8,78026E+14	7,08705E+13
TC0200004676.mm.2	Gpcpd1	glycerophosphocholine phosphodiesterase 1	0.892945796994114	6,50393E+14	1,7768E+14	0.122619201899116	0.263553683753367	-4,51309E+14	6,79877E+13	6,715E+14	6,5699E+14	5,15798E+14	7,10662E+14	6,67533E+13
TC1100003071.mm.2	Tmem95	transmembrane protein 95	-0.95897292134554	4,65797E+14	-1,77665E+14	0.122644400925672	0.263553683753367	-4,51327E+14	4,1441E+14	4,30306E+14	4,62279E+14	5,74231E+14	3,97477E+13	5,16082E+14
TC1500001676.mm.2	Col22a1	collagen, type XXII, alpha 1	-0.646586899403284	7,24358E+14	-1,77664E+14	0.122646889296003	0.263553683753367	-4,51328E+14	6,58075E+14	6,89604E+13	7,02637E+14	7,74359E+14	7,52387E+13	7,69088E+14
TC0X00001163.mm.2	H2afb1	H2A histone family member B1	-0.556544463226555	7,306E+12	-1,77653E+14	0.12266529560976	0.263553683753367	-4,51341E+14	6,87947E+14	6,87199E+14	7,31745E+14	7,54709E+13	7,41787E+14	7,80212E+14
TC1500000165.mm.2	Retreg1	reticulophagy regulator 1	1,85038E+14	6,84734E+14	1,77638E+14	0.122692849452201	0.263553683753367	-4,51361E+14	8,31343E+14	8,15302E+14	7,60312E+14	5,16257E+14	7,24045E+14	4,61146E+14
TC0200003203.mm.2	Med22	mediator complex subunit 22	0.61996405545351	6,97624E+14	1,7763E+14	0.122706137818362	0.263553683753367	-4,5137E+14	7,22354E+14	7,53327E+14	7,03416E+14	6,48273E+14	6,62992E+14	6,95382E+14
TC1700000352.mm.2	Hs3st6	heparan sulfate (glucosamine) 3-O-sulfotransferase 6	-0.574380339935242	5,21178E+14	-1,77623E+14	0.12271752974119	0.263553683753367	-4,51378E+14	5,0088E+13	5,09804E+14	5,72333E+14	5,53227E+14	5,19837E+14	4,70988E+14
TC0400003670.mm.2	Phactr4	phosphatase and actin regulator 4	-0.532897385430531	6,03894E+14	-1,7762E+14	0.122723471060728	0.263553683753367	-4,51382E+14	5,90379E+14	5,70129E+14	6,45116E+14	6,21972E+14	5,99703E+14	5,96066E+14
TC1000003143.mm.2	Slc39a5	solute carrier family 39 (metal ion transporter), member 5	-0.574404925277822	5,34009E+14	-1,77566E+14	0.122817334241135	0.263709708845433	-4,51448E+14	5,11911E+14	4,97509E+13	5,57363E+14	5,66937E+14	5,60374E+14	5,09959E+13
TC0X00000691.mm.2	Abcd1	ATP-binding cassette, sub-family D (ALD), member 1	0.735240100546955	5,58E+14	1,77557E+14	0.122832559282947	0.263709708845433	-4,51458E+14	5,96899E+14	6,11691E+14	4,83872E+14	5,77671E+14	5,14347E+14	5,6352E+13
TC0300001871.mm.2	Nudt6	nudix (nucleoside diphosphate linked moiety X)-type motif 6	-0.521310143348199	7,45225E+14	-1,7755E+14	0.122845222776257	0.263709708845433	-4,51467E+14	7,07755E+14	7,06907E+14	7,49341E+14	7,69583E+13	7,72729E+14	7,65033E+14
TC0400001517.mm.2	Sync	syncoilin	-0.75201957111768	6,49725E+14	-1,77535E+14	0.122872045645282	0.263732152854993	-4,51486E+13	5,85811E+14	6,08358E+14	6,29286E+14	7,15285E+14	6,46061E+14	7,13546E+14
TC1800001113.mm.2	Slc25a46	solute carrier family 25, member 46	1,81623E+14	8,58793E+14	1,77456E+14	0.123009751251447	0.263928396982793	-4,51582E+14	9,54789E+14	9,67111E+14	9,29296E+14	6,29357E+14	9,28287E+14	7,43916E+14
TC0X00000801.mm.2	Tmem47	transmembrane protein 47	1,75002E+14	1,10161E+14	1,77444E+14	0.123030855341298	0.263928396982793	-4,51596E+14	1,21763E+13	1,20168E+14	1,1876E+14	8,81701E+14	1,13238E+14	9,88683E+14
TC0400001112.mm.2	Fam151a	family with sequence simliarity 151, member A	-0.628496833262804	5,7199E+14	-1,77443E+12	0.123032969677579	0.263928396982793	-4,51598E+14	4,83494E+14	5,4407E+12	5,61827E+14	5,91436E+14	6,09973E+14	6,41138E+14
TC0700004321.mm.2	Sephs2	selenophosphate synthetase 2	0.582709505231344	6,10587E+14	1,7744E+14	0.123036760856029	0.263928396982793	-4,51601E+14	6,45164E+14	6,4486E+14	6,05415E+14	5,68067E+14	6,21557E+14	5,78458E+13
TC0700001447.mm.2	Art1	ADP-ribosyltransferase 1	-0.554144042565359	5,38027E+14	-1,77435E+14	0.123045374426534	0.263928396982793	-4,51607E+12	4,9775E+14	5,36855E+13	5,64976E+13	5,80459E+14	5,17784E+14	5,30338E+14
TC1100000925.mm.2	Tmem256	transmembrane protein 256	0.802408608245567	6,01653E+14	1,77416E+14	0.123078789476333	0.263964932095321	-4,5163E+14	6,66491E+14	6,53489E+14	6,41564E+14	5,17935E+14	5,5752E+14	5,72919E+13
TC1800001227.mm.2	Pcdh1	protocadherin 1	0.834928464901022	9,34707E+14	1,77381E+14	0.123139636818564	0.26406028321234	-4,51672E+14	1,00103E+14	1,00455E+14	9,81141E+14	8,57446E+14	9,07359E+14	8,56721E+14
TC0400001307.mm.2	Elovl1	elongation of very long chain fatty acids (FEN1/Elo2, SUR4/Elo3, yeast)-like 1	1,09657E+14	7,19077E+14	1,77309E+13	0.12326585890808	0.264277826956932	-4,5176E+14	7,68954E+14	7,75539E+14	7,59436E+13	5,65743E+14	7,38318E+14	7,06475E+14
TC0200003745.mm.2	Sp3	trans-acting transcription factor 3	1,17865E+14	7,62727E+14	1,77305E+14	0.123273887383374	0.264277826956932	-4,51766E+14	8,4988E+14	8,60161E+14	8,10802E+14	6,63509E+13	7,74133E+13	6,17874E+14
TC0200001764.mm.2	Zfyve19	zinc finger, FYVE domain containing 19	1,10075E+14	5,4932E+14	1,77295E+14	0.123291494097744	0.264280410322862	-4,51778E+14	6,31452E+14	6,26321E+14	5,61983E+14	4,75641E+14	5,88971E+14	4,11553E+14
TC0200003378.mm.2	Lhx6	LIM homeobox protein 6	0.763697654433539	6,23988E+14	1,77271E+14	0.123331962982015	0.264331992488589	-4,51806E+14	7,14586E+14	6,10952E+14	6,08673E+14	5,64124E+14	6,3428E+14	6,11313E+14
TC1400002666.mm.2	Spry2	sprouty RTK signaling antagonist 2	0.619189461489678	7,2674E+14	1,77204E+14	0.123450851848277	0.264551612692018	-4,51889E+14	7,29451E+14	7,72984E+14	7,16133E+14	6,62464E+14	7,48505E+14	7,30903E+14
TC0900001595.mm.2	Rpsa	ribosomal protein SA	0.546106913903877	9,02146E+14	1,77179E+14	0.123493458570436	0.26459346785891	-4,51918E+14	9,17546E+14	9,49212E+13	8,90584E+14	8,66953E+14	8,9743E+14	8,91153E+14
TC0200000894.mm.2	Dapl1	death associated protein-like 1	-0.561169731479041	5,82328E+14	-1,77174E+14	0.123503225455648	0.26459346785891	-4,51925E+14	5,61581E+14	5,25024E+14	6,08259E+14	5,9058E+14	6,16869E+14	5,91655E+14
TC0600003603.mm.2	Grcc10/Rnu7	gene rich cluster, C10 gene/U7 small nuclear RNA	0.913456339785693	9,92365E+13	1,77154E+14	0.123538414412102	0.264619168081947	-4,51949E+14	1,05108E+14	1,03962E+14	1,00991E+14	8,98109E+14	1,03005E+13	9,25418E+13
TC1000002461.mm.2	Ccdc105	coiled-coil domain containing 105	-0.723409444582933	5,51761E+14	-1,77148E+14	0.123548066870259	0.264619168081947	-4,51956E+14	4,80932E+14	5,27581E+14	5,4651E+14	6,06686E+14	5,38958E+14	6,09899E+14
TC0400003776.mm.2	Pithd1	PITH (C-terminal proteasome-interacting domain of thioredoxin-like) domain containing 1	1,34759E+14	6,58019E+14	1,77113E+14	0.12360939550207	0.264708161600476	-4,51999E+13	7,43614E+14	7,33962E+13	7,18556E+14	4,89503E+14	6,84342E+12	5,78135E+13
TC1300002775.mm.2	Zfp58	zinc finger protein 58	1,07362E+14	5,96094E+14	1,77106E+14	0.123622473533394	0.264708161600476	-4,52008E+14	6,65218E+14	6,23308E+14	6,18333E+13	4,5547E+14	6,52846E+14	5,61389E+14
TC0100001479.mm.2	Vamp4	vesicle-associated membrane protein 4	1,33781E+14	8,01632E+13	1,77056E+14	0.123709760082196	0.264859867685944	-4,52068E+14	8,84568E+14	8,80875E+14	8,44492E+14	6,53388E+14	8,54284E+14	6,92187E+14
TC1300000120.mm.2	Mplkip	M-phase specific PLK1 intereacting protein	1,25232E+14	5,46109E+14	1,7704E+14	0.123738329134501	0.264885837298287	-4,52088E+14	6,36741E+14	6,50584E+14	5,47326E+14	4,89533E+14	5,88267E+14	3,64202E+14
TC1200002271.mm.2	Serpina10	serine (or cysteine) peptidase inhibitor, clade A (alpha-1 antiproteinase, antitrypsin), member 10	-1,09152E+14	4,772E+14	-1,76979E+14	0.1238456894967	0.265080445650948	-4,52162E+14	4,30749E+14	3,63035E+14	4,17751E+14	5,94337E+14	5,03481E+14	5,53848E+14
TC0100001863.mm.2	Kcnh1	potassium voltage-gated channel, subfamily H (eag-related), member 1	0.708206488113549	7,59391E+13	1,76961E+14	0.123876124571502	0.265093668163666	-4,52183E+14	8,06026E+14	8,07408E+14	7,76106E+14	6,95688E+14	7,6519E+13	7,05925E+14
TC0800001447.mm.2	Cox4i1	cytochrome c oxidase subunit 4I1	0.861082548407081	1,00352E+14	1,76956E+14	0.123884771381642	0.265093668163666	-4,52189E+14	1,06789E+14	1,05519E+14	1,02403E+14	9,26822E+14	1,02417E+13	9,23052E+14
TC1100000565.mm.2	Clk4	CDC like kinase 4	2,11638E+14	6,40504E+14	1,76872E+14	0.124033507736727	0.265354868918838	-4,52292E+14	7,73099E+14	7,87457E+14	7,024E+14	4,34879E+14	7,32087E+14	4,131E+14
TC0600001254.mm.2	Fancd2	Fanconi anemia, complementation group D2	-0.564561387057481	4,95455E+14	-1,76868E+14	0.124039773642195	0.265354868918838	-4,52296E+14	4,58339E+14	4,85661E+13	5,07785E+14	5,49128E+13	4,83146E+14	4,88674E+14
TC1500001996.mm.2	5031439G07Rik/Mir1249	RIKEN cDNA 5031439G07 gene/microRNA 1249	0.696761926847707	8,25989E+14	1,76836E+14	0.12409713877742	0.265399961435734	-4,52336E+13	8,83028E+14	8,72639E+14	8,45514E+14	7,70801E+14	8,22221E+14	7,61729E+14
TC0700003572.mm.2	Ap3s2	adaptor-related protein complex 3, sigma 2 subunit	1,02192E+14	8,23852E+14	1,76827E+14	0.124113184585731	0.265399961435734	-4,52347E+14	9,02167E+14	8,82628E+14	8,72971E+14	7,0744E+14	8,22704E+14	7,55204E+14
TC0X00000614.mm.2	Fmr1nb	Fmr1 neighbor	-0.588243443104297	5,29291E+14	-1,76808E+14	0.124145276831786	0.265399961435734	-4,52369E+13	5,17221E+14	4,94655E+14	5,91296E+13	5,38228E+14	5,08616E+14	5,25729E+14
TC0400001504.mm.2	A3galt2	alpha 1,3-galactosyltransferase 2 (isoglobotriaosylceramide synthase)	-0.967490620881503	5,44926E+13	-1,76808E+14	0.124145609859685	0.265399961435734	-4,5237E+14	4,69933E+14	4,8831E+14	5,14205E+14	6,37536E+14	5,24233E+14	6,35338E+14
TC0600000301.mm.2	1700012A03Rik	RIKEN cDNA 1700012A03 gene	-0.921240233327979	5,29389E+14	-1,76804E+14	0.124152954839671	0.265399961435734	-4,52375E+14	4,747E+14	4,8107E+14	4,93604E+14	6,46414E+14	5,55345E+14	5,25204E+14
TC0600002689.mm.2	Podxl2	podocalyxin-like 2	-0.690019335823322	7,16785E+14	-1,768E+14	0.124159679097782	0.265399961435734	-4,52379E+14	7,07056E+14	6,42298E+14	7,09485E+14	7,77873E+14	7,37052E+14	7,26949E+14
TC0100001189.mm.2	Klhdc8a	kelch domain containing 8A	0.720420976515901	6,55152E+14	1,76767E+14	0.124218003973014	0.265478646628228	-4,5242E+14	7,27046E+13	6,79566E+14	6,3862E+14	6,23908E+14	5,85405E+14	6,76367E+14
TC1100000509.mm.2	Olfr1389	olfactory receptor 1389	-0.898969395116	4,91077E+14	-1,76761E+14	0.124229441790904	0.265478646628228	-4,52427E+14	4,26605E+14	4,37128E+14	4,46969E+14	5,96558E+14	5,13255E+14	5,25945E+14
TC1300001298.mm.2	Arl15	ADP-ribosylation factor-like 15	0.853912327099462	6,51676E+13	1,76644E+14	0.124434819172096	0.26588227573531	-4,52569E+13	7,16683E+14	7,05335E+14	6,62743E+13	5,88492E+13	6,77127E+14	5,59675E+14
TC1100002837.mm.2	Hist3h2bb-ps	histone cluster 3, H2bb, pseudogene	-0.849176950451384	7,28701E+13	-1,76616E+14	0.124485000651604	0.265954231702372	-4,52603E+14	6,81736E+14	6,46438E+14	6,82449E+14	8,1556E+14	7,66889E+13	7,79132E+13
TC0900002875.mm.2	Atp1b3	ATPase, Na+/K+ transporting, beta 3 polypeptide	1,84991E+14	8,19969E+14	1,76596E+14	0.12452000713485	0.265962810306874	-4,52628E+14	9,30575E+14	9,45839E+14	9,00219E+14	6,06214E+14	8,73178E+14	6,6379E+14
TC0700001282.mm.2	Crebzf	CREB/ATF bZIP transcription factor	-0.550701461028229	7,81914E+14	-1,76592E+14	0.124526758376478	0.265962810306874	-4,52632E+14	7,46066E+14	7,3007E+14	7,85347E+14	8,00928E+14	7,95888E+14	8,33186E+14
TC1100000940.mm.2	Asgr2	asialoglycoprotein receptor 2	0.543168260400036	4,94942E+14	1,76569E+14	0.124567346270129	0.265962810306874	-4,5266E+13	5,22928E+14	4,92069E+14	4,65276E+14	4,41088E+14	5,22163E+14	5,2613E+14
TC0200001940.mm.2	Zc3h6	zinc finger CCCH type containing 6	1,10613E+14	5,91941E+14	1,76554E+14	0.124594026779926	0.265962810306874	-4,52678E+14	6,85484E+13	6,80756E+13	6,39757E+14	5,05257E+14	5,93939E+14	4,46452E+14
TC0200005463.mm.2	Npdc1	neural proliferation, differentiation and control 1	0.890257967028411	6,9872E+14	1,76551E+13	0.124599413151831	0.265962810306874	-4,52682E+14	7,89496E+14	7,59089E+14	7,27773E+14	6,42761E+14	6,9562E+14	5,77583E+14
TC1700002772.mm.2	Tbc1d22b	TBC1 domain family, member 22B	0.946461912575929	7,89244E+14	1,7655E+14	0.124600964493842	0.265962810306874	-4,52683E+14	8,45386E+14	8,23972E+14	8,20107E+14	6,59959E+14	7,85298E+14	8,00742E+14
TC0200003869.mm.2	Ctnnd1	catenin (cadherin associated protein), delta 1	0.964311951365935	5,53337E+14	1,76548E+14	0.124604558741798	0.265962810306874	-4,52686E+14	6,18217E+14	6,49614E+13	5,8759E+14	4,87379E+14	5,53274E+14	4,23951E+14
TC0200000888.mm.2	Pkp4	plakophilin 4	0.962476079740811	8,30539E+14	1,76518E+14	0.124658114064856	0.266041879725433	-4,52722E+14	8,98954E+13	8,89583E+13	8,75722E+14	7,2032E+14	8,28406E+14	7,70249E+14
TC1000003078.mm.2	Slc16a7	solute carrier family 16 (monocarboxylic acid transporters), member 7	2,33361E+14	6,48726E+14	1,76501E+13	0.124687035395418	0.266068361982039	-4,52742E+14	7,38603E+14	8,01501E+14	7,35865E+14	3,37518E+14	7,52981E+14	5,25887E+14
TC1300001445.mm.2	Mtr	5-methyltetrahydrofolate-homocysteine methyltransferase	0.618226010828688	5,90559E+14	1,76485E+14	0.12471652072317	0.26609501778745	-4,52763E+14	6,18021E+14	6,48131E+14	5,38671E+14	6,03836E+14	5,6903E+14	5,65666E+14
TC1100003472.mm.2	Ppm1e	protein phosphatase 1E (PP2C domain containing)	0.51750101512547	8,59515E+14	1,76476E+14	0.12473255565994	0.26609501778745	-4,52774E+14	8,91627E+14	8,95465E+14	8,32827E+14	8,50765E+14	8,48332E+14	8,38075E+13
TC0800002812.mm.2	Tradd	TNFRSF1A-associated via death domain	-0.629170971821004	6,22106E+14	-1,76434E+14	0.124806077458035	0.266216617167238	-4,52824E+14	5,67911E+14	6,12553E+14	6,43698E+14	6,626E+14	5,93837E+14	6,52037E+14
TC0300002760.mm.2	Fam102b	family with sequence similarity 102, member B	0.899735717916655	7,73241E+14	1,76417E+14	0.124836902072308	0.266247121520993	-4,52845E+14	8,24724E+14	8,22234E+14	8,0856E+14	6,58451E+14	7,71349E+14	7,54132E+13
TC0200003112.mm.2	Pax8	paired box 8	-0.560001297677378	6,21818E+13	-1,76402E+14	0.124862164795468	0.266265757192876	-4,52863E+13	5,84313E+14	5,73393E+14	6,21466E+14	6,48241E+14	6,32335E+14	6,71162E+14
TC1900000980.mm.2	Ehbp1l1	EH domain binding protein 1-like 1	-0.700203387799911	5,36908E+13	-1,76371E+14	0.124918598544451	0.266350850647975	-4,52901E+14	4,57355E+14	5,04049E+14	5,24001E+14	5,77444E+14	5,45326E+13	6,13274E+13
TC0800001875.mm.2	Ap3m2	adaptor-related protein complex 3, mu 2 subunit	0.596512123879757	8,03939E+14	1,76342E+14	0.124968771501121	0.266422574119815	-4,52936E+14	8,57644E+14	8,59485E+14	8,26694E+14	7,71134E+14	7,70216E+14	7,38458E+14
TC0600000871.mm.2	Lrrtm1	leucine rich repeat transmembrane neuronal 1	0.558009644069823	7,09961E+14	1,7631E+14	0.125025619977425	0.266477267495948	-4,52975E+14	7,35596E+14	7,55899E+14	6,73301E+14	7,06592E+13	6,80547E+14	7,07834E+14
TC0600001370.mm.2	Slc6a13	solute carrier family 6 (neurotransmitter transporter, GABA), member 13	-0.508678046290694	6,66276E+14	-1,76309E+14	0.125027502157846	0.266477267495948	-4,52976E+14	6,31917E+14	6,30155E+14	6,8087E+14	6,82938E+14	6,90376E+14	6,81397E+14
TC0400001975.mm.2	Rere	arginine glutamic acid dipeptide (RE) repeats	1,04087E+13	6,82388E+14	1,76289E+14	0.125062717523847	0.266512361148811	-4,53E+14	7,75532E+14	7,36384E+14	7,09511E+14	5,94231E+14	7,08971E+14	5,69701E+14
TC0300001102.mm.2	Chia1	chitinase, acidic 1	-0.744148910477103	4,8269E+14	-1,76281E+14	0.125077048036201	0.266512361148811	-4,5301E+14	4,42197E+14	4,09386E+14	4,47245E+14	5,53167E+13	5,23199E+14	5,20948E+14
TC0800000592.mm.2	Gpm6a	glycoprotein m6a	1,52371E+13	1,13337E+14	1,76269E+14	0.125099174185754	0.266524262019708	-4,53025E+14	1,22186E+14	1,22318E+14	1,19406E+14	9,46236E+14	1,18499E+14	1,02986E+14
TC0600002174.mm.2	Olfr455	olfactory receptor 455	-0.624264468507625	5,4388E+14	-1,76257E+14	0.125119184389563	0.26653103636611	-4,53039E+14	4,83395E+13	4,90422E+14	5,39475E+13	5,59194E+14	5,59768E+14	6,31024E+14
TC0800000259.mm.2	Defb1	defensin beta 1	-0.88330487601877	4,9624E+14	-1,76248E+14	0.125135436610788	0.26653103636611	-4,5305E+14	4,22013E+14	4,45287E+14	4,62964E+14	5,80998E+14	5,75553E+14	4,90627E+14
TC0800000691.mm.2	Naf1	nuclear assembly factor 1 ribonucleoprotein	1,33754E+13	6,64343E+14	1,76226E+14	0.125174433204035	0.266578858342138	-4,53076E+13	7,64284E+14	7,79765E+14	7,38235E+14	5,38306E+14	6,58992E+14	5,06476E+14
TC0400001147.mm.2	Tut4	terminal uridylyl transferase 4	-0.631765719351677	7,98458E+14	-1,76213E+14	0.125198662718156	0.266595222991629	-4,53093E+14	7,46025E+14	7,66244E+14	7,88052E+14	8,50569E+14	7,99949E+14	8,39911E+12
TC0500001302.mm.2	Fbxo21	F-box protein 21	0.510331372768542	7,52691E+14	1,76195E+14	0.125230996483716	0.266596384393414	-4,53115E+14	7,72529E+13	7,80966E+14	7,20714E+14	7,30715E+14	7,5597E+14	7,55249E+14
TC0X00001067.mm.2	Atp7a	ATPase, Cu++ transporting, alpha polypeptide	0.921788732419261	5,37884E+14	1,76194E+14	0.125232298980559	0.266596384393414	-4,53116E+14	5,70983E+13	6,3754E+14	4,67094E+14	5,57071E+14	5,52214E+14	4,42403E+14
TC1400002106.mm.2	Sdr39u1	short chain dehydrogenase/reductase family 39U, member 1	0.577407315005945	7,98076E+14	1,76136E+13	0.125335174989996	0.266754168418701	-4,53186E+14	8,53223E+12	8,13384E+14	7,90092E+14	7,61034E+14	7,71469E+14	7,99257E+14
TC0100002552.mm.2	Mreg	melanoregulin	-0.578164133208461	6,21556E+14	-1,76133E+14	0.125339527654564	0.266754168418701	-4,53189E+14	5,71292E+14	5,91196E+13	6,52136E+14	6,25984E+14	6,21127E+14	6,67604E+14
TC0800000032.mm.2	Snapc2	small nuclear RNA activating complex, polypeptide 2	0.712439912500218	6,76983E+14	1,76117E+14	0.125369006497894	0.266760886812946	-4,53209E+14	7,38889E+14	7,21822E+13	7,04322E+14	6,13901E+14	6,19338E+14	6,63626E+14
TC1200002406.mm.2	Gm266	predicted gene 266	-0.605789028092845	6,80122E+14	-1,76112E+14	0.125377494980986	0.266760886812946	-4,53215E+14	6,47219E+14	6,38294E+14	6,90299E+14	7,16372E+14	7,2526E+14	6,63289E+14
TC0200002328.mm.2	Bpifb6	BPI fold containing family B, member 6	-0.939015821171915	5,51206E+14	-1,76097E+14	0.125403640311969	0.266760886812946	-4,53233E+14	5,00881E+14	4,7813E+13	5,28268E+14	6,38546E+14	5,23098E+14	6,38309E+14
TC1400000965.mm.2	Nefl	neurofilament, light polypeptide	1,14014E+14	8,95525E+14	1,76094E+14	0.125408906945203	0.266760886812946	-4,53237E+14	9,94197E+14	9,38434E+14	9,34463E+14	7,7014E+14	9,25371E+14	8,10542E+14
TC0X00001617.mm.2	Eif1ax	eukaryotic translation initiation factor 1A, X-linked	0.573531181902084	8,06279E+14	1,76077E+14	0.125439097885062	0.266789887040919	-4,53257E+14	8,24978E+14	8,36319E+14	7,97232E+14	7,49359E+14	8,27214E+14	8,02571E+13
TC1000003144.mm.2	Nabp2	nucleic acid binding protein 2	0.66628089936491	9,11606E+14	1,76064E+14	0.125462796722543	0.266804222437623	-4,53273E+14	9,6865E+14	9,62803E+14	9,27214E+14	8,70983E+14	9,03923E+14	8,36064E+13
TC1100004159.mm.2	Lgals3bp	lectin, galactoside-binding, soluble, 3 binding protein	0.631859541639111	6,92528E+14	1,76055E+14	0.125478954734209	0.266804222437623	-4,53284E+14	6,87819E+14	7,46992E+14	6,77885E+14	6,30554E+14	7,13152E+14	6,98768E+14
TC0400001950.mm.2	Lzic	leucine zipper and CTNNBIP1 domain containing	-0.672047302270302	7,01545E+14	-1,76037E+14	0.125511157877981	0.266837483426298	-4,53306E+14	6,63087E+14	6,78251E+14	7,14434E+14	7,61313E+14	6,58347E+14	7,33841E+14
TC1800000209.mm.2	AW554918	expressed sequence AW554918	0.579674744215186	6,63248E+14	1,76001E+14	0.125574680129194	0.266937311467244	-4,5335E+13	7,0336E+14	6,87183E+14	6,46374E+13	6,28234E+14	6,83355E+14	6,30982E+14
TC1500001031.mm.2	Larp4	La ribonucleoprotein domain family, member 4	1,04077E+14	7,78162E+14	1,75955E+14	0.125657798335408	0.267078763300149	-4,53406E+14	8,60172E+13	8,53992E+14	8,15448E+14	6,90563E+14	7,92857E+14	6,55942E+14
TC1000001031.mm.2	Btbd11	BTB (POZ) domain containing 11	0.717662820864701	6,1774E+14	1,75924E+14	0.12571161927303	0.26715791629469	-4,53443E+14	7,23917E+14	6,22551E+14	6,05172E+14	5,97764E+13	5,89187E+14	5,67848E+14
TC1800001234.mm.2	Spry4	sprouty RTK signaling antagonist 4	-0.572329982236704	6,68607E+14	-1,7591E+14	0.125736986659874	0.267173678666208	-4,5346E+14	6,55247E+14	6,01264E+14	6,84205E+14	6,86772E+14	6,87726E+14	6,96428E+12
TC1900001159.mm.2	Olfr262	olfactory receptor 262	-0.855024145588322	5,40714E+14	-1,75894E+14	0.125765602754968	0.267173678666208	-4,5348E+14	4,69875E+14	4,98392E+14	5,15301E+14	6,2397E+14	5,26091E+14	6,10653E+13
TC0200004670.mm.2	Tmem230	transmembrane protein 230	1,08599E+14	7,66121E+14	1,75892E+12	0.125768780033711	0.267173678666208	-4,53482E+14	8,46182E+14	8,22235E+14	8,06267E+14	6,44952E+14	7,92891E+14	6,84198E+14
TC0200004837.mm.2	Zfp442	zinc finger protein 442	0.576389330539761	6,07553E+14	1,75875E+14	0.125799987348158	0.267196437691704	-4,53503E+14	6,64881E+14	6,3603E+14	5,89486E+13	5,96147E+14	6,03466E+14	5,55309E+14
TC0100003241.mm.2	Hmcn1	hemicentin 1	-0.869713136694974	5,07748E+14	-1,75868E+13	0.125812658894493	0.267196437691704	-4,53512E+14	4,90298E+14	4,18175E+14	4,889E+14	5,93516E+14	4,9356E+14	5,62039E+13
TC0700002182.mm.2	Zfp784	zinc finger protein 784	-0.847253253788415	7,25888E+14	-1,75854E+14	0.125837201547479	0.267213340608136	-4,53529E+14	6,69458E+14	6,80028E+14	6,91342E+14	8,27595E+14	7,31621E+14	7,55285E+14
TC0100000353.mm.2	Dnah7b	dynein, axonemal, heavy chain 7B	-0.548934752761569	5,16629E+14	-1,75819E+14	0.125900056398592	0.267311583706748	-4,53571E+14	4,86843E+14	5,12599E+14	5,4213E+14	5,67099E+14	4,82977E+14	5,08125E+14
TC1500002259.mm.2	Krt87/Krt81/Krt83/Krt85	keratin 87/keratin 81/keratin 83/keratin 85	-0.766285658437265	6,27764E+14	-1,75803E+14	0.125929097904696	0.267338017725741	-4,53591E+14	5,73191E+14	5,97969E+14	6,38397E+12	6,86019E+14	5,83218E+14	6,87792E+14
TC1100003781.mm.2	Rab5c	RAB5C, member RAS oncogene family	0.73745916814546	8,55346E+14	1,75779E+14	0.125970823995936	0.267391369654396	-4,53619E+14	8,82291E+14	9,06569E+13	8,69022E+14	7,72347E+14	8,73751E+14	8,28099E+14
TC1900001146.mm.2	Olfr1418	olfactory receptor 1418	-0.97001190880424	4,63041E+14	-1,7576E+14	0.126005981284974	0.267394305097922	-4,53643E+13	3,93071E+14	4,35024E+14	4,68211E+14	5,53886E+14	3,94283E+14	5,33769E+13
TC0100002604.mm.2	Tuba4a	tubulin, alpha 4A	1,02477E+13	8,49477E+14	1,75746E+14	0.126030828140239	0.267394305097922	-4,5366E+14	9,68713E+14	8,73489E+14	8,53826E+14	7,83422E+14	8,77783E+14	7,39629E+13
TC0700004287.mm.2	Il27	interleukin 27	-0.741194865028325	5,26228E+14	-1,75739E+14	0.126043620522631	0.267394305097922	-4,53669E+14	4,8238E+14	4,7366E+14	5,00104E+14	6,04175E+13	5,30132E+14	5,66919E+14
TC0700002449.mm.2	Psg18	pregnancy specific glycoprotein 18	-0.799993512029163	6,38776E+14	-1,75738E+14	0.12604425685238	0.267394305097922	-4,53669E+14	5,67055E+14	6,16339E+14	6,13486E+13	7,29907E+13	6,49355E+14	6,56515E+14
TC1400002368.mm.2	Fndc3a	fibronectin type III domain containing 3A	1,37858E+14	8,07509E+14	1,75732E+14	0.126055181625012	0.267394305097922	-4,53677E+14	8,79989E+14	8,65501E+14	8,62597E+13	6,07177E+14	8,42655E+14	7,87134E+14
TC0600000444.mm.2	Ephb6	Eph receptor B6	0.92032705827906	6,8714E+14	1,75692E+14	0.126127580984668	0.267498911391255	-4,53726E+14	6,85969E+14	7,45511E+14	6,8718E+14	5,60235E+14	6,8657E+14	7,57375E+14

TC0900002106.mm.2	Ubash3b	ubiquitin associated and SH3 domain containing, B	-0.558921066125549	8,80392E+14	-1,75686E+14	0.126137698054413	0.267498911391255	-4,53733E+14	8,44748E+14	8,38747E+14	8,75525E+14	9,19755E+14	9,11875E+14	8,91703E+14
TC1900000322.mm.2	Carnmt1	carnosine N-methyltransferase 1	-0.544083912870863	7,20424E+14	-1,75648E+13	0.126204941996609	0.267606294300745	-4,53778E+14	6,4742E+14	6,87138E+14	7,16293E+12	7,27082E+14	7,74981E+14	7,69632E+14
TC0400001487.mm.2	Smim12	small integral membrane protein 12	-0.538564586478012	6,91634E+13	-1,75626E+14	0.126244618002724	0.267655201299723	-4,53805E+14	6,43773E+14	6,56395E+14	6,88943E+14	7,18937E+14	7,10662E+14	7,31096E+14
TC0800002512.mm.2	Lsm6	LSM6 homolog, U6 small nuclear RNA and mRNA degradation associated	0.554517716729294	7,61346E+14	1,75597E+14	0.126296712796443	0.26771178672539	-4,5384E+14	7,86914E+14	7,79047E+14	7,04382E+14	7,50676E+14	7,74375E+14	7,72682E+14
TC0900001555.mm.2	Azi2	5-azacytidine induced gene 2	0.974242093705877	6,8743E+14	1,75593E+14	0.126304536876213	0.26771178672539	-4,53846E+13	7,9137E+14	7,47853E+13	7,09973E+14	6,34402E+14	6,93581E+14	5,47397E+14
TC1900000903.mm.2	Aip	aryl-hydrocarbon receptor-interacting protein	0.734325052781243	6,45935E+13	1,75564E+14	0.126356380798168	0.267743998198948	-4,53881E+14	7,30883E+14	7,04932E+14	6,88655E+14	6,00295E+14	5,4626E+14	6,04584E+14
TC1600001042.mm.2	Cbr1	carbonyl reductase 1	1,14482E+13	7,31122E+14	1,7556E+14	0.126362648905932	0.267743998198948	-4,53885E+14	8,16686E+14	7,78201E+14	7,65055E+14	6,00868E+14	7,75478E+14	6,50443E+14
TC1000002729.mm.2	Nudt4	nudix (nucleoside diphosphate linked moiety X)-type motif 4	0.834881530960741	8,94656E+14	1,75531E+14	0.12641542928841	0.267743998198948	-4,53921E+14	9,39897E+14	9,59129E+14	9,09798E+14	8,22252E+14	9,19526E+14	8,17331E+14
TC1500002350.mm.2	Cpt1b	carnitine palmitoyltransferase 1b, muscle	-0.953345050955969	5,11264E+14	-1,75527E+14	0.126421794770108	0.267743998198948	-4,53925E+14	4,54278E+14	4,72374E+14	4,86106E+14	6,31214E+14	4,73276E+14	5,50337E+14
TC0900001849.mm.2	Angptl6	angiopoietin-like 6	-0.682063066484079	6,85361E+14	-1,75524E+14	0.12642781272976	0.267743998198948	-4,53929E+13	6,52589E+14	6,35307E+14	6,72239E+14	7,52069E+14	6,76899E+14	7,23064E+14
TC1700001653.mm.2	Telo2	telomere maintenance 2	-0.715677368804458	6,29988E+14	-1,75515E+13	0.126444136844974	0.267743998198948	-4,5394E+14	5,85391E+14	5,89726E+14	6,15245E+14	7,03007E+13	6,15686E+14	6,70871E+14
TC1300000612.mm.2	Syk	spleen tyrosine kinase	-0.592725650258164	6,56674E+14	-1,7551E+14	0.126452383069816	0.267743998198948	-4,53946E+14	6,01688E+14	6,19115E+14	6,51756E+14	6,87592E+14	6,65134E+14	7,1476E+12
TC0900000214.mm.2	Pigyl	phosphatidylinositol glycan anchor biosynthesis, class Y-like	-0.558380039752959	7,90949E+13	-1,7551E+14	0.126452667181406	0.267743998198948	-4,53946E+14	7,52237E+14	7,47709E+13	7,89442E+14	8,22179E+14	7,98983E+14	8,35148E+14
TC1500000710.mm.2	Adsl	adenylosuccinate lyase	0.621749847641443	5,89736E+14	1,75475E+14	0.126515032348537	0.267840850904215	-4,53988E+14	6,47565E+14	6,45999E+14	5,63866E+13	6,05348E+14	5,68665E+14	5,06974E+13
TC1000000123.mm.2	Txlnb	taxilin beta	-0.891838040633068	4,96515E+14	-1,75459E+14	0.126543976997584	0.267841076878513	-4,54008E+14	4,6874E+14	4,34134E+14	4,71039E+14	6,10202E+14	4,74868E+14	5,20107E+14
TC0100001168.mm.2	Eif2d	eukaryotic translation initiation factor 2D	0.56915805627864	6,15593E+14	1,75447E+14	0.126564942466194	0.267841076878513	-4,54022E+13	6,52722E+14	6,92047E+14	5,98444E+14	6,32493E+14	5,66194E+14	5,51656E+14
TC0300001624.mm.2	Tpd52	tumor protein D52	0.638778827047979	7,83214E+14	1,75444E+14	0.126571466131823	0.267841076878513	-4,54026E+14	8,16276E+14	8,29039E+14	7,98365E+14	7,19194E+14	7,68608E+14	7,67803E+14
TC0800002235.mm.2	Neil3	nei like 3 (E. coli)	-0.590303011173362	4,84667E+14	-1,75438E+14	0.12658162976664	0.267841076878513	-4,54033E+14	4,89878E+14	4,51833E+14	5,07615E+14	5,52156E+14	4,64571E+14	4,41948E+14
TC1100003724.mm.2	Krtap4-2	keratin associated protein 4-2	-0.540766716729089	7,75307E+14	-1,75412E+14	0.126628155906292	0.26790434297769	-4,54065E+14	7,43156E+14	7,35417E+14	7,74542E+14	8,12184E+14	7,87603E+14	7,98942E+14
TC0600003038.mm.2	B4galnt3	beta-1,4-N-acetyl-galactosaminyl transferase 3	0.78163789999199	5,12495E+14	1,75402E+14	0.126646412926956	0.267907791977425	-4,54077E+14	5,46013E+14	5,12509E+14	4,97073E+14	4,05122E+14	5,2057E+14	5,93681E+14
TC1100002912.mm.2	Rnf112	ring finger protein 112	0.519335801206264	7,16967E+14	1,75385E+14	0.126677231030119	0.2679231529112	-4,54098E+14	7,7498E+13	7,70041E+14	7,32989E+13	7,08164E+14	6,5997E+14	6,5566E+14
TC1700001665.mm.2	Sox8	SRY (sex determining region Y)-box 8	0.520863437462141	7,96225E+14	1,75378E+14	0.126688814465911	0.2679231529112	-4,54106E+14	8,42398E+11	8,27954E+14	7,94417E+14	7,71762E+14	7,71491E+14	7,69329E+14
TC1700001678.mm.2	Wfikkn1	WAP, FS, Ig, KU, and NTR-containing protein 1	-0.648330835809259	7,51126E+14	-1,75363E+14	0.126717023825621	0.2679231529112	-4,54125E+14	7,03188E+14	7,21122E+14	7,50234E+14	8,03742E+14	7,33507E+14	7,94963E+14
TC0200002366.mm.2	Trp53inp2	transformation related protein 53 inducible nuclear protein 2	-0.655209498532576	9,43773E+14	-1,75356E+14	0.126728900147649	0.2679231529112	-4,54133E+13	8,95114E+14	9,03283E+14	1,00511E+14	9,24325E+14	9,8104E+14	9,5376E+14
TC1000002464.mm.2	2610008E11Rik	RIKEN cDNA 2610008E11 gene	0.603223499725484	5,90706E+14	1,75345E+13	0.126748746962017	0.2679231529112	-4,54146E+14	6,49544E+14	6,285E+14	6,06568E+14	5,50831E+14	5,7063E+14	5,38163E+14
TC0600000227.mm.2	Fam71f2	family with sequence similarity 71, member F2	-0.594562194124488	5,05344E+14	-1,75342E+14	0.126753440988151	0.2679231529112	-4,54149E+14	5,00127E+14	4,44493E+14	5,15378E+14	5,48154E+14	5,12331E+14	5,1158E+14
TC1100001545.mm.2	Cisd3	CDGSH iron sulfur domain 3	0.54776313218199	7,73072E+14	1,75331E+14	0.12677441090702	0.267932329872091	-4,54163E+14	8,08909E+14	8,01271E+14	7,70959E+14	7,29668E+14	7,70373E+14	7,57254E+14
TC0600002550.mm.2	Hk2	hexokinase 2	-0.594632781954112	5,6349E+14	-1,7532E+14	0.126793943604328	0.267938467317578	-4,54177E+14	5,03469E+14	5,05953E+14	5,78727E+14	5,49622E+14	5,92757E+14	6,50412E+14
TC0700004636.mm.2	Tnni3	troponin I, cardiac 3	-0.59084032498799	5,53214E+14	-1,75296E+14	0.12683770974076	0.267940057056647	-4,54206E+14	5,37412E+13	5,02753E+14	5,53351E+14	6,04983E+14	5,54778E+14	5,66009E+14
TC1000000037.mm.2	Gm3213	predicted gene 3213	0.744448213048308	4,93203E+14	1,75292E+14	0.126844248436681	0.267940057056647	-4,54211E+13	5,29676E+14	5,51049E+14	5,08092E+14	4,23743E+14	5,08712E+14	4,37947E+14
TC1100002364.mm.2	Ahsa2	AHA1, activator of heat shock protein ATPase 2	1,45403E+14	6,1873E+14	1,75292E+14	0.126844582338987	0.267940057056647	-4,54211E+14	7,02327E+14	7,10914E+14	6,47714E+14	4,74721E+14	6,97755E+14	4,78951E+14
TC1000002057.mm.2	Armc2	armadillo repeat containing 2	-0.662104739582039	6,26413E+13	-1,75271E+14	0.126881356787775	0.267982606097971	-4,54236E+14	6,07091E+13	5,73382E+14	6,17466E+14	6,95428E+14	6,21142E+14	6,43972E+14
TC0800001303.mm.2	Phlpp2	PH domain and leucine rich repeat protein phosphatase 2	0.583101099026824	6,86074E+14	1,75251E+14	0.126918526069525	0.268001325073547	-4,54261E+14	7,43378E+14	7,1142E+14	6,63032E+14	6,75145E+14	6,36974E+14	6,86494E+14
TC0700000085.mm.2	Olfr1350	olfactory receptor 1350	-0.941437556461271	5,50103E+14	-1,75248E+14	0.126923484865403	0.268001325073547	-4,54264E+13	4,78908E+12	4,89073E+14	5,11034E+14	6,45235E+14	5,424E+13	6,33971E+14
TC0X00000702.mm.2	Tktl1	transketolase-like 1	-0.774697820154217	5,63841E+14	-1,75172E+14	0.127060859589591	0.268238119356429	-4,54356E+13	5,004E+14	5,10838E+14	5,26748E+14	6,39429E+14	5,82014E+14	6,2362E+13
TC1400002634.mm.2	Fbxl3	F-box and leucine-rich repeat protein 3	1,05499E+14	7,98934E+14	1,75167E+14	0.127068923542954	0.268238119356429	-4,54362E+14	8,6581E+14	8,49839E+14	8,43695E+14	6,60955E+14	8,11076E+13	7,62228E+14
TC1100003766.mm.2	Hap1	huntingtin-associated protein 1	0.690835353428335	8,76181E+14	1,75131E+14	0.127134505724521	0.268331931193248	-4,54406E+14	9,78859E+14	8,9715E+14	8,94944E+14	8,42898E+14	8,20034E+14	8,23202E+14
TC0200000833.mm.2	Fmnl2	formin-like 2	1,39069E+14	7,58776E+14	1,75124E+14	0.127146670058986	0.268331931193248	-4,54414E+14	8,18956E+14	8,36336E+14	8,02377E+14	5,74778E+14	8,27902E+14	6,92309E+14
TC1500002012.mm.2	Cerk	ceramide kinase	0.694217399126052	6,86098E+14	1,75107E+14	0.127178120815641	0.26836315619466	-4,54435E+14	7,37222E+14	7,26754E+14	7,09682E+14	6,1545E+14	6,76067E+14	6,51411E+14
TC1400000226.mm.2	E330034G19Rik	RIKEN cDNA E330034G19 gene	-0.657486309976629	5,56871E+14	-1,75087E+14	0.127213152828196	0.268401928966965	-4,54459E+14	4,98964E+14	4,99777E+14	5,68996E+14	5,61243E+14	5,62943E+14	6,49301E+14
TC1100001331.mm.2	Trim37	tripartite motif-containing 37	1,46781E+14	8,19726E+14	1,75033E+14	0.127311416972895	0.268574085059473	-4,54525E+14	9,28921E+13	9,28405E+14	8,69974E+14	6,9379E+14	8,59071E+14	6,38194E+14
TC0500001049.mm.2	Zfp326	zinc finger protein 326	1,96235E+14	5,96803E+14	1,75009E+13	0.127353925611182	0.268581648403667	-4,54553E+14	7,29695E+14	7,34341E+14	6,86779E+14	3,84786E+14	6,44943E+14	4,0027E+13
TC0900000095.mm.2	Mtmr2	myotubularin related protein 2	1,21025E+14	7,18416E+14	1,74991E+14	0.127386074351645	0.268581648403667	-4,54575E+14	8,09099E+14	8,23939E+13	7,71995E+14	6,18994E+14	7,25803E+13	5,60664E+14
TC1700002822.mm.2	H2-T22/H2-T9	histocompatibility 2, T region locus 22/histocompatibility 2, T region locus 9	1,12709E+14	4,82257E+14	1,74989E+14	0.127390688126133	0.268581648403667	-4,54578E+14	4,21868E+13	5,84307E+14	4,27691E+14	3,53066E+14	5,01383E+14	6,05228E+14
TC1700001866.mm.2	Olfr1564	olfactory receptor 1564	-0.613271164298018	4,4445E+14	-1,74988E+14	0.127391825226293	0.268581648403667	-4,54579E+14	3,68412E+14	3,89535E+14	4,06647E+14	4,73954E+14	5,06954E+14	5,21196E+14
TC0300002916.mm.2	Ap1ar	adaptor-related protein complex 1 associated regulatory protein	1,05767E+14	9,10471E+14	1,74985E+14	0.127398345357738	0.268581648403667	-4,54583E+14	9,88091E+14	9,69047E+14	9,54481E+14	7,91123E+13	9,28317E+14	8,31768E+14
TC0900003059.mm.2	Ccdc36	coiled-coil domain containing 36	-0.740117518711192	4,91208E+14	-1,74972E+14	0.127420725538043	0.26859368793753	-4,54598E+14	4,34818E+14	4,54704E+14	5,05899E+13	5,31647E+14	4,56855E+14	5,63323E+14
TC1400000867.mm.2	Kcnrg	potassium channel regulator	-0.823255487346612	5,5666E+14	-1,74946E+14	0.127467177952951	0.2686564602166	-4,54629E+14	5,00152E+14	5,04421E+14	5,35308E+14	6,33915E+14	5,38024E+14	6,2814E+14
TC0700001644.mm.2	Mical2	microtubule associated monooxygenase, calponin and LIM domain containing 2	0.902024086756788	7,95365E+14	1,74927E+14	0.127502057646562	0.268676013919113	-4,54653E+14	8,23078E+14	8,41294E+13	8,18221E+14	6,65746E+14	8,12308E+14	8,11543E+14
TC0200002812.mm.2	Col20a1	collagen, type XX, alpha 1	-0.521608844857538	7,1577E+14	-1,74923E+14	0.127509804408829	0.268676013919113	-4,54658E+14	6,69465E+14	6,69761E+14	7,31795E+14	7,11752E+14	7,45719E+14	7,66129E+14
TC0600002595.mm.2	Exoc6b	exocyst complex component 6B	1,22999E+14	8,04159E+14	1,74796E+14	0.127738707281468	0.269053586264533	-4,54811E+14	8,85942E+14	8,77775E+14	8,62618E+14	6,55101E+14	8,216E+13	7,2192E+14
TC0500003538.mm.2	Actb	actin, beta	0.91835708015274	1,18345E+14	1,74794E+14	0.127742794644428	0.269053586264533	-4,54814E+14	1,23411E+14	1,24155E+14	1,21961E+14	1,07238E+14	1,1946E+14	1,13845E+14
TC0500003138.mm.2	Rnft2	ring finger protein, transmembrane 2	0.838539296538563	8,1003E+14	1,74786E+13	0.127758325815317	0.269053586264533	-4,54825E+14	8,77028E+14	8,444E+14	8,36304E+12	7,17416E+14	8,23279E+14	7,61755E+14
TC1800000160.mm.2	Rnf125	ring finger protein 125	-0.741983732302835	5,54263E+14	-1,74784E+14	0.127761457320929	0.269053586264533	-4,54827E+14	4,94198E+14	5,22042E+14	5,39688E+14	6,24948E+14	5,38753E+14	6,0595E+14
TC0500003097.mm.2	Cox6a1	cytochrome c oxidase subunit 6A1	0.561204842496053	1,09816E+14	1,74778E+14	0.127772484459517	0.269053586264533	-4,54834E+14	1,1346E+14	1,13353E+14	1,09753E+14	1,05836E+13	1,09909E+14	1,06582E+14
TC1400001790.mm.2	Sftpd	surfactant associated protein D	-0.638499462951022	5,42682E+14	-1,74767E+14	0.127791478999329	0.269056222297261	-4,54847E+14	5,09982E+14	4,86161E+14	5,35622E+14	5,88221E+14	5,39704E+14	5,96404E+14
TC1700000694.mm.2	Cdsn	corneodesmosin	-0.650447354387016	5,68191E+13	-1,74756E+14	0.127811339302905	0.269056222297261	-4,5486E+14	5,46135E+13	5,16525E+14	5,64016E+14	6,28734E+13	5,53769E+14	5,99968E+14
TC0200002663.mm.2	Mc3r	melanocortin 3 receptor	0.689266099846442	5,35298E+14	1,74749E+14	0.127823830552072	0.269056222297261	-4,54868E+14	6,27942E+14	5,88423E+14	5,49574E+14	5,28937E+14	5,01477E+14	4,15432E+13
TC1300002036.mm.2	Nop16	NOP16 nucleolar protein	0.541811168434103	7,28658E+14	1,74731E+12	0.127856597768416	0.269070743749711	-4,5489E+14	7,67044E+14	7,66319E+14	7,07097E+13	7,17904E+13	7,26985E+14	6,86598E+14
TC0200002579.mm.2	Ddx27	DEAD (Asp-Glu-Ala-Asp) box polypeptide 27	0.562627156265775	5,17718E+13	1,74727E+14	0.127864127405917	0.269070743749711	-4,54895E+14	5,48203E+14	5,71663E+14	4,86675E+14	5,20665E+14	4,73645E+14	5,05455E+13
TC0100003587.mm.2	Cadm3	cell adhesion molecule 3	0.760592549123484	8,46708E+14	1,74708E+14	0.127898309166511	0.269107528806476	-4,54918E+14	8,91314E+14	8,94724E+14	8,74284E+13	7,59636E+14	8,42419E+14	8,17871E+14
TC0400002796.mm.2	Ambp	alpha 1 microglobulin/bikunin precursor	-0.84685299476079	4,84462E+14	-1,74692E+13	0.127928533486449	0.269135978596051	-4,54938E+13	4,43173E+13	4,46558E+14	4,739E+14	5,85202E+14	4,36523E+14	5,21417E+14
TC1600002011.mm.2	2310061N02Rik	RIKEN cDNA 2310061N02 gene	-0.881969935177086	5,0102E+14	-1,74681E+14	0.127947732823476	0.269141229632464	-4,54951E+14	4,56172E+14	4,33635E+14	4,63518E+13	6,02682E+13	5,49009E+14	5,01106E+14
TC0900001430.mm.2	Pfkfb4	6-phosphofructo-2-kinase/fructose-2,6-biphosphatase 4	-0.641186579056074	6,75801E+14	-1,7465E+14	0.12800425309819	0.269147338091724	-4,54989E+14	6,29066E+14	6,30531E+14	6,5305E+14	7,34784E+14	7,00759E+14	7,06614E+14
TC0100002335.mm.2	Dnah7a	dynein, axonemal, heavy chain 7A	-0.715354790090317	6,37792E+14	-1,74648E+14	0.12800704616708	0.269147338091724	-4,54991E+14	5,86469E+14	6,17724E+14	6,41789E+14	7,05475E+14	5,99156E+14	6,76142E+14
TC0600003347.mm.2	Igbp1b	immunoglobulin (CD79A) binding protein 1b	-0.774054246516477	5,51768E+14	-1,74638E+14	0.128025639820205	0.269147338091724	-4,55003E+14	5,04092E+14	5,22496E+14	5,32081E+14	6,49318E+14	5,39415E+14	5,63205E+14
TC0200002381.mm.2	Ergic3	ERGIC and golgi 3	0.8520374009872	9,14037E+14	1,74635E+14	0.128031977208838	0.269147338091724	-4,55007E+14	9,72824E+14	9,58309E+13	9,40123E+14	8,20603E+14	9,3327E+14	8,59095E+14
TC0900003015.mm.2	Grm2	glutamate receptor, metabotropic 2	0.849543078654414	6,8682E+14	1,74633E+14	0.128034155431829	0.269147338091724	-4,55009E+13	7,25505E+14	7,69378E+13	6,19394E+14	7,0558E+14	7,01685E+14	5,9938E+14
TC1000000036.mm.2	Vip	vasoactive intestinal polypeptide	0.768387576031165	6,69068E+14	1,7462E+14	0.128057698889796	0.269161714350546	-4,55025E+14	6,85403E+14	7,25274E+14	5,92589E+14	6,6441E+14	7,16775E+14	6,29955E+13
TC1100004194.mm.2	Cep131	centrosomal protein 131	-0.539707531609912	7,61639E+14	-1,74593E+14	0.128107135194944	0.269198523354335	-4,55058E+14	7,46082E+14	7,09672E+14	7,76125E+14	7,8757E+14	7,67854E+14	7,82529E+14
TC1500001074.mm.2	Krt88	keratin 88	-0.529760052093301	5,82329E+14	-1,74592E+14	0.128108625152426	0.269198523354335	-4,55059E+14	5,53504E+14	5,49838E+14	6,13504E+14	5,9579E+14	5,75703E+14	6,05637E+14
TC0600001734.mm.2	Med21	mediator complex subunit 21	1,49082E+14	6,59009E+14	1,74571E+14	0.12814694814115	0.269221694689873	-4,55084E+14	7,38728E+14	7,2515E+13	7,20399E+14	4,45314E+14	7,05024E+14	6,1944E+13
TC1700000110.mm.2	Prkn	parkin RBR E3 ubiquitin protein ligase	-0.588397245263319	6,64803E+14	-1,74568E+14	0.128153068843252	0.269221694689873	-4,55088E+14	6,5597E+13	6,00547E+14	6,74594E+14	6,99602E+14	6,87663E+14	6,7044E+14
TC1300000278.mm.2	BC005537	cDNA sequence BC005537	1,25779E+14	1,04812E+14	1,74538E+14	0.128207529578599	0.269300993886059	-4,55125E+14	1,11536E+14	1,14134E+14	1,09687E+14	9,08274E+14	1,08403E+14	9,42849E+14
TC1700000658.mm.2	Vars	valyl-tRNA synthetase	1,10447E+14	5,28288E+14	1,74517E+14	0.128245154558123	0.269329429342339	-4,5515E+14	5,98329E+14	5,97167E+14	4,63093E+14	5,11511E+14	4,01643E+12	5,97983E+14
TC0400001527.mm.2	Tmem234	transmembrane protein 234	0.708929899415632	8,11722E+14	1,74492E+14	0.128291255650383	0.269329429342339	-4,5518E+14	8,67164E+14	8,7247E+13	8,49331E+14	7,48517E+14	7,56177E+14	7,76674E+14
TC1500000614.mm.2	Rpl8	ribosomal protein L8	0.66446875392351	9,36507E+14	1,7449E+14	0.128294822217242	0.269329429342339	-4,55183E+14	9,71185E+14	9,76155E+14	9,37979E+14	8,76467E+13	9,61125E+14	8,9613E+14
TC0900001851.mm.2	Dnmt1	DNA methyltransferase (cytosine-5) 1	0.526475099730964	6,36909E+14	1,74487E+14	0.128300109928027	0.269329429342339	-4,55186E+14	6,78546E+14	6,6867E+14	6,33558E+14	6,08363E+14	6,06687E+14	6,25628E+13
TC0200005189.mm.2	Spata2	spermatogenesis associated 2	0.530120764143274	6,53277E+14	1,74484E+14	0.12830464219151	0.269329429342339	-4,55189E+14	6,84308E+14	6,56239E+14	6,06551E+13	6,27972E+14	6,70943E+14	6,7365E+14
TC1100001937.mm.2	Aanat	arylalkylamine N-acetyltransferase	-1,03097E+14	5,15881E+14	-1,74438E+13	0.128388891933871	0.269444416127574	-4,55245E+14	4,87462E+14	4,56777E+14	4,85607E+14	6,64826E+14	5,17961E+13	4,82652E+14
TC0100002776.mm.2	Arl4c	ADP-ribosylation factor-like 4C	0.570330365476261	6,60556E+14	1,74429E+14	0.128406046567292	0.269444416127574	-4,55257E+14	7,07408E+13	6,76949E+14	6,58006E+14	6,12286E+14	6,59683E+14	6,49002E+13
TC1600001296.mm.2	Med15	mediator complex subunit 15	1,40016E+14	7,86217E+14	1,74427E+14	0.128409586758744	0.269444416127574	-4,55259E+14	8,6641E+14	8,62629E+14	8,53373E+14	5,95635E+14	8,07745E+14	7,31509E+14
TC1400000163.mm.2	Nudt13	nudix (nucleoside diphosphate linked moiety X)-type motif 13	0.549428634634943	6,52247E+14	1,74417E+14	0.128426554686613	0.269444931727264	-4,5527E+14	6,78502E+13	6,60013E+14	6,28145E+13	6,00484E+14	6,57064E+14	6,89273E+14
TC1400001656.mm.2	Cacna1d	calcium channel, voltage-dependent, L type, alpha 1D subunit	0.730798638166465	5,62967E+14	1,74397E+13	0.128464402847969	0.269488856581775	-4,55296E+14	6,09135E+14	6,41414E+14	5,0651E+14	5,97878E+14	4,86052E+14	5,3681E+14
TC0400003836.mm.2	Otud3	OTU domain containing 3	-0.56454680756871	5,8283E+14	-1,74383E+14	0.128489171125866	0.269488856581775	-4,55312E+13	5,46275E+13	5,412E+14	6,08859E+14	5,91526E+14	5,79382E+14	6,29737E+14
TC1100003764.mm.2	Krt42	keratin 42	-0.965072837596984	5,95441E+14	-1,74364E+14	0.128523636007327	0.269488856581775	-4,55335E+14	5,32144E+14	5,14823E+13	5,69611E+13	6,70371E+14	5,75156E+14	7,10541E+13
TC1200000652.mm.2	Hif1a	hypoxia inducible factor 1, alpha subunit	1,37133E+14	7,71708E+14	1,74351E+14	0.128548347943188	0.269488856581775	-4,55351E+14	8,57074E+14	8,46811E+14	8,25506E+14	6,04113E+14	8,18777E+14	6,77968E+14
TC0100001501.mm.2	Ccdc181	coiled-coil domain containing 181	0.576602334514595	6,51176E+14	1,74337E+14	0.128573886911792	0.269488856581775	-4,55368E+13	6,95829E+14	6,75969E+13	6,32586E+14	6,23892E+14	6,6669E+14	6,1209E+14
TC1700002807.mm.2	Arhgdig	Rho GDP dissociation inhibitor (GDI) gamma	0.584353867532863	8,05473E+14	1,74333E+14	0.128581135305545	0.269488856581775	-4,55373E+14	8,49754E+13	8,17591E+14	7,79465E+14	7,71009E+13	8,33619E+14	7,81402E+14
TC0300001103.mm.2	Dennd2d	DENN/MADD domain containing 2D	-0.682009972215196	5,5784E+14	-1,74329E+14	0.128586911840763	0.269488856581775	-4,55377E+14	5,23015E+14	5,11709E+14	5,39131E+14	6,31996E+14	5,61719E+14	5,79468E+14
TC1400000158.mm.2	Nid2	nidogen 2	-0.566266643767713	5,68296E+14	-1,74317E+13	0.12860976867111	0.269488856581775	-4,55392E+13	5,17117E+14	5,42952E+14	5,62751E+12	6,10571E+13	5,88377E+14	5,88006E+14
TC0700001862.mm.2	Orai3	ORAI calcium release-activated calcium modulator 3	-0.59259183996245	7,47954E+14	-1,74316E+14	0.12861105956891	0.269488856581775	-4,55393E+14	6,9674E+14	7,17695E+14	7,94896E+14	7,38057E+14	7,58169E+13	7,82167E+14
TC0400002136.mm.2	Sdr16c5	short chain dehydrogenase/reductase family 16C, member 5	-0.741664832513096	5,85695E+14	-1,74311E+14	0.128620890098282	0.269488856581775	-4,554E+14	5,32072E+14	5,5461E+13	6,0923E+14	6,25784E+14	5,41731E+14	6,50745E+14
TC0900003138.mm.2	Arpp21	cyclic AMP-regulated phosphoprotein, 21	0.897052274078876	1,05886E+14	1,74305E+14	0.128631465026403	0.269488856581775	-4,55407E+14	1,12736E+13	1,12372E+14	1,09649E+14	9,75172E+14	1,06188E+14	9,68532E+14
TC0200000405.mm.2	Tprn	taperin	0.61797410505052	6,05333E+14	1,74256E+14	0.128721570931026	0.269642573116436	-4,55466E+14	6,47397E+14	6,33549E+14	6,13975E+14	5,43375E+12	6,06614E+14	5,87087E+14
TC1100003310.mm.2	Ccnq	cyclin Q	0.588316789869947	6,79152E+13	1,74232E+14	0.128764940159782	0.269698359650925	-4,55495E+12	7,46871E+14	6,89259E+14	6,69737E+14	6,48729E+14	6,51707E+11	6,68608E+14
TC0100002121.mm.2	Ptp4a1/Gm13363	protein tyrosine phosphatase 4a1/predicted gene 13363	1,49072E+14	8,8026E+14	1,74184E+14	0.128852527784728	0.269846735143661	-4,55553E+14	9,58245E+14	9,70007E+14	9,42379E+14	6,87729E+14	9,29792E+14	7,9341E+12
TC0400001790.mm.2	Hspb7	heat shock protein family, member 7 (cardiovascular)	-0.907836986461007	5,61205E+13	-1,74167E+14	0.128883787869235	0.269877124618192	-4,55574E+14	5,14401E+14	5,14946E+14	5,68553E+14	6,42361E+14	4,94784E+14	6,32182E+14
TC1200002524.mm.2	Serpina3f	serine (or cysteine) peptidase inhibitor, clade A, member 3F	-0.793267927671694	5,32543E+14	-1,74136E+14	0.128939770613625	0.269959267658178	-4,55611E+14	5,03902E+14	4,62111E+14	5,11364E+14	6,13302E+14	5,20928E+14	5,83652E+14
TC1600000269.mm.2	Yeats2	YEATS domain containing 2	0.638666268413804	6,36165E+14	1,74123E+14	0.128963534613382	0.269973942214555	-4,55627E+14	7,20124E+14	6,49231E+14	6,41153E+14	6,00469E+14	6,04027E+14	6,01988E+14
TC0600000441.mm.2	Prss2	protease, serine 2	-0.88096586667417	4,73811E+14	-1,74107E+14	0.128993770733603	0.269995356055404	-4,55647E+14	4,39962E+14	3,87496E+14	4,32636E+14	5,71015E+14	4,95762E+13	5,15995E+14
TC1900001522.mm.2	Sfrp5	secreted frizzled-related sequence protein 5	-0.727957017162976	5,95999E+14	-1,74098E+13	0.12900904925629	0.269995356055404	-4,55657E+14	5,27083E+14	5,89722E+13	5,87865E+14	6,74531E+14	5,87987E+14	6,08806E+14
TC0500003588.mm.2	Gm4871	predicted gene 4871	-0.952202389222164	5,28591E+13	-1,7409E+14	0.129024032869522	0.269995356055404	-4,55667E+14	4,79498E+14	4,25395E+14	4,94519E+13	6,00813E+14	5,27281E+14	6,44043E+14
TC0700004310.mm.2	Kif22	kinesin family member 22	-0.601946256548461	5,49047E+14	-1,7408E+14	0.129041624396671	0.269997103480529	-4,55679E+14	5,06612E+14	5,03741E+14	5,45648E+14	5,85094E+14	5,48326E+14	6,04859E+14
TC1100002504.mm.2	Spdl1	spindle apparatus coiled-coil protein 1	-0.684183630846173	5,99264E+14	-1,74064E+13	0.129072079999758	0.270025762793573	-4,55699E+14	5,17926E+14	5,80844E+14	5,82177E+14	6,5343E+14	6,37973E+14	6,23233E+14
TC0700002643.mm.2	Hnrnpul1	heterogeneous nuclear ribonucleoprotein U-like 1	1,19673E+14	8,03992E+14	1,74021E+14	0.129149433788544	0.270120284048376	-4,5575E+14	8,9387E+14	8,88384E+14	8,61283E+14	6,81624E+14	8,16288E+14	6,82501E+14
TC0200004942.mm.2	E2f1	E2F transcription factor 1	0.732343322653908	5,98049E+14	1,74021E+14	0.129150789319723	0.270120284048376	-4,55751E+14	5,98902E+14	6,21276E+14	4,95991E+14	5,77719E+14	6,11919E+14	6,82488E+14
TC0500001610.mm.2	Ift22	intraflagellar transport 22	0.849106125200764	7,62363E+14	1,73956E+14	0.129268632039399	0.270314995455479	-4,55829E+14	8,43356E+14	8,2327E+14	7,95991E+13	7,00814E+14	7,56761E+13	6,53985E+14
TC0700002008.mm.2	5830411N06Rik	RIKEN cDNA 5830411N06 gene	-0.751514543806642	6,30309E+14	-1,73951E+14	0.129277437781911	0.270314995455479	-4,55835E+14	5,87973E+14	5,90684E+14	6,09787E+14	7,19173E+12	6,55925E+14	6,18313E+14
TC1500000380.mm.2	Med30	mediator complex subunit 30	0.937333382440237	6,98392E+14	1,73915E+14	0.129343759132412	0.270418579330551	-4,55878E+13	7,69984E+14	7,45319E+14	7,17177E+14	6,10659E+14	7,34408E+14	6,12806E+14
TC1600000750.mm.2	Tomm70a	translocase of outer mitochondrial membrane 70A	1,69382E+14	8,85791E+14	1,73879E+14	0.129409970562948	0.270444981174456	-4,55922E+14	9,82395E+14	9,89783E+14	9,67314E+14	6,66101E+14	9,26417E+14	7,82734E+14
TC1500000852.mm.2	Creld2	cysteine-rich with EGF-like domains 2	0.55093526186835	6,22206E+14	1,73869E+14	0.129427855770928	0.270444981174456	-4,55934E+14	6,4335E+14	6,70483E+14	5,90559E+14	6,13087E+13	5,91662E+14	6,24094E+14

TC0100002461.mm.2	Klf7	Kruppel-like factor 7 (ubiquitous)	0.525735730881012	8,03847E+14	1,73866E+14	0.129433648428674	0.270444981174456	-4,55938E+14	8,47326E+14	8,44872E+14	7,92861E+14	7,94189E+14	7,88752E+12	7,55079E+14
TC0500003108.mm.2	Prkab1	protein kinase, AMP-activated, beta 1 non-catalytic subunit	-0.583532261387996	6,73176E+14	-1,73859E+14	0.129446079862538	0.270444981174456	-4,55946E+13	6,13957E+14	6,35949E+14	7,0735E+14	6,59262E+14	6,94881E+13	7,27658E+14
TC0900001630.mm.2	Ackr2	atypical chemokine receptor 2	0.55208020359504	5,12259E+14	1,73848E+13	0.129467626878824	0.270444981174456	-4,5596E+14	5,26554E+14	5,39407E+14	4,60832E+14	4,94714E+14	5,09905E+13	5,42144E+14
TC0200000036.mm.2	Sephs1	selenophosphate synthetase 1	0.552862945916522	5,63632E+14	1,73843E+13	0.129476063895164	0.270444981174456	-4,55966E+14	6,15641E+14	5,8926E+14	5,4679E+14	5,47539E+14	5,61856E+14	5,20704E+14
TC0700002637.mm.2	Erich4	glutamate rich 4	-0.789482902727631	6,28595E+14	-1,73841E+14	0.129480366908539	0.270444981174456	-4,55968E+14	5,42706E+14	5,76407E+14	5,8409E+14	6,9292E+14	6,60666E+14	7,14781E+14
TC1100001583.mm.2	Rapgefl1	Rap guanine nucleotide exchange factor (GEF)-like 1	0.74340709043754	6,29387E+14	1,73835E+14	0.129490661562771	0.270444981174456	-4,55975E+14	6,82653E+14	6,89907E+14	6,6627E+14	5,57609E+14	5,73786E+14	6,06096E+14
TC1900000373.mm.2	Klf9	Kruppel-like factor 9	1,18682E+14	8,74676E+14	1,73809E+14	0.129539332403688	0.270511568561512	-4,56007E+14	9,34479E+14	9,53791E+13	9,28041E+13	7,22866E+14	8,95913E+14	8,12968E+14
TC0900001857.mm.2	Fdx2/Zglp1/Raver1/Gm38431/ Mir7082	ferredoxin 2/zinc finger, GATA-like protein 1/ribonucleoprotein, PTB-binding 1/predicted gene, 38431/microRNA 7082	0.503677629624636	7,18159E+13	1,73798E+14	0.129559529510782	0.270518685889235	-4,56021E+14	7,64772E+13	7,51454E+14	7,07258E+13	7,08232E+14	6,8982E+14	6,87417E+14
TC1100004290.mm.2	Commd1	COMM domain containing 1	0.725308294985842	6,23083E+14	1,73775E+14	0.129600642779874	0.270565450411267	-4,56048E+14	6,7825E+14	6,97139E+14	6,66319E+14	5,64009E+14	5,81912E+14	5,50869E+14
TC1700000014.mm.2	Cldn20	claudin 20	-0.599862486841994	4,76409E+14	-1,7376E+14	0.129629518423076	0.270565450411267	-4,56067E+13	4,56349E+14	4,08773E+14	4,75393E+14	5,09701E+14	5,21935E+14	4,863E+14
TC0500000263.mm.2	Rbm33	RNA binding motif protein 33	0.580486476002882	7,59407E+14	1,73758E+14	0.129632301692732	0.270565450411267	-4,56069E+14	8,13461E+14	7,93047E+14	7,56418E+14	7,33993E+14	7,563E+13	7,03226E+14
TC0700000766.mm.2	Zfp819	zinc finger protein 819	-0.598694643169428	5,80214E+14	-1,73674E+14	0.129787622132116	0.270827776248658	-4,56171E+14	5,65249E+14	5,19912E+14	5,90904E+14	6,13995E+14	5,72824E+14	6,18401E+14
TC1600001267.mm.2	Spag6l	sperm associated antigen 6-like	-0.546580623546793	7,03897E+14	-1,73671E+14	0.129791602320619	0.270827776248658	-4,56173E+14	6,60721E+14	6,66939E+14	6,99329E+14	7,37647E+14	7,16979E+14	7,41764E+14
TC0400001087.mm.2	C8b	complement component 8, beta polypeptide	-0.619220563297662	5,47357E+14	-1,73646E+14	0.129838248579582	0.270890029698667	-4,56204E+14	4,94895E+14	5,10284E+12	5,89239E+14	5,39785E+14	5,47337E+14	6,02604E+14
TC1900000443.mm.2	Ric1	RAB6A GEF complex partner 1	0.947436448296808	5,62432E+14	1,73633E+14	0.129862416013254	0.270905374057685	-4,5622E+14	6,19839E+14	6,62969E+14	6,15413E+14	4,77908E+14	5,40788E+14	4,57676E+14
TC0400001111.mm.2	Pars2	prolyl-tRNA synthetase (mitochondrial)(putative)	-0.605259499114537	6,35804E+14	-1,7354E+14	0.13003357380591	0.271206441437061	-4,56332E+14	5,91416E+14	6,07323E+14	6,25394E+14	6,94397E+14	6,4638E+14	6,49915E+14
TC1700002042.mm.2	Znrd1	zinc ribbon domain containing, 1	1,05201E+14	6,7006E+14	1,73536E+14	0.130040400083332	0.271206441437061	-4,56337E+13	7,10611E+14	7,38877E+14	7,14192E+14	5,24894E+14	6,67818E+14	6,63968E+14
TC0600001636.mm.2	Strap	serine/threonine kinase receptor associated protein	1,18786E+14	8,31711E+14	1,73526E+14	0.130059828961482	0.271211857649327	-4,5635E+14	9,12285E+13	9,12431E+14	8,75865E+14	7,11279E+14	8,64305E+13	7,14099E+14
TC1300001346.mm.2	Paip1	polyadenylate binding protein-interacting protein 1	1,12537E+14	7,47472E+14	1,73497E+13	0.1301131601073	0.271245021403348	-4,56385E+14	8,28748E+14	8,1731E+14	7,88477E+14	6,32507E+14	7,77882E+14	6,39909E+14
TC0400002942.mm.2	Zdhhc21	zinc finger, DHHC domain containing 21	1,14865E+14	7,69647E+14	1,73495E+14	0.13011538281833	0.271245021403348	-4,56386E+14	8,46285E+14	8,42542E+14	8,02964E+14	6,56134E+14	8,12735E+14	6,57224E+14
TC1300001784.mm.2	Slc22a23	solute carrier family 22, member 23	0.939601689871203	7,96085E+14	1,73481E+14	0.130142048549392	0.271245021403348	-4,56404E+14	8,43374E+14	8,60895E+14	8,31285E+14	6,85064E+14	8,14766E+14	7,41124E+14
TC0300002372.mm.2	Snapin	SNAP-associated protein	1,31436E+14	8,42013E+14	1,7348E+14	0.130143744594069	0.271245021403348	-4,56405E+14	9,15998E+14	8,94143E+14	8,96178E+14	6,51091E+14	8,73142E+14	8,21529E+14
TC1700001096.mm.2	Lama1	laminin, alpha 1	-0.502937759359049	5,17393E+14	-1,73471E+14	0.130159902283292	0.271245021403348	-4,56415E+14	4,85285E+13	4,71785E+14	5,28215E+14	5,29443E+14	5,43297E+14	5,46334E+14
TC0200004978.mm.2	Gdf5	growth differentiation factor 5	-0.720960883744377	5,66675E+14	-1,73407E+14	0.130279314388078	0.271458760214031	-4,56494E+14	5,31025E+14	5,26747E+14	5,54792E+14	6,47172E+14	5,97825E+14	5,42491E+14
TC1600001663.mm.2	Tagln3	transgelin 3	0.988905039428097	9,33683E+14	1,73347E+14	0.130389791146396	0.271625210562067	-4,56566E+14	1,00503E+14	9,82221E+14	9,58229E+14	8,3124E+14	9,73088E+14	8,52289E+14
TC1600001743.mm.2	Nxpe3	neurexophilin and PC-esterase domain family, member 3	0.620556031562119	5,92789E+14	1,73345E+13	0.130392912784558	0.271625210562067	-4,56568E+14	6,34469E+14	6,40689E+14	5,95682E+14	5,55364E+14	5,9767E+14	5,3286E+14
TC0800002465.mm.2	Slc35e1	solute carrier family 35, member E1	0.591523474290439	7,58385E+14	1,73332E+14	0.13041678367619	0.271629081901485	-4,56584E+14	8,06222E+14	8,01982E+14	7,71508E+14	7,18391E+14	7,06602E+13	7,45607E+14
TC1600000142.mm.2	Gm9961	predicted gene 9961	-0.510953569588429	7,16276E+14	-1,73326E+13	0.13042848672946	0.271629081901485	-4,56591E+13	6,79592E+14	6,73781E+14	7,1754E+14	7,38023E+14	7,39219E+14	7,49502E+14
TC1300001043.mm.2	Homer1	homer scaffolding protein 1	0.79751306535942	9,36537E+14	1,73288E+14	0.130497981952955	0.271738689998445	-4,56637E+14	9,73401E+14	9,96105E+14	9,57344E+14	8,5266E+14	9,55735E+14	8,83978E+14
TC0800001927.mm.2	Fnta	farnesyltransferase, CAAX box, alpha	0.825304719573085	6,98649E+14	1,73274E+14	0.130525047501233	0.27175992898144	-4,56654E+14	7,87225E+13	7,40289E+14	7,42919E+14	6,19534E+14	6,67793E+14	6,34131E+14
TC0200000969.mm.2	B3galt1	UDP-Gal:betaGlcNAc beta 1,3-galactosyltransferase, polypeptide 1	1,06045E+14	7,72746E+14	1,7326E+14	0.130550968337302	0.271778779433973	-4,56671E+14	8,39905E+14	8,50705E+14	7,99653E+14	6,78867E+14	8,09264E+14	6,58079E+14
TC0800000621.mm.2	Hpgd	hydroxyprostaglandin dehydrogenase 15 (NAD)	0.644467999278125	5,98893E+14	1,73233E+14	0.130599279706884	0.271841819931028	-4,56703E+14	6,35081E+14	6,50564E+14	5,81955E+14	5,74796E+13	6,16988E+14	5,33973E+14
TC0400003870.mm.2	Crocc	ciliary rootlet coiled-coil, rootletin	-0.559814703834418	6,67784E+14	-1,73225E+14	0.130614992236456	0.271841819931028	-4,56713E+14	6,23822E+14	6,17994E+14	6,5158E+14	7,02199E+14	6,98168E+13	7,12943E+14
TC0100002963.mm.2	Tmem37	transmembrane protein 37	-0.583262078596616	6,10146E+14	-1,73181E+13	0.130695893440545	0.271975065350318	-4,56766E+14	5,54845E+14	6,01608E+14	6,38732E+14	6,34374E+14	5,94652E+14	6,36663E+14
TC1700002346.mm.2	Slc25a23	solute carrier family 25 (mitochondrial carrier; phosphate carrier), member 23	1,2947E+13	9,75619E+14	1,73162E+13	0.130731295908576	0.271987499445593	-4,56789E+14	1,06468E+14	1,06506E+14	1,04478E+14	8,26022E+14	9,84526E+14	8,6865E+14
TC1300001669.mm.2	Acot13	acyl-CoA thioesterase 13	1,1316E+14	8,86025E+14	1,7316E+14	0.130735628573582	0.271987499445593	-4,56792E+13	9,43053E+14	9,31156E+14	9,11271E+13	7,36619E+14	9,55471E+14	8,3858E+14
TC0200000392.mm.2	Dph7	diphthamine biosynethesis 7	0.777193861367013	6,3632E+13	1,73146E+14	0.130761768996049	0.272006762694724	-4,56809E+14	6,85259E+14	6,76921E+13	6,62528E+14	5,44214E+14	6,0698E+14	6,42017E+14
TC1800001005.mm.2	Zfp521	zinc finger protein 521	0.783019910380872	7,0999E+14	1,73129E+14	0.13079280647894	0.272029073972481	-4,56829E+14	7,96904E+14	7,67992E+14	7,63423E+14	6,44868E+14	6,57512E+14	6,29241E+14
TC0600002480.mm.2	Atoh8	atonal bHLH transcription factor 8	-0.594108449384272	6,12468E+14	-1,73122E+14	0.130806259860906	0.272029073972481	-4,56838E+14	5,453E+14	5,84562E+14	6,01869E+14	6,46815E+14	6,3258E+14	6,63679E+14
TC0X00000102.mm.2	Fthl17e	ferritin, heavy polypeptide-like 17, member E	-0.536722464362231	5,38288E+14	-1,73098E+14	0.130850852765685	0.272060246697325	-4,56867E+14	4,80414E+14	5,08923E+14	5,47391E+14	5,49291E+14	5,56599E+14	5,87112E+12
TC0300002836.mm.2	Rwdd3	RWD domain containing 3	-0.516842961371175	6,83623E+12	-1,73095E+14	0.13085501842638	0.272060246697325	-4,5687E+14	6,62214E+14	6,41704E+13	7,01468E+14	7,05818E+14	7,05379E+14	6,85152E+14
TC0900000204.mm.2	Ldlr	low density lipoprotein receptor	-0.582566699931617	6,92561E+14	-1,73056E+14	0.130927210192175	0.27217522098136	-4,56917E+14	6,74789E+14	6,51079E+14	6,97941E+14	7,44441E+14	7,11112E+14	6,76004E+14
TC1000000142.mm.2	Ifngr1	interferon gamma receptor 1	-0.531409117688993	7,38402E+13	-1,72999E+14	0.131033965849967	0.272362008738457	-4,56986E+14	7,03829E+14	6,83698E+14	7,49639E+14	7,4417E+14	7,58086E+14	7,90987E+13
TC1100001054.mm.2	Smg6	Smg-6 homolog, nonsense mediated mRNA decay factor (C. elegans)	0.530960698137115	6,68222E+14	1,72975E+14	0.131078684547943	0.272419817376628	-4,57016E+14	7,08626E+14	7,18842E+14	6,69199E+14	6,52076E+14	6,13689E+14	6,469E+14
TC1400001464.mm.2	Gm5087	predicted gene 5087	-0.768830229154215	5,72258E+14	-1,72947E+14	0.131130728032008	0.272492832187226	-4,57049E+14	5,26784E+14	5,21983E+14	5,59608E+14	6,42924E+14	5,4687E+14	6,35382E+14
TC0300002944.mm.2	Ostc	oligosaccharyltransferase complex subunit (non-catalytic)	0.885273520236394	7,0466E+13	1,72874E+14	0.131264971218606	0.272731857966745	-4,57137E+14	7,61359E+14	7,5973E+14	6,929E+14	6,51134E+14	7,54498E+14	6,0834E+14
TC1100001155.mm.2	Spag5	sperm associated antigen 5	-0.516379910811478	5,14724E+14	-1,72867E+14	0.131279605932481	0.272731857966745	-4,57146E+14	5,25584E+14	5,05608E+14	5,7752E+14	5,56948E+14	4,58336E+14	4,64351E+14
TC0700004641.mm.2	Sptbn4	spectrin beta, non-erythrocytic 4	0.673782663115816	7,18466E+14	1,72849E+13	0.131312426118274	0.272764873281391	-4,57168E+14	7,49344E+14	7,54593E+14	7,30678E+14	6,38504E+14	7,08819E+14	7,28859E+14
TC0300001300.mm.2	Pitx2	paired-like homeodomain transcription factor 2	-0.603150217258408	6,22353E+14	-1,72821E+14	0.131364139309652	0.272837119966026	-4,57201E+14	5,99727E+14	5,93834E+14	6,21863E+14	6,92329E+14	6,16623E+14	6,09741E+14
TC0400001780.mm.2	Fbxo42	F-box protein 42	0.958537186150513	7,04196E+14	1,72811E+14	0.131382365535167	0.272839806143593	-4,57213E+13	7,72578E+14	7,55076E+14	7,52573E+14	5,83373E+14	6,82839E+14	6,78737E+13
TC0300001142.mm.2	Psrc1	proline/serine-rich coiled-coil 1	-0.566757146074115	6,56621E+14	-1,72796E+14	0.131410069951999	0.272862172311413	-4,57231E+14	6,1139E+14	6,4424E+14	6,61542E+14	7,0744E+14	6,60397E+13	6,54714E+14
TC1700001835.mm.2	Ephx3	epoxide hydrolase 3	-1,07698E+14	5,78906E+14	-1,72734E+14	0.131525538167762	0.273066743524951	-4,57306E+14	5,09891E+13	5,58736E+14	5,61459E+14	7,22563E+14	5,0786E+14	6,12926E+14
TC0X00003423.mm.2	Rhox3g	reproductive homeobox 3G	-0.570685026727891	5,02521E+14	-1,72704E+14	0.131582693226676	0.273126762988352	-4,57343E+14	4,63727E+14	4,46768E+14	4,85355E+13	5,39277E+14	5,3241E+14	5,47592E+14
TC1500000433.mm.2	Nsmce2	NSE2/MMS21 homolog, SMC5-SMC6 complex SUMO ligase	1,17962E+14	7,01409E+14	1,72701E+14	0.131588348605385	0.273126762988352	-4,57347E+14	7,58074E+14	7,71383E+14	7,42772E+14	5,50762E+14	7,48278E+13	6,37185E+14
TC0600000226.mm.2	Hilpda	hypoxia inducible lipid droplet associated	-0.560753950737556	6,64891E+14	-1,72676E+14	0.131634378722225	0.273140453773022	-4,57376E+14	6,20545E+12	6,36537E+14	6,58688E+14	7,10545E+14	6,81139E+14	6,81891E+14
TC0400003448.mm.2	Smap2	small ArfGAP 2	1,04608E+14	8,67322E+13	1,72669E+14	0.131647024128782	0.273140453773022	-4,57385E+14	9,3687E+14	9,1239E+11	9,05495E+14	7,34549E+14	8,98439E+14	8,1619E+14
TC0200005092.mm.2	Hnf4aos	hepatic nuclear factor 4 alpha, opposite strand	-0.569486479357994	5,58922E+13	-1,72662E+14	0.131659974893124	0.273140453773022	-4,57393E+14	5,13015E+14	5,49554E+14	5,72703E+14	6,03764E+14	5,40866E+14	5,73632E+14
TC1900000204.mm.2	Cyb561a3	cytochrome b561 family, member A3	0.604805458909679	6,49775E+13	1,72661E+13	0.131662750850559	0.273140453773022	-4,57395E+14	6,58344E+14	7,22401E+14	6,46126E+14	6,13657E+14	6,27885E+14	6,30239E+14
TC0900002009.mm.2	Foxred1	FAD-dependent oxidoreductase domain containing 1	0.538890453581072	6,1065E+14	1,72643E+14	0.13169537735276	0.273172967982109	-4,57416E+13	6,66182E+14	6,24095E+14	5,94551E+14	5,87948E+14	6,0052E+14	5,90606E+14
TC1300002079.mm.2	Cxcl14	chemokine (C-X-C motif) ligand 14	0.555118193114037	8,09486E+14	1,72619E+14	0.131740016940208	0.27318807367827	-4,57445E+14	8,51746E+14	8,63659E+14	8,27298E+14	7,77083E+14	7,70773E+14	7,66355E+14
TC1100000516.mm.2	Olfr1382	olfactory receptor 1382	-1,18736E+14	5,5461E+13	-1,72617E+14	0.131745178183663	0.27318807367827	-4,57448E+14	4,85302E+14	4,89033E+14	4,96988E+14	7,14819E+14	5,44434E+14	5,9708E+14
TC1300001522.mm.2	Epdr1	ependymin related protein 1 (zebrafish)	0.618681274350339	7,23994E+14	1,72612E+14	0.131753523276475	0.27318807367827	-4,57454E+14	7,29095E+14	7,79189E+13	7,09173E+14	6,75375E+13	7,493E+14	7,01834E+14
TC1900000201.mm.2	Cpsf7	cleavage and polyadenylation specific factor 7	0.686281277020535	6,37668E+13	1,72573E+14	0.13182611247645	0.273293031188582	-4,57501E+14	7,00948E+14	7,00562E+14	6,57957E+14	6,06296E+14	6,22114E+14	5,38133E+14
TC0500000812.mm.2	Enam	enamelin	-0.829220079836286	5,39933E+14	-1,72567E+14	0.131838064384587	0.273293031188582	-4,57508E+14	4,94371E+14	4,89128E+14	5,06756E+14	6,42586E+14	5,41113E+14	5,65643E+14
TC0300000974.mm.2	Notch2	notch 2	0.620733135349941	6,5332E+14	1,7255E+14	0.131869867193194	0.273323793424741	-4,57529E+12	6,7563E+14	7,09227E+13	5,95641E+14	6,6507E+14	6,41573E+14	6,3278E+14
TC1200001791.mm.2	Nkx2-1	NK2 homeobox 1	-0.61016806182908	5,078E+14	-1,72511E+14	0.131942963633748	0.273440125148629	-4,57576E+14	4,78023E+14	4,73756E+14	5,19004E+14	5,54808E+14	5,42901E+14	4,78311E+13
TC0500003589.mm.2	Pdap1	PDGFA associated protein 1	0.692870913033603	8,85084E+14	1,7247E+14	0.132019425629597	0.273563400870589	-4,57625E+13	9,51669E+14	9,30009E+14	8,69954E+14	8,73149E+14	8,92003E+14	7,93718E+14
TC0300002153.mm.2	Slitrk3	SLIT and NTRK-like family, member 3	1,00348E+14	7,17198E+14	1,72443E+14	0.132069825436459	0.273632647197848	-4,57658E+14	7,89483E+13	8,13676E+14	7,69924E+14	6,3254E+14	7,06824E+14	5,90743E+14
TC1600001150.mm.2	Rogdi	rogdi homolog	0.726553232705827	9,23147E+14	1,7241E+14	0.13213060472368	0.273723377977971	-4,57697E+13	9,8809E+14	9,68169E+14	9,57368E+14	8,53581E+14	9,02063E+14	8,69608E+14
TC0600003433.mm.2	Itpr2	inositol 1,4,5-triphosphate receptor 2	0.662391937985635	5,6818E+14	1,72368E+14	0.132209279769338	0.273851153737414	-4,57748E+14	6,12644E+14	6,32331E+14	5,55184E+14	5,57313E+14	5,70478E+14	4,81128E+13
TC1100001643.mm.2	Tubg2	tubulin, gamma 2	0.810311501758921	8,5223E+14	1,72338E+14	0.13226513417109	0.273929726251639	-4,57784E+14	9,32869E+13	8,98578E+14	8,85109E+14	7,84275E+14	8,46499E+14	7,6605E+14
TC1100001157.mm.2	Pigs	phosphatidylinositol glycan anchor biosynthesis, class S	-0.775539345654001	7,56407E+14	-1,72322E+14	0.13229497184621	0.273929726251639	-4,57803E+14	7,33895E+14	7,16755E+14	7,80151E+14	8,25607E+14	6,8385E+14	7,98185E+14
TC0100002543.mm.2	Abca12	ATP-binding cassette, sub-family A (ABC1), member 12	-0.772712061143433	5,66512E+14	-1,72321E+14	0.132298214465975	0.273929726251639	-4,57805E+14	5,22164E+13	4,97816E+14	5,26988E+14	6,47534E+14	5,96831E+14	6,07738E+12
TC1300000157.mm.2	Aoah	acyloxyacyl hydrolase	-0.6430897374624	5,54057E+14	-1,72296E+14	0.132344088675736	0.273988367483673	-4,57835E+13	5,07189E+14	5,49083E+14	5,55466E+14	6,29423E+14	5,37263E+14	5,45919E+14
TC0700000852.mm.2	Hsd17b14	hydroxysteroid (17-beta) dehydrogenase 14	-0.589170569755591	5,46548E+14	-1,72284E+14	0.132367075244025	0.273988367483673	-4,5785E+14	5,22344E+14	5,1368E+14	5,48565E+14	6,05294E+14	5,27896E+11	5,61511E+14
TC1500000907.mm.2	Pdzrn4	PDZ domain containing RING finger 4	0.590487201015061	5,41892E+14	1,72278E+14	0.132377548616669	0.273988367483673	-4,57856E+14	6,03904E+14	5,42518E+14	5,2485E+13	5,03475E+14	5,46463E+14	5,30142E+14
TC0700003061.mm.2	Mrgprx1	MAS-related GPR, member X1	-0.645142883991039	5,0702E+14	-1,72263E+14	0.132406728838161	0.273989826819796	-4,57875E+14	4,55166E+14	4,63818E+14	4,89936E+14	5,58077E+14	5,11566E+14	5,63559E+14
TC1700001110.mm.2	Zbtb14	zinc finger and BTB domain containing 14	1,04784E+14	5,38869E+13	1,72246E+14	0.132437553359049	0.273989826819796	-4,57895E+14	6,19557E+14	6,18884E+14	5,89519E+14	4,39354E+14	5,4301E+14	4,2289E+12
TC0300001589.mm.2	Wls	wntless WNT ligand secretion mediator	0.762111413789959	6,3882E+14	1,72246E+14	0.132438588122669	0.273989826819796	-4,57896E+13	7,06852E+14	6,78086E+14	6,09577E+14	6,22938E+14	6,65944E+14	5,49521E+14
TC1900000904.mm.2	Cabp4	calcium binding protein 4	-0.533249134145568	6,75605E+14	-1,72236E+13	0.13245614273231	0.273989826819796	-4,57907E+13	6,42196E+13	6,56931E+14	6,89335E+12	7,16442E+14	6,88131E+13	6,60593E+14
TC1000001947.mm.2	Ncoa7	nuclear receptor coactivator 7	1,08249E+13	6,89601E+14	1,72233E+14	0.132463275052827	0.273989826819796	-4,57912E+14	7,98997E+14	7,85409E+14	7,1947E+14	6,48439E+14	6,86129E+14	4,99162E+14
TSUnmapped00000021.mm.2	Rbm4	RNA binding motif protein 4	0.688037172403117	5,8497E+14	1,72213E+14	0.132499522203386	0.2740275458493	-4,57935E+14	6,3212E+14	6,15428E+14	6,03824E+14	5,06117E+14	5,71523E+12	5,80809E+14
TC1200001490.mm.2	Adam17	a disintegrin and metallopeptidase domain 17	1,37177E+14	6,49958E+14	1,72205E+14	0.132515523940777	0.2740275458493	-4,57945E+14	7,02417E+14	7,42119E+14	6,8833E+13	4,81851E+14	7,24346E+14	5,60682E+14
TC0800000028.mm.2	Evi5l	ecotropic viral integration site 5 like	0.625398015339443	6,46677E+14	1,7218E+14	0.132561169615749	0.274086760685046	-4,57975E+14	6,86743E+14	6,95205E+14	6,5555E+14	6,01318E+14	5,91738E+14	6,49508E+14
TC1500001765.mm.2	BC024139/Eppk1	cDNA sequence BC024139/epiplakin 1	-0.556235455331848	5,81233E+14	-1,72156E+14	0.132607037891071	0.274146420520763	-4,58004E+14	5,53753E+14	5,4307E+14	5,85685E+14	6,22385E+14	5,72899E+14	6,09607E+14
TC1200000472.mm.2	Insm2	insulinoma-associated 2	-0.755229043503995	5,55983E+14	-1,72138E+14	0.132641245155938	0.274181960641133	-4,58026E+14	5,13635E+13	5,07782E+14	5,40655E+14	6,31808E+14	5,32943E+14	6,09072E+14
TC1000001351.mm.2	Lrrc10	leucine rich repeat containing 10	-0.84810440288415	5,74261E+14	-1,72083E+14	0.132744660843264	0.274360533628465	-4,58092E+14	4,98669E+14	5,43845E+14	5,67247E+14	6,44888E+14	5,39438E+14	6,51478E+14
TC0300002126.mm.2	Trim59	tripartite motif-containing 59	-0.72017229780776	5,79469E+14	-1,72073E+13	0.132763239365261	0.274363739373151	-4,58104E+14	5,56312E+14	5,4184E+13	5,6716E+14	6,75027E+14	5,72298E+14	5,64177E+14
TC1200000358.mm.2	Gm7008	predicted gene 7008	-0.525156643320368	6,2044E+14	-1,72057E+14	0.132793176308308	0.274390414190275	-4,58124E+13	5,68915E+14	5,74671E+14	6,12737E+14	6,3588E+14	6,51118E+14	6,79316E+14
TC0700000820.mm.2	Pnkp	polynucleotide kinase 3'- phosphatase	-0.944103470416109	6,83829E+14	-1,7202E+14	0.13286225666605	0.274497953796649	-4,58168E+14	6,15605E+14	6,10616E+14	6,39151E+14	7,75891E+14	6,82378E+14	7,79332E+14
TC1600001578.mm.2	Casr	calcium-sensing receptor	-0.594784535639916	5,33831E+14	-1,71919E+14	0.133051844684487	0.274837436427736	-4,58289E+14	4,73454E+14	5,15652E+14	5,3582E+14	5,72244E+14	5,32483E+14	5,73334E+14
TC0900001054.mm.2	Mto1	mitochondrial tRNA translation optimization 1	0.727010787195921	5,38282E+14	1,71915E+14	0.133060686499893	0.274837436427736	-4,58295E+13	6,1278E+14	5,92513E+14	5,22666E+14	5,37225E+14	5,36475E+14	4,28031E+14
TC1800000771.mm.2	Mex3c	mex3 RNA binding family member C	0.561373934137503	8,43377E+14	1,71895E+13	0.133098339857372	0.274848955539801	-4,58319E+13	8,75894E+14	8,72491E+14	8,3988E+14	7,9623E+14	8,20689E+14	8,55076E+14
TC0600002742.mm.2	Rbsn	rabenosyn, RAB effector	0.955049516938067	6,47407E+14	1,71894E+14	0.133100378581088	0.274848955539801	-4,5832E+14	7,33319E+14	6,87493E+14	6,72418E+14	5,57384E+14	6,75547E+14	5,58279E+14
TC1300000248.mm.2	Hist1h1d	histone cluster 1, H1d	-0.510459649340356	7,48931E+14	-1,7184E+14	0.133201981069294	0.275023516269801	-4,58385E+14	7,14127E+14	7,17138E+14	7,55573E+14	7,77784E+14	7,56917E+14	7,72044E+14
TC1100002157.mm.2	Gck	glucokinase	-0.639906460911847	6,32855E+14	-1,71823E+14	0.13323406576953	0.275039374143776	-4,58406E+13	5,87611E+14	5,93083E+14	6,40549E+13	6,68126E+14	6,13785E+14	6,93974E+14
TC1100001998.mm.2	Ccdc40	coiled-coil domain containing 40	-0.50891070481528	1,03262E+13	-1,71802E+14	0.133273751600174	0.275039374143776	-4,58431E+14	9,86645E+14	1,00874E+14	1,04673E+14	1,05044E+14	1,04515E+14	1,05802E+14
TC1000002076.mm.2	Sobp	sine oculis binding protein	0.62390899058328	7,58482E+14	1,71797E+14	0.133282327411537	0.275039374143776	-4,58437E+14	8,02517E+14	8,04236E+14	7,80763E+14	7,01208E+13	7,32878E+14	7,29287E+14
TC0900001638.mm.2	Abhd5	abhydrolase domain containing 5	-0.499510335842857	1,03366E+14	-1,71794E+14	0.133288123988231	0.275039374143776	-4,58441E+14	1,00908E+14	9,9512E+14	1,0515E+12	1,0526E+14	1,04298E+14	1,05069E+14
TC0400001005.mm.2	Foxd3	forkhead box D3	-0.638248221386749	6,44994E+14	-1,71783E+14	0.133308510857415	0.275039374143776	-4,58454E+14	5,98708E+14	6,06808E+14	6,35813E+14	6,97353E+14	6,36989E+14	6,94293E+14
TC0500002483.mm.2	Rfc1	replication factor C (activator 1) 1	0.714723117530359	6,77707E+14	1,71781E+14	0.133312077953385	0.275039374143776	-4,58456E+14	7,23152E+14	7,16239E+14	6,20912E+14	6,75535E+14	7,1161E+14	6,18793E+14
TC1000003056.mm.2	Rxylt1/Gm9079	ribitol xylosyltransferase 1/transmembrane emp24 domain trafficking protein 2 pseudogene	1,5489E+14	6,15128E+14	1,71738E+14	0.133393567327475	0.275143420140288	-4,58508E+14	7,4257E+14	6,6428E+14	6,92754E+13	4,04315E+14	6,27602E+13	5,59247E+14
TC1700001132.mm.2	Lpin2	lipin 2	0.529656540520766	7,18076E+14	1,71737E+13	0.133396660965427	0.275143420140288	-4,5851E+14	7,60577E+14	7,57795E+14	7,02375E+14	7,10066E+14	6,69144E+14	7,08498E+14
TC1500000766.mm.2	Serhl	serine hydrolase-like	-0.645870101041289	7,25716E+14	-1,71707E+13	0.133451704899109	0.275221722902771	-4,58545E+14	6,78576E+14	6,91852E+14	7,23377E+14	7,76225E+12	7,08184E+14	7,7608E+13
TC0100001831.mm.2	Angel2	angel homolog 2	0.972689115501234	6,95303E+14	1,71681E+14	0.133501177145611	0.275288516425292	-4,58577E+14	7,91188E+14	7,92701E+14	7,27032E+14	6,6232E+14	6,80647E+14	5,17929E+14
TC1000002482.mm.2	Plppr3	phospholipid phosphatase related 3	0.584548127555075	8,15815E+14	1,71667E+12	0.133528805723764	0.275310255486502	-4,58594E+14	8,85579E+14	8,24663E+12	8,07856E+14	7,85476E+14	7,91102E+14	8,00216E+14
TC0200000514.mm.2	Cercam	cerebral endothelial cell adhesion molecule	-0.556483806662004	5,91899E+14	-1,71558E+14	0.133735034080762	0.275669298520857	-4,58726E+14	5,72936E+13	5,37574E+14	5,91141E+14	6,30666E+13	6,04495E+14	6,14584E+14
TC1000002442.mm.2	Gatd3a	glutamine amidotransferase like class 1 domain containing 3A	0.56258389047253	8,04162E+13	1,71556E+14	0.133737162945295	0.275669298520857	-4,58727E+14	8,55986E+14	8,197E+14	7,97252E+14	7,65918E+13	8,08178E+14	7,77939E+14
TC0600002796.mm.2	Eogt	EGF domain-specific O-linked N-acetylglucosamine (GlcNAc) transferase	1,62903E+14	6,40986E+14	1,71538E+14	0.13377182680465	0.275705480340665	-4,58749E+14	7,73918E+14	6,89949E+14	7,21947E+14	4,16113E+14	6,56594E+14	5,87394E+14
TC0800002620.mm.2	Itfg1	integrin alpha FG-GAP repeat containing 1	1,6887E+14	8,24893E+14	1,71519E+14	0.133807709882885	0.275744165410272	-4,58772E+13	9,37008E+14	9,25594E+14	9,10823E+13	6,14039E+14	8,61327E+14	7,00569E+14
TC0700003983.mm.2	Trim3	tripartite motif-containing 3	-0.601403351872969	7,52441E+14	-1,71501E+14	0.133842661401168	0.275776726320187	-4,58794E+14	7,24835E+14	6,96824E+14	7,44764E+13	7,97176E+14	7,55115E+14	7,95932E+14

TC0900003038.mm.2	Gnai2	guanine nucleotide binding protein (G protein), alpha inhibiting 2	0.952232083177099	9,601E+12	1,71493E+14	0.133857740740407	0.275776726320187	-4,58804E+14	1,01846E+14	1,02363E+14	9,98596E+14	8,53047E+14	9,7766E+14	8,89205E+14
TC0200002338.mm.2	Bpifb5	BPI fold containing family B, member 5	-0.765429217538023	6,12054E+14	-1,71466E+14	0.133907929731223	0.27582792685017	-4,58836E+14	5,73889E+14	5,48017E+14	5,84108E+14	6,90884E+14	6,10596E+14	6,64829E+14
TC0100000135.mm.2	Gsta3	glutathione S-transferase, alpha 3	-0.91300986168443	5,42857E+14	-1,71462E+14	0.133916829376831	0.27582792685017	-4,58841E+14	4,8487E+14	4,88733E+14	5,15992E+14	6,40213E+14	5,12667E+13	6,14666E+14
TC1700002534.mm.2	Dpy30	dpy-30, histone methyltransferase complex regulatory subunit	0.83028703364606	7,88915E+14	1,71416E+13	0.1340041304345	0.27595137048724	-4,58897E+14	8,452E+14	8,38196E+14	8,1309E+14	7,04249E+14	8,10143E+14	7,22613E+14
TC0500002177.mm.2	Zfp513	zinc finger protein 513	-0.605151094118431	7,00576E+14	-1,71405E+14	0.134024586960697	0.27595137048724	-4,5891E+14	6,54642E+14	6,71077E+14	6,97591E+14	7,49158E+14	6,90573E+14	7,40414E+14
TC0300002706.mm.2	Rbm15	RNA binding motif protein 15	1,02227E+14	5,77649E+14	1,71388E+14	0.134056209845227	0.27595137048724	-4,5893E+14	6,72903E+14	6,63168E+14	5,76929E+14	5,54689E+14	5,92039E+14	4,06164E+14
TC0500001577.mm.2	Bcl7b	B cell CLL/lymphoma 7B	0.554522683859831	8.03173E+13	1,7138E+14	0.134071092269073	0.27595137048724	-4,58939E+14	8,48486E+13	8,40057E+14	8,15055E+14	7,62584E+14	7,7404E+14	7,78816E+14
TC0900002052.mm.2	Nrgn	neurogranin	0.624800564000843	1,02643E+14	1,7138E+14	0.134071273229251	0.27595137048724	-4,5894E+14	1,03134E+14	1,06651E+13	1,00365E+14	9,69238E+14	1,07575E+14	1,01208E+14
TC1200000145.mm.2	Hpcal1	hippocalcin-like 1	0.621635484020784	7,92577E+14	1,7137E+14	0.13409049934655	0.27595137048724	-4,58952E+14	8,68132E+14	8,18833E+14	7,95257E+14	7,67381E+14	7,80761E+14	7,25101E+14
TC0300000354.mm.2	Noct	nocturnin	0.714086077269312	6,9897E+14	1,71358E+14	0.134112411206825	0.27595137048724	-4,58966E+14	7,50332E+14	7,37038E+14	7,24534E+14	6,20019E+12	6,93364E+13	6,68532E+14
TC0X00003313.mm.2	Syap1	synapse associated protein 1	1,60041E+14	6,89444E+13	1,71353E+13	0.134122566454169	0.27595137048724	-4,58972E+14	7,8485E+14	7,69711E+14	7,66807E+14	4,67672E+14	7,28273E+14	6,19349E+14
TC1500002175.mm.2	Lalba	lactalbumin, alpha	-0.663661071298089	5,09371E+14	-1,71338E+14	0.134152042005104	0.27595137048724	-4,58991E+14	4,41008E+14	4,59153E+13	4,92493E+14	5,404E+14	5,22681E+14	6,00489E+14
TC1500000008.mm.2	Oxct1	3-oxoacid CoA transferase 1	2,0244E+14	8,55161E+13	1,71332E+13	0.134162674442902	0.27595137048724	-4,58998E+14	9,64116E+14	9,77698E+14	9,47268E+14	5,89666E+14	9,27198E+14	7,25021E+14
TC1500001344.mm.2	9430069I07Rik	RIKEN cDNA 9430069I07 gene	-0.766516459092681	5,54975E+14	-1,71331E+14	0.13416514841414	0.27595137048724	-4,58999E+13	4,85062E+14	5,1124E+14	5,24563E+13	6,25042E+14	5,59017E+14	6,24924E+14
TC1400000325.mm.2	Sema3g	sema domain, immunoglobulin domain (Ig), short basic domain, secreted, (semaphorin) 3G	-0.818299793484124	5,90548E+14	-1,71306E+14	0.134211539837418	0.27601155601791	-4,59029E+14	5,44389E+14	5,29584E+14	5,51203E+14	6,8643E+14	6,08364E+14	6,23317E+14
TC0200001445.mm.2	Syt13	synaptotagmin XIII	0.520329985843498	6,96452E+14	1,71294E+14	0.134234025827853	0.276022569954594	-4,59043E+14	7,32977E+14	7,391E+13	7,01394E+13	6,66617E+14	6,68491E+14	6,70131E+14
TC0100002488.mm.2	Kansl1l	KAT8 regulatory NSL complex subunit 1-like	0.505687125296716	6,96463E+14	1,71262E+14	0.134295895006635	0.276114553559004	-4,59082E+14	7,49242E+13	7,48361E+14	6,99061E+14	6,97404E+14	6,54519E+14	6,30193E+14
TC0500002881.mm.2	Hsd17b13	hydroxysteroid (17-beta) dehydrogenase 13	-0.675482497261659	6,2541E+14	-1,71241E+14	0.13433469170229	0.276159082342307	-4,59107E+14	5,93271E+14	5,83707E+14	6,23917E+14	6,88159E+14	5,95732E+14	6,67676E+14
TC0500002932.mm.2	Gfi1	growth factor independent 1 transcription repressor	-1,13901E+14	7,19817E+14	-1,71206E+14	0.134401411325579	0.276260995112777	-4,59149E+14	6,57579E+14	6,41755E+14	7,25728E+13	8,01409E+14	6,45806E+14	8,46627E+14
TC0700000284.mm.2	Dmwd	dystrophia myotonica-containing WD repeat motif	0.542927119374028	8,3633E+14	1,71196E+14	0.134420626858011	0.276265250071828	-4,59161E+14	8,72518E+14	8,67672E+14	8,32553E+14	7,99051E+14	8,40207E+14	8,05977E+13
TC0200002199.mm.2	Cstdc1	cystatin domain containing 1	-1,02763E+13	4,59062E+14	-1,7118E+14	0.134451498269236	0.276293456397424	-4,59181E+14	4,13945E+14	4,08548E+14	4,49764E+14	5,78254E+14	3,82661E+14	5,21201E+14
TC1900001114.mm.2	Cd6	CD6 antigen	-0.692457692069677	5,26173E+13	-1,71168E+14	0.134473934776084	0.276304324285519	-4,59195E+14	4,67977E+14	5,22545E+13	5,30818E+14	5,98196E+14	4,91764E+14	5,45739E+14
TC0700001906.mm.2	Tacc2	transforming, acidic coiled-coil containing protein 2	0.701686876418471	7,07393E+14	1,71154E+14	0.134500785191581	0.27632425752798	-4,59212E+14	7,52386E+13	7,41497E+14	7,23961E+14	6,29585E+13	6,76249E+14	7,20678E+14
TC1700000695.mm.2	2300002M23Rik	RIKEN cDNA 2300002M23 gene	-0.699355434255065	5,08795E+14	-1,71091E+14	0.134621529852668	0.276537061514028	-4,59288E+14	4,54088E+14	4,70876E+14	5,04504E+14	5,60331E+14	4,88263E+14	5,74711E+14
TC0300001554.mm.2	Cryz	crystallin, zeta	0.721071074506077	6,35855E+14	1,71081E+14	0.1346399790259	0.276539704530827	-4,593E+14	7,07501E+14	6,85193E+14	6,04248E+14	6,44231E+13	5,36648E+14	6,37312E+14
TC1300002252.mm.2	Zfp748	zinc finger protein 748	1,17045E+14	6,55113E+14	1,71045E+14	0.134709043552059	0.276646293494051	-4,59344E+13	7,32189E+13	6,99008E+14	7,06082E+14	4,91025E+14	6,74937E+14	6,27435E+14
TC0600000691.mm.2	Vmn1r15	vomeronasal 1 receptor 15	-0.772108736414255	5.03265E+13	-1,71027E+14	0.134743594141188	0.276681984503244	-4,59365E+14	4,68229E+14	4,25259E+14	4,72656E+14	5,75254E+14	5,10713E+14	5,67479E+14
TC0400001770.mm.2	Padi2	peptidyl arginine deiminase, type II	0.838278237092037	6,34023E+14	1,71013E+14	0.134769699493239	0.276687359495702	-4,59382E+14	7,17527E+13	6,83489E+14	6,78112E+14	5,55249E+14	5,53132E+14	6,16629E+14
TC0600001327.mm.2	Rasgef1a/Mir7044	RasGEF domain family, member 1A/microRNA 7044	0.898796024438882	8,50271E+14	1,71007E+14	0.134780555122061	0.276687359495702	-4,59389E+14	9,11089E+14	9,09507E+14	8,69689E+14	7,71148E+14	8,81736E+14	7,58455E+14
TC1700000346.mm.2	Nthl1	nth (endonuclease III)-like 1 (E.coli)	-0.733594483168588	6,73328E+14	-1,70985E+14	0.134822187614201	0.276735660018103	-4,59415E+14	6,22054E+13	6,16893E+14	6,42515E+14	7,4315E+14	6,80738E+14	7,34615E+14
TC1100002497.mm.2	Dock2	dedicator of cyto-kinesis 2	0.689431082463529	5,74789E+14	1,70977E+14	0.134838432743879	0.276735660018103	-4,59425E+14	5,79859E+14	6,3898E+14	5,05941E+14	5,75012E+14	5,96987E+14	5,51955E+14
TC0500002344.mm.2	Fam184b	family with sequence similarity 184, member B	-0.616805125772289	6,75953E+14	-1,70964E+14	0.134863953045924	0.276738819977932	-4,59441E+14	6,61289E+14	6,40326E+14	7,49806E+14	6,7517E+14	6,59249E+14	6,69877E+13
TC0500001098.mm.2	Dr1	down-regulator of transcription 1	0.932568731024228	6,33362E+14	1,70958E+13	0.134874322180022	0.276738819977932	-4,59448E+14	6,88994E+14	6,71193E+14	6,68743E+14	5,04931E+14	6,44355E+14	6,21955E+14
TC0400000920.mm.2	Zfp352	zinc finger protein 352	-1,01106E+13	5,19742E+14	-1,70941E+14	0.134906281941701	0.276740151012478	-4,59468E+14	4,34019E+14	4,63094E+14	4,72283E+14	6,27042E+14	5,1207E+14	6,09946E+14
TC1500001777.mm.2	Dgat1	diacylglycerol O-acyltransferase 1	-0.894752261472314	5,22237E+13	-1,70938E+14	0.134912853061101	0.276740151012478	-4,59472E+14	4,8512E+14	4,36274E+14	4,8235E+14	6,17994E+14	5,24879E+14	5,86808E+14
TC0400003697.mm.2	Wdtc1	WD and tetratricopeptide repeats 1	0.534940916548475	6,99184E+14	1,70931E+14	0.134926495770746	0.276740151012478	-4,59481E+14	7,53677E+14	7,43373E+14	6,78287E+14	7,11775E+14	6,40292E+14	6,677E+12
TC0900002064.mm.2	Olfr919	olfactory receptor 919	-0.964924278647843	4,81384E+14	-1,70911E+14	0.134963545863793	0.276780910589704	-4,59504E+14	4,35802E+14	3,92817E+14	4,26362E+14	5,95242E+13	5,2053E+13	5,17554E+13
TC0900001946.mm.2	Jam3	junction adhesion molecule 3	0.8074637728536	7,27966E+14	1,70892E+14	0.135001021394964	0.276822532163025	-4,59528E+14	7,80404E+14	7,86693E+14	7,01288E+14	7,04316E+14	7,64716E+14	6,30376E+14
TC0800000132.mm.2	Upf3a	UPF3 regulator of nonsense transcripts homolog A (yeast)	-0.613975206288458	6,83514E+14	-1,7085E+13	0.135080796818483	0.276950868957402	-4,59578E+14	6,30838E+14	6,46281E+14	6,65356E+14	7,34558E+14	6,96498E+14	7,27553E+14
TC1700001357.mm.2	Tfb1m	transcription factor B1, mitochondrial	0.766353781512502	5,40937E+13	1,70826E+14	0.135126428249438	0.276993181273759	-4,59607E+14	5,80738E+14	5,97156E+13	5,73263E+14	4,51361E+14	5,27398E+14	5,15706E+13
TC1800001412.mm.2	Cdx1	caudal type homeobox 1	-0.685987156661388	5,95729E+14	-1,70818E+13	0.135141126042555	0.276993181273759	-4,59616E+13	5,18686E+14	5,67775E+14	5,73423E+14	6,50235E+14	6,10831E+14	6,53425E+14
TC0700001391.mm.2	Arrb1	arrestin, beta 1	1,09024E+12	8,57038E+14	1,70812E+14	0.135153006341109	0.276993181273759	-4,59624E+14	9,39079E+14	9,193E+14	9,02054E+14	7,38276E+14	8,82098E+14	7,61419E+13
TC0600001388.mm.2	Dppa3	developmental pluripotency-associated 3	-0.852364581497634	5,21107E+14	-1,70782E+14	0.13521081277901	0.277073480379645	-4,5966E+14	4,83888E+14	4,68252E+14	5,30596E+14	5,92017E+14	4,59986E+14	5,91904E+14
TC1800000614.mm.2	Rbm22	RNA binding motif protein 22	1,2485E+14	6,65754E+14	1,70774E+14	0.135226577900176	0.277073480379645	-4,5967E+14	7,61281E+14	7,49349E+13	7,43238E+14	5,17692E+14	6,53643E+14	5,69321E+14
TC0600000772.mm.2	Mad2l1	MAD2 mitotic arrest deficient-like 1	0.557666413089074	6,25093E+14	1,70748E+14	0.13527668687911	0.277126495897781	-4,59701E+14	6,56611E+14	6,71508E+14	5,97956E+14	6,1863E+14	5,78169E+14	6,27686E+14
TC1200001031.mm.2	Ppp4r4	protein phosphatase 4, regulatory subunit 4	0.917580532492103	5,99947E+14	1,70742E+14	0.135286850166446	0.277126495897781	-4,59708E+14	6,80852E+14	6,97759E+14	6,77623E+14	5,17472E+14	5,25157E+14	5,00818E+14
TC0600000657.mm.2	Znrf2	zinc and ring finger 2	0.849306355603573	7,56084E+14	1,70678E+14	0.135410708279681	0.277336300823855	-4,59786E+13	7,68818E+14	8,02427E+14	7,67624E+14	6,3376E+14	8,05279E+14	7,58595E+14
TC0700002166.mm.2	Ppp6r1	protein phosphatase 6, regulatory subunit 1	0.737316273656616	7,69189E+14	1,70671E+14	0.13542369607659	0.277336300823855	-4,59794E+14	8,09824E+14	8,16958E+13	7,96737E+14	6,82583E+14	7,6323E+14	7,45803E+14
TC1100001469.mm.2	Phospho1/Zfp652	phosphatase, orphan 1/zinc finger protein 652	1,02509E+14	6,73293E+13	1,70641E+14	0.135480659325937	0.277417697765767	-4,5983E+14	7,33444E+13	7,37851E+14	6,99879E+14	5,66399E+14	7,18099E+14	5,84088E+14
TC0500002828.mm.2	Hnrnpdl	heterogeneous nuclear ribonucleoprotein D-like	0.969961410711562	8,63526E+14	1,70601E+13	0.135558128611231	0.277522971059434	-4,59878E+14	9,13854E+14	9,03978E+14	8,976E+14	7,2624E+14	8,89077E+14	8,50404E+13
TC1400001153.mm.2	Pibf1	progesterone immunomodulatory binding factor 1	0.751174441016464	6,21384E+14	1,70596E+14	0.135566518041879	0.277522971059434	-4,59883E+14	6,54253E+14	6,79702E+14	6,28957E+14	5,54764E+14	6,50497E+14	5,6013E+14
TC1500001075.mm.2	Krt86	keratin 86	-0.538877332370227	6,4912E+14	-1,70563E+13	0.135631155918107	0.277587303422421	-4,59924E+14	6,06114E+14	5,842E+14	6,33201E+14	6,64888E+14	7,16721E+14	6,89593E+14
TC1200000608.mm.2	Dact1	dishevelled-binding antagonist of beta-catenin 1	-0.558774659172832	6,31558E+14	-1,70562E+14	0.135632398674656	0.277587303422421	-4,59925E+13	6,14052E+14	6,01418E+14	6,40711E+14	6,86514E+14	6,0809E+14	6,3856E+14
TC0200002534.mm.2	Zswim3	zinc finger SWIM-type containing 3	0.598162655563121	6,25155E+13	1,70544E+14	0.135666681075129	0.277622203729179	-4,59946E+14	6,61943E+14	6,77082E+14	6,42593E+14	5,768E+14	6,08963E+14	5,83549E+13
TC1700000418.mm.2	Atp6v0e	ATPase, H+ transporting, lysosomal V0 subunit E	-1,16211E+14	7,87444E+14	-1,70511E+14	0.135729438908748	0.277715358620527	-4,59986E+14	6,88892E+14	7,09564E+13	9,00033E+14	7,30846E+14	9,18504E+14	7,76828E+14
TC0500001730.mm.2	Grid2ip	glutamate receptor, ionotropic, delta 2 (Grid2) interacting protein 1	-0.632774460046556	5,87384E+14	-1,70474E+14	0.135800637884025	0.277822789129404	-4,6003E+14	5,42999E+14	5,54428E+13	5,8638E+13	6,37601E+14	5,68477E+14	6,34417E+14
TC0400000662.mm.2	Snx30	sorting nexin family member 30	0.831174998424157	7,77652E+14	1,70466E+14	0.135816778034033	0.277822789129404	-4,6004E+14	8,40929E+14	8,14434E+14	8,0708E+14	6,82049E+14	7,90812E+14	7,30609E+14
TC1400002228.mm.2	Prss55	protease, serine 55	-0.739844642291328	5,59131E+14	-1,70457E+14	0.135833670499686	0.277822789129404	-4,60051E+13	5,04577E+14	5,12875E+14	5,86241E+14	5,7918E+14	5,27877E+13	6,44034E+14
TC1300000778.mm.2	Habp4	hyaluronic acid binding protein 4	0.552165864090647	8,30173E+14	1,70437E+14	0.13587186763711	0.277865643258885	-4,60075E+14	8,77866E+14	8,56711E+14	8,35289E+14	7,88855E+14	8,02833E+14	8,19484E+14
TC1400001906.mm.2	Ddhd1/Mir5131	DDHD domain containing 1/microRNA 5131	1,33978E+14	8,04314E+14	1,70396E+14	0.135951738732291	0.277985190117005	-4,60125E+14	8,69055E+14	8,79271E+14	8,47732E+14	6,32637E+14	8,73637E+14	7,23553E+14
TC1500001831.mm.2	Tst	thiosulfate sulfurtransferase, mitochondrial	0.974418318384879	6,70171E+14	1,70389E+14	0.135964828604985	0.277985190117005	-4,60133E+14	7,17386E+14	7,27219E+14	7,04529E+14	5,45193E+14	7,01951E+14	6,24749E+12
TC1800000685.mm.2	Grp	gastrin releasing peptide	-0.743025498897875	6,5094E+14	-1,70361E+14	0.136018000149887	0.278057139145425	-4,60166E+14	6,10519E+14	5,89982E+14	6,30484E+14	7,18621E+14	6,40409E+13	7,15626E+14
TC0800001926.mm.2	Pomk	protein-O-mannose kinase	0.715079999041015	6,78314E+14	1,70345E+14	0.13604961477366	0.278057139145425	-4,60186E+14	7,61355E+14	7,5711E+14	6,63276E+14	7,12173E+14	6,37071E+14	5,38901E+14
TC1300002121.mm.2	Spata31d1a	spermatogenesis associated 31 subfamily D, member 1A	-0.554947242362775	5,23377E+14	-1,70344E+14	0.13605178955021	0.278057139145425	-4,60187E+14	4,86749E+14	5,03337E+14	5,39353E+14	5,61723E+14	5,48304E+13	5,00799E+14
TC0X00003043.mm.2	Slc25a53	solute carrier family 25, member 53	0.828433443181656	5,09435E+14	1,70263E+14	0.13620739447178	0.27827967944408	-4,60285E+14	5,01142E+14	5,56198E+14	4,96552E+14	3,95101E+14	5,13474E+14	5,94141E+14
TC1900001448.mm.2	Cpeb3	cytoplasmic polyadenylation element binding protein 3	-0.67787422764141	8,19086E+14	-1,70263E+14	0.136207807834931	0.27827967944408	-4,60285E+14	7,97308E+14	7,70598E+12	8,09151E+14	8,9433E+14	8,00352E+14	8,42779E+14
TC0100001164.mm.2	Fcamr	Fc receptor, IgA, IgM, high affinity	-0.780004713987082	5,45784E+13	-1,7026E+14	0.13621248878393	0.27827967944408	-4,60288E+14	4,77213E+14	5,11813E+14	5,13598E+14	6,3143E+14	5,5234E+14	5,88311E+14
TC0700002593.mm.2	Zfp109	zinc finger protein 109	0.795206622972385	7,32892E+13	1,70222E+14	0.136284944533166	0.278382089539707	-4,60333E+14	7,89234E+13	7,72434E+14	7,65455E+14	6,37172E+14	7,29863E+14	7,03193E+14
TC0100003886.mm.2	Gm7694	predicted gene 7694	-0.847588700054009	5,54843E+13	-1,70216E+14	0.13629717025872	0.278382089539707	-4,60341E+14	5,01957E+14	4,8923E+14	5,14022E+14	6,46683E+14	5,58953E+14	6,18209E+14
TC1000000831.mm.2	Grin3b	glutamate receptor, ionotropic, NMDA3B	-0.704839133246881	6,50716E+14	-1,70164E+14	0.136397182111173	0.278551051376088	-4,60403E+14	6,02557E+14	6,31268E+14	6,36268E+14	7,38525E+14	6,54316E+13	6,41365E+14
TC0400002075.mm.2	C030017K20Rik	RIKEN cDNA C030017K20 gene	-0.688282908549585	6,39421E+14	-1,70143E+14	0.136437585627678	0.278598253354298	-4,60428E+14	5,79803E+13	5,8494E+14	6,00205E+14	7,02194E+14	6,84158E+14	6,85225E+14
TC0300002376.mm.2	S100a9	S100 calcium binding protein A9 (calgranulin B)	-0.712192335643986	4,87584E+14	-1,70114E+14	0.136495056928384	0.278680290457052	-4,60464E+14	4,45494E+14	4,28648E+14	4,57238E+14	5,59342E+14	4,99957E+14	5,34826E+14
TC1600001057.mm.2	Ttc3	tetratricopeptide repeat domain 3	1,29375E+14	9,58895E+14	1,70071E+14	0.136577711666721	0.278792264151225	-4,60515E+14	1,06621E+14	1,05417E+14	1,00568E+14	8,55942E+14	9,87223E+14	7,84144E+14
[truncated: 2,137,218 more chars]
