# Supplementary material for: Functional involvement of septal miR-132 in extinction and oxytocin-mediated reversal of social fear
Source: Mol Psychiatry. 2023 Nov 8;29(6):1754–66. doi: 10.1038/s41380-023-02309-3 (PMC11371636; doi:10.1038/s41380-023-02309-3)
Supplement: Supplementary file 20 — Supplementary Table S14 [file 41380_2023_2309_MOESM20_ESM.pdf]

PROBEID	SYMBOL	GENENAME	logFC	AveExpr	t	P.Value	adj.P.Val	B	P234_01_N_Scr_1.CEL	P234_02_N_Inh_3.CEL	P234_03_6F4_Scr_1.CEL	P234_04_6F4_Scr_3.CEL	P234_05_6F4_Inh_2.CEL	P234_06_6F4_Inh_3.CEL	cluster.kmeans.10
**TC0100001943.mm.2**	Mybl1	myeloblastosis oncogene-like 1	1.69579115422811	4.62876862918358	5.34246235813202	0.00141016287661733	0.999692817443324	-4.43766569630989	5.62661754592971	5.35088772527242	5.00795465384154	5.08538975233624	3.54582996196477	3.15593213575678	7
**TC0700000567.mm.2**	Igflr1/U2af1l4	IGF-like family receptor 1/U2 small nuclear RNA auxiliary factor 1-like 4	-1.49243528625599	7.18092040453183	-4.8084663081112	0.00246126787214191	0.999692817443324	-4.44565964283307	7.99550848033733	7.81536568785403	6.25680157132756	5.88808727191625	7.5179994928336	7.61175992292219	9
**TC0600002800.mm.2**	Lmod3	leiomodin 3 (fetal)	1.39420865056523	4.28085044894486	4.70471422249897	0.00275501488057704	0.999692817443324	-4.44743940531988	3.77759812837637	3.93444730886002	5.1116090454742	5.26912823330741	3.86374398604452	3.72857599160662	4
**TC1400002839.mm.2**	Gm3500/Gm3373	predicted gene 3500/predicted gene 3373	1.92298270151909	6.02616070292699	4.68992771663218	0.0027999880195338	0.999692817443324	-4.4476997423882	6.80657879722064	6.3087708939325	6.92831307126662	6.51547689345687	5.21189360633927	4.38593095534604	10
**TC1700000656.mm.2**	Hspa1l	heat shock protein 1-like	-1.43972088999033	4.94690909722892	-4.56968386589299	0.00319776366257534	0.999692817443324	-4.44988133226867	4.5121591010245	4.73228171247983	4.5004966684168	4.27828932652746	5.65806604609076	6.00016172883417	2
**TC0100003015.mm.2**	Map3k19	mitogen-activated protein kinase kinase kinase 19	-1.4753706568876	4.50778521114331	-4.44127198001764	0.00369366962755002	0.999692817443324	-4.45234382586308	4.73538798946824	4.88046774930269	3.90723385349461	3.33282325366225	5.05503563804667	5.1357627828854	9
**TC1400002152.mm.2**	Micu2	mitochondrial calcium uptake 2	1.29774376683843	6.54998023485023	4.32446508122745	0.00422011033694046	0.999692817443324	-4.45471038135808	6.72549247020135	7.0328667672064	7.05227203186905	7.01623282081621	5.72349655860322	5.74952076040518	10
**TC1100002799.mm.2**	Fam114a2	family with sequence similarity 114, member A2	1.54340548133979	5.24322620420382	4.19500403058348	0.00490328696417272	0.999692817443324	-4.45748334602648	6.30547699059583	6.27146064483826	5.58368390204748	5.40093137418673	4.3503341286089	3.54747018494573	7
**TC1000002017.mm.2**	Fam229b	family with sequence similarity 229, member B	-1.21348506430691	5.88834518416059	-4.17649027998922	0.00501066340112131	0.999692817443324	-4.45789336586	6.27021288072475	6.14074462961073	5.11202565397082	5.13404607903629	6.37751339102898	6.29552847059195	9
**TC1300002536.mm.2**	Ocln	occludin	-1.40796720923798	5.76480573884737	-3.99981807554704	0.00617736904561707	0.999692817443324	-4.46198490336862	6.06081318561691	6.65147250435668	4.78604363243492	4.74426352988241	6.38597804974355	5.96026353104975	9
**TC0200001982.mm.2**	Vps16	VSP16 CORVET/HOPS core subunit	1.29449479145915	6.61409629666815	3.98199750303823	0.00631084375600092	0.999692817443324	-4.46241621324805	7.75246206758948	7.51696431063032	6.98416387871155	6.51790661364216	5.378176847214	5.5349040622214	6
**TC0300001982.mm.2**	Nhlrc3	NHL repeat containing 3	1.21145836874721	6.10751765728377	3.95280818239692	0.00653641124713772	0.999692817443324	-4.46313031362257	5.57961334884182	5.91998027180231	6.95597921890486	6.82823531137159	5.62124774106163	5.74005005172041	4
**TC0200003182.mm.2**	Qsox2	quiescin Q6 sulfhydryl oxidase 2	1.19989144693042	5.97665542899142	3.89334437684078	0.00702400310140548	0.999692817443324	-4.46461488984629	6.70809614330795	6.47916797975747	6.280285863952	6.25593980841996	4.90482031278533	5.23162246572578	3
**TC0800002011.mm.2**	Smim18	small integral membrane protein 18	1.23793594102995	5.86870791077281	3.88543792490703	0.00709180505396655	0.999692817443324	-4.46481534281552	6.24995839643121	6.19524562314875	6.54650032760904	6.07495733594938	5.14069840259322	5.00488737890528	10
**TC0300000935.mm.2**	Hist2h2bb	histone cluster 2, H2bb	-1.35912771408822	7.14975442447736	-3.86253717730152	0.00729229928210654	0.999692817443324	-4.46540005835011	7.31770724802153	7.81342025832504	6.52777073204002	5.99680107413054	7.64874661016423	7.59408062418277	9
**TC0900001858.mm.2**	Tyk2	tyrosine kinase 2	1.49814872372129	5.499316510598	3.82714549326425	0.00761455550836834	0.999692817443324	-4.46631584589585	5.43214623300457	5.59289970354397	5.82308030484571	6.6604949823953	4.53037422525326	4.95690361454518	1
**TC1700001563.mm.2**	Zfp942	zinc finger protein 942	-1.12181443273906	5.9468847100818	-3.79603166007425	0.00791085182933185	0.999692817443324	-4.46713329311992	6.705385903144	6.74883199960761	5.08287279929679	4.90885794683377	6.03213171582535	6.20322789578331	9
**TC0900001802.mm.2**	Zfp558	zinc finger protein 558	1.68382395672565	5.37957676723081	3.64900434049848	0.00949364497026842	0.999692817443324	-4.47115766847567	6.30606558172728	6.01832289245392	5.84381657779513	5.81654344353235	3.51522742090505	4.77748468697113	3
**TC0900003121.mm.2**	Tdgf1	teratocarcinoma-derived growth factor 1	-1.12314128339833	4.35721985654719	-3.60200294795702	0.0100706168834449	0.999692817443324	-4.47250235728696	3.94127898829992	3.96187684288453	3.80903923656169	4.18790113408933	5.01589452821356	5.2273284092341	2
**TC1100001155.mm.2**	Spag5	sperm associated antigen 5	1.05890734880431	5.14724276452529	3.54486456940766	0.0108243362386275	0.999692817443324	-4.47417644396982	5.25583889555339	5.05607831828476	5.77520130902872	5.56947572643238	4.58335559772287	4.64350674012961	10
**TC1600001443.mm.2**	Atp13a4	ATPase type 13A4	1.14802854514686	4.74855452658009	3.5394453454857	0.0108989661499158	0.999692817443324	-4.47433750003119	5.49768213618651	4.99037870158972	5.11091894962007	5.03874275637895	4.01153774180386	3.84206687390144	7
**TC0500003461.mm.2**	Nyap1	neuronal tyrosine-phosphorylated phosphoinositide 3-kinase adaptor 1	1.28925873985983	6.62319495037957	3.49958426362514	0.0114655106100991	0.999692817443324	-4.47553445851347	6.93057273503931	6.90816649474072	6.71845927765996	7.52101469844857	5.92943879036692	5.73151770602194	1
**TC0900000232.mm.2**	Npsr1	neuropeptide S receptor 1	1.11509082164757	5.06120624354448	3.49791676333153	0.0114899019839436	0.999692817443324	-4.47558500607087	5.94612257817604	5.45385547849797	5.27236210299005	5.32635842095396	4.18288339412877	4.1856554865201	7
**TC0600001279.mm.2**	Pparg	peroxisome proliferator activated receptor gamma	-1.24762421109217	5.45280479649623	-3.42621956492044	0.0125941913381988	0.999692817443324	-4.47779507160096	5.17485700020236	5.23965423945632	5.03574404984839	4.86779050871879	6.5902869002835	5.808496080468	2
**TC1400000341.mm.2**	Parg	poly (ADP-ribose) glycohydrolase	1.68971716114537	6.07217460298203	3.42428652493407	0.0126255240822206	0.999692817443324	-4.47785566029417	7.02694294285954	7.00662808832322	6.69335311393212	6.19610234056796	5.42995604250763	4.08006508970171	5
**TC1400000477.mm.2**	Ptger2	prostaglandin E receptor 2 (subtype EP2)	-1.33393766338588	4.38775330648245	-3.39678508050556	0.0130806208360774	0.999692817443324	-4.47872343898154	4.33622229334404	3.41568770863436	4.11273716743381	3.84063008763846	5.30288366004093	5.3183589218031	2
**TC0600001192.mm.2**	Setmar	SET domain without mariner transposase fusion	1.01044283648215	6.58639279294505	3.3598773702649	0.0137196761908732	0.999692817443324	-4.47990514702696	6.84270835199749	6.87241182920074	6.9497332510462	6.962327873672	6.10401339079909	5.7871620609548	10
**TC0100003823.mm.2**	Traf5	TNF receptor-associated factor 5	1.41688409778879	5.12944601988829	3.35695530965391	0.0137717041600077	0.999692817443324	-4.47999955110022	4.71091827536525	4.88950272207992	5.46400582952304	6.54100582920804	4.68257519991591	4.48866826323758	4
**TC0700001311.mm.2**	Ccdc90b	coiled-coil domain containing 90B	-1.02037378069259	6.72648447927156	-3.29906713236721	0.0148479098998629	0.999692817443324	-4.48189566801659	6.5866686704002	6.58907612671096	6.10809693086432	6.46311032770217	7.20289316986688	7.4090616500848	2
**TC0400002232.mm.2**	Slc26a7	solute carrier family 26, member 7	-1.0530792563721	4.66556375604553	-3.29373501043891	0.0149515422454052	0.999692817443324	-4.48207282184931	4.04720701079943	4.04569548943529	4.19765815081966	4.69950261082746	5.48525359076714	5.51806568362419	2
**TC0300000784.mm.2**	Jtb	jumping translocation breakpoint	-1.40735464885952	7.91148162256767	-3.26810327591657	0.0154607847582916	0.999692817443324	-4.48293036128159	7.96617568610833	8.30434340021676	7.6456392688134	6.54619140686756	8.50935810147802	8.49718187192198	9
**TC0900000922.mm.2**	Bnip2	BCL2/adenovirus E1B interacting protein 2	-1.09163563487317	6.12027143661299	-3.26705826569705	0.0154819420137401	0.999692817443324	-4.48296553324489	6.72631714578388	6.98046626422552	5.22027372900551	5.19551324095558	6.57743257999684	6.0216256597106	9
**TC0600002663.mm.2**	Rab43	RAB43, member RAS oncogene family	1.04806369362716	5.14242575841052	3.25257900990952	0.0157783424617458	0.999692817443324	-4.48345456343767	5.19697115066206	5.27514407328483	5.36671543714877	5.87256791973649	4.62542726518563	4.51772870444532	1
**TC1200001055.mm.2**	Tcl1b1	T cell leukemia/lymphoma 1B, 1	-1.25208365759082	4.76976305642054	-3.23567547984942	0.0161321736999543	0.999692817443324	-4.48402950257205	4.36057046601351	4.34801079706356	4.8142482645509	3.88866661558136	5.56108761448636	5.64599458082753	2
**TC0200003834.mm.2**	Frzb	frizzled-related protein	-1.09926887925888	5.45027564892314	-3.23424403003827	0.0161625298365645	0.999692817443324	-4.48407839059051	5.50108865607184	6.05456764086595	4.83541996735783	4.63830995168381	5.97647484455375	5.69579283300564	9
**TC0700003399.mm.2**	Mkrn3	makorin, ring finger protein, 3	1.06132667015428	4.88553788265429	3.21110503538336	0.0166619081915405	0.999692817443324	-4.48487300921303	4.6076733789605	4.80796827120701	5.23846295495766	5.77165653807574	4.5122847735667	4.37518137915814	4
**TC0900000366.mm.2**	Msantd2	Myb/SANT-like DNA-binding domain containing 2	-1.01140190422451	5.4992355651005	-3.19957021035842	0.0169170662865879	0.999692817443324	-4.48527220587179	5.64079951612409	5.52961996644536	4.76472524913699	5.13636980065529	5.82697446004529	6.09692439819601	2
**TC1900000467.mm.2**	Papss2	3'-phosphoadenosine 5'-phosphosulfate synthase 2	0.945582311013706	6.6159400923185	3.19307135124617	0.0170626849879193	0.999692817443324	-4.48549802427872	7.64819297504627	7.592062624292	6.63853749525907	6.53473729304101	5.52684024817951	5.75526991809316	6
**TC0300000220.mm.2**	4930429B21Rik	RIKEN cDNA 4930429B21 gene	-1.72127814041509	5.33011899425717	-3.18473674867934	0.0172514244574854	0.999692817443324	-4.48578858879026	6.34353657726335	6.47878988577504	4.74464430492934	3.1132713059079	5.58788188389371	5.7125900077737	9
**TC1500001073.mm.2**	Krt7	keratin 7	1.11172346218756	5.845294754255	3.15306987262105	0.0179893750330381	0.999692817443324	-4.4869024482089	5.33588461060006	5.5447526790589	6.25810543162495	6.94918364849812	5.55490433043636	5.4289378253116	4
**TC0300000550.mm.2**	Gfm1	G elongation factor, mitochondrial 1	1.09649201345388	7.04693144233886	3.12633231050448	0.0186390258107047	0.999692817443324	-4.48785518597145	7.65316162804541	7.71277617021526	7.00320562274216	7.55111181859795	6.41333269983899	5.94800071459336	7
**TC1000002453.mm.2**	Pdxk	pyridoxal (pyridoxine, vitamin B6) kinase	1.09180562773057	8.47328005853803	3.12567443943333	0.0186553261143752	0.999692817443324	-4.48787877028537	9.63403438246774	9.50278400202761	8.73062894098548	8.2126076701115	7.61533441129346	7.14429094434238	6
**TC0800000015.mm.2**	Pet100	PET100 homolog	1.709282667281	7.00385719216965	3.11619067225157	0.0188920254079944	0.999692817443324	-4.48821952120948	7.86184490427384	7.41058441833924	6.8926889086215	8.19195067386193	5.35447021439779	6.31160403352363	8
**TC1700002656.mm.2**	Lrpprc	leucine-rich PPR-motif containing	1.21709959269822	5.75688246040184	3.11213459689334	0.0189942455826994	0.999692817443324	-4.48836569202489	7.0863799219516	6.64717847155721	5.53146682964848	6.08950094750086	4.91000500180749	4.27676358994541	7
**TC0400000952.mm.2**	Tek	TEK receptor tyrosine kinase	-0.949013050663617	5.30311642415911	-3.09454226810038	0.019444556263273	0.999692817443324	-4.4890027115664	5.21483623453894	5.53153216494327	4.85363329658924	4.73351872548337	5.83352604044976	5.65165208295009	2
**TC0900001888.mm.2**	Tmem205	transmembrane protein 205	0.951141175555284	6.10391065105911	3.09383949574035	0.0194627825002721	0.999692817443324	-4.48902826173657	6.14369032026656	6.32405591915344	6.62002256725288	6.40897744176974	5.42487305943059	5.70184459848146	10
**TC0200002264.mm.2**	Sdcbp2	syndecan binding protein (syntenin) 2	-0.972181067668635	5.13894057517157	-3.09207669571868	0.0195085813585623	0.999692817443324	-4.4890923853397	5.14917131128081	4.80070406578764	4.84835264457345	4.6213503247384	5.61131480996218	5.80275029468693	2
**TC1500000102.mm.2**	Gm2573	predicted gene 2573	0.889145094107861	7.48176956348378	3.05430857619091	0.0205182358639697	0.999692817443324	-4.49047824027737	8.31837176830917	8.25557992455055	7.50317686995916	7.5443010681702	6.5595498460057	6.70963790390793	6
**TC0200004443.mm.2**	Ndufaf1	NADH:ubiquinone oxidoreductase complex assembly factor 1	1.1273381797675	5.82220950298882	3.03515727150157	0.0210515697458448	0.999692817443324	-4.49118978773453	6.73008379716008	6.86288365733528	6.30668938171627	5.49079357977001	4.85686502625126	4.68594157570003	6
**TC0600001793.mm.2**	Tfpi2	tissue factor pathway inhibitor 2	0.917346466743172	5.32975961745536	3.03496258403955	0.0210570674264153	0.999692817443324	-4.49119705177259	5.02110111582171	5.29796521899352	5.96144458185889	5.78564756984274	4.99387452728487	4.91852469093041	4
**TC0X00000063.mm.2**	Gpkow	G patch domain and KOW motifs	0.882748268511654	7.12322919667483	3.03257814483092	0.0211245257415333	0.999692817443324	-4.49128606834831	7.29771692705445	7.3705086745482	7.53318710458972	7.38513595314509	6.5902673128371	6.5625592078744	10
**TC0100000971.mm.2**	Gin1	gypsy retrotransposon integrase 1	-0.89585887392227	5.86964714852682	-3.01228143003473	0.0217082324958432	0.999692817443324	-4.49204754959283	6.30776739395546	6.16461689509194	5.16334622830995	5.31354419882455	6.04385873208483	6.2247494428942	9
**TC1400001393.mm.2**	Gm3500	predicted gene 3500	1.8469618148673	5.31201956441469	2.97641313912215	0.0227825306805588	0.999692817443324	-4.49340977199779	6.47890724183294	5.6016258835803	6.36975840055193	5.37299554485283	4.75370724654523	3.29512306912492	5
**TC0800001497.mm.2**	Acsf3	acyl-CoA synthetase family member 3	-0.915019485225712	5.7380505744814	-2.96732219577565	0.0230637808442171	0.999692817443324	-4.49375840622801	6.15439763424675	5.88407814448661	5.25032882032942	5.02956552852238	5.95574603527853	6.1541872840247	9
**TC1400000524.mm.2**	Peli2	pellino 2	1.05562665933542	6.18263466104549	2.96631867098149	0.0230950546447686	0.999692817443324	-4.49379697498442	6.76615958750056	6.69712293975368	6.66295677188638	6.2089326069584	5.08193736230683	5.67869869786712	3
**TC1000001531.mm.2**	Lrig3	leucine-rich repeats and immunoglobulin-like domains 3	1.13577951864388	5.75445059621641	2.96550378168024	0.0231204832557085	0.999692817443324	-4.49382830615181	5.66689208906712	5.54492568009983	5.95537001531045	6.83785240739919	5.34908403405379	5.1725793513681	1
**TC1100003137.mm.2**	Pitpnm3	PITPNM family member 3	-0.965485962887138	5.86611893474405	-2.96382046480344	0.0231731062006308	0.999692817443324	-4.4938930618432	6.54799630042019	6.70957580950983	5.18211914624402	4.82196564013598	5.77568721349491	6.15936949865938	9
**TC1100000816.mm.2**	Tvp23b	trans-golgi network vesicle protein 23B	-0.977498975439653	6.9863936773755	-2.96145384442561	0.0232473074040374	0.999692817443324	-4.49398418317775	7.12766183110839	7.16317280618106	6.52992370300279	6.30634103503933	7.12721376460117	7.66404892432024	9
**TC0700002217.mm.2**	Peg3	paternally expressed 3	1.27555880952628	7.64650074474297	2.95148869775844	0.023562551274164	0.999692817443324	-4.49436888986633	9.55020351959442	8.88025456138102	7.67778930997522	7.32204269329225	6.66168063253922	5.78703375167568	6
**TC0400002505.mm.2**	Ccl19	chemokine (C-C motif) ligand 19	-0.935587479822949	5.28705175762998	-2.94645119624285	0.0237236505208652	0.999692817443324	-4.49456399277606	5.31572445653826	5.08458204860615	5.06685420599483	4.65856033449994	5.85919137048031	5.73739812966036	2
**TC0800001977.mm.2**	Mak16	MAK16 homolog	0.982254562631746	6.04862670440964	2.93469238300259	0.0241043048318608	0.999692817443324	-4.49502106023008	6.70985871376243	6.94159706602111	5.93790945620847	6.36449732976043	5.35825829936746	4.97963936133795	7
**TC0700003476.mm.2**	Nr2f2	nuclear receptor subfamily 2, group F, member 2	1.49763740214914	7.26654287937979	2.92991729381273	0.0242607450151973	0.999692817443324	-4.49520732863234	8.6423544375503	8.14194834273548	7.66806049547218	7.23705415467345	6.63727472346501	5.27256512238233	5
**TC0600000827.mm.2**	Cd8a	CD8 antigen, alpha chain	1.29673456157438	5.06977170993624	2.91878702490135	0.0246296246938223	0.999692817443324	-4.49564298469218	4.9202480643684	4.47479496463741	5.35896031448081	6.4495678623994	4.45844694057651	4.75661211315492	4
**TC1700002361.mm.2**	Nudt12	nudix (nucleoside diphosphate linked moiety X)-type motif 12	0.952040545850572	5.70252548413405	2.91532705564322	0.0247455138427234	0.999692817443324	-4.4957788366647	6.18063684917845	6.04542811643481	5.77375311277084	6.17283140267525	4.83941845453219	5.20308496921275	8
**TC1000000292.mm.2**	Rsph4a	radial spoke head 4 homolog A (Chlamydomonas)	0.980499418000202	7.28216156943768	2.90828111719887	0.0249833189444741	0.999692817443324	-4.49605610972523	7.91804181383741	8.11072042659667	7.49168912469185	7.32091388140435	6.71467611676482	6.13692805333098	7
**TC0500002758.mm.2**	Nup54	nucleoporin 54	1.19129639817416	5.70952945036606	2.9074302969259	0.0250121995941131	0.999692817443324	-4.49608964785195	6.88155280045197	6.83900525248537	5.45003752822051	6.00956819458315	4.98083085619597	4.09618207025937	7
**TC0200005018.mm.2**	Ghrh	growth hormone releasing hormone	1.04437651820639	5.75461406855191	2.88653251765401	0.0257328805556647	0.999692817443324	-4.49691723826648	5.63080393139458	5.52623164386514	5.99534526786803	6.73435566836424	5.19321774325471	5.44773015656477	4
**TC0400003777.mm.2**	Eloa	elongin A	0.891684641455737	7.44267164479203	2.88194714600139	0.0258939597402984	0.999692817443324	-4.49709981387952	7.78975757049902	7.83932644041315	7.8924302737922	7.51272729658356	6.88640072221415	6.73538756525013	5
**TC1100002102.mm.2**	Slc35e4	solute carrier family 35, member E4	1.03022659345212	5.53809835632878	2.87893918776986	0.0260002105660557	0.999692817443324	-4.49721977488678	6.0764951597743	5.85221407409851	5.76821122421229	5.91195582128977	5.16840412534301	4.45130973325481	7
**TC0500000110.mm.2**	Speer4d	spermatogenesis associated glutamate (E)-rich protein 4D	-0.829070254995343	4.87503624421666	-2.84555357492755	0.0272112353454252	0.999692817443324	-4.49856154110322	4.4703039234525	4.4112296146893	4.69023344577871	4.66503826280502	5.58712486189896	5.42628735667545	2
**TC0600002625.mm.2**	Nat8f4	N-acetyltransferase 8 (GCN5-related) family member 4	0.890272098485874	5.9585874882209	2.84468904701954	0.0272433830375957	0.999692817443324	-4.49859653826698	6.30891427807167	6.14929335847223	6.0685495097701	6.46838123510652	5.43421706350399	5.32216948440088	1
**TC0500001762.mm.2**	1700018F24Rik	RIKEN cDNA 1700018F24 gene	-0.898769649700882	5.29913316213643	-2.83647032474392	0.0275510272471775	0.999692817443324	-4.49892987901478	5.00865528495929	4.84038013332509	5.04508675936074	5.02902536820547	5.71169564992473	6.15995577704325	2
**TC0600002934.mm.2**	Ghrl	ghrelin	-0.892055676838059	5.73056597423739	-2.82194446438935	0.0281038466831127	0.999692817443324	-4.49952185089899	5.65193093936533	5.66768046592064	5.46026418569857	5.17957235753256	6.40196402359499	6.02198387331226	2
**TC0800000114.mm.2**	F7	coagulation factor VII	0.907491603600102	5.27933518770982	2.81113902685376	0.0285227299375843	0.999692817443324	-4.49996454706575	4.40273935922999	4.44927569629178	5.89758623539724	6.42190340357145	5.27132098341925	5.23318544834923	4
**TC1800000891.mm.2**	Fbxo15	F-box protein 15	0.885436261088841	4.67122503796457	2.80558610313183	0.0287405739014612	0.999692817443324	-4.50019282783507	4.30371326127341	4.62650913132699	5.38608158370178	5.04791859498058	4.3185703318364	4.34455732466827	4
**TC0100000817.mm.2**	Neu2	neuraminidase 2	0.821393267245065	6.10580322809641	2.79446762411112	0.0291820935793143	0.999692817443324	-4.50065150040801	6.25244109477931	6.25423131196024	6.46725853087283	6.4182082172917	5.72973613519212	5.51294407848227	10
**TC1900000665.mm.2**	Kazald1	Kazal-type serine peptidase inhibitor domain 1	0.891439661431535	4.72161179585875	2.79401025949117	0.029200409449117	0.999692817443324	-4.50067041365897	4.49597932647069	4.6014668572561	5.02214621037629	5.48540574676811	4.43599272415416	4.28867991012717	4
**TC1000002269.mm.2**	Tet1	tet methylcytosine dioxygenase 1	1.30769659381275	5.20615440732732	2.78613178242436	0.0295178405353253	0.999692817443324	-4.50099677486222	6.19111180194062	5.54183429312591	5.8350744775848	5.22461229067664	4.71644486185167	3.72784871878427	5
**TC1200001244.mm.2**	Apopt1	apoptogenic, mitochondrial 1	-1.07441469393577	5.88337471229241	-2.78397541978527	0.0296053600716713	0.999692817443324	-4.50108628719136	6.07329630746781	6.28937937482692	5.63892571891528	4.75544588287881	6.35309131770597	6.19010967195965	9
**TC0400000459.mm.2**	Ccin	calicin	1.0817408710478	4.83753055800836	2.77733651533473	0.0298765460982607	0.999692817443324	-4.50136237654509	3.92082322726779	4.29829104773946	5.31110005305778	6.17367535451147	4.63428079771758	4.68701286775607	4
**TC1100002834.mm.2**	Fam183b	family with sequence similarity 183, member B	0.861868048879469	6.41615842240089	2.76853935232198	0.0302399633903759	0.999692817443324	-4.5017293916634	6.56673080103382	6.53342839178371	6.567734373814	6.99252934585938	5.94600886823381	5.89051875368063	1
**TC1000002775.mm.2**	Tmtc3	transmembrane and tetratricopeptide repeat containing 3	-1.09353928408478	6.62122798904414	-2.76501390579376	0.0303869168033191	0.999692817443324	-4.50187684731177	6.20005835202344	6.3791290519516	6.58585858573939	5.89469239532073	7.00210480839497	7.6655247408347	2
**TC1200001373.mm.2**	Klhl29	kelch-like 29	0.992441525340855	6.34277096886617	2.76394513687449	0.0304316165500152	0.999692817443324	-4.50192159215404	7.32921124731038	6.93613366398994	6.61574546995837	6.27233650633082	5.73619410825427	5.16700481735321	5
**TC1900001524.mm.2**	Crtac1	cartilage acidic protein 1	-0.836068041543964	5.95946500222981	-2.7584518219528	0.0306624702996924	0.999692817443324	-4.50215188560052	5.93971734308098	5.92232623202768	5.40612265727876	5.70518252031236	6.47830474513203	6.30513651554702	2
**TC0400000435.mm.2**	Rusc2	RUN and SH3 domain containing 2	0.968930326060839	6.46333124037411	2.74417533433385	0.0312711636054454	0.999692817443324	-4.50275283319917	7.02372705510477	6.84368424338136	6.35718954662957	7.06802885131053	5.71234240393273	5.7750153418857	8
**TC1500000860.mm.2**	Trabd	TraB domain containing	0.860718053218111	7.16640276610515	2.7429265473611	0.0313250130242266	0.999692817443324	-4.50280556691925	6.96395848417965	6.90297758753294	7.65612250146286	7.7703358142144	6.63578797191981	7.06923423732123	4
**TC0400003445.mm.2**	Exo5	exonuclease 5	1.53710836096319	5.96820587599273	2.73532733799848	0.0316548274933737	0.999692817443324	-4.50312704880405	6.28832842896894	6.18607261266898	6.85127710986361	6.35324835825879	5.89297422994536	4.23733451625066	10
**TC1200002398.mm.2**	Ankrd9	ankyrin repeat domain 9	1.0383562565596	5.09722337313586	2.73113731262796	0.0318382515255382	0.999692817443324	-4.50330473476505	5.88769655898327	5.82445949987051	5.12226501772047	5.35168332881983	4.62891944150552	3.76831639191557	7
**TC0500000465.mm.2**	Cc2d2a	coiled-coil and C2 domain containing 2A	0.855518373073846	5.39249364477158	2.7212329934926	0.0322763172593322	0.999692817443324	-4.50372595791938	6.19318654623637	6.39171052745373	5.48279630871174	5.25775446183178	4.68749708004523	4.34201694435061	6
**TC0800000962.mm.2**	Gadd45gip1	growth arrest and DNA-damage-inducible, gamma interacting protein 1	-0.808162846527774	6.48003580827544	-2.71412381191341	0.0325946868722496	0.999692817443324	-4.50402935679316	6.49416063191385	6.33911817842194	6.22324700683943	5.99205816629123	6.89968294106439	6.93194792512183	2
**TC1700000487.mm.2**	Mapk13	mitogen-activated protein kinase 13	1.23575730515615	6.4006727067556	2.70563919043568	0.0329790086737015	0.999692817443324	-4.50439260592724	5.76751611546188	6.72300874307786	6.67653888468975	7.51597411146332	5.89030161441402	5.83069677142675	4
**TC1200001353.mm.2**	Ncoa1	nuclear receptor coactivator 1	0.841379986092502	7.45395719024585	2.70243331020051	0.0331254685882413	0.999692817443324	-4.50453018428978	8.84418234277222	8.60726170197585	7.32361107559519	7.15391845886082	6.55548478957825	6.23928477269276	6
**TC1400001965.mm.2**	Armh4	armadillo-like helical domain containing 4	-0.87105294437487	5.4466681999537	-2.69976826339059	0.0332477443072477	0.999692817443324	-4.50464468934135	5.46645472211544	5.45693490698146	5.08184180350285	4.92541503743493	6.12591542006972	5.62344730961781	2

**TC0800002674.mm.2**	Tox3	TOX high mobility group box family member 3	0.900102839655155	6.99037439815273	2.69023862487113	0.033688895373138	0.999692817443324	-4.50505514724067	7.33764408989981	7.1821855440579	7.56794291119395	7.04336830594053	6.26535280976257	6.54575272806161		3
**TC0200002749.mm.2**	Gm14403	predicted gene 14403	-1.73578790786585	8.00731155474893	-2.6862752911234	0.0338741861075147	0.999692817443324	-4.50522632089696	7.69431382936625	9.18454391949176	6.88523368344862	6.9614841985033	9.38054851531025	7.93774518237338		9
**TC1700000292.mm.2**	Zfp946	zinc finger protein 946	0.951948029487838	5.9265371088044	2.67958638825018	0.0341893471094711	0.999692817443324	-4.50551583136638	7.06569380856893	6.83447170126025	6.09862999699729	5.68284660398914	5.22822699888478	4.64935354312598		6
**TC1800000677.mm.2**	Zfp532	zinc finger protein 532	1.33860853519742	6.33963853067177	2.67586266833872	0.0343661384631872	0.999692817443324	-4.50567734044959	6.75054848055662	6.76787280910899	7.28608781112001	6.31222567125989	6.01011713932188	4.91097927266319		5
**TC0400002626.mm.2**	Tex10	testis expressed gene 10	-0.961593913463928	5.63969366037609	-2.67449336866039	0.0344313913923727	0.999692817443324	-4.50573679201279	6.38406880118498	6.38431929018486	5.17138630790266	4.40190671407677	5.79490795081205	5.70157289809523		9
**TC1000001038.mm.2**	Pwp1	PWP1 homolog, endonuclein	0.881544154602516	5.98235090638704	2.66244684092063	0.0350111288665436	0.999692817443324	-4.50626123316606	6.7399188545164	6.80354162124994	6.1848715761358	5.87199505974467	5.39500849251961	4.89876983415582		6
**TC1700000050.mm.2**	Tulp4	tubby like protein 4	0.873263079300269	6.9934962235545	2.65826676261074	0.035214694753298	0.999692817443324	-4.50644380382632	8.26739899544632	8.15637775803662	6.87844817985465	6.76341519336764	6.22181412204133	5.67352309258042		6
**TC1700001987.mm.2**	Mrps18b	mitochondrial ribosomal protein S18B	1.09460391066932	6.52239430486839	2.6559435949717	0.0353283703701843	0.999692817443324	-4.50654540339344	7.31995545360893	7.17170801871698	6.89233087554116	6.52362421357037	5.12224033521566	6.10450693255723		3
**TC1200002154.mm.2**	Ston2	stonin 2	-0.771299428705515	5.98806675150941	-2.65563512289383	0.0353434933983567	0.999692817443324	-4.50655890096146	7.13252081268818	7.11150526926612	4.97909362632872	5.09179415851685	5.85670312064836	5.75678352160823		9
**TC0700002944.mm.2**	Zfp715	zinc finger protein 715	1.05319127429771	6.41828970243885	2.65011065053514	0.0356154932636851	0.999692817443324	-4.50680091261715	6.72739895146192	6.69616536115299	6.85797468837524	6.73830353693156	6.23336703789842	5.25652863881297		10
**TC0900001132.mm.2**	Zfp949	zinc finger protein 949	1.15158822304817	6.43457910911777	2.64974514677692	0.0356335666922399	0.999692817443324	-4.50681694313505	6.93025958980285	6.14898812046844	6.5247442029886	7.39095749227722	5.7651573708884	5.84736787828109		1
**TC1100001163.mm.2**	Sebox	SEBOX homeobox	-0.913160364106825	5.28420694617327	-2.64666413140624	0.0357863016253896	0.999692817443324	-4.50695216530792	4.75824834568978	5.30427353302869	4.84947720713415	5.0587223279196	6.045665374451	5.6888548888164		2
**TC1300000267.mm.2**	Slc17a1	solute carrier family 17 (sodium phosphate), member 1	-0.778933875480327	5.40255325782094	-2.6361312573198	0.0363136805149914	0.999692817443324	-4.50741569516428	4.27464386655116	4.51062691893627	5.49389044846801	5.54220005677076	6.32491978120483	6.26903847499459		2
**TC1700002303.mm.2**	Zfp119a	zinc finger protein 119a	-0.794616044694393	5.18890834978207	-2.6327272492107	0.0364858646125883	0.999692817443324	-4.50756591358293	5.69701594625423	6.01696838805917	4.4993105188187	4.41580631867641	5.24102888962056	5.26332003726334		9
**TC0800002458.mm.2**	Eps15l1	epidermal growth factor receptor pathway substrate 15-like 1	0.871927939584313	7.36645923899957	2.62475207456339	0.0368926472764134	0.999692817443324	-4.50791865132722	7.70237302551279	7.51251238457267	7.67212888733224	7.69173406420805	7.09331941221835	6.52668766015332		10
**TC0200004942.mm.2**	E2f1	E2F transcription factor 1	-1.10348268599225	5.98049078736779	-2.62211412037511	0.0370282479085387	0.999692817443324	-4.50803557175747	5.98902471950682	6.21275949477248	4.95990729098177	5.77719027798971	6.11918565304623	6.82487728790975		2
**TC1600000278.mm.2**	Dvl3	dishevelled segment polarity protein 3	0.792951700743965	8.01627948110226	2.621949975708	0.0370367028759686	0.999692817443324	-4.50804285106869	8.39018785918719	8.47522560861567	8.17016164885846	8.23892176129086	7.2514429497999	7.57173705886149		3
**TC1100000428.mm.2**	Ccnjl	cyclin J-like	0.806949412788435	7.26573311879571	2.61982014457206	0.0371465933189883	0.999692817443324	-4.50813734531489	6.42518491559757	6.45992897342405	8.24008796471414	7.92150385995061	7.15581694668864	7.39187605239924		4
**TC0X00001749.mm.2**	Mycs	myc-like oncogene, s-myc protein	-0.909378864048207	5.60679450512148	-2.61969608584809	0.0371530048030095	0.999692817443324	-4.5081428518786	4.90714684782533	4.86093291486648	5.21649824522494	5.81046652474538	6.6052584241631	6.24046407390364		2
**TC1600001353.mm.2**	Thpo	thrombopoietin	-0.984107569392427	6.12542875165531	-2.61369490778141	0.0374645470109405	0.999692817443324	-4.50840954661567	5.52162123514877	5.8182629190966	6.09208531407846	5.63015129437236	6.50605383460223	7.18439791263344		2
**TC0200003579.mm.2**	Acvr1	activin A receptor, type 1	-0.832934383859234	5.92882154433626	-2.60966594407975	0.0376752460341674	0.999692817443324	-4.50858894933274	6.52240198602328	6.07618088695762	5.33839983584321	5.31583897681589	6.04960876582205	6.27049881455551		9
**TC0300001559.mm.2**	Lrriq3	leucine-rich repeats and IQ motif containing 3	0.948184037867607	5.28711935625663	2.60384684405363	0.0379817651158267	0.999692817443324	-4.50884856574121	5.20476740960002	4.93893333850023	5.49093527413157	6.24675645845578	4.89981660414294	4.9415070527092		4
**TC0900000365.mm.2**	Robo4	roundabout guidance receptor 4	-0.797935350717674	5.22459481759933	-2.59281919071028	0.0385698582733332	0.999692817443324	-4.50934218715715	4.95755933030023	5.03046515573907	5.01618552713879	4.86565133192187	5.55974403571563	5.91796352478038		2
**TC0200000697.mm.2**	Lhx2	LIM homeobox protein 2	0.804953552409561	7.03839963368793	2.5907483987783	0.038681354711535	0.999692817443324	-4.50943511794547	7.64997017748692	7.9190527698425	7.11701473443577	7.01862624537288	6.41916410320797	6.10656977178156		6
**TC0500003704.mm.2**	Vmn2r18	vomeronasal 2, receptor 18	-1.17849285442423	4.48168852966793	-2.58275186399607	0.0391150960952197	0.999692817443324	-4.50979468345284	4.0739747691219	4.03193293149076	3.5311538578157	4.68246502645755	5.016642160245	5.5539624328767		2
**TC0700001354.mm.2**	Ints4	integrator complex subunit 4	0.767778541231494	6.45340053579737	2.58080391344008	0.0392215270190652	0.999692817443324	-4.50988244315514	7.33099673450469	7.1024977149465	6.39320620272992	6.5180267211681	5.73447056031835	5.64120528111668		6
**TC1100001232.mm.2**	Lig3	ligase III, DNA, ATP-dependent	-1.31415844970976	5.89672423006804	-2.57931881632722	0.0393028731181712	0.999692817443324	-4.50994939492061	5.9847725601808	6.53805595594294	5.7421787524626	4.37242122996989	6.52517803151978	6.21773885033224		9
**TC0200000082.mm.2**	Kin	Kin17 DNA and RNA binding protein	-0.94750264563813	5.58081096457185	-2.56976371027166	0.0398305071321351	0.999692817443324	-4.51038108680629	6.29318038551893	5.84106363798006	5.17315709152822	4.55465114479969	5.96931196356232	5.65350156404185		9
**TC1600000199.mm.2**	Top3b	topoisomerase (DNA) III beta	-0.754417461370967	6.42987679368314	-2.56719933283399	0.0399733739233511	0.999692817443324	-4.51049721568904	7.40028736349936	7.20127286224957	5.61480697944566	5.61962582735832	6.32350184999925	6.41976587954666		9
**TC1800000373.mm.2**	Pcdhb16	protocadherin beta 16	-1.12422389431578	5.80740222526566	-2.55808875099907	0.0404853061956767	0.999692817443324	-4.51091072436598	6.45491290540475	6.60323479200215	5.04975893953224	4.7191499932455	6.57678224680287	5.44057447460644		9
**TC1500000658.mm.2**	Lgals1	lectin, galactose binding, soluble 1	-1.48805156332439	7.32620833544216	-2.5562059879119	0.0405919550745854	0.999692817443324	-4.51099636011629	7.47955989564477	8.91377295899006	6.6997011872331	5.59420582845159	7.70128535390221	7.56872478843126		9
**TC1600001320.mm.2**	Cdc45	cell division cycle 45	1.01121090276413	5.47893392017953	2.5522237122967	0.0408185020602003	0.999692817443324	-4.5111776949455	5.83734715546776	6.01256587670645	5.57705734554638	5.94599880166922	4.30580117765375	5.1948331640336		8
**TC1300002015.mm.2**	Ror2	receptor tyrosine kinase-like orphan receptor 2	-0.831195047426947	6.34386947781657	-2.54934377972261	0.0409831635831801	0.999692817443324	-4.5113090072338	5.85373105560816	5.74481898689848	6.35053171136726	6.05060665340219	7.25737847505799	6.80614998456535		2
**TC1800001620.mm.2**	Katnal2	katanin p60 subunit A-like 2	0.892944499975503	5.57572279925383	2.5334982848252	0.0419016704275388	0.999692817443324	-4.5120340918245	6.29808399999583	5.82502773138554	5.64584187203864	5.91271516000766	4.64092300448807	5.13174502760723		3
**TC0200000492.mm.2**	Ak8	adenylate kinase 8	0.803191871848555	5.75633786393598	2.53142207298808	0.0420236087889152	0.999692817443324	-4.51212942451342	6.13847356260912	5.85125336948614	5.8770429290996	6.20029906850926	5.34272032922996	5.12823792468179		1
**TC1700002822.mm.2**	H2-T22/H2-T9	histocompatibility 2, T region locus 22/histocompatibility 2, T region locus 9	-1.62927316515133	4.82257064403442	-2.52955263378593	0.0421337209514726	0.999692817443324	-4.51221532748937	4.2186842203246	5.84306786799818	4.27690531287457	3.53065740991596	5.01383354216132	6.05227551093188		9
**TC0900002806.mm.2**	Bcl2a1d	B cell leukemia/lymphoma 2 related protein A1d	-0.975793340122796	5.12706904589415	-2.52946771181977	0.0421387301197296	0.999692817443324	-4.51221923120899	4.74368085046758	5.35192744970935	4.43153167919882	4.92607796827238	5.41083421858425	5.89836210913255		2
**TC0100002503.mm.2**	Erbb4	erb-b2 receptor tyrosine kinase 4	0.853670955967085	5.82270569334682	2.52753091583851	0.0422531424408674	0.999692817443324	-4.51230829674084	7.09678168451244	6.95308275721053	5.67624509604327	5.62061071910278	5.10363346079052	4.48588044242137		6
**TC0600001639.mm.2**	Mgst1	microsomal glutathione S-transferase 1	0.83816269851461	7.83069592958158	2.52749168966272	0.0422554630040846	0.999692817443324	-4.51231010127509	8.49748504922033	8.24327630577586	8.01096204508132	7.94890776467994	6.87526125598083	7.40828315675121		3
**TC0200003423.mm.2**	Nr5a1	nuclear receptor subfamily 5, group A, member 1	-0.757065899499259	5.58834885787548	-2.52262570319749	0.042544364509811	0.999692817443324	-4.51253416176557	5.17582336854267	5.34484786542196	5.38573398266176	5.3619110744831	6.26074210780179	6.00103474834158		2
**TC0X00000108.mm.2**	Lancl3	LanC lantibiotic synthetase component C-like 3 (bacterial)	1.28513371843807	5.21613889968831	2.52164166353796	0.0426030393293984	0.999692817443324	-4.51257952350812	5.80587345240223	5.26006993340189	5.2931490039636	6.10742972063735	4.9891335353151	3.84117775240971		1
**TC1700002526.mm.2**	Xdh	xanthine dehydrogenase	-0.867556601246095	4.88690073625166	-2.51698007799025	0.0428821456800015	0.999692817443324	-4.51279464117007	4.2874799273169	4.86121368902855	4.52554726023337	4.69325153910279	5.31194422902977	5.64196777279857		2
**TC0300002411.mm.2**	Lce1k	late cornified envelope 1K	0.788757881303129	4.91101977043835	2.51504084221215	0.0429988174912378	0.999692817443324	-4.51288424288568	4.61920958572595	4.55230756124913	5.31157145846864	5.62448716066199	4.52052557859263	4.83801727793174		4
**TC0500001521.mm.2**	Crcp	calcitonin gene-related peptide-receptor component protein	0.759793948746209	7.07499739824867	2.50943614079665	0.043337887089664	0.999692817443324	-4.51314357609858	7.72067578174981	7.51012053346168	7.05177411291589	7.3176138729706	6.4012318015396	6.44856828685448		7
**TC0800003077.mm.2**	Galns	galactosamine (N-acetyl)-6-sulfate sulfatase	1.0787321540057	6.09765333119936	2.50363957215356	0.0436915051939759	0.999692817443324	-4.51341236519545	6.12544030043169	6.58187836807123	6.08088035967876	6.93715245367355	5.12031423947323	5.74025426586768		8
**TC0700004180.mm.2**	Gpr139	G protein-coupled receptor 139	1.03709565470658	6.29687708142006	2.49683163580763	0.0441106742455123	0.999692817443324	-4.51372880206129	6.3541822908842	6.23028002689019	6.82064633277193	6.81484940730763	6.31492052906964	5.24638390159677		10
**TC1000002419.mm.2**	Gm10941	predicted gene 10941	1.1550625840535	4.35337716564852	2.49296886113096	0.0443503732994326	0.999692817443324	-4.51390870632819	3.53781504043128	3.74613305475427	5.33342891013431	5.23979112327198	3.4898628305315	4.7732320347678		4
**TC0700001893.mm.2**	Sec23ip	Sec23 interacting protein	1.25316428028858	5.86155567325944	2.46888747589399	0.0458756859323804	0.999692817443324	-4.51503614457442	6.97027942402758	6.90294536077511	6.5092491453004	5.39196976236515	5.19718781145587	4.19770253563253		5
**TC1000000048.mm.2**	Lats1	large tumor suppressor	0.928231320911622	6.94178800144539	2.46692434893553	0.0460024181343218	0.999692817443324	-4.51512849992262	7.72317449200219	7.59970423746345	7.44257180376094	6.64958415675403	6.28743806479823	5.9482552538935		5
**TC0800002408.mm.2**	Arrdc2	arrestin domain containing 2	0.935628835490346	5.63471955986011	2.46351762863556	0.0462232100173409	0.999692817443324	-4.51528892856459	5.76061133219877	5.4092106825217	5.71955853080363	6.53531797690681	5.22509882554673	5.15852001118301		1
**TC1400002162.mm.2**	Phf11b	PHD finger protein 11B	-1.03607798296608	5.39133967040309	-2.459527997786	0.0464831850398366	0.999692817443324	-4.51547706457942	4.93849174592081	5.63857056532303	5.3513430325532	4.49806684006808	5.98475978650965	5.93680605204379		2
**TC1600001884.mm.2**	Robo2	roundabout guidance receptor 2	-0.773955255649103	6.74977410777287	-2.45925653883403	0.0465009292836014	0.999692817443324	-4.51548987563802	7.21001903045184	7.14185122184233	6.30038174666796	5.99905019485447	7.09439930221743	6.75294315060321		9
**TC0X00001447.mm.2**	Htr2c	5-hydroxytryptamine (serotonin) receptor 2C	0.714522856650072	8.34086001463635	2.45513043090342	0.0467715075754207	0.999692817443324	-4.51568475847807	8.82318380548732	8.94173885341955	8.37924869639325	8.47539287471243	7.68013119115485	7.74546466665069		6
**TC1300000887.mm.2**	Slc12a7	solute carrier family 12, member 7	-0.799772932801911	5.2238716034677	-2.45232975264644	0.0469561016728553	0.999692817443324	-4.51581720794271	5.66021650063782	5.21420289024559	4.72349907120241	4.71113311095706	5.35349159545191	5.68068645231139		9
**TC1100004277.mm.2**	Ramp2	receptor (calcitonin) activity modifying protein 2	-1.30934382543242	6.01488944716513	-2.44978619179852	0.0471244062620268	0.999692817443324	-4.5159376160905	5.89073187148707	5.97922352698614	5.96787758306213	4.83246923376422	7.28293326794118	6.13610119975002		2
**TC0200001045.mm.2**	Itga6	integrin alpha 6	-0.829945249674896	6.63705374573576	-2.44744090593321	0.0472801476389998	0.999692817443324	-4.51604873793155	6.86766632973341	7.30129142890514	6.16920204753621	5.8275350606769	6.99596047593317	6.66066713162973		9
**TC0400000484.mm.2**	Polr1e	polymerase (RNA) I polypeptide E	0.927554156639651	5.74819915405744	2.44331383495579	0.0475555124501358	0.999692817443324	-4.51624451464118	6.33689872568166	6.35380784705034	5.67775290530679	6.14904542713918	5.36312853968365	4.60856147948302		7
**TC0800000965.mm.2**	Farsa	phenylalanyl-tRNA synthetase, alpha subunit	0.750067769966904	6.37897723289043	2.43792612725125	0.0479175019384635	0.999692817443324	-4.51650053785043	7.44752052783617	7.31441627761256	6.10079276217088	6.40523830374294	5.39659294329424	5.60930258268578		6
**TC0900001016.mm.2**	Leo1	Leo1, Paf1/RNA polymerase II complex component	-0.817870618534815	6.46544942167035	-2.43285455602341	0.0482608695464819	0.999692817443324	-4.51674199888055	6.81129135982014	6.7993387958546	5.64333348058447	6.12982908805439	6.5076522593234	6.90125154638508		9
**TC1700000688.mm.2**	H2-Q10	histocompatibility 2, Q region locus 10	-0.838109008619161	5.02258138864466	-2.43261935943061	0.0482768553230838	0.999692817443324	-4.51675320758566	5.24030940478744	4.73870469027921	4.47490763434955	4.76522047543195	5.27860860169506	5.63773752532476		2
**TC0800001245.mm.2**	Slc7a6	solute carrier family 7 (cationic amino acid transporter, y+ system), member 6	0.797732973771685	6.14237503755449	2.42721238906147	0.0486458764721352	0.999692817443324	-4.517011150831	5.83592124195587	6.09567499713732	6.40551112609826	6.85354884079029	5.95352533956412	5.71006867978106		4
**TC0200001854.mm.2**	AA467197/Mir147	expressed sequence AA467197/microRNA 147	-0.785333565855289	4.77974302506901	-2.42550132656605	0.0487632647046301	0.999692817443324	-4.51709288384811	4.59920896350287	4.77585070665574	4.65466124409341	4.21170443017903	5.09694738888692	5.3400854170961		2
**TC1100003422.mm.2**	Usp32	ubiquitin specific peptidase 32	-0.838984706483751	6.32066553363134	-2.42484591925786	0.0488083071555671	0.999692817443324	-4.5171242043978	7.36396603876411	7.37075480688267	5.15754253216111	5.59810893942577	6.48314378736742	5.95047709718697		9
**TC1500001065.mm.2**	Grasp	GRP1 (general receptor for phosphoinositides 1)-associated scaffold protein	0.72725270287372	7.93128105594771	2.42216759678039	0.0489928228853222	0.999692817443324	-4.51725227321443	8.91159738726056	9.21254640014046	7.7479031696955	7.71112080732083	7.04010897211347	6.96440959915542		6
**TC0X00000706.mm.2**	Taz	tafazzin	-1.6896151323985	5.73529980591953	-2.42200061392614	0.0490043506848071	0.999692817443324	-4.51726026191657	6.57719285242667	6.61117181634661	5.60040192540718	3.32170002556627	6.18114579808922	6.12018641768124		9
**TC1900001569.mm.2**	Mrpl43	mitochondrial ribosomal protein L43	0.770463731442481	6.45624495822692	2.4208468077246	0.049084081684472	0.999692817443324	-4.51731547487929	6.72221400538987	6.55265444597693	6.67109675482552	6.83066762561433	6.19648268065507	5.76435423689982		10
**TC0600002275.mm.2**	Hoxa1	homeobox A1	0.832111444802107	5.80864434278599	2.42039034130057	0.0491156618851456	0.999692817443324	-4.51733732448316	5.0128968946861	4.68206867502619	6.54076080503532	6.86980088326862	5.62790347829472	6.11843532040501		4
**TC0500001865.mm.2**	Brca2	breast cancer 2, early onset	-0.722844349872619	5.33472189042932	-2.41955372995937	0.049173596850624	0.999692817443324	-4.51737737978938	5.44200986184403	5.57989248638804	4.97800205425492	4.7923680930444	5.6983684148071	5.51769043223745		9
**TC0200004842.mm.2**	Apmap	adipocyte plasma membrane associated protein	-0.959804773478413	6.96981750564629	-2.41867989862112	0.0492341850703824	0.999692817443324	-4.51741923005677	8.1769156864384	7.70166818630772	6.34388147505129	5.6664743320361	6.7009736338232	7.22899172022102		9
**TC0200000087.mm.2**	Sfmbt2	Scm-like with four mbt domains 2	0.758369771106509	4.98863722769098	2.4125026248174	0.04966471124613	0.999692817443324	-4.51771545421895	5.1019933864376	5.15577652152902	5.51921129409319	5.07618520610296	4.58920936317511	4.48944759480802		10
**TC0900003043.mm.2**	Rbm5	RNA binding motif protein 5	0.754715414029896	7.31235552954136	2.40718303976283	0.050038592099376	0.999692817443324	-4.51797107780306	8.07811229255423	8.18909678361975	7.16980885861622	7.38836860595077	6.71304084132036	6.33570579518685	NA	
**TC0X00001331.mm.2**	Kir3dl1	killer cell immunoglobulin-like receptor, three domains, long cytoplasmic tail, 1	-1.00250615213483	4.83965665613977	-2.40637375883062	0.0500957269363329	0.999692817443324	-4.51801000928157	4.14824084861228	3.81573138221877	4.58338060511182	4.95109709575712	5.28895281595171	6.2505371891869	NA	
**TC0600000667.mm.2**	Adcyap1r1	adenylate cyclase activating polypeptide 1 receptor 1	0.977838992436682	8.13547446221294	2.4057055153653	0.0501429556175682	0.999692817443324	-4.51804216450425	9.43620818917215	9.27905271053555	8.37582783462063	7.65080409460103	7.39350880977965	6.67744513456865	NA	
**TC0200000380.mm.2**	Il1f10	interleukin 1 family, member 10	-0.81085340023024	5.25746245480313	-2.40374993099733	0.0502814338073813	0.999692817443324	-4.51813630962902	4.25825506209835	4.60050172606054	5.04407872460886	5.48807684549085	5.92907507288628	6.22478729767391	NA	
**TC1600001604.mm.2**	Pla1a	phospholipase A1 member A	-0.943231951552978	5.14168497112789	-2.39978863558944	0.0505631567493754	0.999692817443324	-4.51832721540838	5.00369151240426	5.01184872593084	5.19175208608706	4.28230075657608	5.83674602874224	5.52377071702686	NA	
**TC1600000092.mm.2**	Pmm2	phosphomannomutase 2	1.1746889991874	5.46227423268733	2.39567638691732	0.0508573475242643	0.999692817443324	-4.51852568248493	6.32092636362128	6.62048843104237	5.64402949686562	5.44677480305193	5.06024769481344	3.68117860672932	NA	
**TC0100003662.mm.2**	Coq8a	coenzyme Q8A	-0.879402291286372	6.54566165378303	-2.38887077972622	0.0513481259583538	0.999692817443324	-4.51885477800359	7.22348021893119	7.26086303588254	6.16990029717817	5.34551074547769	6.64244969131099	6.63176593391762	NA	
**TC1500000300.mm.2**	Cthrc1	collagen triple helix repeat containing 1	0.807442355694511	5.67144258302695	2.38772327262925	0.0514313594442814	0.999692817443324	-4.51891034605899	5.31512289458591	5.11965906346101	6.04922369844406	6.55515542730785	5.66415750321136	5.32533691115153	NA	
**TC0700000840.mm.2**	Hrc	histidine rich calcium binding protein	-0.816960372624815	5.03590619506306	-2.3801797800711	0.05198201137186	0.999692817443324	-4.51927620387748	4.28795585791605	4.29140907207027	4.66529769910213	5.33577804846909	5.78645773784728	5.84853875497357	NA	
**TC0600003038.mm.2**	B4galnt3	beta-1,4-N-acetyl-galactosaminyl transferase 3	-1.06028405320791	5.12494767826638	-2.37931156389456	0.0520457791568257	0.999692817443324	-4.51931837496983	5.46013359210169	5.12508959561513	4.97072504789624	4.05122233983659	5.20570312810364	5.93681236604501	NA	
**TC0400001437.mm.2**	Meaf6	MYST/Esa1-associated factor 6	0.753812253437368	7.70217609189844	2.37820763356818	0.0521269764020472	0.999692817443324	-4.51937201390075	7.79506230661455	7.6988931502268	8.23894400558697	7.87441879512505	7.45139251264021	7.15434578119707	NA	
**TC0800001926.mm.2**	Pomk	protein-O-mannose kinase	0.9973845685703	6.78314214292283	2.37594631258243	0.052293712801339	0.999692817443324	-4.51948195471032	7.61354901780519	7.57109831247536	6.63275591738081	7.12173141481771	6.37070709347891	5.38901110157902	NA	
**TC1100004255.mm.2**	B3gntl1	UDP-GlcNAc:betaGal beta-1,3-N-acetylglucosaminyltransferase-like 1	-0.964919525155165	6.31644561826565	-2.37483648879778	0.0523757465078613	0.999692817443324	-4.51953594420055	6.41173982003748	6.29948640071318	6.15118316206228	5.47762105720417	6.39358233947057	7.16506093010621	NA	
**TC0900000081.mm.2**	Ccdc82	coiled-coil domain containing 82	-0.869780372548862	6.14089154265037	-2.3696248462739	0.0527627551580773	0.999692817443324	-4.51978975715766	7.02332757227888	7.05825455367889	5.65039985946203	4.86170333296132	6.23508430622393	6.01657963129715	NA	
**TC1100002065.mm.2**	Fn3k	fructosamine 3 kinase	-0.732577624075443	6.88868749044913	-2.36720860905288	0.0529431840424816	0.999692817443324	-4.51990758871708	7.29545462437289	6.91155259365506	6.34573581027259	6.48424542798539	7.11844962254654	7.17668686386233	NA	
**TC1900000582.mm.2**	Zfp518a/Mir8092	zinc finger protein 518A/microRNA 8092	1.04315227600921	5.57321524267529	2.36379294311074	0.0531993332952879	0.999692817443324	-4.52007432963268	6.0937097222496	5.99430957142294	5.26914670497768	6.44964165222115	4.71685607954499	4.91562772563541	NA	
**TC0500002576.mm.2**	Zar1	zygote arrest 1	-0.967380698207774	5.75336096784276	-2.36304623017102	0.0532555016836009	0.999692817443324	-4.52011080817263	5.82152803197805	5.39515427895892	5.00527395079286	5.67908709905915	5.97124130877584	6.64788113749173	NA	
**TC0200003447.mm.2**	Lrp1b	low density lipoprotein-related protein 1B	-0.935240654238147	6.70093540099388	-2.36194886050635	0.0533381580834279	0.999692817443324	-4.52016443437224	6.31033306026766	6.37388394160561	6.25351171218425	6.5719453356226	7.80874261791594	6.8871957383672	NA	
**TC1500001658.mm.2**	St3gal1	ST3 beta-galactoside alpha-2,3-sialyltransferase 1	0.707751533732824	5.89393460304013	2.35903024868295	0.0535586408128491	0.999692817443324	-4.52030716121958	6.44039748414141	6.27772725069316	6.11354141979651	5.91695155563943	5.39519229137937	5.21979761659092	NA	

**TC1000002031.mm.2**	Mfsd4b2-ps	major facilitator superfamily domain containing 4B2, pseudogene	-0.68302237809688	4.77047073302232	-2.35826438724977	0.0536166527406541	0.999692817443324	-4.52034463774375	4.46489028870641	4.4739612235247	4.5689405429714	4.59002352188311	5.19815997674062	5.32684884430765	NA
**TC0200003202.mm.2**	Surf6	surfeit gene 6	0.708312541493059	5.78506990907928	2.35778785771047	0.0536527813406217	0.999692817443324	-4.52036796121547	5.93069108785604	5.85084227333715	5.93476856214888	6.23798702598543	5.36568103170332	5.39044947344488	NA
**TC0700004315.mm.2**	Cd2bp2	CD2 cytoplasmic tail binding protein 2	0.764860469793347	6.79506058407528	2.35424024533192	0.0539225396162863	0.999692817443324	-4.5205417191294	7.40380904650252	7.51562822719432	7.00604876838006	6.68427481679071	6.29225689897406	5.86834574661001	NA
**TC0500002170.mm.2**	Slc5a6	solute carrier family 5 (sodium-dependent vitamin transporter), member 6	0.73055570410333	6.65812424946495	2.35376759999239	0.0539585849506144	0.999692817443324	-4.5205648849801	6.64143982748721	6.79991830237953	7.186650865253	6.79759852231179	6.27498753326129	6.24815044609684	NA
**TC0200004108.mm.2**	Madd	MAP-kinase activating death domain	0.942481506270366	6.27782503808029	2.35158084733701	0.0541256775560778	0.999692817443324	-4.52067211433675	7.45501464507747	7.41201725659618	6.51945813557469	5.82298253409971	5.58832282607877	4.86915483105489	NA
**TC1800001000.mm.2**	Osbpl1a	oxysterol binding protein-like 1A	-1.4718336292329	8.32467565666825	-2.35093179219614	0.0541753755254541	0.999692817443324	-4.52070395704353	9.11567973921441	9.38743608488188	7.93811906229553	6.31251636642816	9.15903933515987	8.03526335202962	NA
**TC0800001544.mm.2**	Cog2	component of oligomeric golgi complex 2	1.1144551603305	5.4680900405962	2.3494470909114	0.0542892360286105	0.999692817443324	-4.52077682366264	6.50360987687759	6.75629263294123	5.02865402443429	5.8601200027754	3.81105988252194	4.84880382402674	NA
**TC1800000727.mm.2**	Rnmt	RNA (guanine-7-) methyltransferase	-0.828728144189762	6.53406373613565	-2.34779599148449	0.0544161478264264	0.999692817443324	-4.5208579009867	7.24955506040238	7.37119831617078	6.0782210822353	5.38486529369531	6.66650753480009	6.45403512951006	NA
**TC1700001626.mm.2**	Dnase1l2	deoxyribonuclease 1-like 2	-1.11486602433927	5.50653186390792	-2.34749970300814	0.0544389544583547	0.999692817443324	-4.52087245516839	4.92239003045111	4.69290703073049	5.31735915607724	5.27972188071645	5.74539480664206	7.08141827883017	NA
**TC0300002321.mm.2**	Rxfp4	relaxin family peptide receptor 4	-0.743216128371366	5.91707920380546	-2.34700658459694	0.0544769338510157	0.999692817443324	-4.5208966812821	5.73830862492512	5.76412662346408	5.4399626597266	5.81684119912381	6.51756922372893	6.22566689186422	NA
**TC1900001435.mm.2**	Htr7	5-hydroxytryptamine (serotonin) receptor 7	1.06908641619706	6.17553895646943	2.34154980682542	0.0548990397038574	0.999692817443324	-4.52116504065514	6.8370124263291	5.98018554088072	6.51961873657908	6.66748556542138	5.98902270743081	5.05990876217552	NA
**TC0500000661.mm.2**	Guf1	GUF1 homolog, GTPase	1.19710414030457	5.16282483944107	2.3406946294075	0.0549654969074759	0.999692817443324	-4.52120714347262	5.64234749977384	5.85826907818266	5.79427949700194	5.1409908726476	4.94670572499725	3.59435636404316	NA
**TC0100000637.mm.2**	Plcd4	phospholipase C, delta 4	0.924771118994365	6.12327269010914	2.33946730948906	0.0550610190521546	0.999692817443324	-4.52126758967587	6.72637361984653	6.44018552795606	6.29984800714348	6.41146160827701	5.89281080337433	4.96895657405742	NA
**TC1100004032.mm.2**	Slc39a11	solute carrier family 39 (metal ion transporter), member 11	-0.730152848347188	5.6495452838141	-2.33898905275129	0.0550982880913302	0.999692817443324	-4.52129115103224	6.00935156274402	6.13341859634843	5.1925920811359	4.95450584241299	5.63089954292198	5.97650407732128	NA
**TC0400002444.mm.2**	Aptx	aprataxin	0.890408913538252	6.58524934668637	2.33813605881881	0.0551648238647528	0.999692817443324	-4.52133318350587	7.22636604991528	7.04163030235086	6.97423303156084	6.53792574590345	6.24610622318903	5.48523472719876	NA
**TC0600000850.mm.2**	Ggcx	gamma-glutamyl carboxylase	0.831274119776562	6.31492930208495	2.33807794713178	0.0551693597455902	0.999692817443324	-4.52133604749116	6.75008748121654	6.63979168201648	6.6381542360983	6.4429682083166	6.06765607338636	5.35091813147541	NA
**TC1700001084.mm.2**	Ddx11	DEAD/H (Asp-Glu-Ala-Asp/His) box helicase 11	-0.858465763368879	5.30568034841589	-2.33722174732434	0.0552362346587722	0.999692817443324	-4.52137825123083	4.87854210955973	4.82907789439277	5.26764781959104	4.93711746031151	5.58613202373523	6.33556478290507	NA
**TC0700003564.mm.2**	Rhcg	Rhesus blood group-associated C glycoprotein	-0.69951859978368	4.55632321739889	-2.33068360180749	0.0557496736168228	0.999692817443324	-4.52170093987092	4.0805397072272	3.98967010577499	4.34028085221391	4.59406529369797	5.08905786054289	5.24432548493635	NA
**TC1800001415.mm.2**	Slc26a2	solute carrier family 26 (sulfate transporter), member 2	0.806642905710682	4.98632669800484	2.32972357497003	0.055825477803394	0.999692817443324	-4.52174838289476	5.14232523683181	4.54713550394058	5.48194600498849	5.43894662435051	4.82984132070186	4.47776549721578	NA
**TC1200002002.mm.2**	Erh	ERH mRNA splicing and mitosis factor	-1.18171109837225	5.97384768023219	-2.32953762864187	0.0558401725016525	0.999692817443324	-4.52175757387937	7.33807213090657	7.18900728393275	5.37149213149892	4.10480010340572	6.31388944794535	5.5258249837038	NA
**TC0700001841.mm.2**	Itgal	integrin alpha L	-0.732767270524789	5.01465568551456	-2.32466973056109	0.0562262881867352	0.999692817443324	-4.52199839374247	4.65432710346783	4.62035827526612	4.75153302520982	4.92232407144211	5.35418079738894	5.78521084031257	NA
**TC0300001549.mm.2**	Slc44a5	solute carrier family 44, member 5	0.930474721960352	5.98398976762236	2.3198531790866	0.0566110408173063	0.999692817443324	-4.52223706861306	6.55415833737922	6.34982319193234	6.5327197290669	5.89773353110476	5.65969267775611	4.90981113849484	NA
**TC1600001419.mm.2**	Cldn1	claudin 1	1.10934648874723	5.24466191131841	2.31658936235071	0.0568733003564216	0.999692817443324	-4.52239902386169	4.59902936996004	5.61174947956635	5.90526886229603	5.83267393564325	5.22240783301361	4.29684198743121	NA
**TC0400001452.mm.2**	Lsm10	U7 snRNP-specific Sm-like protein LSM10	0.762119864404495	6.37073194742218	2.31624227543106	0.0569012635983702	0.999692817443324	-4.52241625740535	6.40579299119712	6.40766990305215	6.61116993743334	6.85641432211304	5.71163952093803	6.23170500979936	NA
**TC1000002054.mm.2**	Smpd2	sphingomyelin phosphodiesterase 2, neutral	0.755588314831154	6.45716410518444	2.31323990004475	0.0571437435439963	0.999692817443324	-4.52256541616338	6.43000717123594	6.68004794676493	6.5449912196682	7.02706185171585	5.96819926819145	6.0926771735303	NA
**TC0700001417.mm.2**	Fam168a	family with sequence similarity 168, member A	0.679100565876019	8.01703805415717	2.31203273410209	0.0572415373072167	0.999692817443324	-4.52262543137677	8.18698089703184	8.09961738285334	8.27460908785298	8.31230650055195	7.71279414890425	7.51592030774864	NA
**TC0900000069.mm.2**	Pgr	progesterone receptor	-0.898495460495829	6.9205793260021	-2.3115433434071	0.0572812324441186	0.999692817443324	-4.52264976883104	7.53254371202734	7.33863626691345	6.6264163056228	5.80123622241729	7.31411225162941	6.91053119740234	NA
**TC1000002510.mm.2**	Mob3a	MOB kinase activator 3A	0.913658702407807	6.17024377883395	2.31148955348976	0.0572855971455065	0.999692817443324	-4.52265244405621	6.60539272342866	6.2582218402627	6.63469533236505	6.35788742469894	6.01578514685888	5.1494802053895	NA
**TC1100000940.mm.2**	Asgr2	asialoglycoprotein receptor 2	-0.7096491882923	4.94942311697518	-2.30687459704008	0.0576613488690476	0.999692817443324	-4.52288214921914	5.22928357963653	4.92068754265234	4.65275714371886	4.41087745776994	5.22162815078452	5.26130482728887	NA
**TC0700002169.mm.2**	Cox6b2	cytochrome c oxidase subunit 6B2	-0.86930851271402	5.96764065890396	-2.3067304801825	0.0576731237018258	0.999692817443324	-4.52288932828283	5.74207661660549	5.56884126519865	5.56685999607641	5.81129452701938	6.14758050409983	6.96919104442401	NA
**TC0600000341.mm.2**	Stra8	stimulated by retinoic acid gene 8	0.85670687004098	4.68278072289099	2.29587708712358	0.0585670314019709	0.999692817443324	-4.52343098558663	4.07246802189179	4.06831864274814	5.001628570589	5.83302713580497	4.46340875647064	4.65783320984137	NA
**TC1700000341.mm.2**	Caskin1	CASK interacting protein 1	0.727404065708937	7.13176560251653	2.29585917570684	0.0585685183462892	0.999692817443324	-4.52343188112328	8.50358763268153	8.05364426199944	6.9618185442556	6.88226638166245	6.26622424963251	6.12305254486767	NA
**TC1100004207.mm.2**	Oxld1	oxidoreductase like domain containing 1	0.748875190082495	5.70131379749532	2.2952818134881	0.058616469779325	0.999692817443324	-4.5234607510172	5.1682249249511	5.1245713073228	6.08293614959721	6.62348231683429	5.66180443309215	5.54686365317436	NA
**TC1000001995.mm.2**	Dse	dermatan sulfate epimerase	-0.769039314517549	5.50385565679833	-2.2940041648086	0.0587227254130122	0.999692817443324	-4.52352465728551	5.16569722282671	5.25383990511981	5.25105883179048	5.28170026011369	5.72926169104023	6.34157602989903	NA
**TC1100003418.mm.2**	Dhrs11	dehydrogenase/reductase (SDR family) member 11	-0.716039487759297	6.66828562765081	-2.29268706832817	0.0588324690836849	0.999692817443324	-4.52359056534815	6.64928674087177	6.62168365164533	6.10997479710677	6.54335740182782	7.07484106221948	7.01057011223369	NA
**TC0500001361.mm.2**	Tmem116	transmembrane protein 116	-0.764214435433176	5.59281681964299	-2.2899837364826	0.0590583783511177	0.999692817443324	-4.52372593232853	5.77087222871989	5.83271267500822	5.21804971766788	4.99439385396385	5.58904374630554	6.15182869619254	NA
**TC0700004659.mm.2**	Rnf141	ring finger protein 141	0.900279090609275	7.47494095562493	2.28732901210977	0.0592810935847985	0.999692817443324	-4.52385898448675	8.43778757399265	8.40542124995345	7.92931824301607	6.97417930249493	6.61725174403882	6.48568762025364	NA
**TC1400001863.mm.2**	Gm8165	predicted gene 8165	-0.692884635832036	6.10046022850347	-2.28722714610161	0.0592896567019648	0.999692817443324	-4.52386409226101	5.98329541962329	5.78054349280838	5.91613546922544	5.81044112423709	6.68484450288335	6.42750136224326	NA
**TC1100002754.mm.2**	Slc22a4	solute carrier family 22 (organic cation transporter), member 4	-0.710216754947689	6.02690939361317	-2.28318995425072	0.0596300589136485	0.999692817443324	-4.52406666526239	6.13816820222902	6.26539913167717	5.52979076231451	5.63893699662422	6.09799646707151	6.4911648017626	NA
**TC1100003908.mm.2**	Cyb561	cytochrome b-561	-0.733489817627205	6.20175039220503	-2.28019853737642	0.0598835796742974	0.999692817443324	-4.52421694043767	6.94055517340335	6.99396800029356	5.70324402383731	5.20125574830212	6.12611929653343	6.2453601108604	NA
**TC0800002235.mm.2**	Neil3	nei like 3 (E. coli)	0.766262945416279	4.84666968275224	2.27733465054371	0.0601273297618872	0.999692817443324	-4.52436094906076	4.89877642557098	4.51833422197986	5.07615433139398	5.52156233850358	4.64570967231537	4.41948110674963	NA
**TC0700003614.mm.2**	Homer2	homer scaffolding protein 2	0.801138196293367	6.01604770293808	2.27489988341638	0.0603353580139288	0.999692817443324	-4.52448348726709	6.58154527325996	6.40122697531308	5.93879998437704	6.41909519644406	5.1179658714988	5.63765291673556	NA
**TC1200001527.mm.2**	9030624G23Rik	RIKEN cDNA 9030624G23 gene	-0.970743731711481	6.95266129937744	-2.27370860565149	0.0604374107405549	0.999692817443324	-4.52454347849551	6.9640908120207	7.13547771045797	6.9530263330459	5.8844295721356	7.55628978057659	7.22265358802788	NA
**TC1500001701.mm.2**	Mroh5	maestro heat-like repeat family member 5	-0.745348434234459	6.58550316361539	-2.27125814064407	0.0606478912912261	0.999692817443324	-4.52466695508448	6.08519324100216	6.37930264970076	6.52048475565909	6.25842835560115	6.92979855391577	7.33981142581339	NA
**TC1900000232.mm.2**	Mrpl16	mitochondrial ribosomal protein L16	0.679586818578648	5.80302322025326	2.27032796904629	0.0607279844464275	0.999692817443324	-4.52471385170722	6.69179322284842	6.84587431665807	5.57052799629261	5.74929471329258	4.88691290977165	5.07373616265624	NA
**TC1100000469.mm.2**	Fndc9	fibronectin type III domain containing 9	0.727448325949088	6.65461080260883	2.27006338829008	0.0607507862051395	0.999692817443324	-4.52472719375011	7.30599406808335	7.01003304140935	6.69456564439928	6.83870153462996	6.23320656133072	5.84516396580035	NA
**TC0100001112.mm.2**	Gpr39	G protein-coupled receptor 39	0.769694479700092	6.58433780885138	2.26872943794708	0.0608658808916381	0.999692817443324	-4.52479447873951	7.01359312511699	6.88747134130894	7.06161556237285	6.51056011066841	6.17369245685409	5.85909425678699	NA
**TC0400003793.mm.2**	C1qc	complement component 1, q subcomponent, C chain	-0.883564638130527	6.97126258300528	-2.2563881836949	0.061941364441936	0.999692817443324	-4.52541837714857	6.90151149093945	7.79919404370263	6.34137325165536	6.33849709190891	7.0618655737318	7.38513404609352	NA
**TC1800000076.mm.2**	Greb1l	growth regulation by estrogen in breast cancer-like	1.27919643659518	4.92362117082368	2.25408316149177	0.0621443862921993	0.999692817443324	-4.52553518456047	5.15226174106159	4.404836858094	5.13781808747463	6.13369256201379	4.97500794485366	3.7381098314444	NA
**TC0500001390.mm.2**	Gpn3	GPN-loop GTPase 3	-0.914556755597001	5.03948771471199	-2.25148409560332	0.0623741251470959	0.999692817443324	-4.52566699799265	5.24928291432023	5.19671649191842	4.00477321632927	4.97613346909037	5.23616702913517	5.57385316747847	NA
**TC1700000390.mm.2**	Wdr24	WD repeat domain 24	0.773270894207254	6.30661743117246	2.24566372829085	0.0628917655921546	0.999692817443324	-4.52596258621428	6.90054197490701	6.81556834408708	6.46015302025767	6.3749150079699	5.30933994513402	5.97918629467904	NA
**TC0200003017.mm.2**	Nebl	nebulette	-0.940839372447982	6.18268571206157	-2.24504562033725	0.0629469955075349	0.999692817443324	-4.52599400970624	6.60696442122424	6.47059020001055	6.03833970104554	5.0301007520738	6.67619179539098	6.27392740262433	NA
**TC1800001437.mm.2**	Adrb2	adrenergic receptor, beta 2	0.663485267879345	5.81383694261107	2.243598393175	0.0630765042352635	0.999692817443324	-4.52606760871896	5.34491655255846	5.52014531023999	6.38972759323264	6.2827375710807	5.74488859702505	5.60060603152959	NA
**TC1600001667.mm.2**	Phldb2	pleckstrin homology like domain, family B, member 2	-0.681591428636163	5.88860579708089	-2.24305249257372	0.0631254263502641	0.999692817443324	-4.52609537954852	6.07993907627892	6.18882028629801	5.36174001346726	5.48810626785078	5.95003005726931	6.26299908132107	NA
**TC0100000999.mm.2**	Cdh20	cadherin 20	-0.712268952054951	5.94205973538656	-2.24212648228398	0.0632085017155851	0.999692817443324	-4.5261424983686	6.29250474986206	6.18122090111824	5.67454691118529	5.2025005174293	6.16612132011463	6.13546401260986	NA
**TC0500003184.mm.2**	Ptpn11	protein tyrosine phosphatase, non-receptor type 11	0.731429323736833	7.62982303125558	2.23542328017632	0.0638132144382277	0.999692817443324	-4.52648400203325	8.35280364440585	8.18412176301006	7.90336172434517	7.44907398945044	7.08648493480171	6.80309213152023	NA
**TC0500001136.mm.2**	Ankle2	ankyrin repeat and LEM domain containing 2	0.921369212151995	5.87897755183715	2.23520990822136	0.0638325601968076	0.999692817443324	-4.52649488466159	7.00464793815931	6.87278625264272	6.28049767961456	5.33908709264788	5.12289348770617	4.65395286025228	NA
**TC1700001787.mm.2**	Tmem217	transmembrane protein 217	0.944633767339832	5.72175054758192	2.23428171936745	0.0639167859902843	0.999692817443324	-4.52654223384025	5.54151220268157	5.1537851092573	5.8625035897738	6.89973316434236	5.38397041276938	5.48899880666711	NA
**TC0300000322.mm.2**	Jade1	jade family PHD finger 1	0.676776105282225	6.24701199279339	2.23057509356672	0.0642542680740019	0.999692817443324	-4.52673145852948	6.47196115244019	6.61932240193261	6.56549087855869	6.30667942791729	5.66780845153607	5.85080964437546	NA
**TC0900002907.mm.2**	4930579K19Rik	RIKEN cDNA 4930579K19 gene	-0.649724820507274	5.64451315196828	-2.23049523752713	0.0642615588559186	0.999692817443324	-4.52673553768534	5.12475850832761	5.11734527692635	5.59179228655339	5.57097045621719	6.31628268968933	6.1459296940958	NA
**TC0500001369.mm.2**	Brap	BRCA1 associated protein	-0.645668332653083	7.67091411373097	-2.22691562448422	0.0645892446054439	0.999692817443324	-4.52691849627838	8.13541833331067	8.05220840167835	7.18966444931556	7.08359619172976	7.76485246416201	7.79974484218948	NA
**TC1500002183.mm.2**	Rnd1	Rho family GTPase 1	0.800153237387931	6.68283955416183	2.21961099619736	0.0652632364387874	0.999692817443324	-4.52729249381009	7.01732057771624	6.72842788892223	7.07820509399734	6.89759257255684	6.53900369269976	5.83648749907856	NA
**TC0X00003043.mm.2**	Slc25a53	solute carrier family 25, member 53	-1.07980781560572	5.09434716456108	-2.21926134592014	0.0652956780496933	0.999692817443324	-4.52731041772761	5.01141978002398	5.56198059626992	4.96551923192066	3.95101425800993	5.13474246751176	5.94140665363027	NA
**TC1200000788.mm.2**	Rbm25	RNA binding motif protein 25	0.655687545594968	6.70587661905469	2.21817836149901	0.065396265207916	0.999692817443324	-4.52736594674229	7.47780877879697	7.55721945830376	6.50990847259404	6.74589481161462	5.9739750248013	5.97045316821742	NA
**TC0500002461.mm.2**	Rell1	RELT-like 1	-0.785829077536892	6.19053575847974	-2.21782589836717	0.065429035975289	0.999692817443324	-4.5273840230737	6.13771509777019	6.41759629805825	6.0502061291799	5.4579163708082	6.71578657773961	6.36399407732227	NA
**TC0900000025.mm.2**	Kbtbd3	kelch repeat and BTB (POZ) domain containing 3	-0.662716181941031	5.85448420008762	-2.21232960291078	0.065942237702129	0.999692817443324	-4.52766616570582	5.84918848094046	5.97120232440583	5.60025223787319	5.39028877777548	6.06669549991566	6.24927787961508	NA
**TC0700000502.mm.2**	Lrfn1/Mir466f-2	leucine rich repeat and fibronectin type III domain containing 1/microRNA 466f-2	0.736845852040259	5.82516405815637	2.20928083944675	0.0662286782804105	0.999692817443324	-4.52782287986495	6.67083680812717	6.3890764436804	5.57818494173936	6.10419645886623	5.04777404798656	5.16091564853852	NA
**TC0300002237.mm.2**	Tmem131l	transmembrane 131 like	0.753860159079412	5.64745358999786	2.2079170351205	0.0663572227025951	0.999692817443324	-4.52789303158037	6.0734134744208	6.05604716108971	6.08834840402682	5.54314220729091	4.87479531926177	5.24897497389713	NA
**TC0700000917.mm.2**	Gas2	growth arrest specific 2	-0.915372093218671	7.09128804164159	-2.20668765338703	0.0664733154467711	0.999692817443324	-4.52795629461793	8.16674302584651	7.317334408209	6.59306961678964	6.02338369788871	7.38582838034773	7.06136912076796	NA
**TC1300002249.mm.2**	Zfp874a	zinc finger protein 874a	-0.701429398841189	5.89519157713309	-2.20500002942264	0.066633018484616	0.999692817443324	-4.52804317823649	5.88656314334895	6.32635757145868	5.54212617988571	5.33555879526856	6.1102608100708	6.17028296276585	NA
**TC0100003879.mm.2**	Als2	alsin Rho guanine nucleotide exchange factor	0.679711081287505	5.57568022327059	2.20345033647554	0.066780013661835	0.999692817443324	-4.52812300129694	6.26895519910219	6.03097051228002	5.46889963716292	5.78788925824525	4.91838200377888	4.97898472905428	NA
**TC0700003556.mm.2**	Rlbp1	retinaldehyde binding protein 1	-0.69524753986662	6.25640702843684	-2.20281052350726	0.0668407991857852	0.999692817443324	-4.52815596869919	6.47994404446522	6.43595250171605	5.6443543073391	5.97167096501416	6.33718483029198	6.66933552179452	NA
**TC0800002960.mm.2**	Adat1	adenosine deaminase, tRNA-specific 1	-0.634346153760777	5.65348800109737	-2.2016916609764	0.0669472325757464	0.999692817443324	-4.52821363578049	5.72544889395241	5.79137641850572	5.27129082518294	5.29641436811934	5.93300989921301	5.90338760161082	NA
**TC0X00000708.mm.2**	Atp6ap1	ATPase, H+ transporting, lysosomal accessory protein 1	1.02029376093212	7.98539070813306	2.1979273778571	0.0673065861864606	0.999692817443324	-4.5284077981129	9.35111013876752	9.1819210526977	8.13004423656465	7.57990605303404	7.42171014871575	6.24765261901869	NA
**TC1900000928.mm.2**	Gm960	predicted gene 960	-0.661788389179946	4.78328287454252	-2.19659454239237	0.0674342950559058	0.999692817443324	-4.52847660066999	4.689150291154	4.53124467293088	4.64797071964666	4.42989203275853	5.29173350132855	5.10970602943652	NA
**TC1700000912.mm.2**	Mrps10	mitochondrial ribosomal protein S10	-1.01606091367416	6.00323728611585	-2.19513723888758	0.0675742129081371	0.999692817443324	-4.52855186110973	7.07701496391729	7.13396328173711	5.59410870634018	4.29405311550601	5.96941249055695	5.95087115863757	NA
**TC0200003299.mm.2**	Swi5	SWI5 recombination repair homolog (yeast)	-0.672050701197131	8.15022565992139	-2.19462484226906	0.0676234791573718	0.999692817443324	-4.52857833124038	8.81020078877162	8.66992477054911	7.55928803367547	7.4792754652312	8.01943421530145	8.36323068599948	NA
**TC0100003878.mm.2**	Mpp4	membrane protein, palmitoylated 4 (MAGUK p55 subfamily member 4)	-1.05211904671968	4.68736826017856	-2.19278851879178	0.0678003401361163	0.999692817443324	-4.52867322934813	3.91953574623033	4.17394586412026	4.02834846069087	4.93489646794983	5.03061191021519	6.03687111186487	NA
**TC1600001198.mm.2**	Socs1	suppressor of cytokine signaling 1	-0.718026244788577	6.2644409339667	-2.19239319306265	0.067838476595403	0.999692817443324	-4.52869366619795	5.76425940921116	5.31457690964506	6.32547752209877	6.21040087558463	6.82605680760584	7.14587407965471	NA
**TC1000000216.mm.2**	Med23	mediator complex subunit 23	-0.860775807835702	7.02231658270693	-2.19090362067302	0.0679823698185992	0.999692817443324	-4.5287706939958	7.43872591863913	7.54417241171765	6.8212177709854	5.89350700412131	7.10442711522698	7.33184927555114	NA
**TC1500001470.mm.2**	Kcnv1	potassium channel, subfamily V, member 1	-0.906550346485878	7.56159029222313	-2.190823258566	0.0679901416638958	0.999692817443324	-4.52877485064039	8.01814329035705	8.3636314755579	7.2981028488881	6.28923029833794	7.72860771253398	7.67182612766382	NA
**TC0900001234.mm.2**	Rbp2	retinol binding protein 2, cellular	-0.660470323603075	5.27143984418612	-2.18952736052604	0.0681155934719539	0.999692817443324	-4.5288418938827	5.02834667973184	4.91142968566773	5.07899726021032	5.10496376604519	5.59902709316531	5.90587458029636	NA
**TC1300002462.mm.2**	F2r	coagulation factor II (thrombin) receptor	-0.683505890515849	7.00365671120942	-2.18902690717937	0.0681641038922585	0.999692817443324	-4.52886779202228	6.78083142474531	7.15560732361912	6.68396816237977	6.67527670655043	7.24955333170077	7.47670331826112	NA
**TC1400000541.mm.2**	Ap5m1	adaptor-related protein complex 5, mu 1 subunit	-0.744370138834941	6.80955391647399	-2.18875071205937	0.0681908913730691	0.999692817443324	-4.52888208666039	7.09772301002337	7.06522311416815	6.6272561314761	5.97556241701516	7.04490325976336	7.04665556639779	NA
**TC1100003217.mm.2**	Hic1	hypermethylated in cancer 1	-0.739424353408311	6.17093284003092	-2.1856378121866	0.068493546451781	0.999692817443324	-4.52904328102113	5.57079155623282	5.81861832550098	6.27041051837565	5.8082587074419	6.59488125864483	6.96263667398934	NA
**TC1400000946.mm.2**	Stmn4	stathmin-like 4	-0.771722655003618	8.22712164557661	-2.17744016685663	0.0692971331101979	0.999692817443324	-4.52946851765472	9.03725922342183	9.04163532711924	7.74721700299871	7.12297800345696	8.40933919503721	8.00430112142569	NA
**TC0400001239.mm.2**	Mknk1	MAP kinase-interacting serine/threonine kinase 1	-0.883763921819432	5.8364425544963	-2.17657188254682	0.0693828082085084	0.999692817443324	-4.52951362090325	6.51061656469969	6.56934594457743	5.46158465564172	4.6239978313892	5.62923753002112	6.22387280064866	NA
**TC0800001955.mm.2**	Adrb3	adrenergic receptor, beta 3	0.734852619906563	4.64564237879163	2.17644078342432	0.0693957533467775	0.999692817443324	-4.52952043192244	3.70974775078784	3.90642430009471	5.21020520677647	5.65348852406371	4.49130535395054	4.9026831370765	NA
**TC1200000657.mm.2**	Syt16	synaptotagmin XVI	0.901664859879091	6.54426185392952	2.17468875444704	0.0695689902706122	0.999692817443324	-4.52961148166493	7.4016414322333	7.1635352838626	6.63789618993507	6.61396587368464	5.2017920130926	6.24674033076892	NA
**TC0X00000122.mm.2**	Sytl5	synaptotagmin-like 5	0.786476616607659	5.53920294979817	2.1743141411925	0.0696060883049844	0.999692817443324	-4.52963095596102	6.61364836731544	6.49146341343835	5.25086637913448	5.60066319649079	4.98701417306876	4.2915621693412	NA

**TC1400000306.mm.2**	Tkt/Mir3076	transketolase/microRNA 3076	0.635122815090508	8.26737907932064	2.1695222112838	0.0700824132925673	0.999692817443324	-4.52988026122809	9.28535453466444	9.16276238911364	8.08390614305907	8.12929544810433	7.39506327047777	7.54789269050461	NA
**TC0300002410.mm.2**	Lce1i	late cornified envelope 1J	0.719748645289223	6.97275469759496	2.16699301235947	0.070335154758802	0.999692817443324	-4.53001199221315	6.37291539619115	6.56734410136259	7.3270424450985	7.84084054419873	6.74665424463828	6.98173145408051	NA
**TC1000001735.mm.2**	Samd5	sterile alpha motif domain containing 5	-0.677247107723419	6.78306613403201	-2.1650423699815	0.0705307142486738	0.999692817443324	-4.53011365872745	6.92281779763145	6.84181034046136	6.51060093660673	6.27903628871948	6.88732733588218	7.25680410489088	NA
**TC0600003168.mm.2**	Tigar	Trp53 induced glycolysis repulatory phosphatase	0.719162834204465	6.16351445611573	2.1635808207068	0.0706776024101582	0.999692817443324	-4.53018987334662	6.29208232626636	6.12722818194233	6.62476117534137	6.37528977310595	5.52115014609202	6.04057513394637	NA
**TC0900002017.mm.2**	Ddx25	DEAD (Asp-Glu-Ala-Asp) box polypeptide 25	0.872251850234732	6.5082969753871	2.16032068480519	0.0710063728295566	0.999692817443324	-4.53035999932465	7.47569086334569	7.1663201631422	7.02069806382616	6.05543919932591	5.62260645799484	5.70902710468777	NA
**TC1700001439.mm.2**	Pacrg	PARK2 co-regulated	0.646831500908763	7.35922563559903	2.15916240688976	0.0711235538717631	0.999692817443324	-4.53042048288541	7.77261457829919	7.64360193602015	7.62065695919235	7.39574319135385	6.78195307116023	6.94078407756844	NA
**TC1000002092.mm.2**	Qrsl1	glutaminyl-tRNA synthase (glutamine-hydrolyzing)-like 1	0.747061600513268	5.74916460291879	2.15774184283873	0.071267538248049	0.999692817443324	-4.53049469147163	6.0981399369066	6.3009498244504	5.57895012742411	6.21606040116703	5.05978794056504	5.24109938699956	NA
**TC1900000730.mm.2**	Cfap58	cilia and flagella associated protein 58	-0.688240256274788	4.95670726345873	-2.15612857517877	0.0714314136401023	0.999692817443324	-4.53057900515225	4.39404876114702	4.64247962534875	4.61653284782856	5.04708449302496	5.53980165745477	5.50029619594832	NA
**TC0600002946.mm.2**	Tamm41	TAM41 mitochondrial translocator assembly and maintenance homolog	0.868156713502971	7.52095022903778	2.15245398938113	0.0718061073830572	0.999692817443324	-4.53077120140507	7.00120122062147	7.2647691644936	7.71349560473703	8.58452660332175	7.05596051061546	7.50574827043738	NA
**TC1300001131.mm.2**	Mrps27	mitochondrial ribosomal protein S27	0.713332971203072	6.35965501702143	2.15123895954846	0.0719304412853011	0.999692817443324	-4.53083479915014	7.06475282569199	6.8393188240661	6.38444058541224	6.45582161197609	5.97903047519093	5.43456577979125	NA
**TC0400002153.mm.2**	Nsmaf	neutral sphingomyelinase (N-SMase) activation associated factor	-1.06319145619539	6.15789433320123	-2.15038667807678	0.0720177855820882	0.999692817443324	-4.53087942352517	6.91188398456226	6.47732860571867	6.02848287453538	4.68740237373245	6.5751132960849	6.26715486457372	NA
**TC0100001473.mm.2**	Pigc	phosphatidylinositol glycan anchor biosynthesis, class C	0.786401196277608	5.85833164831302	2.1474713827048	0.0723173683456014	0.999692817443324	-4.53103215062178	5.52080547863315	5.75344021663956	6.49482983705983	6.22944345652047	5.94261110878018	5.20885979224491	NA
**TC1100001899.mm.2**	Nup85	nucleoporin 85	0.742507193674355	5.09666558334315	2.14667901027852	0.0723990128277244	0.999692817443324	-4.53107368455545	6.06051250658688	5.47083595913536	5.26028551991161	5.00654419093107	4.26361946257493	4.51819586091904	NA
**TC0400003439.mm.2**	Kcnq4	potassium voltage-gated channel, subfamily Q, member 4	-0.647920254935542	5.57183166791559	-2.14369804192002	0.0727070045835203	0.999692817443324	-4.53123002632478	5.26568194317869	5.41742317593417	5.23681054846833	5.48921164078647	5.92971467383401	6.09214802529187	NA
**TC0500003549.mm.2**	Rbak	RB-associated KRAB zinc finger	0.671313844963712	6.31519366399421	2.14339834240503	0.072738042793898	0.999692817443324	-4.53124575222417	6.42846727587716	6.64292665910043	6.56224991305142	6.51894795640612	6.06420458642474	5.67436559310537	NA
**TC0900001724.mm.2**	Yap1	yes-associated protein 1	-0.768508903135868	5.86078659155298	-2.14309446651039	0.0727695272521012	0.999692817443324	-4.53126169869665	5.97145614849817	5.90673819628287	5.17440629376133	5.70034740537122	5.92692226067084	6.48484924473344	NA
**TC0900000641.mm.2**	4930550C14Rik	RIKEN cDNA 4930550C14 gene	0.663837683542242	6.17170190867118	2.14223880318025	0.0728582564696041	0.999692817443324	-4.53130660900564	5.75511856293552	6.09562278452039	6.63141583531007	6.62215690051776	5.84336065459969	6.08253671414365	NA
**TC1100001345.mm.2**	Mtmr4	myotubularin related protein 4	-0.645882899219239	7.41134636490885	-2.14156574400027	0.0729281273943291	0.999692817443324	-4.53134194317276	8.46856432703719	8.25315248293175	6.60542882953425	6.6218689609886	7.38341945386482	7.13564413509651	NA
**TC0200004843.mm.2**	Acss1	acyl-CoA synthetase short-chain family member 1	0.637646218248668	7.15691839502374	2.13835409370105	0.0732624688576869	0.999692817443324	-4.53151064498952	7.93177436300764	7.80995997382275	7.02250424446742	7.21502999043727	6.56763777348643	6.39460402492092	NA
**TC0900002525.mm.2**	Fbxl22	F-box and leucine-rich repeat protein 22	-0.78341053245067	6.53342665022397	-2.13783214114457	0.0733169521680904	0.999692817443324	-4.53153807729764	5.43061709684579	5.91356157677638	6.43961055442172	6.70516952698845	7.05515144506501	7.6564497012465	NA
**TC1700002150.mm.2**	Tmem151b	transmembrane protein 151B	-0.644338026646412	7.04815511585639	-2.13735341895046	0.073366959052065	0.999692817443324	-4.5315632412628	7.1751359704483	7.17170064504049	6.72271008301528	6.60399893016309	7.46014336499403	7.15524170147716	NA
**TC0700001147.mm.2**	Gdpgp1	GDP-D-glucose phosphorylase 1	0.843634648104428	6.76454505510856	2.1369775596431	0.0734062452040566	0.999692817443324	-4.53158300074764	7.22653641271334	6.98909615974996	7.24731625784572	6.78213726935275	6.58032809155203	5.76185613943759	NA
**TC1400000071.mm.2**	Flnb	filamin, beta	-0.677673569458795	7.06952159191342	-2.136558484826	0.073450073541163	0.999692817443324	-4.53160503472117	6.91633807693262	7.28494316460543	6.58372642903111	6.84652415648132	7.47521897264332	7.3103787517867	NA
**TC0200001700.mm.2**	BC052040	cDNA sequence BC052040	-0.818871969821896	6.44212148419082	-2.13390465459224	0.0737282370907306	0.999692817443324	-4.53174463013265	6.71832966036614	6.22312285154841	6.39524422048282	5.64152200631047	6.90588003857823	6.76863012785885	NA
**TC0100000338.mm.2**	Gulp1	GULP, engulfment adaptor PTB domain containing 1	0.812782385634524	6.28841657318006	2.1333465679706	0.0737868691730836	0.999692817443324	-4.53177400020401	7.30202143322934	7.02185083673417	6.43024557577278	6.08585039442016	5.83512871527753	5.05540248364636	NA
**TC0800000585.mm.2**	Aga	aspartylglucosaminidase	0.682825843435939	5.58266246900992	2.13329962128808	0.0737918035038016	0.999692817443324	-4.53177647105723	6.31418498897434	5.91819035084699	5.7353333969125	5.57929218364253	5.11115266860208	4.83782122508107	NA
**TC0200003188.mm.2**	Snapc4	small nuclear RNA activating complex, polypeptide 4	0.635899674629977	6.36967620140982	2.13300962863851	0.07382229059232	0.999692817443324	-4.53179173443126	6.66744221086966	6.59508646843318	6.41968020038592	6.69398373882208	5.90663013991293	5.93523445003513	NA
**TC0600001601.mm.2**	Ddx47	DEAD (Asp-Glu-Ala-Asp) box polypeptide 47	-0.617608315027495	7.3306697386891	-2.12642837020545	0.0745176237040812	0.999692817443324	-4.53213847957843	7.96650716945732	8.0622178347504	6.63051294595351	6.72952545298244	7.26521684333614	7.3300381856548	NA
**TC0800001221.mm.2**	Ripor1/Mir1966	RHO family interacting cell polarization regulator 1/microRNA 1966	0.858029659686158	6.98989650934612	2.12585826808488	0.0745781683452817	0.999692817443324	-4.53216854785934	7.23057938756058	7.0709226430943	7.30273649619044	7.37423167620664	5.97975518245915	6.98115367056561	NA
**TC0200005314.mm.2**	Ctsz	cathepsin Z	-0.912572933480332	7.92025273032821	-2.12472483808461	0.0746986862114556	0.999692817443324	-4.53222834200079	8.48402833606699	9.37361357888897	7.20175734743346	6.71760695259285	8.1391855742976	7.60532459268938	NA
**TC0200004683.mm.2**	Lrrn4	leucine rich repeat neuronal 4	-0.668341289391552	5.00696034475795	-2.12463323235397	0.0747084352784562	0.999692817443324	-4.53223317552815	4.68396575885194	4.52197688981325	4.97703537675604	4.77253304379367	5.35416845701924	5.73208254231357	NA
**TC0800000623.mm.2**	Fbxo8	F-box protein 8	0.619231367235695	8.6985606545321	2.12419528562589	0.0747550612156643	0.999692817443324	-4.53225628533705	8.47035086462952	8.31731270345004	9.12547228287399	9.19560926391821	8.51470937180733	8.56790944051348	NA
**TC0400003389.mm.2**	Szt2	seizure threshold 2	0.714051017099094	6.70413919703983	2.12369352362383	0.074808517453767	0.999692817443324	-4.53228276621122	7.00780197450573	6.87997396515477	6.80779187770349	7.07478876068484	6.50318107009198	5.95129753409816	NA
**TC0X00002671.mm.2**	Las1l	LAS1-like (S. cerevisiae)	0.932300218262842	5.9310921777852	2.12317959640953	0.0748633098580378	0.999692817443324	-4.53230989312718	6.99666515337011	6.99257452262419	6.41271512051132	5.31824179310998	5.16779136220034	4.69856511489528	NA
**TC0800000150.mm.2**	Kbtbd11	kelch repeat and BTB (POZ) domain containing 11	1.01412749699221	7.08160340933107	2.12199893201451	0.0749893404487718	0.999692817443324	-4.53237222819056	8.25606332952032	8.05244601799228	7.62280340774016	6.48187964348895	6.40310237137627	5.67332568586841	NA
**TC0X00000110.mm.2**	Xk	X-linked Kx blood group	0.618716425554311	5.5919623471181	2.12089948722005	0.0751068943285014	0.999692817443324	-4.53243029438699	6.38656363172022	6.21880966012045	5.53667566227274	5.55524115871555	4.95987962219223	4.89460434768743	NA
**TC0200000033.mm.2**	Bend7	BEN domain containing 7	0.862246419853983	5.54183775649432	2.12052201345973	0.0751472972478284	0.999692817443324	-4.53245023460572	6.06479539437681	5.98418228944143	6.10058201543102	5.36268883199681	5.22776115304759	4.51101685467227	NA
**TC1100002912.mm.2**	Rnf112	ring finger protein 112	0.627617138099536	7.16967381889034	2.11952595452242	0.07525401609448 6	0.999692817443324	-4.5325028623545	7.7497991624694	7.70040763565266	7.3298922683628	7.08164292734673	6.59970234075653	6.55659857875393	NA
**TC1100003872.mm.2**	Plekhm1	pleckstrin homology domain containing, family M (with RUN domain) member 1	-1.02610595165263	6.33786475051907	-2.11700454731574	0.0755248484423729	0.999692817443324	-4.53263615133616	5.96083170154293	6.34706827151479	6.50753142431589	5.32600688905981	6.6209386977611	7.26481151891987	NA
**TC0200005512.mm.2**	Nfs1	nitrogen fixation gene 1 (S. cerevisiae)	0.676120308951318	6.02791443441552	2.11678708575554	0.0755482528260513	0.999692817443324	-4.53264765155226	6.95480925891429	7.12039487203832	6.07444724461649	5.64781430210508	5.28615177287604	5.08386915594288	NA
**TC1700000573.mm.2**	Zfp563	zinc finger protein 563	0.856983112661903	5.37532030974611	2.11671226991623	0.0755563066058337	0.999692817443324	-4.53265160827317	6.32043529336296	6.37814900437214	5.16290432730843	5.47074756572425	4.9467451362988	3.97294053141008	NA
**TC1800001280.mm.2**	Mcc	mutated in colorectal cancers	0.761523479244095	6.83660739396704	2.11451156743841	0.075793596889871	0.999692817443324	-4.53276803322309	7.30696086585508	7.24983231496451	7.06638526178733	6.9265638089481	5.85300146701658	6.61690064523066	NA
**TC0300000494.mm.2**	Arhgef26	Rho guanine nucleotide exchange factor (GEF) 26	1.13437003376868	5.61626056474173	2.1140597641809	0.0758424056735696	0.999692817443324	-4.5327919443721	6.15461544680939	5.82388773398988	5.63708076800054	6.35681936959369	5.57213659553396	4.15302347452291	NA
**TC0500003554.mm.2**	E130309D02Rik	RIKEN cDNA E130309D02 gene	0.828156479081001	7.03665609650087	2.11378642260442	0.0758719504871181	0.999692817443324	-4.53280641216286	7.19812692152907	6.85679374992497	7.48396389825085	7.42670053460573	6.18203022532179	7.07232124937279	NA
**TC0900002209.mm.2**	Rnf214	ring finger protein 214	1.55795600997646	6.5808225352424	2.11037766033561	0.0762413743081938	0.999692817443324	-4.5329869314657	7.7140693714217	7.82831609808633	7.72974527267987	5.79948560826977	5.94972848650508	4.46359037449165	NA
**TC1600001837.mm.2**	4930453N24Rik	RIKEN cDNA 4930453N24 gene	0.883555146650129	7.40853920645986	2.10750590462465	0.0765540113662019	0.999692817443324	-4.53313914964373	7.63615566465141	7.772079404699	7.57582541761441	7.82922981374008	7.34719771982527	6.29074721822896	NA
**TC1000000506.mm.2**	Pkib	protein kinase inhibitor beta, cAMP dependent, testis specific	1.11446680395689	5.86171395849314	2.1056317956162	0.0767587369238922	0.999692817443324	-4.53323855489478	6.08006011287163	5.70812689609122	6.40072301918212	6.40479215577275	6.06333915709012	4.51324240995098	NA
**TC1700001866.mm.2**	Olfr1564	olfactory receptor 1564	-0.737747143021706	4.44449734668659	-2.10505596126484	0.0768217514753595	0.999692817443324	-4.53326910862831	3.68412215723291	3.89534622172843	4.06647432537424	4.73953638218314	5.06954116285158	5.21196383074921	NA
**TC0100003193.mm.2**	Cfh	complement component factor h	-1.00332366250828	6.5786632930024	-2.10480282272607	0.0768494694037305	0.999692817443324	-4.53328254173462	7.28741824593458	7.19845163771298	6.37693890498262	5.11279236969253	7.000517216981	6.49586138271072	NA
**TC1200000057.mm.2**	Apob	apolipoprotein B	0.619096303687438	6.0883531936929	2.1035770833332	0.0769838272801253	0.999692817443324	-4.53334760083858	5.89859887585889	5.95809686280421	6.37809694348905	6.57771107194553	5.90578777316713	5.81182763489258	NA
**TC1300000284.mm.2**	Gpld1	glycosylphosphatidylinositol specific phospholipase D1	0.989157910209911	6.89848618722026	2.1019984398322	0.0771572180286164	0.999692817443324	-4.53343142472617	7.74174975195739	7.3038632756533	6.97794383989253	7.18386611817282	6.71467449184418	5.46881964580134	NA
**TC0100003483.mm.2**	Mgst3	microsomal glutathione S-transferase 3	-0.672995022347735	7.2476118723721	-2.1013245980925	0.0772313497164268	0.999692817443324	-4.53346721632209	7.28300054733431	7.30913554077591	7.13682275483278	6.63694979588066	7.51835705851209	7.60140553689682	NA
**TC1800000994.mm.2**	Npc1	NPC intracellular cholesterol transporter 1	0.85434914493144	5.47049547066436	2.10106286593735	0.0772601631832324	0.999692817443324	-4.53348112026882	6.44159361193865	7.0484988513917	5.47787469879067	5.04291462646869	4.04993505257344	4.76215598282304	NA
**TC1400002310.mm.2**	Slc25a37	solute carrier family 25, member 37	-1.34972537078457	5.03817865585365	-2.10031208625039	0.0773428749831065	0.999692817443324	-4.53352100954073	5.18007602085384	5.72254210240256	5.00869923354155	3.30480230160663	4.99361668351087	6.01933559320646	NA
**TC0300001792.mm.2**	Phc3	polyhomeotic 3	0.9612164879701	5.88086483743312	2.09990195541946	0.0773880959911381	0.999692817443324	-4.53354280358004	6.31999847674072	6.27655965513994	6.0597839490093	6.24574798531984	5.82332764075625	4.55977131763269	NA
**TC0600003048.mm.2**	Bid	BH3 interacting domain death agonist	0.612661565975981	5.07295932461242	2.09709794745402	0.077697981960122	0.999692817443324	-4.53369187447147	5.13547228357094	5.09285033833779	5.28446076684108	5.43291746201779	4.80096591436699	4.69108918253992	NA
**TC0600002163.mm.2**	Olfr459	olfactory receptor 459	0.652323715828312	4.59896340287559	2.09681670700207	0.0777291324112897	0.999692817443324	-4.53370683274525	4.05654027394966	4.24902826869304	5.01506488256259	5.28136477057114	4.63284295881049	4.35893926266662	NA
**TC0200004586.mm.2**	Zc3h8	zinc finger CCCH type containing 8	-0.671546580642952	4.78498941326822	-2.09632929080166	0.0777831489445873	0.999692817443324	-4.53373275966668	4.58151759298069	5.04219652577533	4.3752439974265	4.4963206023572	5.01906288513118	5.19559487593843	NA
**TC1400002790.mm.2**	2610042L04Rik	RIKEN cDNA 2610042L04 gene	1.2029900781052	6.89677519136048	2.09287193717846	0.078167389754105	0.999692817443324	-4.53391676787481	7.88145510408895	6.88480900622851	7.97188278054256	6.53830081648535	6.23860816736984	5.86559527344766	NA
**TC0700002883.mm.2**	Gm5591	predicted gene 5591	-0.682872183892921	5.23252925928759	-2.09228757444696	0.0782325232485833	0.999692817443324	-4.53394788674565	4.747564224669	5.16739222549892	4.90706673274968	5.1501706361362	5.84645775428491	5.57652398238681	NA
**TC0100000836.mm.2**	Ugt1a2/Ugt1a6a/Ugt1a6b/Ugt1a10/Ugt1a7c/Ugt1a5/Ugt1a9/Ugt1a1	UDP glucuronosyltransferase 1 family, polypeptide A2/UDP glucuronosyltransferase 1 family, polypeptide A6A/UDP glucuronosyltransferase 1 family, polypeptide A6B/UDP glycosyltransferase 1 family, polypeptide A10/UDP glucuronosyltransferase 1 family, polypeptide A7C/UDP glucuronosyltransferase 1 family, polypeptide A5/UDP glucuronosyltransferase 1 family, polypeptide A9/UDP glucuronosyltransferase 1 family, polypeptide A1	1.20581040698488	5.32033298208357	2.09166973632165	0.0783014475042195	0.999692817443324	-4.5339807938487	5.51608736025614	5.91259296358422	5.03028921655679	6.42217997475861	5.04907340625649	3.99177497108917	NA
**TC0200002834.mm.2**	Oprl1	opioid receptor-like 1	0.76562466226586	7.37231812938467	2.0867196318416	0.078855883583278	0.999692817443324	-4.53424465144219	8.28182686363109	8.00161638740172	7.56965452012598	7.17120290477748	6.93355297010192	6.27605513026982	NA
**TC0900002594.mm.2**	Rnf111	ring finger 111	0.635016504064169	7.63665013782039	2.08556316939073	0.0789859827274733	0.999692817443324	-4.53430634772455	7.07661896096216	7.09198928065965	8.40865460577936	8.05200819093507	7.62335993829139	7.56726985029469	NA
**TC0500000299.mm.2**	Dpysl5	dihydropyrimidinase-like 5	0.854400000414654	7.56034562867303	2.08550576078054	0.0789924466898759	0.999692817443324	-4.53430941094551	7.97615958419414	7.78113951769896	8.27683066295813	7.37995667252905	7.22621843572737	6.72176889893051	NA
**TC0500002993.mm.2**	Noc4l	NOC4 like	0.628064147457976	6.39380370251798	2.08411215213295	0.0791495247837102	0.999692817443324	-4.53438378651431	7.05767056212849	6.92753947624694	6.43716216920132	6.37970806662289	5.92700206619753	5.63373987471073	NA
**TC1700000352.mm.2**	Hs3st6	heparan sulfate (glucosamine) 3-O-sulfotransferase 6	0.673677350377006	5.21178238026377	2.083303450777	0.0792408204802447	0.999692817443324	-4.53442695942751	5.0088027678481	5.09803977301711	5.72333368325964	5.53226953747606	5.19837291523956	4.70987560474212	NA
**TC1400002032.mm.2**	Mettl3	methyltransferase like 3	0.916551959631811	5.82434296333291	2.07983193187693	0.0796339338535011	0.999692817443324	-4.53461239885458	6.4111279110793	6.37768911765275	5.83803393722996	6.15713839803456	5.65831084741972	4.50375756858119	NA
**TC0600000388.mm.2**	Tbxas1	thromboxane A synthase 1, platelet	-0.880337202800697	5.88423825126653	-2.07786486562167	0.0798575555038256	0.999692817443324	-4.53471755394316	6.37432908070408	6.40844587328438	5.73054472839379	4.65044534561087	6.21140632950769	5.93025815009837	NA
**TC0X00000338.mm.2**	Rhox2a	reproductive homeobox 2A	0.759355543321441	5.95989657683142	2.07728563517956	0.0799235245828976	0.999692817443324	-4.53474852929631	5.7577114200553	5.39692693598276	6.23072226369921	6.83100383209747	5.56757824141029	5.97543676774351	NA
**TC0200000501.mm.2**	Setx	senataxin	-0.772026210026459	5.76564503735031	-2.07610050698323	0.0800586710629049	0.999692817443324	-4.53481192159432	6.77453424560268	6.69967110062639	4.66831222743446	5.11949400147547	6.02214015160165	5.30971849873612	NA
**TC0900003114.mm.2**	Nradd	neurotrophin receptor associated death domain	0.798772822992509	6.89108398166192	2.07514139314157	0.0801682124676745	0.999692817443324	-4.5348632396684	6.31517415578452	6.54911031439894	7.0910491392528	7.94883339363375	6.60571207975938	6.83662480714215	NA
**TC1900000011.mm.2**	Tesmin	testis expressed metallothionein like	-0.66204904518597	5.05772921603087	-2.07495275892491	0.0801897743428068	0.999692817443324	-4.53487333428123	4.60744147928771	4.40025204934941	4.78457128334214	5.22272055524594	5.60947145378562	5.7219184751744	NA
**TC0300002612.mm.2**	Vangl1	VANGL planar cell polarity 1	0.612031957039421	6.44907710327031	2.07316821606207	0.0803940461622341	0.999692817443324	-4.53496885876112	5.96499438856118	6.19019309419081	6.93063950982066	6.95103001565369	6.28414118299076	6.37346442840474	NA
**TC1400000797.mm.2**	Khnyn	KH and NYN domain containing	0.646701222984354	5.36078402390304	2.07216815015243	0.0805087500838608	0.999692817443324	-4.53502241170667	5.19497915359527	5.42983328506547	5.83475699719303	5.58189007817006	4.92763989381287	5.19560473558152	NA
**TC0600001072.mm.2**	Zxdc	ZXD family zinc finger C	0.663773520159839	6.66310605593431	2.07172939893946	0.080559125253916	0.999692817443324	-4.53504591124214	7.3792928938291	7.31207605278357	6.80311794024883	6.50428927440761	5.78703936871982	6.19282080561694	NA
**TC0300000860.mm.2**	Them4	thioesterase superfamily member 4	-0.906592315565344	5.83124038849376	-2.07101481489678	0.0806412380938891	0.999692817443324	-4.53508419048234	6.1439978225222	5.88267369848545	5.81350205570299	4.7602910337091	5.9561812153607	6.43079650518209	NA
**TC1200001583.mm.2**	Sntg2	syntrophin, gamma 2	-0.683822747929702	5.64531853672987	-2.0692963844552	0.0808390480879803	0.999692817443324	-4.53517627517801	5.76166392105094	5.49797409241071	5.1336617831657	5.48865207236339	6.18680214223391	5.8031572091546	NA
**TC1200000022.mm.2**	Ptrhd1	peptidyl-tRNA hydrolase domain containing 1	-0.642754584932439	5.70139633602786	-2.06865450873716	0.0809130602019706	0.999692817443324	-4.53521068222749	6.26878206445106	5.89288758570038	5.28415344057165	5.09644615750379	5.8041718467073	5.86193692123301	NA
**TC1400002035.mm.2**	Olfr1513	olfactory receptor 1513	-0.626925069353314	5.30115890883193	-2.06739695885863	0.0810582610285835	0.999692817443324	-4.5352781094096	4.7697673669484	5.0604838493054	5.08535489775294	5.27607115126264	5.80930401387004	5.80597217385218	NA
**TC1500001971.mm.2**	Arfgap3	ADP-ribosylation factor GTPase activating protein 3	0.633012494413837	6.965066834605	2.06540728444714	0.0812885312903288	0.999692817443324	-4.53538483909379	7.4319468664632	7.31096643497388	7.0398420155278	7.11691433198248	6.62265569682097	6.26807566186164	NA

**TC1500001520.mm.2**	Samd12	sterile alpha motif domain containing 12	-0.872981212180227	7.80856770249909	-2.06494915734524	0.0813416447221349	0.999692817443324	-4.53540942208822	8.33107018533887	8.81059982277881	7.27602486679781	6.70586202446039	8.27679767682908	7.45105163878956	NA
**TC0700002049.mm.2**	Taldo1	transaldolase 1	-0.720629129137841	7.48258724887577	-2.06299000776366	0.0815691749851383	0.999692817443324	-4.53551458435402	8.02988211913003	7.97995406743451	7.16423654798085	6.55797797622635	7.76714611520852	7.39632666727437	NA
**TC1100000908.mm.2**	Kcnab3	potassium voltage-gated channel, shaker-related subfamily, beta member 3	-0.658505671339251	5.99158322324048	-2.06274194387076	0.0815980300825608	0.999692817443324	-4.53552790382013	6.41712029054642	6.08171063023616	5.41232830594135	5.65450023204954	6.05245365204916	6.33138622862023	NA
**TC1000002929.mm.2**	Lyz2	lysozyme 2	-1.51827021469681	5.26098496004995	-2.06003395268644	0.0819136956292884	0.999692817443324	-4.53567336447395	4.43220298328243	6.85469358148595	4.39288817888558	4.22834820418327	5.74401449519939	5.91376231726307	NA
**TC1000003166.mm.2**	Gdf11	growth differentiation factor 11	0.629259841141496	6.47887482457949	2.05999026076044	0.0819187987729287	0.999692817443324	-4.53567571227989	6.71912702922038	6.61924501831798	6.85533833635416	6.54135995475662	6.15533039710488	5.98284821172291	NA
**TC0700000546.mm.2**	Zfp260	zinc finger protein 260	-0.721109515006472	6.05813506018583	-2.05858678961327	0.0820828920484192	0.999692817443324	-4.53575114329345	6.72852692367902	6.80377616120265	5.69686000477035	4.99028411833983	6.11820735537609	6.01115579774703	NA
**TC1400001183.mm.2**	Cln5	ceroid-lipofuscinosis, neuronal 5	0.840092744243678	6.39766020609578	2.05534625635106	0.0824630376098848	0.999692817443324	-4.53592541891655	6.44261641774127	6.19598921370936	7.10240765382419	6.6153628929815	6.4646158650996	5.56496919321873	NA
**TC1100003819.mm.2**	Mpp3	membrane protein, palmitoylated 3 (MAGUK p55 subfamily member 3)	-0.898346359563184	5.66812927607456	-2.05531080838325	0.0824672057676774	0.999692817443324	-4.53592732615154	6.63193575952737	6.80492285263283	5.27729605043868	4.11031611214173	5.62185359355164	5.56245128815513	NA
**TC0700001879.mm.2**	Tgfb1i1	transforming growth factor beta 1 induced transcript 1	0.652024274370878	7.23531426774832	2.05383310961457	0.0826411498842673	0.999692817443324	-4.53600684821263	6.69702081165286	6.89141346436059	7.89595830110636	7.66779163850276	6.93971975808389	7.31998163278347	NA
**TC1700001952.mm.2**	Aif1	allograft inflammatory factor 1	-0.998820126691518	5.74009296329345	-2.05343599279261	0.0826879584689687	0.999692817443324	-4.53602822438786	6.02591066315186	6.85029758239237	5.44077910814022	4.3425755322765	5.73595716134632	6.04503773245343	NA
**TC0700000946.mm.2**	Atp10a	ATPase, class V, type 10A	0.864638610441752	5.30989709769314	2.05293602554316	0.082746928037275	0.999692817443324	-4.53605514009905	4.99550381662249	5.64870853294346	5.54601063415376	5.92621309458443	4.46885563107615	5.27409087677854	NA
**TC0500000857.mm.2**	Areg	amphiregulin	0.790620630115663	5.5840472087087	2.05093065709291	0.0829838799759092	0.999692817443324	-4.53616313547941	4.98480207128296	5.25856869191691	5.77378352243179	6.64729335221004	5.47356415463088	5.36627145977963	NA
**TC0500002223.mm.2**	Nelfa	negative elongation factor complex member A, Whsc2	0.775046140843973	7.01056907053258	2.05051144770368	0.0830334993520303	0.999692817443324	-4.53618571859182	7.16796064382003	7.1302351817111	7.75730712733341	6.90034831234275	6.65361886289592	6.45394429509228	NA
**TC1700000585.mm.2**	Myo1f	myosin IF	-0.619163996104086	5.60898947712321	-2.04945119563404	0.0831591282959907	0.999692817443324	-4.53624284650117	5.15066376663805	5.41696445970941	5.51493499694727	5.40905532514455	6.16770838891545	5.99460992538454	NA
**TC0200002663.mm.2**	Mc3r	melanocortin 3 receptor	0.808009460170966	5.35297529869517	2.04854299739335	0.0832668922560181	0.999692817443324	-4.53629179447434	6.27941929178383	5.88422574551575	5.49574257762762	5.28937025997907	5.01476932650336	4.1543245907614	NA
**TC1200001953.mm.2**	Sptb	spectrin beta, erythrocytic	0.641025172450529	7.07917673103063	2.04836860222666	0.0832876014899277	0.999692817443324	-4.53630119498862	8.1863737736601	8.08907421608949	6.86269320816199	6.87813816250562	6.44622651547408	6.01255451029247	NA
**TC0100001787.mm.2**	Eprs	glutamyl-prolyl-tRNA synthetase	1.26552546287681	7.30185154572803	2.04733320225613	0.0834106606783553	0.999692817443324	-4.53635701575418	8.8325338766699	8.92879067739246	7.57657449536268	6.71384332766704	6.75985894070331	4.99950795657278	NA
**TC0600000294.mm.2**	Mkln1	muskelin 1, intracellular mediator containing kelch motifs	1.16596579622638	7.95049787271613	2.04732090408872	0.0834121234350699	0.999692817443324	-4.53635767886946	8.67071678618641	8.6453537555828	8.41750708586774	7.94191705762242	7.86269200303721	6.16480054800019	NA
**TC0300002714.mm.2**	Strip1	striatin interacting protein 1	-0.893515847795617	7.23215842217494	-2.04675103170484	0.0834799329086228	0.999692817443324	-4.53638840869683	8.09148484062549	8.04140761819848	6.74827928571008	5.98823390360713	7.71153332257032	6.81201156233813	NA
**TC1300001096.mm.2**	Gcnt4	glucosaminyl (N-acetyl) transferase 4, core 2 (beta-1,6-N-acetylglucosaminyltransferase)	-0.895929321515863	6.26468237105069	-2.0467238106341	0.0834831733460479	0.999692817443324	-4.53638987668463	5.68614351309268	6.52644437027718	6.28985075489756	5.5019730950537	6.92948586894376	6.65419662403923	NA
**TC0500000609.mm.2**	Klb	klotho beta	0.892770023218461	4.59160638123995	2.04609713966057	0.0835578081454761	0.999692817443324	-4.53642367498319	3.48783119325235	4.47093460894481	5.13360919463147	5.55459707120827	4.69784227142427	4.20482394797854	NA
**TC0300002351.mm.2**	Lenep/Flad1	lens epithelial protein/flavin adenine dinucleotide synthetase 1	0.730938190146054	7.5166266365018	2.04511616607522	0.083674773980769	0.999692817443324	-4.5364765932949	7.84675150049944	7.97266979101827	7.32728935116226	8.04381810273034	6.8407651795636	7.06846589403688	NA
**TC1100001069.mm.2**	Prpf8	pre-mRNA processing factor 8	-0.626306364972728	6.5928031565513	-2.04469841527289	0.0837246341655335	0.999692817443324	-4.53649913294454	8.38078188628598	8.45677491102609	5.54834187746928	5.18498282855587	6.03514123553411	5.9507962004365	NA
**TC1800000124.mm.2**	Taf4b	TATA-box binding protein associated factor 4b	0.611758600068497	5.76822865551565	2.04409376853514	0.083796853949519	0.999692817443324	-4.53653176097732	6.06940973521415	6.142229073982	6.00400724325624	5.80661791876114	5.40070722475263	5.18640073712776	NA
**TC0200004676.mm.2**	Gpcpd1	glycerophosphocholine phosphodiesterase 1	-1.02703447539136	6.50393447461251	-2.0436105371058	0.0838546166984521	0.999692817443324	-4.53655784096704	6.7987703846924	6.71500331026387	6.56990080064686	5.15798130032118	7.10662357997226	6.6753274717785	NA
**TC0600000470.mm.2**	Olfr449	olfactory receptor 449	0.869949747759647	4.92732603277246	2.0417350443752	0.0840791813643334	0.999692817443324	-4.53665909310687	4.56806273124953	4.8061129313301	5.12780264744337	5.83703736734383	4.19013270218105	5.03480781708686	NA
**TC0200003754.mm.2**	Wipf1	WAS/WASL interacting protein family, member 1	-0.589787188844713	6.2030306410679	-2.04160950771298	0.0840942342053462	0.999692817443324	-4.53666587225537	5.55656486276553	5.55265621165806	6.19048875905971	6.27420543808749	6.8557296546438	6.78853892019283	NA
**TC1700000882.mm.2**	Xpo5	exportin 5	0.984128479524099	6.96205300544568	2.04129374633138	0.0841321084581413	0.999692817443324	-4.53668292479576	8.02628839589288	8.02761116804825	7.04891767071875	6.79442004317182	6.61996496949565	5.25511578534671	NA
**TC0300001846.mm.2**	Qrfpr	pyroglutamylated RFamide peptide receptor	-0.601981657085087	5.39820980655746	-2.0410915587197	0.0841650099995562	0.999692817443324	-4.53669773332699	5.17733338533692	5.07541011106912	5.1465690610095	5.31970695337476	5.77057693088984	5.8996623976646	NA
**TC0600000270.mm.2**	Cpa4	carboxypeptidase A4	-0.643383462300386	4.72941525177725	-2.03955687609753	0.084340745044935	0.999692817443324	-4.53677674923284	4.21040488573759	4.32188853568231	4.63140846427525	4.64730711804616	5.05615341148884	5.50932909543334	NA
**TC1100001241.mm.2**	Slfn4	schlafen 4	-0.666148682293537	4.80340084133004	-2.03937406297862	0.0843627351299357	0.999692817443324	-4.53678662717981	4.32023030123575	4.17879704891781	4.80633782586144	4.68820234075837	5.15049799821701	5.67633953298988	NA
**TC0500000435.mm.2**	Tmem128	transmembrane protein 128	0.662344664421318	6.36742576731477	2.03668365377864	0.0846870231453551	0.999692817443324	-4.53693205353385	6.70461445829601	6.63190536284354	6.73819399350786	6.358168062288	5.69301446052514	6.07865826642808	NA
**TC1600001045.mm.2**	Cbr3	carbonyl reductase 3	0.645266922430003	5.66814424447465	2.0363078816194	0.0847324162079781	0.999692817443324	-4.53695237362627	5.59331110478278	5.96645005380376	5.91737341452996	5.95244566203072	5.1403087857497	5.43897644595098	NA
**TC0400000890.mm.2**	Gm13286	predicted gene 13286	-0.643735733678727	6.67962985406421	-2.03599834648899	0.0847698262083205	0.999692817443324	-4.53696911343055	6.08060250608545	6.36888899042017	6.64627743632678	6.52413064393431	7.05152418653225	7.40635536108629	NA
**TC0600001421.mm.2**	C1rl	complement component 1, r subcomponent-like	0.756604115759737	5.67927092471651	2.03576431217849	0.0847981222798419	0.999692817443324	-4.5369817710246	5.46870842696143	5.05090619386144	5.96448148610249	6.57012809339534	5.7188849671307	5.30251638084766	NA
**TC0800001919.mm.2**	Bag4	BCL2-associated athanogene 4	0.665603268814911	6.72244951875606	2.03411247543713	0.0849981082797152	0.999692817443324	-4.53707113179574	6.77103540687864	7.0400100692154	7.11549183626237	6.81193725077369	6.49229319742127	6.10392935198498	NA
**TC0100000133.mm.2**	Tmem14a	transmembrane protein 14A	-0.744677456598529	7.2028480126106	-2.03252722717443	0.0851904772760504	0.999692817443324	-4.53715692688823	7.59062991751589	7.53196119149724	7.01234416788956	6.29022685883714	7.58288098285939	7.20904495706436	NA
**TC0600001366.mm.2**	Pex26	peroxisomal biogenesis factor 26	-0.691240362792085	5.43295843177802	-2.0323365724193	0.0852136425164365	0.999692817443324	-4.53716724771179	6.13503238091396	6.11159687081856	4.5232063547643	4.96111395191143	5.19167166499732	5.67512936726257	NA
**TC1300001037.mm.2**	Mtx3	metaxin 3	0.771484177115846	5.58257474947688	2.02971327248263	0.0855330249656694	0.999692817443324	-4.53730930885013	6.025964200528	6.04957967667576	5.43793319463227	6.04350329231234	5.29674381279303	4.64172431991989	NA
**TC0400000254.mm.2**	Bach2	BTB and CNC homology, basic leucine zipper transcription factor 2	0.607819411879855	6.43175268738763	2.0265570391362	0.0859188819430074	0.999692817443324	-4.53748035981295	6.4916201935443	6.22890489688358	6.70174016161608	6.84107476721271	6.20462999709697	6.12254610797211	NA
**TC0700000047.mm.2**	Isoc2a	isochorismatase domain containing 2a	-0.630940783236452	6.57847088421323	-2.02558986924006	0.0860374695585581	0.999692817443324	-4.53753280350891	6.51254909863613	6.1512656476355	6.2864791290322	6.48608536723522	6.95459474817889	7.07985131456143	NA
**TC1300002204.mm.2**	Ctsl	cathepsin L	-0.896177792190403	7.53355562703319	-2.02349859993802	0.086294447348534	0.999692817443324	-4.53764624545793	8.13811513380548	8.09262821091126	7.36386338858167	6.22525402796913	7.89225337111465	7.48921962981696	NA
**TC0500001386.mm.2**	Pptc7	PTC7 protein phosphatase homolog	0.701442711297359	7.46613556279553	2.02314961905878	0.0863374053569634	0.999692817443324	-4.53766518211568	7.92068856544602	7.6940117290416	7.82255566440688	7.46994358803326	7.2225275949646	6.66708623488081	NA
**TC0200000404.mm.2**	Tmem203	transmembrane protein 203	-0.704734103156212	6.6331005957364	-2.02260803212727	0.0864041147422521	0.999692817443324	-4.53769457350856	7.11015473972193	7.26166109446867	6.30115013385369	5.707509633104	6.88530835539185	6.53281961787828	NA
**TC0900001844.mm.2**	Fbxl12	F-box and leucine-rich repeat protein 12	-0.639024423129761	6.79975388078436	-2.02196958070758	0.0864828216128843	0.999692817443324	-4.53772922695607	6.52747598585094	6.4656149358544	6.8491908256816	6.41450093268903	7.18420550915596	7.3575350954742	NA
**TC0200005281.mm.2**	Bmp7	bone morphogenetic protein 7	-0.742688011567565	5.81401704864289	-2.01992285081944	0.0867356222965316	0.999692817443324	-4.53784035673783	6.34546549159841	5.82040909488611	5.01877188798713	5.59765395313173	5.92230672937111	6.17949513488288	NA
**TC1400000913.mm.2**	Rp1l1	retinitis pigmentosa 1 homolog like 1	-0.641786043691172	6.1552794075247	-2.01917842419859	0.0868277531334236	0.999692817443324	-4.53788079094181	5.64484976342028	5.64112983902096	5.85242634324373	6.32863603441859	6.78415284081351	6.68048162423115	NA
**TC0200000714.mm.2**	Olfml2a	olfactomedin-like 2A	-0.671224996390527	5.33203463703171	-2.01873719561932	0.0868824061795304	0.999692817443324	-4.53790476034765	5.21639394208947	5.07914890696564	4.87729475800101	5.29981273217602	5.55807587929023	5.96148160366785	NA
**TC0900001982.mm.2**	Tmem45b	transmembrane protein 45b	-0.655427511173848	4.76347342220453	-2.01765454021883	0.0870166559288607	0.999692817443324	-4.53796358636384	4.31896699666466	4.44699983097391	4.78265446500156	4.46935487661889	5.06879474608596	5.49406961788219	NA
**TC1400000165.mm.2**	Fam149b	family with sequence similarity 149, member B	0.682947985245404	6.38171584347518	2.01738338275843	0.0870503120808823	0.999692817443324	-4.5379783222649	6.80098400798343	6.72031138448364	6.21641438028433	6.85103343915307	5.87779922789645	5.82375262105014	NA
**TC1100003638.mm.2**	Tbkbp1	TBK1 binding protein 1	0.862591394815688	6.31179154338868	2.01634533211088	0.0871792757337359	0.999692817443324	-4.5380347440802	7.55353389135848	6.94399123978262	6.72439648771426	5.82480697169693	5.24527837489896	5.57874229488085	NA
**TC1900000448.mm.2**	Il33	interleukin 33	0.79488462134853	7.22318688873072	2.01607936345633	0.0872123494816151	0.999692817443324	-4.53804920287174	7.72270625433465	7.91229435548847	7.5068759640461	7.14006901858303	6.96648932309327	6.0906864168388	NA
**TC0200001164.mm.2**	Itprid2	ITPR interacting domain containing 2	-0.720352807841965	5.76217139782695	-2.01570946290517	0.087258368253637	0.999692817443324	-4.53806931333634	6.28333984662043	6.47180156630135	5.29047170685525	4.89811897232274	6.12742398361019	5.50187231125173	NA
**TC0500003253.mm.2**	Cdk2ap1	CDK2 (cyclin-dependent kinase 2)-associated protein 1	-0.582222500767354	7.3510336394088	-2.01249445877558	0.0876593669407906	0.999692817443324	-4.53824418480583	7.82405514104042	7.88702756937377	6.85906935853691	6.75626770371505	7.39562011895696	7.3841619448297	NA
**TC1000000224.mm.2**	Epb41l2	erythrocyte membrane protein band 4.1 like 2	-0.732495797469334	7.6586181271009	-2.01212678986323	0.0877053426128818	0.999692817443324	-4.53826419236637	7.56510286912769	7.83882853160538	7.57785531116342	6.96353757230343	7.78504142668136	8.22134305172415	NA
**TC1500001977.mm.2**	Ttll1	tubulin tyrosine ligase-like 1	0.894489998127907	6.52763190090413	2.00888512024159	0.0881117470317292	0.999692817443324	-4.53844067691784	8.20139589553194	8.07472608516286	6.65695720273938	5.6823675077535	5.63651381269553	4.91383090154154	NA
**TC1100001072.mm.2**	Slc43a2	solute carrier family 43, member 2	0.66349222609981	6.60756841058467	2.00851674319822	0.0881580490593064	0.999692817443324	-4.53846074153513	7.72398378283847	7.4318154409781	6.7049331035777	6.20336474236785	5.75614871315243	5.8251646805935	NA
**TC0X00000342.mm.2**	Rhox4a/Rhox4a2	reproductive homeobox 4A/reproductive homeobox 4A2	-0.858303835113522	5.2794843218047	-2.00846067378236	0.0881650986666256	0.999692817443324	-4.53846379566764	5.13846707554053	5.308352880378	5.33127367716934	4.42546547517198	5.40590000011043	6.06744682245793	NA
**TC1600001177.mm.2**	Carhsp1	calcium regulated heat stable protein 1	0.610597645718491	7.30554718743623	2.00656586032913	0.0884036652488048	0.999692817443324	-4.53856703289068	7.51512601020894	7.54979092164643	7.49247716181639	7.5023035802831	7.06557625703392	6.7080091936286	NA
**TC0400000836.mm.2**	Ccdc171	coiled-coil domain containing 171	-0.749115901540767	5.95501780823558	-2.00624105649651	0.0884446244470254	0.999692817443324	-4.53858473454088	6.30212015966258	6.10966917434018	5.04310526587045	5.86693759029414	6.14161906177019	6.26665559747593	NA
**TC1900001109.mm.2**	Tkfc	triokinase, FMN cyclase	-0.57813382919919	6.14692045399071	-2.00553695072966	0.0885334804498054	0.999692817443324	-4.53862311298305	6.17623276211397	6.0998786870544	5.85760383577432	5.86696797241443	6.4241517559071	6.45668771068004	NA
**TC0200003728.mm.2**	Dlx2	distal-less homeobox 2	0.661924287933955	8.35456688615701	2.00516295489488	0.0885807138696373	0.999692817443324	-4.53864350103431	8.44212248781417	8.07911954932317	8.95164351119854	8.51336041663777	8.0075612259866	8.1335941259818	NA
**TC1300002722.mm.2**	Isl1	ISL1 transcription factor, LIM/homeodomain	0.826509013802284	6.85070908136013	2.00044431308104	0.0891788178867853	0.999692817443324	-4.53890089974833	7.18161532337155	6.54758870794508	7.65735716865329	6.85667707357108	6.58101849011097	6.27999772450883	NA
**TC1500000488.mm.2**	Tg	thyroglobulin	0.676827448490433	5.08752009173933	1.99860137944533	0.0894135103266251	0.999692817443324	-4.53900151392272	4.97649706575788	4.65430228919035	5.30652226334645	5.81746578288785	4.77524178951896	4.99509135973448	NA
**TC0700002789.mm.2**	Fxyd3	FXYD domain-containing ion transport regulator 3	0.68718489246514	5.44926716868187	1.99844560120262	0.0894333765078451	0.999692817443324	-4.5390100207112	5.5203874128884	5.22664089559332	5.60342092188653	6.05805132238336	5.34724022388232	4.93986223545728	NA
**TC0400002007.mm.2**	Dnajc11	DnaJ heat shock protein family (Hsp40) member C11	0.591023219096369	7.95620551697735	1.99324220464378	0.0900994972165547	0.999692817443324	-4.53929436078637	8.25813729559165	8.35373857505373	8.1774029057207	7.97629892898502	7.41618082675794	7.55547456975504	NA
**TC1900000787.mm.2**	Tcf7l2	transcription factor 7 like 2, T cell specific, HMG box	0.869451198282031	7.9900737748755	1.99303730026489	0.0901258294993385	0.999692817443324	-4.53930556539184	8.91602528602476	7.87778828309966	8.36726227954324	8.07550345880309	7.58793419433776	7.11592914744451	NA
**TC1600000048.mm.2**	Alg1	asparagine-linked glycosylation 1 (beta-1,4-mannosyltransferase)	0.828765101827833	5.63239369276936	1.99232282876142	0.0902177064141192	0.999692817443324	-4.53934463869392	6.78912090225457	5.83366524949419	5.52699896698801	5.88755413727351	5.05231619426585	4.70470670634	NA
**TC0300002431.mm.2**	Gm10697/Tdpoz5	predicted gene 10697/TD and POZ domain containing 5	0.638022250514656	4.64636499867409	1.99221060329064	0.0902321464559951	0.999692817443324	-4.53935077675863	4.24616772402335	4.2510453152012	5.05404653625884	5.27446419066583	4.29779538269739	4.75467084319796	NA
**TC0300002433.mm.2**	Gm10697/Tdpoz5	predicted gene 10697/TD and POZ domain containing 5	0.638022250514656	4.64636499867409	1.99221060329064	0.0902321464559951	0.999692817443324	-4.53935077675863	4.24616772402335	4.2510453152012	5.05404653625884	5.27446419066583	4.29779538269739	4.75467084319796	NA
**TC0700003763.mm.2**	Usp35	ubiquitin specific peptidase 35	1.09604523823738	5.61359514348342	1.99172712047608	0.0902943825314558	0.999692817443324	-4.53937722235402	5.85056202660432	5.79769046959333	5.79331456140315	6.31938985918566	5.75928413569857	4.16132980841549	NA
**TC0100002544.mm.2**	Fn1	fibronectin 1	-0.87555314850892	6.12684158512491	-1.98767769179147	0.0908173279459065	0.999692817443324	-4.5395988434922	5.98498325450733	7.05369775884965	5.57498946947282	5.41064163071452	6.62554920685245	6.11118819035272	NA
**TC0700000832.mm.2**	Slc17a7	solute carrier family 17 (sodium-dependent inorganic phosphate cotransporter), member 7	-0.895032714305131	9.93944607529459	-1.98733413322573	0.0908618341071921	0.999692817443324	-4.53961765636781	10.3379260124741	11.2790710603916	9.43346169156069	8.68134528358509	10.1069306637809	9.7979417399751	NA
**TC1100002104.mm.2**	4930556J24Rik	RIKEN cDNA 4930556J24 gene	-0.665809530341181	5.43065129014742	-1.98650525856053	0.0909692999444192	0.999692817443324	-4.53966305119486	4.76608742491008	4.60160251094801	5.21494279250876	5.72735657966329	5.98416213895838	6.28975629389603	NA
**TC1100001480.mm.2**	Gip	gastric inhibitory polypeptide	0.638199699546329	5.82480740631187	1.98610798941635	0.0910208518980735	0.999692817443324	-4.53968481166494	5.75276629271038	5.62786041124451	5.97921261909696	6.44309594740755	5.47886551497932	5.66704365243253	NA
**TC0900000210.mm.2**	Prkcsh	protein kinase C substrate 80K-H	0.66445578230945	7.35167331328536	1.98575949652392	0.0910660983538718	0.999692817443324	-4.53970392017089	8.16114516408869	7.78747598783655	7.40677685238279	7.33838829382013	6.94995164444735	6.46630193713668	NA
**TC1600001048.mm.2**	Morc3	microrchidia 3	-0.618500014097583	6.02398569062029	-1.9855743449604	0.0910901465688832	0.999692817443324	-4.53971404547768	6.43350285759224	6.11065974002088	5.45878487618633	5.72259088277038	6.15366539747889	6.26471038967299	NA
**TC1400000940.mm.2**	Pbk	PDZ binding kinase	-0.863323776625948	5.3790055979761	-1.98423008041916	0.0912649349736771	0.999692817443324	-4.53978770332128	5.51953044057951	6.35794055369106	4.79569253389754	4.53926498626951	5.14213355083549	5.91947152258346	NA
**TC1400002466.mm.2**	Fam216b	family with sequence similarity 216, member B	0.590633262244657	5.69770470904568	1.98393996891697	0.0913027006692505	0.999692817443324	-4.53980360294471	5.68969266400782	5.71584698529073	5.86523099497192	6.11574656976051	5.33930364457173	5.4604073956714	NA
**TC0500000723.mm.2**	Exoc1	exocyst complex component 1	0.711382212111983	7.11673899753405	1.9823388728901	0.091511406208788	0.999692817443324	-4.53989137181833	7.33812824053199	7.2654392302305	7.21249847716963	7.54731699216326	7.01389414726571	6.32315689784321	NA
**TC1000000778.mm.2**	Icosl	icos ligand	0.876101595632695	5.49446380942609	1.98194434543889	0.0915629065237924	0.999692817443324	-4.53991300433739	6.19329127749458	6.35285914830706	5.59525216330215	5.491165647708	5.26188387425062	4.07233074549414	NA
**TC1100001209.mm.2**	Tmem98	transmembrane protein 98	-0.727455765153648	7.07334222383742	-1.98152777487103	0.0916173156365719	0.999692817443324	-4.53993584779176	6.90290726573121	7.07642799208593	7.14154685421151	6.36135642323853	7.56947471842559	7.38834008933175	NA
**TC1900000967.mm.2**	Ctsw	cathepsin W	0.752622259559637	5.4323180243879	1.98094113410993	0.0916939926162882	0.999692817443324	-4.53996802134197	4.77468888650557	5.01092829142121	5.64890818593588	6.50785955782406	5.34248538977755	5.30903783486311	NA
**TC1300001342.mm.2**	Fgf10	fibroblast growth factor 10	-0.809935398821055	5.33252232980416	-1.98073842623535	0.0917205024518337	0.999692817443324	-4.53997913967002	5.23254182525537	5.25094612186097	5.41170600496694	4.53418161206631	5.50079068324561	6.06496773142975	NA
**TC1700002250.mm.2**	Dazl	deleted in azoospermia-like	0.700351413265886	6.29620398049865	1.98061810601165	0.0917362413655478	0.999692817443324	-4.53998573937803	6.5009940679503	6.60653956330912	6.60193760628207	6.43325893285004	5.46131987987814	6.17317383272221	NA
**TC0700002046.mm.2**	Tmem80	transmembrane protein 80	0.609808434046293	6.28824622808819	1.97948716119014	0.0918843102482511	0.999692817443324	-4.54004778257826	6.61310108735134	6.84113432150762	6.47668121959759	6.27074819428379	5.89078191206741	5.63703063372138	NA
**TC0800001419.mm.2**	Mlycd	malonyl-CoA decarboxylase	0.640538352857426	5.69470659491808	1.97948630191592	0.0918844228391891	0.999692817443324	-4.54004782972426	5.25984136619769	5.45089860250541	5.94561976239703	6.42366839086307	5.47360339422836	5.6146080533169	NA

**TC0800002441.mm.2**	Fcho1	FCH domain only 1	0.816098131374392	5.95924507863175	1.97794089389115	0.0920871407399916	0.999692817443324	-4.54013263804571	6.52674959242759	6.66019570659385	6.38952921815269	5.71083149960623	5.63697041058915	4.831194044421	NA
**TC0200000572.mm.2**	Pomt1	protein-O-mannosyltransferase 1	0.784614423888892	5.95150395882453	1.97739650863397	0.0921586562250809	0.999692817443324	-4.54016252025519	6.64957607567691	6.54858766743465	5.78975686940549	6.25028755940122	5.66577536187868	4.80504021915025	NA
**TC0200003397.mm.2**	Pdcl	phosducin-like	-0.64170966379213	5.89203594760628	-1.97552981677842	0.0924043026097903	0.999692817443324	-4.54026501624251	6.07059135973218	6.25585860558376	5.56344357822903	5.3077296181397	5.85619037140387	6.29840215254912	NA
**TC0300001521.mm.2**	Adgrl4	adhesion G protein-coupled receptor L4	-0.656060870959672	7.34304760828468	-1.97550180842403	0.0924079933181344	0.999692817443324	-4.54026655447574	7.38507797673823	7.4748336799999	7.15835326681212	6.78477285871319	7.85624143756994	7.39900642987471	NA
**TC0600000830.mm.2**	Chmp3	charged multivesicular body protein 3	-0.587030568563816	6.88103931470221	-1.97505429134286	0.0924669833245347	0.999692817443324	-4.54029113376818	7.68284527052066	7.43178048707568	6.26631228277016	6.23246221397447	6.88696461466787	6.7858710192044	NA
**TC0200003308.mm.2**	Cdk9/Mir2861/Mir3960	cyclin-dependent kinase 9 (CDC2-related kinase)/microRNA 2861/microRNA 3960	-0.657732427473113	6.1831968362236	-1.9748422645716	0.0924949449854993	0.999692817443324	-4.54030277999778	7.17330745913913	7.17313076797798	5.65884073248432	5.05979823515481	6.05584671712975	5.97825710545561	NA
**TC1600001639.mm.2**	Gramd1c	GRAM domain containing 1C	0.984582365097186	5.21342592065978	1.97360000560787	0.0926589408953386	0.999692817443324	-4.54037102701027	4.46496847035766	4.9509522067958	5.38471559088131	6.53218419761849	4.59103074108094	5.35670431722448	NA
**TC1300001274.mm.2**	Ccno	cyclin O	0.598780683434426	5.30372173110657	1.97289821004778	0.0927517159080142	0.999692817443324	-4.5404095912481	5.25405304849231	5.20139709452564	5.47170289887748	5.81051790636767	5.07892149613893	5.00573794223738	NA
**TC1500002181.mm.2**	Adcy6	adenylate cyclase 6	0.673220212297482	6.28527763244126	1.97282661389734	0.092761185877124	0.999692817443324	-4.54041352588443	6.73280694513839	6.89245729745439	6.08115834644264	6.63526264188222	5.84575956736173	5.52422099636817	NA
**TC1300002474.mm.2**	Hmgcr	3-hydroxy-3-methylglutaryl-Coenzyme A reductase	-1.56814604187139	6.63500145957471	-1.97102320156687	0.0930000397050688	0.999692817443324	-4.54051265671114	7.76268030124472	8.0308287335374	6.53570960131445	3.90439421814721	6.93266093036764	6.64373497283681	NA
**TC0200004599.mm.2**	Il1a	interleukin 1 alpha	-0.716784566696715	4.94765192508623	-1.96904745964458	0.0932624207948132	0.999692817443324	-4.54062130993029	4.54512127072362	4.83038128119158	4.6796064375236	4.75881349508078	5.06354048934223	5.80844857665559	NA
**TC1900001703.mm.2**	Nrap	nebulin-related anchoring protein	0.651210558542394	5.10335405034486	1.96804342753831	0.0933960398198992	0.999692817443324	-4.5406765451739	4.67853674299485	4.88017667237575	5.32276042747779	5.85915557441388	5.00686782259957	4.87262706220731	NA
**TC1100000058.mm.2**	Ap1b1	adaptor protein complex AP-1, beta 1 subunit	1.25555317103398	6.04156924833422	1.96735022759701	0.0934884037217937	0.999692817443324	-4.54071468827953	8.0756126324561	7.84264482553947	6.45227967229994	4.96885251473891	5.14814553064769	3.76188031432319	NA
**TC1500002056.mm.2**	Arsa	arylsulfatase A	0.627442571198169	5.33313764174285	1.96354556667136	0.0939969683024859	0.999692817443324	-4.54092415177901	6.71969228015137	6.29198890624696	5.120710373985	5.00030452924254	4.31682121250467	4.54930854832653	NA
**TC1700001738.mm.2**	Grm4	glutamate receptor, metabotropic 4	0.592916208310045	7.06324258031179	1.96337569481915	0.0940197389534159	0.999692817443324	-4.54093350843895	7.09051120155275	7.3489200314513	7.32632567453029	7.23660265821311	6.59328339173043	6.78381252439288	NA
**TC1200002053.mm.2**	Numb	NUMB endocytic adaptor protein	0.61890951743394	6.75098287332922	1.96326551923029	0.0940345104895103	0.999692817443324	-4.54093957719055	7.37619851200418	7.66309885708418	6.85864479971835	6.49356465315907	6.04332077929743	6.07106963871211	NA
**TC1000000820.mm.2**	Ptbp1	polypyrimidine tract binding protein 1	0.696239973534411	7.32364200590806	1.96033427508039	0.0944283590268499	0.999692817443324	-4.54110109627312	7.61477816661102	7.61627698568386	7.44702298783175	7.60461542727939	6.46361319924027	7.19554526880205	NA
**TC0700004303.mm.2**	Tmem219	transmembrane protein 219	-0.649129115263268	7.38176026557056	-1.95977756104285	0.0945033455330958	0.999692817443324	-4.54113178541272	7.47375285028849	7.19857076550532	7.29101157144053	6.86897830211098	7.57468789720991	7.88356020686814	NA
**TC0300000283.mm.2**	Spata5	spermatogenesis associated 5	0.59575090199393	5.57790988835802	1.95829189386479	0.0947037471850657	0.999692817443324	-4.54121370346477	6.07146662594387	5.71461814909509	5.70659068728165	5.72984749226687	5.13514997445534	5.10978640110532	NA
**TC0X00001146.mm.2**	Ube2dnl1	ubiquitin-conjugating enzyme E2D N-terminal like 1	-0.724771745148785	4.95091219429187	-1.95811337196212	0.0947278564223814	0.999692817443324	-4.54122354891308	4.49606186546866	4.51569855524563	4.54985005930097	5.0722345680687	5.20909814015449	5.86252997751276	NA
**TC0100001555.mm.2**	Lmx1a	LIM homeobox transcription factor 1 alpha	-0.620426588368138	6.42357664939537	-1.95777416747509	0.0947736825237	0.999692817443324	-4.54124225712478	6.00981895566655	6.04495670661865	6.54671152745964	6.07620400121573	6.88933281416195	6.9744358912497	NA
**TC0200002741.mm.2**	Gm14295	predicted gene 14295	-1.18566018622547	7.1465074431321	-1.9573422081748	0.0948320715866922	0.999692817443324	-4.54126608324141	8.1275911444266	8.82413093048299	6.71340612470093	5.0645949810151	7.41145921016092	6.73786226800606	NA
**TC1200000562.mm.2**	Lrr1	leucine rich repeat protein 1	0.607280346769754	4.7485367981151	1.9555505632763	0.095074634856308	0.999692817443324	-4.54136493327771	4.33316069043929	4.01489983111977	5.41489095493074	5.26396952540478	4.84881774832137	4.61548203847464	NA
**TC0500003191.mm.2**	Mapkapk5/Adam1a	MAP kinase-activated protein kinase 5/a disintegrin and metallopeptidase domain 1a	0.894797977239598	5.97266438450646	1.95541663597365	0.0950927914611208	0.999692817443324	-4.54137232410431	5.77801628270802	5.74450206475665	5.95062853633226	7.10090342069438	5.35388719419582	5.90804880835162	NA
**TC0200001838.mm.2**	B2m	beta-2 microglobulin	-0.619557141800069	9.12165657150007	-1.95518410459587	0.0951243240654106	0.999692817443324	-4.5413851569885	10.0559594388881	10.3224006352991	8.40322242717317	8.15301010843333	8.74271002184072	9.05263679736592	NA
**TC1900001028.mm.2**	Dnajc4	DnaJ heat shock protein family (Hsp40) member C4	1.03741395142262	7.33531095583948	1.95448762270515	0.095218833236709	0.999692817443324	-4.54142359847944	7.97812947055973	7.40627249424605	7.88046645047857	7.4706793860596	7.35696796998885	5.91934996370408	NA
**TC0400001465.mm.2**	Clspn	claspin	-0.629668722690021	5.13531763658986	-1.95444350169899	0.0952248233822213	0.999692817443324	-4.54142603389903	4.80454107010023	4.79916159800998	5.12806577491514	4.84636707810932	5.39678393966593	5.83698635873858	NA
**TC1200000817.mm.2**	Vsx2	visual system homeobox 2	0.670925605815156	5.10147462725472	1.95402999843837	0.0952809813904308	0.999692817443324	-4.54144885995037	4.85077839190132	4.38358442972994	5.44561009838938	5.9125579783743	4.94077942738418	5.07553743774918	NA
**TC1500000676.mm.2**	Kdelr3	KDEL (Lys-Asp-Glu-Leu) endoplasmic reticulum protein retention receptor 3	-0.602415930513296	4.91058706474529	-1.95388372316102	0.0953008549605798	0.999692817443324	-4.54145693511663	4.19565307208604	4.38442851682854	5.09094973650979	4.7483547327555	5.5662215979503	5.47791473234158	NA
**TC1900000019.mm.2**	Unc93b1	unc-93 homolog B1, TLR signaling regulator	0.683800031837825	6.72772181702306	1.95248391424376	0.0954912472619676	0.999692817443324	-4.5415342260346	6.91881470037283	6.86329093390257	6.63085144532464	7.34506122044467	6.27897364034831	6.32933896174535	NA
**TC1500000659.mm.2**	Nol12	nucleolar protein 12	0.671226052232456	6.86434531871706	1.94864495324794	0.0960153373248635	0.999692817443324	-4.54174632615664	7.34051716789314	7.1992390863065	6.66393776738147	7.33044611390235	6.32892754876302	6.32300422805589	NA
**TC0600002472.mm.2**	Kdm3a	lysine (K)-specific demethylase 3A	-0.764291904678504	5.64078469954135	-1.9485201917434	0.09603241744865	0.999692817443324	-4.54175322235325	6.05056733508456	6.17360029190403	5.49059309923416	4.5553852812171	5.70476815324812	5.86979403656015	NA
**TC0X00001669.mm.2**	Asb11	ankyrin repeat and SOCS box-containing 11	-0.632817772759155	6.06042024815536	-1.94801885473488	0.0961010820103033	0.999692817443324	-4.54178093580383	5.51121150764304	5.98892786719491	5.89192408207369	5.90644920221425	6.40255201411668	6.66145681568957	NA
**TC0200001767.mm.2**	Vps18	VPS18 CORVET/HOPS core subunit	0.613739469428367	6.53297247074542	1.94756312235839	0.0961635427272556	0.999692817443324	-4.54180613109098	7.40456417332343	6.9590923277845	6.55345615974817	6.47737247136249	5.84872248659147	5.95462720566246	NA
**TC1500001826.mm.2**	Ift27	intraflagellar transport 27	0.811633572774764	6.31105400100654	1.94720465368888	0.096212701218846	0.999692817443324	-4.54182595101304	6.76323277866623	6.98409381222869	6.4195331934884	6.45159908685853	6.15377288169365	5.09409225310375	NA
**TC1400002856.mm.2**	Gm3383	predicted gene 3383	1.15727858099264	6.78692013631704	1.94619323372297	0.0963515365292145	0.999692817443324	-4.54188188189892	7.87510046494718	6.68399084195176	7.64864217188919	6.58985116460509	6.44831363260973	5.47562254189927	NA
**TC0500000855.mm.2**	Epgn	epithelial mitogen	-0.675647229847453	5.61962645646708	-1.94605993372017	0.0963698491432469	0.999692817443324	-4.54188925428907	5.15276327080042	5.51479266473292	5.33921182132195	5.51024235046518	5.81393461672059	6.38681401476145	NA
**TC0500002569.mm.2**	Corin	corin, serine peptidase	0.766294094386712	6.20047485546461	1.94534715598379	0.0964678284429809	0.999692817443324	-4.54192867959645	5.96908463408597	5.66316534245067	6.42258063050691	7.12901304200531	5.71742986094281	6.30157562279599	NA
**TC0X00003335.mm.2**	Mospd2	motile sperm domain containing 2	0.692615629030414	6.42902794996548	1.94471222662376	0.0965551898743474	0.999692817443324	-4.54196380444897	6.543887611192	6.70686359847063	6.31381488842677	7.04050898566878	5.9233744428851	6.04571817314961	NA
**TC1100002567.mm.2**	Pttg1	pituitary tumor-transforming gene 1	-0.972290008908448	5.77364357028521	-1.94470675773539	0.0965559426926134	0.999692817443324	-4.54196410701524	5.58363178716065	6.24966010116204	5.47852646491391	4.95346829287193	5.58956854501737	6.78700623058537	NA
**TC0900001635.mm.2**	Snrk	SNF related kinase	0.794964719946637	7.3112168304512	1.9434407448621	0.0967303721860305	0.999692817443324	-4.54203415953316	8.23738891059741	8.06043636261683	7.74391818850719	6.83578438618593	6.73684886146835	6.25292427333149	NA
**TC1000002264.mm.2**	Ddx21	DEAD (Asp-Glu-Ala-Asp) box polypeptide 21	-0.769673859686593	5.90616093333366	-1.9430502861288	0.0967842321158594	0.999692817443324	-4.54205576901225	6.67497264041701	6.74827970014885	5.46450549870935	4.77267727132212	5.54465768243819	6.23187280696646	NA
**TC0200001166.mm.2**	Ppp1r1c	protein phosphatase 1, regulatory inhibitor subunit 1C	0.573129071540987	4.93600614594943	1.94244583660893	0.0968676686602964	0.999692817443324	-4.54208922541964	4.63231314854382	4.5646424045392	5.34692153610862	5.43574819673916	4.71164404983359	4.92476753993222	NA
**TC0500001344.mm.2**	Rasal1	RAS protein activator like 1 (GAP1 like)	0.814305183057741	7.16629293971375	1.93924185443152	0.097311130650107	0.999692817443324	-4.54226664467599	7.76817339738125	7.76718763259673	7.35653081159252	7.18897267561747	5.91579143789584	7.00110168319867	NA
**TC1100003035.mm.2**	Cntrob	centrobin, centrosomal BRCA2 interacting protein	0.82408566813934	6.12685738526187	1.93814928266409	0.0974628130741949	0.999692817443324	-4.54232717541531	6.42159535410934	5.92134170554899	6.21202824506534	6.82116104903045	5.29185310375227	6.09316485406484	NA
**TC0500001607.mm.2**	Myl10	myosin, light chain 10, regulatory	0.607141397745841	5.35047319638126	1.93787003095509	0.0975016193738685	0.999692817443324	-4.54234264898203	5.13455569082807	5.07822555245428	5.55453353671193	5.99763682853653	5.18594994017397	5.1519376295828	NA
**TC0700002148.mm.2**	Cdc42ep5	CDC42 effector protein (Rho GTPase binding) 5	-0.688634064905922	5.3799441473982	-1.93741896727565	0.0975643339430396	0.999692817443324	-4.54236764489071	4.99064463005916	5.3003934941071	4.96289113730056	5.34278817790498	5.55739216068257	6.12555528433482	NA
**TC1500000605.mm.2**	Gpt	glutamic pyruvic transaminase, soluble	-0.748413779859396	5.81990105211156	-1.93509251376728	0.0978884329164932	0.999692817443324	-4.54249660754392	5.47781227071477	5.5234736747983	5.39456746898096	5.81607893473779	5.94073355457826	6.76674040885928	NA
**TC1100003233.mm.2**	Doc2b	double C2, beta	0.920549507656051	7.06899008613921	1.9348252973979	0.097925727186379	0.999692817443324	-4.54251142459436	7.91758309348396	7.54675511565575	7.83320138091932	6.56214928058451	6.43244825563279	6.12180339055895	NA
**TC0400001544.mm.2**	Snrnp40	small nuclear ribonucleoprotein 40 (U5)	-0.898770583392944	6.63507252216916	-1.93372115230906	0.0980799774874603	0.999692817443324	-4.54257265864393	6.96389902144935	7.06161858318399	6.6492477431578	5.34444043764008	6.86186082515701	6.92936852242675	NA
**TC0300002360.mm.2**	Hax1	HCLS1 associated X-1	-1.16965621025936	6.47153260932054	-1.93331923292305	0.0981361858416855	0.999692817443324	-4.54259495226138	7.16323859986868	6.91837194126426	6.50327126658872	4.70086508054709	7.06204979270745	6.48139897494707	NA
**TC1200001366.mm.2**	Fkbp1b	FK506 binding protein 1b	-1.12169859907575	6.27027926056996	-1.93270528888213	0.0982221074424861	0.999692817443324	-4.54262901038708	6.19429466534846	5.86510494881694	6.68377006802354	4.97566930752788	7.19862225457483	6.7042143191281	NA
**TC0400003662.mm.2**	Gmeb1	glucocorticoid modulatory element binding protein 1	0.612979396103314	6.30980846705901	1.93169235916135	0.09836403017297	0.999692817443324	-4.54268521239588	6.52025850315483	6.40553144924015	6.59765566617914	6.48185415490371	5.6994335482642	6.15411748061203	NA
**TC0X00002154.mm.2**	Smarca1	SWI/SNF related, matrix associated, actin dependent regulator of chromatin, subfamily a, member 1	0.76445574746891	6.55224632962808	1.93165055956989	0.0983698911249234	0.999692817443324	-4.54268753190853	7.62722169716452	7.43384927971966	6.38790111916669	6.50275812874435	6.15573594955226	5.20601180342097	NA
**TC0500001168.mm.2**	C130026L21Rik	RIKEN cDNA C130026L21 gene	-0.615018946977354	6.97003341852172	-1.93055842323601	0.0985231482945702	0.999692817443324	-4.54274814376762	7.1785698811582	7.20460782880372	6.58545770944536	6.51803474416149	7.41081975973401	6.92271058782755	NA
**TC1000002422.mm.2**	Fam207a	family with sequence similarity 207, member A	0.665432407582803	7.00623495001938	1.93015333552361	0.0985800535194105	0.999692817443324	-4.54277062932966	7.18368903908467	6.7016644201104	7.13637550734747	7.60508502069594	6.76620068829524	6.64439502458256	NA
**TC1400001346.mm.2**	Itgbl1	integrin, beta-like 1	-0.719212588679405	5.22547914144117	-1.92993433444338	0.0986108315414242	0.999692817443324	-4.54278278647815	4.75715426541434	4.94607507550112	4.99189157348058	5.11371859170578	5.36158393737345	6.18245140517172	NA
**TC1500002171.mm.2**	Olfr285/Olfr257	olfactory receptor 285/olfactory receptor 257	-0.662371517150567	4.70833851904891	-1.92917845714329	0.0987171343410208	0.999692817443324	-4.54282475124581	3.99107018459272	4.09745239559472	4.49065210046665	4.92773064943578	5.12055572604301	5.62257005816056	NA
**TC0X00002048.mm.2**	Akap17b	A kinase (PRKA) anchor protein 17B	1.000286600812	6.18497264927736	1.92886741298003	0.0987609109960194	0.999692817443324	-4.54284202187383	6.6064443531081	6.39336616223609	6.57630465535426	6.47899463561773	6.29257371150748	4.7621523778405	NA
**TC0900001656.mm.2**	Exosc7	exosome component 7	0.746741049365021	5.99886661844162	1.92716899459361	0.099000287409744	0.999692817443324	-4.54293634747675	6.83616002219641	6.53134634844634	6.47012579548705	5.58946192388144	5.26931168755403	5.29679393308442	NA
**TC0700003596.mm.2**	Crtc3	CREB regulated transcription coactivator 3	0.645326641031969	7.27139278483483	1.9268155234408	0.0990501781036217	0.999692817443324	-4.54295598287281	7.13346627137723	6.90204067260694	7.6768564289134	7.76489509463097	6.79289683395643	7.35820140752401	NA
**TC1100000005.mm.2**	Eif4enif1	eukaryotic translation initiation factor 4E nuclear import factor 1	1.04689538546476	5.78501804680689	1.92641233703683	0.0991071162559679	0.999692817443324	-4.54297838187554	6.66117645764897	6.78563853632802	6.22697679259405	5.4515652363029	5.52035454320841	4.06439671475901	NA
**TC1000000869.mm.2**	Izumo4	IZUMO family member 4	-0.655554441717774	6.34880788894686	-1.92603140272212	0.0991609417362782	0.999692817443324	-4.54299954653754	6.61584565519863	6.45143949692448	5.80190229719578	6.05532435186548	6.87439629872695	6.29393923376985	NA
**TC0600000584.mm.2**	Ccdc126	coiled-coil domain containing 126	-0.694781609244152	5.41015631424284	-1.92551170523614	0.0992344209429782	0.999692817443324	-4.54302842379692	6.15315037913621	6.0481926278112	5.08342051304883	4.35159531696184	5.54088911835626	5.28368993014271	NA
**TC0600000914.mm.2**	Loxl3	lysyl oxidase-like 3	-0.929415909246552	7.31160505019315	-1.92526885579685	0.0992687755218298	0.999692817443324	-4.54304191901141	7.02308410520921	7.08908263252399	7.14992478347069	6.79939108899562	7.2317188239821	8.57642886697731	NA
**TC1200000236.mm.2**	Adi1	acireductone dioxygenase 1	0.673703830993905	7.27125278507069	1.92353534904785	0.0995143474760972	0.999692817443324	-4.54313827192197	8.31461828968492	8.04644912748862	7.27994838987144	7.02698008774777	6.1715376567438	6.7879831588876	NA
**TC0500000011.mm.2**	Fam133b	family with sequence similarity 133, member B	-1.47728890406092	6.62708052650702	-1.92196406577537	0.0997374580693449	0.999692817443324	-4.54322564044961	7.453828659772	7.36756588367212	6.76662688300133	4.22662852073674	7.11327406463275	6.83455914722716	NA
**TC0700001998.mm.2**	Utf1	undifferentiated embryonic cell transcription factor 1	0.686225130957166	5.0399393395447	1.92120804186817	0.0998449842079244	0.999692817443324	-4.54326768883637	4.7943436906041	5.25038615748352	5.55482827223404	5.2288499533134	4.44830338202899	4.96292458160412	NA
**TC0700001623.mm.2**	Zfp143	zinc finger protein 143	-0.783643185703548	6.4563316148926	-1.92073368451317	0.0999125086461732	0.999692817443324	-4.54329407518164	6.67017169984157	7.08636479879429	6.28351826761231	5.42356514204403	6.84111043112993	6.4332593499335	NA
**TC0900002268.mm.2**	Pts	6-pyruvoyl-tetrahydropterin synthase	0.830458224711958	6.30177268462361	1.91985202011762	0.10003813302743	0.999692817443324	-4.5433431256011	7.38285376813447	7.01176100902828	6.30126653414379	6.23720235585761	5.98716159558324	4.89039084499423	NA
**TC0800001132.mm.2**	Usb1	U6 snRNA biogenesis 1	-0.88618934122995	6.11926572732396	-1.9196127891363	0.100072246919329	0.999692817443324	-4.54335643661699	5.85468817076392	6.30027613842452	6.28276552021999	5.11136016592771	6.41855655355315	6.74794781505445	NA
**TC0700002694.mm.2**	Supt5	suppressor of Ty 5	0.624280821573899	6.46970595387835	1.9195124175751	0.100086563144213	0.999692817443324	-4.54336202158792	7.75631122021719	7.46514163141663	6.36384343067875	6.0588288267133	5.41327886342642	5.76083175081783	NA
**TC1000000834.mm.2**	Cnn2	calponin 2	0.56165533629836	5.60645228509715	1.91693753375189	0.100454518192388	0.999692817443324	-4.54350533845731	6.05683779257942	5.98741834802811	5.73317602085215	5.6257081004339	5.1983295595942	5.03724388909512	NA
**TC1700000277.mm.2**	Gm10509	predicted gene 10509	-1.21036964653838	5.76878622432719	-1.91623656716143	0.100554918952181	0.999692817443324	-4.54354436815334	6.85069816688477	6.71906086072157	5.56655424394879	3.74455526869123	6.29384951838788	5.4379992873289	NA
**TC0600002730.mm.2**	Xpc	xeroderma pigmentosum, complementation group C	0.618532325489148	7.26043516608721	1.91586799581584	0.100607749921209	0.999692817443324	-4.54356489257624	6.99410139152741	7.23818412444638	7.41253659234825	7.87115847341562	7.07033519069432	6.97629522409126	NA
**TC0200005490.mm.2**	Gm14305	predicted gene 14305	-1.37702268652875	7.44079287463718	-1.91580301600416	0.100617066965003	0.999692817443324	-4.543568511244	8.51599125531463	9.12472717653913	7.08185077602465	5.04314594543128	7.95331200408553	6.9257300904279	NA
**TC1100003992.mm.2**	Wipi1	WD repeat domain, phosphoinositide interacting 1	-0.854695157771669	7.17362148571333	-1.91379827012974	0.100904934381461	0.999692817443324	-4.5436801793427	6.97284030398863	6.8011541443764	7.46573234956032	6.31343972562549	7.55314291090878	7.93541947982036	NA
**TC1700001211.mm.2**	Gemin6	gem nuclear organelle associated protein 6	0.719370184552802	5.14283247676357	1.91360395918754	0.100932879319359	0.999692817443324	-4.54369100545815	5.23615732158137	5.02553489875126	5.15163109566932	5.86539040900788	5.01383695042992	4.56444418514167	NA
**TC0700002607.mm.2**	Zfp575	zinc finger protein 575	0.834670471006666	5.40870598486714	1.91158107092301	0.101224256943934	0.999692817443324	-4.54380373909351	5.24835426227061	4.87276294670303	5.50840905039495	6.4918207707263	5.4310142426457	4.89987463646222	NA
**TC1100001891.mm.2**	Cdr2l	cerebellar degeneration-related protein 2-like	-0.552507414247165	5.52277030574322	-1.9106800471126	0.101354308268025	0.999692817443324	-4.54385396845995	5.62270058104829	5.60494670193627	5.25646247219523	5.14551738929499	5.73745494552462	5.76953974445993	NA
**TC1500000346.mm.2**	Trhr	thyrotropin releasing hormone receptor	1.45804258241908	5.6642905160886	1.91005939737489	0.101443987198474	0.999692817443324	-4.54388857360088	7.29401091506875	6.13416337420142	5.90600634759474	5.83082063845506	5.53749884055417	3.28324298065747	NA
**TC0800001608.mm.2**	Insr	insulin receptor	-0.877451263212832	6.87262384835328	-1.90974798848041	0.101489012834618	0.999692817443324	-4.5439059338389	7.99641618310787	7.94974435614919	6.37150261739328	5.3958373948252	7.17920687293338	6.34303566571077	NA
**TC1900000813.mm.2**	Fam160b1	family with sequence similarity 160, member B1	0.612779932945046	6.99191261883539	1.90901712395914	0.101594763821384	0.999692817443324	-4.54394669752706	7.62133530557674	7.53233717863404	7.16082243209529	6.85085911525055	6.19343742038723	6.59268426106852	NA
**TC1400001914.mm.2**	Bmp4	bone morphogenetic protein 4	0.576017207234788	5.82794839295427	1.90548512287351	0.102107355958513	0.999692817443324	-4.54414376339407	6.03700237033813	6.0043297383893	6.1466845532544	5.89251177847948	5.55293630063641	5.33422561662789	NA
**TC1200001272.mm.2**	Pld4	phospholipase D family, member 4	-0.664271130779747	6.45090930579124	-1.90525633725302	0.102140647108194	0.999692817443324	-4.54415653358624	7.01067148249028	7.30741935888822	6.07511077462448	5.45430059128024	6.34508686865827	6.51286675880595	NA
**TC1200001243.mm.2**	Trmt61a	tRNA methyltransferase 61A	0.674415065336897	7.06956088376315	1.90426349567043	0.102285242128773	0.999692817443324	-4.54421195867128	6.97956873015179	7.14724209990896	7.74465448453492	7.07503781706106	6.87227782822298	6.59858434269921	NA

**TC0400004136.mm.2**	Ski	ski sarcoma viral oncogene homolog (avian)	0.645829160004214	7.59438739732884	1.90423450070178	0.102289467921567	0.999692817443324	-4.54421357748651	8.02778110931204	8.17709292445688	7.87648450021283	7.45006983489343	7.24846416226896	6.78643185282888	NA
**TC0900002678.mm.2**	Tmod3	tropomodulin 3	-0.574121486907544	7.21793206744402	-1.89991768697877	0.102920537140082	0.999692817443324	-4.54445470258657	7.7432004768544	7.77760227412614	6.75996260057835	6.55931073935588	7.36716597244931	7.10035034130001	NA
**TC0500001077.mm.2**	Lpcat2b	lysophosphatidylcholine acyltransferase 2B	-0.790535475728895	4.62105800816914	-1.89921094898657	0.103024219670285	0.999692817443324	-4.54449420040974	4.33654251153537	3.65514580481564	4.14762352502623	4.92917086557679	5.48013197860606	5.17773336345474	NA
**TC1800000060.mm.2**	Fzd8	frizzled class receptor 8	0.762991519767666	5.83192257710331	1.89892515015734	0.103066177266968	0.999692817443324	-4.54451017470004	6.00997043125947	6.06870795676573	6.39163714672583	5.82778291033917	5.76302058982434	4.93041642770533	NA
**TC1100002732.mm.2**	Gm9837	predicted gene 9837	-0.642200938481184	5.6708849929049	-1.89681543385649	0.103376422859548	0.999692817443324	-4.54462812443709	5.51365598516086	5.24692703526451	5.39688918966544	5.59327334035539	6.41240773316026	5.86215667382294	NA
**TC0400002779.mm.2**	Mup20	major urinary protein 20	-0.678762405680554	4.62909174449474	-1.89500370063346	0.103643583556611	0.999692817443324	-4.5447294571552	4.16584739724064	4.26227912710917	4.83934993187512	4.15509963375363	5.00956557676582	5.34240880022404	NA
**TC1900001286.mm.2**	Pgm5	phosphoglucomutase 5	-0.563364574400154	6.66162732186874	-1.89497987535924	0.103647101397562	0.999692817443324	-4.54473078999693	6.61606877150241	6.76452943099381	6.31053019339275	6.4206880965652	6.82802283697315	7.02992460178511	NA
**TC0500003726.mm.2**	Prr14l	proline rich 14-like	-1.2701739624242	4.89783588713636	-1.89384172040969	0.103815289099384	0.999692817443324	-4.54479446895074	5.21217435183876	5.39370889427268	5.10354406936039	3.01684800656879	5.62031282909273	5.04042717168484	NA
**TC0100002488.mm.2**	Kansl1l	KAT8 regulatory NSL complex subunit 1-like	0.558764924802946	6.96463391874206	1.89237796878112	0.104031986635324	0.999692817443324	-4.54487638751242	7.4924205716668	7.48360671608157	6.99060930292795	6.97404373422699	6.54519481092358	6.30192837662546	NA
**TC0900002158.mm.2**	Hmbs	hydroxymethylbilane synthase	-0.697675965165756	6.34434191039663	-1.89181193754588	0.104115902871651	0.999692817443324	-4.54490807217329	6.14932798552196	6.37325468776733	6.43316656032831	5.64089186905118	6.68580155671509	6.78360880299592	NA
**TC0100000353.mm.2**	Dnah7b	dynein, axonemal, heavy chain 7B	0.590631058629477	5.16628605688286	1.89173810732787	0.104126853395023	0.999692817443324	-4.54491220523917	4.86842741134546	5.12598573782449	5.42129623011157	5.67098642458152	4.82977155413573	5.08124898329841	NA
**TC0500003722.mm.2**	Medag	mesenteric estrogen dependent adipogenesis	-0.694681061177826	6.87177048273286	-1.89127313696847	0.104195844056576	0.999692817443324	-4.54493823608496	6.44898602033659	6.2901005180332	6.38159927945857	7.1694878383773	7.38746203763963	7.55298720255189	NA
**TC1100001169.mm.2**	Lyrm9	LYR motif containing 9	0.721809975763492	6.48831989084025	1.89116586190534	0.104211767548545	0.999692817443324	-4.54494424212403	6.81525969890998	6.40394912314669	6.55232393866992	7.02484129858598	6.39055838799837	5.74298689773055	NA
**TC0600000813.mm.2**	Eif2ak3	eukaryotic translation initiation factor 2 alpha kinase 3	-0.575507154659359	7.85886930561472	-1.88981604530506	0.104412334164765	0.999692817443324	-4.54501982635372	7.6720613675717	7.81740412033431	7.67963168431192	7.57673633391988	8.04617219515052	8.3612101324	NA
**TC1200000464.mm.2**	1110008L16Rik	RIKEN cDNA 1110008L16 gene	0.591502628001995	5.33766328356044	1.8894922822587	0.104460498031575	0.999692817443324	-4.5450379589766	5.4606071656919	5.35706571431084	5.79453747588382	5.40111856279812	4.91533525752803	5.09731552514992	NA
**TC0900003269.mm.2**	Sec22c	SEC22 homolog C, vesicle trafficking protein	0.704632621862812	6.90700152886256	1.88811142384542	0.104666164227473	0.999692817443324	-4.54511530904587	7.41527398228054	7.12615067276391	6.76315413021408	7.39177075071418	6.1224723877076	6.62318724949502	NA
**TC1800000816.mm.2**	Smad2	SMAD family member 2	0.743263695870771	6.32864494303834	1.8879245999205	0.10469402056601	0.999692817443324	-4.54512577589304	7.12180796708244	7.05686447054565	6.65159111179957	5.98827119437216	5.92575318851177	5.22758172591841	NA
**TC1600002056.mm.2**	Urb1	URB1 ribosome biogenesis 1 homolog (S. cerevisiae)	-0.605395750332979	6.21189842879399	-1.88788707202846	0.104699617034824	0.999692817443324	-4.54512787845029	6.12461015895744	6.20824698104952	6.18352576614004	5.68034519990547	6.54937635359701	6.52528611311447	NA
**TC0600002657.mm.2**	Prokr1	prokineticin receptor 1	0.708087676050443	5.43235287363752	1.88704285852838	0.104825591046821	0.999692817443324	-4.54517518118946	4.8358544525712	5.26214792313533	5.72978895902555	6.22635615008419	5.55318973280926	4.98678002419958	NA
**TC1300002084.mm.2**	Trpc7	transient receptor potential cation channel, subfamily C, member 7	1.10487325707355	5.87136851068242	1.88642080236043	0.104918510167882	0.999692817443324	-4.54521004144848	6.70916540649839	5.989449581797	6.49790366717216	5.87176762780095	4.29890975041851	5.86101503040751	NA
**TC1400002264.mm.2**	Gulo	gulonolactone (L-) oxidase	-0.581400230964335	5.00731316434226	-1.88547933187036	0.105059295898227	0.999692817443324	-4.54526281047342	4.13796476991396	4.34410859491659	4.93985201950952	5.25965056013763	5.74509059143378	5.61721245014204	NA
**TC0X00002993.mm.2**	Timm8a1	translocase of inner mitochondrial membrane 8A1	-0.590230228012996	6.88226231303417	-1.8853243588654	0.105082488101756	0.999692817443324	-4.54527149764483	6.75516599765967	6.99754637202296	6.73932805566181	6.4408724705864	7.29316924237061	7.06749173990359	NA
**TC0900003042.mm.2**	Sema3f	sema domain, immunoglobulin domain (Ig), short basic domain, secreted, (semaphorin) 3F	0.591239833822487	6.3948874541138	1.88465355334392	0.105182934456	0.999692817443324	-4.54530910359485	6.70456772671134	6.74245232204175	6.32519292561019	6.72719924617714	5.83380457532047	6.03610792882188	NA
**TC0200003122.mm.2**	Arrdc1	arrestin domain containing 1	0.696716429549155	7.18330942291887	1.88391447748881	0.105293713103033	0.999692817443324	-4.54535054295884	7.03342597851576	6.9633408035574	7.4340530761568	7.81420823111238	7.29749069994952	6.55733774822135	NA
**TC0300002071.mm.2**	Plch1	phospholipase C, eta 1	-0.685081438730576	6.45305676740965	-1.88136589459425	0.105676597199111	0.999692817443324	-4.54549348895725	6.79330240755203	6.80411362381811	5.92890989617024	5.94647095164306	7.02376478233013	6.22177894294432	NA
**TC1400002116.mm.2**	Gzmd/Gzme	granzyme D/granzyme E	-0.562257829986053	5.45991082140749	-1.88123659725389	0.105696058567491	0.999692817443324	-4.54550074306596	4.89383337456947	4.79892925930164	5.62273070694412	5.34836261035673	6.02136105595008	6.07424792132288	NA
**TC0X00003021.mm.2**	Tceal8	transcription elongation factor A (SII)-like 8	0.86152471288198	7.12654422610713	1.88105176946854	0.105723884304957	0.999692817443324	-4.5455111129983	7.14126143620944	7.39494899143293	6.92311816476406	8.04993401261812	6.89977969629007	6.35022305532815	NA
**TC1300001812.mm.2**	Nrn1	neuritin 1	-0.815721884403626	8.72276045068227	-1.87864954297614	0.106086194992487	0.999692817443324	-4.5456459283417	8.11015014573048	8.71591733838784	8.33111362686637	8.60841209871766	9.76485573197921	8.80611376241207	NA
**TC0100000456.mm.2**	Nop58	NOP58 ribonucleoprotein	1.13190819328869	6.13888429292703	1.87861805628485	0.106090952010528	0.999692817443324	-4.54564769585215	7.81320684317939	7.70865021830643	6.25824826913069	5.52938427219618	5.63728445220893	3.88653170254056	NA
**TC0200004650.mm.2**	Cenpb/Spef1	centromere protein B/sperm flagellar 1	-0.545838300265179	6.99767648693541	-1.87844161177452	0.106117613180688	0.999692817443324	-4.54565760080618	7.13720460270435	7.10862813419025	6.67142707246638	6.65284771962736	7.28384301469631	7.13210837792779	NA
**TC1700001847.mm.2**	Zfp871	zinc finger protein 871	-1.65557664232559	6.37199045395914	-1.87824467147393	0.106147379099129	0.999692817443324	-4.54566865674666	7.69829032195081	8.27775850609511	6.15072767088412	3.32164263464474	6.77627015224653	6.00725343793351	NA
**TC1200001947.mm.2**	Zbtb25	zinc finger and BTB domain containing 25	1.02199436118208	5.97359506238641	1.87801314202411	0.106182383393421	0.999692817443324	-4.54568165504753	7.01792733288591	6.67401480122546	6.85870329921356	5.23810518207207	5.001967276863	5.05085248205846	NA
**TC1400001315.mm.2**	Farp1	FERM, RhoGEF (Arhgef) and pleckstrin domain protein 1 (chondrocyte-derived)	0.674934229365076	7.63653485787467	1.87646706138056	0.106416422393059	0.999692817443324	-4.54576846954443	7.73167909495215	7.8604251564944	7.74221163964921	8.04627503761661	7.57040912441772	6.86820909411795	NA
**TC0600000444.mm.2**	Ephb6	Eph receptor B6	-0.982648142075203	6.87140085995257	-1.87588717499315	0.106504333910462	0.999692817443324	-4.54580103808282	6.85969079806762	7.45511490482614	6.87179983941777	5.60235174691786	6.86569935898167	7.57374851150437	NA
**TC0900002983.mm.2**	Dnajc13	DnaJ heat shock protein family (Hsp40) member C13	0.634507735562605	6.42567493561453	1.87442448483999	0.106726397124336	0.999692817443324	-4.54588320537774	6.70110736545031	7.22763586267128	6.46544655777149	6.4817143705739	6.02242244046063	5.65572301675955	NA
**TC1200000459.mm.2**	1700047I17Rik2/Fam177a	RIKEN cDNA 1700047I17 gene 2/family with sequence similarity 177, member A	-0.648670052713528	8.30261633036697	-1.87352156449593	0.10686370428572	0.999692817443324	-4.54593393967147	8.19972983973904	8.56240881495084	8.18422071651812	7.69388889452432	8.75205092861068	8.42339878785883	NA
**TC1200000461.mm.2**	1700047I17Rik2/Fam177a	RIKEN cDNA 1700047I17 gene 2/family with sequence similarity 177, member A	-0.648670052713528	8.30261633036697	-1.87352156449593	0.10686370428572	0.999692817443324	-4.54593393967147	8.19972983973904	8.56240881495084	8.18422071651812	7.69388889452432	8.75205092861068	8.42339878785883	NA
**TC1100003672.mm.2**	Neurod2	neurogenic differentiation 2	-0.718890825490714	7.01357019513093	-1.87263736260284	0.106998333298489	0.999692817443324	-4.54598363129356	6.56689535038212	7.13381240181886	7.05580772045892	6.41565816334265	7.29458763815617	7.61465989662683	NA
**TC0200000222.mm.2**	Il15ra	interleukin 15 receptor, alpha chain	0.582198608612625	6.29392166810563	1.87150593508877	0.10717084829033	0.999692817443324	-4.54604722996169	6.1543709987597	5.97888317535431	6.51554376242142	6.8817927634511	6.04952600672569	6.18341330192158	NA
**TC0800000653.mm.2**	Sh3rf1	SH3 domain containing ring finger 1	0.584895556564363	6.37774718058742	1.87025578239486	0.107361784013901	0.999692817443324	-4.54611751937649	7.03500028713673	6.76372611303065	6.53880485919751	6.27996903904542	5.70668599164963	5.94229679346457	NA
**TC1500000719.mm.2**	Xpnpep3	X-prolyl aminopeptidase 3, mitochondrial	-0.61096529688913	6.0811120959742	-1.86936972737333	0.107497313596787	0.999692817443324	-4.54616734836303	6.05271674103665	6.26710720283418	5.55233854269144	5.92012047640663	6.16636061984527	6.52802899303106	NA
**TC1400000163.mm.2**	Nudt13	nudix (nucleoside diphosphate linked moiety X)-type motif 13	-0.588541175494719	6.52246790446157	-1.86833833120019	0.107655286156324	0.999692817443324	-4.54622536219059	6.7850198046212	6.60012673348539	6.2814532362067	6.00483603263001	6.57064180023579	6.89272981959036	NA
**TC0800001323.mm.2**	Cog4	component of oligomeric golgi complex 4	-0.6065015062811	5.68006604080517	-1.86831551105236	0.107658783955745	0.999692817443324	-4.54622664591244	6.48111749821223	6.3252354050577	4.76191408342108	5.26860608107838	5.67771192914419	5.56581124791746	NA
**TC1700002165.mm.2**	Mad2l1bp	MAD2L1 binding protein	-0.56862505974189	6.65851079353942	-1.86807796622138	0.107695200702545	0.999692817443324	-4.5462400090836	6.63979945674963	6.74622588177821	6.32984791593105	6.38404673568139	6.75557412793947	7.09557064315675	NA
**TC0600003443.mm.2**	1700034J05Rik	RIKEN cDNA 1700034J05 gene	-0.56247864673171	4.88258625168337	-1.86794910634955	0.107714960595419	0.999692817443324	-4.54624725841204	4.77437546772285	4.53631225594884	4.72199797936963	4.70793826711293	5.16844183064421	5.38645170930177	NA
**TC0200002352.mm.2**	Raly/a	hnRNP-associated with lethal yellow/nonagouti	-0.784869618074579	6.83615697470887	-1.86776930887278	0.107742537420548	0.999692817443324	-4.54625737367695	7.83722947779351	7.72155182383866	6.51316549516736	5.43104516006858	6.83594302069513	6.67800687068997	NA
**TC1700000284.mm.2**	Zfp943	zinc finger prtoein 943	0.864910275231054	6.16452128057903	1.86763004801452	0.107763901622689	0.999692817443324	-4.54626520863681	6.68417512945107	6.90274775725036	6.00864068118539	6.55637199243203	5.99827844514328	4.83691367801202	NA
**TC1100003860.mm.2**	Dcakd	dephospho-CoA kinase domain containing	0.896633000015135	7.46225506903062	1.86762858987159	0.107764125340449	0.999692817443324	-4.54626529067461	7.84584653920425	7.38388640767726	8.46453784052849	7.20399389313774	7.09592601142741	6.77933972220854	NA
**TC1600000234.mm.2**	Dgcr6	DiGeorge syndrome critical region gene 6	0.81950383082259	6.61559894781976	1.86727599842669	0.107818235610939	0.999692817443324	-4.54628512882791	7.37673756823995	7.21335255362008	7.10766699086488	6.26358862248698	6.2773151351381	5.45493281656857	NA
**TC1100000989.mm.2**	Xaf1	XIAP associated factor 1	-0.885107488592373	5.88585971503456	-1.86522998652668	0.108132753153583	0.999692817443324	-4.54640027326303	6.44036458301034	6.31654916766829	5.86295630326499	4.53105847790699	5.97600520847503	6.18822454988171	NA
**TC1300002246.mm.2**	Zfp429	zinc finger protein 429	-0.556742381576576	6.71377966133199	-1.86458336590248	0.10823234045516	0.999692817443324	-4.54643667334312	6.36823090544319	6.52677084918833	6.59823028100218	6.53886544410145	7.00675267144112	7.24382781681567	NA
**TC0700003420.mm.2**	Tjp1	tight junction protein 1	1.14699616962474	6.86874750170469	1.86256015906137	0.108544520777245	0.999692817443324	-4.54655059589161	7.85733653079548	7.92063063778559	7.22944372054882	6.63481136989947	6.7149225582207	4.8553401929781	NA
**TC1800000332.mm.2**	Ankhd1/Eif4ebp3	ankyrin repeat and KH domain containing 1/eukaryotic translation initiation factor 4E binding protein 3	0.702072416039998	6.58892089301262	1.86067733240171	0.108835834768454	0.999692817443324	-4.54665665545385	7.18671272479644	7.49699320159402	6.80682508954373	6.32015704233891	6.1916376227201	5.53119967708254	NA
**TC0600002646.mm.2**	2610306M01Rik	RIKEN cDNA 2610306M01 gene	-0.658975066194208	5.90362103275765	-1.85850675652997	0.109172622403082	0.999692817443324	-4.54677897341376	5.66058124305193	5.3595046001321	5.4964364945327	6.04540861595402	6.22794179673182	6.63185344614332	NA
**TC1400000858.mm.2**	Sacs	sacsin	-0.813852827390012	6.28591073528752	-1.85762740183413	0.109309354494778	0.999692817443324	-4.54682854252642	6.9402277814788	6.77744554170201	6.08733017405266	5.09771254282949	6.72301038222768	6.0897379894345	NA
**TC0500003563.mm.2**	Usp42	ubiquitin specific peptidase 42	0.585603281610063	6.94584524498165	1.855286753102	0.109674124767085	0.999692817443324	-4.5469605267235	6.87564819586187	6.72131093925822	7.52147287391864	7.10318657507632	6.6546328309102	6.79882005486464	NA
**TC1400000941.mm.2**	Ccdc25	coiled-coil domain containing 25	0.616962937057318	6.26538775836064	1.8551681066475	0.109692646582826	0.999692817443324	-4.54696721857178	6.67666825075681	6.82680826973755	6.47789318017975	6.18349477171231	5.46300497445322	5.9644571033242	NA
**TC1500001321.mm.2**	Ropn1l	ropporin 1-like	-0.627505170925387	5.51696715617196	-1.85421934544885	0.109840867459435	0.999692817443324	-4.54702073584479	5.53959551240239	5.40021340747382	4.97287058818252	5.48062124946987	6.03725525133861	5.67124692816455	NA
**TC1500001776.mm.2**	Bop1	block of proliferation 1	0.712807173289261	6.29867605826779	1.85403044144364	0.109870402564002	0.999692817443324	-4.54703139264766	6.3397735955784	6.69982777986241	6.30184398467611	6.78719067569612	6.17597709586191	5.4874432179318	NA
**TC1300002360.mm.2**	Polr3g	polymerase (RNA) III (DNA directed) polypeptide G	-0.674575805899338	5.78962121604259	-1.85300346937513	0.110031105790976	0.999692817443324	-4.54708933503346	5.14697544781731	5.29604457085638	6.0962983724976	5.37647946039398	6.5709236417729	6.25100580291735	NA
**TC0600002656.mm.2**	Arhgap25	Rho GTPase activating protein 25	-0.609173681185187	5.49597397037082	-1.85246500621017	0.110115457996444	0.999692817443324	-4.54711972012682	5.45911213828478	5.68502748997136	4.88976672805144	5.41691168774776	5.7573551502692	5.76767062790038	NA
**TC1500000698.mm.2**	Atf4	activating transcription factor 4	-0.638133779778483	8.12599732233844	-1.85196475319223	0.110193881270827	0.999692817443324	-4.54714795192723	8.68000312952417	8.42666751597178	7.86453969738283	7.32198316710603	8.06808092711057	8.39470949693526	NA
**TC0900001900.mm.2**	Acp5	acid phosphatase 5, tartrate resistant	0.745080582672616	5.63364211283939	1.8514959342314	0.1102674264316	0.999692817443324	-4.54717441225955	5.25727347882376	5.68276424957354	5.63322923505454	6.5427588219376	5.28650371810894	5.39932317353797	NA
**TC0600001391.mm.2**	Nanog	Nanog homeobox	0.786249095040839	5.33549312699885	1.85072678310286	0.110388189951858	0.999692817443324	-4.54721782872487	4.78776592642952	5.10146105752791	5.49830549238194	6.34980949167674	4.80765549402435	5.46796129995266	NA
**TC0200001127.mm.2**	Osbpl6	oxysterol binding protein-like 6	0.744456275372222	6.48720549159268	1.84999030497887	0.110503945049558	0.999692817443324	-4.54725940701086	7.66752110887653	6.9868403930132	6.77451791768748	6.10437408151791	5.85968086203411	5.53029858642684	NA
**TC0400004193.mm.2**	Emc1	ER membrane protein complex subunit 1	0.551310049075711	7.18575710779977	1.84990055850378	0.110518058980019	0.999692817443324	-4.54726447410746	8.13815773844665	8.07758323898826	7.07739107653366	6.9233198072239	6.3338189141294	6.56427187147674	NA
**TC0900001517.mm.2**	Dync1li1	dynein cytoplasmic 1 light intermediate chain 1	-0.856297748562001	8.05658260618334	-1.8493873640444	0.110598800179704	0.999692817443324	-4.54729345083235	8.46834598064428	8.48766980528004	8.06856566247331	6.76687651455254	8.28748996210576	8.26054771204409	NA
**TC0800003211.mm.2**	Ccdc7a	coiled-coil domain containing 7A	0.87127891298949	5.44035216514955	1.84894261111577	0.110668820201269	0.999692817443324	-4.54731856545903	5.33544512874602	5.34307290962986	5.26501342305348	6.58806296619673	4.95077000393033	5.1597485593409	NA
**TC1400001779.mm.2**	Sh2d4b	SH2 domain containing 4B	0.672937418053404	5.83677599040834	1.8487830984949	0.110693943780117	0.999692817443324	-4.54732757346225	5.37273578000912	5.33806074141857	6.12773676983452	6.70013035873007	5.46110554620275	6.02088674625503	NA
**TC0700001737.mm.2**	Polr3e	polymerase (RNA) III (DNA directed) polypeptide E	0.572137042438031	6.65336100785474	1.84779629308268	0.110849492058019	0.999692817443324	-4.54738330659568	6.69658212094292	6.51834262676338	6.82408729652344	7.10067039562567	6.26475416571071	6.51572944156234	NA
**TC1800000829.mm.2**	Loxhd1	lipoxygenase homology domains 1	-0.734688980318078	5.27459196319455	-1.84658038473015	0.111041448024661	0.999692817443324	-4.54745199378278	4.53911115975583	5.25322865362592	4.90316069365588	5.28975630891882	6.11163733551175	5.55065762769911	NA
**TC0800001518.mm.2**	Spire2	spire type actin nucleation factor 2	0.679720616920848	6.57901044169411	1.84613567330835	0.111111736131001	0.999692817443324	-4.54747711976885	7.18211888055307	6.63664810746531	6.80040368509321	6.70696476290077	6.38032846431919	5.76759874983309	NA
**TC0700001855.mm.2**	Rnf40	ring finger protein 40	-0.716682398151995	7.032078881063	-1.84441529457629	0.111384058414581	0.999692817443324	-4.54757434071953	7.8285311562152	7.59352315821361	6.71551779058379	5.95300929723881	7.29764793016518	6.8042439539614	NA
**TC1200001938.mm.2**	Wdr89/Rplp2-ps1	WD repeat domain 89/ribosomal protein, large P2, pseudogene 1	0.846426309307186	5.314703733216	1.84348709927156	0.111531255801229	0.999692817443324	-4.54762680771485	5.55243082300338	5.52479435720229	6.080421853484	5.17150306536835	5.233040269349	4.32603203088898	NA
**TC0100000794.mm.2**	Dis3l2	DIS3 like 3'-5' exoribonuclease 2	1.00255765537834	6.01312282864912	1.84183567020319	0.111793618057023	0.999692817443324	-4.54772017926143	7.50949793431902	7.44255453845059	6.04096248756127	5.52493741737963	5.56749870527784	3.99328588890636	NA
**TC0100000424.mm.2**	Spats2l	spermatogenesis associated, serine-rich 2-like	0.983488250084229	6.80260469682038	1.83998408529386	0.112088497767541	0.999692817443324	-4.54782490276718	7.60843345072122	7.431432808134	7.69013227942456	6.18123693169321	6.22815509985689	5.67623761109242	NA
**TC0400001596.mm.2**	Rpa2	replication protein A2	0.557134725316075	6.96871760992606	1.83890305546023	0.112261012371376	0.999692817443324	-4.54788606170833	6.81662302024478	6.73572770447355	7.17615086364856	7.51096132908654	6.79219905577749	6.78064368632547	NA
**TC0900001442.mm.2**	Cdc25a	cell division cycle 25A	-0.61680713756619	5.15674867120463	-1.83819569739197	0.112374035749424	0.999692817443324	-4.54792608710362	5.55296799660854	5.19285909011028	4.78995473396851	4.69057059871979	5.1086908990747	5.60544870874597	NA
**TC0800003236.mm.2**	Usp10	ubiquitin specific peptidase 10	0.66353872093318	7.51593848964143	1.83758871866547	0.112471109059761	0.999692817443324	-4.54796043689513	8.52341609818271	7.86416917347964	7.62209069996082	7.39547085406548	6.66551330409623	7.02497080806371	NA
**TC0900001489.mm.2**	Dclk3	doublecortin-like kinase 3	0.784498301113385	6.39391755766875	1.83691183189783	0.11257945946573	0.999692817443324	-4.54799874755557	7.59851032240167	7.58061529834083	6.68814016872564	5.68854799502276	5.67055114469081	5.13714041683082	NA
**TC0300001906.mm.2**	1700034I23Rik	FUN14 domain containing 2 pseudogene	-0.718717398340434	5.00685450074423	-1.8360671466622	0.112714812933188	0.999692817443324	-4.54804656223036	4.98036089558427	4.57339891268007	4.74050398554081	4.78446221421927	5.0506810569503	5.91171993949064	NA
**TC1100002224.mm.2**	Fignl1	fidgetin-like 1	-0.668400379892704	5.24165996387824	-1.83490278393145	0.112901653003851	0.999692817443324	-4.54811248526713	5.05947564485265	5.2721413454536	4.7504982004084	5.14027281618049	5.27924480130824	5.94832697506606	NA
**TC0300003067.mm.2**	Lmo4	LIM domain only 4	-0.613935142716587	8.48175087684406	-1.83270898799675	0.113254505191762	0.999692817443324	-4.54823673137091	8.56640605095065	9.16269609945961	8.04903575319258	7.91773065941786	8.64387459770556	8.55076210033806	NA
**TC0100003558.mm.2**	Vangl2	VANGL planar cell polarity 2	-0.600475761055045	6.96038183601006	-1.83204350915326	0.113361754495268	0.999692817443324	-4.54827443104861	7.19396684695226	7.03449819044927	6.77977360339005	6.38666362488431	6.99666347881131	7.37072527157314	NA
**TC0400003008.mm.2**	C87499	expressed sequence C87499	-0.642887022219046	5.75778954504349	-1.8313594347677	0.113472104200574	0.999692817443324	-4.54831318908003	5.5396613687828	5.23516904122285	5.30186207370481	5.94120433420378	6.33801405421341	6.19082639813327	NA
**TC0900001739.mm.2**	Cntn5	contactin 5	0.761182005268109	6.29964388500754	1.8299128815788	0.113705797223609	0.999692817443324	-4.54839516370373	6.16340797589252	6.13665928194208	6.3897126222575	7.12036740911592	6.39041069740304	5.59730532343415	NA
**TC1500000800.mm.2**	Upk3a	uroplakin 3A	-0.660403400395561	5.81246270941803	-1.82975174508288	0.113731858196793	0.999692817443324	-4.54840429650907	5.55758139776297	5.27307260528344	5.36007929137189	6.00157843496345	6.16719479411461	6.51526973301185	NA
**TC1800001122.mm.2**	Proc	protein C	-0.643786484027036	5.61738404766298	-1.82973701643008	0.113734240586675	0.999692817443324	-4.54840513130523	5.17873957825364	5.05807864452243	5.62733659515074	5.46261995242312	5.83922737053612	6.53830214509181	NA
**TC1200001785.mm.2**	Mbip	MAP3K12 binding inhibitory protein 1	1.20168297846168	6.27246338077382	1.82954353687398	0.113765540833109	0.999692817443324	-4.5484160976258	6.68923569812575	6.6237854145969	7.01899380899261	6.34356875542921	6.48288171129138	4.47631489620707	NA
**TC0700000451.mm.2**	BC024978/Mir1191	cDNA sequence BC024978/microRNA 1191	-0.553381180943451	5.72088208541854	-1.82870035958258	0.113902044578174	0.999692817443324	-4.54846389309622	5.62861007636594	5.48044291885014	5.52533374731127	5.52940483039286	6.23403471068182	5.92746622890922	NA

**TC0500001976.mm.2**	Gm21149	predicted gene, 21149	-0.633246653803795	5.15736829743332	-1.8286493447976	0.113910308603527	0.999692817443324	-4.54846678510817	4.99733012806603	4.82563223771202	5.17323987193752	4.75413718366961	5.85932582074966	5.33454454246506	NA
**TC1500002053.mm.2**	Odf3b	outer dense fiber of sperm tails 3B	0.630611686873833	6.34768010658563	1.82754223553315	0.11408979671335	0.999692817443324	-4.54852955354297	6.18183956578291	6.20897399744826	6.40549226017824	7.07275296483691	6.18531360940087	6.03170824186661	NA
**TC0500000816.mm.2**	Dck	deoxycytidine kinase	-0.546466102975737	6.56495847117723	-1.82712329518171	0.11415778873369	0.999692817443324	-4.54855330906549	6.67158037885965	6.58894929892008	6.30352949240474	6.21461497926135	6.66956620950361	6.94151046811395	NA
**TC1100003856.mm.2**	Gfap	glial fibrillary acidic protein	-0.781480633015173	9.25643831022272	-1.82684466156841	0.114203031565087	0.999692817443324	-4.54856910968124	9.73304140563604	10.7492657500133	8.62599713057172	8.1206835892566	9.12443947358734	9.18520251227133	NA
**TC0400001467.mm.2**	5730409E04Rik	RIKEN cDNA 5730409E04Rik gene	0.57787897674674	7.43330913950768	1.8263672342645	0.114280594044152	0.999692817443324	-4.54859618528793	7.11477022232487	7.37089397759552	7.78815433340468	7.84681996190491	7.0416303789539	7.43758596286222	NA
**TC1100003204.mm.2**	Sgsm2	small G protein signaling modulator 2	0.538154080296801	6.71594477398587	1.82630940681978	0.114289992141477	0.999692817443324	-4.54859946493025	7.58856251352474	7.40495979198022	6.53218433833725	6.65704291116469	6.01887670723998	6.09404238166836	NA
**TC0900001408.mm.2**	Lamb2	laminin, beta 2	0.905218752663029	6.89886955168017	1.82607591051861	0.114327947565454	0.999692817443324	-4.54861270786379	6.71420427558146	7.23516726341573	6.93472838352614	7.69241325467881	5.84861310320994	6.96809102966896	NA
**TC1600002097.mm.2**	Runx1	runt related transcription factor 1	0.624099187364487	6.21483141916582	1.82505758575211	0.11449362290826	0.999692817443324	-4.54867046967283	5.65240928737564	6.02905550686941	6.44501409452908	6.98284695321035	6.03724406816669	6.14241860484377	NA
**TC1500000649.mm.2**	Cyth4	cytohesin 4	-0.55181335044045	7.08590948846998	-1.82478922675582	0.114537322348774	0.999692817443324	-4.54868569344454	6.99774293204107	7.32957585518068	6.79630477628371	6.74595094507488	7.30252645596273	7.34335596627676	NA
**TC1600000952.mm.2**	N6amt1	N-6 adenine-specific DNA methyltransferase 1 (putative)	-0.647571840144397	6.1097080136273	-1.82352524334998	0.114743367959698	0.999692817443324	-4.5487574082518	6.47140418325229	6.4283407349924	5.89599200481796	5.33568773679719	6.51058248578532	6.01624093611863	NA
**TC1800001229.mm.2**	Pcdh12	protocadherin 12	0.527251648519308	5.94753745773727	1.82275962396414	0.114868349830348	0.999692817443324	-4.54880085542318	5.8331810143305	5.87491846546711	6.28248905661799	6.23332522521433	5.68020742516366	5.78110355963004	NA
**TC0500002248.mm.2**	Cpz	carboxypeptidase Z	0.637080501260114	8.14213630569387	1.8224282670774	0.114922482680299	0.999692817443324	-4.54881966107327	8.05255799240565	8.13338606255017	8.24977571125591	8.72074167960791	8.1135025400702	7.58285384827339	NA
**TC1100000037.mm.2**	Sf3a1	splicing factor 3a, subunit 1	0.810597810129777	7.57032299611085	1.82118375769357	0.115126017293547	0.999692817443324	-4.54889030149459	8.54811982232071	8.39885068745369	7.97055505529045	7.07752648828469	7.12160316732583	6.30528275598975	NA
**TC1900000432.mm.2**	Rcl1	RNA terminal phosphate cyclase-like 1	0.804142039619441	6.30199118047298	1.82117319691825	0.115127745972112	0.999692817443324	-4.54889090101065	6.98156303636922	7.00215951085832	6.95090399524635	5.76735031217825	5.66051073600604	5.44945949217967	NA
**TC0900000492.mm.2**	Nectin1	nectin cell adhesion molecule 1	0.588276973299112	6.33537747228507	1.82114613814314	0.115132175300891	0.999692817443324	-4.54889243709354	7.04258293787443	6.66566367080896	6.3926683295867	6.34761775622591	5.59738021383309	5.96635192538131	NA
**TC0400001766.mm.2**	Rcc2	regulator of chromosome condensation 2	0.539969885906348	8.23979313468307	1.81967121449985	0.115373861673852	0.999692817443324	-4.54897617761468	8.64459931714434	8.50890959174349	8.31460730672794	8.36798752878371	7.69013517711818	7.91251988658077	NA
**TC0X00001787.mm.2**	Hdac6	histone deacetylase 6	0.651773745962324	7.06318117009285	1.81911593752617	0.115464979667257	0.999692817443324	-4.54900770995608	7.31468032225874	7.54546258451333	6.83958747255801	7.57165833029683	6.55811336892231	6.54958494200788	NA
**TC0900000252.mm.2**	B3gat1	beta-1,3-glucuronyltransferase 1 (glucuronosyltransferase P)	0.550896088009863	7.21217297301148	1.81833350187328	0.115593492429147	0.999692817443324	-4.54905214729741	7.62390562270553	7.6017212864112	7.38860648222972	7.18599507025623	6.59747702930607	6.87533234716015	NA
**TC1400001115.mm.2**	Pcdh17	protocadherin 17	0.942965965899067	7.4078121213888	1.81808848558373	0.115633764333684	0.999692817443324	-4.54906606395553	8.4873464278134	8.31690719936516	7.69646506312015	7.06781045335605	7.14162172122284	5.73672186345522	NA
**TC1100003941.mm.2**	Kpna2	karyopherin (importin) alpha 2	0.884168906826527	6.40252645345664	1.81626546043115	0.11593383465663	0.999692817443324	-4.54916962922096	7.38572901215042	7.58483168165026	6.68396171346551	5.72250620683061	5.80554945500934	5.03258065163372	NA
**TC1000002998.mm.2**	Dyrk2	dual-specificity tyrosine-(Y)-phosphorylation regulated kinase 2	0.632735479709561	7.07299869619832	1.81608968349275	0.115962807711393	0.999692817443324	-4.54917961683949	7.08897335128465	7.0029986570709	7.05341719955618	7.75232836457057	6.79987350286497	6.74040110184266	NA
**TC1700000382.mm.2**	Gng13	guanine nucleotide binding protein (G protein), gamma 13	-0.60949952357025	7.25680700194707	-1.81355683293955	0.116381078861432	0.999692817443324	-4.54932356818584	7.53193555332227	7.6942738089907	7.0663263089804	6.48149049213409	7.45842406764802	7.30839178060696	NA
**TC1100000284.mm.2**	Efemp1	epidermal growth factor-containing fibulin-like extracellular matrix protein 1	0.671204834452941	5.84621205041491	1.81350078910555	0.116390350478884	0.999692817443324	-4.54932675410823	6.33668346734096	6.44884139061661	6.10850118255171	5.70857737416717	5.60048529474958	4.87418359306342	NA
**TC0900001732.mm.2**	Arhgap42	Rho GTPase activating protein 42	-1.01514310319369	5.35660450832276	-1.81241544873549	0.11657004600833	0.999692817443324	-4.54938845874394	6.28939193352393	5.90005556441693	5.2504701146028	3.70947655820136	5.82933573095151	5.16089714824002	NA
**TC1000001149.mm.2**	Tmcc3	transmembrane and coiled coil domains 3	0.67288693948776	6.90960603606767	1.81207295682364	0.116626807186669	0.999692817443324	-4.54940793285893	7.10688794186807	6.82150061006114	6.86155644466479	7.57595432706138	6.37276765704676	6.71896923570388	NA
**TC0200005189.mm.2**	Spata2	spermatogenesis associated 2	-0.55034766029828	6.53277339839676	-1.81141970385583	0.116735145528887	0.999692817443324	-4.54944508017949	6.84308469236306	6.56239134975597	6.0655111205482	6.27972339328429	6.70942719064239	6.73650264378666	NA
**TC1900001360.mm.2**	Dkk1	dickkopf WNT signaling pathway inhibitor 1	0.552103708836966	4.73100653287775	1.81068859627151	0.116856511996285	0.999692817443324	-4.54948665985606	4.48769127504083	4.36993008317672	5.02212661723577	5.29418601112567	4.71338163344981	4.49872357723771	NA
**TC0500002420.mm.2**	Sel1l3	sel-1 suppressor of lin-12-like 3 (C. elegans)	-0.793902071108013	6.31324864213963	-1.80927844932291	0.117090948992577	0.999692817443324	-4.54956687328528	6.92948412650722	7.35496116236531	5.8438268027345	5.15979440814011	6.73321161963266	5.85821373345797	NA
**TC0X00001520.mm.2**	Phf8	PHD finger protein 8	-0.559878109731137	5.62906706215553	-1.80884190178935	0.117163617934668	0.999692817443324	-4.54959170951035	6.44982153206008	6.10099518423165	4.93809073013467	5.11382398845493	5.519080174297	5.65259076375487	NA
**TC0X00000933.mm.2**	Stard8	START domain containing 8	0.531173465440521	6.42289147687518	1.80875816965072	0.117177561247985	0.999692817443324	-4.54959647345052	6.39457658704132	6.57294931111183	6.62152380377295	6.69456114321653	6.17744343743309	6.07629457867534	NA
**TC1500001867.mm.2**	Baiap2l2	BAI1-associated protein 2-like 2	0.784509859454016	5.93411328949574	1.80680906858834	0.11750258845548	0.999692817443324	-4.54970738753944	5.14340584377475	5.4561940479235	6.08295675645344	7.20409302563866	5.8139106395794	5.90411942360467	NA
**TC0100000816.mm.2**	Snorc	secondary ossification center associated regulator of chondrocyte maturation	0.605941100332397	5.43926859668091	1.80669435985685	0.117521744346828	0.999692817443324	-4.54971391626066	5.21116243101174	5.18740397567515	5.57956924168691	6.14489444534478	5.12905639642641	5.38352508994049	NA
**TC0800000530.mm.2**	Cfap97	cilia and flagella associated protein 97	0.609642984914395	6.91005855113233	1.8065413238571	0.11754730547411	0.999692817443324	-4.54972262660894	7.7866134558363	7.30773185811608	6.84630592549085	6.94634005584433	6.08446984514441	6.48889016636198	NA
**TC0700002641.mm.2**	Tmem91	transmembrane protein 91	0.595998266830068	7.98672790727526	1.80621000003757	0.117602664023287	0.999692817443324	-4.54974148536921	8.02683173515592	7.78653463319217	8.17879008837765	8.47070871610415	7.50760930994268	7.94989296087899	NA
**TC1600000196.mm.2**	Yars2	tyrosyl-tRNA synthetase 2 (mitochondrial)	-0.57276352194116	6.36429021003816	-1.80419839767537	0.117939314078075	0.999692817443324	-4.54985600824242	6.8310863056369	6.56929352295091	5.70845142141054	6.11146577246888	6.45336869898417	6.51207553877757	NA
**TC0500000219.mm.2**	Chpf2/Mir671	chondroitin polymerizing factor 2/microRNA 671	-0.521629548616126	6.20598158254495	-1.80407607018344	0.117959816347283	0.999692817443324	-4.54986297379572	6.6547102940844	6.62101317028018	5.69260375024525	5.76584971659119	6.20919991322531	6.29251265084339	NA
**TC1700000335.mm.2**	Abca3	ATP-binding cassette, sub-family A (ABC1), member 3	0.819546979211571	7.5639726702105	1.8021745542652	0.118278959960663	0.999692817443324	-4.54997126881991	8.89143552613698	8.889870742337	7.62563521978994	6.99517663581615	7.03869209483569	5.94302580234726	NA
**TC0700001701.mm.2**	Ccp110	centriolar coiled coil protein 110	1.02539157880517	6.05860572206419	1.80165353185525	0.118366553160375	0.999692817443324	-4.55000094834128	6.37417204022081	6.21822329904654	6.70575600695168	6.1992550684124	6.26726255825352	4.58696535950022	NA
**TC0500001734.mm.2**	Daglb	diacylglycerol lipase, beta	0.637821800724842	7.22197924773497	1.80162135124317	0.118371965368354	0.999692817443324	-4.55000278156617	7.73705559322077	7.79703773101993	7.58804046473134	6.94867241707805	6.81614098898683	6.44492829137288	NA
**TC0200001729.mm.2**	Thbs1	thrombospondin 1	0.646212724561475	5.32667695352891	1.8015892695783	0.118377361175021	0.999692817443324	-4.55000460916459	4.97686007019947	5.65177480644595	5.79943864700845	5.51248749981705	4.89239578456472	5.12710491313783	NA
**TC0300002611.mm.2**	Slc22a15	solute carrier family 22 (organic anion/cation transporter), member 15	0.675326265261012	5.33119180729283	1.80145577173312	0.118399816713899	0.999692817443324	-4.55001221425533	5.08165426890359	5.11908575152461	5.60705323795773	5.96147843896767	5.50221019947642	4.71566894692696	NA
**TC1400001971.mm.2**	Olfr724	olfactory receptor 724	0.538421643597905	4.81690883889044	1.8007835045296	0.118512961153955	0.999692817443324	-4.55005051458822	4.6886080621196	4.54008503946034	5.11908981448365	5.25571179499561	4.75766567368733	4.54029264859612	NA
**TC0800001229.mm.2**	Tsnaxip1	translin-associated factor X (Tsnax) interacting protein 1	0.698852635630478	4.92552579087829	1.80076414620041	0.118516220773414	0.999692817443324	-4.5500516175352	4.36285291195132	4.8628186122362	5.03591412253338	5.82668012363823	4.73323294996765	4.731656024943	NA
**TC0800000542.mm.2**	Irf2	interferon regulatory factor 2	0.55564663268079	6.71983557565875	1.79970066743524	0.118695427012621	0.999692817443324	-4.5501122152796	6.90849282283479	7.13386354760539	6.67979160170717	7.01418357272977	6.3112300401719	6.27145186890346	NA
**TC1700002216.mm.2**	Foxp4	forkhead box P4	0.550454426426691	7.63507978569177	1.79959268192221	0.118713638339404	0.999692817443324	-4.55011836899349	7.39656090106399	7.34750036334734	7.85986590324624	8.2237972480501	7.53223815003503	7.45051614840792	NA
**TC0300002681.mm.2**	Atp5f1	ATP synthase, H+ transporting, mitochondrial F0 complex, subunit B1	-1.05570487877652	8.51018584476633	-1.79765241889716	0.119041319795069	0.999692817443324	-4.55022895735777	9.54321597508724	9.5808854116531	8.27308078339814	6.63972117875416	8.93181383772309	8.09239788198226	NA
**TC0300001555.mm.2**	Erich3	glutamate rich 3	0.63208886448037	6.38377536372748	1.79717396347257	0.119122258971768	0.999692817443324	-4.55025623338156	7.23577406963433	6.63684100353007	6.23219272637751	6.61491469270311	5.86890630175775	5.71402338836213	NA
**TC1100000755.mm.2**	Myo15	myosin XV	0.733581017399224	5.15438084474279	1.79675060228144	0.11919392264174	0.999692817443324	-4.55028037044188	4.622650325669	4.82609552478809	5.52201655550741	5.95033407089165	4.53964664310624	5.46554194849436	NA
**TC0500000825.mm.2**	Npffr2	neuropeptide FF receptor 2	0.642360038600678	5.19732993946204	1.79614270844213	0.119296896095055	0.999692817443324	-4.55031503313248	5.18094405154751	4.74925918757341	5.38934253494932	5.87990570247702	4.79465689175915	5.18987126846584	NA
**TC0400003685.mm.2**	Stx12	syntaxin 12	-0.591285908661448	7.92712232638558	-1.79503636847404	0.119484525379213	0.999692817443324	-4.55037812189313	8.60463093108125	8.53461543639311	7.5794190885984	7.04103879815972	8.00022431829322	7.80280538578779	NA
**TC0400000272.mm.2**	Lyrm2	LYR motif containing 2	-0.60117964590229	6.50681477054349	-1.79478147077821	0.119527795325612	0.999692817443324	-4.55039265948415	7.45718910768079	7.21763489377708	6.0751828350308	5.50666982996845	6.4047385485742	6.37947340822963	NA
**TC0100003409.mm.2**	Prrx1	paired related homeobox 1	0.533233732536645	6.04470476988637	1.79472198882243	0.11953789482886	0.999692817443324	-4.55039605201239	5.77839876993701	6.02016888453745	6.36373482354143	6.40432939141711	5.79280016127113	5.90879658861412	NA
**TC0500000418.mm.2**	Jakmip1	janus kinase and microtubule interacting protein 1	-0.628066209727839	7.01278323867969	-1.79410593819248	0.119642543536849	0.999692817443324	-4.55043119023151	7.3122859378678	7.50388846957792	6.83163745823721	6.17055884435116	7.22996159427552	7.02836712776853	NA
**TC0400003998.mm.2**	Zfp984	zinc finger protein 984	-0.574931123542756	5.24649655988753	-1.79008072342548	0.120328504135333	0.999692817443324	-4.55066087077332	5.27665259456541	4.96536519369796	4.88917714375863	5.1543725182295	5.75297885768709	5.44043305138656	NA
**TC0300003142.mm.2**	Miga1	mitoguardin 1	0.789718450026639	7.34499378218016	1.78860759838253	0.120580502538405	0.999692817443324	-4.5507449671284	8.22461552595114	8.23252093758998	7.8446779954215	6.75145356937507	6.75848898241174	6.25820568233155	NA
**TC1200001010.mm.2**	Slc24a4	solute carrier family 24 (sodium/potassium/calcium exchanger), member 4	1.10564868706608	6.68580535785942	1.78854392704471	0.120591405957085	0.999692817443324	-4.55074860240809	7.92033862963219	8.13966718229311	6.6802731978935	6.4527886567882	6.4295602222461	4.49220425830344	NA
**TC1900001418.mm.2**	Ch25h	cholesterol 25-hydroxylase	-0.826778014552824	6.16104918206243	-1.7882381997798	0.120643773668202	0.999692817443324	-4.5507660582796	5.70996754317277	6.83921697836548	5.38338717741293	5.99839009345241	6.42542991720937	6.60990338276162	NA
**TC1300000818.mm.2**	Rsl1	regulator of sex limited protein 1	-0.942055150246147	5.1995092126498	-1.78755168998473	0.12076144577093	0.999692817443324	-4.55080525866683	5.40711055083837	4.7942714407782	5.50546425359672	4.05031723829825	5.78567567299666	5.6542161193906	NA
**TC1100000680.mm.2**	Mrpl22	mitochondrial ribosomal protein L22	-0.983916116606879	5.44560038959287	-1.78735941844679	0.120794422310795	0.999692817443324	-4.55081623837235	5.66990058353684	5.53007342673201	5.7069176013007	4.04598044573662	5.74058203599281	5.98014824425826	NA
**TC0X00000975.mm.2**	Itgb1bp2	integrin beta 1 binding protein 2	0.742351559899252	4.8854725062233	1.78688984269411	0.120874996149889	0.999692817443324	-4.55084305508155	4.16769845900605	4.29120309561798	5.12061889079697	6.04869941046018	5.10754809192215	4.57706708953649	NA
**TC1600002035.mm.2**	Krtap8-1	keratin associated protein 8-1	0.665517223412024	5.52791209043749	1.78665195371178	0.120915835110037	0.999692817443324	-4.55085664134139	5.57460588800949	5.42039732706413	5.86559174535193	5.88616014183577	4.79246329249311	5.62825414787053	NA
**TC0500002910.mm.2**	Barhl2	BarH like homeobox 2	-0.543378798361557	5.82204856590744	-1.78662418917572	0.120920602386665	0.999692817443324	-4.55085822705838	5.64598631869372	5.40402550431736	5.5674978556354	5.83026313221983	6.25075670883921	6.23376187573913	NA
**TC0500003084.mm.2**	Hnf1a	HNF1 homeobox A	0.77612734773272	6.42854526524797	1.78188366928992	0.121737250113863	0.999692817443324	-4.55112908028691	5.9720995357975	6.11835161442497	6.43797953190336	7.57855803646203	6.11389430071404	6.35038857218591	NA
**TC0700004513.mm.2**	Ifitm2	interferon induced transmembrane protein 2	-0.683834104513186	7.91364374570874	-1.78154349771079	0.121796056866145	0.999692817443324	-4.55114852445178	7.60629955404153	8.14580629663594	7.66570880387565	7.51533540339864	8.63472877401159	7.91398364228907	NA
**TC0800001740.mm.2**	Rasa3	RAS p21 protein activator 3	-0.584013272216798	6.06745694947704	-1.78095808261704	0.121897324371813	0.999692817443324	-4.55118198926398	5.91759072616597	6.97277011241569	5.46397114176331	5.20920601516018	6.17223174975528	5.66897195160181	NA
**TC0X00002454.mm.2**	Gm6880	predicted gene 6880	-0.572039697602147	4.9616745056739	-1.78089364889884	0.121908475372976	0.999692817443324	-4.55118567276643	4.42287972497593	4.20356605951926	4.76700786751517	5.23275305965678	5.56538016621778	5.57846015615846	NA
**TC0800000012.mm.2**	C330021F23Rik	RIKEN cDNA C330021F23 gene	-0.549284201329613	5.46336489489156	-1.78005231866329	0.122054168221151	0.999692817443324	-4.55123377294499	4.87908967976916	5.00684643876191	5.51825633695463	5.3795860871249	5.81979561585028	6.17661521088847	NA
**TC1200002150.mm.2**	Cep128	centrosomal protein 128	-1.1475885443452	5.79337367172358	-1.77925116466357	0.122193060847619	0.999692817443324	-4.55127958237587	6.63564342694435	6.96441461882183	5.67732253082548	3.75518091711698	6.21236711675311	5.51531341987974	NA
**TC0500001746.mm.2**	Lmtk2	lemur tyrosine kinase 2	-0.52344243787959	7.66799717018147	-1.77856397834472	0.122312317591419	0.999692817443324	-4.55131888001593	8.27089884909296	8.34342870275752	7.11946917190247	7.05391612483711	7.50686991772348	7.71340025477527	NA
**TC1200002120.mm.2**	Vipas39	VPS33B interacting protein, apical-basolateral polarity regulator, spe-39 homolog	-0.696799694338255	7.25640007229579	-1.77812545074738	0.122388480250483	0.999692817443324	-4.55134396009876	8.10695442800497	8.08576407819869	6.96537746566667	6.01066380378061	7.20309202350548	7.16654863461831	NA
**TC0X00002428.mm.2**	Haus7	HAUS augmin-like complex, subunit 7	0.654792273768013	6.17797034401139	1.77764673515172	0.122471675225121	0.999692817443324	-4.55137134065172	5.97415656880949	6.02115895053324	6.21963041915763	6.97141512697319	5.77054402975062	6.11091696884417	NA
**TC0500002252.mm.2**	Htra3	HtrA serine peptidase 3	-0.605169848199645	6.81135144535004	-1.77753013258596	0.122491947648372	0.999692817443324	-4.55137801016104	6.44973466562872	6.81547014510167	6.83671008193405	6.35957200055123	7.07336089174253	7.33326088714204	NA
**TC0100002950.mm.2**	Epb41l5	erythrocyte membrane protein band 4.1 like 5	0.812213190030669	5.56239764125541	1.77720058470111	0.122549260169988	0.999692817443324	-4.5513968605421	6.61184095352429	6.37102080250306	5.94376694693728	5.06420828884596	5.13699746327665	4.24655139244525	NA
**TC0200002980.mm.2**	C1ql3	C1q-like 3	-0.90117925499203	8.1265698740505	-1.77496633411495	0.122938511084131	0.999692817443324	-4.55152468801863	8.52444684774445	9.12139516359812	7.99598937339906	6.65961998808914	8.39581691330183	8.06215095817043	NA
**TC0900001562.mm.2**	Golga4	golgi autoantigen, golgin subfamily a, 4	-0.631344393309006	6.60319872596123	-1.7746865380832	0.122987341534986	0.999692817443324	-4.55154069917176	6.58857053649767	7.2936867206658	6.04825072262749	6.18887243336545	6.86053597559339	6.63927596701756	NA
**TC1600001939.mm.2**	Btg3/Gm7334	B cell translocation gene 3/B-cell translocation gene 3 pseudogene	-0.556378912538861	6.4537343708064	-1.77271824752209	0.12333138270066	0.999692817443324	-4.55165335386135	7.27852262103575	7.03408954698152	5.75787077499363	5.89064734087809	6.20192514177871	6.55935079917073	NA
**TC1400002072.mm.2**	Efs	embryonal Fyn-associated substrate	-0.71368677385841	5.73689199160464	-1.77198699776347	0.123459436798356	0.999692817443324	-4.55169521590232	5.17043548732368	5.69379624686771	5.10634581380846	5.95852752005135	6.16799828104628	6.32424860053035	NA
**TC1200001261.mm.2**	Inf2	inverted formin, FH2 and WH2 domain containing	-0.579399171701006	7.85671458211778	-1.77063886292077	0.123695855760744	0.999692817443324	-4.55177240582048	7.64923111349944	7.90030321855272	7.77696362327622	7.43901378535003	8.0015661627401	8.37320958928817	NA
**TC1900000112.mm.2**	Cdc42bpg	CDC42 binding protein kinase gamma (DMPK-like)	-0.616587293433187	5.95108774103051	-1.76735722658009	0.124273183369637	0.999692817443324	-4.55196037146434	5.32752900504429	5.66565074748967	5.67608205002801	6.06400400336336	6.24228954363365	6.7309710966241	NA
**TC1100004287.mm.2**	Mrps24	mitochondrial ribosomal protein S24	-0.519001921681244	7.14337384128247	-1.76715829484467	0.124308264587259	0.999692817443324	-4.55197176903751	6.81875891351976	6.72635936391428	7.07022650008318	7.06833396336595	7.48949776007603	7.68706654673559	NA
**TC0400000458.mm.2**	Glipr2	GLI pathogenesis-related 2	0.656239034876187	6.30429223684711	1.76690718355111	0.124352561265025	0.999692817443324	-4.55198615669403	6.19665340088618	6.3849715871393	6.95038111353747	6.32792213786731	6.25189954203646	5.71392563961594	NA
**TC1700001221.mm.2**	Tmem178	transmembrane protein 178	-0.865818751791602	7.39767024370648	-1.7661218944478	0.124491186981749	0.999692817443324	-4.55203115426123	7.95491795816283	8.2175103374557	7.26497075291688	5.9760070786017	7.75963767361584	7.21297766148595	NA
**TC0200005000.mm.2**	5430405H02Rik	RIKEN cDNA 5430405H02 gene	0.705015945942033	6.96221544613916	1.76536124440489	0.124625605914595	0.999692817443324	-4.55207474532178	6.73165649374803	6.42102404999989	7.13593700871433	7.87938500377123	6.51098983342121	7.09430028718028	NA
**TC0100003524.mm.2**	Fcgr3	Fc receptor, IgG, low affinity III	-0.891171166378927	7.09270543408218	-1.76473472437795	0.124736427505719	0.999692817443324	-4.55211065363659	7.26995999901759	8.13612811129597	6.8640920868629	5.81980899384793	7.54023070450569	6.92601270896299	NA
**TC0800003095.mm.2**	Spata2l	spermatogenesis associated 2-like	-1.02789793324184	6.45001455849826	-1.76367157827212	0.124924699817167	0.999692817443324	-4.55217159480368	7.157088185356	6.99808826360025	6.52638502916243	4.7181724886124	6.59541087121075	6.70494251304775	NA
**TC1400002823.mm.2**	Trav3-3	T cell receptor alpha variable 3-3	-0.535218833396766	4.77863821863063	-1.76346550312962	0.124961225442036	0.999692817443324	-4.55218340852493	4.55934052744801	4.82264128297883	4.47794648000289	4.63175843727882	5.0035469253641	5.17659565871114	NA
**TC1200000641.mm.2**	Slc38a6	solute carrier family 38, member 6	0.936902732986558	5.70732056548942	1.76330397085208	0.124989863330974	0.999692817443324	-4.55219266899323	6.18328668957056	5.91718390250788	5.93475962113549	6.07386951228011	5.85465626601703	4.28016740142545	NA
**TC1200000037.mm.2**	Wdcp	WD repeat and coiled coil containing	0.55066500400084	6.70018857401405	1.76193536629205	0.12523275691379	0.999692817443324	-4.5522711389925	7.62800430970454	7.33636525474395	6.6118676960205	6.55717824779824	6.19697907763915	5.87073685817791	NA
**TC1100002897.mm.2**	Shmt1	serine hydroxymethyltransferase 1 (soluble)	0.701445005882045	5.30565175180214	1.76061890121908	0.125466828110408	0.999692817443324	-4.55234663535941	5.12439822604904	5.13867424035352	5.24978320584437	6.23708082224282	5.07635568942678	5.00761832689633	NA
**TC0100001707.mm.2**	Itpkb	inositol 1,4,5-trisphosphate 3-kinase B	0.521943769285807	6.83398742033611	1.76061589277765	0.125467363504384	0.999692817443324	-4.55234680790462	7.23182343207855	7.05994774757697	6.88533562659681	6.99268481386957	6.33418762808443	6.49994527381033	NA

TC1900000685.mm.2	Gbf1	golgi-specific brefeldin A-resistance factor 1	0.516812959821547	7.1504534084098	1.76041289469092	0.125503494944443	0.999692817443324	-4.55235845077951	8.3587969124516	8.2799415255517	6.78629475177343	6.86250921427586	6.40066954182987	6.21450850457633	NA
TC0700000057.mm.2	U2af2	U2 small nuclear ribonucleoprotein auxiliary factor (U2AF) 2	0.666182601962429	7.11944153881615	1.7603988189925	0.12550600063815	0.999692817443324	-4.55235925809924	8.21275820051271	8.01490749327665	7.36683202789237	6.54384234362382	6.16306591939341	6.41524324819791	NA
TC1700002332.mm.2	Micos13	mitochondrial contact site and cristae organizing system subunit 13	0.766639536296557	7.47535159095507	1.75992724218638	0.125589976646314	0.999692817443324	-4.55238630667693	8.11013706781004	8.08765566313634	7.83126483006489	7.26253311362368	7.29376674024353	6.26675213085193	NA
TC1100003397.mm.2	Ccl9	chemokine (C-C motif) ligand 9	-0.690308690994147	5.21900301253581	-1.75719392440146	0.126077783848496	0.999692817443324	-4.55254312250157	6.04840173287477	5.61392623409824	4.22503138576824	4.91050497735853	5.00095347128681	5.51520027382827	NA
TC1700001308.mm.2	Cript	cysteine-rich PDZ-binding protein	0.715833072177637	7.04555927663067	1.75718998152455	0.126078488844349	0.999692817443324	-4.55254334875997	8.67785676689191	8.55718798966866	6.9417422304993	6.29324629329006	6.30087652774643	5.50244585168766	NA
TC0500001370.mm.2	Atxn2	ataxin 2	0.683098657768545	9.2691330979629	1.75674973131643	0.126157230625056	0.999692817443324	-4.55256861298024	9.93009412411346	9.64804265071113	9.64678140999736	9.0546481542476	9.00401419868064	8.33121805002723	NA
TC0400000602.mm.2	Zfp462	zinc finger protein 462	0.560534018723144	6.18073162386484	1.75640468920487	0.12621897702822 6	0.999692817443324	-4.55258841477902	6.99164221799736	6.94178043128362	6.16200228917956	5.97401528549762	5.73676544061174	5.27818409661915	NA
TC0500003566.mm.2	Aimp2	aminoacyl tRNA synthetase complex-interacting multifunctional protein 2	0.670095799769877	7.53781655910412	1.75572228931268	0.12634118077449 8	0.999692817443324	-4.55262758046446	8.32720358001282	8.18089301437663	7.38449788765694	7.64499929223057	6.41967485941527	7.26963072093248	NA
TC1100004215.mm.2	P4hb	prolyl 4-hydroxylase, beta polypeptide	-0.868274372767322	7.64212785841391	-1.75525742451743	0.126424493984665	0.999692817443324	-4.55265426327767	8.46898005412475	8.21638867496823	7.53588074430903	6.17954409361889	7.93925137251666	7.51272221094591	NA
TC0300002657.mm.2	Tafa3	TAFA chemokine like family member 3	0.603399241534687	5.95402758307578	1.75384728519498	0.126677544697453	0.999692817443324	-4.5527352155809	5.22285544530284	5.35664441878748	6.25612209924074	6.91960995947613	5.95976552302047	6.00916805262703	NA
TC0300000926.mm.2	Sf3b4	splicing factor 3b, subunit 4	-0.572602080144144	8.00953592628294	-1.75317741823533	0.126797924395486	0.999692817443324	-4.55277367693693	8.13470261292362	7.92544091152415	7.61439533303266	7.81153860344815	8.04707674115104	8.52406135561806	NA
TC1000000548.mm.2	Vsir	V-set immunoregulatory receptor	0.642433223074581	6.86002483009694	1.75281187307089	0.126863662005266	0.999692817443324	-4.55279466688011	6.92550585851033	7.22509796001304	7.01260873958276	7.13459706452096	6.05657271191576	6.8057666460388	NA
TC0800001386.mm.2	Dynlrb2	dynein light chain roadblock-type 2	0.59777192779299	5.89164993216525	1.75052321269496	0.127275991329008	0.999692817443324	-4.55292611031502	6.12408716519463	6.21631559971228	6.01958798015781	6.08293236167747	5.12735376561218	5.77962272063712	NA
TC0700000849.mm.2	Tulp2	tubby-like protein 2	0.586360641746241	5.59343865659504	1.74614757466655	0.128067921584769	0.999692817443324	-4.55317754002053	4.77232907518137	4.80627653029545	5.99022449784806	6.58714931094489	5.7904021299758	5.61425039532466	NA
TC0100000509.mm.2	Nrp2	neuropilin 2	0.958978680216508	6.77434936942926	1.74601937272296	0.128091195984944	0.999692817443324	-4.55318490914389	7.69513260706098	7.59576562273508	7.40834056402812	6.22823710957812	6.45417899358649	5.26444131958674	NA
TC0100003393.mm.2	Eef1aknmt	EEF1A lysine and N-terminal methyltransferase	-1.25614385811503	6.26899811785382	-1.74523379284818	0.128233903165708	0.999692817443324	-4.55323006780174	6.64476908888744	6.28691886077748	6.70038495529176	4.38462156532223	6.63264091743504	6.96465331940899	NA
TC1200002090.mm.2	Mlh3	mutL homolog 3	0.728166407827556	5.74820964048364	1.74475024796771	0.128321819463767	0.999692817443324	-4.55325786674707	6.1608479564072	6.17583234869884	5.4336889568817	6.37076621984375	5.44794470298688	4.90017765808346	NA
TC1100003750.mm.2	Krt34	keratin 34	-0.870526325255276	4.87731441291977	-1.7446291803839	0.128343840620252	0.999692817443324	-4.55326482722143	4.64796963692948	4.39640346950942	5.29603053953743	3.94319982074715	5.24167126249995	5.73861174829518	NA
TC1600000493.mm.2	Sema5b	sema domain, seven thrombospondin repeats (type 1 and type 1-like), transmembrane domain (TM) and short cytoplasmic domain, (semaphorin) 5B	-0.669100598132685	6.78992349875994	-1.74387496068992	0.128481108899795	0.999692817443324	-4.55330819197906	7.00089123150746	6.92394261945925	6.82275983034661	5.91549314231718	7.01300590291426	7.06344826601489	NA
TC0300000700.mm.2	Arhgef11	Rho guanine nucleotide exchange factor (GEF) 11	-0.637684267072252	7.32556117355237	-1.74346569490033	0.128555654738148	0.999692817443324	-4.55333172522252	7.74959106313814	7.65387296959114	7.19286694764473	6.44440028957548	7.60582939700038	7.30680637436433	NA
TC0100001168.mm.2	Eif2d	eukaryotic translation initiation factor 2D	0.565434649371634	6.15592704949142	1.74299673645764	0.128641124638222	0.999692817443324	-4.55335869260529	6.52722152713273	6.92046641313605	5.98443858214334	6.32493324556816	5.66193977032199	5.51656275864625	NA
TC0700004134.mm.2	Copb1	coatomer protein complex, subunit beta 1	1.02303246125062	6.22895116813448	1.74229332619509	0.12876942732779	0.999692817443324	-4.55339914559119	7.45745581227818	7.59717241295371	6.51154339425886	5.67102845877926	5.88165884722795	4.25484808330892	NA
TC0500002936.mm.2	Dipk1a	divergent protein kinase domain 1A	0.884320867011055	7.93041357199094	1.74227452186714	0.128772858949075	0.999692817443324	-4.55340022708175	8.4635574773895	8.31206987289732	8.22570795832612	8.06203994951433	8.0014892983303	6.51761687548804	NA
TC1100002706.mm.2	Ube2b	ubiquitin-conjugating enzyme E2B	-0.548542155698367	8.05042903685732	-1.7392693460197	0.129322411972958	0.999692817443324	-4.55357310155446	8.25874997272186	7.97982302536526	7.92114059067297	7.56231786515707	8.23792661093327	8.3426161562935	NA
TC1100001612.mm.2	Krtap31-2	keratin associated protein 31-2	-0.652017018107983	5.0158358733013	-1.7375682522116	0.129634491072484	0.999692817443324	-4.55367099147035	4.9094089957424	4.68610307642553	4.38991259356069	5.20782197215126	5.36373203516634	5.53803656676158	NA
TC0100002071.mm.2	Fam135a	family with sequence similarity 135, member A	0.546176322316678	5.86785645774766	1.73750965644931	0.129645253857635	0.999692817443324	-4.55367436380923	6.72827062655637	6.2946621186321	5.89080646580167	5.74747285716374	5.28427641676633	5.26165026156573	NA
TC1000001153.mm.2	Cep83	centrosomal protein 83	-1.30462846118095	5.79920660522469	-1.73713140605228	0.129714751082455	0.999692817443324	-4.55369613379233	6.70246704332829	6.8792231239069	5.76960041147475	3.53254585940077	6.49348988454628	5.41791330869114	NA
TC0500000130.mm.2	Speer4f1	spermatogenesis associated glutamate (E)-rich protein 4F1	-0.594228913139357	5.02980529091625	-1.73654810084409	0.129821994139094	0.999692817443324	-4.5537297079148	4.63768602052241	4.38695805014031	4.95732890742371	5.02553601685432	5.89253302884602	5.27878972171072	NA
TC0200002722.mm.2	Gm14308/Gm14430/Gm14432/Gm14434/Gm2026/Gm4724	predicted gene 14308/predicted gene 14430/predicted gene 14432/predicted gene 14434/predicted gene 2026/predicted gene 4724	-2.12390275781745	8.70204193163711	-1.7365271460853	0.129825848347819	0.999692817443324	-4.55373091408998	9.70713264175252	10.4945284296506	8.87153183635275	5.00986066503959	9.8778058740489	8.25139214297834	NA
TC0200002724.mm.2	Gm14308/Gm14430/Gm14432/Gm14434/Gm2026/Gm4724	predicted gene 14308/predicted gene 14430/predicted gene 14432/predicted gene 14434/predicted gene 2026/predicted gene 4724	-2.12390275781745	8.70204193163711	-1.7365271460853	0.129825848347819	0.999692817443324	-4.55373091408998	9.70713264175252	10.4945284296506	8.87153183635275	5.00986066503959	9.8778058740489	8.25139214297834	NA
TC0500003576.mm.2	Baiap2l1	BAI1-associated protein 2-like 1	-0.60011277238879	4.92918374628062	-1.73567554914534	0.129982575948949	0.999692817443324	-4.55377993586898	4.66057843030243	5.27831164268187	4.45609964742183	4.76189378253909	5.19676893361992	5.22145004111858	NA
TC0700004545.mm.2	Chid1	chitinase domain containing 1	0.620766703086559	6.22947919060934	1.73415392776158	0.130263068833002	0.999692817443324	-4.55386754220273	7.20643395006382	7.07069437963096	6.16507927847796	6.00556083158925	5.83278681732914	5.09631988656495	NA
TC1700001686.mm.2	Rab11fip3	RAB11 family interacting protein 3 (class II)	0.514703550767277	7.25938315722787	1.73337155848456	0.130407516353846	0.999692817443324	-4.55391259400857	7.40879526508843	7.56678008155884	7.41757489711788	7.38749045200937	6.78017287298648	6.99548537460621	NA
TC1900000046.mm.2	Ctsf	cathepsin F	0.609219271005167	7.06004064635476	1.73295439108004	0.130484600354084	0.999692817443324	-4.55393661815135	8.02996344921226	7.63507435345812	7.18583231713744	6.77098999159681	6.59261876072347	6.14576500600044	NA
TC1300001548.mm.2	Olfr1366	olfactory receptor 1366	-0.863109722831961	4.47278669691311	-1.73188797165628	0.130681852472437	0.999692817443324	-4.55399803837129	4.3116065988642	4.0078137850471	3.48487768278498	4.91066249316674	5.01789706010639	5.10386256150926	NA
TC0400000427.mm.2	1700022I11Rik	RIKEN cDNA 1700022I11 gene	-0.579147579542617	4.98864911829283	-1.73154290780503	0.130745739304853	0.999692817443324	-4.55401791424625	4.87009196769592	4.65856851548644	4.74137781557538	4.88109171816933	5.11076452226272	5.67000017056722	NA
TC1500001643.mm.2	Oc90	otoconin 90	-0.525374724908744	5.08016288608296	-1.73120618713767	0.130808110445623	0.999692817443324	-4.55403731048492	4.54832613678292	4.67712490315671	4.92457801042804	5.17781040294229	5.67900825484088	5.47412960834693	NA
TC0300002633.mm.2	Dclre1b	DNA cross-link repair 1B	0.541327189200195	6.68753372034485	1.73063887683075	0.130913258792676	0.999692817443324	-4.5540699915473	6.77043925402282	6.42239866088219	6.90808348913516	7.09942590364707	6.56090931772819	6.36394569665365	NA
TC0X00000515.mm.2	Zfp449	zinc finger protein 449	1.01599764728989	5.88870750261445	1.73050834043385	0.130937464631463	0.999692817443324	-4.55407751173197	6.30409508727828	6.254867052406	6.87621777095316	5.52642131433794	5.80723873118367	4.56340505952766	NA
TC1900000614.mm.2	Golga7b	golgi autoantigen, golgin subfamily a, 7B	-0.927423615353663	7.21988686855065	-1.73025125360362	0.130985149784363	0.999692817443324	-4.55409232287524	7.62507298395752	7.5268995231347	7.35592248867728	5.8003282480749	7.73126676899966	7.27983119845985	NA
TC1300002119.mm.2	Golm1	golgi membrane protein 1	0.615211492376485	5.92419569871305	1.72939915255756	0.131143319391 89	0.999692817443324	-4.55414141747996	6.41680576552776	6.80253393614518	6.16023078846815	5.61789794921102	5.44913276933004	5.09857298359616	NA
TC0100003723.mm.2	Brox	BRO1 domain and CAAX motif containing	-0.803886516106401	7.10703111282772	-1.72892811987888	0.131230832852558	0.999692817443324	-4.55416855899522	7.73029563359461	7.6361068138943	7.03789314611192	5.79611245252037	7.42571946606205	7.01605916478304	NA
TC0800000704.mm.2	Ints10	integrator complex subunit 10	-0.523142889996509	7.31295929108171	-1.72892290034801	0.131231802907496	0.999692817443324	-4.55416885976142	7.49128893851075	7.25548642092707	7.12066439666595	7.47262891960352	7.6160041639192	6.92168290686375	NA
TC1800001700.mm.2	Tshz1	teashirt zinc finger family member 1	0.62323184414843	6.55966740420808	1.72829686808137	0.131348201714649	0.999692817443324	-4.55420493535572	7.10896460878661	7.10372284799797	6.98593665267698	6.20995367570342	5.92869377424619	6.02073286583735	NA
TC0900000532.mm.2	Mpzl3	myelin protein zero-like 3	0.523487407141028	5.47322097214992	1.72803186849181	0.131397503296594	0.999692817443324	-4.55422020711489	4.97051671112843	4.95580743813253	6.13378605387986	5.84620219508045	5.37216714377392	5.56084629090434	NA
TC0100001567.mm.2	Sh2d1b2	SH2 domain containing 1B2	-0.53729545935323	5.70950964035038	-1.72796549257403	0.131409854927649	0.999692817443324	-4.5542240324058	5.26204148553547	5.49321829572912	5.70609111856501	5.50751245250061	5.99507754746607	6.293116942306	NA
TC1300001654.mm.2	Carmil1	capping protein regulator and myosin 1 linker 1	0.846218511792967	6.19321314265498	1.72607711708894	0.131761723887149	0.999692817443324	-4.55433287565322	6.51168792074695	6.32799313640897	6.90008138544912	6.10593602573083	6.24096409989756	5.07261628769646	NA
TC1700002816.mm.2	Cfb	complement factor B	0.788976972952741	5.39629981834823	1.72392664370417	0.13216353512565	0.999692817443324	-4.55445686059693	4.90206284243979	5.30001036715492	5.53537741964725	6.34146240355282	4.69558231563678	5.60330356165781	NA
TC1700000468.mm.2	Snrpc	U1 small nuclear ribonucleoprotein C	-0.558050566438114	6.56118371803911	-1.72255902177076	0.13241968539445	0.999692817443324	-4.55453572959	6.62936216767571	6.4509695365159	6.41978199528195	6.16555274030145	6.63185381957697	7.06958204888266	NA
TC1100002030.mm.2	Slc25a10	solute carrier family 25 (mitochondrial carrier, dicarboxylate transporter), member 10	0.598252949375059	6.48337233689007	1.72232457282168	0.132463644701831	0.999692817443324	-4.55454925144558	6.98103095805137	7.01176296994751	6.65627106705815	6.39570192898767	6.25220952893608	5.60325756835963	NA
TC1000002330.mm.2	Cabcoco1	ciliary associated calcium binding coiled-coil 1	0.511970994916937	7.3080758980969	1.72157278559683	0.132604699922496	0.999692817443324	-4.55459261374111	7.89590765259662	8.03265611625606	7.22888620812931	7.24303059665198	6.84309509194653	6.60487972300088	NA
TC0900001781.mm.2	Panx1	pannexin 1	0.567786030502887	6.69989609287228	1.72120464347494	0.132673825835394	0.999692817443324	-4.55461384941637	7.36228087422799	6.80982782651757	6.72033524814193	6.86108471060501	6.16645231856766	6.27939557917352	NA
TC0200004456.mm.2	Pla2g4d	phospholipase A2, group IVD	-0.569290097117428	4.84911398164724	-1.72022804685654	0.132857368988363	0.999692817443324	-4.55467018795694	4.50224270868596	4.78150249882527	4.41892348063543	4.91725576343325	5.30892089812947	5.16583854017407	NA
TC0200005408.mm.2	Gata5	GATA binding protein 5	0.93365677234274	5.97372688446972	1.71803744152848	0.133269963451006	0.999692817443324	-4.5547965881919	5.13983360677835	5.65584722792776	6.24098609927007	7.21601090912878	5.17847454790219	6.41120891581118	NA
TC1700000429.mm.2	Zbtb9/Ggnbp1	zinc finger and BTB domain containing 9/gametogenetin binding protein 1	-0.537428933602635	6.52059338143115	-1.71656542739125	0.133547904938128	0.999692817443324	-4.55488154595652	6.42791237639704	6.66773805936529	6.3028238682583	6.17370212455134	6.59911865255939	6.95226520745552	NA
TC0700000087.mm.2	Usp29	ubiquitin specific peptidase 29	1.48026267677643	6.92538376282074	1.71577161362226	0.133698021725924	0.999692817443324	-4.55492736813916	9.0287059054151	8.63990496949279	7.19245492115847	6.22965360662622	6.59397003144674	3.86761314278509	NA
TC0700000369.mm.2	Zfp112	zinc finger protein 112	0.757386844385051	5.85312629234801	1.71522079501403	0.133802281392274	0.999692817443324	-4.55495916650537	4.94541779808246	5.20348789771217	6.03982487916501	7.20248799436675	5.78075208605607	5.94678709870559	NA
TC0100001220.mm.2	Chit1	chitinase 1 (chitotriosidase)	-0.566003369247209	5.43354817517818	-1.71505765878895	0.133833175023071	0.999692817443324	-4.55496858469242	5.03114735908296	5.37882793693542	5.03486157521677	5.49479193306137	5.87134584779545	5.79031439897711	NA
TC0200005107.mm.2	Wfdc15a	WAP four-disulfide core domain 15A	-0.915336669219181	4.92505854687226	-1.71347896585178	0.134132491452249	0.999692817443324	-4.55505973642472	4.5733865307936	4.91642964100597	3.80604637085938	5.30888451463843	5.23185236504098	5.7137518588952	NA
TC0800002856.mm.2	Smpd3	sphingomyelin phosphodiesterase 3, neutral	0.54740323423864	8.42006976008192	1.71337597107904	0.134152041347329	0.999692817443324	-4.55506568387676	8.36643128679348	8.70136067541054	8.80961835091883	8.46409818246358	8.01973649203907	8.15917357286605	NA
TC1500001075.mm.2	Krt86	keratin 86	-0.541127605789508	6.49119624445686	-1.71274748637944	0.134271396141698	0.999692817443324	-4.55510197759904	6.06113523120177	5.84200241069197	6.33201201995923	6.64888028667497	7.16721423413133	6.89593328408188	NA
TC1100000035.mm.2	Rnf215	ring finger protein 215	-0.704527102557678	6.44711246374265	-1.71271082790844	0.134278361056405	0.999692817443324	-4.55510409464487	6.57369058921591	6.92452390742379	5.53506368579383	6.3526393545566	6.36422340684588	6.9325338386199	NA
TSUnmapped00000031.mm.2	Cd99	CD99 antigen	-0.545558212470274	7.11752130741933	-1.71185474775208	0.134441110443699	0.999692817443324	-4.555153536635	6.8004673358702	6.91959159103729	6.8727886115656	7.07418763476838	7.73684000047981	7.30125267079472	NA
TC1100001150.mm.2	Proca1	protein interacting with cyclin A1	-0.87212708322888	6.2257396003944	-1.71174713976762	0.134461581194599	0.999692817443324	-4.55515975181356	7.07523141276079	6.46948234529169	6.17309797680729	4.85963686212078	6.60339326169903	6.1735957436868	NA
TC0700002223.mm.2	Zfp772	zinc finger protein 772	-0.50792058813851	5.78094562183404	-1.71166060242704	0.134478045753717	0.999692817443324	-4.55516475006627	5.83461882663857	5.78187118520186	5.40425370308575	5.62241756835763	6.09257661654777	5.94993583117263	NA
TC1000003163.mm.2	Tmem198b	transmembrane protein 198b	0.519213356212209	6.80983756599004	1.710763599167	0.134648823308526	0.999692817443324	-4.55521656283244	6.7932958601897	6.77817552941965	6.91325199600893	7.24973836336872	6.61084324808154	6.51372039887169	NA
TC1800000710.mm.2	Seh1l	SEH1-like (S. cerevisiae	-0.686355065125816	7.52612753642226	-1.71049457612015	0.134700082319541	0.999692817443324	-4.55523210335059	8.10283873264147	7.65806748476707	7.45546308566142	6.55611134977527	7.71649731390227	7.66778725178606	NA
TC0200003190.mm.2	Inpp5e	inositol polyphosphate-5-phosphatase E	-0.606122860916208	6.75086882652433	-1.71022786482108	0.134750919350832	0.999692817443324	-4.55524751086816	6.90191947803478	6.60424204692882	6.11093374683069	6.7824691093443	6.99341947955478	7.11222909845263	NA
TC1100001256.mm.2	Ccl4	chemokine (C-C motif) ligand 4	0.723165582575616	4.96558889481116	1.7102007829675	0.134756082370857	0.999692817443324	-4.55524907537692	3.98205662551337	4.83401174104409	5.33408809157514	5.87780999215521	5.1153973626033	4.65016955597582	NA
TC0900000351.mm.2	Fez1	fasciculation and elongation protein zeta 1 (zygin I)	0.508165671835027	8.33160974596221	1.70954967412285	0.134880270208011	0.999692817443324	-4.55528669136506	8.63831143786121	8.59835172887338	8.52660930444468	8.35805402190969	7.82855944539984	8.03977253728448	NA
TC1600001276.mm.2	Ppil2	peptidylprolyl isomerase (cyclophilin)-like 2	-0.562529312593538	7.40004629648274	-1.70734840596652	0.135300938416425	0.999692817443324	-4.55541388710473	7.76816374463128	7.33290646241254	7.18013397901143	6.90694049432134	7.47351393118264	7.73861916733722	NA
TC0100000224.mm.2	Ptpn18	protein tyrosine phosphatase, non-receptor type 18	-0.615939422031439	5.45147731440994	-1.70679982077062	0.135405970350444	0.999692817443324	-4.55544559162492	4.54621992769247	4.89408251376526	5.24636527873314	5.77197602173638	5.89280025082597	6.35741989370643	NA
TC1700000614.mm.2	H2-Oa	histocompatibility 2, O region alpha locus	-0.808179184335432	5.58803956598505	-1.70530479795471	0.135692604186744	0.999692817443324	-4.55553200524359	4.673568327079	5.04007658201205	4.90540702890806	6.19371003016613	6.26713703975189	6.44833838799316	NA
TC0200001664.mm.2	Katnbl1/Slc12a6	katanin p80 subunit B like 1/solute carrier family 12, member 6	0.64455506750754	5.5268975111133	1.7052344110979	0.13570611347346	0.999692817443324	-4.55553607407495	5.79937016929395	5.7392724388707	5.71832806709859	5.73759822966652	5.52266256293803	4.644153598812	NA
TC1200002448.mm.2	Brf1	BRF1, RNA polymerase III transcription initiation factor 90 kDa subunit	0.637820609933605	5.7076956133357	1.70433669074004	0.135878525347449	0.999692817443324	-4.55558797154173	6.43033738206522	6.02573304226024	5.85692958011778	5.6759426576602	5.49705764857135	4.76017336933942	NA
TC1100000029.mm.2	Gal3st1	galactose-3-O-sulfotransferase 1	-0.512024653883237	6.82039505966324	-1.70248088615015	0.136235608620643	0.999692817443324	-4.55569527500676	6.79499217090692	6.88328869218169	6.43554102468203	6.67447906888016	6.9713048735342	7.16276452779447	NA
TC0500001622.mm.2	Ufsp1	UFM1-specific peptidase 1	0.653162851994275	6.19350593626894	1.70171519850654	0.136383199931456	0.999692817443324	-4.55573955479663	6.90454608948447	6.49753203367461	6.03152443794812	6.50111716127345	5.2789412056363	5.94737468959671	NA
TC0100001227.mm.2	Cyb5r1	cytochrome b5 reductase 1	0.636704382517527	6.47269253038447	1.70104428893898	0.136512648151022	0.999692817443324	-4.55577835709095	6.43533250447738	6.6718945542987	6.62943886827459	6.87172957600831	5.71845319007131	6.50930648917653	NA
TC0800001328.mm.2	Pdpr	pyruvate dehydrogenase phosphatase regulatory subunit	0.513935772352849	5.77500195036272	1.70062465759246	0.1365936735228	0.999692817443324	-4.55580262829151	5.86647184608141	6.08977960563133	6.05584022268024	5.80497567490441	5.41764290395692	5.41530144892204	NA
TC0100002831.mm.2	Mterf4	mitochondrial transcription termination factor 4	0.52997216617352	6.08880467588911	1.70024742062821	0.136666552437843	0.999692817443324	-4.55582444852504	6.58209646489337	6.43894666219639	5.94806593301873	6.33779869727724	5.67505166056115	5.55086863738778	NA
TC1700000709.mm.2	Dhx16	DEAH (Asp-Glu-Ala-His) box polypeptide 16	-0.53786604898464	6.58861387240303	-1.70024223177655	0.136667555138879	0.999692817443324	-4.55582474866711	6.76933503402399	7.00763406379632	6.20494021541103	6.13455080390327	6.50539006027507	6.90983305700851	NA
TC1300000674.mm.2	Txndc15	thioredoxin domain containing 15	0.497508427545595	8.17396513441536	1.69941047009788	0.136828377134959	0.999692817443324	-4.55587286332157	8.63779818369556	8.47296366410007	8.20727747502288	8.25674543187097	7.78663675407368	7.68236929772898	NA
TC1900001415.mm.2	Acta2	actin, alpha 2, smooth muscle, aorta	0.698524429299442	5.94825830284541	1.69899798231578	0.136908199346011	0.999692817443324	-4.55589672623512	5.84599972352409	6.09504889937726	6.00724536388774	6.56552966249727	6.01755216349159	5.15817400429453	NA

**TC1600001758.mm.2**	Tbc1d23	TBC1 domain family, member 23	-0.660093922804716	6.83938551777969	-1.69859186133212	0.136986833089748	0.999692817443324	-4.55592022202616	7.89145482822227	7.71868734028427	6.37986607933858	5.67312546694249	6.98290028371806	6.39027910817244	NA
**TC0400001774.mm.2**	Mfap2	microfibrillar-associated protein 2	0.811167757506522	6.9089694707525	1.69856046864346	0.136992913190172	0.999692817443324	-4.5559220382737	6.47457405888238	6.28106974121508	7.5572066406807	7.60304762903459	6.0922207488272	7.44569800587504	NA
**TC1700000363.mm.2**	Ccdc154	coiled-coil domain containing 154	-0.531375893437033	5.05663274551894	-1.697320820172	0.137233213730805	0.999692817443324	-4.5559937647548	4.80962869390296	4.74881695041901	4.75697258204946	5.10232693890934	5.59868589377071	5.32336541406215	NA
**TC0200000561.mm.2**	Aif1l	allograft inflammatory factor 1-like	-0.563091186149554	6.71413438129508	-1.69715953201587	0.137264508424233	0.999692817443324	-4.55600309775654	6.08446382145557	6.49680668957535	6.57677142522445	6.71190527699578	7.00388927762843	7.41096979689091	NA
**TC1000001622.mm.2**	Dnajc14	DnaJ heat shock protein family (Hsp40) member C14	0.584462245232658	6.90723970067067	1.69583669494773	0.137521436221731	0.999692817443324	-4.55607965128647	7.6630762327724	7.63292731697591	7.04821925440696	6.60996031796356	6.5058314615722	5.983423620333	NA
**TC1400001182.mm.2**	Acod1	aconitate decarboxylase 1	0.567198748177746	5.18578281804764	1.69387449376042	0.137903392951414	0.999692817443324	-4.5561932283158	4.5794936845471	4.80096511201988	5.53964896202436	5.89466884201281	4.92345311620022	5.37646719148146	NA
**TC1100002740.mm.2**	Rad50	RAD50 double strand break repair protein	0.731257429188831	7.1587164465831	1.69322503897929	0.138030037661749	0.999692817443324	-4.55623082638571	7.42233180178608	7.24300305103184	7.20880747590341	7.66593186662577	6.18268244491855	7.22954203923296	NA
**TC1300001430.mm.2**	Chrm3	cholinergic receptor, muscarinic 3, cardiac	0.590862417499475	6.96213203965012	1.69305271485229	0.138063659828476	0.999692817443324	-4.55624080303153	6.95521395971775	6.95796571626652	6.96877896139631	7.55188973706139	6.87235370137002	6.46659016208872	NA
**TC0600002550.mm.2**	Hk2	hexokinase 2	-0.574100264881396	5.63489924268478	-1.69266202994934	0.138139915416373	0.999692817443324	-4.55626342237682	5.03468828138289	5.05953298479359	5.78726521810043	5.49622161198427	5.92756784297867	6.50411951686882	NA
**TC0900003249.mm.2**	Gorasp1	golgi reassembly stacking protein 1	0.596393781278946	6.58410010547683	1.69257466993251	0.138156972241869	0.999692817443324	-4.55626848037628	6.81733391500928	6.61105765227244	6.73824659348974	6.89625172057885	6.56273330328889	5.87897744822181	NA
**TC0500000021.mm.2**	Akap9	A kinase (PRKA) anchor protein (yotiao) 9	-0.674653165468934	6.69690645067107	-1.69158493628731	0.138350356134118	0.999692817443324	-4.55632578806431	6.97719516841644	7.110105079012	6.66723354251769	5.70518252031236	6.96184750438926	6.75987488937866	NA
**TC0900003052.mm.2**	Bsn	bassoon	0.493626444755003	6.28519667110118	1.69106617069883	0.138451821124837	0.999692817443324	-4.55635582845088	6.62697661804111	6.53279928801913	6.33742928425654	6.43189922077188	5.95486550438416	5.82721011113425	NA
**TC1200002339.mm.2**	Bcl11b	B cell leukemia/lymphoma 11B	0.714024659641471	7.48790817634204	1.69075833947217	0.138512063280746	0.999692817443324	-4.55637365506174	8.37262654617986	8.51112684877753	7.30281819668294	7.43305429450596	7.20068438378869	6.10713878811726	NA
**TC1000001040.mm.2**	Fbxo7	F-box protein 7	-0.663997190044658	6.37481636283384	-1.69037564388927	0.138586991270197	0.999692817443324	-4.55639581801869	6.91034127615779	6.79628893654619	6.27946619921481	5.32767059289007	6.49838190961959	6.43674926257461	NA
**TC0200005090.mm.2**	Oser1	oxidative stress responsive serine rich 1	0.614284227178361	6.65751688801265	1.68878891671012	0.138898070670287	0.999692817443324	-4.55648772068022	7.3266579736722	7.26638650669341	6.76661852934469	6.52369412168882	6.4107522456869	5.65099195098989	NA
**TC0100001590.mm.2**	Arhgap30	Rho GTPase activating protein 30	-0.596415669800891	6.04873657481474	-1.68863463993511	0.138928352393928	0.999692817443324	-4.55649665727021	6.11288030622186	6.17340010537612	5.36000453667471	6.04664931216963	6.16986573517882	6.42961945326731	NA
**TC1200000848.mm.2**	Gpatch2l	G patch domain containing 2 like	-0.564303680269702	6.99152936735697	-1.68772368413027	0.139107285230166	0.999692817443324	-4.55654942838803	7.26241983146878	7.42614656267697	6.77169038577873	6.2943108389496	6.92312608883439	7.27148249643334	NA
**TC0900003073.mm.2**	Trex1/Atrip	three prime repair exonuclease 1/ATR interacting protein	0.531733780614211	7.46712991369722	1.68765575574368	0.13912063676866	0.999692817443324	-4.55655336366862	7.23352704301617	7.26977845678336	8.00662265822892	7.67484811357718	7.14808374465582	7.46991946592186	NA
**TC0900000589.mm.2**	Usp28	ubiquitin specific peptidase 28	0.865336580509946	5.65686722171428	1.6876122336278	0.139129191821998	0.999692817443324	-4.55655588504275	5.82482886294466	5.95297587310968	6.24531615058816	5.70171972703747	5.8166683820142	4.39969433459153	NA
**TC0800001296.mm.2**	Pmfbp1	polyamine modulated factor 1 binding protein 1	-0.512735798388228	4.79152459380393	-1.68715819188576	0.139218471892719	0.999692817443324	-4.5565821899009	4.41607547393151	4.38212546017752	4.56722431359183	4.89551320237723	5.17864907228161	5.30956004046391	NA
**TC0600002912.mm.2**	Lhfpl4	lipoma HMGIC fusion partner-like protein 4	0.517994433162498	7.86333049544712	1.68670125389876	0.139308376785715	0.999692817443324	-4.55660866398926	8.21631844179476	7.95638724240439	7.87493275985361	8.14670031755066	7.54229257661087	7.44335163446841	NA
**TC0200004028.mm.2**	Olfr1200	olfactory receptor 1200	-0.5147213122233	4.63985109848073	-1.68546646127	0.139551606416148	0.999692817443324	-4.5566802126332	4.11152192308778	4.00392126101659	4.85026210107525	4.49684829009144	5.20564314063675	5.17090987497655	NA
**TC1200001044.mm.2**	Serpina3m/Serpina3k	serine (or cysteine) peptidase inhibitor, clade A, member 3M/serine (or cysteine) peptidase inhibitor, clade A, member 3K	-0.653692186479995	5.47281108659277	-1.68527648020028	0.139589064942798	0.999692817443324	-4.55669122179742	5.92835888914493	5.84946140585719	5.35852717073192	4.51730375506534	5.7912177248977	5.39199757385954	NA
**TC0500003297.mm.2**	Stx2	syntaxin 2	0.567839533897605	6.86227471417712	1.68451295698948	0.139739705630664	0.999692817443324	-4.55673546949828	6.80743075884712	6.99918765037368	7.12014787884701	7.13120659297155	6.85899683246449	6.25667857155886	NA
**TC0200005485.mm.2**	Sys1	SYS1 Golgi-localized integral membrane protein homolog (S. cerevisiae)	0.5103477319916	8.11211218307257	1.6842539126805	0.139790849546733	0.999692817443324	-4.5567504825399	8.57290015141871	8.22913248804408	8.23453841898426	8.21112954249364	7.70296481643828	7.72200768105643	NA
**TC0500002950.mm.2**	Gak	cyclin G associated kinase	0.666588728455266	7.26634657879937	1.68407151352603	0.139826871909489	0.999692817443324	-4.55676105384799	8.34165491543988	7.9885958339049	7.34736044541667	6.95314264476431	6.89390235486428	6.07342327840617	NA
**TC1300002715.mm.2**	Itga2	integrin alpha 2	0.727533486380189	4.99191188028942	1.68377896082307	0.139884667241429	0.999692817443324	-4.55677800979864	4.52275480627729	4.88124749779554	5.00623856267306	5.99502941253898	5.01453299105368	4.53166801139799	NA
**TC0100001831.mm.2**	Angel2	angel homolog 2	0.953879883101206	6.95302885381009	1.68361408530575	0.139917249331701	0.999692817443324	-4.55678756601252	7.91188334666651	7.92701310368946	7.27032119729736	6.62319702205614	6.80646538759468	5.17929306555641	NA
**TC0800002404.mm.2**	Pik3r2	phosphoinositide-3-kinase regulatory subunit 2	0.535977821765779	7.12976462121421	1.68216475111423	0.14020397371199	0.999692817443324	-4.5568715776138	7.73021147452582	7.67183557649431	7.32472524732019	6.89952291257817	6.69832129956826	6.45397121679854	NA
**TC1800000617.mm.2**	Cd74/Mir5107	CD74 antigen (invariant polypeptide of major histocompatibility complex, class II antigen-associated)/microRNA 5107	-0.638825022207286	5.7020924106929	-1.68067426216467	0.140499425433311	0.999692817443324	-4.55695798957701	5.55692193632277	5.64428035182435	5.79343849905972	5.07341256673813	5.8122956554708	6.33220545474163	NA
**TC0100001577.mm.2**	Cfap126	cilia and flagella associated protein 126	0.697907557852282	7.28573847330493	1.68026348251794	0.140580956615519	0.999692817443324	-4.55698180739076	7.77880593481512	7.53155916068644	7.97337273900338	6.92656769101292	6.75780094083494	6.74632437347679	NA
**TC0100000271.mm.2**	Eif5b	eukaryotic translation initiation factor 5B	0.527254596518351	6.32910155107591	1.67957305220845	0.14071809445484	0.999692817443324	-4.55702184244693	7.35931378618361	7.25113565796651	6.09014836303874	6.11918616463229	5.79713569911049	5.35768963552383	NA
**TC1000002776.mm.2**	4930430F08Rik	RIKEN cDNA 4930430F08 gene	-0.879524830604113	7.28648323973365	-1.67916457196625	0.140799289664808	0.999692817443324	-4.55704552994239	8.10271330318725	7.85521062444324	7.27570161299218	5.72526131178942	7.40029406642608	7.35971851956375	NA
**TC0X00003349.mm.2**	Egfl6	EGF-like-domain, multiple 6	-0.688923191556769	5.83321097434914	-1.67865636513959	0.140900370389952	0.999692817443324	-4.55707500206828	5.6305451481052	5.22704752553046	5.75019765722802	5.63171573744481	5.89832073765423	6.86143904013213	NA
**TC1000000908.mm.2**	Zfp433	zinc finger protein 433	-0.679674072720587	6.31522379956036	-1.67788279708223	0.141054363672141	0.999692817443324	-4.55711986642111	6.6437275804437	6.81119201474285	6.26855450004494	5.26998302832227	6.52546963493429	6.3724160388741	NA
**TC0200001780.mm.2**	Itpka	inositol 1,4,5-trisphosphate 3-kinase A	-0.544163502768526	6.92238567677409	-1.67770824233344	0.141089134300063	0.999692817443324	-4.55712999056009	7.26225781600481	7.58774572860813	6.60059066670512	6.19740108854215	7.01858197232345	6.86773678846087	NA
**TC1000003093.mm.2**	Tsfm	Ts translation elongation factor, mitochondrial	-0.501408300608071	7.79927709765626	-1.67757543895356	0.141115593699859	0.999692817443324	-4.55713769326358	7.44997115557072	7.43602884898368	7.66998900756074	7.78343398252276	8.09200458577582	8.36423500552383	NA
**TC0500000285.mm.2**	Garem2	GRB2 associated regulator of MAPK1 subtype 2	0.680132086359291	6.38618169690885	1.67721202061979	0.14118802445028	0.999692817443324	-4.55715877241764	6.22864023259392	6.56526679871052	6.26162116284929	7.18010249858433	5.88562878628749	6.19583070242755	NA
**TC0900002374.mm.2**	Mpi	mannose phosphate isomerase	-0.523483067338141	7.24512358595182	-1.67513832459444	0.141602000102723	0.999692817443324	-4.55727906852576	7.6610960928339	7.62628543320426	6.99598523384791	6.57221169365034	7.24966126992183	7.3655017922527	NA
**TC0200003085.mm.2**	Enkur	enkurin, TRPC channel interacting protein	0.585186152690439	5.81368238628156	1.67502302718858	0.141625051101858	0.999692817443324	-4.55728575781257	7.10129359424872	6.80372886888822	5.84551369153627	5.22820838843038	5.04960608221215	4.85374369237362	NA
**TC1100001828.mm.2**	9930022D16Rik	RIKEN cDNA 9930022D16 gene	0.673837919220919	5.34204264501364	1.67469286441654	0.141691079182274	0.999692817443324	-4.55730491356822	5.03792575015619	4.53900211846876	5.51997674626903	6.39152517368032	5.22733317702458	5.33649290448294	NA
**TC0600002843.mm.2**	Prok2	prokineticin 2	0.714357483803099	6.44825905115788	1.67361088576482	0.141907666218617	0.999692817443324	-4.55736769400996	6.13642864594001	6.29449139042699	6.69772097906638	7.14595364002686	6.72117141497715	5.69378823650989	NA
**TC1900001030.mm.2**	Fermt3	fermitin family member 3	-0.521936940393083	5.34419386386639	-1.67356904592241	0.141916047926802	0.999692817443324	-4.55737012186694	5.53623785138151	5.89687141276565	4.83948211814311	4.95460790098941	5.31193217012024	5.52603172979844	NA
**TC0900002812.mm.2**	Trim43b	tripartite motif-containing 43B	0.548143961811886	6.05790861757863	1.67284361832488	0.142061446791425	0.999692817443324	-4.55741221834618	5.54665418137352	5.7885631822868	6.27438513200374	6.77987600071387	6.01462726309334	5.94334594600049	NA
**TC0500002122.mm.2**	Gm10471	predicted gene 10471	-0.587791882398826	5.84187121298543	-1.6727844527412	0.14207331173177	0.999692817443324	-4.55741565186864	5.04390975307747	5.33393012800115	6.19323091399683	5.55567090202132	6.55662331248658	6.36786226832922	NA
**TC1400001715.mm.2**	Zfp488	zinc finger protein 488	-0.514895930746964	5.84644715406838	-1.67238395800925	0.142153650958916	0.999692817443324	-4.55743889414601	5.86593933608001	5.60510886823869	5.49534866176042	5.79357276753841	6.14958602354066	6.16912726725209	NA
**TC1100002488.mm.2**	Ranbp17	RAN binding protein 17	0.740862750350399	5.31822517433216	1.67221781160626	0.142186992639823	0.999692817443324	-4.55744853657669	6.12572334285034	5.35343949789552	5.7381444649882	5.21781238798575	5.12297651359854	4.35125483867462	NA
**TC0700000424.mm.2**	Tgfb1	transforming growth factor, beta 1	-0.824299809652125	6.86154983420532	-1.67208504143045	0.142213641858993	0.999692817443324	-4.55745624212126	7.30002475622264	7.67996329517696	6.81760535293294	5.4527503143311	7.07852042849203	6.84043485807626	NA
**TC1000000842.mm.2**	Midn	midnolin	0.530434011921852	8.10142021892979	1.67178573851074	0.142273734495369	0.999692817443324	-4.55747361309615	8.34422075752583	8.20259155526623	8.50983388559078	8.05145462672439	7.77666056804794	7.72375992042353	NA
**TC0200003806.mm.2**	Fkbp7	FK506 binding protein 7	-0.878918706390989	5.52691785588134	-1.67082464009664	0.142466863147509	0.999692817443324	-4.55752939732908	5.91152022063969	5.71691729415019	5.53345139747344	4.35416470638465	5.30586486501362	6.33958865162645	NA
**TC0400002603.mm.2**	Coro2a	coronin, actin binding protein 2A	-0.539457182806388	7.31876739752765	-1.67033226202396	0.142565901279584	0.999692817443324	-4.55755797831646	7.30704356478815	7.23705077572375	7.32348453263174	6.82131330688888	7.68710631551307	7.53660588962034	NA
**TC0700000976.mm.2**	Trpm1	transient receptor potential cation channel, subfamily M, member 1	0.604600210826995	5.50484507216669	1.6702143264012	0.14258963288568	0.999692817443324	-4.55756482433632	4.9082649985172	5.16202567656013	5.72294752051456	6.36104256927384	5.24248472333373	5.63230494480068	NA
**TC0600002027.mm.2**	Chchd3	coiled-coil-helix-coiled-coil-helix domain containing 3	-0.724412946543415	7.72757023862829	-1.66991601728652	0.142649676982092	0.999692817443324	-4.55758214121577	8.65014650396623	8.66123421824411	7.41268543300349	6.3899219752328	7.90515838180923	7.3462749195139	NA
**TC1000000199.mm.2**	Taar7b	trace amine-associated receptor 7B	-0.626542358689644	5.43132644592528	-1.66979063949994	0.142674920403532	0.999692817443324	-4.55758941958089	5.48256164721796	5.63310968179662	4.88637440239599	5.22322691218293	5.28991928018898	6.07276675176923	NA
**TC1500000880.mm.2**	Acr	acrosin prepropeptide	0.522888666249333	5.97871242568198	1.66978231482255	0.142676596635454	0.999692817443324	-4.55758990284423	5.60778989103025	5.71894531546698	6.24905995388606	6.54659838616058	6.03188924709491	5.71799176045307	NA
**TC0400003937.mm.2**	C87977	expressed sequence C87977	0.505275867137047	5.27766489309951	1.66957833505021	0.142717675255475	0.999692817443324	-4.55760174439569	5.60049792652987	5.72804390103109	5.18663515245542	5.48736448019966	4.78308048935313	4.88036740902785	NA
**TC0200000694.mm.2**	Crb2	crumbs family member 2	0.487186550622065	5.82626387367207	1.66936471846126	0.142760706683509	0.999692817443324	-4.55761414567374	5.52824159485093	5.68608521940828	6.21561126387666	6.14320350063202	5.72162867420532	5.66281298905923	NA
**TC1100003166.mm.2**	Olfr75-ps1	olfactory receptor 75, pseudogene 1	-0.550586130793257	6.10054640786875	-1.66837255431888	0.142960732790721	0.999692817443324	-4.55767174848826	5.637061537128	5.74006693843123	6.23309107256551	5.82939778246786	6.37851219219177	6.78514892442812	NA
**TC1100004288.mm.2**	Urgcp	upregulator of cell proliferation	-0.491259979844977	7.08568999434265	-1.66806271607809	0.143023252731584	0.999692817443324	-4.55768973827922	7.74810693487653	7.58284808917128	6.49515830228894	6.60517418887013	7.10129379593219	6.98155865491682	NA
**TC0600001852.mm.2**	Ica1	islet cell autoantigen 1	-1.20174767236674	6.3850858783426	-1.66721270724939	0.14319490352657	0.999692817443324	-4.5577390945099	7.74531945949941	7.4467467853308	6.35266081526162	4.00481602498433	6.44956464169933	6.31140754328012	NA
**TC1100003829.mm.2**	Hdac5/Mir8101	histone deacetylase 5/microRNA 8101	0.893229477313547	6.51112495344915	1.66564868820267	0.143511254754907	0.999692817443324	-4.55782992203176	7.53442590632187	7.38409325795909	7.07543387714296	5.89191087837756	6.14125262891102	5.0396331719824	NA
**TC1600001253.mm.2**	Ube2v2	ubiquitin-conjugating enzyme E2 variant 2	1.03361907498634	8.38273802287064	1.66501433590044	0.14363975378475	0.999692817443324	-4.55786676524379	9.1591176261049	9.11492962419403	8.69249501505622	8.35231450339259	8.46051207339461	6.51705929508151	NA
**TC0X00000678.mm.2**	F8a	factor 8-associated gene A	0.518798416613656	6.85207169834499	1.66453705939119	0.143736506664574	0.999692817443324	-4.55789448714236	7.14343612362013	6.99292314830618	7.1760731705075	6.83076070517796	6.59151131175608	6.37772573070208	NA
**TC1000000183.mm.2**	Slc2a12	solute carrier family 2 (facilitated glucose transporter), member 12	0.519389265893964	4.95478159043288	1.66411731295874	0.143821648437457	0.999692817443324	-4.55791886866408	5.13743673659573	5.24682213777892	5.06760620787103	5.12399839213424	4.78613493968279	4.36669112853455	NA
**TC0100003796.mm.2**	Vash2	vasohibin 2	0.506855482357363	5.50903237100771	1.66400510868763	0.143844416198266	0.999692817443324	-4.55792538638178	5.09484285277179	5.05264081710201	6.09835107948476	5.86185968095884	5.33742672842845	5.60907306730042	NA
**TC1500001111.mm.2**	Hoxc13	homeobox C13	-0.569912205691968	5.05535858160676	-1.66358796922217	0.143929089541042	0.999692817443324	-4.55794961784953	4.43336753825566	5.02082894587798	4.94587548301814	4.92318981404334	5.34474944019882	5.6641402682466	NA
**TC1200002088.mm.2**	Rps6kl1	ribosomal protein S6 kinase-like 1	0.794719777219399	5.43530310137625	1.66342627814108	0.143961923284988	0.999692817443324	-4.5579590107111	5.88079653142489	5.84877417227359	5.41487887480296	5.82096485469594	4.16975775959055	5.47664641546955	NA
**TC0800000168.mm.2**	Mcph1	microcephaly, primary autosomal recessive 1	0.548604660747966	6.14937458071182	1.66255628180412	0.144138711306654	0.999692817443324	-4.55800955279399	6.19513635382876	6.35343728660063	6.29402405779762	6.42841752487111	6.08441310466224	5.54081915651057	NA
**TC0100003507.mm.2**	Uap1	UDP-N-acetylglucosamine pyrophosphorylase 1	-0.974032196118853	6.42613588242002	-1.66244239391647	0.144161869250469	0.999692817443324	-4.55801616941036	6.99146802030546	7.25082826055481	6.39819988917131	4.78502742153977	6.97908360639373	6.15220809655505	NA
**TC0200001133.mm.2**	Pjvk	pejvakin	-0.623722469547274	5.76185055228448	-1.6616356233876	0.144326019326625	0.999692817443324	-4.55806304315227	5.01309786662151	4.84719846595678	6.1928348783648	5.53884614265223	6.21792201829483	6.76120394181674	NA
**TC0400001273.mm.2**	Hectd3	HECT domain E3 ubiquitin protein ligase 3	0.54356960208531	6.91108665413346	1.66084309765744	0.14448744437816	0.999692817443324	-4.55810909315495	6.86604458549966	7.07031968896138	7.04562233905068	7.26302508820449	6.37259821316046	6.84891000992409	NA
**TC0500003448.mm.2**	Pop7	processing of precursor 7, ribonuclease P family, (S. cerevisiae)	0.639765635018936	7.10884903353771	1.66047462639186	0.144562554725716	0.999692817443324	-4.55813050462376	7.06818898437237	6.7181319789748	7.11445508965449	7.95869716430398	6.95200237529148	6.84161860862912	NA
**TC0500003489.mm.2**	Cox19	cytochrome c oxidase assembly protein 19	-0.541778029937141	6.98803060428988	-1.65972729704712	0.144715006878964	0.999692817443324	-4.55817393367076	6.80136157062423	7.19757586322173	6.58985419586532	6.83299087014418	7.10109995201618	7.40530117386761	NA
**TC0200002398.mm.2**	Dlgap4	DLG associated protein 4	0.498051255889463	7.87542251236945	1.6596746452745	0.144725753396678	0.999692817443324	-4.55817699351597	8.35558704189375	8.29927785074997	8.05110852822771	7.74577781844823	7.39773378198747	7.40305005290955	NA
**TC0300000408.mm.2**	Postn	periostin, osteoblast specific factor	-0.668055919391534	5.23503975373069	-1.65939879532013	0.144782068314478	0.999692817443324	-4.55819302474553	5.61953253174126	5.8972858302675	4.26478791981922	5.01386624097695	5.61741997035329	4.99734602922595	NA
**TC1100003061.mm.2**	Polr2a	polymerase (RNA) II (DNA directed) polypeptide A	0.660812879736747	6.2977386389781	1.65842285340744	0.144981474880668	0.999692817443324	-4.55824974605413	7.17641412042133	7.11838872487366	5.96779600802738	6.43883136599617	5.10805843469268	5.97694317985738	NA
**TC0300000305.mm.2**	Intu	inturned planar cell polarity protein	-0.603390463392695	6.9612375956889	-1.65832308307334	0.145001874893265	0.999692817443324	-4.55825554498603	6.84914042132653	7.18238272324998	6.96520978635543	6.29935096503031	7.38274466646751	7.08859701170362	NA
**TC0X00002403.mm.2**	Gabre	gamma-aminobutyric acid (GABA) A receptor, subunit epsilon	0.525909783848802	5.90991018720989	1.65827678164934	0.145011343060728	0.999692817443324	-4.55825823617519	6.0332518641161	5.59770664874957	6.27326271849121	6.16689837055444	5.606443023708	5.78189849764004	NA
**TC0500001239.mm.2**	Oasl2	2'-5' oligoadenylate synthetase-like 2	-0.663653782602783	6.01905896078811	-1.65809457766212	0.145048607625945	0.999692817443324	-4.55826882658837	5.45137783058106	6.16557026542835	5.54122434444632	6.04382470731052	6.21331269245738	6.69904392450503	NA
**TC1000002696.mm.2**	Snrpf	small nuclear ribonucleoprotein polypeptide F	-0.600216951739415	6.75766938932534	-1.65807951279759	0.145051689116952	0.999692817443324	-4.55826970222658	7.05663817383503	6.87060276276681	6.66082629431432	6.04834445362138	6.72359393278306	7.18601071863147	NA
**TC1500000051.mm.2**	Gdnf	glial cell line derived neurotrophic factor	-0.599213767478002	5.3088797501344	-1.65696237164918	0.145280372024282	0.999692817443324	-4.55833463933937	4.91631742404921	5.12633974125282	5.26811453907033	5.03798236120386	5.39062047518309	6.1139039600471	NA
**TC0900000124.mm.2**	Smco4	single-pass membrane protein with coiled-coil domains 4	0.662434227949412	4.90580387064952	1.65695408037673	0.145282070560215	0.999692817443324	-4.55833512132197	4.61278736893396	4.45095253639859	5.25887441638904	5.58910147084265	5.22486617113752	4.29824126019534	NA
**TC0X00000686.mm.2**	Atp2b3	ATPase, Ca++ transporting, plasma membrane 3	0.666219072472337	7.21445097030029	1.65635319761639	0.14540521669635	0.999692817443324	-4.55837005252541	7.92929786467531	8.07860037748998	7.43214410823402	6.87347875405655	6.90027766840721	6.07290704893869	NA
**TC0800001253.mm.2**	Cdh3	cadherin 3	-0.579425170707511	4.9515854274593	-1.65561010105505	0.14555764577534	0.999692817443324	-4.5584132540475	4.6138260204843	4.46843972332998	4.51922278265316	5.21497545711008	5.40094150818354	5.49210707299473	NA
**TC0200002527.mm.2**	Wfdc13	WAP four-disulfide core domain 13	0.595090905530148	6.51300642213368	1.65497727768277	0.145687574699449	0.999692817443324	-4.55845004719199	6.65053661656409	6.65435681820847	6.46177177912217	7.01989167542272	5.87666247690873	6.41481916657587	NA
**TC0400004176.mm.2**	Palm2	paralemmin 2	0.544763410377062	7.60981558067874	1.6544597001435	0.145793923788232	0.999692817443324	-4.55848014156604	8.18910527525997	7.95406528000592	7.39018774119646	7.91243713358386	7.14058249188863	7.07251556213758	NA
**TC0700000421.mm.2**	Exosc5	exosome component 5	-0.539591857968931	6.15584035845678	-1.65401934314671	0.145884464142212	0.999692817443324	-4.55850574722557	6.53864652046111	6.11468548859012	5.95023977527395	5.65102343760185	6.4373410132281	6.24310591558556	NA
**TC0400002034.mm.2**	A430005L14Rik	RIKEN cDNA A430005L14 gene	-0.61940201463176	7.05005019704562	-1.65334912255587	0.146022368631414	0.999692817443324	-4.55854472107533	7.32464230439174	7.39882702739961	6.95486934016833	6.21414457044109	7.42136197663687	6.98645596323607	NA
**TC0300002444.mm.2**	Riiad1	regulatory subunit of type II PKA R-subunit (RIIa) domain containing 1	-0.653129413537956	5.90344700659868	-1.65223258941936	0.146252381638531	0.999692817443324	-4.55860965421117	5.72686363190563	5.92118738303101	5.95751612291967	5.27566997587009	6.60337338451799	5.93607154134769	NA
**TC1100000935.mm.2**	Phf23	PHD finger protein 23	0.475059840531666	6.28978100696834	1.65108333796115	0.146489494573755	0.999692817443324	-4.55867649772882	6.81756855103667	6.79345615263229	6.25827172021425	6.28061878938796	5.81603604698323	5.77273478155564	NA

**TC1900000903.mm.2**	Aip	aryl-hydrocarbon receptor-interacting protein	0.690532437096403	6.4593478724444	1.65093728730247	0.146519653824849	0.999692817443324	-4.5586849929739	7.30883072154144	7.04932005178663	6.88655272859795	6.00294793916762	5.46259764341032	6.04583815016245	NA
**TC1300002139.mm.2**	Ctsll3	cathepsin L-like 3	0.615849386323895	5.29470423296164	1.65064392889248	0.146580249749357	0.999692817443324	-4.5587020569589	4.86986673387399	4.95758940116065	5.45457343648949	6.13166058120201	5.43338478763271	4.921150457411	NA
**TC0800002920.mm.2**	Calb2	calbindin 2	0.773228128946463	8.38634682797709	1.64998013407935	0.146717450718474	0.999692817443324	-4.55874067021973	9.57637695319135	8.72922605202534	8.62059863671702	8.15886847355238	8.07254167779623	7.16046917458024	NA
**TC0600000257.mm.2**	Nrf1	nuclear respiratory factor 1	0.74897932296971	6.24466307353019	1.64994782878168	0.146724131073441	0.999692817443324	-4.55874254949934	6.52356457250198	6.58079189045887	6.46433336414171	6.46645694793814	6.3459356410426	5.08689602509784	NA
**TC0300001176.mm.2**	Olfm3	olfactomedin 3	0.948794530654997	6.87264682264021	1.64913673099907	0.14689195130947	0.999692817443324	-4.55878973505055	8.44536540558916	7.85312676283057	7.41594218304777	6.00154673131799	6.21932367464823	5.30057617840753	NA
**TC1600000532.mm.2**	Rabl3	RAB, member RAS oncogene family-like 3	1.07094597119752	6.1001828401144	1.64806594318747	0.147113782086126	0.999692817443324	-4.55885203384195	6.70377846005414	6.65432389705602	6.92717982028436	5.76526349270128	6.14369935726279	4.40685201332782	NA
**TC1100003385.mm.2**	Slfn10-ps	schlafen 10, pseudogene	-0.616305448901968	5.06863480584345	-1.64728300513869	0.147276181593337	0.999692817443324	-4.55889758959029	4.49600159617862	4.62930492156253	4.72504702766253	5.30189868209527	5.31834008300062	5.94121652456112	NA
**TC1100002025.mm.2**	Fscn2	fascin actin-bundling protein 2	-0.525887602848107	5.83692668739809	-1.64691802196047	0.147351945754987	0.999692817443324	-4.55891882756239	5.52634420884835	5.53370522964268	5.49813264802431	5.95673509207633	6.35234403217623	6.15429891362062	NA
**TC0100003294.mm.2**	Cacna1e	calcium channel, voltage-dependent, R type, alpha 1E subunit	0.613109045365954	7.42386040508932	1.64687152504877	0.14736160036367	0.999692817443324	-4.55892153322124	8.61405524255593	8.60020544092151	7.44442541527908	6.83313450361612	6.82016160014359	6.2311802280197	NA
**TC0200003545.mm.2**	Prpf40a	pre-mRNA processing factor 40A	-0.635203688416819	6.53012096767442	-1.6465760476483	0.14742296727008	0.999692817443324	-4.55893872736067	7.02703494707703	7.13696453098666	6.39479566108831	5.47836381448628	6.56292811603451	6.58063873637371	NA
**TC0300002876.mm.2**	Mettl14	methyltransferase like 14	0.804152479257761	6.28665607721483	1.64635981006325	0.147467892453481	0.999692817443324	-4.55895131076609	7.42853625308296	7.05453312089321	6.31550426953097	6.10708175438319	6.08229106258506	4.73199000281358	NA
**TC0700003086.mm.2**	Fancf	Fanconi anemia, complementation group F	0.547533739742457	7.1987468396343	1.64495406336744	0.147760265358744	0.999692817443324	-4.55903312112338	6.78007520721063	6.82255030284327	7.37251806296995	7.96994344064845	7.15703419038018	7.0903598337533	NA
**TC0200002835.mm.2**	Myt1	myelin transcription factor 1	-0.561047494847602	6.19563612911369	-1.64488430323863	0.147774788680267	0.999692817443324	-4.5590371812494	6.19940435251292	6.23446676209901	6.11931081105683	5.68961452413067	6.21619483876525	6.71482548611746	NA
**TC1500001201.mm.2**	Dnajc21	DnaJ heat shock protein family (Hsp40) member C21	-0.714486876951309	7.13219874294653	-1.64481277458126	0.147789681598182	0.999692817443324	-4.55904134433437	7.46055289409771	7.03299440825997	7.23137092773688	6.20396477297256	7.61012369324617	7.25418576136589	NA
**TC0400002570.mm.2**	Fbxo10	F-box protein 10	0.856103021658182	5.92294702017607	1.64470901787667	0.147811287222089	0.999692817443324	-4.55904738319552	7.09510852211562	6.68790101072515	6.45040807945934	5.28303123630667	5.48345197963609	4.53778129281356	NA
**TC0400001863.mm.2**	Smarca5-ps	SWI/SNF related, matrix associated, actin depenent ragulator of chromatin, subfamily a, member 5, pseudogene	1.07452123379668	7.01776591192747	1.64140050978406	0.148501803873878	0.999692817443324	-4.55923997690719	8.67815735845654	8.81412337546158	6.97504599928849	6.40663260333154	6.62977360864881	4.60286252637786	NA
**TC0400002065.mm.2**	Pex10	peroxisomal biogenesis factor 10	-0.514603767366093	5.65783035794905	-1.64136150811393	0.148509962117694	0.999692817443324	-4.55924224762058	5.81479791876295	5.93536088958779	5.14910891503375	5.43469898727194	5.64345928452742	5.96955615251045	NA
**TC1500001279.mm.2**	Basp1	brain abundant, membrane attached signal protein 1	-0.49290933838066	8.33760111312662	-1.64106609756021	0.148571768958389	0.999692817443324	-4.55925944696881	8.67762237118927	8.51576128735647	8.07521919179058	7.84798297993577	8.38262163016732	8.52639921832034	NA
**TC1400000930.mm.2**	Ints9	integrator complex subunit 9	0.892475989971289	6.14347750187542	1.6400509469518	0.14878434845894	0.999692817443324	-4.55931855456523	7.19650846848342	7.04866819696495	6.5819677229618	5.61835243991158	5.87783436029602	4.53753382263478	NA
**TC1200001001.mm.2**	Kif4-ps	kinesin family member 4, pseudogene	-0.589246012260409	4.99927619315864	-1.6389475172454	0.149015741495226	0.999692817443324	-4.5593828086395	4.36249604167874	4.09157159788042	4.84669659370043	5.3348521537355	5.41123970357867	5.94880106837808	NA
**TC0600003307.mm.2**	Dusp16	dual specificity phosphatase 16	0.682561967851704	5.53805782159713	1.63836852100814	0.14913729552752	0.999692817443324	-4.55941652695331	5.80183221243089	6.03096043443719	5.28408461557544	6.09625449363361	5.34612269632086	4.66909247718478	NA
**TC0100002376.mm.2**	Kctd18	potassium channel tetramerisation domain containing 18	-0.600573354520265	5.61361714723169	-1.63771258020661	0.149275116859595	0.999692817443324	-4.55945472838796	5.43913259713536	5.230701827422	5.58395834829368	5.32140252660246	5.68061943707057	6.4258881468661	NA
**TC0400000450.mm.2**	Hrct1	histidine rich carboxyl terminus 1	-0.615132498684612	6.57786923106776	-1.63692844335352	0.14944003227733	0.999692817443324	-4.55950039889522	5.87844359323256	6.39977254876067	6.22209468928756	6.75727243423449	6.88333326600863	7.32629885488265	NA
**TC0400000552.mm.2**	Zfp189	zinc finger protein 189	0.51496842061028	5.49695368546202	1.63692153487182	0.149441485999266	0.999692817443324	-4.55950080128082	6.16245499535527	5.78986019521896	5.42025172266349	5.60942015904575	4.90435020831091	5.09538483217777	NA
**TC0700000678.mm.2**	Tshz3	teashirt zinc finger family member 3	-0.486707595587182	8.53800071072291	-1.63663949863788	0.149500845113431	0.999692817443324	-4.55951722874418	7.79485760189918	8.04747120013286	8.55085654914767	8.65527358641786	9.10481174990481	9.07473357683509	NA
**TC1100003410.mm.2**	Aatf	apoptosis antagonizing transcription factor	-0.609413997559431	7.15438646434706	-1.63587349167264	0.149662176571093	0.999692817443324	-4.5595618476825	7.82615664701671	7.42032177382	6.97771293841284	6.25279324665056	7.31535286685855	7.1339813133237	NA
**TC1700000584.mm.2**	Adamts10	a disintegrin-like and metallopeptidase (reprolysin type) with thrombospondin type 1 motif, 10	0.611305318127955	5.98125198537536	1.63565602096577	0.149708008924598	0.999692817443324	-4.55957451564748	6.08028763838676	5.55094135625481	6.10457192588456	6.63487485104871	5.96653531515688	5.55030082552047	NA
**TC0X00002985.mm.2**	Sytl4	synaptotagmin-like 4	0.713194698640734	5.40606304531067	1.63470722526754	0.149908125068978	0.999692817443324	-4.5596297872323	6.01359214865661	5.36475367344406	5.16553552906768	6.07667539445474	4.72412214061412	5.09169938562684	NA
**TC0600001530.mm.2**	Clec2d	C-type lectin domain family 2, member d	0.808736831207752	5.53516092494899	1.63462497544762	0.149925484805367	0.999692817443324	-4.55963457887642	6.03872840521308	5.9898551662512	5.65430634998942	5.74562147033315	5.61159308513616	4.1708610727709	NA
**TC1200000967.mm.2**	Spata7	spermatogenesis associated 7	0.59457504555478	6.3196830728065	1.63443278707208	0.149966055720741	0.999692817443324	-4.55964577537242	6.85988908041042	6.61295604482544	6.45940095695557	6.35780074440079	6.19116623585817	5.43688537438863	NA
**TC1900000322.mm.2**	Carnmt1	carnosine N-methyltransferase 1	-0.50618782169492	7.20424351770808	-1.6341419676213	0.150027467416304	0.999692817443324	-4.55966271828149	6.47420061419987	6.87138265625854	7.162928036041	7.27082306015914	7.74980580602904	7.69632093356093	NA
**TC1800000613.mm.2**	Dctn4	dynactin 4	0.540557732368187	6.86818528196116	1.63412698142085	0.150030632664521	0.999692817443324	-4.55966359137766	7.68418584393066	7.26251711213784	6.65191933294744	7.01984276726999	6.38529688055594	6.20534975492511	NA
**TC0600000657.mm.2**	Znrf2	zinc and ring finger 2	-0.812448944978073	7.56083868139095	-1.63270603969173	0.1503310386184	0.999692817443324	-4.55974638082466	7.68818152697599	8.02427167995863	7.67624356748226	6.33759692824521	8.05279170197726	7.58594668370636	NA
**TC0900001325.mm.2**	Aste1	asteroid homolog 1	0.699474306579321	6.26648683395608	1.63214205577907	0.150450430235521	0.999692817443324	-4.55977924359715	5.95805290782651	5.94671519934522	6.28909800887958	7.25745274598212	5.77273512654348	6.37486701515957	NA
**TC0200005023.mm.2**	Blcap	bladder cancer associated protein	0.639416057643714	8.29098465213489	1.63209536870163	0.150460317602159	0.999692817443324	-4.55978196408036	8.54569364492898	8.1592891925478	8.24131867232664	8.91855992298335	7.66469695203069	8.21634952799187	NA
**TC0300000272.mm.2**	4932438A13Rik	RIKEN cDNA 4932438A13 gene	-0.877545353243077	7.0088823676811	-1.63138298680004	0.150611261873507	0.999692817443324	-4.55982347639631	8.70417561538956	8.59188208106471	6.05935783806864	5.44171506350446	7.38067593059147	5.87548767746779	NA
**TC0300001888.mm.2**	Ankrd50	ankyrin repeat domain 50	-0.560761246158368	5.94499698514745	-1.63081887043079	0.150730892403656	0.999692817443324	-4.5598563507694	6.55878406906234	6.52646686408868	5.68833578378103	5.04326845892745	6.02868254327296	5.82444419175226	NA
**TC0900001134.mm.2**	Trim43a	tripartite motif-containing 43A	-0.698941533825469	5.18802048549493	-1.63042994846662	0.150813422371592	0.999692817443324	-4.55987901649458	5.09700693437159	4.96522229706086	5.39076935548504	4.44323595145806	5.30506859630482	5.92681977828922	NA
**TC1900000050.mm.2**	Mrpl11	mitochondrial ribosomal protein L11	-0.685358266488417	7.41122275802587	-1.63039209294923	0.150821457668351	0.999692817443324	-4.55988122269288	7.91295847012396	7.87548073427184	7.37385695479327	6.28023345059804	7.57044592903906	7.45436100932908	NA
**TC1100004195.mm.2**	Tepsin	TEPSIN, adaptor related protein complex 4 accessory protein	0.588686924287701	7.19010279218469	1.6298915557163	0.150927740951084	0.999692817443324	-4.55991039441597	7.2442072712929	6.94787545163403	7.31834600193493	7.74460793744337	6.64847172058091	7.23710837022199	NA
**TC0900000989.mm.2**	Rab27a	RAB27A, member RAS oncogene family	0.867719121827447	6.94917367833767	1.62969982339385	0.150968471874015	0.999692817443324	-4.55992156907541	7.34898904742975	7.12000646274795	7.64207726937492	6.83866513237668	7.05938796417238	5.68591619392432	NA
**TC0900001288.mm.2**	Ky	kyphoscoliosis peptidase	-0.595670792435909	5.51156434985654	-1.62941735698773	0.151028496982398	0.999692817443324	-4.55993803229759	4.53047034087066	4.53019159996098	5.29905764957305	6.10963363714483	6.29798590298726	6.30204696860244	NA
**TC0500003051.mm.2**	Coro1c	coronin, actin binding protein 1C	0.63916501304193	8.97668143623739	1.62800268078593	0.151329460821304	0.999692817443324	-4.56002049117487	9.68425336007116	9.50199638386344	9.33702310496653	8.63906134482027	8.66522224178146	8.03253218192148	NA
**TC1300002691.mm.2**	Gzma	granzyme A	-0.553269290337091	5.36641136605663	-1.62718374612168	0.151503943623611	0.999692817443324	-4.56006823002468	4.32923255910633	4.44097504224893	5.46230956539416	5.69855144176102	5.83502242742873	6.43237716040063	NA
**TC1600000989.mm.2**	Eva1c	eva-1 homolog C (C. elegans)	0.492141563022216	4.93908100297884	1.62694317587822	0.151555235866881	0.999692817443324	-4.56008225443389	4.64786886912654	4.72352814608063	5.15421956894746	5.4694664954077	4.86959950430669	4.76980343400404	NA
**TC0900002328.mm.2**	Chrnb4	cholinergic receptor, nicotinic, beta polypeptide 4	0.775704717398254	5.28372197303399	1.62678334469619	0.151589322734094	0.999692817443324	-4.56009157219788	5.263587452681	5.06557536363353	5.06908924558039	6.39319998276259	4.8231273609367	5.08775243260977	NA
**TC1700000489.mm.2**	Pnpla1	patatin-like phospholipase domain containing 1	0.629249353418231	5.76291568129647	1.62654721983733	0.151639693875367	0.999692817443324	-4.56010533793083	5.42239548008356	5.45875524964726	5.80309134354355	6.67432968889869	5.44923130153771	5.76969102406806	NA
**TC0X00000649.mm.2**	Mtm1	X-linked myotubular myopathy gene 1	-0.629380757182881	5.62123568124835	-1.62574316664415	0.151811336696277	0.999692817443324	-4.56015221515498	5.01843014073433	4.87664872454914	5.20357020173545	6.08321665218499	6.13870132132902	6.40684704695718	NA
**TC1100001389.mm.2**	Gm525	predicted gene 525	0.62725629480354	6.02348117279561	1.62552236956671	0.151858502850189	0.999692817443324	-4.56016508844531	5.65241417636592	5.43088405747204	6.16018953375785	6.99586116251353	6.11347158826149	5.78806651840282	NA
**TC0200003298.mm.2**	Uck1	uridine-cytidine kinase 1	0.505682720019011	7.34908168784447	1.62521058801868	0.151925128508455	0.999692817443324	-4.56018326688353	7.70731616350588	7.80244590791954	7.23640270806365	7.56164403977605	6.76244376308469	7.02423754471699	NA
**TC0500001350.mm.2**	Oas1f	2'-5' oligoadenylate synthetase 1F	0.703592234856759	6.28323955727417	1.62517116062631	0.151933555851622	0.999692817443324	-4.56018556573412	5.22553053270814	5.89640918638715	6.70924836543732	7.28309268169432	5.91526221152568	6.66989436589244	NA
**TC1100002697.mm.2**	N4bp3	NEDD4 binding protein 3	-0.569139889692456	5.68539608718469	-1.62510106822383	0.151948538729092	0.999692817443324	-4.56018965255563	5.77313708805222	5.63904919382974	5.05794580775447	5.72300942316617	5.84192705416339	6.07730795614217	NA
**TC1400002054.mm.2**	Slc7a7	solute carrier family 7 (cationic amino acid transporter, y+ system), member 7	0.889497324558473	5.09311018861113	1.62489050661493	0.151993556563196	0.999692817443324	-4.56020192974984	5.38791091344348	6.15684204219086	5.38142319483703	5.01502821773766	3.58989922579713	5.02755753766061	NA
**TC0900002944.mm.2**	Ppp2r3a	protein phosphatase 2, regulatory subunit B'', alpha	0.51598381639531	6.11699559021345	1.62475865727021	0.152021752228797	0.999692817443324	-4.56020961758854	7.36796856068014	7.18239566478	5.9727287827013	5.61905969160429	5.41758385260197	5.14223698891299	NA
**TC0200003214.mm.2**	Sardh	sarcosine dehydrogenase	-0.719211134420045	5.64024350437396	-1.62187990064133	0.152638602180677	0.999692817443324	-4.56037749284318	5.88866781954907	5.64698066717335	5.04126827490956	5.39242686043106	5.36914428505888	6.50297311912183	NA
**TC0900001254.mm.2**	Dzip1l	DAZ interacting protein 1-like	0.544623216391551	6.10389144465139	1.62181435119233	0.152652675415201	0.999692817443324	-4.56038131584612	6.2114098294266	6.16205348104445	6.02957252864641	6.6399933664638	5.84831015246784	5.73200930985927	NA
**TC0800003230.mm.2**	Zfp882	zinc finger protein 882	-0.470500954448217	6.25510165766497	-1.62116695679673	0.152791734613809	0.999692817443324	-4.56041907458296	6.29078978009226	6.31875327462605	5.92877094763923	6.06126154354829	6.49373278143576	6.4373016186482	NA
**TC0100000627.mm.2**	Pnkd	paroxysmal nonkinesiogenic dyskinesia	0.537854012851289	7.5944672807815	1.62109455350466	0.152807294160708	0.999692817443324	-4.56042329757119	7.4029901760317	7.55751694162772	8.15114454724503	7.68985774912104	7.55392056203772	7.21137370862577	NA
**TC1600001334.mm.2**	Lamp3	lysosomal-associated membrane protein 3	0.715092168110765	5.29212547398252	1.62078372651649	0.152874108243837	0.999692817443324	-4.56044142712865	4.57733382426616	5.27950524123472	5.47239014390301	6.19065891340487	4.78748112084532	5.44538360024103	NA
**TC0100001600.mm.2**	Slamf1	signaling lymphocytic activation molecule family member 1	-0.54519752924989	5.00952280755725	-1.62057973027139	0.152917973443615	0.999692817443324	-4.56045332584047	4.55252410157415	4.25316045166186	5.0364544916535	5.04407412515036	5.30970163494513	5.86122204035851	NA
**TC1400002848.mm.2**	Gm10406	predicted gene 10406	0.622978038689217	6.26834737964547	1.62016749090854	0.153006653368043	0.999692817443324	-4.56047737159047	6.80467447005933	6.03586074181698	6.72252898099365	6.28522359069382	6.00076620729258	5.76103028701645	NA
**TC0100003576.mm.2**	Fcrl6	Fc receptor-like 6	-0.514655179487174	4.25699984409888	-1.62015001929887	0.153010412891724	0.999692817443324	-4.5604783907203	3.05389023333313	3.21177548815665	4.34207560679546	4.78143588526911	5.00923512395132	5.1435867270876	NA
**TC0100003327.mm.2**	Tex35	testis expressed 35	0.588000670262135	5.0081440144884	1.62003157112348	0.153035902755779	0.999692817443324	-4.5604852999147	5.14944984496137	4.5094324647093	5.16251001129468	5.62048154759733	4.74610871983183	4.86088149853591	NA
**TC0600001016.mm.2**	Gp9	glycoprotein 9 (platelet)	0.68871745454214	7.24018573782573	1.62002437994438	0.153037450415636	0.999692817443324	-4.56048571938517	6.58955973862849	6.67041113234202	7.33572808718163	8.44356114535244	7.11598059994478	7.28587372350501	NA
**TC0600001879.mm.2**	2610001J05Rik	RIKEN cDNA 2610001J05 gene	0.721725645098757	6.13073043160747	1.61929597499072	0.153194291795759	0.999692817443324	-4.56052820944699	6.9865147603499	6.43589365036004	6.48639482584381	5.91631790872238	5.94464987873776	5.01461156563092	NA
**TC1600002136.mm.2**	Brwd1	bromodomain and WD repeat domain containing 1	-0.646904635352209	5.73221157572876	-1.61928603910287	0.153196432257854	0.999692817443324	-4.56052878905498	6.4982453911032	6.40797965828363	5.47121701655548	4.62540055058518	5.95373577405135	5.43669106379373	NA
**TC0400003323.mm.2**	Tex38	testis expressed 38	-0.586292533203451	5.40282804421056	-1.61897515134428	0.153263420268253	0.999692817443324	-4.56054692486781	4.62226443331357	5.2725192800574	5.13066298501093	5.54413675773181	5.83550169663369	6.01188311251595	NA
**TC1100003561.mm.2**	Abcc3	ATP-binding cassette, sub-family C (CFTR/MRP), member 3	0.490747757982206	5.94383906416099	1.61890201054002	0.153279184180817	0.999692817443324	-4.56055119164516	6.09221770351056	6.24340966790982	5.9222862743158	6.23216499043918	5.60363568605613	5.56932006273443	NA
**TC0400001924.mm.2**	Mthfr	methylenetetrahydrofolate reductase	0.486696869772528	7.20042411117521	1.61864319999779	0.15333497748049	0.999692817443324	-4.56056628994762	6.7243185390395	6.98107024659362	7.5392039968563	7.69607081362531	7.07976979843425	7.18211127250231	NA
**TC0100003092.mm.2**	Snrpe	small nuclear ribonucleoprotein E	0.735272706178714	6.92671428665171	1.61838803144431	0.153390004444881	0.999692817443324	-4.56058117609955	7.21099024075343	7.1896415386418	6.75977975293221	7.55531992350402	6.91207125521546	5.93248300886335	NA
**TC0900000821.mm.2**	Megf11	multiple EGF-like-domains 11	0.614017265485878	5.23696228037956	1.61802582223755	0.153468146716962	0.999692817443324	-4.56060230737607	5.51200998871932	5.3448294395283	5.07204246412708	5.82444192837367	5.05576092113458	4.61268894039441	NA
**TC1300000547.mm.2**	G630093K05Rik	RIKEN cDNA G630093K05 gene	-0.546738245085448	5.98091455393555	-1.61795943760053	0.153482472480996	0.999692817443324	-4.56060618032192	5.40542979177451	5.52211957867234	5.83342758629523	6.09880314520253	6.22877838493057	6.7969288367381	NA
**TC0100002356.mm.2**	Rftn2	raftlin family member 2	-0.479222863393799	7.59250723446673	-1.617652061077	0.153548820619251	0.999692817443324	-4.56062411324735	7.94632460982939	7.99525809149145	7.28748332591961	7.04002416342635	7.61418907302601	7.67176414310755	NA
**TC1000000355.mm.2**	Ddo	D-aspartate oxidase	-0.517181084764454	5.07953490280211	-1.61700010562177	0.153689636856853	0.999692817443324	-4.56066215103891	5.26181041435208	5.19812514009968	4.72838692589048	4.76306892052553	5.01359078353606	5.51222723240886	NA
**TC0600001001.mm.2**	Nfu1	NFU1 iron-sulfur cluster scaffold	-0.63128020860997	7.23124120586923	-1.61663070909025	0.153769477169793	0.999692817443324	-4.56068370405953	8.45743683755598	8.11719693284513	6.80738882566559	5.96773769813157	7.15900436750665	6.87868257351045	NA
**TC1100000604.mm.2**	Gdf9	growth differentiation factor 9	-0.655024253501311	6.13323797720614	-1.61645564682099	0.153807328313174	0.999692817443324	-4.56069391856704	5.41652203951622	5.19765901630519	5.74620756183779	6.69139158836862	6.70886701399618	7.03878064321285	NA
**TC0100003320.mm.2**	Tor3a	torsin family 3, member A	-0.692771574241619	6.67361818668952	-1.61498919926324	0.154124742564775	0.999692817443324	-4.56077948827771	6.01709776213649	7.05994869227567	6.37052879303254	6.41903096558831	6.85574373286619	7.3193591742379	NA
**TC0300002648.mm.2**	Lrig2	leucine-rich repeats and immunoglobulin-like domains 2	-0.734315472594357	5.22696742581824	-1.61473716179115	0.154179358631301	0.999692817443324	-4.56079419610423	5.75477984407158	5.53377252544038	5.27280811419909	4.02950250590531	5.41444941672962	5.3564921485635	NA
**TC0100000428.mm.2**	Aox3	aldehyde oxidase 3	0.569554231737821	5.04745325149172	1.61449299461074	0.154232286649903	0.999692817443324	-4.5608084449358	4.4238500449071	5.02334439566003	5.28255266678021	5.70576409914921	4.93351858293954	4.91568971951424	NA
**TC0500000589.mm.2**	Pgm1	phosphoglucomutase 1	-0.587302356509372	5.9069495818964	-1.61439903652369	0.154252658476994	0.999692817443324	-4.56081392810907	5.88176179108262	6.29331613668961	5.8496394421227	5.19636798317101	6.21540802281383	6.00520411549864	NA
**TC0500003061.mm.2**	Mmab	methylmalonic aciduria (cobalamin deficiency) cblB type homolog (human)	-0.48153021283764	6.77752895656036	-1.61425523406207	0.154283842394327	0.999692817443324	-4.5608223201622	6.98295023987294	7.19466703545933	6.39615774577308	6.36609027340424	6.76658916690891	6.95871927794368	NA
**TC0700004431.mm.2**	Adam12	a disintegrin and metallopeptidase domain 12 (meltrin alpha)	-0.5409672273921	5.73560889246543	-1.61394144613455	0.154351908746669	0.999692817443324	-4.56084063259389	5.5589231764085	5.21306589485694	5.47673527649432	5.80312963787716	5.98277551071684	6.37902385843884	NA
**TC1700001677.mm.2**	Mcrip2	MAPK regulated corepressor interacting protein 2	-0.553066979891977	6.13837621474855	-1.61378384204795	0.154386106658135	0.999692817443324	-4.5608498304237	5.88180548577011	5.43194267479815	6.30884882688271	5.89633875718683	6.51756809024964	6.79375345360385	NA
**TC1600001605.mm.2**	Adprh	ADP-ribosylarginine hydrolase	-0.475326586563587	6.9507873372709	-1.61342803421545	0.15446333835589	0.999692817443324	-4.56087059591507	7.67704885784208	7.45872508796077	6.37024838804748	6.43890006430022	6.85693568249505	6.90286594297981	NA
**TC0800003226.mm.2**	Borcs8	BLOC-1 related complex subunit 8	-0.610720415015028	6.87387821766299	-1.61321237930411	0.154510166194556	0.999692817443324	-4.56088318215058	7.14288983075151	6.74999077599832	6.59710756297931	6.46736637161972	6.75235556134308	7.533559203286	NA
**TC0700000287.mm.2**	Six5	sine oculis-related homeobox 5	-0.794290251235468	5.79746427228565	-1.61236383274655	0.154694551837738	0.999692817443324	-4.56093270781945	4.8652963403623	4.95436507838565	5.13252527568088	6.55574658056663	6.55794826718004	6.71890409153841	NA
**TC0200001976.mm.2**	Ebf4	early B cell factor 4	0.538983952153963	5.77850149088511	1.61101150716573	0.154988835913687	0.999692817443324	-4.56101164348952	6.30363580808551	5.81988604385044	5.78641667503747	6.02631082380385	5.51421893707084	5.22054065746255	NA
**TC1300000904.mm.2**	Erap1	endoplasmic reticulum aminopeptidase 1	0.585505661100314	5.60459654304942	1.61082215468675	0.155030083684055	0.999692817443324	-4.56102269671554	6.1794953316363	6.17993024001007	5.25659507088639	5.96298743353898	5.21033588886316	4.83823529336158	NA
**TC1100002195.mm.2**	Igfbp3	insulin-like growth factor binding protein 3	-0.504185806342503	5.95927819259976	-1.61064426837472	0.15506884314885	0.999692817443324	-4.56103308076525	5.47678261549698	5.92198660823974	5.8334574361371	5.84080672345132	6.33363542986328	6.34900034241014	NA
**TC0500000308.mm.2**	Abhd1	abhydrolase domain containing 1	0.687051931244707	6.63733429053394	1.61054460104544	0.155090563557266	0.999692817443324	-4.56103889887229	7.12651561964868	6.3485141726498	6.60865194084808	7.25288796584922	6.49790866463953	5.98952737956835	NA
**TC0100000064.mm.2**	Sulf1	sulfatase 1	0.670315058848322	6.79601852082569	1.61007518166569	0.155192902295162	0.999692817443324	-4.56106630195594	7.027243243441	7.27795208960386	7.13990190924058	6.76587104556237	6.7736614286517	5.79148140845461	NA

**TC1100003641.mm.2**	Npepps	aminopeptidase puromycin sensitive	0.543564844987203	6.33094875080334	1.60943672540815	0.155332195257288	0.999692817443324	-4.56110357441752	7.10383146126679	6.66726584978148	6.0989032736361	6.551959168237	5.73338392648047	5.83034882541822	NA
**TC1100002702.mm.2**	Jade2	jade family PHD finger 2	0.543087739537797	7.34396559390061	1.60902529061358	0.155422021095508	0.999692817443324	-4.56112759455099	7.82186630053568	7.72257343971742	7.53439666661147	7.26836798450161	7.14237021731296	6.57421895472453	NA
**TC0700004515.mm.2**	Ifitm3	interferon induced transmembrane protein 3	-0.737775851064661	8.27463750060855	-1.60872843071476	0.155486862986917	0.999692817443324	-4.56114492611207	7.55588005997374	8.38458552940899	8.5104194887583	7.60548436731134	8.98118210118295	8.61027345701602	NA
**TC0900002740.mm.2**	Filip1	filamin A interacting protein 1	0.675603079538785	5.4445075753144	1.60797254891626	0.155652082383387	0.999692817443324	-4.56118905850032	5.97071062217025	5.23708935611629	5.83090390901389	5.57432190732484	4.63821871512927	5.41580094213189	NA
**TC0200005130.mm.2**	Tnnc2	troponin C2, fast	-0.642186669820762	5.92073138298463	-1.60692502213132	0.155881322826027	0.999692817443324	-4.5612502228441	5.66924191942605	5.98920918253058	5.9113581213723	5.37942380678251	5.89803807819675	6.67711718959958	NA
**TC0800000941.mm.2**	Samd1	sterile alpha motif domain containing 1	0.61028995021279	6.43945233805857	1.60677320824755	0.155914572134413	0.999692817443324	-4.56125908754913	6.16745819119476	6.67830375852385	6.47839545161741	7.02737053791179	6.38307684675965	5.90210924234396	NA
**TC0300001486.mm.2**	Syde2	synapse defective 1, Rho GTPase, homolog 2 (C. elegans)	-0.513895593276232	5.44176973418575	-1.60557546564427	0.156177128686702	0.999692817443324	-4.56132902957905	5.44196769388739	5.48789901084194	5.36690197430573	4.97957828261063	5.84811466413446	5.52615677933436	NA
**TC1200000324.mm.2**	Crppa	CDP-L-ribitol pyrophosphorylase A	-0.636139943687567	5.56954287773521	-1.6054860796259	0.156196739657788	0.999692817443324	-4.56133424951497	6.13225981736845	5.64624811658046	5.50928160208139	4.67395312046223	5.80429353696846	5.65122107295029	NA
**TC1000001131.mm.2**	Lta4h	leukotriene A4 hydrolase	-0.490591070668323	6.72394931591535	-1.60540216951572	0.156215151346925	0.999692817443324	-4.56133914970212	7.3833117140434	7.25935229202491	6.06915279535629	6.29077207868729	6.53383022428279	6.80727679109743	NA
**TC0900002166.mm.2**	Foxr1	forkhead box R1	0.652828755963742	5.35547024803747	1.60513372795674	0.156274066962488	0.999692817443324	-4.56135482637178	4.61287716617858	5.11790436402399	5.48550023083833	6.36834850413655	5.18576709625919	5.3624241267882	NA
**TC0300000930.mm.2**	Hist2h2be	histone cluster 2, H2be	-0.571523737243171	6.12086298123753	-1.60416991235599	0.156485770909528	0.999692817443324	-4.5614111146287	6.43565015340893	6.02874976443969	6.05409234794934	5.50477289959579	6.19017841984168	6.51173430218978	NA
**TC0300002257.mm.2**	Fam160a1	family with sequence similarity 160, member A1	0.615995460663743	5.30864779340221	1.60370927564977	0.156587046091373	0.999692817443324	-4.5614380179049	4.71033548750232	5.17729033530665	5.60408871926625	5.99403721019964	4.84187661489512	5.52425839324328	NA
**TC0900001367.mm.2**	Cacna2d2	calcium channel, voltage-dependent, alpha 2/delta subunit 2	0.819982003610788	7.75172339363113	1.60368673531297	0.156592003375054	0.999692817443324	-4.5614393343863	8.46643402136278	8.34846895780015	8.26047470641797	7.40722598850474	6.38962440918934	7.6381122785118	NA
**TC1400000950.mm.2**	Gm5464	predicted gene 5464	-0.636546624196706	5.29844989800273	-1.60326560389532	0.156684649773546	0.999692817443324	-4.56146393120949	4.87822073118589	5.06675878010747	4.87572445588949	5.41058885827533	5.37846383832959	6.18094272422863	NA
**TC1500000726.mm.2**	L3mbtl2	L3MBTL2 polycomb repressive complex 1 subunit	-0.583799999366984	6.54190611299074	-1.60291263421136	0.156762340786448	0.999692817443324	-4.56148454752538	6.78762233834584	6.64299748442297	6.47699173349912	5.84961669472171	6.98677109187698	6.50743733507781	NA
**TC0300002460.mm.2**	Zfp687	zinc finger protein 687	0.546788787939668	5.90829615408949	1.60223594352138	0.156911386496479	0.999692817443324	-4.56152407327569	6.61487091006766	6.33403142153206	6.16960613110609	5.62761995330219	5.22586911774554	5.47777939078341	NA
**TC0600000711.mm.2**	Abcg2	ATP binding cassette subfamily G member 2 (Junior blood group)	-0.539847467932315	5.78142175525953	-1.60205937436572	0.156950299009428	0.999692817443324	-4.56153438706366	5.90241482942412	6.12382156734651	5.64820807560907	5.14309152385189	6.08915937368569	5.7818351616399	NA
**TC0900001469.mm.2**	Prss42	protease, serine 42	0.582421754797819	5.10563902180915	1.60109794419864	0.157162339669167	0.999692817443324	-4.56159054859456	4.61997137508177	4.61938760567432	5.91275524885948	5.36690408098773	5.34986651893256	4.76494930131901	NA
**TC1600000726.mm.2**	Cblb	Casitas B-lineage lymphoma b	0.67915464981446	7.35735815989304	1.59980794924197	0.157447268311411	0.999692817443324	-4.56166590917905	7.8527144193374	7.84694638142818	7.94537962130686	6.95601910780394	7.03480007823732	6.50828935124455	NA
**TC0900003266.mm.2**	Cck	cholecystokinin	-0.912295528342423	9.04527837735426	-1.59966368029334	0.157479164058755	0.999692817443324	-4.56167433769859	9.24252392798959	10.0111036619336	8.5673308190524	8.02939498970635	9.95919887781378	8.46211798762983	NA
**TC1000002056.mm.2**	Cep57l1	centrosomal protein 57-like 1	-0.528439984116358	5.88544997459727	-1.59861871416226	0.157710372452017	0.999692817443324	-4.56173538956094	5.83423468438422	6.02148348989017	5.53197662481353	5.66807422772473	5.8592118761945	6.39771894457647	NA
**TC0100000725.mm.2**	Fbxo36	F-box protein 36	0.559744194948774	6.25974898182127	1.59851849722879	0.157732563162264	0.999692817443324	-4.56174124494442	6.48784026083242	6.59024993903077	6.71372252137883	6.08622351910215	6.00678662683408	5.67367102374936	NA
**TC1000001572.mm.2**	Zbtb39	zinc finger and BTB domain containing 39	0.509208979925194	5.8072341633785	1.59760862402344	0.157934167980035	0.999692817443324	-4.56179440806738	5.95535540160281	6.04754321823842	5.88684839834853	6.04261376179154	5.68575931783944	5.22528488245025	NA
**TC0200003954.mm.2**	Olfr1087	olfactory receptor 1087	-0.663591914895961	4.61500520140469	-1.59690468097058	0.158090310313796	0.999692817443324	-4.56183554118095	4.07122722124651	4.20251775225556	4.01017527867759	5.03437592388948	5.03346996106518	5.33826507129381	NA
**TC1700002244.mm.2**	Rftn1	raftlin lipid raft linker 1	0.502905947302567	6.12060432162465	1.5965821268842	0.158161904902523	0.999692817443324	-4.56185438947604	6.7065835802723	6.25721195636867	6.20511242152525	6.17770872233079	5.63463984992504	5.74236939932586	NA
**TC0400002542.mm.2**	Olfr159	olfactory receptor 159	0.853014360834171	5.00423213203621	1.59622917430937	0.158240281751361	0.999692817443324	-4.56187501457387	4.12763182149513	4.1360835740217	5.12196446950594	6.61188858967845	4.70119363524426	5.32663070227179	NA
**TC1000001075.mm.2**	Washc3	WASH complex subunit 3	-1.00245088202245	6.65038709581384	-1.59610690121905	0.158267442307284	0.999692817443324	-4.5618821598278	7.38329077120763	7.39836377422197	6.53422067718591	5.02366245551838	7.43894292475927	6.12384197198991	NA
**TC1600000834.mm.2**	Robo1	roundabout guidance receptor 1	-0.520967798564252	5.69222104985079	-1.59586124954855	0.158322022243931	0.999692817443324	-4.56189651511877	6.11035441170201	6.14252515178787	5.42479267015249	5.00446289909069	5.91665068026495	5.55454048610673	NA
**TC0100001381.mm.2**	Rgs16	regulator of G-protein signaling 16	0.801494640479755	6.38051830232646	1.59545402230119	0.15841254075469	0.999692817443324	-4.56192031303009	7.13581207336358	6.26484677972045	7.18366174442053	6.0590583764966	6.02029385286656	5.61943698709105	NA
**TC0X00000076.mm.2**	Timm17b	translocase of inner mitochondrial membrane 17b	-0.530318612157546	7.49860619765069	-1.59542049578631	0.158419995200954	0.999692817443324	-4.56192227231225	7.80537622263402	7.35499269225887	7.37245061394225	7.01286490940584	7.80531356870442	7.64063917895876	NA
**TC0600001867.mm.2**	Thsd7a	thrombospondin, type I, domain containing 7A	-0.642963749337523	7.65026624858605	-1.59411939345156	0.158709543627996	0.999692817443324	-4.56199831190686	8.34705411264758	8.34908549173534	7.33238999312365	6.62737520110552	7.25766299766628	7.98802969523793	NA
**TC1700002798.mm.2**	Rab26	RAB26, member RAS oncogene family	-0.472460757766362	7.40660404726639	-1.59393693260179	0.158750188407078	0.999692817443324	-4.56200897590113	6.91248276789718	7.06955737799279	7.40191503529231	7.35441627579553	7.74904334387294	7.95220948274762	NA
**TC1700002549.mm.2**	Fez2	fasciculation and elongation protein zeta 2 (zygin II)	-0.951714177701454	6.43809469641945	-1.59388072580973	0.158762710943239	0.999692817443324	-4.56201226095513	7.10767325061365	7.23805679839273	6.53108130955964	4.65862357749405	6.55920087007729	6.53393237237931	NA
**TC1300002697.mm.2**	Snx18	sorting nexin 18	0.475583806153325	6.66577838875733	1.59359976875978	0.158825320421792	0.999692817443324	-4.56202868191686	6.52129788954755	6.77150829475514	6.85094207950888	6.9755738007651	6.40045987025757	6.47488839770975	NA
**TSUnmapped00000049.mm.2**	Aopep	aminopeptidase O	0.592176007020191	5.30466991849876	1.59259974046917	0.159048358823194	0.999692817443324	-4.56208713260695	4.74363995000047	4.89417581142695	5.95339143626168	5.73388644554109	4.84973320728672	5.65319266047567	NA
**TC1900000705.mm.2**	Sfxn2	sideroflexin 2	0.885177373482128	4.93429418431389	1.59250510934184	0.159069479852003	0.999692817443324	-4.56209266390789	4.9997747223206	4.98780403393574	4.94208121218165	5.75218933611396	5.21285979214055	3.7110560091908	NA
**TC1800001253.mm.2**	Plac8l1	PLAC8-like 1	-0.562451289857281	5.49745975394015	-1.59189231891837	0.159206314396744	0.999692817443324	-4.56212848307461	4.64957833782377	4.96976438363404	5.44220552748927	5.67805108374499	6.4330793450787	5.81207984587012	NA
**TC1100002510.mm.2**	Fbll1	fibrillarin-like 1	0.495302502131655	7.47758108573057	1.59184852812825	0.159216097005511	0.999692817443324	-4.5621310428141	7.50110970128085	7.52236595041271	7.68094888302919	7.73535905044739	7.00074473005203	7.42495819916123	NA
**TC1200001432.mm.2**	Mycn	v-myc avian myelocytomatosis viral related oncogene, neuroblastoma derived	-0.472485159680945	5.65445867265867	-1.59163278090076	0.159264301939208	0.999692817443324	-4.56214365417481	5.12080939811613	5.23491501978354	5.58162236179302	5.73140628755221	6.2240532539653	6.03394571474182	NA
**TC1000002519.mm.2**	Lmnb2	lamin B2	-0.517369824340571	5.94181163044991	-1.59120495367102	0.159359933022188	0.999692817443324	-4.56216866306355	6.15614523242734	6.15944155582398	5.49583046294859	5.6544412099349	5.83139083288379	6.35362048868084	NA
**TC1500002052.mm.2**	Sco2/Tymp	SCO2 cytochrome c oxidase assembly protein/thymidine phosphorylase	-0.501298625073506	7.27249695515556	-1.59118533515113	0.159364319592227	0.999692817443324	-4.56216980989244	7.16906305322439	7.13915327572282	7.11619307826579	7.04589099765377	7.35379848580995	7.81088284025663	NA
**TC0700000753.mm.2**	Zfp936	zinc finger protein 936	0.586006328087921	4.88379037651208	1.59110723315784	0.159381783802046	0.999692817443324	-4.56217437547191	4.94457069407942	4.55719555199106	5.37432852619237	5.11216580839654	4.99783246697156	4.31664921144151	NA
**TC0800001360.mm.2**	Vat1l	vesicle amine transport protein 1 like	0.51510240805594	8.73640581520742	1.59071424169463	0.159469687043258	0.999692817443324	-4.56219734878545	9.43740343418048	9.06542196031797	8.84598159435852	8.62692556207046	8.37984109524788	8.06286124506922	NA
**TC0500002119.mm.2**	Gm1979	predicted gene 1979	0.545048573376774	5.96938539814632	1.59038607758851	0.159543124801101	0.999692817443324	-4.56221653290565	4.895590375711	5.13741772241015	6.52555452305871	6.91114619569644	6.41909639921578	5.92750717278583	NA
**TC0500002320.mm.2**	Prom1	prominin 1	-0.493344748459127	5.0690706773481	-1.58923563708009	0.159800825727572	0.999692817443324	-4.56228378966641	5.25926072447071	5.09206732256689	4.95555313213682	4.58265012792956	5.31392598381665	5.21096677316799	NA
**TC1300000514.mm.2**	Jarid2	jumonji, AT rich interactive domain 2	0.768268049614425	7.55631975426547	1.58910555385672	0.159829989233189	0.999692817443324	-4.56229139487527	7.40294332379863	7.29963439753042	8.51403883295671	7.57189961878961	7.78608185153004	6.76332050098742	NA
**TC1100004107.mm.2**	Acox1	acyl-Coenzyme A oxidase 1, palmitoyl	0.917374990864944	7.05296298631008	1.58909303836939	0.159832795358291	0.999692817443324	-4.56229212658634	8.39824728959508	8.52859713332977	7.70111165914635	5.91173007918639	5.92658129922299	5.85151045737986	NA
**TC0900002640.mm.2**	Tex9	testis expressed gene 9	0.619289557383622	5.89664350206489	1.58904517448043	0.159843527453895	0.999692817443324	-4.5622949249277	6.47852945881957	6.381569556442	5.72035735590404	6.15881320004346	5.73563189390997	4.90495954727028	NA
**TC0100001063.mm.2**	Tfcp2l1	transcription factor CP2-like 1	0.520849596927261	7.33299852823655	1.58842416968935	0.159982831265198	0.999692817443324	-4.56233123248558	6.95585745108771	6.95957204277649	7.57732575300907	7.98480468169575	7.45707851657507	7.06335272427523	NA
**TC1400002740.mm.2**	Dock9	dedicator of cytokinesis 9	-0.463040529275732	6.59199129987996	-1.5879623095756	0.160086509709957	0.999692817443324	-4.56235823645933	7.09890284010105	6.97139106648755	6.10313464432622	6.17465177274364	6.65292855439367	6.55093892122765	NA
**TC1700002293.mm.2**	Slc5a7	solute carrier family 5 (choline transporter), member 7	0.721870957348774	5.66409524646631	1.58738575549516	0.16021602327956	0.999692817443324	-4.56239194746475	7.29311060200925	6.50936230831301	5.65427627845353	5.15864396313306	5.11413934196493	4.25503898492411	NA
**TC1100003434.mm.2**	Brip1	BRCA1 interacting protein C-terminal helicase 1	0.643274479600453	6.50445013592832	1.58725316628649	0.160245821225893	0.999692817443324	-4.56239970010545	6.16989486931813	6.45298415477644	6.51257029785328	7.33261507748487	6.55228971262392	6.00634670351332	NA
**TC0700002632.mm.2**	Lipe	lipase, hormone sensitive	0.669531355123585	6.02205339627529	1.58709448945423	0.160281488919278	0.999692817443324	-4.56240897820492	6.22622616890109	6.25229329685809	5.72450115311401	6.77193065795587	5.7584805915213	5.3988885093014	NA
**TC0100000822.mm.2**	Inpp5d	inositol polyphosphate-5-phosphatase D	0.501358544153885	6.47193864455167	1.5856179891526	0.160613737358869	0.999692817443324	-4.56249531611021	6.72344674678248	7.15741168981509	6.47744301656427	6.49930224294584	5.89314301517549	6.08088515602686	NA
**TC0800001617.mm.2**	Clec4g	C-type lectin domain family 4, member g	0.809865071340058	5.20447485780028	1.58552021845816	0.160635760962566	0.999692817443324	-4.56250103350496	4.27077521614789	4.57028683729744	5.49384219173377	6.50891642628447	4.64982208810292	5.7332063872352	NA
**TC0300002722.mm.2**	Gstm7	glutathione S-transferase, mu 7	-0.538312421315061	7.14492831143007	-1.58463789801116	0.160834638785308	0.999692817443324	-4.56255263105862	8.37105282803318	8.09778680818669	6.62239120313342	6.0396614917318	6.91454552442452	6.82413201307083	NA
**TC1300000637.mm.2**	Eif4e1b	eukaryotic translation initiation factor 4E family member 1B	0.56087544813608	5.93849392070139	1.58296709507487	0.161211876685367	0.999692817443324	-4.56265034630306	5.4506800138316	5.27155632854024	6.178884312597	6.83635472645733	6.09548931928257	5.7979988234996	NA
**TC1100003437.mm.2**	Ints2	integrator complex subunit 2	0.500945759343545	5.90114853124635	1.5829074616325	0.161225356208996	0.999692817443324	-4.56265383409076	5.32925574292545	5.3288672011568	6.20056460001197	6.6747652810295	5.91756330827125	5.95587505408313	NA
**TC0200000040.mm.2**	Ucma	upper zone of growth plate and cartilage matrix associated	-0.515143653825117	7.29288370138243	-1.58279669910548	0.161250395741797	0.999692817443324	-4.56266031230455	5.87578626929117	6.13516451246078	7.45742809153406	7.90060396791214	8.09812029812861	8.29019906896783	NA
**TC1900000206.mm.2**	Vwce	von Willebrand factor C and EGF domains	0.760546935245739	4.96242312482024	1.58219019269279	0.161387570360959	0.999692817443324	-4.56269578607073	4.52784800635071	4.05352841858418	5.08501509626806	6.27211300097093	4.65541076008181	5.18062346666571	NA
**TC0700000561.mm.2**	Tyrobp	TYRO protein tyrosine kinase binding protein	-0.796269251187369	6.02887177377131	-1.58216355224879	0.16139359818753	0.999692817443324	-4.56269734426538	5.88220038165791	6.50167410863765	5.08197185045508	6.01643697452371	5.8609919005532	6.82995542680032	NA
**TC0600002329.mm.2**	Neurod6	neurogenic differentiation 6	-0.916656749978796	7.74246088857472	-1.58153084552719	0.161536820325048	0.999692817443324	-4.56273435190256	7.95555598934842	9.09501238979639	7.53865262252497	6.246789103648	8.07935722224205	7.5393980038885	NA
**TC1200002270.mm.2**	Ifi27l2b	interferon, alpha-inducible protein 27 like 2B	0.705058110453026	5.65377565155294	1.58066928584088	0.161732038384493	0.999692817443324	-4.56278474763982	5.21423273986226	5.58678257783616	5.63724303879351	6.62863436746911	5.12150159793492	5.73425958742165	NA
**TC0700004569.mm.2**	Mir675/H19	microRNA 675/H19, imprinted maternally expressed transcript	-0.894354921057316	5.29705213673152	-1.58041089090202	0.16179063041113	0.999692817443324	-4.56279986259921	4.6527886764671	4.85904648993281	4.32433537975191	5.91654852618537	6.36095244254861	5.6686413055033	NA
**TC1700001662.mm.2**	Sstr5	somatostatin receptor 5	0.503038197800817	5.65163389589501	1.58025152583284	0.161826776970986	0.999692817443324	-4.56280918486586	5.98883263988326	5.88934156686981	5.51943165003039	5.99942113207893	5.2390741909445	5.27370219556319	NA
**TC1800000169.mm.2**	4930426D05Rik	RIKEN cDNA 4930426D05 gene	0.691077362201267	5.8320396959368	1.57767434526758	0.162412375904092	0.999692817443324	-4.56295995255372	5.77825455227564	5.52277053995527	5.77029829524402	6.76638560865221	5.28208378469793	5.87244539479576	NA
**TC1700000048.mm.2**	Gtf2h5	general transcription factor IIH, polypeptide 5	-0.643610861153571	8.25294652601791	-1.57711914139035	0.162538791850681	0.999692817443324	-4.56299243551234	9.20353445057339	9.37515296579225	7.63060558335587	7.19527942536148	8.52055186241862	7.59255486860587	NA
**TC0900002052.mm.2**	Nrgn	neurogranin	-0.574707985042949	10.2642766871891	-1.57639974555	0.162702730368165	0.999692817443324	-4.56303452628871	10.3133573572897	10.665124779061	10.0364997395724	9.69238126877662	10.7575327170335	10.1207642614014	NA
**TC0100002899.mm.2**	Kdsr	3-ketodihydrosphingosine reductase	-0.530513746737374	6.71823530445006	-1.57565766476005	0.162872000846494	0.999692817443324	-4.56307794614036	7.72291627921424	7.4144713815863	6.28995503505167	5.76554330116086	6.63898206191342	6.47754376777385	NA
**TC1200001275.mm.2**	Btbd6	BTB (POZ) domain containing 6	0.577955465125155	6.49069686510308	1.57517317595365	0.162982603014178	0.999692817443324	-4.56310629502699	7.44897331060176	6.79419602169566	6.26024898906408	6.66821240522162	5.74977319556319	6.02277726847219	NA
**TC0900000126.mm.2**	Slc36a4	solute carrier family 36 (proton/amino acid symporter), member 4	0.670937394178146	7.48633299034738	1.57501230151672	0.163019344015239	0.999692817443324	-4.56311570844214	7.80738892887866	7.37034646068279	7.73755985546373	7.80350881497583	7.61843277642335	6.58076110565991	NA
**TC1300000432.mm.2**	Rreb1	ras responsive element binding protein 1	-0.586497315074432	6.2437687170085	-1.57499791049518	0.163022631059492	0.999692817443324	-4.56311655052328	6.48632235415227	6.68007288207615	6.19434364718402	5.36726757065283	6.36341394332115	6.37119190466456	NA
**TC1100000106.mm.2**	Ramp3	receptor (calcitonin) activity modifying protein 3	-0.546106711607166	6.76711009788459	-1.57387960442749	0.163278252712812	0.999692817443324	-4.5631819895437	6.94612163416558	6.88087272619506	6.57795976855682	6.26376663330946	6.65725493740784	7.27668488767277	NA
**TC0600000228.mm.2**	Fam71f1	family with sequence similarity 71, member F1	-0.493015005136463	4.65931730590959	-1.57385569394811	0.163283722253695	0.999692817443324	-4.56318338873869	3.84656259187489	4.26334774446718	4.63372873400238	4.79625301041888	5.20209604086004	5.21391571383415	NA
**TC0400000439.mm.2**	Creb3	cAMP responsive element binding protein 3	-0.556362703049443	8.54315181036376	-1.57366594527628	0.163327133499224	0.999692817443324	-4.56319449252976	9.31107518307531	9.16811977571497	8.25451534786539	7.57897990078133	8.60447542522137	8.34174522952423	NA
**TC1200001031.mm.2**	Ppp4r4	protein phosphatase 4, regulatory subunit 4	0.845597766506645	5.99946988898496	1.573477417882	0.163370276048504	0.999692817443324	-4.56320552496939	6.80851833655126	6.97759399574588	6.77623078698382	5.17472048032912	5.25157079714571	5.00818493715394	NA
**TC0800002644.mm.2**	Cbln1	cerebellin 1 precursor protein	0.557583855713348	6.4438877193073	1.57304785661024	0.163468616602652	0.999692817443324	-4.56323066290677	7.0089677466505	6.27121451583764	6.64045374169049	6.60870214070068	6.01993710013163	6.11405107083284	NA
**TC0800001872.mm.2**	Polb	polymerase (DNA directed), beta	-0.584606181177929	6.25359496909731	-1.57183137641589	0.163747409524294	0.999692817443324	-4.56330185458115	6.80449461640435	7.01596121359546	5.87809019617049	5.3878606149436	6.54631982183741	5.88884335163254	NA
**TC1000002671.mm.2**	Tmpo	thymopoietin	-0.491552492168271	5.78932776033285	-1.57181517161522	0.163751126345012	0.999692817443324	-4.56330280296135	6.16124659385738	6.08357111011999	5.500943594207	5.2530783426346	5.68927601764874	6.0478509035294	NA
**TC1100003245.mm.2**	Abr	active BCR-related gene	0.615555137477806	8.79660795926747	1.57137575157268	0.163851944234477	0.999692817443324	-4.56332852018127	9.62691487605789	9.57804781706762	8.99285348906653	8.41004417965093	7.70847961134574	8.4633077824161	NA
**TC0200004508.mm.2**	Shf	Src homology 2 domain containing F	0.631382351943178	6.61591663094605	1.57101862267522	0.163933924564393	0.999692817443324	-4.56334942173214	6.27778041149701	6.13434701252342	6.70017508142443	7.5728934513467	6.26362030606074	6.74668352282402	NA
**TC0X00001335.mm.2**	Tceal1	transcription elongation factor A (SII)-like 1	-0.820362833780827	6.75661885276946	-1.56942618113004	0.164299943484985	0.999692817443324	-4.56344262684264	7.86732280857172	7.31898604894419	6.66155701261598	5.19478228315363	6.75626399875941	6.74080096457186	NA
**TC0500000303.mm.2**	Agbl5	ATP/GTP binding protein-like 5	0.600444493829215	6.03812556372634	1.56884579746	0.164433533465517	0.999692817443324	-4.56347659847984	6.66085789092564	6.30412755818021	6.23491323486742	5.99741522558788	5.91525474265267	5.1161847301442	NA
**TC1600002135.mm.2**	Psmg1	proteasome (prosome, macropain) assembly chaperone 1	-0.472641437887621	6.12798076423767	-1.56865231269497	0.164478091475322	0.999692817443324	-4.56348792396759	6.62056984148056	6.70562328350834	5.6564816549857	5.59172263734526	5.94312760817297	6.25035955993322	NA
**TC0300001531.mm.2**	Zzz3	zinc finger, ZZ domain containing 3	0.602537934598481	6.11769752119866	1.56792773167969	0.164645057193243	0.999692817443324	-4.563530337801	6.96344840384591	6.9485843017071	5.69378757960616	6.30582656581179	5.73739561819516	5.05714265802584	NA
**TC0500001183.mm.2**	Gm6588	predicted gene 6588	-0.524219267051105	5.32271797536217	-1.56773867932043	0.164688646753552	0.999692817443324	-4.56354140437053	4.78680080811823	4.92959462582226	5.03649008060411	5.54924686146103	5.66467042730778	5.96950504885958	NA
**TC1300000175.mm.2**	Hist1h2bm	histone cluster 1, H2bm	-0.48702711513384	7.97421603833766	-1.5673536534952	0.164777455068137	0.999692817443324	-4.56356394298632	7.64483551430748	7.8464301039053	7.98951581076127	7.70047238001148	8.2138620077855	8.45018041325493	NA
**TC0200005142.mm.2**	1700025C18Rik	RIKEN cDNA 1700025C18 gene	-0.555915699277637	5.20458941379334	-1.56682274629112	0.164899985236163	0.999692817443324	-4.56359502193131	5.04936617805894	4.86996538632151	4.73902926319456	5.35915749671761	5.41756817616613	5.79244998230132	NA

**TC1500000515.mm.2**	Chrac1	chromatin accessibility complex 1	-0.50300913354825	7.40171183377308	-1.56655340671795	0.164962179776857	0.999692817443324	-4.56361078921011	6.88700619823941	6.64607817994887	7.24135481518586	7.69422936349101	7.95433723840133	7.98726520737204	NA
**TC1700000356.mm.2**	Spsb3	splA/ryanodine receptor domain and SOCS box containing 3	-0.469314543733333	6.07787295031773	-1.56620904514238	0.165041729996227	0.999692817443324	-4.56363094863246	6.71014748206725	6.65463720710746	5.6209290549737	5.46098290765879	6.13712685624998	5.88341419384918	NA
**TC0800002972.mm.2**	Adamts18	a disintegrin-like and metallopeptidase (reprolysin type) with thrombospondin type 1 motif, 18	0.601053600355669	5.0513729074456	1.56602816158323	0.165083529892815	0.999692817443324	-4.56364153795821	5.18237914396362	4.80124623004834	5.69205787107253	5.07130176461397	5.05548878912049	4.50576364585467	NA
**TC0100003869.mm.2**	Tnni1	troponin I, skeletal, slow 1	-0.864850672656932	4.97083334881617	-1.56547273691983	0.165211943404386	0.999692817443324	-4.56367405436088	4.45837796139586	4.80471022381132	3.9902669036267	5.4258383775613	5.15975874502575	5.98604788147611	NA
**TC0800001425.mm.2**	Wfdc1	WAP four-disulfide core domain 1	-0.498585367614279	6.95297844425888	-1.56540776672713	0.165226970538526	0.999692817443324	-4.56367785799124	6.66187057782352	6.48568607784422	6.75765678867839	7.02891484865011	7.20593398899088	7.57780838356617	NA
**TC1100000873.mm.2**	Pik3r6	phosphoinositide-3-kinase regulatory subunit 5	0.743680834856161	5.42425021578979	1.56536239794893	0.165237464765982	0.999692817443324	-4.56368051407915	5.19139755238638	5.28705584958737	5.38006370522056	6.39714107601808	5.59143759281371	4.69840551871262	NA
**TC0800001236.mm.2**	Dus2	dihydrouridine synthase 2	-0.616860164087509	6.78391762719363	-1.56517185397728	0.165281546198271	0.999692817443324	-4.56369166942801	6.76790039067296	6.91331485916758	6.13656497768208	6.75772011489102	6.70035394289923	7.42765147784889	NA
**TC1600001722.mm.2**	Alcam	activated leukocyte cell adhesion molecule	0.494508197739606	9.16455440979398	1.56506956900781	0.16530521387237	0.999692817443324	-4.56369765771995	10.3948751354841	10.2289719054932	9.03062871100558	8.64561919562731	8.45138853950344	8.23584297165024	NA
**TC0600000398.mm.2**	Tmem178b	transmembrane protein 178B	0.688695866780486	7.80623734308985	1.56494541845162	0.1653339452747	0.999692817443324	-4.56370492617796	8.92544964017243	8.70927121703332	8.06724401443822	7.22280345300894	7.3653595430408	6.5472961908454	NA
**TC0700004575.mm.2**	Th	tyrosine hydroxylase	0.604734389195706	6.5382550716582	1.56449847841456	0.165437416553365	0.999692817443324	-4.56373109288628	7.50160260769509	6.78588410825322	6.59131826172621	6.48443798446993	5.64325168401514	6.22303578378959	NA
**TC1200000618.mm.2**	Jkamp	JNK1/MAPK8-associated membrane protein	-0.658976932855641	7.80534371458112	-1.56441822929306	0.16545600147518	0.999692817443324	-4.56373579124143	8.74856852811042	8.6911839333871	7.49115484462717	6.54602313551179	7.96202947443485	7.3931023714154	NA
**TC0400004190.mm.2**	Tesk2	testis-specific kinase 2	-0.74283829022137	5.65096205914367	-1.56389979635032	0.165576112608162	0.999692817443324	-4.5637661444508	5.79099161821511	6.03806013356852	5.76863278197518	4.52688922934264	6.13400192764419	5.64719666411638	NA
**TC0300002701.mm.2**	Cym	chymosin	0.83429368414732	6.18842994909473	1.56369624843112	0.165623293113388	0.999692817443324	-4.56377806198683	5.48573843020419	5.23062041245432	6.34033762178282	7.70106648831942	5.77327410001197	6.59954264179563	NA
**TC1900000952.mm.2**	Rab1b	RAB1B, member RAS oncogene family	-0.491956568026429	6.86393839160135	-1.56173263827211	0.166079085699314	0.999692817443324	-4.56389303559698	6.82561152915123	6.51553366610088	6.68362700669701	6.74565900245454	7.03745495236903	7.37574419283539	NA
**TC1800000047.mm.2**	Rab18	RAB18, member RAS oncogene family	-1.63638049664676	7.51512446219942	-1.56096160589968	0.166258377949022	0.999692817443324	-4.56393818419744	9.04965754443028	9.1342122005585	7.62814505299305	4.18891296446404	8.10100479700296	6.98881421374767	NA
**TC0800000357.mm.2**	Tex24	testis expressed gene 24	-0.526545757388165	5.27414428096375	-1.56087789642572	0.166277854241979	0.999692817443324	-4.56394308599286	4.61932703724198	4.91643293984373	5.04919448014953	5.47881261681071	5.61099123535455	5.97010737638201	NA
**TC1800000599.mm.2**	Chsy3	chondroitin sulfate synthase 3	0.497127042084797	6.67661396012282	1.56018226207806	0.166439786759261	0.999692817443324	-4.56398382117265	6.73798013110213	6.70505394943595	6.73821563836919	7.06723624381503	6.22212152424673	6.58907627376789	NA
**TC0800003068.mm.2**	Cyba	cytochrome b-245, alpha polypeptide	-0.501017506359811	7.29059233136684	-1.55930448950986	0.16664432846353	0.999692817443324	-4.56403522397102	6.90316232088919	7.19323302531385	7.14152326546019	7.18103854917899	7.44998760859578	7.87460921876303	NA
**TC0200004141.mm.2**	Slc35c1	solute carrier family 35, member C1	0.564501144132489	6.14824203658631	1.55916532685339	0.166676778208589	0.999692817443324	-4.56404337359971	6.11076398760343	6.09562733201877	6.62016135845619	6.28587023562412	5.53069686794337	6.24633243787196	NA
**TC0900003204.mm.2**	Rbms3	RNA binding motif, single stranded interacting protein	0.502922225944776	7.405307144526	1.55877724558522	0.16676730162631	0.999692817443324	-4.56406610065283	8.1052285275583	7.70636657770812	7.52613957710469	7.28690652978489	6.78041105586817	7.02679059913185	NA
**TC0700001447.mm.2**	Art1	ADP-ribosyltransferase 1	0.486563390031559	5.38026976069124	1.55796274125871	0.166957441425037	0.999692817443324	-4.56411380148303	4.97750217794887	5.3685542133675	5.6497566942538	5.80458778219329	5.17783541387949	5.30338228250447	NA
**TC0500000234.mm.2**	Cct8l1	chaperonin containing TCP1, subunit 8 (theta)-like 1	0.459743104393977	5.07823360687263	1.55792398717374	0.166966493315115	0.999692817443324	-4.56411607113133	4.5807182007133	4.58618706816826	5.43494464771396	5.67604664285713	5.09989151286397	5.09161356891916	NA
**TC0200002052.mm.2**	Bmp2	bone morphogenetic protein 2	-0.769522271300717	5.06726838916116	-1.55787975919786	0.166976824315985	0.999692817443324	-4.56411866136514	4.90437277252226	5.28425206249049	4.04288980349001	5.29508067518637	5.13685096461613	5.74016405666169	NA
**TC0800000845.mm.2**	Hmox1	heme oxygenase 1	-0.81727643579945	7.73034120512683	-1.5577612657544	0.167004505574628	0.999692817443324	-4.56412560101956	8.09138364923599	8.97831019190669	7.26963631286469	6.56926394614502	7.18872616017795	8.28472697043065	NA
**TC1200000751.mm.2**	Smoc1	SPARC related modular calcium binding 1	0.51032560881718	6.08784713869402	1.55680597357747	0.16722782806211	0.999692817443324	-4.56418154976847	6.23769857156805	6.30746325416638	6.53044878708028	5.97083732495174	5.71689618820105	5.76373870619661	NA
**TC0400003740.mm.2**	Mtfr1l	mitochondrial fission regulator 1-like	-0.633366355582409	7.87532061297146	-1.55591205687953	0.167437055149106	0.999692817443324	-4.56423390611817	8.24204786640603	8.09370193500558	7.91570852957657	6.90901205304959	7.95355597336862	8.13789732042236	NA
**TC0100003832.mm.2**	Syt14	synaptotagmin XIV	0.724004412977573	6.4871891419064	1.55570636146208	0.167485234101614	0.999692817443324	-4.56424595391456	7.07766496870528	6.90907578084059	6.80095445498487	6.39124700893898	6.4900571465491	5.25413549141961	NA
**TC0700001408.mm.2**	Ucp3	uncoupling protein 3 (mitochondrial, proton carrier)	-0.498767173547488	4.94082611773486	-1.5551109959425	0.167624756395143	0.999692817443324	-4.56428082571819	4.98430800410662	4.5276232160599	4.87729173508707	4.69045383448676	5.34923975477946	5.21604016188934	NA
**TC0700004520.mm.2**	Ano9	anoctamin 9	0.893900563625039	5.41180099292694	1.55416915377785	0.167845696192864	0.999692817443324	-4.56433599325225	4.8774341017056	4.94035916579983	5.22002658737995	7.00038032127319	5.28576038220252	5.14684539920055	NA
**TC0700001990.mm.2**	Pwwp2b	PWWP domain containing 2B	-0.465842428185832	7.13415017791631	-1.55336272315664	0.168035086801368	0.999692817443324	-4.56438323098013	7.13822039402431	7.2751365151646	6.95700494571617	6.77292470525247	7.22800213013598	7.43361237720431	NA
**TC1300002740.mm.2**	Nnt	nicotinamide nucleotide transhydrogenase	-0.60503233382884	6.71981948700772	-1.55310848988448	0.168094834946168	0.999692817443324	-4.56439812336739	6.69805439440857	6.81940239000584	5.97522200587616	6.82047572911095	6.80234628703268	7.20341611561211	NA
**TC0200005414.mm.2**	Tcfl5	transcription factor-like 5 (basic helix-loop-helix)	0.488480959788163	4.89098729157679	1.55274174464639	0.168181059807437	0.999692817443324	-4.56441960672705	4.63886363248156	4.64120770398453	5.05555095462011	5.46585621166537	4.87557222910693	4.66887301760223	NA
**TC0900002007.mm.2**	Dcps	decapping enzyme, scavenger	0.493380009342	6.7640450694348	1.55250490951377	0.168236763653416	0.999692817443324	-4.56443348033568	7.38136552313263	7.05835796062984	6.9580873849088	6.60756609085637	6.23960373948386	6.33928971759731	NA
**TC0100001365.mm.2**	Colgalt2	collagen beta(1-O)galactosyltransferase 2	-0.55479892605941	5.44470731124373	-1.55188903467567	0.168381698604545	0.999692817443324	-4.56446955843362	5.36389819433462	5.22892968841301	4.8741330588633	5.60877600743468	5.87379281096294	5.71871410745386	NA
**TC1200001942.mm.2**	Esr2	estrogen receptor 2 (beta)	-0.474346737515577	5.05408306531257	-1.55025689441454	0.168766356811849	0.999692817443324	-4.56456517411404	4.78646175026742	4.44080887655877	5.00747270147249	5.06679444353655	5.44194472682277	5.58101589321742	NA
**TC0300000925.mm.2**	Mtmr11	myotubularin related protein 11	-0.568615286952317	5.60303604729757	-1.54939612774017	0.168969549863569	0.999692817443324	-4.56461560300728	5.22489764906255	5.73201683836303	5.59067912925519	5.17135648197242	5.70600679583489	6.19325938929736	NA
**TC1100003645.mm.2**	4933428G20Rik	RIKEN cDNA 4933428G20 gene	0.585970014409123	5.42281835665232	1.54923943714233	0.169006562870902	0.999692817443324	-4.56462478307596	4.8557317964098	5.71541320199135	5.73433151124838	5.83452107391713	5.28367610072556	5.1132364556217	NA
**TC1400001306.mm.2**	Rap2a	RAS related protein 2a	-0.965675303181975	7.09472028523903	-1.54719217205312	0.169490857231255	0.999692817443324	-4.56474473197466	7.84439654859196	7.96150221890907	7.17043166459701	5.24510450418759	7.41213030095038	6.93475647419818	NA
**TC0400004009.mm.2**	Clcn6	chloride channel, voltage-sensitive 6	0.528242495888721	7.74675308760957	1.54712153866043	0.169507589103746	0.999692817443324	-4.56474887054628	8.41477310681876	8.03311862727445	7.92648639648205	7.61806949518878	7.46583864418804	7.02223225570535	NA
**TC1800000370.mm.2**	Pcdhb14	protocadherin beta 14	-0.541844928968238	5.58367611989136	-1.54600043668717	0.169773365702088	0.999692817443324	-4.56481455999313	6.4666294706699	6.56016154501477	5.14234623582855	4.55344168703495	5.59130279396579	5.18817498683418	NA
**TC0800002807.mm.2**	Rrad	Ras-related associated with diabetes	-0.472053818406029	5.16930564943081	-1.54585622783429	0.169807581082648	0.999692817443324	-4.56482300992166	4.80128774113626	4.66937339872745	5.07502990532461	5.22550265462993	5.46621230367518	5.77842789309142	NA
**TC0800002945.mm.2**	Rfwd3	ring finger and WD repeat domain 3	0.467578614080105	6.61630007034274	1.5455953059584	0.169869504442499	0.999692817443324	-4.56483829877728	7.15660778470785	7.03767911390041	6.76633850860275	6.45299686720143	6.11488407866539	6.16929406897859	NA
**TC0600001844.mm.2**	Rpa3	replication protein A3	0.599613758227606	7.01624913266709	1.54485252756106	0.170045899780044	0.999692817443324	-4.56488182310369	7.29795822025017	7.23008748167434	6.78501095384144	7.59932735142519	6.36448788353186	6.82062290527956	NA
**TC0700001451.mm.2**	Stim1	stromal interaction molecule 1	-0.49573079060657	5.64362062215978	-1.54473853549984	0.170072985766549	0.999692817443324	-4.56488850276509	6.25565825150789	6.49757844310579	5.0098522521476	5.04866047641834	5.29985873883909	5.75011557093999	NA
**TC1500001089.mm.2**	Igfbp6	insulin-like growth factor binding protein 6	-0.527463287978268	7.24193469316981	-1.54471814858082	0.170077830385655	0.999692817443324	-4.56488969739268	6.85353875524164	7.40917265026885	6.85628857585733	7.21069651291859	7.53150625654414	7.59040540818831	NA
**TC0200002532.mm.2**	Ube2c	ubiquitin-conjugating enzyme E2C	-0.756496226709441	6.66220362901047	-1.54458292980523	0.170109966179145	0.999692817443324	-4.56489762093191	6.7294641981352	7.18160513027557	5.50566008695155	6.76891990916505	6.65760793522972	7.12996451430576	NA
**TC0600002681.mm.2**	Eefsec	eukaryotic elongation factor, selenocysteine-tRNA-specific	0.514146575181827	7.06294761131978	1.54420564083359	0.170199661754069	0.999692817443324	-4.56491972949415	7.39556284975339	7.40996960849867	7.24373413806943	7.0564890419457	6.92263422729307	6.3492958023584	NA
**TC0600002404.mm.2**	Hpgds	hematopoietic prostaglandin D synthase	0.743052764773164	5.73512795185897	1.54419160058361	0.170203000492201	0.999692817443324	-4.56492055223755	6.40993160155006	7.1373042765782	5.42356949128398	5.75124919000196	4.27872320290238	5.40998994883724	NA
**TC0300000309.mm.2**	Abhd18/Gm2011	abhydrolase domain containing 18/predicted gene 2011	-0.582493475422337	5.7542935481429	-1.54405817892178	0.170234730891934	0.999692817443324	-4.56492837062358	5.89148306029112	5.75853920924789	5.82708620063007	5.0282898336068	6.1781434538323	5.84221953124925	NA
**TC1100001776.mm.2**	Ddx42	DEAD (Asp-Glu-Ala-Asp) box polypeptide 42	0.702194744746885	7.14430785546627	1.54402203706118	0.170243327114066	0.999692817443324	-4.56493048850989	8.21628000066844	8.13614089642033	7.28782637003553	6.67108149256577	6.82760005627218	5.72691831683535	NA
**TC1000001577.mm.2**	Sdr9c7	4short chain dehydrogenase/reductase family 9C, member 7	-0.528991625960948	4.79382930524595	-1.54393559909269	0.17026388773944	0.999692817443324	-4.56493555372249	4.57374145389834	4.04720168190649	4.79476489994546	4.74725982192902	5.0947392600419	5.50526871375448	NA
**TC0400001547.mm.2**	Pum1	pumilio RNA-binding family member 1	0.549461739867663	7.51337446298313	1.54382863849059	0.170289333197188	0.999692817443324	-4.56494182157151	7.85151667411908	7.67548959188371	7.7968680495146	7.52921394630105	7.45444124273035	6.77271727334998	NA
**TC0100002033.mm.2**	Mcm3	minichromosome maintenance complex component 3	0.617142935767413	5.63693631896117	1.54268841544409	0.170560807500821	0.999692817443324	-4.56500863972856	5.66376530199223	5.93576087659371	5.65884457512839	6.06934422822957	4.81278848686533	5.6811144449578	NA
**TC0200001368.mm.2**	Spi1	spleen focus forming virus (SFFV) proviral integration oncogene	-1.04297703256707	7.81356957745258	-1.54194389292236	0.170738287426814	0.999692817443324	-4.56505227095732	8.32776403679035	9.26498781430853	7.79361732281925	5.80773845142198	7.832713969169	7.85459587020637	NA
**TC0200005175.mm.2**	Prex1	phosphatidylinositol-3,4,5-trisphosphate-dependent Rac exchange factor 1	-0.529356028837637	6.84420472779299	-1.54099155807125	0.170965556518736	0.999692817443324	-4.5651080823039	7.43520074860842	7.56588838398551	5.93201939296747	6.5706941952769	6.86862449948639	6.69280114643326	NA
**TC0700004191.mm.2**	Dnah3	dynein, axonemal, heavy chain 3	-0.452846998421246	5.03303724520606	-1.54051522439552	0.171079336383712	0.999692817443324	-4.56513599842653	4.78128630013273	4.9706458495358	4.88356713099776	4.8867315313649	5.27944714377758	5.39654551542757	NA
**TC1700000819.mm.2**	Mmut	methylmalonyl-Coenzyme A mutase	0.925475766609874	5.82897307115903	1.53895178356337	0.171453284626996	0.999692817443324	-4.56522762905607	6.93425597258346	6.89096062967726	6.23827195612222	5.26151472283438	5.63582236727609	4.01301277846076	NA
**TC1400000001.mm.2**	Gm2888	predicted gene 2888	-0.527429533128652	5.50392013599633	-1.53829625522807	0.171610302006886	0.999692817443324	-4.5652660499246	4.71078325130871	4.94842760528144	5.31468930171445	5.84003614485081	5.93326074882207	6.27632376400049	NA
**TC0700004413.mm.2**	Fam53b	family with sequence similarity 53, member B	0.553129723095334	7.16530008356766	1.53801402726877	0.171677944699025	0.999692817443324	-4.56528259171851	7.18936870939638	7.09373312389714	7.27692222200698	7.63055683514458	7.2424793402467	6.55874027071419	NA
**TC0900001940.mm.2**	Glb1l3	galactosidase, beta 1 like 3	0.684331351035829	5.15987581223456	1.53791539239117	0.171701590760328	0.999692817443324	-4.56528837288995	4.47311172336759	4.35240953864526	5.35398618222221	6.39721197451087	4.88228109175315	5.50025436290827	NA
**TC1100000417.mm.2**	Gabrb2	gamma-aminobutyric acid (GABA) A receptor, subunit beta 2	-0.465591310157051	8.44452021436106	-1.53789224702053	0.171707139914588	0.999692817443324	-4.56528972948542	9.39778631444889	9.13952926647907	7.85487122951765	7.7444403129445	8.36288448341764	8.16760967935861	NA
**TC1900000943.mm.2**	Peli3	pellino 3	-0.835916057639836	6.16445824167787	-1.53696090233017	0.171930570376716	0.999692817443324	-4.56534431828437	6.4732169977057	5.94910223190635	6.50583841477183	4.94046063781593	6.625241549561	6.49288961830643	NA
**TC0100003138.mm.2**	Pkp1	plakophilin 1	0.635004779844007	6.30394235593197	1.53690997923276	0.171942794670834	0.999692817443324	-4.56534730308231	5.89444824017518	5.49997566786474	6.43057833859129	7.41904155502866	6.27323028654868	6.30638004738326	NA
**TC0700000220.mm.2**	Kptn	kaptin	-0.610282373180441	5.69469557185993	-1.53686212327974	0.171954283421582	0.999692817443324	-4.5653501081077	6.42065523835323	6.57345550425466	5.458105224858	4.51864374623741	5.47584833854933	5.72146537890696	NA
**TC0400003823.mm.2**	Fam43b	family with sequence similarity 43, member B	-0.477159401529355	7.22982137298971	-1.5361491417404	0.172125533076593	0.999692817443324	-4.56539189927413	7.47906060146822	7.15505488943829	6.83881562087488	7.05643135111163	7.34292243667241	7.50664333837281	NA
**TC0200003210.mm.2**	Mymk	myomaker, myoblast fusion factor	-0.513544444087755	5.41927310355358	-1.53530871551328	0.172327597118064	0.999692817443324	-4.5654411617999	5.55565402931005	5.64929608795832	5.24558654480761	4.8962132631312	5.33627574869037	5.83261294742395	NA
**TC0500003368.mm.2**	Ncf1	neutrophil cytosolic factor 1	-0.767181904077679	7.05598153248256	-1.53515179576447	0.17236534983339	0.999692817443324	-4.56545035997311	7.01867355554971	7.45641356094971	5.88314234105483	7.28007679406546	7.3063246807123	7.39125826256335	NA
**TC0600003185.mm.2**	Tspan9	tetraspanin 9	0.458794517711507	7.8627220923577	1.53498719363186	0.172404959088909	0.999692817443324	-4.56546000851405	8.05720184860242	8.1787430357194	8.0008711130155	7.92811723960819	7.37615300051649	7.63524631668419	NA
**TC0900002989.mm.2**	Nudt16l2	nudix (nucleoside diphosphate linked moiety X)-type motif 16-like 2	0.882040253727648	5.40408692302089	1.53494080496646	0.172416123416531	0.999692817443324	-4.56546272770377	5.03161660709512	5.3217272145546	5.07884752136826	6.8387815905972	5.04649069655072	5.10705790795944	NA
**TC0900002283.mm.2**	Layn	layilin	0.503288087204269	5.82135047672905	1.53467859690415	0.172479241487843	0.999692817443324	-4.56547809777144	5.35307722992909	5.78054520754265	6.09085713049822	6.30967116815734	5.84764484746331	5.54630727678371	NA
**TC0600003074.mm.2**	Slc2a3	solute carrier family 2 (facilitated glucose transporter), member 3	1.01194585046624	7.80909151700259	1.53464492002559	0.172487349658924	0.999692817443324	-4.56548007184614	9.11597269732697	9.03824575291642	8.4370958448738	6.9250153314785	7.41711210806874	5.92110736735108	NA
**TC0X00000204.mm.2**	Rp2	retinitis pigmentosa 2 homolog	-0.764227929467411	6.31138820656731	-1.5345795830628	0.172503081440988	0.999692817443324	-4.56548390178084	7.05068173782879	7.3208035616765	6.05760765911563	4.92658638136623	6.69671413694813	5.81593576246856	NA
**TC1300001330.mm.2**	Hcn1	hyperpolarization-activated, cyclic nucleotide-gated K+ 1	-0.809916696070892	6.91384178569499	-1.5343119779117	0.172567529120384	0.999692817443324	-4.56549958839274	8.12252035915343	7.89840400469323	6.54520522473305	5.37594125435769	7.29598525913992	6.2449946120926	NA
**TC1000001044.mm.2**	Timp3	tissue inhibitor of metalloproteinase 3	0.631610296471973	6.93869360888605	1.53429641543218	0.172571277740307	0.999692817443324	-4.56550050064593	7.6343827844491	7.43452388253602	6.63376305002132	7.27947473961623	5.91853721214479	6.73147998454882	NA
**TC0400001612.mm.2**	Nr0b2	nuclear receptor subfamily 0, group B, member 2	0.592139054330987	6.36367191703507	1.53410247477314	0.1726179996434	0.999692817443324	-4.56551186924306	6.05969899843019	6.04751497060692	6.35344313555193	7.2761046853657	6.19574269528451	6.24952701697115	NA
**TC0200003473.mm.2**	Zeb2/Mir5129	zinc finger E-box binding homeobox 2/microRNA 5129	-0.509108246556283	8.11134459123623	-1.53383787206754	0.172681763584859	0.999692817443324	-4.56552738008495	8.66217021088621	9.25686814691877	7.41871085156334	7.44669549668656	7.92865369217323	7.95496914918924	NA
**TC0300000478.mm.2**	Gm8298	predicted gene 8298	-0.499639330297151	4.93395809696327	-1.53354588272649	0.172752152570331	0.999692817443324	-4.56554449646054	4.67775894528896	4.92457538853495	4.87553611212376	4.62553168155697	5.0380273674576	5.46231908681742	NA
**TC1100001786.mm.2**	Cep95	centrosomal protein 95	-0.474175664692331	6.04872730985437	-1.53340926799935	0.172785095027195	0.999692817443324	-4.5655525048499	5.75131259926945	5.56281853477424	6.16926348269517	5.84567721515377	6.56503524847222	6.39825677876138	NA
**TC0X00000650.mm.2**	Mtmr1	myotubularin related protein 1	-0.523932513059139	6.29560487988643	-1.53336019812449	0.172796928868251	0.999692817443324	-4.56555538134635	7.14061448187834	6.93390117151612	5.977812163982	5.34781213592093	6.19315012878898	6.18033919723223	NA
**TC1100001021.mm.2**	Spata22/Olfr20	spermatogenesis associated 22/olfactory receptor 20	0.622165725833413	5.57992237468169	1.53330255927998	0.172810830190498	0.999692817443324	-4.56555876016485	5.46069434914696	5.07558235566703	5.62983324257226	6.46396125489922	5.19466103556981	5.65480201023484	NA
**TC0300000750.mm.2**	Gba	glucosidase, beta, acid	-0.662064479666588	6.82957248909004	-1.53301226227996	0.172880859753565	0.999692817443324	-4.56557577761323	7.40733517246939	7.55092883151942	6.60853805122909	5.73898293438005	7.21008450410659	6.46156544083573	NA
**TC0100003026.mm.2**	Mcm6	minichromosome maintenance complex component 6	-0.76878543827149	5.56127645063428	-1.5328538524549	0.172919084744052	0.999692817443324	-4.56558506379027	5.50053465887911	6.54328402184171	4.94066167130781	4.95247290196313	5.19431777686739	6.23638767294653	NA
**TC1100000339.mm.2**	Hba-a1/Hba-a2	hemoglobin alpha, adult chain 1/hemoglobin alpha, adult chain 2	-0.71328654026374	12.1934155166055	-1.53280409602457	0.17293109281169	0.999692817443324	-4.56558798058203	11.251195895135	12.0741839106698	12.2552596929148	11.9490104137354	13.303331035864	12.3275121513137	NA
**TC1100001914.mm.2**	Sap30bp	SAP30 binding protein	-0.560346046671276	7.93829540459629	-1.53267266852773	0.172962814857199	0.999692817443324	-4.56559568506732	8.2613780222564	7.96961272705692	7.94652553260915	7.19251925985177	8.16011016809758	8.09962671770589	NA
**TC0700003568.mm.2**	Pex11a	peroxisomal biogenesis factor 11 alpha	0.577585579534316	6.78655021344708	1.53202707378993	0.173118717615268	0.999692817443324	-4.56563353128001	6.82192033405261	7.0283219395177	6.58124616273679	7.43086892035362	6.44966443721786	6.40727948680393	NA

TC1700001003.mm.2	Uhrf1	ubiquitin-like, containing PHD and RING finger domains, 1	-0.457793732666884	6.30614722607395	-1.53192984352015	0.173142208777349	0.999692817443324	-4.56563923119965	6.43468175701266	6.58207489871782	6.06199377172817	5.89027584596156	6.33965962313106	6.52819745989244	NA
TC0700000798.mm.2	2410002F23Rik/Snord88a	RIKEN cDNA 2410002F23 gene/small nucleolar RNA, C/D box 88A	0.57856385406236	7.5337478551103	1.53179011783132	0.173175972163793	0.999692817443324	-4.56564742235235	8.87283700226836	8.84706328393044	7.58344257729343	6.73641469900043	6.69852028445913	6.46420928371001	NA
TC0800000936.mm.2	Adgrl1	adhesion G protein-coupled receptor L1	0.97164129137121	7.78347859607063	1.53157448190322	0.173228090546745	0.999692817443324	-4.56566006366459	9.30498676077099	9.46442330074699	8.26075365186858	6.67661839695552	7.12546602366528	5.86862344241641	NA
TC1100002855.mm.2	Olfr222	olfactory receptor 222	-0.728440171104741	5.59699961183369	-1.53122765476422	0.173311947921691	0.999692817443324	-4.56568039601986	5.19317301134066	5.32738009034876	5.21336777506474	5.58891433848687	5.47960949886717	6.77955295689393	NA
TC0300000186.mm.2	Egfem1	EGF-like and EMI domain containing 1	-0.598880869755668	6.31960312337274	-1.53064021515127	0.173454067681278	0.999692817443324	-4.56571483448139	7.09829103265911	6.28549891944565	6.06931256493821	5.59872095937196	6.35381549731502	6.51197976650648	NA
TC0200005350.mm.2	Gm14308/Gm14430/Gm14432/Gm14434/Gm2026/Gm4724	predicted gene 14308/predicted gene 14430/predicted gene 14432/predicted gene 14434/predicted gene 2026/predicted gene 4724	-1.6235692962446	8.91825805271045	-1.53047843519801	0.173493226253355	0.999692817443324	-4.56572431888243	10.0105176204127	10.6948960270494	9.00383382121418	5.77466421694152	9.80588991040677	8.21974672023813	NA
TC0400002733.mm.2	Svep1	sushi, von Willebrand factor type A, EGF and pentraxin domain containing 1	-0.598839157038635	5.10700833507557	-1.52997864792921	0.173614250824927	0.999692817443324	-4.56575361934635	4.73392015773314	4.01946056477617	5.05296346535491	5.2925320215785	5.47974516588322	6.06342863512747	NA
TC1100003785.mm.2	Ghdc	GH3 domain containing	0.548342564409623	5.55771500072272	1.52975163901296	0.173669247423882	0.999692817443324	-4.56576692807901	5.32197542080294	5.38177968725364	5.73767096559524	6.13193904695426	5.71498829291998	5.05793659081028	NA
TC0400004219.mm.2	B4galt2	UDP-Gal:betaGlcNAc beta 1,4-galactosyltransferase, polypeptide 2	0.458811025623271	7.24154898622437	1.52955727895756	0.173716347164558	0.999692817443324	-4.56577832279169	7.33885331001419	7.46157662047875	7.50960093868482	7.2736420803651	7.01141180969285	6.85420915811052	NA
TC0900001329.mm.2	Pik3r4	phosphoinositide-3-kinase regulatory subunit 4	0.532538577656629	6.25774740628404	1.529481137992	0.173734801822633	0.999692817443324	-4.5657827867116	7.30967226056256	6.87150177992639	6.01647545823247	6.19871831803182	5.83592348297681	5.31419313797422	NA
TC0200002811.mm.2	Arfgap1	ADP-ribosylation factor GTPase activating protein 1	-0.853507620633533	8.17072799556827	-1.52913760121281	0.173818089156761	0.999692817443324	-4.56580292737631	9.20727392023836	9.01727909344789	8.05922096818863	6.48717889103951	8.45750354245737	7.79591155803783	NA
TC0600003314.mm.2	Hebp1	heme binding protein 1	-0.5081868312894	7.04466389524198	-1.5290454412297	0.173840438805471	0.999692817443324	-4.56580833050834	7.35403963826481	7.55287003938132	6.41800059774087	6.75434941787259	6.86730634619576	7.32141733199651	NA
TC0600000570.mm.2	Tmem176a	transmembrane protein 176A	-0.444381263487543	7.52617697839299	-1.52880050340779	0.17389985146782	0.999692817443324	-4.56582269072763	8.21277183289706	8.36052255787378	6.89965666729401	6.94784580901199	7.38160650934584	7.35465849393525	NA
TC0400000404.mm.2	Gm21953/Gm20878/Gm21586	predicted gene, 21953/predicted gene, 20878/predicted gene, 21586	-0.597847970898906	9.34708960979984	-1.52854450218509	0.173961967847839	0.999692817443324	-4.5658376996758	10.0808166165388	10.2678970513882	8.86459174103212	8.404472283505	9.63827180829311	8.82648815804183	NA
TC0700000302.mm.2	Exoc3l2	exocyst complex component 3-like 2	-0.641917374901432	5.65978048837497	-1.52827197796693	0.174028116025917	0.999692817443324	-4.56585367745682	5.45604658069785	5.24679672065553	5.69658350934083	5.28941893020596	5.63549539429591	6.63434179505375	NA
TC0200000843.mm.2	Arl6ip6	ADP-ribosylation factor-like 6 interacting protein 6	-0.899071486050528	6.22667771548231	-1.52784280693593	0.174132333506499	0.999692817443324	-4.56587883950011	6.77134388956449	6.72115107237086	6.42072039264967	4.61399378677907	6.59328161548938	6.23957553604041	NA
TC1400000795.mm.2	Nfatc4	nuclear factor of activated T cells, cytoplasmic, calcineurin dependent 4	0.628299949287721	5.78578290043663	1.52756570335422	0.174199654568389	0.999692817443324	-4.56589508607373	5.56279412069617	5.0359546185471	5.91562639933019	6.77064788164579	5.55250820157578	5.87716618082476	NA
TC1800001207.mm.2	Taf7	TATA-box binding protein associated factor 7	-0.684582839032353	6.05049867366684	-1.52718224294309	0.174292854399995	0.999692817443324	-4.56591756854329	5.84622385607263	5.71562255393619	6.42571663865318	5.26027333831057	6.71946612210686	6.3356895329216	NA
TC0200005134.mm.2	Spata25	spermatogenesis associated 25	-0.551614708393927	4.42139369219927	-1.52540734112267	0.174724846570467	0.999692817443324	-4.56602163484175	3.84072561690768	3.29505994572533	4.28853815301358	4.85613543387382	5.11796587149881	5.12993713217644	NA
TC1500000409.mm.2	Wdyhv1	WDYHV motif containing 1	-0.617392138523416	6.24131575004619	-1.52492272669566	0.174842969148318	0.999692817443324	-4.56605004964814	7.43559500562257	7.20006540565591	5.88061451978845	4.90811038618746	5.90547353660691	6.11803564641584	NA
TC0700000275.mm.2	Pglyrp1	peptidoglycan recognition protein 1	-0.485100446495317	6.44240169204282	-1.5246988091653	0.174897573062703	0.999692817443324	-4.56606317891041	5.86355707224847	5.74092264714409	6.38742238522055	6.65244238471631	6.80827779547023	7.20178786745727	NA
TC0200004102.mm.2	Ndufs3	NADH:ubiquinone oxidoreductase core subunit S3	-1.1384149729784	9.26139514509202	-1.52456430099799	0.174930381462692	0.999692817443324	-4.56607106574669	9.97854328508017	9.94326111498281	9.54890777855043	7.13596021371573	9.7482170881892	9.21348085003377	NA
TC0700003065.mm.2	Mrgprb4	MAS-related GPR, member B4	-0.566948651295899	4.96933584650742	-1.52309782708093	0.175288445400898	0.999692817443324	-4.56615705358846	4.24176154026026	4.16504674175274	4.64630362833527	5.49135111888459	5.64395421275495	5.62759783705671	NA
TC0100002002.mm.2	Ube2w	ubiquitin-conjugating enzyme E2W (putative)	0.597821122451031	7.85775776226179	1.52289282365781	0.175338554522387	0.999692817443324	-4.5661690743668	7.91844397982542	7.8519228796906	7.71026539806669	8.57564558141171	7.74936840279771	7.34090033177863	NA
TC1800000417.mm.2	2900055J20Rik	RIKEN cDNA 2900055J20 gene	0.660380850934341	6.78279525155541	1.52230495157923	0.175482322173226	0.999692817443324	-4.56620354572429	7.25606022142406	7.62131844656773	6.56204683169842	7.00803043990626	6.63116978062466	5.61814578911134	NA
TC1100001777.mm.2	Psmc5	protease (prosome, macropain) 26S subunit, ATPase 5	-0.909588896946894	7.26057879984526	-1.52222950535475	0.175500780915352	0.999692817443324	-4.56620796973784	8.48933491705752	8.72342989812262	6.88521329789752	5.3805517971013	7.59282847431786	6.49211441457476	NA
TC0100001258.mm.2	Tmem9	transmembrane protein 9	0.525172434134332	6.7283385060711	1.52211907028772	0.175527803306831	0.999692817443324	-4.56621444544017	7.33671033745529	7.2064707423293	6.38548126760733	7.05311614484799	6.15357431838356	6.23467822580311	NA
TC0500002272.mm.2	Evc	EvC ciliary complex subunit 1	-0.4976646520163	5.16464118385101	-1.52193611753	0.175572578566812	0.999692817443324	-4.56622517347855	4.89076243531073	5.18141195556824	4.77803465395053	5.18213705014671	5.3496476147155	5.60585339341434	NA
TC0100001244.mm.2	Ptpn7	protein tyrosine phosphatase, non-receptor type 7	-0.595490381747973	5.77995184559912	-1.52187716645911	0.175587008315572	0.999692817443324	-4.56622863027872	5.56980340335191	5.39641919406728	5.7771119484414	5.48414190789839	5.78129649442277	6.67093812541297	NA
TC0400002576.mm.2	Exosc3	exosome component 3	-0.499165088880376	8.24851123196919	-1.52185997091866	0.175591217561291	0.999692817443324	-4.56622963859967	8.43400327178878	8.54214608672451	8.15135001495426	7.60694391281629	8.31960140920902	8.43702269632228	NA
TC0400004035.mm.2	Kif1b	kinesin family member 1B	-0.515717728786639	9.12531053894228	-1.52111421375043	0.17577385932016	0.999692817443324	-4.56627336907461	9.71515117208094	9.60202047598652	8.87643488910846	8.32519317489801	8.95847297437501	9.27459054720474	NA
TC0500000670.mm.2	Atp10d	ATPase, class V, type 10D	-0.513609575134908	6.23260228175002	-1.52054478020257	0.175913436635735	0.999692817443324	-4.56630676060125	5.76701866243791	5.7240257504325	6.15167740514363	6.28699765853631	6.4225148337401	7.04337938020966	NA
TC0100000858.mm.2	Agap1	ArfGAP with GTPase domain, ankyrin repeat and PH domain 1	0.488183753687314	6.91782288887364	1.52024185774113	0.175987729666936	0.999692817443324	-4.56632452411704	7.76837359954418	7.61526366488499	6.93329214035833	6.61654164773532	6.47428079846913	6.09918548224989	NA
TC0400003609.mm.2	Txlna	taxilin alpha	-0.63625665801264	6.49851270683211	-1.51994369247577	0.176060884381127	0.999692817443324	-4.56634200878446	6.5247432250304	6.51783050313285	6.67887030040799	5.65912429799409	7.00018114810528	6.61032676632208	NA
TC0500000324.mm.2	Gpn1	GPN-loop GTPase 1	0.550378788325221	6.37331822914434	1.51987481000255	0.176077788673139	0.999692817443324	-4.56634604812813	6.29483985381569	6.19032557679388	6.33460898084132	7.09314177961213	6.06545407269325	6.26153911110976	NA
TC1000000752.mm.2	Slc19a1	solute carrier family 19 (folate transporter), member 1	0.461590765178725	7.23236064321717	1.51982145953921	0.176090882323655	0.999692817443324	-4.56634917666189	7.61772953242926	7.77296528846479	7.14731693714666	7.31600834723654	6.63589393276246	6.90424982126328	NA
TC0900001630.mm.2	Ackr2	atypical chemokine receptor 2	-0.482518010769643	5.12259341067239	-1.51942889495189	0.176187256078255	0.999692817443324	-4.56637219722125	5.26554409210445	5.39407099352062	4.60831682098934	4.94713785744564	5.0990528413384	5.42143785863587	NA
TC0100001452.mm.2	Mrps14	mitochondrial ribosomal protein S14	-0.476399735525454	7.43012878220876	-1.51866267715014	0.176375501739151	0.999692817443324	-4.56641712990754	8.02240595727919	8.05496529356053	6.90218808180883	6.87311290387213	7.58873748672363	7.13936297000823	NA
TC0800001213.mm.2	Elmo3	engulfment and cell motility 3	-0.445183618123635	5.52263157971948	-1.51788794025688	0.176566029893572	0.999692817443324	-4.56646256290765	5.37254392509156	5.37862918149275	5.27106293362113	5.47606163412153	5.79383380898051	5.84365799500942	NA
TC1500000246.mm.2	Osr2	odd-skipped related 2	0.509317154227264	5.11446671856273	1.51783775980016	0.176578377160531	0.999692817443324	-4.56646550567203	4.24438534923791	4.75585899121098	5.49984624396442	5.85274889572661	5.15967002247848	5.17429080875802	NA
TC0600002728.mm.2	Chchd4	coiled-coil-helix-coiled-coil-helix domain containing 4	-0.525561553432122	7.04865212029475	-1.51737995913966	0.17669105929854	0.999692817443324	-4.56649235290586	7.22175200056296	6.88841997671892	6.56031468096862	7.00499413784256	7.10067740367753	7.5157545219979	NA
TC0400003559.mm.2	Zmym1	zinc finger, MYM domain containing 1	0.462700344287239	5.29281527805485	1.51719362641921	0.17673694193596	0.999692817443324	-4.56650328026172	5.46833999270297	5.28825305986592	5.33002790761039	5.63282174455695	4.96798533607395	5.0694636275189	NA
TC1100000464.mm.2	Adam19	a disintegrin and metallopeptidase domain 19 (meltrin beta)	0.591303532006651	6.61262969183208	1.51690337975754	0.176808434370468	0.999692817443324	-4.56652030166139	6.14371734546157	6.30597178583409	7.20119642898448	7.00315161287059	6.05618152358475	6.96555945405531	NA
TC0X00001380.mm.2	Frmpd3	FERM and PDZ domain containing 3	0.640399683671573	6.33895084889889	1.51680195454951	0.176833423351688	0.999692817443324	-4.56652624972544	7.07133518889705	6.80243578303053	5.83812364556721	6.88224309883724	5.85049102843065	5.58907634863064	NA
TC0100003532.mm.2	Tomm40l	translocase of outer mitochondrial membrane 40-like	0.650053519290279	6.10972336340892	1.51666586733326	0.176866957443549	0.999692817443324	-4.56653423055583	6.23239114122401	6.27348303892252	5.79489234847483	6.93139417096895	5.75659586533088	5.66958361553234	NA
TC0500003514.mm.2	Iqce	IQ motif containing E	0.784877965964766	5.8893178747938	1.5163394012974	0.176947427976061	0.999692817443324	-4.56655337623305	6.14193000106634	5.95133179157563	5.54201312629223	6.86418756773296	5.80539451644765	5.03105024564802	NA
TC1200001956.mm.2	Rab15	RAB15, member RAS oncogene family	-0.822849327935749	8.66185465506778	-1.51623643321597	0.176972815587629	0.999692817443324	-4.56655941484569	8.73336851358983	8.83607111811422	8.97976107240818	7.39823374900738	9.24330871823301	8.78038475905405	NA
TC0400001212.mm.2	Gm12830	predicted gene 12830	0.602052254908465	4.8735273814431	1.51584314215649	0.177069815731224	0.999692817443324	-4.56658247969982	4.10194180982574	4.08765987440314	5.09489366151043	6.03293989561289	4.81445901993044	5.10927002737595	NA
TC0800002877.mm.2	Nqo1	NAD(P)H dehydrogenase, quinone 1	-0.461211255207245	5.01939734845227	-1.51456033773042	0.177386545169386	0.999692817443324	-4.56665771194092	5.24847009430539	5.400943693122	4.4750899978786	4.79718389855726	5.06624355407282	5.12845285277753	NA
TC1500001810.mm.2	Apol7b	apolipoprotein L 7b	0.504423481039214	5.43998170570008	1.51441178441585	0.177423257441206	0.999692817443324	-4.56666642421791	5.230950706129	5.31317122113348	5.69960205657801	5.85270557793021	4.9823786027855	5.56108206964429	NA
TC1100000336.mm.2	Hba-a2/Hba-a1	hemoglobin alpha, adult chain 2/hemoglobin alpha, adult chain 1	-0.724082772251105	12.1905594461503	-1.51419937496299	0.177475762827493	0.999692817443324	-4.56667888153724	11.2802055460207	12.1408315458531	12.2228556104798	11.914221409783	13.303331035864	12.2819115289011	NA
TC0200001624.mm.2	Bdnf	brain derived neurotrophic factor	-0.4967388326142838	5.47551192946601	-1.51398160953015	0.177529607091167	0.999692817443324	-4.56669165302206	5.62718164320935	5.37218781354692	4.96174217243973	5.46837005496588	5.72797050549337	5.69561938714081	NA
TC1800001163.mm.2	Hspa9	heat shock protein 9	0.627187046793676	7.42884215502042	1.51334688957494	0.177686633029367	0.999692817443324	-4.56672887829665	8.32492188809781	8.17264040312069	7.64412529558314	7.02080707066255	7.13409753116698	6.27646074149135	NA
TC0100003864.mm.2	Psmd1	proteasome (prosome, macropain) 26S subunit, non-ATPase, 1	0.4925518376995	7.99079453579941	1.51270463581509	0.177845653653715	0.999692817443324	-4.56676654583846	9.11131050387506	8.92445801086226	7.96314137549986	7.48390981222922	7.32743724343008	7.1345102689	NA
TC1100003379.mm.2	Slfn9	schlafen 9	0.624954920549048	5.74832371825797	1.51222210115295	0.177965214833897	0.999692817443324	-4.56679484628785	5.00363615853513	5.55218645740547	5.88889137404011	6.70312339331255	5.46432610043869	5.87777882581587	NA
TC0X00003168.mm.2	Maged2	melanoma antigen, family D, 2	0.485640886118822	6.84387082037479	1.5117743338223	0.178076227996568	0.999692817443324	-4.56682110785375	7.88364490666552	7.48592443736413	6.76877678974093	6.56369188548744	6.30825686287606	6.05293004011467	NA
TC0100000657.mm.2	Des	desmin	-0.610814515975471	4.72828690499176	-1.51080554139266	0.178316636182467	0.999692817443324	-4.56687792821586	4.265573180426	4.58061316942071	4.12460851406031	5.02634451001614	5.00733212164977	5.36524993437761	NA
TC0900001645.mm.2	Tcaim	T cell activation inhibitor, mitochondrial	0.540800566258072	5.83795199599254	1.51073035200909	0.178335307162797	0.999692817443324	-4.56688233816347	6.14721917377882	6.18336463389444	5.56342042276118	6.32594422763788	5.43378702363463	5.37397649424828	NA
TC1100001250.mm.2	Rasl10b	RAS-like, family 10, member B	0.544322300830609	8.1623049405926	1.51060413729413	0.178366652787896	0.999692817443324	-4.56688974081982	9.28183653659176	9.21926059488042	8.06768781651595	7.71300074035635	7.69186223168116	7.00018172352993	NA
TC1400001047.mm.2	Esd	esterase D/formylglutathione hydrolase	-0.660992770680395	6.52710173783152	-1.51047238050817	0.178399380233261	0.999692817443324	-4.5668974685415	7.35678468994152	7.07835000337332	6.42120676616533	5.28153832999143	6.59108838165321	6.43364225586433	NA
TC1400000075.mm.2	Rpp14	ribonuclease P 14 subunit	0.850144586984525	7.05746840231382	1.5097207907816	0.17858617601494	0.999692817443324	-4.5669415506434	7.79917651283648	7.91453082207545	7.13443566621599	7.03126046025402	7.09765528481276	5.3677516676882	NA
TC0300000753.mm.2	Muc1	mucin 1, transmembrane	-0.539692844860963	5.23666632444156	-1.50918388008813	0.178719727479034	0.999692817443324	-4.56697304173901	4.61031618138588	4.73047711137206	5.10779734536205	5.39211213672268	5.44047942708514	6.13881574472152	NA
TC0400000407.mm.2	Gm21953/Gm20878/Gm21586	predicted gene, 21953/predicted gene, 20878/predicted gene, 21586	-0.576377267938089	9.09199429024564	-1.50901201715676	0.178762496273834	0.999692817443324	-4.56698312196398	9.84050419363371	10.0112257432036	8.6196201951795	8.15412043920067	9.34031943511961	8.58617573513675	NA
TC1100000315.mm.2	Gpr75	G protein-coupled receptor 75	0.543365350940127	7.54466253229915	1.50839398575213	0.17891637415978	0.999692817443324	-4.56701937138371	8.12923506126196	8.04381710395591	7.72508110901634	7.36574575621231	7.34509910827553	6.65899705507286	NA
TC1400000851.mm.2	Spata13	spermatogenesis associated 13	0.547993002249106	6.2123900839183	1.50765777861932	0.179099835383317	0.999692817443324	-4.56706255258624	6.74240709261421	6.71536496930086	6.07147684652904	6.38480037651743	5.31324706882329	6.04704414972496	NA
TC0900003328.mm.2	Rsl24d1	ribosomal L24 domain containing 1	0.547905188980536	7.53102519357599	1.50745885363061	0.179149436902217	0.999692817443324	-4.56707422032999	7.61128126817677	7.77264218545647	7.62213280426349	7.82688623862839	7.55386536993245	6.79934329499836	NA
TC0300001932.mm.2	Pcdh18	protocadherin 18	0.600605643025975	5.41259465729234	1.50730150405274	0.179188680678212	0.999692817443324	-4.56708344953263	5.55763113417283	5.23929526461085	5.25299355140166	6.18693286410951	5.16021857196429	5.07849655749493	NA
TC0700004119.mm.2	Dkk3	dickkopf WNT signaling pathway inhibitor 3	-0.555559391286135	7.87202662610327	-1.50694708059132	0.179277104787326	0.999692817443324	-4.56710423800449	8.46018605400907	9.22858069293214	7.20100177571433	7.01513533783874	7.78739985304993	7.53985604307541	NA
TC1700001661.mm.2	Cacna1h	calcium channel, voltage-dependent, T type, alpha 1H subunit	-0.441219575937179	7.99042136829121	-1.50678603135012	0.179317297836562	0.999692817443324	-4.56711368427297	7.70596431617599	7.81713330429668	7.80422238385929	7.96427333484083	8.29795678985537	8.35297808071912	NA
TC0100000476.mm.2	Cd28	CD28 antigen	-0.495141008091902	5.0545549467367	-1.50581981531343	0.179558611268972	0.999692817443324	-4.56717035764317	4.57176145121016	4.94575587728218	4.74260964235136	5.16685552552065	5.4971597786162	5.40258740543961	NA
TC1200002518.mm.2	Asap2	ArfGAP with SH3 domain, ankyrin repeat and PH domain 2	0.49315128617822	6.87646672837722	1.50544954885927	0.179651165217439	0.999692817443324	-4.56719207579474	7.15755450543682	6.89294475344055	6.86485457039656	7.23244727147462	6.72371269317277	6.38728657634197	NA
TC1100003838.mm.2	Fam171a2	family with sequence similarity 171, member A2	-0.51716108657619	8.61626995530476	-1.50476221866838	0.17982309115266	0.999692817443324	-4.56723239172951	9.12782852466851	9.13646715993062	8.33793221084684	7.86156872619168	8.37692574356317	8.85689736662773	NA
TC1500000244.mm.2	Kcns2	K+ voltage-gated channel, subfamily S, 2	0.508838993249427	5.50314989137999	1.50398103377104	0.180018678107649	0.999692817443324	-4.56727821319123	6.15231540878354	6.01128592005327	5.62864876471023	5.30783923826076	5.22754243329374	4.6912675831784	NA
TC0900001863.mm.2	Keap1	kelch-like ECH-associated protein 1	-0.636608452488259	6.34077263318322	-1.50375473389535	0.180075374003885	0.999692817443324	-4.56729148719487	7.32133830422828	7.35004295001528	6.07127900598743	4.97873981395219	6.05118259145232	6.27205313346382	NA
TC0100003739.mm.2	Marc2	mitochondrial amidoxime reducing component 2	-0.480248975363162	7.21631606783639	-1.50365054310067	0.180101482924484	0.999692817443324	-4.5672975986943	7.29936538768798	7.60939508572175	7.04354264464666	6.6707763467945	7.3988792075422	7.27593773462528	NA
TC0700001948.mm.2	Bccip	BRCA2 and CDKN1A interacting protein	-0.534281293257436	6.78104748609667	-1.50359849141905	0.180114527740126	0.999692817443324	-4.56730065188246	7.57878708995151	7.60923884293871	6.48139051445948	5.73345768412797	6.6628925277363	6.62051825736602	NA
TC1000002780.mm.2	Nts	neurotensin	1.01319828231405	6.59682836742741	1.503235520067	0.180205517261276	0.999692817443324	-4.56732194269077	8.08371760296868	6.63268224339089	6.9103258713092	6.53515759010729	6.50559252961714	4.91349436717125	NA
TC0Y00000547.mm.2	Gm20871	Sycp3 like Y-linked pseudogene	-0.468668873359258	4.91483416103834	-1.50311962477931	0.180234578790489	0.999692817443324	-4.56732874077774	4.10685595386594	3.9849008189059	5.25457212370436	4.97538309966548	5.42893461243807	5.73835835765029	NA
TC0700001187.mm.2	Efl1	elongation factor like GTPase 1	0.451923698789058	5.39078466823904	1.50162930050858	0.180608673658134	0.999692817443324	-4.56741615968559	5.69885022195125	5.99738423241556	5.32952141723818	5.4466390590846	4.94452582647418	4.92778725227048	NA
TC0X00002450.mm.2	Lage3	L antigen family, member 3	-0.595174821028467	7.73763886099723	-1.50114921819722	0.180729334500157	0.999692817443324	-4.56744432046065	7.13460608079767	7.2635825538592	8.16640250624194	7.25224493839285	8.4685472848295	8.14044980186221	NA
TC0X00003164.mm.2	Apex2	apurinic/apyrimidinic endonuclease 2	-0.515716533647142	5.4859342491073	-1.49969594446789	0.181095044910209	0.999692817443324	-4.56752956767953	5.52871924910016	5.74821392989068	4.83707501421571	5.46654460996364	5.72816629308933	5.6068863983843	NA
TC0300001067.mm.2	Rsbn1	rosbin, round spermatid basic protein 1	0.756274228050635	6.58799770762057	1.49946280855784	0.18115377615806	0.999692817443324	-4.56754324324185	7.17449947807092	6.96817279861431	7.27105798363838	6.17787322893136	6.46121701998843	5.47516573648004	NA
TC0X00001892.mm.2	Maob	monoamine oxidase B	0.537012351610666	6.02096311874506	1.49918529322922	0.181223710321729	0.999692817443324	-4.56755952209944	7.51076043555617	6.88490152383881	5.81254521214572	5.58952551600265	5.35573010358137	4.97231592134567	NA
TC0100002395.mm.2	Tmem237	transmembrane protein 237	-0.607715490983354	5.83825496426521	-1.49784312176966	0.181562290779529	0.999692817443324	-4.56763825345781	5.96165274147052	6.27913229151022	5.7852042252478	5.0014526600741	5.7093594736676	6.29272839362101	NA

**TC0500002945.mm.2**	Mfsd7a	major facilitator superfamily domain containing 7A	0.820773961739356	4.94053119794731	1.49767346191756	0.181605131247449	0.999692817443324	-4.56764820570856	4.08665447275151	4.87055735698117	4.90370624657665	6.26005539413829	4.46665448787442	5.05555922936182	NA
**TC1100000223.mm.2**	Tmem17	transmembrane protein 17	0.453221640630383	6.2657173696902	1.49708564547648	0.181753631738738	0.999692817443324	-4.56768267712678	7.00938530707607	6.86442536302303	6.19031191835944	6.12315649629199	5.85469082254419	5.55233431084647	NA
**TC0300002526.mm.2**	Nudt17	nudix (nucleoside diphosphate linked moiety X)-type motif 17	0.45902135118208	6.00894000968105	1.49692600880162	0.181793980206077	0.999692817443324	-4.56769205146695	6.25460806599494	6.18586983889644	6.07090098477621	6.19470144300332	5.49523730977214	5.85232241564323	NA
**TC0X00000525.mm.2**	Fhl1	four and a half LIM domains 1	0.548134403865686	7.94511971413426	1.49690410907498	0.181799516047343	0.999692817443324	-4.56769333611294	9.05125271775812	8.6098048597771	7.89772862743115	7.65523613006971	7.54814197042861	6.90855397934088	NA
**TC0700004330.mm.2**	1700008J07Rik	RIKEN cDNA 1700008J07 gene	0.46389539743859	5.82260982522413	1.49542062759021	0.182174874649926	0.999692817443324	-4.56778035809597	5.9609151055339	5.67505529051602	6.05506618630447	6.05867348878156	5.43956365831674	5.7463852218921	NA
**TC0800003246.mm.2**	Agrp	agouti related neuropeptide	0.559322866245415	5.31847633788723	1.49542013723993	0.182174998839122	0.999692817443324	-4.56778038686036	4.91680914832794	5.07240069017975	5.5018241693001	6.01832279135316	4.86666283347396	5.53483839468846	NA
**TC0700002175.mm.2**	Isoc2b	isochorismatase domain containing 2b	0.645785016069401	6.81034963848454	1.49483642277018	0.182322889413913	0.999692817443324	-4.56781462813651	6.87583768444154	6.45659084993337	6.76245552765937	7.64816413667619	6.25198563174298	6.86706400045378	NA
**TC1500001963.mm.2**	Nfam1	Nfat activating molecule with ITAM motif 1	0.637111926884297	5.14234648533502	1.4947315219327	0.182349478944596	0.999692817443324	-4.56782078173625	5.07292736690824	4.91324719975831	5.57948064908727	5.49158345046882	4.34127760744139	5.45556263834611	NA
**TC1600001742.mm.2**	Nfkbiz	nuclear factor of kappa light polypeptide gene enhancer in B cells inhibitor, zeta	-0.605311400987699	5.64590383135915	-1.49470007553297	0.18235745045466	0.999692817443324	-4.56782262641767	5.6182275645545	5.95089383903457	4.78998880344898	5.75785058784624	5.88373181299775	5.87473038027287	NA
**TC1900001124.mm.2**	Ms4a8a	membrane-spanning 4-domains, subfamily A, member 8A	0.645924271650681	5.60206890110462	1.49467941112312	0.182362688957449	0.999692817443324	-4.56782383861552	5.00509296157728	5.13613425194846	5.73882568654309	6.64269168165859	5.18917890335986	5.90048992154046	NA
**TC0100003834.mm.2**	Utp25	UTP25 small subunit processome component	0.493183819963835	5.6239224141444	1.49448899195068	0.182410967438629	0.999692817443324	-4.5678350088274	5.81656677950269	5.70103185998223	5.60655955769821	5.99959218495634	5.51665568693285	5.10312841579404	NA
**TC1000001934.mm.2**	Rnf146	ring finger protein 146	-0.6332228654535	6.54977254413836	-1.49419217402963	0.182486245539394	0.999692817443324	-4.56785242053591	7.35637651216831	7.08138415970774	6.4113571320714	5.38585729895217	6.7056195295366	6.35804063239396	NA
**TC1300000359.mm.2**	Wrnip1	Werner helicase interacting protein 1	-0.45554009458177	6.64310594530257	-1.49414894428086	0.182497211732271	0.999692817443324	-4.56785495644887	7.18263571601288	6.97585974051369	6.27918627019086	6.1153437428718	6.77941290179417	6.52619730043202	NA
**TC1600000534.mm.2**	Hgd	homogentisate 1, 2-dioxygenase	-0.516772615762918	5.05655205121512	-1.49383206917106	0.182577612743274	0.999692817443324	-4.56787354476844	4.48807572543749	4.80426168914877	5.13963485214104	4.86707997844827	5.79540589297135	5.24485416914379	NA
**TC1700000999.mm.2**	Tnfaip8l1	tumor necrosis factor, alpha-induced protein 8-like 1	0.559099482996727	5.24409552403676	1.49311936529637	0.182758567080239	0.999692817443324	-4.56791535303227	4.63620847544488	4.68839142776116	5.53404108553474	6.09504501796924	5.5810574336219	4.92982970388863	NA
**TC1700000376.mm.2**	Tekt4	tektin 4	0.521141159624961	5.53205699841091	1.49309408221858	0.182764989447317	0.999692817443324	-4.56791683617737	5.47755312765599	5.35937874326485	5.5019791702223	6.19686704917498	5.28296947749594	5.37359442265142	NA
**TC1900001547.mm.2**	Dnmbp	dynamin binding protein	0.605558404128725	5.97484711547222	1.49257605880557	0.18289662273204	0.999692817443324	-4.56794722427828	5.94526616980366	5.50692985551594	6.64056892427313	6.16343281361246	5.39786970941843	6.19501522020971	NA
**TC0X00000941.mm.2**	Eda	ectodysplasin-A	0.675907080084092	5.8447861611395	1.49179149145383	0.183096153041748	0.999692817443324	-4.56799324839366	5.29942238837636	5.23070695946505	5.84924001065276	7.09596087892911	5.72700242421966	5.86638430519403	NA
**TC0900002008.mm.2**	Tirap	toll-interleukin 1 receptor (TIR) domain-containing adaptor protein	0.476040220337588	7.08535967311613	1.49153843802743	0.183160552083843	0.999692817443324	-4.56800809298325	6.52705330973907	7.01231004344953	7.43486726874964	7.52757029434203	7.03775969320844	6.97259742920806	NA
**TC1000003105.mm.2**	Pip4k2c	phosphatidylinositol-5-phosphate 4-kinase, type II, gamma	-0.922951919483217	8.14445670034037	-1.49098974387644	0.183300259817151	0.999692817443324	-4.56804028045205	8.51471393225515	8.47165525021162	8.44687538923259	6.57035820107191	8.72454189439984	8.13859553487109	NA
**TC1700000523.mm.2**	Glp1r	glucagon-like peptide 1 receptor	0.526362212196156	7.51940283058801	1.49033337580618	0.183467512301133	0.999692817443324	-4.5680787843448	8.77753785005308	8.33034099315176	7.55794056720918	6.97269071514857	6.73650124675947	6.74140561120597	NA
**TC0600003148.mm.2**	Cd9	CD9 antigen	-0.519996308218809	8.80758486179154	-1.49021597260075	0.183497443227059	0.999692817443324	-4.56808567146315	9.04717243924388	9.59727386185285	8.06845711463979	8.51207801196765	8.77565517648994	8.84487256655513	NA
**TC0700000997.mm.2**	Lins1	lines homolog 1	0.608981043049052	5.60719798392853	1.49001667026417	0.183548263923438	0.999692817443324	-4.56809736296033	5.68063590477515	5.43437658670858	6.2144614380272	5.65860731106559	5.75092709130135	4.90417957169334	NA
**TC0500002266.mm.2**	Man2b2	mannosidase 2, alpha B2	0.439724146160508	7.2075792150924	1.48999768637857	0.18355310535721	0.999692817443324	-4.56809847659552	7.49389567918325	7.56561989624619	7.24963068066479	7.28307332305819	6.94075356259655	6.71250214880542	NA
**TC1100000822.mm.2**	Pmp22	peripheral myelin protein 22	0.536402692901614	7.7439948377097	1.48986834102287	0.183586095253452	0.999692817443324	-4.56810606427088	8.34004333514022	8.39234726612339	8.07562509492259	7.32656681047632	7.26659018353351	7.06279633606218	NA
**TC0600000637.mm.2**	Creb5	cAMP responsive element binding protein 5	0.521234489352757	6.4301405616245	1.48880646977274	0.183857135021043	0.999692817443324	-4.56816835597478	6.4463505532996	6.34901731911573	6.35197365351485	7.06199858450375	6.201877913875	6.16962534543809	NA
**TC1700000646.mm.2**	Dxo	decapping exoribonuclease	-0.460767198124861	6.3690282107082	-1.48845525960267	0.183946861536638	0.999692817443324	-4.56818895875615	6.33449635801752	6.69018697239497	6.16819863637323	5.96577713242025	6.49948424412112	6.55602592092209	NA
**TC1100001443.mm.2**	Mrpl27	mitochondrial ribosomal protein L27	-0.48050591445483	8.25293081282041	-1.48840433402866	0.183959875253573	0.999692817443324	-4.56819194616591	9.10198737764209	8.71208825387697	7.5741150716645	7.79713363658239	8.02749587102653	8.30476466613002	NA
**TC0600001579.mm.2**	Etv6	ets variant 6	0.541642538545762	7.5659692569311	1.48678959984981	0.184372950462932	0.999692817443324	-4.56828667018444	7.95534034771532	8.29579832698845	7.90163015291862	7.21235081906856	7.13166185149725	6.8990340433984	NA
**TC1200001273.mm.2**	Clba1	clathrin binding box of aftiphilin containing 1	0.563960789803737	6.5542194676554	1.48630963943397	0.184495896647104	0.999692817443324	-4.56831482576882	6.58829127711858	6.37677262738151	6.48286923087626	7.26121800964362	6.12145042559938	6.49471523531302	NA
**TC1900000127.mm.2**	Ppp1r14b	protein phosphatase 1, regulatory inhibitor subunit 14B	-0.955483925478473	7.89700441857863	-1.48481972024339	0.184878033696156	0.999692817443324	-4.56840222780246	7.85798249665249	8.22408433164076	8.35803204418967	6.33646387192113	8.19872592298421	8.40673784408353	NA
**TC0400004135.mm.2**	Rer1	retention in endoplasmic reticulum sorting receptor 1	-0.461041110512505	7.78806262966715	-1.48470562385963	0.184907327336041	0.999692817443324	-4.56840892094839	7.91962838450116	7.83572827760979	7.53307502257457	7.49239342485889	7.76780243423517	8.1797482342233	NA
**TC0900001017.mm.2**	Gm7972	predicted gene 7972	-0.483024758867253	6.08999987253234	-1.4846633754121	0.18491817548372	0.999692817443324	-4.56841139933536	5.82090261874108	5.92190301087628	5.75119714348038	6.16437490044071	6.2685868202034	6.61303474145219	NA
**TC0500001636.mm.2**	Zcwpw1	zinc finger, CW type with PWWP domain 1	0.877486300771505	5.17309283174562	1.48462486414685	0.184928064544048	0.999692817443324	-4.56841365849084	5.56115014127868	5.74184277158399	5.1237322191333	5.62153612044373	3.60763215791616	5.38266358011786	NA
**TC1700000862.mm.2**	Aars2	alanyl-tRNA synthetase 2, mitochondrial	0.518741914303437	6.40912877462292	1.48442632845017	0.184979052970126	0.999692817443324	-4.56842530502896	6.45834956493757	6.22156772077148	6.36683666017308	7.03933293514459	6.17294751173139	6.19573825497941	NA
**TC0500002999.mm.2**	Pus1	pseudouridine synthase 1	-0.438755773293608	6.02078371471132	-1.48397424981616	0.185095205178083	0.999692817443324	-4.56845182493459	6.36477565803827	6.40119325270302	5.61823943284528	5.62237148262441	5.93540679307752	6.1827156689794	NA
**TC1200000201.mm.2**	Klf11	Kruppel-like factor 11	-0.514632919296182	6.40564722837644	-1.48335530840081	0.185254338121868	0.999692817443324	-4.56848813332924	5.77321866024426	5.44012236830019	6.51139704497181	6.5842412065891	6.75830307320607	7.36660101694721	NA
**TC0200000019.mm.2**	Frmd4a	FERM domain containing 4A	0.525167794473475	5.42608545867792	1.48320199299917	0.185293775723271	0.999692817443324	-4.56849712712228	5.98069670333744	5.80478877452898	5.69792272518595	5.21275870638808	5.1967814434616	4.66356439916548	NA
**TC0300002756.mm.2**	Prpf38b	PRP38 pre-mRNA processing factor 38 (yeast) domain containing B	0.873076112020627	8.0305753880015	1.4826830450113	0.185427322975409	0.999692817443324	-4.56852756963856	8.88708161815013	9.22636809605182	8.30164915298347	7.60642826594069	7.91752648248867	6.24439871239423	NA
**TC0400001430.mm.2**	Yrdc	yrdC domain containing (E.coli)	-0.47696820209486	5.55398235931347	-1.48168177845988	0.185685241365234	0.999692817443324	-4.56858630578028	5.97075506478704	5.70550042587246	5.32135247327993	5.02549865723588	5.48224643632342	5.81854109838211	NA
**TC0700002950.mm.2**	Cd33	CD33 antigen	0.561166781421654	6.14785066051094	1.47991125665108	0.18614212131401	0.999692817443324	-4.56869016730624	5.91166585269638	6.3627749529259	6.1862583571892	6.68124000295413	5.58147553693313	6.16368926036689	NA
**TC1200002025.mm.2**	Med6	mediator complex subunit 6	1.39166747262821	6.58602676953684	1.47951974636383	0.18624328924521	0.999692817443324	-4.56871313378812	7.78548859262595	7.8961194110843	7.40678447953496	5.90215929984864	6.68067418047668	3.84493465365051	NA
**TC1200002239.mm.2**	Ppp4r3a	protein phosphatase 4 regulatory subunit 3A	-1.38686483574797	6.78346202235427	-1.47921668803702	0.186321635506859	0.999692817443324	-4.56873091153625	7.58732059311883	7.98409309731634	7.11327723352786	4.06453715256939	7.42061378776387	6.53093026982932	NA
**TC1700001310.mm.2**	Socs5	suppressor of cytokine signaling 5	0.476723591976481	6.99638042078056	1.47853599276526	0.186497718422869	0.999692817443324	-4.56877084179351	7.10148410940166	7.29729312772816	7.00396263866017	7.26251359709309	6.86272964700809	6.4502994047922	NA
**TC0700001576.mm.2**	Syt9	synaptotagmin IX	0.733440999795552	5.80453428460801	1.47835243453173	0.18654522761455	0.999692817443324	-4.56878160947451	6.71694766584699	6.06503342387546	5.46168483994085	6.29436846881752	5.64094740261997	4.64822390654729	NA
**TC0600001044.mm.2**	4933427D06Rik	RIKEN cDNA 4933427D06 gene	0.497775640460711	5.15545325298132	1.47825686398696	0.186569967923018	0.999692817443324	-4.56878721571877	4.63563963728232	4.8777966932163	5.38071398132122	5.82670325283413	5.29234015601444	4.91952579721949	NA
**TC1600001184.mm.2**	Grin2a	glutamate receptor, ionotropic, NMDA2A (epsilon 1)	-0.679093132103728	7.56200095352502	-1.47824999477326	0.186571746269728	0.999692817443324	-4.56878761867219	8.38952569646514	8.77145374743184	7.16399240579873	6.26242760072412	7.80876931522151	6.9758369555088	NA
**TC1100001893.mm.2**	Kctd2	potassium channel tetramerisation domain containing 2	-1.30151718110538	6.16458748204635	-1.47823578242943	0.18657542570316	0.999692817443324	-4.56878845237929	6.67704133959671	6.56301726366776	6.77599798425982	3.79621797914163	6.65133493966053	6.52391538595168	NA
**TC1100001006.mm.2**	Zzef1	zinc finger, ZZ-type with EF hand domain 1	0.493509991473528	6.50095473722059	1.47799960116841	0.186636580425729	0.999692817443324	-4.56880230694457	7.74794466374436	7.45071207167511	6.1239617590637	6.27308407636188	5.96072977409655	5.44929607838197	NA
**TC0500000663.mm.2**	Gabrb1	gamma-aminobutyric acid (GABA) A receptor, subunit beta 1	0.625715021888412	7.19678500392374	1.47750133344111	0.186765657951011	0.999692817443324	-4.56883153562675	8.97082862694747	8.817430658744	7.08271423404697	6.23922615676692	6.3922826617184	5.67822768531867	NA
**TC0800002815.mm.2**	Exoc3l	exocyst complex component 3-like	0.872867728050284	5.26635927711522	1.47703720630142	0.186885965034739	0.999692817443324	-4.56885876151676	4.6692083315945	4.71644149356912	5.37016531842292	6.6089553283912	4.4287635744533	5.80462161626026	NA
**TC1700000344.mm.2**	Pkd1	polycystin 1, transient receptor poteintial channel interacting	-0.43013479854992	7.30051619912954	-1.47677256072521	0.18695459606944	0.999692817443324	-4.56887428569747	7.15224306278	7.25865423240259	7.19977199144395	7.06619315980344	7.55689030614012	7.56934444220712	NA
**TC0200004459.mm.2**	Tmem87a	transmembrane protein 87A	-1.27901073424311	6.19589711108225	-1.4758862890182	0.187184603176549	0.999692817443324	-4.56892627442087	7.56073880642722	7.67214369425769	6.27738077451494	3.41485857414623	6.43767099561636	5.81258982153105	NA
**TC0400001495.mm.2**	Csmd2	CUB and Sushi multiple domains 2	-0.570850703433591	6.0519621112008	-1.4755420864473	0.187274001393707	0.999692817443324	-4.56894646526053	6.82652577113115	6.40907457041323	5.87082304769001	5.09641241170662	6.20382279990159	5.90511406636223	NA
**TC0200004506.mm.2**	Duox2	dual oxidase 2	-0.576431583400414	5.97660442229435	-1.47548480093372	0.187288883712629	0.999692817443324	-4.56894982560968	5.48922987131897	5.08201434235744	5.86503260127474	6.2027269753697	6.21619278853463	7.00442995491064	NA
**TC0500001656.mm.2**	Zfp157	zinc finger protein 157	-0.562277141508001	6.48107607444436	-1.47431218443101	0.187593758821481	0.999692817443324	-4.56901861055909	6.69613667390727	6.10139902842391	5.90710743076781	6.57507579989167	6.85090408062535	6.75583343305014	NA
**TC1600001770.mm.2**	St3gal6	ST3 beta-galactoside alpha-2,3-sialyltransferase 6	-0.568965718772018	6.3835037463452	-1.47372823040087	0.187745754163331	0.999692817443324	-4.5690528646794	6.17800408635185	6.66361367785238	6.20595287639869	5.95478376176278	7.02226832321944	6.27639975248607	NA
**TC1200001868.mm.2**	Vcpkmt	valosin containing protein lysine (K) methyltransferase	0.578458212404955	5.88165084498386	1.47369716027745	0.187753844462913	0.999692817443324	-4.56905468721466	5.88657589685245	5.89708681267302	6.07884202476234	6.25273736783145	6.05888626605087	5.11577670173301	NA
**TC0800001033.mm.2**	Nkd1	naked cuticle 1	-0.511666378939974	5.67532589966996	-1.47273671435716	0.188004091309824	0.999692817443324	-4.56911102553182	6.31024001943023	5.77024875463971	5.4038557475671	5.07021118546784	5.8967936593853	5.60060603152959	NA
**TC1600000280.mm.2**	Abcf3	ATP-binding cassette, sub-family F (GCN20), member 3	-0.880977572816113	6.63479592258265	-1.47221783752489	0.188139413419686	0.999692817443324	-4.56914146185081	7.48799968905309	7.2745231593505	6.75623222503692	4.88591654569313	6.70429466748533	6.69980924887694	NA
**TC1200002108.mm.2**	Angel1	angel homolog 1	-0.495401906166467	5.78739737218184	-1.4718937236255	0.188223987062958	0.999692817443324	-4.56916047366916	6.03287857160722	5.75713075642152	5.74026673744258	5.23151880892211	6.1040915366258	5.85849782207182	NA
**TC0500000172.mm.2**	Fam185a	family with sequence similarity 185, member A	0.514779999326955	6.83956200631191	1.47144297740405	0.188341661880809	0.999692817443324	-4.56918691335848	7.5087635705429	7.1525822835244	6.88364395530628	6.81914913592277	6.64537512020221	6.02785797237292	NA
**TC0300001070.mm.2**	Phtf1	putative homeodomain transcription factor 1	1.22793065015872	5.88149497300102	1.47139423629283	0.188354390603639	0.999692817443324	-4.56918977238729	6.57015951411434	6.24002408132192	6.95236796422868	5.51495580721497	6.2015962590555	3.80986621207069	NA
**TC0X00001799.mm.2**	Ebp	phenylalkylamine Ca2+ antagonist (emopamil) binding protein	-0.6419775110813	6.81958190811905	-1.47103445313548	0.188448372254295	0.999692817443324	-4.56921087629983	7.48258940127303	7.67895802679775	6.5487887815836	5.68720571765686	6.37047275372837	7.14947676767469	NA
**TC1700001004.mm.2**	Kdm4b	lysine (K)-specific demethylase 4B	-0.446588647735699	6.53085483403998	-1.47040265639783	0.18861351270464	0.999692817443324	-4.56924793559515	7.19401980093029	7.03324478546915	5.86042305228399	6.17192050890054	6.44197563570066	6.48354522095527	NA
**TC1400000744.mm.2**	Mrpl52	mitochondrial ribosomal protein L52	-0.579259145969828	7.265250821202	-1.47029038101554	0.188642873382393	0.999692817443324	-4.56925452130359	7.9735536158272	7.99384540325389	7.01973708900725	6.21305671908836	7.46181912750744	6.92949297252783	NA
**TC0800001748.mm.2**	Tdrp	testis development related protein	0.498374696932267	6.00286149445696	1.47014666225442	0.188680462807133	0.999692817443324	-4.56926295136588	5.55399896164812	5.34951279278978	6.33511516109162	6.72008814199258	5.79142257638925	6.26703133283042	NA
**TC1700002354.mm.2**	Gpr108	G protein-coupled receptor 108	0.531871343338528	7.11739487564221	1.46899863348991	0.188980974668741	0.999692817443324	-4.56933029038386	7.91010981981929	7.44332914163126	6.89566386025352	7.31167262928635	6.81328386740241	6.33030993546041	NA
**TC1400000177.mm.2**	Vcl	vinculin	-0.840890654168463	5.14524352051801	-1.46879777800549	0.189033596361384	0.999692817443324	-4.56934207170911	5.98641424507917	5.63469343995149	5.19673933720888	3.58754672766134	5.56486206806695	4.9012053051402	NA
**TC1300001448.mm.2**	Lgals8	lectin, galactose binding, soluble 8	0.594785838426724	7.01349253015589	1.46673038546435	0.189576009258914	0.999692817443324	-4.56946333435053	7.83730159777308	7.2200373582154	7.42701317025805	6.67958078064211	6.62698171253646	6.29004056151026	NA
**TC0900003237.mm.2**	Slc22a13b	solute carrier family 22 (organic cation transporter), member 13b	-0.460779119776311	5.36697134460977	-1.46643468079943	0.189653708524976	0.999692817443324	-4.56948067859198	5.13412870452308	5.3218840577949	5.16003480006458	5.25209373282942	5.46416214370098	5.86952462874563	NA
**TC1300002313.mm.2**	Exoc3	exocyst complex component 3	0.504845787519054	6.74174434858972	1.46630036704821	0.189689010401643	0.999692817443324	-4.56948855659776	7.97045182073881	7.76350893899201	6.67517386626898	6.18792458715384	6.14035257343471	5.71305430495	NA
**TC0400000057.mm.2**	Chd7	chromodomain helicase DNA binding protein 7	0.426073013747571	8.00253319857544	1.46607703715605	0.189747721848577	0.999692817443324	-4.56950165570102	7.61159006126791	7.55133765364628	8.42601640762437	8.42619234439242	8.07224560659949	7.92781711792215	NA
**TC1000002664.mm.2**	Apaf1	apoptotic peptidase activating factor 1	0.554100853746617	5.202060074932	1.46596301850101	0.189777702761356	0.999692817443324	-4.56950834328995	5.74682068610818	5.38317382627878	5.07082673772988	5.52445708461924	4.41276326657954	5.07431884827635	NA
**TC0300002784.mm.2**	Amy2a5/Amy2a4/Amy2a3/Amy2a2	amylase 2a5/amylase 2a4/amylase 2a3/amylase 2a2	-0.451277338201467	4.99684630087446	-1.46593568456294	0.18978489079521	0.999692817443324	-4.56950994651842	5.05374915692438	5.10602415377693	4.89995666799137	4.5594182410799	5.08305544737644	5.27887413809776	NA
**TC1800001654.mm.2**	Rbfa	ribosome binding factor A	-0.491623029171236	6.43746876522046	-1.46584420524878	0.189808949019191	0.999692817443324	-4.56951531208686	6.74953197264683	6.82529432040521	6.29910078290574	5.73426933705838	6.3857029444754	6.63091323383118	NA
**TC0200001751.mm.2**	Chst14	carbohydrate sulfotransferase 14	-0.505461740667309	6.45864580967877	-1.46579401244516	0.189822150455224	0.999692817443324	-4.56951825606001	5.97943751830885	5.96886751196827	6.18265662575725	6.71366654747319	6.73826523081287	7.16898142375218	NA
**TC0700002591.mm.2**	Zfp111	zinc finger protein 111	0.667360779377188	5.92877666593128	1.46571479725642	0.189842986911434	0.999692817443324	-4.56952290228719	6.33990873196979	6.28023550152397	6.41488857863323	5.72873008179093	5.93598925500165	4.87290784666813	NA
**TC0700004279.mm.2**	Atp2a1	ATPase, Ca++ transporting, cardiac muscle, fast twitch 1	0.554579509347773	4.82212636867939	1.46524578100156	0.189966398068313	0.999692817443324	-4.56955041149724	4.30080034457675	4.71368485911028	5.04994051823654	5.46377549530589	5.03353393891931	4.37102305592757	NA
**TC0100003273.mm.2**	Lamc1	laminin, gamma 1	-0.537633588725816	6.08052518368543	-1.46467699781942	0.190116159272699	0.999692817443324	-4.56958377207708	6.30142120786234	6.26308795971149	6.07282553344259	5.34886184510097	6.43921888424658	6.05773567174862	NA
**TC0200004470.mm.2**	Ubr1	ubiquitin protein ligase E3 component n-recognin 1	-0.464198628843353	7.2618003772312	-1.46452196123222	0.190156999320857	0.999692817443324	-4.56959286531683	6.99009654899721	7.33818133310183	6.91704684318594	7.24001671861479	7.57758314947452	7.50787767001291	NA
**TC0500001599.mm.2**	Alkbh4	alkB homolog 4, lysine demethylase	-0.53121603892555	7.1657998321964	-1.46368413102916	0.190377841311304	0.999692817443324	-4.56964200553512	6.29401365073813	6.44734754011044	7.67846242461452	6.91704043762486	7.7437910971424	7.91414384294807	NA
**TC0400002069.mm.2**	Faap20	Fanconi anemia core complex associated protein 20	0.428716294281864	6.3650059938494	1.4636241018092	0.190393673307877	0.999692817443324	-4.56964552632958	6.75881308439391	6.62492240044976	6.47173212537691	6.36013440803131	5.93402232975209	6.0404116150924	NA
**TC1100001372.mm.2**	Ccdc182	coiled-coil domain containing 182	-0.428772261467699	5.57281556409283	-1.46356403384339	0.1904095167301	0.999692817443324	-4.56964904939311	5.03867502483523	4.85211848085845	5.65462035751056	5.6896573204534	6.06202906943226	6.1397931314671	NA

**TC0200004567.mm.2**	Mtln	mitoregulin	-0.554472167346598	7.07848233450218	-1.46332919279965	0.190471469583272	0.999692817443324	-4.56966282308982	7.51475908155037	7.69197435307594	6.95810128241422	6.11950683643257	6.98133631071451	7.20521614282547	NA
**TC1900001033.mm.2**	Flrt1	fibronectin leucine rich transmembrane protein 1	-0.483528102330142	6.99391572585903	-1.46310969824815	0.190529390603246	0.999692817443324	-4.56967569664942	7.03096508291069	7.23509744467093	6.86892899123393	6.49625882022219	6.96466733526541	7.367576680851	NA
**TC0700001178.mm.2**	Tm6sf1	transmembrane 6 superfamily member 1	-0.882898878447293	6.29755031224977	-1.46280127035387	0.190610806931164	0.999692817443324	-4.56969378615354	7.02572134545699	6.79598450709317	6.34559019906082	4.75330893296612	6.94111874054644	5.92357814837508	NA
**TC1000000512.mm.2**	Gcc2	GRIP and coiled-coil domain containing 2	-1.26171225490954	6.8524204503038	-1.46255151959146	0.190676757478919	0.999692817443324	-4.56970843413611	8.46632801896074	8.4743168879849	6.58245028674496	4.24277635578408	7.54548957705591	5.8031615752922	NA
**TC1500000829.mm.2**	Gramd4	GRAM domain containing 4	0.448831790225235	5.69016401668551	1.46254597293807	0.190678222395569	0.999692817443324	-4.56970875944886	5.97797795211585	5.75891162620465	5.9535768466559	5.69730220446563	5.47120966195139	5.28200580871967	NA
**TC1900001316.mm.2**	Rfx3	regulatory factor X, 3 (influences HLA class II expression)	0.853268458191032	8.17046279761091	1.46225375289418	0.190755414678733	0.999692817443324	-4.56972589819186	8.83085737000763	8.84003039087619	8.69261710028576	7.83659587029609	8.21519492861532	6.60748112558447	NA
**TC0500003023.mm.2**	Myo18b	myosin XVIIIb	0.56767459615316	5.08898379933602	1.46054246515094	0.191208039723051	0.999692817443324	-4.56982626368719	4.58662172201609	4.78265482878101	5.11950680568723	6.03048091307544	4.95065922696594	5.0639792994904	NA
**TC0200005114.mm.2**	Sdc4	syndecan 4	-0.425622273383451	7.89538912620988	-1.46042258745261	0.191239783445681	0.999692817443324	-4.56983329429172	8.65068634051609	8.74396452921946	7.26961979920396	7.29359987117444	7.78025119833709	7.63421301880821	NA
**TC1900001025.mm.2**	Plcb3	phospholipase C, beta 3	-0.42746152111566	6.02298991196321	-1.46005520027827	0.191337097940776	0.999692817443324	-4.56985484077099	5.96906410359663	5.88426937162808	5.94848503323302	5.76635644392859	6.29291631561281	6.27684820378012	NA
**TC1100001049.mm.2**	Mettl16	methyltransferase like 16	0.456844249034006	5.82967787579089	1.45989591689441	0.191379303428665	0.999692817443324	-4.56986418235825	6.90270428197639	6.57545771945827	5.50737897078281	5.69941790490653	5.03070105363733	5.262407323984	NA
**TC0700003908.mm.2**	Hbb-bt/Hbb-b2/Hbb-b1	hemoglobin, beta adult t chain/hemoglobin, beta adult minor chain/hemoglobin, beta adult major chain	-0.979487814928746	10.4548770721583	-1.45976432054317	0.191414179080053	0.999692817443324	-4.56987190014664	10.6190710807418	11.6375720076672	10.0883766270824	9.16844523025934	11.4512772801585	9.76452020704073	NA
**TC0700001149.mm.2**	Ngrn	neugrin, neurite outgrowth associated	-0.472906909598442	7.54621634671693	-1.45958055804908	0.191462889527169	0.999692817443324	-4.56988267731347	7.07628004138715	7.3112342611275	7.26527571978446	7.70670925951057	7.86105420583742	8.0567445926545	NA
**TC0200005499.mm.2**	Gm13889	predicted gene 13889	-0.466662186036298	6.92270758688708	-1.45918052049302	0.191568967840391	0.999692817443324	-4.56990613829002	6.82311966065501	6.60162485521067	6.77402108153242	6.81506723515968	7.03597536907674	7.48643731968795	NA
**TC1500000183.mm.2**	Ctnnd2	catenin (cadherin associated protein), delta 2	0.540394064273066	8.80745145227007	1.45916144288434	0.191574028009809	0.999692817443324	-4.56990725712881	9.42149181239225	9.25182881674638	9.21184829578852	8.41423981072546	8.36916289431443	8.17613708365341	NA
**TC1900000051.mm.2**	Slc29a2	solute carrier family 29 (nucleoside transporters), member 2	0.4772186284154	6.31037518379507	1.4589364302459	0.191633719878047	0.999692817443324	-4.56992045334638	6.72214984709586	6.62730352687041	6.5072719229187	6.22634556989878	5.65168631784362	6.12749391814306	NA
**TC0200002945.mm.2**	Itih2	inter-alpha trypsin inhibitor, heavy chain 2	-0.480867533692265	5.78220973957451	-1.45789337272693	0.191910646863558	0.999692817443324	-4.56998162432338	5.62892348206148	5.28204707530323	5.66482591954635	5.74545048680256	5.95654491913293	6.41546655460051	NA
**TC0800003060.mm.2**	Car5a	carbonic anhydrase 5a, mitochondrial	-0.524356422797146	5.69816293643396	-1.4578876616197	0.191912164143375	0.999692817443324	-4.56998195925252	5.20502147532033	5.24291256735555	5.39987663443812	5.94628873072866	5.92267769865515	6.47220051210592	NA
**TC1100003207.mm.2**	Srr	serine racemase	-0.422732359754493	8.51020986705035	-1.45743604462955	0.192032180758502	0.999692817443324	-4.5700084443081	8.60370740477676	8.6086236625969	8.2951551851666	8.20657652254313	8.61798891209786	8.72920751512086	NA
**TC0300000968.mm.2**	Prkab2	protein kinase, AMP-activated, beta 2 non-catalytic subunit	-0.809184987150331	6.00241281439515	-1.45732880585301	0.192060689403649	0.999692817443324	-4.57001473328492	6.60339360853863	6.95542691275604	5.74204207553358	4.6766011198542	6.65966706181232	5.37734610787613	NA
**TC0800002512.mm.2**	Lsm6	LSM6 homolog, U6 small nuclear RNA and mRNA degradation associated	-0.459998563711356	7.61345811395323	-1.4566602603237	0.192238504637281	0.999692817443324	-4.57005393956607	7.86913567573593	7.79047179866868	7.04381596856846	7.50675607237757	7.74374752600836	7.72682164236038	NA
**TC1700001614.mm.2**	Kctd5	potassium channel tetramerisation domain containing 5	-0.871146824306427	6.68906023047708	-1.45665078266444	0.192241026522994	0.999692817443324	-4.57005449537137	6.93418765622427	7.15367372052197	6.86110279856099	5.29100038019071	7.45336852202487	6.44102830533969	NA
**TC0900000804.mm.2**	Aagab	alpha- and gamma-adaptin binding protein	0.57058543926004	7.11400742881557	1.45586539395344	0.192450113703662	0.999692817443324	-4.57010055310732	8.21451932480633	8.10043911821706	6.91927809942362	6.83585040477142	6.77967811888606	5.8342795067889	NA
**TC0100000317.mm.2**	Gpr45	G protein-coupled receptor 45	0.565038630000552	7.37846337536072	1.45476868753879	0.192742428114815	0.999692817443324	-4.5701648662109	6.86575677385068	6.68404525399701	7.63964422962091	8.28588351253795	7.72476038174076	7.07069010041699	NA
**TC0900002333.mm.2**	Etfa	electron transferring flavoprotein, alpha polypeptide	0.626003309175366	6.84409958217895	1.45461261246973	0.192784061089674	0.999692817443324	-4.5701740186448	7.84098149965005	7.78014866839781	7.23181821355601	6.11591875813228	5.91435296982251	6.18137738351504	NA
**TC0200002550.mm.2**	Eya2	EYA transcriptional coactivator and phosphatase 2	-0.492067899438813	5.25388238292987	-1.45287304076663	0.193248647844466	0.999692817443324	-4.57027602705993	4.84285949058221	4.59186529782182	5.05668296691413	5.49553388823463	5.96687715267696	5.56947550134944	NA
**TC1000001947.mm.2**	Ncoa7	nuclear receptor coactivator 7	0.913092770353108	6.89601203267426	1.45280854128107	0.193265893348433	0.999692817443324	-4.5702798092277	7.98997197884657	7.85409435711041	7.19470291962462	6.48439278077277	6.86128670383536	4.99162345585582	NA
**TC0X00000884.mm.2**	Arx	aristaless related homeobox	0.648475006989314	7.3400006726341	1.45248346416727	0.193352832010904	0.999692817443324	-4.57029887124768	6.934164913832	6.95980399618299	7.25687397921491	8.4646185906692	7.109176346096	7.3153662098095	NA
**TC0200003365.mm.2**	Ggta1	glycoprotein galactosyltransferase alpha 1, 3	0.483034999073937	5.25163128452533	1.45233253286811	0.19339320923853	0.999692817443324	-4.5703077215756	5.47098957164716	5.90788549198645	5.47749313042083	5.0709981904123	4.75660172069056	4.8258196019947	NA
**TC0300002783.mm.2**	Amy2a3/Amy2a2/Amy2a5/Amy2a4	amylase 2a3/amylase 2a2/amylase 2a5/amylase 2a4	-0.451004782286179	5.00408662643386	-1.45160481059273	0.193587998029071	0.999692817443324	-4.57035039338913	5.01163651756431	5.1347240552926	4.91890823172496	4.56916657886198	5.09225867332795	5.29782570183135	NA
**TC1600001311.mm.2**	Dgcr8/Mir1306	DGCR8, microprocessor complex subunit/microRNA 1306	-0.645410188883434	6.31154235480231	-1.45137742972647	0.193648897599611	0.999692817443324	-4.57036372627839	6.02114279800685	6.11542772416617	5.62667955916995	6.59425205526704	6.38966929655917	7.12208269564469	NA
**TC1900001219.mm.2**	Pcsk5	proprotein convertase subtilisin/kexin type 5	-0.437072004494603	5.41406568715975	-1.45109490277736	0.193724591357755	0.999692817443324	-4.57038029265356	5.53295104749861	5.49178162013925	5.20938975627529	5.08336896689043	5.43559333694572	5.73130939520921	NA
**TC1100000056.mm.2**	Thoc5	THO complex 5	0.5065661789776	6.71166277481232	1.45084552665708	0.193791425895244	0.999692817443324	-4.57039491508503	7.17566409510919	7.10271215713075	6.43924483984307	7.06312153745152	6.41162211430712	6.07761190503226	NA
**TC1100001504.mm.2**	Skap1	src family associated phosphoprotein 1	-0.536665983083212	5.64851983864862	-1.45064602198471	0.193844909696464	0.999692817443324	-4.57040661318567	5.33740574955246	5.21687339859795	5.35300066867928	5.77875329010816	5.7480502975376	6.45703562741627	NA
**TC0400002174.mm.2**	Car8	carbonic anhydrase 8	-0.470983598385192	7.01075893640952	-1.44991569014718	0.194040814204255	0.999692817443324	-4.57044943621495	7.17410238988133	7.49326868694383	6.79036407905287	6.43724359337791	7.21589226411213	6.95368260508903	NA
**TC0200004939.mm.2**	Cdk5rap1	CDK5 regulatory subunit associated protein 1	0.506775989122881	5.65439651655013	1.44991331557291	0.194041451455724	0.999692817443324	-4.57044957544688	5.87460247860955	5.62367799876014	5.6101385068311	6.11068679325733	5.13232648790572	5.57494683393695	NA
**TC0600002221.mm.2**	Zfp467	zinc finger protein 467	0.4356026618157	6.75055656791876	1.44955201362485	0.194138434341063	0.999692817443324	-4.57047076009888	6.94410577171447	7.00008245013283	6.88485106151605	6.83032719313228	6.26905309783681	6.57491983318011	NA
**TC0400001419.mm.2**	Fhl3	four and a half LIM domains 3	-0.462170402421803	6.30795464919067	-1.44942930899382	0.194171381535369	0.999692817443324	-4.57047795474194	5.67148930992845	5.92281516582888	6.44855444212337	6.21598686514816	6.97376331548578	6.61511879662936	NA
**TC1400002523.mm.2**	Pcdh20	protocadherin 20	-0.505694125483596	5.57076380151928	-1.44856925505073	0.194402456329024	0.999692817443324	-4.57052838235047	5.69158900454084	6.13039707715671	5.4149171973386	4.88068704088687	5.73667287028052	5.57031961891214	NA
**TC1400002457.mm.2**	Gm6994	predicted gene 6994	-0.45907229631496	5.95728595640086	-1.44847394598435	0.194428078912287	0.999692817443324	-4.57053397054329	5.25716562675803	5.22795309277374	5.98512600353842	6.18510020958333	6.32666251210845	6.76170829364322	NA
**TC1300001836.mm.2**	Bloc1s5	biogenesis of lysosomal organelles complex-1, subunit 5, muted	0.465570036353856	6.10782106829913	1.4480053697635	0.194554094267346	0.999692817443324	-4.57056144405135	6.88415521412501	6.63588986839992	5.81787061055532	6.21114008943346	5.66240501741729	5.43546560986378	NA
**TC0700001349.mm.2**	Ndufc2	NADH:ubiquinone oxidoreductase subunit C2	-0.985091702733863	9.39126178698704	-1.44784683218483	0.194596747039941	0.999692817443324	-4.5705707393298	9.96166388018114	9.96834657182029	9.61216256057978	7.61152587164677	10.0684866660691	9.1253851716252	NA
**TC0400003545.mm.2**	Ago4	argonaute RISC catalytic subunit 4	-0.708381340461771	5.21973887336683	-1.44750015835637	0.194690045509192	0.999692817443324	-4.57059106515763	5.38930615252188	5.06867973307836	4.17029269723876	5.55154963959985	5.61340112471743	5.52520389304472	NA
**TC0600003321.mm.2**	Grin2b	glutamate receptor, ionotropic, NMDA2B (epsilon 2)	-0.567231204985863	8.25518883401232	-1.44663307665107	0.194923577285132	0.999692817443324	-4.57064190215859	9.25372594340507	9.51721878198206	7.62915422154613	7.18370871281141	8.37607533725367	7.5712500070756	NA
**TC0500000359.mm.2**	Gm9903	predicted gene 9903	0.708802122803209	5.2639414693361	1.44628608815344	0.195017103535637	0.999692817443324	-4.5706622457484	5.09494071569231	4.6431477292812	5.31862287244385	6.31295943588089	4.65024270893394	5.56373535378438	NA
**TC0300001903.mm.2**	Gm10731	FUN14 domain containing 2 pseudogene	-0.480796570943578	5.08531458037673	-1.44589111268331	0.195123613867656	0.999692817443324	-4.57068540252256	5.06032821476371	4.60191209053012	4.89295182128431	5.05107519625541	5.27589863836278	5.62972152106409	NA
**TC0700000019.mm.2**	Rps9	ribosomal protein S9	-0.439481941940371	7.83646095707978	-1.44588539216034	0.195125156871662	0.999692817443324	-4.57068573790567	8.56508203951913	8.62317679168889	7.30782221822591	7.16794929546906	7.51650769985243	7.83822769772328	NA
**TC0200000953.mm.2**	Scn2a	sodium channel, voltage-gated, type II, alpha	-0.732689843726408	7.99044163057076	-1.44555572629962	0.195214096870428	0.999692817443324	-4.57070506547899	9.29226574306038	9.2558827955499	7.73054975856174	6.234011020119	7.73366175901807	7.69627870711548	NA
**TC0100003449.mm.2**	Mpzl1	myelin protein zero-like 1	0.44613553948551	7.17963501977276	1.44517895093527	0.195315791737801	0.999692817443324	-4.5707271547516	6.74559152779423	6.66500145296661	7.8276779663464	7.45206614207696	7.25759738401778	7.12987564543456	NA
**TC1600001764.mm.2**	Col8a1	collagen, type VIII, alpha 1	-0.506356801176159	6.3587460622711	-1.44492036980807	0.195385612931485	0.999692817443324	-4.57074231449038	6.47948364953924 8	5.23229694535032	6.49573664851152	7.06057801675423	7.24188698425706	7.32714128336101	NA
**TC1000000929.mm.2**	Hcfc2	host cell factor C2	-0.83407537406807	5.77637115216207	-1.44400517644109	0.195632913184734	0.999692817443324	-4.57079596825488	6.68498576048192	6.33961299792929	5.84342856353821	4.13931013967434	6.05234283972468	5.59854661162401	NA
**TC0500001717.mm.2**	Wipi2	WD repeat domain, phosphoinositide interacting 2	-0.617229981737463	7.88429204590331	-1.44391280663171	0.195657888870537	0.999692817443324	-4.57080138341382	8.66721524480934	8.37581918102632	7.80467703686609	6.70945190618857	7.90572919866595	7.84285970786362	NA
**TC0100000469.mm.2**	Cyp20a1	cytochrome P450, family 20, subfamily a, polypeptide 1	0.431357222424147	6.659773631867	1.44262829566357	0.196005506581796	0.999692817443324	-4.57087668594475	7.25698467317958	7.3665900905247	6.41279681959592	6.68609391657708	6.13669948491049	6.09947680641421	NA
**TC1400002666.mm.2**	Spry2	sprouty RTK signaling antagonist 2	-0.50405657311986	7.26739885075066	-1.44254133606427	0.196029060148079	0.999692817443324	-4.57088178371353	7.29450870250161	7.72983723223937	7.16132956615457	6.62463744560705	7.48505360714599	7.30902655085536	NA
**TC1100003712.mm.2**	Krtap3-3/Krtap3-2	keratin associated protein 3-3/keratin associated protein 3-2	0.464980061246147	6.97539131619511	1.44206602283409	0.196157847379487	0.999692817443324	-4.57090964740804	6.77336443906497	6.75802394787706	7.05717845603769	7.56828136032276	6.90673267828912	6.78876701557903	NA
**TC1100003713.mm.2**	Krtap3-2	keratin associated protein 3-2	0.464980061246147	6.97539131619511	1.44206602283409	0.196157847379487	0.999692817443324	-4.57090964740804	6.77336443906497	6.75802394787706	7.05717845603769	7.56828136032276	6.90673267828912	6.78876701557903	NA
**TC0800001494.mm.2**	Trappc2l	trafficking protein particle complex 2-like	0.573346967455659	8.82682177624201	1.44206301027021	0.196158663886562	0.999692817443324	-4.57090982400849	9.57311826950293	8.95301024013826	9.03718656776249	8.75356147359861	8.67780482890203	7.96624927754775	NA
**TC0700000733.mm.2**	Zfp788	zinc finger protein 788	0.931993004994935	6.21513341718116	1.44189099303099	0.196205291535396	0.999692817443324	-4.57091990785721	7.5360510217434	7.1033493272732	6.53554549794471	5.7221475840854	6.12727700118391	4.26643007085633	NA
**TC1300001081.mm.2**	F2rl2	coagulation factor II (thrombin) receptor-like 2	0.638465461304361	6.11397692891091	1.44180035705085	0.19622986373243	0.999692817443324	-4.57092522102077	6.24985999600426	6.17654491672383	5.88424193665452	6.88295185501851	5.40695444462065	6.08330842444366	NA
**TC0X00003097.mm.2**	Kcne1l	potassium voltage-gated channel, Isk-related family, member 1-like, pseudogene	-0.705541632165374	5.49438969522604	-1.44176108127797	0.196240512604514	0.999692817443324	-4.57092752339757	6.154524046408	5.84759468577097	5.42849973480902	4.34806835261424	5.15889992926543	6.02875142248858	NA
**TC1100004159.mm.2**	Lgals3bp	lectin, galactoside-binding, soluble, 3 binding protein	-0.517404286749871	6.92528267460299	-1.44164399200663	0.196272262238423	0.999692817443324	-4.57093438724603	6.87818969157273	7.46991885531882	6.77885380710765	6.30553565650567	7.13151791641041	6.98768012070266	NA
**TC1900000758.mm.2**	Smc3	structural maintenance of chromosomes 3	-0.479466368370325	6.5246954035163	-1.44128340128771	0.196370068520256	0.999692817443324	-4.57095552514562	7.52282565431698	7.53596800828371	5.9036884149658	5.66153459591243	6.53718091146167	5.98697483615721	NA
**TC0700000099.mm.2**	Aurkc	aurora kinase C	-0.490950711545416	6.19505820280188	-1.44103790778769	0.196436681348133	0.999692817443324	-4.57096991588398	5.62446619559196	5.82789782909564	5.90357716046366	6.46446472405277	6.81196423698529	6.53797907062198	NA
**TC1900000654.mm.2**	Pax2	paired box 2	0.443722729550311	5.84208990438088	1.44097427257859	0.196453951646449	0.999692817443324	-4.57097364613793	5.38308170733047	5.30622588264816	6.29834424565913	6.32699440204453	5.67463462636876	6.06325856223427	NA
**TC0200001295.mm.2**	Srp9	signal recognition particle 9	-0.435454286869855	8.33944213275131	-1.44089844787413	0.196474531922988	0.999692817443324	-4.57097809092207	8.64030143987084	8.700992587843	7.80627178651708	8.10595331101007	8.32199647133763	8.46113719992923	NA
**TC0X00003067.mm.2**	Rbm41	RNA binding motif protein 41	-0.438204502134938	6.04398466413946	-1.44068258986098	0.196533130675855	0.999692817443324	-4.57099074428827	5.99139837460232	5.90483120799082	5.70009976763582	6.04553493135107	6.29317477646146	6.3288689267953	NA
**TC1500000239.mm.2**	Erich5	glutamate rich 5	0.519256505606643	5.65822212469575	1.44037500065357	0.196616659110032	0.999692817443324	-4.571008774686	5.51856707663271	5.37730992197262	5.65522952348259	6.39075485690865	5.39335531962951	5.61411604954844	NA
**TC1100000085.mm.2**	Ykt6	YKT6 v-SNARE homolog (S. cerevisiae)	-0.568313188658702	7.52969529614695	-1.44020496801243	0.196662846780658	0.999692817443324	-4.57101874165557	7.96998716328979	7.80197434502399	7.47151086952617	6.66328107609908	7.89038519349229	7.38103312945037	NA
**TC0400004012.mm.2**	Agtrap	angiotensin II, type I receptor-associated protein	-0.627823615595753	6.53603010293622	-1.440015496885	0.196714326364613	0.999692817443324	-4.57102984800446	7.02654960786338	6.88960395635853	6.50979391782563	5.51239599327631	6.9427977674925	6.33503937480094	NA
**TC1400001000.mm.2**	Reep4	receptor accessory protein 4	-0.586602010187787	5.95551260669974	-1.43995639944493	0.196730385737108	0.999692817443324	-4.57103331214233	5.7307727015973	5.8657374754917	6.2542480256841	5.22743269568282	6.32406205585902	6.33082268588347	NA
**TC0500003462.mm.2**	Mepce	methylphosphate capping enzyme	0.47926868035145	7.30614807978195	1.43978445960939	0.196777116140713	0.999692817443324	-4.57104339076883	7.74996739527755	7.48941752624453	7.64102230376828	7.13699815516799	6.80800585979972	7.01147723843364	NA
**TC1400002327.mm.2**	Chmp7	charged multivesicular body protein 7	-0.856197093033607	8.37969570293166	-1.43966981206119	0.196808281066077	0.999692817443324	-4.57105011105177	9.13187009723834	8.83681727249967	8.4927772442095	6.80576908668286	8.87765615762756	8.13328435933201	NA
**TC1700002333.mm.2**	Lonp1	lon peptidase 1, mitochondrial	0.445720212270535	6.54858808107764	1.43962985056057	0.19681914495571	0.999692817443324	-4.57105245346474	7.02116557999198	6.75799725081811	6.56681529555722	6.63508774454117	6.31106903633587	5.99939357922145	NA
**TC1300002237.mm.2**	Mterf3	mitochondrial transcription termination factor 3	-0.498899377397291	6.87437766253623	-1.43955535332897	0.196839399149392	0.999692817443324	-4.57105682024143	7.757380580126	7.79384806462362	6.49293372299033	5.85568556484625	6.53578684370373	6.81063119892744	NA
**TC1600000226.mm.2**	B830017H08Rik	RIKEN cDNA B830017H08 gene	0.522288256404904	5.93732055543637	1.4386915570717	0.197074385402772	0.999692817443324	-4.57110745227335	5.8248506288498	5.81290604464275	5.8948650192139	6.62050656675383	5.57220811448839	5.89858695866954	NA
**TC0200005010.mm.2**	Dsn1	DSN1 homolog, MIS12 kinetochore complex component	-0.486837737322643	5.20086935125005	-1.43771547216068	0.197340225930357	0.999692817443324	-4.57116466440082	5.01655936934955	5.0460283612154	4.82326306346228	5.26121338768277	5.76373670884544	5.29441521694489	NA
**TC0900002241.mm.2**	Zbtb16	zinc finger and BTB domain containing 16	0.51896135322774	6.88859057972131	1.43747443823363	0.197405922703402	0.999692817443324	-4.5711787920397	6.82666139608172	7.52944714762841	7.14260313320652	6.86407568733008	6.58811572790453	6.38064038617658	NA
**TC0900003122.mm.2**	Rtp3	receptor transporter protein 3	-0.510960909770856	5.82773505553632	-1.43684981132306	0.197576265138555	0.999692817443324	-4.57121540253048	5.11813793426375	5.22981646815274	5.60367606685896	6.19459098877088	6.18481397757881	6.63537489759274	NA
**TC0800002423.mm.2**	Ushbp1	USH1 protein network component harmonin binding protein 1	0.456867396615234	6.23013428514298	1.43665404660055	0.197629679801029	0.999692817443324	-4.57122687648281	5.98484247507165	6.06159466094234	6.35162348126726	6.77242820276991	5.98694135153175	6.22337553927495	NA
**TC1100000985.mm.2**	4930563E22Rik	RIKEN cDNA 4930563E22 gene	0.44162740513095	5.03726537988212	1.43630994559825	0.197723600067739	0.999692817443324	-4.57124704436976	5.02586778695326	4.97654757558035	5.20540423088429	5.34681163262621	4.65303079435431	5.01593025889429	NA
**TC1100001123.mm.2**	Coro6	coronin 6	-0.459600218988375	6.35029630240625	-1.43594361794791	0.19782363160101	0.999692817443324	-4.57126851469731	6.28559603105855	6.01752183646869	6.18814354356911	6.25158621089764	6.89153193984578	6.46739825259773	NA
**TC1900001428.mm.2**	Pank1	pantothenate kinase 1	0.578430077025288	6.02365730936487	1.43534406408652	0.197987448591154	0.999692817443324	-4.57130365371702	6.46608349361505	6.19418192875308	5.83479191138873	6.48447738254711	5.945516995614	5.21689214427126	NA
**TC0800002658.mm.2**	Snx20	sorting nexin 20	0.723131164593097	6.58106452569112	1.43531847928471	0.197994441909705	0.999692817443324	-4.5713051531898	6.34925505759669	6.09798070889514	7.01144839403783	7.23125846438272	5.6751524939692	7.12129203526516	NA
**TC1500001457.mm.2**	Rspo2	R-spondin 2	-0.688367338243443	6.1607051452754	-1.43492870644353	0.198101009739462	0.999692817443324	-4.57132799680424	5.33803585467264	6.55785080729791	5.60750909452646	6.23829567201101	6.66650788519614	6.55603155788822	NA
**TC0200002261.mm.2**	Nsfl1c	NSFL1 (p97) cofactor (p47)	0.543663476191755	8.75010353633492	1.43483052910801	0.198127860640482	0.999692817443324	-4.57133375068182	9.6831067601283	9.49203333004756	8.84824961561044	8.35815442449816	8.41590783661547	7.70316925110962	NA

**TC0700000387.mm.2**	Xrcc1	X-ray repair complementing defective repair in Chinese hamster cells 1	0.528385025781165	6.60097565190773	1.43462677021976	0.198183598017408	0.999692817443324	-4.57134569230969	6.70697856156091	6.2837188621313	6.56798784623485	7.2679754234234	6.44820133761787	6.33099188047805	NA
**TC1100001859.mm.2**	Sstr2	somatostatin receptor 2	0.745895481280691	6.91657298221685	1.43370877556578	0.198434888480706	0.999692817443324	-4.57139949179408	7.39084864678094	7.39539618360853	6.83082388921627	7.27166812352024	7.0511701985782	5.55953085159693	NA
**TC0500002672.mm.2**	Epha5	Eph receptor A5	-0.570698560797421	7.52143569566557	-1.43334113255011	0.198535607768567	0.999692817443324	-4.571421037159	8.05879636997768	7.90179914086693	6.97008119362377	7.04322957715322	8.06395946195113	7.09074843042071	NA
**TC1100002818.mm.2**	Olfr30	olfactory receptor 30	-0.415664295413685	5.17535089170281	-1.43283882745945	0.198673294162197	0.999692817443324	-4.57145047378136	4.60432440400812	4.55028771505591	5.24163861795934	5.2914437022034	5.62059123893344	5.74381967205666	NA
**TC0600000014.mm.2**	Peg10	paternally expressed 10	0.780711589472411	6.73569724158115	1.43262514497834	0.198731892794955	0.999692817443324	-4.57146299606104	8.7317472983005	7.685293940592	7.01621742687807	5.76306526789154	5.76077794291485	5.45708157290994	NA
**TC0200000055.mm.2**	Nudt5	nudix (nucleoside diphosphate linked moiety X)-type motif 5	-0.514792419424933	6.36752219347559	-1.43201764585464	0.198898574520978	0.999692817443324	-4.57149859633133	6.10542572942766	6.1777747713921	6.47039186715946	5.97578204343251	6.44189427867273	7.0338644707691	NA
**TC1600000738.mm.2**	Rpl24/Zbtb11	ribosomal protein L24/zinc finger and BTB domain containing 11	-0.460080305921499	9.26560457150432	-1.43163834839226	0.19900270817579	0.999692817443324	-4.57152082325163	9.98398650112657	9.82096271797836	8.95291046979439	8.48134832924462	9.11099500990143	9.24342440098058	NA
**TC1000001962.mm.2**	Rnf217	ring finger protein 217	-0.435279206639127	6.65817743906452	-1.43143998179325	0.199057188173108	0.999692817443324	-4.57153244745011	6.37022842773525	6.49391728730013	6.665282055303	6.44189819773374	7.11364009983239	6.8640985664826	NA
**TC0X00000614.mm.2**	Fmr1nb	Fmr1 neighbor	0.475891137241715	5.29290750681822	1.43038573087199	0.19934695836135	0.999692817443324	-4.57159422458403	5.17220511343299	4.94654606755887	5.9129625898418	5.38227547735865	5.08616129489596	5.25729449782107	NA
**TC0700002482.mm.2**	A930016O22Rik	RIKEN cDNA A930016O22 gene	0.48828420839725	5.46117587498899	1.43036643676422	0.199352265088023	0.999692817443324	-4.57159535515862	5.2274207612383	5.13019412723537	5.59281962667478	6.1001847624526	5.15199076491879	5.5644452074141	NA
**TC0700002004.mm.2**	Mtg1	mitochondrial ribosome-associated GTPase 1	0.433373583469893	6.06038236624275	1.4291467244388	0.199688000209596	0.999692817443324	-4.57166682471948	6.91703147904659	6.89406317624292	5.70894401990946	6.00002933464393	5.32676244102365	5.51546374658996	NA
**TC1900000413.mm.2**	Gm815	predicted gene 815	0.481507627096946	6.83670932942742	1.42872362869301	0.19980458049788	0.999692817443324	-4.57169161536684	6.11929064033491	6.28154332875286	7.1487424556801	7.64247617515523	6.73456533799569	7.09363803864574	NA
**TC1200002213.mm.2**	Efcab11	EF-hand calcium binding domain 11	0.485404804365757	5.83525427244419	1.42850363292614	0.199865222814641	0.999692817443324	-4.57170450551167	5.27561179345838	5.62518363704474	6.18649945552596	6.3542704509208	5.52378842606961	6.04617187164564	NA
**TC0200004619.mm.2**	Idh3b	isocitrate dehydrogenase 3 (NAD+) beta	-0.77846606356988	9.17856349717062	-1.42776217532501	0.200069730381924	0.999692817443324	-4.57174794864562	9.89839488600918	9.81319544168311	9.26081529475363	7.64061396934222	9.04935569460156	9.40900569663405	NA
**TC1000000356.mm.2**	Mettl24	methyltransferase like 24	0.462121057610979	5.67879730874317	1.4269053541395	0.200306294034089	0.999692817443324	-4.5717981494288	5.26312292559522	5.40446457691458	5.87280684920167	6.29191238338391	5.4689336731547	5.77154344420892	NA
**TC0800000525.mm.2**	Ufsp2	UFM1-specific peptidase 2	0.683669709970069	7.60964468605095	1.42610919946278	0.200526335621792	0.999692817443324	-4.57184479415089	8.56518753480098	8.48689566740205	7.87762971718914	7.10893244983225	7.37903721364974	6.24018553343151	NA
**TC0500001066.mm.2**	Cdc7	cell division cycle 7 (S. cerevisiae)	-0.856835394620497	6.62774920610075	-1.42575618273636	0.200623972753404	0.999692817443324	-4.57186547601246	6.73896390285776	6.87985627235799	6.99939269070213	5.21760944537173	7.27705557800545	6.65361734730941	NA
**TC1100004206.mm.2**	Pde6g	phosphodiesterase 6G, cGMP-specific, rod, gamma	-0.566981912434625	4.51790482640429	-1.42497419808049	0.200840407245463	0.999692817443324	-4.57191128828908	3.11816385585789	3.74454804935028	4.42426312008743	5.13111349408674	5.20116432059029	5.48817611845313	NA
**TC1100003089.mm.2**	0610010K14Rik/Rnasek	RIKEN cDNA 0610010K14 gene/ribonuclease, RNase K	0.492212951274936	7.44387824228908	1.42379706732534	0.20116660826823	0.999692817443324	-4.57198024707994	7.503410934193	6.98598665315493	7.56208594963469	8.01706293483351	7.30920626044736	7.28551672147097	NA
**TC0100000893.mm.2**	Espnl	espin-like	-0.478651056845607	6.99881950709887	-1.42366434648739	0.201203417395578	0.999692817443324	-4.57198802191866	6.82976692502329	6.87711417658076	6.7898449463901	6.87452196725887	7.00155437282005	7.62011465452013	NA
**TC0500003629.mm.2**	Flt1	FMS-like tyrosine kinase 1	-0.516533503571865	6.54089304523377	-1.42330299318174	0.201303666992319	0.999692817443324	-4.57200918990213	6.29540745612478	6.68712606506225	6.6333918562308	5.98148701530512	6.93983325501702	6.70811262366263	NA
**TC1400002141.mm.2**	Gjb6	gap junction protein, beta 6	0.899497492201286	6.56650999829813	1.42302715597906	0.201380222508314	0.999692817443324	-4.57202534814216	7.70312865874605	7.37749673302159	6.4638356055875	6.59487918562435	6.62406769824912	4.63565210856016	NA
**TC0600003108.mm.2**	C1s1	complement component 1, s subcomponent 1	0.450749739119444	6.50199441974457	1.42287593939881	0.201422202178913	0.999692817443324	-4.57203420615748	5.60430574732823	5.76858851038065	6.92246545781426	7.34782041168447	6.61195131360661	6.75683507765323	NA
**TC0700003860.mm.2**	Olfr544	olfactory receptor 544	0.563484358662548	5.35022850381097	1.4225673705509	0.201507889494991	0.999692817443324	-4.5720522814175	4.67434030838831	4.49060161330782	5.57162925911798	6.4600696501294	5.26998394760328	5.634746244319	NA
**TC0500001576.mm.2**	Tbl2	transducin (beta)-like 2	-0.431428855628781	7.26737863967103	-1.42214250908681	0.201625924529194	0.999692817443324	-4.57207716841697	7.51356072095603	7.26219017422243	7.0015293376959	6.98130227809919	7.30216603720898	7.54352328984367	NA
**TC0400003219.mm.2**	Dio1	deiodinase, iodothyronine, type I	0.455548027866098	5.55928318346162	1.42120643575702	0.201886205787146	0.999692817443324	-4.57213199882064	5.29026632933213	4.971004297133	5.82234834650963	6.18041391850877	5.45996628491313	5.63169992437306	NA
**TC1900001498.mm.2**	Blnk	B cell linker	-0.531120376513334	5.94691546385563	-1.42061563858034	0.202050637576086	0.999692817443324	-4.57216660347661	6.1267734935466	6.56436498558269	5.19319424614096	5.77086252934794	5.78361897023097	6.2426785582846	NA
**TC0200002837.mm.2**	Polr3k	polymerase (RNA) III (DNA directed) polypeptide K	-0.487848452331866	7.69185991128372	-1.42027751292504	0.202144799976311	0.999692817443324	-4.57218640801656	7.61112487327643	7.46449421326586	7.4150423000935	7.63487943815465	7.70223770411526	8.32338093879662	NA
**TC1700000461.mm.2**	Hmga1/Hmga1b	high mobility group AT-hook 1/high mobility group AT-hook 1B	0.49713846496604	8.22776157910418	1.41968045280715	0.202311168494861	0.999692817443324	-4.57222137797091	7.78766218004479	7.82263153287179	8.57872620183222	8.79655014398807	7.84894212149853	8.53205729438968	NA
**TC0X00000318.mm.2**	Zcchc12	zinc finger, CCHC domain containing 12	0.889314309863979	6.8351541897655	1.41960919859943	0.202331031495709	0.999692817443324	-4.57222555128046	8.62245740087804	7.87003393286816	7.14459912299701	6.00393208929039	6.41266452846363	4.9572380640958	NA
**TC1700000954.mm.2**	Plcl2	phospholipase C-like 2	0.549249540170768	7.30592898950348	1.41959405398972	0.202335253472711	0.999692817443324	-4.57222643828792	7.9060252899055	7.93818181832905	7.38245917078406	7.16247378377987	7.17434976259347	6.27208411162893	NA
**TC0200003291.mm.2**	Qrfp	pyroglutamylated RFamide peptide	-0.586578202189975	6.14024240693	-1.41930847222566	0.202414882206177	0.999692817443324	-4.57224316445875	5.12804747460781	5.131567200565	5.86935537150505	6.83498630950858	6.71373189197597	7.16376619341761	NA
**TC0700000435.mm.2**	Cyp2b9	cytochrome P450, family 2, subfamily b, polypeptide 9	0.659241664776181	4.92868510405872	1.41856562081334	0.202622144440396	0.999692817443324	-4.57228667126924	4.04747177660563	3.94378430771408	5.14274990419144	6.30691903060105	4.76986369769311	5.36132190754703	NA
**TC1100002998.mm.2**	Adprm	ADP-ribose/CDP-alcohol diphosphatase, manganese dependent	-0.429404885297853	7.99877129006374	-1.41855320583948	0.202625609963431	0.999692817443324	-4.57228739836759	7.6851418389158	7.47476056319773	7.85899132358026	8.12796646025633	8.42796677578744	8.41780077864486	NA
**TC1100003104.mm.2**	Chrne	cholinergic receptor, nicotinic, epsilon polypeptide	0.501482191921958	5.8280007801771	1.41839607569082	0.202669476006993	0.999692817443324	-4.57229660085167	5.81411129981675	5.55710718769081	5.86806206713316	6.43181322156631	5.44718582491311	5.84972507994244	NA
**TC0300003175.mm.2**	Lhx8	LIM homeobox protein 8	0.540201298390496	7.19678245986238	1.41807966204788	0.202757835357734	0.999692817443324	-4.57231513171856	7.42243014563088	6.76551941980941	7.33185322385323	7.70472067140425	6.73620899109161	7.21996230738488	NA
**TC1300001672.mm.2**	Mrs2	MRS2 magnesium transporter	-0.652041882105735	7.06146362760945	-1.41807119643498	0.202760199882034	0.999692817443324	-4.57231562750597	7.69496017752093	7.23268349376924	6.98087580904139	6.08765135603614	7.58813540692271	6.78447552236629	NA
**TC1100003393.mm.2**	Gas2l2	growth arrest-specific 2 like 2	0.465719967550065	6.37852151988191	1.41800348765157	0.20277911247316	0.999692817443324	-4.57231959285376	5.85612576912957	6.11555151985504	6.90377941937222	6.71166646333127	6.10676918720794	6.57723676039543	NA
**TC1600002039.mm.2**	Tiam1	T cell lymphoma invasion and metastasis 1	0.493301506458628	6.7316330759472	1.41763185356237	0.202882946727678	0.999692817443324	-4.57234135727539	7.75401603473515	7.30902627458137	6.81725399068921	6.33942558895276	5.95348858451328	6.21658798221143	NA
**TC1200002525.mm.2**	Serpina3g	serine (or cysteine) peptidase inhibitor, clade A, member 3G	0.590394892597484	5.29838806683289	1.41749659982379	0.202920748464644	0.999692817443324	-4.572349278192	5.22257175827058	5.49186063963134	5.07224777571618	6.056095118429	4.79427186788644	5.15328124106377	NA
**TC1500000170.mm.2**	Zfp622	zinc finger protein 622	0.446791238647149	5.98095782438414	1.41682658625439	0.203108103037766	0.999692817443324	-4.57238851567086	7.38394314019988	6.98819647420063	5.68858970079833	5.51500520380098	5.0677685594686	5.24224386783641	NA
**TC0900002972.mm.2**	Tmem108	transmembrane protein 108	0.432894412774593	6.74366285716942	1.41639926305901	0.203227676407762	0.999692817443324	-4.57241353997905	6.62932977447603	6.36617826170797	7.12100703131697	7.04512193487388	6.77723447066324	6.52310566997843	NA
**TC0300000864.mm.2**	Rorc	RAR-related orphan receptor gamma	0.607358945896034	4.74421646510055	1.4159614449807	0.20335025257785	0.999692817443324	-4.5724391783167	4.0558755402519	4.25278268784995	4.88939095544448	5.79628827170228	4.40257431110706	5.06838702424764	NA
**TC1300002319.mm.2**	Lrrc14b	leucine rich repeat containing 14B	0.542504181066944	5.77474969977172	1.4159123356972	0.203364005906997	0.999692817443324	-4.57244205408858	5.57555079702809	5.62806274837819	5.70915491711277	6.55579159056621	5.75332564377748	5.42661250176762	NA
**TC0300000809.mm.2**	S100a6	S100 calcium binding protein A6 (calcyclin)	-0.576292013275711	6.4378008914425	-1.4156358238028	0.203441460334209	0.999692817443324	-4.57245824611	6.54398487496401	7.03297870555329	5.5281648627891	6.42046400800404	6.45797743488776	6.6432354624568	NA
**TC1600002009.mm.2**	Krtap26-1	keratin associated protein 26-1	0.760385495241157	4.9939711050552	1.4147414067752	0.203692180682784	0.999692817443324	-4.57251061994189	4.133177342963	4.59401694086426	4.98818441612402	6.39051725236909	4.58448662100797	5.27344405700283	NA
**TC1300002540.mm.2**	Cdk7	cyclin-dependent kinase 7	0.719948774230878	6.21682989132288	1.41416544922769	0.203853779456414	0.999692817443324	-4.57254434466174	6.77851965030655	6.80924465176669	6.57448884626925	6.00206745089365	6.26529114975365	4.8713675989475	NA
**TC0700000101.mm.2**	Zfp418	zinc finger protein 418	-0.494293767966663	6.14545291933754	-1.41359144900796	0.20401494451692	0.999692817443324	-4.57257795376854	6.2905603174594	6.19211097330574	6.08952896361781	5.61120038104557	6.60556517723667	6.08375170336004	NA
**TC1500000222.mm.2**	Cpq	carboxypeptidase Q	-0.474143612771464	6.03296430192864	-1.41323803257287	0.204114232466701	0.999692817443324	-4.57259864665729	6.22651942242325	6.07069371899924	5.59283022533293	5.88331249697028	5.94894614975252	6.47548379809362	NA
**TC0300000724.mm.2**	Smg5	Smg-5 homolog, nonsense mediated mRNA decay factor (C. elegans)	-0.79104819649388	6.00402791795579	-1.41318755008654	0.204128418462295	0.999692817443324	-4.57260160242642	6.73872536218818	6.85070192446421	5.98692513915023	4.43939677489707	6.37861697053033	5.62980133650473	NA
**TC0500002754.mm.2**	Sdad1	SDA1 domain containing 1	0.551334587058043	5.94589019371894	1.41300323057908	0.204180221343369	0.999692817443324	-4.57261239433823	6.52378827246646	6.30183563341496	6.30413863737716	5.67205457789697	5.77124020327699	5.10228383788106	NA
**TC0300002262.mm.2**	Prss48	protease, serine 48	-0.81589963085889	5.3941878099912	-1.41290493298417	0.204207852684572	0.999692817443324	-4.57261814962198	4.87410131634446	4.77407815752936	5.55691743048536	4.98565663169244	5.23708932962867	6.9372839942669	NA
**TC0700000810.mm.2**	Napsa	napsin A aspartic peptidase	-0.471742254197557	6.50699863738099	-1.41290449262931	0.204207976475431	0.999692817443324	-4.57261817540451	6.21861279382714	6.01320158745153	6.68934724930849	6.24399921799759	6.75715282075943	7.11967815494177	NA
**TC0600000763.mm.2**	C130060K24Rik	RIKEN cDNA C130060K24 gene	-0.422406387009708	6.30505335131914	-1.41288987023618	0.204212087104763	0.999692817443324	-4.57261903153704	6.13727094580524	6.04796434088758	6.30711333549287	6.09302268810845	6.71461501476771	6.53033378285302	NA
**TC1100002064.mm.2**	Fn3krp	fructosamine 3 kinase related protein	-0.511388695444786	6.68224282818431	-1.41272503199079	0.204258431411936	0.999692817443324	-4.57262868267366	7.59097831815328	7.56182027759325	6.25640326064365	5.70253723059123	6.77187565601894	6.2098422261055	NA
**TC0700004637.mm.2**	Dnaaf3	dynein, axonemal assembly factor 3	-0.596355478914934	5.46258084229028	-1.41255730035893	0.204305598965259	0.999692817443324	-4.57263850312901	5.24210603948562	5.01352846687715	5.14670607596689	5.51686371880761	5.41791449323812	6.43836625936625	NA
**TC0200005335.mm.2**	Gm14308/Gm14430/Gm14432/Gm14434/Gm2026/Gm4724	predicted gene 14308/predicted gene 14430/predicted gene 14432/predicted gene 14434/predicted gene 2026/predicted gene 4724	-1.59628425962136	8.31406473312074	-1.41251373620547	0.204317851185978	0.999692817443324	-4.57264105373571	9.19606170597305	9.91507094768752	8.57900407847592	5.21133953443465	9.42763660095208	7.5552755312012	NA
**TC0200001340.mm.2**	Olfr1261	olfactory receptor 1261	0.854531577943418	4.71050671157319	1.4123512195917	0.20436356411414	0.999692817443324	-4.57265056875343	4.15553346772202	4.02109819454682	4.82411907616943	6.07361680535913	3.87546010197881	5.31321262366291	NA
**TC0X00000516.mm.2**	Smim10l2a	small integral membrane protein 10 like 2A	0.408385043587876	6.98549090653182	1.41231689560395	0.204373220002951	0.999692817443324	-4.57265257834268	6.8186988847501	6.72078611653414	7.29047869959913	7.30463656294209	6.85833291134133	6.92001226402414	NA
**TC0500003728.mm.2**	D5Ertd579e	DNA segment, Chr 5, ERATO Doi 579, expressed	0.477660386999983	6.56936817599612	1.41190029170487	0.204490450285683	0.999692817443324	-4.57267696923351	7.74392408763959	7.66063181487606	6.26876999468018	6.21471696905035	6.0804628904546	5.44770329927596	NA
**TC1100000767.mm.2**	Dhrs7b	dehydrogenase/reductase (SDR family) member 7B	-0.524443534614852	6.49565889666002	-1.41177218098902	0.204526512239284	0.999692817443324	-4.57268446961678	7.16079688645616	7.13561021960231	6.3211760357589	5.49315356657709	6.34978484591058	6.51343182565511	NA
**TC0200003632.mm.2**	Kcnh7	potassium voltage-gated channel, subfamily H (eag-related), member 7	-0.468144729827054	7.12370517531337	-1.41142346904702	0.2046247004973	0.999692817443324	-4.57270488508063	6.67257041521805	7.25679273003003	7.00849256522661	6.92979665826239	7.39978928004436	7.47478940309875	NA
**TC0900002359.mm.2**	Snx33	sorting nexin 33	-0.496739394987927	5.50321336504965	-1.41106963093164	0.204724375741301	0.999692817443324	-4.57272560026532	5.63307541044904	5.99029471504659	5.0978623030478	5.10335333436542	5.27937491387548	5.91531951351359	NA
**TC1500001178.mm.2**	Nipbl	NIPBL cohesin loading factor	1.04183624775439	7.03439947695746	1.41069902078774	0.204828822712597	0.999692817443324	-4.57274729692941	8.21125340831824	8.07627229729122	7.44320868954452	6.55806313627751	7.09362906958362	4.82397026072963	NA
**TC0700002754.mm.2**	Lrfn3	leucine rich repeat and fibronectin type III domain containing 3	-0.926879528902764	6.09494394730649	-1.4086098345843	0.205418508401855	0.999692817443324	-4.5728695960856	6.50675346149114	7.05079053121927	6.24141188476464	4.33776843189687	5.83336925802875	6.59957011643828	NA
**TC0300000631.mm.2**	Pdgfc	platelet-derived growth factor, C polypeptide	-0.998321582147096	5.91022751504127	-1.40844218620747	0.205465894657259	0.999692817443324	-4.57287940946096	6.34042510924893	6.7804999943119	6.22671389680181	3.94518451439449	6.09948070617995	6.06906086931055	NA
**TC1800001216.mm.2**	Diaph1	diaphanous related formin 1	0.556541047222577	7.14783230750959	1.4079822474315	0.205595948312684	0.999692817443324	-4.57290633171459	7.28498457220654	7.34847741348757	7.50311275699783	7.18019421990647	6.32701844260282	7.24320643985632	NA
**TC1400000337.mm.2**	Oxnad1	oxidoreductase NAD-binding domain containing 1	-0.84856641869509	7.15076636223563	-1.40783303572362	0.205638155842347	0.999692817443324	-4.57291506558645	8.15146631358465	8.23393100495712	7.15468540499601	5.25634860374489	7.06290259184427	7.04526425428682	NA
**TC0900000254.mm.2**	Thyn1	thymocyte nuclear protein 1	-0.457219867300767	7.0383950204446	-1.40781408131959	0.205643518037005	0.999692817443324	-4.57291617504729	7.62047982774648	7.85153077722073	6.37806521367699	6.54389467787245	7.14945237827866	6.6869472478723	NA
**TC1800001252.mm.2**	Grxcr2	glutaredoxin, cysteine rich 2	0.560844310618128	6.25267602785092	1.40763238757185	0.205694925551165	0.999692817443324	-4.57292681009289	5.72067288527833	5.32880641065007	6.44346022268989	7.35067252351679	6.30739113429649	6.36505299067393	NA
**TC1300002380.mm.2**	Rasa1	RAS p21 protein activator 1	1.15389978607082	6.6074763430853	1.40747940089789	0.205738219837802	0.999692817443324	-4.57293576474766	7.75885555545046	7.87519039828307	7.35893758512046	5.80036825333949	6.55933577371104	4.29217049260727	NA
**TC0200002720.mm.2**	Zfp967/Zfp966	zinc finger protein 967/zinc finger protein 966	-1.07664162088619	8.96901781969943	-1.4073733788259	0.205768228262781	0.999692817443324	-4.57294197041268	9.96921220505393	10.4461113748701	8.73843347999992	6.88431656825013	9.72465375846329	8.05137953155914	NA
**TC0200002738.mm.2**	Zfp966/Zfp967	zinc finger protein 966/zinc finger protein 967	-1.07664162088619	8.96901781969943	-1.4073733788259	0.205768228262781	0.999692817443324	-4.57294197041268	9.96921220505393	10.4461113748701	8.73843347999992	6.88431656825013	9.72465375846329	8.05137953155914	NA
**TC0200005341.mm.2**	Zfp967/Zfp966	zinc finger protein 967/zinc finger protein 966	-1.07664162088619	8.96901781969943	-1.4073733788259	0.205768228262781	0.999692817443324	-4.57294197041268	9.96921220505393	10.4461113748701	8.73843347999992	6.88431656825013	9.72465375846329	8.05137953155914	NA
**TC0100003315.mm.2**	Fam163a	family with sequence similarity 163, member A	-0.603063052322581	5.47493453346837	-1.40692907547704	0.205894026650329	0.999692817443324	-4.57296797588842	5.820691558416	6.26695063655641	5.4131239037673	4.36479554682903	5.51984955878409	5.4641959964574	NA
**TC0900002415.mm.2**	Tmem202	transmembrane protein 202	-0.449130435092764	4.93130782017888	-1.40645726371501	0.206027689753237	0.999692817443324	-4.5729955907307	4.48233809244935	4.7948206736728	4.7225275750234	4.9836860673594	5.15741487574379	5.44705963682453	NA
**TC1100004283.mm.2**	Syngr2	synaptogyrin 2	0.457287571815355	8.38387641691243	1.40615117968352	0.206114444528643	0.999692817443324	-4.57301350523245	8.34890896921828	8.47950006237424	8.37175655277754	8.82295575397884	8.279508003747	8.00062915937867	NA
**TC0200005476.mm.2**	Nup35	nucleoporin 35	0.546053267583532	6.15883560345551	1.40594654714621	0.206172462858687	0.999692817443324	-4.57302548179883	7.31354268677735	7.27818579134444	6.12747351729216	5.59922232159701	5.70394436577003	4.93064493795208	NA
**TC0800002745.mm.2**	Csnk2a2	casein kinase 2, alpha prime polypeptide	1.01411844407041	6.62750029452376	1.40524214708606	0.206372290334209	0.999692817443324	-4.57306670726036	7.56316655918845	7.61386146720209	7.24714613623629	6.06095917821012	6.66790225920635	4.61196616709923	NA
**TC0500001025.mm.2**	Spp1	secreted phosphoprotein 1	-1.48680774450431	6.65947943931118	-1.40522311241451	0.206377692605143	0.999692817443324	-4.57306782125306	6.11818545095748	9.33206830662474	4.82304027663276	5.94346341800535	6.2082484325264	7.53187075112034	NA
**TC1100002269.mm.2**	Meis1	Meis homeobox 1	0.701507724098534	5.94907588970705	1.40515995716701	0.206395617746387	0.999692817443324	-4.57307151736701	6.11223448278719	6.14008241722283	5.88804020073338	6.5345367424813	4.85308146627405	6.16648002874355	NA
**TC0100001382.mm.2**	Rnasel	ribonuclease L (2', 5'-oligoisoadenylate synthetase-dependent)	-0.543532917394344	5.42877687369782	-1.40501783504557	0.206435960933103	0.999692817443324	-4.57307983490846	5.45547444141935	6.36403001275267	4.86790249297629	4.96514298363682	5.37633494671205	5.54377636468975	NA
**TC0400001631.mm.2**	Trim63	tripartite motif-containing 63	0.59002819428798	6.10556875161372	1.40483278973838	0.206488499091234	0.999692817443324	-4.57309066437814	5.34323782191581	5.98017487431196	6.32926178968099	6.91576631133427	5.6943774656265	6.3705942468128	NA
**TC0500001394.mm.2**	Anapc7	anaphase promoting complex subunit 7	0.62891305331839	5.49202887637041	1.40458111779118	0.206559973301773	0.999692817443324	-4.57310539287176	6.337513360371	6.01690275816025	5.95034615277083	4.97744547039317	5.17682739730431	4.49313811922291	NA
**TC1200001544.mm.2**	Id2	inhibitor of DNA binding 2	-0.902394303208951	8.51107935042043	-1.40451083706544	0.206579936844967	0.999692817443324	-4.57310950584258	9.06104244397926	9.55341544509852	8.42024843891411	6.90336636459934	9.21690035030885	7.91150305962251	NA
**TC0200000990.mm.2**	4932414N04Rik	RIKEN cDNA 4932414N04 gene	-0.436904794628196	4.56547260728718	-1.40373640118781	0.2068000341143	0.999692817443324	-4.57315482626658	3.60418009431829	3.84665991780423	4.70878805591531	4.82530496525679	5.37110133344091	5.03680127698758	NA

**TC0700003017.mm.2**	Sphk2	sphingosine kinase 2	-0.42554235637036	6.51202131548939	-1.40314081579648	0.206969445499712	0.999692817443324	-4.57318967883311	6.26272896177369	6.14419681081768	6.57740389008291	6.32965481371921	6.77213538957436	6.98600802696849	NA
**TC0500003716.mm.2**	Pms2	PMS1 homolog2, mismatch repair system component	-1.02530894863032	6.59828664563439	-1.40311228400751	0.206977564373508	0.999692817443324	-4.57319134842985	7.41629309298396	7.63234897617579	6.77075383245376	4.47447612123923	6.99662017407702	6.2992276768766	NA
**TC0900001005.mm.2**	Fam214a	family with sequence similarity 214, member A	1.01410744892428	7.60263650190843	1.40290506910864	0.207036537113841	0.999692817443324	-4.57320347395273	7.85131351762981	7.79821987051906	8.52610893356636	7.47114132700876	8.05022132971754	5.91881403300902	NA
**TC0600003267.mm.2**	Styk1	serine/threonine/tyrosine kinase 1	-0.528081608533232	6.28610725763501	-1.40286733766457	0.207047277002429	0.999692817443324	-4.57320568185432	6.33379746577134	6.36914632678196	6.35817883027665	5.6205894378185	6.28542281719647	6.74950866796513	NA
**TC0100002827.mm.2**	Kif1a	kinesin family member 1A	0.461098849303618	8.28920949615109	1.40284252336285	0.207054340426239	0.999692817443324	-4.57320713389079	9.99599847067912	9.85099511001629	7.93512198520724	7.47010856220196	7.3918857160436	7.09114713275836	NA
**TC0100003536.mm.2**	Ppox	protoporphyrinogen oxidase	-0.496897722665436	6.92476962003456	-1.40250246010058	0.207151161804884	0.999692817443324	-4.57322703285108	6.69004505538839	6.80713293636593	6.40483091723618	7.1239912243249	7.33499797009527	7.18761961679667	NA
**TC0200005443.mm.2**	Arfrp1	ADP-ribosylation factor related protein 1	-0.468772637427045	6.48227144358364	-1.40237848142605	0.207186470655878	0.999692817443324	-4.57323428741889	7.18868253873817	6.75263269637447	5.80168138157886	6.20570269418869	6.39843924190734	6.5464901087143	NA
**TC1000002470.mm.2**	Theg	testicular haploid expressed gene	-0.425956937926976	5.71939139495144	-1.40222853154337	0.207229183308267	0.999692817443324	-4.57324306160957	5.1782649599481	5.41774568923574	5.7906904715836	5.64352145075181	6.03581306000832	6.25031273818105	NA
**TC0700000288.mm.2**	Fbxo46	F-box protein 46	0.521105426426663	5.62306508109478	1.40189214438432	0.207325030824681	0.999692817443324	-4.57326274472808	6.1009708760932	6.45664857215769	5.93887295472945	5.17261799085612	5.01434787946363	5.05493221326861	NA
**TC0300001359.mm.2**	Cxxc4	CXXC finger 4	0.481539201777514	6.63530389804283	1.40175154581268	0.2073651037339	0.999692817443324	-4.57327097149276	7.23020548359303	6.59145786437342	6.67016304977968	6.8064561721431	6.34252462799437	6.17101619037339	NA
**TC1100000081.mm.2**	Aebp1	AE binding protein 1	-0.458573667797888	6.00294571881749	-1.40153873554588	0.207425771464483	0.999692817443324	-4.5732834234046	6.50529573336536	6.42840388952463	5.54519129285326	5.53822238435631	5.72228454769937	6.27827646510598	NA
**TC1000002036.mm.2**	Slc16a10	solute carrier family 16 (monocarboxylic acid transporters), member 10	0.502002801008337	5.43134584656542	1.40142509686032	0.207458174023808	0.999692817443324	-4.57329007254202	5.1589507223325	5.3459137732477	6.06270002323565	5.48090806967884	5.03792739748588	5.50167509341195	NA
**TC0900002728.mm.2**	Slc17a5	solute carrier family 17 (anion/sugar transporter), member 5	0.436737158933328	6.86880055718281	1.40125384352931	0.207507013262061	0.999692817443324	-4.5733000926944	7.7264989218018	7.90272629069388	6.73722114943803	6.49130507479588	6.02256006178732	6.33249184457993	NA
**TC1000002522.mm.2**	Diras1	DIRAS family, GTP-binding RAS-like 1	0.411971045884299	7.43757924665521	1.40090796460402	0.207605685106217	0.999692817443324	-4.57332032998972	7.2215487284108	7.02139125635067	7.79217207744988	7.81106671601933	7.43974230685881	7.3395543948418	NA
**TC1300000660.mm.2**	Grk6	G protein-coupled receptor kinase 6	0.619831983235458	7.06307848271911	1.40081894472386	0.207631087419974	0.999692817443324	-4.57332553845209	7.37423906211361	7.17555341017074	7.54625395452212	6.98791724072849	7.17286898878267	6.12163823999703	NA
**TC1600000211.mm.2**	Rimbp3	RIMS binding protein 3	-0.449461926012449	5.1393198468606	-1.40040369272199	0.207749618927734	0.999692817443324	-4.57334983404277	5.29318991143823	5.03199066085823	4.91693119500334	4.88897613341777	5.13215805840384	5.57267312204217	NA
**TC1100000617.mm.2**	P4ha2	procollagen-proline, 2-oxoglutarate 4-dioxygenase (proline 4-hydroxylase), alpha II polypeptide	0.418339836868365	5.94774102046315	1.39998484731526	0.207869237998581	0.999692817443324	-4.5733743392397	5.77736194418169	5.9472204469865	6.24738827403721	6.1518834286365	5.89158265779749	5.6710093711395	NA
**TC0900001887.mm.2**	Rab3d	RAB3D, member RAS oncogene family	-0.511942766894886	5.79607854753617	-1.39995220585551	0.207878562761345	0.999692817443324	-4.57337624895172	4.88703125387561	5.22510174705623	5.54872645458511	6.27149992066259	6.34887855071684	6.49523335832063	NA
**TC1700002801.mm.2**	Rhbdl1	rhomboid like 1	-0.579294720641597	7.73180241299633	-1.39968071979422	0.207956133447389	0.999692817443324	-4.57339213228698	8.16393070795437	8.0574276695232	7.78375809856061	6.721675231048	7.85391763984726	7.81010513104454	NA
**TC0500001514.mm.2**	Sumf2	sulfatase modifying factor 2	0.529946288543332	6.94559943252424	1.39943516212239	0.208026318202105	0.999692817443324	-4.57340649844421	7.04293547127029	7.0928253711196	7.22531837642877	7.07354578849233	7.05452776140847	6.18444382642596	NA
**TC0100003560.mm.2**	Ncstn	nicastrin	0.489922768651494	7.58075011452042	1.3990769875541	0.208128729183812	0.999692817443324	-4.57342745276478	8.4313303872764	8.25834442410127	7.48831833021516	7.39901737630874	7.29922018615971	6.60826998306121	NA
**TC0900003248.mm.2**	Cx3cr1	chemokine (C-X3-C motif) receptor 1	0.446657876115424	6.94946792289591	1.39904180148443	0.208138792205468	0.999692817443324	-4.57342951123313	7.24589414222937	7.49745671245637	7.17400940085152	6.74937681660876	6.4778434778696	6.55222698735983	NA
**TC1400000298.mm.2**	Selenok	selenoprotein K	-0.446973653210883	8.52704543651194	-1.39890506920789	0.208177901047859	0.999692817443324	-4.57343751035131	9.43199743916093	9.62502971935105	7.98177153032406	7.62387754674489	8.39633383482232	8.1032625486684	NA
**TC0400002043.mm.2**	Megf6	multiple EGF-like-domains 6	0.5548745088877	6.555304479465	1.39875370526539	0.208221202641247	0.999692817443324	-4.57344636537096	6.27551272241208	6.12689101094075	6.62039481290394	7.39919126770234	6.16616100869044	6.74367605414043	NA
**TC1900000062.mm.2**	Eif1ad	eukaryotic translation initiation factor 1A domain containing	-0.732716571807224	7.0455566889513	-1.39873155517953	0.208227539930831	0.999692817443324	-4.57344766117735	7.01969004555532	6.92817570781377	7.49890996906288	5.93111064929926	7.43069713481752	7.46475662715907	NA
**TC0700002970.mm.2**	Nr1h2	nuclear receptor subfamily 1, group H, member 2	0.412895759687062	7.41060048716314	1.39860779845861	0.208262950768049	0.999692817443324	-4.57345490105926	7.92158587958486	7.9488122654198	7.3953509337199	7.31414721495425	7.05565022729436	6.82805640200567	NA
**TC1300001540.mm.2**	Zscan26	zinc finger and SCAN domain containing 26	0.597615461311947	8.23158327682292	1.39844446405609	0.208309694385621	0.999692817443324	-4.57346445618453	8.62005743010721	8.54033227656914	8.5177310642651	8.19443937417742	8.30071235936711	7.21622715645152	NA
**TC1300000101.mm.2**	Psma2	proteasome (prosome, macropain) subunit, alpha type 2	-1.13743367233442	7.79815924030617	-1.39844347052414	0.208309978747059	0.999692817443324	-4.57346451430623	8.51506702041594	8.75500129564697	8.00758447815861	5.61442541239402	8.59300732097655	7.30386991424492	NA
**TC1000000492.mm.2**	Msl3l2	MSL3 like 2	0.773923729278152	5.83326445350788	1.3983864579882	0.208326297044064	0.999692817443324	-4.57346784953782	6.12829816419701	6.00815107282777	5.36630582128258	6.83918665000682	5.74529360105004	4.91235141168306	NA
**TC0800001594.mm.2**	Nrp1/Mir1903	neuropilin 1/microRNA 1903	0.483177746678208	6.71336162890147	1.39822996535254	0.208371094755157	0.999692817443324	-4.57347700428975	7.10126557352648	7.22233210240877	6.89642068749906	6.56504310791592	6.54317857628289	5.95192972577568	NA
**TC0400002021.mm.2**	Icmt	isoprenylcysteine carboxyl methyltransferase	-0.547483160906466	6.07956440807649	-1.39799337295871	0.208438838383606	0.999692817443324	-4.57349084466902	6.27401314617852	6.52837340807783	5.18800176244377	6.10201502375106	6.24396742783474	6.14101568017302	NA
**TC0900001683.mm.2**	4930433N12Rik	RIKEN cDNA 4930433N12 gene	-0.794046484651384	5.01902786144147	-1.39754503069952	0.208567266942919	0.999692817443324	-4.57351707159182	4.39615812351877	5.07607042926158	4.2085707577227	5.31835206556015	4.96669805329449	6.14831773929112	NA
**TC1500001832.mm.2**	Tmprss6	transmembrane serine protease 6	0.534183162912107	4.76804453082474	1.3972194403573	0.208660577679126	0.999692817443324	-4.5735361173533	4.3386016220446	4.45092560247494	4.96897981073083	5.47457333239572	4.31682204283661	5.05836477446572	NA
**TC1200001058.mm.2**	Tcl1b4	T cell leukemia/lymphoma 1B, 4	-0.454198033006024	5.51303619863308	-1.39676893185687	0.208789750699332	0.999692817443324	-4.57356246967593	4.63426233082592	5.10861485697638	5.48030377927158	5.7331681897205	6.01290390977287	6.10896412523125	NA
**TC0300002450.mm.2**	Tuft1	tuftelin 1	-0.824400950363693	5.48931044202375	-1.3966774679916	0.208815984674814	0.999692817443324	-4.57356781972554	5.14300652433255	5.74801944765621	5.96616468984783	4.23185269986535	6.02983528491178	5.81698400552879	NA
**TC1600001271.mm.2**	Vpreb1	pre-B lymphocyte gene 1	0.95385575249044	4.81106025738671	1.39655814264363	0.208850214441947	0.999692817443324	-4.57357479944528	4.5618002329577	4.19492946357793	4.93143897941717	6.07723269696558	3.61632938456287	5.48463078683899	NA
**TC1200000825.mm.2**	Ylpm1	YLP motif containing 1	1.10580359056909	7.80763273532821	1.39612057359295	0.208975779141898	0.999692817443324	-4.57360039378737	9.13193061872835	8.95765332800529	8.37202175012206	7.11188807306485	7.79680804627792	5.47549459577081	NA
**TC0800003069.mm.2**	Mvd	mevalonate (diphospho) decarboxylase	-0.431085247471481	7.53098881897065	-1.39594839084477	0.209025207310527	0.999692817443324	-4.5736104649204	7.48849805609835	7.21884682064484	7.52788401038186	7.28032476068702	7.74753468596445	7.92284458004739	NA
**TC0800001130.mm.2**	Katnb1	katanin p80 (WD40-containing) subunit B 1	0.444558578999097	6.46740081357915	1.39525230935541	0.209225137493069	0.999692817443324	-4.57365117822596	6.44650905173199	6.69156547478361	6.63961344318043	6.6381103132983	6.40868877248589	5.97991782599465	NA
**TC0700000563.mm.2**	Nphs1	nephrosis 1, nephrin	0.42258322407093	8.6292787153433	1.39510895161726	0.209266334437773	0.999692817443324	-4.57365956288423	8.39925726876966	8.26129991547226	8.98241424736129	8.99772653061857	8.4101815425518	8.7247927872862	NA
**TC0100000280.mm.2**	Pdcl3	phosducin-like 3	0.46429047982493	6.17643073473656	1.39462028229754	0.209406819057247	0.999692817443324	-4.57368814340773	7.35959818886578	7.11252935824849	5.76362738660494	5.99389152387255	5.16466632955085	5.66427162127678	NA
**TC0800002463.mm.2**	Cherp	calcium homeostasis endoplasmic reticulum protein	0.431570687965179	7.76240850873048	1.39461711003184	0.209407731310468	0.999692817443324	-4.57368832893918	7.94100929231168	7.6348593278462	7.83773409316516	8.09312781091251	7.58603573264861	7.48168479549871	NA
**TC0900001038.mm.2**	Gclc	glutamate-cysteine ligase, catalytic subunit	0.410143193363179	7.36220875792814	1.3945269895219	0.209433648896964	0.999692817443324	-4.57369359966322	8.02813917466696	8.13782447919066	7.26675676899436	7.14703087122443	6.87478311203735	6.71871814145508	NA
**TC0X00000172.mm.2**	H2al1n	H2A histone family member L1N	-0.597033652681199	5.59506498920712	-1.39437307577827	0.209477919341222	0.999692817443324	-4.57370260127953	4.62910498754903	4.79656638211534	5.31393908297992	6.16138654712807	5.96391698617314	6.70547594929725	NA
**TC0500002046.mm.2**	AI506816	expressed sequence AI506816	0.454165653992403	6.21729588845273	1.39411081678821	0.209553372715389	0.999692817443324	-4.57371793923345	6.69995942003102	7.04816577943912	5.93009104521771	6.3018996743978	5.76633487084067	5.55732454079004	NA
**TC0X00001286.mm.2**	Armcx1	armadillo repeat containing, X-linked 1	-0.533217965399353	6.67973786450172	-1.39373576462912	0.209661319856293	0.999692817443324	-4.57373987331322	7.68254285798054	7.42700744066982	6.37302438497465	5.57819609380597	6.68465833304869	6.33299807653065	NA
**TC0400000390.mm.2**	Gm21953/Gm21586/Gm20878	predicted gene, 21953/predicted gene, 21586/predicted gene, 20878	-0.538817542820412	9.39377668294732	-1.39271287678332	0.20995598105034	0.999692817443324	-4.57379969177597	9.92888752583253	10.2788739334415	8.97951926600285	8.55911251038168	9.68365089066556	8.9326159713598	NA
**TC0100001589.mm.2**	Nectin4	nectin cell adhesion molecule 4	-0.622424794645945	6.25105149042711	-1.39196296966628	0.210172242123543	0.999692817443324	-4.57384354368168	5.73063722011314	5.80429160935811	6.33933389159793	6.02393137030184	6.2114104289814	7.39670442221026	NA
**TC0X00000073.mm.2**	Otud5	OTU domain containing 5	-0.631442890825944	6.61732435507481	-1.39157029473375	0.210285563208501	0.999692817443324	-4.57386650501634	7.36059553551963	7.43495125878465	6.52919749781399	5.29355927943236	6.42658095810581	6.65906160079243	NA
**TC1900001769.mm.2**	Tmem179b	transmembrane protein 179B	0.488555948061231	7.25118909601908	1.39146616230753	0.210315623753867	0.999692817443324	-4.57387259396751	6.28482568810159	6.65562689090813	8.18755042880044	7.58434651781317	7.4721891181842	7.32259593230694	NA
**TC1100002825.mm.2**	Olfr329-ps	olfactory receptor 329, pseudogene	-0.685924154089654	4.48152173083399	-1.39130331697001	0.210362641070422	0.999692817443324	-4.57388211595971	3.55211101695567	3.83615612105472	3.93010931486218	5.13439815454493	4.85305152369747	5.58330425388895	NA
**TC0700004508.mm.2**	Bet1l	Bet1 golgi vesicular membrane trafficking protein like	0.414820960560855	6.46593676327783	1.39096804649148	0.210459471416092	0.999692817443324	-4.57390171976165	6.1472230773499	6.14179907029604	6.93292386157787	6.73519631499351	6.52285915379219	6.31561910165749	NA
**TC1700001075.mm.2**	Ppp4r1	protein phosphatase 4, regulatory subunit 1	-1.07029537586816	7.4642686661645	-1.39084323884618	0.210495527685828	0.999692817443324	-4.57390901734791	7.65087648481178	7.95606755666234	7.93116306354334	5.58787553834495	7.33730098366802	8.32232836995659	NA
**TC0500001662.mm.2**	A430033K04Rik	RIKEN cDNA A430033K04 gene	-0.839854921179525	6.30102736545403	-1.39026372778858	0.210663018226349	0.999692817443324	-4.57394290091333	6.28891571962369	6.14558069748218	6.85495400869026	4.99102495793938	6.57778456523715	6.94790424375154	NA
**TC0800001612.mm.2**	Xab2	XPA binding protein 2	0.543478201592174	6.12500142612908	1.39015443913346	0.210694618321542	0.999692817443324	-4.57394929078285	7.36045426714305	7.02395150760002	5.53120938007669	6.1950702125312	5.61546688865776	5.02385630076579	NA
**TC1200002493.mm.2**	Wdr60	WD repeat domain 60	-0.410862656575044	6.36689769121359	-1.38988020285244	0.210773930720155	0.999692817443324	-4.57396532456466	6.59415426521223	6.53285424144701	6.11938897350674	6.00693719022938	6.36713517049856	6.58091630638764	NA
**TC0200004383.mm.2**	Meis2	Meis homeobox 2	0.529579546688897	9.25614806851841	1.38983981816108	0.21078561272326	0.999692817443324	-4.57396768571163	10.3177470595049	10.2533012560317	9.38807877262719	8.62442082184867	8.71023534057696	8.24310516052111	NA
**TC1000001625.mm.2**	Cd63	CD63 antigen	-0.76044662288295	8.30922317864458	-1.38949372413187	0.210885750581799	0.999692817443324	-4.57398792030317	8.94841795642133	9.30818383481964	8.19354651049042	6.84537550693989	8.73910305446914	7.82071220872707	NA
**TC0400000880.mm.2**	Focad	focadhesin	0.416043939047907	5.83727881163834	1.38941914805875	0.210907333815019	0.999692817443324	-4.57399228037249	6.19433129990358	6.20911017668975	5.99550974442711	5.73064989123916	5.51953819542809	5.37453356214235	NA
**TC1400000958.mm.2**	Ebf2	early B cell factor 2	-0.4353285683081	5.60610132183859	-1.38928304040872	0.21094673016791	0.999692817443324	-4.57400023780898	5.74276989118789	5.33428064755664	5.3577579009678	5.48669222686761	5.79133621320749	5.92377105124412	NA
**TC0600003139.mm.2**	Tapbpl	TAP binding protein-like	0.522556790238129	5.7389881706608	1.38873433708204	0.211105619480031	0.999692817443324	-4.57403231657723	5.93734948336981	6.15112997615046	6.09895849091864	5.59632308154177	5.66452901806017	4.98563897392398	NA
**TC1100000211.mm.2**	Vps54	VPS54 GARP complex subunit	-0.579553346005335	6.00830894348005	-1.38838891343619	0.211205699835095	0.999692817443324	-4.5740525103899	6.22593249542417	6.1611354467444	5.87188859686479	5.37995091648573	6.68844873727955	5.72249746808164	NA
**TC0100002214.mm.2**	Rev1	REV1, DNA directed polymerase	0.664218325012934	6.90917378415803	1.38786329931659	0.211358069059207	0.999692817443324	-4.57408323736006	7.3554243096269	7.54178225798252	7.59433316506696	6.34880322861535	6.58030696404005	6.0343927796164	NA
**TC0600003201.mm.2**	Rhno1	RAD9-HUS1-RAD1 interacting nuclear orphan 1	0.445670777212301	6.30259898820009	1.38783452245038	0.211366413981427	0.999692817443324	-4.57408491959854	6.32662336913806	6.0309670477433	6.5226696072204	6.65200292615148	5.94143693469135	6.34189404425593	NA
**TC0300000367.mm.2**	Mgst2	microsomal glutathione S-transferase 2	0.490809774190947	5.87597748830568	1.38646314372218	0.21176443993103	0.999692817443324	-4.57416508361762	5.4473225624802	5.71024773005231	5.92512285210559	6.61483424073615	5.81497004886446	5.74336749559539	NA
**TC0400003611.mm.2**	Tmem39b	transmembrane protein 39b	0.435806587769031	6.59381444666549	1.38636406765665	0.211793221639371	0.999692817443324	-4.57417087480482	6.86571147536591	6.62879287611998	6.54446463026664	6.92553312175593	6.34333047925787	6.25505409722664	NA
**TC0200001786.mm.2**	Mapkbp1	mitogen-activated protein kinase binding protein 1	0.435826378595042	6.03382748568528	1.38621446712835	0.211836687425882	0.999692817443324	-4.57417961916484	6.96261274847839	7.08731887339993	5.97194267349223	5.5404003512195	5.27634020650553	5.36435006101612	NA
**TC0700003699.mm.2**	Folh1	folate hydrolase 1	-0.527366932690613	5.50745658726875	-1.38586840751398	0.211937264285543	0.999692817443324	-4.57419984646563	6.22884095633185	5.66068458126173	5.34170012888937	4.70853993142946	5.69114210026284	5.41383182543722	NA
**TC1000000905.mm.2**	BC025920	zinc finger protein pseudogene	-0.412663715639331	5.98678727653077	-1.38542294795768	0.212066793392389	0.999692817443324	-4.57422588296862	5.65444974804016	5.65567019831545	5.90837311115695	5.98426502961823	6.22340400066443	6.49456157138942	NA
**TC0200000587.mm.2**	St6galnac6/St6galnac4	ST6 (alpha-N-acetyl-neuraminyl-2,3-beta-galactosyl-1,3)-N-acetylgalactosaminide alpha-2,6-sialyltransferase 6/ST6 (alpha-N-acetyl-neuraminyl-2,3-beta-galactosyl-1,3)-N-acetylgalactosaminide alpha-2,6-sialyltransferase 4	-0.563700088465896	7.99969081140084	-1.38518245104486	0.212136753785215	0.999692817443324	-4.57423993932904	8.6495856676927	8.38731900048646	7.93299297534497	6.98392703630207	8.17920477134993	7.8651154172289	NA
**TC0200001030.mm.2**	Dcaf17	DDB1 and CUL4 associated factor 17	-0.51977883901677	6.45800510085857	-1.38494808916805	0.212204949457698	0.999692817443324	-4.57425363687322	7.26245000449278	6.85197404322224	6.26020091557498	5.53682352412645	6.29784860209704	6.53873351563793	NA
**TC1200002017.mm.2**	Gm4787	predicted gene 4787	-0.456717713087451	5.2985421666402	-1.38478590231596	0.212252154852782	0.999692817443324	-4.57426311592721	4.76969886096096	4.99626986877158	5.01903837087323	5.53688605109364	5.80121915269248	5.66814069544929	NA
**TC1000002156.mm.2**	Gopc	golgi associated PDZ and coiled-coil motif containing	-0.450435888882849	6.32868140257159	-1.38457965863432	0.212312196867285	0.999692817443324	-4.57427516972925	6.60945680479484	6.61144770338247	5.73710241010998	6.18805365463328	6.2580662814919	6.56796156101705	NA
**TC0200000955.mm.2**	Csrnp3	cysteine-serine-rich nuclear protein 3	0.612602586545753	8.02347843037437	1.38441636172082	0.212359746972057	0.999692817443324	-4.57428471339892	8.88690581164431	9.08008015571336	8.04658354798766	7.65296134600238	7.79937102742399	6.67496869347454	NA
**TC0500002573.mm.2**	Nfxl1	nuclear transcription factor, X-box binding-like 1	-0.497169223074982	8.17095780866363	-1.38414889394001	0.212437651061093	0.999692817443324	-4.57430034494418	7.20967552318778	7.54544452596809	7.97117664294013	8.66696753539785	8.80334746585472	8.82913515863323	NA
**TC0600002490.mm.2**	Vamp8	vesicle-associated membrane protein 8	0.589331852105304	5.94603473599603	1.38374812126126	0.21255443031489	0.999692817443324	-4.57432376660449	5.51524202777906	5.87727714838039	5.87479645037614	6.85638002163753	5.56618532535964	5.98632744244342	NA
**TC1700001450.mm.2**	Slc22a1	solute carrier family 22 (organic cation transporter), member 1	-0.438269401611364	6.23986014212783	-1.38316160108273	0.212725437629362	0.999692817443324	-4.57435804231001	5.74637831107685	5.39299046515316	6.20274238305469	6.50888425360242	6.74189468711703	6.84627075276281	NA
**TC0900000017.mm.2**	Cwf19l2	CWF19-like 2, cell cycle control (S. pombe)	-0.474362070563204	5.84239645409728	-1.38308024533766	0.212749167676524	0.999692817443324	-4.57436279654586	6.0138650287967	5.80128005488567	5.63356471600881	5.51169003387865	5.73431047795094	6.35966841306292	NA
**TC1700000348.mm.2**	Noxo1	NADPH oxidase organizer 1	-0.449713898094225	7.21970358280016	-1.38180637258648	0.213121044313919	0.999692817443324	-4.57443723479982	6.78655051846397	6.80064464887159	7.40262737824792	7.01317188839055	7.46486142258884	7.85036564023809	NA
**TC0X00003066.mm.2**	Morc4	microrchidia 4	-0.430917578862206	5.27722970361836	-1.38160423447995	0.213180107333467	0.999692817443324	-4.57444904599464	4.88404467743818	5.23695208767025	5.14884844053864	5.19142470890001	5.47555410274671	5.72655420441635	NA
**TC0400001112.mm.2**	Fam151a	family with sequence simliarity 151, member A	-0.489233960004085	5.71989826910747	-1.38124651667838	0.213284665428975	0.999692817443324	-4.57446994746535	4.83494170379913	5.440703083396	5.61827346007594	5.91436499364481	6.09972668746376	6.41137968626516	NA
**TC1600002172.mm.2**	Ifnar2	interferon (alpha and beta) receptor 2	-0.585816003145164	8.3267576737971	-1.38123729625274	0.213287361095443	0.999692817443324	-4.57447048620771	8.7605715125079	8.77976681097971	8.3371797889287	7.28710806757363	8.59077598078485	8.20514388200781	NA
**TC0200001716.mm.2**	Spred1	sprouty protein with EVH-1 domain 1, related sequence	-0.722674174781512	8.54918252426184	-1.38123200439449	0.213288908227086	0.999692817443324	-4.57447079540674	9.19240393182023	9.27600541732548	8.61542812325846	7.07524060017269	8.7092780035678	8.42673906942638	NA
**TC0X00001370.mm.2**	Radx	RPA1 related single stranded DNA binding protein, X-linked	0.625596954266497	5.60698579673192	1.38114902430281	0.213313169666805	0.999692817443324	-4.57447564385028	5.63846563700315	5.20897371585112	5.44052538461691	6.5823092834182	5.23681412446641	5.53482663503571	NA
**TC1700000580.mm.2**	Zfp955b	zinc finger protein 955B	-0.494094922818022	7.23934837455891	-1.38051042066074	0.213499965379342	0.999692817443324	-4.57451295577945	8.05114233908833	8.15287268399863	6.91793760307587	6.20400508623935	7.1832014555082	6.92693107944306	NA

**TC0700002976.mm.2**	Ptov1	prostate tumor over expressed gene 1	-0.516719060561567	8.41685229867786	-1.38037891137579	0.213538450930246	0.999692817443324	-4.57452063929117	8.95407649871379	8.9218381770341	8.31960518540134	7.47627531219673	8.3307658146076	8.49855280411361	NA
**TC1100003688.mm.2**	Nr1d1	nuclear receptor subfamily 1, group D, member 1	0.522963764618531	8.0038789074183	1.38014047270302	0.213608244728281	0.999692817443324	-4.57453457001826	8.43397558850665	8.54254515060024	8.42200556700756	7.62433455031243	7.68513313899486	7.31527944908807	NA
**TC0500001849.mm.2**	Tex26	testis expressed 26	0.670850707994372	5.04356539940329	1.38008426559149	0.213624700191916	0.999692817443324	-4.5745378538687	5.40760553598702	5.05024015660215	4.74106033878882	5.83156372112083	5.02175096972183	4.20917167419908	NA
**TC1600002171.mm.2**	Popdc2	popeye domain containing 2	-0.534714149937511	5.18323312341153	-1.38005398523814	0.213633565685096	0.999692817443324	-4.57453962296541	4.38480333043377	4.38589870116354	5.06466043964427	5.56497376485417	5.4562432171392	6.24281928723426	NA
**TC0900002167.mm.2**	Upk2	uroplakin 2	0.586176703556468	5.78719854696434	1.37990759474758	0.213676430605885	0.999692817443324	-4.57454817561161	5.03240164851138	5.14806367738977	5.88033981336559	6.97719986813335	5.73943342405676	5.94575285032923	NA
**TC0800001390.mm.2**	Cenpn	centromere protein N	0.496717140836861	5.1793579452552	1.37936024246763	0.21383677043416	0.999692817443324	-4.57458015298437	5.6511353482142	5.04723692241342	5.09722437040995	5.58838047087869	4.85125823930084	4.84091232031408	NA
**TC1300002774.mm.2**	Zfp456	zinc finger protein 456	-0.509733071201246	4.79881287664468	-1.37928300569214	0.213859404649071	0.999692817443324	-4.57458466519473	5.24131588237472	4.40837276104395	4.53593587575602	4.52592536126744	5.05557384098931	5.02575353843664	NA
**TC1200001225.mm.2**	Rcor1	REST corepressor 1	0.639248982801738	6.99047914879608	1.37915201854058	0.213897795311836	0.999692817443324	-4.57459231746577	7.07922999315499	7.16998287670518	7.77450907366132	6.71157092059858	6.97905095514189	6.22853107351453	NA
**TC0400003626.mm.2**	Hcrtr1	hypocretin (orexin) receptor 1	0.540589518852475	5.86760546379354	1.37913294476557	0.213903386107792	0.999692817443324	-4.57459343174936	5.91262973287795	5.6551369162591	5.76172581634416	6.59779676932041	5.44616905408878	5.83217449387084	NA
**TC0100002586.mm.2**	Zfp142	zinc finger protein 142	-0.574152293205739	5.92197709291373	-1.37798243930137	0.214240858310858	0.999692817443324	-4.57466064078787	7.22322831340144	6.93532583672216	5.56427794709291	4.54822396338074	5.51231406205863	5.74849243482649	NA
**TC1900000114.mm.2**	Map4k2	mitogen-activated protein kinase kinase kinase kinase 2	-0.830920673379601	6.88683739642336	-1.37765720377635	0.214336344648306	0.999692817443324	-4.57467963894637	7.60208683076705	7.37448220369764	7.11212643953003	5.22918055912811	6.93154931079937	7.07159903461798	NA
**TC0400003590.mm.2**	Rbbp4	retinoblastoma binding protein 4, chromatin remodeling factor	-0.917958652919483	8.23137170729789	-1.37745925472427	0.214394479467261	0.999692817443324	-4.57469120160963	9.02735860681758	9.00496741039447	8.44214347618184	6.31784998418633	8.48297691817202	8.11293384803512	NA
**TC1600001395.mm.2**	Masp1	mannan-binding lectin serine peptidase 1	-0.404105850497018	6.5924080487258	-1.37727740255865	0.214447899326199	0.999692817443324	-4.57470182385484	7.09688815992629	7.26265572480823	6.12243829285654	6.07090806045659	6.56262124985784	6.43893680444932	NA
**TC1000003097.mm.2**	Os9	amplified in osteosarcoma	0.585905550205831	7.65554807728284	1.37644833510786	0.214691592797906	0.999692817443324	-4.57475024890273	8.89228846240948	8.54514317204419	7.64564268979533	7.1881912750322	7.31546945663829	6.34655340777758	NA
**TC1300000191.mm.2**	Hist1h2bk	histone cluster 1, H2bk	-0.499357777063295	8.3869798515477	-1.37595321353252	0.214837245700641	0.999692817443324	-4.57477916693976	8.23461349422772	8.56476755539333	8.49259454302169	7.76929670974758	8.62863564205854	8.63197116483733	NA
**TC0500003384.mm.2**	Cldn4	claudin 4	0.563965473853968	5.05526698862755	1.37570982329845	0.214908877757146	0.999692817443324	-4.57479338194687	4.54922125711601	4.82669519802264	5.14684143558579	5.8949667765815	4.61673809655913	5.29713916790022	NA
**TC1000001684.mm.2**	Syne1	spectrin repeat containing, nuclear envelope 1	-0.837824078765963	7.50102253913903	-1.37560866178415	0.214938656851371	0.999692817443324	-4.5747992901193	8.34358091828035	8.51613150403253	7.49243066816415	5.74295665933055	7.84880942712729	7.06222605789933	NA
**TC0500002068.mm.2**	Fam126a	family with sequence similarity 126, member A	1.46291962146121	6.14891184159295	1.37548496181699	0.214975075658253	0.999692817443324	-4.57480651454687	7.09641188581053	7.10457487551443	7.44239454027881	5.36676722529876	4.45645304505768	3.42686947759748	NA
**TC1700001036.mm.2**	Cntnap5c	contactin associated protein-like 5C	-0.486768287074146	6.47266594327304	-1.37547692095724	0.214977443178755	0.999692817443324	-4.57480698415327	6.35899999314277	5.84065783424057	6.27203392866965	6.55936670038365	6.68014494738042	7.12479225582117	NA
**TC0200005383.mm.2**	Zfp931	zinc finger protein 931	-1.07904931873789	7.9487445558114	-1.37528771565804	0.215033158822992	0.999692817443324	-4.57481803412885	8.90676680051952	9.27975147504513	7.95401325363829	5.7199119572757	8.5727646020986	7.25925924629117	NA
**TC1600000109.mm.2**	Rpl39l	ribosomal protein L39-like	-0.443862425155309	5.83887190819096	-1.37528340208624	0.215034423669893	0.999692817443324	-4.57481828495169	5.15036506047868	4.8157098631252	5.86870791095	6.22100792666563	6.38652378924825	6.590916898678	NA
**TC1100001157.mm.2**	Pigs	phosphatidylinositol glycan anchor biosynthesis, class S	0.618613772155051	7.56406993513176	1.37454077002311	0.215252329785208	0.999692817443324	-4.57486165560473	7.33894668605449	7.16754990477373	7.80150817543943	8.25606710669679	6.83850256554359	7.98184517228253	NA
**TC0X00000974.mm.2**	Nono	non-POU-domain-containing, octamer binding protein	0.405164284574527	9.11478614499216	1.37436116190695	0.215306189842953	0.999692817443324	-4.57487214428316	9.37330230504612	9.37648995656045	9.23359747525185	9.14102911349588	8.89099428140097	8.6733037381977	NA
**TC1700001213.mm.2**	Morn2	MORN repeat containing 2	0.530737529270306	8.1984576977816	1.3740929525501	0.215385282064093	0.999692817443324	-4.57488780676961	7.63323616793331	7.52487102075056	8.9817943539797	8.56526267429348	8.65257844436495	7.83300352536762	NA
**TC0700001258.mm.2**	Ctsc	cathepsin C	-0.646733185190205	8.05217229781607	-1.37375216618861	0.215485814073931	0.999692817443324	-4.5749077069972	8.29137179534566	9.36406907690602	7.58909931642683	7.09296395570534	7.67476278040979	8.30076686210279	NA
**TC0200002719.mm.2**	Gm14288/Gm14440	predicted gene 14288/predicted gene 14440	-1.32024506203261	8.19105846009185	-1.37373714557791	0.215490246123095	0.999692817443324	-4.57490858411317	9.46257117593026	9.86154739763457	8.32724689812583	5.26362413333469	8.67970870129623	7.55165245422952	NA
**TC0200002737.mm.2**	Gm14288/Gm14435	predicted gene 14288/KRAB box containing protein	-1.32024506203261	8.19105846009185	-1.37373714557791	0.215490246123095	0.999692817443324	-4.57490858411317	9.46257117593026	9.86154739763457	8.32724689812583	5.26362413333469	8.67970870129623	7.55165245422952	NA
**TC0900003278.mm.2**	Pomgnt2	protein O-linked mannose beta 1,4-N-acetylglucosaminyltransferase 2	-0.473553022532252	7.60043509612226	-1.37364438363373	0.215517618691128	0.999692817443324	-4.57491400084491	7.36850997146849	7.80831391677887	7.62312102057126	7.11621930113959	7.90719686302211	7.77924950375324	NA
**TC0500000310.mm.2**	Atraid	all-trans retinoic acid induced differentiation factor	-0.566961190618893	7.78452040065919	-1.3726954554352	0.215798077173576	0.999692817443324	-4.57496946260876	9.026626297445	9.04232925064699	7.30210709154495	6.45001514576772	7.76159424896428	7.12445036958618	NA
**TC0500001342.mm.2**	Ddx54	DEAD (Asp-Glu-Ala-Asp) box polypeptide 54	-0.458999949376682	6.92513708644405	-1.37244983322711	0.215870389962369	0.999692817443324	-4.57498375155808	6.89404084233141	7.2455340230983	6.73547452029198	6.51114935694863	6.85710446004926	7.30751931594471	NA
**TC1000001183.mm.2**	Btg1	B cell translocation gene 1, anti-proliferative	0.407635409006092	7.67090944715272	1.37236525704197	0.215895386366042	0.999692817443324	-4.57498868976175	7.91895627664458	7.96649665834845	7.76487376224525	7.71276352072248	7.20083538680776	7.46153107814779	NA
**TC0900000829.mm.2**	Igdcc4	immunoglobulin superfamily, DCC subclass, member 4	-0.404504458239959	7.20487751515	-1.37209179072607	0.215976226800426	0.999692817443324	-4.57500465657337	7.23516378791777	7.15814020191288	6.89866149228165	7.11481460001305	7.38224155751206	7.44024345126256	NA
**TC1500001091.mm.2**	Zfp740	zinc finger protein 740	-0.76116345376944	6.62294672678942	-1.37193903327376	0.216021395825237	0.999692817443324	-4.57501357542553	7.61291599149091	7.51905442061971	6.60913864267481	4.93255292318303	6.67107319015466	6.39294528324206	NA
**TC0700004111.mm.2**	Galnt18	polypeptide N-acetylgalactosaminyltransferase 18	0.408771211032166	6.24877876430144	1.371780291357	0.216068343369371	0.999692817443324	-4.57502284356296	6.70130442235291	6.46139164041527	6.35969519558749	6.2140642769649	5.89732384361895	5.85889320686911	NA
**TC1000001466.mm.2**	Grip1	glutamate receptor interacting protein 1	0.583342861561018	5.53699805197243	1.37158389749385	0.216126439035697	0.999692817443324	-4.57503430983482	6.05115388308598	6.22514836462394	4.98463386604019	6.07155202758315	4.82966502440947	5.05983514609183	NA
**TC1000002932.mm.2**	Cpsf6	cleavage and polyadenylation specific factor 6	0.638165740496738	8.09647539255721	1.37134491230379	0.216197152601259	0.999692817443324	-4.5750482625504	8.4078124660867	8.49718557549126	8.43795539813009	8.03713749924931	8.22283851266762	7.97592290371831	NA
**TC1800000708.mm.2**	Psmg2	proteasome (prosome, macropain) assembly chaperone 2	0.618287971967649	6.71071440403603	1.3713322803154	0.216200890869444	0.999692817443324	-4.57504899998934	7.69745409599011	7.61686886744308	6.60012065918876	6.4931490431704	6.54777084073363	5.30892291769022	NA
**TC1300000983.mm.2**	Tmem161b	transmembrane protein 161B	0.780415337445621	5.59564453018852	1.37123118782266	0.216230809927705	0.999692817443324	-4.57505490197946	6.75751467271196	6.77571653363689	5.88691546216269	4.91381786267407	5.3542787833856	3.88562386655991	NA
**TC0200002755.mm.2**	Zfp971	zinc finger protein 971	-1.33391672173672	8.94306798415362	-1.37060060373679	0.216417519594875	0.999692817443324	-4.57509171564382	10.0972895144518	10.5745589565383	9.04242771250494	6.11693352269059	9.67747871692664	8.14971772174233	NA
**TC1400002334.mm.2**	Sorbs3	sorbin and SH3 domain containing 3	0.422881942552086	6.502749552833	1.3699309197417	0.216615964409686	0.999692817443324	-4.57513080979404	6.60013845848261	6.87529280866327	6.61323685849092	6.58017810898723	6.02638952038386	6.32126156199011	NA
**TC0800002169.mm.2**	Stox2	storkhead box 2	-0.629622211335002	8.04305320812236	-1.3696492255921	0.216699486417655	0.999692817443324	-4.57514725358861	9.23014957691806	9.28587936333712	7.76238948360909	6.47913345929539	7.71091752299971	7.78984984257477	NA
**TC0500001615.mm.2**	Plod3/Mir702	procollagen-lysine, 2-oxoglutarate 5-dioxygenase 3/microRNA 702	-0.419794071791014	6.66956847365527	-1.36942311186763	0.216766549737116	0.999692817443324	-4.57516045260431	6.95050645066858	7.05230608671271	6.37646329014785	6.21104179033628	6.54840401500607	6.87868920906009	NA
**TC0700003714.mm.2**	Prss23	protease, serine 23	0.474378983514788	5.83662611862737	1.36920267685326	0.2168319466876	0.999692817443324	-4.57517331988629	6.3899608736899	6.54761192809741	5.58387964530642	5.93159129319682	5.5733111550502	4.99340181642347	NA
**TC1900000857.mm.2**	Gm19956	predicted gene, 19956	0.517333574781383	5.26875156498654	1.36867609461122	0.216988240518151	0.999692817443324	-4.57520405666715	4.70592968893324	4.76089992723932	5.47490571792488	6.11526774372984	4.97666121699244	5.5788450950995	NA
**TC1100002895.mm.2**	Top3a	topoisomerase (DNA) III alpha	-0.521605351384722	5.87948871931219	-1.36736386435509	0.217378159781728	0.999692817443324	-4.57528064583298	5.98234834639108	5.5512456622507	5.93841001698424	5.41165378525467	5.90233853647315	6.49093596852724	NA
**TC1700000428.mm.2**	Syngap1	synaptic Ras GTPase activating protein 1 homolog (rat)	0.610218704936819	8.35756847032717	1.36592227909808	0.217807238488411	0.999692817443324	-4.57536477468986	9.12176006732235	9.35839261877814	8.822051307653	7.62079646521509	7.57219072897226	7.65021963402219	NA
**TC0600002562.mm.2**	Wdr54	WD repeat domain 54	-0.475080526380439	7.61305109368309	-1.36514341605255	0.218039377224243	0.999692817443324	-4.57541022352657	8.58218747088466	8.42433112798412	7.04595876627249	6.81485469296194	7.72856469875437	7.08240980924093	NA
**TC0500000334.mm.2**	Fosl2	fos-like antigen 2	-0.470461764034281	6.08856848243266	-1.36479899408249	0.218142102113074	0.999692817443324	-4.57543032049847	5.77625561944533	5.71519062797196	5.79649252363806	6.25301573591701	6.24305874589381	6.74737304172982	NA
**TC0X00000337.mm.2**	Rnf113a1	ring finger protein 113A1	0.62745892876009	5.83778333979819	1.36468898262745	0.218174922478405	0.999692817443324	-4.57543673951948	5.81226191514347	5.89637707198303	5.6053389982342	6.6811504563572	5.86673083502671	5.16484076204451	NA
**TC1100003627.mm.2**	Cdk5rap3	CDK5 regulatory subunit associated protein 3	0.420548712759873	7.51899973302689	1.36464664921792	0.218187553523021	0.999692817443324	-4.57543920960024	7.51282510706696	7.69252278262996	7.52254227483164	7.85233169216043	7.33511316264815	7.19866337882417	NA
**TC0300000979.mm.2**	Hmgcs2	3-hydroxy-3-methylglutaryl-Coenzyme A synthase 2	0.479532195500631	7.17029695094487	1.36432961976364	0.218282164088655	0.999692817443324	-4.57545770741673	7.53052185671387	7.11349661081204	7.33371685361911	7.33469696094879	6.55754068835171	7.15180873981482	NA
**TC0700003107.mm.2**	Gabrg3	gamma-aminobutyric acid (GABA) A receptor, subunit gamma 3	-1.1712414519667	7.51202608726115	-1.36423529838366	0.218310319425277	0.999692817443324	-4.57546321071332	8.55122915819171	8.44187352680217	7.66487927203634	5.20340619528347	8.37142368815179	6.83934468310142	NA
**TC1100004229.mm.2**	Cbr2	carbonyl reductase 2	0.564961832597702	5.92856705062783	1.3640726807413	0.218358869113898	0.999692817443324	-4.57547269872862	5.68803209624143	6.02942416561081	6.47312249551497	6.01881235804011	5.22782411766614	6.13418707069353	NA
**TC0200004479.mm.2**	Lcmt2	leucine carboxyl methyltransferase 2	0.492374891098311	7.02430162765318	1.36325462095718	0.218603248266025	0.999692817443324	-4.57552042674665	7.06972646849601	7.05652631722266	7.4288356803844	7.07331770512411	6.40892137726066	7.10848222605124	NA
**TC0500003717.mm.2**	Rsph10b	radial spoke head 10 homolog B (Chlamydomonas)	0.442504490903029	5.34125642674112	1.36317466307051	0.21862714721	0.999692817443324	-4.57552509153432	5.40326175711289	5.4598253764343	5.35223855503563	5.68249164931717	4.86202584014856	5.28769538239818	NA
**TC0400000963.mm.2**	Hook1	hook microtubule tethering protein 1	-1.09441562104196	7.27768425344867	-1.36306987186303	0.21865847222632	0.999692817443324	-4.57553120506012	7.91398476793049	7.99513512582454	7.61411457556126	5.16996261686526	8.04523010798481	6.92767832653562	NA
**TC1300000157.mm.2**	Aoah	acyloxyacyl hydrolase	0.508541921978362	5.54057178847704	1.36248263389056	0.218834088276223	0.999692817443324	-4.57556546347461	5.07188927141533	5.49082927024111	5.55466387085321	6.29423414572804	5.37262876908826	5.45918540353626	NA
**TC0900001434.mm.2**	Plxnb1	plexin B1	0.511754865618455	6.44166805526374	1.36247023022664	0.218837799003819	0.999692817443324	-4.57556618706216	7.36768398898989	7.02353665466479	5.97584931456407	6.66529939501828	6.00815163096119	5.60948734738424	NA
**TC1200001931.mm.2**	Kcnh5	potassium voltage-gated channel, subfamily H (eag-related), member 5	-0.761437719749494	6.72998055909972	-1.36219383625167	0.218920500068563	0.999692817443324	-4.57558231073464	7.21601159070354	7.79808524935776	6.67993706407267	5.24151847344636	7.09263422231668	6.35169675470134	NA
**TC0X00000088.mm.2**	Slc38a5	solute carrier family 38, member 5	0.401668505536797	6.6402277293247	1.36130320849095	0.219187181361127	0.999692817443324	-4.57563426344423	6.39173664170737	6.34692876484017	7.00361048930327	6.9494071989298	6.45973841333651	6.68994226382701	NA
**TC0700000382.mm.2**	Smg9	smg-9 homolog, nonsense mediated mRNA decay factor (C. elegans)	-0.495627410012076	7.18106687155779	-1.3608575038914	0.219320747625835	0.999692817443324	-4.57566026097796	7.07079689565009	7.04567475134503	6.72719233347233	7.26214504769141	7.19138968653501	7.78920251465288	NA
**TC0400002800.mm.2**	Whrn	whirlin	0.396215173725876	7.58387301440592	1.36056447887502	0.219408599334015	0.999692817443324	-4.57567735226508	7.83589598647783	7.7416502944291	7.73260874972544	7.62645232676474	7.23648193975813	7.3301487892803	NA
**TC0200002075.mm.2**	Ankef1/Paral1	ankyrin repeat and EF-hand domain containing 1/PPARG activating RBM14 associated lncRNA 1	0.549870401158532	5.3555164503607	1.36034307679042	0.219474998646342	0.999692817443324	-4.57569026568662	5.4591324431507	4.80891487520301	5.57824800180487	5.9041480912589	5.54861920221584	4.83403608853087	NA
**TC1500002159.mm.2**	Vdr	vitamin D (1,25-dihydroxyvitamin D3) receptor	0.446142561814473	7.78917117890414	1.36025826623437	0.21950043840286	0.999692817443324	-4.57569521224687	7.5805527370678	7.59270717831069	7.83851563082126	8.3885105100164	7.59825326972049	7.73648774748822	NA
**TC0700004493.mm.2**	Fuom	fucose mutarotase	-0.397789873995714	8.24874478789595	-1.35996787281814	0.219587564698911	0.999692817443324	-4.5757121490918	8.74790346738514	7.69708074679145	8.32243151689719	8.24002086570666	8.68542520827843	8.67260692231684	NA
**TC0800000231.mm.2**	Defa27	defensin, alpha, 27	-0.425917532114254	6.36808483190984	-1.35930691702313	0.219785985418079	0.999692817443324	-4.57575069681335	5.71614522126528	5.60938518044987	6.30150066289525	6.71407109986243	6.86422827181746	7.00317855516872	NA
**TC1900000994.mm.2**	Slc22a20	solute carrier family 22 (organic anion transporter), member 20	-0.492239753133596	5.60755980837333	-1.35928690544532	0.219791995444189	0.999692817443324	-4.57575186387466	5.18301505704265	5.14616549397376	5.41177327924401	5.75407611723417	5.71938847471052	6.43094042803485	NA
**TC1100000531.mm.2**	Rnf130	ring finger protein 130	-0.766982076491099	7.7444535742867	-1.3588452098713	0.219924686117638	0.999692817443324	-4.57577762269043	8.63081715209649	8.6770850310146	7.6899706145419	6.12245694027156	8.05282161236881	7.29357009542684	NA
**TC1900000733.mm.2**	Sorcs3	sortilin-related VPS10 domain containing receptor 3	0.590121616518836	6.90688568487664	1.35838170013108	0.220060400630362	0.999692817443324	-4.57580465025038	7.13775280149789	7.11851386877699	6.72488578859867	7.45775954736263	6.95618893176969	6.04621317115394	NA
**TC0300002374.mm.2**	S100a1	S100 calcium binding protein A1	-0.715149568929973	6.63377763529332	-1.35833735492723	0.220077340250121	0.999692817443324	-4.57580723845508	7.31153109408222	7.41743597954112	6.22428536737343	5.59741443276488	7.35040984629557	5.90158909170268	NA
**TC0100000824.mm.2**	Atg16l1	autophagy related 16-like 1 (S. cerevisiae)	0.663896403272024	6.04431531440732	1.3578652861093	0.220219322390225	0.999692817443324	-4.57583476605147	6.43178528408796	6.49413535926213	6.23167855228815	6.10220347249078	6.20874670987789	4.79734250839699	NA
**TC0400001453.mm.2**	Stk40	serine/threonine kinase 40	0.460809307593369	8.40421046353167	1.35772190517699	0.220262462651337	0.999692817443324	-4.57584312673134	8.17475772401341	8.34022450282667	8.9835751292164	8.43237445555193	8.3959364514279	8.09839451815369	NA
**TC1300000655.mm.2**	Prelid1	PRELI domain containing 1	-0.743170878396545	8.52798749102378	-1.35675860922858	0.220552493446218	0.999692817443324	-4.57589929445737	9.24917384706462	9.27362330508449	8.53028753662049	7.0491054819797	8.92099749059558	8.14473728479775	NA
**TC0200005373.mm.2**	Gm14325	predicted gene 14325	-0.876860105279932	7.61028573006144	-1.35672719908603	0.220561956200494	0.999692817443324	-4.5759011258278	8.71682935743992	9.00608941859359	7.35460419209765	5.73789750478997	8.05815252158405	6.78810538586344	NA
**TC0500003715.mm.2**	Smok3b/Smok3a	sperm motility kinase 3B/sperm motility kinase 3A	0.453607068663766	5.40577741017733	1.35644165976237	0.220647995619005	0.999692817443324	-4.57591777396168	4.99634622120815	5.12468584149809	5.985376871731	5.62504639597663	5.11171631213764	5.59149281824246	NA
**TC0700001644.mm.2**	Mical2	microtubule associated monooxygenase, calponin and LIM domain containing 2	-0.69942114299885	7.95365087733014	-1.35636892550361	0.220669916871762	0.999692817443324	-4.57592201459887	8.23078322202224	8.4129365529812	8.18220731928065	6.65746428220921	8.12308073556584	8.11543315192172	NA
**TC0200000473.mm.2**	Wdr5	WD repeat domain 5	0.492986307215514	6.46157951190822	1.35560477131644	0.220900341453074	0.999692817443324	-4.57596656539255	6.60208698222009	6.37743367657642	7.06131016011865	6.32665435342328	6.09035035976075	6.31164153935016	NA
**TC1100000861.mm.2**	Rcvrn	recoverin	0.449182628411125	5.39217837158826	1.35528791614309	0.220995949531786	0.999692817443324	-4.5759850373269	5.20374080838916	5.08054663064744	5.53393994185736	5.94963404580024	5.09073961967803	5.49446911151731	NA
**TC0X00002756.mm.2**	Gm9112	predicted gene 9112	-0.550367875877672	5.6746835591377	-1.35496400404847	0.221093725121063	0.999692817443324	-4.57600392007186	4.82523711656233	4.59623842190997	5.65363814788136	6.10930688441792	5.98459866557972	6.8790821184749	NA
**TC1500000849.mm.2**	Zdhhc25	zinc finger, DHHC domain containing 25	0.74755481245929	4.9760245980837	1.35486663700514	0.221123123723026	0.999692817443324	-4.57600959605344	4.59530853088975	4.81549359099213	4.6901352033059	6.28009216246354	4.47430684893007	5.00081089192079	NA
**TC1900001156.mm.2**	Olfr76	olfactory receptor 76	0.60104774852134	4.92760278232328	1.354768784219	0.221152672500413	0.999692817443324	-4.57601530029654	4.51193170389025	4.32343095772835	5.00529069647055	5.96088406821133	4.52113694909835	5.24294231854085	NA
**TC0X00002721.mm.2**	Zmym3	zinc finger, MYM-type 3	0.544598360071214	6.70454609855691	1.35408823712671	0.221358275929584	0.999692817443324	-4.57605497067876	7.8491321589055	7.38914832970766	6.93323058294936	6.10586582848601	6.14994261313395	5.79995707815899	NA

**TC0100002631.mm.2**	Farsb	phenylalanyl-tRNA synthetase, beta subunit	-1.16238347070908	5.98168015853554	-1.35405369659453	0.221368715683306	0.999692817443324	-4.57605698404096	7.1417898985809	6.98819589429463	6.28816042200229	3.42950368645748	6.25608845375743	5.7863425961205	NA
**TC1300000670.mm.2**	Caml	calcium modulating ligand	-1.20046622951692	6.49193623445985	-1.35401265159509	0.221381121960093	0.999692817443324	-4.57605937653861	7.69031135005497	7.64657933866506	6.79522705433394	3.81167007516869	6.693966490191	6.31386309834546	NA
**TC0800002395.mm.2**	Rex1bd	required for excision 1-B domain containing	-0.43685226672876	8.19223550122584	-1.35356706195983	0.221515845951978	0.999692817443324	-4.57608534916614	7.89649171210642	7.9027165646746	7.87259941391004	8.3676506846482	8.46647447554959	8.64748015646617	NA
**TC1500002288.mm.2**	Itgb7	integrin beta 7	-0.423439591445325	8.81812347043361	-1.35229637423065	0.221900439802465	0.999692817443324	-4.5761594089741	8.20399848607155	8.46338501756475	8.66698870980841	9.03025035822896	9.27121468999411	9.27290356093391	NA
**TC1000000879.mm.2**	Thop1	thimet oligopeptidase 1	-0.456401251330733	7.48254321472157	-1.35196238131018	0.222001626769369	0.999692817443324	-4.57617887360446	7.46624828361444	7.41952427391883	7.51867080208225	7.02967131198509	7.93220893952563	7.52893567720318	NA
**TC1400002336.mm.2**	Slc39a14	solute carrier family 39 (zinc transporter), member 14	-0.473003634810864	6.88437866219817	-1.35175230239366	0.222065293688566	0.999692817443324	-4.57619111636896	6.55565466082202	7.05162804007842	6.86898393875504	6.50750706257837	6.97662569408263	7.34587257687251	NA
**TC0700003909.mm.2**	Hbb-bs/Hbb-b1	hemoglobin, beta adult s chain/hemoglobin, beta adult major chain	-1.011672651629	10.5743001525386	-1.35154066494819	0.222129449360559	0.999692817443324	-4.57620344969793	10.6435560438943	11.7195959375633	10.3351502136268	9.19450160163121	11.7363843919085	9.81661272660754	NA
**TC1200001020.mm.2**	Tmem251	transmembrane protein 251	-0.817273115146973	6.88212589260595	-1.35091241977239	0.222319992589846	0.999692817443324	-4.57624005959852	7.34446203794189	7.2192573818628	7.02774421290547	5.51950063986307	7.67291590794295	6.50887517511953	NA
**TC1100002769.mm.2**	Lyrm7	LYR motif containing 7	-1.00845134653042	7.1450746594048	-1.35036646465852	0.222485695954287	0.999692817443324	-4.5762718722949	7.7777745832042	6.42495138708939	7.63311074293367	5.6922989036035	8.02970282478702	7.312609514811	NA
**TC1100003559.mm.2**	Luc7l3	LUC7-like 3 (S. cerevisiae)	-0.541626072879176	7.82293371936088	-1.35034192455114	0.222493146729235	0.999692817443324	-4.57627330220063	8.31684144934879	8.17021598021238	7.74651107938077	6.9371352910421	8.17102804510752	7.5958704710737	NA
**TC0200002291.mm.2**	Defb36	defensin beta 36	0.608449616914696	7.72441296607643	1.34999798354676	0.222597596191727	0.999692817443324	-4.57629334261713	7.21352817885802	7.27517603591345	7.75924825704362	8.77808815071462	7.30351102546065	8.0169261484682	NA
**TC0200005344.mm.2**	Zfp967/Zfp966	zinc finger protein 967/zinc finger protein 966	-0.846074952327995	8.50201397542373	-1.34994542012723	0.222613562720828	0.999692817443324	-4.57629640526885	9.33908944671951	9.54295101816047	8.279982785918	6.9389639555852	9.18876581189572	7.72233083426347	NA
**TC0200004776.mm.2**	Ralgapa2	Ral GTPase activating protein, alpha subunit 2 (catalytic)	-0.417188378462196	5.05621617386088	-1.34871805891942	0.222986672895656	0.999692817443324	-4.57636791380826	5.17478426692654	5.07837288742658	4.91499250893621	4.70988905700767	5.05829161822079	5.40096670464748	NA
**TC1000002487.mm.2**	Tmem259	transmembrane protein 259	0.591078588580309	9.10888016699529	1.3475059073642	0.223355705851581	0.999692817443324	-4.57643852731537	9.40665466210798	9.29148307454018	9.87210361145912	8.69654660978298	8.75972590850242	8.62676713557906	NA
**TC0500003131.mm.2**	Rfc5	replication factor C (activator 1) 5	0.465619829701957	6.00850067293112	1.34649815559755	0.223662924167507	0.999692817443324	-4.57649722679307	5.78422394791558	5.90052590414696	6.08523008816129	6.56351683430276	5.61695627960912	6.10055098345102	NA
**TC1300002351.mm.2**	Nr2f1	nuclear receptor subfamily 2, group F, member 1	-0.426615218373693	6.96838755840565	-1.34633403566613	0.223712992568346	0.999692817443324	-4.57650678585631	7.17451719504395	7.55737551116055	6.68587846136527	6.42672264237575	7.04586194769282	6.91996959279558	NA
**TC0200002526.mm.2**	Wfdc10	WAP four-disulfide core domain 10	-0.427458812377247	5.89512723106075	-1.34622223286241	0.223747106188009	0.999692817443324	-4.57651329764657	5.82769128117734	5.49680861595347	5.82434478776892	5.77132814447068	6.05020629673851	6.40038426025558	NA
**TC0700003032.mm.2**	Ush1c	USH1 protein network component harmonin	-0.527631209898691	6.53782625663517	-1.34597700206851	0.223821947983502	0.999692817443324	-4.57652758048595	6.03594463867053	5.63097969854221	6.27598440739289	6.97640098400756	6.8988617948933	7.40878601630452	NA
**TC1100001267.mm.2**	Wfdc21	WAP four-disulfide core domain 21	-0.508983352891621	4.86682757936469	-1.34592740993724	0.223837085675039	0.999692817443324	-4.57653046880773	4.42524078970131	4.54620904314633	5.23237064973404	4.37340381904458	5.23273809885186	5.39100307571	NA
**TC1500001021.mm.2**	Asic1	acid-sensing (proton-gated) ion channel 1	0.468123990142698	7.66767979356316	1.34592387476355	0.223838164799737	0.999692817443324	-4.57653067470109	8.00583120443213	7.81857377162865	8.10457604521912	7.45438483758267	7.40655104063242	7.21616186188396	NA
**TC1000000549.mm.2**	Gm17455	predicted gene, 17455	0.603887371800055	6.69839560016892	1.34589135481008	0.223848091851637	0.999692817443324	-4.57653256870414	6.53053513152814	6.469210942014	6.57240117780293	7.62679995773281	6.17478655873139	6.81663983320424	NA
**TC1600000670.mm.2**	Atg3	autophagy related 3	-0.662245397659797	7.54174082549766	-1.34534847738237	0.224013868657193	0.999692817443324	-4.57656418560845	8.37596086690624	8.28931848423835	7.45807783544145	6.17225956781945	7.79117937678758	7.16364882179291	NA
**TC1800001428.mm.2**	Pcyox1l	prenylcysteine oxidase 1 like	-0.390711750258574	7.61645137190162	-1.34532010632095	0.224022535243174	0.999692817443324	-4.57656583787466	7.47375707768466	7.37231619231408	7.4675157154563	7.56809001499061	7.88300574119578	7.93402348976828	NA
**TC0400004153.mm.2**	Tmem88b	transmembrane protein 88B	0.67802160701357	7.2190120067549	1.34471495915486	0.224207462253587	0.999692817443324	-4.57660107908695	8.40437471374701	8.44679216175495	7.16367179223784	6.74580239728945	6.98937669476945	5.5640542807307	NA
**TC1800001744.mm.2**	Arhgap26	Rho GTPase activating protein 26	0.59073076555322	8.06079686193833	1.34426408449062	0.224345333368429	0.999692817443324	-4.57662733464556	8.87424757653406	8.78575216498343	8.12191159152321	7.82120988908625	7.9560795671755	6.80558038232753	NA
**TC0X00003376.mm.2**	Hccs	holocytochrome c synthetase	-0.460376375569569	6.28852373489925	-1.34377939427788	0.224493628893085	0.999692817443324	-4.57665555795739	7.00390347331196	6.7014464586577	6.05576304019537	5.49675682294797	6.13196205548459	6.34131055879789	NA
**TC1100001238.mm.2**	Slfn2	schlafen 2	-0.4355499639926	5.77184863110972	-1.34366982370203	0.224527165118614	0.999692817443324	-4.57666193800387	5.09673418185859	5.47971952984662	5.87217464388427	5.71959442959969	6.40437515616827	6.05849384530089	NA
**TC1800001005.mm.2**	Zfp521	zinc finger protein 521	0.607688764149492	7.09990058334513	1.34362451517661	0.224541033984108	0.999692817443324	-4.57666457619477	7.96903780690373	7.67991574972736	7.63432252932798	6.44868120654136	6.57512332504455	6.29241288252581	NA
**TC0800002368.mm.2**	Zfp868	zinc finger protein 868	-0.449984539640555	5.92048746351427	-1.34351028102807	0.224576004251585	0.999692817443324	-4.57667122767827	7.00800696204636	6.87891568366892	5.30257882531369	5.06543770273092	5.91152266144035	5.35646294588537	NA
**TC0400002514.mm.2**	Fancg	Fanconi anemia, complementation group G	0.450284552255938	5.6571333126912	1.34324419303387	0.224657480006626	0.999692817443324	-4.57668672080134	5.56013336909228	5.67258508367378	5.93608789629302	5.86923736765348	5.75502238713446	5.14973377230016	NA
**TC0500003221.mm.2**	Kdm2b	lysine (K)-specific demethylase 2B	0.436011281394716	6.24203845513431	1.343100849798	0.224701382388018	0.999692817443324	-4.57669506685792	6.07141279084667	6.14990139011392	6.26137245683361	6.79009709948375	6.13414755697084	6.04529943655709	NA
**TC1100000038.mm.2**	Tbc1d10a	TBC1 domain family, member 10a	0.414995437614406	5.46300826678247	1.34213581180994	0.224997147521502	0.999692817443324	-4.5767512521354	5.83734390104021	5.63837358086228	5.49408278408495	5.57207871292562	4.94330957822439	5.29286104355736	NA
**TC1700001648.mm.2**	Jpt2	Jupiter microtubule associated homolog 2	0.426809928159708	6.08910148798637	1.34208389773056	0.225013067968724	0.999692817443324	-4.57675427444779	6.53307500226095	6.28185820971359	6.08957213851392	6.19707564761761	5.91918541935441	5.51384251045771	NA
**TC0500001911.mm.2**	Cfap69	cilia and flagella associated protein 69	-0.420749051096658	5.65147060903291	-1.34179595903481	0.225101388066765	0.999692817443324	-4.57677103723398	6.0261131046314	5.92332817995105	5.34166814243625	5.2172739912746	5.48998614148534	5.91045409441882	NA
**TC1300000050.mm.2**	Larp4b	La ribonucleoprotein domain family, member 4B	0.863591727987201	7.38171370475059	1.34178803314514	0.225103819628807	0.999692817443324	-4.5767714986442	8.21170284528625	8.10457699557733	8.10182753448357	6.74876538732361	7.34010399371767	5.7833054721151	NA
**TC1700000316.mm.2**	Cldn6	claudin 6	-0.464827814562889	5.53711726486471	-1.34164771524858	0.22514687123819	0.999692817443324	-4.57677966726511	5.70311459706235	5.5540021463766	5.19515506052077	5.322810547791	5.39083442854329	6.05678680889426	NA
**TC0100002204.mm.2**	Mgat4a	mannoside acetylglucosaminyltransferase 4, isoenzyme A	-0.755864600759294	7.21794992934726	-1.34151408044836	0.22518787917773	0.999692817443324	-4.57678744671273	7.70230500229754	7.48724921247583	7.50914680628554	5.79406127361026	7.31595627199629	7.4989810094181	NA
**TC1300002082.mm.2**	Il9	interleukin 9	0.49904685884824	5.00145060284252	1.34128864144966	0.225257073723656	0.999692817443324	-4.57680057021435	4.26726580381331	4.42392537885971	5.1624604885039	5.99534258753539	5.06975013962722	5.08995921871559	NA
**TC1500001742.mm.2**	Top1mt	DNA topoisomerase 1, mitochondrial	0.605598199708377	6.38242206917081	1.34055623274897	0.225482004146472	0.999692817443324	-4.57684320375632	7.41292771132835	7.35229763043907	6.72738401545184	5.64286772088524	5.88900344604261	5.27005189087772	NA
**TC0800003184.mm.2**	Sipa1l2	signal-induced proliferation-associated 1 like 2	-0.756090789455779	6.35784365635081	-1.34047755369983	0.225506179177799	0.999692817443324	-4.57684778346336	7.24419220215742	7.11787940078364	6.40078358894538	4.73562078918073	6.56990857123741	6.07867738580026	NA
**TC1200000358.mm.2**	Gm7008	predicted gene 7008	-0.409086293958001	6.20439786648398	-1.34028844743286	0.225564293645164	0.999692817443324	-4.57685879069536	5.68914808800403	5.74671459123144	6.12737338991254	6.35880257596367	6.51118423977546	6.79316431401674	NA
**TC0400003530.mm.2**	Thrap3	thyroid hormone receptor associated protein 3	0.443870944523368	7.02753577117764	1.33967460272261	0.225753026680273	0.999692817443324	-4.57689451892477	8.32822019768265	8.2721661547882	6.81137425351259	6.41491082830828	5.9514533709045	6.38708982186962	NA
**TC0100002656.mm.2**	Dock10	dedicator of cytokinesis 10	0.500315096264267	6.12365265930324	1.33862014849433	0.226077557210845	0.999692817443324	-4.57695588672923	6.89797682534269	7.08036015836744	5.69739362780885	6.18471095451006	5.78136304312382	5.10011134666656	NA
**TC0300000711.mm.2**	Nes	nestin	0.568687045005943	5.95760789963504	1.33852365454814	0.226107275940459	0.999692817443324	-4.57696150218638	5.54599325006926	5.94033558108188	5.9766690585448	6.72167726979069	6.15103943127462	5.40993280704898	NA
**TC1100002314.mm.2**	Lgalsl	lectin, galactoside binding-like	-0.426206146108505	8.46325081005625	-1.33818168137623	0.226212626629838	0.999692817443324	-4.57698140279943	8.73932764472899	8.44476554527482	8.38282520665422	7.98867448240412	8.62561516637795	8.5982968148974	NA
**TC0900002735.mm.2**	Col12a1	collagen, type XII, alpha 1	-0.500546763407832	7.33155227823465	-1.33788809945626	0.226303104333703	0.999692817443324	-4.57699848674926	6.43437037884423	6.67953957917941	7.18066779691091	7.7564872953734	7.67467465073089	8.26357396836909	NA
**TC0500002985.mm.2**	Pgam5	phosphoglycerate mutase family member 5	0.395452955529771	7.36793692385661	1.33783843756272	0.226318412588289	0.999692817443324	-4.57700137658996	7.58817361740443	7.46267853828617	7.46886660829596	7.50497104095833	6.98550919740807	7.19742254078668	NA
**TC0200004925.mm.2**	Tspyl3	TSPY-like 3	0.62464223568968	6.87930865553287	1.33689267763009	0.226610118141923	0.999692817443324	-4.57705640756946	7.24600526561951	6.97280955840832	7.61037992161515	6.54278086865923	6.81647512501235	6.08740119388268	NA
**TC0800001507.mm.2**	Spg7	SPG7, paraplegin matrix AAA peptidase subunit	0.530585053954964	5.53175990673257	1.33652545855952	0.226723471311257	0.999692817443324	-4.57707777338143	5.93143338521832	6.68158038465052	5.61580602557873	5.20355186363951	5.12017811312387	4.63800966818444	NA
**TC1100002167.mm.2**	Tmed4	transmembrane p24 trafficking protein 4	0.472090879277244	8.46998600110091	1.3365146978141	0.226726793696583	0.999692817443324	-4.5770783994577	9.45435522189022	8.92424830400148	8.58415233671106	8.10859478292308	7.96879721163609	7.77976814944355	NA
**TC1200000155.mm.2**	5730507C01Rik	RIKEN cDNA 5730507C01 gene	0.910646471976353	4.60138956123753	1.33623837865183	0.226812122172704	0.999692817443324	-4.57709447585854	5.06345499739174	4.89909509500432	4.61004081103534	5.12349749845557	5.03093910158159	2.88130986395662	NA
**TC1100001719.mm.2**	Rprml	reprimo-like	-0.555606342928571	7.18997343558316	-1.33615823027984	0.22683687764611	0.999692817443324	-4.57709913884062	7.27539999424322	6.83758438905873	6.55906120824214	7.39876056392779	7.27698753582494	7.79204692220213	NA
**TC0200002314.mm.2**	Kif3b	kinesin family member 3B	0.533663373404401	6.75214090481281	1.33603375003271	0.226875330685165	0.999692817443324	-4.57710638093928	6.88956458809764	7.21556338191394	7.07958816701231	6.65793393582474	6.75527338187784	5.91492197415041	NA
**TC0900003119.mm.2**	Tmie	transmembrane inner ear	0.545506596550118	6.84869893880081	1.33603022049661	0.226876421074123	0.999692817443324	-4.5771065862816	7.03028237882491	6.74073139518485	6.63348056738839	7.57261595855927	6.72823461814116	6.38684871470627	NA
**TC0800001076.mm.2**	Irx6	Iroquois homeobox 6	0.526697458955504	5.89224841693714	1.33576772868365	0.22695752640143	0.999692817443324	-4.57712185737132	5.99527458394886	5.75289800224373	5.86941003636225	6.45994638030837	6.00210758561783	5.27385391314178	NA
**TSUnmapped00000005.mm.2**	Amot	angiomotin	0.401069933343356	5.05977348490887	1.33571629430735	0.226973421728228	0.999692817443324	-4.57712484963585	5.52174436090303	5.2676394698042	5.15666154993064	5.02903692278571	4.75683378945972	4.62672481656992	NA
**TC0400002549.mm.2**	Gne	glucosamine (UDP-N-acetyl)-2-epimerase/N-acetylmannosamine kinase	-0.405116888661436	8.08625860874287	-1.33557825781212	0.227016085536288	0.999692817443324	-4.57713288000931	8.60803818350161	8.54207677916269	7.80890847366226	7.46969298257275	8.06750143458644	8.02133379897144	NA
**TC0700004095.mm.2**	Sbf2	SET binding factor 2	1.0957054078701	5.95284325225056	1.33557339058095	0.227017590011916	0.999692817443324	-4.57713316316171	7.46710241330812	7.4508919936092	6.15431565426412	5.340922306899	5.96842318227836	3.33540396314456	NA
**TC1800000171.mm.2**	Asxl3	additional sex combs like 3, transcriptional regulator	0.439343088279482	5.64289568169045	1.33541601330403	0.22706624056406	0.999692817443324	-4.57714231853988	6.33254836112567	5.80594940124546	5.53444773561144	5.76433351655382	5.21571772106996	5.20437735453633	NA
**TC0100001317.mm.2**	Uchl5	ubiquitin carboxyl-terminal esterase L5	-0.877120775695811	7.01586869585014	-1.33488356786443	0.227230905760079	0.999692817443324	-4.57717329218046	8.34906973706986	8.38448509774041	6.91461299545435	4.88909489899513	7.09007417767998	6.46787526816113	NA
**TC0100002702.mm.2**	Slc16a14	solute carrier family 16 (monocarboxylic acid transporters), member 14	0.884465987469532	5.53703019098827	1.33487047123422	0.227234957386089	0.999692817443324	-4.57717405401936	5.84892325471961	5.86509878194833	5.50116037984323	5.53738516225715	5.70520836224337	3.56440520491795	NA
**TC1500001823.mm.2**	Foxred2	FAD-dependent oxidoreductase domain containing 2	0.559870584035594	5.43021096728758	1.33465774610977	0.227300775858245	0.999692817443324	-4.57718642820833	5.72080892329506	5.49981385955771	5.20293119469974	6.0372608997722	4.7224141604349	5.39803676596584	NA
**TC1100002597.mm.2**	Nipal4	NIPA-like domain containing 4	0.489115486478858	6.28971405133431	1.33400092770471	0.227504106275607	0.999692817443324	-4.57722463332843	6.10158484015405	6.4478901252553	6.51906003114052	6.56446012663658	5.68344485525695	6.42184432956245	NA
**TC0500003027.mm.2**	2900026A02Rik	RIKEN cDNA 2900026A02 gene	0.461537351963489	6.11297246326719	1.33357133319543	0.227637182535917	0.999692817443324	-4.5772496199637	6.15630739764899	6.74445604957247	5.99817775552862	6.35189526262572	5.66962480335872	5.75737351086864	NA
**TC0600001254.mm.2**	Fancd2	Fanconi anemia, complementation group D2	0.425464024916895	4.95455323182894	1.33291199096625	0.227841562439524	0.999692817443324	-4.57728796706753	4.58339192030307	4.8566087105561	5.07784561914043	5.4912777858337	4.83146028679981	4.88673506834054	NA
**TC0600003387.mm.2**	C2cd5	C2 calcium-dependent domain containing 5	-0.545859640119562	7.01993456252483	-1.33286747679215	0.227855366602161	0.999692817443324	-4.5772905558899	7.25512898643291	7.6602418067531	7.00450026770956	6.05175838315235	7.15064246296096	6.99733546814008	NA
**TC0700001931.mm.2**	Gpr26	G protein-coupled receptor 26	-0.41203720392842	6.93197859863771	-1.33273560196409	0.22789626627888	0.999692817443324	-4.57729822528979	7.13134849083721	7.19764172345136	6.61005722289155	6.60934626194886	6.81977649766362	7.22370139503364	NA
**TC1400001746.mm.2**	2610528A11Rik	RIKEN cDNA 2610528A11 gene	-0.438181940099138	5.69425048558456	-1.33185693667656	0.228168941843599	0.999692817443324	-4.57734932251937	5.99923316226263	5.97513604946197	5.36742504147357	5.28995986931865	5.48237348943689	6.05137530155361	NA
**TC1100004052.mm.2**	Cd300c	CD300C molecule	-0.395953576860233	4.9210709029669	-1.33150482818507	0.228278292560716	0.999692817443324	-4.57736979729312	5.01864020664764	4.84995938381087	4.75803318489108	4.67492615192014	5.01219582184491	5.21267066868677	NA
**TC0700001711.mm.2**	Acsm1	acyl-CoA synthetase medium-chain family member 1	0.429995282430578	4.91665199043351	1.33147054315753	0.228288942590787	0.999692817443324	-4.57737179088965	4.39879882419259	4.7934929161816	5.27033403312187	5.31347135042213	5.03651071362801	4.68730410505484	NA
**TC0700003415.mm.2**	Nsmce3	NSE3 homolog, SMC5-SMC6 complex component	0.424894631616703	7.55141794441903	1.33136638056892	0.228321301535753	0.999692817443324	-4.57737784765846	8.14997914796695	7.80375825505694	7.5058404506548	7.59643931270705	6.95298032978145	7.29951017034699	NA
**TC1600001735.mm.2**	Zpld1	zona pellucida like domain containing 1	0.408467568324135	5.21028712982602	1.33122541705043	0.228365099458482	0.999692817443324	-4.57738604418229	4.98011422033467	4.88242393059519	5.43309303351187	5.67496684882539	5.28789884001103	5.00322590567796	NA
**TC0200000398.mm.2**	Nrarp	Notch-regulated ankyrin repeat protein	0.597878547029185	6.11972066732371	1.33052264962552	0.228583563185812	0.999692817443324	-4.57742690556012	6.89895590195501	6.61903959920802	6.65411276855791	5.54393002986088	5.73796227327306	5.26432343108737	NA
**TC0100001730.mm.2**	Cnih4	cornichon family AMPA receptor auxiliary protein 4	0.405657243055852	8.30274894611142	1.33052036404286	0.228584273988767	0.999692817443324	-4.57742703844643	8.22107454198367	8.48417439101032	8.45505160154409	8.50622801334902	7.95057822875581	8.1993869000256	NA
**TC0700004201.mm.2**	Cdr2	cerebellar degeneration-related 2	-0.460968821929042	6.1144719616895	-1.33017755164744	0.228690908835248	0.999692817443324	-4.57744696953194	6.43226318949092	6.49008225444976	5.46450164094866	5.95677270022047	6.41508058942493	5.92813139560229	NA
**TC0100000912.mm.2**	Olfr12	olfactory receptor 12	0.508011834199137	5.53287233669872	1.33003597779604	0.228734959499755	0.999692817443324	-4.57745520038819	4.91095792024705	5.12401478180645	5.60646544757259	6.48267704569596	5.5552448504253	5.51787397444498	NA
**TC1000000934.mm.2**	Eid3	EP300 interacting inhibitor of differentiation 3	-0.624826264575416	5.31493296971084	-1.32990168894653	0.228776750385055	0.999692817443324	-4.57746300757936	5.08723018057064	5.10375248079782	5.68545961129708	4.53902170257579	6.07715094721573	5.39698289580797	NA
**TC0500000582.mm.2**	Nwd2	NACHT and WD repeat domain containing 2	-0.511787426434575	5.49785462393628	-1.32983105071199	0.228798735821262	0.999692817443324	-4.57746711424564	5.89403504297559	6.26445483305964	4.58785393892922	5.31467756842742	5.6737863403801	5.25232001984569	NA
**TC0700003036.mm.2**	Tph1	tryptophan hydroxylase 1	-0.471461481178143	4.95793103293383	-1.32954449344368	0.228887943072547	0.999692817443324	-4.57748377335503	4.53222906192854	4.66763066390932	4.57896032011093	5.22344143459349	5.54713254464077	5.19819217241993	NA
**TC0300000890.mm.2**	Lysmd1	LysM, putative peptidoglycan-binding, domain containing 1	-0.42652399757671	6.97343073505704	-1.32860578561951	0.229180385093597	0.999692817443324	-4.57753834148277	6.722500953348	6.69573622922052	7.09928397834582	6.68536563796432	7.47023604974633	7.16746156171722	NA
**TC0200004486.mm.2**	Strc	stereocilin	0.828762383193356	4.65625915644855	1.32854906911236	0.229198064968857	0.999692817443324	-4.57754163827996	3.70237911179361	3.85921742204593	4.67169549073409	6.34504609488515	4.15084157394605	5.20837524528648	NA
**TC1100001625.mm.2**	Klhl10	kelch-like 10	0.504032848226065	5.06208519265808	1.32842410790995	0.229237022590414	0.999692817443324	-4.57754890190166	4.61207890243373	4.44446417962392	5.2123464163593	5.94967046881217	5.31042939229305	4.84352179642629	NA
**TC0500002344.mm.2**	Fam184b	family with sequence similarity 184, member B	0.479250638405054	6.75952671154805	1.32836729550575	0.229254736235946	0.999692817443324	-4.57755220420112	6.61288734772026	6.40325966661616	7.49805829840295	6.75169896747804	6.59248824317259	6.6987677458983	NA
**TC0400002613.mm.2**	Alg2	asparagine-linked glycosylation 2 (alpha-1,3-mannosyltransferase)	0.583530085419047	8.77313861632032	1.32770741842183	0.229460569360542	0.999692817443324	-4.57759055881505	9.49692617652737	9.09019492838192	9.03174269750366	8.5776426844217	8.73373049838407	7.70859471270319	NA
**TC0900002083.mm.2**	AW551984	expressed sequence AW551984	0.706973605971053	6.63337351166864	1.32742104838179	0.229549946657606	0.999692817443324	-4.57760720280256	8.37229213072711	6.98794559645206	6.79763108166902	6.12934419571835	5.99282482053706	5.52020324490821	NA

**TC0900003332.mm.2**	Mthfs	5, 10-methenyltetrahydrofolate synthetase	-0.451241240880017	5.30156823529753	-1.32730688295964	0.229585586759577	0.999692817443324	-4.57761383800042	6.08368036335593	5.47013401523368	4.84155121529744	4.83500506042034	5.39931373367967	5.17972502379815	NA
**TC0900001898.mm.2**	Ecsit	ECSIT signalling integrator	-0.42740659411285	6.87336504357585	-1.32717970103558	0.229625296114806	0.999692817443324	-4.57762122959845	7.24081009190121	7.22322361492095	6.48808027899463	6.47259140420898	7.16828092912731	6.64720394230201	NA
**TC0100003142.mm.2**	Inava	innate immunity activator	0.653450630318234	6.31431282105491	1.32684629867117	0.229729421475553	0.999692817443324	-4.57764060583862	5.9610830730913	6.10430124782641	6.10354465719944	7.46015227582467	5.90663156819353	6.35016410419411	NA
**TC0200005482.mm.2**	Hypk	huntingtin interacting protein K	-0.490357021285135	8.32729786686225	-1.32661360599636	0.229802118814918	0.999692817443324	-4.57765412870102	8.73731316880702	8.50896256872515	8.14048621174155	7.72791249879399	8.76845242490226	8.08066032820355	NA
**TC0300003173.mm.2**	Acadm	acyl-Coenzyme A dehydrogenase, medium chain	0.539012755951482	7.26984491491973	1.3251867808515	0.230248329783376	0.999692817443324	-4.57773703981344	7.39377436627135	7.60732320100899	6.93252648048054	7.91547223658997	7.00142099322134	6.7685522119462	NA
**TC1900000767.mm.2**	Adra2a	adrenergic receptor, alpha 2a	-0.524019005796226	6.47866400608963	-1.32499731762534	0.230307638235012	0.999692817443324	-4.57774804820698	7.29032515627113	7.08306885274986	6.32590949491772	5.39936651304443	6.4966790049788	6.27663501457581	NA
**TC0700001633.mm.2**	Ctr9	CTR9 homolog, Paf1/RNA polymerase II complex component	0.458173609826341	7.06907242289233	1.32479279100276	0.230371677214117	0.999692817443324	-4.57775993154218	7.43885759970689	7.43397143378759	6.83582010093315	7.39315626082294	6.4526602699959	6.8599688721075	NA
**TC1500002276.mm.2**	Krt77	keratin 77	-0.523183127808441	5.87103810958567	-1.32440292199301	0.230493792065955	0.999692817443324	-4.57778258274763	6.65542496225832	6.02039271375137	5.29307744294986	5.45894491999386	6.25782580883482	5.54056280972578	NA
**TC0X00001795.mm.2**	Was	Wiskott-Aldrich syndrome	-0.393343478659021	6.0319773458331	-1.32434391024303	0.230512280727317	0.999692817443324	-4.57778601120753	5.50840180821574	5.43480062296134	6.21996584122468	6.01102150202706	6.43186167522911	6.58581262534067	NA
**TC0700000592.mm.2**	Scgb1b7	secretoglobin, family 1B, member 7	0.585723456552598	6.06784049504336	1.32394213679084	0.230638193131048	0.999692817443324	-4.57780935274463	5.22638537954019	5.40557443078064	6.27954191203722	7.19372312448504	5.76760979166425	6.53420833175281	NA
**TC0900000648.mm.2**	Sln	sarcolipin	-0.582077926112216	6.97998647263186	-1.32366941362918	0.230723696922946	0.999692817443324	-4.57782519628034	7.17897327555836	6.76504290011195	7.04974436565077	6.33612903829744	7.7064978078182	6.84353144835444	NA
**TC1700000172.mm.2**	1600012H06Rik	RIKEN cDNA 1600012H06 gene	-0.64381782253587	6.14433768069824	-1.32343050530323	0.230798622169336	0.999692817443324	-4.57783907494195	6.14270634932896	6.51109935976654	6.32783552364358	5.13445684136751	6.69981427599662	6.05011373408622	NA
**TC1700002343.mm.2**	Gtf2f1	general transcription factor IIF, polypeptide 1	0.436434145545366	7.95257665908749	1.32312765515005	0.230893631461629	0.999692817443324	-4.57785666751969	8.47738930242575	8.25324769448979	7.84844657023437	8.08039905411571	7.7695991828612	7.28637815039815	NA
**TC0200003278.mm.2**	Ptges	prostaglandin E synthase	-0.484250309560245	5.49456572045567	-1.32283467242567	0.230985578084405	0.999692817443324	-4.57787368626674	5.54444027929148	5.83391357801452	5.34032902009122	4.96994090306253	5.30744125534992	5.97132928692432	NA
**TC0200004791.mm.2**	Nkx2-2	NK2 homeobox 2	-0.447500322409756	6.60532086098198	-1.32271361170058	0.23102357996353	0.999692817443324	-4.57788071824675	6.55273340061731	6.47189555554487	6.14507025541896	6.71107752703613	6.7311541306793	7.01999429659531	NA
**TC1200001348.mm.2**	Efr3b	EFR3 homolog B	-0.560777785541625	6.81669592691227	-1.32230609533781	0.231151543065531	0.999692817443324	-4.57790438861782	8.25549532191908	7.97916475440785	6.4278161156793	5.34416384135243	6.46005993664298	6.433475591472	NA
**TC0700000240.mm.2**	Dact3	dishevelled-binding antagonist of beta-catenin 3	-0.434456212186134	7.4001207721267	-1.32226428669921	0.231164674825816	0.999692817443324	-4.57790681698209	7.62356868195346	7.4337037952243	6.98736387071601	7.24990599488909	7.31765353950097	7.7885287504764	NA
**TC0300003147.mm.2**	Ak5	adenylate kinase 5	-0.559066999729874	7.85985643756316	-1.32170172563935	0.231341434924868	0.999692817443324	-4.57793949088522	8.01315763251429	8.08128147642042	7.89763157154101	7.07565118695125	7.67184816114927	8.41956859680273	NA
**TC0600000245.mm.2**	Ahcyl2	S-adenosylhomocysteine hydrolase-like 2	-0.973031826652523	6.90515896398599	-1.3216600035904	0.231354549003769	0.999692817443324	-4.57794191403634	7.87539777261061	7.81240090603444	7.13466441790013	4.7638813080828	7.18911277455244	6.65549660473553	NA
**TC0200003590.mm.2**	Wdsub1	WD repeat, SAM and U-box domain containing 1	-0.635446697419654	5.16093588834162	-1.32162860775273	0.231364417780119	0.999692817443324	-4.57794373744893	5.91407360027096	5.96484652675752	5.13800257505015	3.76989832904081	4.99303451494706	5.18575978398321	NA
**TC0600003039.mm.2**	Ccdc77	coiled-coil domain containing 77	0.806039090931804	5.6204400215059	1.32159419347646	0.231375235781564	0.999692817443324	-4.57794573615861	5.51298779389937	5.40152806458409	5.26597464981369	6.94412657639408	4.82632476088463	5.77169828345953	NA
**TC1500000710.mm.2**	Adsl	adenylosuccinate lyase	0.467875009252237	5.89736338429084	1.32047459800338	0.231727420456682	0.999692817443324	-4.57801075520609	6.47565258869951	6.45999064957226	5.6386636781671	6.05347986482178	5.68665038436181	5.0697431401226	NA
**TC1100003921.mm.2**	Scn4a	sodium channel, voltage-gated, type IV, alpha	-0.426083934316726	6.25614770014766	-1.32039821583864	0.231751464806131	0.999692817443324	-4.57801519066524	5.87468636624009	5.9986420040689	5.97774938835296	6.4279455926188	6.50703233878186	6.75083051082335	NA
**TC0700003647.mm.2**	Tlnrd1	talin rod domain containing 1	0.49470302747153	6.22740734883382	1.32015451715986	0.231828193476	0.999692817443324	-4.57802934179053	6.48380475400047	6.54765214360064	6.68062483613957	5.98057178903286	6.08594443227492	5.58584613795444	NA
**TC1000001079.mm.2**	Gnptab	N-acetylglucosamine-1-phosphate transferase, alpha and beta subunits	0.428195336171202	7.88202858372798	1.31994364595497	0.231894604510073	0.999692817443324	-4.57804158633241	8.2860093537735	8.19023139667785	8.02207373396512	7.81408697816435	7.73446889081851	7.24530114896856	NA
**TC1300001954.mm.2**	Card19	caspase recruitment domain family, member 19	0.395944120283592	6.49870080663453	1.31960173509253	0.232002320434028	0.999692817443324	-4.57806143918342	6.46724845260291	6.23382735950136	6.70794449470324	6.83356413943182	6.29443634316667	6.45518405040121	NA
**TC0900000340.mm.2**	Pate2	prostate and testis expressed 2	-0.495375451315662	5.4652577406159	-1.31956865454266	0.232012744506904	0.999692817443324	-4.57806335994	5.03497324903001	5.27979748528535	5.20723219900503	5.53578020436933	5.48514619975812	6.24861710624757	NA
**TC1800000929.mm.2**	Map3k8	mitogen-activated protein kinase kinase kinase 8	0.658767524970249	5.68788011647715	1.31923246349615	0.232118705757217	0.999692817443324	-4.57808287974687	5.25058889226948	5.4329401588871	5.47638654869529	6.90425680012814	5.66685161194801	5.39625668693491	NA
**TC0X00001128.mm.2**	Zfp711	zinc finger protein 711	-0.454802451903149	5.69684527208827	-1.31913668941021	0.232148899814866	0.999692817443324	-4.57808844039488	6.73835845940725	6.42746441195316	5.06844964746384	4.98437228121764	5.78258350267133	5.17984332981644	NA
**TC0600000989.mm.2**	C87436	expressed sequence C87436	0.477047794806247	5.49158895592517	1.31842947506109	0.232371965979794	0.999692817443324	-4.57812949918212	6.68040657786666	6.21144273355753	5.07766001058463	5.42822999628503	5.04009915223861	4.51169526501856	NA
**TC0500001078.mm.2**	Btbd8	BTB (POZ) domain containing 8	-0.433499764293326	5.01756217169167	-1.31826725881826	0.23242315813312	0.999692817443324	-4.57813891645504	5.14366165442721	4.94068736104744	4.52559972220537	5.05141252083898	5.27518405354969	5.16882771808131	NA
**TC0100003611.mm.2**	Opn3	opsin 3	-0.589982342969342	5.56302253324016	-1.31814856562082	0.232460621606363	0.999692817443324	-4.57814580692431	5.5942951304624	5.69957996830705	5.76451830509939	4.68762940226701	6.10930342087104	5.52280897243404	NA
**TC0200002223.mm.2**	Cst7	cystatin F (leukocystatin)	-0.42971887477914	5.30651962064733	-1.31811051376346	0.232472633151672	0.999692817443324	-4.57814801591787	5.17718720152952	5.20659745128367	5.11242550187081	5.18552215888546	5.30531022359889	5.85207518671566	NA
**TC1000002252.mm.2**	Fam241b	family with sequence similarity 241, member B	-0.56283502508864	7.86756588474448	-1.31800933495934	0.232504574174491	0.999692817443324	-4.57815388951611	7.80552073291565	7.45789482762872	8.2293396969812	7.17881515189142	8.14531622750599	8.38850867154391	NA
**TC0800001875.mm.2**	Ap3m2	adaptor-related protein complex 3, mu 2 subunit	0.44576329859889	8.03938614089408	1.3177757724012	0.23257832207434	0.999692817443324	-4.57816744791937	8.57644433959231	8.59485297310145	8.26693701557281	7.71133604936144	7.70216357415433	7.38458289358213	NA
**TC0600002900.mm.2**	Ssu2	ssu-2 homolog (C. elegans)	0.397093979150497	6.66046273779691	1.31760156056932	0.23263334329791	0.999692817443324	-4.57817756072227	6.130067229869	6.17420440586743	6.99938638057944	7.22695999409356	6.60136070439898	6.83079771197303	NA
**TC0800002844.mm.2**	Slc12a4	solute carrier family 12, member 4	-0.387890393138488	5.50824996795916	-1.31723216532335	0.232750047209402	0.999692817443324	-4.57819900295157	6.05628306958267	6.13045167527095	4.95020400799958	5.0932881303126	5.32670920937805	5.49256371521111	NA
**TC1000002385.mm.2**	Gstt2	glutathione S-transferase, theta 2	-0.754355715655397	5.13887454193173	-1.317037052546	0.232811710508275	0.999692817443324	-4.57821032821913	4.98167489154842	4.61951106348235	4.17988456233955	5.68179037028485	5.24625627944508	6.12413008449012	NA
**TC0400004151.mm.2**	Atad3a	ATPase family, AAA domain containing 3A	0.412982951353558	7.51862709791162	1.31681007364418	0.232883462901099	0.999692817443324	-4.57822350278644	7.4532875997514	7.25074427085766	7.69614757120964	7.92070073857425	7.56262766740886	7.22825473966792	NA
**TC1000000422.mm.2**	Prep	prolyl endopeptidase	0.626961403208266	7.29149851524995	1.31629260939629	0.233047116279797	0.999692817443324	-4.57825353658101	7.6701156370019	7.81557111026032	7.94035971735874	6.81825385796825	7.13174978037728	6.37294098853319	NA
**TC0700004173.mm.2**	Coq7	demethyl-Q 7	0.523461264018769	6.9819558450708	1.31615768478653	0.233089804253167	0.999692817443324	-4.5782613673169	7.68319770396446	7.48861184427176	6.49405886028816	7.3893651648249	6.60072947770113	6.23577201937439	NA
**TC0200004965.mm.2**	Gss	glutathione synthetase	-0.464405417228329	6.50185789309037	-1.31584886479922	0.233187535898803	0.999692817443324	-4.5782792900497	6.22965814162026	6.46098630926201	6.06301561409539	6.63283042250627	7.01359695803382	6.61105991302449	NA
**TC1600000036.mm.2**	4930562C15Rik	RIKEN cDNA 4930562C15 gene	0.6187015192552	5.09614922643905	1.31541370246079	0.233325312238054	0.999692817443324	-4.57830454398162	4.49099904979674	4.62096665200287	5.14501602296014	6.20615032471241	4.65669596223065	5.45706734693149	NA
**TC1100003836.mm.2**	Slc25a39	solute carrier family 25, member 39	0.412630060260664	8.79641114266633	1.31519734125954	0.233393840887286	0.999692817443324	-4.57831709961274	8.99517363105576	8.61055168983121	9.10154600982446	8.89745481799172	8.52644690218757	8.64729380510727	NA
**TC1600001373.mm.2**	Tmem41a	transmembrane protein 41a	0.492695070984339	6.65358997000108	1.31502773174522	0.233447574169675	0.999692817443324	-4.57832694195293	6.98991212942166	6.74707954219022	7.20547820750566	6.37949093767599	6.27534409461113	6.32423490860184	NA
**TC1500002335.mm.2**	Pheta2	PH domain containing endocytic trafficking adaptor 2	-0.46142906293603	6.34832083132831	-1.31463822097607	0.233571014768056	0.999692817443324	-4.57834954420245	5.79736192439913	5.90811801375576	6.52454491912829	6.20624854284318	6.50701976308259	7.14663182476094	NA
**TC1200000225.mm.2**	Cmpk2	cytidine monophosphate (UMP-CMP) kinase 2, mitochondrial	0.426417680522767	6.4265631187337	1.31435327414898	0.233661354277208	0.999692817443324	-4.57836607815651	7.2986728686941	6.99633489834626	6.46285795405171	6.09574519915197	5.98110713820095	5.7246606539572	NA
**TC0700003961.mm.2**	Olfr670	olfactory receptor 670	-0.613391409336111	5.19239404419657	-1.31334984450453	0.233979726917363	0.999692817443324	-4.57842429690124	4.62435934456891	4.80153221096657	5.27080020048001	4.98004474500584	5.10090150521829	6.37672625893979	NA
**TC0900002325.mm.2**	Wdr61	WD repeat domain 61	-0.583920299465888	6.63614431574615	-1.31305223879688	0.234074226119623	0.999692817443324	-4.57844156242201	7.73056564765442	7.47308328284163	6.45471785521254	5.26797032731199	6.48720923277905	6.40331954867725	NA
**TC1400002833.mm.2**	Rnf31	ring finger protein 31	-0.453258362962748	5.68723270020238	-1.3128675339594	0.234132892658199	0.999692817443324	-4.57845227768306	6.59341323740767	6.43726980518824	5.37317072394629	4.71992749240016	5.57066894386456	5.42894599840739	NA
**TC0700003028.mm.2**	Emp3	epithelial membrane protein 3	-0.507693982928768	7.91642803169266	-1.31266166567969	0.234198296475405	0.999692817443324	-4.57846422038562	7.59830580350395	8.40227411267561	7.43523950569776	7.80606064836169	7.98847617815144	8.26821194176554	NA
**TC1100003218.mm.2**	Ovca2/Dph1	candidate tumor suppressor in ovarian cancer 2/diphthamide biosynthesis 1	-0.456130466063673	6.79260022349793	-1.3125329026807	0.234239212334005	0.999692817443324	-4.57847168993783	6.29353774259245	6.464797252845	6.6369075063539	6.90559520035748	6.91515154642869	7.53961209241004	NA
**TC0500001830.mm.2**	8430423G03Rik	RIKEN cDNA 8430423G03 gene	-0.542127498164574	5.846954779695	-1.3119440545155	0.234426405636602	0.999692817443324	-4.57850584742968	5.05570556635996	4.92173421200625	5.78120523759384	6.22881171414348	6.06858367925038	7.02568826881609	NA
**TC0X00000207.mm.2**	Jade3	jade family PHD finger 3	0.476886968968047	6.87438146962784	1.31145687874043	0.234581377226882	0.999692817443324	-4.57853410514254	7.43574495657549	7.32608328375883	7.20610474891741	6.51301250876701	6.57400882666732	6.191334493081	NA
**TC0400003697.mm.2**	Wdtc1	WD and tetratricopeptide repeats 1	0.410344027796666	6.99183762883678	1.31118052030564	0.234669327534783	0.999692817443324	-4.57855013397006	7.53676603858523	7.43372645968407	6.78286532716966	7.11774533800269	6.40291788693202	6.677004722647	NA
**TC1100001908.mm.2**	Llgl2	LLGL2 scribble cell polarity complex component	0.381124327428542	5.72654243194615	1.31082310198413	0.234783118026683	0.999692817443324	-4.57857086339691	5.68821554322955	5.74141576975172	5.87246882188609	5.97346714489027	5.48608364589532	5.59760366602396	NA
**TC1800001319.mm.2**	Dtwd2	DTW domain containing 2	-0.787563393078082	6.14190707938445	-1.31054002425133	0.234873275372069	0.999692817443324	-4.57858728053348	5.47632833407802	5.49607375793834	7.02022305467546	5.13173374439161	6.92705186219884	6.80003172302441	NA
**TC0700000559.mm.2**	Syne4	spectrin repeat containing, nuclear envelope family member 4	0.481246092056428	6.49634861814623	1.30993618981179	0.235065692378984	0.999692817443324	-4.57862229790194	5.54726623160344	6.03227389279692	6.75923278315988	7.42128910113506	6.59732210405246	6.62070759612963	NA
**TC1000001830.mm.2**	Pde7b	phosphodiesterase 7B	0.488060776265856	8.74812968005513	1.30960528727164	0.235171196202391	0.999692817443324	-4.57864148627594	9.33094414760394	8.778783395107	9.13218642067073	8.54539962440506	8.48794576149454	8.21351873104954	NA
**TC1300000998.mm.2**	Edil3	EGF-like repeats and discoidin I-like domains 3	-0.422161523097748	7.71370754905072	-1.3094710425995	0.235214010204162	0.999692817443324	-4.57864927060865	8.65894423447423	8.39691762945689	7.18215402734062	7.00887616474825	7.72760827406948	7.30774496421488	NA
**TC0200005502.mm.2**	Ttbk2	tau tubulin kinase 2	-0.499905182345391	6.93694218030555	-1.30938315748971	0.235242042694675	0.999692817443324	-4.57865436665137	8.55536816352574	8.41413821005373	6.32834337874424	5.49782479303729	6.25237140977711	6.5736071266952	NA
**TC1100003487.mm.2**	Lpo	lactoperoxidase	0.596338280517335	5.94820672735876	1.30929999861468	0.235268570379961	0.999692817443324	-4.57865918858585	5.5757517897929	5.63320560992577	5.86231597263454	6.97416379009973	5.51909887422191	6.12470432747769	NA
**TC1700001835.mm.2**	Ephx3	epoxide hydrolase 3	0.816177927656705	5.78905939168668	1.30905061784177	0.235348138660041	0.999692817443324	-4.57867364850331	5.0989070307863	5.58735825109083	5.61459187794631	7.22563158383188	5.07860445412616	6.12926315233861	NA
**TC1700001864.mm.2**	Zfp955a	zinc finger protein 955A	-0.47196312540192	7.64366395814272	-1.30902812090362	0.235355317775376	0.999692817443324	-4.57867495292557	8.51563063417066	8.58021777120234	7.32419790700851	6.58690663933122	7.55233762501841	7.30269317212517	NA
**TC1200002311.mm.2**	Atg2b	autophagy related 2B	0.433963292944211	6.16030423051432	1.30885641045026	0.235410119549438	0.999692817443324	-4.57868490894555	6.94815395312577	6.70502131661794	6.01182544158338	6.07646290803195	5.34148573696313	5.87887602676377	NA
**TC1700000203.mm.2**	Lix1	limb and CNS expressed 1	-0.740958516999463	6.93992514927492	-1.30882205955181	0.235421084064316	0.999692817443324	-4.57868690063218	7.79324851529466	7.92504525900117	6.89441805247602	5.32525199120137	7.23178484884499	6.46980222883133	NA
**TC0X00002758.mm.2**	Dmrtc1c1/Dmrtc1c2	DMRT-like family C1c1/DMRT-like family C1c2	0.635328738699638	6.2538176753372	1.30859679033752	0.235492999230769	0.999692817443324	-4.57869996165044	5.68662560104434	5.35206581292191	6.37855624617987	7.49887981154825	5.91520041845558	6.69157816187326	NA
**TC0800001666.mm.2**	Fam155a	family with sequence similarity 155, member A	0.495599226370267	7.49903372212123	1.30835457157601	0.235570346973385	0.999692817443324	-4.57871400494693	8.07285381947695	8.21355653333645	7.74699933022322	7.10249588610403	7.2243005896861	6.63399617390063	NA
**TC1200002526.mm.2**	Serpina3h	serine (or cysteine) peptidase inhibitor, clade A, member 3H	-0.446501153126134	5.699919330945	-1.30832603228788	0.235579461900504	0.999692817443324	-4.57871565955966	5.36808172317322	5.03924959657847	5.93844397648106	5.51114720335196	5.98060166573312	6.36199182035217	NA
**TC0600000824.mm.2**	Gm1070	predicted gene 1070	0.573671307458356	6.93674912529764	1.30814866219576	0.235636117630665	0.999692817443324	-4.5787259427406	6.26563359538174	6.51992307981578	7.14050618110519	7.85063416464732	6.46290663822667	7.38089109260913	NA
**TC1500000967.mm.2**	Tmem106c	transmembrane protein 106C	0.485926872147996	5.59452363393455	1.30803888101156	0.235671190056352	0.999692817443324	-4.57873230727199	6.38353529891251	5.7660157341352	5.88436270394316	5.31035955348464	5.08715533381157	5.13571317932024	NA
**TC1900000201.mm.2**	Cpsf7	cleavage and polyadenylation specific factor 7	0.520033747978223	6.3766819905482	1.30768382577138	0.235784653071181	0.999692817443324	-4.57875289083558	7.00947965720812	7.00561519190114	6.57957219957745	6.06296009549074	6.22113707234062	5.38132772677112	NA
**TC0600001306.mm.2**	Olfr214	olfactory receptor 214	-0.416299592571638	5.67253536582457	-1.30707094734894	0.235980620368081	0.999692817443324	-4.5787884187731	5.64745463552728	5.22919264705117	5.57686458020491	5.58611828340794	5.86754856634655	6.12803348240957	NA
**TC0300002414.mm.2**	2310050C09Rik	RIKEN cDNA 2310050C09 gene	-0.438928348215076	5.02287115105567	-1.30699300237699	0.236005553474362	0.999692817443324	-4.57879293694808	4.23147279950282	4.40929921962499	4.88902636304071	5.42027273234733	5.45673860498248	5.73041718683571	NA
**TC0800002039.mm.2**	Tnks	tankyrase, TRF1-interacting ankyrin-related ADP-ribose polymerase	0.527644992614412	6.97541795652332	1.30685456874742	0.236049841462831	0.999692817443324	-4.57880096130141	8.59585280938744	8.45475724444355	6.57119338969463	6.35740044557425	6.42889171529308	5.44441213474698	NA
**TC0800000561.mm.2**	Cldn22	claudin 22	-0.397651925311235	6.03602277307179	-1.30664120047324	0.236118117034432	0.999692817443324	-4.57881332896606	5.49651378770641	5.62592639117012	5.93981277623567	6.20938352823021	6.57222935864701	6.37227079644134	NA
**TC0200000636.mm.2**	Gsn	gelsolin	-0.404064065103657	7.07000062005147	-1.30650936877379	0.236160310459733	0.999692817443324	-4.57882097026717	8.00831374592208	8.27808313867359	6.38142968712039	6.28130966563254	6.58916877268135	6.8816987102789	NA
**TC1100000933.mm.2**	Ctdnep1	CTD nuclear envelope phosphatase 1	0.403022165321421	7.62649768746037	1.30635547194544	0.236209574340712	0.999692817443324	-4.57882989034247	8.06666741536291	8.01662054192935	7.78490319649917	7.45596805255724	7.10381025686113	7.33101666155245	NA
**TC1800000259.mm.2**	Ercc3	excision repair cross-complementing rodent repair deficiency, complementation group 3	0.402036727297335	7.39416518395864	1.30573164114471	0.236409361340137	0.999692817443324	-4.57886604649243	7.97597250580557	7.97884909107984	7.42265634641842	7.18446513431214	7.06024371173757	6.74280431439832	NA
**TC0700002783.mm.2**	Hamp	hepcidin antimicrobial peptide	-0.472770617662195	6.55780538568416	-1.30365357572354	0.237075952088565	0.999692817443324	-4.57898646479633	5.91901924167832	6.40775452818999	6.22599906221284	6.8112595922433	6.88152840444595	7.10127148533459	NA
**TC0100003700.mm.2**	Enah	ENAH actin regulator	0.485073314980181	8.32944392982531	1.3036182575186	0.237087295548358	0.999692817443324	-4.5789885110871	8.82105570966679	8.6128058895521	8.42260057237187	8.3338737324748	8.30173247033917	7.48459520454714	NA
**TC1200000970.mm.2**	Zc3h14	zinc finger CCCH type containing 14	-0.776258255565834	7.73518046002481	-1.30346846801264	0.237135410072367	0.999692817443324	-4.57899718958238	8.53030169419315	8.61544588650629	7.72764500585469	6.12876432830417	8.18818585721646	7.22073998807407	NA
**TC0200000510.mm.2**	Coq4	coenzyme Q4	-0.415944085232785	7.65959038389495	-1.30306937334567	0.237263646888937	0.999692817443324	-4.57902031140688	7.56316135407776	7.3567230841587	7.74766259427058	7.35522225306327	8.08144699630027	7.85332602149915	NA
**TC0X00001336.mm.2**	BC065397	cDNA sequence BC065397	0.585072733939276	5.95736815746674	1.30260450987852	0.237413093369683	0.999692817443324	-4.57904724194234	5.88648805051374	5.94935783980967	6.30900264260171	6.23025161857608	6.30154834340531	5.06756044989394	NA
**TC0600003208.mm.2**	A2ml1	alpha-2-macroglobulin like 1	0.561530403486175	6.48956575861547	1.30178541662667	0.237676620697399	0.999692817443324	-4.57909468943256	7.58003528596663	7.06763335544574	6.87006924344617	5.83632411518023	5.79654200187639	5.78679054977766	NA
**TC0400004198.mm.2**	Zfp991/Znf41-ps	zinc finger protein 991/ZNF41, pseudogene	-0.847661713417868	6.5652648875716	-1.30171389431822	0.237699643812106	0.999692817443324	-4.57909883223119	7.51006028286471	7.57614733316795	6.64455760216527	4.66047153911535	6.8359879486745	6.16436461944186	NA
**TC1400000947.mm.2**	Adra1a	adrenergic receptor, alpha 1a	-0.48978424154474	5.0178919338122	-1.30166723112615	0.237714665804909	0.999692817443324	-4.57910153508823	4.97626603315512	5.70038236248044	4.38210847828065	4.84345888379344	5.17667585891911	5.02845998624446	NA
**TC0700001646.mm.2**	Parva	parvin, alpha	0.543957787892492	7.03523549802096	1.30159909156762	0.237736603050502	0.999692817443324	-4.57910548188152	7.34904142988971	7.0775805056254	7.0064985303499	7.42985478384792	7.15680735349988	6.19163038491295	NA
**TC0900001486.mm.2**	Epm2aip1	EPM2A (laforin) interacting protein 1	-0.657469055704348	7.45116598080753	-1.30124735649352	0.237849870987685	0.999692817443324	-4.57912585453931	8.43078793886807	8.33142933290979	7.38995066332351	5.92496958750582	7.44292245926139	7.18693590297663	NA
**TC0600002174.mm.2**	Olfr455	olfactory receptor 455	-0.460616670532818	5.43879645468226	-1.30052479340455	0.238082704094951	0.999692817443324	-4.57916770250121	4.8339473517502	4.90421515258228	5.3947465377156	5.59194490363212	5.59768346336541	6.31024131904795	NA

**TC1900000094.mm.2**	Syvn1	synovial apoptosis inhibitor 1, synoviolin	0.404751686014376	7.6576765742553	1.30046921421001	0.238100621795031	0.999692817443324	-4.57917092124557	7.76954188745791	7.53519379835195	7.7838334525168	7.94158011335854	7.61939666703718	7.2965135268094	NA
**TC1100000585.mm.2**	Cdkl3	cyclin-dependent kinase-like 3	-0.411881702947658	6.42288852132116	-1.30021161336988	0.238183682995128	0.999692817443324	-4.57918583928362	7.59219140201406	7.32820107718926	5.85580714702222	5.54078047439195	5.98778791566465	6.23256311164484	NA
**TC0200004975.mm.2**	Uqcc1	ubiquinol-cytochrome c reductase complex assembly factor 1	0.426626512739746	6.71037164878825	1.30017729166613	0.238194751658462	0.999692817443324	-4.57918782686139	7.66507169963707	7.2472869569397	6.74118419382129	6.36037793699482	6.09489924841343	6.15340985692318	NA
**TC0400003749.mm.2**	Tmem50a	transmembrane protein 50A	-0.440663367623737	7.45911347028167	-1.2998700299647	0.238293862921265	0.999692817443324	-4.57920562001849	8.42926819556621	8.32456919262412	6.80591536152033	6.75384298760579	6.90327740204227	7.53780768233132	NA
**TC0900000271.mm.2**	Snx19	sorting nexin 19	-0.546517521830586	7.06617328456019	-1.29976270365253	0.23832849095809	0.999692817443324	-4.57921183497046	7.73919128346405	7.67877974093236	7.01662553189133	5.92639128776045	7.08843596410501	6.94761589920795	NA
**TC1800001230.mm.2**	Gnpda1	glucosamine-6-phosphate deaminase 1	0.430298964372694	7.40008590110558	1.29969996119934	0.238348736391769	0.999692817443324	-4.57921546815722	7.78758088301417	7.70176409585568	7.63871212168151	7.24717205657299	7.22902874836124	6.79625750114787	NA
**TC0800003177.mm.2**	Exoc8	exocyst complex component 8	-0.518530842462002	6.85326203827362	-1.29966729523856	0.238359277485292	0.999692817443324	-4.57921735971088	7.52528445978232	7.53533382166673	6.73937192470343	5.77157420693089	6.88438243200832	6.66362538455001	NA
**TC1200002158.mm.2**	Sel1l	sel-1 suppressor of lin-12-like (C. elegans)	-0.456840008426618	7.26231503152214	-1.29912761837173	0.238533486990065	0.999692817443324	-4.5792486089193	8.06964623314156	8.07149592587998	6.94660791632318	6.31292609030584	7.2648449004254	6.90836912305685	NA
**TC1100001698.mm.2**	Adam11	a disintegrin and metallopeptidase domain 11	-0.456483130325307	7.44129230275779	-1.29912242339612	0.238535164488982	0.999692817443324	-4.57924890971496	7.95579888489722	7.67292722974051	7.32110512507169	6.73192559555751	7.64032855592306	7.32566842535675	NA
**TC0800002146.mm.2**	Tlr3	toll-like receptor 3	-0.723499968645618	6.7977888339885	-1.29900522938881	0.238573010119197	0.999692817443324	-4.5792556953359	7.2807532269486	7.64188403084654	6.88018218057181	5.3283657238505	6.51577021616179	7.13977762555176	NA
**TC0200004376.mm.2**	Dph6	diphthamine biosynthesis 6	0.441678065757481	6.39538754725544	1.29881648729416	0.238633971963423	0.999692817443324	-4.57926662340176	7.55302116858249	7.43295527117348	6.3845050664417	5.75034742120409	5.67306425739642	5.57843209873442	NA
**TC0900003036.mm.2**	Lsmem2	leucine-rich single-pass membrane protein 2	0.788230319071314	4.71985517867067	1.29824801054132	0.238817667042308	0.999692817443324	-4.57929953608557	4.36255754474397	4.39320175221004	4.59933257117544	5.97058363543089	3.82627329924878	5.16718226921492	NA
**TC1200001549.mm.2**	Rnf144a	ring finger protein 144A	-0.516750522863877	7.4594937652562	-1.29798007814437	0.238904288587014	0.999692817443324	-4.57931504742542	7.35718852268217	7.41205384642574	7.73178573381578	6.74532385453498	7.7676059498807	7.74300468419781	NA
**TC1300000569.mm.2**	Ogn	osteoglycin	-0.706825888359893	5.95368927087697	-1.2978378026769	0.238950296928653	0.999692817443324	-4.57932328389466	5.58807785643769	6.77400157303089	5.06454002751058	5.90866218202614	6.59156414356826	5.79528984268824	NA
**TC1700000846.mm.2**	Clic5	chloride intracellular channel 5	-0.457542196670291	6.38018263277188	-1.29778841636304	0.238966269051058	0.999692817443324	-4.57932614287772	5.76008667810341	6.11036143681906	6.06554511604021	6.6822365281439	6.92338054512971	6.73948549239498	NA
**TC1500001207.mm.2**	Rai14	retinoic acid induced 14	-0.662584643268456	6.48215391418915	-1.2969598733764	0.239234369537964	0.999692817443324	-4.57937410430043	6.23316107418246	6.99349021387766	6.72091174378476	5.44963971148418	6.90653795369685	6.58918278810899	NA
**TC1700001628.mm.2**	E4f1	E4F transcription factor 1	0.51433917238914	6.04529987052005	1.29678873875507	0.23928977828294	0.999692817443324	-4.57938400995066	6.39297170757987	6.63411969200648	6.11235927157268	6.02433381258343	6.0271757917386	5.08083894763923	NA
**TC0X00002177.mm.2**	Igsf1	immunoglobulin superfamily, member 1	0.59241846050398	6.56648962131744	1.29666830809336	0.239328777214781	0.999692817443324	-4.57939098059394	7.6704090563854	6.49273755872607	6.51831771706734	6.69199629983323	6.23955432138513	5.78592277450747	NA
**TC0200001552.mm.2**	Cd59a	CD59a antigen	0.504772009986276	6.80816713855701	1.29643950666863	0.239402885102837	0.999692817443324	-4.57940422350094	8.17310828661241	8.36305941849155	6.77040487510577	5.89078469799954	5.9655751337487	5.68607041938405	NA
**TC0X00002667.mm.2**	Amer1	APC membrane recruitment 1	-0.390902324106224	5.48351017676351	-1.29561423886256	0.239670353480365	0.999692817443324	-4.57945198585047	5.42942148078802	5.33461939824613	5.46999611973372	5.20761164693351	5.64146051351566	5.81795190136402	NA
**TC1800001478.mm.2**	Lman1	lectin, mannose-binding, 1	0.617858493882306	7.31516424483226	1.29557443154334	0.239683261615187	0.999692817443324	-4.5794542895503	7.67678653812887	7.48537433344909	7.49442990589034	7.48784088669976	7.5490412285846	6.19751257624089	NA
**TC1300001278.mm.2**	Cdc20b	cell division cycle 20B	0.628740005226818	7.17176568470031	1.29470419522017	0.239965601581684	0.999692817443324	-4.57950464781958	6.320275778527	6.3684032563403	7.34271497987382	8.45698256202028	6.84466543247643	7.69755209896402	NA
**TC1300002259.mm.2**	Zfp65	zinc finger protein 65	0.576313820935733	5.51031141036219	1.29449217865753	0.240034432543165	0.999692817443324	-4.57951691566725	6.64108243481102	6.17736049760735	5.69455638685981	5.0034701989533	5.21845419304741	4.32694475089423	NA
**TC1200001562.mm.2**	Sox11	SRY (sex determining region Y)-box 11	0.425898309435393	7.09214572520596	1.2943949732407	0.240065995973022	0.999692817443324	-4.57952254010387	7.1163880849269	7.37351893462388	6.9801120788473	7.4772698964306	6.86862010007854	6.73696525632857	NA
**TC0600002698.mm.2**	Chchd6	coiled-coil-helix-coiled-coil-helix domain containing 6	-0.729281356524521	7.48315291367098	-1.29432112275974	0.240089978289195	0.999692817443324	-4.57952681313778	8.02507068449754	7.72945379125171	7.73294858094902	6.10996656566478	7.92454832615967	7.37692953350317	NA
**TC0300002712.mm.2**	Ubl4b	ubiquitin-like 4B	-0.461269050746038	5.75865784141947	-1.29296792823672	0.240529788870267	0.999692817443324	-4.57960510142764	5.53024902418765	5.41459796760269	5.35429559599217	5.98798538162503	5.93230559855531	6.33251348055397	NA
**TC0700000512.mm.2**	Rinl	Ras and Rab interactor-like	0.515585861215131	5.49153298068523	1.29251057199059	0.240678596867338	0.999692817443324	-4.57963155790718	5.42082519010448	5.70088279780869	5.2461222072801	6.18320860203416	5.28303213362604	5.11512695325795	NA
**TC0400001607.mm.2**	Map3k6	mitogen-activated protein kinase kinase kinase 6	-0.564669662552817	4.57560793314802	-1.29232479499461	0.240739065362217	0.999692817443324	-4.57964230394308	3.55266016482355	3.68846466173211	4.22292831870499	5.31866340490843	5.12654353984101	5.54438750887805	NA
**TC0900002181.mm.2**	Kmt2a	lysine (K)-specific methyltransferase 2A	0.407722340433852	6.49956602306899	1.29200031170991	0.240844713269338	0.999692817443324	-4.57966107254478	7.25087599916117	6.91240236504096	6.45280993396707	6.37197129357269	6.16114491999773	5.84819162667433	NA
**TC1500000568.mm.2**	Zfp707	zinc finger protein 707	-0.411526560735673	6.81794735690052	-1.29179331193519	0.240912131171065	0.999692817443324	-4.57967304524136	7.12796276026387	7.4624095195786	6.54974054643417	6.19738882361048	6.74189468711702	6.82828780439897	NA
**TC0700002956.mm.2**	Acp4	acid phosphatase 4	0.509433996177879	6.69728511997666	1.29169463519411	0.240944275089902	0.999692817443324	-4.57967875249053	6.14263181569471	6.02584779694158	6.78337926010218	7.73367028968752	6.6748577484519	6.82332380898204	NA
**TC0600002331.mm.2**	Pde1c	phosphodiesterase 1C	0.53167372933138	7.16846991845824	1.29132265326923	0.241065481887999	0.999692817443324	-4.57970026635111	7.37130514610589	7.44869477624453	7.80810888452518	6.81897463900572	6.59877300365271	6.96496306121543	NA
**TC1700000948.mm.2**	Mocs1	molybdenum cofactor synthesis 1	0.420758886020869	7.93117204717609	1.2910761964802	0.241145816934391	0.999692817443324	-4.57971451969859	7.63491368012023	7.40618154222537	8.17728123082131	8.51644618555503	7.74113362325358	8.11107602108102	NA
**TC0900000791.mm.2**	Itga11	integrin alpha 11	-0.497772516125813	5.27407152941394	-1.29050897039896	0.241330799067454	0.999692817443324	-4.57974732207763	4.93900858706853	4.58022110518697	4.86717313111291	5.69765409487536	5.68897498278837	5.87139727545152	NA
**TC1500002048.mm.2**	Sbf1	SET binding factor 1	0.382335077509307	6.93841689860058	1.28980000331497	0.241562180175282	0.999692817443324	-4.57978831726271	7.84340619611454	7.69264859141931	6.82069221000899	6.60886616953514	6.34612141495536	6.31876680957016	NA
**TC0300002156.mm.2**	Bche	butyrylcholinesterase	-0.510997608358066	6.07750850811588	-1.28964135625972	0.241613983415211	0.999692817443324	-4.57979749023133	5.46345490257564	5.3704923132165	5.6785348743471	6.62601943374641	6.55027202757708	6.77627749723256	NA
**TC1200001263.mm.2**	Siva1	SIVA1, apoptosis-inducing factor	0.614397837047259	6.37856457993892	1.28934397164888	0.241711115037301	0.999692817443324	-4.57981468439934	6.69700329260469	6.66288674588063	6.69130577814951	6.37884077947184	6.58397390861091	5.25737697491592	NA
**TC1600001642.mm.2**	Sidt1	SID1 transmembrane family, member 1	-0.517062394707438	6.43507224179318	-1.28922108347564	0.241751262700873	0.999692817443324	-4.57982178931167	6.42524157485953	6.86183839676547	6.51064824280492	5.63396610205467	6.6987113481357	6.48002778613877	NA
**TC0500002027.mm.2**	Slc26a5	solute carrier family 26, member 5	-0.52438713072099	5.15865202668779	-1.28858300442368	0.241959817541306	0.999692817443324	-4.57985867837683	5.36320747147668	5.15195105385005	4.41819345174659	5.27579623493243	5.10303723333503	5.63972671478597	NA
**TC0400003216.mm.2**	Tceanc2	transcription elongation factor A (SII) N-terminal and central domain containing 2	0.473475058964293	6.43514178143998	1.2885823585133	0.241960028735607	0.999692817443324	-4.57985871571681	6.2556839939319	6.33935471056756	6.54379741150883	6.93758363952567	6.62518612981024	5.90924480329567	NA
**TC0100002350.mm.2**	Ankrd44	ankyrin repeat domain 44	-0.715940278025619	5.8560418704877	-1.28843202343814	0.242009188415232	0.999692817443324	-4.57986740646166	6.26702721683393	5.71757843847873	6.1825007586117	4.67738174716946	6.43832860567943	5.85343445615297	NA
**TC1700000419.mm.2**	Crebrf	CREB3 regulatory factor	0.641243627105838	5.83608376665425	1.28826583496458	0.242063542345871	0.999692817443324	-4.57987701344297	6.82868985371507	6.66505012151176	6.29113095657949	5.11149398287569	5.48032294280425	4.63981474243925	NA
**TC0200002040.mm.2**	Shld1	shieldin complex subunit 1	0.500771267363334	6.04247809781638	1.28764864944298	0.242265493834036	0.999692817443324	-4.57991268938191	5.93113503934199	6.02227770178446	5.91321257719987	6.73828661304938	5.60453061456758	6.045426040955	NA
**TC0400001233.mm.2**	Efcab14	EF-hand calcium binding domain 14	0.563287036077699	7.65052085368835	1.28678575127555	0.24254809311191	0.999692817443324	-4.5799625628144	8.08971184514305	7.95457563379601	7.70492008707803	7.78778577059517	7.76992502963033	6.59620675588748	NA
**TC1300002076.mm.2**	Tifab	TRAF-interacting protein with forkhead-associated domain, family member B	0.470181978360757	5.1419031027239	1.2863855936752	0.242679242685377	0.999692817443324	-4.57998568867938	5.15547051158734	4.96457750786376	5.03681412327935	5.79905315352756	4.84904113495233	5.04646218513307	NA
**TC0800001247.mm.2**	Prmt7	protein arginine N-methyltransferase 7	0.392800905299978	7.42736976135111	1.28619179173845	0.242742782567464	0.999692817443324	-4.57999688833961	8.31334298735146	8.27719527965632	7.31256835311646	7.06707270273296	6.93503421813868	6.65900502711079	NA
**TC1700000278.mm.2**	Zfp760	zinc finger protein 760	0.418044123996618	6.16512433242162	1.28592473736633	0.242830362837771	0.999692817443324	-4.58001232064263	6.64141764946628	6.38307544321461	6.25280224475843	6.14836833016259	6.01696422358803	5.54811810333976	NA
**TC0400003695.mm.2**	Tmem222	transmembrane protein 222	-0.534248416701962	7.6453178512412	-1.28588021625856	0.242844966188655	0.999692817443324	-4.58001489332635	8.22253481312605	8.10524270591503	7.58221251058702	6.65560386691408	7.92052884421344	7.38578436669159	NA
**TC0X00002452.mm.2**	Fam3a	family with sequence similarity 3, member A	0.780143298390968	5.09370322159121	1.28546357030349	0.242981667312947	0.999692817443324	-4.58003896863558	6.10089844133322	5.2320803738886	5.29554974920455	5.09921380634915	5.26605612400689	3.56842083476487	NA
**TC1600001168.mm.2**	Tmem114	transmembrane protein 114	-0.455832221582485	5.28789159174772	-1.28544146309437	0.242988922546955	0.999692817443324	-4.58004024602597	4.75027017218946	4.95365310332018	5.52304428947535	5.03283662643051	5.47211902204706	5.99542633702376	NA
**TC0100003670.mm.2**	Mixl1	Mix1 homeobox-like 1 (Xenopus laevis)	-0.688300079073025	5.76762315456333	-1.28471810276844	0.243226422469233	0.999692817443324	-4.58008204050335	5.00186104491705	5.06462024104871	5.02572247483093	6.55560626680314	6.21183263157948	6.74609626820065	NA
**TC0X00000346.mm.2**	Rhox4c	reproductive homeobox 4C	0.463782998735486	5.66830654697838	1.2844702773793	0.243307837335775	0.999692817443324	-4.58009635831728	5.41943353640279	4.94558335513096	5.90858467904925	6.37760951485451	5.88573872138225	5.47288947505053	NA
**TC1100000062.mm.2**	Rhbdd3	rhomboid domain containing 3	-0.625102631169492	5.88442419623543	-1.28382677242144	0.243519350998559	0.999692817443324	-4.58013353341876	5.84933797607806	6.04600350518359	6.23810866133133	4.84239055557463	6.17804504685718	6.15265943238776	NA
**TC1500001424.mm.2**	Slc25a32	solute carrier family 25, member 32	-0.537411253508093	5.32471455678338	-1.28351033029638	0.243623421337036	0.999692817443324	-4.58015181279917	5.63900321656278	5.3897633945357	5.4836167911752	4.43873232011761	5.39974673684109	5.59742488146791	NA
**TC1900000164.mm.2**	Ints5	integrator complex subunit 5	0.477878978258644	6.94310031744854	1.28345402843837	0.243641941762749	0.999692817443324	-4.58015506499758	6.77140392162171	7.05402977518242	6.80168443463714	7.59277864756506	6.65700394942857	6.78170117625633	NA
**TC0700000978.mm.2**	Mtmr10	myotubularin related protein 10	0.654912663471876	6.28123828007672	1.28317869018356	0.243732531669806	0.999692817443324	-4.58017096910847	6.79615276585003	6.28109801797604	6.25891802029916	6.70108409148984	5.14912782099357	6.50104896385167	NA
**TC0200002287.mm.2**	Defb20	defensin beta 20	0.444469028968744	5.80203460939235	1.28312182801477	0.243751243739853	0.999692817443324	-4.58017425349881	5.14045455054824	5.25034516422917	6.00806611735093	6.64710688240615	5.75962120788835	6.00661373393124	NA
**TC0400002501.mm.2**	Fam205a2	family with sequence similarity 205, member A2	-0.486002353102047	5.78392090825353	-1.2826111955594	0.24391933777019	0.999692817443324	-4.58020374657641	6.45084938362344	6.57958957032407	5.09254664115136	5.25799425353343	6.09164154248459	5.2309040584043	NA
**TC0700002159.mm.2**	Ppp1r12c	protein phosphatase 1, regulatory subunit 12C	-0.476851135148198	7.36811812477616	-1.28255138993819	0.243939031685678	0.999692817443324	-4.58020720066835	7.43943447059174	7.45675148118108	7.51262182116728	6.66678844212661	7.57605399349447	7.55705854009581	NA
**TC1400002332.mm.2**	9930012K11Rik	RIKEN cDNA 9930012K11 gene	-0.590639823687483	5.35633220281406	-1.2824562041101	0.243970379125307	0.999692817443324	-4.58021269808679	4.9442680554551	5.23979650815532	5.82011236276339	4.56621214018611	5.82451486469881	5.74308928562566	NA
**TC0100001222.mm.2**	Chil1	chitinase-like 1	0.378835716880446	6.62615439586707	1.2823037539904	0.244020592684791	0.999692817443324	-4.58022150260638	7.47189151525791	7.69755867006767	6.34041970878361	6.33215410303524	5.90802894048436	6.0068734375736	NA
**TC1100001609.mm.2**	Krtap31-1	keratin associated protein 31-1	-0.564630859297173	4.91424071196849	-1.28147241545342	0.244294575434093	0.999692817443324	-4.58026951148285	4.51200510955103	3.78703663444929	4.71190658577079	5.31666381883736	5.21020777941066	5.94762434379183	NA
**TC0400004118.mm.2**	Arhgef16	Rho guanine nucleotide exchange factor (GEF) 16	-0.402769098589631	5.64102107459534	-1.2810104345273	0.244446945838436	0.999692817443324	-4.58029618759329	4.74619477782101	5.05876562880942	5.6930201031305	5.92479381875069	6.33232629613885	6.0910258229216	NA
**TC0500001076.mm.2**	Ephx4	epoxide hydrolase 4	-1.1646166819893	7.96337137491813	-1.28076054589754	0.244529398630555	0.999692817443324	-4.58031061605477	9.06286337257626	9.29665280463042	8.01084792752791	5.53489142663384	8.8577734918238	7.01719922631655	NA
**TC0100000247.mm.2**	Cnnm3	cyclin M3	-0.384551035526288	5.51766077282445	-1.28031474721147	0.244676553888053	0.999692817443324	-4.58033635483099	5.82901502447984	5.75861353724617	5.06882540937939	5.30579159270468	5.47519258132286	5.66852649181379	NA
**TC1700001241.mm.2**	Eml4	echinoderm microtubule associated protein like 4	0.532476169570779	6.22950703394369	1.27986906149807	0.244823749182856	0.999692817443324	-4.5803620852267	7.16076010525446	6.92546405268738	6.43793834296024	5.73994684947069	5.9507430406666	5.16218981262278	NA
**TC1600001648.mm.2**	Gtpbp8	GTP-binding protein 8 (putative)	0.988103243011784	5.34286036452584	1.27911062818376	0.245074412507678	0.999692817443324	-4.58040586693162	6.58073378038016	6.30550834978958	5.83975744907046	4.73380582243397	5.4513160304333	3.14604075504756	NA
**TC1100003827.mm.2**	Tmem101	transmembrane protein 101	0.726138980945882	6.5263253878487	1.27888366257501	0.245149468535078	0.999692817443324	-4.58041896781148	6.77316754570814	6.8548911764769	6.99102440630635	6.5000613770931	6.86154833489954	5.17725948660815	NA
**TC0500002077.mm.2**	Cdk5	cyclin-dependent kinase 5	-0.549415504892515	7.82073918372028	-1.27812715920569	0.245399784168605	0.999692817443324	-4.58046263109306	8.94112159365603	8.71631609326586	7.5786489618579	6.50543424094949	7.75908898737247	7.42382522521995	NA
**TC0100000771.mm.2**	Cab39	calcium binding protein 39	-0.689230453257216	8.51655288299635	-1.27795077969397	0.245458177576641	0.999692817443324	-4.58047281045281	8.97614386828597	8.97003352034684	8.73921239694244	7.148127104473	8.82501088267549	8.44078952525438	NA
**TC0100003872.mm.2**	Irf6	interferon regulatory factor 6	-0.507342435116427	6.07191678438109	-1.27779864802083	0.245508553072463	0.999692817443324	-4.58048159016465	5.38741977096213	5.31435988622993	5.98480826014682	6.37270982928399	6.23612247386664	7.13608048579703	NA
**TC1000000940.mm.2**	D10Wsu102e	DNA segment, Chr 10, Wayne State University 102, expressed	0.474551357034195	6.42421265529708	1.27778554937132	0.24551289086186	0.999692817443324	-4.58048234609407	6.88429499406683	6.58603264122162	6.389872866644	6.62215263863721	6.40869871695717	5.65422407425565	NA
**TC0400001772.mm.2**	Sdhb	succinate dehydrogenase complex, subunit B, iron sulfur (Ip)	0.370831763010106	8.18055791842011	1.27734543014031	0.245658681007322	0.999692817443324	-4.58050774464237	8.64663512533528	8.53608642597791	8.1237825707393	8.19736217187452	7.82351531335373	7.75596590323989	NA
**TC1300000652.mm.2**	Nsd1	nuclear receptor-binding SET-domain protein 1	0.649621791806809	7.73714326743307	1.27719111563213	0.245709815805686	0.999692817443324	-4.58051664944019	8.84068079832902	8.70994618896329	7.84474824661319	7.24098985384667	7.57952955622961	6.20696496061663	NA
**TC0X00001617.mm.2**	Eif1ax	eukaryotic translation initiation factor 1A, X-linked	-0.415969194780796	8.06278928677736	-1.2770500940453	0.245756553887124	0.999692817443324	-4.58052478696513	8.24978157757546	8.36319244199484	7.97232390261919	7.49358775314694	8.27213583830332	8.0257142070244	NA
**TC0600001245.mm.2**	Brpf1	bromodomain and PHD finger containing, 1	-0.402039047734011	6.39531341226928	-1.27688752032697	0.245810444512535	0.999692817443324	-4.58053416790173	6.86546996305228	6.96469897410609	6.13629725992776	5.73251946056689	6.23600872084823	6.43688609511444	NA
**TC0100001801.mm.2**	D1Pas1	DNA segment, Chr 1, Pasteur Institute 1	-0.403974943850327	5.43078963133379	-1.2763176813965	0.245999418571944	0.999692817443324	-4.58056704713666	5.41078293642255	5.26276149316607	5.09742163209861	5.45420010325812	5.54059912582249	5.81897249723489	NA
**TC0X00002116.mm.2**	Tenm1	teneurin transmembrane protein 1	0.57521748060825	6.428174899704	1.27620910715304	0.246035439113722	0.999692817443324	-4.5805733114291	8.08695074224867	7.69480503495054	6.38430835853431	5.58455593258634	5.84442712218015	4.974002207724	NA
**TC1100001628.mm.2**	Cnp	2',3'-cyclic nucleotide 3' phosphodiesterase	-0.512429775135375	8.58759379693453	-1.27586207547349	0.246150601050394	0.999692817443324	-4.58059333299007	9.94307110371543	9.70899544341287	8.12488036151361	7.29943798059045	8.48761582599833	7.96156206637648	NA
**TC0200004109.mm.2**	Nr1h3	nuclear receptor subfamily 1, group H, member 3	-0.409607523144938	6.74744747776825	-1.27583476652467	0.246159665479447	0.999692817443324	-4.58059490849697	6.16625395740547	6.33672188640178	6.57604067880298	7.0052063094532	7.31274143706638	7.08772059747968	NA
**TC1100001076.mm.2**	Inpp5k	inositol polyphosphate 5-phosphatase K	-0.695577927971573	6.52841491095232	-1.27577085583411	0.246180879954212	0.999692817443324	-4.58059859560231	7.25506363960464	7.10048962395018	6.46415406008977	5.2477361130182	7.11230490597587	5.99074112307524	NA
**TC0900000844.mm.2**	Mtfmt	mitochondrial methionyl-tRNA formyltransferase	-0.42029365611328	7.53192513701188	-1.27555282974496	0.246253263401007	0.999692817443324	-4.58061117356578	7.23802516351902	7.20348611725994	7.42995512328077	7.5247709912521	8.18242993284939	7.61288349391004	NA
**TC1100003506.mm.2**	Scpep1	serine carboxypeptidase 1	0.479585771733766	6.29496875349838	1.27538219507434	0.246309926111949	0.999692817443324	-4.58062101719189	5.97066190022893	6.68010052742039	6.75539833250028	6.28371248590398	6.12804437869629	5.95189489624043	NA
**TC0400004058.mm.2**	H6pd	hexose-6-phosphate dehydrogenase (glucose 1-dehydrogenase)	0.390637433826722	5.90602421568268	1.27522682339319	0.246361530329811	0.999692817443324	-4.58062998007872	6.05859591309832	5.97218610250763	6.05811344102527	6.03520563204653	5.47089528365043	5.84114892176792	NA
**TC0700000800.mm.2**	Shank1	SH3 and multiple ankyrin repeat domains 1	-0.400083727858583	8.50610721544683	-1.27498344195927	0.246442384535982	0.999692817443324	-4.58064401949549	9.49830232734224	9.47763496360029	8.02576342545747	7.60450584755316	8.29699802097059	8.13343870775721	NA
**TC0700001554.mm.2**	Smpd1	sphingomyelin phosphodiesterase 1, acid lysosomal	-0.523871417270551	7.86532971832663	-1.27481594958868	0.246498040937426	0.999692817443324	-4.58065368093345	8.86646312608517	8.61664823486731	7.65113757945673	6.67942447777637	7.84999955640022	7.52830533537398	NA
**TC0700004309.mm.2**	Maz	MYC-associated zinc finger protein (purine-binding transcription factor)	-0.426672879288415	7.11406236086194	-1.27478740159171	0.246507528306441	0.999692817443324	-4.58065532763645	7.62224948773909	7.3943958474112	6.98882321015986	6.41836832556241	7.15069101249932	7.10984628179978	NA
**TC0900003317.mm.2**	2010315B03Rik	RIKEN cDNA 2010315B03 gene	-1.04170457560686	6.40539061948815	-1.27429406313486	0.246671530016084	0.999692817443324	-4.58068378310003	6.78472575730754	6.86013133718069	6.82393298913181	4.52810574648167	7.46881284935876	5.96663503746842	NA
**TC1800001384.mm.2**	Fbn2	fibrillin 2	-0.383317338681282	4.7765519054141	-1.27424956537375	0.246686327192989	0.999692817443324	-4.58068634958849	4.56862771871847	4.40171640960385	4.60707228455987	4.85409402883999	5.05650661233234	5.17129437843009	NA
**TC1400001527.mm.2**	Saysd1	SAYSVFN motif domain containing 1	-0.410834616243132	7.22393182220216	-1.27410036353265	0.246735948058363	0.999692817443324	-4.58069495493359	6.88848764305648	6.96897851573534	6.90680092035154	7.4254268506159	7.57876777636696	7.57512922708675	NA
**TC0400003016.mm.2**	Klhl9	kelch-like 9	0.558106407116378	7.47384488687165	1.27320016992714	0.247035515028648	0.999692817443324	-4.58074686980547	8.71521470388349	8.57861897544008	7.57896988738356	6.75375434068597	7.02952962472127	6.1869817891155	NA

**TC0200002496.mm.2**	Ccn5	cellular communication network factor 5	-0.439596921195298	8.25808345929374	-1.27218520494057	0.247373655931868	0.999692817443324	-4.58080539419098	7.45740312993285	7.50494735121394	8.18698117380179	8.66649704231073	8.62053243570504	9.11213962279807	NA
**TC0600001964.mm.2**	Zfp800	zinc finger protein 800	-0.768696181959977	7.00170548130312	-1.27211369258078	0.247397495855075	0.999692817443324	-4.58080951732092	8.25139268425553	8.03109586088655	6.89300307546838	5.20217291390998	7.24259371837019	6.38997463492812	NA
**TC1900000727.mm.2**	Gsto1	glutathione S-transferase omega 1	-0.905596081673671	6.79333673387018	-1.27208219274473	0.247407997523603	0.999692817443324	-4.58081133346549	7.74675828449605	7.42728868626822	7.09757182105962	4.7898188134951	6.76472230928624	6.93386048861583	NA
**TC0900001792.mm.2**	Fat3	FAT atypical cadherin 3	-0.422365960843653	5.64059382594463	-1.27172729869004	0.24752634180326	0.999692817443324	-4.58083179445446	6.23528779271692	6.12463510086642	5.02382639958656	5.29562767061199	5.32192640887358	5.84225958301228	NA
**TC1700002101.mm.2**	Pgk2	phosphoglycerate kinase 2	0.523800580880425	5.33023870463199	1.27150190346295	0.247601528537213	0.999692817443324	-4.58084478870464	4.95778846552032	4.71717592619524	5.46981988248729	6.20721461643132	4.95443145425144	5.67500188290632	NA
**TC0500003136.mm.2**	Fbxw8	F-box and WD-40 domain protein 8	0.476956267037053	7.86779493288029	1.27139892781373	0.247635885499642	0.999692817443324	-4.58085072518356	7.85297567813641	7.86393354488848	7.88299557264412	8.33889088152137	7.26647526334865	8.00149865674273	NA
**TC0900002652.mm.2**	Pigb	phosphatidylinositol glycan anchor biosynthesis, class B	0.446115845762434	6.49809347350755	1.27103724918462	0.247756589460733	0.999692817443324	-4.58087157489604	6.99885976875276	6.91404057108991	6.62811644173346	6.35582965463029	6.37800201058643	5.71371239425245	NA
**TC0800003035.mm.2**	Fam92b	family with sequence similarity 92, member B	0.659053303697105	4.96126588335448	1.27103602709537	0.247756997398674	0.999692817443324	-4.58087164534372	4.48227472572562	4.28440528633161	4.82403797231903	6.3354729754129	4.78604799537766	5.05535634496006	NA
**TC0200001092.mm.2**	Hoxd12	homeobox D12	-0.507063488762435	5.47556985160234	-1.27101285892326	0.247764731131475	0.999692817443324	-4.58087298087687	4.65764445162596	4.94910354676938	5.22526987439372	5.8910021924532	5.72717723180352	6.40322181256826	NA
**TC1100002240.mm.2**	Sec61g	SEC61, gamma subunit	0.676075638382748	8.55892006382305	1.27062470200266	0.247894332493133	0.999692817443324	-4.58089535546252	8.93947302474871	9.04320286339803	8.61932574455545	8.74217214122309	8.80366515208591	7.20568145692713	NA
**TC1100003372.mm.2**	Rad51d	RAD51 paralog D	-0.392148786002614	6.08914725393411	-1.27051998871738	0.247929305230017	0.999692817443324	-4.5809013912117	6.26354022182223	6.05779889406842	5.95360282887186	5.76102058898253	6.10450658933649	6.39441440052312	NA
**TC1400002346.mm.2**	Dmtn	dematin actin binding protein	0.495608562673578	9.06104396189931	1.27026162844146	0.24801561224465	0.999692817443324	-4.58091628282199	9.46907684793099	9.41192876254748	9.46998918142158	8.76824846171068	8.93987686290852	8.30714365487659	NA
**TC0500001757.mm.2**	Trrap	transformation/transcription domain-associated protein	0.391482621617357	6.99508568760895	1.2700702448773	0.248079562102883	0.999692817443324	-4.58092731354175	7.62295088836789	7.96385892993566	6.68725234856551	6.89608242672692	6.40942238448607	6.39094714757165	NA
**TC1700001650.mm.2**	Cramp1l	cramped chromatin regulator homolog 1	0.378640942266569	6.22916953812165	1.27004045455913	0.248089517679955	0.999692817443324	-4.58092903052529	6.2892344293188	6.40564810709153	6.3938560642646	6.32485222416176	6.10626919634885	5.85515720754437	NA
**TC1100003295.mm.2**	Foxn1	forkhead box N1	-0.587694701245377	4.57303556113256	-1.26993496331451	0.248124774422062	0.999692817443324	-4.58093511050839	4.3197519822925	4.15454118935812	3.84182674790719	5.0524386484198	4.81136483564007	5.25828996317767	NA
**TC0500001692.mm.2**	Eif3b	eukaryotic translation initiation factor 3, subunit B	0.391768158919934	6.94312506964215	1.26975822735974	0.248183851985016	0.999692817443324	-4.5809452964307	7.11426181610807	7.12828471986767	7.01620827808643	7.0836618217721	6.45954181390316	6.85679196811549	NA
**TC0600003017.mm.2**	Adipor2	adiponectin receptor 2	-0.844796760924618	6.57799742367999	-1.2679894467606	0.248775777649547	0.999692817443324	-4.5810472204934	6.85689256574969	6.97922690391067	7.05347688449858	4.91765889078661	6.9631597251835	6.69756957195093	NA
**TC1300001794.mm.2**	Eci2	enoyl-Coenzyme A delta isomerase 2	0.831714405706611	7.42662871055049	1.26747156385227	0.248949320542014	0.999692817443324	-4.58107705703806	8.06514033402671	7.97504116110448	8.1568301450822	6.93467964471029	7.57638138513468	5.85169959324458	NA
**TC1100001937.mm.2**	Aanat	arylalkylamine N-acetyltransferase	0.749095202272314	5.15880996360707	1.2674603833946	0.248953068282101	0.999692817443324	-4.58107770114294	4.87462167133542	4.56777446442567	4.85607018237191	6.64825684284109	5.1796132865983	4.82652333407006	NA
**TC0500003388.mm.2**	Bud23	BUD23, rRNA methyltransferase and ribosome maturation factor	-0.446493966531369	6.365554082197	-1.26729598132953	0.249008182269044	0.999692817443324	-4.58108717218266	5.86365079014988	6.22530594907656	6.22593919158046	6.37975071886594	6.44102326448868	7.05765457902046	NA
**TC1500001401.mm.2**	Rrm2b	ribonucleotide reductase M2 B (TP53 inducible)	0.413600998865891	6.98189705556563	1.26685428981482	0.249156307086256	0.999692817443324	-4.58111261625115	7.516258736083	7.39396881299201	6.68602889237835	7.21814949864691	6.48610520341098	6.5908711898825	NA
**TC0800001835.mm.2**	AY761185	cDNA sequence AY761185	-0.505376506380875	5.54128451958395	-1.26625667270889	0.249356844852558	0.999692817443324	-4.58114703944683	5.01231144423323	5.24229539097789	5.63156842237798	5.35960521238745	5.53727332818735	6.46465331933983	NA
**TC1100002052.mm.2**	Slc16a3	solute carrier family 16 (monocarboxylic acid transporters), member 3	-0.404990367175923	6.34599705314289	-1.26616585568812	0.249387331908457	0.999692817443324	-4.58115227026122	5.69069794805281	6.13470406934473	6.4678166753793	6.25248310817468	6.71386325573062	6.81641726217521	NA
**TC1700000070.mm.2**	Rps6ka2	ribosomal protein S6 kinase, polypeptide 2	0.421494498766325	7.71381175932064	1.26589602328236	0.249477933130264	0.999692817443324	-4.58116781138663	8.7383446814315	8.65852712687454	7.68962872954506	7.17486514303017	7.1629956983074	6.85850917673518	NA
**TC1900000159.mm.2**	Hnrnpul2	heterogeneous nuclear ribonucleoprotein U-like 2	0.465901085938841	8.61911274505174	1.26581133288734	0.249506375400133	0.999692817443324	-4.58117268901874	9.47132262127659	9.27935395721417	8.72874821565987	8.21915281618882	8.31789899597243	7.69819986399857	NA
**TC0200003582.mm.2**	Ccdc148	coiled-coil domain containing 148	0.392080733422573	7.25873772324478	1.26541396087509	0.24963986576995	0.999692817443324	-4.5811955741711	7.49206582036588	7.50044443881969	7.24064574703429	7.43139302652984	7.12866039618602	6.75921691053296	NA
**TC1300001622.mm.2**	Abt1	activator of basal transcription 1	-0.434913446533539	6.70020034983247	-1.26484094881874	0.249832468793305	0.999692817443324	-4.58122857184196	7.0933026998389	6.50539301891282	6.60021574687945	6.26612399670856	6.84822739243324	6.88793924422185	NA
**TC1200000140.mm.2**	Nol10	nucleolar protein 10	-1.10743307700569	6.00439478436897	-1.264357573576	0.249995043214757	0.999692817443324	-4.58125640506766	7.43508520778711	7.20862035323196	6.2145918267603	3.36930666883139	6.30503833961807	5.493726309985	NA
**TC1400001613.mm.2**	Tmem254b/Tmem254c/Tmem254a	transmembrane protein 254b/transmembrane protein 254c/transmembrane protein 254a	-1.13820377814552	8.61826352832928	-1.26417824211024	0.250055381463085	0.999692817443324	-4.58126673054753	8.80358587337889	9.54431453823559	9.19247477216024	6.35016182887484	9.33865868101674	8.4803854763094	NA
**TC0500001301.mm.2**	Nos1	nitric oxide synthase 1, neuronal	0.587944319604786	6.17809694190685	1.26397875280383	0.250122516937772	0.999692817443324	-4.58127821628355	7.18899477572322	7.13680097093251	5.86971402590764	6.08962324608984	4.7455981231552	6.03785050963271	NA
**TC0500003104.mm.2**	Bicdl1	BICD family like cargo adaptor 1	0.595317328950513	6.53326828671975	1.26363470343164	0.250238338999459	0.999692817443324	-4.58129802421373	7.82912595964531	7.64427062390549	6.65222937420943	5.80619452312494	6.14449222016873	5.12329701926461	NA
**TC1900000169.mm.2**	Tut1	terminal uridylyl transferase 1, U6 snRNA-specific	-0.428786320908658	5.69378924614805	-1.26338089168121	0.250323813013732	0.999692817443324	-4.58131263612575	6.22110209262093	5.90387038044459	5.01336821693172	5.57672696407102	5.70323245964334	5.74443536317671	NA
**TC1000002906.mm.2**	Kcnmb4	potassium large conductance calcium-activated channel, subfamily M, beta member 4	-0.371052655860808	8.00103920707623	-1.26329865938155	0.250351511125078	0.999692817443324	-4.58131737008828	8.01930262392447	7.98925069694484	7.74945030303168	7.87833800190155	8.27190001921242	8.09799359744242	NA
**TC0200000552.mm.2**	Fubp3	far upstream element (FUSE) binding protein 3	0.37442743575001	7.67667262691787	1.2631378710581	0.25040567677319	0.999692817443324	-4.5813266261772	7.53141335911737	7.4748491657729	7.84122857048284	8.06008548357566	7.64184825196061	7.51061093059787	NA
**TC0300002126.mm.2**	Trim59	tripartite motif-containing 59	0.528559737458855	5.79468867163523	1.26290323628848	0.250484737757636	0.999692817443324	-4.58134013290422	5.56312414812311	5.4183966230429	5.67159584288677	6.75026952389477	5.72297566600905	5.64177022585478	NA
**TC0500002608.mm.2**	Lnx1	ligand of numb-protein X 1	-0.512411380325513	6.1504764749645	-1.26249023814179	0.250623951589798	0.999692817443324	-4.58136390573138	6.85046029552728	6.40192244752044	6.07322261737754	5.23960405566659	6.36932529333462	5.96832414036053	NA
**TC1400002788.mm.2**	Gm5796/Gm3373	predicted gene 5796/predicted gene 3373	0.538370225761631	8.13881655233274	1.26234968900813	0.250671343365684	0.999692817443324	-4.58137199556305	8.5201973528556	7.73086437371235	8.69116127777622	8.13812774169966	8.17150347335207	7.58104509460055	NA
**TC0300000376.mm.2**	Foxo1	forkhead box O1	0.521576879012169	7.34532165823953	1.26223240527118	0.250710896240414	0.999692817443324	-4.58137874611245	8.09535178528341	8.04133790608397	7.54182941500535	6.94736759304171	7.1640360133684	6.28200723665432	NA
**TC0100002840.mm.2**	Pdcd1	programmed cell death 1	0.48694427802453	5.99593236600413	1.26181281989213	0.250852442003368	0.999692817443324	-4.58140289520691	5.56473373786706	5.88154779183178	6.05794572094701	6.6936548902405	5.59343331857063	6.18427873656782	NA
**TC1600000040.mm.2**	Nudt16l1	nudix (nucleoside diphosphate linked moiety X)-type motif 16-like 1	-0.362856495357587	8.37845779379349	-1.26158489688853	0.250929360185264	0.999692817443324	-4.58141601247874	8.13572803568137	8.17376234821517	8.32228435215017	8.29548734192445	8.67252298018947	8.67096170460032	NA
**TC0400002786.mm.2**	Fkbp15	FK506 binding protein 15	-0.740640267183274	6.94590030293428	-1.26157535000415	0.250932582461527	0.999692817443324	-4.58141656190294	8.04315821724293	7.78259581237894	6.85650788900602	5.32767573780261	7.32409946844935	6.34136469272582	NA
**TC1100000956.mm.2**	Pld2	phospholipase D2	0.41421790177249	5.68073620473536	1.26147511158821	0.250966417226298	0.999692817443324	-4.58142233057766	6.78063115232318	6.55212456704526	5.54109968336917	5.24894897292519	5.19723165763883	4.76438119511054	NA
**TC0600000340.mm.2**	Tmem140	transmembrane protein 140	-0.518715803537328	4.9598516918616	-1.26121404809463	0.251054555955764	0.999692817443324	-4.5814373541751	4.61125356063471	5.46188572836759	4.97442171915576	4.34984790839056	5.18564392730452	5.17605730731647	NA
**TC0200002753.mm.2**	Gm14327	predicted gene 14327	-1.15515809305037	7.78610514630957	-1.26110113090063	0.251092686727781	0.999692817443324	-4.58144385207925	9.04660662719591	9.47413135832551	7.93386513384967	5.00892321926797	8.12439134298954	7.12871319622884	NA
**TC0700004416.mm.2**	Eef1akmt2	EEF1A lysine methyltransferase 2	-0.370610159020472	6.38892023829322	-1.2610474816906	0.251110805180571	0.999692817443324	-4.5814469393169	6.76975677598252	6.68345633657659	6.11943136481083	5.95011263476882	6.34847708646881	6.46228723115178	NA
**TC0900003064.mm.2**	Wdr6	WD repeat domain 6	0.415960526651045	7.5089000910394	1.26102130340814	0.251119646542667	0.999692817443324	-4.58144844573237	8.97551425163575	8.5074353852182	7.1540463640502	7.04713961729207	6.84826244669156	6.52100248134863	NA
**TC1000001203.mm.2**	Poc1b	POC1 centriolar protein B	0.398824967201929	6.19217952802015	1.26085565325338	0.251175598919057	0.999692817443324	-4.58145797781895	6.62520918468533	6.67619740251531	6.27351890432266	6.05114135333941	5.97146481689229	5.55554550636593	NA
**TC1000000733.mm.2**	Zfp280b	zinc finger protein 280B	0.664655830953383	6.87787723806021	1.2606228435856	0.251254254360731	0.999692817443324	-4.58147137401783	6.79620484833693	6.44151971187171	7.99757398220001	6.68185128282967	7.08020183992899	6.26991176319393	NA
**TC1200002194.mm.2**	Ptpn21	protein tyrosine phosphatase, non-receptor type 21	0.507121766750783	5.04919779656033	1.2603765854484	0.25133747668852	0.999692817443324	-4.5814855434498	4.87161164717994	5.07313891188958	5.00721420316682	5.67512567373018	4.46879543517927	5.19930090821616	NA
**TC0800002252.mm.2**	Sap30	sin3 associated polypeptide	-0.417675868623864	5.87724318373369	-1.26011408723482	0.251426213668126	0.999692817443324	-4.5815006466261	5.80665724348882	6.214047459613	5.8136714820471	5.39002984897919	5.98149693863021	6.05755612964381	NA
**TC1200002078.mm.2**	Aldh6a1	aldehyde dehydrogenase family 6, subfamily A1	0.619460039840602	6.3543490274641	1.25965913887335	0.251580072459053	0.999692817443324	-4.58152682097699	7.40755403490933	7.21785572031129	6.55721550056702	5.81258674405557	6.16919908618173	4.96168307875966	NA
**TC0200005132.mm.2**	Acot8	acyl-CoA thioesterase 8	0.384952260891026	6.82707380922141	1.25941227235924	0.251663594349682	0.999692817443324	-4.58154102294213	7.03262247494867	6.78828438020089	6.89010872418244	7.06561153679804	6.70395115325937	6.48186458593907	NA
**TC0400003401.mm.2**	Olfr1338	olfactory receptor 1338	-0.426998374037583	5.62479326181235	-1.2592423436151	0.251721100014814	0.999692817443324	-4.58155079839115	5.17673460494374	5.67000532381458	5.49188570758081	5.5321257394395	5.73157038347904	6.14643781161643	NA
**TC0100000320.mm.2**	AI597479	expressed sequence AI597479	0.395730920482271	7.33654445225065	1.25910769680969	0.251766674003057	0.999692817443324	-4.58155854397095	7.36627770828293	7.02366429166045	7.59460906587625	7.61578421138628	7.3601636122882	7.05876782400979	NA
**TC1400000347.mm.2**	Ercc6	excision repair cross-complementing rodent repair deficiency, complementation group 6	0.502712916253166	5.5060300013082	1.25865586719935	0.251919657402873	0.999692817443324	-4.58158453416088	5.87385731058432	5.96710181929627	5.67408071506535	5.42624264017211	5.52697738236153	4.5679201403696	NA
**TC1600000266.mm.2**	Klhl24	kelch-like 24	0.995284334327508	7.25638357246142	1.2583044457533	0.252038699733042	0.999692817443324	-4.58160474718607	8.32608001129505	8.44161166505395	7.67538746115894	6.70520175237833	7.42283050826043	4.96719003662182	NA
**TC0300001300.mm.2**	Pitx2	paired-like homeodomain transcription factor 2	0.439137495783027	6.22352905523496	1.25826421556034	0.25205233063204	0.999692817443324	-4.58160706105997	5.99727351242276	5.93834263889131	6.21862607279444	6.92329051303644	6.16623458795446	6.09740700631037	NA
**TC1700002558.mm.2**	Eif2ak2	eukaryotic translation initiation factor 2-alpha kinase 2	0.4320053047422	5.82339578674956	1.25822218040689	0.252066573775967	0.999692817443324	-4.5816094787296	5.92571181550511	6.23413821910128	5.68975558502065	6.13251206266705	5.68113750139513	5.27711953680817	NA
**TC1600000244.mm.2**	Gnb1l	guanine nucleotide binding protein (G protein), beta polypeptide 1-like	0.474900430282696	5.32567936920725	1.25821294554383	0.252069703000123	0.999692817443324	-4.58161000987418	4.95145528694461	4.78691484509571	5.41763795188532	6.16511551999898	5.10161460688304	5.53133800443586	NA
**TC0500001590.mm.2**	Hspb1	heat shock protein 1	-0.412391126759228	6.52612081660504	-1.25798138090876	0.252148179468583	0.999692817443324	-4.58162332805758	6.1773025956112	6.55861739548378	6.60520438981277	6.19280693769563	6.8463794587912	6.77641412223566	NA
**TC1300001858.mm.2**	Gcm2	glial cells missing homolog 2	-0.564507964795657	5.72525679672198	-1.2572588020069	0.252393195318674	0.999692817443324	-4.5816648827623	4.68707934197309	5.02149441808626	5.30343775568319	6.45353778965742	6.28767139311542	6.5983200818165	NA
**TC1300002453.mm.2**	Pde8b	phosphodiesterase 8B	0.449644004286873	6.78378262695975	1.25689439016635	0.25251684034128	0.999692817443324	-4.58168583759537	7.39038865845294	7.15631378766568	6.40851491603055	7.11912574607625	6.23600581802762	6.39234683550544	NA
**TC1400001605.mm.2**	Tmem254c/Tmem254a/Tmem254b	transmembrane protein 254c/transmembrane protein 254a/transmembrane protein 254b	-1.01862015147203	8.67070767275991	-1.25686256187502	0.252527642182873	0.999692817443324	-4.58168766775628	8.70493888845116	9.46184832976664	9.18952432976691	6.7205849279319	9.39714345010032	8.55020611054255	NA
**TC0200001218.mm.2**	Med19	mediator complex subunit 19	0.407860005020042	6.93113579724959	1.25616461852303	0.25276461002026	0.999692817443324	-4.58172779755799	6.74790785234863	7.0027453754868	7.03044309469205	7.29549768815904	6.95440865027177	6.55581212253923	NA
**TC0200005342.mm.2**	Gm14440/Gm14288	predicted gene 14440/predicted gene 14288	-1.18116049812298	8.26813656000591	-1.25601900540714	0.252814073336129	0.999692817443324	-4.58173616925755	9.50302634774664	9.85728326710383	8.50329470217472	5.43979967229479	8.60039917668762	7.70501619402786	NA
**TC0600000964.mm.2**	Alms1	ALMS1, centrosome and basal body associated	0.553698800343433	5.69979554109754	1.25523582364257	0.253080256350646	0.999692817443324	-4.5817811926795	6.2637006656716	6.13484352331702	6.07665238183819	5.37716094730355	5.65665267833852	4.68976305011636	NA
**TC1400002293.mm.2**	Dock5	dedicator of cytokinesis 5	-0.390901492049289	6.25103011792098	-1.25505191487256	0.253142797357766	0.999692817443324	-4.58179176425118	6.02500171152639	6.04598663029984	5.94808889272439	6.37860579807614	6.54946033420714	6.55903734069197	NA
**TC1500001729.mm.2**	Ly6c1	lymphocyte antigen 6 complex, locus C1	-0.732178784373933	8.12969157882726	-1.25500181052478	0.253159838434753	0.999692817443324	-4.58179464432172	8.22991269263602	8.25041678449187	8.43691940121544	6.97981181232847	8.9864837846397	7.89460499765208	NA
**TC1100002451.mm.2**	Chac2	ChaC, cation transport regulator 2	-0.378652466840345	6.01082240166776	-1.25444405890658	0.253349603478053	0.999692817443324	-4.58182670288682	6.59425519266974	6.83526597081146	5.54363845777837	5.39541569864397	5.93476540035248	5.76159368975055	NA
**TC1100002045.mm.2**	Rac3	Rac family small GTPase 3	0.510334155309685	7.18464272690887	1.25385127636959	0.253551422324573	0.999692817443324	-4.58186077132559	8.11846069144934	7.18094879439212	7.30885435298205	7.10570324013352	6.504184932936	6.8897043495602	NA
**TC1400001609.mm.2**	Tmem254b/Tmem254c/Tmem254a	transmembrane protein 254b/transmembrane protein 254c/transmembrane protein 254a	-1.00905589171966	8.90390587086557	-1.25383763901309	0.253556066940682	0.999692817443324	-4.58186155504845	9.05269634028939	9.6955428523836	9.4043220689168	6.92422005562377	9.59281266974546	8.75384123823442	NA
**TC0700004261.mm.2**	Gtf3c1	general transcription factor III C 1	0.371322962594104	7.66813697854485	1.25375854487886	0.253583006305374	0.999692817443324	-4.58186610045617	8.15517246667446	7.99777611567835	7.72974913874791	7.56951046830433	7.34135500025813	7.2152586816059	NA
**TC0100001176.mm.2**	Slc41a1	solute carrier family 41, member 1	0.639618101860929	8.15343891502173	1.25364198746857	0.253622710137137	0.999692817443324	-4.58187279869362	8.94813719758312	9.10732862705963	8.59693143000285	7.47527050460188	7.89716222653956	6.89580350434331	NA
**TC0900003234.mm.2**	Acaa1b	acetyl-Coenzyme A acyltransferase 1B	0.444036605797388	7.41243768542867	1.25327517642439	0.253747694914007	0.999692817443324	-4.58189387737642	7.33489372353105	7.45188500896214	7.34096281930869	7.94699747652812	7.40714100942184	6.9927460748202	NA
**TC0600003117.mm.2**	Lrrc23	leucine rich repeat containing 23	-0.418211508434099	5.72718277072442	-1.25294234523399	0.25386114780276	0.999692817443324	-4.58191300216745	5.96397500457473	5.89069810296972	5.40318369967573	5.43281655029121	5.53419059672544	6.13823267010969	NA
**TC1800000265.mm.2**	Wdr36	WD repeat domain 36	0.375779379617748	6.58366242335965	1.25292579349018	0.253866790978621	0.999692817443324	-4.58191395321503	6.95285366601579	7.11409813292576	6.67618668040516	6.41710406982076	6.14848138604085	6.19325060494958	NA
**TC1900000501.mm.2**	Ifit3	interferon-induced protein with tetratricopeptide repeats 3	-0.487284158574282	7.04165206821806	-1.25288669003878	0.253880123398538	0.999692817443324	-4.58191620005073	6.75506796345426	7.431441229455	6.458305685414	7.08611176421127	7.169942999284	7.34904276748984	NA
**TC0200002696.mm.2**	Stx16	syntaxin 16	0.387678768254495	8.10232428051865	1.25236580084219	0.254057779750893	0.999692817443324	-4.58194612812638	8.34637160045187	8.00480425956492	8.23834158773154	8.2807220920705	7.98608395535301	7.75762218794005	NA
**TC1800001049.mm.2**	Trappc8	trafficking protein particle complex 8	-0.627810288683745	6.07558470443442	-1.25231635196078	0.254074650561404	0.999692817443324	-4.58194896909664	6.92460661137907	6.36537499098548	5.35432595849004	5.59962706494721	6.77516597891352	5.43440762189122	NA
**TC0200004623.mm.2**	Cpxm1	carboxypeptidase X 1 (M14 family)	0.411267904920357	5.18867654301877	1.25229235517639	0.254082838056989	0.999692817443324	-4.58195034776646	4.74709117987737	5.18405843760499	5.66288516828418	5.3488375569513	5.18919879199435	4.99998812340042	NA
**TC1900001105.mm.2**	Tmem216	transmembrane protein 216	0.405015508672025	5.52883187664736	1.25228007406004	0.254087028356244	0.999692817443324	-4.58195105334212	6.51979779809589	6.34176449040677	5.0648115772071	5.49591841715568	5.00378993115031	4.74690904586842	NA
**TC1800000201.mm.2**	Fhod3	formin homology 2 domain containing 3	-0.394769218789722	6.12168800833793	-1.25207423804252	0.25415726822037	0.999692817443324	-4.5819628788068	6.32091615206931	6.19882546463459	5.7300236679216	5.98040032995051	6.43649786597949	6.06346456947206	NA
**TC0700002418.mm.2**	Bicra	BRD4 interacting chromatin remodeling complex associated protein	0.404175875468634	6.41434965513147	1.25201384409173	0.25417788035545	0.999692817443324	-4.58196634840689	6.88208098635587	6.89939210302362	6.51579137151853	6.24069692465476	6.19910036647488	5.74903617876114	NA
**TC1500001942.mm.2**	Naga	N-acetyl galactosaminidase, alpha	0.565449254608563	6.38991201501186	1.251950935237	0.254199352353633	0.999692817443324	-4.58196996244497	6.43865534150084	6.59165104782156	5.99107012735639	7.22896197762655	6.00570082051442	6.08343277525139	NA

**TC0400000114.mm.2**	Triqk	triple QxxK/R motif containing	0.676971827892618	6.27571023102791	1.25165644108773	0.254299889747369	0.999692817443324	-4.58198688021098	7.61129578719247	7.49832378894685	6.14076098223079	5.80853175067591	6.10039121003585	4.49495786708561	NA
**TC1300001139.mm.2**	Taf9/Ak6	TATA-box binding protein associated factor 9/adenylate kinase 6	-0.390493307761697	8.10035993489841	-1.25165412885322	0.254300679257644	0.999692817443324	-4.58198701303789	8.91087934958686	8.93161795539143	7.64601830596396	7.34331953848043	8.04167494852749	7.72864951144029	NA
**TC0300000866.mm.2**	Tdrkh	tudor and KH domain containing protein	0.385518561193329	6.31968588405153	1.25145337194345	0.254369235608213	0.999692817443324	-4.58199854535036	6.88313127718404	6.63218902673498	6.17778730228539	6.40912875910301	5.8051660234859	6.01071291551584	NA
**TC1600000763.mm.2**	Cpox	coproporphyrinogen oxidase	0.427136813767455	7.98470077453021	1.25135499016752	0.25440283778274	0.999692817443324	-4.58200419664969	7.84174107606798	7.99594503142962	8.15530049587277	8.30709558773652	8.11627190506294	7.49185055101145	NA
**TC0200005110.mm.2**	Svs3b	seminal vesicle secretory protein 3B	-0.509238012693133	4.74988127684682	-1.25099854273819	0.25452461416118	0.999692817443324	-4.58202467101747	3.88029887195311	4.25248774339597	4.40042163763489	5.27359087253788	5.14105000230006	5.55143853325898	NA
**TC0900002857.mm.2**	Dipk2a	divergent protein kinase domain 2A	-0.981797473809499	7.45702654020904	-1.25076406441823	0.254604748645132	0.999692817443324	-4.58203813871834	7.74687938561175	7.92676976698019	8.07691756420472	8.47553999631694	7.88387127554395	7.63218123259672	NA
**TC1600001342.mm.2**	Cyp2ab1	cytochrome P450, family 2, subfamily ab, polypeptide 1	0.364043266667639	5.53605340076708	1.25048695125649	0.254699482027392	0.999692817443324	-4.58205405446073	5.5437319773829	5.56004125305695	5.77207675415352	5.64824009959544	5.40230629596195	5.28992402445173	NA
**TC1400001486.mm.2**	Oxsm	3-oxoacyl-ACP synthase, mitochondrial	0.487424707363358	5.79463759146132	1.24905225049848	0.255190434253477	0.999692817443324	-4.58213644180694	6.75215932457174	6.41481176060716	5.95314766559742	5.33470427356044	5.47293249756383	4.84007002686732	NA
**TC1900000168.mm.2**	Mta2	metastasis-associated gene family, member 2	0.432033161880502	7.64343948098114	1.2486334023869	0.255333917763893	0.999692817443324	-4.58216048981298	8.34190299394625	8.09938094735102	7.41988807267724	7.72182156149805	7.42517137661084	6.85247193380345	NA
**TC0800000983.mm.2**	Man2b1	mannosidase 2, alpha B1	-0.522751823753819	6.64619214980012	-1.24755606809144	0.255703297600225	0.999692817443324	-4.58222233569755	6.92954219853875	7.46547515409274	6.02787004221498	6.19044590711582	6.16213989630937	7.10167970052907	NA
**TC0600000543.mm.2**	Zfp956/Zfp783	zinc finger protein 956/zinc finger protein 783	0.494861952967296	5.91430863048903	1.24728354291259	0.255796810076625	0.999692817443324	-4.58223797836026	5.75214116267808	5.62051353521991	5.78964555658611	6.76181493889929	5.77212191191546	5.78961467763534	NA
**TC0900001116.mm.2**	A330041J22Rik	RIKEN cDNA A330041J22 gene	-0.439222923486697	6.38148701312243	-1.24725614702566	0.255806212155075	0.999692817443324	-4.58223955081039	5.76114214583809	5.7837962272208	6.2289719191213	6.70379701022985	6.62848618690196	7.18272858942259	NA
**TC0600002337.mm.2**	Lsm5	LSM5 homolog, U6 small nuclear RNA and mRNA degradation associated	-0.455235062870288	5.37456337647238	-1.24724401048394	0.25581037742759	0.999692817443324	-4.58224024741255	5.6854275599626	5.30538953578555	4.7495135346322	5.42353298404057	5.42679282375065	5.6567238206627	NA
**TC0X00003437.mm.2**	Il2rg	interleukin 2 receptor, gamma chain	-0.516830867737903	5.12572442774719	-1.24723564122333	0.255813249799868	0.999692817443324	-4.5822407277828	4.86505147355379	5.32815199633058	4.75006175474643	5.01367892581505	4.93429208367042	5.86311033236686	NA
**TC0X00001549.mm.2**	Gpr143	G protein-coupled receptor 143	-0.494437440071171	5.48657857810363	-1.24710130330527	0.25585935906589	0.999692817443324	-4.58224843826701	4.9704350773048	4.90112153849665	5.32909008562365	5.70042990071534	5.55721118828668	6.46118367819465	NA
**TC0700003597.mm.2**	Iqgap1	IQ motif containing GTPase activating protein 1	-0.585477294160752	6.2508454967093	-1.24706631885897	0.255871368077531	0.999692817443324	-4.58225044620807	6.26052469554604	6.57541880504124	5.8311591402809	5.9179283053926	7.10545955734006	5.81458247665494	NA
**TC0400001971.mm.2**	Slc2a7	solute carrier family 2 (facilitated glucose transporter), member 7	-0.502110659425383	5.87218232668304	-1.2466591407265	0.256011174820023	0.999692817443324	-4.58227381530286	4.88508722735098	4.96112598938757	5.59484624333406	6.59648346892041	6.53548695445662	6.66006407664862	NA
**TC1100001894.mm.2**	Trim80	tripartite motif-containing 80	-0.40893801042008	5.16593383668132	-1.24658104731424	0.256037996150851	0.999692817443324	-4.58227829709292	4.9582423719883	4.73256081684743	5.28637376330009	4.95708814190594	5.32884645173314	5.73249147431304	NA
**TC0600001474.mm.2**	D6Wsu163e	DNA segment, Chr 6, Wayne State University 163, expressed	-0.50493794409478	7.34949080331492	-1.24655065425611	0.256048435361308	0.999692817443324	-4.5822800413358	8.4479192623597	8.29789352201877	6.97441720386863	6.1962108697921	7.41444133343506	6.76606262841523	NA
**TC0700000557.mm.2**	Clip3	CAP-GLY domain containing linker protein 3	0.36514603027207	8.03100909480602	1.24649288106324	0.256068279937661	0.999692817443324	-4.58228335688312	8.66946991595592	8.64237932031803	7.81702261897805	7.9852260775751	7.47928209709881	7.59267453891019	NA
**TC1100002948.mm.2**	Zfp286	zinc finger protein 286	0.609174580605518	6.23295433035494	1.24626672145825	0.256145976538849	0.999692817443324	-4.58229633560662	5.93455239462513	6.26520345027423	5.93000467445514	7.27815497476552	6.14005717491373	5.84975331309589	NA
**TC1800001178.mm.2**	Spata24	spermatogenesis associated 24	0.415067161350206	6.4383865220377	1.24621876263977	0.256162455297884	0.999692817443324	-4.58229908776806	6.21153017569643	6.08414906823655	6.55539402245339	7.02699308304343	6.5538115739723	6.19844120882411	NA
**TC0200003666.mm.2**	Scn3a	sodium channel, voltage-gated, type III, alpha	0.447734786250056	7.34921564066426	1.24608741279022	0.256207592098197	0.999692817443324	-4.58230662527069	7.94210359365423	7.9488155432616	7.45402157602211	7.0959005637628	7.17015069281404	6.48430187447076	NA
**TC1900000335.mm.2**	Aldh1a1	aldehyde dehydrogenase family 1, subfamily A1	1.01646968110716	5.06221138582038	1.24581380772543	0.256301635278878	0.999692817443324	-4.58232232546398	5.74508617556386	5.40655302377201	4.80842230677752	5.81886193212283	5.55857263864091	3.03577223804513	NA
**TC0800003231.mm.2**	Zfp617	zinc finger protein 617	0.39719345631585	7.10140552763882	1.24476296615091	0.256663106664278	0.999692817443324	-4.58238261782398	7.68368308620387	7.68678802650786	7.2255914358506	6.79058304702583	6.73183437464179	6.48995319560294	NA
**TC1500001743.mm.2**	Mafa	v-maf musculoaponeurotic fibrosarcoma oncogene family, protein A (avian)	-0.398382668684551	5.18238954101036	-1.24475082016763	0.256667287247897	0.999692817443324	-4.58238331463146	5.05237607806795	5.10581786725232	5.25758427305864	4.81210470862774	5.31669449194227	5.54975982711321	NA
**TC1900000954.mm.2**	Pacs1	phosphofurin acidic cluster sorting protein 1	0.792157278728697	6.18707006324549	1.24472441175593	0.256676377087527	0.999692817443324	-4.58238482965989	7.58282260188862	7.46405629647366	6.6048373468438	5.22509067244023	5.86938585991303	4.37622760191361	NA
**TC1100004296.mm.2**	Nme1	NME/NM23 nucleoside diphosphate kinase 1	0.445747711120781	9.04074506443886	1.24429542313832	0.256824074987576	0.999692817443324	-4.58240943928401	9.79363637265601	9.65703552974366	9.02689270932686	8.81575424391068	8.83802678081006	8.11312475018593	NA
**TC0700001880.mm.2**	Slc5a2	solute carrier family 5 (sodium/glucose cotransporter), member 2	0.483916518345472	6.51165951346388	1.2440177583673	0.256919712252931	0.999692817443324	-4.58242536687259	5.89514412836224	6.18734870785361	6.59799264047843	7.37965600015076	6.28812777370936	6.72168783022889	NA
**TC1900001493.mm.2**	Tctn3	tectonic family member 3	-0.37744579703707	6.6786206654407	-1.24333894074772	0.257153649897828	0.999692817443324	-4.58246430200943	7.41903823726361	7.29509914533691	6.27235899043801	6.02898851754677	6.63867540234448	6.41756369971444	NA
**TC0500001972.mm.2**	Gm9758	predicted gene 9758	-0.403483729671913	5.51256137774874	-1.24320843057438	0.257198648076468	0.999692817443324	-4.582471787125	5.42940129653838	5.69468827612525	5.51462603314124	5.05752958410124	5.63120487542283	5.74791820116347	NA
**TC0400001510.mm.2**	Rnf19b	ring finger protein 19B	-0.527308840751764	7.15738092580896	-1.24300750102556	0.257267939220675	0.999692817443324	-4.58248331060942	7.53323385544567	7.50886101161867	7.24566875785749	6.17811774528545	7.07148188869302	7.40692229595345	NA
**TC1100002255.mm.2**	Etaa1	Ewing tumor-associated antigen 1	-0.386741253752056	5.76812086873885	-1.24273543728081	0.257361786900071	0.999692817443324	-4.58249891297653	5.9839234940493	5.91400180675939	5.43837574895298	5.53028295310716	5.66464128298196	6.07749992658229	NA
**TC1200001976.mm.2**	Pigh/9230116L04Rik	phosphatidylinositol glycan anchor biosynthesis, class H/RIKEN cDNA 9230116L04 gene	0.694291580172774	5.76382271300182	1.24268268498022	0.257379987090947	0.999692817443324	-4.58250193812957	5.76628017487491	5.69108674153738	5.76507738792298	6.4919988730491	4.65661842287744	6.2118746777491	NA
**TC0300003243.mm.2**	Bcl9	B cell CLL/lymphoma 9	0.621649694470553	7.01470349642485	1.24264520535677	0.257392918696351	0.999692817443324	-4.58250408743089	7.61497368066037	7.35394613321826	7.81322868390498	6.36807159290082	6.45994793307418	6.47805295479052	NA
**TC0200001049.mm.2**	Rapgef4	Rap guanine nucleotide exchange factor (GEF) 4	-0.510577190273976	8.93693232322564	-1.2420116174694	0.257611610651484	0.999692817443324	-4.58254041867214	9.48195445947899	9.91006337634232	8.57202823209116	8.03218262940113	9.21174299882287	8.41362224321737	NA
**TC0800001274.mm.2**	Wwp2	WW domain containing E3 ubiquitin protein ligase 2	-0.461279551652191	6.69100278458362	-1.24129416342221	0.257859443921995	0.999692817443324	-4.58258155348374	7.23003092564361	7.10826924199524	6.63935746215234	5.8032212561269	6.69201126163793	6.6731265599457	NA
**TC1800001149.mm.2**	Fam13b	family with sequence similarity 13, member B	0.617572016695933	7.51572306878888	1.24115161146196	0.257908710772845	0.999692817443324	-4.58258972592187	8.31345778920755	8.07402540648066	7.83313665775611	7.13786296746235	7.49677022447636	6.23908536735023	NA
**TC1600000982.mm.2**	Hunk	hormonally upregulated Neu-associated kinase	0.490970682991151	6.59506747131428	1.24099443887601	0.257963040022914	0.999692817443324	-4.58259873628593	7.13086571441334	6.68921546551205	6.62828349468511	6.7378490122862	6.62343682887091	5.7607543121181	NA
**TC0400003405.mm.2**	Olfr1333	olfactory receptor 1333	0.441693592118575	5.28108617223119	1.240738665662	0.258051473298552	0.999692817443324	-4.5826133986096	4.86787900581933	4.66589987326391	5.46565410626249	6.05240856300802	5.53126766200451	5.10340782302886	NA
**TC0400003568.mm.2**	Gjb5	gap junction protein, beta 5	0.457685709066108	5.41790981628936	1.24055208470926	0.258115999941635	0.999692817443324	-4.58262409398328	4.41290961873501	5.07943247428382	5.85243435808544	6.11280975333935	5.31470832011289	5.73516437317968	NA
**TC1800000689.mm.2**	Pmaip1	phorbol-12-myristate-13-acetate-induced protein 1	-0.444275984749487	6.05925381157779	-1.240320718534	0.258196034325556	0.999692817443324	-4.58263735602753	5.45868581532733	5.6055870659025	5.8741619341855	6.32718707518347	6.24430330340228	6.84559767546567	NA
**TC1600001340.mm.2**	Parl	presenilin associated, rhomboid-like	0.391312962910887	7.62634160199732	1.24002548243179	0.258298193752987	0.999692817443324	-4.58265427824604	8.0626137910549	7.84853891016023	7.71457951980835	7.60018189848692	7.46611488413779	7.06602060833571	NA
**TC0200001990.mm.2**	Mrps26	mitochondrial ribosomal protein S26	-0.383245915889046	7.82561676855471	-1.23977884396043	0.25838356386907	0.999692817443324	-4.5826684142024	7.52621757646714	7.5998362217448	7.70848537588355	7.82209211478557	8.34146585721491	7.95560346523229	NA
**TC0700003769.mm.2**	Aqp11	aquaporin 11	0.806082997407378	6.46277724425445	1.23892500749971	0.258679294310362	0.999692817443324	-4.58271734601723	7.11186751934817	6.82428633749544	7.44078879960736	5.78554900214157	6.43162151527465	5.18255029165953	NA
**TC0200005135.mm.2**	Neurl2	neuralized E3 ubiquitin protein ligase 2	0.384753429842119	6.58807122153016	1.23880368913343	0.258721337206799	0.999692817443324	-4.58272429787353	6.51832537139238	6.35063718134655	7.03809995891022	6.67638585915291	6.40195900652193	6.54301995185697	NA
**TC1400000743.mm.2**	Oxa1l	oxidase assembly 1-like	0.58291055254999	8.56666317623507	1.23862852488466	0.258782050801449	0.999692817443324	-4.58273433493977	9.20985992063695	8.79568292392674	9.19760863192685	8.08252002704652	8.36198967772686	7.75231787614653	NA
**TC1600001509.mm.2**	Slc51a	solute carrier family 51, alpha subunit	-0.534599661137561	5.24709023948874	-1.23862379153975	0.258783691594354	0.999692817443324	-4.58273460615973	4.51790032617731	4.54566514697701	5.02412536385597	5.65076295689553	5.38710815101472	6.35697949201191	NA
**TC0700001197.mm.2**	Tmc3	transmembrane channel-like gene family 3	0.446878310574069	6.01656583025416	1.23852352357265	0.258818451146678	0.999692817443324	-4.58274035143859	5.67650295207682	5.57712035286154	6.05893353080285	6.81083061806451	6.08466995876608	5.89133756895314	NA
**TC0300002659.mm.2**	Mov10	Moloney leukemia virus 10	-0.468087902135495	6.37271866043644	-1.23850780854861	0.258823899385242	0.999692817443324	-4.58274125188713	6.20988926740078	6.00896223130002	6.49262022868315	6.04802210114028	6.3718978364684	7.10492029762602	NA
**TC0700000544.mm.2**	Zfp568	zinc finger protein 568	0.474737155415952	4.96374525494973	1.23841445495589	0.258856266163249	0.999692817443324	-4.58274660085667	5.16605015074733	4.79503436710165	5.20847470844862	5.17695595289203	4.30277804648779	5.13317830402096	NA
**TC1700000668.mm.2**	Gpank1	G patch domain and ankyrin repeats 1	-0.493897999017778	6.79545500202066	-1.23833817970609	0.258882714272547	0.999692817443324	-4.5827509711977	7.72792512310067	6.8515714181992	6.29497287008071	6.30774586631354	6.56290678114701	7.0276079532828	NA
**TC0500001698.mm.2**	Amz1	archaelysin family metallopeptidase 1	-0.37077675932938	5.44978481218042	-1.23768125927128	0.259110595057936	0.999692817443324	-4.58278860797497	5.96354599629127	5.78171148278272	5.17427080903131	4.93167812864357	5.41222669693927	5.43527575939436	NA
**TC0800002597.mm.2**	Junb	jun B proto-oncogene	-0.385753335948501	7.75256360742401	-1.23718623482397	0.259282429613133	0.999692817443324	-4.58281696599346	7.36157949428925	7.4117607939429	7.79192351331114	7.69334382889632	7.9184237480235	8.33835026608096	NA
**TC0800002023.mm.2**	Leprotl1	leptin receptor overlapping transcript-like 1	0.412144362235834	8.21781497869307	1.23711272981315	0.259307953297292	0.999692817443324	-4.58282117656689	8.10213377488417	8.14749002099467	8.45151991739049	8.48925748298512	7.75732875782177	8.35915991808217	NA
**TC1100001498.mm.2**	Hoxb4/Mir10a	homeobox B4/microRNA 10a	-0.378961791107653	4.67507075964101	-1.23637678238252	0.259563621170833	0.999692817443324	-4.58286333024932	4.25146359215208	4.2541162052042	4.88685040439686	4.50661018474039	5.05375348493577	5.09763068641679	NA
**TC1300000572.mm.2**	Iars	isoleucine-tRNA synthetase	-0.513460583990424	7.36529587515302	-1.23614801550591	0.259643138963813	0.999692817443324	-4.58287643230382	7.91334361160993	7.84485150963265	7.36311713106427	6.34021234978307	7.20218141501727	7.52806923381092	NA
**TC1800000802.mm.2**	Dym	dymeclin	0.486086189790055	8.14746055312414	1.23569555208246	0.25980047397551	0.999692817443324	-4.582902344229	9.21254869570478	9.07948917484378	8.14840753931926	7.63404137456893	7.00323431326481	7.80704222104326	NA
**TC1800001340.mm.2**	Cep120	centrosomal protein 120	0.388425654916404	7.16461379395562	1.23525084993455	0.259955190221862	0.999692817443324	-4.58292780935204	7.37674580278735	7.18593131333301	7.11416623914515	7.48676223957794	6.81890416997708	7.0051729989132	NA
**TC1600001317.mm.2**	Tbx1	T-box 1	-0.412683651097802	5.83917696013368	-1.23488730118824	0.260081731383143	0.999692817443324	-4.58294862564589	5.54929451399782	5.35460151726819	5.57065177245902	6.08224744121121	6.10225027404226	6.37601624182357	NA
**TC1000002456.mm.2**	Gm9978	predicted gene 9978	0.427693487342317	4.96945158706457	1.23455013824572	0.260199135817186	0.999692817443324	-4.5829679297436	4.30639672811748	4.48157589174088	5.14520862925753	5.79685330934933	5.12470593189319	4.96196903202903	NA
**TC0500001635.mm.2**	Ppp1r35	protein phosphatase 1, regulatory subunit 35	-0.496417326971965	6.63722322052683	-1.23418525681703	0.26032624362443	0.999692817443324	-4.58298881934886	7.37846916589552	7.22669959842633	6.44317894956143	5.92150382288618	6.39735755085213	6.9601598755394	NA
**TC1600000166.mm.2**	Rrn3	RRN3 RNA polymerase I transcription factor homolog (yeast)	-0.966740178774601	6.48353170092537	-1.23356521655479	0.260542359638665	0.999692817443324	-4.58302431331295	7.78749602353972	7.80721123042408	6.59723799915858	4.08926329786103	6.67981657740732	5.94016507716149	NA
**TC0900001142.mm.2**	Bcl2a1a	B cell leukemia/lymphoma 2 related protein A1a	-0.375666969245282	5.40041191304181	-1.23354845963897	0.260548202432181	0.999692817443324	-4.58302527249336	5.78575941033706	5.91956307861752	5.07774159138158	4.89516593402126	5.50337340766632	5.22086805622709	NA
**TC0700004628.mm.2**	Prr14	proline rich 14	0.951110415003515	7.04435085971025	1.23314772776078	0.260687963057004	0.999692817443324	-4.58304820974893	8.57583571152916	8.43805058906733	7.58456714737519	5.99265269646084	6.84199638817696	4.83300262565205	NA
**TC0500002221.mm.2**	Tmem129	transmembrane protein 129	0.370127548809921	7.69955680557108	1.23250472182868	0.260912354919073	0.999692817443324	-4.58308501042836	7.98161449451721	7.74098145523965	7.89693512732816	7.71056486331657	7.3967048368357	7.47054005618919	NA
**TC0900002152.mm.2**	Nlrx1	NLR family member X1	0.413688017246647	5.78867416397258	1.23187546444243	0.261132109893605	0.999692817443324	-4.58312101951453	5.92720420651725	6.24784847338779	5.82721427418941	5.86496989502246	5.16575279401537	5.6990553407032	NA
**TC1400002062.mm.2**	Cdh24	cadherin-like 24	-0.476368615491309	6.15699080209702	-1.23153043341342	0.261252672381783	0.999692817443324	-4.58314076183103	5.9835243268447	5.92436254225514	6.06269664322002	5.97796371302982	6.03521263425084	6.95818495298162	NA
**TC0900001056.mm.2**	Cd109	CD109 antigen	0.521157359256113	6.5453109711342	1.23115760588958	0.261383001515034	0.999692817443324	-4.58316209304481	5.9763594922955	6.20568699432642	6.48777524992108	7.57829177942667	6.49601852335852	6.52773378747701	NA
**TC0800002705.mm.2**	Ces1f	carboxylesterase 1F	0.499711173487011	5.31195302004712	1.23061174139797	0.261573920029717	0.999692817443324	-4.58319332153283	5.15147712233116	5.34518296540032	5.0898076024233	6.09743258733934	5.0548536960624	5.13296414672622	NA
**TC1000002616.mm.2**	Syn3	synapsin III	0.986102717405757	6.89074307661835	1.23034305671585	0.261667937743976	0.999692817443324	-4.58320869147268	8.83787929774508	8.85371602807999	7.1411543827546	5.67137990159367	6.54261902482669	4.29770982471006	NA
**TC1000000580.mm.2**	Tacr2	tachykinin receptor 2	0.73560948071419	5.06585992092115	1.23031231050528	0.261678698262605	0.999692817443324	-4.58321045023556	4.40891615939402	4.94606549614509	4.73510481654363	6.52059359916445	4.77024891047434	5.01423054380536	NA
**TC0400003736.mm.2**	Pdik1l	PDLIM1 interacting kinase 1 like	-0.396212783343102	7.21553231272382	-1.23012664098703	0.261743686730027	0.999692817443324	-4.58322107077299	7.29549740634734	7.08842816882268	7.24805393535546	6.81036743188789	7.5229375012222	7.32790943270735	NA
**TC1700000531.mm.2**	Ubash3a	ubiquitin associated and SH3 domain containing, A	0.584417626695062	6.93652827747861	1.22999303415283	0.261790460689347	0.999692817443324	-4.58322871300247	6.51504413196065	6.44185194634884	6.97574358899551	7.93981083098064	6.39718339607482	7.34953577051119	NA
**TC0600000754.mm.2**	Atoh1	atonal bHLH transcription factor 1	0.643295859990537	4.97623864155704	1.22984288449038	0.261843034652359	0.999692817443324	-4.58323730121691	4.64289391667727	4.26526295434623	4.98050574829387	6.13742760085605	4.42169175705486	5.40964987211398	NA
**TC1200000608.mm.2**	Dact1	dishevelled-binding antagonist of beta-catenin 1	0.402875816738029	6.31557565718607	1.22974881109301	0.261875978490612	0.999692817443324	-4.58324268186086	6.14052211999175	6.01418019330864	6.40711422265064	6.86513740899542	6.08090400371594	6.38559599445406	NA
**TC0700003403.mm.2**	Chrna7	cholinergic receptor, nicotinic, alpha polypeptide 7	0.439586679318582	6.70750421888383	1.22963969467533	0.261914194754548	0.999692817443324	-4.58324892277621	7.02305760030788	7.15971962960398	6.57557565191961	6.89513506909453	6.6352935770134	5.95624378536357	NA
**TC1700000991.mm.2**	Zfp959	zinc finger protein 959	-0.491380346725714	5.44982838527377	-1.22928759675146	0.262037544086546	0.999692817443324	-4.58326906005019	5.84234815969602	6.03028510680055	5.33283866486726	4.58894951098006	5.77029136021705	5.1342575090817	NA
**TC1300001814.mm.2**	F13a1	coagulation factor XIII, A1 subunit	-0.394134919368045	5.68904098433346	-1.22923208610103	0.262056995513138	0.999692817443324	-4.58327223469379	5.09411708500852	5.31610911462029	5.57611830825674	5.89175662556121	5.96108477327306	6.29505999928098	NA
**TC1900001516.mm.2**	Mms19	MMS19 cytosolic iron-sulfur assembly component	0.52539159840848	6.65559358361609	1.22877202333029	0.262218253410721	0.999692817443324	-4.5832985441729	7.44321142717526	7.32377302011434	6.16704087465092	6.94163925096104	5.61807771188291	6.43981921691209	NA
**TC0500000750.mm.2**	Adgrl3	adhesion G protein-coupled receptor L3	-1.02008968915944	7.31596106234725	-1.22862106200984	0.262271185880631	0.999692817443324	-4.5833071766009	8.64294850919025	8.42678220027201	7.3677739957326	5.02515414741857	7.96981876110904	6.46328876036102	NA
**TC1800000276.mm.2**	Gm10549	predicted gene 10549	-0.496414590870867	5.86637219147	-1.22746066803281	0.262678368586619	0.999692817443324	-4.58337352229576	5.11488888310868	5.12161548205257	5.60393205260142	6.3805177483571	6.15747815612656	6.81980082657369	NA
**TC1500001039.mm.2**	Tmprss12	transmembrane (C-terminal) protease, serine 12	0.598332032887989	4.86602605214829	1.22716327086204	0.262782812820896	0.999692817443324	-4.58339052340465	4.63648388796851	4.60529632900398	4.89612986645775	5.67939021438885	4.10179551331407	5.27706050175655	NA

**TC0700003049.mm.2**	Spty2d1	SPT2, Suppressor of Ty, domain containing 1 (S. cerevisiae)	0.562847930508211	5.79005205223994	1.22692701191233	0.262865811092434	0.999692817443324	-4.58340402870181	6.59274700196239	6.28294552067787	6.01207291447521	5.48308491143269	5.7479929681882	4.62146899670328	NA
**TC1600000277.mm.2**	Eif2b5	eukaryotic translation initiation factor 2B, subunit 5 epsilon	-0.473096170328076	5.35305089931427	-1.22685333512025	0.2628916985145	0.999692817443324	-4.5834082401583	5.64725406508335	5.4548732706346	5.38986766016768	4.64512519958807	5.7450807578265	5.2361044425854	NA
**TC1400000392.mm.2**	Lrit1	leucine-rich repeat, immunoglobulin-like and transmembrane domains 1	-0.370966015032614	5.74771370434288	-1.22680031153379	0.262910330485571	0.999692817443324	-4.5834112710113	5.37832264873388	5.14878594146937	5.78323190216551	5.82538890072889	6.05306032939332	6.2974925035663	NA
**TC0700000988.mm.2**	Pcsk6	proprotein convertase subtilisin/kexin type 6	0.463046227145322	6.37488187869112	1.22675085802427	0.26292770898928	0.999692817443324	-4.58341409776632	6.64169239002815	6.24147294562637	6.33264314268634	6.81346605270509	5.80208902420851	6.41792771689227	NA
**TC0700000381.mm.2**	Kcnn4	potassium intermediate/small conductance calcium-activated channel, subfamily N, member 4	0.530412101376215	5.71933245331092	1.22674671351822	0.262929165458836	0.999692817443324	-4.5834143346643	4.99309833131183	5.18703092133854	5.79765186001088	6.80069297497291	5.49540281214986	6.04211782008151	NA
**TC0500002896.mm.2**	Gbp11	guanylate binding protein 11	0.554425300282674	5.72435485507627	1.22664983382757	0.262963213064418	0.999692817443324	-4.58341987220169	5.39269888900989	5.90850283099232	5.54375489038801	6.53313411512235	5.20085701622834	5.76718138871668	NA
**TC0X00001111.mm.2**	Pou3f4	POU domain, class 3, transcription factor 4	0.399922787248811	7.37343973820188	1.22664342227655	0.26296546648729	0.999692817443324	-4.58342023867494	8.06076071717634	8.21050254102952	7.44464506635764	6.93996530639387	6.87633840775765	6.70842639049624	NA
**TC0700003991.mm.2**	Dchs1	dachsous cadherin related 1	-0.423071855944578	6.26471083444099	-1.22662808678972	0.262970856410614	0.999692817443324	-4.583421115223	6.91950420519412	6.78225695945031	5.54689071364061	5.97328935141558	5.92453468697197	6.44178908997337	NA
**TC0100002073.mm.2**	Col19a1	collagen, type XIX, alpha 1	-0.526266053683203	6.26061759788386	-1.22607883127865	0.263163964407106	0.999692817443324	-4.58345250776889	5.32579455827803	5.52408890708686	5.92921133356909	6.90143367371683	6.65733539621502	7.2258417184373	NA
**TC1700002724.mm.2**	Nrxn1	neurexin I	-1.49542907198133	8.21173413959987	-1.22605570206654	0.263172098879774	0.999692817443324	-4.58345382963211	9.82997197569698	9.82659886203906	8.60915415508632	4.70233377286394	8.98912574470772	7.31322032720519	NA
**TC1100003659.mm.2**	Plxdc1	plexin domain containing 1	0.657844107413471	5.59437079933634	1.22548090454349	0.263374322806626	0.999692817443324	-4.58348667793271	6.40227673851494	6.28723076864938	5.0795482133928	6.01665453844753	5.55224966419493	4.22826487281846	NA
**TC0600000565.mm.2**	Gimap9	GTPase, IMAP family member 9	-0.479923518852404	4.7676064275942	-1.22522396302544	0.263464762540229	0.999692817443324	-4.58350136022192	4.53631729779963	4.06547559742988	4.45317525549323	5.06882406082222	4.96216299632042	5.51968335769982	NA
**TC0200002010.mm.2**	Cdc25b	cell division cycle 25B	0.485008680110142	5.48329091482278	1.22519599667775	0.263474607903698	0.999692817443324	-4.58350295824124	4.43898840598057	4.82904134092709	5.78352579545062	6.51734075567403	5.40898248739127	5.9218667035131	NA
**TC1800001221.mm.2**	Fchsd1	FCH and double SH3 domains 1	-0.448897499633418	6.69213871948667	-1.2250726787129	0.263518024928139	0.999692817443324	-4.58351000461379	6.83744378739788	6.88491643620316	6.70570261554768	6.06063593147838	6.5837174693953	7.08041607689759	NA
**TC1000000529.mm.2**	Sowahc	sosondowah ankyrin repeat domain family member C	-0.65110829077504	5.97830104170301	-1.22494166848251	0.263564156930152	0.999692817443324	-4.58351749031784	6.23967261988419	6.76528784265668	6.11147235533446	4.66984224772911	5.8086021580216	6.27492902659205	NA
**TC0600000456.mm.2**	Casp2	caspase 2	0.557944986432368	6.53488193523356	1.22485616183697	0.263594269714846	0.999692817443324	-4.58352237591048	6.67640565714358	6.25388863838295	6.9344432068986	6.76300043747118	6.88217341127773	5.69938026022731	NA
**TC0900000735.mm.2**	Insyn1	inhibitory synaptic factor 1	-0.47440691054242	7.67003261997213	-1.22451820917347	0.263713315067577	0.999692817443324	-4.58354168463419	7.96005956125129	8.11296549337415	7.70895930989982	6.79021911216142	7.74130600998983	7.70668623315625	NA
**TC1700000351.mm.2**	Msrb1	methionine sulfoxide reductase B1	0.376682696291266	7.32632058258747	1.22417812499226	0.263833157865487	0.999692817443324	-4.5835611137288	7.51493863883578	7.62633684102501	7.300025563629	7.48498114049428	7.18113670340134	6.8505046081394	NA
**TC0500000366.mm.2**	Nat8l	N-acetyltransferase 8-like	-0.387132186396952	7.73200443992446	-1.22412784281822	0.263850880847709	0.999692817443324	-4.58356398624169	7.99196846668262	7.74950553266754	7.64080537806502	7.29733875563631	7.9655001918352	7.74690831466003	NA
**TC0700004320.mm.2**	Dctpp1	dCTP pyrophosphatase 1	-0.370322604151539	8.23362078463855	-1.22381041908479	0.263962786943899	0.999692817443324	-4.58358211926337	7.51971679725387	7.75236153234598	8.28530104402737	8.40919954093681	8.6098760663671	8.82526972690016	NA
**TC0600002137.mm.2**	E330009J07Rik	RIKEN cDNA E330009J07 gene	-0.619453213837913	6.06861216616069	-1.22377814508762	0.263974167256878	0.999692817443324	-4.58358396286557	6.9051049969922	6.62167844397705	6.05043177556833	4.77255978859121	6.39159277801449	5.67030521382088	NA
**TC0900002864.mm.2**	U2surp	U2 snRNP-associated SURP domain containing	0.859771340366907	7.71643201751943	1.2235041433797	0.264070801488839	0.999692817443324	-4.58359961427386	8.9243692096137	9.00211212137064	7.96990522435243	7.07592150308059	7.72191131181566	5.60437273488354	NA
**TC0300002549.mm.2**	Phgdh	3-phosphoglycerate dehydrogenase	0.364255077528814	7.16529141726838	1.22343252831951	0.26409606351377	0.999692817443324	-4.58360370488648	8.04365686550617	7.97522097249426	7.01235323364915	6.83833717668458	6.45700687428102	6.66517338099509	NA
**TC0300001530.mm.2**	Usp33	ubiquitin specific peptidase 33	-0.610117541153585	7.41511307251655	-1.22275011530788	0.264336887008401	0.999692817443324	-4.58364268077148	8.44270897980527	8.3948687069143	7.15722966229128	6.059203170745	7.70113607926031	6.73553183608314	NA
**TC0400000543.mm.2**	Plppr1	phospholipid phosphatase related 1	0.61301825105292	7.34273985898776	1.22259650887559	0.264391120688635	0.999692817443324	-4.58365145318081	8.18427204477556	7.99571536258379	8.00070969036682	6.5505344339697	6.57161842709028	6.7535891951404	NA
**TC0800002861.mm.2**	Chtf8	CTF8, chromosome transmission fidelity factor 8	-0.436932657872975	7.2536057152176	-1.22225230343813	0.264512683660719	0.999692817443324	-4.58367110957395	7.90933288410802	7.88112157231777	7.09337529613526	6.33528196343167	7.11156393846202	7.19095863685085	NA
**TC0400003422.mm.2**	Ppih	peptidyl prolyl isomerase H	-0.576570919599102	6.9745247342821	-1.22216929496247	0.264542006926929	0.999692817443324	-4.58367584968435	7.67487587661561	7.68839707514591	6.8124055652566	5.85296124210983	7.37826771806439	6.44024092850024	NA
**TC1100002060.mm.2**	Hexdc	hexosaminidase (glycosyl hydrolase family 20, catalytic domain) containing	-0.414393586633985	6.30539226583688	-1.22212494194842	0.26455767605165	0.999692817443324	-4.58367838238093	6.17570208297557	6.49253823742926	6.35459170836565	5.8130713423086	6.42192291645386	6.57452730748836	NA
**TC1400002347.mm.2**	Fgf17	fibroblast growth factor 17	0.510638198531124	5.50677904826504	1.22188653153556	0.264641915823902	0.999692817443324	-4.583691995948	4.8877559075091	4.89742107975357	5.54590199541292	6.592484854282	5.40938944490895	5.70772100772372	NA
**TC1900001107.mm.2**	Tmem138	transmembrane protein 138	-0.882385098419988	5.72979281599345	-1.2217700345731	0.264683087151853	0.999692817443324	-4.58369864783098	6.5948275840712	6.72988591328411	5.99208661481965	3.65254998606305	5.90157514975445	5.50783164796823	NA
**TC0100003421.mm.2**	Blzf1	basic leucine zipper nuclear factor 1	0.58252876172733	7.84412554619349	1.22162704870949	0.264733627465973	0.999692817443324	-4.58370681197901	8.20639021389167	8.05292723434134	8.63375923646059	7.35148743973069	7.63082883926047	7.18936031347614	NA
**TC0800003093.mm.2**	Chmp1a	charged multivesicular body protein 1A	-0.394395952975896	7.45853472579756	-1.22158983322575	0.264746783146993	0.999692817443324	-4.58370893685181	7.61395548070999	7.67786623924423	7.42564723752654	6.90965012691313	7.51600273755972	7.60808653283174	NA
**TC0500001043.mm.2**	Lrrc8c	leucine rich repeat containing 8 family, member C	-0.61670304929889	6.85372162494644	-1.22131691040135	0.264843278562193	0.999692817443324	-4.58372451925695	7.54995190632309	7.5343625403017	6.90241731159636	5.49988729063169	6.56775957437294	7.06795112645288	NA
**TC1900001693.mm.2**	Gucy2g	guanylate cyclase 2g	-0.418944997858103	5.7365689168176	-1.22122354267147	0.264876296864317	0.999692817443324	-4.58372984983293	5.65948194580331	5.47742052433796	5.29414135019643	5.92816916732764	5.96553929633591	6.09466121690437	NA
**TC0700000527.mm.2**	Ppp1r14a	protein phosphatase 1, regulatory inhibitor subunit 14A	0.36488092564016	7.45126566594355	1.22056447251378	0.265109469045825	0.999692817443324	-4.58376747456387	7.80832285191298	7.73799175203436	7.33792806932142	7.60759255217572	7.05803790081285	7.15772086940397	NA
**TC1900001777.mm.2**	Ndufb8	NADH:ubiquinone oxidoreductase subunit B8	-0.357778852388886	8.69549454599446	-1.22028398534969	0.26520875589072	0.999692817443324	-4.58378348526108	7.99510260471299	8.1076245068998	8.79602971776781	8.88131151202029	9.11920365199683	9.27369528256904	NA
**TC1200000134.mm.2**	Rock2	Rho-associated coiled-coil containing protein kinase 2	-1.18652296160634	6.45878367130805	-1.22004485952929	0.265293426829839	0.999692817443324	-4.58379713420568	8.13892043080175	8.247215978761	6.51383960027948	3.48292024725695	6.87489373179256	5.49491203895655	NA
**TC1000002253.mm.2**	Tspan15	tetraspanin 15	-0.395020292636717	6.40637078206089	-1.21986126607197	0.265358450306543	0.999692817443324	-4.58380761296148	7.14773362844768	7.04398696366331	6.10126320849136	5.62696854899909	6.37222917465813	6.14604316810575	NA
**TC0X00000713.mm.2**	Ikbkg	inhibitor of kappaB kinase gamma	-0.53406109055222	5.05889156766418	-1.21961246340654	0.265446590803032	0.999692817443324	-4.58382181291717	5.53014033820684	5.50529528561129	5.11029018297637	4.01460561755488	4.87109055088323	5.32192743075247	NA
**TC0100001580.mm.2**	Apoa2	apolipoprotein A-II	-0.392550106325509	6.06042379378404	-1.2195948286682	0.265452839014264	0.999692817443324	-4.58382281935813	5.73776940320461	5.94681879901575	5.77704456772638	6.16938260619005	6.23065676993117	6.50087061663628	NA
**TC0800002702.mm.2**	Ces1d	carboxylesterase 1D	0.692911482388753	5.28572538189568	1.21951606050083	0.265480749113668	0.999692817443324	-4.58382731472805	4.63314013216961	5.00249882285707	5.81360213903871	5.91866601152374	4.31548580924061	6.03095937654433	NA
**TC1000000604.mm.2**	Dnajc12	DnaJ heat shock protein family (Hsp40) member C12	-0.390011837394048	6.99874652868639	-1.21944241577657	0.2655068460881	0.999692817443324	-4.58383151762825	7.38786564083491	7.18458193464048	6.45308454855514	6.86691941237229	6.94850531910336	7.15152231661216	NA
**TC1000003049.mm.2**	Tbk1	TANK-binding kinase 1	-0.703472941041329	7.34082065066553	-1.21926413664147	0.265570030746703	0.999692817443324	-4.58384169172714	8.21517857402254	8.13531983625652	7.43797110996918	5.70576869584657	7.50594993716427	7.04473575073413	NA
**TC0600003071.mm.2**	Apobec1	apolipoprotein B mRNA editing enzyme, catalytic polypeptide 1	-0.546237777318793	5.54436431523265	-1.21920938394598	0.265589438467479	0.999692817443324	-4.58384481629408	5.52082684716653	6.16186317662272	4.84658783981923	5.39892231666531	5.22070664530023	6.1172790658219	NA
**TC0400000103.mm.2**	Rbm12b2	RNA binding motif protein 12 B2	0.566436864467318	5.92461303399182	1.21830155879625	0.265911404760356	0.999692817443324	-4.58389661758521	7.03959855536134	7.04508654277881	6.27433637894243	5.02359703843026	5.27878430629331	4.88627538214475	NA
**TC0800002425.mm.2**	Abhd8	abhydrolase domain containing 8	-0.383771779173009	6.560094473002	-1.21821682645394	0.265941472718776	0.999692817443324	-4.58390145195787	7.08272677100845	6.72268958197738	6.33573936236156	6.05806410097851	6.62503636556147	6.53631065612463	NA
**TC1100001748.mm.2**	Mrc2	mannose receptor, C type 2	-0.384410980126656	5.89663116127427	-1.21741081671259	0.266227637528721	0.999692817443324	-4.58394743402565	5.61687100752846	5.73428426573204	5.76173966039728	5.86816520666863	5.97831928346521	6.42040754385401	NA
**TC0200001382.mm.2**	Ambra1	autophagy/beclin 1 regulator 1	-0.619577018683146	7.98915801630716	-1.21736025623943	0.26624559725539	0.999692817443324	-4.58395031817808	9.06690940225614	8.75464328699542	7.8183837658388	6.61873691977374	8.26622881941814	7.41004590356071	NA
**TC0100000678.mm.2**	Sgpp2	sphingosine-1-phosphate phosphatase 2	-0.494030148859321	6.20874528236447	-1.21674921621173	0.266462728582022	0.999692817443324	-4.58398517155849	6.88658589099176	6.68283833314093	5.74989068270185	5.59760290346589	6.66621979289344	5.66933409099294	NA
**TC0100001339.mm.2**	Pdc	phosducin	-0.684848699851778	4.37346891753184	-1.21634734751315	0.266605614154776	0.999692817443324	-4.58400809134724	3.65346234288496	3.57383918060497	3.6226912420577	5.19921604894107	4.73398834935793	5.4576163413444	NA
**TC0300001484.mm.2**	Bcl10	B cell leukemia/lymphoma 10	-0.384047372563474	6.51861252674547	-1.21632278827666	0.266614348389665	0.999692817443324	-4.58400949196848	6.43736079967084	6.39580431266476	6.49297325906996	6.26223439243518	6.55827159887169	6.96503079776039	NA
**TC0400003792.mm.2**	C1qb	complement component 1, q subcomponent, beta polypeptide	-0.782879252256393	7.73510153235664	-1.21616219801592	0.266671466673035	0.999692817443324	-4.58401865029463	8.29131344321008	9.09963261070976	7.74070388959542	5.9862484282582	8.01252229439977	7.28018852796663	NA
**TC0300002920.mm.2**	Fam241a	family with sequence similarity 241, member A	-0.539908597548999	8.07123513563684	-1.21610153238695	0.266693046777384	0.999692817443324	-4.58402210991874	7.46408032216072	7.63336938977174	8.63813118425151	7.4869407691438	8.77519661049553	8.42969253799777	NA
**TC0300002955.mm.2**	Hadh	hydroxyacyl-Coenzyme A dehydrogenase	0.438783899738823	6.4283311215713	1.2155722830079	0.266881375923026	0.999692817443324	-4.58405228983473	6.92610268486278	7.17455173836589	6.32072462300802	6.35272542983038	5.52670039035017	6.26918186301058	NA
**TC0100001724.mm.2**	Tmem63a	transmembrane protein 63a	0.600159047978356	6.66900170811365	1.21537694618928	0.266950913732113	0.999692817443324	-4.58406342782111	8.37198844003522	8.60330680260628	6.63210981502523	5.48740673597333	5.88106041634871	5.03813803869314	NA
**TC0700002715.mm.2**	Spred3	sprouty-related EVH1 domain containing 3	0.399106570033254	7.130765763219	1.21524638876101	0.266997399423996	0.999692817443324	-4.58407087185521	7.13351514985185	7.11538061601472	7.17938216914957	7.48757380760739	7.17421033434617	6.69453250234429	NA
**TC0600000553.mm.2**	Sspo	SCO-spondin	-0.558638251116015	6.30772650018972	-1.21495016630267	0.267102896767455	0.999692817443324	-4.58408776085893	5.43733434694534	5.88398505471453	5.76587792249309	6.93800362613011	6.76568459822142	7.05547345263381	NA
**TC0800002907.mm.2**	Dhodh	dihydroorotate dehydrogenase	-0.404110662960431	7.48247377308246	-1.2147978749809	0.267157148036594	0.999692817443324	-4.58409644325143	7.36851646514487	7.6558534015802	7.16106647146662	7.37005925145781	7.42717835177702	7.91216869706827	NA
**TC1100000368.mm.2**	Insyn2b	inhibitory synaptic factor family member 2B	0.405359748787995	5.71188345325797	1.21478650835623	0.26716119758729	0.999692817443324	-4.58409709127055	5.8337126866799	5.69913968908106	5.92374729039461	5.85083663028682	5.18745426357336	5.77641015953207	NA
**TC1400002102.mm.2**	Adcy4	adenylate cyclase 4	-0.514982397198231	4.77311643201825	-1.21464897937966	0.267210198756745	0.999692817443324	-4.58410493176173	4.50330643368564	4.52079167049626	4.14562008071399	5.14669776605157	4.91231553226841	5.40996710889361	NA
**TC1100003634.mm.2**	Lrrc46	leucine rich repeat containing 46	-0.415477048846237	5.15297244867052	-1.21396720965443	0.267453224925401	0.999692817443324	-4.58414379570587	5.10132023987578	5.4345635878241	5.16736559844504	4.60813278487035	5.2309208106001	5.37553167040777	NA
**TC0300000624.mm.2**	4930579G24Rik	RIKEN cDNA 4930579G24 gene	-0.462985315600805	6.18153777850458	-1.21347120222127	0.267630152442872	0.999692817443324	-4.58417206662173	5.77896999211802	5.66732039294133	5.88060565582095	6.4778771715623	6.31009060250916	6.9743628560757	NA
**TC0300000955.mm.2**	Ankrd35	ankyrin repeat domain 35	0.655951755714229	5.22612350467754	1.21341389764529	0.267650599630812	0.999692817443324	-4.58417533260495	5.16443516686235	5.02408058492498	5.0495206878322	6.19054370602097	4.37776382486044	5.55039705756427	NA
**TC0100003395.mm.2**	Myocos	myocilin opposite strand	-0.471457520130341	5.35296618697873	-1.21337352227281	0.267665007011332	0.999692817443324	-4.5841776337098	5.02746223756579	4.58772799484959	5.01393753736758	5.76590838723057	5.68739017543701	6.03537078942182	NA
**TC0200000507.mm.2**	Rapgef1	Rap guanine nucleotide exchange factor (GEF) 1	0.357028848764994	7.5414341319954	1.21323556758875	0.26771423919669	0.999692817443324	-4.58418549597322	7.84828021635351	7.99741863924834	7.44346655569835	7.61501526125193	7.18129051038709	7.1631336090332	NA
**TC0500001766.mm.2**	Bud31	BUD31 homolog	0.429232688174094	6.55943540606462	1.21314672537459	0.267745948610861	0.999692817443324	-4.58419055910707	6.94619391487967	7.04269837036916	6.79524028770128	6.31785247604224	6.4061836826892	5.84844370470613	NA
**TC1100002124.mm.2**	Gas2l1	growth arrest-specific 2 like 1	-0.360009276204286	8.20767562015233	-1.21296551556187	0.267810635676284	0.999692817443324	-4.58420088597247	7.93845700861174	8.15102072350398	8.17808406475447	8.04019465344036	8.42737071185898	8.51092655874442	NA
**TC0400003002.mm.2**	Ifna9/Ifna15/Ifna6	interferon alpha 9/interferon alpha 15/interferon alpha 6	0.408228840372099	5.55149928315411	1.21291742098441	0.267827806401843	0.999692817443324	-4.58420362673671	5.23920416571675	5.58844477830216	5.69020611210248	5.9586961057225	5.19202210225569	5.6404224348251	NA
**TC1200000874.mm.2**	Samd15	sterile alpha motif domain containing 15	-0.433218106980542	5.74220955381697	-1.21288175417881	0.26784054077271	0.999692817443324	-4.58420565926067	5.75490931182713	6.10964240066423	5.18093202276084	5.68020267546384	5.63799021983544	6.08958069235032	NA
**TC0600002572.mm.2**	Dguok	deoxyguanosine kinase	-0.350086974791353	7.70431184469296	-1.2125499192708	0.267959042960287	0.999692817443324	-4.58422456856273	7.52224475898472	7.5746908393043	7.57954477376182	7.63483598638121	7.99086962463833	7.92368508508741	NA
**TC1100003303.mm.2**	Nlk	nemo like kinase	-0.394174678075075	8.80862963494846	-1.21194314787121	0.268175844229774	0.999692817443324	-4.58425914119246	9.05473747699456	9.29387541744567	8.51990210784865	8.33750567170154	9.05105628821756	8.59470084748278	NA
**TC0400003381.mm.2**	Ipo13	importin 13	0.423109267685183	5.65474438517183	1.21193926466576	0.268177232193969	0.999692817443324	-4.5842593624345	6.69688172806949	6.71468441381564	5.46871373939098	5.21284561286714	4.58600466969766	5.24933614719009	NA
**TC0400001599.mm.2**	Fgr	FGR proto-oncogene, Src family tyrosine kinase	0.82433700562564	4.84624853892561	1.2115980686565	0.268299209000872	0.999692817443324	-4.58427880099988	3.85608743266874	4.27428699171017	4.71167373022481	6.58622167998821	4.27620459097455	5.37301680798719	NA
**TC1100003644.mm.2**	Gpr179	G protein-coupled receptor 179	0.360035011559641	6.49498779447364	1.21155676688337	0.26831397751108	0.999692817443324	-4.58428115393585	6.12745855777283	5.96922737654046	6.9050335697395	6.89162185808443	6.42788430211546	6.64870110258918	NA
**TC0600000577.mm.2**	Gpnmb	glycoprotein (transmembrane) nmb	-0.578394071199821	6.45306683180605	-1.21144651955049	0.268353402682565	0.999692817443324	-4.58428743454874	6.0305883120578	7.20223422433911	6.20383915371992	5.96055600229995	6.32967679748594	6.99150650093357	NA
**TC0X00003435.mm.2**	Slc10a3	solute carrier family 10 (sodium/bile acid cotransporter family), member 3	-0.464538370412475	5.38293412153687	-1.21109342716359	0.268479704186502	0.999692817443324	-4.58430754859359	5.347440796842	5.25011943584669	5.3381575081766	5.04732636967718	6.08634685825619	5.22821376042255	NA
**TC0100002936.mm.2**	Tsn	translin	0.45003727039055	7.19681192824985	1.21108855035119	0.268481448983066	0.999692817443324	-4.58430782639179	8.10444502709864	7.90460495898656	7.35668617591586	6.67926188618163	6.7999541633526	6.33591935796379	NA
**TC1500000983.mm.2**	Ccdc65	coiled-coil domain containing 65	0.42876035962778	6.74474107018086	1.21105727928599	0.268492637186823	0.999692817443324	-4.58430960768029	6.96605997878627	6.6873708680138	6.66775360307942	7.16851454369091	6.25386162328167	6.7248858042331	NA
**TC0400003650.mm.2**	Ptpru	protein tyrosine phosphatase, receptor type, U	0.359306571307068	6.57520564788151	1.21036853134936	0.268739159454441	0.999692817443324	-4.58434883749636	6.15804876038685	6.17111442103404	6.88524345004133	7.03509847419983	6.49020653863894	6.71152224298808	NA
**TC1500001060.mm.2**	Ankrd33	ankyrin repeat domain 33	0.629493481604408	5.48317025712249	1.21019327250531	0.268801920404101	0.999692817443324	-4.58435881893392	4.7543503414395	4.91054280045365	5.38028384994742	6.86627383207787	5.27843727076948	5.709133448047	NA
**TC0600002279.mm.2**	Hoxa5	homeobox A5	0.4426589816198	5.1146095870499	1.20982617554601	0.268933420021148	0.999692817443324	-4.58437972474484	4.64847274658052	4.60839037154809	5.21053307668664	5.94752310701856	5.30610337171236	4.96663484875324	NA
**TC0100003747.mm.2**	Iars2	isoleucine-tRNA synthetase 2, mitochondrial	-0.640356959269615	6.49728086149967	-1.2094525708868	0.269067307247646	0.999692817443324	-4.58440099935939	7.14411564889043	7.40426683020398	6.45505171272197	5.12224267296022	6.84720219526613	6.01080610895529	NA
**TC1500000974.mm.2**	Olfr282	olfactory receptor 282	-0.397591313363254	5.09999321668343	-1.20940063655844	0.269085923257083	0.999692817443324	-4.58440395657344	5.04552138864953	4.77701348984158	5.02101516675597	4.97010573068553	5.14138748927444	5.64491603489357	NA
**TC1700000998.mm.2**	Hdgfl2	HDGF like 2	-0.368938125266074	8.10470486929707	-1.20916896283375	0.26916898077835	0.999692817443324	-4.58441714797366	8.29527368136224	8.00978093547914	7.88615975169885	7.90648942250558	8.14946251805519	8.38106290668139	NA
**TC1600001592.mm.2**	Gtf2e1	general transcription factor II E, polypeptide 1 (alpha subunit)	-0.572655923853547	6.09207404305208	-1.20911513499518	0.269188281773519	0.999692817443324	-4.58442021280677	6.37287265921239	6.49867697353686	6.30368723328255	4.96410415564554	6.26325464750291	6.14984858913227	NA
**TC0200003347.mm.2**	Gapvd1	GTPase activating protein and VPS9 domains 1	0.623529108246486	6.86951781858303	1.2090980438273	0.269194410384165	0.999692817443324	-4.58442118593057	7.89096156537328	8.05911473870426	7.19718366391626	6.05986074804055	6.5141199991723	5.49586619629153	NA
**TC0600001126.mm.2**	Tafa1	TAFA chemokine like family member 1	-0.484322775501816	7.70043702896039	-1.20897621762218	0.269238098704258	0.999692817443324	-4.58442812226688	8.10543563322225	8.60754822151712	7.56077439725453	6.69972198675513	7.56782840775059	7.66131352726271	NA
**TC0700002185.mm.2**	Rfpl4	ret finger protein-like 4	0.483533509607436	6.88011256717362	1.20831551367596	0.269475140506455	0.999692817443324	-4.58446573693714	6.02857270506267	6.24129204586203	7.03624543264074	7.95269340302521	7.17876771828424	6.84310409816684	NA

**TC1900001023.mm.2**	Gpr137	G protein-coupled receptor 137	-0.628905823239838	7.30664604779111	-1.20826842341269	0.269492041959855	0.999692817443324	-4.58446841762408	8.15513448573837	7.86820220458594	7.37729084037877	5.90207313459256	7.47110036226851	7.06607525918249	NA
**TC0700000855.mm.2**	Fut1	fucosyltransferase 1	0.647340659258913	6.1577138248276	1.20743922849936	0.269789801758302	0.999692817443324	-4.58451561607113	5.72087312587737	5.41559045340364	6.27115403777497	7.28109630632623	5.51197755109206	6.74559147449131	NA
**TC0400000394.mm.2**	Gm13304/Gm10591/Gm21541/Ccl21b/Ccl21c/Ccl21a	predicted gene 13304/predicted gene 10591/predicted gene, 21541/chemokine (C-C motif) ligand 21B (leucine)/chemokine (C-C motif) ligand 21C (leucine)/chemokine (C-C motif) ligand 21A (serine)	-0.534401042892615	4.63344334450576	-1.20740352659474	0.269802628443536	0.999692817443324	-4.58451764804949	4.24584927658787	4.44639716874234	3.94885991701512	5.07094585094444	5.24332090422846	4.84528694951634	NA
**TC0100001560.mm.2**	Rgs5	regulator of G-protein signaling 5	0.433677986076342	6.59166346022374	1.2073261784793	0.269830419221739	0.999692817443324	-4.58452205026959	7.1223371452017	7.03522851515307	6.3710628200391	6.75882271653108	6.46283233697665	5.79969722744084	NA
**TC1400000747.mm.2**	Lrp10	low-density lipoprotein receptor-related protein 10	-0.372389504314575	7.10875130992863	-1.20729383398717	0.269842041155543	0.999692817443324	-4.58452389111297	7.31603173001644	7.55864770355553	6.67495523792475	6.84156947076059	6.99350730870602	7.26779640860847	NA
**TC1000002540.mm.2**	Fzr1	fizzy and cell division cycle 20 related 1	0.460378580120206	8.11998476344806	1.20692685118552	0.269973934349608	0.999692817443324	-4.58454477648133	8.74582726182709	8.62319506008484	8.43991515426074	7.69590655524768	7.85297890362869	7.36208564563931	NA
**TC1700000621.mm.2**	Tap1	transporter 1, ATP-binding cassette, sub-family B (MDR/TAP)	0.601029020426714	6.0425385968251	1.20682002278932	0.270012338695435	0.999692817443324	-4.58455085586142	5.73381165908745	6.02313294049375	5.72154895905121	7.12862355206019	5.951204212445	5.69691025781298	NA
**TC0X00000695.mm.2**	Ssr4	signal sequence receptor, delta	-0.481163049386016	6.85550329551279	-1.20677308439722	0.270029214319263	0.999692817443324	-4.5845535269793	7.69260242809208	7.30302777620448	6.5949294359621	5.99260229904197	6.43276572981406	7.11709210396204	NA
**TC1500001262.mm.2**	Hnrnpa1l2-ps2	heterogeneous nuclear ribonucleoprotein A1-like 2, pseudogene 2	0.36911542875936	7.77600176755935	1.20676378026623	0.270032559513413	0.999692817443324	-4.584554056445	8.42446550420052	8.58300137499391	7.6864815970543	7.5069056947859	7.37258711395602	7.08256932036545	NA
**TC0800002238.mm.2**	Wdr17	WD repeat domain 17	0.586329812125642	6.34035465257388	1.20675063217711	0.270037286819198	0.999692817443324	-4.58455480465503	7.61793373924781	7.66456191138505	5.76201013148748	6.20413581304336	6.06322720828066	4.73025911199889	NA
**TC0800002561.mm.2**	Gm10644	predicted gene 10644	0.511413940705213	6.2943797969685	1.20625505716464	0.27021551896978	0.999692817443324	-4.58458300436498	5.73536516967751	5.37503580769684	6.39753717727019	7.44181566565336	6.32212674282331	6.49439821868981	NA
**TC0700004078.mm.2**	BC051019	cDNA sequence BC051019	-0.521245608640504	5.56309528117862	-1.2061207546006	0.270263837801121	0.999692817443324	-4.58459064602595	4.89049690601922	4.47406848164226	5.2116011434761	6.27415639758851	6.16682013243598	6.36142862590963	NA
**TC0500001112.mm.2**	Idua	iduronidase, alpha-L	-0.371968819168593	6.56919226060764	-1.20593907088382	0.270329214969938	0.999692817443324	-4.58460098323995	6.84616057942755	6.67150851260359	6.34850061070775	6.22827280593099	6.83129467924945	6.48941637572648	NA
**TC0100001086.mm.2**	C1ql2	complement component 1, q subcomponent-like 2	0.467820822880471	6.05513117960401	1.20556089145115	0.270465342564327	0.999692817443324	-4.5846224990202	6.47910153167697	5.81009414445528	6.4541190415415	6.03449748208487	6.02712843042708	5.52584644743834	NA
**TC0900000705.mm.2**	Cox5a	cytochrome c oxidase subunit 5A	-0.706924678537362	9.16467318238451	-1.20523487580662	0.270582740497703	0.999692817443324	-4.58464104552108	9.42798285175911	9.29002741420342	9.49418486329123	7.93390487234368	9.89780131390886	8.94413777880077	NA
**TC0200004897.mm.2**	Nrsn2	neurensin 2	0.55959687927784	8.25057631506247	1.2051052251633	0.270629439660633	0.999692817443324	-4.58464842074352	9.39464343424725	8.69210701004023	8.63670697568758	7.63124362663392	7.80183245072155	7.34692439304427	NA
**TC0700004496.mm.2**	Olfr522	olfactory receptor 522	0.451427502828971	5.40926953974664	1.2050686201371	0.270642625754191	0.999692817443324	-4.58465050299288	5.26590326966937	5.23881270556647	5.36375410884593	6.06312402560505	5.01041504795672	5.51360808083633	NA
**TC0700001280.mm.2**	Sytl2	synaptotagmin-like 2	-0.513849018101615	6.92737695168128	-1.20503328288804	0.270655355681215	0.999692817443324	-4.58465251310879	7.53285053561814	7.84918878662424	6.6586003707575	5.91866180506354	7.19910841434921	6.40585179767506	NA
**TC0300002702.mm.2**	Prok1	prokineticin 1	-0.377325062263452	6.10096598817426	-1.20476003646348	0.270753807507983	0.999692817443324	-4.58466805583468	5.54826698404121	5.76140894169312	5.93928878585878	6.33144615353337	6.50063854170496	6.5247465222141	NA
**TC1300002017.mm.2**	Sptlc1	serine palmitoyltransferase, long chain base subunit 1	0.366433146012295	5.46805778659551	1.20473627713734	0.270762369536324	0.999692817443324	-4.58466940725901	6.17500819786042	6.20499681844628	5.41688630857241	5.16371768907306	4.79830199115434	5.04943571446653	NA
**TC0900003050.mm.2**	Rnf123	ring finger protein 123	0.404747482680042	6.54152682399863	1.2039714710495	0.271038102282045	0.999692817443324	-4.58471290521441	7.65379648518931	7.36551809919726	6.1711295126062	6.34854114987646	6.11831710823772	5.59185858888486	NA
**TC0100000659.mm.2**	Gmppa	GDP-mannose pyrophosphorylase A	0.648947451897286	6.56909340347727	1.20393320763778	0.271051903540474	0.999692817443324	-4.58471508122148	7.85935753400076	7.9344166087445	6.69875316956111	5.76058742139536	6.24953514557072	4.91191054159117	NA
**TC1000001022.mm.2**	Polr3b	polymerase (RNA) III (DNA directed) polypeptide B	0.445263287762477	6.03965581404483	1.20373671002672	0.27112278785509	0.999692817443324	-4.58472625556233	6.37412605716017	6.16904036578357	5.78840519460692	6.50424232381819	5.51164906784631	5.89047187505384	NA
**TC1700001976.mm.2**	Ddr1	discoidin domain receptor family, member 1	-0.457223829166791	5.83921696849266	-1.2032656917845	0.271292766801232	0.999692817443324	-4.58475303912169	6.54092602467114	6.85081362005289	5.46540070916762	4.89915654478156	5.33185362758087	5.94715128470189	NA
**TC0900000255.mm.2**	Ncapd3	non-SMC condensin II complex, subunit D3	-0.354751291581866	7.26724436978504	-1.20320870877654	0.271313336735171	0.999692817443324	-4.58475627915111	7.82249029698386	7.74460938807243	6.83359919742577	6.82983277781934	7.29844954468383	7.07448501372502	NA
**TC1400002027.mm.2**	Chd8	chromodomain helicase DNA binding protein 8	-0.492757497962976	5.46129612800202	-1.20292756484337	0.27141484457	0.999692817443324	-4.58477226423796	6.28270555474939	6.32867943187046	5.30704866298505	4.2783897297481	5.38044363594925	5.19050975270986	NA
**TC0100003786.mm.2**	Smyd2	SET and MYND domain containing 2	0.393660708588933	7.65131667388836	1.20282868092109	0.271450554596206	0.999692817443324	-4.58477788625961	8.36064984203489	8.15769934388433	7.69498102083613	7.39345511645827	7.36486205845844	6.93625266165809	NA
**TC1900001214.mm.2**	Foxb2	forkhead box B2	0.618810263924654	5.71659929431118	1.20281310112574	0.27145618130494	0.999692817443324	-4.58477877203318	5.02799224083237	5.80873176104816	5.71783789616614	6.6324082497518	5.0829342383844	6.02969137968423	NA
**TC0400001688.mm.2**	Luzp1	leucine zipper protein 1	0.458971278331492	6.00717232523408	1.20237278678227	0.271615243816526	0.999692817443324	-4.58480380431445	6.34949258070804	6.53469646893803	6.0558678282804	5.9825259009303	5.99970728794524	5.12074388460248	NA
**TC0600001097.mm.2**	Nr2c2	nuclear receptor subfamily 2, group C, member 2	0.453786960096483	7.87057479045022	1.20219973396348	0.27167778048866	0.999692817443324	-4.58481364181579	8.77162769188021	8.53431390222358	7.6073649829065	7.80517555148876	7.66334800557317	6.84161860862912	NA
**TC0600002217.mm.2**	Zfp746	zinc finger protein 746	0.448143712303042	6.92258744999995	1.20148334036516	0.271936796637598	0.999692817443324	-4.58485436222202	6.93637262559631	6.68145408111329	7.08583850711158	7.3211542018365	6.36574791009503	7.14495737424697	NA
**TC0400001506.mm.2**	Trim62	tripartite motif-containing 62	-0.559527565427253	6.60380563453158	-1.20131962643529	0.271996017874437	0.999692817443324	-4.58486366688676	6.95708520269289	6.76361521129117	6.84396879027585	5.54757034089961	6.80577010375828	6.70482415827167	NA
**TC1000000870.mm.2**	Dot1l	DOT1-like, histone H3 methyltransferase (S. cerevisiae)	0.393220376736947	7.05380696299676	1.20031485535968	0.272359720639927	0.999692817443324	-4.58492076501936	6.93251022022188	6.726267822221	7.27808457902815	7.44716766547763	7.21808777896795	6.72072371206393	NA
**TC0200004364.mm.2**	Scg5	secretogranin V	-0.587372849278321	9.74934991192392	-1.20020966151377	0.272397822215897	0.999692817443324	-4.58492674208071	10.748614805535	10.5696186803228	9.59162575275324	8.40993439081134	9.97225376240087	9.20405207972035	NA
**TC1500000821.mm.2**	Ppara	peroxisome proliferator activated receptor alpha	0.480947976175546	5.82591554497831	1.20014406095003	0.272421585266864	0.999692817443324	-4.58493046939577	5.46849262989377	5.47587822259896	5.80611288037541	6.6803963044887	6.00687003218027	5.51774320033275	NA
**TC1700001458.mm.2**	Acat3	acetyl-Coenzyme A acetyltransferase 3	0.383724565070398	6.39208947433847	1.19984060480975	0.27253153175308	0.999692817443324	-4.58494771051016	6.94979114584716	6.78751801204769	6.15528581558013	6.53605259355824	6.10543653408174	5.81845274491584	NA
**TC0500000734.mm.2**	Srp72	signal recognition particle 72	0.809081008219604	8.81756665255457	1.19968834579539	0.272586711606301	0.999692817443324	-4.58495636076254	9.72608637257469	9.67104468137027	9.54094242961838	8.02227300929244	8.75969591114928	7.18535751132233	NA
**TC1000003095.mm.2**	March9	membrane-associated ring finger (C3HC4) 9	0.420634456876345	5.9141265027147	1.19958192965518	0.272625283295251	0.999692817443324	-4.58496240636852	6.00327536334636	6.04621397116752	6.34827434308713	5.78999495467637	5.421523089489	5.87547729452181	NA
**TC0800000036.mm.2**	Cers4	ceramide synthase 4	0.378235346558905	7.4563657141444	1.19932309381977	0.272719120571035	0.999692817443324	-4.58497711044538	7.9611697099969	8.01734072697172	7.58414194438168	7.17393532612611	7.10802689928187	6.89357967810811	NA
**TC1100003766.mm.2**	Hap1	huntingtin-associated protein 1	0.473029885103132	8.76181092621236	1.19915822161951	0.272778907001001	0.999692817443324	-4.58498647611354	9.78858517145777	8.97150374227347	8.94944060993365	8.42897759694092	8.20033515345277	8.23202328321555	NA
**TC0100002751.mm.2**	Nppc	natriuretic peptide type C	-0.423051398526535	7.08582744436411	-1.19914773500155	0.272782710066059	0.999692817443324	-4.5849870717999	6.39835601698685	6.85052584162162	6.81983036886557	7.39015963639601	7.47997116768586	7.57612163462878	NA
**TC1100000053.mm.2**	Nipsnap1	nipsnap homolog 1	-0.351093894919978	8.08317645174889	-1.19875322116653	0.272925816820538	0.999692817443324	-4.58500948084559	8.99907449198167	8.9193683959757	7.38764864383409	7.55156537251393	7.77531604368456	7.86608576250342	NA
**TC1400001961.mm.2**	Slc35f4	solute carrier family 35, member F4	0.393841772946963	6.58696478028423	1.19800984164084	0.273195645575899	0.999692817443324	-4.58505170027708	6.74278225126777	6.69866949326657	6.63438452684296	6.79962571468954	6.59654254018066	6.0497841554579	NA
**TC1000003225.mm.2**	Ddt	D-dopachrome tautomerase	0.389055811431045	7.68668675520904	1.19787082186183	0.273246131590589	0.999692817443324	-4.58505959491617	8.07588408057196	7.80815386794148	7.94860379665894	7.5584933061425	7.49778523739046	7.2312002425489	NA
**TC0100001358.mm.2**	Edem3	ER degradation enhancer, mannosidase alpha-like 3	-0.544277148377773	6.61632629213344	-1.19679944738186	0.273635475336562	0.999692817443324	-4.58512042712388	7.54789606000575	7.299567886569	6.53873395560182	5.34223579913336	6.64674692940313	6.3227771220876	NA
**TC1400002161.mm.2**	Phf11a	PHD finger protein 11A	-0.393551626135863	4.98281984993291	-1.19674554934292	0.273655074667747	0.999692817443324	-4.58512348701653	4.26840940431136	4.43461378676623	4.82759305633166	5.37580327179241	5.48005269595939	5.5104468844364	NA
**TC1500001827.mm.2**	Pvalb	parvalbumin	-0.638789714977977	6.72952381527568	-1.19603203442743	0.273914647836744	0.999692817443324	-4.58516399082928	6.91380750633097	6.88817291034037	5.69081076871042	6.95798075380297	7.46333081389064	6.46304013857871	NA
**TC0800001358.mm.2**	Nudt7	nudix (nucleoside diphosphate linked moiety X)-type motif 7	0.45867745400027	5.69198576421325	1.19545799675342	0.274123631928158	0.999692817443324	-4.58519657190172	5.74741423878773	5.60483179769894	5.55562153476838	6.30289019362831	5.21916641460651	5.72199040578964	NA
**TC0800002535.mm.2**	Usp38	ubiquitin specific peptidase 38	0.37522657208244	6.64140281485051	1.19504153419389	0.274275334130136	0.999692817443324	-4.58522020652076	7.3203474127825	7.13094117412194	6.69299401470865	6.3807967084731	6.29661033891058	6.0267272401063	NA
**TC1100000526.mm.2**	Gfpt2	glutamine fructose-6-phosphate transaminase 2	0.418662561043678	6.47094402348325	1.19502648443795	0.274280817546941	0.999692817443324	-4.58522106056284	6.11267971013696	6.11422663644721	6.55160479822799	7.16643665997335	6.25411379530624	6.62660254080773	NA
**TC1500001838.mm.2**	Rac2	Rac family small GTPase 2	-0.367055639040271	6.07013482967111	-1.19500311647771	0.274289331907412	0.999692817443324	-4.58522238663934	6.07858904739958	6.39947738332164	5.86825499017767	5.73606064443478	6.08090894842639	6.2575179642666	NA
**TC0200004549.mm.2**	1810024B03Rik	RIKEN cDNA 1810024B03 gene	-0.358250210662066	6.47649802457299	-1.19478751528908	0.274367899059433	0.999692817443324	-4.58523462114059	5.895153467211	5.97343769280904	6.58481356473602	6.55213471831087	6.77896107090713	7.07448763346389	NA
**TC0200005349.mm.2**	Gm14308/Gm14430/Gm14432/Gm14434/Gm2026/Gm4724	predicted gene 14308/predicted gene 14430/predicted gene 14432/predicted gene 14434/predicted gene 2026/predicted gene 4724	-0.898755679817448	9.19884143953957	-1.19472991968582	0.274388890691978	0.999692817443324	-4.58523788935073	10.1696523484437	10.8099064387952	9.05325533702561	7.15473390815616	9.74474575520262	8.26075484961405	NA
**TC1000000875.mm.2**	Tmprss9	transmembrane protease, serine 9	-0.443923442035898	5.82643014473414	-1.19470828175486	0.274396777332625	0.999692817443324	-4.58523911716358	5.46027877953419	5.40652991098175	5.3855522715291	6.21641037537945	6.17290100665142	6.31690852432892	NA
**TC1100002421.mm.2**	Rps27a	ribosomal protein S27A	-0.48983707721756	10.5539651241479	-1.1944177573313	0.274502686986313	0.999692817443324	-4.58525560192002	10.8359310985307	10.7560041597905	10.704507219055	9.67158344701062	10.7615471428403	10.5942176776604	NA
**TC1500000593.mm.2**	Hsf1	heat shock factor 1	0.372077854084405	7.69445362298581	1.19440052738165	0.27450896919554	0.999692817443324	-4.58525657953436	7.5149676187984	7.52956758871178	7.997577023303	7.93559409598376	7.80797388171275	7.38104152940519	NA
**TC0400003606.mm.2**	Iqcc	IQ motif containing C	-0.343483460386144	7.47161018731128	-1.19437694292755	0.274517568517856	0.999692817443324	-4.58525791769182	7.3142708353495	7.27519969541648	7.37955220344162	7.3970596327231	7.72762099191639	7.73595776502062	NA
**TC0400003785.mm.2**	Kdm1a	lysine (K)-specific demethylase 1A	-0.890553282426335	6.94132227773219	-1.1942725021274	0.274555652292858	0.999692817443324	-4.58526384346171	7.68505031206435	7.67799896795468	7.31278317098699	4.93910573977372	7.35390519628312	6.67909027933026	NA
**TC1700001358.mm.2**	Nox3	NADPH oxidase 3	0.955727220135938	4.59884084251936	1.1940901641984	0.2746221516051	0.999692817443324	-4.58527418860182	4.36461476633412	4.06053079493932	4.29866005696357	6.24101691009374	3.40414901333312	5.22407351345232	NA
**TC0100001671.mm.2**	Zbtb18	zinc finger and BTB domain containing 18	-0.615981394102898	7.91394889560322	-1.19408973995827	0.274622306343003	0.999692817443324	-4.585274212671	8.57278515579402	9.28104616329483	7.4793722482113	6.71957738495105	8.28154619176855	7.14936622959959	NA
**TC0400001240.mm.2**	Kncn	kinocilin	-0.410827325628053	6.07317604689852	-1.19407069824865	0.274629251717511	0.999692817443324	-4.58527529299631	5.72751313302532	6.07514310637012	5.74029667941897	6.16707601595081	6.1659668962064	6.56306045041948	NA
**TC0200002721.mm.2**	Zfp973/Zfp965	zinc finger protein 973/zinc finger protein 965	-1.15675995055205	8.22563047373388	-1.19401889374353	0.274648147924351	0.999692817443324	-4.58527823208286	9.48069911099944	9.90214407334368	8.48382128747759	5.34488859100042	8.56582108252578	7.57640869705633	NA
**TC1400000910.mm.2**	Pinx1	PIN2/TERF1 interacting, telomerase inhibitor 1	0.401766003975548	7.37700565431751	1.19324425977562	0.274930835223561	0.999692817443324	-4.58532217586151	7.76746980760938	7.79924492015225	7.51470895731714	7.23471664573012	7.25440003511648	6.69149355997968	NA
**TC1100001684.mm.2**	BC030867	cDNA sequence BC030867	0.502024332896777	5.30100012600357	1.19315106744754	0.27496486057829	0.999692817443324	-4.58532746195421	4.49297159490934	5.28992396211203	5.38124542632682	6.13233150606997	5.19179830573056	5.31772996087268	NA
**TC1100001632.mm.2**	Stat5a	signal transducer and activator of transcription 5A	0.43276441072579	6.96467852877248	1.19230302983922	0.275274651230283	0.999692817443324	-4.58537555911585	6.38618768338861	6.19966370140034	7.18800528626287	7.84586901838588	6.88104754401449	7.28729793918268	NA
**TC0300002696.mm.2**	Olfr266	olfactory receptor 266	-0.894702082790429	4.20029568023579	-1.19205038675883	0.275366999824676	0.999692817443324	-4.58538988603629	3.3326706388085	3.57734382521885	3.03184889490746	5.21932883099579	4.4418098417513	5.59877204973281	NA
**TC1200002434.mm.2**	Akt1	thymoma viral proto-oncogene 1	-0.531695057917186	8.59681528369867	-1.19178850890688	0.275462751789537	0.999692817443324	-4.58540473570035	9.58642304718491	9.43472536634653	8.48062005762101	7.26755652879208	8.43546744822754	8.37609925401992	NA
**TC1500001656.mm.2**	Sla	src-like adaptor	-0.613978013151938	5.59214548892966	-1.19168439430455	0.275500827692878	0.999692817443324	-4.58541063920442	6.08507807908462	6.44003226555534	5.54052055311439	4.35938272820267	5.10808195259887	6.01977735502206	NA
**TC1600000192.mm.2**	Cebpd	CCAAT/enhancer binding protein (C/EBP), delta	-0.350728682980841	6.41990558657007	-1.19164307354015	0.275515940409495	0.999692817443324	-4.58541298213144	6.09977992004737	6.14470505322233	6.40256869292679	6.38417689716773	6.63231188586331	6.85589107019289	NA
**TC0500003714.mm.2**	Smok3a	sperm motility kinase 3A	0.442763808399064	5.34828668915311	1.19135385878928	0.275621737952956	0.999692817443324	-4.58542938021515	4.82263507875026	4.97041355819103	5.69755854920062	5.89354100818712	4.94831068786404	5.75726125272558	NA
**TC0200005480.mm.2**	Capn3	calpain 3	-0.493225219378293	5.5019435410988	-1.19117275640921	0.275688004520329	0.999692817443324	-4.58543964787649	6.07813088809351	6.53866648696867	5.2733218754565	4.43088484093051	5.58065739534085	5.10999975980275	NA
**TC1900000961.mm.2**	Sart1	squamous cell carcinoma antigen recognized by T cells 1	0.34367929991515	6.47878684495821	1.19115040746995	0.275696183084186	0.999692817443324	-4.58544091492531	6.72600582732098	6.72949963622565	6.57217162195785	6.4801154810586	6.17238607604485	6.1925424271413	NA
**TC1700000403.mm.2**	Mrpl28	mitochondrial ribosomal protein L28	-0.377152656713486	8.21825514579005	-1.19067884594125	0.275868798441583	0.999692817443324	-4.58546764795674	7.79227451281002	7.8818464778296	8.00189538091063	8.43865690442622	8.68381502223844	8.51104257652538	NA
**TC1800000672.mm.2**	Malt1	MALT1 paracaspase	-0.484434610639621	5.84791405240366	-1.19066283039444	0.275874662552763	0.999692817443324	-4.58546855583025	5.48526237566701	5.1743121909924	5.41901530105124	6.31050496219043	6.13955873275342	6.55883075176749	NA
**TC0900002452.mm.2**	Paqr5	progestin and adipoQ receptor family member V	0.612053032247283	6.30632692231118	1.19047894536761	0.275941999856792	0.999692817443324	-4.58547897946437	6.09104990458819	5.90422935773016	6.1070051026658	7.42638906535583	5.77978028004569	6.52950782348138	NA
**TC1600000487.mm.2**	Mylk	myosin, light polypeptide kinase	-0.436194326215338	5.61833742152846	-1.19045040522248	0.27595245229309	0.999692817443324	-4.58548059723709	5.55042712461512	5.49860247107161	5.06156475647198	5.83273838405468	6.01329531613215	5.75339647682519	NA
**TC1500000418.mm.2**	Fer1l6	fer-1-like 6 (C. elegans)	-0.42377182026307	5.06430926410589	-1.19026965563104	0.27601865715773	0.999692817443324	-4.58549084260032	4.41980938816208	4.33899695254887	5.09573920128458	5.29401360041453	5.25717837208345	5.98011807014181	NA
**TC0600001443.mm.2**	Lpar5	lysophosphatidic acid receptor 5	-0.555535894115013	4.83797375530858	-1.18989627099978	0.276155462995903	0.999692817443324	-4.58551200556663	5.20828404304912	4.71878193429455	3.92383364946967	5.07101873366921	5.26861215421385	4.83731201715506	NA
**TC0500000677.mm.2**	Slc10a4	solute carrier family 10 (sodium/bile acid cotransporter family), member 4	0.397533622400987	6.88896936471363	1.18966581441329	0.276239929607553	0.999692817443324	-4.58552506657096	7.3356665698156	6.73359535171781	7.02426522324369	7.00554553253149	6.56381904497613	6.67092446599708	NA
**TC0400001047.mm.2**	Pde4b	phosphodiesterase 4B, cAMP specific	0.620133995141585	8.25660353933376	1.18927864390474	0.276381884159342	0.999692817443324	-4.58554700756367	9.20465596913734	9.13578989627684	8.52053925668733	7.69918242374843	8.15226128040879	6.82719240974381	NA
**TC0900000669.mm.2**	Ube2q2	ubiquitin-conjugating enzyme E2Q family member 2	1.16161156828628	7.02884758998907	1.18920630988206	0.276408412010566	0.999692817443324	-4.58555110650513	8.37352213075795	8.24033164599871	7.99535696349208	5.94587048638308	7.1310752084892	4.48692910481339	NA
**TC1100000902.mm.2**	Aloxe3	arachidonate lipoxygenase 3	0.501687986453215	6.86478154152587	1.18914050978969	0.276432545479176	0.999692817443324	-4.58555483512499	7.23239506048658	6.84616729741794	7.30853157821566	6.7482198538629	6.08956221588653	6.9638132432856	NA
**TC1300001135.mm.2**	Serf1	small EDRK-rich factor 1	0.443522973977827	6.65563822585885	1.18888158816691	0.276527527408084	0.999692817443324	-4.58556950655017	6.14493501456261	5.73028386942084	7.37704398161725	7.09578422794539	7.13826204212228	6.44752021948471	NA
**TC1500002213.mm.2**	Bcdin3d	BCDIN3 domain containing	0.46724307192363	5.83093995184845	1.18873934119585	0.276579720593262	0.999692817443324	-4.58557756636797	5.61371261534325	6.06250653587627	5.66600268671761	6.4559506651416	5.75167832167522	5.43578888633673	NA
**TC0400003670.mm.2**	Phactr4	phosphatase and actin regulator 4	0.3565975920406	6.03894291394369	1.18857510697167	0.276639991730039	0.999692817443324	-4.58558687164279	5.90379305666996	5.70129465200378	6.45116480717919	6.21971767235562	5.99702951463066	5.96065778082294	NA

**TC0X00002878.mm.2**	Chm	choroidermia (RAB escort protein 1)	0.598920818538031	5.63549247751418	1.18840106543517	0.27670387414738	0.999692817443324	-4.58559673216812	6.35653394911008	6.72963506216019	5.74525832012815	5.21705542531728	5.55056251562473	4.21390959274464	NA
**TC0500003619.mm.2**	Lnx2	ligand of numb-protein X 2	0.375402079295098	7.15307464664387	1.18698639479765	0.277223597413309	0.999692817443324	-4.58567686599417	7.16675551235528	7.16878140626829	7.41682921577761	7.25002834413731	7.17985013236037	6.73620326896435	NA
**TC0100000244.mm.2**	Cnnm4	cyclin M4	0.3893402638476	6.19763511003323	1.1864314890456	0.277427685315003	0.999692817443324	-4.58570829076211	5.92954256242557	5.92034950162479	6.3039693277901	6.75333023413201	6.31340375992368	5.96521527430323	NA
**TC0700003442.mm.2**	Mef2a	myocyte enhancer factor 2A	0.613133070298661	9.556865304678	1.18642954425912	0.277428400808957	0.999692817443324	-4.58570840088923	10.1459733304755	10.3515974124164	10.1591834527864	8.87576016010031	9.31775345559698	8.49092401669237	NA
**TC0600003336.mm.2**	Erp27	endoplasmic reticulum protein 27	0.674990708823899	4.91643316227166	1.18631819605769	0.277469368823541	0.999692817443324	-4.58571470609573	4.35504151548322	4.34983149888588	5.12567860415839	5.94617508429593	4.08172453389771	5.64014773690881	NA
**TC0100001481.mm.2**	Myoc	myocilin	0.359382571179719	5.72030143365615	1.18602983644006	0.277575487974408	0.999692817443324	-4.5857310339308	5.49923121886406	5.38458459173884	5.87854533926242	6.1998336275843	5.70594059891183	5.65367322557546	NA
**TC0800003099.mm.2**	Fanca	Fanconi anemia, complementation group A	-0.455043903859309	6.02034098007474	-1.18598721853861	0.277591174696616	0.999692817443324	-4.58573344699037	5.44143680237082	5.45476591458797	5.68100919988138	6.47686847800413	6.31149429835371	6.75647118725042	NA
**TC1100002004.mm.2**	Endov	endonuclease V	-0.391348576051825	7.24295620843346	-1.18585006784352	0.27764166198576	0.999692817443324	-4.5857412123954	7.38652161103054	7.67312267164337	6.6508130068443	7.1568849010673	7.30837080968245	7.2820242503328	NA
**TC0400004186.mm.2**	Nfia	nuclear factor I/A	0.882802902070006	8.74303795186527	1.18584603373237	0.277643147122255	0.999692817443324	-4.58574144080071	9.94066284504039	9.91446783214688	9.35917809198046	7.82517332709172	8.67272704434666	6.74601857058551	NA
**TC0200002306.mm.2**	Xkr7	X-linked Kx blood group related 7	-0.353605866583997	6.25876871875488	-1.18571609453489	0.2776909871476	0.999692817443324	-4.58574879763795	5.82106844602285	5.87231846733277	6.37129554588372	6.20471128711912	6.75526657074	6.52795199543083	NA
**TC1500001012.mm.2**	Prpf40b	pre-mRNA processing factor 40B	0.356604750639113	6.58234995243654	1.18570331900747	0.277695691121481	0.999692817443324	-4.58574952094377	6.48886685232257	6.2835917263781	6.74962248897064	6.96780282962775	6.5564611749387	6.44775464238146	NA
**TC0900001785.mm.2**	4931406C07Rik	RIKEN cDNA 4931406C07 gene	0.636169402192433	6.33936063258888	1.18542316496919	0.277798861366865	0.999692817443324	-4.58576538169958	7.09572090886029	7.22054786664218	6.84122731050457	5.65488960170327	6.16401291844463	5.05976518937833	NA
**TC1700002595.mm.2**	Thumpd2	THUMP domain containing 2	-0.542358722227991	5.21180368065633	-1.18532173495722	0.277836222255652	0.999692817443324	-4.58577112382207	4.66545094447051	5.53388333283131	5.44924150040194	5.44143688068815	5.65898449612138	5.41911812942469	NA
**TC1600000464.mm.2**	Osbpl11	oxysterol binding protein-like 11	-0.766928330467155	5.86403325590148	-1.18521522415463	0.277875459196078	0.999692817443324	-4.5857771534175	6.03508200891616	6.45992793062952	6.30321198193709	4.27445448552736	5.97349366612873	6.13802946227003	NA
**TC0500003308.mm.2**	Psph	phosphoserine phosphatase	-0.370287575770528	8.8265824144788	-1.18427011276195	0.278223829501403	0.999692817443324	-4.58583064919041	7.8004543303159	8.00048879091008	8.90759567122695	9.30139243582594	9.46367645748408	9.48588680110986	NA
**TC1100003965.mm.2**	Cacng5	calcium channel, voltage-dependent, gamma subunit 5	-0.557763852190843	7.54739547409402	-1.18412276554473	0.278278175376931	0.999692817443324	-4.58583898826781	8.23406699859787	8.24426274569332	7.59189578170847	6.25336191623716	7.47703072571656	7.48375467661076	NA
**TC1300001859.mm.2**	Elovl2	elongation of very long chain fatty acids (FEN1/Elo2, SUR4/Elo3, yeast)-like 2	-0.620574026440441	6.87156055783888	-1.18373627592597	0.278420766568929	0.999692817443324	-4.58586086005771	6.6201331740834	6.92288776508187	7.3295269253847	5.89307025210886	7.51380479161097	6.94994043876347	NA
**TC1200000830.mm.2**	Zc2hc1c	zinc finger, C2HC-type containing 1C	0.383537973145487	7.11235502098553	1.18363052291863	0.278459793791235	0.999692817443324	-4.58586684433797	6.6638167237296	6.38830709385512	7.38018723478489	7.81435389252483	7.13589779555771	7.29156738546103	NA
**TC1200000197.mm.2**	Taf1b	TATA-box binding protein associated factor, RNA polymerase I, B	0.456364982373746	6.60477235639935	1.18358118248802	0.278478004031375	0.999692817443324	-4.58586963632584	7.49304984164857	7.44012696099475	6.76436833171476	6.03972531853536	6.22959458454476	5.66176910095787	NA
**TC1700001612.mm.2**	Prss22	protease, serine 22	-0.388744163492722	5.50325992492442	-1.18343692517611	0.278531251354838	0.999692817443324	-4.58587779909812	4.92213245017367	5.27908716807443	5.53315544791642	5.48727035424006	5.67732576012169	6.12058836902023	NA
**TC1900001472.mm.2**	Noc3l	NOC3 like DNA replication regulator	0.518474869800585	6.12792340123633	1.18294399399408	0.278713264018015	0.999692817443324	-4.58590568924065	7.35990463834575	7.02819415578574	6.38145265189582	5.32674302454803	5.1197321165506	5.55151382029208	NA
**TC0400002449.mm.2**	B4galt1	UDP-Gal:betaGlcNAc beta 1,4-galactosyltransferase, polypeptide 1	-0.471134640736923	5.64400169051959	-1.18268676245226	0.278808285632592	0.999692817443324	-4.58592024205305	6.14676831854006	6.5704617077694	5.47706289281169	4.62519252485544	5.65826612704664	5.38625857209434	NA
**TC0500000947.mm.2**	Prdm8	PR domain containing 8	-0.500642969558598	6.27990368327131	-1.18257610861761	0.278849169721315	0.999692817443324	-4.58592650197171	6.49127516854006	7.56835978833433	5.53368493602258	5.77556566579556	6.0767339068371	6.23380263409824	NA
**TC0400003331.mm.2**	Rad54l	RAD54 like (S. cerevisiae)	0.552021456258759	5.60164560390603	1.18255470044126	0.278857080144431	0.999692817443324	-4.58592771305646	5.48103264824669	5.51765795374013	5.37055360722304	6.48705935976039	5.02576594944564	5.72780410502028	NA
**TC1400002165.mm.2**	Cdadc1	cytidine and dCMP deaminase domain containing 1	-0.496174685613683	7.33558393641331	-1.18242005374056	0.278906837098846	0.999692817443324	-4.58593533002004	7.88803235325814	7.34114078543681	7.41598910095279	6.48000145332599	7.52747675414443	7.36086317136172	NA
**TC1700000359.mm.2**	Ift140	intraflagellar transport 140	-0.471663701377394	6.61064480620753	-1.18235037488149	0.278932588976434	0.999692817443324	-4.58593927164954	6.85769162700394	6.56404670816718	6.42173458244818	6.22766696721146	7.2603279109431	6.33240104147133	NA
**TC1500002258.mm.2**	Krt80	keratin 80	-0.464522321922662	5.29316103667196	-1.18212143828012	0.279017213428399	0.999692817443324	-4.58595222175604	4.75686366066335	4.9832115720039	4.87085768145913	5.67406549030047	5.49833062946637	5.97563718613855	NA
**TC1600000305.mm.2**	Map3k13	mitogen-activated protein kinase kinase kinase 13	0.772037291417508	5.1127790359207	1.18204688583793	0.279044775792312	0.999692817443324	-4.58595643875061	5.83754458630953	5.92043486802146	5.49478729017799	4.73659738183612	5.32383123556715	3.36347885361194	NA
**TC1700000705.mm.2**	Mdc1	mediator of DNA damage checkpoint 1	0.379845350551991	5.14464132527473	1.18169972733574	0.279173152177423	0.999692817443324	-4.58597607440648	5.13708048911297	5.2997451821801	5.11252053879873	5.48283595193092	5.07868978438621	4.75697600523945	NA
**TC0100001712.mm.2**	Lin9	lin-9 homolog (C. elegans)	-0.44103922416137	5.69206718073674	-1.18164422534879	0.279193680996197	0.999692817443324	-4.58597921349546	5.26063315432881	5.53974889340709	5.85675887249633	5.37821242168457	5.7333284373728	6.38372130513083	NA
**TC0600002154.mm.2**	Try5	trypsin 5	-0.587810748917112	4.7515839838231	-1.18160063789792	0.279209803817439	0.999692817443324	-4.5859816788	3.89857414681655	3.47748206528387	4.7504402274781	5.22847286902388	4.92242688660527	6.23210770773093	NA
**TC1500001061.mm.2**	Acvrl1	activin A receptor, type II-like 1	-0.37730731799514	7.20431097139249	-1.18151295498928	0.279242239757739	0.999692817443324	-4.58598663772379	6.88335128397654	7.1201915941389	7.04866806481756	7.18518609230706	7.28502283636768	7.70344595674721	NA
**TC0100001554.mm.2**	Rxrg	retinoid X receptor gamma	0.56019182866534	7.35621987220742	1.18125440430593	0.279337902223192	0.999692817443324	-4.58600125978215	8.17584201209153	7.84360115465169	7.9616425961231	6.65748726579288	6.8192144805263	6.679531724059	NA
**TC0100003791.mm.2**	Prox1	prospero homeobox 1	-0.380882494567303	6.27740228670147	-1.18075051715089	0.279524417690696	0.999692817443324	-4.58602975377939	6.71908950814905	6.78656718077172	6.55569028645552	6.0428057346212	6.40484267919362	6.05541833101772	NA
**TC0800001964.mm.2**	Unc5d	unc-5 netrin receptor D	0.397384829235929	6.60657744969583	1.18060020170555	0.279580077852469	0.999692817443324	-4.5860382531543	7.35934462510098	6.77413585530382	6.67257246975098	6.47780446837003	6.11665568895294	6.23895159069621	NA
**TC1100004171.mm.2**	Cbx8	chromobox 8	-0.341516163676965	5.8444376156673	-1.18052776723359	0.279606902892963	0.999692817443324	-4.58604234874138	5.79162452760367	5.70457268255707	5.69774228632198	5.74595579192257	6.02920718642591	6.09752321917257	NA
**TC1900000111.mm.2**	Ehd1	EH-domain containing 1	-0.50893031345894	6.18080393621218	-1.18008710937882	0.279770140985944	0.999692817443324	-4.58606726274571	7.02252315939331	6.99325526354864	6.0882639377126	4.93732834599401	5.98714678437095	6.05630612625355	NA
**TC1400000195.mm.2**	Kat6b	K(lysine) acetyltransferase 6B	0.421765515777706	7.90906536161581	1.18007315043027	0.279775313281179	0.999692817443324	-4.58606805191313	8.82828245736893	8.76672043843003	7.70541032025329	7.64604983247236	7.63271213559355	6.87521698557669	NA
**TC0300000730.mm.2**	Ubqln4	ubiquilin 4	-0.483570175066835	7.19020205428597	-1.17992980303767	0.279828433363648	0.999692817443324	-4.58607615587488	7.76465255264149	7.80778081712793	7.12724649483217	6.17357280807418	6.9177288343347	7.35023081870533	NA
**TC1000002551.mm.2**	Sirt6	sirtuin 6	-0.390724939949993	6.97482296687027	-1.17982085615906	0.279868811394109	0.999692817443324	-4.58608231484597	6.72976566853894	6.86223226640922	6.58231138892295	7.15543360426378	7.28592713300722	7.2332677400795	NA
**TC0X00000902.mm.2**	Zxdb	zinc finger, X-linked, duplicated B	0.56060640063131	5.35540366120081	1.17966435282523	0.279926823486727	0.999692817443324	-4.58609116196779	6.63480799876069	6.63069951755751	5.0414796015525	4.95258402452213	5.1137167511611	3.75913407365091	NA
**TC1300001066.mm.2**	Tbca	tubulin cofactor A	-0.36816488386632	7.61182157313723	-1.17963652891606	0.27993713821851	0.999692817443324	-4.58609273481367	8.0993044772888	8.09004797233114	7.20289826743458	7.16972534330083	7.33664282305005	7.772310555418	NA
**TC0800002518.mm.2**	Smad1	SMAD family member 1	0.456435066711968	7.58428724691697	1.17962042960688	0.279943106615757	0.999692817443324	-4.58609364487945	7.98457636312851	7.81947827531764	7.9353217558419	7.37194773239791	7.55745645492992	6.83694289988595	NA
**TC0900001383.mm.2**	Mst1r	macrophage stimulating 1 receptor (c-met-related tyrosine kinase)	-0.404156440015986	5.53423625593885	-1.17930911896585	0.280058538055626	0.999692817443324	-4.58611124197683	5.36840107397389	5.67229422440439	5.52994934617791	5.14825533243351	5.51374575845639	5.97277180018701	NA
**TC0500000819.mm.2**	Slc4a4	solute carrier family 4 (anion exchanger), member 4	0.39899438718665	8.22343786275091	1.17914318839398	0.28012008020592	0.999692817443324	-4.58612062076089	8.52651964233308	8.16670671981574	8.42418521147645	8.29850958288853	7.70468998523423	8.22001603475745	NA
**TC1400001501.mm.2**	Ube2e1	ubiquitin-conjugating enzyme E2E 1	0.358419786722638	8.16979047674286	1.17904585647995	0.280156185057491	0.999692817443324	-4.58612612199993	8.73907848719951	8.49717833787378	8.20636868296796	8.04329412144661	7.86842920846324	7.66439402250606	NA
**TC1300000763.mm.2**	Mir23b/Mir27b/Mir24-1/Aopep	microRNA 23b/microRNA 27b/microRNA 24-1/aminopeptidase O	-0.371204431389562	5.61262414960399	-1.17873768732421	0.28027052503614	0.999692817443324	-4.58614353892531	5.567241677522	5.9689236299563	5.23624629591228	5.46233906777099	5.70288740734717	5.73810681911522	NA
**TC0400003440.mm.2**	Nfyc	nuclear transcription factor-Y gamma	0.610708352094523	7.88561593460683	1.17873021483841	0.280273298042127	0.999692817443324	-4.58614396123356	8.25361929565783	8.37669877015154	8.56254320590631	7.38985391710403	7.86556642015077	6.86541399867053	NA
**TC1000002524.mm.2**	Sgta	small glutamine-rich tetratricopeptide repeat (TPR)-containing, alpha	0.343724428027311	9.10461559987102	1.17850810956803	0.280355730909696	0.999692817443324	-4.58615651315842	9.45505461471482	9.4165205988511	9.02651979153911	9.1952638293183	8.7431094541872	8.79122531061559	NA
**TC1600001380.mm.2**	Dgkg	diacylglycerol kinase, gamma	0.545949683483785	8.30522275525829	1.17838590606442	0.280401094657972	0.999692817443324	-4.58616341898425	9.60126267220203	9.65631985057544	8.35449335872233	7.47833332914759	7.85218282541613	6.88874449548623	NA
**TC0500000985.mm.2**	Mrps18c	mitochondrial ribosomal protein S18C	0.438531389511929	6.37177323405386	1.17807183690185	0.280517710259317	0.999692817443324	-4.58618116629388	6.80838838161686	6.81277611226535	6.26230114413343	6.48096770059895	6.34314272292748	5.52306334278106	NA
**TC0600001427.mm.2**	Phb2	prohibitin 2	-0.587618372516975	8.82711931658508	-1.1777058075981	0.280653670699953	0.999692817443324	-4.58620184791065	9.35874600331313	9.32549743277999	9.00565638902988	7.54596147016183	8.90890625424563	8.81794834998004	NA
**TC1900000703.mm.2**	Trim8	tripartite motif-containing 8	-0.576550422619913	7.0316166642146	-1.17764793433602	0.280675172643315	0.999692817443324	-4.5862051177206	7.88056504384117	8.03503968063123	6.90590954132512	6.65458766646258	7.1851439596194	6.52845409340813	NA
**TC1100002830.mm.2**	Olfr323	olfactory receptor 323	0.559893958700087	4.58358163954503	1.17708612291858	0.280883977661138	0.999692817443324	-4.58623685720437	4.49589919526444	4.38973969009661	5.13186661357563	4.73595282107903	3.72591780182504	5.02211371542944	NA
**TC0500002124.mm.2**	Gm10220	predicted gene 10220	-0.371957369538495	5.16327685205319	-1.17686201746517	0.28096730620131	0.999692817443324	-4.58624951671638	4.70823497413424	4.42081481109493	5.15911614881502	5.39423214519149	5.50278744616826	5.79447558691524	NA
**TC0400001639.mm.2**	Stmn1	stathmin 1	-0.722854095342354	9.15482130271497	-1.17658433825155	0.281070583919549	0.999692817443324	-4.58626520152753	10.4579275344804	10.3616684396455	9.03181663552034	7.29999519021923	9.33154127228915	8.44597874413513	NA
**TC0700001721.mm.2**	Tmem159	transmembrane protein 159	-0.358019711703855	5.39099743290811	-1.17654345469832	0.281085792521252	0.999692817443324	-4.58626751075319	5.8787556507177	6.16762885765144	4.96261193277416	4.82916840006176	5.33740012439619	5.17041963184744	NA
**TC0600000124.mm.2**	Tes	testis derived transcript	-0.432291433320993	6.50653287242515	-1.1759366815297	0.281311592731694	0.999692817443324	-4.58630178020072	5.58116696366489	5.84138473106135	6.30611445092097	7.06991688567036	7.04094223421959	7.19967196901372	NA
**TC0600003125.mm.2**	Lag3	lymphocyte-activation gene 3	-0.480969797728971	5.17981163365483	-1.17576158108934	0.281376781847468	0.999692817443324	-4.58631166853743	5.29427482404802	4.9943503328804	5.02436064639612	4.88979187837519	4.9424765168417	5.93361560338755	NA
**TC0700004313.mm.2**	Spn	sialophorin	-0.418819900588102	5.20728688413374	-1.17548356363213	0.281480312736328	0.999692817443324	-4.58632736790358	4.75092110935309	4.72197760701391	4.85609526708268	5.61049612654694	5.66761879733586	5.63661239746997	NA
**TC0500002989.mm.2**	Fbrsl1	fibrosin-like 1	0.360505933093043	8.53448769160644	1.17544247375557	0.281495616920619	0.999692817443324	-4.58632968810985	8.03860063623838	8.11131787722356	9.12799210415662	8.76101764702478	8.62883308152485	8.53916480347046	NA
**TC0200002882.mm.2**	Gm13199	predicted gene 13199	-0.432845428977704	6.10861676065089	-1.17467156733116	0.28178287624459	0.999692817443324	-4.5863732139149	4.89096293038715	5.29255774442324	6.03929768590439	6.76194683166538	6.84940152987444	6.81753384365073	NA
**TC1000000208.mm.2**	Stx7	syntaxin 7	0.482319672342603	7.95608791930542	1.17405957141308	0.282011097762924	0.999692817443324	-4.58640776121697	8.52401939653068	8.39168398782311	8.05106330021839	7.84166843786359	7.97576842136047	6.9523239720363	NA
**TC0800000972.mm.2**	Dnase2a/Gm38426	deoxyribonuclease II alpha/predicted gene, 38426	-0.369148392718208	6.58391567167533	-1.17404452691175	0.282016710025526	0.999692817443324	-4.58640861041168	6.36197057211382	6.67379137070324	6.33546822865359	6.52924942224565	6.66463327567826	6.9383811606574	NA
**TC0500001725.mm.2**	Spdye4b	speedy/RINGO cell cycle regulator family, member E4B	0.401929855285312	6.29806683915912	1.17392215861908	0.282062362300197	0.999692817443324	-4.58641551742759	5.64186437017548	5.86829043797865	6.55083249985868	6.99022046882694	6.14411933345157	6.59307392466343	NA
**TC1900000557.mm.2**	Tbc1d12	TBC1D12: TBC1 domain family, member 12	0.641636457069689	6.80832904382801	1.17329183208656	0.282297618828111	0.999692817443324	-4.58645109232756	6.87006853460752	7.37409503993489	7.01396482146772	6.9305769798148	7.12492769608571	5.53634119105743	NA
**TC1800000809.mm.2**	Zbtb7c	zinc finger and BTB domain containing 7C	0.364077534680558	5.94888108224263	1.17311051988927	0.28236532058448	0.999692817443324	-4.58646132427267	6.07790301741991	5.82687484739496	6.26632779629967	5.99200405270134	5.66668849080274	5.86348828883715	NA
**TC0400002129.mm.2**	Rps20	ribosomal protein S20	-0.342059334272289	8.67367961758963	-1.17307307454813	0.282379304337579	0.999692817443324	-4.58646343735448	8.71964323292446	8.68626383900457	8.54050636360388	8.4355196189282	8.76002382696123	8.90012082411543	NA
**TC1600000264.mm.2**	B3gnt5	UDP-GlcNAc:betaGal beta-1,3-N-acetylglucosaminyltransferase 5	-0.392169204851543	5.28891018982605	-1.17302278434866	0.282398085851052	0.999692817443324	-4.5864662752526	4.89844964737774	5.39214164970978	5.1709315584566	5.15833415762624	5.73836800195641	5.37523612382951	NA
**TC1100004208.mm.2**	Arl16	ADP-ribosylation factor-like 16	0.390890412917128	7.39791321926541	1.1727870963523	0.282486120579072	0.999692817443324	-4.58647957472287	8.29620864427515	8.08402591144489	7.30973409672683	7.08477869612649	7.0668189650974	6.54591300192167	NA
**TC1800000297.mm.2**	Kdm3b	KDM3B lysine (K)-specific demethylase 3B	0.37667159642946	6.83199778559806	1.17269078056558	0.282522103343371	0.999692817443324	-4.58648500941713	7.30823396862047	7.3237884669521	6.6508086792008	6.90584505623656	6.62517750271015	6.17813303986829	NA
**TC0200001794.mm.2**	Snap23	synaptosomal-associated protein 23	0.429646258131962	8.39414309100017	1.17267953450597	0.282526305028491	0.999692817443324	-4.58648564439758	8.63101509756356	9.03168094193841	8.73193003450094	8.04879747688055	8.057470305373	7.86396468974457	NA
**TC0700003977.mm.2**	Fam160a2	family with sequence similarity 160, member A2	0.383663635772563	6.38244197921783	1.17261827385105	0.282549193792045	0.999692817443324	-4.5864891005735	6.98789662421741	7.30030838731269	6.37618608090445	6.01070098675656	5.66500085229068	5.95455894382521	NA
**TC0700000406.mm.2**	D930028M14Rik	RIKEN cDNA D930028M14 gene	0.50772634334549	4.93603695760916	1.17238729439989	0.282635508514722	0.999692817443324	-4.58650213294925	4.2402454393639	4.48276201951058	5.16987737040875	5.78445611632698	4.49146512339761	5.44741567664713	NA
**TC0800003163.mm.2**	Abcb10	ATP-binding cassette, sub-family B (MDR/TAP), member 10	-0.45876892354128	6.93017967912926	-1.17132723807528	0.283031926504903	0.999692817443324	-4.58656193336002	7.11791064101535	6.97619470228789	6.51976841501341	6.76494902718146	6.64533805028448	7.55691723899296	NA
**TC1700002205.mm.2**	1700001C19Rik	RIKEN cDNA 1700001C19 gene	-0.421609999071512	5.03803362372569	-1.1711572338283	0.28309554481944	0.999692817443324	-4.58657152214051	4.61700200043369	5.00438482418147	4.72164973219788	5.1601477276001	5.61875393881586	5.10626351912515	NA
**TC0X00001282.mm.2**	Rpl36a	ribosomal protein L36A	-0.537450274595008	8.04187243563625	-1.17072244872841	0.283258303259058	0.999692817443324	-4.58659604341105	9.2177151182257	9.5577613552625	7.55322556491179	6.64720323065785	8.05963781341982	7.21569153133984	NA
**TC1600000480.mm.2**	Itgb5	integrin beta 5	-0.373458894725832	7.6768966517862	-1.17070194209158	0.283265981708961	0.999692817443324	-4.58659719988584	7.90108590450703	8.39559216359527	7.27391427353009	7.23497775305152	7.6435474494426	7.61226236659068	NA
**TC0300000507.mm.2**	Gmps	guanine monophosphate synthetase	0.568534691303726	7.35762185274277	1.17070092426364	0.283266362826289	0.999692817443324	-4.58659725728623	8.91427436899071	8.8834568085332	7.41161182834827	6.3309228324218	6.75068129128969	5.85478398687292	NA
**TC1500001724.mm.2**	Cyp11b2	cytochrome P450, family 11, subfamily b, polypeptide 2	0.416052884132529	4.5893009977561	1.17065282957485	0.283284371981367	0.999692817443324	-4.58659996956746	3.73636283506122	3.93109881830751	5.14565247480087	5.20457257591559	4.39803729286589	5.12008198958551	NA
**TC1600001158.mm.2**	Eef2kmt	eukaryotic elongation factor 2 lysine methyltransferase	0.623805919394588	5.537727459311	1.17063230269543	0.283292058607636	0.999692817443324	-4.58660112716214	5.2019346637364	5.34636434520511	5.21895232995616	6.74388646290066	5.11060566734369	5.60462128672396	NA
**TC0800002934.mm.2**	Ddx19a	DEAD (Asp-Glu-Ala-Asp) box polypeptide 19a	-0.414089860662302	6.26919085759726	-1.16983244391902	0.283591715847788	0.999692817443324	-4.58664622948439	6.79239065341421	7.00336010310813	5.43929585537973	6.05631147848857	5.9854777591968	6.33830929599611	NA
**TC0600001949.mm.2**	Tmem229a	transmembrane protein 229A	0.448016837031521	6.57641950893163	1.16915148023647	0.283847041123119	0.999692817443324	-4.58668461989217	6.92116602148119	7.10073100148267	6.61095352258187	6.55537332976262	6.57996511302074	5.6903280652607	NA
**TC1100000872.mm.2**	Pik3r5	phosphoinositide-3-kinase regulatory subunit 5	0.397177031456561	6.35987794196308	1.16886563815832	0.283954274486678	0.999692817443324	-4.58670073258486	5.81727471180122	5.87267164080854	6.56818266849292	7.06365501254802	6.20978691934482	6.62769669878299	NA
**TC1100000918.mm.2**	Fxr2	fragile X mental retardation, autosomal homolog 2	0.505145271754579	8.11793857730736	1.16861705926405	0.284047556367306	0.999692817443324	-4.58671474376647	8.92987422924359	9.00683053657214	8.43597151681113	7.45463710395766	7.74527876648265	7.13503931077698	NA
**TC1100004290.mm.2**	Commd1	COMM domain containing 1	0.487732562041258	6.23082991582086	1.16854931278918	0.284072983433348	0.999692817443324	-4.58671856214149	6.78250467955655	6.97138792009379	6.66318750308585	5.64008850659281	5.81911806240212	5.50869282319402	NA
**TC1100001940.mm.2**	1810032O08Rik/Snord1b/Snord1c/Snord1a	RIKEN cDNA 1810032O08 gene/small nucleolar RNA, C/D box 1B/small nucleolar RNA, C/D box 1C/small nucleolar RNA, C/D box 1A	-0.461178027184459	6.52676301514976	-1.16826509104842	0.284179680373811	0.999692817443324	-4.58673458088421	6.36878508182273	6.4608574848943	6.51055706495548	6.19373266995082	6.35682556312463	7.26982022615058	NA
**TC0700003852.mm.2**	Art5	ADP-ribosyltransferase 5	-0.442966464144033	6.86779618108644	-1.16824703872703	0.284186458362244	0.999692817443324	-4.58673559827163	6.4525499098306	6.22310535475678	6.89008465291166	6.93250979390994	6.92622659262159	7.78230078248808	NA
**TC0700000792.mm.2**	Klk1b24	kallikrein 1-related peptidase b24	-0.366331794436168	4.85985980348994	-1.16813688852537	0.284227818698764	0.999692817443324	-4.58674180597613	4.26706021550802	4.06083567924454	4.9780875940172	5.07121207464019	5.19611711957528	5.58584613795444	NA
**TC0300000956.mm.2**	Pias3	protein inhibitor of activated STAT 3	0.420119261381418	6.14731332781245	1.16761259898236	0.28442475390473	0.999692817443324	-4.58677135066817	6.57514223055708	5.85800248415091	6.34747104509231	6.29801584237247	6.04972527702729	5.75552308767465	NA
**TC0700001651.mm.2**	Tead1	TEA domain family member 1	-1.1047722060835	4.77985256951934	-1.16754784435814	0.284449085206546	0.999692817443324	-4.58677499941927	5.84923228115693	5.71832188100125	5.32931849927893	2.12168992211652	4.63810353753997	5.02244929602247	NA

**TC0100003056.mm.2**	Rassf5	Ras association (RalGDS/AF-6) domain family member 5	0.360051733481632	6.43427382237423	1.16742795918571	0.284494136229937	0.999692817443324	-4.58678175446044	6.27328056754247	5.94164318559928	6.78307088615692	6.77234043787653	6.53109903622086	6.30420882084934	NA
**TC1900001002.mm.2**	Sac3d1	SAC3 domain containing 1	-0.449582473194869	5.84399102107205	-1.16724930179908	0.284561284112615	0.999692817443324	-4.58679182066689	5.45047192674935	5.7258588584921	5.44297043490733	6.05125476249321	5.88032297618015	6.51306716761013	NA
**TC1500002096.mm.2**	Yaf2	YY1 associated factor 2	-0.4105681385412	7.75513736944528	-1.16686504098577	0.284705752719496	0.999692817443324	-4.58681346965506	8.18548033615054	8.07505406105946	7.6828733504006	7.04170342078906	7.60915926092764	7.93655378734441	NA
**TC1900000763.mm.2**	Pdcd4	programmed cell death 4	-0.70839605312872	8.16390127820448	-1.16679561308012	0.284731861769934	0.999692817443324	-4.58681738093291	9.2245617957467	9.21241021049537	8.07672899287263	6.48809278549106	8.52818119908432	7.45343268553681	NA
**TC0700001539.mm.2**	Olfr676	olfactory receptor 676	-0.350241158287385	6.18789547866564	-1.16672182481602	0.284759612787305	0.999692817443324	-4.58682153777378	5.8336210537689	5.65485676773571	6.31948510351161	6.14972126344562	6.48939380089955	6.68029488263245	NA
**TC0200004636.mm.2**	Slc4a11	solute carrier family 4, sodium bicarbonate transporter-like, member 11	-0.394340356806703	6.27914092962334	-1.16632901622744	0.284907382462724	0.999692817443324	-4.5868436651223	6.25961293723261	6.09951316807667	6.06565358725562	6.19786579215305	6.22388475266948	6.82831534035259	NA
**TC0400004177.mm.2**	Akap2	A kinase (PRKA) anchor protein 2	0.344166689339873	6.09406876769915	1.16630753693687	0.284915464565209	0.999692817443324	-4.58684487500626	7.14942387099117	6.92740132989658	5.82079199132672	5.76716840066673	5.48851423127497	5.41111278203873	NA
**TC0100001859.mm.2**	Slc30a1	solute carrier family 30 (zinc transporter), member 1	-0.387716113093077	7.23371667133186	-1.16565188071085	0.285162264000355	0.999692817443324	-4.58688180333807	7.35131176624824	7.32336416472727	7.28500957707614	6.69108635833862	7.41933599208501	7.33219216951591	NA
**TC1100001658.mm.2**	Rnd2	Rho family GTPase 2	0.381911689454009	8.24383221541224	1.16562352042595	0.285172943323647	0.999692817443324	-4.5868834005162	9.11205389973367	8.63939919581965	8.04277632535401	8.19490546256005	7.87142454543643	7.60243386356961	NA
**TC1500002175.mm.2**	Lalba	lactalbumin, alpha	-0.451381616270813	5.09370595458168	-1.1653334013225	0.285282209656677	0.999692817443324	-4.58689973856173	4.41007626486673	4.5915331383853	4.92493082970896	5.40400071613926	5.22680713695941	6.00488764143043	NA
**TC0200005339.mm.2**	Gm14434/Gm2026/Gm4724/Gm14308/Gm14430/Gm14432	predicted gene 14434/predicted gene 2026/predicted gene 4724/predicted gene 14308/predicted gene 14430/predicted gene 14432	-0.866201823554952	9.0227851665458	-1.16399433616257	0.285786993013784	0.999692817443324	-4.58697513106048	9.97990319853092	10.5897751475997	8.86651176659607	7.05080273642106	9.5981365763552	8.05158157377184	NA
**TC0800000955.mm.2**	Cacna1a	calcium channel, voltage-dependent, P/Q type, alpha 1A subunit	0.33640777262013	7.22955997454564	1.16394088874681	0.285807156517262	0.999692817443324	-4.58697813969716	8.42085441259072	8.40168024797741	6.83104196284599	6.78277840312699	6.52023147053506	6.42077335019765	NA
**TC1000002658.mm.2**	Slc17a8	solute carrier family 17 (sodium-dependent inorganic phosphate cotransporter), member 8	0.458762088513083	5.63583338497958	1.16276673958803	0.286250416633266	0.999692817443324	-4.58704422312311	5.39161549610738	5.4931651804051	6.21244245681868	5.7114294483769	5.09118323497628	5.91516449319313	NA
**TC1000001560.mm.2**	Arhgap9	Rho GTPase activating protein 9	0.435511215774181	5.10746739590714	1.16233266793956	0.286414431995542	0.999692817443324	-4.58706864808327	4.32080442016089	4.58410528165482	5.26903221015352	6.03642634243423	5.07092669567488	5.3635094253645	NA
**TC0100001223.mm.2**	Mybph	myosin binding protein H	-0.497257105111022	5.97810357055516	-1.16232255724729	0.286418253294861	0.999692817443324	-4.58706921697096	5.41575027704115	5.53469585451418	5.55688617047238	6.40494437030441	6.10440638277193	6.85193836822691	NA
**TC0100002261.mm.2**	Fhl2	four and a half LIM domains 2	-0.495099491572064	6.59070152214979	-1.16227955561307	0.286434506084932	0.999692817443324	-4.58707163648082	6.49013660690427	6.99024057515702	6.57061803904706	5.9661984447996	7.16863645114126	6.35837901584952	NA
**TC1800001237.mm.2**	Fgf1	fibroblast growth factor 1	0.372717034095331	7.32513778076337	1.16201168302239	0.286535768006337	0.999692817443324	-4.58708670782323	8.15647936904568	7.6707943728538	7.16264506537426	7.27184844006144	6.90282401671638	6.78623542052866	NA
**TC1100001100.mm.2**	Timm22	translocase of inner mitochondrial membrane 22	-0.344383990151256	7.37902927224515	-1.16188248608752	0.286584618142518	0.999692817443324	-4.58709397644169	7.59587546184009	7.47478066307712	7.03932785785058	7.21804790627502	7.54643418431547	7.39970956011264	NA
**TC1400002160.mm.2**	Phf11-ps	PHD finger protein 11, pseudogene	-0.544406936480362	4.50404999477614	-1.16182902669977	0.286604833506285	0.999692817443324	-4.58709698398991	3.33375787664366	4.03690802929632	4.15514232369032	5.12726777118774	4.90159182082044	5.46963214701835	NA
**TC0600001600.mm.2**	Apold1	apolipoprotein L domain containing 1	-0.365112288095806	6.30023941580639	-1.16171536286682	0.286647818824313	0.999692817443324	-4.5871033784047	6.36641335002771	5.97731022852516	6.07151477940627	6.29222939064065	6.59306798799178	6.50090075824674	NA
**TC1500002337.mm.2**	Pfdn5	prefoldin 5	-0.472984659009009	8.28969855195212	-1.16139663050825	0.286768385747525	0.999692817443324	-4.58712130833047	9.30600812809262	9.13578298186454	7.94608860972887	7.22912683213991	8.42402916387514	7.69715559601166	NA
**TC0400003943.mm.2**	1700012P22Rik	RIKEN cDNA 1700012P22 gene	0.485117077994556	5.98548253429289	1.16138432790266	0.28677304031003	0.999692817443324	-4.58712200036759	6.07189539575088	5.7364540941013	5.75779118306821	6.77959875287893	5.70353227312874	5.86362350682929	NA
**TC0600001532.mm.2**	Clec12a	C-type lectin domain family 12, member a	-0.437717690435134	5.06461994674579	-1.1611831319305	0.286849169721958	0.999692817443324	-4.58713331755816	5.51803182690092	5.30715296558451	4.2907074519897	5.05284230156983	5.2968837063006	4.9221014281292	NA
**TC1400000329.mm.2**	Eaf1	ELL associated factor 1	0.449505063091724	6.64714744784432	1.16108795731292	0.286885188229783	0.999692817443324	-4.58713867086971	6.39256776783193	6.44903201418456	7.20775658095761	6.76239093465884	6.94309591030235	6.12804147913065	NA
**TC1800001200.mm.2**	Hars	histidyl-tRNA synthetase	-0.99050433653488	7.70228651129853	-1.16056817338246	0.287081965770291	0.999692817443324	-4.58716790477958	8.79337210901263	8.52224940447837	7.98797551170048	5.47056892891474	8.40818665077309	7.03136646291189	NA
**TC0500000997.mm.2**	Arhgap24	Rho GTPase activating protein 24	-0.391886708737615	6.7176579130923	-1.16036163019505	0.287160189485616	0.999692817443324	-4.58717952008945	6.92945782066539	6.56256164465032	6.5951293114266	6.41994798645483	6.65453731002117	7.14431340533548	NA
**TC0400003581.mm.2**	Azin2	antizyme inhibitor 2	0.520421431066268	6.51443379882937	1.1602959255122	0.287185077453125	0.999692817443324	-4.58718321496394	6.7005824826799	6.60378167052517	6.14424999857176	7.26729075238009	5.93286108471482	6.4378368041045	NA
**TC0600003302.mm.2**	Lrp6	low density lipoprotein receptor-related protein 6	0.358946710578644	6.76706391688821	1.15996780643724	0.287309391267293	0.999692817443324	-4.58720166558019	7.71028319355608	7.73601751787058	6.57625535488493	6.36073275064501	6.28465973778994	5.93443494658271	NA
**TC1300000924.mm.2**	Ttc37	tetratricopeptide repeat domain 37	0.470510743043338	5.4433216865071	1.15967979881182	0.28741854540338	0.999692817443324	-4.58721785927129	6.09805461011432	6.09683761905205	5.38250224314315	5.32052744483832	5.39372042070452	4.36828778119026	NA
**TC0200003526.mm.2**	Rbm43	RNA binding motif protein 43	0.396339478762783	5.72727097401356	1.1586216771782	0.287819869955444	0.999692817443324	-4.58727734260226	6.89137298575567	6.52509528776682	5.67433518262339	5.19558308141882	5.17991292656122	4.89732637995543	NA
**TC1300002775.mm.2**	Zfp58	zinc finger protein 58	-0.702161539437935	5.96094199686269	-1.15829843992475	0.287942561263406	0.999692817443324	-4.58729551016144	6.65218052498075	6.23308423904346	6.1833298798917	4.55470218924634	6.52846190018137	5.61389324783254	NA
**TC0500002586.mm.2**	Fryl	FRY like transcription coactivator	0.447102411719193	6.31824027787454	1.15816030474393	0.287995006691508	0.999692817443324	-4.5873032735477	7.08057868152583	7.09843857701206	5.74707767347629	6.56523694259757	5.92425884031813	5.49385095231735	NA
**TC0300000908.mm.2**	Ensa	endosulfine alpha	0.450671311802405	7.68612520327637	1.15812921922923	0.288006809954554	0.999692817443324	-4.58730502055443	8.07230575308437	8.04748248105532	7.69357760830279	7.75557519625888	7.74329867024378	6.80451151071307	NA
**TC0200004713.mm.2**	Jag1	jagged 1	0.509105249465735	5.63953752760701	1.15778177244154	0.28813876419048	0.999692817443324	-4.58732454602834	5.8282903079838	5.78485471947276	5.49418216254451	6.12696315601398	5.80712546682413	4.79580935280289	NA
**TC1000002172.mm.2**	Fam184a	family with sequence similarity 184, member A	0.412918591713877	5.75879593283751	1.15741242742316	0.288279090658371	0.999692817443324	-4.58734530001308	6.31931688544651	5.95054599238953	6.0867701788348	5.46760477247358	5.24613775409593	5.4824000137847	NA
**TC1500000976.mm.2**	Olfr279	olfactory receptor 279	-0.389092135749779	5.17754935762689	-1.15736996240717	0.288295228209061	0.999692817443324	-4.58734768603931	4.96045868062692	4.84580844918885	5.41487027596906	4.82555209625395	5.42073196814013	5.59787467558244	NA
**TC1700002820.mm.2**	H2-T-ps	histocompatibility 2, T region locus, pseudogene	-0.470866072547589	5.82424221737631	-1.15732701073756	0.288311551469382	0.999692817443324	-4.58735009938046	5.86575233996209	6.45803478976854	5.82474810251723	5.01521891219881	6.01438829974668	5.76731086006454	NA
**TC0700004366.mm.2**	Tial1	Tia1 cytotoxic granule-associated RNA binding protein-like 1	0.469516684846012	8.58001373784417	1.15677831342382	0.288520145500126	0.999692817443324	-4.58738092665068	8.77093093978538	8.93292162158488	8.4962747312365	8.86135688645689	8.68297653178237	7.73562171621899	NA
**TC0700004606.mm.2**	Ppfia1	protein tyrosine phosphatase, receptor type, f polypeptide (PTPRF), interacting protein (liprin), alpha 1	0.382199096836643	5.87527651934975	1.1562816392595	0.288709071518657	0.999692817443324	-4.58740882698089	6.46104710165987	6.14756268358127	6.07478950240703	5.62893425985829	5.36201175246614	5.57731381612589	NA
**TC1400001177.mm.2**	Lmo7	LIM domain only 7	0.78798746454858	7.38786690494128	1.15610903548891	0.288774751230786	0.999692817443324	-4.58741852195644	8.97463032499199	9.20973149892319	7.66203339394735	6.1973738734675	6.96521248598086	5.31821985233682	NA
**TC0300000878.mm.2**	Pogz	pogo transposable element with ZNF domain	0.767402097481424	6.55472838791399	1.15587326401768	0.288864487971773	0.999692817443324	-4.58743176422658	7.70379055946199	7.50377235266365	7.2692189176652	5.55858688749538	6.28701430188499	5.00598730831275	NA
**TC1800000158.mm.2**	Ttr	transthyretin	-1.84598419668052	10.814938972179	-1.15565342501437	0.288948181753891	0.999692817443324	-4.58744411083695	8.55466928751189	12.728694781916	8.10752969826037	11.8496209868822	12.1112204640497	11.537898614454	NA
**TC0400003892.mm.2**	Dnajc16	DnaJ heat shock protein family (Hsp40) member C16	0.361233123096643	6.25317485349974	1.15556895474278	0.288980345409649	0.999692817443324	-4.58744885465523	6.49035053510828	6.78237073990865	6.07890454282772	6.40549250325969	5.85318123592554	5.90874956396859	NA
**TC1900001555.mm.2**	Bloc1s2	biogenesis of lysosomal organelles complex-1, subunit 2	-0.519508280604738	8.26641279874499	-1.15495282251204	0.289215040305182	0.999692817443324	-4.58748345294639	8.50638720201862	8.50024954283883	8.39344942039451	7.38296232280698	8.76802953908683	8.04739876532414	NA
**TC0700002007.mm.2**	Cd163l1	CD163 molecule-like 1	0.445687487498811	6.54030236758959	1.15459637629662	0.289350889527926	0.999692817443324	-4.5875034660459	6.08370754872962	5.99068764282941	6.63960529913803	7.38979169535004	6.30293487597186	6.83508714351858	NA
**TC1800000956.mm.2**	Mpp7	membrane protein, palmitoylated 7 (MAGUK p55 subfamily member 7)	-0.437068383368102	6.21820174294942	-1.15447989788336	0.289395293526683	0.999692817443324	-4.58751000542318	6.52733769305163	6.16415808076357	6.33866315325791	5.53312580531464	6.38705761732955	6.3588681079792	NA
**TC1900000417.mm.2**	Kcnv2	potassium channel, subfamily V, member 2	-0.474202125686305	4.79609726769146	-1.15428654098023	0.289469017827163	0.999692817443324	-4.58752086046134	4.39655715973559	4.4806138766726	4.45753547305022	5.01796868613375	4.76819281266431	5.65571559789226	NA
**TC0300001081.mm.2**	Rhoc	ras homolog family member C	-0.42634381508428	6.97403869020794	-1.15374061274942	0.28967725770192	0.999692817443324	-4.58755150557118	6.61830249030701	7.14574311920322	6.79685983555258	6.81688961523184	6.91077302412876	7.55566405682422	NA
**TC0700002763.mm.2**	Arhgap33	Rho GTPase activating protein 33	0.49812450382159	7.23799481195951	1.15363067294976	0.289719208535502	0.999692817443324	-4.58755767634571	8.40920510074708	8.54503611553106	7.37774174935234	6.3572465822087	6.10657563460705	6.63216368931081	NA
**TC0100002236.mm.2**	Tbc1d8	TBC1 domain family, member 8	0.403886037059562	7.08985461724899	1.15294754490172	0.289979990745042	0.999692817443324	-4.58759601504064	6.50367402162867	7.01994845025263	7.30123826062518	7.6104003922407	7.24838735521254	6.85547922353422	NA
**TC0400004225.mm.2**	Epha8	Eph receptor A8	0.479906121191402	5.08234234535171	1.15290349814236	0.289996812222811	0.999692817443324	-4.58759848678588	4.60730820984468	4.52282517674299	5.06872239480243	6.09314406915027	4.93508946500824	5.26696475656166	NA
**TC0700001626.mm.2**	Swap70	SWA-70 protein	-0.398440978047733	5.85669102026412	-1.15256175706318	0.290127351093972	0.999692817443324	-4.58761766299603	6.03742363219516	6.11805414292962	5.44804603153169	5.64584716365056	5.6176566978154	6.27311845346232	NA
**TC1100001161.mm.2**	Slc46a1	solute carrier family 46, member 1	0.421369351937817	6.37709577433577	1.15189245225825	0.290383156036616	0.999692817443324	-4.58765521439823	6.00162013263842	6.38155041532424	6.32181122626752	7.03926017469628	6.2655762236762	6.25275647341196	NA
**TC1100003771.mm.2**	Nt5c3b	5'-nucleotidase, cytosolic IIIB	0.549275540248408	7.49130923291956	1.15175535546163	0.290435577062154	0.999692817443324	-4.58766290532895	8.59988468245746	8.54746219312246	7.70743497168302	6.74209482953412	7.15734187658665	6.19363684413367	NA
**TC1100001050.mm.2**	Mnt	max binding protein	-0.571582868587012	7.16287176149011	-1.15172318307373	0.290447879808067	0.999692817443324	-4.58766471010888	7.2316487515597	6.78282143421517	7.4695523733033	6.44024494969256	7.06685319842537	7.98610986174452	NA
**TC0200004181.mm.2**	Api5	apoptosis inhibitor 5	-0.627899710969535	6.54758078195191	-1.15163314757334	0.290482311773841	0.999692817443324	-4.58766976075633	7.41083099813179	7.69093715653539	6.48620247312406	4.97775608442856	6.67911797057313	6.04064000891856	NA
**TC0800003233.mm.2**	Trmt1	tRNA methyltransferase 1	0.508699307133214	5.97377155423881	1.15160791975692	0.290491960174052	0.999692817443324	-4.5876711759168	7.33078879408731	7.4142922227554	6.04343313615638	5.01404032527191	5.32873034828317	4.71134449887869	NA
**TC1100004061.mm.2**	Nat9	N-acetyltransferase 9 (GCN5-related, putative)	-0.503729028706336	5.49060803431145	-1.15157084047761	0.29050614166388	0.999692817443324	-4.58767325586917	6.17051218536159	5.65422185137846	4.51014809411866	5.54557996173932	5.65454716623757	5.40863894703309	NA
**TC0300001449.mm.2**	Gtf2b	general transcription factor IIB	0.341163965218299	5.82998205019287	1.1514572826627	0.290549577049838	0.999692817443324	-4.58767962572583	6.55695864510843	6.37560635675421	5.72692926714167	5.63789834772392	5.42257834240063	5.25992134202837	NA
**TC1300002416.mm.2**	Zcchc9	zinc finger, CCHC domain containing 9	-0.355263115414447	6.9151310582723	-1.15052464529467	0.290906512798657	0.999692817443324	-4.58773193269422	6.55049639749692	6.67554828923373	7.01805593673935	6.75905177929778	7.10082560888024	7.38680833798577	NA
**TC0X00003395.mm.2**	Med12	mediator complex subunit 12	0.372556614730278	6.59098335685855	1.14993058466246	0.291134061057814	0.999692817443324	-4.58776524319423	6.9470145812701	6.7083367956467	6.46681265289859	6.85101834394893	6.14177913483104	6.43093863255593	NA
**TC0200002963.mm.2**	Rbm17	RNA binding motif protein 17	-0.802260099901964	6.95021987982211	-1.14979000322709	0.29118793100401	0.999692817443324	-4.58777312511177	7.99608712944526	7.84753011026799	7.06152438434643	5.06506653536132	7.41141183069518	6.3196992888165	NA
**TC1600001545.mm.2**	Sec22a	SEC22 homolog A, vesicle trafficking protein	-0.466835828388039	6.70315502173691	-1.14958161312435	0.291267800173183	0.999692817443324	-4.58778480823361	7.11028014299451	7.37817236511439	6.60523650201219	5.79316648075605	6.95237839520587	6.37969624433844	NA
**TC1700002806.mm.2**	Pdia2	protein disulfide isomerase associated 2	0.397349133390936	4.9666226875358	1.14916828908164	0.291426268146616	0.999692817443324	-4.58780797860954	4.33480597415431	4.7915526272261	5.23286299300333	5.5011749023048	5.19062054310951	4.74871908541674	NA
**TC1400001993.mm.2**	Osgep	O-sialoglycoprotein endopeptidase	-0.340687754291437	6.74850479805427	-1.14907984606044	0.291460186475005	0.999692817443324	-4.58781293623998	6.60977770711045	6.54861372093899	6.53532990248508	6.79030102336157	7.00646950229644	7.00053693213309	NA
**TC0200005295.mm.2**	Ankrd60	ankyrin repeat domain 60	0.469706854510345	5.2193639509796	1.14897526645158	0.291500297527358	0.999692817443324	-4.58781879823357	4.51648072150777	4.54192955660955	5.31190762140332	6.28668594698716	5.51451109443551	5.14466876493428	NA
**TC0200000480.mm.2**	Olfm1	olfactomedin 1	-0.705794266858312	9.61010599375285	-1.1484934981132	0.291685137349597	0.999692817443324	-4.58784580044449	10.2097186059261	10.3523586300186	9.70204254785236	8.14144254857546	10.2062427015511	9.04883092859334	NA
**TC1900000160.mm.2**	Bscl2	Berardinelli-Seip congenital lipodystrophy 2 (seipin)	0.454389274020604	8.25325042341254	1.14819941272176	0.29179801715931	0.999692817443324	-4.58786228150562	8.73312232828761	8.41555294236818	8.4811013000007	8.15870160892963	8.29784543908523	7.43317892180389	NA
**TC0800001862.mm.2**	Nek3	NIMA (never in mitosis gene a)-related expressed kinase 3	0.520227620890142	6.16368316580548	1.14781710216909	0.291944815388707	0.999692817443324	-4.587883704734	5.4128806190142	5.25110243963786	6.24947955528534	7.42980603369522	6.11888805214633	6.51994229505395	NA
**TC1700002773.mm.2**	Rnf8	ring finger protein 8	-0.729116301262428	6.13983747141973	-1.14763648604629	0.292014189208415	0.999692817443324	-4.5878938249376	6.92978424876235	6.57151182638721	6.39775887168957	4.54198920373242	6.57222910886625	5.8257515690806	NA
**TC0500001680.mm.2**	Mafk	v-maf musculoaponeurotic fibrosarcoma oncogene family, protein K (avian)	0.388613932020735	6.58357270393719	1.14739385968431	0.292107402584072	0.999692817443324	-4.58790741882612	6.38844955706508	6.47335941696707	6.86794096705601	6.84048658976022	6.14716207911413	6.78403761366063	NA
**TC1700002225.mm.2**	Nfya	nuclear transcription factor-Y alpha	0.360195727690958	6.6225649989449	1.14739017059915	0.292108820066862	0.999692817443324	-4.58790762551098	7.09644994812295	6.72093853969927	6.78133768954678	6.53785879106777	6.26265161039442	6.33615341483821	NA
**TC1500000417.mm.2**	Fam91a1	family with sequence similarity 91, member A1	-0.894557208325508	7.16414824126556	-1.14723818122097	0.292167225003187	0.999692817443324	-4.58791614068009	8.3209860158587	8.24763226535459	7.41579651899669	4.89778185586783	7.37238117096949	6.73031162054605	NA
**TC0900002387.mm.2**	Islr	immunoglobulin superfamily containing leucine-rich repeat	-0.610959275953243	6.84859615270935	-1.14711885169146	0.292213086590026	0.999692817443324	-4.58792282582191	7.63520730491387	7.65963515381581	6.89974298829027	5.38766496451969	6.4809963995789	7.02833010513755	NA
**TC0400003721.mm.2**	Hmgn2	high mobility group nucleosomal binding domain 2	-0.490750242293562	8.62595178233412	-1.14708562448913	0.292225857780346	0.999692817443324	-4.58792468725194	9.29242067413549	9.48601431736366	8.5008714688254	7.49701614013383	8.73822123810802	8.24116685543833	NA
**TC1600001136.mm.2**	Cdip1	cell death inducing Trp53 target 1	0.371373856722222	7.89430294384137	1.14682316650017	0.292326752408633	0.999692817443324	-4.58793938984108	8.2464308134618	7.98485959729448	7.94745949914037	7.99117798372783	7.36525153708657	7.83063823233719	NA
**TC0800000747.mm.2**	Comp	cartilage oligomeric matrix protein	0.395144004886621	5.26234426922016	1.14638848842377	0.292493916319975	0.999692817443324	-4.58796373749499	4.61592987624371	4.53612839444793	5.51144058642923	6.09470709077205	5.23495469975117	5.58090496767687	NA
**TC0800002087.mm.2**	Sgcz	sarcoglycan zeta	0.334496955030859	6.14322649615933	1.1463811207598	0.292496750387402	0.999692817443324	-4.58796415015355	6.29359814615363	6.20345000310756	6.33618267935368	6.17946968952457	5.91189442417479	5.93476403464175	NA
**TC0200001411.mm.2**	Pex16	peroxisomal biogenesis factor 16	-0.536829543011924	6.8293673715279	-1.14637672884503	0.292498439805263	0.999692817443324	-4.58796439614171	7.26852066050041	7.59560797673867	6.89917431045457	5.62003394249767	6.85846523946661	6.73440209950947	NA
**TC1400002843.mm.2**	LOC100861615	alpha takusan-like	0.699017747178018	7.29939279064452	1.14612243570359	0.292596271506476	0.999692817443324	-4.58797863838338	8.09451102771642	7.1644433192038	8.24141039003708	6.72630855561439	7.1595987263986	6.41008472489684	NA
**TC0500001079.mm.2**	A830010M20Rik/1700028K03Rik	RIKEN cDNA A830010M20 gene/RIKEN cDNA 1700028K03 gene	0.515035217762092	5.61840061422129	1.14604552288858	0.292625866807293	0.999692817443324	-4.58798294584241	5.01358042605208	5.13450611665128	5.76742513857777	6.52876865049653	5.14791834464561	6.1182050089045	NA
**TC0700001804.mm.2**	Tufm	Tu translation elongation factor, mitochondrial	-0.376209889726872	6.60027179687419	-1.14604196971146	0.292627234095674	0.999692817443324	-4.58798314483374	7.05146172945078	7.20783206264247	6.34482961363676	5.95012899121231	6.334007025045	6.71337135925782	NA
**TC1000001286.mm.2**	Osbpl8	oxysterol binding protein-like 8	-0.605382586693802	6.58262661369418	-1.14590072915064	0.292681588817099	0.999692817443324	-4.58799105466862	7.22833149821742	7.24995133217991	6.69860618920191	5.20474964998818	6.82899676836619	6.2851242442115	NA

**TC0200004970.mm.2**	Edem2	ER degradation enhancer, mannosidase alpha-like 2	0.504951921751624	7.24621342105385	1.14573995718522	0.292743470237383	0.999692817443324	-4.5880000579102	7.63701237332647	7.77646897989761	7.64873661738216	6.88811489091899	7.2212809821521	6.3056666826458	NA
**TC0700003720.mm.2**	Eed	embryonic ectoderm development	-0.448319332214239	7.56344778745336	-1.14573586637999	0.292745044938426	0.999692817443324	-4.58800028699	7.10531750297902	7.02162071914274	7.51430959616829	7.66424532291667	7.56910434432396	8.50608923918948	NA
**TC1900000452.mm.2**	Uhrf2	ubiquitin-like, containing PHD and RING finger domains 2	-0.487270320908688	6.95202735994498	-1.14568505062777	0.292764606376558	0.999692817443324	-4.58800313258321	7.29626768642675	7.13233073372117	7.06065272429421	6.09385982455809	7.33980551825128	6.7892476724184	NA
**TC1700000415.mm.2**	Ergic1	endoplasmic reticulum-golgi intermediate compartment (ERGIC) 1	0.536402977257865	8.97952789976542	1.14561839320214	0.292790267697235	0.999692817443324	-4.5880068652175	9.50113329431524	9.2060429773036	9.5580496786343	8.56334886211041	8.93700643453773	8.11158615169124	NA
**TC0100002278.mm.2**	Col5a2	collagen, type V, alpha 2	-0.369196723494149	6.25745778529179	-1.14552626900108	0.292825736138865	0.999692817443324	-4.58801202380057	5.85116271062853	5.37219836266447	6.30028800247488	6.49120809325985	6.71936184584681	6.81052769687622	NA
**TC0200002812.mm.2**	Col20a1	collagen, type XX, alpha 1	-0.341507796581578	7.15770098877683	-1.14525512865933	0.29293014785354	0.999692817443324	-4.58802720574713	6.69464579373254	6.69760585962335	7.31794619670595	7.11752314636502	7.45719100130653	7.66129393492759	NA
**TC0100003799.mm.2**	Tatdn3	TatD DNase domain containing 3	0.547493534470886	6.051365603415	1.14500778138579	0.293025424391787	0.999692817443324	-4.58804105438503	5.94633502668446	5.88044441548669	5.71007138787821	7.07812923575211	5.87324145314973	5.81997210153883	NA
**TC0X00000340.mm.2**	Rhox4a	reproductive homeobox 4A	0.388760778906486	5.37862237292017	1.14499225693088	0.293031405173292	0.999692817443324	-4.58804192354427	5.595842079338	5.23726797966541	5.72495222109199	5.38312064707331	4.95583284611123	5.3747184642411	NA
**TC1800000347.mm.2**	Zmat2	zinc finger, matrin type 2	0.43958695258103	8.55116554067174	1.14438881663073	0.293263959053549	0.999692817443324	-4.58807570492365	9.54033428290574	9.52853995596391	8.32800427791605	8.23064217724536	8.2942420171727	7.38523053282666	NA
**TC0500002087.mm.2**	Smarcd3	SWI/SNF related, matrix associated, actin dependent regulator of chromatin, subfamily d, member 3	-0.418652907889811	8.35185159210764	-1.14386218678511	0.293467037737282	0.999692817443324	-4.58810518139808	8.68253929120677	8.82240154022785	8.32581350400184	7.55861794871396	8.47588273502491	8.24585453347053	NA
**TC0700004576.mm.2**	Ascl2	achaete-scute family bHLH transcription factor 2	-0.449925483313557	5.1219679382986	-1.14386048857671	0.293467692789412	0.999692817443324	-4.58810527644255	3.96215147471183	3.86930818498105	5.22106065041823	5.77918785131758	5.5601957022618	6.33990376610112	NA
**TC1900000972.mm.2**	1700020D05Rik	RIKEN cDNA 1700020D05 gene	-0.579734500069057	4.94302981138611	-1.14382978086246	0.293479537926659	0.999692817443324	-4.5881069950682	4.35053064990793	4.80987420541606	5.20187759610207	4.46727491032522	4.80368725892702	6.02493424763838	NA
**TC0700002080.mm.2**	Mrpl23	mitochondrial ribosomal protein L23	-0.3471346395571	7.08761519179789	-1.14373629814734	0.293515600236568	0.999692817443324	-4.58811222693975	7.67413673932592	7.38479428562244	6.74529804950849	6.64094737385389	7.12446658367596	6.95604811880062	NA
**TC0200000403.mm.2**	Rnf208	ring finger protein 208	0.381840462522331	7.35962913039981	1.14372413839494	0.293520291309103	0.999692817443324	-4.58811290746407	7.6658630966538	7.17536498593621	7.4501797367953	7.58993407563144	6.97116341688003	7.30526947050205	NA
**TC0500000488.mm.2**	Lap3	leucine aminopeptidase 3	-0.735288008370635	7.30305847686353	-1.14368231093349	0.293536428271921	0.999692817443324	-4.58811524833208	8.32471145156542	8.70579762181956	7.31350126695844	5.34513161856903	7.30677660340067	6.82243229886806	NA
**TC1300000814.mm.2**	Gm10767	predicted gene 10767	-0.413906852364947	5.62810330494808	-1.1436653169554	0.293542984731249	0.999692817443324	-4.58811619938933	5.61243631456983	5.46227805813794	5.14456936927137	5.78847650685405	5.66500085229068	6.09585872856464	NA
**TC0600001207.mm.2**	Edem1	ER degradation enhancer, mannosidase alpha-like 1	-0.377628775755471	6.59625556488178	-1.14355993043668	0.293583646714644	0.999692817443324	-4.58812209717166	6.96906392129292	7.17716255768827	6.42969143772744	5.90833324167183	6.62661058533273	6.46667164557749	NA
**TC0Y00000233.mm.2**	Uty	ubiquitously transcribed tetratricopeptide repeat gene, Y chromosome	0.77569205655404	5.323954722472	1.14335111216467	0.293664230361525	0.999692817443324	-4.58813378279317	6.50548503800695	6.38166366605471	5.83788021506826	4.46610165687095	5.23666283844451	3.51593492038663	NA
**TC1100000296.mm.2**	Mtif2	mitochondrial translational initiation factor 2	0.399893929486195	5.35254133759198	1.14323375892267	0.29370952546726	0.999692817443324	-4.58814034964564	6.03059316697572	6.07801672577119	5.45037969750629	4.95283329838237	4.54575626202096	5.05766887489531	NA
**TC0800002525.mm.2**	Hhip	Hedgehog-interacting protein	0.447143090561506	6.9845454936287	1.14321938809991	0.293715072608481	0.999692817443324	-4.58814115379229	7.63589675255779	8.06650496432482	7.07663829354215	6.47294041946414	6.62591872569791	6.02937380618536	NA
**TC1900001545.mm.2**	Cox15	cytochrome c oxidase assembly protein 15	0.483892845949767	6.00548813728321	1.1429737607337	0.293809898366414	0.999692817443324	-4.58815489780349	6.78747673091942	7.04232493416785	6.32639651224122	5.25905991301456	5.20973832238265	5.40793241097359	NA
**TC0700002747.mm.2**	Tbcb	tubulin folding cofactor B	-0.765726221611554	7.93877775755758	-1.14263563876805	0.293940473987562	0.999692817443324	-4.58817381567278	8.36753802750371	8.36493041481799	8.34411824123505	6.3402545886653	8.52583267397796	7.6899925991455	NA
**TC1100003095.mm.2**	Pelp1	proline, glutamic acid and leucine rich protein 1	-0.574029965815125	5.34516704165681	-1.14242748854209	0.294020881345206	0.999692817443324	-4.58818546069196	6.32917680997814	6.2422494529569	5.254860677742	3.92089734994578	4.84634424654994	5.4774737127681	NA
**TC1200001900.mm.2**	Ccdc175	coiled-coil domain containing 175	0.333944537152115	5.09474160883689	1.14238654548041	0.294036699600366	0.999692817443324	-4.58818775117685	4.9390064180735	4.76315696234815	5.34380199329264	5.42328568015933	5.04950250917521	5.04969608997253	NA
**TC1500000660.mm.2**	Triobp	TRIO and F-actin binding protein	0.339946650592915	6.37611937982413	1.14231129508036	0.294065774268164	0.999692817443324	-4.58819196084989	7.31927377894597	7.54818581962321	6.0961064990426	5.93846849173811	5.67804917269757	5.67663251689731	NA
**TC1100000459.mm.2**	Clint1	clathrin interactor 1	0.440610782409054	8.04826556843931	1.1420039953015	0.294184531302959	0.999692817443324	-4.58820915089001	8.65904272282126	8.58610656275503	8.37533443361233	7.58749841132652	7.77888985217023	7.30272142795052	NA
**TC1000003118.mm.2**	Nxph4	neurexophilin 4	0.46076675431406	6.65651617243372	1.14191649600807	0.294218353023684	0.999692817443324	-4.58821404522245	6.54719489155497	6.49456298020944	6.51524258688825	7.39419374884477	6.24052175791773	6.74738106918716	NA
**TC0700002037.mm.2**	B4galnt4	beta-1,4-N-acetyl-galactosaminyl transferase 4	0.363875847410518	6.3743453998843	1.14159758744444	0.294341650488343	0.999692817443324	-4.58823188250113	7.05692574404654	6.95052379030572	6.01803682558021	6.46515045430709	5.96944238753027	5.785993197536	NA
**TC1000003215.mm.2**	Sumo3	small ubiquitin-like modifier 3	0.330996377292958	8.99271181162884	1.14111178810071	0.294529554713933	0.999692817443324	-4.58825905107269	9.64279672341761	9.50589677810744	8.84802497923016	8.88676008218679	8.54121556166675	8.53157674516427	NA
**TC0400000576.mm.2**	Nipsnap3b	nipsnap homolog 3B	-0.751496577338763	7.51864321176893	-1.14092977151963	0.29459998340101	0.999692817443324	-4.58826922941806	7.76961666896602	7.59286874506253	8.12051949477831	6.00267085617545	7.83281365744042	7.79336984819086	NA
**TC0300002139.mm.2**	B3galnt1	UDP-GalNAc:betaGlcNAc beta 1,3-galactosaminyltransferase, polypeptide 1	-0.555682918053467	8.01600276482602	-1.14076194755367	0.294664932915789	0.999692817443324	-4.58827861361946	8.45909876863603	8.37449478710931	8.07608631499827	6.99944228355364	8.54722559062324	7.63966884403561	NA
**TC0900002010.mm.2**	Fam118b	family with sequence similarity 118, member B	-0.363496182452466	6.21456151383727	-1.14007888894733	0.294929406589801	0.999692817443324	-4.58831680325949	5.88727047057568	6.16878673788761	6.20001586311276	6.05214389171495	6.29949777257936	6.67965434715328	NA
**TC0700004194.mm.2**	Crym	crystallin, mu	0.364827079176628	7.5988215131683	1.14001188990001	0.294955358636054	0.999692817443324	-4.58832054873559	7.99244531411042	7.91636606487436	7.42210665381167	7.78477927537747	7.06681412644838	7.4104176443875	NA
**TC0500000238.mm.2**	Actr3b	ARP3 actin-related protein 3B	-0.371001602537165	7.78415014828064	-1.13997603349655	0.2949692483781	0.999692817443324	-4.58832255320012	8.06624821220542	8.16205335573483	7.64023112587369	7.22706693246095	7.9782504965381	7.63105076687087	NA
**TC0100001602.mm.2**	Cd84	CD84 antigen	0.521598897714489	5.60248644609781	1.13980831723586	0.295034224092753	0.999692817443324	-4.58833192867884	5.30880596080956	6.45362848707733	5.51538075690386	5.93246025516062	5.01060941293405	5.39403380370145	NA
**TC1500001410.mm.2**	Klf10	Kruppel-like factor 10	0.540669311852955	6.5334795173408	1.13965396581233	0.295094032627368	0.999692817443324	-4.58834055663164	6.6850903759286	6.82711780629403	6.0288219156888	7.35618185707522	6.26459784902328	6.03906730003483	NA
**TC0700004360.mm.2**	9130023H24Rik	RIKEN cDNA 9130023H24 gene	0.34891420057696	5.69970151563219	1.13942633051546	0.29518225586731	0.999692817443324	-4.58835328027866	5.88148296747814	5.71605544584802	5.99546143810491	5.65378810270553	5.48110740105068	5.47031373860585	NA
**TC1100003071.mm.2**	Tmem95	transmembrane protein 95	0.614758801853501	4.65797367755303	1.13894101721663	0.295370419278889	0.999692817443324	-4.58838040387539	4.14410069356931	4.30305530097837	4.62279059944043	5.74231123779833	3.9747675635228	5.16081667000896	NA
**TC1900000606.mm.2**	Ankrd2	ankyrin repeat domain 2 (stretch responsive muscle)	0.559759446827978	5.58075964144303	1.13865697717779	0.295480592364051	0.999692817443324	-4.58839627669037	5.08843255438163	4.6476335608767	5.60381087489979	6.8301944386281	5.36226044979756	5.95222597007437	NA
**TC1700001173.mm.2**	Rasgrp3	RAS, guanyl releasing protein 3	-0.508207310623921	6.1886302316837	-1.13862432372822	0.295493260138345	0.999692817443324	-4.58839810135297	6.4269859797939	7.25065687705319	5.85221883164251	5.36664312436114	6.50044569016563	5.73483088708586	NA
**TC0200003192.mm.2**	Notch1	notch 1	0.430602626687056	6.79787656600705	1.1381824416466	0.295664731052247	0.999692817443324	-4.58842279177792	6.68841064161281	7.03913696559624	6.66097072605502	7.29948779504865	6.30045721429595	6.79879605343361	NA
**TC1000002224.mm.2**	Ddit4	DNA-damage-inducible transcript 4	-0.380483443222736	6.9062655171871	-1.13790027547879	0.295774268122687	0.999692817443324	-4.58843855624407	7.11689293643203	7.08033764345242	6.88315244755794	6.35654537083841	7.15648223719527	6.84418246764654	NA
**TC0600001736.mm.2**	Stk38l	serine/threonine kinase 38 like	-0.347845364074881	6.82761675647615	-1.13787724826045	0.295783208795366	0.999692817443324	-4.58843984270209	7.14636672991766	7.18161879042143	6.39911938591734	6.57189275926668	6.6682617606275	6.99844111270629	NA
**TC1400000316.mm.2**	Mustn1	musculoskeletal, embryonic nuclear protein 1	-0.369971993752871	5.67643710202651	-1.13783910563084	0.295798018755344	0.999692817443324	-4.58844197359059	5.72406839946434	5.60416403078229	5.34864034619077	5.64658275101257	5.64688756812875	6.08827951658034	NA
**TC0700000843.mm.2**	Kcna7	potassium voltage-gated channel, shaker-related subfamily, member 7	-0.382590822969506	6.51625782527788	-1.13777249823766	0.295823882451247	0.999692817443324	-4.5884456946414	6.11458839816432	5.96924528028561	6.33809846188415	6.78616735175503	6.73820994020901	7.15123751936918	NA
**TC1300000635.mm.2**	Cdhr2	cadherin-related family member 2	-0.454004043012981	5.57398694619395	-1.13721838863418	0.296039116674933	0.999692817443324	-4.58847664728375	5.46652017018522	5.12673364489378	5.13838128106067	5.83294860696869	5.6273518092514	6.25198616480392	NA
**TC0800000758.mm.2**	Lrrc25	leucine rich repeat containing 25	-0.402266292421165	5.58496436595985	-1.13719382519943	0.296048660934187	0.999692817443324	-4.58847801927958	5.6631550587375	5.12757316353985	5.30712747651181	5.65013521780789	5.69179432254073	6.0700009566213	NA
**TC0500003390.mm.2**	Vps37d	vacuolar protein sorting 37D	0.368534832740568	7.53492814206189	1.13699179247084	0.296127171605745	0.999692817443324	-4.58848930346961	6.9862807773656	7.29505996539855	7.69635119236056	8.13629769518359	7.53612518942479	7.55945403263823	NA
**TC0200003705.mm.2**	Mettl5	methyltransferase like 5	0.901964843443207	5.86978475661505	1.13633780396447	0.296381432988958	0.999692817443324	-4.58852582608954	6.22775394057796	6.51085942759584	6.37682604472585	5.7651863844756	6.45230629194605	3.88577645036898	NA
**TC0200000545.mm.2**	Tor1b	torsin family 1, member B	0.32714917537621	7.73313831724926	1.13622898785399	0.296423756785952	0.999692817443324	-4.58853190232064	7.15380035029194	7.18586378923379	8.14929051171671	8.2074415456444	7.85352764532464	7.84890606128405	NA
**TC1500000081.mm.2**	Rad1	RAD1 checkpoint DNA exonuclease	-0.381856370903626	5.13318513000798	-1.13601388133059	0.296507436832581	0.999692817443324	-4.58854391315288	5.26435804169834	5.50317418134058	5.05206924515058	4.58186366245028	5.03054747149819	5.36709817790991	NA
**TC0300000783.mm.2**	Rab13	RAB13, member RAS oncogene family	0.456858626397922	6.11858195594017	1.13596088716298	0.296528055474467	0.999692817443324	-4.58854687204914	7.0414638807012	6.11689841441419	5.9371633699186	6.29625997674215	5.75303461158341	5.5666714822815	NA
**TC0200005334.mm.2**	Gm14391/Gm6710	predicted gene 14391/predicted gene 6710	-0.84833001403883	8.80587677008135	-1.13595248688692	0.296531323911433	0.999692817443324	-4.58854734106889	9.53553643197606	10.3850239708349	8.74045683794553	6.86856325685422	9.33941385071397	7.96626627216344	NA
**TC0200002794.mm.2**	Mrgbp	MRG/MORF4L binding protein	0.497747710369429	7.13276897322072	1.13587002372205	0.296563410838272	0.999692817443324	-4.58855194524064	7.09535102838302	6.16693386974481	7.32625992188994	7.93865225907772	7.34144123823217	6.92797552199663	NA
**TC0100000440.mm.2**	Casp8	caspase 8	-0.622499587079552	6.76916500735001	-1.13570801529792	0.29662645774088	0.999692817443324	-4.58856099032912	7.35750849579122	7.9280341979527	6.56890127543886	5.47332281265966	7.2084959061695	6.07872735608813	NA
**TC0800003242.mm.2**	Trim61/Trim60	tripartite motif-containing 61/tripartite motif-containing 60	0.462237384009295	6.59885753125604	1.13566221297996	0.29664428411189	0.999692817443324	-4.58856354743565	5.7737054735523	5.73684606506407	6.80245643800247	7.70107777046676	6.53457056323236	7.04448887721827	NA
**TC1100003918.mm.2**	Smarcd2	SWI/SNF related, matrix associated, actin dependent regulator of chromatin, subfamily d, member 2	0.539851260637214	7.08091829372466	1.13560143293241	0.29666794122883	0.999692817443324	-4.58856694068122	5.90698824324554	5.82771828593244	7.51616074639782	8.39909213082439	6.90324863442194	7.93230172152585	NA
**TC0900002296.mm.2**	Zc3h12c	zinc finger CCCH type containing 12C	0.475050292231887	6.44309820100655	1.13543586326608	0.296732393062349	0.999692817443324	-4.58857618382961	7.02724759537407	6.56630740336255	6.93829294666924	6.06927444921399	6.26426126449234	5.79320554692711	NA
**TC0700001134.mm.2**	Wdr93	WD repeat domain 93	-0.555206284672556	4.65074645306537	-1.1347636773801	0.296994176820351	0.999692817443324	-4.58861370467207	4.00386895642728	4.06043379569694	4.02093687005059	5.34394482841086	4.98099103081928	5.49430323698729	NA
**TC0800001808.mm.2**	Defb8	defensin beta 8	-0.454308488867512	6.16456318928579	-1.13471128190837	0.297014590390443	0.999692817443324	-4.5886166290163	5.95902785378616	5.83454884044963	6.08952474890294	6.05306798296903	6.02985971798998	7.02134999161702	NA
**TC0900000594.mm.2**	Gm11149	predicted gene 11149	0.506671247183085	7.51477768570351	1.1343063415473	0.297172396844048	0.999692817443324	-4.58863922832634	7.94178854859572	7.64558782238876	8.14395994190386	7.11335617689752	7.41967190125963	6.82430172317558	NA
**TC0600001171.mm.2**	Chl1	cell adhesion molecule L1-like	0.494878618271581	7.4430384633252	1.13425310021528	0.297193150340451	0.999692817443324	-4.58864219946094	9.02521444488302	8.93852136680886	7.34802874293533	6.4940973594659	6.8292346482201	6.02313421763797	NA
**TC1200001118.mm.2**	Slc25a47	solute carrier family 25, member 47	0.65813154408258	5.66844980244572	1.13414483281656	0.297235356745898	0.999692817443324	-4.58864824117669	5.48112004401909	5.34734772254913	5.61499094480898	6.63425612332665	4.72280301736448	6.21018096260599	NA
**TC1300002326.mm.2**	Cast	calpastatin	0.490873917568035	6.11029492058983	1.13397796228354	0.297300418469329	0.999692817443324	-4.58865755276537	5.79139966260182	6.23003230531058	5.8733876311228	6.93765506425853	5.99072840569085	5.83856645455441	NA
**TC0700001169.mm.2**	Pde8a	phosphodiesterase 8A	0.877332516827488	5.41021366415829	1.13392016396156	0.297322956419449	0.999692817443324	-4.58866077787354	6.99453309234782	7.21718283935004	5.70330159256415	4.29881395088929	5.19789384450876	3.0495566652897	NA
**TC0500000400.mm.2**	Ablim2	actin-binding LIM protein 2	0.379978470072016	8.37050310551248	1.13314073378295	0.297627027347543	0.999692817443324	-4.58870426392999	8.40136812035377	8.28663022476129	8.87832977799921	8.26915883605272	8.18692540200713	8.20060627190077	NA
**TC1800001198.mm.2**	Ndufa2	NADH:ubiquinone oxidoreductase subunit A2	-0.405087675015009	8.30015948019262	-1.13298211706226	0.297688938513212	0.999692817443324	-4.58871311220631	8.59785868396171	8.33083496177272	8.3729474686051	7.65809647409054	8.41749260334786	8.4237266893778	NA
**TC1900000186.mm.2**	Fads3	fatty acid desaturase 3	-0.478698673912252	5.98038162914572	-1.13286768962413	0.297733608404418	0.999692817443324	-4.58871949515709	6.29962691369111	6.0966607055628	6.13683520220432	5.12746720169362	6.31978971622218	5.90191003550026	NA
**TC1500002328.mm.2**	Agxt2	alanine-glyoxylate aminotransferase 2	-0.724847144235342	5.20879606916473	-1.13219369773282	0.297996832841679	0.999692817443324	-4.5887570869321	4.12795455866764	4.68058422527394	4.48906988361452	6.00820178767352	5.34169803418818	6.60526792557055	NA
**TC1500001848.mm.2**	Card10	caspase recruitment domain family, member 10	0.580801195959768	4.67457430967559	1.13218840259156	0.29799890160114	0.999692817443324	-4.58875738223647	4.61389403805522	4.03719424925344	4.68728294188865	5.59169703944356	4.01733712414549	5.10004046526718	NA
**TC1600000298.mm.2**	Ephb3	Eph receptor B3	-0.426008436975501	6.02986475849484	-1.13210365111399	0.298032014793671	0.999692817443324	-4.58876210866952	5.71171105298827	5.89283315425653	5.55590594167073	6.30540779321588	6.15367131360515	6.55965929523247	NA
**TC0100001806.mm.2**	Esrrg	estrogen-related receptor gamma	0.36627192678254	7.10708692868233	1.13193729440643	0.298097020812305	0.999692817443324	-4.58877138571274	7.23336768206684	7.09138298145747	7.51963249994596	7.0055248811214	6.90603863135201	6.88657489615027	NA
**TC0Y00000235.mm.2**	Ddx3y	DEAD (Asp-Glu-Ala-Asp) box polypeptide 3, Y-linked	0.669775903185396	7.22586465148373	1.13183962326679	0.298135192559368	0.999692817443324	-4.58877683221477	8.36375845313916	8.19938838994484	7.49350348294334	6.57229295315124	7.1641126211593	5.56213200856449	NA
**TC0700002102.mm.2**	Dhcr7	7-dehydrocholesterol reductase	0.42554458992127	7.78150274601698	1.13109494859521	0.298426359346364	0.999692817443324	-4.58881835256278	8.34158180087318	8.25806928095694	8.0507753571881	7.41945192986905	7.01244965017715	7.60668845703745	NA
**TC1900001114.mm.2**	Cd6	CD6 antigen	0.457555453734743	5.2617326372959	1.13102870920531	0.298452270308756	0.999692817443324	-4.58882204536272	4.67977262209322	5.2254526988955	5.30817777791784	5.98196292721024	4.91763573646104	5.45739406119755	NA
**TC0200000438.mm.2**	Kcnt1	potassium channel, subfamily T, member 1	0.498944422267901	6.79184657323568	1.13101233527454	0.298458675612443	0.999692817443324	-4.5888229581862	7.27324279295663	7.47096947396204	6.72598052006097	6.77639748845462	6.84375785371008	5.66073131026971	NA
**TC1700002147.mm.2**	Spats1	spermatogenesis associated, serine-rich 1	-0.357991132775014	6.76632839647617	-1.13100379882891	0.298462015022497	0.999692817443324	-4.58882343407911	6.71908176695863	6.65277812504927	6.73182018702413	6.52324392362543	6.77405967343701	7.19698670276258	NA
**TC0500002172.mm.2**	Slc30a3	solute carrier family 30 (zinc transporter), member 3	-0.372525389168423	8.07578270546117	-1.1302655425883	0.298750934321056	0.999692817443324	-4.58886458585553	7.86585255981798	8.10785417449663	8.14953887717056	7.71843048288723	8.45727913629293	8.1557410021017	NA
**TC0400001594.mm.2**	Eya3	EYA transcriptional coactivator and phosphatase 3	0.452975886918867	7.86968072660699	1.13010607451021	0.298813373272067	0.999692817443324	-4.5888734736541	7.69412168568714	7.68220895199186	8.46542017925774	7.90843256864259	8.15053217601836	7.31736879804424	NA
**TC0600000962.mm.2**	Smyd5	SET and MYND domain containing 5	-0.424107396666669	6.5691342231324	-1.12984566799317	0.298915357501325	0.999692817443324	-4.5888879862036	6.82657544252314	7.2111948419079	6.48649093369222	5.77791919682279	6.39789644332271	6.71472848052564	NA
**TC1900000465.mm.2**	Minpp1	multiple inositol polyphosphate histidine phosphatase 1	-0.341984047371048	7.52713694593588	-1.12968117552764	0.298979793369714	0.999692817443324	-4.58889715281742	7.5981106703473	7.56708207997459	7.46957571650955	7.1872546987661	7.57526963977624	7.7655288702415	NA
**TC1700001633.mm.2**	Tsc2	tuberous sclerosis 2	-0.347896299926418	6.7373046141027	-1.12951353523946	0.299045474199249	0.999692817443324	-4.5889064943619	8.32098146843349	8.47482028859554	5.85603870894148	5.6100779549257	6.21712667723892	5.94478258648109	NA
**TC0600003415.mm.2**	Casc1	cancer susceptibility candidate 1	-0.371309259234521	4.88093359532528	-1.12950197166293	0.299050005206849	0.999692817443324	-4.58890713870955	4.51841921273264	4.79810455484788	4.56908491093316	5.04414473201792	5.10012803095332	5.2557201304668	NA
**TC1100000711.mm.2**	Btnl10	butyrophilin-like 10	-0.509351211902241	4.77382058586103	-1.12908242719942	0.299214435774028	0.999692817443324	-4.58893051505658	4.42285313701252	4.37575771263827	4.25965491220335	5.15315020865211	4.78128172705904	5.6502258176009	NA
**TC1000000877.mm.2**	Gadd45b	growth arrest and DNA-damage-inducible 45 beta	-0.356864054943815	6.768221941459	-1.12892244693827	0.299277156053414	0.999692817443324	-4.58893942808889	6.67963811229591	7.08625670237714	6.5490098498676	6.51584451222907	6.76996622691098	7.00861624507331	NA
**TC0900001386.mm.2**	Traip	TRAF-interacting protein	-0.41596340263622	5.4883909457834	-1.12792946241766	0.299666700134244	0.999692817443324	-4.58899474051185	5.52835018932667	5.17049688259247	5.32823902107562	5.37154687767879	5.39254249112316	6.1391702129037	NA
**TC1000002721.mm.2**	Cradd	CASP2 and RIPK1 domain containing adaptor with death domain	-0.409181489013449	6.19180432917555	-1.1278317259572	0.29970506453937	0.999692817443324	-4.58900018380946	6.12023235689112	6.24630398211092	6.08268333420395	5.90027999480824	6.01923714225212	6.78208916478697	NA

**TC0900001054.mm.2**	Mto1	mitochondrial tRNA translation optimization 1	0.47692440694843	5.38281591685887	1.12777352794062	0.29972791089255	0.999692817443324	-4.58900342498803	6.12779774987817	5.92513140473307	5.22665921069854	5.37224836952086	5.36475166500145	4.28030710132109	NA
**TC0600002178.mm.2**	Tcaf1	TRPM8 channel-associated factor 1	0.37833107481518	7.80249469767636	1.1274786422001	0.299843694183164	0.999692817443324	-4.58901984692233	9.02847834862454	8.98127946925369	7.55718676273102	7.22374949617414	7.27336306837696	6.75091104089784	NA
**TC1300002689.mm.2**	Gpx8	glutathione peroxidase 8 (putative)	0.448813272211489	5.56150865414272	1.12746651975827	0.29984845470701	0.999692817443324	-4.58902052197796	6.20197528432114	6.93426424538681	5.0934248853948	5.47179458439088	4.56138395278882	5.10620897257388	NA
**TC0900001768.mm.2**	Endod1	endonuclease domain containing 1	-0.467915098919558	7.455299776806	-1.12717591877391	0.299962593485338	0.999692817443324	-4.58903670373704	8.01141438060375	7.6494491635817	7.53735023048064	6.53020222892508	7.5496338515149	7.45374880572993	NA
**TC1700002315.mm.2**	Lrg1	leucine-rich alpha-2-glycoprotein 1	0.412625873467348	5.86151710397786	1.12683333784931	0.300097194660196	0.999692817443324	-4.58905577802256	5.58993222645231	6.08338140857985	5.90255808605069	6.25796228183416	5.93827090981227	5.39699771113788	NA
**TC1000003177.mm.2**	Olfr771	olfactory receptor 771	-0.591234848933428	5.09060001310407	-1.12676683822561	0.300123328393512	0.999692817443324	-4.58905948036113	4.69362485193851	4.63719414208367	4.44961929850156	5.56553639486613	5.04409058920462	6.15353480202993	NA
**TC0300000906.mm.2**	Golph3l	golgi phosphoprotein 3-like	0.461952461445683	6.61410044792333	1.12660927186231	0.300185258051539	0.999692817443324	-4.58906825249036	7.22181926498223	6.97313777405352	7.0732242408366	6.13355104486121	6.33877958887029	5.94409077393616	NA
**TC0X00000396.mm.2**	Xiap	X-linked inhibitor of apoptosis	-0.523705663490637	7.47258877091099	-1.12634365199435	0.300289680923594	0.999692817443324	-4.58908303924616	8.11006615922663	8.02212639893987	7.39536387727932	6.43260049287977	7.88042000956051	6.99495568757986	NA
**TC1200001698.mm.2**	Gm1818	predicted gene 1818	0.45882956529713	7.44260368548977	1.12620432267695	0.300344467375536	0.999692817443324	-4.58909079505195	8.32222120045562	8.39457741400726	7.17325909034254	7.25498222419246	7.27033721430187	6.24024496963888	NA
**TC1500000654.mm.2**	Cdc42ep1	CDC42 effector protein (Rho GTPase binding) 1	-0.373110524952994	6.10532200690226	-1.12594648016949	0.300445876941756	0.999692817443324	-4.58910514702199	5.8024088303085	6.22105172937603	6.02238191449056	5.90874330142096	6.13721946010052	6.54012680571699	NA
**TC0400000269.mm.2**	Casp8ap2	caspase 8 associated protein 2	-0.355558708753854	5.58998056648352	-1.12574756033737	0.300524131615706	0.999692817443324	-4.58911621844742	6.25347843882544	6.40261639592052	4.99094858377816	5.09538698954555	5.18189449606915	5.61555849476227	NA
**TC1900000604.mm.2**	Ubtd1	ubiquitin domain containing 1	0.349136618934694	7.18150148493662	1.12560380648743	0.300580694630101	0.999692817443324	-4.58912421902341	7.17124997910565	6.83081379990768	7.44645498815531	7.44615419608258	7.21934044176371	6.97499550460479	NA
**TC0400003079.mm.2**	Jun	jun proto-oncogene	-0.532212805076782	7.71768045494963	-1.12547676846424	0.300630687800674	0.999692817443324	-4.58913128898037	8.03902492812789	8.56188974622356	7.75556307537319	6.5648081472232	7.86768457097312	7.51711226177683	NA
**TC0400003234.mm.2**	Scp2	sterol carrier protein 2, liver	-0.422057503857518	5.9654010718584	-1.12542238708258	0.300652090571091	0.999692817443324	-4.58913431534135	7.24958215675623	7.66555744384758	4.85507152981364	5.16150438160214	5.77679586347679	5.08389505565403	NA
**TC0600003165.mm.2**	Ndufa9	NADH:ubiquinone oxidoreductase subunit A9	-0.879043030498547	7.6939627885864	-1.12532494942053	0.30069044207748	0.999692817443324	-4.58913973768252	8.79665083425255	8.59304165099709	8.0310047847688	5.47699430786704	7.86844686205862	7.39763829157431	NA
**TC1900000255.mm.2**	Lpxn	leupaxin	0.388507600063175	5.2436162955673	1.12523989040586	0.30072392466184	0.999692817443324	-4.58914447102249	5.13273717974019	5.31561384171396	5.35238128545931	5.54279969057868	4.74218464363872	5.37598113227293	NA
**TC1000000169.mm.2**	1700020N01Rik	RIKEN cDNA 1700020N01 gene	-0.354966134422844	5.68554527981684	-1.12519969517776	0.300739748166109	0.999692817443324	-4.58914670775049	5.23177441789684	4.97080313933844	5.9767096608826	5.62367126552743	6.07540001251101	6.23491318274471	NA
**TC1200001047.mm.2**	Serpina3n	serine (or cysteine) peptidase inhibitor, clade A, member 3N	-0.557406545620508	7.66111507923917	-1.12509171396656	0.300782260144683	0.999692817443324	-4.58915271639637	8.66105546669967	8.93374910942208	7.5077136835839	6.12082272045221	7.21575305690886	7.52759643836827	NA
**TC1900000350.mm.2**	Cemip2	cell migration inducing hyaluronidase 2	0.356678458074956	5.6145058743134	1.12503275890779	0.300805472733378	0.999692817443324	-4.58915599688039	5.91479568456472	5.90710313626852	5.48828376815942	5.8009629024391	5.47037396261669	5.10551579183192	NA
**TC1200000740.mm.2**	Galnt16	polypeptide N-acetylgalactosaminyltransferase 16	0.415000780893442	8.48981915849813	1.12497005368882	0.30083016351757	0.999692817443324	-4.58915948596987	9.54435499435531	9.21831830142387	8.63079707669699	7.87232453180124	7.87807188994827	7.79504815676308	NA
**TC0200002489.mm.2**	Hnf4a	hepatic nuclear factor 4, alpha	-0.369956056441836	6.4059547272612	-1.12467296903848	0.300947166320087	0.999692817443324	-4.58917601562096	5.95599331337044	6.25740849375691	6.5794038492649	6.16180337251319	6.61344229131985	6.86767704334192	NA
**TC1100000754.mm.2**	Drg2	developmentally regulated GTP binding protein 2	0.554737460061888	7.13398928135354	1.12403193939821	0.301199755866881	0.999692817443324	-4.58921167686298	8.24413529242308	8.14740657007693	7.59706587046199	6.1638685024105	6.36874695978631	6.2827124929624	NA
**TC1700002431.mm.2**	Mtcl1	microtubule crosslinking factor 1	-0.329979011821826	6.95867069478558	-1.12400237326123	0.301211410267227	0.999692817443324	-4.58921332148589	7.1633719721955	7.12140332025309	6.70795557134345	6.69568985496716	7.13759885513957	6.9260045948147	NA
**TC0X00000305.mm.2**	Btbd35f8	BTB domain containing 35, family member 8	0.421469619658023	4.96477723800885	1.1239818981238	0.301219481390288	0.999692817443324	-4.5892144604108	4.96334581879654	4.80214626544158	4.858877491024	5.57417780054152	4.56647591421581	5.02364013803366	NA
**TC0500000080.mm.2**	Sema3d	sema domain, immunoglobulin domain (Ig), short basic domain, secreted, (semaphorin) 3D	-0.551790089189763	5.95361778671832	-1.12380134341824	0.301290662268213	0.999692817443324	-4.5892245034023	6.81149645205459	6.60228646797201	5.31150867418678	5.29066313676512	6.55904215786067	5.14670983147075	NA
**TC1000000366.mm.2**	Ppil6	peptidylprolyl isomerase (cyclophilin)-like 6	-0.7207570360452	6.24100849962456	-1.1237789480002	0.301299492285147	0.999692817443324	-4.58922574906177	6.33441039430937	6.33012084390637	6.81104689638473	4.85895594733588	6.879068828283	6.23244808752801	NA
**TC0800003024.mm.2**	Hsdl1	hydroxysteroid dehydrogenase like 1	-0.625710972030843	6.47879015063094	-1.12372773882703	0.301319683733591	0.999692817443324	-4.58922859734255	7.18394724445582	7.0488726563329	6.69883817873327	4.99541135073436	6.51129185511119	6.43437961841812	NA
**TC0700003657.mm.2**	Fah	fumarylacetoacetate hydrolase	0.424449949058057	7.02708073516648	1.12357271970547	0.301380813619557	0.999692817443324	-4.58923721930146	7.51813183683509	7.40453101739365	6.59208702003621	7.45227370740692	6.52559331378051	6.6698675155465	NA
**TC1200000247.mm.2**	Myt1l	myelin transcription factor 1-like	0.437320862155008	6.72258351996486	1.1231131813756	0.301562087351766	0.999692817443324	-4.5892627756966	7.41542105719611	7.57873313717788	6.83818935971964	6.26980496514295	6.48359134434669	5.74976125620588	NA
**TC1800000787.mm.2**	Cxxc1	CXXC finger 1 (PHD domain)	0.339504882976856	6.69857285684322	1.12307167596948	0.301578464408392	0.999692817443324	-4.58926508375917	6.60278613461541	6.81702562244545	6.84384874441469	6.8814688305614	6.6518382897668	6.39446951925559	NA
**TC0800000215.mm.2**	Defa25	defensin, alpha, 25	-0.355136113067209	4.99797485778076	-1.12291792980001	0.301639135454834	0.999692817443324	-4.58927363311884	4.44152915114744	4.49656443923173	5.2675024103513	4.90223925473419	5.58848148771125	5.29153240350866	NA
**TC0600002978.mm.2**	Rassf4	Ras association (RalGDS/AF-6) domain family member 4	0.496395997660311	5.40171233833895	1.12282805399938	0.301674606774246	0.999692817443324	-4.58927863064467	6.21251601463692	5.4575338390287	4.97930755159629	5.88720053424805	5.05692749033151	4.81678860019221	NA
**TC0100001018.mm.2**	Phlpp1	PH domain and leucine rich repeat protein phosphatase 1	0.412200537241106	8.31401730557966	1.12268948191667	0.301729303852284	0.999692817443324	-4.58928633563438	8.93169887137409	8.71912066075735	7.87771106842124	8.65113161949312	7.93675858779342	7.76768302563873	NA
**TC0100000399.mm.2**	Mars2	methionine-tRNA synthetase 2 (mitochondrial)	0.401878108208325	5.89564211218904	1.12266984951159	0.301737053806761	0.999692817443324	-4.58928742722243	6.56758724000395	6.46164567396645	6.1652045893443	5.40898339844595	5.35705414330221	5.41337762807139	NA
**TC0800001855.mm.2**	Defb9	defensin beta 9	0.448086541727283	5.28961628508	1.12262774215141	0.301753676377968	0.999692817443324	-4.58928976842486	5.01807919837106	4.88281410334527	5.19120328238144	6.17528546372767	5.24617606076859	5.22413960188596	NA
**TC1700001015.mm.2**	Ndufa11	NADH:ubiquinone oxidoreductase subunit A11	-0.498432192316685	9.90958435320269	-1.1225550643046	0.301782368936336	0.999692817443324	-4.5892938092958	9.61612434198003	9.42095766636776	10.36210010744	9.34967975567748	10.6747698302663	10.0338744174845	NA
**TC0400004157.mm.2**	Tas1r3	taste receptor, type 1, member 3	-0.512328533811938	5.66193192419818	-1.1223587820463	0.301859870704509	0.999692817443324	-4.58930472206998	5.09776087890225	5.30727840164523	5.19254619822021	6.07840140028865	5.70353613142563	6.59206853470711	NA
**TC1000000760.mm.2**	Pttg1ip	pituitary tumor-transforming 1 interacting protein	0.398552935885746	8.60840567864715	1.1221605231796	0.301938169682523	0.999692817443324	-4.58931574403783	8.67069276816786	8.6268091457093	9.00051569578306	8.57450331910555	8.69602591972317	8.08188722339395	NA
**TC0400002480.mm.2**	Ccl27a	chemokine (C-C motif) ligand 27A	-0.36916198131374	9.54530068262145	-1.12209298994974	0.301964844630383	0.999692817443324	-4.58931949830726	10.2820375464131	10.493845160156	9.1813018572012	8.69749685606486	9.42187348103905	9.19524919485448	NA
**TC1600000502.mm.2**	Wdr5b	WD repeat domain 5B	-0.449581179081878	5.40808374568183	-1.12205514270264	0.301979794767154	0.999692817443324	-4.58932160225444	5.69665372704148	5.90156370177134	5.31792935084787	4.65763199270935	5.07507153681346	5.79965216490751	NA
**TC0700002987.mm.2**	Prrg2	proline-rich Gla (G-carboxyglutamic acid) polypeptide 2	-0.357604872304758	5.68070711728049	-1.12130377780265	0.302276720320455	0.999692817443324	-4.58936336568092	5.66887640473055	5.2958785284253	5.49928357232519	5.70285544063359	5.83045447619412	6.08689428137418	NA
**TC0200001904.mm.2**	Ap4e1	adaptor-related protein complex AP-4, epsilon 1	0.345380504479046	6.3343359036859	1.12043614849526	0.302619892356431	0.999692817443324	-4.58941157885599	5.7643788509523	6.00361207142802	6.59983518709486	6.86455756725173	6.29597912756927	6.47765261781923	NA
**TC1500001320.mm.2**	Ankrd33b	ankyrin repeat domain 33B	-0.386921195626584	9.82904857664569	-1.11983378680136	0.302858333281911	0.999692817443324	-4.5894450434344	8.72485835145597	8.66141414145059	9.86169827308572	10.5453900147715	10.6137249007186	10.5672057783918	NA
**TC0X00000329.mm.2**	Ube2a	ubiquitin-conjugating enzyme E2A	0.676244362269492	6.14888615950856	1.11982757711147	0.302860792157007	0.999692817443324	-4.58944538838348	7.13204167444804	6.68888617614156	6.57001429603546	5.64242461946493	6.23178797984311	4.62816221111829	NA
**TC0700003981.mm.2**	Apbb1	amyloid beta (A4) precursor protein-binding, family B, member 1	-0.550370217551812	8.32466007698856	-1.11979258525696	0.302874648327029	0.999692817443324	-4.58944733217256	9.41472503091517	9.37590156491248	8.22823994295678	6.80005677254328	8.09558462227552	8.03345252832817	NA
**TC0500001421.mm.2**	Lrrc43	leucine rich repeat containing 43	0.44400871796763	5.16810322072732	1.11970133633109	0.302910783795285	0.999692817443324	-4.58945240092436	4.39918924064852	4.54023309130989	5.53545204255014	5.94315517162024	4.8552691271028	5.73532065113232	NA
**TC0700003609.mm.2**	Ap3b2	adaptor-related protein complex 3, beta 2 subunit	0.467569379042661	6.7795904075887	1.11962659202114	0.302940385934021	0.999692817443324	-4.58945655275549	8.12231953881312	8.02377115218832	6.49513165971157	6.23816359659646	6.42478884412799	5.37336765409472	NA
**TC0700001874.mm.2**	Trim72	tripartite motif-containing 72	0.402357597251307	6.04839628710443	1.11915683565513	0.303126485617474	0.999692817443324	-4.58948264405672	4.87835982521139	5.13337033408872	6.45753950739343	7.08414187152112	6.17945399464743	6.55751218976452	NA
**TC1100000560.mm.2**	Zfp354a	zinc finger protein 354A	0.345899227860447	6.12754677378617	1.1188679498378	0.303240978237022	0.999692817443324	-4.58949868742751	5.26674029227849	5.41276702348105	6.52168355817969	6.8671023331595	6.26665547438179	6.4303319612365	NA
**TC0300002706.mm.2**	Rbm15	RNA binding motif protein 15	0.667078805323666	5.77648648786031	1.11839112937274	0.303430032332789	0.999692817443324	-4.58952516450489	6.72903434889825	6.63167969935899	5.76928630708523	5.54689493769075	5.92038569574431	4.06163793838433	NA
**TC1300001669.mm.2**	Acot13	acyl-CoA thioesterase 13	-0.730803808151723	8.86025065698865	-1.11829201129845	0.303469343810413	0.999692817443324	-4.58953066785538	9.43053350931422	9.31156384964803	9.1127063315281	7.36619315180499	9.55471057841587	8.38579652122066	NA
**TC0800001200.mm.2**	Ces3a	carboxylesterase 3A	-0.478351669191449	4.70198488413418	-1.11814390685994	0.303528091752991	0.999692817443324	-4.58953889075227	3.70676672839701	4.70799536347641	4.51005405084902	4.91016788642534	5.0084948635109	5.36843041214637	NA
**TC1800000700.mm.2**	Impa2	inositol (myo)-1(or 4)-monophosphatase 2	0.364258637606205	6.89661764318817	1.11806420745628	0.303559709663739	0.999692817443324	-4.58954331557289	6.71622587477242	6.58344763553394	6.9874066416461	7.41686817037143	6.67876277590058	6.99699476090453	NA
**TC1400002735.mm.2**	Stk24	serine/threonine kinase 24	0.533001407793685	7.35720814981224	1.1170818202541	0.303949660837039	0.999692817443324	-4.58959784711528	7.9138875135974	7.5535428435033	7.82269320582212	7.04821747285794	7.43550449140838	6.36940337168431	NA
**TC0900003321.mm.2**	Ppp2r3d	protein phosphatase 2 (formerly 2A), regulatory subunit B'', delta	-0.412358289573994	4.99847999279053	-1.11634259223766	0.304243364996837	0.999692817443324	-4.58963886949401	5.30097663498187	4.82797283889279	5.08556093544094	4.43304601641934	5.06730690635899	5.27601662464928	NA
**TC1400002331.mm.2**	Ccar2	cell cycle activator and apoptosis regulator 2	0.386493789274587	7.06716406594651	1.1160868308296	0.304345036715868	0.999692817443324	-4.58965306027768	7.48009403497175	7.49208672082101	7.36473929888216	6.73715631033558	6.81153653216587	6.51737149850269	NA
**TC1500002341.mm.2**	9130401M01Rik	RIKEN cDNA 9130401M01 gene	-0.527536297644686	6.58968631320136	-1.11581278603144	0.304454007733649	0.999692817443324	-4.58966826417882	7.06256083615027	7.04317915604475	6.76572726493508	5.42292538092682	6.57304901286701	6.67067622828426	NA
**TC1900000748.mm.2**	Add3	adducin 3 (gamma)	-0.893366503548108	7.90670709808911	-1.11558402184831	0.304544998099986	0.999692817443324	-4.58968095487689	9.1789219854138	9.21345344348337	8.1140540582673	5.51651301800333	8.03943798044562	7.37786210292123	NA
**TC0500001938.mm.2**	Dmtf1	cyclin D binding myb-like transcription factor 1	0.522575525983529	7.37647720532931	1.11555940191216	0.30455479195642	0.999692817443324	-4.58968232061132	8.13612599824043	7.93931712111898	7.30978680519147	7.30449877710029	7.44064085615967	6.12849367416503	NA
**TC0500001233.mm.2**	Ankrd13a	ankyrin repeat domain 13a	0.444307236125637	7.73731599856645	1.11546786186603	0.304591209041466	0.999692817443324	-4.58968739848785	7.67549931116443	7.95055077798138	8.10776456156303	7.73546562568906	7.91447693963634	7.04013877536446	NA
**TC1800001150.mm.2**	Nme5	NME/NM23 family member 5	-0.506811861453576	6.73758780296883	-1.11540875910631	0.304614723616868	0.999692817443324	-4.58969067693339	7.51658988896115	7.49529808780364	6.730469506374	5.4695380526965	6.57895244602542	6.63467883595224	NA
**TC1700002780.mm.2**	H2-DMb1	histocompatibility 2, class II, locus Mb1	0.418358714145001	5.71717489579329	1.11504675698149	0.304758782236111	0.999692817443324	-4.58971075588973	5.54318640684793	6.19806786034011	5.68458927918747	6.01466698874338	5.20165505753439	5.66088378210646	NA
**TC0X00001789.mm.2**	Gata1	GATA binding protein 1	0.377138220067175	7.25248152680828	1.11475475053671	0.304875027133813	0.999692817443324	-4.5897269506869	7.10842236396285	7.04232648451606	7.27603246691333	7.78317590933922	7.34528822177702	6.95964371434118	NA
**TC1000002374.mm.2**	Gucd1	guanylyl cyclase domain containing 1	0.363624223534883	6.53172951257177	1.11451553814643	0.304970282545319	0.999692817443324	-4.58974021633833	6.21295300203989	6.01421959138178	6.7691406724983	7.07608579204106	6.76728828881357	6.35068972865602	NA
**TC1100004064.mm.2**	Fads6	fatty acid desaturase domain family, member 6	0.37722541915974	6.3113783246465	1.11445064872513	0.30499612603541	0.999692817443324	-4.58974381463484	7.16232478452064	6.60685930354355	6.09831895294308	6.32844939612408	5.94826089788709	5.72405661286059	NA
**TC0X00001082.mm.2**	Lpar4	lysophosphatidic acid receptor 4	0.49630869730739	5.15438420326033	1.1144023578495	0.305015360002495	0.999692817443324	-4.58974649244624	5.96424940946955	5.13109352676612	5.50196784916572	4.90982198980481	4.37626715922121	5.04290528513454	NA
**TC0600002548.mm.2**	Pole4	polymerase (DNA-directed), epsilon 4 (p12 subunit)	-1.03797227735528	8.10683503756116	-1.11359311857729	0.30533782434108	0.999692817443324	-4.58979135976477	9.08935540807449	9.05952264082632	8.67540253619332	5.53269127468448	8.38420925693996	7.89982910864839	NA
**TC1800001500.mm.2**	Mppe1	metallophosphoesterase 1	0.374203640142577	6.31787447723584	1.11358333231124	0.305341725679552	0.999692817443324	-4.58979190227902	6.30965959551913	6.88822737138707	6.27930218133902	6.44958140705797	6.07206104437285	5.90841526373899	NA
**TC1200000792.mm.2**	Psen1	presenilin 1	0.61671869469689	7.29235317835295	1.11323956478088	0.305478796269138	0.999692817443324	-4.58981095835475	8.11248072211858	8.11476805242563	8.00466370079318	6.37549014169045	6.81375264329296	6.33296380979688	NA
**TC0700003412.mm.2**	Fan1	FANCD2/FANCI-associated nuclease 1	-0.546491079288604	5.48871931597513	-1.11319783992042	0.305495436701377	0.999692817443324	-4.58981327114195	5.21548620385233	5.28877079830462	4.89105419012507	5.77648417743325	5.3215837216862	6.43893680444932	NA
**TC0600001687.mm.2**	5330439B14Rik	RIKEN cDNA 5330439B14 gene	-0.41616650468928	5.68961901143997	-1.1131290661105	0.305522866250663	0.999692817443324	-4.5898170831682	5.04108487072108	5.58277649562242	5.87613503068222	5.46462481577666	6.34788694730988	5.82520590852756	NA
**TC0X00003108.mm.2**	Ammecr1	Alport syndrome, mental retardation, midface hypoplasia and elliptocytosis chromosomal region gene 1	0.627657322053092	5.5341439752942	1.11307907338757	0.305542806481983	0.999692817443324	-4.589819854133	5.20523595640418	4.61025096904025	6.30188016568806	6.0204656195254	6.33890512387723	4.72812601723006	NA
**TC1500002285.mm.2**	Csad	cysteine sulfinic acid decarboxylase	0.401379788925267	6.05447255102631	1.11299675554744	0.305575642338582	0.999692817443324	-4.58982441669335	6.7001656841355	6.66114248854215	5.66716721634091	6.21697613932445	5.81776094330117	5.26362283451366	NA
**TC0500000291.mm.2**	Drc1	dynein regulatory complex subunit 1	0.360757350484022	6.83165590903161	1.11287040147852	0.305626049527402	0.999692817443324	-4.58983141976872	7.44299397632997	6.98832187016538	6.77436701850209	6.86570013582909	6.59591500332873	6.32263745003441	NA
**TC0400004004.mm.2**	Plod1	procollagen-lysine, 2-oxoglutarate 5-dioxygenase 1	-0.515788269623892	6.18339407933128	-1.11237435759392	0.305824005709572	0.999692817443324	-4.5898589097622	5.74267721976001	6.74199504425354	5.649577125125	6.14248071123818	6.07202560675893	6.75160876885203	NA
**TC0900002468.mm.2**	Pias1	protein inhibitor of activated STAT 1	-0.837902305982297	6.91288686745869	-1.11188757523255	0.306018368826209	0.999692817443324	-4.58988588207379	7.47604792972585	7.88465030247934	7.32853265601451	4.89187652427666	7.13373244707365	6.76248134518211	NA
**TC0800000798.mm.2**	Colgalt1	collagen beta(1-O)galactosyltransferase 1	0.326913079379774	6.84661880505106	1.11171406934225	0.306087671143852	0.999692817443324	-4.5898954948683	6.69911964892595	6.8646196030985	7.08523981464678	6.99966005387394	6.7780905355052	6.65298317425597	NA
**TC1000002032.mm.2**	Mfsd4b5/Mfsd4b3-ps	major facilitator superfamily domain containing 4B5/major facilitator superfamily domain containing 4B3, pseudogene	0.388024651409625	6.13051009683203	1.11168494128426	0.306099306842869	0.999692817443324	-4.58989710860343	5.98319195151842	5.6026338459569	6.39414297853435	6.5924990646337	5.82203615222642	6.38855658812238	NA
**TC1900001266.mm.2**	Mamdc2	MAM domain containing 2	0.375351367152281	6.13793896143758	1.11165157448417	0.306112636228405	0.999692817443324	-4.58989895715149	6.22051556484812	6.39589302961436	5.93492873242769	6.54603522180607	5.8562908250593	5.8739703948699	NA
**TC0500000374.mm.2**	Fam193a	family with sequence homology 193, member A	0.734152514073801	7.69903722510571	1.11129303091804	0.306255897629849	0.999692817443324	-4.58991881945642	8.70589625722555	8.80779504192413	7.66235519479716	7.41206334501894	7.86115478358897	5.74495872807954	NA
**TC0400000341.mm.2**	Aco1	aconitase 1	-0.565373230021622	6.50375673342974	-1.11111110147469	0.306328611402973	0.999692817443324	-4.58992889692395	7.12525304298469	6.96927393062266	6.63907979556403	5.25955368789988	6.2228079919111	6.80657195159606	NA
**TC0400001530.mm.2**	Ptp4a2	protein tyrosine phosphatase 4a2	-1.86220839999911	7.98199065877083	-1.11093765315114	0.30639794870143	0.999692817443324	-4.58993850403177	9.63448325655902	9.69025618640762	8.97872500203146	3.44266885279858	9.12472050298475	7.02109015184352	NA
**TC1000002477.mm.2**	Polrmt	polymerase (RNA) mitochondrial (DNA directed)	0.439785277204922	5.90092983728253	1.11089335979009	0.306415657388749	0.999692817443324	-4.58994095730174	5.67796619159946	5.92216033283344	5.72377528916757	6.61873623766849	5.5787319310431	5.88420904138311	NA

**TC1300001348.mm.2**	4833420G17Rik	RIKEN cDNA 4833420G17 gene	-0.663149859461613	6.60764223422639	-1.11075205811153	0.306472156089004	0.999692817443324	-4.58994878331378	7.26837826016079	7.23068365496587	6.86546339034737	5.04478249530687	6.84534692720707	6.3911986773704	NA
**TC0300001161.mm.2**	Vav3	vav 3 oncogene	-0.363726023470977	4.90250909018363	-1.11060460369555	0.306531124094972	0.999692817443324	-4.5899569497004	4.39231006355995	4.85734189504761	4.96524440237835	4.75373086539777	5.34227584997321	5.10415146474487	NA
**TC1500001979.mm.2**	Ttll12	tubulin tyrosine ligase-like family, member 12	0.361864009662443	7.45087898446505	1.11054894678059	0.306553384101148	0.999692817443324	-4.58996003201157	7.06192759466965	7.08770117465197	7.75936744890087	7.88031912949593	7.18944097716286	7.72651758190905	NA
**TC0300000769.mm.2**	Adar	adenosine deaminase, RNA-specific	0.36247563259259	8.01879203411701	1.11046544228436	0.306586784261226	0.999692817443324	-4.58996465642937	8.96788307427588	8.71636435229126	7.97382914259642	7.60289887906363	7.26178477227037	7.5899919842045	NA
**TC1400002223.mm.2**	Mtmr9	myotubularin related protein 9	-0.710264399892206	6.97064447556214	-1.11035227323174	0.306632054454659	0.999692817443324	-4.58997092344158	8.09912638610657	8.01462418710988	7.06533536582359	5.0794583743624	7.03267817495504	6.53264436501536	NA
**TC0400003951.mm.2**	Gm13178	predicted gene 13178	0.430089706165695	5.18498538049618	1.11018005661348	0.306700955600554	0.999692817443324	-4.58998045989947	5.19624535388985	4.36737854777859	5.66951651045126	5.53371738636875	4.96964748097863	5.37340700351	NA
**TC0300001041.mm.2**	Nras	neuroblastoma ras oncogene	-0.518460796126661	7.33471065596206	-1.11006263124591	0.306747942958179	0.999692817443324	-4.5899869619872	6.89080001759672	7.31410061152329	7.75232594677135	6.63089491042818	7.98711418486543	7.43302826458742	NA
**TC0800000209.mm.2**	Defb33	defensin beta 33	-0.423347948394935	6.01898408919669	-1.10990614034701	0.306810571486282	0.999692817443324	-4.58999562681581	5.60421990837647	5.6429874100215	5.86398495106018	6.14601570893597	6.00163948794258	6.85505706884344	NA
**TC0200004844.mm.2**	Vsx1	visual system homeobox 1	-0.359803355123987	6.47341152655037	-1.10963168936868	0.306920433769661	0.999692817443324	-4.59001082193752	5.8712403178734	6.05515273936101	6.61697886920304	6.48025582670689	6.66451866790984	7.15232273824806	NA
**TC0900000591.mm.2**	Tmprss5	transmembrane protease, serine 5 (spinesin)	0.389142354874592	5.45635280825656	1.10924846990334	0.30707389019632	0.999692817443324	-4.59003203673325	5.44884572225085	5.15161723172254	5.40373198221949	6.05423732043808	5.38879057370366	5.29089401920472	NA
**TC1200001885.mm.2**	Pygl	liver glycogen phosphorylase	-0.340500875797934	5.23489161444538	-1.10911074947878	0.307129054428989	0.999692817443324	-4.59003966018238	5.0439601446604	4.98706701996717	5.01614002469648	5.33252036052793	5.62331594088754	5.40634619593273	NA
**TC1200001644.mm.2**	Bzw2	basic leucine zipper and W2 domains 2	0.331662592750279	7.6242623611004	1.10908329195541	0.307140053580326	0.999692817443324	-4.59004118003829	7.47066872922488	7.56626552736045	7.72562227319308	7.96036027456572	7.5889700316386	7.43368733061965	NA
**TC0400004000.mm.2**	Zfp933	zinc finger protein 933	0.646884782875211	6.00444287913692	1.10889652590717	0.307214878418704	0.999692817443324	-4.590051517723	6.70550289785027	6.70814750037647	5.51863817959892	6.43475004157369	6.1165384666677	4.54308018875448	NA
**TC0700000498.mm.2**	Paf1	Paf1, RNA polymerase II complex component	0.392122678545527	7.54453821179237	1.1085806234741	0.307341473909734	0.999692817443324	-4.59006900174972	8.63037226999644	8.50047145410894	7.45724239971805	7.0030730521519	7.12273539580832	6.55333469897058	NA
**TC1600000115.mm.2**	Nubp1	nucleotide binding protein 1	0.390335004356659	7.8269252146216	1.10851260139023	0.307368738869409	0.999692817443324	-4.59007276627528	7.69174111985834	7.77694637759421	7.70777838401292	8.42898851548226	7.64091969562386	7.71517719515799	NA
**TC1500000398.mm.2**	Mtbp	Mdm2, transformed 3T3 cell double minute p53 binding protein	-0.446811003987785	5.43539883561984	-1.10844309721937	0.307396599946962	0.999692817443324	-4.5900766127341	4.99819385711192	5.30866909570341	5.40904968824163	5.29690433822244	5.32045079220626	6.27912524223337	NA
**TC0900001853.mm.2**	S1pr2	sphingosine-1-phosphate receptor 2	0.594482670819762	5.75254758816831	1.10832010135175	0.307445908528927	0.999692817443324	-4.59008341927727	5.51881327372131	5.75217009497804	6.03725576847408	6.17937798250094	6.31443480863327	4.71323360070222	NA
**TC1100004293.mm.2**	Vps53	VPS53 GARP complex subunit	0.343844779920853	7.06356661910871	1.10825150595762	0.307473410997932	0.999692817443324	-4.59008721519651	7.30925154442233	7.12935711216047	7.07530063788542	7.23993967107015	6.64290659114252	6.98464415797134	NA
**TC1700001407.mm.2**	Gm1604b/Gm1604a	predicted gene 1604b/predicted gene 1604A	0.377728629531062	6.71134890361799	1.10810041499665	0.307533996192092	0.999692817443324	-4.59009557592935	6.84719121871301	6.62901191497539	6.66611631730926	7.10755745623156	6.29104071576094	6.72717579871775	NA
**TC1100000571.mm.2**	Phykpl	5-phosphohydroxy-L-lysine phospholyase	-0.455543010895633	5.03161733447281	-1.10804189067572	0.307557466205696	0.999692817443324	-4.5900988143021	5.33962942199735	5.20036310454001	5.15562267461117	4.21369005464294	4.91356638608734	5.36683236495804	NA
**TC1700001658.mm.2**	Ube2i	ubiquitin-conjugating enzyme E2I	0.332452501652346	8.63268025305052	1.10802128874574	0.307565728550924	0.999692817443324	-4.59009995426978	8.90953533047366	8.75395790506779	8.68263111161143	8.71611553142176	8.49989565303247	8.23394598669603	NA
**TC1400001551.mm.2**	Camk2g	calcium/calmodulin-dependent protein kinase II gamma	-0.736618311114158	8.26871558215911	-1.10789474628992	0.307616482055903	0.999692817443324	-4.59010695607572	9.30761923305599	8.84832645078203	8.49040955626643	6.50114603717772	8.56991800425216	7.89487421142031	NA
**TC0300002463.mm.2**	Pip5k1a	phosphatidylinositol-4-phosphate 5-kinase, type 1 alpha	-0.382439772688756	7.04760884254477	-1.1076540639129	0.307713033726733	0.999692817443324	-4.590120272606	7.53543339848337	7.5313451097955	6.95414746563941	6.27285003516672	7.03851184419105	6.95336520199259	NA
**TC1200000878.mm.2**	Slirp	SRA stem-loop interacting RNA binding protein	-0.352717057420901	7.21396824325505	-1.10740881434285	0.307811443259861	0.999692817443324	-4.59013384071304	7.49223146283941	7.46734792325131	6.66910393726207	7.14029404203681	7.32867592536773	7.18615616877296	NA
**TC1000000097.mm.2**	Adat2	adenosine deaminase, tRNA-specific 2	-0.341017295579007	5.67109227269156	-1.10727905308556	0.307863522120712	0.999692817443324	-4.59014101912564	5.99958370900881	6.10510922746811	5.17839587713052	5.4415171771267	5.79038915140095	5.51155849401428	NA
**TC0200002801.mm.2**	Gid8	GID complex subunit 8	-0.49644988370054	8.56714045887539	-1.10702088937271	0.307967156093729	0.999692817443324	-4.59015529984105	8.85231989228744	8.84445493474223	8.79295869984854	7.56362537956225	8.61670482397432	8.73277902283756	NA
**TC1200001262.mm.2**	Adssl1	adenylosuccinate synthetase like 1	0.363022401057791	6.23406462162124	1.10696490024507	0.307989635453915	0.999692817443324	-4.59015839679885	6.61540783963674	6.62119013074929	5.94583910171558	6.50107817901292	5.80322782216805	5.91764465644487	NA
**TC1500002223.mm.2**	Lima1	LIM domain and actin binding 1	0.854737398054757	5.82607542787254	1.10660138446164	0.308135618151227	0.999692817443324	-4.59017850271584	7.3941020759279	7.19987601815492	6.30727136024082	4.72870327439014	5.67341179188499	3.65308804663645	NA
**TC1100002100.mm.2**	4921536K21Rik	RIKEN cDNA 4921536K21 gene	-0.388646746659629	5.66596487969101	-1.10639628685209	0.308218007551152	0.999692817443324	-4.59018984548769	5.29456306922316	5.15487855791142	5.99525001683633	5.38927706200979	5.89483762535836	6.26698294680702	NA
**TC1700002814.mm.2**	C4b	complement component 4B (Chido blood group)	0.372819131293043	7.22698400422715	1.10607223472284	0.308348218956297	0.999692817443324	-4.59020776533641	7.38823162098868	7.93357195379237	7.10844266376169	7.28442669282229	6.69032289592113	6.95690819807676	NA
**TC1100001321.mm.2**	Ptrh2	peptidyl-tRNA hydrolase 2	-0.389427221862327	6.63356013000023	-1.10600152784244	0.308376636587352	0.999692817443324	-4.59021167511278	7.03883380087739	6.74324779526558	6.57965978653897	6.0405525835279	6.89620411877324	6.50286269501828	NA
**TC0700001775.mm.2**	Hs3st4	heparan sulfate (glucosamine) 3-O-sulfotransferase 4	-0.331794762130037	7.23692500130367	-1.10598976360588	0.308381364932524	0.999692817443324	-4.59021232561365	7.68611490504775	7.60010825055653	7.00302478834612	6.73284387563273	7.26162333018012	7.13783485805879	NA
**TC1100004270.mm.2**	Tlcd2	TLC domain containing 2	0.366163716707544	5.12087355592853	1.10578801629391	0.308462461591419	0.999692817443324	-4.59022348078062	4.66589740774297	4.63663066683459	5.50136648214045	5.57615386506391	4.88019074197713	5.46500217181215	NA
**TC1700001604.mm.2**	Flywch1	FLYWCH-type zinc finger 1	0.354858386954905	7.94408717752693	1.10540445247329	0.308616691745178	0.999692817443324	-4.59024468696673	8.63576722680832	8.35702279208141	8.0471567399199	7.64356817017094	7.5664197067052	7.41458842947583	NA
**TC0500003645.mm.2**	Katnal1/5930430L01Rik	katanin p60 subunit A-like 1/RIKEN cDNA 5930430L01 gene	0.495175825655718	7.186026679139	1.10537322486816	0.308629251090919	0.999692817443324	-4.59024641333261	7.89085744387987	8.08858788184931	7.4654385065125	6.59809469369563	6.96315586279017	6.11002568610652	NA
**TC0900001459.mm.2**	Scap	SREBF chaperone	-0.740103536909794	7.33687679603171	-1.10525332143769	0.308677478637515	0.999692817443324	-4.59025304182185	8.80698234723747	8.64048538131625	7.24126043882533	5.30553254808315	7.49579525647391	6.53120480425416	NA
**TC1000003227.mm.2**	Rdh5	retinol dehydrogenase 5	-0.656664899329058	6.65742267550695	-1.10522403587271	0.308689258817799	0.999692817443324	-4.59025466074219	6.17597212615115	6.90757759493885	5.61446060398655	7.15936766266023	6.67061027641035	7.41654778889454	NA
**TC1100002680.mm.2**	Zfp879	zinc finger protein 879	-0.322494513328712	6.75952002585941	-1.10503265160217	0.308766252660794	0.999692817443324	-4.59026524015892	6.85610125657357	6.76393970282531	6.62280676900037	6.52323831554971	6.95721204794055	6.83382206326696	NA
**TC0100001379.mm.2**	Rgs8	regulator of G-protein signaling 8	0.349971629306481	8.43154287741776	1.1049891225984	0.308783766575262	0.999692817443324	-4.59026764627602	8.36708043807017	8.38605447913767	8.77702614622279	8.49100665673306	8.47521453092675	8.09287501341614	NA
**TC1400001937.mm.2**	Atg14	autophagy related 14	-0.349221664595268	6.18586691065633	-1.10473218946605	0.30888716040421	0.999692817443324	-4.59028184782808	6.31104115447069	6.52663810174477	6.10490251179349	5.6846369274725	6.23505228429547	6.25293048416105	NA
**TC1500001101.mm.2**	Prr13	proline rich 13	0.378557310580034	8.1536998517371	1.1046719912931	0.308911389192992	0.999692817443324	-4.59028517500142	8.36410440617757	7.96252437698245	8.49185891526981	8.1844835589415	8.17917958861104	7.7400482644402	NA
**TC0200005328.mm.2**	Gm14393	predicted gene 14393	-0.957889444076929	6.78476338656732	-1.10458798713383	0.308945202120847	0.999692817443324	-4.59028981782496	7.46383235857874	7.89806182374012	7.1121261798052	4.6033274446604	7.54493461740192	6.08629789521754	NA
**TC0200003701.mm.2**	Fastkd1	FAST kinase domains 1	-0.528360607413013	5.56320075899769	-1.104526715602	0.308969866748831	0.999692817443324	-4.59029320415526	5.89485391087693	5.85556000639301	4.95217617074277	5.3338585402023	6.32978969079673	5.01296623497437	NA
**TC1100004059.mm.2**	Cd300e	CD300E molecule	-0.402194029072905	4.92245119038569	-1.10424085187212	0.309084961561376	0.999692817443324	-4.59030900221642	4.01676321295557	3.99648685055189	4.84085983799238	5.51767467233807	5.36586603798541	5.79705653049085	NA
**TC0100003827.mm.2**	Hhat	hedgehog acyltransferase	0.515585559097652	5.74469179051906	1.10398114243158	0.309189556699831	0.999692817443324	-4.59032335353569	5.63418251637249	5.29823785654242	6.22476584449693	6.05868489970045	4.99309532507384	6.25918430092823	NA
**TC0200004512.mm.2**	Slc30a4	solute carrier family 30 (zinc transporter), member 4	-0.590514958523412	7.51473512380379	-1.10398008815301	0.309189981358374	0.999692817443324	-4.59032341179161	7.92994281308296	8.11574320424925	7.74082654431581	6.19002085990605	7.76719318630792	7.34468413496076	NA
**TC1100001288.mm.2**	1700125H20Rik	RIKEN cDNA 1700125H20 gene	-0.432732719511142	5.2554297928164	-1.10369002795099	0.309306834508104	0.999692817443324	-4.59033943874893	4.92013564323806	4.19659010374974	5.09003346256751	5.68516032287665	5.72333114177076	5.91732808269568	NA
**TC0600001648.mm.2**	Pik3c2g	phosphatidylinositol-4-phosphate 3-kinase catalytic subunit type 2 gamma	-0.360514268547443	5.22239450454879	-1.10362315208609	0.309333781159051	0.999692817443324	-4.59034313367464	4.88209742117636	4.60086520691604	5.27274723851301	5.29244069253972	5.40271153768903	5.88350493045859	NA
**TC0100003401.mm.2**	Fmo1	flavin containing monooxygenase 1	0.385174183371451	7.42593957598179	1.10354366303288	0.309365812622838	0.999692817443324	-4.59034752537495	7.51864780739601	7.37798939994385	7.25904785333679	7.9556264543101	7.2251926213528	7.21913331955119	NA
**TC1100004148.mm.2**	Tha1	threonine aldolase 1	-0.453836692255314	6.4031528844496	-1.1035162157647	0.309376873599676	0.999692817443324	-4.5903490417845	5.72289419300645	5.87254652657368	6.28722543362052	6.67067616768289	6.44695551544111	7.41861947037293	NA
**TC0300002047.mm.2**	Clrn1	clarin 1	-0.386045172311478	5.2205714421282	-1.10334373700859	0.309446388277028	0.999692817443324	-4.59035857058125	5.0968041055836	4.7747980199585	4.85162197981997	5.48824611148209	5.52544389367248	5.58651454225254	NA
**TC0200001288.mm.2**	Olfr1121	olfactory receptor 1121	-0.397466127154702	4.81298019333066	-1.10310680607222	0.309541900284203	0.999692817443324	-4.5903716591942	4.426207208234	4.32613077197311	4.47398501379869	5.19132044893503	5.08574167386192	5.3744960431812	NA
**TC1900000402.mm.2**	Kank1	KN motif and ankyrin repeat domains 1	-0.343103674718251	7.05503747469047	-1.10234218340315	0.309850301770499	0.999692817443324	-4.59041389142992	6.85387153227848	7.01757583327466	6.99967181628973	6.88661325028686	7.09611994013737	7.47637247587573	NA
**TC1000000830.mm.2**	Wdr18	WD repeat domain 18	-0.349017969330387	6.88196555809544	-1.1022933068813	0.309870024129044	0.999692817443324	-4.59041659063724	8.10848363128645	7.9107282484483	6.35023394012935	5.93703882495921	6.42599203647927	6.55931666727007	NA
**TC0600002176.mm.2**	Tcaf3	TRPM8 channel-associated factor 3	-0.447973986676108	5.02317165289697	-1.10206352159841	0.309962759537589	0.999692817443324	-4.59042927992533	4.61989922392867	4.60917872736691	5.0753100821063	4.93169191426072	4.94094300674192	5.96200696297731	NA
**TC0300000635.mm.2**	Ctso	cathepsin O	-0.681024503061237	6.30915344252083	-1.10179664917558	0.310070491010473	0.999692817443324	-4.59044401598823	7.15722086882757	7.19503180230418	6.47149887730403	4.59881061163134	6.50923993725608	5.92311855780176	NA
**TC0700001815.mm.2**	Gdpd3	glycerophosphodiester phosphodiesterase domain containing 3	0.881074985413297	4.8894381999221	1.10172941692017	0.310097636287582	0.999692817443324	-4.59044772817958	5.33262104892754	3.28262084206026	5.43693679859701	5.8048318410887	3.89929043159735	5.58032823726176	NA
**TC1400001455.mm.2**	Fezf2	Fez family zinc finger 2	-0.439285620784398	7.68000416593767	-1.10164573470275	0.310131426029375	0.999692817443324	-4.59045234852573	7.90812636738818	8.36194124241299	7.51481046775926	6.95088260436876	8.01097865217515	7.33328566152166	NA
**TC0600002385.mm.2**	Gprin3	GPRIN family member 3	0.596927789451739	5.88318534343802	1.10159565940808	0.310151647200773	0.999692817443324	-4.5904551132689	7.21015353720823	7.03741272948197	6.11047124896854	5.01222943745217	5.57243424823348	4.35641085928375	NA
**TC1800000884.mm.2**	Zadh2	zinc binding alcohol dehydrogenase, domain containing 2	-0.38344528596521	6.92769410677997	-1.10151992911371	0.310182230314155	0.999692817443324	-4.59045929437769	6.39223414351597	6.76743432921342	6.66386721902125	7.15593557898877	7.12466543680681	7.46202793313362	NA
**TC1100000696.mm.2**	Olfr322	olfactory receptor 322	0.510951197912371	6.00761212815855	1.10148094412513	0.310197975077399	0.999692817443324	-4.59046144671636	5.7814824438658	5.72681692515049	5.91449014724192	6.86514775063796	5.43193291163506	6.32580259042008	NA
**TC0200004425.mm.2**	Ppp1r14d	protein phosphatase 1, regulatory inhibitor subunit 14D	-0.364184186787499	5.92284496587399	-1.1014443603559	0.310212750664371	0.999692817443324	-4.59046346645866	5.22273418008171	5.44837854916924	5.76520194796033	6.30359239824866	6.42812943703701	6.36903328274698	NA
**TC1300002667.mm.2**	Map3k1	mitogen-activated protein kinase kinase kinase 1	0.333599533634516	5.83978908219879	1.10087166026142	0.310444130336437	0.999692817443324	-4.59049508114767	6.03379845590439	6.1577672981734	5.8256052523271	5.93157865086489	5.39279847952735	5.6971863563956	NA
**TC1900000894.mm.2**	Ndufv1	NADH:ubiquinone oxidoreductase core subunit V1	-0.614855832435026	8.99804611473987	-1.10084286420014	0.310455768135269	0.999692817443324	-4.59049667060709	10.0530799686039	9.96156920293306	9.00723385143401	7.36472407458211	9.04792155345194	8.55374803743423	NA
**TC0900003047.mm.2**	Inka1	inka box actin regulator 1	-0.322464692042445	5.99612700552861	-1.10070350459683	0.310512094761616	0.999692817443324	-4.59050436263025	5.74785188758592	5.81359267149035	5.97718512174347	5.90800892326177	6.17429407852973	6.3558293505604	NA
**TC0500000286.mm.2**	Hadhb	hydroxyacyl-Coenzyme A dehydrogenase/3-ketoacyl-Coenzyme A thiolase/enoyl-Coenzyme A hydratase (trifunctional protein), beta subunit	0.446355828023644	7.48885159346641	1.10061133917151	0.310549350971942	0.999692817443324	-4.59050944954262	8.01938240943285	8.06139369982161	7.86290668408279	7.00961586971285	7.27437376525669	6.70543713249166	NA
**TC0500003504.mm.2**	Mad1l1	MAD1 mitotic arrest deficient 1-like 1	0.424100010793956	6.5787247248845	1.10041321507866	0.310629451502792	0.999692817443324	-4.59052038411029	6.73112205952289	6.28020476025258	7.14202857786663	6.51258219769308	6.15369134189228	6.65271941207952	NA
**TC0200002748.mm.2**	Zfp970	zinc finger protein 970	-1.21557010945612	7.13524156197451	-1.10017759936424	0.310724731807534	0.999692817443324	-4.59053338687905	8.3865770748902	8.97078512690325	7.50586336906039	4.0056101065103	7.68649144194416	6.25612225253878	NA
**TC0700004338.mm.2**	Ctf2	cardiotrophin 2	-0.406324333973916	6.02894423661886	-1.10002647069613	0.3107858591606	0.999692817443324	-4.59054172655603	5.13780374496857	5.48476987184567	5.82123042839291	6.54799113908264	6.4855305254483	6.69633970997509	NA
**TC0700001673.mm.2**	Insc	INSC spindle orientation adaptor protein	-0.470440076796659	5.0875095776098	-1.09999464326377	0.310798733733796	0.999692817443324	-4.59054348282147	5.20622881714115	4.70849618176139	4.40979433002065	5.42493182656081	5.33214936182831	5.44345694834647	NA
**TC0400003890.mm.2**	Ddi2/Rsc1a1	DNA-damage inducible protein 2/regulatory solute carrier protein, family 1, member 1	-1.18725689515445	7.49227447151538	-1.09976796792751	0.310890439269756	0.999692817443324	-4.59055599040104	8.47720596529153	8.31816354617662	8.2364406429215	4.6554411207361	8.15243826337185	7.11395729059466	NA
**TC1200002251.mm.2**	Lgmn	legumain	-0.441276343552424	9.32216160779291	-1.09918548761114	0.311126194028661	0.999692817443324	-4.59058812619457	10.065208839573	10.6224419134067	9.0091586740941	8.17222442924237	9.03665079721804	9.02728499322328	NA
**TC1500000585.mm.2**	Gpaa1	GPI anchor attachment protein 1	0.348643614289637	8.22185920079888	1.09902623931423	0.3111906742075	0.999692817443324	-4.59059691088333	8.29549470836682	8.45746023752375	8.09677610859002	8.54096763515096	8.01758412381638	7.92287239134533	NA
**TC0600001813.mm.2**	Slc25a13	solute carrier family 25 (mitochondrial carrier, adenine nucleotide translocator), member 13	0.69028697023961	5.25227862607547	1.0988209666299	0.311273806015113	0.999692817443324	-4.59060823371627	5.2383306049358	4.55006840890486	5.77119136111941	5.78173198042628	4.14542201832613	6.02692738274035	NA
**TC0700000827.mm.2**	Nosip	nitric oxide synthase interacting protein	0.346465250674155	8.26769201569999	1.09826478815843	0.311499140148062	0.999692817443324	-4.59063890841502	8.48876307960078	8.52384591168702	8.11811310224597	8.52512369988426	7.86193822368289	8.08836807709903	NA
**TC0700004318.mm.2**	Sept1	septin 1	0.425337335876888	5.55192784817101	1.09815160549279	0.311545012188368	0.999692817443324	-4.59064515000425	5.26100132906482	5.15179546323641	5.8689457670804	6.0057767171589	5.05458660017464	5.96946121231089	NA
**TC1500001813.mm.2**	1700025B11Rik	RIKEN cDNA 1700025B11 gene	-0.424002470805366	5.78045792337224	-1.09736256429361	0.311864958527681	0.999692817443324	-4.59068865571554	5.43525210304815	5.50122208582395	5.26454089536678	6.18459330950853	6.19176219786553	6.10537694862052	NA
**TC0900002956.mm.2**	Ephb1	Eph receptor B1	0.452478180133793	8.65048163757639	1.09729551194366	0.311892159850657	0.999692817443324	-4.59069235225512	8.98719045552337	9.04095148206585	8.9718284727172	8.41802365135116	8.69398801179674	7.79090775200404	NA
**TC0700003592.mm.2**	Man2a2	mannosidase 2, alpha 2	0.351550333279237	7.92783579075439	1.09706093083602	0.311987338383513	0.999692817443324	-4.5907052838288	8.73515460008571	8.94452632002992	7.84855425330079	7.44666299218381	7.41175944919932	7.18035712972681	NA
**TC0100000644.mm.2**	Wnt6	wingless-type MMTV integration site family, member 6	0.441961891146457	5.13998833254697	1.09695324372401	0.312031039151291	0.999692817443324	-4.59071121985694	4.68173316149228	4.72551845744726	5.56484951385082	5.59345156546676	4.63141280716327	5.6429644898614	NA
**TC0700003071.mm.2**	Mrgprb3	MAS-related GPR, member B3	0.629433059763428	4.90962762708321	1.09615963814551	0.312353249142253	0.999692817443324	-4.59075495877674	4.48719863410236	4.5166106005089	4.61385655024185	6.24255477346559	4.43634902823679	5.16119617594379	NA
**TC1800000232.mm.2**	Pik3c3	phosphatidylinositol 3-kinase catalytic subunit type 3	-0.517619223019457	6.18820652562626	-1.09592289996347	0.312449419534762	0.999692817443324	-4.59076800403867	7.27711633155333	7.30283720214775	5.9688124801176	4.7882111068912	6.2173969261237	5.57486510692401	NA
**TC1100000894.mm.2**	Tmem107	transmembrane protein 107	0.518634020286589	6.79172977177291	1.09587337034094	0.312469543077039	0.999692817443324	-4.59077073318976	6.81786216377144	6.52270827087584	6.67520825847118	7.5483298598105	6.10084639777033	7.08542367993817	NA
**TC1300001926.mm.2**	Nhlrc1	NHL repeat containing 1	-0.464524628251231	6.63401829966724	-1.09581498263862	0.312493266961586	0.999692817443324	-4.59077395037211	6.55074865003892	6.36553988618495	5.99090141940818	6.98848458323038	6.71975076066274	7.18868449847828	NA
**TC0200002729.mm.2**	Gm14391/Gm6710	predicted gene 14391/predicted gene 6710	-0.843648959061205	8.89964583758609	-1.09553356137104	0.312607633785481	0.999692817443324	-4.59078945584979	9.61342952548624	10.4208696482631	8.90790193941826	6.93023702740417	9.47021428664786	8.05522259829698	NA
**TC0900001850.mm.2**	Eif3g	eukaryotic translation initiation factor 3, subunit G	0.434352338513219	7.02846415301327	1.09551902245567	0.312613543189944	0.999692817443324	-4.59079025685906	7.92080559561467	7.79466301586547	7.28816561965126	6.37384487216169	6.56103588258791	6.23226993219859	NA
**TC0300001708.mm.2**	Agtr1b	angiotensin II receptor, type 1b	-0.459129718516526	5.86614481916527	-1.0954870585261	0.312626535388366	0.999692817443324	-4.5907920178703	5.16947306869561	5.19303683094797	5.48038712065274	6.47766266850476	6.21329897926708	6.66301024692347	NA

**TC0300003218.mm.2**	Itga10	integrin, alpha 10	-0.6589243113231	5.46763102280534	-1.09525578476996	0.312720553128811	0.999692817443324	-4.59080475900513	5.04080098705943	4.86872807506691	4.67385362204708	6.11535060398268	5.44406491705944	6.66298793161652	NA
**TC0300000752.mm.2**	Thbs3	thrombospondin 3	-0.484352556250604	5.18296094104091	-1.09519485844266	0.312745324847128	0.999692817443324	-4.59080811533253	5.26770902630097	5.29852224764228	5.47833202678956	4.30308260311094	5.24129623016901	5.5088235122327	NA
**TC0700000859.mm.2**	Ntn5	netrin 5	-0.383805688875292	5.4675211309748	-1.09474547486244	0.312928087105894	0.999692817443324	-4.5908328688717	5.12554815477248	5.47615594377798	5.2763271707978	5.44157848397607	5.43911322078344	6.04640381174102	NA
**TC1500000718.mm.2**	Mchr1	melanin-concentrating hormone receptor 1	0.440437503309046	5.87207983206285	1.09474209184124	0.312929463297402	0.999692817443324	-4.5908330552048	6.74584481820049	6.81389451926833	6.13599238164283	5.14081494912035	5.27697580848413	5.11895651566096	NA
**TC0200004543.mm.2**	Trpm7	transient receptor potential cation channel, subfamily M, member 7	-1.33602463992057	6.82909398281536	-1.09447040358234	0.313040000561779	0.999692817443324	-4.59084801876676	7.717587750796	7.74264047925984	7.64187119943365	3.77927199406395	7.93863133207564	6.15456114126309	NA
**TC0400001950.mm.2**	Lzic	leucine zipper and CTNNBIP1 domain containing	0.417797324668844	7.01545494257696	1.09438399783565	0.31307516173589	0.999692817443324	-4.59085277736647	6.63087074464989	6.78250762058951	7.14434376755576	7.61312920222425	6.58347050688649	7.33840781355583	NA
**TC0600001087.mm.2**	Tmem43	transmembrane protein 43	-0.325209368900977	8.19689564127459	-1.09437876027536	0.313077293165481	0.999692817443324	-4.59085306580848	8.17749647721792	8.2822372220493	7.91698216798657	8.11862853730263	8.26832202087797	8.41770742221318	NA
**TC1100000588.mm.2**	Skp1a	S-phase kinase-associated protein 1A	-0.725787236444328	9.23949097829855	-1.09395860252433	0.313248315488898	0.999692817443324	-4.59087620291703	10.7374828154166	10.6530382825017	8.884514964732	7.41291018476016	9.65337855579945	8.09562106658137	NA
**TC0400002804.mm.2**	Gm11213	predicted gene 11213	-0.40094025512816	6.20277780325714	-1.09351089587585	0.313430635697468	0.999692817443324	-4.59090085327898	5.66429187575007	6.26532999421398	5.84511564837869	6.39746657128253	6.53718142490703	6.50728130501052	NA
**TC0200001071.mm.2**	Sp9	trans-acting transcription factor 9	0.391107926205823	7.73476534757234	1.09319469192939	0.313559456289431	0.999692817443324	-4.59091826084438	7.66181417668693	7.68507935904882	7.86833153777594	8.05362566327903	7.19921362088751	7.94052772775582	NA
**TC0200002668.mm.2**	Cass4	Cas scaffolding protein family member 4	0.59053005043457	5.91754551333872	1.09303404237751	0.313624921086642	0.999692817443324	-4.5909271041248	5.18520810731812	5.40955183816946	5.95491938600601	7.09086723170092	5.35107384796904	6.51365266886876	NA
**TC0200002443.mm.2**	Dhx35	DEAH (Asp-Glu-Ala-His) box polypeptide 35	-0.317239077487101	6.09058735407048	-1.09234479573579	0.313905916690656	0.999692817443324	-4.59096503935608	6.12518045107834	6.06047590551547	5.99173185326033	5.86996295316711	6.21656505095396	6.27960791044767	NA
**TC1300002064.mm.2**	Fam193b	family with sequence similarity 193, member B	0.316429508004505	6.60197451648461	1.09202411180556	0.314036725068184	0.999692817443324	-4.59098268619738	6.6450846233016	6.7170626708451	6.67644562660676	6.76483378377823	6.36850335862868	6.4399170357473	NA
**TC1200000823.mm.2**	Fcf1	FCF1 rRNA processing protein	0.327321536444428	5.99915831122692	1.0919102742393	0.314083170649428	0.999692817443324	-4.59098895005182	6.25125648604807	6.29960897062004	5.88172473235455	6.16763900943657	5.74703823976925	5.64768242913302	NA
**TC0300002660.mm.2**	Capza1	capping protein (actin filament) muscle Z-line, alpha 1	0.612003490269913	7.91862980236005	1.09189567447662	0.314089127741077	0.999692817443324	-4.59098975337791	8.31234954990957	8.26641534519639	8.70870139343539	7.36980905636169	7.97129905281887	6.88320441643839	NA
**TC1400000226.mm.2**	E330034G19Rik	RIKEN cDNA E330034G19 gene	-0.410027103989112	5.56870608272697	-1.09189313229298	0.314090165029106	0.999692817443324	-4.59098989325664	4.98964454040248	4.99776780908987	5.68995663554919	5.61242833389642	5.62942714405935	6.49301203336448	NA
**TC0300001406.mm.2**	Trmt10a	tRNA methyltransferase 10A	0.491903365313002	5.73390443886551	1.09177156631154	0.314139770912906	0.999692817443324	-4.59099658204058	5.88120523138784	5.89992249339606	6.29851271778924	5.50454010172835	5.88877012039413	4.93047596849746	NA
**TC0700000149.mm.2**	Zfp128	zinc finger protein 128	0.32272056051967	5.76923295409392	1.09157059447567	0.314221793010223	0.999692817443324	-4.59100763924254	6.0766289908199	6.0128056479681	5.69872253697187	5.88697956643555	5.54575896744965	5.39450201491843	NA
**TC0X00003146.mm.2**	Lrch2	leucine-rich repeats and calponin homology (CH) domain containing 2	-0.449777062810542	6.21823652912854	-1.09125910239281	0.314348956108082	0.999692817443324	-4.59102477554831	7.26434962020866	6.9327215777208	6.00219747535384	5.10419945025652	5.77565051689514	6.23030053433629	NA
**TC1200000486.mm.2**	Prps1l3/Mipol1	phosphoribosyl pyrophosphate synthetase 1-like 3/mirror-image polydactyly 1	0.532395269198383	7.1287866428686	1.09119489318177	0.314375174027312	0.999692817443324	-4.59102830769139	7.45806975607783	6.85516235609035	7.39314159972248	7.36899754199762	7.4881566277289	6.20919197559443	NA
**TC1500000262.mm.2**	Polr2k	polymerase (RNA) II (DNA directed) polypeptide K	-0.41900022585669	6.50301417153268	-1.09080170342535	0.314535760421628	0.999692817443324	-4.5910499352514	7.3738333234883	6.99079071889216	5.8769766451863	6.03075362236482	6.78076468286218	5.96496603640232	NA
**TC1400000027.mm.2**	Gm3047	predicted gene 3047	-0.369976339598782	4.99668182677535	-1.09052556422922	0.314648581228435	0.999692817443324	-4.59106512257391	4.578513471553	4.63588070996493	5.1751249962702	4.83774705369809	5.65093129224102	5.10189343692484	NA
**TC1700001844.mm.2**	Rasal3	RAS protein activator like 3	0.492057133376873	5.0937552119788	1.08989066966567	0.314908102597196	0.999692817443324	-4.59110003529136	5.39421453189377	5.6629687205284	4.7999313492124	5.44479979388978	4.11270727535068	5.14790960099775	NA
**TC1700001856.mm.2**	Cyp4f14	cytochrome P450, family 4, subfamily f, polypeptide 14	-0.389458623274428	5.83693843028033	-1.08896747071019	0.315285784749795	0.999692817443324	-4.59115078756229	6.18895042494965	6.35671557031547	5.72745803070998	5.12106563922404	5.59726734985982	6.03017356662305	NA
**TC0100002583.mm.2**	Tmbim1	transmembrane BAX inhibitor motif containing 1	-0.375884610427766	7.00556079929029	-1.08875282882343	0.315373648179936	0.999692817443324	-4.59116258493977	6.94747718391487	6.98084630328712	6.56385515422607	7.11278088961603	7.00879921013219	7.41960605456544	NA
**TC1300000113.mm.2**	Inhba	inhibin beta-A	0.333217801375692	5.93932384545619	1.08872957889232	0.31538316671736	0.999692817443324	-4.59116386277248	5.81458652569483	5.92462794841089	6.08805402915644	6.19352807153495	5.98059411807406	5.63455237986595	NA
**TC1300000607.mm.2**	Gadd45g	growth arrest and DNA-damage-inducible 45 gamma	-0.481914453300011	6.26089612841589	-1.08850430843056	0.315475404769528	0.999692817443324	-4.59117624324064	6.74464165084296	7.30366617237762	6.13435742985384	5.14226259048353	5.91871959881242	6.32172932812496	NA
**TC0800000987.mm.2**	Orc6	origin recognition complex, subunit 6	-0.380292420406933	6.32542876226285	-1.088262831948	0.315574302963337	0.999692817443324	-4.59118951324282	6.8565714145069	6.68742577766539	6.13340999668222	5.69058527361325	6.5587804548924	6.02579965621693	NA
**TC0400000849.mm.2**	Cntln	centlein, centrosomal protein	-0.352313402009346	5.71185266762254	-1.08825814754222	0.315576221741611	0.999692817443324	-4.59118977065631	5.7499265166351	5.65364096539779	5.65438612743913	5.4270747324027	6.13034310360442	5.65574456025611	NA
**TC0800001199.mm.2**	Ces2h	carboxylesterase 2H	0.414615862983737	5.7902699333253	1.08823705981529	0.315584859598327	0.999692817443324	-4.5911909294458	5.35950936300151	5.58056300873938	5.73263914470251	6.58275033238669	5.8347369504846	5.65142080063713	NA
**TC0X00000089.mm.2**	Ssxb10	synovial sarcoma, X member B10	-0.344795730617257	5.0178052539689	-1.08817663026022	0.315609613543241	0.999692817443324	-4.591194250055	4.78811764152437	4.84485239181652	4.80297408948126	5.08916092513775	5.10089989012537	5.48082658572815	NA
**TC1100001546.mm.2**	Psmb3	proteasome (prosome, macropain) subunit, beta type 3	-0.376517628472593	7.93939994890586	-1.08815718073332	0.315617581050795	0.999692817443324	-4.5911953187927	8.10615526894636	7.99319166304087	7.99970851396351	7.39230023828785	7.91732719184451	8.22771681735204	NA
**TC1200000258.mm.2**	Sh3yl1	Sh3 domain YSC-like 1	0.334974210163477	5.37997683708528	1.08812065206223	0.31563254548229	0.999692817443324	-4.59119732599691	5.57577480724304	5.78580079988811	5.24249765675145	5.55161926110228	4.99611261918637	5.12805587834041	NA
**TC1000002121.mm.2**	Grik2	glutamate receptor, ionotropic, kainate 2 (beta 2)	0.530750313820542	7.16896853925584	1.0878483852365	0.315744101313203	0.999692817443324	-4.59121228587725	8.21060867385974	8.0200869234539	6.94191004056329	6.98039809236795	7.13074480333406	5.73006270195609	NA
**TC0600000995.mm.2**	Asprv1	aspartic peptidase, retroviral-like 1	0.336172439275108	5.90293037787202	1.08766861337931	0.31581777687622	0.999692817443324	-4.59122216275125	5.71492035839518	5.56024482989704	6.01579027769077	6.39159070105428	5.84701316848034	5.8880229317145	NA
**TC0800002508.mm.2**	Pou4f2	POU domain, class 4, transcription factor 2	-0.458489482368556	5.60069768488931	-1.0873438495912	0.315950909856316	0.999692817443324	-4.59124000401679	4.60236126229768	4.8073350814015	5.36007409636619	6.27868130408358	5.98671676276674	6.56901760242015	NA
**TC0200002710.mm.2**	Edn3	endothelin 3	-0.379330878276653	5.54011473391745	-1.08732254232634	0.315959646115655	0.999692817443324	-4.59124117448193	5.85844298183462	5.21562719547369	5.24260963431644	5.46136860050511	5.83436929946849	5.62827069190635	NA
**TC1100000530.mm.2**	Rasgef1c	RasGEF domain family, member 1C	0.378344610086172	6.33666494043659	1.08729429226685	0.315971229316875	0.999692817443324	-4.59124272631941	6.41590740379754	6.83654374363827	6.22347110312632	6.53864275455172	5.76860345161557	6.23682118589014	NA
**TC0X00001508.mm.2**	Fgd1	FYVE, RhoGEF and PH domain containing 1	-0.356873448291448	6.78361818897434	-1.08718699036297	0.316015228832936	0.999692817443324	-4.59124862050227	6.60874499559096	6.37706414393981	6.61620569305816	6.88487085580802	7.32999590589549	6.8848275395536	NA
**TC0400003938.mm.2**	Pramel5	preferentially expressed antigen in melanoma like 5	-0.318186387468502	5.46226432844242	-1.08715919803258	0.31602662599088	0.999692817443324	-4.59125014712064	5.37183926457084	5.4279054753026	5.39119109978824	5.2775431281338	5.72977542498175	5.57533157787729	NA
**TC1700001483.mm.2**	4930474M22Rik	RIKEN cDNA 4930474M22 gene	-0.39324068615344	5.28226431418493	-1.08711790052301	0.316043562011974	0.999692817443324	-4.59125241554305	4.62282693872913	4.939117622402	5.01488919060214	5.65769078523366	5.56532927730443	5.89373207083825	NA
**TC0400000280.mm.2**	Gabrr1	gamma-aminobutyric acid (GABA) C receptor, subunit rho 1	0.417253655132037	6.74478546718205	1.08664328517757	0.316238254041716	0.999692817443324	-4.59127848314684	5.78935032236605	5.89132808193972	7.04696536937239	7.76430548515292	6.71018965815051	7.26657388611073	NA
**TC1300001554.mm.2**	Olfr1359/Olfr1360	olfactory receptor 1359/olfactory receptor 1360	-0.48666255702543	4.78989445849018	-1.0866002791485	0.316255900395626	0.999692817443324	-4.59128084497222	4.44578245286383	3.95238352932079	4.61627511948119	5.06766270787161	4.81120448085232	5.84605846055134	NA
**TC0100001804.mm.2**	Gpatch2	G patch domain containing 2	0.386998197367888	7.2021916782423	1.0864467944189	0.316318885242157	0.999692817443324	-4.59128927381747	6.98056210957496	6.93028007901616	7.40277840155431	7.63537373624491	6.77379655284262	7.49035919022081	NA
**TC1400000975.mm.2**	Entpd4	ectonucleoside triphosphate diphosphohydrolase 4	0.326681650828831	8.77666942432355	1.08634264863784	0.316361628923489	0.999692817443324	-4.59129499287168	9.55949338710346	9.56318988602251	8.62998570515584	8.46536258208065	8.08483233515754	8.35715265042128	NA
**TC0200002731.mm.2**	Gm2026	predicted gene 2026	-0.875661443097856	9.60152390072728	-1.08619533990526	0.316422095676452	0.999692817443324	-4.59130308180264	10.4486345000432	11.1077557535076	9.53492226445041	7.61579286785816	10.3425795047296	8.55945851377471	NA
**TC0900000189.mm.2**	Pde4a	phosphodiesterase 4A, cAMP specific	0.465022102603421	7.91769657773304	1.08619440453528	0.316422479653902	0.999692817443324	-4.59130313316374	8.00185660308471	7.99248043040692	8.65200120395524	7.56894211510149	7.820400491132	7.47049862271788	NA
**TC1700002764.mm.2**	Sft2d1	SFT2 domain containing 1	-1.21001577675644	7.90282229194621	-1.08604013848504	0.316485812411157	0.999692817443324	-4.59131160366236	8.48570681985691	8.51991248043987	8.7123476465388	5.28329380239499	9.09439859752453	7.32127440492215	NA
**TC0700002361.mm.2**	Mzf1	myeloid zinc finger 1	0.522519066827776	5.42727369430625	1.08587654963635	0.316552983892127	0.999692817443324	-4.59132058554008	5.7921292534602	5.89778936654146	5.11510455917532	5.84427628057039	4.37121100021754	5.54313170587262	NA
**TC0300000233.mm.2**	Ttc14	tetratricopeptide repeat domain 14	-0.755010775154691	6.45394904808583	-1.0858405389317	0.316567771870429	0.999692817443324	-4.59132256264286	7.0901820203964	6.53124007584926	6.80491111312237	4.9912142078576	7.27646310927712	6.02968376201223	NA
**TC0700004565.mm.2**	Ctsd/Ifitm10	cathepsin D/interferon induced transmembrane protein 10	-0.48653082833879	9.69411056537378	-1.0858373294277	0.316569089896912	0.999692817443324	-4.59132273885363	10.6341288457988	11.2330830552183	9.35405002083159	8.30814489644241	9.1875507439705	9.44770582998108	NA
**TC0200001032.mm.2**	Cybrd1	cytochrome b reductase 1	0.638929807549328	5.35024628569989	1.08561835813808	0.31665902402833	0.999692817443324	-4.59133476050375	5.28998103827643	6.59579350956674	5.8506186743602	4.89616271636721	4.29372419165338	5.17519758397537	NA
**TC0700003539.mm.2**	Ntrk3	neurotrophic tyrosine kinase, receptor, type 3	0.407502741211463	8.76744305263407	1.08514941444967	0.31685169505934	0.999692817443324	-4.59136050254791	9.62978303250097	9.67527706300347	8.6495293013194	8.40777255004205	8.53690381314287	7.70539255579565	NA
**TC1000001480.mm.2**	Llph	LLP homolog, long-term synaptic facilitation (Aplysia)	-0.324316652903369	7.52799850847105	-1.08512134342078	0.316863231409932	0.999692817443324	-4.59136204332995	7.77440366924704	7.72016921448742	7.1438241669878	7.36856826365473	7.67219751214269	7.48882822430658	NA
**TC0400003705.mm.2**	Nudc	nudC nuclear distribution protein	-0.446602960313426	7.5635889863434	-1.08507320856674	0.316883014189484	0.999692817443324	-4.59136468535227	8.66363918590361	8.55367014677501	7.34306944101248	6.29243989136497	7.24799457546997	7.28072067753433	NA
**TC0400001933.mm.2**	Exosc10	exosome component 10	-0.378933875309405	7.61965003736781	-1.08477552548289	0.317005380365386	0.999692817443324	-4.59138102352546	8.15407137632085	7.84103321147094	7.56105815549669	6.92140578740144	7.63095183837591	7.60937985514103	NA
**TC0200002150.mm.2**	Sec23b	SEC23 homolog B, COPII coat complex component	-0.515821326862241	6.70949429433886	-1.08447351709578	0.317129563956602	0.999692817443324	-4.59139759727038	7.4777389176658	7.54917538828585	6.65178247783282	5.44742192534569	6.87860216964823	6.25224488725476	NA
**TC1100003879.mm.2**	Gosr2	golgi SNAP receptor complex member 2	-0.709967768268783	7.1563502191462	-1.08444152197767	0.317142722437826	0.999692817443324	-4.59139935300464	7.48503128707374	7.18481421396632	7.75495619527682	5.66920394337297	7.47271852641868	7.37137714876868	NA
**TC0X00002211.mm.2**	Gpc3	glypican 3	0.366237795995016	5.46652907317441	1.08402237657297	0.317315143584682	0.999692817443324	-4.59142235173389	6.71240926939321	6.17436912990087	5.15603687459386	5.16639894127734	4.62420732021358	4.96575290366758	NA
**TC0700002637.mm.2**	Erich4	glutamate rich 4	-0.492185407647906	6.28594976524414	-1.08377139068755	0.317418426677569	0.999692817443324	-4.5914361217679	5.42706336731231	5.76406868777385	5.84090269723494	6.92919516330649	6.60665873878109	7.14780993705615	NA
**TC0500003618.mm.2**	Mtif3	mitochondrial translational initiation factor 3	-0.499091513265345	6.19655301620818	-1.08373394553342	0.317433838070051	0.999692817443324	-4.5914381760417	6.40911120772228	6.57746888335543	6.43018188526407	5.16709560455627	6.40046395714426	6.19499655920677	NA
**TC0300000656.mm.2**	Tmem154	transmembrane protein 154	-0.49529241317999	5.38840707458464	-1.0834144350582	0.317565364621016	0.999692817443324	-4.59145570351487	4.51032607265353	4.90655298315951	5.076594505342	5.88489477732541	5.52442369509355	6.42765041393385	NA
**TC1300000781.mm.2**	Cdk20	cyclin-dependent kinase 20	0.362880303984811	5.88229961495231	1.08325056518821	0.317632838948281	0.999692817443324	-4.59146469216984	6.38279006854738	6.10129249282846	5.83802866392781	5.92970920422602	5.24994294291072	5.79203431727349	NA
**TC0300002813.mm.2**	Agl	amylo-1,6-glucosidase, 4-alpha-glucanotransferase	0.683821837852246	6.25357222216817	1.08271698110458	0.317852626266493	0.999692817443324	-4.59149395677698	7.90791426805603	7.67632934596728	5.61091896621019	6.04149773113492	6.12084098437715	4.16393203726346	NA
**TC1900000020.mm.2**	Aldh3b3	aldehyde dehydrogenase 3 family, member B3	-0.386516760743309	6.13479828973462	-1.0826450830895	0.317882251091998	0.999692817443324	-4.59149789960901	5.44587670565174	6.12332537537647	6.20937638629209	6.02390068165436	6.35811430633724	6.64819628309582	NA
**TC0700002876.mm.2**	Ccne1	cyclin E1	0.374966036023159	6.64526479500404	1.08254030259322	0.317925428835957	0.999692817443324	-4.59150364550388	6.91502743760054	6.87472431169952	6.87860439477658	6.53728015160867	6.63428370316672	6.03166877117221	NA
**TC0700001174.mm.2**	Whamm	WAS protein homolog associated with actin, golgi membranes and microtubules	-0.666342083479679	5.84465321762537	-1.0823675401246	0.317996630913094	0.999692817443324	-4.59151311887145	6.41992616077698	6.43837553205102	6.19277260831555	4.24569411466688	5.81262904825051	5.95852184169128	NA
**TC0X00001800.mm.2**	Porcn	porcupine O-acyltransferase	0.311539887229586	8.72992223129189	1.0821555032378	0.318084037282413	0.999692817443324	-4.59152474501173	8.62094402082625	8.68374635941919	8.91987281192877	8.92908857905376	8.61857713631585	8.60730448020751	NA
**TC1800001171.mm.2**	Sil1	endoplasmic reticulum chaperone SIL1 homolog (S. cerevisiae)	0.347701745252775	6.91177994981336	1.08128657665176	0.318442433174484	0.999692817443324	-4.59157237938466	6.77501373029616	6.80844891322126	6.88736552827518	7.40394474465896	6.80386215983824	6.79204462259036	NA
**TC1900001464.mm.2**	Rbp4	retinol binding protein 4, plasma	-0.472173544155228	7.36262933612087	-1.08118619665476	0.318483856948406	0.999692817443324	-4.59157788120788	7.50287021378024	8.08296162128821	7.32638075322077	6.49641779345238	7.08115650959516	7.68598912538845	NA
**TC1600000965.mm.2**	Gm5965	predicted gene 5965	0.479628858876129	5.16322614638188	1.0811608800742	0.318494305025914	0.999692817443324	-4.59157926877624	4.96159404459502	5.15195904575154	5.03004704389665	5.88248370895185	4.55637729109852	5.39689574399772	NA
**TC0X00000557.mm.2**	C230004F18Rik	RIKEN cDNA C230004F18 gene	-0.435192749610986	7.01257230978035	-1.08099655960413	0.318562126392883	0.999692817443324	-4.59158827464769	7.16655958630302	7.5467630891226	6.65395332400236	6.59190951801489	7.52564625599103	6.59060208524819	NA
**TC0800001181.mm.2**	Cmtm2b	CKLF-like MARVEL transmembrane domain containing 2B	-0.349492256726439	5.45128036311132	-1.08085920729686	0.318618826002966	0.999692817443324	-4.59159580206067	4.82744786759157	4.72827450988338	5.36290629252407	5.86358135134599	5.88791022106443	6.03756193625851	NA
**TC0700000878.mm.2**	Saa2	serum amyloid A 2	0.534790651638501	6.35895508884695	1.08076046715693	0.318659591433844	0.999692817443324	-4.59160121314751	5.67987088030122	5.6251881580203	6.25972870059997	7.69939769841861	6.3905851132282	6.49895998251338	NA
**TC0800000885.mm.2**	Anapc10	anaphase promoting complex subunit 10	-0.564622505597966	6.06030004315475	-1.08071742643026	0.318677362375123	0.999692817443324	-4.59160357177272	6.86601869404392	6.52136176046468	6.04283385517601	4.87975354143597	6.52019674472787	5.53163566308004	NA
**TC1200001813.mm.2**	Sec23a	SEC23 homolog A, COPII coat complex component	-0.873410212059029	6.44526169107894	-1.08058535115875	0.318731899537258	0.999692817443324	-4.59161080924207	7.95525798357543	8.14018255704486	6.41721438439549	3.99744020647216	6.68305818638743	5.47841682859828	NA
**TC1700000257.mm.2**	Zfp160	zinc finger protein 160	-0.614355577484196	6.2509969896591	-1.08034048697768	0.318833030182612	0.999692817443324	-4.59162422638635	7.24817054505498	6.70333087254574	6.18268150347715	4.98020317921559	6.77772780905783	5.6138680286033	NA
**TC1700001236.mm.2**	Pkdcc	protein kinase domain containing, cytoplasmic	-0.346605189803717	5.42378473262543	-1.08023531141584	0.318876476469972	0.999692817443324	-4.59162998902552	5.67296581350453	5.53606040543001	5.40503228618634	4.91520361241896	5.47657853353547	5.53686774467727	NA
**TC0100003170.mm.2**	Nek7	NIMA (never in mitosis gene a)-related expressed kinase 7	-0.400952860473424	6.64378199305826	-1.08014712383346	0.318912909024817	0.999692817443324	-4.59163482070831	7.41474201633904	7.39058954710698	6.26151461280808	5.86621272417029	6.83681959526856	6.09281346265665	NA
**TC0600002070.mm.2**	Mtpn	myotrophin	-0.745375053121219	9.43214034384222	-1.0795579540154	0.319156397303792	0.999692817443324	-4.59166709648817	9.95813313855806	10.165335375967	9.78186740789077	7.70744431325214	9.91525064448392	9.06481118290144	NA
**TC1300002098.mm.2**	2210016F16Rik	RIKEN cDNA 2210016F16 gene	0.378773324566493	6.30833492928678	1.07936732133352	0.319235213189089	0.999692817443324	-4.59167753817227	7.13250358896031	6.85046283404384	5.86706758047302	6.44522732045175	5.94185913201368	5.6128891197781	NA
**TC0700003574.mm.2**	Arpin	actin-related protein 2/3 complex inhibitor	0.335310367901421	7.03652716762296	1.0791027671369	0.319344617749127	0.999692817443324	-4.59169202759209	7.7510821077531	7.33647128694224	6.93052998401224	6.97058518941037	6.57488918126622	6.65560525635355	NA

**TC0200003765.mm.2**	Atp5g3	ATP synthase, H+ transporting, mitochondrial F0 complex, subunit C3 (subunit 9)	-0.542018176639738	9.02193285985685	-1.07909960512559	0.319345925561868	0.999692817443324	-4.59169220076425	9.48691178946352	9.49211034880724	9.22708475449587	7.80718457929955	9.263419395809	8.85488629126591	NA
**TC0700001869.mm.2**	Bckdk	branched chain ketoacid dehydrogenase kinase	-0.421087597488954	6.76447701017842	-1.07903623535707	0.319372136316076	0.999692817443324	-4.59169567125895	7.61617350843784	7.41756199776295	6.44411476989316	5.91136091005276	6.22014256087544	6.97750831404839	NA
**TC0200003603.mm.2**	Pla2r1	phospholipase A2 receptor 1	0.355165629817757	5.47852287731625	1.07894075429588	0.319411632128283	0.999692817443324	-4.59170090019858	4.90303330279002	4.8427359652733	5.71389822161987	6.20395140611497	5.45537343640832	5.75214493169101	NA
**TC1100003990.mm.2**	Slc16a6	solute carrier family 16 (monocarboxylic acid transporters), member 6	-0.893983634740519	4.92637798212822	-1.07864522124104	0.319533904828617	0.999692817443324	-4.59171708363511	5.52477042248632	5.91852866595821	5.35584209937286	2.80765866804902	5.5059657960051	4.44550224089782	NA
**TC0800000932.mm.2**	Ddx39	DEAD (Asp-Glu-Ala-Asp) box polypeptide 39	-0.358162908151509	5.85485508034703	-1.07852421732443	0.319583979525997	0.999692817443324	-4.59172370931302	5.44455986974828	6.03487501822987	5.68902652707606	5.77765836182445	6.08359677820178	6.09941392700175	NA
**TC1900001454.mm.2**	Ide	insulin degrading enzyme	-0.5124478396924	6.8327795973497	-1.07838216977134	0.319642770809925	0.999692817443324	-4.59173148687168	7.82220466924955	7.90966611669653	6.6191581996502	5.50079735973346	6.95456774046473	6.19028349830374	NA
**TC0200002196.mm.2**	Nxt1	NTF2-related export protein 1	0.385740071697939	6.47636208728066	1.07833197750472	0.319663546719536	0.999692817443324	-4.59173423496032	6.12941387463977	6.09388953947101	6.71119216451043	6.9919824619741	6.11092265944222	6.82077182364643	NA
**TC0200005486.mm.2**	Dbndd2	dysbindin (dystrobrevin binding protein 1) domain containing 2	-0.421014257834601	8.66093699928119	-1.07817563508977	0.319728268041436	0.999692817443324	-4.59174279457008	8.91633879089661	9.21058649515824	8.69887725568941	7.79945684129213	8.639432065592	8.70093054705875	NA
**TC0100003883.mm.2**	Obsl1	obscurin-like 1	0.345370290111667	5.22019022467037	1.07797848039731	0.319809899709619	0.999692817443324	-4.59175358790213	5.25428696349603	4.99466668087552	5.26796197701978	5.61350216491723	4.96156948095017	5.2291540807635	NA
**TC1500002262.mm.2**	Krt82	keratin 82	0.324363627692433	5.12384176181304	1.077746817223	0.319905841273081	0.999692817443324	-4.5917662693999	4.5115979636654	4.63014463096846	5.41412869805057	5.71088891776405	5.25449428778694	5.22179607264281	NA
**TC1200001638.mm.2**	Ahr	aryl-hydrocarbon receptor	-0.429421965527192	5.90322690500154	-1.07761633735049	0.31995988885424	0.999692817443324	-4.59177341152744	6.28266966639685	5.82545805495435	5.89828274690318	5.32791214189865	5.70853454829042	6.37650427156581	NA
**TC0700002052.mm.2**	Rplp2/Snora52	ribosomal protein, large P2/small nucleolar RNA, H/ACA box 52	-0.342015679682447	9.48523870624606	-1.07756422453082	0.319981477188343	0.999692817443324	-4.5917762639495	9.65180641997367	9.65383390632654	9.45656189334284	9.00431838256279	9.65811182697013	9.48679980830039	NA
**TC1900001276.mm.2**	Fxn	frataxin	-0.33895110359922	7.328326307484	-1.0773184527132	0.320083306981286	0.999692817443324	-4.59178971564514	7.16228721105834	6.98215207779099	7.09161282455787	7.48219534987023	7.54470993266667	7.70700044895987	NA
**TC0500001516.mm.2**	Nupr1l	nuclear protein transcriptional regulator 1 like	-0.480270665208665	6.8225519375763	-1.07681541942524	0.3202918093368	0.999692817443324	-4.5918172440239	6.29154382092093	6.13874857153747	6.87770754363753	6.89453140765349	6.7595474280053	7.97323285370306	NA
**TC1900001120.mm.2**	Ms4a15	membrane-spanning 4-domains, subfamily A, member 15	-0.34854130227409	6.49998666511768	-1.07676597418162	0.320312309873847	0.999692817443324	-4.59181994962243	6.55329353419082	6.29000619364477	6.20865551821103	6.52111331095012	6.88578863320188	6.54106280050745	NA
**TC1600000442.mm.2**	Tctex1d2	Tctex1 domain containing 2	0.537320562075656	6.64139981525548	1.07649208931516	0.320425884878462	0.999692817443324	-4.59183493544082	6.97252564983587	7.06892321446155	6.85726504841089	6.58353052728251	6.90041346492269	5.4657409866194	NA
**TC0300001413.mm.2**	Adh6-ps1	alcohol dehydrogenase 6 (class V), pseudogene 1	-0.443830110187866	5.33595077070873	-1.07642407130552	0.320454095775655	0.999692817443324	-4.5918386568585	4.65903558003582	4.62293250817898	5.29304353322259	5.62999462460835	5.40288155689871	6.40781682130795	NA
**TC1000000595.mm.2**	Pbld2	phenazine biosynthesis-like protein domain containing 2	-0.378037695239408	5.6389186692251	-1.07577712385641	0.320722522403273	0.999692817443324	-4.59187404803738	4.74233907058016	4.83112930632437	5.54796230852604	6.20402181545759	6.10558692175754	6.4024725927049	NA
**TC1500001940.mm.2**	Cenpm	centromere protein M	-0.420630596238275	6.46803459844449	-1.07572392617319	0.320744602932909	0.999692817443324	-4.59187695782595	6.49181078745494	6.42945429488793	6.0298243984107	6.49301625951306	6.26785099378459	7.09625085661573	NA
**TC0700003019.mm.2**	Sult2b1	sulfotransferase family, cytosolic, 2B, member 1	-0.494563921415811	6.76324056782122	-1.07572230184812	0.320745277153878	0.999692817443324	-4.5918770466718	6.73891801183744	7.41854677108473	5.85694920579057	6.85947618479619	6.63496238973285	7.07059084368553	NA
**TC0700002135.mm.2**	Mboat7	membrane bound O-acyltransferase domain containing 7	0.496221726491346	7.76625960255037	1.07555295557932	0.320815575328681	0.999692817443324	-4.59188630912119	8.71340025900554	8.58533873826524	8.07569279227879	7.06993824322826	7.47905604171116	6.6741315408132	NA
**TC0100002814.mm.2**	Olfr1414	olfactory receptor 1414	0.606234906000308	5.65703155211948	1.07479008398676	0.321132410267257	0.999692817443324	-4.59192802728659	4.20739089379565	5.05157108439769	5.71677696004218	7.2310716132199	5.78965725981995	5.94572150144151	NA
**TC1300002197.mm.2**	Zfp367	zinc finger protein 367	-0.360233482032239	6.69506299408571	-1.07470877720337	0.321166193516031	0.999692817443324	-4.59193247289519	6.60875773169924	6.75743410809426	6.43535753613284	6.60650204419531	6.59313749787103	7.1691890465216	NA
**TC0200003302.mm.2**	1110008P14Rik	RIKEN cDNA 1110008P14 gene	-0.368450069485702	8.70113448182171	-1.07414141851759	0.321402013505606	0.999692817443324	-4.59196349053943	8.53565102399668	8.76418729710583	8.78538913610157	8.29964507932662	9.11303319962572	8.70890115477387	NA
**TC0700003718.mm.2**	Ccdc81	coiled-coil domain containing 81	0.349063582231318	5.87889421334699	1.07409948161188	0.321419449974332	0.999692817443324	-4.59196578297609	5.73770361492715	5.89069136389663	5.82476447658179	6.34678425627861	5.72409175455207	5.74932981384569	NA
**TC0500003240.mm.2**	Zcchc8	zinc finger, CCHC domain containing 8	0.442393748174998	7.35294139838388	1.07383503632309	0.321529418397091	0.999692817443324	-4.59198023775846	8.15502691026486	7.95169460876716	7.72327941591548	6.72457776789514	6.63805680177554	6.92501288568508	NA
**TC0400002139.mm.2**	Penk	preproenkephalin	0.370091624436949	11.1386021040234	1.07376882515807	0.321556956812017	0.999692817443324	-4.59198385668448	11.8688799432673	11.7712352714855	11.2726364424279	10.693203886703	10.4390496653485	10.7866074149085	NA
**TC0300000805.mm.2**	S100a2	S100 calcium binding protein A2	-0.317821969758925	5.55267532080147	-1.0737376808691	0.321569910945626	0.999692817443324	-4.59198555891694	4.81956250936472	5.04136473854842	5.71009969023074	5.69964067845819	5.95997190681875	6.08541240138803	NA
**TC0X00000350.mm.2**	Rhox2e	reproductive homeobox 2E	0.553965249778718	5.8164649250087	1.07358977334795	0.321631437290511	0.999692817443324	-4.59199364272408	5.3205398668023	5.08622793402691	6.10437325943386	6.69560286495635	5.1463642049681	6.54568141986467	NA
**TC0800001269.mm.2**	Nfat5	nuclear factor of activated T cells 5	0.413959571404193	7.0092635503509	1.07355059919495	0.321647734498528	0.999692817443324	-4.59199578369057	7.95608039038266	7.94551221383969	6.95476454027742	6.53618938006831	6.74237122384431	5.92066355369303	NA
**TC0900002529.mm.2**	Aph1b	aph1 homolog B, gamma secretase subunit	-0.585440957395115	7.19189457336769	-1.07348021811126	0.321677016082705	0.999692817443324	-4.59199963011466	7.40874193971625	7.46199340307015	7.60518253795881	5.94969255335593	7.43364245707345	7.29211454903152	NA
**TC1100003906.mm.2**	1700052K11Rik	RIKEN cDNA 1700052K11 gene	-0.34481678676841	7.20811490947021	-1.07329202966992	0.321755321286845	0.999692817443324	-4.59200991436933	7.05216973499777	6.79683363082973	6.95926061678101	7.39576564194746	7.47243478133857	7.57222505092671	NA
**TC0500001719.mm.2**	Slc29a4	solute carrier family 29 (nucleoside transporters), member 4	0.422492182714878	6.90013478402578	1.07321581338036	0.32178703929247	0.999692817443324	-4.59201407928153	7.36645550112509	7.02608574336868	7.06147816190738	6.86514775063796	6.10007923202661	6.98156231508897	NA
**TC0900000845.mm.2**	Spg21	SPG21, maspardin	-0.573739078697015	6.42260296301554	-1.07320179226331	0.321792874567003	0.999692817443324	-4.59201484546576	7.40647611639099	7.67759195878086	6.2465711443988	4.90546462836489	5.71907267635533	6.58044125380239	NA
**TC1100003276.mm.2**	Pipox	pipecolic acid oxidase	-0.351202147093649	6.0488433163803	-1.07309101247956	0.321838981654851	0.999692817443324	-4.59202089888567	6.253254990177	5.98152554454209	5.86480759503971	5.81312993964798	5.94556921046563	6.43477261840937	NA
**TC1100003342.mm.2**	5730455P16Rik	RIKEN cDNA 5730455P16 gene	-0.417821581905957	5.93914035285641	-1.07296833032354	0.321890048836853	0.999692817443324	-4.59202760239779	7.40873559681443	7.23407896073408	5.35162582535051	4.72656637253852	5.79870676675186	5.11512859494909	NA
**TC1500000678.mm.2**	Tomm22	translocase of outer mitochondrial membrane 22	-0.409601685405073	7.97859874982624	-1.07286425867546	0.321933374448602	0.999692817443324	-4.59203328876221	8.72146833035754	8.52937394545275	7.67552386635604	7.22524955981247	8.23854429420212	7.48143250277653	NA
**TC1400000790.mm.2**	Gmpr2	guanosine monophosphate reductase 2	-0.460461409942574	6.87113594078185	-1.07281872851178	0.321952330402408	0.999692817443324	-4.59203577641119	7.21998291666259	7.29651376325243	7.00958612168184	5.88511195076364	7.0042705857857	6.81135030654493	NA
**TC0200002836.mm.2**	Pcmtd2	protein-L-isoaspartate (D-aspartate) O-methyltransferase domain containing 2	-0.339790974080741	7.54713608302239	-1.07279705767379	0.32196135312238	0.999692817443324	-4.59203696043391	8.04925258542486	8.06889844875707	7.35992205296539	6.88261970493006	7.46916451414898	7.45295919190795	NA
**TC0900000607.mm.2**	Alg9	asparagine-linked glycosylation 9 (alpha 1,2 mannosyltransferase)	-0.798128792207761	6.99913607354648	-1.07276241994735	0.32197577507312	0.999692817443324	-4.59203885290413	7.52167629468238	7.47057200949427	7.34621940935737	5.35693586698597	7.87383951996375	6.42557334079512	NA
**TC0X00002400.mm.2**	Cd99l2	CD99 antigen-like 2	0.786246338948087	7.2816158052452	1.072688907692	0.322006384772818	0.999692817443324	-4.59204286924418	8.10615668778698	7.75105869220028	7.99114537553796	6.71134068915209	7.5754283230764	5.55456506371747	NA
**TC1000002612.mm.2**	Rtcb	RNA 2',3'-cyclic phosphate and 5'-OH ligase	-0.316270385061801	7.94113064439596	-1.07249170009103	0.322088511549277	0.999692817443324	-4.59205364312036	8.88612704300829	8.68970861759508	7.39027109184313	7.32893262598128	7.61604503068526	7.73569945726275	NA
**TC1100004147.mm.2**	Tk1	thymidine kinase 1	0.601756620429219	6.29108177844079	1.07225272619816	0.322188054647349	0.999692817443324	-4.59206669769359	5.32066472806008	5.90785251337545	6.20011793315773	7.66062540187609	5.97116368829468	6.6860664058807	NA
**TC1800001186.mm.2**	Nrg2	neuregulin 2	0.325477418132917	6.847874417642	1.07197176279929	0.322305120197806	0.999692817443324	-4.59208204453101	6.31493453288101	6.26646177053211	7.15125302642952	7.42714949292284	7.07026910730213	6.85717857578441	NA
**TC0600002994.mm.2**	Csgalnact2	chondroitin sulfate N-acetylgalactosaminyltransferase 2	-0.82502282985381	5.80343092530974	-1.07144994538171	0.322522631145464	0.999692817443324	-4.59211054297391	6.31797043231863	6.47681679307495	6.10483923607909	4.08303709729951	6.70173300856046	5.13618898452577	NA
**TC0800000426.mm.2**	Mfhas1	malignant fibrous histiocytoma amplified sequence 1	0.41822382199847	7.42700154409362	1.07135299792001	0.322563055226115	0.999692817443324	-4.59211583701894	7.36844930093011	7.4190136271211	7.24103116908391	8.06446582116981	7.46772829735857	7.00132104889821	NA
**TC1200001210.mm.2**	Dync1h1	dynein cytoplasmic 1 heavy chain 1	0.37461380547837	8.47070587143435	1.0713461501571	0.322565910686248	0.999692817443324	-4.59211621094977	9.41932642475819	9.24431132176054	7.92535485703107	8.52955768949099	8.02242716510785	7.68325777045747	NA
**TC0100003104.mm.2**	Adora1	adenosine A1 receptor	-0.579966953595968	8.53011827922498	-1.07128039510483	0.322593331041271	0.999692817443324	-4.59211980153848	9.32711030413679	9.30688192960217	8.5777659019689	7.11562586524059	8.80398345293196	8.04934222146946	NA
**TC0200000546.mm.2**	Usp20	ubiquitin specific peptidase 20	0.656372254924504	7.11310994536643	1.07041752571236	0.322953329632809	0.999692817443324	-4.59216691058978	8.255615621153	8.09877766204367	7.66904441599871	6.14946103342675	6.85296958721068	5.65279135236577	NA
**TC0800002440.mm.2**	B3gnt3	UDP-GlcNAc:betaGal beta-1,3-N-acetylglucosaminyltransferase 3	-0.522184161925194	6.28042950727827	-1.07036967784686	0.322973301834365	0.999692817443324	-4.59216952242655	5.38533138859965	6.25109652985864	5.79282745106141	6.70806294961907	6.45168001658136	7.0935787079495	NA
**TC1100003022.mm.2**	Arhgef15	Rho guanine nucleotide exchange factor (GEF) 15	-0.347791858644245	7.26779788234131	-1.07025749694435	0.323020131254565	0.999692817443324	-4.59217564577611	7.26841936561075	6.96952365358364	6.9657605657321	7.37086971305039	7.41252850607016	7.61968549000083	NA
**TC0300002292.mm.2**	Prcc	papillary renal cell carcinoma (translocation-associated)	-0.331184930202391	7.85789935654871	-1.06986001425281	0.323186103030999	0.999692817443324	-4.59219734008585	8.40118047004163	8.03493399259597	7.59248663660006	7.43196927152487	7.89530865143278	7.79151711709694	NA
**TC1100000876.mm.2**	Ccdc42	coiled-coil domain containing 42	0.653843496723055	4.76686454223865	1.06984116253892	0.323193976420566	0.999692817443324	-4.59219836891607	4.7139856728696	4.01840322860333	4.74411411799209	5.84412855471045	3.92468815792336	5.35586752133306	NA
**TC1000003018.mm.2**	Helb	helicase (DNA) B	0.325557076008792	6.36709104232352	1.06950837762848	0.323332989176638	0.999692817443324	-4.59221652938788	6.28151711291551	6.30062726221669	6.52419905913803	6.61155895627522	6.41691457379473	6.06772928960093	NA
**TC0700001448.mm.2**	Pgap2	post-GPI attachment to proteins 2	-0.385361950394466	6.47941572550127	-1.0694793929013	0.323345099135568	0.999692817443324	-4.59221811100912	6.04432531781468	5.90386164983	6.44550643108303	6.63328531120396	6.55291105524071	7.29660458783521	NA
**TC0200005003.mm.2**	Sla2	Src-like-adaptor 2	-0.37299132222013	6.18667126884294	-1.06937992577054	0.323386659783366	0.999692817443324	-4.5922235385378	5.4234870550804	5.53173582777635	6.01728523196203	6.69212581091827	6.6331642527879	6.82222943453266	NA
**TC1400001405.mm.2**	Gm3453	predicted gene 3453	-0.365485381232242	5.65877640266187	-1.06907669835325	0.323513384964475	0.999692817443324	-4.5922400831779	5.60313910706643	4.95288221238274	5.59872170875619	5.7341114582726	6.03934043918461	6.02446349030867	NA
**TC1300001886.mm.2**	Mcur1	mitochondrial calcium uniporter regulator 1	-0.31740597799253	6.41079340923947	-1.06906047235038	0.323520167292108	0.999692817443324	-4.59224096844368	6.11187693890877	6.36427996430233	6.35779356785846	6.31910223026187	6.69691610106226	6.61479165304313	NA
**TC1000002546.mm.2**	Gna15	guanine nucleotide binding protein, alpha 15	0.324273870276404	5.52197195688496	1.06899397058855	0.323547965654387	0.999692817443324	-4.59224459661975	5.54340177517367	5.66288299865294	5.74136871154894	5.54567864246904	5.18779719838999	5.45070241507518	NA
**TC0200004913.mm.2**	Bcl2l1	BCL2-like 1	0.537918146373822	7.0216304365102	1.06894103627069	0.323570094083383	0.999692817443324	-4.59224748452224	8.40294396127811	8.28946906962521	7.03729841201523	6.21930452843754	6.705440364237	5.47532628346812	NA
**TC0400004133.mm.2**	Plch2	phospholipase C, eta 2	0.35657654434506	5.8433989792533	1.06878205334278	0.323636561992122	0.999692817443324	-4.59225615769403	5.41766691055977	5.56291951059291	6.01932345911216	6.37715681241645	5.60679987137202	6.07652731146647	NA
**TC1600000999.mm.2**	Olig1	oligodendrocyte transcription factor 1	-0.404473388212739	7.93869653612111	-1.06873841191417	0.323654809628818	0.999692817443324	-4.59225853842019	8.32717700052932	8.57882034553309	7.90268789017382	7.05592965694556	7.88084490173433	7.88671942181054	NA
**TC0600003001.mm.2**	Zfp248	zinc finger protein 248	0.349254005293753	6.9038177673188	1.06864118538732	0.32369546561653	0.999692817443324	-4.59226384217517	7.14924508537756	6.89053388864631	7.14816717116919	6.89265064906903	6.88313113477062	6.4591786748801	NA
**TC1400002635.mm.2**	Mycbp2	MYC binding protein 2, E3 ubiquitin protein ligase	1.185042518027	6.8219097794052	1.06859860954901	0.323713270321669	0.999692817443324	-4.59226616464549	8.78517785572152	8.78863226595931	6.46978319740356	6.39408359799861	7.16714833195596	3.32663342739222	NA
**TC0700004525.mm.2**	Hras	Harvey rat sarcoma virus oncogene	-0.454825854341758	8.57933654265993	-1.06842029133652	0.323787849496765	0.999692817443324	-4.59227589131471	9.00815550299544	8.87702059883666	8.55257948894626	7.78801623377571	9.03353395420847	8.21671347719702	NA
**TC0600001970.mm.2**	Rbm28	RNA binding motif protein 28	0.491602743161106	5.40342074785949	1.06813681122333	0.323906439916546	0.999692817443324	-4.59229135283895	5.66603955218712	5.99413743492073	5.1018981802617	5.76987831292394	5.47076240551808	4.41780860134534	NA
**TC0400002790.mm.2**	Hdhd3	haloacid dehalogenase-like hydrolase domain containing 3	0.454153396037136	6.63125794047616	1.06771071688965	0.324084757546189	0.999692817443324	-4.59231458961591	6.29661707128568	6.53115462179501	6.4628533800942	7.47118799083109	6.73275667522073	6.29297790363028	NA
**TC0400002290.mm.2**	Tstd3	thiosulfate sulfurtransferase (rhodanese)-like domain containing 3	0.869866476008056	6.12522643649094	1.06765254021888	0.324109110272929	0.999692817443324	-4.59231776194548	6.38826449648914	6.35123970328497	6.29448110569221	6.58131257990159	6.92745251131493	4.20860822226277	NA
**TC0500003095.mm.2**	Dynll1	dynein light chain LC8-type 1	-0.333453145183073	9.11559510593387	-1.06756166344207	0.32414715421956	0.999692817443324	-4.59232271724395	9.80155649224074	9.89369693691238	8.74469414482156	8.42101131322042	9.05749691479361	8.77511483361452	NA
**TC0Y00000005.mm.2**	Kdm5d	lysine (K)-specific demethylase 5D	0.468516531932788	5.51045979679701	1.06745640041064	0.324191225244537	0.999692817443324	-4.59232845677328	5.9294731484656	6.02361146642435	5.9717669433217	5.05158667155714	5.41740965901602	4.66891089199725	NA
**TC0600000846.mm.2**	Sftpb	surfactant associated protein B	0.338136299215905	5.32576790037199	1.06734103063949	0.324239533294725	0.999692817443324	-4.59233474710973	4.8702595182357	4.68198169587244	5.55065248970178	5.98866690357602	5.41077773974906	5.45226905509694	NA
**TC0400001137.mm.2**	Czib	CXXC motif containing zinc binding protein	-0.347390678805844	8.10200617873186	-1.06728987925368	0.32426095345751	0.999692817443324	-4.59233753596023	8.21802287827169	8.36067619293374	7.62921518528634	8.04006313650068	8.34908852816819	8.01497115123052	NA
**TC1800001701.mm.2**	Zfp407	zinc finger protein 407	-0.683187069096678	5.34879243164695	-1.06724256640469	0.324280767215941	0.999692817443324	-4.59234011547869	6.69592732895974	6.555410883487	5.30483105424186	3.43269006537893	5.46544464936846	4.63845060844568	NA
**TC1700002561.mm.2**	Cebpz	CCAAT/enhancer binding protein zeta	-0.515451721405509	6.2016628837636	-1.06707906193923	0.324349247466444	0.999692817443324	-4.59234902945362	6.80017088259653	6.77238099410584	6.323741439237	4.9795195522971	6.34842178247891	5.9857426518662	NA
**TC1800000098.mm.2**	Riok3	RIO kinase 3	0.520894769728008	6.75074876947804	1.0670093791907	0.324378436118397	0.999692817443324	-4.5923528282623	7.15221034003973	7.13739615527256	7.50648482249866	6.12185300800731	6.43984454231153	6.14670374873842	NA
**TC1100000802.mm.2**	Trpv2	transient receptor potential cation channel, subfamily V, member 2	-0.325738302422525	6.20077653729452	-1.06670792695295	0.324504732728193	0.999692817443324	-4.59236926097961	6.75538476469336	6.77427678791287	5.5728248768246	5.93893565633333	6.05363228893944	6.10960484906354	NA
**TC1100003566.mm.2**	Rsad1	radical S-adenosyl methionine domain containing 1	0.446632565075929	7.15198708140071	1.06670310579106	0.324506752929043	0.999692817443324	-4.59236952377441	6.96768383409491	6.51862124003981	7.33376164931043	7.82567962290027	7.56590857420768	6.70026756785117	NA
**TC1500001958.mm.2**	Tcf20	transcription factor 20	-0.404790100047751	7.25195575689414	-1.06656318245516	0.324565389140302	0.999692817443324	-4.59237715058555	7.70755763826818	7.59008635416573	7.24879942960039	6.45345574481733	7.44592217014289	7.06591320437033	NA
**TC0900001654.mm.2**	Tmem42	transmembrane protein 42	-0.433820172656946	6.32223021239292	-1.06639357532882	0.324636476147512	0.999692817443324	-4.59238639481721	6.28085771934558	6.44133014660822	5.58114606229198	6.59063046925294	6.59368798614782	6.44572889071099	NA
**TC0500003223.mm.2**	Morn3	MORN repeat containing 3	-0.333673194627269	5.83264809677873	-1.06612727243537	0.324748116482242	0.999692817443324	-4.59240090810498	6.00326566402098	5.93290415786484	5.78785752776	5.408328657006	5.82103350044061	6.04249907357993	NA
**TC0200005290.mm.2**	Zbp1	Z-DNA binding protein 1	-0.403584049616165	5.29465534175321	-1.06606968711193	0.324772261675496	0.999692817443324	-4.59240404625985	4.63720845083075	4.76734877113932	4.99483562807525	5.78326773658319	5.60362599503384	5.98164546885693	NA
**TC0200003327.mm.2**	Zbtb43	zinc finger and BTB domain containing 43	0.39345206051241	6.04864805989019	1.06584959126431	0.324864560005883	0.999692817443324	-4.59241603989795	6.41929647686688	6.10785593364663	5.77062125458125	6.50519878034498	5.86085877201528	5.62805714188614	NA
**TC0200003576.mm.2**	Acvr1c	activin A receptor, type IC	-0.360523558769982	6.50948423278572	-1.06584816544077	0.324865158001672	0.999692817443324	-4.59241611759171	7.01756459618925	7.48991361833548	5.98732832155552	5.92686171076929	6.53148364570884	6.10375350415594	NA
**TC0400002471.mm.2**	Fam219a	family with sequence similarity 219, member A	-0.636527165408221	7.91789706366944	-1.06556577497705	0.324983611223421	0.999692817443324	-4.59243150432338	8.47417424696823	8.26740209675769	8.30119531749409	6.44518053624307	8.10986417885064	7.90956600570296	NA

**TC0600000044.mm.2**	Tac1	tachykinin 1	0.635614443252933	9.21404288521974	1.06522368670327	0.325127152546712	0.999692817443324	-4.59245014156688	9.96376168367974	9.84448115121708	9.90622952290293	8.46739215856082	9.14973035882535	7.95266243613252	NA
**TC1500000221.mm.2**	Sdc2	syndecan 2	0.342283107578167	7.47157383187777	1.0650870438189	0.325184502695077	0.999692817443324	-4.59245758528564	8.07362864065027	7.68917124731481	7.39585133700554	7.47975332222338	6.92845756440862	7.26258087966397	NA
**TC1900001144.mm.2**	Stx3	syntaxin 3	-0.321046550693184	6.70472006185446	-1.06480733097241	0.325301926059622	0.999692817443324	-4.59247282160428	6.86931577991997	6.8250241226278	6.60390158790008	6.34204209569624	6.8900100934438	6.69802669153889	NA
**TC0100002337.mm.2**	Stk17b	serine/threonine kinase 17b (apoptosis-inducing)	0.424944623277953	5.83896069398135	1.06456707360126	0.325402813459512	0.999692817443324	-4.59248590740305	5.37073429758754	6.24952103648716	6.07683032377284	6.05486871441182	5.87261842966235	5.40919136196641	NA
**TC0X00000226.mm.2**	Agtr2	angiotensin II receptor, type 2	0.315940268065765	4.73047270043678	1.06444264811877	0.325455071408495	0.999692817443324	-4.59249268384758	4.03898728450262	4.18906966183233	5.12364233216403	5.2696875640446	4.79793628949602	4.96351307058108	NA
**TC0X00002442.mm.2**	Irak1/Mir718/Mir5132	interleukin-1 receptor-associated kinase 1/microRNA 718/microRNA 5132	0.323645952959371	7.28057527325611	1.06431686336327	0.325507907155693	0.999692817443324	-4.5924995339854	7.67632130011884	7.54579957474117	7.15728047847317	7.39703085682451	7.06859502849959	6.83842440087935	NA
**TC0200002478.mm.2**	Mybl2	myeloblastosis oncogene-like 2	-0.338007383827343	5.06720622787953	-1.0636477074341	0.325789102196743	0.999692817443324	-4.59253597001418	4.71335731776796	4.84900286870367	5.05845874338784	5.0239724631876	5.14607786745092	5.6123681067792	NA
**TC1000002473.mm.2**	Odf3l2	outer dense fiber of sperm tails 3-like 2	0.453269699007239	6.02655488086504	1.06339397093893	0.325895779672741	0.999692817443324	-4.59254978365247	5.71212244950917	5.26912229517237	6.01686420248409	7.0254477667775	5.9439185963631	6.19185397488401	NA
**TC1200000083.mm.2**	Osr1	odd-skipped related transcription factor 1	-0.371979037801132	5.98969748519778	-1.06318016289401	0.325985692143553	0.999692817443324	-4.59256142248281	5.42971900972307	5.56522298717293	5.74545505963991	6.35418735970431	6.59763946920208	6.2459610257444	NA
**TC1700001266.mm.2**	Ppm1b	protein phosphatase 1B, magnesium dependent, beta isoform	-0.840216739185387	8.87271436459735	-1.06251671836533	0.326264817654964	0.999692817443324	-4.59259753144919	9.75623150911337	9.86827466262592	9.25255621905955	6.71311704967746	9.18345263729883	8.46265410980896	NA
**TC0200003161.mm.2**	Aim1	apical junction component 1	0.440699478529013	6.92621569148147	1.06240951321455	0.326309939344534	0.999692817443324	-4.59260336536641	7.57563550178213	7.10153469524675	6.83349297460851	7.04726847985048	6.9689295712002	6.03043292620077	NA
**TC0300001158.mm.2**	Slc25a54	solute carrier family 25, member 54	-0.388045271939617	5.44742976648203	-1.06225681757862	0.326374216294994	0.999692817443324	-4.59261167437126	5.11696173647328	5.47073966593219	5.32099074948346	5.33940257682027	5.35457574620557	6.0819081239774	NA
**TC1200001324.mm.2**	Cdca7l	cell division cycle associated 7 like	-0.36806803030586	6.2641694344003	-1.06188246448937	0.326531842915432	0.999692817443324	-4.59263204285567	5.80168981289272	6.12916556635903	5.99690207502221	6.46211050824695	6.39771665582179	6.79743198805908	NA
**TC0500003258.mm.2**	Rilpl1	Rab interacting lysosomal protein-like 1	0.32637464411831	7.34321810722521	1.06184201708445	0.326548877550803	0.999692817443324	-4.59263424341177	7.65430794406536	7.29667482102447	7.36346960437752	7.51706797887151	7.13500987890446	7.09277841610795	NA
**TC0600002484.mm.2**	Usp39	ubiquitin specific peptidase 39	0.375409897414855	6.63750005185767	1.06163094224012	0.326637784492674	0.999692817443324	-4.59264572644738	7.5050697410692	7.5393177216408	6.74822515202913	6.01749116960373	6.06061868561175	5.95427784119141	NA
**TC0500003632.mm.2**	Slc46a3	solute carrier family 46, member 3	0.397195512529754	6.47832982910164	1.06147240299871	0.326704575769465	0.999692817443324	-4.59265435077669	6.23616797686566	6.33614085989173	7.19979809483923	6.34623248661674	6.38695910897412	6.36468044742234	NA
**TC0700000447.mm.2**	Cyp2f2	cytochrome P450, family 2, subfamily f, polypeptide 2	0.592961481519196	6.1406996753024	1.06133736189371	0.326761476191461	0.999692817443324	-4.59266169641201	5.64248634325642	5.67409993997881	5.83183142118293	7.52493594462585	6.2313067241639	5.93953767860649	NA
**TC1300000054.mm.2**	Dip2c	disco interacting protein 2 homolog C	-0.554911326889545	5.68736484127355	-1.06121113733288	0.326814668957983	0.999692817443324	-4.5926685621122	7.29392401774324	6.91014989378744	5.3669066658471	4.03823957531865	5.62566868579509	4.88930020914975	NA
**TC1400001536.mm.2**	Cfap70	cilia and flagella associated protein 70	0.418613955012347	5.53807894471076	1.06096933646759	0.326916586736961	0.999692817443324	-4.59268171336807	5.27922705682209	5.2388087897886	5.50378741204482	6.27004545379447	5.17180900582404	5.76479594999056	NA
**TC0600001622.mm.2**	Atf7ip	activating transcription factor 7 interacting protein	-0.817472599699419	6.18075649688663	-1.06068175989825	0.327037832209714	0.999692817443324	-4.59269735267147	6.96036085391014	7.06790663851482	6.59089872339061	4.11976442135737	6.51178167186315	5.83382667228368	NA
**TC1100003841.mm.2**	Gpatch8	G patch domain containing 8	0.542219842665451	5.81845387837141	1.06057538334206	0.3270826909627	0.999692817443324	-4.5927031373066	7.2041405630683	7.14638045152335	5.65976378857076	5.1625571819131	5.58471505942073	4.15316622573222	NA
**TC1500001754.mm.2**	Tsta3	tissue specific transplantation antigen P35B	-0.358532428057343	6.23082861965626	-1.06047944564692	0.327123151940524	0.999692817443324	-4.59270835407945	6.25730053937226	6.30598314936755	6.33477211626049	5.71753947028104	6.30572620574247	6.46365023691375	NA
**TC0900001410.mm.2**	Qars	glutaminyl-tRNA synthetase	-0.361821712527358	7.47404367308965	-1.06042573629908	0.327145805208642	0.999692817443324	-4.59271127452862	7.78630403112568	7.63846167602458	6.8635504394168	7.48437601374966	7.45174083359892	7.61982904462225	NA
**TC1400002714.mm.2**	Abcc4	ATP-binding cassette, sub-family C (CFTR/MRP), member 4	-0.34023349255633	5.78184853452211	-1.06039512634363	0.327158716294821	0.999692817443324	-4.59271293891908	5.67221105303121	5.6363374643179	5.74601903340925	5.60501881892618	5.77024416564111	6.26126067180698	NA
**TC0600001243.mm.2**	Mtmr14	myotubularin related protein 14	0.336236221758829	7.27672651858481	1.05993627564091	0.327352306029671	0.999692817443324	-4.5927378861177	7.14238602492014	7.12094225276948	7.57336263392429	7.46138900474417	6.94690776519829	7.41537142995251	NA
**TC0700000242.mm.2**	Ptgir	prostaglandin I receptor (IP)	-0.441316762596357	4.86150457004399	-1.05986998788547	0.327380280581359	0.999692817443324	-4.59274148973334	4.38004037645664	4.21587673629742	4.38889430377652	5.45634408738207	5.34331837058968	5.38455354576162	NA
**TC0400004170.mm.2**	Agrn	agrin	-0.324218341503451	7.1041489040631	-1.05986935510917	0.327380547632954	0.999692817443324	-4.59274152413264	7.61488417670496	7.50081559688092	6.73604702082441	6.69433146306849	6.86122878459884	7.21758638230096	NA
**TC0900000535.mm.2**	Scn4b	sodium channel, type IV, beta	0.369488425531294	9.34687334024746	1.05972408617965	0.327441860374881	0.999692817443324	-4.59274942108541	9.24736901723527	9.49090502798782	9.81035715884441	9.23061426481771	9.31403007881666	8.98796449378287	NA
**TC0400002531.mm.2**	Gba2	glucosidase beta 2	0.334474176485834	7.31801817166605	1.05945309653999	0.327556260082862	0.999692817443324	-4.59276415111412	7.82073822015468	7.96525056715355	7.01424918890241	7.38128510892746	6.99027322239083	6.73631272246737	NA
**TC1100000756.mm.2**	Alkbh5	alkB homolog 5, RNA demethylase	-0.533528897245861	7.11688118511012	-1.05922176740912	0.327653942465431	0.999692817443324	-4.59277672408419	7.88130579265956	7.92478515902648	7.19838241871503	5.71568676352643	6.97452675553781	7.00660022119538	NA
**TC0700001689.mm.2**	Xylt1	xylosyltransferase 1	0.481600895217833	7.38325244498749	1.05917024343646	0.327675702484328	0.999692817443324	-4.59277952430624	7.50496522460684	7.76651707684422	7.5751208275308	7.42049625192396	7.62959321396461	6.40282207505449	NA
**TC1100003943.mm.2**	Bptf	bromodomain PHD finger transcription factor	0.420303755813391	7.05695490892896	1.05901324131261	0.327742016089658	0.999692817443324	-4.59278805669516	8.19457877475252	8.18466635307763	6.80280413162857	6.59874178705664	6.76066166253767	5.80027674452075	NA
**TC1500002059.mm.2**	Rabl2	RAB, member RAS oncogene family-like 2	0.485830646488417	7.23040325780036	1.05899003384171	0.327751819240896	0.999692817443324	-4.59278931787598	8.07549461640144	7.29016894435013	7.75016754576918	6.74404109374454	6.69784545012921	6.82470189640768	NA
**TC0800000945.mm.2**	Rfx1	regulatory factor X, 1 (influences HLA class II expression)	0.315221403377524	6.9754231462269	1.05893712257452	0.327774170565885	0.999692817443324	-4.5927921932287	6.64591980911748	6.5638512263522	7.25429064373968	7.38231468058371	6.88608483513809	7.12007768243026	NA
**TC1600000291.mm.2**	Polr2h	polymerase (RNA) II (DNA directed) polypeptide H	-0.336063823749591	6.50189166188618	-1.05868909554497	0.327878961137235	0.999692817443324	-4.59280567093401	6.61874790293775	6.52655319623032	6.45502747333588	6.14193313898902	6.45590386567366	6.81318439415043	NA
**TC0400001970.mm.2**	Slc2a5	solute carrier family 2 (facilitated glucose transporter), member 5	0.553992300291116	5.91637947372139	1.05868475094107	0.327880796958833	0.999692817443324	-4.59280590700646	7.14080215814823	6.70192171754594	6.3567724872372	5.024996296371	4.7855578072519	5.48822637577407	NA
**TC0800000571.mm.2**	4930555F03Rik	RIKEN cDNA 4930555F03 gene	0.443344469672159	5.30057975079361	1.05855864653122	0.327934086265607	0.999692817443324	-4.59281275895517	4.66257547930362	4.91458426244973	5.27018298138278	6.28632086979354	5.17824549714355	5.49156941468846	NA
**TC0700002657.mm.2**	Egln2	egl-9 family hypoxia-inducible factor 2	-0.45492555278426	6.78571617872522	-1.05846362517454	0.327974245094626	0.999692817443324	-4.5928179217625	7.34969014172563	7.0668564023738	6.84337200766307	5.85057770367862	7.05110427430185	6.55269654260836	NA
**TC0400001677.mm.2**	Id3	inhibitor of DNA binding 3	-0.305975611438269	6.04924837817918	-1.05833515145337	0.328028548200169	0.999692817443324	-4.59282490183037	6.63792641972296	6.72278920989312	5.5435253847387	5.61788632355252	5.89779039113133	5.87557254003643	NA
**TC1600001312.mm.2**	Tango2	transport and golgi organization 2	-0.318413977020525	6.93936472975301	-1.05830752572683	0.32804022595562	0.999692817443324	-4.59282640270886	8.22519805125654	8.23331437246672	6.21306786990336	6.05735613047351	6.59340454107334	6.31384741334458	NA
**TC0800000674.mm.2**	Tmem192	transmembrane protein 192	0.312492955377973	7.28151047124967	1.05790411905445	0.328210789503228	0.999692817443324	-4.59284831751088	7.167278848723	7.18925074698205	7.52968228608035	7.44907728519411	7.29247525091421	7.0612984096043	NA
**TC0200004114.mm.2**	1110051M20Rik	RIKEN cDNA 1110051M20 gene	0.332930080360118	6.36651309664498	1.05777803704643	0.328264112675649	0.999692817443324	-4.59285516610902	6.6912327082065	6.47020844789815	6.4985238735132	6.35322491872954	5.90429849201281	6.28159013950969	NA
**TC0900000369.mm.2**	Vsig2	V-set and immunoglobulin domain containing 2	-0.391028328646343	5.9912310459392	-1.05731354741745	0.32846061712682	0.999692817443324	-4.59288039355766	5.63896593789449	5.79433722914574	5.60568388838395	6.26032933726718	6.57133677094203	6.07673311200178	NA
**TC0500001179.mm.2**	Tpst2	protein-tyrosine sulfotransferase 2	0.318141735194264	7.43452212647312	1.05714717002236	0.328531026953763	0.999692817443324	-4.5928894287381	7.66129495702915	7.68118603673146	7.34315391200906	7.60731370572427	7.24771632519732	7.06646782214749	NA
**TC0900001392.mm.2**	Amigo3	adhesion molecule with Ig like domain 3	0.507003719443651	5.10183908856091	1.0571167771501	0.328543890335809	0.999692817443324	-4.59289107916826	4.85374369237362	5.21529555866194	5.11193820665134	5.66606315295725	5.48591840144375	4.27807551927754	NA
**TC0100001330.mm.2**	Brinp3	bone morphogenetic protein/retinoic acid inducible neural specific 3	-0.589670416512378	6.3926166941721	-1.05708990629518	0.328555263409366	0.999692817443324	-4.59289253832489	7.51916328595255	7.40467734835029	6.21298614269782	4.91327320615469	6.70910479519151	5.59649538668575	NA
**TC1400001523.mm.2**	Rtraf	RNA transcription, translation and transport factor	-0.597674884061558	7.87768011961376	-1.05703040717111	0.328580447510423	0.999692817443324	-4.59289576922469	8.33069027434762	8.41421816495363	8.12731876492989	6.53559249019921	8.28604185893263	7.57221916431959	NA
**TC1000001374.mm.2**	Mdm1	transformed mouse 3T3 cell double minute 1	-0.388867739102905	6.46660819360582	-1.05687445971251	0.328646462546556	0.999692817443324	-4.59290423706151	6.46853595441637	5.88956505517928	6.24975637631247	6.58214996060427	7.0465339168844	6.56310789823815	NA
**TC1600002082.mm.2**	Gart	phosphoribosylglycinamide formyltransferase	0.484039698573342	6.75894808055707	1.0567881786742	0.328682991283522	0.999692817443324	-4.59290892183359	7.61786828810769	7.54177515612414	7.16783671303873	6.01322550508992	6.37920472920508	5.8337780917769	NA
**TC0400000499.mm.2**	Tmod1	tropomodulin 1	-0.485895444519191	7.50764464031088	-1.05675969175599	0.328695052488311	0.999692817443324	-4.59291046854249	7.81951663951448	7.76350379288825	7.72784737653126	6.51768088368081	7.82639932058032	7.39091982867014	NA
**TC0800002603.mm.2**	Wdr83	WD repeat domain containing 83	-0.343307907760688	6.67582636520401	-1.05665399443544	0.328739807277495	0.999692817443324	-4.59291620726748	6.79516331792829	6.43260597550821	6.35514770755252	6.71513883358058	6.78514577370849	6.97175658294599	NA
**TC1600000219.mm.2**	Lztr1	leucine-zipper-like transcriptional regulator, 1	0.44844135756585	7.76771993335752	1.05636442119343	0.328862444768939	0.999692817443324	-4.59293192809639	8.47160081084716	8.29820873130404	7.87549644958498	7.49119993697782	7.75409780430825	6.71571586712285	NA
**TC1700000618.mm.2**	H2-DMa	histocompatibility 2, class II, locus DMa	0.363132734944378	5.97855771754603	1.05621634144123	0.328925172468495	0.999692817443324	-4.59293996658787	6.36022023067786	5.8898540482233	6.19235858903396	5.98141015909793	5.9432440284945	5.50425924974863	NA
**TC0300000418.mm.2**	Sohlh2	spermatogenesis and oogenesis specific basic helix-loop-helix 2	-0.518210844444646	4.69237992254173	-1.05560511361323	0.329184195434778	0.999692817443324	-4.59297314195671	3.97777966503637	4.34251784822656	4.11855168202921	5.28022848451988	4.84503336380328	5.59016849163511	NA
**TC1000001545.mm.2**	Cyp27b1	cytochrome P450, family 27, subfamily b, polypeptide 1	0.520293110818538	5.83103958331094	1.05560257934533	0.329185269736431	0.999692817443324	-4.59297327949119	5.31850063269416	5.88036238184663	5.69897548634794	6.71500486713302	5.26399057956048	6.10940355228341	NA
**TC0300000722.mm.2**	Glmp	glycosylated lysosomal membrane protein	0.528536432044955	7.42017572089301	1.05536368885205	0.329286550528355	0.999692817443324	-4.59298624342571	7.92947660400996	8.09696964951955	8.01773271456776	6.75810775339146	6.48882005577904	7.22994754809027	NA
**TC0100003855.mm.2**	Cr1l	complement component (3b/4b) receptor 1-like	0.519081732041144	6.90585145541201	1.05515846069141	0.329373579813802	0.999692817443324	-4.59299737960179	7.22174857227959	7.49393355950075	6.4228375611798	7.45595747120721	6.89712604449366	5.94350552381106	NA
**TC1000001597.mm.2**	Pan2	PAN2 poly(A) specific ribonuclease subunit	-0.311019901214682	6.33825758912986	-1.05478391812198	0.329532456616456	0.999692817443324	-4.59301770081321	6.48867524636782	6.3262021927621	6.14020500058441	6.15610914602552	6.37285806217019	6.54549588686911	NA
**TC0500000201.mm.2**	Klhl7	kelch-like 7	-1.15803516758788	6.84984248908634	-1.05474842931084	0.329547513783705	0.999692817443324	-4.59301962613781	8.18741601257664	8.06112564942823	7.39908219319719	3.86813927547153	7.49134925873719	6.09194254510728	NA
**TC0500003088.mm.2**	Acads	acyl-Coenzyme A dehydrogenase, short chain	0.327530393990584	6.67961302211894	1.05447119806205	0.329665156368552	0.999692817443324	-4.59303466542633	6.31081409742907	6.46664048595877	6.82070400909026	7.15693815956323	6.55915102438225	6.76343035629008	NA
**TC0900002018.mm.2**	Hyls1	HYLS1, centriolar and ciliogenesis associated	-0.348437400277982	7.29117925213851	-1.05443504721203	0.329680499414473	0.999692817443324	-4.59303662641984	7.47878505524036	7.3100539322878	6.89459858936023	7.23608227301324	7.191545044143	7.63601061878644	NA
**TC1100000694.mm.2**	Trim58	tripartite motif-containing 58	-0.438640223345734	5.94278101695711	-1.05443146406852	0.329682020193933	0.999692817443324	-4.59303682078497	5.25424958542881	5.0367747888698	5.6126749817376	6.63151565863869	6.70090007961136	6.4205710074564	NA
**TC0900000662.mm.2**	Crabp1	cellular retinoic acid binding protein I	0.479524684574742	6.08987416558568	1.05419604058809	0.329781952456851	0.999692817443324	-4.59304959055198	6.10878811303394	5.91198696299619	6.32298362741182	6.41577601590489	5.26695799968372	6.5127522744835	NA
**TC0700002027.mm.2**	Ric8a	RIC8 guanine nucleotide exchange factor A	-0.337544558221796	6.84296995549378	-1.05416205829834	0.329796379234792	0.999692817443324	-4.59305143370844	6.86322685156076	7.15516785684081	6.73011407989613	6.45205387416261	7.08219378004634	6.775063290456	NA
**TC1100003741.mm.2**	2300003K06Rik	RIKEN cDNA 2300003K06 gene	-0.48732991363798	5.21561466565355	-1.0539574605455	0.329883249545343	0.999692817443324	-4.59306253029697	4.94590321134032	5.16781178938375	4.60313939847883	5.49951718448181	5.0817150649788	5.9956013452578	NA
**TC1200001337.mm.2**	Tmem196	transmembrane protein 196	-0.44343804727459	7.3527962071777	-1.05386962056921	0.329920551249253	0.999692817443324	-4.59306729411491	8.61741665502094	8.42192529353239	7.08982148773028	6.00545811225155	7.00866212266153	6.97349357186948	NA
**TC0700000853.mm.2**	Bcat2	branched chain aminotransferase 2, mitochondrial	-0.431924102966269	6.11414206594315	-1.0536231708976	0.33002522555302	0.999692817443324	-4.59308065889519	5.88945998633941	5.80286029478713	6.52375689098301	5.5405850633169	6.28698831150646	6.64120184872599	NA
**TC0700004612.mm.2**	Tpcn2	two pore segment channel 2	0.40560848350089	5.15300587699499	1.05340451714022	0.330118116553377	0.999692817443324	-4.59309251520778	4.89110776683762	4.88608551980271	5.291117025297	5.68491244586869	4.66709407905686	5.49771842510705	NA
**TC0900003172.mm.2**	Cnot10	CCR4-NOT transcription complex, subunit 10	0.309976493104773	6.44247440101507	1.05328027104073	0.330170909603646	0.999692817443324	-4.59309925187819	6.4347616549216	6.63796445246888	6.51525930534006	6.58577733711467	6.27886074354549	6.20222291269969	NA
**TC0400003224.mm.2**	Dmrtb1	DMRT-like family B with proline-rich C-terminal, 1	-0.322352856899859	6.76681545514927	-1.0532146369781	0.330198800731589	0.999692817443324	-4.59310281044493	6.3134298248176	6.32134539223703	6.66761971407389	6.99308618594675	7.24891334615506	7.05649826766529	NA
**TC0300000092.mm.2**	Car13	carbonic anhydrase 13	0.450841105675642	5.73128381798737	1.05306742446772	0.330261365448242	0.999692817443324	-4.59311079171142	5.70323867110588	4.94752183995949	5.73414122248614	6.58517108161892	5.70191375755673	5.71571633519705	NA
**TC1100003796.mm.2**	Ccr10	chemokine (C-C motif) receptor 10	0.390738457151366	5.03567133166842	1.05246725118355	0.330516535585968	0.999692817443324	-4.59314332574495	4.58978861965222	4.67241115432783	5.12541521772163	5.74123734744496	4.75759796043046	5.3275776904334	NA
**TC0200005503.mm.2**	Ell3	elongation factor RNA polymerase II-like 3	-0.311212271309659	5.45592395426682	-1.05240950556546	0.33054109513833	0.999692817443324	-4.59314645558593	5.19518139554904	5.35649868081895	5.36355572102048	5.41716383228633	5.60937832617765	5.79376576974847	NA
**TC0800002339.mm.2**	Psd3	pleckstrin and Sec7 domain containing 3	-1.08931459297911	7.84616809570935	-1.05238185396815	0.330552856046892	0.999692817443324	-4.59314795429014	9.28738751392535	9.32323691982803	8.16164969998767	4.98222777728459	8.4687425917152	6.85376407151528	NA
**TC0200001801.mm.2**	Tmem62	transmembrane protein 62	-0.309820085828069	5.78284118672698	-1.05226265219408	0.330603559372917	0.999692817443324	-4.5931544147802	5.94345888850008	5.77708984793776	5.66892500649563	5.50950409963832	5.90331763931367	5.89475163847642	NA
**TC0200004410.mm.2**	Gpr176	G protein-coupled receptor 176	0.460757595771568	5.34279352543148	1.05203982137687	0.330698358867727	0.999692817443324	-4.59316649091074	6.36081780721358	5.21354360234741	5.28332896504207	5.41862850224345	4.75731046055824	5.02313181518415	NA
**TC1100000942.mm.2**	Clec10a	C-type lectin domain family 10, member A	-0.309013296308409	6.71906122888628	-1.05182878979883	0.330788158783055	0.999692817443324	-4.59317792658209	6.51322244124046	6.43716575469819	6.5834213726863	6.78955491969482	6.99414709556395	6.99685578943398	NA
**TC0900000055.mm.2**	Tmem123	transmembrane protein 123	0.337357226313962	7.5926606704663	1.05171766078276	0.330835455227627	0.999692817443324	-4.59318394820024	6.76345127209367	7.02638995556875	7.89142534068892	8.32899328319272	7.78818533204426	7.75751883920946	NA
**TC1300002096.mm.2**	Gkap1	G kinase anchoring protein 1	0.62604849875758	7.43763430713641	1.05143070232388	0.330957609789086	0.999692817443324	-4.59319949602245	7.91316632674376	7.68385863828478	8.04582882956239	7.09461010809015	7.74113007635683	6.14721186378055	NA
**TC0400003392.mm.2**	Mpl	myeloproliferative leukemia virus oncogene	0.316265300959167	5.54523010398483	1.05115071058891	0.331076833737516	0.999692817443324	-4.5932146646248	5.32972255660405	5.23586029607298	5.68651984814545	5.98264433842968	5.47959260516223	5.55704097949457	NA
**TC1900000635.mm.2**	Cutc	cutC copper transporter	-0.310217651320255	5.70246379087607	-1.05099345327529	0.331143811025385	0.999692817443324	-4.5932231833082	5.2498859530362	5.3451967189984	5.70711141289332	5.79252097239733	5.95713479651745	6.16293289141371	NA
**TC1400002242.mm.2**	Hmbox1	homeobox containing 1	0.652287058425885	6.48178351554277	1.05089965730892	0.331183764758847	0.999692817443324	-4.59322826400804	7.82040880661168	7.61782613756001	6.53369375141152	5.84482638155684	6.45588840586935	4.61805761024724	NA
**TC0800002382.mm.2**	Ncan	neurocan	-0.591826748506455	7.14764247465995	-1.0508427208165	0.331208019566531	0.999692817443324	-4.59323134802474	8.36942749535204	8.6700373611828	7.02100786583386	5.31036038137211	6.77314384486236	6.74187789935652	NA
**TC1600002023.mm.2**	Krtap16-3	keratin associated protein 16-3	0.451278800357131	6.51984180726744	1.05043358894606	0.331382350843822	0.999692817443324	-4.59325350691379	6.64533628277867	6.38690683012799	6.61510240042163	6.87958026528449	5.75401671373085	6.83810835126101	NA
**TC0200005287.mm.2**	Ctcfl	CCCTC-binding factor (zinc finger protein)-like	0.411917421220841	5.64521011840703	1.05040353674254	0.331395159015191	0.999692817443324	-4.5932551344176	5.16544616663009	4.9322048297176	5.77345706750531	6.52526521076279	5.46520625792547	6.00968117790095	NA
**TC0700000825.mm.2**	Irf3	interferon regulatory factor 3	0.341324315603433	6.08816262637126	1.05035259091379	0.331416872906584	0.999692817443324	-4.59325789338858	6.55877634850509	6.50241989862365	6.20008362439358	5.87513044675927	5.91284773470297	5.47971770524302	NA
**TC0700000396.mm.2**	Ceacam10	carcinoembryonic antigen-related cell adhesion molecule 10	-0.342659902502903	5.17632932641074	-1.05015500804071	0.331501096590408	0.999692817443324	-4.59326859294377	4.81231047587555	5.22206588456406	4.90775233425677	5.26138756225274	5.36685672552707	5.48760297598825	NA

**TC1000001145.mm.2**	Fgd6	FYVE, RhoGEF and PH domain containing 6	0.622248567026614	5.37660168169369	1.04994931950577	0.331588793790427	0.999692817443324	-4.59327973051965	5.97858179358492	5.41169166059522	5.19501290228526	5.86190398273235	4.08624015507463	5.72617959588974	NA
**TC0700003790.mm.2**	Uvrag	UV radiation resistance associated gene	-0.721924159777394	6.48340440574908	-1.04992686272572	0.331598369576562	0.999692817443324	-4.59328094644746	7.47465207084535	7.40244515142397	6.69481969790904	4.59492074842615	6.7554170381666	5.97817172772337	NA
**TC0400002367.mm.2**	Ankrd6	ankyrin repeat domain 6	0.326125809870054	6.49654986599431	1.0498451032094	0.331633234498993	0.999692817443324	-4.59328537324188	6.9890775946422	6.80416014016149	6.62106751630583	6.29808902414529	6.23444975518087	6.03245516553014	NA
**TC0600000231.mm.2**	Ccdc136	coiled-coil domain containing 136	0.492221978605066	7.33226770237806	1.04976768351586	0.331666251500815	0.999692817443324	-4.5932895649239	7.86503413406196	7.4619051941554	7.63154235903616	7.19401306259441	7.51467265125247	6.32643881316796	NA
**TC1100003469.mm.2**	Smg8	smg-8 homolog, nonsense mediated mRNA decay factor (C. elegans)	-0.459306479533618	5.4599227089817	-1.04947344286159	0.331791759797324	0.999692817443324	-4.59330549458314	6.01592130459728	6.02451161613155	5.40216172264411	4.49808346440296	5.02724344999319	5.79161469612111	NA
**TC0600003119.mm.2**	Tpi1	triosephosphate isomerase 1	-0.542892696242005	9.75778907490888	-1.04935843951168	0.331840824848712	0.999692817443324	-4.5933117201351	10.734020026757	10.7301410461517	9.69108403571606	8.30730995631425	9.88334361483795	9.20083576967637	NA
**TC0700000548.mm.2**	Zfp382	zinc finger protein 382	-0.604538818158463	5.66723685587412	-1.04917838405508	0.331917655476946	0.999692817443324	-4.59332146660559	6.65384110435067	6.33730636928119	5.6661980964205	4.23539991622745	6.07034426622919	5.04033138273569	NA
**TC0400001822.mm.2**	Lrrc38	leucine rich repeat containing 38	0.332181843822901	5.0282549065298	1.04913107974348	0.331937842855648	0.999692817443324	-4.59332402708654	4.40812760344926	4.55980393066012	5.39246004847103	5.54052074788658	4.92061567875541	5.3480014299564	NA
**TC0900002724.mm.2**	Cgas	cyclic GMP-AMP synthase	-0.384304069565868	4.89181467412909	-1.04893275682512	0.332022489026493	0.999692817443324	-4.59333476133953	4.76279517068604	4.50899373679608	4.46958123441525	5.1856642646651	5.3597636843806	5.06408995383148	NA
**TC1000002086.mm.2**	1700021F05Rik	RIKEN cDNA 1700021F05 gene	0.529038247767504	6.78671446132182	1.04888489840588	0.332042918072665	0.999692817443324	-4.59333735155156	6.52828577835716	6.34422929490045	7.72889950815826	6.72402458694592	7.23744832181419	6.15739927775497	NA
**TC1600001080.mm.2**	Sh3bgr	SH3-binding domain glutamic acid-rich protein	0.509074644644858	5.12662955084851	1.04880940793951	0.332075144307613	0.999692817443324	-4.59334143717249	5.54232491430145	4.95937875599882	4.73439152907226	5.90371993296798	4.55446280713408	5.06549936561644	NA
**TC1400000310.mm.2**	Sfmbt1	Scm-like with four mbt domains 1	0.334679173008626	6.46971401113852	1.04877599599671	0.332089408384227	0.999692817443324	-4.5933432454204	6.82433433526447	6.68275212613285	6.32103604905147	6.66924192667406	5.99718116463249	6.32373846507579	NA
**TC1200001658.mm.2**	Arl4a	ADP-ribosylation factor-like 4A	-0.553453869810303	6.85182784278017	-1.04853429082793	0.332192610759484	0.999692817443324	-4.59335632571795	7.40455862980199	7.60003135736799	6.89486176756949	5.60487289737574	7.25588242529816	6.35075997926767	NA
**TC1100002496.mm.2**	Foxi1	forkhead box I1	-0.536239770886121	6.16738730417819	-1.04836570332985	0.332264608904329	0.999692817443324	-4.59336544835529	5.59884545917869	5.88165732611617	5.38280045055181	6.84287029844921	6.49792518901134	6.80022510176192	NA
**TC1900000709.mm.2**	As3mt	arsenic (+3 oxidation state) methyltransferase	0.377770650072247	7.1813721915632	1.04785989721234	0.332480697335659	0.999692817443324	-4.59339281482891	8.21640907366115	7.70015638849226	6.83427430316533	7.12933019051982	6.85713974538924	6.35092344815142	NA
**TC0200000465.mm.2**	Adamtsl2	ADAMTS-like 2	0.327955762341617	6.87319612385057	1.04772789409151	0.332537109775488	0.999692817443324	-4.59339995587602	6.4531816805248	6.70243309143065	7.08372429500088	7.28601245291473	6.70255156035213	7.01127366288025	NA
**TC1000000160.mm.2**	Ahi1	Abelson helper integration site 1	0.425137073444233	8.80237895252749	1.04751984874943	0.332626035014438	0.999692817443324	-4.59341120983291	9.5163988122581	8.92621193505336	9.22345715065939	8.38751140671158	8.33149066780281	8.42920374267969	NA
**TC0100002308.mm.2**	Myo1b	myosin IB	-0.335658744319153	6.42531243400771	-1.04745983431789	0.332651690658898	0.999692817443324	-4.59341445606027	6.75446916251616	7.11656681658427	6.17323471236421	5.83152585578954	6.39058360011889	6.28549445667317	NA
**TC0600002733.mm.2**	Grip2	glutamate receptor interacting protein 2	0.44771190262532	5.60154780849627	1.04728071842854	0.332728270600949	0.999692817443324	-4.59342414410019	5.25017961927051	4.77685044756655	5.64923862563265	6.58960166906295	5.88406309511348	5.45935339433148	NA
**TC1500001818.mm.2**	Apol8	apolipoprotein L 8	0.319759955839054	6.80597076155316	1.04717636978622	0.332772890785066	0.999692817443324	-4.59342978779078	6.6128220166969	6.69741059159873	7.09796548229728	6.98459045405344	6.55019045701164	6.89284556766097	NA
**TC0200000897.mm.2**	Tanc1	tetratricopeptide repeat, ankyrin repeat and coiled-coil containing 1	-0.616563582315116	5.7163594351114	-1.04694392629054	0.33287230249706	0.999692817443324	-4.59344235860997	6.07582238034524	5.42918037812298	4.5268787356437	6.25313460814128	6.02016540982699	5.99297509858823	NA
**TC0600002555.mm.2**	Htra2	HtrA serine peptidase 2	0.332931983303267	6.87776932702048	1.04688200156721	0.332898790566051	0.999692817443324	-4.59344570736856	8.12586458895712	7.97370590849249	6.54617070386703	6.37028401177288	6.34029476836151	5.91029598067186	NA
**TC0700001860.mm.2**	Ctf1	cardiotrophin 1	-0.322908654457585	7.71652644056846	-1.0468324086757	0.33292000496259	0.999692817443324	-4.59344838918585	7.16519534974639	7.42938591749461	7.76275014685909	7.76662988676819	7.93581965428787	8.23937768825459	NA
**TC1100002690.mm.2**	BC049762	cDNA sequence BC049762	-0.372348561788703	5.05263116078796	-1.04649854121219	0.333062852054344	0.999692817443324	-4.59346644218715	4.72744591462559	4.43227471639522	4.77945964748352	5.42622495758124	5.59866712012762	5.35171460851455	NA
**TC0400002013.mm.2**	Tnfrsf25	tumor necrosis factor receptor superfamily, member 25	-0.329803591921145	7.05046151926272	-1.04629493343909	0.333149990990849	0.999692817443324	-4.59347745051534	6.2374474117388	6.38435514327727	7.03514805307042	7.47553163528857	7.57320686341001	7.59708000879126	NA
**TC1000002443.mm.2**	Pwp2	PWP2 periodic tryptophan protein homolog (yeast)	-0.336902771972739	6.02635742308633	-1.04627499599971	0.333158524692053	0.999692817443324	-4.59347852840993	6.2327159144865	6.43674876487409	5.91824304452769	5.48919411307826	5.93334362696614	6.14789907458529	NA
**TC0500000060.mm.2**	9330182L06Rik	RIKEN cDNA 9330182L06 gene	0.667385490377185	6.93268938926595	1.04618848749993	0.333195554436328	0.999692817443324	-4.59348320528878	7.7967355380065	7.64510097812843	7.08085612260105	6.66367927750652	7.20175634592025	5.20800807343295	NA
**TC0900001240.mm.2**	7420426K07Rik	RIKEN cDNA 7420426K07 gene	-0.358869165906696	5.10208945926068	-1.04617080376496	0.333203124321691	0.999692817443324	-4.59348416129774	4.41638356873026	4.94258284025016	4.96010147240845	5.30781453497669	5.37682839233881	5.60882594685973	NA
**TC1600002081.mm.2**	Dnajc28	DnaJ heat shock protein family (Hsp40) member C28	0.40932987255135	6.70334313239223	1.04595821294001	0.333294139007975	0.999692817443324	-4.59349565372495	6.83100839753119	6.30614798915164	6.62142171837401	7.32935935801262	6.38489660795737	6.74722472332656	NA
**TC0900000066.mm.2**	Trpc6	transient receptor potential cation channel, subfamily C, member 6	0.420109582065629	5.10609986416942	1.04581184466849	0.333356814018387	0.999692817443324	-4.59350356564472	5.76254695209661	5.26917778691145	5.11922162127574	5.10332518379412	5.12990134930761	4.252426291631	NA
**TC0800002313.mm.2**	Msmo1	methylsterol monoxygenase 1	-0.73353677370894	6.65699747354686	-1.04577650567928	0.333371947621677	0.999692817443324	-4.59350547581766	7.88277678222485	7.76918080078211	6.78393060037001	4.62754625505814	6.82758179049971	6.05096861234632	NA
**TC0700004533.mm.2**	Deaf1	DEAF1, transcription factor	0.415497964986157	7.84600568978555	1.04564607800026	0.333427806879683	0.999692817443324	-4.59351252556249	8.12516076576409	7.91331845464533	8.36239138786731	7.57188403627078	7.825125840181	7.27815365398478	NA
**TC1300002079.mm.2**	Cxcl14	chemokine (C-X-C motif) ligand 14	0.336262930437428	8.09485798396346	1.04564195936361	0.333429570922199	0.999692817443324	-4.59351274817265	8.51745839191338	8.63659045379055	8.27297919008985	7.77083326938601	7.70773407134156	7.66355252725944	NA
**TC0700000873.mm.2**	Myod1	myogenic differentiation 1	0.487308584910207	5.54045521239299	1.04558835419524	0.33345253109627	0.999692817443324	-4.59351564546934	5.03323241399266	5.08268536301077	5.4006790846487	6.65003624893875	5.74331475052498	5.33278341324206	NA
**TC0700003045.mm.2**	Uevld	UEV and lactate/malate dehyrogenase domains	-0.61943773103751	5.20815000910949	-1.04551959519587	0.333481983822308	0.999692817443324	-4.59351936171851	6.54900635364041	6.4563600062202	5.09648841432839	3.40584070203227	5.25333294754392	4.48787163089176	NA
**TC0700001437.mm.2**	Rpl31	ribosomal protein L31	0.42746873258684	5.93913901228718	1.04550441061686	0.33348848837637	0.999692817443324	-4.59352018239212	6.57851714919014	6.17885424945868	6.39631026388858	5.4698898062354	5.63815951036786	5.37310309458243	NA
**TC1100002995.mm.2**	Shisa6	shisa family member 6	0.37712067547066	7.29211128231728	1.04538754631712	0.33353855245646	0.999692817443324	-4.59352649832731	7.41557013492152	7.53789770761204	7.04552312196003	7.73119747919569	6.83315394042879	7.18932530978561	NA
**TC0900000096.mm.2**	Fam76b	family with sequence similarity 76, member B	0.570946790859745	6.37694707784934	1.0453853143417	0.333539508682332	0.999692817443324	-4.59352661895152	6.73868687287613	6.24844405617554	7.01964762471216	6.18857493516976	6.71206262100708	5.35426635715534	NA
**TC1100003600.mm.2**	Abi3	ABI gene family, member 3	0.365725786575307	6.3363712725309	1.04530472290267	0.333574037249835	0.999692817443324	-4.59353097433605	6.14889279619162	6.36431979823821	6.25153044568188	6.86670286127121	6.04527273939826	6.34150899440422	NA
**TC0500003626.mm.2**	Flt3	FMS-like tyrosine kinase 3	-0.430107300834304	5.20497692656343	-1.04497709084434	0.333714437395037	0.999692817443324	-4.593548678978	5.08164732718385	4.98377443528592	4.55837474897374	5.59373784864736	5.41194852541128	5.60037867387843	NA
**TC1200002289.mm.2**	Serpina3c	serine (or cysteine) peptidase inhibitor, clade A, member 3C	0.34549403650385	5.23175884245377	1.04466287127857	0.333849134498078	0.999692817443324	-4.59356565656723	5.01976151827217	5.39709802826907	5.25033156443304	5.58200922616151	4.91855401793582	5.22279869965103	NA
**TC1100002654.mm.2**	Btnl9	butyrophilin-like 9	-0.442618084648108	4.56587793947357	-1.04456981740796	0.333889032462905	0.999692817443324	-4.59357068393243	3.74255903561797	3.63773495064821	4.48374420755154	5.08112453308798	4.75611515014063	5.69398975979511	NA
**TC1200002242.mm.2**	Fbln5	fibulin 5	0.317085699738183	5.640615490912	1.04455855227354	0.333893862784595	0.999692817443324	-4.59357129253382	6.26426281166812	5.98442921837852	5.47201863310025	5.64256752435062	5.30339567618202	5.17701908179248	NA
**TC1100002684.mm.2**	Zfp354b	zinc finger protein 354B	-0.360114585746614	6.1339694079437	-1.04450715343857	0.333915902551298	0.999692817443324	-4.59357406933106	6.30091626770298	6.08854806558471	5.60095988281466	6.246101588626	6.3213470257816	6.24594361715228	NA
**TC0100001952.mm.2**	Cops5	COP9 signalosome subunit 5	-0.947237418176679	7.03674166781748	-1.04430615712384	0.334002100784662	0.999692817443324	-4.59358492748915	8.40426248677777	8.15690707862788	7.41761805742938	4.46478474514356	7.31837028607486	6.45850735285144	NA
**TC1000001640.mm.2**	Olfr784	olfactory receptor 784	0.584516939899854	5.69213752513337	1.04422849407861	0.334035411742217	0.999692817443324	-4.59358912273357	5.17875270325664	4.86754486974399	5.47178913240869	7.16599159639097	5.7494235118056	5.71932333719435	NA
**TC0300001366.mm.2**	Slc9b2	solute carrier family 9, subfamily B (NHA2, cation proton antiporter 2), member 2	-0.419063533384896	6.56023001088089	-1.04412890001127	0.334078133181977	0.999692817443324	-4.59359450246106	5.68770325895459	5.44817109557532	6.45839308314946	7.23529623884335	6.97052110327583	7.56129528548677	NA
**TC0200004959.mm.2**	Ncoa6	nuclear receptor coactivator 6	0.569752995916427	6.00690827147134	1.04387666815694	0.334186349101777	0.999692817443324	-4.5936081261551	6.8151162551526	7.02915801104856	6.42193181937826	5.24640885785162	5.8436380964302	4.68519658896683	NA
**TC0100002210.mm.2**	Mitd1	MIT, microtubule interacting and transport, domain containing 1	-0.546164989431843	5.40150145507513	-1.04374290501196	0.334243749399777	0.999692817443324	-4.59361535046636	6.40696223895473	6.03576187254922	5.45439095988947	3.98258636015209	5.06724606622129	5.46206123268395	NA
**TC1400001067.mm.2**	Nufip1	nuclear fragile X mental retardation protein interacting protein 1	-0.752599981141218	6.19835990435965	-1.04362965728116	0.334292352335585	0.999692817443324	-4.59362146646099	7.38701183022399	7.34098254470724	6.22344532373693	4.2550372207352	6.65325609644107	5.3304264103135	NA
**TC0X00001551.mm.2**	Usp51	ubiquitin specific protease 51	-0.422867542178889	5.42737141844247	-1.04356712177569	0.334319193356057	0.999692817443324	-4.59362484359514	5.37024890570653	5.51054677583678	4.75127343138304	5.66757544099383	5.41392851934146	5.85065543739318	NA
**TC0300001816.mm.2**	Ccdc39	coiled-coil domain containing 39	-0.385782459627407	5.83580493122404	-1.04355735795492	0.334323384266182	0.999692817443324	-4.59362537086736	6.2153909727498	5.52208492909413	5.40873309879527	5.84416128432749	5.93219980092604	6.09225950145154	NA
**TC0400002796.mm.2**	Ambp	alpha 1 microglobulin/bikunin precursor	0.505811161217893	4.84461960856837	1.04340367497507	0.334389354940002	0.999692817443324	-4.59363366987256	4.4317281579082	4.46558117369275	4.73899563274338	5.85201968837915	4.36522744339798	5.21416555528877	NA
**TC1900000166.mm.2**	B3gat3	beta-1,3-glucuronyltransferase 3 (glucuronosyltransferase I)	-0.303226511709735	8.25267851670892	-1.04330187134153	0.334433061399775	0.999692817443324	-4.59363916705766	8.0215894493494	8.08930487176077	8.17339968578783	8.22596219207412	8.43715869266663	8.56865620861479	NA
**TC0200003272.mm.2**	Ier5l	immediate early response 5-like	0.338084079487147	6.52364930780937	1.04310654350639	0.334516932629359	0.999692817443324	-4.59364971370064	6.15290775964265	6.34462584508468	6.94034579528418	6.71991940526741	6.26624907287507	6.71784796870222	NA
**TC0400000294.mm.2**	Cnr1	cannabinoid receptor 1 (brain)	-0.981435675406288	7.26603154154652	-1.04307787604844	0.334529243483927	0.999692817443324	-4.59365126151528	8.55826116631441	8.61742401139572	7.58602102418039	4.64279533619781	7.73972124574785	6.45196646544293	NA
**TC1400002335.mm.2**	Ppp3cc	protein phosphatase 3, catalytic subunit, gamma isoform	0.423556926564986	7.25377230041176	1.04282977071496	0.334635804185424	0.999692817443324	-4.59366465645369	7.73675507850461	7.75249095911085	7.51716620124231	6.92308460775022	7.21690499027021	6.37623196559235	NA
**TC0700000660.mm.2**	Cep89	centrosomal protein 89	-0.312493779468237	6.66157905654774	-1.04278538994626	0.334654868502586	0.999692817443324	-4.59366705237669	6.88038612386766	6.66168979707171	6.55420635146216	6.34699907824312	6.73838642642424	6.78780656221752	NA
**TC0800002814.mm.2**	4931428F04Rik	RIKEN cDNA 4931428F04 gene	-0.334103748717768	7.5062417470898	-1.0427812723852	0.334656637296915	0.999692817443324	-4.59366727466349	7.45890635002297	7.18269166277868	7.64522833832219	7.2185941478286	7.75946803002181	7.77256195356452	NA
**TC0500001735.mm.2**	Fam220a	family with sequence similarity 220, member A	0.302678930553949	6.85583761345178	1.04274717121369	0.334671286538499	0.999692817443324	-4.59366911560272	7.41763859990948	7.29555642545524	6.79271946783123	6.7208747903957	6.47536538414642	6.43287101297261	NA
**TC1000002260.mm.2**	Srgn	serglycin	0.31749479672181	8.42675165212626	1.04268555726315	0.334697756073908	0.999692817443324	-4.59367244174258	8.3486735050594	8.48870881579922	8.66531232813621	8.51374626453507	8.42045569859933	8.12361330062832	NA
**TC0900002491.mm.2**	Map2k1	mitogen-activated protein kinase kinase 1	-0.64716893058548	8.52306143902871	-1.04226570580329	0.334878170255454	0.999692817443324	-4.5936951045295	9.21452099861661	9.24313027316735	8.83325463083479	6.85993511977387	8.47094512126817	8.51658249051146	NA
**TC1700002583.mm.2**	Srsf7	serine/arginine-rich splicing factor 7	0.447862238081365	6.71024855702888	1.04220947155139	0.334902340577957	0.999692817443324	-4.59369813964507	7.0741005636584	7.31950347749297	6.71483725519892	6.66696863339341	6.8022168152608	5.6838645971688	NA
**TC1400001557.mm.2**	Dupd1	dual specificity phosphatase and pro isomerase domain containing 1	0.316184890863656	5.97640485996028	1.04210103862706	0.334948950624042	0.999692817443324	-4.59370399186289	5.63713069695409	5.72392475438399	6.2061079240366	6.35876382103885	5.81511519815729	6.11738676519084	NA
**TC0X00002760.mm.2**	1700031F05Rik	RIKEN cDNA 1700031F05 gene	0.463425867204659	5.2856249755959	1.04204964913006	0.334971042290702	0.999692817443324	-4.59370676530508	4.82518456932512	5.08965205669119	5.27311985194214	6.08976262904207	4.78642136665446	5.64960937992043	NA
**TC1100002851.mm.2**	Snap47	synaptosomal-associated protein, 47	-0.691120249646545	8.65814951488074	-1.04197114887432	0.335004790772583	0.999692817443324	-4.59371100177363	10.0790552128824	9.69321908622309	8.72444873297652	6.6727424124664	8.63369897230697	8.14573267242904	NA
**TC0X00001710.mm.2**	Mid1	midline 1	-0.814512036566271	6.91550236250672	-1.04184875992342	0.335057413132048	0.999692817443324	-4.59371760653021	6.93817879948317	7.27173257426348	7.53886740112934	5.28817196295121	7.86486833537946	6.59119510183364	NA
**TC1900000888.mm.2**	Tcirg1	T cell, immune regulator 1, ATPase, H+ transporting, lysosomal V0 protein A3	-0.304086538661883	6.3571828450092	-1.04120047567682	0.335336260102922	0.999692817443324	-4.59375258572163	5.95767253850962	6.083056111471	6.38330424323813	6.36379342813729	6.60848999677827	6.74678075192092	NA
**TC0300000961.mm.2**	Acp6	acid phosphatase 6, lysophosphatidic	0.412138836874421	6.39343307156146	1.04115772595907	0.335354654612684	0.999692817443324	-4.59375489201448	6.31193535640406	6.43225281463052	7.06698884262373	6.15335512341778	6.35952303677941	6.03654325551327	NA
**TC1100001219.mm.2**	Ccl2	chemokine (C-C motif) ligand 2	-0.379933146817721	6.38543336390901	-1.04095425266543	0.335442216965946	0.999692817443324	-4.59376586857092	5.59696349871606	6.37049397509536	6.35905720743593	6.43358100056766	6.88537059561305	6.66713390602598	NA
**TC1100001863.mm.2**	D11Wsu47e	DNA segment, Chr 11, Wayne State University 47, expressed	-0.348625148287288	6.46029313620495	-1.04087047785243	0.335478273807818	0.999692817443324	-4.59377038760801	6.54367997294426	6.66160702341902	6.43666085228144	5.99294990986447	6.76973224882359	6.35712880989691	NA
**TC1000001293.mm.2**	Bbs10	Bardet-Biedl syndrome 10 (human)	-0.325459238951458	5.59444764051025	-1.0407403315464	0.335534295197525	0.999692817443324	-4.59377740773099	4.82493088113021	4.96345790934518	5.59687316737877	5.96681611996281	6.20719982639834	6.00740793884616	NA
**TC1500001675.mm.2**	Fam135b	family with sequence similarity 135, member B	-0.509209465226354	6.2374158086396	-1.04047383012189	0.335649033973683	0.999692817443324	-4.59379178168048	7.50357993874965	7.51488608786758	6.02272599870655	4.67107894867728	5.63724455618485	6.07497932165169	NA
**TC0300002298.mm.2**	Ttc24	tetratricopeptide repeat domain 24	-0.379640289958648	6.65708772130653	-1.04026019393046	0.335741035025878	0.999692817443324	-4.59380330313857	6.0245756928384	5.91085700556948	6.42942382278562	7.19448270197139	7.07656461594072	7.30662248873358	NA
**TC1100003783.mm.2**	Kcnh4	potassium voltage-gated channel, subfamily H (eag-related), member 4	-0.39516205203338	5.524488585752	-1.04003393864232	0.335838492462934	0.999692817443324	-4.5938155040156	5.6642693919724	6.18466330144948	5.31047567858301	4.94336167992866	5.83406343420863	5.2100980283698	NA
**TC0500001821.mm.2**	Mtus2	microtubule associated tumor suppressor candidate 2	-0.331546178064187	6.90560449532019	-1.03983812747078	0.335922854682452	0.999692817443324	-4.59382606224464	7.10290936554695	6.92133447219655	6.81402915558308	6.55911623344156	7.21032112286485	6.82591662228817	NA
**TC1400000600.mm.2**	Tmem253	transmembrane protein 253	0.322358301258394	5.988158043176	1.03979459723264	0.335941611322054	0.999692817443324	-4.59382840929658	5.64662188045717	5.30529735066365	6.29029482929274	6.52057798593324	6.11780497834362	6.04835123436558	NA
**TC0700004635.mm.2**	Lilra6	leukocyte immunoglobulin-like receptor, subfamily A (with TM domain), member 6	-0.67749825709123	4.92613664279729	-1.03966329855358	0.335998191380914	0.999692817443324	-4.59383548836275	4.08159919655652	4.00380635140607	4.23456874658825	5.82364015073109	5.02311980022839	6.39008561127341	NA
**TC1300002208.mm.2**	Cntnap3	contactin associated protein-like 3	0.541814692120127	5.70003604718628	1.03868195712748	0.336421319244004	0.999692817443324	-4.59388838566539	6.09351899216358	6.15137387495773	5.85223264805943	5.66724375205889	5.99256660049802	4.44328041538004	NA
**TC1200001446.mm.2**	Lratd1	LRAT domain containing 1	-0.610819020448473	7.48116959102546	-1.03862975540228	0.33644383917438	0.999692817443324	-4.59389119888204	8.12664848613259	7.93053765966661	7.79910789505953	6.00498878466878	7.69256092341622	7.33317379720904	NA
**TC0200004025.mm.2**	Olfr1197	olfactory receptor 1197	0.487537224014492	5.29166667469852	1.03850579658419	0.336497320101517	0.999692817443324	-4.59389787892915	4.9804478966232	4.3355745040572	5.30415759706848	6.40036845070136	5.16773071972043	5.56172088002043	NA
**TC0200005500.mm.2**	Alkbh3	alkB homolog 3, alpha-ketoglutarate-dependent dioxygenase	0.399430876050914	5.79233701239075	1.03847841541146	0.33650913438311	0.999692817443324	-4.59389935443251	5.40259905116137	5.80617676262061	5.68858353062974	6.48347047570242	5.79408345228405	5.57910880194629	NA
**TC0500000302.mm.2**	Tmem214	transmembrane protein 214	0.45672613409762	7.33380244560451	1.03842848530208	0.336530678814782	0.999692817443324	-4.59390204499791	7.9446097993104	7.89376855421035	7.80233169473491	6.73661259941586	7.08100144042561	6.54449058552992	NA
**TC1100003079.mm.2**	Elp5	elongator acetyltransferase complex subunit 5	1.15314058884421	7.32133406179103	1.03813397302769	0.336657780930665	0.999692817443324	-4.59391791411226	8.79240229385702	8.69617924588561	8.07308499185908	6.2997670124869	7.74012081423537	4.32645001242219	NA
**TC1300002090.mm.2**	Klhl3	kelch-like 3	-0.565008751901844	6.22264671620447	-1.03806020216417	0.336689624109243	0.999692817443324	-4.59392188877423	6.57306752726713	6.80903201176517	6.52323587186192	4.88864575533349	6.34321935002659	6.19867978097251	NA
**TC0700001345.mm.2**	Gab2	growth factor receptor bound protein 2-associated protein 2	0.470885806775144	7.01333109837958	1.03800225739057	0.336714637651566	0.999692817443324	-4.59392501066319	7.05484479658798	6.95910910697838	6.94291521660056	7.56098693353014	6.23475059303116	7.32737994354925	NA
**TC1600001761.mm.2**	Cmss1	cms small ribosomal subunit 1	0.480960333054774	7.81959326551677	1.03768725494637	0.336850643644169	0.999692817443324	-4.59394198069621	6.87952385047299	7.31507992078827	8.067005177032	8.77543306694244	7.43786899488425	8.44264858298065	NA

**TC0800000759.mm.2**	Lsm4	LSM4 homolog, U6 small nuclear RNA and mRNA degradation associated	0.354154212810352	7.6809152533582	1.03745601340623	0.336950512897309	0.999692817443324	-4.59395443685609	7.69108062354736	7.94493715862019	7.80804996734674	7.77084111445444	7.73989974470464	7.13068291147583	NA
**TC1300002766.mm.2**	Nsun2	NOL1/NOP2/Sun domain family member 2	-0.588368408710481	6.00493074655082	-1.03739116750469	0.336978522987525	0.999692817443324	-4.59395792965429	7.15372545049237	7.02165321573805	5.9256758536563	4.41305864417048	6.19645731005576	5.31901400519198	NA
**TC0600001239.mm.2**	Setd5	SET domain containing 5	0.45979724402947	8.08868062555372	1.03728178421398	0.337025775163127	0.999692817443324	-4.59396382115372	8.67668337415731	8.8696001957385	8.20265982139717	7.75003751434556	8.06955502862309	6.9635478190607	NA
**TC1400001064.mm.2**	Kctd4	potassium channel tetramerisation domain containing 4	-0.762850387416629	6.152499333562	-1.03723952455825	0.337044032212521	0.999692817443324	-4.59396609722988	7.58630630573571	7.34182728646599	6.27047205032934	3.96010876683918	6.23078935050238	5.52549224149939	NA
**TC1100003136.mm.2**	Aipl1	aryl hydrocarbon receptor-interacting protein-like 1	-0.389547880491252	5.19570591015073	-1.03699525864545	0.337149575685915	0.999692817443324	-4.59397925242206	4.38604851551259	4.56814895975316	5.01524689928732	5.70522421304076	5.50285570745414	5.99671116585644	NA
**TC1300000834.mm.2**	Gm7969	predicted gene 7969	0.319747800783902	5.82653399440641	1.03684458382659	0.337214693113582	0.999692817443324	-4.59398736648668	5.19447013016408	5.34662051325	6.15184591245229	6.3769585498438	6.0925748061877	5.79673405454059	NA
**TC1700001950.mm.2**	Prrc2a	proline-rich coiled-coil 2A	0.33270077515361	7.38497473424184	1.03681494272509	0.337227504351285	0.999692817443324	-4.59398896264302	8.28321549851339	8.20717440629739	6.91745405307406	7.32497597239968	6.63795493761302	6.9390735375535	NA
**TC0700001729.mm.2**	E130201H02Rik	Y box protein 1 pseudogene	-0.313676734319246	7.61713199899166	-1.03646414041218	0.337379154902375	0.999692817443324	-4.59400785161395	6.95423923722903	7.24832254128743	7.65907758982841	7.7773607835691	7.96918833299083	8.09460350904517	NA
**TC0300000440.mm.2**	Tm4sf4	transmembrane 4 superfamily member 4	0.394703414894456	6.05218967793465	1.03627819145448	0.337459562096027	0.999692817443324	-4.59401786289954	5.88228645841352	5.57324955567472	5.99887656260735	6.82462787904692	5.93536282369737	6.09873478816799	NA
**TC0100002497.mm.2**	Myl1	myosin, light polypeptide 1	0.305631852607855	5.6014613022367	1.03623753333838	0.337477145335843	0.999692817443324	-4.59402005178148	5.90377143415853	5.68261941024624	5.65147774030425	5.66534259681131	5.30821320732937	5.39734342457048	NA
**TC0500001689.mm.2**	Nudt1	nudix (nucleoside diphosphate linked moiety X)-type motif 1	0.400832694718443	5.68849273295388	1.03616899268635	0.337506788476117	0.999692817443324	-4.59402374166952	5.67909511367156	5.7655638295483	5.65431721049295	6.08966421147721	5.06315676746596	5.8791592650673	NA
**TC0800000844.mm.2**	Tom1	target of myb1 trafficking protein	0.322103298250527	7.36426430990647	1.03613601602219	0.337521051293323	0.999692817443324	-4.59402551693071	7.59411912009711	7.59482456740505	7.50903651176371	7.31138787245515	6.89964975822697	7.27656802949085	NA
**TC1000002164.mm.2**	Cep85l	centrosomal protein 85-like	0.656504849662323	5.93581328165235	1.03579848528353	0.337667065282356	0.999692817443324	-4.59404368607516	6.30067561284989	6.49711876092212	6.31138578821839	5.75366171951498	6.34494385357307	4.40709395483565	NA
**TC0X00000808.mm.2**	Dmd	dystrophin, muscular dystrophy	0.501022551867245	6.47027564344111	1.03563960827232	0.337735812126765	0.999692817443324	-4.59405223745321	6.58804253127112	6.37836825656903	6.04603145663842	7.38261263163207	6.38500067227609	6.04159831225991	NA
**TC1300002471.mm.2**	Polk	polymerase (DNA directed), kappa	0.43987349683947	5.35178850391887	1.03496705805426	0.338026951926982	0.999692817443324	-4.59408843027612	5.47417910006223	5.69214393775904	5.47208053591797	5.43999695376748	5.5668218968555	4.46550859915101	NA
**TC1000002041.mm.2**	Rpf2	ribosome production factor 2 homolog	-0.512174802313574	6.82833999609118	-1.03479671736643	0.338100722457503	0.999692817443324	-4.59409759538613	7.35932141854189	7.27393582150999	6.88658951277064	5.76962705316338	7.29691879954258	6.38364737101859	NA
**TC0500003709.mm.2**	Babam2	BRISC and BRCA1 A complex member 2	-0.458862034293947	8.21415499084894	-1.03457458857649	0.338196940544278	0.999692817443324	-4.59410954592716	9.21827076338182	9.13052718447531	7.92669375651746	7.08251020780686	8.39587307408042	7.5310549588318	NA
**TC0400000523.mm.2**	Sec61b	Sec61 beta subunit	0.305349017475913	7.55023284573498	1.03450531288254	0.338226952728538	0.999692817443324	-4.59411327273016	7.53010904328918	7.39588059416804	7.67885308760142	7.81419964835084	7.36637440007921	7.51598030092122	NA
**TC0200002672.mm.2**	Tfap2c	transcription factor AP-2, gamma	0.320323873734212	5.09643805287774	1.03448511815666	0.338235702054414	0.999692817443324	-4.59411435911868	4.76948651325149	5.11565734571818	5.27525083658598	5.3918152662966	5.1106039726329	4.91581438278127	NA
**TC1500000392.mm.2**	Deptor	DEP domain containing MTOR-interacting protein	-0.650624061320906	5.84470493126355	-1.03434349810257	0.338297063756041	0.999692817443324	-4.59412197739765	6.85272419202694	7.17725613830717	4.63591220643717	5.23258836086553	6.52902770401785	4.64072098592666	NA
**TC0700004179.mm.2**	Gprc5b	G protein-coupled receptor, family C, group 5, member B	-0.393156761902866	7.74150466997786	-1.03433203426517	0.338302031242822	0.999692817443324	-4.59412259406059	8.27186676040513	7.99645271176559	7.7729642660366	6.92423324590876	7.73399090301077	7.74952013274033	NA
**TC0800001901.mm.2**	Adam9	a disintegrin and metallopeptidase domain 9 (meltrin gamma)	-0.486303609202555	7.72041352650582	-1.03420745061893	0.338356019338804	0.999692817443324	-4.59412929547039	8.37553182184593	8.24781059557606	7.82259322533931	6.54067253626461	7.84279348002727	7.49307949998176	NA
**TC0600001742.mm.2**	Mrps35	mitochondrial ribosomal protein S35	-0.30706749395871	5.7589989418425	-1.03414152498509	0.338384590880492	0.999692817443324	-4.59413284149441	6.26098233759544	6.28153090385755	5.27363526930096	5.42503744154135	5.76687799920982	5.5459296995499	NA
**TC1000001957.mm.2**	Supt4b	suppressor of Ty 4B	0.401532524908645	7.62053842352523	1.03409032017983	0.338406783886935	0.999692817443324	-4.59413559564157	8.21075285848793	7.75078106840393	8.04252755177662	7.23985328026177	7.31297731531	7.16633846691109	NA
**TC0200002199.mm.2**	Cstdc1	cystatin domain containing 1	0.620782423604636	4.59062192144731	1.03408610977553	0.33840860879735	0.999692817443324	-4.59413582210342	4.13944850617365	4.08548013606833	4.49763919062893	5.78254467619666	3.82660612765605	5.21201289196026	NA
**TC1000003090.mm.2**	Atp23	ATP23 metallopeptidase and ATP synthase assembly factor homolog	-0.316765770930577	6.25870741003465	-1.03384960768425	0.338511128251365	0.999692817443324	-4.59414854200506	6.35400479931463	6.40808766874792	6.12321850829752	5.95509171684456	6.18679614154422	6.52504562545901	NA
**TC0700002053.mm.2**	Pnpla2	patatin-like phospholipase domain containing 2	0.390146027732937	7.09733074753106	1.03366299227891	0.338592040246325	0.999692817443324	-4.59415857791431	7.27699884866564	7.13904637293528	7.58380087357684	6.89031478594882	7.10696132165246	6.58686228240733	NA
**TC0300002296.mm.2**	Hapln2	hyaluronan and proteoglycan link protein 2	0.490564046187131	6.30187846552596	1.03334189481027	0.338731296663015	0.999692817443324	-4.59417584418878	6.51657867838672	6.50973392405993	6.25309679092251	6.62994635061917	6.60187338452455	5.30004166464286	NA
**TC0500001641.mm.2**	Zkscan1	zinc finger with KRAB and SCAN domains 1	-0.491928993580698	6.3071942692939	-1.03311064201755	0.338831616805806	0.999692817443324	-4.59418827779351	6.89063384097645	6.8586910839795	6.434955932388	5.12003541943501	6.09317930430791	6.4456700346765	NA
**TC0500000357.mm.2**	Uvssa	UV stimulated scaffold protein A	-0.46945727868365	5.84268102443701	-1.03310124558945	0.33883569358748	0.999692817443324	-4.59418878297838	5.6967816263589	5.53386447126439	6.01834390205489	5.42491884376085	5.63988393860965	6.74229336457339	NA
**TC1000000322.mm.2**	Lama4	laminin, alpha 4	-0.321054646098841	5.92800972677425	-1.03289342929285	0.338925867839765	0.999692817443324	-4.59419995538652	5.47439473989069	5.60248023477343	5.76738599345744	6.15715105343441	6.33921511801123	6.2274312210783	NA
**TC1600000376.mm.2**	Il1rap	interleukin 1 receptor accessory protein	-0.348479428051764	6.14909885865214	-1.03287608496462	0.338933394641643	0.999692817443324	-4.59420088778934	6.72657634199366	7.1130579393237	5.73856419903896	5.44043580820702	6.14044897835013	5.73550988499938	NA
**TC0700003004.mm.2**	Snrnp70	small nuclear ribonucleoprotein 70 (U1)	-0.381302194544467	8.33467780734814	-1.0326762198439	0.339020138411536	0.999692817443324	-4.59421163170881	8.73728407986668	8.96997687745892	7.71704320510747	8.05205754372968	8.62823951664872	7.90346562127736	NA
**TC1900001519.mm.2**	Morn4	MORN repeat containing 4	-0.420169349546953	8.59189593509469	-1.03224466432867	0.339207499019113	0.999692817443324	-4.594234827179	9.00207961998406	8.80283062111184	8.68311824018245	7.76994509500672	8.86781394993769	8.42558808434538	NA
**TC0500001302.mm.2**	Fbxo21	F-box protein 21	-0.298949637045574	7.52690658524655	-1.03213897667648	0.339253396118599	0.999692817443324	-4.59424050707428	7.7252916335369	7.80966360928387	7.20713775610292	7.30715474118077	7.55970430615768	7.55248746521716	NA
**TC1200001109.mm.2**	Eml1	echinoderm microtubule associated protein like 1	0.372268581626805	6.79157027219193	1.03205327729473	0.339290616532685	0.999692817443324	-4.59424511256372	7.01458589993587	7.20090334406735	6.99034104839863	6.64889372780236	6.78657409711474	6.10812351583264	NA
**TC1100000028.mm.2**	Pes1	pescadillo ribosomal biogenesis factor 1	-0.374894348654291	7.87541696770317	-1.03201307835504	0.339308076606661	0.999692817443324	-4.5942472727981	8.67589717830031	8.74324492015491	7.19286621978686	7.34891928544073	7.25638722710778	8.0351869754284	NA
**TC1700001395.mm.2**	Tagap1	T cell activation GTPase activating protein 1	-0.304170392093159	8.07557940900768	-1.0315047618507	0.339528921590236	0.999692817443324	-4.59427458576763	7.76210928966546	7.66332643307898	8.05510917757504	8.15474079598263	8.31307715334338	8.50511360440061	NA
**TC1100003083.mm.2**	Acadvl	acyl-Coenzyme A dehydrogenase, very long chain	-0.342798900351996	7.35715873187176	-1.03131338538032	0.339612097442659	0.999692817443324	-4.59428486728994	7.1549644644418	7.26280442846419	7.27624993989335	7.24354290891693	7.30838214493988	7.89700850457439	NA
**TC0200003304.mm.2**	Slc25a25	solute carrier family 25 (mitochondrial carrier, phosphate carrier), member 25	0.342442301583222	6.7625495549357	1.03114335816339	0.339686008159538	0.999692817443324	-4.59429400112778	7.32495856917236	6.99860747941991	6.94049199005911	6.52781595203508	6.53303268543151	6.25039065349624	NA
**TC1700000163.mm.2**	Gm7168	predicted gene 7168	-0.574327970479157	4.84306227242387	-1.03113147877686	0.339691172601127	0.999692817443324	-4.59429463926155	4.40086022601418	4.62683383731018	4.35249505865785	5.08851675647241	4.53072011954565	6.05894763654293	NA
**TC0500002025.mm.2**	Dnajc2	DnaJ heat shock protein family (Hsp40) member C2	-0.656963906927335	6.65348531671246	-1.03094475695244	0.339772356243109	0.999692817443324	-4.59430466910335	7.66205279155018	7.79371467782448	6.6574903501212	4.91811795840151	6.97349719751513	5.91603892486226	NA
**TC0900001836.mm.2**	Zfp426/Zfp26	zinc finger protein 426/zinc finger protein 26	-0.734698256863138	6.51218016977731	-1.03091495148934	0.339785316612808	0.999692817443324	-4.59430627004117	8.06437296902947	7.82574763455347	6.58381484402781	4.2729671066495	6.29860734312074	6.02757112128285	NA
**TC0300000641.mm.2**	Fga	fibrinogen alpha chain	-0.359124387922051	5.0381322199392	-1.03079981048504	0.339835387317623	0.999692817443324	-4.59431245440368	4.70299460294621	4.63674411118533	5.38053963656207	4.70486327826771	5.4997732260655	5.30387846460838	NA
**TC1200000038.mm.2**	Atad2b	ATPase family, AAA domain containing 2B	0.440189426077752	6.97417615551924	1.03066201650455	0.33989531673843	0.999692817443324	-4.59431985507839	7.25094448997806	7.23621859793518	7.15121987216587	6.96791647651299	7.17849119942098	6.06026629710237	NA
**TC0500002651.mm.2**	Igfbp7	insulin-like growth factor binding protein 7	-0.423362407152959	8.19212939867765	-1.03058998925221	0.339926646223129	0.999692817443324	-4.59432372336035	7.99450923141198	8.32844233436336	8.22473199549208	7.76681801050023	8.86281980114029	7.97545501915794	NA
**TC1000002525.mm.2**	Creb3l3	cAMP responsive element binding protein 3-like 3	-0.337552938252982	6.44298088404011	-1.03053821620306	0.339949167220655	0.999692817443324	-4.59432650379912	5.70970398329056	5.82952437989398	6.35589375726346	6.86588177501161	6.84397700863498	7.05290440014606	NA
**TC0500002095.mm.2**	Prkag2	protein kinase, AMP-activated, gamma 2 non-catalytic subunit	-1.23896966507597	7.62006640775083	-1.03044602544653	0.339989272653474	0.999692817443324	-4.59433145469031	8.15863760781803	8.31393948281092	8.77825041966294	4.6066905931991	8.09390199953887	7.76897834347512	NA
**TC0200005148.mm.2**	Zfp334	zinc finger protein 334	0.344954899781845	7.31649530989888	1.03037875220177	0.340018540690192	0.999692817443324	-4.59433506731896	7.62295111764005	7.02080527672689	7.42241550383202	7.550147128463	7.15570625844821	7.12694657428312	NA
**TC1400002867.mm.2**	Xpo4	exportin 4	0.327529107740265	7.10538241099315	1.03030788940339	0.340049372581567	0.999692817443324	-4.59433887259531	6.90606938657184	6.85929878788218	7.13860099387735	7.62239125961537	7.032354465019	7.07357957299319	NA
**TC0500003208.mm.2**	Fam216a	family with sequence similarity 216, member A	-0.384242828885668	8.5122231959067	-1.03017548560366	0.340106986500855	0.999692817443324	-4.59434598226054	8.90694342837939	8.76866455263828	8.52704707019803	7.78757569812757	8.74251703307231	8.34059139302462	NA
**TC0400002847.mm.2**	Cdk5rap2	CDK5 regulatory subunit associated protein 2	-0.341920603703702	5.86027654109364	-1.0301409327812	0.340122023028016	0.999692817443324	-4.59434783757042	5.56275358622494	5.53956597240698	5.55010447224859	6.13764476801268	6.23423759309282	6.13735285457586	NA
**TC1700002171.mm.2**	Tjap1	tight junction associated protein 1	0.314730183900193	6.69157033748378	1.02963131173909	0.340343859061313	0.999692817443324	-4.59437519837296	6.05097106608769	6.17082519354565	6.96502240898066	7.31352065755421	6.79249959266131	6.85658310607317	NA
**TC1400000895.mm.2**	Ctsb	cathepsin B	-0.59613374782508	9.77444296584039	-1.02961256018307	0.340352023742768	0.999692817443324	-4.5943762050005	10.9969504851853	11.3475652805663	9.58697640160623	7.96796086521403	9.72911576648768	9.01808899598274	NA
**TC1600002120.mm.2**	Vps26c	VPS26 endosomal protein sorting factor C	0.309551602069781	6.2717966808325	1.02943767089355	0.340428180438472	0.999692817443324	-4.59438559307177	6.79936297503677	6.81263280755279	6.15830621389086	6.16063753938164	6.00878959326426	5.69105095586868	NA
**TC0400000535.mm.2**	Tmeff1	transmembrane protein with EGF-like and two follistatin-like domains 1	-0.427338973481088	8.52906394490899	-1.02925014666642	0.34050985421374	0.999692817443324	-4.59439565859203	8.76276684298243	8.56813226603508	8.77550230332774	7.71890100340937	8.69073392277586	8.65834733092343	NA
**TC0X00000436.mm.2**	Rab33a	RAB33A, member RAS oncogene family	0.321657875986944	6.7719547101292	1.02924993456391	0.340509946601127	0.999692817443324	-4.59439566997634	7.15418085813963	7.10462798606955	6.6407813257668	6.86733625850317	6.61985481480278	6.24494701749329	NA
**TC0700003025.mm.2**	Grin2d	glutamate receptor, ionotropic, NMDA2D (epsilon 4)	0.491555612459658	6.14194444190449	1.0289656533512	0.340633791496583	0.999692817443324	-4.59441092743675	6.17322845056231	6.25276745781904	5.7629287028432	6.94146228113925	5.51491655962482	6.20636319943831	NA
**TC1100002712.mm.2**	Olfr1373	olfactory receptor 1373	0.457156645807169	5.67682120682032	1.02889352870228	0.340665217754862	0.999692817443324	-4.59441479808772	5.19918377977423	5.38113866788035	5.53996235446367	6.65749668797717	5.42060788742512	5.86253786340139	NA
**TC0700004399.mm.2**	Ikzf5	IKAROS family zinc finger 5	0.801331076245446	6.81028039301779	1.02828575672452	0.340930128966035	0.999692817443324	-4.59444741001342	7.4837622217666	7.55830740161787	7.72833228000311	5.98280516360348	7.0155366959741	5.0929385951416	NA
**TC1300002505.mm.2**	Tmem174	transmembrane protein 174	-0.462733600361302	5.68782649531849	-1.02820368093589	0.340965916155913	0.999692817443324	-4.59445181338477	4.82233150945471	4.53775353557529	5.45961908037519	6.46108428270398	6.08426566353125	6.76190490027053	NA
**TC0700002858.mm.2**	Zfp507	zinc finger protein 507	0.313066021284154	6.11158558505347	1.02816092383634	0.340984560556652	0.999692817443324	-4.59445410724323	6.42610925950456	6.16129535559557	6.29542565375046	6.05869481514404	5.89747356237629	5.8305148639499	NA
**TC0200002072.mm.2**	Lamp5	lysosomal-associated membrane protein family, member 5	-0.567591875652389	7.40157861216358	-1.02775399563145	0.341162043904143	0.999692817443324	-4.59447593622151	8.13574199168838	8.25779716964379	7.42572590584855	6.0146484743237	7.75006753886095	6.82549059261609	NA
**TC1700002779.mm.2**	H2-DMb2	histocompatibility 2, class II, locus Mb2	0.588749768727698	4.91985110646774	1.02751943958009	0.341264379930755	0.999692817443324	-4.59448851681729	5.73254244162173	4.86186596792761	5.04524054677025	5.00585833658599	3.66202930099315	5.2115700449077	NA
**TC0400002987.mm.2**	Slc24a2	solute carrier family 24 (sodium/potassium/calcium exchanger), member 2	-0.694347180479792	7.49285602423591	-1.02718529572003	0.341410208070768	0.999692817443324	-4.59450643664692	8.66409760720216	8.75838711139792	7.25934700820143	5.81363152472648	8.05486039898964	6.40681249489785	NA
**TC1000000656.mm.2**	Rhobtb1	Rho-related BTB domain containing 1	-0.388762731745734	5.29830720537618	-1.02683308646552	0.341563974163293	0.999692817443324	-4.59452532246102	4.56713982137257	4.51751097349913	5.18563279672798	5.77820069021897	5.54350618245852	6.19785276797989	NA
**TC0600001335.mm.2**	Cacna2d4	calcium channel, voltage-dependent, alpha 2/delta subunit 4	0.380566838002946	5.53132547256259	1.02662506391018	0.341654817746116	0.999692817443324	-4.59453647546629	5.69283099516319	5.58308099829419	5.83689761790908	5.49968964105296	4.90699774759647	5.66845583535967	NA
**TC0700002736.mm.2**	Zfp27	zinc finger protein 27	0.300053219819831	5.74313946972853	1.02660814633277	0.341662206509943	0.999692817443324	-4.59453738244726	6.59318847279884	6.68263823566684	5.3797223602398	5.51183591453277	5.09223996093251	5.19921187420039	NA
**TC0100003783.mm.2**	Cenpf	centromere protein F	0.470756556224176	5.49827337798798	1.02637006564319	0.341766201931256	0.999692817443324	-4.5945501456542	5.46891111574783	5.1275684635505	5.37240796557315	6.2949289349658	5.77410086223696	4.95172292585363	NA
**TC0600002870.mm.2**	Cntn3	contactin 3	-0.358087766044738	7.01497608419046	-1.02626039126689	0.341814117006413	0.999692817443324	-4.59455602470993	6.91673475321585	7.31819572106815	6.9932481955061	6.57612705387855	7.34826457122762	6.93728621024649	NA
**TC0900002236.mm.2**	Rbm7	RNA binding motif protein 7	0.359937750566897	7.52132918694819	1.02607941552358	0.341893194278573	0.999692817443324	-4.59456572522915	7.81024427321894	7.77200808540689	7.90086181274025	7.23193731935829	7.34055267141839	7.07237095954636	NA
**TC0700002413.mm.2**	Crx	cone-rod homeobox	-0.386490847998123	5.35696083064153	-1.02600109516516	0.341927420845801	0.999692817443324	-4.59456992305554	4.99229309853588	4.78209794813928	5.39054065960153	5.40665546098735	5.36117865277248	6.20899916381265	NA
**TC0200002558.mm.2**	Ncoa3	nuclear receptor coactivator 3	-0.592038817057596	6.52374607900832	-1.02577650063108	0.342025585438919	0.999692817443324	-4.59458196010377	6.7361298731891	6.8146641024611	6.99755764557816	5.20624478656409	6.65044392158611	6.73743614467134	NA
**TC0300003219.mm.2**	Zfp697	zinc finger protein 697	0.400389294866997	6.70626244479392	1.02573299276082	0.34204460422599	0.999692817443324	-4.5945842917517	6.31931818574424	6.28504704681426	6.66265785931945	7.55433615365005	6.5487188036863	6.86749661954921	NA
**TC0400002527.mm.2**	Arhgef39	Rho guanine nucleotide exchange factor (GEF) 39	0.437780417070138	5.48967662277085	1.02553272936215	0.342132157116884	0.999692817443324	-4.5945950235718	5.47098413903251	5.41859849162986	5.80749048298001	5.65452848707149	4.73292466513704	5.85353347077419	NA
**TC1100004077.mm.2**	Sumo2/Mir684-1	small ubiquitin-like modifier 2/microRNA 684-1	-0.82089152758514	9.06960075191069	-1.02515404030726	0.342297764500005	0.999692817443324	-4.59461531436698	9.59351497963379	9.69923586534186	9.60967168116443	7.13186362449467	9.70014162253037	8.68317673829901	NA
**TC1600000938.mm.2**	Jam2	junction adhesion molecule 2	-0.45219167245328	7.66628761961912	-1.02488591405379	0.342415059443542	0.999692817443324	-4.59462967896935	8.49378411568867	8.42365630916933	7.56699134282823	6.52095963114685	7.7889155093701	7.20341880951154	NA
**TC0100001254.mm.2**	Phlda3	pleckstrin homology like domain, family A, member 3	-0.300590985827383	5.19830584237906	-1.02439728675994	0.3426288974758	0.999692817443324	-4.5946558523197	5.42869446425831	5.47959845688052	4.8168223345338	5.02335774620657	5.21124114994732	5.23012090244781	NA
**TC1300000469.mm.2**	Tmem170b	transmembrane protein 170B	0.55977733794218	7.22302065021828	1.0243210327017	0.342662278143694	0.999692817443324	-4.59465993636231	7.82501023654558	7.68430326766655	7.79454760463043	6.67963493186053	7.29302444218976	6.06160341841684	NA
**TC0900001252.mm.2**	Dbr1	debranching RNA lariats 1	0.337376017197544	5.9674677655338	1.02355460440532	0.342997930557163	0.999692817443324	-4.59470097734347	6.16475705833669	6.5589474715615	5.86116764961623	6.01675939923364	5.40414859991764	5.79902641453713	NA
**TC1700002153.mm.2**	Mymx	myomixer, myoblast fusion factor	0.353514600708252	6.05309005851586	1.02324553880542	0.343133357928008	0.999692817443324	-4.59471752335206	5.52815916243494	5.65519837816225	6.17402554167246	6.74708046428479	5.92759512001961	6.28648168452114	NA
**TC1500000566.mm.2**	Tigd5	tigger transposable element derived 5	0.332877882698813	6.01015213690763	1.02313729158395	0.343180800112704	0.999692817443324	-4.59472331789355	5.67140542319194	5.47790676130041	6.15134180874118	6.63733639243435	6.14206706325752	5.98085537252038	NA
**TC0700001607.mm.2**	Tub	tubby bipartite transcription factor	0.309538797380302	8.76335866827676	1.0230305899571	0.34322757001041	0.999692817443324	-4.59472902942554	8.97314428104444	8.70149631702195	8.93726349258164	8.82503101059576	8.65714400933775	8.48607289907904	NA
**TC0900000040.mm.2**	Dcun1d5	DCN1, defective in cullin neddylation 1, domain containing 5 (S. cerevisiae)	-0.935119633553648	7.133497967929	-1.02296100501217	0.34325807350417	0.999692817443324	-4.59473275402727	8.21446556504188	8.13970037791742	7.60879458456896	4.67949671418474	7.6034907313118	6.55503983454919	NA

**TC1100001071.mm.2**	Scarf1	scavenger receptor class F, member 1	0.352571300374398	6.64376248227194	1.02265041980367	0.343394249006645	0.999692817443324	-4.59474937699651	6.31156282146093	6.34204006805291	7.04401448527698	6.91304281715632	6.29097818494873	6.96093651673578	NA
**TC1700002677.mm.2**	Atp6v1e2	ATPase, H+ transporting, lysosomal V1 subunit E2	-0.368889172412957	5.90154351367419	-1.02246224838158	0.343476773328615	0.999692817443324	-4.59475944708522	5.8433903894615	5.82096337416596	6.12142094715995	5.38214353963594	5.99291294499538	6.24842988662643	NA
**TC0900001357.mm.2**	Dcaf1	DDB1 and CUL4 associated factor 1	-0.87397194816412	6.10403325489523	-1.02243596574941	0.343488301076794	0.999692817443324	-4.59476085354639	7.83768160233462	7.84436638940679	6.15245344905245	3.44465037159843	6.17338054810135	5.17166716887777	NA
**TC0900002712.mm.2**	Fbxo9	f-box protein 9	-0.368305301343727	7.69061778479794	-1.02230115792444	0.343547433581213	0.999692817443324	-4.59476806725194	8.1749355048802	7.92270044682856	7.69471899536841	6.96001108182732	7.72782377465199	7.66351690523119	NA
**TC0100000655.mm.2**	Stk16	serine/threonine kinase 16	-0.510343073362508	6.83212381752631	-1.0222407883408	0.343573916900612	0.999692817443324	-4.59477129755007	7.46980847117292	7.15625000662908	6.84142087930209	5.83157826101334	7.35322253930351	6.34046274773693	NA
**TC0X00002272.mm.2**	Rbmx	RNA binding motif protein, X chromosome	-0.576158318190556	8.00965687404455	-1.02222108388678	0.343582561329603	0.999692817443324	-4.59477235189104	8.91131263932881	8.81645850715241	8.13106886446066	6.45785786624181	8.10268915295963	7.63855421412396	NA
**TC0700000051.mm.2**	Zfp524	zinc finger protein 524	0.361369649307052	9.02305542479972	1.02212784349652	0.343623468638268	0.999692817443324	-4.5947773408488	8.3450671767991	8.65050183699149	9.1686876596199	9.76406375719102	8.94843668915648	9.26157542904033	NA
**TC1500002301.mm.2**	Calcoco1	calcium binding and coiled coil domain 1	0.597895652481397	7.86323781451592	1.02168287178143	0.343818744231961	0.999692817443324	-4.59480114682682	8.85121439028866	8.69852664942762	8.58653356922343	6.8262050069476	6.87355974372168	7.34338752748655	NA
**TC1200001843.mm.2**	Mdga2	MAM domain containing glycosylphosphatidylinositol anchor 2	0.435676047092373	6.66567662257511	1.02149104084885	0.343902956362379	0.999692817443324	-4.59481140831961	6.51722043819619	6.18506278383308	6.62878400951472	7.45278029428834	6.25562996324283	6.95458224637548	NA
**TC0300002802.mm.2**	Vcam1	vascular cell adhesion molecule 1	-0.428993927416207	5.84934944350926	-1.02137577057305	0.343953566914211	0.999692817443324	-4.59481757397793	6.322376927271	7.04575924297814	5.08817537213941	5.34681094584758	5.25375510588197	6.03921906693743	NA
**TC0700002692.mm.2**	Dll3	delta like canonical Notch ligand 3	0.364635840918046	5.96312529054414	1.02121592977983	0.344023756395019	0.999692817443324	-4.59482612313021	5.60983482103152	5.38994201910031	6.04302644312559	6.71109684935895	5.86814812736779	6.15670348328065	NA
**TC1100001565.mm.2**	Stard3	START domain containing 3	-0.475713389793836	6.25992134843539	-1.02111422798734	0.344068421738405	0.999692817443324	-4.59483156237552	7.31116429511505	7.10242727201648	6.11852799274538	4.97872687920118	6.33536399837234	5.7133176531619	NA
**TC1700001034.mm.2**	Adgre1	adhesion G protein-coupled receptor E1	-0.467534233673486	5.22480216528191	-1.02095904950582	0.344136581835508	0.999692817443324	-4.59483986120023	5.0647955211496	5.52825451677045	5.39352624149133	4.5168210017209	5.0190035736313	5.8264121369279	NA
**TC1100001952.mm.2**	Sec14l1	SEC14-like lipid binding 1	-0.312940954297654	7.33779050058337	-1.02089184010919	0.344166106007281	0.999692817443324	-4.59484345532779	7.53476546586698	7.51164237881105	7.23855180927927	6.93867481583416	7.28295206253639	7.52015647117235	NA
**TC0800000368.mm.2**	Rnf122	ring finger protein 122	0.424301691395199	6.74731768547033	1.02037834723029	0.34439174291981	0.999692817443324	-4.59487091159325	6.98477779125721	6.7428728553435	7.42311047864458	6.37931894586126	6.41756618041425	6.5362598613012	NA
**TC1800001652.mm.2**	Adnp2	ADNP homeobox 2	-0.355866549127841	5.75362556612385	-1.02026519863683	0.34444147801719	0.999692817443324	-4.59487696075558	6.43816623446657	6.32306096619971	5.60831210512897	4.9160844437816	5.66929509438655	5.56683455277971	NA
**TC0400002873.mm.2**	Dmac1	distal membrane arm assembly complex 1	-0.411677331483538	8.91737922941047	-1.0201779372034	0.344479838168633	0.999692817443324	-4.5948816257262	9.14870775937066	9.38771086752453	8.885249098108	8.18700194519227	9.29704855550631	8.59855715076104	NA
**TC1200002269.mm.2**	Ifi27l2a	interferon, alpha-inducible protein 27 like 2A	-0.423662832156726	5.66252880931069	-1.02007107749848	0.344526818352216	0.999692817443324	-4.59488733816601	5.25531869071142	5.82179726597047	5.82535479540323	5.20001082203116	5.60287976535966	6.26981151638818	NA
**TC0400001158.mm.2**	Kti12	KTI12 homolog, chromatin associated	0.326855540236185	7.13778823963187	1.02004950152374	0.344536304709099	0.999692817443324	-4.59488849152786	7.47696561468114	7.07699139434023	7.34323562308872	7.1200061315324	7.01539373453563	6.79413693961311	NA
**TC1400002368.mm.2**	Fndc3a	fibronectin type III domain containing 3A	-0.800075501117574	8.07508881656691	-1.01988170133318	0.344610088880161	0.999692817443324	-4.59489746104691	8.79988699468231	8.65501421234709	8.6259720611258	6.07176828394267	8.42654745947242	7.87134388783119	NA
**TC0600003145.mm.2**	Ltbr	lymphotoxin B receptor	0.31094687058711	7.50752828887998	1.01973156825019	0.344676115214755	0.999692817443324	-4.59490548562344	7.35087020477495	7.32495293398703	7.6707877152053	7.82483245264077	7.27126609367527	7.60246033299658	NA
**TC1000000036.mm.2**	Vip	vasoactive intestinal polypeptide	-0.448654942354547	6.69067503513094	-1.01959353666015	0.344736828361783	0.999692817443324	-4.59491286290083	6.85402791972759	7.25273562367282	5.92589243477294	6.64409595656513	7.16774718423727	6.2995510918099	NA
**TC0300003133.mm.2**	Ifi44	interferon-induced protein 44	0.449051467112476	4.80719376122905	1.01949748670663	0.344779080915736	0.999692817443324	-4.59491799614531	4.55888898607466	4.93931673449376	4.68025041117147	5.44127947934394	4.19054484243924	5.03288211385122	NA
**TC0300002045.mm.2**	Siah2	siah E3 ubiquitin protein ligase 2	0.414998514639082	5.55034236399834	1.01937219044324	0.344834205162585	0.999692817443324	-4.59492469208157	5.68856938353337	5.93303878592318	5.13177738752285	6.12344413438298	5.29429442004723	5.13093007258044	NA
**TC0300001431.mm.2**	Unc5c	unc-5 netrin receptor C	0.390794118692368	7.54213393382728	1.01936809139932	0.344836008660369	0.999692817443324	-4.59492491113151	7.28153555722123	7.26828634951065	7.63022201532354	8.11206295148473	7.09430314034115	7.86639358908238	NA
**TC0X00001333.mm.2**	Tceal3	transcription elongation factor A (SII)-like 3	-0.37466254432027	7.7624114569073	-1.01918126629991	0.344918215944958	0.999692817443324	-4.59493489450099	8.11716214534634	7.87183502666633	7.74402827023562	7.17404497015966	7.56388189510039	8.10351643393543	NA
**TC0300002054.mm.2**	P2ry12	purinergic receptor P2Y, G-protein coupled 12	0.386783740069782	8.51581287068468	1.01894241323342	0.345023339398756	0.999692817443324	-4.59494765686616	9.49957536060101	9.6294735400967	8.55145544274037	7.8182424590346	8.04145038938406	7.55468003225134	NA
**TC1100001529.mm.2**	Mrpl10	mitochondrial ribosomal protein L10	0.404070123513525	7.35371111647587	1.018941213461	0.345023867504047	0.999692817443324	-4.59494772096877	8.20299981468556	7.87106627706193	7.57208195967543	6.85608846739197	7.10068237604486	6.51934780399549	NA
**TC0100003881.mm.2**	D630023F18Rik	RIKEN cDNA D630023F18 gene	0.54026975514246	6.19932675625599	1.01832915464039	0.345293361930361	0.999692817443324	-4.59498041813211	6.90727030093517	6.69026661349953	6.75045499845775	5.58902656823532	6.16854530014515	5.090396756263	NA
**TC1300002776.mm.2**	Zfp87	zinc finger protein 87	0.619947958165753	6.87725969390767	1.01772907402567	0.345557744569563	0.999692817443324	-4.59501246664092	7.86651992104142	7.60677109233785	7.18916861802324	6.32591291517589	6.98875340397087	5.28643221289676	NA
**TC1100000644.mm.2**	Slc36a1	solute carrier family 36 (proton/amino acid symporter), member 1	0.305635698606907	7.43488391602152	1.01764048593085	0.345596788198299	0.999692817443324	-4.5950171971303	7.70099528843539	7.69273256051556	7.43761459192329	7.47580893027271	7.30665011600682	6.99550200897537	NA
**TC0700001666.mm.2**	Far1	fatty acyl CoA reductase 1	0.737551934645322	6.53960342896068	1.01754995266254	0.345636692747113	0.999692817443324	-4.5950220312938	7.54516350044254	7.87037047312136	6.75449306156816	5.89410217317724	6.68780158426651	4.48568978118824	NA
**TC1700000643.mm.2**	Atf6b	activating transcription factor 6 beta	-0.346269263131448	8.17440423571634	-1.01718809699116	0.345796225210163	0.999692817443324	-4.59504135115975	8.28360093574957	8.29689793107376	8.13312625749399	7.75356775311192	8.02296915726681	8.55626337960199	NA
**TC0500002828.mm.2**	Hnrnpdl	heterogeneous nuclear ribonucleoprotein D-like	-0.578205657311734	8.63525743705422	-1.01697057628471	0.345892152389062	0.999692817443324	-4.5950529633038	9.13854255226154	9.03978376458782	8.97600291004273	7.26240058538351	8.89076981970901	8.5040449903407	NA
**TC1800001463.mm.2**	Atp8b1	ATPase, class I, type 8B, member 1	0.560317568777048	5.20393547864637	1.0169058120333	0.345920717668104	0.999692817443324	-4.59505642046326	4.66041069730374	4.87968469321327	4.87437456890925	6.52770174054839	5.01682366527665	5.2646175066269	NA
**TC0500000844.mm.2**	Cxcl3	chemokine (C-X-C motif) ligand 3	-0.310577858390057	7.48946400206083	-1.01680210243482	0.345966464298001	0.999692817443324	-4.59506195634045	7.35648796946393	7.40971302712571	7.31630131212244	7.45841233737516	7.52829512466556	7.86757424161216	NA
**TC1400002791.mm.2**	Gm3264	predicted gene 3264	0.866295647424133	6.54667108211938	1.01665018927893	0.346033482338028	0.999692817443324	-4.59507006478844	7.21214135552063	6.65907542557045	7.44849471028226	6.12220579295449	7.16522704726204	4.67288216112644	NA
**TC1100000593.mm.2**	9530068E07Rik	RIKEN cDNA 9530068E07 gene	0.355611532823126	8.95014572697032	1.01651474549643	0.346093243435268	0.999692817443324	-4.5950772937039	8.79747693368566	9.03100063020289	9.22312211133034	9.06868782045946	8.46095609400232	9.11963077214123	NA
**TC1000001568.mm.2**	Stat6	signal transducer and activator of transcription 6	-0.495009244430356	5.54863382424106	-1.01641532160145	0.346137116894099	0.999692817443324	-4.5950825998808	5.33665757937565	5.80938063500994	4.65169424739466	5.92617887370536	5.59623730876986	5.97165430119087	NA
**TC0800000736.mm.2**	Hapln4	hyaluronan and proteoglycan link protein 4	-0.328386745711097	7.00873943089395	-1.01640607972933	0.346141195342138	0.999692817443324	-4.59508309310026	7.94990103075042	8.14478004635393	6.55427190801577	6.09621910040281	6.56287124324548	6.7443932565953	NA
**TC0800000784.mm.2**	Ocel1	occludin/ELL domain containing 1	-0.361161572192199	6.59481445963084	-1.01627487037207	0.346199102286415	0.999692817443324	-4.5950900952473	6.42441711348937	6.22113944009847	6.20069212553952	6.89981140436687	6.82137486792854	7.00145180636225	NA
**TC0700002429.mm.2**	Npas1	neuronal PAS domain protein 1	-0.359214865664761	6.15078723319236	-1.01625308906424	0.346208715827951	0.999692817443324	-4.59509125759296	6.11267821580831	5.59959276700771	5.97450948847379	6.26250185403052	6.70072648651316	6.25471458732067	NA
**TC0400000412.mm.2**	Ccl19	chemokine (C-C motif) ligand 19	-0.365728896727151	7.56143199838019	-1.01611267586505	0.34627069460797	0.999692817443324	-4.59509875037847	7.62860085170837	7.50139546160511	7.60545231239598	7.14811662936071	7.43467639690408	8.05035033830691		2
**TC0400002025.mm.2**	Nphp4	nephronophthisis 4 (juvenile) homolog (human)	-0.418602549325636	5.48493818007499	-1.01586003274567	0.346382234163931	0.999692817443324	-4.59511223081933	5.46437864812938	5.7287068874849	4.70713235630792	5.73253686678427	5.65203954712995	5.62483477461351	NA
**TC0800002959.mm.2**	Tmem231	transmembrane protein 231	0.525832740113654	6.86611547763557	1.01582027492502	0.346399789463038	0.999692817443324	-4.595114352062	7.42933378178112	7.17828478715328	7.61875665575438	6.20161323279878	6.66415486275982	6.10454954556603	NA
**TC1100004291.mm.2**	Psme2b	protease (prosome, macropain) activator subunit 2B	-0.36431455089558	6.17141718632543	-1.0156041155775	0.346495248245759	0.999692817443324	-4.59512588437855	6.04820437792646	5.91906701858808	5.75515958168391	6.41114172813951	6.25628681301101	6.63864359860358	NA
**TC0100003352.mm.2**	Rabgap1l	RAB GTPase activating protein 1-like	0.306136872314053	9.13196328490633	1.01550041105455	0.346541052921658	0.999692817443324	-4.59513141671597	9.5597128402554	9.45884667815078	8.94117458521924	9.25157238261072	8.80183662402558	8.77863659917627	NA
**TC0600003332.mm.2**	Wbp11	WW domain binding protein 11	0.457169283944748	6.67780492843813	1.01546790014223	0.346555413475354	0.999692817443324	-4.59513315102574	7.71782106204755	7.54111693173885	6.91207200795865	5.9490430644073	6.35626245762181	5.59051404685465	NA
**TC1700002145.mm.2**	Cdc5l	cell division cycle 5-like (S. pombe)	0.347114049459227	6.1179045605187	1.01531153951219	0.346624486887889	0.999692817443324	-4.59514149179815	6.88664775405684	6.65286847112683	5.85529389490622	6.07577572351726	5.90919640384376	5.32764511566126	NA
**TC1700002331.mm.2**	Safb2	scaffold attachment factor B2	-0.322977634076188	6.74250548698866	-1.01489058838506	0.346810499301172	0.999692817443324	-4.59516394371803	7.20546606878241	6.85898317470332	6.4856388047967	6.38667540035021	6.92565363540154	6.59261583789775	NA
**TC0700000841.mm.2**	Mtag2	metastasis associated gene 2	0.409377153591702	6.60372734274552	1.01483709044419	0.346834144962322	0.999692817443324	-4.59516679678493	6.05835104160099	6.07933579894018	6.56845330826384	7.58326245329384	6.65232602029958	6.6806354340747	NA
**TC0900003324.mm.2**	Bcl9l	B cell CLL/lymphoma 9-like	0.324865671393749	6.80791685690864	1.01472864563414	0.346882080622354	0.999692817443324	-4.5951725799773	7.23375950988002	7.0132267558977	6.78164687623798	6.84347623299284	6.26186706460859	6.71352470183473	NA
**TC1500000604.mm.2**	Ppp1r16a	protein phosphatase 1, regulatory subunit 16A	0.319303511468764	7.17557146562048	1.01460410155809	0.346937139093527	0.999692817443324	-4.59517922136575	7.11946107179952	7.37139466188681	7.205947095638	7.39464294584905	6.81296306423323	7.1490199543163	NA
**TC0700003923.mm.2**	Olfr642	olfactory receptor 642	-0.430949470156549	5.0964238788198	-1.0145998328254	0.346939026338605	0.999692817443324	-4.59517944899182	4.16726450994157	4.1624254548602	5.13735000433097	5.556127179571	5.25731619021168	6.29805993400338	NA
**TC0300001797.mm.2**	Zmat3	zinc finger matrin type 3	-0.773220095634353	7.36852209385453	-1.01416207903087	0.347132604453183	0.999692817443324	-4.59520278944009	7.86437643627972	7.88108414488309	7.9406844825534	5.51893141279443	7.87357351655788	7.13248257005866	NA
**TC1800001175.mm.2**	Slc23a1	solute carrier family 23 (nucleobase transporters), member 1	-0.30797711185835	4.90277074632788	-1.01413606101398	0.347144112517393	0.999692817443324	-4.59520417653924	4.0535586466209	4.33816590335288	5.04410582571567	5.16036702642272	5.4965568244817	5.32387025137339	NA
**TC1900000237.mm.2**	Osbp	oxysterol binding protein	-0.642467886986863	7.23353692714473	-1.0141243776207	0.34714928031328	0.999692817443324	-4.5952047994109	8.24399518740728	8.15606816362919	7.34076494328664	5.51734627564243	7.50956070220428	6.63348629069853	NA
**TC1100002379.mm.2**	Papolg	poly(A) polymerase gamma	0.833159569185488	6.48214840067442	1.01405466973979	0.347180114755134	0.999692817443324	-4.59520851564777	7.80158622102433	7.52328779347128	7.13316390029181	5.48400386366914	6.57349814311007	4.3773504824799	NA
**TC0X00003411.mm.2**	Ppp1r3f	protein phosphatase 1, regulatory subunit 3F	0.499694725429318	6.66967856155848	1.013869151659	0.347262187024093	0.999692817443324	-4.59521840533225	7.05313766146576	7.01188444268666	6.26041943310405	7.2157999249245	6.77411944751605	5.70271045965386	NA
**TC1300002125.mm.2**	Isca1	iron-sulfur cluster assembly 1	-0.52006960530796	8.52810979666348	-1.01380987129938	0.347288415600577	0.999692817443324	-4.59522156530062	9.59683195592516	9.3415952789712	8.53554490458369	7.05950126265062	8.45802375901685	8.17716161883337	NA
**TC1100004092.mm.2**	Caskin2	CASK-interacting protein 2	-0.336491170082377	7.4775205896725	-1.0137618351395	0.347309670336323	0.999692817443324	-4.59522412582868	7.62894347679841	7.71916190409991	7.09080004101283	7.33121786747313	7.27859156614689	7.81640868250383	NA
**TC0X00000836.mm.2**	5430427O19Rik	RIKEN cDNA 5430427O19 gene	0.329676567719824	5.16247540157753	1.01369402557968	0.347339676036157	0.999692817443324	-4.59522774026588	4.87307168077822	5.00643077675181	5.33995354922968	5.53739799445772	5.35485910372065	4.86313930452709	NA
**TC0900000689.mm.2**	Imp3	IMP3, U3 small nucleolar ribonucleoprotein	-0.348206202481816	7.80084044474707	-1.01364369388318	0.347361949118034	0.999692817443324	-4.59523042301214	7.91230012105082	8.13836816441399	7.79054945651183	7.23843153251516	7.70459653649359	8.02079685749703	NA
**TC1200001025.mm.2**	Fam181a	family with sequence similarity 181, member A	0.410087754152192	6.49656191429165	1.01343081792491	0.347456164774863	0.999692817443324	-4.59524176890056	5.92646952076015	5.70418003659538	6.70655011900302	7.37789859934636	6.26333365959045	7.00093955045455	NA
**TC0800002951.mm.2**	Ctrb1	chymotrypsinogen B1	-0.333489964325064	5.27098884486377	-1.01336838376028	0.347483801026406	0.999692817443324	-4.59524509631463	5.15215546101319	4.66363929699913	5.15451216877733	5.41706702248275	5.54259588240082	5.69596323750939	NA
**TC0200002032.mm.2**	Cds2	CDP-diacylglycerol synthase (phosphatidate cytidylyltransferase) 2	-0.832739698330833	9.08462604236739	-1.01336522007195	0.347485201467697	0.999692817443324	-4.59524526492013	10.4287145711458	10.2340399639846	9.30813980161977	6.78162135958638	9.42582352528803	8.32941703257978	NA
**TC0400004220.mm.2**	Tmem269	transmembrane protein 269	0.339165138295283	5.11053125268601	1.0133450296619	0.347494139079227	0.999692817443324	-4.59524634094137	5.14622115044459	4.99621866272215	5.55901583782214	5.04052315194781	4.81257876514005	5.10862994803933	NA
**TC1400000776.mm.2**	Dhrs2	dehydrogenase/reductase member 2	0.342420416216277	5.74404839141022	1.01331327476533	0.34750819626637	0.999692817443324	-4.59524803325653	5.52369071649751	5.52675603788917	6.34337111969997	5.70597109355362	5.6942416071422	5.67025977367884	NA
**TC0500002486.mm.2**	Ugdh	UDP-glucose dehydrogenase	-0.381605799934923	6.34925744014993	-1.01319605858301	0.347560089168295	0.999692817443324	-4.59525427985093	7.02062711922652	6.89636706606711	6.22931923200758	5.47835019586046	6.44948416690572	6.02139686083217	NA
**TC0100002495.mm.2**	Acadl	acyl-Coenzyme A dehydrogenase, long-chain	0.332587347086195	6.45282067579956	1.0129844531595	0.347653784783255	0.999692817443324	-4.5952655557151	6.99086441053169	7.33418097309874	6.47459211217327	6.05393457049639	6.0179983217978	5.84535366669948	NA
**TC1100002862.mm.2**	Flcn	folliculin	0.340066326553574	7.02883831223015	1.0129387709156	0.347674014802568	0.999692817443324	-4.59526798985131	7.8082261463862	7.56294010107369	7.15145684964758	6.5895412898665	6.5849674172866	6.47589806912032	NA
**TC0500000547.mm.2**	Stim2	stromal interaction molecule 2	-0.431146457227943	7.75193737917021	-1.0128053050317	0.34773312445462	0.999692817443324	-4.59527510116588	8.09491047159725	8.02442992510564	7.94461697939711	6.82037850253414	7.81115965143183	7.81612874495531	NA
**TC1900000939.mm.2**	Actn3	actinin alpha 3	-0.361323946994294	6.75771928959731	-1.01275037220813	0.347757455532557	0.999692817443324	-4.59527802796418	6.1702416793927	6.58322696362668	6.54147379150625	6.9936258087817	6.9050907492819	7.35265674499464	NA
**TC0500000043.mm.2**	Steap4	STEAP family member 4	-0.386361826361163	4.94576612946326	-1.01250128423786	0.347867799554089	0.999692817443324	-4.59529129834402	5.07080997395118	4.84651298087646	4.31398112772187	5.17829395689293	5.19088545660241	5.07411328073472	NA
**TC0400001124.mm.2**	Ldlrad1	low density lipoprotein receptor class A domain containing 1	-0.325548550514866	6.21764726936039	-1.01247498026619	0.347879453626931	0.999692817443324	-4.59529269962265	6.09205563965247	5.89930825871418	6.15998546005416	6.17172584832883	6.25129463719119	6.73151377222153	NA
**TC1000001010.mm.2**	Washc4	WASH complex subunit 4	0.392556384393576	5.24191933540112	1.01223165494729	0.347987274483936	0.999692817443324	-4.59530566137148	6.22491266049356	6.03166918118821	4.83059103646484	5.15943243329122	4.17611456274744	5.02879613822146	NA
**TC0200000391.mm.2**	Zmynd19	zinc finger, MYND domain containing 19	0.486973401308219	6.28410143500174	1.01205866421941	0.348063945198486	0.999692817443324	-4.59531487557117	6.85375574449379	6.58253960859011	6.90673197692592	5.71439805284557	6.14791077264775	5.4992724545073	NA
**TC1900000520.mm.2**	Hectd2	HECT domain E3 ubiquitin protein ligase 2	0.296892098858748	7.87611560593495	1.01201623010754	0.348082754340763	0.999692817443324	-4.59531713567517	7.21332394399348	7.24599346141341	8.42624832163452	8.26933189232562	7.98121458660401	8.12058142963864	NA
**TC0500001300.mm.2**	Ksr2	kinase suppressor of ras 2	0.320175361301216	7.02138644826761	1.01160190818925	0.348266447010979	0.999692817443324	-4.59533920076142	7.30273439071986	7.28771121255074	6.95415770013441	7.13495420433433	6.94442421889702	6.50433696296929	NA
**TC0X00002073.mm.2**	Rhox8	reproductive homeobox 8	-0.484852715654029	6.17620088458739	-1.0115582865949	0.348285791432137	0.999692817443324	-4.59534152362405	5.34631120668866	4.49673932646492	6.1152689373196	7.00695573421176	6.73932407741862	7.35260602542079	NA
**TC1100001283.mm.2**	Myo19	myosin XIX	0.380869052296785	6.65683706488407	1.01148199263163	0.348319626779397	0.999692817443324	-4.59534558618779	6.09540698544588	6.22186922436576	6.67595768572473	7.51678445631845	6.61701558241706	6.81398845503255	NA
**TC0400000374.mm.2**	Nudt2	nudix (nucleoside diphosphate linked moiety X)-type motif 2	0.29583646872528	6.26240966828826	1.0113762577247	0.348366523093112	0.999692817443324	-4.59535121621131	6.92236384593137	6.75957705387558	6.14274701826304	6.09934800542354	5.8758669207536	5.77455516548241	NA
**TC0500001026.mm.2**	Pkd2	polycystic kidney disease 2	-0.455198340970279	7.15021244343425	-1.01131775637918	0.348392472183127	0.999692817443324	-4.59535433109088	7.85423853473973	7.92166973986257	7.1378849855023	5.96959986652902	7.21775214112275	6.80012939284913	NA
**TC0300000747.mm.2**	Pklr	pyruvate kinase liver and red blood cell	0.299707360654555	6.59373621313688	1.01122999758401	0.348431401694362	0.999692817443324	-4.59535900361375	6.62735052572897	6.37965724819815	6.74832750683318	6.82908460626845	6.49363754601284	6.48435984577968	NA
**TC0300002334.mm.2**	Fdps	farnesyl diphosphate synthetase	-0.306607639082003	7.81455663249459	-1.0112012755256	0.34844414345259	0.999692817443324	-4.59536053281518	7.85918937804554	8.1203268181376	7.47543385688257	7.67187030342761	7.93913910025936	7.82138033821483	NA
**TC0500003369.mm.2**	Gtf2i	general transcription factor II I	0.49429406920642	8.47443970031753	1.01106642932793	0.348503969224547	0.999692817443324	-4.59536771193961	9.35687233616537	9.59239476213743	8.67891279337991	7.7640668276277	8.25628985618692	7.19810162640785	NA
**TC1100002907.mm.2**	Aldh3a2	aldehyde dehydrogenase family 3, subfamily A2	0.925313868026516	6.34057593728251	1.01103217716649	0.348519166810343	0.999692817443324	-4.59536953543152	7.43961322895494	7.51282289407304	6.34471230584286	6.12611131251718	6.86396795119007	3.75622793111694	NA
**TC1900001289.mm.2**	Cbwd1	COBW domain containing 1	0.561448925417771	5.15152118726252	1.01102775456674	0.348521129143419	0.999692817443324	-4.5953697708766	6.28250883490743	6.36059956742985	5.30237173325801	4.39208655277866	5.0051134110225	3.56644702417863	NA
**TC0500001597.mm.2**	Rasa4/Mir7035	RAS p21 protein activator 4/microRNA 7035	-0.306657695972714	6.26633970800576	-1.01100633865121	0.348530631632694	0.999692817443324	-4.59537091098475	5.92633989712376	5.82250691754337	6.14383291839562	6.4741051023154	6.63580380592625	6.5954496067302	NA
**TC0200000484.mm.2**	Gbgt1	globoside alpha-1,3-N-acetylgalactosaminyltransferase 1	0.374442386562603	5.50148401874084	1.01092631137358	0.348566142476361	0.999692817443324	-4.59537517125643	5.43359887511546	5.08225135912623	5.43081046768757	6.19015885797672	5.46174364172139	5.41034091081769	NA
**TC1500001905.mm.2**	Rps19bp1	ribosomal protein S19 binding protein 1	-0.327794513724986	7.61305909402321	-1.01090671480208	0.348574838582834	0.999692817443324	-4.59537621446075	7.33239711455776	7.37713723256508	7.7792488208932	7.37736677389004	7.72792730163676	8.08427732059645	NA

**TC0700002879.mm.2**	Plekhf1	pleckstrin homology domain containing, family F (with FYVE domain) member 1	-0.533420835378067	6.31664155215041	-1.01085598591663	0.348597350654618	0.999692817443324	-4.5953789149196	6.20951863661875	5.97940868733245	5.39395485010154	6.92808530899603	6.52630979804698	6.86257203180672	NA
**TC1700002520.mm.2**	Galnt14	polypeptide N-acetylgalactosaminyltransferase 14	-0.308688763143437	6.89107045945086	-1.01070888000237	0.348662638686286	0.999692817443324	-4.59538674547502	6.668858690631	6.54740490927762	6.82132398666006	6.93506682859476	7.35289100614385	7.02087733539784	NA
**TC0500003742.mm.2**	Tmem184a	transmembrane protein 184a	-0.352522323452459	5.7233328539792	-1.01057349718585	0.348722732367186	0.999692817443324	-4.59539395153192	4.87770129818303	5.43496374942546	5.69385975957645	5.96728395510445	6.01137792820847	6.35481043337736	NA
**TC0300002052.mm.2**	Gpr87	G protein-coupled receptor 87	-0.402271248451698	5.37406479849849	-1.01032898794069	0.348831285827534	0.999692817443324	-4.59540696494932	4.69320052510299	4.96528272199712	5.02952117579559	5.86116034769813	5.61767920100888	6.07754481938823	NA
**TC0800002596.mm.2**	Rnaseh2a	ribonuclease H2, large subunit	-0.327275077645357	5.99149487250888	-1.01002434078437	0.348966575815558	0.999692817443324	-4.5954231770069	6.77139363596505	6.65299911769344	5.66316866880295	5.27184449424909	5.62055605117153	5.96900726717123	NA
**TC1600001143.mm.2**	Ubald1	UBA-like domain containing 1	0.371892836135187	8.55090778513237	1.00980756811733	0.349062867127267	0.999692817443324	-4.59543471135904	8.96205616538459	8.81496068181156	8.8909608555722	8.24514691236202	8.44309047837254	7.94923161729131	NA
**TC0300000894.mm.2**	Cdc42se1	CDC42 small effector 1	-0.332424356034014	7.4984685089937	-1.00978460512303	0.34907306861545	0.999692817443324	-4.59543593313938	7.30354844958548	7.60831901012416	7.59754234063988	7.10950510045239	7.7017054892958	7.6701906638645	NA
**TC0300001423.mm.2**	Tspan5	tetraspanin 5	-0.789647672192429	8.67851313725872	-1.00974443912542	0.349090913238441	0.999692817443324	-4.59543807019996	9.5070604907837	9.88478374640503	8.82650912188707	6.72346049910227	9.31268274175264	7.81658222362157	NA
**TC0600000250.mm.2**	Strip2	striatin interacting protein 2	-0.469411146875533	7.24886365949382	-1.00969891555533	0.349111138952875	0.999692817443324	-4.595440492266	8.34325490679361	8.72193162637235	6.99912836757878	5.74545819744418	6.81474774117577	6.86866111759826	NA
**TC1300000045.mm.2**	Idi1	isopentenyl-diphosphate delta isomerase	0.515204311237528	6.20003335122199	1.00964413744455	0.349135477606036	0.999692817443324	-4.59544340664912	7.42099285795745	7.23790198777373	6.37735417786635	5.40850276417156	5.95108598577658	4.80436233378629	NA
**TC0200002170.mm.2**	Naa20	N(alpha)-acetyltransferase 20, NatB catalytic subunit	-0.295535363709629	8.36786649435301	-1.00961465304095	0.349148578479898	0.999692817443324	-4.5954449752898	8.31626810294577	8.39601419594569	8.31366111598338	8.13826185392027	8.54942307613094	8.49357062119197	NA
**TC0700001988.mm.2**	Dpysl4	dihydropyrimidinase-like 4	0.332278143745866	7.18730673065212	1.00960055251423	0.349154843937334	0.999692817443324	-4.59544572546393	7.75990988874516	7.35304600438942	6.96902635379756	7.36869403533738	6.79763307036683	6.87553103127639	NA
**TC1000000839.mm.2**	Stk11	serine/threonine kinase 11	0.294931865974563	7.96995673624008	1.00929228713048	0.349291841453688	0.999692817443324	-4.59546212452959	8.89816633420784	8.80758402422542	7.63729751775799	7.71462937772018	7.3064268631601	7.45563630036895	NA
**TC1900000669.mm.2**	Btrc	beta-transducin repeat containing protein	-0.880812587719527	6.75286290670094	-1.00920660126811	0.349329929020919	0.999692817443324	-4.59546668242027	8.41176552535412	8.25490385557442	6.97798954695868	4.06645189496035	6.64468827651654	6.16137834084154	NA
**TC0900003040.mm.2**	Slc38a3	solute carrier family 38, member 3	-0.465346419841042	8.50195504523647	-1.00880829782172	0.349507018973663	0.999692817443324	-4.59548786701174	9.2030971336361	9.2435289940837	8.49253023027364	7.32467542173483	8.65205207761653	8.09584641407402	NA
**TC1500001056.mm.2**	Scn8a	sodium channel, voltage-gated, type VIII, alpha	-0.318840180360096	7.72424887699158	-1.00820669448852	0.349774632834654	0.999692817443324	-4.59551985710712	7.53113352727324	7.56330041581066	7.51058973254202	7.79609974653067	8.1605567644479	7.78381307534498	NA
**TC1100003107.mm.2**	Pfn1	profilin 1	-0.457981051926819	10.0316840836568	-1.00817922971016	0.349786853979679	0.999692817443324	-4.59552131732601	10.2770529700821	10.4514915898213	10.2460297450056	9.0267691740864	10.2315146399438	9.9572463830019	NA
**TC1700001301.mm.2**	Tmem247	transmembrane protein 247	-0.358946360070425	5.32334590677832	-1.00808091555121	0.349830604109115	0.999692817443324	-4.59552654423864	5.08645669607676	4.92167423415551	5.00855174331645	5.59847415183194	5.44241399603614	5.88250461925309	NA
**TC0800000353.mm.2**	Eif4ebp1	eukaryotic translation initiation factor 4E binding protein 1	-0.468316446280054	6.26129679484692	-1.00784506208407	0.349935577331221	0.999692817443324	-4.59553908251112	4.7096628257037	5.35971476652245	6.27493140311779	7.00595373902985	7.54415204316787	6.67336599153987	NA
**TC0900002472.mm.2**	Map2k5	mitogen-activated protein kinase kinase 5	-0.985106344328592	6.90292245694107	-1.00783157512325	0.349941580835348	0.999692817443324	-4.59553979945367	7.97383344408612	7.8751845328253	7.38782252249546	4.41132951554344	7.65228925895257	6.11707546774352	NA
**TC0X00003236.mm.2**	Sat1	spermidine/spermine N1-acetyl transferase 1	-1.25098877017936	6.27510788008408	-1.00776662633425	0.349970492886578	0.999692817443324	-4.59554325195135	7.77192991342427	8.30682707969028	6.78245916140915	2.75249721210646	6.76515668974903	5.27177722412529	NA
**TC1300001940.mm.2**	Zfp169	zinc finger protein 169	0.30837994483687	5.88900654538639	1.0074161656392	0.350126533581584	0.999692817443324	-4.59556187967025	6.0802006626255	5.78592274528514	5.90377830487298	6.13855957216773	5.73303828375605	5.69253970361093	NA
**TC0800001613.mm.2**	Pcp2	Purkinje cell protein 2 (L7)	-0.344603419979403	7.45412312708821	-1.00740176122766	0.350132948243034	0.999692817443324	-4.59556264522982	6.524780326949	6.62818966353565	7.57919042432852	7.86209054171438	7.76841437348449	8.36207343251723	NA
**TC1600000415.mm.2**	Dlg1	discs large MAGUK scaffold protein 1	-0.416344932319582	7.9127192435146	-1.00725640149826	0.350197685933858	0.999692817443324	-4.59557037045978	8.95294107587177	8.86359832135994	7.67003855176564	6.74350454784272	7.88253302305985	7.36369994118767	NA
**TC0700002997.mm.2**	Dkkl1	dickkopf-like 1	-0.491766236688669	6.49810126086529	-1.00706287680653	0.35028388814797	0.999692817443324	-4.59558065463352	6.30579623439702	6.83447032344508	5.60285087359177	6.82955339339439	6.47304268210079	6.9428940582627	NA
**TC0300001666.mm.2**	Rbis	ribosomal biogenesis factor	-0.5127700568721	8.21348163719849	-1.00702899529627	0.350298982978557	0.999692817443324	-4.595582455491	8.84570805034697	8.89632689116902	8.34089500519707	6.91576237876831	8.38568285511134	7.89651464259824	NA
**TC0400000704.mm.2**	4933430I17Rik	RIKEN cDNA 4933430I17 gene	-0.477452205158326	4.98496539550973	-1.00696403126377	0.350327925159834	0.999692817443324	-4.59558590706645	3.57653858729108	4.66431186499373	4.84350707871035	5.51351167651811	5.43374045271557	5.87818271282955	NA
**TC1400002738.mm.2**	Slc15a1	solute carrier family 15 (oligopeptide transporter), member 1	0.404315483326841	5.43117239087243	1.00689497244964	0.350358693682982	0.999692817443324	-4.59558957655501	4.76906540536633	5.06621705695214	5.41690543474544	6.36328599003947	5.40205172349966	5.56950873463157	NA
**TC1400000791.mm.2**	Nop9	NOP9 nucleolar protein	0.327367089032882	6.05329642919804	1.00680123276433	0.350400461959425	0.999692817443324	-4.59559455728987	5.98389108726978	6.26098855389035	6.32703871292606	6.03777784312086	5.66960973795681	6.04047264002435	NA
**TC0600002639.mm.2**	Pcbp1	poly(rC) binding protein 1	-0.547168564896425	8.58902919780663	-1.00676798710666	0.350415276415256	0.999692817443324	-4.59559632370183	9.34825350069369	9.21076426339091	8.77868718728346	7.16172295919769	8.67842495907566	8.35632231719834	NA
**TC0200002372.mm.2**	Myh7b	myosin, heavy chain 7B, cardiac muscle, beta	-0.339276856398081	6.57317342653318	-1.00621062560725	0.35066371360684	0.999692817443324	-4.59562593341234	6.10921868238484	5.84193226487154	6.52465096846721	6.88001698110604	6.81648799615744	7.26673366621198	NA
**TC0100000442.mm.2**	Stradb	STE20-related kinase adaptor beta	-0.548028708944985	8.97378547083045	-1.00608151856796	0.350721281364944	0.999692817443324	-4.59563279109643	8.64860612957429	8.76008791194092	9.55204300151392	8.11693768127484	9.79556476597198	8.96947333470675	NA
**TC1100003622.mm.2**	Snx11	sorting nexin 11	0.335905409658533	6.62450476115556	1.0058576508851	0.350821119804628	0.999692817443324	-4.595644681131	6.54115483490284	6.5888618249326	6.53049334423955	7.11391801896794	6.57272569053043	6.39987485336	NA
**TC0700000822.mm.2**	Tsks	testis-specific serine kinase substrate	0.30199153131399	5.72351982899167	1.00585209501095	0.350823597847489	0.999692817443324	-4.59564497619812	5.54025000266878	5.66319275196245	5.81642990711043	6.05439973386295	5.55311208770264	5.71373449064276	NA
**TC1400002266.mm.2**	Ephx2	epoxide hydrolase 2, cytoplasmic	0.482541459543938	5.35423302324335	1.00559746083348	0.350937185166078	0.999692817443324	-4.59565849875023	5.56939411744553	6.26320516214588	5.31182612903615	4.81711476044214	3.95718951425715	5.20666845613326	NA
**TC1400001659.mm.2**	Prkcd	protein kinase C, delta	0.477340481458823	7.88480579644227	1.00558402378492	0.350943179976998	0.999692817443324	-4.59565921229069	8.89088345933943	9.14345132088536	8.19048509031792	6.8741053903553	7.19649821754744	6.91341130020814	NA
**TC1400000364.mm.2**	Gdf2	growth differentiation factor 2	-0.375113649247739	5.37064385205295	-1.00557901468487	0.350945414759851	0.999692817443324	-4.59565947828513	5.27517635407816	4.93924548967871	5.57187141520741	5.05773556982527	5.39380594569861	5.98602833782955	NA
**TC0400001574.mm.2**	Tmem200b	transmembrane protein 200B	-0.330325951006643	6.31972515036323	-1.0054585031619	0.350999183711213	0.999692817443324	-4.5956658775289	6.24654499301256	6.30188548903964	5.91874306043292	6.43589119862404	6.38944708432464	6.62583907674561	NA
**TC0800001654.mm.2**	Efnb2	ephrin B2	0.549396071539917	9.23319053356481	1.00543762696313	0.351008498762269	0.999692817443324	-4.59566698603254	9.8907828605742	9.80620580786439	9.81272801936609	8.58774531864896	9.21445113299971	8.08723006193551	NA
**TC0100001037.mm.2**	Cdh7	cadherin 7, type 2	-0.415893779830098	6.53158344895446	-1.00539901860837	0.351025726493175	0.999692817443324	-4.59566903606598	7.40233083472131	6.93245146703272	6.47962254320938	5.53184287294688	6.49218058811552	6.35107238770094	NA
**TC1100001162.mm.2**	Vtn	vitronectin	-0.47027680123497	8.42537147295413	-1.00538500299211	0.351031980674306	0.999692817443324	-4.59566978026054	9.38125989252607	9.12099643198866	8.2268675777567	7.32784187761335	8.71567634218244	7.77958671565755	NA
**TC0300001173.mm.2**	Col11a1	collagen, type XI, alpha 1	0.396068124205452	6.00005026147837	1.00520580490039	0.351111951900778	0.999692817443324	-4.59567929480535	5.25341316235289	5.63001942760385	6.08844385977215	6.86605875389005	5.86414991625281	6.29821644899848	NA
**TC0500001974.mm.2**	Gm10354	predicted gene 10354	-0.359912773736519	7.38526893868837	-1.00491120416438	0.351243455450575	0.999692817443324	-4.59569493493071	7.13611953336016	7.07503002182147	7.69964749595769	6.99067176878008	7.84791306205395	7.56223175015686	NA
**TC1900000797.mm.2**	Casp7	caspase 7	-0.445087666031057	5.62521052346299	-1.00488916595315	0.351253294406082	0.999692817443324	-4.59569610483543	5.39869853719004	5.46472163831387	5.20983877313295	5.788995043473	5.41498232932829	6.47402681933978	NA
**TC1100003047.mm.2**	Wrap53	WD repeat containing, antisense to Trp53	0.299056729591672	5.8691133557412	1.00465303491959	0.35135872872138	0.999692817443324	-4.59570863915928	6.7642164584812	6.92555274273682	5.5831620003458	5.47835019586046	5.33272030946052	5.13067842756239	NA
**TC1800001179.mm.2**	Dnajc18	DnaJ heat shock protein family (Hsp40) member C18	0.472496119840003	6.69228917397926	1.00460204758689	0.351381498235728	0.999692817443324	-4.59571134549001	7.51663238789745	7.08935555007859	7.08627943881617	6.1600902339736	6.58164934739151	5.71972808571825	NA
**TC0300002598.mm.2**	Cd2	CD2 antigen	-0.457339856633359	4.9839232176158	-1.0043762473323	0.351482348306038	0.999692817443324	-4.595723329848	4.98386378799468	5.03406218147972	5.21382567396447	4.27164113751237	4.77738230276176	5.6227642219818	NA
**TC1600000301.mm.2**	Vps8	VPS8 CORVET complex subunit	-0.333600182648267	7.01595411483765	-1.00398570398783	0.351656832174016	0.999692817443324	-4.59574405495051	7.67917327178391	7.59534967152821	6.74302320055685	6.33397748965177	7.08649157220567	6.65770948329949	NA
**TC0600003123.mm.2**	Gpr162	G protein-coupled receptor 162	-0.293496925934446	7.598967919739	-1.00354212601965	0.351855093341227	0.999692817443324	-4.59576758984198	8.18774425627902	8.32016260294701	7.13507816865373	7.11437523501581	7.34694236356593	7.4895048919725	NA
**TC0200002426.mm.2**	Lbp	lipopolysaccharide binding protein	0.534373749885956	6.71052920816867	1.00353876931423	0.351856593987138	0.999692817443324	-4.59576776791978	6.70689339919019	7.93372595751512	6.23057362595164	7.11507807008766	6.41118107050917	5.86572312575822	NA
**TC1200000432.mm.2**	Akap6	A kinase (PRKA) anchor protein 6	0.595493319745159	7.42945460690118	1.00351547197199	0.351867009416601	0.999692817443324	-4.59576900386775	8.95766538167313	8.83529880250735	7.34776423089985	6.6396108174586	7.25706876536144	5.5393196435067	NA
**TC0200001681.mm.2**	Fmn1	formin 1	0.514849352803751	6.41377780749039	1.00349500374441	0.351876160248288	0.999692817443324	-4.59577008971708	5.59967336194112	5.74123835178562	6.43025397461547	7.45694281506608	5.88004565517645	6.97745248289976	NA
**TC0500000057.mm.2**	Tmem243	transmembrane protein 243, mitochondrial	0.54938310201729	6.93842944273984	1.00345457753138	0.351894234346897	0.999692817443324	-4.5957722343164	6.90887297677742	6.9865554557553	8.03008244207873	6.38687477189172	6.51144033486106	6.80675067507481	NA
**TC0300002250.mm.2**	Fbxw7as1	F-box and WD-40 domain protein 7 antisense transcript 1	-0.384003703567672	5.26012793003259	-1.00338606923819	0.351924865295675	0.999692817443324	-4.59577586856895	5.35053330832466	5.3382755272311	5.19866649182282	4.85330917692939	5.82907793448507	4.99090514140249	NA
**TC0400003712.mm.2**	Arid1a/Mir7227	AT rich interactive domain 1A (SWI-like)/microRNA 7227	0.46752013422136	8.87731771930567	1.00318240587925	0.352015938255188	0.999692817443324	-4.59578667188305	9.26335044510369	9.19116819372094	9.48444406635124	8.38776990637481	8.83041403513108	8.10675966915225	NA
**TC0400003446.mm.2**	Zfp69	zinc finger protein 69	0.460237810269802	5.66027056481947	1.00311126546057	0.352047754782593	0.999692817443324	-4.59579044527904	5.29432752757059	5.54463109187206	5.44767623404488	6.57389396096201	5.83099414011018	5.27010043435711	NA
**TC1900001649.mm.2**	Sorcs1	sortilin-related VPS10 domain containing receptor 1	0.427184746156836	6.66638068569141	1.00285615400583	0.352161868360655	0.999692817443324	-4.5958039757373	7.59241464354437	7.22782572596429	6.99252311536904	6.02368350310769	6.30470271998649	5.85713440617655	NA
**TC0500000229.mm.2**	Galnt11	polypeptide N-acetylgalactosaminyltransferase 11	0.593366280172672	7.31429332432143	1.00230118638086	0.352410210991183	0.999692817443324	-4.59583340417079	7.76689208597299	7.75362342136466	7.73244862922331	7.04353987024482	7.65645040344821	5.93280553567458	NA
**TC1500001345.mm.2**	Rpl30/Gm12191	ribosomal protein L30/ribosomal protein L30 pseudogene	-0.523663863761389	8.62421811323434	-1.00229225893776	0.352414207063915	0.999692817443324	-4.59583387750591	9.4916728832107	9.80137856256447	8.57326914728213	7.12919560577192	8.63360865612327	8.11618382445356	NA
**TC0X00000940.mm.2**	Tmem28	transmembrane protein 28	0.29394856316779	6.79273980059886	1.00211105785737	0.352495323419388	0.999692817443324	-4.59584348439766	6.70068987600357	6.61701128531538	7.0793207193426	6.9339966649623	6.77734028047666	6.64807997749266	NA
**TC0900001431.mm.2**	Shisa5	shisa family member 5	-0.325155102992555	6.98611041264692	-1.00208219570612	0.352508245190123	0.999692817443324	-4.59584501453086	7.40478663025239	7.42580621345757	6.87316881030271	6.34471090279051	6.87789190155429	6.99029801752404	NA
**TC1700001598.mm.2**	Tnfrsf12a	tumor necrosis factor receptor superfamily, member 12a	-0.425203916681931	6.49918266584678	-1.00205578408584	0.352520070169679	0.999692817443324	-4.59584641473033	6.05173537335398	6.55230091052038	6.50095235975002	6.26937357917123	6.32307824059676	7.29765553168835	NA
**TC0700000566.mm.2**	Hspb6	heat shock protein, alpha-crystallin-related, B6	-0.341118588638856	7.86411929678968	-1.00195843496661	0.352563657903968	0.999692817443324	-4.59585157549641	7.81118982034038	8.36803326826057	7.65049019247953	7.51113756495018	8.07960747592541	7.764257458782	NA
**TC1700001197.mm.2**	Rmdn2	regulator of microtubule dynamics 2	0.542535506836853	5.07206214625585	1.00178149608866	0.352642892560989	0.999692817443324	-4.59586095494329	5.71804313786333	6.10541507651833	5.15256149321145	4.69443134520212	5.14752772024382	3.61439410449605	NA
**TC0500003305.mm.2**	Zfp11	zinc finger protein 11	0.344424965128225	7.19386297092522	1.00112315004574	0.352937828533068	0.999692817443324	-4.59589584665459	7.14499153955973	7.20713520529147	7.04016100036622	7.70978950511208	6.98513292996299	7.07596764525886	NA
**TC1000000874.mm.2**	Sppl2b	signal peptide peptidase like 2B	-0.692568071428484	6.808256652822	-1.00095613853474	0.353012679825882	0.999692817443324	-4.59590469637321	7.2274984231271	7.11689399518874	7.40097104674473	5.15903463113486	7.09182771236898	6.85331410836758	NA
**TC0400002962.mm.2**	Bnc2	basonuclin 2	0.359934897915946	4.90449962569449	1.00077502539356	0.353093865333488	0.999692817443324	-4.59591429252571	4.85390004320219	4.64924524136709	4.84660630710766	5.47525482560713	4.59803046117679	5.0039608757061	NA
**TSUnmapped00000061.mm.2**	Defa-rs4	defensin, alpha,, related sequence 4	-0.364668021676229	5.46112060917043	-1.00067941060177	0.353136731412156	0.999692817443324	-4.59591935827542	4.6729061228957	4.821874200918	5.75184430248594	5.51945934144226	5.6228162911024	6.37782339617827	NA
**TC0500002803.mm.2**	Gk2	glycerol kinase 2	-0.37502759442439	6.94564883006573	-1.00063151930741	0.353158203606318	0.999692817443324	-4.59592189550882	6.14611817472402	6.17446449834197	6.98310932154452	7.31851823769528	7.12849945461623	7.92318329347236	NA
**TC0500002102.mm.2**	Kmt2c	lysine (K)-specific methyltransferase 2C	0.399512593519188	6.73465409928435	1.00057134916599	0.353185182513809	0.999692817443324	-4.59592508318155	7.20631531102644	7.16992024796494	6.57827366872684	6.83708344314972	6.787845043217	5.82848688162119	NA
**TC0200003528.mm.2**	Nmi	N-myc (and STAT) interactor	0.412096811301295	5.95031872177475	1.00035941208711	0.353280223160213	0.999692817443324	-4.59593631038416	5.91204424713017	5.81087408961886	6.12048443779158	6.28110937045946	6.30946236296947	5.26793782267899	NA
**TC0X00002820.mm.2**	Atrx	ATRX, chromatin remodeler	-0.642075132756983	7.66982372229082	-1.0003115842164	0.35330167378308	0.999692817443324	-4.59593884387214	8.73307930118822	8.88957125921822	7.49902836749806	6.0570423864142	8.15040756068704	6.68981345873919	NA
**TC1500000240.mm.2**	Pop1	processing of precursor 1, ribonuclease P/MRP family, (S. cerevisiae)	-0.449834878467466	5.98819017288807	-1.00010939427279	0.353392366562904	0.999692817443324	-4.59594955342979	5.46168372220149	5.66231941450946	5.71350547436629	6.23922859747498	5.87313769330344	6.97926613547276	NA
**TC1100003790.mm.2**	Stat3	signal transducer and activator of transcription 3	-0.355432297013826	6.85035903685464	-0.999952649194036	0.353462687560871	0.999692817443324	-4.59595785516377	7.69454436609633	8.10601827010734	6.41335736750911	5.88200612793916	6.33613795162089	6.67009013785504	NA
**TC1000000907.mm.2**	Gm10778	predicted gene 10778	0.758822095127181	5.78955974969291	0.999882018635686	0.353494378355095	0.999692817443324	-4.59596159578761	6.6373501635879	6.55300173774305	6.63396330219436	4.89836209134606	5.91924236124956	4.0954388420365	NA
**TC1000000909.mm.2**	Gm10778	predicted gene 10778	0.758822095127181	5.78955974969291	0.999882018635686	0.353494378355095	0.999692817443324	-4.59596159578761	6.6373501635879	6.55300173774305	6.63396330219436	4.89836209134606	5.91924236124956	4.0954388420365	NA
**TC0600000977.mm.2**	Figla	folliculogenesis specific basic helix-loop-helix	-0.381214390633088	5.07307334982755	-0.999676071127502	0.353586796463959	0.999692817443324	-4.59597250213538	4.64218659508289	4.77649575785954	4.94397990590696	5.18468457647141	5.01761083776867	5.87348242587587	NA
**TC1700001573.mm.2**	Zfp944	zinc finger protein 944	-0.493776043299003	6.07598172407595	-0.999270509454394	0.353768846258684	0.999692817443324	-4.59599397630775	6.92134228296433	6.94202429730392	6.12198351754511	4.68050232124961	5.88650898806748	5.90352893732523	NA
**TC0200002084.mm.2**	Btbd3	BTB (POZ) domain containing 3	0.509580104477543	9.17732819122509	0.999218319562663	0.353792278780798	0.999692817443324	-4.59599673942002	9.40468290488273	9.35496730431957	9.70753773538918	8.95420183816248	9.47573419539214	8.16684516920445	NA
**TC1800000995.mm.2**	Ankrd29	ankyrin repeat domain 29	0.299111593541367	7.02428433873742	0.999207542711817	0.353797117586427	0.999692817443324	-4.59599730997498	7.04584354431018	7.1829116476623	7.25499588202807	7.00259113173931	6.87500883028744	6.78435499639721	NA
**TC1500000705.mm.2**	Tnrc6b/Mir5113	trinucleotide repeat containing 6b/microRNA 5113	0.471663070174538	6.43440376644809	0.999108486112583	0.35384159643691	0.999692817443324	-4.59600255415568	7.57189270901763	7.51541487419092	6.62143676825515	5.6097838096594	6.08494270578591	5.20295173177956	NA
**TC1300002031.mm.2**	Thoc3	THO complex 3	-0.294261252316369	7.5020739095735	-0.998830363149417	0.353966504029025	0.999692817443324	-4.59601727700923	7.68478408585461	7.57728608253976	7.25803339296571	7.32289199924123	7.48633763920427	7.68311025763541	NA
**TC0700001524.mm.2**	Olfr652	olfactory receptor 652	-0.477894208979897	4.97243918418543	-0.998545989045125	0.354094254960322	0.999692817443324	-4.59603232875442	4.17354461862968	4.78785139276674	4.42328934273308	5.53543599514512	5.19752283035601	5.71699092548198	NA
**TC1100002106.mm.2**	Mtfp1	mitochondrial fission process 1	-0.442375396633074	7.95030289105757	-0.998143517826275	0.354275121338837	0.999692817443324	-4.59605362780994	8.52878079520151	8.32102027102746	8.04938860663386	6.93424413679128	8.14501788096015	7.72336565573114	NA
**TC0100000082.mm.2**	Kcnb2	potassium voltage gated channel, Shab-related subfamily, member 2	0.423183487401403	5.46424216004159	0.998026165510725	0.354327871939269	0.999692817443324	-4.59605983740355	6.31693982470444	6.18720582041541	5.29408699982475	5.2697501451415	5.41397767804359	4.30349249211986	NA
**TC1500000389.mm.2**	Ccn3	cellular communication network factor 3	-0.955047140470629	7.55537205990569	-0.998015046864122	0.354332870161111	0.999692817443324	-4.59606042571878	8.54046041184739	8.72372713655427	8.17328057689286	4.90569468815274	7.53087125774644	7.45819828824043	NA
**TC1300000442.mm.2**	Dsp	desmoplakin	-0.301826859656109	4.92342294512845	-0.997957314436843	0.354358823836122	0.999692817443324	-4.59606348043828	4.52805074189144	4.77074564739818	4.82945212008399	4.98959166100045	5.12455880679324	5.29813869360341	NA
**TC1100003868.mm.2**	1700023F06Rik	RIKEN cDNA 1700023F06 gene	-0.445098240780463	7.13004423223613	-0.997722157518279	0.354464554185646	0.999692817443324	-4.59607592209414	6.42541720476691	6.46878442617983	6.82513058974973	7.67280305070481	7.25320562094059	8.13492450107488	NA
**TC0200000966.mm.2**	Xirp2	xin actin-binding repeat containing 2	0.583677323947644	5.89183232380617	0.997694528690009	0.354476978186443	0.999692817443324	-4.59607738378551	5.17268197980872	5.48831151418671	5.66515495385748	7.26352259451097	5.46662976266679	6.29469313780637	NA
**TC0700004630.mm.2**	Hsd3b7	hydroxy-delta-5-steroid dehydrogenase, 3 beta- and steroid delta-isomerase 7	-0.299942542060843	5.83874749596998	-0.997630701858616	0.354505680844308	0.999692817443324	-4.59608076044279	5.88007281267451	6.11496529718701	5.53307844135272	5.68570244956561	5.9840393264684	5.83462664857162	NA
**TC1000002376.mm.2**	Lrrc75b	leucine rich repeat containing 75B	-0.315894784399341	6.3803434747839	-0.997606548321903	0.354516543065266	0.999692817443324	-4.59608203822041	5.97386464101177	6.1732964112267	6.20071965439272	6.5508354594404	6.81959030536874	6.56375437726307	NA

**TC0600000902.mm.2**	Eva1a	eva-1 homolog A (C. elegans)	-0.32554774105258	7.1209592891937	-0.997491284324572	0.35456838268396	0.999692817443324	-4.5960881357471	7.03941593159962	6.66334209811692	6.89227798518173	7.29367312648851	7.38113667489339	7.455909918882	NA
**TC1400002021.mm.2**	Zfp219	zinc finger protein 219	0.307008936619921	7.13819690757511	0.997339938089755	0.354636459232624	0.999692817443324	-4.59609614153164	7.02144716548381	7.04824941807461	7.17437484593083	7.5123765216352	7.13879209775682	6.93394139656937	NA
**TC0200003231.mm.2**	1700007K13Rik	RIKEN cDNA 1700007K13 gene	0.621573076978774	7.06605526313058	0.997253562393441	0.354675316207892	0.999692817443324	-4.59610071029937	7.34224878322638	7.2816494358153	7.89331680890705	6.61447294794262	7.38987367333666	5.87476992955546	NA
**TC1900001753.mm.2**	Sfxn4	sideroflexin 4	-0.504587280036685	6.27376184680489	-0.997035994959295	0.354773205950332	0.999692817443324	-4.59611221750144	6.84125389794173	6.59240590435821	6.04963602847399	5.55023233075404	6.99752120125502	5.61152171804638	NA
**TC0200004899.mm.2**	Zcchc3	zinc finger, CCHC domain containing 3	-0.298022148402687	7.34754776172054	-0.996880557602636	0.354843154629801	0.999692817443324	-4.59612043789045	6.61962015678763	6.56191736024809	7.72201956622815	7.43183281201293	7.88093612336824	7.86896055167822	NA
**TC1100001708.mm.2**	Fmnl1	formin-like 1	0.488803195394355	5.9356382725302	0.996682543155484	0.35493227922511	0.999692817443324	-4.59613090910316	6.50012245357677	6.60132487182278	6.10200200913887	5.64299234114631	6.05650909683549	4.71087886266097	NA
**TC0200002427.mm.2**	Snhg11	small nucleolar RNA host gene 11	-0.390149922434224	8.92269486177553	-0.996629002205186	0.35495638056912	0.999692817443324	-4.59613374023395	8.59189592421861	8.47392817189094	8.51917944213962	9.32584317269797	9.55993303608386	9.06538942362218	NA
**TC0X00003418.mm.2**	Rpgr	retinitis pigmentosa GTPase regulator	0.484808910664144	5.97519785041012	0.996570019042926	0.354982933203356	0.999692817443324	-4.59613685905291	7.23017635333135	7.18867201083897	6.22565934724283	4.97531893256653	5.43852540989193	4.79283504858915	NA
**TC0100000648.mm.2**	Cdk5r2	cyclin-dependent kinase 5, regulatory subunit 2 (p39)	-0.35554901420023	9.64612790389102	-0.996362589413114	0.355076324855189	0.999692817443324	-4.59614782648848	9.74586767033914	9.65961251822719	9.78472934632985	9.09536525685983	9.94833168425054	9.64286094733959	NA
**TC0700000870.mm.2**	Nomo1	nodal modulator 1	-0.315477942949717	7.33354133237891	-0.996111674543556	0.35518932081776	0.999692817443324	-4.59616109165856	8.20316667965355	8.30920911861463	6.93511580010107	6.49384235495186	7.1046246772003	6.95528936375206	NA
**TC1700002090.mm.2**	Olfr137	olfactory receptor 137	-0.304387132809707	4.77562393622344	-0.995823177152374	0.355319276482527	0.999692817443324	-4.59617634173182	4.32613077197311	4.19410544015375	4.70769051000189	5.0546760597953	5.16307941404263	5.20806142137397	NA
**TC1900001353.mm.2**	Ranbp6	RAN binding protein 6	-0.464645587476468	5.94402251210084	-0.995754632559807	0.355350158368885	0.999692817443324	-4.59617996471147	7.22366687623892	6.91572441127718	5.72419720977723	4.57352909529078	5.89625712692036	5.33076035310059	NA
**TC0500000591.mm.2**	Tbc1d1	TBC1 domain family, member 1	-0.335595918971842	5.94976894873234	-0.995604929287076	0.355417612594349	0.999692817443324	-4.59618787698246	5.83612295902	6.03206263407934	5.77000654542843	5.80961158524706	5.82558263836898	6.4252273302502	NA
**TC0500003028.mm.2**	Sgsm1	small G protein signaling modulator 1	0.378150347890627	7.53660808166854	0.995428486681644	0.35549712811752	0.999692817443324	-4.59619720177502	8.81895833345095	8.68373402764455	7.55542665026066	6.68120176208786	6.68600186274393	6.79432585382333	NA
**TC0600002127.mm.2**	Braf	Braf transforming gene	0.725339165981712	7.79583697852738	0.995413797876943	0.355503748396347	0.999692817443324	-4.59619797802599	9.22076346503132	9.06666687579917	7.98718672382075	6.98194820732785	7.8470068007484	5.67144979843678	NA
**TC0200003774.mm.2**	Evx2	even-skipped homeobox 2	0.419253997378599	6.54871145939792	0.995235370590841	0.355584173732891	0.999692817443324	-4.59620740683292	6.09305249402761	5.89357570910792	6.65112654079525	7.42094773320935	6.23331627201282	7.00025000723459	NA
**TC0700002842.mm.2**	Cebpg	CCAAT/enhancer binding protein (C/EBP), gamma	0.37419651508297	7.00331371284493	0.995053592304512	0.355666124225143	0.999692817443324	-4.59621701188515	7.43941620308431	7.20294329232915	7.37490975850626	6.68804814740475	6.8967348895096	6.41782998623547	NA
**TC1100000930.mm.2**	Ybx2	Y box protein 2	-0.303223415222439	5.06126484183598	-0.994817198792245	0.355772718919841	0.999692817443324	-4.59622950151115	4.86715464059689	4.56420805422923	5.00989554246942	5.15499422040303	5.45684612878723	5.3144904645301	NA
**TC0700001718.mm.2**	Lyrm1	LYR motif containing 1	-0.370885343126747	7.24302331143298	-0.994768111416682	0.355794856574485	0.999692817443324	-4.59623209481704	7.40689944780337	7.21052310201077	7.42091072342065	6.62856259284447	7.27350711075699	7.51773689176163	NA
**TC0700003617.mm.2**	3110040N11Rik	RIKEN cDNA 3110040N11 gene	-0.495105589644085	6.92002477911566	-0.994480222089909	0.355924712044652	0.999692817443324	-4.59624730288798	7.01571922703416	6.80320887758757	7.26319117100919	6.09231352438285	7.59413419727608	6.75158167740413	NA
**TC0500003592.mm.2**	Zkscan14	zinc finger with KRAB and SCAN domains 14	0.395345268310616	6.37360016283191	0.994387810349624	0.355966403214715	0.999692817443324	-4.59625218419116	6.08686632976482	5.86841990512137	6.58543352510653	6.95306911425672	5.92997576579439	6.81783633694763	NA
**TC0300003234.mm.2**	Pear1	platelet endothelial aggregation receptor 1	0.409231332063908	5.50214603712959	0.994252760038333	0.356027337498908	0.999692817443324	-4.59625931732425	4.8474983584126	5.06687862708673	5.46242667126118	6.49605427944183	5.58192946214147	5.55808882443372	NA
**TC1000002506.mm.2**	Abhd17a	abhydrolase domain containing 17A	-0.306881979692846	7.83857112617124	-0.993964133229578	0.356157592498638	0.999692817443324	-4.59627456054978	7.97615083583661	7.88490436239079	7.44189189143959	7.8364119082676	7.93840039252303	7.95366736656984	NA
**TC0600001509.mm.2**	Nrip2	nuclear receptor interacting protein 2	0.309751303309489	6.96052160138703	0.993666448034766	0.356291974691376	0.999692817443324	-4.59629027994293	7.44321930285725	7.06584814678853	6.89715769146263	7.03962469118505	6.57914016329862	6.73813961273009	NA
**TC0100002644.mm.2**	Wdfy1	WD repeat and FYVE domain containing 1	0.481892347210469	6.75587722619876	0.993605590760407	0.356319452018431	0.999692817443324	-4.59629349325794	6.2984047618584	6.32398719422846	6.84594959471334	7.59237845305	6.14447355424243	7.33006979909997	NA
**TC1000000762.mm.2**	Tspear	thrombospondin type laminin G domain and EAR repeats	0.424956923496933	6.29992798234467	0.993417985646397	0.356404167026648	0.999692817443324	-4.59630339836823	5.60905367404673	5.39655990979234	6.36069487074954	7.46123920786185	6.39199654177502	6.58002368984251	NA
**TC0400002101.mm.2**	C1qtnf12	C1q and tumor necrosis factor related 12	0.427687893455642	5.96144680501393	0.993288028455008	0.35646285979943	0.999692817443324	-4.59631025927485	5.66344686115443	6.17057056873587	5.68173889716165	6.71328069639062	5.78868359302584	5.75096021361515	NA
**TC0600000664.mm.2**	Aqp1	aquaporin 1	-0.454392105187196	6.38199074965452	-0.992217495615645	0.356946635070416	0.999692817443324	-4.59636676007056	5.78043018922096	5.95534538466544	5.85910364396219	6.96458871287097	6.55686153022363	7.17561503698393	NA
**TC0800002503.mm.2**	Tmem184c	transmembrane protein 184C	-0.899285300783253	8.58964693715483	-0.992169261925497	0.356968444070329	0.999692817443324	-4.5963693050654	9.69022755829493	9.76841754395412	9.00687467459159	6.13345828496513	9.02658530233652	7.91231825878671	NA
**TC1500001713.mm.2**	Jrk	jerky	0.314751398150077	5.55885630252028	0.99203226189319	0.35703039472819	0.999692817443324	-4.59637653338758	5.70661097043243	5.5687654140354	5.88795072330455	5.46568139017244	5.26772067868418	5.45640863849266	NA
**TC0200001222.mm.2**	Ube2l6	ubiquitin-conjugating enzyme E2L 6	-0.323288544182704	5.27941624010715	-0.992016851266183	0.35703736385657	0.999692817443324	-4.59637734644463	5.33196216904975	5.59406956296481	5.26810818473334	4.78383612539812	5.36446787073475	5.33405352776211	NA
**TC1700000055.mm.2**	Tmem181a	transmembrane protein 181A	0.642459762825141	7.90880609067268	0.991978596124545	0.357054664392436	0.999692817443324	-4.59637936474059	8.63727039561924	7.55577793187764	8.44979155760419	7.82256231349056	8.32587755774632	6.66155678769814	NA
**TC0500000177.mm.2**	Psmc2	proteasome (prosome, macropain) 26S subunit, ATPase 2	-0.537510052118396	9.03490661904891	-0.991782855245622	0.357143196700441	0.999692817443324	-4.59638969120887	9.52625895826508	9.65741451723736	9.23944061203293	7.73593245524419	9.35616998089155	8.69422319062237	NA
**TC1300001946.mm.2**	Ptpdc1	protein tyrosine phosphatase domain containing 1	0.352736756816636	7.54594335129258	0.991471877938906	0.357283885109086	0.999692817443324	-4.59640609504194	7.38711690354401	7.39283150016638	7.68274156391007	7.91785104492911	7.80491175539427	7.09020733981164	NA
**TC0900001626.mm.2**	Klhl40	kelch-like 40	-0.351414900332832	5.36846940729949	-0.991382257906834	0.357324437938594	0.999692817443324	-4.59641082197344	5.36234036899093	4.8297551584724	5.07025738440942	5.58768817342456	5.70705219668839	5.65372316181125	NA
**TC1500000041.mm.2**	Rictor	RPTOR independent companion of MTOR, complex 2	-0.416470587752726	7.11510968395316	-0.991348096316942	0.357339896919139	0.999692817443324	-4.5964126237427	7.88014030052337	8.00621773777188	7.02478674876786	5.96089269619128	7.03212042112853	6.78650019933605	NA
**TC0X00002739.mm.2**	Cited1	Cbp/p300-interacting transactivator with Glu/Asp-rich carboxy-terminal domain 1	-0.547421399916547	4.97603024298808	-0.991186808757389	0.357412890692962	0.999692817443324	-4.59642113005363	5.44539774668486	4.78434553939366	5.12778750030113	4.1380101857073	4.57936718456198	5.78127330127954	NA
**TC1100004275.mm.2**	Msl1	male specific lethal 1	-0.610808321216294	8.4034299268787	-0.990868623507571	0.357556925823383	0.999692817443324	-4.5964379091892	8.56783126143499	8.74769194023952	8.91442614534844	7.02729371323412	8.82242326648001	8.34091323453514	NA
**TC1000001024.mm.2**	Rfx4	regulatory factor X, 4 (influences HLA class II expression)	0.396387721811062	6.69892166053054	0.990686179893729	0.357639534380641	0.999692817443324	-4.59644752896798	6.91799986175499	6.82931896669707	6.32896065109133	7.29053263808532	6.28615611559127	6.54056172996327	NA
**TC1400000784.mm.2**	Psme1	proteasome (prosome, macropain) activator subunit 1 (PA28 alpha)	-0.503217260082602	8.9784679571472	-0.990650276432126	0.357655792854776	0.999692817443324	-4.59644942196339	9.89340798064442	9.91527393733551	8.95722943167178	7.57061622069725	9.05595641713357	8.47832375540066	NA
**TC1300001777.mm.2**	Serpinb6a	serine (or cysteine) peptidase inhibitor, clade B, member 6a	0.50133605688286	6.4002643697406	0.990650122494441	0.357655862564954	0.999692817443324	-4.59644943007962	6.49390013575814	6.49504655114206	7.17293625664887	6.03471956600568	6.58636432828376	5.61861938060508	NA
**TC1700001378.mm.2**	Serac1	serine active site containing 1	0.342928483476039	6.42790470645373	0.990539172530222	0.35770610866563	0.999692817443324	-4.59645527966087	6.50432070873039	6.51873180763353	6.86853567017878	6.24658067447648	6.36686455739386	6.06239482030933	NA
**TC0700002424.mm.2**	Ccdc9	coiled-coil domain containing 9	-0.295976456282292	5.91872597932988	-0.990515712167509	0.357716733909447	0.999692817443324	-4.59645651651378	6.05568082731992	5.98232624955479	5.77628180069406	5.66491614257593	6.1477138028752	5.88543705295937	NA
**TC0400001235.mm.2**	Atpaf1	ATP synthase mitochondrial F1 complex assembly factor 1	0.517271370104739	7.65808554244643	0.990511463649586	0.357718658097936	0.999692817443324	-4.59645674049822	7.84608861940255	7.61017599214097	8.21842040545922	7.54497528621305	8.06023368681577	6.66861926464703	NA
**TC0100003378.mm.2**	Fasl	Fas ligand (TNF superfamily, member 6)	-0.341465460103407	4.7791594500578	-0.990437302813252	0.357752247454433	0.999692817443324	-4.59646065022744	3.97813583144765	4.13798892026767	4.68998027197606	5.24797024223628	5.13014856688238	5.49073286753677	NA
**TC1900001342.mm.2**	Plgrkt	plasminogen receptor, C-terminal lysine transmembrane protein	-0.324063675315089	6.10094995779983	-0.989880559660716	0.358004489788161	0.999692817443324	-4.59648999696848	6.52623035700549	6.68228311753997	5.93441735440124	5.44011210641042	6.1208078006697	5.90184901077214	NA
**TC0X00000656.mm.2**	Vma21	VMA21 vacuolar H+-ATPase homolog (S. cerevisiae)	-0.638122255051366	6.34842990735192	-0.989743704386462	0.358066515839627	0.999692817443324	-4.59649720958343	7.57304525765088	7.18188490072279	6.3080601614347	4.72164222638286	6.78833584266056	5.51761105525973	NA
**TC0500002940.mm.2**	Tmed5	transmembrane p24 trafficking protein 5	0.332232488027135	6.61724090570168	0.989443627282199	0.358202547380576	0.999692817443324	-4.59651302270258	6.77143133128744	6.73765356982587	6.84660054788708	6.58281220668844	6.66423471999575	6.1007130585255	NA
**TC1500002092.mm.2**	Gxylt1	glucoside xylosyltransferase 1	-0.397058576850983	7.16063122079459	-0.989302062253834	0.358266735979796	0.999692817443324	-4.59652048192591	7.13723463253318	6.91031196738934	7.50120678321648	6.55985500235503	7.56206103456791	7.29311790470557	NA
**TC1300001155.mm.2**	Cd180	CD180 antigen	-0.954219314947346	6.69224103814591	-0.989214568770128	0.358306411897071	0.999692817443324	-4.59652509179849	7.44748428215642	8.57247555759019	6.92718719179559	4.18533668782148	7.2388964730363	5.78206603647546	NA
**TC1100001220.mm.2**	Ccl7	chemokine (C-C motif) ligand 7	0.511731292539833	6.39518101723694	0.989177739841724	0.358323113845264	0.999692817443324	-4.59652703218855	5.61337184232771	6.36101965248162	6.19983406458626	7.51024453225973	6.18194412397207	6.50467188779425	NA
**TC0300001765.mm.2**	Tmem212	transmembrane protein 212	0.320154513479011	6.30354910979852	0.988937308372634	0.358432164702919	0.999692817443324	-4.59653969883635	5.8474513546675	5.90355467794472	6.45162132942569	6.90367749714278	6.49771192441043	6.21727787520002	NA
**TC0X00000897.mm.2**	Apoo/Apoo-ps	apolipoprotein O/apolipoprotein O, pseudogene	-1.05228769212408	8.53802186524003	-0.988650339447909	0.358562357284285	0.999692817443324	-4.59655481525713	9.46506201255321	9.45257446333642	9.21733258180067	5.88562708385053	9.37968466858326	7.8278503813161	NA
**TC1100001054.mm.2**	Smg6	Smg-6 homolog, nonsense mediated mRNA decay factor (C. elegans)	0.303434559306884	6.68221836333273	0.98851966374732	0.358621654751174	0.999692817443324	-4.5965616980443	7.08625769402133	7.18841633636488	6.69199479262813	6.52075784148385	6.13688769489069	6.46899582060751	NA
**TC1700002772.mm.2**	Tbc1d22b	TBC1 domain family, member 22B	-0.529875719771577	7.89244012851964	-0.988413693597737	0.358669747079713	0.999692817443324	-4.59656727924664	8.45385914151608	8.23971985244339	8.20106574377914	6.59958942502847	7.85298336060548	8.00742324774529	NA
**TC0300002273.mm.2**	Dclk2	doublecortin-like kinase 2	0.496219193402515	7.43040973699855	0.988341583254462	0.358702475734978	0.999692817443324	-4.59657107696498	8.34332323138391	8.04817438225641	7.75524896154231	6.83645063603568	7.35841589435163	6.24084531642133	NA
**TC1100002484.mm.2**	Fgf18	fibroblast growth factor 18	0.563176682903501	6.66044416103851	0.98827449001834	0.358732929381829	0.999692817443324	-4.59657461033442	6.01675473968258	5.71702972036824	6.61954636369407	8.05807057229954	6.2868543495451	7.26440922064152	NA
**TC0X00002710.mm.2**	Slc7a3	solute carrier family 7 (cationic amino acid transporter, y+ system), member 3	-0.909604183220023	5.97613990381894	-0.988208786174667	0.358762754346098	0.999692817443324	-4.59657807042037	7.61676393234104	7.02612276537665	6.38809275394099	3.30927942543697	5.71654889417371	5.8000316516443	NA
**TC0200001221.mm.2**	Ypel4	yippee like 4	-0.361587261542868	7.72024033226785	-0.987971981320083	0.358870263359114	0.999692817443324	-4.59659054007306	8.07133652717783	7.90707416211916	7.80211978682001	7.00780860379218	7.7941944206522	7.73890849304573	NA
**TC1700002770.mm.2**	Mrps34	mitochondrial ribosomal protein S34	-0.320790550040893	7.55985158441715	-0.987878863186383	0.358912545750994	0.999692817443324	-4.59659544308238	7.46486321884818	7.48189846437204	7.17617794739106	7.70920541420938	7.71348293698049	7.81348152470174	NA
**TC1100002725.mm.2**	Hspa4	heat shock protein 4	-1.25798750999502	8.23554533385774	-0.987857306718108	0.358922334508565	0.999692817443324	-4.59659657807697	9.79185753965819	9.67036964162425	8.96837746964396	4.74915743129302	8.8200764899892	7.41343343093784	NA
**TC0400002530.mm.2**	Tln1	talin 1	0.311695372982313	6.72696843381118	0.987617256918222	0.359031354868121	0.999692817443324	-4.59660921640203	7.46449611273368	7.90682729337762	6.45760367245083	6.34933529890936	6.03724226660793	6.14630595878764	NA
**TC0700003485.mm.2**	Mctp2	multiple C2 domains, transmembrane 2	0.401798013793238	5.91953636041541	0.987472291744106	0.359097204414665	0.999692817443324	-4.59661684791446	4.94768560051734	4.99758911510637	6.28338336747844	6.90438636974916	5.77994135240967	6.60423235723145	NA
**TC0300002532.mm.2**	Gja8	gap junction protein, alpha 8	0.390529611307499	5.47035579799311	0.98710553965747	0.359263841550129	0.999692817443324	-4.59663615268658	4.85578983714403	4.70672764225557	5.60683167472976	6.41350659085726	5.35583094127821	5.88344810169381	NA
**TC0200003354.mm.2**	Phf19	PHD finger protein 19	0.3045976784927	4.94223799273565	0.987100341978366	0.359266203597854	0.999692817443324	-4.59663642625229	4.88764609783401	5.01259861016765	5.15574940871119	5.02543989398762	4.60552323126075	4.96647071445266	NA
**TC0500003173.mm.2**	Cfap73	cilia and flagella associated protein 73	0.356506311587967	5.32961928493109	0.987099859239887	0.359266422975486	0.999692817443324	-4.59663645165988	5.2201406178448	5.20928177718383	5.58625085792941	5.54440211093753	4.81703431416141	5.60060603152959	NA
**TC0100000898.mm.2**	Asb1	ankyrin repeat and SOCS box-containing 1	-0.306821801985652	6.44588555785393	-0.986677157994024	0.359458557305401	0.999692817443324	-4.59665869703331	6.59509567907312	6.6279521995784	6.11075692263043	6.30855400961996	6.70468569488485	6.32826884133684	NA
**TC0700002697.mm.2**	Med29	mediator complex subunit 29	0.444433870276438	7.42965980329669	0.9864750290198	0.359550461296162	0.999692817443324	-4.59666933277171	7.23515855615296	6.84962430034236	8.43710563960966	7.25391621230918	7.40764400818234	7.39451010318362	NA
**TC0700004610.mm.2**	Ccnd1/Mir3962	cyclin D1/microRNA 3962	-0.368097775449902	7.87439568667345	-0.98625357335165	0.359651173862775	0.999692817443324	-4.59668098423179	7.68771543475268	8.30161008148215	7.8341410060399	7.42628552041314	7.78233681370408	8.21428526364876	NA
**TC0200003265.mm.2**	Kyat1	kynurenine aminotransferase 1	-0.396201120450336	6.64046723326614	-0.986117789375165	0.359712935980221	0.999692817443324	-4.59668812761026	7.60636217122908	7.11184397259145	6.51961446266033	5.64648304477747	6.43604680509392	6.52245294324455	NA
**TC1600000307.mm.2**	Senp2	SUMO/sentrin specific peptidase 2	-0.58163889100303	7.06192661030523	-0.986092949352613	0.359724235504594	0.999692817443324	-4.59668943435224	7.70547437161239	7.71539106549459	7.30475899592766	5.58894922543152	7.37082211495904	6.6861638884062	NA
**TC0900002368.mm.2**	Ppcdc	phosphopantothenoylcysteine decarboxylase	0.335235124534413	7.46144019581203	0.986023333397545	0.359755904717087	0.999692817443324	-4.59669309650522	7.70275182401796	7.794902473073	7.31023252161395	7.66049604181107	7.42109744989347	6.87916086446273	NA
**TC0X00003287.mm.2**	Nhs	NHS actin remodeling regulator	0.401732123116795	5.61794591053559	0.985939951854806	0.359793838948221	0.999692817443324	-4.59669748263178	6.32464368804911	6.07642793760672	6.02806505766488	5.02696898423075	5.07651544480415	5.1750543508579	NA
**TC0400001670.mm.2**	Hmgcl	3-hydroxy-3-methylglutaryl-Coenzyme A lyase	-0.530689831418631	6.84450827158502	-0.985733078362451	0.359887969061407	0.999692817443324	-4.59670836403322	6.93164141665822	6.88815775484803	7.3602249017773	5.73271049580601	7.13900157008811	7.01531349033247	NA
**TC0400003747.mm.2**	Maco1	macoilin 1	-0.408242759335887	7.00505520301278	-0.985660172855892	0.359921146601577	0.999692817443324	-4.59671219854601	8.00205705380825	7.97236564740719	6.82108209157907	5.79862940751565	6.56137632361059	6.8748206941559	NA
**TC1000002408.mm.2**	Pcbp3	poly(rC) binding protein 3	0.347054683012951	7.691269393108	0.985453569687795	0.360015179729252	0.999692817443324	-4.59672306422131	8.35728563960663	8.0028390359758	7.9333939746491	7.30740654989663	7.21029394168449	7.33639721683534	NA
**TC0900002766.mm.2**	Lca5	Leber congenital amaurosis 5 (human)	-0.406072399752257	7.85564068894044	-0.984881181385834	0.360275796278207	0.999692817443324	-4.59675316144969	8.82514536868586	8.3037685618827	7.75687636800185	6.83951633378294	7.75721428234514	7.65132321894416	NA
**TC1100001802.mm.2**	Helz	helicase with zinc finger domain	0.373412043277678	6.89933265304271	0.984372808337952	0.360507389494381	0.999692817443324	-4.59677988545799	7.05214236414412	7.12382782728018	6.63692517271744	7.34649973397621	6.35357732530854	6.88302349482976	NA
**TC0600002965.mm.2**	Plxnd1	plexin D1	-0.355830024705825	6.84776171593586	-0.984220107570976	0.360576976226547	0.999692817443324	-4.59678791126749	6.87597306009533	7.14968762957275	6.32780363274769	6.84682114552003	6.68094470964637	7.205340118033	NA
**TC0100000131.mm.2**	Efhc1	EF-hand domain (C-terminal) containing 1	0.314221641646439	6.75674953200422	0.984120373017875	0.360622431581061	0.999692817443324	-4.59679315289278	6.33180360061875	6.22116040463457	6.92310549126445	7.38488274376797	6.76245438921029	6.91709056252925	NA
**TC0800000564.mm.2**	Dctd	dCMP deaminase	-0.519722898905545	7.17921423058915	-0.983425365557701	0.360939314924249	0.999692817443324	-4.59682967230307	8.22517478625976	8.09915506154921	7.17881664549034	5.67693822346708	6.69324242904761	7.2019582377209	NA
**TC0Y00000223.mm.2**	Erdr1	erythroid differentiation regulator 1	-0.416102870791466	10.3680710644354	-0.983094727812305	0.361090143068354	0.999692817443324	-4.59684704133306	9.58754672934656	10.5556252590045	10.0573021170907	10.5592222112485	10.8396843171146	10.6090457528075	NA
**TC0300002636.mm.2**	Magi3	membrane associated guanylate kinase, WW and PDZ domain containing 3	0.40941508493442	5.74314168573106	0.982768523533669	0.361238997053211	0.999692817443324	-4.59686417465244	6.94056002984653	6.55796505204159	5.603100321942	5.28647727924156	5.51591419558672	4.554833235728	NA
**TC0600002421.mm.2**	4930597O21Rik	RIKEN cDNA 4930597O21 gene	-0.372788304049877	5.35095243616108	-0.982592356640827	0.361319405711111	0.999692817443324	-4.59687342635356	5.59412750515196	5.09936638074406	4.85220642710176	5.48111563438359	5.34635235724353	5.73254631234158	NA
**TC0100001207.mm.2**	Etnk2	ethanolamine kinase 2	0.499650272372575	5.69607338162682	0.982559860389102	0.361334239651171	0.999692817443324	-4.59687513285987	6.16646273733971	5.4146913919018	6.44933199841983	5.34796135421245	5.05047205749476	5.74752075039237	NA
**TC1900001509.mm.2**	Slit1	slit guidance ligand 1	0.296136429199122	7.52804867197351	0.98230791449947	0.361449264453901	0.999692817443324	-4.59688836258999	8.32144174722508	8.04184485399841	7.40163938794797	7.29699975655994	6.9861351580192	7.12023112809047	NA
**TC0700004622.mm.2**	Tm2d3	TM2 domain containing 3	-0.331982845558066	7.6393059686952	-0.98194790011138	0.361613677103608	0.999692817443324	-4.59690726412186	8.68274001184063	8.25181345649351	7.26451054638288	6.85414777997757	7.50771398506116	7.27491003241543	NA
**TC1900000953.mm.2**	Klc2	kinesin light chain 2	0.357629742558391	8.63077417919245	0.981614355796489	0.361766053435682	0.999692817443324	-4.59692477287348	9.19292995140719	9.09903961774843	8.69478200571361	8.40918548984431	8.56684034906757	7.82186766137357	NA
**TC1200001043.mm.2**	Serpina3j	serine (or cysteine) peptidase inhibitor, clade A (alpha-1 antiproteinase, antitrypsin), member 3J	-0.289045356440018	5.81196915567879	-0.981548486519787	0.361796151074583	0.999692817443324	-4.59692823020494	5.45597946977453	5.68673181897085	5.78539354799923	5.79011291822445	6.0688678632842	6.08472931581951	NA
**TC0700002996.mm.2**	Ccdc155	coiled-coil domain containing 155	-0.388403552682854	5.70882121412506	-0.981444103873544	0.361843850626817	0.999692817443324	-4.5969337087832	5.04343347333313	5.10579176270708	5.38302487399357	6.28042259767864	6.0308572554897	6.40939732154822	NA
**TC0500003047.mm.2**	Sart3	squamous cell carcinoma antigen recognized by T cells 3	0.323815926293419	5.93864288612203	0.981258320548677	0.361928759851791	0.999692817443324	-4.59694345900868	6.48476882353577	6.26462447583222	5.61733787724092	6.14771005773458	5.51111197969215	5.60630410269651	NA

**TC0200002530.mm.2**	Dnttip1	deoxynucleotidyltransferase, terminal, interacting protein 1	-0.328681942491631	7.44097237724536	-0.981252920861552	0.361931227922994	0.999692817443324	-4.59694374237986	7.94170242150031	7.78077515890535	7.35840058890285	6.77459581013876	7.45543075404147	7.3349295299834	NA
**TC0900001720.mm.2**	Birc2	baculoviral IAP repeat-containing 2	0.622734330783686	6.04509171112099	0.981221638334772	0.361945526695904	0.999692817443324	-4.59694538404622	6.79636275830611	6.65885632709869	6.09425547634421	5.93614444510004	6.40175417240564	4.38317708747124	NA
**TC0700004589.mm.2**	Cars	cysteinyl-tRNA synthetase	-0.379944186511717	6.56389807220631	-0.981094879522258	0.362003470759461	0.999692817443324	-4.59695203591992	7.13314518117386	7.12239315939552	6.22544202060302	5.95853883921951	6.91795502136996	6.02591421147601	NA
**TC1100004151.mm.2**	Dnah17	dynein, axonemal, heavy chain 17	-0.488796195827208	4.74542098619814	-0.980898899248881	0.362093071634329	0.999692817443324	-4.59696231946761	4.18148107229775	4.17631039329998	4.15951345408873	5.40905757587962	4.89541519655803	5.65074822506474	NA
**TC0300002122.mm.2**	Ift80	intraflagellar transport 80	0.36441376436467	6.2862389825899	0.980697799366279	0.362185031159208	0.999692817443324	-4.59697287059982	6.55881427525235	6.31181534532878	6.14779829927794	6.64001760256585	5.70903619707277	6.34995217604168	NA
**TC0600001463.mm.2**	Ano2	anoctamin 2	0.454566189791034	6.97878924381164	0.980677507942452	0.362194311091672	0.999692817443324	-4.59697393517316	7.56838762413253	7.81300932374183	7.49071293146291	6.20952251582588	6.38795906770564	6.40314400000108	NA
**TC1600001081.mm.2**	B3galt5	UDP-Gal:betaGlcNAc beta 1,3-galactosyltransferase, polypeptide 5	0.475757155237015	6.53065598180823	0.980598493565224	0.362230448722053	0.999692817443324	-4.59697808049574	7.22040990642254	6.95132642170482	7.03648796228391	5.94536897431411	6.43430907569441	5.59603355042958	NA
**TC1700001689.mm.2**	Nme4	NME/NM23 nucleoside diphosphate kinase 4	-0.308318635386433	6.65200922944876	-0.980517093145815	0.36226768056449	0.999692817443324	-4.59698235082461	5.68338193406305	5.72257907173063	6.86458759177114	7.08014095829188	7.07950718509763	7.48185863573825	NA
**TC0100000127.mm.2**	Il17a	interleukin 17A	0.410545493319275	6.72251277316296	0.980453977687701	0.362296551081074	0.999692817443324	-4.59698566178985	6.37362172420721	6.24967665778746	6.68834186131467	7.57809276049617	6.41193125382916	7.03341238134313	NA
**TC0100003304.mm.2**	Xpr1	xenotropic and polytropic retrovirus receptor 1	-1.17930432247185	7.75344589023594	-0.980295635768472	0.36236898835797	0.999692817443324	-4.59699396776394	9.31287576034003	9.327390560318	8.44254825551697	4.31835193238999	7.92799174982987	7.19151708302078	NA
**TC1400000120.mm.2**	Nek10	NIMA (never in mitosis gene a)- related kinase 10	-0.316226502001738	5.53548274242847	-0.980260678863596	0.362384981748759	0.999692817443324	-4.59699580137195	5.92619822661903	5.52947213387618	5.14319175768233	5.41919478735373	5.70120620133734	5.49363334770218	NA
**TC1000000133.mm.2**	Perp	PERP, TP53 apoptosis effector	-0.341654449749159	6.67608378290068	-0.980254673038345	0.362387729573525	0.999692817443324	-4.59699611639457	6.44928251767203	6.70724922151753	6.23055374041332	6.87777728894477	6.95733120053122	6.83430872832519	NA
**TC1500000932.mm.2**	Irak4	interleukin-1 receptor-associated kinase 4	0.303614106173785	5.00095517013108	0.980058686033312	0.362477407764801	0.999692817443324	-4.59700639594733	5.01367518870403	5.38561707700305	5.03095772247989	5.07587576123358	4.66012306771836	4.83948220364755	NA
**TC0700004617.mm.2**	Coq8b	coenzyme Q8B	0.313212513916097	7.25115424206626	0.979921802639591	0.362540052062932	0.999692817443324	-4.59701357490304	7.50568009303744	7.71063760205391	7.2317914475126	7.2267249450566	6.68684127231186	7.14525009242514	NA
**TC0X00002269.mm.2**	Arhgef6	Rac/Cdc42 guanine nucleotide exchange factor (GEF) 6	0.524381327522254	6.00160996613916	0.979887332423752	0.362555828591389	0.999692817443324	-4.59701538264214	6.60249845846081	6.60563143765559	6.49191490810653	5.43323136977499	6.0506924372962	4.82569118554081	NA
**TC0700004519.mm.2**	Sigirr	single immunoglobulin and toll-interleukin 1 receptor (TIR) domain	-0.340048886449384	5.67439232427463	-0.979734912777401	0.362625595322022	0.999692817443324	-4.59702337568662	5.58540274650106	5.17751250340125	5.92295914093222	5.37871132049112	5.91183936409609	6.06992887022603	NA
**TC0700003970.mm.2**	Olfr684	olfactory receptor 684	-0.370986721707978	4.5418742479824	-0.979649578815559	0.362664659634744	0.999692817443324	-4.59702785041978	4.09884866520306	3.77260995218707	4.25059171294991	5.06831500059424	4.98702519648966	5.07385496047044	NA
**TC1000000809.mm.2**	Tpgs1	tubulin polyglutamylase complex subunit 1	0.283065252608736	7.6710537247795	0.979604934259378	0.362685098396888	0.999692817443324	-4.59703019140947	7.93799306127342	7.99187495281284	7.62973975704788	7.70155266285621	7.35081459995933	7.4143473147273	NA
**TC1000003228.mm.2**	Bloc1s1	biogenesis of lysosomal organelles complex-1, subunit 1	-0.535439678764638	9.15655220423253	-0.979459102689476	0.362751867934575	0.999692817443324	-4.59703783789134	9.79800917055362	9.57666164196339	9.37942332126048	7.86745820641398	9.49308072446065	8.82468016074309	NA
**TC1600001156.mm.2**	AU021092	expressed sequence AU021092	0.314608159398245	6.21052504807105	0.979417274583809	0.362771020861322	0.999692817443324	-4.59704003098781	5.85381912316417	5.733936254111	6.3653000133281	6.7870056016457	6.39669867401602	6.1263906221613	NA
**TC1900000021.mm.2**	Aldh3b2	aldehyde dehydrogenase 3 family, member B2	0.360704215135025	6.10356853043966	0.979330240260352	0.362810876068036	0.999692817443324	-4.59704459415062	5.70842998345051	5.48441119510909	6.72132699499596	6.35366222217825	5.82408943977959	6.52949134712457	NA
**TC0900001775.mm.2**	Fut4	fucosyltransferase 4	-0.654280056915717	4.76311528154983	-0.979317430191494	0.362816742409291	0.999692817443324	-4.59704526575871	4.19726953381886	3.89139600448944	4.27363731798055	5.31709570059907	4.51468802081633	6.38460511159472	NA
**TC1700002320.mm.2**	Ticam1	toll-like receptor adaptor molecule 1	0.335753082547038	5.67413982297678	0.979192589645135	0.362873916722089	0.999692817443324	-4.59705181068888	5.79607693300007	6.26215433040693	5.6414808183834	5.68757610139047	5.5690847903523	5.0884659643275	NA
**TC0500002343.mm.2**	Qdpr	quinoid dihydropteridine reductase	-0.335478947887403	9.0421981785947	-0.9791693134108	0.362884577519329	0.999692817443324	-4.59705303093047	9.70001344783048	9.29505101176926	8.63174063085775	8.66184272723907	9.24828293416418	8.71625831970744	NA
**TC1300000385.mm.2**	Bphl	biphenyl hydrolase-like (serine hydrolase, breast epithelial mucin-associated antigen)	0.421650178690865	6.01151655076726	0.979037097208701	0.362945138777039	0.999692817443324	-4.59705996200739	6.44908578821674	6.43207572755737	6.57607615375923	5.43954291934636	5.49700722504947	5.67531149067439	NA
**TC1700001939.mm.2**	Sapcd1	suppressor APC domain containing 1	-0.341802088954342	6.44153961174564	-0.978885769496112	0.363014463683733	0.999692817443324	-4.59706789438502	5.45888387469586	5.422884554539	6.41572760120454	7.12620493046062	7.10513878449538	7.12039792507846	NA
**TC1500002039.mm.2**	Mapk11	mitogen-activated protein kinase 11	0.357916814724792	7.19804255565283	0.978827801494615	0.363041022205258	0.999692817443324	-4.59707093282217	7.04784029893175	7.20644305205533	7.02457871609224	7.80032409009749	6.96661334884634	7.1424558278938	NA
**TC0200003362.mm.2**	Stom	stomatin	0.430371371820805	5.20921955575604	0.978537056913675	0.363174252114436	0.999692817443324	-4.59708617107754	5.73911881370429	5.8909092196584	5.57226689211991	4.67074913028767	4.30699460912431	5.07527866964167	NA
**TC1000001822.mm.2**	Pex7	peroxisomal biogenesis factor 7	0.40604891223598	5.94288445821258	0.978508253236831	0.363187453099079	0.999692817443324	-4.59708768058904	6.78889763704692	6.72556733988409	5.82296956635868	5.65450023204954	5.86195894498181	4.80341302895445	NA
**TC0Y00000561.mm.2**	Gm21693/Gm21677/Gm21704	predicted gene, 21693/predicted gene, 21677/predicted gene, 21704	-0.367661922088882	4.60990333566753	-0.978265357239313	0.363298789407039	0.999692817443324	-4.59710040914042	3.89495237256706	4.0632727299025	4.4569385439199	5.02599698975903	5.42642360320285	4.79183577465385	NA
**TC0700001400.mm.2**	Lipt2	lipoyl(octanoyl) transferase 2 (putative)	-0.358980227370512	6.61489606234253	-0.978232457413717	0.363313871755918	0.999692817443324	-4.59710213307936	6.36616947176432	6.51772862587091	6.84838366985632	6.19537524098314	7.11580971101966	6.64590965456082	NA
**TC1000000469.mm.2**	Nus1	NUS1 dehydrodolichyl diphosphate synthase subunit	-0.711366757202068	8.50582941758676	-0.978164317129376	0.363345111028759	0.999692817443324	-4.59710570351417	9.29142976572062	9.22601276615654	8.89330986914144	6.65409036047819	8.90993637250734	8.06019737151643	NA
**TC0100001302.mm.2**	Zbtb41	zinc finger and BTB domain containing 41	-0.556359789668988	7.25909022772224	-0.978140212652631	0.36335616235478	0.999692817443324	-4.59710696651817	7.86033109788852	7.79612059900503	7.55619226434166	5.8364927807093	7.43825124424442	7.06715338014452	NA
**TC1700001975.mm.2**	Gtf2h4	general transcription factor II H, polypeptide 4	-0.420990543703772	6.03668646337404	-0.978112586783455	0.363368828477324	0.999692817443324	-4.59710841401384	6.02285144204089	6.04085981772752	6.39785155830298	5.25936165823116	6.17158195607232	6.32761234786937	NA
**TC1200000895.mm.2**	Adck1	aarF domain containing kinase 1	0.366578215910462	6.86598604384419	0.978062296835	0.363391886686302	0.999692817443324	-4.5971110489733	7.10809497040147	6.89065192439727	7.3233308937178	6.64183200632585	6.37455183262155	6.85745463560117	NA
**TC0300003100.mm.2**	Spata1	spermatogenesis associated 1	-0.411826856260017	5.15002833615116	-0.977986525980659	0.363426630180582	0.999692817443324	-4.59711501888689	5.35747211172755	5.84313932860023	5.16705165373238	4.2709007782972	5.34285369247237	4.91875245207724	NA
**TC0X00001760.mm.2**	Nudt10	nudix (nucleoside diphosphate linked moiety X)-type motif 10	0.294618283157413	7.70339960400716	0.977881135814131	0.363474959435611	0.999692817443324	-4.59712054041151	7.67424154178919	7.70181184557038	7.98072961344692	7.73606078805218	7.66987487530383	7.45767895988045	NA
**TC0300000467.mm.2**	Med12l	mediator complex subunit 12-like	0.456385577169923	8.43034651662786	0.977715423393511	0.363550961078213	0.999692817443324	-4.5971292216987	8.06344287577233	7.93783251303526	9.39618828746375	8.35059914518597	8.81435947266643	8.01965680564344	NA
**TC1700002655.mm.2**	Abcg5	ATP binding cassette subfamily G member 5	-0.292743800007377	5.8527373849758	-0.977553382942046	0.363625290603949	0.999692817443324	-4.5971377099141	5.41848742564507	5.42389051815612	5.81613749353736	6.02814188948207	6.106902231912	6.32286475112218	NA
**TC1300000033.mm.2**	Pitrm1	pitrilysin metallepetidase 1	0.352721439769233	7.49517006892877	0.977520635575158	0.36364031357742	0.999692817443324	-4.59713942524481	8.02853164016855	8.21804760296265	7.684128240142	7.03081378484794	7.19841725804562	6.81108188740585	NA
**TC0300000829.mm.2**	Sprr2i	small proline-rich protein 2I	0.570968527660899	6.24884152644358	0.977403790766424	0.363693920500556	0.999692817443324	-4.59714554542776	5.52346370550014	5.39048488866419	6.19190983874906	7.66860897116041	5.82804921804596	6.89053253654171	NA
**TC1500001790.mm.2**	C030006K11Rik/Lrrc24	RIKEN cDNA C030006K11 gene/leucine rich repeat containing 24	0.391402005257225	7.18228483579323	0.97732381188715	0.363730617351675	0.999692817443324	-4.5971497344107	6.60525189345434	6.96583536159254	7.22279679419531	7.92991609091817	6.88041794121816	7.48949093338088	NA
**TC0X00003098.mm.2**	Acsl4	acyl-CoA synthetase long-chain family member 4	0.646744376231688	7.25784537448864	0.977320193273701	0.363732277754747	0.999692817443324	-4.59714992393558	8.55312361109481	8.47539906962519	7.84897092809835	6.05704823123927	6.88794351295549	5.72458689391876	NA
**TC0400002316.mm.2**	Ufl1	UFM1 specific ligase 1	0.38978259502566	6.87254422602096	0.977303641714726	0.363739872523368	0.999692817443324	-4.59715079081908	7.76318435373532	6.85487489760097	6.91521513601622	6.78317051140416	6.64755674776367	6.2712637096054	NA
**TC1900000723.mm.2**	Slk	STE20-like kinase	0.930806757420348	8.20073528489992	0.977272702753497	0.363754069357536	0.999692817443324	-4.5971524112194	9.64269967037682	9.85673827023901	9.05863486359901	6.72465877821317	8.09136949159831	5.83031063537317	NA
**TC0700001115.mm.2**	Aen	apoptosis enhancing nuclease	-0.335168501713484	6.67179297551188	-0.977253175375778	0.36376303002752	0.999692817443324	-4.59715343393516	6.27251216368671	6.21868103745708	6.43951294245532	6.99510088179495	6.86572857292719	7.23922225475005	NA
**TC1700002502.mm.2**	Pcare	photoreceptor cilium actin regulator	-0.327302350326964	5.22193522332645	-0.977155897698302	0.363807671106877	0.999692817443324	-4.59715852854967	4.94697489184387	5.24882596466602	4.8527578841262	5.38784500727123	5.41698538943321	5.47822220261816	NA
**TC1100000996.mm.2**	Ube2g1	ubiquitin-conjugating enzyme E2G 1	-0.42975198675112	7.14521260923465	-0.976958461407465	0.363898288464207	0.999692817443324	-4.59716886788426	7.3468248985706	7.43163077076222	7.40369296600916	6.21296504027727	7.25974530974197	7.2164166700467	NA
**TC0500001645.mm.2**	Cnpy4	canopy FGF signaling regulator 4	-0.507326809382961	6.47030571641011	-0.976757143120073	0.363990705652521	0.999692817443324	-4.5971794094422	7.35108710545287	6.94102109793278	6.02693622983711	5.73060000831745	5.65262263159228	7.1195672253282	NA
**TC0200003303.mm.2**	Lcn2	lipocalin 2	-0.9056475615918	6.21581993260332	-0.97673689949089	0.36399999970638	0.999692817443324	-4.59718046939243	5.94813606525455	8.39365583893042	5.69216340091227	4.8787528832134	5.27549700673618	7.10671440057309	NA
**TC0X00003133.mm.2**	Amot	angiomotin	0.300897351788326	5.94635012573548	0.976699084373573	0.364017361502586	0.999692817443324	-4.59718244935114	5.98355164874147	6.24653326548565	6.04087632991136	5.98402894196984	5.85861056800328	5.56450000030126	NA
**TC0600000927.mm.2**	Mob1a	MOB kinase activator 1A	-0.295836962739565	7.46255489553508	-0.976687295174171	0.364022774329023	0.999692817443324	-4.5971830666131	6.89401212312891	7.14789147315472	7.64781366313484	7.42306226258902	7.80281563030172	7.85973422090126	NA
**TC0200002784.mm.2**	Adrm1	adhesion regulating molecule 1	0.301994676393336	8.10176844179175	0.976387739022069	0.364160331891633	0.999692817443324	-4.59719874960893	8.08383453052186	8.08016690159617	8.42054850830609	8.10475077740348	7.82945460691992	8.09185532600299	NA
**TC1500000110.mm.2**	Drosha	drosha, ribonuclease type III	0.407822565610099	8.64243582193984	0.976054381154513	0.364313458962698	0.999692817443324	-4.59721619945048	9.27838270447265	9.10816326783007	8.46476819486227	8.677088850416	8.68976882515534	7.63644308890273	NA
**TC0600000921.mm.2**	Ccdc142/Mrpl53	coiled-coil domain containing 142/mitochondrial ribosomal protein L53	-0.403174607389932	8.60155637244746	-0.975811298675128	0.36442514988667	0.999692817443324	-4.59722892189669	7.3905280991555	7.45464648750304	9.47027567789051	8.50863153873266	9.61894875544888	9.16630767595415	NA
**TC1200002125.mm.2**	Alkbh1/Nrp	alkB homolog 1, histone H2A dioxygenase/neural regeneration protein	-0.296973539219413	6.53009342344911	-0.975757765630778	0.364449750697887	0.999692817443324	-4.5972317234962	6.29457574219156	5.9580558458888	6.56174290884911	6.60524802823864	6.82042698002671	6.94051103549986	NA
**TC0100003119.mm.2**	Lgr6	leucine-rich repeat-containing G protein-coupled receptor 6	-0.331748375217663	5.77244246820506	-0.975710956626966	0.364471262573786	0.999692817443324	-4.59723417313692	5.59901282951874	5.03172371197807	5.75680649509963	5.91340426354947	6.02658174196353	6.3071257671209	NA
**TC1400002252.mm.2**	Fzd3	frizzled class receptor 3	-0.294912687003828	7.54407168140611	-0.975575100209709	0.364533703307185	0.999692817443324	-4.5972412825362	8.52929649101092	8.41406939492211	7.0905003474132	6.7751190668348	7.24167297912104	7.21377180913461	NA
**TC1600002167.mm.2**	Cluap1	clusterin associated protein 1	-0.312725967712549	5.84279097974128	-0.97541291919972	0.364608253949291	0.999692817443324	-4.59724976886215	6.18950707741535	6.53678503349999	5.46099771219737	5.39150320385624	5.55540128163903	5.92255156983969	NA
**TC0400001107.mm.2**	Dhcr24	24-dehydrocholesterol reductase	0.406396525606809	7.73560690346761	0.975411088318602	0.364609095628311	0.999692817443324	-4.5972498646613	8.45029208455854	8.29128810980228	8.0896755597622	7.15275157906702	7.47351946199143	6.95611462562417	NA
**TC1000000653.mm.2**	Tmem26	transmembrane protein 26	0.346865648152985	6.6236802253331	0.975023941322513	0.364787105946037	0.999692817443324	-4.59727011975499	6.33469408931932	6.28096811458201	6.60353560661584	7.30653961558577	6.73934761660606	6.47699630928958	NA
**TC0300002743.mm.2**	5330417C22Rik	RIKEN cDNA 5330417C22 gene	-0.5836967019228	6.77506747602933	-0.974934038576898	0.364828452937998	0.999692817443324	-4.59727482279164	7.28439514603686	7.41533505376322	7.0395365080539	5.35210411821126	7.19296341886152	6.36607061124924	NA
**TC0200001879.mm.2**	Slc12a1	solute carrier family 12, member 1	0.441452920975484	5.40686212617664	0.974661908838121	0.364953629818555	0.999692817443324	-4.59728905725828	5.2781507779741	4.43956388116357	5.43907998332381	6.36410198661276	5.3934976746963	5.5267784532893	NA
**TC0100000056.mm.2**	A830018L16Rik	RIKEN cDNA A830018L16 gene	-0.817420469040123	7.00730341393346	-0.974498686342348	0.365028726526772	0.999692817443324	-4.59729759409205	8.16881049772975	7.86507656083144	7.2156918712691	4.97185437221057	7.75684079755851	6.0655463840014	NA
**TC1700000595.mm.2**	Cd320	CD320 antigen	-0.289991613425	7.88056282401091	-0.974254725140815	0.365140992565945	0.999692817443324	-4.59731035237686	7.51471611662037	7.57588485182068	8.00129116155752	7.80510521282967	8.28736569399985	8.09901390723735	NA
**TC1700000649.mm.2**	Zbtb12	zinc finger and BTB domain containing 12	0.349817887138685	6.59513598479674	0.974222327852146	0.365155903167772	0.999692817443324	-4.59731204651746	6.44156217029685	6.48132052874008	6.46186162754328	7.21192286446715	6.3972023706419	6.57694634709115	NA
**TC1000000027.mm.2**	Armt1	acidic residue methyltransferase 1	0.3359132483637	5.6941826885497	0.974095280121425	0.36521438047719	0.999692817443324	-4.59731868991054	5.8297802277352	5.90758692169363	6.11376626437666	5.4360114749217	5.41516831656603	5.46278292600494	NA
**TC1100003406.mm.2**	Dusp14	dual specificity phosphatase 14	-0.285182329509054	8.43301257327494	-0.97402778059425	0.365245452007068	0.999692817443324	-4.59732221932055	8.45843719776719	8.60615135161717	8.28130891431177	8.20025220131183	8.47040010823133	8.58152566641038	NA
**TC0200003376.mm.2**	Ndufa8	NADH:ubiquinone oxidoreductase subunit A8	0.316501777565957	9.2281632041001	0.973551242582267	0.365464871554305	0.999692817443324	-4.59734713302632	9.52079602306855	9.36623850705906	9.42340731781482	9.13406680698763	9.18013735895911	8.74433321071142	NA
**TC0200004525.mm.2**	Fbn1	fibrillin 1	-0.367293173472144	5.52123404396767	-0.973507967295232	0.365484802522645	0.999692817443324	-4.59734939518364	5.53964687321568	5.15055522290021	5.21796977015925	5.63333814021369	5.45938353552362	6.12651072179361	NA
**TC1300000596.mm.2**	Nxnl2	nucleoredoxin-like 2	-0.456115740355963	5.32083088792606	-0.973485022536384	0.365495370357978	0.999692817443324	-4.5973505945696	5.08750431815574	5.4582657624608	5.52922553683146	4.70426634628248	5.08715790099232	6.05856546283355	NA
**TC0800000277.mm.2**	Slc20a2	solute carrier family 20, member 2	0.394563596747673	6.85323139065374	0.973145853452433	0.365651611672479	0.999692817443324	-4.59736832222613	7.87351816656427	7.88538098459389	6.93105523735272	6.14375295577711	6.47167182618275	5.81400917345173	NA
**TC1400000792.mm.2**	Ltb4r2	leukotriene B4 receptor 2	0.420825163655513	6.1348733071809	0.973107248436266	0.365669398716159	0.999692817443324	-4.59737033983348	5.6175338239869	5.88775366638785	6.29191145386683	6.780889886144	5.6077878244011	6.6233631882987	NA
**TC1100002833.mm.2**	Olfr317	olfactory receptor 317	-0.400772542967356	5.71960648283808	-0.973056510242608	0.365692777076552	0.999692817443324	-4.59737299149411	5.00662447437814	5.66056554802799	5.3975190482598	6.02693284608401	5.8408903155479	6.38510666473063	NA
**TC0300002372.mm.2**	Snapin	SNAP-associated protein	-0.737007047423589	8.42013326064681	-0.97276572598749	0.365826782573744	0.999692817443324	-4.59738818701831	9.15997555680026	8.94142758038899	8.96177829690295	6.51091286901927	8.73141961456665	8.21528564620276	NA
**TC1400001656.mm.2**	Cacna1d	calcium channel, voltage-dependent, L type, alpha 1D subunit	0.407630528699294	5.62966629957687	0.972763268933296	0.365827915049359	0.999692817443324	-4.597388315407	6.09134577816058	6.41413869101464	5.06510476418919	5.97878242865311	4.86052274153182	5.36810339391188	NA
**TC0600002466.mm.2**	Rmnd5a	required for meiotic nuclear division 5 homolog A	0.543352238345602	7.43255386614466	0.972366396723062	0.366010872362407	0.999692817443324	-4.59740905107786	8.08132061257544	8.38439616042193	7.83878834448022	6.76936710580067	7.41621505084764	6.10523592274204	NA
**TC0700000050.mm.2**	Ssc5d	scavenger receptor cysteine rich family, 5 domains	0.626932679701381	4.47693524790041	0.972329117733755	0.366028061558491	0.999692817443324	-4.59741099860257	4.15796015208786	4.07616723521984	4.35905045520717	5.5816242745416	3.50917678075204	5.17763258959397	NA
**TC1100000763.mm.2**	Smcr8	Smith-Magenis syndrome chromosome region, candidate 8 homolog (human)	0.733979011061922	6.45824979039682	0.972041633363354	0.366160640561643	0.999692817443324	-4.59742601607154	7.59133839743022	7.6429431851632	7.03468303948597	5.4569045514697	6.4838260893365	4.53980347949533	NA
**TC0600002773.mm.2**	Lrig1	leucine-rich repeats and immunoglobulin-like domains 1	-0.286786634652317	6.89791033659727	-0.971982911635131	0.366187725818878	0.999692817443324	-4.59742908327493	7.18706242650343	7.26162733750664	6.65445320584261	6.52814628729185	6.97342924158508	6.78274352085402	NA
**TC1100002813.mm.2**	Gemin5	gem nuclear organelle associated protein 5	0.36170184486836	5.67945765757926	0.971582253549342	0.366372569975669	0.999692817443324	-4.59745000831011	6.37682396115796	6.50830385419587	5.80239569014782	5.15511521978141	5.38372392651402	4.85038329367849	NA
**TC0900002936.mm.2**	Nck1	non-catalytic region of tyrosine kinase adaptor protein 1	-1.09709459517482	5.92254566830234	-0.971425033290531	0.366445123567905	0.999692817443324	-4.59745821821748	6.65180417599489	6.62796772736337	6.73388700043977	3.29676945761332	7.05159189376071	5.17325375464202	NA
**TC1500000933.mm.2**	Tmem117	transmembrane protein 117	0.367044607285085	6.2034446020798	0.971329136255498	0.366489383362528	0.999692817443324	-4.59746322555125	6.18280878853946	5.74616266790323	6.84411000711568	6.16878267818746	6.31827975666192	5.96052371407105	NA
**TC0100003127.mm.2**	Shisa4	shisa family member 4	-0.386487581180202	7.62767971984694	-0.971211290297866	0.366543779032833	0.999692817443324	-4.59746937862426	7.65720855669646	7.63526693771685	7.89647359718717	6.9538402339668	7.95817980773163	7.66510918578273	NA
**TC0900001409.mm.2**	Usp19	ubiquitin specific peptidase 19	-0.364123287478875	6.72851174119817	-0.971195207829638	0.366551202910517	0.999692817443324	-4.59747021830667	7.26092790014386	7.26346451683487	6.54419918706072	6.01501654056555	6.98507288523371	6.30238941735031	NA

**TC0700002347.mm.2**	Zscan18	zinc finger and SCAN domain containing 18	-0.300328216832249	6.34176660976729	-0.971143552430476	0.36657504850878	0.999692817443324	-4.59747291524159	6.74788083948323	6.79844689552198	6.15571289047202	5.79609485449501	6.37156002896921	6.18090414966232	NA
**TC0500003585.mm.2**	Smurf1	SMAD specific E3 ubiquitin protein ligase 1	-0.650192788566589	6.68176823081488	-0.970952386725737	0.366663306509116	0.999692817443324	-4.59748289540072	7.30310527643185	7.60150545538624	6.96760692470248	4.97519961326652	6.21946761982013	7.02372449528206	NA
**TC0100002409.mm.2**	Ica1l	islet cell autoantigen 1-like	-0.377300811118959	6.6627132561937	-0.970848027424718	0.366711494415277	0.999692817443324	-4.59748834325466	7.50472088129589	7.50021712149624	6.31627222383969	5.79209773222637	6.82069286937653	6.04227870892745	NA
**TC0700004072.mm.2**	Lmo1	LIM domain only 1	0.299412699152239	7.26368170317064	0.970788420639618	0.366739020055528	0.999692817443324	-4.59749145476732	7.33650307570447	7.22951042174669	7.21064748737024	7.59680357256833	7.12070363987505	7.08792202175904	NA
**TC1000000707.mm.2**	Rab36	RAB36, member RAS oncogene family	0.284018853783609	7.68591004181617	0.970684000828063	0.366787243639402	0.999692817443324	-4.59749690531755	7.67446036848861	7.63461307528171	7.87228347210969	7.81492878523727	7.65236450664732	7.46681004313242	NA
**TC0800002690.mm.2**	Irx3	Iroquois related homeobox 3	0.317104314377493	5.88162766011779	0.970676161444586	0.366790864254309	0.999692817443324	-4.59749731450917	5.88486796736599	5.9378779353756	5.76121728568113	6.28939705767895	5.62302228891318	5.79338342569191	NA
**TC0900002908.mm.2**	Mrps22	mitochondrial ribosomal protein S22	0.34702045005336	6.47464980307921	0.97059004887494	0.366830637124524	0.999692817443324	-4.59750180921022	7.17071211791588	6.52344232876794	6.36220976977934	6.56168286616974	6.29067693185751	5.93917480398484	NA
**TC1800000394.mm.2**	Dele1	DAP3 binding cell death enhancer 1	-0.385550444846231	7.17436769207647	-0.970334158670604	0.3669488450758	0.999692817443324	-4.59751516438162	7.64784585421464	7.38737034567632	7.28439791763001	6.3355466138077	7.20739795208839	7.18364746904178	NA
**TC0100000697.mm.2**	Nyap2	neuronal tyrosine-phophorylated phosphoinositide 3-kinase adaptor 2	0.592321244897478	7.01966539968464	0.970170353259873	0.367024530178933	0.999692817443324	-4.59752371262406	7.68502741245885	7.33596410771938	7.27478684434851	6.86603483951378	7.40590676158569	5.55027243248163	NA
**TC1900000025.mm.2**	Cabp2	calcium binding protein 2	-0.346661308145376	5.25783276349224	-0.970140355412123	0.36703839178063	0.999692817443324	-4.59752527799357	4.85539060386297	5.20492746630146	5.52712048738904	4.86955745986009	5.46460066456457	5.62539989897532	NA
**TC0X00000760.mm.2**	Cldn34b4	claudin 34B4	0.341121037933402	4.84262774182139	0.970028760058473	0.367089962061637	0.999692817443324	-4.59753110112975	4.18494876486855	4.31681394149165	4.97058509116349	5.64753781905398	5.08311684939972	4.85276398495095	NA
**TC0600001296.mm.2**	March8	membrane-associated ring finger (C3HC4) 8	0.293878252672158	8.11400001602326	0.969929891975734	0.36713565553631	0.999692817443324	-4.59753625986508	8.27844260277812	8.167205596192	8.36483380807955	8.04822039317734	7.87296984413614	7.95232785177643	NA
**TC0900000826.mm.2**	Dpp8	dipeptidylpeptidase 8	0.558313164497552	7.78964224878488	0.969659280656506	0.367260745501708	0.999692817443324	-4.59755037845967	8.75353252933398	8.8789732118941	7.90736046712163	7.20362657311652	7.81741127853439	6.17694943270866	NA
**TC1400002422.mm.2**	Spert	spermatid associated	-0.317962730966753	6.54678519208479	-0.969644382487548	0.367267633134988	0.999692817443324	-4.59755115568372	5.76047689208978	5.93253598805668	6.61584383186199	6.8600425733524	7.29671157730113	6.81510028984677	NA
**TC0500001504.mm.2**	Sfswap	splicing factor SWAP	-0.40658885497571	6.90135887378014	-0.969367695036929	0.367395567869596	0.999692817443324	-4.59756558912586	6.82382153822165	6.76641134392324	7.26000747697398	6.24236384831829	7.35901242549386	6.95653660974983	NA
**TC0700000847.mm.2**	Gys1	glycogen synthase 1, muscle	-0.31848410013722	7.19124654045533	-0.969150929125812	0.367495820255519	0.999692817443324	-4.59757689530263	6.99564889539092	7.00904185261608	7.24068669127637	7.01222345594891	7.18284931637957	7.70702903112015	NA
**TC1100002301.mm.2**	Slc1a4	solute carrier family 1 (glutamate/neutral amino acid transporter), member 4	0.473820622330897	6.1779012276275	0.969095811063031	0.367521315276146	0.999692817443324	-4.59757976997294	7.25058500965467	7.7984598581866	6.30884812119706	5.1741537500957	5.59366579200168	4.94169483462928	NA
**TC1700002120.mm.2**	Cd2ap	CD2-associated protein	-0.608083566675618	5.60975476964525	-0.96892823323939	0.367598837320622	0.999692817443324	-4.59758850945102	5.77627618308928	5.82406054724306	6.12997073513785	4.29104164195611	6.21224842009388	5.42493109035132	NA
**TC0100002667.mm.2**	Irs1	insulin receptor substrate 1	0.456232043560363	5.16823983738601	0.968892521736092	0.367615359218464	0.999692817443324	-4.59759037177024	5.83966095513306	4.95442316698368	5.59469060840071	4.9692188862593	4.43358857261669	5.21785683492259	NA
**TC0500000852.mm.2**	Mthfd2l	methylenetetrahydrofolate dehydrogenase (NADP+ dependent) 2-like	0.343703114790264	7.1161898370467	0.968841651162278	0.367638895443297	0.999692817443324	-4.59759302456008	7.67906056956691	7.11891304420475	7.39140019127786	6.90188562776667	6.83232886918008	6.77355072028393	NA
**TC1700001791.mm.2**	Ccdc167	coiled-coil domain containing 167	0.32795981147954	7.57493530387921	0.968526814031043	0.367784586766956	0.999692817443324	-4.59760944106975	6.88902985020247	6.77905395472726	7.83861805580129	8.38010576485101	7.6173805687861	7.94542362890712	NA
**TC1400000827.mm.2**	Mrpl57	mitochondrial ribosomal protein L57	-0.305002736259992	8.7399881174753	-0.968293071851517	0.367892780268615	0.999692817443324	-4.59762162731566	8.75812539741586	8.8215809849482	8.78956089118791	8.33554753379596	8.89974769936726	8.83536619813659	NA
**TC0300003091.mm.2**	Wdr63	WD repeat domain 63	0.478907000103832	5.44886910657014	0.968136278214694	0.367965370023978	0.999692817443324	-4.59762980098552	4.89564190978577	5.0987665685241	5.2304526769969	6.59785740366243	5.22650060269353	5.64399547775814	NA
**TC0400004031.mm.2**	Cort	cortistatin	-0.29194187346822	8.97147468870889	-0.967925703981207	0.368062875726398	0.999692817443324	-4.59764077719267	8.34756491967519	8.52557520862112	9.13047123215667	9.05544089635363	9.25174360429671	9.51805227115003	NA
**TC1800000240.mm.2**	Sap130	Sin3A associated protein	0.318242508409911	7.08484040000041	0.967807566503778	0.36811758767244	0.999692817443324	-4.59764693459494	7.84374782202731	7.85637592131773	7.09557805879878	6.62712377793984	6.70839342374052	6.37782339617826	NA
**TC0400004104.mm.2**	Ajap1	adherens junction associated protein 1	-0.743610571404603	7.98514941267793	-0.96756635220505	0.368229318672383	0.999692817443324	-4.59765950566243	9.29123022944317	9.15022032858804	8.18941053943444	5.80170184817915	8.19020471770475	7.28812881271805	NA
**TC1100001047.mm.2**	Cluh	clustered mitochondria (cluA/CLU1) homolog	0.300163355905809	7.54420105376294	0.967446216961445	0.368284975397803	0.999692817443324	-4.59766576601184	8.59052908930686	8.57602732916618	7.03541239133065	7.31407591662745	7.03144644711925	6.71771514902723	NA
**TC0200005225.mm.2**	Zfp64/Gm38422	zinc finger protein 64/predicted gene, 38422	0.294789746632816	8.11430691705409	0.96736884773044	0.368320822772082	0.999692817443324	-4.59766979758011	7.93101265053553	7.67251064352936	8.3019492906829	8.53399956007974	8.17768051703848	8.06868884045853	NA
**TC0100002405.mm.2**	Sumo1	small ubiquitin-like modifier 1	0.297386696714535	8.31201963824836	0.967340054293639	0.368334164284687	0.999692817443324	-4.59767129791152	9.18272771365733	8.79611955669195	8.12798488936703	8.11603708691795	7.79241664256038	7.85683194029553	NA
**TC1100001454.mm.2**	Dlx3	distal-less homeobox 3	-0.463637392808321	6.29768732320829	-0.967331134767531	0.368338297245972	0.999692817443324	-4.59767176267414	5.71741631788115	5.64456594224936	6.13359068048293	6.61484276626837	6.19445124326413	7.48125698910381	NA
**TC1200000833.mm.2**	Fos	FBJ osteosarcoma oncogene	0.305979260895804	5.39916479329324	0.96714067264201	0.368426558567873	0.999692817443324	-4.59768168645415	5.32537936840906	5.62102640238765	5.40721815209154	5.62305260328563	5.05786599138489	5.36044624220067	NA
**TC0400001307.mm.2**	Elovl1	elongation of very long chain fatty acids (FEN1/Elo2, SUR4/Elo3, yeast)-like 1	-0.598068990762026	7.19077341435452	-0.96704320836409	0.368471730458733	0.999692817443324	-4.59768676426156	7.68953518683396	7.75539395981771	7.5943613208664	6.55742535810931	7.38317763457034	7.06474702592941	NA
**TC1200002354.mm.2**	Degs2	delta(4)-desaturase, sphingolipid 2	0.382921099788268	5.16153690359998	0.966973225707367	0.368504168057529	0.999692817443324	-4.59769041016796	4.65770440823794	4.69323101289467	5.30393301204373	5.88813108797819	5.60443163393766	4.82179026650772	NA
**TC1500002351.mm.2**	Chkb	choline kinase beta	-0.494238775835317	5.56705132549219	-0.966929538481684	0.368524418607254	0.999692817443324	-4.59769268608614	6.10523756759061	6.29136078077586	5.70436652344862	4.30424950300939	5.77399693789136	5.22309664023729	NA
**TC0700000921.mm.2**	Luzp2	leucine zipper protein 2	-0.681773600672503	7.9995104865505	-0.96645100109739	0.368746293842951	0.999692817443324	-4.59771761243709	9.30077086395258	9.40067378919779	8.03725297111105	5.92878256129276	8.13463227120769	7.19495046254112	NA
**TC1100000264.mm.2**	Fancl	Fanconi anemia, complementation group L	0.329199572983835	6.25415309830412	0.966381189280119	0.368778670948465	0.999692817443324	-4.59772124831613	6.81177668184768	6.76845853017679	6.01924675244857	6.28229450943541	6.12141319992421	5.5217289159921	NA
**TC1100002686.mm.2**	Prop1	paired like homeodomain factor 1	0.376029076664535	4.96190624274267	0.966348145511345	0.368793996652334	0.999692817443324	-4.59772296922692	4.50740240979292	4.30738102670292	5.44068496417855	5.41367112246607	4.59155467646228	5.51074325685326	NA
**TC0200001366.mm.2**	Psmc3	proteasome (prosome, macropain) 26S subunit, ATPase 3	-0.423065043266023	8.62436850102151	-0.966347006968432	0.368794524717471	0.999692817443324	-4.59772302852141	9.41423116275586	9.52079794226612	8.56784628462917	7.41467962265833	8.5389943137918	8.28966168002775	NA
**TC0200003195.mm.2**	Agpat2	1-acylglycerol-3-phosphate O-acyltransferase 2 (lysophosphatidic acid acyltransferase, beta)	0.293469227356404	5.91326130401387	0.966276270749085	0.368827333880702	0.999692817443324	-4.59772671234274	5.7201648596631	5.84547826707253	6.14831396712586	6.10211760890435	5.66841077628549	5.99508234503191	NA
**TC1700002132.mm.2**	Slc25a27	solute carrier family 25, member 27	0.346636094892017	6.48967603170525	0.966101906223642	0.368908218013264	0.999692817443324	-4.5977357923658	7.43654198169668	7.37208536338002	6.26734275768113	6.14400775978829	6.23804199323628	5.4800363344491	NA
**TC1400002439.mm.2**	Gpalpp1	GPALPP motifs containing 1	-0.339837522662371	6.67983391455763	-0.966087990770024	0.368914673699665	0.999692817443324	-4.59773651697652	7.28612598280137	7.33237735654371	6.50782990557057	5.88258264576742	6.71984133991904	6.35024625674369	NA
**TC0200001760.mm.2**	Rad51	RAD51 recombinase	-0.304962220628203	5.32388624975732	-0.965905663736928	0.36899926732983	0.999692817443324	-4.59774601068959	4.92486312527466	5.10250951838567	5.35338303219055	5.29962717462304	5.84700564728213	5.41592900078787	NA
**TC1000000330.mm.2**	Traf3ip2	TRAF3 interacting protein 2	0.288468138702235	5.33390377290546	0.965462887736496	0.369204763138812	0.999692817443324	-4.59776906212008	5.03600869883358	5.07943538713914	5.4827619470617	5.74969546737057	5.3853270833259	5.2701940537019	NA
**TC1600001262.mm.2**	Fgd4	FYVE, RhoGEF and PH domain containing 4	0.499962810551346	7.46620678334068	0.965368293426334	0.369248676594503	0.999692817443324	-4.59777398611445	7.84418461036922	7.74439046085832	7.9599924046491	7.1443032203105	7.70651065737335	6.39785934648356	NA
**TC0300002295.mm.2**	Bcan	brevican	0.308096068455779	7.30040356648434	0.965296552779198	0.36928198340766	0.999692817443324	-4.59777772032538	8.41894638631356	8.11832488832682	7.04203761925121	6.8986335113374	6.47685219290674	6.84762680077031	NA
**TC0200005463.mm.2**	Npdc1	neural proliferation, differentiation and control 1	0.486652322205774	6.98720402851332	0.965101444785369	0.369372577380579	0.999692817443324	-4.59778787528416	7.89496335819773	7.59089020300398	7.27773145694621	6.42760617019868	6.95620174409016	5.77583123864318	NA
**TC1000000385.mm.2**	Ostm1	osteopetrosis associated transmembrane protein 1	-0.345858188371817	7.67965662658149	-0.96506030875025	0.36939168016367	0.999692817443324	-4.59779001619491	7.92736188125041	7.71752628703932	7.80051939660015	7.07014821062764	7.83732261597965	7.72506136799178	NA
**TC1000001336.mm.2**	Ptprb	protein tyrosine phosphatase, receptor type, B	-0.441766857844527	5.30677384812085	-0.964946561891845	0.369444506000372	0.999692817443324	-4.59779593586978	5.58705955216475	5.30826880710594	4.98648948770667	5.04440101917599	6.0802703132519	4.83415390931981	NA
**TC1300001500.mm.2**	Cdk13	cyclin-dependent kinase 13	0.287039152384368	7.31005300965279	0.964449673154503	0.369675337602054	0.999692817443324	-4.59782179106505	7.70560947026973	7.90405142318384	7.3019192934189	7.11044844119706	6.90045504506561	6.9378343847816	NA
**TC0400003822.mm.2**	Cda	cytidine deaminase	0.31872451154424	5.61495978126568	0.964276613713797	0.369755759265679	0.999692817443324	-4.59783079448182	5.47622255945677	5.07867704454877	5.73375476476403	6.15239928857449	5.57427817917082	5.67442685107921	NA
**TC0400001413.mm.2**	Rragc	Ras-related GTP binding C	-0.505855220587081	7.66593843094788	-0.964233390945146	0.369775847236064	0.999692817443324	-4.59783304301807	8.60260769703687	8.72437551588139	7.47190634414059	6.35656212165683	7.97216879989437	6.86801010707722	NA
**TC0400001712.mm.2**	Rap1gap	Rap1 GTPase-activating protein	0.287753547033173	8.17054971980835	0.964132062269084	0.369822943493879	0.999692817443324	-4.59783831414085	8.62399837074797	8.35351992242099	8.10944211745482	8.20120144241892	7.89150236861309	7.8436340971943	NA
**TC1500001698.mm.2**	Slc45a4	solute carrier family 45, member 4	0.313163076103202	7.43814557716583	0.963992076674089	0.369888014629177	0.999692817443324	-4.59784559573523	7.62095030876454	7.41678127626203	7.52397279541586	7.58476121967155	6.99113008527951	7.4912777776015	NA
**TC1100000335.mm.2**	Hba-x	hemoglobin X, alpha-like embryonic chain in Hba complex	-0.359445491457523	6.83620138266922	-0.963512120018659	0.370111185786246	0.999692817443324	-4.59787055743347	6.10186895174303	6.33451394535975	6.58711016649943	7.34385704149933	7.48411740448544	7.16574078642836	NA
**TC1700001053.mm.2**	Fer	fer (fms/fps related) protein kinase	-0.42743591487173	6.91836177920453	-0.963037602402583	0.370331930356567	0.999692817443324	-4.59789523003731	7.44726297681656	7.54605000104088	6.91134181542181	5.91965111839133	7.1888480309037	6.4970167326529	NA
**TC1300002422.mm.2**	Fam151b	family with sequence similarity 151, member B	-0.323314694966384	6.16271889944896	-0.962957766520516	0.37036907985002	0.999692817443324	-4.59789938050593	6.27457387579263	6.55642019690199	5.73044550877802	6.01889945825515	5.96059531729731	6.43537903966863	NA
**TC1700001384.mm.2**	Tmem181b-ps	transmembrane protein 181B, pseudogene	0.74746886246498	8.41173896908029	0.962895788156444	0.370397921813606	0.999692817443324	-4.59790260248583	9.33452758411689	8.46710414740272	9.50935953919653	7.57251036474951	8.53249367788252	7.05443850113355	NA
**TC1700002807.mm.2**	Arhgdig	Rho GDP dissociation inhibitor (GDI) gamma	-0.32274100348709	8.05473307542316	-0.962845918250366	0.370421130303526	0.999692817443324	-4.59790519492407	8.4975378048738	8.17590616702495	7.79464737711112	7.7100888597219	8.33619365455371	7.81402458925348	NA
**TC0900000746.mm.2**	Adpgk	ADP-dependent glucokinase	0.35555305594524	5.84358496987799	0.962783732942609	0.370450071719935	0.999692817443324	-4.59790842747057	6.21073479193336	5.84090641138316	5.75576018996483	6.10472717395612	5.24541244221516	5.90396880981531	NA
**TC0800002556.mm.2**	Pkn1	protein kinase N1	0.40041627843903	6.69850735967323	0.962712732315705	0.370483117978631	0.999692817443324	-4.59791211812914	5.98637095631205	5.83343944381661	6.78007911169286	7.80595404570154	6.7448343773053	7.04036622321104	NA
**TC0X00001047.mm.2**	Uprt	uracil phosphoribosyltransferase	0.584248981333225	7.3073689310017	0.962431679905118	0.370613952320958	0.999692817443324	-4.59792672605406	8.75998756762841	8.60989694058555	7.7209040712345	6.10050944899685	6.83381065817647	5.81910489938844	NA
**TC0600003342.mm.2**	Eps8	epidermal growth factor receptor pathway substrate 8	-0.288226903053831	8.12941580217366	-0.961831951809454	0.37089325476998	0.999692817443324	-4.59795789013049	7.97953810527316	7.79149500618556	7.98800899972383	8.22649494801396	8.38039272406401	8.41056502978145	NA
**TC0300002497.mm.2**	Bola1	bolA-like 1 (E. coli)	-0.301950332892782	6.94874931888505	-0.961473989022665	0.371060041040169	0.999692817443324	-4.59797648646799	6.98529826834294	6.96329699966388	6.66593398663442	6.90406600312454	6.89362532356782	7.2802753319767	NA
**TC0700001413.mm.2**	Rab6a	RAB6A, member RAS oncogene family	-0.756911642312536	9.84461060065582	-0.961417439477215	0.37108639458077	0.999692817443324	-4.59797942392053	10.8656713734116	10.8693401096344	10.1387435601016	7.77067085803032	10.282186057357	9.1410516454	NA
**TC0400003871.mm.2**	Necap2	NECAP endocytosis associated 2	-0.290783154288102	7.84496330037474	-0.961354933562017	0.37111552562696	0.999692817443324	-4.5979826706726	7.38053242398846	7.38225902086093	7.76436870454218	8.09834231986925	8.25107170286221	8.19320563012542	NA
**TC1800001745.mm.2**	Ier3ip1	immediate early response 3 interacting protein 1	0.402635144484255	7.82889156869086	0.961353207155789	0.371116330248223	0.999692817443324	-4.59798276034599	8.45021498089611	8.09684595323102	8.16557983011818	7.45019955337508	7.77861834458499	7.03189074993976	NA
**TC1700001771.mm.2**	4930539E08Rik	RIKEN cDNA 4930539E08 gene	0.377247447240896	5.64192156925736	0.961345103512054	0.371120107108542	0.999692817443324	-4.59798318126622	5.80528610399269	5.02562347110117	6.02223745344638	5.86531991401966	5.2990225325562	5.83403994042805	NA
**TC0200004245.mm.2**	Tcp11l1	t-complex 11 like 1	-0.756933808464278	5.76273676940174	-0.961326199229042	0.371128917931816	0.999692817443324	-4.59798416318724	7.08422569977338	6.96332700516209	5.72133794690359	3.7861622003696	6.39843781049711	4.62292995370464	NA
**TC0600000651.mm.2**	Wipf3	WAS/WASL interacting protein family, member 3	0.302428105947921	8.50249573700373	0.961069508510342	0.37124857120046	0.999692817443324	-4.59799749516882	7.7094009771094	7.79321398368139	9.17505020473132	8.88355763183239	8.55382573875796	8.8999258859099	NA
**TC0200002304.mm.2**	Ttll9	tubulin tyrosine ligase-like family, member 9	0.318201619816046	6.57649018625522	0.961045786955234	0.371259630221589	0.999692817443324	-4.59799872712517	6.4077831255309	6.29931468756401	6.63073172599467	7.06339154603958	6.33572717826569	6.72199285413648	NA
**TC1100000638.mm.2**	Gpx3	glutathione peroxidase 3	0.514331730742275	8.55317698930218	0.960712487646878	0.371415041745706	0.999692817443324	-4.59801603506349	9.97567308796839	8.67877416845526	8.8989076416151	7.9477314288219	8.00619876666648	7.81177684228596	NA
**TC1900000442.mm.2**	Pdcd1lg2	programmed cell death 1 ligand 2	0.306386613675176	5.40742213867812	0.960558650730372	0.371486790120114	0.999692817443324	-4.59802402264302	5.0278719019968	5.14620459276172	5.4796020838151	5.96201269851518	5.41343886576	5.41540268921992	NA
**TC0800000882.mm.2**	Otud4	OTU domain containing 4	0.682611047587693	6.1459571504796	0.960484046586208	0.371521588786674	0.999692817443324	-4.5980278960325	6.83517571889866	6.93187020821246	6.96249407677313	5.27446545869781	6.2382932593894	4.63344418090615	NA
**TC1400001235.mm.2**	Slitrk5	SLIT and NTRK-like family, member 5	-0.339432439596898	8.32577302670474	-0.960188876186143	0.371659294001145	0.999692817443324	-4.59804321954202	8.47584992960294	8.73736689448296	8.3265578382481	7.70472039022628	8.5053857822106	8.20475732545758	NA
**TC0400002797.mm.2**	Kif12	kinesin family member 12	0.538561896126484	5.56560627271225	0.959810980299217	0.37183565054141	0.999692817443324	-4.59806283415247	4.98820564144362	5.23270159521816	5.35418231884631	6.77074477708605	5.04401797636448	6.0037853273149	NA
**TC1100000668.mm.2**	Sap30l	SAP30-like	0.401613138288594	7.15993813161609	0.959641695508171	0.371914673378321	0.999692817443324	-4.5980716195632	7.31320234431504	7.12302175357759	7.17918711088977	7.48412837330077	6.41486064790481	7.44522855970854	NA
**TC0100000951.mm.2**	Gal3st2c	galactose-3-O-sulfotransferase 2C	0.34633788221452	6.59071298544355	0.95952203615633	0.371970538684138	0.999692817443324	-4.59807782907246	6.43129815406255	6.51620164659447	6.56054053276254	7.08418640545412	6.187744324899	6.76430684888861	NA
**TC1100002550.mm.2**	Ccng1	cyclin G1	-0.958476500098826	7.69901184065136	-0.959379561018307	0.372037064441518	0.999692817443324	-4.59808522204883	8.61395708871044	9.20339392173828	8.04851325249921	5.18137026413167	8.46888628579626	6.67795023103227	NA
**TC0600002326.mm.2**	Inmt	indolethylamine N-methyltransferase	-0.314755550907472	5.00920396131361	-0.959307410786639	0.372070756974949	0.999692817443324	-4.59808896568008	4.58181299207599	4.91432407164078	4.79796478646017	5.1668230147148	5.43514565187547	5.15915325111445	NA
**TC1100001490.mm.2**	Gm53	predicted gene 53	-0.283396786788445	6.58969691159774	-0.959268095149106	0.372089117484597	0.999692817443324	-4.59809100557465	6.11552307048136	6.10791569031875	6.56539127426518	6.80858329333954	6.9899909238775	6.95077721730411	NA
**TC0900001655.mm.2**	Tgm4	transglutaminase 4 (prostate)	0.416132332320832	5.74654455201158	0.958443958890984	0.372474152491829	0.999692817443324	-4.59813375606951	5.33001162447021	5.36455655611235	5.70922909454511	6.5992528035192	5.36083350865557	6.11538372476707	NA
**TC0X00003046.mm.2**	Esx1	extraembryonic, spermatogenesis, homeobox 1	-0.319779661682357	5.04710807609344	-0.958142423514347	0.372615106153589	0.999692817443324	-4.59814939292853	4.63758740832689	4.73758940567024	4.89903889628792	5.23491726331149	5.1377554182685	5.63576006469562	NA
**TC0200003148.mm.2**	Paxx	non-homologous end joining factor	0.434171425422232	5.9827509515889	0.957938258905733	0.372710566925499	0.999692817443324	-4.59815997895177	6.6659282447063	6.41398180814103	5.62177180837381	6.22069744539145	6.02512334981413	4.94900305310667	NA
**TC0500001399.mm.2**	P2rx7	purinergic receptor P2X, ligand-gated ion channel, 7	-0.311233220134598	5.57035427239699	-0.957849429783363	0.372752106446438	0.999692817443324	-4.59816458441828	6.4514596566347	6.21348412739837	5.05016700015306	5.01719070488677	5.10079072482644	5.58903342048258	NA
**TC1100001515.mm.2**	Cbx1	chromobox 1	-0.352485780642621	6.90854195427534	-0.957831939062053	0.372760286124197	0.999692817443324	-4.59816549122262	7.68501326655556	7.10524007130294	6.58370116646591	6.39431224678825	6.56401273266464	7.11897224187477	NA
**TC0600001657.mm.2**	Aebp2	AE binding protein 2	-0.745687219904306	8.13861749284402	-0.957721755848122	0.372811817384473	0.999692817443324	-4.59817120346241	8.86586284276929	8.8926377373538	8.63635528601312	6.15455968255308	8.52031925033342	7.7619701580414	NA
**TC1800000253.mm.2**	Iws1	IWS1, SUPT6 interacting protein	-0.312999840795743	6.59258174210932	-0.957667452635333	0.372837216315929	0.999692817443324	-4.59817401858523	7.13364512095482	6.80291248734037	6.09681337150334	6.39965320988129	6.72780573655495	6.39466052642117	NA
**TC1100003651.mm.2**	Pcgf2	polycomb group ring finger 2	-0.474532423970446	6.7898465498906	-0.957442322581862	0.372942529358183	0.999692817443324	-4.59818568863531	7.12939260447879	6.97237831126833	7.11405623011697	5.7300655377108	7.09499362084372	6.69819299492495	NA
**TC1100003062.mm.2**	Slc35g3	solute carrier family 35, member G3	0.450787759919565	5.89980403895845	0.957387326732761	0.37296825922261	0.999692817443324	-4.59818853923666	5.3078848115879	5.41536208021666	5.9965694200546	6.79200701083804	5.40840065508758	6.47860025596593	NA
**TC0100003279.mm.2**	Dhx9	DEAH (Asp-Glu-Ala-His) box polypeptide 9	0.565428256142519	6.43409952623736	0.957284526699753	0.37301635799778	0.999692817443324	-4.59819386744773	7.8747724757346	7.7836333987132	6.59647900101507	5.44204489661564	6.17567738253209	4.73199000281358	NA

**TC0300001956.mm.2**	Ndufc1	NADH:ubiquinone oxidoreductase subunit C1	-0.433863815601262	9.3157749917545	-0.95719657214983	0.373057514568176	0.999692817443324	-4.59819842597143	10.7131358790698	10.5239132533254	8.90726592444842	7.98767066901624	9.2993963756106	8.46326784905659	NA
**TC1300002711.mm.2**	Fst	follistatin	-0.343932806465452	5.56776150477884	-0.957104619611482	0.373100545664397	0.999692817443324	-4.59820319147314	5.73366581088216	5.60251859074844	5.06748739438295	5.6237721126728	5.42862570282293	5.95049941716373	NA
**TC0500002268.mm.2**	Wfs1	wolframin ER transmembrane glycoprotein	0.368880018824235	8.51267894734051	0.956994330018234	0.373152163018057	0.999692817443324	-4.59820890699421	9.59114379241292	9.48507165943437	8.61881463017057	7.74999450475154	7.67146271002596	7.95958638724768	NA
**TC0200004424.mm.2**	Dnajc17	DnaJ heat shock protein family (Hsp40) member C17	-0.301308263182871	6.53773613596597	-0.956986063292472	0.37315603220444	0.999692817443324	-4.59820933538584	6.44869971988768	6.54572347554217	6.27886017268724	6.53582837431289	6.89421161158105	6.52309346178481	NA
**TC0500002952.mm.2**	Dgkq	diacylglycerol kinase, theta	0.339406110466735	6.45144767896782	0.956882693525251	0.373204416355168	0.999692817443324	-4.5982146919702	6.52151303464439	6.10611819126232	6.38347613340178	6.99645740101507	6.30576618619346	6.39535512728992	NA
**TC1300000686.mm.2**	Gm3045	predicted gene 3045	-0.42818933420016	6.38496468891455	-0.956768493534415	0.373257875422787	0.999692817443324	-4.59822060942655	5.48974678018859	5.91850768334134	5.95159003022765	7.07098747055086	6.80044398016836	7.07851218901047	NA
**TC0100001765.mm.2**	Dusp10	dual specificity phosphatase 10	-0.348178935930197	6.88498427635198	-0.95620696706041	0.37352082201169	0.999692817443324	-4.59824970053255	7.38031109767705	7.08925657141278	6.6914661721135	6.38052388646731	7.22405065397522	6.54429727646599	NA
**TC0100002954.mm.2**	Ptpn4	protein tyrosine phosphatase, non-receptor type 4	-0.282201103997569	7.21704903565081	-0.956130463484668	0.373556657479237	0.999692817443324	-4.59825366328629	7.64656943495039	7.52430622374259	6.93857752408487	6.84493064952349	7.26734290887898	7.08056747272452	NA
**TC1500001778.mm.2**	Scrt1	scratch family zinc finger 1	0.292790704994429	6.48708812262478	0.95607514923472	0.37358256918817	0.999692817443324	-4.59825652836758	6.5416495143089	6.6281595676726	6.40295901792922	6.7661915139488	6.32708372156988	6.25648540031928	NA
**TC0500003608.mm.2**	Rnf6	ring finger protein (C3H2C3 type) 6	-0.585210345514135	6.92373264327957	-0.955686660004893	0.373764594264516	0.999692817443324	-4.59827664831885	7.929399054583	7.73552431416264	7.1189899122208	5.23453598773097	6.9693678315917	6.55457875938833	NA
**TC1100001660.mm.2**	Nbr1	NBR1, autophagy cargo receptor	1.08377140939427	5.82929294091108	0.955510189810557	0.373847301257964	0.999692817443324	-4.59828578635975	7.71006983392708	7.52473628801686	6.41206705013304	4.5421801210225	6.12203431989667	2.66467003247032	NA
**TC1400001107.mm.2**	Sugt1	SGT1, suppressor of G2 allele of SKP1 (S. cerevisiae)	-0.505590792083275	7.25646695469414	-0.955478137634847	0.373862324794029	0.999692817443324	-4.59828744600321	8.36531586879679	8.42979659694253	7.12114326745802	5.74511057167147	7.33926847353705	6.53816694975899	NA
**TC1400002490.mm.2**	Naa16	N(alpha)-acetyltransferase 16, NatA auxiliary subunit	0.300686784729185	5.16819670331804	0.955414410111839	0.373892196621705	0.999692817443324	-4.59829074569281	5.03517515654043	5.20147588447278	5.43071023509921	5.25624113907749	4.84657074452968	5.23900706018864	NA
**TC0700000486.mm.2**	9530053A07Rik	RIKEN cDNA 9530053A07 gene	-0.341942999733993	5.31357291531333	-0.955400019896692	0.37389894218844	0.999692817443324	-4.59829149077499	4.82877056473534	4.95932244148296	5.14494484577255	5.5597843973243	6.00917689442532	5.37943834813952	NA
**TC1800001507.mm.2**	Spire1	spire type actin nucleation factor 1	-0.525548194416571	8.73686118138379	-0.955133809424775	0.374023748110792	0.999692817443324	-4.59830527330983	9.4720319578237	9.54012507166633	8.91229578352037	7.26666105146942	8.78776629792946	8.44228692589347	NA
**TC1500001084.mm.2**	Krt18	keratin 18	-0.385383581192791	5.50314388539587	-0.955105078740863	0.374037219672	0.999692817443324	-4.59830676066714	4.90902747691269	5.84936242337294	5.27142968094064	5.47342344391137	5.92148258164062	5.59413770559697	NA
**TC1000000831.mm.2**	Grin3b	glutamate receptor, ionotropic, NMDA3B	0.395555599078189	6.50716406628284	0.954961588442705	0.374104506591654	0.999692817443324	-4.5983141886632	6.02556727838591	6.31267907590273	6.36267916350378	7.38524545727861	6.5431597752336	6.41365364739241	NA
**TC0800000008.mm.2**	Tex45	testis expressed 45	0.625715456289683	4.77386977070718	0.95488210466393	0.374141782943945	0.999692817443324	-4.59831830301521	4.32579056734561	3.98189588750705	4.55123631886391	6.242245222121	4.16212968666205	5.37992094174349	NA
**TC0700000818.mm.2**	Il4i1b/Il4i1/Nup62	interleukin 4 induced 1B/interleukin 4 induced 1/nucleoporin 62	0.381072035147064	7.85063303642594	0.954545633415822	0.374299613156341	0.999692817443324	-4.59833571795689	7.28252519185635	7.22542081811656	7.91318530855347	8.76581283088495	7.70457640774591	8.21227766139838	NA
**TC1700000994.mm.2**	Shd	src homology 2 domain-containing transforming protein D	0.320836341717224	7.12137362066446	0.953939663827304	0.374583987696173	0.999692817443324	-4.59836707348014	7.62787313959196	7.11591911594623	7.33726150556267	6.97579957037884	6.84420917302585	6.82717921948121	NA
**TC0200000821.mm.2**	Rif1	replication timing regulatory factor 1	0.4174234526636	5.50205231665345	0.953857982001714	0.374622332765023	0.999692817443324	-4.59837129926813	6.45501638901882	6.09486643897137	4.80560178770204	5.84303720092682	5.12785050760162	4.68594157570003	NA
**TC1600000790.mm.2**	Cggbp1	CGG triplet repeat binding protein 1	0.346740733367488	7.15238232617985	0.953710471362189	0.374691588459188	0.999692817443324	-4.59837893021822	7.34009911733079	7.54194538958407	6.79671338556729	7.56615207288231	6.79763881759274	6.87174517412187	NA
**TC1900000717.mm.2**	Taf5	TATA-box binding protein associated factor 5	-0.39544259783893	6.1771005048509	-0.95313599101522	0.374961398848349	0.999692817443324	-4.5984086431373	6.25203020830003	6.21072173245584	6.22121022242211	5.68327272391372	6.81162355559912	5.88374458641456	NA
**TC1500000104.mm.2**	Golph3	golgi phosphoprotein 3	-0.281796896775743	8.78797892874967	-0.952871035314891	0.375085888334796	0.999692817443324	-4.59842234388753	8.27655826459796	8.52423844430851	8.82542370457263	8.85631783044741	9.13637869111184	9.10895663745969	NA
**TC0X00000666.mm.2**	Gabrq	gamma-aminobutyric acid (GABA) A receptor, subunit theta	0.616050308551136	5.58249305612735	0.952633023996587	0.3751977451356	0.999692817443324	-4.59843464967124	7.49283090985096	5.85558567591021	5.91466493191397	4.77465625123862	4.43851826593963	5.0187023010107	NA
**TC0300002730.mm.2**	Ampd2	adenosine monophosphate deaminase 2	-0.654888575420548	6.18696016367589	-0.952102043243378	0.37544737965234	0.999692817443324	-4.59846209696455	7.3596783096752	7.44410392708104	6.34757891448104	4.15652188274795	6.13893135420558	5.67494659386451	NA
**TC1100004049.mm.2**	Btbd17	BTB (POZ) domain containing 17	-0.308174755168754	6.25872134479147	-0.951880033438038	0.375551792918777	0.999692817443324	-4.59847357067233	6.15929108223309	6.24338212609977	6.16810204119269	6.09855063384655	6.17959187767525	6.7034103077015	NA
**TC1400000835.mm.2**	Cab39l	calcium binding protein 39-like	-0.59254725750618	8.06128988886401	-0.951874384103435	0.375554450144729	0.999692817443324	-4.59847386261795	8.72803286274037	9.05446585540725	7.7937389959649	6.90633405404714	8.81265892925085	7.07250863577355	NA
**TC0700002787.mm.2**	Fxyd7	FXYD domain-containing ion transport regulator 7	-0.450691369754592	8.64386522964147	-0.951775457358427	0.375600983760436	0.999692817443324	-4.59847897479632	8.55127252472901	8.35408366359482	8.91128000778106	8.11694621722684	9.5015167274519	8.42809223706519	NA
**TC1400001545.mm.2**	Myoz1	myozenin 1	0.40182738867489	6.26144748404801	0.951411560631906	0.375772193357354	0.999692817443324	-4.59849777729537	5.01164540532026	5.01190745024963	6.85152555972039	7.32286785331359	6.182816148847	7.1879224868372	NA
**TC0500003101.mm.2**	Sirt4	sirtuin 4	-0.399553633629353	6.85195547709866	-0.951335035057551	0.375808205480315	0.999692817443324	-4.59850173088693	6.57597308671362	6.28265789510421	6.6236252020062	7.10337210475152	6.79117139038503	7.73493318363139	NA
**TC0500001447.mm.2**	Tmed2	transmembrane p24 trafficking protein 2	-0.537633049382784	8.5984065048836	-0.95131379800663	0.375818199881818	0.999692817443324	-4.59850282804149	9.14028276382919	9.35291183503411	8.78632584447424	7.22466332136213	8.9179726739645	8.16828259063743	NA
**TC1300000883.mm.2**	Slc6a3	solute carrier family 6 (neurotransmitter transporter, dopamine), member 3	-0.346518616420384	5.43787275135647	-0.950574636373247	0.376166185337937	0.999692817443324	-4.59854100688358	5.42360774825316	5.39656338693419	5.6019216380799	4.95509243197545	5.38617763895126	5.86387366394487	NA
**TC1900001097.mm.2**	Myrf	myelin regulatory factor	0.288774974050945	7.74048046567754	0.95021876718081	0.376333811224258	0.999692817443324	-4.59855938257098	7.81195542289731	8.0980965182048	7.78965299018637	7.76553741034614	7.59321763776098	7.38442281466964	NA
**TC0800003051.mm.2**	1700018B08Rik	RIKEN cDNA 1700018B08 gene	0.353506521723084	6.60756427148251	0.950085728021223	0.376396491717727	0.999692817443324	-4.59856625127064	6.24979370939785	6.1416383007204	6.64882967310945	7.33165365800204	6.39136573378743	6.88210455387789	NA
**TC1000002435.mm.2**	Krtap10-4	keratin associated protein 10-4	-0.317264371538908	6.87271282796811	-0.949960660296119	0.37645542384 54	0.999692817443324	-4.59857270795505	6.32650893662853	6.61467062141406	7.1364152782368	6.69386905510732	7.07920243501028	7.38561064141165	NA
**TC0100002844.mm.2**	St8sia4	ST8 alpha-N-acetyl-neuraminide alpha-2,8-sialyltransferase 4	-0.361174842239484	6.37250597842776	-0.949955891532836	0.376457671035306	0.999692817443324	-4.59857295413609	6.40762585922517	6.24214537488677	6.45205831540431	5.97939916058351	6.94655704672998	6.2072501137368	NA
**TC0600002616.mm.2**	Nat8f7	N-acetyltransferase 8 (GCN5-related) family member 7	-0.46309665299308	5.78755769150547	-0.949927899432897	0.376470861993329	0.999692817443324	-4.59857439917795	5.3703172092865	5.27157521706303	5.22235528649184	6.35627492185672	6.6705517432145	5.83427177112022	NA
**TC1500001836.mm.2**	Sstr3	somatostatin receptor 3	0.29083990124199	6.25815564656232	0.949923468736896	0.376472949940897	0.999692817443324	-4.59857462790264	6.11848382252124	6.08898297463143	6.49996429076385	6.46160915158875	6.0018578535716	6.37803578629702	NA
**TC1700000350.mm.2**	Rpl3l	ribosomal protein L3-like	0.444440227535948	5.82756261710364	0.94947715545736	0.376683318896633	0.999692817443324	-4.59859766497007	5.39288915870555	5.43935542928137	5.65780153368403	6.85320425116937	5.5259786873122	6.0961466424693	NA
**TC1600001473.mm.2**	Acap2	ArfGAP with coiled-coil, ankyrin repeat and PH domains 2	-0.553845847908536	8.12788858914697	-0.949340307760375	0.376747839911704	0.999692817443324	-4.59860472741964	8.72333525104357	8.87654507009102	8.38706542781992	6.64281433114516	8.31867573836927	7.81889571641288	NA
**TC0700003820.mm.2**	Dnajb13	DnaJ heat shock protein family (Hsp40) member B13	0.337482936712466	5.33880087995275	0.948907031678268	0.376952177149978	0.999692817443324	-4.59862708447267	5.44367152224935	5.10180446155398	5.51742586926934	5.56372171539971	4.86916357180946	5.53701813943466	NA
**TC0400002572.mm.2**	Tomm5	translocase of outer mitochondrial membrane 5	0.332795658558686	8.40169175487426	0.948895117765588	0.376957797070868	0.999692817443324	-4.59862769915553	8.35298126741267	8.51073693699934	8.54009922346167	8.5659125975138	8.57186932026101	7.86855118359708	NA
**TC0700000647.mm.2**	Pepd	peptidase D	-0.623970582503656	6.32639199343712	-0.948459868269684	0.377163153095629	0.999692817443324	-4.59865015252608	7.29500089775172	7.47613657028429	6.37495105854691	4.59468560524278	6.67942493860488	5.53815289019212	NA
**TC0100003457.mm.2**	Pou2f1	POU domain, class 2, transcription factor 1	0.667167963437437	6.43238463826803	0.948208711223964	0.377281691164751	0.999692817443324	-4.59866310660589	6.52854476152929	6.2389040410054	7.69602196968063	5.88457550729356	6.81958718523391	5.4266743648654	NA
**TC1700002138.mm.2**	Runx2	runt related transcription factor 2	-0.374063879434426	7.24105570526537	-0.948178034980631	0.377296171328989	0.999692817443324	-4.59866468869033	6.10201669680817	6.58558314221814	7.22117961869208	7.78412369815643	7.56479845924497	8.1886326164724	NA
**TC0600002427.mm.2**	Tacstd2	tumor-associated calcium signal transducer 2	0.316753115632151	5.73263864775606	0.947993515431646	0.377383279443765	0.999692817443324	-4.59867420446422	5.1052934020071	4.94242129535466	6.00948976450847	6.48132194571099	5.75191971952889	6.10538575942626	NA
**TC0400003885.mm.2**	Fblim1	filamin binding LIM protein 1	-0.331597915393878	7.57638682947746	-0.947829622590018	0.377460663043662	0.999692817443324	-4.59868265569577	6.83883090217658	7.04909930101132	7.51334006764942	7.94025740379511	7.79104635365017	8.32574694858212	NA
**TC0100003440.mm.2**	Tiprl	TIP41, TOR signalling pathway regulator-like (S. cerevisiae)	-0.519945685547055	7.06886364177225	-0.947705954961247	0.377519061986315	0.999692817443324	-4.59868903218462	7.90606502380514	8.00528725024617	7.00534719510761	5.72562190763642	7.42440261389129	6.34645785994686	NA
**TC0700001766.mm.2**	Rbbp6	retinoblastoma binding protein 6, ubiquitin ligase	-1.31003679337558	7.49812985266011	-0.947664439269998	0.377538668288419	0.999692817443324	-4.59869117269853	9.1040659800415	8.80825650547124	8.32520291250997	3.90298860933842	8.40808871622628	6.44017639237326	NA
**TC1700001656.mm.2**	Baiap3	BAI1-associated protein 3	-0.351333033860691	6.20639924749214	-0.947495573049181	0.377618425524394	0.999692817443324	-4.59869987879248	6.10381226272996	5.85338751760932	5.7817288684284	6.50753595001768	6.32489286373903	6.66703802242843	NA
**TC1800000363.mm.2**	Pcdhb8	protocadherin beta 8	0.349395647636463	6.45406000261233	0.947494153910723	0.377619095852773	0.999692817443324	-4.59869995195437	6.63264858547149	6.6344431260925	6.38174454931151	6.69628525037996	5.8061518991019	6.57308660531664	NA
**TC0X00000343.mm.2**	Rhox2b	reproductive homeobox 2B	0.332083502998516	6.56539911119665	0.947145227290141	0.377783938668314	0.999692817443324	-4.59871793868538	5.84993991756745	5.87748116372526	6.73546505908943	7.42910523685269	6.84686719757828	6.6535360923668	NA
**TC0500001487.mm.2**	Glt1d1	glycosyltransferase 1 domain containing 1	0.512646751649027	6.23214905438581	0.947098178473903	0.377806170088599	0.999692817443324	-4.59872036372774	6.38358604649969	6.21489328492072	6.47671873354179	6.43313551555445	6.76021296821028	5.12434777758791	NA
**TC0700000507.mm.2**	Fbxo27	F-box protein 27	-0.548850787648805	5.76361284464105	-0.946832589978912	0.377931684318037	0.999692817443324	-4.59873405180204	6.04592607230656	5.76438567968837	6.12112081096963	4.71571105930724	6.516225237713	5.41830820786147	NA
**TC1100003629.mm.2**	Pnpo	pyridoxine 5'-phosphate oxidase	-0.314478583990643	7.5544420147608	-0.946829107809315	0.377933330166064	0.999692817443324	-4.59873423125503	8.20130939857463	7.74155428159554	7.3342472413675	7.04316837883918	7.62660168903739	7.37977109915057	NA
**TC1000001624.mm.2**	Sarnp	SAP domain containing ribonucleoprotein	-0.711505753837205	5.58726054242731	-0.94682502574549	0.377935259561203	0.999692817443324	-4.59873444162303	7.47886670088426	7.44705856042942	5.34913093791181	3.23818230487609	5.66544875302333	4.34487599743898	NA
**TC1600001112.mm.2**	Slx4	SLX4 structure-specific endonuclease subunit homolog (S. cerevisiae)	-0.299159760920025	5.94616927382725	-0.94665136365524	0.378017348279086	0.999692817443324	-4.5987433908093	5.40471472091636	5.84317841506885	5.88994564314106	6.02545584942805	6.2020062378378	6.31171477657136	NA
**TC0600000572.mm.2**	Aoc1	amine oxidase, copper-containing 1	0.397110497407793	4.83837068498058	0.9464891637635	0.378094031267255	0.999692817443324	-4.59875174854672	4.64008201944631	4.21920167265846	4.9614897658107	5.52109094048645	4.42230014673473	5.26605956474683	NA
**TC0600003379.mm.2**	Abcc9	ATP-binding cassette, sub-family C (CFTR/MRP), member 9	-0.375360521991074	5.2844874538419	-0.94634542170132	0.378161997956105	0.999692817443324	-4.59875915457306	5.62310736687604	5.35740981649615	5.00936784772773	4.97847540012079	5.84100172716844	4.89756256466223	NA
**TC1800001636.mm.2**	Siglec15	sialic acid binding Ig-like lectin 15	-0.377267468196605	6.90125031077156	-0.946270186442154	0.37819757577369	0.999692817443324	-4.5987630306864	5.93727681955339	5.86835079257696	6.86868985484276	7.55497980321013	7.23314628530064	7.94505830914546	NA
**TC1500000696.mm.2**	Mief1	mitochondrial elongation factor 1	0.273538534083969	7.38746956256568	0.946179266786582	0.378240573975457	0.999692817443324	-4.59876771464275	7.42480881280309	7.4000664148144	7.56765539801806	7.4558542099542	7.23589310834272	7.24053943146161	NA
**TC0600001074.mm.2**	Klf15	Kruppel-like factor 15	-0.320845559518467	6.29172342939151	-0.946114686704653	0.378271117806526	0.999692817443324	-4.59877104150592	6.25226818521829	6.01096666443554	6.46825517415457	5.95445212967458	6.37803128858468	6.68636713428139	NA
**TC0600002099.mm.2**	Zc3hav1	zinc finger CCCH type, antiviral 1	-0.473275973820531	4.8780274033347	-0.945870990655392	0.378386393473747	0.999692817443324	-4.59878359451171	4.61175513684486	4.97841605814499	4.3481764004196	5.01754423826905	4.52850310965456	5.78376947667515	NA
**TC0800002634.mm.2**	N4bp1	NEDD4 binding protein 1	-0.539995138251482	7.35204507373854	-0.945657512488626	0.378487397310642	0.999692817443324	-4.59879458957286	7.97223968424947	7.38473806972293	7.65906740323569	6.17858380274226	7.84448335215694	7.07315813032398	NA
**TC0400003122.mm.2**	Kank4	KN motif and ankyrin repeat domains 4	-0.365099290024556	5.8538138584998	-0.94550770834906	0.378558287134673	0.999692817443324	-4.59880230436494	5.63101046056242	5.22199979732483	5.48123886795878	6.28859288857243	6.1541202970751	6.34591543950522	NA
**TC1200000647.mm.2**	Prkch	protein kinase C, eta	0.410493060242606	7.73420226003257	0.945483223344783	0.378569874816741	0.999692817443324	-4.59880356526187	8.46399472848449	8.52104798954796	7.90931518799239	7.21126329333171	7.61670296382499	6.6828893970139	NA
**TC0100001968.mm.2**	Prdm14	PR domain containing 14	-0.378499221234613	4.77196856863552	-0.945469674647818	0.37857628693964	0.999692817443324	-4.59880426296769	4.31807108871591	3.96701036103013	4.75425258460222	5.04061317519671	4.83034975554341	5.72151444672475	NA
**TC1300002078.mm.2**	Neurog1	neurogenin 1	0.385946553325036	5.56153360015191	0.945304047510967	0.37865467918798	0.999692817443324	-4.5988127917044	4.71733334398561	5.55337103216585	5.67149874426559	6.26369642143944	5.67168585805455	5.49161620100041	NA
**TC0900002128.mm.2**	Oaf	out at first homolog	-0.314776809120295	5.76209942287105	-0.94511282322377	0.378745202199058	0.999692817443324	-4.59882263755453	5.80010620343181	6.1310879743947	5.3122314876377	5.69369288294191	5.65203969627458	5.98343829254561	NA
**TC0300002068.mm.2**	Gpr149	G protein-coupled receptor 149	0.383355882242512	5.54234038135849	0.944833849732124	0.378877294280006	0.999692817443324	-4.59883699960206	5.97823886170398	5.41937284350474	6.03659205102753	5.27497912268609	5.07049065162509	5.47436875760351	NA
**TC0300000655.mm.2**	Tigd4	tigger transposable element derived 4	0.515732723771201	4.62793373797884	0.944660714114841	0.378959290603981	0.999692817443324	-4.59884591180863	4.56851689958893	4.57139587783518	4.41311116898197	5.4164663800137	3.73481003242563	5.06330206902764	NA
**TC1300000555.mm.2**	Barx1	BarH-like homeobox 1	0.315770526451506	5.08398912731164	0.944545147282411	0.379014030159355	0.999692817443324	-4.59885186016736	4.58521524228988	4.52091060272606	5.21101794753061	5.80365703834784	5.26276877147384	5.12036516150159	NA
**TC0500001264.mm.2**	Pxn	paxillin	0.307478428605606	7.29970438859099	0.944315558017068	0.379122795736406	0.999692817443324	-4.59886367625756	7.0324575904682	7.06834767938406	7.30846096655521	7.84772799289723	7.22137514413444	7.31985695810679	NA
**TC1100003726.mm.2**	Gm11555	predicted gene 11555	0.314180422664863	5.87557254985345	0.943973208265097	0.379285024895977	0.999692817443324	-4.59888129289664	5.83872651615599	5.3035063609087	6.31168094467716	6.05810068901572	5.92583754676409	5.81558324159906	NA
**TC0600003468.mm.2**	Ipo8	importin 8	-0.454749932600191	6.69970618227406	-0.943625470117684	0.379449861931682	0.999692817443324	-4.59889918337011	7.49764011389807	7.40907748460495	6.6247728312102	5.56623698376028	6.99658927877942	6.10392040139144	NA
**TC1000001226.mm.2**	1700017N19Rik	RIKEN cDNA 1700017N19 gene	0.527110841421729	5.07342918136659	0.943536994802289	0.379491810322362	0.999692817443324	-4.59890373470479	4.40897105604576	4.12008982664631	5.18116496713878	6.30170297703668	4.57928004614901	5.84936621518299	NA
**TC0200003217.mm.2**	Brd3/Mir7578	bromodomain containing 3/microRNA 7578	0.358502094569698	7.39853028411954	0.943535638167744	0.379492453564872	0.999692817443324	-4.59890380449084	7.88776596018378	7.6841755683794	7.72361956101297	7.04450262163374	7.29588978487218	6.75522820863514	NA
**TC0300001391.mm.2**	Emcn	endomucin	-0.380220653424616	6.24801545472929	-0.943335363704342	0.379587422019729	0.999692817443324	-4.59891410614343	6.71784082667759	5.92106473387286	5.7853641578623	6.25900877262573	6.1971977695753	6.60761646776196	NA
**TSUnmapped00000028.mm.2**	Defa-rs10	defensin, alpha, related sequence 10	-0.323681009702286	6.34032475912118	-0.94332373483864	0.379592936888868	0.999692817443324	-4.59891470426985	5.88723688909642	5.85322077284815	6.18846852284687	6.6385959138421	6.48786507703411	6.98656137905944	NA
**TC1600001844.mm.2**	Htr1f	5-hydroxytryptamine (serotonin) receptor 1F	0.328868028891115	5.62411109181896	0.943171039525758	0.379665356756807	0.999692817443324	-4.59892255773652	6.01472485412743	6.03114556775744	5.2483763014474	5.92988979195816	5.3566586187823	5.16387141684103	NA
**TC0700003439.mm.2**	Asb7	ankyrin repeat and SOCS box-containing 7	0.371566052027818	5.1057660198189	0.942722064991275	0.379878356367311	0.999692817443324	-4.59894564563433	5.34397334046297	4.89300344811059	4.89125628868547	5.67911942851228	4.74622027287126	5.08102334027086	NA
**TC1300001835.mm.2**	Txndc5	thioredoxin domain containing 5	0.402919025459873	7.4343698582809	0.942712299549565	0.379882990242547	0.999692817443324	-4.59894614774433	8.22557945931173	8.09922799227367	7.5341348873694	7.00948998714046	7.36800149978404	6.36978532380608	NA
**TC1400001104.mm.2**	Kbtbd6	kelch repeat and BTB (POZ) domain containing 6	-0.384597626523001	5.15906373856838	-0.942406390178504	0.380028171590864	0.999692817443324	-4.59896187530623	4.63076138859437	4.83660644581328	4.86003720661802	5.49887246536028	5.16726807835521	5.96083684666909	NA
**TC0100000386.mm.2**	Ormdl1	ORM1-like 1 (S. cerevisiae)	-0.349369000043176	6.27239560764885	-0.942355798678173	0.380052185876754	0.999692817443324	-4.59896447608162	6.05165836453359	6.29785731993181	6.1389403988935	6.15411958177717	6.09429643079962	6.89750154995741	NA
**TC0700000852.mm.2**	Hsd17b14	hydroxysteroid (17-beta) dehydrogenase 14	0.322254096914901	5.46548446175262	0.942328389033392	0.380065196908322	0.999692817443324	-4.59896588510829	5.22343995478083	5.13680427366023	5.48564751663811	6.05293785131413	5.27896370736	5.61511346676244	NA
**TC0200004227.mm.2**	Cat	catalase	0.503654162737734	8.46038928997773	0.942327831753462	0.380065461446	0.999692817443324	-4.59896591375573	9.8177548438397	9.85851943671134	8.79147307443459	7.2552118179608	7.76995263294032	7.2694239339796	NA
**TC0100002207.mm.2**	2010300C02Rik	RIKEN cDNA 2010300C02 gene	0.294678917011416	7.84351021772881	0.94198795855671	0.380226823633189	0.999692817443324	-4.59898338355478	8.19567647671562	8.4520862353671	7.61592942931138	7.88539878484511	7.57688889350603	7.33508148662763	NA

**TC1000000626.mm.2**	Jmjd1c	jumonji domain containing 1C	-1.00296066367469	6.77597191482286	-0.94195666302464	0.380241684505102	0.999692817443324	-4.59898499200701	7.56494638904126	7.61735098014101	7.54912651159101	4.18467988461174	7.61100835298685	6.12871937056528	NA
**TC0500002306.mm.2**	Bod1l	biorientation of chromosomes in cell division 1-like	-0.56503246052252	6.43015553778754	-0.94176775047799	0.380331400213049	0.999692817443324	-4.59899470067882	7.82223510173023	7.68378482314881	5.8493105370096	5.12311365339099	6.9203412686848	5.18214784276083	NA
**TC0400001608.mm.2**	Slc9a1/Mir5122	solute carrier family 9 (sodium/hydrogen exchanger), member 1/microRNA 5122	0.531054899390167	7.38668766826384	0.941611315063282	0.380405704604195	0.999692817443324	-4.59900273949386	8.62193132941654	8.72105284408556	7.81325510211279	6.20637071531785	6.80938192053984	6.14813409811047	NA
**TC1700001634.mm.2**	Slc9a3r2	solute carrier family 9 (sodium/hydrogen exchanger), member 3 regulator 2	0.310826110993013	7.01847645424799	0.941576437044707	0.380422272636443	0.999692817443324	-4.59900453168966	7.32378293117185	7.51749921781173	7.1003864989089	6.84522790033629	6.41849767958767	6.9054644976715	NA
**TC0300002770.mm.2**	Prmt6	protein arginine N-methyltransferase 6	0.362735566850254	6.24006590716033	0.941568625300491	0.380425983508704	0.999692817443324	-4.59900493308883	6.49326596726828	5.87093520282116	6.76650386994818	6.13432883333834	5.96512027162426	6.21024129796176	NA
**TC0700000652.mm.2**	Cebpa	CCAAT/enhancer binding protein (C/EBP), alpha	-0.321652684060466	6.26925562804762	-0.941530188446118	0.380444242863653	0.999692817443324	-4.59900690810498	5.92315874457872	6.28335004276592	5.92788091579311	6.45497889061696	6.6020820392708	6.4240831352602	NA
**TC0X00000901.mm.2**	Gspt2	G1 to S phase transition 2	0.411087226885502	6.88653180830867	0.941466383097211	0.380474554955512	0.999692817443324	-4.59901018654649	7.10821269246248	6.58221890857933	6.88035932296045	7.34510752833016	7.17537966453506	6.22791273298454	NA
**TC0X00003329.mm.2**	Siah1b	siah E3 ubiquitin protein ligase 1B	0.367022319096405	5.73077083516191	0.94131336775884	0.380547255690466	0.999692817443324	-4.59901804829033	6.0890396154439	5.94630312511732	5.72213996727776	5.81952348702378	5.86846269063463	4.93915612547409	NA
**TC0700004623.mm.2**	Dctn5	dynactin 5	-0.387074459497126	8.13924624124812	-0.941308678158973	0.380549483983802	0.999692817443324	-4.59901828922566	8.78365199880689	8.75674112665612	8.14162941639567	7.11883828512006	8.14590646772183	7.88871015278816	NA
**TC0100001980.mm.2**	Lactb2	lactamase, beta 2	0.52915942184613	6.15468853752904	0.941239528718448	0.380582341941867	0.999692817443324	-4.59902184180969	7.3815627779775	7.73746296384597	6.0745932043569	5.35911895916462	5.95656612935613	4.41882719047313	NA
**TC0700004444.mm.2**	Insyn2a	inhibitory synaptic factor 2A	-0.29930240804485	7.6830074413813	-0.941233890064109	0.380585021374836	0.999692817443324	-4.59902213149204	7.23346630485621	7.00772564062194	7.80092580461785	7.82819813874213	7.89763856607191	8.33009019337777	NA
**TC1500000583.mm.2**	Smpd5	sphingomyelin phosphodiesterase 5	-0.310583625713117	6.30958545865721	-0.941220004497209	0.380591619720949	0.999692817443324	-4.59902284485047	5.75370413446947	6.13396429668638	6.54003511600067	6.13430341867992	6.56198182330856	6.73352396279826	NA
**TC0200003599.mm.2**	Cd302	CD302 antigen	0.300149990216975	6.47337294112803	0.941059712189106	0.38066779609787	0.999692817443324	-4.59903107931989	7.03236001500079	7.50157574332076	6.29207350988078	6.16122742455951	5.9747012859153	5.87829966809104	NA
**TC1900000047.mm.2**	Zdhhc24	zinc finger, DHHC domain containing 24	0.290993014775607	7.64237121964949	0.940953169904416	0.380718435085713	0.999692817443324	-4.59903655215526	7.48670693537636	7.35640473048022	7.92532773783632	7.87122310295945	7.79985460878393	7.41471020246063	NA
**TC0200002212.mm.2**	Syndig1	synapse differentiation inducing 1	0.397220557984686	7.94277323759491	0.940833160305287	0.380775481192608	0.999692817443324	-4.59904271638348	8.53641676289693	8.28852018803896	8.3754216996545	7.43765009564698	7.77854045006308	7.24009022926903	NA
**TC0700004666.mm.2**	Fgfr2	fibroblast growth factor receptor 2	-0.414946940136057	7.25813909024401	-0.940656043631307	0.380859684883693	0.999692817443324	-4.59905181312576	7.25662996861721	7.65735437738532	6.51431808134229	7.38816007625243	7.0067617519797	7.72561028588713	NA
**TC0X00001341.mm.2**	H2bfm	H2B histone family, member M	0.651790036580023	4.44709131167848	0.940492220338544	0.380937581406024	0.999692817443324	-4.59906022631087	3.69214106154296	3.60194876654181	4.36747994240325	5.97853911516982	3.72878969026395	5.31364929414907	NA
**TC1200000588.mm.2**	Tmx1	thioredoxin-related transmembrane protein 1	0.61992027315346	6.99070692062909	0.94044277045897	0.380961096780597	0.999692817443324	-4.59906276566908	8.39600375079675	8.04109202841678	7.32213067412493	6.05136247130903	6.90137540339495	5.23227719573209	NA
**TC1000002544.mm.2**	Ncln	nicalin	0.386038329590381	7.43822336652558	0.940383996497467	0.380989047569924	0.999692817443324	-4.59906578374712	8.07060024804831	7.83326218735148	7.83718037018601	6.9115968412812	7.20776449253745	6.76893605974901	NA
**TC1600001303.mm.2**	Slc25a1	solute carrier family 25 (mitochondrial carrier, citrate transporter), member 1	-0.607574382738015	8.68648570620213	-0.940369941940153	0.380995731646098	0.999692817443324	-4.59906650544224	9.19030404711149	8.94238998104057	9.18282073347837	7.20271498831396	9.08533255437856	8.5153519328898	NA
**TC0600001450.mm.2**	Mrpl51	mitochondrial ribosomal protein L51	-0.3936465299075	7.89312614827308	-0.940148148468293	0.381101224222394	0.999692817443324	-4.59907789367982	8.3356749131604	8.44771015311643	7.99009156930557	6.90394781246775	7.85435048335375	7.82698195823457	NA
**TC1700001836.mm.2**	Brd4	bromodomain containing 4	0.490822244646369	9.02392293246545	0.940085817926975	0.3811308747868	0.999692817443324	-4.59908109385612	9.59577983163813	9.38661196593184	9.68777601326966	8.38361912998807	8.96306995669561	8.12668069726939	NA
**TC1100001077.mm.2**	Myo1c	myosin IC	0.272906625874396	5.68257795264982	0.940030950255863	0.381156976733442	0.999692817443324	-4.59908391078054	6.03962611988872	5.95865117626112	5.69831564426362	5.62318619148532	5.33781107396584	5.43787751003431	NA
**TC0700003818.mm.2**	Ppme1	protein phosphatase methylesterase 1	0.316892127710523	8.05028306048549	0.939929366621094	0.38120530626241	0.999692817443324	-4.59908912588809	8.66497621603016	8.71094636116975	8.07092354311881	7.70885647744823	7.83131539831031	7.31468036683568	NA
**TC1800000789.mm.2**	Cfap53	cilia and flagella associated protein 53	-0.396483354540513	4.81347230221641	-0.939815191788979	0.381259631787695	0.999692817443324	-4.59909498704696	4.9739707689947	5.06287393630915	3.97344122615427	5.05206997330253	5.02745714883367	4.79102075970416	NA
**TC0600000591.mm.2**	Mpp6	membrane protein, palmitoylated 6 (MAGUK p55 subfamily member 6)	-0.807649125493454	7.02481497667935	-0.939529991467005	0.381395358824035	0.999692817443324	-4.59910962614148	8.20584517480215	8.13065833174037	7.45023277719429	4.64831127407905	7.23964636783972	6.47419593442053	NA
**TC0400000133.mm.2**	Tmem64	transmembrane protein 64	-0.509878008091206	8.1451906962489	-0.939446719437148	0.38143499501287	0.999692817443324	-4.599113899981	8.2649016272691	8.31917175094789	8.54149674588832	7.09216064565868	8.72144248939492	7.93197091833449	NA
**TC0200005092.mm.2**	Hnf4aos	hepatic nuclear factor 4 alpha, opposite strand	0.309838605416958	5.5892244013175	0.939397827356378	0.381458268349565	0.999692817443324	-4.59911640921626	5.13015273292082	5.49553983418151	5.72703044067553	6.03763508514278	5.40866401034349	5.73632430464091	NA
**TC1400002469.mm.2**	Tnfsf11	tumor necrosis factor (ligand) superfamily, member 11	-0.293583084548878	5.44518166771923	-0.939287280492206	0.381510894257971	0.999692817443324	-4.5991220824378	4.81630197180038	5.03374457563799	5.47154940519192	5.64538923969773	5.68089482320902	6.02320999077838	NA
**TC0700001430.mm.2**	Stard10	START domain containing 10	0.301629805638403	7.76707225037712	0.938731294746416	0.381775655835918	0.999692817443324	-4.59915061003617	8.47594967682205	8.17053350193229	7.58365285651177	7.69595211088081	7.5382870202661	7.13805833584967	NA
**TC0200003605.mm.2**	Itgb6	integrin beta 6	-0.308487170479264	7.14874459187105	-0.93871873396552	0.381781638928628	0.999692817443324	-4.5991512544256	6.02244997279137	6.09049094663374	7.29581708159641	7.78545906382493	7.70525538908531	7.99299509729456	NA
**TC0600000841.mm.2**	St3gal5	ST3 beta-galactoside alpha-2,3-sialyltransferase 5	-0.526203648687483	8.76431255964171	-0.93859264281285	0.381841704059649	0.999692817443324	-4.59915772286258	9.27208083180549	9.52361064574206	9.00020767260185	7.36868061886202	8.96337273927678	8.45792284956206	NA
**TC0700003778.mm.2**	Tsku	tsukushi, small leucine rich proteoglycan	-0.311109049315102	6.15265575351968	-0.938574736064796	0.381850234753653	0.999692817443324	-4.59915864143579	5.76013004748458	6.18935726262584	6.00494846166722	6.16716609452151	6.20957215517388	6.58476049964505	NA
**TC0200005489.mm.2**	Zfp965	zinc finger protein 965	-0.703486330596688	8.2362603692508	-0.938153717124734	0.382050848265891	0.999692817443324	-4.59918023601458	9.34648563938735	9.71839841608006	8.36297741460457	6.10987533481745	8.4286100556365	7.45121535497889	NA
**TC1500001760.mm.2**	Scrib	scribbled planar cell polarity	0.317410238920592	6.06720818460985	0.938039006784725	0.382105521158024	0.999692817443324	-4.59918611875647	6.00382091939795	6.09011183974129	5.91962989752002	6.55243851566051	5.89157598675863	5.94567194858071	NA
**TC0500003261.mm.2**	Eif2b1	eukaryotic translation initiation factor 2B, subunit 1 (alpha)	-0.667906032919075	7.2892051809019	-0.937937578850151	0.382153868411989	0.999692817443324	-4.59919132001231	7.68398795942653	7.58007364184565	7.95062611469345	5.61705259445709	7.42135571179461	7.48213506319408	NA
**TC1100004039.mm.2**	Cdc42ep4	CDC42 effector protein (Rho GTPase binding) 4	-0.299753014226697	6.65257377532905	-0.937591179052781	0.382319020688708	0.999692817443324	-4.59920908124635	6.57526098845129	6.4099625524695	6.38674448230056	6.77861205899949	6.74835326375512	7.01650930599832	NA
**TC0400003930.mm.2**	Pramef6	PRAME family member 6	-0.338761448308621	5.19123171439202	-0.937509228572355	0.382358100017597	0.999692817443324	-4.59921328264853	4.51137538379656	4.86094911536406	5.02690477658528	5.52186666870185	5.36299433239304	5.86330000951132	NA
**TC1600000958.mm.2**	Bach1	BTB and CNC homology 1, basic leucine zipper transcription factor 1	-0.29289353675131	6.91589196276208	-0.937457523129442	0.382382758112283	0.999692817443324	-4.59921593336054	6.80728659102223	6.76628253331862	7.05281566116588	6.61518212819863	7.1160746682157	7.13771019465143	NA
**TC0X00001759.mm.2**	AU022751	expressed sequence AU022751	0.355656944248107	4.83851163599991	0.937436775121323	0.382392653086255	0.999692817443324	-4.59921699699835	4.39708672878746	4.28361040145942	4.94949767297968	5.58134561414473	4.60261818499066	5.21691121363753	NA
**TC0400001569.mm.2**	Mecr	mitochondrial trans-2-enoyl-CoA reductase	-0.298063043417903	5.75971879304934	-0.937431118005351	0.382395351066672	0.999692817443324	-4.59921728700582	6.05844245800855	5.96946038260639	5.40794063031599	5.55920128510665	5.55378722511503	6.00948077714341	NA
**TC1200000073.mm.2**	Matn3	matrilin 3	0.470150559489842	4.77618777781784	0.93734143956797	0.382438122270249	0.999692817443324	-4.59922188417391	4.1288464712934	3.96299500401969	4.82464734004143	5.92814581524537	4.4244064595883	5.38808557671882	NA
**TC1100002392.mm.2**	5730522E02Rik	RIKEN cDNA 5730522E02 gene	-0.338101553958849	5.80661557467625	-0.937234719042265	0.382489026284776	0.999692817443324	-4.59922735466225	5.74381385097313	5.53319387651339	6.09219458745269	5.35104671887395	6.12732013734954	5.9921242768948	NA
**TC1800001682.mm.2**	Zfp236	zinc finger protein 236	0.305979052253547	6.21408373288516	0.937166033564258	0.382521790916864	0.999692817443324	-4.59923087530079	6.48687553551632	6.54632883839675	5.94482146618912	6.48680659776338	5.85522660176852	5.96444335767688	NA
**TC1000000811.mm.2**	Gzmm	granzyme M (lymphocyte met-ase 1)	-0.356389878781823	6.27358774238475	-0.936942131002577	0.382628612734497	0.999692817443324	-4.59924235100869	5.64558590353107	5.56232834019787	6.29952345638963	6.56089277011834	6.36120990029029	7.21198608378133	NA
**TC0700000528.mm.2**	Dpf1	D4, zinc and double PHD fingers family 1	-0.361417737806021	7.3806569354383	-0.936720575901247	0.382734337031013	0.999692817443324	-4.59925370496464	7.45402133259161	7.449054741813	7.48088253425452	6.84813249705206	7.18960347827091	7.86224702864772	NA
**TC0600002084.mm.2**	Dgki	diacylglycerol kinase, iota	0.656094184479582	6.79352798328132	0.93645139850221	0.382862816322032	0.999692817443324	-4.59926749747902	8.29704913685493	8.39399170530875	6.85099350640698	5.84016420683472	6.7166455885906	4.66232375569194	NA
**TC1800001665.mm.2**	Nfatc1	nuclear factor of activated T cells, cytoplasmic, calcineurin dependent 1	-0.39356853724241	5.35839912142905	-0.936436390207672	0.382869980801588	0.999692817443324	-4.59926826643431	5.55353963379116	5.36266665129589	4.57636560517951	5.6471600793217	5.58746778871031	5.42319497027572	NA
**TC0800002558.mm.2**	Adgre5/Mir1668	adhesion G protein-coupled receptor E5/microRNA 1668	0.525824117578052	6.20055069941353	0.936265380190366	0.382951622741642	0.999692817443324	-4.59927702772932	5.99980028790221	6.049424104161	5.85850223265564	7.24436178713139	5.5079638821884	6.54325190244252	NA
**TC1400001225.mm.2**	Frg2f3	FSHD region gene 2 family member 3	0.285486818413781	5.68647592806468	0.936220302010233	0.382973145742122	0.999692817443324	-4.59927933706154	5.47480655308954	5.58767907282694	5.9208160262539	5.89285576339568	5.79499485354606	5.44770329927596	NA
**TC1100003161.mm.2**	Aspa	aspartoacylase	-0.869389883219422	6.24128363152735	-0.936035752828755	0.383061270113982	0.999692817443324	-4.599288790804	7.65357149740786	7.39931357409121	6.58792394185061	3.74009453376247	6.7150430172866	5.35175522476532	NA
**TC1500000777.mm.2**	Mpped1	metallophosphoesterase domain containing 1	-0.369106465477049	8.23565535540648	-0.935976590161878	0.383089524249937	0.999692817443324	-4.59929182126878	8.42837050460476	8.20456030971226	8.41891139285532	7.60248280007285	8.61523172649757	8.1443753980404	NA
**TC0400001151.mm.2**	Orc1	origin recognition complex, subunit 1	0.32874617017011	5.10539429518372	0.935806677627667	0.383170677712134	0.999692817443324	-4.59930052406007	4.58784374688486	4.91148057777686	5.77021197047002	5.12505492292038	5.08549679541434	5.15227775763584	NA
**TC0700001865.mm.2**	Stx4a	syntaxin 4A (placental)	-0.373592301917781	6.51022050419274	-0.935777711677663	0.383184513710884	0.999692817443324	-4.59930200759	7.96460174951575	8.01211268826556	6.03140489050118	5.13730710126858	5.74203912395596	6.17385747164938	NA
**TC1500001212.mm.2**	Rxfp3	relaxin family peptide receptor 3	0.419555980268361	5.39168737676192	0.935664526801658	0.38323858173677	0.999692817443324	-4.59930780427013	5.4578735436358	5.12029461374785	5.52375947071162	5.78177456115067	4.65362702830219	5.81279504302338	NA
**TC0400003275.mm.2**	Elavl4	ELAV like RNA binding protein 4	-0.597542278611101	7.46314638728569	-0.935441857159483	0.383344967257677	0.999692817443324	-4.59931920703983	8.11192588890031	8.1819351458798	7.79886961731526	5.84609674854066	7.72332320754961	7.11672771552851	NA
**TC0600000678.mm.2**	Fkbp9	FK506 binding protein 9	-0.663131091218951	6.9060860389133	-0.935367229012079	0.383380627607408	0.999692817443324	-4.59932302837533	8.20708459793271	8.34942840047716	6.63439567718375	5.14247484913225	7.4398838551535	6.66324885360041	NA
**TC0700002506.mm.2**	Ceacam19	carcinoembryonic antigen-related cell adhesion molecule 19	0.297756686141901	5.53339710850839	0.9352528358199	0.383435294220334	0.999692817443324	-4.59932888556463	5.22344313573824	5.38481214550632	5.57808727682007	6.01573309422472	5.42127496424592	5.57703203451507	NA
**TC1100003496.mm.2**	Msi2	musashi RNA-binding protein 2	-0.382098540009701	8.28050829621152	-0.935046380321428	0.383533970943191	0.999692817443324	-4.59933945558331	8.87446173442729	8.52019497789602	8.29069994902616	7.47139804343705	8.53200700725766	7.99428806522494	NA
**TC0100002764.mm.2**	Ngef	neuronal guanine nucleotide exchange factor	0.356633593870125	8.40080838921645	0.935009902470389	0.383551407779303	0.999692817443324	-4.59934132303088	9.2538881215602	9.2923224068544	8.34697575024835	7.93897774706383	8.18677472116751	7.38591158840442	NA
**TC0500001669.mm.2**	Get4	golgi to ER traffic protein 4	-0.384104053142385	6.81771983743076	-0.93487336544737	0.383616679430858	0.999692817443324	-4.59934831256349	7.50129601584987	7.89819128145905	6.52498368593548	5.84432812455994	6.91822098019642	6.21929893658376	NA
**TC0100003321.mm.2**	Fam20b	family with sequence similarity 20, member B	0.30267025384982	8.01271592663124	0.934703441445448	0.383697923597056	0.999692817443324	-4.59935701046328	8.3960758123796	8.23217397679336	7.98811242160485	8.03858071755222	7.9643428770022	7.45700975445523	NA
**TC1900000921.mm.2**	Rhod	ras homolog family member D	0.289355070104608	4.97694171133658	0.93459650625839	0.383749058243545	0.999692817443324	-4.59936248372144	4.63029610727006	4.73430311829601	5.10949314942866	5.42838744190265	5.10054334947496	4.85862710164713	NA
**TC0100003875.mm.2**	Ankrd39	ankyrin repeat domain 39	0.405048136673587	5.93508306796302	0.934472027979278	0.383808588256583	0.999692817443324	-4.59936885446515	5.96253510907986	5.49273692894096	5.81334788060599	6.66931344094625	5.5195730188162	6.15299202938887	NA
**TC0100002243.mm.2**	Gm3646	predicted gene 3646	0.385994750131896	5.77547655848796	0.934393241555033	0.383846270413574	0.999692817443324	-4.59937288648532	5.09915774145199	4.87833632780834	5.84140696154624	6.88227042941936	5.96142815671005	5.99025973399176	NA
**TC0200000228.mm.2**	Pter	phosphotriesterase related	-0.315800217865399	5.96391824505464	-0.934207383800095	0.383935174081578	0.999692817443324	-4.59938239733034	5.93637069087509	6.42393881467147	5.69415968112973	5.7016400833955	5.80747129519096	6.21992890506508	NA
**TC1500000591.mm.2**	Mroh1/Mir6954	maestro heat-like repeat family member 1/microRNA 6954	-0.310085322590997	5.96284605732565	-0.934155739386077	0.383959880374324	0.999692817443324	-4.59938503991228	6.15687574845882	6.37067577659553	5.45511122290181	5.85956586395696	6.15850708555274	5.77634064648803	NA
**TC1900001621.mm.2**	Atp5md	ATP synthase membrane subunit DAPIT	-0.576489560219613	10.0428556209841	-0.934076682969675	0.383997703253386	0.999692817443324	-4.59938908503999	11.4352009226684	11.3106575535443	9.92696882222351	8.25217924240278	10.1702356778856	9.16189150717992	NA
**TC1300000588.mm.2**	Nutm2	NUT family member 2	-0.429841728413183	5.56661061804151	-0.934059057108984	0.384006136315016	0.999692817443324	-4.59938998688302	5.48163293139929	5.02555072388702	5.04336573678617	5.97303256128201	5.55411415175782	6.32196760313673	NA
**TC0700001509.mm.2**	Olfr631	olfactory receptor 631	-0.307945851169065	4.89658969083607	-0.934055877722857	0.38400765750168	0.999692817443324	-4.5993901495582	4.47866538740793	4.11091866701423	4.82889366707788	5.25813752705017	5.28690881365635	5.41601408280983	NA
**TC0100002725.mm.2**	Sp110	Sp110 nuclear body protein	-0.28361089248488	6.7860168811534	-0.933925582812882	0.384070001439357	0.999692817443324	-4.59939681591995	6.35716975451605	6.07256211691189	6.86625705374072	6.99331676152063	7.13104236357754	7.29575323665357	NA
**TC1800000968.mm.2**	Usp14	ubiquitin specific peptidase 14	0.3407098824482	6.59238898090681	0.933895806970671	0.384084249766047	0.999692817443324	-4.59939833929053	8.09830423376857	8.00413350048103	5.68379409601088	6.38286386203293	5.88785698693815	5.49738120620927	NA
**TC0500000843.mm.2**	Pf4	platelet factor 4	-0.370115200990174	6.93932788107815	-0.933684579215865	0.384185337979273	0.999692817443324	-4.59940914522929	6.09253846708487	6.29509958756506	6.69132107270915	7.56272834221017	7.29788890558357	7.69639091131609	NA
**TC1700002813.mm.2**	Ppt2	palmitoyl-protein thioesterase 2	0.323635102578508	6.72618619378572	0.933660694334573	0.384196769950741	0.999692817443324	-4.59941036704397	7.00355390985957	6.58110013303315	7.12078022657987	6.58908643590945	6.60608480683999	6.45651165049231	NA
**TC1000000593.mm.2**	Rufy2	RUN and FYVE domain-containing 2	-0.300036987435966	7.93726804420785	-0.933367219891009	0.384337256194713	0.999692817443324	-4.59942537816115	8.01270503670276	7.75198176443186	8.0114537175269	7.61797002709339	8.22013525401627	8.00936246547595	NA
**TC1000002380.mm.2**	Susd2	sushi domain containing 2	0.404915959297878	6.55827346525258	0.933345692118356	0.384347563083399	0.999692817443324	-4.59942647919984	7.29891783170763	6.29709408053287	6.64096080536586	6.6407695935695	6.53394720161309	5.93795127872653	NA
**TC1100001880.mm.2**	Gprc5c	G protein-coupled receptor, family C, group 5, member C	-0.434602745840545	5.69999195524777	-0.933265383694841	0.384386014344804	0.999692817443324	-4.59943058645698	5.00010249582754	5.16212850722326	5.1546702963966	6.42958732198076	6.36423840580435	6.08922470425409	NA
**TC0300000054.mm.2**	Zbtb10	zinc finger and BTB domain containing 10	0.374224681155045	5.86447561421852	0.932965566456799	0.38452959121518	0.999692817443324	-4.59944591849904	6.08705262921831	6.24177114883803	6.32527922637864	5.47796040840378	5.78508305851636	5.26970721395598	NA
**TC1800001009.mm.2**	Ss18	SS18, nBAF chromatin remodeling complex subunit	-0.41490318899966	7.04607314775709	-0.932955727744021	0.384534303483551	0.999692817443324	-4.59944642158601	6.84242090519875	6.90502398230736	7.52759253066126	6.3220012798573	7.26143627820022	7.41796391031765	NA
**TC0X00002238.mm.2**	Mospd1	motile sperm domain containing 1	-0.790062589088986	7.0873263102144	-0.932920925182272	0.384550972583509	0.999692817443324	-4.59944820113696	7.67163148877589	7.83581065003138	7.63220621354629	5.08598905860431	7.79182514008227	6.50649531024629	NA
**TC0300002554.mm.2**	Hsd3b5	hydroxy-delta-5-steroid dehydrogenase, 3 beta- and steroid delta-isomerase 5	-0.302156460943134	5.8236130919726	-0.932809209237525	0.384604483983003	0.999692817443324	-4.59945391324084	5.4200306183108	5.7516600578257	5.61524156797771	5.96759590892872	5.97786491275764	6.20928548603506	NA
**TC1800000188.mm.2**	Zfp397	zinc finger protein 397	0.381300787360505	6.50635912063721	0.932715695790851	0.384649280837615	0.999692817443324	-4.599458694358	7.05478608352968	7.00707104368101	6.71636089483196	6.15308869083483	6.49162864953638	5.61521936140941	NA
**TC0800000017.mm.2**	Stxbp2	syntaxin binding protein 2	-0.348938208334897	6.49546526038556	-0.932437424530454	0.384782607943078	0.999692817443324	-4.59947292017637	6.75124268496007	7.13407197402416	5.78497369433577	6.40982654899389	6.2214265656336	6.67125009436585	NA
**TC1500001464.mm.2**	Tmem74	transmembrane protein 74	-0.354123007403845	6.01843064276121	-0.932316985085423	0.384840324579951	0.999692817443324	-4.5994790765908	6.76062776600413	7.10069596787571	5.73770791384867	5.03279914009121	5.9522145332213	5.52653853552627	NA
**TC0700004429.mm.2**	Dhx32	DEAH (Asp-Glu-Ala-His) box polypeptide 32	-0.48069587176302	7.45993542484836	-0.93198968472362	0.384997205823051	0.999692817443324	-4.59949580480672	7.82438105673436	8.1815366076946	7.69118821524629	6.20496335532131	7.35260623309238	7.50493708100126	NA
**TSUnmapped00000046.mm.2**	Egr3	early growth response 3	0.318008405871756	8.10401767394687	0.931951345099946	0.385015585916553	0.999692817443324	-4.59949776412623	8.36622562002154	8.49406594126105	8.1568596085318	8.04305603853929	8.10071843307639	7.46318040225118	NA
**TC1700002318.mm.2**	Dpp9	dipeptidylpeptidase 9	0.330014871345162	7.0836892138397	0.931852210529104	0.385063114330626	0.999692817443324	-4.59950283012817	7.25365212345024	7.22833334458017	6.84595719689219	7.49413258195688	6.65938226168359	7.02067777447515	NA
**TC1000002594.mm.2**	Ckap4	cytoskeleton-associated protein 4	-0.288474785966375	6.01175882382895	-0.931843390563862	0.385067343132173	0.999692817443324	-4.59950328083442	5.69665723181044	5.61864733262588	5.99603561641703	6.09311378688529	6.13601758353798	6.5300813916971	NA
**TC0900001555.mm.2**	Azi2	5-azacytidine induced gene 2	0.516987202931262	6.87429507903896	0.931792738746247	0.385091629228677	0.999692817443324	-4.5995058691326	7.91370302238284	7.4785347292571	7.09973199324346	6.34402157098472	6.93580536275263	5.47397379561303	NA

**TC1000000569.mm.2**	Npffr1	neuropeptide FF receptor 1	0.324768350737278	6.1671790113194	0.931787205870221	0.385094282155035	0.999692817443324	-4.59950615185693	6.08840450412413	6.43255542906818	6.01523246256708	6.55059295553226	5.80355437772686	6.11273433889793	NA
**TC1500002139.mm.2**	Scaf11	SR-related CTD-associated factor 11	-0.379072767671606	6.21675304017152	-0.931474318778419	0.385244329214542	0.999692817443324	-4.59952213860123	7.30958205236227	7.34339022186255	5.79979955273046	5.14490066300008	6.25086946096721	5.45197629010654	NA
**TC1000000047.mm.2**	Nup43	nucleoporin 43	-0.285985252919581	5.85535043700192	-0.931102849518419	0.385422527554669	0.999692817443324	-4.59954111481403	5.594499531195	5.91010232375961	5.73722384036891	5.79054129023998	5.93908286730219	6.16065276914585	NA
**TC0200003811.mm.2**	Sestd1	SEC14 and spectrin domains 1	-0.548682659590224	6.24735964934649	-0.931025683787198	0.385459552772011	0.999692817443324	-4.59954505625387	6.8885468182093	6.95505453007387	6.22934261825757	5.04225299605009	6.89008513241978	5.47887580106832	NA
**TC0600001020.mm.2**	Hmces	5-hydroxymethylcytosine (hmC) binding, ES cell specific	0.399572595955464	6.70937957829419	0.930824889067562	0.385555909633969	0.999692817443324	-4.59955531154168	6.63462517605782	6.37591972670222	6.78097553031091	7.24146334914711	6.10439851216456	7.11889517538254	NA
**TC1500002110.mm.2**	Pus7l	pseudouridylate synthase 7-like	0.297988914336674	4.97123298394135	0.930761262513902	0.385586446410189	0.999692817443324	-4.59955856092385	4.7152133720755	4.79271146687834	5.01444175180623	5.44328369487758	4.80184976104608	5.05989785696438	NA
**TC1100000780.mm.2**	Mfap4	microfibrillar-associated protein 4	0.444651942138876	6.5885821343106	0.930634589905969	0.385647246842055	0.999692817443324	-4.59956502968657	6.27009139978815	6.24747625286831	6.30083631697745	7.65077820176501	6.41774501279456	6.64456562167014	NA
**TC0500000790.mm.2**	Ythdc1	YTH domain containing 1	0.355316151820865	7.30080646665002	0.930522713901724	0.385700951257138	0.999692817443324	-4.59957074244039	7.58135475695287	7.55858838160746	7.40419017391077	7.28357380858	7.43533536148531	6.54179631736373	NA
**TC1700001449.mm.2**	Slc22a3	solute carrier family 22 (organic cation transporter), member 3	-0.281541750551304	5.99103204193255	-0.930514986155782	0.385704661057095	0.999692817443324	-4.59957113703067	6.07350015387101	6.14494076890682	5.80218653521428	5.78014737864315	5.90685754512856	6.23855986983148	NA
**TC0700001552.mm.2**	Cckbr	cholecystokinin B receptor	0.320373913925226	7.68912029407005	0.930384365262563	0.38576737134189	0.999692817443324	-4.59957780646221	7.65246579850221	8.2845224606367	7.83584211542724	7.58339855113867	7.39094918177451	7.38754365694095	NA
**TC0400001800.mm.2**	Agmat	agmatine ureohydrolase (agmatinase)	0.464853995954197	5.44354051128718	0.929535621163117	0.386175037226813	0.999692817443324	-4.59962113059048	4.48068226520487	5.0677013642392	5.37450729033133	6.64677642476237	5.32699096653618	5.76458475664912	NA
**TC0600001734.mm.2**	Med21	mediator complex subunit 21	-0.793758966199825	6.59009102047067	-0.929468767597578	0.386207162028786	0.999692817443324	-4.59962454222447	7.38727979227743	7.2514956502807	7.20398523073489	4.45314114319822	7.05024479828246	6.1943995080503	NA
**TC1700000657.mm.2**	Lsm2	LSM2 homolog, U6 small nuclear RNA and mRNA degradation associated	0.31801967997585	6.3129107967182	0.929031023743799	0.386417559094042	0.999692817443324	-4.59964687765611	6.24043329146457	6.29434612237391	6.22889050267576	6.76047186053544	5.9767864075777	6.37653659568179	NA
**TC0100003682.mm.2**	Nvl	nuclear VCP-like	0.464737702516343	5.65656158486732	0.929025344712575	0.386420289234674	0.999692817443324	-4.59964716738558	6.47580093093476	6.73114736980468	6.02477701097881	4.80617129576977	5.34011745510953	4.56135544660637	NA
**TC1400001537.mm.2**	Anxa7	annexin A7	-0.372447242672806	6.88171270481423	-0.928925225474866	0.386468423022559	0.999692817443324	-4.59965227505286	7.08529992763687	7.33507812617426	7.01674475174857	6.04575709311577	6.91544321335585	6.8919531168541	NA
**TC0400002488.mm.2**	4933409K07Rik	RIKEN cDNA 4933409K07 gene	-0.335533521665472	5.66077186847713	-0.928882789659703	0.386488826037646	0.999692817443324	-4.59965443986211	5.57535128745977	5.88837869305271	5.64755299741625	5.26736409609344	6.0987109920839	5.48727314475673	NA
**TC4_JH584293_random00000004.mm.2**	4933409K07Rik	RIKEN cDNA 4933409K07 gene	-0.335533521665472	5.66077186847713	-0.928882789659703	0.386488826037646	0.999692817443324	-4.59965443986211	5.57535128745977	5.88837869305271	5.64755299741625	5.26736409609344	6.0987109920839	5.48727314475673	NA
**TC1300001522.mm.2**	Epdr1	ependymin related protein 1 (zebrafish)	-0.332929912972699	7.23994402149985	-0.928874627563155	0.386492750443611	0.999692817443324	-4.59965485623499	7.29095083376922	7.7918922997158	7.09172762716695	6.7537529576174	7.49300059300929	7.01833981772045	NA
**TC0700000058.mm.2**	Epn1	epsin 1	0.346783015011786	8.33631520930144	0.928828194531052	0.386515076420084	0.999692817443324	-4.59965722488483	9.47578058727228	9.46174809909386	8.36819119196263	7.51877310777042	7.64492842752684	7.54846984218263	NA
**TC0100002382.mm.2**	Ppil3	peptidylprolyl isomerase (cyclophilin)-like 3	0.272485136095965	6.59949553738317	0.928731786892141	0.386561434369491	0.999692817443324	-4.59966214264387	6.96914776368729	7.02867880510858	6.60137221503741	6.47068624881014	6.18496104195225	6.34212714970337	NA
**TC0600002342.mm.2**	Nt5c3	5'-nucleotidase, cytosolic III	0.798305990756838	5.71594542604287	0.928646341861618	0.386602524445574	0.999692817443324	-4.59966650096935	6.94318554750775	7.21113869798639	6.11468593061371	4.75429421552466	5.91291540962083	3.35945275500386	NA
**TC0300003182.mm.2**	Fpgt	fucose-1-phosphate guanylyltransferase	-0.559959004730906	6.48045980879643	-0.928598827570364	0.386625375261371	0.999692817443324	-4.5996689244543	6.89660697330761	6.98321098524199	6.92040612918361	5.02110531319997	6.5360539225231	6.52537552932229	NA
**TC1000000661.mm.2**	Ank3	ankyrin 3, epithelial	0.41809846725956	7.68504723975145	0.928582428394906	0.386633262275113	0.999692817443324	-4.5996697608851	8.91381041423911	8.98215564953939	7.53571940147621	6.98953775314845	7.40189035966421	6.28716986044132	NA
**TC0900001243.mm.2**	Faim	Fas apoptotic inhibitory molecule	0.419538292865002	7.72725702534531	0.928572676763294	0.386637952279449	0.999692817443324	-4.59967025825785	7.62176324890781	7.35425405591027	8.57198240352074	7.54131831297117	7.96490849323141	7.30931563753049	NA
**TC0200004763.mm.2**	Rbbp9	retinoblastoma binding protein 9, serine hydrolase	-0.599291007518191	5.74388544133189	-0.928339855379745	0.386749939549219	0.999692817443324	-4.59968213225499	7.52485438920112	7.28980173828677	5.40636103325435	3.81867621947919	5.86793958109282	4.55567968667709	NA
**TC0700004265.mm.2**	Xpo6	exportin 6	0.538264953660936	7.80810219460321	0.928084536444076	0.386872776515769	0.999692817443324	-4.59969515178891	9.27769627109434	8.99571699183059	7.97359884679502	6.85226605921308	7.56883847449535	6.18049652419088	NA
**TC0400000084.mm.2**	Trp53inp1	transformation related protein 53 inducible nuclear protein 1	-0.509923623653939	4.90278686076301	-0.928081428812525	0.386874271816871	0.999692817443324	-4.59969531024512	4.59110583238856	4.53233379796107	5.64453034128169	3.99218680217859	5.47588390770982	5.18068048305833	NA
**TC0500002305.mm.2**	Nkx3-2	NK3 homeobox 2	-0.353321176620068	6.6670175147529	-0.928064915534079	0.386882217595567	0.999692817443324	-4.59969615224218	5.98301981836288	5.67086664785671	6.71212141134113	7.10866672318771	6.89370633878466	7.63372414898432	NA
**TC0600002325.mm.2**	Crhr2	corticotropin releasing hormone receptor 2	0.310991165883406	6.74044756851899	0.927903515165272	0.386959885970605	0.999692817443324	-4.59970438147411	6.70407695864748	6.50512391075013	6.79754955828163	7.13018387845995	6.41168947982832	6.89406162514644	NA
**TC0600002133.mm.2**	Mrps33	mitochondrial ribosomal protein S33	0.401667442921655	8.1919776673697	0.92733574577115	0.387233199659982	0.999692817443324	-4.59973332387198	8.91280011423461	8.87333802259172	8.28878800324039	7.79574337337719	8.16655018091546	7.11464630985881	NA
**TC1200000714.mm.2**	Plekhh1	pleckstrin homology domain containing, family H (with MyTH4 domain) member 1	0.276624785193989	7.14784705037221	0.927306688448505	0.387247191264014	0.999692817443324	-4.59973480482938	7.33683871527007	7.309525181262	7.21021316165843	7.18677082638615	6.77918514675552	7.06454927090109	NA
**TC1100000326.mm.2**	4930524B15Rik	RIKEN cDNA 4930524B15 gene	-0.338617562832246	5.28505045943707	-0.927034842954371	0.387378108190523	0.999692817443324	-4.59974865869552	4.58736667169106	4.70038491393192	5.08933149093733	5.78332653173014	5.9748191651223	5.57507398320966	NA
**TC1600001459.mm.2**	Lrrc15	leucine rich repeat containing 15	0.397262231076926	5.82673102370403	0.926979194435086	0.387404911873643	0.999692817443324	-4.5997514943999	5.22297379466286	5.04113193067138	5.85582401595265	6.88957842356924	5.75260682990279	6.19827114746526	NA
**TC1400002433.mm.2**	Gtf2f2	general transcription factor IIF, polypeptide 2	0.336026409730636	5.76024990867747	0.926882887298459	0.387451302529252	0.999692817443324	-4.59975640174439	5.6849260623805	5.5390121268426	6.14056200789101	5.86424503326048	5.30204585118058	6.03070837050963	NA
**TC1900001070.mm.2**	Polr2g	polymerase (RNA) II (DNA directed) polypeptide G	-0.527842492654024	8.493319592402	-0.926588180746906	0.387593287392438	0.999692817443324	-4.59977141684584	8.77369562868566	8.95989521966558	8.8139864141758	7.27133444620054	8.9880795301314	8.15292631555299	NA
**TC0600002493.mm.2**	Sh2d6	SH2 domain containing 6	0.35749508665295	5.82489365153023	0.925897949071089	0.387925984593785	0.999692817443324	-4.59980657356332	5.45426417733379	5.5599912695361	5.85687397674056	6.46817434106813	5.46199337871468	6.14806476578811	NA
**TC0600002248.mm.2**	Gsdme	gasdermin E	-0.298863718022785	5.63937616465584	-0.925813281301218	0.38796681006917	0.999692817443324	-4.59981088510982	6.28334127321426	6.24389660906892	5.43418457798501	4.92146125681813	5.4174782499875	5.5358950208612	NA
**TC0800002431.mm.2**	Bst2	bone marrow stromal cell antigen 2	-0.423107543560947	5.35369187599541	-0.925764461200308	0.387990351842019	0.999692817443324	-4.5998133710842	5.15036977267972	6.28301248433773	4.76586004581838	5.1554169100982	5.16575279401537	5.6017392490231	NA
**TC0400002104.mm.2**	Tnfrsf18	tumor necrosis factor receptor superfamily, member 18	0.419769132303481	6.51890681442495	0.925764253285518	0.387990452103918	0.999692817443324	-4.5998133816713	5.59179313354989	5.88333075706275	6.67133053018562	7.56759710008639	6.28310002427731	7.11628934138773	NA
**TC0900001845.mm.2**	Olfm2	olfactomedin 2	-0.332849798818472	6.31392821685503	-0.925529546084366	0.388103646546364	0.999692817443324	-4.59982533223161	6.93307257187341	6.83750970809523	6.23129446995826	5.49234924180403	6.30244902802506	6.08689428137418	NA
**TC0400001957.mm.2**	Clstn1	calsyntenin 1	-0.457177913565959	9.04699794258635	-0.925489666739097	0.388122882024367	0.999692817443324	-4.59982736260041	10.1566616994321	10.1412097264615	8.95070622930738	7.5841739719389	8.95320575799846	8.49603027037973	NA
**TC0200000406.mm.2**	Anapc2	anaphase promoting complex subunit 2	0.329559634932044	8.08849854936271	0.925473379236348	0.388130738377126	0.999692817443324	-4.59982819182899	8.7101139524917	8.69307217690012	8.29568676432481	7.59777545399944	7.47421989712546	7.76012305133471	NA
**TC1200000029.mm.2**	Itsn2	intersectin 2	0.600393464967527	6.55472556817848	0.925389658745722	0.388171123251081	0.999692817443324	-4.59983245407775	6.54981468225316	6.89763853599989	7.25465662484986	6.28618693552658	7.07738672932416	5.26266990111723	NA
**TC0600002166.mm.2**	Fam131b	family with sequence similarity 131, member B	-0.390280960189386	7.4783897368765	-0.925143178691675	0.388290038186902	0.999692817443324	-4.599845001276	7.63557695196328	7.9381398940638	7.60019367055276	6.65783615687381	7.7680504875056	7.27054126029974	NA
**TC0800003169.mm.2**	Pgbd5	piggyBac transposable element derived 5	-0.290221139277333	8.00587094547196	-0.925033254300873	0.388343080408995	0.999692817443324	-4.59985059645059	7.94395640694213	8.23428898348173	7.95210847740384	7.68616052452278	7.99847265584282	8.22023862463847	NA
**TC1400002100.mm.2**	Dhrs1	dehydrogenase/reductase (SDR family) member 1	-0.623279578205199	9.4398855594498	-0.925013546082329	0.388352590869951	0.999692817443324	-4.59985159956489	9.98791587323506	10.4454690228164	9.75737427582782	7.72231037629066	9.68970434214541	9.03653946638347	NA
**TC0500002083.mm.2**	Asb10	ankyrin repeat and SOCS box-containing 10	0.444806654199676	5.31786073305333	0.924887881295628	0.388413236229331	0.999692817443324	-4.59985799541272	4.72472683864633	5.36097740467629	5.06379108513291	6.29174564656544	5.24138191416132	5.22454150913768	NA
**TC1000002714.mm.2**	Plxnc1	plexin C1	-0.283301311306134	8.28981971226341	-0.924875703093739	0.388419113766429	0.999692817443324	-4.59985861521065	8.39545931036171	8.08467185391642	8.28053789184509	8.06555435149992	8.47955608554123	8.43313878041607	NA
**TC0900000794.mm.2**	Calml4	calmodulin-like 4	-0.753752315349912	4.84084475124838	-0.924799688503448	0.388455802036301	0.999692817443324	-4.59986248380029	4.06361367224621	5.2434751232501	3.34241167642567	5.77282586422142	5.52526268975614	5.09747948159078	NA
**TC1400001604.mm.2**	Plac9a/Plac9b	placenta specific 9a/placenta specific 9b	-0.276550573723856	5.96472548092441	-0.9247924746016	0.388459283946354	0.999692817443324	-4.59986285092644	5.5117408477527	5.61557873859668	5.96967900264952	6.08428707322516	6.17662300478394	6.43044421853845	NA
**TC0100000282.mm.2**	Npas2	neuronal PAS domain protein 2	0.476208994524091	7.04087561799191	0.924702957128312	0.388502493018957	0.999692817443324	-4.59986740647384	8.23462253259239	8.12262847177588	7.47140110887223	5.94880923744346	6.30163692152587	6.16615543574163	NA
**TC0700001847.mm.2**	Fbrs	fibrosin	0.341928034350561	8.18474385907811	0.92462991120484	0.388537754163532	0.999692817443324	-4.59987112360599	7.90501061700762	7.74108915291169	8.86014987420987	8.21295985241535	8.44527773279923	7.94397592512488	NA
**TC1300002605.mm.2**	Shisal2b	shisa like 2B	0.346427850175186	5.47674313791595	0.92459111877449	0.388556481254181	0.999692817443324	-4.59987309759504	5.53008731970499	5.36467203037006	5.96326368095832	5.36601390792719	5.61808919457782	5.01833269395732	NA
**TC0200000007.mm.2**	Phxr1	per-hexamer repeat gene 1	-0.525795553957961	4.71401041378321	-0.924495085933146	0.388602844166683	0.999692817443324	-4.59987798412226	4.59025335949777	4.22920222734193	3.93389116708304	5.27261672688878	4.58686686169074	5.671232140197	NA
**TC0900001414.mm.2**	Impdh2	inosine monophosphate dehydrogenase 2	0.432554262412298	6.50490558583081	0.924470057342357	0.388614928205672	0.999692817443324	-4.59987925762972	7.3394443837243	7.38096776251153	6.88004680061026	5.70701814617656	6.16764116752653	5.55431525443569	NA
**TC0200004204.mm.2**	Prr5l	proline rich 5 like	0.308826813369661	5.95535164601096	0.924426121335558	0.388636141612623	0.999692817443324	-4.59988149314103	5.71484806429928	6.17697531493492	6.11135633577145	6.117613726014	5.60565226301052	6.00566417203562	NA
**TC0500001642.mm.2**	Zscan21	zinc finger and SCAN domain containing 21	-0.325133368052114	5.72412469508124	-0.924323430223933	0.388685726886509	0.999692817443324	-4.59988671795013	6.2532029590424	5.98585568817303	5.47565428902128	5.2520571045626	5.36958394372008	6.00839418596803	NA
**TC1400001661.mm.2**	Itih3	inter-alpha trypsin inhibitor, heavy chain 3	0.343304430343927	5.92130045857528	0.924159564705169	0.388764860684331	0.999692817443324	-4.59989505459131	6.43712937143745	5.79453017236571	6.15299092632114	5.83838510784704	5.42815618756142	5.87661098591892	NA
**TC0700002216.mm.2**	Zim1	zinc finger, imprinted 1	0.620926853679808	5.9047995288505	0.924037575839984	0.388823779381855	0.999692817443324	-4.5999012602403	6.41044084203939	5.78424318566175	6.84583214683419	5.39215127954655	6.24646329490306	4.74966642411807	NA
**TC1200000009.mm.2**	Asxl2	additional sex combs like 2, transcriptional regulator	-0.311767566026117	7.23900367069681	-0.923972106847173	0.38885540266634	0.999692817443324	-4.59990459050475	8.1831382572197	8.18323390259058	6.92380583518785	6.29825153097131	6.86988837682901	6.97570412138238	NA
**TC0100001193.mm.2**	Dstyk	dual serine/threonine and tyrosine protein kinase	0.287552542095614	6.13114857900055	0.923311864711317	0.389174426451775	0.999692817443324	-4.59993816840197	6.57655867548032	6.40417050006094	6.28968484354806	5.90094884777856	5.77300849349872	5.84252011363667	NA
**TC1000001698.mm.2**	Pcmt1	protein-L-isoaspartate (D-aspartate) O-methyltransferase 1	-0.580334413764001	8.70727217163746	-0.923247728985444	0.389205426904325	0.999692817443324	-4.59994142945281	8.64902744745323	8.5383797790678	9.41361378545034	7.53416470243754	9.39894285995543	8.70950445546046	NA
**TC1700001020.mm.2**	Clpp	caseinolytic mitochondrial matrix peptidase proteolytic subunit	-0.321961116508904	7.78190171953562	-0.923219145032754	0.389219243761529	0.999692817443324	-4.59994288279528	8.38901299220198	8.11655260051115	7.62526885644954	7.14569238929185	7.93001449891934	7.48486897983985	NA
**TC0600002020.mm.2**	Podxl	podocalyxin-like	0.294967933725063	6.26939043963745	0.92315530843475	0.389250102319163	0.999692817443324	-4.59994612845943	5.95581134539676	6.10958416515854	6.57442920900868	6.49601228835108	6.00494266239346	6.47556296751618	NA
**TC1500000062.mm.2**	Ranbp3l	RAN binding protein 3-like	-0.296637431797216	5.87654709999006	-0.923129013828265	0.389262813649308	0.999692817443324	-4.59994746532881	5.65607081643084	5.32418943742609	5.78676344933433	6.05611029191018	6.36261433564021	6.07353426919873	NA
**TC1900000163.mm.2**	Lbhd1/1810009A15Rik	LBH domain containing 1/RIKEN cDNA 1810009A15 gene	-0.292278088815049	8.18044792524483	-0.923069350615139	0.389291657188027	0.999692817443324	-4.59995049864669	7.96196603269402	8.09305293782081	8.07174173455106	8.14981446711098	8.62910977211373	8.17700260717841	NA
**TC0400000488.mm.2**	Dcaf10	DDB1 and CUL4 associated factor 10	-0.406217126723702	5.87258101330591	-0.922866658094054	0.389389658814048	0.999692817443324	-4.59996080287312	6.77109932862056	6.68780830561839	5.83155405482151	4.65051804125304	5.73136787118642	5.56313847833553	NA
**TC1100001235.mm.2**	Unc45b	unc-45 myosin chaperone B	-0.394510671588675	5.75095269858548	-0.922293131435399	0.389667059720403	0.999692817443324	-4.59998995241544	5.65386024434177	4.99607006470592	5.36117846819991	6.17220380144401	5.93703380009641	6.38536981272486	NA
**TC1400002096.mm.2**	Tinf2	Terf1 (TRF1)-interacting nuclear factor 2	-0.330569290701653	6.12119924088014	-0.922285524802551	0.38967073987029	0.999692817443324	-4.59999033895684	5.78798363307923	6.14321272611359	5.69325180044113	6.37417845190123	6.37687303262775	6.35169580111792	NA
**TC0900001048.mm.2**	Gsta4	glutathione S-transferase, alpha 4	0.394302764429718	7.85212547719828	0.92191403973903	0.389850499383213	0.999692817443324	-4.60000921435934	8.54547289863186	8.29201016700505	7.59388427165195	7.93805339155416	7.89802465575415	6.84530747859251	NA
**TC0100003778.mm.2**	Kcnk2	potassium channel, subfamily K, member 2	-0.472505625513722	6.61915033154374	-0.921619566305425	0.3899930380576	0.999692817443324	-4.60002417380153	7.74667743266461	7.01020504214612	6.65461191872897	5.35189221298316	6.33488995609066	6.61662542664892	NA
**TC1100001361.mm.2**	Vezf1	vascular endothelial zinc finger 1	0.418664956788361	7.83610946593829	0.921440093205267	0.390079930653937	0.999692817443324	-4.60003328987231	8.63020084966093	8.09745993090292	8.29161089660814	7.27155206771315	7.62224856283616	7.10358448790841	NA
**TC1400000745.mm.2**	Mmp14	matrix metallopeptidase 14 (membrane-inserted)	-0.313528849693293	5.28044348066755	-0.921422126667547	0.390088630030469	0.999692817443324	-4.60003420240265	5.35242850819401	5.8521366947607	4.97222845958157	4.95329053125043	5.065784380155	5.48679231006359	NA
**TC1900000904.mm.2**	Cabp4	calcium binding protein 4	0.28526641534643	6.75604643028129	0.921394405726551	0.390102052766036	0.999692817443324	-4.60003561034544	6.4219634878344	6.56930813743171	6.893347036152	7.16442285740525	6.8813059837537	6.60593107911068	NA
**TC0100000214.mm.2**	1700001G17Rik	RIKEN cDNA 1700001G17 gene	0.433160795875594	5.41259158883317	0.921350621216986	0.390123254342719	0.999692817443324	-4.60003783410755	4.96827735211776	4.51254095640937	5.41853852769997	6.51198788041157	5.22485009304146	5.8393547233189	NA
**TC0400000961.mm.2**	Fggy	FGGY carbohydrate kinase domain containing	0.437500860541375	6.18633202282558	0.921326804982691	0.390134787137635	0.999692817443324	-4.60003904368096	6.64483990835287	6.60514501683945	6.20489970504628	6.16660476137569	6.42561794779476	5.07088479754446	NA
**TC0700002342.mm.2**	Zfp551	zinc fingr protein 551	-0.465219914872915	5.40468253717851	-0.921200962335233	0.390195729584007	0.999692817443324	-4.60004543466474	5.67974705752264	5.75310247243261	5.72171866847838	4.31068426320661	5.71699230104019	5.24585046039064	NA
**TC1900001404.mm.2**	Rnls	renalase, FAD-dependent amine oxidase	0.369688698364622	5.69321686889016	0.921085203226411	0.390251795193338	0.999692817443324	-4.60005131312968	5.41909981703094	4.78178334957617	5.78612134191815	6.56277637981339	5.7914912943069	5.81802903069539	NA
**TC0400004088.mm.2**	Zbtb48	zinc finger and BTB domain containing 48	-0.3739438278165	5.9699016101364	-0.92087297279949	0.390354600607272	0.999692817443324	-4.60006208954087	6.35711749637229	5.6339745329044	6.00260899195268	5.53760599600168	5.86127396961267	6.42682867397469	NA
**TC1400001105.mm.2**	Elf1	E74-like factor 1	-0.47332745797885	6.20466368828459	-0.920700513816035	0.390438155654997	0.999692817443324	-4.60007084548033	6.71072490508412	6.87578373370915	6.36516697178798	4.9822423156903	6.46233603263633	5.83172817079965	NA
**TC0500001328.mm.2**	Tbx5	T-box 5	-0.415383044705946	5.06219110390478	-0.92064604529429	0.390464548048522	0.999692817443324	-4.60007361072307	4.62193341028832	4.35709134329161	4.67602939863959	5.60564849157885	5.16186847553941	5.95057550409092	NA
**TC0200004309.mm.2**	Fibin	fin bud initiation factor homolog (zebrafish)	-0.394546136899414	5.83726467033004	-0.920469035017424	0.390550326640826	0.999692817443324	-4.60008259651373	5.4852994318592	5.6263340219353	5.2649014119599	6.29652973523356	5.94230873758929	6.408214683403	NA
**TC1100001641.mm.2**	Mlx	MAX-like protein X	0.442728152435625	6.76537854861167	0.920213194805906	0.39067433117641	0.999692817443324	-4.60009558237533	7.90456268992043	7.77677805288753	7.03477948684746	5.8634139400192	6.36928259433831	5.6434545276571	NA
**TC1100000883.mm.2**	Rnf222	ring finger protein 222	-0.294670949857299	6.13378542580364	-0.92010266527368	0.390727913537752	0.999692817443324	-4.60010119198961	5.30844762947354	5.42284771992834	6.14339062649268	6.59764702636	6.56783804584437	6.76254150672291	NA
**TC0200001853.mm.2**	Spata5l1	spermatogenesis associated 5-like 1	-0.289952370259886	5.94973653788421	-0.919969477940028	0.390792487310617	0.999692817443324	-4.60010795104672	5.72662608944842	5.88621276267861	6.07304300439438	5.67979481293485	6.07190576388199	6.260836793967	NA
**TC1600000168.mm.2**	Mpv17l	Mpv17 transgene, kidney disease mutant-like	-0.549275331315235	8.4808853392083	-0.919757036183719	0.390895503059262	0.999692817443324	-4.60011873103821	8.74619814422968	8.78011683432213	9.00215141999177	7.128071777042	8.66872541278468	8.56004844687955	NA
**TC1300001670.mm.2**	Aldh5a1	aldhehyde dehydrogenase family 5, subfamily A1	0.373631159891922	7.29451336838743	0.919751563072244	0.390898157313255	0.999692817443324	-4.60011900874382	8.41909758018505	8.03450976589443	7.48247268474252	6.54789490727196	6.54243999117303	6.74066528105761	NA

**TC1700002164.mm.2**	Rsph9	radial spoke head 9 homolog (Chlamydomonas)	0.572572135220185	6.58398588326685	0.919465703053482	0.391036807678354	0.999692817443324	-4.60013351202003	7.28586176296769	6.858989123164	5.66144312036591	7.59066122158896	6.12686376635022	5.98009630516429	NA
**TC1600000850.mm.2**	Rbm11	RNA binding motif protein 11	0.361982168649189	6.48805896326113	0.919242833611643	0.391144931616878	0.999692817443324	-4.60014481771891	6.9880496824324	6.90930626195194	6.89730021664282	5.98018086959758	6.22274789790546	5.93076885103655	NA
**TC1300001806.mm.2**	Lyrm4	LYR motif containing 4	0.334984884350074	7.92065907220904	0.919093630466006	0.391217329382672	0.999692817443324	-4.60015238564197	8.93445106790742	8.931638291311	7.94410136290347	7.21981605846451	7.42170677949396	7.07224087317386	NA
**TC1100003683.mm.2**	Ormdl3	ORM1-like 3 (S. cerevisiae)	-0.328043489661561	7.26050106536498	-0.918878700710801	0.391321637462753	0.999692817443324	-4.60016328618452	6.90994431657806	6.87503646040971	7.1650647438493	7.39590457409019	7.24696484959696	7.97009144766565	NA
**TC0700002964.mm.2**	Emc10	ER membrane protein complex subunit 10	0.343902624647061	8.75855903804542	0.91879187543994	0.391363780812674	0.999692817443324	-4.60016768928526	9.49954645156927	9.19132368237917	8.98427881602876	8.28986585578034	8.49495706901324	8.09138235350174	NA
**TC1100000712.mm.2**	Hist3h2ba	histone cluster 3, H2ba	0.394841485945457	6.79442217381482	0.918742101161191	0.391387941858014	0.999692817443324	-4.60017021334569	6.04394150272347	5.73499791087344	7.06426670974245	7.824371590849	6.64933560335347	7.44961972534706	NA
**TC0900001800.mm.2**	Naalad2	N-acetylated alpha-linked acidic dipeptidase 2	0.341926218344482	5.31729655662168	0.918658963309875	0.391428300507504	0.999692817443324	-4.60017442911016	5.02132652594202	4.56775060817682	5.48469592166199	6.0145813994881	5.1661354907877	5.64928939367343	NA
**TC1500000617.mm.2**	Apol6	apolipoprotein L 6	0.381519785325925	6.20154837535241	0.918571460661593	0.391470781416064	0.999692817443324	-4.60017886597926	5.20113655444133	5.86354587818716	6.36646228206269	7.08736141300624	6.12267346677861	6.56811065763846	NA
**TC1500001018.mm.2**	Aqp5	aquaporin 5	-0.372372818747889	6.35664287734837	-0.918375289510538	0.391566031537224	0.999692817443324	-4.60018881210098	5.80567111153525	5.43510773537376	6.08731629985381	6.98985008998891	6.75220821873626	7.06970380860223	NA
**TC1000000864.mm.2**	Adat3/Scamp4	adenosine deaminase, tRNA-specific 3/secretory carrier membrane protein 4	0.288082562880333	7.20396248067512	0.918138931694217	0.391680817419721	0.999692817443324	-4.60020079418819	8.01561205855501	7.6605795981974	7.11626997415668	6.94560420237279	6.63190228840757	6.85380676236124	NA
**TC1200000600.mm.2**	Actr10	ARP10 actin-related protein 10	0.517309019766698	7.54673337737145	0.91807244656303	0.391713110146813	0.999692817443324	-4.6002041643265	8.5135856360148	8.47133220384618	8.18386118573883	6.48118904621172	6.98903000432503	6.64140218809213	NA
**TC1300001100.mm.2**	Enc1	ectodermal-neural cortex 1	-0.374175043851595	7.96515929348757	-0.917812607130347	0.391839336986677	0.999692817443324	-4.60021733432946	8.48368948286216	8.90414219354967	7.84347812391139	6.9839088744938	7.98798335370466	7.58775373240372	NA
**TC0200000407.mm.2**	Tmem210	transmembrane protein 210	-0.401179168419295	5.71918929082235	-0.91779041373833	0.391850119692702	0.999692817443324	-4.60021845911016	5.11605595599449	5.34206628347829	5.18579238317189	6.34153520113948	6.11794868095947	6.21173724019048	NA
**TC1900001622.mm.2**	Calhm2	calcium homeostasis modulator family member 2	0.334744904690941	5.71681160287702	0.91773395412515	0.391877551725647	0.999692817443324	-4.60022132046586	5.70606652207953	5.88455938411326	5.45236326257921	6.2375034976464	5.52624009949868	5.49413685134504	NA
**TC1700001973.mm.2**	Mucl3	mucin like 3	0.361415567899681	5.87204184644723	0.917639507822085	0.391923443598681	0.999692817443324	-4.60022610675907	5.43413639465128	5.68279095242758	6.30834018015346	6.11073725354849	5.39194360525495	6.30430269264764	NA
**TC0100002576.mm.2**	Tns1	tensin 1	0.34958547219672	6.58051771114305	0.917617612351853	0.391934083284003	0.999692817443324	-4.60022721632596	7.21161191834793	7.03938323227245	6.51320450399475	6.45243652632094	5.6940486964451	6.57242138947715	NA
**TC1500000160.mm.2**	Myo10	myosin X	0.317241696010841	7.05680084838733	0.91753969232052	0.391971948805829	0.999692817443324	-4.60023116485515	7.8676147555894	7.30171627317468	6.78311656542853	7.11986216136228	6.73681773342067	6.53167760134845	NA
**TC1100002515.mm.2**	Tenm2	teneurin transmembrane protein 2	0.275230132151982	8.22632001216881	0.917356183612046	0.392061136456706	0.999692817443324	-4.60024046326991	8.2293738448113	8.38023644152396	8.44344561538097	8.20593941010982	7.98473087611735	8.11419388506947	NA
**TC1500002109.mm.2**	Adamts20	a disintegrin-like and metallopeptidase (reprolysin type) with thrombospondin type 1 motif, 20	0.277340330942612	5.74528713467299	0.917018802639136	0.392225148089542	0.999692817443324	-4.60025755574847	5.73145388350236	5.68193699251936	5.78478253418391	6.02172376276681	5.50756788100395	5.74425775406156	NA
**TC0800001635.mm.2**	Elavl1	ELAV (embryonic lethal, abnormal vision)-like 1 (Hu antigen R)	0.396949533706307	7.90388319788206	0.916901111071674	0.392282373908586	0.999692817443324	-4.60026351745448	8.61981463855466	8.66664012025151	8.10541617611513	7.35995557183428	7.7803821609155	6.8910905196213	NA
**TC0500000868.mm.2**	Uso1	USO1 vesicle docking factor	-0.423159038102571	8.1226846326154	-0.916618705419759	0.392419715304511	0.999692817443324	-4.6002778210911	8.9510870064259	9.02605220280997	8.11180768949533	6.84451756563036	8.03134010672025	7.77130322461058	NA
**TC0700001399.mm.2**	Chrdl2	chordin-like 2	-0.31714060421176	4.82803868288239	-0.916541087031524	0.392457469561784	0.999692817443324	-4.6002817519801	4.22409355437807	4.20293759126764	4.89088439341004	5.06257547820252	4.95914450484428	5.62859657519179	NA
**TC1800001731.mm.2**	Dok6	docking protein 6	-0.569514783704641	5.70364053424672	-0.916308048752274	0.392570837895114	0.999692817443324	-4.60029355281987	7.25352148071676	7.30082477755939	5.46470451321169	3.79952917668576	5.73350168293458	4.66976157437215	NA
**TC1700000473.mm.2**	Def6	differentially expressed in FDCP 6	-0.275889707646472	5.87050830831971	-0.916053226985371	0.392694831769986	0.999692817443324	-4.60030645486392	5.44399876134625	5.44171847362041	5.78487766603495	6.10789893379438	6.2116892140963	6.23286680102598	NA
**TC1100003466.mm.2**	Gdpd1	glycerophosphodiester phosphodiesterase domain containing 1	0.732994231737355	6.9234431870381	0.915835521485865	0.392800788655484	0.999692817443324	-4.60031747608483	7.96965203035024	7.80737032831751	7.6817157567353	5.93309685678247	7.08594764784315	5.06287650219991	NA
**TC1600002026.mm.2**	Krtap6-5	keratin associated protein 6-5	0.302172882896584	6.56466511439278	0.915825523831621	0.392805655014964	0.999692817443324	-4.6003179821759	5.98770598772312	6.05341389423395	6.86281000899192	7.11279827610449	6.42508230040326	6.94618021889998	NA
**TC0300002487.mm.2**	Prpf3	pre-mRNA processing factor 3	-0.497364150068773	5.30336670946416	-0.915812205479598	0.392812137795261	0.999692817443324	-4.60031865635924	6.15606504307524	6.07605664363644	5.16619617859459	4.13047895637328	5.78844124887325	4.50296218623216	NA
**TC0600001193.mm.2**	Itpr1	inositol 1,4,5-trisphosphate receptor 1	-0.330104301586938	8.32195555160695	-0.915756424640141	0.392839290300479	0.999692817443324	-4.60032147996162	9.56753124830372	9.90047437237869	7.63713162101366	7.26462792187906	8.08090307695902	7.48106506910758	NA
**TC1100001467.mm.2**	Phb	prohibitin	-0.338111453137237	8.49981974344426	-0.915452068904529	0.392987466875235	0.999692817443324	-4.60033688465004	9.36306748159844	9.28675879727827	8.24835602865305	7.58807860910414	8.50675381423	8.00590372980166	NA
**TC0X00000181.mm.2**	Maoa	monoamine oxidase A	-0.623321966003696	6.23097714847685	-0.915209555164833	0.393105565691386	0.999692817443324	-4.60034915724125	7.00955125329298	6.8271155062632	6.58999567881549	4.56128042083328	6.63343698988063	5.76448304177553	NA
**TC1000000095.mm.2**	Fuca2	fucosidase, alpha-L- 2, plasma	-0.344576369059741	7.04964470850512	-0.914532467596156	0.393435434202041	0.999692817443324	-4.6003834122738	7.12429357346793	7.06107511565252	6.42699515476905	7.28467825712633	7.27855206041154	7.12227408960332	NA
**TC0X00002057.mm.2**	Rpl39	ribosomal protein L39	0.285819862627511	8.3974100517138	0.914474420831562	0.393463723575722	0.999692817443324	-4.60038634830891	9.40224627107555	9.42504622165066	8.14055536133794	7.92384841006786	7.55459336555517	7.93817068059562	NA
**TC0300000843.mm.2**	Flg2	filaggrin family member 2	-0.474617230176388	4.74894273817386	-0.914327082638011	0.393535536457987	0.999692817443324	-4.60039380028817	4.21287409407695	4.1920176910839	4.26956098913598	5.30020410262879	4.6804715487467	5.83852800337085	NA
**TC0400000090.mm.2**	Virma	vir like m6A methyltransferase associated	-0.438604118052484	6.28051131686452	-0.914315245456643	0.393541306349351	0.999692817443324	-4.60039439895297	7.31522952942474	7.29880420309243	6.1758833822225	4.92002958405998	6.26959193096466	5.7035292714228	NA
**TC1000002003.mm.2**	Amd2	S-adenosylmethionine decarboxylase 2	-0.427144095868295	6.97903659199769	-0.914124418462806	0.39363433147966	0.999692817443324	-4.60040404942696	7.4491343350024	7.50075395972678	7.09792885515912	5.93709267760105	7.25937326881881	6.62993645567795	NA
**TC1200002413.mm.2**	Bag5	BCL2-associated athanogene 5	-0.292974740113317	7.40658256219565	-0.913920780033299	0.393733620286011	0.999692817443324	-4.60041434657148	7.99354334946516	7.87521905008972	7.12766116160602	6.86473058509017	7.49939077948905	7.07895044743377	NA
**TC0900001425.mm.2**	Slc26a6	solute carrier family 26, member 6	0.418607471817823	6.09212071660419	0.913896041629769	0.393745683376121	0.999692817443324	-4.60041559740282	6.39581161416058	5.73318383824086	5.76889772002185	6.86157417540782	5.92664120770434	5.86661574408969	NA
**TC1900000148.mm.2**	Slc22a8	solute carrier family 22 (organic anion transporter), member 8	-0.437922557787186	6.29790592880293	-0.913651836883983	0.393864778939179	0.999692817443324	-4.6004279439598	6.84262755924281	6.78537631038195	6.42155185922595	5.22024143458328	6.59293604505152	5.92470236433208	NA
**TC0800001251.mm.2**	Zfp90	zinc finger protein 90	-0.286896478169439	6.33463832906961	-0.913638989469895	0.393871045212952	0.999692817443324	-4.60042859345166	6.53729187389249	6.22456328836113	6.03006785557777	6.30602307233482	6.36485594966365	6.54502793458782	NA
**TC1500000395.mm.2**	Col14a1	collagen, type XIV, alpha 1	-0.383768989740921	4.64268018645105	-0.913445588254394	0.393965384956228	0.999692817443324	-4.60043837010216	4.13086491627636	3.88402776667138	4.23730918276524	5.29951604537313	5.22144004379631	5.08292316382391	NA
**TC0200003505.mm.2**	Mmadhc	methylmalonic aciduria (cobalamin deficiency) cblD type, with homocystinuria	0.405959985518708	6.49994982226588	0.913331217244898	0.394021182346296	0.999692817443324	-4.60044415114762	7.26400542955168	7.22845869768084	6.93317142712885	5.72640596157124	5.85030734098831	5.99735007667437	NA
**TC0100003803.mm.2**	Fam71a	family with sequence similarity 71, member A	0.280609412281362	4.88033103671609	0.912888572894917	0.394237188359553	0.999692817443324	-4.60046652146178	4.41496391806087	4.45369572503226	5.04936857216336	5.43790412871971	4.94742380898879	4.97863006733156	NA
**TC0600002658.mm.2**	Aplf	aprataxin and PNKP like factor	-0.602209802542935	5.04937934961314	-0.912539106362749	0.394407787615535	0.999692817443324	-4.60048417852466	5.2274218923802	5.65725624046981	5.56579462102453	3.53779455884694	5.39990072118293	4.90810806377441	NA
**TC0900002599.mm.2**	Mindy2	MINDY lysine 48 deubiquitinase 2	0.375717061416591	6.60047938699107	0.912481423244026	0.394435952165574	0.999692817443324	-4.60048709264822	7.33326472651831	7.11169406267723	6.78908813125844	6.16558769653358	6.51420118934726	5.68904051561157	NA
**TC1700000061.mm.2**	Dynlt1c/Dynlt1f	dynein light chain Tctex-type 1C/dynein light chain Tctex-type 1F	0.746340722234613	8.57091879005971	0.912470663135934	0.394441206099327	0.999692817443324	-4.60048763623244	8.17855025834826	6.90532728377432	10.3965013592525	8.52065696209997	9.48728585328674	7.93719102359649	NA
**TC0900002969.mm.2**	Cdv3	carnitine deficiency-associated gene expressed in ventricle 3	0.334528612238131	8.4252790178036	0.911909607752513	0.394715230723138	0.999692817443324	-4.60051597497239	8.51719815308172	8.51995237106506	8.87805430541432	8.21373609816121	8.44995516504955	7.97277801404972	NA
**TC0400003535.mm.2**	Adprhl2	ADP-ribosylhydrolase like 2	-0.269753959675007	6.40883501398475	-0.911708684547407	0.394813398363093	0.999692817443324	-4.60052612119397	6.34725727984198	6.43213778931154	6.21250334462995	6.35455020307255	6.45742088802021	6.6491405790323	NA
**TC1200001281.mm.2**	Crip2	cysteine rich protein 2	-0.310639454859682	8.17163672528593	-0.911701556138645	0.39481688151966	0.999692817443324	-4.60052648114164	8.70889191283845	8.98281791690871	7.97641137532243	7.3820044308021	7.96256666672641	8.01712804911748	NA
**TC1100001988.mm.2**	C1qtnf1	C1q and tumor necrosis factor related protein 1	0.270679067547198	6.11690882552156	0.91150769497409	0.394911616840918	0.999692817443324	-4.60053626952649	5.78686733128993	5.78979313820045	6.527911493114	6.3051638162527	6.07044430757065	6.22127286670166	NA
**TC0400000167.mm.2**	Slc7a13	solute carrier family 7, (cationic amino acid transporter, y+ system) member 13	-0.496783222818237	5.14834715930641	-0.91147530666323	0.394927445905114	0.999692817443324	-4.60053790475565	4.7263614735068	4.77941641273524	4.44968042158439	5.74568889039556	5.07572931627343	6.113206441343	NA
**TC1200000678.mm.2**	Zbtb1	zinc finger and BTB domain containing 1	0.329215602372701	5.51838125863816	0.911385952536744	0.394971118219938	0.999692817443324	-4.60054241592206	6.17738736724834	6.32860598045281	5.43381021829131	5.1975524861453	5.35311442992974	4.61981706976146	NA
**TC0100001114.mm.2**	Mgat5	mannoside acetylglucosaminyltransferase 5	-0.267803213907261	7.75865132949005	-0.911316116362122	0.395005253563657	0.999692817443324	-4.60054594152689	8.39425639421995	8.32692295018719	7.38672014022261	7.26084096213671	7.50718924188553	7.67597828828831	NA
**TC0700001145.mm.2**	Sema4b	sema domain, immunoglobulin domain (Ig), transmembrane domain (TM) and short cytoplasmic domain, (semaphorin) 4B	-0.298485241355008	8.65085198109956	-0.911051367566394	0.395134680772394	0.999692817443324	-4.60055930572604	8.25539353089644	8.30769332779322	8.81982479277532	8.55270247982353	8.73743561489224	9.23206214041662	NA
**TC1600000918.mm.2**	Ncam2	neural cell adhesion molecule 2	-0.32655835041497	8.19311987866277	-0.910756084544687	0.395279072879417	0.999692817443324	-4.60057420871303	9.60771919183861	9.51231794421277	7.69647825546641	6.99630446208124	7.82491025552206	7.52098916285553	NA
**TC1100003759.mm.2**	Krt9	keratin 9	-0.393777071520619	5.71400551784319	-0.910734769630648	0.395289497317402	0.999692817443324	-4.60057528437674	6.80986028070612	6.43448244325866	5.59179755369785	4.53427056632871	5.3124793675466	5.6011428955212	NA
**TC1600002060.mm.2**	Synj1	synaptojanin 1	0.339152820080022	9.19430492356246	0.910705479565984	0.395303822482568	0.999692817443324	-4.60057676248609	9.82571593503743	9.80832512433787	9.28173415529461	8.82331290578514	9.07095298337916	8.35578843754055	NA
**TC0500001783.mm.2**	Wasf3	WAS protein family, member 3	0.328585693801763	8.22810973606893	0.910583142910344	0.395363659040186	0.999692817443324	-4.60058293586244	7.78237436879851	8.13710349408731	8.24112073852877	8.81205523203687	7.99728848834216	8.39871609461995	NA
**TC1900000259.mm.2**	Gm5512	required for meiotic nuclear division 1 pseudogene	0.302052099762264	6.47681857919775	0.910548761777305	0.395380476558117	0.999692817443324	-4.600584670727	7.25350744812828	7.13331495205854	6.3938560642646	6.14524057299749	6.23076500863046	5.7042274291071	NA
**TC0900002302.mm.2**	Ddx10	DEAD (Asp-Glu-Ala-Asp) box polypeptide 10	-0.290230401665315	6.32710732571257	-0.91038272041961	0.395461703209651	0.999692817443324	-4.6005930486258	6.67697625083698	6.67379347834457	6.25541249293028	5.76029421795134	6.29088898696999	6.30527852724226	NA
**TC0700001466.mm.2**	Olfr558	olfactory receptor 558	-0.39396867940183	4.66342030930366	-0.910320159962359	0.395492310751162	0.999692817443324	-4.60059620499975	4.1779777684264	4.45038958969024	4.07850546259634	5.20360310685449	4.99295498932898	5.07709093892551	NA
**TC0600001998.mm.2**	Tmem209	transmembrane protein 209	0.495589152984747	6.28527939543968	0.910029885380949	0.39563435018387	0.999692817443324	-4.60061084869765	6.74029059840729	6.68160930401682	6.10907303402071	6.53140435407102	5.02297192665172	6.62632715547053	NA
**TC0700001269.mm.2**	Me3	malic enzyme 3, NADP(+)-dependent, mitochondrial	-0.287094985656248	8.22105667917495	-0.910029825177041	0.395634379647303	0.999692817443324	-4.60061085173453	8.70833646710837	8.77626192931733	8.02794665914096	7.60582919451478	8.19814262734846	8.00982319761977	NA
**TC0X00002470.mm.2**	Mpp1	membrane protein, palmitoylated	0.343724351947488	6.53742051215909	0.909972163862602	0.39566259950836	0.999692817443324	-4.60061376030857	7.61941075948354	7.48361961023308	6.34103119505661	6.06343950850982	6.27014816918644	5.44687383048502	NA
**TC0600002216.mm.2**	Zfp777	zinc finger protein 777	0.389115690143871	6.44894129597649	0.909901553753172	0.395697158667248	0.999692817443324	-4.60061732191109	6.52881090182527	5.70746255431351	6.48094951835596	7.13685333164798	6.20444308125755	6.63512838845865	NA
**TC0500003725.mm.2**	Pisd	phosphatidylserine decarboxylase	0.314465809540551	6.84660969922247	0.909574562173214	0.39585722989147	0.999692817443324	-4.60063381349449	7.58690986255457	7.58972676676699	6.95747333683418	6.30850325571299	6.43507367152822	6.20197130193785	NA
**TC0300000258.mm.2**	Atp11b	ATPase, class VI, type 11B	-0.549795361324314	6.18934929173632	-0.909407103846873	0.395939224147436	0.999692817443324	-4.60064225785688	6.30858110817167	7.28920477966694	6.13150736185727	5.08785220810806	6.78959154418391	5.52935874843005	NA
**TC1700000202.mm.2**	Riok2	RIO kinase 2	0.37144426697269	6.18931391035773	0.909309299691475	0.395987118865603	0.999692817443324	-4.6006471893925	7.43462153836887	7.28802594691384	6.29420539464178	5.28385686076273	5.31508816866362	5.52008555279551	NA
**TC1400001060.mm.2**	Erich6b	glutamate rich 6B	0.422411439381802	4.81164777116348	0.909250146632275	0.396016088249322	0.999692817443324	-4.60065017189774	4.3146822513792	4.15857894905889	4.93613748871096	5.68458666394223	4.35656292406153	5.41933834982805	NA
**TC0900001204.mm.2**	Xrn1	5'-3' exoribonuclease 1	0.490311884184385	5.67320141837811	0.909133631225019	0.396073154707091	0.999692817443324	-4.60065604630439	6.8113198900284	6.73272875667147	5.89926955974038	4.83862225622839	5.50072112585475	4.25654692174524	NA
**TC0900002332.mm.2**	Nrg4	neuregulin 4	0.377868611037575	6.70526745969114	0.908872576404448	0.396201035580379	0.999692817443324	-4.60066920649406	6.2356085786359	6.00884913322684	6.67955414860242	7.6918879855772	6.6619997454224	6.95370516668207	NA
**TC1600001466.mm.2**	Lsg1	large 60S subunit nuclear export GTPase 1	0.308451748374959	6.87503058784593	0.908828138912225	0.396222806920268	0.999692817443324	-4.60067144664923	6.89918998218494	7.25482293565372	6.87965607280864	6.97688098018478	6.88616777372282	6.35346578252068	NA
**TC1900001217.mm.2**	Gcnt1	glucosaminyl (N-acetyl) transferase 1, core 2	-0.296198433167588	5.23226349056296	-0.908745609012106	0.396263243330427	0.999692817443324	-4.60067560636182	5.99030451861338	5.45266583650543	4.89801201248385	4.78109484847803	5.15728622783876	5.1142174994583	NA
**TC1000002305.mm.2**	Reep3	receptor accessory protein 3	0.321754257146741	7.29288556889434	0.908686700671481	0.39629210800352	0.999692817443324	-4.60067857550298	7.75915222687307	7.69928487746195	7.57105703354797	6.90013537811428	7.06958199137812	6.75810190599065	NA
**TC0800003172.mm.2**	2310022B05Rik	RIKEN cDNA 2310022B05 gene	-0.35626652902347	7.60960080299302	-0.908640781280108	0.396314609280125	0.999692817443324	-4.60068088989127	7.89365642493655	7.29614719448951	7.80887302093683	7.06876104930574	7.84665546264781	7.7435116656417	NA
**TC0700000773.mm.2**	Klk10	kallikrein related-peptidase 10	0.321209418551508	5.89155657927787	0.908546486295864	0.396360818427749	0.999692817443324	-4.6006856422588	5.39966859805429	5.14111121841028	6.07388397679184	6.651605271361	5.85103220405079	6.23203820699903	NA
**TC1600000343.mm.2**	Rtp1	receptor transporter protein 1	0.399471526168498	5.30751722067271	0.908213200489619	0.396524177239416	0.999692817443324	-4.60070243730813	4.99624141516556	4.67133475920665	5.16948748981199	6.31874761118852	5.34158075982532	5.34771128883819	NA
**TC1100003592.mm.2**	Nxph3	neurexophilin 3	0.360017714387147	5.55905399512113	0.908103940873632	0.396577741461834	0.999692817443324	-4.60070794240733	5.89728646532315	5.95719269687696	5.46396565536827	5.64597446328223	4.71460102212771	5.67530366774849	NA
**TC1200001605.mm.2**	Pik3cg	phosphatidylinositol-4,5-bisphosphate 3-kinase catalytic subunit gamma	0.380847722171585	5.1562419695217	0.908072526738437	0.39659314316163	0.999692817443324	-4.60070952515554	5.45621394390337	5.60276620350675	4.68770285561707	5.63238070141455	5.04687452008263	4.51151359260582	NA
**TC1800001139.mm.2**	Nrep	neuronal regeneration related protein	-0.387302370828351	8.51464829865916	-0.908032437416336	0.396612798779412	0.999692817443324	-4.60071154494406	7.84353433710046	8.06480683922906	8.9879483307727	8.21452360621169	9.38568362251798	8.59139305612311	NA
**TC0800000120.mm.2**	Cul4a	cullin 4A	0.470242007609683	6.30844924298143	0.908031216945052	0.396613397182594	0.999692817443324	-4.60071160643331	7.3380086292065	7.24102449771182	6.65066450611914	5.45540866697568	6.06999368048009	5.09559547739537	NA
**TC0200002544.mm.2**	Cd40	CD40 antigen	-0.374050294383494	6.52254608922707	-0.907822686390608	0.396715650729786	0.999692817443324	-4.60072211185268	5.82281802480321	6.04900076090875	6.18084799562165	7.07683058482009	6.74417736369964	7.26160180550909	NA
**TC1100000519.mm.2**	Flt4	FMS-like tyrosine kinase 4	-0.455393292010963	5.54113957233334	-0.907796159124569	0.396728659871357	0.999692817443324	-4.60072344815531	4.82127355592326	4.9716917087732	5.23832600157162	6.03321679106921	5.47466295877081	6.70766641789195	NA
**TC0600002697.mm.2**	Plxna1	plexin A1	0.513878362733515	6.54358950546676	0.90771691063456	0.396767525750816	0.999692817443324	-4.60072744014316	7.5571220977285	7.26993553514805	6.60724046197024	6.12387760072529	6.67994542553144	5.02341591169705	NA
**TC0400000391.mm.2**	Fam205a2/Fam205a4	family with sequence similarity 205, member A2/family with sequence similarity 205, member A4	-0.381994746555866	5.84495836831658	-0.907696073913897	0.396777745190379	0.999692817443324	-4.60072848971996	6.51794689574661	6.63492136283628	5.25826094532044	5.31818528378199	6.21765893611647	5.12277678609769	NA
**TC0400001701.mm.2**	Wnt4	wingless-type MMTV integration site family, member 4	-0.271781215695508	6.23822891356045	-0.907682422676764	0.396784440592777	0.999692817443324	-4.60072917734592	6.19032982452182	6.27012907999673	6.00126402029208	6.2114120524345	6.2771774265331	6.47906107758449	NA
**TC0900002392.mm.2**	Tbc1d21	TBC1 domain family, member 21	0.455555539959287	4.88343412073412	0.907633448826381	0.396808461066372	0.999692817443324	-4.60073164415842	4.64003051674751	3.90573101058774	4.93336870811938	5.89960843037464	4.54098434844191	5.38088171013353	NA
**TC1800001581.mm.2**	Me2	malic enzyme 2, NAD(+)-dependent, mitochondrial	-0.624338247285688	5.49749479772829	-0.907509824697956	0.396869100537029	0.999692817443324	-4.60073787077447	5.92627192657995	6.11807723509298	5.60953790811519	4.23643365694753	6.42813466671466	4.66651339291944	NA

**TC0900002155.mm.2**	Hinfp	histone H4 transcription factor	0.319317324123986	5.88935925225773	0.907502592378415	0.396872648312932	0.999692817443324	-4.60073823503236	6.17687043013608	5.85151937864665	6.29259867145098	5.68060150505484	5.77926428921517	5.55530123904268	NA
**TC1200001466.mm.2**	Kcnf1	potassium voltage-gated channel, subfamily F, member 1	0.397631841105015	6.50263140000808	0.907499932970946	0.396873952877126	0.999692817443324	-4.60073836897379	6.91705331808104	6.77456709313653	7.1186977775235	5.94101805799698	6.13894935592804	6.12550279738241	NA
**TC1200001397.mm.2**	Rhob	ras homolog family member B	-0.311063426016815	7.77060055077739	-0.907272925148634	0.396985322756664	0.999692817443324	-4.6007498014469	8.0132200560866	7.91398760944274	7.84987969478459	7.1872546987661	7.8127213943743	7.84653985121001	NA
**TC0500000206.mm.2**	Nupl2	nucleoporin like 2	0.305900208228662	5.31123969566564	0.907109406276652	0.397065559561341	0.999692817443324	-4.60075803552647	5.35432514926195	5.44530806048041	5.72162044183168	5.11818224852272	5.19826483728956	5.02973743660752	NA
**TC1900001754.mm.2**	Prdx3	peroxiredoxin 3	0.531557610556342	6.83055591789702	0.907051303447939	0.397094072878206	0.999692817443324	-4.60076096112564	7.62734025509341	7.34302206706144	7.57545450101456	5.96258970215542	6.647651365792	5.8272776162653	NA
**TC1400001662.mm.2**	Itih1	inter-alpha trypsin inhibitor, heavy chain 1	-0.330398633196136	5.68736667613286	-0.907045356547729	0.397096991340179	0.999692817443324	-4.60076126055862	5.47914006770531	5.58174535873001	5.82371042795673	5.37754825402806	5.6012097370174	6.26084621135966	NA
**TC0700001348.mm.2**	Alg8	asparagine-linked glycosylation 8 (alpha-1,3-glucosyltransferase)	-0.516934945654626	4.87625221472187	-0.906974011294086	0.397132005529019	0.999692817443324	-4.60076485278529	5.27814969051491	4.86321763044689	3.72443925291454	5.31669878511556	4.71783052311145	5.3571774062279	NA
**TC1300000775.mm.2**	Ercc6l2	excision repair cross-complementing rodent repair deficiency, complementation group 6 like 2	-0.300526930307313	5.58870863536069	-0.906867150345279	0.397184454136802	0.999692817443324	-4.60077023292851	5.72939024813687	5.57597078909904	5.54412624336774	5.26879221378906	5.95763206714055	5.45634025063087	NA
**TC0400003886.mm.2**	Tmem82	transmembrane protein 82	-0.346811768052174	5.59599203423181	-0.906597978551412	0.39731658989098	0.999692817443324	-4.60078378338867	5.25227138368761	5.11881263796078	5.20272287448364	6.05289944933543	5.83345670452345	6.11578915539997	NA
**TC0200002130.mm.2**	Pcsk2	proprotein convertase subtilisin/kexin type 2	-0.377904695086412	8.99168503339502	-0.906477390419159	0.397375797017511	0.999692817443324	-4.6007898532237	9.435064852741	9.79092811824657	8.85605408804352	8.12809983156134	9.22948350825861	8.51047980151907	NA
**TC0100002960.mm.2**	Tmem177	transmembrane protein 177	0.322176671254376	5.94294037251285	0.90647242481834	0.397378235201875	0.999692817443324	-4.60079010315883	5.8976734588982	6.0244200459696	6.40615536901848	5.78379566734055	5.57311870177702	5.97247899207325	NA
**TC0200004163.mm.2**	Ext2	exostoses (multiple) 2	0.277013912675073	7.6493837832888	0.906405351404513	0.397411170354268	0.999692817443324	-4.60079347911086	7.63995865982597	7.54736614032272	7.784360774291	7.84714208817614	7.36056704723665	7.71690798988033	NA
**TC0700000552.mm.2**	Cox7a1	cytochrome c oxidase subunit 7A1	-0.281170138364955	5.43655736073186	-0.906393403286446	0.397417037470713	0.999692817443324	-4.60079408047119	5.687409593399979	5.63974129411038	5.33705262745775	5.0278738726178	5.60325823686342	5.32400853994204	NA
**TC1500001123.mm.2**	Hoxc4	homeobox C4	0.543482829876392	5.75695492677726	0.906319854558602	0.39745315496468	0.999692817443324	-4.60079778215214	5.07734399290949	5.07195791847848	5.47627128050362	7.26342537401055	5.52259826771325	6.13013272704814	NA
**TC1400001995.mm.2**	Rnase9	ribonuclease, RNase A family, 9 (non-active)	-0.402571001751494	5.98940446106647	-0.906112524752589	0.397554981466226	0.999692817443324	-4.60080821608222	5.30958467825776	5.39585065709478	5.50518498880978	6.70773972496186	6.43269113180196	6.58537558547267	NA
**TC0100000257.mm.2**	Vwa3b	von Willebrand factor A domain containing 3B	-0.313472906778495	6.20142415101939	-0.905854743356991	0.397681613745784	0.999692817443324	-4.60082118714011	5.66357880695023	5.81069038911064	5.94088202582977	6.61278292241948	6.62668337287052	6.55392738893572	NA
**TC1300000490.mm.2**	Sirt5	sirtuin 5	0.280338250514139	5.74826303583925	0.90573501566951	0.397740438963832	0.999692817443324	-4.60082721090183	5.81714550912365	5.5135848888763	5.79471801693222	6.06504414209969	5.68523440652748	5.61385125147615	NA
**TC1100000134.mm.2**	Ikzf1	IKAROS family zinc finger 1	0.329685483760174	6.17547830238734	0.905317981607921	0.397945389310432	0.999692817443324	-4.60084818931679	6.47879258022021	6.51032889440624	6.08089977956471	6.28065987404426	6.23934089637979	5.46284778970883	NA
**TC1700000888.mm.2**	Dlk2	delta like non-canonical Notch ligand 2	-0.275284426401568	6.57362369890943	-0.905184041509063	0.398011230665522	0.999692817443324	-4.60085492587022	6.52720089128922	6.3797760252873	6.57040166201727	6.4216965500212	6.62650890039708	6.91615816444452	NA
**TC0800001698.mm.2**	Rab20	RAB20, member RAS oncogene family	-0.523786625576295	5.38373444478154	-0.905168242784214	0.398018997433786	0.999692817443324	-4.60085572043454	4.3721133595591	4.80745019138448	4.83545358831308	6.20218134498345	5.4977690760702	6.58743910837892	NA
**TC0400003604.mm.2**	Eif3i	eukaryotic translation initiation factor 3, subunit I	0.263834545626278	7.39348798399657	0.90501325928239	0.398095194488555	0.999692817443324	-4.6008635145985	7.51454731718257	7.65686921247627	7.47454269800229	7.38404753478428	7.19199776732832	7.1389233742057	NA
**TC0200001972.mm.2**	Nop56/Snord57	NOP56 ribonucleoprotein/small nucleolar RNA, C/D box 57	-0.59047751363724	8.32932956231797	-0.904990086657555	0.398106588163686	0.999692817443324	-4.60086467989206	9.30849864864401	9.07270982559695	8.54251719039786	6.66438974579831	8.64960014121657	7.73826182225409	NA
**TC1900000056.mm.2**	Yif1a	Yip1 interacting factor homolog A (S. cerevisiae)	-0.331469192662468	6.22686393722266	-0.904912646653584	0.398144666172824	0.999692817443324	-4.60086857403561	5.98779728916126	6.03319317565551	5.76961001049741	6.5690173760997	6.42532067258196	6.57624509934009	NA
**TC1200002389.mm.2**	Hsp90aa1	heat shock protein 90, alpha (cytosolic), class A member 1	-1.04173676741339	9.57102634227331	-0.904894571160714	0.398153554463587	0.999692817443324	-4.60086948295191	11.3185995825154	11.2900590899642	10.0541162329293	6.31289669023741	9.9298882273451	8.5205982306484	NA
**TC1900000098.mm.2**	Tmem262	transmembrane protein 262	-0.279814370498304	6.18101880675303	-0.904891811034061	0.398154911718156	0.999692817443324	-4.60086962174248	5.4498271021122	5.68136060967785	6.50764644130269	6.19000175256307	6.57053740206838	6.68673953279399	NA
**TC1100001694.mm.2**	Fzd2	frizzled class receptor 2	0.270717997947827	6.1466664429334	0.904474563533401	0.398360127440148	0.999692817443324	-4.60089059994045	6.54699329151342	6.70266691548086	6.1189638528453	5.96692337040557	5.6684685856776	5.87598264167762	NA
**TC0200001095.mm.2**	Hoxd9	homeobox D9	0.474976185661589	4.95259540427351	0.90408713458102	0.398550748538432	0.999692817443324	-4.60091007407512	4.6887551467032	4.34640825052367	4.66086636373114	6.15431433613753	4.7452119906815	5.120016337864	NA
**TC1400000517.mm.2**	Ktn1	kinectin 1	-0.470032158249589	6.89861490487946	-0.904035598628743	0.398576110191403	0.999692817443324	-4.60091266417944	8.14520590622036	8.16082543681236	6.8058445027812	5.26695238209123	6.61426570862184	6.39859549274976	NA
**TC1200000775.mm.2**	Rgs6	regulator of G-protein signaling 6	-0.360350458735624	6.46528547932951	-0.903855431749506	0.398664782667746	0.999692817443324	-4.60092171839167	7.25888665893559	7.3072916890558	6.2204032241895	5.5320135810677	6.5923199744823	5.88079774824614	NA
**TC1500002291.mm.2**	Aaas	achalasia, adrenocortical insufficiency, alacrimia	-0.316418595671683	6.64448305275557	-0.903778298456064	0.398702749777103	0.999692817443324	-4.60092559438386	6.59432480005261	6.97225805394643	6.13480305241068	6.69893608318483	6.61752173228401	6.84905459465486	NA
**TC0100003090.mm.2**	Sox13	SRY (sex determining region Y)-box 13	0.296311669097618	6.69745311180201	0.903608807226246	0.398786187513215	0.999692817443324	-4.60093411076284	6.50574182071828	6.74185711401842	7.05606349133556	6.70880804579974	6.39580916294485	6.77643903599521	NA
**TC1000000774.mm.2**	Cfap410	cilia and flagella associated protein 410	0.464664817279147	7.78482775607526	0.903463376663866	0.398857791024913	0.999692817443324	-4.60094141745709	7.8886739035115	7.5206497172072	7.40773711127812	8.70674916386747	7.23291567955746	7.95224096102984	NA
**TC0900000655.mm.2**	Gldn	gliomedin	0.367671619671383	6.08440934559517	0.903458495220276	0.398860194596997	0.999692817443324	-4.60094166269818	5.72854774492861	6.21901305400544	5.9448445505241	6.70227470646577	5.70389040644572	6.20788561120139	NA
**TC1700000488.mm.2**	Brpf3	bromodomain and PHD finger containing, 3	-0.265617299969282	7.27082380395227	-0.903401722198284	0.398888149841202	0.999692817443324	-4.60094451488946	7.15007638721106	7.14962916703328	7.08776781669366	7.30923351807171	7.47986805751113	7.44836787719281	NA
**TC0300001987.mm.2**	Exosc8	exosome component 8	-0.36843377269746	6.24053305825228	-0.903391497008445	0.398893184918415	0.999692817443324	-4.60094502857693	6.2259717085705	6.11459739414346	5.62540424448507	6.55747660621732	6.2383668641123	6.68138153198501	NA
**TC1700001794.mm.2**	Mdga1	MAM domain containing glycosylphosphatidylinositol anchor 1	0.400262537023881	6.77113389144106	0.903364992872383	0.398906236279204	0.999692817443324	-4.60094636006195	6.62155520990935	6.68175288166015	6.60915399243185	7.45285617313046	7.06308863608352	6.19839645543102	NA
**TC1000002042.mm.2**	Gtf3c6	general transcription factor IIIC, polypeptide 6, alpha	0.316574990677071	7.46230735796539	0.903363018434274	0.398907208559303	0.999692817443324	-4.60094645925068	7.97354999640554	7.90565221922405	7.63896150341686	7.12493445334159	7.31480391557493	6.81594205982938	NA
**TC1000002794.mm.2**	Tmtc2	transmembrane and tetratricopeptide repeat containing 2	0.373056275219326	5.86179737456649	0.903231086909591	0.398972180133002	0.999692817443324	-4.60095308674377	5.79737013766947	5.77126142808315	5.83260707861734	6.34152553742515	6.18105929226239	5.24696077334145	NA
**TC1600001280.mm.2**	Sdf2l1	stromal cell-derived factor 2-like 1	-0.308056413134683	6.74927936231054	-0.903161501047005	0.399006451888378	0.999692817443324	-4.60095658212457	6.77654185336292	7.10340669852094	6.21604890074346	6.78375849711154	6.77132036506968	6.84459985905469	NA
**TC1500000407.mm.2**	Tbc1d31	TBC1 domain family, member 31	0.308375735305279	5.91799613546588	0.902959474833077	0.399105964398415	0.999692817443324	-4.60096672928451	5.9268602601893	5.7962816276392	5.91104598579644	6.28974721199225	5.52848056325791	6.05556116392021	NA
**TC1300002499.mm.2**	Utp15	UTP15 small subunit processome component	0.313553279598583	7.16925309272069	0.902842881298149	0.399163403612896	0.999692817443324	-4.6009725848411	7.14056838277331	7.16403703091366	7.54028153061731	7.12872832029985	7.30359432702344	6.73830896469655	NA
**TC0600000215.mm.2**	Snd1	staphylococcal nuclease and tudor domain containing 1	0.310302199223421	6.27096235962368	0.902831102512859	0.399169206716681	0.999692817443324	-4.60097317637121	6.2451384846898	6.47468083899933	6.1792487059293	6.58403091032061	5.82589218767038	6.31678303013268	NA
**TC0X00000225.mm.2**	Gm5124	nucleolar and coiled-body phosphoprotein 1 pseudogene	0.360278959815203	7.11625849367947	0.902697207158198	0.399235177953988	0.999692817443324	-4.60097990028543	7.64074191982264	7.2009713426521	7.45462520135731	6.83357260825894	6.46451649070821	7.10312339927765	NA
**TC0300000144.mm.2**	Tbl1xr1	transducin (beta)-like 1X-linked receptor 1	-0.716946165401909	7.17484826700309	-0.902695091442473	0.399236220448497	0.999692817443324	-4.60098000652727	8.20084777787736	8.31228898638328	7.48949682557116	5.06153342790587	7.52718868295816	6.45773390132269	NA
**TC0400001256.mm.2**	Ccdc17	coiled-coil domain containing 17	-0.278078957690031	5.04693670477527	-0.902379924926496	0.399391537919675	0.999692817443324	-4.60099583122403	4.98785494690425	4.90803065570336	4.76174306964665	5.15304528568532	5.24004707700466	5.23089919370737	NA
**TC0700004276.mm.2**	Nfatc2ip	nuclear factor of activated T cells, cytoplasmic, calcineurin dependent 2 interacting protein	-0.387642341270731	5.8403054429135	-0.90234272504145	0.39940987341547	0.999692817443324	-4.60099769884709	5.84674727532538	5.85604570128105	6.1389406341917	5.14293686497487	6.31128989888038	5.74587228282765	NA
**TC0100000018.mm.2**	Oprk1	opioid receptor, kappa 1	0.401300724235233	6.90352210476174	0.902298819411397	0.399431514926143	0.999692817443324	-4.60099990307686	7.43893979927425	7.49918544513548	7.42919193371078	6.21361248260482	6.40426221187211	6.43594075597302	NA
**TC0100001186.mm.2**	Lemd1	LEM domain containing 1	0.375399391097833	5.34351579855129	0.902258344669644	0.399451466097687	0.999692817443324	-4.60100193500951	5.05117654551047	5.01491219068066	5.15066683440662	6.22223558424951	5.372462628083	5.24964100837746	NA
**TC1400002345.mm.2**	Fam160b2	family with sequence similarity 160, member B2	0.287242027364698	6.18607375082014	0.902154077724616	0.399502865734589	0.999692817443324	-4.60100716923307	6.62604182114372	6.39834633577378	6.09460104549886	6.2386681558675	5.67357571162229	6.08520943501468	NA
**TC0100001447.mm.2**	Tnr	tenascin R	-0.306511334589861	6.93312729395231	-0.902118158410179	0.39952057373851	0.999692817443324	-4.60100897231171	8.23708467448978	8.37737419494205	6.40991379600678	5.77572731654438	6.38284275586786	6.41582102586302	NA
**TC1700002777.mm.2**	Zbtb22	zinc finger and BTB domain containing 22	0.301834729531401	6.3493244748137	0.902029824270834	0.399564124436312	0.999692817443324	-4.60101340633914	6.51954025023758	6.23416460965731	6.21878564512752	6.75417007889753	6.2351549971388	6.13413126782343	NA
**TC1800001462.mm.2**	Nars	asparaginyl-tRNA synthetase	0.309332964900213	7.57523786974149	0.901987985604391	0.399584753072801	0.999692817443324	-4.60101550639085	8.69483759604286	8.71331387843879	7.47622167048937	6.8547491663945	6.98293007876652	6.72937482831692	NA
**TC0900001507.mm.2**	Ubp1	upstream binding protein 1	-0.295210406631939	7.79266484473457	-0.901925889713058	0.399615371045798	0.999692817443324	-4.60101862313343	7.53876917637072	7.75582853842819	7.95063843759361	7.48484683257869	8.12697710605203	7.89892897738415	NA
**TC1100003711.mm.2**	Krt40	keratin 40	0.422802168938484	5.58137329001521	0.901816650242072	0.399669238639833	0.999692817443324	-4.60102410583278	4.71707674461868	5.20095986947126	5.77019065752821	6.43771307441094	5.16021797344317	6.202081420619	NA
**TC0800001432.mm.2**	Crispld2	cysteine-rich secretory protein LCCL domain containing 2	0.300022632260065	5.9740948435241	0.90171594013906	0.399718905099541	0.999692817443324	-4.60102916011416	5.7491515372663	5.94633500263294	6.04899337444712	6.32557051843562	5.63893659877006	6.13558202959254	NA
**TC1000002445.mm.2**	Trappc10	trafficking protein particle complex 10	-0.348949748943049	7.50788652975439	-0.90170917514149	0.399722241509404	0.999692817443324	-4.6010294996139	8.07812559168833	8.21868642758504	7.46061747713185	6.56568635355157	7.46657380886235	7.25762951970717	NA
**TC0800001963.mm.2**	Gm8096	3-phosphoglycerate dehydrogenase pseudogene	0.306610419723812	6.64769200286832	0.901683015532392	0.399735143288883	0.999692817443324	-4.60103081241395	5.92048324835011	6.03910100234124	6.81671284454591	7.45318145843718	6.7889884049532	6.86768505858227	NA
**TC1100003652.mm.2**	Pip4k2b	phosphatidylinositol-5-phosphate 4-kinase, type II, beta	0.660635986633102	6.83234234591495	0.901615609273937	0.399768389134089	0.999692817443324	-4.60103419504683	8.14816752066855	8.39675054604425	7.40542347146555	5.47978051955601	6.50193245509539	5.06199956265997	NA
**TC0100001341.mm.2**	Tpr	translocated promoter region, nuclear basket protein	0.754949061035997	6.81210969152113	0.901560465793031	0.399795588322624	0.999692817443324	-4.60103696219361	9.10553515979301	8.81633286552761	6.9965786714688	5.23376545147028	6.45172914891481	4.26871685195227	NA
**TC0300002153.mm.2**	Slitrk3	SLIT and NTRK-like family, member 3	0.524487987917015	7.17198377673106	0.901306969840429	0.399920641514569	0.999692817443324	-4.60104968160941	7.8948326021811	8.13676405765631	7.69924498983763	6.32539599835385	7.06823559905337	5.90742941330408	NA
**TC0200002759.mm.2**	Fam217b	family with sequence similarity 217, member B	-0.638356122189719	5.50642172386806	-0.90120430633352	0.399971295236452	0.999692817443324	-4.60105483228124	6.45898964364189	6.40956890176213	5.8250598393117	3.62156993740075	5.72687096307709	4.99647105801479	NA
**TC0700003039.mm.2**	Saa3	serum amyloid A 3	0.431634934141478	6.11867398355239	0.901118127082491	0.400013819411326	0.999692817443324	-4.60105915567563	5.65578078187031	5.81205831359867	5.85669585803114	7.19704147903302	5.96467659354125	6.22579087523995	NA
**TC0800002409.mm.2**	Kcnn1	potassium intermediate/small conductance calcium-activated channel, subfamily N, member 1	0.263701643219387	6.90135692135561	0.901115223514881	0.400015252202862	0.999692817443324	-4.60105930133619	7.23872155016642	7.22844805548659	6.7889433918547	6.94524421260502	6.66481875164134	6.54196556637961	NA
**TC0500002781.mm.2**	A430089I19Rik	RIKEN cDNA A430089I19 gene	0.268582443131968	5.05933668444288	0.900840513099367	0.400150827922624	0.999692817443324	-4.60107308127993	4.99681351511115	5.07044929566629	5.11463237351638	5.29832871755552	4.83505354821939	5.04074265658858	NA
**TC1900001205.mm.2**	Cep78	centrosomal protein 78	-0.304114360012545	6.48015396183189	-0.900792141004869	0.400174704195478	0.999692817443324	-4.6010755074615	6.21048686881779	6.13249394782683	6.75231564143975	6.21254147572107	6.62322020527072	6.94986563191519	NA
**TC1800000835.mm.2**	Epg5	ectopic P-granules autophagy protein 5 homolog (C. elegans)	0.289820828889108	6.1056633119097	0.900758919637188	0.400191102748341	0.999692817443324	-4.60107717369101	6.72560322646885	6.50346637182506	5.82591438309811	6.16636158237315	5.54474286740284	5.8678914402902	NA
**TC0200000833.mm.2**	Fmnl2	formin-like 2	-0.715281710676615	7.58776257530406	-0.900727918696504	0.400206405720123	0.999692817443324	-4.60107872852296	8.18955511353021	8.36336417462311	8.02376592541233	5.74778044574658	8.27901590428979	6.92309388822235	NA
**TC0200003326.mm.2**	Zbtb34	zinc finger and BTB domain containing 34	-0.302338506571196	6.72438571490951	-0.900707416708326	0.400216526342484	0.999692817443324	-4.60107975677021	6.28141212628516	6.54509854469028	6.82880263712378	6.62876066554585	7.29464965273657	6.76759066307545	NA
**TC0100002032.mm.2**	Il17f	interleukin 17F	-0.361976436359561	5.57662671495306	-0.900559715436617	0.400289443416053	0.999692817443324	-4.60108716412143	4.59907233751661	4.74267419043292	5.37524494884819	6.32178549567668	6.05273998831967	6.36824332892432	NA
**TC0500003381.mm.2**	Tmem270	transmembrane protein 270	-0.410295018098977	6.31077137983009	-0.900412237989267	0.400362259922317	0.999692817443324	-4.60109455956383	5.58455468168575	5.61744916570338	5.84350327170775	7.07751392598897	6.72513701742416	7.01647021647051	NA
**TC0500003710.mm.2**	Lrrc8d	leucine rich repeat containing 8D	0.355932067176576	8.16804554260488	0.900398591610626	0.400368998278607	0.999692817443324	-4.60109524384413	7.74842797375716	7.65147383266391	8.23154455305657	8.92857323872411	7.88769039595675	8.56056326147077	NA
**TC0300001803.mm.2**	Mrpl47	mitochondrial ribosomal protein L47	-0.475282581092412	5.82498225680574	-0.900329212031701	0.400403258082308	0.999692817443324	-4.60109872270436	6.26289815502253	6.44210462616221	6.10625242194359	4.54091037678884	5.69890002345183	5.89882793746543	NA
**TC0400003001.mm.2**	Ifnb1	interferon beta 1, fibroblast	0.459176806098595	5.80999341412185	0.899966270377881	0.400582515340868	0.999692817443324	-4.60111691901204	5.52912796835806	5.48386240544215	5.43878023162958	6.94388162993446	5.80090249470297	5.66340575466388	NA
**TC0200002148.mm.2**	Zfp133-ps/Gm38402	zinc finger protein 133, pseudogene/zinc finger protein pseudogene	-0.300836966225829	6.38744615867542	-0.899925106600029	0.400602849967989	0.999692817443324	-4.60111898252233	5.96084857052544	5.93507792985067	6.17790183912779	6.73563642048459	6.88384383396852	6.63136835809552	NA
**TC1100001647.mm.2**	Cntd1	cyclin N-terminal domain containing 1	0.346518120451036	5.40172675981233	0.899855414975155	0.400637278920472	0.999692817443324	-4.60112247599134	5.22551497283793	4.75076986797033	5.42480928210919	6.13874669737471	5.6013250366763	5.26919470190552	NA
**TC1000000142.mm.2**	Ifngr1	interferon gamma receptor 1	-0.276322734084241	7.3840167421625	-0.89956126687057	0.400782617942206	0.999692817443324	-4.60113721922544	7.03829298864894	6.83697984936791	7.49639206608866	7.44169900730619	7.58086471769143	7.9098718238719	NA
**TC0800002730.mm.2**	Pllp	plasma membrane proteolipid	-0.263721266017019	7.59968729501261	-0.899461426964537	0.400831957963647	0.999692817443324	-4.60114222276272	7.32939452447682	7.24658699639919	7.70360245131538	7.54374740726742	7.83392615020788	7.94086624040897	NA
**TC0600003449.mm.2**	Pthlh	parathyroid hormone-like peptide	-0.311475793734794	5.93177238981975	-0.899212568983518	0.400954961229945	0.999692817443324	-4.60115469306467	5.49923383849448	5.70790596996384	5.80343782038416	6.07683365111114	5.95033009201747	6.55289296694742	NA
**TC1700000494.mm.2**	Srsf3	serine/arginine-rich splicing factor 3	-0.609703201624226	7.40363695603241	-0.898619426781112	0.401248248113886	0.999692817443324	-4.60118440761996	8.40650401585702	8.67357939177277	7.21948305361366	5.84168290904444	7.99017565727565	6.29039670863091	NA
**TC0800000211.mm.2**	Defa21	defensin, alpha, 21	-0.372597335149838	4.57870547991157	-0.898557807785317	0.401278725632835	0.999692817443324	-4.60118749390145	4.19405104851598	3.51271735937582	5.01736661467793	4.49276828596104	4.80741862749079	5.44791094344785	NA
**TC0700000620.mm.2**	Scgb1b24	secretoglobin, family 1B, member 24	-0.262390497401179	5.08680969070198	-0.89837003899337	0.401371609087416	0.999692817443324	-4.60119689784948	5.24865200354387	5.11493041872552	4.85681680964895	4.9594305539211	5.12878109508924	5.21224726328317	NA
**TC1200000142.mm.2**	Odc1	ornithine decarboxylase, structural 1	0.337185554823228	6.47287320237547	0.898069781237238	0.401520170808185	0.999692817443324	-4.60121193322437	7.1741751487488	7.34556996134135	6.66064875915523	5.83528384774932	6.0115748952361	5.809986602022	NA
**TSUnmapped00000035.mm.2**	Pemt	phosphatidylethanolamine N-methyltransferase	0.27912411243112	5.88610785089217	0.897896210699299	0.401606068910906	0.999692817443324	-4.60122062345212	5.62669748052305	5.76113324404148	5.92921258764244	6.31431971518292	5.78199704167448	5.90328703628864	NA

**TC1100001109.mm.2**	Blmh	bleomycin hydrolase	-0.337356136639148	8.32504396707049	-0.897847486420711	0.401630184478243	0.999692817443324	-4.60122306277964	8.72053406717568	8.83871049019951	8.32769021642954	7.53046326945519	8.42801757701979	8.10484818214323	NA
**TC0200003026.mm.2**	A930004D18Rik	RIKEN cDNA A930004D18 gene	0.325461555811371	6.732279628485	0.897718514897817	0.401694022800654	0.999692817443324	-4.60122951923536	6.19211532668268	6.24723049546441	6.76092178276484	7.54170574742799	6.88912760041193	6.76257681815816	NA
**TC0900000563.mm.2**	Bud13	BUD13 homolog	-0.347744431874912	6.35553661818207	-0.89760578172266	0.401749829682592	0.999692817443324	-4.60123516235113	6.08572168804902	6.20750515146182	5.94557248068925	6.62667952222664	6.32169076328506	6.94605010338065	NA
**TC0400000405.mm.2**	Fam205a2/Fam205a3	family with sequence similarity 205, member A2/family with sequence similarity 205, member A3	-0.348172396589328	5.81948840566486	-0.897439371414649	0.401832219229074	0.999692817443324	-4.60124349166393	6.5358750386012	6.56198795384441	5.25937457166629	5.30198675251614	6.09563375634027	5.16207236102081	NA
**TC0300001007.mm.2**	Trim45	tripartite motif-containing 45	-0.27410754778279	5.08480563379701	-0.897437673638065	0.401833059861368	0.999692817443324	-4.60124357663801	5.26841336674311	4.93595712267084	4.95925550239894	4.91886860650232	5.13272614382267	5.29361306064417	NA
**TC0400004066.mm.2**	Slc45a1	solute carrier family 45, member 1	0.311259004741013	6.59413752627188	0.897270346502277	0.401915916198635	0.999692817443324	-4.60125195094894	6.46166591706003	6.6039426696715	6.67056076928847	6.89030652090242	6.79219619067033	6.14615309003853	NA
**TC1800000534.mm.2**	Snx2	sorting nexin 2	-0.680037147718974	7.88802616683789	-0.897191821548659	0.401954804254814	0.999692817443324	-4.60125588062417	8.86344707293643	8.62143991210193	8.35599878957033	5.8855990707052	8.0043755764007	7.59729657931277	NA
**TC1000000011.mm.2**	Plekhg1	pleckstrin homology domain containing, family G (with RhoGef domain) member 1	-0.286714711786386	6.71817424942395	-0.897116506988537	0.401992105065454	0.999692817443324	-4.60125964945635	6.87302790856682	6.6754104037002	6.44635006108408	6.64723881926786	6.62585484088225	7.04116346304247	NA
**TC1100003043.mm.2**	Kdm6b	KDM1 lysine (K)-specific demethylase 6B	0.293597203634008	7.67935107712381	0.896839115405647	0.402129510264302	0.999692817443324	-4.60127352892073	7.31876176614696	6.8699816340397	8.20294817327455	8.03433056163756	7.67778988227702	7.97229444536706	NA
**TC0200000293.mm.2**	Mllt10	myeloid/lymphoid or mixed-lineage leukemia; translocated to, 10	0.682904540801949	6.91040062318074	0.896698265968011	0.402199293107422	0.999692817443324	-4.60128057547975	7.52612722538826	7.87260270596776	7.54666732638454	6.16807411828162	7.22055876012716	5.12837360293509	NA
**TC0X00002762.mm.2**	Dmrtc1a	DMRT-like family C1a	0.338579806973034	6.74914278892899	0.896652687156862	0.402221876741055	0.999692817443324	-4.60128285560839	6.57963527805816	6.30039157827866	6.81325269708908	7.33274204850251	6.4087634209854	7.06007171066012	NA
**TC0800001407.mm.2**	Plcg2	phospholipase C, gamma 2	0.421023925725203	5.39103802877782	0.896609084096648	0.402243482306258	0.999692817443324	-4.60128503683642	4.94389743040406	5.31407847636111	5.10624708804142	6.35890297063465	5.44189256104479	5.18120964618089	NA
**TC1800000632.mm.2**	Csnk1a1	casein kinase 1, alpha 1	0.530314729245491	8.97848296794636	0.896021466576102	0.402534734771746	0.999692817443324	-4.60131442632487	9.85970088432576	9.91049432999311	9.28129249641807	8.29937352950707	9.046896447261	7.47314012017316	NA
**TC0200004826.mm.2**	Zfp120	zinc finger protein 120	0.425310929401016	6.24672114879517	0.895625142156602	0.402731261840285	0.999692817443324	-4.60133424217361	6.4528597024159	6.70674940866872	6.68703485540806	5.89863496483614	6.404299280041	5.33074868140117	NA
**TC0700004494.mm.2**	Echs1	enoyl Coenzyme A hydratase, short chain, 1, mitochondrial	-0.832013380485084	6.48747586075056	-0.895614203499878	0.402736687054817	0.999692817443324	-4.60133478902551	7.67611978287101	7.69945142374227	7.03588810006146	3.90674049839848	6.55115826530913	6.05549709412099	NA
**TC0200004159.mm.2**	Cd82	CD82 antigen	0.353730010755048	8.06541374442719	0.895603637659222	0.402741927416666	0.999692817443324	-4.60133531723577	9.1066053788366	9.30347612682796	7.82617932071426	7.51875117049006	7.76908439429224	6.86838607540199	NA
**TC1100000517.mm.2**	Olfr1381	olfactory receptor 1381	0.365909907100941	5.75154993734717	0.895122953462925	0.402980387232767	0.999692817443324	-4.60135934397756	5.1830971013262	5.22478932215942	5.71323098086084	6.7033855265388	5.68019893091542	6.00459776228234	NA
**TC1300002236.mm.2**	Uqcrb	ubiquinol-cytochrome c reductase binding protein	-0.573995806927861	10.1037483495487	-0.895019103448461	0.403031919431396	0.999692817443324	-4.60136453390107	10.8263623011107	10.7259164564594	10.297253065689	8.6638567972443	10.677036414019	9.43206506276998	NA
**TC0600003045.mm.2**	Atp6v1e1	ATPase, H+ transporting, lysosomal V1 subunit E1	-0.59810961967135	8.628926713605	-0.894912438214989	0.403084853714817	0.999692817443324	-4.60136986415899	9.77589633231529	9.73508727247267	8.69636648366389	6.83681223508577	8.91777655783108	7.81162140026129	NA
**TC0200003869.mm.2**	Ctnnd1	catenin (cadherin associated protein), delta 1	0.488722126350714	5.53337360963403	0.894761577870126	0.40315972937561	0.999692817443324	-4.60137740231067	6.18216879147084	6.4961424471856	5.87589995721909	4.87378737870549	5.53273725453395	4.23950582868919	NA
**TC1400000096.mm.2**	Ptprg	protein tyrosine phosphatase, receptor type, G	0.361818006623235	7.37915265399041	0.894669975115384	0.403205199120259	0.999692817443324	-4.60138197914108	8.3055070897526	7.93275417434877	7.28553559728551	7.09460973925828	7.29363383816883	6.36287548512849	NA
**TC1300000425.mm.2**	Ly86	lymphocyte antigen 86	-0.684070768187487	8.29649198145445	-0.894569909016315	0.403254874273423	0.999692817443324	-4.60138697852827	9.51128977750096	9.78414940770289	8.40165736161791	6.15602822195603	8.54206378511968	7.38376333482923	NA
**TC0200005399.mm.2**	Psma7	proteasome (prosome, macropain) subunit, alpha type 7	-0.442472678409835	7.60853238559567	-0.894233855437281	0.403421732576938	0.999692817443324	-4.60140376571973	8.3077049004971	8.12026456176265	7.71092700611255	6.45821274113473	7.87441828903595	7.179666815031	NA
**TC1300001169.mm.2**	Sgtb	small glutamine-rich tetratricopeptide repeat (TPR)-containing, beta	-0.600819798823534	7.63358563810042	-0.894210853471023	0.403433155464946	0.999692817443324	-4.6014049146266	8.56521399244749	8.71596484927917	7.86154673772072	5.79780095689368	7.77060251553505	7.09038477672642	NA
**TC0400002737.mm.2**	Lpar1	lysophosphatidic acid receptor 1	0.494153999652191	7.96617654564886	0.894044676894707	0.403515686732397	0.999692817443324	-4.60141321434771	8.64094090662413	8.52539994611669	7.99761187013341	7.81190134009495	8.25016788081945	6.57103733010453	NA
**TC1000002338.mm.2**	A930033H14Rik	RIKEN cDNA A930033H14 gene	-0.279387191621643	6.8472197382205	-0.89394168335406	0.403566844596469	0.999692817443324	-4.60141835793876	6.71143170096241	6.46018321308524	7.00424458985995	6.6722199761561	7.04497142691728	7.19026752234205	NA
**TC0200000555.mm.2**	Prdm12	PR domain containing 12	-0.46447224563435	5.15913203750338	-0.893719407140841	0.403677267788613	0.999692817443324	-4.60142945746394	5.74406416162719	5.81885419101585	5.17260102012393	4.05886367043034	4.54029580333779	5.62011337848518	NA
**TC1400001988.mm.2**	Tep1	telomerase associated protein 1	-0.265731529794169	5.60774393857467	-0.89366481899421	0.403704389737071	0.999692817443324	-4.60143218312228	5.60797581235794	5.68301454908994	5.35056657895468	5.56143852625121	5.64223718407579	5.80123098071844	NA
**TC0500002275.mm.2**	Msx1	msh homeobox 1	-0.265296281823812	6.95867896851353	-0.893592855812753	0.40374014649497	0.999692817443324	-4.60143577619404	6.55554938135448	6.74151791193543	6.99663751268248	6.96556946438933	7.15115308253475	7.34164645818469	NA
**TC0600000764.mm.2**	Tnip3	TNFAIP3 interacting protein 3	0.287313713932404	8.14116349525403	0.893505895165421	0.403783358283302	0.999692817443324	-4.60144011785874	7.90163858068082	7.94025994518044	8.23010108744542	8.55975384931846	7.90892715310124	8.30630035579782	NA
**TC1000000751.mm.2**	Gm10787	predicted gene 10787	0.416896919710646	4.70646910002578	0.89337879184837	0.403846523654417	0.999692817443324	-4.60144646328399	4.48778544673363	4.30669428608652	4.95381495433345	5.18524939904445	4.00986774443587	5.29540276952074	NA
**TC1000002586.mm.2**	Appl2	adaptor protein, phosphotyrosine interaction, PH domain and leucine zipper containing 2	-0.818135462135539	6.79942056059036	-0.892989053144581	0.404040254494965	0.999692817443324	-4.60146591714085	7.78569758955845	7.68543407553378	7.41266532567876	4.43189506141066	7.1983311134454	6.2825001979151	NA
**TC0100002624.mm.2**	Pax3	paired box 3	0.430749074019499	6.36613127194863	0.89297004750139	0.404049703569732	0.999692817443324	-4.60146686568608	5.39905846807267	5.83481562623607	6.401129088483	7.51107675422801	6.16861940827507	6.88208828639694	NA
**TC1000001991.mm.2**	Rwdd1	RWD domain containing 1	-0.87677526941835	7.58547642166616	-0.892916020583559	0.404076565144166	0.999692817443324	-4.60146956203151	8.47638675206638	8.61826785630108	8.24485300784239	5.08747368355401	8.1238107243124	6.96206650592071	NA
**TC0500000425.mm.2**	Cytl1	cytokine-like 1	0.353308684720132	6.53543839012105	0.89282666605325	0.40412099413294	0.999692817443324	-4.60147402128345	5.746331583547	5.52042295370831	6.80612838484353	7.5201182016121	6.4970027312337	7.12262648578167	NA
**TC1700000747.mm.2**	Trim10	tripartite motif-containing 10	-0.326581162542364	5.27819829900453	-0.89280773174174	0.404130409146596	0.999692817443324	-4.60147496617055	5.04440479471246	4.98765709731107	5.03817848330177	5.45380430515768	5.22503192021617	5.92011319332801	NA
**TC0600003330.mm.2**	Gucy2c	guanylate cyclase 2c	-0.409770447963349	5.48270686855128	-0.8927616737975	0.404153311965536	0.999692817443324	-4.60147726457221	5.31203104819148	5.22226891186069	4.90855357426801	5.86264660339639	5.36834200189157	6.22239907169953	NA
**TC0200003791.mm.2**	Nfe2l2	nuclear factor, erythroid derived 2, like 2	0.310156970861489	6.02373571341034	0.892258929035066	0.404403370287963	0.999692817443324	-4.60150234833563	5.707415812425	5.98454881075921	5.96976385111913	6.56561794838127	5.83462266806407	6.08044518971334	NA
**TC0600002121.mm.2**	Slc37a3	solute carrier family 37 (glycerol-3-phosphate transporter), member 3	0.337130760238206	7.08730558057858	0.891967911853257	0.404548170943364	0.999692817443324	-4.60151686455169	7.60202106667871	7.47445904503853	7.21243566438587	6.84837178172946	7.08910114636843	6.29744477927048	NA
**TC1000000771.mm.2**	1700009J07Rik	RIKEN cDNA 1700009J07 gene	0.440701854498214	6.1198790927832	0.891952061239548	0.404556058801667	0.999692817443324	-4.60151765511778	5.7511595705789	5.60322874426757	5.89869986463914	7.22444511078545	5.84770800459197	6.39403326183619	NA
**TC0200001765.mm.2**	Spint1	serine protease inhibitor, Kunitz type 1	0.331635581216328	6.22425062295565	0.891838547987842	0.40461255059311	0.999692817443324	-4.60152331647637	5.96381389861695	6.63284892427352	6.27959615509438	6.42645988354367	6.27012185073434	5.77266302547105	NA
**TC0200004725.mm.2**	Tasp1	taspase, threonine aspartase 1	-0.336030608086258	7.44979603184694	-0.891737256392434	0.404662965035879	0.999692817443324	-4.60152836794443	8.09790707165348	7.70708308025429	7.37873546598472	6.73212694551595	7.6114964524778	7.17142717519539	NA
**TC0400001165.mm.2**	Ttc39a	tetratricopeptide repeat domain 39A	-0.401966296955175	5.40081716478975	-0.891700828650814	0.40468109684887	0.999692817443324	-4.60153018453591	4.83170271591475	4.82748790205577	4.90537893615723	6.06551095227157	5.66057289479084	6.11424958754832	NA
**TC1100003144.mm.2**	Smtnl2	smoothelin-like 2	-0.276167627906349	7.69869107699513	-0.891308720351265	0.404876306076097	0.999692817443324	-4.6015497356437	7.45175564394523	7.29969031926909	7.63962947182118	7.8045531496507	7.82316654090348	8.17335133638111	NA
**TC1600000553.mm.2**	Cd80	CD80 antigen	-0.484495866296721	4.58462132974019	-0.891224840998108	0.404918074119607	0.999692817443324	-4.6015539173553	4.14631504141239	4.03156110204742	3.79730026269095	5.38312978850301	4.84495758788341	5.30446419590399	NA
**TC0200005113.mm.2**	Matn4	matrilin 4	0.445153650202784	5.98133722413584	0.891222034098609	0.404919471881439	0.999692817443324	-4.60155405728625	6.06215683111038	5.51297659461968	6.08394902408777	6.51764958565751	5.21197185753417	6.49931945180555	NA
**TC0300000749.mm.2**	Fam189b	family with sequence similarity 189, member B	-0.264681411226842	7.11249515688514	-0.890933577988182	0.405063134581884	0.999692817443324	-4.60156843619886	6.63484079662258	6.62703175377814	7.15538857361106	7.28647921061715	7.60403079048573	7.36719981619617	NA
**TC0500001936.mm.2**	Crot	carnitine O-octanoyltransferase	0.571646967142146	5.62630345356035	0.890887167279234	0.405086252521369	0.999692817443324	-4.6015707494234	6.74377005303159	6.83736671465625	6.28781166699513	4.37217727698413	5.13090193884598	4.38579307084899	NA
**TC1900000915.mm.2**	Ankrd13d	ankyrin repeat domain 13 family, member D	-0.345769477289606	7.42577815131578	-0.890450601443107	0.405303761285338	0.999692817443324	-4.60159250556697	8.11314771835398	7.64282074244662	7.32288008248779	6.73070066376965	7.09615022297224	7.64896947786441	NA
**TC0500001466.mm.2**	Tmem132b	transmembrane protein 132B	0.272213313241885	8.40079011945414	0.890418757996547	0.405319629945407	0.999692817443324	-4.60159409223774	8.57921459426543	8.57081768076469	8.30150703879755	8.59806049529172	8.29184156775537	8.06329933985012	NA
**TC0900001433.mm.2**	Ccdc51	coiled-coil domain containing 51	-0.297664918504336	7.03435786293436	-0.890397057897242	0.405330444099676	0.999692817443324	-4.60159517347497	6.23371115046138	6.3191515813053	6.96659296570981	7.5623843387056	7.50441518018961	7.61989196123449	NA
**TC0400003814.mm.2**	Alpl	alkaline phosphatase, liver/bone/kidney	0.257018063748888	5.59164019392379	0.890242309505256	0.405407568549273	0.999692817443324	-4.60160288358827	5.36962760665921	5.33572663532337	5.83234101968337	5.8469205048456	5.53555845706827	5.62966693996292	NA
**TC0400003691.mm.2**	Gpr3	G-protein coupled receptor 3	-0.411952010537511	6.748718787648	-0.890207016847935	0.405425159450327	0.999692817443324	-4.60160464188599	6.9078744500454	6.65694233354538	7.16131044137796	5.89048551923314	6.99799298841608	6.87770699327004	NA
**TC1700001101.mm.2**	Tmem200c	transmembrane protein 200C	-0.287981234514538	7.90887653901659	-0.890026309939863	0.405515238010119	0.999692817443324	-4.60161364416793	7.72231947122387	7.86076123309548	7.65820540468872	7.98890262568684	7.91392778830875	8.30914271109589	NA
**TC1200001747.mm.2**	Sptssa/Mir1892	serine palmitoyltransferase, small subunit A/microRNA 1892	0.493074537682237	7.42206502534085	0.889774112963814	0.405640977769796	0.999692817443324	-4.60162620612141	7.95928012920776	7.99774932469313	7.44654187775756	7.33421300899676	7.7449045917091	6.04970121968076	NA
**TC1300000673.mm.2**	B230219D22Rik	RIKEN cDNA B230219D22 gene	-0.758590128597662	7.41146258325551	-0.889604703037749	0.405725458063368	0.999692817443324	-4.60163464329838	8.40342844643884	8.73609668976862	7.76577419112776	5.14026086193737	7.77868309246786	6.64453221779259	NA
**TC1100003008.mm.2**	Cfap52	cilia and flagella associated protein 52	-0.298777937567253	7.16721355425417	-0.889494309769253	0.405780515345187	0.999692817443324	-4.60164014075389	7.19771990636661	7.18196299632518	7.1126856347054	6.90033563914397	7.01204298725949	7.59853416172438	NA
**TC0X00002158.mm.2**	Zdhhc9	zinc finger, DHHC domain containing 9	-0.452097339705328	7.98310558830436	-0.889062388932218	0.405995983999916	0.999692817443324	-4.60166164614346	8.53414894254186	8.68723240212944	8.0800368307512	6.80649192212091	8.28861413629053	7.50210929599223	NA
**TC0200004580.mm.2**	Anapc1	anaphase promoting complex subunit 1	0.525781852861908	6.19812523184171	0.889006758311164	0.406023742162645	0.999692817443324	-4.60166441556283	7.8327880268289	8.16999511744356	6.03729112978576	5.08147484646504	5.80975108849932	4.25745118202766	NA
**TC0400003453.mm.2**	Tmco2	transmembrane and coiled-coil domains 2	0.603995600842918	5.28542394282988	0.888916918547235	0.406068572737593	0.999692817443324	-4.6016688877814	4.69168344671416	4.52839355726955	4.86586655456601	6.98436237277469	5.02052637108635	5.62171135456852	NA
**TC1100003664.mm.2**	Cacnb1	calcium channel, voltage-dependent, beta 1 subunit	0.342647128905481	8.92404215061574	0.88886757073369	0.406093199156883	0.999692817443324	-4.60167134420245	9.16531158102086	9.22874528335229	9.30453213083201	8.61321301773413	8.91932221221307	8.31312867854211	NA
**TC0500002833.mm.2**	Sec31a	Sec31 homolog A (S. cerevisiae)	0.307876358231072	8.62192574921134	0.888742608377144	0.406155565055292	0.999692817443324	-4.6016775641919	8.83082904569358	8.94441587631162	8.91383020435133	8.37220094051115	8.54453124533635	8.12574718306399	NA
**TC0600003347.mm.2**	Igbp1b	immunoglobulin (CD79A) binding protein 1b	0.393891277822292	5.51767829006932	0.888676811018272	0.406188405900351	0.999692817443324	-4.60168083904734	5.04092072322218	5.22495771344268	5.32081182457567	6.49317510512215	5.39414963374595	5.63205474030729	NA
**TC0100000640.mm.2**	Ttll4	tubulin tyrosine ligase-like family, member 4	0.280492441528044	5.78369149944176	0.888629303370487	0.406212119194706	0.999692817443324	-4.60168320350368	5.54305185181972	5.96108144631073	6.04155856913041	5.83794172165768	5.68846581960141	5.63004958813059	NA
**TC1400000351.mm.2**	Fam170b	family with sequence similarity 170, member B	0.266194309691373	6.8878179464116	0.888580885580474	0.406236287843797	0.999692817443324	-4.6016856131831	6.65522519390451	6.73752920153141	7.0436562746347	7.18961467657352	6.72359636441549	6.97728596740998	NA
**TC0400001515.mm.2**	Yars	tyrosyl-tRNA synthetase	0.283171523159655	7.80277508617208	0.888471778416313	0.4062907546565	0.999692817443324	-4.60169104300341	8.53296547985418	8.15889924969017	7.81120092601178	7.53436349089194	7.47344832259961	7.30577304798479	NA
**TC1500001885.mm.2**	Sun2	Sad1 and UNC84 domain containing 2	-0.389701029020086	7.01159564496409	-0.888452006892531	0.406300625276339	0.999692817443324	-4.60169202691082	6.48100707558228	6.35684167691593	6.81263028611634	7.41353124350674	6.99381607780476	8.01174750985849	NA
**TC0600001442.mm.2**	Acrbp/Ing4	proacrosin binding protein/inhibitor of growth family, member 4	0.294731520803506	7.30480714820721	0.888028574483937	0.406512060028563	0.999692817443324	-4.60171309552281	6.37637209591581	6.95491610439495	7.80163999533872	7.74186886993103	7.54154627063059	7.41249955303215	NA
**TC1700001279.mm.2**	Six3	sine oculis-related homeobox 3	0.359151813316306	8.08335992821893	0.888027353175013	0.406512669989608	0.999692817443324	-4.6017131562828	8.09290125514482	8.09924386268173	7.96428618006839	8.54887285899144	7.47904894393066	8.31580646849656	NA
**TC0600002143.mm.2**	Tas2r138	taste receptor, type 2, member 138	-0.465070901657378	5.11743649116939	-0.887951500658401	0.406550554515857	0.999692817443324	-4.6017169298432	4.61527410637005	4.16920136658274	4.77333238596801	5.7216684494064	5.1277546856131	6.29738795307606	NA
**TC0200003263.mm.2**	Zer1	zyg-11 related, cell cycle regulator	-0.337874931113822	7.66318530368367	-0.887923509514813	0.406564535351113	0.999692817443324	-4.60171832231802	7.91984895393789	7.91702251105027	7.81382354659046	6.91942170085265	7.69285800558384	7.71613710408692	NA
**TC0900000704.mm.2**	Rpp25	ribonuclease P/MRP 25 subunit	-0.258744294695383	6.6248837459293	-0.887801452252333	0.406625503902969	0.999692817443324	-4.60172439400302	7.07755204686726	7.10815775468291	6.34684108577827	6.17621095653915	6.53249295954426	6.50804767216393	NA
**TC1100002297.mm.2**	Cep68	centrosomal protein 68	0.305032204260314	6.7964288268036	0.887753963960995	0.406649226511552	0.999692817443324	-4.60172675615752	6.67485668049327	6.46639585787949	6.74572116447731	7.37797125100743	6.81477918774175	6.69884881922236	NA
**TC0800001207.mm.2**	D230025D16Rik	RIKEN cDNA D230025D16 gene	0.636014324925411	7.39525778073784	0.887746665575901	0.406652872485372	0.999692817443324	-4.60172711918611	8.01597453151167	8.10541228927841	8.30941110128763	6.45168315545626	7.46574200615361	6.02332360073946	NA
**TC1600000797.mm.2**	Vgll3	vestigial like family member 3	0.545675887515578	5.10443710404854	0.887525329870335	0.406763454275531	0.999692817443324	-4.60173812781813	5.26359146713819	5.61103410868875	4.65011012938956	5.77156428235816	5.4432389898382	3.88708364687836	NA
**TC0200001260.mm.2**	Olfr1029	olfactory receptor 1029	0.37976435246276	5.41764667431127	0.887507797122575	0.406772214788025	0.999692817443324	-4.60173899978149	4.53961878586317	4.77678994140939	5.50127190590519	6.47322810585509	5.33722810230336	5.8777432045314	NA
**TC1100002914.mm.2**	Mapk7	mitogen-activated protein kinase 7	-0.380466059500963	7.47650141339594	-0.88742901643681	0.406811580525573	0.999692817443324	-4.60174291769145	7.32974879751158	7.58101834929708	7.80270365121317	6.79095095606934	7.90937949311372	7.44520723317072	NA
**TC0300002755.mm.2**	Fndc7	fibronectin type III domain containing 7	-0.34532987504643	6.78979805238199	-0.887406731341564	0.406822716627045	0.999692817443324	-4.60174402593436	5.95360062244978	5.78274395573662	6.83694295697619	7.31894903603014	7.02820121165126	7.81835053144794	NA
**TC1700002686.mm.2**	Mcfd2	multiple coagulation factor deficiency 2	-0.272191741182114	7.17538821540226	-0.887384522079401	0.406833815059468	0.999692817443324	-4.60174513039012	7.22978832236214	7.44613099708121	6.893028719742	7.02298452556101	7.08477153846441	7.37562518920283	NA
**TC0800002683.mm.2**	Rpgrip1l	Rpgrip1-like	0.402280032333056	5.88750863977818	0.887278172479024	0.406886963297953	0.999692817443324	-4.60175041888337	6.29509853575203	6.13025236625909	5.79767375777598	6.05445674288606	4.91368761008733	6.13388282590861	NA
**TC1200002358.mm.2**	Slc25a29	solute carrier family 25 (mitochondrial carrier, palmitoylcarnitine transporter), member 29	-0.314195668333403	7.47536891635023	-0.887267219612684	0.406892437288813	0.999692817443324	-4.60175096352064	6.89017637288555	7.05190890503044	7.26932670744391	7.8715417343154	7.68326948744425	8.08599029098185	NA
**TC1700001813.mm.2**	Tff1	trefoil factor 1	0.511037029773921	5.78109048409861	0.887248805640342	0.406901640292401	0.999692817443324	-4.60175187915677	4.88568977039702	5.49634660965541	5.79708680791416	6.86620348412938	5.17078288058692	6.47043335190878	NA
**TC0400001128.mm.2**	Yipf1	Yip1 domain family, member 1	-0.290265644060962	7.16082431416316	-0.887085671793424	0.406983178693323	0.999692817443324	-4.6017599905215	6.94332653417949	7.03092816117733	6.82617708036917	7.37890287038094	7.37003853664534	7.41557270222668	NA
**TC0100003042.mm.2**	Pfkfb2/C4bp-ps1	6-phosphofructo-2-kinase/fructose-2,6-biphosphatase 2/complement component 4 binding protein, pseudogene 1	-0.359590583875486	7.23528966284643	-0.887059322773739	0.406996349735877	0.999692817443324	-4.60176130057072	7.78245544829849	7.84327251955135	7.27731698859366	6.25609743214522	7.09057144484877	7.16202414364108	NA
**TC0400003870.mm.2**	Crocc	ciliary rootlet coiled-coil, rootletin	-0.286663229670795	6.67784235023641	-0.887029523841229	0.407011245662772	0.999692817443324	-4.60176278211946	6.23821557184176	6.17994070373797	6.51579760895283	7.02198807429574	6.9816807047035	7.12943143788665	NA
**TC0500002091.mm.2**	Wdr86	WD repeat domain 86	-0.343194421977471	6.30367580426002	-0.886806750720722	0.407122618606355	0.999692817443324	-4.60177385708584	5.56102666742834	5.6415719062557	6.12282945386286	6.84370425009771	6.5356433290095	7.11727921890602	NA
**TC0X00000376.mm.2**	6030498E09Rik	RIKEN cDNA 6030498E09 gene	-0.533021445789158	4.67168925232863	-0.88674179330677	0.407155097609091	0.999692817443324	-4.60177708608385	3.81339417818181	3.98527361090465	4.05272986344988	5.5299825532036	4.73839558944288	5.91035971878892	NA
**TC0100000854.mm.2**	Sh3bp4	SH3-domain binding protein 4	-0.472776238509887	7.52311480868932	-0.886739696145615	0.407156146231315	0.999692817443324	-4.60177719033033	7.40357674655768	7.08375602077249	6.85294436100699	7.99995744288602	7.35694262676199	8.44151165415079	NA
**TC0400000777.mm.2**	Kdm4c	lysine (K)-specific demethylase 4C	-0.300714328773408	6.98373849915486	-0.886592021875985	0.407229991347257	0.999692817443324	-4.60178453062162	7.44978122917679	7.42719341873901	6.833116482096	6.37889736263727	7.13639358663372	6.67704891564637	NA

**TC0900000194.mm.2**	Ilf3	interleukin enhancer binding factor 3	-0.464140837522573	6.76012787990174	-0.886319040855794	0.407366522821995	0.999692817443324	-4.60179809755043	7.76865934538919	7.32506040940331	6.87762795585632	5.39175496893009	6.47572170093657	6.72194289889498	NA
**TC0700004633.mm.2**	Gm15448	predicted gene 15448	-0.275154008749084	6.58878911017781	-0.885925028890576	0.407563647919478	0.999692817443324	-4.60181767536908	7.09142616457186	7.27076276804196	6.70662480691267	6.60349404856476	6.74578828702523	7.11463858595037	NA
**TC0900002879.mm.2**	Rnf7	ring finger protein 7	-0.345370021895987	8.31211497528036	-0.885922755569403	0.407564785472956	0.999692817443324	-4.60181778831218	8.71200706084429	8.66742930010503	8.18962053687173	7.71163618659869	8.7029339816041	7.88906278565829	NA
**TC1100001111.mm.2**	Slc6a4	solute carrier family 6 (neurotransmitter transporter, serotonin), member 4	0.392349876006765	5.84977180744484	0.885687808011927	0.407682364254255	0.999692817443324	-4.60182946007033	5.44351961776419	5.07306480892378	5.77828420092745	6.90508888406985	5.83187240629887	6.0668009266849	NA
**TC1200002296.mm.2**	Clmn	calmin	0.321081997589128	6.6886147342407	0.885603796647594	0.407724413599338	0.999692817443324	-4.60183363316533	7.72157239872265	7.72911009731328	6.49799213507942	6.16359281721386	6.36919928114395	5.65022167597107	NA
**TC0600003122.mm.2**	P3h3	prolyl 3-hydroxylase 3	-0.315461451806538	5.89730164687826	-0.8855587862039	0.407746943539911	0.999692817443324	-4.60183586887432	5.54182106741984	6.04524037993473	5.88837066949158	5.69454209565937	5.84267174908204	6.37116391968199	NA
**TC1000000848.mm.2**	Dazap1	DAZ associated protein 1	0.260311492861548	7.32444611481828	0.88553272977869	0.407759986484813	0.999692817443324	-4.60183716309029	7.24994730610164	7.39063325058074	7.4470475922089	7.4663119667663	7.11058958446885	7.28214698878325	NA
**TC1600001117.mm.2**	Crebbp	CREB binding protein	0.344860582157457	7.8405963712072	0.885423776927409	0.407814527870087	0.999692817443324	-4.60184257451247	9.0269352185364	9.18928193459404	7.61414445102672	7.1443966681871	7.43511502971627	6.63370492518264	NA
**TC1100001705.mm.2**	Acbd4	acyl-Coenzyme A binding domain containing 4	0.273770937592671	6.30058226808867	0.885394494863761	0.40782918728438	0.999692817443324	-4.60184402881557	6.37811886788062	6.66127950109166	6.19387053179692	6.46194802557562	5.99122159638801	6.11705508579919	NA
**TC1100000192.mm.2**	Rab1a	RAB1A, member RAS oncogene family	-0.792546207273118	8.5923881378062	-0.885232079629016	0.407910503968548	0.999692817443324	-4.60185209471634	10.036489068464	10.07203836491	8.91970898650615	6.01064550295232	8.70028495059362	7.81516195341109	NA
**TC1700001918.mm.2**	Rnf5	ring finger protein 5	0.291327077480339	9.65820117057505	0.884540241129121	0.408257022717688	0.999692817443324	-4.60188644337666	10.2698864909002	9.88415767526606	9.80682047378758	9.3820880323348	9.33535013422786	9.27090421693384	NA
**TC0200002833.mm.2**	Tcea2	transcription elongation factor A (SII), 2	-0.415897575193767	6.31078338107001	-0.884407692779659	0.408323436693082	0.999692817443324	-4.60189302241613	6.52111472142276	6.45357394561918	6.56331065840863	5.46579757608664	6.80902876800278	6.05187461688003	NA
**TC0700000811.mm.2**	Kcnc3	potassium voltage gated channel, Shaw-related subfamily, member 3	0.27626668643841	7.72610455860021	0.884198337445169	0.408428351510096	0.999692817443324	-4.60190341261047	7.33505529091329	7.28520122481998	7.87514407172723	8.26930803264519	7.70116855326145	7.89075017823415	NA
**TC1900000140.mm.2**	Atl3	atlastin GTPase 3	-0.682672059498401	5.64842103840996	-0.884082508458188	0.408486405808391	0.999692817443324	-4.60190916053058	6.09060549838818	6.24976709067575	6.07345347699073	4.01895128420878	6.49709993673565	4.96064894346066	NA
**TC0300001554.mm.2**	Cryz	crystallin, zeta	0.372598793248114	6.35855425468791	0.884026240873742	0.4085146097257	0.999692817443324	-4.60191195260694	7.05501046248098	6.85192534173501	6.04248127440834	6.4423123807955	5.36647507669804	6.37312099200957	NA
**TC1000000473.mm.2**	Slc35f1	solute carrier family 35, member F1	0.438535396399377	7.27017051001942	0.883487499154854	0.408784725182859	0.999692817443324	-4.60193868805208	8.56535655556878	8.57898491898662	7.33298160845785	6.34389458072208	6.93106673833885	5.86873865804232	NA
**TC1100001271.mm.2**	Hnf1b	HNF1 homeobox B	-0.283349653459998	5.4546529386003	-0.883370504368725	0.408843401787974	0.999692817443324	-4.60194448358941	5.28769149901804	5.19139673233813	5.23434342134803	5.60672162531479	5.53540570391829	5.87235864966453	NA
**TC0600003474.mm.2**	Sinhcaf	SIN3-HDAC complex associated factor	-0.331210779486073	6.33885177125817	-0.883192179358769	0.40893284947219	0.999692817443324	-4.60195332784785	6.01157040137594	6.12929263905855	5.88159101287062	6.73332200120056	6.68250500570777	6.59482956733556	NA
**TC1800000006.mm.2**	Cul2	cullin 2	0.737164137363962	6.56264170133339	0.882947945869156	0.40905538035695	0.999692817443324	-4.60196543924419	7.88395157846131	7.73474670252432	7.20633743658761	5.4094026642837	6.64911736274877	4.49229446339462	NA
**TC1500001019.mm.2**	Aqp6	aquaporin 6	-0.6019261343281	4.96864009424853	-0.88280968576738	0.409124756923985	0.999692817443324	-4.60197229462186	4.55727621180999	4.40756457618195	3.99308973144232	5.82848402297919	4.89499910744604	6.13042691563167	NA
**TC0100000948.mm.2**	Gal3st2	galactose-3-O-sulfotransferase 2	-0.263321057442001	5.98513271879094	-0.882783595281533	0.409137849664809	0.999692817443324	-4.60197358820177	5.6681228770043	5.72669291242623	5.99396356931153	6.00070563490402	6.12006696999107	6.40124434910848	NA
**TC0300001960.mm.2**	Maml3	mastermind like transcriptional coactivator 3	0.37610654053091	6.55625211499091	0.882595238761325	0.409232380050089	0.999692817443324	-4.60198292635899	6.2231310365113	6.0260374300063	6.51350817525799	7.40677047698686	6.25351018879529	6.91455538238774	NA
**TC1800000103.mm.2**	Lama3	laminin, alpha 3	0.285713454348754	5.38659128783842	0.88253015573026	0.40926504700125	0.999692817443324	-4.60198615271404	5.32421267852618	5.23958190247573	5.33501012386069	5.82857990350235	5.39020365675769	5.20195946190785	NA
**TC1900000382.mm.2**	Ptar1	protein prenyltransferase alpha subunit repeat containing 1	0.353066614376638	6.38116706864192	0.882427969346905	0.409316341040445	0.999692817443324	-4.60199121811185	7.14697850869522	7.27378390118937	5.86772007691409	6.41846653844601	6.20165224927921	5.37840113732762	NA
**TC1000001768.mm.2**	Phactr2	phosphatase and actin regulator 2	0.506932655592252	6.17074348316765	0.882427273164561	0.409316690516315	0.999692817443324	-4.60199125262057	7.03254851627828	7.10126071012	6.62486658206761	5.32739190982846	6.08046293268236	4.85793024802921	NA
**TC1900000105.mm.2**	Majin	membrane anchored junction protein	-0.278902673260205	4.85764315302256	-0.882427052864763	0.409316801104434	0.999692817443324	-4.6019912635405	4.60013646047503	4.63393578976897	5.0735201288725	4.60347053181298	5.10223418876212	5.13256181844376	NA
**TC0700003570.mm.2**	Anpep	alanyl (membrane) aminopeptidase	-0.318079620453213	5.46919868682499	-0.882353489674187	0.409353730270497	0.999692817443324	-4.60199490986867	5.40059362155779	5.48397507043504	5.61150078279458	5.03573131123077	5.90204271987041	5.38134861506137	NA
**TC1200000261.mm.2**	6030469F06Rik	RIKEN cDNA 6030469F06 gene	0.343666456638549	6.65192474856354	0.882329631032867	0.409365707983183	0.999692817443324	-4.60199609243907	6.17118574845662	6.35559641538041	6.56107549102334	7.47497412938732	6.60332225602414	6.74539445110942	NA
**TC0700003771.mm.2**	Capn5	calpain 5	0.327818052149069	6.58117056451515	0.882308150374859	0.409376492103324	0.999692817443324	-4.60199715712719	6.88265204596646	6.7044812348549	6.77780503372799	6.49995807155584	5.92224042817388	6.69988657281181	NA
**TC0500001674.mm.2**	C130050O18Rik	RIKEN cDNA C130050O18 gene	0.313675601426651	5.56781402072434	0.882298650049852	0.409381261700677	0.999692817443324	-4.60199762800057	5.67024464095483	5.79992195619047	5.89095136659911	5.39108299842789	5.05478856622007	5.59989459595364	NA
**TC1100000717.mm.2**	Mrpl55	mitochondrial ribosomal protein L55	0.292161108813509	7.09875332919839	0.882069149135426	0.409496494170382	0.999692817443324	-4.60200900220179	7.30524570029513	7.13941458854939	6.91704958839582	7.4490413635906	6.79252955237988	6.98923918197952	NA
**TC0700000781.mm.2**	Klk15	kallikrein related-peptidase 15	0.264649417090588	5.9161155638812	0.88184789408557	0.409607609156691	0.999692817443324	-4.6020199661031	5.38122169209301	5.50247038305059	6.37755051995689	6.19359955120551	5.91135113452842	6.1305001024528	NA
**TC1700002799.mm.2**	Eme2	essential meiotic structure-specific endonuclease subunit 2	-0.357316720548043	6.7399414907358	-0.881615651694437	0.409724266064009	0.999692817443324	-4.60203147274575	7.11231744033233	6.60579052292611	6.07855095392357	6.92490281610657	6.9987026064954	6.71938460463082	NA
**TC0500001610.mm.2**	Ift22	intraflagellar transport 22	0.43029688624476	7.62362923863228	0.881548811817815	0.409757844744959	0.999692817443324	-4.60203478405711	8.43356435453374	8.23270021382836	7.9599100357665	7.00814228219407	7.5676121120828	6.53984643338825	NA
**TC0100000523.mm.2**	Zdbf2	zinc finger, DBF-type containing 2	0.449952513758171	5.82965201632759	0.881349821711314	0.409857824469434	0.999692817443324	-4.60204464135427	7.266986556086	6.82979171664608	4.86251227193709	6.02800715443781	5.42567038932567	4.56494400953289	NA
**TC0700001660.mm.2**	Arntl	aryl hydrocarbon receptor nuclear translocator-like	0.514288490647132	6.25144467436217	0.881319904911926	0.409872857299483	0.999692817443324	-4.60204612321965	6.97207975801057	7.16077247577303	6.54369153194859	5.65850486489325	6.37716518144238	4.79645423410519	NA
**TC0500000466.mm.2**	Bst1	bone marrow stromal cell antigen 1	-0.35280070513657	6.58854130139137	-0.881282365235428	0.409891721111227	0.999692817443324	-4.6020479826268	6.05483229861975	6.20967670174724	6.16274418891095	7.11782450994308	7.11881953232691	6.86735057680026	NA
**TC1100002738.mm.2**	Il13	interleukin 13	0.41820118082105	6.56210154912211	0.881238088402654	0.409913971193302	0.999692817443324	-4.6020501756782	6.0209893634908	6.00254145993889	6.46820006619004	7.6245403502825	6.27559480648975	6.9807432483407	NA
**TC1900000896.mm.2**	Gstp1	glutathione S-transferase, pi 1	0.497486936940136	8.87050512048675	0.881215885608599	0.409925128922496	0.999692817443324	-4.60205127536846	10.0676264863037	8.95730624169837	9.21009503948221	8.38644089491716	8.83136406875194	7.77019799176716	NA
**TC0400001668.mm.2**	Fuca1	fucosidase, alpha-L- 1, tissue	-0.266071997793421	7.39068855870395	-0.881213545707456	0.409926304822894	0.999692817443324	-4.60205139126134	7.62780067175446	7.82030737750288	7.1188155487121	7.06312410497765	7.48987441614659	7.22420923313	NA
**TC1300001201.mm.2**	Smim15	small integral membrane protein 15	-0.708766412465857	6.08176069165073	-0.881103039835545	0.409981841596495	0.999692817443324	-4.60205686429907	7.41070989369456	7.68286270233887	6.16300080615827	3.82672855831135	6.36094060816148	5.04632158123986	NA
**TC0700000388.mm.2**	Ethe1	ethylmalonic encephalopathy 1	0.270609472059347	6.98699916440599	0.88103330991396	0.410016888529787	0.999692817443324	-4.6020603176162	7.03717399704351	7.17887471640042	7.24288206878284	6.8807005397725	6.80002355630994	6.78234010812671	NA
**TSUnmapped00000019.mm.2**	Ccdc169	coiled-coil domain containing 169	-0.520205232680326	6.56216149078555	-0.880960998514103	0.410053235283852	0.999692817443324	-4.60206389861123	5.25126083874939	5.74251640683721	6.04070337068642	7.6286872461966	6.94876440313045	7.76103667911322	NA
**TC0400003341.mm.2**	Tmem69	transmembrane protein 69	0.268387143141397	5.58658877784887	0.88078713183104	0.410140637770323	0.999692817443324	-4.60207250811381	5.97123207358396	5.63319285979911	5.56648524867065	5.6594557613258	5.39324897523085	5.29591774848282	NA
**TC1000001807.mm.2**	Hebp2	heme binding protein 2	0.471202843133276	6.74114363126039	0.880773807638056	0.410147336388916	0.999692817443324	-4.60207316785832	6.00162379061659	5.7989227044802	6.86366703103004	7.93069345833602	6.32604980456162	7.52590499853788	NA
**TC1700001147.mm.2**	Lbh	limb-bud and heart	0.263661490266438	6.99422013416077	0.880715870054737	0.410176464932169	0.999692817443324	-4.60207603655772	7.31897467795879	7.36446556382897	7.07544269551747	6.82915907633741	6.77255281870493	6.60472597261707	NA
**TC1700002155.mm.2**	Tmem63b	transmembrane protein 63b	-0.265860427213306	7.17389651329737	-0.88043921407683	0.410315576878396	0.999692817443324	-4.60208973328375	7.118564033881	6.99482280617094	6.9887308347272	7.21040485792563	7.25762846039273	7.47322808668672	NA
**TC1700001062.mm.2**	Man2a1	mannosidase 2, alpha 1	0.454707133540347	7.20866981245423	0.880145267720476	0.410463421300099	0.999692817443324	-4.60210428328281	7.2698531688012	7.39979119957667	7.47509552985685	7.27079885685725	7.67855935395337	6.15792076568004	NA
**TC0300002869.mm.2**	Usp53	ubiquitin specific peptidase 53	0.542633846701359	7.46271261098386	0.880080410672898	0.410496047369799	0.999692817443324	-4.60210749324988	8.01129552920742	7.88370816293141	7.84162777359757	7.14164205998594	7.86023175421955	6.03777038596125	NA
**TC0900000546.mm.2**	Pcsk7	proprotein convertase subtilisin/kexin type 7	0.322246353764058	6.33207251348585	0.879939176229289	0.410567101417866	0.999692817443324	-4.60211448288398	6.09201397828579	6.32950449415889	6.52171558186442	6.58598907613485	5.84348402744526	6.61972792302589	NA
**TC0X00001580.mm.2**	Acot9	acyl-CoA thioesterase 9	0.594959945833622	5.56485751481862	0.879869743717871	0.410602035761785	0.999692817443324	-4.6021179188309	6.53517619499074	6.46986681944223	6.38016974175861	4.40684124131438	5.27828108426582	4.31881000713993	NA
**TC0500000949.mm.2**	Cfap299	cilia and flagella associated protein 299	-0.28565807721119	5.27241504519644	-0.879806386569926	0.410633915261872	0.999692817443324	-4.60212105399386	5.41148778552238	5.33471093464312	4.81885196854519	5.33963572975019	5.42996464886913	5.29983920384863	NA
**TC0400003666.mm.2**	Rab42	RAB42, member RAS oncogene family	0.406996603059794	5.63075372974142	0.879665205132199	0.410704960305452	0.999692817443324	-4.60212803973872	5.26868997544525	5.60910285278338	5.71848865616033	6.14187272200943	4.93236819039986	6.11399998165031	NA
**TC0300000916.mm.2**	Anp32e	acidic (leucine-rich) nuclear phosphoprotein 32 family, member E	0.406037988338823	5.90522493506836	0.879501093832035	0.410787555507053	0.999692817443324	-4.60213615924715	6.52196156497231	6.59989828258547	6.36245853919242	5.19832433057258	5.65638835299516	5.0923185400922	NA
**TC1100004262.mm.2**	Wdr92	WD repeat domain 92	0.347183953837618	5.5797613032526	0.879454311261276	0.410811102852687	0.999692817443324	-4.60213847368233	6.10991971462106	5.53794786251251	5.76508999942109	5.49744407560754	5.6527987405587	4.91536742679469	NA
**TC0400003171.mm.2**	Wdr78	WD repeat domain 78	0.342362954374416	5.73482335417354	0.879368733621485	0.410854179733804	0.999692817443324	-4.60214270720815	6.40055843595032	6.71514656581879	5.84872030047365	5.14026021553684	5.41990044429061	4.88435416297105	NA
**TC0700001409.mm.2**	Ucp2	uncoupling protein 2 (mitochondrial, proton carrier)	-0.703249660005661	8.49884318363724	-0.879115586953372	0.41098162475562	0.999692817443324	-4.60215522896909	9.34227523273865	9.93906409182128	8.74621364344145	6.40639658518465	8.85782697299831	7.70128257563911	NA
**TC1200001908.mm.2**	Dhrs7	dehydrogenase/reductase (SDR family) member 7	-0.469713490712445	5.40192829293869	-0.879013140359884	0.41103320913468	0.999692817443324	-4.60216029583665	6.43099521555189	6.19366058629044	5.47706650766842	3.94667697951404	5.41196572800307	4.95120474060428	NA
**TC0200004894.mm.2**	Rbck1	RanBP-type and C3HC4-type zinc finger containing 1	0.298194989897519	7.57818333691694	0.878821998198474	0.411129466724681	0.999692817443324	-4.6021697485451	7.94133973823554	7.71929040001677	7.74242924323583	7.46000068828634	7.57223353223554	7.03380641949159	NA
**TC0200001938.mm.2**	Tmem87b	transmembrane protein 87B	-0.637532795276897	5.96337137203024	-0.878798968413224	0.411141065457497	0.999692817443324	-4.6021708873749	7.14923878020506	6.7086100065554	6.23366193267449	4.08999499475911	6.27831350927001	5.32040900871739	NA
**TC0700001372.mm.2**	Thap12	THAP domain containing 12	0.326440238047764	7.66527145242642	0.878739540987537	0.41117099663526	0.999692817443324	-4.60217382599859	7.5131558734064	7.71739440296044	7.45565447612382	8.25132498101977	7.62491141124894	7.42918756979912	NA
**TC0900000609.mm.2**	Ppp2r1b	protein phosphatase 2, regulatory subunit A, beta	0.364704533758349	6.45133731800226	0.878729608537119	0.41117599936392	0.999692817443324	-4.60217431713652	6.93589568950711	7.19657524740386	6.49624023475469	6.15624078455495	6.45400261038205	5.46906934141089	NA
**TC0200002290.mm.2**	Defb21	defensin beta 21	0.459506126420525	5.19558681246981	0.87860585360183	0.411238335429154	0.999692817443324	-4.60218043627578	4.43377324512911	4.96617724490411	4.95919273048964	6.38709858832371	5.39648500770106	5.03079405827124	NA
**TC0700004641.mm.2**	Sptbn4	spectrin beta, non-erythrocytic 4	-0.342487094852998	7.18466225717673	-0.878599710836175	0.411241429757341	0.999692817443324	-4.60218073999553	7.49344493149543	7.54593173711046	7.30677631632055	6.38503502605371	7.08819294734358	7.28859258473667	NA
**TC1700001740.mm.2**	AI413582	expressed sequence AI413582	-0.333174209020926	7.70842618210186	-0.878550966337891	0.411265984694887	0.999692817443324	-4.60218315004963	7.57746996192865	8.43429149935506	7.41748812765544	7.36873547898735	7.83960973085227	7.61296229383238	NA
**TC0100002335.mm.2**	Dnah7a	dynein, axonemal, heavy chain 7A	0.35982762226506	6.37792442949324	0.878491112273533	0.411296137536717	0.999692817443324	-4.60218610928277	5.86468974707152	6.17723780663791	6.41788519889128	7.05475193499878	5.99156301106008	6.76141887829986	NA
**TC0300001152.mm.2**	Clcc1	chloride channel CLIC-like 1	-0.32264881884231	6.27843421473834	-0.878269523520377	0.411407782132363	0.999692817443324	-4.60219706378583	7.2309985206536	7.01276434272177	5.30895245054244	6.08181994314258	5.93284524504597	6.10322478632367	NA
**TC1700001299.mm.2**	Epas1	endothelial PAS domain protein 1	0.257416931052829	7.5406191812596	0.878263063027782	0.411411037503431	0.999692817443324	-4.60219738314377	7.60023676733823	7.68031659235996	7.69070197369748	7.54829582128506	7.4260947162584	7.29806921661848	NA
**TC0500000701.mm.2**	Fip1l1	FIP1 like 1 (S. cerevisiae)	-0.429026174511335	6.47229207175807	-0.87802460001511	0.411531209723158	0.999692817443324	-4.6022091699945	7.40178963457324	7.43177397941864	6.32529544908752	5.24577278467941	6.67354656360848	5.75557401918112	NA
**TC1900001544.mm.2**	Slc25a28	solute carrier family 25, member 28	-0.345220016536645	7.23610714377054	-0.877995891318513	0.411545679075566	0.999692817443324	-4.60221058889416	7.83030317642471	7.68838968020214	7.26330951440452	6.34044547205703	7.2640914462426	7.03010357329224	NA
**TC0900002971.mm.2**	Bfsp2	beaded filament structural protein 2, phakinin	-0.342241140272026	5.4708284287468	-0.877876236416457	0.411605989904805	0.999692817443324	-4.60221650243082	4.68094786703827	5.18638132343551	5.19587235946346	5.94070719126802	5.76674990998852	6.05431192128702	NA
**TC1600000379.mm.2**	Ccdc50	coiled-coil domain containing 50	0.303795632306138	7.91108028096074	0.877761490315543	0.41166383264355	0.999692817443324	-4.60222217292484	8.15770387150588	7.86411172545134	8.21201802040977	7.81411065629997	7.95129674592076	7.46724066617671	NA
**TC1500001870.mm.2**	Tmem184b	transmembrane protein 184b	-0.302365846271508	7.44158522633529	-0.877737654377426	0.411675848933983	0.999692817443324	-4.60222335078898	7.97237140709627	7.97664904242778	7.3516575106052	6.69622209736714	7.41963087944897	7.2329804210664	NA
**TC0800001176.mm.2**	Cdh5	cadherin 5	-0.683309054458912	5.8594226892157	-0.87772746719399	0.411680984626162	0.999692817443324	-4.60222385418777	6.0960978350059	6.3416481054421	6.23550322971092	4.44058281325327	6.95018687845129	5.09251727343073	NA
**TC0700004631.mm.2**	Pirb/Pira1	paired Ig-like receptor B/paired-Ig-like receptor A1	-0.416064165559462	5.47058259299075	-0.877719170211666	0.411685167441185	0.999692817443324	-4.60222426417991	4.94886807826019	5.47223522296135	4.83097318229519	5.95415878050682	5.53810002977903	6.07916026414191	NA
**TC0900001770.mm.2**	Kdm4d	lysine (K)-specific demethylase 4D	-0.296607157176079	5.07051897870001	-0.877633573897028	0.411728321537503	0.999692817443324	-4.60222849375629	5.0896656946898	5.02909671674793	4.65933979281964	5.19622878038545	5.05461224122663	5.39417064633061	NA
**TC1600000494.mm.2**	Hspbap1	Hspb associated protein 1	-0.386597447067111	5.22387229401465	-0.877627134968744	0.411731567912223	0.999692817443324	-4.60222881191375	5.00243347503737	5.16895915299193	4.58711597553001	5.61220714543217	5.18821210271962	5.78430591237679	NA
**TC1100000343.mm.2**	Stk10	serine/threonine kinase 10	0.279208628126775	6.11421954130338	0.877610804333988	0.411739801566039	0.999692817443324	-4.60222961882968	6.36487387246777	6.457284236407	5.88204593332286	6.32874226427666	5.91552100612458	5.7368499352214	NA
**TC0200004415.mm.2**	Bmf	BCL2 modifying factor	0.387652130070101	6.03872226014761	0.87755844840272	0.411766199438948	0.999692817443324	-4.6022322057389	6.05175305215894	5.01538539093684	6.26786401487311	6.70238567409195	5.88239607286633	6.31254935595852	NA
**TC1300001089.mm.2**	Col4a3bp	collagen, type IV, alpha 3 (Goodpasture antigen) binding protein	-0.4909421967692	7.65564143866857	-0.877418401342888	0.411836817351266	0.999692817443324	-4.60223912502832	8.07625435558972	8.32737805486183	7.97298816420688	6.30117774980387	7.79139916547255	7.4646511420766	NA
**TC0800000734.mm.2**	Tm6sf2	transmembrane 6 superfamily member 2	0.359097768488195	5.49594431748179	0.877287122380565	0.411903022133561	0.999692817443324	-4.6022456105277	5.74425131100328	5.07499963176117	5.3309741751023	6.10633107444905	5.25461957346661	5.46449013910835	NA
**TC0900001155.mm.2**	Ctsh	cathepsin H	0.314694902226388	6.77800774017286	0.877281335873734	0.411905940485361	0.999692817443324	-4.60224589638214	6.47945145684216	7.06757739354533	6.69205706492222	7.18314663262901	6.58380721151373	6.66200668158472	NA
**TC0200005331.mm.2**	Gm14399	predicted gene 14399	-0.653532446476918	8.96625354248095	-0.87723398847558	0.411929820123312	0.999692817443324	-4.60224823531043	9.83940951739644	10.1896185436179	9.0889420748912	7.14177207556753	9.50413330368924	8.03364573972333	NA
**TC0300000880.mm.2**	Selenbp1	selenium binding protein 1	0.401456377213412	5.54739260662851	0.877197748010522	0.411948098676375	0.999692817443324	-4.60225002551419	4.97904834012437	5.00221023601584	5.38901266548361	6.66399224354522	5.6635346293909	5.58655752521111	NA
**TC1500002155.mm.2**	Endou	endonuclease, polyU-specific	0.306739856643925	4.96331948981111	0.877182142044807	0.411955970020366	0.999692817443324	-4.6022507964032	4.81721249574525	4.46945744940866	5.27109540933762	5.28226794416269	4.65774777603975	5.28213586417271	NA
**TC0700002056.mm.2**	Tspan4	tetraspanin 4	0.331488874326928	6.3833144021212	0.877068784727078	0.41201314857268	0.999692817443324	-4.60225639568228	6.62506671849049	6.39725757749738	6.71386110700785	6.25640882568875	6.51220488607141	5.79508729797132	NA
**TC0100002457.mm.2**	Mdh1b	malate dehydrogenase 1B, NAD (soluble)	-0.312665756193713	5.26562917948164	-0.877011844838744	0.412041871829342	0.999692817443324	-4.602259208057	4.75239975064153	4.95009701293472	4.96709460919256	5.6658787912705	5.73583338220116	5.52247153064933	NA
**TC0500001251.mm.2**	Pop5	processing of precursor 5, ribonuclease P/MRP family (S. cerevisiae)	0.424092891133196	7.05919779040626	0.876788628024875	0.41215448755369	0.999692817443324	-4.60227023219463	7.81094875058029	7.40860935261754	7.64962242309516	6.3422847876579	6.43385248059436	6.70986894789231	NA
**TC1600002045.mm.2**	Scaf4	SR-related CTD-associated factor 4	0.42079539481731	7.7573004563423	0.876729727177731	0.412184207574631	0.999692817443324	-4.60227314088896	8.40512692776791	8.40281509067372	8.32989535616369	6.9588303984597	7.29629486272181	7.15084010226696	NA
**TC0400002587.mm.2**	Igfbpl1	insulin-like growth factor binding protein-like 1	-0.317571762125466	7.17180458948891	-0.876660774592476	0.412219001485507	0.999692817443324	-4.60227654582208	7.09827066054274	6.94684358526299	6.78225648950223	7.39302839393617	7.64499927791499	7.16542912977435	NA
**TC0700004068.mm.2**	Ric3	RIC3 acetylcholine receptor chaperone	0.514624822946347	6.62479723152804	0.876543491659193	0.412278188182496	0.999692817443324	-4.60228233698629	7.7356247801571	7.30132161939759	6.66861893314312	6.20192438461002	6.78161103767417	5.05968263418627	NA
**TC1200001988.mm.2**	Zfp36l1	zinc finger protein 36, C3H type-like 1	0.266495127484649	8.54919307999671	0.876390904936788	0.412355200297124	0.999692817443324	-4.60228987069304	8.05620144989784	8.38411806052552	8.85455490357855	8.83935970868454	8.65062559830461	8.51029875898918	NA
**TC0100001438.mm.2**	Astn1	astrotactin 1	0.546112406785794	7.21865480619964	0.876314038295719	0.412393999721436	0.999692817443324	-4.60229366556057	8.54390880831121	8.345470247036	7.28646867573159	6.47091862197953	7.23100676039172	5.43415572374781	NA
**TC0500001288.mm.2**	Taok3	TAO kinase 3	-0.301247877361263	7.0263683704838	-0.876292677635038	0.412404782268286	0.999692817443324	-4.60229472009111	7.03289604962041	7.39309922465423	6.94342048175779	6.62143911519502	6.84662866043774	7.32072669123759	NA
**TC0500003092.mm.2**	Cabp1	calcium binding protein 1	-0.338975468886191	7.64297873958858	-0.876275832055863	0.41241328581544	0.999692817443324	-4.60229555171104	7.26800716225961	7.5433845489533	7.19161736234919	7.9850975319239	8.13683813754156	7.72537769450391	NA
**TC1800001445.mm.2**	2700046A07Rik	RIKEN cDNA 2700046A07 gene	-0.3375946083847	6.05860930019118	-0.87627540156459	0.412413503126546	0.999692817443324	-4.60229557296309	5.26928537341758	5.50685579477473	6.12737524903266	6.32278745906002	6.12892876881121	6.99642315605087	NA

**TC0700002764.mm.2**	Proser3	proline and serine rich 3	0.279190439908885	5.48612656905471	0.875650882007621	0.412728848415297	0.999692817443324	-4.60232639716512	4.99609277100815	5.39618821638706	5.63288549857757	5.90854415479786	5.4425340664549	5.54051470710275	NA
**TC0200000417.mm.2**	Abca2	ATP-binding cassette, sub-family A (ABC1), member 2	0.284442619642302	7.4086670364648	0.875617837818962	0.412745538728959	0.999692817443324	-4.6023280277577	8.10500281751593	7.89636841294737	7.13624478176078	7.37351333204429	6.74930474601432	7.19156812850614	NA
**TC0500003451.mm.2**	Irs3	insulin receptor substrate 3	0.395393491180835	6.54994405946756	0.875547459729167	0.412781087712079	0.999692817443324	-4.60233150050217	6.0542506624638	5.85427818888713	6.77152281038526	7.31943843352279	6.09760566999844	7.20256859154795	NA
**TC1900000444.mm.2**	Mlana	melan-A	0.331819154768755	5.42483677078802	0.875388974666728	0.412861149072386	0.999692817443324	-4.60233932020856	4.9523155060999	5.02756403838477	5.64667509390287	5.96971460098763	5.88751283420976	5.06523855114322	NA
**TC0100001595.mm.2**	Cd244a/LOC677008	CD244 molecule A/natural killer cell receptor 2B4-like	-0.393377700689543	5.04523856683744	-0.874950920969679	0.413082498829269	0.999692817443324	-4.60236092961157	4.66617283495647	4.99264521789352	4.61968216457351	5.29324680882426	4.85543971457654	5.84424466020032	NA
**TC1700000622.mm.2**	Psmb8	proteasome (prosome, macropain) subunit, beta type 8 (large multifunctional peptidase 7)	-0.315120839990653	6.39490865600356	-0.874672705987661	0.413223127132775	0.999692817443324	-4.60237465080096	5.7206853827239	6.09699912229559	6.23358027643991	6.72718259907037	6.5597497379406	7.03125481755099	NA
**TC1200000884.mm.2**	Gm1995	REX1, RNA exonuclease 1 homolog pseudogene	0.466404773569643	5.76081252460854	0.874647673475339	0.413235781957768	0.999692817443324	-4.60237588524541	4.68382706933687	5.6232978175592	5.79968984710566	6.79559005684155	5.40668352433556	6.25578683247237	NA
**TC1500002212.mm.2**	Nckap5l	NCK-associated protein 5-like	0.295459089629136	7.47108655134866	0.874566415417266	0.413276862767064	0.999692817443324	-4.60237989223375	6.65181663447942	6.88384632479671	7.71644803520336	8.22443922883369	7.52151018490033	7.82845889987845	NA
**TC0600002565.mm.2**	Mthfd2	methylenetetrahydrofolate dehydrogenase (NAD+ dependent), methenyltetrahydrofolate cyclohydrolase	-0.289154282507904	6.2918467845408	-0.874465491605851	0.413327889988834	0.999692817443324	-4.60238486867357	6.19679418790845	6.04323029893892	6.51635531703889	5.95001851065192	6.52819101916927	6.51649137353734	NA
**TC0500003171.mm.2**	Rita1	RBPJ interacting and tubulin associated 1	-0.27180573006153	6.6992586305755	-0.874302820035929	0.413410146754239	0.999692817443324	-4.60239288911697	6.75852961853208	6.69010383940423	6.66095500054476	6.44069843215206	7.00154773929697	6.64371715352291	NA
**TC0700001150.mm.2**	Vps33b	vacuolar protein sorting 33B	0.264171802896303	5.77439953115672	0.874295885963955	0.413413653316629	0.999692817443324	-4.6023932309786	7.02456225788132	6.83209878779831	5.45869929895267	5.20034057457399	5.11177589026207	5.01892037747198	NA
**TC1800000029.mm.2**	Zeb1	zinc finger E-box binding homeobox 1	0.631771247190043	7.93593665259841	0.874285840671723	0.413418733263028	0.999692817443324	-4.6023937262259	9.20302282720474	9.13089407164174	8.59145051554674	6.68117224001528	7.71159187001212	6.29748839116981	NA
**TC1700001781.mm.2**	Ppil1	peptidylprolyl isomerase (cyclophilin)-like 1	0.40172673736103	5.09543429209665	0.874273407247973	0.413425020961357	0.999692817443324	-4.60239433920688	5.073059909797	5.04171665166381	4.81288236678975	5.81775896613083	4.51266359237258	5.31452426582594	NA
**TC0100001316.mm.2**	Glrx2	glutaredoxin 2 (thioltransferase)	-0.611354347205348	8.77811921584134	-0.873905775166746	0.413610967832738	0.999692817443324	-4.60241246154666	9.68071124456335	9.62157941402278	9.05177314735771	7.0200848236679	9.17197718295886	8.12258948247745	NA
**TC1500001571.mm.2**	Klhl38	kelch-like 38	0.303134904252463	5.13085061092799	0.873474876439056	0.413828993283646	0.999692817443324	-4.6024336969055	4.90474396989608	4.69288628298847	5.19190478294294	5.70496682765121	4.93368861884523	5.356913183244	NA
**TC0700002032.mm.2**	Nlrp6	NLR family, pyrin domain containing 6	0.385209390104914	5.98078709388224	0.873368929448253	0.413882613158442	0.999692817443324	-4.60243891719577	5.34909376148185	5.97895075975945	5.83876092932994	6.82478748180104	5.81311665966058	6.08001297126058	NA
**TC1100000475.mm.2**	Havcr2	hepatitis A virus cellular receptor 2	0.354259279870122	5.62677607372667	0.873241045611367	0.413947342119383	0.999692817443324	-4.60244521787733	5.78445618873138	6.41170382176869	5.27090281678429	5.8656046790158	4.93659037513297	5.49139856092687	NA
**TC0400002943.mm.2**	Cer1	cerberus 1, DAN family BMP antagonist	0.321676429587503	5.74222020829933	0.873218075430545	0.413958969367547	0.999692817443324	-4.60244634953286	5.10381647410654	5.54792107974783	5.817971375565	6.4044969019933	5.6009370443735	5.97817837400979	NA
**TC0700002034.mm.2**	Ifitm1	interferon induced transmembrane protein 1	-0.321721507083756	6.81107781476125	-0.8731250707141	0.414006049775501	0.999692817443324	-4.60245093135114	6.4021079561877	7.00920305301168	6.88188861853553	6.52396781406476	7.24129545982681	6.80800398694099	NA
**TC0800002422.mm.2**	Nr2f6	nuclear receptor subfamily 2, group F, member 6	0.268759753277953	6.17342327787284	0.872901475780799	0.414119253107549	0.999692817443324	-4.60246194543989	6.23419618232008	6.15429786766033	6.26019591146679	6.33458665043948	5.83789510297719	6.21936795237316	NA
**TC0500000876.mm.2**	Fam47e	family with sequence similarity 47, member E	0.432912646250938	6.67841432243891	0.872591213486123	0.414276372861648	0.999692817443324	-4.60247722593905	5.60728487456009	5.8980798635978	6.72872021885987	7.98675302562885	6.59441572563661	7.25523222635024	NA
**TC0600001740.mm.2**	Ppfibp1	PTPRF interacting protein, binding protein 1 (liprin beta 1)	0.336939300445249	5.55974455496814	0.872562199574691	0.414291068027592	0.999692817443324	-4.60247865471845	5.04802229505898	4.97678094811898	5.54242592297382	6.46134542078688	5.62463934251783	5.70525340035237	NA
**TC0300001643.mm.2**	Impa1	inositol (myo)-1(or 4)-monophosphatase 1	0.408918835252364	6.54454530379039	0.872113716413616	0.414518267763263	0.999692817443324	-4.60250073655196	7.56083671359567	7.00656943863248	6.31153917318107	6.4473124973284	6.57053790154061	5.37047609846412	NA
**TC1300000392.mm.2**	Psmg4	proteasome (prosome, macropain) assembly chaperone 4	0.34435786773289	5.27584345757641	0.871940902738339	0.414605838977391	0.999692817443324	-4.60250924354501	5.4268836849048	5.54764722035655	5.05532119993676	5.6293015878947	4.61059564657113	5.38531140579455	NA
**TC0600000904.mm.2**	Tacr1	tachykinin receptor 1	0.733597290810137	6.02823391451617	0.871718933782052	0.414718339034609	0.999692817443324	-4.60252016881897	7.35424543369724	6.98641378123998	7.20568750003077	4.44228192685928	5.35262376850052	4.82815107676926	NA
**TC1400000833.mm.2**	Rcbtb1	regulator of chromosome condensation (RCC1) and BTB (POZ) domain containing protein 1	-0.553035297259018	5.3963959238154	-0.871288785508391	0.414936414216634	0.999692817443324	-4.60254133597991	6.84668751140983	6.79936765285075	5.41987371443813	3.39325117761877	4.90852037471783	5.0106751118571	NA
**TC1100003696.mm.2**	Ccr7	chemokine (C-C motif) receptor 7	0.253487951289734	6.94727268776113	0.871283583323206	0.41493905212073	0.999692817443324	-4.60254159193651	6.50425269036035	6.42766251891975	7.34324130770936	7.28610710222372	6.99463532867109	7.12773717868251	NA
**TC1800001075.mm.2**	Ino80c	INO80 complex subunit C	-0.273601575337263	6.50395676567045	-0.871202986813437	0.414979922268292	0.999692817443324	-4.60254555731053	6.76201333355574	6.32619325140959	6.33583013298579	6.35833529620563	6.55694477199433	6.68442380787161	NA
**TC0600000735.mm.2**	Grid2	glutamate receptor, ionotropic, delta 2	0.283722171031153	6.77150563344917	0.87118855065142	0.414987243097909	0.999692817443324	-4.60254626755148	6.83865110054066	6.68296731558897	6.99262922271077	6.84480064060306	6.88808863093179	6.38189689031973	NA
**TC1500001002.mm.2**	Tuba1c	tubulin, alpha 1C	-0.460726719681658	9.17871631850516	-0.871166763075919	0.414998292137741	0.999692817443324	-4.60254733945957	10.1521546118649	10.1125788829264	9.10421408922647	7.8388413992117	9.4151458705579	8.44936305724359	NA
**TC0500003481.mm.2**	Zfp68	zinc finger protein 68	0.293582718770508	7.7771197058935	0.871125425457537	0.415019256102822	0.999692817443324	-4.6025493731499	7.62225141057331	7.6037925116189	7.75028094960101	8.2616389257539	7.52719501001795	7.89755942779594	NA
**TC1400002849.mm.2**	Gm3696	predicted gene 3696	0.441174870587655	6.33791591233452	0.87104755905918	0.415058747397177	0.999692817443324	-4.60255320379526	6.94591064311819	6.43217693535498	7.06306542216811	5.70281339618652	6.11735647477844	5.76617260240088	NA
**TC0300001047.mm.2**	Bcas2	breast carcinoma amplified sequence 2	0.345263117367499	7.58159501153715	0.871014309174037	0.415075611496571	0.999692817443324	-4.60255483946524	7.97218166391035	7.96651250017809	7.66484415170775	7.45585691822698	7.69346161841548	6.73671321678426	NA
**TC1600000940.mm.2**	Gabpa	GA repeat binding protein, alpha	-0.462284429016616	7.40962096725555	-0.870962882066079	0.415101695945081	0.999692817443324	-4.60255736925943	8.11379490247622	8.05735151950797	7.5527217469195	6.12828351483844	7.66655966399524	6.93901445579592	NA
**TC0900000858.mm.2**	Csnk1g1	casein kinase 1, gamma 1	-0.78154440026406	6.86787371866605	-0.870169405417533	0.415504309914801	0.999692817443324	-4.60259639067265	7.83547228078687	7.76362892754098	7.31681969842602	4.70570645314414	7.58181352657197	6.00380142552632	NA
**TC0300000658.mm.2**	Fbxw7	F-box and WD-40 domain protein 7	-0.878901130984475	7.03544666204758	-0.869952676568541	0.415614329187876	0.999692817443324	-4.6026070452657	8.16588285270121	8.3441481474017	7.4235176594173	4.54890569568952	7.83598541191002	5.89424020516574	NA
**TC1400002649.mm.2**	Rnf219	ring finger protein 219	0.284727178557658	6.19463604296333	0.869816428887315	0.415683504336956	0.999692817443324	-4.60261374252716	6.55843963335878	6.3839054123943	5.94869721854131	6.4487655660298	6.00862061693425	5.81938781052154	NA
**TC1700001774.mm.2**	Stk38	serine/threonine kinase 38	0.32102027768826	6.51919584198397	0.869803102566836	0.415690270780242	0.999692817443324	-4.60261439754984	6.18510966589635	6.48973015357081	7.00706760743783	6.53412028646876	6.75520505950751	6.14394227902257	NA
**TC1100000503.mm.2**	Olfr1392	olfactory receptor 1392	0.370054177733119	5.3828189352942	0.869794058052093	0.415694863181554	0.999692817443324	-4.60261484210747	5.29066072815471	5.0645158167575	5.37587836456939	5.96504434659022	4.83458405405623	5.76623030163714	NA
**TC1400000207.mm.2**	Lrmda	leucine rich melanocyte differentiation associated	0.406006034807994	5.39810702306252	0.869787563411384	0.415698160893565	0.999692817443324	-4.60261516133154	5.14420793603978	5.04593697231589	5.0981610164721	6.40709363334561	5.28024502247846	5.41299755772326	NA
**TC1900000397.mm.2**	Tmem252	transmembrane protein 252	-0.270706069087708	5.31396403955538	-0.869714447306027	0.415735287580073	0.999692817443324	-4.6026187550315	4.60132594166711	4.92273794205179	5.34061625825225	5.56853784946673	5.64015034921702	5.81041589667737	NA
**TC0400004086.mm.2**	Phf13	PHD finger protein 13	0.263810607449445	6.2056643021613	0.869680022291695	0.415752768660426	0.999692817443324	-4.6026204469796	6.02645840188199	6.08566339269525	6.28658724975173	6.538155366893	6.03143224455127	6.26568915719456	NA
**TC1100003997.mm.2**	Abca8b	ATP-binding cassette, sub-family A (ABC1), member 8b	0.27155053304031	6.06284374294341	0.869609523341582	0.415788569834981	0.999692817443324	-4.60262391179489	7.03865267047056	6.66610360205001	5.9139385800149	5.69376504559534	5.56788139856579	5.49672116096384	NA
**TC0100001369.mm.2**	Arpc5	actin related protein 2/3 complex, subunit 5	-0.656823985753079	8.10092477066196	-0.869592237027534	0.415797348614944	0.999692817443324	-4.60262476134087	8.53921431726895	8.82802091725129	8.6449410585225	6.31739165045017	8.57813873516953	7.6978419453093	NA
**TC1700000693.mm.2**	Psors1c2	psoriasis susceptibility 1 candidate 2 (human)	-0.272193149861369	5.52330905044711	-0.869463832222743	0.415862562711662	0.999692817443324	-4.60263107155761	4.79910545732591	5.00456678374223	5.5162847726067	5.87961310833921	5.89191776259904	6.0483664180696	NA
**TC0700003827.mm.2**	Arhgef17	Rho guanine nucleotide exchange factor (GEF) 17	-0.304693135515316	7.52932574768957	-0.869372254179217	0.415909077871015	0.999692817443324	-4.60263557165495	8.49939353920567	8.36054914875041	7.26412090768336	6.58919185589199	7.12742650873431	7.33527252587167	NA
**TC0200000538.mm.2**	Cstad	CSA-conditional, T cell activation-dependent protein	0.303004841927294	6.39772266778427	0.869210230938871	0.415991383581244	0.999692817443324	-4.60264353270647	6.65035142562945	6.65968590286842	6.5643039403722	6.27685024065896	5.79414760900496	6.4409968881716	NA
**TC1900000443.mm.2**	Ric1	RAB6A GEF complex partner 1	0.47428236948289	5.62432218357621	0.869198067765525	0.415997562799658	0.999692817443324	-4.60264413031158	6.19839238374168	6.62968882746175	6.15412952246718	4.77907879214262	5.40788498705065	4.57675858859338	NA
**TC1400002098.mm.2**	Tgm1	transglutaminase 1, K polypeptide	-0.607522968815455	6.24383045925471	-0.869187981478491	0.416002686955335	0.999692817443324	-4.60264462587067	5.96185169623052	7.25440087740501	4.92543047283383	6.59041164929708	5.92424255886691	6.80664550089491	NA
**TC1500002042.mm.2**	Dennd6b	DENN/MADD domain containing 6B	0.258892893566729	6.81637066520746	0.869155185011865	0.41601934892803	0.999692817443324	-4.60264623720199	6.73850955015029	6.91440907340729	7.04978732426161	6.83175825314871	6.66724316370789	6.69651662656898	NA
**TC0900003098.mm.2**	Camp	cathelicidin antimicrobial peptide	-0.287247099541735	6.12862004829111	-0.868969302664434	0.416113794192121	0.999692817443324	-4.60265536915458	5.02900611363266	5.44236316105877	6.40378249501739	6.45914591296849	6.51276942984033	6.92465317722901	NA
**TC0700002691.mm.2**	Selenov	selenoprotein V	0.324944272727392	6.54920703884086	0.868923858035418	0.416136886627582	0.999692817443324	-4.60265760156424	6.48799112370268	6.56762320059247	6.41007093708076	7.03468729002164	6.10573582186873	6.68913385977888	NA
**TC0400003702.mm.2**	Trnp1	TMF1-regulated nuclear protein 1	-0.430603523719306	9.36350491335114	-0.868893896420019	0.416152111971591	0.999692817443324	-4.60265907335277	9.69408079544463	9.80495659792746	9.60956953189015	8.30082298775791	9.69067931517258	9.08092025191409	NA
**TC0700001698.mm.2**	Tmc5	transmembrane channel-like gene family 5	-0.307534670734977	4.80113553069872	-0.868832577952687	0.416183272943145	0.999692817443324	-4.60266208537376	4.48594617399455	4.90526894106767	4.39651439725877	5.0037499665713	4.97215707110916	5.04317663419086	NA
**TC0X00003364.mm.2**	Frmpd4	FERM and PDZ domain containing 4	0.593717610017215	7.16166301418661	0.868575258797851	0.416314056751248	0.999692817443324	-4.60267472376294	8.84053079372899	8.96534531029583	7.17409378902181	6.00167481154282	6.93723184485217	5.05110153567803	NA
**TC1700000078.mm.2**	Rsph3a	radial spoke 3A homolog (Chlamydomonas)	-0.447211925510208	6.72309036434123	-0.868453005410597	0.416376203269621	0.999692817443324	-4.60268072753763	7.65589962752763	7.58982975079913	6.77444248926942	5.32475198908068	6.74316851588209	6.25044981348841	NA
**TC1800000434.mm.2**	Rbm27	RNA binding motif protein 27	0.525504931277919	7.65563573848933	0.868039013361986	0.416586703454217	0.999692817443324	-4.60270105467137	8.61443579129994	8.37745115569528	8.20469015298577	6.79177852026253	7.60507404327348	6.34038476741898	NA
**TC1100003251.mm.2**	Gosr1	golgi SNAP receptor complex member 1	0.690797543599153	8.39720576762444	0.867832643952133	0.416691664162999	0.999692817443324	-4.60271118532855	9.15357229263382	9.01951355456206	9.31513599526417	7.48073592761036	8.66948716345919	6.74478967221703	NA
**TC1100000924.mm.2**	G630025P09Rik	RIKEN cDNA G630025P09 gene	0.383016568598766	5.90160820061733	0.867805358822021	0.416705542997858	0.999692817443324	-4.60271252464658	5.39383781164238	5.49391918985404	5.80336993631684	6.84059273338571	5.63989599515709	6.23803353734793	NA
**TC0300000787.mm.2**	Dennd4b	DENN/MADD domain containing 4B	0.335404863014055	6.90276332399481	0.867626234841294	0.416796664515169	0.999692817443324	-4.6027213165079	7.36067742661812	7.20525488735928	6.74040750311108	7.02032117489869	6.10971945425726	6.9801994977244	NA
**TC0100001491.mm.2**	Kifap3	kinesin-associated protein 3	-0.649837018930758	8.33623560429243	-0.86752428576993	0.41684853320322	0.999692817443324	-4.60272631994805	9.78743406290064	9.77237562849673	8.38921224300389	6.18975270524396	8.51311692265112	7.36552206345824	NA
**TC1500000801.mm.2**	Fam118a	family with sequence similarity 118, member A	0.328807721640765	6.67048286474721	0.867313429772933	0.416955825588573	0.999692817443324	-4.60273666719958	6.56676251885345	6.76963884806283	6.67531643076478	6.99673920165946	6.9079189946144	6.10652119452831	NA
**TC0400001523.mm.2**	Bsdc1	BSD domain containing 1	0.342983432160231	8.15455636453102	0.867255187379726	0.416985465341969	0.999692817443324	-4.60273952504243	9.14052632087854	9.10245951741888	8.31733721106624	7.36782239553833	7.60773424171389	7.39145850057021	NA
**TC0700001808.mm.2**	Apobr	apolipoprotein B receptor	-0.511070281967133	7.39389465958868	-0.867182084578905	0.417022669799727	0.999692817443324	-4.60274311189609	6.59862492790884	6.71282947550618	6.8843053005593	8.13058119453211	7.34044792738385	8.69657913164182	NA
**TC0400003043.mm.2**	Elavl2/Mir6402	ELAV like RNA binding protein 1/microRNA 6402	0.445964831351962	8.13869283914444	0.867041637713709	0.417094154748983	0.999692817443324	-4.60275000254303	9.06361780883698	8.55924242019001	8.71064462782622	7.33996860644557	7.80718382591475	7.35149974565312	NA
**TC0600000012.mm.2**	Casd1	CAS1 domain containing 1	0.46576336844417	9.07222610289968	0.866973788777646	0.417128691873642	0.999692817443324	-4.60275333113123	9.43764442380759	9.48721682732544	9.51788106643761	8.70212998513907	9.34821120800265	7.94027310668569	NA
**TC1000002604.mm.2**	Mterf2	mitochondrial transcription termination factor 2	-0.522204541425614	6.08353756871857	-0.866896371778147	0.417168101994329	0.999692817443324	-4.60275712892861	6.82117523461367	6.96446133779248	6.34560799223129	4.48998188629573	6.12794709139377	5.75205186998447	NA
**TC0300003237.mm.2**	Mtx1	metaxin 1	-0.313080410911653	6.97287639409279	-0.866733688653216	0.417250926851585	0.999692817443324	-4.60276510891777	7.15212300146186	7.46521554447888	6.97130858864598	6.32557090975037	6.80234654391688	7.12069377630278	NA
**TC0300002474.mm.2**	6330562C20Rik	RIKEN cDNA 6330562C20 gene	0.352366118498388	6.96596436654726	0.866724533673002	0.417255588173138	0.999692817443324	-4.60276555796464	6.14352305802681	6.1086221202849	7.19568913748203	7.92849749150229	6.84718712524861	7.57226726673894	NA
**TC1700000183.mm.2**	Tbp	TATA box binding protein	0.435026734496064	7.2668573190201	0.866652367925436	0.417292333202846	0.999692817443324	-4.60276909755741	7.66879050909392	7.67948177651415	7.92117753568071	6.64028501307161	7.21246132380246	6.47894775595773	NA
**TC0300002358.mm.2**	Atp8b2	ATPase, class I, type 8B, member 2	0.298783637653933	7.5414336217406	0.866572645695232	0.417332928572658	0.999692817443324	-4.60277300757805	6.90633869176798	7.04000004621309	7.72536787887224	8.22454725501295	7.89984055945032	7.45250729912701	NA
**TC0800000297.mm.2**	Sfrp1	secreted frizzled-related protein 1	0.56253411992729	6.09698611527653	0.866492046206834	0.41737397360123	0.999692817443324	-4.60277696040741	7.8606612753438	7.62410039546741	6.54130110312829	4.56981052722299	5.31558411382954	4.67045927666716	NA
**TC0X00001501.mm.2**	Alas2	aminolevulinic acid synthase 2, erythroid	-0.279363036840756	5.55414721171196	-0.866337048991743	0.417452913767835	0.999692817443324	-4.60278456130092	5.08460988634298	5.53343955539916	5.55524995418796	5.5188039232361	5.95130054316264	5.68147940794292	NA
**TC0500003112.mm.2**	Ccdc60	coiled-coil domain containing 60	-0.258767936885998	4.95917193011131	-0.866093672095508	0.417576887888584	0.999692817443324	-4.60279649460883	4.81368632592438	4.74339353847811	4.77793994992279	5.06226797132389	5.16743343712657	5.19031035789211	NA
**TC0900003163.mm.2**	Fbxl2	F-box and leucine-rich repeat protein 2	0.337155715550089	8.3691657692001	0.865929709785554	0.417660424141793	0.999692817443324	-4.60280453292175	8.71878737146062	8.72650844212853	8.31280693800453	8.40919817835129	8.4925228730965	7.55517081215915	NA
**TC1300001209.mm.2**	Pde4d/Mir1904	phosphodiesterase 4D, cAMP specific/microRNA 1904	0.346395486851389	8.38678914404596	0.86588770987149	0.417681824421229	0.999692817443324	-4.60280659183771	8.89756044004761	8.76781986637726	8.6030776422723	8.07099512350454	8.40794370643191	7.57333808564216	NA
**TC0100003406.mm.2**	Fmo3	flavin containing monooxygenase 3	0.443631371039194	5.82427806627134	0.865843928191842	0.417704133423749	0.999692817443324	-4.6028087380361	5.31226909245668	4.8613202751448	5.86436254523891	6.96530834081357	5.49708216345159	6.4453259805225	NA
**TC0300000832.mm.2**	Lce1c	late cornified envelope 1C	-0.385180124567764	5.72053897370833	-0.865766221418933	0.417743731149635	0.999692817443324	-4.60281254709972	5.28600422815719	4.78018591637623	6.41538490807595	5.32795681621456	6.36361978655115	6.15008218687489	NA
**TC1100003024.mm.2**	Rangrf	RAN guanine nucleotide release factor	-0.289785564563621	5.76896288660418	-0.865736465893465	0.417758894665265	0.999692817443324	-4.60281400561496	5.43043697705627	5.84973262508667	5.463504511917	5.91351378226045	5.99338767303746	5.96320175026723	NA
**TC1200001810.mm.2**	Clec14a	C-type lectin domain family 14, member a	0.335113105200242	5.27165445635777	0.865528653903904	0.417864807623026	0.999692817443324	-4.60282419102588	5.12900106419263	5.05891776802983	5.09373515611781	5.96238190204451	5.35631094097914	5.02957990678269	NA
**TC1400002711.mm.2**	Tgds	TDP-glucose 4,6-dehydratase	0.665020054102619	4.94534535518898	0.86550423046329	0.417877256509763	0.999692817443324	-4.6028253879874	4.67093813158212	4.82560407509468	5.97413128637763	4.77865372995354	5.83811754746583	3.5846273606601	NA
**TC0300002695.mm.2**	Cd53	CD53 antigen	-0.797866261038203	6.05442950357334	-0.865465276991068	0.417897112071317	0.999692817443324	-4.60282729700564	7.08764519137896	7.67733450223002	6.25074353567599	3.73218886720135	6.68069070533275	4.897974219621	NA
**TC1600002173.mm.2**	Il10rb	interleukin 10 receptor, beta	-0.279625319183495	6.8202191583263	-0.865411969423459	0.417924285397993	0.999692817443324	-4.60282990940166	6.72961776191233	7.18820107354468	6.58820864196466	6.63391409610224	7.0216166615846	6.75975671484929	NA
**TC1600001606.mm.2**	Timmdc1	translocase of inner mitochondrial membrane domain containing 1	-0.28341663036331	7.44087512885216	-0.865320976549308	0.417970671661613	0.999692817443324	-4.60283436838672	6.82864883972712	7.24521824314365	7.40139776845933	7.60087744629848	7.62285661909779	7.94625185638663	NA
**TC0500001767.mm.2**	Cpsf4	cleavage and polyadenylation specific factor 4	0.25785738851304	6.78301439298689	0.865220102798899	0.418022099407596	0.999692817443324	-4.60283931124536	6.3846619719375	6.56568286377342	6.97048658929108	7.16124156032718	6.86136504346405	6.75464832912813	NA
**TC0100003099.mm.2**	Prelp	proline arginine-rich end leucine-rich repeat	0.287783363568576	7.25975861539935	0.865036928931932	0.418115497513889	0.999692817443324	-4.60284828597068	7.46138701341198	7.75014516018036	7.41399503847951	7.04729808449096	6.75323462240734	7.13249177342597	NA

**TC1600002094.mm.2**	Rcan1	regulator of calcineurin 1	-0.350339284415883	7.14811288922387	-0.864857087167677	0.418207211525717	0.999692817443324	-4.60285709633838	7.59642563693106	7.550742799676	7.25191350934754	6.26850165560464	7.25150917346333	6.96958456032062	NA
**TC1900000106.mm.2**	Gpha2	glycoprotein hormone alpha 2	0.305362182628118	6.22221608743074	0.86484209759737	0.418214856432527	0.999692817443324	-4.60285783062156	5.80868567531009	5.83262490905157	6.2086827232603	6.94267242947919	6.24630943515594	6.29432135232732	NA
**TC0400001152.mm.2**	Cc2d1b	coiled-coil and C2 domain containing 1B	0.275911810652655	7.0924191831389	0.864809518522553	0.418231472605567	0.999692817443324	-4.6028594265229	6.43564658819934	6.57553093173301	7.3576943361011	7.68988626400207	7.07653540475412	7.41922157404374	NA
**TC1100003852.mm.2**	Ccdc43	coiled-coil domain containing 43	0.33671521700611	7.45178643550647	0.864687032782011	0.418293947845591	0.999692817443324	-4.60286542622477	7.51640595556777	7.55180660030663	7.4292904721391	7.72867777344921	6.79572778032817	7.68881003124791	NA
**TC0900001424.mm.2**	Celsr3	cadherin, EGF LAG seven-pass G-type receptor 3	-0.262736666754988	9.1650375003211	-0.86461879262484	0.418328757482256	0.999692817443324	-4.60286876860358	8.99296118837039	9.0208414005654	8.94439757934784	9.28107696039257	9.42956834912527	9.32137952412512	NA
**TC0700002426.mm.2**	Sae1	SUMO1 activating enzyme subunit 1	-0.507491681706773	6.76514637873591	-0.864378992592924	0.418451097509892	0.999692817443324	-4.60288051267988	7.79149320434959	7.55224135663225	6.87038876605673	5.2456914079533	6.97624148631326	6.15482205111031	NA
**TC1100003907.mm.2**	March10	membrane-associated ring finger (C3HC4) 10	0.436723301117943	6.22290338286123	0.864301053081646	0.418490865968461	0.999692817443324	-4.60288432930773	5.92204221865	5.29403848827334	6.09510417799404	7.4022889182459	6.13960642597402	6.48434006803004	NA
**TC1100003532.mm.2**	Stxbp4	syntaxin binding protein 4	-0.568023954507249	5.50354623240272	-0.864140292189151	0.418572902608723	0.999692817443324	-4.6028922009773	6.80317621843014	6.6290278182938	5.42810805840948	3.79840466592945	5.85379516883837	4.50876546451506	NA
**TC0800001555.mm.2**	Disc1	disrupted in schizophrenia 1	0.358982458200395	5.95763194917427	0.863676707604322	0.418809536924658	0.999692817443324	-4.60291489555077	5.55987679840594	5.34998440925246	5.94785475566237	6.82909294623165	5.7381535619316	6.32082922356162	NA
**TC0400003026.mm.2**	Ifne	interferon epsilon	0.418419866538987	5.61950963467824	0.863593368077603	0.418852087553399	0.999692817443324	-4.60291897463047	4.92678938907986	5.06443644760106	5.54706728317836	6.73426856905487	5.35857621313647	6.08591990601879	NA
**TC1200000303.mm.2**	Snx13	sorting nexin 13	-0.482868118849005	7.07078430564618	-0.863357170276619	0.418972700199131	0.999692817443324	-4.60293053413297	8.2165978803989	8.17974321637683	6.82702137416348	5.70429287553817	7.40034192446775	6.09670856293192	NA
**TC0200001969.mm.2**	Tgm6	transglutaminase 6	-0.415876281197919	5.4752388191765	-0.862985187847004	0.419162701896903	0.999692817443324	-4.60294873510403	4.86642763312569	5.03179381650723	5.01878329893619	6.04194615257892	5.48504053863406	6.40744147527689	NA
**TC1800001151.mm.2**	4933408B17Rik	RIKEN cDNA 4933408B17 gene	-0.297844154415909	4.56116813054093	-0.862970802109564	0.419170051132496	0.999692817443324	-4.60294943889926	3.56996993210363	4.16958878930888	4.59941763278142	4.91646324371921	5.10628468096523	5.00528450436722	NA
**TC1400002653.mm.2**	Rbm26	RNA binding motif protein 26	0.85016650545693	5.31774067630641	0.862812710494642	0.419250821553325	0.999692817443324	-4.60295717277418	6.60182240504729	6.51041729735781	5.95268397325611	4.29458470991751	5.74551533637671	2.80142033588304	NA
**TC1500002350.mm.2**	Cpt1b	carnitine palmitoyltransferase 1b, muscle	0.468537136928656	5.11264131373413	0.862657588493018	0.419330085863223	0.999692817443324	-4.60296476055364	4.54277606736374	4.72373791678232	4.86105941976258	6.31214466629543	4.73276376825877	5.50336604394193	NA
**TC0500000274.mm.2**	Ube3c	ubiquitin protein ligase E3C	-0.593078226978508	7.83093125881015	-0.862643253182152	0.419337411481324	0.999692817443324	-4.60296546172308	8.64574479128606	8.48594714427522	8.1814641801932	6.1524054014781	8.22767612975184	7.29234990587648	NA
**TC0700000016.mm.2**	Cnot3/Mir3572	CCR4-NOT transcription complex, subunit 3/microRNA 3572	0.340396349868916	6.69462090197907	0.862595645472739	0.41936174060479	0.999692817443324	-4.60296779026384	6.35084972080642	6.59357226687076	6.5677791102468	7.38426895172073	6.40353700346331	6.86771835876639	NA
**TC1100001586.mm.2**	Cdc6	cell division cycle 6	-0.336437723002982	5.03855326383988	-0.862544559460526	0.419387848407643	0.999692817443324	-4.60297028884661	4.96754842599665	5.00719739530093	4.44561756526394	5.34623159260392	5.0889436681563	5.37578093571753	NA
**TC1000003103.mm.2**	Arhgef25	Rho guanine nucleotide exchange factor (GEF) 25	-0.330820471714229	7.556132662062	-0.86233788318382	0.419493483678542	0.999692817443324	-4.60298039634426	8.28676642252236	8.32482553558531	6.98375305649687	7.04802847892108	7.80099234510267	6.89243013374374	NA
**TC1800000944.mm.2**	Arhgap12	Rho GTPase activating protein 12	0.552770400731236	6.66610889541877	0.862058259601349	0.419636434426159	0.999692817443324	-4.60299406902699	7.89594686513946	7.58896793077059	7.0923239379801	5.71631575105241	6.59966865322065	5.10343023434939	NA
**TC0700002044.mm.2**	Phrf1	PHD and ring finger domains 1	-0.313653962756321	5.39078288495082	-0.862046751628193	0.419642318361999	0.999692817443324	-4.6029946316727	5.42770563667693	5.95542476113611	5.26097290138233	4.90615659180731	5.6403678125246	5.15406960617768	NA
**TC0800002591.mm.2**	Dand5	DAN domain family member 5, BMP antagonist	-0.257842231171745	6.86960914776585	-0.861871581131752	0.419731889118761	0.999692817443324	-4.60300319552277	7.07431554461218	7.04857948400258	6.76361694387066	6.52592075394775	6.81297165014331	6.99225051001859	NA
**TC0300003233.mm.2**	Lrrc71	leucine rich repeat containing 71	0.365101825146715	5.75058750048266	0.861518316611402	0.419912568146176	0.999692817443324	-4.60302046298851	5.46229547869335	5.16830692552326	5.61593607000873	6.68562705447767	5.85164731223637	5.7197121619566	NA
**TC0700002471.mm.2**	Qpctl	glutaminyl-peptide cyclotransferase-like	-0.446374004126501	6.5088205558758	-0.861511536352362	0.419916036503157	0.999692817443324	-4.60302079436422	7.58075696536073	7.19547377085734	6.59309186209688	5.098880433295	6.34194461036602	6.24277569327886	NA
**TC0500003168.mm.2**	Plbd2	phospholipase B domain containing 2	0.475443143792544	6.56265796707626	0.861491860579752	0.419926101518155	0.999692817443324	-4.60302175598135	7.33443961397406	7.29150344958433	7.00508058319215	5.84536493004999	6.5639228045942	5.33563642106285	NA
**TC0100002285.mm.2**	Slc40a1	solute carrier family 40 (iron-regulated transporter), member 1	-0.253494760354415	6.01767741063646	-0.861447535820854	0.419948776211192	0.999692817443324	-4.60302392222418	5.75446857930259	5.75519392544225	6.06153157480566	5.98317464437689	6.38281530936446	6.16888043052691	NA
**TC0600003383.mm.2**	St8sia1	ST8 alpha-N-acetyl-neuraminide alpha-2,8-sialyltransferase 1	0.560435550346583	6.71560709912512	0.861328644142719	0.420009600651344	0.999692817443324	-4.603029732378	7.57973801399685	7.05670384451474	7.01206575643219	6.37697016203395	7.09667555161	5.17148926616296	NA
**TC0700001406.mm.2**	C2cd3	C2 calcium-dependent domain containing 3	-0.24976498299962	6.02162133844638	-0.861300684223143	0.420023905755901	0.999692817443324	-4.60303109869027	6.74322537781203	6.84748495207174	5.55230994250375	5.46743392489389	5.78007943805542	5.73919439534146	NA
**TC0600003002.mm.2**	Zfp9	zinc finger protein 9	-0.325034533452445	7.08011010587158	-0.86116445180763	0.420093611310815	0.999692817443324	-4.60303775555553	7.32612026182607	7.17582473773868	7.25093350852678	6.41338977585314	7.04955323608407	7.26483911520074	NA
**TC0700000303.mm.2**	Trappc6a	trafficking protein particle complex 6A	0.320730969853045	7.24691723986584	0.861108620874996	0.42012218057301	0.999692817443324	-4.60304048349824	6.78998113495913	6.93343341572815	7.22001205861436	7.97976335549256	7.45568587781296	7.10262759658787	NA
**TC0400004173.mm.2**	Plekhn1	pleckstrin homology domain containing, family N member 1	0.454120132282247	5.72855893037437	0.861045105223602	0.420154683915132	0.999692817443324	-4.60304358679372	5.03921002637057	5.11438696083171	5.49242310024874	7.07057532955548	5.89715667006165	5.75760149517808	NA
**TC1700001178.mm.2**	Crim1	cysteine rich transmembrane BMP regulator 1 (chordin like)	0.503778774238731	7.75288787022237	0.861019490418225	0.420167792493332	0.999692817443324	-4.60304483826243	8.92695161324773	8.91044303243683	7.80815535620216	7.03558970586138	7.74634598953611	6.08984152404997	NA
**TC1900001729.mm.2**	Shtn1	shootin 1	0.418059305168051	6.62792571454433	0.860985680200952	0.420185095596679	0.999692817443324	-4.60304649010227	7.37913888980598	7.16657610772398	6.83073117876663	6.19824777126944	6.71394290822292	5.47891743147704	NA
**TC0900001070.mm.2**	Myo6	myosin VI	-0.391058031219215	6.39414339016845	-0.860966228582953	0.420195050615587	0.999692817443324	-4.60304744041747	7.13932568266752	7.39477615328074	6.2436634825266	5.2806577387854	6.54017636453855	5.76626091921188	NA
**TC1100003797.mm.2**	Ezh1	enhancer of zeste 1 polycomb repressive complex 2 subunit	0.319181255828146	6.80304112115208	0.860941693635922	0.420207607445225	0.999692817443324	-4.60304863906208	8.21240292364748	7.59182790144481	6.69528720133847	6.13090200539978	6.0699790437047	6.11784765137725	NA
**TC1100002473.mm.2**	Nprl3	nitrogen permease regulator-like 3	0.266515284495922	6.92369363134863	0.860773709686247	0.420293587942921	0.999692817443324	-4.60305684529921	7.14025126493277	6.78472382256403	6.97797577398998	7.09713286080343	6.85848185231802	6.68359621348356	NA
**TC0700000511.mm.2**	Sirt2	sirtuin 2	-0.268065488713583	9.15517095750649	-0.860643208225443	0.420360392385833	0.999692817443324	-4.6030632198064	9.62601792235876	9.86028996294034	8.9021042239094	8.55218921724692	8.94878311208822	9.04164130649528	NA
**TC0400003894.mm.2**	Efhd2	EF hand domain containing 2	-0.336630315901832	8.40693600123138	-0.860412859589698	0.420478328141125	0.999692817443324	-4.60307447006283	8.32593151301963	8.7347064143932	8.01735083178219	8.3365078923037	8.912920093324	8.11419926256557	NA
**TC0100002215.mm.2**	Aff3	AF4/FMR2 family, member 3	-0.348564328438495	7.30688250281722	-0.860359727491823	0.420505534579846	0.999692817443324	-4.60307706478545	7.75059302370958	7.79298393782923	7.32788942458672	6.47240527465703	7.52959626456413	6.96782709155661	NA
**TC0400003653.mm.2**	Epb41	erythrocyte membrane protein band 4.1	-0.258902868338558	7.04611245181774	-0.860240848882003	0.420566411370701	0.999692817443324	-4.60308286991226	6.94500017158593	7.06020308546247	6.841734522751	7.03509833583946	7.08271633924166	7.31192225602592	NA
**TC1100000316.mm.2**	Asb3	ankyrin repeat and SOCS box-containing 3	0.376886917731715	5.11687858657808	0.860192231815984	0.420591309642394	0.999692817443324	-4.60308524386154	5.72569595106645	5.62620624190673	5.18742679654785	4.86414478443151	5.21740284938437	4.08039489613156	NA
**TC0700000150.mm.2**	Zscan22	zinc finger and SCAN domain containing 22	0.434494982582151	7.34751727231012	0.860158591342035	0.420608538579412	0.999692817443324	-4.60308688646361	7.22169562516498	7.12950585778009	8.36600235454882	6.93544370349117	7.41928143459525	7.01317465828044	NA
**TC0700000283.mm.2**	Rsph6a	radial spoke head 6 homolog A (Chlamydomonas)	0.310684154961176	5.6789042016186	0.860028533254025	0.420675152566269	0.999692817443324	-4.60309323659758	5.34124574745919	5.20260982532459	5.66217148303483	6.41329749039025	5.63592227063126	5.81817839287147	NA
**TC0100001145.mm.2**	Thsd7b	thrombospondin, type I, domain containing 7B	-0.326431268096004	6.22813100028901	-0.860006335237942	0.420686522860183	0.999692817443324	-4.60309432036645	5.86837533072187	5.63149839225049	6.00544187705259	6.60258299423226	6.3228363775078	6.93805102996906	NA
**TC0200002411.mm.2**	Nnat	neuronatin	0.414350035630763	9.77233632048174	0.859897198231013	0.420742428418001	0.999692817443324	-4.60309964849499	10.996973727855	10.2300980686969	10.0375522964559	9.08027080234414	9.46859697104346	8.82052605649505	NA
**TC0400000248.mm.2**	Map3k7	mitogen-activated protein kinase kinase kinase 7	0.258836108687833	7.12427771986368	0.859322174106873	0.42103707498743	0.999692817443324	-4.60312771479913	7.2291492643624	7.22612511453734	7.34375655999332	7.06027551883568	7.02178688459316	6.86457297686017	NA
**TC1300001851.mm.2**	Tfap2a	transcription factor AP-2, alpha	-0.358637239410013	5.29724741111902	-0.859273435323409	0.421062056036011	0.999692817443324	-4.60313009316918	4.48530333400566	3.76529463806834	5.30166991727507	6.10613608760949	6.0053488620221	6.11973162168249	NA
**TC1500002124.mm.2**	Dbx2	developing brain homeobox 2	0.293523403157261	6.49507266704896	0.859071511670502	0.421165563495531	0.999692817443324	-4.60313994583988	5.99599672880367	5.82216903733925	6.63112773785936	7.23853078337332	6.54066130349565	6.74195041142251	NA
**TC1000001772.mm.2**	Pex3	peroxisomal biogenesis factor 3	0.683843933537963	7.42325236467394	0.859046411945671	0.421178431088513	0.999692817443324	-4.60314117045985	8.46064127771891	8.46119947160598	8.1667619268309	6.32591872633134	7.52422808361658	5.60076470246974	NA
**TC0200003141.mm.2**	Ssna1	SS nuclear autoantigen 1	-0.355050317138978	7.49773770398182	-0.858996761410812	0.421203885715639	0.999692817443324	-4.60314359285495	7.67079094461128	7.24440631466444	7.27554174760894	7.4050224175597	7.21136039533313	8.17930440411346	NA
**TC1000000306.mm.2**	Hs3st5	heparan sulfate (glucosamine) 3-O-sulfotransferase 5	-0.328639894335448	6.33320924932549	-0.858932913876991	0.421236620454437	0.999692817443324	-4.60314670778267	6.04780163748814	6.57494660684403	5.8620574445958	6.49755628687915	6.3218368976641	6.69505662248175	NA
**TC0700002054.mm.2**	Cracr2b	calcium release activated channel regulator 2B	-0.267993089949035	9.73239079325496	-0.858908502263291	0.421249136821928	0.999692817443324	-4.60314789871465	9.39202422847887	9.42853074211572	9.79337073038194	9.7255310741366	9.81274647067885	10.2421415137378	NA
**TC0100003463.mm.2**	Dusp27	dual specificity phosphatase 27 (putative)	0.322034554110875	5.30845962786368	0.858807215774394	0.42130107152283	0.999692817443324	-4.60315283980651	4.51001628499513	4.6479779792488	5.43788444726867	6.23053185831127	5.35460282244979	5.6697443749084	NA
**TC0X00003007.mm.2**	Tceal6	transcription elongation factor A (SII)-like 6	-0.28918588486582	8.20567396366029	-0.85880180662841	0.421303845197033	0.999692817443324	-4.60315310367282	8.66918823161721	8.38321194203355	8.16983735663503	7.63179856265465	8.26948900487976	8.11051868414155	NA
**TC0700000495.mm.2**	Rps16	ribosomal protein S16	-0.411376170528495	9.13622980694975	-0.858550665198368	0.42143263892069	0.999692817443324	-4.60316535363327	9.67789170390157	9.71366934499961	9.28751937889955	8.01401334697061	9.32094393090751	8.80334113601965	NA
**TC0500000176.mm.2**	Pmpcb	peptidase (mitochondrial processing) beta	0.334397177708628	7.58515245342306	0.858541373285584	0.421437404676634	0.999692817443324	-4.60316580682496	8.09834273661969	8.0510857747681	7.80505590315461	7.21008437912931	7.53825312720641	6.80809279966025	NA
**TC1400000200.mm.2**	Vdac2	voltage-dependent anion channel 2	-0.72423635260753	9.46482959667394	-0.858517333094627	0.421449734902398	0.999692817443324	-4.60316697931637	10.6274801276164	10.5904134912999	9.87875188606785	7.1825537418883	9.70767906463078	8.80209926854043	NA
**TC0900001099.mm.2**	Sh3bgrl2	SH3 domain binding glutamic acid-rich protein like 2	-0.429383261689545	7.46991064052274	-0.858431844852956	0.421493584000746	0.999692817443324	-4.60317114860076	6.8430474959242	6.84986364823595	6.8624964909282	8.27139659687041	7.78955845615452	8.20310115502319	NA
**TC1200000801.mm.2**	Acot5	acyl-CoA thioesterase 5	-0.412057357360176	4.54367797851542	-0.858384516023611	0.421517861598554	0.999692817443324	-4.60317345673327	4.41958190424091	4.5510181811672	3.71861157706328	5.01506495841874	4.55313166685932	5.00465958334305	NA
**TC1400002464.mm.2**	Dnajc15	DnaJ heat shock protein family (Hsp40) member C15	0.278606037107065	7.58788544770146	0.858349073336194	0.421536042800439	0.999692817443324	-4.60317518515237	8.11006355759502	7.7288511406428	7.4467460170609	7.67605901403164	7.12843760897595	7.43715534790245	NA
**TC1100001302.mm.2**	Tbx2	T-box 2	0.332319550612938	7.08126084531967	0.85827076294093	0.421576216085038	0.999692817443324	-4.60317900393166	6.75647630369079	6.63433135676037	7.00447347369147	7.8762247826549	6.95675650796699	7.25930264715352	NA
**TC1400000971.mm.2**	Nkx2-6	NK2 homeobox 6	0.258156746902212	6.83080070142147	0.858204016677261	0.421610459172334	0.999692817443324	-4.603182258625	6.3851244566636	6.47118437109275	7.17954292081286	7.14286151647559	7.05572519457083	6.75036574891319	NA
**TC0500001912.mm.2**	Steap2	six transmembrane epithelial antigen of prostate 2	0.366347967502833	6.34031919608069	0.858151314392131	0.421637498661214	0.999692817443324	-4.60318482839599	7.01163422200463	6.58863841518504	5.97661928152634	6.61054995562372	6.34597332322796	5.50849997891642	NA
**TC0700002089.mm.2**	Tssc4	tumor-suppressing subchromosomal transferable fragment 4	0.272974955728333	7.62190062051765	0.857883708655607	0.421774816301091	0.999692817443324	-4.60319787542821	7.61982051332797	7.55605893415477	7.53415308477673	8.01658400876319	7.472392391274	7.53239479080926	NA
**TC1900000881.mm.2**	Ppp6r3	protein phosphatase 6, regulatory subunit 3	0.375350323424495	7.0075952526342	0.857617781505501	0.421911304984003	0.999692817443324	-4.60321083820077	7.96920606564726	7.68278408093574	7.33261482183633	6.23952618619927	6.58746106653014	6.23397929465647	NA
**TC0400001393.mm.2**	Heyl	hairy/enhancer-of-split related with YRPW motif-like	0.414423954860696	7.39045213814835	0.857543679090601	0.42194934423776	0.999692817443324	-4.60321444993573	7.43070971125225	6.6185749553983	7.47969257593212	8.08144546004834	6.88648419962838	7.84580592663069	NA
**TC1000000744.mm.2**	Lss	lanosterol synthase	0.282201387913014	7.37978451045926	0.857397196791518	0.42202454588728	0.999692817443324	-4.60322158889824	7.25287732844121	7.13792529813973	7.39891821055567	7.82723539544467	7.14727892682531	7.514471903349	NA
**TC0400002705.mm.2**	Elp1	elongator complex protein 1	-0.530888654407591	6.16871222874914	-0.857376740309847	0.422035048694422	0.999692817443324	-4.60322258580718	6.92424879241042	6.88957657403395	6.47769395934225	4.5906413892754	6.34219571560866	5.78791694182418	NA
**TC0600001874.mm.2**	Tmem168	transmembrane protein 168	-0.424722535618102	7.24206591234868	-0.857231172966742	0.422109791682592	0.999692817443324	-4.60322967935066	6.38539763038695	6.57219111712637	6.73539875740094	8.08728207027034	7.59474789339957	8.07737800550792	NA
**TC1200001106.mm.2**	Hhipl1	hedgehog interacting protein-like 1	0.287234892958073	6.21228751482896	0.857226471573886	0.42211220582037	0.999692817443324	-4.60322990843898	6.60415685861344	6.27388886068996	6.07480578818523	6.41026878960801	5.75492197402901	6.15568281784808	NA
**TC1300000179.mm.2**	Hist1h2bp	histone cluster 1, H2bp	-0.346603675289009	6.96323526426666	-0.857061919144527	0.422196708888549	0.999692817443324	-4.60323792623366	6.5374964071798	6.93768696358375	7.36918895377845	6.43632147835075	7.3023985415258	7.19631924118142	NA
**TC1100004282.mm.2**	Mfsd11	major facilitator superfamily domain containing 11	0.301590439294289	8.44158354051925	0.856986109554982	0.422235643757415	0.999692817443324	-4.60324161973422	8.50700272708645	8.42062629517302	8.91807961925869	8.24444693046363	8.41178905290709	8.14755661822664	NA
**TC0200001906.mm.2**	Blvra	biliverdin reductase A	0.294967876502047	6.12490579614472	0.856904293133221	0.422277666604505	0.999692817443324	-4.60324560567179	6.8286789054975	6.57033701492938	6.1413299641116	5.82884734061116	5.96296667601178	5.41727487570689	NA
**TC1000000850.mm.2**	Rps15	ribosomal protein S15	0.263278184896789	10.9694283734642	0.85685375031216	0.422303628118283	0.999692817443324	-4.60324806790577	11.5968249118408	11.594739692455	10.9785450680765	10.5972359350649	10.5665845806713	10.4826400526766	NA
**TC1700001655.mm.2**	Gnptg	N-acetylglucosamine-1-phosphotransferase, gamma subunit	-0.292202567800935	8.95331370645555	-0.856728907095169	0.42236775931255	0.999692817443324	-4.6032541493687	9.04187983749471	8.93345978272955	9.0436512598368	8.53641748161678	9.28552044006126	8.87895343699419	NA
**TC0600002543.mm.2**	Mrpl19	mitochondrial ribosomal protein L19	-0.727815269076228	7.08991102390757	-0.85663822482162	0.42241434670048	0.999692817443324	-4.60325856642219	7.67317970850612	7.66333654555748	7.77512389909645	5.09853577651822	7.67893870290915	6.65035151085798	NA
**TC0800000262.mm.2**	Defb10	defensin beta 10	-0.291859584778666	4.909031762145	-0.856623135428053	0.422422099135839	0.999692817443324	-4.60325930138593	4.82934163966361	4.76050509840949	4.59660086161116	5.04371147100861	4.87402951516042	5.35000198701668	NA
**TC0700004371.mm.2**	Mcmbp	minichromosome maintenance complex binding protein	0.262526476743221	6.98242400185192	0.856619256304767	0.422424092118909	0.999692817443324	-4.60325949032633	7.29453240166249	7.0730406725694	7.12951003765091	6.89650190753213	6.64699817664272	6.85396081505387	NA
**TC1500002197.mm.2**	Lmbr1l	limb region 1 like	0.282323491128941	6.18780476712948	0.856417252669623	0.422527885325629	0.999692817443324	-4.60326932860282	6.10815031359729	5.97738482143731	6.66383946927128	6.1391307557288	6.01490176092596	6.22342148181624	NA
**TC0900001445.mm.2**	Map4	microtubule-associated protein 4	0.393994273503234	8.29412885233138	0.856380430517813	0.422546807235849	0.999692817443324	-4.60327112181868	9.15732487102582	9.16296555943997	8.57996976293274	7.53626585233174	8.04199518239879	7.28625188585923	NA
**TC0500000707.mm.2**	Gsx2	GS homeobox 2	0.313569408718273	5.89574465353369	0.856366933241729	0.42255374327683	0.999692817443324	-4.603271779116	5.66856172690979	5.52507521124862	5.8063930023654	6.59759189787472	5.82514901680544	5.95169706599813	NA
**TC0700002700.mm.2**	Nccrp1	non-specific cytotoxic cell receptor protein 1 homolog (zebrafish)	0.448483236316723	5.89465746057015	0.856332489898953	0.422571443553691	0.999692817443324	-4.60327345642744	5.1773264265941	5.5395646917075	5.62992507323479	7.14408498564157	6.0410435434135	5.83600004282941	NA
**TC0600000127.mm.2**	Cav2	caveolin 2	-0.49004632206753	6.07044430542989	-0.856194350505105	0.422642438199595	0.999692817443324	-4.60328018308968	6.4669822381326	6.16407776672531	6.56250532444292	4.84325126735026	6.35407281249211	6.03177642343613	NA
**TC1000000892.mm.2**	Pip5k1c	phosphatidylinositol-4-phosphate 5-kinase, type 1 gamma	-0.309761576797595	8.51320947519542	-0.856088163655718	0.422697017241143	0.999692817443324	-4.60328535338696	8.38409558104546	8.6584790964393	8.66088181831175	8.04769769173455	8.87068355584284	8.45741910779865	NA
**TC0900000966.mm.2**	Mns1	meiosis-specific nuclear structural protein 1	-0.292794684943875	6.54281188454765	-0.856061194751402	0.422710879823803	0.999692817443324	-4.60328666645658	6.63629020904979	6.87892799133925	6.35069281611775	6.22733905238679	6.88539850672091	6.27822273167139	NA

**TC0600002918.mm.2**	Tada3	transcriptional adaptor 3	0.277947784879992	6.47921269485893	0.855982352102709	0.422751408503402	0.999692817443324	-4.60329050502685	7.08636425197131	7.23704101942847	6.51553301973935	6.03835021401754	5.89425641518456	6.10373124881234	NA
**TC0100002478.mm.2**	Crygc	crystallin, gamma C	-0.381619246176491	5.14166440218262	-0.855920548132883	0.422783180516951	0.999692817443324	-4.60329351389516	4.20024964578706	4.86124053522154	4.81878975307621	5.69383911679087	5.36761049754955	5.90825686467052	NA
**TC0800000271.mm.2**	Thsd1	thrombospondin, type I, domain 1	-0.309811702283036	5.19821140781416	-0.855897200264013	0.422795183578417	0.999692817443324	-4.60329465053022	4.81764326122294	5.39115947047548	5.05574705136878	5.12467410394146	5.13090635121086	5.66913820866545	NA
**TC1100002832.mm.2**	Olfr318	olfactory receptor 318	-0.302616050573485	4.99748586079805	-0.855789448828708	0.422850581455612	0.999692817443324	-4.60329989590876	4.66127347186453	4.77287757392423	4.85977779156444	5.11298821736183	4.94627842068039	5.63171968939285	NA
**TC0600001877.mm.2**	Gpr85	G protein-coupled receptor 85	-0.866069831330357	5.5477254801344	-0.855734167099721	0.422879005318925	0.999692817443324	-4.6033025868888	6.20321343992066	6.19103219237906	6.45191404651456	3.1280697464084	6.17183005798945	5.14029339759423	NA
**TC1100004099.mm.2**	Galk1	galactokinase 1	0.35904826376692	6.89459309657469	0.85551198426973	0.422993257754291	0.999692817443324	-4.60331340115217	6.31224109354225	6.59049257739395	6.99695913228677	7.59450158573613	6.49818774243763	7.37517644805143	NA
**TC1400000174.mm.2**	Zswim8	zinc finger SWIM-type containing 8	-0.397282173727345	6.42279787298469	-0.855382619977107	0.423059790759996	0.999692817443324	-4.60331969689691	7.40717330618059	7.39487502049626	6.09591784939833	5.37416943248997	6.67810776788382	5.58654386145916	NA
**TC0600002494.mm.2**	Elmod3	ELMO/CED-12 domain containing 3	-0.49904833854955	7.23188587240829	-0.855378425358002	0.42306194821126	0.999692817443324	-4.60331990102595	7.94424582412921	8.03278600343542	7.3987287786108	5.80936458628221	7.54527621928339	6.66091382270872	NA
**TC1400001960.mm.2**	ccdc198	coiled-coil domain containing 198	-0.30194232817782	5.12186970989876	-0.855207000159985	0.423150125545287	0.999692817443324	-4.60332824283138	4.70860783143723	4.99923990523342	4.86618462210808	5.34355831107505	5.16750390725527	5.6461236822835	NA
**TC0400003542.mm.2**	Ago1	argonaute RISC catalytic subunit 1	-0.495355316722968	6.95451768217261	-0.855042679714075	0.4232346609425	0.999692817443324	-4.60333623796302	7.43568519725643	7.19326302900642	7.42067583425654	5.63304778240689	7.01140898574578	7.03302526436359	NA
**TC0700002627.mm.2**	Gsk3a	glycogen synthase kinase 3 alpha	-0.406189686281683	8.61816130616044	-0.855019225233024	0.423246728211544	0.999692817443324	-4.60333737908244	9.02394771155648	9.14239376056122	8.85208379051654	7.51303970562424	8.68065482146512	8.49684804723903	NA
**TC0900001942.mm.2**	Acad8	acyl-Coenzyme A dehydrogenase family, member 8	0.256749165844942	7.36442034467945	0.854777835417781	0.423370937228868	0.999692817443324	-4.60334912220658	7.74071574375726	7.94800292501591	7.15180089290255	7.35384997259415	7.05689293339355	6.93525960041327	NA
**TC1400001741.mm.2**	Ccser2	coiled-coil serine rich 2	-0.751321733613863	6.98683250005522	-0.854737975025216	0.423391450264045	0.999692817443324	-4.60335106114135	7.98047853810146	7.74108658302263	7.50906581201757	4.83932739397217	7.57567605322227	6.27536061999519	NA
**TC0400003513.mm.2**	1110065P20Rik	RIKEN cDNA 1110065P20 gene	-0.263575500472562	7.98315433436678	-0.854668720843661	0.423427091714386	0.999692817443324	-4.60335442975271	7.79120254017406	7.76028677047713	7.8271225586632	8.083020288639	8.06455050386193	8.37274334438539	NA
**TC0100000911.mm.2**	Olfr1410	olfactory receptor 1410	-0.27140869965153	6.54457132300274	-0.854564150867559	0.423480912477308	0.999692817443324	-4.60335951585719	5.78219374136454	6.03487897001563	6.52312893937893	6.93063997428768	6.96084197962032	7.03574433334935	NA
**TC1000003152.mm.2**	Pa2g4	proliferation-associated 2G4	-0.680052121934946	8.17008637964033	-0.854413319102743	0.423558552353112	0.999692817443324	-4.60336685139447	9.26844729131414	9.24102459120332	8.5758517913787	5.99961928434863	8.30252855932809	7.63304676026913	NA
**TC1400000846.mm.2**	Amer2	APC membrane recruitment 2	0.295759071406014	6.16389530938744	0.853977656390022	0.423782865805772	0.999692817443324	-4.60338803498378	6.12761547020378	6.10816298375911	6.30456414376497	6.36499162882193	5.69375026095004	6.38428736882483	NA
**TC1100001494.mm.2**	Hoxb7	homeobox B7	-0.365870504666312	7.40029106147498	-0.853422302839439	0.424068931116993	0.999692817443324	-4.60341502894085	6.4871233544464	6.21340975959838	7.25706034570945	8.22767577702678	7.84130376031461	8.37517337175425	NA
**TC1700000632.mm.2**	BC051142	cDNA sequence BC051142	0.271904502165549	5.26571228453474	0.852971648788856	0.424301168663629	0.999692817443324	-4.6034369259918	5.30556427502631	5.55898711325229	5.12207300966282	5.51469265196766	4.96468178273078	5.1282748745686	NA
**TC0800000184.mm.2**	Defb5	defensin beta 5	0.291584666907134	5.60770676196349	0.852921052352063	0.424327248536714	0.999692817443324	-4.60343938401036	5.30071670786373	5.81084848302881	5.98687309487343	5.57204926247792	5.53540497909416	5.44034804444293	NA
**TC0200004930.mm.2**	Nol4l	nucleolar protein 4-like	0.375800281409274	7.50324045830778	0.852663590517651	0.424459975031265	0.999692817443324	-4.60345189036252	7.59011508498064	7.61355546545009	8.08715615491824	7.19653022619902	7.66690336427322	6.86518245402549	NA
**TC0300002329.mm.2**	Dap3	death associated protein 3	-0.830843704041156	7.32841953649223	-0.852653028411834	0.424465420644592	0.999692817443324	-4.60345240337394	8.41797054685891	8.40448757041293	8.00316575932155	4.74002008747806	7.55661483730107	6.84825841758086	NA
**TC0400003011.mm.2**	Ifna16	interferon alpha 16	0.466856289686632	5.73177338605553	0.852493472633948	0.424547690648791	0.999692817443324	-4.60346015268055	5.42196861344585	4.91545965120606	5.52693315598656	6.96652915954072	5.43839980998164	6.12134992617237	NA
**TC1100001570.mm.2**	Grb7	growth factor receptor bound protein 7	0.307533976610085	6.6870515922943	0.852490621112184	0.42454916105351	0.999692817443324	-4.6034602911653	6.11730560119113	6.41477933336565	6.7926887243225	7.30995756189209	6.49084778003955	6.99673055295487	NA
**TC0900002164.mm.2**	Trappc4	trafficking protein particle complex 4	0.470919583292146	7.17504168078566	0.852248342602136	0.424674107007844	0.999692817443324	-4.60347205644877	7.93558110065562	7.68495579966543	7.52178159139701	6.66399458409158	7.34073304859655	5.90320396030775	NA
**TC0X00000685.mm.2**	Bgn	biglycan	0.336190926738062	6.65139248620623	0.852244599745015	0.424676037454594	0.999692817443324	-4.60347223818973	7.10838998556756	7.44844982926766	6.58485547766847	6.42709300027069	6.61673607831786	5.72283054614517	NA
**TC0900001889.mm.2**	Epor	erythropoietin receptor	-0.248671650706998	6.36787074244806	-0.852095800793149	0.42475278840426	0.999692817443324	-4.60347946299121	6.57373800473217	6.43037712785413	6.12983119233477	6.22305181800926	6.45806401959445	6.39216229216357	NA
**TC1900001146.mm.2**	Olfr1418	olfactory receptor 1418	0.470225462753797	4.63040599229798	0.852016559852392	0.424793665244597	0.999692817443324	-4.60348331015338	3.93070866719002	4.35023774750281	4.68211280237245	5.53885742992885	3.94282716446562	5.3376921423281	NA
**TC0700004326.mm.2**	Zfp764	zinc finger protein 764	0.389006159041219	5.43949834788685	0.851995812880319	0.424804368148497	0.999692817443324	-4.60348431738689	5.30225181091002	5.22451383960106	5.15257790784284	6.29154046960338	5.051935027106	5.61417103225778	NA
**TC0900002776.mm.2**	Ube2cbp	ubiquitin-conjugating enzyme E2C binding protein	0.423903265817601	6.15850903456556	0.851982433848895	0.424811270199001	0.999692817443324	-4.60348496691037	6.39197841741568	6.57850425475543	6.84320642412131	5.57098260930741	6.12552458402474	5.44085791776878	NA
**TC0400001877.mm.2**	Gm13034	SWI/SNF related, matrix associated, actin dependent regulator of chromatin, subfamily a, member 5 pseudogene	-0.387063797982172	5.5707357768568	-0.851918580741082	0.424844212227177	0.999692817443324	-4.60348806675766	5.78978903751759	5.50457698337501	5.06785615187913	5.6101043702628	5.17427817756221	6.27780994054407	NA
**TC0100001996.mm.2**	Rpl7	ribosomal protein L7	-0.437698467784445	9.33917466260635	-0.851597864268216	0.425009699052814	0.999692817443324	-4.60350363430391	10.0710695072327	10.1319953407359	9.13605179775845	8.34224129829186	9.82913609485741	8.52455393676179	NA
**TC0300000705.mm.2**	Insrr	insulin receptor-related receptor	0.500903801444527	6.50208740371978	0.851307938710764	0.425159338438771	0.999692817443324	-4.60351770420624	5.47562719224246	6.19122021924637	6.24492776388363	7.92881454297582	6.48185463773452	6.69008006623587	NA
**TC1600000564.mm.2**	Igsf11	immunoglobulin superfamily, member 11	0.481932635641333	8.15730608940721	0.851204938462574	0.425212509229796	0.999692817443324	-4.60352270204368	8.77512613394378	8.8073527380003	8.83694287140014	7.32566859649077	8.03983317022254	7.15891302638571	NA
**TC0900003023.mm.2**	Dock3	dedicator of cyto-kinesis 3	0.427516118422445	7.82358140508004	0.851189882959509	0.425220281587565	0.999692817443324	-4.60352343254479	8.53764527624709	8.69538670187633	7.97071841640821	7.31102592819264	7.86959032228004	6.55712178547593	NA
**TC0700004599.mm.2**	Nadsyn1	NAD synthetase 1	0.436998261133853	5.5363774702181	0.851172730267731	0.425229136738699	0.999692817443324	-4.60352426479307	5.63735542125609	5.66531708084641	5.00603272384181	6.38876169689509	4.93807048898238	5.58272740948681	NA
**TC0700001989.mm.2**	Lrrc27	leucine rich repeat containing 27	-0.266858919149948	6.1806329894998	-0.85091904591259	0.425360118058526	0.999692817443324	-4.60353657236614	6.66905138079288	6.4711668375259	6.05466424168312	5.65026669850694	6.10851711545513	6.13013166303483	NA
**TC1100003769.mm.2**	Jup	junction plakoglobin	-0.298865355872502	7.93282778030273	-0.850865335810352	0.425387853216798	0.999692817443324	-4.60353917784266	8.33119221537894	8.53082220208811	7.76089405023435	7.30771672606781	7.56870471713362	8.09763677091354	NA
**TC0300000680.mm.2**	Lrba	LPS-responsive beige-like anchor	-0.307620295255938	7.26307243725426	-0.850375810582472	0.425640697989221	0.999692817443324	-4.60356292010884	7.5272291210953	7.85253185619629	7.25248255631084	6.5392339715502	7.24443405513674	7.16252306323617	NA
**TC0300002043.mm.2**	Erich6	glutamate rich 6	-0.490914500456516	4.98328879759761	-0.850361838687868	0.425647916220365	0.999692817443324	-4.60356359763241	5.22051569231909	5.35640123507483	5.25783150169539	3.91266192694397	5.66706231485708	4.48526011469531	NA
**TC0300000725.mm.2**	Paqr6	progestin and adipoQ receptor family member VI	0.438349777456013	6.5291962847865	0.850196604813777	0.425733286939562	0.999692817443324	-4.60357160962288	7.62153329150178	6.23319235858035	6.53090935564528	6.56766645112917	6.43816169727458	5.78371455458784	NA
**TC0500001241.mm.2**	2210016L21Rik	RIKEN cDNA 2210016L21 gene	0.437903881243187	9.05409714224109	0.850184534022946	0.42573952399468	0.999692817443324	-4.60357219488388	10.1364208454888	9.94833077259349	8.83161359051985	8.72620590840545	9.09991938035386	7.58209235608506	NA
**TC1000000860.mm.2**	Onecut3	one cut domain, family member 3	-0.273741996137948	5.4951693512841	-0.850127477617641	0.425769006305508	0.999692817443324	-4.60357496123657	4.73756653612782	4.62975833380374	5.7253616870922	5.80274193565638	5.78573837821503	6.28984923680944	NA
**TC0700004105.mm.2**	Mrvi1	MRV integration site 1	0.46555407408757	6.24095403850639	0.8496198473229	0.426031375546041	0.999692817443324	-4.60359956848735	6.38496082997013	5.69617059054308	6.3693240607985	6.77852641855164	5.37385894064772	6.84288339052728	NA
**TC0400002642.mm.2**	Grin3a	glutamate receptor ionotropic, NMDA3A	0.337686459040301	6.64014597035936	0.849418322426338	0.426135566502404	0.999692817443324	-4.60360933488063	7.44384220432196	6.88159292674717	6.18140968426641	6.9139971203174	6.38184067821784	6.03819320828536	NA
**TC1900000890.mm.2**	Aldh3b1	aldehyde dehydrogenase 3 family, member B1	0.273165902405274	6.29656962756082	0.849384366427939	0.426153124013403	0.999692817443324	-4.60361098033346	6.03613817181718	6.17528779577092	6.39578999850307	6.66137180279063	6.0441335694583	6.46669642702485	NA
**TC1900000340.mm.2**	Zfand5	zinc finger, AN1-type domain 5	-0.756094948346259	9.71927900329874	-0.849382504905636	0.426154086559533	0.999692817443324	-4.60361107053866	9.89081589642758	10.0131315143644	10.5762008316576	7.87356752449635	10.6537388924482	9.30821936039825	NA
**TC0700002704.mm.2**	Mrps12	mitochondrial ribosomal protein S12	0.314905281101778	8.36711664885719	0.849134032508974	0.426282579546395	0.999692817443324	-4.60362310987715	7.77400031414306	8.02799118506691	8.36716667599718	9.14809280207118	8.35228253112168	8.53316638474312	NA
**TC0500001098.mm.2**	Dr1	down-regulator of transcription 1	-0.463180140326835	6.33361919700935	-0.849099771272564	0.426300299332528	0.999692817443324	-4.60362476978344	6.88994187392996	6.71193473456981	6.68743122916011	5.04930791729121	6.44355232082694	6.21954710627805	NA
**TC1900000061.mm.2**	Catsper1	cation channel, sperm associated 1	-0.398177882456335	4.67269838949269	-0.849071261221569	0.426315045034897	0.999692817443324	-4.60362615102226	3.69879141722405	4.0446222651067	4.31060416944649	5.43760627540987	4.96529745581064	5.57926875395839	NA
**TC0200001121.mm.2**	Agps	alkylglycerone phosphate synthase	-0.351972126505554	5.71103075403903	-0.849010586762389	0.426346427746686	0.999692817443324	-4.60362909045028	5.96867360203052	6.57362159568808	5.36459838351054	5.14537415324168	6.03550961684313	5.1784071729202	NA
**TC1200001749.mm.2**	Eapp	E2F-associated phosphoprotein	0.305444113511946	6.6958453840151	0.848924140517999	0.426391143326514	0.999692817443324	-4.60363327819472	7.24519000556517	7.33779706581939	6.57508267255959	6.52640405730538	6.63009158697759	5.86050691586349	NA
**TC1000000123.mm.2**	Txlnb	taxilin beta	0.431328252251393	4.96514882313745	0.848589930753331	0.426564050272223	0.999692817443324	-4.60364946599156	4.68739660456429	4.34133582236745	4.71039173129303	6.10201677690484	4.74867979606033	5.20107220763476	NA
**TC0300000223.mm.2**	Zfp639	zinc finger protein 639	-0.490010967764859	6.32658785409077	-0.848439845322296	0.426641715135002	0.999692817443324	-4.60365673427373	7.30445818857691	7.25868698294112	6.4387994874568	4.76938052129164	6.42544471452841	5.76275722974974	NA
**TC1100003119.mm.2**	Dhx33	DEAH (Asp-Glu-Ala-His) box polypeptide 33	-0.252215553226078	6.19133716162896	-0.848320741308858	0.426703355326101	0.999692817443324	-4.60366250164255	6.39393260321316	6.17685074929689	6.06610675110007	5.9702975043057	6.33893092506195	6.20190443679598	NA
**TC1700000900.mm.2**	Mea1	male enhanced antigen 1	0.267624044708432	7.88664483925074	0.848278012388415	0.426725470506252	0.999692817443324	-4.60366457058326	8.17355779452607	7.87515665721692	7.95527125139353	7.94793008519563	7.5042101403068	7.8637431068655	NA
**TC0500001462.mm.2**	Bri3bp	Bri3 binding protein	-0.375808300062974	7.42800376839509	-0.848274430453338	0.426727324443953	0.999692817443324	-4.60366474401821	7.21198611234096	7.48130709299751	7.46557729580067	7.09597910665238	7.09680706900265	8.21636593357635	NA
**TC0900002583.mm.2**	Foxb1	forkhead box B1	0.354239843447601	6.08131101563222	0.848165796827647	0.426783553809217	0.999692817443324	-4.6036700037755	5.48692376160794	5.51854259083824	6.36295821104429	6.73248150307688	5.68256512239841	6.70439490482756	NA
**TC0600001029.mm.2**	Gata2	GATA binding protein 2	-0.322169164194531	5.77277182984801	-0.84796901892066	0.426885420852201	0.999692817443324	-4.60367953102024	4.86614329153901	5.08969657555034	5.61110695727368	6.40711943453114	6.16462367840075	6.49794104179313	NA
**TC1000001694.mm.2**	Cnksr3	Cnksr family member 3	0.254450649736309	6.10799626244387	0.847864356764151	0.426939609052733	0.999692817443324	-4.60368459657103	6.00917456113842	5.77970681406712	6.29000859791409	6.39399015155105	6.17851227478775	5.99658517520477	NA
**TC1700000373.mm.2**	Prss29	protease, serine 29	0.405164482319774	6.49132925953915	0.847845514876769	0.426949364857198	0.999692817443324	-4.60368550860706	6.06480144364956	5.68691683673782	6.54817623431606	7.45511688642747	6.11952300457853	7.07344115152546	NA
**TC0100002189.mm.2**	Actr1b	ARP1 actin-related protein 1B, centractin beta	-0.411732872060174	8.89224920841909	-0.84772687770396	0.427010975601565	0.999692817443324	-4.60369125092111	9.89879791264629	9.86745861803243	9.03903683837203	7.6428496494857	8.82654807536063	8.67880415661744	NA
**TC0X00002246.mm.2**	Zfp36l3	zinc finger protein 36, C3H type-like 3	-0.316041461157568	5.019704619677	-0.847625664343514	0.427063209311001	0.999692817443324	-4.60369614949545	4.30250055615413	3.99874181763333	4.91715223915003	5.67529897182966	5.49065916421601	5.73387496907882	NA
**TC1000000789.mm.2**	Ilvbl	ilvB (bacterial acetolactate synthase)-like	0.45211348982889	5.4518528734741	0.847533014986038	0.42711119221756	0.999692817443324	-4.60370063327263	5.61679182908197	5.62048387084241	4.80166757721488	6.38736668307411	4.99778672604231	5.28702055458889	NA
**TC1300000028.mm.2**	Klf6	Kruppel-like factor 6	-0.411740737315118	7.0863643441596	-0.847473300323284	0.427142120390844	0.999692817443324	-4.60370352301294	7.25224765806088	7.85380060362778	7.10641616196263	6.18791200235671	7.49724404520093	6.62056559374866	NA
**TC0700000949.mm.2**	Ube3a	ubiquitin protein ligase E3A	0.630618029184808	7.19605173434158	0.847437606406659	0.427160608212404	0.999692817443324	-4.60370525027067	8.55109738368039	8.40937320994779	7.40284945060763	6.33568848478783	7.34559979604919	5.13170208097665	NA
**TC1100000436.mm.2**	Il12b	interleukin 12b	0.278037178333098	5.10297591375	0.847355449429206	0.427203166321017	0.999692817443324	-4.60370922596313	5.11571623810372	4.89096574252475	5.03138835985774	5.55223556941112	5.03062162734525	4.99692794525741	NA
**TC0800002812.mm.2**	Tradd	TNFRSF1A-associated via death domain	0.302120016232855	6.22106104275224	0.847214553829582	0.42727615244445	0.999692817443324	-4.60371604296279	5.67911135198662	6.12552944857843	6.43698014593178	6.62600259827528	5.93837058732036	6.52037212442098	NA
**TC0900002887.mm.2**	Zbtb38/E030011O05Rik	zinc finger and BTB domain containing 38/RIKEN cDNA E030011O05 gene	-0.49719822078453	7.75047725591805	-0.847173473671017	0.427297434993827	0.999692817443324	-4.60371803049147	8.69769568387352	8.50997108957638	7.98794619634156	6.1624539639031	7.56086778158667	7.58392882022705	NA
**TC0100000109.mm.2**	Gdap1	ganglioside-induced differentiation-associated-protein 1	-0.719147016138872	6.83109861090598	-0.8471416657917	0.427313914344792	0.999692817443324	-4.60371956937069	7.98629239852859	7.83033072285999	7.28350268776571	4.58233456811907	7.12773323117369	6.18139805698884	NA
**TC1200000500.mm.2**	Pnn	pinin	0.593338775252295	8.07783820008346	0.847008494488908	0.427382914122666	0.999692817443324	-4.60372601187625	8.93842762773521	9.01534486006498	8.47134422035821	7.37862291124437	8.33781796375804	6.32547161733994	NA
**TC0400002008.mm.2**	Klhl21	kelch-like 21	0.351373901173519	5.98385459488346	0.847001013518969	0.427386790462337	0.999692817443324	-4.60372637376915	6.18000539669657	5.64295143929924	6.0376025736606	6.3538566941654	5.40756045888541	6.28115100659356	NA
**TC1400000999.mm.2**	Lgi3	leucine-rich repeat LGI family, member 3	0.433973847818334	7.55195422790851	0.846990994614838	0.427391981898081	0.999692817443324	-4.60372685843189	8.80404086086426	8.47528005831387	7.88135932319249	6.56881674876231	7.13888720979283	6.4433411665253	NA
**TC0X00001413.mm.2**	Pak3	p21 (RAC1) activated kinase 3	0.502920841591819	8.08973126992737	0.846856861169019	0.427461489435749	0.999692817443324	-4.60373334677743	9.39410053300413	9.02317802720159	8.52308893983927	7.04038643143179	7.81223895008361	6.74539473800382	NA
**TC1400002756.mm.2**	Ggact	gamma-glutamylamine cyclotransferase	-0.346251248687415	5.76117113628319	-0.846848258588912	0.427465947546315	0.999692817443324	-4.60373376288279	6.75726226982174	6.36192055889721	5.59877984517407	4.77889090062862	5.78993912187325	5.28023412130427	NA
**TC0300000767.mm.2**	Kcnn3	potassium intermediate/small conductance calcium-activated channel, subfamily N, member 3	0.335606966184478	6.25805094655767	0.846723067176377	0.427530829247086	0.999692817443324	-4.60373981807867	6.63075040636557	6.36887944644474	6.54526090037757	6.06468397907475	6.37532607301628	5.56340487406708	NA
**TC1100003033.mm.2**	Alox8	arachidonate 8-lipoxygenase	0.287732086394955	6.77292814000088	0.846720974105228	0.427531914062901	0.999692817443324	-4.60373991931065	6.80189811583314	6.5975052415971	7.23600926429477	6.67080556338771	6.54100265287345	6.79034800201912	NA
**TC0X00002421.mm.2**	Spin2d	spindlin family, member 2D	0.321067761342386	4.87279985676706	0.846605736350014	0.427591643611544	0.999692817443324	-4.60374549258205	4.82607696673473	4.60256666345065	4.74209138137062	5.48305413518027	5.00887607409062	4.57413391977549	NA
**TC1000002496.mm.2**	Pcsk4	proprotein convertase subtilisin/kexin type 4	-0.268219658192006	6.46718875401149	-0.846485044807095	0.427654206439887	0.999692817443324	-4.60375132912028	5.74512122793534	5.39170591873017	6.79395696953856	6.77097606097115	7.21199956212311	6.8893727847706	NA
**TC0700002222.mm.2**	Zfp773	zinc finger protein 773	-0.274817217703896	6.34140406702625	-0.846333939786759	0.427732544059149	0.999692817443324	-4.60375863571226	6.23225509798184	6.41723949430288	6.39757270522608	6.0270749820064	6.31191092160453	6.66237120103575	NA
**TC0400001726.mm.2**	Mul1	mitochondrial ubiquitin ligase activator of NFKB 1	-0.265779958692379	6.02345804064569	-0.846163075125885	0.427821138249624	0.999692817443324	-4.60376689681092	6.76001097115119	6.39991981476651	5.49212850873753	5.73250026154832	5.80799621753316	5.94819247013745	NA
**TC0200002172.mm.2**	Insm1	insulinoma-associated 1	0.269455340613797	5.97427277512616	0.846096989818635	0.42785540738741	0.999692817443324	-4.60377009168221	6.06148004901985	5.93375627672573	6.16982314220158	6.02483236091789	6.05442485811102	5.60131996378086	NA
**TC1000001216.mm.2**	Kitl	kit ligand	-0.249164736261522	7.42207526389801	-0.846000004026023	0.427905703860824	0.999692817443324	-4.603774780151	7.97679656071766	7.84239362350189	7.02797816109779	7.07948780222492	7.39389423677779	7.21190119906797	NA
**TC0900001269.mm.2**	Msl2	MSL complex subunit 2	0.579087382946233	5.40068942552083	0.845800849252563	0.428008998242849	0.999692817443324	-4.6037844066247	6.0738863467972	6.0650509538456	5.54937562879904	5.16231138038827	5.87416630743334	3.67934593586152	NA
**TC0700001362.mm.2**	Pak1	p21 (RAC1) activated kinase 1	-0.729329265424129	9.16176228636937	-0.845711229544713	0.428055486648972	0.999692817443324	-4.60378873808929	10.2006158671505	9.91550146124263	9.67613162289378	7.02176730659364	9.66487923056978	8.4916782297659	NA
**TC1600000475.mm.2**	Heg1	heart development protein with EGF-like domains 1	-0.313717135324408	8.52351844792606	-0.845356990406625	0.428239276882667	0.999692817443324	-4.6038058562926	8.48587831832564	8.96492538788782	8.56696067627302	7.96447567907402	8.75046267453561	8.40840795146025	NA
**TC0500001526.mm.2**	Rabgef1	RAB guanine nucleotide exchange factor (GEF) 1	0.287217273943409	7.89031409520645	0.845338587713474	0.428248826333887	0.999692817443324	-4.6038067454621	8.40436500930645	8.32655132803096	7.91134217418594	7.6813592167081	7.81079210198605	7.20747474102117	NA
**TC1600001218.mm.2**	Cpped1	calcineurin-like phosphoesterase domain containing 1	0.318310904182892	6.62547141094748	0.845298987484811	0.428269376048552	0.999692817443324	-4.60380865880039	7.10370722179864	6.68607877041588	6.41749404484554	6.88233809607254	6.02685704504706	6.63635328750524	NA
**TC0300002482.mm.2**	Ecm1/Mir7014	extracellular matrix protein 1/microRNA 7014	-0.271303366432863	6.79676991227985	-0.845261066092294	0.42828905523666	0.999692817443324	-4.60381049097213	6.24796900958506	6.30883888112263	6.74989778272645	7.09070464232638	7.0022926412844	7.38091651663417	NA
**TC1500000235.mm.2**	Laptm4b	lysosomal-associated protein transmembrane 4B	-0.738958377639432	6.59356391534226	-0.84525536461847	0.428292014055409	0.999692817443324	-4.60381076643445	6.89599412821295	7.2111050601166	7.35591312838839	4.63227064583419	7.29221265032413	6.17388787917731	NA
**TC0300002042.mm.2**	Serp1	stress-associated endoplasmic reticulum protein 1	-0.409775480621997	8.28272203820991	-0.845233852872527	0.428303177856289	0.999692817443324	-4.60381180574765	7.64205968842801	7.80248395349784	9.05364850293989	7.66247031010491	8.76954439233108	8.76612538195772	NA
**TC0700004126.mm.2**	Btbd10	BTB (POZ) domain containing 10	-1.09693106064673	6.89228701595454	-0.84520579899129	0.428317737100694	0.999692817443324	-4.60381316111168	7.72440311851825	8.07709894382384	7.86291889635896	3.81626005968687	7.94913068502904	5.92391039231025	NA
**TC1300002609.mm.2**	Rnf180	ring finger protein 180	-0.315252692220178	6.97891610643873	-0.844966037616387	0.428442181747191	0.999692817443324	-4.60382474355467	7.15102184720399	7.22653217809299	7.09682821990557	6.33589039454194	6.84201690249344	7.22120709639442	NA
**TC0200003378.mm.2**	Lhx6	LIM homeobox protein 6	-0.363977092580637	6.23988069329151	-0.844873129101932	0.428490411606928	0.999692817443324	-4.60382923125877	7.14585667386022	6.10951685691377	6.08673481307856	5.64124340882835	6.34280184467706	6.11313056239113	NA

**TC0X00000166.mm.2**	Ddx3x	DEAD/H (Asp-Glu-Ala-Asp/His) box polypeptide 3, X-linked	0.647549412926539	8.81900613866885	0.844730530051902	0.428564444045004	0.999692817443324	-4.60383611854805	9.65530334126014	9.49481733158119	9.85516684274972	7.67434064976268	8.77185761563228	7.46255105102705	NA
**TC1400001061.mm.2**	Tpt1	tumor protein, translationally-controlled 1	-0.275845416222886	9.74476338771465	-0.844605148717516	0.428629545310993	0.999692817443324	-4.60384217366393	10.2239607267753	10.3544324138378	9.42083620088414	9.24841197573036	9.8670442834161	9.35389472564417	NA
**TC0900000709.mm.2**	Scamp2	secretory carrier membrane protein 2	0.288028757977647	6.86173099833799	0.844596858461552	0.428633850081331	0.999692817443324	-4.60384257401085	6.77424973712222	6.91539820494446	7.2411451959427	6.78725258601558	6.95421773973855	6.49812252626444	NA
**TC0700003834.mm.2**	Atg16l2	autophagy related 16-like 2 (S. cerevisiae)	-0.294945391650533	9.27566603948576	-0.844531184977807	0.428667952580789	0.999692817443324	-4.60384574538134	7.84087313318422	7.79182248081332	9.50548226177404	10.2102226580339	10.1085176111002	10.1970780920088	NA
**TC1300001395.mm.2**	Pfkp	phosphofructokinase, platelet	-0.435097235248095	7.92780336500749	-0.844409760679466	0.42873101018217	0.999692817443324	-4.60385160855966	8.94934721926357	9.2730885867788	7.7147269796344	6.52236797711878	8.00817518675285	7.09911420409651	NA
**TC0600003337.mm.2**	Arhgdib	Rho, GDP dissociation inhibitor (GDI) beta	-0.27493263909281	8.15784999251727	-0.843364662047949	0.429274024850108	0.999692817443324	-4.60390205158661	7.83637268330792	8.33036346546473	7.97802613201846	8.13722313205422	8.23814751556167	8.42696702669664	NA
**TC0400002206.mm.2**	Dpy19l4	dpy-19-like 4 (C. elegans)	0.32722308369689	6.85190062494051	0.843282159913155	0.429316912709403	0.999692817443324	-4.60390603202884	6.05207763255526	5.54349021277607	7.16804406727845	7.9170969685743	7.33263184765136	7.09806302080076	NA
**TC0800000714.mm.2**	D130040H23Rik	RIKEN cDNA D130040H23 gene	-0.369613715827776	5.8676190055517	-0.843113241262428	0.429404732963085	0.999692817443324	-4.60391418102431	6.53260025657696	6.68414269113731	5.5541162977553	5.07075552921489	6.21903182671776	5.14506743190799	NA
**TC1100002497.mm.2**	Dock2	dedicator of cyto-kinesis 2	-0.339942911725863	5.74788737697224	-0.843048498605083	0.42943839590877	0.999692817443324	-4.60391730408279	5.79858501690959	6.38980257250236	5.05940834835412	5.75011707613077	5.96986547651803	5.51954577141858	NA
**TC1200000074.mm.2**	Wdr35	WD repeat domain 35	-0.246264183539193	5.85590229671677	-0.843032019900593	0.42944696431666	0.999692817443324	-4.60391809895963	6.75448533869182	6.88264400001379	5.28349453709333	5.21938350016497	5.4361241677633	5.55928223657338	NA
**TC1100000794.mm.2**	Ttc19	tetratricopeptide repeat domain 19	-0.359608943708946	7.68396002363924	-0.842681549866628	0.429629227036717	0.999692817443324	-4.60393500219052	8.57358701121951	8.44347608646725	7.52582895492106	6.65791062344433	7.80861532380344	7.09434214197984	NA
**TC1100004266.mm.2**	Irgm2	immunity-related GTPase family M member 2	-0.30494386689548	4.82697704684136	-0.842659764415173	0.429640558458603	0.999692817443324	-4.60393605276439	4.54129786509704	4.35604796197494	4.48444600941648	5.24286835067613	5.11159002866474	5.22561206521884	NA
**TC0100000608.mm.2**	Smarcal1	SWI/SNF related matrix associated, actin dependent regulator of chromatin, subfamily a-like 1	0.483836074151644	7.01248144170789	0.842605940645239	0.429668555127122	0.999692817443324	-4.60393864827134	8.11733606163991	8.21053569536117	7.17045460750767	6.18688991326711	5.45274432135335	6.93692805111814	NA
**TC1200001284.mm.2**	Tmem121	transmembrane protein 121	-0.295375919169301	6.32320392899056	-0.842519527638765	0.429713505992616	0.999692817443324	-4.60394281509418	6.28574672085134	6.45179666912984	5.80274208565073	6.50272208716104	6.46726684490378	6.4289491662466	NA
**TC1100001924.mm.2**	Ten1	TEN1 telomerase capping complex subunit	0.247614513961476	7.36955539264935	0.842478081754093	0.429735066784111	0.999692817443324	-4.60394481351583	6.99736513433633	6.82984923531502	7.672405711838	7.77026779524585	7.41988804778199	7.52755643137891	NA
**TC1000002356.mm.2**	Tfam	transcription factor A, mitochondrial	0.278712083268299	6.23912319600563	0.842414453529098	0.429768168697444	0.999692817443324	-4.60394788139964	6.32036333905438	6.45502320136374	6.58586911935917	6.02251928171698	5.97416304571127	6.07680118882827	NA
**TC0700002740.mm.2**	Zfp14	zinc finger protein 14	-0.266987777612116	6.75887353379595	-0.841903839125765	0.430033877324119	0.999692817443324	-4.60397249591765	7.08843028927898	6.83514702150908	6.4442763835963	6.60356778478542	6.97940423966787	6.60241548393808	NA
**TC0100002305.mm.2**	Nabp1	nucleic acid binding protein 1	-0.484170921390183	7.60807701065806	-0.841890967430344	0.430040576910112	0.999692817443324	-4.60397311628821	6.88619223967313	6.92992008695533	8.57441359007881	8.65759035719095	8.44643514832035	7.95391064172977	NA
**TC0200000856.mm.2**	Kcnj3	potassium inwardly-rectifying channel, subfamily J, member 3	-0.416006991432214	6.99956746700245	-0.841827967244472	0.430073368953363	0.999692817443324	-4.6039761525922	7.94300073836749	8.19252321638314	6.88010192795564	5.63483150424416	7.01481821952668	6.33212919553755	NA
**TC0400002389.mm.2**	Cfap206	cilia and flagella associated protein 206	-0.420776482753899	7.82348368239357	-0.8416643490329	0.430158541847375	0.999692817443324	-4.60398403754636	6.73272004334654	6.92118727212317	7.50240652780231	8.72031437888966	8.1340523025934	8.93022156960637	NA
**TC0X00003434.mm.2**	Ubl4a	ubiquitin-like 4A	-0.280052635835191	7.35126733534458	-0.84149745785443	0.430245431109104	0.999692817443324	-4.60399207925913	7.34129834267436	7.38028366670931	7.467291819102	6.94566654640472	7.33728960690433	7.63577403027277	NA
**TC0800002843.mm.2**	Lcat	lecithin cholesterol acyltransferase	-0.260094720637229	6.73213622531419	-0.841440687788098	0.430274990420545	0.999692817443324	-4.6039948145224	7.54818289114304	7.32468429802703	6.2996909535291	6.20018940719122	6.67375777607267	6.34631204126809	NA
**TC1100003706.mm.2**	Krt10	keratin 10	0.311127506331936	5.74272232734964	0.841375913148288	0.430308719404772	0.999692817443324	-4.60399793531907	5.83162865404834	5.97105867815191	6.20387778734067	5.43407303494006	5.6534837950075	5.36221201460936	NA
**TC1800000627.mm.2**	Pde6a	phosphodiesterase 6A, cGMP-specific, rod, alpha	0.255994216868932	5.42639665791348	0.841324998941486	0.430335232432477	0.999692817443324	-4.60400038822623	5.43616011592228	5.75887649488758	5.44911034444403	5.48855554076041	5.13658783054174	5.28908962092484	NA
**TC0800002369.mm.2**	Zfp869	zinc finger protein 869	-0.471532601861244	5.57194383860851	-0.841252773373024	0.430372845147284	0.999692817443324	-4.60400386770005	6.16671577273206	6.13905406152406	5.85956766970821	4.23184632712803	5.8038938108988	5.23058538965992	NA
**TC1400000753.mm.2**	Psmb11	proteasome (prosome, macropain) subunit, beta type, 11	0.243840604754339	5.43641460338234	0.841214492076603	0.430392781759568	0.999692817443324	-4.60400571183083	4.66575625848798	4.63975403477842	5.95792811575308	5.94240115251509	5.63934494933403	5.77330310942546	NA
**TC0700002791.mm.2**	Scn1b	sodium channel, voltage-gated, type I, beta	-0.264081720217842	9.76215031157314	-0.841144795183825	0.430429081095047	0.999692817443324	-4.60400906921759	9.64666281337144	9.81798496814832	9.79430204842238	9.4957432753193	10.0569548464524	9.76125391772501	NA
**TC0700000762.mm.2**	Etfb/Mir7051	electron transferring flavoprotein, beta polypeptide/microRNA 7051	-0.275691091789188	7.88395680641745	-0.841098336838978	0.430453278627249	0.999692817443324	-4.6040113070794	7.11620608412794	7.12144203693848	7.88100850327181	8.37634676365814	8.26780006353316	8.54093738697516	NA
**TC0700000055.mm.2**	Zfp580	zinc finger protein 580	-0.298825429774545	7.72576197602803	-0.840927394297558	0.430542321432183	0.999692817443324	-4.60401954059279	7.20608738057736	7.10495081093014	7.61841959778818	8.10452180476761	7.87933383862705	8.44125842347784	NA
**TC0400003856.mm.2**	Igsf21	immunoglobulin superfamily, member 21	-0.300845378393511	7.56794982182113	-0.840855549955518	0.430579748637685	0.999692817443324	-4.60402300069623	8.528919309948	8.6520294173132	7.24130042503496	6.57122929840433	7.38063515201104	7.03358532821527	NA
**TC0900002292.mm.2**	Fdx1	ferredoxin 1	-0.290844031339836	7.47143704062817	-0.840829855593377	0.430593134649733	0.999692817443324	-4.60402423812119	7.32096688313572	7.13509289303257	7.6219899633298	7.27344723913072	7.45527303198713	8.02185223315307	NA
**TC0700001854.mm.2**	Phkg2	phosphorylase kinase, gamma 2 (testis)	0.284834974583287	7.78223708167483	0.840782154381777	0.430617986385749	0.999692817443324	-4.60402653532108	9.02413747130757	8.98787592384518	7.47654349093525	7.14899603109616	7.30437569744355	6.75149387542128	NA
**TC1400001321.mm.2**	Ubac2	ubiquitin associated domain containing 2	0.342838805463464	6.9701150870193	0.840605180275356	0.430710196753171	0.999692817443324	-4.60403505735837	7.20644881857273	7.0828125189947	6.54021505817977	7.56833833955788	6.6752290383919	6.74764674841883	NA
**TC0900002938.mm.2**	Slc35g2	solute carrier family 35, member G2	0.258029181433312	7.05448406679954	0.840493417231577	0.430768436987982	0.999692817443324	-4.60404043864424	7.0872847653009	6.89358390123778	7.07331096037409	7.35773608818849	6.86813222311987	7.04685646257608	NA
**TC1800000094.mm.2**	Cables1	CDK5 and Abl enzyme substrate 1	0.608540689317254	6.27671303956758	0.840115499800389	0.430965413690241	0.999692817443324	-4.60405863175436	6.72230459256325	6.11936069976526	7.12611957242477	5.89172758943097	6.87495487209803	4.9258109111232	NA
**TC0500003225.mm.2**	Rhof	ras homolog family member F (in filopodia)	-0.302438092537022	6.90232856383576	-0.83989694804853	0.431079356135106	0.999692817443324	-4.60406915063358	7.61281149396688	7.29460256934788	6.75753289379229	6.19330767352058	6.56206524754218	6.99365150484474	NA
**TC0200003281.mm.2**	BC005624	cDNA sequence BC005624	0.242020592993903	8.08008988098874	0.839831420999281	0.431113523053068	0.999692817443324	-4.60407230411728	8.90659817882309	8.96486624678576	7.7770637880335	7.76949423512222	7.55793776076611	7.5045790764018	NA
**TC0800002818.mm.2**	Lrrc29	leucine rich repeat containing 29	0.501860019256734	7.60475185879822	0.839736296944349	0.431163125837414	0.999692817443324	-4.60407688168487	6.99656051612558	6.9099215304175	7.28331890081467	9.07955567156519	7.40663752470158	7.95251700916481	NA
**TC0400000948.mm.2**	Gm12657	predicted gene 12657	-0.281023531704808	7.7787199771317	-0.839734580271639	0.431164021040545	0.999692817443324	-4.6040769642918	7.61546027958262	7.40981602666081	8.03523975131123	7.50725849525734	8.17224651805456	7.93229879192363	NA
**TC0800001736.mm.2**	Atp4b	ATPase, H+/K+ exchanging, beta polypeptide	-0.324949585785919	6.06272994912889	-0.839730215637992	0.431166297097052	0.999692817443324	-4.60407717431914	5.56952842285627	5.55484174294364	5.70963596349223	6.59141921520858	6.32852457786958	6.62242977240306	NA
**TC0700004607.mm.2**	Fadd	Fas (TNFRSF6)-associated via death domain	0.25938334802266	6.8600367433248	0.839637234261142	0.431214786819708	0.999692817443324	-4.60408164844815	6.18244806090468	6.4029767589867	7.42072275319511	7.12605841485626	6.92718007604218	7.10083439596387	NA
**TC0X00000437.mm.2**	Slc25a14	solute carrier family 25 (mitochondrial carrier, brain), member 14	0.455304954394489	6.40660363216029	0.839256999873704	0.431413119809055	0.999692817443324	-4.60409994160063	7.87749890205285	7.72072528176644	6.21003118552611	5.66597257343961	6.25776976752457	4.70762408265217	NA
**TC1100001285.mm.2**	Car4	carbonic anhydrase 4	-0.245520683090606	6.13109058553472	-0.839245280537969	0.431419233746066	0.999692817443324	-4.60410050533915	5.99067484432542	5.94280992801707	6.15760064773392	6.02340803960839	6.40431412051387	6.26773593300966	NA
**TC0600001481.mm.2**	Parp11	poly (ADP-ribose) polymerase family, member 11	0.272186781930152	7.52946274617367	0.839099317696674	0.431495387303987	0.999692817443324	-4.60410752622457	7.29559826051402	7.35592678269967	7.60519148746964	7.92962101137467	7.28155032449802	7.70888861048599	NA
**TC1400000810.mm.2**	Mphosph8	M-phase phosphoprotein 8	-0.426412593899853	7.36159331555285	-0.838917292023072	0.431590369624787	0.999692817443324	-4.60411628069592	8.69537134770253	8.67488882919035	7.18701149404365	5.78622577026862	7.18617495284612	6.63988749926585	NA
**TC0900002526.mm.2**	Usp3	ubiquitin specific peptidase 3	0.344003768332809	6.80335251634591	0.838909721962469	0.431594320064698	0.999692817443324	-4.60411664475041	7.68200824788971	7.59579084634615	6.87026843712242	6.24489333313021	6.63259951800632	5.79455471558068	NA
**TC0500001517.mm.2**	Vkorc1l1	vitamin K epoxide reductase complex, subunit 1-like 1	-0.271482109667945	8.72066152275409	-0.838886859337618	0.431606251095533	0.999692817443324	-4.60411774423279	8.67162492570515	8.7886603066958	8.8044026707318	8.35595717166205	8.71900147612949	8.98432258560025	NA
**TC0500003568.mm.2**	Ccz1	CCZ1 vacuolar protein trafficking and biogenesis associated	-0.496530727086479	7.84457580273272	-0.838786329409331	0.431658716214398	0.999692817443324	-4.60412257858036	8.67693888087541	8.52050338722856	8.06133709597514	6.37713845108456	8.0979028388038	7.33363416242885	NA
**TC0700002623.mm.2**	Pou2f2/Mir6537	POU domain, class 2, transcription factor 2/microRNA 6537	0.298207786847544	5.26637530588378	0.838736449486072	0.431684749536955	0.999692817443324	-4.6041249771055	4.75245922473725	5.11287405460364	5.2613138169168	5.90335324791165	5.35815927679069	5.21009221434267	NA
**TC0200003407.mm.2**	Dennd1a	DENN/MADD domain containing 1A	-0.963999610855638	6.26029692130105	-0.838717855551981	0.431694454370692	0.999692817443324	-4.6041258711906	7.2805196569731	7.03219100224743	7.30054997808392	3.35998584535332	6.58544587153997	6.00308917360853	NA
**TC0400004112.mm.2**	Smim1	small integral membrane protein 1	-0.280242097738105	6.3350131529834	-0.838558514954987	0.431777626343811	0.999692817443324	-4.60413353254599	6.44330880937829	6.045166437348	6.47244619435833	6.00811354349061	6.53200781946255	6.5090361138626	NA
**TC0300002842.mm.2**	A730020M07Rik	RIKEN cDNA A730020M07 gene	0.353253334137881	5.64557701846126	0.838545987071118	0.431784166089834	0.999692817443324	-4.604134134869	4.94327336255432	4.90991046078313	5.63448196211333	6.7289105157396	5.90226683906354	5.75461897051363	NA
**TSUnmapped00000045.mm.2**	Aopep	aminopeptidase O	-0.293893885411809	6.58799404601874	-0.838427127175185	0.431846216324479	0.999692817443324	-4.60413984920925	5.49788693948596	5.82348199870859	6.73643382435382	7.07296995919331	7.47210787639277	6.92508367797797	NA
**TSUnmapped00000038.mm.2**	Pemt	phosphatidylethanolamine N-methyltransferase	0.270773154864424	6.05925293097822	0.838395997629127	0.431862468418092	0.999692817443324	-4.60414134571911	5.80832237404553	6.0420943543972	6.02576831902912	6.49755526454861	6.0159248389298	5.96585243491908	NA
**TC1400001500.mm.2**	Rpl15	ribosomal protein L15	-0.446834007569375	10.0822626970975	-0.838382799419221	0.431869359064503	0.999692817443324	-4.60414198019445	10.9903517615605	10.961660753327	10.1013571631358	8.72259066314371	10.27407130094	9.44354454047822	NA
**TC1300001837.mm.2**	Eef1e1	eukaryotic translation elongation factor 1 epsilon 1	0.355481080882429	8.46492832992813	0.838329457174638	0.431897209297856	0.999692817443324	-4.60414454444451	9.38696384062024	8.47179362822248	8.58106480632481	8.23982252992065	8.32734891972507	7.78257625475553	NA
**TC1400001504.mm.2**	Ube2e2	ubiquitin-conjugating enzyme E2E 2	-0.52825141245221	7.38959465696857	-0.838204650686184	0.431962376413184	0.999692817443324	-4.60415054370596	8.32049623977036	7.86379006543286	7.65198583698931	5.8964035688626	7.73712214473724	6.86777008601908	NA
**TC1400001413.mm.2**	Gm3460	predicted gene 3460	-0.270019838128237	4.98304426466798	-0.838133841953955	0.431999352014156	0.999692817443324	-4.60415394713095	4.60071338285857	4.59447061921972	5.21457952754008	4.86694142729648	5.50586287739238	5.11569775370065	NA
**TC0500002045.mm.2**	5031425E22Rik	RIKEN cDNA 5031425E22 gene	0.411489794558212	6.72960079581608	0.838080029521114	0.432027453848956	0.999692817443324	-4.60415653350941	6.17498641561647	6.30791952260526	6.49285999490016	7.86597921799543	6.58380721151373	6.95205241226544	NA
**TC0400003373.mm.2**	Dmap1	DNA methyltransferase 1-associated protein 1	-0.265771632528303	6.43608201334847	-0.837980037586626	0.432079674968479	0.999692817443324	-4.60416133913387	6.59932420113176	6.23444731016071	6.36948292591997	6.25610572595089	6.43199554772315	6.72513636920432	NA
**TC1100000096.mm.2**	Ppia	peptidylprolyl isomerase A	-0.486851411522757	10.9629676467578	-0.837857883413099	0.432143476576916	0.999692817443324	-4.60416720939806	11.6480897144116	11.6574889485244	11.0113586321446	9.73790356513812	11.5054806600205	10.2174843603078	NA
**TC1100001265.mm.2**	Wfdc18	WAP four-disulfide core domain 18	-0.25818694124138	5.35394542798961	-0.837833124245226	0.43215640921752	0.999692817443324	-4.60416839916497	5.6129377352141	5.68563786216906	5.10674475643721	5.04761678759867	5.14121532502593	5.52952010149271	NA
**TC0X00003423.mm.2**	Rhox3g	reproductive homeobox 3G	-0.276846452928605	5.02521464635673	-0.837807427837572	0.432169831709119	0.999692817443324	-4.60416963394667	4.63727411400302	4.46767750778752	4.8535538198029	5.39276785544342	5.32409557728452	5.47591900381902	NA
**TC1100000014.mm.2**	Pla2g3	phospholipase A2, group III	0.300520754001167	5.43023857935521	0.83777552676143	0.432186495621658	0.999692817443324	-4.60417116684691	5.38764107284525	5.37101215428126	5.23740610419748	5.97450377430607	5.19412816346468	5.41674020703653	NA
**TC1500000237.mm.2**	Matn2	matrilin 2	0.357940562516808	7.4299597801735	0.837519728980188	0.432320131459249	0.999692817443324	-4.60418345705667	7.34919843625208	7.17640571197554	7.16328519790124	8.22173263102225	7.14933993096056	7.51979677292931	NA
**TC0300001410.mm.2**	Adh6a	alcohol dehydrogenase 6A (class V)	0.289782860776674	5.13230855106073	0.837304770517483	0.432432454774524	0.999692817443324	-4.60419378328471	4.5916130587266	4.8127947720286	5.20938466539234	5.77431535848992	5.36264792107399	5.04309553065292	NA
**TC0300000060.mm.2**	Fabp5	fatty acid binding protein 5, epidermal	-0.254176746248227	6.6361335729069	-0.837019844528833	0.432581371036342	0.999692817443324	-4.60420746810681	7.16222626011893	7.22679040943019	6.32587951295696	6.13383612474096	6.33983989916435	6.62822923103002	NA
**TC0300002551.mm.2**	Hsd3b4/Gm10681/Gm4450	hydroxy-delta-5-steroid dehydrogenase, 3 beta- and steroid delta-isomerase 4/predicted gene 10681/predicted gene 4450	0.302677250233097	5.41325021375669	0.836930986846032	0.432627819979699	0.999692817443324	-4.6042117352987	5.18469027322758	5.37858366148053	5.49983090090322	5.7609600232459	4.9690439996975	5.68639242398541	NA
**TC0800001125.mm.2**	Adgrg5	adhesion G protein-coupled receptor G5	-0.245375672370228	5.48824431218411	-0.836756803808081	0.432718881841912	0.999692817443324	-4.60422009923805	5.15291974844776	5.36216586603527	5.47115193170385	5.49066252523672	5.72324345748994	5.72932234419109	NA
**TC0200004418.mm.2**	Plcb2	phospholipase C, beta 2	0.290754258197547	6.54693774448963	0.83659368696032	0.432804170906437	0.999692817443324	-4.60422793082428	6.38298175949324	6.35149363612967	6.57278039010598	6.99154940374899	6.21063126551569	6.7721900119442	NA
**TC0700002024.mm.2**	Urah	urate (5-hydroxyiso-) hydrolase	-0.384157715444916	5.92406190927159	-0.836587924301413	0.432807184255321	0.999692817443324	-4.60422820748451	5.6125702600821	5.74206980373418	5.39090891692438	6.31979906353732	5.80060257276514	6.6784208385864	NA
**TC0600000007.mm.2**	Gng11	guanine nucleotide binding protein (G protein), gamma 11	-0.314592448439786	5.07031543358922	-0.836486362371754	0.432860294434119	0.999692817443324	-4.60423308319084	5.70162327365087	5.2773510264811	4.32500310367807	5.08186359858381	5.05159062003254	4.98446097910891	NA
**TC1500001793.mm.2**	Zfp251	zinc finger protein 251	0.272984583508629	5.9514718007656	0.836002044407474	0.433113625405695	0.999692817443324	-4.60425632891661	6.01029576658282	5.99232206225862	6.12956656100886	5.99652451037585	6.06003265956688	5.52008924480058	NA
**TC0200005270.mm.2**	Cbln4	cerebellin 4 precursor protein	0.489939897013733	5.51133972578825	0.835879540126767	0.433177720353419	0.999692817443324	-4.6042622074137	6.58418613573283	5.84775483546382	6.18251255564604	4.62547603313412	4.60175276763345	5.22635602711923	NA
**TC0700004311.mm.2**	Zg16	zymogen granule protein 16	0.355593593879346	5.34268258084447	0.835529591212018	0.4333608533314	0.999692817443324	-4.60427899714452	4.65760828359551	4.79771343391497	5.61946554311189	6.03651493454563	4.95417876394807	5.99061452595077	NA
**TC1100000957.mm.2**	Mink1	misshapen-like kinase 1 (zebrafish)	-0.270154628578858	9.00376487555627	-0.835528679017638	0.433361330767944	0.999692817443324	-4.60427904090378	8.98687876788638	9.1796500144981	9.02501602056904	8.63285958632869	9.25062746000866	8.94755740404678	NA
**TC0700001773.mm.2**	Lcmt1	leucine carboxyl methyltransferase 1	0.327345154455513	7.19958264926458	0.835343251428417	0.433458390224684	0.999692817443324	-4.60428793551589	7.62856330862316	7.87299103076979	7.22017505484646	6.95514087770633	7.20281499125938	6.31781063238238	NA
**TC1300002594.mm.2**	Nln	neurolysin (metallopeptidase M3 family)	-0.259913242164943	7.69334141476538	-0.835092511213616	0.433589661579656	0.999692817443324	-4.60429996110726	7.33315511777026	7.64113333551026	7.51737150659763	7.8155952688933	7.93503594401069	7.91775731581012	NA
**TC0700003930.mm.2**	Olfm5	olfactomedin 5	0.268613589158897	5.48924227930593	0.834978592593895	0.433649311466609	0.999692817443324	-4.60430542394666	5.13009689291361	5.0137083939576	5.75671700090949	5.9077207827316	5.80490719528457	5.32230341003873	NA
**TC0200001940.mm.2**	Zc3h6	zinc finger CCCH type containing 6	0.523113793381572	5.91941005124359	0.834964123172711	0.433656888345355	0.999692817443324	-4.60430611777845	6.8548438134181	6.8075578515247	6.39757364033028	5.05256947431065	5.93939080175965	4.46452472611814	NA
**TC1100003286.mm.2**	Nek8	NIMA (never in mitosis gene a)-related expressed kinase 8	0.242624465639818	6.06369370114125	0.834708242261529	0.433790895554924	0.999692817443324	-4.6043183864448	5.53813101734707	5.6894157102534	6.4121764261289	6.40775577913444	6.14527989512096	6.18940337886274	NA
**TC1000002383.mm.2**	Gstt1	glutathione S-transferase, theta 1	0.285543231151781	6.46001327223616	0.834705509385541	0.433792326948529	0.999692817443324	-4.60431851746483	6.35089590489815	6.15576122993409	6.49821963281409	6.91403484763007	6.17958209215723	6.66158592598337	NA
**TC1300000658.mm.2**	Slc34a1	solute carrier family 34 (sodium phosphate), member 1	-0.304020181866323	5.19115620564079	-0.834681741614489	0.433804775895329	0.999692817443324	-4.60431965693239	4.38523031793889	4.63898143138521	5.04708183242967	5.71026072796432	5.49541160943597	5.86997131469066	NA
**TC0800000267.mm.2**	Ccdc70	coiled-coil domain containing 70	-0.258274101285859	4.76002085717852	-0.834659232127278	0.43381656602283	0.999692817443324	-4.60432073605712	4.19837250013177	4.32481119619001	4.68407689023667	5.07611973185214	5.14031430900536	5.13643051565516	NA
**TC0800003037.mm.2**	Gins2	GINS complex subunit 2 (Psf2 homolog)	0.364230343724007	5.23026660126482	0.83456368667373	0.433866613850722	0.999692817443324	-4.60432531638993	4.91499126244224	5.07787246065083	4.96247765759238	6.09612062837955	5.30316160244478	5.02697599607913	NA
**TC1600001559.mm.2**	Fam162a	family with sequence similarity 162, member A	-0.258910789297891	7.79434955496603	-0.834413931792874	0.433945065583378	0.999692817443324	-4.60433249480273	7.46032533118905	7.38842150909845	7.91905946170509	7.78070499375135	8.30385338165384	7.91373265239838	NA
**TC1700000865.mm.2**	Slc35b2	solute carrier family 35, member B2	-0.329119810025029	5.73894771955827	-0.834399700921514	0.433952521207877	0.999692817443324	-4.60433317690969	5.98752185875814	5.19249193128122	5.56647260900898	5.73124384462113	5.71340810225206	6.24254797142811	NA

**TC0300000139.mm.2**	Hltf	helicase-like transcription factor	-0.252781443728703	6.21851168351226	-0.83433248257294	0.433987738483699	0.999692817443324	-4.60433639868822	6.95389950652604	7.21763351250069	5.65076617711273	5.66622092018199	5.80058804592392	6.02196193882821	NA
**TC0X00002171.mm.2**	Enox2	ecto-NOX disulfide-thiol exchanger 2	0.319215479236997	6.57866329985665	0.834263536955476	0.434023862855775	0.999692817443324	-4.60433970308763	7.15731381538553	7.06068067515843	6.85922410528489	6.08698402825009	5.92177267463395	6.38600450042703	NA
**TC0400000388.mm.2**	Gm13304/Gm21541/Gm10591/Ccl21b/Ccl21c/Ccl21a	predicted gene 13304/predicted gene, 21541/predicted gene 10591/chemokine (C-C motif) ligand 21B (leucine)/chemokine (C-C motif) ligand 21C (leucine)/chemokine (C-C motif) ligand 21A (serine)	-0.419921455070709	5.05051260985433	-0.834217001607066	0.434048246489388	0.999692817443324	-4.60434193332044	4.83561855649573	4.74003229606958	4.5492937466007	5.39449720160893	5.99876930197402	4.78486455637702	NA
**TC0400002098.mm.2**	Acap3	ArfGAP with coiled-coil, ankyrin repeat and PH domains 3	0.277880831805506	8.27064834870751	0.834180410557984	0.434067420193278	0.999692817443324	-4.60434368691291	7.73078799556781	8.03302963249506	8.35534052557372	8.85257653832287	8.40795522580912	8.24420017447646	NA
**TC1000003143.mm.2**	Slc39a5	solute carrier family 39 (metal ion transporter), member 5	0.269838738844657	5.34008934628544	0.834153992777512	0.434081263485365	0.999692817443324	-4.60434495293117	5.11911206647592	4.9750858849543	5.57363333875024	5.66937446323562	5.60374225051705	5.0995880737795	NA
**TC0100000708.mm.2**	Mff	mitochondrial fission factor	-0.571486052839619	9.31068686102034	-0.834132539743015	0.434092505414097	0.999692817443324	-4.6043459810058	10.0576697028975	9.93876491551355	9.62778762529298	7.7345695957229	9.83937262351966	8.66595670317546	NA
**TC1500000066.mm.2**	Lmbrd2	LMBR1 domain containing 2	0.662140880437947	6.95158084436412	0.834057441223189	0.43413186057647	0.999692817443324	-4.60434957975597	8.39278051324509	8.51147061647184	7.40268866968834	5.6620691789835	6.87026073677713	4.87021535101882	NA
**TC0800000740.mm.2**	Tmem161a	transmembrane protein 161A	0.254181406509631	6.96506675752999	0.833903384158348	0.434212601781226	0.999692817443324	-4.60435696160113	6.93935500848726	7.09188827719532	6.9051914776889	7.22856855856942	6.77628590020602	6.84911132303304	NA
**TC0600002642.mm.2**	Snrnp27	small nuclear ribonucleoprotein 27 (U4/U6.U5)	-0.259926126729647	6.47303715483085	-0.833798961124271	0.434267335974406	0.999692817443324	-4.60436196468591	6.56118986521298	6.40092422693172	6.14139859712806	6.53672969456248	6.63806518809141	6.55991535705842	NA
**TC1100002426.mm.2**	Eml6	echinoderm microtubule associated protein like 6	-0.2522610266459	5.39226379944708	-0.833540654850668	0.434402750685706	0.999692817443324	-4.6043743389074	5.57429515985466	5.25310441166051	5.20866034732977	5.30217023860799	5.47499596703984	5.54035667218972	NA
**TC0200001810.mm.2**	Ckmt1	creatine kinase, mitochondrial 1, ubiquitous	0.247502774135952	7.7714131410833	0.833537118704486	0.434404604689127	0.999692817443324	-4.60437450829079	8.8648145697548	8.68954414471414	7.33557177557636	7.44899106457504	7.22931551829104	7.06024177358845	NA
**TC0300000716.mm.2**	Mef2d	myocyte enhancer factor 2D	0.271752627350413	8.92968620822258	0.833493352626623	0.434427551739971	0.999692817443324	-4.60437660467354	9.22248656494167	9.09175699133677	9.2197083615892	8.68398111228972	8.66198365578498	8.69820056339311	NA
**TC1500001119.mm.2**	Hoxc9	homeobox C9	-0.299714505598645	7.47598694999733	-0.83347363270823	0.43443789140205	0.999692817443324	-4.60437754922973	6.58892210324997	6.66291516877524	7.48099466149522	8.02133304688551	7.77525495866247	8.32650176091555	NA
**TC1200001269.mm.2**	Cep170b	centrosomal protein 170B	-0.314585025640037	7.3619903675226	-0.833472331164104	0.434438573841456	0.999692817443324	-4.60437761157136	8.17642522904609	8.1958643427172	7.05838659927922	6.52685469176691	6.7580848811586	7.45632646116759	NA
**TC0700000405.mm.2**	Zfp574	zinc finger protein 574	0.286207596280439	8.19608615294089	0.833163054934595	0.4346007587186	0.999692817443324	-4.60439242363545	8.05084823530371	8.52804260882075	8.17790343388098	8.4071171991599	7.79673769841284	8.21586774206717	NA
**TC0500003108.mm.2**	Prkab1	protein kinase, AMP-activated, beta 1 non-catalytic subunit	-0.279635389897265	6.73176244048638	-0.833154670298531	0.434605156240118	0.999692817443324	-4.60439282515052	6.13956959388209	6.35948867864183	7.07350484005847	6.59261795524144	6.9488115611813	7.27658201391314	NA
**TC0X00000741.mm.2**	Fundc2	FUN14 domain containing 2	-0.567328502783995	7.2806853439763	-0.833045471633024	0.434662430999446	0.999692817443324	-4.60439805411792	8.1684425855871	8.27008827916141	7.52229500585866	5.53316709091199	7.5788915553032	6.61122754703544	NA
**TC0800003096.mm.2**	Vps9d1	VPS9 domain containing 1	-0.355953066601168	5.66398569576645	-0.833002074554672	0.434685194299544	0.999692817443324	-4.60440013206455	5.50462757385627	5.54487511979709	5.48191196883428	5.62934070503723	5.34959568549898	6.47356312157486	NA
**TC0400004074.mm.2**	Per3	period circadian clock 3	-0.298863948436211	7.0793437608446	-0.8327929245626	0.434794912891773	0.999692817443324	-4.60441014567867	7.00177675715338	7.01854198006571	7.34209131679914	6.58691664868892	7.34086337232323	7.18587249003725	NA
**TC0900000834.mm.2**	Cilp	cartilage intermediate layer protein, nucleotide pyrophosphohydrolase	0.362766758801913	4.87254006302635	0.832713245942504	0.434836716970633	0.999692817443324	-4.60441396009432	4.373570542423	4.06242029515532	4.83489611457481	5.927495414517	4.86965766791368	5.16720034357431	NA
**TC0X00002059.mm.2**	Upf3b	UPF3 regulator of nonsense transcripts homolog B (yeast)	0.540515249406841	6.24997324718223	0.832703831330691	0.434841656619466	0.999692817443324	-4.60441441078045	7.27025708582447	6.94213896322936	6.40020210447007	5.78403486195653	6.53520996699529	4.56799650061764	NA
**TC1000001737.mm.2**	Stxbp5	syntaxin binding protein 5 (tomosyn)	0.515740755193409	6.59055161671088	0.832650327491832	0.434869729725594	0.999692817443324	-4.60441697199831	7.74200214058369	8.04440767896665	6.36225320575559	6.03193748979528	6.63947125281752	4.72323793234653	NA
**TC1100004156.mm.2**	Usp36	ubiquitin specific peptidase 36	0.255563021122128	7.23557618525098	0.832492050243811	0.434952784375839	0.999692817443324	-4.6044245480991	6.79989002766746	6.94463336992827	7.44306406030169	7.64696581777552	7.14134649361623	7.43755734221673	NA
**TC0700004190.mm.2**	Dcun1d3	DCN1, defective in cullin neddylation 1, domain containing 3 (S. cerevisiae)	-0.260515438315622	6.21691226811537	-0.832344702949539	0.4350301138858	0.999692817443324	-4.60443160022192	6.25162937740944	5.97943887094193	5.97345267642684	6.30123456542796	6.33340834380168	6.46230977468436	NA
**TC1900000141.mm.2**	Plaat3	phospholipase A and acyltransferase 3	-0.271480775365907	7.21277079959463	-0.832218124670454	0.435096551484129	0.999692817443324	-4.6044376577085	7.86145639622245	7.49281226050472	7.01110889756413	6.67858839749026	7.23919923936539	6.99345960642082	NA
**TC1200000837.mm.2**	Jdp2	Jun dimerization protein 2	0.415116236057241	7.02002783402472	0.832068444385742	0.435175124136949	0.999692817443324	-4.60444482001747	7.20242369521009	6.75949601352965	7.47214823057613	7.02209165318539	7.49167147561015	6.17233593603689	NA
**TC0900002958.mm.2**	Cep63	centrosomal protein 63	0.301938302995008	5.82106890702497	0.832053738798444	0.435182844188045	0.999692817443324	-4.60444552364714	6.91874460382945	6.44336297874918	5.84955603267703	5.23453520010358	5.15702304511529	5.3231915816753	NA
**TC0100003721.mm.2**	Disp1	dispatched RND transporter family member 1	0.2428893876672	7.3023066766707	0.832017756456491	0.435201734397847	0.999692817443324	-4.60444724528963	6.76422985487868	6.60952906971293	7.71579935804318	7.74713059734033	7.44018059657844	7.53697058347067	NA
**TC1700001985.mm.2**	2310061I04Rik	RIKEN cDNA 2310061I04 gene	-0.830964561875517	7.97513983747675	-0.831985476208156	0.43521868156402	0.999692817443324	-4.60444878975912	8.31220783851132	8.3054612033331	8.98563229046949	5.79998813916303	8.86010787641386	7.58744167696969	NA
**TC0300003213.mm.2**	Clk2	CDC-like kinase 2	0.279853675937392	6.62362309525325	0.831951325672392	0.435236611150051	0.999692817443324	-4.60445042367315	6.51001299514861	6.26228791241467	6.59415441988799	7.1704180880275	6.60650092545371	6.59836423058699	NA
**TC1100000361.mm.2**	Kcnmb1	potassium large conductance calcium-activated channel, subfamily M, beta member 1	-0.27090342008801	7.63189751122864	-0.83194402273644	0.43524044537903	0.999692817443324	-4.60445077307278	7.34440755216672	7.55058354694512	7.47075057905418	7.70654298498781	8.08528939856907	7.63381100564893	NA
**TC0700001423.mm.2**	Fchsd2	FCH and double SH3 domains 2	-0.557667544382493	6.67424817725993	-0.831807381254011	0.435312190148734	0.999692817443324	-4.60445731015795	7.52583911495069	7.18945332876199	7.08099642485296	5.02643434068798	6.92150236758295	6.30126348672299	NA
**TC1700001642.mm.2**	Ndufb10	NADH:ubiquinone oxidoreductase subunit B10	0.292033849753402	7.65435614683669	0.831787494609722	0.435322632509256	0.999692817443324	-4.60445826150212	7.66111534157378	7.72005220944483	7.49787666327482	8.06664185147934	7.71192734564414	7.26852346960321	NA
**TC0100002187.mm.2**	Sema4c	sema domain, immunoglobulin domain (Ig), transmembrane domain (TM) and short cytoplasmic domain, (semaphorin) 4C	-0.280052318354907	7.33671400499692	-0.831745104382162	0.435344891971104	0.999692817443324	-4.60446028933327	6.91282421772005	6.95369872160428	7.18261137627026	7.61421685070342	7.4502400087342	7.90669285494928	NA
**TC0900002501.mm.2**	Slc24a1	solute carrier family 24 (sodium/potassium/calcium exchanger), member 1	-0.371593991984618	5.30505301979547	-0.831572438707357	0.435435568616243	0.999692817443324	-4.60446854851679	4.15350145744406	4.81903964586579	5.16225050215209	5.89504401359478	5.53745381501603	6.26302868470008	NA
**TC0100001190.mm.2**	Nuak2	NUAK family, SNF1-like kinase, 2	0.311586141759964	5.47297421026008	0.831297620151626	0.435579919613288	0.999692817443324	-4.60448169181714	5.19720196226957	5.27396352702143	5.410076297599	6.08484973029572	5.16947286851413	5.70228087586066	NA
**TC0900002149.mm.2**	Cbl	Casitas B-lineage lymphoma	-0.513845229505964	7.10815359762604	-0.831225601735887	0.435617753645285	0.999692817443324	-4.60448513567762	8.27015244319003	7.9779807244272	7.14196735631117	5.54458162325235	7.40850513068187	6.30573430789359	NA
**TC1900000119.mm.2**	Nrxn2	neurexin II	0.304072085396814	7.81949213311261	0.830932317660856	0.435771851410128	0.999692817443324	-4.60449915835814	8.66253890415174	8.41480307946065	7.51873420319787	7.70514328973058	7.68349635837034	6.93223696376448	NA
**TC0700000493.mm.2**	Eid2	EP300 interacting inhibitor of differentiation 2	0.27636488274502	7.13327361956345	0.830569582841427	0.435962494301186	0.999692817443324	-4.60451649739686	7.02425393047574	7.42339558928051	7.4004352347978	7.05192574675944	7.09247600564922	6.80715521041798	NA
**TC0700002181.mm.2**	Fiz1	Flt3 interacting zinc finger protein 1	0.384373957956089	7.68777416914803	0.830321569129621	0.436092877614966	0.999692817443324	-4.60452834995153	8.58608708110593	8.70692288769548	8.03709966188091	6.76409181911857	7.13788756251824	6.89455600256907	NA
**TC0700004337.mm.2**	Bcl7c	B cell CLL/lymphoma 7C	-0.24332727454872	6.84735579112833	-0.830215743485375	0.436148519755237	0.999692817443324	-4.60453340667866	6.43084123855965	6.53232927751758	6.92958307723934	6.8875717635583	7.06393649554696	7.23987289434813	NA
**TC0100001356.mm.2**	Fam129a	family with sequence similarity 129, member A	-0.320362454229202	6.71630172707419	-0.830144281109071	0.436186096894623	0.999692817443324	-4.60453682117885	5.83975121085971	5.72054818893767	6.58677317958941	7.46161984750526	7.20848712386359	7.48063081168949	NA
**TC0200003512.mm.2**	Rnd3	Rho family GTPase 3	-0.471916783744679	6.88901890077511	-0.83003620717889	0.436242929947302	0.999692817443324	-4.6045419846452	7.23693528576363	7.41837724801193	7.2068307744735	5.66065287721937	7.31555144306977	6.49576577611247	NA
**TC0600001972.mm.2**	Prrt4	proline-rich transmembrane protein 4	0.307590333075981	6.87780108933019	0.829946422554378	0.436290149213066	0.999692817443324	-4.60454627398151	5.85016707748579	6.44818629985721	7.12826490136187	7.66355201103318	6.96520379037842	7.21143245586468	NA
**TC1400002168.mm.2**	Atp8a2	ATPase, aminophospholipid transporter-like, class I, type 8A, member 2	-0.299819202616769	7.91872279838725	-0.829663026318348	0.43643921621877	0.999692817443324	-4.60455981094636	7.45630557893954	6.97275989190298	8.20280963658823	8.03900682053547	8.7195379200011	8.12191694235615	NA
**TC1300000162.mm.2**	Gpx6	glutathione peroxidase 6	-0.368838131095087	5.02642430227031	-0.829634511911411	0.436454216881641	0.999692817443324	-4.60456117283201	4.87047734174442	4.94009433151046	5.29589433048693	4.50925460860146	4.80864361779807	5.7341815834805	NA
**TC0700002718.mm.2**	Kcnk6	potassium inwardly-rectifying channel, subfamily K, member 6	-0.27926095420885	6.67814879500078	-0.829520095534616	0.436514411986403	0.999692817443324	-4.60456663721523	6.31061351330386	6.60835756024364	6.73644867790945	6.55925121611028	6.66452175206155	7.18970005037589	NA
**TC0400001881.mm.2**	Zfp992/Zfp991	zinc finger protein 992/zinc finger protein 991	-0.631803242505098	6.17147862579662	-0.829139783509568	0.436714539156302	0.999692817443324	-4.60458479706213	7.77133802329482	7.05191690733241	6.24216484487703	4.22884032469411	6.54462124831017	5.18999040627117	NA
**TC1600000014.mm.2**	Zfp174	zinc finger protein 174	0.382263029680368	5.45002749731484	0.828948881100623	0.436815020346371	0.999692817443324	-4.60459391066602	5.57326210275547	5.3653601783269	6.23903744339816	5.02399693768554	5.44525142659398	5.05325689512898	NA
**TC0600002200.mm.2**	Ezh2	enhancer of zeste 2 polycomb repressive complex 2 subunit	0.296347700245088	7.57336096687907	0.828815063257153	0.436885465060168	0.999692817443324	-4.6046002982945	7.62041383652992	7.53865976460532	8.09645466834128	7.34043913197341	7.38952167693938	7.45467672288513	NA
**TC1900000650.mm.2**	Hif1an	hypoxia-inducible factor 1, alpha subunit inhibitor	-0.261927463227458	7.11552074714494	-0.828747398271101	0.436921088531442	0.999692817443324	-4.60460352795334	6.667638648491	6.98689438254337	7.12824274050948	7.12912552218069	7.56453327788267	7.21668991126241	NA
**TC0400003456.mm.2**	Rlf	rearranged L-myc fusion sequence	0.416225868966649	7.32219524517719	0.828703329648108	0.436944290386666	0.999692817443324	-4.60460563126605	8.04610437829239	8.07955998442285	7.8232822767809	6.4966971463597	7.06847977532262	6.41904790988469	NA
**TC0400002665.mm.2**	Abca1	ATP-binding cassette, sub-family A (ABC1), member 1	0.400969254413702	6.36681906786575	0.828690500589874	0.436951044972086	0.999692817443324	-4.6046062435598	7.36301034128654	7.53089353365971	6.31135236233465	5.74312215820319	6.25837542137982	4.99416059033062	NA
**TC0X00000627.mm.2**	1110012L19Rik	RIKEN cDNA 1110012L19 gene	0.318137184301717	6.61469616603138	0.828581865399494	0.437008245135585	0.999692817443324	-4.60461142816507	6.29130805249544	6.07376301328482	6.55069505911069	7.42899509039503	6.73396703797171	6.60944874293058	NA
**TC1100000891.mm.2**	Ctc1	CTS telomere maintenance complex component 1	0.403584331176533	6.65685119164629	0.828400538210218	0.437103732111196	0.999692817443324	-4.60462008104376	6.22281963472045	6.15232215601264	6.52297762281628	7.66358938793257	6.27591043756342	7.10348791083236	NA
**TC0100001412.mm.2**	Nphs2	nephrosis 2, podocin	0.279251635214948	5.57369847962665	0.82831789595328	0.437147256527559	0.999692817443324	-4.60462402431468	5.26286733784385	5.17787157465705	5.60373534671844	6.176242271126	5.48325094458164	5.7382234028329	NA
**TC1000003156.mm.2**	Ikzf4	IKAROS family zinc finger 4	0.298086060048741	8.06354275485049	0.828225733483474	0.437195798533596	0.999692817443324	-4.60462842155205	7.17308192933252	7.28252761475229	8.53552194287286	8.72538760968496	7.95948249440252	8.70525493805781	NA
**TC1400002142.mm.2**	Cryl1	crystallin, lambda 1	0.244788411979892	6.4548372356683	0.828196719085202	0.437211081228347	0.999692817443324	-4.60462980581793	6.00872953196144	5.96407869814056	6.85037747686892	6.77251852706488	6.45076183602179	6.68255734395223	NA
**TC1300002318.mm.2**	Sdha	succinate dehydrogenase complex, subunit A, flavoprotein (Fp)	-0.455180596751362	7.05423767749914	-0.827945464911573	0.437343439863614	0.999692817443324	-4.60464179179073	8.44069276653204	8.23229374284562	6.97682546169266	5.39421371936457	6.90406918219443	6.37733119236553	NA
**TC0400002075.mm.2**	C030017K20Rik	RIKEN cDNA C030017K20 gene	-0.334917364744858	6.39420871981287	-0.827914704672372	0.437359646080634	0.999692817443324	-4.60464325903854	5.7980302958205	5.84939835590923	6.00205486699512	7.02193960183379	6.84158004628681	6.85224915203181	NA
**TC1300000523.mm.2**	Gmpr	guanosine monophosphate reductase	-0.380108059799835	7.77401310200708	-0.827911784043159	0.437361184854103	0.999692817443324	-4.6046433983493	7.58923768927631	7.50846456435067	8.2965422549485	7.09653786445941	8.30175984937383	7.85153638963376	NA
**TC0300002629.mm.2**	Olfml3/LOC269472	olfactomedin-like 3/uncharacterized LOC269472	0.245194064976274	7.00169524446754	0.827911659469729	0.437361250487405	0.999692817443324	-4.6046434042913	7.67679785554344	7.83420733491183	6.66711077623694	6.82766642691432	6.56490897310529	6.43948010009342	NA
**TC1300000232.mm.2**	Vmn1r221	vomeronasal 1 receptor 221	-0.272132322004873	5.35879518331964	-0.827622853776974	0.437513430922572	0.999692817443324	-4.60465717848183	4.63953083064548	4.55308673747801	5.40812645011106	5.79981799378124	5.67125536451296	6.0809537233891	NA
**TC0200000909.mm.2**	March7	membrane-associated ring finger (C3HC4) 7	0.692959503470144	6.70792732242965	0.827308557016897	0.43767908653769	0.999692817443324	-4.60467216501736	7.39207233674117	7.48927907359101	7.69619559603855	5.67987016955446	6.98079101724474	5.00935574140798	NA
**TC0800001959.mm.2**	Chrna6	cholinergic receptor, nicotinic, alpha polypeptide 6	0.352198281277591	5.79158238368169	0.827221988780706	0.437724721746182	0.999692817443324	-4.60467629220368	5.24273827612552	5.03151515684055	6.02147251101607	6.56834620482357	5.46158476348308	6.42383738980137	NA
**TC1000001575.mm.2**	Rdh19	retinol dehydrogenase 19	0.413037622041537	5.87049453861934	0.827134493014367	0.437770849378538	0.999692817443324	-4.60468046333581	4.6616229488195	5.03384867520738	5.88517418247136	7.29161124341476	6.09063693448563	6.26007324731741	NA
**TC0400004175.mm.2**	Samd11	sterile alpha motif domain containing 11	-0.28653802331098	6.28297466201814	-0.82712815472634	0.437774191049595	0.999692817443324	-4.60468076548641	6.43929922735269	5.81199456836505	6.24167231840268	6.1950667464819	6.35825668629927	6.65155842520726	NA
**TC0300001485.mm.2**	2410004B18Rik	RIKEN cDNA 2410004B18 gene	0.33289347332398	7.96892382596819	0.827066969438542	0.43780645008667	0.999692817443324	-4.6046836821571	7.51594170750757	7.7866891403661	8.19238542318107	8.39596410411064	8.4374832590956	7.48507932154815	NA
**TC0800001258.mm.2**	Utp4	UTP4 small subunit processome component	0.271252044955014	7.5607768488912	0.826816331088832	0.437938613242734	0.999692817443324	-4.6046956285461	8.07585787866287	8.09488810863451	7.60484675695493	7.26336284102498	7.38950737906439	6.9361981290055	NA
**TC0500000462.mm.2**	C1qtnf7	C1q and tumor necrosis factor related protein 7	0.366307824120392	4.92221888730617	0.826779243008345	0.437958172447545	0.999692817443324	-4.60469739611427	4.74126173918328	4.28339182864085	4.83312885197171	5.78750885015515	4.65144807484328	5.23657397904278	NA
**TC1100000427.mm.2**	C1qtnf2	C1q and tumor necrosis factor related protein 2	-0.308281247714204	6.79386306609391	-0.826776366655194	0.43795968938103	0.999692817443324	-4.60469753319533	6.2177864518027	6.41885138067034	6.54943201033139	7.20555702399963	6.93307048650723	7.4384810432522	NA
**TC0500001818.mm.2**	Pomp	proteasome maturation protein	0.304843003647681	7.96579322613689	0.826646506272307	0.438028179181414	0.999692817443324	-4.60470372176217	8.76779808748295	8.56436918889259	7.37984457162128	8.15629447224929	7.35955087293901	7.5669021636362	NA
**TC1300002047.mm.2**	Hk3	hexokinase 3	0.306552659311081	6.61672398454198	0.826479597303608	0.438116220094304	0.999692817443324	-4.6047116750051	6.08633115044935	6.60517588389985	6.65582300608443	7.15514808967799	6.81636953468827	6.38149624245199	NA
**TC1700001764.mm.2**	Clps	colipase, pancreatic	-0.26461714442978	5.26472875583665	-0.826441766611432	0.438136176739368	0.999692817443324	-4.60471347750465	4.87149083736115	5.15007713432255	5.10875162071688	5.41003351652143	5.37536104729023	5.67265837880765	NA
**TC0700003547.mm.2**	Mrpl46	mitochondrial ribosomal protein L46	0.271709905510823	6.13325608134254	0.826226262805457	0.438249872868067	0.999692817443324	-4.60472374451759	6.64170994481688	6.16795782358615	6.29305485381502	5.97358941152191	5.88723002382346	5.83599443049182	NA
**TC0900002698.mm.2**	Mlip	muscular LMNA-interacting protein	-0.745241082403377	7.50106414761699	-0.826224232706902	0.438250944013959	0.999692817443324	-4.60472384122738	7.52187593266835	7.84906019058842	8.46064742148495	5.61183587733426	8.3244237327858	7.23854173084016	NA
**TC1600001528.mm.2**	Kalrn	kalirin, RhoGEF kinase	-0.688405057969839	6.95834987608001	-0.826170862109805	0.43827910474658	0.999692817443324	-4.60472638364141	7.66326450470723	8.118035327002	7.09854741329788	5.19744724111769	7.84224747707666	5.83055729327858	NA

**TC0900001725.mm.2**	Cfap300	cilia and flagella associated protein 300	-0.804307903503953	7.1481893129872	-0.826122449710259	0.438304650432669	0.999692817443324	-4.60472868977285	7.11295234679777	7.22207133197576	8.39940953037828	5.07333866569259	7.65759781377435	7.42376618930443	NA
**TC1200000786.mm.2**	Dcaf4	DDB1 and CUL4 associated factor 4	-0.299843140913174	7.12611027579653	-0.826097823477888	0.438317645323124	0.999692817443324	-4.60472986281437	8.05508213070915	7.7548414979683	6.92476119007291	6.24876468206478	6.7404195572359	7.03279259672814	NA
**TC1200001108.mm.2**	Cyp46a1	cytochrome P450, family 46, subfamily a, polypeptide 1	-0.24773163207042	8.1017510292444	-0.825938541121036	0.438401702884226	0.999692817443324	-4.6047374495121	8.76825525004586	8.81096517149874	7.77394251559164	7.49396872929884	7.94304208353875	7.82033242549257	NA
**TC0400001925.mm.2**	Mad2l2	MAD2 mitotic arrest deficient-like 2	0.401341556540503	7.54638909074849	0.825834379651272	0.438456677926471	0.999692817443324	-4.60474241027859	7.29172178110697	7.22640222492188	7.44279761753758	8.33864920823398	6.94878279964174	8.02998091304881	NA
**TC1700000567.mm.2**	Cyp4f15	cytochrome P450, family 4, subfamily f, polypeptide 15	0.353998390521489	5.53832018734174	0.825660006406592	0.438548720870741	0.999692817443324	-4.60475071405426	6.24985303476899	6.59280284205416	5.33172432881278	5.21590668532236	4.37866079249561	5.46097344059655	NA
**TC0300000419.mm.2**	Dclk1	doublecortin-like kinase 1	-0.480597955746759	9.99172118419036	-0.825602254919232	0.438579208054514	0.999692817443324	-4.60475346397798	10.7482454717676	10.7991682788826	10.2055365801531	8.51532214134604	10.1715643635671	9.51049026942565	NA
**TC0900000207.mm.2**	Ccdc159	coiled-coil domain containing 159	-0.332153390582865	5.92043741856731	-0.825185607252229	0.438799202612257	0.999692817443324	-4.60477329970592	6.07983611930202	6.41615698882809	5.19578986344893	5.98537244760508	5.65558071562508	6.18988837659465	NA
**TC0700000476.mm.2**	Zfp607a	zinc finger protein 607A	0.254101632742968	6.90164393689339	0.824844174549415	0.4389795417688	0.999692817443324	-4.60478954992116	7.40477856432101	7.42826699325005	6.81252033467582	6.72999032996178	6.71170779168427	6.32259960746738	NA
**TC1500001544.mm.2**	Sntb1	syntrophin, basic 1	-0.253350302454722	6.25134713899746	-0.824440065694924	0.439193054030263	0.999692817443324	-4.60480877770066	6.07003808494484	5.7797003064826	6.25391774174968	6.32190417707426	6.41493586844033	6.66758665529306	NA
**TC1400000127.mm.2**	Nglv1	N-glycanase 1	0.313046183810466	5.36490935229067	0.824281087387379	0.439277071128423	0.999692817443324	-4.60481634037652	5.98024428754818	5.43238497875042	5.16843789795658	5.53302170957661	5.33775887225658	4.73760836765568	NA
**TC0200004833.mm.2**	3300002I08Rik	RIKEN cDNA 3300002I08 gene	0.296401527766914	6.46907389232589	0.824232485724851	0.439302758505727	0.999692817443324	-4.6048186521985	6.82099094135176	6.63069622652238	6.12846186957326	6.84931775123426	6.29923770527473	6.08573885999897	NA
**TC0200003751.mm.2**	Ola1	Obg-like ATPase 1	-0.415923662520483	7.26745325201983	-0.82418357414122	0.439328610771001	0.999692817443324	-4.60482097867592	8.07175349958334	7.75430839151546	7.38856735303693	6.08483779495269	7.47400925911677	6.83124321391382	NA
**TC0100001550.mm.2**	Aldh9a1	aldehyde dehydrogenase 9, subfamily A1	0.306853604006519	6.59506404703915	0.82409313419417	0.439376415762248	0.999692817443324	-4.60482528021969	6.96088350523024	7.07229182963722	6.17402272880528	6.90143534888497	6.01882700636701	6.4429238633102	NA
**TC0500001888.mm.2**	Ankib1	ankyrin repeat and IBR domain containing 1	0.5028973988899	6.72938394606916	0.82365723927145	0.439606874502424	0.999692817443324	-4.60484600828042	7.68605690720938	7.73755057077507	7.45437390682503	5.52487159128012	6.02912677556346	5.94432392476189	NA
**TC1600001368.mm.2**	Ehhadh	enoyl-Coenzyme A, hydratase/3-hydroxyacyl Coenzyme A dehydrogenase	0.285642121478198	5.18425226378187	0.823294998372644	0.439798457986347	0.999692817443324	-4.60486322863284	5.30307284556305	5.28996835146498	5.4104326972824	5.13144561702741	4.66577280173375	5.30482126961966	NA
**TC0700001827.mm.2**	Cdipt	CDP-diacylglycerol--inositol 3-phosphatidyltransferase (phosphatidylinositol synthase)	-0.430076565176696	8.89461086121997	-0.823189121692683	0.439854465769781	0.999692817443324	-4.60486826094075	9.58085916440966	9.7704116229886	9.02758168125692	7.55053894352717	8.9595603966178	8.47871335851968	NA
**TC0900003326.mm.2**	Ccpg1	cell cycle progression 1	0.258554653895557	7.35611152191499	0.822949500233701	0.439981242146815	0.999692817443324	-4.6048796486206	7.85773584706205	7.84299427957044	7.12115389102862	7.35537026529566	7.17507467771331	6.78434017081986	NA
**TC0900001022.mm.2**	Hmgcll1	3-hydroxymethyl-3-methylglutaryl-Coenzyme A lyase-like 1	-0.59492840939991	6.46799033004837	-0.822870206729014	0.440023199668022	0.999692817443324	-4.60488341647573	6.59846278688561	6.67878949491671	7.17640494892354	4.99401149092049	7.15230811600085	6.207965142643	NA
**TC1300000470.mm.2**	Gm5082	predicted gene 5082	0.42660246791609	5.90168819211878	0.822675902737864	0.440126026164177	0.999692817443324	-4.60489264841316	5.01348829910911	5.38498894738738	5.71489974243696	7.21752867858724	6.05299775689546	6.02622572829656	NA
**TC0700004586.mm.2**	Phlda2	pleckstrin homology like domain, family A, member 2	0.398878163610969	4.95251358539819	0.822598600050939	0.440166939852624	0.999692817443324	-4.60489632090286	4.55330391808632	3.98578118862911	4.87617727097637	6.11069909547144	4.92415109268233	5.26496894654355	NA
**TC0400001347.mm.2**	Scmh1	sex comb on midleg homolog 1	0.259698485384336	7.03931261206103	0.822437818836493	0.440252044604598	0.999692817443324	-4.60490395858751	6.9650586356357	7.17918485196915	7.34620413673608	6.95931044102893	6.97221545111279	6.81390215588354	NA
**TC1400002870.mm.2**	Setdb2/Phf11c	SET domain, bifurcated 2/PHD finger protein 11C	-0.823652145661969	5.2848205229948	-0.822414357567371	0.44026446411313	0.999692817443324	-4.60490507300337	5.96584468054982	6.31760600068033	5.43122158793082	3.45786249477652	6.66644841156361	3.86993996246767	NA
**TC1700001388.mm.2**	Dynlt1f/Dynlt1c	dynein light chain Tctex-type 1F/dynein light chain Tctex-type 1C	0.714592055779356	9.55075266743145	0.822409109917729	0.440267242054664	0.999692817443324	-4.60490532226524	9.16852549600029	7.8410001764633	11.3737025243283	9.4883846975136	10.6290943546015	8.8038087556817	NA
**TC0600000277.mm.2**	Mest	mesoderm specific transcript	0.374283907420502	8.51982351100715	0.822336509251089	0.440305675862393	0.999692817443324	-4.60490877067345	9.55606265755131	9.52086792441398	8.70768156940095	7.68760758005835	8.19778274384455	7.44893859077374	NA
**TC0800000098.mm.2**	Tex29	testis expressed 29	-0.45201875363466	6.65882160403426	-0.822262595943712	0.440344807028963	0.999692817443324	-4.60491228123287	5.35523067287297	5.2852705660946	6.32968149310862	7.8745139458757	7.22491470030068	7.88331824595295	NA
**TC0400001010.mm.2**	Pgm2	phosphoglucomutase 2	0.451183421488175	7.07629502048091	0.822169659941799	0.440394012715242	0.999692817443324	-4.60491669500512	7.94663898706591	7.90015843613349	7.71500445840966	6.04166531292153	6.37920318339917	6.47509974495567	NA
**TC0900003323.mm.2**	C1qtnf5/Mfrp	C1q and tumor necrosis factor related protein 5/membrane frizzled-related protein	-0.278329862565038	6.93955015015467	-0.822115200041465	0.440422848758295	0.999692817443324	-4.60491928130125	6.89822865000867	7.40956565873379	6.58137083278228	6.80505260074546	6.81274844275785	7.13033471589997	NA
**TC1100002641.mm.2**	Irgm1	immunity-related GTPase family M member 1	0.398307123931107	5.92419051062011	0.821840566091254	0.440568285612643	0.999692817443324	-4.60493232199968	6.19280597740854	6.62352224034359	5.37412300145787	6.38859154545749	5.91448172909685	5.05161856995629	NA
**TC1900001104.mm.2**	Sdhaf2	succinate dehydrogenase complex assembly factor 2	0.288082124234846	7.37961059433618	0.82179128231795	0.440594388265675	0.999692817443324	-4.60493466189616	8.16625837986671	7.86228183360133	7.1792576516088	7.23338614890056	7.24358099299529	6.59289855904438	NA
**TC0800001632.mm.2**	Ctxn1	cortexin 1	-0.410412307828121	10.3531021322511	-0.821787516911268	0.440596382620772	0.999692817443324	-4.60493484066661	10.5576844664361	10.7201839055853	10.6103657367216	9.3995941661928	10.8196358433727	10.0111486751979	NA
**TC1300000838.mm.2**	Fastkd3	FAST kinase domains 3	0.266887747764864	5.64843807449905	0.821652919551263	0.440667676616896	0.999692817443324	-4.60494123061499	5.78854367760022	5.84834606224576	5.67760347363478	5.71615362770423	5.16068141619604	5.69930018961324	NA
**TC0300001142.mm.2**	Psrc1	proline/serine-rich coiled-coil 1	0.26935240571317	6.56620713648594	0.821218319721204	0.440897933255155	0.999692817443324	-4.60496185853786	6.11390177292569	6.44240457575615	6.61541584046221	7.07440480036786	6.6039713295679	6.54714449983583	NA
**TC1100003780.mm.2**	Kat2a	K(lysine) acetyltransferase 2A	-0.351165552652438	7.24146186315282	-0.821013649907909	0.441006399756019	0.999692817443324	-4.60497157063747	7.58823711204914	7.80484380320373	7.39404278773501	6.28263679144457	7.23695874843097	7.14205193605349	NA
**TC0900000204.mm.2**	Ldlr	low density lipoprotein receptor	0.276332307841201	6.92561028052575	0.820868483966214	0.441083343232282	0.999692817443324	-4.60497845820214	6.74789430419052	6.51079219551295	6.97941178902937	7.44440811053734	7.11111567358037	6.76003961030394	NA
**TC0500001768.mm.2**	Zkscan5	zinc finger with KRAB and SCAN domains 5	-0.324190843993049	6.74221238298956	-0.820841863102954	0.441097454344	0.999692817443324	-4.60497972117614	6.71001454870124	6.80383606370875	6.55987303832	6.58564796045063	6.41349401368165	7.38040867307507	NA
**TC0400001180.mm.2**	Agbl4	ATP/GTP binding protein-like 4	-0.371649355298104	6.76533967879298	-0.82078272882736	0.441128801227047	0.999692817443324	-4.60498252659195	7.12794612897463	7.32356384610806	6.579129995408	6.11948469813149	7.29662940327085	6.14528400086485	NA
**TC0800000783.mm.2**	Use1	unconventional SNARE in the ER 1 homolog (S. cerevisiae)	-0.25095284745871	8.08672644230704	-0.820774745490493	0.441133033289324	0.999692817443324	-4.60498290532326	8.97967553489572	8.81035535901943	7.66781448612144	7.44639654638339	7.68271417929554	7.93340254812671	NA
**TC1100002142.mm.2**	Ccdc117	coiled-coil domain containing 117	0.284278434927052	6.35263632949459	0.820518883350346	0.4412686842284	0.999692817443324	-4.60499504224951	6.42718796787436	6.39967529260782	6.25801975526156	6.67073603790818	6.45891104384908	5.90128787946655	NA
**TC1200000769.mm.2**	Sipa1l1	signal-induced proliferation-associated 1 like 1	0.602736855671004	7.63516517719064	0.820482646097488	0.441287898613207	0.999692817443324	-4.60499676098542	9.1839409070711	9.35294064750773	8.11016696573567	6.12962464421783	7.2113888694009	5.8229290292106	NA
**TC0600001586.mm.2**	Bcl2l14	BCL2-like 14 (apoptosis facilitator)	-0.286421106005385	6.31085295609265	-0.820435361827247	0.441312971449992	0.999692817443324	-4.60499900361021	5.78987534563599	6.11689448771388	6.65783587222164	6.03491697337599	6.58096861171363	6.68462644589475	NA
**TC0500002065.mm.2**	Tomm7	translocase of outer mitochondrial membrane 7	0.286970238394591	8.45122745696922	0.820187064010686	0.441444649917399	0.999692817443324	-4.60501077867809	8.86188188978367	8.7420714180955	8.31121925445258	8.5274567009101	8.46836266753507	7.79637281103842	NA
**TC1700000424.mm.2**	Kifc5b	kinesin family member C5B	-0.478102940632508	6.5731177871088	-0.820162266020185	0.441457802445388	0.999692817443324	-4.6050119545536	5.72837907697085	6.3465567808473	5.80840858497956	7.39537390680524	6.76057823978843	7.39941013326139	NA
**TC0400004195.mm.2**	Iffo2	intermediate filament family orphan 2	-0.253171303493933	7.57350833588257	-0.820041632065372	0.441521789100021	0.999692817443324	-4.60501767447548	7.37628134158217	7.36057353417552	7.7398232894284	7.35910297684653	7.73392199712238	7.87134687614042	NA
**TC0900002676.mm.2**	Mapk6	mitogen-activated protein kinase 6	-1.06122582904495	5.71843260062185	-0.819854159700317	0.441621241321992	0.999692817443324	-4.60502652652043	6.46979117924926	6.54944997300927	6.92988163951349	2.65456975717784	6.59553125611798	5.11137179866326	NA
**TC0200005508.mm.2**	Nanp	N-acetylneuraminic acid phosphatase	-0.270859404618236	6.35642864873458	-0.819789340197755	0.441655631143052	0.999692817443324	-4.60502963530742	6.73315547754017	6.16374103603365	6.24069297488494	6.10928530991365	6.45927386631015	6.43242322772491	NA
**TC0200003109.mm.2**	Hnmt	histamine N-methyltransferase	-0.316255069820354	6.65540472147273	-0.819769493229234	0.441666161283679	0.999692817443324	-4.60503057612791	8.24463105474599	7.85382438559432	5.75751365585828	5.8432177185694	6.53264314652587	5.70059836754252	NA
**TC1500000208.mm.2**	Atpsckmt	ATP synthase C subunit lysine N-methyltransferase	-0.242934459907356	5.87706783967673	-0.81962486186034	0.441742903284299	0.999692817443324	-4.6050374317607	5.76387270594233	5.68984854271045	5.74878122400874	5.91262721078771	5.97907028704965	6.16820706756151	NA
**TC0700001325.mm.2**	Fam181b	family with sequence similarity 181, member B	0.266387988925897	6.51709473794209	0.819502595619472	0.441807785680052	0.999692817443324	-4.60504322667362	6.70988627178223	6.5024569515669	6.61935435071641	6.59214624036119	6.08765212046913	6.59107249275668	NA
**TC1300000536.mm.2**	Fam8a1	family with sequence similarity 8, member A1	-0.70610682339844	8.46645808205512	-0.819290676221441	0.44192025996664	0.999692817443324	-4.60505326947973	9.1954967602104	9.17442661358016	9.1618032690366	6.34650246683504	8.83613527786345	8.08438410480508	NA
**TC0500002508.mm.2**	Apbb2	amyloid beta (A4) precursor protein-binding, family B, member 2	0.532296303784664	9.24448302605728	0.819104926095757	0.442018861955601	0.999692817443324	-4.60506207077986	10.0235496086715	9.92090844890098	9.89499169300949	8.39852466016077	9.34242287120963	7.8865008743913	NA
**TC0200005467.mm.2**	Phyhd1	phytanoyl-CoA dioxygenase domain containing 1	-0.359076185797567	6.73802947301424	-0.818950147659474	0.442101035191521	0.999692817443324	-4.60506940360059	6.40122449373401	6.1911171145508	6.19506921701557	7.36377221208717	7.12477748869908	7.15221631199879	NA
**TC0400000996.mm.2**	Atg4c	autophagy related 4C, cysteine peptidase	-0.541789087805924	6.42165936801971	-0.818643718020384	0.442263753538067	0.999692817443324	-4.60508391849912	7.39748959837503	7.47218398835073	6.50432202791811	4.78403019497222	6.81969796351204	5.55223243499013	NA
**TC0500000833.mm.2**	Gm9958	predicted gene 9958	0.253945892284489	5.22506272320121	0.818643057261057	0.442264104456503	0.999692817443324	-4.60508394979413	5.27512191929224	4.89790544231088	5.43685381038223	5.40576657070432	5.25757535328967	5.0771532432279	NA
**TC0500000896.mm.2**	Ccng2	cyclin G2	-0.349635849932854	9.77642892938019	-0.8185854045011	0.442294723656078	0.999692817443324	-4.60508668029361	8.6838386644088	9.00999138692269	10.5685937111218	9.56414220142022	10.6217067263755	10.2103008860321	NA
**TC0300002317.mm.2**	Lamtor2	late endosomal/lysosomal adaptor, MAPK and MTOR activator 2	-0.25925613778085	7.83110306048046	-0.818566475091259	0.442304777335906	0.999692817443324	-4.60508757678439	8.30525028203858	8.19844694564661	7.55500310775389	7.42720132206405	7.97430697650347	7.52640972887617	NA
**TC0500000800.mm.2**	Odam	odontogenic, ameloblast asssociated	0.278597955698174	6.2590802965742	0.818465057281859	0.442358644558897	0.999692817443324	-4.60509237967902	6.00384584597279	6.21622679024626	6.2297213213179	6.71608120599336	6.0027614508845	6.3858451650304	NA
**TC0800000627.mm.2**	Scrg1	scrapie responsive gene 1	-0.24617644319541	8.02179834543867	-0.818353297767948	0.442418010111437	0.999692817443324	-4.60509767189467	8.26315820109755	8.16976776699543	7.95266015672937	7.65009544234474	8.06875104285943	8.0263574426055	NA
**TC1100000156.mm.2**	Vstm2a	V-set and transmembrane domain containing 2A	0.331788639674141	9.86883236636574	0.81834280132591	0.442423586009924	0.999692817443324	-4.60509816891551	10.7853115199825	10.4183186774633	10.0767053134736	9.25976532657491	9.58659970743879	9.08629365326139	NA
**TC0300002989.mm.2**	Cisd2	CDGSH iron sulfur domain 2	0.377427419775499	7.35667520178059	0.818333459118264	0.442428548800536	0.999692817443324	-4.60509861127839	6.83979346225752	6.82157722638897	7.27188542684626	8.34488225394776	7.06956453169256	7.79234830955047	NA
**TC0800001256.mm.2**	Tango6	transport and golgi organization 6	0.335412608515664	5.70923764239141	0.818183864017068	0.442508022487925	0.999692817443324	-4.60510569432048	5.86011234562728	5.69508748382671	5.51107149258091	6.17445412838199	5.10974894828474	5.90495145564682	NA
**TC0200003241.mm.2**	Cfap77	cilia and flagella associated protein 77	-0.358047435307272	6.98875765032498	-0.818068792310766	0.442569162251768	0.999692817443324	-4.60511114218778	5.85332691011767	5.75122974610335	6.93289837312776	7.8730488144294	7.40478703488047	8.11725502329124	NA
**TC0300001490.mm.2**	Lpar3	lysophosphatidic acid receptor 3	0.289903689472243	5.04195647571358	0.818004773628779	0.442603179190599	0.999692817443324	-4.60511417282943	4.94938038211172	4.41333338244818	5.13700180156815	5.59741443276488	4.96079711847818	5.19381173691037	NA
**TC1700000060.mm.2**	Dynlt1b	dynein light chain Tctex-type 1B	0.644384102218766	8.94173843096332	0.817846653961826	0.442687205577023	0.999692817443324	-4.60512165756384	8.37450679787861	7.18067963625657	10.8332985653172	8.85870761272394	9.83372244103181	8.5695155325718	NA
**TC1900000660.mm.2**	Sema4g	sema domain, immunoglobulin domain (Ig), transmembrane domain (TM) and short cytoplasmic domain, (semaphorin) 4G	-0.253008369654132	5.82463223646168	-0.817785868728541	0.442719510489898	0.999692817443324	-4.60512453464273	6.03986318244214	5.68979677435757	5.71326710504086	5.6427912562902	5.82886974693468	6.03320535370465	NA
**TC0200001362.mm.2**	Celf1	CUGBP, Elav-like family member 1	0.246194642454086	8.67926528127559	0.817606596395689	0.442814796331279	0.999692817443324	-4.60513301914871	8.81537797660309	8.59302100479857	8.67612745374983	8.9036635418302	8.51564591524508	8.57175579542677	NA
**TC1500002192.mm.2**	Kmt2d	lysine (K)-specific methyltransferase 2D	0.329278305922755	6.853761282414	0.817356341851608	0.44294783469466	0.999692817443324	-4.60514486109154	7.3722115549104	7.45062626675742	6.56640477125718	6.91273847107365	5.93609839371979	6.88448823676552	NA
**TC0800001437.mm.2**	Gse1	genetic suppressor element 1, coiled-coil protein	0.25137494568876	6.00757233327908	0.817329096089028	0.442962320593498	0.999692817443324	-4.60514615021119	5.97167962667288	5.93507910446105	6.00211029185128	6.31860228810775	6.0243872744312	5.79357541415032	NA
**TC1700000550.mm.2**	Rrp1b	ribosomal RNA processing 1 homolog B (S. cerevisiae)	0.277174695227358	7.05875747839867	0.817210322489618	0.443025473526457	0.999692817443324	-4.60515176960642	7.09493665177852	6.94293605958892	7.52587227423976	6.9086385004999	6.88162339505291	6.99853798923204	NA
**TC1900000101.mm.2**	Naaladl1	N-acetylated alpha-linked acidic dipeptidase-like 1	-0.364443842903557	4.62955780511567	-0.817167724544126	0.443048124779792	0.999692817443324	-4.60515378486662	3.97441634739726	3.96235198996333	4.28563283600847	5.27021256775469	4.77945474363807	5.50527834593221	NA
**TC1700000367.mm.2**	Tsr3	TSR3 20S rRNA accumulation	-0.257311591143792	7.60180694610508	-0.817130159889752	0.443068100289573	0.999692817443324	-4.60515556195228	7.56974185820456	7.12473581724971	7.66255336392706	7.53831704551727	7.85333124293864	7.86216234879327	NA
**TC0200000416.mm.2**	Fut7	fucosyltransferase 7	0.315401844506332	6.67287716072792	0.8170995560937	0.443084374743873	0.999692817443324	-4.60515700969963	5.80170664239212	6.26719727111428	6.76772100395808	7.53186036597881	6.71827378402339	6.95050389690083	NA
**TC1000001789.mm.2**	Vta1	vesicle (multivesicular body) trafficking 1	0.245298391200977	7.4274409928538	0.816832031997882	0.443226656563693	0.999692817443324	-4.60516966376662	7.8431437766985	7.55946313478547	7.47238003656683	7.35393787745357	7.19778473721206	7.13793639440639	NA
**TC1600002178.mm.2**	Litaf	LPS-induced TN factor	-0.262980229766272	6.8233855861925	-0.816552321489836	0.443375454498482	0.999692817443324	-4.60518289144771	6.8027594757924	6.72931301381031	6.5887877084639	6.85235257554597	6.76141352711316	7.20568721642926	NA
**TC0700002163.mm.2**	Ptprh	protein tyrosine phosphatase, receptor type, H	0.408038252298107	5.36430275976976	0.816332453317719	0.443492443013752	0.999692817443324	-4.60519328712985	4.66957065216089	4.65095903351517	5.43821079163613	6.40247089713322	4.95971865701916	6.06488652715398	NA
**TC1600002142.mm.2**	Itgb2l	integrin beta 2-like	0.492482289020184	4.69519206891897	0.815703238823193	0.443827359982566	0.999692817443324	-4.60522302746952	3.65689551311833	4.07577986808674	4.68618813525829	6.02553266991627	4.21973890143594	5.50701732569826	NA
**TC1300001561.mm.2**	Hist1h3i	histone cluster 1, H3i	-0.243226086246128	9.3872148236722	-0.815633026879974	0.443864743415072	0.999692817443324	-4.60522634519127	9.40960317757973	9.44207735000706	9.36004564162522	9.13253247935184	9.40420872860808	9.57482156486124	NA
**TC1800000741.mm.2**	Tcf4	transcription factor 4	-0.42280585081482	9.72254745318717	-0.815611281891068	0.443876321705204	0.999692817443324	-4.60522737266944	10.548284457364	10.7895750666341	9.64945086182214	8.42645588492551	9.9191550362586	9.00236341211869	NA
**TC1100003518.mm.2**	Ankfn1	ankyrin-repeat and fibronectin type III domain containing 1	0.370316510017449	5.65070535240332	0.815398679845599	0.443989534662997	0.999692817443324	-4.60523741746731	5.64278371876684	5.84526983588136	5.22559713729603	6.35280865260729	5.15462415665963	5.68314861320878	NA
**TC0700004358.mm.2**	Cox6a2	cytochrome c oxidase subunit 6A2	-0.300717691149455	7.28473460276469	-0.815382863982742	0.443997957607614	0.999692817443324	-4.60523816465206	7.11825966024427	7.01842502937841	6.8409717693938	7.64417200293947	7.63340759828536	7.45317155634681	NA
**TC1900001619.mm.2**	Pcgf6	polycomb group ring finger 6	-0.769800971825696	7.00597652756716	-0.815238491119692	0.444074850522586	0.999692817443324	-4.60524498479632	6.86715926908422	6.98777142239913	8.11585073904795	5.20481252608614	8.1224465942332	6.73781861455228	NA
**TC1400000762.mm.2**	Il25	interleukin 25	-0.412375100573349	5.79924417968417	-0.815019581169246	0.444191459935302	0.999692817443324	-4.60525532459254	4.80634258913575	5.29260606672711	5.5225342333156	6.41334887723213	5.83241425037384	6.9282190613206	NA
**TC0700003031.mm.2**	Abcc8	ATP-binding cassette, sub-family C (CFTR/MRP), member 8	-0.290755963533718	6.04460178859654	-0.814967461149828	0.444219226548667	0.999692817443324	-4.60525778612255	6.28029459886104	6.13562060956885	5.87192346419036	5.76316833385059	5.74002535473993	6.47657837036846	NA

**TC0X00000053.mm.2**	Dgkk	diacylglycerol kinase kappa	0.480094287645195	6.38340295621606	0.814734929050924	0.444343121614872	0.999692817443324	-4.60526876695439	8.2481388619484	6.49463258367671	6.39231504501607	5.86660238846474	5.76441146685917	5.53431739133124	NA
**TC0900001365.mm.2**	Cish	cytokine inducible SH2-containing protein	0.307794064710571	5.6947007683509	0.814732630838656	0.444344346244482	0.999692817443324	-4.60526887547261	5.02713307117414	5.30691549802609	5.80082361281416	6.42404847234899	5.52754924885949	6.08173470688251	NA
**TC0400002132.mm.2**	Plag1	pleiomorphic adenoma gene 1	-0.270266355833583	5.17553035440605	-0.814647592452532	0.444389661643672	0.999692817443324	-4.60527289072386	4.96123963923149	5.17135337333144	5.25165114264794	4.93837705845517	5.13533850602716	5.59522240674312	NA
**TC0400000064.mm.2**	Clvs1	clavesin 1	-0.247499773162834	7.75144362100403	-0.814489637636902	0.444473841599558	0.999692817443324	-4.6052803481572	7.90548552672912	7.90953469623377	7.37340054899081	7.72592042937699	7.81659880706357	7.7777217176299	NA
**TC0500001667.mm.2**	Dnaaf5	dynein, axonemal assembly factor 5	-0.341921180326236	5.86759429371356	-0.814029165988241	0.444719308691134	0.999692817443324	-4.60530208289827	5.37422462559118	5.86449812813535	5.72134180287114	5.92015852108005	5.68115150653905	6.64419117806461	NA
**TC0400003602.mm.2**	Lck/Mir8119	lymphocyte protein tyrosine kinase/microRNA 8119	-0.361522075283919	5.57083867172995	-0.813967469060469	0.444752205261309	0.999692817443324	-4.60530499446306	5.25977407344147	5.19882591458427	5.00130608064682	6.12038786524623	5.68823020747573	6.15650788898517	NA
**TC0800002153.mm.2**	Ankrd37	ankyrin repeat domain 37	-0.41643162731161	6.58848822039228	-0.813797026197805	0.444843093750253	0.999692817443324	-4.60531303717112	6.65388949159257	6.61389155404021	7.11326824042201	5.60187427062684	6.70316461251272	6.84484115315935	NA
**TC1000001911.mm.2**	Tmem200a	transmembrane protein 200A	0.245989309080191	6.34279252893944	0.813719202226406	0.444884597698705	0.999692817443324	-4.60531670910361	6.66530186624109	6.36140957239784	6.30948305243767	6.45152812414136	6.15065674414939	6.11837581426926	NA
**TC1000001260.mm.2**	Lin7a	lin-7 homolog A (C. elegans)	0.671822447991107	7.75666231212999	0.813707015282779	0.444891097311582	0.999692817443324	-4.60531728409428	8.88842411694079	8.68481236094429	8.72011441494983	6.4350767304887	7.68720627984178	6.12433996961453	NA
**TC0900002922.mm.2**	Armc8	armadillo repeat containing 8	0.484804294361193	8.06294460254328	0.813394957396104	0.445057548870893	0.999692817443324	-4.60533200539005	9.07469956685571	8.86341162188434	8.40212933464808	7.30245317297294	8.11802735177599	6.61694656712264	NA
**TC0600000374.mm.2**	Ttc26	tetratricopeptide repeat domain 26	-0.319991514679499	5.35851876708407	-0.813347138266925	0.445083059488279	0.999692817443324	-4.60533426093421	6.0409024837194	5.8187422799883	5.23204523947493	4.59369716524393	4.88931588477725	5.5764095493006	NA
**TC0700002705.mm.2**	Nfkbib	nuclear factor of kappa light polypeptide gene enhancer in B cells inhibitor, beta	0.273883646387308	8.28923103635249	0.813231107634095	0.445144963997665	0.999692817443324	-4.60533973354364	8.33255153695361	8.14759154854148	8.72922316879684	8.17228204390038	8.06219254196199	8.29154537796062	NA
**TC0700004188.mm.2**	Thumpd1	THUMP domain containing 1	0.428547229721633	5.7765304510088	0.813172341349446	0.445176319242879	0.999692817443324	-4.60534250507811	6.91192541884905	6.59967252054317	5.98551665526718	5.01682295778475	5.67974903215534	4.46549612145332	NA
**TC0200004691.mm.2**	Tmx4	thioredoxin-related transmembrane protein 4	-0.338277895062404	8.76810358894627	-0.813102012659076	0.445213845770591	0.999692817443324	-4.60534582175075	8.99033964232246	8.68999029548908	9.08037717589034	8.04549072698028	8.87332449495431	8.92909919804112	NA
**TC0900001398.mm.2**	Nicn1	nicolin 1	-0.521840327918662	8.71814296497304	-0.813093061597031	0.445218622108958	0.999692817443324	-4.6053462438661	9.48718741806003	9.40177774864325	8.93435495020075	7.25375103344808	9.20837260324172	8.02341403624444	NA
**TC0800000509.mm.2**	Fat1	FAT atypical cadherin 1	0.26638297505654	6.69825599350705	0.813066798195996	0.445232636621548	0.999692817443324	-4.6053474823819	7.66636193109428	7.54779304867691	6.14662115373018	6.60745231196192	6.26257692042816	5.95873059515086	NA
**TC0900002113.mm.2**	Sorl1	sortilin-related receptor, LDLR class A repeats-containing	-0.380968611314663	7.77864816762651	-0.812895060900677	0.445324285737271	0.999692817443324	-4.6053555804513	9.11753303517688	9.09500231339702	7.56304746613815	6.28566075113979	7.48618630880077	7.12445913110649	NA
**TC1500000939.mm.2**	Ano6	anoctamin 6	-0.313812553351116	6.42456828078987	-0.812646926956356	0.445456728276174	0.999692817443324	-4.6053672789879	6.83201064005518	6.94161309468222	6.49058253695221	5.58249788469759	6.28058060018831	6.42012492816372	NA
**TC1700000143.mm.2**	Tcp1	t-complex protein 1	0.494371788015059	7.03141539205902	0.812167123505065	0.445712904774202	0.999692817443324	-4.60538989336589	7.87192200353463	7.77943661117265	7.63090780016232	6.13203085667615	6.98886132355083	5.78533375725752	NA
**TC0800001423.mm.2**	Adad2	adenosine deaminase domain containing 2	0.329255224530227	6.24184664445218	0.811680126089224	0.44597302927727	0.999692817443324	-4.60541283809694	5.75737363142949	5.62815056983571	6.19283027919828	7.16920277805589	6.20638524133869	6.49713736685503	NA
**TC1600001019.mm.2**	Slc5a3/Mrps6	solute carrier family 5 (inositol transporters), member 3/mitochondrial ribosomal protein S6	0.25097384977385	7.54134065307209	0.81157210623329	0.446030741544076	0.999692817443324	-4.60541792622797	7.94766503656295	8.19814145033906	7.51782450811701	7.28426805742209	7.2628558612154	7.03728900477601	NA
**TC1100003770.mm.2**	P3h4	prolyl 3-hydroxylase family member 4 (non-enzymatic)	-0.284800087412091	6.42704334349764	-0.811555389203952	0.44603967350234	0.999692817443324	-4.60541871362267	6.46037850614383	6.55013696306163	5.92070467535198	6.57036753312611	6.37867470000926	6.68199768329302	NA
**TC1900000926.mm.2**	Rce1	Ras converting CAAX endopeptidase 1	0.342422680471857	5.70268281897204	0.811457476175026	0.446091991274149	0.999692817443324	-4.60542332525075	6.03202519468551	6.13921599787654	5.65825072191388	5.70659981919308	5.89105230932229	4.78895287084096	NA
**TC0700003548.mm.2**	Det1	de-etiolated homolog 1 (Arabidopsis)	-0.242936702295253	6.02477684010835	-0.811428157667185	0.446107657851104	0.999692817443324	-4.60542470606069	5.6283396037313	5.71955122847926	6.0052475024743	6.15220089945022	6.19802145709397	6.44530034942106	NA
**TC1000003220.mm.2**	Nt5dc3	5'-nucleotidase domain containing 3	-0.279450314486793	7.40332638328263	-0.811238709002977	0.446208900663976	0.999692817443324	-4.60543362773138	7.88986607812814	8.27898882898694	6.71993662535018	7.12616475645338	7.39480912603759	7.01019288473955	NA
**TC0500001307.mm.2**	Hrk	harakiri, BCL2 interacting protein (contains only BH3 domain)	-0.267770801543438	8.01092494938111	-0.811192635477875	0.446233525173494	0.999692817443324	-4.60543579726211	7.04216356046159	7.18658383424109	8.05714290841989	8.59348744082866	8.50491079053058	8.68126116180484	NA
**TC1200000700.mm.2**	Gphn	gephyrin	0.505110031739342	8.22017401002278	0.81116682638756	0.446247319551334	0.999692817443324	-4.60543701253768	8.58160673095139	8.6171697325769	9.2435338857002	7.32270994434334	8.0200763765612	7.53594739000366	NA
**TC0900002477.mm.2**	2300009A05Rik	RIKEN cDNA 2300009A05 gene	-0.258489322421008	7.84065502276045	-0.810956373940834	0.446359812945562	0.999692817443324	-4.60544692121374	7.75606323458215	7.63461039177236	8.00854456941097	7.55959436327211	7.95798905762098	8.12712851990412	NA
**TC0600001146.mm.2**	Gpr27	G protein-coupled receptor 27	0.252644662202797	7.73672098928448	0.810949766589753	0.446363345106611	0.999692817443324	-4.60544723227927	7.49810532309046	7.63241434292469	7.82233749962159	8.07521029742706	7.5344748204677	7.85778365217536	NA
**TC1500000844.mm.2**	Tafa5	TAFA chemokine like family member 5	0.313026324235866	7.90706981073887	0.810930384428085	0.446373706546762	0.999692817443324	-4.60544814475699	8.62661147579854	8.55286464525315	8.17230380247534	7.27219389345128	7.50790860913949	7.31053643831539	NA
**TC0400003867.mm.2**	Padi1	peptidyl arginine deiminase, type I	-0.436910237821501	4.88537279275052	-0.810896097161326	0.446392036471619	0.999692817443324	-4.60544975890643	3.69635358602712	4.28898787251211	4.5539562242041	5.67258118695633	5.03312170472532	6.06723618207811	NA
**TC0400001788.mm.2**	Fam131c	family with sequence similarity 131, member C	0.33395000938263	5.40130782734878	0.810884207177734	0.446398392964976	0.999692817443324	-4.6054503186437	5.41236438106754	5.02453739425984	5.25399209694918	6.0654305068161	5.04703405374764	5.60448853125238	NA
**TC0500002325.mm.2**	Tapt1	transmembrane anterior posterior transformation 1	0.41324178980085	7.12936695518289	0.810425045571619	0.446643914080725	0.999692817443324	-4.6054719302912	7.94925165660993	7.9631811615729	7.55055616479452	6.29457008146359	6.92900787873084	6.08963478792557	NA
**TC1200001814.mm.2**	Trappc6b	trafficking protein particle complex 6B	-0.354592357670716	8.70648391553436	-0.810251766337327	0.446736594181896	0.999692817443324	-4.60548008409899	9.17319416952811	8.97653425744751	8.89157795730537	7.7984172181392	8.90439022158465	8.49478966920134	NA
**TC0600003159.mm.2**	Kcna5	potassium voltage-gated channel, shaker-related subfamily, member 5	0.270852380558678	5.63772904065704	0.810100276584548	0.446817631143044	0.999692817443324	-4.60548721166956	5.88698630559253	5.74000795381668	5.82210417668059	5.54843819614461	5.67872791358234	5.15010969812549	NA
**TC0400003578.mm.2**	Zfp362	zinc finger protein 362	0.302228242897096	9.03674584640696	0.810010877892314	0.446865458401462	0.999692817443324	-4.60549141746477	7.75132289183837	7.85026275552372	10.2035868869425	9.40808607149441	9.65032730362926	9.35688916901347	NA
**TC1100000407.mm.2**	Nudcd2	NudC domain containing 2	-0.324511076474908	7.67949203244139	-0.809780838082975	0.446988543713409	0.999692817443324	-4.60550223840876	7.38179366368495	7.33268213426499	8.15846708910733	7.19826003276696	8.14361906138551	7.8621302134386	NA
**TC0400002717.mm.2**	Ptpn3	protein tyrosine phosphatase, non-receptor type 3	-0.378064233083	6.05935472342853	-0.809599128801101	0.447085786246785	0.999692817443324	-4.60551078452093	6.30452258379149	6.61348727401441	6.19043119660765	5.15056381169198	6.42475229951678	5.67237117494885	NA
**TC0600000487.mm.2**	Arhgef5	Rho guanine nucleotide exchange factor (GEF) 5	-0.316788947625574	6.26615136641287	-0.809388055830191	0.447198761724009	0.999692817443324	-4.60552071011812	5.42239469546248	5.74359449137352	6.00130109055182	6.8973694676432	6.79020214719467	6.7420463062515	NA
**TC0600001744.mm.2**	Klhl42	kelch-like 42	-0.280993642351161	7.0771323757181	-0.809264025801471	0.447265157457099	0.999692817443324	-4.60552654179288	6.75305193374416	6.49814774896981	7.47699521773142	6.84780842571472	7.35979133417542	7.52699959397305	NA
**TC0500001327.mm.2**	Tbx3	T-box 3	0.365196120908545	5.82396420255551	0.80924212879032	0.447276880087137	0.999692817443324	-4.60552757129255	5.21838739755276	4.95990931672825	5.77158574487838	6.9763546265562	5.90975710617126	6.10779102344624	NA
**TC0300002404.mm.2**	Lce1d	late cornified envelope 1D	-0.261799004925653	5.66910607442284	-0.809238933754689	0.447278590577176	0.999692817443324	-4.60552772150737	5.18608730580295	5.3591229645328	5.50677805410424	5.96613602907074	6.10234227324784	5.89416981977845	NA
**TC0800001545.mm.2**	Capn9	calpain 9	-0.336542585911991	5.57786119769644	-0.809081717173045	0.447362763566836	0.999692817443324	-4.60553511258618	5.61610085777786	5.11710460498877	5.047680780545	5.98275749524903	5.75161789919049	5.95190554842752	NA
**TC1800000756.mm.2**	Ccdc68	coiled-coil domain containing 68	-0.457804316907444	5.3053023224199	-0.808927765304854	0.447445199527074	0.999692817443324	-4.6055423492924	4.58764099159999	5.23013620927704	4.51664162275299	6.03257242716076	5.43800206796027	6.02682061576837	NA
**TC1100004123.mm.2**	Cygb	cytoglobin	-0.387532677745327	8.06801483398554	-0.808886620243724	0.447467233123603	0.999692817443324	-4.60554428321966	8.83903183594978	8.32865539327262	8.23470999642575	6.99795821317435	8.16218376523708	7.84554979985367	NA
**TC1400002858.mm.2**	Colq	collagen-like tail subunit (single strand of homotrimer) of asymmetric acetylcholinesterase	-0.292341278822525	7.02476884602648	-0.808876818190896	0.447472482335504	0.999692817443324	-4.6055447439329	6.41259667611841	6.49044954465262	6.78445926662375	7.54598288224765	7.36783154413307	7.54729316238338	NA
**TC0100001257.mm.2**	Tnnt2	troponin T2, cardiac	-0.323051368770655	5.72819240410187	-0.808743459581769	0.447543903100073	0.999692817443324	-4.60555101166007	5.57249193201181	6.02855016017273	5.11293077168757	5.94807402575512	5.70785971006496	5.99924782491904	NA
**TC0200004312.mm.2**	Ano3	anoctamin 3	-0.675372644892909	7.16555217377447	-0.808612851765639	0.447613858497118	0.999692817443324	-4.60555714946056	8.50518623924084	8.99574871481295	6.89760166528251	5.1732147341211	7.79430923488837	5.62725245430107	NA
**TC1100001907.mm.2**	Tsen54	tRNA splicing endonuclease subunit 54	-0.265357797068567	6.22812506878072	-0.808509031728111	0.4476694715087	0.999692817443324	-4.60556202793939	5.56145322284362	5.82900401445958	6.15777847421197	6.56601031641002	6.48114169440908	6.77336269035006	NA
**TC1500000662.mm.2**	H1f0	H1 histone family, member 0	-0.499293346804845	7.03292687042354	-0.808504080333816	0.447672123931637	0.999692817443324	-4.6055622605942	7.91272822451996	7.62890198625457	7.31176464910133	5.51690750997717	6.54583399465129	7.28142485803689	NA
**TC0300001699.mm.2**	Crh	corticotropin releasing hormone	-0.255526312669939	6.86524284584068	-0.808473189514363	0.447688672151874	0.999692817443324	-4.60556371206322	6.90542722494339	6.44274739684838	6.87500956300895	6.79110535094727	7.11022527986707	7.06694225942902	NA
**TC0200002375.mm.2**	Mmp24	matrix metallopeptidase 24	-0.238148035003547	6.90525515403241	-0.808440273179192	0.447706305918485	0.999692817443324	-4.60556525866616	6.97424498201888	6.92131861684213	6.68394421055129	6.84589141711189	7.08249584396033	6.92363585370995	NA
**TC0300002370.mm.2**	Ints3	integrator complex subunit 3	-0.246703154424791	6.85388527851798	-0.808426567643013	0.447713648321653	0.999692817443324	-4.60556590262091	8.39863777795694	8.38666904277191	5.90616051633315	6.01613875443157	6.38370242141788	6.03200315819644	NA
**TC1700001620.mm.2**	Tbc1d24/Ntn3	TBC1 domain family, member 24/netrin 3	0.273860795110423	6.89521708364645	0.808408311531948	0.447723428715679	0.999692817443324	-4.6055667603736	7.2646611125968	7.31897078872033	6.94340421424756	6.72429188114363	6.86133747361342	6.25863703155693	NA
**TC0700004662.mm.2**	9130019O22Rik	RIKEN cDNA 9130019O22 gene	-0.284503475890669	6.47652033397055	-0.808302299523118	0.447780225798124	0.999692817443324	-4.60557174104029	6.68929722505603	6.36593643234699	6.04109487538928	6.57634582193019	6.40579499474845	6.78065265435236	NA
**TC0700000826.mm.2**	Rras	related RAS viral (r-ras) oncogene	0.257297268920121	7.35916230822468	0.80827369125827	0.447795553860761	0.999692817443324	-4.60557308504485	7.62739318891911	7.75177018517051	7.25945657907346	7.38574592747588	7.29447682728659	6.8361311414225	NA
**TC0400003003.mm.2**	Ifna14	interferon alpha 14	-0.247270827942313	4.93927281504628	-0.808141799233891	0.44786622528291	0.999692817443324	-4.60557928088353	4.61349999715217	4.84402657062753	4.8362014309667	5.00558290233996	5.29111088548409	5.0452151037072	NA
**TC0900000824.mm.2**	Dennd4a	DENN/MADD domain containing 4A	-0.915795793254742	6.23984293900005	-0.808087225668406	0.447895469631406	0.999692817443324	-4.60558184437432	7.55582570523786	7.68721306066069	6.77461055276811	3.40760308802803	7.01965059837576	4.99415462892986	NA
**TC0300003209.mm.2**	Iqcj	IQ motif containing J	0.272583042192326	5.91499991776161	0.807986464234356	0.447949468238073	0.999692817443324	-4.6055865771617	6.1186804502459	5.74158583528315	5.83987571240265	6.24757394030998	5.61819109701658	5.92409247131141	NA
**TC1300000255.mm.2**	Hist1h2bc	histone cluster 1, H2bc	-0.43834691902055	8.85200973197856	-0.807969771626243	0.447958414343867	0.999692817443324	-4.60558736118072	8.95534477591506	8.91932583512577	9.20204455158132	7.97830241981341	9.58190914789733	8.47513166153849	NA
**TC0500002263.mm.2**	Bloc1s4	biogenesis of lysosomal organelles complex-1, subunit 4, cappuccino	-0.389878474555034	6.08821256760953	-0.80796412422589	0.447961440996082	0.999692817443324	-4.60558762642567	6.14621008992446	6.45498251133899	6.38619740200976	5.18796552563208	6.4939260483822	5.85999382836971	NA
**TC0500000489.mm.2**	Med28	mediator complex subunit 28	-0.49513672814378	7.43778710571438	-0.807920441902611	0.447984852469712	0.999692817443324	-4.60558967804025	8.01722806794422	7.89493405987164	7.7545683658655	6.10757515922592	7.93642046163046	6.91599651974852	NA
**TC1700001163.mm.2**	Ttc27	tetratricopeptide repeat domain 27	0.254841555068401	6.24616055855622	0.807855837184862	0.448019478857209	0.999692817443324	-4.60559271218017	6.25265484683519	6.55758340206114	6.46488959853809	6.12331450775081	5.99341238014432	6.08510861600778	NA
**TC0600002067.mm.2**	Fam180a	family with sequence similarity 180, member A	-0.275062638573941	5.2534360645376	-0.807836884438368	0.448029637378192	0.999692817443324	-4.60559360226032	5.25677976803544	5.44050250797783	4.88208708805976	5.25451732897246	5.59676166962561	5.08996802455449	NA
**TC0100000682.mm.2**	Utp14b/Acsl3	UTP14B small subunit processome component/acyl-CoA synthetase long-chain family member 3	1.03063456827349	8.20598598172914	0.807808671489125	0.448044759596225	0.999692817443324	-4.60559492720368	9.85651935446983	9.68261674867965	9.15907792808576	6.7199465338004	8.77260759280165	5.04514773253753	NA
**TC0100002997.mm.2**	Lypd1	Ly6/Plaur domain containing 1	0.299277743140962	8.60986742818459	0.807725972507546	0.448089088559926	0.999692817443324	-4.6055988107622	9.86961483724926	9.66911849606869	8.57080441730984	7.78870894372594	7.99449948140216	7.76645839335169	NA
**TC1500001720.mm.2**	Ly6k	lymphocyte antigen 6 complex, locus K	0.398575802766572	4.99141832033573	0.807418768687976	0.448253785589811	0.999692817443324	-4.60561323487205	3.66606974038476	4.99855027778747	5.27390737675512	5.76661337793253	5.0054528008069	5.2379163483476	NA
**TC1200000387.mm.2**	Pnpla8/Gm2027	patatin-like phospholipase domain containing 8/nuclear assembly factor 1 homolog pseudogene	-0.494385517249499	6.380955216382	-0.80705143850508	0.448450773624133	0.999692817443324	-4.60563047746403	7.35812099130798	7.24494268378198	6.45365724871976	4.89329104563174	6.74165964532151	5.59405968352899	NA
**TC0900001247.mm.2**	Cep70	centrosomal protein 70	-0.364794847070544	6.58643173792803	-0.807049412659384	0.44845186019365	0.999692817443324	-4.60563057254388	6.675490688652	6.95261858180304	6.66899184767233	5.9114538838137	7.13337449443118	6.17666093119594	NA
**TC0400001405.mm.2**	Rhbdl2	rhomboid like 2	0.29099635790027	5.27646781638927	0.807043480913886	0.448455041717087	0.999692817443324	-4.60563085094005	4.65366398620182	4.76677793262923	5.35586198640064	6.05431686125191	5.2484873253751	5.5796988064769	NA
**TC1100003830.mm.2**	Atxn7l3	ataxin 7-like 3	-0.287555572723838	8.01495463846049	-0.807030654919382	0.448461921062499	0.999692817443324	-4.60563145290133	7.77004148684137	8.02266076005768	8.15011814797668	7.71083907123144	8.49834434241863	7.93772402223716	NA
**TC0700003517.mm.2**	Slco3a1	solute carrier organic anion transporter family, member 3a1	-0.29858001027809	8.04056747570705	-0.806981485919693	0.448488294020218	0.999692817443324	-4.60563376048874	8.35389055757271	8.23551453833135	8.17787452145166	7.35054534743936	8.05267470567367	8.07290518377354	NA
**TC0900001908.mm.2**	Anln	anillin, actin binding protein	-0.39886699773151	5.81493153498265	-0.80669713162607	0.448640835717713	0.999692817443324	-4.60564710396288	6.84065960242024	6.83656797169895	5.78667848757035	4.42063533258648	5.26992374191126	5.7351240737086	NA
**TC1900000257.mm.2**	Olfr1444	olfactory receptor 1444	0.27870665036411	5.21828737240298	0.8066532157154	0.448664397656384	0.999692817443324	-4.60564916447061	4.36410823362099	4.06673524826811	5.56292812517992	6.15521890144858	5.48773251195565	5.67300121394463	NA
**TC1400000882.mm.2**	Fam124a	family with sequence similarity 124, member A	-0.280050657781298	7.47379928738562	-0.806482310501557	0.448756100736652	0.999692817443324	-4.60565718255275	6.99533827403192	6.66215025303619	7.37613633966028	7.93646660118122	7.80978201917791	8.06292223722618	NA
**TC1900000916.mm.2**	Grk2	G protein-coupled receptor kinase 2	-0.555000107056941	8.07169349425371	-0.806402900796317	0.448798714333946	0.999692817443324	-4.60566090771724	9.14740738488953	8.91706854153203	8.3061732703468	6.32166914214661	8.37858682099604	7.35925580561126	NA
**TC1500002243.mm.2**	Pou6f1	POU domain, class 6, transcription factor 1	-0.250039577855506	7.14547879044081	-0.806392960314927	0.44880404889204	0.999692817443324	-4.60566137401553	7.62707748042633	7.60961114833892	6.99228003682979	6.57577244225451	7.04968337852199	7.01844825627332	NA
**TC1200000990.mm.2**	Psmc1	protease (prosome, macropain) 26S subunit, ATPase 1	-0.277393192401592	8.40360384172265	-0.80630258141047	0.448852552778521	0.999692817443324	-4.60566561343226	9.19278152499568	9.04041566153907	8.17293839776523	7.64388134173377	8.38412899344516	7.98747713085702	NA
**TC0500002428.mm.2**	Cckar/Rnu7	cholecystokinin A receptor/U7 small nuclear RNA	0.396132988919804	6.11407350151636	0.806266848835636	0.448871730497475	0.999692817443324	-4.60566728946121	6.34114374526962	5.20489166203333	6.90901767338118	6.05631811643624	6.0079684581123	6.16510135386551	NA
**TC0X00003430.mm.2**	Arhgap4	Rho GTPase activating protein 4	0.295897677461623	5.92633513024901	0.805982398738586	0.449024415910351	0.999692817443324	-4.60568062982997	5.42804188476204	4.78877764113014	6.35739108222445	6.60910222303811	5.9639236793976	6.41077427094171	NA
**TC1700002588.mm.2**	Map4k3	mitogen-activated protein kinase kinase kinase kinase 3	0.339731396957855	7.75710797863192	0.805897377993406	0.449070059967027	0.999692817443324	-4.60568461661337	8.10449271817594	8.01229740757971	8.06796193383034	7.48469833614545	7.90433523985111	6.96886223620897	NA
**TC0500002154.mm.2**	Hadha	hydroxyacyl-Coenzyme A dehydrogenase/3-ketoacyl-Coenzyme A thiolase/enoyl-Coenzyme A hydratase (trifunctional protein), alpha subunit	0.417384065769888	7.39158018513671	0.805842135772805	0.449099718947487	0.999692817443324	-4.60568720688049	8.25524028679296	8.20591667652953	7.90611499400521	6.45543114551355	7.02872580460002	6.49805220337897	NA
**TC1100002027.mm.2**	Ccdc137	coiled-coil domain containing 137	0.414652373265373	6.66147270297127	0.805775843377348	0.449135312482492	0.999692817443324	-4.6056903151314	6.11760315299461	5.73120477373403	6.75854406694523	7.71612245186963	6.25747833685489	7.38788343542923	NA
**TC1000000230.mm.2**	Gm9767	predicted gene 9767	-0.311335367586479	5.11467117083109	-0.805591874116871	0.449234099216899	0.999692817443324	-4.60569894003454	4.76199103893803	5.03008068441308	5.17953945115012	4.95710283208113	4.95017944451673	5.80913357388748	NA

**TC1600000216.mm.2**	Crkl	v-crk avian sarcoma virus CT10 oncogene homolog-like	-0.241063984006635	8.83814461079457	-0.805424307702794	0.449324091420563	0.999692817443324	-4.60570679483154	8.88451797706001	9.04242724289675	8.74605961434859	8.5638376240501	8.98573798346543	8.80628722294653	NA	
**TC1100002512.mm.2**	Wwc1	WW, C2 and coiled-coil domain containing 1	0.241444000215365	7.21353131026402	0.805420684086168	0.449326037638998	0.999692817443324	-4.60570696467958	7.16134401538467	7.26709225156128	7.2710529145011	7.39676688303333	7.22188944157214	6.96304235553156	NA	
**TC0300000957.mm.2**	Rnf115	ring finger protein 115	-0.637830872796564	7.82152878912053	-0.805402518946513	0.449335794094748	0.999692817443324	-4.60570781611801	8.49610350821813	8.42731585451613	8.39976661269011	5.96527920050779	8.33828773019352	7.3024198285975	NA	
**TC0400001790.mm.2**	Hspb7	heat shock protein family, member 7 (cardiovascular)	0.419739334411513	5.6120451653372	0.805261435451238	0.449411574824127	0.999692817443324	-4.60571442857833	5.14400802897929	5.14945920935478	5.68553115637679	6.42361005487929	4.94784057387286	6.32182196856019	NA	
**TC0700001927.mm.2**	Bub3	BUB3 mitotic checkpoint protein	-0.330663538340881	8.59224244731163	-0.805170115977784	0.449460630427955	0.999692817443324	-4.6057187082448	9.38214432248783	9.21459720550641	8.57673157475378	7.57096146484311	8.53459765633573	8.27442245994293	NA	
**TC0900002053.mm.2**	Spa17	sperm autoantigenic protein 17	-0.280901595825635	5.82183385563697	-0.805068680073425	0.449515124882308	0.999692817443324	-4.60572346164918	6.32197551863601	6.5329954715403	5.24436829199904	5.5127461839981	5.34823892624594	5.97067874140247	NA	
**TC0900000599.mm.2**	Il18	interleukin 18	-0.344624725351175	6.96099543800624	-0.805059070937469	0.449520287444769	0.999692817443324	-4.60572391192447	7.96910425906612	7.79421833310715	6.87552875480906	5.78117153777186	6.80222216809429	6.54372757518897	NA	
**TC0700002711.mm.2**	Ryr1	ryanodine receptor 1, skeletal muscle	0.391523687504134	5.39896604709846	0.805052444971514	0.44952384730675	0.999692817443324	-4.60572422240913	4.77009225897575	5.52057892571475	5.33690845798152	6.10617777847275	4.87582291083242	5.78421595061358	NA	
**TC0900002320.mm.2**	Dmxl2	Dmx-like 2	-0.786861164986093	6.8980171895016	-0.804939194100353	0.44958469547502	0.999692817443324	-4.60572952895204	8.53065230401764	8.26206175981222	7.03821008404533	4.47262328755844	7.61162392902435	5.47293177255161	NA	
**TC0300001601.mm.2**	Pex2	peroxisomal biogenesis factor 2	-0.278595667225919	6.96997831559582	-0.804748255945957	0.449687297159433	0.999692817443324	-4.6057384745628	6.69565429973068	6.76288451275285	7.00326912356966	6.8988007497501	6.88897714803797	7.57028405973364	NA	
**TC0800002060.mm.2**	Lonrf1	LON peptidase N-terminal domain and ring finger 1	-0.398827863087485	6.50267713311188	-0.804507706104754	0.449816581522317	0.999692817443324	-4.60574974257602	6.71643778494372	7.06046304446726	6.79473004051044	5.42602308103221	6.67299307136611	6.34541577635152	NA	
**TC0800002878.mm.2**	Nob1	NIN1/RPN12 binding protein 1 homolog	0.278423759807438	7.27695511189926	0.804457021325185	0.449843825587035	0.999692817443324	-4.60575211651303	7.70870851298865	7.57720422771687	6.92295836606277	7.54337435908969	7.09314102422209	6.8163441813155	NA	
**TC1300000280.mm.2**	Tdp2	tyrosyl-DNA phosphodiesterase 2	0.371517894838915	5.90540235311297	0.804208890549781	0.449977217583316	0.999692817443324	-4.60576373689157	6.46662196705066	6.30389484888576	6.45598701740869	5.24647952880093	5.69972288760894	5.25970786892286	NA	
**TC1000002511.mm.2**	Ap3d1	adaptor-related protein complex 3, delta 1 subunit	0.275821677878524	9.10697326034571	0.803848931800927	0.450170776579684	0.999692817443324	-4.60578059025274	9.6995764364375	9.56832131726815	9.08236195839648	8.88043062366636	9.01960015071616	8.39154907558964	NA	
**TC1100003283.mm.2**	Eral1	Era (G-protein)-like 1 (E. coli)	0.287114945501787	6.68608466424787	0.803830370063674	0.450180759293723	0.999692817443324	-4.60578145918614	6.50500196419446	6.39137654199476	6.63416328216952	7.26301640298127	6.8680531462805	6.45489664786672	NA	
**TC0500001672.mm.2**	Cyp2w1	cytochrome P450, family 2, subfamily w, polypeptide 1	0.38144354429048	5.50721012177814	0.803593211028521	0.450308319892668	0.999692817443324	-4.60579256020872	4.96391389412428	4.96924785029163	5.45556612501751	6.48092691239944	5.14389627209175	6.02970967674424	NA	
**TC1400002632.mm.2**	Kctd12/Mir5130	potassium channel tetramerisation domain containing 12/microRNA 5130	-0.302435228560319	8.28009816500865	-0.803581591272241	0.450314570460625	0.999692817443324	-4.60579310405602	8.64342500331539	8.75472140082433	8.31638626321331	7.52239980118245	8.05067516394315	8.39298135757326	NA	
**TC0400000867.mm.2**	Rraga	Ras-related GTP binding A	-0.644288774841101	7.66235121051051	-0.803289278933773	0.450471833003977	0.999692817443324	-4.60580678367826	8.91476162830131	8.73957418585283	7.99632548763636	5.51927146197701	7.91334119556089	6.89083330373468	NA	
**TC0400003958.mm.2**	Tnfrsf8	tumor necrosis factor receptor superfamily, member 8	-0.294865094157001	5.39322060471133	-0.803063033643199	0.450593578438754	0.999692817443324	-4.60581736929209	5.0284643144513	5.05036078907086	5.07758021247693	5.76780395573898	5.93850138770051	5.4966129688294	NA	
**TC1700001610.mm.2**	Sbpl	spermine binding protein-like	-0.415892541250976	5.05676469359863	-0.803060915117408	0.450594718553904	0.999692817443324	-4.60581746840504	4.60788054874515	4.85269428880545	4.83724758528234	5.18686653548728	4.68540032341128	6.1704988798603	NA	
**TC0100003487.mm.2**	Pbx1	pre B cell leukemia homeobox 1	-0.748392696435104	7.81272285282788	-0.802989660266478	0.450633066564051	0.999692817443324	-4.60582080188825	8.62914161255602	8.74327926573332	8.43230957983308	5.57125584307078	8.44145199941212	7.05889881636195	NA	
**TC0X00000916.mm.2**	Msn	moesin	-0.275693929635933	6.59791831893367	-0.802674698087857	0.450802600842921	0.999692817443324	-4.60583553433238	6.5007455205614	6.98190702669973	6.28506094802411	6.49167380551039	6.45117974721736	6.87694286558901	NA	
**TC1700000496.mm.2**	Cdkn1a	cyclin-dependent kinase inhibitor 1A (P21)	-0.566445028837422	7.02186660201338	-0.802489458244306	0.450902330676453	0.999692817443324	-4.60584419723467	7.25864914853241	7.37939310470003	7.63235277020276	5.54778088038374	7.65305559030747	6.65996811795387	NA	
**TC0300000492.mm.2**	Rap2b	RAP2B, member of RAS oncogene family	-0.277845417346885	7.34774019167919	-0.802399353527401	0.450950847081369	0.999692817443324	-4.60584841059315	7.65654733451844	7.89168820243745	7.29474596203112	6.69651142718161	7.14392182235318	7.40302640155332	NA	
**TC1600001744.mm.2**	Cep97	centrosomal protein 97	-0.350859197411602	5.9834117325686	-0.802266798934847	0.451022227092793	0.999692817443324	-4.60585460838137	6.66031009006146	6.44213455635462	6.08594971415772	4.96220396292844	6.02300284351152	5.72686922839785	NA	
**TC0500001403.mm.2**	Rnf34	ring finger protein 34	-0.578332422785163	7.87834283242719	-0.802181507734161	0.451068160210639	0.999692817443324	-4.60585859594918	8.52494398216102	8.41923693773098	8.39946252769487	6.18514308685554	8.29157658925059	7.44969387087015	NA	
**TC0800001867.mm.2**	Slc25a15	solute carrier family 25 (mitochondrial carrier ornithine transporter), member 15	-0.246712566592103	5.89664983203347	-0.802092309724853	0.451116200850753	0.999692817443324	-4.60586276587656	6.08137250994891	5.90266643410323	5.80150257292735	5.6497148845549	5.81417560435157	6.13046698631488	NA	
**TC0700003551.mm.2**	Hapln3	hyaluronan and proteoglycan link protein 3	0.293495131233666	6.50946560760864	0.801972142809952	0.451180926563069	0.999692817443324	-4.60586838309881	5.82723001246382	6.0582477536387	6.58833361696203	7.2908194540463	6.47733591716623	6.81482689137477	NA	
**TC0X00002631.mm.2**	Pola1	polymerase (DNA directed), alpha 1	-0.262626704685214	5.12541484557556	-0.801607350457741	0.451377455434465	0.999692817443324	-4.60588543204438	4.94127539319204	5.20631121130328	4.80655781238504	5.23326671740877	5.15369536746522	5.41138257169902	NA	
**TC0700001810.mm.2**	Sgf29	SAGA complex associated factor 29	-0.251245455164577	7.22042206874427	-0.801546873251852	0.451410042865549	0.999692817443324	-4.60588825802406	7.42933752145947	7.54829497899455	6.81128634884149	7.10991815199973	7.05490366470169	7.36879174646869	NA	
**TC0500001737.mm.2**	Cyth3	cytohesin 3	-0.544720826719574	6.44499896177345	-0.801058630505494	0.451673187506665	0.999692817443324	-4.60591106758591	7.18952583988398	7.08153911552502	6.84871288422071	4.80603069667558	6.79075844211479	5.95342679222065	NA	
**TC0200002012.mm.2**	Mavs	mitochondrial antiviral signaling protein	0.274900361842887	7.29124838102387	0.801019605839601	0.451694225014905	0.999692817443324	-4.60591289033912	7.49606918545179	7.25134762146708	7.09714755701761	7.67778954443745	6.99428459484534	7.23085178292395	NA	
**TC0500003468.mm.2**	Gjc3	gap junction protein, gamma 3	0.471313028600851	8.72130368871587	0.801010277568326	0.451699253823541	0.999692817443324	-4.60591332603291	9.91472825538989	10.0328561775088	8.97510651714487	7.68632536115425	8.51439736599509	7.20440845510232	NA	
**TC0700000524.mm.2**	Ggn	gametogenetin	-0.286437312915569	6.50616731117105	-0.800938957587209	0.451737703253026	0.999692817443324	-4.6059166570531	6.10536092401504	6.15162429329143	6.49512207599396	6.6084499359504	6.46134200332007	7.21510463445543	NA	
**TC4_JH584294_random00000008.mm.2**	Ccl19	chemokine (C-C motif) ligand 19	-0.385623923183461	7.2228484000077	-0.800720700060405	0.451855382755857	0.999692817443324	-4.60592684964079	7.54572118855014	7.66314703500471	7.52884966243481	6.1496375026274	7.18882347430099	7.26091153712815		2
**TC0500000339.mm.2**	Ppp1cb	protein phosphatase 1 catalytic subunit beta	-0.571771233628329	9.25189315007524	-0.80067047056579	0.451882468418057	0.999692817443324	-4.6059291950947	10.2399794961387	10.2540210224043	9.45539559806933	7.48151235925658	9.67280003242732	8.40765039215525	NA	
**TC1500000803.mm.2**	Ribc2	RIB43A domain with coiled-coils 2	-0.307242764976679	5.81871850404612	-0.800627839078444	0.451905457842509	0.999692817443324	-4.60593118568679	5.93918896199021	5.27305726236886	5.50761716697302	6.03517246800913	5.91265148761201	6.24462367732349	NA	
**TC1100000240.mm.2**	Usp34	ubiquitin specific peptidase 34	0.801059709995957	6.78608963450975	0.800275140522514	0.45209568543354	0.999692817443324	-4.60594765160784	8.86242179648343	8.73372791693671	6.54681272797284	5.8144410288423	7.05824953296858	3.70088480385464	NA	
**TC0900000666.mm.2**	Hykk	hydroxylysine kinase 1	0.239606162060326	5.51505225987484	0.79995996979336	0.452265720175484	0.999692817443324	-4.6059623615457	5.26689462212204	5.50306689615166	5.76630745158835	5.63347473095966	5.52853320610777	5.39203665231958	NA	
**TC0300001112.mm.2**	Kcna2	potassium voltage-gated channel, shaker-related subfamily, member 2	0.241892355118869	7.28070778432825	0.799931804084264	0.452280917780824	0.999692817443324	-4.6059636759388	8.20356268558438	8.05293280348116	6.92826999635423	7.02749796721662	6.87892886895656	6.59305438437655	NA	
**TC0400001527.mm.2**	Tmem234	transmembrane protein 234	0.324987277265276	8.11722056182747	0.799904549860728	0.452295623910912	0.999692817443324	-4.60596494776757	8.67163600364747	8.7247031707385	8.49331067868865	7.48516869686606	7.56176559965553	7.76673922136863	NA	
**TC0700000846.mm.2**	Lhb	luteinizing hormone beta	0.268012401792945	6.31436546862758	0.79971947278528	0.452395498723246	0.999692817443324	-4.60597358371718	6.17263319217339	6.19977464963349	6.4292489088209	6.59565597795137	5.94682600413284	6.54205407905353	NA	
**TC4_JH584293_random00000007.mm.2**	Ccl27b/Gm2506/Gm13306/LOC100861978	chemokine (C-C motif) ligand 27b/predicted gene 2506/predicted gene 13306/c-C motif chemokine 27-like	-0.404391100075234	9.77215860695384	-0.799684756238489	0.452414234857263	0.999692817443324	-4.60597520349449	10.4592766880112	10.4319666937643	9.90360527609051	8.56285775380805	9.96502897780501	9.31021625224402	NA	
**TC4_JH584294_random00000001.mm.2**	Gm13306/Ccl27b/Gm2506/LOC100861978	predicted gene 13306/chemokine (C-C motif) ligand 27b/predicted gene 2506/c-C motif chemokine 27-like	-0.404391100075234	9.77215860695384	-0.799684756238489	0.452414234857263	0.999692817443324	-4.60597520349449	10.4592766880112	10.4319666937643	9.90360527609051	8.56285775380805	9.96502897780501	9.31021625224402	NA	
**TC0600001992.mm.2**	Ube2h	ubiquitin-conjugating enzyme E2H	-0.358539927050565	7.42222681197521	-0.79953903988954	0.45249288231233	0.999692817443324	-4.60598200171594	8.34653468776784	8.38431049961651	7.03204460719316	6.51067330798971	7.68920234212432	6.57059542715969	NA	
**TC0800001418.mm.2**	Hsbp1	heat shock factor binding protein 1	-0.326873922225015	9.63729005100047	-0.799455628029261	0.452537906512976	0.999692817443324	-4.60598589283473	10.3170558355123	10.319186869206	9.58674674125605	8.68012813716119	9.71835781684623	9.20226490602103	NA	
**TC1600001515.mm.2**	Smbd1	somatomedin B domain containing 1	-0.255190881939228	5.09304931336585	-0.799318288436383	0.452612046795667	0.999692817443324	-4.60599229907991	5.03225956753656	5.12142166125938	4.74476057400039	5.20235586975996	5.31790172037152	5.13959648726729	NA	
**TC1300001270.mm.2**	Plpp1	phospholipid phosphatase 1	-0.337069372354986	7.00102562728943	-0.799155110665543	0.452700146469888	0.999692817443324	-4.60599990962649	7.88697702448867	7.64578603644038	6.9267047778635	5.97292120118527	7.04103586263687	6.53272886112187	NA	
**TC1400001288.mm.2**	Cldn10	claudin 10	0.356965562021065	6.98381432994739	0.798998924619516	0.452784482623626	0.999692817443324	-4.60600719313771	7.393452938862	7.2258607938682	7.60659334352115	6.39215834197699	6.66690859703855	6.61791196441745	NA	
**TC0400000140.mm.2**	Nbn	nibrin	-0.633842133656863	5.48573723045978	-0.798998921156164	0.452784484493864	0.999692817443324	-4.60600719329921	6.65503353094323	6.56803187069799	5.60442566151478	3.60741119538708	6.11103408744389	4.36848703677169	NA	
**TC0700004608.mm.2**	Ano1	anoctamin 1, calcium activated chloride channel	-0.258413329068361	7.22834674481479	-0.798902247663341	0.45283669105504	0.999692817443324	-4.60601170106546	7.71430966697511	7.73024208036592	7.09840239388413	6.60594863782137	7.21194063766196	7.00923705218026	NA	
**TC0200003271.mm.2**	Crat	carnitine acetyltransferase	0.279077274542074	7.74215916582781	0.798824473902351	0.452878694275557	0.999692817443324	-4.60601532730353	7.82854903346909	7.73577798927809	8.20649682071429	7.51689443993763	7.5054599174114	7.65977679415638	NA	
**TC0500000297.mm.2**	Slc35f6/Mir5625	solute carrier family 35, member F6/microRNA 5625	-0.256403306481325	7.51925922865708	-0.798777206542589	0.452904223265613	0.999692817443324	-4.60601753105441	8.23680904790638	8.23485881565794	7.13924425425265	6.9262961934551	7.52266073570239	7.055686324968	NA	
**TC0600001834.mm.2**	Dlx5	distal-less homeobox 5	0.413998205537945	9.20133077604396	0.798610695683195	0.452994163461652	0.999692817443324	-4.60602529363278	8.96130349258745	8.61757942225576	10.347525684953	8.88102339129517	9.37758836969148	9.02296429548084	NA	
**TC0100001526.mm.2**	Creg1	cellular repressor of E1A-stimulated genes 1	0.514763548013445	7.33924009795956	0.798286078707912	0.453169540214374	0.999692817443324	-4.60604042394779	8.39262802552734	8.38027572001701	7.41636578711668	6.72966618200328	7.53478167733596	5.58172319575711	NA	
**TC1600001079.mm.2**	Wrb	tryptophan rich basic protein	0.233238899176401	8.72000466576449	0.798184052001073	0.453224670776886	0.999692817443324	-4.60604517856178	9.12913173620841	9.01036388309562	8.59829077750279	8.72521430931507	8.46788055305716	8.3891467354079	NA	
**TC0700000774.mm.2**	Klk9	kallikrein related-peptidase 9	-0.353543750532539	6.21277205810853	-0.79807361680326	0.453284350222965	0.999692817443324	-4.60605032458059	5.96495641679659	5.88448650157133	5.7032539826315	6.6567969819776	6.1639468480284	6.90319161764578	NA	
**TC0700004534.mm.2**	B230206H07Rik	RIKEN cDNA B230206H07 gene	-0.236353455610328	4.93895406459291	-0.798073042176935	0.453284660766874	0.999692817443324	-4.6060503513556	4.66187605762824	4.91303606410633	4.91077706389909	4.88227561340202	5.10858942223432	5.15717016628745	NA	
**TC0600003169.mm.2**	Ccnd2	cyclin D2	0.329159689591541	8.01924222069921	0.797919184361574	0.453367815159406	0.999692817443324	-4.60605751999119	7.85064528616276	8.42056644085866	7.69809262469695	8.55318786348152	7.92961211798693	7.66334899100845	NA	
**TC1000002211.mm.2**	Oit3	oncoprotein induced transcript 3	0.433891518097284	5.75020150890774	0.797876807784734	0.453390719999024	0.999692817443324	-4.60605949426835	4.80690421350312	5.57225418858658	5.54127104002305	6.95364580375261	5.61287894846797	6.01425485911312	NA	
**TC1300002121.mm.2**	Spata31d1a	spermatogenesis associated 31 subfamily D, member 1A	0.259865248153856	5.23377484046157	0.797668119710838	0.453503529258251	0.999692817443324	-4.60606921581766	4.86749290224782	5.03337062096087	5.39352732084603	5.61723068708821	5.4830387400603	5.00798877156623	NA	
**TC1800001404.mm.2**	Ndst1	N-deacetylase/N-sulfotransferase (heparan glucosaminyl) 1	0.341393003500814	7.63572608339825	0.79752987068268	0.453578272579386	0.999692817443324	-4.60607565511629	8.7276002282379	8.63529841748405	7.58927514476055	6.97784678607405	7.41980232005499	6.46453360377798	NA	
**TC0100001249.mm.2**	Lmod1	leiomodin 1 (smooth muscle)	-0.320273076398407	5.43577835216202	-0.797305217689034	0.453699748052045	0.999692817443324	-4.60608611735108	4.91354116779525	5.02632615336089	5.13415350247227	5.88297481703731	5.4971565612511	6.1605179110553	NA	
**TC0900002942.mm.2**	Pccb	propionyl Coenzyme A carboxylase, beta polypeptide	0.309655866055481	6.59510481535996	0.797298058347328	0.453703619662771	0.999692817443324	-4.60608645073473	7.68629140493228	7.60705922305375	6.5479194292359	5.90037556890644	6.25088158157618	5.5781016844552	NA	
**TC0400003423.mm.2**	Ccdc30	coiled-coil domain containing 30	-0.232505663148459	6.80040221449702	-0.797286992058223	0.453709604108368	0.999692817443324	-4.60608696604642	6.88919531175031	6.78366177298693	6.73541622684831	6.59685621112566	6.91889836675386	6.87838539751703	NA	
**TC1400001579.mm.2**	Polr3a	polymerase (RNA) III (DNA directed) polypeptide A	-0.274884078402046	5.74478448966166	-0.797192925205666	0.453760475973879	0.999692817443324	-4.60609134616613	5.91483609890831	5.98804880996455	5.33325565401934	5.67477128212716	6.07275170381592	5.48504338913468	NA	
**TC1100004057.mm.2**	Cd300ld4/Cd300ld5/Cd300ld3	CD300 molecule like family member D4/CD300 molecule like family member D5/CD300 molecule like family member D3	-0.269807364365879	6.03080353336504	-0.797118882955063	0.453800521247404	0.999692817443324	-4.60609479362587	5.64629897007971	5.79457236323557	5.89899457030426	6.20317299876735	6.04831265626448	6.59346964153889	NA	
**TC0400002704.mm.2**	Actl7b	actin-like 7b	-0.448674321612807	5.69012219342218	-0.796733619988749	0.454008928349421	0.999692817443324	-4.60611272838176	5.17866451661081	5.11681587101981	5.25485989461682	6.21909217022161	5.46395777627736	6.90734293178668	NA	
**TC1400000368.mm.2**	Anxa8	annexin A8	0.315650947715347	5.35352409537387	0.796729656679915	0.454011072641756	0.999692817443324	-4.60611291285234	5.50820182590571	5.12230221114224	5.10643602950077	5.9545351858122	5.36379487233015	5.06587444755213	NA	
**TC0X00002689.mm.2**	Ophn1	oligophrenin 1	-0.256673777044045	6.50239706207176	-0.796709038805769	0.454022227766596	0.999692817443324	-4.6061138724932	6.07506334281503	6.33197397019176	6.44467456513353	6.6023241875343	6.56671340339887	6.99363290335705	NA	
**TC0200003680.mm.2**	Scn9a	sodium channel, voltage-gated, type IX, alpha	-0.305822552814363	5.91001212225116	-0.796676613697184	0.454039771484713	0.999692817443324	-4.60611538165875	6.05171591817437	5.42201241501663	5.55719201491785	6.13015763242576	5.98470498673455	6.31428976623779	NA	
**TC1200001939.mm.2**	Sgpp1	sphingosine-1-phosphate phosphatase 1	-0.8091244761883	7.80167995607254	-0.79636137115411	0.454210359404741	0.999692817443324	-4.60613005194519	8.56840950218921	8.4305878182256	8.74453624414826	5.35188048767364	8.27147257601137	7.44319310818714	NA	
**TC0700003726.mm.2**	Tmem126a	transmembrane protein 126A	-0.698367247013518	6.59232922699563	-0.796347506406046	0.45421786310189	0.999692817443324	-4.60613069707537	7.6276561079761	7.90132459214875	7.00976948411463	4.30436059979631	6.95445407142903	5.75641050650894	NA	
**TC0300002451.mm.2**	BC021767	cingulin pseudogene	0.236921930244057	6.11245632239457	0.796211687936898	0.454291373600665	0.999692817443324	-4.60613701635588	5.7663233092771	5.84146649250353	6.4551923319652	6.31520366457226	6.26201820512204	6.0345339309273	NA	
**TC1900000162.mm.2**	Ubxn1	UBX domain protein 1	-0.231448539741666	7.24997279090601	-0.796150973715978	0.454324237319464	0.999692817443324	-4.6061398410039	7.3070293639518	7.41892933174908	7.06136535051427	7.09412513461165	7.25544765165229	7.36293991295696	NA	
**TC0200005030.mm.2**	Tgm2	transglutaminase 2, C polypeptide	0.277390959925477	6.29878116317687	0.796101190310764	0.454351185594539	0.999692817443324	-4.60614215700567	5.8501502712925	6.37377114198365	6.31262028380002	6.74915345901801	6.16002561660819	6.34696620635888	NA	
**TC0500003393.mm.2**	Pom121	nuclear pore membrane protein 121	0.441101977653189	7.12491770707203	0.796059371013834	0.454373823683177	0.999692817443324	-4.60614410243181	8.0926755148783	7.81138785143231	7.76818701818385	6.09563639753013	6.54216560383084	6.43945385657677	NA	
**TC0300001902.mm.2**	Mfsd8	major facilitator superfamily domain containing 8	-0.548586843477141	5.79973383722824	-0.795837255698754	0.454494074911456	0.999692817443324	-4.6061544340807	6.57673068533158	6.52242725097386	6.041124544557	4.25991115549786	6.38353688648237	5.01467250052677	NA	
**TC0700002854.mm.2**	Nudt19	nudix (nucleoside diphosphate linked moiety X)-type motif 19	-0.385082817141404	8.88849760289227	-0.79574433000294	0.454544390679853	0.999692817443324	-4.60615875594347	9.26372672226184	9.20454637002324	9.07122336083641	7.97505008455645	9.33980656030594	8.47663251936973	NA	
**TC1800001120.mm.2**	Myo7b	myosin VIIB	0.407020702140585	4.69467088977818	0.795716208949084	0.454559617944031	0.999692817443324	-4.6061600637552	4.31783583048887	4.43377399347873	4.7804247947732	5.33480366471814	5.35461436076566	3.94657269444451	NA	
**TC0200001042.mm.2**	Dlx1	distal-less homeobox 1	0.277147438229669	8.48407180287376	0.795689727606337	0.454573957649038	0.999692817443324	-4.60616129528218	8.47429382095988	8.25364876396362	8.66877022842411	8.6966213259651	8.07047411015975	8.74062256777012	NA	
**TC1200001028.mm.2**	Otub2	OTU domain, ubiquitin aldehyde binding 2	-0.565404324119798	6.6138304759732	-0.795233842785447	0.454820870110812	0.999692817443324	-4.60618249222737	6.61322288139345	7.11493507047681	7.30903356326843	5.10297456459624	7.05484268996809	6.48797408613618	NA	
**TC0200000523.mm.2**	Pkn3	protein kinase N3	-0.242578920341985	7.44707804300528	-0.795219693486997	0.45482853504069	0.999692817443324	-4.60618314999034	6.87172314070887	7.06775823786238	7.42223438753731	7.70668013185093	7.74445753214853	7.86961482792367	NA	
**TC1300002544.mm.2**	Slc30a5	solute carrier family 30 (zinc transporter), member 5	-0.348351670404687	7.64674403283705	-0.79513218915785	0.454875939727895	0.999692817443324	-4.60618721766266	8.18726759326065	7.66356303545943	7.61427754462742	7.052187569119	7.22976444512386	8.13340400943194	NA	
**TC1000001084.mm.2**	Arl1	ADP-ribosylation factor-like 1	-0.367978317393767	9.43874660200353	-0.7951124004417	0.454886660568892	0.999692817443324	-4.60618813750827	10.2265336137511	10.1809581486646	9.51603775961919	8.22847784778977	9.33110296146927	9.14936928072722	NA	

**TC1300000625.mm.2**	Cplx2	complexin 2	-0.247677045303925	9.34105760154992	-0.794992342926968	0.454951707376488	0.999692817443324	-4.60619371786251	9.67123945393866	9.51448761780692	9.28070110090091	8.90193112257213	9.40702346915383	9.27096284492707	NA
**TC0600001595.mm.2**	Cdkn1b	cyclin-dependent kinase inhibitor 1B	0.302879520610428	7.6636299095034	0.794930899255716	0.454984999903474	0.999692817443324	-4.60619657359324	8.12176643143797	7.97888549379803	7.84541492982755	7.39802835667509	7.70118513503673	6.93649911024505	NA
**TC0100002964.mm.2**	Dbi	diazepam binding inhibitor	-0.33099339702475	9.3847081363726	-0.794828552276861	0.455040459202653	0.999692817443324	-4.60620133007626	10.0050187262902	10.0793092281596	9.36829554982489	8.41267148504328	9.47140492471724	8.97154890420043	NA
**TC1500001730.mm.2**	Ly6c2	lymphocyte antigen 6 complex, locus C2	-0.260226477198255	6.83329920271772	-0.794626273925128	0.455150082805833	0.999692817443324	-4.6062107296044	6.12099288046724	6.59111360844935	6.86400940855971	7.01960847793688	7.33648233457046	7.06758850632265	NA
**TC0200002497.mm.2**	Kcnk15	potassium channel, subfamily K, member 15	-0.274545556933363	6.67187453084272	-0.794598574028657	0.455165096050713	0.999692817443324	-4.60621201664961	6.42697281576788	6.12413121013971	6.59403447909352	6.87149154354748	6.73121449164461	7.28340264486313	NA
**TC1100001222.mm.2**	Ccl12	chemokine (C-C motif) ligand 12	-0.569507373628486	5.36330650277437	-0.794430720946248	0.455256079258557	0.999692817443324	-4.60621981513234	4.77420799988253	6.81002220530283	4.45112294788012	5.27717408422183	5.00912710471936	5.85818467463956	NA
**TC0100001837.mm.2**	Batf3	basic leucine zipper transcription factor, ATF-like 3	0.283004179547047	5.1933881813274	0.794361807264276	0.455293436978094	0.999692817443324	-4.60622301656246	4.91457685159055	4.859622710253	5.15354013644161	5.82252880616585	5.03176646654076	5.37829411697261	NA
**TC0700001867.mm.2**	Zfp646	zinc finger protein 646	-0.255790450048003	6.04513702270485	-0.794119953827986	0.455424561394672	0.999692817443324	-4.60623425058772	6.63036725494434	6.96290662835087	5.72571599195091	5.35726768446849	5.87831728559958	5.71624729091583	NA
**TC0300002114.mm.2**	Rarres1	retinoic acid receptor responder (tazarotene induced) 1	0.327559978079128	5.79527389065974	0.794082698601991	0.455444762222123	0.999692817443324	-4.60623598088422	5.40305210876458	5.59016629373257	5.65055680819028	6.56621564061949	5.52921987263003	6.03243262002149	NA
**TC0600001931.mm.2**	Cadps2	Ca2+-dependent activator protein for secretion 2	0.372975008154948	7.11871426707565	0.794038888633331	0.455468518019313	0.999692817443324	-4.60623801554399	7.94520486430671	7.69274064237136	7.00540679466704	6.90473826137582	7.23440471983762	5.92979031989535	NA
**TC0100000884.mm.2**	Lrrfip1	leucine rich repeat (in FLII) interacting protein 1	-0.232476204590714	6.8910497748036	-0.793971343707546	0.455505145715231	0.999692817443324	-4.60624115237923	7.00929006388882	6.9166296348132	6.76251076356458	6.71520250690448	7.05797624046649	6.88468943918399	NA
**TC0400002515.mm.2**	Pigo	phosphatidylinositol glycan anchor biosynthesis, class O	0.294638777989743	6.1960844947989	0.793776834886514	0.455610633875035	0.999692817443324	-4.60625018453644	6.43371165753378	6.49247145085052	5.87511960036551	6.5446811078288	5.66106070412515	6.16946244808967	NA
**TC1400002598.mm.2**	Mzt1	mitotic spindle organizing protein 1	-0.263796330621582	8.23047284073273	-0.793722512838564	0.455640097466784	0.999692817443324	-4.60625270676174	8.77133047546041	8.85409067070633	8.10178189103912	7.51312972745411	8.10833596263029	8.0341683171061	NA
**TC0200004540.mm.2**	Gabpb1	GA repeat binding protein, beta 1	-0.306359508955985	6.89166233820946	-0.79370533401412	0.455649415322144	0.999692817443324	-4.60625350436774	7.44610416957675	7.06493849899361	6.86332957276291	6.24977659862429	7.1512852328118	6.57453995648737	NA
**TC1900000234.mm.2**	Olfr1420	olfactory receptor 1420	-0.318573037175473	4.60955265222652	-0.79350529358031	0.455757927807048	0.999692817443324	-4.60626279133562	4.34106498544047	4.26897377782911	4.15898038934071	5.04608514852859	4.6870706774987	5.15514093472154	NA
**TC0300000132.mm.2**	Trim55	tripartite motif-containing 55	0.362592943315938	5.98072544961034	0.79333343987395	0.455851164801658	0.999692817443324	-4.60627076850079	5.28878282838278	5.07467925277192	5.93375892521794	7.18927932635167	6.22496112856254	6.1728912363752	NA
**TC1600001706.mm.2**	Bbx	bobby sox HMG box containing	0.577150103149235	6.79935209326199	0.793286555773845	0.455876603483821	0.999692817443324	-4.60627294458728	7.49109853457008	7.58814175653958	6.90772057502207	6.52786566235831	7.32304284044539	4.95824319063652	NA
**TC1700001319.mm.2**	Foxn2	forkhead box N2	-0.557795130718667	6.69160280065285	-0.793233120392188	0.455905598017517	0.999692817443324	-4.6062754246436	7.43108916563434	7.26391186180548	6.95534032404926	5.2141724334707	5.89450734324693	7.39059567571036	NA
**TC0X00002676.mm.2**	Vsig4	V-set and immunoglobulin domain containing 4	-0.457776879725952	4.55185098838625	-0.79320477484744	0.455920979098091	0.999692817443324	-4.60627674017981	3.81415913549251	3.90550985874799	4.12291421095788	5.21502737735468	4.41595397810994	5.83754136965452	NA
**TC1700000604.mm.2**	Vps52	VPS52 GARP complex subunit	-0.441611312676547	8.22527657660922	-0.793077449467049	0.455990073878761	0.999692817443324	-4.60628264905931	8.87161022496583	9.05645164577972	8.38106114172268	6.88912634005567	8.48475121751399	7.66865888961745	NA
**TC1000000889.mm.2**	Matk	megakaryocyte-associated tyrosine kinase	-0.251942264806193	7.70290662428106	-0.793037646576581	0.456011674943925	0.999692817443324	-4.60628449609318	8.09001772306249	7.80244461322583	7.56232968785378	7.34821675203905	7.8713408277035	7.54309014180173	NA
**TC0100001349.mm.2**	3110040M04Rik	RIKEN cDNA 3110040M04 gene	-0.299065737749814	6.66468626341325	-0.792981214432963	0.456042301950124	0.999692817443324	-4.60628711469564	6.08638776529755	6.31197030052284	6.42709527804558	7.06871874153415	6.77084573051819	7.32309976456117	NA
**TC1800001147.mm.2**	Reep5	receptor accessory protein 5	-0.458750497928438	9.13121207003586	-0.792932576607948	0.45606869996009	0.999692817443324	-4.60628937152314	9.99042125068223	9.99730613490502	9.33139197656267	7.60963004282284	9.19319828169735	8.66532473354503	NA
**TC1100003141.mm.2**	Slc13a5	solute carrier family 13 (sodium-dependent citrate transporter), member 5	0.31712658105508	5.5697517200485	0.792746060147712	0.456169941052957	0.999692817443324	-4.60629802517235	6.12017252656875	6.4065161928068	5.12895781224482	5.63407956926797	4.65841931996284	5.4703648994398	NA
**TC0200004671.mm.2**	Pcna	proliferating cell nuclear antigen	-0.747425607339709	8.06609128732876	-0.792651580459095	0.456221230619865	0.999692817443324	-4.60630240816009	9.2256059340769	9.48169966429119	8.50922954931827	5.58796590614428	8.46206557672451	7.12998109341745	NA
**TC0300000708.mm.2**	Mrpl24	mitochondrial ribosomal protein L24	0.27762581835395	7.73492310805358	0.792543629429297	0.456279838234488	0.999692817443324	-4.60630741567592	8.12497215120084	8.17873738520975	7.39372893874722	7.93681143556216	7.62140509129334	7.15388364630814	NA
**TC0600003433.mm.2**	Itpr2	inositol 1,4,5-triphosphate receptor 2	0.304450876720629	5.68179535734851	0.792244791778476	0.456442107496782	0.999692817443324	-4.60632127550765	6.12643735260004	6.32330986389157	5.55183630326571	5.57312703725464	5.70478020624339	4.8112813808357	NA
**TC0300003166.mm.2**	5730460C07Rik	RIKEN cDNA 5730460C07 gene	-0.240365439598413	7.36003769933875	-0.792052604047555	0.456546487075355	0.999692817443324	-4.60633018720163	6.34219793091559	6.4102644900472	7.61096570068937	7.85255074724705	8.09427279363052	7.84997453350273	NA
**TC1000002239.mm.2**	Sgpl1	sphingosine phosphate lyase 1	0.307110788388964	7.77095605980354	0.792014028993804	0.456567439689069	0.999692817443324	-4.60633197574614	7.20849511056814	7.05176770786442	7.79086146388515	8.69898609469815	8.01324169259009	7.86238428921528	NA
**TC0400002097.mm.2**	Ints11	integrator complex subunit 11	-0.315652791935226	7.08449826481254	-0.791991647805506	0.456579596673057	0.999692817443324	-4.60633301343085	6.83789030952377	7.06366385722932	6.58768765728458	7.39937726184128	7.04871285428835	7.56965764870796	NA
**TC1700002662.mm.2**	Six2	sine oculis-related homeobox 2	-0.322039206069024	6.07387517102508	-0.791721175384622	0.456726529458835	0.999692817443324	-4.6063455521366	5.33294778913016	5.41681909785344	5.76978128793642	6.754921575578	6.43554506603915	6.73323620961331	NA
**TC0300001314.mm.2**	Pla2g12a	phospholipase A2, group XIIA	-0.245333881550606	5.3611358808858	-0.791636578260325	0.456772493224251	0.999692817443324	-4.60634947335997	5.37183813765869	5.19724088546918	5.4506870574747	5.10284719206817	5.44798714301303	5.59621486963104	NA
**TC1100002969.mm.2**	F930015N05Rik	RIKEN cDNA F930015N05 gene	-0.281595910135793	5.33953650024623	-0.791602810390142	0.45679084107051	0.999692817443324	-4.60635103848269	5.47702107150685	5.1712003383283	4.95257430689955	5.46032857878576	5.25653803694477	5.71955666901212	NA
**TC0800000979.mm.2**	Fbxw9	F-box and WD-40 domain protein 9	0.483277230429678	6.68997307813223	0.791517222380445	0.456837347816306	0.999692817443324	-4.60635500524536	6.7609311534162	6.9115347400166	7.38201808591095	6.33494543219902	7.18357450447091	5.56683455277971	NA
**TC1800001227.mm.2**	Pcdh1	protocadherin 1	0.372536413184235	9.34706843586902	0.791457652982263	0.456869718547303	0.999692817443324	-4.60635776595307	10.0102656233015	10.0454668104274	9.81141432607741	8.57446117784945	9.07359344786633	8.56720922969205	NA
**TC0200000306.mm.2**	4930426L09Rik	RIKEN cDNA 4930426L09 gene	0.342727127508448	5.26153969959318	0.791381786700098	0.456910947528643	0.999692817443324	-4.60636128173301	4.92940472523749	4.57609439348285	5.19956668761929	6.17502997930853	5.08282296133413	5.60631945057679	NA
**TC1400001499.mm.2**	Nr1d2	nuclear receptor subfamily 1, group D, member 2	-0.47550806535813	7.99843292970261	-0.790888640898955	0.457179007576899	0.999692817443324	-4.60638412961477	8.71951431366309	8.6142064049835	8.29936111614862	6.5535692482778	8.26545413902421	7.53849235611847	NA
**TC0200004608.mm.2**	Pdyn	prodynorphin	0.273896500551137	7.65132323621962	0.7908759043522	0.457185932260728	0.999692817443324	-4.60638471958665	8.82043499625392	8.36122577635646	7.42320932008358	7.21382650282124	6.80689166811155	7.282351153691	NA
**TC1200001758.mm.2**	Baz1a	bromodomain adjacent to zinc finger domain 1A	-0.280465988562568	8.06210481726985	-0.790848764800477	0.457200687903628	0.999692817443324	-4.60638597670196	6.69417100651378	6.85205336444917	8.33143673174895	8.80129954601654	8.56841250322231	9.12525575166833	NA
**TC0800002961.mm.2**	Kars	lysyl-tRNA synthetase	-0.340472614885118	7.99456486710175	-0.790828269507536	0.457211831316227	0.999692817443324	-4.60638692603371	8.62822381264129	8.95845737609126	7.87832086578747	6.97156052626637	8.07799898888929	7.4528276329348	NA
**TC1100000955.mm.2**	Psmb6	proteasome (prosome, macropain) subunit, beta type 6	0.242392089524984	8.31343268880188	0.790768633575931	0.457244256808641	0.999692817443324	-4.60638968824862	9.04941282282516	8.97959323527287	8.10429759358337	8.06388953329824	8.02887999947431	7.65452294835733	NA
**TC0600000838.mm.2**	Immt	inner membrane protein, mitochondrial	0.289067796673658	8.10082183161682	0.790660345116738	0.457303139969087	0.999692817443324	-4.60639470360049	8.63120964862082	8.42777797047435	8.27639602871477	7.78564345326176	8.0481833764988	7.43572051213041	NA
**TC1300001729.mm.2**	E2f3	E2F transcription factor 3	-0.351962638401294	6.54286251724836	-0.790632278339532	0.457318402485358	0.999692817443324	-4.60639600343253	6.3225902655632	6.22267972126232	7.10896113572743	5.89502878420361	6.82492214577853	6.8829930509551	NA
**TC0600003050.mm.2**	Mical3	microtubule associated monooxygenase, calponin and LIM domain containing 3	-0.232922839931015	7.21912956823011	-0.790473397811284	0.457404807324842	0.999692817443324	-4.60640336095684	7.41005248716646	7.2582546076944	7.02449637664351	7.06581594068536	7.19124407647036	7.36491392072054	NA
**TC0X00001797.mm.2**	Rbm3	RNA binding motif (RNP1, RRM) protein 3	-0.298561145495688	9.6596708440502	-0.790450790773863	0.457417102758636	0.999692817443324	-4.60640440777682	10.457313242504	10.7587749455245	9.37879186069343	8.69361543194723	9.56537836077678	9.10415122285525	NA
**TC0800001951.mm.2**	Brf2	BRF2, RNA polymerase III transcription initiation factor 50kDa subunit	-0.460888032138896	6.03095851530292	-0.790253474358849	0.457524428320993	0.999692817443324	-4.60641354368969	6.28341439491333	6.5719104932429	6.50057741036629	4.70374765932547	6.04678954010809	6.07931159386146	NA
**TC1900001196.mm.2**	Tle4	transducin-like enhancer of split 4	-0.539635917125709	7.82998824140397	-0.790244165736917	0.457529491959508	0.999692817443324	-4.60641397464963	8.76876951570948	8.65148707667392	8.21120820914535	6.02899230174915	7.85963738079335	7.45983496435257	NA
**TC0500000313.mm.2**	Trim54	tripartite motif-containing 54	-0.383374695424771	5.39373745791145	-0.789900659520835	0.457716377483435	0.999692817443324	-4.60642987557912	5.14615065313534	4.94933104149299	4.68948449624156	6.06061233475385	5.64761850950185	5.86922771234311	NA
**TC0700004639.mm.2**	Mia	melanoma inhibitory activity	-0.264122257604894	6.7221249269822	-0.789856334092225	0.457740496749056	0.999692817443324	-4.6064319270763	6.17653045713714	6.5735048982593	6.5546616453815	6.97257320026198	6.92272604444552	7.13275331640776	NA
**TC0200001932.mm.2**	Spdye4c	speedy/RINGO cell cycle regulator family, member E4C	0.41117597446229	4.90056709734389	0.789831946993817	0.457753767138891	0.999692817443324	-4.60643305574302	4.68625364362622	4.54901829892151	4.81872584033016	5.67651545488995	4.19046805487373	5.4824212914218	NA
**TC0300000763.mm.2**	Shc1	src homology 2 domain-containing transforming protein C1	-0.413427441015727	6.52012548339959	-0.789762987655009	0.457791293245007	0.999692817443324	-4.60643624714739	6.15256124514466	6.1699953354217	7.09822070524183	5.88745001365803	7.40536242665932	6.40716317427199	NA
**TC1500000297.mm.2**	Baalc	brain and acute leukemia, cytoplasmic	-0.838187226654222	9.88580685248398	-0.789725855165908	0.45781150079401	0.999692817443324	-4.60643796554504	10.6365925290435	10.6636525017835	10.7007917199119	7.46831909547236	10.7854261116033	9.0600591570894	NA
**TC0200002127.mm.2**	Snrpb2	U2 small nuclear ribonucleoprotein B	-0.255774898564713	7.02626557155947	-0.789584581399322	0.457888387867024	0.999692817443324	-4.60644450285292	7.62331170126258	7.6812123040259	6.5506346831265	6.62012513034297	7.10555568420296	6.57675392639594	NA
**TC0X00001796.mm.2**	Wdr13	WD repeat domain 13	-0.3889249846577	7.80356548833213	-0.789554714543961	0.45790464381172	0.999692817443324	-4.60644588481467	8.58691326761475	8.5576715238855	7.92224637682074	6.52723270776781	7.44632315599923	7.78100589790472	NA
**TC1300001204.mm.2**	Elovl7	ELOVL family member 7, elongation of long chain fatty acids (yeast)	-0.536680586182547	6.86845411950967	-0.789553644363602	0.457905226297433	0.999692817443324	-4.60644593433208	7.1140796655336	7.40066929964604	7.4596239609368	5.35168332881983	7.24023768299454	6.64443077912719	NA
**TC1300001099.mm.2**	Gfm2	G elongation factor, mitochondrial 2	-0.548248474258457	6.03745308156505	-0.789509331889717	0.457929345474977	0.999692817443324	-4.60644798463839	6.86781602488287	6.9513847861921	6.17517737082291	4.47933299407628	6.62671701478418	5.12429029863193	NA
**TC0900000019.mm.2**	Gucy1a2	guanylate cyclase 1, soluble, alpha 2	0.286112096211038	5.89341648201436	0.789352974560064	0.458014457511311	0.999692817443324	-4.6064552185761	6.7046268028835	6.41871943469767	5.34894301791239	6.05574540555115	5.52419957016243	5.30826466087903	NA
**TC0400004016.mm.2**	Fbxo44	F-box protein 44	0.293866898226463	8.42668137874547	0.789301785789503	0.45804232417088	0.999692817443324	-4.60645758664176	8.87140628886342	8.41387937852963	8.54067371513936	8.39059448562698	8.52146551770635	7.82206888660707	NA
**TC1500002327.mm.2**	Prlr	prolactin receptor	0.301500909839629	5.45887502012897	0.789097850423514	0.458153356339398	0.999692817443324	-4.60646701998204	6.11432398353918	6.14059612988954	4.9019550533419	5.6487108601703	5.22754229646782	4.72012179736512	NA
**TC0500003591.mm.2**	Atp5j2	ATP synthase, H+ transporting, mitochondrial F0 complex, subunit F2	-0.295534082696932	9.60422732956229	-0.789076523276881	0.458164968945523	0.999692817443324	-4.60646800640916	10.1415974777965	10.1571255101377	9.47507039572578	8.89271601629701	9.79681828120009	9.16203629621657	NA
**TC0100000548.mm.2**	Crygf	crystallin, gamma F	-0.268913106460573	6.10094455423913	-0.789020565547079	0.458195438842002	0.999692817443324	-4.60647059449318	5.93734273876176	5.83616804802407	6.12612787751969	6.02103728534423	6.02199797053193	6.66299340525313	NA
**TC0900002805.mm.2**	Mthfsl	5, 10-methenyltetrahydrofolate synthetase-like	0.392037808534275	5.71463345379487	0.788825211150198	0.458301823608064	0.999692817443324	-4.60647962882516	6.44732709046115	6.17515831376918	5.78566677991121	5.43902868789251	5.91325992521743	4.52735992551775	NA
**TC1400000530.mm.2**	Tmem260	transmembrane protein 260	0.275503263277772	5.19140696702032	0.788735950289878	0.458350438428461	0.999692817443324	-4.60648375628123	5.41486414822493	5.25737396967851	5.53715072242051	4.9764543829665	5.18637507132957	4.7762235075019	NA
**TC0600003324.mm.2**	E330021D16Rik	RIKEN cDNA E330021D16 gene	0.359016054383559	5.65147717609796	0.788718310327117	0.458360046242126	0.999692817443324	-4.60648457192349	4.75879791888584	4.94593715759854	5.7022411844887	6.75883885994655	5.53550583714335	6.20754209852478	NA
**TC1400002093.mm.2**	Mdp1	magnesium-dependent phosphatase 1	0.303254573082302	7.08247536641799	0.788671104884887	0.458385757935442	0.999692817443324	-4.60648675456493	7.3241079345696	7.30446480152658	6.69076362336525	7.54563068092293	6.65017902387814	6.97970613424543	NA
**TC0100001734.mm.2**	Cnih3	cornichon family AMPA receptor auxiliary protein 3	-0.401909145196987	7.70334350984384	-0.788335238904429	0.458568725345264	0.999692817443324	-4.60650228154616	8.59392787412816	8.65462099870531	7.59781891937596	6.48602802854183	7.9540613867265	6.93360385158526	NA
**TC0100000041.mm.2**	Vxn	vexin	-0.311256261176506	8.55449296175392	-0.788334150740422	0.45856931821989	0.999692817443324	-4.60650233184458	8.83360923265992	9.18316513068064	8.34901007385149	7.99482536856347	8.90016370741549	8.06618425735249	NA
**TC1000002534.mm.2**	Mrpl54	mitochondrial ribosomal protein L54	-0.286129302224587	7.33973953760939	-0.788242919408335	0.458619026550031	0.999692817443324	-4.60650654868649	6.94427938764476	6.57352219401439	7.34912670650307	7.62506181327092	7.44945443624767	8.0969926879755	NA
**TC0900002405.mm.2**	Bbs4	Bardet-Biedl syndrome 4 (human)	-0.671786185816677	7.009654856434	-0.788144033458756	0.458672909831345	0.999692817443324	-4.60651111897337	8.6649769187434	8.38035955961486	7.25523951309992	4.57927063120628	7.09907102084956	6.07901149508999	NA
**TC0200000461.mm.2**	Adamts13	a disintegrin-like and metallopeptidase (reprolysin type) with thrombospondin type 1 motif, 13	0.408834834039308	5.05296148588381	0.788080051956405	0.458707775921719	0.999692817443324	-4.60651407585409	3.73307578900675	4.13633463936964	5.14460344562445	6.48841063187808	5.07938001105725	5.73596439836668	NA
**TC1000002940.mm.2**	Mdm2	transformed mouse 3T3 cell double minute 2	0.314529144087325	7.95971748836727	0.788064977159604	0.45871599105362	0.999692817443324	-4.6065147725071	8.309904688451	8.47833664841239	8.29862120835464	7.5009397324028	7.86909885736849	7.30140379521429	NA
**TC1200002535.mm.2**	Serpina11	serine (or cysteine) peptidase inhibitor, clade A (alpha-1 antiproteinase, antitrypsin), member 11	0.248203190280393	7.00508726242902	0.788052567178608	0.45872275404987	0.999692817443324	-4.60651534600415	6.72678020146168	6.73500141179922	7.49635548234795	7.03621868858904	7.04176600432828	6.99440178604793	NA
**TC0900002783.mm.2**	Snap91	synaptosomal-associated protein 91	0.451580242857679	9.08914574667178	0.787919520003336	0.458795264385697	0.999692817443324	-4.60652149408219	9.83849484273484	9.81124622292058	9.5429929183028	8.35115403174249	9.15535462328955	7.83563184104038	NA
**TC0600000912.mm.2**	M1ap	meiosis 1 associated protein	0.318978092536723	6.87246864968052	0.787801557010962	0.458859560584017	0.999692817443324	-4.60652694455279	5.73245757245269	6.28599274516786	7.0610225129184	7.8661363698496	6.99285293973304	7.29634975796152	NA
**TC1000002111.mm.2**	Lin28b	lin-28 homolog B (C. elegans)	0.446196042471828	5.46551701748032	0.78767700140708	0.45892745694609	0.999692817443324	-4.60653269905186	5.33899780331107	5.3423167556919	4.89387476558428	6.60821504982702	5.12714415476016	5.48255357570748	NA
**TC0600002237.mm.2**	Igf2bp3	insulin-like growth factor 2 mRNA binding protein 3	0.2746112405807	6.46193590777185	0.787202613603016	0.459186114220722	0.999692817443324	-4.60655461040106	5.41988704457654	5.08882042216114	6.89035945997127	7.51570577055614	6.90254110559081	6.9543016437752	NA
**TC0X00000363.mm.2**	Zbtb33	zinc finger and BTB domain containing 33	-0.546538492451552	5.4684153293219	-0.787136299723781	0.459222279601309	0.999692817443324	-4.60655767265991	6.97646227228767	7.11540518240558	5.51651940438903	3.2962543637785	5.17079380256959	4.73505695050104	NA
**TC1600001304.mm.2**	Prodh	proline dehydrogenase	-0.238022873624057	6.70576098273314	-0.787052451380711	0.45926801054272	0.999692817443324	-4.60656154438648	6.38439297496051	6.45969243609891	6.81459052339512	6.64262684565054	7.10740313283124	6.82585998346253	NA
**TC0700000933.mm.2**	Cyfip1	cytoplasmic FMR1 interacting protein 1	0.301324758266926	7.92358176706008	0.786815510473868	0.459397255463565	0.999692817443324	-4.60657248374782	8.91917677923422	9.0667660793102	7.95150877208033	7.12758985809462	7.06223620389504	7.41421290974605	NA
**TC0200002296.mm.2**	Mcts2	malignant T cell amplified sequence 2	0.334380469419453	7.373611788026	0.786802722115551	0.459404231893078	0.999692817443324	-4.60657307411383	7.24876797523243	7.16825424999357	7.08684040641656	8.1598643144679	7.44966238370421	7.12828139834135	NA
**TC1500000655.mm.2**	Gga1	golgi associated, gamma adaptin ear containing, ARF binding protein 1	0.281096876709297	6.75432947842175	0.786795233331536	0.459408317281663	0.999692817443324	-4.6065734198256	6.89508336652234	6.54287401794623	6.57531825401477	7.24978836572549	6.61660937596733	6.64630349035433	NA
**TC1000000843.mm.2**	Cirbp	cold inducible RNA binding protein	-0.35572810549707	6.88253379766869	-0.786784838846334	0.459413987871977	0.999692817443324	-4.60657389967239	8.06172616487842	7.78873446125366	6.6978665619914	5.66877641245157	6.86836284370906	6.20973634172805	NA
**TC0400004222.mm.2**	Rcc1	regulator of chromosome condensation 1	0.320226760443767	6.48435150278111	0.786753005085294	0.45943135471356	0.999692817443324	-4.60657536920722	6.15306395886262	6.47608271409388	6.24153163818527	7.21717629412356	6.35276992458007	6.46548448684123	NA
**TC0100002008.mm.2**	Eloc	elongin C	-0.536264836901773	7.70731063197718	-0.786731090144006	0.459443310630277	0.999692817443324	-4.60657638083903	8.58812278898196	8.76949385833669	7.71242686156144	6.19443187380899	8.29231097644637	6.68707743272761	NA
**TC0700002710.mm.2**	Eif3k	eukaryotic translation initiation factor 3, subunit K	-0.361280011018547	8.61113929012594	-0.786474454341036	0.459583337045078	0.999692817443324	-4.60658822621269	9.45650593079301	9.4664398750302	8.62576360702072	7.38490134942693	8.53420567492888	8.19901930355587	NA
**TC1200002152.mm.2**	Gtf2a1	general transcription factor II A, 1	-0.466876907928374	7.93172303534183	-0.786443967999612	0.459599973076269	0.999692817443324	-4.60658963318196	8.27915385003576	8.43955964059279	8.34459433975909	6.62434111302376	8.29988039612914	7.60280887251046	NA

**TC1700002316.mm.2**	Sema6b	sema domain, transmembrane domain (TM), and cytoplasmic domain, (semaphorin) 6B	0.232927221953888	8.18688679606813	0.786325025576963	0.459664882551181	0.999692817443324	-4.60659512212723	8.7025768155806	8.53831358173741	8.01093126800387	8.16221114349541	7.78864532206144	7.91864264553005	NA
**TC1700000628.mm.2**	H2-Eb2	histocompatibility 2, class II antigen E beta2	-0.289535562547381	5.74587134372306	-0.786318188648909	0.459668613806486	0.999692817443324	-4.60659543762061	4.92066638776108	5.08353240126064	5.62151342501725	6.32446564909367	6.06169249643823	6.46335770276746	NA
**TC1900001073.mm.2**	Uqcc3	ubiquinol-cytochrome c reductase complex assembly factor 3	0.227301504368102	6.64898162643635	0.786195421903615	0.459735617389212	0.999692817443324	-4.60660110244424	7.06919957309726	7.16591489070896	6.54228833416004	6.51440081761401	6.27305222281972	6.32903392021812	NA
**TC0700003022.mm.2**	Cyth2	cytohesin 2	-0.258696696368803	8.35650089001719	-0.78610194240124	0.459786641164936	0.999692817443324	-4.60660541547627	8.70689582480126	8.75561115146978	8.25245473413161	7.82709775141565	8.49068444339468	8.10626143489018	NA
**TC1100001419.mm.2**	Mbtd1	mbt domain containing 1	0.324473498360226	6.88453843968179	0.786092119974876	0.459792002754592	0.999692817443324	-4.6066058686516	7.23644185758819	6.89712622129615	7.4533855292551	6.45791924870833	6.55467996860478	6.7076778126382	NA
**TC1800000438.mm.2**	Tcerg1	transcription elongation regulator 1 (CA150)	0.462236704917203	7.00918434682613	0.785770606440869	0.459967525607336	0.999692817443324	-4.606620700197	8.02043092652442	8.0679462254552	7.24327593483946	6.20232523456631	7.01935244791914	5.50177531165222	NA
**TC0800002119.mm.2**	Asah1	N-acylsphingosine amidohydrolase 1	-0.518468859302182	7.16122949553793	-0.785706906359855	0.460002306737943	0.999692817443324	-4.60662363823194	8.31006080922367	8.34316356519799	7.34451567849681	5.29409176160397	7.12638345137654	6.5491617073286	NA
**TC0700002057.mm.2**	Ap2a2	adaptor-related protein complex 2, alpha 2 subunit	-0.620879463501594	6.64363045499102	-0.785673495994269	0.460020549996214	0.999692817443324	-4.60662517915339	8.21043280375251	8.32841917773595	6.82471099754083	4.21587491318642	6.3349259362704	5.94741890146004	NA
**TC1700001386.mm.2**	Tmem181c-ps	transmembrane protein 181C, pseudogene	0.437498894545758	7.62406982422529	0.785517392326322	0.460105794889872	0.999692817443324	-4.6066323782473	8.61354031717373	7.96551858493069	8.27051639426965	6.74966252189978	7.29332095155398	6.85186017552393	NA
**TC1200000484.mm.2**	Pax9	paired box 9	-0.272488124994039	6.34357148395876	-0.785278302040059	0.460236378378901	0.999692817443324	-4.60664340263823	6.10975383934069	5.94105984956579	6.15472305728881	6.57809642514019	6.3747512536368	6.90304447878027	NA
**TC1300000405.mm.2**	Cdyl	chromodomain protein, Y chromosome-like	0.290263398479943	6.919903986	0.785208677764013	0.460274409798539	0.999692817443324	-4.60664661257926	6.93584285082142	6.68639542035737	7.17541854552034	7.06343767537022	7.22317385501581	6.43515556891487	NA
**TC1100004117.mm.2**	Qrich2	glutamine rich 2	0.2455912751265	4.93037636111173	0.784967111353552	0.460406379530561	0.999692817443324	-4.60665774824328	4.44571786099439	4.47960073971994	5.06566138476687	5.50839967333766	5.04160092584318	5.04127758200834	NA
**TC1900001522.mm.2**	Sfrp5	secreted frizzled-related sequence protein 5	0.32801524223222	5.95998741185488	0.784480993421217	0.460672029771474	0.999692817443324	-4.60668015032286	5.27082545803476	5.8972168376125	5.87864995225323	6.74530637771999	5.87986775510159	6.08805809040719	NA
**TC1100000558.mm.2**	Olfr54	olfactory receptor 54	-0.323343749658132	4.79478465920939	-0.784443797659797	0.460692360645458	0.999692817443324	-4.60668186406124	4.01668303982924	4.45160512517111	4.76177471553932	5.06509142993054	4.77790314805601	5.69565049673011	NA
**TC1400001621.mm.2**	Slmap	sarcolemma associated protein	0.437665530194643	6.09534417653013	0.784432223332011	0.46069868719821	0.999692817443324	-4.60668239731991	6.99988298755195	6.92157667741608	6.213234528526	5.54973369877502	6.23368330697948	4.65395385993225	NA
**TC0400003667.mm.2**	Trnau1ap	tRNA selenocysteine 1 associated protein 1	0.29082384587525	7.31290432466822	0.784243015949298	0.460802116954244	0.999692817443324	-4.60669111384774	6.67505213408179	6.79955076387506	7.35985357067544	8.13238180022604	7.60906142751221	7.30152625163876	NA
**TC1300000258.mm.2**	Hist1h1c	histone cluster 1, H1c	0.31774717853289	6.6392455351423	0.78415830640777	0.460848428465197	0.999692817443324	-4.60669501585145	6.67715225458397	6.9173093240483	7.09165885831466	6.346594136329	6.73197271729664	6.07078592028124	NA
**TC1800001153.mm.2**	Brd8	bromodomain containing 8	0.279788632304444	7.92862546201208	0.784139131927425	0.460858911786058	0.999692817443324	-4.60669589905326	8.72898814101042	8.57618985105515	7.88113135392556	7.53194466838234	7.75475706901365	7.09874168868536	NA
**TC0900002054.mm.2**	Tbrg1	transforming growth factor beta regulated gene 1	0.283392602701254	7.02929359583687	0.784069010943548	0.460897250656403	0.999692817443324	-4.60669912879657	8.05696975328897	7.81265880327936	7.08492029861939	6.3515388133083	6.50704202969254	6.36263187683265	NA
**TC0200002437.mm.2**	Slc32a1	solute carrier family 32 (GABA vesicular transporter), member 1	0.415050062487354	8.28851197866819	0.784033940386657	0.460916426428633	0.999692817443324	-4.60670074406023	9.3727691181609	9.01655659501109	8.79052550056234	7.29539764134358	7.84016071262922	7.415662304302	NA
**TC1700000782.mm.2**	Olfr122	olfactory receptor 122	0.409275215882094	6.03693434058712	0.783959841549196	0.460956943793942	0.999692817443324	-4.60670415671358	5.1204069636125	5.34686203048618	6.10951947410626	7.17692426648786	5.67427672539242	6.7936165834375	NA
**TC1500001927.mm.2**	Rangap1	RAN GTPase activating protein 1	-0.31373776964805	7.72298166538515	-0.783955317466178	0.460959417650335	0.999692817443324	-4.60670436506524	8.59958485410607	8.26860803405135	7.45270815286275	6.96840262956593	7.92863726006672	7.11994906165806	NA
**TC1700002789.mm.2**	H2-Q7/H2-Q9	histocompatibility 2, Q region locus 7/histocompatibility 2, Q region locus 9	0.353274089558591	5.57664171545392	0.78393556535633	0.460970218595728	0.999692817443324	-4.60670527471758	5.21071346851818	5.4082918643789	5.67965004255175	6.09404652692007	4.95493098510294	6.11221740525169	NA
**TC0400001062.mm.2**	Tctex1d1	Tctex1 domain containing 1	0.387673907031394	4.8953898587116	0.783916915920972	0.46098041673316	0.999692817443324	-4.60670613357408	4.56712710216415	4.7142448800279	4.63738780345544	5.79576968861473	4.40459567399747	5.25321400400991	NA
**TC1700002418.mm.2**	Twsg1	twisted gastrulation BMP signaling modulator 1	-0.231376600099367	8.31366479301968	-0.783718908704614	0.461088703425369	0.999692817443324	-4.60671525150452	7.68900011442345	7.82431392424789	8.48252951987344	8.47043123975056	8.60691936131861	8.80879459850411	NA
**TC0X00000439.mm.2**	Rbmx2	RNA binding motif protein, X-linked 2	-0.331074167834291	6.49367328209397	-0.78371075762907	0.461093161486252	0.999692817443324	-4.6067156268165	7.14386442960978	6.88706007697669	6.51006502244986	5.62441840270451	6.70780076345735	6.0888309973656	NA
**TC0500001510.mm.2**	Nipsnap2	nipsnap homolog 2	-0.526908765486791	8.56669439109006	-0.783541833916689	0.461185557551692	0.999692817443324	-4.60672340423896	9.44638988521926	9.37729476094793	8.91127753300368	6.85005455169611	8.76145601003837	8.053693605635	NA
**TC1100004113.mm.2**	Exoc7	exocyst complex component 7	0.399900691763281	6.38410191991488	0.783540542190583	0.461186264135771	0.999692817443324	-4.6067234637071	7.20522685328981	7.2875187924792	6.44464603008591	5.86118759853751	6.44965114403562	5.05638110106125	NA
**TC0100003128.mm.2**	Ipo9	importin 9	-0.234349309110145	7.991061327318	-0.783537289057367	0.46118804362791	0.999692817443324	-4.60672361347368	8.54908717333751	8.46739584375073	7.75643857768728	7.47415458661246	7.87401487211174	7.82527691040829	NA
**TC1400000573.mm.2**	Apex1	apurinic/apyrimidinic endonuclease 1	-0.240173821112854	6.45939752161968	-0.783169751893073	0.46138912045522	0.999692817443324	-4.60674053137017	6.67942434116837	6.58730480471355	6.18588070519427	6.31877346561094	6.31812916118183	6.66687265184909	NA
**TC1100001035.mm.2**	Olfr401	olfactory receptor 401	-0.28032683752372	4.78962655890694	-0.783165230844605	0.461391594267153	0.999692817443324	-4.60674073944333	4.4345063419399	4.4869032086547	4.45606722871536	5.17178083518445	4.96098450040629	5.22751723854096	NA
**TC0700000484.mm.2**	Fcgbp	Fc fragment of IgG binding protein	0.290860324619109	5.61820758512953	0.783016810302646	0.461472811645461	0.999692817443324	-4.60674756979169	5.25842405679758	5.44055479465988	5.48584974451072	6.31014390976826	5.5994861287798	5.61478687626096	NA
**TC0700002977.mm.2**	Med25	mediator complex subunit 25	0.346262297242458	7.95265499496851	0.782807215194024	0.46158752138991	0.999692817443324	-4.60675721394935	8.22644620054557	8.37220559589645	8.35863385256516	7.54626753136181	8.04592574509354	7.16645104434852	NA
**TC0200000783.mm.2**	Mbd5	methyl-CpG binding domain protein 5	0.428066545706441	8.44878155808435	0.782744999200934	0.461621575532683	0.999692817443324	-4.60676007638223	9.47245437264151	9.33173217968539	8.72014120719848	7.65217673659755	8.39535105244284	7.12083379994031	NA
**TC0900001624.mm.2**	Nktr	natural killer tumor recognition sequence	1.02432826236876	6.45370899141911	0.78269184759217	0.461650669638651	0.999692817443324	-4.60676252166173	7.75641886715535	7.73186475297477	7.16858799072456	5.47272543583646	7.42295361518683	3.16970328663668	NA
**TC0900003110.mm.2**	Ptpn23	protein tyrosine phosphatase, non-receptor type 23	0.443374791615808	5.52162874824194	0.78264881385417	0.46167422636411	0.999692817443324	-4.60676450138052	6.35151415300272	5.85059466807447	6.20346462036284	4.7037420054402	4.63416162478641	5.38629541778501	NA
**TC1200000145.mm.2**	Hpcal1	hippocalcin-like 1	0.283882125258339	7.92577317734697	0.78259507249861	0.461703645629056	0.999692817443324	-4.60676697359008	8.68132118183984	8.18832723505404	7.95257234803098	7.67380510082133	7.80760620588829	7.25100699244734	NA
**TC0100001231.mm.2**	Rabif	RAB interacting factor	0.247470006174272	6.31768867159912	0.782497657566881	0.461756976150683	0.999692817443324	-4.60677145458482	6.58777630164847	6.80458075515916	6.39659441134388	6.10776308122394	6.15813125630505	5.85128622391422	NA
**TC0300001080.mm.2**	Ppm1j	protein phosphatase 1J	0.391890700274015	5.63890356220891	0.782460882595298	0.461777109994881	0.999692817443324	-4.60677314610274	4.90314807174132	5.09361757018363	5.43455671975405	6.87566184618422	5.64991017324001	5.87652699215023	NA
**TC0300000609.mm.2**	Fstl5	follistatin-like 5	-0.494960871131655	7.121341113937	-0.782416741005695	0.461801277778941	0.999692817443324	-4.60677517638939	8.00223932207927	7.58380292218287	7.46591365315465	5.61012769539362	7.37436416238619	6.69159892842539	NA
**TC0200002475.mm.2**	L3mbtl1	L3MBTL1 histone methyl-lysine binding protein	-0.302644796390664	5.67930688495766	-0.782277200278763	0.461877682952993	0.999692817443324	-4.60678159404815	5.44649380454129	4.99684731070226	5.35476701109383	6.15883828976672	5.99922736961706	6.11966752402481	NA
**TC0100000436.mm.2**	Ndufb3	NADH:ubiquinone oxidoreductase subunit B3	0.226445532984229	6.97019361642368	0.782173237461887	0.461934613252659	0.999692817443324	-4.60678637494008	7.8688766732809	7.83049932329169	6.62196502939179	6.66537335457717	6.47385500044456	6.36059231755595	NA
**TC1200001071.mm.2**	Ak7	adenylate kinase 7	0.240833879834698	5.47750747924686	0.782104531796898	0.461972239328206	0.999692817443324	-4.60678953424608	6.25946834769396	5.97396948372014	5.36855536640509	5.18808203546313	4.9330087496366	5.14196089256222	NA
**TC0700002162.mm.2**	Syt5	synaptotagmin V	-0.499294002184008	9.32712970949012	-0.781854442174566	0.46210921680766	0.999692817443324	-4.60680103261376	10.6833683621514	10.5267644545451	9.44409518404593	7.43293353389216	9.1065923744102	8.7690243478959	NA
**TC1100000555.mm.2**	Olfr1377	olfactory receptor 1377	0.36221192942486	4.58985324848845	0.781788097863932	0.462145559230519	0.999692817443324	-4.60680408251654	4.11881758801305	3.55256327307935	5.09064228244636	5.20543896189765	4.20162230270874	5.37003508278555	NA
**TC0200004129.mm.2**	Mdk	midkine	0.235400571352874	7.73448958052944	0.781695584119649	0.462196240199176	0.999692817443324	-4.60680833516355	7.37980348663466	7.49406740364906	7.88307688439792	8.11885698340142	7.86031147476197	7.67082125033162	NA
**TC0800001490.mm.2**	Ctu2	cytosolic thiouridylase subunit 2	0.253424852673197	7.14088980407107	0.781677113182345	0.462206359429071	0.999692817443324	-4.60680918419072	6.56794520591695	6.50557636939202	7.30519090431025	7.83414257292166	7.27627904944988	7.35620472243564	NA
**TC0700001793.mm.2**	Il4ra	interleukin 4 receptor, alpha	0.306194274702076	6.64728422518818	0.781641846453838	0.462225680594904	0.999692817443324	-4.60681080520908	6.57434342024594	6.60137672432782	6.41189931703973	7.24828756094001	6.29391790442276	6.75388042415282	NA
**TC1400000366.mm.2**	Gm5460	predicted gene 5460	0.362975143586787	5.36476870145253	0.781358285039204	0.462381052463448	0.999692817443324	-4.60682383721276	4.94360099476059	4.88126259180067	5.13223727178386	6.41261218287987	5.54217340579685	5.27672576169331	NA
**TC0900003044.mm.2**	Rbm6	RNA binding motif protein 6	0.353790474479737	7.14235169381903	0.781228226133473	0.462452327822555	0.999692817443324	-4.60682981345195	8.42212297665853	8.116730242792	6.76378774424201	6.74763120196955	7.01068335126236	5.79315464598972	NA
**TC1500002032.mm.2**	Ttll8	tubulin tyrosine ligase-like family, member 8	-0.321849704859229	5.38027375953778	-0.781130428102838	0.46250592849295	0.999692817443324	-4.60683430686145	4.68057225424474	4.80945114257572	5.17742498216259	5.89653489318127	5.4754406148402	6.24221867022212	NA
**TC1700001684.mm.2**	Capn15	calpain 15	0.367140698058295	5.67971363657302	0.781085116053107	0.462530764366452	0.999692817443324	-4.60683638863374	5.47762847936942	5.56753841293346	5.39595967081957	6.48773849080633	5.19401829547911	5.9553984700302	NA
**TC0100000375.mm.2**	Stat1	signal transducer and activator of transcription 1	-0.325573402454598	5.99669580902488	-0.781060935802557	0.462544018122325	0.999692817443324	-4.60683749951464	6.57040429368611	7.21329994429861	4.95325528631887	5.81940661930882	5.66353493675026	5.76027377378662	NA
**TC0500001139.mm.2**	Pole	polymerase (DNA directed), epsilon	0.512597747590425	4.92871220604102	0.780829704533662	0.462670774683426	0.999692817443324	-4.60684812151167	4.47619866664956	4.38876554093192	4.37876124489508	6.48749101702767	5.00209931969884	4.83895744704305	NA
**TC1400001125.mm.2**	Tdrd3	tudor domain containing 3	-0.54659423552626	5.83969806608429	-0.780727687877042	0.462726705944413	0.999692817443324	-4.60685280715512	6.62483512656233	6.63895739203433	5.54684043614352	4.79376326728477	6.79797446761866	4.63581770686214	NA
**TC0400000501.mm.2**	Ncbp1	nuclear cap binding protein subunit 1	-0.294955745859435	7.3166609286259	-0.780706637169081	0.462738247710073	0.999692817443324	-4.60685377396739	7.38803787531011	7.36415644896814	6.77272647638339	7.50620340149573	7.19180373967256	7.67703762992544	NA
**TC0800001906.mm.2**	Tacc1/Mir8108	transforming, acidic coiled-coil containing protein 1/microRNA 8108	0.254019664718054	7.34597829701156	0.780627810737081	0.462781468757354	0.999692817443324	-4.60685739413675	8.03115626921657	8.26401648347439	7.28575545054582	6.85861272886143	6.94406141748674	6.6922674324844	NA
**TC0900000403.mm.2**	Olfr906	olfactory receptor 906	-0.51180116916126	4.56084724153319	-0.78056397967568	0.462816469802024	0.999692817443324	-4.60686032545356	4.0556007905611	4.09583318818548	3.81095163647918	5.28407192958584	4.30171013188328	5.81691577250425	NA
**TC1000000053.mm.2**	Ppil4	peptidylprolyl isomerase (cyclophilin)-like 4	-0.288434888130557	6.47151725382104	-0.780258888561062	0.462983788508191	0.999692817443324	-4.60687433397012	7.42920990686084	7.27913708063177	6.18623465982094	5.5857087197653	6.45395912846224	5.89485402738512	NA
**TC1800000462.mm.2**	Ythdc2	YTH domain containing 2	0.237628252292027	5.87190180298198	0.780156207277979	0.463040110649499	0.999692817443324	-4.6068790478506	6.81843971396763	6.54319387032864	5.51190711871457	5.66060975037526	5.44022277407519	5.25703759043059	NA
**TC0200003398.mm.2**	Rc3h2	ring finger and CCCH-type zinc finger domains 2	0.601605039707819	7.4582971658185	0.780108704399024	0.463066168264291	0.999692817443324	-4.60688122846796	8.7947414381552	8.6568596075573	7.63472835443731	6.61596765986974	7.69956733804856	5.34791859684286	NA
**TC1600002115.mm.2**	Hlcs	holocarboxylase synthetase (biotin- [propriony-Coenzyme A-carboxylase (ATP-hydrolysing)] ligase)	0.266608884839919	5.2308059791707	0.780069718185581	0.463087554839849	0.999692817443324	-4.60688301806234	5.62724579799502	5.13844594073846	5.24989437972547	5.32628657325982	5.24272499491806	4.80023818838739	NA
**TC1300000972.mm.2**	Mef2c	myocyte enhancer factor 2C	-0.490541739751031	9.12437370579972	-0.779974073350451	0.463140025410835	0.999692817443324	-4.60688740822079	10.0927117203696	10.6877222431609	9.00287568940049	7.48948670648233	9.32430968479324	8.14913619059165	NA
**TC0200004463.mm.2**	Zfp106	zinc finger protein 106	0.229671978795174	8.22851055802187	0.779935537845857	0.463161167081745	0.999692817443324	-4.60688917692382	8.83464223351947	8.81881858476637	7.94818324356062	8.14029000015725	7.8784781734652	7.75065111266233	NA
**TC1500001356.mm.2**	Stk3	serine/threonine kinase 3	-0.454495475426474	6.83900016607862	-0.779690507138039	0.463295613571343	0.999692817443324	-4.60690042198851	7.42925972960481	7.34238695852875	7.24501222966188	5.43166944908072	6.84909866718878	6.73657396240678	NA
**TC0200001628.mm.2**	Ccdc34	coiled-coil domain containing 34	-0.25682542278405	7.47730033188656	-0.77967368117027	0.463304846847231	0.999692817443324	-4.60690119408765	8.04912815585459	7.93428303418626	7.10037508358295	7.08299489427225	7.63146084790912	7.06555997551418	NA
**TC0500003482.mm.2**	Pdgfa	platelet derived growth factor, alpha	0.286630974872268	7.80988117077775	0.779435481338659	0.463435573092996	0.999692817443324	-4.60691212326023	8.37643405904872	8.03763350962265	7.90805643508055	7.60118426778928	7.81024269831092	7.12573605481438	NA
**TC1100001956.mm.2**	Sept9	septin 9	-0.322564914836134	7.69646208221962	-0.779061050837314	0.463641115777987	0.999692817443324	-4.60692929853337	7.83704661061832	8.06805491075834	7.92095271296135	6.89331785817304	7.8214346850813	7.63796571572537	NA
**TC1600001194.mm.2**	Tvp23a	trans-golgi network vesicle protein 23A	0.417926132982071	7.26824204834886	0.77894574855405	0.46370442342843	0.999692817443324	-4.60693458638951	8.03791274092996	7.81291020111554	7.94048836758884	6.35675243941705	6.87358471351695	6.5878038275248	NA
**TC1200000509.mm.2**	Lrfn5	leucine rich repeat and fibronectin type III domain containing 5	0.262026110177818	6.57856696064047	0.778767996403761	0.463802031445321	0.999692817443324	-4.60694273722783	6.67015212018371	6.67243967695257	6.34944544023925	6.97698565329184	6.41112385174821	6.39125502142725	NA
**TC0200005514.mm.2**	Zhx3	zinc fingers and homeoboxes 3	-0.487930762013891	5.75352992938482	-0.778517678383892	0.463939511335669	0.999692817443324	-4.60695421348217	7.09717749024625	7.10861213975106	5.7278681058437	3.9418961052982	5.77014613217551	4.87547960299417	NA
**TC0900002921.mm.2**	Mras	muscle and microspikes RAS	-0.495488068195533	9.47083196437496	-0.778491593655745	0.463953839241644	0.999692817443324	-4.60695540923963	9.9625494449841	10.0143291647578	9.89130601219029	8.03726250786812	9.88053194140126	9.03901271504823	NA
**TC0100001680.mm.2**	Desi2	desumoylating isopeptidase 2	-0.419460859855777	7.28795202171911	-0.778460621143279	0.463970852324996	0.999692817443324	-4.60695682902481	6.90626725314285	6.98523868022433	8.01995303199726	6.4786892066207	7.93835434290304	7.39920961542647	NA
**TC1700002775.mm.2**	Zfp414	zinc finger protein 414	0.249699702605545	8.78141430236159	0.77842438293974	0.463990758382336	0.999692817443324	-4.60695849014244	8.85382887189858	8.51856768594429	9.10074751665046	8.80699681411841	8.82959058912058	8.57875433643721	NA
**TC0800000020.mm.2**	Mcemp1	mast cell expressed membrane protein 1	0.2519790270783	5.10376900604367	0.778100304946744	0.46416880447287	0.999692817443324	-4.6069733432183	4.57081392020669	4.883299779331	5.20815341430653	5.62807578113393	5.22318807611894	5.10908306516492	NA
**TC0500000534.mm.2**	Anapc4	anaphase promoting complex subunit 4	-0.51803110661	6.35456421735868	-0.777909101928945	0.464273872261746	0.999692817443324	-4.60698210446529	7.89809152724536	7.76579816954855	6.22245001797222	4.49126667909687	6.51270147878206	5.23707743150703	NA
**TC0500003111.mm.2**	Tmem233	transmembrane protein 233	0.282148974403911	6.85436819086114	0.777639075936942	0.46442228210356	0.999692817443324	-4.60699447507362	6.57287690840982	6.4613194833884	6.91428099741787	7.41387435367034	6.57808102109978	7.18577638118061	NA
**TC0500001020.mm.2**	Dspp	dentin sialophosphoprotein	0.397948371874891	5.07050585663914	0.777524085719508	0.464485492235616	0.999692817443324	-4.60699974221301	4.56558484596121	4.39545897030532	5.03604396394221	6.09290006971685	4.62767789996257	5.70536938994671	NA
**TC1500000175.mm.2**	Ank/Mir7117	progressive ankylosis/microRNA 7117	-0.362611464504975	8.41955769891682	-0.777408284916306	0.464549153979427	0.999692817443324	-4.60700504595722	8.96186016704343	9.01336446563293	8.56502774359166	7.34342157231564	8.56249579586239	8.07117644905487	NA
**TC0400003992.mm.2**	Zfp979	zinc finger protein 979	0.275340594050494	5.32973572118922	0.777263763324295	0.464628613531484	0.999692817443324	-4.60701166439225	6.00717550290904	5.90847396008774	5.34819163639984	4.95853138971994	5.19050566024653	4.56553617777226	NA
**TC0900003233.mm.2**	Plcd1	phospholipase C, delta 1	0.233567251083059	5.60516098956551	0.77709752088979	0.464720027101037	0.999692817443324	-4.60701927652994	5.67756130831818	5.75501949733149	5.78022483414624	5.5525349828085	5.53558017931462	5.33004513547401	NA
**TC1000003078.mm.2**	Slc16a7	solute carrier family 16 (monocarboxylic acid transporters), member 7	-1.02742808062844	6.48725807359695	-0.777091134002434	0.464723539378413	0.999692817443324	-4.6070195689599	7.38602838355833	8.01501089497899	7.35864991534401	3.37517658554975	7.52980884250712	5.25887381964352	NA
**TC0300000764.mm.2**	Pygo2	pygopus 2	-0.29691815642786	6.71808986843519	-0.776926326590037	0.464814176645783	0.999692817443324	-4.60702711427671	6.90550690847918	6.83394946579387	6.94234574462086	6.04527751712033	6.74945125171238	6.83200832288453	NA
**TC0600001082.mm.2**	Hdac11	histone deacetylase 11	0.321904348678892	8.50546881166736	0.776863013288868	0.464848999605193	0.999692817443324	-4.60703001264264	9.32852758234222	9.03483261766657	8.23371352261911	8.42291716105746	8.51248074962243	7.50034123669635	NA
**TC0400003613.mm.2**	Khdrbs1	KH domain containing, RNA binding, signal transduction associated 1	-0.23604324856536	8.31457875935904	-0.776780788936425	0.464894226535849	0.999692817443324	-4.6070337764866	8.77313918630997	8.67324760926196	7.99876901844207	7.98573061328373	8.05824093973767	8.39834518911885	NA
**TC1500002346.mm.2**	Plec	plectin	0.238373234754143	6.54023990445963	0.776715166136772	0.464930324087995	0.999692817443324	-4.60703678019908	7.54667120886819	7.73983843737865	6.13517021606819	6.08066790894142	6.03484962037504	5.70424203512628	NA
**TC0900001987.mm.2**	Fli1	Friend leukemia integration 1	-0.30961303385728	6.49628334288231	-0.776696468486739	0.464940609579467	0.999692817443324	-4.60703763600428	6.29363963245593	6.61261272204489	6.51973204444261	6.20637877309664	7.11482374967318	6.23051313558063	NA
**TC0300003200.mm.2**	Lrrc7	leucine rich repeat containing 7	0.440512603041501	7.53224039098195	0.77668657261843	0.464946053315066	0.999692817443324	-4.60703808893989	8.53922928468342	8.68968596699056	7.82462884050924	6.59814730964113	7.39308441412584	6.14866652994153	NA

**TC1600000097.mm.2**	1810013L24Rik	RIKEN cDNA 1810013L24 gene	0.289761302953929	8.05467419974754	0.776605352467768	0.464990734341426	0.999692817443324	-4.60704180625499	8.4611241978727	8.31030916733589	8.44669681744274	7.62137040214952	7.82296979751482	7.66557481616957	NA
**TC0200000522.mm.2**	Set	SET nuclear oncogene	-0.676176320067633	9.11395724287232	-0.776507819410501	0.465044393398036	0.999692817443324	-4.60704626984304	9.82428805676702	9.8523446219384	9.72844628593485	7.09893278326177	9.75612108615142	8.42361062318047	NA
**TC1800001640.mm.2**	Setbp1	SET binding protein 1	-0.288777378734195	7.43592626553087	-0.776434876633433	0.465084526604263	0.999692817443324	-4.60704960781573	7.76667338273015	7.65640594618364	7.50609964664826	6.80136210675325	7.67082283126912	7.21419367960078	NA
**TC0700003857.mm.2**	Rhog	ras homolog family member G	0.418115977783178	7.75021483924071	0.776329791011413	0.465142349065352	0.999692817443324	-4.60705441632646	8.3110300025715	7.78659057016901	8.49442059083049	7.12552961830456	7.75241327343462	7.03130498013408	NA
**TC0100000027.mm.2**	Pcmtd1	protein-L-isoaspartate (D-aspartate) O-methyltransferase domain containing 1	-0.668587695192243	7.98049887231235	-0.775893456460838	0.465382491713156	0.999692817443324	-4.60707437749159	9.24842473056278	9.20733687286762	8.17662200006542	5.86840611996417	8.57972371992287	6.80247979049121	NA
**TC1500001068.mm.2**	Atg101	autophagy related 101	-0.319645645294857	6.30114812061273	-0.775889340776698	0.465384757244872	0.999692817443324	-4.60707456573774	6.46345078596565	6.65647863716767	6.16099108125278	5.86284292372389	5.83283512872857	6.83029016683782	NA
**TC1900001115.mm.2**	Tmem132a	transmembrane protein 132A	0.327428182876527	6.53681336196421	0.775766647501271	0.465452298860216	0.999692817443324	-4.607080177266	7.42485241513943	7.60013948583281	6.68759928073355	5.73777303754948	5.61300550766945	6.15751044486054	NA
**TC1900001098.mm.2**	Dagla	diacylglycerol lipase, alpha	0.241519901530614	7.38833716918957	0.775750857054752	0.465460991861497	0.999692817443324	-4.60708089941854	7.58181049221631	7.95372883421061	7.321224091281	7.31753765460486	7.18572092100768	6.97000102181694	NA
**TSUnmapped00000043.mm.2**	Rora	RAR-related orphan receptor alpha	0.533958313080591	5.85320992524935	0.775522651321681	0.465586636890137	0.999692817443324	-4.60709133497235	6.8987720370184	6.48520574820826	4.70429132500405	6.69730787121126	5.63907543226718	4.69460713778695	NA
**TC0100001161.mm.2**	AA986860	expressed sequence AA986860	-0.247338646795876	5.30873119529634	-0.775422590904844	0.46564173535298	0.999692817443324	-4.60709590995991	5.00294609996933	4.95967459250389	5.24162975216374	5.45591484069278	5.37307397808248	5.81914790836579	NA
**TC1300000221.mm.2**	Vmn1r213	vomeronasal 1 receptor 213	-0.258474369933784	5.05173957221	-0.775412146932901	0.465647486606789	0.999692817443324	-4.60709638745911	5.22691259516354	4.91229717905323	5.15084820377428	4.67629125581355	5.06823532162661	5.27585287782879	NA
**TC0900001337.mm.2**	Twf2	twinfilin actin binding protein 2	-0.24166044851105	7.00832232917662	-0.775377297348774	0.46566667782291	0.999692817443324	-4.60709798075372	7.33906491031693	7.46957504060928	6.88344623608083	6.49554032747489	7.0019038266568	6.86040363392102	NA
**TC1900001583.mm.2**	Fbxw4	F-box and WD-40 domain protein 4	0.491373926970986	6.28326324996429	0.775312229018768	0.465702511579077	0.999692817443324	-4.60710095549683	6.43265666457108	6.60230724303724	6.86943995941976	5.95424176363995	6.82017414389277	5.02075972522496	NA
**TC1100003118.mm.2**	C1qbp	complement component 1, q subcomponent binding protein	-0.4556093234314	9.10373417644887	-0.775011081101265	0.46586838151846	0.999692817443324	-4.60711472097127	9.84939941262318	9.73606867001597	9.38337334167599	7.67948582291965	9.32871991632086	8.64535789513758	NA
**TC0100002837.mm.2**	Thap4	THAP domain containing 4	0.358058888548738	7.35861600018393	0.775000605117308	0.465874152345702	0.999692817443324	-4.60711519976433	6.87843556228045	6.94378056698446	7.11419981449202	8.40859900997604	7.37256834987219	7.43411269749839	NA
**TC1100004302.mm.2**	Trim65	tripartite motif-containing 65	0.29789011631452	6.65717281826269	0.774825817063388	0.465970443843659	0.999692817443324	-4.60712318761794	6.26120839242105	6.02555552559625	6.74356019850879	7.38246641358514	7.07152079726122	6.45872558220368	NA
**TC0800002832.mm.2**	Enkd1	enkurin domain containing 1	0.304222447700227	6.42337421107208	0.774731864901933	0.466022208192926	0.999692817443324	-4.60712748075565	5.81418189718426	5.72885199591368	6.43245388490608	7.37037424946141	6.5044048742572	6.68997836470984	NA
**TC0900000262.mm.2**	Opcml	opioid binding protein/cell adhesion molecule-like	-0.286164963050126	9.33740526360796	-0.774705837036949	0.466036549339041	0.999692817443324	-4.60712867003567	10.0778404664805	10.2600809796144	9.03635868439627	8.52073142033004	9.37855884205731	8.75086118876925	NA
**TC0200001018.mm.2**	Myo3b	myosin IIIB	0.318016363844074	5.54827783286596	0.774645325279915	0.466069892015995	0.999692817443324	-4.60713143487015	5.48429924687655	5.19643647157526	5.34646843292598	6.27601357029007	5.29391411466352	5.69253516086438	NA
**TC1900001511.mm.2**	Frat2	frequently rearranged in advanced T cell lymphomas 2	0.239742250942119	6.37061447205458	0.774606836158129	0.466091100826102	0.999692817443324	-4.60713319339631	5.91282957085614	6.0606654257535	6.74744143498563	6.6173967338154	6.26139533833604	6.62395832858076	NA
**TC0700004132.mm.2**	Rras2	related RAS viral (r-ras) oncogene 2	-0.363168484955101	6.77752951921903	-0.774551638440621	0.466121517804984	0.999692817443324	-4.60713571521816	6.8140242533331	6.61203059007947	7.10681800509441	6.14957464590128	7.44097509936325	6.54175452154264	NA
**TC0800001700.mm.2**	Cars2	cysteinyl-tRNA synthetase 2 (mitochondrial)(putative)	0.365391372166234	6.35796243439463	0.77445526993823	0.466174625445776	0.999692817443324	-4.60714011772459	6.58828995911069	6.31255717750906	5.84501747417334	7.14383763286692	6.03133365809849	6.2267387046093	NA
**TC0200004487.mm.2**	Catsper2	cation channel, sperm associated 2	-0.407400839966233	5.28802690886483	-0.774297821841364	0.466261402405109	0.999692817443324	-4.60714730980846	5.04958924066635	5.6426617502022	4.33281151770014	5.77774287349384	5.58431802466537	5.34103804646108	NA
**TC0500001741.mm.2**	Eif2ak1	eukaryotic translation initiation factor 2 alpha kinase 1	-0.373553254902619	7.35046423498408	-0.774125300853515	0.466356499574249	0.999692817443324	-4.60715518928581	8.19011944027537	7.96779544134008	7.45652370348711	6.1423583057548	7.37083747120076	6.97515104784639	NA
**TC1200000032.mm.2**	Pfn4	profilin family, member 4	0.498093991415593	7.06746416701542	0.773886332323035	0.466488246193109	0.999692817443324	-4.60716610165418	7.8600526743078	7.58439089843372	7.5474314139347	6.43083329215639	7.34884496876557	5.63323175449433	NA
**TC0200003260.mm.2**	Wdr34	WD repeat domain 34	-0.269562370878131	6.67424438964196	-0.773853903905862	0.466506126407812	0.999692817443324	-4.60716758230666	7.02318255785565	6.51734311916178	6.71626223991238	6.26664571962666	6.86197266666268	6.66006003463263	NA
**TC1100000601.mm.2**	Aff4	AF4/FMR2 family, member 4	0.609863565497557	9.05356924833962	0.773714103436434	0.466583214305127	0.999692817443324	-4.60717396499584	10.3197040628403	10.2750176419662	9.34932210509842	8.12388835301475	9.29136179455798	6.96212153256008	NA
**TC1800001307.mm.2**	Cdo1	cysteine dioxygenase 1, cytosolic	0.260863304453372	8.11313170586094	0.773688335736472	0.466597423930605	0.999692817443324	-4.6071751413542	8.41242654678142	8.46559974677917	8.13598191125575	8.02526336400013	8.13009719325671	7.50942147309243	NA
**TC1300001751.mm.2**	Gmds	GDP-mannose 4, 6-dehydratase	-0.359820179509557	6.38281378389205	-0.773523198454563	0.466688496169127	0.999692817443324	-4.6071826796518	6.82139744974397	6.54353266773091	6.48103541350662	5.62512069992254	6.89023550492497	5.9355609675233	NA
**TC1500002275.mm.2**	Krt1	keratin 1	0.237935497394176	5.08756274241994	0.773332265081849	0.46679381013061	0.999692817443324	-4.60719139416468	4.96008510497128	5.12563827843181	5.04979638776087	5.40796564519159	4.99367749447589	4.98821354368822	NA
**TC1400002222.mm.2**	Tdh	L-threonine dehydrogenase	-0.285548227417294	6.21971122399873	-0.773257573302705	0.466835012678021	0.999692817443324	-4.6071948028285	5.83371925177765	5.38906402136371	6.03094763966134	6.73124616834688	6.63523448708009	6.69805577576272	NA
**TC0500003116.mm.2**	Hspb8	heat shock protein 8	0.313096796814246	5.39639025240467	0.772786985962908	0.467094662654335	0.999692817443324	-4.60721627367174	6.33143749938926	6.52824594856981	4.78908109218304	5.28334473786569	5.16082599685231	4.28540623956793	NA
**TC1700001717.mm.2**	Ip6k3	inositol hexaphosphate kinase 3	-0.290494678459126	4.99390275270037	-0.772775182612616	0.467101176522198	0.999692817443324	-4.60721681209453	4.42185178160036	4.96062853134367	5.31293854836867	4.6870348748013	5.42613137840501	5.15483140168322	NA
**TC1900000154.mm.2**	Stx5a/Mir6992	syntaxin 5A/microRNA 6992	-0.285011277860019	6.58971706287841	-0.772774804047502	0.467101385440458	0.999692817443324	-4.6072168293631	7.20659509896086	7.30845514272226	6.4676331911795	5.75898159875415	6.60755195092003	6.18908539473366	NA
**TC1100003745.mm.2**	Krtap16-1	keratin associated protein 16-1	0.249919740474154	5.31490417096696	0.772749042759095	0.467115602442548	0.999692817443324	-4.60721800447304	5.03212049850286	5.0367596586775	5.36774918348678	5.79244299129808	5.49072247385213	5.16963021998442	NA
**TC1000000035.mm.2**	Myct1	myc target 1	-0.34812511311853	5.51267583828646	-0.772642961941421	0.467174148910136	0.999692817443324	-4.60722284310924	4.91555101418166	4.96871816089994	5.0575592792118	6.19020853498826	5.71537139192003	6.22864664851709	NA
**TC1900000430.mm.2**	1700018L02Rik	RIKEN cDNA 1700018L02 gene	0.39347132017446	6.13478414126385	0.772525856951183	0.467238785559197	0.999692817443324	-4.60722818407148	5.85026038254567	5.24906317445916	5.94878581453598	7.2993761509276	6.08131784012934	6.37990148498532	NA
**TC1000000462.mm.2**	Vgll2	vestigial like family member 2	0.254842857182549	6.56955291183457	0.772525237171903	0.467239127665729	0.999692817443324	-4.60722821233713	5.79364338238447	5.94422745286529	6.88492814175828	7.2096380333031	6.56347088736755	7.02140957332873	NA
**TC0X00001772.mm.2**	Usp27x	ubiquitin specific peptidase 27, X chromosome	0.357949306598191	6.35919706952773	0.772413352511741	0.46730088873631	0.999692817443324	-4.60723331469959	6.70591230121355	6.33811990037955	6.13529887018055	6.77822554420429	5.56224238492547	6.63538341626299	NA
**TC1100000835.mm.2**	Elac2	elaC ribonuclease Z 2	0.287168311041091	6.41435118715269	0.772342271660712	0.467340128766929	0.999692817443324	-4.6072365559973	6.8149181426172	6.57357989198061	6.69072776393076	6.14524509126948	6.43100644404506	5.830629789073	NA
**TC0700002978.mm.2**	Ap2a1	adaptor-related protein complex 2, alpha 1 subunit	-0.585857000240429	7.55627538155982	-0.772341161523423	0.467340741633893	0.999692817443324	-4.60723660661815	8.91222131378839	8.93548610758721	7.76568838108189	5.39342705266934	7.58684153445735	6.74398789977474	NA
**TC1700001881.mm.2**	Kifc1	kinesin family member C1	-0.265926247729424	6.6967093509347	-0.772322497032214	0.467351045712994	0.999692817443324	-4.60723745768787	6.20226620490171	6.46479585492847	6.56105331531721	6.92961745984237	6.75717142412695	7.26535184649148	NA
**TC0700000663.mm.2**	Ankrd27	ankyrin repeat domain 27 (VPS9 domain)	0.459312730425653	6.04003932704367	0.772252806622644	0.467389520986312	0.999692817443324	-4.60724063533271	6.26893519157527	6.36362300816015	6.60570425506277	5.65744735662616	6.47534839386985	4.86917775696778	NA
**TC0500003227.mm.2**	Hpd	4-hydroxyphenylpyruvic acid dioxygenase	-0.336435046475917	5.51004398596092	-0.772017541242826	0.467519424468867	0.999692817443324	-4.60725136121196	5.36886591325704	5.29377461755252	5.65826446777354	5.20411217822851	5.22706263610331	6.30818410285059	NA
**TC1100000907.mm.2**	Trappc1	trafficking protein particle complex 1	0.328285806318508	8.89801996922311	0.771678520102888	0.467706661331947	0.999692817443324	-4.60726681352133	8.33590995323167	8.38463663066136	9.70429621817315	8.95777620386818	9.42292914210077	8.58257166730355	NA
**TC0400000587.mm.2**	Fktn	fukutin	-0.291407859852463	6.47084568298056	-0.77159533709535	0.467752610115848	0.999692817443324	-4.60727060424035	7.11318771341024	7.1227451253384	6.3563532399231	5.64680952979179	6.55238155360729	6.03359693581252	NA
**TC1600000043.mm.2**	Smim22	small integral membrane protein 22	0.260963941437645	5.81813635920015	0.771536611977433	0.467785050683637	0.999692817443324	-4.60727328022794	5.26648150856072	5.44145991035567	5.87378465229421	6.48761765728567	5.94328124257058	5.89619318413401	NA
**TC0100001218.mm.2**	Fmod	fibromodulin	0.398652034454921	5.04516219357309	0.771522648902906	0.467792764307985	0.999692817443324	-4.60727391647756	5.14767615205115	4.93911440542588	5.0369248816826	5.45381845475308	5.58067092239244	4.1127683451334	NA
**TC1400002341.mm.2**	Polr3d	polymerase (RNA) III (DNA directed) polypeptide D	0.262550002035317	6.28487098537313	0.771408247075357	0.467855966644653	0.999692817443324	-4.60727912908733	6.67924950651517	6.64462337323013	6.41139421369639	6.04383230458565	6.23513763763783	5.69498887657358	NA
**TC0400001157.mm.2**	Txndc12	thioredoxin domain containing 12 (endoplasmic reticulum)	0.246662054874299	7.90785383310819	0.771228649995732	0.467955198595183	0.999692817443324	-4.60728731121054	8.68717623494429	8.455233012576	7.70979559111416	7.68922333932456	7.22946716474156	7.67622765594855	NA
**TC0700002826.mm.2**	Uba2	ubiquitin-like modifier activating enzyme 2	0.235859775180218	7.30831037688159	0.771138297406955	0.468005126193468	0.999692817443324	-4.60729142703043	7.54680478673134	7.26526045887234	7.46454673887676	7.2902115441464	7.23058387140821	7.05245486125451	NA
**TC1700001289.mm.2**	Prkce	protein kinase C, epsilon	-0.49259138154866	9.37380496227473	-0.77103870299328	0.468060164960435	0.999692817443324	-4.60729596346715	10.0800382710602	10.3179205421245	9.63971806810082	7.79012603058236	9.6288279271469	8.78619893463361	NA
**TC0400001666.mm.2**	Srsf10	serine/arginine-rich splicing factor 10	-0.52432547821863	7.18372701163882	-0.770950473381603	0.468108926942103	0.999692817443324	-4.60729998191887	7.91710298097602	8.14846981131686	7.41124378109315	5.58282537945823	7.63178339838043	6.41093671860821	NA
**TC1100003697.mm.2**	Smarce1	SWI/SNF related, matrix associated, actin dependent regulator of chromatin, subfamily e, member 1	0.397034125275469	7.91788177156233	0.770863095232501	0.468157221802611	0.999692817443324	-4.60730396128627	8.38984458546668	8.29598783813452	8.5091314974969	7.29863173066496	7.97480391586518	7.03889106174575	NA
**TC1700002308.mm.2**	Stap2	signal transducing adaptor family member 2	-0.304883798259204	5.91398686800874	-0.770437516986128	0.468392492755038	0.999692817443324	-4.60732333860228	4.81954772128265	5.50428718164695	5.8242549957495	6.45090435855271	6.29183624359669	6.59309070722392	NA
**TC1700001747.mm.2**	Taf11	TATA-box binding protein associated factor 11	-0.341827361235667	6.68662578166361	-0.770408087082958	0.468408765404118	0.999692817443324	-4.60732467833126	6.9327145825604	6.6514507383327	5.87463692397816	7.05133039933045	6.84502699244325	6.76459505333669	NA
**TC1000000424.mm.2**	Popdc3	popeye domain containing 3	-0.273179425755928	5.94883541119524	-0.770284236438324	0.468477250303638	0.999692817443324	-4.60733031597179	5.48013469002105	5.43351084586258	5.84116990479421	6.27533413509375	6.02928245876913	6.6335804326307	NA
**TC0100001240.mm.2**	Ube2t	ubiquitin-conjugating enzyme E2T	0.461223538980827	4.90281390551809	0.769931343135941	0.468672425363462	0.999692817443324	-4.60734637622012	4.09297187839148	4.70230066535625	4.56788273099652	6.20414625266472	4.56949467280545	5.28008723289414	NA
**TC0300002311.mm.2**	Slc25a44	solute carrier family 25, member 44	0.350065989217535	8.47725808318807	0.769814807826744	0.468736889990904	0.999692817443324	-4.60735167868063	8.94849962371893	8.87422245841339	8.74029510824537	8.13018408947022	8.6374527793779	7.53289443990262	NA
**TC1100000851.mm.2**	Myh3	myosin, heavy polypeptide 3, skeletal muscle, embryonic	-0.429736169237142	5.22098742043367	-0.769671867529347	0.46881596960039	0.999692817443324	-4.60735818185519	4.42767804159111	4.49405127141314	4.62211708031633	6.1502443552454	5.42603220576606	6.20580156826996	NA
**TC0400003769.mm.2**	Grhl3	grainyhead like transcription factor 3	-0.359511621369406	6.50024611559982	-0.769357614396526	0.468989857872894	0.999692817443324	-4.60737247618018	6.04804504719106	5.93767940324944	5.92238079548193	7.22598370472786	6.84010396187454	7.02728378107406	NA
**TC0700003629.mm.2**	Saxo2	stabilizer of axonemal microtubules 2	-0.275405975100893	6.14541503325074	-0.769298674418387	0.469022476570948	0.999692817443324	-4.60737515672583	5.81283288140351	6.11897996300411	6.37512616634276	5.81980653610474	6.15814319416732	6.58760145848197	NA
**TC1600000429.mm.2**	Cep19	centrosomal protein 19	-0.327542195570549	7.39002516741447	-0.76921272223988	0.469070047228197	0.999692817443324	-4.60737906551844	7.83711213885895	7.90582503247087	7.5200267886952	6.45103793231277	7.48825655253949	7.13789255960957	NA
**TC1300002252.mm.2**	Zfp748	zinc finger protein 748	-0.526329090399257	6.55112823440737	-0.76915359602563	0.469102772855338	0.999692817443324	-4.60738175419324	7.3218935362829	6.99008117012282	7.06082036861559	4.91024789100441	6.74937252011188	6.27435392030663	NA
**TC1600001199.mm.2**	Tnp2	transition protein 2	-0.345821406068047	5.00925218958669	-0.769095134165351	0.469135132319203	0.999692817443324	-4.60738441252091	4.55780302753062	4.46066157592165	4.55496708233675	5.61773577862914	5.11608623145337	5.74825944164861	NA
**TC1000003144.mm.2**	Nabp2	nucleic acid binding protein 2	0.291047269238886	9.11605965502009	0.769089909409203	0.469138024370338	0.999692817443324	-4.60738465008993	9.68649937629519	9.62802597905746	9.27213716000989	8.70982639661294	9.03922546058146	8.3606435575636	NA
**TC1600000571.mm.2**	Lsamp	limbic system-associated membrane protein	0.271289466221591	8.63639072375754	0.769071687229421	0.469148110961374	0.999692817443324	-4.60738547864173	9.46772960820526	9.0456201840522	8.75648017668479	8.1673065646807	8.24821362632601	8.1329941825963	NA
**TC1100003983.mm.2**	E030025P04Rik	RIKEN cDNA E030025P04 gene	-0.371713611745237	5.81268585709112	-0.768898916528971	0.469243752802917	0.999692817443324	-4.60739333376783	4.98443231043497	4.40758459006799	5.71686663848572	6.65346887079092	6.12849057191508	6.98527216085203	NA
**TC0100001268.mm.2**	Ddx59	DEAD (Asp-Glu-Ala-Asp) box polypeptide 59	0.227560839347292	7.01715946953816	0.768852881405613	0.469269239045234	0.999692817443324	-4.60739542658273	7.17935520134374	6.96700459120875	7.05252122471575	7.15333812696978	6.82632556708745	6.92441210590349	NA
**TC1600001573.mm.2**	Cstdc5/Cstdc6	cystatin domain containing 5/cystatin domain containing 6	0.308229852550119	5.04212997701404	0.768382745712364	0.469529573053318	0.999692817443324	-4.60741679471865	4.28668949423543	4.49515158580448	5.03259859678586	6.01110064678642	5.25504787663877	5.17219166183328	NA
**TC0300002023.mm.2**	Tm4sf1	transmembrane 4 superfamily member 1	0.341984826881607	6.23633966152665	0.768271248687492	0.469591328274018	0.999692817443324	-4.60742186107921	7.27890623516828	8.09949885844499	5.68286073419027	5.67894053046465	5.78981743424503	4.88801417664667	NA
**TC0400001116.mm.2**	Ssbp3	single-stranded DNA binding protein 3	-0.377005882344742	9.015308851482	-0.768254422451827	0.469600648362261	0.999692817443324	-4.60742262561068	9.28743389432719	9.26870239988309	9.38326656861189	8.00758595638421	9.26124052489019	8.8836237647954	NA
**TC0400002516.mm.2**	Stoml2	stomatin (Epb7.2)-like 2	0.526221744409876	7.55838152042043	0.768215071847411	0.46962244524476	0.999692817443324	-4.60742441353496	8.66496963515594	8.81931859525274	7.99635027806977	6.46287191239708	7.50924084610543	5.89753785554166	NA
**TC1500000805.mm.2**	Atxn10	ataxin 10	-0.523108967918457	9.02292997717086	-0.768097229562765	0.469687724002581	0.999692817443324	-4.60742976741895	10.1630025841194	9.97230476078796	9.3203957566352	7.15763153450522	8.9798938671021	8.54435135987524	NA
**TC1500001174.mm.2**	Wdr70	WD repeat domain 70	-0.258463093013281	7.88897562424533	-0.767973911517529	0.469756042755809	0.999692817443324	-4.60743536948896	7.47847585491385	7.91096119004255	7.80571146616611	7.90803379107841	8.33752291291957	7.89314853035151	NA
**TC0900002791.mm.2**	Tbx18	T-box18	0.333264389840664	4.96007395496734	0.767853923499508	0.46982252323257	0.999692817443324	-4.60744081970173	4.6462698145633	4.45907017438411	4.75684897749114	5.90396728277786	4.94705926154499	5.04722821904268	NA
**TC1900000118.mm.2**	Rasgrp2	RAS, guanyl releasing protein 2	-0.286872467000659	7.96349154124045	-0.76779286061658	0.469856358179799	0.999692817443324	-4.60744359313935	8.52794484363003	8.49022143944622	7.79095445738696	7.30356455779559	8.18580789175053	7.48245605743334	NA
**TC1700002339.mm.2**	Mllt1	myeloid/lymphoid or mixed-lineage leukemia; translocated to, 1	0.242752788877033	5.93785105045436	0.767751420045168	0.469879321359437	0.999692817443324	-4.60744547525935	5.85036486641275	6.21875867103941	6.15561383829268	5.86613033322136	5.73458287727731	5.80165571648266	NA
**TC1200002236.mm.2**	Gpr68	G protein-coupled receptor 68	-0.305371413932737	6.97428872034495	-0.767727422567466	0.469892619270759	0.999692817443324	-4.60744656512936	5.75611488392512	5.81406371190432	6.95429845521719	7.87810699397022	7.55108254211432	7.89206573493856	NA
**TC0100001262.mm.2**	Kif21b	kinesin family member 21B	-0.22656565250589	8.153804996638	-0.76768019853905	0.469918788650843	0.999692817443324	-4.60744870978995	8.17742556366545	8.14204862318116	8.08347464874311	7.99163759524169	8.15369760511037	8.37454594388622	NA
**TC1300002255.mm.2**	Zfp729a	zinc finger protein 729a	-0.29197951013365	5.59456197338882	-0.767447779504787	0.470047599201387	0.999692817443324	-4.60745926371349	6.31094531774131	6.05891865441589	5.54130792835211	4.76546649560208	5.26206503493531	5.62866840928619	NA
**TC0400001216.mm.2**	Tal1	T cell acute lymphocytic leukemia 1	-0.284844792390217	7.32806160847181	-0.767340677930107	0.470106964888163	0.999692817443324	-4.6074641263677	7.01727993212768	6.56725174003285	7.1409597220091	7.76611447493584	7.56726274380705	7.90950103791832	NA
**TC0500001771.mm.2**	Zscan25	zinc finger and SCAN domain containing 25	0.233077524442828	7.08973565852751	0.76726275190379	0.47015016200963	0.999692817443324	-4.60746766409847	7.24740431920184	7.07792144258078	7.07159970313441	7.26802191599964	6.81656151784879	7.05690505239961	NA
**TC1100002938.mm.2**	Cenpv	centromere protein V	-0.382145562855211	8.78114046501881	-0.76717694900783	0.47019772871809	0.999692817443324	-4.60747155914785	8.70983071758999	8.82603846939224	9.32338404575402	7.86995719295608	9.00821747821944	8.94941488620108	NA
**TC1400000951.mm.2**	Pnma2	paraneoplastic antigen MA2	0.337916485130386	7.01720685497885	0.767076531400079	0.47025340162083	0.999692817443324	-4.60747611726378	6.93286417453539	6.93019868616683	6.68499670310564	7.77300891661018	7.14125765268975	6.6409149967653	NA
**TC0X00000169.mm.2**	Gpr34	G protein-coupled receptor 34	-0.737363110828634	6.76452546623385	-0.766932167865283	0.470333446704927	0.999692817443324	-4.60748266945094	7.54834417448803	8.33155595608232	7.40032910660556	4.21593411598219	6.68075635513491	6.41023308911011	NA
**TC0800002403.mm.2**	Ifi30	interferon gamma inducible protein 30	-0.34124784946435	7.82185247965773	-0.766916859623582	0.470341935198439	0.999692817443324	-4.60748336419305	7.22647554923018	8.24499281561281	7.78476055714397	7.60381484994337	8.35745028359283	7.71362082242322	NA
**TC1200001351.mm.2**	Cenpo	centromere protein O	0.23029214972465	5.91237845565413	0.766696190155711	0.470464309199526	0.999692817443324	-4.60749337791451	5.91040466155672	5.88972629258638	5.88808348653249	6.179278553083	5.74558635280032	5.86119138736587	NA
**TC0200004634.mm.2**	Ddrgk1	DDRGK domain containing 1	-0.402022475578067	7.92667548346833	-0.766583287745175	0.470526928602812	0.999692817443324	-4.60749850054192	8.49470614051762	8.38698872407471	7.70832605529671	7.22883048723404	8.61792479699911	7.12327669668778	NA
**TC0500003089.mm.2**	Unc119b	unc-119 lipid binding chaperone B	0.227677329359579	6.95039572330982	0.766578543229209	0.470529560193601	0.999692817443324	-4.60749871579976	6.927698037122	6.95550702461157	7.0036157056398	7.13364626278245	6.72363737032568	6.95826993937741	NA
**TC1100003356.mm.2**	1700071K01Rik	RIKEN cDNA 1700071K01 gene	-0.254342675139934	7.28149574066499	-0.766560225461149	0.470539720412516	0.999692817443324	-4.60749954686522	7.07550001737026	7.09760371150339	6.97960261546633	7.52399006695188	7.62579081841638	7.3864872142817	NA
**TC1300002596.mm.2**	Trappc13	trafficking protein particle complex 13	0.667364564545111	5.99348761780003	0.766544053813737	0.470548690379655	0.999692817443324	-4.60750028055141	7.58573594410774	7.58499126190243	6.49823981706003	4.56422399788008	5.97588565089301	3.75184903495688	NA
**TC0400000309.mm.2**	Rars2	arginyl-tRNA synthetase 2, mitochondrial	0.235188126295336	5.95119123317408	0.766492227482492	0.470577437810507	0.999692817443324	-4.60750263177299	6.15203279747146	5.76752047470434	6.07815894706076	6.05082624266892	5.81815259898228	5.84045633815673	NA
**TC0700000224.mm.2**	Bbc3	BCL2 binding component 3	0.393633809871613	7.83144300929949	0.766273476107671	0.47069878983967	0.999692817443324	-4.60751255475429	6.86781116220786	7.08735280552694	7.86950253228616	9.04087832161653	7.58410608878693	8.53900714537254	NA

**TC0700003567.mm.2**	Plin1	perilipin 1	-0.312876839735915	5.76991497245804	-0.766222074668951	0.470727307843632	0.999692817443324	-4.60751488614474	4.89525170923538	4.97272278242505	5.65714171076161	6.40573912104638	5.98504525644469	6.70358925483513	NA
**TC1300002024.mm.2**	Drd1	dopamine receptor D1	0.383494653770328	8.22133894719363	0.766131390704676	0.470777623063917	0.999692817443324	-4.60751899899657	8.79815880276983	8.94286556886416	8.79134961512097	7.38564969441327	7.90738380969649	7.5026261922971	NA
**TC0800000823.mm.2**	Klf2	Kruppel-like factor 2 (lung)	-0.318568905093827	5.72124571299906	-0.765889048961101	0.470912102466458	0.999692817443324	-4.60752998847534	5.34549705221948	5.00252544072383	5.40058373196525	6.27057325546645	5.88541616909929	6.42287862852007	NA
**TC0500002152.mm.2**	Tyms	thymidylate synthase	0.447929388013253	5.49804987234711	0.765810417181084	0.470955742208905	0.999692817443324	-4.60753355368895	6.10973078185133	5.77136735327463	5.19170178638345	5.80982815110817	5.89571130477531	4.2099598566898	NA
**TC1300002405.mm.2**	Atp6ap1l	ATPase, H+ transporting, lysosomal accessory protein 1-like	-0.44042800903042	6.32177918157119	-0.765442675727952	0.471159871466638	0.999692817443324	-4.60755022403355	6.20527802997244	6.45628340407825	5.74565941727619	6.44846940138161	5.72519180687048	7.34979302984815	NA
**TC0600001907.mm.2**	Cttnbp2	cortactin binding protein 2	0.273657784214161	7.56421321842404	0.765413086364569	0.4711762988441	0.999692817443324	-4.60755156513462	8.06914433563887	8.2013419201186	7.63886296413872	7.19219134746883	7.44405075426652	6.8396879889127	NA
**TC1500001105.mm.2**	Tarbp2	TARBP2, RISC loading complex RNA binding subunit	-0.373304433925148	5.45431381626149	-0.765239695407507	0.471272569689432	0.999692817443324	-4.60755942316017	4.42848178745431	5.21846660767562	4.99916012502817	6.16700269226618	6.00874344311429	5.90402824203036	NA
**TC0X00001526.mm.2**	Huwe1	HECT, UBA and WWE domain containing 1	-0.489254233186753	6.06156100836824	-0.765236005514375	0.471274618554201	0.999692817443324	-4.60755959037194	8.03500293303996	7.99981033028829	5.46905310950657	4.20896905074727	6.13549836661339	4.52103226001396	NA
**TC1800000455.mm.2**	Myot	myotilin	0.414319699345242	4.50879131839088	0.765202112188549	0.471293438584016	0.999692817443324	-4.60756112626167	4.30016776525933	3.81456733060091	4.49296888416889	5.39035722241887	3.88165330614449	5.17303340175279	NA
**TC0900002050.mm.2**	Robo3	roundabout guidance receptor 3	-0.24669718852594	6.14257690296776	-0.76516691869128	0.471312981110273	0.999692817443324	-4.60756272102056	5.5789800568516	5.31359610242823	6.2544423072812	6.48030313345622	6.41805833622993	6.81008148155938	NA
**TC0400003343.mm.2**	Nasp	nuclear autoantigenic sperm protein (histone-binding)	-0.325466380596231	5.16197077436497	-0.765128176542094	0.471334494804799	0.999692817443324	-4.60756447652584	5.76003533449194	6.11794548966745	5.00628103891606	4.21517449150292	4.57861587576207	5.29377241584937	NA
**TC1400002460.mm.2**	Gm1587	predicted gene 1587	0.388413755339451	6.60952739019662	0.765044134806044	0.471381165891026	0.999692817443324	-4.60756828446416	5.90740923472091	6.11561229484895	6.48468650145867	7.72079865968571	6.30139909694933	7.12725855351615	NA
**TC0900001406.mm.2**	Ccdc71	coiled-coil domain containing 71	0.309228372270391	6.66750670832383	0.76500996222506	0.47140014393615	0.999692817443324	-4.60756983274593	7.34525176334294	7.17159743802694	7.01641582211531	6.03690807444163	6.3292760213543	6.10559113066186	NA
**TC0400003380.mm.2**	Dph2	DPH2 homolog	-0.235743511474123	7.63411318674626	-0.764787412935823	0.471523751506601	0.999692817443324	-4.60757991480388	7.02290821126371	7.03133476847674	7.68385832308649	7.95561623580794	7.90857387791945	8.20238770392323	NA
**TC1200001666.mm.2**	Zfp277	zinc finger protein 277	-0.727267861448519	6.12464683298568	-0.764784589070989	0.471525320071199	0.999692817443324	-4.60758004271952	6.63190938539259	7.10249590533719	6.91921665659272	3.86025333555092	6.61449691780189	5.61950879723879	NA
**TC1600000329.mm.2**	Eif4a2/Snord2/Snora81	eukaryotic translation initiation factor 4A2/small nucleolar RNA, C/D box 2/small nucleolar RNA, H/ACA box 81	-0.242120929493951	7.90118208158717	-0.764635837030518	0.471607952039206	0.999692817443324	-4.60758678044915	8.90360191022806	8.5139938208008	7.48959057056694	7.26303687918621	7.69694029754716	7.53992901119389	NA
**TC1700002042.mm.2**	Znrd1	zinc ribbon domain containing, 1	-0.463492527921117	6.70059953952774	-0.764564520222218	0.471647572157436	0.999692817443324	-4.60759001043265	7.10610906535143	7.38877083819735	7.14192494969952	5.24894118918819	6.67817612174306	6.63967507298688	NA
**TC1400002572.mm.2**	Klhl1	kelch-like 1	-0.315541670051102	5.05366994179644	-0.764564001987034	0.471647860071843	0.999692817443324	-4.6075900339031	4.80904106032796	5.10329416083908	4.58605298424277	5.30324756051194	5.62347860997143	4.89690527488548	NA
**TC0400000127.mm.2**	Pip4p2	phosphatidylinositol-4,5-bisphosphate 4-phosphatase 2	0.312483344168307	7.58962223954722	0.764550124323132	0.471655570089954	0.999692817443324	-4.60759066240735	8.21818956873116	8.09252147673513	7.87416087067479	7.05183366940203	7.46462620392366	6.83640164781654	NA
**TC0900001868.mm.2**	Ap1m2	adaptor protein complex AP-1, mu 2 subunit	0.322367712047578	4.92867016506249	0.764447204527543	0.471712751977418	0.999692817443324	-4.60759532329181	4.37273920636088	4.11360809433358	4.9113903099571	5.95381424693072	4.97647275487134	5.24399637792133	NA
**TC1100004192.mm.2**	Aatk	apoptosis-associated tyrosine kinase	-0.311344565347539	7.39159216524429	-0.764330401651937	0.471777653030045	0.999692817443324	-4.6076006123794	8.03043595574475	8.1705244523812	7.39350034206997	6.3694513842524	7.16129580765451	7.22434504936293	NA
**TC0700001203.mm.2**	Mesd	mesoderm development LRP chaperone	-0.283051457350677	7.75630395745348	-0.764276199109271	0.471807772542018	0.999692817443324	-4.60760306660246	8.58295493064223	8.13545360939819	7.66512349706459	6.96153264792497	7.63818242521947	7.55457663447145	NA
**TC0500000689.mm.2**	Dcun1d4	DCN1, defective in cullin neddylation 1, domain containing 4 (S. cerevisiae)	-0.754165505865227	7.8806009261956	-0.764266566933771	0.471813125130805	0.999692817443324	-4.60760350272296	8.83426832576683	8.6473054451597	8.68531932006577	5.46153106719255	8.33604652017155	7.31913487881722	NA
**TC1400001708.mm.2**	3425401B19Rik	RIKEN cDNA 3425401B19 gene	-0.242422262568565	4.7521648797958	-0.764228048751328	0.471834530057436	0.999692817443324	-4.60760524669149	4.34559786614749	4.06505815813393	4.70841543223936	5.10032893243877	5.14270973844306	5.15087915137221	NA
**TC1800000682.mm.2**	Sec11c	SEC11 homolog C, signal peptidase complex subunit	-0.364943753002898	7.63902733814751	-0.763608143790646	0.472179109047627	0.999692817443324	-4.60763330564016	8.33645347768983	8.31290877028193	7.57612460925305	6.65133252820069	7.98366101804193	6.97368362541761	NA
**TC0700003815.mm.2**	Pold3	polymerase (DNA-directed), delta 3, accessory subunit	0.307463677666624	7.9987464718273	0.76356483069558	0.472203191428979	0.999692817443324	-4.60763526555825	7.82680790835465	7.5828356588582	7.85950200973331	8.73937929980878	7.77410387343352	8.20985008077532	NA
**TC1100001201.mm.2**	Rhbdl3	rhomboid like 3	0.247012870428739	5.88481505890054	0.763288390821311	0.47235691379574	0.999692817443324	-4.60764777269318	6.02248355832561	6.28060652734868	5.94003840580392	5.80987459848929	5.40345361986965	5.85243364356609	NA
**TC0100003319.mm.2**	Soat1	sterol O-acyltransferase 1	0.310094196658261	7.04191027076516	0.763166186545277	0.472424879964155	0.999692817443324	-4.60765330067722	7.82342695009876	8.06679324038204	7.2301555769005	6.26055933681283	6.56005259278939	6.31047392760743	NA
**TC1700001019.mm.2**	1700061G19Rik	RIKEN cDNA 1700061G19 gene	-0.403525877298589	5.58171915653901	-0.763143204940743	0.472437662358901	0.999692817443324	-4.60765434019699	4.5665428159709	4.098749030053	5.43976899585015	6.56921667345635	5.89818629487848	6.91785112902519	NA
**TC0600001462.mm.2**	Vwf	Von Willebrand factor	-0.279457654770463	5.3904692790487	-0.763131155930389	0.472444364127166	0.999692817443324	-4.60765488519766	5.06727536903564	5.61217356720845	5.5770046177499	4.97522109650369	5.52441464396781	5.58672637982671	NA
**TC0800001619.mm.2**	Cd209a	CD209a antigen	0.296707643385335	6.49616047777184	0.762853024829176	0.472599081303302	0.999692817443324	-4.60766746400002	6.08290377348021	5.79766392204873	6.51296591532752	7.33193931360886	6.80724867118094	6.44424127098477	NA
**TC1300002175.mm.2**	Fbp2	fructose bisphosphatase 2	0.263351804563358	4.76647003784877	0.762682946072373	0.472693708880958	0.999692817443324	-4.60767515447917	4.27768434186671	4.55373647994192	5.25963265243561	4.88741885476975	5.06361057975412	4.55673731832453	NA
**TC1000000045.mm.2**	Lrp11	low density lipoprotein receptor-related protein 11	-0.281892550025446	8.18103749947924	-0.762437112916144	0.472830507305443	0.999692817443324	-4.6076862683042	8.97039972509347	8.65959712922104	8.10673104731421	7.3394904739408	7.95240539065477	8.05760123065113	NA
**TC0400001460.mm.2**	Col8a2	collagen, type VIII, alpha 2	-0.267361850355627	8.2066791674666	-0.76233963351189	0.472884759054052	0.999692817443324	-4.60769067456004	7.26980219639632	7.60631061082598	8.34028728872926	8.57433195970377	8.42796756852264	9.02137538062164	NA
**TC1700001381.mm.2**	Dynlt1a	dynein light chain Tctex-type 1A	0.575908889937955	10.182801403204	0.762297178681419	0.472908388445347	0.999692817443324	-4.60769259348037	9.59074182924551	8.66043917414057	11.951385954275	10.0473366435818	11.0920703575142	9.75483446046672	NA
**TC0300002627.mm.2**	Atg4a	autophagy related 4A, cysteine peptidase	0.248840778855493	5.36429917611622	0.762265180736869	0.47292619830779	0.999692817443324	-4.60769403971094	4.76074094070306	5.1074784180646	5.5012634644924	5.90636516332793	5.37671143045024	5.53323563965911	NA
**TC0800002797.mm.2**	Tk2	thymidine kinase 2, mitochondrial	0.240258692850876	7.06909508523763	0.762165960238691	0.472981426764045	0.999692817443324	-4.60769852397855	7.3683099345361	7.14707003139436	7.20220320619106	6.98765075940748	6.68054460688082	7.02879197301597	NA
**TC0700003035.mm.2**	Sergef	secretion regulating guanine nucleotide exchange factor	-0.292220762517698	6.787133956976	-0.761909345395365	0.473124285131579	0.999692817443324	-4.60771011984382	7.66120591788436	7.25955771333242	6.67115580183256	5.93764349096936	6.77743095102675	6.41580986681056	NA
**TC0100003399.mm.2**	Fmo4	flavin containing monooxygenase 4	0.335162752269238	5.74871349571507	0.761806774008555	0.473181395257991	0.999692817443324	-4.60771475408048	5.60637805917079	5.18311070974128	5.89085400027623	6.2957048546822	5.23738755232851	6.27884579809145	NA
**TC1100004285.mm.2**	Sfi1	Sfi1 homolog, spindle assembly associated (yeast)	-0.295827046084536	6.30163505634714	-0.761790218372439	0.473190613617747	0.999692817443324	-4.60771550203437	6.4845218374835	5.91162977569781	6.53008175149403	5.88092056487221	6.68028818707608	6.32236822145923	NA
**TC0X00001039.mm.2**	Zcchc13	zinc finger, CCHC domain containing 13	0.349248069137768	4.72046476358816	0.761582165444974	0.473306470273532	0.999692817443324	-4.60772490055199	4.23162547667016	4.72146867201977	4.69647075498469	5.3376245305726	4.16982756901037	5.16577157827138	NA
**TC1500002191.mm.2**	Prkag1	protein kinase, AMP-activated, gamma 1 non-catalytic subunit	-0.336246829223376	7.13414054228383	-0.76156670575236	0.473315079957138	0.999692817443324	-4.60772559885379	8.26641343843787	8.07388408323401	6.67093364141097	6.22509239538121	7.33416055152099	6.23435914371794	NA
**TC1100001702.mm.2**	Fam187a/Ccdc103	family with sequence similarity 187, member A/coiled-coil domain containing 103	-0.260060164741151	5.05497644269383	-0.761455404900063	0.473377067874502	0.999692817443324	-4.60773062593933	4.72109859159281	5.35358791819307	5.00382367504887	4.86370223339852	5.12540721871229	5.2622390192174	NA
**TC1000002500.mm.2**	Uqcr11	ubiquinol-cytochrome c reductase, complex III subunit XI	-0.236223313936089	7.67040366643051	-0.761423909695896	0.473394609830213	0.999692817443324	-4.60773204838141	7.61273350078181	7.54361053733592	7.40007709172028	7.79673857457631	7.77552224498802	7.89374004918075	NA
**TC1600000392.mm.2**	Hes1	hes family bHLH transcription factor 1	-0.266761422346393	7.04457777944457	-0.761305582793597	0.473460518619417	0.999692817443324	-4.60773739211336	6.14922239245775	6.45989930927877	7.52543809314241	7.03697297197665	7.33453486838458	7.76139904142728	NA
**TSUnmapped00000008.mm.2**	Marc1	mitochondrial amidoxime reducing component 1	0.27099404889917	5.17389728975056	0.761194590380467	0.473522347775027	0.999692817443324	-4.60774240410204	4.97666853780489	4.87830326564462	5.08120390179754	5.78399611462855	5.03440285176866	5.2888090668591	NA
**TC0100000658.mm.2**	Speg	SPEG complex locus	-0.32286840047472	6.39887571868911	-0.760981157052268	0.47364125797207	0.999692817443324	-4.60775204053296	6.675162500802	6.93390766623763	5.56533206796081	6.50389160411199	6.08108395810314	6.6338765149191	NA
**TC0800000996.mm.2**	Phkb	phosphorylase kinase beta	-0.85005835903283	7.05510480401123	-0.760939800944361	0.473664301085358	0.999692817443324	-4.60775390753284	8.49057984613635	8.31037228423852	7.79318790646254	4.12159208135089	7.41479457506891	6.20010213081018	NA
**TC1300000969.mm.2**	Cetn3	centrin 3	-0.453856804763574	7.57785657704422	-0.760887246892664	0.473693584663937	0.999692817443324	-4.60775627995866	8.94490023164184	8.78944733016738	7.53120678444441	5.88133236102006	7.59828913972428	6.72196361526734	NA
**TC0X00001402.mm.2**	Nxt2	nuclear transport factor 2-like export factor 2	-0.7188438880421	8.07820653623818	-0.760794727434096	0.473745140334048	0.999692817443324	-4.60776045625602	9.37886267440923	9.46565087735864	8.46484489862353	5.62867404616498	8.53516995365872	6.99603676721399	NA
**TC0100002836.mm.2**	Stk25	serine/threonine kinase 25 (yeast)	0.357923170928625	7.88807471125283	0.760705750553788	0.473794725561966	0.999692817443324	-4.60776447231733	8.40703307591286	8.22329421267557	8.03185694565042	7.67512671474249	8.13284762986036	6.8582896886753	NA
**TC1100003669.mm.2**	Med1	mediator complex subunit 1	0.451350530102157	6.82461071099658	0.760534391431758	0.47389023096795	0.999692817443324	-4.60777220588561	7.88066685371323	8.10786206037248	6.92612590487201	6.00479230117702	6.81528356373507	5.21293358210964	NA
**TC1700002135.mm.2**	Enpp4	ectonucleotide pyrophosphatase/phosphodiesterase 4	-0.536456896440293	6.93529844139541	-0.760275826207847	0.474034364858627	0.999692817443324	-4.60778387289206	7.66070381528698	7.36799867353865	7.46470671655419	5.29038046677892	7.27742339239226	6.55057758382144	NA
**TC1300000087.mm.2**	Nid1	nidogen 1	-0.321593108123215	6.43086218958625	-0.760231640209617	0.474058998786287	0.999692817443324	-4.60778586638756	6.381310770697	5.78491671100133	6.65011738463608	6.2377623351503	6.34304687609167	7.18801905994114	NA
**TC0700000758.mm.2**	Lim2	lens intrinsic membrane protein 2	0.354946630346695	5.73731815647893	0.760098411891702	0.47413327958868	0.999692817443324	-4.60779187663988	5.11943556388695	5.1915012892383	5.58956913901624	6.82186353420462	5.61673568086696	6.08480373166051	NA
**TC0300001802.mm.2**	Gnb4	guanine nucleotide binding protein (G protein), beta 4	0.260693347524509	6.16536935339928	0.759931335896208	0.474226443272149	0.999692817443324	-4.60779941283132	6.96081456481932	6.92126506356805	6.22851842618116	5.58724316734751	5.54848530909027	5.74588958938939	NA
**TC1700001602.mm.2**	Paqr4	progestin and adipoQ receptor family member IV	-0.223713070368754	7.06885354484139	-0.759860904368	0.47426572053926	0.999692817443324	-4.60780258940444	7.06729049789791	7.28208578373012	6.94503453834109	6.8631248850003	7.13443162140309	7.12115394267581	NA
**TC1200002093.mm.2**	Nek9	NIMA (never in mitosis gene a)-related expressed kinase 9	0.271301423195816	6.06971839449701	0.759711770284777	0.474348894900029	0.999692817443324	-4.60780931492654	6.1894636347541	6.33024991673039	6.16647662253528	6.05412320840931	6.20844235704649	5.46955462750647	NA
**TC1300000174.mm.2**	Hist1h3h	histone cluster 1, H3h	-0.227171852399573	9.54118636275661	-0.759681249596513	0.474365917986803	0.999692817443324	-4.60781069121198	9.56548920323558	9.59565088234356	9.527398688073	9.28841850500769	9.54806992699032	9.72209097088951	NA
**TC0600000650.mm.2**	Prr15	proline rich 15	-0.387029979803786	4.98804225631551	-0.759641686163734	0.474387985338447	0.999692817443324	-4.60781247521071	5.48541035857002	5.16260919604036	5.02900129210148	4.22408571973606	5.63096345094927	4.39618352049585	NA
**TC0500001087.mm.2**	Rpl5	ribosomal protein L5	-0.437277325381142	8.83573029315284	-0.759635040712522	0.474391692050116	0.999692817443324	-4.60781277486194	9.96472541093844	10.0038119785801	8.76331650862583	7.32232835069225	9.00972739355591	7.95047211652445	NA
**TC0600002726.mm.2**	Wnt7a	wingless-type MMTV integration site family, member 7A	0.272326220093594	7.47374167308681	0.759617598783087	0.474401420935107	0.999692817443324	-4.60781356133063	6.94903285507094	7.04533275790658	7.56175616671687	8.13461226614838	7.32397228336531	7.82774370931276	NA
**TC0600000588.mm.2**	Npy	neuropeptide Y	-0.246854727077021	7.84978331836712	-0.759498591443723	0.474467805353565	0.999692817443324	-4.60781892712638	8.13497950730097	8.25626303821748	7.81856136261335	7.28831259265175	7.81621338897247	7.78437002044668	NA
**TC0800002310.mm.2**	Cpe	carboxypeptidase E	-0.688870218533447	10.2178592663067	-0.759420133163775	0.474511574263003	0.999692817443324	-4.60782246433592	10.9423208453424	11.0491546508553	10.9887406976812	7.98022913460676	10.5118821863305	9.83482808302427	NA
**TC0100001088.mm.2**	En1	engrailed 1	-0.229369845332255	5.20774556518377	-0.759332924034143	0.474560228186146	0.999692817443324	-4.60782639577676	4.60262681371019	4.80310599890533	5.44324337155324	5.24775707235807	5.48357823708211	5.66616189749371	NA
**TC0800002452.mm.2**	Cib3	calcium and integrin binding family member 3	0.296029824782443	4.79771919337899	0.759278042309021	0.474590848420605	0.999692817443324	-4.60782886972103	4.91615956435163	4.58251669394827	4.69776632618208	5.24208294958738	4.32544929586133	5.02234033034325	NA
**TC1600001375.mm.2**	Igf2bp2	insulin-like growth factor 2 mRNA binding protein 2	0.226961421203645	4.81442683656105	0.75925151607468	0.47460564872314	0.999692817443324	-4.6078300654202	4.31576019198686	4.04674305098748	5.18696148254985	5.30202882684977	5.03912087823263	4.9959465887597	NA
**TC1500000679.mm.2**	Gtpbp1	GTP binding protein 1	0.270317508227034	6.40874020026794	0.75915730301236	0.474658217421096	0.999692817443324	-4.60783431194786	7.67907865986989	7.27939655971438	6.32764337252216	5.68965712671657	5.76141474519043	5.71525073759424	NA
**TC1600002183.mm.2**	Atp5o	ATP synthase, H+ transporting, mitochondrial F1 complex, O subunit	-0.353870162636447	9.72125987337463	-0.759129635390515	0.474673656068132	0.999692817443324	-4.60783555896079	9.64747103602435	9.47027483993389	10.2030595626016	9.04797695690674	10.2824355131684	9.67634133161285	NA
**TC0400002799.mm.2**	Akna	AT-hook transcription factor	-0.241650876518	6.08432025020783	-0.759047304143442	0.474719599277496	0.999692817443324	-4.60783926954604	5.74180086100267	5.99860521150439	5.88477631230887	6.2563305255431	6.21505068297051	6.40935790791745	NA
**TC0700001443.mm.2**	Lamtor1	late endosomal/lysosomal adaptor, MAPK and MTOR activator 1	0.26199204570063	7.09527289094017	0.759023413221358	0.474732931670751	0.999692817443324	-4.60784034623425	7.05835996213509	6.7917387361248	7.03207499544822	7.59068637394299	6.90156264505311	7.19721463293684	NA
**TC1400000580.mm.2**	Rnase6	ribonuclease, RNase A family, 6	0.239909486419801	8.62781825833366	0.758776039651559	0.474870994227266	0.999692817443324	-4.60785149322239	7.99738064834081	8.01358036045499	8.88938916496158	9.22849459206131	8.98388125333797	8.65418353084531	NA
**TC1500000125.mm.2**	Cdh10	cadherin 10	-0.430686280398537	6.76427472760557	-0.758641179536084	0.474946273070487	0.999692817443324	-4.60785756916043	8.01285174066721	7.9234657896418	6.81714478928812	5.07683434797554	6.48567879932402	6.26967289873672	NA
**TC1200000368.mm.2**	Immp2l	IMP2 inner mitochondrial membrane peptidase-like (S. cerevisiae)	-0.23332434998709	8.19587397285404	-0.758624041840302	0.47495583990797	0.999692817443324	-4.60785834122324	7.73440755029199	7.81005129882075	8.11789664024088	8.46417150377778	8.43891855548258	8.60979828851025	NA
**TC0600003503.mm.2**	Herc6	hect domain and RLD 6	-0.315415210696467	5.39515474235427	-0.758481407615438	0.475035468241498	0.999692817443324	-4.60786476651447	5.53801494963362	6.39390600644515	4.69277017660398	5.21131836172299	5.46802912182814	5.06688983789176	NA
**TC1300002597.mm.2**	Ppwd1	peptidylprolyl isomerase domain and WD repeat containing 1	-0.284742901807081	7.31163554031142	-0.758477147754451	0.47503784653237	0.999692817443324	-4.60786495839719	7.05086616875289	7.28310599037715	7.65879383208104	6.82438380748111	7.51347505634759	7.53918838682872	NA
**TC1800000538.mm.2**	Prdm6	PR domain containing 6	-0.287406381426025	5.46901641033383	-0.758462480124021	0.475046035568876	0.999692817443324	-4.60786561908565	5.03399023894362	5.14318245615799	5.14078549813513	5.89027100388952	5.57308373661301	6.03278552826369	NA
**TC0X00000102.mm.2**	Fthl17e	ferritin, heavy polypeptide-like 17, member E	-0.235148527092407	5.38288494068882	-0.758373968287581	0.475095454372024	0.999692817443324	-4.60786960582642	4.80414344494033	5.08923079922607	5.47390681767917	5.49291235521169	5.56599243650768	5.871123790568	NA
**TC0100000929.mm.2**	Ppp1r7	protein phosphatase 1, regulatory subunit 7	-0.816102511295319	8.9648597132162	-0.758336403980103	0.475116428710872	0.999692817443324	-4.60787129769824	10.4531399733086	10.4072561678309	9.49532114688644	6.15295741089706	9.45574068597354	7.8247428944006	NA
**TC1000002727.mm.2**	Mrpl42	mitochondrial ribosomal protein L42	-0.264499219329529	8.68638104496939	-0.75827812138722	0.475148972528538	0.999692817443324	-4.60787392259513	9.0634765961348	9.28643954668698	8.64268350073075	7.97700234343701	8.62493748583914	8.52374679698769	NA
**TC0900000667.mm.2**	Psma4	proteasome (prosome, macropain) subunit, alpha type 4	-0.290368628924019	8.14418415143326	-0.758255987276957	0.475161332166436	0.999692817443324	-4.60787491942208	8.88575650885812	8.61831506810588	7.9991253314631	7.39102270543065	8.29485948982082	7.67602580492097	NA

**TC1100003340.mm.2**	Tefm	transcription elongation factor, mitochondrial	-0.326020157229646	5.03659187143715	-0.75821643255286	0.47518341998424	0.999692817443324	-4.60787670075065	5.28507410784559	4.86405992994021	5.37963062859402	4.32955780959487	5.27572650458382	5.08550224806437	NA
**TC1700001113.mm.2**	Dlgap1	DLG associated protein 1	-0.239316357306455	9.10624181435874	-0.75807490595384	0.475262455837162	0.999692817443324	-4.60788307381682	9.21095325079138	9.45517571817768	8.67393585225106	9.07240874903418	9.13216276444244	9.09281455145571	NA
**TC0200004277.mm.2**	Dnajc24	DnaJ heat shock protein family (Hsp40) member C24	-0.284622861976767	6.66861250667292	-0.75802817765344	0.475288553338645	0.999692817443324	-4.60788517785502	6.69955536587586	6.83628123243513	6.36286442077229	6.59043193811422	6.34866539080574	7.17387669203429	NA
**TC1900001448.mm.2**	Cpeb3	cytoplasmic polyadenylation element binding protein 3	0.30174957733303	8.19086445090283	0.757908199332879	0.47535556506871	0.999692817443324	-4.607890579722	7.97308111729446	7.705981865878	8.09151329450875	8.94329814394652	8.00352163785316	8.42779064593606	NA
**TC1000000392.mm.2**	Pdss2	prenyl (solanesyl) diphosphate synthase, subunit 2	0.240380380908189	6.00004775186318	0.75784786097187	0.475389268416397	0.999692817443324	-4.60789329615768	5.92439585414326	5.79982738595848	6.1099658803444	6.26844613610245	6.16765105013704	5.73000020449343	NA
**TC0700004441.mm.2**	D7Ertd443e	DNA segment, Chr 7, ERATO Doi 443, expressed	0.557529430711948	5.06566063663543	0.757831320692267	0.475398507646827	0.999692817443324	-4.60789404077608	4.84422850887291	5.37995501077132	4.24309855699587	6.39932102380025	4.27301282236695	5.25434789700527	NA
**TC1800001220.mm.2**	Hdac3	histone deacetylase 3	-0.448302368912204	8.94005475373331	-0.757827040088786	0.47540089876801	0.999692817443324	-4.60789423348057	9.47086537525235	9.38605672435593	9.26006124676685	7.68333959571674	9.40625513085797	8.43375044945002	NA
**TC0400003675.mm.2**	Atpif1	ATPase inhibitory factor 1	-0.364902111443735	9.78915728341515	-0.757810684210617	0.475410035145933	0.999692817443324	-4.60789496978377	10.4764320582161	10.282874025946	9.90259004559548	8.7203266511252	10.024597600815	9.32812331879314	NA
**TC1000002000.mm.2**	Nt5dc1	5'-nucleotidase domain containing 1	0.376479133425718	5.08900047649053	0.757651623145025	0.475498892769748	0.999692817443324	-4.60790212977449	5.41670784404741	4.78534813228681	5.02839018112881	5.51406239360139	4.286604274026	5.50289003385276	NA
**TC0600001123.mm.2**	Kbtbd8	kelch repeat and BTB (POZ) domain containing 8	-0.534240470651218	5.33357566652925	-0.757597214879649	0.475529289925249	0.999692817443324	-4.60790457867853	4.82831610830744	5.04305550928167	6.23518779052426	4.29561292961771	6.41725627206814	5.18202538937628	NA
**TC1500000804.mm.2**	Fbln1	fibulin 1	-0.303472602595051	5.80295112913359	-0.75753966061309	0.475561446155387	0.999692817443324	-4.60790716905296	6.31927861436302	6.16091414269934	5.62410870964568	5.24117569662886	5.28044988958267	6.19177972188197	NA
**TC1400002067.mm.2**	Slc7a8	solute carrier family 7 (cationic amino acid transporter, y+ system), member 8	0.360943600913084	8.20348192089102	0.757504695464612	0.475580982311918	0.999692817443324	-4.60790874268186	8.98397348632425	9.51680400019027	8.16999626635623	7.55100435397266	8.04527574963018	6.95383766887255	NA
**TC0800000625.mm.2**	Hand2	heart and neural crest derivatives expressed 2	0.357857504918641	6.57559341548332	0.757354242350725	0.475665051583455	0.999692817443324	-4.60791551335584	6.22410294911181	6.50215837119427	6.6738822169195	7.04762487429606	5.87188136328859	7.1339107180897	NA
**TC0100000233.mm.2**	Plekhb2	pleckstrin homology domain containing, family B (evectins) member 2	0.240823297063606	8.3357987831579	0.757319303148544	0.475684576151473	0.999692817443324	-4.6079170855547	8.95642234227146	8.91369291571868	8.39764857886797	7.91551343867426	7.94921576195698	7.88229966145804	NA
**TC0200005120.mm.2**	Wfdc6a	WAP four-disulfide core domain 6A	-0.256577600023184	5.08400166022151	-0.757280780914792	0.475706103608378	0.999692817443324	-4.60791881892598	4.47512558688884	5.10396461295187	5.09106696182981	5.1148153188912	5.2912703324814	5.42776714828598	NA
**TC1600001807.mm.2**	Arl6	ADP-ribosylation factor-like 6	-0.85241711282749	6.60880435976408	-0.757030269302518	0.475846113767313	0.999692817443324	-4.60793008964161	7.75969419910589	7.82481986616398	7.0277706498017	4.15396828402811	7.76445212562157	5.12212103386322	NA
**TC0100002589.mm.2**	Prkag3	protein kinase, AMP-activated, gamma 3 non-catalytic subunit	0.262214590469144	7.48722218908075	0.757017541132823	0.475853228253778	0.999692817443324	-4.60793066222416	6.63413750786813	6.62997831131195	7.69914324428916	8.3926800038322	7.77529320945906	7.79210085772401	NA
**TC0900001101.mm.2**	Ttk	Ttk protein kinase	-0.354825790494909	5.0258704054628	-0.757010314490392	0.475857267661092	0.999692817443324	-4.60793098731507	4.44454750476431	5.22309902356352	4.86903058775577	5.01993157397379	4.76664356428433	5.83197017843505	NA
**TC1500000485.mm.2**	Phf20l1	PHD finger protein 20-like 1	-0.290336548471998	7.03124469346285	-0.75690839980759	0.475914236434932	0.999692817443324	-4.60793557172726	7.03824399910633	6.46493902201376	6.76600357501714	7.28580244633937	7.07556187930755	7.55691723899296	NA
**TC1100003090.mm.2**	Alox12	arachidonate 12-lipoxygenase	-0.309214569616707	4.87340985300176	-0.756905995259382	0.475915580597496	0.999692817443324	-4.60793567988559	4.4518783583714	4.11964742422002	4.75768851886494	5.26756357922794	4.89925217255369	5.74442906477261	NA
**TC1600001692.mm.2**	Dzip3	DAZ interacting protein 3, zinc finger	0.451861930588825	7.1596094723288	0.756661181862103	0.476052446919031	0.999692817443324	-4.60794669054423	8.50353175736539	8.39008613162146	7.06555059140992	6.41833081167189	7.16943998019391	5.41071756171024	NA
**TC0200004169.mm.2**	Hsd17b12	hydroxysteroid (17-beta) dehydrogenase 12	-0.467916189924168	7.20461963109009	-0.756658784104536	0.476053787551662	0.999692817443324	-4.60794679837308	8.33281542436349	8.30197794682716	7.1719214101395	5.65662460761128	7.50659562528699	6.25778277231212	NA
**TC1000000836.mm.2**	Arhgap45	Rho GTPase activating protein 45	-0.232555682684906	6.69279638153151	-0.756639945230937	0.476064320820044	0.999692817443324	-4.60794764556247	6.16777150245681	6.33410213784735	6.77286357222942	6.82203306952813	7.20542394116045	6.85458406596691	NA
**TC0300000334.mm.2**	Pcdh10	protocadherin 10	-0.407623644544603	7.54803603100562	-0.756630003922531	0.476069879309447	0.999692817443324	-4.60794809262008	9.00262545445664	9.043920925362	7.41645650073438	5.79675475782855	7.12136307965185	6.90709546800029	NA
**TC0100002179.mm.2**	Kansl3	KAT8 regulatory NSL complex subunit 3	0.26513360853044	7.35382779248373	0.756538673956014	0.476120946761244	0.999692817443324	-4.60795219951329	7.83258954608583	7.69407302663048	7.60667818039764	6.95660751922582	6.87861511708534	7.15440336547724	NA
**TC0800002811.mm.2**	B3gnt9	UDP-GlcNAc:betaGal beta-1,3-N-acetylglucosaminyltransferase 9	-0.280947493544512	6.46207144995529	-0.75647512922892	0.476156480213614	0.999692817443324	-4.60795505677028	6.04956176632482	6.19240784868064	6.08160964626464	6.90267240255399	6.70668025188826	6.8394967840194	NA
**TC1800000368.mm.2**	Pcdhb12	protocadherin beta 12	0.247895552382671	5.18157531357125	0.756334945365345	0.476234875765244	0.999692817443324	-4.60796135948873	5.32324737300783	5.72926100479614	5.26758496313952	4.99878234105493	5.02912253130356	4.74145366812554	NA
**TC1100001877.mm.2**	Dnaic2	dynein, axonemal, intermediate chain 2	-0.358204159545425	5.60272974148548	-0.756217056036464	0.476300810287828	0.999692817443324	-4.60796665921961	5.33642997843696	5.67561161403502	5.44593022534058	5.49803404333444	5.17708160995629	6.48329097780958	NA
**TC0900002817.mm.2**	Tmed3	transmembrane p24 trafficking protein 3	-0.298532427362733	7.17755076974315	-0.755911844134619	0.476471541748917	0.999692817443324	-4.60798037744256	7.33261282442058	7.55945708859242	7.34708673621461	6.44099818914561	7.32649936272044	7.05865041736526	NA
**TC1200001715.mm.2**	Strn3	striatin, calmodulin binding protein 3	0.551922641856784	9.17055663269104	0.755654181430326	0.476615707368179	0.999692817443324	-4.60799195554969	10.4161525211294	10.2306850954524	9.48095167430994	8.25922205732905	9.3516481798861	7.28468026803932	NA
**TC0900000095.mm.2**	Mtmr2	myotubularin related protein 2	0.522609201567434	7.18415530732224	0.755645996470093	0.476620287447649	0.999692817443324	-4.60799232329777	8.09099291043518	8.2393909964951	7.71994741430987	6.18993575575915	7.2580280858752	5.60663668105895	NA
**TC0100003533.mm.2**	Fcer1g	Fc receptor, IgE, high affinity I, gamma polypeptide	-0.379408044359887	7.13324782197632	-0.755630960058753	0.476628701489765	0.999692817443324	-4.60799299887263	8.26166228390612	8.85425999051248	6.90636525471582	5.55600902964396	6.64960048858218	6.57158988449738	NA
**TC1400001438.mm.2**	Acox2	acyl-Coenzyme A oxidase 2, branched chain	-0.428266316337501	6.06986509817803	-0.755600182017515	0.476645924515036	0.999692817443324	-4.60799438167867	5.22152097412701	5.74858376291424	5.36596655003842	6.93031005963755	6.28273400255505	6.87007523979592	NA
**TC0500001495.mm.2**	Fzd10	frizzled class receptor 10	-0.262552120049313	5.44786783742429	-0.755537945438991	0.476680752661918	0.999692817443324	-4.60799717774677	5.17557102898854	5.18589485776855	5.27271789524866	5.62760055359636	5.41051721893926	6.01490547000438	NA
**TC0900001231.mm.2**	Nmnat3	nicotinamide nucleotide adenylyltransferase 3	0.315638691930155	5.92952210865775	0.755535366433663	0.476682195933908	0.999692817443324	-4.60799729360891	5.70403949356161	5.34320851746426	5.78338736265948	6.797193649731	5.89573935760677	6.0535642709234	NA
**TC1200002260.mm.2**	Prima1	proline rich membrane anchor 1	-0.289702420412323	6.89450831434458	-0.755423520850176	0.476744790222287	0.999692817443324	-4.60800231802604	6.71717422049486	7.05112106601072	7.06009045006826	6.44958442930035	6.75064733230542	7.33843238788785	NA
**TC0600001019.mm.2**	Copg1	coatomer protein complex, subunit gamma 1	0.298978130533852	8.23089427065405	0.755413945009306	0.476750149594585	0.999692817443324	-4.60800274817604	9.10914970478362	8.87898895875137	8.34327479131036	7.65431681941816	8.02585760977859	7.37377773988223	NA
**TC0600002315.mm.2**	Fkbp14	FK506 binding protein 14	-0.472886711097283	5.28673111038867	-0.755388981235922	0.47676412142325	0.999692817443324	-4.60800386953965	6.76966499231293	6.13022159603301	5.01790135090688	3.91946197498889	4.17294898724199	5.71018776084834	NA
**TC1900001113.mm.2**	Cd5	CD5 antigen	-0.327941334864667	6.16974939728646	-0.755286531571938	0.47682146380651	0.999692817443324	-4.60800847127609	5.43893625416889	5.38662742922984	5.86957648903918	6.89894852625615	6.43808782771158	6.98631985731309	NA
**TC1100003037.mm.2**	Chd3	chromodomain helicase DNA binding protein 3	0.301471351312456	8.74519556970636	0.755230492640125	0.476852831503769	0.999692817443324	-4.60801098819905	9.19947417168225	9.14201749648135	8.20707725549181	9.15923497085791	8.24233298804237	8.52103653568245	NA
**TC1000001598.mm.2**	Cnpy2	canopy FGF signaling regulator 2	-0.400265369495863	7.72820977607443	-0.755215623663063	0.476861154625054	0.999692817443324	-4.60801165600031	8.66743668104155	8.28521932642147	7.78065747667189	6.52737847832403	8.01095449104776	7.0976122029399	NA
**TC0300002709.mm.2**	Kcnc4	potassium voltage gated channel, Shaw-related subfamily, member 4	-0.308430581019382	7.05490724778199	-0.755124177445593	0.476912345126354	0.999692817443324	-4.60801576287075	7.6727716084659	7.47038548220402	6.8465223912438	6.43819022574783	7.41881424137289	6.48275953765751	NA
**TC0X00001559.mm.2**	Cypt3	cysteine-rich perinuclear theca 3	-0.309897900237904	4.70783863672859	-0.755039637121778	0.47695967312849	0.999692817443324	-4.60801955929362	3.87310242094807	3.84641643211232	4.55248449369086	5.40137408972682	4.97718330841728	5.5964710754762	NA
**TC1800000498.mm.2**	Eno1b	enolase 1B, retrotransposed	-0.381084061119066	7.2977244069169	-0.754951881416268	0.477008804581767	0.999692817443324	-4.60802349980125	8.16216659646915	8.40475927158263	7.320427990547	5.90819823505874	7.19668903868122	6.79410530916265	NA
**TC0900001124.mm.2**	Mrap2	melanocortin 2 receptor accessory protein 2	0.358841242995896	6.32598838975501	0.754754885783156	0.477119108361469	0.999692817443324	-4.60803234438557	6.99328842042986	6.14943681829854	6.56962630987494	6.19581748302177	6.52228533632221	5.52547597058271	NA
**TC0700002360.mm.2**	Ube2m	ubiquitin-conjugating enzyme E2M	-0.474029339172173	9.49122145438682	-0.754743709282296	0.477125366942342	0.999692817443324	-4.60803284613369	9.9789960297698	9.99813516588282	9.74476818693458	8.26630123922741	10.1550972402758	8.80403086423056	NA
**TC1100001348.mm.2**	Rnf43/1110028F11Rik	ring finger protein 43/RIKEN cDNA 1110028F11 gene	-0.223157913683965	7.76038887738199	-0.7545807860094	0.477216606553254	0.999692817443324	-4.60804015969268	7.50477857097632	7.35358313886003	7.78369212670513	7.84513573683871	7.94212596485334	8.13301772605843	NA
**TC1400001669.mm.2**	Smim4	small integral membrane protein 4	-0.303650968701705	7.08285619785739	-0.754544769984618	0.477236777704183	0.999692817443324	-4.60804177629158	6.64812601654054	6.69837836801402	6.66352044767855	7.60814498491462	7.26181694329567	7.61715042670091	NA
**TC0900003000.mm.2**	Col6a4	collagen, type VI, alpha 4	-0.251812080303264	5.62145320837981	-0.754435207307848	0.477298143003405	0.999692817443324	-4.60804669374764	4.95159736402839	5.23022820167766	5.53819125805061	5.98344350393254	6.16687107600962	5.85838784658006	NA
**TC1700001683.mm.2**	A930017K11Rik	RIKEN cDNA A930017K11 gene	0.297904154971484	6.88330426900313	0.754317745241879	0.477363938677112	0.999692817443324	-4.60805196520626	6.0812000529646	6.12097433350368	7.17709908817511	7.66963068057162	6.70711703656161	7.54380442224215	NA
**TC1100000713.mm.2**	Trim17/Hist3h2a	tripartite motif-containing 17/histone cluster 3, H2a	-0.279141389850222	7.76926320633373	-0.753953900479006	0.477567783420405	0.999692817443324	-4.60806829025595	8.07834545012477	8.18567935983782	7.85053190079412	7.04610392337555	7.62430756856958	7.83061103530054	NA
**TC1000002267.mm.2**	Ccar1	cell division cycle and apoptosis regulator 1	0.45754439495111	7.69402486362352	0.753832908305336	0.477635582679012	0.999692817443324	-4.60807371775973	7.71719318575666	7.82846827046403	8.56380381754615	7.20298444016519	8.0989204187638	6.75277904904532	NA
**TC1900001117.mm.2**	Zp1	zona pellucida glycoprotein 1	0.342026817221086	5.39695644308908	0.753343311699295	0.477910000408577	0.999692817443324	-4.60809567414575	5.12602301612097	4.90410594295127	5.23888431258177	6.27894735437044	5.07839708110748	5.75538095140256	NA
**TC0500003377.mm.2**	Eif4h	eukaryotic translation initiation factor 4H	-0.419611091853834	9.05311203253668	-0.753285159636294	0.477942601643303	0.999692817443324	-4.60809828137677	9.80694058372646	9.73844823598708	9.33353957016321	7.63349102573624	8.99556562552691	8.81068715408021	NA
**TC1100003470.mm.2**	Prr11	proline rich 11	0.228506213006336	5.24805000728897	0.753033468142694	0.478083722522059	0.999692817443324	-4.6081095643032	5.07696832222789	5.23020194723986	5.26983119904977	5.54923990108959	5.10867364695388	5.25338502717281	NA
**TC0800001244.mm.2**	Pla2g15	phospholipase A2, group XV	0.249986876166104	7.15627569891953	0.753029411404959	0.478085997326546	0.999692817443324	-4.60810974613911	7.63756405790472	7.6309900872053	7.32641416317715	6.75812273719252	6.8826001116534	6.70196303638407	NA
**TC0400001246.mm.2**	Pomgnt1	protein O-linked mannose beta 1,2-N-acetylglucosaminyltransferase	-0.417532615505523	6.79943901301937	-0.752998650641616	0.4781032465798	0.999692817443324	-4.60811112491267	7.5069587517245	7.32886843542377	7.11684835346234	5.4460224765161	6.56740948517515	6.83052657581434	NA
**TC0900001403.mm.2**	Usp4	ubiquitin specific peptidase 4 (proto-oncogene)	-0.325657274426527	7.76282869794124	-0.752986128374707	0.478110268625664	0.999692817443324	-4.60811168618062	8.21194666238002	8.31638546439873	7.80571564981795	6.89294710618987	8.02847897138625	7.32149833347462	NA
**TC0400003842.mm.2**	Micos10	mitochondrial contact site and cristae organizing system subunit 10	-0.612943175742455	9.87767969952041	-0.752949359575247	0.47813088767859	0.999692817443324	-4.60811333417983	10.9818585522585	10.8639973972636	10.2297713077523	7.86739664030538	10.3446427098596	8.97841158968301	NA
**TC1700000835.mm.2**	Pla2g7	phospholipase A2, group VII (platelet-activating factor acetylhydrolase, plasma)	0.376129558095069	7.32503345866542	0.752848923046544	0.478187213142674	0.999692817443324	-4.60811783552315	9.14114898858509	8.76867020987337	7.18666210943231	6.20965822542979	6.83565567186268	5.80840554680929	NA
**TC1200001704.mm.2**	Prkd1	protein kinase D1	0.257007087951819	6.14765774290882	0.752436587109221	0.478418501238722	0.999692817443324	-4.60813631119681	6.45552880402591	6.03808252234543	6.11039277561997	6.34278187787265	5.74020591857036	6.19895455901863	NA
**TC1200000460.mm.2**	Srp54b/Srp54a	signal recognition particle 54B/signal recognition particle 54A	-0.460070417414683	7.90781286562025	-0.751989732600637	0.478669237602397	0.999692817443324	-4.60815632572166	8.54713989598704	9.11985564063853	7.81402467047181	6.61584574066146	8.38010235724341	6.96990888871923	NA
**TC0700000764.mm.2**	Zfp658	zinc finger protein 658	-0.238845123554436	6.9798640761223	-0.751883246899609	0.478729001436479	0.999692817443324	-4.60816109399221	6.46983113763328	6.62770975451023	6.91682593899525	7.23515071974544	7.14916190830056	7.48050499754901	NA
**TC0800001422.mm.2**	Dnaaf1	dynein, axonemal assembly factor 1	-0.257570378349498	4.78538821166478	-0.751623849030758	0.478874606677291	0.999692817443324	-4.60817270750279	4.28920169042307	3.97642225657932	4.72830179612016	5.2374804870235	5.08924300989899	5.39168002994366	NA
**TC0800000133.mm.2**	Champ1	chromosome alignment maintaining phosphoprotein 1	0.222041209452073	7.8467760862886	0.751387439478115	0.4790073343807	0.999692817443324	-4.6081832894081	8.16733294310752	7.93511094121819	7.87438420203449	7.83676332412054	7.67222626152036	7.59483884573052	NA
**TC1200001064.mm.2**	D430019H16Rik	RIKEN cDNA D430019H16 gene	0.248120324587385	7.80489617529471	0.750694602692102	0.479396458839216	0.999692817443324	-4.60821428824824	8.60089394488947	8.20692739785343	7.84378997639746	7.41510820270261	7.41397103893591	7.34868649098939	NA
**TC1900001760.mm.2**	Gm7102	predicted gene 7102	0.246867039170717	6.72155562572112	0.750549059974096	0.479478228705944	0.999692817443324	-4.60822079761154	6.24953130913342	6.50575201558568	6.76062864571581	7.27326360825872	6.78723630893186	6.75292186670124	NA
**TC1300000476.mm.2**	Hivep1	human immunodeficiency virus type I enhancer binding protein 1	0.521629112375203	6.58194203480668	0.75051264244981	0.479498690548364	0.999692817443324	-4.60822242624102	7.60537824866199	7.86209556485963	6.80590493043751	5.72781337959692	6.7355506649513	4.75490942033272	NA
**TC0800002854.mm.2**	Slc7a6os	solute carrier family 7, member 6 opposite strand	-0.248900360857278	7.55233152446971	-0.750304283761878	0.479615772057474	0.999692817443324	-4.60823174321397	7.18236880050749	7.35928646329273	7.2904221957382	7.84684438491354	7.87377820058823	7.76128910177807	NA
**TC1200000270.mm.2**	Cog5	component of oligomeric golgi complex 5	0.283683976348599	7.32460572119823	0.750255328034777	0.479643284221255	0.999692817443324	-4.60823393206178	7.93223420232859	7.92026066161348	7.27943934536159	7.05181436261066	7.30769881559551	6.45618693967954	NA
**TC1200000604.mm.2**	Arid4a	AT rich interactive domain 4A (RBP1-like)	-0.497783423103681	6.15299489296997	-0.750165066107288	0.479694012478727	0.999692817443324	-4.60823796748399	7.13081329591461	7.00257517816553	6.50955675322383	4.38495026554235	5.81188116044725	6.07819270452628	NA
**TC0800001798.mm.2**	Defb12/Defb37	defensin beta 12/defensin beta 37	0.456445426137007	4.37029800059738	0.750052957918095	0.479757023672132	0.999692817443324	-4.60824297914237	3.74614188409343	3.64575490855024	4.29661663670585	5.57477439490146	3.76205824346638	5.19644193586691	NA
**TC0500001134.mm.2**	Golga3	golgi autoantigen, golgin subfamily a, 3	0.28359915753544	5.74589522476862	0.750026297013224	0.479772009449228	0.999692817443324	-4.60824417090949	6.77767180739128	6.78754620693313	5.76872227945285	4.96995354522625	5.27210068925152	4.8993768203567	NA
**TC1100000502.mm.2**	Olfr1393	olfactory receptor 1393	0.246060811448597	5.80564241296938	0.749835165520619	0.479879451508243	0.999692817443324	-4.60825271381151	6.10916992514273	5.79935625765801	5.6302221939239	6.07850276503248	5.68929728186963	5.52730605418956	NA
**TC0800000843.mm.2**	Hmgxb4	HMG box domain containing 4	0.328458403705341	7.86433437554772	0.74970359063835	0.479953424110359	0.999692817443324	-4.60825859387378	7.2287909200006	7.28814747546697	7.78570118753151	8.8772911450832	7.77291648521295	8.23315903999108	NA
**TC1400002859.mm.2**	Hacl1	2-hydroxyacyl-CoA lyase 1	0.287692034133124	6.76626878928819	0.749699405742585	0.479955777024271	0.999692817443324	-4.60825878088452	6.71201222663534	6.64177377575239	6.54050235530443	7.3691030454994	6.47959458344254	6.85462674909505	NA
**TC0200001053.mm.2**	Map3k20	mitogen-activated protein kinase kinase kinase 20	0.225499491278373	5.3396123573333	0.7496242011364	0.479998061365312	0.999692817443324	-4.60826214143587	5.4378027912176	5.48828414653693	5.46344178652914	5.31785130787186	5.30505744693943	5.02523666490483	NA
**TC0700002172.mm.2**	Tmem238	transmembrane protein 238	-0.237113207236408	6.60514536437945	-0.74949849986444	0.480068743482425	0.999692817443324	-4.60826775793569	6.37589799442133	6.58449865700522	6.56401955564487	6.5341050045438	6.57343478922405	6.99891618543744	NA
**TC1300000867.mm.2**	8030423J24Rik	RIKEN cDNA 8030423J24 gene	-0.346219105559293	5.68282549880808	-0.749331014477308	0.480162931900964	0.999692817443324	-4.60827524039881	5.47031547178944	5.42589341622129	5.09281894754513	6.16133399931445	5.60582309970818	6.34076805826998	NA
**TC1100003056.mm.2**	Eif4a1	eukaryotic translation initiation factor 4A1	-0.34164431764756	8.67325517733409	-0.749029788509975	0.480332363354759	0.999692817443324	-4.60828869487022	9.81914298318701	10.0236091944719	8.35690991337314	7.39983521215212	8.62502132340274	7.81501243741764	NA
**TC1100003075.mm.2**	2810408A11Rik	RIKEN cDNA 2810408A11 gene	-0.265097075771414	6.09039916700289	-0.748869601263996	0.480422480894829	0.999692817443324	-4.60829584823122	5.43394437189818	5.66448273743468	5.89573097495104	6.56115589561978	6.3773783972714	6.60970262484225	NA
**TC0900001866.mm.2**	Kri1	KRI1 homolog	0.393658348282836	6.0560707673153	0.748834923477165	0.480441991307075	0.999692817443324	-4.60829739667236	6.48869135711923	6.0777355675153	5.82651106207235	6.45214612583912	6.44068988780108	5.05065060354472	NA
**TC1600001607.mm.2**	Poglut1	protein O-glucosyltransferase 1	-0.252770332362512	7.53954669231028	-0.748792367210897	0.48046593504436	0.999692817443324	-4.60829929683787	7.87120562615634	7.56905074037351	7.59968166700015	7.04605989430324	7.53684676323098	7.61443546279744	NA
**TC1600001825.mm.2**	Arl13b	ADP-ribosylation factor-like 13B	0.22059436975594	6.38459396727105	0.748662881080403	0.480538793742135	0.999692817443324	-4.60830507802238	6.23514234815643	6.07171771469166	6.55305095057424	6.6678952895708	6.45096972981699	6.32878777081617	NA

**TC0900002764.mm.2**	Hmgn3	high mobility group nucleosomal binding domain 3	-0.345043419510452	8.36426688935549	-0.748594179512145	0.48057745349276	0.999692817443324	-4.60830814507101	8.19336732759843	7.99855415695419	8.9158663786108	7.73593012766892	8.91609244075706	8.42579090454356	NA
**TC0300000948.mm.2**	Hjv	hemojuvelin BMP co-receptor	0.302830508671848	5.01141028005493	0.748431896034393	0.480668782053004	0.999692817443324	-4.60831538913359	4.18968537085193	4.05324617237057	5.11088893049345	6.10470664673194	5.40185855396134	5.20807600592036	NA
**TC1600000651.mm.2**	Usf3	upstream transcription factor family member 3	0.248054967589097	7.64785121242891	0.748298087247481	0.480744094730467	0.999692817443324	-4.60832136132028	8.08682234557139	8.18876861856638	7.54863982853343	7.50517329427353	7.57006737538481	6.98763581224395	NA
**TC1700001486.mm.2**	Wdr27	WD repeat domain 27	0.259363005819535	5.20283432895566	0.748114967577138	0.480847174441127	0.999692817443324	-4.60832953317071	5.13563361271117	5.50330139181563	5.3725102423641	5.17588824805902	4.73634996908683	5.29332250969723	NA
**TC1100001785.mm.2**	Milr1	mast cell immunoglobulin like receptor 1	-0.505841442394133	5.02373403545468	-0.748076280260946	0.480868953800455	0.999692817443324	-4.6083312594445	4.95197410992694	4.78966736282323	3.87342967097805	5.82111025661677	4.85334895883985	5.85287385354323	NA
**TC0700000401.mm.2**	Rps19	ribosomal protein S19	0.220785343975615	8.46748115109501	0.747829356248727	0.481007977594817	0.999692817443324	-4.60834227603709	8.61345076037285	8.5957533508514	8.4851385306462	8.53348821100232	8.40748539830714	8.16957065539014	NA
**TC0400001266.mm.2**	Mutyh	mutY DNA glycosylase	-0.303875661571382	6.42650598533286	-0.747746920164238	0.481054397048821	0.999692817443324	-4.60834595339109	5.94289445192694	5.52083005676062	6.41942048590091	6.82435955418252	6.50145078897282	7.35008057425337	NA
**TC0100002832.mm.2**	Pask	PAS domain containing serine/threonine kinase	-0.269737198170405	5.63177287205837	-0.747699005231203	0.481081379168122	0.999692817443324	-4.6083480906783	4.9786385070803	4.90834968424365	5.4526926016282	6.22939472071453	6.07975815282456	6.14180356585898	NA
**TC0900003033.mm.2**	Cyb561d2	cytochrome b-561 domain containing 2	0.295960559878029	6.97658633005587	0.747682582333739	0.481090627556497	0.999692817443324	-4.6083488232142	7.02824700738417	6.87191046247066	7.0507070927242	7.22493372239402	7.31583924978623	6.36788044557593	NA
**TC0200002876.mm.2**	Camk1d	calcium/calmodulin-dependent protein kinase ID	-0.273002444552342	7.75101690678387	-0.747184882593854	0.481370959388948	0.999692817443324	-4.60837101762484	7.95403568143205	8.12132117013124	7.51524233562204	7.42712751439559	8.13770934385098	7.35066539527134	NA
**TC0400001455.mm.2**	Eva1b	eva-1 homolog B (C. elegans)	-0.259876492243936	6.66648549868461	-0.74714524340735	0.481393291122691	0.999692817443324	-4.60837278485579	6.14608282859549	6.48411956859952	6.50303705453493	6.92144175067745	6.74579469509104	7.19843709460921	NA
**TC0700000161.mm.2**	Lig1	ligase I, DNA, ATP-dependent	-0.280122554845947	6.26624727103877	-0.747036350678557	0.481454642202343	0.999692817443324	-4.60837763927962	6.38230302203635	6.27825897765705	5.98511589069074	6.20322236773292	6.78909231026852	5.95949105784704	NA
**TC1300001112.mm.2**	Ankra2	ankyrin repeat, family A (RFXANK-like), 2	0.389382187247435	7.47208565750355	0.746952666113592	0.481501794396464	0.999692817443324	-4.60838136959519	8.17569627339337	7.694415212577	7.63352913213756	7.23705428463534	7.76082887653862	6.33099016573941	NA
**TC0100003478.mm.2**	Uck2	uridine-cytidine kinase 2	-0.369053986855038	8.32529947794537	-0.746952108803529	0.481502108424163	0.999692817443324	-4.60838139443683	7.82347649047706	8.01735456878813	8.99947151711126	7.68695740023723	8.99404109741826	8.4304957936403	NA
**TC1800000805.mm.2**	Smad7	SMAD family member 7	-0.216179599505159	5.83868197137817	-0.746818064809794	0.481577642277949	0.999692817443324	-4.60838736896611	5.54694885206846	5.62157937868073	5.90696971848091	5.80863248077385	6.08666206783415	6.06129933043093	NA
**TC1700001949.mm.2**	Apom	apolipoprotein M	-0.346802979718421	5.07975415193564	-0.746806494997656	0.481584162249506	0.999692817443324	-4.60838788461424	4.1592868892917	4.15766388744154	4.73815218834292	5.99583189937894	5.53351308421574	5.89407696294297	NA
**TC0200003134.mm.2**	Stpg3	sperm tail PG rich repeat containing 3	-0.28497165779918	4.98025177893846	-0.746749207138225	0.481616446734432	0.999692817443324	-4.60839043776183	4.23589730307915	4.53665110713208	4.75267578814177	5.51683368576881	5.23477015401648	5.60468263549245	NA
**TC0X00000519.mm.2**	Ints6l	integrator complex subunit 6 like	-0.593724337038084	7.23495584082	-0.746676011529883	0.481657698143637	0.999692817443324	-4.60839369967335	7.15459989190427	7.39342657227702	8.12393364146682	5.71319631186444	8.06033988104011	6.96423874636731	NA
**TC1900000197.mm.2**	Syt7	synaptotagmin VII	-0.224836256798135	8.82782592206268	-0.746282282605539	0.481879636097113	0.999692817443324	-4.6084112421448	9.01078584136604	9.03921696488606	8.77070153997915	8.4629385662847	8.89182531036851	8.79148730949161	NA
**TC1000001767.mm.2**	Ltv1	LTV1 ribosome biogenesis factor	-0.411106392060708	6.30540720418839	-0.746221352556838	0.481913987473313	0.999692817443324	-4.60841395629393	6.63685019452932	6.4929290008359	6.67801507121258	5.26221055160925	6.8330361167153	5.92940229022795	NA
**TC0X00002053.mm.2**	Sept6	septin 6	-0.298923962159225	7.6056879342792	-0.746128356362625	0.481966420425985	0.999692817443324	-4.60841809854521	7.92407718764567	7.83766546671906	7.80142735360284	6.83584115989317	7.74278775167326	7.4923286861412	NA
**TC0200004530.mm.2**	Secisbp2l	SECIS binding protein 2-like	0.297509694165646	7.61775975551191	0.74608527107246	0.481990714010984	0.999692817443324	-4.60842001753627	8.74677014008686	8.94377742991138	7.23116390608471	7.07435126961755	7.33480500682264	6.37569078054833	NA
**TC1700000148.mm.2**	Sod2	superoxide dismutase 2, mitochondrial	-0.430613784798261	9.25250591906985	-0.746027121083395	0.482023503123748	0.999692817443324	-4.60842260737802	9.69226428087789	9.49619808545089	9.63755470631438	8.09511808293251	9.74575198106808	8.84814837777533	NA
**TC1700001033.mm.2**	Vav1	vav 1 oncogene	-0.443390193221377	5.35161687646738	-0.745854441830146	0.482120880923038	0.999692817443324	-4.60843029721913	5.05470417641925	6.21868029627401	5.12844926209481	4.84631893773932	4.71506501818658	6.1464835680903	NA
**TC0X00001062.mm.2**	Fgf16	fibroblast growth factor 16	0.302460572453572	6.57008621055377	0.745829162655974	0.482135137549627	0.999692817443324	-4.60843142286116	6.10896329862981	6.32404576930534	6.70102859728152	7.09518607286579	6.13419121096878	7.05710231427138	NA
**TC0700002744.mm.2**	Zfp146	zinc finger protein 146	-0.267108692459152	6.87720815280991	-0.745705773623001	0.482204729027421	0.999692817443324	-4.60843691680336	7.47018273675267	7.63520204902074	6.57664980431268	6.2351735687712	7.00639030131014	6.33965045669204	NA
**TC1700001154.mm.2**	Ehd3	EH-domain containing 3	0.326378590330765	6.77137179343284	0.745671515114902	0.482224052051491	0.999692817443324	-4.60843844206474	7.91242634327075	7.79667778497478	6.97212503225447	5.81381687425205	5.9580717013827	6.17511302446229	NA
**TC1300001722.mm.2**	Sox4	SRY (sex determining region Y)-box 4	-0.277084590574915	6.75844842352463	-0.745665877264137	0.482227232051309	0.999692817443324	-4.60843869306916	6.75033334196698	6.78802794840163	6.80832887570446	6.42075115911021	6.5166266012328	7.2666226147317	NA
**TC0400003451.mm.2**	Zmpste24	zinc metallopeptidase, STE24	-0.217842136558037	7.81650113942651	-0.745532771327251	0.482302313879319	0.999692817443324	-4.60844461873727	7.85469306841609	8.01747516919553	7.64599451956127	7.64958264335442	7.91509856916013	7.81616286687163	NA
**TC0900000714.mm.2**	Edc3	enhancer of mRNA decapping 3	-0.461182792838922	6.98221168439934	-0.74550359087184	0.482318774925015	0.999692817443324	-4.60844591770786	7.36548152350647	7.03919859824475	7.52548784352264	5.75762435596085	7.49907436940538	6.70640341575595	NA
**TC1900000282.mm.2**	Olfr1494	olfactory receptor 1494	-0.345118275147702	4.64984110507713	-0.745328253132469	0.482417693071427	0.999692817443324	-4.60845372214346	4.23579549433627	4.5377563286164	4.17499360044624	5.04263552816111	4.49246536039862	5.41540031850413	NA
**TC0400004042.mm.2**	Nmnat1	nicotinamide nucleotide adenylyltransferase 1	-0.221608044631525	6.41388833199523	-0.745174371306535	0.482504518027246	0.999692817443324	-4.60846057051266	6.71469032141944	6.89361656800705	6.16880519456969	6.04709831207124	6.41450042236755	6.24461917353642	NA
**TC1100004248.mm.2**	Ogfod3	2-oxoglutarate and iron-dependent oxygenase domain containing 3	-0.248536942824455	6.37115734804995	-0.745156114631736	0.48251481971894	0.999692817443324	-4.60846138294435	6.50219605294807	6.74566473891366	6.39141362196512	5.84959108342942	6.30550240534429	6.43257618569915	NA
**TC0400004018.mm.2**	Disp3	dispatched RND transporter family member 3	0.22868332395409	6.23363882380408	0.745154958164503	0.482515472283587	0.999692817443324	-4.60846143440729	6.28944548627546	6.10385076190358	6.33279328513011	6.4001583861467	6.30404417446269	5.97154084890593	NA
**TC1400001253.mm.2**	Gpc6	glypican 6	-0.222020183526237	6.19659124793617	-0.745009650345525	0.482597470499036	0.999692817443324	-4.6084679001848	6.39470570109641	6.20631217612704	6.12063982419253	5.94660479747801	6.19513168311214	6.31615330561088	NA
**TC1100001247.mm.2**	Ap2b1	adaptor-related protein complex 2, beta 1 subunit	-0.501841104575621	7.25541115745521	-0.744923985115314	0.482645816410211	0.999692817443324	-4.60847171163135	8.77046404844024	8.82289246156775	7.31684352906254	5.15087058372348	6.87809865553993	6.59329766639733	NA
**TC1500002256.mm.2**	A330009N23Rik	RIKEN cDNA A330009N23 gene	-0.225490822703909	5.67658354015911	-0.744756668254139	0.482740252587835	0.999692817443324	-4.60847915507633	5.88707530281457	5.93413133167836	5.51302167017472	5.38063481035223	5.82842494690358	5.51621317903119	NA
**TC1100002183.mm.2**	Wap	whey acidic protein	0.322247981673314	7.07105179312399	0.744716703208811	0.482762811318654	0.999692817443324	-4.608480932835	6.52205803741638	6.44526019167948	7.14171545454666	7.91002879195068	6.77056250523831	7.6366857779124	NA
**TC0900003003.mm.2**	Ppm1m	protein phosphatase 1M	0.255880875441917	6.2908679344987	0.744229825056743	0.483037692656669	0.999692817443324	-4.60850258526022	6.3609067859849	6.54243650656257	6.66232846132844	6.01448457133582	6.12100997302497	6.04404130875546	NA
**TC1600001548.mm.2**	Pdia5	protein disulfide isomerase associated 5	0.294522069859818	5.50002052014501	0.744055811422851	0.483135962866608	0.999692817443324	-4.60851032161157	5.02769888100395	5.33705530909485	5.44498113638287	6.16722539886259	5.22173453453495	5.80142786099087	NA
**TC0200004482.mm.2**	Trp53bp1	transformation related protein 53 binding protein 1	-0.504346945638472	6.58389182059642	-0.744021494006652	0.483155344438751	0.999692817443324	-4.60851184715801	8.22268780488491	7.88590932185083	6.60811134896587	4.58491860381706	6.501274148381	5.70044969567886	NA
**TC0100001889.mm.2**	Xkr4	X-linked Kx blood group related 4	0.507434262185725	6.40080866846842	0.743752467087645	0.48330730199337	0.999692817443324	-4.60852380479387	7.76905566827294	8.12935144636775	6.21158330529837	5.54907339597228	6.41647198161634	4.32931619528286	NA
**TC1200001791.mm.2**	Nkx2-1	NK2 homeobox 1	0.2630047619802	5.07800374059102	0.743583962500259	0.483402496847594	0.999692817443324	-4.60853129291593	4.78022642042324	4.73756015297348	5.19003970299671	5.54808299405818	5.42900765158828	4.7831055215062	NA
**TC1500000088.mm.2**	Slc45a2	solute carrier family 45, member 2	0.415928648793087	5.93589514002302	0.743520416629079	0.483438399699779	0.999692817443324	-4.60853411650583	5.28005675899202	5.14236752823188	5.72079744804071	7.29160447720949	5.74510083506349	6.43544379260054	NA
**TC1700001783.mm.2**	Mtch1	mitochondrial carrier 1	-0.596838291134748	8.92691614343893	-0.743490720516579	0.483455178358139	0.999692817443324	-4.6085354359622	9.58061922323303	9.48553697740171	9.55427820230994	7.09655383655473	9.46464864628717	8.379859974847	NA
**TC0700000236.mm.2**	Slc1a5	solute carrier family 1 (neutral amino acid transporter), member 5	-0.224155348403468	5.24991550981913	-0.7433231729089	0.483549852144128	0.999692817443324	-4.60854287974648	4.75377059643534	4.77406967197518	5.54106511562298	5.22060593122567	5.63921294373088	5.57076879992471	NA
**TC1100003192.mm.2**	Olfr411	olfactory receptor 411	0.230479852643496	5.95175849128245	0.743311070068753	0.48355669141285	0.999692817443324	-4.60854341740497	5.2839671966607	5.64754700954336	6.20728076085902	6.4127174625298	6.06994473699325	6.08909378110858	NA
**TC0100003865.mm.2**	Armc9	armadillo repeat containing 9	0.307930603066777	5.92357094055965	0.743260829411479	0.483585082916284	0.999692817443324	-4.60854564923911	6.65956882008728	6.28879081604084	5.6850912104959	5.91937239618576	5.0060908606933	5.9825115398548	NA
**TC1100001039.mm.2**	Olfr406	olfactory receptor 406	-0.396897736976723	5.13181253516275	-0.743187657028768	0.48362643538772	0.999692817443324	-4.60854889957941	4.63348544806443	4.17935024098624	4.76086290216482	5.83125912182136	5.13296305041474	6.25295444752489	NA
**TC0600002253.mm.2**	Cycs	cytochrome c, somatic	-0.638250788271995	9.78684352735064	-0.743172983108522	0.483634728461198	0.999692817443324	-4.6085495513729	10.6443162017746	10.7112306455258	10.2274600667572	7.81704630337251	10.4682483427997	8.85275960387398	NA
**TC1500000404.mm.2**	Zhx2	zinc fingers and homeoboxes 2	0.294292501775002	7.73417428642568	0.743075735627044	0.4836896910197	0.999692817443324	-4.60855387073463	7.64391857351237	7.76716958878659	8.30054949308458	7.49072178681797	7.33463095553145	7.8680553208211	NA
**TC1700002782.mm.2**	H2-Eb1	histocompatibility 2, class II antigen E beta	-0.258199444972497	5.84502207341465	-0.74302112151014	0.483720559807089	0.999692817443324	-4.60855629631347	5.31823912725291	5.46037877441274	5.7430855675888	6.14447225684982	5.92359822867451	6.48035848570911	NA
**TC0800000865.mm.2**	Prmt9	protein arginine methyltransferase 9	-0.29461448773347	6.14721054777457	-0.74300773671655	0.483728125313565	0.999692817443324	-4.60855689075397	6.41757005995718	6.63944636069476	6.27381921435579	5.34468973090847	5.99451924603801	6.2132186746932	NA
**TC1500001646.mm.2**	Kcnq3	potassium voltage-gated channel, subfamily Q, member 3	-0.303847699449904	7.11786507582254	-0.742914913754108	0.483780593975453	0.999692817443324	-4.60856101296898	8.45771353438364	8.67728779526922	6.73363069253978	5.7486161706515	6.67833569162772	6.41160657046336	NA
**TC1400001906.mm.2**	Ddhd1/Mir5131	DDHD domain containing 1/microRNA 5131	-0.584101112031122	8.04314398386921	-0.742868282478161	0.483806953999596	0.999692817443324	-4.60856308370281	8.69055023724539	8.79271465993667	8.47732341253672	6.32637497844877	8.73637215447955	7.23552846056818	NA
**TC0200002277.mm.2**	Csnk2a1	casein kinase 2, alpha 1 polypeptide	0.360972158628689	9.19960349450471	0.742217144877489	0.484175134716879	0.999692817443324	-4.60859198906372	9.5346383287174	9.55570440467046	9.47307374367174	8.94153753177715	9.49774213939437	8.19492481879715	NA
**TC1900001077.mm.2**	Rom1	rod outer segment membrane protein 1	0.40071936559731	5.46388213776083	0.742200949218432	0.484184294841583	0.999692817443324	-4.608592707799	5.60202489666651	5.51829998111018	4.79962289192496	6.43258044806649	5.15678306721314	5.27398154158368	NA
**TC0800000264.mm.2**	Defb35	defensin beta 35	0.294198540624487	6.49034667784112	0.742166303269915	0.484203890683948	0.999692817443324	-4.60859424528964	5.83745762742975	6.20123327147514	6.52129060710588	7.22460251758952	6.86694448908866	6.29055155435776	NA
**TC1800000385.mm.2**	Rell2	RELT-like 2	-0.220035846240621	7.52780516483119	-0.742148742791901	0.484213823142699	0.999692817443324	-4.60859502455571	7.85440088382877	7.59388343818484	7.33332130039437	7.30591618685176	7.53360978177342	7.54569939795396	NA
**TC1100002207.mm.2**	Tns3	tensin 3	0.430469959063108	5.38920938700176	0.741954120843756	0.484323913338589	0.999692817443324	-4.60860366026877	6.36580114203285	6.46271901439209	4.2603900420644	5.92344799979152	5.01229506383272	4.31060305989697	NA
**TC1500001993.mm.2**	Phf21b	PHD finger protein 21B	-0.317698050832279	7.78256871873118	-0.74146297189442	0.484601812757241	0.999692817443324	-4.60862544641425	7.05804723281178	7.10306001178482	7.48693063933251	8.46252384373046	7.99847419683752	8.58637638789001	NA
**TC0200004978.mm.2**	Gdf5	growth differentiation factor 5	0.308237905108773	5.66675286010615	0.741378417311073	0.484649665876924	0.999692817443324	-4.6086291960354	5.31025079574674	5.26746568287891	5.54792096554892	6.47171728056549	5.97825175766779	5.42491067822907	NA
**TC1300000182.mm.2**	Hist1h4m/Hist1h4n	histone cluster 1, H4m/histone cluster 1, H4n	-0.222543238358046	9.69770353806712	-0.741366523722628	0.484656397232585	0.999692817443324	-4.60862972343955	9.8532997399086	9.83367744376454	9.62218230521989	9.40489647878685	9.84735160814085	9.62481365258198	NA
**TC1300001564.mm.2**	Hist1h4n	histone cluster 1, H4n	-0.222543238358046	9.69770353806712	-0.741366523722628	0.484656397232585	0.999692817443324	-4.60862972343955	9.8532997399086	9.83367744376454	9.62218230521989	9.40489647878685	9.84735160814085	9.62481365258198	NA
**TC0X00000316.mm.2**	Il13ra1	interleukin 13 receptor, alpha 1	-0.320059181891598	6.33468573250261	-0.741352478182955	0.484664346598848	0.999692817443324	-4.60863034626133	6.78843472592927	7.09818653482069	6.32107743690431	5.41960994833695	6.51704853066454	5.86375721835991	NA
**TC1100001151.mm.2**	Sdf2	stromal cell derived factor 2	-0.240020349836241	7.06742747726108	-0.741325297431848	0.484679730362366	0.999692817443324	-4.60863155151493	7.34458496276557	7.16766379599176	6.63105933260696	7.07507836996136	7.21349678147251	6.9726816207683	NA
**TC1100002126.mm.2**	Ewsr1	Ewing sarcoma breakpoint region 1	-0.250739042265403	8.91376446510249	-0.741310617982235	0.484688038775573	0.999692817443324	-4.60863220242089	9.39477025819053	9.42496542184285	8.60363900276244	8.47704751026295	9.10440061842362	8.47776397913257	NA
**TC0100002381.mm.2**	Clk1	CDC-like kinase 1	0.44322304731632	6.956126462602	0.741296084630861	0.484696264593537	0.999692817443324	-4.60863284683986	7.09556166105225	7.28152571973376	7.19839334742776	6.92466539730154	7.54384523362035	5.69276741647631	NA
**TC1600000472.mm.2**	Slc12a8	solute carrier family 12 (potassium/chloride transporters), member 8	-0.242982656742373	5.25275442719449	-0.740999171358204	0.484864336953094	0.999692817443324	-4.60864601026252	5.38060247756022	5.2402843249572	4.87673337981436	5.32810384376801	5.18909791005529	5.50170462701183	NA
**TC0700004388.mm.2**	Nsmce4a	NSE4 homolog A, SMC5-SMC6 complex component	0.271920964107862	6.87096556894655	0.74097022563169	0.484880724236492	0.999692817443324	-4.60864729335333	7.56285852607962	7.34918069615835	7.07768248098881	6.35111557883973	6.28695793281681	6.59799819879601	NA
**TC1000001304.mm.2**	Krr1	KRR1, small subunit (SSU) processome component, homolog (yeast)	-0.21856505784079	7.19057251248887	-0.740845086002887	0.484951574874537	0.999692817443324	-4.60865284007601	7.06640118449037	6.87913586287075	7.17909904186907	7.2012849140762	7.33447407639328	7.48303999523357	NA
**TC0800001923.mm.2**	Ash2l	ASH2 like histone lysine methyltransferase complex subunit	-0.80177982971839	6.96972094732629	-0.740795210914242	0.484979814735845	0.999692817443324	-4.60865505057162	8.04423713281657	7.7900181032189	7.93358854509015	4.25666684915259	7.22092973443016	6.57288531924936	NA
**TC0700003111.mm.2**	Gabra5	gamma-aminobutyric acid (GABA) A receptor, subunit alpha 5	-0.407641132609621	8.42593058451375	-0.74056800903602	0.485108473165434	0.999692817443324	-4.60866511899557	9.37077560531816	9.12220338339188	8.54311666530918	7.08054446126742	8.61764115245374	7.82130223934211	NA
**TC0700003851.mm.2**	Rnf121	ring finger protein 121	0.386999792004312	5.86921619584394	0.740474248242983	0.485161574148021	0.999692817443324	-4.60866927336865	5.71060963935033	5.18626976459207	5.85108677205651	6.69512190550841	5.28113095288581	6.49107814067048	NA
**TC0700004301.mm.2**	Ino80e	INO80 complex subunit E	0.591451967330702	6.90419197628652	0.740381946765997	0.485213852488104	0.999692817443324	-4.60867336272517	7.87523882496546	7.68350782961258	7.80623832538203	5.7184162435192	7.00003578701014	5.34171484722969	NA
**TC0200000578.mm.2**	Ptges2	prostaglandin E synthase 2	-0.246610597936292	7.15895074204849	-0.74037659011817	0.485216886539625	0.999692817443324	-4.60867360003707	7.09304782731923	7.25683580102518	6.75489452652481	7.30040528751216	7.17333851002663	7.37518249988293	NA
**TC1300002311.mm.2**	Cep72	centrosomal protein 72	-0.219936114685511	5.3037163655619	-0.740294400318772	0.485263441157149	0.999692817443324	-4.60867724108598	5.30781243874602	5.3598275227717	5.05096629850854	5.30642670290409	5.43551787384846	5.36174735693519	NA
**TC1100001789.mm.2**	1810010H24Rik	RIKEN cDNA 1810010H24 gene	-0.270466860213035	6.56164096025497	-0.740208306505491	0.485312210352551	0.999692817443324	-4.6086810547835	6.29193337904556	6.27109311046153	6.64432917864586	6.48861359715246	6.43986258326762	7.23401391295678	NA
**TC0900000863.mm.2**	Ciao2a	cytosolic iron-sulfur assembly component 2A	0.382114008824378	7.66612958684298	0.739943547415432	0.485462208042385	0.999692817443324	-4.60869278088529	7.99770865837191	8.07267384443403	8.40806301818689	6.93724849976347	7.51367585133043	7.06740764897118	NA
**TC0500001894.mm.2**	Cyp51	cytochrome P450, family 51	-0.922845460697742	7.09858603280773	-0.739834746385377	0.485523857692351	0.999692817443324	-4.60869759880555	8.68677291627843	8.47744818326551	7.96631289226376	3.82448919568972	7.47988062824656	6.1566123811024	NA
**TC0800002399.mm.2**	Gdf15	growth differentiation factor 15	-0.244598035979982	5.99495950491813	-0.739778144901348	0.485555931736505	0.999692817443324	-4.60870010503413	5.21090370860798	5.66913224031125	6.20190683595452	6.09835566836027	6.21899502948295	6.5704635467918	NA
**TC1800001710.mm.2**	Cndp1	carnosine dipeptidase 1 (metallopeptidase M20 family)	0.341880140142496	5.8306732336761	0.739692987556422	0.485604190064018	0.999692817443324	-4.60870387542216	5.71004598337332	4.96836890478487	5.71693614004972	6.77775625704197	5.95609277458428	5.85483934222241	NA
**TC0900000658.mm.2**	Sh2d7	SH2 domain containing 7	0.249096855071248	5.29346194626718	0.739401441798668	0.485769432394037	0.999692817443324	-4.60871678148495	4.84601933616171	5.23435959147845	5.49423855516453	5.59505467488819	5.05216883416185	5.53893068574838	NA
**TC1100001813.mm.2**	Apoh	apolipoprotein H	0.413656600045553	5.46436054641684	0.739259102169419	0.485850121444987	0.999692817443324	-4.60872308124893	5.05321822700103	4.759919615487	5.47278462447267	6.42738469357938	4.83502650800649	6.23782960995446	NA
**TC0100002681.mm.2**	Slc19a3	solute carrier family 19, member 3	0.333954114702432	5.33383103698483	0.738962241711712	0.486018433883721	0.999692817443324	-4.60873621718619	5.25378823256678	5.27346618204621	5.70885548777927	5.36296453057116	5.8029800554954	4.60093173345017	NA
**TC1900000905.mm.2**	Rps6kb2	ribosomal protein S6 kinase, polypeptide 2	-0.286354243858797	9.15918575616024	-0.738936574150019	0.486032988595192	0.999692817443324	-4.60873735279147	8.2726055442113	8.50763913854482	9.29713020587291	9.50395047737096	10.1261073156043	9.24768185535713	NA
**TC0800003000.mm.2**	Cdyl2	chromodomain protein, Y chromosome-like 2	-0.259470972303939	6.47831164689043	-0.738706974344693	0.486163195532482	0.999692817443324	-4.60874750971318	6.76680946578615	6.31633029521719	6.11778359604148	6.5161104918242	6.37429889796349	6.77853713451007	NA
**TC0600002629.mm.2**	Dusp11	dual specificity phosphatase 11 (RNA/RNP complex 1-interacting)	-0.30118304390961	8.25418433732038	-0.738514252199441	0.486272507166353	0.999692817443324	-4.60875603356359	7.79957887510838	8.27780540044054	8.60021231191448	7.82246551836258	8.75936108586918	8.2656828322271	NA
**TC0200000567.mm.2**	Prrc2b	proline-rich coiled-coil 2B	0.21699950633821	7.59746589900767	0.738513344598001	0.486273021995372	0.999692817443324	-4.60875607370196	8.06236120208489	8.17992186830104	7.53008036003149	7.35817530813676	7.19086444952581	7.26339220596602	NA
**TC0X00002845.mm.2**	Itm2a	integral membrane protein 2A	-0.824593521747656	6.98925567299873	-0.738344136695044	0.486369010127742	0.999692817443324	-4.60876355626521	7.75164069883819	7.5605990189441	7.63311936523501	4.85393427312238	8.41157948469188	5.72466119716082	NA
**TC1900000864.mm.2**	Nanos1	nanos C2HC-type zinc finger 1	-0.273566218091956	6.33051204682948	-0.73828420581614	0.486403010735479	0.999692817443324	-4.60876620618963	6.42004286948273	6.29383675378997	5.76660987059566	6.59442024016447	6.42711486547786	6.48104768146617	NA

TC1400000914.mm.2	Prss51	protease, serine 51	0.227689628508026	6.78311426451077	0.738263493054972	0.486414762086586	0.999692817443324	-4.6087671219974	6.22911795943835	6.29283305951844	6.96857163716228	7.34748527539967	6.87133495849766	6.98934269704823	NA
TC1100000821.mm.2	Tekt3	tektin 3	0.296818858432945	5.44593198721507	0.738259181308263	0.486417208373087	0.999692817443324	-4.60876731263758	4.81359878875892	4.92359189160376	5.64343252501303	6.12258695488379	5.14587431978714	6.02650744324378	NA
TC0500002539.mm.2	Kctd8	potassium channel tetramerisation domain containing 8	-0.298887190731862	6.52612474140878	-0.738241294562119	0.486427356577636	0.999692817443324	-4.60876810347644	6.71411034194182	6.82835160568888	5.75051502671616	6.75774103296298	6.48441457570325	6.6216158654396	NA
TC0600002797.mm.2	Tmf1	TATA element modulatory factor 1	0.517638688975544	5.68009299894376	0.738230152402835	0.486433678252984	0.999692817443324	-4.60876859610555	7.34408034139353	7.20794337304702	5.77728162011692	4.50462420846964	5.57509180313801	3.67153664749746	NA
TC0700000250.mm.2	Psg16	pregnancy specific glycoprotein 16	0.285467063981462	5.12438576860026	0.738099130826497	0.486508019513628	0.999692817443324	-4.60877438858381	4.93983607181406	5.05825940149806	5.03638555135267	5.62319108177352	5.39917708860845	4.68946541655481	NA
TC1800000577.mm.2	Prrc1	proline-rich coiled-coil 1	0.745288485314077	6.68909619221591	0.737918597163394	0.486610466335033	0.999692817443324	-4.60878236882384	7.24055052792095	7.28599928796072	8.07603494003913	5.47326721398183	7.17841863666685	4.88030654672595	NA
TC1900001779.mm.2	Itprip	inositol 1,4,5-triphosphate receptor interacting protein	-0.275891682482632	7.16907192669048	-0.737773658335547	0.486692724799382	0.999692817443324	-4.60878877465956	6.28560417327588	6.14009014344951	7.08790583894229	7.93057110028382	7.74810271748664	7.82215758670473	NA
TC0800001526.mm.2	Afg3l1	AFG3-like AAA ATPase 1	0.269720127511108	5.80652266388347	0.737618969659258	0.486780527022821	0.999692817443324	-4.60879561044055	6.29772863863113	6.43372390371982	5.26619731507479	6.05736453291126	5.33405354735704	5.4500680456068	NA
TC1500001528.mm.2	Tnfrsf11b	tumor necrosis factor receptor superfamily, member 11b (osteoprotegerin)	-0.361255807670544	4.79663588983181	-0.737394603383379	0.486907897639446	0.999692817443324	-4.60880552353953	4.67984746873666	4.81623761439511	5.33703746627898	3.94357185398003	5.12876598732216	4.87435494827794	NA
TC0300001909.mm.2	Pgrmc2	progesterone receptor membrane component 2	0.338654312883801	8.84414818275272	0.737370768404738	0.486921429851176	0.999692817443324	-4.60880657650881	8.91925292375934	8.74821890547769	9.40139293111495	8.6359700154085	9.18893665460244	8.17111766615341	NA
TC1000001687.mm.2	Mtrf1l	mitochondrial translational release factor 1-like	0.360614255205799	5.82166433278794	0.737327613286196	0.486945931638121	0.999692817443324	-4.60880848293282	6.68797124444932	6.49019690743202	5.38985332400632	5.84666985362264	5.92226798089695	4.59302668632042	NA
TC0400001736.mm.2	Pla2g2e	phospholipase A2, group IIE	-0.307314074716536	4.89689136547905	-0.737274532400298	0.486976070026424	0.999692817443324	-4.60881082773167	4.36719654556843	4.39721792320148	4.80689336194244	5.19425942539322	4.80246326187027	5.81331767489847	NA
TC0400001445.mm.2	Grik3	glutamate receptor, ionotropic, kainate 3	0.370417131973838	6.66721446521204	0.737224040503573	0.487004739598702	0.999692817443324	-4.60881305805514	8.02779932212499	7.90325535735832	6.80428740603415	5.60224578183415	5.4072002003558	6.25849872356482	NA
TC0900002997.mm.2	Col6a6	collagen, type VI, alpha 6	0.327401745472211	4.82885624060228	0.737108725150293	0.48707022054434	0.999692817443324	-4.60881815135515	4.80433064155067	4.56185387226942	4.66997112409203	5.46090708627699	4.29328428913119	5.18279043029341	NA
TC1100000535.mm.2	Tbc1d9b	TBC1 domain family, member 9B	0.220933122469096	8.06507662855106	0.736976125075522	0.487145523818148	0.999692817443324	-4.60882400740788	8.52501180245534	8.64180239916268	7.90014240980547	7.93261349750779	7.55458373153649	7.83630593083859	NA
TC1800001026.mm.2	Cdh2	cadherin 2	0.500618077913865	7.96052306226761	0.736909090486496	0.487183595589313	0.999692817443324	-4.6088269675949	9.20313982468281	9.15431687287808	8.53700292040835	6.6664559955279	7.68690040490071	6.5153223552078	NA
TC1600000179.mm.2	Abcc1	ATP-binding cassette, sub-family C (CFTR/MRP), member 1	0.232586827653801	8.23162837477657	0.736850122489742	0.487217087659174	0.999692817443324	-4.60882957141325	7.63943185860639	7.80086591665147	8.82363127873253	8.38369178562206	8.36770918367546	8.37444022537152	NA
TC0100003719.mm.2	Ccdc185	coiled-coil domain containing 185	0.348879161664069	6.01962101108773	0.736737452801777	0.487281085005771	0.999692817443324	-4.60883454610445	5.51793770223698	5.87194131076977	6.16857570826428	6.54422698015961	5.38384552369186	6.63119884140388	NA
TC0500001308.mm.2	2410131K14Rik	RIKEN cDNA 2410131K14 gene	0.329978796995055	6.05224191791151	0.73671502700783	0.487293823724082	0.999692817443324	-4.60883553620453	6.43509568735587	6.26004365377685	6.02511179495288	6.11402308521034	6.34784534118756	5.13133194498555	NA
TC0600000671.mm.2	Itprid1	ITPR interacting domain containing 1	-0.278798428902379	5.14367602641305	-0.736686342065108	0.487310118207867	0.999692817443324	-4.60883680261575	4.56827663302298	5.16245896560202	4.97598675780239	5.31087509322186	5.14157669079646	5.70288201803255	NA
TC0700004587.mm.2	Nap1l4	nucleosome assembly protein 1-like 4	0.262764170379938	7.86402728674603	0.736584762687435	0.487367823330921	0.999692817443324	-4.60884128696602	8.59793577308634	8.46159581350492	7.91571094940796	7.40927928791445	7.67310978710214	7.12653210946039	NA
TC0200000319.mm.2	Etl4	enhancer trap locus 4	-0.599392558846565	7.25643979360312	-0.736523837491067	0.487402435869987	0.999692817443324	-4.60884397637904	8.06643010235885	8.40446245630233	7.53710495989424	5.39737558273798	7.89781990186173	6.23544575846362	NA
TC0500000349.mm.2	Ywhah	tyrosine 3-monooxygenase/tryptophan 5-monooxygenase activation protein, eta polypeptide	-0.634223626890316	10.2942072427177	-0.736231378824052	0.487568609168097	0.999692817443324	-4.60885688418327	11.3109785597843	11.2208781177061	10.76411316363	8.21835659888764	10.8314177582558	9.41949925804251	NA
TC0700003956.mm.2	Usp17lb	ubiquitin specific peptidase 17-like B	-0.293040310012686	5.24753807364611	-0.73621624490852	0.487577209207844	0.999692817443324	-4.60885755202859	4.70503334397826	4.78901412935907	5.30294631935151	5.39960385490549	5.15455178610305	6.13407900817932	NA
TC0200004737.mm.2	Kif16b	kinesin family member 16B	-0.217687117507849	7.27308907838538	-0.736171814910686	0.487602457708454	0.999692817443324	-4.60885951262685	6.70812897123735	6.68492406616028	7.32870364364128	7.5763499553082	7.68548907703084	7.65493875693434	NA
TC0200002011.mm.2	Ap5s1	adaptor-related protein 5 complex, sigma 1 subunit	-0.225957225152279	7.1804149636981	-0.736098953751626	0.48764386486016	0.999692817443324	-4.60886272765109	7.64334830949623	7.43803933674192	6.8063071988895	6.96828664393344	7.25619003291761	6.97031826020989	NA
TC1600000538.mm.2	Lrrc58	leucine rich repeat containing 58	-0.386079859224798	9.04001202921975	-0.736057448887985	0.487667453220588	0.999692817443324	-4.6088645589685	9.35433409563868	9.38574780372558	9.34605049801289	8.01786478073943	9.48983152638344	8.64624347081847	NA
TC1900000247.mm.2	Fam111a	family with sequence similarity 111, member A	0.273550668089978	5.17525255186664	0.7360232371633	0.487686897266126	0.999692817443324	-4.60886606843685	4.94029818668954	5.53929545831844	5.23043005431561	5.3290814468703	4.71199392699418	5.30041623801177	NA
TC0200004422.mm.2	Ccdc32	coiled-coil domain containing 32	-0.361897396845635	7.72680213062448	-0.735929663212344	0.487740082171811	0.999692817443324	-4.60887019679811	8.5008784287481	8.58230287106567	7.84399917344862	6.4329191716723	7.61284722844677	7.38786591036542	NA
TC0900000074.mm.2	Gm16833	predicted gene, 16833	-0.334354811782851	4.86912829353991	-0.735850592186001	0.487785027049825	0.999692817443324	-4.60887368502356	4.05238281381545	4.10995072515755	4.68330042655618	5.5085628727942	4.95352695977237	5.90704596314371	NA
TC0200003269.mm.2	Sh3glb2	SH3-domain GRB2-like endophilin B2	-0.242373211059535	7.57272012820118	-0.735827179631572	0.487798335549029	0.999692817443324	-4.6088747178203	7.94780067812344	7.69491719692671	7.10868911469248	7.54573912132644	7.70982592834623	7.42934872979176	NA
TC1600001795.mm.2	Olfr198	olfactory receptor 198	-0.471032096214257	3.98050504039911	-0.735791308858422	0.487818726201172	0.999692817443324	-4.60887630014127	3.04682931943776	3.2038848905283	3.26774695994186	5.07737896005817	4.16672738885936	5.12046272356919	NA
TC1000000465.mm.2	Dcbld1	discoidin, CUB and LCCL domain containing 1	0.239541116484997	7.44296332042893	0.7357086298393	0.487865727069442	0.999692817443324	-4.60887994704865	7.46064534853841	7.44481654955185	7.28702555255912	7.82867457616755	7.36307288561978	7.2735450101369	NA
TC0600003114.mm.2	Ptpn6	protein tyrosine phosphatase, non-receptor type 6	-0.38403435252086	5.44308308733773	-0.73565086834413	0.487898564782576	0.999692817443324	-4.60888249469362	5.30725014453066	5.8803731576388	4.62470148640674	5.72670177200086	5.08531376314171	6.03415820030761	NA
TC0500002623.mm.2	Kdr	kinase insert domain protein receptor	-0.308490853962122	5.23204078207556	-0.735650696233963	0.487898662630331	0.999692817443324	-4.60888250228455	5.38220880874705	5.47494267879026	5.45819055463899	4.50086519385691	5.55012881100789	5.02590864541226	NA
TC1500000875.mm.2	Mapk8ip2	mitogen-activated protein kinase 8 interacting protein 2	0.303293554062881	9.17563936943999	0.735631264146201	0.487909710210018	0.999692817443324	-4.60888335933021	10.1506260687805	9.91650386268142	9.37975664982411	8.41689004682777	8.78377789186071	8.40628169666541	NA
TC1800000543.mm.2	Csnk1g3	casein kinase 1, gamma 3	0.490180046900533	7.94514849587714	0.735523789565857	0.487970814970109	0.999692817443324	-4.60888809917474	8.62283187721957	8.63798081888159	8.61146395662208	7.08375522985928	8.11840183163439	6.5964572610459	NA
TC0200002863.mm.2	Prpf18	pre-mRNA processing factor 18	-0.359581727968172	6.72476421772969	-0.735479717083187	0.487995873904539	0.999692817443324	-4.6088900427198	7.62540590794776	7.54471980905173	6.72716179632634	5.50248627039482	6.83012197558503	6.11868954707246	NA
TC1800001595.mm.2	Lipg	lipase, endothelial	-0.218610536528125	5.64539871429955	-0.735431368262349	0.488023365294803	0.999692817443324	-4.60889217475282	5.63889220361399	5.43626960310033	5.68121338679351	5.49879131621984	5.7997690516821	5.81745672438751	NA
TC0900002326.mm.2	AY074887	cDNA sequence AY074887	-0.419427525532885	6.56906966700358	-0.735164296305859	0.488175242567478	0.999692817443324	-4.60890395003363	5.9952854877844	5.57704454806845	6.13714332125289	7.36447313629855	6.56955499798855	7.77091651062866	NA
TC1100001276.mm.2	Acaca	acetyl-Coenzyme A carboxylase alpha	0.236814434659742	6.65611307131238	0.734833051242945	0.488363657675918	0.999692817443324	-4.60891855057405	7.27984256320388	6.87920568802601	6.41900326525479	6.70662625772714	6.41364565425969	6.23835499940275	NA
TC1200002428.mm.2	A730018C14Rik	RIKEN cDNA A730018C14 gene	0.405536770913825	4.62659230981968	0.734733124385499	0.488420506552516	0.999692817443324	-4.60892295422328	4.49214498906328	4.14318131467973	4.32960540880021	5.63804513970115	5.08391711128496	4.07265989538875	NA
TC1100000093.mm.2	Ogdh	oxoglutarate (alpha-ketoglutarate) dehydrogenase (lipoamide)	0.264702241969138	8.71678172540852	0.734689028059156	0.488445594582659	0.999692817443324	-4.60892489735906	9.75198290735965	9.69000646118265	8.66714438887321	8.02690834505033	8.30255249963975	7.86209575034551	NA
TC0500001448.mm.2	Ddx55	DEAD (Asp-Glu-Ala-Asp) box polypeptide 55	0.373200926152387	6.45861715477844	0.734616322464294	0.488486961375282	0.999692817443324	-4.60892810100446	6.61574636522837	6.08867134785016	6.03068136367445	7.366162170274	6.10372888539975	6.54671279624393	NA
TC0700002849.mm.2	Gpatch1	G patch domain containing 1	-0.363260111226382	7.58202588535846	-0.734525117642935	0.488538856873739	0.999692817443324	-4.60893211947267	7.83666516038597	7.76159600531221	7.95154058049112	6.63214638150879	7.94554260430264	7.36466458015004	NA
TC0X00002434.mm.2	Idh3g	isocitrate dehydrogenase 3 (NAD+), gamma	-0.547547219371214	9.26005007151071	-0.734174770368312	0.48873823876359	0.999692817443324	-4.60894755246744	9.89557343156686	9.78648918413381	9.87135092589354	7.52022076141704	9.61489037254924	8.87177575350376	NA
TC1800000788.mm.2	Mbd1	methyl-CpG binding domain protein 1	0.219827825804878	7.70898041163611	0.73403080144997	0.488820187022374	0.999692817443324	-4.60895389288666	8.19852208187375	8.26877303249734	7.58728816620038	7.5258333373273	7.48079485769859	7.19267099421933	NA
TC1000000131.mm.2	Nhsl1	NHS-like 1	-0.330223667881448	7.75752388776174	-0.734027507316633	0.488822062177449	0.999692817443324	-4.60895403795076	7.84118877315114	8.03095189661864	8.0445923191273	6.96168534139157	8.12169546432842	7.54502953195335	NA
TC0500000019.mm.2	Krit1	KRIT1, ankyrin repeat containing	0.646513134576562	5.33059984632837	0.733946598006532	0.48886812057486	0.999692817443324	-4.60895760081961	6.29501781967485	6.36604665935569	5.59337359835358	4.71440683569282	5.93392827444295	3.08082589045032	NA
TC0700000005.mm.2	Myadm	myeloid-associated differentiation marker	0.252441436971204	7.15259467207941	0.733719327317374	0.488997512185313	0.999692817443324	-4.60896760729147	7.22977120925327	7.2817702826855	7.29628135226342	7.15814635497664	6.64367651949923	7.30592231379842	NA
TC1100000327.mm.2	Nsg2	neuron specific gene family member 2	0.230607980362143	9.07900856898654	0.733599848190163	0.489065544247979	0.999692817443324	-4.60897286695293	9.87407643974817	9.77786673060155	9.0378683115695	8.60379379057741	8.53671802664469	8.64372811477794	NA
TC0900000690.mm.2	Snupn	snurportin 1	0.243539043917215	5.64996497688867	0.733392498152574	0.489183625575731	0.999692817443324	-4.60898199340895	6.22171702347952	6.32768983307257	5.51932329530366	5.39940725100352	5.50342245424897	4.92823000422377	NA
TC1400002780.mm.2	Gm2897	predicted gene 2897	0.339380673627871	6.58878651359298	0.733155931186108	0.489318368693964	0.999692817443324	-4.60899240363465	6.9880352060708	6.17998009996307	7.16175660364375	6.35997595774611	6.73705691198806	6.10591430214606	NA
TC1700002809.mm.2	March2	membrane-associated ring finger (C3HC4) 2	-0.31732780718503	7.43587639984426	-0.733079421794066	0.489361952022711	0.999692817443324	-4.60899576995841	7.2921021019396	7.27325076100813	6.79992689018682	7.90769807068705	7.84478735222929	7.49749322301465	NA
TC0X00000470.mm.2	Phf6	PHD finger protein 6	-0.546283651087604	6.4725658691116	-0.73303936716039	0.489384770055391	0.999692817443324	-4.60899753221734	7.03209841507322	6.83884591892559	7.01914230930037	4.91679947994741	7.15140376835624	5.87710532306676	NA
TC1300001052.mm.2	Arsb	arylsulfatase B	-0.566259360232318	8.58540455524845	-0.733037535003413	0.489385813802353	0.999692817443324	-4.60899761282401	9.59999566511746	9.43155617309463	9.0143918112993	6.65978657510768	8.91336238826154	7.89333471861008	NA
TC0500002830.mm.2	Tmem150c	transmembrane protein 150C	0.520888590059531	7.01319105939618	0.732861825892089	0.489485919076951	0.999692817443324	-4.60900534257883	8.6002091269713	8.22689455245027	7.50253103855453	5.64437888998274	6.73142980153462	5.37370294688359	NA
TC0600003164.mm.2	Kcna6	potassium voltage-gated channel, shaker-related, subfamily, member 6	0.290425848000689	6.13663537739004	0.732735024099529	0.489558169336656	0.999692817443324	-4.60901092000791	7.18040064709914	6.51563457342404	6.21108067811928	5.64123369178994	5.85487476160456	5.41658791230327	NA
TC0200002689.mm.2	1700021F07Rik	RIKEN cDNA 1700021F07 gene	-0.32435047022218	5.91292943589211	-0.732639897799279	0.489612375951159	0.999692817443324	-4.60901510373362	4.79877925684969	5.53617563598371	5.77189662374627	6.47506376729118	6.12348664687393	6.77217468460789	NA
TC0X00000074.mm.2	Pim2	proviral integration site 2	0.252897703921505	7.67790385569536	0.732611986813817	0.489628281465755	0.999692817443324	-4.60901633120739	8.15948016005218	8.08420635573416	7.49201857153521	7.67274744157921	7.00066837776438	7.65830222750703	NA
TC1300000881.mm.2	Mrpl36	mitochondrial ribosomal protein L36	-0.241204227282625	6.19151271353637	-0.732461048886403	0.489714301830357	0.999692817443324	-4.60902296861204	6.00028405069916	6.03571504157962	6.23447626146958	6.08085810571751	6.12134670965191	6.67639611210043	NA
TC1500001403.mm.2	Ubr5	ubiquitin protein ligase E3 component n-recognin 5	-0.455887232337989	6.11711421454265	-0.732409227261004	0.489743837602109	0.999692817443324	-4.60902524721591	7.42284697059948	7.5321254357494	5.58771586926227	4.83025333885324	6.57804843444934	4.75169523834214	NA
TC1200000239.mm.2	Eipr1	EARP complex and GARP complex interacting protein 1	-0.223002418877658	7.49366635125634	-0.732383835690614	0.489758309981911	0.999692817443324	-4.60902636364545	7.42063388749961	7.58998706456261	7.26636544917848	7.48632070968176	7.71506983651295	7.48362116010262	NA
TC1600000284.mm.2	Ece2	endothelin converting enzyme 2	-0.239708928533268	7.0447078594852	-0.732378585464967	0.489761302477758	0.999692817443324	-4.60902659448667	7.15477437286377	6.74598285620638	7.07554085950164	6.86849517588561	7.37453548129986	7.04891841115393	NA
TC1600001302.mm.2	Gsc2	goosecoid homebox 2	-0.221766812823429	5.54929148331573	-0.732293097438479	0.489810030217733	0.999692817443324	-4.60903035304965	5.42123782887182	5.21583726035333	5.68762799059883	5.41994210191236	5.74433018014326	5.80677353801479	NA
TC0X00002050.mm.2	C330007P06Rik	RIKEN cDNA C330007P06 gene	-0.258404662322338	7.44342618107786	-0.732149417551422	0.489891934351535	0.999692817443324	-4.60903666938085	8.03293972584514	7.74132115442522	7.4044267961767	6.78031664459936	7.47898702147595	7.22256574394479	NA
TC1900000913.mm.2	Ssh3	slingshot protein phosphatase 3	-0.260338271984121	6.44572482774786	-0.732146487501192	0.489893604710342	0.999692817443324	-4.60903679818015	6.692919703908	6.34401694684678	6.09302995520516	6.4653379306769	6.26382323763819	6.81522119221211	NA
TC0800000729.mm.2	Tssk6	testis-specific serine kinase 6	0.289712750531533	4.81968546729255	0.731962606809518	0.489998438795688	0.999692817443324	-4.6090448804944	4.65938495760377	4.50210896591283	4.9059530965887	5.26206909406218	4.34666114814318	5.24193554144464	NA
TC0700000009.mm.2	Cacng8	calcium channel, voltage-dependent, gamma subunit 8	-0.212840405082263	7.99401575765574	-0.731894540073056	0.490037248829907	0.999692817443324	-4.60904787194709	8.31333023553015	8.28048425647393	7.77318366182922	7.69911596005369	8.01848544130634	7.8794949907411	NA
TC1900000940.mm.2	Bbs1	Bardet-Biedl syndrome 1 (human)	-0.216836158465991	7.66155956176791	-0.731884642244728	0.490042892507961	0.999692817443324	-4.60904830692872	7.83516218744213	7.57937768866036	7.52673562825779	7.5338369605287	7.76749294863429	7.72675195708418	NA
TC1300000161.mm.2	Trim27	tripartite motif-containing 27	-0.269092908864159	5.57096660879189	-0.73179638961684	0.490093215516636	0.999692817443324	-4.60905218520034	6.7473368224556	6.52605956502963	5.29688002948901	4.51022869662989	5.22131396658782	5.1239805772594	NA
TC0200003923.mm.2	Olfr1048	olfactory receptor 1048	-0.319983948390172	4.59457509315382	-0.731783008256235	0.490100846077387	0.999692817443324	-4.60905277321711	4.11401144960002	4.10272100232517	4.24152694660579	5.11384815850289	4.59884313709155	5.39649986479747	NA
TC1400000036.mm.2	Gm8297	predicted gene 8297	-0.239259999592429	5.00308476154969	-0.731745473860963	0.49012250042445	0.999692817443324	-4.60905442254978	4.61466318193988	4.7615238464154	5.21476943556131	4.86713133531771	5.4868531878252	5.07356758223867	NA
TC0800000691.mm.2	Naf1	nuclear assembly factor 1 ribonucleoprotein	0.555368296051089	6.64343082095012	0.731721054751889	0.490136175360575	0.999692817443324	-4.60905549553999	7.64284114132194	7.79765131837863	7.38234978781749	5.38306474123369	6.58991531196373	5.06476262498527	NA
TC0900003061.mm.2	Klhdc8b	kelch domain containing 8B	0.289359030806628	6.36103616448168	0.731532245284302	0.490243855402561	0.999692817443324	-4.60906379109291	6.52861254989646	6.04200353065097	6.58024928204338	6.50691020193457	5.83340239899706	6.67503902336764	NA
TC0100000101.mm.2	Tmem70	transmembrane protein 70	-0.256607825525392	6.50633422873752	-0.731478123710757	0.490274724435929	0.999692817443324	-4.60906616870727	6.74786792718659	6.76437762361869	6.46467847422454	6.04159361105999	6.21745490559144	6.80203283074388	NA
TC1300001036.mm.2	Serinc5	serine incorporator 5	0.325137988734318	7.40846378462248	0.731435292626902	0.490299154696973	0.999692817443324	-4.60906805023176	8.12335905458587	8.26426926677791	7.77662073158058	6.58009445033929	6.76945095209042	6.93698825236082	NA
TC0200005516.mm.2	Tmem189	transmembrane protein 189	-0.230455456820044	7.38185158803974	-0.731390445488184	0.49032473576299	0.999692817443324	-4.60907002023661	7.53588630753536	7.52519387502795	7.40980520563993	6.97475401037759	7.34251107077906	7.50295905887855	NA
TC0200005119.mm.2	Spint3	serine peptidase inhibitor, Kunitz type, 3	-0.294984091390136	6.10161808488179	-0.730920953809656	0.490592590250435	0.999692817443324	-4.60909063855791	5.4761604299549	5.49423597991282	6.00564977378725	6.51902218453412	6.11881730006495	6.9958228410367	NA
TC0200005479.mm.2	Ganc	glucosidase, alpha; neutral C	0.234715126674561	6.11329968701898	0.730887819362354	0.490611497827556	0.999692817443324	-4.60909209334812	6.38810221879613	6.68020337763686	6.15550050810798	5.88496088140704	5.6226692538448	5.9483618823211	NA
TC0100003715.mm.2	Capn2	calpain 2	-0.227185092785812	6.79226030613681	-0.730789194773758	0.49066777906841	0.999692817443324	-4.60909642325312	7.1490198611771	7.39984472735354	6.56917472188511	6.30598880947419	6.55408010787237	6.77545360905855	NA
TC0900002175.mm.2	Arcn1	archain 1	-0.500828428552656	7.90776138677008	-0.730759615552876	0.490684659630196	0.999692817443324	-4.60909772178662	8.70310130312449	8.62851403202436	8.25806788852579	6.29858017565736	8.3116379950574	7.24666692623107	NA
TC0400001615.mm.2	Gpn2	GPN-loop GTPase 2	0.251133474358808	6.44242987610671	0.730748227317564	0.490691158884545	0.999692817443324	-4.6090982217225	6.8112221187283	6.1446673926805	6.50415071705058	6.59632762992397	6.26582666482832	6.33238473342861	NA
TC0200003316.mm.2	Lrsam1	leucine rich repeat and sterile alpha motif containing 1	-0.227869315838966	6.3582588419083	-0.730707388215329	0.490714466193307	0.999692817443324	-4.60910001448671	7.10760213177447	6.98857997543893	5.99424433830419	5.80457181797503	6.31357189816194	5.94098288979522	NA
TC1100004251.mm.2	Rab40b	Rab40B, member RAS oncogene family	-0.242777479674167	7.6988592032538	-0.730629824209312	0.490758734837239	0.999692817443324	-4.60910341921546	7.42106072319193	7.46782616076525	7.58978522910849	7.81957145900016	7.67209684400752	8.22281480344947	NA
TC0200004126.mm.2	Atg13	autophagy related 13	0.334858483971559	6.42925041617782	0.730595719443978	0.490778200534093	0.999692817443324	-4.60910491618875	6.50119283276357	6.19578983639084	6.28082637646353	6.9932920214643	5.79250735798684	6.81189407199786	NA
TC1500002353.mm.2	Olfr287	olfactory receptor 287	-0.254018668593632	6.68374527280157	-0.73013070352322	0.4910436654823	0.999692817443324	-4.60912532241166	6.62702168994691	6.51492641818837	6.64265083145197	6.58359226429146	7.21315390822425	6.52112652470645	NA
TC1100003121.mm.2	Derl2	Der1-like domain family, member 2	-0.379099646418501	6.84985932804145	-0.729717791177898	0.491279466544886	0.999692817443324	-4.6091434345312	7.25110338133335	7.67257938171606	7.08498599899259	5.62365095718857	6.8885853743374	6.57825087468075	NA
TC0100000393.mm.2	Coq10b	coenzyme Q10B	-0.772315784979709	6.42060429165028	-0.729696964021127	0.491291362278456	0.999692817443324	-4.60914434790956	7.20709296422692	7.20433224199771	7.41905093670395	3.86473355015487	6.83630450063179	5.99211155618645	NA
TC1700000270.mm.2	Zfp54	zinc finger protein 54	-0.318891688721742	5.85747846675648	-0.729695075108448	0.491292441167953	0.999692817443324	-4.60914443074723	4.96247118339398	5.69525645703322	5.49969959275667	6.42498029857745	6.26221362835558	6.30024964042202	NA
TC1300000466.mm.2	Smim13	small integral membrane protein 13	-0.481184565392248	9.08129784891036	-0.729524614194887	0.491389809792816	0.999692817443324	-4.60915190563621	9.74583281654028	9.85229295356962	9.29948050208695	7.66416559419693	9.64271763863819	8.28329758843019	NA

**TC0800001228.mm.2**	4933405L10Rik	RIKEN cDNA 4933405L10 gene	-0.270884980862104	5.18361081140498	-0.729337996774897	0.491496421960115	0.999692817443324	-4.6091600875973	4.01896842243712	4.29427536192703	5.16378173340427	5.95954382776648	5.80353465920409	5.86156086369087	NA
**TC1300001997.mm.2**	Shc3	src homology 2 domain-containing transforming protein C3	-0.288662923296674	8.18588866330584	-0.729228686725855	0.491558876598681	0.999692817443324	-4.60916487944913	9.13991846475564	9.15759516394122	7.95314471312466	7.16710153914776	8.13833084126715	7.55924125759862	NA
**TC0600000193.mm.2**	Ptprz1	protein tyrosine phosphatase, receptor type Z, polypeptide 1	-0.669103059802317	8.68696061412288	-0.729153202575533	0.491602007805646	0.999692817443324	-4.60916818817185	10.4285395375565	10.4449112155143	8.93372490321864	6.02132850281228	8.71365808431465	7.5796014413209	NA
**TC0100000652.mm.2**	Retreg2	reticulophagy regulator family member 2	0.257014595437101	8.35422269726021	0.728973249117836	0.491704842390667	0.999692817443324	-4.60917607516213	9.12215608385234	8.78114409039933	8.46547266619379	7.90255993389811	8.08808986480499	7.7659135444127	NA
**TC0600003417.mm.2**	Kras	Kirsten rat sarcoma viral oncogene homolog	-0.700796019822617	7.00828324564875	-0.728908454116191	0.491741873085475	0.999692817443324	-4.60917891465868	8.48938942998877	8.44047329617191	7.24183787211357	4.61728448192972	7.6223228490939	5.63839154459462	NA
**TC1900000073.mm.2**	Cfl1	cofilin 1, non-muscle	-0.503288999120505	11.0570044298968	-0.728311015133663	0.492083400655592	0.999692817443324	-4.60920508772377	11.902048881171	11.9460682397437	11.3725710375949	9.37109469251766	11.3928257041086	10.357418024245	NA
**TC1000000246.mm.2**	Ptprk	protein tyrosine phosphatase, receptor type, K	-0.272665333003331	7.13847105011098	-0.728236251484944	0.492126150663505	0.999692817443324	-4.60920836196384	7.10692638405897	7.34995172042241	7.09012490065381	6.82418386443511	7.6207971437662	6.83884228732938	NA
**TC0200002694.mm.2**	Vapb	vesicle-associated membrane protein, associated protein B and C	0.21692336662885	7.97049867308992	0.728193172390993	0.492150784506227	0.999692817443324	-4.60921024848578	8.50083904959632	8.23459522930852	7.91705328579124	7.84364896065494	7.6349042164894	7.69195129669908	NA
**TC0700004241.mm.2**	Zkscan2	zinc finger with KRAB and SCAN domains 2	-0.235201157169442	6.30108650509848	-0.728159430220875	0.492170079808281	0.999692817443324	-4.6092117260698	6.14771845322552	6.07991306362747	6.48991603945007	6.06432656024944	6.66591692826487	6.35872798577351	NA
**TC0500002153.mm.2**	3110082J24Rik	RIKEN cDNA 3110082J24 gene	-0.236891868685071	7.78856659520009	-0.728097202749095	0.492205665624037	0.999692817443324	-4.60921445091093	6.98453658381636	6.88709633336313	7.85596845086022	8.33702300746523	8.21217001265592	8.45460518303967	NA
**TC0900001784.mm.2**	Med17	mediator complex subunit 17	0.227925904889871	6.7928336244852	0.727984164393765	0.492270312892546	0.999692817443324	-4.60921940025974	6.92894505000218	6.6322344930067	6.78446511013282	7.04137189670821	6.57901228490517	6.79097291215612	NA
**TC1200001685.mm.2**	Dnajb9	DnaJ heat shock protein family (Hsp40) member B9	-0.723956221299439	7.62107474868413	-0.727819220622825	0.492364655348723	0.999692817443324	-4.60922662130009	8.58031187573389	8.53949958216214	8.22095008375306	5.35841221205187	8.44471798982968	6.58255674857412	NA
**TC0900002862.mm.2**	Chst2	carbohydrate sulfotransferase 2	-0.221100687611923	8.47075372924275	-0.72730475321517	0.492658991593733	0.999692817443324	-4.60924913666278	8.4582437859648	8.530496347676	8.23475503849337	8.46203539480256	8.43727416070318	8.7017176478166	NA
**TC1300002426.mm.2**	Thbs4	thrombospondin 4	-0.219385226948939	7.46745647286365	-0.727233172359782	0.492699953635538	0.999692817443324	-4.60925226846789	7.12173182013478	7.32729931474515	7.36596273895221	7.59250588524984	7.63720851231871	7.76003056578122	NA
**TC1000000540.mm.2**	Dnajb12	DnaJ heat shock protein family (Hsp40) member B12	0.329263094798553	6.83455994980253	0.727188413979909	0.492725567711094	0.999692817443324	-4.60925422662587	7.23125497981005	6.97621330687761	7.42139957195049	6.30780922891182	6.7950991193212	6.27558349194399	NA
**TC0500003740.mm.2**	Gal3st4	galactose-3-O-sulfotransferase 4	-0.28191369385278	5.80407770314607	-0.72716876419528	0.49273681306123	0.999692817443324	-4.60925508626784	5.76135830353049	6.06386029603861	5.48985057297894	5.72785954282193	5.46558270665525	6.31595479685118	NA
**TC0300000228.mm.2**	Usp13	ubiquitin specific peptidase 13 (isopeptidase T-3)	0.360516068623038	6.09345935006415	0.727102224334414	0.492774894347301	0.999692817443324	-4.60925799714292	6.72448738150703	6.4907631259152	6.70511731898216	5.32815154612221	5.84940097987167	5.46283574798663	NA
**TC1100004062.mm.2**	Grin2c	glutamate receptor, ionotropic, NMDA2C (epsilon 3)	-0.226832456647152	6.26986709184389	-0.727067251834982	0.492794910172678	0.999692817443324	-4.60925952698636	7.35883801155923	7.10603664598	5.58158848874016	5.76874300137474	5.75107270831987	6.05292369508933	NA
**TC0800001984.mm.2**	Nrg1	neuregulin 1	-0.278193024502046	6.73311459475615	-0.726930397200192	0.492873241467654	0.999692817443324	-4.6092655130821	6.86067114296283	6.89803036976329	6.10048812716838	6.94131187623497	6.63067809910002	6.96750795330742	NA
**TC1100002685.mm.2**	4933414I15Rik	RIKEN cDNA 4933414I15 gene	0.347071955510418	5.99073210800182	0.726874993589973	0.49290495512044	0.999692817443324	-4.60926793624012	5.70917653755245	5.1719807205121	5.81125153059313	7.06743811989048	6.12357068207555	6.06097505738722	NA
**TC1200002523.mm.2**	Fntb	farnesyltransferase, CAAX box, beta	0.224312166546915	7.09428802698053	0.726867599209518	0.492909187850553	0.999692817443324	-4.60926825963433	7.6407681412108	7.61269614663592	6.76570266328808	7.11474144027705	6.61535830216938	6.81646146830193	NA
**TC0X00002727.mm.2**	Cxcr3	chemokine (C-X-C motif) receptor 3	0.258974277972174	5.40601513132275	0.726377216752974	0.493189949374035	0.999692817443324	-4.60928970139859	5.32017667482312	5.02247909481036	5.99029022539305	5.31540156173063	5.47895318826859	5.30879004291074	NA
**TC1000002418.mm.2**	Col18a1	collagen, type XVIII, alpha 1	-0.311774854606097	6.18990347219433	-0.726337535683949	0.493212672879307	0.999692817443324	-4.6092914359902	5.28175418150189	5.67020024758696	5.85315729541262	6.92880105201985	6.70141830369362	6.70408975295105	NA
**TC1100003369.mm.2**	Rffl	ring finger and FYVE like domain containing protein	0.247515320418044	7.90789838517677	0.726327447754631	0.493218449879406	0.999692817443324	-4.60929187695651	7.68296170717025	7.75227126244547	8.44722870485111	7.80636528628937	7.94306681329595	7.81549653700844	NA
**TC0400002216.mm.2**	Pdp1	pyruvate dehyrogenase phosphatase catalytic subunit 1	-0.601840056133614	8.28644687629495	-0.726139455055917	0.493326114914762	0.999692817443324	-4.60930009375419	9.08823327651984	9.43026765141373	8.80130412259344	6.19694598619102	8.56830059025826	7.63362963079343	NA
**TC1300002541.mm.2**	Mrps36	mitochondrial ribosomal protein S36	-0.25458954558555	7.8454503209868	-0.725862949533708	0.493484500527698	0.999692817443324	-4.60931217654976	7.490423602459	7.4411581727148	8.15864340828866	7.65732712149929	8.4207692778149	7.90438034314416	NA
**TC1200001067.mm.2**	Bdkrb1	bradykinin receptor, beta 1	0.247781245359257	5.07438267442454	0.725804415702077	0.493518033749232	0.999692817443324	-4.60931473395681	4.40037663845818	4.7901574563932	5.17251078096336	5.70315144024385	5.15986549172034	5.22023423876835	NA
**TC0100002649.mm.2**	Fam124b	family with sequence similarity 124, member B	0.272220712058314	5.86346084976471	0.725656327405241	0.493602878277868	0.999692817443324	-4.60932120344639	5.70472621882888	5.14461275749602	5.92475553111283	6.51317824207717	5.79825618287769	6.09523616619568	NA
**TC0100002888.mm.2**	Pign	phosphatidylinositol glycan anchor biosynthesis, class N	-0.315984523268739	5.16204486677437	-0.725573067295493	0.493650584938502	0.999692817443324	-4.60932484039697	5.51011414537704	6.13626379908263	5.13701723065559	4.20994387416894	5.1669017824564	4.81202836890561	NA
**TC0X00001798.mm.2**	Tbc1d25	TBC1 domain family, member 25	0.246262389630603	5.691937326079	0.725545752163957	0.493666236726006	0.999692817443324	-4.60932603350663	6.81787193135124	6.38931216450579	5.26471384402001	5.45376847591909	4.8891018284834	5.3368557121945	NA
**TC1700002352.mm.2**	C3	complement component 3	0.224129129917189	5.07384912062484	0.72533738973534	0.493785640956468	0.999692817443324	-4.6093351336174	4.78018863732194	4.63930947424936	5.25812851982411	5.47779891618196	4.98721125307997	5.30045792309173	NA
**TC0300001354.mm.2**	Ppa2	pyrophosphatase (inorganic) 2	0.983350901122078	6.85215093686653	0.725335136334256	0.493786932396478	0.999692817443324	-4.60933523202333	7.74997291668175	7.77657897805863	8.05592476868439	5.7206029956671	7.97389133365968	3.83593462844765	NA
**TC1400001233.mm.2**	Frg2f8	FSHD region gene 2 family member 8	-0.231093178523845	5.36901247251145	-0.725310988490542	0.493800771837087	0.999692817443324	-4.60933628654506	4.77477176328544	4.57503918660742	5.5827696228788	5.61826914118527	5.60652053148156	6.0567045896302	NA
**TC0900002991.mm.2**	Atp2c1	ATPase, Ca++-sequestering	-0.541934037610677	7.06868254181018	-0.725280526809897	0.493818230188358	0.999692817443324	-4.60933761675288	8.13090302724827	8.41012587200314	7.27151633963412	5.12208279856003	7.33474336138347	6.14272385203204	NA
**TC0X00002803.mm.2**	Zdhhc15	zinc finger, DHHC domain containing 15	0.495394800015514	6.10560648593347	0.725135759091173	0.493901205821312	0.999692817443324	-4.60934393796435	6.55879848807239	5.92332497199099	7.31830531343738	5.25284721434683	6.05048931853762	5.52987360921557	NA
**TC1900000075.mm.2**	Ap5b1	adaptor-related protein complex 5, beta 1 subunit	-0.253880571620898	6.93161406362472	-0.72507910575299	0.493933680003806	0.999692817443324	-4.609346411462	6.71559049202926	6.29040240273374	6.76687662302859	7.27108854884316	7.13144897286475	7.4142773422488	NA
**TC0200003758.mm.2**	Chrna1	cholinergic receptor, nicotinic, alpha polypeptide 1 (muscle)	-0.472032983835827	4.58598959632559	-0.724960652785271	0.494001582871729	0.999692817443324	-4.60935158270454	3.76755472775613	4.20849650497476	4.13008615894004	5.16782402983547	4.23788560744936	6.00409054899779	NA
**TC0X00001653.mm.2**	Ctps2	cytidine 5'-triphosphate synthase 2	-0.299451185365326	7.49349205445413	-0.724911379841073	0.494029830298417	0.999692817443324	-4.60935373361331	8.03531580924171	8.16439203640655	6.53573920850735	7.54543184666558	7.49555454328746	7.18451888261613	NA
**TC1500002240.mm.2**	Csrnp2	cysteine-serine-rich nuclear protein 2	-0.264113265316616	6.20867131874184	-0.724744502634363	0.494125506459805	0.999692817443324	-4.60936101752652	6.30981736688903	6.58250025678378	6.31339541344504	5.60234646562747	6.09323675774467	6.35073165196107	NA
**TC0200002542.mm.2**	Mmp9	matrix metallopeptidase 9	-0.339586520984674	5.01615425544277	-0.724677174634494	0.494164111304463	0.999692817443324	-4.6093639559466	4.59599318503068	4.20960677484811	4.74420154305098	5.56187472235327	5.0066844008486	5.978564906525	NA
**TC1400000314.mm.2**	Stimate	STIM activating enhancer	0.260649293245677	6.60197491037066	0.724646773661728	0.494181543416377	0.999692817443324	-4.60936528268397	6.39661831880519	5.81637268191161	6.89186355328003	7.06821497071923	6.47731336113079	6.96146657637712	NA
**TC0200000422.mm.2**	Edf1	endothelial differentiation-related factor 1	-0.252085452800378	9.05477451834078	-0.724576108577986	0.494222064811727	0.999692817443324	-4.60936836644673	9.11232199000352	9.19550511020512	9.18167090259014	8.5766536495275	9.3058765499448	8.95661890777359	NA
**TC0300001038.mm.2**	Sike1	suppressor of IKBKE 1	0.43846561313068	8.10674766867269	0.72447237580928	0.494281552185567	0.999692817443324	-4.60937289286957	8.81001025621706	8.61575955589622	8.68279317224821	7.36303054084389	8.26813581001859	6.90075667681215	NA
**TC0800000782.mm.2**	Myo9b	myosin IXb	-0.2287660926175	7.3150264123044	-0.724382406413333	0.494333150579771	0.999692817443324	-4.609376818351	6.6750379285069	6.9984582335707	7.27414821534583	7.60541684791106	7.62793030113597	7.70916694735592	NA
**TC0200002300.mm.2**	Cox4i2	cytochrome c oxidase subunit 4I2	-0.26551887368635	6.90015262631614	-0.724371636117812	0.494339327698241	0.999692817443324	-4.60937728824987	6.37614292827707	6.42654440267249	6.64807245244263	7.38552288734467	7.10919584196553	7.45543724519447	NA
**TC0800002609.mm.2**	Mylk3	myosin light chain kinase 3	0.221582558687705	4.98499003730427	0.724326341905498	0.494365305981237	0.999692817443324	-4.60937926434394	4.74165731489167	4.77886585729814	5.03678979473617	5.37950128976944	4.91074103034823	5.06238493678197	NA
**TC1900000907.mm.2**	Tbc1d10c	TBC1 domain family, member 10c	0.304909060779109	7.20470497623186	0.72429932746728	0.49438080041904	0.999692817443324	-4.60938044288719	6.29917101875813	6.70166771766389	7.27759604416377	8.14100857709993	7.68277066914871	7.12601583055677	NA
**TC0200003817.mm.2**	Cwc22	CWC22 spliceosome-associated protein	-0.919215045432554	6.81975519698875	-0.724296558449446	0.494382388638964	0.999692817443324	-4.60938056368777	5.95371568849143	8.96159469733228	5.19482623499356	6.88756911762826	5.56930688435687	8.35151855913007	NA
**TC0200001980.mm.2**	1700020A23Rik	RIKEN cDNA 1700020A23 gene	-0.304069442624341	5.4069340086137	-0.724152821505143	0.494464836218648	0.999692817443324	-4.60938683387676	4.97551649229151	4.9314371649026	5.14531740938051	5.81793834523919	5.35225736442679	6.21913727544159	NA
**TC0800003245.mm.2**	Kxd1	KxDL motif containing 1	0.346714497525121	8.32088678237429	0.724063084093623	0.494516314289798	0.999692817443324	-4.60939074801601	8.78646009323759	8.48206979622451	9.0035770381174	7.67153286179956	7.91970212619544	8.06197877867127	NA
**TC1900000681.mm.2**	Pprc1	peroxisome proliferative activated receptor, gamma, coactivator-related 1	0.32346610917645	5.63372366651309	0.723965081785297	0.494572537620485	0.999692817443324	-4.60939502225925	4.81007526521066	5.16724161789226	5.83531299419863	6.40066567296564	5.28010226477351	6.30894418403786	NA
**TC0800000269.mm.2**	Vps36	vacuolar protein sorting 36	-0.567608899968245	6.96051731322086	-0.723887090976755	0.494617283520522	0.999692817443324	-4.60939842343464	7.71783093205023	8.01411657827233	7.32929329295532	5.11867599157775	7.47555135462442	6.10763572984514	NA
**TC0700004592.mm.2**	Tnfrsf22	tumor necrosis factor receptor superfamily, member 22	0.264579260492139	5.80360852040994	0.723694600800262	0.494727733037556	0.999692817443324	-4.60940681681294	5.552460048751	5.3427165063858	6.23318776612447	5.99462877802908	6.22227572938454	5.47638229378473	NA
**TC1700002637.mm.2**	Haao	3-hydroxyanthranilate 3,4-dioxygenase	0.343775846414683	6.8765578683714	0.723607928414803	0.494777470414317	0.999692817443324	-4.60941059557669	6.07295403425511	6.30074125706513	6.87732020603129	7.90928159983746	6.62400286873438	7.47504724430501	NA
**TC1700002107.mm.2**	9130008F23Rik	RIKEN cDNA 9130008F23 gene	0.359668732558451	5.44121092385049	0.723605160171635	0.494779059039271	0.999692817443324	-4.60941071626195	4.59224042880285	5.0404142810796	5.3220742585822	6.54489989058648	5.24994401732876	5.89769266672303	NA
**TC1100002024.mm.2**	0610009L18Rik	RIKEN cDNA 0610009L18 gene	-0.23311451714213	7.81791297869707	-0.723521453713098	0.494827097671053	0.999692817443324	-4.60941436540273	7.19862293799379	7.23575656581651	7.7728969693571	8.23053769768683	8.10980681513592	8.35985688619227	NA
**TC1100003584.mm.2**	Itga3	integrin alpha 3	-0.222335915576859	5.9871694148211	-0.723259561843516	0.494977415914357	0.999692817443324	-4.60942578051755	6.46434181993449	6.30653480631029	5.76763519847662	5.58609881728743	5.74448647774798	6.05391936916979	NA
**TC0300003045.mm.2**	Pdlim5	PDZ and LIM domain 5	-0.212116460432717	7.34287458943489	-0.723148320701378	0.495041274271137	0.999692817443324	-4.6094306283148	7.77908685976126	7.76150468790871	7.00725243136881	7.03895910266816	7.33947152233928	7.13097293256312	NA
**TC0600001042.mm.2**	Tpra1	transmembrane protein, adipocyte asscociated 1	-0.267665578505083	6.46445423191448	-0.72310576932209	0.495065702487014	0.999692817443324	-4.6094324825293	7.38329194770299	7.42280738618931	6.27932570076795	5.44332174952426	6.12835294234902	6.12962566495336	NA
**TC1600001328.mm.2**	Olfr165	olfactory receptor 165	-0.457501751056914	4.61151074155635	-0.723059164713153	0.495092458533087	0.999692817443324	-4.60943451327831	4.07243511746755	3.77097040686308	3.73844277100467	5.71688494044214	5.04187575977287	5.32845545378777	NA
**TC1500002242.mm.2**	Tfcp2	transcription factor CP2	0.309302566656212	6.50392249549445	0.722936330112005	0.495162983390318	0.999692817443324	-4.6094398652288	7.17196122980089	6.85638012831343	6.11783523291197	6.68906414117043	6.55959767369073	5.62869656707925	NA
**TC0800002594.mm.2**	Gcdh	glutaryl-Coenzyme A dehydrogenase	0.224712973701934	7.05862543236946	0.722920771690368	0.495171916655996	0.999692817443324	-4.60944054306925	7.15543478283363	7.26846022066371	6.89025579775891	7.29838597130275	6.88931487785988	6.84990094379792	NA
**TC1400000111.mm.2**	Olfr31	olfactory receptor 31	-0.372468607706968	4.70861798708941	-0.722819682629392	0.495229962140925	0.999692817443324	-4.60944494700836	3.74230418527947	4.03838799671095	4.19067366427527	5.67236559829078	5.17145296539714	5.43652351258285	NA
**TC0200004563.mm.2**	Mal	myelin and lymphocyte protein, T cell differentiation protein	-0.470482816989565	10.3735012049371	-0.722797723996724	0.495242571418798	0.999692817443324	-4.60944590357729	11.1530005747156	11.4072301070846	10.5956429894904	8.77426246743136	10.6567995192387	9.65407157166214	NA
**TC1100003323.mm.2**	AU040972	expressed sequence AU040972	0.238322495891638	5.08171511564584	0.722725104595863	0.495284273088761	0.999692817443324	-4.60944906690016	4.50858833269025	4.49342064278777	5.2140926928493	5.76837066224083	5.33847212777061	5.16734623553625	NA
**TC1300002258.mm.2**	Zfp738	zinc finger protein 738	-0.279348150652055	5.58571662599967	-0.722649899789643	0.49532746189249	0.999692817443324	-4.60945234260689	6.91150839420866	6.65914705992337	5.24192861335566	4.45054538692527	5.33180290924794	4.9193673923371	NA
**TC0200003307.mm.2**	Fpgs/Mir8093	folylpolyglutamyl synthetase/microRNA 8093	0.2237616441929	7.98048441844506	0.722575334121496	0.495370286127058	0.999692817443324	-4.6094555902363	7.97259423533977	8.02669189205742	8.14381606103736	8.02175577479212	8.05508034265528	7.6629682047884	NA
**TC1900001683.mm.2**	Bbip1	BBSome interacting protein 1	0.533787279102206	6.54908298321894	0.722561185361169	0.495378412260733	0.999692817443324	-4.60945620644392	7.09569845015957	7.14718959946796	7.40945011887954	5.65014208506571	6.84149532415005	5.15052232159079	NA
**TC0800001118.mm.2**	Ccl22	chemokine (C-C motif) ligand 22	0.242124187141237	5.92385754299672	0.722554840005598	0.49538205665179	0.999692817443324	-4.60945648279443	5.41604751068377	5.42391585288254	5.98818983084064	6.6055253035076	6.0372133662332	6.07225339383256	NA
**TC0900000842.mm.2**	Rasl12	RAS-like, family 12	0.332617987679218	5.34700085140109	0.72253940959795	0.495390919025511	0.999692817443324	-4.60945715480651	4.68754111420484	5.2415663439238	5.22621933955827	6.18284747325991	5.03961642283677	5.70421441462296	NA
**TC0200004352.mm.2**	Emc4	ER membrane protein complex subunit 4	-0.425551172662564	9.7464043229903	-0.722446523079689	0.495444270141328	0.999692817443324	-4.60946119990696	10.3298374602043	10.2147543637693	10.0426264603311	8.49873942399045	10.1972071852599	9.1952610443868	NA
**TC0100000430.mm.2**	Aox4	aldehyde oxidase 4	0.344545604528208	5.23497103529168	0.722355964866828	0.495496287635022	0.999692817443324	-4.60946514325783	4.59202645540863	4.66423298213058	5.11357027576112	6.30775871587252	5.03712845664395	5.69510932593327	NA
**TC1100001592.mm.2**	Igfbp4	insulin-like growth factor binding protein 4	-0.251707319784419	7.60662403375393	-0.722227873569061	0.495569870697864	0.999692817443324	-4.60947072038631	7.3769371515317	7.67057290427925	7.28624765367024	7.75816209990167	7.55512955252945	7.9926948406113	NA
**TC0200002778.mm.2**	Lsm14b	LSM family member 14B	0.311760267106573	8.31578304999446	0.722194206731902	0.495589212087262	0.999692817443324	-4.60947218613291	8.625593753142	8.5478739708852	8.88386777357139	7.78850778150497	8.20407813020559	7.84477689065762	NA
**TC1100004101.mm.2**	Unc13d	unc-13 homolog D	-0.31715346823174	5.83138709644348	-0.722124020640716	0.495629535178046	0.999692817443324	-4.60947524165559	5.2363727233831	5.40928429071683	5.39618311694865	6.45799619710008	5.98844599066127	6.50004025985094	NA
**TC1500001736.mm.2**	9030619P08Rik	lymphocyte antigen 6 complex pseudogene	0.248882131212074	4.89087356454016	0.722062996189176	0.495664596527972	0.999692817443324	-4.60947789815907	4.58223500985222	4.63121961430502	4.83165283037787	5.48312268237607	4.80925547749255	5.00775577283723	NA
**TC0900000742.mm.2**	Hcn4	hyperpolarization-activated, cyclic nucleotide-gated K+ 4	-0.253095586682893	5.80834803273316	-0.721957949893274	0.495724954316989	0.999692817443324	-4.60948247063957	6.10338799887383	5.61258932262453	5.64432819613322	5.66963165463419	5.64676682172762	6.17338420240557	NA
**TC0400003200.mm.2**	Pcsk9	proprotein convertase subtilisin/kexin type 9	0.404390600358946	5.53265589724598	0.721878900480175	0.49577037797215	0.999692817443324	-4.60948591121066	5.41974665910685	5.21585942388144	5.5725097577952	6.11204549280753	4.62523181586731	6.25054223401753	NA
**TC1500002061.mm.2**	Syt10	synaptotagmin X	0.25660991449774	8.37794278948199	0.721864791335351	0.495778485710576	0.999692817443324	-4.60948652527337	9.09205189624912	8.84822760584796	8.5490259002056	7.87127263168956	8.05921509077213	7.84786361212755	NA
**TC0800003027.mm.2**	Kcng4	potassium voltage-gated channel, subfamily G, member 4	-0.214466381402064	5.89688842646819	-0.721801644957135	0.495814773492923	0.999692817443324	-4.60948927344598	5.98441092427338	5.78724518718703	5.87647651330548	5.71389432896683	5.96394076345558	6.05536284162086	NA
**TC1000001813.mm.2**	Tnfaip3	tumor necrosis factor, alpha-induced protein 3	0.271613927365259	5.22271503775016	0.721691077190377	0.495878316772019	0.999692817443324	-4.60949408501879	5.47387472246014	5.85815237277945	4.83127528036688	5.44247021262906	5.10955991812838	4.62095772013704	NA
**TC0500001411.mm.2**	Psmd9	proteasome (prosome, macropain) 26S subunit, non-ATPase, 9	0.265186608337983	6.90228616739778	0.721665600214752	0.495892959154478	0.999692817443324	-4.60949519362521	7.29294680660514	6.92899455423586	6.72923769975	7.13183673036083	6.97370848682449	6.35699272661038	NA
**TC1300001185.mm.2**	Htr1a	5-hydroxytryptamine (serotonin) receptor 1A	0.240109599753229	6.82904212688464	0.721640427973233	0.495907426679732	0.999692817443324	-4.60949628894416	7.25060372188668	6.95382875618694	7.01756747396007	6.60745226741028	6.7350286024954	6.4097719393685	NA
**TC1000000888.mm.2**	Zfr2	zinc finger RNA binding protein 2	0.28177065214022	7.39573418617487	0.72155621974292	0.495955826664064	0.999692817443324	-4.6094999528974	7.07053743944024	6.90347942467218	7.63634336374147	7.84562141486715	7.004299552893	7.91412392143518	NA
**TC0500003694.mm.2**	Stard13	StAR-related lipid transfer (START) domain containing 13	0.335104596558857	5.61362185848044	0.721521325901123	0.495975883361215	0.999692817443324	-4.60950147106166	5.58620568979752	5.85406616233734	5.15367657611437	6.30215766981839	5.67641904521401	5.10920600760104	NA

**TC0X00000044.mm.2**	Nudt11	nudix (nucleoside diphosphate linked moiety X)-type motif 11	-0.362071244747028	7.97916533284395	-0.721448021573251	0.49601801985223	0.999692817443324	-4.60950466022312	8.29926403466927	8.24547228895069	8.27040555439835	7.03265103757647	8.41443876760332	7.61276031386557	NA
**TC0800001324.mm.2**	St3gal2	ST3 beta-galactoside alpha-2,3-sialyltransferase 2	-0.275689928971978	7.61728568586607	-0.721399607321122	0.496045850438924	0.999692817443324	-4.60950676639664	7.41027992661717	7.52911859168029	7.19028792743519	7.91617994204231	7.56647262682286	8.0913751005986	NA
**TC1500002169.mm.2**	H1fnt	H1 histone family, member N, testis-specific	-0.217813177732081	6.34016235023039	-0.721292006820363	0.496107707538441	0.999692817443324	-4.60951144700081	6.43402799325857	6.21108159456454	6.36324350488638	6.11687557416117	6.44529036086282	6.47045507364889	NA
**TC0200005378.mm.2**	Gm14326	predicted gene 14326	-0.595252748840826	8.77790653319002	-0.721217058616739	0.496150796601748	0.999692817443324	-4.6095147069429	9.78918287294351	10.259664634013	8.79448936495657	6.9195537322944	9.44758501050905	7.45696358442357	NA
**TC0400000726.mm.2**	Tmem268	transmembrane protein 268	-0.29689652699727	6.06604163138227	-0.721212294920103	0.496153535420343	0.999692817443324	-4.60951491413622	5.81632328422738	6.01160854204638	5.47932227792437	6.50794017608827	6.36400007917619	6.217055428831	NA
**TC0700000938.mm.2**	Oca2	oculocutaneous albinism II	0.442968716770834	4.59443626460305	0.721160938507048	0.496183062687247	0.999692817443324	-4.60951714778219	3.92119896619391	4.51276574561034	4.20566639881674	5.80362875586112	4.09069079201022	5.03266692912598	NA
**TC0200001843.mm.2**	Sord	sorbitol dehydrogenase	0.334724815567871	6.97595276095753	0.721081126448237	0.496228952790965	0.999692817443324	-4.60952061882653	7.28278130887172	7.4341214286196	7.2814044780216	6.62272725167319	6.05769271642786	7.17698938213119	NA
**TC1000002212.mm.2**	Mcu	mitochondrial calcium uniporter	0.318696579520366	6.89152181591503	0.721011834870761	0.496268796148918	0.999692817443324	-4.60952363211183	7.29341739194312	7.33728452924744	7.30297559538009	6.37493547129009	6.90360789339755	6.13691001423191	NA
**TC0900001483.mm.2**	Lrrfip2	leucine rich repeat (in FLII) interacting protein 2	-0.444339809738123	6.29386055308447	-0.720941126157023	0.496309456568428	0.999692817443324	-4.60952670681244	6.94173225652165	6.66633955732814	6.78456808930454	4.84863785328586	6.26708411420007	6.25480144786658	NA
**TC0700002437.mm.2**	Fkrp	fukutin related protein	0.248550507693256	6.41452769750722	0.720925153997593	0.496318641524053	0.999692817443324	-4.60952740131687	6.45914403904946	6.38234426203851	6.23912620488251	6.8322632447884	6.12187922034706	6.45240921393734	NA
**TC1200001490.mm.2**	Adam17	a disintegrin and metallopeptidase domain 17	-0.574231413107371	6.49957583768975	-0.720858363201866	0.496357051487577	0.999692817443324	-4.60953030540849	7.02417066314519	7.42119074772881	6.8833020440092	4.81851335051566	7.24345740374623	5.60682081699338	NA
**TC0700002460.mm.2**	Psg19	pregnancy specific glycoprotein 19	-0.286084713020903	5.45023819130153	-0.7208094450714	0.496385184516472	0.999692817443324	-4.60953243226809	5.00294941540602	4.64759930293024	5.23537708655546	6.00397841516009	5.65718050449593	6.15434442326143	NA
**TC1100003781.mm.2**	Rab5c	RAB5C, member RAS oncogene family	-0.302406264423146	8.55346451040541	-0.720809297822228	0.49638526920171	0.999692817443324	-4.60953243867003	8.82291000412284	9.0656937312665	8.69021846330634	7.72346693579208	8.73750719919144	8.28099072875327	NA
**TC1700001958.mm.2**	Nfkbil1	nuclear factor of kappa light polypeptide gene enhancer in B cells inhibitor like 1	-0.275140822035728	7.29041818691309	-0.720731425076726	0.496430056348306	0.999692817443324	-4.60953582420494	6.949332528186	6.92963396081035	6.93628890452805	7.72034158967731	7.38128869969526	7.82562343858156	NA
**TC1400002037.mm.2**	Olfr1511	olfactory receptor 1511	0.32103296469712	4.89461726555494	0.720665429692834	0.496468014542223	0.999692817443324	-4.60953869316576	4.48781545955186	4.32517320720895	4.8562798734058	5.74211055457572	4.56606705500123	5.39025744358606	NA
**TC0100001994.mm.2**	Sbspon	somatomedin B and thrombospondin, type 1 domain containing	0.229479783652563	5.34169791036035	0.72060296984761	0.496503940998115	0.999692817443324	-4.60954140825745	5.20121506842735	4.99357136717966	5.57163011066376	5.58555018626635	5.57228090669843	5.12593982292655	NA
**TC0X00000653.mm.2**	Gpr50	G-protein-coupled receptor 50	-0.350051706402091	5.37865670695767	-0.720565287191022	0.496525616628633	0.999692817443324	-4.60954304621897	6.0469103744469	4.6821605254417	5.21198333617169	5.20939962835492	5.39416917322975	5.72731720410103	NA
**TC0500000815.mm.2**	Mob1b	MOB kinase activator 1B	0.221949270735315	6.4787257630884	0.720522572269081	0.496550187654771	0.999692817443324	-4.60954490284574	6.15598442094244	6.42964096086593	6.56715945003301	6.79815441906333	6.37359251521444	6.54782281241127	NA
**TC0100000558.mm.2**	Unc80	unc-80, NALCN activator	0.385971976660335	7.44276375458603	0.720520464753984	0.49655139998761	0.999692817443324	-4.60954499444798	9.13840120917353	8.73278188287196	7.2003023647038	6.57836932969189	7.22405177765256	5.78267596342245	NA
**TC0500000653.mm.2**	Shisa3	shisa family member 3	0.226179867265242	7.82211945567173	0.720505560225447	0.496559973766195	0.999692817443324	-4.60954564226151	7.23064296253211	7.42096432926128	8.0225014442314	8.34423314415233	7.82883574250123	8.08553911135202	NA
**TC0200001829.mm.2**	Ctdspl2	CTD (carboxy-terminal domain, RNA polymerase II, polypeptide A) small phosphatase like 2	0.529356653899663	6.36699494990286	0.72045532909069	0.496588869778	0.999692817443324	-4.60954782544795	7.0055212646769	6.72638724215601	7.06296447525593	5.70142277493587	6.84655101645359	4.85912292593889	NA
**TC1500000603.mm.2**	Kifc2	kinesin family member C2	-0.245208051534861	6.91137435838382	-0.720401938178653	0.496619584712208	0.999692817443324	-4.60955014584877	7.76025523835369	7.68515985165711	6.69290221413464	6.07330526447657	6.52989684644073	6.7267267352402	NA
**TC0100000364.mm.2**	Tmeff2	transmembrane protein with EGF-like and two follistatin-like domains 2	-0.388602620697301	8.21370481706078	-0.720122433262789	0.496780400016402	0.999692817443324	-4.60956229130671	8.92558131973382	8.77829239885705	8.44446789302845	6.95610707816115	8.42302967651126	7.75475053607295	NA
**TC0100001138.mm.2**	Ubxn4	UBX domain protein 4	-0.541898950134872	7.00503866354533	-0.720089196180715	0.49679952553024	0.999692817443324	-4.60956373535061	8.04591410550215	7.87491038925585	7.43638976657835	5.07641502654375	7.1930111445383	6.40359154885355	NA
**TC1900000980.mm.2**	Ehbp1l1	EH domain binding protein 1-like 1	-0.285774646505498	5.3690795306204	-0.719822939238347	0.496952754624371	0.999692817443324	-4.60957530164699	4.57354514929271	5.04049296387787	5.24000888876082	5.77443600000958	5.4532557417087	6.1327384400727	NA
**TC1800001243.mm.2**	Nr3c1	nuclear receptor subfamily 3, group C, member 1	-0.314729702145107	6.52276525526867	-0.719808667883707	0.496960968580621	0.999692817443324	-4.60957592151395	7.184694712862	7.17385557863036	6.39454474661457	5.67974617130014	6.82250984454099	5.88124047766394	NA
**TC1700001460.mm.2**	Wtap	Wilms tumour 1-associating protein	-0.37638036630808	8.22423332421298	-0.719592478300113	0.497085408714444	0.999692817443324	-4.60958531049958	8.64385058664593	8.79399936410546	8.52125311658818	7.05614151436699	8.43326660361183	7.8968887599595	NA
**TC1100004298.mm.2**	Ccdc47	coiled-coil domain containing 47	-0.512351075025368	8.38303552726683	-0.7195415205221	0.497114743358981	0.999692817443324	-4.60958752327397	9.17519668195673	9.1348472722562	8.82800521752636	6.6537283121423	8.68043542755123	7.82600025216817	NA
**TC0700004351.mm.2**	Pycard	PYD and CARD domain containing	0.273293606314943	5.77146492002031	0.719402763368579	0.497194626922171	0.999692817443324	-4.60959354805631	5.34354614791501	5.63705238405176	5.94297321525148	6.15441588514101	5.36813567680574	6.18266621095686	NA
**TC0300002394.mm.2**	Sprr4	small proline-rich protein 4	-0.358559778524802	5.01888657493374	-0.719274674232899	0.497268376395242	0.999692817443324	-4.60959910890465	4.50315257589808	4.56402043934609	4.82383962765789	5.34067381099644	4.75826715754522	6.12336583815871	NA
**TC1400000796.mm.2**	Nynrin	NYN domain and retroviral integrase containing	0.271434533156435	5.47274145729326	0.719079818860046	0.497380581587634	0.999692817443324	-4.60960756698604	6.24794420687066	5.7027865562886	5.5626055233275	5.15168800012908	4.80969481865938	5.36172963848433	NA
**TC0300000967.mm.2**	Fmo5	flavin containing monooxygenase 5	0.219854884993511	5.8937499039143	0.718914166165289	0.497475983983161	0.999692817443324	-4.60961475618677	5.60916185654288	5.74135351871159	6.06958535970566	6.15626154940353	6.06340360505597	5.72273353406619	NA
**TC1700001400.mm.2**	Unc93a2	unc-93 homolog A2	0.382230500502209	6.06457870169816	0.718906617328975	0.497480331784616	0.999692817443324	-4.60961508377246	5.4512315133822	5.29161170537496	5.84293988386167	7.36160511235644	5.97400716199893	6.46607683321477	NA
**TC1700002811.mm.2**	Slc39a7	solute carrier family 39 (zinc transporter), member 7	0.229062199066767	7.39894036266495	0.718840141429406	0.497518620101662	0.999692817443324	-4.60961796842328	7.90942632541062	7.73497798282288	7.10985883938611	7.49382229355878	6.9440256594121	7.20153107539924	NA
**TC1100003757.mm.2**	Krt15	keratin 15	0.35213268668935	6.26961502639046	0.718677839679544	0.497612109733678	0.999692817443324	-4.60962501053802	5.63776590701859	5.64033566847865	6.27725230808124	7.24467467003086	5.88682162575149	6.93083997898191	NA
**TC1200000655.mm.2**	Snapc1	small nuclear RNA activating complex, polypeptide 1	0.278152749473426	6.13669079331942	0.71858193938169	0.497667356047268	0.999692817443324	-4.60962917102708	6.9615497671195	6.87923099502532	6.18068410520424	5.58715064315503	5.96151742655509	5.25001182285733	NA
**TC1900000005.mm.2**	Mrpl21	mitochondrial ribosomal protein L21	-0.234887416160586	7.6168390291811	-0.718516325777303	0.49770515712666	0.999692817443324	-4.60963201734639	7.55696295930354	7.59927572611221	7.70539041369166	7.33211991498319	7.96073774745338	7.54654741354264	NA
**TC1200002101.mm.2**	Erg28	ergosterol biosynthesis 28	-0.411194709391268	8.11033852128308	-0.718507694598694	0.497710129832849	0.999692817443324	-4.60963239175331	9.0712130196686	8.83268939350911	8.32184603042789	6.64602361744122	8.16216973036573	7.62808933628592	NA
**TC1700002058.mm.2**	Olfr94	olfactory receptor 94	0.384042428847875	5.54971669420142	0.718431599276222	0.49775397228103	0.999692817443324	-4.60963569251005	5.07484388614882	4.86282852373373	5.55507388461827	6.50928242189258	5.00433891415752	6.29193253465757	NA
**TC0200000261.mm.2**	Arl5b	ADP-ribosylation factor-like 5B	0.312067028946789	6.19557682636011	0.718323418411636	0.497816305281644	0.999692817443324	-4.60964038460068	6.21348915718632	6.3155827818734	6.53518253769372	6.09907900080354	6.54474229688171	5.46538518372197	NA
**TC0700001818.mm.2**	Fam57b	family with sequence similarity 57, member B	0.241753110665393	7.06587718213158	0.718257072490497	0.497854535865799	0.999692817443324	-4.60964326195038	7.15391612659256	7.32254116417491	7.3116109430628	6.88954506861358	6.6416400754143	7.07600971493129	NA
**TC1500002031.mm.2**	Alg12	asparagine-linked glycosylation 12 (alpha-1,6-mannosyltransferase)	-0.218678122533189	7.44487412811777	-0.718184580707845	0.49789631011457	0.999692817443324	-4.60964640562321	7.30861259867303	7.37526292161222	7.30765068478869	7.46635581688881	7.44678586090482	7.76457688583906	NA
**TC0400000659.mm.2**	Hsdl2	hydroxysteroid dehydrogenase like 2	0.453543650827012	6.80649027472493	0.718024759192681	0.497988417375312	0.999692817443324	-4.60965333563279	7.80878683392232	7.61801716890845	7.56227794379568	5.59733452979073	6.33383613362608	5.91868903830631	NA
**TC0400001034.mm.2**	Ak4	adenylate kinase 4	0.30837881755762	7.36598558091922	0.71782108157519	0.498105815877673	0.999692817443324	-4.6096621656933	6.9756419050937	6.83068294045936	8.21395715401738	7.28921598352137	7.77326160881358	7.11315389360993	NA
**TC0400001611.mm.2**	Kdf1	keratinocyte differentiation factor 1	0.269214764915617	7.21514565979431	0.717631207077483	0.498215274887173	0.999692817443324	-4.6096703957398	6.88279880169834	6.64905994580182	7.30654197314247	7.84218039740601	6.98628727367387	7.62400556704339	NA
**TC0500000992.mm.2**	Cds1	CDP-diacylglycerol synthase 1	0.315323326629859	6.94680021018434	0.717618862837322	0.498222391657538	0.999692817443324	-4.60967093074311	7.76534482003501	7.98822809598687	6.92101319113176	6.35792430804017	6.83763020190468	5.81066064400754	NA
**TC1400001634.mm.2**	Ccdc66	coiled-coil domain containing 66	0.333789680561417	6.67004174903367	0.717552615553169	0.498260586064482	0.999692817443324	-4.60967380180962	7.25006651742124	7.4596373320927	6.79647276446678	6.19259023843868	6.73360693335588	5.58787670842675	NA
**TC0500000171.mm.2**	Fgl2	fibrinogen-like protein 2	-0.219111427221572	6.42035145550405	-0.717511825517224	0.498284104239783	0.999692817443324	-4.60967556950002	5.70231442400591	6.03323000597182	6.50758828195332	6.66658244234839	6.83271370752417	6.77967987122069	NA
**TC1300002753.mm.2**	B020031M17Rik	RIKEN cDNA B020031M17 gene	0.380918813075819	4.99331637015947	0.717394937137428	0.498351502262667	0.999692817443324	-4.60968063461705	3.88777169556537	4.5515987632354	4.87424516560129	6.26693752855254	4.97518500413515	5.40416006386705	NA
**TC1400000281.mm.2**	Erc2	ELKS/RAB6-interacting/CAST family member 2	0.431831254677487	8.84380830379526	0.717323995593159	0.498392410216892	0.999692817443324	-4.60968370843616	9.49694914597187	9.4598561991437	9.31675696961572	8.16809652388975	9.06532698551533	7.55586399863517	NA
**TC1900000700.mm.2**	Sufu	SUFU negative regulator of hedgehog signaling	0.243257740861115	6.68593042741958	0.717294269368594	0.498409552302289	0.999692817443324	-4.60968499637643	6.95784068327791	6.86346161250222	6.99311047413674	6.39728740109304	6.33629665093915	6.56758574256839	NA
**TC1900000760.mm.2**	Rbm20	RNA binding motif protein 20	-0.224042449777637	6.87325298414075	-0.717245081813749	0.49843791792255	0.999692817443324	-4.60968712742915	6.4598088690836	6.63085012030618	6.92064324691518	6.92974376103456	6.94688548999435	7.35158641751067	NA
**TC1500001630.mm.2**	Asap1	ArfGAP with SH3 domain, ankyrin repeat and PH domain1	-0.443137667079723	8.04060411593276	-0.717175051956895	0.498478304788703	0.999692817443324	-4.60969016129587	8.62499858901797	8.90173406287935	8.26430884717017	6.65099950759974	8.4414480279664	7.36013566096296	NA
**TC1400000336.mm.2**	Galnt15	polypeptide N-acetylgalactosaminyltransferase 15	-0.259336558318527	5.42769048134757	-0.716942303438744	0.498612548604484	0.999692817443324	-4.60970024302188	5.30324137343102	5.42230071242815	5.40465718671385	5.25630665608074	5.2074307367909	5.97220622264075	NA
**TC0100003738.mm.2**	Marc1	mitochondrial amidoxime reducing component 1	0.225523631376734	5.11712842532439	0.716786149987811	0.49870262764127	0.999692817443324	-4.60970700565232	5.00786384556909	5.12032255880882	5.33081463097114	5.1820010741898	5.241006110379	4.82076233202847	NA
**TC1100002865.mm.2**	Rasd1	RAS, dexamethasone-induced 1	0.254079090633622	6.92654227625308	0.716764147889694	0.498715320689104	0.999692817443324	-4.60970795842593	8.05551042061018	7.53485254588458	6.8733575492475	6.36516688689799	6.28615049911903	6.44421575575922	NA
**TC0500000911.mm.2**	Gm3286/D5Ertd577e	predicted gene 3286/DNA segment, Chr 5, ERATO Doi 577, expressed	-0.384139795665248	5.19972678873632	-0.716762555730988	0.49871623921648	0.999692817443324	-4.60970802737159	4.38225206299014	5.10385201451892	4.7534922344327	5.71849629735648	5.07689593282865	6.16337219029103	NA
**TC0900001385.mm.2**	Camkv	CaM kinase-like vesicle-associated	-0.263978752438691	9.0811296302766	-0.716633091870679	0.498790931567041	0.999692817443324	-4.60971363321281	10.0258830693382	10.0685612294615	8.8788121093248	8.05337587966645	8.72318910263057	8.73695639123806	NA
**TC0800001524.mm.2**	Def8	differentially expressed in FDCP 8	0.223752683523124	7.37855000441369	0.716476914706946	0.498881045650339	0.999692817443324	-4.60972039479139	7.46383484794456	7.41837389433064	7.24092090284415	7.67737742278245	7.23593568967839	7.23485726890197	NA
**TC0600002648.mm.2**	Antxr1	anthrax toxin receptor 1	-0.416181975738702	6.80522188309411	-0.716299186175389	0.498983607980681	0.999692817443324	-4.60972808814334	7.31017454173667	7.51454822850859	7.12191292007666	5.46520936834435	7.06100165452243	6.35848458537599	NA
**TC0300001271.mm.2**	Camk2d	calcium/calmodulin-dependent protein kinase II, delta	-0.748215864571669	8.66998346414915	-0.71622214508533	0.499028070652253	0.999692817443324	-4.6097314226057	9.91195316084747	9.81280595996643	9.3684010473865	6.03095392008233	9.12352595146357	7.77226074514859	NA
**TC0800000141.mm.2**	Dlgap2	DLG associated protein 2	0.243516332915148	7.84711875623124	0.71620595936265	0.499037412241054	0.999692817443324	-4.60973212311733	7.59089008559415	7.87109960959829	7.73601660807372	8.31786114593893	7.82814870255668	7.73869638562568	NA
**TC1100000839.mm.2**	Zkscan6	zinc finger with KRAB and SCAN domains 6	-0.213360604378799	5.72096983148217	-0.716138518664621	0.499076336882966	0.999692817443324	-4.6097350418024	5.62393326323527	5.63817638753869	5.532529724225	5.78596434045572	5.93465287038409	5.81056240305423	NA
**TC0400003773.mm.2**	Pnrc2	proline-rich nuclear receptor coactivator 2	-0.262095306978667	6.55567239518175	-0.716135134256334	0.499078290309604	0.999692817443324	-4.60973518826698	7.12828451796102	7.19441370876754	6.4482198200809	5.79535294512141	6.62384373098034	6.1439196481793	NA
**TC0500003018.mm.2**	Asphd2	aspartate beta-hydroxylase domain containing 2	-0.412797853388757	7.49253909217271	-0.71607488774524	0.499113064485526	0.999692817443324	-4.60973779542897	7.79909256051352	8.14422794598866	7.87795695669815	6.21520221318013	7.74592953321135	7.17282534344445	NA
**TC1200002529.mm.2**	Itpk1	inositol 1,3,4-triphosphate 5/6 kinase	-0.214841108508985	8.517778996657	-0.716026454669614	0.499141021132267	0.999692817443324	-4.60973989125228	8.33566114050186	8.19127348394253	8.65738299039554	8.41764557884428	8.81900613781814	8.68570464843966	NA
**TC0X00002741.mm.2**	Hdac8	histone deacetylase 8	-0.256787711626799	6.20207175077972	-0.715988985242508	0.499162650029482	0.999692817443324	-4.60974151258108	6.64841670519726	6.82228140028616	6.18154675378538	5.43253173418527	6.08179876834349	6.04585514288076	NA
**TC0X00002084.mm.2**	Tmem255a	transmembrane protein 255A	0.499387149961528	7.63618694031007	0.715951093837209	0.499184523141418	0.999692817443324	-4.60974315210765	9.56590121549086	8.55752490647364	7.98160381822243	6.36463109168706	7.29907292016019	6.04838768982624	NA
**TC0500003637.mm.2**	Slc7a1	solute carrier family 7 (cationic amino acid transporter, y+ system), member 1	0.238523756948361	6.70485235769957	0.715890239805726	0.499219652938969	0.999692817443324	-4.60974578507666	7.30962797899952	7.30471122962014	6.733306851828	6.3126043739114	6.50178174432566	6.06708196751702	NA
**TC0400002633.mm.2**	Acnat2	acyl-coenzyme A amino acid N-acyltransferase 2	0.354761118088251	5.44052506769161	0.715780510361715	0.499283001659839	0.999692817443324	-4.60975053233289	4.78924174450295	5.02916600576491	5.31406731062168	6.45306513540747	5.09790587378191	5.95970433607074	NA
**TC1500000869.mm.2**	Adm2	adrenomedullin 2	0.387906116273491	5.44957040861753	0.715720899659761	0.499317418189519	0.999692817443324	-4.60975311107015	4.87606481508709	5.05663457339839	5.23146604470179	6.53880160318155	5.00366782466718	5.99078759066918	NA
**TC0600001629.mm.2**	BC049715	cDNA sequence BC049715	0.251331118270139	4.72225539430923	0.715705209861161	0.499326477032567	0.999692817443324	-4.60975378977966	4.05021627431487	4.33977662624643	4.91836391771631	5.30473693320089	4.59044549583981	5.12999311853711	NA
**TC1100000738.mm.2**	Nt5m	5',3'-nucleotidase, mitochondrial	0.259859476661354	6.25130543511384	0.715550734528399	0.499415672504628	0.999692817443324	-4.60976047150897	6.32433227459269	6.14047891488086	6.26405279785294	6.51731738941315	5.76459782262271	6.49705341132068	NA
**TC1500002339.mm.2**	Hoxc10	homeobox C10	-0.245453865461643	5.52463885331258	-0.715481847267624	0.499455452042294	0.999692817443324	-4.6097634508506	5.03026625992207	5.0784834151313	5.30198156990701	5.9721062870424	5.89349439294812	5.87150119492458	NA
**TC1200001757.mm.2**	Cfl2	cofilin 2, muscle	-0.721187195192541	8.15106750698288	-0.715446601900517	0.499475805592369	0.999692817443324	-4.60976497511702	8.98263493237713	8.86143015228897	8.91252412914263	5.89745865428041	8.97786952477259	7.27448764903553	NA
**TC1300000829.mm.2**	Zfp493	zinc finger protein 493	0.217094407759182	5.94962796986884	0.715363888299303	0.499523573330271	0.999692817443324	-4.60976855204529	5.73914603459791	6.05361043052758	6.09827356338368	6.07132652141929	5.79173885115776	5.94367241812685	NA
**TC0900000026.mm.2**	Msantd4	Myb/SANT-like DNA-binding domain containing 4 with coiled-coils	-0.383111450769099	7.01217243263763	-0.71535676673095	0.499527686231966	0.999692817443324	-4.60976886000187	7.88944760879804	7.76023441391584	7.1741644702751	5.65440036551177	7.07157203463215	6.52321570269291	NA
**TC0900002358.mm.2**	Odf3l1	outer dense fiber of sperm tails 3-like 1	-0.368757960971873	6.36385544648279	-0.71530762057414	0.499556070101334	0.999692817443324	-4.60977098515986	6.42631067075936	5.35588503882592	5.97852063667702	6.85318988700685	6.45090188779232	7.1183245578353	NA
**TC1400002863.mm.2**	Ipo4	importin 4	0.259986642275231	7.24447088716101	0.715287699047507	0.499567575883212	0.999692817443324	-4.60977184656868	7.87307714581822	7.6698155910685	7.49653827394204	6.72541466137286	6.86969032633298	6.83228932443145	NA
**TC0500000871.mm.2**	Art3	ADP-ribosyltransferase 3	0.215239734718141	5.95443893886387	0.715227374983133	0.499602417432266	0.999692817443324	-4.60977445488292	6.1138158301378	5.99560359368845	5.86564427391612	6.1582025654805	5.7674189020826	5.82594846787774	NA
**TC1200000679.mm.2**	Hspa2	heat shock protein 2	0.262637173761846	6.09133602814801	0.715093498193527	0.499679746795267	0.999692817443324	-4.60978024293634	6.73489548946418	6.95897583977023	5.46496135808248	6.22474823550618	5.68901313779618	5.47542210826879	NA
**TC0200002961.mm.2**	Pfkfb3	6-phosphofructo-2-kinase/fructose-2,6-biphosphatase 3	0.212524734518516	6.75043404058843	0.714738250696235	0.499884981812421	0.999692817443324	-4.60979559802222	6.94422921830414	7.05733644399987	6.61010209553184	6.85294192959997	6.54891854985032	6.48907600624444	NA
**TC0100002461.mm.2**	Klf7	Kruppel-like factor 7 (ubiquitous)	0.216094795850122	8.03846622636056	0.714647890364861	0.499937194048112	0.999692817443324	-4.60979950285465	8.47325561557155	8.44872100889101	7.92861184431576	7.94189331838477	7.887524522131	7.55079104886929	NA
**TC0800000960.mm.2**	G430095P16Rik	RIKEN cDNA G430095P16 gene	0.302343494189323	5.6375859594045	0.71464626714075	0.499938132016584	0.999692817443324	-4.60979957299746	4.73606983991944	4.98632852100977	5.72164919718858	6.63225299474963	5.5794855298548	6.16972967370477	NA
**TC0800002599.mm.2**	Asna1	arsA arsenite transporter, ATP-binding, homolog 1 (bacterial)	0.275584490988811	8.0803159709164	0.714571790851639	0.499981168861632	0.999692817443324	-4.60980279114648	8.58068253120 16	8.75126103747574	8.11053506091946	7.74002555847989	8.06345703581416	7.23593460160757	NA
**TC1900001254.mm.2**	1110059E24Rik	RIKEN cDNA 1110059E24 gene	-0.522443491910066	6.72944345370373	-0.714510912294149	0.500016349947038	0.999692817443324	-4.6098054215544	7.2521819966062	6.96282905785633	7.39178066068687	5.16660068128298	7.24301333752435	6.36025498826563	NA

**TC0400002096.mm.2**	Dvl1	dishevelled segment polarity protein 1	-0.284862023158427	6.94660251274906	-0.714503132153142	0.500020846127697	0.999692817443324	-4.60980575770301	7.67393598106534	7.45825684432665	6.0771111850584	6.91173791733437	6.53463961730241	7.02393353140721	NA
**TC1700000850.mm.2**	Supt3	suppressor of Ty 3	0.342087099714393	5.85903458684264	0.71437806444792	0.500093127024213	0.999692817443324	-4.6098111610169	5.82710081429382	5.38274943919534	5.92662867888551	6.38763705461222	5.20729636443592	6.42279516963303	NA
**TC0400002391.mm.2**	Smim8	small integral membrane protein 8	-0.257450817909214	7.30475130171717	-0.714342250559639	0.500113826366299	0.999692817443324	-4.60981270816385	7.76040124360708	7.59031059642529	6.96765259659675	7.01379457062935	7.62821226362403	6.8681365394205	NA
**TC0X00000922.mm.2**	Gpr165	G protein-coupled receptor 165	-0.54619161881593	7.31410906119574	-0.714289944039385	0.500144058968512	0.999692817443324	-4.60981496768696	8.8826291016991	7.77226399301425	7.67013288551996	5.39855613189465	7.20843070341054	6.95264155163594	NA
**TC1800001366.mm.2**	Aldh7a1	aldehyde dehydrogenase family 7, member A1	-0.23658382359992	6.83855783950357	-0.714202416152129	0.500194651844254	0.999692817443324	-4.60981874842895	7.42594288570621	7.04689430655012	6.57229243769734	6.4703786610853	6.97895744541712	6.53688130056536	NA
**TC0500003589.mm.2**	Pdap1	PDGFA associated protein 1	0.286915728217958	8.85083709694689	0.714191835346925	0.500200767991449	0.999692817443324	-4.60981920544137	9.51668926635568	9.30008996372821	8.69954249844734	8.73149490556934	8.92002516695274	7.93718078062802	NA
**TC0900000183.mm.2**	Mrpl4	mitochondrial ribosomal protein L4	-0.205349088010903	7.81073019022629	-0.714186025634354	0.500204126268936	0.999692817443324	-4.60981945637587	7.82824677851308	7.81514004589957	7.69464974382874	7.71049832663291	7.891543550398	7.92430269608545	NA
**TC1300002053.mm.2**	Mxd3	Max dimerization protein 3	-0.372396927804732	5.8624936626123	-0.714142062648811	0.500229539352987	0.999692817443324	-4.60982135518842	5.55557885383705	5.72381582204119	5.67594953408146	5.89943718801158	5.39242472988687	6.92775584781564	NA
**TC1500002265.mm.2**	Gm5476	type II keratin Kb17P pseudogene	0.229287243219077	6.17897498292068	0.714066869614221	0.500273007145908	0.999692817443324	-4.60982460266962	5.87004789925038	5.74146503325267	6.25442846994677	6.70602725578283	6.14628157224079	6.35559966705066	NA
**TC1000000829.mm.2**	Arid3a	AT rich interactive domain 3A (BRIGHT-like)	-0.223431416383868	7.52203784745734	-0.713899379514158	0.500369839258098	0.999692817443324	-4.6098318354528	6.83234938583554	7.01925075358548	7.5076541755057	7.90922788077195	7.95318645811816	7.91055843092723	NA
**TC0700003865.mm.2**	Trim68	tripartite motif-containing 68	-0.223971494574938	5.92065435646078	-0.713880360832233	0.500380835432912	0.999692817443324	-4.6098326566665	5.61848183980092	5.65343522243766	5.86401773290893	6.03801531077918	5.96721469528106	6.38276133755693	NA
**TC0400003855.mm.2**	Klhdc7a/Mir2139	kelch domain containing 7A/microRNA 2139	0.245175198184093	6.76883018678159	0.713841832722019	0.500403112010965	0.999692817443324	-4.60983432023608	6.62660929534772	6.55476161863092	6.75872820188814	7.2022520996514	6.48356482780752	6.98706507736383	NA
**TC1500001531.mm.2**	Enpp2	ectonucleotide pyrophosphatase/phosphodiesterase 2	-0.493990049089042	9.96418533787905	-0.713736693550814	0.500463905793172	0.999692817443324	-4.60983885961649	10.1481645906106	11.6426299077967	8.88864333713269	9.61452537821177	10.5102369340826	8.98091187943993	NA
**TC0X00002457.mm.2**	Gm5640	predicted gene 5640	-0.207298912977358	6.78663725296576	-0.713653954023787	0.500511751042838	0.999692817443324	-4.60984243155697	6.45451254717483	6.50180345142388	6.7828916547268	6.89156319189376	7.09288592210762	6.99616675046765	NA
**TC1100003488.mm.2**	Epx	eosinophil peroxidase	-0.427714408195147	5.35760059803228	-0.713557435009875	0.500567568289618	0.999692817443324	-4.60984659799638	5.07320927009941	4.67154838203024	4.57732377420895	6.19538478562791	5.37254437073146	6.25559300549569	NA
**TC0100001755.mm.2**	Aida	axin interactor, dorsalization associated	-0.949513670953303	7.24747099675613	-0.713488243276023	0.500607584618228	0.999692817443324	-4.60984958455031	8.74034309552705	8.55296916627228	8.23471420941711	3.91152897899832	7.88739345139019	6.15787707893185	NA
**TC0600001293.mm.2**	Washc2	WASH complex subunit 2`	-0.38637031844677	7.74533034713465	-0.713359768241602	0.500681892445113	0.999692817443324	-4.60985512942687	8.63619988931772	8.49340473633238	7.85227142400249	6.43254698612964	7.93960966830283	7.11794937872284	NA
**TC0700004645.mm.2**	Zfp566	zinc finger protein 566	0.225391538359463	5.9748437552558	0.71334647301383	0.50068958260051	0.999692817443324	-4.60985570319718	5.89483545902862	5.76734891566462	6.00833041421469	6.31050020256554	6.10331670186524	5.76473083819606	NA
**TC1100001722.mm.2**	Arf2	ADP-ribosylation factor 2	-0.784339437816952	8.0642403688609	-0.713312848017936	0.500709032141488	0.999692817443324	-4.60985715428707	8.29822106721438	8.16742411361916	9.4245297183208	5.75102936002818	8.86625789209426	7.87798006188862	NA
**TC0600000788.mm.2**	Serbp1	serpine1 mRNA binding protein 1	-0.459670655113706	9.49772679514355	-0.713144305522536	0.500806528874951	0.999692817443324	-4.60986442701745	10.3319147379514	10.4435219314022	9.73185533614872	7.91393605949145	9.77359376854076	8.79153893732681	NA
**TC0500000627.mm.2**	Rhoh	ras homolog family member H	0.276549939773108	4.81384129317561	0.713086174939103	0.500840158569029	0.999692817443324	-4.60986693510906	4.40569775605707	4.82732854745128	5.31955356711274	4.78200710043302	4.45567868154183	5.09278210645771	NA
**TC0200002405.mm.2**	Tldc2	TBC/LysM associated domain containing 2	0.259798511141543	5.66025572038726	0.712882348613475	0.500958087929664	0.999692817443324	-4.60987572820748	5.52841876977667	4.92784612463094	5.81404236273355	6.19839086236598	5.56071322967205	5.93212297314439	NA
**TC0600003533.mm.2**	Ttll3	tubulin tyrosine ligase-like family, member 3	0.476940220858054	6.35625975580216	0.712864219737741	0.500968577781084	0.999692817443324	-4.60987651020284	5.9555205088312	6.30409845079486	6.42737806752766	6.98853194092384	5.19916125673232	7.26286831000307	NA
**TC1100002480.mm.2**	Smim23	small integral membrane protein 23	-0.317606526705942	5.59227526927965	-0.712808137293948	0.501001029499294	0.999692817443324	-4.60987892924848	4.80377623204834	5.45339308569033	5.53209045269364	5.79854416957002	5.48326794984651	6.48257972582903	NA
**TC0700000806.mm.2**	Josd2/Mir7052	Josephin domain containing 2/microRNA 7052	-0.207444326741117	7.33444896343845	-0.71250793301555	0.50117476425026	0.999692817443324	-4.60989187586698	7.39533652728068	7.48532640356357	7.14786711107422	7.20770398707789	7.44992825186973	7.32053149976461	NA
**TC0900002686.mm.2**	Hcrtr2	hypocretin (orexin) receptor 2	0.258148108501073	6.01180336727498	0.712461857772959	0.501201432519546	0.999692817443324	-4.60989386256381	6.19028571477261	5.4384945814531	6.21573389388955	6.26343416832361	5.85973027424508	6.10314157096593	NA
**TC1400000764.mm.2**	Mhrt	myosin heavy chain associated RNA transcript	-0.218090324249799	5.86728189852866	-0.712334774151756	0.501274993155026	0.999692817443324	-4.60989934174647	6.03671499449165	5.97548241497883	5.70573314345091	5.67192352315003	5.72058166509449	6.09325565000604	NA
**TC0700002458.mm.2**	Psg20	pregnancy-specific glycoprotein 20	0.267275532663905	4.73948324833395	0.712236723661951	0.501331753229331	0.999692817443324	-4.60990356869389	4.49005799432644	4.22918779268561	4.88729521897266	5.23880716518708	4.42235975228076	5.16919156655118	NA
**TC0500002879.mm.2**	Klhl8	kelch-like 8	-0.20794626717232	7.70307214267425	-0.712229164314306	0.501336129407552	0.999692817443324	-4.60990389455937	7.38192598779041	7.44947962640473	7.74773522969514	7.73783212405771	7.86427107759404	8.03718881050345	NA
**TC0900000105.mm.2**	Cwc15	CWC15 spliceosome-associated protein	0.287540559356051	8.58343211078954	0.712191068611275	0.501358183756252	0.999692817443324	-4.60990553673673	9.44606309434021	9.3399460899579	8.76094305944286	7.88388924013275	8.28697459511003	7.78277658575348	NA
**TC1700000313.mm.2**	Zscan10	zinc finger and SCAN domain containing 10	-0.284856320965984	5.96269912460813	-0.712128040833714	0.501394673178525	0.999692817443324	-4.60990825351373	5.52000914166834	5.91727338895002	5.98623519253112	5.89836459501811	5.7714796652607	6.68283276422049	NA
**TC0100001158.mm.2**	Yod1	YOD1 deubiquitinase	0.278554391211881	6.66226818006125	0.712073064040415	0.501426502980929	0.999692817443324	-4.60991062311701	5.79990891714021	6.14465426399195	7.13237993632159	7.16069740450796	7.31146765041975	6.42450090798604	NA
**TC1300002201.mm.2**	Prxl2c	peroxiredoxin like 2C	-0.331218785315606	6.4570290576945	-0.711916373618455	0.501517229038915	0.999692817443324	-4.60991737605052	6.89196191747744	6.8409474659199	5.83473868330487	6.33867501276435	7.02323424771712	5.81261701898331	NA
**TC0300002765.mm.2**	Ntng1	netrin G1	0.383134632289489	6.95232407982017	0.711747886949961	0.501614797392592	0.999692817443324	-4.60992463618301	8.01193020206859	7.4489002726531	7.27811139126863	6.23158024312052	6.93135341446099	5.81206895534918	NA
**TC1900001586.mm.2**	Fgf8	fibroblast growth factor 8	-0.367306473343903	5.8477728666887	-0.711602026199387	0.501699273504643	0.999692817443324	-4.60993092036517	5.48787719406686	4.96795127841511	5.3858596406825	6.56223824979872	5.91984727132382	6.76286356584519	NA
**TC0600001418.mm.2**	Cd163	CD163 antigen	0.227295541060879	4.81555151063401	0.711557660860688	0.501724969814576	0.999692817443324	-4.60993283159339	4.76563411851779	4.46131863877116	5.03149033884424	5.02898335547419	4.60423054226798	5.0016520699287	NA
**TC0200003339.mm.2**	Pbx3	pre B cell leukemia homeobox 3	0.555200929024156	7.40713973527338	0.711478508087877	0.501770817090525	0.999692817443324	-4.6099362412283	7.71600189091879	7.28811402892701	8.77147054187975	6.50309163304164	7.68237873327505	6.48178158359803	NA
**TC0600003602.mm.2**	Eif4e3	eukaryotic translation initiation factor 4E member 3	0.268839428358962	7.86403343923226	0.711215344066076	0.501923268191821	0.999692817443324	-4.60994757549447	8.03639505115698	7.56590419800661	8.22713730994237	7.83265281153158	8.076854627961	7.44525663679502	NA
**TC0X00003159.mm.2**	Tmem29	transmembrane protein 29	0.311828217801104	7.06806715556876	0.711209753501707	0.501926507141273	0.999692817443324	-4.60994781624306	6.98614909970964	6.61670528714661	6.81373673136252	7.90086575971674	7.19737455916318	6.89357149631388	NA
**TC1300000332.mm.2**	Agtr1a	angiotensin II receptor, type 1a	0.247854948342629	5.70340894873858	0.711097278266452	0.50199167372591	0.999692817443324	-4.60995265951868	5.32805941054372	5.5416288438093	5.63866320257642	6.28457446480543	5.61640717108629	5.8111205996103	NA
**TC1700001675.mm.2**	Stub1/Mir3082	STIP1 homology and U-Box containing protein 1/microRNA 3082	-0.333247233663302	8.30433491796622	-0.711015249280875	0.5020392036987	0.999692817443324	-4.60995619140771	8.95237040305856	8.75922104509094	8.45410866368608	7.26985313247455	8.47971519401731	7.91074106946991	NA
**TC1800000706.mm.2**	Prelid3a	PRELI domain containing 3A	-0.218287597115451	7.48807109719457	-0.711000143740064	0.502047956610856	0.999692817443324	-4.60995684176906	7.29593049186993	7.31332253176999	7.63356172882262	7.30773745382569	7.79385964833432	7.5840147285449	NA
**TC0100000329.mm.2**	1500015O10Rik	RIKEN cDNA 1500015O10 gene	-0.72707060422802	5.71926019740432	-0.710983791166845	0.502057432230004	0.999692817443324	-4.6099575458096	4.807384400018	7.03483890208131	3.89304958617788	6.61654875075741	5.93394264335424	6.02979690203709	NA
**TC0300001206.mm.2**	Plppr5	phospholipid phosphatase related 5	-0.552089536958296	8.08387597460663	-0.710930812911186	0.502088131681504	0.999692817443324	-4.60995982664551	8.93939126994153	8.94536765695528	8.54204232533293	6.21511659808026	8.49632072393007	7.36501727339972	NA
**TC0700001356.mm.2**	Rsf1	remodeling and spacing factor 1	-0.602825948673287	6.54021571341759	-0.710857875711213	0.502130398822499	0.999692817443324	-4.60996296656061	8.04765897159617	7.86519563853104	6.90450262666847	4.1568912598474	6.5223643875274	5.74468139633506	NA
**TC0800002958.mm.2**	Chst5	carbohydrate (N-acetylglucosamine 6-O) sulfotransferase 5	0.249569668576696	4.56687943992024	0.710553468953602	0.502306828025135	0.999692817443324	-4.60997606864729	3.83421600271249	3.73651185586476	4.78055030910305	5.38429374994574	4.63869089388672	5.02701382800868	NA
**TC0200001886.mm.2**	Eid1	EP300 interacting inhibitor of differentiation 1	-0.223374574236015	7.85660689918339	-0.710520299001607	0.502326055260154	0.999692817443324	-4.60997749608466	8.32320632537448	8.17521179496231	7.46751371555631	7.62972334758945	7.97271712081499	7.5712690908028	NA
**TC1100000853.mm.2**	Myh2	myosin, heavy polypeptide 2, skeletal muscle, adult	-0.38912967980462	5.2664905156648	-0.710504887331517	0.502334988926456	0.999692817443324	-4.60997815929498	4.83071311169716	5.31373508055644	4.86579193419821	5.47232583686478	4.81743904524889	6.29893808542334	NA
**TC1000003158.mm.2**	Suox	sulfite oxidase	0.209853058634831	6.37352092363343	0.710409768691945	0.502390128566021	0.999692817443324	-4.60998225230661	6.38182063627985	6.22592793764955	6.49908867032479	6.52745287224563	6.39746553675696	6.20936988854379	NA
**TC0400001733.mm.2**	Pla2g2d	phospholipase A2, group IID	-0.338050810358973	5.13230795387875	-0.710330531000583	0.502436065174117	0.999692817443324	-4.60998566165155	4.78131445683516	4.71902044599213	4.72170546457048	5.58700013529314	6.01645722307821	4.96834999750336	NA
**TC1700000934.mm.2**	Treml4	triggering receptor expressed on myeloid cells-like 4	0.541040169853659	5.04224696900572	0.709830812862337	0.502725831484925	0.999692817443324	-4.61000715664253	4.63321491719339	4.76476654905222	4.50656715039025	6.46222319335777	4.14205633423992	5.74465366980079	NA
**TC0600000229.mm.2**	Calu	calumenin	-0.580481085274504	6.74773051572109	-0.709781668612955	0.502754334194752	0.999692817443324	-4.6100092699584	7.65655243863599	7.93588125431654	7.28711498026413	4.57937863514838	6.6944526439224	6.33300314203911	NA
**TC0100000601.mm.2**	Tmem169	transmembrane protein 169	-0.287231231827198	6.23233766281439	-0.709753200791156	0.502770845465622	0.999692817443324	-4.61001049409226	6.40451373679371	5.9994305841859	6.39479215146473	5.81301744466145	6.02302149726294	6.75925056251763	NA
**TC1500000725.mm.2**	Aco2	aconitase 2, mitochondrial	0.22461709419456	9.03293905623346	0.70968023905853	0.502813164734205	0.999692817443324	-4.61001363133077	9.9062649919685	9.69558641706919	8.96872299834254	8.55378556003355	8.48653193236111	8.58674243762586	NA
**TC0700000759.mm.2**	Nkg7	natural killer cell group 7 sequence	0.319920910140385	5.93668559989298	0.709465700950884	0.502937614743737	0.999692817443324	-4.61002285478727	5.36642624763059	5.4540934251882	5.76131134194433	6.9584065314656	5.87206993951907	6.2078061136101	NA
**TC0400003379.mm.2**	Atp6v0b	ATPase, H+ transporting, lysosomal V0 subunit B	-0.209842773900276	7.7597871230368	-0.709044773900517	0.503181846553893	0.999692817443324	-4.61004094553232	8.29030224920612	8.1959131680373	7.52795634650778	7.29845454008063	7.65098308549394	7.59511334889502	NA
**TC1500001944.mm.2**	Cyp2d22	cytochrome P450, family 2, subfamily d, polypeptide 22	0.23082112090014	7.30330093017727	0.708973466601804	0.503223228471324	0.999692817443324	-4.61004400943783	8.31009463455065	8.07472439408645	7.21905127365903	6.72926312345436	6.78263608444492	6.70403607086818	NA
**TC0800002376.mm.2**	Ndufa13	NADH:ubiquinone oxidoreductase subunit A13	-0.266428269935993	9.69592697073066	-0.708882120497174	0.503276242827799	0.999692817443324	-4.61004793403996	10.1972561954727	10.1612837369545	9.70236031772965	8.93972235831275	9.79487618820636	9.38006302770802	NA
**TC1100003353.mm.2**	Asic2	acid-sensing (proton-gated) ion channel 2	-0.691513201482455	8.43745523123109	-0.708760348105588	0.503346921349431	0.999692817443324	-4.6100531653149	9.27566146732334	8.89643975462447	9.31913609466667	6.21566578657023	9.05177427416216	7.86605401003965	NA
**TC0600001388.mm.2**	Dppa3	developmental pluripotency-associated 3	0.353612856313267	5.21107036124702	0.708507756863829	0.503493549939593	0.999692817443324	-4.61006401443697	4.83887911515229	4.68251740288709	5.30596012008313	5.92016556095151	4.59986057064103	5.91903939776708	NA
**TC1700001014.mm.2**	Ranbp3	RAN binding protein 3	0.226938180813132	8.11776983461189	0.708411951910317	0.503549171835739	0.999692817443324	-4.61006812865739	8.23339640670712	8.01928799704017	8.01047998182142	8.44342550095373	8.07874610923541	7.92128301191347	NA
**TC1700000722.mm.2**	2410017I17Rik	RIKEN cDNA 2410017I17 gene	-0.267169224960717	5.86533407427814	-0.708366838108552	0.503575365152068	0.999692817443324	-4.61007006587308	5.11785535753391	5.29827583265765	5.68475019943366	6.43601720334426	6.12091013667518	6.53419571602418	NA
**TC1800000681.mm.2**	Oacyl	O-acyltransferase like	-0.306757670395388	4.98674669038494	-0.708082607912346	0.503740411368092	0.999692817443324	-4.61008226886144	5.00024567412434	4.71001290466714	4.37596898346409	5.4223841278996	4.99806272288232	5.41380572927215	NA
**TC0500003487.mm.2**	Adap1	ArfGAP with dual PH domains 1	-0.319872868452526	7.89686216660019	-0.708052207456004	0.503758066351797	0.999692817443324	-4.6100835738501	8.24286700315989	8.03857271319261	8.18904097313161	7.04095280004019	8.16029276628743	7.70944674378943	NA
**TC0X00003361.mm.2**	Prps2	phosphoribosyl pyrophosphate synthetase 2	-0.213098928481844	7.39278853639306	-0.707876214359354	0.503860281877242	0.999692817443324	-4.61009112784621	7.91490443046439	7.77803342619483	7.19281958905039	6.92597816331735	7.33130593520439	7.21368967412703	NA
**TC1800001083.mm.2**	Slc39a6	solute carrier family 39 (metal ion transporter), member 6	-0.34890002297614	7.64795177982919	-0.707749001596063	0.503934174636004	0.999692817443324	-4.61009658724647	8.39240201401729	8.06575932101033	7.87655101448837	6.48932363450924	7.62225107875077	7.44142361619912	NA
**TC0500003014.mm.2**	Srrd	SRR1 domain containing	0.214586609594818	6.29270195074283	0.707663574539608	0.503983799767454	0.999692817443324	-4.61010025299591	6.43657665526812	6.2934633936836	6.36572012687883	6.36195231046862	5.99088516384015	6.30761405431766	NA
**TC1000002425.mm.2**	Gm9508	predicted gene 9508	-0.259873980609572	4.84376446485923	-0.707321591737901	0.504182492003332	0.999692817443324	-4.61011492458863	4.07308530805181	4.40427727060574	4.64350222417088	5.38923590046845	5.3223986405234	5.23008744533508	NA
**TC1700001673.mm.2**	Fam173a	family with sequence similarity 173, member A	-0.209314110096298	7.28068189547385	-0.707296183629334	0.504197256188074	0.999692817443324	-4.6101160144323	7.63105065979843	7.43614673490595	7.11331943798217	6.98581344099089	7.2080467335992	7.30971436556645	NA
**TC0700000720.mm.2**	Vstm2b	V-set and transmembrane domain containing 2B	-0.282291935021822	7.70926527103124	-0.707280819179612	0.504206184325339	0.999692817443324	-4.61011667345424	8.3132516765697	7.94165987894809	7.76043439152579	6.95761370878721	7.86737687883641	7.41525509152024	NA
**TC0100001500.mm.2**	Slc19a2	solute carrier family 19 (thiamine transporter), member 2	0.211173652548545	7.00248398567546	0.707090196333135	0.504316962119701	0.999692817443324	-4.61012484891635	7.08559999824257	7.09269507475734	7.20169516253761	6.92778291053738	6.90424498914806	6.80288577882983	NA
**TC0600002254.mm.2**	4921507P07Rik	RIKEN cDNA 4921507P07 gene	0.227101972419879	5.75895925339422	0.706917689403951	0.504417225902702	0.999692817443324	-4.61013224605327	5.83480051462047	5.44268858117822	6.04669226217105	5.81854292253214	5.82775989525012	5.58327134461331	NA
**TC1100003421.mm.2**	Znhit3	zinc finger, HIT type 3	0.285404542719168	6.37454444218924	0.706872065605489	0.504443745375451	0.999692817443324	-4.61013420219506	6.51507480646076	6.53608086155178	6.16500061118522	6.7184594240954	5.72967848015111	6.58297246969118	NA
**TC1600001240.mm.2**	Ifitm7	interferon induced transmembrane protein 7	0.289608365158536	6.25197715081433	0.706816462816031	0.504476066516488	0.999692817443324	-4.61013658606793	5.97489293181502	6.28384587838835	6.37853763701751	6.53763277548233	6.662608599148	5.67434508303478	NA
**TC0300001265.mm.2**	Ndst4	N-deacetylase/N-sulfotransferase (heparin glucosaminyl) 4	0.37778820383887	5.91322224083801	0.70680716036769	0.504481474035475	0.999692817443324	-4.61013698488103	7.21788864999202	6.00832792374605	6.15748281718017	5.34686382230371	5.72525455457881	5.02351567722733	NA
**TC0200000048.mm.2**	Ccdc3	coiled-coil domain containing 3	0.260463393536395	5.63475427980965	0.706668935144388	0.504561828935094	0.999692817443324	-4.61014291040597	6.33691833186109	5.66000739523125	5.4014514626124	5.76481190680679	5.16839898187303	5.47693760047336	NA
**TC1100000820.mm.2**	Cdrt4	CMT1A duplicated region transcript 4	0.310188475606704	5.97553769472949	0.706467038454847	0.504679213291146	0.999692817443324	-4.61015156393956	5.36451231065389	4.96702304999274	6.03107144805475	7.03996243141709	5.9794626213964	6.47119430686203	NA
**TC1000002464.mm.2**	2610008E11Rik	RIKEN cDNA 2610008E11 gene	0.24302952039763	5.90706146010223	0.706438583980698	0.504695758394409	0.999692817443324	-4.61015278338921	6.49543886530399	6.28500173479953	6.06568277333743	5.50831082731512	5.70630189737937	5.38163266247793	NA
**TC0200001948.mm.2**	Chchd5	coiled-coil-helix-coiled-coil-helix domain containing 5	0.342181278127286	5.78026583538079	0.70642956666375	0.504701001666758	0.999692817443324	-4.61015316982946	5.87359133958161	5.93131695307943	5.22632774025087	6.55419689768827	5.76559341549229	5.33056866619229	NA
**TC0400004047.mm.2**	Pik3cd	phosphatidylinositol-4,5-bisphosphate 3-kinase catalytic subunit delta	0.217301072207724	5.90217967826537	0.706427486933179	0.504702210966562	0.999692817443324	-4.61015325895653	5.88789612751426	5.80972201471974	5.86984359216643	6.20518744372042	5.91587300788001	5.72455588359139	NA
**TC0600002553.mm.2**	Sema4f	sema domain, immunoglobulin domain (Ig), TM domain, and short cytoplasmic domain	-0.225662904513033	8.0433356684084	-0.706412716942329	0.504710799319926	0.999692817443324	-4.6101538919206	7.67780285515897	7.79229040217733	7.93222473314902	8.23707273889502	8.12015094440913	8.50047233666097	NA
**TC0600000214.mm.2**	Fscn3	fascin actin-bundling protein 3	-0.537368401972189	6.18200829382049	-0.706291575437761	0.50478124348021	0.999692817443324	-4.61015908304822	5.13826331803787	4.8960133176897	5.33061700086387	7.66090116076163	6.56029328841433	7.50596167715555	NA
**TC0200003310.mm.2**	6330409D20Rik	RIKEN cDNA 6330409D20 gene	-0.255272599902541	5.61226483229382	-0.706261478459975	0.504798745968456	0.999692817443324	-4.61016037265748	5.14939688259622	4.94468279857169	5.58204692836583	5.95243512802912	5.69110384178508	6.35392341441496	NA
**TC1900000725.mm.2**	Sfr1	SWI5 dependent recombination repair 1	0.254799568152747	8.07796326338848	0.70618875431055	0.504841039355483	0.999692817443324	-4.61016348861221	8.71321656119915	8.68730818649549	7.68810585727655	8.10032112719433	7.97181275065793	7.30701509750746	NA
**TC1700001488.mm.2**	Phf10	PHD finger protein 10	-0.409425473368598	7.31905790660084	-0.706065473187189	0.504912739929068	0.999692817443324	-4.61016877021406	7.86155227988326	8.04487840196234	7.66257908506893	5.93195382044218	7.44863889435494	6.96474495789337	NA
**TC1700000272.mm.2**	Zfp53	zinc finger protein 53	-0.228268733203349	6.98296802283883	-0.70602452443582	0.504936557294117	0.999692817443324	-4.61017052439084	6.99369089141793	6.81486244326873	6.81839838636914	6.99796028160069	6.90276791420957	7.37012822016696	NA
**TC1100002227.mm.2**	Grb10	growth factor receptor bound protein 10	0.774031677061736	6.55425020594631	0.705952838225146	0.504978254522821	0.999692817443324	-4.61017359513308	7.98077743111722	7.40747993591221	7.98454934775409	4.75810426363185	6.55400492800262	4.64058532925985	NA

**TSUnmapped00000039.mm.2**	Pemt	phosphatidylethanolamine N-methyltransferase	0.211995927835657	5.83236269333078	0.70592625192648	0.504993719368917	0.999692817443324	-4.61017473392359	5.78501874223277	5.71051677894188	6.04487152991132	5.91644471732937	5.90657018360488	5.63075420796449	NA
**TC0700004664.mm.2**	Prss53	protease, serine 53	-0.452283834671886	5.35042292355828	-0.705844429114277	0.50504131640897	0.999692817443324	-4.61017823850648	4.57897716609783	5.20031959455279	4.80335478169693	5.90598177398071	4.99808524542778	6.61581897959363	NA
**TC0300003210.mm.2**	Schip1	schwannomin interacting protein 1	-0.507863161801071	9.89390388997013	-0.70544400603252	0.505274288504875	0.999692817443324	-4.61019538495255	9.54520123745367	9.8461575509727	10.8098459920572	8.66832312183893	10.7284490421259	9.76544639537235	NA
**TC1300002459.mm.2**	S100z	S100 calcium binding protein, zeta	0.244788669855776	5.13832118191323	0.705366756508553	0.505319241548464	0.999692817443324	-4.61019869203381	4.42611656770125	4.63201477057888	5.37801608913948	5.75267045731593	5.04468789729258	5.59642130945128	NA
**TC1100002695.mm.2**	Rmnd5b	required for meiotic nuclear division 5 homolog B	0.258365246666578	6.39488220410012	0.705321894073131	0.505345349103133	0.999692817443324	-4.61020061249175	6.53622137539267	6.65717912595466	6.42732169954944	6.41898990874382	6.56767767236441	5.7619034425957	NA
**TC0700002714.mm.2**	Fam98c	family with sequence similarity 98, member C	0.29846617312342	6.42657877999571	0.705295863869185	0.505360497705506	0.999692817443324	-4.6102017267447	6.62421446025503	6.45469733347551	6.24691688275267	6.7918297334926	5.74242970643294	6.69938456356549	NA
**TC0100000462.mm.2**	Bmpr2	bone morphogenetic protein receptor, type II (serine/threonine kinase)	-0.711571033727856	7.89001350137626	-0.705190653831622	0.505421729040507	0.999692817443324	-4.6102062300798	9.85198390762458	9.93163613275648	8.15392077061509	4.91273867959531	7.86170259140122	6.6280989262649	NA
**TC0600002557.mm.2**	Tlx2	T cell leukemia, homeobox 2	-0.315906028302074	6.27772503619744	-0.70518801017265	0.505423267690002	0.999692817443324	-4.61020634323085	5.55261566317615	5.79740325806142	6.10171690626277	6.74054271340869	6.22438974916458	7.24968192711103	NA
**TC0500001312.mm.2**	Med13l	mediator complex subunit 13-like	-0.292190470241454	7.58691673425132	-0.705171196264621	0.505433053710712	0.999692817443324	-4.61020706287449	8.324537073591	8.34154440923089	7.47250857043793	6.66301042066362	7.71023329127858	7.00966664030589	NA
**TC1700001215.mm.2**	Arhgef33	Rho guanine nucleotide exchange factor (GEF) 33	0.326068475050187	5.2136698306673	0.70491247680872	0.505583649143694	0.999692817443324	-4.61021813463617	5.34618051176977	5.33342942867478	4.78470891135682	5.842564085473	4.60977706716694	5.3653589795625	NA
**TC1700002154.mm.2**	Capn11	calpain 11	-0.210377244703013	5.88153523342088	-0.704878712373432	0.505603304921234	0.999692817443324	-4.61021957935109	5.62864379339657	5.52467209894767	5.85409571786201	6.00347479152549	6.02652745613483	6.25179754265869	NA
**TC0200004269.mm.2**	Elp4	elongator acetyltransferase complex subunit 4	-0.898343252615755	6.11081773730384	-0.704864981165886	0.505611298610591	0.999692817443324	-4.61022016686865	7.46633741429241	7.34700834461538	7.0518806678272	2.97555641201466	6.80629576915458	5.01782781591879	NA
**TC1400000261.mm.2**	Dennd6a	DENN/MADD domain containing 6A	-0.452844202499098	7.49808006814901	-0.70481446291233	0.505640708772274	0.999692817443324	-4.61022232832351	8.02674181065581	7.87074511581388	8.06757291663921	6.02507962207386	7.38583688980548	7.61250405390579	NA
**TC1400001058.mm.2**	Siah3	siah E3 ubiquitin protein ligase family member 3	-0.289496858555967	5.7417605358905	-0.704744466459432	0.505681460394397	0.999692817443324	-4.61022532298055	5.87988464968361	5.44196601321485	6.11573236915083	5.15912704851547	5.90689858711642	5.94695454766182	NA
**TC0600003412.mm.2**	Bcat1	branched chain aminotransferase 1, cytosolic	0.316358017781171	8.78862758069058	0.704737666302038	0.505685419530572	0.999692817443324	-4.61022561390013	8.82880503649142	8.21523482380378	9.50287968330802	8.65734114639729	9.09486409279152	8.43264070135145	NA
**TC0200002474.mm.2**	Srsf6	serine/arginine-rich splicing factor 6	-0.207744146204154	7.46196100811543	-0.704708541704877	0.505702376463363	0.999692817443324	-4.61022685986539	7.80772366194196	7.88167632784386	7.17771127467406	7.15572760857517	7.4862213893549	7.26270578630264	NA
**TC0200005444.mm.2**	Zbtb46	zinc finger and BTB domain containing 46	-0.285015618214659	6.45698743162876	-0.704705668938964	0.50570404906633	0.999692817443324	-4.61022698276179	6.35896174971813	5.92391895674475	6.06529863747137	6.87920768596882	6.5391382719648	6.97539928790471	NA
**TC0600003110.mm.2**	Emg1	EMG1 N1-specific pseudouridine methyltransferase	-0.205896407916581	7.37657429260566	-0.704701833457323	0.505706282194415	0.999692817443324	-4.61022714684242	7.42990157801906	7.46739329418689	7.18987450730152	7.28530452649592	7.5235264491747	7.36344540045591	NA
**TC0300000706.mm.2**	Sh2d2a	SH2 domain containing 2A	-0.278051453967149	5.61668674991624	-0.704598908135469	0.505766210704753	0.999692817443324	-4.61023154971285	5.23231462728717	5.16505325526816	5.58919765910074	5.78412719540318	5.49075080275596	6.43867695968225	NA
**TC1600001379.mm.2**	Etv5	ets variant 5	-0.557794456999897	8.31290367870771	-0.704407957797106	0.505877404335545	0.999692817443324	-4.61023971682953	8.12447007254727	8.33832836097934	9.36267536731419	6.78684199504574	8.91638730436663	8.3487189719931	NA
**TC0900003342.mm.2**	Syncrip	synaptotagmin binding, cytoplasmic RNA interacting protein	-0.433814200936498	7.88194405527541	-0.704321624808685	0.505927682770793	0.999692817443324	-4.61024340884428	8.88298298447038	9.2068410797179	7.87908109341282	6.28802483938278	8.06076340005904	6.97397093460956	NA
**TC1500000907.mm.2**	Pdzrn4	PDZ domain containing RING finger 4	-0.241403021791793	5.41892098466786	-0.70430861082268	0.50593526211472	0.999692817443324	-4.61024396535674	6.03904155643722	5.42518133305739	5.2484966451748	5.03475184228969	5.46463238947882	5.30142214156926	NA
**TC0200001724.mm.2**	Fam98b	family with sequence similarity 98, member B	-0.908740175638054	7.25547615187437	-0.704289366656486	0.50594647005144	0.999692817443324	-4.61024478827457	9.00097058948939	9.04326789575972	8.13111712541923	3.70445191194126	7.26052976686102	6.39251962177558	NA
**TC0200004221.mm.2**	Pdhx	pyruvate dehydrogenase complex, component X	-0.233107448557607	6.52337972649888	-0.704144055844493	0.506031105343475	0.999692817443324	-4.61025100152253	6.93921727901847	6.7761021179419	6.36786431040031	6.11150772205855	6.72549015164417	6.22009677792991	NA
**TC1000003069.mm.2**	Usp15	ubiquitin specific peptidase 15	-0.334148465706223	8.24372633826594	-0.703916821538799	0.506163475039954	0.999692817443324	-4.61026071582735	9.10366322060208	9.02985619921814	8.27305775187142	7.05721308731007	8.2962075825088	7.70236018808513	NA
**TC0700003854.mm.2**	Nup98	nucleoporin 98	0.236209304041208	6.59787008886873	0.703882212215034	0.506183637834784	0.999692817443324	-4.61026219518314	7.41373493146705	7.43726533323246	6.48748483473822	6.11683460355941	6.31527511100132	5.8166257192139	NA
**TC0100002479.mm.2**	Crygb	crystallin, gamma B	-0.275584937367391	5.22773020435505	-0.703871750249008	0.506189732899392	0.999692817443324	-4.61026264236358	5.10239386830735	4.72241838670762	4.91805320557551	5.57714634261477	5.81084399341138	5.23552542951367	NA
**TC1400000866.mm.2**	Trim13	tripartite motif-containing 13	0.310659728948298	5.6653624044356	0.703843964866528	0.506205920693026	0.999692817443324	-4.61026382998315	5.9706054229923	5.51446062128705	5.32905635548682	6.2351575646286	5.79342638625954	5.14946807595929	NA
**TC1700000596.mm.2**	Smim40	small integral membrane protein 40	0.22404662866677	6.88125368201733	0.703795345246269	0.506234247353074	0.999692817443324	-4.61026590803083	6.16201407890741	6.43583406009442	7.14713879361006	7.42174481160779	6.90939407503187	7.21139627285244	NA
**TC1900001336.mm.2**	Ak3	adenylate kinase 3	0.483631810477199	8.12165959735802	0.703621974682022	0.506335264609087	0.999692817443324	-4.6102733172058	8.71986262396926	8.64023779901338	9.02330282484797	7.14525756621198	8.20915248858712	6.99214428151842	NA
**TC0600001875.mm.2**	Bmt2	base methyltransferase of 25S rRNA 2	0.311513871457437	8.05748390489494	0.703581119269332	0.506359071623004	0.999692817443324	-4.61027506301329	8.04382656503295	8.07374999800716	8.74390688043592	7.68127042418629	8.18802321778086	7.61412634392647	NA
**TC0200005146.mm.2**	Elmo2	engulfment and cell motility 2	-0.646794305217719	7.11006889513632	-0.70354700978313	0.506378948254674	0.999692817443324	-4.61027652050206	7.96321275307065	8.05411862503501	7.83338729458468	4.84135939655372	7.44455493231035	6.52378036926349	NA
**TC0400004075.mm.2**	Vamp3	vesicle-associated membrane protein 3	0.247746987473698	7.60549039081589	0.703438042564006	0.506442450185667	0.999692817443324	-4.61028117629882	7.02382587517745	7.38712907363404	7.80976173137605	8.04897895413958	7.97502296177751	7.38822374879073	NA
**TC1900000977.mm.2**	Sipa1	signal-induced proliferation associated gene 1	0.215501053384352	7.50475208012263	0.70326612750804	0.506542646330131	0.999692817443324	-4.61028852058225	7.22626666139285	7.32612892657797	7.64441573412043	7.80914376564642	7.34637875531591	7.67617863768223	NA
**TC1300000262.mm.2**	Hist1h1a	histone cluster 1, H1a	-0.2290615425187	7.26374394328432	-0.70321328920351	0.506573444350358	0.999692817443324	-4.61029077759628	7.31073535434064	7.84315065587356	7.02091734803009	6.96430993419707	7.28527180522301	7.15807856204155	NA
**TC0700003087.mm.2**	Svip	small VCP/p97-interacting protein	0.204297063360675	8.45272212528531	0.7031560515507	0.506606808017357	0.999692817443324	-4.61029322239239	8.05479025069461	7.95944655730853	8.7207941834779	8.83455085173713	8.55172666927265	8.59502423922103	NA
**TC0500002559.mm.2**	Gabra2	gamma-aminobutyric acid (GABA) A receptor, subunit alpha 2	0.353969884973412	8.81479071900433	0.703055322578711	0.506665526151089	0.999692817443324	-4.61029752448727	10.3992648919386	10.2473569481176	8.87879890169553	7.5962322202628	8.24766610865451	7.51942524335699	NA
**TC0300000923.mm.2**	Otud7b	OTU domain containing 7B	-0.246768956532312	6.86960438647963	-0.703048725433858	0.506669371993144	0.999692817443324	-4.6102978062332	6.66169863820249	6.70096575000734	6.64096249010002	7.03974951870166	6.78622761559034	7.38802230627596	NA
**TC0100000561.mm.2**	Rpe	ribulose-5-phosphate-3-epimerase	-0.232102306913954	7.11787903073638	-0.702919462444655	0.506744730428695	0.999692817443324	-4.61030332631486	6.80336820126607	7.02354069320497	7.01627967877175	7.19180065928792	7.59168502495962	7.08059992692796	NA
**TC0900001428.mm.2**	Col7a1/Mir711	collagen, type VII, alpha 1/microRNA 711	-0.228249020489031	8.6795070725339	-0.702780726335161	0.506825619730295	0.999692817443324	-4.61030925012306	7.9314024041375	8.08354505668739	8.67550267289115	9.12729579380906	8.99965922989904	9.25963727777923	NA
**TC1100000789.mm.2**	Specc1	sperm antigen with calponin homology and coiled-coil domains 1	0.316527578385003	6.50179801724322	0.702774275528404	0.5068293810425	0.999692817443324	-4.61030952554155	7.36964104439079	7.49067510111059	6.74048526469556	5.65127829266841	6.17355597952773	5.58515242106625	NA
**TC1800000595.mm.2**	Minar2	membrane integral NOTCH2 associated receptor 2	0.592955848395232	8.45362608145356	0.702708398241332	0.5068677935715	0.999692817443324	-4.61031233808091	9.82243745209014	8.62276376526335	9.39760290883094	7.33363057524822	8.54671247703767	6.99860931025103	NA
**TC0300002454.mm.2**	Psmb4	proteasome (prosome, macropain) subunit, beta type 4	-0.288157567942103	7.47274438610396	-0.702682284924271	0.50688302056984	0.999692817443324	-4.6103134529	8.34677207142848	8.10332113482657	7.18045191877848	6.72457706846377	7.69728620784011	6.78405791528634	NA
**TC0500003177.mm.2**	Oas1e	2'-5' oligoadenylate synthetase 1E	-0.338537471280191	4.48516890258855	-0.702568433001197	0.506949412538492	0.999692817443324	-4.61031831306999	4.0866456100469	4.14132266825663	3.87977671603554	5.12320838129814	4.53370806153897	5.1463519783551	NA
**TC0800000260.mm.2**	Defb50	defensin beta 50	0.395163940837393	4.87486868199526	0.702495057348032	0.506992204067917	0.999692817443324	-4.61032144506626	4.11996398797381	4.13687473636885	4.72217996823692	6.16917065641493	4.57255211862244	5.52847062435463	NA
**TC0800001657.mm.2**	Arglu1/Mir7654	arginine and glutamate rich 1/microRNA 7654	-0.219630128978368	8.37922258965609	-0.702342362400121	0.507081260953742	0.999692817443324	-4.6103279620014	8.53673298676519	8.21525290122615	8.21390347754789	8.32814121844635	8.63163114337383	8.34967381057714	NA
**TC0X00003185.mm.2**	Ribc1	RIB43A domain with coiled-coils 1	-0.234984849422934	6.96245902954531	-0.702224550376998	0.507149979937045	0.999692817443324	-4.61033298945267	6.26650041055987	6.5201265065139	6.85463859249014	7.40444018818598	7.31026416317608	7.4187843163459	NA
**TC0500002567.mm.2**	Commd8	COMM domain containing 8	0.561898502873908	7.38453714359271	0.702219205009561	0.507153097999649	0.999692817443324	-4.61033321754374	8.57492585556996	8.21104551919681	8.31606733808421	6.00645690818444	7.25250937159266	5.94621786892818	NA
**TC1100001378.mm.2**	C030037D09Rik	RIKEN cDNA C030037D09 gene	0.246295614042941	6.25919381282024	0.70221451080478	0.507155836236061	0.999692817443324	-4.61033341784815	6.39924206361048	6.04513574525647	6.33866605087266	6.46302209719751	6.46258814270239	5.8465087772819	NA
**TC1700001497.mm.2**	Dll1	delta like canonical Notch ligand 1	0.339447169801443	5.33639629455396	0.702188827229519	0.507170818221144	0.999692817443324	-4.61033451376387	5.61713819592875	5.75815747229941	5.63495847178874	5.02602974756049	4.36793353364907	5.61416034609728	NA
**TC0300000623.mm.2**	Ppid	peptidylprolyl isomerase D (cyclophilin D)	-0.358438711319762	8.80708109183228	-0.702184969628685	0.507173068498252	0.999692817443324	-4.61033467836483	9.04104534299633	9.06206972286252	9.19088779486641	7.82035923638124	9.18450267050734	8.54362178337983	NA
**TC1200001077.mm.2**	Vrk1	vaccinia related kinase 1	0.332499480359627	6.55052058978579	0.702119454271401	0.50721128695806	0.999692817443324	-4.61033747375652	7.08012271658366	6.93562913670253	6.23957345565544	6.73661186741845	5.5321687632025	6.77901759915213	NA
**TC1100000497.mm.2**	Olfr1395	olfactory receptor 1395	-0.263749916378214	5.5009948414538	-0.702111863839901	0.507215714967062	0.999692817443324	-4.61033779761082	4.52268170560015	5.08146058424287	5.40477469248852	6.03238877057316	5.87392793315877	6.09073536265934	NA
**TC0X00003041.mm.2**	Rab9b	RAB9B, member RAS oncogene family	0.302920111904541	7.45196557238921	0.702111439781742	0.507215962349443	0.999692817443324	-4.61033781570366	7.97350593129041	7.48601068001544	7.60696837558406	7.32209014783519	7.66495908841865	6.65825921119151	NA
**TC1900000036.mm.2**	Pold4	polymerase (DNA-directed), delta 4	-0.202186905600358	6.17661885490135	-0.702080276338625	0.507234142352197	0.999692817443324	-4.61033914529991	6.25249344984159	6.19746412224674	6.07260243012797	6.03008844293155	6.25571582543155	6.25134885882869	NA
**TC0500001528.mm.2**	Tmem248	transmembrane protein 248	0.296250004090472	7.77278451912217	0.701913044192856	0.507331708853588	0.999692817443324	-4.61034627957285	8.40839479670964	8.10338584079084	8.11502112007378	7.24369212263295	7.69012123965207	7.07609199487372	NA
**TC1600002139.mm.2**	Hmgn1	high mobility group nucleosomal binding domain 1	-0.273135254619223	7.68395785279171	-0.701774285419087	0.507412672764753	0.999692817443324	-4.61035219821296	7.67378276328512	7.75538266312362	7.66924750198703	7.3949080885645	7.35672809507929	8.25369800471069	NA
**TC0300000903.mm.2**	Ctss	cathepsin S	0.218526682741477	7.82949371346353	0.701701755261856	0.507454996520991	0.999692817443324	-4.61035529158959	8.28119387920067	8.70822453125055	7.60532493534502	7.60697368256145	7.35929273011291	7.4159525223106	NA
**TC1600002013.mm.2**	2310057N15Rik	RIKEN cDNA 2310057N15 gene	-0.35890848641012	5.14494597073404	-0.701605995893771	0.507510878834784	0.999692817443324	-4.61035937532662	4.59987372762985	4.85584642123805	4.87599812065859	5.47207123069945	4.84682511770968	6.2190612064686	NA
**TC1400000813.mm.2**	Zmym2	zinc finger, MYM-type 2	0.479548465908717	7.9960573900475	0.701593431799594	0.507518211164831	0.999692817443324	-4.61035991110283	9.0634127699095	8.92532381906014	8.42143231225284	7.05192002931357	8.13066859981479	6.38358680993419	NA
**TC0900002537.mm.2**	Lactb	lactamase, beta	-0.216314788096512	7.19200572867546	-0.701587717088084	0.507521546259165	0.999692817443324	-4.61036015479549	7.02864668788436	6.88735278484512	7.02137436313442	7.38032829843071	7.47200750939323	7.36232472836493	NA
**TC0700001118.mm.2**	Acan	aggrecan	-0.208206077223547	6.05271015190257	-0.701338441744736	0.507667036823179	0.999692817443324	-4.61037078325556	5.77498851293367	5.90715432004007	5.93978195564626	6.16907100635104	6.28301467289013	6.24225044355427	NA
**TC0100003776.mm.2**	Kctd3	potassium channel tetramerisation domain containing 3	0.578819580002985	6.99421416062808	0.701336863472292	0.507667958075277	0.999692817443324	-4.61037085054033	7.89841134359733	7.89336789286649	7.58177301585972	6.08379942779558	7.42172075493486	5.08621252871448	NA
**TC1700001606.mm.2**	Elob	elongin B	-0.26836735572167	10.0614317201278	-0.701305582513175	0.507686217283769	0.999692817443324	-4.61037218408483	10.5535420814986	10.4064441675172	10.0899512099743	9.34598347017954	10.234548224417	9.73812116718023	NA
**TC1200001816.mm.2**	Fbxo33	F-box protein 33	-0.563504234296432	7.18565442447993	-0.701157189772739	0.507772842416185	0.999692817443324	-4.61037850965722	8.12481075400554	7.90216776112304	7.33669682910153	5.64327295247755	8.09369260830211	6.01328564186983	NA
**TC1000000852.mm.2**	Reep6	receptor accessory protein 6	0.465677713046558	6.50322531290843	0.701093877485744	0.507809804279706	0.999692817443324	-4.6103812081901	7.73198389669946	7.42794233468849	7.19840825587054	5.19698228020733	6.09058587205974	5.37344923792501	NA
**TC1700000869.mm.2**	Mrpl14	mitochondrial ribosomal protein L14	-0.267542366115849	6.20320722484233	-0.701028724207425	0.50784784275711	0.999692817443324	-4.61038398500657	6.26382591340346	6.40539998096157	5.74170103857366	6.26576532265497	5.91183281785897	6.63071827560136	NA
**TC1400000112.mm.2**	Il3ra	interleukin 3 receptor, alpha chain	0.235395878934701	7.13734156594577	0.701007530831495	0.507860216500454	0.999692817443324	-4.61038488822269	6.94103332645697	6.81448335376862	7.26648492403474	7.50317731262446	6.86818289564672	7.43068758314308	NA
**TC1100000238.mm.2**	Xpo1	exportin 1	0.640280846303297	6.48729768182108	0.700985325560412	0.507873181250045	0.999692817443324	-4.61038583454239	8.04583940579778	8.22312299741372	6.59689202004976	5.37080067011103	6.76955280062712	3.91757819692708	NA
**TC0600000337.mm.2**	Agbl3	ATP/GTP binding protein-like 3	0.345781124403379	6.45044982510817	0.700724227457852	0.508025642002083	0.999692817443324	-4.61039696010451	6.82914118071459	6.55240831605388	6.80526681423451	6.20108903710914	6.79544309542698	5.51935050710991	NA
**TC0100003313.mm.2**	Tor1aip1	torsin A interacting protein 1	-0.424286272231872	5.92259072607636	-0.700686833758347	0.508047479432687	0.999692817443324	-4.61039855322894	6.44709862067067	6.23443382872914	6.32974565878455	4.67297402251275	6.39038199101727	5.46091023474378	NA
**TC0700004215.mm.2**	Usp31	ubiquitin specific peptidase 31	0.357028773159971	7.13282806187288	0.700670935746918	0.508056763849168	0.999692817443324	-4.61039923053054	8.34809415049012	8.55957824757279	7.06370669155836	6.23797006818881	6.91651020972988	5.67110900369735	NA
**TC1000002517.mm.2**	Lsm7	LSM7 homolog, U6 small nuclear RNA and mRNA degradation associated	-0.253557517622527	7.97873286859071	-0.700650384504494	0.508068765910584	0.999692817443324	-4.61040010605685	8.17369912766206	8.26487576829813	8.08412210642909	7.37923153374043	8.15655065925072	7.81391801616385	NA
**TC0500002756.mm.2**	Cxcl10	chemokine (C-X-C motif) ligand 10	-0.309476900627979	5.78389863820843	-0.700633363675122	0.508078706327906	0.999692817443324	-4.61040083116603	5.68566998169013	6.72904771150221	5.15330160673545	5.68155856066568	5.58559643171832	5.86821753693878	NA
**TC0400003124.mm.2**	Dock7	dedicator of cytokinesis 7	-0.379621508358111	5.31486476207438	-0.700476311904501	0.508170432900572	0.999692817443324	-4.61040752117096	6.26535498141733	6.00137405240772	5.23287192891604	4.19873633203645	5.72323331445769	4.46761796321102	NA
**TC1200000348.mm.2**	Etv1	ets variant 1	-0.256450920817382	8.09863729469383	-0.700366732049089	0.508234439771142	0.999692817443324	-4.61041218834983	8.28505705178591	8.50721479736739	8.12181087985615	7.52151415883131	8.32957299279562	7.8266538875266	NA
**TC1100000537.mm.2**	Mrnip	MRN complex interacting protein	0.227659457509479	6.20554141523396	0.700345533036755	0.508246822970153	0.999692817443324	-4.61041309118812	7.40038120917985	7.20159744633253	6.01789732144753	5.52539705400764	5.60559998352294	5.48237547691327	NA
**TC1300002247.mm.2**	Zfp459	zinc finger protein 459	0.229318026039377	5.23671340321439	0.700196956717166	0.50833361793664	0.999692817443324	-4.61041941830413	5.67093044472073	5.54486255335204	5.22538045051264	5.10618128613352	5.20461100881992	4.66831467574749	NA
**TC1700001718.mm.2**	Lemd2	LEM domain containing 2	-0.21794483746852	6.61346059664211	-0.700103575954936	0.508388173853266	0.999692817443324	-4.61042339442191	6.65800465547661	6.70255505645405	6.50575092091184	6.43640617558064	6.47818440234615	6.89986236908337	NA
**TC0800000483.mm.2**	Pdgfrl	platelet-derived growth factor receptor-like	0.270349102361462	5.60597543597197	0.699866699582422	0.508526581501626	0.999692817443324	-4.61043347880475	5.69496457674216	5.8613471695508	5.2102313043756	6.09988823275529	5.28701491755881	5.48240641484914	NA
**TC1800000566.mm.2**	Phax	phosphorylated adaptor for RNA export	-0.379857697679653	6.59354460573157	-0.699845918296264	0.508538725251566	0.999692817443324	-4.61043436339483	7.90665238964993	7.796844398791	6.35652088784843	5.19250683744616	6.73751486738371	5.57122825327019	NA
**TC0700003526.mm.2**	Sv2b	synaptic vesicle glycoprotein 2 b	0.338688224081953	7.39997625803996	0.699828915375428	0.508548661217444	0.999692817443324	-4.61043508713832	8.61861406469453	9.35982024598686	7.15863321175934	6.39076663110179	6.8864406941679	5.98558270052932	NA
**TC1600000745.mm.2**	Impg2	interphotoreceptor matrix proteoglycan 2	-0.382007392051519	4.87886950021471	-0.699808499184857	0.508560591957271	0.999692817443324	-4.61043595615364	4.52136165582826	4.44434289171104	4.18391195991391	5.58783687490906	4.822435238155	5.71332838077101	NA
**TC1500001657.mm.2**	Ndrg1	N-myc downstream regulated gene 1	0.205346518899399	7.38035633585182	0.69972814015725	0.508607553654032	0.999692817443324	-4.61043937645778	7.99994723950876	8.071073772456	7.07302672693975	7.23787829353273	6.89173875070022	7.00847323197346	NA
**TC0200001551.mm.2**	Cd59b	CD59b antigen	0.219114106104498	5.68031225197196	0.699629494102373	0.508665206139216	0.999692817443324	-4.61044357471939	6.80892988264027	6.56449686781649	5.46044349212587	5.11289399466614	5.13680988631342	4.9982993882696	NA
**TC0100001726.mm.2**	9130409I23Rik	RIKEN cDNA 9130409I23 gene	-0.276157820078164	5.1403694303675	-0.69961916230763	0.508671244677991	0.999692817443324	-4.61044401440376	4.33754245125537	4.48043293545467	4.94426995658805	5.79169282108126	5.41854010093393	5.86973831689171	NA
**TC0700002716.mm.2**	Psmd8	proteasome (prosome, macropain) 26S subunit, non-ATPase, 8	-0.346003585883983	8.40381050126255	-0.699609767068372	0.508676735876601	0.999692817443324	-4.61044441422757	8.80918100031984	8.74910904916829	8.76061429666882	7.32566859649077	8.46224773794386	8.3160423269837	NA
**TC0900003264.mm.2**	Ulk4	unc-51-like kinase 4	-0.354877197901565	5.72393643956921	-0.699599454786957	0.508682763099858	0.999692817443324	-4.61044485307255	5.02756427929659	6.30446378146702	5.20055102338068	5.95036706704358	6.0474694958978	5.81320299032959	NA
**TC0400004117.mm.2**	Tprgl	transformation related protein 63 regulated like	-0.317416486766534	8.00603023974621	-0.699598777993537	0.508683158667213	0.999692817443324	-4.61044488187371	8.63152298307205	8.50983240812521	8.14737877239856	6.98261776447492	8.11388275584506	7.65094675456149	NA
**TC0200005453.mm.2**	Sox18	SRY (sex determining region Y)-box 18	-0.294311770904321	6.07527586453714	-0.69937912512571	0.508811550421038	0.999692817443324	-4.61045422820534	5.34928008796294	5.92417336307358	5.68406366199385	6.610725435195	6.5232632613502	6.36014937764729	NA
**TC0600002056.mm.2**	Wdr91	WD repeat domain 91	-0.497299659378702	6.38373673673801	-0.699319736437851	0.508846268004817	0.999692817443324	-4.61045675485606	7.52262541807496	7.32170676977206	6.75732742393165	4.47441703298017	6.33114105123829	5.89520272443093	NA

**TC0200002513.mm.2**	Svs5	seminal vesicle secretory protein 5	-0.429604877243132	4.61281569195401	-0.699151564814679	0.508944586581901	0.999692817443324	-4.61046390875732	3.59918609858213	4.25503089246314	4.18738837803098	5.29434532506528	4.43924368549015	5.90169977209236	NA	
**TC0X00000660.mm.2**	Prrg3	proline rich Gla (G-carboxyglutamic acid) 3 (transmembrane)	0.206017187744221	6.28907471112338	0.699033877244596	0.509013397930648	0.999692817443324	-4.61046891436049	6.29983959423899	6.19747159449125	6.4907072972586	6.33387842949063	6.27742732218939	6.1351240290714	NA	
**TC1500002347.mm.2**	Parp10	poly (ADP-ribose) polymerase family, member 10	0.225887352578241	6.37925455677456	0.698907693143336	0.50908718391355	0.999692817443324	-4.61047428066727	6.19931353817185	6.52648562179993	6.63316905612398	6.36758238679207	6.11315534857051	6.43582138918906	NA	
**TC1300000726.mm.2**	Spata31d1b	spermatogenesis associated 31 subfamily D, member 1B	-0.373604543060065	4.82132301609102	-0.698626823199257	0.509251447324333	0.999692817443324	-4.61048622286747	4.23893546307945	4.32072959666484	4.38530893962151	5.42522303571935	4.66077240139857	5.89696866006242	NA	
**TC1400002784.mm.2**	Gm3020/Gm10409	predicted gene 3020/predicted pseudogene 8348	0.353907259498019	7.13228211670472	0.698528333819989	0.50930905585276	0.999692817443324	-4.61049040967416	7.80419620730698	6.89801252663894	7.70288361305117	6.69676562958806	7.16457512132608	6.52725960231712	NA	
**TC1500001548.mm.2**	Has2	hyaluronan synthase 2	0.272835046163293	4.98534863163309	0.698426338584804	0.50936871951753	0.999692817443324	-4.61049474506396	4.67602510650265	4.73502793486657	4.92112603226536	5.6022283881126	4.65964032183948	5.31804400621189	NA	
**TC0200000242.mm.2**	Stam	signal transducing adaptor molecule (SH3 domain and ITAM motif) 1	0.314095950165165	7.16868046350925	0.697855601792616	0.509702664924925	0.999692817443324	-4.61051899620877	7.82487049482142	7.55246920761455	7.60938663362237	6.52208085585257	7.01959968790154	6.48367590124307	NA	
**TC1700001145.mm.2**	Ypel5	yippee like 5	-0.442168899721116	8.46189306111979	-0.697837027763376	0.509713538154751	0.999692817443324	-4.61051978540727	9.24488927507845	9.07964580942735	8.7809942425058	7.00024849887956	8.80805108419828	7.85752945662931	NA	
**TC0200000408.mm.2**	Lrrc26	leucine rich repeat containing 26	0.373305365878416	5.19857531353916	0.697826762112612	0.509719543184315	0.999692817443324	-4.61052022125181	4.04829730970943	4.28359291166611	5.10250047223199	6.70058572357614	5.47927562622607	5.57719983782522	NA	
**TC0700004509.mm.2**	Sirt3	sirtuin 3	-0.204692691207619	8.78581023406049	-0.697785765621268	0.509743536776687	0.999692817443324	-4.61052196262484	8.90869448843405	8.84674032476748	8.62206103592643	8.65295956844665	8.74200010370672	8.94240588308161	NA	
**TC1300002637.mm.2**	Ndufaf2	NADH:ubiquinone oxidoreductase complex assembly factor 2	-0.2240564313567	6.18358531441328	-0.69771999095797	0.509782033573818	0.999692817443324	-4.61052475632389	6.44104734634693	5.96452043499167	6.01385935486554	6.11005626634832	6.20209083272537	6.36993765120189	NA	
**TC1200001692.mm.2**	Nova1	neuro-oncological ventral antigen 1	0.323170075606857	9.28550473731138	0.697597562456726	0.509853693957123	0.999692817443324	-4.61052995581651	9.56639839739933	9.21537909436965	9.74707487164943	9.04172067000707	9.59028154057064	8.55217384987214	NA	
**TC0400000395.mm.2**	Ccl19	chemokine (C-C motif) ligand 19	-0.343881757534575	7.21606847102411	-0.69756423397108	0.509873203069975	0.999692817443324	-4.61053137115002	7.6032731812806	7.67411800239167	7.5459091751996	6.11971888850201	7.09795227838225	7.25543930038851		2
**TC0100001011.mm.2**	Tnfrsf11a	tumor necrosis factor receptor superfamily, member 11a, NFKB activator	0.273449337169337	6.14723671403073	0.697503671553483	0.509908655043877	0.999692817443324	-4.61053394287886	5.53440034267529	5.63919779284242	6.13002025491191	6.99834015659077	6.09709303583363	6.48436870133038	NA	
**TC1500000388.mm.2**	Mal2	mal, T cell differentiation protein 2	-0.452350857411153	8.91778912281119	-0.697491231248774	0.509915937537507	0.999692817443324	-4.61053447112517	9.51254904005072	9.67317416981484	9.17306876450916	7.53508614158048	9.44654002911461	8.16631659179733	NA	
**TC0100000227.mm.2**	Cfc1	cripto, FRL-1, cryptic family 1	0.373652181152117	6.04922624654525	0.697439125885608	0.50994644050025	0.999692817443324	-4.61053668357424	5.08385866438717	5.3402576658315	6.01727023912002	7.29200251655853	5.79645117612904	6.76551721724527	NA	
**TC1100002134.mm.2**	Znrf3	zinc and ring finger 3	0.212569910917297	6.73533546546315	0.697432296412521	0.509950438625391	0.999692817443324	-4.61053697355202	6.76135185371716	6.94197908807593	6.64308620290471	6.92382463350548	6.64427940423918	6.49749161033641	NA	
**TC1200000206.mm.2**	Mboat2	membrane bound O-acyltransferase domain containing 2	0.426700529579006	6.25175189209324	0.697335431575271	0.510007147653988	0.999692817443324	-4.61054108618744	5.92961976650341	5.92000086949016	6.14457681654348	7.11256907131846	5.36271938104107	7.04102544766286	NA	
**TC1100000916.mm.2**	Trp53	transformation related protein 53	-0.204701984677256	6.98382999651203	-0.697241452368357	0.510062171238035	0.999692817443324	-4.61054507590952	7.43007586407656	7.44672945543905	6.67152728027778	6.63685806482326	6.96532015900284	6.75246915545271	NA	
**TC0500000277.mm.2**	Dnajb6	DnaJ heat shock protein family (Hsp40) member B6	-0.510148513254837	9.33154722208188	-0.697132875054277	0.510125746643681	0.999692817443324	-4.61054968488196	10.3620143534451	10.2543323916952	9.5474271850337	7.62889259538694	9.82857448916926	8.36804231776106	NA	
**TC0100002900.mm.2**	Vps4b	vacuolar protein sorting 4B	0.352922528497434	6.83957775486149	0.696823816722122	0.510306738243031	0.999692817443324	-4.61056280116856	7.67561794815453	7.17653413479954	7.38969907343844	6.05588067816642	6.59855484265443	6.14117985195556	NA	
**TC1500001796.mm.2**	Zfp647	zinc finger protein 647	0.345600398508876	5.49855243155046	0.696725335355304	0.510364419974251	0.999692817443324	-4.61056697978181	5.37657372574826	5.49077347191861	5.32488593301256	6.08269816131426	4.74267433628367	5.9737089610254	NA	
**TC0100001882.mm.2**	Cd34	CD34 antigen	-0.444739725053446	6.9034994823711	-0.696720622783868	0.510367180291123	0.999692817443324	-4.61056717972779	6.78604351394157	7.37646337426879	6.8606770928672	6.32382818508748	7.97800551117277	6.0959792168888	NA	
**TC1100000442.mm.2**	Ebf1	early B cell factor 1	0.24163153116019	7.0578701311018	0.696607400626241	0.510433501371092	0.999692817443324	-4.61057198324574	7.46661462109328	7.1749571499633	7.34804029285392	6.74641574608338	6.90389523338215	6.70729774323478	NA	
**TC0500003724.mm.2**	Gtf3c2	general transcription factor IIIC, polypeptide 2, beta	0.237868619653254	7.38487378368237	0.696596446140645	0.510439918375447	0.999692817443324	-4.61057244796624	7.570617311727	7.7917813444466	7.31223041387897	7.3990602287346	7.42658057458679	6.80897282872027	NA	
**TC0700004165.mm.2**	Arl6ip1	ADP-ribosylation factor-like 6 interacting protein 1	-0.387439900352931	8.83182155525723	-0.69652271493169	0.510483110589162	0.999692817443324	-4.61057557571596	10.1212627076723	10.1398973296168	8.62850925394324	7.34893549283093	8.94185418928837	7.81047035819166	NA	
**TC1600000250.mm.2**	Ufd1	ubiquitin recognition factor in ER-associated degradation 1	0.232892463706292	7.36186193333747	0.696357010481235	0.510580190012976	0.999692817443324	-4.61058260418142	8.27432293438829	7.94287040378785	7.03816134698599	7.17172024764464	6.62250247677635	7.1215941904417	NA	
**TC0X00003270.mm.2**	Pdha1	pyruvate dehydrogenase E1 alpha 1	-0.251174767951692	8.28001133200488	-0.696273331198672	0.510629218837943	0.999692817443324	-4.61058615303205	8.35014081932828	8.11528707560784	7.86677420016493	8.48937108042995	8.22361664265698	8.63487817384129	NA	
**TC0400003841.mm.2**	Nbl1	NBL1, DAN family BMP antagonist	0.215461634502896	7.31374345700866	0.696043382033678	0.510763964974703	0.999692817443324	-4.61059590361184	7.71027188175715	7.47625360207381	7.1679542739117	7.39547498970171	7.19283121069003	6.93967478391759	NA	
**TC1100004284.mm.2**	Afmid	arylformamidase	0.321755997298839	6.0938570964026	0.695992645715066	0.510793698679622	0.999692817443324	-4.61059805467837	5.94415664083187	5.85777907971451	5.87128175483356	6.83107767139989	5.59403078320842	6.46481664842735	NA	
**TC1100003784.mm.2**	Hcrt	hypocretin	0.247540505945682	5.2886455445889	0.6959800248612	0.51080109522801	0.999692817443324	-4.61059858974669	4.78467632208168	4.92157070397033	5.27155703557649	5.98879659110989	5.29314694915402	5.47212566564099	NA	
**TC1100004104.mm.2**	Mrpl38	mitochondrial ribosomal protein L38	0.212686500306418	7.90443618173546	0.695644923133155	0.510997509705232	0.999692817443324	-4.61061279399317	8.32023738157057	8.1967053286435	8.00758240080618	7.65994128959958	7.65400589946875	7.58814479032417	NA	
**TC0400002407.mm.2**	Lingo2	leucine rich repeat and Ig domain containing 2	0.436560350450478	6.69928314639879	0.695471275030101	0.511099310100031	0.999692817443324	-4.61062015260643	7.75436332358764	7.56508828328198	7.21470216068829	5.65998182552374	6.62388113699493	5.37768214831615	NA	
**TC1500001348.mm.2**	Nipal2	NIPA-like domain containing 2	0.485206501138704	5.68150036980063	0.695405928804182	0.511137622443568	0.999692817443324	-4.61062292140981	6.43397386763306	5.91522555698808	6.62075418273581	4.73435371549422	5.77798800073714	4.60670689521548	NA	
**TC1000002451.mm.2**	Rrp1	ribosomal RNA processing 1 homolog (S. cerevisiae)	0.368173459146227	9.37990677051819	0.695284819598234	0.511208633442679	0.999692817443324	-4.61062805246121	10.633138246816	10.3191484537354	9.64801456575154	8.38373585467358	9.1080179311512	8.18738557098147	NA	
**TC0100002063.mm.2**	Ogfrl1	opioid growth factor receptor-like 1	0.264927044051165	8.67493920796802	0.695282510324505	0.511209987521384	0.999692817443324	-4.61062815029222	9.06337738870338	8.98693962635642	9.05262254160043	8.21196361882491	8.51982470278778	8.21490736953523	NA	
**TC0800002729.mm.2**	Fam192a	family with sequence similarity 192, member A	-0.207697131166958	8.41888805014607	-0.69527241727628	0.511215905765211	0.999692817443324	-4.61062857787545	8.90947661613548	8.714485852532	8.16851536492976	8.06847042000775	8.42353719919234	8.2288428480791	NA	
**TC0900001885.mm.2**	Dock6	dedicator of cytokinesis 6	-0.239250556024347	6.19338284406666	-0.695246410475947	0.511231155534953	0.999692817443324	-4.61062967961016	5.92894778504083	5.68889241378993	6.10279369520309	6.42918418155717	6.23270111996848	6.77777786884047	NA	
**TC0400003311.mm.2**	Cyp4x1	cytochrome P450, family 4, subfamily x, polypeptide 1	0.242779611744558	6.21319884653382	0.695228430950419	0.511241698474747	0.999692817443324	-4.61063044126522	6.87100610243528	6.20932346748903	6.11259983220424	6.22961153417963	5.81096504619469	6.04568709670007	NA	
**TC0300000824.mm.2**	Sprr2d	small proline-rich protein 2D	-0.376535031008451	5.74703564475107	-0.695121042411997	0.511304672546034	0.999692817443324	-4.61063499019795	4.94791003918079	5.06843545548314	5.30515740491739	6.5512417509954	5.7697704724905	6.83969874543919	NA	
**TC1300002429.mm.2**	Cmya5	cardiomyopathy associated 5	0.34697288843073	6.14739394608564	0.695057200033745	0.511342112962886	0.999692817443324	-4.6106376942913	5.78681962233732	5.86552294861942	5.83336442639228	7.129619014817	5.79657985188782	6.47245781246	NA	
**TC0800002377.mm.2**	Gatad2a	GATA zinc finger domain containing 2A	0.204806751214242	7.15204837760529	0.694952402541689	0.51140357541222	0.999692817443324	-4.61064213267795	7.05165383790389	7.27489934624878	7.28539033398421	7.21228495796955	7.04788274503251	7.04017904449277	NA	
**TC1000001565.mm.2**	Stac3	SH3 and cysteine rich domain 3	0.211977689725213	7.93370930213526	0.694911270573109	0.511427700124271	0.999692817443324	-4.61064387456714	7.78937537453449	7.73200870587139	8.30248830043109	7.94992525549697	7.95719292481252	7.87126525166512	NA	
**TC0700001119.mm.2**	Abhd2	abhydrolase domain containing 2	0.231709745150934	8.35476039591217	0.694844628459087	0.511466788613693	0.999692817443324	-4.61064669662074	8.83753057444809	9.22931512478972	8.3350818332985	7.92748624997003	8.01002044994173	7.78912814302494	NA	
**TC0400003807.mm.2**	Cela3a	chymotrypsin-like elastase family, member 3A	0.254510925915542	5.33945430553243	0.694749053098159	0.511522851102742	0.999692817443324	-4.61065074354898	4.77165884478172	4.47713711429212	5.51207504844034	6.13640081453557	5.35310588373532	5.78634812740951	NA	
**TC0500003588.mm.2**	Gm4871	predicted gene 4871	-0.379955572572442	5.2859146319194	-0.694667921916472	0.511570444081114	0.999692817443324	-4.61065417855193	4.79497558145757	4.25394678170339	4.9451946244213	6.00813251718398	5.27280834065915	6.44042994609102	NA	
**TC0900001237.mm.2**	Prr23a1	proline rich 23A, member 1	0.290221412910546	4.83303906155127	0.69444033032543	0.511703968699827	0.999692817443324	-4.61066381296351	4.28859752771597	4.68335092856016	4.75806669088234	5.54455767854396	4.54078918042262	5.18213236318259	NA	
**TC0X00001277.mm.2**	Drp2	dystrophin related protein 2	-0.200835580205681	6.62651168592922	-0.69443000811846	0.511710025123716	0.999692817443324	-4.61066424986882	7.08538201179691	7.04947893312862	6.30858659073226	6.30268241438695	6.56267690704877	6.4502632584818	NA	
**TC0700001198.mm.2**	Stard5	StAR-related lipid transfer (START) domain containing 5	0.21248549086669	6.65450621882782	0.694376549021798	0.511741392316624	0.999692817443324	-4.61066651254209	7.21622181809106	6.96802844649343	6.68328328896423	6.40059572609367	6.31320271284584	6.34570532047867	NA	
**TC1700002027.mm.2**	H2-M10.4	histocompatibility 2, M region locus 10.4	-0.214075994651539	5.05579678798803	-0.694362860227638	0.511749424435274	0.999692817443324	-4.61066709190424	4.2915828157428	4.38945330057537	5.18314653399115	5.42964977716232	5.37016475118518	5.67078354927136	NA	
**TC0300000951.mm.2**	Ankrd34a	ankyrin repeat domain 34A	-0.263930550529824	6.4210343499835	-0.694258424354154	0.511810706553742	0.999692817443324	-4.61067151175595	7.27851867506929	6.57891121036925	5.87042725592748	6.20003030077392	6.52214280687159	6.07617585088946	NA	
**TC1400001752.mm.2**	Nrg3	neuregulin 3	0.241077735301241	6.20957935164651	0.693899615597769	0.512021288998465	0.999692817443324	-4.61068669328037	6.64594178961368	6.81153108068809	5.73541325932816	6.40566609576173	5.88855576438851	5.7703681200989	NA	
**TC0900002934.mm.2**	Il20rb	interleukin 20 receptor beta	0.213791993129365	5.12391249797432	0.693803803639766	0.512077529928324	0.999692817443324	-4.61069074620319	4.29246760862126	4.55067091607714	5.55636521370408	5.60759501099905	5.21806739457311	5.51830884387129	NA	
**TC0700002974.mm.2**	Atf5	activating transcription factor 5	-0.249909609880048	7.31904182535651	-0.693771921074335	0.512096245656062	0.999692817443324	-4.61069209477082	6.86195786444855	6.30379398139649	7.36652902848883	7.75781091477812	7.62338547898712	8.00077368403992	NA	
**TC1500001881.mm.2**	Fam227a	family with sequence similarity 227, member A	0.206531515818655	5.5263303377952	0.693689677751667	0.512144526248419	0.999692817443324	-4.6106955732873	5.41899603051278	5.24320445210718	5.63439787778426	5.82002441011003	5.57750147251898	5.46385778373799	NA	
**TC1700000913.mm.2**	Guca1b	guanylate cyclase activator 1B	0.207452710877756	6.77131347386035	0.693620695245923	0.51218502441826	0.999692817443324	-4.61069849069989	6.08570992695175	6.12990502907703	7.09502783091186	7.31855782353254	6.90345281247033	7.09522742021856	NA	
**TC1200001893.mm.2**	Timm9	translocase of inner mitochondrial membrane 9	-0.291705465159958	7.50344856878544	-0.693606645432686	0.512193273021761	0.999692817443324	-4.61069908486956	7.18412373514366	7.19843890152548	6.96321974230531	8.06413918055649	7.7910820724142	7.81968778076753	NA	
**TCX_GL456233_random00000005.mm.2**	Vamp7	vesicle-associated membrane protein 7	0.480768729423103	6.99898590773409	0.693500240121207	0.512255746068714	0.999692817443324	-4.61070358448836	8.24525509982494	8.38100271188785	7.03349741082555	6.13110013594342	7.13673279740635	5.06632729051642	NA	
**TC0400002271.mm.2**	Wwp1	WW domain containing E3 ubiquitin protein ligase 1	0.46312209144415	6.73521263213564	0.693498030360553	0.512257043523411	0.999692817443324	-4.61070367792836	7.33202997228738	7.516779803029	7.3321209921257	5.91223410806719	6.96895106930506	5.34915984799953	NA	
**TC1900000889.mm.2**	Ndufs8	NADH:ubiquinone oxidoreductase core subunit S8	-0.429976744465165	8.82616950681978	-0.693490479137956	0.512261477218463	0.999692817443324	-4.61070399723111	9.68557128509928	9.4843205004143	9.07194640201496	7.39163948122242	9.16636959530936	8.15716977685836	NA	
**TC0800002012.mm.2**	Rbpms	RNA binding protein gene with multiple splicing	-0.215893184750303	5.996160582923	-0.693393511900232	0.512318413682392	0.999692817443324	-4.61070809725627	5.56735405001785	5.94709175407296	6.10608384794102	5.90928181403226	6.24650954721816	6.20064248425572	NA	
**TC0300000312.mm.2**	Larp1b	La ribonucleoprotein domain family, member 1B	-0.350881064847328	5.13217915542688	-0.693359108007882	0.512338615679947	0.999692817443324	-4.61070955184112	5.78362424973655	5.50266894716127	5.24057959453507	4.16193020844934	5.54951490796168	4.55475702471738	NA	
**TC0200005386.mm.2**	Ppp1r3d	protein phosphatase 1, regulatory subunit 3D	0.289525765813501	6.42232231436639	0.693276146718989	0.512387332756916	0.999692817443324	-4.61071305919956	5.87229465122501	5.73418903642386	7.37485891094871	6.37839195413954	6.7928270633425	6.38137227011875	NA	
**TC0600002480.mm.2**	Atoh8	atonal bHLH transcription factor 8	-0.237879495215823	6.12467511246547	-0.693174504395433	0.512447023941209	0.999692817443324	-4.61071735591954	5.45300099113034	5.84561830447769	6.01868956980235	6.46814662457422	6.32580316655564	6.63679201825257	NA	
**TC0700003566.mm.2**	Kif7	kinesin family member 7	0.325986413854195	5.71847275925171	0.693058523088	0.512515141461565	0.999692817443324	-4.6107222582289	4.92696330992763	5.08989248435446	5.59971906809161	6.87325772637669	5.72666344079369	6.09434052596621	NA	
**TC0300002219.mm.2**	Gucy1b1	guanylate cyclase 1, soluble, beta 1	0.396538226199715	8.37744636004399	0.69301789578344	0.512539003864877	0.999692817443324	-4.61072397532622	9.6516981743587	9.55069276238722	8.64754913108143	7.28013270687729	8.13055715315792	7.00404823240138	NA	
**TC1100003234.mm.2**	Rph3al	rabphilin 3A-like (without C2 domains)	-0.264155599401553	5.90732611844624	-0.692957144592467	0.512574687358812	0.999692817443324	-4.61072654281475	5.45558186595898	5.24477781827282	6.08730576833668	6.02033714548458	5.88259708379311	6.75335702883125	NA	
**TC1700000542.mm.2**	Cryaa	crystallin, alpha A	-0.28692419850803	5.77550466881424	-0.692799228051079	0.512667450527745	0.999692817443324	-4.61073321597137	5.37248269045384	5.1902871190998	5.60445209429099	6.15375280886686	5.71622338524397	6.61582991492995	NA	
**TC0600002560.mm.2**	Wbp1	WW domain binding protein 1	0.43686071031979	6.53787178969574	0.692671297513106	0.512742607351521	0.999692817443324	-4.61073862118063	6.84594097025509	6.25424330936516	7.63259018114644	5.86779375845043	6.69721463145165	5.92944788750565	NA	
**TC1100003816.mm.2**	Sost	sclerostin	-0.279471095348531	6.10264697210613	-0.6925249848989	0.512828572084015	0.999692817443324	-4.61074480215982	5.33768449964434	5.79614387365974	6.02447707488675	6.43707855943108	6.10641298449767	6.91408484051722	NA	
**TC0500000468.mm.2**	Cd38	CD38 antigen	-0.275534453503305	5.97787619831709	-0.692328164584252	0.512944226961553	0.999692817443324	-4.6107531153336	5.95592031739192	5.74071087553103	6.35572830624322	5.45405023874326	6.35070721844048	6.01014023355261	NA	
**TC1100001567.mm.2**	Pnmt	phenylethanolamine-N-methyltransferase	0.316269594152047	5.56971953690169	0.692282195748516	0.51297124145251	0.999692817443324	-4.61075505668801	4.81923452619083	5.40566940058262	5.40156449634477	6.51141174512564	5.53196523411832	5.74847181904799	NA	
**TC1100000919.mm.2**	Sox15	SRY (sex determining region Y)-box 15	-0.339852112432425	6.99651983368774	-0.69222650447783	0.513003970762167	0.999692817443324	-4.61075740851385	5.91402047122101	5.93385131779037	6.66150691653758	8.06426457758751	7.57030351863661	7.83517220035333	NA	
**TC0800002066.mm.2**	Dlc1	deleted in liver cancer 1	-0.274491827865818	6.34753338869141	-0.692203523446878	0.513017476923799	0.999692817443324	-4.61075837895569	6.31638050940625	7.0115582579997	6.03235108075435	6.07178787737511	5.98165649012097	6.67146612011612	NA	
**TC1600000450.mm.2**	Muc4	mucin 4	0.250284395139485	6.45946675792169	0.691542979802126	0.513405783203254	0.999692817443324	-4.61078626229689	5.95379740471526	5.93671093687681	6.4741058611741	7.20932463693442	6.44821020617828	6.73465150165127	NA	
**TC1100003693.mm.2**	Top2a	topoisomerase (DNA) II alpha	-0.211665006386177	5.7006717988586	-0.691467610218343	0.513450101993002	0.999692817443324	-4.61078944261505	5.53275739541128	5.84023361375782	5.59008044423786	5.61377444136721	5.70375290202953	5.92343199634789	NA	
**TC0600002718.mm.2**	Iqsec1	IQ motif and Sec7 domain 1	-0.206442260068048	7.83348023059422	-0.69141755094088	0.513479539200429	0.999692817443324	-4.61079155479169	8.0402405960229	7.81078292049534	7.7359720096628	7.63251466379268	7.81748362610372	7.96388756748785	NA	
**TC1500001570.mm.2**	Fbxo32	F-box protein 32	0.242703041911303	6.76872042792612	0.691387729507428	0.513497076125478	0.999692817443324	-4.61079281300951	6.69488300539832	7.05479105079549	6.95461503857046	6.71941225902231	6.88509802870525	6.30352318506491	NA	
**TC1800000658.mm.2**	St8sia3	ST8 alpha-N-acetyl-neuraminide alpha-2,8-sialyltransferase 3	-0.629704369536094	9.89814644555015	-0.691383345322729	0.513499654341459	0.999692817443324	-4.6107929979825	10.658131894514	10.8923398388758	10.6035450269818	7.68595407343768	10.2843588975652	9.26454894192644	NA	
**TC1000002248.mm.2**	H2afy2	H2A histone family, member Y2	0.219861886314595	6.65865368161093	0.691365848278846	0.513509943945632	0.999692817443324	-4.61079373619113	7.31875951895414	7.21435799686981	6.57147135669976	6.35779281653566	6.4582735750337	6.03126682557253	NA	
**TC0100000895.mm.2**	Erfe	erythroferrone	0.233239217397727	5.71457627094221	0.691133203703259	0.513646769528189	0.999692817443324	-4.61080355027853	5.1896633231988	5.55129816763026	5.74731718465268	6.25917010015715	5.82049924697701	5.71950960303737	NA	
**TC0200004053.mm.2**	Olfr1232	olfactory receptor 1232	0.23830595166013	5.92256573749005	0.691059595089311	0.513690065994737	0.999692817443324	-4.61080665494731	5.10042178895864	5.70586548098389	6.14645541987592	6.4564041092831	6.02777583886315	6.0984717869756	NA	
**TC1900000711.mm.2**	Cnnm2	cyclin M2	-0.21287735848821	7.07843355168826	-0.691013350561198	0.51371726816237	0.999692817443324	-4.61080860532825	7.63346647670688	7.67268539138939	6.82332422701664	6.54602313551179	7.03930449784823	6.75579758165661	NA	
**TC0X00001922.mm.2**	Slc9a7	solute carrier family 9 (sodium/hydrogen exchanger), member 7	-0.214998691161745	6.77703674789662	-0.690935358849801	0.513763146921413	0.999692817443324	-4.61081189444273	7.92199463151915	7.56770291461633	6.29756884645869	6.07369393300169	6.44506148583209	6.35619867595177	NA	
**TC0X00002441.mm.2**	Hcfc1	host cell factor C1	0.224946850676046	6.53643268366595	0.690934389350716	0.513763717247661	0.999692817443324	-4.61081193532733	7.84722255689156	7.70994225945723	6.15660434446705	5.89905814903244	5.57035579241319	6.03541299997342	NA	
**TC1400000815.mm.2**	B020004C17Rik	RIKEN cDNA B020004C17 gene	-0.309188962190747	4.95279970895774	-0.690908848577216	0.513778742240703	0.999692817443324	-4.61081301238843	4.51725607398409	4.32779338563028	4.54689174332935	5.57979369154593	5.05193830853921	5.69312505071756	NA	

TC0700002179.mm.2	Sbk3	SH3 domain binding kinase family, member 3	0.276865758630255	5.38719327062324	0.690817984247022	0.513832197747136	0.999692817443324	-4.6108168439249	5.22202794385282	5.03586440528127	5.16246468815788	6.14703470777506	5.38038945643988	5.37537842223255	NA
TC1000001708.mm.2	Ust	uronyl-2-sulfotransferase	0.290119025577368	6.9997953239552	0.690571033343282	0.513977497255906	0.999692817443324	-4.61082725540491	7.58342449136848	7.43720090797022	7.23786442912973	6.54132786864387	7.01830053046761	6.18065371615126	NA
TC0800000968.mm.2	Syce2	synaptonemal complex central element protein 2	0.209780229909097	6.90129681913765	0.690557059702323	0.513985719780858	0.999692817443324	-4.61082784445389	6.8491984274488	6.7028295085152	6.91637242306445	7.2212842962756	6.8072706732336	6.91082558628826	NA
TC1100001841.mm.2	Map2k6	mitogen-activated protein kinase kinase 6	-0.488591101981097	5.37169487384852	-0.690468955457892	0.514037565024124	0.999692817443324	-4.61083155822532	6.30450726982765	6.36723919914172	5.73389033578729	3.55672994929249	5.53212144291574	4.73568104612624	NA
TC1900000668.mm.2	Tlx1	T cell leukemia, homeobox 1	-0.320678381524659	6.00728946598413	-0.690365928691639	0.514098195779882	0.999692817443324	-4.61083590057129	5.28614126529216	5.56345390268025	5.64253931556955	6.63385311687197	6.05463495350967	6.86311424198116	NA
TC1900001529.mm.2	Pyroxd2	pyridine nucleotide-disulphide oxidoreductase domain 2	0.375834082209606	5.73603286117248	0.690066740253767	0.514274293032437	0.999692817443324	-4.61084850800118	5.10712185624704	4.95100273990924	5.47269779011405	7.08217257753486	6.08765002918635	5.71555217404335	NA
TC0300003239.mm.2	Gm128	predicted gene 128	0.412394738669924	4.90989246581931	0.689862312018099	0.514394638529577	0.999692817443324	-4.61085712005295	4.10261473361382	3.8782471406955	4.88661209849731	6.26502910047588	4.53415399863072	5.79269772300263	NA
TC1100003778.mm.2	Zfp385c	zinc finger protein 385C	-0.300281380338261	7.01686911160515	-0.689768184008955	0.514450057181599	0.999692817443324	-4.61086108480308	6.50396104487733	6.27728120173552	6.64825462499436	7.71145020617641	7.25996063369123	7.70030695815607	NA
TC1400000319.mm.2	Glt8d1	glycosyltransferase 8 domain containing 1	0.339710652596827	5.69774432177791	0.689749410154241	0.51446111090854	0.999692817443324	-4.61086187552609	6.42373283192755	6.08422635976964	6.19504219912167	4.98392182296029	5.59470188454666	4.90484083234164	NA
TC0400004089.mm.2	Tas1r1	taste receptor, type 1, member 1	-0.209698954263432	6.15689599121164	-0.68962668300595	0.51453337436388	0.999692817443324	-4.61086704419772	5.96608684804565	6.16759997876541	6.09636426076542	6.09778134520054	6.45407405486468	6.15946945962815	NA
TC0200004406.mm.2	Fsip1	fibrous sheath-interacting protein 1	-0.224713149453165	5.93821255570187	-0.68958924399332	0.51455542028629	0.999692817443324	-4.61086862081308	4.82349625136432	5.19115429671904	6.11170557112954	6.47089367248123	6.41632949241517	6.61569605010193	NA
TC0300000978.mm.2	Reg4	regenerating islet-derived family, member 4	-0.227877148814382	4.76271707073371	-0.689559579289504	0.514572888750154	0.999692817443324	-4.61086986999562	4.42747988810975	4.2991793644505	4.57763237724417	5.11931205986245	5.16705298937054	4.98564574536484	NA
TC0500001525.mm.2	Kctd7	potassium channel tetramerisation domain containing 7	-0.281219905811198	6.34811015680075	-0.68929991309282	0.514725813129272	0.999692817443324	-4.6108808028752	6.60397510240607	6.25027398835992	5.69450193309541	6.64148408611265	6.32976936045529	6.56865647037517	NA
TC0100003731.mm.2	1700056E22Rik	RIKEN cDNA 1700056E22 gene	-0.25084297179467	6.74023502113082	-0.689246188007398	0.514757456948937	0.999692817443324	-4.61088306451764	6.35706704254905	6.26527035048821	6.81137177837815	6.847321616701	6.68269974618873	7.47767959247976	NA
TC0200004427.mm.2	Rhov	ras homolog family member V	0.292294555729887	6.65002862989019	0.68911235824158	0.514836287526422	0.999692817443324	-4.61088869773171	6.44051296160935	6.16713081837239	6.53454401866482	7.40401453674477	6.35240165106615	7.00156779288367	NA
TC0200002390.mm.2	Cnbd2	cyclic nucleotide binding domain containing 2	0.256862805797534	6.26657363390088	0.689041843642623	0.51487782632527	0.999692817443324	-4.61089166553696	6.24126543714823	6.0941530164156	6.05499271740523	6.83388176331301	6.03168564848601	6.34346322063717	NA
TC1300000261.mm.2	Hist1h4b	histone cluster 1, H4b	-0.201428615154903	9.49527039152202	-0.688947818945425	0.514933217851109	0.999692817443324	-4.61089562248459	9.67240565935459	9.56914695927532	9.41022081541098	9.25338543468522	9.55748799910761	9.5089754812984	NA
TC0300002680.mm.2	I830077J02Rik	RIKEN cDNA I830077J02 gene	-0.199603121789942	5.78296407304525	-0.688915631130743	0.51495218111969	0.999692817443324	-4.61089697698986	5.89422270764192	5.94842569444577	5.66379912047492	5.56416577582705	5.84898456646595	5.7781865734159	NA
TC0800001215.mm.2	Slc9a5	solute carrier family 9 (sodium/hydrogen exchanger), member 5	-0.298094808606717	5.71388766789787	-0.68888708696068	0.514968998134265	0.999692817443324	-4.61089817812701	6.21048392904279	6.3364703482156	4.84694298559968	5.72314807085801	5.95825544400829	5.20802522966283	NA
TC0100002707.mm.2	C130026I21Rik/A530032D15Rik	RIKEN cDNA C130026I21 gene/RIKEN cDNA A530032D15Rik gene	-0.240928060285787	6.01912871221783	-0.688837066305765	0.514998469041178	0.999692817443324	-4.61090028290552	5.35016683896384	5.79938332056523	5.8466983378779	6.39498465872527	6.31391364670252	6.40962547047223	NA
TC0500001165.mm.2	Mn1	meningioma 1	0.258008883878205	6.98029986802577	0.688791308250312	0.515025429489152	0.999692817443324	-4.61090220822346	7.00517158453939	7.46098950764349	7.01682834481361	6.94899959705047	7.0890622353496	6.36074793875806	NA
TC0400000536.mm.2	Cavin4	caveolae associated 4	-0.301428848447368	5.38022871375498	-0.688726033951899	0.515063890405507	0.999692817443324	-4.61090495454544	5.08509932244043	5.467148080618	5.83361920812486	4.72951438316349	5.48712123487548	5.6788700533076	NA
TC0700004219.mm.2	Cog7	component of oligomeric golgi complex 7	0.362739180928116	6.50985888511167	0.688721468183887	0.515066580716561	0.999692817443324	-4.61090514663641	7.52223253779522	7.31380705657307	6.70877374737837	5.76552229170062	6.50145635654255	5.2473613206802	NA
TC1500001749.mm.2	Naprt	nicotinate phosphoribosyltransferase	0.211028367107904	5.37906823511462	0.688710625808726	0.515072969461726	0.999692817443324	-4.61090560279301	5.49210041599588	5.56757324825514	5.25481625091599	5.56357998941026	5.09245555877572	5.30388394733473	NA
TC0700003783.mm.2	Emsy	EMSY, BRCA2-interacting transcriptional repressor	0.517173988404713	7.15397971440107	0.688613747234941	0.515130056328488	0.999692817443324	-4.61090967840075	8.13987693914507	7.96730497536336	7.8802560524577	6.045266121896	7.28161626712164	5.60955793042264	NA
TC1400002290.mm.2	Cdca2	cell division cycle associated 2	-0.229169196705499	6.22914099933586	-0.688572506588965	0.515154359119714	0.999692817443324	-4.61091141323574	5.33463674911245	5.61803794562284	6.22519627355116	6.75672018038326	6.71959791650517	6.72065693084025	NA
TC1000001901.mm.2	Akap7	A kinase (PRKA) anchor protein 7	0.473356360678238	7.94955792447265	0.688554541231223	0.515164946197241	0.999692817443324	-4.61091216894526	8.78602953834138	8.34656004403258	8.46619945020284	7.28953589270636	8.3317268921648	6.47729572938792	NA
TC1600000218.mm.2	Aifm3	apoptosis-inducing factor, mitochondrion-associated 3	0.213231379305337	6.34808130807538	0.688548720598847	0.515168376356005	0.999692817443324	-4.61091241378601	6.98563620704229	7.06473809343914	6.31936102173033	5.91292713156041	5.91017039883027	5.8956549958498	NA
TC1300002463.mm.2	Iqgap2	IQ motif containing GTPase activating protein 2	0.224132380309909	5.5252480612799	0.688545303442529	0.515170390128401	0.999692817443324	-4.61091255752554	5.89852096989833	6.14061900141432	5.54857555745476	5.23173102103851	5.3544395851936	4.97760223267985	NA
TC1100000566.mm.2	Col23a1	collagen, type XXIII, alpha 1	-0.204435430813662	6.85912309590074	-0.688321906308008	0.515302051879821	0.999692817443324	-4.61092195338553	6.26291545045818	6.49670675858182	6.94559441212892	7.04752834023962	7.24484990097307	7.1571437130228	NA
TC0800001770.mm.2	Csmd1	CUB and Sushi multiple domains 1	-0.222770605003085	9.47526526821408	-0.68810460390587	0.515430142525108	0.999692817443324	-4.61093109076192	8.63796728293187	8.74384241673979	9.52555382226346	9.98656652753986	10.1038120636441	9.85384949616538	NA
TC0500001596.mm.2	Upk3bl	uroplakin 3B-like	0.240817129432298	6.11572499531585	0.688100917104801	0.515432315927617	0.999692817443324	-4.61093124577043	5.35656286300783	5.91309499666368	6.60623400827936	6.34692917726472	6.05101888321271	6.42051004346678	NA
TC0X00000988.mm.2	Nhsl2	NHS-like 2	0.255087789372017	7.60079858892824	0.688075494145662	0.515447303084102	0.999692817443324	-4.61093231464121	8.04624927673263	8.28903291923471	7.60769752107938	7.28214493709367	7.55757150578638	6.82209537364264	NA
TC1300002601.mm.2	Cwc27	CWC27 spliceosome-associated protein	0.224408025116819	10.0443444576727	0.687939816271509	0.515527291680895	0.999692817443324	-4.61093801852729	9.6324330591326	9.76593385378832	10.0982172170456	10.5600407246288	10.2422571829313	9.96718470850946	NA
TC0400003599.mm.2	Hdac1	histone deacetylase 1	-0.205767672758895	6.43073367419173	-0.687918928872362	0.515539606515622	0.999692817443324	-4.61093889655844	6.4385514520188	6.73457951548388	6.23455634940204	6.26531151666291	6.44641814886868	6.46498506271405	NA
TC0700002642.mm.2	Ccdc97	coiled-coil domain containing 97	0.225183836584136	7.0781765583066	0.687913887610256	0.515542578781586	0.999692817443324	-4.61093910847205	7.55037013639094	7.31773856895201	7.15626932926464	6.86938982956782	6.57555269080318	6.999738794861	NA
TC0100001615.mm.2	Slamf9	SLAM family member 9	-0.258027734328245	5.43874876145876	-0.687851071206446	0.515579615488938	0.999692817443324	-4.61094174891575	4.4125275656934	4.95359153677941	5.436900680914	5.93825831789762	5.71982856757702	6.17138589889109	NA
TC0500001017.mm.2	Nudt9	nudix (nucleoside diphosphate linked moiety X)-type motif 9	-0.210773085920136	7.98258124062384	-0.687437299037805	0.515823619622894	0.999692817443324	-4.61095913711908	8.17417275310406	8.18069657448888	7.90850216579051	7.6510338063644	8.1327975193956	7.84828462459958	NA
TC1400001057.mm.2	Zc3h13	zinc finger CCCH type containing 13	0.201134439046246	6.39230328618406	0.687419408901514	0.515834171237667	0.999692817443324	-4.61095988875415	7.32387708849534	7.31984363124059	6.11388133262062	5.94230260510985	5.87652747226433	5.77738758737364	NA
TC1400000078.mm.2	Kctd6	potassium channel tetramerisation domain containing 6	0.203374232545025	7.04245958785382	0.686903302532638	0.516138631264946	0.999692817443324	-4.61098156623212	7.23814285005225	6.98881432732383	7.09187497671358	7.12539943070485	6.92948688290527	6.88103905942311	NA
TC1200001282.mm.2	Crip1	cysteine-rich protein 1 (intestinal)	-0.238139455806146	6.55684899735108	-0.686869848371646	0.516158370461228	0.999692817443324	-4.61098297095982	6.30973830555076	6.12246545858791	6.27307709442875	6.94322855974901	6.84758494753892	6.84499961825113	NA
TC1000000498.mm.2	Gm9956	predicted gene 9956	0.26337867522508	5.04606186127656	0.686857102538776	0.516165891104006	0.999692817443324	-4.61098350613936	3.98823065424451	3.90472537054544	5.3033204260412	6.15176582061858	5.30409902723821	5.6242298689714	NA
TC0700002076.mm.2	Tnni2	troponin I, skeletal, fast 2	0.270082511594256	6.24547524337704	0.686841445379776	0.516175129663579	0.999692817443324	-4.61098416355139	5.47228790688015	5.47637252410929	6.60144584837164	6.93073217785901	6.04366378198289	6.94834922105925	NA
TC1600002124.mm.2	Kcnj6	potassium inwardly-rectifying channel, subfamily J, member 6	0.228167105778663	6.59022700798069	0.686492921618362	0.516380804973353	0.999692817443324	-4.61099879450056	7.39003580058157	7.20916637390538	6.30042699468838	6.39882004778888	6.40278238596448	5.84013044495544	NA
TC0300001137.mm.2	Sort1	sortilin 1	0.318485835833071	8.94537253086473	0.686469225906451	0.516394790514426	0.999692817443324	-4.61099978904317	9.73618356136092	9.82003507052208	9.19023491434296	8.1862591981428	8.78557128439403	7.95395115642558	NA
TC0700001282.mm.2	Crebzf	CREB/ATF bZIP transcription factor	-0.213988170050784	7.81914154429397	-0.68619048336402	0.516559326653135	0.999692817443324	-4.61101148636924	7.46065670306059	7.30069899078918	7.85347479976795	8.00928381613829	7.95887533291405	8.33185962309375	NA
TC1300000015.mm.2	Tubal3	tubulin, alpha-like 3	-0.234229260284973	5.68138244627506	-0.686011004722443	0.516665287234494	0.999692817443324	-4.6110190162754	4.9430807293743	5.17162520605295	5.56999931311326	6.18256579771332	6.06024878017948	6.16077485121704	NA
TC1200002346.mm.2	Ccdc85c	coiled-coil domain containing 85C	0.200567582156854	7.44758733502707	0.685885268608907	0.51673952767	0.999692817443324	-4.6110242905874	7.84926539551777	7.82581086452866	7.44558260817185	7.26020884904301	7.12459505766249	7.18006123523866	NA
TC0900000719.mm.2	Ubl7	ubiquitin-like 7 (bone marrow stromal cell-derived)	-0.476111551746479	8.8623226035191	-0.685850518327623	0.516760047072121	0.999692817443324	-4.61102574814848	9.89754318821957	9.90954302496953	9.17535197384913	7.03196117836713	8.94782600787435	8.21171024783487	NA
TC0500002579.mm.2	Txk	TXK tyrosine kinase	-0.272544009990431	5.62955586476925	-0.685776644356905	0.516803670030304	0.999692817443324	-4.61102884652791	4.92944588119935	5.18010733687302	5.8614747988745	5.69987217640663	5.58399911488062	6.52243588038138	NA
TC0200001878.mm.2	Ctxn2	cortexin 2	0.218128379976184	5.69494850499481	0.685677964090794	0.516861944945455	0.999692817443324	-4.61103298493588	6.16854274412265	5.76713909489428	5.57946150750029	5.75567146795186	5.55854429238319	5.34033192311659	NA
TC0100000420.mm.2	1700066M21Rik	RIKEN cDNA 1700066M21 gene	-0.20416537754454	6.81415155129671	-0.685550725675968	0.516937090935015	0.999692817443324	-4.61103832035617	6.76591814706295	6.87989349936582	6.80927616898776	6.60610728414343	6.99034971031085	6.83336449790942	NA
TC0700001402.mm.2	Pgm2l1	phosphoglucomutase 2-like 1	-0.335210175142332	7.86288861042211	-0.685546306673242	0.516939700889495	0.999692817443324	-4.61103850564276	8.65762681261649	8.95817287414973	7.78483018026182	6.66072563247906	7.96397293286281	7.15200323016274	NA
TC0900002312.mm.2	Elmod1	ELMO/CED-12 domain containing 1	-0.585599156612069	9.94973103717737	-0.68541130558264	0.517019439431082	0.999692817443324	-4.61104416574826	11.0097722827213	11.0929074107831	10.5005065605663	7.71174754760156	10.0599669219285	9.3234854994635	NA
TC0100001230.mm.2	Klhl12	kelch-like 12	0.252287226060489	6.07616419513877	0.685144119028281	0.517177276884977	0.999692817443324	-4.61105536549326	6.27502276222689	6.28982739204654	5.74593762860696	6.45241710573312	6.06063523148726	5.63314505073184	NA
TC1400000923.mm.2	Mir124a-1/Mir3078/Mir124a-1hg	microRNA 124a-1/microRNA 3078/Mir124-1 host gene (non-protein coding)	-0.231673555542382	6.1111869371752	-0.685064398426971	0.517224376961998	0.999692817443324	-4.61105870654526	5.93185448294014	6.47033775116615	5.77783696282536	6.12295417610471	6.18436241571187	6.17977583430295	NA
TC0800002725.mm.2	Bbs2	Bardet-Biedl syndrome 2 (human)	-0.297890380939238	6.01607725900193	-0.685031392806176	0.517243877968019	0.999692817443324	-4.6110600897112	6.7255560765804	7.25143862386216	5.85471489008287	4.90712915576241	5.45754966516727	5.90007514255648	NA
TC0600001425.mm.2	Lpcat3	lysophosphatidylcholine acyltransferase 3	-0.555016825181523	8.63557437182013	-0.684826675941107	0.517364843269601	0.999692817443324	-4.61106866768195	8.98263571548963	9.21649280408331	9.35859334184436	6.89354868864805	9.24117296756698	8.12100271328847	NA
TC1400002273.mm.2	Gm4583	predicted gene 4583	-0.299865816143043	5.76006215344475	-0.684596580981712	0.517500826054388	0.999692817443324	-4.61107830678428	5.67887856649202	5.59092750094734	5.65018875830041	5.69522885217113	5.38180586948048	6.56334337327714	NA
TC0100002784.mm.2	Iqca	IQ motif containing with AAA domain	-0.199104518181676	5.401057605547	-0.684489709036794	0.517563993671179	0.999692817443324	-4.61108278303641	5.11291753024351	5.12930210078778	5.37246363115296	5.51049521179073	5.68272127424887	5.59844588505817	NA
TC1700000579.mm.2	Olfr63	olfactory receptor 63	0.280120790103834	5.39167782106685	0.684284179235898	0.517685487883457	0.999692817443324	-4.61109139005367	4.7904359322318	5.09245768612331	5.25986303271697	6.25384441140987	5.41697984827675	5.53648601564242	NA
TC0200001761.mm.2	Gchfr	GTP cyclohydrolase I feedback regulator	0.252016967877975	5.48244219365378	0.684223327295838	0.517721462639021	0.999692817443324	-4.61109393799882	5.14499158875568	5.22979173507369	5.37878451456371	6.13316737236091	5.34059721205739	5.66732073911128	NA
TC0700004624.mm.2	Plk1	polo like kinase 1	-0.33779854141148	6.28885600399985	-0.684192909164757	0.517739445987339	0.999692817443324	-4.61109521158062	6.39471206609352	7.33881250877301	5.41289411481876	6.24911306833605	5.8214248583322	6.51617940764558	NA
TC0300002838.mm.2	Tmem56	transmembrane protein 56	-0.775809689458606	8.45773087181317	-0.684173181729505	0.51775110915934	0.999692817443324	-4.61109603752961	9.30284609316039	9.47746433494271	9.21281727044441	5.99441044148494	9.50713528514755	7.2517111805699	NA
TC1700000539.mm.2	Ndufv3	NADH:ubiquinone oxidoreductase core subunit V3	0.218091812186365	6.72229387102361	0.684133153970811	0.517774774724956	0.999692817443324	-4.61109771335941	7.08051514509967	6.59562052770476	6.72885409348312	6.81805149537185	6.57859295965723	6.53212900482501	NA
TC1700001914.mm.2	Btnl6	butyrophilin-like 6	0.309105920995131	5.78739812298835	0.684033699665902	0.517833578000794	0.999692817443324	-4.6111018768695	4.92261000847713	4.92655851642717	5.75542435851493	6.9912916689931	5.96788777573036	6.16061640978741	NA
TC0500000174.mm.2	Armc10	armadillo repeat containing 10	-0.315030651485203	5.80790273721635	-0.683992942156908	0.517857677499038	0.999692817443324	-4.61110358299468	6.21082369464205	6.44088958982574	5.2075203504811	5.57530056744885	5.09851342316679	6.31436879773357	NA
TC1300001615.mm.2	Vmn1r217	vomeronasal 1 receptor 217	0.349049629471683	5.24415787308244	0.68394486482474	0.517886106064169	0.999692817443324	-4.61110559543426	4.26410447477004	4.61975646860982	5.0906704896789	6.54892228735016	5.37716384779613	5.56432967028957	NA
TC0500003723.mm.2	Mpv17	MpV17 mitochondrial inner membrane protein	-0.472440354994593	8.4919137740166	-0.683761555228781	0.51799450793917	0.999692817443324	-4.61111326752236	9.22965048013753	9.13606524264863	8.9453152969512	6.87512780871094	8.82533350427134	7.93999031137998	NA
TC1700000374.mm.2	Tpsb2	tryptase beta 2	0.30621820376849	6.23775538492814	0.683721118032238	0.518018422830971	0.999692817443324	-4.61111495974337	5.32340191127516	5.28334483219366	6.30132927676054	7.41478171005797	6.28884369674788	6.81483088253365	NA
TC1100000674.mm.2	Larp1	La ribonucleoprotein domain family, member 1	-0.234100089072287	8.88736953830979	-0.683642662419948	0.518064824159136	0.999692817443324	-4.61111824275368	8.99708477781833	9.00349540332217	9.04735014586709	8.38036828941973	8.92917341491698	8.96674519851442	NA
TC1300000891.mm.2	Brd9	bromodomain containing 9	-0.266188944977932	6.32344421976265	-0.683621291203439	0.518077464290975	0.999692817443324	-4.61111913699373	6.9674008149829	6.47596134203942	6.05295842068703	5.92950421511181	5.86009656847678	6.65474395727793	NA
TC0300000823.mm.2	Sprr2b	small proline-rich protein 2B	0.328063372407025	6.28091740154892	0.683449529310305	0.518179061098175	0.999692817443324	-4.61112632331271	5.48603071039573	5.4229022916796	6.36227461310409	7.35407446291204	6.0521054667746	7.00811686442748	NA
TC0900001158.mm.2	Adamts7	a disintegrin-like and metallopeptidase (reprolysin type) with thrombospondin type 1 motif, 7	-0.269453077845964	5.73435567970088	-0.683421558536803	0.518195606969864	0.999692817443324	-4.61112749345155	5.37158043685232	5.15233084541047	5.4415804619456	6.23007785817969	5.84658124916475	6.36398322665246	NA
TC0700002479.mm.2	Cd3eap	CD3E antigen, epsilon polypeptide associated protein	-0.212266469953276	6.68606793475147	-0.68333102712887	0.518249162385545	0.999692817443324	-4.61113128053128	6.18184380552822	6.28969255830275	6.74897549764517	6.86119365474048	6.82048068658841	7.21422140570379	NA
TC1400000241.mm.2	Zmiz1	zinc finger, MIZ-type containing 1	-0.205310668382304	7.60708736876295	-0.683161231626509	0.518349617468006	0.999692817443324	-4.61113838236321	7.96016417856836	7.92723756251381	7.49059228390495	7.18165828346051	7.51269025741865	7.57018164671141	NA
TC1900001465.mm.2	Fra10ac1	FRA10AC1 homolog (human)	0.457895520660667	5.69868461169026	0.683077608050093	0.518399095791029	0.999692817443324	-4.61114187950774	6.56742254225857	6.72911792450044	6.10570408668796	4.79997503566397	5.85901954137071	4.13086853965989	NA
TC1400001573.mm.2	Dlg5	discs large MAGUK scaffold protein 5	0.348687666857377	6.67216815264147	0.683045808171472	0.518417911913939	0.999692817443324	-4.61114320929834	6.31440729430851	6.98291502313803	6.29876630276506	7.41776466329347	6.12525380850801	6.89390182383576	NA
TC0700002933.mm.2	Zfp975	zinc finger protein 975	0.521914143272584	5.22208409705502	0.682965354884398	0.518465518430941	0.999692817443324	-4.61114657344783	5.61375613858169	5.59484892570592	5.99464713781601	4.58921676447783	5.82754550927794	3.71249010647074	NA
TC1500001939.mm.2	Tnfrsf13c	tumor necrosis factor receptor superfamily, member 13c	0.222767190620404	6.73524684308577	0.682874147852169	0.518519491655812	0.999692817443324	-4.61115038691123	6.2856807416414	6.38270949579922	6.80229053570375	7.29202206545365	6.94445085464608	6.7043273652705	NA
TC1900001341.mm.2	Rln1	relaxin 1	-0.332789442421165	4.98168148431031	-0.682819270438757	0.518551967990982	0.999692817443324	-4.61115268121356	4.44573244360231	4.19667836194391	4.49687915515443	5.79417045258222	5.22919301810174	5.72743547447724	NA
TC1100001945.mm.2	Mgat5b	mannoside acetylglucosaminyltransferase 5, isoenzyme B	0.229143789199209	6.72001505571122	0.68268921677904	0.518628938702443	0.999692817443324	-4.6111581179243	7.01004430056999	7.26255176372225	6.4465664554181	6.80632446876864	6.17708134305304	6.61752200273529	NA
TC0700000301.mm.2	Ckm	creatine kinase, muscle	0.225780223411226	8.2707354253394	0.682683589978961	0.518632269023321	0.999692817443324	-4.61115835312755	7.82833335525043	8.01738862695801	8.3103333997637	8.80479210856152	8.20456260547009	8.45900245603268	NA
TC0200000798.mm.2	Epc2	enhancer of polycomb homolog 2	0.838255799847428	6.37870097799682	0.682629659396808	0.518664189488793	0.999692817443324	-4.61116060738217	7.36255593552096	7.33550728388108	7.72575950170111	4.89956762243574	7.08253282481938	3.86628269962261	NA
TC0800002359.mm.2	Lzts1	leucine zipper, putative tumor suppressor 1	0.234504374682665	8.82717947215953	0.682599458901561	0.518682065129167	0.999692817443324	-4.61116186968103	8.75235340374332	8.68049091716533	8.72662032368623	9.27300030702069	8.57007225707952	8.96053962426207	NA
TC1500001121.mm.2	Hoxc6	homeobox C6	0.260241030741487	5.22478524756438	0.682429323355179	0.518782775593133	0.999692817443324	-4.61116898011625	4.92504245463906	5.02309616158087	5.0540687888816	5.90645867644307	5.08550449956063	5.35454090428107	NA
TC0500001853.mm.2	B3glct	beta-3-glucosyltransferase	0.227651110448035	6.24658502678352	0.682363449726779	0.518821772378373	0.999692817443324	-4.61117173280637	6.54910922607216	6.60519869927525	6.29711531140414	6.09314069672077	6.2525072216398	5.68244278558903	NA
TC0200000718.mm.2	Wdr38	WD repeat domain 38	-0.290125719192554	7.56259369283169	-0.682281212899968	0.518870458727821	0.999692817443324	-4.61117516899754	6.80286594101545	6.95223935405481	7.53275252783514	7.98735018393223	7.54154433002592	8.55880982012656	NA
TC1600001497.mm.2	Pak2	p21 (RAC1) activated kinase 2	-0.235070562116697	6.57063059854687	-0.681887456067551	0.519103613843388	0.999692817443324	-4.61119161753544	7.26055955500615	7.34068667527276	6.14146423374218	6.03473388464228	6.65815993863762	5.98817930398024	NA
TC0400000460.mm.2	Clta	clathrin, light polypeptide (Lca)	-0.279809778964566	9.38081939106681	-0.681800389812816	0.519155177467878	0.999692817443324	-4.61119525363773	9.87642613361831	9.81162012758002	9.45491161865704	8.56371364497965	9.56569879940733	9.0125460221585	NA

**TC0400003426.mm.2**	Rimkla	ribosomal modification protein rimK-like family member A	0.219770453630543	6.92922784573576	0.681779121366279	0.519167773873236	0.999692817443324	-4.61119614180863	7.12087237917928	6.68154439175632	6.99397408942163	7.11227151594841	6.70333958291844	6.96336511519051	NA
**TC1800001752.mm.2**	Tmed7	transmembrane p24 trafficking protein 7	-0.393338548592157	9.29763816290596	-0.681744750741111	0.51918813056338	0.999692817443324	-4.61119757708362	9.99432940639432	10.0719036518985	9.56693114070839	7.89952827027089	9.45568626317769	8.79745024498591	NA
**TC0700002663.mm.2**	Ltbp4	latent transforming growth factor beta binding protein 4	-0.291451808881749	8.00667333709653	-0.681687087535729	0.519222283904579	0.999692817443324	-4.61119998490814	7.21521682480397	7.16517270320159	7.73890014443338	8.79947329397168	8.35946613956076	8.76181091660781	NA
**TC0100000908.mm.2**	Olfr1413	olfactory receptor 1413	-0.270676747277228	4.93829605888449	-0.681648918352492	0.519244891926752	0.999692817443324	-4.61120157864416	4.5574740883022	4.47549439147661	5.3570856213579	4.67064156812895	4.93515373993314	5.63392694410816	NA
**TC0700004135.mm.2**	Psma1	proteasome (prosome, macropain) subunit, alpha type 1	-0.348501744364417	7.84760212963526	-0.681639050357215	0.519250736950757	0.999692817443324	-4.61120199066687	8.84573775725161	8.75756690841746	7.65925928053487	6.73339303117194	8.14381407397935	6.94584172645629	NA
**TC1100004105.mm.2**	Fbf1	Fas (TNFRSF6) binding factor 1	0.201447058277746	7.65853380184619	0.681524585406203	0.519318540077885	0.999692817443324	-4.61120676965018	7.76492122670037	7.77713173655623	7.81342783846554	7.59259414372249	7.44459383399262	7.55853403163992	NA
**TC1200000130.mm.2**	Ntsr2	neurotensin receptor 2	0.345023479679192	9.48391219293985	0.681387183950873	0.519399937134154	0.999692817443324	-4.61121250546442	10.077543536645	9.74132448653536	9.99703685368686	8.89028919322172	9.5724194362226	8.6248596513276	NA
**TC0300000775.mm.2**	4933434E20Rik/1700094D03Rik	RIKEN cDNA 4933434E20 gene/RIKEN cDNA 1700094D03 gene	-0.35148137871449	7.18329222928731	-0.681362847422583	0.519414355026614	0.999692817443324	-4.6112135213021	7.79013670476764	7.65585526033456	7.49546781980019	5.97993150679613	7.19614488974741	6.98221719427789	NA
**TC0900002982.mm.2**	Ackr4	atypical chemokine receptor 4	-0.235156209362047	4.98968315479959	-0.681345140144959	0.51942484565874	0.999692817443324	-4.61121426040958	4.39782664493467	4.73855742803008	4.93185436615219	5.23384685240215	5.0616190447706	5.57439459250784	NA
**TC0400002945.mm.2**	Ttc39b	tetratricopeptide repeat domain 39B	0.299120856460926	7.59242174475916	0.681251246255611	0.519480475136752	0.999692817443324	-4.61121817933382	8.20412183049988	7.98116836928497	7.6279886392268	7.35575235161918	7.76149875692937	6.62400052099475	NA
**TC1000000896.mm.2**	Dohh	deoxyhypusine hydroxylase/monooxygenase	0.215291891043588	7.10774229367818	0.681171209038915	0.519527897955386	0.999692817443324	-4.61122151959618	7.00261924293138	7.12968252584687	7.04955850930639	7.42280937838261	7.15082747578184	6.89095662981998	NA
**TC0X00002136.mm.2**	Dcaf12l1	DDB1 and CUL4 associated factor 12-like 1	0.528471849713131	7.01250237672214	0.681161986212201	0.519533362747733	0.999692817443324	-4.61122190448175	8.46246929056521	8.09600094720771	7.80329525768092	5.48344860331217	6.65430305748282	5.57549710408401	NA
**TC0800001093.mm.2**	Mt3	metallothionein 3	-0.209458024316518	8.92668991066624	-0.681089447725253	0.51957634519522	0.999692817443324	-4.61122493151251	9.72543756505279	9.46183683392556	8.39083461542733	8.58613989276569	8.58958115905734	8.80630939776872	NA
**TC0800002589.mm.2**	Nfix	nuclear factor I/X	-0.339450270601576	10.1664646117667	-0.681062980343472	0.519592028927205	0.999692817443324	-4.61122603593696	10.5751799707995	10.8973748136369	10.2834139736303	9.14025219885003	10.4762956768243	9.62627103685914	NA
**TC1600000667.mm.2**	Ccdc80	coiled-coil domain containing 80	-0.244155708796757	5.7741864998585	-0.681031172948489	0.519610877384965	0.999692817443324	-4.61122736314624	4.66211756106826	5.22409661438919	5.92018740619169	6.21510929685832	6.09474096171657	6.52886715892696	NA
**TC0200002851.mm.2**	Hspa14	heat shock protein 14	0.216383167259377	6.39061650835452	0.68099858268368	0.519630190212936	0.999692817443324	-4.6112287229744	6.81860488700744	6.70392467502125	6.48889887659457	6.13806903471404	5.93861041851997	6.25559115826987	NA
**TC0400001728.mm.2**	Camk2n1	calcium/calmodulin-dependent protein kinase II inhibitor 1	-0.228537183661743	9.65026559468854	-0.680973360926417	0.519645136817283	0.999692817443324	-4.61122977531874	9.53850055688481	9.92666812198178	9.48761129767291	9.50206396329767	9.96622349170866	9.48052613658541	NA
**TC0200005466.mm.2**	Lrrc8a	leucine rich repeat containing 8A	-0.258691323452018	7.4297567530684	-0.680840611773408	0.519723809531014	0.999692817443324	-4.6112353136264	8.18444030456274	7.88941291177776	7.40052003165886	6.59313229592407	7.37391672067114	7.13711825381583	NA
**TC1000001566.mm.2**	Ndufa4l2	Ndufa4, mitochondrial complex associated like 2	0.223799762070573	6.13126378398317	0.680743318325281	0.519781474574571	0.999692817443324	-4.61123937221207	5.72322022151279	5.58404605304589	6.20459712262033	6.75936085412043	6.27519446280082	6.24116398979879	NA
**TC0700002827.mm.2**	Pdcd2l	programmed cell death 2-like	0.22997872554161	6.92479770605107	0.680700101950527	0.519807089892435	0.999692817443324	-4.61124117484103	7.16354142905426	7.13717779485781	6.75118901946065	7.10282321227813	6.43153107585052	6.96252370480505	NA
**TC0200001640.mm.2**	Olfr1278	olfactory receptor 1278	-0.322637256233945	4.63970324568912	-0.680670928670578	0.519824382011179	0.999692817443324	-4.61124239166067	4.24408305572247	4.42269649465486	4.19967483571741	5.06340786992733	4.44185476892683	5.4665024491858	NA
**TC0700002496.mm.2**	Apoc1	apolipoprotein C-I	-0.285411111231313	5.45245858440481	-0.680663890897169	0.519828553624048	0.999692817443324	-4.61124268520094	5.53540324672582	4.97483614803089	5.21266076149552	5.60418418310924	5.26604453126465	6.12162263580273	NA
**TC0400003421.mm.2**	Ybx1	Y box protein 1	-0.32862469806353	9.68118822960786	-0.68064440645348	0.519840103064774	0.999692817443324	-4.61124349787084	9.79308600917522	10.2121267247858	9.98704982718625	8.72528379659332	9.8901394132747	9.47944360663193	NA
**TC1900001512.mm.2**	Rrp12	ribosomal RNA processing 12 homolog (S. cerevisiae)	-0.227294189781747	5.97329890114189	-0.68023199000975	0.52008460241639	0.999692817443324	-4.61126069517228	6.18921194021789	6.34544242958632	5.58084013139561	5.84443519734619	6.20465105428979	5.67521265401551	NA
**TC1700001188.mm.2**	Gpatch11	G patch domain containing 11	0.196764251415869	7.00598553981821	0.680136951592616	0.52014095602628	0.999692817443324	-4.61126465707509	7.61661874359765	7.49594198924069	6.83159203237075	6.82684847208057	6.63624685995899	6.6286651416606	NA
**TC0200000467.mm.2**	Dbh	dopamine beta hydroxylase	-0.316588799130487	4.77831795953115	-0.679997472348648	0.520223668206185	0.999692817443324	-4.61127047085865	4.64471551186573	4.20560267017683	4.30707384858885	5.28613213985283	4.73416307249523	5.49222051420742	NA
**TC0500001128.mm.2**	Zfp932	zinc finger protein 932	0.359652397916439	7.01473056801206	0.679919539032416	0.520269886897758	0.999692817443324	-4.61127371889668	7.72746855040546	8.12480814285266	7.17530656260746	6.30239919271609	7.0150081382907	5.74339282119997	NA
**TC0400000918.mm.2**	Dmrta1	doublesex and mab-3 related transcription factor like family A1	0.326064058885229	5.83415157454078	0.679875763476174	0.520295849339471	0.999692817443324	-4.61127554321611	5.18911702957813	4.87492752701705	5.96248771527567	6.8340087889343	5.56272544703396	6.58164293940555	NA
**TC0500003615.mm.2**	Usp12	ubiquitin specific peptidase 12	-0.319361324052383	7.46233138732375	-0.679840955885968	0.520316493647252	0.999692817443324	-4.61127699373908	7.84433158024555	8.02485167685117	7.72675204282324	6.40628916654727	7.37945170122875	7.39231215624654	NA
**TC0800002437.mm.2**	Unc13a	unc-13 homolog A	0.209419616314171	7.30921056891771	0.67979165961729	0.520345732060531	0.999692817443324	-4.61127904794907	7.93746370567287	7.69410704146494	7.06697237457965	7.25429357491874	6.82423892961552	7.07818778725453	NA
**TC1600001064.mm.2**	Dyrk1a	dual-specificity tyrosine-(Y)-phosphorylation regulated kinase 1a	-0.504415455695889	7.37750019690381	-0.679715184148977	0.520391092986565	0.999692817443324	-4.61128223451732	8.50025052406725	8.47914951698037	7.78966900933624	5.34871610515549	7.19851376557481	6.9487022603087	NA
**TC0800001233.mm.2**	Pskh1	protein serine kinase H1	0.270768103566057	5.26314635449193	0.679638202056667	0.520436756980682	0.999692817443324	-4.61128544192776	4.46030552578724	4.49197735054033	6.2476591392617	5.33640658961637	5.71376187166496	5.32876765008099	NA
**TC1000003122.mm.2**	Gpr182	G protein-coupled receptor 182	-0.274144643445556	5.61004669487575	-0.679570505545849	0.52047691511137	0.999692817443324	-4.61128826223826	5.34735068334733	5.72975180934956	5.96981925233173	5.04762494250153	5.68988872682675	5.87584475489762	NA
**TC0400000902.mm.2**	Gm13287	predicted gene 13287	-0.222511214546129	6.32942928900021	-0.679562728152961	0.520481528852781	0.999692817443324	-4.61128858623963	5.56369993600198	5.8811263179372	6.4011214421669	6.64224208331802	6.54531531731543	6.94307063726174	NA
**TC1700002337.mm.2**	Rfx2	regulatory factor X, 2 (influences HLA class II expression)	-0.2536238143239	7.24398343246465	-0.679526903140901	0.520502781473553	0.999692817443324	-4.61129007865201	6.0437195648966	5.98509701528348	7.33084393417366	8.13307425880637	7.84166065021276	8.12950517141507	NA
**TC0200003318.mm.2**	Garnl3	GTPase activating RANGAP domain-like 3	0.208790075584374	7.48472216102138	0.679519359134263	0.520507256907056	0.999692817443324	-4.61129039291578	7.67468691130732	7.6556512747287	7.35381171494852	7.64397575068198	7.15982578832144	7.42038152614032	NA
**TC0600000406.mm.2**	Agk	acylglycerol kinase	-0.52517143459947	5.99301077883657	-0.679471760929014	0.520535494807062	0.999692817443324	-4.61129237567415	7.07444725991196	6.21412560431521	6.38223355365979	4.42734091613688	6.64754847594393	5.21236886305168	NA
**TC0500003651.mm.2**	Gm8615	glucosamine-6-phosphate deaminase 1 pseudogene	0.205532966792566	7.25334327632467	0.679369624412267	0.520596091186864	0.999692817443324	-4.6112966299423	7.50208201114083	7.42639551861582	7.18987855025786	7.3114454806304	7.19913394455304	6.89112415275008	NA
**TC1000002505.mm.2**	Klf16	Kruppel-like factor 16	0.213345503948677	8.3201506243652	0.679236352035367	0.520675166918842	0.999692817443324	-4.61130218039362	8.53129857395684	8.42798198745372	8.56066730204497	8.13348979429402	8.07475925896122	8.19270682948042	NA
**TC0700004475.mm.2**	Stk32c	serine/threonine kinase 32C	-0.225542100867199	7.17848293091892	-0.679128465412955	0.520739185939193	0.999692817443324	-4.61130667300355	7.75001177782537	8.09789807638434	6.68546019791774	6.70049156686696	7.16115460473395	6.67588136178514	NA
**TC0700001817.mm.2**	Tbx6	T-box 6	-0.209044698808339	5.53063106233528	-0.67907595838928	0.520770344998045	0.999692817443324	-4.61130885930766	5.1402351287505	5.00451192400101	5.48648357841446	5.8239913834073	5.94281233482604	5.7857520246124	NA
**TC1700002006.mm.2**	H2-T3	histocompatibility 2, T region locus 3	0.320695748461558	6.21152059616668	0.678969375037008	0.520833598066587	0.999692817443324	-4.61131329687443	5.94852125060301	6.0173995440234	5.81728977295697	7.15500736669144	6.10787135684917	6.22303428587612	NA
**TC1100003100.mm.2**	Vmo1	vitelline membrane outer layer 1 homolog (chicken)	-0.289123367823346	5.6264420925827	-0.678848162889817	0.520905538753402	0.999692817443324	-4.61131834288034	4.80106098578494	4.54917693993284	5.48412190554975	6.43096204151611	5.96772726779251	6.52560341492004	NA
**TC1700001938.mm.2**	D17H6S56E-5	DNA segment, Chr 17, human D6S56E 5	-0.233999022764124	6.8624944325149	-0.678804764275506	0.520931297840205	0.999692817443324	-4.61132014938258	6.60409715590743	7.19815449341964	6.88209080136302	6.57026764875402	7.02427322288491	6.89608327276037	NA
**TC0100003612.mm.2**	Chml	choroideremia-like	0.228639827461354	5.93886610898034	0.678767694718572	0.520953300986383	0.999692817443324	-4.61132169236502	6.36614289595866	6.14569988562733	5.60208233982496	6.18723442378442	5.62562551799524	5.70641159069143	NA
**TC0200002528.mm.2**	Spint4	serine protease inhibitor, Kunitz type 4	0.22206771578836	6.51876431506261	0.678684557979455	0.521002650113862	0.999692817443324	-4.61132515262005	6.15636661334269	6.01083303266293	6.7788197730167	6.91594106495668	6.36301947712051	6.88760592927615	NA
**TC0600001257.mm.2**	Vhl	von Hippel-Lindau tumor suppressor	-0.330854191705336	6.90496288836979	-0.678672439086013	0.521009844017178	0.999692817443324	-4.61132565699735	7.33880832515744	7.46884455977003	7.18127362448143	5.79893440645889	6.69148577952173	6.95043063482925	NA
**TC1300000464.mm.2**	Sycp2l	synaptonemal complex protein 2-like	0.355901178654878	4.80721518480074	0.67856636281958	0.521072814733942	0.999692817443324	-4.61133007151027	4.00578317092127	4.57260200345977	4.52473527569928	5.9636188701673	4.7428681598286	5.03368362872822	NA
**TC0800000010.mm.2**	Mcoln1	mucolipin 1	0.303621020931082	9.04855056407807	0.678381243825347	0.521182719786601	0.999692817443324	-4.61133777427366	9.61107289054008	9.62069753342065	9.2555420100976	8.57784549108733	9.12668435466641	8.09946110465636	NA
**TC0400002082.mm.2**	Cdk11b	cyclin-dependent kinase 11B	0.416278832606987	5.23714816267032	0.678374763883831	0.521186567194213	0.999692817443324	-4.61133804387451	5.78870961138078	5.59060042445353	4.83252184771977	5.60554645498101	3.91612906468262	5.68938157280419	NA
**TC0100001692.mm.2**	Kif26b	kinesin family member 26B	0.30764240875583	6.21768533453973	0.678267703309533	0.521250136097269	0.999692817443324	-4.61134249790129	6.68748357725767	6.06541978944635	5.90434360114713	6.67990312787588	5.59857922666625	6.3703826848451	NA
**TC1500002231.mm.2**	2310068J16Rik	RIKEN cDNA 2310068J16 gene	-0.204971960117013	5.12110072799814	-0.678134423718572	0.521329279910549	0.999692817443324	-4.61134804198686	4.76895279248243	4.45424332452379	5.21385676026812	5.33287540510617	5.47605099389292	5.48062509171539	NA
**TC1300001793.mm.2**	Eci3	enoyl-Coenzyme A delta isomerase 3	-0.278185659611365	5.16914273807069	-0.678117809912075	0.52133914602779	0.999692817443324	-4.61134873302159	5.0396459492973	5.08997430315954	5.59908675871086	4.56534566966143	5.27165717167685	5.44914657591817	NA
**TC0400002222.mm.2**	Fam92a	family with sequence similarity 92, member A	-0.525285366029252	8.20192450914872	-0.677984727151656	0.521418181616857	0.999692817443324	-4.61135426801389	9.07167517748089	8.84288684212239	8.6827481005881	6.44045905102717	8.71505774291176	7.45872014076201	NA
**TC1300000275.mm.2**	Ripor2	RHO family interacting cell polarization regulator 2	0.269209499976866	7.62959980907032	0.677751238571917	0.521556865098164	0.999692817443324	-4.61136397700022	8.58567273819554	8.38593494838817	7.81749850191988	6.85470658197611	7.05506278281908	7.07872330112317	NA
**TC0700001851.mm.2**	Srcap	Snf2-related CREBBP activator protein	-0.202079356613405	8.96720724332848	-0.677705209516921	0.521584207428024	0.999692817443324	-4.61136589070067	8.91201991083066	8.90963519983176	9.02899625895729	8.75971855908353	9.04840253296238	9.14447099830524	NA
**TC1300000177.mm.2**	Hist1h2bn	histone cluster 1, H2bn	-0.212993989598194	8.94783026503112	-0.677608356683226	0.521641743269335	0.999692817443324	-4.61136991713214	8.69220032581521	8.93936505303685	9.09731726666333	8.71739684940581	9.09058901123985	9.15011308402568	NA
**TSUnmapped00000047.mm.2**	Cd83	CD83 antigen	0.221474206911559	5.6072044990372	0.67753714462068	0.521684049698578	0.999692817443324	-4.61137287733609	6.30227802733206	5.78884209764809	5.38867116561403	5.60885647591906	5.26770352201074	5.28687570569923	NA
**TC0900001605.mm.2**	Rpl14/Rpl14-ps1	ribosomal protein L14/ribosomal protein L14, pseudogene 1	-0.269304971698954	9.79640519513879	-0.677480558005686	0.521717668854152	0.999692817443324	-4.61137522941254	10.4033687933551	10.3149615800466	9.8013350502702	8.95941037674639	9.90158679556804	9.39776857484646	NA
**TC0800002665.mm.2**	Sall1	spalt like transcription factor 1	-0.206051355574705	6.69717776596655	-0.677404296604291	0.521762979363637	0.999692817443324	-4.61137839905986	6.33522374356587	6.26535581889549	6.96804478847103	6.61714737262324	6.99180998779225	7.00548488445143	NA
**TC1700000009.mm.2**	Tiam2	T cell lymphoma invasion and metastasis 2	-0.327359469935955	7.4623183734814	-0.677370100107129	0.521783297941645	0.999692817443324	-4.61137982028061	8.25885067665106	8.68916580309924	7.34603023054244	6.23955718009067	7.48506964976128	6.75523670074373	NA
**TC0200003311.mm.2**	Ttc16	tetratricopeptide repeat domain 16	0.386877318071693	5.92400975108869	0.677283498372061	0.521834756479839	0.999692817443324	-4.61138341924714	5.70445364700466	5.7351463842951	5.38632737720913	7.05277917847875	6.13132495664901	5.53402696289548	NA
**TC0200002269.mm.2**	Rspo4	R-spondin 4	0.230241464864941	6.30352283039135	0.677246130779751	0.521856961216793	0.999692817443324	-4.61138497205196	5.64478981463063	5.7412084703042	6.40240650494252	7.04540430862906	6.51577440833453	6.47155347550717	NA
**TC1600000692.mm.2**	Dppa4	developmental pluripotency associated 4	-0.218978015775119	5.65751996416863	-0.677236550004223	0.521862654445475	0.999692817443324	-4.61138537016945	5.08031935703569	4.87787657581401	5.80586829807202	5.96861561223391	5.8741718537457	6.33826808811047	NA
**TC1500002154.mm.2**	Rpap3	RNA polymerase II associated protein 3	0.226389021613326	6.31060457752429	0.677234804400589	0.52186369174791	0.999692817443324	-4.61138544270544	6.90886119574319	6.36754192775739	6.33789860093562	6.1821025915003	6.15415105803956	5.91307209116971	NA
**TC1100001721.mm.2**	Wnt3	wingless-type MMTV integration site family, member 3	-0.205540566673304	4.99624170867045	-0.677105602123956	0.521940472205455	0.999692817443324	-4.61139081113109	5.0167310573642	4.88694704808581	4.7545825004851	5.07676300612794	5.09520480767757	5.14722183228208	NA
**TC0500001682.mm.2**	Elfn1	leucine rich repeat and fibronectin type III, extracellular 1	0.359183882229939	7.49311148000848	0.677098148653755	0.521944901766815	0.999692817443324	-4.61139112080371	8.18675541458409	7.70196959254007	8.19345706583892	6.70069875285437	7.24128213127462	6.9345059229588	NA
**TC0400001502.mm.2**	Phc2	polyhomeotic 2	0.245151331116144	8.45808595818089	0.676972320002461	0.522019684766268	0.999692817443324	-4.6113963482808	7.92837372771795	7.80040319559736	8.59858914529305	9.15643159870812	8.35975424303832	8.90496383873056	NA
**TC0500003391.mm.2**	Fzd9	frizzled class receptor 9	-0.29705236277607	7.15263667005658	-0.676893222473078	0.522066697851333	0.999692817443324	-4.61139963397225	6.83544453007103	6.63301413584498	7.07670132333899	7.34992699109669	6.94038684347769	8.08034619651013	NA
**TC1700002131.mm.2**	Tdrd6	tudor domain containing 6	-0.242282107662674	4.80757541826262	-0.676577387326335	0.522254447399153	0.999692817443324	-4.61141275084646	4.17888111172573	4.63364168719516	4.6011200021615	5.17306274550325	5.0136502149146	5.2450967480755	NA
**TC0600000963.mm.2**	Cct7	chaperonin containing Tcp1, subunit 7 (eta)	0.230796098532237	6.19095514096835	0.676456804935401	0.522326139532243	0.999692817443324	-4.61141775752729	6.07308275131565	6.28802401322542	6.26863288488926	6.3544752542775	5.77181312821974	6.38970281388255	NA
**TC1400001045.mm.2**	Htr2a	5-hydroxytryptamine (serotonin) receptor 2A	-0.291058370212089	6.92055439596436	-0.676255774453366	0.522445676018548	0.999692817443324	-4.61142610300762	7.15367062113276	7.53351208705336	6.65495319402426	6.47206026956368	7.38910738922005	6.32002281479206	NA
**TC1400000076.mm.2**	Pxk	PX domain containing serine/threonine kinase	0.377199975098579	8.03703638103312	0.676179700439071	0.522490915623687	0.999692817443324	-4.61142926062674	8.34294888566704	8.5026708230045	8.65227549672671	7.41322376713547	8.24285779611993	7.06824151754509	NA
**TC1700000047.mm.2**	Synj2	synaptojanin 2	0.212083654687231	6.68728356837586	0.676165891198736	0.522499127955679	0.999692817443324	-4.61142983378134	7.18839824330752	7.31695812510198	6.30699346456149	6.71426271104858	6.22588215383496	6.37120671240065	NA
**TC1800001259.mm.2**	Gpr151	G protein-coupled receptor 151	0.229909941484337	5.48262998692467	0.676146480168522	0.522510671802555	0.999692817443324	-4.61143063942438	5.47511609246992	5.42080359613626	5.52164445912001	5.70819559883525	5.07376482051167	5.69625535447491	NA
**TC1100000334.mm.2**	Mpg	N-methylpurine-DNA glycosylase	-0.437283760307378	6.37188370470302	-0.676029666174559	0.522580145181398	0.999692817443324	-4.61143548735595	6.96822699499896	6.46314097343015	6.98814522875216	4.97453814083498	6.46795095632088	6.36929993388101	NA
**TC1700000366.mm.2**	Unkl	unkempt family like zinc finger	0.416239457787048	6.90438149786026	0.675822179820269	0.522703559186367	0.999692817443324	-4.61144409677477	7.50552909215503	7.26036771977062	7.7993643622528	5.94707118315221	6.74240560527462	6.1715510245563	NA
**TC1100002602.mm.2**	Itk	IL2 inducible T cell kinase	0.392881201594213	5.30099720137509	0.675754144654944	0.52274403093563	0.999692817443324	-4.61144691939229	4.71735756210938	4.71652023568911	4.95396629791872	6.62496760890152	5.04537499002245	5.74779651360936	NA
**TC1200001070.mm.2**	Gskip	GSK3B interacting protein	-0.253195276310461	6.94088758590717	-0.675653390436317	0.52280396979749	0.999692817443324	-4.61145109905815	7.73983674075449	7.32651625033079	6.81872665272131	6.21756433314711	6.54105966451012	7.00162187397922	NA
**TC1100000078.mm.2**	Dbnl	drebrin-like	0.228391539112064	7.10347937776723	0.675639956818254	0.522811961813132	0.999692817443324	-4.61145165630045	7.55952893581868	7.64239992454656	7.14772222591099	6.79014301632013	6.47922740933586	7.00185475467113	NA
**TC0100002534.mm.2**	Ikzf2	IKAROS family zinc finger 2	0.220103871073811	6.03561097070327	0.675628097581941	0.522819017252361	0.999692817443324	-4.61145214822871	5.58881362392268	5.67074170103451	6.18875093658202	6.50840818412302	5.90807806221447	6.34887331634295	NA
**TC1600001479.mm.2**	Apod	apolipoprotein D	-0.406836132695823	9.77189849253777	-0.675485409678443	0.522903911634371	0.999692817443324	-4.61145806650617	10.5089135388703	10.5969871809618	9.96369594942517	8.39221303557627	10.1154408113538	9.0541404390393	NA
**TC0200002793.mm.2**	Ntsr1	neurotensin receptor 1	0.29668964361138	6.11832552389745	0.675375526900962	0.522969294115185	0.999692817443324	-4.61146262349019	6.5169411890598	6.34297928505586	6.68358845768886	5.53811752055704	5.67466107844045	5.95366561258269	NA
**TC0800001420.mm.2**	Osgin1	oxidative stress induced growth inhibitor 1	-0.273806152657678	5.42063234123648	-0.675358861557092	0.522979210789978	0.999692817443324	-4.61146331457595	5.2223845794628	4.88369547671797	5.06056583663309	5.87448500632829	5.47911299937437	6.00355014890236	NA
**TC0600003232.mm.2**	Clec12b	C-type lectin domain family 12, member B	0.211543275336354	4.74272155972493	0.675126258031212	0.523117633556664	0.999692817443324	-4.61147295895853	4.18621409135641	4.32007019896676	5.00468494886346	5.1818808604861	4.68703494472339	5.07644431395346	NA
**TC1900000609.mm.2**	Pi4k2a	phosphatidylinositol 4-kinase type 2 alpha	0.211741719679091	7.32818440487112	0.675089827812181	0.523139315368684	0.999692817443324	-4.61147446923221	7.01413797452998	6.92236075226271	7.44473968157343	7.78330588932266	7.26049589510795	7.54406623642997	NA
**TC1500000408.mm.2**	Fam83a	family with sequence similarity 83, member A	-0.271836969765602	5.17360872534814	-0.674942903618295	0.523226764623038	0.999692817443324	-4.61148055959796	4.47499766065804	4.82191506779069	4.83769606679779	5.76283677525667	5.46228174921738	5.68192503236828	NA
**TC1400002328.mm.2**	Rhobtb2	Rho-related BTB domain containing 2	0.301976114637306	8.31881038723875	0.674713125198839	0.523363547459538	0.999692817443324	-4.6114900824986	8.64455588720597	8.59567034951279	8.71077957005739	7.9275145879368	8.49114479352311	7.54319713519647	NA
**TC1000000891.mm.2**	Apba3/Mir3057	amyloid beta (A4) precursor protein-binding, family A, member 3/microRNA 3057	0.225850783200886	6.87285792420283	0.674704726485952	0.523368547491291	0.999692817443324	-4.61149043052789	6.80224867742235	6.73858658831517	6.79948093566281	7.2745259872778	6.6177494205925	7.00455593594635	NA

**TC1400002215.mm.2**	Fdft1	farnesyl diphosphate farnesyl transferase 1	-0.328821156275543	8.32902199600519	-0.674548541686827	0.523461535042348	0.999692817443324	-4.61149690199074	9.3836241888121	9.07944214185497	8.32776021816728	7.0989514482392	8.3551886660974	7.72916531286016	NA
**TC0100000519.mm.2**	Eef1b2	eukaryotic translation elongation factor 1 beta 2	0.198058280422371	9.51388704777447	0.674510362739215	0.523484267203397	0.999692817443324	-4.6114984837528	10.4512898067182	10.3193750383962	9.15025126801966	9.20413573316893	8.89725976036878	9.06101067997507	NA
**TC1300000560.mm.2**	Ninj1	ninjurin 1	0.210258073851138	8.32631374905154	0.674410125092552	0.523543952796097	0.999692817443324	-4.6115026363035	8.00774077485148	7.85475345232887	8.77678379167859	8.481168415737	8.55792642911976	8.27950963059356	NA
**TC0100003502.mm.2**	Rgs4	regulator of G-protein signaling 4	-0.499668240716515	8.82628968685313	-0.674303896055117	0.523607210660429	0.999692817443324	-4.6115070365592	9.36469056058963	9.44448154041685	9.44851593638022	7.12609883295941	9.17440989448359	8.39954135628906	NA
**TC1300001558.mm.2**	Hist1h2ak	histone cluster 1, H2ak	-0.22517412240355	9.01923930467323	-0.674020801524635	0.523775813263211	0.999692817443324	-4.6115187604816	9.01357091956127	9.36572677602928	8.89754704714142	8.64534789667945	8.86944366729221	9.22379952133575	NA
**TC0600001033.mm.2**	Ruvbl1	RuvB-like protein 1	-0.435409985771403	6.46549597863069	-0.67376040036834	0.523930931034732	0.999692817443324	-4.61152954136056	7.59215369464802	7.74193429481633	6.67513710270186	4.61889685268663	5.96110129547377	6.20375263145753	NA
**TC0700004075.mm.2**	Trim66	tripartite motif-containing 66	0.257912399142619	6.05222406435728	0.673504553734484	0.52408336434338	0.999692817443324	-4.6115401306598	7.04261082842645	6.48934483714654	5.58941040624209	6.05919635318588	5.29369701427364	5.83908494686909	NA
**TC0100000137.mm.2**	Khdc1a	KH domain containing 1A	0.335682699516575	5.31422860200272	0.673444482812357	0.524119158681903	0.999692817443324	-4.61154261651627	4.24083041305556	4.9064229006257	5.29150452367579	6.41323732500832	5.18788904011381	5.84548740953716	NA
**TC0500001432.mm.2**	Hip1r	huntingtin interacting protein 1 related	0.206691363082109	6.46838422598039	0.673270577574703	0.524222792055253	0.999692817443324	-4.61154981213723	6.57687046885913	6.86420604399784	6.40036765796537	6.49093812662942	6.11691117199657	6.361011886434	NA
**TC0300001631.mm.2**	Pag1	phosphoprotein associated with glycosphingolipid microdomains 1	-0.200436274961192	6.60447022443105	-0.673140470763569	0.524300333695276	0.999692817443324	-4.61155519462051	6.41155529830779	6.56839149912715	6.51209428391174	6.61090671570275	6.86702400972535	6.65684953981153	NA
**TC1500001831.mm.2**	Tst	thiosulfate sulfurtransferase, mitochondrial	-0.384894811619684	6.70171071068672	-0.673034913308128	0.524363249686958	0.999692817443324	-4.61155956093481	7.17385911641287	7.27219018839395	7.04528686702286	5.45192580101419	7.01950854376843	6.247493747508	NA
**TC0700002850.mm.2**	Faap24	Fanconi anemia core complex associated protein 24	-0.21128219281503	6.1543891556223	-0.672987105682643	0.524391746316923	0.999692817443324	-4.61156153829793	6.39685427129159	6.39926426911433	5.89888533027831	5.95494067357059	6.36331346759025	5.91307692188871	NA
**TC0900001779.mm.2**	Izumo1r	IZUMO1 receptor, JUNO	0.281428978517687	6.6610700024188	0.672984108875519	0.524393532652865	0.999692817443324	-4.61156166224488	6.34337720080547	6.17039447485316	6.94567291261878	7.06208023532599	6.18768788680465	7.25720730410475	NA
**TC1500001938.mm.2**	Shisa8	shisa family member 8	-0.27977197512485	6.47308567538448	-0.672941717813805	0.524418801521509	0.999692817443324	-4.61156341548116	5.91263956236139	6.5912879127335	6.05269191969512	6.83482939378603	6.57443883260378	6.87262643112707	NA
**TC1500000433.mm.2**	Nsmce2	NSE2/MMS21 homolog, SMC5-SMC6 complex SUMO ligase	-0.459645311436371	7.01408859878444	-0.67293724250517	0.524421469251604	0.999692817443324	-4.611563600569	7.58073953452206	7.71383496076958	7.42771615552476	5.50761708174636	7.4827784243927	6.37184543575116	NA
**TC0600002233.mm.2**	Tmem176b	transmembrane protein 176B	-0.522923492185492	8.03138880389541	-0.672931375288964	0.524424966710511	0.999692817443324	-4.61156384322135	9.06700398198281	9.28082381524305	8.35926225733527	6.03806676355252	8.28527956235318	7.15789644290559	NA
**TC0800000488.mm.2**	Pcm1	pericentriolar material 1	-0.570339660831542	6.3451197849799	-0.67274696170999	0.524534903598137	0.999692817443324	-4.61157146927051	8.08664853545084	7.87263834592035	6.55589914573612	3.92947710768646	6.36938577853256	5.2566697965531	NA
**TC0300000778.mm.2**	Tpm3	tropomyosin 3, gamma	-0.443569190383853	8.98120304958016	-0.67267649520285	0.524576915610381	0.999692817443324	-4.61157438285985	9.96863245934986	10.1796881525643	9.13458937974157	7.291290272658	9.17811978803479	8.13489824513249	NA
**TC0100003725.mm.2**	Mia3	melanoma inhibitory activity 3	0.473715902782176	6.50563202213301	0.672573850708881	0.524638115930476	0.999692817443324	-4.61157862650851	8.42247132868559	8.31340143382103	6.59283836375505	5.02983722417286	6.18374249678626	4.4915012855773	NA
**TC0200002751.mm.2**	Gm14322	predicted gene 14322	-0.6361712595128	7.78947696681224	-0.672456688446546	0.524707977838224	0.999692817443324	-4.6115834697794	8.69860649892912	8.97376256101431	8.26850506023263	5.62757005071959	8.50600172031112	6.66241590966669	NA
**TC1200001051.mm.2**	Glrx5	glutaredoxin 5	-0.336071621806122	7.61638957303713	-0.672452952702349	0.524710205498097	0.999692817443324	-4.6115836241978	7.97332189398443	7.8383047592766	7.96990598598157	6.6373777846932	7.93834948262299	7.34107753166402	NA
**TC1500000053.mm.2**	Nup155	nucleoporin 155	0.251261265114917	5.69158406185175	0.672338555804599	0.524778424383723	0.999692817443324	-4.6115883525278	6.51315426085548	6.863250038997	5.39235377409028	5.24545752665364	5.45179835086479	4.6834904196493	NA
**TC1200000183.mm.2**	Iah1	isoamyl acetate-hydrolyzing esterase 1 homolog	-0.264079490752841	6.28120088483666	-0.672338406877538	0.524778513197849	0.999692817443324	-4.61158835868296	6.16225554273198	5.95972996657605	6.45671933961593	6.0618110694872	6.09748648122892	6.94920290937989	NA
**TC0900003311.mm.2**	Fyco1	FYVE and coiled-coil domain containing 1	-0.420019347425631	5.50661001963539	-0.672233539114438	0.524841054509512	0.999692817443324	-4.61159269262124	6.38297696410452	6.26797825247811	5.58502383817864	4.18930926501059	6.01392441853659	4.60044737950391	NA
**TC0200004413.mm.2**	Srp14	signal recognition particle 14	-0.202512467871083	8.23845433150565	-0.672173932197535	0.524876605160804	0.999692817443324	-4.61159515581063	8.53578800601454	8.38773188318238	7.98964668694113	8.06144389510626	8.08791885325558	8.36819666453398	NA
**TC1500001347.mm.2**	Rida	reactive intermediate imine deaminase A homolog	-0.619046832751483	8.04711205279708	-0.672155309720294	0.524887712260823	0.999692817443324	-4.61159592533039	9.40361880338404	9.19134741125309	8.38122500031903	5.84358121800215	8.64834443210672	6.81455545171742	NA
**TC0100002054.mm.2**	Rims1	regulating synaptic membrane exocytosis 1	-0.262867407142229	8.63301221105118	-0.672068563489767	0.524939452739987	0.999692817443324	-4.61159950965699	9.08060654400713	9.31678781978334	8.57061389088272	7.86685815323334	8.77940495435412	8.1838019040464	NA
**TC1300000894.mm.2**	Slc9a3	solute carrier family 9 (sodium/hydrogen exchanger), member 3	0.242709210964167	6.3374969641927	0.671909130771619	0.52503455614641	0.999692817443324	-4.61160609646729	5.57855300926629	5.8335286059869	6.45600044578004	7.09315885013562	6.34120833371512	6.7225325402722	NA
**TC0700000411.mm.2**	Prr19	proline rich 19	0.406918503080172	6.75728234604908	0.671884977844204	0.525048964598301	0.999692817443324	-4.61160709422083	6.15148191552342	5.64235610749677	6.62266116321848	8.15918536649883	6.47518170187732	7.49282782167964	NA
**TC1900001762.mm.2**	Csf2ra	colony stimulating factor 2 receptor, alpha, low-affinity (granulocyte-macrophage)	0.257919689842621	6.23614363916928	0.671869148998819	0.525058407448058	0.999692817443324	-4.6116077480934	6.12801576554894	6.40841350414736	6.04249311516266	6.65564285733967	6.39895285705219	5.7833437357649	NA
**TC1100001640.mm.2**	Coasy	Coenzyme A synthase	0.204539392371523	6.0832182098455	0.671637672429797	0.525196509405047	0.999692817443324	-4.61161730882987	6.07466040298301	6.12073654156851	6.08622584123617	6.27026970839609	5.81957808755269	6.12783867733652	NA
**TC0400001707.mm.2**	1700013G24Rik	RIKEN cDNA 1700013G24 gene	-0.361095017100614	5.42971376190117	-0.671471037412644	0.525295940394632	0.999692817443324	-4.61162418987903	4.54467532492122	4.5910757187637	4.89719539017235	6.46297535658809	5.8154493812659	6.26691139969578	NA
**TC1800000890.mm.2**	Cyb5a	cytochrome b5 type A (microsomal)	-0.636980229414764	9.72426101458071	-0.671464873801402	0.525299618447246	0.999692817443324	-4.61162444437563	9.24478072285236	9.22692523395819	11.0779507104417	8.22199912548039	11.0543679006636	9.51954239408798	NA
**TC0700000931.mm.2**	Siglech	sialic acid binding Ig-like lectin H	-0.267947096655111	6.15551139631599	-0.671451953707655	0.525307328393813	0.999692817443324	-4.61162497784293	6.92781469924812	6.35578516401762	6.18103420312142	5.37575295753857	5.99477459857047	6.09790675539975	NA
**TC1000000261.mm.2**	Soga3	SOGA family member 3	0.469189791727791	5.47640909063602	0.671386989311869	0.525346096391309	0.999692817443324	-4.61162766008994	6.52232049695578	6.70207493423753	5.8463403190269	4.4398790290123	5.56959189311563	3.77824787146799	NA
**TC0100003534.mm.2**	Ndufs2	NADH:ubiquinone oxidoreductase core subunit S2	-0.193547805113848	8.60543581052233	-0.67129834709556	0.525398997253797	0.999692817443324	-4.61163131963362	8.98835985100586	8.92230677430826	8.31607129003804	8.35135502375804	8.50642069761865	8.54810122640513	NA
**TC0500003741.mm.2**	Gpc2	glypican 2 (cerebroglycan)	-0.373579909807697	5.20639530855536	-0.671204229474515	0.525455169508405	0.999692817443324	-4.611635204832	5.10185489967854	5.30203459865882	4.92124625220785	5.12241501448184	4.55251252800224	6.23830855830284	NA
**TC0500003382.mm.2**	Cldn13	claudin 13	0.251991458803662	5.89102230171446	0.671144668270698	0.52549071942359	0.999692817443324	-4.61163766332319	5.35817088053241	5.51879269214927	5.81844222261201	6.66813435499419	6.02111014733022	5.96148351266866	NA
**TC1500000317.mm.2**	Zfpm2	zinc finger protein, multitype 2	-0.223946345928865	6.6726168796689	-0.671105944265145	0.525513833196587	0.999692817443324	-4.6116392616359	6.12398164421217	6.56072728072942	6.91966522147811	6.53188460912893	7.03524176557815	6.86420075688662	NA
**TC0100001060.mm.2**	Clasp1	CLIP associating protein 1	0.328532547970571	7.51867462058824	0.670863800702836	0.525658379734324	0.999692817443324	-4.61164925442295	8.47816520251204	8.22640217134498	7.86161396332539	6.67065875948138	7.30532895665556	6.56987867021007	NA
**TC1200001364.mm.2**	Fam228b	family with sequence similarity 228, member B	-0.259637278618731	6.35820627386511	-0.67083465638691	0.525675779015828	0.999692817443324	-4.61165045697004	7.17388177681961	6.92647910953376	6.31257984401351	5.4522212557864	6.01615981156626	6.2679158454711	NA
**TC0500003711.mm.2**	D830014E11Rik	RIKEN cDNA D830014E11 gene	0.388400680485811	4.47737228347381	0.670661354540442	0.525779248547738	0.999692817443324	-4.61165760691228	3.79643352563121	3.84998322623846	4.52692302897339	5.47038612599901	3.84232934838955	5.37817844561123	NA
**TC1700002334.mm.2**	Vmac	vimentin-type intermediate filament associated coiled-coil protein	0.20942093425597	6.60867970834847	0.670414433446783	0.525926694745007	0.999692817443324	-4.61166779179505	6.0044566058046	5.65715351962068	7.13029575165948	7.07435924492927	6.76113652637797	7.02467660169884	NA
**TC0900002915.mm.2**	Pik3cb	phosphatidylinositol-4,5-bisphosphate 3-kinase catalytic subunit beta	0.311058014324824	6.86354576859391	0.670328805462306	0.525977832704541	0.999692817443324	-4.61167132308359	7.46537061119698	7.37885446112222	7.00202723780742	6.47755554613955	7.02562360455134	5.83184315074598	NA
**TC0300003246.mm.2**	Gstm3	glutathione S-transferase, mu 3	0.204046303817894	6.43793541556616	0.670320672730041	0.525982689825168	0.999692817443324	-4.61167165845907	6.64371988573022	6.97352422423314	6.29801368682939	6.4112168087053	6.19628235503264	6.10485553286626	NA
**TC0400001076.mm.2**	Dab1	disabled 1	0.391802965841489	8.02143485764928	0.670257598082616	0.526020360937139	0.999692817443324	-4.61167425941167	8.65527788913181	8.74283848264382	8.48902870500607	7.26802064789544	8.16393812530993	6.80950529590861	NA
**TC0900000696.mm.2**	Man2c1	mannosidase, alpha, class 2C, member 1	-0.243910502606625	7.01174709560463	-0.670137081989109	0.526092343534453	0.999692817443324	-4.61167922851772	6.32907765909332	6.35002571404119	6.90283568231424	7.54894341532576	7.23309021721088	7.70650988564237	NA
**TC0500002933.mm.2**	Evi5	ecotropic viral integration site 5	0.236613999007405	8.29223443118466	0.669998388650931	0.526175190920151	0.999692817443324	-4.6116849462805	8.3542818287832	8.47226671974947	8.56142442255274	8.1386185957423	8.40903636096236	7.81777865931787	NA
**TC0200003283.mm.2**	Fnbp1	formin binding protein 1	-0.396317832923056	7.69125512634361	-0.669683635383858	0.526363237182301	0.999692817443324	-4.61169791900118	8.65021841310434	8.42793903368573	7.91517691665357	6.22319190605917	7.8296363697876	7.10136811877125	NA
**TC0600002624.mm.2**	Nat8f2	N-acetyltransferase 8 (GCN5-related) family member 2	0.26311395131822	4.91649364757892	0.669625720750828	0.526397842378126	0.999692817443324	-4.61170030548735	4.84787493507943	5.13123854668694	5.37257368832186	4.65046446484994	4.4646087249343	5.03220152560106	NA
**TC0X00002417.mm.2**	Xlr4a	X-linked lymphocyte-regulated 4A	-0.251486642033762	5.26643186861976	-0.669622809102885	0.526399582186505	0.999692817443324	-4.61170042546345	4.84431984838252	5.24975002734152	4.95879690861156	5.54197711735194	5.75078085203047	5.25296645800056	NA
**TC1800000567.mm.2**	Tex43	testis expressed 43	0.203217951189315	6.01398988476434	0.669598093089884	0.526414350989953	0.999692817443324	-4.61170144388507	5.83109540756796	5.65563282075442	6.1617683225202	6.34005516880095	6.17044111140285	5.92494647753966	NA
**TC0X00001682.mm.2**	Gpm6b	glycoprotein m6b	-0.389635391134773	10.7840298068685	-0.669538228314868	0.52645012367642	0.999692817443324	-4.61170391049268	11.6539269143632	11.7566211708611	10.8769293905014	9.38025059635727	11.0289294486187	10.0075213205095	NA
**TC1600001111.mm.2**	Nlrc3	NLR family, CARD domain containing 3	-0.202306086012041	5.94905060325866	-0.669530485447969	0.526454750601044	0.999692817443324	-4.6117042295099	5.33387352229711	5.40842576997049	6.13410819856391	6.13958787906624	6.17568957440031	6.50261867525392	NA
**TC1800000327.mm.2**	Cystm1	cysteine-rich transmembrane module containing 1	0.19937245680606	7.49035070476017	0.669400975009049	0.526532146353579	0.999692817443324	-4.61170956511734	7.06161744092883	6.84738976834788	7.82111067789372	7.89481028855449	7.74192170581078	7.57525434702532	NA
**TC1600001130.mm.2**	Srl	sarcalumenin	0.372151233699761	5.19999269361535	0.669281282190599	0.526603681517389	0.999692817443324	-4.61171449557022	5.5697835896646	5.46452991300591	5.71686707538257	4.73810548782798	4.1578872886156	5.55278280719543	NA
**TC0500003024.mm.2**	Grk3	G protein-coupled receptor kinase 3	0.260961380612901	6.69348106336528	0.669279899971524	0.526604507645449	0.999692817443324	-4.61171455250351	7.63614057503586	7.21631304315298	6.71990111923326	6.19527664238106	6.5290706859523	5.86418431443622	NA
**TC0200002171.mm.2**	Cfap61	cilia and flagella associated protein 61	-0.275073175413606	6.39013686407057	-0.669193707102215	0.526656025240198	0.999692817443324	-4.61171810259458	6.74930662792525	7.21909398803609	5.72027310153235	6.19086400728508	5.82163152798222	6.63965193166242	NA
**TC0300002957.mm.2**	Cyp2u1	cytochrome P450, family 2, subfamily u, polypeptide 1	0.210509297181569	7.80868201533752	0.669184961568019	0.526661252637221	0.999692817443324	-4.61171846278444	7.88029754106921	7.90259733225399	7.91729453720176	7.82781336933076	7.88701116171458	7.43707815045481	NA
**TC1700001834.mm.2**	Notch3	notch 3	-0.207327366956187	6.07804283973313	-0.669003542019506	0.526769698521732	0.999692817443324	-4.61172593385992	6.02512507799778	6.12639966700302	5.99903507183757	5.95200370790525	5.98224762539177	6.38344588826342	NA
**TC1400002343.mm.2**	Bmp1	bone morphogenetic protein 1	-0.230572686864027	6.27455715338035	-0.668922631108776	0.526818068651752	0.999692817443324	-4.61172926538065	6.37298204131461	6.77005759887199	5.7908752490455	6.23070370413823	6.4110609066427	6.07166342026909	NA
**TC0300001192.mm.2**	Dbt	dihydrolipoamide branched chain transacylase E2	0.284008391916618	6.95846863199745	0.668909263681782	0.526826060234136	0.999692817443324	-4.61172981575778	7.29161276633984	6.92298470287748	7.54232582403805	6.50978972926226	6.62506903234417	6.8590297371229	NA
**TC0300001582.mm.2**	Lrrc40	leucine rich repeat containing 40	0.417057697985955	6.07408211971401	0.668811452655036	0.526884537926134	0.999692817443324	-4.61173384268158	7.70594834821721	7.6459790936316	5.85908315123147	5.10425718497211	5.98195009374451	4.14727484648716	NA
**TC0X00002995.mm.2**	Gla	galactosidase, alpha	-0.384638945981813	5.63840657710681	-0.66864288471926	0.526985328326022	0.999692817443324	-4.61174078166628	6.37298359995085	6.24197295928322	6.00263101849304	4.22047148722855	5.48042191301712	5.51195848466809	NA
**TC1000003216.mm.2**	Ube2g2/Mir1930	ubiquitin-conjugating enzyme E2G 2/microRNA 1930	-0.194376220213539	7.09939767651677	-0.66845944913309	0.527095022360207	0.999692817443324	-4.61174833118234	7.32329683644273	7.27612271986125	6.96875353977648	6.83535349140831	7.13409411524758	7.05876535636428	NA
**TC0400000437.mm.2**	Ccdc107	coiled-coil domain containing 107	0.2832657234696	7.60237251102802	0.668410296937354	0.527124417722601	0.999692817443324	-4.61175035383808	7.07067396852611	6.83612559971387	7.69356122748282	8.44342224495085	7.38811600738446	8.18233601811002	NA
**TC0700000939.mm.2**	Gm9962	predicted gene 9962	0.296724294999928	6.65605454419405	0.668090442019572	0.527315731738879	0.999692817443324	-4.61176351343071	5.7235521203617	6.38585906916556	6.64336248921832	7.56681984360013	6.59400574789972	7.02272799491887	NA
**TC0300000729.mm.2**	Mex3a	mex3 RNA binding family member A	-0.208864936650649	5.87994845891238	-0.667868339557188	0.527448603315092	0.999692817443324	-4.61177264848376	5.92345403462236	5.85417995008631	5.68828249056688	5.85388095716528	5.77754785644778	6.18234546458567	NA
**TC1700000704.mm.2**	Flot1	flotillin 1	-0.241345664554788	6.93047956739647	-0.667810340636762	0.527483304357429	0.999692817443324	-4.61177503359974	7.46356268841997	7.39922047205096	6.68493732194792	6.43376413545124	7.17177333324143	6.42961945326731	NA
**TC0X00002504.mm.2**	Prkx	protein kinase, X-linked	-0.388371305456339	6.76873174139317	-0.667689863780625	0.527555390900983	0.999692817443324	-4.61177998752909	6.79728149367165	6.92654218961482	7.27491127457371	5.78100080250622	7.42651545424839	6.40613923374422	NA
**TC0700004403.mm.2**	Cpxm2	carboxypeptidase X 2 (M14 family)	-0.286591126003379	5.13301012570965	-0.667613623809436	0.527601011829503	0.999692817443324	-4.6117831221213	5.03469880712215	4.73658975772023	4.77377253466651	5.45302243403787	4.9646614376158	5.83531578309534	NA
**TC1100003058.mm.2**	Senp3	SUMO/sentrin specific peptidase 3	-0.197441881153349	7.53254422769024	-0.667595797807693	0.527611679020658	0.999692817443324	-4.61178385499541	7.82641613682672	7.90132871590404	7.17220703729177	7.36411133826021	7.53759936225847	7.39360277560021	NA
**TC1600000529.mm.2**	Hcls1	hematopoietic cell specific Lyn substrate 1	-0.228876454490949	5.85788487078681	-0.667564583201364	0.52763035836712	0.999692817443324	-4.61178513827523	5.72036166055822	6.00623565160572	5.72003946880887	5.76144003297863	6.27235480220385	5.66687760856555	NA
**TC0900002180.mm.2**	Ttc36	tetratricopeptide repeat domain 36	-0.23539745520118	7.45481724530312	-0.667564270080656	0.52763054574596	0.999692817443324	-4.61178515114787	7.18802924954621	7.23326410380069	7.23094903977486	7.68745856425986	7.40869459697797	7.98050791745911	NA
**TC0300000459.mm.2**	Selenot	selenoprotein T	-0.244774466627124	9.16950499028352	-0.66751059937377	0.527662664192044	0.999692817443324	-4.61178735752633	10.0523654428947	9.8171443040757	8.99482818351968	8.33415744721854	9.11892586378408	8.69960870020839	NA
**TC0800000786.mm.2**	Babam1	BRISC and BRCA1 A complex member 1	-0.225522586827201	7.79254078697309	-0.667398583842951	0.527729702246704	0.999692817443324	-4.61179196200648	8.54313277807493	8.49755219308804	7.62582586972964	7.00593141878096	7.6128875117513	7.4699149504137	NA
**TC0400000091.mm.2**	Rad54b/Fsbp	RAD54 homolog B (S. cerevisiae)/fibrinogen silencer binding protein	-0.360899282460657	4.74829986132291	-0.667387424794615	0.52773638091097	0.999692817443324	-4.61179242067564	3.78015731618508	4.55659462632842	4.20235761275516	5.51326671749615	4.91135344813601	5.52606944703662	NA
**TC1100004264.mm.2**	Mgat4b/Mir6919	mannoside acetylglucosaminyltransferase 4, isoenzyme B/microRNA 6919	0.203576389075099	7.22319987798348	0.667248807936579	0.527819347256207	0.999692817443324	-4.61179811775095	6.76635127477725	6.98053891452657	7.52735746284588	7.47237346552776	7.14922617051647	7.44335197970697	NA
**TC0900002401.mm.2**	Neo1	neogenin	0.493946831355194	7.10061234032046	0.667142655600383	0.527882888219138	0.999692817443324	-4.61180247995376	9.05302749530089	8.93079772027051	7.51802351902549	5.28584772550537	6.40573694959045	5.41024063223002	NA
**TC1500000432.mm.2**	Sqle	squalene epoxidase	-0.31057521936243	6.83488900585425	-0.667122487198383	0.527894961227519	0.999692817443324	-4.61180330869131	7.58114480085695	7.46187875361588	6.92956462339943	5.74301539756447	6.92234476644041	6.37138569324834	NA
**TC0500001851.mm.2**	Wdr95	WD40 repeat domain 95	-0.220537066588636	6.12376200352023	-0.667119688165541	0.527896636770616	0.999692817443324	-4.61180342370457	5.88354394437755	5.96797277562515	5.84978378014904	6.37520680382167	6.20999569644037	6.45606920207761	NA
**TC1100002249.mm.2**	Plek	pleckstrin	-0.25819126253357	7.29927278172197	-0.667034033216716	0.527947912744315	0.999692817443324	-4.61180694312248	7.6385604875775	7.97497414143117	7.30572147523661	6.52713829289141	7.36554832694473	6.98369396625043	NA
**TC0200002879.mm.2**	Cdc123	cell division cycle 123	0.280169961999485	8.97828331961016	0.667024507088447	0.527953615605244	0.999692817443324	-4.61180733451418	9.71463875428399	9.57561173172025	9.06892231505604	8.5009723627718	8.96990653928267	8.0396482145462	NA
**TC0200001743.mm.2**	Pak6	p21 (RAC1) activated kinase 6	-0.266077155294875	6.37701742822824	-0.666965782255167	0.527988772364255	0.999692817443324	-4.61180974719764	5.94440448941124	5.82086097252962	6.1179449393502	6.86439745906921	6.43605876027523	7.07843794873393	NA
**TC0300003020.mm.2**	Metap1	methionyl aminopeptidase 1	0.22840678536977	6.49559732607441	0.666823643276549	0.52807387277748	0.999692817443324	-4.61181558625564	7.70864448716748	7.41166743827051	5.78122179504315	6.37382100583084	5.89333232308776	5.80489690704669	NA
**TC0100001914.mm.2**	Rgs20	regulator of G-protein signaling 20	0.311649598751786	6.62414810727212	0.666731075144182	0.528129299182535	0.999692817443324	-4.6118193884462	6.62080506281909	6.79998055694924	6.53276927993535	6.94090908074862	5.80276909325788	7.04761006992252	NA
**TC0500002315.mm.2**	Fbxl5	F-box and leucine-rich repeat protein 5	-0.431477285081597	6.81393041189255	-0.666553909200988	0.528235389965819	0.999692817443324	-4.61182666434815	7.89054619318686	7.93792021250507	7.03910640009454	5.05697434765555	6.79433000505878	6.1647053128545	NA
**TC1200002195.mm.2**	Eml5	echinoderm microtubule associated protein like 5	0.209316244487382	6.86208784611764	0.666522505584052	0.528254196541099	0.999692817443324	-4.61182795388985	7.10718872261611	7.28285552253327	6.68753431660163	6.91302334366397	6.76768796291441	6.41423720837643	NA
**TC0900002779.mm.2**	Pgm3	phosphoglucomutase 3	0.313860566096564	5.50636457093875	0.666458614943675	0.52829245982091	0.999692817443324	-4.61183057732123	6.52284752897559	6.3262717026442	5.7134881213151	4.69490654178783	4.46769973717338	5.31297379373642	NA

**TC0200000486.mm.2**	Ralgds	ral guanine nucleotide dissociation stimulator	-0.331747097484902	8.50254320591969	-0.666440907297495	0.528303065013314	0.999692817443324	-4.61183130438649	8.88938973305118	8.77054667563727	8.87912732958377	7.46678698634618	8.67525930460137	8.33414920629838	NA
**TC0300000807.mm.2**	S100a4	S100 calcium binding protein A4	-0.253218619993098	6.06398542058974	-0.666368503409181	0.52834642944917	0.999692817443324	-4.61183427709589	5.49317573021438	6.08151646723487	5.83213157487424	6.31925996817725	6.09873317579059	6.5590956072471	NA
**TC0300001795.mm.2**	Slc7a14	solute carrier family 7 (cationic amino acid transporter, y+ system), member 14	0.50246397282925	7.29319991722281	0.666310387501865	0.528381238104543	0.999692817443324	-4.61183666300411	9.1589774708956	8.86636745605897	7.89634902336058	5.47304223765981	6.45333927639973	5.91112403896216	NA
**TC0100000279.mm.2**	Nms	neuromedin S	-0.225451078181108	5.25054321054962	-0.666094294426562	0.528510680325482	0.999692817443324	-4.61184553318786	4.71920534283286	5.07442438025358	5.11932976554831	5.51003392637621	5.35793819482672	5.72232765346002	NA
**TC1700001187.mm.2**	Vit	vitrin	0.309805847951051	5.87852126316742	0.665991588804382	0.52857220922797	0.999692817443324	-4.61184974829024	6.31890003683383	6.97845817645111	5.41837031902555	5.8783202117853	5.85688845317622	4.82019038173253	NA
**TC1100003314.mm.2**	Lgals9	lectin, galactose binding, soluble 9	0.292813244679621	7.02075765677966	0.66595963769515	0.52859135143202	0.999692817443324	-4.61185105948425	7.10192348893371	7.35753907489597	6.53735915254239	7.58795578056139	7.02257267299078	6.51715577075375	NA
**TC0300000853.mm.2**	S100a10	S100 calcium binding protein A10 (calpactin)	0.20996318416394	6.93862149322221	0.665831163819021	0.528668325759834	0.999692817443324	-4.61185633125657	6.98225933899944	6.9504218986519	6.97705684068578	7.08243020431913	6.59338525307876	7.04617542359826	NA
**TC1100001700.mm.2**	Higd1b	HIG1 domain family, member 1B	0.330511999946498	5.52985394525255	0.665624502397039	0.528792160605888	0.999692817443324	-4.61186480976236	5.34881972524717	5.50301301550629	5.24490609710953	6.24925136821789	4.89772707149835	5.93540639393607	NA
**TC0500000015.mm.2**	Pex1	peroxisomal biogenesis factor 1	0.350395425335456	6.22679901961072	0.665587546729149	0.528814306977263	0.999692817443324	-4.61186632570023	6.40244763289035	6.46776376685369	6.50634900238669	6.08933778190891	6.70976500976023	5.18513092386447	NA
**TC1900001541.mm.2**	Got1	glutamic-oxaloacetic transaminase 1, soluble	0.340952930545521	9.7746823905505	0.665586142868546	0.528815148278451	0.999692817443324	-4.61186638328597	10.8341365135607	10.5695589913834	10.1565587287553	8.80659362096973	9.46495260785892	8.81629388077502	NA
**TC0900002355.mm.2**	Lingo1	leucine rich repeat and Ig domain containing 1	-0.422478722976674	9.37471164769953	-0.66546127236618	0.528889983681773	0.999692817443324	-4.61187150505446	9.79801860686117	10.0512953630206	9.78172704697329	7.99527218820771	9.77640378604645	8.84555289508789	NA
**TC0700002116.mm.2**	LTO1	ABCE maturation factor	-0.204618212596096	7.17333510262661	-0.665406627750691	0.528922734538336	0.999692817443324	-4.61187374616639	7.44642861115853	7.32177556951491	6.8587750173159	7.07250998763113	7.01763394963884	7.32288748050038	NA
**TC1100002908.mm.2**	Slc47a2	solute carrier family 47, member 2	-0.322167305751171	5.24383605488869	-0.66539373705895	0.528930460669224	0.999692817443324	-4.61187427482569	4.50961777997692	4.23314067052288	4.93862487688645	6.09933675677854	5.4772940588498	6.20500218631753	NA
**TC0300003170.mm.2**	Rabggtb/Snord45c	Rab geranylgeranyl transferase, b subunit/small nucleolar RNA, C/D box 45C	-0.378622723978364	8.07222746460647	-0.6653557065732	0.528953254937986	0.999692817443324	-4.61187583444667	8.97071862178539	9.0090874947437	8.08732812635451	6.760828485222	8.38190922298121	7.22349283655203	NA
**TC0700001800.mm.2**	Sbk1	SH3-binding kinase 1	-0.209227849448855	6.16320600540459	-0.665123805108175	0.529092262825428	0.999692817443324	-4.61188534322297	5.71208757273519	6.04370540315863	6.04548570600596	6.35700797281203	6.37933051654345	6.44161886117226	NA
**TC1200001803.mm.2**	Foxa1	forkhead box A1	0.21648921916872	5.16394665254229	0.664993939295395	0.529170117963649	0.999692817443324	-4.61189066709265	5.09117712499631	4.70137562714655	5.21392420972834	5.5981285909582	5.21040568819082	5.1686686741959	NA
**TC0600003540.mm.2**	Hoxa2	homeobox A2	0.243870865653213	5.73688620850382	0.664896510295313	0.529228531859687	0.999692817443324	-4.61189466069815	5.45312257235068	5.37297712420481	5.86929872470037	6.17218091818654	5.39929087969357	6.15444703188692	NA
**TC1700002422.mm.2**	Ndufv2	NADH:ubiquinone oxidoreductase core subunit V2	-0.55106154718935	8.39011822791293	-0.664776741266796	0.529300345403635	0.999692817443324	-4.6118995694179	9.64108378939423	9.55205437049023	8.81118184607219	6.21154221053503	8.56486746012236	7.55997969086356	NA
**TC0500003507.mm.2**	Snx8	sorting nexin 8	-0.249439036884851	6.32968502953722	-0.664750232079531	0.52931624115558	0.999692817443324	-4.61190065580421	6.09107894123308	6.45297186017801	6.30994687723841	6.15764377378284	6.09756359279062	6.86890513200034	NA
**TC0800000754.mm.2**	Ell	elongation factor RNA polymerase II	0.21715220035554	6.44445005879177	0.664725701733381	0.529330950601242	0.999692817443324	-4.6119016610657	6.71156104184633	6.31547732267496	6.59150017725724	6.44548301721296	6.11929170660883	6.48338708715029	NA
**TC0300003015.mm.2**	Gm5105	predicted gene 5105	-0.379834511054929	4.28010547687974	-0.664658618003185	0.52937117820196	0.999692817443324	-4.61190441003644	3.34232810353073	3.58265733943972	3.83053978955542	5.16744940854366	4.31018714023515	5.44747107997378	NA
**TC0500002722.mm.2**	Cox18	cytochrome c oxidase assembly protein 18	-0.394225516584548	7.29365299223124	-0.664594278226859	0.529409762178334	0.999692817443324	-4.61190704636934	7.78632333673145	7.79606483717725	7.77083091441953	5.9247084587353	7.35970285421537	7.12428755210856	NA
**TC1300000934.mm.2**	2210408I21Rik	RIKEN cDNA 2210408I21 gene	0.335366758344085	5.61939636542865	0.664574301949032	0.529421742132667	0.999692817443324	-4.61190786486166	6.77160440684255	6.60202297373694	4.98560099425391	5.52114117008636	5.60682034287018	4.22918830478193	NA
**TC1300000460.mm.2**	Gcnt2	glucosaminyl (N-acetyl) transferase 2, I-branching enzyme	-0.421916355433252	6.95536958561197	-0.664565580666458	0.529426372701681	0.999692817443324	-4.61190818122247	7.10068213833734	7.82082506783593	7.1473824896562	5.83605630865981	7.59993195601588	6.22733955316663	NA
**TC1100003073.mm.2**	Acap1	ArfGAP with coiled-coil, ankyrin repeat and PH domains 1	-0.421604186795492	5.10669222791878	-0.664426777452311	0.529510219230643	0.999692817443324	-4.61191390884294	4.41151391505779	4.87909545254125	4.54400513905062	5.70916267411071	4.75124427121311	6.34513191553921	NA
**TC0700004323.mm.2**	Zfp768	zinc finger protein 768	0.20545580928823	6.87641950367593	0.664305215781369	0.529583132297856	0.999692817443324	-4.61191888838772	6.26657569187403	6.59032203049664	7.28900789286866	7.11725756626202	6.90529531197973	7.09005852857449	NA
**TC0400003537.mm.2**	Ago3	argonaute RISC catalytic subunit 3	0.217182880532616	6.93006657229796	0.66418815096224	0.529653354157478	0.999692817443324	-4.61192368308113	6.8356301246397	7.01132996383686	6.87835679765066	7.20554575553755	7.03420623955068	6.6153305525723	NA
**TC1400000924.mm.2**	Kif13b	kinesin family member 13B	0.266916829475153	5.08335466905571	0.664092940766076	0.529710470776741	0.999692817443324	-4.6119275821941	4.99499929568595	4.66491798481698	5.14912188291116	5.53790031347965	4.64190974581108	5.51127879162944	NA
**TC0700001397.mm.2**	Olfr521	olfactory receptor 521	-0.308614416518275	5.60387211706789	-0.664019721548601	0.529754397659124	0.999692817443324	-4.61193058043209	4.84148326745315	4.50142876877539	5.32529662573574	6.50624929083537	5.99740207993111	6.45137266967656	NA
**TC1100000701.mm.2**	Olfr315	olfactory receptor 315	-0.234364108664509	5.72500385290393	-0.663927658237646	0.529809633078891	0.999692817443324	-4.61193434996062	5.41379061047702	5.27836852133254	5.54296867896302	6.05159920517948	5.77485190261372	6.28844419885779	NA
**TC0800002612.mm.2**	4921524J17Rik	RIKEN cDNA 4921524J17 gene	-0.196200664993123	7.17836936086681	-0.663650564026476	0.529975903911135	0.999692817443324	-4.61194569320389	6.71855576015514	6.51525463439694	7.29148218380503	7.43052003652625	7.53683955729649	7.57756399302103	NA
**TC1100002107.mm.2**	Sec14l2	SEC14-like lipid binding 2	0.250238512557947	6.7742624187244	0.663628639046966	0.529989061442659	0.999692817443324	-4.61194659058213	6.54532342882391	6.83620007960458	7.21329154972225	6.66897246479465	7.00155034205083	6.38023664735017	NA
**TC0600001983.mm.2**	Tnpo3	transportin 3	0.30934496160092	7.09838973138408	0.663584950252666	0.530015280405481	0.999692817443324	-4.61194837867583	7.50778359488232	7.29703103819073	6.7424458379838	7.45966100123285	7.33355451662946	6.24986239938535	NA
**TC0400001737.mm.2**	Rnf186	ring finger protein 186	-0.237362400952996	6.79407832384842	-0.663404510074006	0.530123576706105	0.999692817443324	-4.61195576278962	6.11458347041493	6.39560626501243	6.72016076590937	7.16961693696922	6.90670726227807	7.45779524250651	NA
**TC0300002346.mm.2**	Efna3	ephrin A3	0.228074048730946	9.38335774909983	0.663295380014155	0.530189081016873	0.999692817443324	-4.61196022796313	9.18779712724164	9.23051333555369	9.7074333253659	9.46155873926687	9.67128981827076	9.04155414890012	NA
**TC1200002402.mm.2**	Cdc42bpb	CDC42 binding protein kinase beta	0.197901189732805	8.29382002816123	0.663258334254384	0.530211318558038	0.999692817443324	-4.61196174360469	8.63754620272286	8.50920366903109	8.23457581549562	8.27141052284389	7.927208969832	8.1829749890419	NA
**TC1400001902.mm.2**	Fermt2	fermitin family member 2	-0.199393374769935	8.09978068063333	-0.663207323988179	0.530241939571814	0.999692817443324	-4.61196383046767	8.1244361238905	8.03221074932366	8.15974832267353	7.86187690784944	8.18052799261533	8.2398839874475	NA
**TC1000000446.mm.2**	Ascc3	activating signal cointegrator 1 complex subunit 3	-0.372762035256589	5.90394659830292	-0.663204903839192	0.530243392393786	0.999692817443324	-4.61196392947453	6.65799239552372	6.99971802754409	5.89627848290877	4.61394406520949	6.1846799127938	5.07106670583764	NA
**TC0200000482.mm.2**	Mrps2	mitochondrial ribosomal protein S2	0.20312125058938	8.1660691124698	0.663139711338842	0.530282528576763	0.999692817443324	-4.61196659635904	8.07523882993952	7.84173290361141	8.52140658567898	8.22143613554435	8.17793477300783	8.15866544703674	NA
**TC0200003860.mm.2**	Zswim2	zinc finger SWIM-type containing 2	0.309652260513474	6.1047780817492	0.663129861581283	0.530288441715329	0.999692817443324	-4.61196699927425	5.17411095999231	5.73323166734712	5.99026207155806	7.18005312053331	6.33547430920359	6.21553636186083	NA
**TC0200005426.mm.2**	Nkain4	Na+/K+ transporting ATPase interacting 4	-0.342756231165276	6.90032595506485	-0.663129753494976	0.530288506603382	0.999692817443324	-4.61196700369561	7.90031761064833	7.36524975840645	7.07786774602699	6.54757020347489	6.60272975997987	6.80822065185256	NA
**TC0700003041.mm.2**	Saa1	serum amyloid A 1	-0.23917433255227	5.36058876279031	-0.66302305532776	0.530352563760241	0.999692817443324	-4.61197136801317	4.70011538338967	5.23528232839114	5.30669822491737	5.56819487501088	5.42311763103742	5.93012413399537	NA
**TC0700003985.mm.2**	Arfip2	ADP-ribosylation factor interacting protein 2	-0.224664818936072	8.05489610882297	-0.662975854056293	0.530380903010875	0.999692817443324	-4.61197329853719	8.33507525816559	8.43749343063774	7.99108457337417	7.56265458975701	8.23664392572147	7.76642487528185	NA
**TC0700000787.mm.2**	Egfbp2/Klk1b26	epidermal growth factor binding protein type B/kallikrein 1-related petidase b26	0.309430600378463	5.98610566584951	0.662961852374479	0.530389309688599	0.999692817443324	-4.61197387118375	5.25965896699139	5.12229975161315	6.01806870566274	7.05869953296199	5.82950710639268	6.62839993147512	NA
**TC0700000879.mm.2**	Gtf2h1	general transcription factor II H, polypeptide 1	-0.579244723169001	7.17853876953619	-0.662952972072515	0.530394641508743	0.999692817443324	-4.6119742343693	7.70045113398898	7.79700533188126	8.02203221011805	5.18561114238639	7.60203378351816	6.76409901532429	NA
**TC0400001325.mm.2**	Slc2a1	solute carrier family 2 (facilitated glucose transporter), member 1	-0.300178919405759	9.06738276630465	-0.662859325115709	0.530450870124177	0.999692817443324	-4.61197806410897	9.70285585509382	9.67980719680856	9.19778686469931	8.01285098885769	9.11236953070702	8.6986261616615	NA
**TC0200003204.mm.2**	Surf1	surfeit gene 1	0.259532229837509	9.11928164137134	0.662491659966076	0.530671664495539	0.999692817443324	-4.61199309602927	8.72353218052545	8.65892798068193	9.24089087655567	9.68525619679216	9.62496860625952	8.7821140074133	NA
**TC0700004288.mm.2**	Nupr1	nuclear protein transcription regulator 1	0.228675434789567	6.32410749972599	0.662485375794412	0.530675438842058	0.999692817443324	-4.61199335290195	6.19835407891583	6.63309225516604	6.44529672046297	6.33997804646363	5.90390093753266	6.4240229598148	NA
**TC0500003241.mm.2**	Rsrc2	arginine/serine-rich coiled-coil 2	0.412087665536889	7.5555464779571	0.662476777063452	0.530680603367324	0.999692817443324	-4.61199370438192	8.90956741749116	8.73806413627973	7.72435647358384	6.53055484893891	7.49961180439022	5.93112418705875	NA
**TC0700001352.mm.2**	Kctd14	potassium channel tetramerisation domain containing 14	-0.268626592910417	5.4561076544471	-0.662467926201748	0.530685919359289	0.999692817443324	-4.61199406616435	5.31889388829003	4.81269759639917	5.09508906127268	5.9388115668136	5.6399383994956	5.93121541441152	NA
**TC0200002363.mm.2**	Map1lc3a	microtubule-associated protein 1 light chain 3 alpha	0.200182348522432	8.29876352677192	0.662445907413738	0.530699144398688	0.999692817443324	-4.61199496617514	8.79528785008463	8.65948832191416	8.23737893678582	8.13170590605297	8.12846180922926	7.84025833656466	NA
**TC1300000630.mm.2**	Arl10	ADP-ribosylation factor-like 10	0.232989490821213	7.12690803989214	0.662357729903848	0.530752108125968	0.999692817443324	-4.61199857017681	7.7693541944242	7.07691524057343	7.20720853686329	6.98337035613552	6.86445443277099	6.86014547858539	NA
**TC1100002770.mm.2**	Tnip1	TNFAIP3 interacting protein 1	0.203135851604911	7.29240368079326	0.662219021226317	0.530835430117158	0.999692817443324	-4.61200423876436	7.35205476827536	7.32938998660997	7.52699197504355	7.21263254149848	7.05591242084858	7.27744039248363	NA
**TC1200000856.mm.2**	Vash1	vasohibin 1	0.247035778075111	6.36205932610198	0.661927736752359	0.531010430995334	0.999692817443324	-4.61201613973909	5.75301041296109	5.69267633299482	6.40311204125624	7.20725834214685	6.41735543347873	6.69894339377414	NA
**TC1700000005.mm.2**	Scaf8	SR-related CTD-associated factor 8	0.206566288855447	6.51229246587352	0.661807903312872	0.531082436358704	0.999692817443324	-4.61202103461623	7.32229110120544	7.51744000564844	6.26557781296128	6.05800032008778	6.12106411536162	5.78938143997654	NA
**TC1500001017.mm.2**	Aqp2	aquaporin 2	-0.214489580898297	5.98994746115593	-0.661787796408007	0.531094518775187	0.999692817443324	-4.61202185586448	5.58009265060259	5.45354056236487	5.93608332712447	6.3024528689613	6.14735115068509	6.52016420719728	NA
**TC0800000384.mm.2**	Purg	purine-rich element binding protein G	-0.243473819109603	6.97578744145808	-0.661739354079064	0.53112362891223	0.999692817443324	-4.61202383437029	7.28922411896702	7.30109256954703	6.56958434757692	6.81914581343069	7.33054293214549	6.54513486708132	NA
**TC1900001102.mm.2**	Ppp1r32	protein phosphatase 1, regulatory subunit 32	0.255147681854187	6.67854645789721	0.661574107667326	0.531222936970573	0.999692817443324	-4.61203058262706	6.61195243353392	6.54035805795452	6.42113190366758	7.29349990613402	6.4563488132635	6.74798763282973	NA
**TC1100001153.mm.2**	2610507B11Rik	RIKEN cDNA 2610507B11 gene	-0.362365013443905	7.13566135616156	-0.661519627332939	0.531255680566252	0.999692817443324	-4.6120328071919	8.47188954289177	8.59739305115774	7.05045778231424	5.45951997570177	6.86763046529664	6.36707731960718	NA
**TC1500002254.mm.2**	Fignl2	fidgetin-like 2	0.26172746530649	5.26646129071213	0.661404500231131	0.531324878086969	0.999692817443324	-4.61203750765864	4.79528040558969	4.76830256283827	5.99748825806072	5.28183159516816	5.04654049052348	5.70932443209242	NA
**TC1700001905.mm.2**	H2-Aa	histocompatibility 2, class II antigen A, alpha	-0.279365751959107	7.1245160654349	-0.661322944336966	0.531373900973349	0.999692817443324	-4.61204083709084	6.25334743300254	6.51931046818784	6.90355515193555	7.80429834181486	7.33695546220964	7.92962953545898	NA
**TC0300001469.mm.2**	Selenof	selenoprotein F	-0.350313476248827	8.98198199198185	-0.661274393010642	0.53140308631878	0.999692817443324	-4.61204281900012	9.94782070309972	10.001937358575	8.93183246855136	7.68892100030802	9.16065951232868	8.16072090902836	NA
**TC0900002197.mm.2**	Il10ra	interleukin 10 receptor, alpha	0.257876740538951	6.29755077646208	0.661257094260701	0.531413485249642	0.999692817443324	-4.61204352512436	6.04228709041492	6.34153738977326	6.09785154779536	6.86076528203576	5.98551190198245	6.45735144677076	NA
**TC1500001227.mm.2**	Pdzd2	PDZ domain containing 2	-0.214487594858213	6.94008829569192	-0.661236661863283	0.531425768099944	0.999692817443324	-4.61204435914424	7.06912983707205	7.15511786815325	6.63032028565608	6.86333315394882	7.2009863389478	6.72164229037353	NA
**TC0X00003000.mm.2**	Armcx2	armadillo repeat containing, X-linked 2	0.526482320443472	5.96361523417181	0.660886510004316	0.531636288327886	0.999692817443324	-4.61205864880431	7.32685169162206	7.20900737368344	6.49714630376656	4.65225218653959	6.00774584154745	4.08868800787176	NA
**TC0500003206.mm.2**	Rad9b	RAD9 checkpoint clamp component B	0.20287819678145	5.72393204654785	0.660858913760975	0.531652882134513	0.999692817443324	-4.61205977476183	6.05122728516425	5.9428573172294	5.73049702095378	5.64713501427441	5.66733706759917	5.30453857406611	NA
**TC0800000974.mm.2**	Prdx2	peroxiredoxin 2	-0.473855645637547	9.56124384935959	-0.660831386706332	0.531669434663729	0.999692817443324	-4.61206089786111	10.6608361521932	10.5982362865396	9.88865498421382	7.69168469886099	9.68847036625963	8.83958060809028	NA
**TC0900001423.mm.2**	Nckipsd	NCK interacting protein with SH3 domain	0.216856267168279	7.51825899321263	0.660808011495056	0.531683490868216	0.999692817443324	-4.61206185153821	7.86283469664277	7.49925682378477	7.7108388959018	7.37974859069061	7.1856971203455	7.47117783191035	NA
**TC0400003172.mm.2**	Slc35d1	solute carrier family 35 (UDP-glucuronic acid/UDP-N-acetylgalactosamine dual transporter), member D1	-0.578352346241275	7.06599569281116	-0.660654599636727	0.531775747777173	0.999692817443324	-4.6120681099048	7.04435180716797	7.12936674231612	8.14274582823412	5.39002962921605	7.89475432462767	6.79472582530506	NA
**TC1200001021.mm.2**	Ubr7	ubiquitin protein ligase E3 component n-recognin 7 (putative)	0.430760607856406	7.3802935273658	0.660623400554903	0.531794511129813	0.999692817443324	-4.61206938252318	8.11029978905981	7.92111721215282	8.27030774061977	6.28562494872771	7.17574334107384	6.51866813256083	NA
**TC1500000549.mm.2**	Ly6e	lymphocyte antigen 6 complex, locus E	-0.626796562019422	10.0016195149949	-0.660601871053369	0.531807459371209	0.999692817443324	-4.61207026069056	10.7184509816072	10.8399180481868	10.7210601994586	7.87781726860971	10.7037584915494	9.14871210055775	NA
**TC0400003393.mm.2**	Tie1	tyrosine kinase with immunoglobulin-like and EGF-like domains 1	-0.235600092255257	5.61840372432631	-0.660585827204682	0.531817108568854	0.999692817443324	-4.6120709150895	5.37988798968171	5.40182129339725	5.49303337449368	5.73572306469051	5.49122426469579	6.20873235899892	NA
**TC0800003102.mm.2**	Dbndd1	dysbindin (dystrobrevin binding protein 1) domain containing 1	-0.200693969960756	7.46023315104567	-0.660440938563179	0.531904253466382	0.999692817443324	-4.61207682428726	7.04568149170277	6.80519043198223	7.49706580925694	7.7575037120768	7.83495951938709	7.82099794186817	NA
**TC1700002810.mm.2**	H2-Ke6	H2-K region expressed gene 6	0.235491351656144	5.84005372764988	0.660310022105873	0.531983002397826	0.999692817443324	-4.61208216279812	6.1092064731549	6.03078655410051	5.58267212957795	6.10298389140014	5.8799073289692	5.3347659886966	NA
**TC1800001197.mm.2**	Cd14	CD14 antigen	-0.354842054047085	6.47009197019124	-0.660197661844579	0.532050595271854	0.999692817443324	-4.61208674389875	6.28943775872402	7.37220507029653	6.53869376679082	6.58591867522552	6.08503994425168	6.84925660585884	NA
**TC0900001857.mm.2**	Fdx2/Zglp1/Raver1/Gm38431/Mir7082	ferredoxin 2/zinc finger, GATA-like protein 1/ribonucleoprotein, PTB-binding 1/predicted gene, 38431/microRNA 7082	0.191269115286604	7.1815878413113	0.659988069872079	0.53217669459706	0.999692817443324	-4.61209528797397	7.6477231258404	7.51453547313945	7.0725783469487	7.08232499278188	6.89819994408165	6.87416516507573	NA
**TC0200005105.mm.2**	Wfdc5	WAP four-disulfide core domain 5	0.203237354379351	4.84863334811992	0.659873009772219	0.532245927607776	0.999692817443324	-4.61209997751253	4.24281305877961	4.50967180786157	5.32501350344624	5.04788146197229	5.03378976922184	4.93263048743799	NA
**TC1400001303.mm.2**	Mbnl2	muscleblind like splicing factor 2	0.363973836801968	9.35552427628106	0.659779211787697	0.532302371163692	0.999692817443324	-4.61210380000888	10.3248706357616	10.4604652915355	9.68673183392407	8.35114686807254	9.16734400298575	8.14258702540692	NA
**TC1300000273.mm.2**	Cmah	cytidine monophospho-N-acetylneuraminic acid hydroxylase	-0.243852456286935	5.39744741925581	-0.659736957494876	0.532327799198248	0.999692817443324	-4.61210552184045	4.48051367546439	4.77120196219904	5.51785140952992	5.80478057311886	5.54167948639825	6.2686574088244	NA
**TC1300001739.mm.2**	Hus1b	HUS1 checkpoint clamp component B	0.30923530096198	5.80883441900608	0.659657177834818	0.532375811558609	0.999692817443324	-4.61210877257671	4.83067553223816	5.6898570269558	5.7329567000833	6.74251557829993	5.88613547524418	5.97086620121508	NA
**TC0600001748.mm.2**	Ccdc91	coiled-coil domain containing 91	-0.322927350781138	7.52150970208232	-0.659582879179966	0.532420527843368	0.999692817443324	-4.61211179971512	8.14909376964284	7.73138588572516	7.824559770667	6.47680215711482	7.38675564191577	7.56046098742833	NA
**TC1900000026.mm.2**	Cdk2ap2	CDK2-associated protein 2	-0.216382915884135	7.29373434071718	-0.659471641954067	0.532487479861359	0.999692817443324	-4.61211633135545	7.12987684233343	7.21952177587213	7.06234640089526	7.42777439626936	7.24855268134473	7.67433394758816	NA
**TC0700002065.mm.2**	Brsk2/Mir3104	BR serine/threonine kinase 2/microRNA 3104	0.211509730816159	8.58003879070572	0.659467551526721	0.532489941929568	0.999692817443324	-4.61211649798243	8.75507622367673	8.74649031293327	8.83046055050047	8.37038228412785	8.50011931414136	8.27770405885465	NA
**TC0200001150.mm.2**	Ube2e3	ubiquitin-conjugating enzyme E2E 3	-0.195147495510659	8.50791167453773	-0.659045481260754	0.532744028782765	0.999692817443324	-4.6121336871799	8.58422361539909	8.52195513068198	8.48181895587095	8.29367919919105	8.66445533526122	8.5013378108221	NA
**TC0600000556.mm.2**	Atp6v0e2	ATPase, H+ transporting, lysosomal V0 subunit E2	0.248986958828088	8.71594792876783	0.658892606962729	0.532836078186902	0.999692817443324	-4.61213991107813	9.83343557376164	9.76447117032674	8.57941309768673	8.01846427540066	8.38735993414065	7.71254352129056	NA
**TC0100003665.mm.2**	Gm5069	glyceraldehyde-3-phosphate dehydrogenase pseudogene	-0.261786335011953	6.67990150783877	-0.658883461970503	0.532841584931567	0.999692817443324	-4.61214028335931	6.44959599230311	6.52299353356494	7.08548402697147	6.20613939859886	6.68971335576796	7.12548273982628	NA

**TC1400002493.mm.2**	Cnmd	chondromodulin	-0.287806182544002	6.11499376497957	-0.658716231996932	0.532942290395254	0.999692817443324	-4.61214709039472	6.0961344876066	6.09815434567065	6.44137505370885	5.51865564204723	5.89971186727898	6.6359311935651	NA
**TC0500003378.mm.2**	Limk1	LIM-domain containing, protein kinase	-0.201721023415031	8.34785673702988	-0.658541122475433	0.533047753773642	0.999692817443324	-4.61215421676715	8.67800904920966	8.53290742696135	8.28919716436901	7.94719378522007	8.3616222659539	8.27821073046525	NA
**TC0200002215.mm.2**	Gm14124	predicted gene 14124	0.249698999739283	4.63942852993492	0.658411847187415	0.533125620972344	0.999692817443324	-4.61215947692217	4.38657300322153	4.27504099517286	4.64430365538863	5.19287393495821	5.01336256103431	4.32441702983396	NA
**TC1500001748.mm.2**	Mroh6	maestro heat-like repeat family member 6	0.222211192942674	5.00492000252821	0.658387222475201	0.533140454143946	0.999692817443324	-4.6121604788026	4.5964693804036	4.52328323922004	5.05594471985077	5.6211501708647	5.25639012528345	4.97628237954668	NA
**TC0900001371.mm.2**	Nprl2	NPR2 like, GATOR1 complex subunit	-0.231998176950231	6.6462888588189	-0.658365125974976	0.53315376461978	0.999692817443324	-4.61216137779619	7.12661888517899	7.31382099998466	6.5067169501285	5.97993150679614	6.71129324772145	6.23935156310365	NA
**TC1100003391.mm.2**	Pex12	peroxisomal biogenesis factor 12	-0.20735151207059	7.26044798738104	-0.658290898721846	0.533198479120363	0.999692817443324	-4.61216439755681	7.57859403115388	7.19997345310222	7.04637403528119	7.13833467266328	7.42465087345182	7.17476085863383	NA
**TC0300002473.mm.2**	Anxa9	annexin A9	0.221073410746276	5.84736589211885	0.658221959212697	0.533240010402317	0.999692817443324	-4.61216720196789	5.40876195511472	5.7384685736352	6.21428737313838	5.97526844958949	5.63748380856957	6.10992519266574	NA
**TC1900001340.mm.2**	Insl6	insulin-like 6	-0.253559441100563	5.48496605352853	-0.658137498644834	0.53329089482866	0.999692817443324	-4.61217063746265	5.22691270011819	4.74244823229388	5.4702963305835	5.74636192269548	5.49571688538875	6.22806025009135	NA
**TC1500000031.mm.2**	Dab2	disabled 2, mitogen-responsive phosphoprotein	-0.208057084252347	7.08938042899265	-0.65790929018026	0.533428397459218	0.999692817443324	-4.61217991834289	7.26467867417758	7.6613687548365	6.69660553213309	6.90045495608548	6.92670064542706	7.08647401129621	NA
**TC0600000822.mm.2**	Krcc1/Mir8112	lysine-rich coiled-coil 1/microRNA 8112	-0.473028379698722	5.19676623427282	-0.657734545127712	0.533533701848041	0.999692817443324	-4.61218702330729	6.27982644645654	6.24166349657762	5.09533622655468	3.76118912504799	5.87711602705449	3.92546608394562	NA
**TC0500002157.mm.2**	Otof	otoferlin	-0.267685679967949	6.96632349676757	-0.65759060115475	0.533620454817439	0.999692817443324	-4.61219287485921	6.34493979840899	6.21721391912598	6.66977397975041	7.68043397181685	7.35380289075836	7.5317764207448	NA
**TC0800000039.mm.2**	Zfp958	zinc finger protein 958	-0.351946066838875	6.13131425169498	-0.657503824102869	0.533672758398655	0.999692817443324	-4.61219640201738	6.96461579806373	6.72097199502905	6.2038208846858	4.99538190701386	6.47680285651499	5.42629206886243	NA
**TC1900001213.mm.2**	Vps13a	vacuolar protein sorting 13A	-0.510650372421128	5.92150673007919	-0.65747921160782	0.533687593803276	0.999692817443324	-4.612197402358	7.02269314160953	7.35963818179486	6.18414770180342	3.87855645431084	6.10487617373392	4.97912872722259	NA
**TC0800000711.mm.2**	Atp6v1b2	ATPase, H+ transporting, lysosomal V1 subunit B2	-0.60246899992607	8.96431902065517	-0.657339841632328	0.533771605223184	0.999692817443324	-4.61220306632325	10.7017716343139	10.7167257058738	9.19251451135925	6.38872488058637	9.07875804719345	7.70741934460431	NA
**TC0800000937.mm.2**	Asf1b	anti-silencing function 1B histone chaperone	-0.221400900773765	5.54022740232483	-0.6573358822289	0.533773992050552	0.999692817443324	-4.61220322721929	5.90682684399158	5.6178544121421	5.06575391113414	5.57118676699973	5.66059524295172	5.41914723672968	NA
**TC0700004327.mm.2**	Zfp688	zinc finger protein 688	0.20874112837225	7.19977787567305	0.657012606859711	0.533968893217163	0.999692817443324	-4.61221636150747	7.01122407735304	6.69393670607073	7.2935725914924	7.66192177218711	7.27402517226793	7.26398693466708	NA
**TC0X00002482.mm.2**	Pls3	plastin 3 (T-isoform)	0.369109700335791	7.87753854816552	0.656830123987644	0.534078931059444	0.999692817443324	-4.6122237734108	9.14638890913644	9.33690769890887	8.04750325223657	6.71257378857313	7.55439746278188	6.46746017735624	NA
**TC0900001561.mm.2**	Eomes	eomesodermin	-0.239271190302155	5.94151136071543	-0.656783996554874	0.53410674833244	0.999692817443324	-4.61222564672151	6.19513334912178	5.89341604815573	5.61967435104518	5.92131384216019	5.66948975078876	6.35004082302093	NA
**TC0500002644.mm.2**	Hopx	HOP homeobox	0.233628782280746	7.19719105413644	0.656651253350622	0.53418680457234	0.999692817443324	-4.61223103708598	6.99694305337012	7.01272201846792	7.07849827240024	7.74187113637079	6.99910289080505	7.35400895340449	NA
**TC1600001810.mm.2**	Epha6	Eph receptor A6	0.417921111019051	6.21150434202366	0.656577367400819	0.534231367802009	0.999692817443324	-4.61223403705004	6.8301820801959	6.8809865118845	6.09292560534995	6.10392423569987	6.69862830253091	4.6623793164808	NA
**TC1600000130.mm.2**	Snn	stannin	-0.251606202936751	9.16661549141912	-0.656569101927707	0.534236353146793	0.999692817443324	-4.61223437263414	8.9713259449484	8.90759502861228	9.51238488047482	8.79639490406545	9.68448956524624	9.12750262516753	NA
**TC1000001549.mm.2**	Agap2	ArfGAP with GTPase domain, ankyrin repeat and PH domain 2	-0.379116997853262	8.12435725619843	-0.65631117930456	0.534391934667635	0.999692817443324	-4.6122448428717	9.27878027005291	9.48398008039494	8.17769760153421	6.4348769939839	7.87920448245671	7.49160410876793	NA
**TC0300002828.mm.2**	Ptbp2	polypyrimidine tract binding protein 2	-0.309663039911285	8.28137478713711	-0.656224616913175	0.534444156366076	0.999692817443324	-4.61224835613082	9.48997727080939	9.30970588054337	8.06816274706591	7.06645699875775	8.31464318720542	7.4393026384408	NA
**TC1300001762.mm.2**	Serpinb1a	serine (or cysteine) peptidase inhibitor, clade B, member 1a	0.22362171139175	6.68268713565421	0.656214343276089	0.534450354499366	0.999692817443324	-4.6122487730779	7.52551741502501	6.94365132677566	6.4038094522307	6.63328929522336	6.395606940639	6.19424838403155	NA
**TC0300003241.mm.2**	Cd160	CD160 antigen	-0.397510393790427	5.79543072035466	-0.656211848607486	0.534451859551426	0.999692817443324	-4.61224887432122	5.30546158484675	5.16593891213676	5.29378360857509	6.45929791020671	5.51909644857686	7.0290058577858	NA
**TC1500000356.mm.2**	A930017M01Rik/2310069G16Rik	Smg-5 homolog, nonsense mediated mRNA decay factor pseudogene/RIKEN cDNA 2310069G16 gene	0.382748019080887	5.99122479922533	0.656196733786023	0.534460978492318	0.999692817443324	-4.61224948773302	7.00742482304298	6.4924888279339	6.22903739762775	5.3774281936407	4.65833447485789	6.18263507824878	NA
**TC1000000743.mm.2**	Mcm3ap	minichromosome maintenance complex component 3 associated protein	-0.258651788329779	6.5873140325564	-0.656018255485674	0.534568663854541	0.999692817443324	-4.61225673019265	6.79639167927805	6.64109344258709	5.94066510123232	6.84388264717452	6.49469611464842	6.80715521041798	NA
**TC0200000759.mm.2**	Zeb2os	zinc finger E-box binding homeobox 2, opposite strand	-0.270605273354883	5.23012820487453	-0.655967452265526	0.53459931861083	0.999692817443324	-4.61225879146089	4.6303319394669	5.07598453251233	4.80512591961754	5.76149518566155	5.49537438082891	5.61245727115995	NA
**TC0800003228.mm.2**	Pgls	6-phosphogluconolactonase	0.318154500575524	7.07633381061288	0.655935446563422	0.534618631478315	0.999692817443324	-4.61226008998464	6.78905691109875	6.8721544464709	6.82036438282687	7.89618587080247	6.59298454937863	7.48725670309966	NA
**TC1100003719.mm.2**	Krtap9-3	keratin associated protein 9-3	-0.206782005749005	7.01694697376735	-0.655817345063328	0.534689900036211	0.999692817443324	-4.61226488114125	6.43208470348746	6.68521054068607	7.13110032550748	7.15431096795878	7.1544011211323	7.54457418383198	NA
**TC1100000478.mm.2**	Gm12171	predicted gene 12171	-0.532616071734327	3.90450038887541	-0.65556799535945	0.534840390190029	0.999692817443324	-4.61227499464808	3.12201933697206	3.22434590876204	2.91349667465322	5.09420579737162	3.72072798580502	5.35220662968847	NA
**TC1100001782.mm.2**	Prr29	proline rich 29	0.229456942450411	6.35440628535946	0.655543668539766	0.534855073594646	0.999692817443324	-4.61227598117668	5.572933737445	5.37480121476753	6.77086689344244	7.04794142898009	6.36453846475379	6.99535597276791	NA
**TC0200002788.mm.2**	Gata5os	GATA binding protein 5, opposite strand	0.216972854360652	6.46008172707704	0.65547807672754	0.534894665365212	0.999692817443324	-4.6122786409917	5.60376068772054	6.0269057906927	6.9809780823617	6.80090671402346	6.48960402191241	6.85833506575144	NA
**TC0900002159.mm.2**	Vps11	VPS11, CORVET/HOPS core subunit	0.275972227876519	7.19241598684046	0.655446779393107	0.534913557349777	0.999692817443324	-4.61227991005964	8.36238446363883	8.28630275928185	7.03314759403224	6.49572898290531	6.96899916061851	6.007932960566	NA
**TC1400000990.mm.2**	Egr3	early growth response 3	-0.217939900299853	6.27650588028398	-0.655347354075727	0.534973576158257	0.999692817443324	-4.61228394132822	6.31668472060897	6.48531667273631	6.18656650673852	6.02401053714092	6.04676141818692	6.59969542629223	NA
**TC0X00003241.mm.2**	Ptchd1	patched domain containing 1	-0.376598943442349	7.36095886098242	-0.655301094273322	0.535001502664941	0.999692817443324	-4.61228581680631	7.63653807576539	7.78802052297559	7.68234840108796	6.31164993904647	7.9402218084886	6.80697441853052	NA
**TC1500001102.mm.2**	Pcbp2	poly(rC) binding protein 2	0.442221175566521	9.74345559272964	0.655298992994418	0.53500277120444	0.999692817443324	-4.61228590199458	10.7665644981908	10.7030490754861	10.2527665945866	8.68501457233035	9.79045924485768	8.26287957092627	NA
**TC0100000925.mm.2**	Agxt	alanine-glyoxylate aminotransferase	-0.283001269601805	5.76210556588659	-0.655079481923204	0.535135300191272	0.999692817443324	-4.61229480008758	5.3632598533312	5.29745205827463	5.63219667506455	6.04076279719051	5.57926967048214	6.65969234097653	NA
**TC0200002821.mm.2**	Rtel1	regulator of telomere elongation helicase 1	0.297652282958951	5.93133311091008	0.654936391670822	0.535221701510195	0.999692817443324	-4.61230059917408	5.53949880084471	5.7138516981151	5.72658617300224	6.73839019320705	5.56722483721917	6.30244696307222	NA
**TC1900000838.mm.2**	Pnlip	pancreatic lipase	-0.207668752340025	5.5693108843837	-0.654463392310323	0.535507372261415	0.999692817443324	-4.61231976181759	5.30787904625923	5.05347774087894	5.61588924928349	5.7036962579585	5.6669947086543	6.06792830326774	NA
**TC1100000573.mm.2**	Nhp2	NHP2 ribonucleoprotein	-0.193682910535795	8.24901878282477	-0.654402163969558	0.535544358483794	0.999692817443324	-4.6123222415976	8.2155817674049	8.41015131077869	8.14842170863484	8.09208519021189	8.39108348052216	8.23678923939616	NA
**TC0400003240.mm.2**	Shisal2a	shisa like 2A	0.299743745294976	5.53370159992733	0.654399120585474	0.535546196943627	0.999692817443324	-4.61232236485167	5.19804411932001	5.03647065523066	5.30506039938095	6.47853075842067	5.3504697915908	5.83363387562087	NA
**TC0700000763.mm.2**	Vsig10l	V-set and immunoglobulin domain containing 10 like	-0.189408583029084	5.71407998502132	-0.654349713372629	0.53557604360857	0.999692817443324	-4.61232436573463	5.90276233360444	5.85531871968845	5.55657688159016	5.51721396379827	5.78115030200695	5.67145770943965	NA
**TC0300000605.mm.2**	Serpini1	serine (or cysteine) peptidase inhibitor, clade I, member 1	-0.503736243797531	7.84799463675124	-0.654312147087372	0.535598737924449	0.999692817443324	-4.6123258870096	9.15823230309594	9.44380248884169	7.98944530590998	5.74978496457739	7.97823809032357	6.76846466775885	NA
**TSUnmapped00000023.mm.2**	Rbm4	RNA binding motif protein 4	-0.223797198590636	7.63257835297225	-0.654098801611847	0.535727634357845	0.999692817443324	-4.61233452534025	7.77478842700175	7.54047798914569	7.81617353677306	7.20013111547934	7.7804311941546	7.68346785527907	NA
**TC0400003097.mm.2**	Cyp2j6	cytochrome P450, family 2, subfamily j, polypeptide 6	-0.227485721964319	6.65420273008338	-0.654024939562577	0.535772263959999	0.999692817443324	-4.61233751550772	6.94729233648451	6.98991218750321	6.73182874730693	6.03469145898505	6.65069699735862	6.57079465286199	NA
**TC0700000703.mm.2**	Zfp939	zinc finger protein 939	0.293249129561358	5.41847024451208	0.653858648277946	0.535872750548408	0.999692817443324	-4.61234424656415	6.05331123426416	5.10443346382194	5.8325013154961	5.13728619855846	5.37972615598239	5.00356309894945	NA
**TC0300000411.mm.2**	Supt20	suppressor of Ty 20	-0.219699951455392	6.00084939745359	-0.653854332164768	0.53587535884927	0.999692817443324	-4.61234442125238	6.09893992869387	5.77741995929531	5.66694337007705	6.17772492683375	6.22265632958283	6.06141187023875	NA
**TC1900000572.mm.2**	Entpd1	ectonucleoside triphosphate diphosphohydrolase 1	0.249408526819595	7.58561973445957	0.653651596883167	0.535997884225409	0.999692817443324	-4.61235262567522	7.53486019569594	7.63610869829431	7.46393760390303	7.95684567930017	7.08765963954059	7.83430659002342	NA
**TC1700002404.mm.2**	AU016765	expressed sequence AU016765	0.21225621652722	5.38969955871258	0.653477125249934	0.536103342229744	0.999692817443324	-4.61235968476255	5.13831042944654	5.06564411430177	5.55346880727541	5.7259088135154	5.17243607311049	5.68242911462589	NA
**TC0600003124.mm.2**	Cd4	CD4 antigen	0.353870759054828	6.99106094052499	0.653441541448683	0.536124852177773	0.999692817443324	-4.61236112430064	8.24166969048224	8.63914786807442	7.16734758520986	5.71929721614161	6.42683160841926	5.75207167482256	NA
**TC0300003217.mm.2**	Pex11b	peroxisomal biogenesis factor 11 beta	0.324707855200393	7.0603137939823	0.653398640446951	0.536150785999504	0.999692817443324	-4.61236285977613	8.49846360394253	7.95430480492045	6.88192250687304	6.39734252584277	6.94412040770365	5.68572891461137	NA
**TC0X00002418.mm.2**	Xlr3a	X-linked lymphocyte-regulated 3A	-0.343542388959631	5.36778894145611	-0.653395251776454	0.536152834497514	0.999692817443324	-4.61236299685443	6.26772414457897	5.42134712172656	5.49263467090788	4.42265413134807	5.69882441330119	4.90354916687402	NA
**TC0400003869.mm.2**	Gm13031	predicted gene 13031	0.220831186215244	5.63907647325585	0.653277534096687	0.53622399950779	0.999692817443324	-4.61236775842876	5.53253782049034	5.50108469116643	5.6121233658202	6.00912598433421	5.34016581874091	5.83942115898301	NA
**TC1800000962.mm.2**	Ccny	cyclin Y	-0.452009736039454	8.01678122786249	-0.65324177397569	0.536245619094881	0.999692817443324	-4.6123692047647	8.65262698967209	8.83049657569052	8.41609629564265	6.44067586922407	8.41942891947476	7.34136271747086	NA
**TC1500000746.mm.2**	1500009C09Rik	RIKEN cDNA 1500009C09 gene	0.20622019489109	8.84395630233234	0.653222941648195	0.536257004822892	0.999692817443324	-4.61236996642345	9.08679329886873	9.00090819888	9.06998165075906	8.62425670225469	8.70002310404648	8.5817748591851	NA
**TC0700002731.mm.2**	Zfp940	zinc finger protein 940	-0.242800930280738	5.87155656192753	-0.653194388979947	0.536274267605025	0.999692817443324	-4.61237112118202	6.14019477890077	5.82210137934295	5.53633072973544	5.85438994664455	5.58006561209658	6.29625692484488	NA
**TC1600000446.mm.2**	Zdhhc19	zinc finger, DHHC domain containing 19	0.230871030565111	6.48793148343325	0.653164826135192	0.536292141501788	0.999692817443324	-4.61237231675494	5.56902642959958	5.73905999982675	6.75385744375566	7.28676482239604	6.54851557793017	7.03036462709131	NA
**TC1200000016.mm.2**	Dnmt3a	DNA methyltransferase 3A	0.212121741072883	7.19573276402499	0.652971815768125	0.536408846072314	0.999692817443324	-4.6123801214218	6.82732434446168	6.93542355013072	7.29682537576331	7.62112071008833	7.0355248867104	7.45817771699548	NA
**TC0500002073.mm.2**	Kcnh2	potassium voltage-gated channel, subfamily H (eag-related), member 2	0.257854342058724	7.9056821065414	0.652922681298903	0.536438557991506	0.999692817443324	-4.61238210796923	8.81926584966956	8.16791917189779	8.07896230118273	7.40234584971653	7.3582358177002	7.60736636490816	NA
**TC0100000159.mm.2**	B3gat2	beta-1,3-glucuronyltransferase 2 (glucuronosyltransferase S)	-0.214605805083457	7.45740397980224	-0.652812979752695	0.536504898926573	0.999692817443324	-4.61238654288476	7.53941298110083	7.23391626722307	7.35529742809836	7.41564408206293	7.35967605062893	7.84047706969928	NA
**TC0900001195.mm.2**	Paqr9	progestin and adipoQ receptor family member IX	0.285869596309959	6.57694026217296	0.652786164172753	0.536521116168893	0.999692817443324	-4.61238762687496	7.51596895566472	7.21198685405175	6.89826155852483	5.75445091944576	6.00884286459114	6.07213042075953	NA
**TC1400000770.mm.2**	Ngdn	neuroguidin, EIF4E binding protein	0.210905416249116	5.96957354795162	0.652782029253781	0.536523616868755	0.999692817443324	-4.61238779402149	6.51478707405168	6.3361930376034	5.64600530797082	6.04813069630562	5.7837659964331	5.4885591753451	NA
**TC1900001588.mm.2**	Oga	O-GlcNAcase	0.385218036537305	8.90257383006935	0.652647639760694	0.536604896394429	0.999692817443324	-4.61239322600334	9.85048435475086	9.77544233615144	9.49179012725498	7.78818605403921	8.61218672881184	7.89735337940774	NA
**TC0400002517.mm.2**	Fam214b	family with sequence similarity 214, member B	-0.229606628373148	7.52620783208287	-0.652601599857114	0.536632743365427	0.999692817443324	-4.61239508676728	7.01391255213616	7.16715353367102	7.50569884542968	7.7527849795422	7.5250304933154	8.19266658840277	NA
**TC0700001942.mm.2**	Zranb1	zinc finger, RAN-binding domain containing 1	0.527144871236265	6.70883462122031	0.652567019645734	0.536653659603775	0.999692817443324	-4.61239648428445	7.69738342040774	7.810730583009	7.79302643633926	5.10656529684956	5.76382038617169	6.0814816045446	NA
**TC0500001436.mm.2**	Ogfod2	2-oxoglutarate and iron-dependent oxygenase domain containing 2	-0.191168596623267	6.90407760522525	-0.6525337115145	0.536673806893469	0.999692817443324	-4.61239783033893	6.68866322504399	6.70966032389154	6.81162541043514	7.01027703414958	7.07883919500529	7.12540044282596	NA
**TC0600002960.mm.2**	Rpl32	ribosomal protein L32	-0.200404411002003	9.27631719613541	-0.65246175067493	0.536717335899543	0.999692817443324	-4.61240073825598	9.87131268538453	9.80559479938187	9.05624989726478	8.73384353775624	9.19950789024022	8.9913943667848	NA
**TC0300000490.mm.2**	Ube2v1	ubiquitin-conjugating enzyme E2 variant 1	0.392218266852694	9.1921193559177	0.652425323882939	0.536739371256886	0.999692817443324	-4.61240221015951	10.0235715333788	9.89249475867093	9.6871698158411	8.32337337273985	9.26674583833335	7.95936081654221	NA
**TC1100000021.mm.2**	Morc2a	microrchidia 2A	0.315036115217135	5.78812120865539	0.652407041392887	0.536750430945967	0.999692817443324	-4.61240294887968	7.00225429024117	6.48818521269673	5.84047611574785	5.09370387396649	5.68007779942572	4.62402995985435	NA
**TC0600002878.mm.2**	Crbn	cereblon	0.368485517358602	8.07368177067513	0.652164409007721	0.536897220920791	0.999692817443324	-4.61241275116791	8.9203645855134	8.95061409225851	8.4130144930176	7.24102699748045	8.10461905386374	6.81245140191711	NA
**TC1900000325.mm.2**	D030056L22Rik	RIKEN cDNA D030056L22 gene	0.222913162416357	7.27226322598152	0.652075305045162	0.536951134177462	0.999692817443324	-4.61241635025121	6.79713483429193	6.3441357668776	7.5226987492455	7.94636879053065	7.39275011331068	7.63049110163277	NA
**TC0100003861.mm.2**	Tcea1	transcription elongation factor A (SII) 1	0.340564283729325	8.12467952063649	0.652040667062653	0.536972093156718	0.999692817443324	-4.61241774924631	8.92873775022869	9.11953709520666	8.36394883129317	7.32651659162794	8.08219800026716	6.9271388551953	NA
**TC0700000280.mm.2**	Mypop	Myb-related transcription factor, partner of profilin	0.227805921287496	6.76794573008337	0.651931777453386	0.537037984142331	0.999692817443324	-4.61242214682644	6.54453922141273	6.55427855633569	6.6360790123871	7.34615521027629	6.79616489324973	6.73045748683867	NA
**TC1500000859.mm.2**	Panx2	pannexin 2	0.250046416724787	6.73968456371541	0.651828986569121	0.537100189335997	0.999692817443324	-4.61242629759389	6.5148772519183	5.60579115782243	7.18060342375882	7.22816247924185	6.97736991355231	6.93130315599878	NA
**TC0400000811.mm.2**	Lurap1l	leucine rich adaptor protein 1-like	-0.233160761226867	5.77513947451677	-0.651790781468881	0.537123310783515	0.999692817443324	-4.61242784021576	6.56548478610611	6.27312110801231	5.59106900856457	5.08188570669966	5.81422173466827	5.3250545030497	NA
**TC1300000650.mm.2**	Fgfr4	fibroblast growth factor receptor 4	0.335207918983751	5.62493606663214	0.651758918027814	0.537142594782529	0.999692817443324	-4.61242912672555	5.09767374529955	4.92060656357871	5.42129717047476	6.77957879396629	5.42464576385558	6.10581436261796	NA
**TC0400001425.mm.2**	Inpp5b/Mir698	inositol polyphosphate-5-phosphatase B/microRNA 698	-0.2523432070763	6.77323420541063	-0.651627923229512	0.537221878400047	0.999692817443324	-4.61243441523484	6.66952417006198	6.87200667179506	6.24536066140342	7.05123332682365	6.69057472197482	7.11070568040486	NA
**TC0600001953.mm.2**	Gpr37	G protein-coupled receptor 37	0.273049159180679	7.82464777069689	0.651605100926772	0.537235692177723	0.999692817443324	-4.61243533653207	8.75690922278354	8.96112473592355	7.71606257805153	7.17191291386628	7.63429710064253	6.70758007291392	NA
**TC0500001796.mm.2**	Rasl11a	RAS-like, family 11, member A	-0.26432449624807	5.49820077365908	-0.651592013704508	0.537243613651442	0.999692817443324	-4.61243586482974	5.10788180009117	5.6885743637074	5.14385637654166	5.68819336628822	5.3558622732595	6.00483646206651	NA
**TC0100001711.mm.2**	Parp1/Mir6904	poly (ADP-ribose) polymerase family, member 1/microRNA 6904	0.229257617617563	6.74105496467339	0.651565104571607	0.537259901523349	0.999692817443324	-4.61243695105725	7.30102058293901	6.90154298880412	6.95723342674934	6.39390729901681	6.33058549014752	6.5620400003835	NA
**TC0700000298.mm.2**	Ercc1	excision repair cross-complementing rodent repair deficiency, complementation group 1	0.257692520738062	6.24420634903745	0.651460620673534	0.537323147682799	0.999692817443324	-4.61244116838358	6.27072239495384	6.18058875680238	6.55100573947684	6.21365025249548	6.57979472351313	5.66947622698306	NA
**TC1700002267.mm.2**	Satb1	special AT-rich sequence binding protein 1	-0.524573740931804	7.79114466112492	-0.651284041014917	0.537430045432257	0.999692817443324	-4.61244829457301	8.4217751290135	8.52563994622001	8.00011771239072	6.37503499243547	8.78805137517816	6.63624881151164	NA
**TC0600002919.mm.2**	Rpusd3	RNA pseudouridylate synthase domain containing 3	0.262055857109377	6.32239034875407	0.651160907950624	0.537504595582823	0.999692817443324	-4.61245326296071	6.26752951260472	6.27395380586906	6.20446528730713	6.75401995682757	5.79315741845199	6.64121611146397	NA
**TC0100002597.mm.2**	Ihh	Indian hedgehog	-0.247127640165976	5.82617986584547	-0.651066146060996	0.537561972993943	0.999692817443324	-4.61245708609283	5.02508072595839	5.24486769982359	5.66335407322764	6.4330836712518	6.10141391450316	6.48927911030823	NA
**TC0200004733.mm.2**	Flrt3	fibronectin leucine rich transmembrane protein 3	0.190283796324238	7.32290539267098	0.650384442328606	0.537974851126747	0.999692817443324	-4.61248457670683	7.25954061108183	7.24808384022521	7.53271582500588	7.37247192367779	7.3199894418637	7.20463071417149	NA

**TC1700000647.mm.2**	Nelfe	negative elongation factor complex member E, Rdbp	-0.379412454469441	6.02783229295969	-0.650258949359778	0.538050878367307	0.999692817443324	-4.61248963499273	6.44930130963443	6.61377743971553	6.48294861428835	4.6895964354463	6.10140105987746	5.82996889879608	NA
**TC0500003679.mm.2**	Zar1l	zygote arrest 1-like	-0.203086855962721	5.49669718392269	-0.650222175742351	0.538073158154102	0.999692817443324	-4.61249111709829	4.7231753559613	4.74579632808098	5.57433657669319	5.97818227709103	6.06489013165578	5.89380243405388	NA
**TC1900000931.mm.2**	Rbm4/Rbm14	RNA binding motif protein 4/RNA binding motif protein 14	-0.248515925981447	8.75458883264526	-0.650073082652139	0.538163494110234	0.999692817443324	-4.61249712541898	9.1811319739867	8.78077125691719	8.79303151821264	8.24126743828974	9.06277839309412	8.46855241537116	NA
**TC1900000612.mm.2**	Zfyve27	zinc finger, FYVE domain containing 27	-0.198979399082493	6.94034146978965	-0.649977368119804	0.538221492839802	0.999692817443324	-4.61250098207842	7.06478285071266	7.03045367609253	6.92758165927666	6.64684508760719	7.11224006646344	6.86014547858539	NA
**TC0300002491.mm.2**	Car14	carbonic anhydrase 14	0.20911979020848	6.19773020417271	0.649859343115602	0.538293016105021	0.999692817443324	-4.61250573710698	6.59620588729527	6.8630490344321	5.86594957856348	6.20673336329944	5.97098445376445	5.6834589076815	NA
**TC0200004789.mm.2**	Nkx2-4	NK2 homeobox 4	0.229184421417398	6.86364565823613	0.649828975088344	0.538311420120734	0.999692817443324	-4.61250696047741	5.9983298125262	6.16650399116934	7.04481413271205	7.69289036156597	6.98991640286747	7.28941924857575	NA
**TC0800003007.mm.2**	1700030J22Rik	RIKEN cDNA 1700030J22 gene	-0.204568450593363	6.72628075919517	-0.649716871661582	0.538379361866379	0.999692817443324	-4.61251147616692	6.13550226968925	6.3704514906493	6.70223085935091	7.01906608747197	6.94517258202982	7.18526126597979	NA
**TC0400001874.mm.2**	Gm13238	carnitine deficiency-associated gene expressed in ventricle 3 pseudogene	0.213095716235152	8.21430607116242	0.64965061639136	0.538419519261009	0.999692817443324	-4.61251414474721	7.62481328488319	7.47514801359747	8.37936246076721	8.92667081971487	8.43556007132226	8.44428177668951	NA
**TC0X00001316.mm.2**	Arxes2	adipocyte-related X-chromosome expressed sequence 2	0.58986281979664	7.26924177177228	0.649433342909452	0.538551222116522	0.999692817443324	-4.61252289447353	8.31441605254401	7.94556286593578	8.31935923396951	5.94823944190406	7.51734270319941	5.57053033308088	NA
**TC0400000532.mm.2**	Msantd3	Myb/SANT-like DNA-binding domain containing 3	0.244552982987302	6.88620524746802	0.649273289870481	0.538648253024358	0.999692817443324	-4.61252933847577	6.55481058876568	6.71823360096704	7.4205282694901	6.84611836103491	7.20743992151654	6.57010074303387	NA
**TC1100003567.mm.2**	Acsf2	acyl-CoA synthetase family member 2	0.255327213045123	5.93522197719153	0.649271467406535	0.538649357941894	0.999692817443324	-4.61252941184425	6.58142271302957	6.14739062353541	5.76680253039019	5.92978394594703	6.01493680782394	5.17099524242304	NA
**TC0200005151.mm.2**	Trp53rka	transformation related protein 53 regulating kinase A	0.187781809975528	6.86115783152592	0.649255225853254	0.538659204880806	0.999692817443324	-4.61253006568733	6.70806551422806	6.82145521691797	7.02050963732289	6.98598530165739	6.8186214929933	6.81230982603593	NA
**TC0600003121.mm.2**	Gnb3	guanine nucleotide binding protein (G protein), beta 3	0.272643718997895	6.86524852526817	0.649224200262006	0.538678015408344	0.999692817443324	-4.61253131466307	5.86777614598889	6.47703814540883	7.06340574960944	7.63257639949412	6.71782266581313	7.43287204529464	NA
**TC0X00000618.mm.2**	Aff2	AF4/FMR2 family, member 2	0.202334917000271	5.66035492030475	0.64913223103852	0.538733777907739	0.999692817443324	-4.61253501673755	6.8111435309122	6.52572306908663	5.16891601070949	5.3460503672056	4.91826816659395	5.1920283773206	NA
**TC1200000528.mm.2**	Fancm	Fanconi anemia, complementation group M	0.265180647606276	5.0438514340595	0.648968826283439	0.538832861886542	0.999692817443324	-4.61254159335081	5.24963599908364	5.37241931544491	5.36125026987211	4.7244570226484	4.36991045872272	5.18543553858524	NA
**TC0400003849.mm.2**	Mrto4	mRNA turnover 4, ribosome maturation factor	0.339776473623674	8.06846895715733	0.648899143140251	0.538875119221394	0.999692817443324	-4.61254439753144	7.60034836996919	7.6184239009432	8.54693292033111	8.38886428930835	8.9090195217224	7.34722474066971	NA
**TC1300002131.mm.2**	Gas1	growth arrest specific 1	-0.286823341850939	6.6854833410297	-0.648893477226439	0.538878555242708	0.999692817443324	-4.61254462552838	6.12134461136907	5.68237168676441	6.61920323575135	7.24856529642007	6.75641112597627	7.68500408989703	NA
**TC0800000005.mm.2**	Arhgef18	rho/rac guanine nucleotide exchange factor (GEF) 18	-0.19731991363455	6.46575085245145	-0.64876459013215	0.538956720880942	0.999692817443324	-4.6125498115487	6.76384357135997	7.06754893770747	6.19641808844697	6.0878183007391	6.26209644558668	6.4167797708685	NA
**TC0400001770.mm.2**	Padi2	peptidyl arginine deiminase, type II	0.318000391105217	6.34023042234672	0.648736638534936	0.538973673509187	0.999692817443324	-4.61255093613153	7.1752724396125	6.83489298194047	6.78111552750594	5.55249341986296	5.53132232873639	6.16628583642208	NA
**TC0600000131.mm.2**	Met	met proto-oncogene	-0.240538318875379	5.23479657201	-0.648658923651072	0.539020809295315	0.999692817443324	-4.61255406265789	5.11589296182435	5.74044976246194	4.75567175405482	5.28000828095665	5.15617245111621	5.36058422164601	NA
**TC0700004464.mm.2**	Ebf3	early B cell factor 3	0.197601012432857	5.95986469545675	0.648623446123295	0.539042328052889	0.999692817443324	-4.61255548984985	6.20774320626129	6.1382583365651	5.8372381839453	6.0669561434446	5.61823276530579	5.8907595372184	NA
**TC0400003741.mm.2**	Selenon	selenoprotein N	-0.206985508657872	7.46163393248247	-0.648609148391272	0.539051000438908	0.999692817443324	-4.612556065003	7.5193803544528	7.54675721195668	7.57072384902639	7.07412365655839	7.51678820871128	7.54203031418925	NA
**TC0500001625.mm.2**	Gigyf1	GRB10 interacting GYF protein 1	0.22862803416171	7.16202302845719	0.648583866493226	0.539066335558569	0.999692817443324	-4.61255708199126	7.07923465574522	6.94727320298832	7.70931350045238	6.99212968971412	7.15593569192397	7.08825142991911	NA
**TC0700001070.mm.2**	Fam174b	family with sequence similarity 174, member B	-0.219335575971423	7.05696658130568	-0.648560248131004	0.539080661881676	0.999692817443324	-4.61255803203495	7.03563635373043	6.94949562826202	7.13326350234671	6.82573467460269	7.47595813594265	6.9217111929496	NA
**TC1900001485.mm.2**	Sorbs1	sorbin and SH3 domain containing 1	-0.257546493853257	8.07706991049528	-0.64849471078604	0.539120416484954	0.999692817443324	-4.612560668123	9.06664418011614	9.07365868277699	7.88979745910183	7.01371434708418	7.93245616332756	7.48614863056497	NA
**TC0600002177.mm.2**	Tcaf2	TRPM8 channel-associated factor 2	0.242399747830479	5.09314723711677	0.648384957147022	0.539186996560067	0.999692817443324	-4.61256508225483	4.64122819597968	4.75247691919622	5.42454251093751	5.40044639065532	4.7449994552683	5.59518995066357	NA
**TC1100003925.mm.2**	Ern1	endoplasmic reticulum (ER) to nucleus signalling 1	-0.211599873869548	7.79485756733958	-0.648343650748015	0.539212055676634	0.999692817443324	-4.61256674339079	7.42685423875165	7.40641366959707	7.60081798124356	8.15552089273128	8.09352078300343	8.08601783871051	NA
**TC0800000842.mm.2**	Isx	intestine specific homeobox	0.271876226116189	5.2639193316495	0.648304534003137	0.539235787079307	0.999692817443324	-4.61256831639555	4.29362099945548	4.77575121304892	5.344943022899	6.1840050919135	5.74932130663414	5.23587435594598	NA
**TC0900001354.mm.2**	Iqcf6	IQ motif containing F6	0.299732206457863	4.83902743757971	0.648177477716523	0.539312874272201	0.999692817443324	-4.61257342522135	4.50664848447276	4.23002320659507	4.78486365368223	5.66361501998084	4.4663316089236	5.38268265182374	NA
**TC1900001661.mm.2**	Xpnpep1	X-prolyl aminopeptidase (aminopeptidase P) 1, soluble	0.193313802608186	7.25976166211962	0.648095893499297	0.539362376423838	0.999692817443324	-4.61257670525158	7.33530918733223	7.52916590219713	7.24625583007153	7.29410541413082	6.97363671873673	7.18009692024925	NA
**TC1600000386.mm.2**	Plaat1	phospholipase A and acyltransferase 1	-0.463492449932567	6.39460971765462	-0.648064742050017	0.539381278668958	0.999692817443324	-4.61257795758849	7.84630235223422	7.36360514773094	6.58780904757768	4.52757390547104	5.51145660471368	6.53091124820017	NA
**TC1100002063.mm.2**	Foxk2	forkhead box K2	0.246534570659218	8.9842955862569	0.647956149797975	0.539447174100111	0.999692817443324	-4.61258232280804	9.12099547782978	9.1642107178139	9.119444593194	8.93737363841407	9.21921703598992	8.34453205429972	NA
**TC0900000867.mm.2**	Herc1	HECT and RLD domain containing E3 ubiquitin protein ligase family member 1	0.335536548291954	7.61960730177626	0.64791618920927	0.539471424059417	0.999692817443324	-4.61258392901389	8.80894524326402	8.6502889555327	7.471075774913	6.99366557930939	7.62731475313469	6.16635352250379	NA
**TC1100000440.mm.2**	Rnf145	ring finger protein 145	-0.48713456098633	7.55944008888376	-0.647837252059685	0.539519328825191	0.999692817443324	-4.61258710165103	8.37724965617727	8.27504205849399	7.99663370627272	5.8684061420566	8.06587634372404	6.77343262657794	NA
**TC0X00000398.mm.2**	Stag2	stromal antigen 2	0.398240723323243	6.08456751319974	0.647767798988311	0.539562087083619	0.999692817443324	-4.61258993304786	7.44020161324476	7.73077992305844	6.01184207095533	5.05461042381554	5.97144563628759	4.2985254118368	NA
**TC1700001879.mm.2**	Rps28	ribosomal protein S28	-0.212695368640683	10.152744136482	-0.647752610092766	0.539570698638693	0.999692817443324	-4.61259050324861	10.2882572135439	10.079225418246	9.88641828943215	10.1753774354781	10.0212608585793	10.4659256036123	NA
**TC0600000974.mm.2**	Tprkb	Tp53rk binding protein	-0.355292581774727	6.99590780988497	-0.647663599794185	0.539624722919604	0.999692817443324	-4.61259408003567	8.0456613574195	7.92082596990245	6.93343600774813	5.71575117647106	7.25223132657657	6.10754102119208	NA
**TC1400000170.mm.2**	Sec24c	Sec24 related gene family, member C (S. cerevisiae)	-0.238771038046094	6.35226475435705	-0.647588547060898	0.539670278363131	0.999692817443324	-4.61259709566127	7.26265397189594	7.41006171773981	5.80023813502842	5.68142724517876	6.38296833943227	5.5762391168671	NA
**TC1600000861.mm.2**	Usp25	ubiquitin specific peptidase 25	-0.318310669515818	7.15015076137328	-0.647460147821415	0.539748219592526	0.999692817443324	-4.6126022541367	8.00612390130221	8.02439889419321	6.95118617257757	6.16569404427875	7.47455343202241	6.27894812386554	NA
**TC1700001893.mm.2**	Ring1	ring finger protein 1	0.226343110550172	6.31987992530791	0.64739543299522	0.539787505613399	0.999692817443324	-4.61260485377748	6.54493824623041	6.23417161031885	6.15330314096553	6.64312481723374	6.38032846431919	5.96341327277973	NA
**TC1300000893.mm.2**	Tppp	tubulin polymerization promoting protein	-0.221669224859257	9.50513115550816	-0.647200656040446	0.5399057584035	0.999692817443324	-4.61261267691153	9.58595721212213	9.72976368642433	9.52468888962285	9.11117490276913	9.79223554734085	9.28696669476964	NA
**TC1500002294.mm.2**	Map3k12	mitogen-activated protein kinase kinase kinase 12	0.207724020065405	7.38195149079893	0.647126600597224	0.539950723113717	0.999692817443324	-4.61261565084621	7.86606271960406	7.62575133739241	7.08089058329508	7.52678088066889	7.05474425631665	7.13747916751651	NA
**TC0X00002435.mm.2**	Pdzd4	PDZ domain containing 4	0.215778618306711	6.93559807097548	0.649698980506763	0.540046434080394	0.999692817443324	-4.61262197972441	7.08822794821245	7.09579646110773	6.85824438704855	7.0723162395245	7.03366227566746	6.46534111429217	NA
**TC0500000690.mm.2**	Spata18	spermatogenesis associated 18	-0.219242315627225	5.51073134190316	-0.646852212805549	0.540117345255387	0.999692817443324	-4.61262666750926	5.76265686308689	6.33298723726462	5.10183084666084	5.16329881324567	5.204625149807	5.49898914135396	NA
**TC1100004294.mm.2**	Glod4/Gemin4	glyoxalase domain containing 4/gem nuclear organelle associated protein 4	-0.443790340376768	6.89170480485043	-0.646777334527193	0.540162820705549	0.999692817443324	-4.61262967325146	8.33417376987185	8.28236142526063	6.93353489631761	4.98952158029066	6.95568504150637	5.85495211585543	NA
**TC0800001458.mm.2**	Foxl1	forkhead box L1	-0.253027676739936	6.43116445307644	-0.646761155929268	0.5401726466864	0.999692817443324	-4.61263032265319	5.9925684539331	5.94608971995036	6.37593990582292	6.69519668972472	6.34064888161585	7.23654306741166	NA
**TC1600000282.mm.2**	Vwa5b2	von Willebrand factor A domain containing 5B2	-0.207560639863497	7.42174323857273	-0.646752037976484	0.540178184473114	0.999692817443324	-4.61263068863831	6.43760891708474	6.57879257931089	7.64829949078781	7.90116883686905	7.77076096742531	8.19382863995854	NA
**TC1400002267.mm.2**	Ptk2b	PTK2 protein tyrosine kinase 2 beta	-0.195440100316716	7.35255786378745	-0.646745526866523	0.540182139016068	0.999692817443324	-4.61263094998507	8.0804035688536	8.30855369035749	6.94521049544467	6.72254436599541	7.08190859160221	6.9767264704713	NA
**TC1300000259.mm.2**	Hist1h2bb	histone cluster 1, H2bb	-0.20997455266451	7.36874776999899	-0.646358960983061	0.540416953387138	0.999692817443324	-4.61264464625967	6.94957802435523	7.244392162972	7.60466810274365	7.1946155609252	7.71016892393714	7.50906384506073	NA
**TC0600003534.mm.2**	Vamp1	vesicle-associated membrane protein 1	0.353684024983124	8.66331154462392	0.646302294428455	0.540451380092742	0.999692817443324	-4.61264873599024	9.53960016617881	8.90932839295359	8.69281882689083	8.42633555239784	8.96773430875018	7.44405202057224	NA
**TC0500001437.mm.2**	Arl6ip4	ADP-ribosylation factor-like 6 interacting protein 4	-0.271745325943019	7.09114163032149	-0.645951441467278	0.540664564704179	0.999692817443324	-4.61266280839984	6.60750843577052	6.63690198933404	6.86040014628056	7.51907420618862	7.01997855259122	7.90298645176399	NA
**TC1100001577.mm.2**	Gsdma	gasdermin A	-0.266705766852385	5.29614456081898	-0.645881362096689	0.540707152481327	0.999692817443324	-4.61266561852416	3.8543012064945	4.78698757508119	5.44911446643884	5.85196905837785	5.75988163762792	6.07461342089355	NA
**TC0X00003327.mm.2**	Car5b	carbonic anhydrase 5b, mitochondrial	0.199948216131214	5.4867727103023	0.645873789093501	0.540711754779348	0.999692817443324	-4.61266592218134	5.67973633006011	5.94251950279166	5.40031233189836	5.44882609844338	5.38227209873306	5.06696989934624	NA
**TC0200002580.mm.2**	Zfas1/Snord12	zinc finger, NFX1-type containing 1, antisense RNA 1/small nucleolar RNA, C/D box 12	-0.214911455736057	7.90128525372848	-0.645844579815104	0.540729506196264	0.999692817443324	-4.61266709336985	7.56394342689482	7.79568141952687	7.62849754910464	8.1806343331339	8.08871451635874	8.15024027735191	NA
**TC1800000714.mm.2**	Ldlrad4	low density lipoprotein receptor class A domain containing 4	-0.303711437690168	6.52453990578831	-0.645819360417659	0.540744833126657	0.999692817443324	-4.61266810454575	6.75642763315461	7.05354783637272	6.82948454976599	5.53543599514512	6.55643796684381	6.41590545344764	NA
**TC0100000936.mm.2**	Sept2	septin 2	0.437406156271297	7.01241866853974	0.645787613001725	0.540764127804419	0.999692817443324	-4.61266937742078	7.665384940449	7.69776765577819	7.82741220195583	5.96567366182111	7.0446805370164	5.87359301421794	NA
**TC0800001392.mm.2**	Atmin	ATM interactor	0.245996582152427	7.78470663211934	0.645545945668119	0.54091101661028	0.999692817443324	-4.61267906522033	8.63257654338513	8.47681466768739	7.81450362280283	7.23091725017138	7.60875582747668	6.94467188119266	NA
**TC0900001402.mm.2**	Gpx1	glutathione peroxidase 1	-0.248764279487101	9.98307471541619	-0.645338479680598	0.541037137196316	0.999692817443324	-4.61268737977038	10.2863142535641	10.3012535987534	10.0978759833116	9.30879995729116	10.1900455713834	9.71415892819354	NA
**TC1300001621.mm.2**	Zfp322a	zinc finger protein 322A	0.482236316905293	6.07355416164977	0.6448773314186	0.541317539333057	0.999692817443324	-4.61270585375506	6.78626137436647	6.42012905572597	6.37658980149144	5.72311378531694	6.74757182760469	4.3876591253931	NA
**TC1900000386.mm.2**	Apba1	amyloid beta (A4) precursor protein binding, family A, member 1	0.240607969577812	8.09579613912942	0.644856801284943	0.541330024827275	0.999692817443324	-4.61270667597481	8.72719609719365	8.62940966150195	8.29835875519115	7.5513347524271	7.88494403095772	7.48353353750491	NA
**TC1000000279.mm.2**	Hddc2	HD domain containing 2	0.311073603115304	5.18544496228061	0.64484257698746	0.541338675503735	0.999692817443324	-4.61270724563781	5.55750183059525	4.93733276799573	5.04313148999551	5.57685970066612	4.43728740767119	5.56055657675983	NA
**TC0400001458.mm.2**	Trappc3	trafficking protein particle complex 3	-0.36199563514033	8.00374897257614	-0.64482474739913	0.541349518902522	0.999692817443324	-4.61270795967405	8.95273253328306	8.75977937084992	7.95064204363265	6.84235328688893	8.41537917027791	7.10160743052433	NA
**TC1500001690.mm.2**	Ptk2	PTK2 protein tyrosine kinase 2	-0.494312217701397	7.16665168868841	-0.644790244730433	0.54137050273233	0.999692817443324	-4.61270934138807	8.04239189040308	7.86904328186698	7.65056854600746	5.39935671622135	7.61067534134807	6.42787435628353	NA
**TC0200005496.mm.2**	Fam78a	family with sequence similarity 78, member A	-0.199292889646749	6.22415979079125	-0.644779123221353	0.54137726671866	0.999692817443324	-4.61270987675448	5.1171607825876	5.40104132900221	6.52733467309656	6.68675075383552	6.68292839607608	6.9297428101495	NA
**TC0100001351.mm.2**	Ivns1abp	influenza virus NS1A binding protein	-0.377161154580112	7.17201831954261	-0.644686780578109	0.541433430588493	0.999692817443324	-4.61271348443524	8.76514916071722	8.38463705550241	7.16140875075503	5.40259194518289	6.81878925403141	6.49953375106672	NA
**TC1300001371.mm.2**	Tasor2	transcription activation suppressor family member 2	0.398305646624254	6.27177298075291	0.644529387137285	0.541529167474292	0.999692817443324	-4.61271978601536	6.97629473794154	7.09991019940683	6.3453869372479	5.8301351829609	6.63406953742714	4.74484128953316	NA
**TC0400002475.mm.2**	Cntfr	ciliary neurotrophic factor receptor	-0.27696058957574	8.45510945219993	-0.644482503756906	0.541557687020896	0.999692817443324	-4.6127216628636	8.95979371501696	8.64193399074572	8.62691816732065	7.66058574682207	8.68461735277936	8.15680774051483	NA
**TC1100004212.mm.2**	Mcrip1	MAPK regulated corepressor interacting protein 1	0.195652901090689	7.97674745098834	0.644471433156386	0.541564421494923	0.999692817443324	-4.61272210602967	8.01141637988819	7.74210072523407	8.03040662959612	8.21873007189845	7.86650612442068	7.99132477489252	NA
**TC1100003857.mm.2**	Kif18b	kinesin family member 18B	-0.204885732310996	6.97841096764636	-0.644462030754871	0.541570141210135	0.999692817443324	-4.61272248241166	6.43108111265651	6.77037126294608	7.13318975993618	6.9964312228906	7.10821255774916	7.43117988969962	NA
**TC1500002208.mm.2**	Fam186b	family with sequence similarity 186, member B	-0.239046295761264	5.38993093626061	-0.644244771741097	0.541702315760599	0.999692817443324	-4.61273117821025	4.94675439987045	5.2112179096424	5.82263412646559	5.02912623179855	5.61662086398533	5.71323208580134	NA
**TC0X00002704.mm.2**	P2ry4	pyrimidinergic receptor P2Y, G-protein coupled, 4	0.249798544271909	5.5795219226654	0.644166262879494	0.541750083383867	0.999692817443324	-4.61273431997852	5.1861429099285	5.5417240940113	5.40900314325872	6.21542766703948	5.40788688147519	5.71694684027918	NA
**TC0700002683.mm.2**	Zfp780b	zinc finger protein 780B	-0.254520136037655	6.1597002782607	-0.644135393337371	0.541768866245031	0.999692817443324	-4.61273555523594	7.28726602273151	6.86094785370287	5.85488506294752	5.29558869757973	6.16580550180886	5.49370853079371	NA
**TC0200002709.mm.2**	Zfp831	zinc finger protein 831	0.330187768207478	5.55039284908153	0.644111762766138	0.541783244760711	0.999692817443324	-4.61273650079238	5.89799555825597	5.52055279058054	5.27877876217395	5.99331337885987	5.95102449540094	4.66069210921793	NA
**TC0200001043.mm.2**	Platr26	pluripotency associated transcript 26	0.255056311668566	5.55212314467323	0.643908367362037	0.541907014773475	0.999692817443324	-4.61274463838327	4.91471983898708	5.01602640754383	5.58403372098221	6.36201890144057	5.42614747628567	6.00979252279999	NA
**TC0200005494.mm.2**	Phpt1	phosphohistidine phosphatase 1	0.247454839285275	7.73302942237132	0.643896545660496	0.541914209047223	0.999692817443324	-4.61274511129406	7.45120955691743	7.55852586211486	7.57963281799223	8.36204257889087	7.49204813260607	7.95471758570648	NA
**TC0800002056.mm.2**	Cldn23	claudin 23	-0.243762656920714	5.19285238500768	-0.643721395604078	0.542020806205269	0.999692817443324	-4.61275211715137	5.6788281063273	5.2736107720955	5.32115762659264	4.53741743229831	5.20481730136864	5.14128307136373	NA
**TC0400001555.mm.2**	Sdc3	syndecan 3	0.233090845554576	9.5439241487559	0.643387192445259	0.542224240037436	0.999692817443324	-4.61276548095656	10.0316080388241	10.0030928644616	9.80892987580435	9.03858296437506	9.10116292094972	9.28016822812053	NA
**TC1200000808.mm.2**	Zfp410	zinc finger protein 410	0.283301949004501	6.15416015628706	0.643340125305395	0.54225289423643	0.999692817443324	-4.6127673626071	6.77389615489823	6.80878999317884	6.56694648104458	5.38749286278255	5.79383448734503	5.5940009584731	NA
**TC0200005013.mm.2**	Samhd1	SAM domain and HD domain, 1	-0.217915920164647	6.00040158482831	-0.643192788512647	0.542342598102088	0.999692817443324	-4.61277325215789	6.3695215955537	6.30011712805579	5.56887678416139	5.87959268835414	5.66233943648872	6.22196187635611	NA
**TSUnmapped00000025.mm.2**	Gm21992	predicted gene 21992	-0.190315712327777	7.10086798525933	-0.643141202302492	0.542374007798166	0.999692817443324	-4.61277531398953	7.84464749188444	7.77968383355484	6.7012550620991	6.59886751863147	6.95187627403948	6.72887773134664	NA
**TC1100000786.mm.2**	Fam83g	family with sequence similarity 83, member G	0.22168420020653	6.62063806281625	0.643047149385794	0.542431277439175	0.999692817443324	-4.6127790728328	5.73425103694474	5.88110441443905	6.99241370701822	7.28350695594518	6.6160972327713	7.21645502977904	NA
**TC1200000503.mm.2**	Mia2	MIA SH3 domain ER export factor 2	-0.415985831238869	7.01834441619247	-0.64302320916244	0.542445855450357	0.999692817443324	-4.61278002954136	7.91545458795909	7.97695505319772	7.18344910318329	5.50939349357685	7.37774415552876	6.14707010370912	NA
**TC1100001704.mm.2**	Nmt1	N-myristoyltransferase 1	-0.293139127379826	8.02087582825799	-0.642915376358726	0.542511521509583	0.999692817443324	-4.61278433846008	9.27817416133167	9.36258991470618	7.84112165786975	6.60798466150545	7.37256385414655	7.66282071998831	NA
**TC1100001063.mm.2**	Rtn4rl1	reticulon 4 receptor-like 1	0.208183289910884	7.12878502094983	0.642872836084166	0.542537428271778	0.999692817443324	-4.61278603818571	7.02330773272998	7.31533099364023	6.99400818424221	7.43121080533305	7.05573079772153	6.95312161203196	NA
**TC1000002218.mm.2**	Anapc16	anaphase promoting complex subunit 16	0.18775682928656	8.55636502742491	0.642600153601489	0.542703508472859	0.999692817443324	-4.61279693135541	8.44169960304782	8.51992873603168	8.6239291306861	8.75210861133543	8.55867239649874	8.44185168694967	NA

[truncated: 3,028,077 more chars]
