# Supplementary material for: Functional involvement of septal miR-132 in extinction and oxytocin-mediated reversal of social fear
Source: Mol Psychiatry. 2023 Nov 8;29(6):1754–66. doi: 10.1038/s41380-023-02309-3 (PMC11371636; doi:10.1038/s41380-023-02309-3)
Supplement: Supplementary file 18 — Supplementary Table S12 [file 41380_2023_2309_MOESM18_ESM.pdf]

PROBEID	SYMBOL	GENENAME	logFC	AveExpr	t	P.Value	adj.P.Val	B	P234_01_N_Scr_1.CEL	P234_02_N_Inh_3.CEL	P234_03_6F4_Scr_1.CEL	P234_04_6F4_Scr_3.CEL	P234_05_6F4_Inh_2.CEL	P234_06_6F4_Inh_3.CEL
TC0500000096.mm.2	Pclo	piccolo (presynaptic cytomatrix protein)	3,14814E+14	7,85637E+14	9,0868E+14	6,67E+09	0.141570560577024	1,74505E+14	9,8049E+14	1,00368E+14	7,06161E+13	6,68958E+13	7,04185E+14	6,50352E+14
TC0100002827.mm.2	Kif1a	kinesin family member 1A	2,68198E+14	8,28921E+14	8,15963E+14	0.000126925501829928	0.141570560577024	1,31727E+14	9,996E+14	9,851E+14	7,93512E+14	7,47011E+14	7,39189E+13	7,09115E+14
TC1100001069.mm.2	Prpf8	pre-mRNA processing factor 8	2,42581E+14	6,5928E+13	7,91953E+14	0.000151458621806074	0.141570560577024	1,19378E+14	8,38078E+14	8,45677E+14	5,54834E+14	5,18498E+14	6,03514E+14	5,9508E+13
TC1700001633.mm.2	Tsc2	tuberous sclerosis 2	2,31695E+14	6,7373E+13	7,52242E+14	0.00020492519574682	0.141570560577024	0.976738147139796	8,32098E+14	8,47482E+14	5,85604E+14	5,61008E+13	6,21713E+14	5,94478E+14
TC1200001353.mm.2	Ncoa1	nuclear receptor coactivator 1	2,32834E+14	7,45396E+14	7,4784E+13	0.000212080518062083	0.141570560577024	0.951646852141301	8,84418E+14	8,60726E+14	7,32361E+14	7,15392E+14	6,55548E+14	6,23928E+14
TC0300002370.mm.2	Ints3	integrator complex subunit 3	2,1848E+14	6,85389E+14	7,15942E+14	0.000273339243491855	0.141570560577024	0.763343405764932	8,39864E+14	8,38667E+14	5,90616E+14	6,01614E+14	6,3837E+14	6,032E+14
TC0100001605.mm.2	Copa	coatomer protein complex subunit alpha	2,47186E+14	7,09309E+14	7,00874E+14	0.000309129989242284	0.141570560577024	0.670289288385286	8,72847E+14	8,61881E+14	6,69745E+14	6,11025E+13	6,41458E+14	5,98897E+14
TC0700002217.mm.2	Peg3	paternally expressed 3	2,99087E+14	7,6465E+14	6,92052E+14	0.000332552370737172	0.141570560577024	0.614529890021609	9,5502E+14	8,88025E+14	7,67779E+14	7,32204E+14	6,66168E+14	5,78703E+14
TC1700000050.mm.2	Tulp4	tubby like protein 4	2,26422E+14	6,9935E+13	6,89242E+14	0.000340428840265665	0.141570560577024	0.596576125358347	8,2674E+14	8,15638E+14	6,87845E+14	6,76342E+14	6,22181E+14	5,67352E+14
TC1500001065.mm.2	Grasp	GRP1 (general receptor for phosphoinositides 1)-associated scaffold protein	2,05981E+14	7,93128E+14	6,86035E+14	0.000349681893496401	0.141570560577024	0.575959037308716	8,9116E+14	9,21255E+14	7,7479E+13	7,71112E+14	7,04011E+14	6,96441E+14
TC1900000685.mm.2	Gbf1	golgi-specific brefeldin A-resistance factor 1	2,01178E+14	7,15045E+13	6,8527E+14	0.000351932689110936	0.141570560577024	0.571018764894354	8,3588E+13	8,27994E+13	6,78629E+14	6,86251E+14	6,40067E+14	6,21451E+14
TC0500000663.mm.2	Gabrb1	gamma-aminobutyric acid (GABA) A receptor, subunit beta 1	2,85887E+13	7,19679E+14	6,75066E+14	0.000383563111115232	0.141570560577024	0.504465167345887	8,97083E+14	8,81743E+12	7,08271E+14	6,23923E+14	6,39228E+13	5,67823E+14
TC0100001943.mm.2	Mybl1	myeloblastosis oncogene-like 1	2,13787E+14	4,62877E+14	6,7352E+14	0.000388631716033943	0.141570560577024	0.494266833852262	5,62662E+14	5,35089E+14	5,00795E+14	5,08539E+14	3,54583E+14	3,15593E+14
TC0200000040.mm.2	Ucma	upper zone of growth plate and cartilage matrix associated	-2,18868E+14	7,29288E+14	-6,72481E+13	0.000392082897415152	0.141570560577024	0.48739179515275	5,87579E+14	6,13516E+14	7,45743E+14	7,9006E+14	8,09812E+14	8,2902E+14
TC0800000955.mm.2	Cacna1a	calcium channel, voltage-dependent, P/Q type, alpha 1A subunit	1,94076E+14	7,22956E+14	6,71487E+14	0.000395413761636016	0.141570560577024	0.480808403752845	8,42085E+14	8,40168E+14	6,83104E+14	6,78278E+14	6,52023E+14	6,42077E+14
TC0200001982.mm.2	Vps16	VSP16 CORVET/HOPS core subunit	2,17817E+13	6,6141E+14	67002806398	0.000400364897944963	0.141570560577024	0.471115155850526	7,75246E+14	7,51696E+14	6,98416E+14	6,51791E+14	5,37818E+12	5,5349E+13
TC0700003834.mm.2	Atg16l2	autophagy related 16-like 2 (S. cerevisiae)	-2,33645E+14	9,27567E+14	-6,69007E+14	0.000403871572748206	0.141570560577024	0.464315578158772	7,84087E+14	7,79182E+14	9,50548E+14	1,02102E+14	1,01085E+14	1,01971E+14
TC1900000467.mm.2	Papss2	3'-phosphoadenosine 5'-phosphosulfate synthase 2	1,97907E+13	6,61594E+13	6,68299E+14	0.00040632159660717	0.141570560577024	0.459596682401652	7,64819E+14	7,59206E+12	6,63854E+14	6,53474E+14	5,52684E+14	5,75527E+14
TC1700001308.mm.2	Cript	cysteine-rich PDZ-binding protein	2,71586E+14	7,04556E+14	6,66676E+14	0.000412008649493992	0.141570560577024	0.448742088531435	8,67786E+14	8,55719E+14	6,94174E+13	6,29325E+14	6,30088E+14	5,50245E+14
TC1200001722.mm.2	Hectd1	HECT domain E3 ubiquitin protein ligase 1	2,22047E+14	6,64012E+14	6,63805E+14	0.000422284273964771	0.141570560577024	0.429470776255372	8,02684E+14	8,17002E+14	6,21498E+14	5,67293E+13	6,00347E+14	5,75246E+14
TC1000000100.mm.2	Hivep2	human immunodeficiency virus type I enhancer binding protein 2	2,14303E+14	7,01306E+14	6,61302E+14	0.000431484604036831	0.141570560577024	0.41257508501616	8,42991E+14	8,34355E+14	6,65991E+14	6,15757E+14	6,32893E+14	6,15848E+14
TC0100002503.mm.2	Erbb4	erb-b2 receptor tyrosine kinase 4	2,23018E+14	5,82271E+14	6,60306E+14	0.000435208813495033	0.141570560577024	0.405829033081744	7,09678E+14	6,95308E+14	5,67625E+14	5,62061E+14	5,10363E+14	4,48588E+14
TC1700000341.mm.2	Caskin1	CASK interacting protein 1	2,08398E+13	7,13177E+14	6,57753E+13	0.000444921085315991	0.141570560577024	0.388480613038288	8,50359E+14	8,05364E+14	6,96182E+13	6,88227E+14	6,26622E+14	6,12305E+14
TC1600002145.mm.2	Dscam	DS cell adhesion molecule	1,94933E+14	6,87544E+12	6,53188E+14	0.000462904492786512	0.141570560577024	0.357250449166425	8,19642E+14	8,06077E+14	6,34966E+14	6,28727E+14	6,30314E+14	6,0554E+14
TC1500002056.mm.2	Arsa	arylsulfatase A	2,07278E+14	5,33314E+14	6,48663E+14	0.000481553053814925	0.141570560577024	0.326013634325519	6,71969E+14	6,29199E+14	5,12071E+12	5,0003E+14	4,31682E+14	4,54931E+14
TC1600001764.mm.2	Col8a1	collagen, type VIII, alpha 1	-2,27095E+14	6,35875E+13	-6,48029E+14	0.000484235184183336	0.141570560577024	0.321612203335496	4,79484E+14	5,2323E+14	6,49574E+14	7,06058E+14	7,24189E+14	7,32714E+14
TC1800000968.mm.2	Usp14	ubiquitin specific peptidase 14	2,3586E+14	6,59239E+14	6,46499E+14	0.00049077234886062	0.141570560577024	0.310977195992065	8,0983E+14	8,00413E+14	5,68379E+14	6,38286E+14	5,88786E+14	5,49738E+14
TC1300000267.mm.2	Slc17a1	solute carrier family 17 (sodium phosphate), member 1	-1,90434E+12	5,40255E+14	-6,44483E+14	0.000499539713606886	0.141570560577024	0.29691546328447	4,27464E+14	4,51063E+14	5,49389E+14	5,5422E+14	6,32492E+14	6,26904E+14
TC1500000170.mm.2	Zfp622	zinc finger protein 622	2,03106E+14	5,98096E+14	6,44074E+14	0.000501343673862157	0.141570560577024	0.294050158154248	7,38394E+14	6,9882E+14	5,68859E+14	5,51501E+14	5,06777E+13	5,24224E+14
TC0400003530.mm.2	Thrap3	thyroid hormone receptor associated protein 3	2,13092E+14	7,02754E+14	6,43147E+14	0.000505451984445354	0.141570560577024	0.287559785924953	8,32822E+14	8,27217E+13	6,81137E+14	6,41491E+14	5,95145E+13	6,38709E+14
TC1500001977.mm.2	Ttll1	tubulin tyrosine ligase-like 1	2,86289E+14	6,52763E+14	6,4296E+13	0.000506283504344781	0.141570560577024	0.286252011799265	8,2014E+14	8,07473E+14	6,65696E+14	5,68237E+13	5,63651E+14	4,91383E+14
TC0800002039.mm.2	Tnks	tankyrase, TRF1-interacting ankyrin-related ADP-ribose polymerase	2,58865E+14	6,97542E+14	6,41149E+14	0.000514434380514454	0.141570560577024	0.273535717766112	8,59585E+14	8,45476E+14	6,57119E+14	6,3574E+14	6,42889E+14	5,44441E+14
TC1100002799.mm.2	Fam114a2	family with sequence similarity 114, member A2	2,33957E+14	5,24323E+14	6,35898E+14	0.000538927171089109	0.141570560577024	0.236404337068357	6,30548E+14	6,27146E+14	5,58368E+14	5,40093E+14	4,35033E+13	3,54747E+14
TC0X00000151.mm.2	Atp6ap2	ATPase, H+ transporting, lysosomal accessory protein 2	2,22792E+13	8,83989E+14	6,32324E+11	0.000556357357705891	0.141570560577024	0.210910083502114	1,02641E+14	1,02411E+14	8,60781E+14	7,87687E+13	8,02841E+14	8,02098E+14
TC0900002944.mm.2	Ppp2r3a	protein phosphatase 2, regulatory subunit B'', alpha	1,99527E+14	6,117E+14	6,28282E+14	0.000576839676906149	0.141570560577024	0.18187127242835	7,36797E+14	7,1824E+11	5,97273E+13	5,61906E+14	5,41758E+14	5,14224E+14
TC0600002714.mm.2	Cfap100	cilia and flagella associated protein 100	-2,02234E+14	8,73004E+13	-6,27388E+14	0.00058148560021857	0.141570560577024	0.175416312341331	7,47849E+14	7,35337E+14	9,14593E+14	9,52591E+14	9,26906E+14	9,60748E+14
TC0200001552.mm.2	Cd59a	CD59a antigen	2,44226E+13	6,80817E+14	6,27262E+14	0.000582143979413596	0.141570560577024	0.174505404235626	8,17311E+14	8,36306E+14	6,7704E+14	5,89078E+14	5,96558E+13	5,68607E+14
TC1000002453.mm.2	Pdxk	pyridoxal (pyridoxine, vitamin B6) kinase	2,1886E+14	8,47328E+14	6,26562E+14	0.000585815949733901	0.141570560577024	0.169442285241533	9,63403E+14	9,50278E+14	8,73063E+14	8,21261E+13	7,61533E+14	7,14429E+14
TC1000000191.mm.2	Slc18b1	solute carrier family 18, subfamily B, member 1	2,16596E+14	5,58976E+14	6,25084E+14	0.000593653217970278	0.141570560577024	0.158732556470955	7,19467E+14	6,79305E+13	5,1376E+14	4,75747E+14	5,03804E+14	4,61775E+14
TC0900003064.mm.2	Wdr6	WD repeat domain 6	2,05684E+14	7,5089E+13	6,2355E+14	0.00060191749666342	0.141570560577024	0.147579042114991	8,97551E+14	8,50744E+13	7,15405E+13	7,04714E+14	6,84826E+14	6,521E+14
TC1600001722.mm.2	Alcam	activated leukocyte cell adhesion molecule	1,96831E+14	9,16455E+14	6,2295E+14	0.000605184994611521	0.141570560577024	0.143207972263752	1,03949E+14	1,0229E+14	9,03063E+14	8,64562E+14	8,45139E+14	8,23584E+14
TC0700002694.mm.2	Supt5	suppressor of Ty 5	2,02367E+14	6,46971E+14	6,2223E+14	0.000609132372458669	0.141570560577024	0.137956224452865	7,75631E+14	7,46514E+14	6,36384E+14	6,05883E+13	5,41328E+14	5,76083E+14
TC0700001150.mm.2	Vps33b	vacuolar protein sorting 33B	1,86298E+14	5,7744E+14	6,16568E+14	0.000641210194499642	0.141570560577024	0.0964022877226123	7,02456E+14	6,8321E+14	5,4587E+14	5,20034E+14	5,11178E+14	5,01892E+14
TC1100004233.mm.2	Fasn	fatty acid synthase	1,82714E+14	7,45493E+14	6,15519E+14	0.000647358020351889	0.141570560577024	0.0886573160583648	8,76359E+14	8,69743E+14	6,62878E+14	6,83303E+14	6,98309E+14	6,82365E+14
TC0100001724.mm.2	Tmem63a	transmembrane protein 63a	3,02805E+14	6,669E+14	6,13207E+14	0.000661149614148524	0.141570560577024	0.0715261513798087	8,37199E+14	8,60331E+14	6,63211E+14	5,48741E+14	5,88106E+14	5,03814E+14
TC0100003576.mm.2	Fcrl6	Fc receptor-like 6	-1,94358E+14	4,257E+14	-6,11844E+14	0.000669438908003683	0.141570560577024	0.0613872628877843	3,05389E+14	3,21178E+14	4,34208E+14	4,78144E+14	5,00924E+14	5,14359E+13
TC0800000965.mm.2	Farsa	phenylalanyl-tRNA synthetase, alpha subunit	1,87802E+14	6,37898E+14	6,10408E+14	0.000678298013136893	0.141570560577024	0.0506784427315567	7,44752E+14	7,31442E+14	6,10079E+14	6,40524E+14	5,39659E+14	5,6093E+14
TC1200001953.mm.2	Sptb	spectrin beta, erythrocytic	1,90833E+14	7,07918E+14	6,098E+14	0.000682091648221413	0.141570560577024	0.0461320750861329	8,18637E+13	8,08907E+14	6,86269E+14	6,87814E+14	6,44623E+14	6,01255E+14
TC0X00002441.mm.2	Hcfc1	host cell factor C1	1,9757E+13	6,53643E+14	6,06845E+14	0.000700863505935379	0.141570560577024	0.0239728266403576	7,84722E+14	7,70994E+14	6,1566E+14	5,89906E+14	5,57036E+14	6,03541E+13
TC0600002555.mm.2	Htra2	HtrA serine peptidase 2	1,92449E+14	6,87777E+14	6,05143E+14	0.000711938369286089	0.141570560577024	0.0111547758693726	8,12586E+14	7,97371E+14	6,54617E+14	6,37028E+14	6,34029E+14	5,9103E+14
TC0700000798.mm.2	2410002F23Rik/Snord88a	RIKEN cDNA 2410002F23 gene/small nucleolar RNA, C/D box 88A	2,27859E+14	7,53375E+13	6,03272E+14	0.000724341345110364	0.141570560577024	-0.00298393178198886	8,87284E+14	8,84706E+14	7,58344E+14	6,73641E+14	6,69852E+14	6,46421E+14
TC1300000571.mm.2	Nol8	nucleolar protein 8	2,39259E+14	6,216E+13	5,98921E+14	0.000754146476144663	0.141570560577024	-0.0360672939764468	7,81677E+14	7,76559E+14	5,93585E+14	4,98061E+14	5,26111E+14	5,53608E+14
TC1400000306.mm.2	Tkt/Mir3076	transketolase/microRNA 3076	1,75258E+14	8,26738E+14	5,98666E+14	0.000755940833191103	0.141570560577024	-0.0380202658522482	9,28535E+14	9,16276E+14	8,08391E+14	8,1293E+14	7,39506E+14	7,54789E+14
TC1900000232.mm.2	Mrpl16	mitochondrial ribosomal protein L16	1,78851E+13	5,80302E+14	5,97496E+14	0.000764212698082639	0.141570560577024	-0.0469683305985642	6,69179E+14	6,84587E+14	5,57053E+14	5,74929E+14	4,88691E+14	5,07374E+14
TC1900000040.mm.2	Pcx	pyruvate carboxylase	1,90797E+14	5,94759E+14	5,95495E+14	0.000778601490590378	0.141570560577024	-0.0623222926484281	7,46733E+14	7,04425E+13	5,16641E+14	5,31193E+14	5,22705E+14	5,4686E+14
TC0400001930.mm.2	Mtor	mechanistic target of rapamycin kinase	1,83899E+14	6,60884E+14	5,94623E+13	0.000784960524773751	0.141570560577024	-0.069024483597385	7,7471E+14	7,84793E+14	6,23106E+14	5,90987E+14	5,84121E+14	6,07584E+14
TC0600002178.mm.2	Tcaf1	TRPM8 channel-associated factor 1	1,99274E+14	7,80249E+14	5,93864E+14	0.000790547975374628	0.141570560577024	-0.0748721572397333	9,02848E+14	8,98128E+14	7,55719E+14	7,22375E+14	7,27336E+14	6,75091E+14
TC1500000660.mm.2	Triobp	TRIO and F-actin binding protein	1,75639E+14	6,37612E+14	5,90193E+14	0.000818220201951472	0.141570560577024	-0.103281426464262	7,31927E+14	7,54819E+14	6,09611E+13	5,93847E+14	5,67805E+14	5,67663E+14
TC1600001312.mm.2	Tango2	transport and golgi organization 2	1,77563E+14	6,93936E+14	5,90163E+14	0.000818451159097969	0.141570560577024	-0.103514772774744	8,2252E+14	8,23331E+14	6,21307E+14	6,05736E+14	6,5934E+14	6,31385E+14
TC0700000165.mm.2	Cabp5	calcium binding protein 5	-2,26592E+14	6,95049E+13	-5,89477E+14	0.000823746846935333	0.141570560577024	-0.10884859463659	5,6299E+14	5,37448E+14	7,14283E+14	8,01952E+14	7,80263E+14	7,73359E+14
TC0400000085.mm.2	Ccne2	cyclin E2	-1,76521E+14	6,25954E+14	-5,87816E+14	0.000836723733893518	0.141570560577024	-0.121785950049838	5,10286E+14	5,09094E+14	6,77794E+14	6,86129E+14	6,71132E+14	7,0129E+14
TC0600001171.mm.2	Chl1	cell adhesion molecule L1-like	2,55568E+14	7,44304E+13	5,85758E+14	0.000853126949780385	0.141570560577024	-0.137875958134518	9,02521E+14	8,93852E+14	7,34803E+14	6,4941E+13	6,82923E+13	6,02313E+14

TC1200001758.mm.2	Baz1a	bromodomain adjacent to zinc finger domain 1A	-2,07372E+14	8,0621E+14	-5,84741E+14	0.000861365403814984	0.141570560577024	-0.145849311803517	6,69417E+14	6,85205E+14	8,33144E+14	8,8013E+14	8,56841E+14	9,12526E+14
TC0700001854.mm.2	Phkg2	phosphorylase kinase, gamma 2 (testis)	1,97807E+14	7,78224E+14	5,83892E+14	0.000868318205553379	0.141570560577024	-0.152523602669254	9,02414E+14	8,98788E+14	7,47654E+14	7,149E+14	7,30438E+14	6,75149E+14
TC1700002656.mm.2	Lrpprc	leucine-rich PPR-motif containing	2,27339E+14	5,75688E+14	5,81309E+14	0.000889843623078975	0.141570560577024	-0.172877231588771	7,08638E+13	6,64718E+14	5,53147E+14	6,0895E+14	4,91001E+14	4,27676E+14
TC0200005512.mm.2	Nfs1	nitrogen fixation gene 1 (S. cerevisiae)	1,85259E+14	6,02791E+14	5,80007E+14	0.000900929270416546	0.141570560577024	-0.183181988223112	6,95481E+14	7,12039E+14	6,07445E+14	5,64781E+14	5,28615E+14	5,08387E+14
TC1500002346.mm.2	Plec	plectin	1,77371E+14	6,54024E+14	5,77945E+14	0.000918792796027295	0.141570560577024	-0.199542326320512	7,54667E+14	7,73984E+14	6,13517E+14	6,08067E+14	6,03485E+14	5,70424E+14
TC1800000994.mm.2	Npc1	NPC intracellular cholesterol transporter 1	2,339E+14	5,4705E+14	5,7522E+14	0.00094302687918355	0.141570560577024	-0.221271605953005	6,44159E+14	7,0485E+13	5,47787E+14	5,04291E+14	4,04994E+14	4,76216E+14
TC0500003728.mm.2	D5Ertd579e	DNA segment, Chr 5, ERATO Doi 579, expressed	1,93819E+14	6,56937E+14	5,72905E+14	0.000964187077831025	0.141570560577024	-0.239824878187259	7,74392E+14	7,66063E+14	6,26877E+14	6,21472E+14	6,08046E+13	5,4477E+14
TC0200003085.mm.2	Enkur	enkurin, TRPC channel interacting protein	2,00084E+14	5,81368E+14	5,72715E+14	0.000965945522435351	0.141570560577024	-0.241349618424155	7,10129E+14	6,80373E+14	5,84551E+14	5,22821E+14	5,04961E+14	4,85374E+14
TC0800003162.mm.2	Nup133	nucleoporin 133	1,73296E+14	6,30436E+14	5,72305E+14	0.000969749040537899	0.141570560577024	-0.244638833266754	7,30368E+14	7,5054E+14	5,72097E+13	5,95297E+14	5,59581E+14	5,74734E+14
TC0600000667.mm.2	Adcyap1r1	adenylate cyclase activating polypeptide 1 receptor 1	2,32215E+13	8,13547E+14	5,71302E+14	0.000979141938154395	0.141570560577024	-0.252710582563024	9,43621E+14	9,27905E+14	8,37583E+14	7,6508E+14	7,39351E+14	6,67745E+14
TC0600002293.mm.2	Hibadh	3-hydroxyisobutyrate dehydrogenase	2,20007E+14	6,71698E+14	5,70598E+14	0.000985795262007505	0.141570560577024	-0.258384675395744	8,2434E+14	8,1429E+13	5,90988E+14	6,01955E+14	6,44809E+14	5,53806E+14
TC0900000508.mm.2	Hyou1	hypoxia up-regulated 1	1,83127E+14	8,13991E+14	5,69608E+14	0.000995243320172372	0.141570560577024	-0.26638132449596	9,37054E+13	9,38092E+13	7,61522E+14	7,38386E+14	7,77572E+14	7,31319E+14
TC0X00001892.mm.2	Maob	monoamine oxidase B	2,03381E+14	6,02096E+14	5,67781E+14	0.00101294034092564	0.141570560577024	-0.281171679275122	7,51076E+14	6,8849E+14	5,81255E+14	5,58953E+14	5,35573E+14	4,97232E+14
TC1500000102.mm.2	Gm2573	predicted gene 2573	1,65238E+14	7,48177E+14	5,67611E+14	0.00101460779405143	0.141570560577024	-0.282552848941084	8,31837E+14	8,25558E+14	7,50318E+14	7,5443E+13	6,55955E+13	6,70964E+14
TC1100001006.mm.2	Zzef1	zinc finger, ZZ-type with EF hand domain 1	1,89432E+14	6,50095E+14	5,67323E+14	0.00101743064062109	0.141570560577024	-0.284886239375902	7,74794E+14	7,45071E+14	6,12396E+13	6,27308E+14	5,96073E+14	5,4493E+14
TC0500002758.mm.2	Nup54	nucleoporin 54	2,32177E+12	5,70953E+14	5,66643E+14	0.0010241502033365	0.141570560577024	-0.29041655686084	6,88155E+14	6,83901E+14	5,45004E+14	6,00957E+14	4,98083E+14	4,09618E+14
TC1700000292.mm.2	Zfp946	zinc finger protein 946	2,01129E+14	5,92654E+13	5,66148E+14	0.00102906514574739	0.141570560577024	-0.294440310672054	7,06569E+14	6,83447E+14	6,09863E+14	5,68285E+14	5,22823E+14	4,64935E+14
TC1900000042.mm.2	Sptbn2	spectrin beta, non-erythrocytic 2	1,98765E+14	8,05616E+14	5,6593E+14	0.00103123145711455	0.141570560577024	-0.296208148284663	9,34458E+14	9,41034E+14	7,68552E+14	7,11689E+14	7,61176E+14	7,16786E+14
TC0500000465.mm.2	Cc2d2a	coiled-coil and C2 domain containing 2A	1,77769E+14	5,39249E+14	5,65448E+14	0.0010360594016323	0.141570560577024	-0.300135638424345	6,19319E+14	6,39171E+14	5,4828E+14	5,25775E+14	4,6875E+14	4,34202E+14
TC1300002313.mm.2	Exoc3	exocyst complex component 3	1,94028E+14	6,74174E+14	5,63544E+14	0.0010553695557038	0.141570560577024	-0.315675888322731	7,97045E+14	7,76351E+14	6,67517E+14	6,18792E+14	6,14035E+14	5,71305E+11
TC0100001447.mm.2	Tnr	tenascin R	1,9079E+14	6,93313E+14	5,61529E+13	0.00107625391670618	0.141570560577024	-0.332187895629779	8,23708E+14	8,37737E+14	6,40991E+14	5,77573E+14	6,38284E+14	6,41582E+14
TC1400002473.mm.2	Akap11	A kinase (PRKA) anchor protein 11	1,99948E+14	7,91047E+14	5,61074E+14	0.00108103399497534	0.141570560577024	-0.335925234826925	9,33555E+13	9,21341E+14	7,53602E+14	6,82782E+14	7,39237E+14	7,15763E+14
TC0100003294.mm.2	Cacna1e	calcium channel, voltage-dependent, R type, alpha 1E subunit	2,08146E+14	7,42386E+14	5,59101E+14	0.00110203626161823	0.141570560577024	-0.352165741755831	8,61406E+14	8,60021E+14	7,44443E+14	6,83313E+14	6,82016E+14	6,23118E+13
TC1000001679.mm.2	Syne1	spectrin repeat containing, nuclear envelope 1	1,83945E+14	7,6986E+14	5,58597E+12	0.00110747492459898	0.141570560577024	-0.356324307466606	8,94125E+14	8,90145E+14	6,91965E+13	7,26546E+14	7,31195E+14	6,85185E+14
TC1300002520.mm.2	Map1b	microtubule-associated protein 1B	2,89995E+14	9,5519E+14	5,57797E+14	0.00111616934356951	0.141570560577024	-0.362932960235881	1,15727E+14	1,14228E+13	9,13021E+14	7,99011E+13	9,12034E+13	8,07526E+13
TC0400004193.mm.2	Emc1	ER membrane protein complex subunit 1	1,65883E+14	7,18576E+14	5,56613E+14	0.00112917975669071	0.141570560577024	-0.372733144879295	8,13816E+14	8,07758E+14	7,07739E+14	6,92332E+13	6,33382E+13	6,56427E+14
TC1600000528.mm.2	Golgb1	golgi autoantigen, golgin subfamily b, macrogolgin 1	1,70189E+14	5,53599E+14	5,56025E+14	0.00113570612980305	0.141570560577024	-0.377609604209534	6,62158E+14	6,67683E+13	5,11207E+14	4,91081E+14	4,78952E+14	5,1051E+14
TC0400003605.mm.2	Dcdc2b	doublecortin domain containing 2b	-1,81331E+14	7,99593E+14	-5,54474E+14	0.0011531117340937	0.141570560577024	-0.390488304738054	6,68098E+14	6,94402E+14	8,30397E+14	8,79496E+14	8,65531E+14	8,59632E+14
TC0100003080.mm.2	Nfasc	neurofascin	1,64003E+14	8,41344E+14	5,54417E+14	0.00115376040378895	0.141570560577024	-0.390964754316116	9,46875E+14	9,47898E+14	7,97565E+14	7,88961E+14	7,95229E+14	7,71537E+14
TC0100001222.mm.2	Chil1	chitinase-like 1	1,62727E+14	6,62615E+14	5,50809E+14	0.0011954863040399	0.141570560577024	-0.421099366418506	7,47189E+14	7,69756E+14	6,34042E+14	6,33215E+14	5,90803E+14	6,00687E+13
TC1100001047.mm.2	Cluh	clustered mitochondria (cluA/CLU1) homolog	1,7087E+14	7,5442E+14	5,50724E+14	0.00119647913675387	0.141570560577024	-0.421804370581187	8,59053E+14	8,57603E+14	7,03541E+14	7,31408E+14	7,03145E+14	6,71772E+14
TC0X00002116.mm.2	Tenm1	teneurin transmembrane protein 1	2,48166E+14	6,42817E+12	5,50595E+13	0.00119800293848551	0.141570560577024	-0.422885350965536	8,08695E+14	7,69481E+14	6,38431E+14	5,58456E+14	5,84443E+14	4,974E+12
TC0100000271.mm.2	Eif5b	eukaryotic translation initiation factor 5B	1,72781E+14	6,3291E+14	5,50396E+14	0.0012003674486496	0.141570560577024	-0.424560189113815	7,35931E+14	7,25114E+14	6,09015E+14	6,11919E+14	5,79714E+14	5,35769E+14
TC0200004108.mm.2	Madd	MAP-kinase activating death domain	2,20478E+14	6,27783E+14	5,50113E+14	0.00120372480260398	0.141570560577024	-0.426932998609884	7,45501E+14	7,41202E+14	6,51946E+14	5,82298E+14	5,58832E+14	4,86915E+14
TC0200001810.mm.2	Ckmt1	creatine kinase, mitochondrial 1, ubiquitous	1,6324E+14	7,77141E+13	5,49758E+14	0.00120794993611836	0.141570560577024	-0.429910346620096	8,86481E+13	8,68954E+14	7,33557E+14	7,44899E+14	7,22932E+14	7,06024E+14
TC1500001320.mm.2	Ankrd33b	ankyrin repeat domain 33B	-1,89733E+13	9,82905E+14	-5,49128E+14	0.00121549498065747	0.141570560577024	-0.435203052254113	8,72486E+14	8,66141E+14	9,8617E+14	1,05454E+14	1,06137E+14	1,05672E+14
TC0200004376.mm.2	Dph6	diphthamine biosynthesis 6	1,86724E+14	6,39539E+14	5,49088E+14	0.00121597528339174	0.141570560577024	-0.435538936090358	7,55302E+14	7,43296E+14	6,38451E+13	5,75035E+14	5,67306E+14	5,57843E+14
TC1100000058.mm.2	Ap1b1	adaptor protein complex AP-1, beta 1 subunit	3,50412E+14	6,04157E+14	5,49067E+14	0.00121623514834373	0.141570560577024	-0.435720612410518	8,07561E+13	7,84264E+14	6,45228E+14	4,96885E+14	5,14815E+14	3,76188E+14
TC0100003864.mm.2	Psmd1	proteasome (prosome, macropain) 26S subunit, non-ATPase, 1	1,78691E+14	7,99079E+14	5,48788E+12	0.0012195851906651	0.141570560577024	-0.438059448044033	9,11131E+14	8,92446E+14	7,96314E+14	7,48391E+14	7,32744E+14	71345102689
TC0700000282.mm.2	Sympk	symplekin	1,72628E+14	6,99289E+14	5,47927E+14	0.001230034245227	0.141570560577024	-0.445316119110764	8,24284E+14	8,05678E+14	6,30317E+14	6,50747E+14	6,6096E+14	6,23747E+14
TC0100000280.mm.2	Pdcl3	phosducin-like 3	1,82159E+14	6,17643E+14	5,47165E+14	0.0012393559974507	0.141570560577024	-0.451741469479025	7,3596E+14	7,11253E+14	5,76363E+14	5,99389E+14	5,16467E+14	5,66427E+14
TC1600001287.mm.2	Pi4ka	phosphatidylinositol 4-kinase alpha	1,62293E+14	7,64782E+14	5,47135E+14	0.00123971879858655	0.141570560577024	-0.451990629378529	8,70744E+14	8,85684E+14	7,01809E+14	6,9861E+14	7,26701E+14	7,05141E+13
TC0200002228.mm.2	Pygb	brain glycogen phosphorylase	2,10687E+14	7,22852E+14	5,46775E+14	0.0012441495485905	0.141570560577024	-0.455028039525882	8,81661E+14	8,34461E+14	6,79271E+14	6,46972E+14	6,83454E+14	6,11293E+14
TC0200003605.mm.2	Itgb6	integrin beta 6	-1,79265E+14	7,14874E+14	-5,455E+14	0.00125999338643393	0.141570560577024	-0.465807395365205	6,02245E+14	6,09049E+14	7,29582E+14	7,78546E+14	7,70526E+14	7,993E+14
TC0200004443.mm.2	Ndufaf1	NADH:ubiquinone oxidoreductase complex assembly factor 1	2,02508E+14	5,82221E+14	5,45217E+14	0.00126354774386003	0.141570560577024	-0.468208200932838	6,73008E+14	6,86288E+14	6,30669E+14	5,49079E+14	4,85687E+14	4,68594E+14
TC1200000523.mm.2	Togaram1	TOG array regulator of axonemal microtubules 1	1,74634E+12	6,74104E+14	5,43726E+14	0.0012824279722645	0.141570560577024	-0.480856071313792	7,85729E+14	7,82712E+14	6,16249E+14	6,4076E+14	6,32168E+14	5,87006E+14
TC0200005502.mm.2	Ttbk2	tau tubulin kinase 2	2,07176E+14	6,93694E+14	5,42649E+14	0.00129625321933086	0.141570560577024	-0.490007679064892	8,55537E+14	8,41414E+14	6,32834E+14	5,49782E+14	6,25237E+14	6,57361E+13
TC0200001786.mm.2	Mapkbp1	mitogen-activated protein kinase binding protein 1	1,70462E+14	6,03383E+14	5,42181E+14	0.00130231724566343	0.141570560577024	-0.493992991511299	6,96261E+14	7,08732E+14	5,97194E+14	5,5404E+13	5,27634E+14	5,36435E+14
TC1000002484.mm.2	Med16	mediator complex subunit 16	1,82387E+14	7,17153E+14	5,42023E+14	0.00130438291573859	0.141570560577024	-0.495346599719971	8,42467E+14	8,25246E+14	6,62235E+14	6,70029E+14	6,81417E+14	6,21522E+14
TC1100003047.mm.2	Wrap53	WD repeat containing, antisense to Trp53	1,61319E+14	5,86911E+13	5,41934E+14	0.00130553082189453	0.141570560577024	-0.496097942180346	6,76422E+13	6,92555E+14	5,58316E+13	5,47835E+14	5,33272E+14	5,13068E+14
TC0600001193.mm.2	Itpr1	inositol 1,4,5-trisphosphate receptor 1	1,95302E+14	8,32196E+14	5,41795E+14	0.00130734556024061	0.141570560577024	-0.497284488761604	9,56753E+14	9,90047E+14	7,63713E+14	7,26463E+14	8,0809E+14	7,48107E+14
TC1100001072.mm.2	Slc43a2	solute carrier family 43, member 2	1,78724E+14	6,60757E+14	5,41032E+14	0.00131734344057107	0.141570560577024	-0.503793998178647	7,72398E+14	7,43182E+13	6,70493E+13	6,20336E+14	5,75615E+14	5,82516E+13
TC0400003234.mm.2	Scp2	sterol carrier protein 2, liver	2,02722E+14	5,9654E+13	5,40562E+14	0.00132354575729003	0.141570560577024	-0.507809070167698	7,24958E+14	7,66556E+14	4,85507E+14	5,1615E+14	5,7768E+14	5,0839E+14
TC1100000537.mm.2	Mrnip	MRN complex interacting protein	1,757E+14	6,20554E+14	5,40504E+14	0.00132431853029588	0.141570560577024	-0.508308091328295	7,40038E+14	7,2016E+14	6,0179E+14	5,5254E+14	5,6056E+14	5,48238E+14
TC0500002254.mm.2	Sorcs2	sortilin-related VPS10 domain containing receptor 2	2,02366E+14	5,67606E+14	5,38969E+14	0.00134481113314474	0.141570560577024	-0.521442830159978	7,06973E+14	6,98039E+14	5,25661E+14	4,7468E+14	4,65374E+14	5,34906E+14
TC1800001462.mm.2	Nars	asparaginyl-tRNA synthetase	1,84792E+13	7,57524E+14	5,38838E+14	0.00134657742918638	0.141570560577024	-0.522566154198259	8,69484E+14	8,71331E+14	7,47622E+14	6,85475E+13	6,98293E+14	6,72937E+14
TC0400004177.mm.2	Akap2	A kinase (PRKA) anchor protein 2	1,5886E+14	6,09407E+14	5,38342E+14	0.00135328695580187	0.141570560577024	-0.526820728753369	7,14942E+14	6,9274E+14	5,82079E+14	5,76717E+14	5,48851E+14	5,41111E+14
TC1200001233.mm.2	Amn	amnionless	-1,6818E+14	7,59187E+14	-5,37191E+14	0.00136901038545429	0.141570560577024	-0.53671425943802	6,44461E+14	6,43939E+13	7,98696E+14	8,43267E+14	8,118E+14	8,12961E+14
TC0300000507.mm.2	Gmps	guanine monophosphate synthetase	2,59613E+14	7,35762E+14	5,34584E+14	0.00140537303755992	0.141570560577024	-0.559192935225359	8,91427E+14	8,88346E+13	7,41161E+14	6,33092E+13	6,75068E+14	5,85478E+14
TC1800000282.mm.2	Apc	APC, WNT signaling pathway regulator	2,26385E+14	7,4449E+14	5,33731E+14	0.0014175060978531	0.141570560577024	-0.566572252482342	8,88334E+14	8,98517E+14	6,98772E+13	6,47236E+14	7,1644E+14	6,17642E+14
TC0X00002057.mm.2	Rpl39	ribosomal protein L39	1,66726E+14	8,39741E+13	5,33438E+14	0.00142170721398691	0.141570560577024	-0.569113580108564	9,40225E+14	9,42505E+14	8,14056E+14	7,92385E+14	7,55459E+14	7,93817E+14

TC0800003233.mm.2	Trmt1	tRNA methyltransferase 1	2,3525E+14	5,97377E+14	5,32566E+14	0.00143426378972239	0.141570560577024	-0.576667536730354	7,33079E+14	7,41429E+13	6,04343E+14	5,01404E+14	5,32873E+14	4,71134E+14
TC1600001117.mm.2	Crebbp	CREB binding protein	2,0737E+14	7,8406E+13	5,32419E+14	0.00143640447973943	0.141570560577024	-0.577949174888767	9,02694E+13	9,18928E+14	7,61414E+14	7,1444E+13	7,43512E+14	6,6337E+14
TC0500003028.mm.2	Sgsm1	small G protein signaling modulator 1	2,01118E+14	7,53661E+14	5,29416E+14	0.00148072219843151	0.141570560577024	-0.604087823403662	8,81896E+14	8,68373E+14	7,55543E+14	6,6812E+14	6,686E+14	6,79433E+14
TC0X00000122.mm.2	Sytl5	synaptotagmin-like 5	1,91327E+14	5,5392E+14	5,28947E+14	0.00148778012205626	0.141570560577024	-0.608182723144448	6,61365E+14	6,49146E+14	5,25087E+14	5,60066E+14	4,98701E+14	4,29156E+13
TC1100001250.mm.2	Rasl10b	RAS-like, family 10, member B	1,90453E+14	8,1623E+13	5,28545E+14	0.00149387009921717	0.141570560577024	-0.611701417297193	9,28184E+14	9,21926E+14	8,06769E+14	7,713E+14	7,69186E+14	7,00018E+14
TC1700002337.mm.2	Rfx2	regulatory factor X, 2 (influences HLA class II expression)	-1,97117E+14	7,24398E+14	-5,28131E+14	0.00150015429866142	0.141570560577024	-0.615318251437198	6,04372E+13	5,9851E+14	7,33084E+14	8,13307E+14	7,84166E+14	8,12951E+14
TC0300002023.mm.2	Tm4sf1	transmembrane 4 superfamily member 1	2,35029E+14	6,23634E+14	5,27994E+14	0.00150225167532727	0.141570560577024	-0.616522221184129	7,27891E+14	8,0995E+14	5,68286E+14	5,67894E+14	5,78982E+14	4,88801E+14
TC0700002780.mm.2	Mag	myelin-associated glycoprotein	1,73571E+14	8,03886E+14	5,27858E+14	0.00150432338787207	0.141570560577024	-0.617709911598894	9,0038E+14	9,2606E+14	7,84421E+14	7,33158E+14	7,40121E+14	7,39178E+14
TC1700000335.mm.2	Abca3	ATP-binding cassette, sub-family A (ABC1), member 3	2,39979E+14	7,56397E+13	5,27712E+14	0.00150655392881134	0.141570560577024	-0.618986942256452	8,89144E+14	8,88987E+12	7,62564E+14	6,99518E+14	7,03869E+14	5,94303E+14
TC0700003526.mm.2	Sv2b	synaptic vesicle glycoprotein 2 b	2,55321E+14	7,39998E+14	5,27567E+14	0.00150877733923828	0.141570560577024	-0.620258126305314	8,61861E+14	9,35982E+14	7,15863E+14	6,39077E+14	6,88644E+13	5,98558E+14
TC1700000835.mm.2	Pla2g7	phospholipase A2, group VII (platelet-activating factor acetylhydrolase, plasma)	2,63288E+14	7,32503E+14	5,26989E+14	0.00151767366170585	0.141570560577024	-0.625326870628627	9,14115E+14	8,76867E+14	7,18666E+14	6,20966E+14	6,83566E+14	5,80841E+14
TC1600000918.mm.2	Ncam2	neural cell adhesion molecule 2	1,88707E+14	8,19312E+14	5,26295E+14	0.00152842738871623	0.141570560577024	-0.63141679408377	9,60772E+14	9,51232E+14	7,69648E+14	6,9963E+14	7,82491E+14	7,52099E+14
TC0700001455.mm.2	Rrm1	ribonucleotide reductase M1	2,71382E+14	5,42837E+14	5,2599E+14	0.00153316945401469	0.141570560577024	-0.634089497819823	6,9729E+13	7,42085E+14	5,12082E+14	4,08957E+14	5,00692E+14	3,95918E+14
TC0300003166.mm.2	5730460C07Rik	RIKEN cDNA 5730460C07 gene	-1,59589E+14	7,36004E+14	-5,25879E+14	0.00153491529514538	0.141570560577024	-0.635071528007114	6,3422E+14	6,41026E+13	7,61097E+14	7,85255E+14	8,09427E+14	7,84997E+14
TC0700000936.mm.2	Herc2	HECT and RLD domain containing E3 ubiquitin protein ligase 2	1,94966E+14	6,49865E+13	5,25515E+14	0.00154061465698111	0.141570560577024	-0.638270119090801	7,60866E+14	8,01933E+14	6,18444E+14	5,45079E+14	5,88418E+14	5,84449E+14
TC0900002728.mm.2	Slc17a5	solute carrier family 17 (anion/sugar transporter), member 5	1,63709E+14	6,8688E+14	5,25253E+14	0.00154473688261164	0.141570560577024	-0.640576676221915	7,7265E+13	7,90273E+14	6,73722E+14	6,49131E+14	6,02256E+14	6,33249E+14
TC1100001291.mm.2	Ppm1d	protein phosphatase 1D magnesium-dependent, delta isoform	2,12964E+14	6,71938E+14	5,25179E+13	0.00154590258304529	0.141570560577024	-0.641227886211833	8,1534E+14	7,99519E+14	6,54714E+14	5,73122E+14	6,24563E+14	5,64368E+14
TC0900000173.mm.2	Zfp846	zinc finger protein 846	2,06316E+14	5,90578E+13	5,25161E+14	0.00154617555802499	0.141570560577024	-0.641380315064191	7,3673E+14	7,09203E+14	5,60543E+14	5,0369E+14	5,52853E+14	4,80448E+14
TC0X00000708.mm.2	Atp6ap1	ATPase, H+ transporting, lysosomal accessory protein 1	2,43183E+14	7,98539E+14	5,23868E+14	0.00156671151796092	0.141570560577024	-0.652775731952872	9,35111E+14	9,18192E+13	8,13004E+14	7,57991E+14	7,42171E+14	6,24765E+14
TC0700000459.mm.2	Sertad3	SERTA domain containing 3	-1,95696E+14	7,89866E+14	-5,23727E+14	0.00156897937386783	0.141570560577024	-0.654025555053831	6,59038E+14	6,65997E+14	8,15519E+14	8,82211E+13	8,31552E+14	8,84875E+14
TC1700002050.mm.2	Mog	myelin oligodendrocyte glycoprotein	2,08909E+14	6,93174E+13	5,22936E+14	0.00158170853740829	0.141570560577024	-0.661009310332422	8,40403E+14	8,12914E+14	6,6891E+14	6,01315E+14	6,51601E+14	5,83899E+14
TC1200000788.mm.2	Rbm25	RNA binding motif protein 25	1,5453E+14	6,70588E+14	5,22772E+14	0.00158436003842833	0.141570560577024	-0.662457390182696	7,47781E+14	7,55722E+14	6,50991E+14	6,74589E+14	5,97398E+13	5,97045E+14
TC0600003302.mm.2	Lrp6	low density lipoprotein receptor-related protein 6	1,6136E+12	6,76706E+14	5,2145E+14	0.00160594262237808	0.141570560577024	-0.674160446189832	7,71028E+14	7,73602E+14	6,57626E+14	6,36073E+14	6,28466E+14	5,93443E+14
TC0600001093.mm.2	Ccdc174	coiled-coil domain containing 174	1,65124E+14	6,31018E+14	5,20742E+14	0.00161763991195487	0.141570560577024	-0.68044164300137	7,55727E+14	7,24296E+14	5,60981E+14	5,95329E+14	5,80934E+14	5,68841E+14
TC0700003609.mm.2	Ap3b2	adaptor-related protein complex 3, beta 2 subunit	2,17397E+14	6,77959E+13	5,20571E+13	0.00162047233818876	0.141570560577024	-0.681956176285804	8,12232E+14	8,02377E+14	6,49513E+14	6,23816E+14	6,42479E+14	5,37337E+14
TC0200002830.mm.2	Prpf6	pre-mRNA splicing factor 6	2,83522E+13	6,58631E+14	5,19787E+14	0.00163355717731761	0.141570560577024	-0.688920664927045	8,56121E+13	8,25805E+14	6,48625E+14	5,06355E+14	5,97715E+14	5,17167E+14
TC1000002008.mm.2	Marcks	myristoylated alanine rich protein kinase C substrate	1,78138E+14	9,30287E+14	5,19373E+14	0.00164052844421657	0.141570560577024	-0.692609785482864	1,04639E+14	1,05325E+14	9,01761E+14	8,36973E+14	8,63496E+14	8,79859E+14
TC0500001868.mm.2	Pds5b	PDS5 cohesin associated factor B	2,22376E+14	7,52302E+14	5,18763E+14	0.00165083145422859	0.141570560577024	-0.698035134247061	9,01898E+14	8,97839E+14	7,14779E+14	6,44308E+13	7,22306E+14	6,3268E+14
TC0X00000618.mm.2	Aff2	AF4/FMR2 family, member 2	1,61329E+14	5,66035E+14	5,17575E+14	0.00167112888324715	0.141570560577024	-0.70863083105575	6,81114E+13	6,52572E+14	5,16892E+14	5,34605E+13	4,91827E+14	5,19203E+13
TC0700004576.mm.2	Ascl2	achaete-scute family bHLH transcription factor 2	-2,03432E+14	5,12197E+13	-5,17192E+14	0.00167773696623608	0.141570560577024	-0.712054290991307	3,96215E+14	3,86931E+14	5,22106E+14	5,77919E+14	5,5602E+13	6,3399E+14
TC0200001551.mm.2	Cd59b	CD59b antigen	1,61916E+14	5,68031E+14	5,16996E+14	0.00168112547437819	0.141570560577024	-0.713804865128214	6,80893E+14	6,5645E+14	5,46044E+14	5,11289E+14	5,13681E+14	4,9983E+13
TC0200003135.mm.2	Tubb4b	tubulin, beta 4B class IVB	1,60002E+14	7,90182E+14	5,16333E+14	0.00169266048513141	0.141570560577024	-0.719739315796029	8,8612E+14	9,02436E+14	7,53374E+14	7,30607E+14	7,18882E+14	7,4967E+14
TC1700002597.mm.2	Slc8a1	solute carrier family 8 (sodium/calcium exchanger), member 1	2,14878E+14	7,41699E+14	5,16125E+14	0.0016962834574304	0.141570560577024	-0.72159538796712	8,88949E+14	8,8603E+14	7,15003E+13	6,14991E+14	6,93007E+14	6,52214E+14
TC0800000105.mm.2	Sox1	SRY (sex determining region Y)-box 1	-1,57169E+14	9,16823E+14	-5,1428E+14	0.00172890898901318	0.141570560577024	-0.738143639130818	8,23766E+14	8,10374E+14	9,42395E+14	9,75922E+14	9,79006E+14	9,69473E+14
TC1300002691.mm.2	Gzma	granzyme A	-1,7486E+14	5,36641E+14	-5,14268E+14	0.00172912581173996	0.141570560577024	-0.738252629357951	4,32923E+14	4,44098E+14	5,46231E+14	5,69855E+14	5,83502E+14	6,43238E+14
TC0900002869.mm.2	Trpc1	transient receptor potential cation channel, subfamily C, member 1	1,55819E+13	7,03491E+14	5,14262E+14	0.00172922973544118	0.141570560577024	-0.738304864099789	7,98286E+14	8,03888E+14	6,8052E+14	6,47718E+14	6,49804E+14	6,40732E+14
TC0700001354.mm.2	Ints4	integrator complex subunit 4	1,52891E+14	6,4534E+14	5,13926E+14	0.00173524794253476	0.141570560577024	-0.741324742562301	7,331E+14	7,1025E+13	6,39321E+14	6,51803E+13	5,73447E+14	5,64121E+14
TC1700000523.mm.2	Glp1r	glucagon-like peptide 1 receptor	1,81499E+13	7,5194E+14	5,13892E+14	0.00173585884317977	0.141570560577024	-0.741630735334528	8,77754E+14	8,33034E+14	7,55794E+14	6,97269E+14	6,7365E+14	6,74141E+14
TC0200002276.mm.2	Tcf15	transcription factor 15	-2,00082E+14	6,71515E+14	-5,13842E+14	0.00173676447461941	0.141570560577024	-0.742084168763022	5,3128E+14	5,6116E+14	6,81719E+14	7,62324E+14	7,26985E+14	7,65619E+14
TC1100000956.mm.2	Pld2	phospholipase D2	1,68557E+14	5,68074E+14	5,1333E+14	0.0017459777811586	0.141570560577024	-0.746684499818107	6,78063E+14	6,55212E+14	5,5411E+14	5,24895E+14	5,19723E+14	4,76438E+14
TC0900003043.mm.2	Rbm5	RNA binding motif protein 5	1,60923E+14	7,31236E+14	5,13268E+14	0.001747102524017З	0.141570560577024	-0.747244533637595	8,07811E+14	8,1891E+14	7,16981E+14	7,38837E+14	6,71304E+14	6,33571E+14
TC0400003381.mm.2	Ipo13	importin 13	1,78811E+14	5,65474E+14	5,12181E+14	0.00176689449228614	0.141570560577024	-0.75704424564998	6,69688E+14	6,71468E+14	5,46871E+14	5,21285E+14	4,586E+14	5,24934E+14
TC0900001489.mm.2	Dclk3	doublecortin-like kinase 3	2,18572E+14	6,39392E+14	5,11788E+14	0.0017740999421202	0.141570560577024	-0.760586276586828	7,59851E+14	7,58062E+14	6,68814E+14	5,68855E+14	5,67055E+14	5,13714E+14
TC1000002438.mm.2	Pfkl	phosphofructokinase, liver, B-type	1,73103E+14	7,58052E+14	5,11467E+14	0.00178001244611684	0.141570560577024	-0.763482621878111	8,63687E+14	8,76771E+14	7,28707E+14	6,84894E+14	7,19617E+14	6,74635E+13
TC1400001057.mm.2	Zc3h13	zinc finger CCCH type containing 13	1,4949E+14	6,3923E+14	5,10915E+14	0.00179025479354015	0.141570560577024	-0.768478643681671	7,32388E+14	7,31984E+14	6,11388E+14	5,9423E+14	5,87653E+14	5,77739E+14
TC0700002736.mm.2	Zfp27	zinc finger protein 27	1,49219E+14	5,74314E+14	5,1054E+14	0.00179723249553335	0.141570560577024	-0.771866840220317	6,59319E+14	6,68264E+14	5,37972E+13	5,51184E+14	5,09224E+14	5,19921E+14
TC0100002997.mm.2	Lypd1	Ly6/Plaur domain containing 1	1,88889E+14	8,60987E+14	5,09795E+14	0.00181119745973449	0.141570560577024	-0.778610821653641	9,86961E+14	9,66912E+14	8,5708E+14	7,78871E+14	7,9945E+14	7,76646E+14
TC1900000941.mm.2	Dpp3	dipeptidylpeptidase 3	1,73195E+14	6,34159E+14	5,0974E+12	0.00181223728762356	0.141570560577024	-0.779111015515759	7,38197E+13	7,48411E+13	6,12647E+13	5,65479E+14	5,91908E+13	5,4831E+14
TC0200005476.mm.2	Nup35	nucleoporin 35	1,97857E+14	6,15884E+14	5,09431E+14	0.00181807670662457	0.141570560577024	-0.781914969099589	7,31354E+14	7,27819E+14	6,12747E+14	5,59922E+14	5,70394E+14	4,93064E+14
TC1100001049.mm.2	Mettl16	methyltransferase like 16	1,59253E+14	5,82968E+14	5,08909E+14	0.00182796589780084	0.141570560577024	-0.786644233931758	6,9027E+14	6,57546E+14	5,50738E+14	5,69942E+14	5,0307E+14	5,26241E+12
TC0700001865.mm.2	Stx4a	syntaxin 4A (placental)	2,03041E+14	6,51022E+14	5,08579E+14	0.00183426814161074	0.141570560577024	-0.789645567527736	7,9646E+14	8,01211E+14	6,0314E+14	5,13731E+14	5,74204E+14	6,17386E+14
TC1100002028.mm.2	Hgs	HGF-regulated tyrosine kinase substrate	1,90402E+14	7,45664E+14	5,08536E+14	0.00183509587048804	0.141570560577024	-0.790039036799702	8,83484E+14	8,69137E+14	7,12346E+14	6,37201E+14	7,05651E+14	6,66165E+14
TC1100000294.mm.2	Ccdc88a	coiled coil domain containing 88A	1,61843E+14	7,27883E+14	5,07464E+14	0.00185569804386335	0.141570560577024	-0.799778982136226	8,2262E+14	8,56517E+14	6,57901E+14	6,74808E+14	6,93765E+14	6,61688E+14
TC1700000070.mm.2	Rps6ka2	ribosomal protein S6 kinase, polypeptide 2	1,68768E+14	7,71381E+14	5,06871E+14	0.00186722667232058	0.141570560577024	-0.805184908679636	8,73834E+13	8,65853E+14	7,68963E+14	7,17487E+14	7,163E+13	6,85851E+14
TC0700003434.mm.2	Lrrk1	leucine-rich repeat kinase 1	-1,89433E+14	7,25239E+14	-5,05374E+14	0.0018966513680094	0.141570560577024	-0.818841175206441	5,81012E+14	6,22261E+14	7,49675E+14	8,16345E+14	7,72997E+14	8,09143E+14
TC0400002530.mm.2	Tln1	talin 1	1,59389E+14	6,72697E+14	5,05029E+14	0.00190351363697772	0.141570560577024	-0.82199727656833	7,4645E+14	7,90683E+14	6,4576E+14	6,34934E+14	6,03724E+14	6,14631E+14
TC0400003884.mm.2	Spen	spen family transcription repressor	1,74379E+14	5,86558E+14	5,04563E+14	0.00191280180936132	0.141570560577024	-0.826252053677401	7,19646E+13	6,95964E+14	5,35191E+14	5,01696E+14	5,6109E+14	5,05761E+14
TC0600003020.mm.2	Erc1	ELKS/RAB6-interacting/CAST family member 1	1,74683E+14	7,0073E+14	5,0379E+14	0.00192835971746591	0.141570560577024	-0.833335439106943	8,06152E+14	8,15616E+14	6,89093E+14	6,21116E+14	6,30403E+14	6,41999E+14
TC1000000839.mm.2	Stk11	serine/threonine kinase 11	1,47184E+14	7,96996E+14	5,03683E+14	0.00193053211870024	0.141570560577024	-0.834320222968006	8,89817E+14	8,80758E+14	7,6373E+14	7,71463E+14	7,30643E+13	7,45564E+14
TC1700001160.mm.2	Birc6	baculoviral IAP repeat-containing 6	2,09396E+14	6,01385E+14	5,03636E+14	0.00193147281830719	0.141570560577024	-0.834746332668623	7,31614E+14	7,49142E+14	5,46987E+14	5,18601E+14	5,82221E+14	4,79743E+14
TC0500003308.mm.2	Psph	phosphoserine phosphatase	-1,57431E+14	8,82658E+13	-5,03503E+14	0.00193417262288229	0.141570560577024	-0.835968179627693	7,80045E+13	8,00049E+14	8,9076E+14	9,30139E+14	9,46368E+14	9,48589E+14
TC0300002295.mm.2	Bcan	brevican	1,6064E+14	7,3004E+14	5,033E+14	0.00193828189452287	0.141570560577024	-0.837824822399731	8,41895E+14	8,11832E+14	7,04204E+14	6,89863E+13	6,47685E+14	6,84763E+14
TC0500001230.mm.2	Tchp	trichoplein, keratin filament binding	-1,51301E+14	1,04251E+14	-5,02316E+14	0.00195840108556894	0.141570560577024	-0.846861767465436	9,4231E+14	9,50723E+14	1,07004E+14	1,09637E+14	1,10628E+14	1,08935E+14
TC0200004580.mm.2	Anapc1	anaphase promoting complex subunit 1	2,96779E+14	6,19813E+14	5,01802E+14	0.00196899571710591	0.141570560577024	-0.851585408250098	7,83279E+13	8,17E+14	6,03729E+14	5,08147E+14	5,80975E+14	4,25745E+14
TC1700001689.mm.2	Nme4	NME/NM23 nucleoside diphosphate kinase 4	-1,5777E+13	6,65201E+14	-5,01742E+14	0.00197024411479247	0.141570560577024	-0.852140428611111	5,68338E+14	5,72258E+14	6,86459E+14	7,08014E+14	7,07951E+14	7,48186E+14

TC0700000058.mm.2	Epn1	epsin 1	1,87207E+14	8,33632E+14	5,01416E+14	0.00197699718213151	0.141570560577024	-0.855137007194361	9,47578E+14	9,46175E+14	8,36819E+14	7,51877E+14	7,64493E+14	7,54847E+14
TC0200003109.mm.2	Hnmt	histamine N-methyltransferase	1,93261E+14	6,6554E+14	5,00954E+14	0.00198663318213127	0.141570560577024	-0.859396160679079	8,24463E+14	7,85382E+14	5,75751E+14	5,84322E+13	6,53264E+14	5,7006E+14
TC0200005496.mm.2	Fam78a	family with sequence similarity 78, member A	-1,54723E+14	6,22416E+14	-5,00582E+14	0.00199441900432123	0.141570560577024	-0.862823307500788	5,11716E+13	5,40104E+14	6,52733E+14	6,68675E+14	6,68293E+14	6,92974E+13
TC1500001750.mm.2	Eef1d	eukaryotic translation elongation factor 1 delta (guanine nucleotide exchange protein)	2,518E+14	6,98629E+14	4,99891E+14	0.00200897309134665	0.141570560577024	-0.86919595797044	8,59585E+14	8,52577E+14	6,35238E+14	6,35811E+14	6,78384E+14	5,30178E+14
TC0300003020.mm.2	Metap1	methionyl aminopeptidase 1	1,71104E+14	6,4956E+14	4,99531E+14	0.00201661249743958	0.141570560577024	-0.872523522897251	7,70864E+14	7,41167E+14	5,78122E+14	6,37382E+14	5,89333E+14	5,8049E+14
TC0200004530.mm.2	Secisbp2l	SECIS binding protein 2-like	1,99003E+14	7,61776E+14	4,99052E+14	0.00202681325827651	0.141570560577024	-0.876948249631576	8,74677E+14	8,94378E+14	7,23116E+14	7,07435E+14	7,33481E+14	6,37569E+14
TC0100000456.mm.2	Nop58	NOP58 ribonucleoprotein	2,99902E+14	6,13888E+14	4,97745E+14	0.00205496962549486	0.141570560577024	-0.889053209846705	7,81321E+14	7,70865E+14	6,25825E+14	5,52938E+14	5,63728E+14	3,88653E+14
TC0500001136.mm.2	Ankle2	ankyrin repeat and LEM domain containing 2	2,05029E+14	5,87898E+14	4,97394E+14	0.00206259289757953	0.141570560577024	-0.892303648568833	7,00465E+14	6,87279E+14	6,2805E+14	5,33909E+14	5,12289E+14	4,65395E+14
TC1100003292.mm.2	Supt6	suppressor of Ty 6	1,77533E+14	7,44021E+14	4,97184E+14	0.00206717845322786	0.141570560577024	-0.894253392137219	8,68343E+14	8,55215E+14	7,07294E+14	6,64785E+14	7,15074E+14	6,53417E+14
TC0X00003173.mm.2	Gnl3l	guanine nucleotide binding protein-like 3 (nucleolar)-like	1,62626E+14	7,02267E+14	4,9669E+14	0.00207800299576886	0.141570560577024	-0.898839745966058	8,29062E+13	7,95274E+14	6,22571E+14	6,67609E+14	6,4953E+14	6,49554E+14
TC0100000794.mm.2	Dis3l2	DIS3 like 3'-5' exoribonuclease 2	2,69563E+13	6,01312E+14	4,95225E+14	0.00211048070315785	0.141570560577024	-0.91246628481588	7,5095E+14	7,44255E+14	6,04096E+14	5,52494E+14	5,5675E+14	3,99329E+14
TC0800001977.mm.2	Mak16	MAK16 homolog	1,65678E+14	6,04863E+14	4,94998E+14	0.00211556827929629	0.141570560577024	-0.914582882902096	6,70986E+14	6,9416E+14	5,93791E+14	6,3645E+14	5,35826E+14	4,97964E+14
TC0200003182.mm.2	Qsox2	quiescin Q6 sulfhydryl oxidase 2	1,52541E+14	5,97666E+14	4,94957E+14	0.0021164752328686 4	0.141570560577024	-0.914959699871276	6,7081E+14	6,47917E+14	6,28029E+12	6,25594E+14	4,90482E+14	5,23162E+14
TC0100000436.mm.2	Ndufb3	NADH:ubiquinone oxidoreductase subunit B3	1,43246E+14	6,97019E+14	4,94792E+14	0.002120175644213 6	0.141570560577024	-0.916495549863113	7,86888E+13	7,8305E+14	6,62197E+14	6,66537E+14	6,47386E+14	6,36059E+14
TC1100002007.mm.2	Rptor	regulatory associated protein of MTOR, complex 1	1,71786E+13	6,28415E+14	4,94524E+14	0.00212620652478404	0.141570560577024	-0.918993235507337	7,55242E+14	7,29085E+14	5,71843E+14	5,73569E+14	6,02838E+14	5,37917E+14
TC1500001646.mm.2	Kcnq3	potassium voltage-gated channel, subfamily Q, member 3	2,02253E+14	7,11787E+14	4,94513E+14	0.00212645827557194	0.141570560577024	-0.919097352265031	8,45771E+14	8,67729E+14	6,73363E+14	5,74862E+13	6,67834E+14	6,41161E+14
TC0Y00000547.mm.2	Gm20871	Sycp3 like Y-linked pseudogene	-1,53777E+14	4,91483E+14	-4,93195E+14	0.00215643113442112	0.141570560577024	-0.931410710445104	4,10686E+14	3,9849E+13	5,25457E+14	4,97538E+14	5,42893E+14	5,73836E+14
TC0900003050.mm.2	Rnf123	ring finger protein 123	1,65457E+14	6,54153E+14	4,92172E+14	0.00217999481781215	0.141570560577024	-0.940977961258334	7,6538E+14	7,36552E+14	6,17113E+13	6,34854E+14	6,11832E+14	5,59186E+14
TC1000001543.mm.2	Avil	advillin	-2,30207E+14	7,2754E+14	-4,91711E+13	0.00219072508558501	0.141570560577024	-0.945302259669443	5,62219E+13	5,90916E+13	7,36331E+14	8,62227E+14	7,91779E+14	8,21769E+14
TC0100001581.mm.2	Adamts4	a disintegrin-like and metallopeptidase (reprolysin type) with thrombospondin type 1 motif, 4	1,49794E+14	6,37144E+14	4,91544E+14	0.00219461532409131	0.141570560577024	-0.946865078619594	7,19323E+14	7,43388E+14	6,08003E+14	5,89022E+14	5,7173E+14	5,91394E+14
TC1100001890.mm.2	Otop3	otopetrin 3	-1,82848E+14	5,0658E+14	-4,91323E+14	0.00219978069104693	0.141570560577024	-0.948936107605404	3,55685E+14	4,10566E+14	5,38978E+14	6,02307E+14	5,72538E+14	5,59409E+14
TC0X00002637.mm.2	Pdk3	pyruvate dehydrogenase kinase, isoenzyme 3	1,6002E+14	5,39833E+14	4,91238E+14	0.00220176689268071	0.141570560577024	-0.949731241126011	6,50468E+14	6,45956E+14	5,01647E+14	4,6454E+14	5,08393E+14	4,67991E+14
TC1000001038.mm.2	Pwp1	PWP1 homolog, endonuclein	1,62484E+14	5,98235E+14	4,90736E+14	0.00221358758476183	0.141570560577024	-0.954449412161845	6,73992E+13	6,80354E+14	6,18487E+13	5,872E+14	5,39501E+14	4,89877E+14
TC0800001247.mm.2	Prmt7	protein arginine N-methyltransferase 7	1,49825E+14	7,42737E+14	4,90589E+14	0.00221706500580666	0.141570560577024	-0.955832869977939	8,31334E+14	8,2772E+14	7,31257E+14	7,06707E+14	6,93503E+14	6,65901E+14
TC0200000697.mm.2	Lhx2	LIM homeobox protein 2	1,52164E+14	7,0384E+14	4,89742E+14	0.00223716341534848	0.141570560577024	-0.963788819183236	7,64997E+14	7,91905E+13	7,11701E+14	7,01863E+14	6,41916E+14	6,10657E+14
TC1700001366.mm.2	Tmem242	transmembrane protein 242	1,55967E+14	6,55541E+14	4,89586E+14	0.00224091002427699	0.141570560577024	-0.965264429564125	7,61592E+14	7,58174E+14	6,19713E+14	5,85933E+14	6,21817E+14	5,86015E+14
TC0700002004.mm.2	Mtg1	mitochondrial ribosome-associated GTPase 1	1,48443E+14	6,06038E+14	4,89526E+14	0.00224234501225109	0.141570560577024	-0.965828984472067	6,91703E+14	6,89406E+14	5,70894E+14	6,00003E+14	5,32676E+14	5,51546E+14
TC0800000619.mm.2	Glra3	glycine receptor, alpha 3 subunit	2,6374E+14	5,53438E+14	4,89254E+14	0.00224885750819002	0.141570560577024	-0.968386849572822	7,46872E+14	7,03873E+14	5,16176E+14	4,30444E+14	5,27885E+14	3,9538E+14
TC0X00003230.mm.2	Magea8	melanoma antigen, family A, 8	-1,72238E+14	5,12449E+13	-4,89105E+13	0.002252428589885 8	0.141570560577024	-0.969786462295968	4,22283E+13	3,8547E+14	5,27834E+14	5,86881E+14	5,87177E+14	5,6505E+14
TC1500000679.mm.2	Gtpbp1	GTP binding protein 1	1,7409E+13	6,40874E+14	4,88914E+13	0.00225702995583381	0.141570560577024	-0.971586780397547	7,67908E+14	7,2794E+14	6,32764E+14	5,68966E+14	5,76141E+14	5,71525E+14
TC0200000924.mm.2	Psmd14	proteasome (prosome, macropain) 26S subunit, non-ATPase, 14	1,53843E+14	7,27378E+14	4,88883E+14	0.00225778510160988	0.141570560577024	-0.971881904794079	8,09159E+14	8,41162E+14	6,95133E+13	6,7618E+14	6,84871E+14	6,57763E+14
TC1100003204.mm.2	Sgsm2	small G protein signaling modulator 2	1,4403E+14	6,71594E+14	4,88789E+14	0.00226005735892494	0.141570560577024	-0.972769380108172	7,58856E+14	7,40496E+14	6,53218E+14	6,65704E+14	6,01888E+14	6,09404E+14
TC0400000353.mm.2	Chmp5	charged multivesicular body protein 5	1,50276E+14	6,95333E+14	4,88421E+14	0.00226895800602798	0.141570560577024	-0.97623758993748	7,89356E+14	7,88843E+14	6,75598E+14	6,40556E+14	6,29752E+13	6,47895E+14
TC0200000380.mm.2	Il1f10	interleukin 1 family, member 10	-1,64755E+14	5,25746E+14	-4,88412E+14	0.00226917476787512	0.141570560577024	-0.976321892210102	4,25826E+14	4,6005E+14	5,04408E+14	5,48808E+14	5,92908E+14	6,22479E+14
TC0700000498.mm.2	Paf1	Paf1, RNA polymerase II complex component	1,72739E+14	7,54454E+14	4,88354E+14	0.00227057606480057	0.141570560577024	-0.976866695915849	8,63037E+14	8,50047E+14	7,45724E+14	7,00307E+13	7,12274E+14	6,55333E+14
TC0700002763.mm.2	Arhgap33	Rho GTPase activating protein 33	2,10775E+14	7,23799E+14	4,88144E+14	0.00227567800183246	0.141570560577024	-0.978847568621645	8,40921E+14	8,54504E+14	7,37774E+14	6,35725E+13	6,10658E+14	6,63216E+14
TC0200003526.mm.2	Rbm43	RNA binding motif protein 43	1,66961E+14	5,72727E+14	4,88079E+14	0.00227725553238602	0.141570560577024	-0.979459208011247	6,89137E+14	6,5251E+14	5,67434E+14	5,19558E+14	5,17991E+14	4,89733E+14
TC1100001176.mm.2	Nf1	neurofibromin 1	3,24362E+13	6,39123E+14	4,88002E+14	0.00227913974142232	0.141570560577024	-0.980189227143238	8,63883E+14	8,55205E+14	6,22355E+14	4,22927E+13	5,75044E+14	4,9532E+14
TC0X00002211.mm.2	Gpc3	glypican 3	1,64841E+14	5,46653E+14	4,8791E+14	0.002281375908666 7	0.141570560577024	-0.98105486806744	6,71241E+14	6,17437E+14	5,15604E+14	5,1664E+14	4,62421E+14	4,96575E+14
TC1100003797.mm.2	Ezh1	enhancer of zeste 1 polycomb repressive complex 2 subunit	1,8082E+14	6,80304E+14	4,87734E+14	0.00228567702391803	0.141570560577024	-0.982717613082307	8,2124E+14	7,59183E+14	6,69529E+14	6,1309E+14	6,06998E+13	6,11785E+14
TC1600001380.mm.2	Dgkg	diacylglycerol kinase, gamma	2,25833E+14	8,30522E+14	4,87441E+14	0.002292867432129 8	0.141570560577024	-0.985490709308605	9,60126E+14	9,65632E+14	8,35449E+14	7,47833E+14	7,85218E+14	6,88874E+14
TC1700002293.mm.2	Slc5a7	solute carrier family 5 (choline transporter), member 7	2,21665E+14	5,6641E+14	4,87438E+14	0.00229293241572471	0.141570560577024	-0.985515733707627	7,29311E+14	6,50936E+14	5,65428E+14	5,15864E+14	5,11414E+14	4,25504E+14
TC0200003281.mm.2	BC005624	cDNA sequence BC005624	1,40447E+14	8,08009E+14	4,87364E+14	0.00229475266129191	0.141570560577024	-0.986216415607688	8,9066E+14	8,96487E+14	7,77706E+13	7,76949E+14	7,55794E+14	7,50458E+13
TC1600000955.mm.2	Usp16	ubiquitin specific peptidase 16	-1,60659E+14	9,60862E+13	-4,86886E+14	0.002306530037526	0.141570560577024	-0.990737297621735	8,59885E+14	8,59454E+13	9,76359E+14	1,02882E+13	1,03291E+14	1,00774E+14
TC1200002236.mm.2	Gpr68	G protein-coupled receptor 68	-1,93648E+14	6,97429E+14	-4,86847E+14	0.00230748231422633	0.141570560577024	-0.991101884189717	5,75611E+14	5,81406E+14	6,9543E+14	7,87811E+14	7,55108E+14	7,89207E+14
TC0300002549.mm.2	Phgdh	3-phosphoglycerate dehydrogenase	1,44835E+14	7,16529E+14	4,8646E+14	0.00231706443944	0.141570560577024	-0.9947625654545	8,04366E+14	7,97522E+14	7,01235E+14	6,83834E+14	6,45701E+14	6,66517E+14
TC1200000236.mm.2	Adi1	acireductone dioxygenase 1	1,70077E+14	7,27125E+14	4,85599E+14	0.00233857087970942	0.141570560577024	-1,00293E+13	8,31462E+14	8,04645E+14	7,27995E+14	7,02698E+14	6,17154E+13	6,78798E+13
TC0100001787.mm.2	Eprs	glutamyl-prolyl-tRNA synthetase	3,00098E+14	7,30185E+14	4,8549E+14	0.00234129360428263	0.141570560577024	-1,00396E+14	8,83253E+13	8,92879E+14	7,57657E+14	6,71384E+14	6,75986E+14	4,99951E+14
TC1400000410.mm.2	Dydc1	DPY30 domain containing 1	-1,89533E+14	5,33137E+14	-4,85049E+14	0.00235239872516276	0.141570560577024	-1,00814E+14	4,04798E+14	4,12155E+14	5,46806E+13	6,39041E+14	5,87305E+14	6,08714E+14
TC0800001544.mm.2	Cog2	component of oligomeric golgi complex 2	2,30002E+14	5,46809E+13	4,8488E+14	0.00235667282298537	0.141570560577024	-1,00974E+14	6,50361E+14	6,75629E+14	5,02865E+14	5,86012E+13	3,81106E+14	4,8488E+14
TC0500002559.mm.2	Gabra2	gamma-aminobutyric acid (GABA) A receptor, subunit alpha 2	2,43977E+14	8,81479E+14	4,84586E+12	0.00236412386085947	0.141570560577024	-1,01254E+14	1,03993E+14	1,02474E+14	8,8788E+14	7,59623E+13	8,24767E+14	7,51943E+14
TC1500002048.mm.2	Sbf1	SET binding factor 1	1,43558E+14	6,93842E+14	4,84291E+14	0.00237163261546236	0.141570560577024	-1,01534E+14	7,84341E+14	7,69265E+14	6,82069E+14	6,60887E+14	6,34612E+14	6,31877E+14
TC0900001288.mm.2	Ky	kyphoscoliosis peptidase	-1,76969E+14	5,51156E+14	-4,84086E+14	0.00237688145539185	0.141570560577024	-1,0173E+14	4,53047E+14	4,53019E+14	5,29906E+14	6,10963E+14	6,29799E+14	6,30205E+14
TC0300002752.mm.2	Stxbp3	syntaxin binding protein 3	1,76029E+14	6,06171E+14	4,82572E+14	0.00241590132978713	0.141570560577024	-1,03171E+14	7,04959E+14	7,34335E+14	5,82207E+14	5,28289E+14	5,6972E+14	5,17516E+14
TC0700000057.mm.2	U2af2	U2 small nuclear ribonucleoprotein auxiliary factor (U2AF) 2	1,82468E+14	7,11944E+14	4,82174E+13	0.00242627685966571	0.141570560577024	-1,0355E+14	8,21276E+14	8,01491E+14	7,36683E+14	6,54384E+14	6,16307E+14	6,41524E+14
TC0700003592.mm.2	Man2a2	mannosidase 2, alpha 2	1,54378E+14	7,92784E+14	4,81758E+14	0.00243717929250242	0.141570560577024	-1,03947E+14	8,73515E+14	8,94453E+14	7,84855E+14	7,44666E+14	7,41176E+14	7,18036E+14
TC1200002273.mm.2	Serpina16	serine (or cysteine) peptidase inhibitor, clade A (alpha-1 antiproteinase, antitrypsin), member 16	-1,6014E+14	4,95978E+14	-4,81436E+14	0.00244566481682573	0.141570560577024	-1,04255E+14	3,79463E+14	4,00959E+14	5,2408E+14	5,70666E+14	5,66004E+14	5,34699E+14
TC1000000404.mm.2	F930017D23Rik	RIKEN cDNA F930017D23 gene	-1,74811E+14	6,17974E+14	-4,81415E+14	0.002446213298209 1	0.141570560577024	-1,04275E+14	4,93799E+14	5,13967E+12	6,33013E+14	7,09677E+14	6,82451E+14	6,74937E+14
TC1100003943.mm.2	Bptf	bromodomain PHD finger transcription factor	1,90915E+14	7,05695E+14	4,81038E+14	0.00245620289242968	0.141570560577024	-1,04636E+13	8,19458E+14	8,18467E+14	6,8028E+14	6,59874E+14	6,76066E+14	5,80028E+14
TC1900001105.mm.2	Tmem216	transmembrane protein 216	1,55543E+14	5,52883E+14	4,80929E+14	0.00245908701624113	0.141570560577024	-1,0474E+14	6,5198E+14	6,34176E+14	5,06481E+13	5,49592E+14	5,00379E+14	4,74691E+14
TC1100004206.mm.2	Pde6g	phosphodiesterase 6G, cGMP-specific, rod, gamma	-1,91331E+14	4,5179E+14	-4,80866E+14	0.00246075232528173	0.141570560577024	-1,048E+14	3,11816E+14	3,74455E+14	4,42426E+14	5,13111E+14	5,20116E+14	5,48818E+14

TC0600002237.mm.2	Igf2bp3	insulin-like growth factor 2 mRNA binding protein 3	-1,67407E+14	6,46194E+14	-4,79889E+14	0.00248684430607734	0.141570560577024	-1,05735E+14	5,41989E+14	5,08882E+14	6,89036E+14	7,51571E+14	6,90254E+14	6,9543E+13
TC1100001905.mm.2	Tmem94	transmembrane protein 94	1,68154E+14	6,15108E+14	4,79637E+14	0.00249362837495363	0.141570560577024	-1,05977E+13	7,31696E+14	7,32601E+14	5,84154E+14	5,1421E+14	5,72612E+14	5,55377E+14
TC1000000830.mm.2	Wdr18	WD repeat domain 18	1,51695E+14	6,88197E+14	4,79094E+14	0.00250831557277706	0.141570560577024	-1,06498E+14	8,10848E+14	7,91073E+13	6,35023E+14	5,93704E+14	6,42599E+14	6,55932E+14
TC0800001612.mm.2	Xab2	XPA binding protein 2	1,87254E+14	6,125E+14	4,78974E+14	0.00251157388492368	0.141570560577024	-1,06613E+14	7,36045E+14	7,02395E+14	5,53121E+14	6,19507E+13	5,61547E+14	5,02386E+14
TC0100000519.mm.2	Eef1b2	eukaryotic translation elongation factor 1 beta 2	1,4062E+14	9,51389E+14	4,78897E+13	0.00251368688204516	0.141570560577024	-1,06687E+14	1,04513E+14	1,03194E+14	9,15025E+14	9,20414E+14	8,89726E+14	9,06101E+14
TC1700001939.mm.2	Sapcd1	suppressor APC domain containing 1	-1,67188E+14	6,44154E+14	-4,7881E+14	0.00251603863514627	0.141570560577024	-1,0677E+14	5,45888E+14	5,42288E+12	6,41573E+14	7,1262E+14	7,10514E+14	7,1204E+14
TC1100003342.mm.2	5730455P16Rik	RIKEN cDNA 5730455P16 gene	1,86449E+14	5,93914E+14	4,78802E+14	0.00251626323320328	0.141570560577024	-1,06778E+14	7,40874E+14	7,23408E+14	5,35163E+14	4,72657E+14	5,79871E+14	5,11513E+14
TC1200002294.mm.2	Gsc	goosecoid homeobox	-1,52898E+14	5,87123E+14	-4,78701E+14	0.00251901835029989	0.141570560577024	-1,06875E+14	4,86978E+14	4,91577E+13	6,07575E+14	6,52256E+14	6,31159E+13	6,53192E+14
TC0400001863.mm.2	Smarca5-ps	SWI/SNF related, matrix associated, actin depenent ragulator of chromatin, subfamily a, member 5, pseudogene	3,12982E+14	7,01777E+14	4,78101E+14	0.00253544823221648	0.141570560577024	-1,07452E+14	8,67816E+14	8,81412E+14	6,97505E+14	6,40663E+14	6,62977E+14	4,60286E+14
TC0200004159.mm.2	Cd82	CD82 antigen	1,88631E+14	8,06541E+14	4,77591E+14	0.00254949115699381	0.141570560577024	-1,07943E+14	9,10661E+13	9,30348E+14	7,82618E+14	7,51875E+14	7,76908E+14	6,86839E+14
TC0600000014.mm.2	Peg10	paternally expressed 10	2,59959E+14	6,7357E+14	4,77031E+14	0.00256500446851375	0.141570560577024	-1,08481E+14	8,73175E+13	7,68529E+12	7,01622E+14	5,76307E+14	5,76078E+14	5,45708E+14
TC0600000921.mm.2	Ccdc142/Mrpl53	coiled-coil domain containing 142/mitochondrial ribosomal protein L53	-1,97004E+14	8,60156E+14	-4,76813E+13	0.00257109376135952	0.141570560577024	-1,08692E+14	7,39053E+13	7,45465E+14	9,47028E+14	8,50863E+14	9,61895E+14	9,16631E+14
TC0700003476.mm.2	Nr2f2	nuclear receptor subfamily 2, group F, member 2	2,43723E+14	7,26654E+14	4,7681E+14	0.00257116904917938	0.141570560577024	-1,08694E+14	8,64235E+13	8,14195E+14	7,66806E+14	7,23705E+14	6,63727E+14	5,27257E+14
TC1700000202.mm.2	Riok2	RIO kinase 2	1,94374E+14	6,18931E+14	4,75834E+14	0.00259856422400923	0.141570560577024	-1,09636E+14	7,43462E+14	7,28803E+14	6,29421E+14	5,28386E+14	5,31509E+14	5,52009E+14
TC0200002772.mm.2	Cdh4	cadherin 4	1,78273E+14	5,47271E+14	4,75793E+14	0.00259971873216388	0.141570560577024	-1,09676E+13	6,78423E+14	6,45568E+14	5,2207E+14	4,70121E+14	5,13971E+14	4,53474E+14
TC0900000232.mm.2	Npsr1	neuropeptide S receptor 1	1,51572E+14	5,06121E+14	4,75464E+14	0.00260901872372613	0.141570560577024	-1,09993E+13	5,94612E+14	5,45386E+14	5,27236E+14	5,32636E+14	4,18288E+14	4,18566E+13
TC0700004659.mm.2	Rnf141	ring finger protein 141	1,87013E+14	7,47494E+14	4,75143E+14	0.00261815274568498	0.141570560577024	-1,10304E+14	8,43779E+14	8,40542E+14	7,92932E+14	6,97418E+14	6,61725E+14	6,48569E+14
TC0200000990.mm.2	4932414N04Rik	RIKEN cDNA 4932414N04 gene	-1,47853E+14	4,56547E+14	-4,75039E+13	0.00262111634614251	0.141570560577024	-1,10404E+14	3,60418E+14	3,84666E+14	4,70879E+14	4,8253E+14	5,3711E+14	5,0368E+14
TC1100002920.mm.2	Prpsap2	phosphoribosyl pyrophosphate synthetase-associated protein 2	1,69327E+14	5,74109E+14	4,74763E+14	0.00262899495764975	0.141570560577024	-1,10671E+14	6,88825E+14	6,77609E+14	5,43204E+14	5,07233E+14	5,46763E+14	4,81018E+14
TC0200004383.mm.2	Meis2	Meis homeobox 2	1,80885E+14	9,25615E+14	4,74719E+14	0.00263024304850977	0.141570560577024	-1,10713E+14	1,03177E+14	1,02533E+14	9,38808E+14	8,62442E+14	8,71024E+14	8,24311E+14
TC1600001193.mm.2	Tekt5	tektin 5	1,6892E+14	6,35592E+14	4,74212E+14	0.00264480645398684	0.141570560577024	-1,11204E+14	7,57479E+14	7,32621E+14	6,21189E+14	5,50001E+14	5,78116E+14	5,74144E+13
TC1200002296.mm.2	Clmn	calmin	1,71563E+14	6,68861E+13	4,73203E+14	0.00267404815909675	0.141570560577024	-1,12183E+13	7,72157E+14	7,72911E+14	6,49799E+14	6,16359E+14	6,3692E+14	5,65022E+14
TC0200002882.mm.2	Gm13199	predicted gene 13199	-1,74171E+14	6,10862E+14	-4,72671E+14	0.00268960708109849	0.141570560577024	-1,12699E+14	4,89096E+14	5,29256E+14	6,0393E+14	6,76195E+14	6,8494E+14	6,81753E+14
TC1100001273.mm.2	Synrg	synergin, gamma	2,79415E+14	6,41425E+14	4,72559E+14	0.00269290190466729	0.141570560577024	-1,12808E+14	8,28159E+14	8,18882E+14	6,2519E+14	4,88107E+14	6,06294E+14	4,81918E+14
TC1100004051.mm.2	Cd300lb	CD300 molecule like family member B	-1,48498E+14	5,79376E+14	-4,72538E+14	0.00269351499385956	0.141570560577024	-1,12828E+14	4,98436E+14	4,683E+13	6,08112E+14	6,3768E+14	6,23188E+14	6,40543E+14
TC0900000804.mm.2	Aagab	alpha- and gamma-adaptin binding protein	1,8505E+14	7,11401E+14	4,72161E+14	0.00270462525628556	0.141570560577024	-1,13195E+14	8,21452E+14	8,10044E+14	6,91928E+14	6,83585E+14	6,77968E+14	5,83428E+13
TC1000002440.mm.2	Aire	autoimmune regulator (autoimmune polyendocrinopathy candidiasis ectodermal dystrophy)	-1,75783E+14	7,07336E+14	-4,71561E+14	0.00272239665722619	0.141570560577024	-1,13778E+14	5,79428E+14	6,12965E+14	7,28668E+14	7,78999E+14	7,41997E+14	8,01962E+14
TC0X00000110.mm.2	Xk	X-linked Kx blood group	1,37544E+13	5,59196E+13	4,71489E+14	0.00272453478920559	0.141570560577024	-1,13848E+13	6,38656E+14	6,21881E+14	5,53668E+14	5,55524E+14	4,95988E+14	4,8946E+14
TC1700001852.mm.2	Zfp799	zinc finger protein 799	1,76879E+14	5,60118E+14	4,71402E+14	0.0027271385997694	0.141570560577024	-1,13933E+14	6,74668E+14	6,75283E+14	5,48195E+14	4,66368E+14	5,12182E+14	4,84011E+14
TC0600000839.mm.2	Polr1a	polymerase (RNA) I polypeptide A	1,66045E+14	5,53012E+14	4,71302E+14	0.00273010581916211	0.141570560577024	-1,1403E+14	6,86222E+14	6,5093E+14	5,13597E+14	4,62262E+14	4,83391E+14	5,2167E+14
TC1000000292.mm.2	Rsph4a	radial spoke head 4 homolog A (Chlamydomonas)	1,58858E+14	7,28216E+14	4,71192E+14	0.00273339303388953	0.141570560577024	-1,14137E+14	7,91804E+14	8,11072E+14	7,49169E+14	7,32091E+14	6,71468E+14	6,13693E+14
TC0100002783.mm.2	Asb18	ankyrin repeat and SOCS box-containing 18	-1,8808E+14	6,1257E+14	-4,70983E+14	0.00273964213001268	0.141570560577024	-1,14341E+14	5,09192E+14	4,6211E+14	6,37866E+14	7,18789E+13	6,56948E+14	6,90513E+14
TC0400003237.mm.2	Zyg11b	zyg-ll family member B, cell cycle regulator	1,73439E+14	8,60533E+14	4,70728E+14	0.00274729430694239	0.141570560577024	-1,14589E+14	9,75123E+14	9,62469E+14	8,31823E+14	8,03072E+14	8,33617E+14	7,57096E+14
TC0500003019.mm.2	Sez6l	seizure related 6 homolog like	2,44052E+14	7,79888E+14	4,70321E+14	0.00275953915603579	0.141570560577024	-1,14986E+14	9,47956E+14	9,26074E+14	7,85816E+14	6,33558E+14	6,83587E+14	7,02339E+14
TC0X00002671.mm.2	Las1l	LAS1-like (S. cerevisiae)	2,06144E+14	5,93109E+13	4,69464E+14	0.00278557740084336	0.141570560577024	-1,15823E+14	6,99667E+14	6,99257E+14	6,41272E+14	5,31824E+14	5,16779E+14	4,69857E+14
TC1700000005.mm.2	Scaf8	SR-related CTD-associated factor 8	1,46464E+14	6,51229E+14	4,6925E+14	0.00279210793268158	0.141570560577024	-1,16032E+14	7,32229E+14	7,51744E+14	6,26558E+14	6,058E+14	6,12106E+14	5,78938E+14
TC0X00003168.mm.2	Maged2	melanoma antigen, family D, 2	1,50419E+14	6,84387E+14	4,68247E+14	0.00282298938629158	0.141570560577024	-1,17013E+12	7,88364E+14	7,48592E+14	6,76878E+14	6,56369E+14	6,30826E+14	6,05293E+14
TC1200001373.mm.2	Klhl29	kelch-like 29	1,68107E+14	6,34277E+14	4,68178E+13	0.00282511780615204	0.141570560577024	-1,1708E+14	7,32921E+14	6,93613E+14	6,61575E+14	6,27234E+14	5,73619E+14	5,167E+14
TC0100003096.mm.2	Atp2b4/Mir6903	ATPase, Ca++ transporting, plasma membrane 4/microRNA 6903	2,77791E+14	7,5036E+14	4,67909E+14	0.00283347941370843	0.141570560577024	-1,17344E+13	9,25164E+14	9,24922E+14	7,26511E+13	6,31058E+14	7,27256E+14	5,67248E+14
TC0100003397.mm.2	Prrc2c	proline-rich coiled-coil 2C	2,16138E+14	8,41982E+14	4,67873E+14	0.00283458639593954	0.141570560577024	-1,17379E+13	9,88337E+14	9,80006E+14	7,81432E+14	7,66052E+14	8,32305E+13	7,03763E+14
TC1000002496.mm.2	Pcsk4	proprotein convertase subtilisin/kexin type 4	-1,48227E+13	6,46719E+14	-4,67796E+14	0.0028369806687978	0.141570560577024	-1,17454E+14	5,74512E+14	5,39171E+14	6,79396E+14	6,77098E+14	7,212E+14	6,88937E+13
TC0700004545.mm.2	Chid1	chitinase domain containing 1	1,67401E+14	6,22948E+14	4,67646E+14	0.00284165664816165	0.141570560577024	-1,17601E+14	7,20643E+14	7,07069E+14	6,16508E+14	6,00556E+14	5,83279E+14	5,09632E+14
TC0X00002154.mm.2	Smarca1	SWI/SNF related, matrix associated, actin dependent regulator of chromatin, subfamily a, member 1	1,84966E+14	6,55225E+14	4,67378E+14	0.00285003384389521	0.141570560577024	-1,17864E+14	7,62722E+14	7,43385E+14	6,3879E+14	6,50276E+14	6,15574E+14	5,20601E+14
TC1000000661.mm.2	Ank3	ankyrin 3, epithelial	2,10345E+14	7,68505E+14	4,6717E+14	0.0028565704373152	0.141570560577024	-1,18069E+14	8,91381E+14	8,98216E+14	7,53572E+14	6,98954E+14	7,40189E+14	6,28717E+14
TC1400002264.mm.2	Gulo	gulonolactone (L-) oxidase	-1,44011E+14	5,00731E+14	-4,67029E+14	0.00286099571915153	0.141570560577024	-1,18207E+14	4,13796E+14	4,34411E+14	4,93985E+14	5,25965E+14	5,74509E+14	5,61721E+14
TC1000000886.mm.2	Dapk3	death-associated protein kinase 3	2,68729E+14	6,74337E+14	4,66728E+12	0.00287047965746254	0.141570560577024	-1,18503E+13	8,70795E+14	8,28452E+14	6,76672E+14	5,08316E+14	6,02589E+14	5,592E+14
TC1700000573.mm.2	Zfp563	zinc finger protein 563	1,88945E+14	5,37532E+14	4,66686E+14	0.00287179619151324	0.141570560577024	-1,18544E+14	6,32044E+14	6,37815E+14	5,1629E+14	5,47075E+14	4,94675E+13	3,97294E+14
TC1600000109.mm.2	Rpl39l	ribosomal protein L39-like	-1,50568E+14	5,83887E+14	-4,66528E+14	0.00287680356270949	0.141570560577024	-1,18699E+14	5,15037E+14	4,81571E+13	5,86871E+11	6,22101E+14	6,38652E+14	6,59092E+12
TC0200004843.mm.2	Acss1	acyl-CoA synthetase short-chain family member 1	1,38975E+14	7,15692E+14	4,66053E+14	0.00289185958876966	0.141570560577024	-1,19165E+14	7,93177E+14	7,80996E+14	7,0225E+14	7,21503E+14	6,56764E+14	6,3946E+14
TC0300002054.mm.2	P2ry12	purinergic receptor P2Y, G-protein coupled 12	1,76646E+14	8,51581E+14	4,65356E+14	0.00291414384594772	0.141570560577024	-1,19851E+14	9,49958E+14	9,62947E+13	8,55146E+14	7,81824E+13	8,04145E+14	7,55468E+14
TC1100003855.mm.2	Eftud2	elongation factor Tu GTP binding domain containing 2	2,60167E+14	6,30856E+14	4,65353E+14	0.00291424501067107	0.141570560577024	-1,19854E+14	7,92354E+14	7,92578E+14	6,46593E+14	4,89015E+14	4,97833E+14	5,66764E+14
TC0400003016.mm.2	Klhl9	kelch-like 9	2,03866E+13	7,47384E+14	4,65077E+14	0.00292310907380761	0.141570560577024	-1,20126E+14	8,71521E+14	8,57862E+14	7,57897E+14	6,75375E+14	7,02953E+14	6,18698E+13
TC1700001877.mm.2	Angptl4	angiopoietin-like 4	-1,52864E+14	7,42995E+14	-4,64809E+13	0.00293175751618462	0.141570560577024	-1,2039E+14	6,59013E+14	6,31302E+14	7,90393E+14	7,81219E+14	7,7332E+14	8,22724E+14
TC0600003397.mm.2	Sox5	SRY (sex determining region Y)-box 5	-1,62692E+14	7,97795E+14	-4,64724E+14	0.00293448519639382	0.141570560577024	-1,20473E+14	6,82486E+14	7,05225E+14	8,41276E+14	8,44688E+14	8,90518E+13	8,22577E+14
TC1300001851.mm.2	Tfap2a	transcription factor AP-2, alpha	-1,93724E+14	5,29725E+14	-4,64151E+14	0.00295308143349135	0.141570560577024	-1,21038E+14	4,4853E+13	3,76529E+14	5,30167E+14	6,10614E+14	6,00535E+13	6,11973E+14
TC0700000006.mm.2	Prkcg	protein kinase C, gamma	1,97591E+14	7,71049E+14	4,6404E+14	0.00295671124174245	0.141570560577024	-1,21148E+14	8,83699E+14	9,16449E+14	7,54981E+14	6,66201E+14	7,33088E+14	6,71878E+14
TC1200002053.mm.2	Numb	NUMB endocytic adaptor protein	1,46245E+14	6,75098E+14	4,6391E+14	0.00296095096384642	0.141570560577024	-1,21276E+14	7,3762E+14	7,6631E+14	6,85864E+14	6,49356E+14	6,04332E+14	6,07107E+14
TC1200000037.mm.2	Wdcp	WD repeat and coiled coil containing	1,44833E+14	6,70019E+14	4,63414E+14	0.00297721527150875	0.141570560577024	-1,21766E+13	7,628E+14	7,33637E+14	6,61187E+13	6,55718E+14	6,19698E+14	5,87074E+14
TC0200004406.mm.2	Fsip1	fibrous sheath-interacting protein 1	-1,50869E+14	5,93821E+14	-4,62979E+14	0.00299155011893598	0.141570560577024	-1,22196E+13	4,8235E+14	5,19115E+14	6,11171E+14	6,47089E+14	6,41633E+14	6,6157E+14
TC0200002667.mm.2	Cstf1	cleavage stimulation factor, 3' pre-RNA, subunit 1	1,98309E+14	6,36544E+14	4,62948E+14	0.00299258244333869	0.141570560577024	-1,22227E+14	7,61393E+13	7,68097E+14	6,3532E+14	5,21584E+14	5,65873E+14	5,66998E+14
TC0600000556.mm.2	Atp6v0e2	ATPase, H+ transporting, lysosomal V0 subunit E2	1,749E+14	8,71595E+14	4,62837E+14	0.00299624034453793	0.141570560577024	-1,22336E+14	9,83344E+14	9,76447E+14	8,57941E+14	8,01846E+14	8,38736E+14	7,71254E+14
TC1300001806.mm.2	Lyrm4	LYR motif containing 4	1,68607E+13	7,92066E+14	4,62605E+14	0.00300393684091455	0.141570560577024	-1,22566E+14	8,93445E+14	8,93164E+12	7,9441E+14	7,21982E+14	7,42171E+14	7,07224E+14

TC0X00000902.mm.2	Zxdb	zinc finger, X-linked, duplicated B	2,19633E+13	5,3554E+14	4,62166E+14	0.0030185647410434	0.141570560577024	-1,23001E+14	6,63481E+14	6,6307E+14	5,04148E+13	4,95258E+14	5,11372E+13	3,75913E+14
TC0800002238.mm.2	Wdr17	WD repeat domain 17	2,2445E+14	6,34035E+14	4,61951E+14	0.00302573424471468	0.141570560577024	-1,23213E+14	7,61793E+14	7,66456E+14	5,76201E+14	6,20414E+14	6,06323E+14	4,73026E+14
TC1100002301.mm.2	Slc1a4	solute carrier family 1 (glutamate/neutral amino acid transporter), member 4	2,25684E+14	6,1779E+13	4,61587E+14	0.00303793751919845	0.141570560577024	-1,23573E+14	7,25059E+14	7,79846E+13	6,30885E+14	5,17415E+13	5,59367E+14	4,94169E+14
TC0600000989.mm.2	C87436	expressed sequence C87436	1,67003E+14	5,49159E+14	4,6155E+13	0.00303919976238222	0.141570560577024	-1,23611E+14	6,68041E+14	6,21144E+14	5,07766E+14	5,42823E+14	5,0401E+14	4,5117E+14
TC1000000165.mm.2	Hbs1l	Hbs1-like (S. cerevisiae)	1,58432E+14	6,46818E+14	4,61544E+14	0.00303939268087452	0.141570560577024	-1,23616E+14	7,68769E+14	7,38965E+13	6,0899E+14	5,73316E+14	6,19806E+14	5,71064E+14
TC1600002015.mm.2	Krtap14	keratin associated protein 14	-1,92767E+14	5,25798E+14	-4,61514E+14	0.00304041086493557	0.141570560577024	-1,23646E+14	3,94195E+14	4,15085E+14	5,44205E+14	6,06492E+14	5,54094E+14	6,40719E+14
TC0300002488.mm.2	Mrps21	mitochondrial ribosomal protein S21	1,41936E+14	6,6466E+14	4,61513E+14	0.0030404308100491	0.141570560577024	-1,23647E+14	7,70193E+14	7,54012E+14	6,10282E+14	6,13142E+14	6,38033E+13	6,02301E+14
TC1800001505.mm.2	Afg3l2	AFG3-like AAA ATPase 2	1,9033E+14	6,8993E+14	4,60647E+14	0.00306972303952851	0.141570560577024	-1,24506E+14	8,30393E+14	8,12503E+14	6,58014E+14	5,76434E+14	6,64146E+14	5,98091E+14
TC0300001112.mm.2	Kcna2	potassium voltage-gated channel, shaker-related subfamily, member 2	1,39226E+14	7,28071E+14	4,60416E+14	0.00307762430480279	0.141570560577024	-1,24736E+14	8,20356E+14	8,05293E+14	6,92827E+14	7,0275E+14	6,87893E+14	6,59305E+14
TC0300001999.mm.2	Nbea	neurobeachin	2,4795E+14	7,47362E+14	4,60004E+13	0.00309170447081932	0.141570560577024	-1,25145E+14	9,09319E+14	9,06406E+14	7,23481E+14	6,25143E+14	7,25239E+14	5,94585E+14
TC0200000620.mm.2	Hspa5	heat shock protein 5	2,04471E+14	9,21659E+14	4,59828E+14	0.00309773716859555	0.141570560577024	-1,2532E+14	1,04343E+14	1,0849E+14	8,98329E+14	7,83908E+14	8,59902E+14	8,59486E+14
TC0900002525.mm.2	Fbxl22	F-box and leucine-rich repeat protein 22	-1,68371E+14	6,53343E+14	-4,59464E+14	0.0031102634773469	0.141570560577024	-1,25682E+14	5,43062E+14	5,91356E+14	6,43961E+14	6,70517E+14	7,05515E+14	7,65645E+13
TC1600000282.mm.2	Vwa5b2	von Willebrand factor A domain containing 5B2	-1,47409E+14	7,42174E+14	-4,59323E+14	0.0031151532578705	0.141570560577024	-1,25822E+14	6,43761E+14	6,57879E+14	7,6483E+14	7,90117E+14	7,77076E+14	8,19383E+14
TC0500000289.mm.2	Selenoi	selenoprotein I	1,39344E+14	7,64041E+14	4,58451E+14	0.00314547215019099	0.141570560577024	-1,26691E+14	8,61909E+14	8,59367E+14	7,27207E+14	6,93174E+13	7,16316E+13	7,26271E+13
TC1400000341.mm.2	Parg	poly (ADP-ribose) glycohydrolase	2,26177E+14	6,07217E+14	4,58359E+14	0.00314868960854479	0.141570560577024	-1,26783E+14	7,02694E+14	7,00663E+14	6,69335E+14	6,1961E+14	5,42996E+14	4,08007E+14
TC0X00003166.mm.2	Tro	trophinin	2,20297E+14	6,86035E+14	4,58342E+14	0.00314926841444331	0.141570560577024	-1,26799E+14	8,49699E+14	7,97826E+14	6,63617E+14	5,98138E+14	6,585E+14	5,48431E+13
TC0700000933.mm.2	Cyfip1	cytoplasmic FMR1 interacting protein 1	1,75475E+14	7,92358E+14	4,58197E+14	0.00315434120189857	0.141570560577024	-1,26944E+13	8,91918E+14	9,06677E+13	7,95151E+14	7,12759E+14	7,06224E+14	7,41421E+14
TC1200000416.mm.2	Scfd1	Sec1 family domain containing 1	2,3506E+14	5,91416E+14	4,58138E+14	0.00315641061538937	0.141570560577024	-1,27002E+14	7,44851E+14	7,39615E+14	5,60741E+13	4,88942E+14	5,74361E+14	4,39985E+14
TC0700004166.mm.2	Smg1	SMG1 homolog, phosphatidylinositol 3-kinase-related kinase (C. elegans)	1,38934E+14	6,87551E+14	4,5771E+14	0.00317149427150556	0.141570560577024	-1,2743E+13	7,86554E+14	7,76922E+14	6,49529E+14	6,26694E+14	6,55235E+14	6,30373E+14
TC0400000602.mm.2	Zfp462	zinc finger protein 462	1,45924E+14	6,18073E+14	4,57244E+14	0.00318795671956458	0.141570560577024	-1,27895E+14	6,99164E+14	6,94178E+14	6,162E+14	5,97402E+14	5,73677E+14	5,27818E+14
TC0800000509.mm.2	Fat1	FAT atypical cadherin 1	1,49642E+14	6,69826E+14	4,56746E+14	0.0032057074197467	0.141570560577024	-1,28393E+14	7,66636E+14	7,54779E+14	6,14662E+14	6,60745E+14	6,26258E+14	5,95873E+14
TC0400003578.mm.2	Zfp362	zinc finger protein 362	-1,70282E+14	9,03675E+14	-4,56377E+14	0.00321891046882951	0.141570560577024	-1,28762E+13	7,75132E+14	7,85026E+14	1,02036E+14	9,40809E+14	9,65033E+14	9,35689E+14
TC0700004164.mm.2	Rps15a	ribosomal protein S15A	1,91835E+14	7,50776E+14	4,55802E+14	0.00323959611162904	0.141570560577024	-1,29338E+14	8,72112E+14	8,72029E+14	6,69337E+14	7,30705E+14	6,34415E+14	7,26056E+13
TC0200000636.mm.2	Gsn	gelsolin	1,40776E+14	7,07E+14	4,5519E+13	0.00326179835515755	0.141570560577024	-1,29951E+14	8,00831E+14	8,27808E+14	6,38143E+14	6,28131E+14	6,58917E+14	6,8817E+13
TC0800000936.mm.2	Adgrl1	adhesion G protein-coupled receptor L1	2,88766E+14	7,78348E+14	4,55175E+14	0.00326233669997838	0.141570560577024	-1,29966E+14	9,30499E+14	9,46442E+14	8,26075E+14	6,67662E+14	7,12547E+14	5,86862E+14
TC0400002232.mm.2	Slc26a7	solute carrier family 26, member 7	-1,45521E+13	4,66556E+14	-4,55148E+14	0.00326330884498748	0.141570560577024	-1,29993E+14	4,04721E+14	4,0457E+14	4,19766E+14	4,6995E+14	5,48525E+14	5,51807E+14
TC1100000093.mm.2	Ogdh	oxoglutarate (alpha-ketoglutarate) dehydrogenase (lipoamide)	1,63867E+14	8,71678E+14	4,54818E+13	0.00327535972918751	0.141570560577024	-1,30324E+14	9,75198E+14	9,69001E+14	8,66714E+14	8,02691E+14	8,30255E+14	7,8621E+14
TC1400001314.mm.2	Ipo5	importin 5	1,33934E+14	7,88974E+14	4,54494E+14	0.0032872260540974	0.141570560577024	-1,30649E+14	8,7416E+14	8,81256E+14	7,54474E+14	7,36406E+14	7,50373E+14	7,37176E+13
TC0400002513.mm.2	Vcp	valosin containing protein	2,35795E+14	6,75854E+14	4,54435E+14	0.00328939722826873	0.141570560577024	-1,30708E+14	8,46228E+13	8,38586E+14	6,33681E+14	5,234E+14	6,609E+14	5,52325E+13
TC0100000558.mm.2	Unc80	unc-80, NALCN activator	2,43223E+14	7,44276E+14	4,54041E+14	0.00330392118150532	0.141570560577024	-1,31104E+14	9,1384E+14	8,73278E+14	7,2003E+13	6,57837E+14	7,22405E+14	5,78268E+14
TC1700001950.mm.2	Prrc2a	proline-rich coiled-coil 2A	1,45668E+14	7,38497E+14	4,53954E+14	0.00330712545672412	0.141570560577024	-1,31192E+14	8,28322E+14	8,20717E+14	6,91745E+14	7,32498E+14	6,63795E+14	6,93907E+13
TC1200002154.mm.2	Ston2	stonin 2	1,31527E+14	5,98807E+14	4,52856E+14	0.00334798735678276	0.141570560577024	-1,32296E+14	7,13252E+14	7,11151E+14	4,97909E+14	5,09179E+14	5,8567E+14	5,75678E+14
TC0200001096.mm.2	Hoxd8	homeobox D8	-1,60376E+14	6,42863E+14	-4,52186E+14	0.00337322244677937	0.141570560577024	-1,32972E+14	5,34295E+14	5,52262E+14	6,65655E+13	6,97658E+14	6,71199E+14	7,36109E+14
TC1200000074.mm.2	Wdr35	WD repeat domain 35	1,32086E+14	5,8559E+14	4,52168E+14	0.00337387457516257	0.141570560577024	-1,32989E+13	6,75449E+14	6,88264E+14	5,28349E+14	5,21938E+14	5,43612E+13	5,55928E+14
TC0500001757.mm.2	Trrap	transformation/transcription domain-associated protein	1,39322E+14	6,99509E+14	4,51996E+14	0.00338037732014108	0.141570560577024	-1,33162E+14	7,62295E+14	7,96386E+14	6,68725E+14	6,89608E+14	6,40942E+14	6,39095E+14
TC0800001228.mm.2	4933405L10Rik	RIKEN cDNA 4933405L10 gene	-1,67593E+13	5,18361E+14	-4,51231E+14	0.00340951914328188	0.141570560577024	-1,33935E+14	4,01897E+14	4,29428E+14	5,16378E+14	5,95954E+14	5,80353E+14	5,86156E+14
TC1100003841.mm.2	Gpatch8	G patch domain containing 8	2,30632E+14	5,81845E+14	4,51113E+14	0.00341401371570302	0.141570560577024	-1,34053E+14	7,20414E+13	7,14638E+14	5,65976E+14	5,16256E+13	5,58472E+14	4,15317E+14
TC0100000338.mm.2	Gulp1	GULP, engulfment adaptor PTB domain containing 1	1,71667E+14	6,28842E+14	4,50582E+14	0.00343442272114633	0.141570560577024	-1,3459E+14	7,30202E+14	7,02185E+14	6,43025E+14	6,08585E+14	5,83513E+14	5,0554E+14
TC0700002828.mm.2	Gpi1	glucose phosphate isomerase 1	1,66081E+14	1,0185E+13	4,5058E+14	0.00343452263994745	0.141570560577024	-1,34593E+14	1,13802E+14	1,1169E+14	1,00373E+14	9,29577E+14	9,76473E+14	9,46292E+13
TC0900000892.mm.2	Rora	RAR-related orphan receptor alpha	1,61374E+14	6,26473E+14	4,50518E+14	0.00343689170877107	0.141570560577024	-1,34655E+14	7,30813E+14	7,27593E+13	6,03669E+14	5,61102E+14	5,99694E+14	5,35964E+14
TC0700001615.mm.2	Akip1	A kinase (PRKA) interacting protein 1	-1,73833E+14	6,77911E+14	-4,50376E+13	0.00344238002732347	0.141570560577024	-1,34799E+14	5,87093E+14	5,47274E+14	7,51388E+14	6,99679E+14	7,73739E+14	7,08294E+14
TC0X00003432.mm.2	Mecp2	methyl CpG binding protein 2	1,51466E+14	7,10291E+14	4,49777E+14	0.00346561109069217	0.141570560577024	-1,35405E+14	8,17241E+14	7,94756E+14	6,89574E+14	6,51109E+14	6,76795E+14	6,3227E+14
TC0200001229.mm.2	Ssrp1	structure specific recognition protein 1	1,99496E+14	6,48673E+13	4,49431E+14	0.00347914010706116	0.141570560577024	-1,35756E+14	7,72814E+14	7,96148E+14	6,06031E+14	5,47075E+14	6,36714E+14	5,33258E+14
TC0900003344.mm.2	Trf	transferrin	1,72172E+14	9,15956E+14	4,49023E+14	0.00349513666897133	0.141570560577024	-1,36169E+14	1,0009E+14	1,04571E+14	9,13032E+12	8,33831E+14	8,49547E+14	8,52718E+14
TC0500001134.mm.2	Golga3	golgi autoantigen, golgin subfamily a, 3	1,69687E+14	5,7459E+14	4,48766E+14	0.00350524395221502	0.141570560577024	-1,36429E+14	6,77767E+14	6,78755E+14	5,76872E+14	4,96995E+14	5,2721E+14	4,89938E+13
TC0900003098.mm.2	Camp	cathelicidin antimicrobial peptide	-1,48303E+14	6,12862E+14	-4,4864E+14	0.00351023801057633	0.141570560577024	-1,36558E+14	5,02901E+14	5,44236E+14	6,40378E+14	6,45915E+14	6,51277E+14	6,92465E+14
TC1100003776.mm.2	Dnajc7	DnaJ heat shock protein family (Hsp40) member C7	2,08096E+14	7,67436E+14	4,48335E+14	0.00352230411382308	0.141570560577024	-1,36867E+14	9,08402E+14	9,03064E+14	7,62231E+14	6,35645E+14	7,13745E+14	6,81528E+14
TC0200004913.mm.2	Bcl2l1	BCL2-like 1	2,25582E+13	7,02163E+13	4,48273E+14	0.00352475904875884	0.141570560577024	-1,3693E+14	8,40294E+14	8,28947E+14	7,0373E+14	6,2193E+14	6,70544E+12	5,47533E+14
TC0X00002470.mm.2	Mpp1	membrane protein, palmitoylated	1,693E+14	6,53742E+14	4,48204E+14	0.00352749555606123	0.141570560577024	-1,37E+14	7,61941E+14	7,48362E+14	6,34103E+14	6,06344E+14	6,27015E+14	5,44687E+14
TC0500001307.mm.2	Hrk	harakiri, BCL2 interacting protein (contains only BH3 domain)	-1,47871E+14	8,01092E+14	-4,47965E+14	0.00353699278269539	0.141570560577024	-1,37242E+14	7,04216E+14	7,18658E+14	8,05714E+14	8,59349E+14	8,50491E+14	8,68126E+14
TC0700002444.mm.2	Ppp5c	protein phosphatase 5, catalytic subunit	1,71583E+14	8,20185E+14	4,47808E+14	0.00354325001243146	0.141570560577024	-1,37402E+14	9,52599E+14	9,25306E+14	7,90767E+14	7,17698E+14	7,90716E+14	7,44023E+14
TC0200003241.mm.2	Cfap77	cilia and flagella associated protein 77	-1,95874E+14	6,98876E+14	-4,47535E+14	0.00355420183610312	0.141570560577024	-1,3768E+14	5,85333E+14	5,75123E+14	6,9329E+14	7,87305E+13	7,40479E+14	8,11726E+14
TC0600002745.mm.2	Prickle2	prickle planar cell polarity protein 2	1,91946E+13	6,95443E+14	4,47245E+14	0.00356583149647547	0.141570560577024	-1,37975E+14	8,1438E+14	8,30493E+14	6,29149E+14	6,37655E+14	6,86925E+14	5,74055E+14
TC0500002493.mm.2	Pds5a	PDS5 cohesin associated factor A	1,35078E+14	7,06608E+14	4,47165E+14	0.00356904999243243	0.141570560577024	-1,38056E+14	8,04013E+14	8,00916E+14	6,50647E+14	6,49299E+14	6,8402E+13	6,50754E+14
TC0500002268.mm.2	Wfs1	wolframin ER transmembrane glycoprotein	1,72258E+14	8,51268E+14	4,46894E+14	0.00357997235028051	0.141570560577024	-1,38332E+14	9,59114E+14	9,48507E+14	8,61881E+14	7,74999E+14	7,67146E+14	7,95959E+14
TC0200004608.mm.2	Pdyn	prodynorphin	1,54621E+14	7,65132E+14	4,46468E+13	0.00359722997822862	0.141570560577024	-1,38766E+14	8,82043E+14	8,36123E+14	7,42321E+14	7,21383E+14	6,80689E+14	7,28235E+12
TC0800000353.mm.2	Eif4ebp1	eukaryotic translation initiation factor 4E binding protein 1	-2,07407E+13	6,2613E+14	-4,46352E+14	0.00360191712720523	0.141570560577024	-1,38883E+14	4,70966E+13	5,35971E+14	6,27493E+14	7,00595E+14	7,54415E+14	6,67337E+14
TC0700001926.mm.2	Hmx2	H6 homeobox 2	-1,52497E+14	7,1498E+14	-4,46347E+14	0.00360212695823752	0.141570560577024	-1,38888E+14	6,16178E+14	6,14072E+14	7,39504E+14	7,84882E+14	7,43479E+14	7,91764E+14
TC0500003104.mm.2	Bicdl1	BICD family like cargo adaptor 1	2,1028E+14	6,53327E+14	4,46346E+12	0.00360217087861144	0.141570560577024	-1,3889E+12	7,82913E+14	7,64427E+14	6,65223E+14	5,80619E+14	6,14449E+14	5,1233E+14
TC1100003687.mm.2	Med24	mediator complex subunit 24	2,9834E+14	7,466E+14	4,46052E+14	0.00361416986798702	0.141570560577024	-1,3919E+14	9,23862E+14	9,38696E+14	7,3835E+14	6,12813E+14	7,20886E+14	5,44992E+14
TC0200003329.mm.2	Lmx1b	LIM homeobox transcription factor 1 beta	-1,43877E+14	8,10176E+14	-4,46046E+14	0.00361440940865444	0.141570560577024	-1,39196E+14	7,13087E+14	7,17928E+14	8,29478E+14	8,81792E+14	8,60326E+14	8,58443E+14
TC0900002942.mm.2	Pccb	propionyl Coenzyme A carboxylase, beta polypeptide	1,73218E+14	6,5951E+14	4,46E+14	0.00361625485325173	0.141570560577024	-1,39242E+14	7,68629E+14	7,60706E+14	6,54792E+13	5,90038E+14	6,25088E+14	5,5781E+13
TC1100004107.mm.2	Acox1	acyl-Coenzyme A oxidase 1, palmitoyl	2,57438E+12	7,05296E+14	4,45938E+14	0.0036188087964804	0.141570560577024	-1,39306E+14	8,39825E+14	8,5286E+14	7,70111E+14	5,91173E+14	5,92658E+14	5,85151E+14
TC1400000975.mm.2	Entpd4	ectonucleoside triphosphate diphosphohydrolase 4	1,34035E+14	8,77667E+14	4,45718E+14	0.0036278183212731	0.141570560577024	-1,3953E+13	9,55949E+14	9,56319E+14	8,62999E+14	8,46536E+14	8,08483E+14	8,35715E+14

TC0400003349.mm.2	Mmachc	methylmalonic aciduria cblC type, with homocystinuria	1,51626E+13	7,15617E+14	4,45545E+14	0.00363489498330501	0.141570560577024	-1,39706E+13	8,24278E+14	8,15246E+14	6,69405E+14	6,485E+14	6,98791E+14	6,37481E+14
TC0X00000888.mm.2	Pcyt1b	phosphate cytidylyltransferase 1, choline, beta isoform	1,89776E+14	5,31236E+14	4,45413E+14	0.00364034878894518	0.141570560577024	-1,39841E+14	6,31064E+14	6,76601E+14	5,09451E+14	4,42187E+14	5,0321E+14	4,24904E+14
TC0700000840.mm.2	Hrc	histidine rich calcium binding protein	-1,52782E+14	5,03591E+14	-4,45123E+14	0.00365230169308961	0.141570560577024	-1,40137E+14	4,28796E+14	4,29141E+14	4,6653E+14	5,33578E+14	5,78646E+14	5,84854E+14
TC1500001944.mm.2	Cyp2d22	cytochrome P450, family 2, subfamily d, polypeptide 22	1,44907E+12	7,3033E+14	4,45087E+14	0.00365378033684237	0.141570560577024	-1,40174E+14	8,31009E+14	8,07472E+14	7,21905E+14	6,72926E+14	6,78264E+14	6,70404E+14
TC1700000318.mm.2	Pkmyt1	protein kinase, membrane associated tyrosine/threonine 1	-1,65075E+14	6,57427E+14	-4,44975E+14	0.0036583868612644	0.141570560577024	-1,40288E+13	5,6558E+13	5,4151E+13	6,6025E+14	7,39979E+14	7,12571E+14	7,24669E+14
TC0100001252.mm.2	Csrp1	cysteine and glycine-rich protein 1	1,66629E+14	8,99559E+14	4,44601E+14	0.0036739177244821	0.141570560577024	-1,4067E+14	1,01614E+14	9,98909E+14	8,80111E+14	8,20395E+13	8,70833E+14	8,10961E+14
TC0700003010.mm.2	Ppp1r15a	protein phosphatase 1, regulatory subunit 15A	-1,40407E+14	6,41807E+14	-4,44578E+14	0.00367486679896693	0.141570560577024	-1,40694E+13	5,62838E+14	5,29536E+14	6,7684E+14	7,0844E+14	6,91662E+14	6,81525E+14
TC0900002271.mm.2	Sdhd	succinate dehydrogenase complex, subunit D, integral membrane protein	1,70174E+14	7,85813E+14	4,4382E+14	0.00370654726364088	0.141570560577024	-1,41469E+14	9,05642E+14	8,8282E+14	7,79207E+14	6,99097E+13	7,42444E+14	7,0567E+14
TC1000003097.mm.2	Os9	amplified in osteosarcoma	1,8877E+13	7,65555E+14	4,43472E+14	0.00372117704044331	0.141570560577024	-1,41825E+14	8,89229E+14	8,54514E+14	7,64564E+14	7,18819E+13	7,31547E+14	6,34655E+14
TC0100003840.mm.2	Camk1g	calcium/calmodulin-dependent protein kinase I gamma	1,57678E+14	7,27237E+14	4,43364E+14	0.00372573097750715	0.141570560577024	-1,41936E+14	8,26171E+14	8,35547E+14	7,05463E+14	6,49876E+14	6,48325E+14	6,98037E+14
TC0X00001749.mm.2	Mycs	myc-like oncogene, s-myc protein	-1,53882E+14	5,60679E+14	-4,43296E+14	0.00372859005419351	0.141570560577024	-1,42005E+13	4,90715E+14	4,86093E+14	5,2165E+14	5,81047E+14	6,60526E+13	6,24046E+14
TC0X00002073.mm.2	Rhox8	reproductive homeobox 8	-2,12444E+14	6,1762E+14	-4,43226E+14	0.00373155680098825	0.141570560577024	-1,42077E+14	5,34631E+14	4,49674E+14	6,11527E+13	7,00696E+14	6,73932E+14	7,35261E+14
TC1900000165.mm.2	Ganab	alpha glucosidase 2 alpha neutral subunit	2,0347E+13	6,33262E+14	4,43202E+14	0.00373257974762003	0.141570560577024	-1,42102E+14	7,77445E+14	7,69865E+14	6,07988E+13	5,039E+14	6,07593E+14	5,32778E+14
TC0800000150.mm.2	Kbtbd11	kelch repeat and BTB (POZ) domain containing 11	2,11604E+14	7,0816E+14	4,42768E+13	0.00375097892447072	0.141570560577024	-1,42546E+14	8,25606E+14	8,05245E+14	7,6228E+14	6,48188E+14	6,4031E+14	5,67333E+14
TC1800000802.mm.2	Dym	dymeclin	1,74088E+14	8,14746E+14	4,42555E+14	0.00376007068462246	0.141570560577024	-1,42765E+14	9,21255E+14	9,07949E+14	8,14841E+14	7,63404E+14	7,00323E+14	7,80704E+14
TC0700001684.mm.2	Nucb2	nucleobindin 2	1,84072E+14	5,89363E+14	4,42322E+14	0.00377000207650193	0.141570560577024	-1,43004E+14	7,12675E+14	6,99059E+14	5,85238E+14	4,95616E+14	5,51113E+14	4,92478E+14
TC0X00001873.mm.2	Cask	calcium/calmodulin-dependent serine protein kinase (MAGUK family)	2,60242E+13	6,19534E+13	4,42291E+14	0.00377136107026948	0.141570560577024	-1,43036E+13	7,79454E+14	7,87185E+14	6,12663E+14	4,91747E+14	5,92412E+14	4,53743E+14
TC1600001198.mm.2	Socs1	suppressor of cytokine signaling 1	-1,44655E+14	6,26444E+13	-4,41683E+13	0.00379746530591925	0.141570560577024	-1,4366E+14	5,76426E+14	5,31458E+14	6,32548E+14	6,2104E+14	6,82606E+14	7,14587E+14
TC0200004729.mm.2	Esf1	ESF1 nucleolar pre-rRNA processing protein homolog	2,26585E+14	6,2302E+14	4,41565E+14	0.00380255288716689	0.141570560577024	-1,43781E+14	7,71512E+14	7,67697E+14	6,19183E+14	4,93691E+14	5,86277E+14	4,99761E+14
TC0200000503.mm.2	Med27	mediator complex subunit 27	2,34126E+14	7,26117E+14	4,41308E+14	0.00381369283557167	0.141570560577024	-1,44046E+14	8,93358E+14	8,50521E+14	7,32921E+14	6,04274E+13	6,8023E+14	5,95398E+13
TC1800000347.mm.2	Zmat2	zinc finger, matrin type 2	1,6947E+14	8,55117E+14	4,41186E+14	0.00381896438774277	0.141570560577024	-1,44171E+14	9,54033E+14	9,52854E+14	8,328E+14	8,23064E+14	8,29424E+13	7,38523E+14
TC0700001893.mm.2	Sec23ip	Sec23 interacting protein	2,23917E+14	5,86156E+14	4,41143E+14	0.00382081827514105	0.141570560577024	-1,44215E+14	6,97028E+14	6,90295E+14	6,50925E+13	5,39197E+14	5,19719E+14	4,1977E+14
TC0500000395.mm.2	Hmx1	H6 homeobox 1	-1,80367E+14	7,21758E+14	-4,41074E+14	0.00382381786115881	0.141570560577024	-1,44286E+13	6,01382E+14	6,11248E+14	7,27365E+14	8,17188E+14	7,60589E+14	8,12775E+14
TC1700000611.mm.2	Rxrb	retinoid X receptor beta	1,6467E+13	6,09139E+14	4,41049E+14	0.00382492846073299	0.141570560577024	-1,44312E+14	7,00759E+14	7,27065E+14	6,0494E+14	5,23587E+13	5,49048E+14	5,49437E+14
TC0900001009.mm.2	Myo5a	myosin VA	2,11006E+14	7,95572E+14	4,41E+14	0.00382706108360388	0.141570560577024	-1,44362E+13	9,22226E+14	9,34696E+14	7,88599E+14	6,92999E+14	7,6627E+14	6,68639E+14
TC1000000484.mm.2	Asf1a	anti-silencing function 1A histone chaperone	1,35037E+14	5,88752E+14	4,40712E+14	0.0038396079115163	0.141570560577024	-1,44659E+14	6,95116E+14	6,69944E+13	5,48114E+14	5,24354E+14	5,39449E+14	5,55537E+13
TC1600000491.mm.2	Adcy5	adenylate cyclase 5	1,85352E+14	7,98578E+14	4,40557E+14	0.00384639195701933	0.141570560577024	-1,44818E+14	9,32839E+14	9,2255E+14	7,80564E+14	6,7083E+14	7,4258E+14	7,42105E+14
TC0200001909.mm.2	Snrnp200	small nuclear ribonucleoprotein 200 (U5)	2,5437E+14	6,52073E+14	4,40297E+14	0.00385777830903838	0.141570560577024	-1,45086E+14	8,12127E+14	8,17901E+14	6,65411E+14	4,9571E+14	5,90127E+14	5,31162E+14
TC0600002351.mm.2	Ppm1k	protein phosphatase 1K (PP2C domain containing)	1,48728E+14	7,17957E+14	4,40257E+13	0.00385953809481957	0.141570560577024	-1,45127E+14	8,24155E+13	8,15277E+14	6,74028E+14	6,52307E+14	7,00608E+14	6,41369E+13
TC0700002980.mm.2	Prmt1	protein arginine N-methyltransferase 1	1,54175E+14	8,10218E+14	4,39378E+14	0.00389832564776586	0.141570560577024	-1,46033E+14	9,24897E+14	8,95192E+14	7,97536E+13	7,31942E+14	7,52997E+14	7,58741E+13
TC0200001378.mm.2	Ckap5/Snord67	cytoskeleton associated protein 5/small nucleolar RNA, C/D box 67	2,60657E+14	6,7911E+14	4,3921E+14	0.00390579237700486	0.141570560577024	-1,46206E+14	8,5932E+14	8,68193E+14	6,56374E+14	4,84574E+14	6,38758E+14	5,67441E+14
TC0X00001798.mm.2	Tbc1d25	TBC1 domain family, member 25	1,49061E+14	5,69194E+12	4,39169E+14	0.00390763333628706	0.141570560577024	-1,46249E+14	6,81787E+14	6,38931E+14	5,26471E+14	5,45377E+14	4,8891E+13	5,33686E+13
TC0500002649.mm.2	Spink2	serine peptidase inhibitor, Kazal type 2	-2,32361E+14	5,79589E+14	-4,38777E+14	0.00392513720745237	0.141570560577024	-1,46653E+14	4,07763E+14	4,46323E+14	5,75221E+13	7,2942E+14	6,66229E+14	6,52579E+13
TC0200003754.mm.2	Wipf1	WAS/WASL interacting protein family, member 1	-1,26752E+14	6,20303E+13	-4,38766E+14	0.00392560119093701	0.141570560577024	-1,46664E+14	5,55656E+14	5,55266E+14	6,19049E+14	6,27421E+14	6,85573E+13	6,78854E+14
TSUnmapped00000045.mm.2	Aopep	aminopeptidase O	-1,53791E+13	6,58799E+14	-4,38739E+14	0.00392683968736431	0.141570560577024	-1,46693E+13	5,49789E+14	5,82348E+14	6,73643E+14	7,07297E+14	7,47211E+14	6,92508E+14
TC1900000102.mm.2	Batf2	basic leucine zipper transcription factor, ATF-like 2	-1,49185E+14	7,81123E+14	-4,38463E+14	0.00393921802449141	0.141570560577024	-1,46978E+14	6,82816E+14	6,75442E+14	8,06897E+14	8,64952E+14	8,13766E+14	8,42863E+14
TC0300000307.mm.2	Hspa4l	heat shock protein 4 like	2,36596E+14	7,4917E+14	4,38404E+14	0.00394185699087199	0.141570560577024	-1,47038E+14	8,96677E+14	8,94141E+14	7,26171E+14	6,60404E+14	7,33828E+14	5,83799E+14
TC0700003798.mm.2	Rps3/Snord15b	ribosomal protein S3/small nucleolar RNA, C/D box 14B	1,90257E+14	9,48085E+14	4,38184E+13	0.00395177539147597	0.141570560577024	-1,47266E+14	1,06129E+14	1,0791E+14	9,2343E+14	8,64822E+14	9,29627E+14	8,30244E+14
TC1300001273.mm.2	Dhx29	DEAH (Asp-Glu-Ala-His) box polypeptide 29	1,61938E+14	7,52344E+14	4,3775E+14	0.00397139277661698	0.141570560577024	-1,47715E+14	8,53906E+14	8,78616E+14	7,18002E+14	6,54894E+14	7,28766E+14	6,7988E+14
TC0200003291.mm.2	Qrfp	pyroglutamylated RFamide peptide	-1,80894E+14	6,14024E+11	-4,37699E+14	0.00397370250747576	0.141570560577024	-1,47768E+14	5,12805E+14	5,13157E+12	5,86936E+14	6,83499E+14	6,71373E+14	7,16377E+14
TC1000000473.mm.2	Slc35f1	solute carrier family 35, member F1	2,17227E+14	7,27017E+14	4,37632E+14	0.00397673365197617	0.141570560577024	-1,47837E+14	8,56536E+14	8,57898E+14	7,33298E+14	6,34389E+14	6,93107E+14	5,86874E+14
TC0100000858.mm.2	Agap1	ArfGAP with GTPase domain, ankyrin repeat and PH domain 1	1,40509E+14	6,91782E+14	4,37554E+14	0.00398026029141244	0.141570560577024	-1,47917E+14	7,76837E+14	7,61526E+14	6,93329E+14	6,61654E+14	6,47428E+14	6,09919E+14
TC0X00000525.mm.2	Fhl1	four and a half LIM domains 1	1,60218E+14	7,94512E+14	4,37541E+14	0.00398088612034204	0.141570560577024	-1,47931E+14	9,05125E+14	8,6098E+13	7,89773E+14	7,65524E+14	7,54814E+14	6,90855E+14
TC1800000462.mm.2	Ythdc2	YTH domain containing 2	1,33219E+14	5,8719E+14	4,3737E+14	0.00398867270639651	0.141570560577024	-1,48108E+14	6,81844E+14	6,54319E+14	5,51191E+14	5,66061E+14	5,44022E+14	5,25704E+14
TC0900001038.mm.2	Gclc	glutamate-cysteine ligase, catalytic subunit	1,28623E+13	7,36221E+14	4,37331E+14	0.00399042102370084	0.141570560577024	-1,48148E+14	8,02814E+14	8,13782E+14	7,26676E+14	7,14703E+14	6,87478E+14	6,71872E+14
TC0900001802.mm.2	Zfp558	zinc finger protein 558	2,01584E+14	5,37958E+14	4,36851E+14	0.00401237123286622	0.141570560577024	-1,48645E+14	6,30607E+14	6,01832E+14	5,84382E+14	5,81654E+14	3,51523E+14	4,77748E+14
TC0900002159.mm.2	Vps11	VPS11, CORVET/HOPS core subunit	1,83588E+14	7,19242E+14	4,36029E+14	0.00405024394125405	0.141570560577024	-1,49497E+14	8,36238E+14	8,2863E+14	7,03315E+14	6,49573E+14	6,969E+14	6,00793E+12
TC1200002398.mm.2	Ankrd9	ankyrin repeat domain 9	1,65746E+14	5,09722E+14	4,35954E+14	0.00405375897048196	0.141570560577024	-1,49576E+14	5,8877E+14	5,82446E+14	5,12227E+14	5,35168E+14	4,62892E+14	3,76832E+14
TC0200000868.mm.2	Gpd2	glycerol phosphate dehydrogenase 2, mitochondrial	1,61017E+14	6,76338E+14	4,35854E+14	0.00405840012722152	0.141570560577024	-1,4968E+13	7,73615E+14	7,94346E+14	6,5855E+14	5,85591E+14	6,40156E+14	6,05772E+14
TC1300002689.mm.2	Gpx8	glutathione peroxidase 8 (putative)	1,73432E+14	5,56151E+14	4,3568E+14	0.00406645076168683	0.141570560577024	-1,4986E+14	6,20198E+14	6,93426E+14	5,09342E+13	5,47179E+14	4,56138E+14	5,10621E+14
TC0600002668.mm.2	Cnbp	cellular nucleic acid binding protein	1,6657E+14	8,58339E+14	4,35614E+14	0.00406954206772007	0.141570560577024	-1,49929E+14	9,6944E+14	9,62145E+14	8,48468E+14	7,71534E+14	8,22756E+14	7,75688E+12
TC0300002901.mm.2	Ank2	ankyrin 2, brain	1,31835E+14	7,27313E+14	4,35356E+14	0.00408158560832292	0.141570560577024	-1,50197E+14	8,27359E+14	8,07214E+14	6,93044E+12	6,65357E+14	6,86403E+14	6,845E+14
TC0900001329.mm.2	Pik3r4	phosphoinositide-3-kinase regulatory subunit 4	1,51553E+14	6,25775E+14	4,35268E+14	0.00408567275123301	0.141570560577024	-1,50288E+14	7,30967E+14	6,8715E+14	6,01648E+14	6,19872E+14	5,83592E+14	5,31419E+14
TC0800001613.mm.2	Pcp2	Purkinje cell protein 2 (L7)	-1,48876E+14	7,45412E+14	-4,35219E+14	0.00408800501017099	0.141570560577024	-1,50339E+14	6,52478E+12	6,62819E+14	7,57919E+14	7,86209E+14	7,76841E+14	8,36207E+14
TC1100000745.mm.2	Rai1	retinoic acid induced 1	-1,3536E+14	1,03489E+14	-4,34704E+14	0.00411215771799947	0.141570560577024	-1,50874E+14	9,51356E+14	9,41869E+14	1,05704E+13	1,09513E+14	1,0908E+14	1,07314E+14
TC0600003124.mm.2	Cd4	CD4 antigen	2,35096E+14	6,99106E+14	4,34117E+14	0.00413994551633066	0.141570560577024	-1,51485E+14	8,24167E+14	8,63915E+14	7,16735E+14	5,7193E+14	6,42683E+14	5,75207E+14
TC0300000769.mm.2	Adar	adenosine deaminase, RNA-specific	1,41624E+14	8,01879E+14	4,33872E+14	0.00415159364657604	0.141570560577024	-1,5174E+14	8,96788E+14	8,71636E+14	7,97383E+14	7,6029E+14	7,26178E+14	7,58999E+13
TC0200004227.mm.2	Cat	catalase	2,31845E+14	8,46039E+14	4,33778E+14	0.00415609454879297	0.141570560577024	-1,51839E+14	9,81775E+13	9,85852E+14	8,79147E+14	7,25521E+13	7,76995E+14	7,26942E+13
TC1500000053.mm.2	Nup155	nucleoporin 155	1,62056E+13	5,69158E+14	4,33638E+14	0.00416277315019914	0.141570560577024	-1,51985E+14	6,51315E+14	6,86325E+12	5,39235E+14	5,24546E+14	5,4518E+14	4,68349E+13
TC1100002104.mm.2	4930556J24Rik	RIKEN cDNA 4930556J24 gene	-1,45311E+14	5,43065E+14	-4,3355E+14	0.00416694952291766	0.141570560577024	-1,52076E+14	4,76609E+14	4,6016E+14	5,21494E+14	5,72736E+14	5,98416E+14	6,28976E+14
TC1700000882.mm.2	Xpo5	exportin 5	2,08941E+14	6,96205E+14	4,33388E+14	0.00417470093221673	0.141570560577024	-1,52244E+14	8,02629E+14	8,02761E+14	7,04892E+14	6,79442E+14	6,61996E+14	5,25512E+14

TC1300002407.mm.2	Atg10	autophagy related 10	2,53932E+14	5,92396E+14	4,33209E+14	0.0041833003114524	0.141570560577024	-1,52431E+14	7,45742E+14	7,65934E+14	5,97729E+14	4,41162E+14	5,48412E+14	4,554E+13
TC1400001177.mm.2	Lmo7	LIM domain only 7	2,95046E+14	7,38787E+14	4,32882E+14	0.00419902755449207	0.141570560577024	-1,52772E+14	8,97463E+14	9,20973E+14	7,66203E+14	6,19737E+13	6,96521E+14	5,31822E+14
TC1100004059.mm.2	Cd300e	CD300E molecule	-1,57484E+13	4,92245E+14	-4,32378E+14	0.00422343434908012	0.141570560577024	-1,53299E+14	4,01676E+14	3,99649E+14	4,84086E+14	5,51767E+14	5,36587E+14	5,79706E+14
TC0300003211.mm.2	5830417I10Rik	gon-4-like pseudogene	1,84769E+14	6,10259E+14	4,3234E+14	0.00422529915917199	0.141570560577024	-1,53339E+14	7,24684E+14	7,40336E+13	6,02799E+14	4,98255E+14	5,27134E+14	5,68347E+14
TC0X00001922.mm.2	Slc9a7	solute carrier family 9 (sodium/hydrogen exchanger), member 7	1,34422E+14	6,77704E+14	4,31988E+14	0.00424242211727773	0.141570560577024	-1,53706E+14	7,92199E+14	7,5677E+14	6,29757E+14	6,07369E+14	6,44506E+14	6,3562E+14
TC0900000313.mm.2	Kirrel3	kirre like nephrin family adhesion molecule 3	1,73928E+14	6,71852E+14	4,31975E+14	0.00424307067187357	0.141570560577024	-1,5372E+14	7,92125E+14	7,76526E+12	6,31032E+14	6,10631E+14	6,59318E+14	5,61478E+14
TC1400000195.mm.2	Kat6b	K(lysine) acetyltransferase 6B	1,54354E+14	7,90907E+14	4,31872E+14	0.00424808696275326	0.141570560577024	-1,53828E+14	8,82828E+14	8,76672E+14	7,70541E+14	7,64605E+14	7,63271E+14	6,87522E+14
TC0800000736.mm.2	Hapln4	hyaluronan and proteoglycan link protein 4	1,39371E+14	7,00874E+14	4,31374E+14	0.0042725108825855	0.141570560577024	-1,54349E+13	7,9499E+14	8,14478E+14	6,55427E+14	6,09622E+14	6,56287E+14	6,74439E+13
TC0600001833.mm.2	Dlx6os2	distal-less homeobox 6, opposite strand 2	-1,60357E+14	5,84061E+13	-4,3085E+14	0.00429835062733808	0.141570560577024	-1,54897E+14	4,72324E+14	4,90781E+14	5,91627E+14	6,65814E+14	6,23512E+14	6,60307E+14
TC0600000416.mm.2	Mgam	maltase-glucoamylase	-1,4896E+14	4,68222E+13	-4,30845E+14	0.0042985863045924	0.141570560577024	-1,54902E+14	3,7794E+14	3,69533E+14	4,74329E+14	5,42139E+14	5,27367E+14	5,18026E+14
TC0500002484.mm.2	Rpl9	ribosomal protein L9	1,81592E+14	9,90923E+13	4,30694E+14	0.004306072544656	0.141570560577024	-1,5506E+14	1,11519E+14	1,10939E+14	9,76337E+14	8,83223E+14	9,60455E+14	9,00941E+13
TC1700002132.mm.2	Slc25a27	solute carrier family 25, member 27	1,54527E+14	6,48968E+14	4,3068E+14	0.00430676291639473	0.141570560577024	-1,55075E+14	7,43654E+14	7,37209E+14	6,26734E+14	6,14401E+14	6,23804E+14	5,48004E+13
TC0X00001784.mm.2	Pqbp1	polyglutamine binding protein 1	2,02034E+14	7,7591E+14	4,30582E+14	0.00431164140651391	0.141570560577024	-1,55178E+14	9,17056E+14	8,98569E+14	7,79913E+14	6,48365E+14	7,09354E+14	7,02202E+14
TC0X00003418.mm.2	Rpgr	retinitis pigmentosa GTPase regulator	2,09374E+14	5,9752E+14	4,30389E+14	0.00432124978427664	0.141570560577024	-1,5538E+14	7,23018E+14	7,18867E+14	6,22566E+14	4,97532E+14	5,43853E+14	4,79284E+14
TC0200005134.mm.2	Spata25	spermatogenesis associated 25	-1,55606E+14	4,42139E+14	-4,30305E+14	0.00432543741572439	0.141570560577024	-1,55468E+14	3,84073E+14	3,29506E+14	4,28854E+14	4,85614E+14	5,11797E+14	5,12994E+14
TC0100003725.mm.2	Mia3	melanoma inhibitory activity 3	3,03031E+14	6,50563E+14	4,30239E+14	0.00432871135894781	0.141570560577024	-1,55537E+14	8,42247E+14	8,3134E+14	6,59284E+14	5,02984E+14	6,18374E+14	4,4915E+13
TC0200003424.mm.2	Nr6a1	nuclear receptor subfamily 6, group A, member 1	-1,28567E+14	7,58057E+14	-4,30156E+14	0.00433285353071742	0.141570560577024	-1,55624E+14	6,85751E+14	6,60121E+14	8,02831E+14	7,96632E+14	7,94867E+14	8,08139E+14
TC0100001341.mm.2	Tpr	translocated promoter region, nuclear basket protein	3,60071E+14	6,81211E+14	4,29997E+13	0.0043407988907965	0.141570560577024	-1,5579E+14	9,10554E+14	8,81633E+14	6,99658E+13	5,23377E+14	6,45173E+14	4,26872E+14
TC0100000659.mm.2	Gmppa	GDP-mannose pyrophosphorylase A	2,31616E+14	6,56909E+14	4,29697E+14	0.00435585038651613	0.141570560577024	-1,56105E+14	7,85936E+14	7,93442E+13	6,69875E+14	5,76059E+14	6,24954E+14	4,91191E+14
TC1100004196.mm.2	Slc38a10	solute carrier family 38, member 10	1,8011E+14	8,42551E+14	4,29625E+14	0.00435945483843131	0.141570560577024	-1,56181E+14	9,56888E+14	9,71537E+14	8,29078E+14	7,29597E+14	8,05754E+14	7,62451E+14
TC0200004899.mm.2	Zcchc3	zinc finger, CCHC domain containing 3	-1,28418E+14	7,34755E+14	-4,29557E+14	0.00436290778729728	0.141570560577024	-1,56253E+14	6,61962E+14	6,56192E+14	7,72202E+14	7,43183E+14	7,88094E+14	7,86896E+14
TC0500002153.mm.2	3110082J24Rik	RIKEN cDNA 3110082J24 gene	-1,39757E+14	7,78857E+14	-4,29549E+14	0.00436326683200931	0.141570560577024	-1,5626E+14	6,98454E+14	6,8871E+14	7,85597E+14	8,33702E+14	8,21217E+14	8,45461E+14
TC1300002258.mm.2	Zfp738	zinc finger protein 738	1,65974E+13	5,58572E+14	4,29361E+14	0.00437274819115977	0.141570560577024	-1,56458E+13	6,91151E+14	6,65915E+14	5,24193E+14	4,45055E+14	5,3318E+14	4,91937E+13
TC1100003638.mm.2	Tbkbp1	TBK1 binding protein 1	1,83675E+14	6,31179E+14	4,29349E+14	0.00437337240587838	0.141570560577024	-1,56471E+14	7,55353E+14	6,94399E+14	6,7244E+14	5,82481E+14	5,24528E+14	5,57874E+14
TC0X00001331.mm.2	Kir3dl1	killer cell immunoglobulin-like receptor, three domains, long cytoplasmic tail, 1	-1,78776E+14	4,83966E+14	-4,29126E+14	0.00438463048960749	0.141570560577024	-1,56705E+14	4,14824E+14	3,81573E+14	4,58338E+14	4,9511E+14	5,28895E+14	6,25054E+13
TC0200003674.mm.2	Scn1a	sodium channel, voltage-gated, type I, alpha	1,3783E+14	6,89278E+14	4,29109E+13	0.00438549871782558	0.141570560577024	-1,56723E+14	7,79697E+14	7,78047E+14	6,50132E+14	6,45706E+14	6,66678E+14	6,15406E+14
TC1600000205.mm.2	Ypel1	yippee like 1	-1,32589E+14	9,20426E+14	-4,29106E+14	0.00438562753743707	0.141570560577024	-1,56725E+14	8,24968E+14	8,35175E+14	9,51319E+14	9,85769E+14	9,53038E+13	9,72284E+14
TC1400002839.mm.2	Gm3500/Gm3373	predicted gene 3500/predicted gene 3373	1,75876E+14	6,02616E+14	4,28941E+13	0.00439399103850033	0.141570560577024	-1,56899E+14	6,80658E+14	6,30877E+13	6,92831E+14	6,51548E+14	5,21189E+14	4,38593E+14
TC1900000024.mm.2	Doc2g/Nudt8	double C2, gamma/nudix (nucleoside diphosphate linked moiety X)-type motif 8	-1,9457E+14	7,62037E+14	-4,28826E+14	0.00439984193663139	0.141570560577024	-1,5702E+14	6,27031E+14	6,4159E+14	7,65245E+14	8,80598E+14	8,04687E+14	8,53073E+14
TC1100001889.mm.2	Otop2	otopetrin 2	-1,68616E+14	8,56921E+14	-4,2863E+14	0.00440984017266469	0.141570560577024	-1,57226E+14	7,43499E+13	7,37303E+14	8,76818E+14	9,65874E+14	8,9088E+14	9,27155E+14
TC0100002353.mm.2	Sf3b1	splicing factor 3b, subunit 1	2,16305E+14	7,00305E+14	4,28492E+13	0.00441686463929365	0.141570560577024	-1,57371E+14	8,43929E+14	8,32448E+14	6,72046E+14	6,09643E+13	6,89208E+14	5,54558E+14
TC0600002484.mm.2	Usp39	ubiquitin specific peptidase 39	1,51475E+14	6,6375E+14	4,28359E+14	0.00442366790619371	0.141570560577024	-1,57511E+14	7,50507E+13	7,53932E+13	6,74823E+14	6,01749E+14	6,06062E+14	5,95428E+14
TC0300000550.mm.2	Gfm1	G elongation factor, mitochondrial 1	1,5023E+14	7,04693E+14	4,28338E+14	0.00442470372312126	0.141570560577024	-1,57533E+14	7,65316E+14	7,71278E+14	7,00321E+14	7,55111E+14	6,41333E+14	5,948E+14
TC0800000277.mm.2	Slc20a2	solute carrier family 20, member 2	1,73661E+14	6,85323E+14	4,28315E+14	0.00442591295655946	0.141570560577024	-1,57557E+14	7,87352E+14	7,88538E+14	6,93106E+14	6,14375E+14	6,47167E+14	5,81401E+14
TC1600000092.mm.2	Pmm2	phosphomannomutase 2	2,09999E+14	5,46227E+14	4,28276E+14	0.00442791235298976	0.141570560577024	-1,57599E+14	6,32093E+14	6,62049E+14	5,64403E+14	5,44677E+14	5,06025E+14	3,68118E+14
TC1400000945.mm.2	Trim35	tripartite motif-containing 35	1,63564E+14	7,70392E+14	4,28173E+12	0.00443318001128809	0.141570560577024	-1,57707E+14	8,80783E+13	8,81485E+14	7,46176E+14	6,78772E+14	7,47597E+14	6,87542E+13
TC0X00002789.mm.2	Rlim	ring finger protein, LIM domain interacting	2,06164E+14	6,28831E+14	4,27513E+14	0.00446714221671223	0.141570560577024	-1,58402E+14	7,89892E+14	7,61907E+14	5,97996E+14	4,83718E+14	6,06646E+14	5,32826E+13
TC0700000502.mm.2	Lrfn1/Mir466f-2	leucine rich repeat and fibronectin type III domain containing 1/microRNA 466f-2	1,42561E+14	5,82516E+14	4,2744E+14	0.00447087854003342	0.141570560577024	-1,58478E+14	6,67084E+14	6,38908E+13	5,57818E+14	6,1042E+14	5,04777E+14	5,16092E+14
TC1000002616.mm.2	Syn3	synapsin III	3,42563E+14	6,89074E+14	4,2741E+14	0.00447243776203966	0.141570560577024	-1,5851E+14	8,83788E+14	8,85372E+14	7,14115E+13	5,67138E+14	6,54262E+14	4,29771E+14
TC0Y00000434.mm.2	Ssty2	spermiogenesis specific transcript on the Y 2	-1,62442E+13	5,38958E+14	-4,27323E+14	0.00447692878689596	0.141570560577024	-1,58601E+14	4,21051E+14	4,51491E+14	5,45264E+14	6,18513E+14	5,78247E+14	6,1918E+14
TC0X00000437.mm.2	Slc25a14	solute carrier family 25 (mitochondrial carrier, brain), member 14	2,31642E+14	6,4066E+14	4,26981E+14	0.00449468517017501	0.141570560577024	-1,58962E+14	7,8775E+14	7,72073E+14	6,21003E+14	5,66597E+14	6,25777E+14	4,70762E+14
TC1100004062.mm.2	Grin2c	glutamate receptor, ionotropic, NMDA2C (epsilon 3)	1,33044E+14	6,26987E+14	4,26446E+14	0.00452261948562439	0.141570560577024	-1,59526E+12	7,35884E+14	7,10604E+11	5,58159E+14	5,76874E+14	5,75107E+14	6,05292E+14
TC0400004153.mm.2	Tmem88b	transmembrane protein 88B	2,14887E+14	7,21901E+13	4,26183E+14	0.00453642216322897	0.141570560577024	-1,59804E+14	8,40437E+14	8,44679E+14	7,16367E+14	6,7458E+14	6,98938E+14	5,56405E+13
TC1100000585.mm.2	Cdkl3	cyclin-dependent kinase-like 3	1,35002E+14	6,42289E+14	4,26169E+14	0.00453716773860311	0.141570560577024	-1,59819E+14	7,59219E+14	7,3282E+14	5,85581E+14	5,54078E+14	5,98779E+14	6,23256E+14
TC0800001414.mm.2	Cdh13	cadherin 13	2,03453E+14	6,94205E+14	4,26085E+14	0.00454160776608775	0.141570560577024	-1,59908E+14	8,42269E+14	8,19169E+14	6,72975E+14	5,76286E+14	6,74233E+14	5,80298E+14
TC0X00003364.mm.2	Frmpd4	FERM and PDZ domain containing 4	2,90877E+14	7,16166E+14	4,25537E+14	0.00457054635193269	0.141570560577024	-1,60487E+14	8,84053E+14	8,96535E+14	7,17409E+14	6,00167E+14	6,93723E+14	5,0511E+14
TC1500001584.mm.2	Tatdn1	TatD DNase domain containing 1	1,36324E+14	6,82625E+14	4,25214E+14	0.00458770169887848	0.141570560577024	-1,60828E+14	7,78042E+14	7,58409E+14	6,69997E+14	6,25498E+14	6,39583E+13	6,2422E+14
TC0100001397.mm.2	Stx6	syntaxin 6	1,45956E+14	7,80186E+14	4,24882E+14	0.00460542380200035	0.141570560577024	-1,6118E+14	8,82776E+14	8,59737E+14	7,75388E+14	7,12613E+14	7,2999E+14	7,20611E+14
TC0X00003396.mm.2	Nlgn3	neuroligin 3	2,36403E+14	7,82316E+14	4,24857E+14	0.00460675666991507	0.141570560577024	-1,61206E+14	9,52901E+14	9,22312E+14	7,79896E+14	6,36378E+14	7,44992E+14	6,57416E+14
TC0500002950.mm.2	Gak	cyclin G associated kinase	1,68146E+14	7,26635E+14	4,24805E+14	0.00460950821258317	0.141570560577024	-1,6126E+14	8,34165E+14	7,9886E+13	7,34736E+14	6,95314E+14	6,8939E+14	6,07342E+14
TC0200003140.mm.2	Ndor1	NADPH dependent diflavin oxidoreductase 1	-1,45163E+14	7,00724E+14	-4,24735E+14	0.00461323838988118	0.141570560577024	-1,61334E+14	5,92636E+14	6,2584E+14	7,13948E+14	7,63115E+14	7,69279E+14	7,39524E+14
TC0700004315.mm.2	Cd2bp2	CD2 cytoplasmic tail binding protein 2	1,37942E+14	6,79506E+14	4,24585E+14	0.00462132497078132	0.141570560577024	-1,61494E+12	7,40381E+14	7,51563E+14	7,00605E+14	6,68427E+14	6,29226E+14	5,86835E+14
TC0400001445.mm.2	Grik3	glutamate receptor, ionotropic, kainate 3	2,13268E+14	6,66721E+14	4,24457E+14	0.00462817773049214	0.141570560577024	-1,61629E+14	8,0278E+14	7,90326E+14	6,80429E+14	5,60225E+14	5,4072E+13	6,2585E+14
TC1100003941.mm.2	Kpna2	karyopherin (importin) alpha 2	2,06622E+14	6,40253E+14	4,24443E+14	0.00462891476749724	0.141570560577024	-1,61643E+14	7,38573E+14	7,58483E+14	6,88396E+14	5,72251E+14	5,80555E+14	5,03258E+14
TC1000000884.mm.2	Eef2	eukaryotic translation elongation factor 2	1,48165E+14	1,05109E+13	4,2428E+14	0.0046376962206906	0.141570560577024	-1,61816E+14	1,14934E+14	1,14107E+14	1,04042E+14	9,81635E+14	1,01668E+14	9,77398E+14
TC0100003566.mm.2	Atp1a2	ATPase, Na+/K+ transporting, alpha 2 polypeptide	1,78407E+14	8,40638E+13	4,24252E+14	0.00463921569321754	0.141570560577024	-1,61846E+14	9,74137E+13	9,47614E+14	8,22565E+14	7,34577E+14	8,12803E+14	7,52134E+14
TC0600001027.mm.2	Rpn1	ribophorin I	1,79107E+13	7,10206E+14	4,24217E+14	0.0046411222315326	0.141570560577024	-1,61884E+13	8,20434E+14	8,23994E+13	7,14652E+14	6,15944E+14	6,68369E+14	6,17846E+14
TC0X00002333.mm.2	Slitrk4	SLIT and NTRK-like family, member 4	1,67103E+13	7,32843E+14	4,24038E+14	0.00465074740120185	0.141570560577024	-1,62073E+14	8,30702E+14	8,53124E+14	7,27468E+12	6,36144E+14	6,86128E+14	6,63491E+14
TC1900001472.mm.2	Noc3l	NOC3 like DNA replication regulator	1,85843E+13	6,12792E+14	4,24016E+14	0.00465196720642846	0.141570560577024	-1,62096E+14	7,3599E+14	7,02819E+14	6,38145E+14	5,32674E+14	5,11973E+13	5,55151E+14
TC0700004215.mm.2	Usp31	ubiquitin specific peptidase 31	2,16003E+14	7,13283E+14	4,23906E+14	0.00465787384903952	0.141570560577024	-1,62212E+14	8,34809E+14	8,55958E+14	7,06371E+14	6,23797E+14	6,91651E+14	5,67111E+14
TC1400001659.mm.2	Prkcd	protein kinase C, delta	2,01221E+14	7,88481E+14	4,23901E+13	0.00465819161993526	0.141570560577024	-1,62218E+14	8,99088E+14	9,14345E+14	8,19049E+14	6,87411E+13	7,1965E+14	6,91341E+14

TC0X00002756.mm.2	Gm9112	predicted gene 9112	-1,7211E+14	5,67468E+13	-4,23722E+14	0.00466784450756245	0.141570560577024	-1,62407E+14	4,82524E+14	4,59624E+14	5,65364E+14	6,10931E+14	5,9846E+14	6,87908E+13
TC0800000297.mm.2	Sfrp1	secreted frizzled-related protein 1	2,74936E+14	6,09699E+14	4,23494E+14	0.00468026037573597	0.141570560577024	-1,6265E+14	7,86066E+13	7,6241E+14	6,5413E+14	4,56981E+14	5,31558E+14	4,67046E+14
TC1200002339.mm.2	Bcl11b	B cell leukemia/lymphoma 11B	1,78797E+14	7,48791E+14	4,23377E+14	0.00468662229029335	0.141570560577024	-1,62774E+14	8,37263E+14	8,51113E+14	7,30282E+14	7,43305E+14	7,20068E+14	6,10714E+14
TC0300002629.mm.2	Olfml3/LOC269472	olfactomedin-like 3/uncharacterized LOC269472	1,25331E+14	7,0017E+14	4,23187E+13	0.00469701900106846	0.141570560577024	-1,62976E+14	7,6768E+14	7,83421E+14	6,66711E+14	6,82767E+14	6,56491E+14	6,43948E+14
TC0600001072.mm.2	Zxdc	ZXD family zinc finger C	1,35575E+14	6,66311E+14	4,2315E+14	0.00469902923596843	0.141570560577024	-1,63015E+13	7,37929E+13	7,31208E+14	6,80312E+14	6,50429E+14	5,78704E+14	6,19282E+14
TC0300001366.mm.2	Slc9b2	solute carrier family 9, subfamily B (NHA2, cation proton antiporter 2), member 2	-1,69797E+14	6,56023E+14	-4,23062E+13	0.00470380351698212	0.141570560577024	-1,63107E+13	5,6877E+14	5,44817E+14	6,45839E+14	7,2353E+14	6,97052E+14	7,5613E+14
TC1200001210.mm.2	Dync1h1	dynein cytoplasmic 1 heavy chain 1	1,47898E+13	8,47071E+14	4,22968E+14	0.00470898949743888	0.141570560577024	-1,63208E+14	9,41933E+14	9,24431E+14	7,92535E+14	8,52956E+14	8,02243E+14	7,68326E+14
TC1800001636.mm.2	Siglec15	sialic acid binding Ig-like lectin 15	-1,68629E+14	6,90125E+14	-4,22958E+14	0.00470950204441614	0.141570560577024	-1,63218E+11	5,93728E+14	5,86835E+14	6,86869E+14	7,55498E+14	7,23315E+14	7,94506E+14
TC0600000398.mm.2	Tmem178b	transmembrane protein 178B	1,86103E+14	7,80624E+14	4,22888E+14	0.00471334494073157	0.141570560577024	-1,63292E+14	8,92545E+14	8,70927E+14	8,06724E+14	7,2228E+14	7,36536E+13	6,5473E+13
TC0100000228.mm.2	Amer3	APC membrane recruitment 3	2,01073E+14	5,4958E+14	4,22822E+14	0.00471700829761023	0.141570560577024	-1,63363E+14	7,00904E+14	6,70414E+14	5,3854E+14	4,18453E+14	5,12502E+14	4,5667E+13
TC1100003997.mm.2	Abca8b	ATP-binding cassette, sub-family A (ABC1), member 8b	1,32008E+14	6,06284E+14	4,2274E+14	0.0047215139487779	0.141570560577024	-1,6345E+14	7,03865E+14	6,6661E+14	5,91394E+13	5,69377E+14	5,56788E+14	5,49672E+14
TC0500000316.mm.2	Snx17	sorting nexin 17	1,9567E+14	7,0804E+14	4,22356E+13	0.00474265404755434	0.141570560577024	-1,63858E+14	8,40497E+13	8,28395E+14	6,98516E+14	6,03278E+14	6,8285E+14	5,94703E+14
TC0100001980.mm.2	Lactb2	lactamase, beta 2	2,37182E+13	6,15469E+14	4,21886E+14	0.00476869911103578	0.141570560577024	-1,64358E+14	7,38156E+13	7,73746E+14	6,07459E+13	5,35912E+14	5,95657E+14	4,41883E+14
TC0400000103.mm.2	Rbm12b2	RNA binding motif protein 12 B2	1,95981E+14	5,92461E+14	4,2152E+14	0.00478907540773259	0.141570560577024	-1,64747E+13	7,0396E+14	7,04509E+14	6,27434E+14	5,0236E+14	5,27878E+14	4,88628E+14
TC0900001939.mm.2	Glb1l2	galactosidase, beta 1-like 2	-1,52656E+13	4,90397E+14	-4,21441E+14	0.00479349266948544	0.141570560577024	-1,64832E+14	3,9046E+14	3,82696E+14	5,06998E+14	5,83761E+14	5,47957E+14	5,30511E+13
TC0700004302.mm.2	Taok2	TAO kinase 2	1,7203E+14	8,4315E+14	4,21412E+14	0.0047950801637336	0.141570560577024	-1,64862E+14	9,49582E+14	9,55255E+14	8,37204E+14	7,56083E+14	8,12801E+14	7,47976E+14
TC0500003116.mm.2	Hspb8	heat shock protein 8	1,70673E+14	5,39639E+14	4,21255E+14	0.00480388668856652	0.141570560577024	-1,65029E+14	6,33144E+14	6,52825E+14	4,78908E+14	5,28334E+14	5,16083E+14	4,28541E+14
TC1200000432.mm.2	Akap6	A kinase (PRKA) anchor protein 6	2,49829E+14	7,42945E+14	4,21007E+14	0.0048177718609731	0.141570560577024	-1,65293E+14	8,95767E+14	8,8353E+14	7,34776E+14	6,63961E+13	7,25707E+14	5,53932E+13
TC0500003399.mm.2	Ccl24	chemokine (C-C motif) ligand 24	-1,85504E+14	6,54583E+14	-4,20623E+14	0.00483943156662017	0.141570560577024	-1,65703E+13	5,66005E+14	4,96313E+14	6,72555E+14	7,593E+14	6,94214E+14	7,39113E+14
TC0X00001526.mm.2	Huwe1	HECT, UBA and WWE domain containing 1	2,68914E+14	6,06156E+14	4,20605E+14	0.0048404403483602	0.141570560577024	-1,65722E+14	8,035E+14	7,99981E+14	5,46905E+14	4,20897E+14	6,1355E+14	4,52103E+14
TC1000000860.mm.2	Onecut3	one cut domain, family member 3	-1,35413E+14	5,49517E+13	-4,20536E+14	0.00484432640305826	0.141570560577024	-1,65795E+14	4,73757E+14	4,62976E+14	5,72536E+13	5,80274E+14	5,78574E+14	6,28985E+14
TC1900001779.mm.2	Itprip	inositol 1,4,5-triphosphate receptor interacting protein	-1,57228E+14	7,16907E+14	-4,20451E+14	0.00484915572015579	0.141570560577024	-1,65886E+13	6,2856E+14	6,14009E+14	7,08791E+14	7,93057E+14	7,7481E+14	7,82216E+14
TC0200003599.mm.2	Cd302	CD302 antigen	1,34047E+13	6,47337E+14	4,20276E+14	0.00485903665329166	0.141570560577024	-1,66072E+14	7,03236E+14	7,50158E+14	6,29207E+14	6,16123E+14	5,9747E+13	5,8783E+14
TC0800001269.mm.2	Nfat5	nuclear factor of activated T cells 5	1,61928E+14	7,00926E+13	4,19939E+14	0.00487822269621243	0.141570560577024	-1,66432E+14	7,95608E+14	7,94551E+14	6,95476E+14	6,53619E+14	6,74237E+14	5,92066E+14
TC1800000503.mm.2	Dmxl1	Dmx-like 1	1,54037E+14	6,51465E+14	4,19659E+14	0.00489419800807691	0.141570560577024	-1,66731E+14	7,60905E+14	7,61285E+14	5,95638E+14	5,7685E+14	6,46922E+14	5,67193E+14
TC1200000806.mm.2	Ptgr2	prostaglandin reductase 2	1,24922E+14	5,89435E+14	4,19654E+13	0.00489446740742908	0.141570560577024	-1,66736E+14	6,84724E+14	6,67241E+14	5,4719E+14	5,35334E+14	5,6018E+14	5,41942E+14
TC1200000201.mm.2	Klf11	Kruppel-like factor 11	-1,45578E+14	6,40565E+14	-4,19608E+14	0.00489712150482292	0.141570560577024	-1,66785E+14	5,77322E+14	5,44012E+14	6,5114E+14	6,58424E+13	6,7583E+14	7,3666E+14
TC0300002813.mm.2	Agl	amylo-1,6-glucosidase, 4-alpha-glucanotransferase	2,64974E+14	6,25357E+14	4,19541E+14	0.00490095765761841	0.141570560577024	-1,66857E+14	7,90791E+14	7,67633E+14	5,61092E+14	6,0415E+14	6,12084E+14	4,16393E+14
TC1600001254.mm.2	Mcm4	minichromosome maintenance complex component 4	1,24431E+13	5,34393E+13	4,19501E+14	0.00490327751601033	0.141570560577024	-1,669E+14	6,14708E+14	6,1197E+14	4,96801E+14	5,05059E+14	5,01379E+14	4,76438E+14
TC0200002887.mm.2	Proser2	proline and serine rich 2	-1,50076E+14	6,92597E+14	-4,1944E+14	0.00490672456039465	0.141570560577024	-1,66964E+14	6,15762E+14	5,62415E+14	7,23942E+14	7,75135E+14	7,29408E+14	7,48921E+14
TC0500000611.mm.2	Lias	lipoic acid synthetase	1,4001E+14	6,01043E+14	4,19409E+14	0.00490852873632716	0.141570560577024	-1,66998E+14	7,1806E+14	6,77863E+14	5,39068E+14	5,55364E+14	5,79361E+14	5,36541E+14
TC0700002644.mm.2	Axl	AXL receptor tyrosine kinase	1,35765E+14	6,82764E+14	4,19332E+14	0.00491295205931208	0.141570560577024	-1,6708E+14	7,74412E+13	7,81935E+14	6,26415E+14	6,29005E+14	6,68869E+14	6,15948E+14
TC1700002556.mm.2	Heatr5b	HEAT repeat containing 5B	1,77467E+14	6,11906E+14	4,19072E+14	0.00492791950532308	0.141570560577024	-1,67358E+14	7,36467E+14	7,37402E+14	5,91316E+14	4,87318E+14	5,39278E+14	5,79656E+14
TC0200002834.mm.2	Oprl1	opioid receptor-like 1	1,53692E+14	7,37232E+14	4,18889E+14	0.00493847284641721	0.141570560577024	-1,67554E+13	8,28183E+14	8,00162E+14	7,56965E+14	7,1712E+14	6,93355E+14	6,27606E+14
TC0700004134.mm.2	Copb1	coatomer protein complex, subunit beta 1	2,45906E+14	6,22895E+14	4,18795E+14	0.00494391669292803	0.141570560577024	-1,67655E+14	7,45746E+14	7,59717E+14	6,51154E+14	5,67103E+14	5,88166E+14	4,25485E+14
TC0300001582.mm.2	Lrrc40	leucine rich repeat containing 40	2,61135E+14	6,07408E+14	4,18767E+14	0.00494549216166841	0.141570560577024	-1,67684E+14	7,70595E+14	7,64598E+13	5,85908E+14	5,10426E+14	5,98195E+14	4,14727E+14
TC1200000740.mm.2	Galnt16	polypeptide N-acetylgalactosaminyltransferase 16	1,54478E+14	8,48982E+14	4,18753E+14	0.00494633538771909	0.141570560577024	-1,67699E+14	9,54435E+14	9,21832E+14	8,6308E+14	7,87232E+14	7,87807E+14	7,79505E+14
TC0200005072.mm.2	Ptprt	protein tyrosine phosphatase, receptor type, T	1,35981E+14	6,3808E+14	4,18402E+14	0.00496666235731496	0.141570560577024	-1,68074E+14	7,05463E+14	7,55829E+14	5,84279E+14	5,93577E+13	5,85392E+14	6,03937E+14
TC1200000576.mm.2	Dmac2l	distal membrane arm assembly complex 2 like	1,78114E+14	5,47037E+14	4,18397E+14	0.00496699672404751	0.141570560577024	-1,6808E+14	6,96721E+14	6,50092E+14	5,15481E+14	4,29341E+14	5,23113E+14	4,67473E+14
TC1100000884.mm.2	Rpl26/Gm15772	ribosomal protein L26/ribosomal protein L26 pseudogene	1,4132E+14	9,83374E+14	4,18396E+14	0.00496700508539621	0.141570560577024	-1,68081E+12	1,07495E+14	1,07546E+14	9,64032E+14	9,18032E+14	9,55902E+14	9,11868E+14
TC1300001781.mm.2	Tubb2b	tubulin, beta 2B class IIB	1,53364E+14	8,36723E+14	4,1779E+14	0.00500239903242688	0.141570560577024	-1,6873E+14	9,4077E+14	9,5259E+14	8,10673E+14	7,29671E+14	7,91598E+13	7,95034E+14
TC0700000087.mm.2	Usp29	ubiquitin specific peptidase 29	3,60351E+14	6,92538E+14	4,17683E+13	0.00500866491233438	0.141570560577024	-1,68844E+14	9,02871E+13	8,6399E+14	7,19245E+14	6,22965E+14	6,59397E+14	3,86761E+14
TC1100001776.mm.2	Ddx42	DEAD (Asp-Glu-Ala-Asp) box polypeptide 42	1,89895E+14	7,14431E+14	4,17551E+14	0.00501640929778732	0.141570560577024	-1,68986E+14	8,21628E+14	8,13614E+14	7,28783E+14	6,67108E+14	6,8276E+14	5,72692E+14
TC1000000595.mm.2	Pbld2	phenazine biosynthesis-like protein domain containing 2	-1,4673E+14	5,63892E+13	-4,17546E+14	0.00501668810863617	0.141570560577024	-1,68991E+14	4,74234E+14	4,83113E+14	5,54796E+14	6,20402E+14	6,10559E+14	6,40247E+13
TC0400000114.mm.2	Triqk	triple QxxK/R motif containing	2,25714E+14	6,27571E+14	4,17323E+14	0.00502985239114572	0.141570560577024	-1,69231E+14	7,6113E+14	7,49832E+14	6,14076E+14	5,80853E+14	6,10039E+14	4,49496E+14
TC1000000817.mm.2	Fstl3	follistatin-like 3	-1,31255E+14	7,23682E+14	-4,17291E+14	0.00503172349958536	0.141570560577024	-1,69265E+14	6,22785E+14	6,48885E+13	7,86875E+14	7,4937E+14	7,69251E+14	7,64928E+13
TC1500001262.mm.2	Hnrnpa1l2-ps2	heterogeneous nuclear ribonucleoprotein A1-like 2, pseudogene 2	1,27616E+14	7,776E+14	4,17219E+14	0.00503600485769546	0.141570560577024	-1,69342E+14	8,42447E+14	8,583E+14	7,68648E+13	7,50691E+13	7,37259E+14	7,08257E+14
TC0500002859.mm.2	Wdfy3	WD repeat and FYVE domain containing 3	1,82375E+14	6,47192E+14	4,17218E+14	0.00503602776212657	0.141570560577024	-1,69343E+14	7,95323E+14	7,49423E+14	6,31791E+14	5,26622E+13	6,04048E+14	5,75947E+14
TC1000002330.mm.2	Cabcoco1	ciliary associated calcium binding coiled-coil 1	1,24029E+14	7,30808E+13	4,17066E+14	0.00504501318131199	0.141570560577024	-1,69506E+14	7,89591E+14	8,03266E+14	7,22889E+14	7,24303E+14	6,8431E+14	6,60488E+14
TC0200001809.mm.2	Map1a	microtubule-associated protein 1 A	1,88224E+14	8,20445E+14	4,16969E+14	0.00505076382754758	0.141570560577024	-1,6961E+13	9,55053E+14	9,22989E+14	7,6263E+13	7,80403E+14	8,10475E+14	6,91119E+13
TC0900002401.mm.2	Neo1	neogenin	3,08392E+14	7,10061E+14	4,16526E+14	0.00507706534971406	0.141570560577024	-1,70085E+14	9,05303E+14	8,9308E+14	7,51802E+14	5,28585E+14	6,40574E+14	5,41024E+14
TC0400004118.mm.2	Arhgef16	Rho guanine nucleotide exchange factor (GEF) 16	-1,3092E+14	5,64102E+14	-4,16391E+14	0.00508512580914369	0.141570560577024	-1,70231E+14	4,74619E+14	5,05877E+14	5,69302E+13	5,92479E+14	6,33233E+14	6,09103E+13
TC1300002017.mm.2	Sptlc1	serine palmitoyltransferase, long chain base subunit 1	1,26613E+14	5,46806E+14	4,16272E+13	0.00509224441372249	0.141570560577024	-1,70359E+14	6,17501E+14	6,205E+14	5,41689E+14	5,16372E+14	4,7983E+14	5,04944E+14
TC1100000722.mm.2	Jmjd4	jumonji domain containing 4	1,83325E+14	6,17599E+14	4,16264E+14	0.00509268362986195	0.141570560577024	-1,70366E+14	7,28254E+14	7,32888E+14	6,21172E+14	5,28789E+14	5,85231E+14	5,0926E+14
TC1900000046.mm.2	Ctsf	cathepsin F	1,46333E+14	7,06004E+14	4,16251E+14	0.00509349888725604	0.141570560577024	-1,70381E+14	8,02996E+14	7,63507E+14	7,18583E+14	6,77099E+14	6,59262E+14	6,14577E+14
TC1500000090.mm.2	Adamts12	a disintegrin-like and metallopeptidase (reprolysin type) with thrombospondin type 1 motif, 12	-1,33081E+14	7,24454E+13	-4,15972E+14	0.0051101868351112	0.141570560577024	-1,7068E+13	6,38511E+14	6,3604E+14	7,56985E+14	7,74472E+14	7,46722E+14	7,93991E+13
TC0400001971.mm.2	Slc2a7	solute carrier family 2 (facilitated glucose transporter), member 7	-1,67467E+14	5,87218E+14	-4,15793E+13	0.00512093125524344	0.141570560577024	-1,70873E+14	4,88509E+14	4,96113E+14	5,59485E+14	6,59648E+14	6,53549E+14	6,66006E+14
TC1600002039.mm.2	Tiam1	T cell lymphoma invasion and metastasis 1	1,44648E+14	6,73163E+13	4,15685E+14	0.00512743625556653	0.141570560577024	-1,70989E+14	7,75402E+14	7,30903E+14	6,81725E+14	6,33943E+14	5,95349E+14	6,21659E+14
TC1200001010.mm.2	Slc24a4	solute carrier family 24 (sodium/potassium/calcium exchanger), member 4	2,56912E+14	6,68581E+14	4,15592E+14	0.00513305050237495	0.141570560577024	-1,71089E+14	7,92034E+14	8,13967E+14	6,68027E+13	6,45279E+13	6,42956E+13	4,4922E+14
TC1100003775.mm.2	Acly	ATP citrate lyase	2,07447E+14	7,6319E+14	4,15461E+14	0.0051409371344291	0.141570560577024	-1,7123E+14	9,19713E+13	8,67352E+14	7,75738E+14	6,44167E+14	6,67409E+14	7,04761E+14
TC0200001133.mm.2	Pjvk	pejvakin	-1,55941E+14	5,76185E+14	-4,15438E+14	0.00514234112442852	0.141570560577024	-1,71255E+14	5,0131E+14	4,8472E+14	6,19283E+13	5,53885E+14	6,21792E+14	6,7612E+14
TC0800001007.mm.2	Gm10638	predicted gene 10638	-1,57162E+14	7,3434E+14	-4,15372E+13	0.00514630874818972	0.141570560577024	-1,71325E+14	6,24477E+14	6,42634E+14	7,35583E+14	8,21912E+14	7,88981E+14	7,92454E+13

TC0300001176.mm.2	Olfm3	olfactomedin 3	2,3893E+14	6,87265E+14	4,15293E+14	0.00515110774767553	0.141570560577024	-1,71411E+14	8,44537E+14	7,85313E+14	7,41594E+14	6,00155E+14	6,21932E+14	5,30058E+14
TC1400001785.mm.2	Prxl2a	peroxiredoxin like 2A	2,50863E+14	8,407E+14	4,1517E+14	0.00515856704431947	0.141570560577024	-1,71543E+14	1,01974E+14	9,85277E+14	8,53922E+14	6,81959E+14	7,89E+14	7,14295E+14
TC1100004208.mm.2	Arl16	ADP-ribosylation factor-like 16	1,38375E+14	7,39791E+14	4,15166E+14	0.00515876884912612	0.141570560577024	-1,71547E+14	8,29621E+14	8,08403E+14	7,30973E+14	7,08478E+14	7,06682E+13	6,54591E+14
TC1000003007.mm.2	Cand1	cullin associated and neddylation disassociated 1	1,99526E+14	6,98983E+14	4,15149E+14	0.00515981033858517	0.141570560577024	-1,71565E+14	8,5885E+14	8,17415E+14	6,6923E+13	5,71191E+14	6,82657E+14	5,94555E+14
TC0900002054.mm.2	Tbrg1	transforming growth factor beta regulated gene 1	1,49998E+14	7,02929E+14	4,15002E+14	0.00516873014572711	0.141570560577024	-1,71723E+14	8,05697E+14	7,81266E+14	7,08492E+14	6,35154E+13	6,50704E+14	6,36263E+14
TC1700001929.mm.2	Skiv2l/Mir6970	superkiller viralicidic activity 2-like (S. cerevisiae)/microRNA 6970	1,29073E+14	6,98255E+13	4,14961E+14	0.0051712574533716	0.141570560577024	-1,71768E+14	7,70091E+14	7,92184E+14	6,79738E+14	6,43385E+14	6,54061E+14	6,50068E+14
TC0900002123.mm.2	Arhgef12	Rho guanine nucleotide exchange factor (GEF) 12	1,34009E+14	6,60062E+14	4,14756E+14	0.00518368959199845	0.141570560577024	-1,71988E+13	7,37618E+14	7,50221E+14	6,13898E+14	6,38813E+14	6,32434E+14	5,87388E+14
TC1100001012.mm.2	Camkk1	calcium/calmodulin-dependent protein kinase kinase 1, alpha	1,29282E+13	7,30802E+14	4,14532E+14	0.00519741754096133	0.141570560577024	-1,7223E+14	8,11909E+13	8,18344E+14	7,09576E+14	6,73294E+14	6,97399E+14	6,74289E+13
TC0100002656.mm.2	Dock10	dedicator of cytokinesis 10	1,54843E+14	6,12365E+14	4,14291E+14	0.00521213408678561	0.141570560577024	-1,72489E+14	6,89798E+14	7,08036E+14	5,69739E+14	6,18471E+14	5,78136E+14	5,10011E+14
TC0700003007.mm.2	Bax	BCL2-associated X protein	-1,29232E+14	8,33647E+14	-4,1405E+14	0.00522692298809302	0.141570560577024	-1,72749E+13	7,34919E+14	7,673E+14	8,55042E+13	8,83943E+14	8,77725E+14	8,82957E+14
TC1600000439.mm.2	Ubxn7	UBX domain protein 7	1,30102E+14	7,26927E+14	4,13772E+14	0.00524410188791272	0.141570560577024	-1,73049E+13	8,15829E+14	8,21038E+14	6,95722E+14	6,52312E+13	6,95151E+14	6,81511E+14
TC0700002226.mm.2	Clcn4	chloride channel, voltage-sensitive 4	1,51415E+14	7,40105E+14	4,1311E+14	0.00528510379167806	0.141570560577024	-1,73763E+14	8,45367E+14	8,44576E+14	7,18948E+13	6,44627E+14	6,76706E+14	7,10408E+14
TC1700002787.mm.2	Ddx39b/Mir8094	DEAD (Asp-Glu-Ala-Asp) box polypeptide 39B/microRNA 8094	1,3295E+14	8,93347E+13	4,12995E+14	0.00529228559444156	0.141570560577024	-1,73887E+14	9,7362E+14	9,78935E+13	8,77047E+14	8,43827E+14	8,63193E+14	8,23462E+14
TC1600000288.mm.2	Eif4g1	eukaryotic translation initiation factor 4, gamma 1	1,29942E+14	8,42038E+14	4,12774E+14	0.00530606922017444	0.141570560577024	-1,74126E+14	9,30169E+14	9,21168E+14	8,23554E+14	7,8588E+14	8,0825E+14	7,83204E+14
TC1500002284.mm.2	Spryd3	SPRY domain containing 3	1,51409E+14	7,58158E+14	4,12756E+14	0.00530721479275129	0.141570560577024	-1,74146E+14	8,65933E+13	8,51067E+14	7,24581E+14	6,93183E+14	7,4411E+14	6,70072E+14
TC0900003044.mm.2	Rbm6	RNA binding motif protein 6	1,86751E+14	7,14235E+14	4,12377E+14	0.00533101112576433	0.141570560577024	-1,74556E+14	8,42212E+14	8,11673E+12	6,76379E+14	6,74763E+14	7,01068E+14	5,79315E+14
TC1400002744.mm.2	Gpr18	G protein-coupled receptor 18	-1,51761E+14	4,97713E+14	-4,12171E+14	0.00534394893002471	0.141570560577024	-1,74778E+14	4,1231E+14	3,90223E+14	5,04554E+14	5,73135E+14	5,32859E+14	5,73197E+14
TC0X00001111.mm.2	Pou3f4	POU domain, class 3, transcription factor 4	1,34325E+14	7,37344E+14	4,12001E+14	0.00535466369558522	0.141570560577024	-1,74962E+14	8,06076E+14	8,2105E+14	7,44465E+14	6,93997E+14	6,87634E+14	6,70843E+14
TC0200002968.mm.2	Fbh1	F-box DNA helicase 1	1,5492E+14	6,78775E+14	4,11531E+14	0.00538449556972744	0.141570560577024	-1,75471E+14	7,86798E+14	7,62309E+14	6,83003E+13	6,01276E+14	6,28707E+14	6,10559E+14
TC1400002252.mm.2	Fzd3	frizzled class receptor 3	1,24396E+14	7,54407E+14	4,11504E+13	0.00538621182627728	0.141570560577024	-1,755E+14	8,5293E+14	8,41407E+14	7,0905E+13	6,77512E+13	7,24167E+14	7,21377E+14
TC0300002636.mm.2	Magi3	membrane associated guanylate kinase, WW and PDZ domain containing 3	1,71389E+13	5,74314E+14	4,11405E+14	0.00539247085387093	0.141570560577024	-1,75607E+14	6,94056E+14	6,55797E+14	5,6031E+12	5,28648E+14	5,51591E+14	4,55483E+12
TC1000003183.mm.2	Olfr796	olfactory receptor 796	-1,21623E+14	4,82816E+14	-4,11293E+14	0.00539963550548566	0.141570560577024	-1,75729E+14	4,02067E+14	4,0758E+14	5,27047E+14	5,07307E+14	5,33665E+14	5,19228E+14
TC0300002407.mm.2	Lce1g	late cornified envelope 1G	-1,36405E+14	6,26445E+14	-4,11115E+14	0.00541102815984462	0.141570560577024	-1,75922E+14	5,37882E+14	5,3198E+14	6,44198E+14	7,01938E+14	6,64776E+14	6,77896E+14
TC1100000863.mm.2	Gsg1l2	GSG1-like 2	-2,04509E+14	7,17085E+14	-4,1092E+14	0.00542345833752021	0.141570560577024	-1,76132E+14	5,90827E+14	5,91148E+14	7,02551E+14	8,26989E+14	7,5757E+14	8,33423E+14
TC1200000563.mm.2	Mgat2	mannoside acetylglucosaminyltransferase 2	2,45135E+14	6,87713E+14	4,1086E+14	0.00542732617973003	0.141570560577024	-1,76198E+13	8,62787E+14	8,22719E+14	7,09252E+14	5,36282E+14	6,27791E+14	5,67446E+14
TC0100000790.mm.2	Ptma	prothymosin alpha	1,34156E+14	9,95763E+14	4,10822E+14	0.00542977258683274	0.141570560577024	-1,76239E+14	1,08451E+14	1,09253E+14	9,71669E+14	9,17132E+14	9,58193E+14	9,50539E+13
TC1400002457.mm.2	Gm6994	predicted gene 6994	-1,30163E+14	5,95729E+14	-4,10692E+14	0.00543814073377571	0.141570560577024	-1,7638E+14	5,25717E+14	5,22795E+14	5,98513E+14	6,1851E+14	6,32666E+14	6,76171E+14
TC0300002815.mm.2	Palmd	palmdelphin	1,60956E+14	6,91997E+14	4,10633E+14	0.00544192788996148	0.141570560577024	-1,76444E+13	7,87762E+14	8,04884E+14	6,65749E+14	6,22855E+14	6,77228E+14	5,93506E+14
TC0800000088.mm.2	Col4a2	collagen, type IV, alpha 2	-1,68152E+14	6,7093E+14	-4,10565E+14	0.00544625407041119	0.141570560577024	-1,76517E+14	5,7228E+14	5,51968E+14	6,72626E+14	7,68151E+14	7,11446E+14	7,49106E+14
TC0800002927.mm.2	Sf3b3	splicing factor 3b, subunit 3	1,79789E+14	6,53456E+14	4,10353E+12	0.00545998180960341	0.141570560577024	-1,76748E+14	7,83771E+14	7,70705E+14	6,44272E+14	5,27089E+14	6,03939E+14	5,90958E+14
TC1000000048.mm.2	Lats1	large tumor suppressor	1,54359E+14	6,94179E+14	4,10235E+14	0.00546760148933267	0.141570560577024	-1,76876E+14	7,72317E+14	7,5997E+14	7,44257E+14	6,64958E+14	6,28744E+14	5,94826E+13
TC0400001523.mm.2	Bsdc1	BSD domain containing 1	1,6219E+14	8,15456E+14	4,10107E+14	0.00547587848424705	0.141570560577024	-1,77015E+14	9,14053E+14	9,10246E+14	8,31734E+14	7,36782E+14	7,60773E+14	7,39146E+14
TC1100001494.mm.2	Hoxb7	homeobox B7	-1,75797E+14	7,40029E+14	-4,10061E+14	0.00547883765561149	0.141570560577024	-1,77064E+14	6,48712E+13	6,21341E+14	7,25706E+14	8,22768E+14	7,8413E+14	8,37517E+14
TC1700000117.mm.2	Agpat4	1-acylglycerol-3-phosphate O-acyltransferase 4 (lysophosphatidic acid acyltransferase, delta)	1,47264E+14	8,27382E+13	4,10055E+14	0.00547922842680433	0.141570560577024	-1,77071E+14	9,33304E+14	9,33144E+14	7,72626E+14	7,53296E+14	8,23365E+14	7,48555E+14
TC0900002017.mm.2	Ddx25	DEAD (Asp-Glu-Ala-Asp) box polypeptide 25	1,65519E+14	6,5083E+13	4,09943E+14	0.00548647326092049	0.141570560577024	-1,77192E+14	7,47569E+14	7,16632E+13	7,0207E+14	6,05544E+14	5,62261E+14	5,70903E+14
TC0600003000.mm.2	Bms1	BMS1, ribosome biogenesis factor	1,58178E+14	6,19988E+14	4,09883E+14	0.00549037541909725	0.141570560577024	-1,77257E+14	7,39787E+14	7,1492E+13	5,60778E+14	5,66093E+14	6,13587E+14	5,24765E+14
TC0300002306.mm.2	Tmem79	transmembrane protein 79	-1,3761E+14	6,4082E+14	-4,09717E+14	0.00550120013163665	0.141570560577024	-1,77438E+14	5,51543E+14	5,54881E+14	6,54201E+14	7,02651E+13	6,71252E+14	7,10392E+14
TC0700000537.mm.2	Zfp420	zinc finger protein 420	-1,36563E+12	8,40973E+14	-4,09708E+14	0.00550176902599497	0.141570560577024	-1,77448E+14	7,50087E+14	7,52923E+14	8,53086E+13	9,13603E+14	8,87602E+14	8,88535E+14
TC0100003620.mm.2	Cep170	centrosomal protein 170	2,39153E+13	6,47785E+14	4,09653E+14	0.00550534438829526	0.141570560577024	-1,77507E+14	8,17447E+14	7,92226E+14	6,54226E+14	4,91447E+14	6,04679E+14	5,26688E+14
TC0900002333.mm.2	Etfa	electron transferring flavoprotein, alpha polypeptide	1,7627E+14	6,8441E+14	4,0959E+14	0.00550947750903897	0.141570560577024	-1,77576E+14	7,84098E+14	7,78015E+14	7,23182E+14	6,11592E+14	5,91435E+14	6,18138E+14
TC0400001539.mm.2	Col16a1	collagen, type XVI, alpha 1	-1,48032E+14	7,98978E+14	-4,09562E+14	0.00551129242471827	0.141570560577024	-1,77606E+14	6,89676E+14	7,23925E+14	7,99907E+14	8,70694E+13	8,5465E+14	8,55015E+14
TC1800001441.mm.2	Fbxo38	F-box protein 38	1,45467E+14	6,26083E+14	4,09229E+14	0.00553305957553838	0.141570560577024	-1,77968E+14	7,26686E+13	7,25167E+14	5,80929E+14	5,62798E+14	6,16867E+14	5,44052E+14
TC0X00000318.mm.2	Zcchc12	zinc finger, CCHC domain containing 12	2,56129E+14	6,83515E+13	4,08858E+14	0.00555739530526701	0.141570560577024	-1,78371E+14	8,62246E+14	7,87003E+14	7,1446E+14	6,00393E+14	6,41266E+14	4,95724E+13
TC0X00002721.mm.2	Zmym3	zinc finger, MYM-type 3	1,64419E+13	6,70455E+14	4,08811E+14	0.00556051237522641	0.141570560577024	-1,78423E+14	7,84913E+13	7,38915E+14	6,93323E+14	6,10587E+14	6,14994E+14	5,79996E+14
TC0800001906.mm.2	Tacc1/Mir8108	transforming, acidic coiled-coil containing protein 1/microRNA 8108	1,32942E+14	7,34598E+14	4,08545E+14	0.00557810135813127	0.141570560577024	-1,78713E+13	8,03116E+14	8,26402E+14	7,28576E+14	6,85861E+14	6,94406E+14	6,69227E+13
TC1700001154.mm.2	Ehd3	EH-domain containing 3	1,78796E+14	6,77137E+14	4,08492E+14	0.00558157949901437	0.141570560577024	-1,7877E+14	7,91243E+14	7,79668E+14	6,97213E+14	5,81382E+14	5,95807E+13	6,17511E+14
TC0X00000321.mm.2	Gm6268	predicted gene 6268	-1,23721E+14	4,62093E+14	-4,08062E+14	0.0056101076830109	0.141570560577024	-1,79238E+14	3,89446E+14	3,74312E+14	4,83453E+14	5,1415E+14	5,08494E+14	5,02705E+14
TC0800001926.mm.2	Pomk	protein-O-mannose kinase	1,71246E+14	6,78314E+14	4,07939E+14	0.00561828032387842	0.141570560577024	-1,79372E+14	7,61355E+14	7,5711E+14	6,63276E+14	7,12173E+14	6,37071E+14	5,38901E+14
TC0X00002794.mm.2	Nexmif	neurite extension and migration factor	2,15207E+14	5,91343E+13	4,0793E+14	0.00561889362444298	0.141570560577024	-1,79382E+14	7,37058E+14	7,10388E+14	5,24743E+14	5,58837E+14	5,84844E+14	4,32188E+14
TC0300000221.mm.2	Pik3ca	phosphatidylinositol-4,5-bisphosphate 3-kinase catalytic subunit alpha	2,14516E+14	7,26476E+14	4,07911E+14	0.005620156508281	0.141570560577024	-1,79402E+13	8,62656E+14	8,56793E+14	7,15926E+14	6,33062E+14	7,12385E+14	5,78032E+14
TC0700003977.mm.2	Fam160a2	family with sequence similarity 160, member A2	1,33432E+13	6,38244E+14	4,07818E+14	0.00562634218511237	0.141570560577024	-1,79503E+13	6,9879E+14	7,30031E+14	6,37619E+14	6,0107E+14	5,665E+14	5,95456E+14
TC0700003539.mm.2	Ntrk3	neurotrophic tyrosine kinase, receptor, type 3	1,53138E+14	8,76744E+14	4,07796E+14	0.00562786912695517	0.141570560577024	-1,79528E+14	9,62978E+14	9,67528E+14	8,64953E+13	8,40777E+14	8,5369E+14	7,70539E+14
TC0500003480.mm.2	1700123K08Rik	RIKEN cDNA 1700123K08 gene	-1,86028E+14	6,14279E+14	-4,07725E+14	0.0056326106291315	0.141570560577024	-1,79606E+14	5,08314E+14	4,91404E+14	6,00071E+14	7,14111E+14	6,59124E+14	7,1265E+14
TC0200001838.mm.2	B2m	beta-2 microglobulin	1,29151E+14	9,12166E+14	4,07571E+14	0.00564290676884014	0.141570560577024	-1,79773E+14	1,0056E+14	1,03224E+14	8,40322E+14	8,15301E+14	8,74271E+14	9,05264E+14
TC1100000223.mm.2	Tmem17	transmembrane protein 17	1,23339E+14	6,26572E+13	4,07415E+14	0.00565331747672564	0.141570560577024	-1,79943E+14	7,00939E+14	6,86443E+14	6,19031E+14	6,12316E+14	5,85469E+14	5,55233E+14
TC1000001622.mm.2	Dnajc14	DnaJ heat shock protein family (Hsp40) member C14	1,40337E+14	6,90724E+14	4,07194E+14	0.00566821220848995	0.141570560577024	-1,80184E+14	7,66308E+13	7,63293E+14	7,04822E+14	6,60996E+14	6,50583E+13	5,98342E+12
TC0100003079.mm.2	Cntn2	contactin 2	1,4778E+14	7,5985E+14	4,07145E+14	0.0056715018169595	0.141570560577024	-1,80238E+14	8,76205E+14	8,51621E+14	7,29871E+14	6,69136E+14	7,38679E+14	6,93586E+14
TC1900000598.mm.2	Frat1	frequently rearranged in advanced T cell lymphomas	-1,63885E+14	8,10927E+14	-4,07113E+14	0.00567366402850986	0.141570560577024	-1,80273E+14	7,17928E+14	6,95404E+14	8,0628E+14	9,04851E+14	8,70874E+14	8,70228E+14
TC1600000988.mm.2	Mrap	melanocortin 2 receptor accessory protein	-1,23196E+14	6,96916E+14	-4,06737E+14	0.00569901633310375	0.141570560577024	-1,80682E+13	6,03486E+14	6,29696E+14	7,23426E+14	7,45313E+14	7,37836E+11	7,41738E+14
TC0900002268.mm.2	Pts	6-pyruvoyl-tetrahydropterin synthase	1,75853E+14	6,30177E+14	4,06537E+14	0.00571260089645665	0.141570560577024	-1,80901E+14	7,38285E+14	7,01176E+14	6,30127E+14	6,2372E+14	5,98716E+14	4,89039E+14
TC0700001435.mm.2	Clpb	ClpB caseinolytic peptidase B	1,93088E+14	6,2437E+14	4,06511E+14	0.00571435680286935	0.141570560577024	-1,80929E+14	7,4568E+14	7,59827E+13	6,23412E+14	4,97969E+14	5,8448E+14	5,3485E+14
TC1400000344.mm.2	Ogdhl	oxoglutarate dehydrogenase-like	1,73344E+14	7,31261E+14	4,0641E+13	0.00572122656228511	0.141570560577024	-1,8104E+13	8,60848E+14	8,14829E+14	7,15908E+14	6,66991E+14	7,09963E+14	6,19027E+14

TC0800000098.mm.2	Tex29	testis expressed 29	-2,23387E+14	6,65882E+14	-4,0636E+14	0.00572461298087645	0.141570560577024	-1,81094E+14	5,35523E+14	5,28527E+13	6,32968E+14	7,87451E+13	7,22491E+14	7,88332E+14
TC1800001404.mm.2	Ndst1	N-deacetylase/N-sulfotransferase (heparan glucosaminyl) 1	1,73928E+14	7,63573E+14	4,06314E+14	0.00572773234170024	0.141570560577024	-1,81144E+14	8,7276E+13	8,6353E+14	7,58928E+14	6,97785E+14	7,4198E+14	6,46453E+14
TC1400001094.mm.2	Vwa8	von Willebrand factor A domain containing 8	1,20415E+14	6,32106E+14	4,06301E+14	0.00572867573462771	0.141570560577024	-1,81159E+14	7,07507E+14	7,05012E+14	6,09302E+14	5,99129E+14	5,97773E+14	5,73915E+13
TC0200001767.mm.2	Vps18	VPS18 CORVET/HOPS core subunit	1,28015E+12	6,53297E+14	4,06228E+14	0.00573363818414473	0.141570560577024	-1,81239E+14	7,40456E+14	6,95909E+13	6,55346E+14	6,47737E+14	5,84872E+14	5,95463E+14
TC1600001443.mm.2	Atp13a4	ATPase type 13A4	1,31723E+14	4,74855E+14	4,0611E+14	0.00574167543245631	0.141570560577024	-1,81368E+14	5,49768E+14	4,99038E+14	5,11092E+14	5,03874E+14	4,01154E+14	3,84207E+14
TC0200004975.mm.2	Uqcc1	ubiquinol-cytochrome c reductase complex assembly factor 1	1,33202E+14	6,71037E+14	4,05945E+14	0.00575295036794433	0.141570560577024	-1,81548E+14	7,66507E+14	7,24729E+13	6,74118E+14	6,36038E+14	6,0949E+14	6,15341E+14
TC0100001586.mm.2	Pfdn2	prefoldin 2	1,42005E+14	8,0106E+14	4,05877E+14	0.0057575815153631	0.141570560577024	-1,81622E+14	9,05504E+14	8,8074E+14	7,70744E+14	7,47139E+14	7,82563E+14	7,19672E+14
TC1100000883.mm.2	Rnf222	ring finger protein 222	-1,29954E+13	6,13379E+14	-4,05779E+14	0.00576432480629106	0.141570560577024	-1,8173E+11	5,30845E+14	5,42285E+14	6,14339E+14	6,59765E+11	6,56784E+14	6,76254E+14
TC0300001531.mm.2	Zzz3	zinc finger, ZZ domain containing 3	1,55875E+12	6,1177E+14	4,05618E+14	0.00577535138000315	0.141570560577024	-1,81905E+14	6,96345E+14	6,94858E+13	5,69379E+14	6,30583E+14	5,7374E+14	5,05714E+14
TC0800000231.mm.2	Defa27	defensin, alpha, 27	-1,27094E+14	6,36808E+14	-4,05617E+14	0.00577540851662237	0.141570560577024	-1,81906E+14	5,71615E+14	5,60939E+14	6,3015E+14	6,71407E+14	6,86423E+14	7,00318E+14
TC0200001127.mm.2	Osbpl6	oxysterol binding protein-like 6	1,63219E+14	6,48721E+14	4,05603E+14	0.00577638422841948	0.141570560577024	-1,81922E+14	7,66752E+14	6,98684E+13	6,77452E+14	6,10437E+14	5,85968E+14	5,5303E+14
TC0700000800.mm.2	Shank1	SH3 and multiple ankyrin repeat domains 1	1,27275E+14	8,50611E+14	4,05599E+14	0.0057766656702011	0.141570560577024	-1,81926E+14	9,4983E+14	9,47763E+14	8,02576E+14	7,60451E+14	8,297E+14	8,13344E+14
TC0100001356.mm.2	Fam129a	family with sequence similarity 129, member A	-1,56441E+14	6,7163E+14	-4,0538E+12	0.00579173483853669	0.141570560577024	-1,82166E+14	5,83975E+14	5,72055E+14	6,58677E+14	7,46162E+14	7,20849E+14	7,48063E+14
TC1100001011.mm.2	P2rx1	purinergic receptor P2X, ligand-gated ion channel, 1	-1,48221E+14	5,74235E+14	-4,0526E+14	0.00580001554605031	0.141570560577024	-1,82297E+14	4,70251E+14	4,94943E+14	5,72699E+14	6,45883E+13	6,18359E+14	6,43276E+14
TC0700003854.mm.2	Nup98	nucleoporin 98	1,35955E+14	6,59787E+14	4,05133E+14	0.00580875836831024	0.141570560577024	-1,82436E+14	7,41373E+14	7,43727E+14	6,48748E+14	6,11683E+14	6,31528E+14	5,81663E+13
TC0800002544.mm.2	Zfp330	zinc finger protein 330	1,7983E+14	5,9793E+14	4,05047E+14	0.0058147625792474	0.141570560577024	-1,82531E+14	7,24143E+14	7,09385E+14	5,92569E+14	4,87616E+14	5,66516E+14	5,07352E+13
TC0400000588.mm.2	Tal2	T cell acute lymphocytic leukemia 2	-1,6099E+14	6,88779E+14	-4,04755E+14	0.00583502326386284	0.141570560577024	-1,8285E+14	5,73974E+14	5,84059E+14	7,1074E+14	7,83891E+13	7,07309E+14	7,72703E+14
TC0500003184.mm.2	Ptpn11	protein tyrosine phosphatase, non-receptor type 11	1,32367E+14	7,62982E+14	4,04547E+14	0.00584949704561485	0.141570560577024	-1,83078E+14	8,3528E+14	8,18412E+14	7,90336E+14	7,44907E+14	7,08648E+14	6,80309E+14
TC1600000618.mm.2	Zbtb20/Mir568	zinc finger and BTB domain containing 20/microRNA 568	2,49299E+14	7,65805E+14	4,04449E+14	0.00585631864333511	0.141570560577024	-1,83185E+13	9,38958E+14	9,14261E+13	7,81635E+14	6,05354E+14	7,17815E+14	6,36806E+13
TC0900001635.mm.2	Snrk	SNF related kinase	1,65403E+13	7,31122E+13	4,04358E+14	0.00586266756330125	0.141570560577024	-1,83285E+14	8,23739E+14	8,06044E+14	7,74392E+14	6,83578E+14	6,73685E+14	6,25292E+14
TC0400002013.mm.2	Tnfrsf25	tumor necrosis factor receptor superfamily, member 25	-1,27424E+13	7,05046E+14	-4,04251E+14	0.0058701573859438	0.141570560577024	-1,83403E+14	6,23745E+13	6,38436E+14	7,03515E+14	7,47553E+14	7,57321E+14	7,59708E+14
TC0800000011.mm.2	Pnpla6	patatin-like phospholipase domain containing 6	1,41059E+14	6,38147E+14	4,04248E+14	0.00587031740584239	0.141570560577024	-1,83405E+14	7,5143E+14	7,14332E+14	6,18335E+14	5,61143E+14	6,01758E+14	5,81886E+14
TC1200001001.mm.2	Kif4-ps	kinesin family member 4, pseudogene	-1,45299E+14	4,99928E+14	-4,04138E+14	0.00587802281307597	0.141570560577024	-1,83526E+14	4,3625E+14	4,09157E+14	4,8467E+14	5,33485E+13	5,41124E+14	5,9488E+14
TC1100002865.mm.2	Rasd1	RAS, dexamethasone-induced 1	1,43E+14	6,92654E+14	4,03406E+12	0.00592954022857065	0.141570560577024	-1,84328E+14	8,05551E+14	7,53485E+14	6,87336E+13	6,36517E+14	6,28615E+14	6,44422E+14
TC0200002261.mm.2	Nsfl1c	NSFL1 (p97) cofactor (p47)	1,52803E+14	8,7501E+14	4,03276E+14	0.0059387542186223	0.141570560577024	-1,84471E+14	9,68311E+13	9,49203E+14	8,84825E+14	8,35815E+14	8,41591E+14	7,70317E+14
TC1100003041.mm.2	Cyb5d1	cytochrome b5 domain containing 1	1,49376E+13	6,22198E+14	4,03171E+14	0.00594619798474247	0.141570560577024	-1,84587E+14	7,27979E+14	7,30345E+14	5,89783E+14	5,25513E+14	6,06716E+14	5,52855E+14
TC1500002033.mm.2	Mlc1	megalencephalic leukoencephalopathy with subcortical cysts 1 homolog (human)	1,4925E+14	8,05866E+14	4,03166E+14	0.00594657809867502	0.141570560577024	-1,84592E+14	8,89361E+14	9,16012E+14	8,01031E+14	7,21919E+14	7,50324E+14	7,5655E+14
TC1100001442.mm.2	Lrrc59	leucine rich repeat containing 59	1,56792E+14	7,56815E+14	4,03156E+14	0.00594731367761148	0.141570560577024	-1,84604E+14	8,60696E+14	8,56244E+14	7,13766E+14	7,06827E+14	7,48495E+14	6,54861E+14
TC0600000016.mm.2	Ppp1r9a	protein phosphatase 1, regulatory subunit 9A	2,26992E+14	7,80139E+14	4,03148E+14	0.00594787706875765	0.141570560577024	-1,84613E+14	9,26046E+14	9,51899E+14	7,57037E+13	6,21889E+13	7,64949E+14	6,59012E+14
TC0600001953.mm.2	Gpr37	G protein-coupled receptor 37	1,68808E+14	7,82465E+14	4,02843E+14	0.00596952250978338	0.141570560577024	-1,84947E+14	8,75691E+14	8,96112E+14	7,71606E+14	7,17191E+14	7,6343E+14	6,70758E+14
TC1100003061.mm.2	Polr2a	polymerase (RNA) II (DNA directed) polypeptide A	1,6049E+14	6,29774E+13	4,02777E+14	0.00597423921165227	0.141570560577024	-1,8502E+14	7,17641E+14	7,11839E+14	5,9678E+14	6,43883E+14	5,10806E+14	5,97694E+14
TC0900002522.mm.2	Snx1	sorting nexin 1	1,31285E+14	6,20287E+14	4,0273E+14	0.00597759980020377	0.141570560577024	-1,85071E+14	7,14939E+14	7,05374E+14	5,98551E+14	5,45113E+14	5,83932E+14	5,73812E+14
TC0400000271.mm.2	Mdn1	midasin AAA ATPase 1	1,28254E+14	5,83356E+14	4,0249E+14	0.00599475756644873	0.141570560577024	-1,85335E+14	6,66407E+14	6,60698E+14	5,48634E+13	5,53801E+14	5,59702E+13	5,10896E+14
TC1500000705.mm.2	Tnrc6b/Mir5113	trinucleotide repeat containing 6b/microRNA 5113	1,89971E+14	6,4344E+14	4,02409E+14	0.00600059520559704	0.141570560577024	-1,85425E+14	7,57189E+14	7,51541E+14	6,62144E+14	5,60978E+13	6,08494E+14	5,20295E+14
TC0500001715.mm.2	Foxk1	forkhead box K1	1,22019E+14	6,61936E+14	4,02336E+13	0.00600581902067536	0.141570560577024	-1,85505E+14	7,59522E+14	7,27172E+14	6,2134E+14	6,20929E+14	6,14908E+14	6,27748E+14
TC0500002993.mm.2	Noc4l	NOC4 like	1,21223E+14	6,3938E+14	4,02257E+14	0.00601147885752382	0.141570560577024	-1,85592E+14	7,05767E+14	6,92754E+14	6,43716E+14	6,37971E+14	5,927E+14	5,63374E+14
TC0100003279.mm.2	Dhx9	DEAH (Asp-Glu-Ala-His) box polypeptide 9	2,37537E+14	6,4341E+14	4,02156E+14	0.0060187272879995	0.141570560577024	-1,85702E+14	7,87477E+13	7,78363E+13	6,59648E+14	5,44204E+14	6,17568E+14	4,73199E+14
TC0100000523.mm.2	Zdbf2	zinc finger, DBF-type containing 2	2,05308E+14	5,82965E+14	4,0215E+14	0.00601917615358597	0.141570560577024	-1,85709E+13	7,26699E+12	6,82979E+14	4,86251E+14	6,02801E+14	5,42567E+14	4,56494E+14
TC1400002116.mm.2	Gzmd/Gzme	granzyme D/granzyme E	-1,20142E+14	5,45991E+14	-4,0198E+14	0.00603144251991541	0.141570560577024	-1,85897E+14	4,89383E+14	4,79893E+14	5,62273E+14	5,34836E+14	6,02136E+14	6,07425E+14
TC0X00001447.mm.2	Htr2c	5-hydroxytryptamine (serotonin) receptor 2C	1,16966E+14	8,34086E+14	4,01901E+14	0.00603708735690192	0.141570560577024	-1,85983E+14	8,82318E+14	8,94174E+14	8,37925E+14	8,47539E+14	7,68013E+14	7,74546E+14
TC0300002722.mm.2	Gstm7	glutathione S-transferase, mu 7	1,36508E+14	7,14493E+14	4,01841E+14	0.00604144736402815	0.141570560577024	-1,86049E+13	8,37105E+14	8,09779E+14	6,62239E+14	6,03966E+13	6,91455E+14	6,82413E+14
TC0500001599.mm.2	Alkbh4	alkB homolog 4, lysine demethylase	-1,45829E+14	7,1658E+13	-4,01809E+14	0.00604378136578507	0.141570560577024	-1,86085E+14	6,29401E+14	6,44735E+14	7,67846E+14	6,91704E+14	7,74379E+13	7,91414E+14
TC0X00003402.mm.2	Bhlhb9	basic helix-loop-helix domain containing, class B9	1,17715E+14	7,10028E+14	4,01791E+14	0.00604502557701655	0.141570560577024	-1,86104E+14	7,92277E+14	7,84067E+14	6,62112E+13	6,80796E+14	6,71597E+14	6,69316E+14
TC0500002406.mm.2	Dhx15	DEAH (Asp-Glu-Ala-His) box polypeptide 15	2,38503E+14	6,56301E+13	4,01361E+14	0.00607620481267032	0.141570560577024	-1,86578E+13	8,13192E+14	8,02778E+14	6,64188E+14	5,18683E+13	6,27614E+14	5,1135E+14
TC0600002404.mm.2	Hpgds	hematopoietic prostaglandin D synthase	1,92926E+14	5,73513E+14	4,00934E+14	0.00610735195537024	0.141570560577024	-1,87048E+14	6,40993E+14	7,1373E+13	5,42357E+14	5,75125E+14	4,27872E+14	5,40999E+14
TC0200002496.mm.2	Ccn5	cellular communication network factor 5	-1,38516E+14	8,25808E+14	-4,00863E+14	0.00611253005903843	0.141570560577024	-1,87127E+14	7,4574E+14	7,50495E+14	8,18698E+14	8,6665E+14	8,62053E+14	9,11214E+14
TC0400004146.mm.2	Mmp23	matrix metallopeptidase 23	-1,55588E+14	6,56649E+14	-4,0086E+14	0.00611275981013556	0.141570560577024	-1,8713E+14	5,87193E+14	5,28228E+14	6,6314E+13	7,34736E+14	7,1952E+14	7,07078E+14
TC1100000251.mm.2	Bcl11a	B cell CLL/lymphoma 11A (zinc finger protein)	1,37491E+14	8,01553E+14	4,00731E+14	0.00612219150195166	0.141570560577024	-1,87272E+14	8,92154E+14	8,90881E+13	7,92726E+14	7,25504E+14	7,54689E+14	7,53365E+14
TC0500002573.mm.2	Nfxl1	nuclear transcription factor, X-box binding-like 1	-1,43868E+14	8,17096E+14	-4,00537E+14	0.00613638989158634	0.141570560577024	-1,87485E+14	7,20968E+14	7,54544E+14	7,97118E+14	8,66697E+14	8,80335E+14	8,82914E+14
TC1600000387.mm.2	Opa1	OPA1, mitochondrial dynamin like GTPase	1,84103E+14	7,77246E+14	4,00344E+14	0.00615059100616635	0.141570560577024	-1,87698E+14	8,82943E+14	8,97333E+14	7,90313E+14	6,80818E+14	7,39141E+14	6,7293E+14
TC1300002498.mm.2	Arhgef28	Rho guanine nucleotide exchange factor (GEF) 28	1,40513E+14	6,40737E+14	4,00339E+14	0.00615099120281751	0.141570560577024	-1,87704E+14	7,40938E+14	7,22366E+14	6,34027E+14	5,6481E+13	5,86392E+12	5,95887E+14
TC0900002188.mm.2	Cd3g	CD3 antigen, gamma polypeptide	-1,77683E+14	4,73978E+14	-4,00254E+14	0.00615728836833971	0.141570560577024	-1,87799E+14	3,86794E+14	3,27203E+14	4,77476E+14	5,83032E+14	5,31736E+14	5,37627E+14
TC1400000298.mm.2	Selenok	selenoprotein K	1,27872E+14	8,52705E+14	4,00203E+14	0.00616102961489243	0.141570560577024	-1,87855E+14	9,432E+14	9,62503E+14	7,98177E+14	7,62388E+14	8,39633E+14	8,10326E+13
TC1800001175.mm.2	Slc23a1	solute carrier family 23 (nucleobase transporters), member 1	-1,21435E+14	4,90277E+14	-3,99873E+14	0.00618542638482211	0.141570560577024	-1,88219E+14	4,05356E+13	4,33817E+14	5,04411E+14	5,16037E+14	5,49656E+13	5,32387E+14
TC0900000285.mm.2	Arhgap32	Rho GTPase activating protein 32	2,08884E+14	8,84074E+14	3,99785E+14	0.00619194393672846	0.141570560577024	-1,88316E+14	1,01403E+14	1,03929E+14	8,69225E+14	7,46346E+14	8,64562E+14	7,70989E+14
TC0300002755.mm.2	Fndc7	fibronectin type III domain containing 7	-1,5551E+13	6,7898E+14	-3,99621E+14	0.00620416745711195	0.141570560577024	-1,88497E+14	5,9536E+14	5,78274E+14	6,83694E+14	7,31895E+14	7,0282E+14	7,81835E+14
TC1500000676.mm.2	Kdelr3	KDEL (Lys-Asp-Glu-Leu) endoplasmic reticulum protein retention receptor 3	-1,23203E+14	4,91059E+14	-3,99597E+14	0.00620589674094833	0.141570560577024	-1,88523E+14	4,19565E+14	4,38443E+14	5,09095E+14	4,74835E+13	5,56622E+13	5,47791E+14
TC1500000725.mm.2	Aco2	aconitase 2, mitochondrial	1,26429E+14	9,03294E+14	3,99453E+14	0.00621661924648982	0.141570560577024	-1,88682E+14	9,90626E+13	9,69559E+14	8,96872E+14	8,55379E+14	8,48653E+14	8,58674E+14

TC0100000469.mm.2	Cyp20a1	cytochrome P450, family 20, subfamily a, polypeptide 1	1,1937E+14	6,65977E+12	3,9922E+13	0.00623404216563802	0.141570560577024	-1,8894E+14	7,25698E+14	7,36659E+13	6,4128E+14	6,68609E+14	6,1367E+14	6,09948E+14
TC1500001742.mm.2	Top1mt	DNA topoisomerase 1, mitochondrial	1,80309E+14	6,38242E+14	3,99132E+13	0.00624061772704076	0.141570560577024	-1,89037E+14	7,41293E+14	7,3523E+14	6,72738E+14	5,64287E+14	5,889E+14	5,27005E+14
TC1000002256.mm.2	Hk1	hexokinase 1	2,3599E+14	8,17284E+14	3,99101E+14	0.00624295509337158	0.141570560577024	-1,89072E+14	9,83337E+14	9,70364E+13	8,25699E+14	6,42583E+14	7,53615E+14	7,28107E+14
TC1200000225.mm.2	Cmpk2	cytidine monophosphate (UMP-CMP) kinase 2, mitochondrial	1,29462E+13	6,42656E+13	3,99043E+14	0.00624732483005064	0.141570560577024	-1,89136E+13	7,29867E+13	6,99633E+14	6,46286E+14	6,09575E+14	5,98111E+14	5,72466E+13
TC0700002846.mm.2	Lrp3	low density lipoprotein receptor-related protein 3	1,36583E+14	7,81886E+14	3,98897E+14	0.00625823777784932	0.141570560577024	-1,89297E+14	8,63941E+14	8,6836E+13	7,27613E+14	7,72268E+14	7,54301E+14	7,04834E+14
TC1100001628.mm.2	Cnp	2',3'-cyclic nucleotide 3' phosphodiesterase	1,60144E+14	8,58759E+14	3,98732E+14	0.00627063897827748	0.141570560577024	-1,8948E+13	9,94307E+14	9,709E+14	8,12488E+14	7,29944E+14	8,48762E+14	7,96156E+14
TC1700000271.mm.2	Zfp51	zinc finger protein 51	1,21093E+14	5,60548E+14	3,98702E+14	0.00627289541888992	0.141570560577024	-1,89513E+14	6,47181E+14	6,3705E+13	5,2955E+14	5,07465E+14	5,08219E+14	5,33825E+14
TC0900003120.mm.2	Fam240a	family with sequence similarity 240 member A	-1,68212E+14	7,03866E+14	-3,9865E+14	0.00627680200577477	0.141570560577024	-1,8957E+14	5,99023E+14	5,93583E+14	7,03245E+14	7,98311E+13	7,3617E+14	7,92862E+14
TC0900002322.mm.2	Acsbg1	acyl-CoA synthetase bubblegum family member 1	1,30244E+14	9,00323E+14	3,98608E+14	0.00628001884335254	0.141570560577024	-1,89617E+14	9,82194E+13	9,79178E+14	8,64381E+14	8,75302E+14	8,7776E+14	8,23124E+14
TC0X00000398.mm.2	Stag2	stromal antigen 2	2,45051E+14	6,08457E+14	3,98592E+14	0.00628118793745461	0.141570560577024	-1,89635E+14	7,4402E+14	7,73078E+14	6,01184E+14	5,05461E+14	5,97145E+14	4,29853E+13
TC0500003679.mm.2	Zar1l	zygote arrest 1-like	-1,24486E+14	5,4967E+14	-3,98566E+14	0.00628312750359841	0.141570560577024	-1,89663E+14	4,72318E+13	4,7458E+14	5,57434E+14	5,97818E+14	6,06489E+14	5,8938E+14
TC0600002385.mm.2	Gprin3	GPRIN family member 3	2,15936E+14	5,88319E+14	3,98497E+14	0.00628832334853455	0.141570560577024	-1,89739E+13	7,21015E+14	7,03741E+14	6,11047E+14	5,01223E+14	5,57243E+14	4,35641E+14
TC1300000161.mm.2	Trim27	tripartite motif-containing 27	1,46405E+14	5,57097E+14	3,98148E+14	0.00631479133860594	0.141570560577024	-1,90127E+14	6,74734E+13	6,52606E+14	5,29688E+14	4,51023E+14	5,22131E+14	5,12398E+13
TC1100003771.mm.2	Nt5c3b	5'-nucleotidase, cytosolic IIIB	1,89818E+13	7,49131E+14	3,98023E+14	0.00632424548328099	0.141570560577024	-1,90264E+14	8,59988E+14	8,54746E+14	7,70743E+14	6,74209E+14	7,15734E+14	6,19364E+14
TC0700001875.mm.2	Itgam	integrin alpha M	1,54996E+12	6,63941E+13	3,9795E+14	0.00632978361722791	0.141570560577024	-1,90345E+14	7,30963E+14	7,87411E+14	6,35339E+14	6,21554E+14	5,67055E+14	6,41327E+14
TC0800001770.mm.2	Csmd1	CUB and Sushi multiple domains 1	-1,28793E+14	9,47527E+14	-3,97821E+14	0.00633963749212196	0.141570560577024	-1,90489E+14	8,63797E+14	8,74384E+14	9,52555E+14	9,98657E+14	1,01038E+14	9,85385E+14
TC0600003277.mm.2	Gm8882/Gm4736/Prb1/LOC100502592	predicted gene 8882/predicted gene 4736/proline-rich protein BstNI subfamily 1/proline-rich proteoglycan 2-like	-1,42257E+14	6,44417E+14	-3,97606E+14	0.00635598951008325	0.141570560577024	-1,90726E+14	5,5293E+14	5,56011E+14	6,49059E+14	7,15048E+14	6,77481E+14	7,15973E+14
TC1100001899.mm.2	Nup85	nucleoporin 85	1,37477E+14	5,09667E+14	3,97462E+14	0.00636704573390721	0.141570560577024	-1,90886E+14	6,06051E+14	5,47084E+14	5,26029E+14	5,00654E+14	4,26362E+14	4,5182E+14
TC1800000240.mm.2	Sap130	Sin3A associated protein	1,30695E+14	7,08484E+14	3,97458E+14	0.00636735812808333	0.141570560577024	-1,90891E+14	7,84375E+14	7,85638E+14	7,09558E+14	6,62712E+14	6,70839E+14	6,37782E+14
TC0100003560.mm.2	Ncstn	nicastrin	1,39109E+14	7,58075E+14	3,97256E+14	0.00638285565081763	0.141570560577024	-1,91115E+14	8,43133E+13	8,25834E+14	7,48832E+14	7,39902E+14	7,29922E+14	6,60827E+14
TC0900002958.mm.2	Cep63	centrosomal protein 63	1,44095E+14	5,82107E+14	3,97083E+14	0.00639613057427167	0.141570560577024	-1,91307E+14	6,91874E+14	6,44336E+14	5,84956E+14	5,23454E+14	5,15702E+14	5,32319E+13
TC0X00002482.mm.2	Pls3	plastin 3 (T-isoform)	2,23072E+13	7,87754E+14	3,96956E+14	0.00640587459558124	0.141570560577024	-1,91447E+14	9,14639E+14	9,33691E+14	8,0475E+14	6,71257E+14	7,5544E+14	6,46746E+14
TC1700000323.mm.2	Srrm2	serine/arginine repetitive matrix 2	2,61874E+14	9,04288E+14	3,96848E+13	0.00641418696111071	0.141570560577024	-1,91567E+14	1,07019E+14	1,07198E+14	8,94461E+14	7,70668E+14	8,95901E+14	7,22525E+14
TC1000001809.mm.2	Arfgef3	ARFGEF family member 3	1,32855E+13	6,50832E+14	3,96376E+14	0.00645073820889583	0.141570560577024	-1,92091E+13	7,47973E+14	7,27014E+13	6,20534E+14	6,00194E+14	6,31923E+13	5,77354E+14
TC0300002098.mm.2	4931440P22Rik	RIKEN cDNA 4931440P22 gene	-1,7315E+14	8,76493E+14	-3,96348E+14	0.00645289664073071	0.141570560577024	-1,92122E+14	7,563E+14	7,68479E+14	8,80229E+14	9,82869E+14	9,07058E+14	9,64021E+14
TC1500000793.mm.2	Arhgap8	Rho GTPase activating protein 8	-1,45146E+14	7,01375E+14	-3,96333E+13	0.00645408210495256	0.141570560577024	-1,92139E+14	6,14411E+14	6,10239E+14	6,93754E+14	7,74902E+14	7,60173E+14	7,54769E+14
TC0800000587.mm.2	Vegfc	vascular endothelial growth factor C	-1,4163E+14	8,60109E+13	-3,96295E+14	0.00645708026496588	0.141570560577024	-1,92182E+13	7,71796E+13	7,69347E+14	8,62148E+14	9,32963E+14	8,98162E+14	9,26241E+14
TC1500001119.mm.2	Hoxc9	homeobox C9	-1,42496E+14	7,47599E+14	-3,96266E+13	0.00645930641094779	0.141570560577024	-1,92214E+13	6,58892E+14	6,66292E+14	7,48099E+14	8,02133E+14	7,77525E+14	8,3265E+14
TC1400001545.mm.2	Myoz1	myozenin 1	-1,67359E+14	6,26145E+14	-3,96259E+14	0.00645987268411018	0.141570560577024	-1,92222E+14	5,01165E+14	5,01191E+14	6,85153E+14	7,32287E+14	6,18282E+12	7,18792E+13
TC1700001987.mm.2	Mrps18b	mitochondrial ribosomal protein S18B	1,63246E+14	6,52239E+14	3,96099E+14	0.0064722878633851	0.141570560577024	-1,92399E+13	7,31996E+14	7,17171E+14	6,89233E+14	6,52362E+14	5,12224E+14	6,10451E+14
TC0100001175.mm.2	Pm20d1	peptidase M20 domain containing 1	-1,8233E+14	5,90616E+14	-3,95819E+14	0.00649417931368887	0.141570560577024	-1,9271E+14	4,76748E+14	4,77574E+14	5,87165E+14	6,83227E+14	6,16352E+14	7,02631E+14
TC1000000498.mm.2	Gm9956	predicted gene 9956	-1,51769E+14	5,04606E+14	-3,95793E+14	0.00649621435892923	0.141570560577024	-1,92739E+14	3,98823E+14	3,90473E+14	5,30332E+13	6,15177E+14	5,3041E+14	5,62423E+13
TC1300002468.mm.2	Sv2c	synaptic vesicle glycoprotein 2c	2,40971E+14	6,62899E+14	3,95759E+14	0.00649884425703118	0.141570560577024	-1,92777E+14	8,02653E+14	8,19338E+14	6,59021E+14	5,56331E+14	6,51019E+13	4,8903E+14
TC0100001779.mm.2	Rab3gap2	RAB3 GTPase activating protein subunit 2	1,4198E+14	6,52961E+14	3,95731E+14	0.00650105839583962	0.141570560577024	-1,92808E+14	7,55804E+14	7,4014E+13	6,41722E+14	5,68114E+13	6,1427E+14	5,97714E+14
TC1200002267.mm.2	Ddx24	DEAD (Asp-Glu-Ala-Asp) box polypeptide 24	1,7752E+14	8,49656E+13	3,95636E+14	0.00650845427694579	0.141570560577024	-1,92913E+14	9,60854E+14	9,58201E+14	8,49813E+14	7,65053E+13	8,26964E+14	7,37052E+14
TC0800001917.mm.2	Ddhd2	DDHD domain containing 2	1,41209E+14	7,53386E+14	3,95601E+14	0.00651123655569119	0.141570560577024	-1,92953E+14	8,47145E+13	8,37481E+14	7,50415E+14	6,83068E+14	7,17986E+14	6,84221E+13
TC1100000037.mm.2	Sf3a1	splicing factor 3a, subunit 1	1,76004E+14	7,57032E+14	3,95432E+14	0.00652453813076918	0.141570560577024	-1,93141E+14	8,54812E+14	8,39885E+14	7,97056E+14	7,07753E+14	7,1216E+14	6,30528E+14
TC0500000710.mm.2	Kit	KIT proto-oncogene receptor tyrosine kinase	1,86515E+14	6,04407E+14	3,95411E+14	0.0065261380098926	0.141570560577024	-1,93163E+14	7,51934E+14	7,07081E+14	5,58578E+14	5,22863E+14	4,82347E+14	6,03637E+14
TC1100001704.mm.2	Nmt1	N-myristoyltransferase 1	1,80269E+14	8,02088E+14	3,95368E+14	0.00652958251423804	0.141570560577024	-1,93212E+14	9,27817E+14	9,36259E+14	7,84112E+14	6,60798E+14	7,37256E+14	7,66282E+14
TC0500003249.mm.2	Pitpnm2	phosphatidylinositol transfer protein, membrane-associated 2	1,17372E+14	7,17692E+14	3,95149E+14	0.00654684239676497	0.141570560577024	-1,93456E+14	7,91521E+14	8,06081E+14	6,6257E+12	6,83122E+14	6,86307E+14	6,76551E+14
TC1900000257.mm.2	Olfr1444	olfactory receptor 1444	-1,36495E+14	5,21829E+14	-3,95052E+14	0.00655443344491346	0.141570560577024	-1,93563E+14	4,36411E+14	4,06674E+14	5,56293E+14	6,15522E+14	5,48773E+14	5,673E+14
TC1500000980.mm.2	4930415O20Rik	RIKEN cDNA 4930415O20 gene	-1,743E+14	5,53812E+14	-3,94814E+14	0.00657332465673155	0.141570560577024	-1,93829E+14	4,50335E+14	4,35742E+14	5,76419E+14	6,257E+12	5,63008E+14	6,71669E+14
TC0900001463.mm.2	Setd2	SET domain containing 2	1,22945E+14	6,26928E+14	3,94676E+14	0.00658427307634902	0.141570560577024	-1,93982E+13	6,98933E+13	7,10721E+14	6,08858E+14	5,79294E+14	5,96455E+14	5,6731E+14
TC0200000955.mm.2	Csrnp3	cysteine-serine-rich nuclear protein 3	1,74632E+14	8,02348E+14	3,9465E+14	0.00658629278578139	0.141570560577024	-1,9401E+14	8,88691E+14	9,08008E+14	8,04658E+14	7,65296E+14	7,79937E+14	6,67497E+14
TC0X00003401.mm.2	Gprasp2	G protein-coupled receptor associated sorting protein 2	2,21363E+14	8,44275E+13	3,94548E+14	0.00659443692055869	0.141570560577024	-1,94125E+14	1,01431E+14	9,7568E+14	8,42209E+14	6,86186E+14	8,05381E+14	7,41885E+14
TC1200000197.mm.2	Taf1b	TATA-box binding protein associated factor, RNA polymerase I, B	1,52091E+14	6,60477E+14	3,94447E+14	0.00660249987857402	0.141570560577024	-1,94237E+14	7,49305E+14	7,44013E+14	6,76437E+14	6,03973E+14	6,22959E+14	5,66177E+14
TC1100001372.mm.2	Ccdc182	coiled-coil domain containing 182	-1,15551E+14	5,57282E+14	-3,94421E+14	0.00660451769397465	0.141570560577024	-1,94266E+14	5,03868E+14	4,85212E+14	5,65462E+14	5,68966E+13	6,06203E+14	6,13979E+13
TC1000002983.mm.2	Iltifb	interleukin 10-related T cell-derived inducible factor beta	-1,24617E+14	5,87727E+14	-3,94264E+14	0.00661708214863888	0.141570560577024	-1,94441E+14	5,1597E+14	5,06583E+14	6,11489E+14	6,20534E+14	6,58594E+14	6,13194E+14
TC1800000509.mm.2	Hsd17b4	hydroxysteroid (17-beta) dehydrogenase 4	1,24087E+14	6,87924E+14	3,94037E+14	0.00663525792585618	0.141570560577024	-1,94694E+14	7,68748E+14	7,70619E+14	6,3449E+14	6,62494E+14	6,6396E+14	6,27231E+14
TC0100002071.mm.2	Fam135a	family with sequence similarity 135, member A	1,2385E+13	5,86786E+14	3,93996E+14	0.00663853796185972	0.141570560577024	-1,9474E+14	6,72827E+14	6,29466E+13	5,89081E+14	5,74747E+14	5,28428E+14	5,26165E+14
TC1200001348.mm.2	Efr3b	EFR3 homolog B	1,67056E+14	6,8167E+14	3,93916E+14	0.0066449076397565	0.141570560577024	-1,94829E+14	8,2555E+14	7,97916E+14	6,42782E+13	5,34416E+14	6,46006E+14	6,43348E+12
TC0200000060.mm.2	Usp6nl	USP6 N-terminal like	1,28993E+14	6,79067E+14	3,93877E+14	0.00664801941868551	0.141570560577024	-1,94872E+14	7,55004E+14	7,64041E+14	6,7495E+14	6,1935E+14	6,29637E+14	6,31422E+14
TC0100002950.mm.2	Epb41l5	erythrocyte membrane protein band 4.1 like 5	1,79966E+14	5,5624E+14	3,93782E+14	0.0066556751490389	0.141570560577024	-1,94978E+14	6,61184E+14	6,37102E+14	5,94377E+14	5,06421E+14	5,137E+14	4,24655E+14
TC0200004906.mm.2	Defb19	defensin beta 19	-1,45731E+14	5,06717E+14	-3,93772E+14	0.00665645952388221	0.141570560577024	-1,94989E+14	4,2041E+14	4,06521E+14	5,07856E+14	5,8712E+14	5,47771E+14	5,70621E+14
TC1200001698.mm.2	Gm1818	predicted gene 1818	1,60311E+14	7,4426E+14	3,93485E+14	0.00667957276184961	0.141570560577024	-1,95309E+14	8,32222E+14	8,39458E+14	7,17326E+14	7,25498E+14	7,27034E+14	6,24024E+14
TC1000001010.mm.2	Washc4	WASH complex subunit 4	1,52584E+14	5,24192E+14	3,93446E+14	0.0066827195247027	0.141570560577024	-1,95353E+14	6,22491E+14	6,03167E+14	4,83059E+14	5,15943E+14	4,17611E+14	5,0288E+14
TC0500001521.mm.2	Crcp	calcitonin gene-related peptide-receptor component protein	1,1905E+13	7,075E+14	3,93196E+14	0.00670297673361188	0.141570560577024	-1,95632E+14	7,72068E+14	7,51012E+14	7,05177E+14	7,31761E+13	6,40123E+13	6,44857E+14
TC1500000718.mm.2	Mchr1	melanin-concentrating hormone receptor 1	1,5819E+14	5,87208E+14	3,93195E+14	0.00670308793886682	0.141570560577024	-1,95634E+12	6,74584E+14	6,81389E+14	6,13599E+14	5,14081E+14	5,27698E+14	5,11896E+14
TC1100000327.mm.2	Nsg2	neuron specific gene family member 2	1,23575E+14	9,07901E+14	3,93111E+14	0.00670987641936362	0.141570560577024	-1,95727E+14	9,87408E+14	9,77787E+14	9,03787E+13	8,60379E+14	8,53672E+14	8,64373E+14
TC1100003790.mm.2	Stat3	signal transducer and activator of transcription 3	1,39717E+14	6,85036E+14	3,93071E+14	0.00671311052521154	0.141570560577024	-1,95772E+14	7,69454E+14	8,10602E+14	6,41336E+14	5,88201E+14	6,33614E+14	6,67009E+14
TC0400000314.mm.2	Cga	glycoprotein hormones, alpha subunit	-1,51963E+14	5,34468E+14	-3,93021E+14	0.00671716473693721	0.141570560577024	-1,95828E+14	4,36043E+14	4,39715E+14	5,29511E+14	6,21855E+14	5,85685E+14	5,93999E+14
TC0X00001178.mm.2	Pcdh11x	protocadherin 11 X-linked	2,16413E+14	6,03485E+14	3,93012E+14	0.00671787918206975	0.141570560577024	-1,95837E+14	7,67928E+14	7,11427E+14	5,57724E+14	5,373E+14	6,01958E+12	4,44572E+14

TC0700000678.mm.2	Tshz3	teashirt zinc finger family member 3	-1,16861E+14	8,538E+14	-3,92965E+13	0.00672171502921769	0.141570560577024	-1,9589E+14	7,79486E+14	8,04747E+14	8,55086E+14	8,65527E+14	9,10481E+14	9,07473E+14
TC0100000591.mm.2	Apol7d	apolipoprotein L 7d	-2,0462E+13	5,67605E+14	-3,92858E+14	0.0067304336103214	0.141570560577024	-1,9601E+14	4,2092E+14	4,54864E+14	5,48403E+13	6,96417E+14	6,251E+14	6,59924E+14
TC1200001990.mm.2	Actn1	actinin, alpha 1	1,19938E+14	6,64983E+14	3,92728E+14	0.00674103418850063	0.141570560577024	-1,96155E+14	7,36764E+14	7,62068E+14	6,23322E+14	6,08786E+14	6,40946E+14	6,18011E+14
TC1700000275.mm.2	Zfp948	zinc finger protein 948	1,68981E+14	5,95364E+14	3,92566E+14	0.00675424090300261	0.141570560577024	-1,96336E+14	7,19349E+14	7,04008E+14	5,83183E+14	4,8025E+14	5,67102E+14	5,18292E+14
TC1700001019.mm.2	1700061G19Rik	RIKEN cDNA 1700061G19 gene	-2,07537E+14	5,58172E+14	-3,92492E+14	0.00676027374481894	0.141570560577024	-1,96419E+13	4,56654E+13	4,09875E+12	5,43977E+14	6,56922E+14	5,89819E+14	6,91785E+14
TC0400001607.mm.2	Map3k6	mitogen-activated protein kinase kinase kinase 6	-1,7149E+13	4,57561E+14	-3,92479E+14	0.00676130547190864	0.141570560577024	-1,96433E+14	3,55266E+14	3,68846E+14	4,22293E+14	5,31866E+14	5,12654E+14	5,54439E+14
TC0300000397.mm.2	Stoml3	stomatin (Epb7.2)-like 3	2,06756E+14	6,15052E+14	3,92421E+14	0.00676608010325363	0.141570560577024	-1,96498E+14	7,37801E+14	7,51844E+14	6,20704E+13	5,03827E+14	4,85088E+14	5,91046E+14
TC1800000332.mm.2	Ankhd1/Eif4ebp3	ankyrin repeat and KH domain containing 1/eukaryotic translation initiation factor 4E binding protein 3	1,48043E+14	6,58892E+14	3,92354E+14	0.00677155003261579	0.141570560577024	-1,96573E+14	7,18671E+14	7,49699E+14	6,80683E+14	6,32016E+14	6,19164E+13	5,5312E+14
TC0700000762.mm.2	Etfb/Mir7051	electron transferring flavoprotein, beta polypeptide/microRNA 7051	-1,28554E+14	7,88396E+14	-3,92203E+14	0.00678392670931749	0.141570560577024	-1,96741E+14	7,11621E+14	7,12144E+14	7,88101E+14	8,37635E+14	8,2678E+14	8,54094E+14
TC0400004001.mm.2	Miip	migration and invasion inhibitory protein	-1,68282E+14	6,01243E+14	-3,92177E+14	0.00678609710226424	0.141570560577024	-1,96771E+14	4,80552E+14	5,0379E+14	6,05308E+14	6,96901E+14	6,28306E+14	6,926E+13
TC0200004564.mm.2	Mall	mal, T cell differentiation protein-like	-1,38971E+14	7,5312E+14	-3,92029E+14	0.00679827636427061	0.141570560577024	-1,96937E+14	6,77863E+14	6,49139E+14	7,80826E+14	8,05951E+14	7,70503E+14	8,3444E+14
TC0400001686.mm.2	Htr1d	5-hydroxytryptamine (serotonin) receptor 1D	1,1469E+14	6,76122E+14	3,92024E+14	0.00679864541985644	0.141570560577024	-1,96942E+14	7,59106E+14	7,40697E+14	6,39688E+14	6,46818E+14	6,34901E+14	6,35523E+14
TC0X00002246.mm.2	Zfp36l3	zinc finger protein 36, C3H type-like 3	-1,46165E+14	5,0197E+12	-3,92015E+14	0.00679943695157376	0.141570560577024	-1,96952E+14	4,3025E+14	3,99874E+14	4,91715E+14	5,6753E+14	5,49066E+14	5,73387E+14
TC0X00002454.mm.2	Gm6880	predicted gene 6880	-1,2587E+14	4,96167E+13	-3,91862E+14	0.00681200861927905	0.141570560577024	-1,97123E+14	4,42288E+14	4,20357E+14	4,76701E+14	5,23275E+14	5,56538E+14	5,57846E+14
TC1100002511.mm.2	Rars	arginyl-tRNA synthetase	1,59426E+14	7,02447E+13	3,9186E+14	0.00681217141072894	0.141570560577024	-1,97125E+14	8,06092E+13	8,1807E+14	6,92143E+14	5,93065E+14	6,65984E+14	6,39325E+14
TC0700004585.mm.2	Cdkn1c	cyclin-dependent kinase inhibitor 1C (P57)	-1,22081E+14	6,89607E+14	-3,91818E+14	0.00681560412089924	0.141570560577024	-1,97172E+14	6,13455E+13	6,05277E+14	7,06991E+14	7,49026E+14	7,34367E+14	7,28527E+14
TC0500000813.mm.2	Utp3	UTP3 small subunit processome component	1,18139E+14	7,08383E+14	3,91792E+12	0.00681781988099273	0.141570560577024	-1,97202E+14	7,98979E+14	7,74086E+14	6,78339E+14	6,62107E+13	6,75338E+14	6,61449E+14
TC1200000139.mm.2	Pdia6	protein disulfide isomerase associated 6	1,63865E+13	7,4016E+14	3,91668E+14	0.0068279885314243	0.141570560577024	-1,9734E+14	8,45518E+14	8,45741E+14	7,29731E+14	6,5644E+14	7,22147E+13	6,41382E+14
TC0X00001004.mm.2	Dmrtc1c2/Dmrtc1c1	DMRT-like family C1c2/DMRT-like family C1c1	-1,9681E+14	6,4724E+14	-3,91622E+14	0.00683183919921278	0.141570560577024	-1,97392E+14	5,16842E+14	5,29659E+13	6,43713E+14	7,53104E+14	6,7073E+14	7,6939E+13
TC0900001471.mm.2	Prss43	protease, serine 43	-1,14069E+14	6,00195E+13	-3,91599E+14	0.00683374016363492	0.141570560577024	-1,97418E+14	5,2258E+14	5,21343E+14	6,43864E+14	6,41323E+14	6,2756E+14	6,44501E+14
TC0200002678.mm.2	Rbm38	RNA binding motif protein 38	-1,32246E+13	7,02909E+14	-3,91395E+14	0.00685067769122213	0.141570560577024	-1,97646E+13	6,19306E+14	6,003E+14	7,43063E+14	7,70686E+14	7,14933E+14	7,69164E+14
TC0700000792.mm.2	Klk1b24	kallikrein 1-related peptidase b24	-1,22703E+14	4,85986E+14	-3,91269E+14	0.00686108842407038	0.141570560577024	-1,97787E+14	4,26706E+14	4,06084E+14	4,97809E+13	5,07121E+14	5,19612E+14	5,58585E+14
TC0800002368.mm.2	Zfp868	zinc finger protein 868	1,30947E+14	5,92049E+14	3,90966E+14	0.00688637742772222	0.141570560577024	-1,98127E+14	7,00801E+14	6,87892E+14	5,30258E+14	5,06544E+14	5,91152E+14	5,35646E+14
TC1700000482.mm.2	Clpsl2	colipase-like 2	-1,69613E+14	7,3617E+14	-3,90587E+14	0.00691807723929549	0.141570560577024	-1,98552E+14	6,36721E+14	6,26914E+14	7,22297E+14	8,28225E+14	7,77356E+14	8,25506E+14
TC1900001777.mm.2	Ndufb8	NADH:ubiquinone oxidoreductase subunit B8	-1,14509E+14	8,69549E+14	-3,90557E+14	0.00692057411099393	0.141570560577024	-1,98585E+14	7,9951E+14	8,10762E+13	8,79603E+14	8,88131E+14	9,1192E+14	9,2737E+14
TC1000002175.mm.2	Man1a	mannosidase 1, alpha	1,4532E+14	7,04272E+14	3,90484E+14	0.00692671988590366	0.141570560577024	-1,98667E+14	7,91509E+14	8,1939E+14	6,87269E+14	6,07206E+14	6,58743E+14	6,61514E+14
TC0500001531.mm.2	Caln1	calneuron 1	1,66224E+14	7,60014E+14	3,90317E+14	0.00694074156999594	0.141570560577024	-1,98854E+14	8,606E+14	8,71638E+13	7,66066E+14	6,61992E+14	6,78948E+14	7,20841E+14
TC1300001670.mm.2	Aldh5a1	aldhehyde dehydrogenase family 5, subfamily A1	1,58525E+14	7,29451E+14	3,90234E+14	0.00694770020486243	0.141570560577024	-1,98947E+14	8,4191E+14	8,03451E+14	7,48247E+14	6,54789E+14	6,54244E+14	6,74067E+14
TC1000000547.mm.2	Psap	prosaposin	1,50171E+14	1,05685E+14	3,90119E+14	0.00695746747554314	0.141570560577024	-1,99077E+13	1,1487E+14	1,1542E+14	1,06103E+14	9,74579E+14	1,01657E+13	9,85994E+14
TC1500000775.mm.2	Bik	BCL2-interacting killer	-1,50889E+14	8,01453E+14	-3,90094E+14	0.00695950257641466	0.141570560577024	-1,99104E+14	6,84394E+13	7,21893E+14	8,07141E+14	8,87227E+14	8,39848E+14	8,68216E+14
TC0700004265.mm.2	Xpo6	exportin 6	2,26204E+14	7,8081E+14	3,90024E+14	0.00696543692166215	0.141570560577024	-1,99182E+14	9,2777E+14	8,99572E+14	7,9736E+14	6,85227E+14	7,56884E+14	6,1805E+14
TC0200001935.mm.2	Mertk	MER proto-oncogene tyrosine kinase	1,68468E+14	6,01443E+14	3,89813E+14	0.00698328976602727	0.141570560577024	-1,99419E+14	7,08402E+13	7,12836E+13	5,72717E+14	5,30401E+14	4,88314E+14	5,95989E+14
TC0100002764.mm.2	Ngef	neuronal guanine nucleotide exchange factor	1,48676E+14	8,40081E+14	3,89794E+14	0.00698489969882017	0.141570560577024	-1,99441E+14	9,25389E+13	9,29232E+13	8,34698E+14	7,93898E+14	8,18677E+14	7,38591E+14
TC0300003142.mm.2	Miga1	mitoguardin 1	1,72022E+14	7,34499E+14	3,89607E+14	0.00700077602359833	0.141570560577024	-1,99651E+14	8,22462E+14	8,23252E+14	7,84468E+13	6,75145E+14	6,75849E+14	6,25821E+14
TC0800002525.mm.2	Hhip	Hedgehog-interacting protein	1,52355E+14	6,98455E+13	3,8953E+14	0.00700733044376	0.141570560577024	-1,99737E+14	7,6359E+14	8,0665E+14	7,07664E+14	6,47294E+14	6,62592E+14	6,02937E+14
TC0100003879.mm.2	Als2	alsin Rho guanine nucleotide exchange factor	1,20128E+14	5,57568E+14	3,89424E+14	0.00701634764816231	0.141570560577024	-1,99856E+14	6,26896E+14	6,03097E+14	5,4689E+14	5,78789E+14	4,91838E+14	4,97898E+14
TC0100000224.mm.2	Ptpn18	protein tyrosine phosphatase, non-receptor type 18	-1,40496E+14	5,45148E+14	-3,89321E+14	0.00702512177502691	0.141570560577024	-1,99972E+13	4,54622E+14	4,89408E+14	5,24637E+14	5,77198E+14	5,8928E+14	6,35742E+14
TC0500002229.mm.2	Poln/Haus3	DNA polymerase N/HAUS augmin-like complex, subunit 3	-1,34397E+14	5,58705E+14	-3,89297E+14	0.00702720717855033	0.141570560577024	-1,99999E+14	4,46327E+14	4,92806E+14	5,91314E+14	6,13854E+14	5,81367E+14	6,26561E+14
TC1300000232.mm.2	Vmn1r221	vomeronasal 1 receptor 221	-1,2798E+14	5,3588E+14	-3,89218E+14	0.00703393570445061	0.141570560577024	-2,00088E+14	4,63953E+14	4,55309E+14	5,40813E+14	5,79982E+14	5,67126E+14	6,08095E+13
TC0700000070.mm.2	Zfp444	zinc finger protein 444	-1,56035E+14	8,02762E+14	-3,89036E+14	0.0070495388926579	0.141570560577024	-2,00293E+14	7,17416E+14	6,91134E+14	8,11186E+13	8,76217E+14	8,24421E+14	8,96198E+14
TC0400001472.mm.2	AU040320	expressed sequence AU040320	1,25377E+14	7,19412E+14	3,8903E+14	0.00705003374823687	0.141570560577024	-2,00299E+14	7,99821E+14	7,96749E+14	7,07368E+14	6,66718E+14	6,8933E+14	6,56486E+14
TC0800003248.mm.2	1190005I06Rik	RIKEN cDNA 1190005I06 gene	-1,17915E+14	7,65016E+14	-3,89027E+14	0.00705026570614177	0.141570560577024	-2,00302E+14	6,87028E+14	6,86945E+14	7,85866E+14	8,20455E+14	8,04275E+14	8,05528E+14
TC0200003187.mm.2	Card9	caspase recruitment domain family, member 9	-1,67791E+14	9,33013E+14	-3,88954E+14	0.00705657289025279	0.141570560577024	-2,00385E+14	8,28894E+13	8,29373E+14	9,18706E+14	1,02725E+14	9,77342E+14	1,01651E+14
TC0400001608.mm.2	Slc9a1/Mir5122	solute carrier family 9 (sodium/hydrogen exchanger), member 1/microRNA 5122	2,19273E+14	7,38669E+14	3,88793E+14	0.00707037369207012	0.141570560577024	-2,00566E+14	8,62193E+14	8,72105E+14	7,81326E+14	6,20637E+14	6,80938E+14	6,14813E+14
TC1100000053.mm.2	Nipsnap1	nipsnap homolog 1	1,13852E+14	8,08318E+14	3,88729E+14	0.00707582424640691	0.141570560577024	-2,00637E+14	8,99907E+14	8,91937E+13	7,38765E+14	7,55157E+14	7,77532E+14	7,86609E+14
TC1700002138.mm.2	Runx2	runt related transcription factor 2	-1,53292E+14	7,24106E+14	-3,88564E+14	0.00709007825081056	0.141570560577024	-2,00823E+13	6,10202E+14	6,58558E+14	7,22118E+14	7,78412E+14	7,5648E+14	8,18863E+13
TC0X00001047.mm.2	Uprt	uracil phosphoribosyltransferase	2,35848E+14	7,30737E+13	3,88512E+14	0.00709450554798557	0.141570560577024	-2,00881E+14	8,75999E+14	8,6099E+14	7,7209E+13	6,10051E+14	6,83381E+14	5,8191E+14
TC1100004274.mm.2	Rpl19	ribosomal protein L19	1,25416E+14	9,64042E+14	3,88306E+14	0.00711235100814184	0.141570560577024	-2,01114E+14	1,06259E+14	1,03889E+14	8,92427E+14	9,39703E+14	9,26024E+14	9,2462E+14
TC1800000616.mm.2	Rps14	ribosomal protein S14	1,49573E+14	8,53738E+14	3,87969E+14	0.00714157206709668	0.141570560577024	-2,01493E+14	9,55517E+14	9,65087E+14	8,17173E+14	7,63194E+14	8,47786E+14	7,73672E+14
TC1000001293.mm.2	Bbs10	Bardet-Biedl syndrome 10 (human)	-1,21311E+14	5,59445E+14	-3,87923E+13	0.00714552797872485	0.141570560577024	-2,01544E+14	4,82493E+14	4,96346E+14	5,59687E+14	5,96682E+14	6,2072E+14	6,00741E+14
TC0100003533.mm.2	Fcer1g	Fc receptor, IgE, high affinity I, gamma polypeptide	1,94737E+14	7,13325E+14	3,87838E+13	0.00715290794181463	0.141570560577024	-2,0164E+14	8,26166E+14	8,85426E+14	6,90637E+14	5,55601E+14	6,6496E+14	6,57159E+14
TC0300000139.mm.2	Hltf	helicase-like transcription factor	1,17449E+13	6,21851E+14	3,87654E+14	0.00716900718947873	0.141570560577024	-2,01848E+14	6,9539E+14	7,21763E+14	5,65077E+14	5,66622E+14	5,80059E+14	6,02196E+14
TC1100002912.mm.2	Rnf112	ring finger protein 112	1,14695E+13	7,16967E+14	3,87337E+14	0.00719664647334555	0.141570560577024	-2,02204E+14	7,7498E+13	7,70041E+14	7,32989E+13	7,08164E+14	6,5997E+14	6,5566E+14
TC1600000998.mm.2	Olig2	oligodendrocyte transcription factor 2	1,17933E+14	8,15581E+14	3,87315E+13	0.00719860389037878	0.141570560577024	-2,02229E+14	8,81092E+14	9,12636E+14	7,71134E+13	7,70759E+14	7,8775E+14	7,70113E+13
TC1300002505.mm.2	Tmem174	transmembrane protein 174	-1,74304E+14	5,68783E+14	-3,87308E+14	0.00719925566762501	0.141570560577024	-2,02238E+14	4,82233E+14	4,53775E+14	5,45962E+14	6,46108E+14	6,08427E+14	6,7619E+14
TC0700000870.mm.2	Nomo1	nodal modulator 1	1,22623E+14	7,33354E+14	3,87179E+14	0.00721058889844194	0.141570560577024	-2,02383E+14	8,20317E+14	8,30921E+14	6,93512E+14	6,49384E+14	7,10462E+13	6,95529E+14
TC1100003245.mm.2	Abr	active BCR-related gene	1,51659E+14	8,79661E+14	3,87151E+14	0.00721299055391234	0.141570560577024	-2,02414E+14	9,62691E+14	9,57805E+14	8,99285E+14	8,41004E+14	7,70848E+14	8,46331E+13
TC1100002158.mm.2	Camk2b	calcium/calmodulin-dependent protein kinase II, beta	2,27497E+14	1,04676E+13	3,86886E+14	0.00723633222083177	0.141570560577024	-2,02713E+14	1,19492E+14	1,1995E+14	1,05887E+14	8,8781E+14	1,00352E+14	9,35914E+14
TC0X00000212.mm.2	Uba1	ubiquitin-like modifier activating enzyme 1	1,88489E+14	8,88758E+14	3,86742E+14	0.00724902349835401	0.141570560577024	-2,02875E+14	1,00264E+14	1,01317E+14	9,02735E+13	7,75184E+14	8,49214E+14	7,89611E+12
TC1100001577.mm.2	Gsdma	gasdermin A	-1,5966E+14	5,29614E+14	-3,86649E+14	0.00725721668092696	0.141570560577024	-2,0298E+14	3,8543E+13	4,78699E+14	5,44911E+14	5,85197E+14	5,75988E+14	6,07461E+14
TC0400003856.mm.2	Igsf21	immunoglobulin superfamily, member 21	1,38336E+14	7,56795E+14	3,86647E+14	0.00725743441704724	0.141570560577024	-2,02983E+13	8,52892E+12	8,65203E+13	7,2413E+14	6,57123E+14	7,38064E+14	7,03359E+14
TC1100000604.mm.2	Gdf9	growth differentiation factor 9	-1,56673E+14	6,13324E+14	-3,86635E+14	0.00725846373995767	0.141570560577024	-2,02996E+14	5,41652E+14	5,19766E+14	5,74621E+14	6,69139E+14	6,70887E+14	7,03878E+14

TC0100002936.mm.2	Tsn	translin	1,43659E+14	7,19681E+14	3,86598E+14	0.00726175306426741	0.141570560577024	-2,03038E+14	8,10445E+14	7,9046E+14	7,35669E+14	6,67926E+14	6,79995E+13	6,33592E+14
TC1500002171.mm.2	Olfr285/Olfr257	olfactory receptor 285/olfactory receptor 257	-1,3273E+14	4,70834E+14	-3,86581E+14	0.00726327730602165	0.141570560577024	-2,03057E+14	3,99107E+14	4,09745E+14	4,49065E+14	4,92773E+14	5,12056E+14	5,62257E+14
TC0500002777.mm.2	Gm16427	predicted gene 16427	-1,63623E+14	5,82398E+14	-3,86489E+14	0.00727137896427289	0.141570560577024	-2,03161E+14	4,4326E+14	5,01766E+13	5,9712E+13	6,79967E+13	6,12968E+14	6,59304E+14
TC1100002066.mm.2	Tbcd	tubulin-specific chaperone d	1,47564E+14	7,50207E+14	3,86426E+13	0.00727700132620732	0.141570560577024	-2,03232E+14	8,60704E+14	8,37897E+14	7,39368E+14	6,59802E+13	7,19669E+14	6,83803E+14
TC1700001450.mm.2	Slc22a1	solute carrier family 22 (organic cation transporter), member 1	-1,2244E+14	6,23986E+14	-3,86415E+13	0.00727793491458995	0.141570560577024	-2,03244E+14	5,74638E+14	5,39299E+14	6,20274E+14	6,50888E+14	6,74189E+14	6,84627E+14
TC1700001996.mm.2	Gm11127	predicted gene 11127	-2,05709E+14	6,01449E+14	-3,86319E+14	0.00728649454146284	0.141570560577024	-2,03353E+14	4,58416E+14	4,79165E+14	5,82269E+14	7,39846E+14	6,62373E+14	6,86625E+14
TC0600001536.mm.2	Gabarapl1	gamma-aminobutyric acid (GABA) A receptor-associated protein-like 1	1,25059E+13	1,01577E+14	3,86308E+14	0.00728743843974699	0.141570560577024	-2,03365E+14	1,09874E+14	1,0957E+14	9,96505E+14	9,5935E+14	9,91341E+12	9,52986E+14
TC1500001183.mm.2	Slc1a3	solute carrier family 1 (glial high affinity glutamate transporter), member 3	1,26185E+14	8,02432E+12	3,86044E+12	0.00731093609898796	0.141570560577024	-2,03663E+14	9,02278E+13	8,84449E+14	7,71905E+14	7,21607E+13	7,7548E+13	7,58876E+14
TC0200002439.mm.2	Ppp1r16b	protein phosphatase 1, regulatory subunit 16B	1,54273E+14	7,93622E+14	3,86011E+14	0.00731386845539208	0.141570560577024	-2,037E+14	8,93265E+14	8,86099E+14	8,02864E+14	7,08684E+14	7,51253E+14	7,19565E+14
TC1500001785.mm.2	Cyhr1	cysteine and histidine rich 1	1,38635E+14	6,311E+14	3,85877E+14	0.00732590715426022	0.141570560577024	-2,03853E+13	7,31304E+14	7,05357E+14	6,23964E+14	5,66587E+14	5,57236E+14	6,02155E+14
TC1400002267.mm.2	Ptk2b	PTK2 protein tyrosine kinase 2 beta	1,16516E+14	7,35256E+14	3,85572E+14	0.00735316495532901	0.141570560577024	-2,04197E+14	8,0804E+13	8,30855E+14	6,94521E+14	6,72254E+14	7,08191E+14	6,97673E+13
TC1900001115.mm.2	Tmem132a	transmembrane protein 132A	1,62724E+14	6,53681E+14	3,85537E+14	0.00735632122049665	0.141570560577024	-2,04236E+14	7,42485E+14	7,60014E+14	6,6876E+14	5,73777E+14	5,61301E+14	6,15751E+14
TC1700001866.mm.2	Olfr1564	olfactory receptor 1564	-1,35102E+14	4,4445E+14	-3,85494E+14	0.00736020869288016	0.141570560577024	-2,04285E+14	3,68412E+14	3,89535E+14	4,06647E+14	4,73954E+14	5,06954E+14	5,21196E+14
TC0700001169.mm.2	Pde8a	phosphodiesterase 8A	2,98213E+13	5,41021E+14	3,8543E+13	0.00736596073316992	0.141570560577024	-2,04358E+14	6,99453E+14	7,21718E+14	5,7033E+14	4,29881E+14	5,19789E+14	3,04956E+13
TC0400000183.mm.2	Pnisr	PNN interacting serine/arginine-rich	1,19253E+14	7,88902E+14	3,85382E+14	0.00737026438383662	0.141570560577024	-2,04412E+14	8,60117E+14	8,6352E+14	7,47223E+14	7,77424E+14	7,56473E+14	7,28659E+14
TC0500000310.mm.2	Atraid	all-trans retinoic acid induced differentiation factor	1,59146E+14	7,78452E+14	3,85314E+14	0.00737635044621529	0.141570560577024	-2,04488E+14	9,02663E+12	9,04233E+14	7,30211E+14	6,45002E+14	7,76159E+14	7,12445E+14
TC0700002414.mm.2	Selenow	selenoprotein W	1,17489E+14	9,69391E+14	3,85311E+14	0.00737668624835547	0.141570560577024	-2,04492E+14	1,05851E+14	1,04E+14	9,15998E+14	9,38318E+13	9,42935E+14	9,20591E+14
TC1600000484.mm.2	Ropn1	ropporin, rhophilin associated protein 1	-1,52712E+14	5,57302E+14	-3,85198E+14	0.00738679836579541	0.141570560577024	-2,04619E+14	4,59092E+14	4,41286E+13	5,82919E+14	6,5471E+14	5,70785E+14	6,35018E+14
TC1100003983.mm.2	E030025P04Rik	RIKEN cDNA E030025P04 gene	-1,86087E+14	5,81269E+14	-3,84926E+13	0.00741137693326224	0.141570560577024	-2,04927E+14	4,98443E+14	4,40758E+14	5,71687E+14	6,65347E+14	6,12849E+14	6,98527E+14
TC1500000543.mm.2	Them6	thioesterase superfamily member 6	-1,29247E+13	8,46686E+14	-3,84854E+14	0.007417938610273	0.141570560577024	-2,05009E+14	7,81167E+14	7,50603E+14	9,03212E+13	8,54872E+14	9,07549E+13	8,82714E+14
TC0700003065.mm.2	Mrgprb4	MAS-related GPR, member B4	-1,43237E+14	4,96934E+14	-3,84804E+14	0.00742242521612751	0.141570560577024	-2,05065E+14	4,24176E+14	4,16505E+14	4,6463E+14	5,49135E+14	5,64395E+14	5,6276E+14
TC0700001101.mm.2	Agbl1	ATP/GTP binding protein-like 1	-1,56588E+14	5,52099E+14	-3,8477E+14	0.00742550184019906	0.141570560577024	-2,05104E+13	4,62882E+14	4,48905E+14	5,38405E+14	6,37437E+14	6,25525E+14	5,99439E+14
TC0300003217.mm.2	Pex11b	peroxisomal biogenesis factor 11 beta	1,91146E+14	7,06031E+13	3,84637E+14	0.00743764563107067	0.141570560577024	-2,05255E+13	8,49846E+14	7,9543E+14	6,88192E+14	6,39734E+14	6,94412E+14	5,68573E+14
TC0900001433.mm.2	Ccdc51	coiled-coil domain containing 51	-1,28572E+13	7,03436E+14	-3,84595E+14	0.00744145550384556	0.141570560577024	-2,05302E+14	6,23371E+14	6,31915E+13	6,96659E+14	7,56238E+13	7,50442E+14	7,61989E+14
TC0700003724.mm.2	Ccdc83	coiled-coil domain containing 83	-1,1541E+14	5,2379E+14	-3,84476E+14	0.00745228485514825	0.141570560577024	-2,05437E+14	4,39874E+14	4,54904E+14	5,49455E+14	5,72909E+14	5,55819E+14	5,6978E+14
TC0X00002166.mm.2	Zfp280c	zinc finger protein 280C	1,55741E+14	6,07622E+14	3,84471E+14	0.00745268087385617	0.141570560577024	-2,05442E+14	7,045E+14	7,25052E+14	5,7045E+14	5,2766E+14	6,04402E+14	5,13667E+14
TC1100003438.mm.2	Med13	mediator complex subunit 13	1,82327E+14	6,23168E+14	3,84436E+13	0.00745587065859683	0.141570560577024	-2,05482E+14	7,28092E+14	7,53004E+14	6,32876E+14	5,08593E+13	5,81911E+14	5,34533E+14
TC0700002461.mm.2	Psg17	pregnancy specific glycoprotein 17	-1,74522E+14	5,3064E+14	-3,84336E+14	0.00746499889978353	0.141570560577024	-2,05595E+14	4,2607E+14	4,03624E+14	5,3265E+14	6,42758E+14	5,6076E+14	6,17978E+14
TC1500001920.mm.2	St13	suppression of tumorigenicity 13	1,2827E+14	7,80119E+14	3,84326E+14	0.00746588683872949	0.141570560577024	-2,05606E+14	8,64913E+14	8,53649E+14	7,55471E+14	7,44661E+14	7,60442E+14	7,0158E+14
TC0900002113.mm.2	Sorl1	sortilin-related receptor, LDLR class A repeats-containing	1,80094E+14	7,77865E+14	3,84278E+14	0.00747028421207372	0.141570560577024	-2,05661E+14	9,11753E+14	9,095E+14	7,56305E+14	6,28566E+14	7,48619E+14	7,12446E+14
TC1800000816.mm.2	Smad2	SMAD family member 2	1,51267E+14	6,32864E+14	3,84225E+14	0.0074751460035763	0.141570560577024	-2,05721E+14	7,12181E+14	7,05686E+14	6,65159E+14	5,98827E+14	5,92575E+14	5,22758E+14
TC0200001608.mm.2	Kcna4	potassium voltage-gated channel, shaker-related subfamily, member 4	1,54485E+14	6,41069E+14	3,84104E+14	0.00748617285740948	0.141570560577024	-2,05858E+14	7,31918E+14	7,57828E+14	6,35251E+14	5,40638E+14	6,02261E+14	5,78515E+14
TC0800000763.mm.2	Pde4c	phosphodiesterase 4C, cAMP specific	-1,4177E+14	7,60404E+14	-3,83946E+14	0.00750064423104829	0.141570560577024	-2,06037E+14	6,69999E+14	6,50532E+14	7,81535E+14	8,56287E+14	7,86619E+14	8,17452E+14
TC1500001480.mm.2	Csmd3	CUB and Sushi multiple domains 3	1,33538E+14	5,50876E+14	3,83873E+14	0.00750741162583431	0.141570560577024	-2,0612E+14	6,59662E+14	6,26954E+14	4,75526E+14	5,23579E+14	5,29564E+14	4,89975E+14
TC0100003102.mm.2	Btg2	B cell translocation gene 2, anti-proliferative	-1,41015E+13	6,10583E+14	-3,83859E+14	0.00750864288730282	0.141570560577024	-2,06135E+14	5,17184E+14	5,14698E+14	6,79263E+14	6,38441E+14	6,21367E+14	6,92545E+14
TC0600003107.mm.2	Clstn3	calsyntenin 3	1,59723E+13	7,87324E+13	3,83812E+14	0.00751298017696083	0.141570560577024	-2,06189E+14	9,09338E+14	8,79185E+14	7,78025E+14	6,88318E+14	7,60514E+14	7,08562E+13
TC0500003025.mm.2	Crybb2	crystallin, beta B2	-1,48229E+14	8,00708E+13	-3,83715E+14	0.00752193628357393	0.141570560577024	-2,06299E+14	6,96194E+14	7,15816E+14	8,09507E+13	8,74263E+14	8,22625E+14	8,85843E+13
TC1300002093.mm.2	Hnrnpa0	heterogeneous nuclear ribonucleoprotein A0	1,71105E+14	7,07414E+14	3,83498E+14	0.00754188651443724	0.141570560577024	-2,06545E+14	8,28445E+14	8,08788E+14	7,11769E+14	6,00458E+14	6,71008E+14	6,24016E+14
TC0900003021.mm.2	Manf/Rbm15b	mesencephalic astrocyte-derived neurotrophic factor/RNA binding motif protein 15B	1,12433E+14	7,86711E+14	3,83494E+14	0.00754228925243311	0.141570560577024	-2,0655E+13	8,58897E+14	8,70742E+14	7,3989E+13	7,45964E+14	7,60447E+14	7,44326E+14
TC1800000636.mm.2	Il17b	interleukin 17B	-1,251E+14	7,81631E+14	-3,83478E+14	0.00754376921229727	0.141570560577024	-2,06568E+14	6,89023E+14	7,20124E+14	7,93337E+14	8,27957E+14	8,14642E+14	8,44706E+14
TC1800000708.mm.2	Psmg2	proteasome (prosome, macropain) assembly chaperone 2	1,72881E+14	6,71071E+14	3,83443E+14	0.00754700529399333	0.141570560577024	-2,06608E+13	7,69745E+14	7,61687E+14	6,60012E+14	6,49315E+13	6,54777E+14	5,30892E+14
TC0600000124.mm.2	Tes	testis derived transcript	-1,40903E+14	6,50653E+14	-3,8329E+14	0.00756107013523397	0.141570560577024	-2,0678E+14	5,58117E+14	5,84138E+14	6,30611E+14	7,06992E+14	7,04094E+14	7,19967E+14
TC0300001956.mm.2	Ndufc1	NADH:ubiquinone oxidoreductase subunit C1	1,73719E+14	9,31577E+13	3,83262E+14	0.00756370780830494	0.141570560577024	-2,06813E+14	1,07131E+14	1,05239E+14	8,90727E+14	7,98767E+14	9,2994E+13	8,46327E+14
TC1600001134.mm.2	Coro7	coronin 7	1,35967E+14	7,10216E+14	3,83232E+14	0.00756646378201378	0.141570560577024	-2,06847E+14	8,05993E+14	7,82993E+14	6,91191E+14	6,64069E+14	6,25712E+14	6,9134E+14
TC1700000656.mm.2	Hspa1l	heat shock protein 1-like	-1,20689E+13	4,94691E+14	-3,83069E+14	0.00758160213601304	0.141570560577024	-2,07032E+14	4,51216E+13	4,73228E+14	4,5005E+13	4,27829E+14	5,65807E+14	6,00016E+14
TC0100002032.mm.2	Il17f	interleukin 17F	-1,53962E+14	5,57663E+14	-3,83041E+14	0.00758417961513701	0.141570560577024	-2,07063E+14	4,59907E+14	4,74267E+14	5,37524E+14	6,32179E+14	6,05274E+14	6,36824E+14
TC1700002027.mm.2	H2-M10.4	histocompatibility 2, M region locus 10.4	-1,17996E+14	5,0558E+14	-3,82723E+14	0.007613784224057	0.141570560577024	-2,07425E+14	4,29158E+13	4,38945E+14	5,18315E+14	5,42965E+14	5,37016E+14	5,67078E+14
TC1100000955.mm.2	Psmb6	proteasome (prosome, macropain) subunit, beta type 6	1,1728E+14	8,31343E+14	3,82609E+14	0.0076243759858709	0.141570560577024	-2,07554E+14	9,04941E+14	8,97959E+14	8,1043E+14	8,06389E+14	8,02888E+14	7,65452E+14
TC1300000045.mm.2	Idi1	isopentenyl-diphosphate delta isomerase	1,95172E+14	6,20003E+14	3,82479E+14	0.0076365963492511	0.141570560577024	-2,07702E+14	7,42099E+14	7,2379E+14	6,37735E+14	5,4085E+14	5,95109E+14	4,80436E+14
TC0700000557.mm.2	Clip3	CAP-GLY domain containing linker protein 3	1,11995E+14	8,03101E+14	3,82314E+14	0.00765198710735702	0.141570560577024	-2,07889E+14	8,66947E+14	8,64238E+14	7,81702E+14	7,98523E+13	7,47928E+14	7,59267E+14
TC1400002266.mm.2	Ephx2	epoxide hydrolase 2, cytoplasmic	1,83437E+13	5,35423E+14	3,82276E+14	0.00765560170803069	0.141570560577024	-2,07933E+14	6,56939E+14	6,26321E+14	5,31183E+14	4,81711E+14	3,95719E+14	5,20667E+14
TC0200003347.mm.2	Gapvd1	GTPase activating protein and VPS9 domains 1	1,97005E+14	6,86952E+14	3,82015E+14	0.00768004858382006	0.141570560577024	-2,08228E+14	7,89096E+14	8,05911E+14	7,19718E+14	6,05986E+14	6,51412E+13	5,49587E+14
TC0400001177.mm.2	Dmrta2	doublesex and mab-3 related transcription factor like family A2	-1,14923E+14	6,26233E+14	-3,82013E+14	0.00768032209933438	0.141570560577024	-2,08232E+14	5,47873E+14	5,54793E+14	6,45999E+14	6,76219E+14	6,70155E+14	6,62357E+14
TC0500001632.mm.2	Sap25/Lrch4/Gm20605	sin3 associated polypeptide/leucine-rich repeats and calponin homology (CH) domain containing 4/predicted gene 20605	-1,5731E+14	8,72472E+13	-3,81877E+14	0.00769308649049426	0.141570560577024	-2,08386E+14	7,60598E+14	7,77233E+14	9,62089E+14	8,8246E+14	9,60099E+14	8,92353E+14
TC1600000667.mm.2	Ccdc80	coiled-coil domain containing 80	-1,3687E+14	5,77419E+13	-3,81775E+14	0.00770272545792462	0.141570560577024	-2,08502E+14	4,66212E+14	5,2241E+14	5,92019E+14	6,21511E+14	6,09474E+14	6,52887E+14
TC1100003455.mm.2	Cltc	clathrin, heavy polypeptide (Hc)	2,40903E+14	9,49515E+14	3,81686E+14	0.00771117151328609	0.141570560577024	-2,08603E+14	1,11356E+14	1,09682E+14	9,55938E+14	8,02201E+14	9,28903E+14	7,99669E+14
TC0500000732.mm.2	Paics	phosphoribosylaminoimidazole carboxylase, phosphoribosylaminoribosylaminoimidazole, succinocarboxamide synthetase	1,57281E+14	8,13914E+14	3,81507E+14	0.007728099217003	0.141570560577024	-2,08807E+14	9,10128E+14	9,10611E+14	8,15734E+14	7,40835E+14	7,9061E+14	7,15567E+14
TC0600003016.mm.2	Lrtm2	leucine-rich repeats and transmembrane domains 2	1,4406E+14	6,59745E+14	3,81349E+14	0.00774310899794001	0.141570560577024	-2,08987E+14	7,73389E+14	7,47652E+14	6,35709E+14	5,68799E+14	6,41939E+14	5,90981E+13

TC1600000212.mm.2	Hic2	hypermethylated in cancer 2	-1,2857E+14	7,29159E+14	-3,81307E+14	0.00774709278961483	0.141570560577024	-2,09035E+14	6,48726E+13	6,29018E+13	7,51464E+14	8,10861E+13	7,62751E+14	7,72133E+14
TC1100001641.mm.2	Mlx	MAX-like protein X	1,8343E+14	6,76538E+14	3,81261E+14	0.00775144956360192	0.141570560577024	-2,09087E+13	7,90456E+14	7,77678E+14	7,03478E+14	5,86341E+13	6,36928E+14	5,64345E+13
TC1100003692.mm.2	Gjd3	gap junction protein, delta 3	-1,45525E+14	6,00773E+14	-3,81252E+14	0.00775231153829397	0.141570560577024	-2,09097E+14	5,0582E+14	5,03298E+14	6,0707E+14	6,88283E+14	6,30597E+14	6,6957E+14
TC0X00001282.mm.2	Rpl36a	ribosomal protein L36A	1,75007E+14	8,04187E+14	3,81217E+14	0.00775563707614107	0.141570560577024	-2,09137E+14	9,21772E+13	9,55776E+13	7,55323E+14	6,6472E+14	8,05964E+14	7,21569E+14
TC0900002907.mm.2	4930579K19Rik	RIKEN cDNA 4930579K19 gene	-1,11005E+14	5,64451E+14	-3,8108E+14	0.00776866708487152	0.141570560577024	-2,09293E+14	5,12476E+14	5,11735E+14	5,59179E+14	5,57097E+14	6,31628E+14	6,14593E+13
TC1900000758.mm.2	Smc3	structural maintenance of chromosomes 3	1,26732E+14	6,5247E+13	3,80958E+14	0.00778029280155329	0.141570560577024	-2,09431E+14	7,52283E+14	7,53597E+14	5,90369E+13	5,66153E+14	6,53718E+14	5,98697E+14
TC1100002818.mm.2	Olfr30	olfactory receptor 30	-1,1049E+14	5,17535E+14	-3,80871E+13	0.00778865639346326	0.141570560577024	-2,09531E+14	4,60432E+14	4,55029E+14	5,24164E+14	5,29144E+13	5,62059E+14	5,74382E+14
TC1500001887.mm.2	Npcd/Cbx6/Nptxr	neuronal pentraxin chromo domain/chromobox 6/neuronal pentraxin receptor	1,35436E+14	8,19247E+14	3,80859E+14	0.00778978498261796	0.141570560577024	-2,09544E+14	9,02165E+14	9,02017E+14	8,13836E+14	7,64152E+14	7,94841E+14	7,38471E+13
TC0700002748.mm.2	Ovol3	ovo like zinc finger 3	-1,14372E+14	6,46571E+14	-3,80856E+14	0.00779005001487789	0.141570560577024	-2,09548E+14	5,83036E+14	5,53874E+14	6,83686E+14	6,93172E+13	6,79652E+14	6,86003E+13
TC0500001301.mm.2	Nos1	nitric oxide synthase 1, neuronal	1,77117E+14	6,1781E+14	3,80772E+14	0.00779810646625734	0.141570560577024	-2,09643E+14	7,18899E+14	7,1368E+14	5,86971E+14	6,08962E+14	4,7456E+13	6,03785E+14
TC1400002152.mm.2	Micu2	mitochondrial calcium uptake 2	1,14267E+14	6,54998E+14	3,80772E+14	0.00779811162444875	0.141570560577024	-2,09644E+14	6,72549E+14	7,03287E+13	7,05227E+14	7,01623E+14	5,7235E+14	5,74952E+14
TC0700002696.mm.2	Zfp36	zinc finger protein 36	-1,50566E+13	6,37028E+14	-3,80621E+14	0.00781258863920259	0.141570560577024	-2,09816E+14	5,19672E+14	5,70183E+14	7,08262E+14	6,33065E+14	6,77451E+14	7,13535E+14
TC1900001203.mm.2	Psat1	phosphoserine aminotransferase 1	1,91002E+14	7,6293E+14	3,80488E+14	0.00782530271933637	0.141570560577024	-2,09967E+14	8,935E+14	9,08087E+14	7,46202E+14	6,10208E+14	7,37564E+14	6,82019E+14
TC0900000752.mm.2	Gramd2	GRAM domain containing 2	-1,56244E+14	5,9379E+14	-3,80357E+14	0.00783788613384776	0.141570560577024	-2,10116E+14	4,86415E+14	4,95195E+13	6,01586E+14	6,85444E+14	6,15523E+14	6,78575E+13
TC1000000517.mm.2	Ranbp2	RAN binding protein 2	2,02307E+14	6,1824E+14	3,80272E+14	0.00784605983280121	0.141570560577024	-2,10212E+14	7,49321E+13	7,646E+14	6,03271E+13	4,82942E+13	6,07263E+14	5,02044E+14
TC0200000354.mm.2	Pdss1	prenyl (solanesyl) diphosphate synthase, subunit 1	-1,33892E+14	5,5612E+14	-3,80135E+14	0.00785928500205915	0.141570560577024	-2,10369E+12	4,87445E+14	4,47373E+14	6,10369E+13	5,88933E+13	6,29905E+14	5,72697E+14
TC0600002154.mm.2	Try5	trypsin 5	-1,88924E+14	4,75158E+13	-3,7977E+12	0.00789468975488109	0.141570560577024	-2,10786E+14	3,89857E+14	3,47748E+14	4,75044E+13	5,22847E+14	4,92243E+14	6,23211E+14
TC1400000524.mm.2	Peli2	pellino 2	1,35132E+14	6,18263E+14	3,79723E+14	0.00789922509813949	0.141570560577024	-2,10839E+14	6,76616E+14	6,69712E+14	6,66296E+14	6,20893E+13	5,08194E+14	5,6787E+14
TC0300003212.mm.2	Gon4l	gon-4-like (C.elegans)	2,31093E+14	5,6382E+14	3,79722E+14	0.00789931113120678	0.141570560577024	-2,1084E+14	6,99803E+14	7,1777E+12	5,7247E+14	4,37488E+14	5,45654E+14	4,09733E+14
TC0700003923.mm.2	Olfr642	olfactory receptor 642	-1,61284E+14	5,09642E+13	-3,79717E+14	0.00789974710622628	0.141570560577024	-2,10845E+14	4,16726E+14	4,16243E+13	5,13735E+14	5,55613E+12	5,25732E+14	6,29806E+14
TC0300001449.mm.2	Gtf2b	general transcription factor IIB	1,12503E+14	5,82998E+14	3,79708E+14	0.00790065918405616	0.141570560577024	-2,10856E+14	6,55696E+14	6,37561E+14	5,72693E+14	5,6379E+14	5,42258E+14	5,25992E+14
TC0200002663.mm.2	Mc3r	melanocortin 3 receptor	1,49728E+14	5,35298E+14	3,79604E+14	0.00791080523826698	0.141570560577024	-2,10975E+13	6,27942E+14	5,88423E+14	5,49574E+14	5,28937E+14	5,01477E+14	4,15432E+13
TC0600000765.mm.2	Ndnf	neuron-derived neurotrophic factor	1,89421E+14	5,82967E+14	3,79564E+14	0.00791464581347867	0.141570560577024	-2,1102E+14	6,96367E+14	7,218E+14	5,70825E+13	4,69483E+14	5,70888E+14	4,68438E+14
TC0X00001322.mm.2	Tceal9	transcription elongation factor A like 9	1,37665E+12	8,1668E+14	3,79412E+14	0.00792947210655522	0.141570560577024	-2,11194E+14	9,27027E+14	8,79523E+14	7,6607E+14	7,96237E+14	7,37558E+14	7,93662E+14
TC1900001545.mm.2	Cox15	cytochrome c oxidase assembly protein 15	1,60607E+14	6,00549E+14	3,79359E+14	0.00793464307035133	0.141570560577024	-2,11254E+13	6,78748E+14	7,04232E+14	6,3264E+14	5,25906E+14	5,20974E+14	5,40793E+14
TC1000001691.mm.2	Ipcef1	interaction protein for cytohesin exchange factors 1	1,37959E+13	6,53247E+14	3,7912E+14	0.00795798568931184	0.141570560577024	-2,11527E+14	7,19072E+14	7,64035E+14	6,16738E+14	6,12445E+14	6,36658E+14	5,70531E+14
TC0300002414.mm.2	2310050C09Rik	RIKEN cDNA 2310050C09 gene	-1,27319E+14	5,02287E+14	-3,79117E+14	0.00795826578966454	0.141570560577024	-2,1153E+14	4,23147E+14	4,4093E+14	4,88903E+14	5,42027E+14	5,45674E+14	5,73042E+14
TC0600002084.mm.2	Dgki	diacylglycerol kinase, iota	2,65604E+14	6,79353E+14	3,79099E+14	0.00796001974179109	0.141570560577024	-2,11551E+14	8,29705E+14	8,39399E+14	6,85099E+14	5,84016E+14	6,71665E+13	4,66232E+14
TC0X00001201.mm.2	Cldn34c1	claudin 34C1	-1,17772E+14	8,20078E+14	-3,79062E+14	0.00796370436658375	0.141570560577024	-2,11594E+14	7,49433E+14	7,40923E+14	8,32853E+13	8,71356E+14	8,55167E+13	8,70733E+14
TC0700001874.mm.2	Trim72	tripartite motif-containing 72	-1,36262E+14	6,0484E+14	-3,79012E+14	0.00796857916026517	0.141570560577024	-2,11651E+14	4,87836E+14	5,13337E+14	6,45754E+14	7,08414E+14	6,17945E+14	6,55751E+14
TC1100000754.mm.2	Drg2	developmentally regulated GTP binding protein 2	1,87004E+14	7,13399E+14	3,78915E+14	0.00797804059653696	0.141570560577024	-2,11761E+14	8,24414E+14	8,14741E+14	7,59707E+14	6,16387E+13	6,36875E+14	6,28271E+13
TC0100002405.mm.2	Sumo1	small ubiquitin-like modifier 1	1,1648E+14	8,31202E+14	3,78886E+13	0.00798091710615849	0.141570560577024	-2,11794E+14	9,18273E+14	8,79612E+14	8,12798E+14	8,11604E+14	7,79242E+14	7,85683E+14
TC0X00000172.mm.2	H2al1n	H2A histone family member L1N	-1,62186E+14	5,59506E+14	-3,78786E+14	0.00799077441533968	0.141570560577024	-2,11909E+14	4,6291E+14	4,79657E+14	5,31394E+14	6,16139E+14	5,96392E+14	6,70548E+14
TC0500000675.mm.2	Nipal1	NIPA-like domain containing 1	-1,28887E+14	6,03752E+14	-3,78716E+14	0.00799763661396743	0.141570560577024	-2,11989E+13	5,12378E+13	5,13173E+14	6,249E+14	6,88735E+14	6,48783E+14	6,34542E+14
TC0500003533.mm.2	Tnrc18	trinucleotide repeat containing 18	1,1316E+14	6,68865E+14	3,78614E+14	0.00800773956820171	0.141570560577024	-2,12106E+14	7,5903E+14	7,37941E+14	6,16146E+14	6,29418E+14	6,27418E+14	6,43234E+14
TC1000000815.mm.2	Fgf22	fibroblast growth factor 22	-1,31373E+14	7,77916E+14	-3,78521E+14	0.00801687001190732	0.141570560577024	-2,12212E+14	6,92483E+14	6,91769E+14	7,9095E+14	8,45299E+14	8,01589E+14	8,45408E+14
TC0900002898.mm.2	Clstn2	calsyntenin 2	1,53241E+14	7,51596E+14	3,78518E+14	0.00801713589232077	0.141570560577024	-2,12215E+14	8,34789E+14	8,75579E+14	7,34979E+14	6,60343E+14	7,30348E+14	6,73537E+14
TC1400001115.mm.2	Pcdh17	protocadherin 17	1,96296E+14	7,40781E+13	3,78468E+14	0.00802208028738445	0.141570560577024	-2,12272E+14	8,48735E+13	8,31691E+14	7,69647E+14	7,06781E+14	7,14162E+14	5,73672E+14
TC0900000210.mm.2	Prkcsh	protein kinase C substrate 80K-H	1,26618E+14	7,35167E+14	3,78405E+14	0.0080282817431962	0.141570560577024	-2,12344E+14	8,16115E+14	7,78748E+14	7,40678E+14	7,33839E+14	6,94995E+14	6,4663E+14
TC0600002797.mm.2	Tmf1	TATA element modulatory factor 1	2,6527E+14	5,68009E+14	3,78314E+14	0.00803728639035008	0.141570560577024	-2,12448E+14	7,34408E+14	7,20794E+14	5,77728E+14	4,50462E+14	5,57509E+14	3,67154E+14
TC1600001054.mm.2	Ripply3	ripply transcriptional repressor 3	-1,89089E+13	7,08152E+14	-3,78264E+14	0.00804222848884018	0.141570560577024	-2,12505E+14	5,67219E+14	6,06596E+13	6,90945E+14	8,3216E+14	7,73325E+14	7,78667E+14
TC0800001522.mm.2	Tubb3	tubulin, beta 3 class III	1,50585E+14	9,14821E+14	3,78262E+14	0.00804243745984137	0.141570560577024	-2,12507E+14	1,02495E+14	9,89125E+14	9,2222E+14	8,39719E+14	8,76916E+14	8,35993E+14
TC0600002055.mm.2	Cyren	cell cycle regulator of NHEJ	-1,91098E+14	7,25711E+14	-3,78221E+14	0.00804656073335558	0.141570560577024	-2,12555E+14	5,84305E+14	6,2324E+14	7,0656E+14	8,50422E+14	7,94056E+14	7,95685E+14
TC0700002703.mm.2	Acp7	acid phosphatase 7, tartrate resistant	-1,30816E+14	6,50713E+14	-3,78207E+14	0.00804793654659644	0.141570560577024	-2,12571E+14	5,68283E+14	5,52535E+14	6,66905E+14	7,34106E+14	6,90097E+14	6,92354E+14
TC1700002527.mm.2	Srd5a2	steroid 5 alpha-reductase 2	-1,16785E+14	5,4566E+14	-3,78205E+14	0.00804814292677103	0.141570560577024	-2,12573E+14	4,77618E+14	4,47836E+14	5,85979E+14	6,03505E+14	5,68891E+14	5,90133E+14
TC0900000888.mm.2	Vps13c	vacuolar protein sorting 13C	2,87957E+14	5,86501E+14	3,78045E+12	0.0080639766122219	0.141570560577024	-2,12756E+14	7,53443E+14	7,85523E+14	5,70505E+14	4,46485E+14	5,90604E+14	3,72448E+14
TC0700000275.mm.2	Pglyrp1	peptidoglycan recognition protein 1	-1,20279E+14	6,4424E+14	-3,78045E+13	0.00806400842315229	0.141570560577024	-2,12756E+14	5,86356E+14	5,74092E+14	6,38742E+14	6,65244E+14	6,80828E+14	7,20179E+14
TC1300002015.mm.2	Ror2	receptor tyrosine kinase-like orphan receptor 2	-1,23249E+14	6,34387E+14	-3,78015E+14	0.00806700776256275	0.141570560577024	-2,12791E+14	5,85373E+14	5,74482E+14	6,35053E+14	6,05061E+14	7,25738E+14	6,80615E+14
TC0100003380.mm.2	Suco	SUN domain containing ossification factor	1,55905E+14	6,35605E+14	3,77952E+14	0.00807321914395158	0.141570560577024	-2,12862E+14	7,57229E+14	7,09739E+14	6,33296E+14	5,58207E+14	6,06617E+14	5,48542E+14
TC0X00001283.mm.2	Hnrnph2	heterogeneous nuclear ribonucleoprotein H2	1,56785E+14	7,82725E+14	3,77916E+14	0.00807676914130597	0.141570560577024	-2,12903E+14	9,03294E+14	8,88967E+14	7,60556E+14	6,64843E+14	7,62542E+14	7,16149E+14
TC1600002171.mm.2	Popdc2	popeye domain containing 2	-1,46418E+14	5,18323E+14	-3,77893E+14	0.0080790929593532	0.141570560577024	-2,1293E+14	4,3848E+14	4,3859E+14	5,06466E+14	5,56497E+14	5,45624E+13	6,24282E+14
TC0700000461.mm.2	Prx	periaxin	-1,27524E+14	6,27554E+14	-3,77863E+14	0.00808208868136686	0.141570560577024	-2,12964E+14	5,49346E+14	5,42892E+14	6,41441E+14	6,84358E+14	6,50473E+14	6,96813E+14
TC0100000282.mm.2	Npas2	neuronal PAS domain protein 2	1,94473E+14	7,04088E+14	3,77628E+14	0.00810555518109921	0.141570560577024	-2,13233E+14	8,23462E+14	8,12263E+14	7,4714E+14	5,94881E+14	6,30164E+14	6,16616E+14
TC0100001889.mm.2	Xkr4	X-linked Kx blood group related 4	2,57631E+14	6,40081E+14	3,77613E+14	0.00810704500976815	0.141570560577024	-2,13251E+14	7,76906E+14	8,12935E+14	6,21158E+14	5,54907E+14	6,41647E+14	4,32932E+14
TC0500003518.mm.2	Card11	caspase recruitment domain family, member 11	-1,31067E+13	5,34331E+14	-3,77542E+14	0.00811413617170016	0.141570560577024	-2,13332E+14	4,29878E+14	4,58143E+14	5,51966E+14	6,15844E+14	5,74172E+14	5,75984E+14
TC0900002735.mm.2	Col12a1	collagen, type XII, alpha 1	-1,41217E+14	7,33155E+14	-3,77452E+14	0.00812310795344541	0.141570560577024	-2,13434E+14	6,43437E+14	6,67954E+14	7,18067E+14	7,75649E+13	7,67467E+14	8,26357E+14
TC1700002719.mm.2	Lhcgr	luteinizing hormone/choriogonadotropin receptor	-1,6881E+14	4,35417E+14	-3,77427E+14	0.00812566600700605	0.141570560577024	-2,13464E+14	3,46985E+14	3,0051E+14	4,41298E+14	5,38595E+14	4,63044E+14	5,22071E+14
TC0500002305.mm.2	Nkx3-2	NK3 homeobox 2	-1,43677E+13	6,66702E+13	-3,77395E+13	0.00812879744716097	0.141570560577024	-2,13499E+14	5,98302E+14	5,67087E+14	6,71212E+14	7,10867E+14	6,89371E+14	7,63372E+14
TC0X00001054.mm.2	Pbdc1	polysaccharide biosynthesis domain containing 1	1,18474E+14	6,91724E+14	3,77356E+14	0.0081327519781147	0.141570560577024	-2,13545E+14	7,64539E+14	7,70817E+13	6,74851E+13	6,41727E+14	6,64393E+14	6,34014E+14
TC0X00000686.mm.2	Atp2b3	ATPase, Ca++ transporting, plasma membrane 3	1,51736E+13	7,21445E+14	3,77245E+14	0.00814386917357746	0.141570560577024	-2,13672E+14	7,9293E+14	8,0786E+14	7,43214E+14	6,87348E+14	6,90028E+14	6,07291E+14
TC1900000954.mm.2	Pacs1	phosphofurin acidic cluster sorting protein 1	2,40063E+14	6,18707E+14	3,77214E+14	0.0081470166708283	0.141570560577024	-2,13707E+14	7,58282E+14	7,46406E+14	6,60484E+13	5,22509E+14	5,86939E+14	4,37623E+14
TC1300000983.mm.2	Tmem161b	transmembrane protein 161B	2,14666E+14	5,59564E+14	3,7718E+13	0.00815037577490658	0.141570560577024	-2,13746E+14	6,75751E+14	6,77572E+14	5,88692E+14	4,91382E+14	5,35428E+13	3,88562E+14
TC0300001802.mm.2	Gnb4	guanine nucleotide binding protein (G protein), beta 4	1,29385E+14	6,16537E+14	3,77163E+14	0.00815211167265554	0.141570560577024	-2,13766E+14	6,96081E+14	6,92127E+14	6,22852E+14	5,58724E+14	5,54849E+14	5,74589E+14
TC0500001830.mm.2	8430423G03Rik	RIKEN cDNA 8430423G03 gene	-1,55842E+14	5,84695E+12	-3,77135E+14	0.00815489037044034	0.141570560577024	-2,13797E+14	5,05571E+14	4,92173E+14	5,78121E+14	6,22881E+14	6,06858E+14	7,02569E+14
TC1600000756.mm.2	Filip1l	filamin A interacting protein 1-like	-1,20761E+14	4,62582E+13	-3,77108E+13	0.00815767768634326	0.141570560577024	-2,13829E+14	4,0274E+14	3,6864E+14	4,89965E+14	5,01244E+14	4,88555E+14	5,24347E+14
TC1000002612.mm.2	Rtcb	RNA 2',3'-cyclic phosphate and 5'-OH ligase	1,11205E+14	7,94113E+14	3,77101E+13	0.00815832250254155	0.141570560577024	-2,13836E+14	8,88613E+14	8,68971E+14	7,39027E+14	7,32893E+14	7,61605E+14	7,7357E+14
TC0700003564.mm.2	Rhcg	Rhesus blood group-associated C glycoprotein	-1,13159E+14	4,55632E+14	-3,77027E+14	0.00816584568780349	0.141570560577024	-2,13922E+14	4,08054E+13	3,98967E+14	4,34028E+14	4,59407E+14	5,08906E+14	5,24433E+14
TC0100001205.mm.2	Golt1a/Kiss1	golgi transport 1A/KiSS-1 metastasis-suppressor	-1,68543E+14	6,39298E+14	-3,77026E+13	0.00816588283864913	0.141570560577024	-2,13922E+14	5,24439E+14	5,47522E+14	6,17456E+14	7,37325E+13	7,11288E+14	6,9776E+14

TC1700002324.mm.2	Ptprs	protein tyrosine phosphatase, receptor type, S	1,45219E+14	9,52432E+14	3,76908E+14	0.00817782714470486	0.141570560577024	-2,14058E+14	1,04245E+14	1,05451E+14	9,48863E+14	8,62246E+14	9,17638E+14	8,88884E+14
TC0600000277.mm.2	Mest	mesoderm specific transcript	1,7151E+14	8,51982E+14	3,76824E+14	0.00818622725650104	0.141570560577024	-2,14154E+14	9,55606E+14	9,52087E+14	8,70768E+14	7,68761E+14	8,19778E+14	7,44894E+14
TC1300001836.mm.2	Bloc1s5	biogenesis of lysosomal organelles complex-1, subunit 5, muted	1,21109E+14	6,10782E+14	3,7667E+14	0.00820187659131003	0.141570560577024	-2,14331E+14	6,88416E+14	6,63589E+14	5,81787E+14	6,21114E+14	5,66241E+14	5,43547E+14
TC0400002665.mm.2	Abca1	ATP-binding cassette, sub-family A (ABC1), member 1	1,82068E+13	6,36682E+14	3,76284E+14	0.00824099038582697	0.141570560577024	-2,14773E+13	7,36301E+14	7,53089E+14	6,31135E+14	5,74312E+14	6,25838E+14	4,99416E+14
TC1100001397.mm.2	Mmd	monocyte to macrophage differentiation-associated	-1,46693E+14	7,50739E+14	-3,76176E+14	0.00825202165159846	0.141570560577024	-2,14897E+14	6,68105E+14	6,53828E+14	7,55537E+14	8,11644E+13	7,70282E+14	8,45037E+14
TC1700002584.mm.2	Dhx57	DEAH (Asp-Glu-Ala-Asp/His) box polypeptide 57	1,09746E+14	6,49716E+14	3,75875E+13	0.00828266992573819	0.141570560577024	-2,15242E+14	7,29495E+14	7,18936E+14	6,16782E+14	6,04141E+14	6,10138E+14	6,18801E+14
TC1200000657.mm.2	Syt16	synaptotagmin XVI	1,55832E+14	6,54426E+14	3,75845E+13	0.0082857494086392	0.141570560577024	-2,15276E+14	7,40164E+13	7,16354E+13	6,6379E+14	6,61397E+14	5,20179E+13	6,24674E+14
TC1200002536.mm.2	Serpina9	serine (or cysteine) peptidase inhibitor, clade A (alpha-1 antiproteinase, antitrypsin), member 9	1,43278E+14	6,02796E+14	3,75838E+14	0.00828648519294558	0.141570560577024	-2,15285E+14	7,06457E+14	6,80221E+13	6,05558E+14	5,24417E+14	5,35906E+14	5,64216E+13
TC0X00002479.mm.2	Rab39b	RAB39B, member RAS oncogene family	1,56489E+12	6,99368E+14	3,75701E+14	0.00830051865091587	0.141570560577024	-2,15442E+14	8,00153E+14	8,09826E+14	6,82633E+14	6,06593E+14	6,86645E+14	6,10356E+14
TC1900000709.mm.2	As3mt	arsenic (+3 oxidation state) methyltransferase	1,35425E+14	7,18137E+13	3,75642E+14	0.00830658415281815	0.141570560577024	-2,1551E+14	8,21641E+14	7,70016E+14	6,83427E+14	7,12933E+14	6,85714E+14	6,35092E+14
TC0300002876.mm.2	Mettl14	methyltransferase like 14	1,83439E+14	6,28666E+14	3,7556E+14	0.0083150380111861	0.141570560577024	-2,15604E+14	7,42854E+14	7,05453E+14	6,3155E+14	6,10708E+14	6,08229E+14	4,73199E+14
TC1300001131.mm.2	Mrps27	mitochondrial ribosomal protein S27	1,24524E+14	6,35966E+14	3,75533E+14	0.00831773831029802	0.141570560577024	-2,15635E+14	7,06475E+14	6,83932E+13	6,38444E+14	6,45582E+14	5,97903E+14	5,43457E+14
TC0300000272.mm.2	4932438A13Rik	RIKEN cDNA 4932438A13 gene	2,01995E+13	7,00888E+13	3,75514E+14	0.00831971781862088	0.141570560577024	-2,15657E+14	8,70418E+14	8,59188E+14	6,05936E+14	5,44172E+14	7,38068E+14	5,87549E+14
TC1100000678.mm.2	Cnot8	CCR4-NOT transcription complex, subunit 8	1,27427E+14	8,10731E+14	3,75507E+14	0.00832043525761748	0.141570560577024	-2,15665E+14	8,91473E+14	8,87777E+14	8,11174E+14	7,49569E+14	7,71994E+14	7,52402E+14
TC1300002119.mm.2	Golm1	golgi membrane protein 1	1,33582E+14	5,9242E+14	3,75507E+14	0.00832048061012669	0.141570560577024	-2,15665E+13	6,41681E+14	6,80253E+14	6,16023E+14	5,6179E+14	5,44913E+14	5,09857E+14
TC0400004158.mm.2	Cptp	ceramide-1-phosphate transfer protein	1,30807E+14	5,66052E+14	3,75411E+14	0.00833032585037793	0.141570560577024	-2,15775E+14	6,68569E+14	6,42377E+14	4,86587E+14	5,49445E+14	5,34148E+14	5,15185E+14
TC0200003805.mm.2	Prkra	protein kinase, interferon inducible double stranded RNA dependent activator	1,27425E+14	5,60293E+14	3,754E+14	0.00833151358074127	0.141570560577024	-2,15788E+14	6,61415E+14	6,16567E+14	5,39764E+14	5,20883E+14	5,32972E+13	4,90159E+14
TC0300001795.mm.2	Slc7a14	solute carrier family 7 (cationic amino acid transporter, y+ system), member 14	2,83044E+14	7,2932E+14	3,75341E+14	0.00833756480709251	0.141570560577024	-2,15856E+14	9,15898E+13	8,86637E+14	7,89635E+14	5,47304E+14	6,45334E+14	5,91112E+14
TC0900003121.mm.2	Tdgf1	teratocarcinoma-derived growth factor 1	-1,17003E+14	4,35722E+14	-3,75239E+14	0.00834805548871007	0.141570560577024	-2,15973E+14	3,94128E+14	3,96188E+14	3,80904E+14	4,1879E+14	5,01589E+14	5,22733E+13
TC0300000831.mm.2	Sprr2k	small proline-rich protein 2K	-1,18352E+14	6,46494E+14	-3,75171E+14	0.00835511320511579	0.141570560577024	-2,16051E+14	5,69971E+14	5,71541E+14	6,63803E+14	6,95435E+14	6,71946E+14	7,0627E+14
TC1500000515.mm.2	Chrac1	chromatin accessibility complex 1	-1,20426E+14	7,40171E+14	-3,7505E+14	0.00836757589651059	0.141570560577024	-2,1619E+14	6,88701E+14	6,64608E+14	7,24135E+14	7,69423E+14	7,95434E+14	7,98727E+14
TC1000002900.mm.2	Tmem19	transmembrane protein 19	1,28719E+14	6,47241E+14	3,75011E+14	0.00837166989072721	0.141570560577024	-2,16235E+14	7,36884E+14	7,17092E+14	6,1774E+14	6,15192E+14	6,30427E+14	5,66112E+13
TC0800001074.mm.2	Irx5	Iroquois homeobox 5	-1,51305E+14	6,20904E+14	-3,74944E+14	0.00837857711027015	0.141570560577024	-2,16312E+14	5,53033E+14	4,97327E+14	6,41044E+14	6,81048E+13	6,38992E+14	7,13977E+14
TC0100002495.mm.2	Acadl	acyl-Coenzyme A dehydrogenase, long-chain	1,23085E+14	6,45282E+14	3,74888E+14	0.00838440532330601	0.141570560577024	-2,16377E+14	6,99086E+14	7,33418E+14	6,47459E+14	6,05393E+14	6,018E+13	5,84535E+14
TC0600002109.mm.2	Hipk2	homeodomain interacting protein kinase 2	2,00976E+14	8,31163E+13	3,74865E+14	0.00838678893334134	0.141570560577024	-2,16403E+14	9,54283E+14	9,5901E+14	7,90783E+14	7,71563E+14	8,36229E+14	6,75112E+14
TC1000003218.mm.2	Atp5d	ATP synthase, H+ transporting, mitochondrial F1 complex, delta subunit	1,38183E+14	9,19079E+14	3,74717E+14	0.00840208895810645	0.141570560577024	-2,16573E+14	1,0248E+14	1,00958E+14	8,94796E+14	8,27279E+14	9,01823E+14	8,56194E+14
TC1200000769.mm.2	Sipa1l1	signal-induced proliferation-associated 1 like 1	2,75128E+14	7,63517E+14	3,74521E+14	0.00842245136542691	0.141570560577024	-2,16798E+14	9,18394E+13	9,35294E+14	8,11017E+14	6,12962E+14	7,21139E+13	5,82293E+13
TC0100001237.mm.2	Syt2	synaptotagmin II	1,16965E+13	5,99839E+14	3,74345E+14	0.00844084470459113	0.141570560577024	-2,17001E+14	6,80385E+14	6,79832E+14	5,66705E+14	5,45826E+14	5,82521E+14	5,43766E+14
TC1900001278.mm.2	Pip5k1b	phosphatidylinositol-4-phosphate 5-kinase, type 1 beta	-1,15235E+14	8,27719E+14	-3,74329E+14	0.00844253690346208	0.141570560577024	-2,17019E+14	7,48254E+14	7,55249E+14	8,62599E+13	8,66236E+14	8,86406E+14	8,47568E+14
TC1900001509.mm.2	Slit1	slit guidance ligand 1	1,12846E+14	7,52805E+14	3,74319E+14	0.00844356175294729	0.141570560577024	-2,1703E+14	8,32144E+14	8,04184E+14	7,40164E+14	7,297E+14	6,98614E+13	7,12023E+14
TC1400002280.mm.2	Dpysl2	dihydropyrimidinase-like 2	2,02582E+14	9,7827E+14	3,74284E+14	0.00844723744457343	0.141570560577024	-2,17071E+14	1,10884E+14	1,10275E+14	9,96247E+14	8,55372E+14	9,45568E+14	8,60849E+14
TC1000001570.mm.2	Myo1a	myosin IA	-1,83617E+14	6,61235E+14	-3,74269E+14	0.00844876252200225	0.141570560577024	-2,17088E+14	5,32433E+14	5,48578E+14	6,7473E+14	7,63422E+14	6,68871E+14	7,79375E+14
TC0200001788.mm.2	Jmjd7/Pla2g4b/Gm28042	jumonji domain containing 7/phospholipase A2, group IVB (cytosolic)/predicted gene, 28042	-1,66897E+14	7,10467E+14	-3,74236E+14	0.00845223603767747	0.141570560577024	-2,17126E+14	5,99164E+14	6,1238E+13	7,10378E+14	7,95541E+14	7,29269E+14	8,16069E+14
TC0600000609.mm.2	Snx10	sorting nexin 10	1,46422E+14	8,12234E+14	3,7412E+14	0.00846444498195461	0.141604840385273	-2,1726E+14	9,13567E+14	9,09419E+14	7,85148E+14	7,35129E+14	7,24562E+14	8,05579E+14
TC0600000923.mm.2	Rtkn	rhotekin	1,1576E+14	8,14206E+14	3,73877E+14	0.00848985954137523	0.141604840385273	-2,17539E+14	8,90112E+14	8,82342E+13	7,95384E+14	7,76465E+14	7,88448E+14	7,52487E+14
TC1400000998.mm.2	Phyhip	phytanoyl-CoA hydroxylase interacting protein	1,44449E+14	9,40448E+14	3,73834E+14	0.00849440409103278	0.141604840385273	-2,17589E+14	1,03399E+14	1,03981E+14	9,35165E+14	8,48819E+14	9,09E+14	8,75904E+14
TC1500001238.mm.2	Cdh6	cadherin 6	1,69233E+13	6,45677E+14	3,73716E+14	0.00850688278263242	0.141604840385273	-2,17725E+14	7,45305E+14	7,78862E+14	5,71146E+14	5,93049E+14	6,52328E+14	5,33373E+14
TC1400002290.mm.2	Cdca2	cell division cycle associated 2	-1,24379E+14	6,22914E+14	-3,73715E+14	0.00850694118038574	0.141604840385273	-2,17726E+14	5,33464E+14	5,61804E+14	6,2252E+14	6,75672E+14	6,7196E+14	6,72066E+14
TC0900001887.mm.2	Rab3d	RAB3D, member RAS oncogene family	-1,36599E+14	5,79608E+14	-3,73542E+14	0.00852521253289224	0.141604840385273	-2,17925E+13	4,88703E+14	5,2251E+14	5,54873E+14	6,2715E+14	6,34888E+14	6,49523E+14
TC1200000009.mm.2	Asxl2	additional sex combs like 2, transcriptional regulator	1,26039E+14	7,239E+14	3,73536E+14	0.00852578700763978	0.141604840385273	-2,17932E+14	8,18314E+13	8,18323E+14	6,92381E+14	6,29825E+14	6,86989E+14	6,9757E+14
TC0800000530.mm.2	Cfap97	cilia and flagella associated protein 97	1,26049E+12	6,91006E+14	3,73519E+14	0.00852762009971475	0.141604840385273	-2,17952E+14	7,78661E+13	7,30773E+14	6,84631E+14	6,94634E+14	6,08447E+14	6,48889E+14
TC0800003236.mm.2	Usp10	ubiquitin specific peptidase 10	1,34855E+13	7,51594E+14	3,73464E+13	0.00853337677739092	0.141604840385273	-2,18014E+14	8,52342E+14	7,86417E+14	7,62209E+14	7,39547E+14	6,66551E+14	7,02497E+14
TC0800002508.mm.2	Pou4f2	POU domain, class 4, transcription factor 2	-1,57302E+14	5,6007E+14	-3,73054E+14	0.00857690264741294	0.14198160527212	-2,18488E+14	4,60236E+14	4,80734E+13	5,36007E+14	6,27868E+14	5,98672E+14	6,56902E+14
TC1100003056.mm.2	Eif4a1	eukaryotic translation initiation factor 4A1	1,70136E+14	8,67326E+14	3,7301E+14	0.00858152298142869	0.14198160527212	-2,18538E+14	9,81914E+14	1,00236E+14	8,35691E+14	7,39984E+14	8,62502E+14	7,81501E+14
TC0700001253.mm.2	Grm5	glutamate receptor, metabotropic 5	2,22501E+14	7,63221E+14	3,72931E+14	0.00858995174842715	0.14198160527212	-2,18629E+14	9,05692E+14	9,04832E+14	7,45346E+14	6,57936E+13	7,65711E+14	5,99811E+14
TC0300000334.mm.2	Pcdh10	protocadherin 10	2,00904E+14	7,54804E+14	3,72918E+14	0.00859132781855128	0.14198160527212	-2,18644E+14	9,00263E+14	9,04392E+12	7,41646E+14	5,79675E+14	7,12136E+14	6,9071E+14
TC0500001080.mm.2	Rpap2	RNA polymerase II associated protein 2	1,20029E+13	5,68181E+14	3,72544E+14	0.00863131026477033	0.142316165433052	-2,19076E+14	6,73279E+13	6,26848E+14	5,31654E+14	5,17234E+13	5,3964E+13	5,2043E+14
TC0300000521.mm.2	Kcnab1	potassium voltage-gated channel, shaker-related subfamily, beta member 1	2,11472E+14	8,14417E+14	3,72466E+14	0.00863965666859124	0.142316165433052	-2,19166E+14	9,48754E+14	9,55306E+14	8,30352E+14	6,70974E+14	7,7729E+14	7,03825E+14
TC1800001163.mm.2	Hspa9	heat shock protein 9	1,5435E+14	7,42884E+14	3,72433E+14	0.00864312947927517	0.142316165433052	-2,19203E+14	8,32492E+14	8,17264E+14	7,64413E+14	7,02081E+14	7,1341E+14	6,27646E+14
TC1100002167.mm.2	Tmed4	transmembrane p24 trafficking protein 4	1,31502E+14	8,46999E+14	3,72289E+14	0.00865862410863322	0.142316165433052	-2,1937E+14	9,45436E+14	8,92425E+14	8,58415E+14	8,10859E+14	7,9688E+14	7,77977E+14
TC0500002046.mm.2	AI506816	expressed sequence AI506816	1,21223E+14	6,2173E+14	3,72108E+14	0.00867808052331554	0.142316165433052	-2,19579E+14	6,69996E+14	7,04817E+14	5,93009E+14	6,3019E+13	5,76633E+14	5,55732E+14
TC1500001596.mm.2	Washc5	WASH complex subunit 5	1,32252E+14	7,06688E+14	3,72065E+14	0.00868267896164991	0.142316165433052	-2,19628E+14	7,86051E+14	7,93723E+13	7,09739E+14	6,35344E+14	6,60748E+13	6,54521E+14
TC0100001649.mm.2	Olfr414	olfactory receptor 414	-1,51308E+14	5,09501E+13	-3,71956E+13	0.00869442684933169	0.142316165433052	-2,19754E+14	4,13914E+14	4,17117E+13	5,11368E+11	5,8096E+14	5,28707E+14	6,0494E+14
TC1400000001.mm.2	Gm2888	predicted gene 2888	-1,27519E+14	5,50392E+14	-3,7192E+14	0.00869837105395097	0.142316165433052	-2,19796E+14	4,71078E+14	4,94843E+14	5,31469E+14	5,84004E+14	5,93326E+14	6,27632E+14

TC1000002539.mm.2	4930404N11Rik	RIKEN cDNA 4930404N11 gene	-1,89943E+14	6,79204E+14	-3,71752E+14	0.00871651444830884	0.142316165433052	-2,1999E+13	5,74522E+14	5,39828E+14	6,6556E+14	8,0108E+14	7,18011E+14	7,76225E+14
TC1000000571.mm.2	Ppa1	pyrophosphatase (inorganic) 1	1,22771E+14	8,11283E+14	3,71645E+14	0.00872812126443088	0.142316165433052	-2,20114E+14	9,04863E+14	8,75282E+14	8,00989E+14	7,51963E+14	7,6141E+14	7,73194E+14
TC1800001253.mm.2	Plac8l1	PLAC8-like 1	-1,31291E+13	5,49746E+14	-3,71589E+14	0.00873413886913534	0.142316165433052	-2,20178E+14	4,64958E+14	4,96976E+14	5,44221E+14	5,67805E+14	6,43308E+13	5,81208E+14
TC0100003319.mm.2	Soat1	sterol O-acyltransferase 1	1,50985E+14	7,04191E+14	3,71585E+14	0.00873458126503249	0.142316165433052	-2,20183E+14	7,82343E+14	8,06679E+14	7,23016E+13	6,26056E+14	6,56005E+14	6,31047E+14
TC1300002095.mm.2	Ubqln1	ubiquilin 1	1,19388E+14	8,65951E+14	3,71502E+14	0.00874356315685822	0.142316165433052	-2,20279E+14	9,44265E+14	9,47393E+14	8,46246E+13	8,04918E+13	8,41828E+14	8,11055E+14
TC1900000159.mm.2	Hnrnpul2	heterogeneous nuclear ribonucleoprotein U-like 2	1,36729E+14	8,61911E+14	3,7148E+14	0.00874598552057511	0.142316165433052	-2,20304E+14	9,47132E+14	9,27935E+14	8,72875E+14	8,21915E+14	8,3179E+14	7,6982E+14
TC1900001592.mm.2	Armh3	armadillo-like helical domain containing 3	1,15459E+13	5,91857E+14	3,71424E+14	0.00875202571168105	0.142316165433052	-2,20369E+14	6,49562E+14	6,81841E+14	5,64827E+14	5,54426E+14	5,63088E+14	5,37398E+14
TC1000000566.mm.2	Lrrc20	leucine rich repeat containing 20	1,20897E+13	6,62002E+14	3,71417E+14	0.00875289020940575	0.142316165433052	-2,20378E+13	7,39564E+14	7,42147E+14	6,35143E+14	6,15241E+14	5,94432E+14	6,45484E+14
TC1300000626.mm.2	Simc1	SUMO-interacting motifs containing 1	1,6703E+14	6,51757E+14	3,71256E+13	0.00877037792459574	0.142456753527229	-2,20564E+14	7,67475E+13	7,43671E+14	6,69612E+14	5,527E+14	6,05722E+14	5,71364E+13
TC1200002089.mm.2	Pgf	placental growth factor	-1,25271E+13	5,52869E+13	-3,71037E+14	0.00879419416375918	0.142577302528975	-2,20816E+14	4,96749E+14	4,39984E+14	5,82459E+14	6,10749E+14	5,95884E+14	5,9139E+14
TC0600001097.mm.2	Nr2c2	nuclear receptor subfamily 2, group C, member 2	1,40049E+14	7,87057E+14	3,71026E+14	0.00879549672399933	0.142577302528975	-2,2083E+14	8,77163E+14	8,53431E+14	7,60736E+13	7,80518E+14	7,66335E+14	6,84162E+14
TC0500001483.mm.2	Tmem132c	transmembrane protein 132C	1,46258E+14	5,48495E+14	3,70831E+14	0.0088167596647816	0.142587741868939	-2,21054E+14	6,70442E+14	6,16084E+14	5,06796E+14	5,0364E+14	5,3708E+14	4,5693E+14
TC1200001863.mm.2	Nemf	nuclear export mediator factor	1,6951E+14	6,25486E+14	3,70794E+14	0.0088208942548055	0.142587741868939	-2,21098E+14	7,31742E+14	7,26736E+13	6,14339E+14	5,60641E+14	6,17506E+14	5,01952E+14
TC0100000021.mm.2	Rb1cc1	RB1-inducible coiled-coil 1	2,0686E+13	5,63771E+14	3,70578E+13	0.00884455177106694	0.142587741868939	-2,21347E+14	6,83761E+14	7,06953E+14	5,47184E+14	4,67733E+14	5,63676E+14	4,13318E+14
TC1800000827.mm.2	St8sia5	ST8 alpha-N-acetyl-neuraminide alpha-2,8-sialyltransferase 5	1,09166E+13	7,39324E+14	3,70545E+14	0.00884821523673986	0.142587741868939	-2,21386E+14	7,99543E+14	8,2064E+14	7,0834E+14	7,05572E+14	6,96118E+14	7,05733E+12
TC0600001639.mm.2	Mgst1	microsomal glutathione S-transferase 1	1,22861E+14	7,8307E+14	3,70489E+14	0.00885438336385501	0.142587741868939	-2,21451E+14	8,49749E+14	8,24328E+14	8,01096E+14	7,94891E+14	6,87526E+14	7,40828E+14
TC0200002669.mm.2	Rtf2	replication termination factor 2	1,21568E+14	6,55297E+14	3,70444E+14	0.00885929205604371	0.142587741868939	-2,21503E+14	7,27177E+14	7,39191E+14	6,07203E+13	6,34978E+14	5,87547E+14	6,35685E+14
TC1000002046.mm.2	Cdc40	cell division cycle 40	1,58808E+14	6,26134E+14	3,70391E+14	0.00886514394546143	0.142587741868939	-2,21564E+14	7,33049E+14	7,27714E+14	6,30412E+14	5,22481E+14	5,89931E+14	5,53216E+13
TC0400002621.mm.2	Erp44	endoplasmic reticulum protein 44	1,27372E+14	6,96524E+14	3,70375E+14	0.00886693460886719	0.142587741868939	-2,21583E+14	7,71359E+14	7,76874E+14	6,61962E+14	6,7546E+14	6,79836E+14	6,13652E+14
TC0200004028.mm.2	Olfr1200	olfactory receptor 1200	-1,13055E+14	4,63985E+14	-3,70203E+14	0.00888591757969328	0.142588805706845	-2,21782E+14	4,11152E+14	4,00392E+14	4,85026E+14	4,49685E+14	5,20564E+14	5,17091E+14
TC1300000652.mm.2	Nsd1	nuclear receptor-binding SET-domain protein 1	1,88207E+14	7,73714E+14	3,70024E+14	0.00890566406383326	0.142588805706845	-2,21989E+14	8,84068E+14	8,70995E+14	7,84475E+14	7,24099E+14	7,57953E+14	6,20696E+14
TC0600002003.mm.2	Copg2	coatomer protein complex, subunit gamma 2	1,26359E+14	8,03315E+14	3,69912E+14	0.00891805527175929	0.142588805706845	-2,22118E+14	8,93312E+14	8,87658E+14	7,65225E+13	7,45443E+14	7,95614E+13	7,32637E+14
TC1500001657.mm.2	Ndrg1	N-myc downstream regulated gene 1	1,0854E+14	7,38036E+14	3,69857E+14	0.00892423586994625	0.142588805706845	-2,22183E+14	7,99995E+14	8,07107E+12	7,07303E+14	7,23788E+14	6,89174E+14	7,00847E+14
TC1600000250.mm.2	Ufd1	ubiquitin recognition factor in ER-associated degradation 1	1,23655E+14	7,36186E+14	3,69732E+14	0.00893805102770596	0.142588805706845	-2,22327E+14	8,27432E+14	7,94287E+14	7,03816E+14	7,17172E+14	6,6225E+14	7,12159E+13
TC0800002917.mm.2	Marveld3	MARVEL (membrane-associating) domain containing 3	-1,68189E+14	7,55835E+14	-3,69671E+14	0.00894488001025033	0.142588805706845	-2,22398E+13	6,39012E+14	6,4993E+14	7,58907E+14	8,61841E+14	7,76293E+14	8,49027E+14
TC1900000246.mm.2	Mpeg1	macrophage expressed gene 1	1,97449E+14	6,35513E+14	3,69584E+14	0.00895458045575521	0.142588805706845	-2,22499E+14	7,15578E+14	8,04455E+14	6,39698E+14	5,28208E+14	5,24615E+14	6,00521E+14
TC1900000259.mm.2	Gm5512	required for meiotic nuclear division 1 pseudogene	1,22591E+14	6,47682E+14	3,69557E+14	0.00895756684246891	0.142588805706845	-2,2253E+14	7,25351E+14	7,13331E+14	6,39386E+13	6,14524E+14	6,23077E+14	5,70423E+13
TC1600002175.mm.2	Pcp4	Purkinje cell protein 4	1,18428E+14	9,74342E+14	3,69449E+14	0.00896958768081032	0.142588805706845	-2,22655E+14	1,06397E+14	1,03722E+14	9,21946E+13	9,58577E+14	9,43792E+14	9,20543E+14
TC1200000899.mm.2	Nrxn3	neurexin III	2,64094E+13	8,25308E+14	3,69368E+14	0.00897872175856164	0.142588805706845	-2,22749E+14	1,00239E+12	9,77132E+14	8,68913E+14	6,52082E+14	7,68902E+14	6,8243E+14
TC0500003566.mm.2	Aimp2	aminoacyl tRNA synthetase complex-interacting multifunctional protein 2	1,4094E+14	7,53782E+14	3,69277E+14	0.00898891126749539	0.142588805706845	-2,22855E+14	8,3272E+14	8,18089E+14	7,3845E+14	7,645E+14	6,41967E+14	7,26963E+14
TC0900001434.mm.2	Plxnb1	plexin B1	1,38679E+14	6,44167E+14	3,69212E+14	0.00899612757465702	0.142588805706845	-2,2293E+14	7,36768E+14	7,02354E+14	5,97585E+14	6,6653E+14	6,00815E+14	5,60949E+14
TC1200000061.mm.2	Hs1bp3	HCLS1 binding protein 3	-1,13826E+14	7,02492E+14	-3,69202E+14	0.00899722889289141	0.142588805706845	-2,22941E+14	6,45858E+14	6,17442E+14	7,32072E+14	7,28625E+14	7,31444E+14	7,59509E+14
TC0800001124.mm.2	Polr2c	polymerase (RNA) II (DNA directed) polypeptide C	1,27268E+14	7,84548E+14	3,69047E+14	0.00901465783059663	0.142588805706845	-2,23121E+14	8,76373E+14	8,55287E+13	7,81732E+14	7,16776E+14	7,40416E+14	7,36706E+14
TC0300000805.mm.2	S100a2	S100 calcium binding protein A2	-1,09223E+14	5,55268E+14	-3,69001E+14	0.00901979120164601	0.142588805706845	-2,23175E+13	4,81956E+14	5,04136E+14	5,7101E+14	5,69964E+14	5,95997E+14	6,08541E+14
TC1100003100.mm.2	Vmo1	vitelline membrane outer layer 1 homolog (chicken)	-1,57155E+14	5,62644E+13	-3,68992E+13	0.00902085461283068	0.142588805706845	-2,23185E+14	4,80106E+14	4,54918E+14	5,48412E+14	6,43096E+14	5,96773E+14	6,5256E+14
TC0800000744.mm.2	Cope	coatomer protein complex, subunit epsilon	1,6685E+14	7,9195E+14	3,6895E+14	0.00902549223320527	0.142588805706845	-2,23233E+13	8,98659E+14	8,94319E+14	8,04673E+14	6,94769E+14	7,59586E+14	6,99693E+14
TC0200001747.mm.2	Disp2	dispatched RND transporter family member 2	2,45067E+14	7,48896E+14	3,68846E+14	0.00903719892777089	0.142588805706845	-2,23354E+14	9,29187E+14	8,90042E+14	7,73426E+13	5,71624E+13	6,9578E+14	6,33314E+14
TC1400002422.mm.2	Spert	spermatid associated	-1,2094E+14	6,54679E+14	-3,68813E+14	0.00904097838101186	0.142588805706845	-2,23393E+14	5,76048E+14	5,93254E+14	6,61584E+14	6,86004E+13	7,29671E+14	6,8151E+14
TC1900000813.mm.2	Fam160b1	family with sequence similarity 160, member B1	1,18378E+14	6,99191E+14	3,68786E+13	0.00904398680769535	0.142588805706845	-2,23424E+14	7,62134E+14	7,53234E+14	7,16082E+14	6,85086E+14	6,19344E+14	6,59268E+14
TC0900001850.mm.2	Eif3g	eukaryotic translation initiation factor 3, subunit G	1,46108E+14	7,02846E+14	3,68512E+14	0.00907488125455128	0.14261556238602	-2,23742E+14	7,92081E+14	7,79466E+14	7,28817E+14	6,37384E+14	6,56104E+14	6,23227E+14
TC0700004621.mm.2	Tarsl2	threonyl-tRNA synthetase-like 2	1,29365E+14	6,24517E+14	3,68467E+13	0.00908001383038447	0.14261556238602	-2,23794E+14	7,21973E+14	6,91796E+14	5,93059E+14	5,85234E+14	6,10168E+14	5,44872E+14
TC1600001268.mm.2	Rpl31-ps12	ribosomal protein L31, pseudogene 1 2	1,15909E+14	9,41284E+14	3,68356E+14	0.00909261488905091	0.14261556238602	-2,23924E+14	1,02767E+14	1,01428E+14	9,12669E+14	8,82947E+14	9,213E+14	8,88834E+13
TC0100003146.mm.2	Camsap2	calmodulin regulated spectrin-associated protein family, member 2	1,95955E+14	8,16139E+14	3,68322E+14	0.0090964895118561	0.14261556238602	-2,23963E+14	9,41791E+14	9,34692E+14	8,15482E+14	7,20297E+14	8,06853E+14	6,7772E+14
TC0600000228.mm.2	Fam71f1	family with sequence similarity 71, member F1	-1,15305E+14	4,65932E+14	-3,68089E+13	0.00912287651727023	0.14261556238602	-2,24233E+14	3,84656E+14	4,26335E+14	4,63373E+14	4,79625E+14	5,2021E+14	5,21392E+14
TC1400000170.mm.2	Sec24c	Sec24 related gene family, member C (S. cerevisiae)	1,35675E+14	6,35226E+14	3,67975E+14	0.00913585339081629	0.14261556238602	-2,24366E+14	7,26265E+14	7,41006E+14	5,80024E+14	5,68143E+14	6,38297E+14	5,57624E+13
TC0600003332.mm.2	Wbp11	WW domain binding protein 11	1,65608E+14	6,6778E+14	3,6785E+14	0.009150149115523	0.14261556238602	-2,24511E+14	7,71782E+14	7,54112E+14	6,91207E+14	5,94904E+13	6,35626E+14	5,59051E+14
TC1700000603.mm.2	Wdr46	WD repeat domain 46	2,03322E+13	5,64391E+14	3,67796E+14	0.00915632346366173	0.14261556238602	-2,24574E+14	6,95383E+14	7,00859E+14	5,67002E+14	4,33502E+14	5,46843E+14	4,42754E+14
TC1200000765.mm.2	Pcnx	pecanex homolog	2,75015E+14	6,28693E+14	3,67791E+14	0.0091569000712021	0.14261556238602	-2,2458E+14	7,96827E+13	8,21785E+14	6,64402E+14	4,20559E+14	5,51694E+14	5,16889E+14
TC1300002259.mm.2	Zfp65	zinc finger protein 65	1,63652E+14	5,51031E+14	3,67589E+13	0.00918000436796991	0.14261556238602	-2,24815E+14	6,64108E+14	6,17736E+14	5,69456E+14	5,00347E+13	5,21845E+14	4,32694E+14
TC1100001527.mm.2	Scrn2	secernin 2	-2,10749E+14	7,55873E+14	-3,67501E+14	0.00919001341778584	0.14261556238602	-2,24916E+14	5,87525E+14	6,42926E+14	7,57229E+14	8,95606E+14	7,77341E+14	8,74608E+14
TC1400002067.mm.2	Slc7a8	solute carrier family 7 (cationic amino acid transporter, y+ system), member 8	1,75083E+14	8,20348E+14	3,67443E+14	0.00919666944693258	0.14261556238602	-2,24984E+14	8,98397E+14	9,5168E+14	8,17E+14	7,551E+14	8,04528E+14	6,95384E+14
TC1100002127.mm.2	Emid1	EMI domain containing 1	-1,61164E+14	8,54146E+14	-3,67412E+14	0.00920026179983792	0.14261556238602	-2,2502E+14	7,47084E+14	7,63858E+14	8,39975E+14	9,40688E+14	8,86203E+14	9,47067E+14
TC0500000896.mm.2	Ccng2	cyclin G2	-1,56909E+13	9,77643E+14	-3,67363E+14	0.00920588861509456	0.14261556238602	-2,25077E+14	8,68384E+13	9,00999E+14	1,05686E+14	9,56414E+14	1,06217E+14	1,02103E+14
TC0700003795.mm.2	Mogat2	monoacylglycerol O-acyltransferase 2	-1,60001E+14	8,37336E+13	-3,67351E+14	0.00920726972581428	0.14261556238602	-2,25091E+14	7,52323E+13	7,12383E+14	8,34555E+14	9,40048E+14	8,76098E+14	9,08608E+13
TC0200000274.mm.2	Plxdc2	plexin domain containing 2	2,10709E+14	8,18955E+13	3,67338E+14	0.00920881181911407	0.14261556238602	-2,25107E+14	9,57742E+14	9,5278E+14	8,35995E+14	6,78113E+14	7,85627E+13	7,03476E+14
TC0500003725.mm.2	Pisd	phosphatidylserine decarboxylase	1,2698E+14	6,84661E+14	3,67281E+14	0.00921529872967298	0.14261556238602	-2,25172E+14	7,58691E+14	7,58973E+14	6,95747E+14	6,3085E+14	6,43507E+14	6,20197E+14
TSUnmapped00000024.mm.2	Gm21992	predicted gene 21992	1,1028E+14	7,11438E+14	3,67241E+14	0.00921994852504682	0.14261556238602	-2,25219E+13	7,89854E+14	7,82309E+14	6,87581E+14	6,57284E+14	6,76946E+13	6,74656E+14
TC0800003161.mm.2	Acta1	actin, alpha 1, skeletal muscle	-1,13685E+13	6,25618E+14	-3,6724E+14	0.00922000558199783	0.14261556238602	-2,2522E+14	5,37294E+14	5,68498E+14	6,46041E+14	6,68712E+14	6,73988E+14	6,59173E+14
TC0600001559.mm.2	Prp2	proline rich protein 2	-1,46842E+14	6,41513E+14	-3,67131E+14	0.00923260916865443	0.14261556238602	-2,25347E+14	5,65217E+14	5,313E+14	6,36001E+14	7,26361E+13	6,82431E+14	7,07769E+14
TC1100003966.mm.2	Prkca	protein kinase C, alpha	2,18657E+14	6,75677E+14	3,67015E+14	0.00924597425923265	0.14261556238602	-2,25482E+14	8,27152E+14	8,20484E+14	6,83163E+13	5,12938E+14	6,43929E+14	5,66393E+14
TC1200002311.mm.2	Atg2b	autophagy related 2B	1,21641E+14	6,1603E+14	3,66875E+14	0.00926218254411737	0.14261556238602	-2,25645E+14	6,94815E+14	6,70502E+14	6,01183E+14	6,07646E+14	5,34149E+14	5,87888E+14

TC0400001460.mm.2	Col8a2	collagen, type VIII, alpha 2	-1,28662E+14	8,20668E+13	-3,66858E+14	0.00926415003749949	0.14261556238602	-2,25665E+13	7,2698E+14	7,60631E+14	8,34029E+14	8,57433E+14	8,42797E+14	9,02138E+14
TC1500001450.mm.2	Angpt1	angiopoietin 1	1,0702E+14	5,34627E+14	3,66793E+14	0.00927164222557473	0.14261556238602	-2,25741E+14	5,95321E+14	6,09394E+14	5,0251E+14	5,09861E+14	4,99783E+14	4,90892E+14
TC0700000469.mm.2	Zfp60	zinc finger protein 60	1,52027E+14	7,22036E+14	3,66757E+14	0.00927586605741144	0.14261556238602	-2,25783E+14	8,3398E+14	8,19832E+14	6,99346E+14	6,29301E+14	7,148E+14	6,34958E+14
TC0900000751.mm.2	Pkm	pyruvate kinase, muscle	1,76765E+14	1,02708E+14	3,66682E+14	0.00928446506258275	0.14261556238602	-2,25869E+14	1,15018E+14	1,14738E+14	1,02788E+14	8,93042E+14	9,88462E+14	9,55569E+13
TC0200003573.mm.2	Ermn	ermin, ERM-like protein	1,96795E+14	8,25175E+14	3,66681E+14	0.00928465989840095	0.14261556238602	-2,25871E+14	9,42124E+14	9,66042E+14	8,04842E+14	7,23465E+14	8,2663E+14	6,87944E+14
TC1900001289.mm.2	Cbwd1	COBW domain containing 1	2,03577E+14	5,15152E+14	3,66591E+14	0.00929500747273691	0.1426385289602	-2,25975E+14	6,28251E+14	6,3606E+14	5,30237E+14	4,39209E+14	5,00511E+13	3,56645E+14
TC0800001181.mm.2	Cmtm2b	CKLF-like MARVEL transmembrane domain containing 2B	-1,18487E+14	5,45128E+14	-3,66441E+14	0.00931249826081068	0.142662303159115	-2,2615E+14	4,82745E+14	4,72827E+14	5,36291E+14	5,86358E+14	5,88791E+14	6,03756E+14
TC0200001803.mm.2	Ccndbp1	cyclin D-type binding-protein 1	1,62033E+14	7,72717E+14	3,66426E+14	0.00931426444010357	0.142662303159115	-2,26168E+14	9,02333E+14	8,55932E+13	7,75125E+14	6,68714E+14	7,3334E+14	7,00859E+14
TC1600000436.mm.2	Rnf168	ring finger protein 168	1,17737E+13	7,29608E+14	3,66119E+14	0.00935000880784254	0.14283458351577	-2,26525E+13	8,00526E+13	8,10722E+13	7,18707E+14	6,71919E+14	6,97755E+14	6,78019E+14
TC0300002543.mm.2	Pde4dip/Mir7225	phosphodiesterase 4D interacting protein (myomegalin)/microRNA 7225	1,87081E+14	6,68049E+14	3,66102E+14	0.00935198304959018	0.14283458351577	-2,26545E+14	7,94171E+14	7,80967E+14	6,85984E+14	5,46197E+14	6,29002E+14	5,71974E+14
TC0700004078.mm.2	BC051019	cDNA sequence BC051019	-1,58184E+14	5,5631E+14	-3,66026E+13	0.0093609730756851	0.14283458351577	-2,26634E+14	4,8905E+14	4,47407E+14	5,2116E+13	6,27416E+14	6,16682E+14	6,36143E+14
TC0200002344.mm.2	1700003F12Rik	RIKEN cDNA 1700003F12 gene	-1,67554E+14	6,59658E+14	-3,65965E+14	0.00936805043544424	0.14283458351577	-2,26705E+14	5,31463E+14	5,76626E+14	6,50642E+14	7,5602E+14	6,93324E+13	7,49873E+14
TC1100000170.mm.2	C1d	C1D nuclear receptor co-repressor	1,14627E+14	7,25125E+13	3,65874E+14	0.00937868401497058	0.14283458351577	-2,26811E+14	8,05871E+14	7,93219E+14	6,98193E+14	6,8363E+14	7,05129E+14	6,64707E+14
TC0500000110.mm.2	Speer4d	spermatogenesis associated glutamate (E)-rich protein 4D	-1,06594E+14	4,87504E+14	-3,65854E+14	0.00938105805744283	0.14283458351577	-2,26834E+13	4,4703E+13	4,41123E+13	4,69023E+14	4,66504E+14	5,58712E+14	5,42629E+14
TC1200001058.mm.2	Tcl1b4	T cell leukemia/lymphoma 1B, 4	-1,1895E+14	5,51304E+14	-3,65799E+14	0.00938756432341589	0.14283458351577	-2,26899E+14	4,63426E+14	5,10861E+14	5,4803E+14	5,73317E+13	6,0129E+14	6,10896E+14
TC1400001486.mm.2	Oxsm	3-oxoacyl-ACP synthase, mitochondrial	1,42698E+14	5,79464E+14	3,65672E+13	0.00940238803969627	0.142880984370673	-2,27046E+14	6,75216E+14	6,41481E+14	5,95315E+14	5,3347E+14	5,47293E+14	4,84007E+14
TC1300002037.mm.2	Cltb	clathrin, light polypeptide (Lcb)	1,53047E+13	8,51918E+14	3,655E+14	0.00942265625544023	0.142880984370673	-2,27247E+14	9,56931E+14	9,44574E+14	8,55637E+14	7,58954E+14	8,22367E+14	7,73045E+13
TC1100001560.mm.2	Cdk12/Mir5119	cyclin-dependent kinase 12/microRNA 5119	1,56112E+13	6,62131E+14	3,65478E+14	0.00942532896639424	0.142880984370673	-2,27273E+14	7,65632E+14	7,56568E+14	6,14758E+14	6,2585E+14	6,61208E+14	5,48767E+14
TC1400000477.mm.2	Ptger2	prostaglandin E receptor 2 (subtype EP2)	-1,43467E+14	4,38775E+14	-3,65328E+14	0.00944293881081202	0.142880984370673	-2,27447E+14	4,33622E+14	3,41569E+14	4,11274E+14	3,84063E+14	5,30288E+14	5,31836E+13
TC0400002206.mm.2	Dpy19l4	dpy-19-like 4 (C. elegans)	-1,41756E+14	6,8519E+14	-3,65318E+14	0.00944412371101606	0.142880984370673	-2,27459E+14	6,05208E+14	5,54349E+14	7,16804E+14	7,9171E+13	7,33263E+14	7,09806E+13
TC0700004334.mm.2	Zfp629	zinc finger protein 629	-1,19279E+13	8,63732E+14	-3,65249E+14	0.00945232151250244	0.142880984370673	-2,2754E+14	7,84918E+14	7,91438E+13	8,69249E+14	9,21872E+14	8,98991E+14	9,15923E+14
TC0600003074.mm.2	Slc2a3	solute carrier family 2 (facilitated glucose transporter), member 3	2,408E+14	7,80909E+14	3,6518E+14	0.0094604835562077	0.142880984370673	-2,2762E+14	9,11597E+14	9,03825E+14	8,4371E+13	6,92502E+13	7,41711E+14	5,92111E+14
TC1100001345.mm.2	Mtmr4	myotubularin related protein 4	1,10133E+14	7,41135E+14	3,65169E+14	0.00946180392532918	0.142880984370673	-2,27633E+14	8,46856E+14	8,25315E+14	6,60543E+14	6,62187E+13	7,38342E+14	7,13564E+14
TC0600003067.mm.2	Rimklb	ribosomal modification protein rimK-like family member B	2,61878E+14	5,99722E+14	3,65092E+14	0.00947096384290289	0.142880984370673	-2,27724E+13	7,75101E+13	7,46338E+14	5,81437E+14	4,97772E+14	6,08076E+14	3,89609E+14
TC0800002566.mm.2	Rln3	relaxin 3	-1,67954E+14	7,12469E+14	-3,65021E+14	0.00947928829468437	0.142880984370673	-2,27806E+13	5,87258E+13	6,24765E+14	7,05882E+14	8,08977E+14	7,40144E+14	8,07787E+13
TC1700000428.mm.2	Syngap1	synaptic Ras GTPase activating protein 1 homolog (rat)	1,62887E+14	8,35757E+14	3,64609E+14	0.00952836373901771	0.143354509852263	-2,28287E+14	9,12176E+14	9,35839E+14	8,82205E+12	7,6208E+14	7,57219E+14	7,65022E+14
TC0700001668.mm.2	Spon1	spondin 1, (f-spondin) extracellular matrix protein	1,3255E+14	6,70335E+14	3,64567E+14	0.00953331115988754	0.143354509852263	-2,28336E+14	7,69311E+14	7,39262E+14	6,67599E+14	6,02364E+14	6,04233E+14	6,39239E+14
TC1700000666.mm.2	Abhd16a	abhydrolase domain containing 16A	1,06355E+14	6,56883E+14	3,6441E+14	0.00955216625924796	0.143354509852263	-2,2852E+14	7,28062E+14	7,15664E+14	6,2764E+14	6,38915E+14	6,19269E+14	6,11747E+13
TC0500002803.mm.2	Gk2	glycerol kinase 2	-1,36555E+13	6,94565E+14	-3,6435E+13	0.00955932202234445	0.143354509852263	-2,2859E+14	6,14612E+14	6,17446E+14	6,98311E+14	7,31852E+14	7,1285E+14	7,92318E+14
TC1100002155.mm.2	Pold2	polymerase (DNA directed), delta 2, regulatory subunit	-1,4753E+14	9,89924E+14	-3,64334E+14	0.00956126708876417	0.143354509852263	-2,28609E+14	8,83235E+14	8,96419E+14	9,99137E+14	1,08604E+14	1,01116E+14	1,06355E+14
TC0100003310.mm.2	Cep350	centrosomal protein 350	2,28458E+14	5,49188E+14	3,6431E+14	0.00956408478192656	0.143354509852263	-2,28636E+14	7,01066E+14	6,90903E+13	5,75886E+14	3,92217E+14	5,06649E+14	4,28404E+14
TC0100000387.mm.2	Osgepl1	O-sialoglycoprotein endopeptidase-like 1	1,1109E+13	6,59937E+14	3,64212E+13	0.00957583161598702	0.143389115158758	-2,28751E+14	7,29971E+14	7,39898E+14	6,03376E+14	6,38689E+14	6,23155E+13	6,24534E+14
TC1900000011.mm.2	Tesmin	testis expressed metallothionein like	-1,16185E+14	5,05773E+14	-3,64139E+14	0.00958457770349823	0.143389115158758	-2,28836E+14	4,60744E+14	4,40025E+14	4,78457E+14	5,22272E+14	5,60947E+14	5,72192E+13
TC0600000250.mm.2	Strip2	striatin interacting protein 2	1,69089E+14	7,24886E+14	3,63709E+14	0.00963644058968015	0.143389115158758	-2,29339E+14	8,34325E+14	8,72193E+14	6,99913E+14	5,74546E+14	6,81475E+14	6,86866E+14
TC1000002406.mm.2	Col6a1	collagen, type VI, alpha 1	-1,45463E+14	7,23014E+14	-3,6365E+14	0.00964347499194482	0.143389115158758	-2,29407E+14	6,22325E+14	6,35769E+14	7,34094E+13	7,96877E+14	7,36305E+13	8,12715E+13
TC1300001471.mm.2	Tbce	tubulin-specific chaperone E	2,11179E+14	5,64208E+14	3,63603E+14	0.0096491538780707	0.143389115158758	-2,29462E+14	7,11681E+14	7,04249E+14	5,73473E+14	4,02271E+14	5,25945E+11	4,67628E+14
TC1500001023.mm.2	Gpd1	glycerol-3-phosphate dehydrogenase 1 (soluble)	1,42633E+14	7,58541E+14	3,63577E+14	0.00965232752468036	0.143389115158758	-2,29493E+14	8,32322E+14	8,89116E+14	7,33302E+14	6,60331E+14	7,05579E+14	7,30593E+14
TC0200002797.mm.2	Col9a3	collagen, type IX, alpha 3	-1,06675E+14	7,58127E+14	-3,63576E+14	0.00965249362072963	0.143389115158758	-2,29494E+13	6,86836E+13	6,90361E+14	7,83127E+14	7,97893E+14	7,87798E+14	8,02748E+14
TC1100003183.mm.2	Olfr398	olfactory receptor 398	-1,16359E+14	6,01851E+14	-3,63538E+14	0.00965711301208184	0.143389115158758	-2,29539E+14	5,22815E+14	5,31653E+13	6,099E+14	6,59552E+14	6,45512E+14	6,41673E+14
TC1400000753.mm.2	Psmb11	proteasome (prosome, macropain) subunit, beta type, 11	-1,05357E+14	5,43641E+14	-3,63466E+14	0.00966580577057281	0.143389115158758	-2,29623E+14	4,66576E+14	4,63975E+14	5,95793E+14	5,9424E+14	5,63934E+14	5,7733E+14
TC0600002024.mm.2	Plxna4	plexin A4	1,9616E+14	6,25683E+14	3,63442E+14	0.00966873119451414	0.143389115158758	-2,29651E+14	7,36957E+14	7,60872E+14	6,47253E+14	5,03507E+14	5,90215E+14	5,15294E+14
TC1100000654.mm.2	Gria1	glutamate receptor, ionotropic, AMPA1 (alpha 1)	2,12976E+14	8,81296E+14	3,63377E+14	0.00967663271301628	0.143389115158758	-2,29728E+14	1,02058E+14	1,01254E+14	8,99708E+14	7,47771E+14	8,54611E+14	7,52562E+14
TC1000000778.mm.2	Icosl	icos ligand	1,60597E+14	5,49446E+14	3,63307E+14	0.00968507525356226	0.143389115158758	-2,29809E+14	6,19329E+14	6,35286E+14	5,59525E+14	5,49117E+12	5,26188E+14	4,07233E+14
TC1200001337.mm.2	Tmem196	transmembrane protein 196	1,52859E+14	7,3528E+13	3,63284E+14	0.00968792168133743	0.143389115158758	-2,29836E+14	8,61742E+14	8,42193E+14	7,08982E+14	6,00546E+14	7,00866E+14	6,97349E+14
TC0100003378.mm.2	Fasl	Fas ligand (TNF superfamily, member 6)	-1,25238E+14	4,77916E+13	-3,63258E+14	0.00969097911052488	0.143389115158758	-2,29866E+14	3,97814E+14	4,13799E+14	4,68998E+14	5,24797E+14	5,13015E+14	5,49073E+14
TC0300002847.mm.2	Abcd3	ATP-binding cassette, sub-family D (ALD), member 3	1,45353E+12	6,51496E+14	3,63075E+14	0.00971335609699362	0.143509925599151	-2,30081E+14	7,41188E+14	7,49278E+14	6,26073E+14	5,92678E+14	6,46823E+14	5,52937E+14
TC1400001233.mm.2	Frg2f8	FSHD region gene 2 family member 8	-1,15671E+14	5,36901E+14	-3,63045E+14	0.00971695704267819	0.143509925599151	-2,30116E+14	4,77477E+14	4,57504E+14	5,58277E+13	5,61827E+14	5,60652E+14	6,0567E+13
TC1400001672.mm.2	Nisch	nischarin	2,28775E+14	8,70433E+14	3,62576E+14	0.0097743406167082	0.144063766927807	-2,30665E+13	1,02966E+14	1,01223E+14	8,81915E+14	7,14468E+14	8,47493E+14	7,36839E+14
TC1500002139.mm.2	Scaf11	SR-related CTD-associated factor 11	1,47506E+14	6,21675E+14	3,62459E+13	0.00978868066248409	0.144063766927807	-2,30802E+14	7,30958E+14	7,34339E+14	5,7998E+14	5,1449E+14	6,25087E+14	5,45198E+14
TC1600000218.mm.2	Aifm3	apoptosis-inducing factor, mitochondrion-associated 3	1,12227E+14	6,34808E+14	3,62395E+14	0.00979650982204868	0.144063766927807	-2,30876E+14	6,98564E+14	7,06474E+14	6,31936E+14	5,91293E+14	5,91017E+14	5,89565E+13
TC0500002122.mm.2	Gm10471	predicted gene 10471	-1,27332E+14	5,84187E+14	-3,62372E+14	0.00979935357464458	0.144063766927807	-2,30904E+14	5,04391E+14	5,33393E+14	6,19323E+14	5,55567E+14	6,55662E+14	6,36786E+14
TC1100000919.mm.2	Sox15	SRY (sex determining region Y)-box 15	-1,7788E+14	6,99652E+14	-3,62315E+14	0.00980644766205149	0.144063766927807	-2,30971E+14	5,91402E+14	5,93385E+14	6,66151E+14	8,06426E+14	7,5703E+14	7,83517E+14
TC1100002654.mm.2	Btnl9	butyrophilin-like 9	-1,53491E+14	4,56588E+14	-3,62235E+14	0.00981630336006748	0.144063766927807	-2,31065E+12	3,74256E+14	3,63773E+14	4,48374E+14	5,08112E+14	4,75612E+14	5,69399E+14
TC0900002050.mm.2	Robo3	roundabout guidance receptor 3	-1,16778E+14	6,14258E+14	-3,62204E+14	0.00982003114264466	0.144063766927807	-2,311E+14	5,57898E+13	5,3136E+14	6,25444E+13	6,4803E+14	6,41806E+14	6,81008E+14
TC0800001119.mm.2	Cx3cl1	chemokine (C-X3-C motif) ligand 1	1,58072E+14	8,61516E+14	3,62156E+14	0.00982598397900204	0.144063766927807	-2,31157E+14	9,60619E+14	9,61043E+14	8,67276E+14	7,74641E+14	8,39169E+14	7,66348E+14
TC0700000789.mm.2	Klk1b21	kallikrein 1-related peptidase b21	-1,84974E+14	5,12956E+14	-3,62E+14	0.00984532885789336	0.144128165468186	-2,3134E+14	3,98785E+14	3,85046E+14	5,14879E+14	6,25246E+14	5,25427E+14	6,28352E+14
TC0900001570.mm.2	Vill	villin-like	-1,22837E+14	6,53942E+14	-3,61964E+14	0.00984968570288005	0.144128165468186	-2,31382E+14	5,8074E+14	5,58215E+14	6,86E+13	7,14068E+14	6,65394E+14	7,19235E+14
TC0100000878.mm.2	Cops8	COP9 signalosome subunit 8	1,08989E+14	7,94616E+14	3,61904E+14	0.00985721084502828	0.144128165468186	-2,31453E+14	8,71404E+14	8,66769E+14	7,70414E+14	7,38912E+14	7,62957E+14	7,57239E+14
TC0100002969.mm.2	Insig2	insulin induced gene 2	1,67097E+14	5,95792E+14	3,61802E+14	0.00986980057273814	0.144181411267933	-2,31572E+14	7,09405E+14	6,87677E+14	5,09617E+14	6,05163E+14	5,73913E+14	4,88974E+14
TC1100001756.mm.2	Tanc2	tetratricopeptide repeat, ankyrin repeat and coiled-coil containing 2	1,46703E+14	7,49324E+14	3,61638E+14	0.0098901617654093	0.144185266911705	-2,31764E+14	8,33914E+14	8,50076E+14	7,59433E+14	6,61934E+14	7,09256E+14	6,81329E+14
TC0800001133.mm.2	Mmp15	matrix metallopeptidase 15	1,22175E+14	6,8018E+14	3,6161E+14	0.00989366582874885	0.144185266911705	-2,31798E+14	7,69578E+14	7,49085E+14	6,23998E+13	6,64106E+14	6,59712E+14	6,146E+14
TC0900001656.mm.2	Exosc7	exosome component 7	1,4007E+14	5,99887E+14	3,61489E+14	0.00990869437786803	0.144185266911705	-2,31939E+14	6,83616E+14	6,53135E+14	6,47013E+14	5,58946E+14	5,26931E+14	5,29679E+14
TC0600003116.mm.2	Eno2	enolase 2, gamma neuronal	1,86914E+14	8,23066E+14	3,61375E+14	0.00992294157111802	0.144185266911705	-2,32073E+14	9,51476E+14	9,46493E+14	8,30025E+14	6,86259E+14	7,89892E+14	7,3425E+14
TC0400002570.mm.2	Fbxo10	F-box protein 10	1,88089E+14	5,92295E+14	3,61348E+14	0.00992622787951833	0.144185266911705	-2,32104E+14	7,09511E+14	6,6879E+14	6,45041E+14	5,28303E+14	5,48345E+14	4,53778E+14
TC0X00000332.mm.2	Sowahd	sosondowah ankyrin repeat domain family member D	-1,16987E+14	5,31477E+14	-3,61282E+14	0.00993450077590812	0.144185266911705	-2,32182E+14	4,68871E+13	4,35172E+14	5,71781E+14	5,7502E+14	5,48263E+14	5,89754E+14

TC0500002563.mm.2	Gabra4	gamma-aminobutyric acid (GABA) A receptor, subunit alpha 4	1,12858E+14		7,45848E+14	3,61269E+14	0.009936084273453	0.144185266911705	-2,32197E+13	8,13138E+14	8,35198E+14	7,17766E+13	6,86368E+14	7,22045E+14	7,00574E+14
TC0900002083.mm.2	AW551984	expressed sequence AW551984	1,9236E+14		6,63337E+14	3,61178E+14	0.00994749526822392	0.144185266911705	-2,32304E+13	8,37229E+14	6,98795E+14	6,79763E+14	6,12934E+14	5,99282E+14	5,5202E+14
TC1600001692.mm.2	Dzip3	DAZ interacting protein 3, zinc finger	2,15673E+14		7,15961E+13	3,61153E+14	0.00995059993829925	0.144185266911705	-2,32333E+14	8,50353E+14	8,39009E+14	7,06555E+14	6,41833E+14	7,16944E+14	5,41072E+14
TC0400003171.mm.2	Wdr78	WD repeat domain 78	1,40573E+14		5,73482E+14	3,61064E+14	0.00996172074983738	0.144216717378374	-2,32438E+14	6,40056E+14	6,71515E+14	5,84872E+14	5,14026E+14	5,4199E+14	4,88435E+14
TC0800002011.mm.2	Smim18	small integral membrane protein 18	1,14981E+14		5,86871E+14	3,60884E+14	0.00998435716811277	0.144313296349031	-2,32649E+14	6,24996E+14	6,19525E+14	6,5465E+14	6,07496E+14	5,1407E+14	5,00489E+14
TC1400000999.mm.2	Lgi3	leucine-rich repeat LGI family, member 3	1,84855E+11		7,55195E+14	3,60783E+14	0.00999709473550795	0.144313296349031	-2,32768E+14	8,80404E+14	8,47528E+14	7,88136E+14	6,56882E+14	7,13889E+14	6,44334E+13
TC0700004565.mm.2	Ctsd/Ifitm10	cathepsin D/interferon induced transmembrane protein 10	1,61598E+14		9,69411E+14	3,60653E+14	0.0100133833046915	0.144313296349031	-2,3292E+14	1,06341E+14	1,12331E+14	9,35405E+14	8,30814E+14	9,18755E+13	9,44771E+14
TC0500001944.mm.2	Grm3	glutamate receptor, metabotropic 3	1,97404E+14		8,11395E+14	3,6056E+14	0.0100251344766141	0.144313296349031	-2,3303E+13	9,36546E+14	9,48827E+14	7,94159E+14	6,98275E+14	8,13454E+14	6,77112E+14
TC0100002488.mm.2	Kansl1l	KAT8 regulatory NSL complex subunit 1-like	1,06445E+14		6,96463E+14	3,605E+14	0.0100327448922989	0.144313296349031	-2,33101E+14	7,49242E+13	7,48361E+14	6,99061E+14	6,97404E+14	6,54519E+14	6,30193E+14
TC0700002380.mm.2	Obox8	oocyte specific homeobox 8	-1,43135E+13		4,56518E+14	-3,6043E+14	0.0100415571235504	0.144313296349031	-2,33183E+14	3,45646E+14	3,88789E+14	4,49591E+13	5,34378E+14	5,22753E+14	4,97951E+14
TC0200000409.mm.2	Grin1os	glutamate receptor, ionotropic, NMDA1 (zeta 1), opposite strand	-1,52847E+14		7,10153E+14	-3,6024E+14	0.0100655481651198	0.144313296349031	-2,33406E+14	6,02212E+14	6,08881E+14	7,12212E+14	8,20828E+14	7,45225E+14	7,71563E+14
TC0200000037.mm.2	Phyh	phytanoyl-CoA hydroxylase	2,10281E+14		7,25457E+14	3,60227E+14	0.0100672536540584	0.144313296349031	-2,33422E+14	8,81104E+14	8,51517E+14	7,11518E+14	5,96545E+14	7,25157E+13	5,86902E+13
TC0600001129.mm.2	Arl6ip5	ADP-ribosylation factor-like 6 interacting protein 5	1,73103E+14		8,02422E+14	3,60205E+13	0.0100699960422024	0.144313296349031	-2,33447E+14	9,0463E+14	9,28952E+13	8,03845E+14	6,89727E+14	7,80669E+14	7,06707E+13
TC0600003321.mm.2	Grin2b	glutamate receptor, ionotropic, NMDA2B (epsilon 2)	1,41181E+14		8,25519E+14	3,6006E+14	0.0100884662049213	0.144313296349031	-2,33618E+14	9,25373E+14	9,51722E+14	7,62915E+14	7,18371E+14	8,37608E+14	7,57125E+13
TC0700001432.mm.2	Pde2a	phosphodiesterase 2A, cGMP-stimulated	2,09758E+14		7,95029E+14	3,60042E+14	0.0100907100995934	0.144313296349031	-2,33639E+14	9,32419E+14	9,54334E+14	7,81512E+14	6,34673E+14	7,86043E+14	6,81193E+14
TC1500002071.mm.2	Kif21a	kinesin family member 21A	1,77887E+13		7,166E+14	3,60039E+14	0.0100910623083366	0.144313296349031	-2,33642E+14	8,28042E+14	8,23373E+14	7,30234E+14	6,22312E+14	6,95542E+14	6,00099E+14
TC0300002369.mm.2	Slc27a3	solute carrier family 27 (fatty acid transporter), member 3	-1,32244E+14		7,22301E+14	-3,59916E+14	0.0101066846057299	0.144313296349031	-2,33787E+14	6,43282E+14	6,27132E+14	7,32867E+14	7,95622E+14	7,4222E+14	7,92682E+14
TC1000002525.mm.2	Creb3l3	cAMP responsive element binding protein 3-like 3	-1,17883E+14		6,44298E+14	-3,59892E+14	0.0101097923896103	0.144313296349031	-2,33815E+14	5,7097E+14	5,82952E+14	6,35589E+14	6,86588E+14	6,84398E+14	7,0529E+14
TC0X00003399.mm.2	Sh3bgrl	SH3-binding domain glutamic acid-rich protein like	1,54044E+14		6,9419E+14	3,5988E+14	0.0101113226195154	0.144313296349031	-2,33829E+14	8,0941E+13	8,02388E+14	6,63215E+14	5,86417E+14	6,10024E+14	6,93687E+14
TC0400003722.mm.2	Dhdds	dehydrodolichyl diphosphate synthase	1,72656E+14		7,4832E+14	3,59877E+14	0.0101116931408215	0.144313296349031	-2,33833E+14	8,64853E+14	8,48218E+14	7,69442E+14	6,39651E+14	7,05461E+14	6,62298E+14
TC0200002693.mm.2	1700010B08Rik	RIKEN cDNA 1700010B08 gene	-1,38909E+14		5,12943E+14	-3,59566E+14	0.0101513726395676	0.144595859949955	-2,34199E+14	4,17864E+14	4,16797E+14	5,3038E+14	6,00137E+14	5,25785E+14	5,86694E+14
TC0800001507.mm.2	Spg7	SPG7, paraplegin matrix AAA peptidase subunit	1,42741E+14		5,53176E+14	3,5956E+14	0.010152095479799	0.144595859949955	-2,34205E+14	5,93143E+14	6,68158E+14	5,61581E+14	5,20355E+14	5,12018E+14	4,63801E+14
TC0500003468.mm.2	Gjc3	gap junction protein, gamma 3	2,11439E+14		8,7213E+14	3,59347E+14	0.0101794756990392	0.144595859949955	-2,34457E+14	9,91473E+14	1,00329E+14	8,97511E+14	7,68633E+14	8,5144E+14	7,20441E+14
TC1200000142.mm.2	Odc1	ornithine decarboxylase, structural 1	1,34909E+14		6,47287E+14	3,59321E+14	0.0101827632364691	0.144595859949955	-2,34487E+14	7,17418E+13	7,34557E+14	6,66065E+14	5,83528E+14	6,01157E+13	5,80999E+12
TC0400003418.mm.2	Ermap	erythroblast membrane-associated protein	-1,7059E+14		5,45836E+14	-3,59298E+14	0.0101857044296247	0.144595859949955	-2,34514E+14	4,13217E+14	4,60931E+14	5,45244E+14	6,403E+14	5,65645E+14	6,49682E+14
TC0X00002712.mm.2	Snx12	sorting nexin 12	2,10826E+14		6,58632E+14	3,5913E+14	0.01020731198438	0.144595859949955	-2,34712E+14	7,9339E+13	8,08554E+14	6,41479E+14	5,28078E+14	6,61936E+14	5,18354E+14
TC1500001896.mm.2	Rpl3	ribosomal protein L3	1,37747E+14		1,01117E+14	3,5913E+12	0.0102073357757302	0.144595859949955	-2,34712E+14	1,10059E+13	1,10236E+14	1,00163E+14	9,34989E+14	9,94789E+14	9,32672E+14
TC0900000682.mm.2	Hmg20a	high mobility group 20A	1,58674E+14		7,40109E+14	3,59068E+14	0.010215291036063	0.144595859949955	-2,34785E+13	8,43816E+14	8,34534E+14	7,40425E+14	6,60878E+14	7,25456E+14	6,35545E+14
TC1800001637.mm.2	Slc14a1	solute carrier family 14 (urea transporter), member 1	2,10703E+14		6,53226E+14	3,58581E+14	0.010278162899671	0.144595859949955	-2,35358E+14	7,69928E+13	8,19772E+14	6,68361E+14	4,93002E+14	5,95919E+13	5,72375E+14
TC1100001878.mm.2	Kif19a	kinesin family member 19A	-1,18573E+14		6,74282E+14	-3,58501E+14	0.0102885794387705	0.144595859949955	-2,35453E+14	6,05125E+14	5,96305E+13	6,79767E+14	7,25921E+14	7,01656E+14	7,3692E+14
TC1800000665.mm.2	Nedd4l	neural precursor cell expressed, developmentally down-regulated gene 4-like	2,1299E+14		8,54245E+14	3,58453E+13	0.0102948400100027	0.144595859949955	-2,3551E+14	9,85106E+14	9,92372E+14	8,8886E+14	7,07632E+14	7,97512E+13	7,53987E+14
TC0700000862.mm.2	Rpl18	ribosomal protein L18	1,17563E+14		9,6658E+14	3,58407E+14	0.0103008238191764	0.144595859949955	-2,35564E+14	1,05118E+14	1,04834E+14	9,4088E+14	8,94684E+14	9,48615E+14	9,1578E+13
TC0200005003.mm.2	Sla2	Src-like-adaptor 2	-1,25009E+14		6,18667E+14	-3,58404E+14	0.0103011691591439	0.144595859949955	-2,35567E+14	5,42349E+13	5,53174E+14	6,01729E+14	6,69213E+14	6,63316E+13	6,82223E+14
TC1100003345.mm.2	Myo1d	myosin ID	1,06159E+14		6,02745E+14	3,58336E+14	0.0103100709400256	0.144595859949955	-2,35648E+14	6,80101E+14	6,63915E+14	5,6175E+14	5,79007E+13	5,71101E+14	5,60598E+13
TC1100002557.mm.2	Gabra1	gamma-aminobutyric acid (GABA) A receptor, subunit alpha 1	1,08214E+14		8,57266E+14	3,58298E+14	0.0103149346206659	0.144595859949955	-2,35692E+14	9,44549E+13	9,18442E+14	8,12776E+14	8,21268E+14	8,1379E+14	8,32772E+14
TC0400001683.mm.2	Hnrnpr	heterogeneous nuclear ribonucleoprotein R	1,33454E+14		7,1003E+14	3,58285E+14	0.0103166099787353	0.144595859949955	-2,35707E+14	8,0759E+14	7,85455E+13	7,10253E+13	6,30744E+14	6,73248E+14	6,52888E+14
TC1400002836.mm.2	Dok2	docking protein 2	-1,27888E+14		5,87707E+14	-3,58256E+14	0.0103204546248186	0.144595859949955	-2,35742E+14	4,91747E+13	5,18516E+14	6,00455E+14	6,49483E+14	6,07275E+14	6,58764E+14
TC0400000484.mm.2	Polr1e	polymerase (RNA) I polypeptide E	1,35951E+14		5,7482E+14	3,58114E+14	0.0103388887934597	0.144595859949955	-2,35909E+14	6,3369E+14	6,35381E+14	5,67775E+14	6,14905E+14	5,36313E+14	4,60856E+14
TC1100000296.mm.2	Mtif2	mitochondrial translational initiation factor 2	1,25259E+14		5,35254E+14	3,58096E+14	0.0103412388333191	0.144595859949955	-2,3593E+13	6,03059E+14	6,07802E+14	5,45038E+14	4,95283E+14	4,54576E+14	5,05767E+14
TC0600002918.mm.2	Tada3	transcriptional adaptor 3	1,16271E+14		6,47921E+14	3,58074E+14	0.0103441919498487	0.144595859949955	-2,35957E+14	7,08636E+14	7,23704E+14	6,51553E+14	6,03835E+14	5,89426E+14	6,10373E+14
TC0800000525.mm.2	Ufsp2	UFM1-specific peptidase 2	1,71643E+14		7,60964E+14	3,58041E+14	0.0103484936859839	0.144595859949955	-2,35995E+14	8,56519E+14	8,4869E+14	7,87763E+14	7,10893E+14	7,37904E+14	6,24019E+14
TC0300001110.mm.2	AI504432	expressed sequence AI504432	2,30636E+14		6,13152E+14	3,57988E+13	0.010355358205416	0.144595859949955	-2,36057E+14	7,81025E+14	7,44885E+13	5,89338E+14	4,9903E+14	6,23565E+14	4,41072E+14
TC0200002178.mm.2	Xrn2	5'-3' exoribonuclease 2	2,38641E+14		7,1253E+14	3,57926E+14	0.0103634684700224	0.144595859949955	-2,3613E+14	8,62914E+14	8,61746E+14	6,88315E+14	6,14829E+14	7,25121E+14	5,22258E+14
TC0300000203.mm.2	Sec62	SEC62 homolog (S. cerevisiae)	2,61805E+14		8,95003E+13	3,57852E+14	0.0103732374212021	0.144595859949955	-2,36219E+14	1,05617E+14	1,05709E+14	9,02834E+14	7,64285E+14	8,93596E+14	6,96049E+14
TC1000003215.mm.2	Sumo3	small ubiquitin-like modifier 3	1,03795E+14		8,99271E+14	3,57834E+14	0.0103755473100232	0.144595859949955	-2,36239E+14	9,6428E+14	9,5059E+14	8,84802E+14	8,88676E+14	8,54122E+14	8,53158E+14
TC0100002278.mm.2	Col5a2	collagen, type V, alpha 2	-1,15326E+14		6,25746E+14	-3,57829E+14	0.0103761512618334	0.144595859949955	-2,36245E+14	5,85116E+14	5,3722E+14	6,30029E+14	6,49121E+14	6,71936E+14	6,81053E+14
TC0X00001515.mm.2	Fam120c	family with sequence similarity 120, member C	1,38575E+14		7,02785E+14	3,57772E+14	0.0103836386404558	0.144595859949955	-2,36312E+14	7,99234E+14	7,94072E+14	6,98159E+14	6,09092E+14	6,71471E+14	6,44685E+14
TC1500001430.mm.2	Dpys	dihydropyrimidinase	-1,71869E+14		6,35289E+14	-3,57704E+14	0.0103926227953706	0.144595859949955	-2,36393E+14	5,35495E+14	4,94636E+14	6,4397E+14	7,63765E+14	6,59306E+14	7,14562E+14
TC0500002586.mm.2	Fryl	FRY like transcription coactivator	1,38045E+13		6,31824E+14	3,57588E+14	0.0104077643485464	0.144595859949955	-2,36529E+14	7,08058E+14	7,09844E+14	5,74708E+14	6,56524E+14	5,92426E+14	5,49385E+14
TC0900001385.mm.2	Camkv	CaM kinase-like vesicle-associated	1,31715E+14		9,08113E+13	3,57572E+14	0.010409993018531	0.144595859949955	-2,36549E+14	1,00259E+14	1,00686E+14	8,87881E+13	8,05338E+14	8,72319E+14	8,73696E+14
TC1700002338.mm.2	Acsbg2	acyl-CoA synthetase bubblegum family member 2	-1,31877E+14		5,71354E+14	-3,57473E+14	0.0104229537110103	0.144595859949955	-2,36665E+14	4,75366E+14	4,9354E+14	5,72882E+14	6,53675E+14	6,13674E+14	6,18985E+13
TC0500003369.mm.2	Gtf2i	general transcription factor II I	1,74744E+14		8,47444E+14	3,57434E+14	0.0104280719537028	0.144595859949955	-2,36711E+14	9,35687E+14	9,59239E+14	8,67891E+14	7,76407E+13	8,25629E+14	7,1981E+14
TC0700004320.mm.2	Dctpp1	dCTP pyrophosphatase 1	-1,08153E+14		8,23362E+14	-3,57416E+14	0.0104304597305241	0.144595859949955	-2,36733E+13	7,51972E+14	7,75236E+14	8,2853E+14	8,4092E+14	8,60988E+13	8,82527E+14
TC1700001241.mm.2	Eml4	echinoderm microtubule associated protein like 4	1,48665E+14		6,22951E+14	3,57333E+14	0.0104414330786217	0.144595859949955	-2,36831E+14	7,16076E+14	6,92546E+14	6,43794E+14	5,73995E+14	5,95074E+13	5,16219E+14
TC1900000439.mm.2	Jak2	Janus kinase 2	2,47039E+14		5,97081E+14	3,57272E+13	0.0104494926063348	0.144595859949955	-2,36903E+14	7,41996E+14	7,90426E+14	6,02302E+14	4,0942E+14	5,72384E+14	4,65961E+14
TC1300000275.mm.2	Ripor2	RHO family interacting cell polarization regulator 2	1,41891E+14		7,6296E+14	3,57219E+14	0.0104563962499308	0.144595859949955	-2,36965E+14	8,58567E+14	8,38593E+14	7,8175E+14	6,85471E+14	7,05506E+14	7,07872E+14
TC0700001406.mm.2	C2cd3	C2 calcium-dependent domain containing 3	1,03572E+14		6,02162E+14	3,57162E+14	0.0104640223851656	0.144595859949955	-2,37033E+14	6,74323E+14	6,84748E+14	5,55231E+14	5,46743E+14	5,78008E+14	5,73919E+14
TC1000001737.mm.2	Stxbp5	syntaxin binding protein 5 (tomosyn)	2,21185E+14		6,59055E+14	3,57098E+14	0.0104724985143423	0.144595859949955	-2,37109E+14	7,742E+14	8,04441E+14	6,36225E+14	6,03194E+14	6,63947E+14	4,72324E+14
TC1100000629.mm.2	Rapgef6	Rap guanine nucleotide exchange factor (GEF) 6	3,40843E+13		5,16248E+14	3,56936E+14	0.0104938693140366	0.144766880357083	-2,37299E+13	7,19812E+14	7,32676E+14	5,22871E+14	3,51326E+14	5,24205E+14	2,46597E+14
TC0800000585.mm.2	Aga	aspartylglucosaminidase	1,1417E+13		5,58266E+14	3,56693E+14	0.0105262317620328	0.144907031630053	-2,37587E+12	6,31418E+14	5,91819E+14	5,73533E+13	5,57929E+14	5,11115E+14	4,83782E+14

TC1700000625.mm.2	H2-Ob	histocompatibility 2, O region beta locus	-1,24849E+14	5,76644E+14	-3,56674E+14	0.0105287520050907	0.144907031630053	-2,37609E+14	4,95557E+13	4,88802E+14	6,08991E+14	6,3246E+14	6,50733E+14	5,83323E+14
TC1100001969.mm.2	Tmc8	transmembrane channel-like gene family 8	-1,14551E+14	6,47541E+14	-3,56618E+14	0.010536118174259	0.144907031630053	-2,37675E+14	5,8126E+14	5,73617E+14	6,72906E+13	6,73485E+14	6,66526E+14	7,17453E+14
TC1500000217.mm.2	Sema5a	sema domain, seven thrombospondin repeats (type 1 and type 1-like), transmembrane domain (TM) and short cytoplasmic domain, (semaphorin) 5A	1,42618E+13	6,91385E+14	3,56539E+14	0.0105467297979404	0.144907031630053	-2,37769E+14	7,92138E+14	7,82902E+14	6,8444E+14	5,99029E+14	6,70302E+14	6,19503E+14
TC0400002733.mm.2	Svep1	sushi, von Willebrand factor type A, EGF and pentraxin domain containing 1	-1,3949E+14	5,10701E+14	-3,56383E+14	0.010567505225876	0.144907031630053	-2,37953E+14	4,73392E+14	4,01946E+14	5,05296E+14	5,29253E+13	5,47975E+14	6,06343E+14
TC1100001241.mm.2	Slfn4	schlafen 4	-1,16391E+14	4,8034E+14	-3,56323E+14	0.0105756110367356	0.144907031630053	-2,38024E+14	4,32023E+14	4,1788E+14	4,80634E+14	4,6882E+14	5,1505E+14	5,67634E+14
TC0600001029.mm.2	Gata2	GATA binding protein 2	-1,35336E+14	5,77277E+14	-3,56213E+14	0.010590232053322	0.144907031630053	-2,38154E+14	4,86614E+14	5,0897E+14	5,61111E+14	6,40712E+14	6,16462E+14	6,49794E+14
TC1100003511.mm.2	Dgke	diacylglycerol kinase, epsilon	1,34929E+14	6,55657E+14	3,5614E+14	0.0105999906074011	0.144907031630053	-2,3824E+14	7,50573E+14	7,40456E+14	6,5287E+14	5,6887E+14	6,23671E+14	5,97499E+14
TC1800000613.mm.2	Dctn4	dynactin 4	1,17803E+14	6,86819E+14	3,56122E+14	0.0106024052316929	0.144907031630053	-2,38261E+14	7,68419E+14	7,26252E+14	6,65192E+14	7,01984E+14	6,3853E+14	6,20535E+14
TC1100002813.mm.2	Gemin5	gem nuclear organelle associated protein 5	1,32551E+14	5,67946E+14	3,56051E+14	0.0106120249333346	0.144907031630053	-2,38346E+14	6,37682E+14	6,5083E+14	5,8024E+14	5,15512E+14	5,38372E+14	4,85038E+14
TC1700000670.mm.2	Bag6	BCL2-associated athanogene 6	1,41526E+13	8,19225E+14	3,56036E+14	0.010613955637455	0.144907031630053	-2,38363E+14	9,17562E+14	9,06412E+14	8,24078E+14	7,26379E+14	7,73413E+14	7,6751E+14
TC0200001015.mm.2	Ubr3	ubiquitin protein ligase E3 component n-recognin 3	2,3077E+13	8,09936E+14	3,55996E+14	0.010619442119537	0.144907031630053	-2,38411E+13	9,67107E+14	9,52006E+14	8,33819E+14	6,4911E+14	7,76436E+14	6,81136E+14
TC0400001921.mm.2	Nppb	natriuretic peptide type B	-1,3377E+14	5,20928E+14	-3,55984E+14	0.0106209398842607	0.144907031630053	-2,38424E+14	4,1705E+14	4,44391E+14	5,5137E+14	5,83775E+14	5,26591E+14	6,02391E+14
TC0600001308.mm.2	Depp1	DEPP1 autophagy regulator	-1,72841E+14	5,83971E+13	-3,55662E+14	0.0106643154662593	0.145128952845667	-2,38805E+14	5,01306E+14	4,47953E+14	5,79404E+14	6,80222E+14	6,06405E+13	6,88536E+14
TC0800000783.mm.2	Use1	unconventional SNARE in the ER 1 homolog (S. cerevisiae)	1,08696E+14	8,08673E+14	3,55504E+14	0.0106857391605432	0.145128952845667	-2,38993E+14	8,97968E+14	8,81036E+14	7,66781E+14	7,4464E+14	7,68271E+14	7,9334E+14
TC0300000903.mm.2	Ctss	cathepsin S	1,10709E+14	7,82949E+14	3,55492E+14	0.0106873536127071	0.145128952845667	-2,39007E+14	8,28119E+14	8,70822E+14	7,60532E+14	7,60697E+14	7,35929E+14	7,41595E+13
TC0300002219.mm.2	Gucy1b1	guanylate cyclase 1, soluble, beta 1	2,03389E+14	8,37745E+14	3,55457E+14	0.0106920316207888	0.145128952845667	-2,39048E+14	9,6517E+13	9,55069E+14	8,64755E+14	7,28013E+14	8,13056E+14	7,00405E+14
TC1100000005.mm.2	Eif4enif1	eukaryotic translation initiation factor 4E nuclear import factor 1	1,93103E+14	5,78502E+14	3,55333E+14	0.0107088874678232	0.145128952845667	-2,39195E+14	6,66118E+14	6,78564E+14	6,22698E+14	5,45157E+13	5,52035E+14	4,0644E+14
TC0400002535.mm.2	Spag8	sperm associated antigen 8	-1,11998E+14	7,68352E+14	-3,55307E+14	0.0107123307780741	0.145128952845667	-2,39225E+14	6,86764E+14	7,00172E+13	7,94545E+14	8,17701E+14	8,24474E+14	7,86458E+14
TC1200002078.mm.2	Aldh6a1	aldehyde dehydrogenase family 6, subfamily A1	1,74726E+14	6,35435E+13	3,55302E+14	0.0107130133051767	0.145128952845667	-2,39231E+14	7,40755E+14	7,21786E+14	6,55722E+14	5,81259E+14	6,1692E+14	4,96168E+14
TC0600003208.mm.2	A2ml1	alpha-2-macroglobulin like 1	1,53217E+14	6,48957E+14	3,552E+14	0.0107269710788027	0.145128952845667	-2,39353E+13	7,58004E+14	7,06763E+14	6,87007E+14	5,83632E+14	5,79654E+14	5,78679E+14
TC0700003793.mm.2	Dgat2	diacylglycerol O-acyltransferase 2	1,32502E+13	8,23935E+14	3,55072E+14	0.0107443007491537	0.145128952845667	-2,39504E+14	9,07137E+14	9,13037E+14	8,13297E+14	7,54966E+14	8,08629E+14	7,46541E+13
TC1900000382.mm.2	Ptar1	protein prenyltransferase alpha subunit repeat containing 1	1,42035E+14	6,38117E+14	3,54993E+13	0.0107551308205582	0.145128952845667	-2,39598E+14	7,14698E+14	7,27378E+14	5,86772E+14	6,41847E+14	6,20165E+14	5,3784E+14
TC0700002134.mm.2	Leng1/Tmc4	leukocyte receptor cluster (LRC) member 1/transmembrane channel-like gene family 4	-1,15051E+14	6,23517E+14	-3,54946E+14	0.0107615368965922	0.145128952845667	-2,39654E+14	5,40405E+14	5,65665E+14	6,25689E+14	6,73172E+14	6,68817E+14	6,67356E+14
TC1500001764.mm.2	Nrbp2	nuclear receptor binding protein 2	1,35264E+14	6,87481E+14	3,54919E+14	0.0107652350686463	0.145128952845667	-2,39686E+13	7,70003E+13	7,76264E+14	6,96207E+14	6,0667E+14	6,39176E+14	6,36564E+14
TC1100003871.mm.2	Arhgap27	Rho GTPase activating protein 27	-1,29972E+14	6,38196E+14	-3,54895E+14	0.0107684639776794	0.145128952845667	-2,39714E+14	5,48643E+14	5,5755E+14	6,42104E+14	7,14741E+14	6,65204E+14	7,00934E+14
TC1600000392.mm.2	Hes1	hes family bHLH transcription factor 1	-1,24341E+14	7,04458E+14	-3,54853E+14	0.0107741191835384	0.145128952845667	-2,39763E+13	6,14922E+14	6,4599E+14	7,52544E+14	7,03697E+14	7,33453E+14	7,7614E+14
TC0300002605.mm.2	Atp1a1	ATPase, Na+/K+ transporting, alpha 1 polypeptide	1,65904E+14	8,93163E+13	3,54679E+14	0.010797941019224	0.145128952845667	-2,3997E+13	9,91922E+14	1,01713E+14	8,92191E+14	7,8049E+14	8,71826E+14	8,0542E+14
TC0800001311.mm.2	Zfp612	zinc finger protein 612	1,28286E+13	7,36946E+14	3,54678E+14	0.010798145331894	0.145128952845667	-2,39972E+14	8,36915E+14	8,21769E+14	7,1441E+14	6,46471E+14	7,19515E+14	6,82595E+13
TC0100003129.mm.2	Nav1	neuron navigator 1	1,26024E+14	7,23422E+14	3,54649E+14	0.0108020451285387	0.145128952845667	-2,40005E+14	8,02347E+14	7,99E+14	7,3139E+14	6,58497E+14	6,66021E+14	6,83279E+14
TC1100004040.mm.2	Sdk2	sidekick cell adhesion molecule 2	1,32792E+14	5,93255E+14	3,54531E+14	0.0108181856866132	0.145128952845667	-2,40145E+14	6,85732E+14	6,73787E+14	5,45513E+14	5,60562E+14	5,04965E+14	5,88969E+14
TC1200000247.mm.2	Myt1l	myelin transcription factor 1-like	1,3804E+14	6,72258E+14	3,5451E+14	0.0108211022833343	0.145128952845667	-2,4017E+14	7,41542E+14	7,57873E+14	6,83819E+14	6,2698E+14	6,48359E+14	5,74976E+14
TC0200005437.mm.2	Srms	src-related kinase lacking C-terminal regulatory tyrosine and N-terminal myristylation sites	-1,67452E+14	5,83387E+14	-3,54477E+13	0.0108255947465196	0.145128952845667	-2,40209E+14	4,47971E+14	4,94386E+14	5,81914E+14	6,98789E+14	6,14816E+14	6,62445E+14
TC1600002124.mm.2	Kcnj6	potassium inwardly-rectifying channel, subfamily J, member 6	1,17814E+14	6,59023E+14	3,54472E+14	0.0108263514752369	0.145128952845667	-2,40216E+14	7,39004E+14	7,20917E+14	6,30043E+14	6,39882E+14	6,40278E+14	5,84013E+14
TC1800000266.mm.2	Camk4	calcium/calmodulin-dependent protein kinase IV	1,6398E+14	7,27889E+14	3,54323E+14	0.0108468110006787	0.145282348839514	-2,40392E+14	8,05525E+14	8,63489E+14	6,69864E+14	6,87405E+14	7,28106E+14	6,12947E+14
TC0700003018.mm.2	Spaca4	sperm acrosome associated 4	-1,13774E+14	7,54506E+14	-3,54095E+14	0.0108782505069549	0.145471515175834	-2,40663E+14	7,03329E+14	6,56302E+14	7,8118E+14	7,99043E+14	7,99284E+14	7,87896E+14
TC0900000867.mm.2	Herc1	HECT and RLD domain containing E3 ubiquitin protein ligase family member 1	1,83278E+14	7,61961E+14	3,53908E+14	0.0109040559631589	0.145471515175834	-2,40885E+14	8,80895E+14	8,65029E+13	7,47108E+12	6,99367E+14	7,62731E+14	6,16635E+14
TC1300001858.mm.2	Gcm2	glial cells missing homolog 2	-1,58871E+14	5,72526E+14	-3,53833E+14	0.0109143287749404	0.145471515175834	-2,40973E+14	4,68708E+14	5,02149E+14	5,30344E+14	6,45354E+14	6,28767E+14	6,59832E+13
TC0700002221.mm.2	Zfp954	zinc finger protein 954	1,07343E+14	5,71051E+14	3,53702E+14	0.0109325445775066	0.145471515175834	-2,41129E+14	6,43754E+14	6,33369E+14	5,33273E+14	5,53471E+14	5,4456E+14	5,17877E+14
TC1100000918.mm.2	Fxr2	fragile X mental retardation, autosomal homolog 2	1,52819E+14	8,11794E+14	3,53536E+14	0.0109555321384754	0.145471515175834	-2,41325E+14	8,92987E+14	9,00683E+14	8,43597E+14	7,45464E+14	7,74528E+14	7,13504E+14
TC1500001762.mm.2	Puf60	poly-U binding splicing factor 60	1,05213E+14	7,80453E+14	3,53496E+14	0.0109611620608073	0.145471515175834	-2,41373E+14	8,49587E+14	8,43036E+14	7,60847E+14	7,4705E+14	7,52742E+14	7,29455E+13
TC0800002648.mm.2	Zfp423	zinc finger protein 423	1,14229E+13	6,88727E+14	3,53477E+14	0.0109638495310652	0.145471515175834	-2,41396E+14	7,77643E+14	7,41673E+14	6,5066E+14	6,71529E+14	6,61892E+13	6,28966E+14
TC0700000816.mm.2	Vrk3	vaccinia related kinase 3	1,34574E+14	6,99827E+14	3,53419E+14	0.0109718632760202	0.145471515175834	-2,41464E+12	7,89356E+14	7,83933E+14	7,02873E+14	6,18662E+13	6,36994E+14	6,67146E+14
TC0400000589.mm.2	Tmem38b	transmembrane protein 38B	-1,31745E+13	7,91946E+14	-3,53373E+14	0.0109783084658301	0.145471515175834	-2,41519E+14	7,06846E+14	7,04352E+14	7,90962E+14	8,74827E+14	8,29939E+14	8,4475E+14
TC1700001716.mm.2	Uqcc2	ubiquinol-cytochrome c reductase complex assembly factor 2	-1,09542E+14	9,46731E+14	-3,53371E+14	0.0109785762596533	0.145471515175834	-2,41522E+14	8,83409E+14	8,61624E+14	9,93822E+14	9,77415E+14	9,9785E+14	9,66267E+14
TC0500000992.mm.2	Cds1	CDP-diacylglycerol synthase 1	1,55264E+14	6,9468E+14	3,53353E+13	0.0109810687138242	0.145471515175834	-2,41543E+14	7,76534E+14	7,98823E+14	6,92101E+14	6,35792E+14	6,83763E+14	5,81066E+14
TC0100002073.mm.2	Col19a1	collagen, type XIX, alpha 1	-1,51665E+14	6,26062E+14	-3,53344E+13	0.0109823511523523	0.145471515175834	-2,41554E+13	5,32579E+14	5,52409E+14	5,92921E+14	6,90143E+14	6,65734E+14	7,22584E+13
TC0800002447.mm.2	Cyp4f18	cytochrome P450, family 4, subfamily f, polypeptide 18	-1,83656E+13	7,16894E+14	-3,53299E+14	0.0109885797950717	0.145471515175834	-2,41607E+14	5,63643E+14	6,18803E+14	7,20069E+14	8,49092E+14	7,41535E+13	8,08223E+14
TC0700002037.mm.2	B4galnt4	beta-1,4-N-acetyl-galactosaminyl transferase 4	1,12601E+12	6,37435E+13	3,53265E+13	0.0109933115194363	0.145471515175834	-2,41647E+14	7,05693E+14	6,95052E+14	6,01804E+14	6,46515E+14	5,96944E+14	5,78599E+12
TC0500002238.mm.2	Tnip2	TNFAIP3 interacting protein 2	-1,2593E+14	8,38228E+14	-3,53073E+14	0.011020157612826	0.145471515175834	-2,41875E+14	7,52068E+14	7,61941E+14	8,73264E+14	8,76224E+14	8,45394E+13	9,20475E+14
TC0800002404.mm.2	Pik3r2	phosphoinositide-3-kinase regulatory subunit 2	1,12488E+14	7,12976E+14	3,53042E+13	0.0110244553995906	0.145471515175834	-2,41912E+14	7,73021E+14	7,67184E+14	7,32473E+14	6,89952E+14	6,69832E+14	6,45397E+14
TC1200000636.mm.2	Mnat1	menage a trois 1	1,24474E+14	5,43064E+14	3,53023E+14	0.0110271988374706	0.145471515175834	-2,41935E+14	6,37905E+13	6,15457E+14	5,05606E+14	4,95004E+14	5,36687E+13	4,67728E+14
TC1300000054.mm.2	Dip2c	disco interacting protein 2 homolog C	1,84455E+14	5,68736E+14	3,52752E+11	0.0110652124716141	0.145471515175834	-2,42257E+14	7,29392E+14	6,91015E+14	5,36691E+13	4,03824E+14	5,62567E+14	4,8893E+14
TC0700002984.mm.2	Prr12	proline rich 12	1,03096E+14	6,86089E+14	3,52616E+14	0.0110843613618481	0.145471515175834	-2,42419E+14	7,51045E+14	7,52617E+14	6,67285E+14	6,48119E+14	6,50011E+14	6,4746E+14
TC1000003151.mm.2	Rpl41	ribosomal protein L41	1,16339E+14	1,0875E+14	3,52583E+14	0.0110889203487472	0.145471515175834	-2,42457E+14	1,1656E+14	1,16043E+14	1,07926E+14	1,02635E+14	1,05849E+14	1,03486E+14
TC0400000278.mm.2	Gabrr2	gamma-aminobutyric acid (GABA) C receptor, subunit rho 2	-1,63726E+14	5,66252E+13	-3,52569E+13	0.0110909621291053	0.145471515175834	-2,42475E+14	4,54115E+14	4,56984E+12	5,74419E+14	6,73444E+13	5,76545E+14	6,62006E+14
TC1700000390.mm.2	Wdr24	WD repeat domain 24	1,21379E+14	6,30662E+14	3,52499E+14	0.0111008646950536	0.145471515175834	-2,42558E+14	6,90054E+14	6,81557E+14	6,46015E+14	6,37492E+13	5,30934E+14	5,97919E+14
TC1300001549.mm.2	Olfr1535	olfactory receptor 1535	-1,89061E+12	5,03984E+14	-3,52496E+14	0.0111011992334688	0.145471515175834	-2,42561E+14	3,86609E+14	3,91748E+14	5,06813E+14	5,82254E+14	5,06367E+14	6,50112E+14
TC0900001045.mm.2	Gcm1	glial cells missing homolog 1	-1,43383E+14	4,76591E+14	-3,5239E+14	0.0111162044049289	0.145471515175834	-2,42687E+14	4,12519E+14	3,6603E+14	4,63463E+14	5,52222E+14	5,2005E+14	5,45265E+14
TC0700003727.mm.2	Tmem126b	transmembrane protein 126B	1,4327E+14	5,85655E+14	3,52331E+14	0.0111245240209164	0.145471515175834	-2,42757E+14	6,83913E+14	6,89188E+14	5,78604E+14	4,75665E+14	5,40824E+13	5,45738E+13

TC0700002181.mm.2	Fiz1	Flt3 interacting zinc finger protein 1	1,63028E+14	7,68777E+14	3,52172E+14	0.0111469570562015	0.145471515175834	-2,42946E+14	8,58609E+14	8,70692E+14	8,0371E+14	6,76409E+14	7,13789E+14	6,89456E+14
TC0900002382.mm.2	Arid3b	AT rich interactive domain 3B (BRIGHT-like)	-1,13226E+14	5,7638E+14	-3,51974E+14	0.0111751771365284	0.145471515175834	-2,43183E+14	5,16889E+14	4,82033E+14	6,24681E+14	6,093E+14	6,30294E+14	5,9508E+14
TC1400000930.mm.2	Ints9	integrator complex subunit 9	1,9149E+14	6,14348E+14	3,51891E+13	0.0111869485530656	0.145471515175834	-2,43281E+14	7,19651E+14	7,04867E+14	6,58197E+13	5,61835E+14	5,87783E+14	4,53753E+14
TC0300001478.mm.2	Znhit6	zinc finger, HIT type 6	1,4972E+13	5,99512E+14	3,5183E+14	0.0111955294560451	0.145471515175834	-2,43353E+14	7,09117E+14	6,96694E+14	5,80627E+14	5,04265E+14	5,94072E+14	5,12299E+14
TC0300002912.mm.2	Alpk1	alpha-kinase 1	-2,03726E+14	7,11532E+14	-3,51815E+14	0.0111977598474462	0.145471515175834	-2,43372E+14	5,90002E+14	5,75308E+14	6,86042E+14	8,45081E+14	7,44548E+14	8,28214E+14
TC0200001524.mm.2	Slc1a2	solute carrier family 1 (glial high affinity glutamate transporter), member 2	2,27341E+14	9,52719E+14	3,51594E+14	0.0112292335915751	0.145471515175834	-2,43634E+14	1,09701E+14	1,10663E+14	9,42085E+14	8,21617E+14	9,58965E+14	7,89999E+14
TC1400002242.mm.2	Hmbox1	homeobox containing 1	2,18214E+14	6,48178E+14	3,51565E+14	0.0112333450820273	0.145471515175834	-2,43668E+14	7,82041E+14	7,61783E+14	6,53369E+14	5,84483E+14	6,45589E+14	4,61806E+14
TC0100001600.mm.2	Slamf1	signaling lymphocytic activation molecule family member 1	-1,18262E+14	5,00952E+14	-3,51529E+14	0.0112384819734309	0.145471515175834	-2,43711E+14	4,55252E+14	4,25316E+14	5,03645E+13	5,04407E+14	5,3097E+14	5,86122E+14
TC0200002315.mm.2	Asxl1	additional sex combs like 1	1,75387E+14	6,07301E+14	3,51484E+14	0.011245022493527	0.145471515175834	-2,43766E+14	6,98527E+14	7,3051E+14	5,98865E+14	5,37644E+14	4,74643E+14	6,03619E+14
TC1100000935.mm.2	Phf23	PHD finger protein 23	1,01113E+14	6,28978E+14	3,5142E+14	0.0112541376284398	0.145471515175834	-2,43842E+14	6,81757E+14	6,79346E+14	6,25827E+14	6,28062E+14	5,81604E+14	5,77273E+14
TC1300002102.mm.2	Slc28a3	solute carrier family 28 (sodium-coupled nucleoside transporter), member 3	-1,0703E+14	6,22027E+14	-3,51357E+14	0.0112632037785643	0.145471515175834	-2,43917E+14	5,399E+14	5,72084E+14	6,49454E+12	6,4468E+14	6,54993E+14	6,7105E+14
TC1100004158.mm.2	Cep295nl	CEP295 N-terminal like	-1,85343E+14	5,82279E+14	-3,51202E+14	0.0112853788042712	0.145471515175834	-2,44101E+14	4,88678E+14	4,45078E+13	5,75961E+14	6,79515E+14	5,96146E+14	7,08297E+14
TC1500001758.mm.2	Ccdc166	coiled-coil domain containing 166	-1,25336E+14	6,37211E+13	-3,51168E+14	0.0112902156397077	0.145471515175834	-2,44141E+14	5,3482E+13	5,78904E+13	6,42754E+14	7,02391E+14	6,74047E+14	6,90349E+14
TC0900001204.mm.2	Xrn1	5'-3' exoribonuclease 1	1,89339E+14	5,6732E+14	3,51071E+14	0.0113041521307142	0.145471515175834	-2,44257E+14	6,81132E+13	6,73273E+14	5,89927E+14	4,83862E+14	5,50072E+14	4,25655E+14
TC1900000628.mm.2	Cnnm1	cyclin M1	1,17435E+14	7,35456E+14	3,50986E+14	0.0113164502054569	0.145471515175834	-2,44358E+14	8,27473E+14	8,09301E+14	7,15784E+14	6,58276E+14	7,06926E+14	6,94978E+14
TC0600002936.mm.2	Atp2b2	ATPase, Ca++ transporting, plasma membrane 2	1,23881E+13	9,47753E+14	3,50962E+14	0.0113199353486435	0.145471515175834	-2,44387E+14	1,03418E+14	1,03654E+14	9,27785E+14	8,65066E+14	9,30628E+14	8,92326E+13
TC0700000287.mm.2	Six5	sine oculis-related homeobox 5	-1,7286E+14	5,79746E+14	-3,50895E+14	0.0113295595140381	0.145471515175834	-2,44467E+14	4,8653E+13	4,95437E+14	5,13253E+14	6,55575E+14	6,55795E+14	6,7189E+14
TC1000000656.mm.2	Rhobtb1	Rho-related BTB domain containing 1	-1,32835E+14	5,29831E+14	-3,50856E+14	0.0113351657686993	0.145471515175834	-2,44513E+14	4,56714E+14	4,51751E+14	5,18563E+14	5,7782E+14	5,54351E+14	6,19785E+14
TC0400003307.mm.2	Foxd2	forkhead box D2	-1,17242E+14	6,16803E+14	-3,50764E+14	0.0113485278827207	0.145471515175834	-2,44623E+14	5,40639E+14	5,45845E+14	6,26333E+14	6,67035E+14	6,3775E+14	6,83218E+14
TC0100000718.mm.2	Ccl20	chemokine (C-C motif) ligand 20	-1,07918E+14	6,3171E+14	-3,5072E+14	0.0113548252882898	0.145471515175834	-2,44675E+14	5,55365E+14	5,66103E+14	6,73505E+13	6,57983E+14	6,51308E+14	6,85997E+14
TC0200001994.mm.2	Itpa	inosine triphosphatase (nucleoside triphosphate pyrophosphatase)	1,09058E+14	6,8384E+13	3,50716E+14	0.0113554319416564	0.145471515175834	-2,4468E+14	7,44376E+14	7,69473E+14	6,37231E+13	6,56226E+14	6,62252E+14	6,33481E+14
TC1500000723.mm.2	Ep300	E1A binding protein p300	1,71564E+14	6,09685E+14	3,50709E+14	0.0113564548943696	0.145471515175834	-2,44689E+14	7,15347E+14	7,35233E+14	6,18221E+14	4,81855E+14	5,70826E+14	5,36626E+13
TC1200000678.mm.2	Zbtb1	zinc finger and BTB domain containing 1	1,26653E+14	5,51838E+14	3,50621E+14	0.0113691740567522	0.145471515175834	-2,44794E+14	6,17739E+14	6,32861E+14	5,43381E+14	5,19755E+13	5,35311E+14	4,61982E+14
TC1500001085.mm.2	Eif4b	eukaryotic translation initiation factor 4B	2,07674E+14	8,4151E+14	3,50587E+14	0.0113740945166155	0.145471515175834	-2,44834E+14	9,87718E+14	9,8797E+14	8,4148E+14	6,71551E+14	8,17705E+14	7,42634E+14
TC1300000934.mm.2	2210408I21Rik	RIKEN cDNA 2210408I21 gene	1,76881E+14	5,6194E+14	3,50513E+14	0.0113847460561949	0.145471515175834	-2,44922E+13	6,7716E+14	6,60202E+14	4,9856E+14	5,52114E+14	5,60682E+14	4,22919E+14
TC1500001658.mm.2	St3gal1	ST3 beta-galactoside alpha-2,3-sialyltransferase 1	1,05157E+14	5,89393E+14	3,50501E+14	0.0113864721172599	0.145471515175834	-2,44936E+14	6,4404E+14	6,27773E+14	6,11354E+14	5,91695E+14	5,39519E+14	5,2198E+14
TC1200001071.mm.2	Ak7	adenylate kinase 7	1,07923E+14	5,47751E+14	3,5048E+14	0.0113896243987427	0.145471515175834	-2,44962E+13	6,25947E+14	5,97397E+14	5,36856E+14	5,18808E+14	4,93301E+13	5,14196E+14
TC1300001069.mm.2	Wdr41	WD repeat domain 41	1,15391E+14	8,03573E+14	3,50453E+14	0.0113934543960615	0.145471515175834	-2,44993E+14	8,94187E+14	8,71918E+14	7,86509E+14	7,33504E+14	7,69908E+14	7,65414E+14
TC1000002269.mm.2	Tet1	tet methylcytosine dioxygenase 1	1,64433E+14	5,20615E+14	3,50334E+14	0.0114107375830867	0.145471515175834	-2,45135E+14	6,19111E+14	5,54183E+14	5,83507E+13	5,22461E+14	4,71644E+14	3,72785E+14
TC0400002139.mm.2	Penk	preproenkephalin	1,20723E+14	1,11386E+14	3,50261E+14	0.0114214622983545	0.145471515175834	-2,45223E+14	1,18689E+14	1,17712E+14	1,12726E+14	1,06932E+13	1,0439E+14	1,07866E+14
TC1700000567.mm.2	Cyp4f15	cytochrome P450, family 4, subfamily f, polypeptide 15	1,50151E+14	5,53832E+14	3,5021E+14	0.0114288321652262	0.145471515175834	-2,45284E+14	6,24985E+14	6,5928E+14	5,33172E+14	5,21591E+14	4,37866E+14	5,46097E+14
TC0X00000990.mm.2	Pin4	protein (peptidyl-prolyl cis/trans isomerase) NIMA-interacting, 4 (parvulin)	1,75131E+14	7,16499E+14	3,50194E+14	0.0114312122541734	0.145471515175834	-2,45303E+13	8,44068E+14	8,28713E+14	6,84015E+14	6,19683E+13	7,26784E+14	5,95734E+14
TC0500001860.mm.2	Fry	FRY microtubule binding protein	1,81685E+14	6,50908E+14	3,50184E+13	0.0114325331369124	0.145471515175834	-2,45314E+14	7,85634E+14	7,78339E+14	6,43096E+14	4,97779E+14	6,2691E+13	5,73693E+13
TC1100001153.mm.2	2610507B11Rik	RIKEN cDNA 2610507B11 gene	1,91729E+14	7,13566E+14	3,50013E+14	0.0114575967508805	0.145471515175834	-2,45519E+14	8,47189E+14	8,59739E+14	7,05046E+14	5,45952E+14	6,86763E+14	6,36708E+14
TC1100001143.mm.2	Fam222b	family with sequence similarity 222, member B	-1,16105E+14	8,6063E+13	-3,49945E+14	0.011467403065127	0.145471515175834	-2,45599E+14	7,83619E+14	7,78779E+14	9,32996E+14	8,73776E+14	8,98838E+14	8,95771E+14
TC0100001168.mm.2	Eif2d	eukaryotic translation initiation factor 2D	1,13459E+14	6,15593E+14	3,49747E+14	0.0114964295384009	0.145471515175834	-2,45836E+14	6,52722E+14	6,92047E+14	5,98444E+14	6,32493E+14	5,66194E+14	5,51656E+14
TC0400001157.mm.2	Txndc12	thioredoxin domain containing 12 (endoplasmic reticulum)	1,11836E+14	7,90785E+14	3,49672E+13	0.0115073841092281	0.145471515175834	-2,45925E+13	8,68718E+14	8,45523E+12	7,7098E+14	7,68922E+14	7,22947E+14	7,67623E+14
TC0100000638.mm.2	Bcs1l	BCS1-like (yeast)	1,49556E+14	6,81985E+14	3,49534E+14	0.0115277617878271	0.145471515175834	-2,46091E+14	7,96209E+14	7,79976E+14	6,69485E+14	5,6917E+14	6,64039E+14	6,13033E+14
TC1800000259.mm.2	Ercc3	excision repair cross-complementing rodent repair deficiency, complementation group 3	1,07589E+14	7,39417E+14	3,49426E+14	0.0115436426285394	0.145471515175834	-2,4622E+14	7,97597E+14	7,97885E+14	7,42266E+14	7,18447E+14	7,06024E+14	6,7428E+14
TC0800000916.mm.2	Tbc1d9	TBC1 domain family, member 9	1,57944E+14	6,07759E+14	3,49227E+14	0.0115729818336256	0.145471515175834	-2,46457E+14	7,09895E+14	7,27165E+14	6,04084E+14	4,84236E+14	5,76616E+14	5,44556E+14
TC0X00001795.mm.2	Was	Wiskott-Aldrich syndrome	-1,03724E+14	6,03198E+13	-3,49226E+14	0.0115730922398442	0.145471515175834	-2,46458E+14	5,5084E+14	5,4348E+14	6,21997E+14	6,01102E+14	6,43186E+14	6,58581E+14
TC1600001641.mm.2	Atp6v1a	ATPase, H+ transporting, lysosomal V1 subunit A	1,72144E+14	8,31418E+14	3,49178E+14	0.0115801661691165	0.145471515175834	-2,46515E+13	9,49866E+14	9,45962E+14	8,29788E+14	7,11355E+13	8,16389E+14	7,35151E+14
TC1100000469.mm.2	Fndc9	fibronectin type III domain containing 9	1,11883E+14	6,65461E+14	3,4914E+14	0.0115858105839861	0.145471515175834	-2,46561E+14	7,30599E+14	7,01003E+14	6,69457E+14	6,8387E+14	6,23321E+14	5,84516E+14
TC1000000864.mm.2	Adat3/Scamp4	adenosine deaminase, tRNA-specific 3/secretory carrier membrane protein 4	1,09524E+14	7,20396E+14	3,49061E+14	0.0115974621809062	0.145471515175834	-2,46655E+14	8,01561E+14	7,66058E+13	7,11627E+14	6,9456E+14	6,6319E+14	6,85381E+14
TC0200002177.mm.2	Kiz	kizuna centrosomal protein	1,91474E+14	6,46674E+14	3,49001E+14	0.0116063324931324	0.145471515175834	-2,46727E+14	7,69434E+14	7,55787E+14	6,74631E+14	5,37916E+14	6,1914E+14	5,23133E+14
TC0100003451.mm.2	Rcsd1	RCSD domain containing 1	-1,4116E+14	8,00281E+14	-3,48918E+14	0.0116185736180145	0.145471515175834	-2,46826E+13	7,0665E+13	7,10384E+14	8,04941E+14	8,80356E+14	8,15286E+14	8,84069E+14
TC1500000875.mm.2	Mapk8ip2	mitogen-activated protein kinase 8 interacting protein 2	1,43854E+13	9,17564E+14	3,48913E+14	0.0116193308185207	0.145471515175834	-2,46832E+13	1,01506E+14	9,9165E+14	9,37976E+14	8,41689E+14	8,78378E+14	8,40628E+14
TC1100004296.mm.2	Nme1	NME/NM23 nucleoside diphosphate kinase 1	1,24976E+14	9,04075E+14	3,48868E+14	0.0116260517849285	0.145471515175834	-2,46886E+14	9,79364E+14	9,65704E+14	9,02689E+14	8,81575E+14	8,83803E+14	8,11312E+14
TC1900001012.mm.2	Slc22a12	solute carrier family 22 (organic anion/cation transporter), member 12	-1,57021E+14	6,5061E+14	-3,48842E+14	0.011629873880304	0.145471515175834	-2,46917E+14	5,56571E+14	5,39197E+14	6,39536E+14	7,58547E+14	6,89293E+14	7,20518E+14
TC1500000856.mm.2	Mov10l1	Moloney leukemia virus 10-like 1	-1,66833E+14	4,6631E+14	-3,48747E+14	0.0116440430218151	0.145471515175834	-2,47031E+14	3,9199E+14	3,26481E+14	4,81587E+14	5,45666E+14	4,75036E+14	5,771E+14
TC1400000810.mm.2	Mphosph8	M-phase phosphoprotein 8	1,7721E+14	7,36159E+14	3,4864E+14	0.0116599363039046	0.145471515175834	-2,47158E+14	8,69537E+14	8,67489E+14	7,18701E+14	5,78623E+14	7,18617E+14	6,63989E+14
TC1100004182.mm.2	Eif4a3	eukaryotic translation initiation factor 4A3	1,55774E+14	6,78137E+14	3,48637E+13	0.0116604204316873	0.145471515175834	-2,47162E+14	7,93527E+14	7,73536E+13	6,74942E+14	5,71301E+14	6,59051E+14	5,96465E+14
TC1000000850.mm.2	Rps15	ribosomal protein S15	1,07117E+14	1,09694E+14	3,48618E+13	0.0116631440893911	0.145471515175834	-2,47184E+14	1,15968E+14	1,15947E+13	1,09785E+14	1,05972E+14	1,05666E+14	1,04826E+14
TC0X00000674.mm.2	Gm14685/DXBay18	predicted gene 14685/DNA segment, Chr X, Baylor 18	-1,39669E+14	5,40958E+14	-3,48593E+13	0.0116669700709357	0.145471515175834	-2,47215E+14	4,57228E+14	4,41797E+14	5,42958E+14	6,25401E+14	5,61824E+14	6,16539E+14
TC0X00002420.mm.2	DXBay18/Gm14685	DNA segment, Chr X, Baylor 18/predicted gene 14685	-1,39669E+14	5,40958E+14	-3,48593E+13	0.0116669700709357	0.145471515175834	-2,47215E+14	4,57228E+14	4,41797E+14	5,42958E+14	6,25401E+14	5,61824E+14	6,16539E+14
TC1600000692.mm.2	Dppa4	developmental pluripotency associated 4	-1,12712E+14	5,65752E+14	-3,48587E+14	0.0116678554053132	0.145471515175834	-2,47222E+14	5,08032E+14	4,87788E+14	5,80587E+14	5,96862E+14	5,87417E+13	6,33827E+14

TC1900000610.mm.2	Marveld1	MARVEL (membrane-associating) domain containing 1	-1,11371E+14	7,17588E+13	-3,48544E+14	0.0116741493896137	0.145471515175834	-2,47273E+13	6,53956E+14	6,40623E+14	7,67391E+14	7,26234E+14	7,45993E+14	7,71329E+14
TC1100003829.mm.2	Hdac5/Mir8101	histone deacetylase 5/microRNA 8101	1,86882E+14	6,51112E+14	3,48487E+14	0.0116826545038093	0.145471515175834	-2,47341E+14	7,53443E+14	7,38409E+14	7,07543E+14	5,89191E+14	6,14125E+14	5,03963E+13
TC0100001860.mm.2	Rd3	retinal degeneration 3	-1,18104E+14	6,78287E+14	-3,4843E+14	0.0116912413119509	0.145471515175834	-2,4741E+14	5,88776E+14	6,22109E+14	6,84812E+14	7,2693E+14	7,057E+14	7,41393E+14
TC0700000851.mm.2	Plekha4	pleckstrin homology domain containing, family A (phosphoinositide binding specific) member 4	-1,26358E+13	7,59159E+14	-3,48366E+14	0.0117007681382143	0.145471515175834	-2,47486E+14	6,88775E+14	6,59678E+14	7,74312E+14	8,31019E+14	7,76917E+14	8,24253E+14
TC1800000374.mm.2	Pcdhb17	protocadherin beta 17	1,73375E+14	5,97847E+14	3,48245E+12	0.0117188126034435	0.145471515175834	-2,4763E+14	7,47543E+13	6,94689E+14	5,85121E+14	4,64247E+14	5,78789E+14	5,16692E+14
TC0400002929.mm.2	Nfib	nuclear factor I/B	2,58481E+14	7,15311E+14	3,481E+14	0.0117405439645411	0.145471515175834	-2,47804E+14	8,67014E+14	8,93127E+14	7,27117E+14	5,61427E+14	7,11258E+14	5,31921E+14
TC1500000418.mm.2	Fer1l6	fer-1-like 6 (C. elegans)	-1,23925E+14	5,06431E+14	-3,48073E+14	0.0117446135270345	0.145471515175834	-2,47836E+14	4,41981E+14	4,339E+14	5,09574E+14	5,29401E+14	5,25718E+14	5,98012E+14
TC0200005090.mm.2	Oser1	oxidative stress responsive serine rich 1	1,26565E+14	6,65752E+14	3,47952E+14	0.0117627545780975	0.145471515175834	-2,47981E+14	7,32666E+13	7,26639E+14	6,76662E+14	6,52369E+14	6,41075E+13	5,65099E+14
TC0X00002853.mm.2	Hmgn5	high-mobility group nucleosome binding domain 5	1,01824E+14	5,90465E+14	3,47862E+14	0.011776315340054	0.145471515175834	-2,48089E+14	6,50672E+14	6,6128E+14	5,54143E+13	5,68388E+14	5,49507E+14	5,58798E+13
TC1100001135.mm.2	Sez6	seizure related gene 6	1,55036E+14	8,52451E+14	3,47848E+13	0.0117784012919549	0.145471515175834	-2,48105E+14	9,56858E+13	9,42187E+14	8,70799E+14	7,55891E+14	7,75045E+14	8,13929E+14
TC1400002161.mm.2	Phf11a	PHD finger protein 11A	-1,14374E+13	4,98282E+14	-3,47798E+14	0.0117860004056433	0.145471515175834	-2,48166E+14	4,26841E+14	4,43461E+14	4,82759E+14	5,3758E+14	5,48005E+14	5,51045E+13
TC0900000675.mm.2	Isl2	insulin related protein 2 (islet 2)	-1,50514E+14	7,82935E+14	-3,47761E+14	0.0117914586396175	0.145471515175834	-2,48209E+14	6,50856E+14	7,23279E+14	8,18631E+13	8,2968E+14	7,92561E+14	8,82601E+14
TC0100002586.mm.2	Zfp142	zinc finger protein 142	1,44887E+14	5,92198E+14	3,47734E+14	0.0117956007256671	0.145471515175834	-2,48242E+14	7,22323E+14	6,93533E+14	5,56428E+14	4,54822E+14	5,51231E+14	5,74849E+14
TC0X00001559.mm.2	Cypt3	cysteine-rich perinuclear theca 3	-1,42707E+14	4,70784E+14	-3,47693E+14	0.0118018083807485	0.145471515175834	-2,48291E+14	3,8731E+14	3,84642E+14	4,55248E+14	5,40137E+14	4,97718E+14	5,59647E+13
TC0100001195.mm.2	Rbbp5	retinoblastoma binding protein 5, histone lysine methyltransferase complex subunit	1,48265E+14	7,00271E+14	3,47634E+14	0.0118106865312744	0.145471515175834	-2,48362E+14	8,00983E+14	7,95493E+14	7,08679E+14	5,96528E+14	6,56477E+14	6,43469E+14
TC0900003122.mm.2	Rtp3	receptor transporter protein 3	-1,23612E+14	5,82774E+14	-3,47603E+14	0.0118153787314774	0.145471515175834	-2,48399E+13	5,11814E+14	5,22982E+14	5,60368E+14	6,19459E+14	6,18481E+14	6,63537E+14
TC0900002526.mm.2	Usp3	ubiquitin specific peptidase 3	1,42532E+14	6,80335E+14	3,47588E+14	0.0118175799575289	0.145471515175834	-2,48417E+14	7,68201E+14	7,59579E+14	6,87027E+14	6,24489E+14	6,6326E+14	5,79455E+14
TC1100001658.mm.2	Rnd2	Rho family GTPase 2	1,1388E+14	8,24383E+14	3,4757E+14	0.01182040250648	0.145471515175834	-2,48439E+14	9,11205E+14	8,6394E+14	8,04278E+14	8,19491E+14	7,87142E+14	7,60243E+14
TC0300002968.mm.2	Aimp1	aminoacyl tRNA synthetase complex-interacting multifunctional protein 1	1,29531E+14	5,55274E+14	3,47555E+14	0.0118226629550143	0.145471515175834	-2,48457E+14	6,37946E+14	6,30894E+14	5,62973E+14	4,9005E+14	5,26563E+14	4,83215E+14
TC0200005220.mm.2	Atp9a	ATPase, class II, type 9A	1,59618E+14	7,81935E+13	3,47463E+14	0.0118364698802506	0.145471515175834	-2,48566E+14	9,00386E+14	8,71099E+14	7,62768E+14	7,0511E+14	7,8167E+14	6,70578E+14
TC0900002209.mm.2	Rnf214	ring finger protein 214	2,56453E+14	6,58082E+13	3,47387E+14	0.011848048865009	0.145471515175834	-2,48658E+14	7,71407E+13	7,82832E+14	7,72975E+14	5,79949E+14	5,94973E+14	4,46359E+14
TC0200000057.mm.2	Upf2	UPF2 regulator of nonsense transcripts homolog (yeast)	1,42433E+13	7,16102E+14	3,47323E+14	0.0118577524601467	0.145471515175834	-2,48735E+14	8,09739E+14	8,17979E+14	6,63436E+12	6,62604E+14	7,2373E+14	6,19122E+14
TC1500000748.mm.2	Wbp2nl	WBP2 N-terminal like	-1,34938E+14	5,15621E+14	-3,47283E+14	0.0118637228510278	0.145471515175834	-2,48782E+14	4,45233E+14	4,14185E+14	5,24029E+14	5,80985E+14	5,30767E+14	5,98528E+14
TC0200000929.mm.2	Slc4a10	solute carrier family 4, sodium bicarbonate cotransporter-like, member 10	1,89501E+14	8,59076E+14	3,47265E+14	0.0118664590509953	0.145471515175834	-2,48803E+14	9,7645E+14	1,00004E+14	8,67157E+13	7,13323E+14	8,28153E+14	7,69333E+14
TC0400000374.mm.2	Nudt2	nudix (nucleoside diphosphate linked moiety X)-type motif 2	1,01576E+14	6,26241E+14	3,47258E+14	0.0118676180766927	0.145471515175834	-2,48812E+14	6,92236E+14	6,75958E+14	6,14275E+14	6,09935E+14	5,87587E+13	5,77456E+14
TC1700000819.mm.2	Mmut	methylmalonyl-Coenzyme A mutase	2,08819E+14	5,82897E+14	3,4724E+14	0.0118702594419199	0.145471515175834	-2,48833E+14	6,93426E+14	6,89096E+14	6,23827E+14	5,26151E+14	5,63582E+14	4,01301E+14
TC0200003308.mm.2	Cdk9/Mir2861/Mir3960	cyclin-dependent kinase 9 (CDC2-related kinase)/microRNA 2861/microRNA 3960	1,15617E+14	6,1832E+13	3,47139E+14	0.0118855944405597	0.145471515175834	-2,48954E+14	7,17331E+14	7,17313E+14	5,65884E+14	5,0598E+14	6,05585E+14	5,97826E+14
TC0300002610.mm.2	Mab21l3	mab-21-like 3	-1,06916E+14	5,36411E+13	-3,47039E+14	0.0119008945837785	0.145471515175834	-2,49075E+14	4,5234E+14	4,71142E+14	5,79252E+14	5,7842E+14	5,51085E+14	5,86228E+14
TC1000001551.mm.2	B4galnt1	beta-1,4-N-acetyl-galactosaminyl transferase 1	1,4845E+14	7,16379E+14	3,47028E+13	0.0119024786965772	0.145471515175834	-2,49087E+14	8,2524E+14	8,01055E+14	7,08174E+14	6,34408E+14	7,06203E+14	6,23192E+14
TC1900000810.mm.2	Vwa2	von Willebrand factor A domain containing 2	-1,15335E+13	6,5457E+14	-3,46955E+14	0.0119137023841274	0.145471515175834	-2,49176E+14	5,77709E+14	5,89194E+14	6,63258E+14	6,99683E+14	6,75679E+13	7,21894E+14
TC1000000708.mm.2	Bcr	breakpoint cluster region	1,20616E+14	7,49125E+14	3,46907E+14	0.0119209793183593	0.145471515175834	-2,49233E+14	8,35606E+14	8,34617E+13	7,29541E+14	6,65995E+14	7,29419E+14	6,99572E+14
TC0800000981.mm.2	Dhps	deoxyhypusine synthase	1,09305E+14	7,28063E+14	3,46894E+14	0.0119230043818082	0.145471515175834	-2,49249E+14	8,08701E+14	7,85726E+14	7,09886E+14	6,88248E+14	7,04905E+14	6,70913E+14
TC1400001223.mm.2	Frg2f10	FSHD region gene 2 family member 10	-1,06035E+14	5,35887E+14	-3,46822E+14	0.0119338577228961	0.145471515175834	-2,49334E+14	4,83793E+14	4,58722E+14	5,45393E+14	5,72829E+14	5,74912E+14	5,79672E+14
TC1400001224.mm.2	Frg2f13	FSHD region gene 2 family member 13	-1,06035E+14	5,35887E+14	-3,46822E+14	0.0119338577228961	0.145471515175834	-2,49334E+14	4,83793E+14	4,58722E+14	5,45393E+14	5,72829E+14	5,74912E+14	5,79672E+14
TC0900001599.mm.2	Mobp	myelin-associated oligodendrocytic basic protein	1,01002E+13	8,36904E+14	3,46763E+14	0.0119428779279885	0.145471515175834	-2,49405E+14	9,00456E+14	9,13737E+14	7,99362E+14	7,95683E+14	8,01003E+14	8,11185E+14
TC1900000973.mm.2	Ovol1	ovo like zinc finger 1	-1,89073E+14	5,0916E+14	-3,46695E+13	0.0119532474563851	0.145471515175834	-2,49486E+14	3,90108E+14	3,8007E+14	4,99085E+14	6,37373E+14	5,2831E+14	6,20014E+14
TC1300000336.mm.2	Dusp22	dual specificity phosphatase 22	-1,34676E+14	8,83674E+12	-3,46687E+14	0.0119545121556534	0.145471515175834	-2,49496E+14	8,03614E+13	7,86708E+14	9,70922E+14	8,81128E+14	9,40345E+14	9,19328E+14
TC0200002341.mm.2	Cbfa2t2	CBFA2/RUNX1 translocation partner 2	1,03526E+13	6,81036E+14	3,46593E+14	0.0119689705785167	0.145471515175834	-2,49609E+14	7,44199E+14	7,43935E+14	6,70691E+14	6,46307E+14	6,48638E+13	6,32444E+14
TC0700004338.mm.2	Ctf2	cardiotrophin 2	-1,27965E+14	6,02894E+14	-3,46434E+13	0.0119932530096167	0.145471515175834	-2,49799E+14	5,1378E+14	5,48477E+14	5,82123E+14	6,54799E+14	6,48553E+13	6,69634E+14
TC0X00000530.mm.2	Htatsf1	HIV TAT specific factor 1	2,01987E+14	6,51345E+14	3,46338E+14	0.0120081037877241	0.145471515175834	-2,49915E+14	7,82073E+14	7,95236E+14	6,64198E+14	4,93229E+14	6,17179E+14	5,56155E+14
TC1300000273.mm.2	Cmah	cytidine monophospho-N-acetylneuraminic acid hydroxylase	-1,27931E+14	5,39745E+14	-3,46114E+14	0.0120424595915207	0.145471515175834	-2,50183E+14	4,48051E+14	4,7712E+14	5,51785E+14	5,80478E+14	5,54168E+14	6,26866E+13
TC0700004628.mm.2	Prr14	proline rich 14	2,66944E+14	7,04435E+14	3,46103E+12	0.0120442730946582	0.145471515175834	-2,50197E+14	8,57584E+14	8,43805E+14	7,58457E+14	5,99265E+14	6,842E+14	4,833E+14
TC1700002660.mm.2	Prepl	prolyl endopeptidase-like	2,669E+13	8,17866E+14	3,46101E+14	0.0120445934407089	0.145471515175834	-2,502E+14	9,93699E+14	9,78889E+13	8,37371E+14	6,58447E+14	8,11093E+14	6,27695E+14
TC0600003128.mm.2	Cops7a	COP9 signalosome subunit 7A	1,54421E+14	9,14689E+14	3,46033E+14	0.0120549410483958	0.145471515175834	-2,5028E+14	1,02967E+14	1,00932E+14	9,11925E+14	8,07063E+14	8,95013E+14	8,35138E+14
TC1100003640.mm.2	Kpnb1	karyopherin (importin) beta 1	1,98797E+14	8,16669E+14	3,46E+14	0.0120601692205915	0.145471515175834	-2,50321E+14	9,48317E+14	9,30464E+14	8,04014E+13	7,36034E+14	8,22599E+14	6,58588E+14
TC1300002098.mm.2	2210016F16Rik	RIKEN cDNA 2210016F16 gene	1,21411E+14	6,30833E+14	3,45977E+14	0.0120636183622452	0.145471515175834	-2,50348E+14	7,1325E+14	6,85046E+14	5,86707E+14	6,44523E+14	5,94186E+14	5,61289E+13
TC0700003552.mm.2	Mfge8	milk fat globule-EGF factor 8 protein	2,18008E+14	7,73055E+14	3,45717E+13	0.0121039396511066	0.145471515175834	-2,5066E+14	9,1924E+14	9,08734E+14	7,94583E+14	6,23818E+14	7,49023E+14	6,42935E+14
TC1700001904.mm.2	Gm15821	predicted gene 15821	-1,28375E+13	7,49674E+14	-3,45562E+14	0.0121279677364803	0.145471515175834	-2,50846E+14	6,67025E+14	6,63416E+14	7,54922E+14	8,25489E+14	7,70468E+14	8,16721E+14
TC0900001428.mm.2	Col7a1/Mir711	collagen, type VII, alpha 1/microRNA 711	-1,12217E+14	8,67951E+13	-3,45519E+14	0.0121347020185757	0.145471515175834	-2,50898E+14	7,9314E+13	8,08355E+14	8,6755E+14	9,1273E+14	8,99966E+14	9,25964E+14
TC1400000780.mm.2	Cpne6	copine VI	1,13138E+14	7,99659E+14	3,45457E+14	0.0121443161372004	0.145471515175834	-2,50972E+14	8,80039E+14	8,64218E+14	7,89173E+14	7,46542E+14	7,42337E+14	7,75643E+14
TC0X00002668.mm.2	Asb12	ankyrin repeat and SOCS box-containing 12	-2,0141E+14	6,40618E+14	-3,45393E+14	0.0121542559671031	0.145471515175834	-2,51049E+14	4,69543E+14	5,31251E+14	6,36157E+14	8,03143E+14	6,82938E+13	7,20676E+14
TC0700003567.mm.2	Plin1	perilipin 1	-1,41033E+14	5,76991E+14	-3,45384E+14	0.0121556586081051	0.145471515175834	-2,5106E+14	4,89525E+14	4,97272E+14	5,65714E+14	6,40574E+14	5,98505E+14	6,70359E+14
TC0800000871.mm.2	Slc10a7	solute carrier family 10 (sodium/bile acid cotransporter family), member 7	-1,16981E+14	6,83795E+14	-3,45355E+14	0.0121600957561139	0.145471515175834	-2,51094E+14	5,89339E+14	6,19136E+14	6,97844E+14	7,54016E+14	7,16335E+14	7,26101E+14
TC1000002121.mm.2	Grik2	glutamate receptor, ionotropic, kainate 2 (beta 2)	1,68494E+14	7,16897E+14	3,45353E+14	0.0121604147416912	0.145471515175834	-2,51097E+14	8,21061E+14	8,02009E+13	6,94191E+14	6,9804E+14	7,13074E+14	5,73006E+14
TC1100002166.mm.2	Ddx56	DEAD (Asp-Glu-Ala-Asp) box polypeptide 56	1,10793E+14	5,72406E+14	3,45327E+14	0.012164507611495	0.145471515175834	-2,51128E+14	6,38409E+14	6,45714E+14	5,27014E+14	5,60763E+14	5,50167E+14	5,12369E+14
TC1000002451.mm.2	Rrp1	ribosomal RNA processing 1 homolog (S. cerevisiae)	1,82844E+14	9,37991E+14	3,45296E+14	0.0121693666592044	0.145471515175834	-2,51166E+14	1,06331E+13	1,03191E+14	9,64801E+14	8,38374E+14	9,10802E+13	8,18739E+14
TC1600000447.mm.2	Tfrc	transferrin receptor	1,64575E+14	6,0095E+14	3,45207E+14	0.0121832643979828	0.145471515175834	-2,51273E+14	7,199E+13	7,07653E+14	5,71695E+14	5,08052E+14	6,09229E+14	4,89173E+14
TC0100003010.mm.2	Tmem163	transmembrane protein 163	1,41174E+14	6,88057E+14	3,45194E+14	0.0121853356431601	0.145471515175834	-2,51289E+14	8,07708E+12	7,55864E+13	6,81572E+14	6,01976E+13	6,62236E+14	6,18988E+14

TC0500003026.mm.2	Crybb3	crystallin, beta B3	-1,4635E+14	7,704E+14	-3,45123E+14	0.0121963225433859	0.145471515175834	-2,51373E+14	6,5828E+14	6,76671E+14	7,7666E+14	8,83138E+14	7,9939E+14	8,28262E+14
TC1900000152.mm.2	Wdr74	WD repeat domain 74	1,56526E+13	7,2732E+14	3,44998E+14	0.012215915443766	0.145471515175834	-2,51524E+14	8,25952E+14	8,18052E+14	7,41376E+14	6,47587E+14	7,07302E+14	6,2365E+14
TC1100002288.mm.2	Actr2	ARP2 actin-related protein 2	1,03946E+14	9,63775E+14	3,44945E+14	0.0122241817438342	0.145471515175834	-2,51587E+14	1,02605E+14	1,04832E+14	9,32743E+14	9,09063E+14	9,32222E+14	9,34254E+14
TC0800002021.mm.2	Dctn6	dynactin 6	2,01593E+14	8,41816E+14	3,44924E+14	0.0122275242418061	0.145471515175834	-2,51613E+14	9,83172E+14	9,69743E+14	8,51998E+14	6,96253E+14	8,21978E+14	7,27751E+14
TC0500000306.mm.2	Khk	ketohexokinase	-1,1809E+14	7,71683E+14	-3,44837E+14	0.0122411436014179	0.145471515175834	-2,51717E+14	6,8969E+14	7,03241E+14	7,75466E+14	8,3259E+14	7,986E+14	8,30512E+14
TC1800001535.mm.2	Rab27b	RAB27B, member RAS oncogene family	1,24183E+14	7,64296E+14	3,44811E+14	0.0122451491315049	0.145471515175834	-2,51748E+13	8,71408E+14	8,17394E+14	7,16234E+14	7,40302E+14	6,94884E+14	7,45553E+14
TC1300002506.mm.2	Tmem171	transmembrane protein 171	-1,35798E+14	7,30335E+14	-3,44657E+14	0.0122694358476615	0.145471515175834	-2,51933E+14	6,47662E+14	6,29648E+14	7,39341E+14	8,16452E+14	7,46713E+14	8,02194E+14
TC0600002550.mm.2	Hk2	hexokinase 2	-1,16873E+14	5,6349E+14	-3,44586E+14	0.0122805768028739	0.145471515175834	-2,52018E+13	5,03469E+14	5,05953E+14	5,78727E+14	5,49622E+14	5,92757E+14	6,50412E+14
TC1100001782.mm.2	Prr29	proline rich 29	-1,20608E+14	6,35441E+14	-3,44569E+14	0.0122832486455984	0.145471515175834	-2,52039E+14	5,57293E+12	5,3748E+14	6,77087E+14	7,04794E+14	6,36454E+14	6,99536E+14
TC0700000155.mm.2	Trim28	tripartite motif-containing 28	1,16937E+13	6,12932E+14	3,44481E+14	0.0122970780196162	0.145471515175834	-2,52144E+14	6,98939E+14	6,89832E+14	5,83523E+14	5,504E+14	5,49961E+14	6,04936E+14
TC1500001791.mm.2	Arhgap39	Rho GTPase activating protein 39	1,84128E+14	7,14576E+14	3,44468E+14	0.0122991532567869	0.145471515175834	-2,5216E+14	8,3877E+13	8,18039E+14	7,51164E+14	5,90928E+14	6,46744E+14	6,41809E+14
TC1600000048.mm.2	Alg1	asparagine-linked glycosylation 1 (beta-1,4-mannosyltransferase)	1,43288E+14	5,63239E+14	3,4446E+14	0.0123004903967177	0.145471515175834	-2,5217E+14	6,78912E+14	5,83367E+14	5,527E+14	5,88755E+14	5,05232E+14	4,70471E+11
TC0400003100.mm.2	Cyp2j9	cytochrome P450, family 2, subfamily j, polypeptide 9	1,98418E+14	6,66268E+14	3,44351E+13	0.0123176662305287	0.145471515175834	-2,52301E+14	8,20469E+14	7,59823E+12	6,77784E+14	5,56075E+14	6,50023E+14	5,33433E+14
TC1200000679.mm.2	Hspa2	heat shock protein 2	1,26472E+14	6,09134E+14	3,4435E+14	0.0123177961901634	0.145471515175834	-2,52302E+14	6,7349E+14	6,95898E+14	5,46496E+14	6,22475E+14	5,68901E+14	5,47542E+14
TC1600000329.mm.2	Eif4a2/Snord2/Snora81	eukaryotic translation initiation factor 4A2/small nucleolar RNA, C/D box 2/small nucleolar RNA, H/ACA box 81	1,09036E+13	7,90118E+14	3,44345E+14	0.0123186532173634	0.145471515175834	-2,52309E+14	8,9036E+14	8,51399E+13	7,48959E+14	7,26304E+14	7,69694E+14	7,53993E+14
TC0500003375.mm.2	Syna	syncytin a	-1,60499E+13	4,92379E+14	-3,44321E+14	0.0123223550649964	0.145471515175834	-2,52337E+14	4,09934E+14	3,78985E+14	4,82784E+14	5,72651E+14	5,09424E+14	6,00493E+13
TC1100000789.mm.2	Specc1	sperm antigen with calponin homology and coiled-coil domains 1	1,5508E+13	6,5018E+14	3,44319E+14	0.0123227040940336	0.145471515175834	-2,5234E+14	7,36964E+14	7,49068E+14	6,74049E+14	5,65128E+14	6,17356E+14	5,58515E+14
TC0800001003.mm.2	Lonp2	lon peptidase 2, peroxisomal	1,47372E+14	8,05411E+14	3,44196E+14	0.0123421944360739	0.145471515175834	-2,52488E+14	9,06092E+14	8,98854E+14	8,02963E+14	7,14355E+14	7,19548E+14	7,90654E+14
TC0600001042.mm.2	Tpra1	transmembrane protein, adipocyte asscociated 1	1,27406E+14	6,46445E+14	3,44191E+14	0.0123430045010883	0.145471515175834	-2,52494E+14	7,38329E+14	7,42281E+14	6,27933E+14	5,44332E+14	6,12835E+14	6,12963E+14
TC0500002167.mm.2	Ost4	oligosaccharyltransferase complex subunit 4 (non-catalytic)	1,21772E+14	1,00209E+14	3,44121E+14	0.0123540168086166	0.145471515175834	-2,52578E+14	1,09332E+12	1,06831E+14	9,99778E+12	9,33056E+14	9,69166E+14	9,48918E+14
TC1100002862.mm.2	Flcn	folliculin	1,15515E+14	7,02884E+14	3,44079E+14	0.0123607381794405	0.145471515175834	-2,52629E+14	7,80823E+13	7,56294E+14	7,15146E+14	6,58954E+13	6,58497E+13	6,4759E+14
TC0500002592.mm.2	Ociad2	OCIA domain containing 2	1,32105E+13	7,22897E+14	3,44017E+14	0.0123704985025823	0.145471515175834	-2,52703E+14	8,0593E+14	8,19021E+14	7,16971E+14	6,34717E+14	6,99149E+14	6,61592E+14
TC0600001007.mm.2	Gkn2	gastrokine 2	-1,42472E+14	6,12017E+13	-3,43962E+14	0.0123792771064227	0.145471515175834	-2,52769E+14	5,28329E+14	5,02765E+14	6,14602E+14	7,10365E+14	6,37869E+14	6,78169E+14
TC0200004485.mm.2	Ppip5k1	diphosphoinositol pentakisphosphate kinase 1	-1,44263E+13	6,75073E+14	-3,43817E+14	0.0124023082584065	0.145471515175834	-2,52943E+14	5,85695E+14	5,82559E+14	6,63757E+14	7,61649E+14	7,03729E+14	7,53051E+14
TSUnmapped00000061.mm.2	Defa-rs4	defensin, alpha,, related sequence 4	-1,25293E+14	5,46112E+14	-3,43814E+14	0.0124027825497033	0.145471515175834	-2,52947E+14	4,67291E+13	4,82187E+12	5,75184E+14	5,51946E+14	5,62282E+13	6,37782E+14
TC1000003119.mm.2	Lrp1	low density lipoprotein receptor-related protein 1	1,25122E+14	7,94878E+14	3,43794E+14	0.0124059434565034	0.145471515175834	-2,52971E+13	8,62047E+14	8,83938E+13	7,34241E+14	7,93303E+14	7,7262E+14	7,23121E+14
TC0200004878.mm.2	Snph	syntaphilin	-1,32583E+14	8,27518E+14	-3,43785E+14	0.0124075135778375	0.145471515175834	-2,52983E+14	7,72674E+12	7,14089E+14	8,41837E+14	8,8458E+14	8,47451E+14	9,04478E+14
TC1900000098.mm.2	Tmem262	transmembrane protein 262	-1,06304E+14	6,18102E+14	-3,43778E+14	0.0124085616152598	0.145471515175834	-2,52991E+13	5,44983E+13	5,68136E+14	6,50765E+14	6,19E+14	6,57054E+14	6,68674E+14
TC0300001955.mm.2	Mgarp	mitochondria localized glutamic acid rich protein	-1,25822E+14	4,68142E+13	-3,43739E+14	0.0124148426291618	0.145471515175834	-2,53038E+14	4,06353E+14	3,61417E+14	4,76954E+14	5,44714E+13	5,10669E+14	5,08745E+14
TC0200004463.mm.2	Zfp106	zinc finger protein 106	1,01217E+14	8,22851E+14	3,43718E+14	0.0124181203175899	0.145471515175834	-2,53063E+14	8,83464E+14	8,81882E+14	7,94818E+14	8,14029E+14	7,87848E+13	7,75065E+14
TC0800000653.mm.2	Sh3rf1	SH3 domain containing ring finger 1	1,07487E+14	6,37775E+14	3,437E+14	0.0124210204437709	0.145471515175834	-2,53085E+14	7,035E+14	6,76373E+14	6,5388E+14	6,27997E+14	5,70669E+14	5,9423E+14
TC1700000978.mm.2	4932415M13Rik	RIKEN cDNA 4932415M13 gene	-1,16412E+14	5,34104E+14	-3,43693E+14	0.0124220370977276	0.145471515175834	-2,53092E+14	4,51243E+14	4,75205E+14	5,38635E+14	5,80267E+14	5,58248E+14	6,01025E+14
TC1600002084.mm.2	Cryzl1	crystallin, zeta (quinone reductase)-like 1	2,62513E+14	5,42099E+13	3,43681E+14	0.0124240833298152	0.145471515175834	-2,53108E+14	7,27212E+14	7,22943E+14	5,29357E+13	3,47952E+13	5,51297E+14	3,73832E+14
TC0200000572.mm.2	Pomt1	protein-O-mannosyltransferase 1	1,36367E+14	5,9515E+14	3,43675E+14	0.0124249699177661	0.145471515175834	-2,53115E+14	6,64958E+14	6,54859E+14	5,78976E+14	6,25029E+14	5,66578E+14	4,80504E+14
TC1800001151.mm.2	4933408B17Rik	RIKEN cDNA 4933408B17 gene	-1,18601E+14	4,56117E+14	-3,43632E+14	0.0124318423879553	0.145471515175834	-2,53166E+14	3,56997E+14	4,16959E+14	4,59942E+14	4,91646E+14	5,10628E+14	5,00528E+14
TC1800001153.mm.2	Brd8	bromodomain containing 8	1,22584E+14	7,92863E+14	3,43555E+14	0.0124440854840672	0.145509106970083	-2,53259E+14	8,72899E+14	8,57619E+14	7,88113E+14	7,53194E+14	7,75476E+14	7,09874E+14
TC1000001822.mm.2	Pex7	peroxisomal biogenesis factor 7	1,42455E+14	5,94288E+14	3,43291E+14	0.0124863535660443	0.145747714841222	-2,53577E+14	6,7889E+14	6,72557E+14	5,82297E+14	5,6545E+14	5,86196E+14	4,80341E+14
TC0600001384.mm.2	Mfap5	microfibrillar associated protein 5	-1,7169E+14	4,4954E+14	-3,4319E+14	0.012502542931636	0.145747714841222	-2,53698E+13	3,69881E+12	3,151E+13	4,28055E+14	5,55842E+14	4,90513E+14	5,37847E+14
TC1200002511.mm.2	Itgb8	integrin beta 8	1,23254E+14	7,07293E+13	3,43185E+14	0.0125033412196633	0.145747714841222	-2,53704E+14	7,84785E+14	7,80677E+14	6,51952E+13	7,07388E+14	6,86317E+14	6,32636E+14
TC1600000116.mm.2	Ciita	class II transactivator	-1,56137E+14	6,01097E+14	-3,43136E+14	0.0125113262995631	0.145747714841222	-2,53764E+14	4,77985E+13	5,32754E+14	5,97207E+14	6,75622E+14	6,20084E+13	7,02929E+14
TC0100000911.mm.2	Olfr1410	olfactory receptor 1410	-1,08976E+14	6,54457E+14	-3,43124E+14	0.0125132866250045	0.145747714841222	-2,53779E+14	5,78219E+14	6,03488E+14	6,52313E+14	6,93064E+14	6,96084E+14	7,03574E+14
TC1100002670.mm.2	Canx	calnexin	2,05809E+14	8,63373E+14	3,43033E+14	0.0125278040832329	0.145747714841222	-2,53888E+14	9,89854E+14	9,88611E+14	8,89665E+14	7,45261E+12	8,443E+14	7,22548E+13
TC0500002124.mm.2	Gm10220	predicted gene 10220	-1,08411E+14	5,16328E+14	-3,43008E+14	0.0125318545347558	0.145747714841222	-2,53918E+14	4,70823E+14	4,42081E+14	5,15912E+14	5,39423E+14	5,50279E+14	5,79448E+14
TC0X00000652.mm.2	Hmgb3	high mobility group box 3	1,44063E+14	6,24516E+14	3,42923E+14	0.0125455076483706	0.145747714841222	-2,5402E+14	7,29531E+14	7,25203E+14	6,10076E+14	5,15677E+14	6,11371E+14	5,55236E+14
TC0100000608.mm.2	Smarcal1	SWI/SNF related matrix associated, actin dependent regulator of chromatin, subfamily a-like 1	1,9691E+14	7,01248E+14	3,42921E+14	0.0125458996142726	0.145747714841222	-2,54023E+14	8,11734E+14	8,21054E+14	7,17045E+14	6,18689E+14	5,45274E+14	6,93693E+14
TC1400000913.mm.2	Rp1l1	retinitis pigmentosa 1 homolog like 1	-1,08933E+14	6,15528E+13	-3,42723E+14	0.0125778956584112	0.145958366134914	-2,54262E+14	5,64485E+14	5,64113E+14	5,85243E+14	6,32864E+14	6,78415E+14	6,68048E+14
TC1900001485.mm.2	Sorbs1	sorbin and SH3 domain containing 1	1,36085E+14	8,07707E+14	3,42658E+14	0.0125883749848803	0.145958366134914	-2,5434E+14	9,06664E+14	9,07366E+14	7,8898E+14	7,01371E+14	7,93246E+14	7,48615E+14
TC1900000212.mm.2	Prpf19	pre-mRNA processing factor 19	1,89365E+14	7,74773E+14	3,42592E+14	0.0125991104720931	0.145958366134914	-2,5442E+14	9,04623E+14	8,83413E+13	8,03199E+14	6,48097E+14	7,36942E+14	6,72364E+14
TC0400002478.mm.2	Arid3c	AT rich interactive domain 3C (BRIGHT-like)	-1,4429E+14	7,11289E+13	-3,42581E+14	0.0126007737437276	0.145958366134914	-2,54432E+14	6,11821E+14	6,25032E+14	7,1423E+13	7,91219E+14	7,23678E+14	8,01755E+14
TC1600001427.mm.2	Fgf12	fibroblast growth factor 12	1,53062E+14	8,77381E+14	3,42436E+14	0.0126244026810955	0.145958366134914	-2,54608E+14	9,85691E+14	9,69693E+14	8,76837E+14	7,82807E+14	8,63297E+13	7,85963E+14
TC1900000819.mm.2	Atrnl1	attractin like 1	1,85214E+14	7,50905E+14	3,4238E+14	0.0126333545732166	0.145958366134914	-2,54674E+14	8,46686E+14	8,82586E+14	7,69696E+14	6,47614E+14	7,31448E+14	6,27398E+14
TC0X00002084.mm.2	Tmem255a	transmembrane protein 255A	2,38798E+14	7,63619E+14	3,42355E+14	0.0126374200897832	0.145958366134914	-2,54705E+12	9,5659E+14	8,55752E+14	7,9816E+14	6,36463E+14	7,29907E+14	6,04839E+14
TC0X00000649.mm.2	Mtm1	X-linked myotubular myopathy gene 1	-1,32523E+14	5,62124E+14	-3,42319E+14	0.0126432986129273	0.145958366134914	-2,54748E+14	5,01843E+14	4,87665E+14	5,20357E+14	6,08322E+14	6,1387E+14	6,40685E+14
TC1700000628.mm.2	H2-Eb2	histocompatibility 2, class II antigen E beta2	-1,26043E+14	5,74587E+14	-3,42305E+14	0.0126455581905505	0.145958366134914	-2,54765E+14	4,92067E+14	5,08353E+14	5,62151E+14	6,32447E+14	6,06169E+14	6,46336E+14
TC0700004095.mm.2	Sbf2	SET binding factor 2	2,80708E+14	5,95284E+14	3,4216E+14	0.0126692026975976	0.145960581339816	-2,5494E+14	7,4671E+14	7,45089E+13	6,15432E+14	5,34092E+12	5,96842E+14	3,3354E+14
TC0700003714.mm.2	Prss23	protease, serine 23	1,18543E+14	5,83663E+14	3,42151E+14	0.0126706303945696	0.145960581339816	-2,54951E+14	6,38996E+13	6,54761E+14	5,58388E+14	5,93159E+14	5,57331E+13	4,9934E+14
TC1400002868.mm.2	Lats2	large tumor suppressor 2	2,7959E+14	5,64035E+14	3,42137E+14	0.0126729257924907	0.145960581339816	-2,54968E+14	7,34686E+14	7,44105E+14	5,84225E+14	4,01583E+14	5,62059E+14	3,57552E+14
TC0700003827.mm.2	Arhgef17	Rho guanine nucleotide exchange factor (GEF) 17	1,19862E+14	7,52933E+14	3,41999E+14	0.0126954134116602	0.145983254809557	-2,55134E+14	8,49939E+14	8,36055E+14	7,26412E+14	6,58919E+14	7,12743E+14	7,33527E+14
TC0800000012.mm.2	C330021F23Rik	RIKEN cDNA C330021F23 gene	-1,05524E+14	5,46336E+14	-3,41968E+14	0.0127004899008319	0.145983254809557	-2,55172E+14	4,87909E+14	5,00685E+14	5,51826E+14	5,37959E+13	5,8198E+14	6,17662E+14
TC1700001178.mm.2	Crim1	cysteine rich transmembrane BMP regulator 1 (chordin like)	2,0006E+14	7,75289E+14	3,41928E+14	0.0127071365228839	0.145983254809557	-2,55221E+14	8,92695E+14	8,91044E+14	7,80816E+14	7,03559E+14	7,74635E+14	6,08984E+14
TC1500001075.mm.2	Krt86	keratin 86	-1,08E+14	6,4912E+14	-3,41837E+14	0.0127219131670534	0.145983254809557	-2,5533E+14	6,06114E+14	5,842E+14	6,33201E+14	6,64888E+14	7,16721E+14	6,89593E+14
TC0800000115.mm.2	F10	coagulation factor X	-1,15888E+14	6,82074E+14	-3,41782E+14	0.0127309000915684	0.145983254809557	-2,55396E+14	5,96824E+14	6,2055E+14	6,90522E+14	7,35397E+14	7,04626E+13	7,44524E+14
TC0500003313.mm.2	Gusb	glucuronidase, beta	1,06493E+14	6,50939E+14	3,41715E+14	0.0127418607195781	0.145983254809557	-2,55477E+14	7,12811E+14	7,38673E+14	6,245E+14	5,91149E+14	6,14454E+13	6,24045E+14
TC0X00003360.mm.2	Tlr7	toll-like receptor 7	1,70459E+14	5,61314E+14	3,417E+13	0.0127443811899071	0.145983254809557	-2,55495E+14	6,2321E+13	7,43867E+14	4,46207E+14	5,28438E+14	5,17216E+14	5,08943E+14
TC0700003420.mm.2	Tjp1	tight junction protein 1	2,10385E+14	6,86875E+14	3,41636E+14	0.012754904600257	0.145983254809557	-2,55573E+14	7,85734E+14	7,92063E+14	7,22944E+14	6,63481E+14	6,71492E+13	4,85534E+13
TC1100000240.mm.2	Usp34	ubiquitin specific peptidase 34	3,41851E+14	6,78609E+14	3,41516E+14	0.012774615821273	0.145983254809557	-2,55718E+14	8,86242E+14	8,73373E+14	6,54681E+14	5,81444E+13	7,05825E+14	3,70088E+14
TC0700000856.mm.2	Izumo1/Rasip1	izumo sperm-egg fusion 1/Ras interacting protein 1	-1,17E+14	6,70698E+14	-3,41495E+14	0.0127781285581996	0.145983254809557	-2,55743E+14	5,92842E+14	6,00103E+14	6,93395E+14	7,109E+14	6,81379E+14	7,45566E+14

TC1100003069.mm.2	Nlgn2	neuroligin 2	1,74716E+13	7,48262E+14	3,41467E+14	0.0127826848634197	0.145983254809557	-2,55777E+14	8,64102E+14	8,48779E+14	7,70062E+14	6,4318E+14	7,22711E+14	6,40738E+14
TC0100001703.mm.2	Cdc42bpa	CDC42 binding protein kinase alpha	2,84215E+14	6,6767E+14	3,41461E+14	0.0127836140095752	0.145983254809557	-2,55784E+13	8,57807E+14	8,6813E+14	6,67965E+14	4,54611E+14	6,6939E+14	4,88117E+14
TC0900002181.mm.2	Kmt2a	lysine (K)-specific methyltransferase 2A	1,07697E+14	6,49957E+14	3,41273E+14	0.0128146061824883	0.146031446234368	-2,56011E+14	7,25088E+14	6,9124E+14	6,45281E+14	6,37197E+14	6,16114E+14	5,84819E+14
TC1700000693.mm.2	Psors1c2	psoriasis susceptibility 1 candidate 2 (human)	-1,06831E+14	5,52331E+14	-3,41248E+14	0.0128187674736616	0.146031446234368	-2,56041E+14	4,79911E+14	5,00457E+14	5,51628E+13	5,87961E+14	5,89192E+14	6,04837E+13
TC0700002974.mm.2	Atf5	activating transcription factor 5	-1,2292E+14	7,31904E+14	-3,41238E+14	0.0128203726222199	0.146031446234368	-2,56053E+14	6,86196E+14	6,30379E+14	7,36653E+14	7,75781E+14	7,62339E+14	8,00077E+14
TC0400003749.mm.2	Tmem50a	transmembrane protein 50A	1,15638E+14	7,45911E+14	3,41108E+14	0.0128418496846217	0.146031446234368	-2,5621E+14	8,42927E+14	8,32457E+14	6,80592E+14	6,75384E+14	6,90328E+14	7,53781E+14
TC0400002389.mm.2	Cfap206	cilia and flagella associated protein 206	-1,70518E+14	7,82348E+14	-3,41082E+14	0.0128462099517051	0.146031446234368	-2,56242E+14	6,73272E+14	6,92119E+14	7,50241E+14	8,72031E+14	8,13405E+13	8,93022E+14
TC0400001970.mm.2	Slc2a5	solute carrier family 2 (facilitated glucose transporter), member 5	1,78447E+13	5,91638E+14	3,41014E+14	0.0128574372557144	0.146031446234368	-2,56324E+14	7,1408E+14	6,70192E+14	6,35677E+13	5,025E+12	4,78556E+13	5,48823E+14
TC1500002223.mm.2	Lima1	LIM domain and actin binding 1	2,63374E+14	5,82608E+14	3,40982E+14	0.0128627531988099	0.146031446234368	-2,56363E+13	7,3941E+13	7,19988E+14	6,30727E+14	4,7287E+14	5,67341E+14	3,65309E+14
TC0500002675.mm.2	Cenpc1	centromere protein C1	1,40753E+14	6,12451E+14	3,40943E+14	0.0128692358049085	0.146031446234368	-2,5641E+14	7,23678E+14	6,86159E+14	6,15046E+14	5,21489E+14	5,81058E+14	5,47274E+14
TC0700002135.mm.2	Mboat7	membrane bound O-acyltransferase domain containing 7	1,57278E+14	7,76626E+14	3,40897E+14	0.012876862720694	0.146031446234368	-2,56466E+14	8,7134E+14	8,58534E+14	8,07569E+14	7,06994E+14	7,47906E+14	6,67413E+13
TC0600003120.mm.2	Usp5	ubiquitin specific peptidase 5 (isopeptidase T)	2,27228E+14	7,3185E+14	3,40868E+14	0.0128816137014167	0.146031446234368	-2,565E+14	8,80263E+14	8,75058E+14	7,75065E+14	5,59846E+14	6,57303E+14	6,43562E+14
TC0600001394.mm.2	Necap1	NECAP endocytosis associated 1	1,56506E+14	8,27815E+14	3,40832E+14	0.0128875266272744	0.146031446234368	-2,56543E+14	9,29948E+14	9,20724E+14	8,50158E+13	7,284E+14	7,88772E+14	7,48887E+14
TC1900000903.mm.2	Aip	aryl-hydrocarbon receptor-interacting protein	1,42486E+14	6,45935E+13	3,40657E+14	0.0129166036878541	0.146258071133094	-2,56755E+13	7,30883E+14	7,04932E+14	6,88655E+14	6,00295E+14	5,4626E+14	6,04584E+14
TC0700001913.mm.2	Htra1	HtrA serine peptidase 1	1,13013E+14	7,89302E+14	3,40601E+14	0.0129260695098387	0.146262470514067	-2,56824E+14	8,69112E+14	8,72504E+14	7,68148E+13	7,10455E+14	7,64898E+14	7,50692E+14
TC0600002567.mm.2	Tet3	tet methylcytosine dioxygenase 3	-1,20211E+14	8,28986E+14	-3,4036E+14	0.0129661474771713	0.146527977081623	-2,57114E+14	7,51131E+14	7,52594E+14	8,26232E+14	8,99814E+14	8,70568E+13	8,73579E+14
TC0700002128.mm.2	Tarm1	T cell-interacting, activating receptor on myeloid cells 1	-1,29071E+14	5,92662E+14	-3,40316E+14	0.0129736198783131	0.146527977081623	-2,57168E+14	5,17299E+14	5,11882E+14	5,84496E+14	6,54973E+14	6,168E+14	6,70522E+14
TC1100000694.mm.2	Trim58	tripartite motif-containing 58	-1,41522E+14	5,94278E+14	-3,402E+14	0.0129928926996648	0.146527977081623	-2,57307E+14	5,25425E+14	5,03677E+13	5,61267E+13	6,63152E+14	6,7009E+14	6,42057E+13
TC0900000113.mm.2	Gpr83	G protein-coupled receptor 83	2,07197E+14	7,14391E+14	3,4014E+13	0.0130030751567767	0.146527977081623	-2,57381E+14	8,56631E+14	8,32103E+14	7,54368E+14	5,68904E+14	6,60126E+14	6,14214E+14
TC0200004763.mm.2	Rbbp9	retinoblastoma binding protein 9, serine hydrolase	2,19552E+14	5,74389E+14	3,401E+14	0.0130097678214854	0.146527977081623	-2,57429E+14	7,52485E+14	7,2898E+14	5,40636E+14	3,81868E+14	5,86794E+14	4,55568E+14
TC0100001831.mm.2	Angel2	angel homolog 2	1,92657E+14	6,95303E+14	3,40043E+14	0.0130193438144468	0.146527977081623	-2,57498E+14	7,91188E+14	7,92701E+14	7,27032E+14	6,6232E+14	6,80647E+14	5,17929E+14
TC0700001119.mm.2	Abhd2	abhydrolase domain containing 2	1,13385E+14	8,35476E+14	3,40015E+14	0.0130239403461316	0.146527977081623	-2,57531E+14	8,83753E+14	9,22932E+14	8,33508E+13	7,92749E+14	8,01002E+14	7,78913E+14
TC1700002334.mm.2	Vmac	vimentin-type intermediate filament associated coiled-coil protein	-1,0621E+14	6,60868E+14	-3,40008E+14	0.0130251457255941	0.146527977081623	-2,5754E+14	6,00446E+13	5,65715E+14	7,1303E+14	7,07436E+14	6,76114E+14	7,02468E+14
TC1100003652.mm.2	Pip4k2b	phosphatidylinositol-5-phosphate 4-kinase, type II, beta	2,49049E+14	6,83234E+14	3,39895E+14	0.0130441882223908	0.146527977081623	-2,57677E+14	8,14817E+14	8,39675E+14	7,40542E+14	5,47978E+14	6,50193E+14	5,062E+14
TC0200005114.mm.2	Sdc4	syndecan 4	0.990093326295124	7,89539E+14	3,39727E+14	0.0130724085251898	0.146527977081623	-2,5788E+14	8,65069E+14	8,74396E+14	7,26962E+14	7,2936E+14	7,78025E+14	7,63421E+14
TC0500003491.mm.2	Mir339/4930432F04Rik	microRNA 339/RIKEN cDNA 4930432F04 gene	-1,46035E+14	7,21322E+14	-3,39629E+14	0.0130890253388939	0.146527977081623	-2,57999E+14	6,17737E+14	6,34618E+14	7,17372E+14	8,1378E+14	7,42289E+14	8,02136E+14
TC1200000655.mm.2	Snapc1	small nuclear RNA activating complex, polypeptide 1	1,31463E+13	6,13669E+14	3,39621E+14	0.0130902547053018	0.146527977081623	-2,58008E+14	6,96155E+13	6,87923E+14	6,18068E+14	5,58715E+14	5,96152E+14	5,25001E+14
TC0500001586.mm.2	Mdh2	malate dehydrogenase 2, NAD (mitochondrial)	1,18555E+14	8,78901E+14	3,39501E+14	0.0131105746936137	0.146527977081623	-2,58154E+14	9,70807E+14	9,45557E+14	8,71179E+14	8,06611E+14	8,47673E+13	8,31582E+14
TC0200002067.mm.2	Plcb4	phospholipase C, beta 4	2,07759E+14	5,84252E+14	3,39301E+14	0.0131445071083164	0.146527977081623	-2,58396E+14	7,37105E+14	6,96313E+14	5,8292E+14	4,71278E+14	5,85558E+14	4,32341E+14
TC0700003027.mm.2	Syngr4	synaptogyrin 4	-1,22463E+14	6,42322E+14	-3,39207E+13	0.0131604084644551	0.146527977081623	-2,5851E+14	5,59088E+14	5,67491E+14	6,53602E+14	7,02247E+14	6,55303E+14	7,16202E+14
TC1100003184.mm.2	Olfr139	olfactory receptor 139	-1,66866E+14	6,06679E+13	-3,39206E+12	0.0131605487567838	0.146527977081623	-2,58511E+14	5,04474E+14	5,05361E+14	5,76524E+14	7,1015E+14	6,46047E+14	6,9752E+14
TC0400002457.mm.2	Nol6	nucleolar protein family 6 (RNA-associated)	2,08968E+14	7,89483E+14	3,39189E+14	0.0131635165562108	0.146527977081623	-2,58532E+14	9,19972E+14	9,14304E+14	8,29576E+14	6,56708E+14	7,53675E+14	6,62665E+13
TC1500000298.mm.2	Fzd6	frizzled class receptor 6	-1,02443E+14	6,02883E+14	-3,39186E+14	0.0131639191372173	0.146527977081623	-2,58535E+13	5,19143E+14	5,5127E+14	6,31631E+14	6,39953E+14	6,39433E+14	6,35865E+14
TC0100002673.mm.2	Col4a4	collagen, type IV, alpha 4	-1,46843E+14	6,63343E+14	-3,39161E+14	0.0131681848829908	0.146527977081623	-2,58565E+14	5,75934E+14	5,60387E+14	6,61253E+14	7,5248E+14	6,79906E+14	7,501E+14
TC1400002349.mm.2	Xpo7	exportin 7	1,97846E+14	6,18505E+13	3,39146E+14	0.013170844095918	0.146527977081623	-2,58584E+14	7,51111E+14	7,48103E+14	6,44183E+14	4,6411E+13	5,65139E+14	5,38383E+14
TC0200004506.mm.2	Duox2	dual oxidase 2	-1,32469E+14	5,9766E+14	-3,39079E+14	0.0131821696041605	0.146527977081623	-2,58665E+14	5,48923E+14	5,08201E+14	5,86503E+14	6,20273E+13	6,21619E+14	7,00443E+14
TC1900000322.mm.2	Carnmt1	carnosine N-methyltransferase 1	-1,05027E+14	7,20424E+14	-3,39063E+13	0.0131849883623101	0.146527977081623	-2,58685E+14	6,4742E+14	6,87138E+14	7,16293E+12	7,27082E+14	7,74981E+14	7,69632E+14
TC0500003241.mm.2	Rsrc2	arginine/serine-rich coiled-coil 2	2,10845E+14	7,55555E+13	3,38956E+14	0.0132030499877975	0.146527977081623	-2,58813E+14	8,90957E+14	8,73806E+14	7,72436E+14	6,53055E+14	7,49961E+14	5,93112E+14
TC0100001438.mm.2	Astn1	astrotactin 1	2,11211E+14	7,21865E+14	3,38917E+14	0.013209699915541	0.146527977081623	-2,58861E+14	8,54391E+14	8,34547E+12	7,28647E+14	6,47092E+14	7,23101E+14	5,43416E+14
TC1700000419.mm.2	Crebrf	CREB3 regulatory factor	1,6868E+14	5,83608E+14	3,3888E+14	0.013216033907108	0.146527977081623	-2,58906E+14	6,82869E+14	6,66505E+14	6,29113E+14	5,11149E+14	5,48032E+14	4,63981E+14
TC0800002535.mm.2	Usp38	ubiquitin specific peptidase 38	1,06398E+14	6,6414E+14	3,38861E+14	0.0132193983627035	0.146527977081623	-2,5893E+13	7,32035E+13	7,13094E+14	6,69299E+14	6,3808E+13	6,29661E+14	6,02673E+13
TC1800000276.mm.2	Gm10549	predicted gene 10549	-1,37039E+13	5,86637E+11	-3,38849E+14	0.0132213515360598	0.146527977081623	-2,58943E+14	5,11489E+14	5,12162E+14	5,60393E+14	6,38052E+13	6,15748E+14	6,8198E+14
TC1500001127.mm.2	Hnrnpa1/Gm10052/Gm5643	heterogeneous nuclear ribonucleoprotein A1/heterogeneous nuclear ribonucleoprotein A1 pseudogene/heterogeneous nuclear ribonucleoprotein A1 pseudogene	1,09243E+14	8,92551E+13	3,38844E+14	0.0132222030503473	0.146527977081623	-2,5895E+14	9,65357E+14	9,52676E+14	8,84936E+14	8,52788E+14	8,69497E+14	8,30049E+14
TC1100003136.mm.2	Aipl1	aryl hydrocarbon receptor-interacting protein-like 1	-1,27268E+14	5,19571E+14	-3,38795E+14	0.0132306261301027	0.146527977081623	-2,59009E+13	4,38605E+14	4,56815E+14	5,01525E+14	5,70522E+14	5,50286E+14	5,99671E+14
TC1600000425.mm.2	Ncbp2	nuclear cap binding protein subunit 2	1,33491E+14	6,33392E+14	3,38771E+14	0.0132346828380667	0.146527977081623	-2,59038E+14	7,15235E+14	7,20001E+13	6,07261E+14	5,89601E+14	6,31607E+14	5,36646E+14
TC0100003786.mm.2	Smyd2	SET and MYND domain containing 2	1,10862E+14	7,65132E+14	3,38738E+12	0.0132404116871086	0.146527977081623	-2,59079E+14	8,36065E+14	8,1577E+14	7,69498E+14	7,39346E+14	7,36486E+14	6,93625E+14
TC1600001993.mm.2	Grik1	glutamate receptor, ionotropic, kainate 1	1,20504E+14	5,52173E+14	3,38737E+14	0.013240534638543	0.146527977081623	-2,5908E+13	6,59342E+14	5,96308E+14	5,38603E+14	5,04143E+14	4,96164E+14	5,18479E+14
TC0900001422.mm.2	Ip6k2	inositol hexaphosphate kinase 2	1,0695E+14	8,7331E+14	3,38647E+14	0.0132559232766904	0.146597592146405	-2,59189E+14	9,51931E+14	9,40571E+14	8,56541E+14	8,12212E+14	8,44683E+14	8,33919E+14
TC1900001445.mm.2	Ppp1r3c	protein phosphatase 1, regulatory subunit 3C	1,06899E+14	7,72061E+14	3,3851E+14	0.01327932693442	0.146645149731067	-2,59354E+13	8,37455E+14	8,39264E+14	7,69471E+14	7,23256E+14	7,27311E+14	7,35611E+14
TC0700004258.mm.2	Nsmce1	NSE1 homolog, SMC5-SMC6 complex component	2,08313E+14	6,39224E+14	3,38478E+13	0.013284782610594	0.146645149731067	-2,59393E+14	7,67958E+14	7,63645E+14	6,86961E+14	5,01808E+14	5,88296E+14	5,2668E+14
TC0500003439.mm.2	Srrt	serrate RNA effector molecule homolog (Arabidopsis)	1,11292E+14	7,07412E+14	3,38462E+14	0.0132875267552509	0.146645149731067	-2,59412E+14	7,5745E+13	8,0005E+14	6,84433E+14	6,67626E+13	6,84711E+14	6,50204E+14
TC0800000732.mm.2	Sugp1	SURP and G patch domain containing 1	1,04322E+13	7,23595E+14	3,38299E+14	0.0133156450409743	0.146704078412634	-2,59611E+14	8,15952E+14	7,77629E+14	6,75643E+14	6,8741E+14	6,93631E+14	6,91305E+14
TC1900000516.mm.2	Rpp30	ribonuclease P/MRP 30 subunit	1,46215E+14	6,91457E+14	3,38203E+14	0.0133321125673843	0.146704078412634	-2,59727E+14	7,9241E+14	7,86834E+14	6,81864E+14	6,0082E+14	6,84705E+14	6,02109E+14
TC1100003252.mm.2	Cpd	carboxypeptidase D	1,6616E+14	7,23885E+14	3,38173E+14	0.0133372600156645	0.146704078412634	-2,59763E+14	8,08638E+14	8,40336E+14	7,41239E+14	6,36441E+14	7,04274E+14	6,1238E+14
TC0600001967.mm.2	Pax4	paired box 4	-1,22445E+14	6,08735E+14	-3,38151E+14	0.0133410962269703	0.146704078412634	-2,5979E+14	5,39534E+14	5,23391E+14	6,03398E+14	6,78272E+14	6,44175E+14	6,63639E+13
TC1100000730.mm.2	Mprip	myosin phosphatase Rho interacting protein	1,95429E+14	8,82543E+14	3,3814E+14	0.0133429636990223	0.146704078412634	-2,59803E+14	1,00876E+14	1,00857E+14	8,8141E+14	7,70037E+13	8,8384E+14	7,42635E+14
TC1700001626.mm.2	Dnase1l2	deoxyribonuclease 1-like 2	-1,60576E+14	5,50653E+14	-3,38114E+14	0.0133474945046187	0.146704078412634	-2,59835E+14	4,92239E+14	4,69291E+14	5,31736E+14	5,27972E+14	5,74539E+14	7,08142E+14
TC1000002642.mm.2	Dram1	DNA-damage regulated autophagy modulator 1	-1,0843E+14	5,19864E+14	-3,37974E+14	0.0133715655347103	0.146868463163454	-2,60004E+14	4,63854E+14	4,36143E+13	5,40193E+14	5,62135E+14	5,39917E+14	5,7694E+14

TC0400003483.mm.2	D830031N03Rik/Macf1	RIKEN cDNA D830031N03 gene/microtubule-actin crosslinking factor 1	1,52646E+14	8,40836E+14	3,37897E+13	0.013384917726887	0.146914972297908	-2,60098E+12	9,54559E+14	9,35357E+14	7,98962E+14	7,71511E+14	8,52536E+14	7,32089E+14
TC1500001675.mm.2	Fam135b	family with sequence similarity 135, member B	1,65312E+14	6,23742E+13	3,37784E+14	0.0134044705249613	0.146947525042419	-2,60235E+14	7,50358E+14	7,51489E+14	6,02273E+14	4,67108E+14	5,63724E+14	6,07498E+14
TC0700002033.mm.2	Pgghg	protein glucosylgalactosylhydroxylysine glucosidase	-1,4168E+14	6,67986E+14	-3,37647E+13	0.0134283387765588	0.146947525042419	-2,60402E+13	5,7508E+14	5,72221E+14	6,61657E+14	7,68302E+14	7,02622E+14	7,28038E+14
TC1600000278.mm.2	Dvl3	dishevelled segment polarity protein 3	1,02112E+14	8,01628E+14	3,37639E+14	0.0134295940747608	0.146947525042419	-2,60411E+14	8,39019E+14	8,47523E+14	8,17016E+14	8,23892E+14	7,25144E+13	7,57174E+14
TC0700004353.mm.2	Armcx3	armadillo repeat containing, X-linked 3	2,1439E+14	5,88913E+14	3,37578E+13	0.0134401736019935	0.146947525042419	-2,60485E+14	7,21086E+14	7,22891E+14	5,99724E+14	4,7458E+12	5,87585E+14	4,27612E+14
TC0500002119.mm.2	Gm1979	predicted gene 1979	-1,1568E+14	5,96939E+14	-3,3754E+14	0.013446915657562	0.146947525042419	-2,60532E+14	4,89559E+12	5,13742E+14	6,52555E+14	6,91115E+14	6,4191E+14	5,92751E+14
TC1100002814.mm.2	Zfp672	zinc finger protein 672	-1,35462E+14	6,47697E+14	-3,37337E+14	0.0134821327846155	0.146947525042419	-2,60777E+14	5,7226E+13	5,52461E+14	6,34018E+14	7,31797E+14	7,04319E+14	6,91327E+14
TC1900001595.mm.2	Pitx3	paired-like homeodomain transcription factor 3	-1,25944E+14	6,08759E+14	-3,37331E+14	0.0134832622304298	0.146947525042419	-2,60785E+14	5,27828E+12	5,1998E+14	6,24463E+14	6,80585E+14	6,17837E+14	6,81859E+14
TC0200004137.mm.2	Large2	LARGE xylosyl- and glucuronyltransferase 2	-1,88975E+14	6,27618E+14	-3,37313E+14	0.0134864156795221	0.146947525042419	-2,60807E+13	5,07468E+13	4,80609E+14	6,29533E+14	7,8207E+14	6,45064E+14	7,20964E+14
TC0X00001797.mm.2	Rbm3	RNA binding motif (RNP1, RRM) protein 3	1,27328E+14	9,65967E+13	3,37105E+14	0.0135227033101403	0.146947525042419	-2,61059E+14	1,04573E+13	1,07588E+14	9,37879E+14	8,69362E+14	9,56538E+14	9,10415E+14
TC1500001585.mm.2	Mtss1	MTSS I-BAR domain containing 1	1,13843E+14	6,25914E+14	3,37058E+14	0.0135308541662194	0.146947525042419	-2,61116E+14	7,25337E+14	6,7948E+14	6,08023E+14	5,6551E+14	5,94565E+14	5,82566E+14
TC0400000435.mm.2	Rusc2	RUN and SH3 domain containing 2	1,19003E+14	6,46333E+14	3,37036E+14	0.0135348169926048	0.146947525042419	-2,61144E+14	7,02373E+14	6,84368E+14	6,35719E+14	7,06803E+14	5,71234E+14	5,77502E+13
TC0X00001275.mm.2	Tmem35a	transmembrane protein 35A	1,32942E+12	6,80408E+14	3,36905E+14	0.0135577439882995	0.146947525042419	-2,61303E+14	7,92397E+14	7,42017E+14	6,72889E+13	6,06614E+14	6,09375E+14	6,59154E+14
TC0700003032.mm.2	Ush1c	USH1 protein network component harmonin	-1,32036E+14	6,53783E+14	-3,36822E+14	0.0135723555903361	0.146947525042419	-2,61404E+14	6,03594E+14	5,63098E+14	6,27598E+14	6,9764E+14	6,89886E+13	7,40879E+14
TC1000001555.mm.2	Dctn2	dynactin 2	1,53671E+13	8,36791E+14	3,3677E+14	0.0135815222431575	0.146947525042419	-2,61467E+14	9,45641E+14	9,35498E+14	8,34017E+14	7,31795E+14	8,24347E+14	7,49451E+14
TC1700002145.mm.2	Cdc5l	cell division cycle 5-like (S. pombe)	1,15134E+14	6,1179E+13	3,36767E+14	0.0135819424629335	0.146947525042419	-2,6147E+14	6,88665E+14	6,65287E+14	5,85529E+14	6,07578E+14	5,9092E+14	5,32765E+14
TC0800003231.mm.2	Zfp617	zinc finger protein 617	1,07434E+13	7,10141E+14	3,36688E+14	0.0135959430921283	0.146947525042419	-2,61567E+14	7,68368E+14	7,68679E+14	7,22559E+13	6,79058E+14	6,73183E+14	6,48995E+14
TC0500003497.mm.2	Ints1	integrator complex subunit 1	1,14239E+14	7,54593E+14	3,36674E+14	0.0135982760204085	0.146947525042419	-2,61583E+14	8,21802E+14	8,33338E+14	7,023E+13	7,43455E+14	6,89024E+14	7,37638E+14
TC1200001275.mm.2	Btbd6	BTB (POZ) domain containing 6	1,23531E+14	6,4907E+14	3,36674E+14	0.0135983090957958	0.146947525042419	-2,61583E+13	7,44897E+14	6,7942E+14	6,26025E+14	6,66821E+14	5,74977E+14	6,02278E+14
TC0500002577.mm.2	Gm5868	predicted gene 5868	-1,25592E+14	6,46358E+14	-3,3665E+14	0.0136024984531188	0.146947525042419	-2,61612E+14	5,73637E+13	5,42857E+14	6,61185E+14	7,3279E+14	6,66464E+13	7,01214E+13
TC0400001045.mm.2	Gm12789	predicted gene 12789	-1,24818E+14	5,28716E+14	-3,36613E+14	0.0136091192266655	0.146947525042419	-2,61658E+14	4,32685E+14	4,71154E+14	5,34503E+14	5,8048E+14	5,47274E+13	6,06201E+14
TC0100001615.mm.2	Slamf9	SLAM family member 9	-1,26255E+14	5,43875E+14	-3,3657E+12	0.0136165826456264	0.146947525042419	-2,61709E+14	4,41253E+13	4,95359E+14	5,4369E+12	5,93826E+14	5,71983E+14	6,17139E+14
TC1200000875.mm.2	Ahsa1	AHA1, activator of heat shock protein ATPase 1	1,16519E+14	7,76676E+14	3,36535E+14	0.0136228391857728	0.146947525042419	-2,61752E+14	8,41902E+14	8,55121E+13	7,58237E+14	7,40809E+14	7,64861E+14	6,99124E+14
TC0400000518.mm.2	Col15a1	collagen, type XV, alpha 1	-1,44987E+14	6,96381E+14	-3,3652E+14	0.0136254558223204	0.146947525042419	-2,6177E+14	6,18619E+13	5,67561E+14	7,06661E+14	8,09291E+14	7,32686E+14	7,43467E+14
TC0700002970.mm.2	Nr1h2	nuclear receptor subfamily 1, group H, member 2	0.993345757852316	7,4106E+14	3,36477E+14	0.013632960505026	0.146947525042419	-2,61822E+14	7,92159E+14	7,94881E+13	7,39535E+13	7,31415E+14	7,05565E+14	6,82806E+14
TC1600001231.mm.2	Parn	poly(A)-specific ribonuclease (deadenylation nuclease)	1,79931E+14	5,6638E+14	3,36411E+13	0.0136447213174456	0.146947525042419	-2,61903E+14	6,90774E+13	6,7188E+14	5,96733E+14	4,36103E+14	4,9941E+14	5,03382E+14
TC0600002954.mm.2	Mkrn2os	makorin, ring finger protein 2, opposite strand	-1,52904E+14	6,29204E+14	-3,36404E+14	0.0136459560040819	0.146947525042419	-2,61912E+14	5,32338E+14	5,35687E+14	6,14918E+14	7,18447E+14	6,5078E+14	7,23053E+14
TC0900000105.mm.2	Cwc15	CWC15 spliceosome-associated protein	1,35813E+14	8,58343E+14	3,36386E+14	0.013649037361156	0.146947525042419	-2,61933E+14	9,44606E+14	9,33995E+13	8,76094E+14	7,88389E+14	8,28697E+14	7,78278E+14
TC1900000119.mm.2	Nrxn2	neurexin II	1,2308E+14	7,81949E+14	3,3634E+14	0.0136572946851039	0.146947525042419	-2,6199E+14	8,66254E+14	8,4148E+14	7,51873E+14	7,70514E+14	7,6835E+14	6,93224E+14
TC0100003872.mm.2	Irf6	interferon regulatory factor 6	-1,33521E+14	6,07192E+14	-3,36288E+14	0.0136664393266373	0.146947525042419	-2,62052E+14	5,38742E+14	5,31436E+14	5,98481E+14	6,37271E+14	6,23612E+14	7,13608E+14
TC0X00000454.mm.2	Stk26	serine/threonine kinase 26	1,0592E+14	6,50447E+14	3,36259E+14	0.0136715944130643	0.146947525042419	-2,62088E+14	7,26625E+14	7,20221E+14	6,28346E+14	5,92482E+14	6,31787E+14	6,03218E+14
TC0300002418.mm.2	Crct1	cysteine-rich C-terminal 1	-1,03376E+14	5,48812E+14	-3,36217E+14	0.0136790457577043	0.146947525042419	-2,62139E+13	4,82814E+14	4,76278E+14	5,93819E+14	5,7412E+14	5,9958E+14	5,66263E+14
TC0600001071.mm.2	Uroc1	urocanase domain containing 1	-1,11314E+14	6,37145E+14	-3,36213E+14	0.0136797174681083	0.146947525042419	-2,62144E+14	5,6039E+14	5,73471E+14	6,46908E+14	6,8561E+14	6,56966E+14	6,99523E+14
TC0500001584.mm.2	Por	P450 (cytochrome) oxidoreductase	1,31399E+14	7,39044E+14	3,36109E+14	0.0136981758505957	0.14704777313834	-2,6227E+14	8,46243E+14	8,22805E+14	7,22372E+14	6,36594E+14	7,19944E+14	6,86306E+14
TC0X00001514.mm.2	Wnk3	WNK lysine deficient protein kinase 3	1,17935E+14	6,99803E+14	3,35992E+14	0.0137189626416684	0.147172866208524	-2,62413E+14	8,05307E+14	7,46846E+14	6,8257E+13	6,47812E+14	6,70412E+14	6,45871E+14
TC0X00000912.mm.2	Zc3h12b	zinc finger CCCH-type containing 12B	1,07568E+14	5,55249E+14	3,35891E+14	0.0137368844375582	0.147202713809244	-2,62535E+14	6,25653E+14	6,4038E+14	5,31514E+14	4,83049E+14	5,2675E+14	5,24147E+14
TC0700001321.mm.2	Prcp	prolylcarboxypeptidase (angiotensinase C)	1,43321E+13	5,82378E+13	3,35868E+14	0.0137410166038584	0.147202713809244	-2,62564E+14	6,72818E+14	6,83571E+14	4,79937E+14	5,8819E+14	5,5042E+14	5,19329E+14
TC1400001958.mm.2	Exoc5	exocyst complex component 5	2,27077E+12	6,14242E+14	3,35804E+14	0.0137522894707556	0.147202713809244	-2,62641E+14	7,49683E+14	7,68215E+14	5,95258E+14	5,08551E+14	6,32286E+12	4,31458E+14
TC0400003549.mm.2	Ncdn	neurochondrin	2,36458E+13	9,32146E+14	3,35771E+14	0.0137582875316032	0.147202713809244	-2,62682E+14	1,07613E+14	1,08759E+14	9,8107E+14	7,573E+14	8,72608E+13	8,18188E+14
TC1700002635.mm.2	Kcng3	potassium voltage-gated channel, subfamily G, member 3	1,23232E+14	5,89015E+14	3,35704E+14	0.0137702688274332	0.147233139758747	-2,62763E+14	6,6021E+14	6,78473E+12	5,12371E+14	5,90817E+14	5,38598E+14	5,53621E+14
TC0300002756.mm.2	Prpf38b	PRP38 pre-mRNA processing factor 38 (yeast) domain containing B	1,97576E+14	8,03058E+13	3,3553E+14	0.0138013355268069	0.147376561350925	-2,62975E+14	8,88708E+14	9,22637E+14	8,30165E+14	7,60643E+14	7,91753E+14	6,2444E+14
TC0200004090.mm.2	Ptprj	protein tyrosine phosphatase, receptor type, J	1,64573E+14	7,85892E+13	3,35526E+14	0.0138019754905074	0.147376561350925	-2,62979E+14	8,81306E+14	9,09692E+14	7,81272E+14	6,81226E+14	7,79684E+14	6,82169E+14
TC0800001392.mm.2	Atmin	ATM interactor	1,27798E+14	7,78471E+14	3,35369E+14	0.013830122545365	0.147504092986005	-2,63171E+14	8,63258E+14	8,47681E+14	7,8145E+14	7,23092E+14	7,60876E+14	6,94467E+14
TC0300002298.mm.2	Ttc24	tetratricopeptide repeat domain 24	-1,22388E+14	6,65709E+14	-3,35357E+14	0.0138322276734223	0.147504092986005	-2,63185E+14	6,02458E+13	5,91086E+14	6,42942E+14	7,19448E+14	7,07656E+14	7,30662E+14
TC1000002381.mm.2	Cabin1	calcineurin binding protein 1	1,3525E+14	7,27805E+14	3,35108E+14	0.0138769433238984	0.14774637272622	-2,63488E+14	8,26966E+14	7,92992E+14	7,46604E+14	6,50808E+14	6,75271E+14	6,74188E+14
TC0200003118.mm.2	Cacna1b	calcium channel, voltage-dependent, N type, alpha 1B subunit	2,19434E+14	7,77431E+14	3,35062E+14	0.0138851840009337	0.14774637272622	-2,63544E+14	9,28201E+14	9,10201E+14	8,14818E+14	6,11835E+14	7,27239E+13	6,72295E+13
TC0500003660.mm.2	Hsph1	heat shock 105kDa/110kDa protein 1	1,44108E+14	8,61472E+14	3,34957E+13	0.0139042472385956	0.14774637272622	-2,63673E+14	9,53912E+14	9,57608E+14	8,6723E+14	7,66776E+14	8,39416E+14	7,83889E+14
TC1700001949.mm.2	Apom	apolipoprotein M	-1,55532E+14	5,07975E+14	-3,34923E+14	0.0139102989013084	0.14774637272622	-2,63714E+14	4,15929E+13	4,15766E+14	4,73815E+14	5,99583E+14	5,53351E+14	5,89408E+14
TC0700000288.mm.2	Fbxo46	F-box protein 46	1,24417E+14	5,62307E+14	3,3471E+14	0.0139487784502744	0.14774637272622	-2,63973E+13	6,10097E+13	6,45665E+14	5,93887E+14	5,17262E+14	5,01435E+14	5,05493E+14
TC0300001382.mm.2	Ppp3ca	protein phosphatase 3, catalytic subunit, alpha isoform	1,79578E+14	1,07278E+14	3,34655E+14	0.0139586405035498	0.14774637272622	-2,6404E+14	1,18976E+13	1,20758E+14	1,07482E+14	9,26326E+14	1,0509E+13	9,87283E+14
TC0700003016.mm.2	Sec1	secretory blood group 1	-1,21766E+14	6,3312E+14	-3,34647E+14	0.0139602381241696	0.14774637272622	-2,64051E+14	5,35449E+14	5,82885E+14	6,36549E+14	6,81973E+14	6,58038E+14	7,03827E+14
TC1900000905.mm.2	Rps6kb2	ribosomal protein S6 kinase, polypeptide 2	-1,29677E+14	9,15919E+14	-3,34632E+14	0.01396291930439	0.14774637272622	-2,64069E+14	8,27261E+13	8,50764E+14	9,29713E+14	9,50395E+14	1,01261E+14	9,24768E+14
TC0800002750.mm.2	Cnot1	CCR4-NOT transcription complex, subunit 1	1,806E+14	6,80594E+14	3,34605E+14	0.0139677830688825	0.14774637272622	-2,64101E+14	7,95323E+14	8,03392E+14	7,03626E+14	5,43709E+13	6,3616E+14	6,01355E+14
TC0700003652.mm.2	Arnt2	aryl hydrocarbon receptor nuclear translocator 2	1,16051E+14	8,54696E+14	3,34602E+14	0.0139683156936997	0.14774637272622	-2,64105E+14	9,39825E+14	9,32617E+14	8,34126E+14	7,81271E+14	8,42575E+14	7,97764E+14
TC1400002333.mm.2	Pdlim2	PDZ and LIM domain 2	-1,14341E+14	8,63391E+14	-3,34557E+14	0.0139764875995937	0.14774637272622	-2,6416E+14	7,83525E+13	7,99618E+14	8,60499E+14	9,2488E+14	9,05396E+14	9,0643E+14
TC1000002537.mm.2	Gipc3	GIPC PDZ domain containing family, member 3	-1,31576E+14	6,86005E+14	-3,34479E+14	0.0139906101993065	0.14774637272622	-2,64255E+14	5,93824E+14	5,9961E+14	6,87731E+14	7,78278E+14	7,12075E+13	7,44511E+14
TC1800001063.mm.2	Nol4	nucleolar protein 4	1,22191E+14	7,90423E+12	3,34385E+14	0.0140077721907072	0.14774637272622	-2,6437E+14	8,66148E+13	8,73447E+14	7,70761E+14	7,36968E+13	7,84216E+14	7,10998E+13
TC1200001947.mm.2	Zbtb25	zinc finger and BTB domain containing 25	1,81956E+14	5,9736E+14	3,34362E+14	0.0140118994138874	0.14774637272622	-2,64398E+14	7,01793E+14	6,67401E+14	6,8587E+14	5,23811E+14	5,00197E+12	5,05085E+14

TC1700002361.mm.2	Nudt12	nudix (nucleoside diphosphate linked moiety X)-type motif 12	1,09178E+14	5,70253E+14	3,34324E+14	0.0140188312431186	0.14774637272622	-2,64444E+14	6,18064E+14	6,04543E+14	5,77375E+14	6,17283E+14	4,83942E+14	5,20308E+14
TC0900000550.mm.2	Sik3	SIK family kinase 3	1,63796E+14	8,80468E+14	3,34141E+14	0.0140521106471357	0.14774637272622	-2,64667E+14	9,89976E+14	9,79541E+14	9,00015E+14	7,71354E+14	8,51066E+14	7,90858E+14
TC0700001397.mm.2	Olfr521	olfactory receptor 521	-1,55293E+14	5,60387E+14	-3,34131E+14	0.0140539118378816	0.14774637272622	-2,64679E+14	4,84148E+14	4,50143E+14	5,3253E+14	6,50625E+14	5,9974E+14	6,45137E+14
TC1000000169.mm.2	1700020N01Rik	RIKEN cDNA 1700020N01 gene	-1,05387E+14	5,68555E+14	-3,34063E+14	0.0140662937408592	0.14774637272622	-2,64762E+14	5,23177E+14	4,9708E+14	5,97671E+13	5,62367E+14	6,0754E+14	6,23491E+14
TC0200005223.mm.2	Sall4	spalt like transcription factor 4	-1,78762E+14	7,81243E+14	-3,33992E+14	0.0140792887035721	0.14774637272622	-2,64848E+14	6,49547E+14	6,57416E+14	7,97758E+14	9,18248E+14	7,78284E+14	8,86203E+14
TC1400000198.mm.2	Samd8	sterile alpha motif domain containing 8	1,51912E+14	7,38398E+14	3,33959E+14	0.0140852864935612	0.14774637272622	-2,64888E+14	8,33567E+14	8,41601E+14	7,23125E+14	6,6075E+14	7,40534E+14	6,3081E+14
TC1800001731.mm.2	Dok6	docking protein 6	2,07554E+14	5,70364E+14	3,3394E+12	0.0140889066449948	0.14774637272622	-2,64913E+14	7,25352E+14	7,30082E+14	5,4647E+14	3,79953E+14	5,7335E+14	4,66976E+14
TC1000003094.mm.2	Eef1akmt3	EEF1A lysine methyltransferase 3	-1,01059E+14	6,59528E+14	-3,33936E+14	0.0140895200336184	0.14774637272622	-2,64917E+14	5,9508E+14	5,94159E+14	6,72577E+14	7,03994E+14	6,89098E+14	7,02258E+14
TC1200001030.mm.2	Ifi27	interferon, alpha-inducible protein 27	1,40874E+13	5,40948E+14	3,33861E+14	0.0141033380529373	0.14774637272622	-2,65009E+14	5,90741E+14	6,79888E+14	4,64262E+14	5,21917E+12	5,1033E+14	4,78552E+14
TC0800000288.mm.2	Kat6a	K(lysine) acetyltransferase 6A	1,08116E+14	5,99696E+14	3,33839E+14	0.0141072969638084	0.14774637272622	-2,65035E+14	6,53573E+14	6,82777E+14	5,84367E+14	5,57342E+14	5,78005E+14	5,42113E+14
TC0800001851.mm.2	AY761184	cDNA sequence AY761184	-1,07253E+14	6,9073E+14	-3,33823E+14	0.0141102330435113	0.14774637272622	-2,65055E+14	6,26961E+14	6,13276E+14	7,0954E+14	7,3986E+14	7,07675E+14	7,47067E+14
TC1400002168.mm.2	Atp8a2	ATPase, aminophospholipid transporter-like, class I, type 8A, member 2	-1,20619E+14	7,91872E+14	-3,3378E+14	0.0141182141175998	0.14774637272622	-2,65108E+14	7,45631E+14	6,97276E+14	8,20281E+14	8,03901E+14	8,71954E+13	8,12192E+14
TC1100000822.mm.2	Pmp22	peripheral myelin protein 22	1,2015E+14	7,74399E+13	3,33719E+14	0.0141292418675813	0.14774637272622	-2,65181E+14	8,34004E+14	8,39235E+14	8,07563E+14	7,32657E+14	7,26659E+14	7,0628E+14
TC1100003669.mm.2	Med1	mediator complex subunit 1	1,98016E+13	6,82461E+14	3,3366E+14	0.0141401250355308	0.14774637272622	-2,65254E+14	7,88067E+14	8,10786E+14	6,92613E+14	6,00479E+14	6,81528E+14	5,21293E+14
TC0X00003098.mm.2	Acsl4	acyl-CoA synthetase long-chain family member 4	2,208E+14	7,25785E+14	3,33659E+14	0.0141403734601979	0.14774637272622	-2,65255E+14	8,55312E+14	8,4754E+14	7,84897E+14	6,05705E+14	6,88794E+14	5,72459E+14
TC0300000896.mm.2	Mindy1	MINDY lysine 48 deubiquitinase 1	-1,26027E+14	7,75751E+14	-3,3363E+14	0.0141456154875248	0.14774637272622	-2,6529E+14	6,92307E+14	6,97911E+14	7,70132E+14	8,51883E+14	8,05172E+14	8,371E+14
TC0X00000709.mm.2	Gdi1	guanosine diphosphate (GDP) dissociation inhibitor 1	1,5704E+14	9,9304E+14	3,33616E+14	0.0141481371868267	0.14774637272622	-2,65307E+12	1,09801E+13	1,08465E+14	1,01174E+14	8,95262E+14	9,66438E+14	9,0214E+14
TC0400003380.mm.2	Dph2	DPH2 homolog	-1,02836E+14	7,63411E+14	-3,33615E+14	0.0141483679709897	0.14774637272622	-2,65308E+14	7,02291E+14	7,03133E+14	7,68386E+14	7,95562E+14	7,90857E+14	8,20239E+14
TC0700000586.mm.2	Lgi4	leucine-rich repeat LGI family, member 4	1,70511E+13	6,47263E+14	3,33514E+14	0.0141669350860339	0.147798126703199	-2,65432E+14	7,41034E+14	7,64332E+14	5,77964E+14	6,35907E+14	5,13071E+14	6,51272E+14
TC0400003073.mm.2	Plaa	phospholipase A2, activating protein	1,49611E+14	8,28945E+14	3,33474E+13	0.0141744276731587	0.147798126703199	-2,65481E+14	9,19502E+14	9,2153E+14	8,53569E+14	7,37258E+14	7,91745E+14	7,50065E+14
TC1800001620.mm.2	Katnal2	katanin p60 subunit A-like 2	1,17522E+14	5,57572E+14	3,33439E+14	0.0141808417975017	0.147798126703199	-2,65524E+14	6,29808E+14	5,82503E+14	5,64584E+14	5,91272E+14	4,64092E+14	5,13175E+14
TC0500001355.mm.2	Hectd4	HECT domain E3 ubiquitin protein ligase 4	1,66858E+13	8,00051E+14	3,33325E+14	0.0142017478508791	0.147848036574745	-2,65662E+14	9,10123E+14	9,20968E+14	7,64177E+14	7,07661E+14	8,16325E+14	6,81051E+14
TC1800001237.mm.2	Fgf1	fibroblast growth factor 1	1,06911E+14	7,32514E+14	3,33313E+14	0.0142039819163226	0.147848036574745	-2,65677E+14	8,15648E+14	7,67079E+13	7,16265E+14	7,27185E+14	6,90282E+14	6,78624E+14
TC1600001071.mm.2	Ets2	E26 avian leukemia oncogene 2, 3' domain	1,72292E+14	6,80351E+13	3,33259E+14	0.0142140310484261	0.147857122197089	-2,65743E+14	8,05054E+14	7,89212E+14	6,91406E+11	5,46749E+14	6,50245E+14	5,99438E+14
TC0400002444.mm.2	Aptx	aprataxin	1,26833E+14	6,58525E+14	3,33052E+14	0.0142523040543965	0.148126830099115	-2,65996E+14	7,22637E+14	7,04163E+14	6,97423E+14	6,53793E+14	6,24611E+14	5,48523E+14
TC0900002308.mm.2	Cul5	cullin 5	2,77234E+14	6,33073E+14	3,33019E+14	0.0142583450309518	0.148126830099115	-2,66036E+14	8,10156E+14	8,06691E+14	6,98977E+13	4,20236E+14	5,45396E+14	5,16983E+14
TC1600001233.mm.2	Pla2g10	phospholipase A2, group X	-1,71448E+14	6,87363E+14	-3,32844E+14	0.0142908104945457	0.148243484929805	-2,6625E+12	5,80973E+14	5,71368E+14	6,68136E+14	8,08464E+13	7,17112E+14	7,78125E+14
TC1400002641.mm.2	Ednrb	endothelin receptor type B	2,05881E+14	8,43102E+14	3,32838E+14	0.0142920368650273	0.148243484929805	-2,66258E+14	9,71927E+14	9,6409E+14	8,58584E+14	7,39755E+12	8,40247E+14	6,84008E+14
TC0200003656.mm.2	Cobll1	Cobl-like 1	-1,46987E+14	7,22216E+14	-3,3281E+14	0.0142971746776465	0.148243484929805	-2,66292E+14	6,38384E+13	6,04055E+14	7,20434E+13	8,34007E+14	7,58306E+14	7,78108E+14
TC1100003422.mm.2	Usp32	ubiquitin specific peptidase 32	1,15055E+13	6,32067E+14	3,32534E+13	0.0143486143714311	0.148431997722027	-2,66629E+14	7,36397E+14	7,37075E+14	5,15754E+14	5,59811E+14	6,48314E+14	5,95048E+14
TC0100002589.mm.2	Prkag3	protein kinase, AMP-activated, gamma 3 non-catalytic subunit	-1,15164E+13	7,48722E+14	-3,3248E+14	0.0143586299110616	0.148431997722027	-2,66695E+14	6,63414E+14	6,62998E+14	7,69914E+14	8,39268E+13	7,77529E+14	7,7921E+14
TC1300002535.mm.2	Gtf2h2	general transcription factor II H, polypeptide 2	1,01924E+14	5,83405E+14	3,32464E+13	0.0143615389359857	0.148431997722027	-2,66714E+14	6,67861E+14	6,3811E+14	5,4395E+14	5,48382E+14	5,39176E+14	5,62948E+14
TC1100003880.mm.2	Wnt9b	wingless-type MMTV integration site family, member 9B	-1,11271E+14	8,19288E+14	-3,32458E+14	0.0143626835180809	0.148431997722027	-2,66721E+14	7,51026E+14	7,45262E+14	8,5041E+14	8,502E+13	8,28772E+14	8,90058E+14
TC0900002983.mm.2	Dnajc13	DnaJ heat shock protein family (Hsp40) member C13	1,1253E+13	6,42567E+14	3,32429E+14	0.0143681495183078	0.148431997722027	-2,66757E+14	6,70111E+14	7,22764E+14	6,46545E+14	6,48171E+13	6,02242E+14	5,65572E+14
TC0700004282.mm.2	Eif3c	eukaryotic translation initiation factor 3, subunit C	1,68669E+14	8,66428E+14	3,32405E+13	0.0143727289967899	0.148431997722027	-2,66787E+14	9,81676E+14	9,81647E+14	8,74556E+14	7,34707E+14	8,40101E+13	7,85884E+14
TC1000000837.mm.2	Gpx4	glutathione peroxidase 4	1,09018E+14	8,93114E+14	3,32366E+14	0.0143798391636619	0.148431997722027	-2,66833E+14	9,69692E+14	9,71624E+14	8,63776E+14	8,30312E+14	8,84529E+14	8,38752E+14
TC0500002830.mm.2	Tmem150c	transmembrane protein 150C	2,36099E+14	7,01319E+14	3,32178E+13	0.014415173049311	0.148692750567899	-2,67064E+14	8,60021E+13	8,22689E+14	7,50253E+14	5,64438E+14	6,73143E+14	5,3737E+14
TC1300001139.mm.2	Taf9/Ak6	TATA-box binding protein associated factor 9/adenylate kinase 6	1,03609E+14	8,10036E+14	3,32098E+13	0.0144300725037315	0.148692750567899	-2,67161E+14	8,91088E+14	8,93162E+14	7,64602E+14	7,34332E+14	8,04167E+14	7,72865E+14
TC1200000989.mm.2	Kcnk13	potassium channel, subfamily K, member 13	-1,4812E+14	8,02741E+14	-3,32015E+14	0.0144456772177209	0.148692750567899	-2,67263E+14	7,16104E+14	6,98325E+14	7,8771E+14	9,03639E+14	8,37841E+14	8,72829E+14
TC1100000426.mm.2	Slu7	SLU7 splicing factor homolog (S. cerevisiae)	2,12592E+14	7,25741E+14	3,3197E+14	0.0144540950745136	0.148692750567899	-2,67318E+14	8,74226E+14	8,59495E+14	7,55436E+14	5,56753E+14	6,78944E+14	6,29593E+14
TC0900000492.mm.2	Nectin1	nectin cell adhesion molecule 1	1,07226E+13	6,33538E+14	3,31942E+14	0.0144594936927661	0.148692750567899	-2,67353E+14	7,04258E+14	6,66566E+14	6,39267E+13	6,34762E+14	5,59738E+14	5,96635E+14
TC1100001644.mm.2	Cntnap1	contactin associated protein-like 1	1,89956E+14	7,71519E+14	3,31937E+14	0.0144604691950535	0.148692750567899	-2,67359E+14	9,07508E+14	9,06644E+14	7,69181E+14	6,11539E+14	6,76346E+14	7,57894E+14
TC1500000244.mm.2	Kcns2	K+ voltage-gated channel, subfamily S, 2	1,1224E+14	5,50315E+14	3,31748E+14	0.0144960530881767	0.148878803750249	-2,6759E+14	6,15232E+14	6,01129E+14	5,62865E+14	5,30784E+14	5,22754E+14	4,69127E+13
TC0500001382.mm.2	Hvcn1	hydrogen voltage-gated channel 1	-1,13367E+14	5,42782E+14	-3,31742E+14	0.014497042331294	0.148878803750249	-2,67596E+14	4,73933E+14	4,57801E+14	5,57278E+14	6,09214E+14	5,60233E+13	5,98234E+14
TC0200001971.mm.2	Tmc2	transmembrane channel-like gene family 2	-1,58686E+14	4,05454E+14	-3,31617E+14	0.0145207175625745	0.148934088239149	-2,6775E+14	2,99935E+14	2,99284E+14	4,07734E+14	5,09178E+14	4,12086E+14	5,04505E+14
TC1600000150.mm.2	Mrtfb	myocardin related transcription factor B	2,07928E+14	7,96691E+14	3,31526E+14	0.0145380291160717	0.148934088239149	-2,67862E+14	9,175E+14	9,32492E+14	8,21783E+14	6,74234E+13	7,8411E+14	6,50026E+14
TC1100000417.mm.2	Gabrb2	gamma-aminobutyric acid (GABA) A receptor, subunit beta 2	1,00341E+14	8,44452E+14	3,31436E+14	0.0145549740617969	0.148934088239149	-2,67971E+14	9,39779E+14	9,13953E+14	7,85487E+14	7,74444E+13	8,36288E+14	8,16761E+14
TC0400001626.mm.2	Crybg2	crystallin beta-gamma domain containing 2	-1,35872E+14	7,3038E+14	-3,31403E+14	0.0145612313856091	0.148934088239149	-2,68012E+13	6,35814E+14	6,51613E+14	7,39263E+14	7,96417E+14	7,36328E+14	8,22844E+14
TC1800000953.mm.2	Mkx	mohawk homeobox	-1,19195E+14	6,94956E+14	-3,31399E+14	0.014561917103613	0.148934088239149	-2,68016E+14	6,14904E+14	6,21753E+14	6,90478E+14	7,67555E+14	7,34684E+14	7,40363E+14
TC0700003740.mm.2	Pcf11	PCF11 cleavage and polyadenylation factor subunit	1,95105E+14	5,7411E+14	3,31381E+14	0.0145653571451612	0.148934088239149	-2,68038E+14	7,25757E+14	6,63017E+14	6,08462E+14	4,48863E+14	5,33035E+14	4,65529E+13
TC1700001756.mm.2	Tulp1	tubby like protein 1	-2,10608E+14	5,74116E+14	-3,31372E+14	0.0145671273546142	0.148934088239149	-2,6805E+14	4,47602E+14	4,36209E+14	5,30027E+14	7,25828E+14	6,25741E+14	6,79286E+14
TC0700000410.mm.2	Cic	capicua transcriptional repressor	1,24004E+14	7,57913E+14	3,31283E+14	0.0145839829664951	0.148991846596596	-2,68159E+14	8,42046E+13	8,31023E+14	7,67303E+14	6,82043E+14	7,14231E+14	7,1083E+14
TC0200003654.mm.2	Grb14	growth factor receptor bound protein 14	1,602E+14	6,7072E+14	3,31245E+14	0.0145912700260304	0.148991846596596	-2,68205E+14	7,69508E+14	7,84451E+12	6,87682E+14	5,4912E+14	6,20022E+14	6,13536E+14
TC0400002189.mm.2	Asph	aspartate-beta-hydroxylase	1,6992E+14	8,78504E+14	3,31166E+14	0.0146062295972514	0.149035447726053	-2,68302E+14	9,96084E+14	9,80936E+14	8,59271E+14	7,97556E+14	8,87879E+14	7,49301E+14
TC0300000958.mm.2	Pdzk1	PDZ domain containing 1	-1,48627E+13	5,67116E+14	-3,31125E+14	0.0146140388138251	0.149035447726053	-2,68352E+14	4,52603E+14	4,8692E+14	5,59318E+14	6,67077E+14	5,93367E+14	6,43411E+14
TC0200004549.mm.2	1810024B03Rik	RIKEN cDNA 1810024B03 gene	-0.99242877217549	6,4765E+14	-3,30981E+14	0.0146414045156885	0.149219708033089	-2,68528E+13	5,89515E+12	5,97344E+14	6,58481E+14	6,55213E+14	6,77896E+14	7,07449E+14
TC0800002806.mm.2	Cdh16	cadherin 16	-1,78042E+14	5,27292E+14	-3,30933E+14	0.0146506285675136	0.149219708033089	-2,68587E+13	4,27902E+14	4,02836E+14	4,97212E+14	6,48983E+14	5,66273E+14	6,20548E+14
TC0700003818.mm.2	Ppme1	protein phosphatase methylesterase 1	1,11496E+14	8,05028E+14	3,30708E+14	0.0146936862158575	0.149474109468378	-2,68863E+14	8,66498E+14	8,71095E+14	8,07092E+14	7,70886E+14	7,83132E+14	7,31468E+14
TC0600001557.mm.2	Prh1	proline rich protein HaeIII subfamily 1	-1,42991E+14	6,87586E+14	-3,30594E+14	0.0147153944078941	0.149474109468378	-2,69002E+14	6,03849E+13	5,81036E+14	6,87452E+14	7,82312E+14	7,04112E+14	7,66756E+14

TC0400001974.mm.2	Eno1/Eno1b	enolase 1, alpha non-neuron/enolase 1B, retrotransposed	1,77538E+14	1,02209E+14	3,3057E+14	0.014720005966608	0.149474109468378	-2,69031E+14	1,14247E+14	1,1609E+14	1,0145E+14	8,6637E+14	1,00655E+14	9,4175E+14
TC1100000266.mm.2	Gm6899	predicted gene 6899	-1,35878E+14	6,68462E+14	-3,30549E+14	0.0147240739341981	0.149474109468378	-2,69057E+14	5,64546E+14	5,75085E+14	6,83159E+14	7,76596E+14	6,81854E+14	7,29533E+14
TC1500002181.mm.2	Adcy6	adenylate cyclase 6	1,12764E+14	6,28528E+14	3,30448E+14	0.0147435309504745	0.149474109468378	-2,69181E+13	6,73281E+14	6,89246E+14	6,08116E+14	6,63526E+14	5,84576E+14	5,52422E+14
TC1100004084.mm.2	Grb2	growth factor receptor bound protein 2	1,0201E+14	8,7127E+13	3,30443E+14	0.0147444864485557	0.149474109468378	-2,69188E+13	9,44387E+14	9,33937E+14	8,50813E+14	8,24177E+14	8,51473E+14	8,22831E+14
TC0100003682.mm.2	Nvl	nuclear VCP-like	1,65274E+14	5,65656E+14	3,30387E+13	0.0147551378197119	0.149474109468378	-2,69255E+13	6,4758E+14	6,73115E+14	6,02478E+14	4,80617E+14	5,34012E+14	4,56136E+14
TC0900000430.mm.2	Olfr148	olfactory receptor 148	-1,38265E+14	4,71478E+14	-3,30379E+14	0.0147568107988771	0.149474109468378	-2,69266E+14	3,79076E+14	3,87267E+14	4,83362E+14	5,36292E+14	4,73898E+14	5,68974E+14
TC1100003298.mm.2	Tmem199	transmembrane protein 199	1,30123E+13	6,37281E+14	3,30359E+14	0.0147605646779277	0.149474109468378	-2,6929E+14	7,52149E+14	6,94857E+14	6,11643E+14	5,78274E+14	6,28522E+14	5,58239E+14
TC0500001241.mm.2	2210016L21Rik	RIKEN cDNA 2210016L21 gene	1,70137E+14	9,0541E+14	3,30319E+14	0.0147683722629962	0.149474109468378	-2,6934E+14	1,01364E+14	9,94833E+14	8,83161E+14	8,72621E+14	9,09992E+14	7,58209E+14
TC1000000508.mm.2	Fabp7	fatty acid binding protein 7, brain	2,60567E+14	8,38416E+14	3,30265E+14	0.0147787106054894	0.149484848704489	-2,69406E+14	1,00909E+14	1,04533E+14	8,03535E+14	6,39251E+14	8,68056E+14	6,65233E+14
TC0100003027.mm.2	Dars	aspartyl-tRNA synthetase	1,4757E+14	7,14098E+14	3,30083E+14	0.0148137676831442	0.149579546733218	-2,69628E+14	8,11478E+14	8,00871E+14	7,19724E+14	6,35302E+14	7,02901E+14	6,14309E+13
TC0700000972.mm.2	E030018B13Rik	RIKEN cDNA E030018B13 gene	-1,48715E+14	5,09632E+14	-3,30061E+13	0.0148179742206138	0.149579546733218	-2,69655E+13	4,1244E+14	4,07191E+14	5,112E+14	6,09895E+14	5,20703E+14	5,96359E+14
TC0100003586.mm.2	Ackr1	atypical chemokine receptor 1 (Duffy blood group)	1,0716E+14	6,12071E+14	3,2998E+14	0.0148337417273881	0.149579546733218	-2,69755E+14	6,76531E+14	6,95296E+14	5,89547E+14	5,53544E+14	5,96763E+14	5,60745E+14
TC0900001859.mm.2	Cdc37	cell division cycle 37	1,11444E+14	6,92881E+14	3,29948E+14	0.0148399641775058	0.149579546733218	-2,69794E+14	7,92656E+14	7,50665E+14	6,67691E+14	6,25838E+13	6,71833E+14	6,486E+14
TC0200003436.mm.2	Scai	suppressor of cancer cell invasion	1,40516E+14	7,1697E+14	3,29935E+14	0.0148425086384	0.149579546733218	-2,69811E+14	8,04909E+14	8,23124E+14	6,7852E+14	6,48268E+14	7,27034E+14	6,19966E+14
TC1100000021.mm.2	Morc2a	microrchidia 2A	1,59317E+14	5,78812E+14	3,29928E+14	0.0148437718132201	0.149579546733218	-2,69819E+14	7,00225E+14	6,48819E+14	5,84048E+14	5,0937E+14	5,68008E+14	4,62403E+14
TC0300000821.mm.2	Sprr2a2/Sprr2a1	small proline-rich protein 2A2/small proline-rich protein 2A1	-1,10646E+14	5,71625E+14	-3,29789E+14	0.0148706584368876	0.14958965289113	-2,69989E+13	5,15436E+14	4,91473E+14	5,81354E+14	6,13286E+14	5,90302E+14	6,379E+14
TC1800001744.mm.2	Arhgap26	Rho GTPase activating protein 26	1,44917E+14	8,0608E+14	3,29772E+14	0.0148739399687113	0.14958965289113	-2,7001E+14	8,87425E+14	8,78575E+14	8,12191E+14	7,82121E+14	7,95608E+13	6,80558E+14
TC0800001732.mm.2	Adprhl1	ADP-ribosylhydrolase like 1	-0.965677805527259	5,61821E+14	-3,2965E+14	0.0148977263325142	0.14958965289113	-2,7016E+14	4,99801E+13	5,062E+14	5,732E+14	5,92589E+14	5,98846E+14	6,00291E+14
TC0900003318.mm.2	Ppp2r3d	protein phosphatase 2 (formerly 2A), regulatory subunit B'', delta	-1,45032E+14	7,85612E+14	-3,29596E+14	0.0149081764037124	0.14958965289113	-2,70226E+14	6,87821E+14	6,86843E+14	7,87743E+13	8,86535E+14	7,98733E+12	8,65996E+14
TC0800000120.mm.2	Cul4a	cullin 4A	1,70672E+14	6,30845E+14	3,29566E+14	0.0149140775120617	0.14958965289113	-2,70263E+14	7,33801E+13	7,24102E+14	6,65066E+14	5,45541E+14	6,06999E+14	5,0956E+14
TC0X00002878.mm.2	Chm	choroidermia (RAB escort protein 1)	1,66085E+14	5,63549E+14	3,29552E+14	0.0149168106017655	0.14958965289113	-2,7028E+14	6,35653E+14	6,72964E+14	5,74526E+14	5,21706E+14	5,55056E+14	4,21391E+14
TC0100003468.mm.2	Pogk	pogo transposable element with KRAB domain	0.980430410864073	6,69045E+14	3,2949E+14	0.0149288715372297	0.14958965289113	-2,70356E+14	7,3102E+14	7,32562E+14	6,32496E+14	6,50694E+14	6,23304E+14	6,44192E+14
TC0800002441.mm.2	Fcho1	FCH domain only 1	1,35939E+14	5,95925E+14	3,29469E+14	0.0149328391483601	0.14958965289113	-2,70381E+14	6,52675E+14	6,6602E+14	6,38953E+14	5,71083E+14	5,63697E+14	4,83119E+12
TC1100001688.mm.2	Grn	granulin	1,00195E+14	8,64127E+14	3,29393E+14	0.0149476585101095	0.14958965289113	-2,70474E+14	9,15613E+14	9,45532E+14	8,37906E+14	8,24956E+14	8,22973E+12	8,37783E+14
TC1000001490.mm.2	Wif1	Wnt inhibitory factor 1	-1,23005E+14	6,31563E+14	-3,29335E+14	0.0149591343740027	0.14958965289113	-2,70547E+14	5,50832E+14	5,45698E+14	6,41645E+14	7,08663E+14	6,44785E+13	6,97756E+14
TC0300000761.mm.2	Dcst2	DC-STAMP domain containing 2	-1,1536E+14	5,46161E+14	-3,29325E+14	0.0149610070469422	0.14958965289113	-2,70558E+12	4,52676E+14	4,73497E+14	5,83357E+14	6,10541E+13	5,46859E+14	6,10035E+14
TC1600000656.mm.2	Cfap44	cilia and flagella associated protein 44	-1,18301E+14	7,18207E+14	-3,29317E+14	0.0149626420088439	0.14958965289113	-2,70569E+14	6,31553E+14	6,52616E+14	7,17519E+14	7,86782E+14	7,46799E+14	7,73971E+14
TC0200004737.mm.2	Kif16b	kinesin family member 16B	-0.973687398283775	7,27309E+14	-3,2928E+14	0.0149696986132461	0.14958965289113	-2,70613E+14	6,70813E+14	6,68492E+14	7,3287E+14	7,57635E+13	7,68549E+14	7,65494E+14
TC1600001270.mm.2	Igll1	immunoglobulin lambda-like polypeptide 1	-1,34423E+14	5,51775E+14	-3,29255E+14	0.0149747477262703	0.14958965289113	-2,70645E+14	4,59726E+14	4,5765E+14	5,51645E+14	6,55408E+14	5,89635E+14	5,96586E+12
TC0200000591.mm.2	Sh2d3c	SH2 domain containing 3C	-1,25702E+14	7,92282E+14	-3,29169E+14	0.0149914694795178	0.149595847121901	-2,7075E+14	6,86817E+14	7,38047E+14	7,9371E+14	8,58849E+14	8,20314E+14	8,55954E+14
TC0800000403.mm.2	Saraf	store-operated calcium entry-associated regulatory factor	1,17672E+14	8,5561E+14	3,29092E+14	0.0150066141049855	0.149595847121901	-2,70845E+14	9,41829E+14	9,28813E+14	8,40766E+14	7,86958E+14	8,43922E+14	7,91375E+14
TC0600002212.mm.2	Pdia4/Mir704	protein disulfide isomerase associated 4/microRNA 704	1,96936E+14	6,85337E+14	3,29031E+14	0.0150184725377567	0.149595847121901	-2,70919E+14	7,93659E+14	8,16527E+14	6,53369E+14	6,32151E+14	7,01006E+14	5,15308E+14
TC0300002724.mm.2	Gstm2	glutathione S-transferase, mu 2	1,23094E+14	5,55291E+14	3,2903E+14	0.0150186931037319	0.149595847121901	-2,7092E+14	6,38288E+14	6,29456E+14	5,59967E+14	4,82479E+14	4,92746E+14	5,28809E+14
TC0300002460.mm.2	Zfp687	zinc finger protein 687	1,12263E+14	5,9083E+14	3,28959E+14	0.015032563831159	0.149595847121901	-2,71007E+14	6,61487E+14	6,33403E+14	6,16961E+14	5,62762E+14	5,22587E+14	5,47778E+14
TC0200003873.mm.2	Tmx2	thioredoxin-related transmembrane protein 2	1,06553E+14	8,70429E+14	3,28936E+14	0.0150372278385959	0.149595847121901	-2,71036E+14	9,59418E+14	9,30107E+14	8,50164E+14	8,06469E+13	8,4344E+14	8,3298E+14
TC0100002725.mm.2	Sp110	Sp110 nuclear body protein	-0.998531864401585	6,78602E+13	-3,28815E+14	0.0150609808483768	0.149595847121901	-2,71185E+14	6,35717E+14	6,07256E+14	6,86626E+14	6,99332E+14	7,13104E+14	7,29575E+14
TC0500000843.mm.2	Pf4	platelet factor 4	-1,30332E+14	6,93933E+14	-3,28787E+13	0.0150664421704429	0.149595847121901	-2,71219E+14	6,09254E+14	6,2951E+14	6,69132E+14	7,56273E+14	7,29789E+14	7,69639E+14
TC0800000015.mm.2	Pet100	PET100 homolog	1,80318E+14	7,00386E+14	3,28737E+14	0.0150762731695151	0.149595847121901	-2,7128E+14	7,86184E+14	7,41058E+14	6,89269E+13	8,19195E+14	5,35447E+14	6,3116E+14
TC0100000082.mm.2	Kcnb2	potassium voltage gated channel, Shab-related subfamily, member 2	1,39334E+13	5,46424E+14	3,28602E+14	0.0151029673746065	0.149595847121901	-2,71447E+14	6,31694E+14	6,18721E+14	5,29409E+14	5,26975E+13	5,41398E+14	4,30349E+14
TC0800001739.mm.2	1700029H14Rik	RIKEN cDNA 1700029H14 gene	-1,04245E+14	7,49576E+14	-3,28585E+14	0.0151061686104988	0.149595847121901	-2,71467E+14	6,75131E+14	6,77674E+14	7,7099E+14	8,12362E+14	7,92987E+14	7,68309E+14
TC0700000733.mm.2	Zfp788	zinc finger protein 788	2,12285E+14	6,21513E+14	3,28427E+14	0.0151375066084245	0.149595847121901	-2,71661E+14	7,53605E+13	7,10335E+13	6,53555E+14	5,72215E+13	6,12728E+14	4,26643E+14
TC1300001997.mm.2	Shc3	src homology 2 domain-containing transforming protein C3	1,29997E+14	8,18589E+14	3,28402E+14	0.0151423027787835	0.149595847121901	-2,71691E+14	9,13992E+14	9,1576E+14	7,95314E+14	7,1671E+14	8,13833E+14	7,55924E+14
TSUnmapped00000025.mm.2	Gm21992	predicted gene 21992	0.971788660026577	7,10087E+14	3,284E+14	0.0151427230104626	0.149595847121901	-2,71694E+14	7,84465E+14	7,77968E+14	6,70126E+13	6,59887E+14	6,95188E+14	6,72888E+14
TC0400002062.mm.2	Hes5	hes family bHLH transcription factor 5	-1,10088E+13	8,77595E+14	-3,28261E+14	0.0151702399508668	0.149595847121901	-2,71865E+14	8,02428E+14	8,17297E+14	8,80719E+14	9,25224E+14	8,99668E+14	9,40234E+14
TC1400000340.mm.2	Ncoa4	nuclear receptor coactivator 4	1,82067E+14	8,08681E+13	3,28131E+14	0.0151961186387343	0.149595847121901	-2,72025E+14	9,25042E+14	9,20267E+14	8,42887E+13	6,82714E+14	7,69312E+14	7,11865E+14
TC1200001031.mm.2	Ppp4r4	protein phosphatase 4, regulatory subunit 4	1,76318E+14	5,99947E+14	3,2809E+14	0.0152042417343841	0.149595847121901	-2,72075E+14	6,80852E+14	6,97759E+14	6,77623E+14	5,17472E+14	5,25157E+14	5,00818E+14
TC0600003317.mm.2	Pbp2	phosphatidylethanolamine binding protein 2	-1,40482E+13	5,46127E+14	-3,28085E+14	0.0152051343809244	0.149595847121901	-2,72081E+14	4,64754E+14	4,50554E+13	5,30753E+14	6,34427E+13	5,75705E+13	6,20567E+14
TC0400002480.mm.2	Ccl27a	chemokine (C-C motif) ligand 27A	1,07938E+14	9,5453E+14	3,28085E+14	0.0152052421374839	0.149595847121901	-2,72081E+14	1,0282E+14	1,04938E+13	9,1813E+13	8,6975E+14	9,42187E+14	9,19525E+14
TC0800002169.mm.2	Stox2	storkhead box 2	1,50763E+14	8,04305E+14	3,27963E+14	0.0152295651414025	0.149595847121901	-2,72232E+14	9,23015E+14	9,28588E+14	7,76239E+14	6,47913E+14	7,71092E+14	7,78985E+14
TC0800002471.mm.2	Large1	LARGE xylosyl- and glucuronyltransferase 1	2,40254E+14	6,82426E+14	3,27879E+12	0.0152461565344832	0.149595847121901	-2,72334E+13	8,4197E+14	8,68769E+14	6,89018E+14	4,6457E+14	6,57961E+14	5,7227E+14
TC0X00002738.mm.2	Rps4x	ribosomal protein S4, X-linked	1,43094E+14	8,22432E+14	3,27837E+14	0.015254588025838	0.149595847121901	-2,72386E+14	9,03686E+14	9,22811E+14	8,20609E+14	7,47179E+14	8,1523E+14	7,25078E+14
TC0800002920.mm.2	Calb2	calbindin 2	1,5363E+14	8,38635E+14	3,27828E+13	0.0152563868847054	0.149595847121901	-2,72397E+14	9,57638E+14	8,72923E+14	8,6206E+14	8,15887E+14	8,07254E+14	7,16047E+14
TC0100001853.mm.2	Ints7	integrator complex subunit 7	1,27027E+14	5,84124E+14	3,27795E+14	0.015262998179656	0.149595847121901	-2,72438E+14	6,50893E+14	6,74121E+14	5,72943E+13	5,35828E+14	5,76648E+14	4,94312E+14
TC0100000259.mm.2	Inpp4a	inositol polyphosphate-4-phosphatase, type I	1,50217E+14	7,81747E+14	3,27701E+14	0.0152817367650633	0.149595847121901	-2,72553E+14	8,89204E+14	8,88636E+14	7,72062E+14	6,63177E+14	7,72433E+14	7,04972E+14
TC1900000008.mm.2	Cpt1a	carnitine palmitoyltransferase 1a, liver	0.993711865446848	6,00035E+13	3,2765E+14	0.0152918656393554	0.149595847121901	-2,72616E+14	6,82733E+14	6,61089E+13	5,69159E+14	5,42153E+14	5,74436E+14	5,70643E+14
TC1500000742.mm.2	Srebf2	sterol regulatory element binding factor 2	1,44848E+14	9,38439E+14	3,2764E+14	0.0152939310888784	0.149595847121901	-2,72628E+14	1,02668E+13	1,03328E+14	9,53262E+14	8,47146E+13	9,13482E+14	8,56782E+14
TC0600001637.mm.2	Dera	deoxyribose-phosphate aldolase (putative)	-1,04512E+14	7,38912E+13	-3,27624E+14	0.0152971494535738	0.149595847121901	-2,72648E+14	6,55424E+14	6,8254E+14	7,6363E+14	7,84892E+14	7,55587E+14	7,914E+14
TC1400000320.mm.2	Pbrm1	polybromo 1	1,24671E+14	7,0079E+13	3,27612E+14	0.015299484730394	0.149595847121901	-2,72663E+14	7,80171E+13	7,71576E+14	6,67835E+14	6,82756E+14	6,95108E+14	6,07298E+14
TC0100001351.mm.2	Ivns1abp	influenza virus NS1A binding protein	1,91573E+14	7,17202E+14	3,27459E+13	0.0153302234587127	0.149595847121901	-2,72851E+14	8,76515E+14	8,38464E+14	7,16141E+14	5,40259E+14	6,81879E+14	6,49953E+14
TC0700002680.mm.2	Zfp974	zinc finger protein 974	1,40977E+14	5,53853E+14	32738916419	0.0153441601160631	0.149595847121901	-2,72937E+14	6,57363E+14	6,36351E+14	5,42333E+14	4,75311E+14	5,51423E+14	4,60337E+14
TC0900002864.mm.2	U2surp	U2 snRNP-associated SURP domain containing	2,3001E+14	7,71643E+14	3,27317E+14	0.0153585901431978	0.149595847121901	-2,73025E+14	8,92437E+13	9,00211E+14	7,96991E+14	7,07592E+14	7,72191E+14	5,60437E+14
TC0200005252.mm.2	Cyp24a1	cytochrome P450, family 24, subfamily a, polypeptide 1	-1,2738E+14	6,33782E+14	-3,27314E+14	0.0153593493516087	0.149595847121901	-2,7303E+14	5,33045E+14	5,74706E+14	6,26416E+14	7,06015E+14	6,6721E+14	6,95302E+14
TC0600002127.mm.2	Braf	Braf transforming gene	2,38449E+14	7,79584E+14	3,27233E+13	0.0153754739861781	0.149595847121901	-2,73129E+14	9,22076E+14	9,06667E+14	7,98719E+14	6,98195E+14	7,84701E+13	5,67145E+14
TC0500003582.mm.2	Tmem130	transmembrane protein 130	1,96823E+14	8,71172E+14	3,27199E+14	0.0153824613694696	0.149595847121901	-2,73171E+14	1,03302E+14	9,4845E+14	9,12244E+14	7,45489E+14	8,09416E+14	7,78411E+14

TC0700004490.mm.2	Msx3	msh homeobox 3	-1,31201E+14	6,93266E+14	-3,27177E+14	0.015386759689623	0.149595847121901	-2,73198E+13	5,90421E+14	6,23686E+14	7,15649E+14	7,53333E+14	6,94845E+14	7,81663E+14
TC1100003995.mm.2	1700012B07Rik	RIKEN cDNA 1700012B07 gene	-1,3684E+14	5,80072E+14	-3,27169E+14	0.0153884358720595	0.149595847121901	-2,73208E+14	4,65696E+14	5,0912E+14	5,78602E+14	6,78517E+13	6,22456E+14	6,26042E+14
TC0900002450.mm.2	Rplp1	ribosomal protein, large, P1	1,26081E+14	1,06479E+14	3,27132E+14	0.0153957773553981	0.149595847121901	-2,73253E+14	1,14908E+14	1,15035E+14	1,05796E+14	9,84115E+14	1,05117E+14	9,96101E+14
TC1000001624.mm.2	Sarnp	SAP domain containing ribonucleoprotein	2,4578E+14	5,58726E+14	3,27068E+14	0.0154087743737371	0.149595847121901	-2,73332E+14	7,47887E+14	7,44706E+14	5,34913E+14	3,23818E+14	5,66545E+14	4,34488E+14
TC0600001019.mm.2	Copg1	coatomer protein complex, subunit gamma 1	1,29425E+14	8,23089E+14	3,27012E+14	0.0154199493008747	0.149595847121901	-2,734E+14	9,10915E+14	8,87899E+14	8,34327E+14	7,65432E+14	8,02586E+14	7,37378E+14
TC0X00000742.mm.2	Brcc3	BRCA1/BRCA2-containing complex, subunit 3	2,20984E+14	7,11241E+14	3,26982E+14	0.0154260453027866	0.149595847121901	-2,73438E+14	8,48128E+14	8,51357E+13	7,45666E+12	5,64777E+14	6,90796E+14	5,66721E+14
TC0700000420.mm.2	Dmac2	distal membrane arm assembly complex 2	1,05026E+14	7,02923E+13	3,26978E+14	0.0154268948240598	0.149595847121901	-2,73443E+11	7,92564E+14	7,6404E+13	6,78402E+14	6,35979E+14	6,70195E+14	6,76357E+14
TC1900000448.mm.2	Il33	interleukin 33	1,28891E+14	7,22319E+14	3,26909E+14	0.0154408263149525	0.149595847121901	-2,73528E+14	7,72271E+14	7,91229E+14	7,50688E+13	7,14007E+14	6,96649E+14	6,09069E+13
TC1300001265.mm.2	Il6st	interleukin 6 signal transducer	1,90082E+14	7,31404E+14	3,26832E+14	0.0154563909914367	0.149595847121901	-2,73623E+14	8,43095E+14	8,50915E+14	7,37892E+14	6,4268E+13	7,33992E+14	5,79853E+14
TC1100003641.mm.2	Npepps	aminopeptidase puromycin sensitive	1,10368E+14	6,33095E+14	3,26788E+14	0.0154652240689873	0.149595847121901	-2,73676E+14	7,10383E+14	6,66727E+14	6,0989E+13	6,55196E+12	5,73338E+14	5,83035E+14
TC1900000262.mm.2	Olfr1451	olfactory receptor 1451	-2,21121E+14	5,10606E+14	-3,26785E+14	0.0154659987268053	0.149595847121901	-2,73681E+14	3,74672E+14	3,73299E+14	4,53017E+14	6,72438E+14	5,89579E+14	6,00634E+14
TC1200002251.mm.2	Lgmn	legumain	1,31186E+14	9,32216E+14	3,26774E+13	0.0154682119307461	0.149595847121901	-2,73694E+14	1,00652E+13	1,06224E+14	9,00916E+13	8,17222E+14	9,03665E+14	9,02728E+14
TC1300002360.mm.2	Polr3g	polymerase (RNA) III (DNA directed) polypeptide G	-1,18945E+14	5,78962E+14	-3,26733E+14	0.0154763791622568	0.149595847121901	-2,73744E+14	5,14698E+14	5,29604E+14	6,0963E+13	5,37648E+14	6,57092E+13	6,25101E+14
TC0300001150.mm.2	Wdr47	WD repeat domain 47	2,09039E+14	6,67746E+14	3,26704E+14	0.0154824110498646	0.149595847121901	-2,73781E+13	8,07321E+14	8,10195E+14	6,72319E+14	5,17204E+14	6,66741E+14	5,32698E+14
TC0900002981.mm.2	Uba5	ubiquitin-like modifier activating enzyme 5	1,09695E+14	7,96969E+14	3,26662E+14	0.0154908390496664	0.149595847121901	-2,73832E+13	8,66922E+14	8,69455E+14	7,75091E+14	7,53359E+14	7,87677E+13	7,29311E+14
TC1300000281.mm.2	D130043K22Rik	RIKEN cDNA D130043K22 gene	1,14609E+14	6,00914E+14	3,2663E+12	0.0154973446872433	0.149595847121901	-2,73871E+14	6,70632E+14	6,93682E+14	5,87247E+13	5,18825E+14	5,68562E+14	5,66535E+14
TC0400003705.mm.2	Nudc	nudC nuclear distribution protein	1,3443E+14	7,56359E+13	3,26612E+13	0.0155008812199804	0.149595847121901	-2,73893E+14	8,66364E+14	8,55367E+14	7,34307E+14	6,29244E+14	7,24799E+14	7,28072E+14
TC0200003927.mm.2	Olfr1053	olfactory receptor 1053	-1,0984E+13	5,77174E+14	-3,26591E+14	0.0155052428395575	0.149595847121901	-2,73919E+14	5,1874E+14	5,00768E+14	5,82371E+14	6,21978E+13	5,9702E+14	6,42167E+14
TC0X00000522.mm.2	Slc9a6	solute carrier family 9 (sodium/hydrogen exchanger), member 6	1,90495E+14	7,5625E+14	3,26585E+14	0.0155064577652151	0.149595847121901	-2,73927E+14	8,83866E+14	8,82273E+14	7,80624E+14	6,05587E+14	7,17575E+14	6,67573E+14
TC0600000829.mm.2	Rnf103	ring finger protein 103	1,17268E+14	7,3052E+14	3,26549E+14	0.0155136752959758	0.149595847121901	-2,73971E+14	7,97252E+14	8,07922E+14	7,12117E+13	6,95192E+14	7,22629E+13	6,48009E+14
TC1900000502.mm.2	Ifit3b	interferon-induced protein with tetratricopeptide repeats 3B	1,46944E+14	6,07577E+14	3,2654E+14	0.0155156445050008	0.149595847121901	-2,73982E+14	6,86175E+14	7,36248E+14	4,94151E+14	6,00354E+14	5,84519E+14	5,44017E+14
TC1300002510.mm.2	Tnpo1	transportin 1	1,01013E+14	7,44077E+14	3,26511E+14	0.0155214210818588	0.149595847121901	-2,74018E+14	8,13661E+13	8,17619E+14	7,17419E+14	6,86508E+14	7,28049E+14	7,01205E+14
TC0200003353.mm.2	Psmd5	proteasome (prosome, macropain) 26S subunit, non-ATPase, 5	2,95185E+13	6,30902E+14	3,26486E+12	0.0155265253373138	0.149595847121901	-2,74048E+14	8,03141E+14	8,24999E+14	7,0638E+14	4,13121E+14	5,63715E+14	4,74055E+14
TC1200000588.mm.2	Tmx1	thioredoxin-related transmembrane protein 1	2,15172E+14	6,99071E+14	3,26424E+13	0.0155390764955896	0.149595847121901	-2,74124E+14	8,396E+14	8,04109E+14	7,32213E+14	6,05136E+14	6,90138E+14	5,23228E+14
TC0100003240.mm.2	Prg4	proteoglycan 4 (megakaryocyte stimulating factor, articular superficial zone protein)	-1,3819E+14	5,60248E+14	-3,26376E+14	0.0155489756757865	0.149595847121901	-2,74184E+14	4,24828E+14	5,26578E+14	5,7247E+14	6,09828E+14	6,27033E+14	6,00753E+14
TC0200004630.mm.2	Avp	arginine vasopressin	-1,16595E+14	7,28676E+14	-3,2636E+14	0.0155522155694969	0.149595847121901	-2,74204E+14	6,79682E+14	6,22161E+14	7,55833E+14	7,79346E+14	7,45517E+13	7,89517E+14
TC1000002199.mm.2	Dux	double homeobox	-1,34787E+14	6,485E+14	-3,26321E+13	0.0155601888450073	0.149595847121901	-2,74252E+14	5,4681E+14	5,70285E+14	6,42747E+14	7,4449E+14	6,83058E+14	7,0361E+14
TC1100000560.mm.2	Zfp354a	zinc finger protein 354A	-1,00874E+14	6,12755E+14	-3,26294E+14	0.015565715144909	0.149595847121901	-2,74286E+13	5,26674E+14	5,41277E+14	6,52168E+14	6,8671E+13	6,26666E+14	6,43033E+13
TC0500001818.mm.2	Pomp	proteasome maturation protein	1,20286E+14	7,96579E+14	3,2618E+14	0.0155888296758106	0.149595847121901	-2,74425E+14	8,7678E+14	8,56437E+14	7,37984E+14	8,15629E+14	7,35955E+14	7,5669E+13
TC0400002531.mm.2	Gba2	glucosidase beta 2	1,0297E+14	7,31802E+14	3,2616E+14	0.0155929989895809	0.149595847121901	-2,7445E+14	7,82074E+14	7,96525E+14	7,01425E+14	7,38129E+14	6,99027E+14	6,73631E+14
TC0X00002061.mm.2	Ndufa1	NADH:ubiquinone oxidoreductase subunit A1	1,37235E+14	7,11337E+14	3,26144E+14	0.015596309550302	0.149595847121901	-2,7447E+14	8,16456E+12	8,06373E+14	7,03129E+14	5,93707E+14	6,78634E+14	6,69724E+14
TC1100003918.mm.2	Smarcd2	SWI/SNF related, matrix associated, actin dependent regulator of chromatin, subfamily d, member 2	-1,55042E+14	7,08092E+14	-3,26138E+14	0.0155974072590327	0.149595847121901	-2,74477E+14	5,90699E+14	5,82772E+14	7,51616E+14	8,39909E+14	6,90325E+14	7,9323E+14
TC0300002347.mm.2	Efna4	ephrin A4	-1,18423E+13	6,85322E+14	-3,2606E+14	0.0156133989792941	0.149660141435673	-2,74573E+14	6,0326E+14	6,24556E+14	6,72114E+14	7,47339E+14	7,24662E+14	7,39999E+14
TC0700004643.mm.2	Pak4	p21 (RAC1) activated kinase 4	-1,07757E+14	7,35021E+14	-3,25893E+14	0.0156476044911903	0.149837786924098	-2,74779E+14	6,78734E+14	6,51013E+14	7,4675E+14	7,88369E+14	7,57277E+14	7,87984E+14
TC1100000473.mm.2	Med7	mediator complex subunit 7	2,27948E+14	5,81227E+14	3,25879E+14	0.0156505303415414	0.149837786924098	-2,74797E+14	7,2979E+14	7,49626E+14	5,92279E+14	3,92147E+14	5,66222E+13	4,57296E+14
TC0900002779.mm.2	Pgm3	phosphoglucomutase 3	1,53422E+12	5,50636E+14	3,2578E+14	0.0156706909883799	0.149855719738408	-2,74918E+14	6,52285E+14	6,32627E+13	5,71349E+13	4,69491E+14	4,4677E+14	5,31297E+14
TC0700002603.mm.2	Irgc1	immunity-related GTPase family, cinema 1	-1,25688E+14	5,79043E+14	-3,25747E+14	0.0156774674855276	0.149855719738408	-2,74959E+14	4,97611E+14	4,92568E+14	5,81654E+14	6,6087E+14	6,44386E+14	5,97171E+14
TC1300001779.mm.2	Tubb2a	tubulin, beta 2A class IIA	1,62472E+13	8,94401E+14	3,25594E+14	0.0157090909270366	0.149855719738408	-2,75148E+14	1,00858E+14	9,98433E+14	9,0487E+14	7,7246E+14	8,71741E+14	8,10325E+14
TC1600000234.mm.2	Dgcr6	DiGeorge syndrome critical region gene 6	1,42892E+14	6,6156E+14	3,25586E+14	0.0157106356008286	0.149855719738408	-2,75158E+13	7,37674E+14	7,21335E+14	7,10767E+14	6,26359E+14	6,27732E+13	5,45493E+14
TC0300000376.mm.2	Foxo1	forkhead box O1	1,34532E+14	7,34532E+14	3,25572E+14	0.0157134351789229	0.149855719738408	-2,75174E+14	8,09535E+14	8,04134E+14	7,54183E+14	6,94737E+14	7,16404E+13	6,28201E+14
TC0300001555.mm.2	Erich3	glutamate rich 3	1,14484E+14	6,38378E+14	3,25505E+14	0.0157273070712208	0.149855719738408	-2,75257E+14	7,23577E+14	6,63684E+14	6,23219E+14	6,61491E+14	5,86891E+14	5,71402E+14
TC1900000432.mm.2	Rcl1	RNA terminal phosphate cyclase-like 1	1,43688E+14	6,30199E+14	3,25415E+14	0.0157458483306794	0.149855719738408	-2,75368E+14	6,98156E+14	7,00216E+14	6,9509E+14	5,76735E+14	5,66051E+14	5,44946E+14
TC1800000916.mm.2	Tmx3	thioredoxin-related transmembrane protein 3	1,75924E+14	7,25371E+14	3,25397E+14	0.015749694890381	0.149855719738408	-2,75391E+14	8,46094E+14	8,29298E+14	7,55452E+13	5,9784E+14	6,8329E+14	6,40253E+14
TC1500000066.mm.2	Lmbrd2	LMBR1 domain containing 2	2,58189E+14	6,95158E+14	3,25224E+14	0.0157853104540574	0.149855719738408	-2,75604E+14	8,39278E+14	8,51147E+14	7,40269E+14	5,66207E+13	6,87026E+14	4,87022E+14
TC0100000509.mm.2	Nrp2	neuropilin 2	1,78614E+14	6,77435E+14	3,25204E+14	0.015789576582938	0.149855719738408	-2,75629E+14	7,69513E+14	7,59577E+14	7,40834E+14	6,22824E+14	6,45418E+14	5,26444E+14
TC0X00002406.mm.2	Magea10	melanoma antigen family A, 10	-1,3515E+14	4,86469E+14	-3,25184E+14	0.0157936598395176	0.149855719738408	-2,75654E+14	4,03991E+14	3,95744E+14	4,79298E+14	5,69744E+14	5,06081E+14	5,63955E+14
TC0900001445.mm.2	Map4	microtubule-associated protein 4	1,49602E+14	8,29413E+14	3,25173E+14	0.01579587582043	0.149855719738408	-2,75667E+14	9,15732E+14	9,16297E+14	8,57997E+14	7,53627E+14	8,042E+14	7,28625E+14
TC1200000566.mm.2	Klhdc1	kelch domain containing 1	-1,13742E+14	6,6824E+14	-3,25161E+14	0.0157983018287643	0.149855719738408	-2,75681E+14	5,88893E+14	6,09133E+13	6,68921E+14	7,16983E+14	6,88261E+14	7,37249E+14
TC0900001236.mm.2	Copb2	coatomer protein complex, subunit beta 2 (beta prime)	1,48206E+14	7,02617E+14	3,25158E+14	0.0157989412950023	0.149855719738408	-2,75685E+14	8,16601E+13	8,05679E+14	6,68524E+14	5,99032E+14	7,15738E+14	6,1013E+14
TC0700003826.mm.2	Relt	RELT tumor necrosis factor receptor	0.970446857743572	5,92864E+14	3,25094E+14	0.0158121867612106	0.149855719738408	-2,75764E+14	6,655E+14	6,44396E+14	5,67808E+14	5,63673E+14	5,48152E+14	5,67654E+14
TC1000000763.mm.2	Gm10272	keratin associated protein 12-4	-1,53407E+14	6,13573E+13	-3,25075E+14	0.0158162688444693	0.149855719738408	-2,75788E+14	5,00487E+14	5,25559E+14	6,38222E+14	6,84311E+14	6,0464E+14	7,2822E+14
TC0300003200.mm.2	Lrrc7	leucine rich repeat containing 7	1,84358E+13	7,53224E+14	3,2505E+14	0.015821437458842	0.149855719738408	-2,75819E+13	8,53923E+14	8,68969E+14	7,82463E+14	6,59815E+14	7,39308E+14	6,14867E+14
TC0200005006.mm.2	Ndrg3	N-myc downstream regulated gene 3	1,71251E+14	9,08937E+14	3,25029E+14	0.0158258580283286	0.149855719738408	-2,75845E+14	1,03124E+14	1,00724E+14	9,28979E+14	7,90185E+14	8,84202E+14	8,11775E+14
TC0X00001413.mm.2	Pak3	p21 (RAC1) activated kinase 3	1,92982E+14	8,08973E+14	3,24958E+14	0.0158404231619163	0.149855719738408	-2,75932E+14	9,3941E+14	9,02318E+14	8,52309E+14	7,04039E+14	7,81224E+14	6,74539E+14
TC0800002821.mm.2	Kctd19	potassium channel tetramerisation domain containing 19	-1,07796E+14	5,23883E+14	-3,24878E+14	0.0158571042955046	0.149855719738408	-2,76031E+13	4,41807E+14	4,54693E+14	5,39047E+14	5,9566E+14	5,62579E+14	5,49513E+14
TC1400001393.mm.2	Gm3500	predicted gene 3500	2,01585E+14	5,31202E+14	3,24858E+14	0.0158612468479274	0.149855719738408	-2,76055E+14	6,47891E+14	5,60163E+13	6,36976E+14	5,373E+14	4,75371E+14	3,29512E+14
TC0600001001.mm.2	Nfu1	NFU1 iron-sulfur cluster scaffold	1,26847E+12	7,23124E+14	3,2484E+14	0.0158649489064041	0.149855719738408	-2,76077E+14	8,45744E+14	8,1172E+14	6,80739E+14	5,96774E+14	7,159E+14	6,87868E+14
TC0800001737.mm.2	Gas6	growth arrest specific 6	1,31883E+14	7,67777E+14	3,24834E+14	0.0158663103006097	0.149855719738408	-2,76086E+14	8,45321E+14	8,56362E+14	7,57342E+14	7,09722E+14	7,64216E+14	6,737E+14
TC0400002086.mm.2	Tmem240	transmembrane protein 240	-1,20346E+14	7,48022E+14	-3,24717E+14	0.0158906669528101	0.149997842185489	-2,7623E+14	6,65122E+14	6,82806E+14	7,34941E+12	8,16644E+14	7,90434E+13	7,98185E+14
TC0700002073.mm.2	Krtap5-4	keratin associated protein 5-4	-1,04053E+14	6,14586E+14	-3,24546E+14	0.015926280932627	0.150072023264332	-2,76441E+14	5,43286E+14	5,57845E+14	6,19521E+14	6,57632E+14	6,39304E+14	6,69931E+14
TC1100001609.mm.2	Krtap31-1	keratin associated protein 31-1	-1,4294E+14	4,91424E+14	-3,24412E+14	0.0159543377875526	0.150072023264332	-2,76606E+14	4,51201E+14	3,78704E+14	4,71191E+14	5,31666E+14	5,21021E+14	5,94762E+14
TC1200000068.mm.2	Pum2	pumilio RNA-binding family member 2	1,82278E+14	7,86884E+14	3,24405E+14	0.0159558096403347	0.150072023264332	-2,76615E+14	9,15277E+14	9,13829E+14	7,96209E+14	6,31436E+14	7,58898E+14	7,05653E+14
TC0400003777.mm.2	Eloa	elongin A	1,00365E+14	7,44267E+14	3,24382E+14	0.0159607306902348	0.150072023264332	-2,76644E+13	7,78976E+14	7,83933E+14	7,89243E+13	7,51273E+14	6,8864E+14	6,73539E+14
TC0500003645.mm.2	Katnal1/5930430L01 Rik	katanin p60 subunit A-like 1/RIKEN cDNA 5930430L01 gene	1,45313E+14	7,18603E+12	3,2438E+13	0.0159609730643716	0.150072023264332	-2,76645E+14	7,89086E+14	8,08859E+14	7,46544E+13	6,59809E+14	6,96316E+14	6,11003E+14

TC1400001696.mm.2	Timm23	translocase of inner mitochondrial membrane 23	1,17298E+12	7,83038E+14	3,2437E+14	0.0159631168873223	0.150072023264332	-2,76658E+14	8,72869E+14	8,54502E+14	7,56148E+14	7,21935E+14	7,80639E+14	7,12137E+14
TC0200001023.mm.2	Gad1	glutamate decarboxylase 1	1,15414E+14	8,85762E+13	3,24334E+13	0.015970770423946	0.150072023264332	-2,76703E+14	9,72573E+14	9,47651E+14	8,82463E+13	8,22492E+14	8,25653E+14	8,63742E+14
TC1300001470.mm.2	Ggps1	geranylgeranyl diphosphate synthase 1	1,75895E+14	6,82591E+14	3,24323E+14	0.0159730354309147	0.150072023264332	-2,76716E+14	8,13216E+14	7,8436E+14	6,88475E+14	5,63709E+14	6,74886E+14	5,709E+14
TC0700000252.mm.2	Psg29	pregnancy-specific glycoprotein 29	-1,34435E+14	4,77261E+14	-3,24275E+14	0.0159830140684037	0.150078266715727	-2,76775E+14	3,88674E+14	3,88331E+14	4,824E+14	5,58284E+13	4,85095E+14	5,60781E+14
TC0Y00000235.mm.2	Ddx3y	DEAD (Asp-Glu-Ala-Asp) box polypeptide 3, Y-linked	1,91845E+14	7,22586E+14	3,24195E+14	0.0159998712485589	0.150149053831118	-2,76874E+14	8,36376E+14	8,19939E+14	7,4935E+14	6,57229E+14	7,16411E+13	5,56213E+14
TC1900000978.mm.2	Pcnx3	pecanex homolog 3	0.969323880124998	7,06538E+14	3,24026E+14	0.0160353068159109	0.150394003914303	-2,77083E+14	7,70278E+14	7,66295E+14	6,93138E+14	6,66807E+14	6,64597E+14	6,78112E+13
TC0400000704.mm.2	4933430I17Rik	RIKEN cDNA 4933430I17 gene	-1,53554E+14	4,98497E+14	-3,2385E+14	0.0160724159825734	0.150654356443982	-2,773E+14	3,57654E+14	4,66431E+14	4,84351E+14	5,51351E+14	5,43374E+14	5,87818E+14
TC0700002988.mm.2	Rcn3	reticulocalbin 3, EF-hand calcium binding domain	-1,04306E+14	6,51342E+14	-3,23794E+14	0.0160843528356162	0.15067859141877	-2,7737E+14	5,70724E+14	5,97744E+14	6,58914E+14	7,03586E+14	6,87661E+14	6,89421E+14
TC1700002308.mm.2	Stap2	signal transducing adaptor family member 2	-1,28055E+14	5,91399E+14	-3,23592E+14	0.0161269167462061	0.150829173769126	-2,77619E+14	4,81955E+14	5,50429E+14	5,82425E+13	6,4509E+14	6,29184E+14	6,59309E+14
TC0100002832.mm.2	Pask	PAS domain containing serine/threonine kinase	-1,16729E+14	5,63177E+14	-3,23566E+14	0.0161324003740846	0.150829173769126	-2,77651E+14	4,97864E+13	4,90835E+14	5,45269E+13	6,22939E+14	6,07976E+14	6,1418E+14
TC1600000536.mm.2	Fstl1	follistatin-like 1	-1,20557E+14	8,88656E+14	-3,23561E+13	0.0161335750551515	0.150829173769126	-2,77658E+14	8,16394E+13	8,10503E+14	8,7791E+14	9,59117E+14	9,21679E+14	9,46331E+14
TC1100002406.mm.2	Ccdc85a	coiled-coil domain containing 85A	1,44591E+14	7,14987E+14	3,23521E+14	0.0161420937390801	0.150829173769126	-2,77707E+14	8,10402E+14	8,03222E+14	7,25008E+14	6,26848E+14	6,98913E+14	6,25528E+14
TC0400001421.mm.2	Sf3a3	splicing factor 3a, subunit 3	1,58134E+14	6,21767E+14	3,23482E+14	0.0161503288329714	0.150829173769126	-2,77755E+14	7,25854E+14	7,15678E+14	6,42381E+14	5,21423E+14	5,25948E+14	5,99317E+14
TC1900000881.mm.2	Ppp6r3	protein phosphatase 6, regulatory subunit 3	1,41527E+14	7,0076E+13	3,23369E+14	0.0161743902464687	0.150829173769126	-2,77896E+14	7,96921E+14	7,68278E+14	7,33261E+14	6,23953E+14	6,58746E+14	6,23398E+14
TC0700003008.mm.2	Dhdh	dihydrodiol dehydrogenase (dimeric)	1,34992E+14	6,96267E+14	3,23303E+14	0.0161883451005078	0.150829173769126	-2,77977E+14	7,9264E+14	7,70801E+14	7,13528E+14	6,07175E+13	6,44675E+14	6,48782E+14
TC0700001925.mm.2	Hmx3	H6 homeobox 3	-1,09344E+14	6,7884E+14	-3,23279E+14	0.0161934209996937	0.150829173769126	-2,78006E+14	5,97544E+14	6,12337E+14	6,86424E+14	7,48166E+14	7,10257E+14	7,18313E+14
TC0800001422.mm.2	Dnaaf1	dynein, axonemal assembly factor 1	-1,10765E+14	4,78539E+14	-3,23227E+14	0.0162045922525226	0.150829173769126	-2,78071E+14	4,2892E+14	3,97642E+14	4,7283E+14	5,23748E+13	5,08924E+14	5,39168E+14
TC0600001617.mm.2	Gm8994	predicted gene 8994	-1,37639E+14	6,61594E+14	-3,23222E+14	0.0162054788739797	0.150829173769126	-2,78076E+14	5,84138E+14	5,73462E+14	6,44488E+14	7,34597E+14	6,83008E+14	7,49869E+14
TC1700002336.mm.2	Nrtn	neurturin	-1,33633E+14	7,91159E+14	-3,23195E+14	0.0162112609365029	0.150829173769126	-2,7811E+14	6,93832E+14	7,15142E+14	7,90181E+14	8,71557E+14	8,05356E+14	8,70884E+14
TC0X00001700.mm.2	Arhgap6	Rho GTPase activating protein 6	2,03059E+13	7,09968E+14	3,23185E+14	0.0162134758127059	0.150829173769126	-2,78123E+14	8,5114E+14	8,15054E+14	7,28694E+14	6,04842E+14	7,06708E+13	5,53369E+14
TC0700002619.mm.2	Grik5	glutamate receptor, ionotropic, kainate 5 (gamma 2)	1,2463E+14	8,75758E+14	3,23144E+14	0.0162221161882887	0.150829173769126	-2,78173E+14	9,41402E+13	9,68188E+14	8,75609E+14	8,09018E+14	8,59153E+14	8,01177E+14
TC0X00003429.mm.2	L1cam	L1 cell adhesion molecule	1,63317E+14	7,03107E+14	3,2306E+14	0.0162401340363038	0.150909619219702	-2,78277E+14	8,23367E+14	7,83136E+14	7,36965E+11	5,95306E+14	6,51824E+14	6,28045E+14
TC1600000184.mm.2	Snai2	snail family zinc finger 2	-1,21965E+14	6,57463E+13	-3,22957E+14	0.016262138905835	0.151017464289389	-2,78405E+14	5,98275E+14	5,67931E+13	7,12096E+14	6,56339E+12	7,35463E+14	6,74673E+14
TC1800000071.mm.2	Thoc1	THO complex 1	1,4171E+14	6,18922E+14	3,22918E+14	0.0162704845780661	0.151017464289389	-2,78453E+14	7,3532E+14	6,96469E+14	6,10552E+14	5,22819E+14	6,09344E+14	5,39025E+14
TC1200001055.mm.2	Tcl1b1	T cell leukemia/lymphoma 1B, 1	-1,24925E+14	4,76976E+14	-3,22835E+14	0.0162880929794733	0.151093864236185	-2,78555E+13	4,36057E+14	4,34801E+14	4,81425E+13	3,88867E+14	5,56109E+14	5,64599E+14
TC0300002828.mm.2	Ptbp2	polypyrimidine tract binding protein 2	1,52287E+14	8,28137E+14	3,2272E+14	0.0163128591931532	0.151100913395532	-2,78698E+13	9,48998E+14	9,30971E+14	8,06816E+14	7,06646E+14	8,31464E+14	7,4393E+13
TC0400001741.mm.2	Capzb	capping protein (actin filament) muscle Z-line, beta	1,88574E+14	9,55214E+14	3,22669E+14	0.0163237542888167	0.151100913395532	-2,78761E+13	1,07668E+14	1,07261E+14	9,78568E+13	8,31293E+14	9,39927E+14	8,32209E+14
TC1100002102.mm.2	Slc35e4	solute carrier family 35, member E4	1,1545E+13	5,5381E+14	3,22621E+14	0.0163340292257709	0.151100913395532	-2,7882E+14	6,0765E+13	5,85221E+14	5,76821E+14	5,91196E+14	5,1684E+14	4,45131E+14
TC1900001730.mm.2	Vax1	ventral anterior homeobox 1	-1,08498E+14	8,18948E+14	-3,226E+14	0.0163385498035347	0.151100913395532	-2,78846E+14	7,36293E+14	7,61721E+14	8,26929E+13	8,73735E+14	8,40977E+14	8,74034E+14
TC1500000409.mm.2	Wdyhv1	WDYHV motif containing 1	1,30608E+14	6,24132E+14	3,22593E+14	0.0163400500296736	0.151100913395532	-2,78855E+14	7,4356E+14	7,20007E+14	5,88061E+14	4,90811E+14	5,90547E+14	6,11804E+14
TC1500000301.mm.2	Dcaf13	DDB1 and CUL4 associated factor 13	1,33027E+14	6,39606E+14	3,2257E+14	0.016345118354646	0.151100913395532	-2,78884E+13	7,21313E+14	7,36282E+14	6,42934E+14	5,45566E+14	6,15463E+14	5,76078E+13
TC0500002745.mm.2	G3bp2	GTPase activating protein (SH3 domain) binding protein 2	1,48992E+14	8,80851E+14	3,22326E+14	0.0163974761658331	0.151275318713727	-2,79185E+14	9,80762E+13	9,71417E+14	8,99884E+14	7,7885E+14	8,50151E+14	8,04044E+14
TC1100001938.mm.2	Prcd	photoreceptor disc component	-1,06591E+14	7,99691E+14	-3,22307E+14	0.0164015504418074	0.151275318713727	-2,79208E+14	7,33842E+14	7,26596E+14	8,0985E+14	8,54242E+14	8,17932E+14	8,55687E+14
TC0400001047.mm.2	Pde4b	phosphodiesterase 4B, cAMP specific	1,6805E+14	8,2566E+14	3,22282E+14	0.016407097522293	0.151275318713727	-2,7924E+14	9,20466E+14	9,13579E+14	8,52054E+14	7,69918E+14	8,15226E+14	6,82719E+14
TC0600000270.mm.2	Cpa4	carboxypeptidase A4	-1,01659E+14	4,72942E+14	-3,22265E+14	0.0164106019362187	0.151275318713727	-2,7926E+14	4,2104E+14	4,32189E+14	4,63141E+14	4,64731E+14	5,05615E+14	5,50933E+14
TC1700002418.mm.2	Twsg1	twisted gastrulation BMP signaling modulator 1	-0.951199960575694	8,31366E+14	-3,2219E+14	0.0164267811191956	0.151275318713727	-2,79353E+14	7,689E+14	7,82431E+14	8,48253E+14	8,47043E+14	8,60692E+14	8,80879E+14
TC1400001052.mm.2	Rubcnl	RUN and cysteine rich domain containing beclin 1 interacting protein like	-1,01049E+14	5,8499E+14	-3,22109E+14	0.0164444522764174	0.151275318713727	-2,79454E+14	5,28038E+14	5,20373E+14	5,91358E+14	6,19664E+14	6,08081E+14	6,42428E+13
TC0500001297.mm.2	Wsb2	WD repeat and SOCS box-containing 2	1,21274E+14	8,28749E+14	3,21971E+14	0.0164743251222776	0.151275318713727	-2,79625E+14	9,11673E+14	8,97936E+14	8,3928E+14	7,56542E+14	7,71338E+14	7,95724E+14
TC1700000957.mm.2	Gm6934	predicted gene 6934	-1,36097E+14	7,7286E+14	-3,21874E+14	0.0164952951527036	0.151275318713727	-2,79745E+14	6,91844E+14	6,66911E+14	7,74429E+14	8,73028E+14	7,92933E+14	8,38015E+14
TC1100001288.mm.2	1700125H20Rik	RIKEN cDNA 1700125H20 gene	-1,26197E+14	5,25543E+13	-3,21866E+14	0.0164970159949945	0.151275318713727	-2,79755E+14	4,92014E+14	4,19659E+14	5,09003E+14	5,58516E+14	5,72333E+14	5,91733E+14
TC1700000644.mm.2	Tnxb	tenascin XB	-1,17303E+14	5,92626E+14	-3,21796E+14	0.0165122121979193	0.151275318713727	-2,79842E+14	5,25171E+14	5,08508E+14	5,9138E+14	6,62412E+14	6,17194E+13	6,51091E+14
TC0900001116.mm.2	A330041J22Rik	RIKEN cDNA A330041J22 gene	-1,13314E+14	6,38149E+14	-3,21776E+14	0.0165166112337102	0.151275318713727	-2,79867E+14	5,76114E+14	5,7838E+13	6,22897E+13	6,7038E+14	6,62849E+14	7,18273E+14
TC1600002082.mm.2	Gart	phosphoribosylglycinamide formyltransferase	1,47333E+14	6,75895E+14	3,21667E+14	0.0165402061181782	0.151275318713727	-2,80001E+14	7,61787E+14	7,54178E+14	7,16784E+14	6,01323E+14	6,3792E+14	5,83378E+13
TC1700001631.mm.2	Mlst8	MTOR associated protein, LST8 homolog (S. cerevisiae)	1,33135E+14	6,88557E+14	3,21655E+14	0.0165428802220458	0.151275318713727	-2,80016E+14	7,79989E+14	7,76517E+14	6,9529E+13	5,89312E+14	6,51691E+14	6,38544E+14
TC1700002286.mm.2	Sgo1	shugoshin 1	-1,30089E+14	5,46251E+14	-3,21631E+14	0.0165481426892788	0.151275318713727	-2,80046E+14	4,31434E+14	4,83111E+14	5,63497E+13	6,24739E+14	5,55734E+13	6,18989E+14
TC1100000786.mm.2	Fam83g	family with sequence similarity 83, member G	-1,1086E+14	6,62064E+14	-3,21575E+14	0.0165603298420715	0.151275318713727	-2,80116E+14	5,73425E+14	5,8811E+14	6,99241E+14	7,28351E+14	6,6161E+13	7,21646E+14
TC0200003171.mm.2	Camsap1	calmodulin regulated spectrin-associated protein 1	2,6098E+14	7,15667E+13	3,21553E+14	0.0165652237904741	0.151275318713727	-2,80143E+14	8,86159E+14	8,67676E+14	7,71732E+14	5,36561E+13	6,83866E+14	5,4801E+14
TC1300000383.mm.2	Nqo2	N-ribosyldihydronicotinamide quinone reductase 2	1,34444E+14	5,5789E+13	3,21542E+14	0.0165674359424997	0.151275318713727	-2,80156E+13	6,48719E+13	6,58833E+13	5,45915E+14	4,55208E+14	4,89596E+14	5,49069E+14
TC1500000603.mm.2	Kifc2	kinesin family member C2	1,0944E+14	6,91137E+14	3,21525E+14	0.0165712711320951	0.151275318713727	-2,80178E+14	7,76026E+14	7,68516E+14	6,6929E+14	6,07331E+14	6,5299E+14	6,72673E+13
TC0200004425.mm.2	Ppp1r14d	protein phosphatase 1, regulatory inhibitor subunit 14D	-1,06302E+14	5,92284E+14	-3,21503E+14	0.0165760496024923	0.151275318713727	-2,80205E+14	5,22273E+14	5,44838E+14	5,7652E+14	6,30359E+14	6,42813E+14	6,36903E+14
TC1000000829.mm.2	Arid3a	AT rich interactive domain 3A (BRIGHT-like)	-1,00607E+14	7,52204E+14	-3,21456E+13	0.0165862216964787	0.151275318713727	-2,80263E+13	6,83235E+14	7,01925E+14	7,50765E+13	7,90923E+14	7,95319E+14	7,91056E+14
TC1300000658.mm.2	Slc34a1	solute carrier family 34 (sodium phosphate), member 1	-1,17059E+14	5,19116E+14	-3,21382E+14	0.0166024390333388	0.151275318713727	-2,80355E+14	4,38523E+14	4,63898E+14	5,04708E+14	5,71026E+14	5,49541E+14	5,86997E+14
TC0200000535.mm.2	Ptpa	protein phosphatase 2 protein activator	1,03945E+14	8,76344E+14	3,21377E+14	0.0166035458493892	0.151275318713727	-2,80361E+14	9,44749E+14	9,33609E+14	8,75375E+14	8,33866E+14	8,50962E+14	8,19506E+14
TC0800001319.mm.2	Vac14	Vac14 homolog (S. cerevisiae)	1,31315E+14	6,35326E+14	3,21319E+14	0.0166163388023923	0.151275318713727	-2,80433E+14	7,2946E+14	7,20036E+14	6,3117E+14	5,44424E+14	5,64933E+14	6,21933E+14
TC1000002248.mm.2	H2afy2	H2A histone family, member Y2	1,02179E+14	6,65865E+14	3,21306E+14	0.0166190494338434	0.151275318713727	-2,80449E+14	7,31876E+14	7,21436E+14	6,57147E+14	6,35779E+14	6,45827E+13	6,03127E+14
TC0900000216.mm.2	Zfp809	zinc finger protein 809	1,20687E+14	6,87687E+14	3,21265E+14	0.01662806695756	0.151275318713727	-2,805E+14	7,74392E+14	7,71459E+14	6,1699E+14	6,58804E+14	6,90236E+14	6,14241E+14
TC1600000446.mm.2	Zdhhc19	zinc finger, DHHC domain containing 19	-1,1354E+14	6,48793E+14	-3,21219E+14	0.0166381521528151	0.151275318713727	-2,80557E+14	5,56903E+14	5,73906E+14	6,75386E+14	7,28676E+14	6,54852E+14	7,03036E+14
TC0400001123.mm.2	Tmem59	transmembrane protein 59	1,78143E+14	8,49416E+13	3,21095E+14	0.0166653063512769	0.151275318713727	-2,80711E+14	9,72592E+14	9,62307E+14	8,43905E+12	7,39081E+14	8,56156E+14	7,22456E+14
TC0500001734.mm.2	Daglb	diacylglycerol lipase, beta	1,13651E+14	7,22198E+14	3,21025E+14	0.0166807856432048	0.151275318713727	-2,80798E+14	7,73706E+14	7,79704E+14	7,58804E+14	6,94867E+14	6,81614E+14	6,44493E+14
TC1000003109.mm.2	Mbd6	methyl-CpG binding domain protein 6	-1,17472E+14	7,00345E+14	-3,21015E+13	0.0166828167042602	0.151275318713727	-2,80809E+14	6,07173E+14	6,23073E+14	7,3988E+13	7,66754E+14	6,95079E+14	7,70112E+14
TC0600002305.mm.2	Tril	TLR4 interactor with leucine-rich repeats	1,50593E+14	6,4438E+14	3,2101E+14	0.0166839536188658	0.151275318713727	-2,80816E+14	7,53207E+14	7,44639E+14	6,5166E+14	5,20114E+14	6,07119E+14	5,89541E+14

TC1000000543.mm.2	Spock2	sparc/osteonectin, cwcv and kazal-like domains proteoglycan 2	1,9207E+14	9,43668E+14	3,20959E+14	0.0166950786822785	0.151275318713727	-2,80879E+14	1,08003E+14	1,05218E+14	9,73054E+14	8,08664E+14	9,17742E+14	8,30334E+14
TC1900000069.mm.2	Fibp	fibroblast growth factor (acidic) intracellular binding protein	1,28399E+14	6,79323E+14	3,20864E+14	0.016716028392274	0.151275318713727	-2,80997E+14	7,58229E+14	7,60294E+14	6,10517E+14	6,85172E+14	6,64028E+14	5,97696E+14
TC0500003509.mm.2	Grifin	galectin-related inter-fiber protein	-1,03741E+14	7,82407E+14	-3,20821E+14	0.0167255078078818	0.151275318713727	-2,8105E+14	7,12221E+14	7,12093E+14	7,9261E+14	8,4572E+14	8,14351E+14	8,17445E+14
TC0300002342.mm.2	Trim46	tripartite motif-containing 46	1,10569E+14	7,27055E+13	3,20815E+14	0.016726768973997	0.151275318713727	-2,81057E+14	7,94369E+14	8,03891E+14	7,26788E+14	6,6016E+14	6,94247E+14	6,82875E+14
TC1100002015.mm.2	Baiap2	brain-specific angiogenesis inhibitor 1-associated protein 2	2,97948E+14	7,00436E+14	3,20745E+13	0.0167422112381034	0.151275318713727	-2,81144E+14	8,94373E+13	9,04139E+14	7,21401E+13	4,80088E+14	7,0382E+14	4,98794E+14
TC1100003106.mm.2	Slc25a11	solute carrier family 25 (mitochondrial carrier oxoglutarate carrier), member 11	1,81107E+14	9,01215E+14	3,20721E+13	0.0167475544848307	0.151275318713727	-2,81174E+14	1,02507E+13	1,01366E+13	9,11806E+14	7,80247E+14	8,95168E+14	7,81344E+14
TC1100003450.mm.2	Vmp1/Mir21a	vacuole membrane protein 1/microRNA 21a	1,47035E+14	7,63655E+14	3,20691E+14	0.0167542081234871	0.151275318713727	-2,81212E+14	8,63682E+14	8,6767E+14	7,55586E+14	6,57711E+14	7,59943E+14	6,7734E+14
TC1800000370.mm.2	Pcdhb14	protocadherin beta 14	1,12366E+14	5,58368E+14	3,20603E+14	0.0167735516549174	0.151275318713727	-2,8132E+14	6,46663E+13	6,56016E+14	5,14235E+14	4,55344E+14	5,5913E+14	5,18817E+14
TC0700000558.mm.2	Alkbh6	alkB homolog 6	1,40597E+14	8,3116E+14	3,20582E+14	0.0167781918796783	0.151275318713727	-2,81346E+14	9,32908E+14	9,09308E+14	8,34319E+14	7,49406E+13	8,20363E+14	7,40659E+14
TC0300000090.mm.2	Lrrcc1	leucine rich repeat and coiled-coil domain containing 1	1,64202E+14	5,50358E+14	3,20565E+14	0.0167821507256633	0.151275318713727	-2,81368E+14	6,84238E+14	6,21291E+14	5,1479E+14	5,04702E+14	5,57598E+14	4,19528E+14
TC0100000233.mm.2	Plekhb2	pleckstrin homology domain containing, family B (evectins) member 2	1,0193E+14	8,3358E+13	3,2054E+14	0.0167875364082418	0.151275318713727	-2,81399E+14	8,95642E+14	8,91369E+14	8,39765E+14	7,91551E+14	7,94922E+14	7,8823E+14
TC0700002663.mm.2	Ltbp4	latent transforming growth factor beta binding protein 4	-1,37044E+13	8,00667E+14	-3,20538E+13	0.0167880238737771	0.151275318713727	-2,81401E+14	7,21522E+14	7,16517E+14	7,7389E+14	8,79947E+14	8,35947E+14	8,76181E+14
TC0700001509.mm.2	Olfr631	olfactory receptor 631	-1,05667E+14	4,89659E+14	-3,20507E+14	0.016794864033953	0.151275318713727	-2,8144E+14	4,47867E+14	4,11092E+14	4,82889E+14	5,25814E+14	5,28691E+14	5,41601E+14
TC0800001666.mm.2	Fam155a	family with sequence similarity 155, member A	1,21406E+14	7,49903E+14	3,20504E+13	0.0167954845359611	0.151275318713727	-2,81443E+14	8,07285E+14	8,21356E+14	7,747E+14	7,1025E+14	7,2243E+13	6,634E+14
TC1100003217.mm.2	Hic1	hypermethylated in cancer 1	-1,08405E+14	6,17093E+14	-3,20432E+14	0.0168115724192397	0.151275318713727	-2,81533E+13	5,57079E+14	5,81862E+14	6,27041E+14	5,80826E+13	6,59488E+14	6,96264E+14
TC0900001414.mm.2	Impdh2	inosine monophosphate dehydrogenase 2	1,49923E+13	6,50491E+14	3,2042E+14	0.016814101691728	0.151275318713727	-2,81548E+14	7,33944E+13	7,38097E+14	6,88005E+14	5,70702E+14	6,16764E+14	5,55432E+14
TC1200001432.mm.2	Mycn	v-myc avian myelocytomatosis viral related oncogene, neuroblastoma derived	-0.951137275403724	5,65446E+14	-3,20404E+14	0.0168176971669493	0.151275318713727	-2,81568E+14	5,12081E+14	5,23492E+14	5,58162E+14	5,73141E+14	6,22405E+13	6,03395E+14
TC1600001111.mm.2	Nlrc3	NLR family, CARD domain containing 3	-0.968004478693319	5,94905E+14	-3,2036E+14	0.0168273671070302	0.151275318713727	-2,81622E+14	5,33387E+14	5,40843E+14	6,13411E+14	6,13959E+14	6,17569E+14	6,50262E+14
TC1200001018.mm.2	Chga	chromogranin A	1,558E+14	8,48386E+14	3,20314E+14	0.0168375751750187	0.151275318713727	-2,81679E+14	9,74024E+14	9,44086E+14	8,51331E+14	7,14367E+14	8,09474E+14	7,97035E+14
TC0700004119.mm.2	Dkk3	dickkopf WNT signaling pathway inhibitor 3	1,18076E+14	7,87203E+14	3,20278E+14	0.0168455843414698	0.151275318713727	-2,81724E+13	8,46019E+14	9,22858E+14	7,201E+14	7,01514E+14	7,7874E+14	7,53986E+14
TC0500001077.mm.2	Lpcat2b	lysophosphatidylcholine acyltransferase 2B	-1,33309E+13	4,62106E+14	-3,20266E+14	0.0168483073454905	0.151275318713727	-2,81739E+14	4,33654E+14	3,65515E+14	4,14762E+14	4,92917E+14	5,48013E+14	5,17773E+14
TC0X00001931.mm.2	Syn1	synapsin I	2,36754E+13	9,16642E+14	3,20227E+14	0.0168569946536568	0.151275318713727	-2,81787E+14	1,07144E+14	1,08177E+14	9,40422E+14	7,26518E+14	8,98634E+14	7,81066E+14
TC1400002160.mm.2	Phf11-ps	PHD finger protein 11, pseudogene	-1,50028E+13	4,50405E+14	-3,20177E+14	0.0168680022561641	0.151275318713727	-2,81849E+14	3,33376E+14	4,03691E+14	4,15514E+14	5,12727E+14	4,90159E+14	5,46963E+14
TC1100001448.mm.2	Col1a1	collagen, type I, alpha 1	-1,52423E+14	7,39704E+14	-3,20144E+14	0.0168755243648464	0.151275318713727	-2,81891E+14	6,33669E+14	6,54679E+13	7,13563E+13	8,43116E+14	7,78494E+14	8,147E+14
TC0400003184.mm.2	C8a	complement component 8, alpha polypeptide	-1,50059E+14	5,23476E+14	-3,20122E+14	0.0168803464933458	0.151275318713727	-2,81918E+14	4,07114E+14	4,34636E+14	5,26217E+14	6,31021E+14	5,32523E+14	6,09344E+14
TC1100000015.mm.2	Selenom	selenoprotein M	1,04737E+14	8,53722E+14	3,19994E+14	0.0169087613248286	0.15128384659421	-2,82076E+14	9,28736E+14	9,08752E+14	8,45625E+14	8,11205E+14	8,3386E+14	7,94154E+14
TC1700001323.mm.2	Ston1	stonin 1	-1,08886E+14	6,43874E+14	-3,19932E+13	0.0169225603446185	0.15128384659421	-2,82153E+13	5,96865E+14	5,59798E+14	6,42452E+14	6,89697E+14	6,7424E+14	7,00196E+14
TC1400002227.mm.2	4930578I06Rik	RIKEN cDNA 4930578I06 gene	-1,19618E+14	4,82676E+14	-3,19916E+14	0.0169263060502512	0.15128384659421	-2,82174E+14	4,17271E+14	3,85808E+14	4,93403E+14	5,57255E+14	4,97199E+14	5,45117E+14
TC0400004136.mm.2	Ski	ski sarcoma viral oncogene homolog (avian)	1,08499E+14	7,59439E+14	3,1991E+14	0.0169275072357906	0.15128384659421	-2,82181E+14	8,02778E+14	8,17709E+14	7,87648E+14	7,45007E+14	7,24846E+14	6,78643E+14
TC1300002253.mm.2	Zfp729b	zinc finger protein 729b	1,39577E+14	5,74536E+14	3,19907E+13	0.0169282427486726	0.15128384659421	-2,82185E+14	6,7681E+14	6,41175E+14	5,51075E+14	5,39323E+14	5,75222E+14	4,63608E+14
TC1300002686.mm.2	Mtrex	Mtr4 exosome RNA helicase	1,9115E+14	6,57928E+14	3,19771E+14	0.0169586733430273	0.151365630067564	-2,82354E+14	7,87875E+14	7,84287E+14	6,60729E+14	5,24816E+14	6,59844E+14	5,30018E+14
TC0100001176.mm.2	Slc41a1	solute carrier family 41, member 1	1,63125E+14	8,15344E+14	3,19723E+14	0.0169694395645827	0.151365630067564	-2,82414E+14	8,94814E+14	9,10733E+14	8,59693E+14	7,47527E+14	7,89716E+14	6,8958E+14
TC0700003641.mm.2	Il16	interleukin 16	-1,15832E+14	5,65661E+14	-3,1966E+14	0.0169834113800665	0.151365630067564	-2,82491E+14	4,69939E+14	5,01353E+14	5,78252E+12	6,41467E+14	6,19414E+14	5,83543E+14
TC0200002844.mm.2	Rpp38	ribonuclease P/MRP 38 subunit	1,65291E+14	5,52271E+14	3,19659E+14	0.0169836324705061	0.151365630067564	-2,82492E+14	6,89391E+14	6,47445E+14	5,53503E+13	4,17036E+14	4,74023E+14	5,32231E+13
TC1400002025.mm.2	Supt16	suppressor of Ty 16	2,42552E+14	6,11925E+14	3,19592E+14	0.0169986330541008	0.151365630067564	-2,82575E+13	7,46318E+13	7,87059E+13	6,23435E+14	4,66464E+13	6,20525E+14	4,27748E+14
TC1100002854.mm.2	Zkscan17	zinc finger with KRAB and SCAN domains 17	-1,25292E+13	7,41896E+14	-3,19592E+14	0.0169986913165034	0.151365630067564	-2,82576E+14	6,53533E+14	6,5494E+14	7,45943E+14	8,37905E+14	7,66392E+14	7,92665E+14
TC0X00001128.mm.2	Zfp711	zinc finger protein 711	1,1017E+14	5,69685E+14	3,19543E+14	0.0170096356634633	0.151365630067564	-2,82636E+14	6,73836E+14	6,42746E+14	5,06845E+14	4,98437E+14	5,78258E+14	5,17984E+14
TC0700004188.mm.2	Thumpd1	THUMP domain containing 1	1,68318E+14	5,77653E+13	3,19384E+14	0.0170453283498267	0.151365630067564	-2,82834E+14	6,91193E+14	6,59967E+14	5,98552E+14	5,01682E+14	5,67975E+14	4,4655E+14
TC1000002067.mm.2	Nr2e1	nuclear receptor subfamily 2, group E, member 1	1,18334E+14	6,35724E+14	3,19377E+14	0.0170469340804528	0.151365630067564	-2,82843E+14	7,04188E+14	7,15408E+14	6,44897E+14	5,66924E+14	6,05893E+14	5,77036E+14
TC1000000156.mm.2	Bclaf1	BCL2-associated transcription factor 1	2,38332E+14	6,94836E+14	3,19365E+13	0.0170497257840921	0.151365630067564	-2,82858E+13	8,44548E+14	8,40932E+14	7,155E+14	5,59223E+14	7,00029E+13	5,08785E+14
TC0800001316.mm.2	Cmtr2	cap methyltransferase 2	-1,00695E+14	7,57235E+13	-3,19349E+14	0.017053249917674	0.151365630067564	-2,82878E+14	6,96421E+14	6,89558E+14	7,66642E+14	8,03418E+14	7,79813E+14	8,07556E+14
TC1700001188.mm.2	Gpatch11	G patch domain containing 11	0.92382436560938	7,00599E+14	3,1933E+14	0.0170575611886501	0.151365630067564	-2,82901E+14	7,61662E+14	7,49594E+14	6,83159E+14	6,82685E+14	6,63625E+14	6,62867E+13
TC0300002955.mm.2	Hadh	hydroxyacyl-Coenzyme A dehydrogenase	1,15239E+14	6,42833E+13	3,19248E+14	0.0170760147565512	0.151365630067564	-2,83003E+14	6,9261E+14	7,17455E+14	6,32072E+14	6,35273E+14	5,5267E+14	6,26918E+14
TC1400002635.mm.2	Mycbp2	MYC binding protein 2, E3 ubiquitin protein ligase	3,54001E+14	6,82191E+13	3,19217E+14	0.017083044209084	0.151365630067564	-2,83042E+14	8,78518E+14	8,78863E+14	6,46978E+14	6,39408E+14	7,16715E+14	3,32663E+14
TC1400000096.mm.2	Ptprg	protein tyrosine phosphatase, receptor type, G	1,29088E+14	7,37915E+14	3,19196E+14	0.0170877616960298	0.151365630067564	-2,83068E+14	8,30551E+13	7,93275E+14	7,28554E+14	7,09461E+14	7,29363E+14	6,36288E+14
TC0500001762.mm.2	1700018F24Rik	RIKEN cDNA 1700018F24 gene	-1,01131E+13	5,29913E+14	-3,19164E+14	0.0170950397870433	0.151365630067564	-2,83108E+14	5,00866E+14	4,84038E+14	5,04509E+14	5,02903E+14	5,7117E+14	6,15996E+14
TC1000000053.mm.2	Ppil4	peptidylprolyl isomerase (cyclophilin)-like 4	1,17977E+14	6,47152E+14	3,19144E+14	0.0170993739774907	0.151365630067564	-2,83132E+14	7,42921E+14	7,27914E+14	6,18623E+14	5,58571E+13	6,45396E+14	5,89485E+14
TC0400004139.mm.2	Prkcz	protein kinase C, zeta	0.972097794340746	9,30533E+14	3,19113E+13	0.017106486213184	0.151365630067564	-2,83171E+14	1,00017E+14	9,89759E+14	9,09135E+14	8,88632E+14	9,11791E+14	8,83714E+14
TCX_GL456233_random00000005.mm.2	Vamp7	vesicle-associated membrane protein 7	2,2116E+14	6,99899E+14	3,19019E+14	0.0171276450092123	0.151396966728259	-2,83288E+14	8,24526E+14	8,381E+14	7,0335E+14	6,1311E+14	7,13673E+14	5,06633E+14
TC1100001935.mm.2	Sphk1	sphingosine kinase 1	-1,20865E+14	6,42E+14	-3,19014E+13	0.0171288196081187	0.151396966728259	-2,83294E+14	5,70878E+14	5,67937E+14	6,42077E+14	6,90565E+13	6,53245E+14	7,27299E+14
TC1400000882.mm.2	Fam124a	family with sequence similarity 124, member A	-1,10761E+14	7,4738E+14	-3,18966E+14	0.0171396833481071	0.151409932997834	-2,83354E+14	6,99534E+14	6,66215E+14	7,37614E+14	7,93647E+14	7,80978E+14	8,06292E+14
TC0500003193.mm.2	Aldh2	aldehyde dehydrogenase 2, mitochondrial	1,63989E+14	7,65853E+14	3,18892E+13	0.0171564059484051	0.151435978667833	-2,83446E+14	8,87984E+14	8,63688E+14	7,81446E+14	6,38305E+14	7,359E+14	6,87795E+14
TC0900000690.mm.2	Snupn	snurportin 1	1,05888E+14	5,64996E+14	3,1887E+14	0.0171614284768487	0.151435978667833	-2,83473E+14	6,22172E+14	6,32769E+14	5,51932E+14	5,39941E+14	5,50342E+14	4,92823E+14
TC1600001471.mm.2	Xxylt1	xyloside xylosyltransferase 1	1,11259E+14	5,41302E+14	3,18626E+14	0.0172167821849735	0.15178807146366	-2,83776E+14	6,25196E+14	5,92225E+14	5,48133E+14	4,87353E+14	5,05856E+14	4,89047E+14
TC0500000837.mm.2	5830473C10Rik	RIKEN cDNA 5830473C10 gene	-1,24297E+14	6,15171E+14	-3,18611E+14	0.0172201697161032	0.15178807146366	-2,83795E+13	5,54339E+14	5,02246E+14	6,27894E+14	7,01366E+14	6,38058E+14	6,67121E+14
TC0400003885.mm.2	Fblim1	filamin binding LIM protein 1	-1,11443E+14	7,57639E+14	-3,18546E+14	0.0172350024302258	0.151835754050426	-2,83876E+14	6,83883E+14	7,0491E+14	7,51334E+14	7,94026E+14	7,79105E+14	8,32575E+14
TC0100002582.mm.2	Aamp	angio-associated migratory protein	1,97349E+14	6,31182E+14	3,18502E+14	0.0172450391026927	0.151841155771414	-2,83931E+14	7,72877E+14	7,51096E+14	6,56994E+14	4,76849E+14	6,02827E+14	5,26448E+14
TC0X00000668.mm.2	Nsdhl	NAD(P) dependent steroid dehydrogenase-like	1,38228E+14	7,44899E+14	3,18417E+14	0.0172642472589002	0.151927261650824	-2,84036E+14	8,43422E+14	8,27286E+14	7,42397E+14	6,62041E+14	7,38665E+14	6,55586E+14
TC1500001018.mm.2	Aqp5	aquaporin 5	-1,29057E+14	6,35664E+14	-3,1829E+14	0.0172933810626854	0.151954329788049	-2,84195E+14	5,80567E+14	5,43511E+14	6,08732E+14	6,98985E+14	6,75221E+14	7,0697E+14
TC0700004622.mm.2	Tm2d3	TM2 domain containing 3	1,07596E+14	7,63931E+13	3,18252E+13	0.0173020537571979	0.151954329788049	-2,84242E+14	8,68274E+14	8,25181E+14	7,26451E+14	6,85415E+14	7,50771E+14	7,27491E+14

TC0600000633.mm.2	Tax1bp1	Tax1 (human T cell leukemia virus type I) binding protein 1	2,56659E+14	7,17724E+14	3,18162E+14	0.0173226648089633	0.151954329788049	-2,84354E+14	8,93451E+14	8,96695E+13	7,44482E+13	4,94891E+14	6,90308E+14	5,8652E+14
TC1700000314.mm.2	Bicdl2	BICD family like cargo adaptor 2	-1,14717E+14	6,0181E+14	-3,18125E+14	0.0173311620092971	0.151954329788049	-2,844E+14	5,0582E+14	5,49935E+14	6,03298E+14	6,66618E+14	6,36448E+14	6,48742E+14
TC0800001360.mm.2	Vat1l	vesicle amine transport protein 1 like	1,03006E+14	8,73641E+14	3,18099E+13	0.0173370971254742	0.151954329788049	-2,84432E+14	9,4374E+14	9,06542E+14	8,84598E+14	8,62693E+14	8,37984E+14	8,06286E+14
TC1300002237.mm.2	Mterf3	mitochondrial transcription termination factor 3	1,10241E+14	6,87438E+14	3,18095E+14	0.0173379452596682	0.151954329788049	-2,84437E+14	7,75738E+12	7,79385E+14	6,49293E+14	5,85569E+14	6,53579E+14	6,81063E+14
TC0800002612.mm.2	4921524J17Rik	RIKEN cDNA 4921524J17 gene	-0.940296577882724	7,17837E+14	-3,18056E+14	0.0173468133822378	0.151954329788049	-2,84485E+14	6,71856E+14	6,51525E+14	7,29148E+14	7,43052E+14	7,53684E+14	7,57756E+14
TC1300001737.mm.2	Exoc2	exocyst complex component 2	1,40407E+14	7,54129E+14	3,17986E+14	0.0173630009257052	0.151954329788049	-2,84573E+14	8,59362E+14	8,30735E+14	7,15876E+14	7,09517E+14	7,62946E+14	6,46338E+14
TC1500000215.mm.2	Tas2r119	taste receptor, type 2, member 119	-1,39011E+14	4,49681E+14	-3,17976E+14	0.0173652205462306	0.151954329788049	-2,84585E+14	3,49867E+14	3,79852E+13	4,40613E+13	5,20015E+14	4,62763E+14	5,44978E+14
TC1600002120.mm.2	Vps26c	VPS26 endosomal protein sorting factor C	0.956077616728308	6,2718E+13	3,17951E+14	0.0173709440840333	0.151954329788049	-2,84616E+14	6,79936E+14	6,81263E+14	6,15831E+14	6,16064E+14	6,00879E+14	5,69105E+14
TC1200002527.mm.2	Rpl36al	ribosomal protein L36A-like	1,39027E+13	6,91447E+14	3,1795E+13	0.0173710591118716	0.151954329788049	-2,84617E+14	8,03356E+14	7,71326E+13	6,50114E+14	6,27255E+14	7,04046E+14	5,92583E+14
TC1300000904.mm.2	Erap1	endoplasmic reticulum aminopeptidase 1	1,15543E+14	5,6046E+14	3,17877E+14	0.0173879336060199	0.152019410848507	-2,84708E+14	6,1795E+13	6,17993E+14	5,2566E+14	5,96299E+14	5,21034E+14	4,83824E+14
TC1500002030.mm.2	Brd1	bromodomain containing 1	-1,3654E+14	8,28605E+14	-3,17792E+14	0.0174074913829088	0.1520546317988	-2,84814E+14	7,4365E+14	7,44417E+13	8,05436E+14	9,16981E+14	8,67521E+14	8,93626E+12
TC0300000721.mm.2	Cct3	chaperonin containing Tcp1, subunit 3 (gamma)	1,55407E+14	7,20584E+14	3,17777E+14	0.0174108357021527	0.1520546317988	-2,84832E+14	8,23408E+14	8,23972E+14	7,30377E+14	6,09184E+14	7,05208E+14	6,31358E+14
TC0900002385.mm.2	Ccdc33	coiled-coil domain containing 33	-1,05936E+14	6,03908E+14	-3,17734E+13	0.0174207561132401	0.152058853332956	-2,84886E+14	5,21214E+14	5,49389E+14	6,12264E+14	6,58106E+13	6,27239E+14	6,55235E+14
TC0200000647.mm.2	Mrrf	mitochondrial ribosome recycling factor	1,46426E+14	6,91707E+14	3,17641E+13	0.0174422590922011	0.152064387647197	-2,85002E+14	7,946E+14	7,76416E+14	7,02173E+14	5,98888E+14	6,76879E+14	6,01284E+14
TC0400000890.mm.2	Gm13286	predicted gene 13286	-1,00419E+14	6,67963E+14	-3,17605E+14	0.0174505257494013	0.152064387647197	-2,85047E+14	6,0806E+14	6,36889E+14	6,64628E+14	6,52413E+14	7,05152E+14	7,40636E+14
TC1500001643.mm.2	Oc90	otoconin 90	-0.96384341162409	5,08016E+14	-3,17604E+14	0.01745075071753	0.152064387647197	-2,85048E+14	4,54833E+14	4,67712E+14	4,92458E+14	5,17781E+14	5,67901E+14	5,47413E+14
TC0400001139.mm.2	Slc1a7	solute carrier family 1 (glutamate transporter), member 7	-0.977556562306879	7,16391E+14	-3,17568E+14	0.0174591396479435	0.152064387647197	-2,85093E+14	6,45299E+14	6,64956E+13	7,26823E+14	7,55503E+14	7,43394E+14	7,62372E+14
TC1000001947.mm.2	Ncoa7	nuclear receptor coactivator 7	1,99558E+14	6,89601E+14	3,17514E+14	0.0174716596349157	0.152091221878659	-2,85161E+14	7,98997E+14	7,85409E+14	7,1947E+14	6,48439E+14	6,86129E+14	4,99162E+14
TC1600001047.mm.2	Dop1b	DOP1 leucine zipper like protein B	1,18849E+14	6,66835E+14	3,17449E+14	0.017486554812449	0.15213869205885	-2,85241E+13	7,57895E+14	7,39534E+13	6,32394E+13	6,11458E+14	5,8914E+14	6,7059E+14
TC0100003338.mm.2	Brinp2	bone morphogenic protein/retinoic acid inducible neural-specific 2	1,69375E+14	7,10094E+14	3,17309E+14	0.0175189116067915	0.152253651290758	-2,85415E+14	8,06919E+14	8,25583E+14	7,2556E+14	6,0875E+13	7,01727E+14	5,92025E+14
TC1100003935.mm.2	Ddx5/Mir3064	DEAD (Asp-Glu-Ala-Asp) box polypeptide 5/microRNA 3064	1,06534E+14	1,00298E+13	3,172E+13	0.0175441784673435	0.152253651290758	-2,85551E+14	1,07763E+14	1,07663E+14	9,72184E+14	9,50228E+14	9,99783E+14	9,41415E+14
TC1000000894.mm.2	Tbxa2r	thromboxane A2 receptor	-0.96001992937732	7,05301E+14	-3,17167E+13	0.0175518189782626	0.152253651290758	-2,85592E+14	6,56499E+14	6,25015E+14	7,32217E+14	7,44557E+14	7,39102E+14	7,34416E+14
TC0200000417.mm.2	Abca2	ATP-binding cassette, sub-family A (ABC1), member 2	1,03025E+14	7,40867E+13	3,17148E+14	0.0175562550972107	0.152253651290758	-2,85616E+14	8,105E+14	7,89637E+14	7,13624E+14	7,37351E+14	6,7493E+14	7,19157E+14
TC0200003435.mm.2	Golga1	golgi autoantigen, golgin subfamily a, 1	1,3124E+14	5,43432E+14	3,17145E+12	0.0175570991871616	0.152253651290758	-2,8562E+14	6,30062E+14	6,14508E+14	5,5478E+14	4,79151E+14	5,2794E+14	4,5415E+14
TC0900003293.mm.2	Zfp445	zinc finger protein 445	1,78357E+14	6,8749E+14	3,17129E+14	0.0175607851796861	0.152253651290758	-2,8564E+14	7,9977E+14	7,92686E+13	7,23648E+14	5,73092E+14	6,60726E+13	5,75017E+14
TC0X00000160.mm.2	Usp9x	ubiquitin specific peptidase 9, X chromosome	3,37551E+14	6,48146E+14	3,1703E+13	0.0175837401621472	0.152253651290758	-2,85763E+14	8,80179E+13	8,68696E+14	6,51492E+14	4,14733E+14	6,7742E+14	3,96353E+14
TC1100001451.mm.2	Ppp1r9b	protein phosphatase 1, regulatory subunit 9B	1,16546E+14	9,13243E+14	3,16922E+14	0.0176089326808939	0.152253651290758	-2,85898E+14	9,82045E+14	9,95479E+14	9,13505E+14	8,43995E+14	8,93211E+14	8,51222E+14
TC0500003051.mm.2	Coro1c	coronin, actin binding protein 1C	1,24425E+14	8,97668E+14	3,16919E+14	0.0176094303196383	0.152253651290758	-2,85901E+14	9,68425E+14	9,502E+14	9,33702E+14	8,63906E+14	8,66522E+14	8,03253E+14
TC0700004587.mm.2	Nap1l4	nucleosome assembly protein 1-like 4	1,12994E+14	7,86403E+14	3,16856E+14	0.0176241117823507	0.152253651290758	-2,85979E+14	8,59794E+14	8,4616E+14	7,91571E+14	7,40928E+14	7,67311E+14	7,12653E+14
TC1300002210.mm.2	Mfsd14b	major facilitator superfamily domain containing 14B	1,05088E+13	7,2242E+14	3,1684E+14	0.0176279120016831	0.152253651290758	-2,86E+14	7,96999E+14	7,81851E+13	7,14853E+14	6,72144E+14	7,03554E+14	6,6512E+14
TC1900001516.mm.2	Mms19	MMS19 cytosolic iron-sulfur assembly component	1,35454E+13	6,65559E+14	3,16797E+14	0.017637957317852	0.152253651290758	-2,86053E+14	7,44321E+14	7,32377E+14	6,16704E+14	6,94164E+14	5,61808E+14	6,43982E+14
TC0500001936.mm.2	Crot	carnitine O-octanoyltransferase	2,03222E+14	5,6263E+14	3,16713E+14	0.017657628412648	0.152253651290758	-2,86158E+14	6,74377E+14	6,83737E+14	6,28781E+14	4,37218E+14	5,1309E+14	4,38579E+14
TC0400000436.mm.2	Tesk1	testis specific protein kinase 1	0.93936594510177	8,71288E+14	3,1671E+14	0.0176581868177792	0.152253651290758	-2,86161E+14	9,27651E+14	9,33407E+14	8,45054E+14	8,48434E+14	8,49359E+14	8,23826E+14
TC1700000614.mm.2	H2-Oa	histocompatibility 2, O region alpha locus	-1,50092E+12	5,58804E+14	-3,16702E+14	0.0176602149754461	0.152253651290758	-2,86172E+14	4,67357E+12	5,04008E+14	4,90541E+14	6,19371E+14	6,26714E+14	6,44834E+14
TC1400000517.mm.2	Ktn1	kinectin 1	1,64659E+14	6,89861E+14	3,16696E+14	0.0176616450146901	0.152253651290758	-2,8618E+14	8,14521E+14	8,16083E+14	6,80584E+13	5,26695E+14	6,61427E+14	6,3986E+14
TC0600001474.mm.2	D6Wsu163e	DNA segment, Chr 6, Wayne State University 163, expressed	1,28265E+14	7,34949E+14	3,16652E+14	0.0176719649483228	0.152253651290758	-2,86235E+13	8,44792E+13	8,29789E+14	6,97442E+14	6,19621E+13	7,41444E+14	6,76606E+14
TC0500002091.mm.2	Wdr86	WD repeat domain 86	-1,22516E+14	6,30368E+14	-3,16579E+14	0.0176888998928972	0.152253651290758	-2,86325E+14	5,56103E+14	5,64157E+13	6,12283E+14	6,8437E+14	6,53564E+13	7,11728E+14
TC1100002847.mm.2	Arf1	ADP-ribosylation factor 1	1,33161E+14	9,58884E+14	3,16532E+14	0.0177000114578306	0.152253651290758	-2,86384E+14	1,04218E+14	1,04792E+14	9,60531E+14	8,78903E+14	9,48792E+12	8,74981E+14
TC0X00002199.mm.2	Hs6st2	heparan sulfate 6-O-sulfotransferase 2	1,08837E+14	7,32998E+14	3,1651E+14	0.0177051256886464	0.152253651290758	-2,86411E+14	8,08782E+14	8,03013E+14	6,84736E+14	7,07337E+14	6,65257E+14	7,28864E+14
TC0700001490.mm.2	Olfr598	olfactory receptor 598	-1,07508E+14	4,97816E+14	-3,1649E+14	0.0177097573292094	0.152253651290758	-2,86436E+14	4,07151E+14	4,39099E+14	5,23662E+13	5,55716E+14	5,07318E+14	5,53948E+14
TC0200003023.mm.2	H2al2a	H2A histone family member L2A	-1,29647E+14	6,37819E+14	-3,16443E+14	0.0177207737743788	0.152253651290758	-2,86495E+13	5,6224E+14	5,5594E+14	6,24175E+14	7,07082E+14	6,56889E+14	7,20587E+14
TC0900002501.mm.2	Slc24a1	solute carrier family 24 (sodium/potassium/calcium exchanger), member 1	-1,41397E+14	5,30505E+14	-3,16426E+14	0.0177248397162629	0.152253651290758	-2,86516E+14	4,1535E+14	4,81904E+14	5,16225E+14	5,89504E+14	5,53745E+14	6,26303E+14
TC0400001525.mm.2	Marcksl1	MARCKS-like 1	-1,04527E+13	7,65422E+14	-3,16392E+14	0.017732694191985	0.152253651290758	-2,86558E+14	6,8911E+14	6,92469E+14	7,89247E+14	8,31073E+14	7,74951E+14	8,15683E+14
TC0200004897.mm.2	Nrsn2	neurensin 2	1,469E+14	8,25058E+14	3,16352E+14	0.0177421556725695	0.152253651290758	-2,86608E+14	9,39464E+14	8,69211E+14	8,63671E+14	7,63124E+14	7,80183E+14	7,34692E+14
TC0200001092.mm.2	Hoxd12	homeobox D12	-1,26183E+14	5,47557E+14	-3,16291E+14	0.0177564639878732	0.152253651290758	-2,86684E+14	4,65764E+14	4,9491E+14	5,22527E+14	5,891E+13	5,72718E+14	6,40322E+14
TC0200000518.mm.2	Sptan1	spectrin alpha, non-erythrocytic 1	1,06175E+14	8,59225E+14	3,1627E+14	0.0177613827604228	0.152253651290758	-2,8671E+14	9,29275E+14	9,381E+14	8,42063E+14	7,90885E+13	8,44688E+14	8,10337E+13
TC0700003614.mm.2	Homer2	homer scaffolding protein 2	1,11358E+14	6,01605E+14	3,1621E+14	0.0177756281854668	0.152253651290758	-2,86786E+14	6,58155E+14	6,40123E+14	5,9388E+14	6,4191E+14	5,11797E+13	5,63765E+14
TC1600000199.mm.2	Top3b	topoisomerase (DNA) III beta	0.929146248101511	6,42988E+14	3,16178E+14	0.0177830064473063	0.152253651290758	-2,86825E+14	7,40029E+14	7,20127E+14	5,61481E+14	5,61963E+14	6,3235E+14	6,41977E+14
TC0200002372.mm.2	Myh7b	myosin, heavy chain 7B, cardiac muscle, beta	-1,06604E+14	6,57317E+14	-3,1616E+13	0.0177874051172256	0.152253651290758	-2,86848E+13	6,10922E+14	5,84193E+14	6,52465E+14	6,88002E+14	6,81649E+14	7,26673E+14
TC1500002285.mm.2	Csad	cysteine sulfinic acid decarboxylase	1,13996E+14	6,05447E+14	3,16103E+14	0.0178006809344671	0.152253651290758	-2,86918E+14	6,70017E+13	6,66114E+14	5,66717E+14	6,21698E+14	5,81776E+14	5,26362E+14
TC0300003150.mm.2	St6galnac5	ST6 (alpha-N-acetyl-neuraminyl-2,3-beta-galactosyl-1,3)-N-acetylgalactosaminide alpha-2,6-sialyltransferase 5	1,51784E+14	8,341E+13	3,16097E+14	0.0178021398269589	0.152253651290758	-2,86926E+14	9,36827E+14	9,459E+14	8,37173E+14	7,05542E+14	8,09755E+14	7,69404E+14
TC0900000019.mm.2	Gucy1a2	guanylate cyclase 1, soluble, alpha 2	1,14544E+14	5,89342E+14	3,16015E+14	0.0178214608532862	0.152296241357227	-2,87028E+14	6,70463E+13	6,41872E+14	5,34894E+14	6,05575E+14	5,5242E+14	5,30826E+14
TC0600000570.mm.2	Tmem176a	transmembrane protein 176A	0.918514693744876	7,52618E+14	3,15996E+14	0.0178260231614057	0.152296241357227	-2,87052E+14	8,21277E+14	8,36052E+14	6,89966E+14	6,94785E+14	7,38161E+14	7,35466E+14
TC0900000826.mm.2	Dpp8	dipeptidylpeptidase 8	1,81907E+14	7,78964E+14	3,1593E+14	0.0178414641237742	0.152347382843865	-2,87134E+14	8,75353E+14	8,87897E+13	7,90736E+14	7,20363E+14	7,81741E+14	6,17695E+14
TC1100002024.mm.2	0610009L18Rik	RIKEN cDNA 0610009L18 gene	-1,01764E+14	7,81791E+14	-3,15847E+14	0.017861088946298	0.152434177008316	-2,87238E+14	7,19862E+14	7,23576E+14	7,7729E+13	8,23054E+14	8,10981E+14	8,35986E+14
TC0300001415.mm.2	Adh5	alcohol dehydrogenase 5 (class III), chi polypeptide	-0.967776978696416	6,78433E+14	-3,15763E+14	0.0178811396722136	0.152524512196071	-2,87343E+13	6,19318E+14	6,10628E+14	6,92807E+14	7,24345E+14	7,21666E+14	7,01835E+14
TC0800002945.mm.2	Rfwd3	ring finger and WD repeat domain 3	0.955054375482142	6,6163E+14	3,15696E+14	0.0178968814913552	0.152549343319272	-2,87426E+14	7,15661E+14	7,03768E+14	6,76634E+14	6,453E+14	6,11488E+14	6,16929E+14
TC1300001242.mm.2	Plk2	polo like kinase 2	1,71865E+14	7,83661E+14	3,15658E+14	0.0179059582421118	0.152549343319272	-2,87474E+14	8,69293E+14	9,06043E+14	8,07265E+13	6,87759E+14	7,70293E+14	6,61314E+14
TC0400001112.mm.2	Fam151a	family with sequence simliarity 151, member A	-1,11773E+14	5,7199E+14	-3,15567E+14	0.0179274752330484	0.152549343319272	-2,87587E+14	4,83494E+14	5,4407E+12	5,61827E+14	5,91436E+14	6,09973E+14	6,41138E+14
TC0200001097.mm.2	Hoxd3/Hoxd4	homeobox D3/homeobox D4	-1,53668E+14	6,07915E+14	-3,15525E+14	0.017937425126576	0.152549343319272	-2,8764E+14	5,02654E+14	5,10358E+14	5,86673E+14	7,27456E+14	6,58669E+14	6,6168E+14
TC0500002240.mm.2	Nop14	NOP14 nucleolar protein	2,20164E+14	5,98506E+14	3,15518E+14	0.0179391644844975	0.152549343319272	-2,87649E+14	7,32702E+12	7,59773E+14	6,17508E+14	4,28905E+14	5,88726E+14	4,63421E+14

TC0100000399.mm.2	Mars2	methionine-tRNA synthetase 2 (mitochondrial)	1,1294E+13	5,89564E+14	3,15505E+13	0.0179423371962349	0.152549343319272	-2,87665E+14	6,56759E+14	6,46165E+14	6,1652E+13	5,40898E+14	5,35705E+14	5,41338E+14
TC0600002562.mm.2	Wdr54	WD repeat domain 54	1,09777E+14	7,61305E+14	3,15445E+14	0.0179565863564112	0.152549343319272	-2,8774E+14	8,58219E+14	8,42433E+14	7,04596E+14	6,81485E+14	7,72856E+14	7,08241E+14
TC0700004165.mm.2	Arl6ip1	ADP-ribosylation factor-like 6 interacting protein 1	1,75442E+14	8,83182E+14	3,15402E+14	0.0179668289910092	0.152549343319272	-2,87794E+14	1,01213E+14	1,01399E+14	8,62851E+14	7,34894E+14	8,94185E+14	7,81047E+14
TC1000000093.mm.2	Plagl1	pleiomorphic adenoma gene-like 1	1,18662E+14	5,92702E+14	3,15384E+14	0.0179709506904278	0.152549343319272	-2,87815E+14	6,86317E+14	6,59525E+14	5,76886E+14	5,24968E+14	5,86568E+14	5,21951E+14
TC0500003024.mm.2	Grk3	G protein-coupled receptor kinase 3	1,2296E+14	6,69348E+14	3,15352E+14	0.0179787254368086	0.152549343319272	-2,87856E+14	7,63614E+14	7,21631E+14	6,7199E+14	6,19528E+14	6,52907E+13	5,86418E+14
TC1300000209.mm.2	Vmn1r201	vomeronasal 1 receptor 201	-1,53674E+14	4,32036E+12	-3,15114E+12	0.0180354221352373	0.152687709321889	-2,88153E+14	3,4032E+14	3,01336E+14	4,41126E+14	5,60429E+14	4,42032E+14	5,06972E+14
TC1900000064.mm.2	4930481A15Rik	RIKEN cDNA 4930481A15 gene	-1,10707E+14	6,50915E+14	-3,15098E+14	0.0180392146760624	0.152687709321889	-2,88173E+14	5,91311E+14	5,68388E+14	6,68345E+14	6,96328E+14	6,59133E+14	7,21981E+14
TC0400001091.mm.2	Plpp3	phospholipid phosphatase 3	1,81142E+14	8,93043E+14	3,15025E+14	0.0180566859476916	0.152687709321889	-2,88264E+14	1,00227E+14	1,00176E+14	9,25336E+14	7,87148E+14	8,77463E+13	7,6428E+13
TC0600003108.mm.2	C1s1	complement component 1, s subcomponent 1	-0.99794606677548	6,50199E+14	-3,1502E+14	0.0180578336098637	0.152687709321889	-2,8827E+14	5,60431E+14	5,76859E+14	6,92247E+14	7,34782E+14	6,61195E+14	6,75684E+14
TC1400001303.mm.2	Mbnl2	muscleblind like splicing factor 2	1,7377E+14	9,35552E+14	3,14995E+14	0.0180638667844557	0.152687709321889	-2,88301E+14	1,03249E+14	1,04605E+14	9,68673E+14	8,35115E+14	9,16734E+14	8,14259E+14
TC1500001683.mm.2	Kcnk9	potassium channel, subfamily K, member 9	1,0532E+14	6,49411E+14	3,1496E+14	0.0180723826777014	0.152687709321889	-2,88346E+14	7,43691E+14	7,01985E+14	5,86954E+12	6,28802E+14	6,09258E+14	6,25778E+14
TC0700002683.mm.2	Zfp780b	zinc finger protein 780B	1,24435E+14	6,1597E+13	3,14918E+14	0.0180823394997311	0.152687709321889	-2,88398E+14	7,28727E+14	6,86095E+14	5,85489E+14	5,29559E+14	6,16581E+14	5,49371E+14
TC0400004003.mm.2	Mfn2	mitofusin 2	1,05923E+13	7,57997E+14	3,14911E+14	0.0180840266844403	0.152687709321889	-2,88407E+14	8,35089E+14	8,17515E+14	7,5388E+14	7,00743E+14	7,34527E+14	7,06231E+14
TC1700002580.mm.2	Hnrnpll	heterogeneous nuclear ribonucleoprotein L-like	-1,05241E+14	8,30239E+14	-3,14878E+14	0.0180920212330596	0.152687709321889	-2,88448E+14	7,47112E+14	7,86292E+14	8,31429E+14	8,72714E+14	8,60653E+14	8,83233E+14
TC1900000067.mm.2	Fosl1	fos-like antigen 1	-1,10322E+14	6,9808E+13	-3,14877E+14	0.0180921682015332	0.152687709321889	-2,88449E+14	6,49414E+14	5,93347E+14	7,21712E+14	7,60601E+14	7,20363E+14	7,43041E+14
TC1600001516.mm.2	Rubcn	RUN domain and cysteine-rich domain containing, Beclin 1-interacting protein	1,18241E+14	6,18671E+14	3,14847E+14	0.0180993636905889	0.152687709321889	-2,88486E+14	6,76439E+14	7,06933E+14	5,71069E+14	6,10698E+14	5,38014E+14	6,08875E+14
TC1300000408.mm.2	Ppp1r3g	protein phosphatase 1, regulatory subunit 3G	-1,35682E+14	5,60077E+14	-3,14808E+14	0.0181087452686731	0.152687709321889	-2,88535E+14	4,79241E+14	4,76726E+14	5,44138E+14	6,33026E+13	5,77039E+14	6,50291E+14
TSUnmapped00000019.mm.2	Ccdc169	coiled-coil domain containing 169	-1,85801E+14	6,56216E+14	-3,14652E+13	0.0181462325476866	0.152857036946384	-2,8873E+14	5,25126E+14	5,74252E+14	6,0407E+14	7,62869E+13	6,94876E+14	7,76104E+14
TC1000000726.mm.2	Derl3	Der1-like domain family, member 3	-1,4409E+13	7,13712E+14	-3,14557E+14	0.0181692075465449	0.152857036946384	-2,88849E+14	6,23567E+14	6,29389E+14	6,8775E+14	8,00428E+14	7,40406E+14	8,0073E+14
TC1200002110.mm.2	Irf2bpl	interferon regulatory factor 2 binding protein-like	1,36509E+13	7,18889E+14	3,14544E+14	0.0181721166172611	0.152857036946384	-2,88864E+14	8,23131E+14	8,00853E+14	7,25202E+14	6,1318E+14	6,63642E+14	6,87325E+14
TC0300002156.mm.2	Bche	butyrylcholinesterase	-1,2463E+14	6,07751E+14	-3,14538E+14	0.0181736836666771	0.152857036946384	-2,88873E+14	5,46345E+14	5,37049E+13	5,67853E+13	6,62602E+14	6,55027E+14	6,77628E+14
TC1200001265.mm.2	Zbtb42	zinc finger and BTB domain containing 42	-1,20035E+14	6,93605E+14	-3,14512E+14	0.0181800565790967	0.152857036946384	-2,88906E+14	6,04665E+14	6,29994E+14	6,92051E+13	7,6019E+14	7,10759E+14	7,63971E+14
TC1100004124.mm.2	St6galnac2	ST6 (alpha-N-acetyl-neuraminyl-2,3-beta-galactosyl-1,3)-N-acetylgalactosaminide alpha-2,6-sialyltransferase 2	-1,41106E+14	7,21798E+14	-3,14488E+14	0.0181858418912949	0.152857036946384	-2,88936E+13	5,90316E+14	6,78918E+14	7,23198E+14	7,86909E+14	7,46718E+13	8,04727E+14
TC0400004031.mm.2	Cort	cortistatin	-0.948327873575208	8,97147E+14	-3,14416E+12	0.0182031889008449	0.152857036946384	-2,89025E+14	8,34756E+14	8,52558E+14	9,13047E+14	9,05544E+14	9,25174E+14	9,51805E+14
TC0200005110.mm.2	Svs3b	seminal vesicle secretory protein 3B	-1,27985E+14	4,74988E+14	-3,14409E+14	0.0182047200335202	0.152857036946384	-2,89033E+14	3,8803E+14	4,25249E+14	4,40042E+14	5,27359E+14	5,14105E+14	5,55144E+14
TC0200002401.mm.2	Tgif2	TGFB-induced factor homeobox 2	-1,03516E+14	6,4697E+14	-3,14191E+14	0.0182576044201117	0.15287771764461	-2,89307E+14	5,73353E+14	5,90057E+14	6,5485E+14	6,93115E+14	6,65222E+14	7,0522E+14
TC1100000497.mm.2	Olfr1395	olfactory receptor 1395	-1,18026E+14	5,50099E+13	-3,1419E+14	0.0182578387045923	0.15287771764461	-2,89308E+14	4,52268E+14	5,08146E+14	5,40477E+14	6,03239E+14	5,87393E+14	6,09074E+14
TC1000000673.mm.2	Mrln	myoregulin	-1,65024E+14	7,04163E+12	-3,14178E+14	0.0182607210286189	0.15287771764461	-2,89323E+13	5,84607E+14	6,17252E+13	6,70096E+14	8,21113E+14	7,50188E+14	7,81721E+14
TC0400000907.mm.2	Ifna5	interferon alpha 5	-1,5424E+14	6,42451E+14	-3,14082E+14	0.0182838599530193	0.15287771764461	-2,89442E+14	5,4379E+14	5,37498E+14	6,33363E+14	7,5029E+14	6,53997E+14	7,35771E+14
TC1300002144.mm.2	Tpbpa	trophoblast specific protein alpha	-1,18052E+14	4,54084E+14	-3,13991E+14	0.0183060235418077	0.15287771764461	-2,89556E+14	3,91653E+14	3,71702E+14	4,39754E+14	5,21936E+14	5,09856E+14	4,89602E+13
TC0400003794.mm.2	C1qa	complement component 1, q subcomponent, alpha polypeptide	1,14402E+13	7,01932E+12	3,13956E+14	0.0183145248212231	0.15287771764461	-2,896E+13	7,3596E+14	8,11622E+13	6,80272E+14	6,6496E+14	6,7057E+14	6,48209E+13
TC1500002191.mm.2	Prkag1	protein kinase, AMP-activated, gamma 1 non-catalytic subunit	1,38589E+14	7,13414E+14	3,13891E+14	0.018330443211672	0.15287771764461	-2,89682E+14	8,26641E+14	8,07388E+14	6,67093E+14	6,22509E+14	7,33416E+14	6,23436E+14
TC1900000730.mm.2	Cfap58	cilia and flagella associated protein 58	-1,00178E+14	4,95671E+14	-3,13841E+14	0.0183426083474303	0.15287771764461	-2,89744E+14	4,39405E+14	4,64248E+14	4,61653E+14	5,04708E+14	5,5398E+14	5,5003E+14
TC1600000089.mm.2	Abat	4-aminobutyrate aminotransferase	1,94853E+14	8,8279E+14	3,13827E+14	0.0183459244375524	0.15287771764461	-2,89761E+14	1,02265E+14	9,90272E+14	9,14647E+14	7,45949E+14	8,60231E+14	7,62987E+14
TC0200000546.mm.2	Usp20	ubiquitin specific peptidase 20	1,92432E+14	7,11311E+14	3,13819E+13	0.0183478097481517	0.15287771764461	-2,89771E+14	8,25562E+12	8,09878E+14	7,66904E+14	6,14946E+14	6,85297E+14	5,65279E+14
TC1800001700.mm.2	Tshz1	teashirt zinc finger family member 1	1,13163E+14	6,55967E+14	3,13815E+14	0.0183488846634411	0.15287771764461	-2,89777E+14	7,10896E+14	7,10372E+14	6,98594E+14	6,20995E+14	5,92869E+14	6,02073E+14
TC1400000372.mm.2	Syt15	synaptotagmin XV	-1,20864E+14	7,21541E+14	-3,138E+14	0.0183525911709328	0.15287771764461	-2,89796E+14	6,24125E+14	6,63889E+14	7,1477E+14	7,96718E+14	7,58441E+14	7,71302E+14
TC0900002757.mm.2	Phip	pleckstrin homology domain interacting protein	2,20323E+14	6,38455E+14	3,13783E+14	0.0183566667177632	0.15287771764461	-2,89817E+13	7,98349E+14	7,88795E+13	6,14761E+14	4,82326E+14	6,67005E+14	4,79494E+14
TC0200004238.mm.2	D430041D05Rik	RIKEN cDNA D430041D05 gene	0.960878732753456	7,95999E+14	3,13731E+14	0.0183692187990829	0.15287771764461	-2,89881E+14	8,52269E+14	8,69727E+14	7,78195E+14	7,45981E+14	7,59808E+14	7,70012E+14
TC0500000130.mm.2	Speer4f1	spermatogenesis associated glutamate (E)-rich protein 4F1	-1,07334E+14	5,02981E+14	-3,13668E+14	0.0183846721151547	0.15287771764461	-2,8996E+13	4,63769E+14	4,38696E+14	4,95733E+14	5,02554E+14	5,89253E+14	5,27879E+14
TC1500001464.mm.2	Tmem74	transmembrane protein 74	1,19129E+14	6,01843E+14	3,13636E+14	0.0183925756974777	0.15287771764461	-2,90001E+14	6,76063E+14	7,1007E+14	5,73771E+14	5,0328E+14	5,95221E+13	5,52654E+14
TC0100002481.mm.2	Idh1	isocitrate dehydrogenase 1 (NADP+), soluble	1,76557E+14	7,02836E+14	3,13634E+14	0.0183929822426069	0.15287771764461	-2,90003E+13	8,20171E+13	8,17698E+14	6,92746E+14	6,01648E+14	7,16261E+14	5,68494E+14
TC0900002435.mm.2	Lrrc49	leucine rich repeat containing 49	1,68829E+14	8,2574E+14	3,13601E+14	0.0184010174576412	0.15287771764461	-2,90044E+14	9,46259E+14	9,09627E+14	8,17032E+13	7,6329E+14	8,33994E+14	6,84236E+14
TC1600001316.mm.2	Comt	catechol-O-methyltransferase	1,22917E+14	9,2927E+14	3,13597E+14	0.0184019613363771	0.15287771764461	-2,90049E+13	1,01436E+14	1,00876E+14	9,33744E+14	8,4148E+14	9,0086E+14	8,7642E+14
TC0400003631.mm.2	Zcchc17	zinc finger, CCHC domain containing 17	1,7328E+14	7,92453E+14	3,13539E+14	0.0184161093315146	0.15287771764461	-2,90121E+14	9,29436E+14	8,77119E+14	7,99064E+14	6,89103E+14	7,88811E+14	6,71184E+14
TC1300000735.mm.2	Dapk1	death associated protein kinase 1	1,43834E+14	7,5677E+13	3,1351E+14	0.0184232028232981	0.15287771764461	-2,90158E+14	8,52614E+14	8,4326E+13	7,60089E+14	6,76451E+14	7,52504E+14	6,55703E+14
TC0800000036.mm.2	Cers4	ceramide synthase 4	0.988451929789318	7,45637E+13	3,13422E+14	0.0184447535984219	0.15287771764461	-2,90268E+14	7,96117E+13	8,01734E+14	7,58414E+14	7,17394E+14	7,10803E+14	6,89358E+14
TC0700003971.mm.2	Olfr685	olfactory receptor 685	-1,17244E+14	5,41639E+14	-3,13403E+14	0.0184494044341861	0.15287771764461	-2,90292E+14	4,50969E+14	4,60654E+14	5,68284E+14	6,23815E+13	5,40636E+14	6,05476E+14
TC1000000813.mm.2	Hcn2	hyperpolarization-activated, cyclic nucleotide-gated K+ 2	1,49492E+14	7,33738E+14	3,13375E+14	0.0184562147231709	0.15287771764461	-2,90326E+14	8,543E+14	8,31986E+14	7,06622E+14	6,22217E+14	7,46158E+14	6,41143E+14
TC1300000998.mm.2	Edil3	EGF-like repeats and discoidin I-like domains 3	1,01025E+14	7,71371E+14	3,13363E+14	0.018459191786358	0.15287771764461	-2,90342E+14	8,65894E+14	8,39692E+14	7,18215E+14	7,00888E+14	7,72761E+14	7,30774E+14
TC1700001604.mm.2	Flywch1	FLYWCH-type zinc finger 1	1,00589E+14	7,94409E+14	3,13341E+14	0.0184646622641113	0.15287771764461	-2,9037E+14	8,63577E+14	8,35702E+14	8,04716E+13	7,64357E+14	7,56642E+13	7,41459E+14
TC1400002496.mm.2	Pcdh8	protocadherin 8	0.916233374780246	6,68888E+14	3,1326E+14	0.0184845661287604	0.15287771764461	-2,90471E+14	7,44613E+14	7,25983E+12	6,29454E+14	6,25928E+14	6,41172E+14	6,46178E+14
TC0700004075.mm.2	Trim66	tripartite motif-containing 66	1,19959E+14	6,05222E+14	3,13256E+13	0.0184853705256469	0.15287771764461	-2,90475E+14	7,04261E+14	6,48934E+14	5,58941E+14	6,0592E+14	5,2937E+14	5,83908E+14
TC0700004575.mm.2	Th	tyrosine hydroxylase	1,2106E+14	6,53826E+13	3,13192E+14	0.0185011330134296	0.15287771764461	-2,90555E+14	7,5016E+14	6,78588E+14	6,59132E+14	6,48444E+14	5,64325E+14	6,22304E+14
TC0600001238.mm.2	Thumpd3	THUMP domain containing 3	1,63247E+14	5,48488E+14	3,13182E+14	0.0185035809713148	0.15287771764461	-2,90568E+14	6,62193E+14	6,32707E+14	5,85813E+14	4,41811E+14	5,10285E+14	4,5812E+14
TC0300000753.mm.2	Muc1	mucin 1, transmembrane	-1,11925E+14	5,23667E+14	-3,12985E+14	0.0185522264462191	0.15287771764461	-2,90815E+14	4,61032E+14	4,73048E+14	5,1078E+14	5,39211E+14	5,44048E+14	6,13882E+14
TC0100001726.mm.2	9130409I23Rik	RIKEN cDNA 9130409I23 gene	-1,23515E+13	5,14037E+13	-3,12914E+14	0.0185697038122477	0.15287771764461	-2,90904E+14	4,33754E+14	4,48043E+14	4,94427E+14	5,79169E+14	5,41854E+14	5,86974E+14
TC0800002382.mm.2	Ncan	neurocan	1,76222E+14	7,14764E+14	3,12899E+14	0.018573426743132	0.15287771764461	-2,90923E+14	8,36943E+14	8,67004E+13	7,02101E+14	5,31036E+14	6,77314E+14	6,74188E+14
TC0800003171.mm.2	Agt	angiotensinogen (serpin peptidase inhibitor, clade A, member 8)	1,37337E+14	8,21197E+14	3,12832E+14	0.0185897932456313	0.15287771764461	-2,91006E+14	9,6184E+14	8,51213E+14	8,05424E+14	7,70324E+14	7,82416E+14	7,55963E+14
TC1200000427.mm.2	Arhgap5	Rho GTPase activating protein 5	2,56919E+14	7,33064E+14	3,12821E+14	0.0185925392951764	0.15287771764461	-2,9102E+14	9,11659E+14	9,20052E+14	7,35591E+14	5,13211E+14	7,40503E+14	5,77371E+14
TC1400001275.mm.2	Gpr180	G protein-coupled receptor 180	0.910060431468665	6,04993E+14	3,12769E+14	0.0186054137107564	0.15287771764461	-2,91085E+14	6,68326E+14	6,53084E+14	5,81471E+12	5,87682E+14	5,69783E+14	5,69614E+14
TC0400001419.mm.2	Fhl3	four and a half LIM domains 3	-0.997288818178903	6,30795E+14	-3,12763E+14	0.0186068305202284	0.15287771764461	-2,91092E+14	5,67149E+14	5,92282E+14	6,44855E+14	6,21599E+14	6,97376E+14	6,61512E+14
TC1800000566.mm.2	Phax	phosphorylated adaptor for RNA export	1,69738E+14	6,59354E+14	3,12723E+14	0.0186168172717846	0.15287771764461	-2,91143E+14	7,90665E+14	7,79684E+12	6,35652E+14	5,19251E+14	6,73751E+14	5,57123E+14
TC0700002046.mm.2	Tmem80	transmembrane protein 80	0.963211431535086	6,28825E+14	3,12666E+14	0.0186308707056739	0.15287771764461	-2,91214E+14	6,6131E+14	6,84113E+14	6,47668E+14	6,27075E+14	5,89078E+14	5,63703E+14

TC1900000168.mm.2	Mta2	metastasis-associated gene family, member 2	1,08182E+14	7,64344E+14	3,1266E+14	0.018632277394311	0.15287771764461	-2,91221E+14	8,3419E+14	8,09938E+14	7,41989E+14	7,72182E+14	7,42517E+14	6,85247E+14
TC1900001104.mm.2	Sdhaf2	succinate dehydrogenase complex assembly factor 2	1,09603E+14	7,37961E+14	3,12657E+14	0.0186332052630297	0.15287771764461	-2,91226E+14	8,16626E+14	7,86228E+14	7,17926E+13	7,23339E+14	7,24358E+14	6,5929E+14
TC0200000821.mm.2	Rif1	replication timing regulatory factor 1	1,36805E+14	5,50205E+14	3,12613E+14	0.0186439763542656	0.15287771764461	-2,9128E+14	6,45502E+14	6,09487E+14	4,8056E+14	5,84304E+14	5,12785E+14	4,68594E+14
TC1300002596.mm.2	Trappc13	trafficking protein particle complex 13	2,7215E+14	5,99349E+14	3,12595E+13	0.0186485517162406	0.15287771764461	-2,91304E+14	7,58574E+14	7,58499E+14	6,49824E+14	4,56422E+14	5,97589E+14	3,75185E+14
TC1700001143.mm.2	Clip4	CAP-GLY domain containing linker protein family, member 4	1,44465E+14	8,57696E+14	3,12592E+14	0.0186492865990287	0.15287771764461	-2,91307E+14	9,61382E+14	9,38006E+14	8,75948E+14	7,60382E+14	8,32386E+14	7,78073E+14
TC0X00000237.mm.2	Wdr44	WD repeat domain 44	1,37263E+14	6,07315E+14	3,12501E+14	0.0186717002924857	0.15287771764461	-2,9142E+14	6,80898E+14	7,1755E+14	5,65482E+14	5,56037E+14	6,18448E+14	5,05474E+14
TC0800001740.mm.2	Rasa3	RAS p21 protein activator 3	1,02458E+14	6,06746E+14	3,12447E+14	0.0186852286005696	0.15287771764461	-2,91489E+14	6,91759E+14	6,97277E+14	5,46397E+14	5,20921E+14	6,17223E+14	5,66897E+14
TC0700004531.mm.2	Cdhr5	cadherin-related family member 5	-1,11427E+14	5,62362E+14	-3,12445E+14	0.0186857759516248	0.15287771764461	-2,91491E+14	4,85589E+14	4,98483E+13	5,61071E+14	6,22106E+14	5,81967E+13	6,24958E+14
TC1900000521.mm.2	Tnks2	tankyrase, TRF1-interacting ankyrin-related ADP-ribose polymerase 2	1,58144E+14	7,75721E+14	3,12411E+14	0.0186942035288174	0.15287771764461	-2,91534E+14	8,60719E+14	8,85643E+13	7,99914E+14	6,77973E+14	7,56413E+14	6,73662E+14
TC0700004444.mm.2	Insyn2a	inhibitory synaptic factor 2A	-0.993268406985764	7,68301E+13	-3,12359E+14	0.0187070847993953	0.15287771764461	-2,91599E+14	7,23347E+14	7,00773E+14	7,80093E+14	7,8282E+14	7,89764E+14	8,33009E+14
TSUnmapped00000027.mm.2	Nkx1-1	NK1 homeobox 1	-1,07598E+14	6,84785E+14	-3,12355E+14	0.0187080221999425	0.15287771764461	-2,91604E+14	6,154E+14	6,19356E+14	6,87157E+14	7,36843E+14	7,01741E+14	7,4821E+13
TC0100001258.mm.2	Tmem9	transmembrane protein 9	1,07746E+14	6,72834E+13	3,12284E+14	0.0187257593415438	0.15287771764461	-2,91693E+14	7,33671E+14	7,20647E+13	6,38548E+14	7,05312E+14	6,15357E+14	6,23468E+14
TC0700001399.mm.2	Chrdl2	chordin-like 2	-1,08035E+14	4,82804E+14	-3,12224E+14	0.0187406225734712	0.15287771764461	-2,91768E+14	4,22409E+14	4,20294E+14	4,89088E+14	5,06258E+14	4,95914E+14	5,6286E+14
TC0100002392.mm.2	Trak2	trafficking protein, kinesin binding 2	1,77801E+14	7,43779E+14	3,12224E+14	0.018740685850793	0.15287771764461	-2,91768E+14	8,58795E+14	8,89756E+14	7,17363E+14	6,03812E+14	7,62122E+14	6,30827E+14
TC1100001156.mm.2	Aldoc	aldolase C, fructose-bisphosphate	1,94375E+14	1,05172E+14	3,12219E+14	0.0187419917567016	0.15287771764461	-2,91775E+14	1,1822E+14	1,17677E+14	1,08188E+13	8,99238E+14	1,02333E+14	9,46885E+14
TC1100000284.mm.2	Efemp1	epidermal growth factor-containing fibulin-like extracellular matrix protein 1	1,15543E+14	5,84621E+14	3,1218E+14	0.0187515511974683	0.15287771764461	-2,91823E+14	6,33668E+14	6,44884E+14	6,1085E+14	5,70858E+14	5,60049E+14	4,87418E+14
TC1600001353.mm.2	Thpo	thrombopoietin	-1,17528E+14	6,12543E+14	-3,12144E+14	0.018760599335196	0.15287771764461	-2,91868E+14	5,52162E+14	5,81826E+13	6,09209E+14	5,63015E+14	6,50605E+14	7,1844E+14
TC1400000941.mm.2	Ccdc25	coiled-coil domain containing 25	1,03801E+14	6,26539E+14	3,12122E+14	0.0187660670662626	0.15287771764461	-2,91896E+14	6,67667E+14	6,82681E+14	6,47789E+14	6,18349E+14	5,463E+14	5,96446E+13
TC1100000156.mm.2	Vstm2a	V-set and transmembrane domain containing 2A	1,26537E+14	9,86883E+14	3,12098E+14	0.0187721323342014	0.15287771764461	-2,91926E+14	1,07853E+14	1,04183E+14	1,00767E+14	9,25977E+14	9,5866E+14	9,08629E+14
TC1900001353.mm.2	Ranbp6	RAN binding protein 6	1,45619E+14	5,94402E+14	3,12067E+14	0.0187798645862363	0.15287771764461	-2,91965E+14	7,22367E+14	6,91572E+14	5,7242E+14	4,57353E+14	5,89626E+14	5,33076E+14
TC0400000902.mm.2	Gm13287	predicted gene 13287	-1,02178E+12	6,32943E+14	-3,12058E+14	0.0187821356877045	0.15287771764461	-2,91976E+14	5,5637E+14	5,88113E+13	6,40112E+13	6,64224E+14	6,54532E+14	6,94307E+14
TC1900001649.mm.2	Sorcs1	sortilin-related VPS10 domain containing receptor 1	1,3292E+14	6,66638E+14	3,12043E+14	0.0187859418442455	0.15287771764461	-2,91995E+14	7,59241E+14	7,22783E+14	6,99252E+14	6,02368E+14	6,3047E+14	5,85713E+14
TC1500000901.mm.2	Cntn1	contactin 1	1,75604E+14	9,15151E+14	3,11831E+14	0.01883887471766	0.15297717631228	-2,92261E+14	1,03969E+14	1,01509E+14	9,24785E+14	8,07756E+14	9,14772E+14	7,88805E+14
TC0800000874.mm.2	Zfp827	zinc finger protein 827	1,19678E+14	7,22001E+14	3,11828E+14	0.0188394834392292	0.15297717631228	-2,92264E+14	7,98808E+14	7,97169E+14	6,98045E+14	6,81365E+14	7,2316E+14	6,33462E+14
TC0300000928.mm.2	Sv2a	synaptic vesicle glycoprotein 2 a	1,5835E+14	9,26003E+14	3,11826E+14	0.018840111511841	0.15297717631228	-2,92267E+14	1,03973E+14	1,01679E+13	9,48857E+14	8,10821E+14	8,97154E+14	8,42666E+14
TC0500001344.mm.2	Rasal1	RAS protein activator like 1 (GAP1 like)	1,30923E+14	7,16629E+14	3,1179E+14	0.0188491513225954	0.15297717631228	-2,92312E+14	7,76817E+14	7,76719E+14	7,35653E+14	7,18897E+14	5,91579E+14	7,0011E+14
TC0700001510.mm.2	Olfr632	olfactory receptor 632	-1,14004E+13	5,57567E+14	-3,1169E+14	0.0188742935240123	0.15297717631228	-2,92438E+14	4,86811E+14	4,9071E+14	5,40782E+14	6,21569E+14	5,99358E+14	6,0617E+13
TC1700002151.mm.2	Hsp90ab1	heat shock protein 90 alpha (cytosolic), class B member 1	1,52317E+14	9,43759E+14	3,11689E+14	0.0188745652421521	0.15297717631228	-2,92439E+14	1,05297E+14	1,04663E+14	9,5239E+14	8,15594E+14	9,15809E+14	8,79158E+14
TC0700001612.mm.2	Rpl27a/Snora3	ribosomal protein L27A/small nucleolar RNA, H/ACA box 3	-1,04273E+14	9,70779E+13	-3,11666E+14	0.0188803284509329	0.15297717631228	-2,92468E+14	9,06925E+14	8,92431E+13	9,80971E+14	1,03645E+14	1,01503E+14	9,92872E+14
TC0200005402.mm.2	Lama5	laminin, alpha 5	-1,67068E+14	6,5318E+13	-3,1164E+14	0.018886802640613	0.15297717631228	-2,925E+14	5,2455E+13	5,39346E+14	6,61035E+14	7,96115E+14	6,54632E+14	7,434E+13
TC0600000158.mm.2	Kcnd2	potassium voltage-gated channel, Shal-related family, member 2	1,90275E+14	7,61174E+14	3,11589E+12	0.0188995249311551	0.15297717631228	-2,92564E+14	8,79506E+14	8,71382E+14	7,96314E+13	6,49502E+14	7,47865E+14	6,22474E+14
TC0100001048.mm.2	Cntnap5a	contactin associated protein-like 5A	1,53396E+14	6,27961E+14	3,11546E+14	0.0189104395634011	0.15297717631228	-2,92618E+14	7,29179E+14	7,24603E+14	6,12561E+13	5,54432E+14	6,38997E+14	5,07993E+14
TC1100001999.mm.2	Gaa	glucosidase, alpha, acid	2,43099E+14	1,01751E+14	3,11507E+14	0.0189200912076507	0.15297717631228	-2,92666E+14	1,18852E+14	1,15026E+14	1,07705E+14	8,36611E+14	9,72212E+14	8,80374E+14
TC1100003945.mm.2	Nol11	nucleolar protein 11	2,06922E+14	6,23519E+14	3,11485E+14	0.0189257873073243	0.15297717631228	-2,92695E+14	7,59753E+14	7,68597E+14	6,50341E+14	4,47917E+14	5,92913E+14	5,21591E+14
TC0500002280.mm.2	Nsg1	neuron specific gene family member 1	1,02571E+14	9,68418E+14	3,11454E+14	0.0189336119902448	0.15297717631228	-2,92733E+14	1,05464E+14	1,0125E+14	9,60303E+14	9,21056E+14	9,32962E+14	9,29042E+13
TC0200001889.mm.2	Galk2	galactokinase 2	1,08534E+14	5,86732E+14	3,11299E+14	0.0189726213909459	0.15297717631228	-2,92927E+14	6,36652E+13	6,67263E+13	5,32906E+14	5,96723E+14	5,4467E+14	5,42177E+14
TC0200003666.mm.2	Scn3a	sodium channel, voltage-gated, type III, alpha	1,11823E+14	7,34922E+14	3,11215E+14	0.0189939349770282	0.15297717631228	-2,93033E+14	7,9421E+14	7,94882E+13	7,45402E+14	7,0959E+13	7,17015E+14	6,4843E+14
TC0900002542.mm.2	Tln2	talin 2	1,12137E+14	6,7067E+14	3,11177E+14	0.0190033933843377	0.15297717631228	-2,9308E+14	7,58453E+14	7,22077E+14	6,42195E+14	6,4504E+14	5,93657E+14	6,62599E+14
TC1200000021.mm.2	Adcy3	adenylate cyclase 3	1,19706E+14	6,41504E+14	3,11135E+14	0.0190140658865959	0.15297717631228	-2,93133E+13	7,46315E+14	7,05415E+14	6,32313E+14	5,52665E+14	6,16204E+14	5,96113E+14
TC0800001055.mm.2	Rbl2	RB transcriptional corepressor like 2	1,43994E+14	7,36201E+14	3,11122E+14	0.0190173842404358	0.15297717631228	-2,9315E+14	8,33951E+14	8,19778E+13	7,56689E+14	6,41049E+14	7,11845E+13	6,53895E+13
TC1100000880.mm.2	Myh10	myosin, heavy polypeptide 10, non-muscle	2,94185E+14	6,99712E+14	3,11002E+14	0.0190478616787185	0.15297717631228	-2,93301E+14	8,95734E+14	8,90315E+14	7,01579E+14	5,12963E+14	7,30043E+14	4,67635E+14
TC0500003056.mm.2	Alkbh2	alkB homolog 2, alpha-ketoglutarate-dependent dioxygenase	-1,38651E+14	7,57275E+14	-3,10919E+14	0.0190689328540157	0.15297717631228	-2,93405E+14	6,50849E+14	6,79919E+14	7,45148E+13	8,59663E+13	7,88224E+14	8,19847E+14
TC1600000415.mm.2	Dlg1	discs large MAGUK scaffold protein 1	1,28515E+14	7,91272E+13	3,10915E+14	0.0190698460535344	0.15297717631228	-2,93409E+13	8,95294E+14	8,8636E+14	7,67004E+14	6,7435E+14	7,88253E+14	7,3637E+14
TC1000000811.mm.2	Gzmm	granzyme M (lymphocyte met-ase 1)	-1,18264E+14	6,27359E+14	-3,10914E+14	0.0190700952311918	0.15297717631228	-2,93411E+14	5,64559E+14	5,56233E+14	6,29952E+14	6,56089E+14	6,36121E+14	7,21199E+14
TC0500001735.mm.2	Fam220a	family with sequence similarity 220, member A	0.902479314122843	6,85584E+14	3,1091E+13	0.0190712226756254	0.15297717631228	-2,93416E+14	7,41764E+14	7,29556E+14	6,79272E+14	6,72087E+13	6,47537E+14	6,43287E+14
TC0400003956.mm.2	Vps13d	vacuolar protein sorting 13D	1,45865E+14	6,11014E+13	3,10905E+14	0.0190723711094769	0.15297717631228	-2,93422E+14	7,10104E+12	7,12111E+14	6,19863E+14	4,9352E+14	5,8601E+14	5,44475E+14
TC1700002587.mm.2	Cdkl4	cyclin-dependent kinase-like 4	1,29178E+13	5,36051E+14	3,10865E+14	0.0190824361677568	0.15297717631228	-2,93472E+14	6,59013E+14	5,89502E+14	5,15912E+14	4,61721E+14	5,25991E+14	4,64168E+14
TC0500001202.mm.2	Aym1	activator of yeast meiotic promoters 1	-1,40453E+14	6,12168E+14	-3,10822E+14	0.0190935810331731	0.15297717631228	-2,93527E+14	4,75746E+14	5,43835E+14	6,27183E+14	7,25759E+14	6,32281E+14	6,68206E+14
TC0100001373.mm.2	Nmnat2	nicotinamide nucleotide adenylyltransferase 2	1,42475E+14	8,5702E+14	3,10797E+14	0.0190999081724093	0.15297717631228	-2,93558E+14	9,56355E+14	9,44568E+14	8,76391E+14	7,48838E+14	8,08572E+14	8,07399E+14
TC1700001864.mm.2	Zfp955a	zinc finger protein 955A	1,12041E+14	7,64366E+14	3,10754E+14	0.0191106322087925	0.15297717631228	-2,93611E+14	8,51563E+14	8,58022E+14	7,3242E+14	6,58691E+14	7,55234E+14	7,30269E+14
TC1900000300.mm.2	Gnaq	guanine nucleotide binding protein, alpha q polypeptide	1,42624E+14	8,00178E+14	3,10727E+14	0.0191176769418908	0.15297717631228	-2,93645E+14	9,02754E+14	8,87396E+14	7,95671E+14	7,10344E+14	7,99785E+14	7,05117E+13
TC0500002846.mm.2	Abraxas1	BRCA1 A complex subunit	-1,05343E+14	8,64675E+12	-3,10708E+14	0.0191223644791393	0.15297717631228	-2,93669E+14	8,07963E+14	7,92431E+14	8,5737E+14	9,19208E+13	9,10561E+14	9,00518E+14
TC1400000895.mm.2	Ctsb	cathepsin B	1,79866E+14	9,77444E+14	3,10655E+14	0.0191360069055189	0.15297717631228	-2,93736E+14	1,0997E+14	1,13476E+14	9,58698E+14	7,96796E+14	9,72912E+14	9,01809E+14
TC0900000013.mm.2	Alkbh8	alkB homolog 8, tRNA methyltransferase	2,49398E+14	6,58977E+14	3,10642E+14	0.0191391701311799	0.15297717631228	-2,93751E+14	8,15538E+14	8,15603E+14	7,03804E+14	4,86569E+14	6,45373E+14	4,86972E+14
TC0900003204.mm.2	Rbms3	RNA binding motif, single stranded interacting protein	1,0022E+13	7,40531E+12	3,10625E+14	0.0191436456108827	0.15297717631228	-2,93773E+14	8,10523E+13	7,70637E+14	7,52614E+14	7,28691E+14	6,78041E+14	7,02679E+14
TC1900000426.mm.2	Slc1a1	solute carrier family 1 (neuronal/epithelial high affinity glutamate transporter, system Xag), member 1	1,39653E+14	7,12679E+14	3,10623E+14	0.0191440791394627	0.15297717631228	-2,93776E+14	8,10903E+14	8,04834E+14	7,09014E+14	6,14889E+14	7,08473E+14	6,27957E+14
TC0300000043.mm.2	Stmn2	stathmin-like 2	1,31486E+14	1,04078E+14	3,10581E+14	0.0191547816657455	0.15297717631228	-2,93828E+14	1,13589E+13	1,11782E+13	1,03834E+14	9,61877E+14	1,03493E+14	9,55809E+14
TC1100004281.mm.2	Mettl23	methyltransferase like 23	-0.94848588010014	9,851E+14	-3,10534E+14	0.0191668722976166	0.15297717631228	-2,93888E+14	9,12662E+14	9,41105E+14	9,95507E+14	1,01786E+14	1,01749E+14	1,02597E+14
TC0900000696.mm.2	Man2c1	mannosidase, alpha, class 2C, member 1	-1,13025E+14	7,01175E+14	-3,10532E+14	0.0191672071281695	0.15297717631228	-2,93889E+14	6,32908E+14	6,35003E+14	6,90284E+14	7,54894E+14	7,23309E+14	7,70651E+14

TC1600001509.mm.2	Slc51a	solute carrier family 51, alpha subunit	-1,34026E+14	5,24709E+14	-3,10528E+14	0.0191684646081363	0.15297717631228	-2,93896E+14	4,5179E+14	4,54567E+14	5,02413E+14	5,65076E+14	5,38711E+14	6,35698E+14
TC0700002435.mm.2	Ceacam15	carcinoembryonic antigen-related cell adhesion molecule 15	-1,26454E+14	6,31604E+14	-3,10523E+13	0.0191695539436011	0.15297717631228	-2,93901E+14	5,3194E+14	5,69912E+14	6,35591E+14	6,97421E+14	6,40481E+14	7,14278E+14
TC1700001607.mm.2	Prss33	protease, serine 33	-1,21345E+14	6,91478E+14	-3,1047E+14	0.0191832607979248	0.15297717631228	-2,93968E+14	6,22958E+14	6,01152E+14	6,86024E+14	7,71931E+14	7,16273E+13	7,50527E+14
TC1300000547.mm.2	G630093K05Rik	RIKEN cDNA G630093K05 gene	-1,04908E+13	5,98091E+14	-3,10453E+14	0.0191874184402109	0.15297717631228	-2,93989E+14	5,40543E+14	5,52212E+14	5,83343E+14	6,0988E+14	6,22878E+14	6,79693E+13
TC0400000552.mm.2	Zfp189	zinc finger protein 189	0.976290075042772	5,49695E+14	3,10332E+14	0.019218528100 2707	0.152987824145964	-2,94141E+14	6,16245E+14	5,78986E+14	5,42025E+14	5,60942E+14	4,90435E+14	5,09538E+14
TC0400002022.mm.2	Rpl22	ribosomal protein L22	1,09218E+14	8,70583E+14	3,10266E+14	0.0192354531864389	0.152987824145964	-2,94224E+13	9,39091E+14	9,45903E+12	8,34693E+14	8,37255E+14	8,69563E+14	7,96994E+14
TC0200002550.mm.2	Eya2	EYA transcriptional coactivator and phosphatase 2	-1,05081E+14	5,25388E+14	-3,10262E+14	0.0192364024811701	0.152987824145964	-2,94229E+14	4,84286E+14	4,59187E+14	5,05668E+14	5,49553E+14	5,96688E+14	5,56948E+14
TC0700001867.mm.2	Zfp646	zinc finger protein 646	0.999354653389431	6,04514E+14	3,10257E+14	0.0192376896620465	0.152987824145964	-2,94235E+14	6,63037E+13	6,96291E+14	5,72572E+14	5,35727E+14	5,87832E+14	5,71625E+14
TC0600000273.mm.2	Cpa1	carboxypeptidase A1, pancreatic	-1,43258E+14	6,57098E+14	-3,10223E+14	0.0192463141341568	0.152987824145964	-2,94278E+14	5,47762E+14	5,78584E+14	6,46517E+13	7,56861E+14	6,76087E+14	7,36775E+14
TC1900000563.mm.2	Cyp2c65	cytochrome P450, family 2, subfamily c, polypeptide 65	-1,10532E+14	4,62131E+13	-3,10184E+14	0.0192564433771949	0.152987824145964	-2,94327E+13	3,61106E+14	4,0369E+14	4,8709E+14	5,35042E+13	4,72924E+14	5,12936E+14
TC0400001312.mm.2	Ebna1bp2/D4Ertd617e	EBNA1 binding protein 2/DNA segment, Chr 4, ERATO Doi 617, expressed	1,34452E+14	7,51493E+13	3,1008E+14	0.0192831653576912	0.152987824145964	-2,94458E+14	8,46444E+14	8,44304E+14	7,40678E+14	6,55687E+14	7,50719E+14	6,71124E+14
TC0600000651.mm.2	Wipf3	WAS/WASL interacting protein family, member 3	-0.975568331938535	8,5025E+14	-3,1002E+14	0.0192983767695081	0.152987824145964	-2,94532E+14	7,7094E+13	7,79321E+14	9,17505E+14	8,88356E+14	8,55383E+14	8,89993E+13
TC1300000033.mm.2	Pitrm1	pitrilysin metallepetidase 1	1,11854E+14	7,49517E+14	3,09989E+14	0.0193065879741839	0.152987824145964	-2,94572E+14	8,02853E+14	8,21805E+14	7,68413E+12	7,03081E+14	7,19842E+14	6,81108E+14
TC0400004097.mm.2	Kcnab2	potassium voltage-gated channel, shaker-related subfamily, beta member 2	0.949370258750587	9,13655E+14	3,09925E+14	0.0193228229298842	0.152987824145964	-2,94652E+14	9,77204E+14	9,7742E+13	8,95779E+14	8,66782E+14	8,94719E+14	8,7003E+14
TC0100003878.mm.2	Mpp4	membrane protein, palmitoylated 4 (MAGUK p55 subfamily member 4)	-1,487E+14	4,68737E+14	-3,09915E+14	0.0193254289743759	0.152987824145964	-2,94664E+14	3,91954E+14	4,17395E+14	4,02835E+14	4,9349E+14	5,03061E+14	6,03687E+14
TC0X00002320.mm.2	Cdr1	cerebellar degeneration related antigen 1	-1,56226E+13	1,15796E+14	-3,09893E+13	0.0193311511911378	0.152987824145964	-2,94692E+13	1,04135E+14	1,08203E+14	1,24633E+14	1,14219E+14	1,26682E+14	1,16901E+14
TC0400003767.mm.2	Nipal3	NIPA-like domain containing 3	1,06701E+13	7,98031E+14	3,09877E+14	0.0193351809508611	0.152987824145964	-2,94712E+14	8,81467E+13	8,48365E+13	7,98858E+14	7,4307E+14	7,68501E+13	7,47927E+14
TC0600000236.mm.2	Irf5	interferon regulatory factor 5	-1,25279E+14	6,16325E+14	-3,09866E+14	0.0193381643057538	0.152987824145964	-2,94727E+14	5,43273E+14	5,12242E+13	6,21234E+14	7,15127E+14	6,40525E+14	6,65548E+14
TC1700000954.mm.2	Plcl2	phospholipase C-like 2	1,19889E+14	7,30593E+14	3,09865E+14	0.0193383799147598	0.152987824145964	-2,94728E+14	7,90603E+13	7,93818E+14	7,38246E+14	7,16247E+14	7,17435E+14	6,27208E+14
TC1200002359.mm.2	Wars	tryptophanyl-tRNA synthetase	1,63888E+14	6,86269E+14	3,09773E+14	0.019362134912094	0.152987824145964	-2,94843E+14	7,98998E+13	7,80259E+14	7,02251E+14	5,84624E+14	6,78642E+14	5,7284E+14
TC1100000621.mm.2	Acsl6	acyl-CoA synthetase long-chain family member 6	1,06036E+14	7,95552E+14	3,09773E+14	0.0193621796765925	0.152987824145964	-2,94844E+14	8,92669E+14	8,38072E+14	7,74968E+14	7,48931E+14	7,73416E+14	7,45254E+14
TC1200000869.mm.2	Gstz1	glutathione transferase zeta 1 (maleylacetoacetate isomerase)	1,42645E+14	6,96665E+14	3,09761E+13	0.0193650976246003	0.152987824145964	-2,94858E+14	8,09226E+14	7,80128E+14	6,93591E+14	5,92984E+13	6,8974E+14	6,14324E+14
TC1100004064.mm.2	Fads6	fatty acid desaturase domain family, member 6	1,04843E+14	6,31138E+13	3,09742E+14	0.0193700050407636	0.152987824145964	-2,94882E+14	7,16232E+14	6,60686E+14	6,09832E+14	6,32845E+14	5,94826E+14	5,72406E+14
TC1600000468.mm.2	Zfp148	zinc finger protein 148	1,28775E+14	7,30604E+14	3,09709E+14	0.0193786476188117	0.152987824145964	-2,94924E+14	8,09179E+14	8,12591E+14	7,35477E+14	6,62157E+14	7,21575E+14	6,42645E+14
TC1100000326.mm.2	4930524B15Rik	RIKEN cDNA 4930524B15 gene	-1,13107E+14	5,28505E+14	-3,09654E+14	0.0193929072940958	0.153025423716829	-2,94993E+14	4,58737E+14	4,70038E+14	5,08933E+14	5,78333E+14	5,97482E+13	5,57507E+14
TC0800003053.mm.2	Zcchc14	zinc finger, CCHC domain containing 14	0.996810648105225	6,25267E+14	3,09565E+14	0.0194158731157172	0.153125004640794	-2,95105E+14	6,76639E+14	6,94921E+14	6,22118E+14	5,85723E+14	6,02368E+14	5,6983E+14
TC1100000900.mm.2	Per1	period circadian clock 1	1,21875E+14	7,38108E+14	3,09476E+14	0.0194388247638529	0.153125004640794	-2,95216E+13	8,28191E+14	8,07372E+14	7,46417E+14	6,54854E+14	6,84599E+14	7,07213E+14
TC1900000397.mm.2	Tmem252	transmembrane protein 252	-0.963251181087742	5,31396E+14	-3,0947E+14	0.0194405258199441	0.153125004640794	-2,95224E+14	4,60133E+14	4,92274E+14	5,34062E+14	5,56854E+14	5,64015E+14	5,81042E+14
TC1100001631.mm.2	Hspb9	heat shock protein, alpha-crystallin-related, B9	-1,32809E+14	5,05219E+14	-3,09442E+14	0.0194477051120969	0.153125004640794	-2,95259E+14	4,10034E+14	4,21174E+14	5,44532E+14	5,58747E+14	4,91962E+14	6,04864E+14
TC1100001529.mm.2	Mrpl10	mitochondrial ribosomal protein L10	1,22702E+14	7,35371E+14	3,09416E+14	0.0194543684290925	0.153125004640794	-2,95291E+14	8,203E+14	7,87107E+14	7,57208E+14	6,85609E+14	7,10068E+14	6,51935E+14
TC1200001064.mm.2	D430019H16Rik	RIKEN cDNA D430019H16 gene	1,02258E+13	7,8049E+14	3,09385E+14	0.0194625463603517	0.153125004640794	-2,95331E+14	8,60089E+14	8,20693E+14	7,84379E+14	7,41511E+14	7,41397E+14	7,34869E+14
TC1700001750.mm.2	Tcp11	t-complex protein 11	-1,29375E+14	6,26692E+13	-3,09306E+14	0.0194831034482491	0.153148813563812	-2,95431E+14	5,29783E+14	5,67006E+14	6,03924E+14	7,03901E+14	6,66559E+14	6,8898E+14
TC1200000647.mm.2	Prkch	protein kinase C, eta	1,34273E+14	7,7342E+14	3,09268E+14	0.0194928653636033	0.153148813563812	-2,95478E+14	8,46399E+14	8,52105E+14	7,90932E+14	7,21126E+14	7,6167E+14	6,68289E+13
TC0100003093.mm.2	Zbed6/Zc3h11a	zinc finger, BED type containing 6/zinc finger CCCH type containing 11A	1,23945E+14	7,68658E+14	3,09263E+14	0.0194940865524346	0.153148813563812	-2,95484E+14	8,38181E+14	8,4829E+14	7,79178E+14	7,07718E+13	7,54044E+14	6,84537E+14
TC1100002825.mm.2	Olfr329-ps	olfactory receptor 329, pseudogene	-1,52404E+14	4,48152E+14	-3,09132E+14	0.0195283956521325	0.153343586326906	-2,95649E+14	3,55211E+14	3,83616E+14	3,93011E+14	5,1344E+14	4,85305E+14	5,5833E+14
TC0400003659.mm.2	Ythdf2	YTH N6-methyladenosine RNA binding protein 2	1,40068E+14	6,9694E+14	3,09032E+14	0.0195543508452592	0.153350982864233	-2,95775E+14	7,81829E+14	7,80041E+14	7,12888E+14	6,2515E+14	6,85874E+14	5,9586E+14
TC0700001620.mm.2	Ipo7/Snora23	importin 7/small nucleolar RNA, H/ACA box 23	2,45839E+14	6,9586E+14	3,09014E+14	0.0195589953362592	0.153350982864233	-2,95797E+14	8,75259E+14	8,55566E+14	7,05983E+14	4,99204E+14	7,02697E+14	5,36449E+14
TC1100003110.mm.2	Camta2	calmodulin binding transcription activator 2	1,01382E+14	8,16746E+14	3,08986E+14	0.0195664323526754	0.153350982864233	-2,95833E+14	8,78648E+14	8,90263E+14	8,05015E+13	7,60401E+14	7,99679E+14	7,66468E+14
TC0200002398.mm.2	Dlgap4	DLG associated protein 4	0.927040528873354	7,87542E+14	3,08921E+14	0.0195832695520301	0.153350982864233	-2,95914E+14	8,35559E+14	8,29928E+14	8,05111E+14	7,74578E+14	7,39773E+14	7,40305E+14
TC1700000995.mm.2	Fsd1	fibronectin type 3 and SPRY domain-containing protein	1,23326E+14	7,38736E+14	3,08876E+14	0.0195951635960603	0.153350982864233	-2,95971E+14	8,28753E+14	8,25803E+14	7,32265E+14	6,3769E+14	7,19222E+13	6,88682E+14
TC1500001365.mm.2	Cox6c	cytochrome c oxidase subunit 6C	1,75823E+14	1,00199E+14	3,08828E+14	0.0196075046837171	0.153350982864233	-2,96031E+14	1,11745E+14	1,12757E+14	1,01599E+14	8,57559E+14	9,84985E+14	9,08391E+14
TC0900001487.mm.2	Trank1	tetratricopeptide repeat and ankyrin repeat containing 1	1,53373E+14	6,60183E+14	3,08822E+14	0.0196091875424919	0.153350982864233	-2,96039E+13	7,78095E+14	7,63127E+14	6,64793E+14	5,20608E+14	6,2201E+14	6,12467E+14
TC1100000781.mm.2	B9d1	B9 protein domain 1	-1,10275E+14	7,38037E+14	-3,08817E+12	0.0196105645924241	0.153350982864233	-2,96045E+14	6,46322E+14	6,94285E+14	7,33732E+14	7,92727E+14	7,81121E+14	7,80036E+14
TC1900001527.mm.2	Loxl4	lysyl oxidase-like 4	-1,02548E+13	7,24215E+14	-3,088E+14	0.0196149925949968	0.153350982864233	-2,96067E+14	6,54572E+14	6,61668E+14	7,2649E+14	7,81223E+14	7,4927E+14	7,72066E+14
TC0700002062.mm.2	Muc5ac	mucin 5, subtypes A and C, tracheobronchial/gastric	-1,04475E+14	5,56042E+14	-3,08722E+14	0.0196354902799965	0.153354060954721	-2,96165E+14	4,7643E+14	5,02989E+14	5,55078E+14	6,13385E+13	5,95416E+14	5,92952E+14
TC0500003240.mm.2	Zcchc8	zinc finger, CCHC domain containing 8	1,27183E+13	7,35294E+14	3,08714E+14	0.0196374717187093	0.153354060954721	-2,96175E+14	8,15503E+14	7,95169E+14	7,72328E+14	6,72458E+14	6,63806E+14	6,92501E+14
TC0700002907.mm.2	AI987944	expressed sequence AI987944	2,50793E+14	5,86536E+14	3,08685E+14	0.0196450882294219	0.153354060954721	-2,96211E+14	7,4162E+14	7,45644E+14	6,37786E+14	4,0849E+13	5,66469E+14	4,1921E+14
TC1000000834.mm.2	Cnn2	calponin 2	0.904341345959108	5,60645E+14	3,08653E+14	0.0196534559592564	0.153354060954721	-2,96251E+14	6,05684E+14	5,98742E+14	5,73318E+14	5,62571E+13	5,19833E+13	5,03724E+14
TC0500003410.mm.2	Ywhag	tyrosine 3-monooxygenase/tryptophan 5-monooxygenase activation protein, gamma polypeptide	1,52211E+14	9,4081E+14	3,08459E+14	0.0197044650031633	0.153495146029602	-2,96496E+14	1,06055E+14	1,04149E+14	9,40423E+13	8,0478E+14	9,20817E+14	8,76799E+14
TC1300002318.mm.2	Sdha	succinate dehydrogenase complex, subunit A, flavoprotein (Fp)	1,69579E+14	7,05424E+14	3,08454E+14	0.0197056217698048	0.153495146029602	-2,96501E+14	8,44069E+14	8,23229E+14	6,97683E+14	5,39421E+14	6,90407E+14	6,37733E+14
TC1300000015.mm.2	Tubal3	tubulin, alpha-like 3	-1,05316E+14	5,68138E+14	-3,08449E+14	0.0197069347718551	0.153495146029602	-2,96508E+14	4,94308E+13	5,17163E+14	5,57E+14	6,18257E+14	6,06025E+14	6,16077E+14
TC1600002059.mm.2	Cfap298	cilia and flagella associate protien 298	1,64083E+14	6,05827E+14	3,08439E+13	0.0197096417262612	0.153495146029602	-2,96521E+14	7,26954E+14	6,92332E+14	6,3904E+14	4,85516E+13	5,29654E+14	5,61465E+14
TC0200002809.mm.2	Birc7	baculoviral IAP repeat-containing 7 (livin)	-1,56942E+14	4,74748E+14	-3,08396E+14	0.0197208979878196	0.153508613177651	-2,96574E+14	3,80384E+14	3,71857E+14	4,76105E+14	5,54015E+14	4,68744E+14	5,97382E+14
TC1500002194.mm.2	Rhebl1	Ras homolog enriched in brain like 1	-1,36575E+14	5,85648E+14	-3,08132E+14	0.0197906191443109	0.153927217040827	-2,96907E+14	5,03134E+13	4,83702E+14	6,92456E+14	5,74611E+14	6,20189E+14	6,39798E+14
TC0800001875.mm.2	Ap3m2	adaptor-related protein complex 3, mu 2 subunit	1,04228E+14	8,03939E+14	3,0812E+14	0.0197937810282749	0.153927217040827	-2,96922E+14	8,57644E+14	8,59485E+14	8,26694E+14	7,71134E+14	7,70216E+14	7,38458E+14
TC0700000467.mm.2	Zfp607b	zinc finger protein 607B	0.948246121119343	7,13793E+14	3,0791E+14	0.0198493386285633	0.154059233271624	-2,97187E+14	7,93564E+14	7,56012E+14	6,86531E+14	6,86723E+14	6,86478E+14	6,73449E+14

TC1800000625.mm.2	Csf1r	colony stimulating factor 1 receptor	1,59532E+14	7,6956E+12	3,07875E+14	0.0198586697552802	0.154059233271624	-2,97231E+14	8,69125E+14	8,96444E+14	7,73015E+14	6,32271E+14	6,94269E+14	7,52236E+14
TC0500003516.mm.2	Gna12	guanine nucleotide binding protein, alpha 12	1,15297E+14	8,74947E+13	3,07748E+13	0.0198923243899273	0.154059233271624	-2,9739E+14	9,50191E+14	9,45024E+14	8,75689E+14	8,14158E+14	8,62401E+14	8,02221E+14
TC1100002030.mm.2	Slc25a10	solute carrier family 25 (mitochondrial carrier, dicarboxylate transporter), member 10	1,06866E+14	6,48337E+14	3,0766E+14	0.0199156874375716	0.154059233271624	-2,97501E+12	6,98103E+14	7,01176E+14	6,65627E+14	6,3957E+14	6,25221E+14	5,60326E+14
TC0700003806.mm.2	Neu3	neuraminidase 3	-1,07011E+13	7,61641E+14	-3,07652E+14	0.0199177976050928	0.154059233271624	-2,97511E+14	6,86715E+14	7,01425E+14	7,56553E+14	8,2299E+14	8,09918E+14	7,92244E+14
TC0X00001801.mm.2	Ftsj1	FtsJ RNA methyltransferase homolog 1 (E. coli)	1,10597E+14	6,79069E+14	3,07614E+14	0.0199278413039373	0.154059233271624	-2,97559E+14	7,55663E+14	7,42722E+14	6,69852E+14	6,28989E+14	6,70883E+14	6,06308E+14
TC0200000953.mm.2	Scn2a	sodium channel, voltage-gated, type II, alpha	1,5591E+14	7,99044E+14	3,07602E+14	0.0199310171191974	0.154059233271624	-2,97574E+14	9,29227E+14	9,25588E+13	7,73055E+14	6,23401E+12	7,73366E+14	7,69628E+14
TC0700001655.mm.2	Rassf10	Ras association (RalGDS/AF-6) domain family (N-terminal) member 10	-1,38798E+14	6,36022E+14	-3,07599E+14	0.0199318960430142	0.154059233271624	-2,97578E+14	5,51807E+14	5,39094E+14	6,47058E+14	7,09678E+14	6,30662E+14	7,37835E+14
TC1500000710.mm.2	Adsl	adenylosuccinate lyase	1,08962E+14	5,89736E+14	3,07523E+14	0.0199522495091739	0.154059233271624	-2,97674E+14	6,47565E+14	6,45999E+14	5,63866E+13	6,05348E+14	5,68665E+14	5,06974E+13
TC1500002141.mm.2	Slc38a1	solute carrier family 38, member 1	1,85973E+14	8,65735E+14	3,07507E+14	0.0199563439438453	0.154059233271624	-2,97693E+14	9,98472E+12	9,76786E+14	8,96428E+14	7,19411E+14	8,35634E+14	7,67677E+14
TC0700001729.mm.2	E130201H02Rik	Y box protein 1 pseudogene	-0.930615031759773	7,61713E+14	-3,07498E+14	0.0199588872801518	0.154059233271624	-2,97705E+14	6,95424E+14	7,24832E+14	7,65908E+14	7,77736E+13	7,96919E+14	8,0946E+14
TC1800001005.mm.2	Zfp521	zinc finger protein 521	1,39071E+14	7,0999E+14	3,07491E+13	0.0199606164922241	0.154059233271624	-2,97714E+14	7,96904E+14	7,67992E+14	7,63423E+14	6,44868E+14	6,57512E+14	6,29241E+14
TC0700000907.mm.2	Slc6a5	solute carrier family 6 (neurotransmitter transporter, glycine), member 5	-1,52205E+14	4,90356E+14	-3,07483E+14	0.0199629562653329	0.154059233271624	-2,97725E+14	3,80128E+14	4,00862E+14	4,66433E+14	6,09312E+14	5,39108E+14	5,46292E+13
TC0200001009.mm.2	Ppig	peptidyl-prolyl isomerase G (cyclophilin G)	2,16934E+14	7,40949E+14	3,07442E+14	0.0199737200709917	0.154059233271624	-2,97776E+14	8,77109E+14	8,9426E+13	7,35655E+14	6,01168E+14	7,6234E+14	5,7516E+14
TC1000001573.mm.2	Rdh1/Rdh9	retinol dehydrogenase 1 (all trans)/retinol dehydrogenase 9	-1,19917E+14	7,87552E+14	-3,07434E+14	0.0199759008173385	0.154059233271624	-2,97786E+14	7,08229E+14	7,00627E+14	8,01274E+14	8,66491E+13	7,9029E+14	8,58399E+14
TC1400000104.mm.2	Synpr	synaptoporin	1,01407E+13	8,92982E+13	3,07408E+14	0.0199828544510797	0.154059233271624	-2,97819E+14	9,50478E+14	9,64761E+14	8,9316E+14	8,37072E+14	8,58854E+14	8,5357E+14
TC1900000409.mm.2	Smarca2	SWI/SNF related, matrix associated, actin dependent regulator of chromatin, subfamily a, member 2	2,0402E+14	8,81348E+14	3,07377E+14	0.0199911011047093	0.154059233271624	-2,97858E+14	1,01186E+14	1,00564E+14	9,13473E+14	7,4765E+14	8,72752E+14	7,3671E+14
TC1100001770.mm.2	Dcaf7	DDB1 and CUL4 associated factor 7	1,50917E+14	8,31021E+14	3,07356E+14	0.0199968242463661	0.154059233271624	-2,97885E+13	9,24636E+14	9,24236E+14	8,53131E+14	7,37086E+14	8,15124E+14	7,31914E+14
TC0600002225.mm.2	Rarres2	retinoic acid receptor responder (tazarotene induced) 2	-1,37653E+13	5,71712E+14	-3,07345E+14	0.019999655278925	0.154059233271624	-2,97898E+14	4,61234E+13	4,89389E+14	5,73789E+14	6,7993E+14	5,85024E+14	6,40904E+14
TC0800000211.mm.2	Defa21	defensin, alpha, 21	-1,27428E+14	4,57871E+14	-3,07306E+14	0.0200100407468256	0.154059233271624	-2,97947E+14	4,19405E+14	3,51272E+14	5,01737E+14	4,49277E+14	4,80742E+14	5,44791E+14
TC0500000171.mm.2	Fgl2	fibrinogen-like protein 2	-0.938424574383565	6,42035E+14	-3,07301E+14	0.0200115419374114	0.154059233271624	-2,97954E+14	5,70231E+14	6,03323E+14	6,50759E+14	6,66658E+14	6,83271E+14	6,77968E+14
TC0900002934.mm.2	Il20rb	interleukin 20 receptor beta	-0.946618856873	5,12391E+14	-3,07199E+14	0.0200386136572077	0.154193974144502	-2,98081E+14	4,29247E+14	4,55067E+14	5,55637E+14	5,6076E+14	5,21807E+14	5,51831E+14
TC0800002389.mm.2	Ddx49	DEAD (Asp-Glu-Ala-Asp) box polypeptide 49	0.883963019546448	6,69328E+14	3,07075E+14	0.0200718470583046	0.154221517613239	-2,98238E+14	7,25344E+14	7,20003E+14	6,50476E+14	6,51594E+14	6,3269E+14	6,35864E+14
TC0800000215.mm.2	Defa25	defensin, alpha, 25	-0.970960150420368	4,99797E+14	-3,07011E+14	0.020089009675554	0.154221517613239	-2,98318E+13	4,44153E+14	4,49656E+14	5,2675E+13	4,90224E+14	5,58848E+14	5,29153E+14
TC0300000875.mm.2	Selenbp2	selenium binding protein 2	-1,71937E+14	6,71048E+14	-3,06998E+13	0.0200925428880992	0.154221517613239	-2,98335E+14	5,33141E+14	5,78239E+14	6,55169E+14	8,04486E+14	6,90905E+13	7,64349E+14
TC0600001035.mm.2	Mgll	monoglyceride lipase	1,33331E+14	7,32864E+14	3,06971E+14	0.0201000056568375	0.154221517613239	-2,9837E+13	7,99247E+14	8,28852E+14	7,51315E+14	6,5633E+14	7,11325E+14	6,50112E+14
TC0500001885.mm.2	Rbm48	RNA binding motif protein 48	-1,08626E+13	7,13802E+14	-3,06961E+14	0.0201026791135647	0.154221517613239	-2,98382E+14	6,51618E+14	6,38474E+14	7,06527E+14	7,7885E+14	7,58279E+14	7,49066E+14
TC0500003637.mm.2	Slc7a1	solute carrier family 7 (cationic amino acid transporter, y+ system), member 1	1,02274E+14	6,70485E+14	3,06958E+13	0.0201033433860218	0.154221517613239	-2,98386E+14	7,30963E+13	7,30471E+14	6,73331E+12	6,3126E+13	6,50178E+14	6,06708E+14
TC0100002974.mm.2	Ddx18	DEAD (Asp-Glu-Ala-Asp) box polypeptide 18	1,56758E+14	6,53216E+14	3,06917E+14	0.0201143076475207	0.154221517613239	-2,98437E+14	7,62892E+14	7,43839E+14	6,81407E+13	5,37944E+14	5,74313E+14	6,18902E+14
TC1000003156.mm.2	Ikzf4	IKAROS family zinc finger 4	-1,10456E+14	8,06354E+14	-3,06901E+14	0.0201187631119611	0.154221517613239	-2,98458E+13	7,17308E+14	7,28253E+14	8,53552E+14	8,72539E+14	7,95948E+14	8,70525E+14
TC0800000816.mm.2	Rab8a	RAB8A, member RAS oncogene family	2,44239E+14	7,09275E+14	3,06798E+14	0.0201464921144948	0.154360640723184	-2,98588E+14	8,67146E+14	8,6136E+14	7,55684E+14	5,31435E+14	6,91728E+14	5,483E+14
TC1000001913.mm.2	L3mbtl3	L3MBTL3 histone methyl-lysine binding protein	1,439E+14	7,06611E+14	3,06712E+14	0.0201695545875745	0.154384906149131	-2,98696E+14	8,12772E+14	7,95146E+14	7,02898E+13	6,0873E+14	7,06196E+14	6,13923E+14
TC1900001731.mm.2	Pdzd8	PDZ domain containing 8	2,00357E+14	7,11623E+14	3,06695E+14	0.0201742954107564	0.154384906149131	-2,98718E+14	8,37005E+14	8,4747E+14	7,33139E+14	5,68365E+14	7,07094E+14	5,76667E+14
TC0300001413.mm.2	Adh6-ps1	alcohol dehydrogenase 6 (class V), pseudogene 1	-1,26437E+14	5,33595E+14	-3,06647E+14	0.0201870346387242	0.154384906149131	-2,98777E+14	4,65904E+14	4,62293E+14	5,29304E+14	5,62999E+14	5,40288E+14	6,40782E+14
TC0200003473.mm.2	Zeb2/Mir5129	zinc finger E-box binding homeobox 2/microRNA 5129	1,01771E+14	8,11134E+14	3,06614E+14	0.020195944032567	0.154384906149131	-2,98819E+14	8,66217E+14	9,25687E+14	7,41871E+14	7,4467E+14	7,92865E+14	7,95497E+14
TC1000000843.mm.2	Cirbp	cold inducible RNA binding protein	1,38618E+14	6,88253E+14	3,0659E+13	0.0202025700845189	0.154384906149131	-2,9885E+14	8,06173E+14	7,78873E+14	6,69787E+13	5,66878E+14	6,86836E+14	6,20974E+14
TC0700001430.mm.2	Stard10	START domain containing 10	0.985068911319281	7,76707E+14	3,06573E+14	0.0202071474628261	0.154384906149131	-2,98871E+14	8,47595E+14	8,17053E+14	7,58365E+14	7,69595E+14	7,53829E+13	7,13806E+14
TC0400003747.mm.2	Maco1	macoilin 1	1,26911E+14	7,00506E+14	3,06414E+14	0.0202500407188158	0.154562073150675	-2,99071E+14	8,00206E+14	7,97237E+14	6,82108E+14	5,79863E+14	6,56138E+14	6,87482E+13
TC0400002105.mm.2	9430015G10Rik	RIKEN cDNA 9430015G10 gene	-1,06979E+14	6,66613E+14	-3,06376E+14	0.0202604272455795	0.154562073150675	-2,9912E+14	5,76477E+14	6,19791E+14	6,81808E+14	7,11375E+14	6,81067E+13	7,29159E+14
TC1100002574.mm.2	Ttc1	tetratricopeptide repeat domain 1	1,01669E+12	6,48961E+14	3,06335E+14	0.0202715443553736	0.154562073150675	-2,99171E+14	7,03257E+14	7,25873E+14	6,38831E+14	6,00015E+14	6,32459E+14	5,93332E+14
TC0400002768.mm.2	Mup1	major urinary protein 1	-1,2527E+14	5,24365E+14	-3,06323E+14	0.0202748489097446	0.154562073150675	-2,99187E+14	4,32967E+14	4,56925E+14	5,0804E+14	6,07828E+14	5,60734E+14	5,79698E+14
TC0600002174.mm.2	Olfr455	olfactory receptor 455	-1,08488E+14	5,4388E+14	-3,0631E+14	0.0202782984323545	0.154562073150675	-2,99203E+13	4,83395E+13	4,90422E+14	5,39475E+13	5,59194E+14	5,59768E+14	6,31024E+14
TC0100000944.mm.2	Atg4b	autophagy related 4B, cysteine peptidase	0.926256890990205	7,57567E+14	3,06159E+14	0.0203193325255389	0.154588608052183	-2,99393E+14	8,34836E+14	8,10671E+13	7,21814E+14	7,17825E+14	7,41901E+14	7,18354E+14
TC0500001716.mm.2	Ap5z1	adaptor-related protein complex 5, zeta 1 subunit	-1,09943E+13	9,16986E+14	-3,06109E+14	0.020333004047165	0.154588608052183	-2,99457E+14	8,54903E+14	8,40419E+13	9,1294E+14	9,78446E+14	9,38419E+14	9,7679E+14
TC1200000358.mm.2	Gm7008	predicted gene 7008	-0.93424293727837	6,2044E+14	-3,06086E+14	0.0203391974529173	0.154588608052183	-2,99485E+14	5,68915E+14	5,74671E+14	6,12737E+14	6,3588E+14	6,51118E+14	6,79316E+14
TC0800001301.mm.2	Ap1g1	adaptor protein complex AP-1, gamma 1 subunit	1,5284E+14	7,70281E+14	3,06036E+14	0.0203527871962116	0.154588608052183	-2,99548E+14	8,85905E+14	8,57806E+13	7,90004E+14	6,49941E+14	7,35246E+14	7,02784E+14
TC0800001058.mm.2	Fto	fat mass and obesity associated	1,73233E+14	7,38974E+14	3,06016E+14	0.0203582647885496	0.154588608052183	-2,99574E+14	8,4925E+14	8,4889E+14	7,79267E+14	6,04764E+14	6,81885E+14	6,69789E+14
TC1000002265.mm.2	Ddx50	DEAD (Asp-Glu-Ala-Asp) box polypeptide 50	1,90983E+14	5,65664E+14	3,05937E+14	0.0203796977834304	0.154588608052183	-2,99673E+14	6,79915E+14	6,9164E+14	6,0045E+14	4,32387E+14	5,47519E+14	4,42071E+14
TC1100001247.mm.2	Ap2b1	adaptor-related protein complex 2, beta 1 subunit	2,06098E+14	7,25541E+14	3,05928E+14	0.0203821211275087	0.154588608052183	-2,99684E+14	8,77046E+14	8,82289E+14	7,31684E+14	5,15087E+14	6,8781E+14	6,5933E+14
TC0200005507.mm.2	Ninl	ninein-like	-1,03126E+14	7,71694E+14	-3,05927E+14	0.0203824508813625	0.154588608052183	-2,99686E+14	6,7423E+14	7,29574E+14	7,937E+14	8,22602E+14	8,00068E+14	8,09988E+14
TC1500002180.mm.2	Ccnt1	cyclin T1	1,7832E+14	6,1279E+14	3,05919E+14	0.0203846440591671	0.154588608052183	-2,99696E+14	7,26993E+14	7,19119E+14	6,37225E+14	5,0393E+14	6,06486E+14	4,82986E+13
TC0600003164.mm.2	Kcna6	potassium voltage-gated channel, shaker-related, subfamily, member 6	1,21229E+14	6,13664E+14	3,05856E+13	0.0204018482471725	0.154588608052183	-2,99776E+14	7,1804E+14	6,51563E+14	6,21108E+14	5,64123E+14	5,85487E+14	5,41659E+14
TC1500000844.mm.2	Tafa5	TAFA chemokine like family member 5	1,18052E+13	7,90707E+14	3,05826E+14	0.0204100113907831	0.154588608052183	-2,99813E+14	8,62661E+14	8,55286E+14	8,1723E+14	7,27219E+14	7,50791E+14	7,31054E+14
TC1800001026.mm.2	Cdh2	cadherin 2	2,07762E+14	7,96052E+14	3,05825E+14	0.0204103094097628	0.154588608052183	-2,99815E+14	9,20314E+14	9,15432E+14	8,537E+14	6,66646E+13	7,6869E+14	6,51532E+13
TC1100003233.mm.2	Doc2b	double C2, beta	1,45504E+14	7,06899E+14	3,05823E+14	0.0204107614167529	0.154588608052183	-2,99817E+14	7,91758E+14	7,54676E+14	7,8332E+14	6,56215E+14	6,43245E+14	6,1218E+14
TC0600000998.mm.2	Aak1	AP2 associated kinase 1	1,43503E+14	8,98058E+14	3,05723E+14	0.0204382337611692	0.154588608052183	-2,99944E+14	1,00743E+14	9,91652E+14	8,97011E+14	7,80173E+14	8,86938E+14	8,2514E+13
TC1200002076.mm.2	Fam161b	family with sequence similarity 161, member B	1,09538E+14	6,29982E+13	3,05713E+14	0.0204407723396913	0.154588608052183	-2,99955E+14	6,97556E+14	6,99386E+14	5,64305E+14	6,40779E+14	5,92178E+14	5,85688E+14
TC0100002597.mm.2	Ihh	Indian hedgehog	-1,16037E+14	5,82618E+14	-3,05704E+14	0.0204433574453934	0.154588608052183	-2,99967E+14	5,02508E+14	5,24487E+14	5,66335E+14	6,43308E+13	6,10141E+14	6,48928E+14

TC0500002882.mm.2	Hsd17b11	hydroxysteroid (17-beta) dehydrogenase 11	0.989581159496343	7,73108E+14	3,05698E+14	0.0204448782820829	0.154588608052183	-2,99974E+14	8,38328E+14	8,53672E+14	7,24109E+14	7,28457E+14	7,72562E+14	7,21522E+14
TC1100000238.mm.2	Xpo1	exportin 1	2,79092E+14	6,4873E+14	3,05552E+14	0.0204849909755269	0.154819258718886	-3,00159E+14	8,04584E+14	8,22312E+14	6,59689E+14	5,3708E+14	6,76955E+14	3,91758E+14
TC1200000336.mm.2	Dgkb	diacylglycerol kinase, beta	1,98834E+14	9,04494E+14	3,05473E+14	0.0205065358663695	0.154850450772857	-3,00258E+14	1,03485E+14	1,04199E+14	9,17844E+14	7,53111E+14	9,04171E+14	7,75002E+14
TSUnmapped00000040.mm.2	Rorb	RAR-related orphan receptor beta	2,05813E+14	6,4419E+14	3,05467E+13	0.0205083387295524	0.154850450772857	-3,00267E+14	7,92632E+12	7,64021E+14	6,73334E+14	4,90129E+12	6,3036E+13	5,14667E+14
TC0X00001793.mm.2	Suv39h1	suppressor of variegation 3-9 1	1,56732E+14	6,4955E+13	3,0543E+14	0.0205185102936245	0.154854686820221	-3,00313E+13	7,66915E+14	7,24086E+14	6,50306E+14	5,78453E+14	6,52511E+14	5,25027E+14
TC0200004550.mm.2	Ciao1	cytosolic iron-sulfur protein assembly 1	1,55343E+14	6,73139E+14	3,0532E+14	0.0205486846993097	0.154861906534596	-3,00452E+14	7,8982E+14	7,56037E+14	6,31038E+14	6,26766E+14	6,90927E+14	5,44244E+14
TC0700004493.mm.2	Fuom	fucose mutarotase	-0.893023958209343	8,24874E+14	-3,05308E+14	0.0205520385866927	0.154861906534596	-3,00467E+14	7,8749E+14	7,69708E+14	8,32243E+14	8,24002E+14	8,68543E+14	8,67261E+14
TC1900000607.mm.2	4933411K16Rik/Hoga1	RIKEN cDNA 4933411K16 gene/4-hydroxy-2-oxoglutarate aldolase 1	-1,5423E+14	6,38659E+14	-3,05307E+14	0.0205523522100401	0.154861906534596	-3,00469E+14	5,53544E+14	5,31836E+14	6,04908E+14	7,47828E+14	6,78849E+14	7,14991E+13
TC1700002348.mm.2	Tubb4a	tubulin, beta 4A class IVA	2,09399E+14	9,91551E+14	3,05287E+14	0.020557910884224	0.154861906534596	-3,00494E+14	1,1345E+14	1,11208E+14	1,04034E+14	8,34605E+14	9,55908E+14	8,71873E+14
TC0700002087.mm.2	Tspan32	tetraspanin 32	-0.902225707858838	6,40989E+14	-3,05205E+14	0.0205803433813246	0.154944139860188	-3,00597E+13	5,92209E+14	5,77243E+14	6,53508E+14	6,7308E+14	6,76833E+14	6,73063E+14
TC1700002354.mm.2	Gpr108	G protein-coupled receptor 108	1,10492E+14	7,11739E+14	3,05173E+14	0.0205890858300954	0.154944139860188	-3,00637E+14	7,91011E+14	7,44333E+14	6,89566E+14	7,31167E+14	6,81328E+14	6,33031E+14
TC0600002278.mm.2	Hoxa4	homeobox A4	-1,07255E+14	6,18416E+14	-3,05079E+14	0.0206150257033213	0.154944139860188	-3,00756E+14	5,29745E+14	5,59653E+14	6,35467E+14	6,8172E+14	6,28265E+14	6,75643E+14
TC0100000828.mm.2	Sag	S-antigen, retina and pineal gland (arrestin)	-1,35352E+14	7,34815E+14	-3,04978E+14	0.0206430516651544	0.154944139860188	-3,00884E+14	6,74806E+13	6,18192E+14	7,35214E+14	8,16974E+13	7,4712E+14	8,16582E+13
TC1900001035.mm.2	Otub1	OTU domain, ubiquitin aldehyde binding 1	1,37945E+14	1,02367E+14	3,0496E+14	0.0206480465134551	0.154944139860188	-3,00907E+14	1,11898E+14	1,11434E+14	1,02567E+14	9,25583E+14	1,01633E+14	9,41101E+14
TC0500003688.mm.2	N4bp2l2	NEDD4 binding protein 2-like 2	1,19229E+14	7,47497E+14	3,04888E+14	0.0206679218892627	0.154944139860188	-3,00998E+14	8,29648E+14	8,28513E+14	7,50945E+14	6,56172E+14	7,13953E+14	7,0575E+14
TC1800001445.mm.2	2700046A07Rik	RIKEN cDNA 2700046A07 gene	-1,17461E+14	6,05861E+14	-3,04886E+14	0.0206685489539863	0.154944139860188	-3,01E+14	5,26929E+14	5,50686E+14	6,12738E+14	6,32279E+14	6,12893E+14	6,99642E+14
TC1000000736.mm.2	S100b	S100 protein, beta polypeptide, neural	1,54158E+14	6,61305E+14	3,04883E+14	0.0206692738854479	0.154944139860188	-3,01004E+14	7,60355E+14	7,48429E+12	6,13248E+13	6,45328E+14	6,7285E+14	5,27619E+14
TC0200002411.mm.2	Nnat	neuronatin	1,46897E+14	9,77234E+14	3,04855E+14	0.0206770483214382	0.154944139860188	-3,01039E+14	1,0997E+13	1,02301E+14	1,00376E+14	9,08027E+14	9,4686E+14	8,82053E+14
TC0400003992.mm.2	Zfp979	zinc finger protein 979	1,0798E+12	5,32974E+14	3,0482E+14	0.0206868373003096	0.154944139860188	-3,01084E+13	6,00718E+14	5,90847E+14	5,34819E+14	4,95853E+14	5,19051E+14	4,56554E+14
TC0400002637.mm.2	Mrpl50	mitochondrial ribosomal protein L50	0.969360378214946	7,50372E+14	3,04777E+14	0.020698593441177	0.154944139860188	-3,01137E+14	8,30756E+14	8,01205E+14	7,11269E+14	7,20913E+14	7,00161E+14	7,37928E+14
TC1900001541.mm.2	Got1	glutamic-oxaloacetic transaminase 1, soluble	1,56122E+14	9,77468E+13	3,04772E+14	0.0207000321100223	0.154944139860188	-3,01144E+13	1,08341E+14	1,05696E+14	1,01566E+14	8,80659E+14	9,46495E+14	8,81629E+14
TC0600001579.mm.2	Etv6	ets variant 6	1,11022E+14	7,56597E+13	3,04752E+13	0.0207056492600094	0.154944139860188	-3,0117E+14	7,95534E+14	8,2958E+14	7,90163E+14	7,21235E+14	7,13166E+14	6,89903E+13
TC0Y00000233.mm.2	Uty	ubiquitously transcribed tetratricopeptide repeat gene, Y chromosome	2,06728E+14	5,32395E+12	3,04711E+14	0.0207168844813677	0.154944139860188	-3,01221E+14	6,50549E+14	6,38166E+14	5,83788E+14	4,4661E+14	5,23666E+14	3,51593E+14
TC0800002374.mm.2	Cilp2	cartilage intermediate layer protein 2	-1,16487E+14	7,27322E+14	-3,04659E+14	0.0207313385564439	0.154944139860188	-3,01286E+14	6,75146E+14	6,39605E+14	7,10103E+13	7,91353E+14	7,82805E+14	7,6492E+13
TC1000000752.mm.2	Slc19a1	solute carrier family 19 (folate transporter), member 1	0.925275533434151	7,23236E+14	3,04654E+14	0.0207328732472287	0.154944139860188	-3,01293E+14	7,61773E+14	7,77297E+14	7,14732E+14	7,31601E+14	6,63589E+14	6,90425E+14
TC0200000406.mm.2	Anapc2	anaphase promoting complex subunit 2	1,08442E+14	8,0885E+14	3,04529E+14	0.0207676698462699	0.154944139860188	-3,01452E+14	8,71011E+13	8,69307E+14	8,29569E+14	7,59778E+14	7,47422E+14	7,76012E+14
TC0700001525.mm.2	Olfr653	olfactory receptor 653	-1,23704E+14	4,98123E+14	-3,04491E+14	0.020778088065147	0.154944139860188	-3,01499E+14	4,04463E+14	4,43473E+14	4,8142E+14	5,6404E+14	5,23891E+14	5,71453E+14
TC0900002127.mm.2	Pou2f3	POU domain, class 2, transcription factor 3	-0.936019085236704	5,82967E+14	-3,0438E+14	0.0208090721404473	0.154944139860188	-3,01639E+14	5,06729E+13	5,45518E+14	6,06059E+14	6,00042E+14	6,20705E+14	6,18747E+14
TC0100001433.mm.2	Cryzl2	crystallin zeta like 2	0.92735285222834	5,53265E+14	3,04379E+14	0.0208094067878571	0.154944139860188	-3,01641E+14	6,19575E+14	6,11195E+14	5,04147E+14	5,39377E+13	5,24167E+14	5,21132E+14
TC1100002438.mm.2	Acyp2	acylphosphatase 2, muscle type	1,17967E+14	6,79875E+14	3,04294E+14	0.0208331123550749	0.154944139860188	-3,01748E+14	7,85952E+14	7,26796E+13	6,13829E+14	6,75858E+14	6,56877E+14	6,19937E+14
TC0200000567.mm.2	Prrc2b	proline-rich coiled-coil 2B	0.894013207447053	7,59747E+14	3,04259E+14	0.0208428103573189	0.154944139860188	-3,01792E+14	8,06236E+14	8,17992E+14	7,53008E+14	7,35818E+14	7,19086E+14	7,26339E+14
TC0400004216.mm.2	Podn	podocan	-1,21669E+14	5,41136E+14	-3,04214E+14	0.0208553113199176	0.154944139860188	-3,01849E+14	4,40286E+14	4,96609E+14	5,23669E+14	6,06021E+14	5,8983E+14	5,90402E+14
TC0900001315.mm.2	Cpne4	copine IV	0.995059918877303	7,0481E+14	3,04212E+14	0.0208560954491102	0.154944139860188	-3,01852E+14	7,74783E+14	7,62168E+14	6,96282E+14	6,57688E+14	6,49643E+14	6,88295E+14
TC1800001122.mm.2	Proc	protein C	-1,07036E+14	5,61738E+14	-3,04211E+14	0.0208562412193469	0.154944139860188	-3,01853E+14	5,17874E+14	5,05808E+14	5,62734E+14	5,46262E+14	5,83923E+14	6,5383E+14
TC1700000047.mm.2	Synj2	synaptojanin 2	0.954133751086948	6,68728E+14	3,04197E+14	0.020860080165526	0.154944139860188	-3,0187E+14	7,1884E+14	7,31696E+14	6,30699E+14	6,71426E+14	6,22588E+14	6,37121E+14
TC1300000284.mm.2	Gpld1	glycosylphosphatidylinositol specific phospholipase D1	1,43106E+14	6,89849E+14	3,04106E+14	0.0208857245649026	0.154944139860188	-3,01986E+14	7,74175E+14	7,30386E+13	6,97794E+14	7,18387E+14	6,71467E+14	5,46882E+14
TC0400002467.mm.2	Kif24	kinesin family member 24	-1,51524E+14	5,79968E+14	-3,04103E+14	0.0208865957629606	0.154944139860188	-3,0199E+14	4,67276E+14	5,02667E+14	5,6078E+14	6,76095E+13	5,95377E+14	6,77614E+14
TC1200000825.mm.2	Ylpm1	YLP motif containing 1	2,40864E+14	7,80763E+14	3,041E+14	0.0208871933053063	0.154944139860188	-3,01993E+14	9,13193E+14	8,95765E+14	8,37202E+14	7,11189E+14	7,79681E+14	5,47549E+14
TC1300001170.mm.2	Trim23	tripartite motif-containing 23	1,52253E+14	7,08499E+14	3,04094E+14	0.0208888369646529	0.154944139860188	-3,02E+14	8,06699E+14	8,02099E+13	7,07258E+14	6,30647E+14	7,1486E+14	5,89432E+14
TC1400001531.mm.2	Ecd	ecdysoneless cell cycle regulator	1,10464E+13	7,32327E+14	3,04073E+14	0.0208949443279012	0.154944139860188	-3,02028E+14	8,0011E+14	8,13827E+14	7,30503E+14	6,5651E+14	7,09664E+14	6,83345E+14
TC1400002182.mm.2	Sgcg	sarcoglycan, gamma (dystrophin-associated glycoprotein)	-1,35682E+14	5,50612E+14	-3,04003E+13	0.0209145708699708	0.154944139860188	-3,02116E+14	4,51719E+14	4,75154E+14	5,36735E+14	6,41826E+14	5,69926E+14	6,28311E+14
TC0500001399.mm.2	P2rx7	purinergic receptor P2X, ligand-gated ion channel, 7	0.987559819362024	5,57035E+14	3,03931E+14	0.0209347263538515	0.154944139860188	-3,02207E+14	6,45146E+13	6,21348E+14	5,05017E+14	5,01719E+14	5,10079E+14	5,58903E+14
TC1400000935.mm.2	Zfp395	zinc finger protein 395	-0.963268106814506	7,3667E+14	-3,03821E+14	0.0209654442719443	0.154944139860188	-3,02346E+14	6,63676E+14	6,87786E+14	7,46721E+14	7,77725E+14	7,85913E+14	7,58202E+13
TC1200000750.mm.2	Srsf5	serine/arginine-rich splicing factor 5	1,17559E+14	8,43115E+14	3,03767E+14	0.0209806268283879	0.154944139860188	-3,02414E+14	9,40538E+14	9,0068E+14	8,40143E+14	7,71229E+13	8,27066E+14	7,79033E+14
TC1200001314.mm.2	Rapgef5	Rap guanine nucleotide exchange factor (GEF) 5	1,41082E+14	7,11619E+14	3,03764E+14	0.0209814866839266	0.154944139860188	-3,02418E+14	7,97011E+14	8,30541E+14	7,00541E+14	5,96235E+14	7,08476E+14	6,36911E+13
TC0500001578.mm.2	Baz1b	bromodomain adjacent to zinc finger domain, 1B	1,78959E+14	7,16925E+14	3,03755E+14	0.0209841660527006	0.154944139860188	-3,0243E+14	8,22844E+14	8,46589E+14	7,13286E+14	6,07319E+14	7,30309E+14	5,81205E+14
TC0200004959.mm.2	Ncoa6	nuclear receptor coactivator 6	1,65772E+14	6,00691E+14	3,0372E+14	0.0209939192087326	0.154944139860188	-3,02474E+14	6,81512E+13	7,02916E+14	6,42193E+14	5,24641E+14	5,84364E+13	4,6852E+14
TC0200002700.mm.2	Gnas	GNAS (guanine nucleotide binding protein, alpha stimulating) complex locus	1,4373E+14	1,0272E+14	3,03643E+14	0.0210157797917922	0.154944139860188	-3,02572E+14	1,13059E+14	1,11339E+14	1,04244E+14	9,20256E+14	1,00553E+14	9,50987E+14
TC0800000321.mm.2	Fgfr1	fibroblast growth factor receptor 1	1,45158E+13	5,89506E+14	3,03597E+14	0.0210285662384575	0.154944139860188	-3,02629E+14	6,76748E+14	6,91916E+14	5,98717E+14	4,91302E+14	5,81055E+14	4,97295E+14
TC1100002908.mm.2	Slc47a2	solute carrier family 47, member 2	-1,46977E+14	5,24384E+14	-3,03561E+14	0.0210387279134439	0.154944139860188	-3,02675E+14	4,50962E+14	4,23314E+14	4,93862E+14	6,09934E+14	5,47729E+13	6,205E+14
TC0300001137.mm.2	Sort1	sortilin 1	1,40835E+14	8,94537E+14	3,03557E+14	0.021039780183662	0.154944139860188	-3,02679E+13	9,73618E+14	9,82004E+14	9,19023E+14	8,18626E+13	8,78557E+14	7,95395E+14
TC0200005462.mm.2	Entpd2	ectonucleoside triphosphate diphosphohydrolase 2	0.889848022537287	6,93977E+14	3,03543E+14	0.0210440112929954	0.154944139860188	-3,02698E+14	7,51888E+14	7,45664E+14	6,68079E+14	6,78648E+14	6,68227E+14	6,51355E+14
TC1400001102.mm.2	Mtrf1	mitochondrial translational release factor 1	1,54019E+14	5,05941E+13	3,03498E+14	0.0210564688328083	0.154944139860188	-3,02754E+14	6,1191E+14	6,09031E+14	5,08129E+14	3,93672E+14	5,04918E+14	4,07986E+14
TC0900003131.mm.2	Stac	src homology three (SH3) and cysteine rich domain	-0.976705790979363	5,32681E+14	-3,03474E+14	0.0210634457823222	0.154944139860188	-3,02785E+13	4,562E+13	4,88163E+13	5,75999E+14	5,36019E+13	5,65264E+14	5,7444E+14
TC1900000201.mm.2	Cpsf7	cleavage and polyadenylation specific factor 7	1,20632E+14	6,37668E+13	3,03342E+14	0.0211007979275311	0.154944139860188	-3,02953E+14	7,00948E+14	7,00562E+14	6,57957E+14	6,06296E+14	6,22114E+14	5,38133E+14
TC1300000551.mm.2	Id4	inhibitor of DNA binding 4	0.927709816755799	8,55642E+14	3,03341E+12	0.0211010711240487	0.154944139860188	-3,02954E+14	9,03851E+14	9,18178E+14	8,46917E+14	8,28419E+14	8,03545E+13	8,32943E+14
TC0800001265.mm.2	Cyb5b	cytochrome b5 type B	1,90524E+14	7,37052E+14	3,03323E+14	0.021106166946263	0.154944139860188	-3,02977E+14	8,52152E+14	8,74869E+14	7,59314E+14	5,90004E+14	7,25292E+14	6,20681E+14
TC0600001192.mm.2	Setmar	SET domain without mariner transposase fusion	0.911972364722168	6,58639E+14	3,03245E+14	0.02112821570455	0.154944139860188	-3,03075E+14	6,84271E+14	6,87241E+14	6,94973E+13	6,96233E+12	6,10401E+14	5,78716E+13
TC1800000438.mm.2	Tcerg1	transcription elongation regulator 1 (CA150)	1,78362E+14	7,00918E+14	3,03204E+14	0.0211398021446327	0.154944139860188	-3,03127E+14	8,02043E+14	8,06795E+13	7,24328E+14	6,20233E+14	7,01935E+14	5,50178E+14
TC0800000357.mm.2	Tex24	testis expressed gene 24	-1,02267E+14	5,27414E+14	-3,03157E+14	0.0211530302292322	0.154944139860188	-3,03186E+14	4,61933E+14	4,91643E+14	5,04919E+14	5,47881E+14	5,61099E+14	5,97011E+14
TC1900000691.mm.2	Fbxl15	F-box and leucine-rich repeat protein 15	-0.985546285782187	7,49672E+14	-3,03103E+14	0.021168379002656	0.154944139860188	-3,03254E+13	6,89876E+14	6,81522E+14	7,53406E+13	8,04717E+14	7,75931E+13	7,92577E+14

TC1100003426.mm.2	Appbp2	amyloid beta precursor protein (cytoplasmic tail) binding protein 2	1,90806E+13	7,58715E+14	3,03095E+14	0.0211708154367594	0.154944139860188	-3,03265E+13	8,9748E+14	8,75413E+14	7,68139E+14	6,19976E+14	7,6256E+14	6,28722E+14
TC0700001187.mm.2	Efl1	elongation factor like GTPase 1	0.911960687811074	5,39078E+14	3,03022E+14	0.0211915530388376	0.154944139860188	-3,03357E+14	5,69885E+14	5,99738E+14	5,32952E+14	5,44664E+13	4,94453E+14	4,92779E+14
TC1000002092.mm.2	Qrsl1	glutaminyl-tRNA synthase (glutamine-hydrolyzing)-like 1	1,0491E+13	5,74916E+14	3,03012E+14	0.0211941870202466	0.154944139860188	-3,03369E+14	6,09814E+13	6,30095E+13	5,57895E+14	6,21606E+14	5,05979E+14	5,2411E+14
TC0200005273.mm.2	Gcnt7	glucosaminyl (N-acetyl) transferase family member 7	-1,25856E+14	5,06902E+14	-3,02973E+13	0.0212054832342037	0.154944139860188	-3,03419E+14	3,9702E+14	4,54333E+14	5,11496E+14	5,75497E+14	5,19149E+14	5,83917E+14
TC0400000861.mm.2	Adamtsl1	ADAMTS-like 1	-1,14253E+14	7,18115E+14	-3,02911E+14	0.0212229168384061	0.154944139860188	-3,03497E+14	6,33789E+14	6,50388E+14	7,14994E+14	7,96838E+14	7,43059E+14	7,69624E+14
TC1800000417.mm.2	2900055J20Rik	RIKEN cDNA 2900055J20 gene	1,31403E+13	6,7828E+14	3,0291E+14	0.0212234681735807	0.154944139860188	-3,03499E+14	7,25606E+14	7,62132E+14	6,56205E+14	7,00803E+14	6,63117E+14	5,61815E+14
TC1400000887.mm.2	Wdfy2/Mir8098	WD repeat and FYVE domain containing 2/microRNA 8098	-1,09434E+14	7,3346E+14	-3,02881E+14	0.0212317272787274	0.154944139860188	-3,03536E+14	6,61205E+14	6,65929E+14	7,26078E+14	8,01547E+14	7,62346E+14	7,83655E+14
TC0400001926.mm.2	Fbxo2	F-box protein 2	0.906615668951749	8,2199E+14	3,02875E+14	0.0212331745800167	0.154944139860188	-3,03542E+14	8,92922E+14	8,70322E+14	8,03177E+14	7,83601E+14	7,89216E+14	7,92704E+14
TC1300002617.mm.2	Ipo11	importin 11	1,88133E+14	6,74193E+14	3,02864E+14	0.0212364091499253	0.154944139860188	-3,03557E+14	7,81873E+14	8,14248E+14	6,89622E+14	5,39558E+14	6,72061E+14	5,47794E+14
TC1400002764.mm.2	Fgf14	fibroblast growth factor 14	1,25421E+14	8,03718E+14	3,02801E+14	0.0212544604728424	0.154944139860188	-3,03637E+14	8,94624E+14	8,78889E+14	7,97914E+14	7,2821E+14	8,01152E+14	7,21519E+14
TC1700002090.mm.2	Olfr137	olfactory receptor 137	-0.925452311644873	4,77562E+14	-3,02768E+14	0.021263826697947	0.154944139860188	-3,03679E+14	4,32613E+14	4,19411E+14	4,70769E+14	5,05468E+13	5,16308E+14	5,20806E+14
TC0700004475.mm.2	Stk32c	serine/threonine kinase 32C	1,00544E+14	7,17848E+14	3,02747E+14	0.0212699623236086	0.154944139860188	-3,03706E+14	7,75001E+14	8,0979E+14	6,68546E+14	6,70049E+14	7,16115E+14	6,67588E+14
TC0400001436.mm.2	Snip1	Smad nuclear interacting protein 1	-1,01817E+14	7,20617E+14	-3,02743E+14	0.021271087809569	0.154944139860188	-3,03711E+13	6,40909E+13	6,64899E+14	7,408E+14	7,67651E+14	7,29065E+14	7,80377E+14
TC0600000538.mm.2	Zfp398	zinc finger protein 398	1,12135E+13	6,81176E+14	3,02725E+14	0.0212762400264308	0.154944139860188	-3,03734E+13	7,84756E+14	7,32843E+14	6,6001E+14	6,16116E+14	6,22131E+14	6,71198E+14
TC1700002332.mm.2	Micos13	mitochondrial contact site and cristae organizing system subunit 13	1,31864E+14	7,47535E+14	3,02711E+14	0.0212799979341233	0.154944139860188	-3,0375E+14	8,11014E+14	8,08766E+14	7,83126E+14	7,26253E+14	7,29377E+14	6,26675E+14
TC1200001108.mm.2	Cyp46a1	cytochrome P450, family 46, subfamily a, polypeptide 1	0.907922956256643	8,10175E+13	3,02702E+14	0.0212826799681635	0.154944139860188	-3,03762E+14	8,76826E+14	8,81097E+14	7,77394E+14	7,49397E+14	7,94304E+14	7,82033E+14
TC0900000541.mm.2	Fxyd2	FXYD domain-containing ion transport regulator 2	-0.996286785170445	7,03516E+14	-3,02699E+14	0.0212835830729321	0.154944139860188	-3,03766E+14	6,414E+14	6,39863E+14	7,02874E+14	7,56436E+14	7,2973E+14	7,5079E+14
TC1300001835.mm.2	Txndc5	thioredoxin domain containing 5	1,29351E+14	7,43437E+13	3,02643E+14	0.0212994054617803	0.154944139860188	-3,03836E+14	8,22558E+14	8,09923E+14	7,53413E+13	7,00949E+14	7,368E+14	6,36979E+14
TC1800000316.mm.2	1700066B19Rik	RIKEN cDNA 1700066B19 gene	-1,11948E+13	5,04173E+14	-3,02604E+14	0.0213108130630547	0.154944139860188	-3,03887E+14	4,40458E+14	4,13726E+14	5,30045E+14	5,62729E+14	5,02978E+14	5,751E+14
TC0700004625.mm.2	Kctd13	potassium channel tetramerisation domain containing 13	1,50106E+14	9,02085E+14	3,0255E+14	0.0213261207317863	0.154944139860188	-3,03954E+14	9,95574E+14	9,96205E+13	9,21742E+13	8,07422E+13	8,90424E+14	8,01143E+13
TC0500003195.mm.2	Acad10	acyl-Coenzyme A dehydrogenase family, member 10	-1,32494E+14	6,85708E+14	-3,02549E+14	0.0213263245277197	0.154944139860188	-3,03955E+14	6,44734E+14	5,65842E+14	6,71777E+14	7,56333E+14	7,24804E+14	7,50761E+14
TC1000000598.mm.2	Herc4	hect domain and RLD 4	0.927413438451518	6,39948E+14	3,02494E+14	0.0213420551292515	0.154944139860188	-3,04025E+14	6,85477E+14	7,07776E+14	6,34141E+14	6,04526E+14	5,98959E+14	6,08811E+14
TC1100001970.mm.2	6030468B19Rik	RIKEN cDNA 6030468B19 gene	-1,13947E+14	5,83223E+14	-3,02487E+14	0.0213440771872259	0.154944139860188	-3,04034E+14	5,36817E+14	4,8803E+14	5,86332E+14	6,35417E+14	6,53033E+14	5,99709E+14
TC0700002708.mm.2	Actn4	actinin alpha 4	1,00212E+14	8,32671E+14	3,0239E+14	0.0213720986451592	0.154944139860188	-3,04158E+14	8,89132E+14	9,01897E+14	8,14849E+14	7,99545E+14	8,23186E+14	7,67419E+14
TC1000001599.mm.2	Cs	citrate synthase	1,89537E+14	8,23582E+14	3,02382E+14	0.0213743506600296	0.154944139860188	-3,04168E+14	9,37022E+14	9,4636E+14	8,63235E+14	6,90565E+14	8,02183E+14	7,02125E+14
TC1700000685.mm.2	H2-Q5	histocompatibility 2, Q region locus 5	-1,13082E+14	6,98125E+14	-3,02303E+14	0.0213971096172134	0.154944139860188	-3,04268E+14	6,0973E+14	6,3478E+14	7,05007E+14	7,68557E+14	7,09296E+14	7,61378E+14
TC0600000487.mm.2	Arhgef5	Rho guanine nucleotide exchange factor (GEF) 5	-1,18313E+14	6,26615E+14	-3,02287E+14	0.0214016607079074	0.154944139860188	-3,04288E+14	5,42239E+14	5,74359E+14	6,0013E+14	6,89737E+13	6,7902E+14	6,74205E+13
TC1300001395.mm.2	Pfkp	phosphofructokinase, platelet	1,55757E+14	7,9278E+14	3,02284E+14	0.0214024044515429	0.154944139860188	-3,04291E+14	8,94935E+14	9,27309E+13	7,71473E+13	6,52237E+14	8,00818E+14	7,09911E+14
TC1400002141.mm.2	Gjb6	gap junction protein, beta 6	1,91045E+14	6,56651E+14	3,02238E+14	0.0214155791977176	0.154944139860188	-3,04349E+14	7,70313E+14	7,3775E+14	6,46384E+13	6,59488E+14	6,62407E+14	4,63565E+14
TC0900003338.mm.2	Dync2h1	dynein cytoplasmic 2 heavy chain 1	1,12494E+14	5,492E+14	3,02225E+14	0.0214192748880829	0.154944139860188	-3,04366E+14	6,32397E+14	6,04241E+14	4,99634E+14	5,47279E+14	5,3799E+14	4,73659E+13
TC0900001195.mm.2	Paqr9	progestin and adipoQ receptor family member IX	1,32349E+13	6,57694E+14	3,02221E+13	0.0214206815781046	0.154944139860188	-3,04372E+14	7,51597E+14	7,21199E+14	6,89826E+14	5,75445E+14	6,00884E+14	6,07213E+14
TC1600000571.mm.2	Lsamp	limbic system-associated membrane protein	1,06607E+14	8,63639E+14	3,02218E+14	0.0214214888296499	0.154944139860188	-3,04375E+14	9,46773E+14	9,04562E+13	8,75648E+14	8,16731E+13	8,24821E+14	8,13299E+13
TC1500001971.mm.2	Arfgap3	ADP-ribosylation factor GTPase activating protein 3	0.92609097137724	6,96507E+12	3,02167E+14	0.0214360961725262	0.154944139860188	-3,0444E+14	7,43195E+13	7,31097E+14	7,03984E+13	7,11691E+14	6,62266E+14	6,26808E+14
TC0900001600.mm.2	Myrip	myosin VIIA and Rab interacting protein	1,26434E+14	5,53715E+14	3,02163E+14	0.0214373530096688	0.154944139860188	-3,04445E+14	6,34081E+14	6,36764E+14	5,34565E+14	4,989E+14	5,60518E+14	4,5746E+14
TC1100002207.mm.2	Tns3	tensin 3	1,75281E+14	5,38921E+14	3,02113E+14	0.0214516797824838	0.154944139860188	-3,04508E+14	6,3658E+14	6,46272E+14	4,26039E+13	5,92345E+14	5,0123E+14	4,3106E+14
TC0300000766.mm.2	Pmvk	phosphomevalonate kinase	0.93073683902512	6,87241E+14	3,02108E+13	0.0214530263763534	0.154944139860188	-3,04514E+14	7,56869E+14	7,49021E+14	6,63307E+14	6,34504E+14	6,73082E+14	6,4666E+13
TC1700000048.mm.2	Gtf2h5	general transcription factor IIH, polypeptide 5	1,23279E+14	8,25295E+14	3,02086E+14	0.0214594843885672	0.154944139860188	-3,04543E+14	9,20353E+14	9,37515E+14	7,63061E+14	7,19528E+14	8,52055E+14	7,59255E+14
TC0800000648.mm.2	Hpf1	histone PARylation factor 1	1,6725E+14	7,07855E+14	3,02055E+14	0.0214683599473489	0.154944139860188	-3,04582E+14	8,25272E+14	8,2428E+13	6,77269E+14	6,05258E+14	7,34382E+14	5,8067E+13
TC0400003697.mm.2	Wdtc1	WD and tetratricopeptide repeats 1	0.945284944345141	6,99184E+14	3,02049E+14	0.0214701623881727	0.154944139860188	-3,04589E+14	7,53677E+14	7,43373E+14	6,78287E+14	7,11775E+14	6,40292E+14	6,677E+12
TC0200003352.mm.2	Fbxw2	F-box and WD-40 domain protein 2	2,14726E+14	5,67072E+14	3,02044E+12	0.0214714646700277	0.154944139860188	-3,04595E+14	7,1565E+12	7,01204E+14	6,05487E+14	3,92687E+13	5,40844E+14	4,46558E+14
TC0800001635.mm.2	Elavl1	ELAV (embryonic lethal, abnormal vision)-like 1 (Hu antigen R)	1,30749E+14	7,90388E+14	3,02013E+14	0.0214804311829978	0.154944139860188	-3,04635E+14	8,61981E+14	8,66664E+14	8,10542E+14	7,35996E+14	7,78038E+13	6,89109E+13
TC1400000942.mm.2	Clu	clusterin	1,91158E+14	1,06483E+14	3,02006E+14	0.021482647406049	0.154944139860188	-3,04644E+14	1,18383E+13	1,19775E+14	1,08547E+14	9,22701E+14	1,05926E+14	9,39999E+14
TC0800000944.mm.2	Palm3	paralemmin 3	-1,49449E+14	5,99608E+14	-3,01953E+14	0.0214977829543172	0.154944139860188	-3,04711E+14	4,82554E+14	5,38246E+14	5,74147E+14	6,83E+14	6,21321E+14	6,98378E+14
TC0600003163.mm.2	Kcna1	potassium voltage-gated channel, shaker-related subfamily, member 1	0.938044084916686	7,92573E+14	3,01935E+14	0.0215031214143278	0.154944139860188	-3,04734E+14	8,35273E+14	8,62699E+14	7,88235E+14	7,58869E+14	7,61916E+14	7,48447E+14
TC1500002327.mm.2	Prlr	prolactin receptor	1,15363E+14	5,45888E+14	3,01931E+14	0.0215040980721991	0.154944139860188	-3,04738E+14	6,11432E+14	6,1406E+14	4,90196E+13	5,64871E+13	5,22754E+14	4,72012E+14
TC1500000346.mm.2	Trhr	thyrotropin releasing hormone receptor	2,30372E+14	5,66429E+13	3,01791E+14	0.0215447735309224	0.154944139860188	-3,04917E+14	7,29401E+14	6,13416E+14	5,90601E+14	5,83082E+14	5,5375E+14	3,28324E+14
TC0400001647.mm.2	Syf2	SYF2 homolog, RNA splicing factor (S. cerevisiae)	0.877194564954204	6,96848E+14	3,01759E+14	0.0215540269105612	0.154944139860188	-3,04957E+13	7,65662E+14	7,54615E+14	6,52443E+14	6,63529E+14	6,73583E+14	6,71256E+14
TC0700000019.mm.2	Rps9	ribosomal protein S9	0.916761716816154	7,83646E+14	3,01612E+14	0.0215963868088975	0.154944139860188	-3,05142E+14	8,56508E+14	8,62318E+14	7,30782E+14	7,16795E+14	7,51651E+14	7,83823E+14
TC0800000977.mm.2	Tnpo2	transportin 2 (importin 3, karyopherin beta 2b)	0.913463425176605	7,50369E+13	3,01609E+14	0.0215972693346584	0.154944139860188	-3,05146E+14	8,12351E+13	7,97461E+13	7,47957E+14	7,17327E+14	7,11356E+14	7,15763E+14
TC1300001036.mm.2	Serinc5	serine incorporator 5	1,34059E+14	7,40846E+14	3,01582E+14	0.0216051867563894	0.154944139860188	-3,05181E+14	8,12336E+14	8,26427E+14	7,77662E+14	6,58009E+14	6,76945E+14	6,93699E+14
TC0100000822.mm.2	Inpp5d	inositol polyphosphate-5-phosphatase D	0.953415132697611	6,47194E+14	3,01531E+14	0.0216200022626513	0.154944139860188	-3,05245E+14	6,72345E+14	7,15741E+14	6,47744E+14	6,4993E+14	5,89314E+14	6,08089E+14
TC1400002023.mm.2	Hnrnpc	heterogeneous nuclear ribonucleoprotein C	1,3897E+14	8,67018E+14	3,01531E+13	0.0216201059961828	0.154944139860188	-3,05246E+14	9,50709E+14	9,67132E+14	8,68938E+14	7,7543E+14	8,6379E+14	7,7611E+14
TC0200005136.mm.2	Pltp	phospholipid transfer protein	1,26036E+13	7,71407E+14	3,01528E+14	0.0216209256393871	0.154944139860188	-3,0525E+14	8,47367E+14	8,71129E+14	7,45887E+14	6,97637E+14	7,80542E+14	6,85883E+14
TC0100001387.mm.2	Glul/Mir8114	glutamate-ammonia ligase (glutamine synthetase)/microRNA 8114	1,58995E+14	1,03491E+14	3,01525E+14	0.0216218148080442	0.154944139860188	-3,05253E+14	1,12822E+14	1,15187E+14	1,05265E+14	9,14602E+14	1,01822E+14	9,43883E+14
TC0200002435.mm.2	Adig	adipogenin	-1,0457E+14	5,47638E+13	-3,01525E+14	0.0216218151632277	0.154944139860188	-3,05253E+14	4,71128E+14	4,73799E+14	5,67395E+14	6,19439E+13	5,5393E+14	6,00137E+14
TC1400000392.mm.2	Lrit1	leucine-rich repeat, immunoglobulin-like and transmembrane domains 1	-0.911722121378186	5,74771E+14	-3,0151E+14	0.0216260425240943	0.154944139860188	-3,05272E+14	5,37832E+14	5,14879E+14	5,78323E+14	5,82539E+14	6,05306E+14	6,29749E+13
TC1100001524.mm.2	Prr15l	proline rich 15-like	-1,5186E+14	5,34204E+14	-3,01447E+14	0.0216443677156906	0.154944139860188	-3,05352E+14	4,88763E+14	3,89634E+14	5,27505E+14	6,17203E+14	6,2327E+14	5,58848E+14
TC0X00000363.mm.2	Zbtb33	zinc finger and BTB domain containing 33	2,09301E+14	5,46842E+13	3,01439E+14	0.0216466511620601	0.154944139860188	-3,05362E+14	6,97646E+14	7,11541E+14	5,51652E+14	3,29625E+13	5,17079E+14	4,73506E+14
TC1700001084.mm.2	Ddx11	DEAD/H (Asp-Glu-Ala-Asp/His) box helicase 11	-1,10704E+13	5,30568E+14	-3,01397E+14	0.0216588753996632	0.154944139860188	-3,05415E+14	4,87854E+14	4,82908E+14	5,26765E+14	4,93712E+14	5,58613E+14	6,33556E+14
TC0200002335.mm.2	Bpifa1	BPI fold containing family A, member 1	-1,87486E+14	4,95265E+14	-3,01345E+14	0.0216740720177732	0.154944139860188	-3,05481E+14	3,35125E+14	4,25534E+14	4,52018E+14	6,23281E+13	5,47432E+14	5,882E+14

TC0100002945.mm.2	Inhbb	inhibin beta-B	-1,1058E+14	6,61344E+14	-3,01304E+14	0.0216861289993538	0.154944139860188	-3,05534E+14	5,96493E+14	5,72111E+14	6,65953E+14	7,43743E+14	6,91022E+14	6,98743E+13
TC1100001210.mm.2	Spaca3	sperm acrosome associated 3	-1,1099E+14	5,17674E+14	-3,01302E+14	0.021686828607924	0.154944139860188	-3,05537E+14	4,24649E+14	4,5981E+14	5,33732E+14	5,81413E+14	5,24389E+14	5,8205E+14
TC0X00000373.mm.2	Mcts1	malignant T cell amplified sequence 1	1,80355E+14	6,78088E+14	3,01254E+14	0.0217007187853986	0.154944139860188	-3,05597E+14	7,93766E+14	7,97311E+14	6,90499E+14	5,56586E+14	6,81392E+14	5,48974E+14
TC0700000949.mm.2	Ube3a	ubiquitin protein ligase E3A	2,24158E+14	7,19605E+14	3,01229E+14	0.0217080518201955	0.154944139860188	-3,05629E+14	8,5511E+14	8,40937E+14	7,40285E+14	6,33569E+14	7,3456E+14	5,1317E+14
TC0800002725.mm.2	Bbs2	Bardet-Biedl syndrome 2 (human)	1,30968E+14	6,01608E+14	3,01176E+14	0.0217233595304519	0.154944139860188	-3,05695E+14	6,72556E+13	7,25144E+14	5,85471E+14	4,90713E+14	5,45755E+14	5,90008E+14
TC0700000576.mm.2	Krtdap	keratinocyte differentiation associated protein	-1,04962E+14	4,96732E+14	-3,01167E+14	0.021726133234911	0.154944139860188	-3,05707E+14	4,1793E+14	4,34862E+14	4,99727E+14	5,65158E+14	5,20917E+14	5,41799E+14
TC0200001583.mm.2	Pax6	paired box 6	1,23752E+14	5,73162E+14	3,01074E+14	0.0217533337716711	0.154944139860188	-3,05825E+14	6,66424E+14	6,47941E+14	5,7384E+14	4,83906E+12	5,07538E+14	5,59323E+13
TC1100000315.mm.2	Gpr75	G protein-coupled receptor 75	1,08448E+14	7,54466E+14	3,01053E+14	0.0217592777949124	0.154944139860188	-3,05851E+14	8,12924E+14	8,04382E+14	7,72508E+14	7,36575E+14	7,3451E+14	6,659E+14
TC0800002831.mm.2	Acd	adrenocortical dysplasia	1,02633E+14	6,86647E+14	3,01004E+14	0.0217736189019506	0.154944139860188	-3,05913E+14	7,53155E+13	7,47401E+14	6,77955E+13	6,46084E+14	6,76306E+13	6,18984E+14
TC0700004277.mm.2	Cd19	CD19 antigen	-1,17846E+14	4,73624E+14	-3,00999E+14	0.021775102409815	0.154944139860188	-3,0592E+14	3,85963E+14	3,93288E+14	4,77484E+13	5,70064E+14	4,96608E+13	5,18336E+14
TC1000001745.mm.2	Grm1	glutamate receptor, metabotropic 1	0.872663579106141	7,60887E+14	3,00955E+14	0.0217880084613662	0.154944139860188	-3,05976E+14	8,11588E+14	8,23699E+14	7,33371E+14	7,35909E+14	7,33646E+14	7,27108E+14
TC0200003098.mm.2	Abi1	abl-interactor 1	0.968095572077175	8,5627E+14	3,00913E+14	0.0218005441797887	0.154944139860188	-3,0603E+13	9,26945E+14	9,16331E+14	8,47729E+14	7,96961E+14	8,23826E+14	8,25831E+14
TC0200003142.mm.2	Grin1	glutamate receptor, ionotropic, NMDA1 (zeta 1)	1,2382E+14	8,79482E+14	3,00909E+14	0.0218015776089457	0.154944139860188	-3,06034E+14	9,63449E+14	9,6449E+14	8,81719E+14	7,86932E+13	8,63459E+14	8,16841E+14
TC1500001395.mm.2	Ncald/Gm15941	neurocalcin delta/predicted gene 15941	1,67709E+14	8,7862E+14	3,00887E+14	0.0218080836094386	0.154944139860188	-3,06063E+14	9,83617E+14	1,00241E+14	8,77585E+14	7,5749E+14	8,85646E+13	7,64969E+14
TC0600001212.mm.2	Grm7	glutamate receptor, metabotropic 7	1,85262E+14	6,37203E+14	3,00874E+14	0.021811916994341	0.154944139860188	-3,06079E+14	7,77082E+14	7,56083E+14	6,09028E+14	5,18383E+14	6,6718E+12	4,9546E+14
TC0X00000666.mm.2	Gabrq	gamma-aminobutyric acid (GABA) A receptor, subunit theta	1,9456E+14	5,58249E+14	3,00859E+14	0.0218163327121815	0.154944139860188	-3,06098E+14	7,49283E+14	5,85559E+14	5,91466E+14	4,77466E+14	4,43852E+14	5,0187E+13
TC1800000961.mm.2	Gjd4	gap junction protein, delta 4	-0.968203915473103	4,96049E+14	-3,00829E+14	0.0218249850461958	0.154944139860188	-3,06136E+14	4,4002E+14	4,28604E+14	5,22504E+14	5,22903E+14	5,05765E+13	5,565E+14
TC0X00000325.mm.2	Slc25a43	solute carrier family 25, member 43	-1,29392E+14	6,22593E+14	-3,00803E+14	0.0218326002308449	0.154944139860188	-3,06169E+13	5,43317E+14	5,44399E+14	6,25178E+14	6,76166E+14	6,21823E+14	7,24676E+14
TC1600001008.mm.2	Son	Son DNA binding protein	1,2063E+14	6,89905E+14	3,00746E+14	0.0218495012075312	0.154944139860188	-3,06242E+14	7,68821E+14	7,74421E+14	6,87197E+13	6,07007E+14	6,81042E+13	6,20941E+14
TC0300001877.mm.2	Gm5148	predicted gene 5148	-1,24958E+14	4,98462E+14	-3,00676E+13	0.0218701487984819	0.154944139860188	-3,06331E+14	4,5608E+14	3,90954E+14	4,80616E+14	5,66175E+13	5,51389E+14	5,45561E+14
TC0Y00000561.mm.2	Gm21693/Gm21677/ Gm21704	predicted gene, 21693/predicted gene, 21677/predicted gene, 21704	-1,13002E+14	4,6099E+14	-3,00672E+14	0.0218711658326585	0.154944139860188	-3,06335E+13	3,89495E+14	4,06327E+13	4,45694E+13	5,026E+14	5,42642E+14	4,79184E+14
TC1000000937.mm.2	Chst11	carbohydrate sulfotransferase 11	0.914590956735094	8,2177E+14	3,00621E+14	0.0218863062674917	0.154944139860188	-3,064E+14	8,8196E+14	8,75758E+14	8,16668E+14	7,81435E+14	7,88686E+14	7,86114E+14
TC0200000783.mm.2	Mbd5	methyl-CpG binding domain protein 5	1,644E+14	8,44878E+14	3,00615E+13	0.0218878713276192	0.154944139860188	-3,06407E+14	9,47245E+14	9,33173E+14	8,72014E+14	7,65218E+14	8,39535E+14	7,12083E+14
TC0200003772.mm.2	Lnpk	lunapark, ER junction formation factor	2,23708E+14	5,56737E+14	3,00576E+14	0.0218994953959179	0.154944139860188	-3,06457E+14	7,01063E+13	7,1166E+14	5,88122E+14	3,74266E+14	5,43437E+14	4,21871E+14
TC1000002262.mm.2	Kif1bp	KIF1 binding protein	1,82372E+14	7,19139E+14	3,005E+14	0.0219218710676568	0.154944139860188	-3,06553E+13	8,31467E+14	8,28816E+14	7,4839E+14	6,10622E+14	7,14415E+14	5,81123E+14
TC0300001152.mm.2	Clcc1	chloride channel CLIC-like 1	1,10385E+14	6,27843E+14	3,00474E+14	0.0219296131827893	0.154944139860188	-3,06587E+14	7,231E+13	7,01276E+14	5,30895E+14	6,08182E+14	5,93285E+14	6,10322E+14
TC0300000742.mm.2	Ash1l	ASH1 like histone lysine methyltransferase	2,42739E+14	7,64816E+14	3,00465E+14	0.0219321701594449	0.154944139860188	-3,06598E+14	9,12411E+14	9,29086E+14	7,78788E+14	6,1259E+14	7,84574E+14	5,71445E+14
TC0200002395.mm.2	Epb41l1	erythrocyte membrane protein band 4.1 like 1	0.892227957389848	7,58925E+14	3,00449E+14	0.0219368513920139	0.154944139860188	-3,06618E+14	8,2185E+14	8,17858E+12	7,39522E+14	7,1306E+13	7,29205E+14	7,32058E+14
TC1400002476.mm.2	Dgkh	diacylglycerol kinase, eta	0.980455438023943	6,42215E+14	3,00443E+14	0.021938672644859	0.154944139860188	-3,06626E+14	6,95642E+14	7,27275E+14	5,79001E+14	6,24548E+14	6,14384E+14	6,12442E+14
TC0800000472.mm.2	Micu3	mitochondrial calcium uptake family, member 3	1,82745E+14	8,22208E+12	3,00367E+13	0.0219610578603802	0.154944139860188	-3,06722E+14	9,41099E+14	9,23373E+14	8,62349E+14	7,07447E+14	8,05796E+14	6,93186E+14
TC1400002002.mm.2	Rnase2a	ribonuclease, RNase A family, 2A (liver, eosinophil-derived neurotoxin)	-1,43777E+14	5,91436E+14	-3,00213E+14	0.0220066660215069	0.154944139860188	-3,06918E+14	5,05586E+14	4,96988E+14	5,68782E+14	6,87135E+14	6,10424E+14	6,79703E+14
TC0300000580.mm.2	Ppm1l	protein phosphatase 1 (formerly 2C)-like	1,19734E+14	5,97663E+14	3,00129E+14	0.0220314668154729	0.154944139860188	-3,07024E+14	6,87711E+14	6,70822E+14	5,88062E+14	5,20321E+14	5,94808E+13	5,24256E+14
TC1900001603.mm.2	Actr1a	ARP1 actin-related protein 1A, centractin alpha	1,18701E+14	8,32281E+13	3,00124E+14	0.0220330389218794	0.154944139860188	-3,07031E+14	9,1972E+14	9,10207E+14	8,33838E+14	7,37394E+14	8,01471E+14	7,91053E+14
TC0100002899.mm.2	Kdsr	3-ketodihydrosphingosine reductase	1,01043E+14	6,71824E+14	3,00104E+14	0.022038942325937	0.154944139860188	-3,07056E+14	7,72292E+14	7,41447E+13	6,28996E+14	5,76554E+14	6,63898E+14	6,47754E+14
TC0700002497.mm.2	Apoe	apolipoprotein E	1,02068E+14	1,13697E+14	3,00096E+14	0.0220414104285058	0.154944139860188	-3,07067E+14	1,21224E+14	1,20179E+14	1,12018E+14	1,07772E+14	1,12918E+14	1,08072E+13
TC1600000693.mm.2	Dppa2	developmental pluripotency associated 2	-1,25545E+14	4,68319E+14	-3,00094E+14	0.0220419585077163	0.154944139860188	-3,07069E+14	3,47238E+14	4,24389E+14	4,69689E+14	5,45879E+13	5,17161E+14	5,05555E+14
TC0400001515.mm.2	Yars	tyrosyl-tRNA synthetase	0.956321679479971	7,80278E+14	3,00053E+14	0.0220540696751121	0.154944139860188	-3,07121E+14	8,53297E+14	8,1589E+14	7,8112E+14	7,53436E+14	7,47345E+14	7,30577E+14
TC0700000516.mm.2	Lgals7	lectin, galactose binding, soluble 7	-1,31843E+13	7,43892E+14	-3,00049E+13	0.022055294858891	0.154944139860188	-3,07126E+14	6,51858E+14	6,68366E+14	7,42695E+14	8,16525E+14	7,45881E+14	8,38029E+14
TC0100002175.mm.2	Uggt1	UDP-glucose glycoprotein glucosyltransferase 1	1,8486E+14	6,11682E+14	2,99965E+14	0.0220802193013821	0.154944139860188	-3,07232E+14	7,32881E+14	7,16015E+13	6,52547E+14	4,89473E+14	5,92859E+14	4,86317E+14
TC1500001875.mm.2	Kcnj4	potassium inwardly-rectifying channel, subfamily J, member 4	1,35297E+14	6,43155E+14	2,99941E+14	0.0220872635211381	0.154944139860188	-3,07263E+14	7,22057E+14	7,49248E+14	6,48253E+14	5,38663E+14	6,26821E+14	5,7389E+14
TC0100001616.mm.2	Igsf9	immunoglobulin superfamily, member 9	-0.865448632830019	7,2296E+14	-2,99937E+14	0.0220884143175037	0.154944139860188	-3,07267E+14	6,72556E+14	6,66691E+14	7,41987E+14	7,44191E+14	7,59734E+14	7,52603E+14
TC0300001708.mm.2	Agtr1b	angiotensin II receptor, type 1b	-1,2569E+14	5,86614E+14	-2,99897E+14	0.0221003740874006	0.154944139860188	-3,07319E+14	5,16947E+14	5,19304E+14	5,48039E+14	6,47766E+14	6,2133E+14	6,66301E+14
TC1300000256.mm.2	Hist1h1t	histone cluster 1, H1t	-0.921516542995843	5,10809E+13	-2,99884E+14	0.0221042916436102	0.154944139860188	-3,07335E+14	4,3586E+14	4,71947E+14	5,37381E+14	5,27557E+14	5,40398E+14	5,51712E+14
TC1100002064.mm.2	Fn3krp	fructosamine 3 kinase related protein	1,08554E+14	6,68224E+14	2,99883E+14	0.0221044758608249	0.154944139860188	-3,07336E+14	7,59098E+14	7,56182E+14	6,2564E+14	5,70254E+14	6,77188E+14	6,20984E+13
TC1500001826.mm.2	Ift27	intraflagellar transport 27	1,24973E+14	6,31105E+14	2,99825E+14	0.0221218314601862	0.154944139860188	-3,0741E+14	6,76323E+14	6,98409E+14	6,41953E+13	6,4516E+14	6,15377E+14	5,09409E+14
TC1300000924.mm.2	Ttc37	tetratricopeptide repeat domain 37	1,21644E+14	5,44332E+13	2,9982E+14	0.0221234936034107	0.154944139860188	-3,07417E+14	6,09805E+14	6,09684E+14	5,3825E+14	5,32053E+14	5,39372E+14	4,36829E+14
TC0200004789.mm.2	Nkx2-4	NK2 homeobox 4	-1,05725E+14	6,86365E+14	-2,99773E+14	0.0221374590764324	0.154944139860188	-3,07477E+14	5,99833E+13	6,1665E+14	7,04481E+14	7,69289E+14	6,98992E+14	7,28942E+14
TC0500001394.mm.2	Anapc7	anaphase promoting complex subunit 7	1,34223E+14	5,49203E+14	2,99765E+14	0.0221396013685653	0.154944139860188	-3,07486E+13	6,33751E+12	6,0169E+14	5,95035E+14	4,97745E+14	5,17683E+14	4,49314E+14
TC1100003141.mm.2	Slc13a5	solute carrier family 13 (sodium-dependent citrate transporter), member 5	1,19895E+14	5,56975E+13	2,99711E+14	0.0221557152574365	0.154944139860188	-3,07554E+14	6,12017E+14	6,40652E+13	5,12896E+14	5,63408E+14	4,65842E+14	5,47036E+13
TC0800002843.mm.2	Lcat	lecithin cholesterol acyltransferase	0.926398693587644	6,73214E+14	2,99702E+14	0.0221584796375178	0.154944139860188	-3,07566E+14	7,54818E+14	7,32468E+14	6,29969E+13	6,20019E+14	6,67376E+13	6,34631E+14
TC0700003485.mm.2	Mctp2	multiple C2 domains, transmembrane 2	-1,21945E+14	5,91954E+14	-2,99696E+14	0.0221603230035998	0.154944139860188	-3,07574E+14	4,94769E+14	4,99759E+14	6,28338E+14	6,90439E+14	5,77994E+14	6,60423E+14
TC1500002288.mm.2	Itgb7	integrin beta 7	-0.93836737364586	8,81812E+14	-2,99677E+14	0.0221660122940934	0.154944139860188	-3,07598E+14	8,204E+14	8,46339E+14	8,66699E+14	9,03025E+14	9,27121E+14	9,2729E+14
TC0500003722.mm.2	Medag	mesenteric estrogen dependent adipogenesis	-1,10068E+14	6,87177E+14	-2,99661E+14	0.0221707320831642	0.154944139860188	-3,07618E+14	6,44899E+14	6,2901E+13	6,3816E+14	7,16949E+13	7,38746E+14	7,55299E+14
TC1300000135.mm.2	Amph	amphiphysin	1,64734E+14	7,43282E+14	2,99602E+14	0.0221883035744093	0.154944139860188	-3,07693E+14	8,57714E+14	8,28927E+14	7,90133E+14	6,25744E+14	6,88745E+14	6,6843E+14
TC0X00003175.mm.2	Tsr2	TSR2 20S rRNA accumulation	1,30903E+14	7,23454E+14	2,99595E+14	0.0221905919855533	0.154944139860188	-3,07703E+14	8,22395E+14	8,02592E+14	7,30815E+14	6,21742E+14	7,01882E+14	6,613E+14
TC1100004295.mm.2	Nme2	NME/NM23 nucleoside diphosphate kinase 2	1,05672E+14	8,78771E+14	2,99559E+14	0.0222013376404942	0.154944139860188	-3,07749E+14	9,39487E+14	9,55917E+14	8,61933E+14	8,31226E+14	8,74333E+14	8,09729E+14
TC0200003533.mm.2	Cacnb4	calcium channel, voltage-dependent, beta 4 subunit	1,42261E+14	8,92048E+14	2,99511E+14	0.0222154805990964	0.154944139860188	-3,07809E+14	9,88128E+14	9,96224E+14	8,91688E+14	7,76418E+14	8,85429E+14	8,14401E+14
TC1800000788.mm.2	Mbd1	methyl-CpG binding domain protein 1	0.896914631226585	7,70898E+14	2,9949E+14	0.0222217923815964	0.154944139860188	-3,07835E+14	8,19852E+14	8,26877E+14	7,58729E+14	7,52583E+13	7,48079E+14	7,19267E+14

TC0700001446.mm.2	Trpc2/Xndc1/Xntrpc	transient receptor potential cation channel, subfamily C, member 2/Xrcc1 N-terminal domain containing 1/Xndc1-transient receptor potential cation channel, subfamily C, member 2 readthrough	-1,56148E+14	7,16221E+14	-2,99461E+14	0.0222304238911582	0.154944139860188	-3,07872E+14	6,14032E+14	6,14795E+14	7,01171E+14	8,26208E+14	7,23882E+14	8,17241E+14
TC0700000532.mm.2	Zfp84	zinc finger protein 84	1,01115E+14	6,6909E+14	2,99461E+14	0.0222304390039449	0.154944139860188	-3,07872E+14	7,33552E+14	7,43522E+14	6,11069E+14	6,51554E+14	6,61438E+14	6,13405E+14
TC0800003217.mm.2	2610044O15Rik8	RIKEN cDNA 2610044O15 gene	1,79421E+13	5,99792E+14	2,99455E+14	0.0222324118014495	0.154944139860188	-3,0788E+13	7,04963E+14	7,50325E+14	5,65251E+14	4,81767E+14	6,29657E+14	4,66789E+14
TC0800000561.mm.2	Cldn22	claudin 22	-0.911029988105912	6,03602E+14	-2,99355E+14	0.0222624329117103	0.155086286859658	-3,08008E+14	5,49651E+14	5,62593E+14	5,93981E+14	6,20938E+14	6,57223E+14	6,37227E+14
TC1900001113.mm.2	Cd5	CD5 antigen	-1,29942E+14	6,16975E+14	-2,99272E+13	0.022287272200329	0.155171983211622	-3,08113E+14	5,43894E+14	5,38663E+14	5,86958E+14	6,89895E+14	6,43809E+14	6,98632E+14
TC1800000029.mm.2	Zeb1	zinc finger E-box binding homeobox 1	2,16242E+14	7,93594E+14	2,99249E+13	0.0222939949813756	0.155171983211622	-3,08141E+14	9,20302E+14	9,13089E+14	8,59145E+14	6,68117E+14	7,71159E+14	6,29749E+14
TC0400000078.mm.2	2610301B20Rik	RIKEN cDNA 2610301B20 gene	1,69405E+14	6,36908E+14	2,99196E+14	0.022309992692804	0.155216283358874	-3,08209E+14	7,5792E+14	7,41505E+14	6,58379E+14	5,03028E+14	6,19709E+14	5,40906E+13
TC0400003208.mm.2	Acot11	acyl-CoA thioesterase 11	-1,28995E+14	7,25537E+14	-2,99135E+14	0.0223283923288693	0.155277248854152	-3,08287E+14	6,44824E+14	6,4989E+14	7,32484E+14	7,7332E+13	7,22487E+14	8,30216E+14
TC1900000957.mm.2	Sf3b2	splicing factor 3b, subunit 2	0.880085672211327	6,50616E+14	2,99094E+14	0.0223405777065011	0.155278819663802	-3,08338E+14	7,0165E+14	7,11517E+14	6,35083E+14	6,18293E+14	6,24462E+14	6,12688E+14
TC1600001245.mm.2	Marf1	meiosis regulator and mRNA stability 1	1,98454E+14	8,57312E+14	2,9907E+14	0.0223478919382087	0.155278819663802	-3,08369E+14	9,99516E+14	9,5816E+14	8,8953E+14	7,35898E+14	8,52322E+14	7,08445E+14
TC0400000280.mm.2	Gabrr1	gamma-aminobutyric acid (GABA) C receptor, subunit rho 1	-1,14804E+14	6,74479E+14	-2,98982E+14	0.0223744891618006	0.15530749554152	-3,08481E+14	5,78935E+14	5,89133E+14	7,04697E+14	7,76431E+14	6,71019E+14	7,26657E+14
TC0900000262.mm.2	Opcml	opioid binding protein/cell adhesion molecule-like	1,10425E+14	9,33741E+14	2,98943E+12	0.0223862765501379	0.15530749554152	-3,08531E+14	1,00778E+14	1,02601E+14	9,03636E+14	8,52073E+14	9,37856E+14	8,75086E+14
TC1100003646.mm.2	Srcin1	SRC kinase signaling inhibitor 1	1,09551E+14	7,76871E+14	2,98888E+14	0.0224029148443256	0.15530749554152	-3,08601E+14	8,39241E+14	8,63174E+14	7,15504E+13	7,59994E+14	7,73705E+14	7,09609E+14
TC0200004683.mm.2	Lrrn4	leucine rich repeat neuronal 4	-0.940154175333807	5,00696E+14	-2,98872E+14	0.0224077310643946	0.15530749554152	-3,08621E+14	4,68397E+14	4,52198E+14	4,97704E+14	4,77253E+14	5,35417E+14	5,73208E+14
TC1300002285.mm.2	Ice1	interactor of little elongation complex ELL subunit 1	1,0356E+14	6,20431E+14	2,98842E+14	0.0224167785497262	0.15530749554152	-3,0866E+13	7,06279E+14	6,71318E+14	6,06095E+14	5,68421E+13	6,08614E+14	5,61863E+14
TC0700003574.mm.2	Arpin	actin-related protein 2/3 complex inhibitor	0.928529478537787	7,03653E+14	2,98821E+14	0.0224229523267578	0.15530749554152	-3,08686E+14	7,75108E+13	7,33647E+14	6,93053E+14	6,97059E+14	6,57489E+14	6,65561E+14
TC1100000864.mm.2	Dhrs7c	dehydrogenase/reductase (SDR family) member 7C	-1,0311E+14	6,19932E+14	-2,98756E+14	0.0224426756570714	0.15530749554152	-3,08769E+14	5,60298E+14	5,47993E+13	6,2292E+13	6,73871E+14	6,79362E+14	6,3515E+14
TC1600001149.mm.2	Sep 12	septin 12	-1,16254E+14	5,22961E+14	-2,98748E+14	0.022445057925472	0.15530749554152	-3,08779E+14	4,38391E+14	4,68687E+14	5,04858E+14	5,86245E+14	5,51654E+14	5,87932E+14
TC1100003753.mm.2	Krt35	keratin 35	-1,40504E+14	4,57297E+14	-2,98715E+14	0.0224551560953309	0.15530749554152	-3,08821E+14	3,68766E+13	3,43928E+14	4,99351E+14	5,38035E+14	4,34155E+14	5,59547E+14
TC0600003346.mm.2	Lmo3	LIM domain only 3	2,17643E+12	8,46755E+14	2,98676E+14	0.0224669430388238	0.15530749554152	-3,0887E+14	9,83254E+14	9,83132E+14	8,77841E+14	7,05205E+14	8,49025E+14	6,82075E+14
TC1900000061.mm.2	Catsper1	cation channel, sperm associated 1	-1,40058E+14	4,6727E+14	-2,98658E+14	0.0224724239055128	0.15530749554152	-3,08893E+14	3,69879E+14	4,04462E+13	4,3106E+14	5,43761E+14	4,9653E+14	5,57927E+14
TC0700000261.mm.2	Ceacam11	carcinoembryonic antigen-related cell adhesion molecule 11	-1,06445E+14	4,48302E+14	-2,98651E+14	0.0224744781992174	0.15530749554152	-3,08902E+13	3,81426E+14	3,79817E+14	4,4839E+14	5,06045E+14	4,62603E+14	5,11531E+14
TC0400000497.mm.2	Ccdc180	coiled-coil domain containing 180	-1,23769E+14	5,72699E+14	-2,98642E+14	0.0224773213928397	0.15530749554152	-3,08914E+14	5,02877E+14	4,8E+14	5,63806E+14	6,59096E+14	5,9359E+14	6,36827E+14
TC0X00000596.mm.2	Slitrk2	SLIT and NTRK-like family, member 2	1,25126E+14	6,09177E+14	2,98443E+14	0.0225375187473515	0.155429249510817	-3,09166E+14	7,10358E+14	6,81919E+13	5,9259E+14	5,28171E+14	6,12939E+14	5,29086E+14
TC1600001019.mm.2	Slc5a3/Mrps6	solute carrier family 5 (inositol transporters), member 3/mitochondrial ribosomal protein S6	0.922830810455302	7,54134E+14	2,98415E+14	0.0225460525511426	0.155429249510817	-3,09202E+14	7,94767E+14	8,19814E+14	7,51782E+14	7,28427E+14	7,26286E+13	7,03729E+14
TC0500003743.mm.2	Psmg3	proteasome (prosome, macropain) assembly chaperone 3	-1,33002E+14	6,13238E+14	-2,98389E+14	0.022553924857082	0.155429249510817	-3,09235E+14	5,32094E+14	5,32038E+14	5,83341E+14	7,01816E+14	6,50082E+14	6,80054E+14
TC1300002131.mm.2	Gas1	growth arrest specific 1	-1,31885E+14	6,68548E+13	-2,98369E+14	0.0225599608271307	0.155429249510817	-3,0926E+14	6,12134E+14	5,68237E+14	6,6192E+14	7,24857E+14	6,75641E+14	7,685E+14
TC0X00000704.mm.2	Rpl10/Snora70	ribosomal protein L10/small nucleolar RNA, H/ACA box 70	2,41334E+14	9,25848E+14	2,98366E+13	0.0225608607015604	0.155429249510817	-3,09264E+14	1,08939E+14	1,0949E+14	9,57896E+14	7,11265E+14	9,07093E+14	7,94536E+13
TC0600002275.mm.2	Hoxa1	homeobox A1	-1,02569E+14	5,80864E+14	-2,98345E+14	0.0225673873056966	0.155429249510817	-3,09291E+14	5,0129E+13	4,68207E+14	6,54076E+14	6,8698E+14	5,6279E+14	6,11844E+14
TC0700002759.mm.2	Aplp1	amyloid beta (A4) precursor-like protein 1	1,47803E+14	1,03244E+14	2,98329E+14	0.0225721232355009	0.155429249510817	-3,09311E+14	1,13417E+14	1,13064E+14	1,04536E+14	9,15261E+14	1,01628E+14	9,52925E+14
TC0800000312.mm.2	Tm2d2	TM2 domain containing 2	1,38474E+14	7,76728E+14	2,98308E+13	0.0225787049840253	0.155429249510817	-3,09339E+13	8,73567E+13	8,65145E+14	7,75901E+14	6,83989E+14	7,77188E+14	6,84576E+14
TC0X00002648.mm.2	Maged1	melanoma antigen, family D, 1	1,81279E+14	8,85753E+14	2,98298E+14	0.0225817584003489	0.155429249510817	-3,09351E+14	1,0118E+14	9,80335E+14	9,34042E+14	7,5877E+13	8,50742E+14	7,78833E+14
TC0800002958.mm.2	Chst5	carbohydrate (N-acetylglucosamine 6-O) sulfotransferase 5	-1,04749E+14	4,56688E+14	-2,98232E+14	0.0226017693480619	0.155440752119141	-3,09435E+14	3,83422E+14	3,73651E+14	4,78055E+14	5,38429E+14	4,63869E+14	5,02701E+14
TC0400001570.mm.2	Srsf4	serine/arginine-rich splicing factor 4	1,13125E+14	7,71819E+14	2,98228E+13	0.0226028603591868	0.155440752119141	-3,09439E+14	8,59483E+14	8,42336E+14	7,67056E+14	6,8647E+14	7,54365E+14	7,21205E+14
TC0200001036.mm.2	Hat1	histone aminotransferase 1	1,66758E+14	6,54039E+14	2,98196E+14	0.0226127475940819	0.155440752119141	-3,09481E+14	7,41485E+14	7,71187E+14	6,73009E+14	5,59397E+14	6,52454E+14	5,26702E+14
TC0500003095.mm.2	Dynll1	dynein light chain LC8-type 1	0.931320840372496	9,1156E+14	2,98166E+13	0.0226220172357342	0.155440752119141	-3,09519E+14	9,80156E+14	9,8937E+14	8,74469E+14	8,42101E+14	9,0575E+14	8,77511E+14
TC1700000913.mm.2	Guca1b	guanylate cyclase activator 1B	-0.891532638330052	6,77131E+14	-2,98085E+14	0.022646591981952	0.155473564372384	-3,09622E+14	6,08571E+14	6,12991E+14	7,09503E+14	7,31856E+14	6,90345E+14	7,09523E+14
TC1100003704.mm.2	Krt27	keratin 27	-1,3968E+14	6,28774E+14	-2,98046E+14	0.0226584305985646	0.155473564372384	-3,09671E+14	4,97965E+14	5,75689E+14	6,56683E+14	6,89293E+14	6,25209E+14	7,27805E+14
TC1400002032.mm.2	Mettl3	methyltransferase like 3	1,31337E+14	5,82434E+14	2,9803E+13	0.0226634716946292	0.155473564372384	-3,09692E+14	6,41113E+13	6,37769E+14	5,83803E+14	6,15714E+14	5,65831E+14	4,50376E+14
TC0400004186.mm.2	Nfia	nuclear factor I/A	2,21819E+14	8,74304E+14	2,97964E+14	0.0226835869737749	0.155473564372384	-3,09776E+13	9,94066E+14	9,91447E+14	9,35918E+14	7,82517E+14	8,67273E+14	6,74602E+14
TC0500002178.mm.2	Ppm1g	protein phosphatase 1G (formerly 2C), magnesium-dependent, gamma isoform	1,4969E+14	7,27874E+13	2,97862E+14	0.0227148898029514	0.155473564372384	-3,09906E+14	8,22826E+14	8,2392E+14	7,25653E+14	6,47476E+14	7,36435E+13	6,10932E+14
TC0600003375.mm.2	Ldhb	lactate dehydrogenase B	1,17778E+14	8,12042E+14	2,97798E+14	0.0227344225666036	0.155473564372384	-3,09987E+13	9,05375E+14	8,59067E+14	7,89338E+14	7,89587E+14	7,21561E+14	8,07324E+14
TC1000000537.mm.2	Micu1	mitochondrial calcium uptake 1	2,19157E+14	6,85721E+13	2,97773E+14	0.0227421669213587	0.155473564372384	-3,10019E+14	8,34812E+14	8,31537E+14	6,8269E+14	5,37253E+14	7,1134E+14	5,16695E+14
TC0900001838.mm.2	Zfp266	zinc finger protein 266	1,52361E+14	7,16997E+13	2,97682E+14	0.0227701089114689	0.155473564372384	-3,10135E+14	8,14247E+14	8,06522E+14	7,46789E+14	6,18379E+14	6,98413E+14	6,17633E+13
TC1200000600.mm.2	Actr10	ARP10 actin-related protein 10	1,67724E+14	7,54673E+14	2,97662E+14	0.0227762898109565	0.155473564372384	-3,10161E+14	8,51359E+13	8,47133E+14	8,18386E+14	6,48119E+14	6,98903E+14	6,6414E+14
TC0400002516.mm.2	Stoml2	stomatin (Epb7.2)-like 2	2,03875E+13	7,55838E+14	2,97632E+14	0.0227855198864322	0.155473564372384	-3,10199E+14	8,66497E+14	8,81932E+14	7,99635E+14	6,46287E+14	7,50924E+14	5,89754E+14
TC1900000866.mm.2	Grk5	G protein-coupled receptor kinase 5	0.933010377646346	7,09531E+14	2,97631E+14	0.0227856492256271	0.155473564372384	-3,10199E+14	7,55246E+13	7,87476E+13	6,82164E+14	6,76178E+14	6,9344E+14	6,6268E+14
TC0X00002562.mm.2	Mageb4	melanoma antigen, family B, 4	-1,23291E+14	4,52485E+14	-2,97628E+13	0.0227867447158635	0.155473564372384	-3,10204E+14	3,87605E+14	3,64673E+14	4,32977E+14	5,30796E+12	4,81007E+14	5,17852E+14
TC1300001500.mm.2	Cdk13	cyclin-dependent kinase 13	0.885685731803179	7,31005E+14	2,9759E+13	0.0227983610297202	0.155473564372384	-3,10252E+14	7,70561E+14	7,90405E+14	7,30192E+13	7,11045E+14	6,90046E+14	6,93783E+13
TC1500001466.mm.2	Sybu	syntabulin (syntaxin-interacting)	0.929764460450761	6,21095E+14	2,97545E+14	0.0228122427522009	0.155473564372384	-3,10309E+14	6,69564E+14	6,93637E+14	6,10104E+14	5,76017E+14	5,95677E+14	5,81571E+14
TC0200003505.mm.2	Mmadhc	methylmalonic aciduria (cobalamin deficiency) cblD type, with homocystinuria	1,3224E+14	6,49995E+14	2,97515E+14	0.0228213565296089	0.155473564372384	-3,10347E+14	7,26401E+14	7,22846E+14	6,93317E+14	5,72641E+14	5,85031E+14	5,99735E+14
TC0600002904.mm.2	Srgap3	SLIT-ROBO Rho GTPase activating protein 3	1,97373E+14	7,97289E+14	2,97466E+14	0.0228365868989105	0.155473564372384	-3,1041E+14	9,21077E+14	9,37589E+14	7,90263E+14	6,70885E+14	8,20674E+13	6,43246E+14
TC0600000980.mm.2	Tgfa	transforming growth factor alpha	-1,17123E+14	7,39072E+14	-2,97458E+14	0.0228390899115077	0.155473564372384	-3,1042E+13	6,6461E+13	6,603E+14	7,28844E+14	8,21521E+14	7,66216E+14	7,92939E+14
TC0800000765.mm.2	Rab3a	RAB3A, member RAS oncogene family	1,04117E+14	1,00211E+14	2,9744E+14	0.0228444627423658	0.155473564372384	-3,10443E+14	1,08439E+14	1,05495E+14	9,97993E+14	9,44223E+14	9,84152E+14	9,46958E+14
TC1500002076.mm.2	Slc2a13	solute carrier family 2 (facilitated glucose transporter), member 13	-1,01297E+14	9,33047E+14	-2,97403E+14	0.0228559075450054	0.155473564372384	-3,1049E+14	8,87663E+13	8,53911E+14	9,29213E+14	9,83327E+14	9,64414E+14	9,79755E+14
TC1700002662.mm.2	Six2	sine oculis-related homeobox 2	-1,20951E+14	6,07388E+14	-2,97353E+14	0.0228714022995883	0.155473564372384	-3,10554E+14	5,33295E+14	5,41682E+14	5,76978E+14	6,75492E+12	6,43555E+14	6,73324E+14
TC1100003562.mm.2	Cacna1g	calcium channel, voltage-dependent, T type, alpha 1G subunit	1,06693E+14	6,30248E+14	2,97317E+14	0.0228825759989467	0.155473564372384	-3,106E+14	6,72011E+14	7,43924E+14	5,76413E+14	5,8659E+14	6,14212E+14	5,88336E+14
TC0400001469.mm.2	Psmb2	proteasome (prosome, macropain) subunit, beta type 2	1,08013E+14	7,92032E+14	2,97299E+13	0.0228878730273211	0.155473564372384	-3,10622E+14	8,62976E+14	8,71895E+14	7,88637E+14	7,09841E+14	7,63616E+14	7,55228E+13
TC0500000425.mm.2	Cytl1	cytokine-like 1	-1,17644E+14	6,53544E+14	-2,97291E+14	0.0228904965215088	0.155473564372384	-3,10632E+14	5,74633E+12	5,52042E+14	6,80613E+14	7,52012E+13	6,497E+13	7,12263E+14

TC0700002112.mm.2	Fgf3	fibroblast growth factor 3	-1,51789E+14	6,85805E+14	-2,9729E+14	0.0228906905881271	0.155473564372384	-3,10633E+14	5,99828E+14	5,62215E+14	6,82856E+14	8,04313E+13	6,91751E+14	7,73871E+13
TC0700002617.mm.2	Atp1a3	ATPase, Na+/K+ transporting, alpha 3 polypeptide	1,7342E+14	1,05415E+14	2,97288E+14	0.022891498166008	0.155473564372384	-3,10637E+14	1,17572E+14	1,15974E+14	1,08837E+14	9,12427E+14	1,01631E+14	9,72305E+14
TC0400001671.mm.2	Gale	galactose-4-epimerase, UDP	-1,11709E+14	7,91388E+12	-2,97285E+14	0.0228923592300976	0.155473564372384	-3,1064E+14	7,13412E+14	7,31759E+14	7,98909E+14	8,35656E+14	7,96035E+14	8,72555E+14
TC1700002680.mm.2	Pigf	phosphatidylinositol glycan anchor biosynthesis, class F	1,1001E+14	6,43041E+14	2,97271E+14	0.0228966831954537	0.155473564372384	-3,10658E+14	7,18344E+14	7,1784E+14	6,34273E+14	5,71624E+14	6,35788E+14	5,80377E+14
TC0400004166.mm.2	Ttll10	tubulin tyrosine ligase-like family, member 10	-0.954765415287222	6,69356E+13	-2,97267E+14	0.0228978700461487	0.155473564372384	-3,10663E+14	5,98483E+14	6,10415E+14	6,81601E+14	7,25785E+14	6,88147E+14	7,11704E+14
TC0300002316.mm.2	Rab25	RAB25, member RAS oncogene family	-1,0634E+14	6,41644E+14	-2,97238E+14	0.0229069460730805	0.155473564372384	-3,107E+14	5,69601E+14	5,78747E+13	6,3721E+14	7,0328E+14	6,61841E+14	6,99187E+14
TC0700003622.mm.2	Hdgfl3	HDGF like 3	2,39326E+13	6,67576E+14	2,97173E+14	0.0229270937211073	0.155473564372384	-3,10783E+14	8,28555E+14	8,05798E+14	7,16278E+14	4,99124E+14	6,57105E+14	4,98597E+14
TC1900000023.mm.2	Tbx10	T-box 10	-1,01073E+14	6,13195E+14	-2,9714E+14	0.0229370502375996	0.155473564372384	-3,10824E+14	5,29104E+14	5,69394E+14	6,21386E+14	6,5864E+14	6,3263E+14	6,68015E+14
TC0800002593.mm.2	Calr	calreticulin	1,98946E+14	9,37175E+14	2,97097E+14	0.0229504648301274	0.155473564372384	-3,10879E+14	1,05485E+14	1,09171E+13	9,56942E+14	7,70868E+14	9,26947E+14	8,21726E+14
TC1700000284.mm.2	Zfp943	zinc finger prtoein 943	1,37587E+14	6,16452E+14	2,97095E+14	0.0229510181755698	0.155473564372384	-3,10882E+14	6,68418E+14	6,90275E+14	6,00864E+14	6,55637E+14	5,99828E+14	4,83691E+14
TC0400003417.mm.2	Zfp691	zinc finger protein 691	-1,19494E+14	7,66687E+14	-2,97083E+13	0.0229548569255819	0.155473564372384	-3,10897E+14	6,85424E+14	6,92719E+14	8,52222E+14	7,52622E+14	8,01208E+14	8,15924E+14
TC1400002668.mm.2	Frg2f4	FSHD region gene 2 family member 4	-1,40547E+14	5,43441E+14	-2,97043E+14	0.0229671376392887	0.155491381841117	-3,10948E+14	4,49193E+14	4,55677E+14	5,18882E+14	6,50932E+14	6,06173E+13	5,79791E+14
TC0800003102.mm.2	Dbndd1	dysbindin (dystrobrevin binding protein 1) domain containing 1	-0.902542768785128	7,46023E+14	-2,97008E+14	0.0229782175866755	0.155501058367956	-3,10993E+13	7,04568E+14	6,80519E+14	7,49707E+14	7,7575E+13	7,83496E+14	7,821E+14
TC1400000609.mm.2	Tox4	TOX high mobility group box family member 4	2,22074E+14	7,65412E+14	2,96957E+14	0.0229937652799089	0.155540948763716	-3,11057E+14	9,26131E+14	9,12658E+14	7,98274E+14	5,60767E+14	7,31668E+14	6,62973E+14
TC0900000074.mm.2	Gm16833	predicted gene, 16833	-1,34912E+14	4,86913E+14	-2,96915E+14	0.0230068489641022	0.155547164944138	-3,11111E+14	4,05238E+14	4,10995E+14	4,6833E+14	5,50856E+13	4,95353E+14	5,90705E+14
TC0200005433.mm.2	Eef1a2	eukaryotic translation elongation factor 1 alpha 2	1,46103E+14	9,31372E+14	2,96857E+14	0.0230249027233172	0.155547164944138	-3,11185E+14	1,03373E+14	1,02914E+14	9,40292E+14	8,14406E+14	9,19491E+14	8,51173E+14
TC1700000722.mm.2	2410017I17Rik	RIKEN cDNA 2410017I17 gene	-1,11949E+13	5,86533E+14	-2,96819E+14	0.0230369269776022	0.155547164944138	-3,11234E+14	5,11786E+14	5,29828E+14	5,68475E+14	6,43602E+14	6,12091E+14	6,5342E+14
TC0800002935.mm.2	Ddx19b	DEAD (Asp-Glu-Ala-Asp) box polypeptide 19b	1,15423E+14	5,71116E+14	2,96805E+14	0.0230411367106163	0.155547164944138	-3,11251E+14	6,14737E+14	6,73945E+14	5,46685E+14	5,33493E+14	5,6481E+13	4,93025E+14
TC1400001708.mm.2	3425401B19Rik	RIKEN cDNA 3425401B19 gene	-0.941466432766921	4,75216E+13	-2,96794E+14	0.0230445084972977	0.155547164944138	-3,11265E+14	4,3456E+14	4,06506E+14	4,70842E+14	5,10033E+14	5,14271E+14	5,15088E+14
TC0500001296.mm.2	Vsig10	V-set and immunoglobulin domain containing 10	-1,38117E+14	6,43846E+14	-2,96768E+14	0.02305260534268	0.155547164944138	-3,11298E+14	5,7199E+14	5,39249E+14	6,31844E+13	7,32522E+14	6,54904E+14	7,32569E+14
TC1500001062.mm.2	Acvr1b	activin A receptor, type 1B	1,08459E+13	7,84032E+14	2,96718E+14	0.0230682201531769	0.155587371840996	-3,11362E+14	8,51533E+14	8,60595E+14	7,87977E+14	7,08878E+14	7,55149E+14	7,4006E+14
TC1100000335.mm.2	Hba-x	hemoglobin X, alpha-like embryonic chain in Hba complex	-1,10674E+14	6,8362E+14	-2,96667E+14	0.0230841891004823	0.155629932625971	-3,11427E+13	6,10187E+14	6,33451E+14	6,58711E+14	7,34386E+14	7,48412E+14	7,16574E+14
TC1400002469.mm.2	Tnfsf11	tumor necrosis factor (ligand) superfamily, member 11	-0.92702913327451	5,44518E+14	-2,96593E+14	0.02310720110513	0.15563305753385	-3,11521E+14	4,8163E+14	5,03374E+14	5,47155E+14	5,64539E+14	5,68089E+14	6,02321E+14
TC0900001692.mm.2	Aasdhppt	aminoadipate-semialdehyde dehydrogenase-phosphopantetheinyl transferase	2,92733E+14	6,61228E+14	2,96549E+14	0.0231208347871479	0.15563305753385	-3,11577E+14	8,41254E+14	8,5198E+14	6,97741E+14	4,68626E+14	6,78481E+14	4,29285E+13
TC1700002199.mm.2	Ubr2	ubiquitin protein ligase E3 component n-recognin 2	1,07891E+14	6,25938E+14	2,96525E+14	0.0231282810884098	0.15563305753385	-3,11608E+14	6,89302E+14	7,16445E+14	6,1404E+14	5,45877E+13	6,10998E+14	5,78968E+14
TC1000001802.mm.2	Abracl	ABRA C-terminal like	1,42575E+14	5,9983E+14	2,96501E+14	0.0231359679909171	0.15563305753385	-3,11639E+14	6,68704E+13	7,32152E+14	5,92938E+14	4,8948E+14	5,90987E+14	5,2472E+14
TC1600001320.mm.2	Cdc45	cell division cycle 45	1,17464E+14	5,47893E+14	2,96471E+14	0.0231454268893377	0.15563305753385	-3,11677E+14	5,83735E+14	6,01257E+14	5,57706E+14	5,946E+14	4,3058E+14	5,19483E+13
TC0600001802.mm.2	Sgce	sarcoglycan, epsilon	2,88829E+14	6,89848E+14	2,96466E+14	0.0231468751572215	0.15563305753385	-3,11683E+13	8,67565E+14	8,61349E+14	7,61661E+14	4,97257E+14	6,77494E+14	4,73761E+14
TC0900001892.mm.2	Elavl3	ELAV like RNA binding protein 3	1,70795E+14	8,48515E+14	2,96426E+14	0.0231593107735839	0.15563305753385	-3,11734E+13	9,64276E+14	9,60136E+14	8,80713E+14	7,03146E+14	7,79872E+14	8,02949E+14
TC1600000279.mm.2	Ap2m1	adaptor-related protein complex 2, mu 1 subunit	1,25823E+14	1,08254E+14	2,9638E+14	0.023173785752854	0.15563305753385	-3,11793E+14	1,16943E+14	1,16125E+14	1,08934E+14	9,96155E+14	1,07038E+14	1,00865E+14
TC0500003373.mm.2	Clip2	CAP-GLY domain containing linker protein 2	0.884616518394764	6,89119E+14	2,96362E+14	0.0231793976623107	0.15563305753385	-3,11816E+14	7,41749E+14	7,59142E+14	6,66344E+13	6,43513E+14	6,64076E+14	6,59892E+14
TC1600002060.mm.2	Synj1	synaptojanin 1	1,10365E+13	9,1943E+14	2,96356E+13	0.0231812411147049	0.15563305753385	-3,11823E+14	9,82572E+14	9,80833E+14	9,28173E+14	8,82331E+14	9,07095E+14	8,35579E+14
TC0500003252.mm.2	Mphosph9	M-phase phosphoprotein 9	-0.921167871076872	7,76228E+14	-2,96295E+14	0.0232002871729212	0.155640092840492	-3,11901E+12	7,28918E+14	7,05194E+14	7,90048E+14	8,14864E+14	8,2177E+14	7,96575E+13
TC0600001380.mm.2	M6pr	mannose-6-phosphate receptor, cation dependent	1,01285E+14	8,64393E+14	2,96278E+14	0.0232056212155651	0.155640092840492	-3,11922E+14	9,37107E+14	9,13692E+14	8,56648E+14	8,30679E+14	8,52355E+14	7,95874E+14
TC0700000844.mm.2	Ntf5	neurotrophin 5	-1,05169E+14	5,34978E+14	-2,96213E+14	0.0232259768809668	0.155640092840492	-3,12005E+14	4,73525E+14	4,61186E+13	5,3424E+14	5,95868E+13	5,52248E+14	5,92801E+14
TC1100000535.mm.2	Tbc1d9b	TBC1 domain family, member 9B	0.88796226962147	8,06508E+14	2,96201E+13	0.0232297230428733	0.155640092840492	-3,1202E+14	8,52501E+14	8,6418E+14	7,90014E+14	7,93261E+14	7,55458E+14	7,83631E+14
TC0800002585.mm.2	Ier2	immediate early response 2	-0.864813321112179	5,11911E+14	-2,9619E+14	0.0232333868827539	0.155640092840492	-3,12035E+14	4,58695E+14	4,56533E+14	5,41955E+14	5,26093E+14	5,48849E+12	5,39341E+14
TC0400001250.mm.2	Pik3r3	phosphoinositide-3-kinase regulatory subunit 3	1,25563E+14	7,80072E+14	2,961E+14	0.0232614782741076	0.155640092840492	-3,12149E+14	8,51114E+14	8,86496E+14	7,27578E+14	7,28757E+14	7,96291E+14	6,90195E+14
TC1100001004.mm.2	Ankfy1	ankyrin repeat and FYVE domain containing 1	1,07101E+14	5,92158E+14	2,96085E+14	0.0232662652451858	0.155640092840492	-3,12169E+14	6,58479E+14	6,58587E+14	6,03371E+14	5,29644E+14	5,6535E+14	5,37515E+14
TC0200002783.mm.2	Osbpl2	oxysterol binding protein-like 2	1,91052E+14	6,63556E+14	2,96082E+14	0.0232673623534127	0.155640092840492	-3,12173E+14	7,99016E+14	7,86486E+14	6,77942E+13	5,14491E+14	6,65063E+14	5,38335E+14
TC0400000179.mm.2	Usp45	ubiquitin specific petidase 45	1,55512E+14	5,99318E+13	2,96076E+14	0.0232692225937283	0.155640092840492	-3,12181E+14	7,00375E+14	7,23982E+14	6,04792E+14	4,5343E+14	5,75353E+14	5,37979E+14
TC0200002437.mm.2	Slc32a1	solute carrier family 32 (GABA vesicular transporter), member 1	1,56675E+14	8,28851E+14	2,95961E+14	0.0233053005818567	0.155780996221165	-3,12327E+14	9,37277E+13	9,01656E+14	8,79053E+14	7,2954E+14	7,84016E+14	7,41566E+12
TC0500001131.mm.2	Zfp605	zinc finger protein 605	1,73721E+14	5,53536E+14	2,95905E+14	0.0233230312301451	0.155780996221165	-3,12399E+14	6,73421E+14	6,6351E+14	5,49265E+14	4,45532E+14	5,70329E+14	4,1916E+14
TC1100001125.mm.2	Git1	GIT ArfGAP 1	1,90846E+14	6,81516E+14	2,95904E+14	0.0233232038246669	0.155780996221165	-3,12399E+14	7,88878E+14	8,15359E+14	7,31525E+13	5,30785E+14	6,32973E+14	5,89573E+14
TC1900000865.mm.2	Fam45a	family with sequence similarity 45, member A	1,03901E+14	9,02765E+14	2,95886E+14	0.0233289607076069	0.155780996221165	-3,12423E+14	9,78519E+14	9,68361E+14	8,66425E+14	8,6421E+14	9,05362E+14	8,33716E+14
TC1500001178.mm.2	Nipbl	NIPBL cohesin loading factor	2,18496E+13	7,0344E+14	2,95855E+14	0.0233386761638056	0.155781312770257	-3,12462E+14	8,21125E+14	8,07627E+14	7,44321E+14	6,55806E+14	7,09363E+14	4,82397E+14
TC1100002000.mm.2	Card14	caspase recruitment domain family, member 14	-1,15492E+14	6,14495E+14	-2,95792E+14	0.023358735581579	0.155785295134991	-3,12543E+14	5,28638E+14	5,3919E+14	6,1351E+14	7,06821E+14	6,4286E+14	6,55953E+14
TC0700001841.mm.2	Itgal	integrin alpha L	-0.93235312948378	5,01466E+14	-2,95785E+14	0.0233608998788058	0.155785295134991	-3,12552E+14	4,65433E+14	4,62036E+14	4,75153E+14	4,92232E+14	5,35418E+14	5,78521E+14
TC0500000374.mm.2	Fam193a	family with sequence homology 193, member A	1,95379E+14	7,69904E+14	2,95747E+14	0.0233728778033948	0.155785295134991	-3,126E+13	8,7059E+14	8,8078E+14	7,66236E+14	7,41206E+14	7,86115E+14	5,74496E+14
TC0500002892.mm.2	Zfp951	zinc finger protein 951	-0.880000032844493	5,61876E+14	-2,95731E+14	0.0233779459837652	0.155785295134991	-3,12621E+13	4,94724E+14	5,08397E+14	5,95037E+14	5,93978E+14	5,77494E+14	6,01627E+13
TC0100002228.mm.2	Lonrf2	LON peptidase N-terminal domain and ring finger 2	2,5473E+14	7,82141E+14	2,95633E+14	0.023408804714421	0.155896605530002	-3,12745E+14	9,54496E+14	9,31431E+14	8,51783E+14	5,7867E+14	7,37595E+14	6,38871E+14
TC0500003461.mm.2	Nyap1	neuronal tyrosine-phosphorylated phosphoinositide 3-kinase adaptor 1	1,08889E+14	6,62319E+14	2,9557E+14	0.0234286493619607	0.155896605530002	-3,12825E+14	6,93057E+14	6,90817E+14	6,71846E+14	7,52101E+14	5,92944E+14	5,73152E+14
TC0600000362.mm.2	Trim24	tripartite motif-containing 24	2,4627E+14	6,85899E+14	2,9556E+14	0.0234319352813931	0.155896605530002	-3,12838E+14	8,53609E+14	8,22597E+13	7,43063E+14	5,12459E+14	6,70152E+14	5,13515E+14
TC0100003025.mm.2	Lct	lactase	-1,25064E+14	5,94365E+13	-2,95556E+14	0.0234333506233268	0.155896605530002	-3,12844E+14	4,97564E+14	5,26114E+14	6,08204E+12	6,60502E+14	5,89837E+14	6,8397E+14
TC0200004109.mm.2	Nr1h3	nuclear receptor subfamily 1, group H, member 3	-0.948743095369401	6,74745E+14	-2,95512E+14	0.0234471459403828	0.155924004348902	-3,12899E+14	6,16625E+14	6,33672E+14	6,57604E+14	7,00521E+13	7,31274E+14	7,08772E+14
TC0400003056.mm.2	Tusc1	tumor suppressor candidate 1	-1,0334E+14	7,88329E+14	-2,9543E+14	0.023473173797876	0.155969922761294	-3,13004E+14	7,2216E+14	7,20032E+14	7,96E+13	8,42906E+14	7,97445E+14	8,51428E+14
TC1100000686.mm.2	Zfp692	zinc finger protein 692	-1,0215E+13	6,72981E+14	-2,95412E+14	0.0234788217001457	0.155969922761294	-3,13027E+14	6,11138E+14	6,01906E+14	6,73619E+14	7,33881E+14	6,92519E+14	7,24824E+14
TC1000001193.mm.2	Ccer1	coiled-coil glutamate-rich protein 1	-1,33594E+14	5,67887E+14	-2,95394E+13	0.023484699507283	0.155969922761294	-3,1305E+14	5,03388E+14	4,53065E+14	5,60742E+13	6,66487E+13	5,89607E+14	6,34035E+14
TC1500001446.mm.2	Abra	actin-binding Rho activating protein	-1,23238E+14	4,85765E+14	-2,9534E+14	0.0235017877359205	0.155969922761294	-3,13119E+14	4,19338E+14	3,94293E+14	5,04953E+14	5,359E+14	4,79577E+14	5,80529E+14
TC0900000840.mm.2	Ubap1l	ubiquitin-associated protein 1-like	-1,26111E+14	6,24864E+14	-2,95319E+14	0.0235083722392777	0.155969922761294	-3,13145E+14	5,60646E+14	5,38313E+14	5,97681E+14	7,01359E+14	6,55668E+14	6,95514E+14

TC1900000031.mm.2	Ptprcap	protein tyrosine phosphatase, receptor type, C polypeptide-associated protein	-1,29393E+14	6,05644E+14	-2,95298E+14	0.0235151840951272	0.155969922761294	-3,13173E+14	5,37153E+14	5,04545E+13	5,90187E+13	7,01493E+14	6,37754E+14	6,6273E+14
TC0200003316.mm.2	Lrsam1	leucine rich repeat and sterile alpha motif containing 1	0.920813659628122	6,35826E+13	2,95277E+13	0.0235218092416027	0.155969922761294	-3,13199E+14	7,1076E+14	6,98858E+14	5,99424E+14	5,80457E+14	6,31357E+14	5,94098E+14
TC0900002105.mm.2	Crtam	cytotoxic and regulatory T cell molecule	-1,21415E+14	5,68888E+14	-2,95222E+13	0.0235393805534846	0.156022229065528	-3,1327E+14	4,86419E+13	4,75726E+14	5,7064E+14	6,75569E+14	6,04186E+14	6,00788E+14
TC1700001905.mm.2	H2-Aa	histocompatibility 2, class II antigen A, alpha	-1,24696E+14	7,12452E+13	-2,95185E+14	0.0235510635758741	0.15603548001565	-3,13317E+14	6,25335E+14	6,51931E+14	6,90356E+14	7,8043E+14	7,33696E+14	7,92963E+14
TC1200002523.mm.2	Fntb	farnesyltransferase, CAAX box, beta	0.910822258687705	7,09429E+14	2,95145E+14	0.0235636359709188	0.156054610110734	-3,13367E+14	7,64077E+13	7,6127E+14	6,7657E+14	7,11474E+14	6,61536E+14	6,81646E+14
TC1800000232.mm.2	Pik3c3	phosphatidylinositol 3-kinase catalytic subunit type 3	1,39385E+14	6,18821E+14	2,9511E+14	0.0235748545397946	0.156064762202018	-3,13412E+14	7,27712E+14	7,30284E+14	5,96881E+13	4,78821E+13	6,2174E+13	5,57487E+14
TC1100003766.mm.2	Hap1	huntingtin-associated protein 1	1,16387E+14	8,76181E+14	2,95047E+13	0.0235951636785352	0.156111366407332	-3,13493E+14	9,78859E+14	8,9715E+14	8,94944E+14	8,42898E+14	8,20034E+14	8,23202E+14
TC0700000814.mm.2	Izumo2	IZUMO family member 2	-1,15005E+14	4,85558E+14	-2,94991E+14	0.0236128956469311	0.156111366407332	-3,13564E+14	4,51483E+14	3,69492E+14	5,00945E+14	5,40441E+14	5,3822E+14	5,12767E+14
TC1300001921.mm.2	Nup153	nucleoporin 153	1,2087E+14	6,87352E+14	2,94939E+14	0.0236295759471962	0.156111366407332	-3,13631E+14	7,67619E+14	7,77669E+14	6,81115E+14	5,94158E+13	6,80584E+12	6,22966E+14
TC0500000297.mm.2	Slc35f6/Mir5625	solute carrier family 35, member F6/microRNA 5625	0.946660401446963	7,51926E+14	2,94915E+14	0.0236373288326815	0.156111366407332	-3,13662E+14	8,23681E+14	8,23486E+14	7,13924E+14	6,9263E+13	7,52266E+14	7,05569E+12
TC1800000944.mm.2	Arhgap12	Rho GTPase activating protein 12	1,89091E+14	6,66611E+14	2,94891E+14	0.0236447173671227	0.156111366407332	-3,13691E+14	7,89595E+14	7,58897E+14	7,09232E+13	5,71632E+14	6,59967E+14	5,10343E+14
TC1100000953.mm.2	Tm4sf5	transmembrane 4 superfamily member 5	-0.955343876166155	5,77293E+13	-2,94887E+14	0.0236461309967679	0.156111366407332	-3,13697E+14	5,1488E+14	5,22328E+14	5,73784E+12	6,2449E+14	6,06164E+14	6,22112E+14
TC1700001780.mm.2	Cpne5	copine V	1,24394E+13	7,96584E+14	2,94876E+13	0.0236497142307638	0.156111366407332	-3,13711E+14	9,02361E+14	8,55387E+14	7,98558E+14	7,14236E+14	7,27307E+14	7,81653E+14
TC1900000971.mm.2	Snx32	sorting nexin 32	1,45793E+14	7,35466E+14	2,94839E+14	0.0236615224198769	0.156125268587169	-3,13758E+14	8,51621E+14	8,11172E+14	7,41014E+14	6,37779E+14	7,318E+14	6,39407E+14
TC0300000878.mm.2	Pogz	pogo transposable element with ZNF domain	1,95728E+14	6,55473E+14	2,94809E+14	0.0236711991037333	0.156125268587169	-3,13797E+14	7,70379E+14	7,50377E+14	7,26922E+13	5,55859E+14	6,28701E+14	5,00599E+14
TC0400004130.mm.2	Tnfrsf14	tumor necrosis factor receptor superfamily, member 14 (herpesvirus entry mediator)	-1,47427E+14	7,56333E+14	-2,94699E+14	0.0237062893610226	0.156262350520866	-3,13936E+14	6,49418E+14	6,72908E+13	7,35734E+14	8,62761E+14	7,73655E+14	8,43524E+14
TC0100001617.mm.2	Tagln2	transgelin 2	1,04525E+14	7,04416E+14	2,94683E+14	0.0237113788260112	0.156262350520866	-3,13957E+14	7,432E+14	7,93783E+14	6,57453E+14	7,04128E+14	6,7834E+14	6,49592E+14
TC0400003369.mm.2	Rnf220	ring finger protein 220	1,29359E+14	8,51584E+14	2,9464E+14	0.0237253058006741	0.15629020947108	-3,14012E+14	9,47654E+14	9,2089E+14	8,68021E+14	7,63113E+13	8,29668E+14	7,80158E+14
TC1700000036.mm.2	Zdhhc14	zinc finger, DHHC domain containing 14	1,09044E+14	6,99918E+14	2,94568E+14	0.023748524040461	0.156379226752737	-3,14104E+14	7,57915E+14	7,74496E+14	6,99272E+14	6,53502E+14	6,91209E+14	6,23114E+14
TC1800000257.mm.2	Map3k2	mitogen-activated protein kinase kinase kinase 2	1,89422E+14	5,23903E+14	2,94465E+14	0.0237813845216968	0.156510721816823	-3,14235E+14	6,73792E+14	6,34782E+14	5,09074E+14	3,96038E+14	5,48984E+14	3,80747E+14
TC1400000742.mm.2	Abhd4	abhydrolase domain containing 4	1,39464E+14	8,75512E+14	2,94445E+14	0.0237879201718736	0.156510721816823	-3,14261E+14	9,66911E+13	9,57462E+14	8,99154E+14	7,84102E+14	8,57757E+14	7,87688E+14
TC0500003742.mm.2	Tmem184a	transmembrane protein 184a	-1,02676E+14	5,72333E+13	-2,94341E+13	0.0238214432779438	0.156644170607628	-3,14394E+14	4,8777E+14	5,43496E+14	5,69386E+14	5,96728E+14	6,01138E+14	6,35481E+14
TC1400002035.mm.2	Olfr1513	olfactory receptor 1513	-0.892512485734209	5,30116E+14	-2,94322E+14	0.0238276461341336	0.156644170607628	-3,14418E+14	4,76977E+13	5,06048E+13	5,08535E+14	5,27607E+14	5,8093E+14	5,80597E+14
TC1800001628.mm.2	8030462N17Rik	RIKEN cDNA 8030462N17 gene	1,64674E+14	6,24408E+14	2,94272E+14	0.0238438405016112	0.156674658633471	-3,14482E+14	7,30067E+14	7,30453E+14	6,38569E+13	5,16187E+14	6,26167E+14	5,05004E+14
TC0400000269.mm.2	Casp8ap2	caspase 8 associated protein 2	0.929320921957276	5,58998E+14	2,94236E+14	0.023855442191727	0.156674658633471	-3,14528E+14	6,25348E+14	6,40262E+14	4,99095E+14	5,09539E+14	5,18189E+14	5,61556E+14
TSUnmapped00000001.mm.2	Plppr2	phospholipid phosphatase related 2	2,32215E+14	8,76303E+14	2,94202E+14	0.0238664335647206	0.156674658633471	-3,14572E+13	1,02628E+14	1,02828E+13	9,0995E+14	7,03176E+14	8,76842E+14	7,13289E+14
TC0200000686.mm.2	Rabgap1/Gpr21	RAB GTPase activating protein 1/G protein-coupled receptor 21	1,89546E+14	7,15755E+14	2,9416E+14	0.0238798047209654	0.156674658633471	-3,14624E+14	8,45977E+14	8,14715E+14	7,3517E+14	6,17071E+14	7,24255E+14	5,57345E+14
TC1400002833.mm.2	Rnf31	ring finger protein 31	1,01553E+14	5,68723E+14	2,9415E+13	0.0238829819712476	0.156674658633471	-3,14637E+14	6,59341E+14	6,43727E+14	5,37317E+14	4,71993E+14	5,57067E+14	5,42895E+14
TC0100001533.mm.2	Ildr2	immunoglobulin-like domain containing receptor 2	1,00848E+14	6,99751E+14	2,94127E+14	0.0238906247292521	0.156674658633471	-3,14667E+14	7,80094E+14	7,67526E+14	6,43166E+14	6,61792E+14	7,04574E+14	6,41351E+14
TC1900000725.mm.2	Sfr1	SWI5 dependent recombination repair 1	1,06085E+14	8,07796E+14	2,94019E+14	0.0239255162534743	0.156678635412881	-3,14805E+14	8,71322E+14	8,68731E+14	7,68811E+14	8,10032E+14	7,97181E+14	7,30702E+14
TC1700001636.mm.2	Syngr3	synaptogyrin 3	1,62315E+14	9,45344E+14	2,93986E+14	0.0239361397090797	0.156678635412881	-3,14847E+14	1,07484E+14	1,04763E+14	9,37514E+14	8,14238E+14	9,55158E+14	8,42683E+14
TC0Y00000240.mm.2	Sry	sex determining region of Chr Y	-1,0533E+14	6,59438E+14	-2,93935E+14	0.0239526463905905	0.156678635412881	-3,14912E+14	5,84352E+14	5,93033E+14	6,7116E+14	7,20042E+14	6,64728E+14	7,23316E+14
TC0X00003430.mm.2	Arhgap4	Rho GTPase activating protein 4	-1,07894E+14	5,92634E+14	-2,93887E+14	0.0239681918208554	0.156678635412881	-3,14973E+12	5,42804E+14	4,78878E+14	6,35739E+14	6,6091E+14	5,96392E+13	6,41077E+14
TC0500000534.mm.2	Anapc4	anaphase promoting complex subunit 4	1,95706E+14	6,35456E+14	2,93884E+14	0.0239692560302322	0.156678635412881	-3,14977E+14	7,89809E+14	7,7658E+14	6,22245E+14	4,49127E+14	6,5127E+14	5,23708E+14
TC1000001044.mm.2	Timp3	tissue inhibitor of metalloproteinase 3	1,20944E+14	6,93869E+14	2,93796E+14	0.0239978263043728	0.156678635412881	-3,1509E+14	7,63438E+13	7,43452E+14	6,63376E+14	7,27947E+14	5,91854E+14	6,73148E+14
TC0400001266.mm.2	Mutyh	mutY DNA glycosylase	-1,1939E+14	6,42651E+14	-2,93784E+14	0.0240018235631857	0.156678635412881	-3,15105E+13	5,94289E+14	5,52083E+14	6,41942E+14	6,82436E+14	6,50145E+14	7,35008E+14
TC1200002448.mm.2	Brf1	BRF1, RNA polymerase III transcription initiation factor 90 kDa subunit	1,09942E+14	5,7077E+13	2,93779E+14	0.0240034842582031	0.156678635412881	-3,15112E+13	6,43034E+14	6,02573E+14	5,85693E+14	5,67594E+13	5,49706E+14	4,76017E+14
TC0100000814.mm.2	Gigyf2	GRB10 interacting GYF protein 2	0.982730869556677	6,63005E+14	2,93774E+14	0.0240049523897999	0.156678635412881	-3,15118E+14	7,2654E+14	7,1847E+14	6,49453E+14	6,35103E+13	6,53826E+14	5,94637E+14
TC1000000704.mm.2	Gnaz	guanine nucleotide binding protein, alpha z subunit	0.880867886119966	7,99946E+14	2,93773E+13	0.0240054192345013	0.156678635412881	-3,15119E+14	8,67069E+13	8,50138E+14	7,76512E+14	7,64925E+13	7,81968E+14	7,59065E+14
TC1700001814.mm.2	Tmprss3	transmembrane protease, serine 3	-1,17175E+14	5,13593E+14	-2,93751E+14	0.0240124398379052	0.156678635412881	-3,15147E+14	4,1711E+14	4,6918E+14	5,05087E+14	5,69544E+14	5,33023E+14	5,87616E+14
TC0300002237.mm.2	Tmem131l	transmembrane 131 like	1,00285E+14	5,64745E+14	2,93715E+14	0.0240242718440605	0.156678635412881	-3,15194E+14	6,07341E+13	6,05605E+14	6,08835E+14	5,54314E+14	4,8748E+14	5,24897E+14
TC0900000273.mm.2	Zbtb44	zinc finger and BTB domain containing 44	1,33486E+14	6,76575E+14	2,93684E+14	0.024034324191919	0.156678635412881	-3,15233E+14	7,70152E+13	7,59745E+14	6,69405E+13	5,97222E+14	6,83023E+14	5,79903E+14
TC1000001549.mm.2	Agap2	ArfGAP with GTPase domain, ankyrin repeat and PH domain 2	1,69598E+14	8,12436E+14	2,936E+14	0.0240616171449988	0.156678635412881	-3,1534E+14	9,27878E+14	9,48398E+14	8,1777E+14	6,43488E+13	7,8792E+14	7,4916E+14
TC0700004173.mm.2	Coq7	demethyl-Q 7	1,16765E+14	6,98196E+13	2,93587E+14	0.0240657284994312	0.156678635412881	-3,15356E+14	7,6832E+14	7,48861E+14	6,49406E+14	7,38937E+13	6,60073E+14	6,23577E+14
TC1400000833.mm.2	Rcbtb1	regulator of chromosome condensation (RCC1) and BTB (POZ) domain containing protein 1	1,86343E+14	5,3964E+13	2,93577E+14	0.0240690857155417	0.156678635412881	-3,15369E+14	6,84669E+14	6,79937E+14	5,41987E+14	3,39325E+14	4,90852E+14	5,01068E+13
TC0200004645.mm.2	Gfra4/Mir6973b	glial cell line derived neurotrophic factor family receptor alpha 4/microRNA 6973b	-1,11359E+14	7,6452E+14	-2,93439E+11	0.024114047056129	0.156678635412881	-3,15545E+14	6,99825E+14	6,87119E+14	7,71351E+14	8,19166E+14	7,67895E+14	8,41766E+14
TC1200001942.mm.2	Esr2	estrogen receptor 2 (beta)	-0.897844996607	5,05408E+14	-2,93433E+14	0.0241161024273237	0.156678635412881	-3,15554E+14	4,78646E+14	4,44081E+14	5,00747E+14	5,06679E+14	5,44194E+14	5,58102E+14
TC0200001998.mm.2	Atrn	attractin	1,59765E+13	8,48209E+14	2,93402E+14	0.0241263780228423	0.156678635412881	-3,15594E+14	9,48991E+14	9,49927E+13	8,61062E+14	7,49888E+14	8,5125E+14	7,28137E+14
TC0300002746.mm.2	Tmem167b	transmembrane protein 167B	1,86956E+14	6,74585E+14	2,93374E+14	0.0241352827888726	0.156678635412881	-3,15629E+14	8,03009E+14	8,04095E+14	6,82781E+14	5,24433E+14	6,80895E+14	5,52297E+14
TC0100001527.mm.2	Cd247	CD247 antigen	-1,32168E+14	6,89322E+14	-2,93317E+14	0.0241542051016251	0.156678635412881	-3,15702E+14	5,94806E+14	5,91592E+14	6,8858E+14	8,10221E+14	7,12244E+14	7,3849E+14
TC1100000231.mm.2	Zrsr1	zinc finger (CCCH type), RNA binding motif and serine/arginine rich 1	0.913968539272199	7,24272E+14	2,93308E+14	0.0241571371177035	0.156678635412881	-3,15714E+14	7,91646E+14	7,6904E+14	7,21372E+14	6,85681E+13	6,93569E+14	6,84323E+14
TC0100000424.mm.2	Spats2l	spermatogenesis associated, serine-rich 2-like	1,56774E+14	6,8026E+14	2,93304E+14	0.0241583060479351	0.156678635412881	-3,15718E+14	7,60843E+14	7,43143E+12	7,69013E+14	6,18124E+14	6,22816E+14	5,67624E+14
TC1600002170.mm.2	Cox17	cytochrome c oxidase assembly protein 17, copper chaperone	2,42871E+13	8,08772E+14	2,93291E+14	0.0241626047452824	0.156678635412881	-3,15735E+14	9,79602E+14	9,54034E+14	8,54728E+14	6,16375E+14	7,94854E+14	6,53041E+14
TC0800001341.mm.2	Gabarapl2	gamma-aminobutyric acid (GABA) A receptor-associated protein-like 2	1,08182E+14	8,28833E+14	2,93239E+14	0.0241797583834277	0.156678635412881	-3,15802E+13	9,11272E+14	9,0484E+13	8,01561E+14	7,55572E+14	8,34263E+14	7,65486E+14
TC0200001906.mm.2	Blvra	biliverdin reductase A	1,00939E+14	6,12491E+14	2,93235E+14	0.0241810034238122	0.156678635412881	-3,15807E+14	6,82868E+13	6,57034E+14	6,14133E+13	5,82885E+14	5,96297E+14	5,41727E+14
TC0400002456.mm.2	Aqp3	aquaporin 3	-1,28099E+14	6,42281E+14	-2,93222E+14	0.0241853240548451	0.156678635412881	-3,15824E+14	5,44924E+14	5,70371E+14	6,2931E+14	7,37589E+14	6,66019E+14	7,05475E+14
TC0800000753.mm.2	Fkbp8	FK506 binding protein 8	0.975067559491277	8,73315E+14	2,93183E+14	0.0241979198524	0.156678635412881	-3,15873E+14	9,32022E+14	9,48845E+14	8,65103E+14	8,08069E+14	8,38683E+14	8,47171E+14

TC1100000472.mm.2	Fam71b	family with sequence similarity 71, member B	-1,25263E+14	5,98141E+14	-2,93159E+14	0.0242056702898796	0.156678635412881	-3,15903E+14	5,12313E+14	5,11122E+14	5,93888E+14	6,97564E+13	6,13994E+14	6,59968E+14
TC1100001741.mm.2	Mettl2	methyltransferase like 2	1,75192E+14	5,39083E+14	2,93138E+13	0.0242125826584793	0.156678635412881	-3,1593E+14	6,68533E+14	6,32086E+14	5,54219E+14	4,29424E+14	5,42974E+14	4,07261E+14
TC1600000117.mm.2	Clec16a	C-type lectin domain family 16, member A	2,3792E+14	6,43729E+14	2,9313E+14	0.0242153220944345	0.156678635412881	-3,15941E+14	7,94812E+14	7,98254E+14	7,09916E+14	4,42163E+14	5,83144E+14	5,34082E+14
TC1100003923.mm.2	Icam2	intercellular adhesion molecule 2	-1,12515E+14	5,28877E+14	-2,93123E+13	0.0242174686929835	0.156678635412881	-3,15949E+14	4,61259E+14	4,5718E+14	5,10504E+14	6,00852E+14	5,7791E+13	5,65559E+14
TC1300002116.mm.2	Agtpbp1/A230056J06 Rik	ATP/GTP binding protein 1/RIKEN cDNA A230056J06 gene	2,39404E+14	6,57383E+14	2,93088E+14	0.0242291536379479	0.156678635412881	-3,15995E+14	8,23072E+14	8,3258E+14	6,72742E+14	4,39061E+14	6,60008E+14	5,16836E+14
TC0400004176.mm.2	Palm2	paralemmin 2	0.965036250619841	7,60982E+14	2,93084E+14	0.0242304797706829	0.156678635412881	-3,16E+14	8,18911E+14	7,95407E+14	7,39019E+14	7,91244E+14	7,14058E+14	7,07252E+14
TC0700004219.mm.2	Cog7	component of oligomeric golgi complex 7	1,54361E+14	6,50986E+14	2,93081E+14	0.024231562058518	0.156678635412881	-3,16004E+14	7,52223E+14	7,31381E+14	6,70877E+14	5,76552E+14	6,50146E+14	5,24736E+13
TC0500001162.mm.2	Pitpnb	phosphatidylinositol transfer protein, beta	1,5938E+14	8,61448E+14	2,93046E+14	0.0242429839535907	0.156689611088731	-3,16049E+14	9,62846E+14	9,61608E+14	8,89512E+14	7,49026E+13	8,46688E+14	7,59007E+14
TC1100003774.mm.2	Klhl11	kelch-like 11	0.944510815084572	7,26334E+14	2,92995E+14	0.0242595992931665	0.156734131279387	-3,16113E+14	8,10541E+14	7,80758E+14	6,60832E+14	7,0348E+14	7,0442E+12	6,97976E+14
TC1100000028.mm.2	Pes1	pescadillo ribosomal biogenesis factor 1	1,06378E+14	7,87542E+14	2,9284E+13	0.0243108275883081	0.156939259843172	-3,16312E+14	8,6759E+14	8,74324E+14	7,19287E+14	7,34892E+14	7,25639E+14	8,03519E+13
TC0700004532.mm.2	Sct	secretin	-0.882866380559047	5,85516E+13	-2,92838E+14	0.0243112465804796	0.156939259843172	-3,16314E+14	5,36089E+14	5,16842E+13	6,09684E+14	6,20976E+14	6,02769E+14	6,26735E+14
TC1900000723.mm.2	Slk	STE20-like kinase	2,78888E+14	8,20074E+14	2,9281E+14	0.0243205692191647	0.156939259843172	-3,1635E+14	9,6427E+14	9,85674E+14	9,05863E+14	6,72466E+14	8,09137E+14	5,83031E+14
TC0900002007.mm.2	Dcps	decapping enzyme, scavenger	0.930415013340649	6,76405E+13	2,92771E+14	0.0243334073268139	0.15695924429822	-3,164E+14	7,38137E+14	7,05836E+14	6,95809E+13	6,60757E+14	6,2396E+14	6,33929E+14
TC1500000351.mm.2	Eny2	ENY2 transcription and export complex 2 subunit	1,02991E+14	7,02213E+12	2,92684E+14	0.0243620954214544	0.156989506625485	-3,16511E+14	7,73233E+14	7,62652E+13	7,04314E+14	6,43175E+14	6,8404E+14	6,45863E+14
TC0800003189.mm.2	Tarbp1	TAR RNA binding protein 1	-0.908868664154141	6,25963E+14	-2,92678E+14	0.0243640628987121	0.156989506625485	-3,16519E+14	5,71958E+14	5,70766E+14	6,25371E+14	6,63185E+13	6,71647E+14	6,5285E+14
TC0700001666.mm.2	Far1	fatty acyl CoA reductase 1	2,12102E+14	6,5396E+14	2,92623E+14	0.0243823563177734	0.156989506625485	-3,16589E+14	7,54516E+14	7,87037E+14	6,75449E+14	5,8941E+14	6,6878E+14	4,48569E+14
TC1700002701.mm.2	Fbxo11	F-box protein 11	2,32628E+14	8,27843E+14	2,92602E+13	0.0243893876424194	0.156989506625485	-3,16617E+14	9,83868E+14	9,83738E+14	8,6135E+14	6,35751E+13	8,21497E+14	6,80852E+14
TC1100004113.mm.2	Exoc7	exocyst complex component 7	1,49336E+14	6,3841E+14	2,92599E+14	0.0243902164443062	0.156989506625485	-3,1662E+13	7,20523E+14	7,28752E+13	6,44465E+14	5,86119E+14	6,44965E+14	5,05638E+14
TC1700000580.mm.2	Zfp955b	zinc finger protein 955B	1,04694E+14	7,23935E+14	2,92517E+14	0.0244172413203978	0.156989506625485	-3,16724E+14	8,05114E+14	8,15287E+14	6,91794E+14	6,20401E+14	7,1832E+13	6,92693E+14
TC1400001219.mm.2	Frg2f7	FSHD region gene 2 family member 7	-1,05604E+14	5,03286E+14	-2,92498E+14	0.024423587050942	0.156989506625485	-3,16749E+14	4,27011E+14	4,37506E+14	5,19704E+14	5,59772E+14	5,04578E+14	5,71146E+14
TC0300000948.mm.2	Hjv	hemojuvelin BMP co-receptor	-1,1835E+13	5,01141E+14	-2,92497E+14	0.024423959767561	0.156989506625485	-3,1675E+13	4,18969E+14	4,05325E+14	5,11089E+14	6,10471E+14	5,40186E+14	5,20808E+14
TC0X00002205.mm.2	1700080O16Rik	RIKEN cDNA 1700080O16 gene	-0.97054118528881	4,91908E+14	-2,92492E+14	0.0244257862043128	0.156989506625485	-3,16757E+14	3,97877E+14	4,45299E+14	5,19875E+14	5,51114E+14	5,09722E+14	5,27563E+14
TC0500001128.mm.2	Zfp932	zinc finger protein 932	1,54694E+14	7,01473E+14	2,92447E+13	0.0244404770717925	0.15702129468014	-3,16814E+14	7,72747E+14	8,12481E+14	7,17531E+14	6,3024E+14	7,01501E+13	5,74339E+14
TC0500000670.mm.2	Atp10d	ATPase, class V, type 10D	-0.987424900539674	6,2326E+14	-2,92328E+14	0.024480054314848	0.157208902746615	-3,16967E+14	5,76702E+14	5,72403E+13	6,15168E+14	6,287E+14	6,42251E+13	7,04338E+14
TC0700003029.mm.2	Abcc6	ATP-binding cassette, sub-family C (CFTR/MRP), member 6	-1,02926E+14	7,36557E+14	-2,92274E+14	0.0244978821111323	0.157208902746615	-3,17035E+13	6,4537E+14	6,86324E+13	7,45706E+14	8,04397E+14	7,61528E+14	7,76016E+14
TC0Y00000565.mm.2	Gm20854	predicted gene, 20854	-1,19325E+14	7,28573E+14	-2,92251E+14	0.0245056368451968	0.157208902746615	-3,17065E+14	6,41405E+14	6,67526E+14	7,23201E+14	7,91728E+14	7,36897E+14	8,10684E+14
TC0X00000053.mm.2	Dgkk	diacylglycerol kinase kappa	1,72202E+14	6,3834E+14	2,92232E+14	0.0245117672829859	0.157208902746615	-3,17089E+14	8,24814E+13	6,49463E+14	6,39232E+14	5,8666E+14	5,76441E+14	5,53432E+14
TC0400001708.mm.2	Hspg2	perlecan (heparan sulfate proteoglycan 2)	-1,10381E+14	5,70648E+14	-2,92201E+13	0.0245222280146386	0.157208902746615	-3,17129E+14	4,80474E+14	5,1877E+14	5,66111E+14	6,38524E+14	5,93996E+14	6,2601E+14
TC0700000508.mm.2	Fbxo17	F-box protein 17	-1,24994E+14	6,14947E+14	-2,92183E+12	0.0245282183023019	0.157208902746615	-3,17152E+14	4,88956E+14	5,76509E+14	6,29995E+14	6,78767E+14	6,30917E+14	6,84536E+14
TC0700002946.mm.2	Iglon5	IgLON family member 5	-1,40374E+14	6,86594E+14	-2,92092E+14	0.0245586161322885	0.1573411458209	-3,17269E+14	6,06816E+13	5,98924E+14	7,7768E+14	6,49658E+14	7,6891E+14	7,17578E+14
TC1500001491.mm.2	Trps1/Mir1907	transcriptional repressor GATA binding 1/microRNA 1907	0.96562340487506	6,53607E+14	2,91955E+13	0.0246040752843157	0.157425926438305	-3,17444E+14	7,14593E+14	7,20965E+14	6,03645E+14	6,40005E+14	6,44068E+14	5,98366E+14
TC0700000476.mm.2	Zfp607a	zinc finger protein 607A	0.899369079209703	6,90164E+14	2,91946E+14	0.0246071785702506	0.157425926438305	-3,17455E+14	7,40478E+14	7,42827E+14	6,81252E+14	6,72999E+14	6,71171E+14	6,3226E+14
TC0800002580.mm.2	Ccdc130	coiled-coil domain containing 130	1,25249E+14	6,40138E+14	2,91939E+14	0.0246093558894938	0.157425926438305	-3,17464E+14	7,53796E+14	7,11808E+14	6,31361E+14	5,28759E+14	5,93726E+14	6,21381E+14
TC1700001811.mm.2	Tff3	trefoil factor 3, intestinal	-1,03597E+14	5,0583E+14	-2,91935E+14	0.0246109296033073	0.157425926438305	-3,1747E+14	4,47102E+14	4,33107E+14	5,14108E+14	5,53259E+14	5,12679E+14	5,74723E+14
TC1500000729.mm.2	Zc3h7b	zinc finger CCCH type containing 7B	1,03656E+14	7,4004E+14	2,91849E+14	0.0246395245475231	0.157532263039598	-3,17579E+14	8,02425E+14	8,06257E+14	7,24672E+14	7,05516E+14	7,36826E+13	6,64544E+14
TC0100001206.mm.2	Ren1/Ren2	renin 1 structural/renin 2 tandem duplication of Ren1	-1,37558E+14	5,39685E+14	-2,91826E+14	0.0246471070019751	0.157532263039598	-3,17608E+14	4,61006E+14	4,52565E+14	5,09005E+14	6,26848E+14	5,6272E+14	6,25967E+13
TC0200002005.mm.2	Hspa12b	heat shock protein 12B	-1,10833E+14	6,44514E+13	-2,91761E+14	0.0246688911838166	0.157608978447596	-3,17692E+14	5,62187E+14	5,80585E+14	6,55498E+14	7,04379E+14	6,46115E+14	7,18322E+14
TC1300002380.mm.2	Rasa1	RAS p21 protein activator 1	2,39127E+14	6,60748E+13	2,91677E+14	0.0246970323655962	0.157704985483388	-3,17799E+13	7,75886E+14	7,87519E+14	7,35894E+14	5,80037E+14	6,55934E+14	4,29217E+14
TC1100002871.mm.2	Tom1l2	target of myb1-like 2 (chicken)	1,15831E+14	8,11229E+14	2,91658E+14	0.0247034930404065	0.157704985483388	-3,17824E+14	8,93349E+14	8,73223E+13	8,27037E+14	7,38853E+14	7,86732E+14	7,48178E+14
TC1500002348.mm.2	Vps28	vacuolar protein sorting 28	1,28374E+14	9,07719E+14	2,91585E+13	0.0247280414542343	0.157716678117479	-3,17918E+14	1,01417E+14	9,88896E+14	9,05628E+14	7,913E+14	8,85424E+14	8,60897E+14
TC1000002043.mm.2	Amd2/Amd1	S-adenosylmethionine decarboxylase 2/S-adenosylmethionine decarboxylase 1	1,49568E+14	8,48205E+14	2,91553E+14	0.0247385828911808	0.157716678117479	-3,17958E+14	9,30179E+14	9,50303E+14	8,79668E+14	7,47736E+14	8,27479E+14	7,53866E+14
TC1100003394.mm.2	Mmp28	matrix metallopeptidase 28 (epilysin)	-1,08037E+14	6,21748E+14	-2,91547E+14	0.0247408088664372	0.157716678117479	-3,17966E+13	5,45615E+13	5,55646E+14	6,36951E+14	6,74941E+13	6,21589E+14	6,95747E+14
TC0100000637.mm.2	Plcd4	phospholipase C, delta 4	1,1524E+14	6,12327E+14	2,91531E+14	0.0247461588076164	0.157716678117479	-3,17987E+14	6,72637E+14	6,44019E+14	6,29985E+14	6,41146E+14	5,89281E+14	4,96896E+14
TC0200004844.mm.2	Vsx1	visual system homeobox 1	-0.945224174461739	6,47341E+14	-2,91507E+14	0.0247542654353072	0.157716678117479	-3,18018E+14	5,87124E+13	6,05515E+14	6,61698E+14	6,48026E+14	6,66452E+14	7,15232E+14
TC1700001677.mm.2	Mcrip2	MAPK regulated corepressor interacting protein 2	-0.998786691642612	6,13838E+14	-2,91434E+14	0.0247786447223965	0.157807772292537	-3,18111E+12	5,88181E+14	5,43194E+14	6,30885E+14	5,89634E+14	6,51757E+14	6,79375E+14
TC1300000462.mm.2	Pak1ip1	PAK1 interacting protein 1	1,00223E+14	7,97957E+14	2,91386E+14	0.0247949059407038	0.157807772292537	-3,18173E+14	8,68835E+14	8,67686E+14	7,75888E+13	7,39261E+14	7,96562E+14	7,39513E+14
TC0900002220.mm.2	4931429L15Rik	RIKEN cDNA 4931429L15 gene	-1,35943E+14	6,59896E+14	-2,91377E+14	0.0247979444823872	0.157807772292537	-3,18184E+14	5,64018E+14	5,87573E+14	6,53739E+13	7,30569E+13	6,59489E+14	7,63989E+14
TC1200002242.mm.2	Fbln5	fibulin 5	0.88413863603607	5,64062E+12	2,91257E+14	0.0248382473575889	0.157900762791384	-3,18337E+14	6,26426E+14	5,98443E+14	5,47202E+14	5,64257E+14	5,3034E+14	5,17702E+14
TC0800001093.mm.2	Mt3	metallothionein 3	0.895691921076146	8,92669E+14	2,9125E+13	0.0248406658278896	0.157900762791384	-3,18347E+14	9,72544E+14	9,46184E+14	8,39083E+14	8,58614E+14	8,58958E+14	8,80631E+14
TC0700004411.mm.2	Nkx1-2	NK1 homeobox 2	-1,21947E+14	5,06541E+14	-2,91246E+14	0.0248419557919791	0.157900762791384	-3,18351E+14	4,63155E+14	3,99448E+14	4,99448E+14	5,70697E+14	5,26814E+14	5,79683E+14
TC1500001403.mm.2	Ubr5	ubiquitin protein ligase E3 component n-recognin 5	1,81261E+13	6,11711E+14	2,91207E+14	0.0248551427833093	0.15791351135366	-3,18401E+14	7,42285E+14	7,53213E+13	5,58772E+14	4,83025E+14	6,57805E+14	4,7517E+14
TC0500000317.mm.2	Nrbp1	nuclear receptor binding protein 1	2,39503E+14	7,07357E+13	2,91166E+14	0.0248689052095176	0.15791351135366	-3,18454E+12	8,71869E+14	8,75883E+14	7,44518E+14	4,83128E+14	6,79271E+14	5,89475E+14
TC0700003610.mm.2	BC048679	cDNA sequence BC048679	-1,13296E+14	6,37459E+14	-2,91153E+14	0.0248733626150059	0.15791351135366	-3,18471E+14	5,62399E+14	5,75988E+14	6,17264E+14	7,0412E+14	6,65695E+14	6,99286E+14
TC0300001549.mm.2	Slc44a5	solute carrier family 44, member 5	1,16724E+14	5,98399E+14	2,91015E+13	0.024919941774905	0.158037548700058	-3,18647E+14	6,55416E+14	6,34982E+14	6,53272E+13	5,89773E+14	5,65969E+14	4,90981E+14
TC1600000376.mm.2	Il1rap	interleukin 1 receptor accessory protein	0.981837708983928	6,1491E+14	2,91012E+14	0.0249210463913131	0.158037548700058	-3,18651E+14	6,72658E+14	7,11306E+13	5,73856E+14	5,44044E+14	6,14045E+14	5,73551E+14
TC1700000535.mm.2	Pde9a	phosphodiesterase 9A	0.94118615025924	6,99217E+14	2,90996E+14	0.0249265130719489	0.158037548700058	-3,18672E+13	7,83523E+14	7,39814E+14	6,73659E+14	6,63209E+14	6,5326E+13	6,81839E+14
TC1100001238.mm.2	Slfn2	schlafen 2	-0.943207644881974	5,77185E+14	-2,90979E+14	0.0249321323648946	0.158037548700058	-3,18693E+14	5,09673E+14	5,47972E+14	5,87217E+14	5,71959E+14	6,40438E+14	6,05849E+14
TC0100003861.mm.2	Tcea1	transcription elongation factor A (SII) 1	1,51947E+14	8,12468E+14	2,90916E+14	0.0249535643968056	0.15805606724347	-3,18774E+14	8,92874E+14	9,11954E+14	8,36395E+14	7,32652E+14	8,0822E+14	6,92714E+13
TC0300002475.mm.2	Setdb1	SET domain, bifurcated 1	1,13288E+13	6,39577E+14	2,90913E+13	0.0249546723184627	0.15805606724347	-3,18779E+14	7,3403E+14	6,95143E+14	6,10538E+14	5,95158E+14	6,43541E+14	5,59055E+14
TC0100002696.mm.2	Dner	delta/notch-like EGF repeat containing	1,72846E+14	7,45484E+14	2,90869E+14	0.0249693337272658	0.158086787562842	-3,18834E+14	8,80621E+14	8,29966E+14	7,83785E+14	6,13637E+14	7,11092E+14	6,53802E+14
TC1600001917.mm.2	Hspa13	heat shock protein 70 family, member 13	1,43074E+14	8,07536E+14	2,90788E+14	0.0249968932668691	0.15817607835497	-3,18938E+13	9,20286E+14	9,04827E+14	8,04758E+14	6,76382E+13	8,00298E+14	7,38667E+14
TC1200001749.mm.2	Eapp	E2F-associated phosphoprotein	1,04619E+14	6,69585E+13	2,9077E+14	0.0250030702892143	0.15817607835497	-3,18961E+14	7,24519E+14	7,3378E+14	6,57508E+14	6,5264E+14	6,63009E+14	5,86051E+14
TC0700001888.mm.2	Inpp5f	inositol polyphosphate-5-phosphatase F	2,21219E+14	7,23917E+14	2,90731E+14	0.0250163938192596	0.158183340647309	-3,19012E+12	8,75145E+14	8,45374E+14	7,49193E+14	5,9571E+14	7,38292E+14	5,39789E+13
TC0X00002805.mm.2	Magee2	melanoma antigen, family E, 2	1,65061E+14	5,58585E+14	2,90655E+14	0.0250422530200067	0.158183340647309	-3,19109E+14	7,11513E+14	6,46897E+14	5,63942E+14	4,00866E+13	5,09356E+14	5,18932E+14
TC1100001490.mm.2	Gm53	predicted gene 53	-0.85866469019075	6,5897E+14	-2,90649E+14	0.0250441932167353	0.158183340647309	-3,19116E+14	6,11552E+14	6,10792E+14	6,56539E+14	6,80858E+14	6,98999E+13	6,95078E+14
TC1100004290.mm.2	Commd1	COMM domain containing 1	1,21304E+13	6,23083E+14	2,9063E+14	0.0250505583287566	0.158183340647309	-3,1914E+14	6,7825E+14	6,97139E+14	6,66319E+14	5,64009E+14	5,81912E+14	5,50869E+14

TC0100000652.mm.2	Retreg2	reticulophagy regulator family member 2	1,02465E+14	8,35422E+14	2,90622E+14	0.0250533038746313	0.158183340647309	-3,19151E+14	9,12216E+14	8,78114E+14	8,46547E+14	7,90256E+14	8,08809E+14	7,76591E+13
TC0800002807.mm.2	Rrad	Ras-related associated with diabetes	-0.886989528451442	5,16931E+14	-2,90467E+14	0.0251063069544599	0.158455904409406	-3,1935E+14	4,80129E+14	4,66937E+14	5,07503E+14	5,2255E+14	5,46621E+14	5,77843E+14
TC0600002282.mm.2	Hoxa9/Mir196b	homeobox A9/microRNA 196b	-1,26903E+14	5,77354E+14	-2,90358E+14	0.0251432634948358	0.158627018282024	-3,19489E+14	5,02974E+14	4,77004E+14	5,7292E+14	6,77441E+14	5,9265E+14	6,41134E+14
TC1300001541.mm.2	Nkapl	NFKB activating protein-like	-0.886963573434016	5,13856E+14	-2,90293E+14	0.0251655398582221	0.1586647388958	-3,19572E+14	4,52986E+14	4,60272E+14	5,21368E+14	5,57857E+14	5,46364E+14	5,44287E+13
TC0X00003185.mm.2	Ribc1	RIB43A domain with coiled-coils 1	-0.971210781224103	6,96246E+14	-2,90235E+14	0.0251854245172829	0.1586647388958	-3,19647E+14	6,2665E+14	6,52013E+13	6,85464E+14	7,40444E+14	7,31026E+14	7,41878E+13
TC1400001567.mm.2	Kcnma1	potassium large conductance calcium-activated channel, subfamily M, alpha member 1	2,47706E+14	7,51653E+14	2,90233E+13	0.0251861423167719	0.1586647388958	-3,1965E+14	9,01763E+14	9,46346E+14	7,65059E+14	5,44053E+13	7,66119E+14	5,86576E+14
TC0700000808.mm.2	Fam71e1	family with sequence similarity 71, member E1	-1,32798E+14	7,18205E+14	-2,90226E+14	0.0251886304285643	0.1586647388958	-3,19659E+14	6,33252E+14	6,30162E+14	7,03941E+14	8,12867E+14	7,31206E+14	7,97804E+14
TC0500000091.mm.2	Speer3	spermatogenesis associated glutamate (E)-rich protein 3	-1,11937E+14	4,68981E+14	-2,9015E+14	0.0252144157093946	0.158765095867712	-3,19756E+14	3,89645E+14	3,96266E+14	4,6434E+14	5,53852E+14	4,94078E+13	5,15706E+14
TC0200003423.mm.2	Nr5a1	nuclear receptor subfamily 5, group A, member 1	-0.870552811089368	5,58835E+14	-2,90078E+14	0.025239297053847	0.158859684933061	-3,19849E+14	5,17582E+14	5,34485E+14	5,38573E+14	5,36191E+13	6,26074E+14	6,00103E+14
TC0100002642.mm.2	Ap1s3	adaptor-related protein complex AP-1, sigma 3	-0.896213711212348	5,9727E+14	-2,90032E+14	0.0252550058512351	0.158878265208963	-3,19907E+13	5,28468E+14	5,58301E+14	6,27219E+14	6,03618E+14	6,25663E+14	6,40348E+14
TC1700002798.mm.2	Rab26	RAB26, member RAS oncogene family	-0.859606340365292	7,4066E+14	-2,90005E+14	0.0252643332731996	0.158878265208963	-3,19942E+14	6,91248E+14	7,06956E+14	7,40192E+14	7,35442E+14	7,74904E+14	7,95221E+14
TC0100002324.mm.2	Mfsd6	major facilitator superfamily domain containing 6	2,37616E+14	7,54742E+14	2,89982E+14	0.02527221835852	0.158878265208963	-3,19972E+14	9,1034E+14	8,9159E+13	7,94204E+14	6,05623E+12	7,63129E+14	5,63568E+14
TC1800001401.mm.2	Myoz3	myozenin 3	-1,40213E+14	6,42643E+12	-2,89954E+14	0.0252816900636618	0.158878265208963	-3,20007E+14	5,40357E+14	5,70059E+14	6,21062E+14	7,33538E+13	6,56937E+14	7,33904E+13
TC0100002499.mm.2	Lancl1	LanC (bacterial lantibiotic synthetase component C)-like 1	1,51321E+13	6,83506E+14	2,89884E+14	0.025305910243454	0.158968472418235	-3,20097E+14	7,86109E+14	7,73172E+14	7,17132E+14	5,67983E+14	6,50766E+14	6,05872E+14
TC1100001566.mm.2	Tcap	titin-cap	-0.915920304744178	6,03677E+14	-2,89792E+14	0.0253375894574272	0.159010776265906	-3,20215E+14	5,47952E+14	5,30269E+14	6,30442E+14	6,51994E+14	6,11645E+14	6,4976E+14
TC1100002664.mm.2	Sqstm1	sequestosome 1	1,13423E+14	9,77461E+14	2,89785E+14	0.0253397283364956	0.159010776265906	-3,20223E+14	1,05842E+14	1,0516E+14	9,80625E+14	8,90942E+14	9,57366E+14	9,25812E+14
TC0600002975.mm.2	Alox5	arachidonate 5-lipoxygenase	-1,2045E+14	6,69631E+14	-2,89778E+14	0.0253422499504032	0.159010776265906	-3,20233E+14	5,95086E+14	5,8916E+14	6,76551E+14	7,31842E+14	6,66445E+14	7,58702E+14
TC1800001280.mm.2	Mcc	mutated in colorectal cancers	1,04345E+14	6,83661E+14	2,89732E+14	0.0253581126682363	0.159048372683259	-3,20292E+14	7,30696E+14	7,24983E+14	7,06639E+14	6,92656E+13	5,853E+14	6,6169E+14
TC0800000786.mm.2	Babam1	BRISC and BRCA1 A complex member 1	0.978941254498978	7,79254E+14	2,89702E+14	0.0253683893895827	0.159050917600913	-3,2033E+14	8,54313E+14	8,49755E+14	7,62583E+14	7,00593E+14	7,61289E+13	7,46991E+13
TC0900002036.mm.2	Chek1	checkpoint kinase 1	-0.867808399706289	6,63995E+14	-2,89672E+14	0.0253789505876053	0.159055243414268	-3,20369E+14	6,23099E+13	5,97772E+14	6,81364E+14	6,87302E+14	7,05099E+14	6,89334E+14
TC0100002348.mm.2	Gtf3c3	general transcription factor IIIC, polypeptide 3	1,20113E+14	6,09617E+14	2,89509E+14	0.0254352839470401	0.159107156145871	-3,20578E+14	6,82384E+14	6,97421E+14	6,06598E+14	5,31716E+13	6,07496E+14	5,32084E+14
TC0400000739.mm.2	Trim32	tripartite motif-containing 32	1,21946E+14	6,78761E+13	2,89485E+14	0.0254432497151872	0.159107156145871	-3,20608E+14	7,61358E+14	7,57929E+14	6,44744E+14	6,33139E+14	6,92603E+14	5,82791E+14
TC0700002996.mm.2	Ccdc155	coiled-coil domain containing 155	-1,14551E+14	5,70882E+14	-2,89456E+14	0.0254533448062005	0.159107156145871	-3,20645E+14	5,04343E+14	5,10579E+14	5,38302E+14	6,28042E+14	6,03086E+13	6,4094E+14
TC0X00001071.mm.2	Pgk1	phosphoglycerate kinase 1	1,27676E+14	8,65502E+14	2,89455E+14	0.0254537713644739	0.159107156145871	-3,20647E+14	9,62437E+14	9,56619E+14	8,44333E+14	7,65918E+14	8,77191E+14	7,86513E+14
TC0200002812.mm.2	Col20a1	collagen, type XX, alpha 1	-0.863116641439116	7,1577E+14	-2,89448E+14	0.0254560941776377	0.159107156145871	-3,20656E+14	6,69465E+14	6,69761E+14	7,31795E+14	7,11752E+14	7,45719E+14	7,66129E+14
TC1500002189.mm.2	Wnt10b	wingless-type MMTV integration site family, member 10B	-1,39444E+14	6,05663E+14	-2,89368E+14	0.0254838084855619	0.159107156145871	-3,20758E+14	5,35809E+14	5,10152E+14	5,81622E+14	6,81545E+14	6,16042E+14	7,08807E+14
TC0600002492.mm.2	Tgoln1/Tgoln2	trans-golgi network protein/trans-golgi network protein 2	1,2901E+14	7,78788E+14	2,89365E+14	0.0254850382534399	0.159107156145871	-3,20763E+14	8,62736E+14	8,58108E+14	7,96619E+14	6,92445E+14	7,63323E+13	6,99501E+14
TC0500000661.mm.2	Guf1	GUF1 homolog, GTPase	1,47978E+14	5,16282E+14	2,8934E+14	0.0254934713880544	0.159107156145871	-3,20794E+13	5,64235E+14	5,85827E+14	5,79428E+14	5,14099E+13	4,94671E+14	3,59436E+14
TC1100001467.mm.2	Phb	prohibitin	1,06858E+14	8,49982E+14	2,89324E+14	0.0254991590488936	0.159107156145871	-3,20815E+14	9,36307E+14	9,28676E+14	8,24836E+14	7,58808E+14	8,50675E+11	8,0059E+14
TC0400003565.mm.2	Gja4	gap junction protein, alpha 4	-1,40397E+13	7,6329E+14	-2,89279E+14	0.0255146927459738	0.159107156145871	-3,20872E+14	6,67334E+14	6,78828E+14	7,37457E+14	8,69163E+14	7,90035E+14	8,36921E+14
TC0900000252.mm.2	B3gat1	beta-1,3-glucuronyltransferase 1 (glucuronosyltransferase P)	0.876408766325251	7,21217E+14	2,89275E+14	0.0255162519873163	0.159107156145871	-3,20878E+14	7,62391E+14	7,60172E+13	7,38861E+14	7,186E+14	6,59748E+14	6,87533E+14
TC0600003499.mm.2	Gimap7	GTPase, IMAP family member 7	-1,0298E+14	5,05395E+14	-2,89265E+14	0.0255195045294718	0.159107156145871	-3,2089E+13	4,36562E+14	4,35199E+14	5,05533E+14	5,77355E+14	5,50287E+14	5,27434E+14
TC0200002689.mm.2	1700021F07Rik	RIKEN cDNA 1700021F07 gene	-1,28035E+14	5,91293E+14	-2,89205E+13	0.0255404664941643	0.159107156145871	-3,20968E+14	4,79878E+14	5,53618E+14	5,7719E+14	6,47506E+14	6,12349E+14	6,77217E+14
TC0600002133.mm.2	Mrps33	mitochondrial ribosomal protein S33	1,25247E+14	8,19198E+13	2,8916E+14	0.0255561622586755	0.159107156145871	-3,21026E+14	8,9128E+14	8,87334E+14	8,28879E+14	7,79574E+14	8,16655E+14	7,11465E+14
TC1300001087.mm.2	Poc5	POC5 centriolar protein	0.895832353175283	6,00525E+14	2,89145E+14	0.0255614388045152	0.159107156145871	-3,21045E+14	6,65426E+14	6,45877E+14	5,69233E+14	5,90476E+14	5,81021E+14	5,51117E+14
TC0X00002643.mm.2	Eif2s3x	eukaryotic translation initiation factor 2, subunit 3, structural gene X-linked	1,54481E+14	8,38626E+14	2,89135E+14	0.0255647244970663	0.159107156145871	-3,21057E+13	9,4262E+14	9,42535E+14	8,61976E+14	7,08431E+14	8,13198E+14	7,62995E+14
TC0500001127.mm.2	Gm15446	predicted gene 15446	1,40796E+13	7,05058E+14	2,89129E+14	0.0255667416206832	0.159107156145871	-3,21065E+14	7,74588E+14	8,06792E+14	7,1636E+14	6,32821E+14	7,04358E+14	5,95431E+14
TC0200000551.mm.2	Ass1/Gm5424	argininosuccinate synthetase 1/argininosuccinate synthase pseudogene	0.91569510532822	7,83282E+14	2,89114E+14	0.0255720453043666	0.159107156145871	-3,21084E+13	8,56463E+14	8,21169E+14	7,71534E+14	7,56033E+14	7,61371E+13	7,33123E+14
TC0700003970.mm.2	Olfr684	olfactory receptor 684	-1,09471E+14	4,54187E+13	-2,89076E+14	0.0255853806040882	0.159107156145871	-3,21134E+14	4,09885E+14	3,77261E+14	4,25059E+14	5,06832E+14	4,98703E+14	5,07385E+14
TC0400003867.mm.2	Padi1	peptidyl arginine deiminase, type I	-1,55751E+13	4,88537E+14	-2,8907E+13	0.0255873370619771	0.159107156145871	-3,21141E+14	3,69635E+14	4,28899E+14	4,55396E+13	5,67258E+14	5,03312E+14	6,06724E+14
TC1900000777.mm.2	Vti1a	vesicle transport through interaction with t-SNAREs 1A	1,92465E+13	5,96198E+14	2,89021E+14	0.0256043637131121	0.159107156145871	-3,21203E+13	7,28246E+14	7,28266E+14	6,15349E+14	4,33748E+14	5,9375E+14	4,77832E+14
TC1000000386.mm.2	Sec63	SEC63-like (S. cerevisiae)	0.969904795698074	7,50719E+14	2,89021E+14	0.0256044719100256	0.159107156145871	-3,21204E+14	8,14422E+14	8,15177E+13	7,24143E+14	7,14953E+14	7,48554E+14	6,87064E+14
TC0100001060.mm.2	Clasp1	CLIP associating protein 1	1,41468E+13	7,51867E+14	2,88878E+14	0.0256543982723416	0.159300924265001	-3,21388E+14	8,47817E+14	8,2264E+14	7,86161E+14	6,67066E+14	7,30533E+14	6,56988E+14
TC0400002849.mm.2	Megf9	multiple EGF-like-domains 9	0.860597696565756	8,05335E+14	2,88861E+14	0.0256604236045063	0.159300924265001	-3,2141E+14	8,65237E+14	8,56673E+14	7,92676E+14	7,67634E+14	7,79834E+14	7,69956E+14
TC0500003712.mm.2	Denr	density-regulated protein	0.999009171371442	8,47158E+12	2,8881E+14	0.0256782193606017	0.159300924265001	-3,21475E+14	9,11648E+14	9,10796E+14	8,27506E+14	8,10353E+14	8,44772E+14	7,7787E+14
TC1000001577.mm.2	Sdr9c7	4short chain dehydrogenase/reductase family 9C, member 7	-0.989532418995775	4,79383E+14	-2,88809E+13	0.0256784840183865	0.159300924265001	-3,21476E+14	4,57374E+14	4,0472E+14	4,79476E+14	4,74726E+14	5,09474E+13	5,50527E+14
TC0800002399.mm.2	Gdf15	growth differentiation factor 15	-0.954711313677759	5,99496E+14	-2,88749E+14	0.0256993544961549	0.159300924265001	-3,21553E+14	5,2109E+14	5,66913E+14	6,20191E+14	6,09836E+14	6,219E+14	6,57046E+13
TC0600000294.mm.2	Mkln1	muskelin 1, intracellular mediator containing kelch motifs	1,64429E+14	7,9505E+14	2,88721E+13	0.0257091984095489	0.159300924265001	-3,21589E+14	8,67072E+14	8,64535E+13	8,41751E+14	7,94192E+14	7,86269E+14	6,1648E+14
TC0100001088.mm.2	En1	engrailed 1	-0.872003660980149	5,20775E+14	-2,88678E+14	0.0257240940297156	0.159300924265001	-3,21644E+14	4,60263E+14	4,80311E+14	5,44324E+14	5,24776E+14	5,48358E+14	5,66616E+14
TC1400000851.mm.2	Spata13	spermatogenesis associated 13	1,04874E+14	6,21239E+13	2,88533E+14	0.0257749556003165	0.159300924265001	-3,2183E+14	6,74241E+14	6,71536E+14	6,07148E+14	6,3848E+14	5,31325E+14	6,04704E+14
TC0800000796.mm.2	Slc27a1	solute carrier family 27 (fatty acid transporter), member 1	1,05855E+13	8,34732E+14	2,88512E+14	0.0257824191604244	0.159300924265001	-3,21857E+13	9,02284E+14	9,10085E+14	8,3785E+14	7,57512E+14	8,07045E+14	7,93614E+13
TC1200001344.mm.2	1110002L01Rik	RIKEN cDNA 1110002L01 gene	0.855402170923979	5,44063E+14	2,88491E+13	0.0257898423589305	0.159300924265001	-3,21884E+14	6,03192E+14	5,86953E+14	5,17305E+14	5,37865E+13	5,08654E+14	5,10411E+14
TC1400000075.mm.2	Rpp14	ribonuclease P 14 subunit	1,62415E+14	7,05747E+14	2,88423E+14	0.0258135987781271	0.159300924265001	-3,21971E+14	7,79918E+14	7,91453E+14	7,13444E+14	7,03126E+14	7,09766E+14	5,36775E+13
TC1900001031.mm.2	Stip1	stress-induced phosphoprotein 1	1,60758E+14	8,78451E+14	2,88412E+14	0.0258175080580297	0.159300924265001	-3,21986E+14	9,96963E+14	9,69074E+14	9,03624E+14	7,56522E+14	8,63864E+14	7,80657E+14
TC0500001370.mm.2	Atxn2	ataxin 2	1,12145E+14	9,26913E+13	2,88408E+14	0.025818912418617	0.159300924265001	-3,21991E+14	9,93009E+14	9,64804E+14	9,64678E+14	9,05465E+13	9,00401E+14	8,33122E+14
TC1400001718.mm.2	A630023A22Rik	RIKEN cDNA A630023A22 gene	-1,10539E+14	5,52259E+14	-2,88334E+14	0.0258449883653631	0.159300924265001	-3,22086E+14	5,06517E+14	4,36479E+14	5,9953E+14	6,06954E+14	5,53208E+14	6,10866E+14
TC0400001957.mm.2	Clstn1	calsyntenin 1	1,42432E+14	9,047E+14	2,88332E+14	0.0258455363275835	0.159300924265001	-3,22088E+14	1,01567E+14	1,01412E+14	8,95071E+14	7,58417E+13	8,95321E+14	8,49603E+14
TC0700004573.mm.2	Ins2	insulin II	-1,47499E+14	7,42687E+14	-2,88318E+14	0.0258505548532956	0.159300924265001	-3,22106E+14	6,45052E+14	6,59766E+14	7,19202E+14	8,32283E+14	7,49811E+14	8,50006E+14
TC0800000661.mm.2	Spock3	sparc/osteonectin, cwcv and kazal-like domains proteoglycan 3	1,34464E+14	8,65387E+14	2,8831E+14	0.0258532361935208	0.159300924265001	-3,22116E+14	9,64134E+14	9,53226E+14	8,73327E+14	7,532E+14	8,50712E+14	7,97722E+14
TC1900001346.mm.2	Ermp1	endoplasmic reticulum metallopeptidase 1	1,7099E+14	6,36786E+14	2,88301E+13	0.0258565420699418	0.159300924265001	-3,22128E+14	7,64393E+14	7,26347E+14	6,81093E+14	5,00125E+14	5,64051E+14	5,84708E+14
TC0800002390.mm.2	Upf1	UPF1 regulator of nonsense transcripts homolog (yeast)	1,29045E+14	5,77339E+14	2,88293E+14	0.0258594618921271	0.159300924265001	-3,22139E+14	6,7347E+14	6,71599E+14	5,77442E+14	4,54543E+14	5,4017E+14	5,46809E+14

TC0500001614.mm.2	Cldn15	claudin 15	-1,34017E+14	5,80584E+13	-2,8829E+14	0.0258604937158132	0.159300924265001	-3,22143E+14	4,7954E+14	5,0272E+14	5,88279E+14	6,62672E+14	5,72459E+14	6,77836E+14
TC0X00001380.mm.2	Frmpd3	FERM and PDZ domain containing 3	1,2171E+14	6,33895E+14	2,88273E+14	0.0258662176813811	0.159300924265001	-3,22163E+14	7,07134E+14	6,80244E+14	5,83812E+14	6,88224E+14	5,85049E+14	5,58908E+14
TC0400001382.mm.2	Ppt1	palmitoyl-protein thioesterase 1	1,62202E+14	8,03528E+14	2,88207E+14	0.0258895764879577	0.159300924265001	-3,22249E+13	9,26738E+14	8,98031E+14	8,03887E+14	6,92149E+14	8,15008E+14	6,85356E+13
TC0200004482.mm.2	Trp53bp1	transformation related protein 53 binding protein 1	1,95344E+14	6,58389E+14	2,88174E+14	0.0259011164377621	0.159300924265001	-3,22291E+13	8,22269E+14	7,88591E+14	6,60811E+14	4,58492E+14	6,50127E+12	5,70045E+14
TC1000001282.mm.2	E2f7	E2F transcription factor 7	-1,58332E+14	7,20405E+14	-2,88155E+14	0.0259078825116623	0.159300924265001	-3,22315E+14	6,08262E+14	6,31121E+14	6,85632E+14	8,41366E+13	7,4871E+14	8,07337E+14
TC0900001785.mm.2	4931406C07Rik	RIKEN cDNA 4931406C07 gene	1,54625E+14	6,33936E+14	2,88124E+14	0.0259189904614667	0.159300924265001	-3,22356E+14	7,09572E+14	7,22055E+14	6,84123E+14	5,65489E+14	6,16401E+14	5,05977E+14
TC1700001981.mm.2	Tubb5	tubulin, beta 5 class I	1,95045E+14	9,8109E+14	2,88066E+13	0.025939283569382	0.159300924265001	-3,2243E+14	1,11253E+14	1,1184E+14	1,00938E+13	8,05376E+14	9,61319E+14	8,79526E+14
TC0200000501.mm.2	Setx	senataxin	1,07117E+14	5,76565E+14	2,88055E+14	0.025943096656267	0.159300924265001	-3,22443E+14	6,77453E+14	6,69967E+14	4,66831E+14	5,11949E+14	6,02214E+14	5,30972E+13
TC0200005290.mm.2	Zbp1	Z-DNA binding protein 1	-1,09036E+14	5,29466E+14	-2,88018E+14	0.0259561525676624	0.159300924265001	-3,22491E+14	4,63721E+14	4,76735E+14	4,99484E+14	5,78327E+14	5,60363E+14	5,98165E+14
TC0X00000963.mm.2	Dlg3	discs large MAGUK scaffold protein 3	1,71239E+14	7,77152E+14	2,88013E+14	0.0259580347710581	0.159300924265001	-3,22498E+14	8,93187E+14	8,81413E+14	8,08161E+14	6,48029E+14	7,63667E+14	6,68455E+14
TC0100001200.mm.2	Lrrn2	leucine rich repeat protein 2, neuronal	-0.958952202759884	7,65864E+14	-2,87983E+14	0.0259687632767831	0.159300924265001	-3,22537E+14	7,00635E+14	7,12185E+14	7,71317E+14	8,06433E+14	8,26162E+14	7,78449E+14
TC0100002831.mm.2	Mterf4	mitochondrial transcription termination factor 4	0.897561414570415	6,0888E+14	2,87954E+14	0.0259789221902037	0.159300924265001	-3,22574E+14	6,5821E+14	6,43895E+14	5,94807E+14	6,3378E+14	5,67505E+14	5,55087E+14
TC1500002175.mm.2	Lalba	lactalbumin, alpha	-1,11504E+13	5,09371E+14	-2,87871E+14	0.0260083476273744	0.159300924265001	-3,2268E+14	4,41008E+14	4,59153E+13	4,92493E+14	5,404E+14	5,22681E+14	6,00489E+14
TC1300001814.mm.2	F13a1	coagulation factor XIII, A1 subunit	-0.922959286462616	5,68904E+14	-2,87854E+14	0.0260145224148037	0.159300924265001	-3,22703E+14	5,09412E+14	5,31611E+14	5,57612E+14	5,89176E+14	5,96108E+14	6,29506E+14
TC0500002077.mm.2	Cdk5	cyclin-dependent kinase 5	1,23726E+14	7,82074E+14	2,87829E+14	0.0260231594044043	0.159300924265001	-3,22734E+14	8,94112E+14	8,71632E+14	7,57865E+13	6,50543E+14	7,75909E+14	7,42383E+14
TC1600001758.mm.2	Tbc1d23	TBC1 domain family, member 23	1,11848E+14	6,83939E+14	2,87814E+14	0.0260284832029672	0.159300924265001	-3,22753E+14	7,89145E+14	7,71869E+14	6,37987E+14	5,67313E+14	6,9829E+14	6,39028E+14
TC1200001047.mm.2	Serpina3n	serine (or cysteine) peptidase inhibitor, clade A, member 3N	1,42573E+14	7,66112E+14	2,87775E+13	0.0260425003348059	0.159300924265001	-3,22804E+14	8,66106E+14	8,93375E+14	7,50771E+13	6,12082E+14	7,21575E+14	7,5276E+14
TC1700000624.mm.2	Notch4	notch 4	-0.928451034343437	6,48726E+13	-2,87755E+14	0.0260493510997028	0.159300924265001	-3,22829E+14	5,81734E+14	5,86648E+14	6,6004E+14	7,0986E+14	6,68892E+13	6,8518E+14
TC1700000899.mm.2	Cul7	cullin 7	2,19681E+14	6,80923E+13	2,87752E+14	0.0260504995139973	0.159300924265001	-3,22833E+13	8,30556E+14	8,10752E+14	7,47412E+14	4,94872E+14	6,07582E+14	5,94364E+14
TC1100002160.mm.2	Nudcd3	NudC domain containing 3	1,54975E+14	6,9962E+14	2,87708E+14	0.0260662387789828	0.159300924265001	-3,2289E+14	7,90203E+14	7,98014E+14	7,00153E+14	6,31084E+14	7,13072E+13	5,65195E+14
TC0300000749.mm.2	Fam189b	family with sequence similarity 189, member B	-0.854679028140591	7,1125E+14	-2,8769E+14	0.0260724666702147	0.159300924265001	-3,22913E+14	6,63484E+14	6,62703E+14	7,15539E+14	7,28648E+14	7,60403E+14	7,3672E+14
TC1700002438.mm.2	Ptprm	protein tyrosine phosphatase, receptor type, M	1,62698E+14	6,51908E+14	2,87687E+14	0.0260734321377468	0.159300924265001	-3,22916E+14	7,79362E+14	7,31623E+14	6,60154E+14	5,54718E+14	6,58219E+14	5,27371E+14
TC1100004121.mm.2	Ube2o	ubiquitin-conjugating enzyme E2O	1,15951E+14	7,33493E+14	2,87671E+14	0.0260791523558756	0.159300924265001	-3,22937E+14	8,17688E+14	8,03809E+14	6,60302E+14	7,29564E+14	7,31155E+14	6,58441E+14
TC0100002606.mm.2	Ptprn	protein tyrosine phosphatase, receptor type, N	1,89997E+14	9,15098E+14	2,87669E+14	0.0260798053124073	0.159300924265001	-3,22939E+14	1,04579E+14	1,03305E+14	9,44329E+14	7,68581E+14	9,09498E+14	7,89345E+14
TC0100001178.mm.2	Nucks1	nuclear casein kinase and cyclin-dependent kinase substrate 1	1,33165E+14	8,52274E+14	2,87649E+14	0.0260872181856219	0.159300924265001	-3,22966E+14	9,39912E+14	9,37227E+14	8,61925E+14	7,63775E+14	8,4814E+14	7,62668E+14
TC1800000265.mm.2	Wdr36	WD repeat domain 36	0.862609903975557	6,58366E+14	2,87612E+14	0.0261002529578703	0.159300924265001	-3,23013E+14	6,95285E+14	7,1141E+14	6,67619E+14	6,4171E+14	6,14848E+14	6,19325E+14
TC0200002310.mm.2	Tm9sf4	transmembrane 9 superfamily protein member 4	2,00539E+14	7,04057E+14	2,87594E+13	0.0261066156444983	0.159300924265001	-3,23036E+14	8,34785E+14	8,40857E+14	7,45585E+14	5,28554E+14	6,71844E+14	6,02719E+14
TC1200001279.mm.2	Tex22	testis expressed gene 22	-1,64793E+14	6,93898E+14	-2,87586E+14	0.0261094693653109	0.159300924265001	-3,23047E+14	5,94854E+14	5,61899E+14	6,89425E+14	8,30872E+14	6,91887E+14	7,94452E+13
TC0900001954.mm.2	Ntm	neurotrimin	1,12876E+13	9,0872E+14	2,87565E+14	0.0261167648850652	0.159300924265001	-3,23073E+14	9,88519E+14	9,91206E+14	9,01986E+14	8,16637E+14	8,98819E+14	8,55154E+12
TC1300001638.mm.2	Hist1h2ac	histone cluster 1, H2ac	-1,1637E+14	9,10602E+14	-2,87555E+14	0.0261203933083661	0.159300924265001	-3,23086E+14	8,36218E+13	8,21681E+14	9,27786E+14	9,87289E+14	9,04413E+13	9,86226E+14
TC1700001946.mm.2	Ly6g5b	lymphocyte antigen 6 complex, locus G5B	-1,61103E+14	5,53335E+13	-2,87524E+14	0.0261314995454655	0.159300924265001	-3,23126E+14	4,56131E+14	4,462E+14	5,23903E+14	6,6924E+14	5,66066E+14	6,58471E+14
TC0600002635.mm.2	Pcyox1	prenylcysteine oxidase 1	1,07071E+14	8,00974E+14	2,87491E+13	0.0261433802417179	0.159300924265001	-3,23169E+14	8,83283E+13	8,61592E+14	7,91053E+14	7,39186E+14	7,9723E+14	7,33502E+13
TC1900000684.mm.2	Elovl3	elongation of very long chain fatty acids (FEN1/Elo2, SUR4/Elo3, yeast)-like 3	-0.966052244036358	4,65742E+14	-2,87473E+14	0.0261497514231322	0.159300924265001	-3,23192E+14	4,17431E+14	3,85183E+14	4,76172E+14	5,19842E+14	4,82425E+14	5,134E+14
TC1400002600.mm.2	Dis3	DIS3 homolog, exosome endoribonuclease and 3'-5' exoribonuclease	1,25254E+14	5,43729E+13	2,87394E+14	0.0261776869426046	0.159337773199879	-3,23293E+14	6,49317E+14	6,1168E+13	5,11369E+12	4,79517E+14	4,51905E+14	5,58584E+14
TC0600003535.mm.2	E130112N10Rik	RIKEN cDNA E130112N10 gene	-0.930024482808771	6,33454E+13	-2,8736E+14	0.0261898918672567	0.159337773199879	-3,23337E+14	5,66078E+14	5,77653E+14	6,4947E+14	6,77786E+14	6,4297E+14	6,86766E+14
TC0200004626.mm.2	Pced1a	PC-esterase domain containing 1A	1,07015E+14	5,36544E+14	2,87334E+13	0.0261991462222779	0.159337773199879	-3,2337E+13	6,24071E+14	5,89593E+13	5,40064E+14	4,659E+14	5,11363E+14	4,88271E+14
TC1500000183.mm.2	Ctnnd2	catenin (cadherin associated protein), delta 2	1,06401E+14	8,80745E+14	2,87302E+14	0.0262106642410971	0.159337773199879	-3,23412E+13	9,42149E+14	9,25183E+14	9,21185E+14	8,41424E+14	8,36916E+14	8,17614E+14
TC0900001372.mm.2	Zmynd10	zinc finger, MYND domain containing 10	0.986845146470151	5,3479E+14	2,87281E+14	0.0262182747415896	0.159337773199879	-3,23439E+14	6,09524E+14	5,93896E+14	4,73485E+14	5,25782E+14	4,83155E+14	5,22896E+14
TC0700003013.mm.2	Fgf21	fibroblast growth factor 21	-0.882181578262257	4,66575E+14	-2,87238E+14	0.0262333927233029	0.159337773199879	-3,23493E+14	4,23589E+14	3,91165E+14	4,89807E+14	5,03698E+14	5,03802E+14	4,87389E+14
TC0700001516.mm.2	Olfr641	olfactory receptor 641	-1,32486E+14	5,00265E+14	-2,87203E+14	0.026246121740319	0.159337773199879	-3,23539E+14	4,14815E+14	4,04099E+14	4,93415E+14	6,05377E+13	5,09927E+14	5,73958E+14
TC1900000054.mm.2	Rin1	Ras and Rab interactor 1	1,19165E+14	6,7056E+13	2,8719E+14	0.0262505311591361	0.159337773199879	-3,23555E+14	7,35493E+14	7,62534E+14	6,77224E+14	5,8841E+14	6,02894E+14	6,56804E+14
TC1200000017.mm.2	Pomc	pro-opiomelanocortin-alpha	-1,6966E+14	6,26917E+14	-2,87179E+14	0.0262546321819461	0.159337773199879	-3,2357E+14	5,37241E+14	5,02459E+14	5,91529E+14	7,51256E+14	6,46068E+14	7,32952E+14
TC0700001741.mm.2	Otoa	otoancorin	-0.986451258370662	5,59239E+14	-2,87128E+14	0.0262730116246751	0.159337773199879	-3,23636E+14	5,00818E+14	4,97658E+14	5,58503E+14	6,02686E+14	5,72386E+14	6,23381E+14
TC0400002392.mm.2	Zfp292	zinc finger protein 292	0.962129185133778	6,40235E+14	2,87121E+14	0.0262755387622117	0.159337773199879	-3,23645E+14	7,02541E+13	7,07791E+14	6,21068E+14	5,92103E+14	6,36145E+14	5,81762E+14
TC0700000564.mm.2	Prodh2	proline dehydrogenase (oxidase) 2	-1,24584E+14	7,04918E+14	-2,87092E+14	0.0262856364825806	0.159337773199879	-3,23681E+14	6,42765E+14	6,1719E+14	6,92852E+14	7,67577E+14	7,11655E+14	7,97468E+14
TC1100002451.mm.2	Chac2	ChaC, cation transport regulator 2	0.866581036689084	6,01082E+14	2,87091E+14	0.0262860865545755	0.159337773199879	-3,23683E+14	6,59426E+14	6,83527E+14	5,54364E+14	5,39542E+14	5,93477E+14	5,76159E+14
TC0400001276.mm.2	Ptch2	patched 2	-1,34684E+14	7,0101E+14	-2,87058E+14	0.0262977577665843	0.159337773199879	-3,23724E+13	5,89153E+14	6,37813E+13	6,94001E+14	7,88762E+14	7,08333E+14	7,88E+13
TC0400004009.mm.2	Clcn6	chloride channel, voltage-sensitive 6	0.979910417099911	7,74675E+14	2,86997E+13	0.0263197762356848	0.159337773199879	-3,23803E+14	8,41477E+14	8,03312E+14	7,92649E+14	7,61807E+14	7,46584E+14	7,02223E+14
TC0400003506.mm.2	Utp11	UTP11 small subunit processome component	1,37193E+14	6,0936E+13	2,86991E+14	0.0263220383957382	0.159337773199879	-3,23812E+14	6,98539E+14	6,95495E+14	6,18247E+14	5,24229E+14	6,09635E+14	5,10014E+14
TC0900003037.mm.2	Sema3b	sema domain, immunoglobulin domain (Ig), short basic domain, secreted, (semaphorin) 3B	-0.854288511778742	6,5172E+14	-2,86969E+14	0.0263298799554394	0.159337773199879	-3,2384E+14	5,92455E+14	5,96826E+14	6,69121E+14	6,91782E+14	6,87934E+14	6,72204E+14
TC0100003439.mm.2	Sft2d2	SFT2 domain containing 2	1,11868E+13	6,50888E+14	2,86958E+14	0.0263337981773275	0.159337773199879	-3,23854E+14	7,29252E+14	7,20782E+14	6,42519E+14	5,86475E+14	6,5095E+14	5,75349E+14
TC0200004217.mm.2	Fjx1	four jointed box 1	1,0568E+14	8,137E+14	2,86905E+13	0.0263529018342045	0.159367872936095	-3,23922E+14	8,87653E+14	8,72561E+14	8,25926E+14	7,47205E+14	7,64334E+14	7,8452E+14
TC1100000109.mm.2	Adcy1	adenylate cyclase 1	1,06374E+14	7,39293E+14	2,86887E+14	0.0263593798325975	0.159367872936095	-3,23945E+14	7,84704E+14	8,32467E+14	6,98429E+14	7,15734E+14	7,35691E+14	6,68732E+14
TC0900000865.mm.2	Dapk2	death-associated protein kinase 2	-1,16924E+14	5,81812E+14	-2,86795E+14	0.026392502584399	0.159367872936095	-3,24064E+14	5,17728E+14	5,03383E+14	5,69033E+13	6,45766E+14	5,93327E+14	6,61632E+14
TC0600003005.mm.2	Cacna1c	calcium channel, voltage-dependent, L type, alpha 1C subunit	1,1255E+14	7,15518E+14	2,86785E+14	0.0263958751993166	0.159367872936095	-3,24076E+14	7,96337E+14	7,9157E+14	7,03253E+14	6,39142E+14	7,16965E+13	6,45842E+14
TC0500001183.mm.2	Gm6588	predicted gene 6588	-0.958890021113431	5,32272E+14	-2,86767E+14	0.0264023547546204	0.159367872936095	-3,24099E+13	4,7868E+14	4,92959E+14	5,03649E+14	5,54925E+14	5,66467E+14	5,96951E+14
TC1500002231.mm.2	2310068J16Rik	RIKEN cDNA 2310068J16 gene	-0.866739984301046	5,1211E+14	-2,86754E+14	0.0264069469881291	0.159367872936095	-3,24115E+13	4,76895E+14	4,45424E+14	5,21386E+14	5,33288E+14	5,47605E+14	5,48063E+14
TC1100002120.mm.2	Cabp7	calcium binding protein 7	1,02679E+13	6,35746E+14	2,86721E+13	0.0264189061282643	0.159367872936095	-3,24158E+14	7,06446E+14	6,95036E+14	5,99452E+13	6,17418E+14	5,61241E+14	6,34884E+14
TC1900001119.mm.2	Ms4a10	membrane-spanning 4-domains, subfamily A, member 10	-1,06457E+14	5,58032E+14	-2,86687E+14	0.0264311222292929	0.159367872936095	-3,24202E+14	4,84704E+11	4,92122E+14	5,63416E+14	6,18207E+14	5,63013E+14	6,26727E+14
TC0600003446.mm.2	Mansc4	MANSC domain containing 4	-1,28305E+14	5,42553E+14	-2,86678E+14	0.0264345968056743	0.159367872936095	-3,24214E+14	4,38602E+14	4,74639E+14	5,37877E+14	6,34348E+14	5,5124E+14	6,18612E+14
TC0100001721.mm.2	Lefty2	left-right determination factor 2	-2,03036E+14	6,6434E+13	-2,86669E+14	0.0264376791632957	0.159367872936095	-3,24225E+14	5,38626E+14	5,39627E+14	6,14659E+14	8,08802E+13	6,7851E+14	8,05815E+14
TC0X00000703.mm.2	Emd	emerin	1,45863E+14	7,64191E+14	2,86601E+14	0.0264621859025386	0.15940548196176	-3,24313E+14	8,70208E+14	8,48088E+14	7,89528E+14	6,5075E+14	7,40429E+14	6,86142E+14
TC0200002517.mm.2	Pigt	phosphatidylinositol glycan anchor biosynthesis, class T	1,55503E+14	8,60741E+14	2,86597E+14	0.0264637041052386	0.15940548196176	-3,24318E+14	9,69219E+14	9,47503E+13	8,99721E+14	7,42288E+14	8,25158E+14	7,80558E+14
TC1100001564.mm.2	Ppp1r1b	protein phosphatase 1, regulatory inhibitor subunit 1B	1,17826E+14	9,46425E+14	2,86489E+14	0.0265025390486556	0.159543547800424	-3,24456E+13	1,01904E+14	1,02505E+14	9,61427E+14	8,64591E+14	9,24977E+14	8,83463E+14

TC0900002727.mm.2	Eef1a1	eukaryotic translation elongation factor 1 alpha 1	1,30155E+14	1,18077E+14	2,86452E+14	0.026516117868845	0.159543547800424	-3,24505E+14	1,26811E+14	1,27375E+14	1,17681E+14	1,0844E+14	1,18367E+14	1,09787E+14
TC0700004506.mm.2	Zfp941	zinc finger protein 941	1,01666E+14	5,47859E+14	2,86451E+14	0.0265163297343472	0.159543547800424	-3,24505E+14	6,46627E+14	5,8835E+14	5,32541E+14	4,87989E+14	5,08716E+14	5,22929E+14
TC1700002488.mm.2	Myl12a	myosin, light chain 12A, regulatory, non-sarcomeric	1,02675E+14	9,70438E+14	2,8642E+14	0.0265276139891006	0.159551864205442	-3,24545E+14	1,03835E+14	1,03952E+14	9,51566E+14	9,20656E+14	9,71452E+14	9,01076E+14
TC0900000605.mm.2	Cryab	crystallin, alpha B	1,15422E+14	8,79012E+14	2,86301E+14	0.0265705928564886	0.159750732349478	-3,24698E+14	9,36886E+14	9,71494E+14	8,73365E+14	8,1479E+14	8,76374E+14	8,01162E+14
TC1400002065.mm.2	Cebpe	CCAAT/enhancer binding protein (C/EBP), epsilon	-1,20869E+14	5,63062E+14	-2,86261E+14	0.0265850572326206	0.159778078026563	-3,2475E+14	4,86029E+14	4,79688E+13	5,56472E+14	6,4873E+14	5,73069E+14	6,34386E+14
TC1300000969.mm.2	Cetn3	centrin 3	1,70705E+13	7,57786E+14	2,86185E+13	0.0266127136052647	0.159884658583755	-3,24848E+14	8,9449E+14	8,78945E+14	7,53121E+14	5,88133E+14	7,59829E+14	6,72196E+14
TC1300000476.mm.2	Hivep1	human immunodeficiency virus type I enhancer binding protein 1	1,98851E+13	6,58194E+14	2,86104E+14	0.0266423211460751	0.160002877609656	-3,24953E+14	7,60538E+14	7,8621E+14	6,8059E+14	5,72781E+14	6,73555E+13	4,75491E+14
TC1900000205.mm.2	Ddb1	damage specific DNA binding protein 1	2,31823E+14	7,04566E+13	2,86031E+14	0.0266688133141879	0.160102305861218	-3,25046E+14	8,67552E+14	8,81159E+14	7,28272E+14	4,65349E+12	6,76269E+14	6,08796E+14
TC1500002061.mm.2	Syt10	synaptotagmin X	1,0166E+13	8,37794E+14	2,85978E+14	0.0266879705470802	0.160128813498043	-3,25114E+14	9,09205E+14	8,84823E+14	8,54903E+13	7,87127E+14	8,05922E+14	7,84786E+14
TC1200001269.mm.2	Cep170b	centrosomal protein 170B	1,07894E+14	7,36199E+13	2,85858E+14	0.0267317736605724	0.160128813498043	-3,25269E+14	8,17643E+14	8,19586E+13	7,05839E+14	6,52685E+14	6,75808E+13	7,45633E+14
TC0700000400.mm.2	Dmrtc2	doublesex and mab-3 related transcription factor like family C2	-1,31452E+14	6,00272E+14	-2,85822E+14	0.0267449766062696	0.160128813498043	-3,25315E+14	5,16327E+14	5,17522E+14	5,86232E+14	6,84796E+14	6,06934E+14	6,89819E+14
TC0X00001056.mm.2	Magee1	melanoma antigen, family E, 1	1,84567E+14	6,70925E+14	2,85802E+14	0.0267522713184937	0.160128813498043	-3,25341E+13	8,18666E+14	7,96006E+14	6,80429E+14	4,84912E+14	6,55475E+14	5,90063E+14
TC1000002418.mm.2	Col18a1	collagen, type XVIII, alpha 1	-1,22678E+14	6,1899E+14	-2,858E+14	0.0267526893293635	0.160128813498043	-3,25343E+14	5,28175E+14	5,6702E+14	5,85316E+14	6,9288E+14	6,70142E+14	6,70409E+14
TC0900001846.mm.2	Col5a3	collagen, type V, alpha 3	-1,28539E+14	6,69771E+14	-2,85774E+14	0.0267622139772276	0.160128813498043	-3,25376E+14	5,81164E+14	5,74248E+14	6,82315E+14	7,68411E+14	6,61686E+14	7,50803E+14
TC0600000493.mm.2	Cntnap2	contactin associated protein-like 2	2,00027E+13	7,85516E+14	2,85763E+14	0.0267664350382471	0.160128813498043	-3,25391E+14	9,43649E+14	8,91943E+14	8,20142E+14	6,21821E+14	7,68745E+14	6,66794E+14
TC1400002222.mm.2	Tdh	L-threonine dehydrogenase	-1,05525E+14	6,21971E+14	-2,8576E+14	0.0267674505216711	0.160128813498043	-3,25395E+14	5,83372E+14	5,38906E+14	6,03095E+14	6,73125E+14	6,63523E+14	6,69806E+14
TC0600003123.mm.2	Gpr162	G protein-coupled receptor 162	0.835729801843797	7,59897E+12	2,85758E+14	0.0267683089369921	0.160128813498043	-3,25398E+13	8,18774E+14	8,32016E+14	7,13508E+14	7,11438E+14	7,34694E+14	7,4895E+13
TC1700001027.mm.2	Tnfsf9	tumor necrosis factor (ligand) superfamily, member 9	-0.923460857413642	4,96379E+14	-2,85746E+13	0.0267726074327393	0.160128813498043	-3,25413E+14	4,32025E+14	4,2948E+14	5,09129E+14	5,6144E+14	5,2734E+14	5,18857E+14
TC0X00001306.mm.2	Gprasp1/Armcx5/Mir 7646	G protein-coupled receptor associated sorting protein 1/armadillo repeat containing, X-linked 5/microRNA 7646	0.879256293553763	8,35591E+14	2,85676E+14	0.0267980093564126	0.160221270782885	-3,25502E+14	9,04453E+14	8,71036E+14	8,23096E+14	8,15323E+14	8,09673E+13	7,89964E+14
TC0100003546.mm.2	Itln1	intelectin 1 (galactofuranose binding)	-0.86565496742492	6,44347E+14	-2,85607E+14	0.0268234375436885	0.160313816447126	-3,25592E+14	5,81478E+14	5,82535E+14	6,9152E+14	6,73406E+13	6,83304E+14	6,53841E+14
TC0200003398.mm.2	Rc3h2	ring finger and CCCH-type zinc finger domains 2	2,20206E+14	7,4583E+13	2,85544E+14	0.0268466398098639	0.16038960875772	-3,25673E+14	8,79474E+13	8,65686E+13	7,63473E+14	6,61597E+14	7,69957E+14	5,34792E+14
TC0100003202.mm.2	Cdc73	cell division cycle 73, Paf1/RNA polymerase II complex component	2,71054E+14	6,09928E+14	2,85518E+13	0.0268560270854793	0.16038960875772	-3,25706E+14	7,74148E+14	7,73098E+14	6,64378E+14	4,42804E+14	6,21101E+14	3,84038E+14
TC1900001749.mm.2	Eif3a	eukaryotic translation initiation factor 3, subunit A	-1,06294E+14	7,81497E+14	-2,85489E+14	0.0268664821313955	0.16039259969736	-3,25743E+14	7,07403E+14	7,10261E+13	7,78447E+14	8,62618E+14	8,09695E+14	8,20556E+14
TC0100001555.mm.2	Lmx1a	LIM homeobox transcription factor 1 alpha	-0.904496521563225	6,42358E+14	-2,85417E+14	0.0268932113867547	0.16044564162987	-3,25837E+14	6,00982E+14	6,04496E+14	6,54671E+14	6,0762E+14	6,88933E+14	6,97444E+13
TC1600000508.mm.2	Cstdc4	cystatin domain containing 4	-1,39114E+14	5,47479E+13	-2,85409E+14	0.0268960022191396	0.16044564162987	-3,25847E+14	4,72646E+14	4,33757E+14	5,34376E+14	6,59464E+14	5,65959E+14	6,18672E+14
TC0500002157.mm.2	Otof	otoferlin	-1,16171E+13	6,96632E+14	-2,85384E+14	0.0269052394764419	0.16044564162987	-3,25879E+14	6,34494E+14	6,21721E+14	6,66977E+14	7,68043E+14	7,3538E+14	7,53178E+13
TC0700001871.mm.2	Fus	fused in sarcoma	1,84964E+14	8,42823E+14	2,85286E+14	0.0269411087057168	0.160600105281249	-3,26005E+14	9,66347E+14	9,55062E+13	8,8901E+14	6,95039E+14	8,13466E+14	7,38015E+14
TC1100003385.mm.2	Slfn10-ps	schlafen 10, pseudogene	-1,06713E+13	5,06863E+14	-2,85225E+14	0.0269636082055801	0.160674785139242	-3,26083E+14	4,496E+14	4,6293E+14	4,72505E+14	5,3019E+14	5,31834E+14	5,94122E+14
TC1000002249.mm.2	Col13a1	collagen, type XIII, alpha 1	-1,01159E+14	7,92444E+14	-2,85161E+14	0.0269873312726658	0.16075669826117	-3,26166E+14	7,03315E+14	7,50913E+14	7,99588E+14	8,44301E+14	8,10228E+14	8,46318E+14
TC0300002349.mm.2	Dcst1	DC-STAMP domain containing 1	-0.834686117808483	6,61703E+14	-2,85065E+14	0.0270227025299076	0.160775375847485	-3,2629E+14	6,02421E+14	6,16373E+14	6,80465E+14	6,85228E+13	6,85947E+14	6,99785E+14
TC1500000960.mm.2	Pced1b	PC-esterase domain containing 1B	-0.998002460634005	6,07549E+14	-2,85059E+14	0.0270248440269893	0.160775375847485	-3,26297E+14	5,44215E+14	5,42225E+14	6,26904E+14	6,45908E+14	6,08446E+14	6,77594E+14
TC1200002421.mm.2	Atp5mpl	ATP synthase membrane subunit 6.8PL	1,07847E+14	9,27737E+14	2,85041E+14	0.0270314277538296	0.160775375847485	-3,2632E+14	9,93446E+14	9,98616E+13	9,19359E+14	8,78634E+13	9,27262E+13	8,49106E+14
TC0300001727.mm.2	Nlgn1	neuroligin 1	1,68063E+12	7,85E+14	2,85003E+13	0.0270453812388629	0.160775375847485	-3,26369E+14	8,97711E+14	8,99198E+14	7,67344E+14	6,84962E+14	8,1303E+14	6,47753E+14
TC0600002098.mm.2	Zc3hav1l	zinc finger CCCH-type, antiviral 1-like	-1,01676E+14	6,82055E+14	-2,84986E+14	0.0270517254692143	0.160775375847485	-3,26391E+13	6,13491E+13	6,01187E+14	7,61635E+14	6,97988E+14	6,89325E+14	7,28705E+14
TC0100003264.mm.2	Rgl1	ral guanine nucleotide dissociation stimulator,-like 1	1,47318E+14	7,46812E+14	2,84949E+14	0.027065219414931	0.160775375847485	-3,26438E+14	8,4579E+14	8,27818E+14	7,773E+14	6,50992E+14	7,32735E+14	6,46237E+14
TC0X00000832.mm.2	Tab3	TGF-beta activated kinase 1/MAP3K7 binding protein 3	1,22037E+14	6,74589E+14	2,84938E+14	0.0270694609927018	0.160775375847485	-3,26453E+14	7,40847E+14	7,52986E+14	7,06856E+14	5,97086E+14	6,38823E+14	6,10936E+14
TC1500002337.mm.2	Pfdn5	prefoldin 5	1,1603E+14	8,2897E+14	2,84908E+14	0.0270804392278359	0.160775375847485	-3,26491E+14	9,30601E+14	9,13578E+14	7,94609E+14	7,22913E+14	8,42403E+14	7,69716E+14
TC0300002472.mm.2	Prune1	prune exopolyphosphatase	1,0716E+14	7,27844E+14	2,84869E+13	0.0270950317401201	0.160775375847485	-3,26542E+14	7,97894E+14	8,0336E+14	7,28737E+14	6,5014E+14	7,12004E+14	6,74932E+14
TC1100001694.mm.2	Fzd2	frizzled class receptor 2	0.852604489819531	6,14667E+13	2,84857E+14	0.0270993978471168	0.160775375847485	-3,26557E+14	6,54699E+14	6,70267E+14	6,11896E+13	5,96692E+14	5,66847E+13	5,87598E+14
TC1800001072.mm.2	Zscan30	zinc finger and SCAN domain containing 30	-0.946399215431891	5,18235E+14	-2,84821E+13	0.0271128584078917	0.160775375847485	-3,26604E+14	4,66499E+14	4,43031E+14	5,25704E+14	5,75364E+13	5,61295E+14	5,37515E+14
TC0200003132.mm.2	Tor4a	torsin family 4, member A	-1,26604E+14	5,66287E+14	-2,8482E+14	0.0271130173457584	0.160775375847485	-3,26605E+13	4,66364E+14	5,0509E+14	5,62505E+14	6,39101E+14	5,69239E+14	6,55422E+14
TC1500002301.mm.2	Calcoco1	calcium binding and coiled coil domain 1	1,6664E+14	7,86324E+14	2,84754E+14	0.0271376858069923	0.160775375847485	-3,2669E+14	8,85121E+14	8,69853E+14	8,58653E+14	6,82621E+13	6,87356E+14	7,34339E+14
TC0900002640.mm.2	Tex9	testis expressed gene 9	1,10975E+14	5,89664E+14	2,84754E+13	0.0271376953721082	0.160775375847485	-3,26691E+14	6,47853E+14	6,38157E+12	5,72036E+14	6,15881E+14	5,73563E+14	4,90496E+14
TC0900001054.mm.2	Mto1	mitochondrial tRNA translation optimization 1	1,20394E+14	5,38282E+14	2,84692E+14	0.0271604595636046	0.160775375847485	-3,2677E+13	6,1278E+14	5,92513E+14	5,22666E+14	5,37225E+14	5,36475E+14	4,28031E+14
TC1800000886.mm.2	Dipk1c	divergent protein kinase domain 1C	-1,78087E+14	6,79514E+13	-2,84682E+12	0.0271641240905305	0.160775375847485	-3,26782E+14	5,80842E+14	5,55062E+14	6,37434E+14	8,11669E+14	6,99428E+14	7,92651E+14
TC0400000543.mm.2	Plppr1	phospholipid phosphatase related 1	1,42739E+14	7,34274E+14	2,84677E+14	0.0271660747708082	0.160775375847485	-3,26789E+13	8,18427E+14	7,99572E+14	8,00071E+14	6,55053E+13	6,57162E+14	6,75359E+13
TC1800000297.mm.2	Kdm3b	KDM3B lysine (K)-specific demethylase 3B	0.914355946497065	6,832E+14	2,84666E+14	0.0271700706530566	0.160775375847485	-3,26803E+14	7,30823E+14	7,32379E+13	6,65081E+13	6,90585E+14	6,62518E+14	6,17813E+14
TC0600001266.mm.2	Slc6a11	solute carrier family 6 (neurotransmitter transporter, GABA), member 11	1,71114E+13	8,74256E+14	2,84608E+14	0.0271918558839369	0.160828887535509	-3,26879E+14	1,02591E+14	9,43734E+14	9,00792E+14	7,4768E+14	8,51379E+14	7,76039E+14
TC1700000662.mm.2	Clic1	chloride intracellular channel 1	1,18327E+14	6,67523E+14	2,84535E+13	0.0272188056570005	0.160828887535509	-3,26972E+13	7,00333E+14	7,89399E+14	6,41474E+14	6,20854E+14	6,55116E+14	5,97963E+14
TC0300002482.mm.2	Ecm1/Mir7014	extracellular matrix protein 1/microRNA 7014	-0.913200633605436	6,79677E+14	-2,84513E+14	0.0272270366553974	0.160828887535509	-3,27001E+14	6,24797E+14	6,30884E+14	6,7499E+14	7,0907E+14	7,00229E+13	7,38092E+14
TC0100001313.mm.2	B3galt2	UDP-Gal:betaGlcNAc beta 1,3-galactosyltransferase, polypeptide 2	1,96978E+14	6,63077E+13	2,84509E+14	0.027228338835819	0.160828887535509	-3,27005E+14	8,06914E+14	7,99214E+14	6,52416E+14	5,07745E+14	6,9275E+14	5,19422E+14
TC0500002085.mm.2	Abcf2	ATP-binding cassette, sub-family F (GCN20), member 2	1,32944E+14	7,18379E+14	2,84497E+14	0.0272328009239675	0.160828887535509	-3,27021E+14	8,12268E+14	8,04871E+14	7,36025E+14	6,05862E+13	6,86462E+14	6,64788E+14
TC1900001691.mm.2	Gpam	glycerol-3-phosphate acyltransferase, mitochondrial	1,72855E+14	7,25539E+14	2,84431E+14	0.0272573780072685	0.160828887535509	-3,27106E+14	8,5536E+14	8,51088E+14	7,38462E+14	5,47588E+14	6,90755E+14	6,69982E+14
TC1100000264.mm.2	Fancl	Fanconi anemia, complementation group L	0.968546548054083	6,25415E+14	2,84322E+14	0.0272982891043063	0.160828887535509	-3,27247E+13	6,81178E+14	6,76846E+14	6,01925E+14	6,28229E+14	6,12141E+14	5,52173E+13
TC1400000226.mm.2	E330034G19Rik	RIKEN cDNA E330034G19 gene	-1,06751E+14	5,56871E+14	-2,84276E+14	0.0273150868608786	0.160828887535509	-3,27305E+14	4,98964E+14	4,99777E+14	5,68996E+14	5,61243E+14	5,62943E+14	6,49301E+14
TC1100001660.mm.2	Nbr1	NBR1, autophagy cargo receptor	3,22405E+14	5,82929E+14	2,84249E+14	0.027325197208125	0.160828887535509	-3,2734E+13	7,71007E+14	7,52474E+14	6,41207E+14	4,54218E+13	6,12203E+14	2,66467E+14
TC1100002598.mm.2	Cyfip2	cytoplasmic FMR1 interacting protein 2	2,58897E+14	8,96899E+14	2,84221E+14	0.0273357201613976	0.160828887535509	-3,27376E+14	1,05061E+14	1,05938E+14	9,52472E+14	7,26745E+13	9,02242E+13	6,89949E+14
TC0400004090.mm.2	Espn	espin	-0.973692928146647	6,435E+14	-2,84196E+14	0.0273451566274905	0.160828887535509	-3,27409E+12	5,5794E+14	6,00386E+14	6,52636E+14	6,96974E+14	6,63193E+14	6,89871E+14

TC1500001641.mm.2	Adcy8	adenylate cyclase 8	1,07969E+14	5,37594E+14	2,84143E+14	0.0273650901438124	0.160828887535509	-3,27478E+13	6,44754E+14	5,65934E+14	5,25266E+14	4,94863E+14	4,83099E+14	5,11651E+14
TC0500000172.mm.2	Fam185a	family with sequence similarity 185, member A	0.99405638074608	6,83956E+14	2,8414E+14	0.0273659564807675	0.160828887535509	-3,27481E+14	7,50876E+13	7,15258E+13	6,88364E+14	6,81915E+14	6,64538E+14	6,02786E+14
TC0200000033.mm.2	Bend7	BEN domain containing 7	1,1551E+14	5,54184E+14	2,84074E+14	0.0273908864005469	0.160828887535509	-3,27567E+14	6,0648E+14	5,98418E+14	6,10058E+14	5,36269E+14	5,22776E+14	4,51102E+14
TC0200001751.mm.2	Chst14	carbohydrate sulfotransferase 14	-0.979470812143967	6,45865E+14	-2,84038E+14	0.027404292402732	0.160828887535509	-3,27613E+14	5,97944E+14	5,96887E+14	6,18266E+14	6,71367E+14	6,73827E+14	7,16898E+14
TC0200005514.mm.2	Zhx3	zinc fingers and homeoboxes 3	1,78008E+14	5,75353E+14	2,84021E+14	0.0274106301517682	0.160828887535509	-3,27635E+14	7,09718E+14	7,10861E+14	5,72787E+13	3,9419E+13	5,77015E+14	4,87548E+14
TC0900000208.mm.2	Plppr2	phospholipid phosphatase related 2	1,82789E+13	8,59086E+14	2,83981E+14	0.0274257176644382	0.160828887535509	-3,27686E+14	9,9158E+14	9,84639E+13	8,75285E+14	6,92371E+14	8,5318E+13	7,57462E+14
TC1100001907.mm.2	Tsen54	tRNA splicing endonuclease subunit 54	-0.932023573727967	6,22813E+14	-2,83975E+14	0.0274278667226732	0.160828887535509	-3,27694E+14	5,56145E+14	5,829E+14	6,15778E+14	6,56601E+14	6,48114E+14	6,77336E+14
TC0300002242.mm.2	Trim2	tripartite motif-containing 2	1,53205E+14	9,04525E+14	2,83961E+14	0.0274329460330818	0.160828887535509	-3,27711E+14	1,01843E+14	1,01544E+14	9,07269E+14	7,58547E+14	8,97985E+14	8,29476E+14
TC0700002418.mm.2	Bicra	BRD4 interacting chromatin remodeling complex associated protein	0.916668272071734	6,41435E+14	2,83956E+14	0.0274349683113267	0.160828887535509	-3,27718E+14	6,88208E+14	6,89939E+14	6,51579E+14	6,2407E+14	6,1991E+14	5,74904E+14
TC0600002342.mm.2	Nt5c3	5'-nucleotidase, cytosolic III	2,44098E+14	5,71595E+14	2,83952E+14	0.0274364651477236	0.160828887535509	-3,27723E+14	6,94319E+14	7,21114E+14	6,11469E+14	4,75429E+14	5,91292E+14	3,35945E+14
TC0200000461.mm.2	Adamts13	a disintegrin-like and metallopeptidase (reprolysin type) with thrombospondin type 1 motif, 13	-1,47297E+14	5,05296E+14	-2,83933E+13	0.0274436672663872	0.160828887535509	-3,27748E+13	3,73308E+14	4,13633E+14	5,1446E+14	6,48841E+14	5,07938E+14	5,73596E+14
TC0100002769.mm.2	Usp40	ubiquitin specific peptidase 40	-1,35603E+14	6,2871E+14	-2,83925E+14	0.0274464766039759	0.160828887535509	-3,27758E+14	5,44329E+14	5,33909E+14	6,14058E+13	7,30523E+14	6,39202E+14	7,10241E+14
TC1900000536.mm.2	Exoc6	exocyst complex component 6	1,14552E+14	6,56006E+14	2,83872E+14	0.0274663249311652	0.160828887535509	-3,27826E+14	7,39768E+14	7,39065E+14	6,54302E+14	5,5317E+14	6,29795E+14	6,19935E+14
TC1900000112.mm.2	Cdc42bpg	CDC42 binding protein kinase gamma (DMPK-like)	-0.990040443861896	5,95109E+14	-2,83781E+14	0.0275007785148995	0.160828887535509	-3,27944E+14	5,32753E+14	5,66565E+14	5,67608E+14	6,064E+14	6,24229E+14	6,73097E+13
TC1100002429.mm.2	Sptbn1	spectrin beta, non-erythrocytic 1	1,73385E+14	9,44814E+14	2,83758E+14	0.0275092402077211	0.160828887535509	-3,27973E+14	1,0614E+14	1,04479E+14	9,67559E+14	8,35707E+14	9,49961E+14	8,09461E+14
TC0500002325.mm.2	Tapt1	transmembrane anterior posterior transformation 1	1,4469E+14	7,12937E+14	2,83756E+14	0.0275098776998796	0.160828887535509	-3,27975E+14	7,94925E+14	7,96318E+13	7,55056E+14	6,29457E+14	6,92901E+14	6,08963E+14
TC1200002507.mm.2	Sp4	trans-acting transcription factor 4	1,8827E+14	6,17333E+12	2,83746E+14	0.0275136537264103	0.160828887535509	-3,27988E+14	7,51644E+14	7,34543E+14	6,43941E+14	4,64226E+14	6,12734E+13	4,96912E+14
TC0700004171.mm.2	Syt17	synaptotagmin XVII	1,53686E+13	6,72847E+14	2,837E+14	0.0275309818994974	0.160828887535509	-3,28048E+14	7,70247E+14	7,75225E+14	6,92115E+14	5,61396E+14	6,69082E+14	5,69017E+14
TC0600002924.mm.2	Emc3	ER membrane protein complex subunit 3	0.85103308261797	7,51772E+14	2,83697E+13	0.0275323254529652	0.160828887535509	-3,28052E+14	8,13449E+13	8,14522E+14	7,26599E+14	6,98301E+14	7,2268E+14	7,35085E+14
TC1100002416.mm.2	Cfap36	cilia and flagella associated protein 36	0.822083008417103	7,21032E+14	2,83678E+14	0.0275394174186786	0.160828887535509	-3,28077E+14	7,78673E+14	7,75881E+14	6,96004E+14	6,85499E+13	6,99101E+14	6,91036E+14
TC0500002306.mm.2	Bod1l	biorientation of chromosomes in cell division 1-like	1,70177E+13	6,43016E+14	2,83642E+14	0.0275530285271093	0.160828887535509	-3,28123E+14	7,82224E+14	7,68378E+14	5,84931E+13	5,12311E+14	6,92034E+13	5,18215E+14
TC0600001082.mm.2	Hdac11	histone deacetylase 11	1,17527E+14	8,50547E+14	2,83632E+14	0.0275567637446052	0.160828887535509	-3,28136E+14	9,32853E+14	9,03483E+14	8,23371E+14	8,42292E+14	8,51248E+14	7,50034E+14
TC1600001497.mm.2	Pak2	p21 (RAC1) activated kinase 2	0.977453493830525	6,57063E+14	2,83538E+14	0.027592293214162	0.160828887535509	-3,28258E+14	7,26056E+14	7,34069E+14	6,14146E+14	6,03473E+14	6,65816E+14	5,98818E+14
TC1200002526.mm.2	Serpina3h	serine (or cysteine) peptidase inhibitor, clade A, member 3H	-0.967631083166797	5,69992E+12	-2,83533E+14	0.0275941031329477	0.160828887535509	-3,28264E+14	5,36808E+14	5,03925E+14	5,93844E+14	5,51115E+14	5,9806E+14	6,36199E+14
TC1000002353.mm.2	Bicc1	BicC family RNA binding protein 1	-0.90431093432858	6,36981E+14	-2,83532E+12	0.0275942226041298	0.160828887535509	-3,28264E+14	5,58916E+14	5,99132E+13	6,58537E+14	6,66392E+14	6,83476E+14	6,55434E+14
TC0800002314.mm.2	Klhl2	kelch-like 2, Mayven	1,35374E+14	7,51439E+13	2,83521E+14	0.0275986380683664	0.160828887535509	-3,28279E+14	8,36818E+14	8,54484E+14	7,63035E+14	6,33746E+13	7,30747E+14	6,89806E+14
TC0100001526.mm.2	Creg1	cellular repressor of E1A-stimulated genes 1	1,8282E+14	7,33924E+14	2,83514E+14	0.0276012126924512	0.160828887535509	-3,28288E+14	8,39263E+14	8,38028E+14	7,41637E+14	6,72967E+14	7,53478E+14	5,58172E+14
TC1700001458.mm.2	Acat3	acetyl-Coenzyme A acetyltransferase 3	0.906709939448632	6,39209E+14	2,83513E+14	0.0276017081556495	0.160828887535509	-3,2829E+14	6,94979E+14	6,78752E+14	6,15529E+14	6,53605E+14	6,10544E+14	5,81845E+14
TC0700000805.mm.2	Aspdh	aspartate dehydrogenase domain containing	-1,11984E+14	5,99768E+14	-2,83501E+13	0.027606174309099	0.160828887535509	-3,28305E+14	5,21225E+14	5,24498E+13	5,93828E+14	6,89363E+14	6,43676E+14	6,26016E+14
TC0600001239.mm.2	Setd5	SET domain containing 5	1,25659E+14	8,08868E+14	2,83481E+14	0.0276135866103601	0.160828887535509	-3,2833E+14	8,67668E+14	8,8696E+13	8,20266E+14	7,75004E+14	8,06956E+14	6,96355E+13
TC1500000489.mm.2	Ccn4	cellular communication network factor 4	-1,22116E+13	5,13894E+14	-2,83434E+14	0.0276313492172903	0.160828887535509	-3,28391E+14	4,54238E+14	4,10876E+14	5,07984E+14	6,00921E+14	5,28507E+14	5,80838E+14
TC0500001622.mm.2	Ufsp1	UFM1-specific peptidase 1	1,08788E+14	6,19351E+14	2,83431E+14	0.0276326315182414	0.160828887535509	-3,28395E+14	6,90455E+14	6,49753E+14	6,03152E+14	6,50112E+14	5,27894E+13	5,94737E+14
TC1100001681.mm.2	Nags	N-acetylglutamate synthase	-1,22277E+14	5,41064E+14	-2,83399E+14	0.0276445179749688	0.160828887535509	-3,28436E+14	4,3159E+14	5,04024E+14	5,24916E+14	6,05686E+14	5,70371E+14	6,09798E+14
TC0X00002415.mm.2	Cetn2	centrin 2	0.948456222282752	6,18993E+14	2,83383E+14	0.0276506212190034	0.160828887535509	-3,28457E+14	6,88578E+14	6,71425E+14	6,14018E+13	5,69625E+11	6,04676E+13	5,65636E+14
TC0800000267.mm.2	Ccdc70	coiled-coil domain containing 70	-0.876780564169375	4,76002E+14	-2,83347E+14	0.0276640987568776	0.160828887535509	-3,28503E+13	4,19837E+14	4,32481E+14	4,68408E+14	5,07612E+14	5,14031E+14	5,13643E+14
TC0200002452.mm.2	Top1	topoisomerase (DNA) I	0.958613861088027	9,25055E+14	2,83341E+14	0.0276666567751194	0.160828887535509	-3,28512E+14	9,84516E+14	9,89149E+13	9,20333E+14	8,74391E+14	9,13172E+14	8,6877E+12
TC0500002154.mm.2	Hadha	hydroxyacyl-Coenzyme A dehydrogenase/3-ketoacyl-Coenzyme A thiolase/enoyl-Coenzyme A hydratase (trifunctional protein), alpha subunit	1,46719E+14	7,39158E+14	2,8327E+13	0.0276934621234143	0.160828887535509	-3,28603E+14	8,25524E+14	8,20592E+14	7,90611E+14	6,45543E+14	7,02873E+14	6,49805E+14
TC0900000449.mm.2	Olfr986	olfactory receptor 986	-1,19462E+14	4,48304E+13	-2,8325E+14	0.0277010371336629	0.160828887535509	-3,28629E+14	3,68392E+14	3,7777E+14	4,48305E+14	5,10271E+14	4,46904E+14	5,38182E+13
TC0300002706.mm.2	Rbm15	RNA binding motif protein 15	1,68935E+13	5,77649E+14	2,83227E+14	0.0277096015904588	0.160828887535509	-3,28658E+14	6,72903E+14	6,63168E+14	5,76929E+14	5,54689E+14	5,92039E+14	4,06164E+14
TC0100002758.mm.2	Ecel1	endothelin converting enzyme-like 1	1,54171E+14	8,66043E+14	2,83186E+14	0.0277252887913866	0.160828887535509	-3,28711E+14	1,01875E+14	9,02828E+14	8,9002E+14	7,71428E+13	8,06793E+14	8,06441E+14
TC1100002334.mm.2	Ehbp1	EH domain binding protein 1	1,70599E+14	6,97203E+14	2,8314E+14	0.0277427708328955	0.160828887535509	-3,28771E+14	7,97546E+14	8,0148E+14	7,10087E+14	6,16274E+14	7,11463E+14	5,46365E+14
TC0800002596.mm.2	Rnaseh2a	ribonuclease H2, large subunit	0.917414717657866	5,99149E+14	2,83129E+14	0.0277467750231299	0.160828887535509	-3,28784E+14	6,77139E+14	6,653E+14	5,66317E+14	5,27184E+14	5,62056E+14	5,96901E+14
TC0100001968.mm.2	Prdm14	PR domain containing 14	-1,13339E+14	4,77197E+14	-2,83115E+14	0.0277522397717727	0.160828887535509	-3,28803E+14	4,31807E+14	3,96701E+14	4,75425E+14	5,04061E+14	4,83035E+14	5,72151E+14
TC0900000368.mm.2	Esam	endothelial cell-specific adhesion molecule	-1,24666E+14	8,39991E+14	-2,83111E+13	0.0277537338186278	0.160828887535509	-3,28808E+14	7,47132E+14	7,48896E+14	8,43472E+13	9,5509E+14	8,51122E+14	8,94237E+13
TC1700000420.mm.2	Bnip1	BCL2/adenovirus E1B interacting protein 1	1,88439E+14	5,46644E+14	2,83105E+14	0.0277561299595213	0.160828887535509	-3,28816E+14	6,42582E+14	6,7509E+13	6,10872E+14	4,10525E+14	5,06343E+14	4,34451E+14
TC0200001359.mm.2	Kbtbd4	kelch repeat and BTB (POZ) domain containing 4	1,28962E+14	7,54132E+13	2,831E+14	0.0277578029411729	0.160828887535509	-3,28822E+14	8,53341E+14	8,30993E+14	7,69301E+14	6,4475E+14	7,17037E+14	7,09372E+14
TC1500001651.mm.2	Lrrc6	leucine rich repeat containing 6 (testis)	1,26015E+14	5,67771E+14	2,8308E+14	0.0277656041112436	0.160828887535509	-3,28848E+14	6,06052E+14	6,78676E+14	5,85182E+14	5,04019E+14	4,8893E+14	5,43768E+13
TC0100000375.mm.2	Stat1	signal transducer and activator of transcription 1	1,17995E+14	5,9967E+14	2,83073E+14	0.0277680112408481	0.160828887535509	-3,28856E+14	6,5704E+14	7,2133E+14	4,95326E+14	5,81941E+14	5,66353E+14	5,76027E+14
TC0800003099.mm.2	Fanca	Fanconi anemia, complementation group A	-1,08588E+14	6,02034E+14	-2,83015E+14	0.0277902157857251	0.16089965754775	-3,28932E+14	5,44144E+14	5,45477E+14	5,68101E+14	6,47687E+14	6,31149E+14	6,75647E+14
TC0400002712.mm.2	Frrs1l	ferric-chelate reductase 1 like	1,35717E+14	9,20671E+14	2,82961E+14	0.0278105297111901	0.160959434352158	-3,29001E+14	9,96915E+14	1,0186E+14	9,02732E+14	8,61694E+13	9,36877E+14	8,07206E+14
TC0300000731.mm.2	Ssr2	signal sequence receptor, beta	1,10208E+14	8,42127E+14	2,82836E+14	0.0278580537872978	0.161082074378772	-3,29162E+14	9,17284E+14	9,1417E+14	8,4799E+13	7,6228E+14	8,25227E+14	7,85812E+14
TC1500001993.mm.2	Phf21b	PHD finger protein 21B	-1,21187E+14	7,78257E+14	-2,82834E+14	0.0278590224912035	0.161082074378772	-3,29165E+14	7,05805E+14	7,10306E+14	7,48693E+14	8,46252E+14	7,99847E+14	8,58638E+14
TC1800000689.mm.2	Pmaip1	phorbol-12-myristate-13-acetate-induced protein 1	-1,01281E+14	6,05925E+14	-2,82755E+14	0.0278889816016659	0.161082074378772	-3,29266E+14	5,45869E+14	5,60559E+13	5,87416E+13	6,32719E+14	6,2443E+14	6,8456E+14
TC0700000055.mm.2	Zfp580	zinc finger protein 580	-1,00478E+14	7,72576E+14	-2,82755E+14	0.0278890384429494	0.161082074378772	-3,29267E+14	7,20609E+14	7,10495E+14	7,61842E+14	8,10452E+14	7,87933E+14	8,44126E+14
TC0500003182.mm.2	Rph3a	rabphilin 3A	1,19965E+13	7,64921E+13	2,82709E+14	0.0279067165967904	0.161082074378772	-3,29326E+14	8,55516E+14	8,38845E+14	7,15991E+14	7,2474E+14	7,82412E+14	6,7202E+14
TC1900000719.mm.2	Neurl1a	neuralized E3 ubiquitin protein ligase 1A	1,02525E+14	7,78417E+14	2,82682E+14	0.0279171027167345	0.161082074378772	-3,29362E+14	8,54627E+14	8,41222E+14	7,80057E+14	7,03799E+14	7,36664E+14	7,54135E+14

TC0800001217.mm.2	Plekhg4	pleckstrin homology domain containing, family G (with RhoGef domain) member 4	-0.94412704768156	5,30553E+14	-2,82655E+14	0.0279274862148166	0.161082074378772	-3,29397E+14	4,82894E+14	4,49158E+13	5,46967E+14	5,83422E+14	5,42659E+14	5,78218E+14
TC1700001197.mm.2	Rmdn2	regulator of microtubule dynamics 2	1,53077E+13	5,07206E+14	2,82653E+14	0.0279279006690029	0.161082074378772	-3,29398E+14	5,71804E+14	6,10542E+14	5,15256E+14	4,69443E+14	5,14753E+14	3,61439E+14
TC1700001439.mm.2	Pacrg	PARK2 co-regulated	0.846739682795331	7,35923E+14	2,82647E+14	0.0279304389396915	0.161082074378772	-3,29407E+14	7,77261E+14	7,6436E+14	7,62066E+14	7,39574E+14	6,78195E+14	6,94078E+14
TC0200005430.mm.2	Kcnq2	potassium voltage-gated channel, subfamily Q, member 2	1,57812E+14	7,7995E+14	2,82541E+13	0.0279707459185084	0.161082074378772	-3,29543E+14	8,89071E+14	8,75243E+14	8,05739E+13	6,60958E+14	7,70044E+14	6,78645E+14
TC0500001888.mm.2	Ankib1	ankyrin repeat and IBR domain containing 1	1,72508E+14	6,72938E+14	2,82537E+14	0.0279722649811641	0.161082074378772	-3,29548E+14	7,68606E+14	7,73755E+14	7,45437E+14	5,52487E+14	6,02913E+14	5,94432E+14
TC1100000952.mm.2	Zmynd15	zinc finger, MYND-type containing 15	-1,14234E+14	5,64039E+14	-2,82535E+14	0.0279733571315069	0.161082074378772	-3,29551E+14	4,72684E+14	5,11154E+14	5,64876E+13	6,23212E+14	5,68991E+14	6,43316E+13
TC1700001886.mm.2	Pfdn6	prefoldin subunit 6	1,74664E+14	7,32517E+14	2,82513E+14	0.0279816633424809	0.161082074378772	-3,29579E+14	8,46402E+14	8,55815E+12	7,35387E+14	6,04608E+14	7,49504E+14	6,03384E+14
TC0X00001382.mm.2	Prps1	phosphoribosyl pyrophosphate synthetase 1	1,41555E+14	8,15322E+14	2,82442E+14	0.0280088838034489	0.161082074378772	-3,29671E+14	9,18637E+14	8,86179E+14	8,53963E+14	7,1145E+14	7,69855E+14	7,51852E+14
TC1900001073.mm.2	Uqcc3	ubiquinol-cytochrome c reductase complex assembly factor 3	0.816514160384189	6,64898E+14	2,82418E+14	0.028018116850121	0.161082074378772	-3,29702E+14	7,0692E+14	7,16591E+14	6,54229E+14	6,5144E+14	6,27305E+14	6,32903E+14
TC0900001942.mm.2	Acad8	acyl-Coenzyme A dehydrogenase family, member 8	0.848283067483178	7,36442E+14	2,82413E+14	0.0280198401081426	0.161082074378772	-3,29708E+14	7,74072E+14	7,948E+14	7,1518E+14	7,35385E+14	7,05689E+14	6,93526E+14
TC1000002425.mm.2	Gm9508	predicted gene 9508	-1,03756E+14	4,84376E+14	-2,82402E+14	0.0280240631850975	0.161082074378772	-3,29722E+13	4,07309E+14	4,40428E+14	4,6435E+14	5,38924E+14	5,3224E+13	5,23009E+14
TC1100000934.mm.2	Gabarap	gamma-aminobutyric acid receptor associated protein	1,10923E+14	9,55811E+14	2,824E+14	0.0280248283964873	0.161082074378772	-3,29725E+14	1,03683E+14	1,02414E+14	9,52891E+14	8,81873E+14	9,51267E+14	8,87862E+14
TC1100001579.mm.2	Csf3	colony stimulating factor 3 (granulocyte)	-1,19324E+14	6,55003E+14	-2,82361E+14	0.0280397676009083	0.161082074378772	-3,29775E+14	5,43906E+14	6,15098E+14	6,46312E+14	7,27051E+14	6,8501E+14	7,12642E+14
TC0900000599.mm.2	Il18	interleukin 18	1,20869E+12	6,961E+14	2,82355E+14	0.0280422979894338	0.161082074378772	-3,29784E+14	7,9691E+14	7,79422E+14	6,87553E+14	5,78117E+14	6,80222E+14	6,54373E+14
TC1000002540.mm.2	Fzr1	fizzy and cell division cycle 20 related 1	1,07698E+14	8,11998E+14	2,8234E+14	0.0280477692647908	0.161082074378772	-3,29802E+14	8,74583E+14	8,6232E+14	8,43992E+14	7,69591E+14	7,85298E+14	7,36209E+14
TC0700002659.mm.2	Snrpa	small nuclear ribonucleoprotein polypeptide A	0.868799492066848	7,64465E+14	2,8233E+14	0.0280516539878877	0.161082074378772	-3,29815E+14	8,30687E+14	8,0967E+14	7,23301E+13	7,56533E+13	7,35236E+14	7,31361E+14
TC1800000835.mm.2	Epg5	ectopic P-granules autophagy protein 5 homolog (C. elegans)	0.908217645300432	6,10566E+13	2,82273E+14	0.0280737574832215	0.161151569051353	-3,29889E+14	6,7256E+14	6,50347E+14	5,82591E+14	6,16636E+14	5,54474E+14	5,86789E+13
TC0700002637.mm.2	Erich4	glutamate rich 4	-1,28167E+14	6,28595E+14	-2,82218E+14	0.028094817796077	0.161164332943608	-3,2996E+14	5,42706E+14	5,76407E+14	5,8409E+14	6,9292E+14	6,60666E+14	7,14781E+14
TC0400000272.mm.2	Lyrm2	LYR motif containing 2	0.945306022327025	6,50681E+14	2,82215E+14	0.0280960324693616	0.161164332943608	-3,29964E+14	7,45719E+14	7,21763E+14	6,07518E+13	5,50667E+14	6,40474E+13	6,37947E+14
TC0900003327.mm.2	Khdc3	KH domain containing 3, subcortical maternal complex member	-1,31813E+14	4,51178E+14	-2,82189E+14	0.0281059874369477	0.161164332943608	-3,29998E+14	3,73574E+14	3,50996E+14	5,00925E+14	4,93381E+14	4,2914E+14	5,59056E+14
TC0200000856.mm.2	Kcnj3	potassium inwardly-rectifying channel, subfamily J, member 3	1,39429E+13	6,99957E+14	2,82147E+14	0.0281221373861417	0.161199572999965	-3,30052E+14	7,943E+14	8,19252E+14	6,8801E+14	5,63483E+14	7,01482E+14	6,33213E+14
TC0500002142.mm.2	Mnx1	motor neuron and pancreas homeobox 1	-1,25087E+14	5,98352E+13	-2,82021E+14	0.0281706758143992	0.161377489747546	-3,30214E+14	5,11275E+12	5,12366E+14	5,98965E+14	6,93693E+14	5,99128E+14	6,74686E+14
TC0100002628.mm.2	Gm2102	predicted gene 2102	-0.910352723020082	5,18806E+14	-2,82006E+14	0.0281765443628508	0.161377489747546	-3,30234E+13	4,64623E+14	4,63329E+14	5,14233E+14	5,60629E+14	5,42457E+14	5,67567E+14
TC0500002819.mm.2	A930011G23Rik	RIKEN cDNA A930011G23 gene	-1,05619E+14	8,39663E+14	-2,81987E+14	0.0281837900625939	0.161377489747546	-3,30258E+14	7,81098E+14	7,70983E+13	8,28814E+14	8,93767E+13	8,53801E+14	9,09518E+14
TC0600001446.mm.2	Chd4/Mir7045	chromodomain helicase DNA binding protein 4/microRNA 7045	2,52643E+14	6,22504E+14	2,81962E+14	0.0281932373635786	0.161377489747546	-3,3029E+14	7,84095E+14	7,79428E+14	6,71717E+14	4,41546E+14	6,28666E+14	4,29571E+14
TC0100003873.mm.2	A130010J15Rik	RIKEN cDNA A130010J15 gene	-0.876571648440181	5,98318E+14	-2,81889E+14	0.0282216824977114	0.161482943922452	-3,30385E+14	5,40183E+13	5,50739E+14	6,06599E+12	6,26151E+14	6,14917E+14	6,51319E+14
TC1700001187.mm.2	Vit	vitrin	1,31014E+14	5,87852E+14	2,81642E+14	0.0283173557385991	0.161786613778811	-3,30704E+14	6,3189E+14	6,97846E+14	5,41837E+14	5,87832E+13	5,85689E+14	4,82019E+14
TC0300002284.mm.2	Fcrls	Fc receptor-like S, scavenger receptor	1,20404E+14	7,55075E+14	2,81619E+14	0.0283261143675624	0.161786613778811	-3,30733E+14	8,10567E+14	8,60943E+14	7,17457E+14	7,10779E+14	7,66119E+14	6,64583E+13
TC0900001345.mm.2	Dusp7	dual specificity phosphatase 7	-0.8120665860051	8,87486E+14	-2,816E+14	0.0283333954335691	0.161786613778811	-3,30758E+14	8,34257E+14	8,38836E+14	9,05867E+14	9,10448E+12	9,20742E+14	9,14764E+14
TC1400002031.mm.2	Rab2b	RAB2B, member RAS oncogene family	2,42072E+14	7,35885E+14	2,81553E+14	0.0283515760436366	0.161786613778811	-3,30818E+14	9,01997E+14	9,0681E+14	7,84903E+13	4,96937E+14	6,81742E+14	6,42922E+14
TC0800000811.mm.2	Olfr373	olfactory receptor 373	-1,60975E+14	5,20324E+14	-2,8152E+14	0.0283645439025497	0.161786613778811	-3,30861E+14	4,47172E+14	3,96994E+14	4,83353E+14	6,28308E+14	5,38605E+14	6,27511E+13
TC1400001824.mm.2	Gm17026	predicted gene 17026	-0.880620746127643	4,70839E+14	-2,8151E+14	0.0283682770625404	0.161786613778811	-3,30874E+14	4,25815E+14	4,11157E+14	4,6659E+14	5,08375E+14	5,07869E+14	5,05227E+14
TC1500000022.mm.2	Ttc33	tetratricopeptide repeat domain 33	1,31314E+14	7,51233E+14	2,81489E+14	0.0283764021048067	0.161786613778811	-3,30901E+14	8,64404E+14	8,32856E+14	7,42597E+14	6,32912E+14	7,50273E+14	6,84359E+14
TC1200001038.mm.2	Serpina3a	serine (or cysteine) peptidase inhibitor, clade A, member 3A	-1,09925E+14	4,80083E+13	-2,8142E+14	0.0284032978849885	0.161786613778811	-3,3099E+14	3,95153E+14	4,21153E+14	5,07241E+14	5,20797E+14	4,72952E+14	5,63204E+14
TC0600002607.mm.2	Sfxn5	sideroflexin 5	1,15853E+14	8,76332E+14	2,81403E+14	0.0284100767667368	0.161786613778811	-3,31013E+14	9,63371E+14	9,35362E+14	8,96979E+14	7,95252E+13	8,36994E+14	8,30033E+14
TC1500000688.mm.2	Apobec3	apolipoprotein B mRNA editing enzyme, catalytic polypeptide 3	-0.867796127591299	6,05195E+14	-2,81402E+14	0.0284105944410562	0.161786613778811	-3,31014E+14	5,50538E+14	5,45785E+14	6,18097E+14	6,46866E+14	6,21129E+14	6,48753E+14
TC1800000324.mm.2	Igip/Pura	IgA inducing protein/purine rich element binding protein A	1,06693E+14	9,86137E+14	2,81381E+14	0.0284184671676887	0.161786613778811	-3,3104E+14	1,0603E+14	1,04788E+14	9,94497E+14	9,19355E+14	9,70159E+14	9,24632E+13
TC1900001066.mm.2	Slc3a2	solute carrier family 3 (activators of dibasic and neutral amino acid transport), member 2	1,38704E+12	9,29861E+14	2,8133E+13	0.0284384117771415	0.161786613778811	-3,31107E+14	1,0215E+14	1,04136E+14	9,22064E+14	8,08787E+14	9,35796E+14	8,49659E+14
TC1100002715.mm.2	Tcf7	transcription factor 7, T cell specific	-1,12807E+14	6,27643E+14	-2,81275E+14	0.0284597822017564	0.161786613778811	-3,31177E+14	5,52869E+14	5,47974E+13	6,54377E+14	6,84184E+14	6,15383E+14	7,11073E+13
TC0400002590.mm.2	Xpa	xeroderma pigmentosum, complementation group A	1,70861E+14	6,35626E+14	2,81267E+14	0.0284629944387689	0.161786613778811	-3,31188E+14	7,67104E+14	7,47259E+14	6,30895E+14	4,95857E+14	6,538E+14	5,18843E+13
TC0200002359.mm.2	Itch	itchy, E3 ubiquitin protein ligase	1,67392E+14	5,63392E+14	2,81233E+14	0.0284762985346422	0.161786613778811	-3,31232E+14	6,88619E+14	6,80326E+14	5,68425E+14	4,0882E+14	5,65567E+14	4,68594E+14
TC0800001258.mm.2	Utp4	UTP4 small subunit processome component	0.92252023961375	7,56078E+13	2,81198E+14	0.028489967195767	0.161786613778811	-3,31277E+14	8,07586E+14	8,09489E+14	7,60485E+14	7,26336E+14	7,38951E+14	6,9362E+13
TC1700000391.mm.2	Jmjd8	jumonji domain containing 8	1,53663E+14	8,25194E+14	2,81154E+14	0.0285069699380962	0.161786613778811	-3,31334E+14	9,32775E+14	9,38345E+14	8,34547E+14	6,81698E+14	8,15087E+14	7,48709E+14
TC0700002964.mm.2	Emc10	ER membrane protein complex subunit 10	1,05227E+14	8,75856E+14	2,8113E+14	0.0285165080447693	0.161786613778811	-3,31365E+14	9,49955E+14	9,19132E+14	8,98428E+14	8,28987E+14	8,49496E+14	8,09138E+14
TC1300001037.mm.2	Mtx3	metaxin 3	1,06854E+14	5,58257E+14	2,81124E+14	0.0285188699096734	0.161786613778811	-3,31373E+14	6,02596E+12	6,04958E+14	5,43793E+14	6,0435E+14	5,29674E+14	4,64172E+14
TC1800000655.mm.2	Wdr7	WD repeat domain 7	2,4153E+14	6,95881E+14	2,81106E+14	0.0285259839668831	0.161786613778811	-3,31397E+14	8,6567E+14	8,54259E+13	7,25928E+14	4,92563E+14	7,04664E+14	5,32204E+14
TC0100003882.mm.2	Chpf	chondroitin polymerizing factor	1,32513E+14	7,65778E+14	2,8109E+14	0.0285320791340472	0.161786613778811	-3,31417E+14	8,48443E+14	8,54168E+13	7,76887E+14	6,77588E+14	7,63716E+14	6,73869E+14
TC1200000013.mm.2	Dtnb	dystrobrevin, beta	-1,24353E+13	1,03764E+14	-2,81078E+14	0.0285368515358214	0.161786613778811	-3,31433E+14	9,5548E+14	9,59941E+14	1,12582E+14	1,0205E+13	1,1114E+14	1,05273E+14
TC0X00000685.mm.2	Bgn	biglycan	1,10864E+14	6,65139E+14	2,8104E+14	0.0285517870004343	0.161786613778811	-3,31482E+14	7,10839E+14	7,44845E+14	6,58486E+14	6,42709E+14	6,61674E+14	5,72283E+14
TC1300002418.mm.2	Rasgrf2	RAS protein-specific guanine nucleotide-releasing factor 2	1,12804E+14	7,27726E+14	2,81011E+14	0.0285628707522693	0.161786613778811	-3,31518E+14	8,24301E+14	7,66315E+14	7,43211E+14	6,67521E+14	6,98438E+14	6,6657E+14
TC0700002884.mm.2	Gm21276	predicted gene, 21276	-1,31633E+14	4,58434E+14	-2,80984E+14	0.0285733433411757	0.161786613778811	-3,31553E+14	3,74323E+14	3,77084E+14	4,50085E+14	5,34436E+14	4,56137E+14	5,58537E+14
TC1100000901.mm.2	Hes7	hes family bHLH transcription factor 7	-1,24172E+14	7,67133E+14	-2,80984E+14	0.0285734336560557	0.161786613778811	-3,31553E+14	6,84003E+14	6,94053E+14	7,46878E+14	8,51466E+14	7,83791E+14	8,42609E+14
TC0100002685.mm.2	Sphkap	SPHK1 interactor, AKAP domain containing	1,45763E+14	7,2314E+14	2,80938E+14	0.028591628623977	0.161786613778811	-3,31613E+14	8,31135E+14	8,29117E+13	7,19004E+14	5,90857E+14	7,27892E+14	6,40833E+14
TC0200000600.mm.2	Rpl12/Snora65	ribosomal protein L12/small nucleolar RNA, H/ACA box 65	1,48441E+14	1,02416E+14	2,80927E+14	0.0285957565364084	0.161786613778811	-3,31627E+14	1,12699E+13	1,12761E+14	1,02193E+14	9,10749E+14	1,03571E+14	9,22E+14
TC1700001100.mm.2	L3mbtl4	L3MBTL4 histone methyl-lysine binding protein	-0.871980841731935	5,81855E+14	-2,80894E+14	0.0286088502480679	0.161786613778811	-3,3167E+14	5,29209E+14	5,19938E+14	5,88692E+14	6,29745E+14	6,10652E+14	6,12891E+14
TC1800000191.mm.2	Galnt1	polypeptide N-acetylgalactosaminyltransferase 1	1,24628E+14	6,26406E+14	2,80824E+14	0.0286364301647441	0.161786613778811	-3,31761E+14	7,17544E+14	7,05111E+13	6,15161E+13	5,47221E+14	6,36556E+14	5,36843E+13

TC1700002328.mm.2	Znrf4	zinc and ring finger 4	-1,06225E+14	6,56805E+14	-2,80822E+14	0.0286369950723413	0.161786613778811	-3,31763E+14	5,95574E+14	5,8825E+14	6,48744E+14	7,11985E+14	6,67594E+14	7,2868E+14
TC0700001008.mm.2	Igf1r	insulin-like growth factor I receptor	0.834456544389331	6,28279E+13	2,80806E+14	0.0286433327888731	0.161786613778811	-3,31784E+14	6,86738E+14	6,92455E+14	5,81821E+14	5,96356E+14	6,17282E+13	5,95019E+14
TC1500001401.mm.2	Rrm2b	ribonucleotide reductase M2 B (TP53 inducible)	0.916625577890765	6,9819E+14	2,80761E+12	0.0286609003968692	0.161786613778811	-3,31842E+14	7,51626E+12	7,39397E+14	6,68603E+14	7,21815E+14	6,48611E+14	6,59087E+13
TC0800002437.mm.2	Unc13a	unc-13 homolog A	0.864572015133877	7,30921E+14	2,80647E+13	0.0287059727394869	0.161786613778811	-3,3199E+14	7,93746E+14	7,69411E+14	7,06697E+14	7,25429E+14	6,82424E+14	7,07819E+14
TC1000000122.mm.2	Cited2	Cbp/p300-interacting transactivator, with Glu/Asp-rich carboxy-terminal domain, 2	1,49445E+14	8,83669E+14	2,8061E+14	0.0287203187231103	0.161786613778811	-3,32037E+14	9,90559E+14	9,62089E+14	9,24324E+14	7,71282E+14	8,47059E+14	8,067E+14
TC1000001466.mm.2	Grip1	glutamate receptor interacting protein 1	1,1934E+14	5,537E+14	2,80598E+14	0.028724978938133	0.161786613778811	-3,32052E+14	6,05115E+14	6,22515E+14	4,98463E+14	6,07155E+14	4,82967E+14	5,05984E+14
TC0200000347.mm.2	Gad2	glutamic acid decarboxylase 2	2,03351E+14	8,58676E+14	2,80593E+14	0.028726918009443	0.161786613778811	-3,32059E+14	1,00812E+14	9,8057E+14	8,98517E+13	6,82858E+14	8,40149E+14	7,4184E+13
TC0400000889.mm.2	Gm13271	predicted gene 13271	-1,3494E+14	6,59892E+14	-2,80576E+14	0.0287338373345141	0.161786613778811	-3,32081E+14	5,49019E+14	5,76448E+14	6,79077E+14	7,59462E+14	6,43092E+14	7,52255E+14
TC1200000786.mm.2	Dcaf4	DDB1 and CUL4 associated factor 4	1,01836E+13	7,12611E+14	2,80567E+14	0.0287371968143961	0.161786613778811	-3,32092E+14	8,05508E+14	7,75484E+13	6,92476E+14	6,24876E+14	6,74042E+13	7,03279E+14
TC1200000334.mm.2	Agmo	alkylglycerol monooxygenase	1,39811E+14	5,80855E+14	2,80536E+14	0.0287493984272916	0.161786613778811	-3,32132E+14	6,7879E+14	6,51521E+14	5,65343E+14	5,38787E+14	5,95838E+14	4,54851E+14
TC1100001493.mm.2	Hoxb8	homeobox B8	-1,44115E+14	6,44506E+14	-2,80531E+14	0.028751266113441	0.161786613778811	-3,32139E+14	5,54782E+14	5,4101E+14	6,38377E+14	7,48843E+14	6,39733E+14	7,44289E+13
TC1200001564.mm.2	Dcdc2c	doublecortin domain containing 2C	-1,25536E+14	6,14082E+14	-2,80527E+14	0.028752928045866	0.161786613778811	-3,32144E+14	5,16071E+14	5,3041E+14	6,37905E+14	7,0255E+14	5,96928E+14	7,00625E+13
TC1800000443.mm.2	Stk32a	serine/threonine kinase 32A	1,70702E+14	5,54599E+14	2,80516E+14	0.028757372482993	0.161786613778811	-3,32159E+14	6,95487E+14	6,40986E+14	5,7007E+14	4,25986E+14	5,5515E+14	4,39919E+14
TC1000001203.mm.2	Poc1b	POC1 centriolar protein B	0.887198131971212	6,19218E+14	2,80481E+14	0.0287711096832056	0.161786613778811	-3,32204E+14	6,62521E+14	6,6762E+14	6,27352E+14	6,05114E+14	5,97146E+14	5,55555E+14
TC0300002491.mm.2	Car14	carbonic anhydrase 14	0.902405780140703	6,19773E+14	2,80431E+14	0.0287908652116538	0.161786613778811	-3,32268E+14	6,59621E+14	6,86305E+13	5,86595E+14	6,20673E+14	5,97098E+14	5,68346E+13
TC0400003567.mm.2	Gjb4	gap junction protein, beta 4	-0.948189397695637	5,90868E+14	-2,80427E+14	0.0287924717344058	0.161786613778811	-3,32274E+14	5,18503E+14	5,39794E+14	5,90857E+14	6,48119E+14	6,14559E+14	6,33375E+14
TC0X00001858.mm.2	1810030O07Rik	RIKEN cDNA 1810030O07 gene	0.919616412379814	6,73265E+14	2,80423E+14	0.0287941893305607	0.161786613778811	-3,32279E+14	7,26569E+14	7,40561E+13	6,19532E+14	6,69722E+14	6,52773E+14	6,30435E+14
TC0600000912.mm.2	M1ap	meiosis 1 associated protein	-1,13538E+14	6,87247E+14	-2,80411E+14	0.0287985973543975	0.161786613778811	-3,32294E+14	5,73246E+14	6,28599E+14	7,06102E+13	7,86614E+13	6,99285E+14	7,29635E+14
TC1300000674.mm.2	Txndc15	thioredoxin domain containing 15	0.820877897996484	8,17397E+14	2,80399E+13	0.0288035295506781	0.161786613778811	-3,3231E+14	8,6378E+14	8,47296E+14	8,20728E+14	8,25675E+14	7,78664E+14	7,68237E+14
TC0700000152.mm.2	Rnf225	ring finger protein 225	-1,21985E+14	8,50959E+14	-2,8039E+14	0.0288069442675686	0.161786613778811	-3,32321E+13	7,68843E+14	7,7054E+14	8,40084E+14	9,42932E+14	8,63638E+14	9,19716E+14
TC1100000459.mm.2	Clint1	clathrin interactor 1	1,08177E+14	8,04827E+14	2,8038E+14	0.0288110182068358	0.161786613778811	-3,32334E+14	8,65904E+14	8,58611E+14	8,37533E+14	7,5875E+14	7,77889E+14	7,30272E+14
TC0300001362.mm.2	Tacr3	tachykinin receptor 3	1,25425E+14	5,91261E+14	2,80371E+14	0.0288146639356898	0.161786613778811	-3,32346E+14	7,32069E+14	6,34533E+14	5,28112E+14	5,37099E+14	5,95048E+14	5,20704E+14
TC0300002803.mm.2	Gpr88	G-protein coupled receptor 88	1,40276E+13	9,38279E+14	2,8037E+14	0.0288151611969567	0.161786613778811	-3,32348E+14	1,03346E+14	1,03583E+14	9,53989E+14	8,17647E+14	9,21679E+13	8,67066E+14
TC0500002780.mm.2	Gm7978/BC061212	predicted gene 7978/cDNA sequence BC061212	-1,09913E+14	5,24042E+14	-2,80365E+14	0.0288169541081851	0.161786613778811	-3,32354E+14	4,33421E+14	4,77015E+14	5,22639E+14	5,80917E+14	5,34097E+13	5,96164E+14
TC1600002160.mm.2	Zbtb21	zinc finger and BTB domain containing 21	0.962215336906771	5,58333E+14	2,8032E+14	0.028834659307753	0.161829629197431	-3,32412E+14	5,99282E+13	6,32308E+13	5,68308E+13	5,10953E+14	5,13336E+14	5,25811E+14
TC1400001501.mm.2	Ube2e1	ubiquitin-conjugating enzyme E2E 1	0.851716797051998	8,16979E+14	2,80178E+14	0.0288909363371597	0.161839222791112	-3,32596E+14	8,73908E+14	8,49718E+14	8,20637E+14	8,04329E+14	7,86843E+14	7,66439E+14
TC1500000125.mm.2	Cdh10	cadherin 10	1,59048E+14	6,76427E+14	2,80159E+14	0.0288984873950852	0.161839222791112	-3,3262E+14	8,01285E+14	7,92347E+13	6,81714E+14	5,07683E+14	6,48568E+14	6,26967E+14
TC0200000927.mm.2	Tbr1	T-box brain gene 1	1,23687E+13	7,66732E+14	2,80158E+14	0.0288988544863823	0.161839222791112	-3,32621E+14	8,18917E+14	8,89801E+14	7,4441E+14	6,85921E+14	7,69418E+13	6,91927E+14
TC1700000009.mm.2	Tiam2	T cell lymphoma invasion and metastasis 2	1,35386E+14	7,46232E+13	2,80139E+14	0.0289064359457743	0.161839222791112	-3,32646E+14	8,25885E+14	8,68917E+14	7,34603E+14	6,23956E+14	7,48507E+14	6,75524E+14
TC0200005066.mm.2	Chd6	chromodomain helicase DNA binding protein 6	1,42652E+14	7,79866E+14	2,80126E+14	0.0289114402016368	0.161839222791112	-3,32662E+14	8,83345E+14	8,70568E+14	7,90825E+14	6,65851E+14	7,75998E+14	6,9261E+14
TC0500001560.mm.2	Rfc2	replication factor C (activator 1) 2	0.888626585801052	6,69727E+14	2,80089E+14	0.0289261810749277	0.161839222791112	-3,32711E+14	7,13601E+14	7,31123E+14	6,66811E+14	6,39827E+12	6,51477E+14	6,15521E+14
TC0500001453.mm.2	Zfp664	zinc finger protein 664	0.840372021606936	6,62314E+14	2,80088E+14	0.0289265806617517	0.161839222791112	-3,32712E+14	7,01507E+13	7,25005E+13	6,4505E+14	6,43883E+14	6,39304E+13	6,19135E+14
TC0300001643.mm.2	Impa1	inositol (myo)-1(or 4)-monophosphatase 1	1,3132E+14	6,54455E+14	2,80069E+14	0.0289339811609242	0.161839222791112	-3,32736E+14	7,56084E+14	7,00657E+14	6,31154E+14	6,44731E+13	6,57054E+14	5,37048E+14
TC0700001539.mm.2	Olfr676	olfactory receptor 676	-0.840605431013695	6,1879E+14	-2,80022E+14	0.0289527247053828	0.161839222791112	-3,32797E+14	5,83362E+13	5,65486E+14	6,31949E+14	6,14972E+14	6,48939E+14	6,68029E+14
TC1000000462.mm.2	Vgll2	vestigial like family member 2	-0.923504812723262	6,56955E+14	-2,79949E+14	0.0289816502469702	0.161839222791112	-3,32891E+14	5,79364E+14	5,94423E+14	6,88493E+14	7,20964E+13	6,56347E+14	7,02141E+14
TC0900002613.mm.2	Tcf12	transcription factor 12	1,07077E+14	7,60192E+14	2,79936E+14	0.0289869671804786	0.161839222791112	-3,32909E+14	8,15896E+14	8,33181E+14	7,68201E+14	7,08949E+13	7,5059E+14	6,84333E+14
TC1900000520.mm.2	Hectd2	HECT domain E3 ubiquitin protein ligase 2	-0.82123930541788	7,87612E+14	-2,79936E+13	0.0289869813291988	0.161839222791112	-3,32909E+14	7,21332E+14	7,24599E+14	8,42625E+14	8,26933E+14	7,98121E+14	8,12058E+14
TC0200000398.mm.2	Nrarp	Notch-regulated ankyrin repeat protein	1,25785E+13	6,11972E+14	2,79924E+14	0.0289917773097567	0.161839222791112	-3,32924E+14	6,89896E+14	6,61904E+14	6,65411E+14	5,54393E+14	5,73796E+14	5,26432E+14
TC1200002432.mm.2	Tmem179	transmembrane protein 179	1,36967E+13	7,73405E+14	2,79862E+14	0.0290163873683607	0.161839222791112	-3,33004E+14	8,88248E+13	8,53147E+14	7,78091E+14	6,53486E+14	7,63012E+14	7,04449E+14
TC0600002713.mm.2	Chst13	carbohydrate sulfotransferase 13	-1,04791E+14	6,64861E+13	-2,79845E+14	0.0290231956547383	0.161839222791112	-3,33026E+14	5,92612E+14	5,9645E+13	6,61673E+14	7,39788E+14	6,81296E+14	7,1735E+14
TC1200002158.mm.2	Sel1l	sel-1 suppressor of lin-12-like (C. elegans)	0.983964067769641	7,26232E+14	2,79812E+14	0.0290361150757053	0.161839222791112	-3,33068E+14	8,06965E+14	8,0715E+14	6,94661E+14	6,31293E+14	7,26484E+13	6,90837E+14
TC1600001922.mm.2	Nrip1	nuclear receptor interacting protein 1	0.992469627767159	5,74225E+14	2,79801E+14	0.029040695392716	0.161839222791112	-3,33083E+13	6,60809E+14	6,07726E+14	5,77098E+14	5,29676E+13	5,46169E+14	5,23872E+14
TC1700001791.mm.2	Ccdc167	coiled-coil domain containing 167	-0.947360196381747	7,57494E+14	-2,79773E+14	0.0290517093967004	0.161839222791112	-3,33119E+14	6,88903E+14	6,77905E+14	7,83862E+14	8,38011E+14	7,61738E+13	7,94542E+14
TC1000002531.mm.2	Atcay	ataxia, cerebellar, Cayman type	1,49543E+14	7,91522E+13	2,79768E+14	0.0290538226384454	0.161839222791112	-3,33126E+14	9,15992E+13	8,85108E+14	7,8924E+14	6,56779E+14	7,94681E+14	7,07333E+14
TC1600001375.mm.2	Igf2bp2	insulin-like growth factor 2 mRNA binding protein 2	-0.836282112008998	4,81443E+14	-2,79761E+14	0.0290567716955922	0.161839222791112	-3,33135E+14	4,31576E+14	4,04674E+14	5,18696E+14	5,30203E+14	5,03912E+14	4,99595E+13
TC0400003881.mm.2	Clcnkb	chloride channel, voltage-sensitive Kb	-1,08348E+14	5,36734E+14	-2,79757E+14	0.029058179238848	0.161839222791112	-3,3314E+13	4,67686E+14	4,61884E+14	5,41796E+14	6,02772E+14	5,38055E+14	6,08211E+14
TC1100001349.mm.2	Supt4a	suppressor of Ty 4A	1,00019E+14	7,79212E+14	2,79734E+14	0.0290671550182685	0.161839222791112	-3,33169E+14	8,53478E+14	8,35703E+14	7,84765E+14	7,12181E+14	7,52316E+14	7,36829E+14
TC1100003625.mm.2	Nfe2l1	nuclear factor, erythroid derived 2,-like 1	1,31924E+13	9,6025E+14	2,79734E+14	0.0290673810437211	0.161839222791112	-3,3317E+14	1,04677E+14	1,03382E+14	9,81398E+13	8,82771E+13	9,53349E+14	8,63392E+14
TC0300000459.mm.2	Selenot	selenoprotein T	1,02549E+14	9,1695E+14	2,79655E+14	0.0290988881319063	0.161897015968125	-3,33272E+14	1,00524E+14	9,81714E+13	8,99483E+14	8,33416E+14	9,11893E+14	8,69961E+14
TC1800000568.mm.2	Lmnb1	lamin B1	-1,24424E+14	7,21972E+14	-2,79642E+14	0.0291040793571687	0.161897015968125	-3,33289E+14	6,55622E+14	6,17539E+14	7,14977E+14	8,21689E+14	7,38609E+14	7,83398E+14
TC0X00003064.mm.2	Ripply1	ripply transcriptional repressor 1	-0.90073421807204	5,10905E+14	-2,79632E+14	0.0291079038825581	0.161897015968125	-3,33301E+14	4,50598E+13	4,64595E+14	5,0261E+14	5,52286E+13	5,43698E+14	5,51641E+14
TC0X00001558.mm.2	Ubqln2	ubiquilin 2	1,90221E+14	8,22916E+14	2,79574E+14	0.0291313427106947	0.161971471738241	-3,33377E+14	9,588E+14	9,38458E+13	8,61371E+11	6,62052E+14	8,05261E+14	7,11555E+14
TC0900000602.mm.2	Pih1d2	PIH1 domain containing 2	-1,04207E+14	6,43068E+14	-2,79534E+14	0.029147211182454	0.161986293308364	-3,33428E+14	5,59164E+14	6,03307E+14	6,30426E+14	6,94624E+14	6,68226E+14	7,0266E+14
TC1100003868.mm.2	1700023F06Rik	RIKEN cDNA 1700023F06 gene	-1,24696E+14	7,13004E+14	-2,79517E+14	0.0291541147268824	0.161986293308364	-3,33451E+14	6,42542E+14	6,46878E+14	6,82513E+14	7,6728E+14	7,25321E+14	8,13492E+14
TC1000002534.mm.2	Mrpl54	mitochondrial ribosomal protein L54	-1,01432E+14	7,33974E+14	-2,79431E+14	0.0291885763587467	0.162068926234067	-3,33562E+14	6,94428E+14	6,57352E+14	7,34913E+14	7,62506E+14	7,44945E+14	8,09699E+13
TC0600001463.mm.2	Ano2	anoctamin 2	1,29515E+14	6,97879E+14	2,79414E+14	0.0291952213539025	0.162068926234067	-3,33584E+14	7,56839E+14	7,81301E+14	7,49071E+14	6,20952E+14	6,38796E+14	6,40314E+14
TC0300002402.mm.2	Lce1b	late cornified envelope 1B	-0.852855206114212	7,46496E+14	-2,79404E+14	0.0291991617239184	0.162068926234067	-3,33596E+14	7,05354E+14	6,74604E+14	7,68751E+14	7,79734E+14	7,83912E+14	7,66618E+14
TC0900003023.mm.2	Dock3	dedicator of cyto-kinesis 3	1,40316E+14	7,82358E+14	2,79371E+14	0.0292124886495749	0.162080645587622	-3,33639E+14	8,53765E+14	8,69539E+14	7,97072E+14	7,31103E+14	7,86959E+14	6,55712E+14
TC0200002334.mm.2	Bpifa3	BPI fold containing family A, member 3	-1,23134E+14	6,44604E+13	-2,79349E+12	0.0292213911395793	0.162080645587622	-3,33668E+14	5,6033E+13	5,59705E+14	6,37226E+14	7,4406E+14	6,55961E+14	7,1034E+14
TC1200000711.mm.2	Mpp5	membrane protein, palmitoylated 5 (MAGUK p55 subfamily member 5)	2,05386E+14	6,77169E+14	2,79291E+14	0.0292445026234099	0.162114679436265	-3,33743E+14	8,10504E+14	8,21708E+14	7,25846E+14	4,83517E+14	6,06087E+13	6,15352E+14
TC0700002609.mm.2	Tex101	testis expressed gene 101	-1,66992E+14	6,11825E+14	-2,79283E+14	0.0292477179810836	0.162114679436265	-3,33753E+14	4,77475E+14	4,99301E+13	6,1579E+14	7,67626E+14	6,00989E+14	7,09773E+14
TC0200002042.mm.2	Chgb	chromogranin B	1,04766E+14	7,96786E+14	2,79258E+14	0.0292577104077861	0.162114679436265	-3,33785E+14	8,7944E+14	8,47789E+14	7,89712E+14	7,46078E+14	7,92679E+14	7,25017E+14
TC0200002594.mm.2	Cebpb	CCAAT/enhancer binding protein (C/EBP), beta	-0.989796791213622	7,37169E+14	-2,79085E+13	0.0293274712653891	0.16218779701038	-3,3401E+14	6,59733E+14	6,75583E+14	7,45511E+14	8,0891E+14	7,48688E+14	7,84588E+14
TC1300001375.mm.2	Ucn3	urocortin 3	-0.920762664887547	5,05089E+14	-2,79064E+14	0.0293357678508165	0.16218779701038	-3,34037E+14	4,24557E+14	4,59717E+14	5,25621E+14	5,52213E+14	5,15238E+14	5,53188E+14
TC1400002259.mm.2	Elp3	elongator acetyltransferase complex subunit 3	1,66326E+14	7,21003E+14	2,79006E+14	0.0293592803592081	0.16218779701038	-3,34112E+13	8,44338E+14	8,2313E+14	7,40789E+14	5,82945E+14	7,16715E+14	6,181E+14
TC0X00002136.mm.2	Dcaf12l1	DDB1 and CUL4 associated factor 12-like 1	2,16434E+14	7,0125E+14	2,78967E+14	0.029374778692534	0.16218779701038	-3,34162E+14	8,46247E+14	8,096E+14	7,8033E+14	5,48345E+14	6,6543E+14	5,5755E+14
TSUnmapped00000029.mm.2	Psd3	pleckstrin and Sec7 domain containing 3	1,6239E+14	8,86194E+14	2,7893E+13	0.0293899476719195	0.16218779701038	-3,34211E+14	9,89572E+14	9,95771E+14	8,89285E+14	7,81971E+14	9,0346E+14	7,57103E+14

TC0700000236.mm.2	Slc1a5	solute carrier family 1 (neutral amino acid transporter), member 5	-0.841070737622531	5,24992E+14	-2,78908E+14	0.0293985962214222	0.16218779701038	-3,34238E+14	4,75377E+14	4,77407E+14	5,54107E+14	5,22061E+14	5,63921E+14	5,57077E+14
TC0X00000069.mm.2	Gripap1	GRIP1 associated protein 1	0.991875837774915	7,52543E+13	2,78898E+14	0.0294028603159737	0.16218779701038	-3,34252E+14	8,0679E+14	8,29758E+14	6,9438E+14	7,46158E+14	7,43788E+14	6,94384E+14
TC0700004626.mm.2	Sez6l2	seizure related 6 homolog like 2	2,08239E+14	8,33759E+14	2,78833E+14	0.0294287153981712	0.16218779701038	-3,34335E+14	9,80999E+14	9,74951E+14	8,65739E+14	6,4139E+14	8,21569E+14	7,17904E+14
TC1100000763.mm.2	Smcr8	Smith-Magenis syndrome chromosome region, candidate 8 homolog (human)	2,10533E+13	6,45825E+14	2,78818E+14	0.0294350235966174	0.16218779701038	-3,34355E+14	7,59134E+14	7,64294E+13	7,03468E+14	5,4569E+13	6,48383E+13	4,5398E+14
TC1500001789.mm.2	Recql4	RecQ protein-like 4	-1,22048E+13	6,29387E+14	-2,78817E+14	0.0294355487135391	0.16218779701038	-3,34357E+14	5,48422E+13	5,6131E+14	6,18286E+14	6,94478E+14	6,32039E+14	7,21789E+14
TC0X00002450.mm.2	Lage3	L antigen family, member 3	-1,1054E+14	7,73764E+14	-2,78805E+14	0.0294402494479433	0.16218779701038	-3,34372E+13	7,13461E+14	7,26358E+13	8,1664E+14	7,25224E+14	8,46855E+13	8,14045E+14
TC1000000430.mm.2	Hace1	HECT domain and ankyrin repeat containing, E3 ubiquitin protein ligase 1	1,54243E+14	6,1065E+14	2,78802E+14	0.0294414514114843	0.16218779701038	-3,34376E+14	7,14542E+14	7,05457E+14	6,3847E+14	4,93914E+14	5,99824E+14	5,1169E+14
TC0900001294.mm.2	Gm5627	predicted gene 5627	-1,12268E+14	6,25929E+14	-2,78801E+14	0.0294418826128241	0.16218779701038	-3,34377E+14	5,30312E+14	5,85067E+14	6,2495E+14	6,7533E+14	6,35676E+14	7,0424E+14
TC0200000490.mm.2	Tsc1	tuberous sclerosis 1	1,36504E+14	7,37577E+14	2,78789E+14	0.029446530163672	0.16218779701038	-3,34392E+14	8,29247E+14	8,31162E+14	7,55959E+14	6,21691E+14	7,17069E+14	6,70333E+13
TC1400001001.mm.2	Hr	lysine demethylase and nuclear receptor corepressor	-1,36076E+14	7,44638E+14	-2,78788E+14	0.0294470222922758	0.16218779701038	-3,34393E+14	6,43603E+14	6,73283E+14	7,28748E+14	8,33155E+14	7,49128E+14	8,39909E+14
TC1000001596.mm.2	Stat2	signal transducer and activator of transcription 2	0.80286909224519	6,26409E+14	2,78756E+14	0.0294598874235518	0.16218779701038	-3,34435E+14	6,80141E+14	6,79061E+14	6,03725E+14	5,96901E+14	6,00244E+14	5,98384E+14
TC1700000374.mm.2	Tpsb2	tryptase beta 2	-1,24846E+14	6,23776E+14	-2,78756E+13	0.0294600874376537	0.16218779701038	-3,34435E+14	5,3234E+14	5,28334E+14	6,30133E+14	7,41478E+14	6,28884E+14	6,81483E+14
TC1800001272.mm.2	Jakmip2	janus kinase and microtubule interacting protein 2	2,48588E+14	6,63833E+14	2,78704E+14	0.029481240962483	0.16218779701038	-3,34503E+14	8,14762E+14	8,2799E+13	6,95069E+14	4,996E+13	6,88243E+14	4,57333E+14
TC0700004361.mm.2	BC017158/Mir3103	cDNA sequence BC017158/microRNA 3103	1,41206E+14	6,42562E+14	2,787E+14	0.0294825823080227	0.16218779701038	-3,34507E+14	7,65173E+14	7,07943E+14	6,56393E+14	5,35154E+14	6,28607E+14	5,62098E+14
TC0500003381.mm.2	Tmem270	transmembrane protein 270	-1,2698E+14	6,31077E+14	-2,78664E+14	0.029497206843825	0.16218779701038	-3,34554E+14	5,58455E+14	5,61745E+14	5,8435E+14	7,07751E+14	6,72514E+14	7,01647E+14
TC0X00003000.mm.2	Armcx2	armadillo repeat containing, X-linked 2	2,21971E+14	5,96362E+14	2,78638E+14	0.0295079138536508	0.16218779701038	-3,34588E+14	7,32685E+14	7,20901E+14	6,49715E+14	4,65225E+14	6,00775E+14	4,08869E+14
TC0500000656.mm.2	Gm5108	predicted gene 5108	-0.921041405863148	6,86241E+14	-2,78588E+14	0.0295280478295862	0.16218779701038	-3,34653E+14	6,23007E+13	6,26427E+14	6,99371E+14	7,34997E+14	6,93588E+14	7,40054E+13
TC0600001354.mm.2	Slc25a18	solute carrier family 25 (mitochondrial carrier), member 18	1,56381E+14	7,35147E+14	2,78584E+14	0.029529672611606	0.16218779701038	-3,34658E+14	8,56367E+14	8,24522E+14	7,36134E+14	6,25734E+14	7,48691E+14	6,19437E+14
TC1300002072.mm.2	Pitx1	paired-like homeodomain transcription factor 1	-1,16424E+14	7,75014E+14	-2,78568E+13	0.0295360151546271	0.16218779701038	-3,34678E+14	7,29597E+14	6,78207E+14	7,53485E+14	8,48142E+14	8,12477E+13	8,28175E+14
TC1200001841.mm.2	Rpl10l	ribosomal protein L10-like	-1,231E+14	8,08493E+14	-2,78547E+14	0.0295444960803587	0.16218779701038	-3,34705E+14	7,24087E+14	7,17382E+14	8,14293E+14	9,07524E+14	8,05873E+14	8,81797E+14
TC1000002448.mm.2	Agpat3	1-acylglycerol-3-phosphate O-acyltransferase 3	1,16137E+14	9,21583E+14	2,78534E+14	0.0295499518773343	0.16218779701038	-3,34723E+14	1,00599E+13	9,98831E+14	9,26792E+14	8,25347E+14	9,04979E+14	8,67562E+14
TC0700000099.mm.2	Aurkc	aurora kinase C	-0.948789641459833	6,19506E+14	-2,78489E+14	0.0295683627552825	0.16218779701038	-3,34781E+14	5,62447E+14	5,8279E+14	5,90358E+14	6,46446E+14	6,81196E+14	6,53798E+14
TC1100002998.mm.2	Adprm	ADP-ribose/CDP-alcohol diphosphatase, manganese dependent	-0.842932576159384	7,99877E+14	-2,78466E+14	0.029577725080534	0.16218779701038	-3,34811E+14	7,68514E+13	7,47476E+14	7,85899E+14	8,12797E+14	8,42797E+14	8,4178E+14
TC1100002486.mm.2	Npm1	nucleophosmin 1	1,67277E+14	9,05253E+14	2,78452E+14	0.0295832041432575	0.16218779701038	-3,34829E+14	1,02189E+14	1,02375E+14	9,139E+14	7,60886E+14	9,11179E+14	7,99911E+14
TC1100004187.mm.2	Nptx1	neuronal pentraxin 1	0.882926800884269	7,18961E+14	2,78412E+14	0.0295995649440899	0.16218779701038	-3,34881E+14	7,59798E+14	7,98616E+14	6,99449E+14	6,74073E+14	6,84469E+14	6,9736E+14
TC1400000309.mm.2	Rft1	RFT1 homolog	1,16409E+14	6,19826E+14	2,78388E+14	0.0296092752114205	0.16218779701038	-3,34912E+14	7,14286E+14	6,87609E+13	5,94437E+14	5,53546E+14	5,35838E+14	6,33238E+14
TC1400002858.mm.2	Colq	collagen-like tail subunit (single strand of homotrimer) of asymmetric acetylcholinesterase	-1,00604E+14	7,02477E+14	-2,7836E+14	0.0296205364934648	0.16218779701038	-3,34947E+13	6,4126E+14	6,49045E+14	6,78446E+14	7,54598E+14	7,36783E+14	7,54729E+14
TC0700002646.mm.2	Cyp2s1	cytochrome P450, family 2, subfamily s, polypeptide 1	0.815553907312578	5,55634E+14	2,78287E+14	0.0296503660187381	0.16218779701038	-3,35042E+14	6,02796E+14	6,07846E+14	5,39729E+13	5,35903E+14	5,33614E+14	5,13916E+14
TC0X00001146.mm.2	Ube2dnl1	ubiquitin-conjugating enzyme E2D N-terminal like 1	-1,02993E+14	4,95091E+14	-2,78257E+14	0.0296625988359228	0.16218779701038	-3,35081E+14	4,49606E+14	4,5157E+14	4,54985E+14	5,07223E+13	5,2091E+14	5,86253E+14
TC0100003649.mm.2	Smyd3	SET and MYND domain containing 3	1,14905E+14	7,34997E+14	2,78217E+14	0.029678756314117	0.16218779701038	-3,35133E+14	8,03627E+14	8,11004E+13	7,2877E+12	6,81759E+14	7,39991E+13	6,44829E+14
TC1000000852.mm.2	Reep6	receptor accessory protein 6	1,84795E+13	6,50323E+14	2,78215E+14	0.0296798322132948	0.16218779701038	-3,35136E+14	7,73198E+14	7,42794E+14	7,19841E+14	5,19698E+14	6,09059E+14	5,37345E+14
TC1900000582.mm.2	Zfp518a/Mir8092	zinc finger protein 518A/microRNA 8092	1,22777E+14	5,57322E+14	2,78213E+14	0.0296803421174546	0.16218779701038	-3,35138E+14	6,09371E+13	5,99431E+14	5,26915E+14	6,44964E+14	4,71686E+14	4,91563E+14
TC1000002470.mm.2	Theg	testicular haploid expressed gene	-0.845057574502755	5,71939E+14	-2,78189E+14	0.0296904065394723	0.16218779701038	-3,3517E+14	5,17826E+13	5,41775E+14	5,79069E+13	5,64352E+14	6,03581E+14	6,25031E+14
TC0800002953.mm.2	Bcar1	breast cancer anti-estrogen resistance 1	0.882912982741804	6,73764E+14	2,78181E+14	0.0296933713804596	0.16218779701038	-3,35179E+14	7,39925E+14	7,25918E+13	6,64331E+14	6,23152E+14	6,3407E+14	6,5519E+14
TC1100002852.mm.2	Zfp867	zinc finger protein 867	0.914290940068994	5,76356E+14	2,78169E+14	0.0296982475985871	0.16218779701038	-3,35195E+14	6,49755E+14	6,26014E+14	5,55034E+14	5,34423E+14	5,70372E+14	5,22539E+14
TC0200005184.mm.2	Ptgis	prostaglandin I2 (prostacyclin) synthase	-0.950663402188372	6,63647E+14	-2,78165E+14	0.0297000261805311	0.16218779701038	-3,352E+14	6,14262E+14	5,93291E+14	6,71971E+14	7,04674E+14	6,71939E+14	7,25746E+14
TC1400001558.mm.2	Dusp13	dual specificity phosphatase 13	-0.856531319230625	6,70954E+13	-2,78135E+13	0.0297122885189267	0.16218779701038	-3,35239E+14	5,99236E+13	6,30642E+14	6,87315E+14	7,07344E+14	6,93666E+14	7,07519E+14
TC1000001768.mm.2	Phactr2	phosphatase and actin regulator 2	1,59771E+14	6,17074E+14	2,78116E+13	0.0297200187706517	0.16218779701038	-3,35264E+14	7,03255E+14	7,10126E+11	6,62487E+14	5,32739E+14	6,08046E+14	4,85793E+14
TC0300002273.mm.2	Dclk2	doublecortin-like kinase 2	1,39612E+14	7,43041E+14	2,78071E+14	0.0297384177124503	0.16218779701038	-3,35322E+14	8,34332E+14	8,04817E+14	7,75525E+14	6,83645E+14	7,35842E+14	6,24085E+14
TC1400001103.mm.2	Kbtbd7	kelch repeat and BTB (POZ) domain containing 7	3,14154E+14	5,82895E+14	2,78064E+13	0.0297412341513899	0.16218779701038	-3,35331E+14	7,76527E+14	7,64434E+14	6,64845E+14	3,78912E+14	5,90159E+13	3,22494E+14
TC0800001296.mm.2	Pmfbp1	polyamine modulated factor 1 binding protein 1	-0.845004089318243	4,79152E+14	-2,78049E+14	0.0297474997132218	0.16218779701038	-3,35351E+14	4,41608E+14	4,38213E+14	4,56722E+14	4,89551E+14	5,17865E+14	5,30956E+14
TC1200000792.mm.2	Psen1	presenilin 1	1,54027E+14	7,29235E+14	2,78034E+14	0.029753695036117	0.16218779701038	-3,3537E+14	8,11248E+14	8,11477E+14	8,00466E+14	6,37549E+14	6,81375E+14	6,33296E+14
TC1700001757.mm.2	Fkbp5	FK506 binding protein 5	1,82254E+14	7,08172E+13	2,78033E+14	0.0297540574668084	0.16218779701038	-3,35372E+13	8,38376E+14	8,1324E+14	7,46593E+14	5,63713E+14	6,94959E+14	5,92149E+13
TC1700001976.mm.2	Ddr1	discoidin domain receptor family, member 1	1,05637E+14	5,83922E+14	2,78002E+14	0.0297666844582055	0.162201754032826	-3,35412E+14	6,54093E+14	6,85081E+14	5,4654E+14	4,89916E+14	5,33185E+14	5,94715E+14
TC0400003871.mm.2	Necap2	NECAP endocytosis associated 2	-0.840742944069118	7,84496E+14	-2,77957E+14	0.0297849848880163	0.162246606322044	-3,3547E+14	7,38053E+14	7,38226E+14	7,76437E+14	8,09834E+14	8,25107E+14	8,19321E+14
TC0600001907.mm.2	Cttnbp2	cortactin binding protein 2	0.993373756289123	7,56421E+14	2,77844E+14	0.0298313578132541	0.16244429484453	-3,35616E+14	8,06914E+14	8,20134E+13	7,63886E+14	7,19219E+14	7,44405E+14	6,83969E+13
TC1600001387.mm.2	Kng2	kininogen 2	-1,02302E+14	4,9909E+14	-2,77807E+14	0.0298466331025397	0.162472567290954	-3,35665E+13	4,39555E+14	4,29743E+14	4,87217E+14	5,64119E+14	5,22694E+14	5,51209E+14
TC0100001944.mm.2	Vcpip1	valosin containing protein (p97)/p47 complex interacting protein 1	2,42813E+13	6,70019E+14	2,7778E+14	0.0298574138031023	0.162476362245656	-3,35699E+14	8,45763E+14	8,47689E+14	6,43043E+14	4,75794E+14	7,26058E+12	4,81767E+14
TC1100004287.mm.2	Mrps24	mitochondrial ribosomal protein S24	-0.815723014688791	7,14337E+14	-2,77747E+14	0.0298711131218087	0.162496031644735	-3,35742E+14	6,81876E+14	6,72636E+14	7,07023E+14	7,06833E+14	7,4895E+14	7,68707E+14
TC0300000269.mm.2	Exosc9	exosome component 9	1,16411E+13	7,1674E+14	2,77661E+14	0.0299063318832718	0.162632711992966	-3,35853E+14	7,75374E+13	7,98037E+13	7,26068E+14	6,60372E+14	7,12602E+13	6,27987E+13
TC1600001648.mm.2	Gtpbp8	GTP-binding protein 8 (putative)	2,14444E+14	5,34286E+14	2,776E+14	0.0299312581916159	0.162713347922236	-3,35931E+14	6,58073E+14	6,30551E+14	5,83976E+14	4,73381E+14	5,45132E+13	3,14604E+14
TC1300002040.mm.2	Gprin1	G protein-regulated inducer of neurite outgrowth 1	1,03073E+13	7,34131E+14	2,77574E+14	0.0299421877205436	0.162713528364225	-3,35966E+14	8,04007E+14	7,94435E+14	7,04789E+13	7,09262E+14	7,37996E+14	6,54299E+14
TC1500002335.mm.2	Pheta2	PH domain containing endocytic trafficking adaptor 2	-0.974085824844317	6,34832E+14	-2,77523E+14	0.0299632614690132	0.162713528364225	-3,36032E+14	5,79736E+14	5,90812E+14	6,52454E+14	6,20625E+14	6,50702E+14	7,14663E+14
TC0X00000979.mm.2	Taf1	TATA-box binding protein associated factor 1	1,98551E+14	6,26566E+14	2,77518E+14	0.0299652286924737	0.162713528364225	-3,36038E+14	7,86712E+14	7,38807E+14	6,16658E+14	4,88798E+14	6,59829E+14	4,68588E+14
TC0400001107.mm.2	Dhcr24	24-dehydrocholesterol reductase	1,15597E+14	7,73561E+14	2,7745E+14	0.0299930355189985	0.162713528364225	-3,36126E+14	8,45029E+14	8,29129E+14	8,08968E+13	7,15275E+14	7,47352E+14	6,95611E+14
TC0100002207.mm.2	2010300C02Rik	RIKEN cDNA 2010300C02 gene	0.867896165974528	7,84351E+14	2,77437E+14	0.0299986592979963	0.162713528364225	-3,36144E+14	8,19568E+14	8,45209E+13	7,61593E+14	7,8854E+14	7,57689E+14	7,33508E+14
TC1100002103.mm.2	Tcn2	transcobalamin 2	1,1575E+14	6,70425E+14	2,77362E+14	0.0300294519452761	0.162713528364225	-3,3624E+14	7,2567E+14	7,746E+14	6,74799E+14	5,78712E+14	6,40604E+14	6,28166E+14
TC1100000936.mm.2	Dvl2/Mir324	dishevelled segment polarity protein 2/microRNA 324	-0.968470065567315	7,94594E+14	-2,77361E+13	0.0300298953543673	0.162713528364225	-3,36242E+14	7,27464E+14	7,29722E+14	8,01773E+14	8,57727E+12	8,03491E+14	8,47389E+14

TC1000000261.mm.2	Soga3	SOGA family member 3	1,93828E+14	5,47641E+14	2,77358E+13	0.0300312394383979	0.162713528364225	-3,36246E+14	6,52232E+14	6,70207E+14	5,84634E+13	4,43988E+13	5,56959E+14	3,77825E+14
TC1100004294.mm.2	Glod4/Gemin4	glyoxalase domain containing 4/gem nuclear organelle associated protein 4	1,90295E+14	6,8917E+14	2,77335E+14	0.0300408072088759	0.162713528364225	-3,36276E+14	8,33417E+14	8,28236E+14	6,93353E+14	4,98952E+14	6,95569E+14	5,85495E+14
TC0900001863.mm.2	Keap1	kelch-like ECH-associated protein 1	1,17407E+14	6,34077E+14	2,77332E+14	0.0300419883528329	0.162713528364225	-3,3628E+14	7,32134E+14	7,35004E+14	6,07128E+14	4,97874E+14	6,05118E+14	6,27205E+14
TC0700000610.mm.2	Scgb1b19	secretoglobin, family 1B, member 19	-0.94532298453109	5,76008E+14	-2,77331E+14	0.0300423724249718	0.162713528364225	-3,36281E+14	4,99143E+14	5,2933E+14	5,91803E+14	6,18235E+14	5,82713E+14	6,34825E+14
TC1400002248.mm.2	Extl3	exostoses (multiple)-like 3	1,61472E+14	7,62677E+14	2,77278E+14	0.0300643322945287	0.162723891060274	-3,3635E+14	8,56491E+14	8,65219E+14	8,05476E+14	6,50108E+14	7,46243E+14	6,52522E+14
TC0300000656.mm.2	Tmem154	transmembrane protein 154	-1,2676E+14	5,38841E+14	-2,77277E+14	0.0300644835652228	0.162723891060274	-3,3635E+14	4,51033E+14	4,90655E+14	5,07659E+12	5,88489E+14	5,52442E+14	6,42765E+14
TC0600000850.mm.2	Ggcx	gamma-glutamyl carboxylase	0.985652479185624	6,31493E+14	2,77229E+14	0.0300844807328527	0.162769703950466	-3,36413E+14	6,75009E+14	6,63979E+14	6,63815E+13	6,44297E+13	6,06766E+14	5,35092E+14
TC1500001825.mm.2	Cacng2	calcium channel, voltage-dependent, gamma subunit 2	1,83455E+14	8,00143E+14	2,77198E+14	0.0300973172853537	0.162769703950466	-3,36453E+14	9,24899E+14	9,1049E+14	8,35315E+14	6,61672E+14	7,962E+14	6,72279E+14
TC1300002079.mm.2	Cxcl14	chemokine (C-X-C motif) ligand 14	0.891381123551465	8,09486E+14	2,77184E+14	0.0301032531355048	0.162769703950466	-3,36472E+14	8,51746E+14	8,63659E+14	8,27298E+14	7,77083E+14	7,70773E+14	7,66355E+14
TC1200002006.mm.2	Ccdc177	coiled-coil domain containing 177	-0.863882898784457	7,63438E+14	-2,7715E+14	0.0301171365475692	0.162778618061429	-3,36515E+14	7,02775E+14	7,12345E+14	7,67925E+14	8,09685E+14	7,97209E+14	7,90688E+14
TC0900003326.mm.2	Ccpg1	cell cycle progression 1	0.870657639049657	7,35611E+14	2,7712E+14	0.0301294594788083	0.162778618061429	-3,36554E+13	7,85774E+14	7,84299E+14	7,12115E+14	7,35537E+14	7,17507E+14	6,78434E+14
TC0200002157.mm.2	Slc24a3	solute carrier family 24 (sodium/potassium/calcium exchanger), member 3	1,44616E+13	9,11849E+14	2,77087E+13	0.0301432613554642	0.162778618061429	-3,36597E+14	1,03195E+14	9,81677E+14	9,28848E+14	8,04227E+14	9,02066E+14	8,22328E+14
TC0700000368.mm.2	Zfp180	zinc finger protein 180	0.817838223741095	7,09552E+14	2,77082E+14	0.0301453110094523	0.162778618061429	-3,36603E+13	7,55863E+14	7,70895E+14	6,90684E+14	6,76679E+14	6,75176E+14	6,88015E+14
TC1100001682.mm.2	G6pc3	glucose 6 phosphatase, catalytic, 3	1,15368E+14	7,40438E+14	2,76999E+14	0.0301796067761119	0.1628914292246	-3,36711E+13	8,41402E+14	7,88159E+14	7,32195E+14	6,82048E+14	6,59591E+14	7,39233E+14
TC0600003102.mm.2	1700013D24Rik	RIKEN cDNA 1700013D24 gene	-0.910838805055104	4,75449E+14	-2,76983E+14	0.0301864213780584	0.1628914292246	-3,36732E+13	4,05936E+14	4,28517E+14	4,91643E+14	5,09977E+14	4,83592E+14	5,33029E+14
TC1500002246.mm.2	Smagp	small cell adhesion glycoprotein	-0.892692039302144	6,96918E+14	-2,76939E+14	0.0302044171318112	0.162933971625334	-3,36788E+14	6,43842E+14	6,28501E+14	7,04861E+14	7,53423E+14	7,19275E+14	7,31607E+14
TC0200003298.mm.2	Uck1	uridine-cytidine kinase 1	0.86154038181187	7,34908E+14	2,7689E+14	0.0302250002335163	0.162990438006241	-3,36852E+14	7,70732E+14	7,80245E+14	7,2364E+14	7,56164E+14	6,76244E+14	7,02424E+14
TC0100000284.mm.2	Rpl31	ribosomal protein L31	0.901035485688892	1,00121E+14	2,76865E+13	0.0302351732316891	0.162990748170694	-3,36884E+14	1,07302E+14	1,05186E+14	9,78773E+14	9,58904E+14	9,95584E+14	9,49095E+14
TC0200004762.mm.2	Dzank1	double zinc ribbon and ankyrin repeat domains 1	2,49192E+14	7,34194E+14	2,76817E+14	0.0302552403919673	0.163044377403267	-3,36947E+14	8,99576E+12	8,79844E+13	7,74774E+14	5,69935E+14	7,5372E+14	5,27316E+14
TC0200003576.mm.2	Acvr1c	activin A receptor, type IC	0.936120532329976	6,50948E+14	2,76754E+14	0.0302816608086437	0.16313202520373	-3,37029E+14	7,01756E+14	7,48991E+14	5,98733E+14	5,92686E+14	6,53148E+14	6,10375E+14
TC1600000266.mm.2	Klhl24	kelch-like 24	2,18884E+14	7,25638E+14	2,76727E+14	0.0302927438370179	0.16313202520373	-3,37063E+14	8,32608E+14	8,44161E+14	7,67539E+14	6,7052E+14	7,42283E+14	4,96719E+14
TC1400000510.mm.2	Socs4	suppressor of cytokine signaling 4	1,11104E+14	5,22072E+14	2,76685E+14	0.030310250060933	0.16313202520373	-3,37118E+14	5,97427E+14	6,01131E+14	5,21069E+14	4,36455E+13	5,15319E+14	4,6103E+14
TC0400002007.mm.2	Dnajc11	DnaJ heat shock protein family (Hsp40) member C11	0.820110237066196	7,95621E+14	2,76584E+14	0.0303522252936668	0.16313202520373	-3,37248E+14	8,25814E+14	8,35374E+14	8,1774E+13	7,9763E+14	7,41618E+14	7,55547E+14
TC0900001982.mm.2	Tmem45b	transmembrane protein 45b	-0.898448768164786	4,76347E+14	-2,76577E+14	0.0303554962734601	0.16313202520373	-3,37259E+14	4,31897E+14	4,447E+14	4,78265E+14	4,46935E+14	5,06879E+14	5,49407E+14
TC0X00002292.mm.2	Fgf13	fibroblast growth factor 13	1,75456E+14	6,17065E+14	2,76568E+14	0.0303588877830515	0.16313202520373	-3,37269E+14	7,41309E+14	7,07468E+13	6,40344E+14	5,15406E+14	6,27019E+14	4,70846E+14
TC1900001621.mm.2	Atp5md	ATP synthase membrane subunit DAPIT	1,70687E+14	1,00429E+14	2,76561E+13	0.030362153329812	0.16313202520373	-3,37279E+14	1,14352E+14	1,13107E+14	9,92697E+14	8,25218E+14	1,01702E+14	9,16189E+14
TC0400002330.mm.2	Manea	mannosidase, endo-alpha	2,32259E+13	7,12532E+14	2,76542E+14	0.0303700201680003	0.16313202520373	-3,37304E+14	8,56054E+14	8,46938E+12	7,66364E+14	5,67362E+14	7,19759E+14	5,18715E+14
TC0700000777.mm.2	Klk6	kallikrein related-peptidase 6	1,38021E+14	6,46332E+14	2,76522E+14	0.0303782090491774	0.16313202520373	-3,37329E+13	6,74081E+14	8,05556E+14	6,03739E+14	5,91022E+14	5,70268E+13	6,33327E+14
TC1700000498.mm.2	Rab44	RAB44, member RAS oncogene family	-1,01445E+14	5,80421E+14	-2,76505E+14	0.0303855503236826	0.16313202520373	-3,37352E+13	5,11679E+14	5,03651E+14	5,90605E+14	6,58369E+14	5,8654E+14	6,31681E+14
TC1100000566.mm.2	Col23a1	collagen, type XXIII, alpha 1	-0.821185702477936	6,85912E+14	-2,76488E+13	0.0303923755527129	0.16313202520373	-3,37373E+14	6,26292E+14	6,49671E+14	6,94559E+14	7,04753E+14	7,24485E+14	7,15714E+13
TC1100003185.mm.2	Olfr399	olfactory receptor 399	-1,13877E+13	4,74975E+14	-2,76364E+14	0.0304445309256772	0.16313202520373	-3,37535E+14	4,14257E+14	3,98503E+14	4,5387E+13	5,42704E+14	4,92509E+14	5,48005E+14
TC1300002126.mm.2	Tut7	terminal uridylyl transferase 7	1,40085E+14	6,00541E+14	2,76361E+12	0.0304456063994647	0.16313202520373	-3,37538E+13	7,23399E+13	6,68762E+14	6,09618E+14	4,89476E+14	5,91381E+13	5,2061E+14
TC0200000561.mm.2	Aif1l	allograft inflammatory factor 1-like	-0.916794281744213	6,71413E+14	-2,76322E+14	0.0304618853779486	0.16313202520373	-3,37588E+14	6,08446E+14	6,49681E+14	6,57677E+14	6,71191E+14	7,00389E+14	7,41097E+14
TC0600001879.mm.2	2610001J05Rik	RIKEN cDNA 2610001J05 gene	1,23157E+14	6,13073E+14	2,76321E+14	0.0304622600375819	0.16313202520373	-3,3759E+14	6,98651E+13	6,43589E+14	6,48639E+14	5,91632E+14	5,94465E+14	5,01461E+14
TC1700000612.mm.2	Col11a2	collagen, type XI, alpha 2	-0.887276790721859	8,08553E+14	-2,76321E+14	0.0304624537251243	0.16313202520373	-3,3759E+14	7,49206E+14	7,60011E+14	8,13045E+14	8,42383E+14	8,2293E+14	8,63743E+14
TC0X00003250.mm.2	Yy2/Mbtps2	Yy2 transcription factor/membrane-bound transcription factor peptidase, site 2	1,91521E+14	6,05593E+14	2,76302E+14	0.0304705642502331	0.16313202520373	-3,37615E+14	7,18256E+14	7,37826E+14	6,41249E+14	4,63186E+14	6,056E+14	4,67441E+14
TC0500002642.mm.2	Ppat	phosphoribosyl pyrophosphate amidotransferase	1,02291E+14	5,27732E+14	2,76282E+14	0.0304788101717898	0.16313202520373	-3,37641E+14	5,88661E+14	6,04465E+14	5,2334E+14	4,6138E+14	5,21307E+14	4,67237E+14
TC1700002214.mm.2	Mdfi	MyoD family inhibitor	-1,03709E+14	6,02772E+14	-2,76276E+14	0.0304810895130074	0.16313202520373	-3,37648E+13	5,43354E+14	5,31803E+14	5,89405E+14	6,69493E+14	6,25815E+14	6,5676E+14
TC0800000982.mm.2	Wdr83os	WD repeat domain 83 opposite strand	1,46716E+14	8,09007E+14	2,76204E+14	0.0305115345426877	0.16313202520373	-3,37742E+14	9,14965E+14	9,10235E+14	8,24603E+14	6,7247E+14	7,90909E+14	7,40859E+14
TC0500000391.mm.2	Adra2c	adrenergic receptor, alpha 2c	1,12925E+14	5,65719E+14	2,76196E+14	0.0305149098150469	0.16313202520373	-3,37752E+14	6,40742E+14	6,43514E+14	5,13293E+14	5,3836E+14	4,78592E+14	5,79814E+14
TC0300000732.mm.2	Arhgef2	rho/rac guanine nucleotide exchange factor (GEF) 2	0.908881545003609	7,06381E+14	2,76187E+14	0.0305184738746204	0.16313202520373	-3,37763E+13	7,62377E+14	7,63314E+14	7,02229E+14	6,66448E+14	6,94481E+14	6,49434E+14
TC1200000270.mm.2	Cog5	component of oligomeric golgi complex 5	1,0443E+14	7,32461E+14	2,76186E+13	0.030519080343809	0.16313202520373	-3,37765E+14	7,93223E+14	7,92026E+14	7,27944E+14	7,05181E+14	7,3077E+14	6,45619E+14
TC0700004388.mm.2	Nsmce4a	NSE4 homolog A, SMC5-SMC6 complex component	1,01354E+14	6,87097E+14	2,76185E+14	0.0305195699199355	0.16313202520373	-3,37767E+14	7,56286E+14	7,34918E+14	7,07768E+14	6,35112E+14	6,28696E+14	6,598E+14
TC0400001446.mm.2	Mir692-3/Ftl1/Mir692-2	microRNA 692-3/ferritin light polypeptide 1/microRNA 692-2	-1,29372E+14	1,10356E+14	-2,76169E+14	0.0305260724618922	0.16313202520373	-3,37787E+14	9,89852E+13	1,03808E+14	1,20996E+14	1,0968E+14	1,16821E+14	1,11847E+14
TC0900003000.mm.2	Col6a4	collagen, type VI, alpha 4	-0.921716678441816	5,62145E+14	-2,76149E+14	0.0305347351836685	0.16313202520373	-3,37814E+14	4,9516E+14	5,23023E+14	5,53819E+14	5,98344E+14	6,16687E+14	5,85839E+14
TC0400001772.mm.2	Sdhb	succinate dehydrogenase complex, subunit B, iron sulfur (Ip)	0.801620167359784	8,18056E+14	2,76121E+14	0.030546159574659	0.16313896891829	-3,37849E+14	8,64664E+14	8,53609E+14	8,12378E+13	8,19736E+14	7,82352E+14	7,75597E+14
TC1300000686.mm.2	Gm3045	predicted gene 3045	-1,23535E+14	6,38496E+14	-2,76033E+14	0.0305832430830659	0.163207323874264	-3,37963E+13	5,48975E+14	5,91851E+14	5,95159E+14	7,07099E+14	6,80044E+14	7,07851E+14
TC0500003532.mm.2	Mmd2	monocyte to macrophage differentiation-associated 2	1,60539E+14	8,7632E+14	2,7603E+14	0.0305844682818932	0.163207323874264	-3,37967E+14	9,8666E+14	9,87226E+13	8,93974E+14	7,37251E+14	8,72023E+14	7,80785E+14
TC0600001648.mm.2	Pik3c2g	phosphatidylinositol-4-phosphate 3-kinase catalytic subunit type 2 gamma	-0.901626920027611	5,22239E+14	-2,7601E+14	0.0305929056102468	0.163207323874264	-3,37993E+13	4,8821E+14	4,60087E+14	5,27275E+14	5,29244E+14	5,40271E+14	5,8835E+14
TC1100004148.mm.2	Tha1	threonine aldolase 1	-1,13507E+14	6,40315E+13	-2,75995E+14	0.0305994740535067	0.163207323874264	-3,38013E+14	5,72289E+14	5,87255E+14	6,28723E+14	6,67068E+14	6,44696E+14	7,41862E+14
TC0X00000672.mm.2	Pnma3	paraneoplastic antigen MA3	1,15085E+13	5,79776E+13	2,7589E+14	0.0306436962172699	0.163344441800934	-3,38149E+14	7,01249E+14	6,17116E+14	5,63769E+14	5,08324E+14	5,64155E+14	5,2404E+14
TC0600002697.mm.2	Plxna1	plexin A1	1,56185E+14	6,54359E+14	2,75886E+14	0.0306454569331734	0.163344441800934	-3,38155E+14	7,55712E+13	7,26994E+14	6,60724E+14	6,12388E+14	6,67995E+14	5,02342E+14
TC0300000866.mm.2	Tdrkh	tudor and KH domain containing protein	0.84972068245864	6,31969E+14	2,75833E+14	0.0306677859203996	0.163394993228286	-3,38223E+14	6,88313E+14	6,63219E+14	6,17779E+14	6,40913E+14	5,80517E+13	6,01071E+14
TC0200004140.mm.2	Cry2	cryptochrome 2 (photolyase-like)	1,14419E+14	8,59167E+14	2,75808E+14	0.0306782495865361	0.163394993228286	-3,38256E+14	9,45236E+14	9,32267E+14	8,58056E+13	7,70774E+14	8,5487E+14	7,93796E+14
TC0200000718.mm.2	Wdr38	WD repeat domain 38	-1,17262E+14	7,56259E+14	-2,75763E+14	0.0306971329822707	0.163394993228286	-3,38314E+14	6,80287E+14	6,95224E+14	7,53275E+14	7,98735E+14	7,54154E+14	8,55881E+14
TC0800000138.mm.2	Fbxo25	F-box protein 25	1,48194E+14	7,65904E+14	2,7576E+14	0.0306983747759484	0.163394993228286	-3,38317E+14	8,70693E+13	8,70504E+14	7,76015E+14	6,33401E+14	7,61733E+14	6,83076E+13
TC1300001762.mm.2	Serpinb1a	serine (or cysteine) peptidase inhibitor, clade B, member 1a	0.939656708565057	6,68269E+14	2,75741E+14	0.0307065623462074	0.163394993228286	-3,38343E+14	7,52552E+14	6,94365E+14	6,40381E+13	6,63329E+14	6,39561E+12	6,19425E+14
TC0300001330.mm.2	Lef1	lymphoid enhancer binding factor 1	-0.886954579880187	6,37592E+14	-2,75712E+14	0.0307189093032226	0.163394993228286	-3,38381E+14	5,9409E+14	5,64684E+14	6,44165E+13	6,8645E+14	6,7122E+14	6,64944E+14
TC1400001107.mm.2	Sugt1	SGT1, suppressor of G2 allele of SKP1 (S. cerevisiae)	1,45884E+14	7,25647E+14	2,75695E+14	0.0307259249973131	0.163394993228286	-3,38402E+14	8,36532E+14	8,4298E+14	7,12114E+14	5,74511E+14	7,33927E+14	6,53817E+14
TC0400000650.mm.2	Zkscan16	zinc finger with KRAB and SCAN domains 16	1,6828E+14	7,348E+14	2,75633E+14	0.0307520638595948	0.163480041230502	-3,38482E+14	8,52402E+14	8,37406E+14	7,65821E+14	5,99926E+14	7,2555E+14	6,27699E+14
TC1800000977.mm.2	Esco1	establishment of sister chromatid cohesion N-acetyltransferase 1	1,8157E+14	5,15714E+14	2,75586E+14	0.0307719184109262	0.163494006627554	-3,38543E+14	6,455E+14	6,37449E+14	5,38048E+14	3,5348E+14	5,12537E+14	4,07271E+14
TC0100002409.mm.2	Ica1l	islet cell autoantigen 1-like	1,07098E+14	6,66271E+13	2,75579E+14	0.0307749843046839	0.163494006627554	-3,38552E+14	7,50472E+14	7,50022E+14	6,31627E+14	5,7921E+14	6,82069E+14	6,04228E+14
TC0800002941.mm.2	Glg1	golgi apparatus protein 1	1,97776E+13	7,5294E+14	2,75524E+13	0.0307984563453846	0.1635189383097	-3,38624E+14	8,92174E+14	8,88071E+14	7,80835E+14	5,7187E+14	7,47047E+14	6,37645E+14

TC0600001992.mm.2	Ube2h	ubiquitin-conjugating enzyme E2H	1,23552E+14	7,42223E+14	2,7552E+14	0.0307999737956891	0.1635189383097	-3,38629E+13	8,34653E+14	8,38431E+14	7,03204E+14	6,51067E+14	7,6892E+14	6,5706E+14
TC0700003045.mm.2	Uevld	UEV and lactate/malate dehyrogenase domains	1,63208E+14	5,20815E+14	2,75471E+14	0.0308206969236525	0.16356391543725	-3,38692E+14	6,54901E+14	6,45636E+13	5,09649E+14	3,40584E+14	5,25333E+14	4,48787E+14
TC1700000890.mm.2	Zfp318	zinc finger protein 318	1,91668E+14	6,30421E+14	2,7542E+14	0.0308424789113348	0.16356391543725	-3,38759E+13	7,61051E+14	7,5452E+14	6,36153E+14	4,98565E+14	6,56527E+13	4,75708E+14
TC0400000585.mm.2	Fsd1l	fibronectin type III and SPRY domain containing 1-like	0.914788901376149	5,70877E+14	2,75388E+14	0.0308558157934043	0.16356391543725	-3,388E+14	6,38572E+14	6,34052E+13	5,46835E+14	5,16134E+14	5,70382E+14	5,19285E+14
TC1100000601.mm.2	Aff4	AF4/FMR2 family, member 4	2,17062E+14	9,05357E+14	2,75379E+14	0.0308596649397079	0.16356391543725	-3,38811E+14	1,03197E+14	1,0275E+14	9,34932E+14	8,12389E+14	9,29136E+14	6,96212E+14
TC0100001112.mm.2	Gpr39	G protein-coupled receptor 39	0.934138876392426	6,58434E+14	2,75344E+14	0.0308746666137305	0.16356391543725	-3,38857E+14	7,01359E+14	6,88747E+14	7,06162E+14	6,51056E+14	6,17369E+14	5,85909E+14
TC0200003169.mm.2	Sohlh1	spermatogenesis and oogenesis specific basic helix-loop-helix 1	-0.920469077866192	6,75257E+14	-2,75329E+14	0.0308808901466248	0.16356391543725	-3,38876E+13	5,97302E+14	6,36959E+14	6,97633E+14	7,01296E+14	6,85882E+14	7,32473E+13
TC0200001959.mm.2	Sirpa	signal-regulatory protein alpha	1,10248E+14	1,0038E+13	2,75294E+14	0.0308958556227659	0.16356391543725	-3,38922E+14	1,07241E+14	1,08784E+14	1,01074E+14	9,12035E+12	9,72524E+14	9,67231E+14
TC0500001669.mm.2	Get4	golgi to ER traffic protein 4	1,13098E+14	6,81772E+14	2,75271E+14	0.0309058143322589	0.16356391543725	-3,38952E+14	7,5013E+14	7,89819E+14	6,52498E+14	5,84433E+14	6,91822E+14	6,2193E+14
TC0400000090.mm.2	Virma	vir like m6A methyltransferase associated	1,32046E+14	6,28051E+14	2,75263E+14	0.0309093099512856	0.16356391543725	-3,38963E+13	7,31523E+14	7,2988E+14	6,17588E+13	4,92003E+14	6,26959E+14	5,70353E+13
TC1900000325.mm.2	D030056L22Rik	RIKEN cDNA D030056L22 gene	-0.940985306886952	7,27226E+14	-2,75261E+14	0.0309099560917537	0.16356391543725	-3,38965E+13	6,79713E+14	6,34414E+13	7,5227E+13	7,94637E+14	7,39275E+14	7,63049E+14
TC0400003937.mm.2	C87977	expressed sequence C87977	0.832546964589991	5,27766E+14	2,75098E+14	0.0309795771498633	0.163760074338319	-3,39177E+14	5,6005E+14	5,72804E+14	5,18664E+14	5,48736E+14	4,78308E+14	4,88037E+14
TC0900001883.mm.2	Spc24	SPC24, NDC80 kinetochore complex component, homolog (S. cerevisiae)	-1,038E+14	6,23205E+14	-2,75082E+14	0.0309861973449757	0.163760074338319	-3,39197E+14	5,31015E+14	5,74746E+14	6,24273E+14	6,95834E+14	6,43819E+13	6,69543E+14
TC0300000832.mm.2	Lce1c	late cornified envelope 1C	-1,22376E+14	5,72054E+14	-2,75063E+14	0.0309945529664659	0.163760074338319	-3,39223E+14	5,286E+14	4,78019E+14	6,41538E+14	5,32796E+14	6,36362E+14	6,15008E+14
TC0100000774.mm.2	Itm2c	integral membrane protein 2C	1,25975E+14	1,02785E+14	2,75062E+14	0.0309949704380036	0.163760074338319	-3,39224E+14	1,12111E+14	1,10125E+14	1,04216E+14	9,3216E+14	1,01628E+14	9,54126E+14
TC0700000504.mm.2	Ifnl3	interferon lambda 3	-0.868268950442768	7,05086E+13	-2,75051E+14	0.0309996210756868	0.163760074338319	-3,39238E+14	6,67659E+14	6,34527E+14	7,0967E+14	7,42817E+14	7,37179E+14	7,38661E+14
TC1500001701.mm.2	Mroh5	maestro heat-like repeat family member 5	-0.902557044513118	6,5855E+14	-2,75031E+14	0.0310080051390933	0.163760074338319	-3,39264E+14	6,08519E+14	6,3793E+14	6,52048E+14	6,25843E+14	6,9298E+14	7,33981E+14
TC0700001332.mm.2	Tenm4	teneurin transmembrane protein 4	-0.937426765302139	7,8995E+14	-2,74891E+14	0.0310678187643837	0.163949756414454	-3,39445E+14	7,38029E+14	7,1248E+14	7,97739E+14	8,53455E+14	8,06936E+14	8,31059E+14
TC1500000160.mm.2	Myo10	myosin X	0.950417846997476	7,0568E+14	2,74884E+14	0.0310709593397452	0.163949756414454	-3,39455E+14	7,86761E+13	7,30172E+14	6,78312E+14	7,11986E+14	6,73682E+14	6,53168E+14
TC0900000203.mm.2	Smarca4	SWI/SNF related, matrix associated, actin dependent regulator of chromatin, subfamily a, member 4	1,19406E+14	7,19788E+14	2,74876E+14	0.0310744464773625	0.163949756414454	-3,39465E+14	8,02353E+13	7,89779E+14	7,34094E+14	6,39178E+14	7,10145E+13	6,43176E+14
TC1200000866.mm.2	Tmem63c	transmembrane protein 63c	1,68344E+14	6,97627E+14	2,74741E+14	0.0311319752335712	0.164109204735071	-3,3964E+13	8,01386E+14	8,25378E+14	7,1119E+14	5,57733E+14	7,03229E+14	5,86846E+14
TC0400000284.mm.2	Rngtt	RNA guanylyltransferase and 5'-phosphatase	1,66695E+14	5,58701E+14	2,7468E+14	0.031158436108161	0.164109204735071	-3,3972E+14	7,01459E+14	6,54073E+14	5,73045E+14	4,01482E+14	5,46674E+14	4,75469E+14
TC1000002050.mm.2	Fig4	FIG4 phosphoinositide 5-phosphatase	1,39598E+14	7,16435E+14	2,7467E+14	0.031162442184353	0.164109204735071	-3,39732E+14	7,92343E+14	8,07132E+14	7,43231E+14	6,35622E+14	7,12114E+13	6,08164E+14
TC0100003721.mm.2	Disp1	dispatched RND transporter family member 1	-0.801696127728746	7,30231E+13	-2,74621E+14	0.0311836099017913	0.164109204735071	-3,39796E+14	6,76423E+14	6,60953E+14	7,7158E+14	7,74713E+14	7,44018E+14	7,53697E+14
TC0400002605.mm.2	Tbc1d2	TBC1 domain family, member 2	-1,14648E+14	6,7811E+14	-2,74607E+14	0.0311895439007775	0.164109204735071	-3,39814E+14	6,03539E+14	6,16522E+14	6,62936E+14	7,36303E+14	6,85154E+14	7,64203E+14
TC0700000279.mm.2	Nanos2	nanos C2HC-type zinc finger 2	-0.992850555714512	5,20587E+14	-2,74575E+14	0.0312035515094106	0.164109204735071	-3,39856E+14	4,29248E+14	4,72887E+14	5,29536E+14	5,91145E+14	5,39188E+14	5,61517E+14
TC1800000632.mm.2	Csnk1a1	casein kinase 1, alpha 1	1,62508E+14	8,97848E+14	2,74574E+13	0.0312038644448975	0.164109204735071	-3,39857E+12	9,8597E+14	9,91049E+14	9,28129E+14	8,29937E+14	9,0469E+12	7,47314E+14
TC1200001715.mm.2	Strn3	striatin, calmodulin binding protein 3	2,00525E+14	9,17056E+14	2,74546E+14	0.0312160661582681	0.164109204735071	-3,39894E+14	1,04162E+14	1,02307E+14	9,48095E+14	8,25922E+14	9,35165E+13	7,28468E+14
TC0300001576.mm.2	Zranb2	zinc finger, RAN-binding domain containing 2	1,20119E+14	6,95573E+14	2,74538E+14	0.031219182999767	0.164109204735071	-3,39903E+14	7,72592E+14	7,85541E+14	6,86529E+14	6,10881E+14	7,03983E+14	6,13911E+14
TC1100001502.mm.2	Hoxb1	homeobox B1	-0.892046003645441	6,2866E+14	-2,74524E+14	0.0312251632593789	0.164109204735071	-3,39921E+14	5,86183E+14	5,60527E+14	6,30293E+14	6,69839E+14	6,48957E+14	6,76161E+14
TC0400001677.mm.2	Id3	inhibitor of DNA binding 3	0.793676349224163	6,04925E+14	2,74524E+14	0.0312254589411674	0.164109204735071	-3,39922E+14	6,63793E+14	6,72279E+14	5,54353E+13	5,61789E+14	5,89779E+14	5,87557E+14
TC0X00000207.mm.2	Jade3	jade family PHD finger 3	0.998242460293	6,87438E+14	2,7452E+14	0.031226886471652	0.164109204735071	-3,39927E+14	7,43574E+14	7,32608E+14	7,2061E+14	6,51301E+14	6,57401E+14	6,19133E+12
TC0200000523.mm.2	Pkn3	protein kinase N3	-0.837295490750473	7,44708E+14	-2,74481E+14	0.0312436885708431	0.164143969332245	-3,39977E+14	6,87172E+14	7,06776E+14	7,42223E+14	7,70668E+14	7,74446E+14	7,86961E+14
TC1100000588.mm.2	Skp1a	S-phase kinase-associated protein 1A	1,82076E+14	9,23949E+14	2,74438E+14	0.0312622943175005	0.164168987295683	-3,40033E+14	1,07375E+14	1,0653E+14	8,88451E+12	7,41291E+14	9,65338E+14	8,09562E+14
TC0700002310.mm.2	Nlrp4d	NLR family, pyrin domain containing 4D	-1,09519E+13	5,11291E+14	-2,74423E+14	0.0312688277794607	0.164168987295683	-3,40053E+14	4,73016E+14	4,11746E+14	5,01461E+14	5,77723E+14	5,43288E+14	5,60512E+14
TC0700000526.mm.2	Yif1b	Yip1 interacting factor homolog B (S. cerevisiae)	1,10099E+14	7,83731E+14	2,74357E+14	0.0312974042291729	0.164209588195552	-3,40139E+13	8,58383E+14	8,43077E+14	8,09302E+14	7,10362E+14	7,36422E+14	7,44839E+14
TC0300001856.mm.2	Ccna2	cyclin A2	1,04774E+14	6,60508E+14	2,7435E+14	0.0313004301164791	0.164209588195552	-3,40148E+14	7,05833E+14	7,49041E+14	6,68518E+14	5,94329E+14	6,36529E+14	6,08798E+14
TC1400000111.mm.2	Olfr31	olfactory receptor 31	-1,41364E+14	4,70862E+14	-2,74334E+14	0.0313071342975694	0.164209588195552	-3,40169E+14	3,7423E+14	4,03839E+14	4,19067E+14	5,67237E+14	5,17145E+14	5,43652E+14
TC1400002151.mm.2	Zdhhc20	zinc finger, DHHC domain containing 20	1,20473E+14	7,9013E+14	2,74287E+14	0.0313274954463978	0.164262913806641	-3,4023E+14	8,90107E+14	8,49178E+13	8,01967E+14	7,01186E+13	7,73469E+13	7,2487E+14
TC0100003541.mm.2	Nit1	nitrilase 1	0.895085976535181	7,35798E+14	2,74199E+14	0.0313655522095159	0.164408959096288	-3,40344E+14	79873218429	7,8352E+14	6,99038E+14	7,30264E+14	6,79934E+14	7,23302E+14
TC1100002826.mm.2	Olfr328	olfactory receptor 328	-1,93196E+14	4,78249E+14	-2,74113E+14	0.0314026111943198	0.164477486126782	-3,40456E+14	3,42614E+14	3,67141E+14	4,35787E+14	6,27805E+14	4,85228E+14	6,10918E+14
TC0100000216.mm.2	Dst	dystonin	2,62324E+14	6,1423E+14	2,74102E+14	0.0314073314553312	0.164477486126782	-3,4047E+14	7,77155E+14	7,79147E+14	6,25473E+14	4,7195E+14	6,60401E+13	3,71252E+14
TC0700004085.mm.2	Dennd5a	DENN/MADD domain containing 5A	1,73064E+14	8,68879E+14	2,74082E+14	0.0314162864527481	0.164477486126782	-3,40497E+14	9,91323E+14	9,9314E+14	8,84851E+14	7,05624E+14	8,65362E+14	7,72972E+12
TC1200002025.mm.2	Med6	mediator complex subunit 6	2,578E+14	6,58603E+14	2,74074E+14	0.0314194564822338	0.164477486126782	-3,40506E+14	7,78549E+14	7,89612E+13	7,40678E+14	5,90216E+14	6,68067E+14	3,84493E+14
TC1500000659.mm.2	Nol12	nucleolar protein 12	0.943912238690364	6,86435E+14	2,74028E+13	0.0314393000009185	0.164507826493406	-3,40566E+14	7,34052E+14	7,19924E+13	6,66394E+14	7,33045E+14	6,32893E+14	6,323E+14
TC0600001839.mm.2	Asns	asparagine synthetase	1,51285E+13	8,98179E+14	2,73988E+14	0.0314568679746307	0.164507826493406	-3,40618E+14	1,00526E+13	1,00162E+14	9,22952E+14	7,54917E+14	8,61449E+14	8,42869E+14
TC0400001707.mm.2	1700013G24Rik	RIKEN cDNA 1700013G24 gene	-1,4733E+14	5,42971E+14	-2,73967E+14	0.0314658591649211	0.164507826493406	-3,40645E+14	4,54468E+14	4,59108E+13	4,8972E+14	6,46298E+14	5,81545E+13	6,26691E+14
TC0600001874.mm.2	Tmem168	transmembrane protein 168	-1,35727E+14	7,24207E+14	-2,73942E+14	0.0314767949301075	0.164507826493406	-3,40678E+14	6,3854E+14	6,57219E+14	6,7354E+14	8,08728E+14	7,59475E+14	8,07738E+14
TC1800000261.mm.2	Bin1	bridging integrator 1	1,05523E+14	8,79345E+14	2,73932E+14	0.031481286130084	0.164507826493406	-3,40691E+14	9,53749E+14	9,43949E+14	8,81452E+14	8,10267E+14	8,72066E+14	8,14586E+14
TC0300002702.mm.2	Prok1	prokineticin 1	-0.857854569092368	6,10097E+14	-2,73904E+14	0.0314931829392598	0.164507826493406	-3,40727E+14	5,54827E+14	5,76141E+14	5,93929E+14	6,33145E+14	6,50064E+14	6,52475E+13
TC1800000396.mm.2	Rnf14	ring finger protein 14	1,87799E+14	8,73682E+14	2,73895E+14	0.0314971179330659	0.164507826493406	-3,40739E+14	9,91095E+13	9,98276E+13	9,13404E+14	7,25547E+13	8,67106E+14	7,46666E+14
TC1500000784.mm.2	Samm50	SAMM50 sorting and assembly machinery component	1,11396E+13	7,17871E+14	2,73848E+14	0.0315175830761818	0.164507826493406	-3,408E+14	7,91742E+14	7,91657E+14	7,32382E+14	6,30835E+14	6,89684E+14	6,70924E+13
TC0X00003278.mm.2	Cdkl5	cyclin-dependent kinase-like 5	1,45906E+14	7,10374E+12	2,73828E+14	0.0315263387685095	0.164507826493406	-3,40826E+14	8,02063E+14	8,00906E+14	7,44012E+14	6,04106E+14	6,95336E+14	6,15822E+14
TC1000002031.mm.2	Mfsd4b2-ps	major facilitator superfamily domain containing 4B2, pseudogene	-0.793078654408583	4,77047E+14	-2,73825E+14	0.0315273486136434	0.164507826493406	-3,40829E+14	4,46489E+14	4,47396E+13	4,56894E+13	4,59002E+14	5,19816E+14	5,32685E+14
TC0X00002433.mm.2	Bcap31	B cell receptor associated protein 31	1,37843E+14	7,90904E+14	2,73719E+14	0.0315738492886251	0.164681562921785	-3,40968E+14	8,99556E+14	8,78971E+14	7,83389E+14	6,80667E+14	8,03917E+14	6,98924E+14
TC0900000055.mm.2	Tmem123	transmembrane protein 123	-0.877931471795656	7,59266E+13	-2,73697E+14	0.0315832976259191	0.164681562921785	-3,40996E+14	6,76345E+14	7,02639E+14	7,89143E+14	8,32899E+14	7,78819E+14	7,75752E+14
TC0200004458.mm.2	Vps39	VPS39 HOPS complex subunit	1,01891E+14	7,70722E+14	2,73678E+14	0.0315913058394611	0.164681562921785	-3,4102E+13	8,41689E+14	8,41973E+14	7,72679E+14	6,88112E+14	7,36146E+14	7,43733E+13
TC1000002907.mm.2	Cnot2	CCR4-NOT transcription complex, subunit 2	1,48669E+14	8,72983E+14	2,73649E+14	0.0316040750239294	0.164688227118026	-3,41058E+14	9,74589E+14	9,78568E+14	8,97024E+12	7,31901E+14	8,31852E+14	8,23966E+14
TC0500003550.mm.2	Spdye4a	speedy/RINGO cell cycle regulator family, member E4A	-1,05959E+14	6,05625E+14	-2,73629E+14	0.0316130259092693	0.164688227118026	-3,41085E+14	5,2588E+14	5,36097E+14	6,30345E+14	6,67534E+14	5,94578E+14	6,79317E+14
TC0900002239.mm.2	Nnmt	nicotinamide N-methyltransferase	-1,38418E+13	6,62802E+14	-2,73528E+14	0.0316568564544056	0.164863260520309	-3,41216E+14	5,57366E+14	5,67307E+14	6,708E+14	7,79832E+14	6,49933E+13	7,51576E+12
TC0100000910.mm.2	Olfr1411	olfactory receptor 1411	-1,36784E+13	5,02791E+14	-2,73481E+14	0.0316773630470031	0.164916753077984	-3,41277E+14	4,2438E+14	3,93031E+14	4,93345E+14	6,1501E+14	5,07506E+14	5,83472E+14
TC1000001089.mm.2	Uhrf1bp1l	UHRF1 (ICBP90) binding protein 1-like	2,2377E+14	7,58384E+14	2,7344E+14	0.0316951869319432	0.164956249042119	-3,4133E+13	8,8928E+14	9,15038E+14	7,70732E+14	6,18473E+14	7,92042E+14	5,64735E+14
TC0200001779.mm.2	Rtf1	RTF1, Paf1/RNA polymerase II complex component	1,30049E+14	7,22543E+14	2,73396E+14	0.0317147087624174	0.164960302891259	-3,41388E+14	8,3147E+13	7,79252E+14	7,30926E+13	6,42987E+13	7,20099E+14	6,30523E+14
TC0X00001286.mm.2	Armcx1	armadillo repeat containing, X-linked 1	1,04595E+14	6,67974E+14	2,73392E+14	0.0317164412807746	0.164960302891259	-3,41393E+14	7,68254E+14	7,42701E+14	6,37302E+14	5,5782E+14	6,68466E+14	6,333E+14

TC0400001856.mm.2	Dhrs3	dehydrogenase/reductase (SDR family) member 3	1,03061E+14	6,81367E+14	2,73334E+13	0.0317414677011531	0.165008902766508	-3,41467E+14	7,67918E+14	7,34596E+14	6,82711E+14	6,06587E+13	6,61996E+13	6,34396E+14
TC1100001538.mm.2	Arhgap23	Rho GTPase activating protein 23	0.824826046227351	7,00183E+14	2,73324E+14	0.0317462669010224	0.165008902766508	-3,41481E+14	7,47503E+14	7,63562E+14	6,79924E+14	6,64008E+14	6,60971E+14	6,85129E+14
TC0700000666.mm.2	E130304I02Rik	RIKEN cDNA E130304I02 gene	-1,00212E+14	8,50102E+14	-2,73219E+14	0.0317919481633354	0.165126172873073	-3,41617E+14	7,87861E+14	7,8932E+14	8,35201E+14	9,10625E+14	8,73043E+14	9,04561E+14
TC0X00000531.mm.2	Vgll1	vestigial like family member 1	-1,64476E+14	4,92873E+14	-2,73217E+14	0.0317930328260529	0.165126172873073	-3,4162E+14	3,87017E+14	3,89215E+14	4,7367E+14	6,0215E+14	4,82807E+14	6,22377E+14
TC1400000174.mm.2	Zswim8	zinc finger SWIM-type containing 8	1,2687E+14	6,4228E+14	2,73162E+14	0.0318172201317681	0.165126172873073	-3,41692E+14	7,40717E+14	7,39488E+14	6,09592E+14	5,37417E+14	6,67811E+14	5,58654E+14
TC0700003049.mm.2	Spty2d1	SPT2, Suppressor of Ty, domain containing 1 (S. cerevisiae)	1,25312E+14	5,79005E+14	2,73161E+13	0.031817506717331	0.165126172873073	-3,41693E+14	6,59275E+14	6,28295E+14	6,01207E+14	5,48308E+14	5,74799E+13	4,62147E+14
TC0X00000689.mm.2	Dusp9	dual specificity phosphatase 9	-0.856926994046508	5,89678E+14	-2,73155E+14	0.0318200686880712	0.165126172873073	-3,417E+14	5,12651E+14	5,52537E+14	6,085E+14	6,27808E+14	6,22651E+14	6,13922E+13
TC1900001493.mm.2	Tctn3	tectonic family member 3	0.828949140270804	6,67862E+13	2,73063E+14	0.0318605443655687	0.165234110246106	-3,4182E+14	7,41904E+14	7,2951E+14	6,27236E+14	6,02899E+14	6,63868E+14	6,41756E+14
TC0200005463.mm.2	Npdc1	neural proliferation, differentiation and control 1	1,37691E+14	6,9872E+14	2,73061E+14	0.0318613778026842	0.165234110246106	-3,41823E+14	7,89496E+14	7,59089E+14	7,27773E+14	6,42761E+14	6,9562E+14	5,77583E+14
TC0500003709.mm.2	Babam2	BRISC and BRCA1 A complex member 2	1,21093E+14	8,21415E+14	2,73024E+14	0.0318777693567174	0.165252192119663	-3,41871E+14	9,21827E+14	9,13053E+14	7,92669E+14	7,08251E+14	8,39587E+14	7,53105E+13
TC1000002392.mm.2	Mmp11	matrix metallopeptidase 11	-1,31459E+14	5,98726E+14	-2,73006E+14	0.0318853761124578	0.165252192119663	-3,41894E+13	5,03944E+14	5,0608E+14	6,09639E+13	6,9975E+14	5,83843E+14	6,89099E+14
TC1400002034.mm.2	Sall2	spalt like transcription factor 2	1,26583E+14	5,24937E+14	2,72922E+14	0.0319226107837277	0.165260661986006	-3,42004E+14	6,14129E+14	5,92837E+14	5,29824E+14	4,59032E+14	5,30715E+14	4,23086E+14
TC0400003224.mm.2	Dmrtb1	DMRT-like family B with proline-rich C-terminal, 1	-0.835318198382861	6,76682E+14	-2,72921E+14	0.0319229026998896	0.165260661986006	-3,42004E+14	6,31343E+13	6,32135E+14	6,66762E+14	6,99309E+14	7,24891E+14	7,0565E+14
TC1300000396.mm.2	Prpf4b	pre-mRNA processing factor 4B	1,277E+14	6,78327E+14	2,72874E+14	0.0319435042356987	0.165260661986006	-3,42065E+12	7,599E+14	7,55102E+14	6,79848E+14	6,15511E+14	6,87652E+14	5,7195E+14
TC0100002977.mm.2	Dpp10	dipeptidylpeptidase 10	1,79175E+14	8,102E+14	2,72865E+14	0.0319475998930705	0.165260661986006	-3,42077E+13	9,35006E+14	9,28887E+13	8,2287E+14	6,68894E+14	8,25387E+14	6,80156E+14
TC0400004183.mm.2	Gm38423/Gm11239/ Gm11237	predicted gene, 38423/predicted gene 11239/predicted gene 11237	-1,23626E+14	4,20454E+13	-2,72821E+14	0.0319671051746796	0.165260661986006	-3,42135E+14	3,30387E+14	3,45818E+14	4,15912E+13	5,07151E+14	4,19218E+14	5,04239E+14
TC1100001108.mm.2	Tmigd1	transmembrane and immunoglobulin domain containing 1	-1,05066E+14	5,29235E+14	-2,72805E+13	0.0319740477876715	0.165260661986006	-3,42155E+14	4,52473E+14	4,79035E+14	5,30833E+14	5,71428E+14	5,31751E+14	6,09889E+14
TC1100000641.mm.2	Gm2a	GM2 ganglioside activator protein	0.939835282290415	8,40075E+14	2,72794E+13	0.0319791160402602	0.165260661986006	-3,4217E+14	8,92507E+14	9,02697E+14	8,4283E+14	7,9518E+14	8,2746E+14	7,79777E+14
TC0900000976.mm.2	Nedd4	neural precursor cell expressed, developmentally down-regulated 4	1,80538E+14	7,72616E+14	2,72762E+14	0.0319933122719726	0.165260661986006	-3,42212E+14	9,08739E+13	9,03288E+14	7,86679E+13	5,86038E+14	7,62356E+14	6,88595E+14
TC0100002739.mm.2	Ncl	nucleolin	1,63527E+13	8,43179E+14	2,72761E+14	0.0319936569011482	0.165260661986006	-3,42213E+14	9,46418E+14	9,51371E+14	8,68149E+14	7,22402E+14	8,45249E+14	7,25486E+14
TC0200000923.mm.2	Tank	TRAF family member-associated Nf-kappa B activator	1,98594E+13	6,96262E+14	2,7271E+14	0.0320159128096492	0.165260661986006	-3,42279E+14	8,26228E+14	8,2121E+14	7,48901E+14	5,30986E+14	6,75313E+14	5,74936E+14
TC1800000969.mm.2	Rock1	Rho-associated coiled-coil containing protein kinase 1	2,22535E+14	6,4769E+14	2,72685E+14	0.0320271470062725	0.165260661986006	-3,42312E+14	7,91164E+14	8,16435E+14	6,75067E+14	4,40948E+14	6,51244E+14	5,11284E+14
TC0300002207.mm.2	Gria2	glutamate receptor, ionotropic, AMPA2 (alpha 2)	1,99714E+14	8,18515E+14	2,72685E+14	0.0320271741881384	0.165260661986006	-3,42312E+14	9,62455E+14	9,6201E+14	8,34016E+14	6,27571E+14	8,25271E+14	6,99766E+14
TC0200003205.mm.2	Surf4	surfeit gene 4	0.866515023708622	7,33732E+14	2,72674E+14	0.0320318491166722	0.165260661986006	-3,42326E+14	8,04078E+14	7,74679E+14	7,28485E+14	6,89694E+14	7,02132E+14	7,03322E+13
TC0600002976.mm.2	Olfr215	olfactory receptor 215	-1,57199E+13	4,8527E+14	-2,72658E+14	0.032039209701041	0.165260661986006	-3,42347E+14	3,56701E+14	3,97363E+13	4,73327E+13	6,15765E+14	4,83755E+14	5,84706E+14
TC0400001010.mm.2	Pgm2	phosphoglucomutase 2	1,49625E+14	7,0763E+14	2,72654E+14	0.0320408557093205	0.165260661986006	-3,42352E+14	7,94664E+14	7,90016E+14	7,715E+14	6,04167E+14	6,3792E+14	6,4751E+14
TC1000001198.mm.2	Atp2b1	ATPase, Ca++ transporting, plasma membrane 1	2,29227E+14	7,77094E+14	2,72604E+14	0.0320627163550668	0.165311138703996	-3,42416E+14	9,04208E+14	9,47565E+14	8,0516E+14	6,12314E+14	7,98213E+14	5,95105E+14
TC0900001367.mm.2	Cacna2d2	calcium channel, voltage-dependent, alpha 2/delta subunit 2	1,39358E+14	7,75172E+14	2,72551E+14	0.0320862788708396	0.165311138703996	-3,42486E+14	8,46643E+14	8,34847E+14	8,26047E+14	7,40723E+14	6,38962E+14	7,63811E+13
TC0200003940.mm.2	Olfr1066	olfactory receptor 1066	-1,5524E+14	4,26558E+14	-2,72541E+14	0.0320908295675326	0.165311138703996	-3,42499E+14	3,23429E+14	3,35014E+13	3,8151E+14	5,50472E+14	4,59822E+13	5,09101E+14
TC0400002236.mm.2	Otud6b	OTU domain containing 6B	1,12214E+14	7,98021E+14	2,72539E+14	0.0320916801257431	0.165311138703996	-3,42501E+14	8,68894E+14	8,77847E+14	8,05527E+14	7,13546E+14	7,8687E+14	7,35444E+14
TC1500000395.mm.2	Col14a1	collagen, type XIV, alpha 1	-1,14474E+14	4,64268E+14	-2,72469E+14	0.0321224805920275	0.16533317275334	-3,42592E+14	4,13086E+14	3,88403E+14	4,23731E+14	5,29952E+14	5,22144E+14	5,08292E+14
TC0700001640.mm.2	Usp47	ubiquitin specific peptidase 47	0.889477960794359	8,08128E+14	2,72464E+14	0.0321247043696469	0.16533317275334	-3,42598E+14	8,48123E+14	8,75161E+14	8,11805E+14	7,68291E+13	7,83469E+14	7,61919E+14
TC0600001279.mm.2	Pparg	peroxisome proliferator activated receptor gamma	-0.992135870546413	5,4528E+14	-2,7246E+14	0.0321267401409239	0.16533317275334	-3,42604E+14	5,17486E+14	5,23965E+14	5,03574E+14	4,86779E+14	6,59029E+13	5,8085E+12
TC0300000897.mm.2	Cers2	ceramide synthase 2	1,25278E+14	6,81448E+13	2,72378E+14	0.0321630422761293	0.165440921842343	-3,42711E+14	7,64299E+14	7,48154E+13	7,03184E+14	6,1115E+14	5,86624E+14	6,75274E+14
TC1500001819.mm.2	Myh9	myosin, heavy polypeptide 9, non-muscle	0.990220439573399	6,99995E+14	2,72366E+14	0.0321683675842094	0.165440921842343	-3,42726E+14	7,46964E+14	7,9723E+14	6,8585E+14	6,23777E+14	6,73821E+14	6,72328E+14
TC0500003212.mm.2	Atp2a2	ATPase, Ca++ transporting, cardiac muscle, slow twitch 2	1,78654E+14	9,68081E+14	2,72342E+14	0.0321790437453706	0.165440921842343	-3,42758E+14	1,0953E+14	1,10041E+14	9,91737E+14	7,82639E+14	9,34853E+14	9,03549E+14
TC0X00001008.mm.2	1700011M02Rik	RIKEN cDNA 1700011M02 gene	-1,23177E+14	6,11613E+14	-2,72259E+14	0.0322157217708383	0.165440921842343	-3,42865E+14	5,38891E+14	5,21808E+14	6,17326E+14	6,84601E+14	6,01349E+14	7,05703E+14
TC0700002460.mm.2	Psg19	pregnancy specific glycoprotein 19	-1,08049E+14	5,45024E+14	-2,72236E+14	0.0322260794993756	0.165440921842343	-3,42895E+14	5,00295E+14	4,6476E+14	5,23538E+14	6,00398E+14	5,65718E+14	6,15434E+14
TC1000001687.mm.2	Mtrf1l	mitochondrial translational release factor 1-like	1,33144E+14	5,82166E+14	2,72231E+14	0.0322282155139536	0.165440921842343	-3,42901E+14	6,68797E+14	6,4902E+14	5,38985E+14	5,84667E+14	5,92227E+14	4,59303E+14
TC0900002599.mm.2	Mindy2	MINDY lysine 48 deubiquitinase 2	1,12086E+14	6,60048E+14	2,72216E+14	0.0322349704994216	0.165440921842343	-3,42921E+14	7,33326E+14	7,11169E+14	6,78909E+14	6,16559E+14	6,5142E+14	5,68904E+14
TC1900001454.mm.2	Ide	insulin degrading enzyme	1,29351E+14	6,83278E+13	2,72203E+13	0.032240875672382	0.165440921842343	-3,42939E+14	7,8222E+14	7,90967E+14	6,61916E+13	5,5008E+14	6,95457E+14	6,19028E+14
TC0700004068.mm.2	Ric3	RIC3 acetylcholine receptor chaperone	1,59783E+14	6,6248E+14	2,72152E+14	0.0322633128007858	0.165440921842343	-3,43004E+14	7,73562E+13	7,30132E+14	6,66862E+14	6,20192E+14	6,78161E+14	5,05968E+14
TC1500002050.mm.2	Lmf2	lipase maturation factor 2	1,09277E+14	6,80502E+13	2,72142E+13	0.0322679067338048	0.165440921842343	-3,43017E+14	7,30655E+14	7,85272E+14	6,76233E+14	5,93476E+14	6,40207E+14	6,57166E+14
TC0300001805.mm.2	Pex5l	peroxisomal biogenesis factor 5-like	1,18665E+14	7,40978E+14	2,72118E+14	0.0322785367083355	0.165440921842343	-3,43049E+14	8,04122E+14	8,3099E+14	6,90789E+14	7,22184E+14	7,53964E+14	6,43818E+14
TC0400003707.mm.2	Sfn	stratifin	-1,12445E+14	5,89329E+14	-2,72106E+14	0.0322841684988939	0.165440921842343	-3,43065E+14	5,14973E+14	5,26963E+14	5,69018E+14	6,58193E+14	6,04912E+14	6,61914E+14
TC0900001455.mm.2	Cspg5	chondroitin sulfate proteoglycan 5	0.982194771607473	9,65166E+14	2,72095E+14	0.0322889139554546	0.165440921842343	-3,43079E+13	1,03303E+14	1,02349E+14	9,7092E+14	9,03478E+14	9,49416E+14	9,10665E+13
TC0400001259.mm.2	Prdx1	peroxiredoxin 1	1,53979E+14	9,50375E+14	2,72074E+14	0.0322984690823221	0.165440921842343	-3,43107E+14	1,05886E+14	1,06913E+14	9,48185E+14	8,06047E+14	9,6068E+14	8,59351E+14
TC0X00003358.mm.2	Tmsb4x	thymosin, beta 4, X chromosome	1,05036E+14	1,15103E+13	2,72066E+14	0.0323016905676169	0.165440921842343	-3,43116E+14	1,21785E+14	1,23115E+14	1,13874E+14	1,07951E+13	1,15434E+14	1,08459E+14
TC0500002219.mm.2	Letm1	leucine zipper-EF-hand containing transmembrane protein 1	1,04103E+14	6,62416E+14	2,71957E+14	0.0323506436080237	0.165620714744396	-3,43259E+14	7,37103E+14	7,20843E+14	6,57988E+14	6,08821E+14	5,87634E+14	6,62106E+14
TC0700000849.mm.2	Tulp2	tubby-like protein 2	-0.913023459911819	5,59344E+14	-2,71893E+14	0.0323790776803157	0.165620714744396	-3,43342E+14	4,77233E+14	4,80628E+14	5,99022E+14	6,58715E+14	5,7904E+13	5,61425E+14
TC0600002625.mm.2	Nat8f4	N-acetyltransferase 8 (GCN5-related) family member 4	0.85091054431952	5,95859E+13	2,71892E+14	0.0323796729273001	0.165620714744396	-3,43343E+14	6,30891E+14	6,14929E+14	6,06855E+13	6,46838E+14	5,43422E+14	5,32217E+14
TC1400001621.mm.2	Slmap	sarcolemma associated protein	1,51691E+14	6,09534E+14	2,71877E+14	0.032386023227697	0.165620714744396	-3,43362E+14	6,99988E+14	6,92158E+14	6,21323E+12	5,54973E+14	6,23368E+14	4,65395E+14
TC0200000019.mm.2	Frmd4a	FERM domain containing 4A	0.962569817619674	5,42609E+14	2,71853E+14	0.0323968845495492	0.165620714744396	-3,43393E+14	5,9807E+14	5,80479E+14	5,69792E+14	5,21276E+14	5,19678E+13	4,66356E+14
TC0600000553.mm.2	Sspo	SCO-spondin	-1,24992E+14	6,30773E+14	-2,71838E+14	0.032403793987009	0.165620714744396	-3,43413E+14	5,43733E+14	5,88399E+14	5,76588E+14	6,938E+14	6,76568E+14	7,05547E+14
TC1100001054.mm.2	Smg6	Smg-6 homolog, nonsense mediated mRNA decay factor (C. elegans)	0.834395257443998	6,68222E+14	2,71827E+14	0.0324087453353864	0.165620714744396	-3,43428E+14	7,08626E+14	7,18842E+14	6,69199E+14	6,52076E+14	6,13689E+14	6,469E+14
TC0100000055.mm.2	Prex2	phosphatidylinositol-3,4,5-trisphosphate-dependent Rac exchange factor 2	1,78603E+14	6,1383E+14	2,71724E+13	0.0324548451809383	0.165803716043265	-3,43562E+14	7,32299E+14	7,12518E+14	6,80351E+14	4,702E+14	5,53725E+14	5,33886E+14
TC1000000039.mm.2	Oprm1	opioid receptor, mu 1	1,52897E+14	6,98304E+14	2,71677E+14	0.0324757865762679	0.165814772742965	-3,43623E+14	8,48757E+14	7,38103E+14	6,99844E+14	6,22057E+14	6,949E+14	5,86165E+14
TC1400001656.mm.2	Cacna1d	calcium channel, voltage-dependent, L type, alpha 1D subunit	1,13843E+14	5,62967E+14	2,71673E+13	0.0324775909375534	0.165814772742965	-3,43628E+14	6,09135E+14	6,41414E+14	5,0651E+14	5,97878E+14	4,86052E+14	5,3681E+14
TC1900000170.mm.2	Eef1g	eukaryotic translation elongation factor 1 gamma	0.981477650825113	9,8139E+14	2,71514E+13	0.0325489255541435	0.16605757996582	-3,43835E+13	1,05641E+14	1,04879E+14	9,76154E+14	8,98093E+14	9,57469E+14	9,5143E+14
TC0200002879.mm.2	Cdc123	cell division cycle 123	1,14035E+14	8,97828E+14	2,71492E+14	0.0325587156882541	0.16605757996582	-3,43863E+14	9,71464E+14	9,57561E+14	9,06892E+14	8,50097E+13	8,96991E+14	8,03965E+13

TC0700003823.mm.2	Plekhb1	pleckstrin homology domain containing, family B (evectins) member 1	1,24363E+14	9,38574E+14	2,71482E+14	0.0325632060390001	0.16605757996582	-3,43876E+14	1,0246E+14	1,02023E+14	9,2976E+14	8,60747E+14	9,5188E+14	8,44227E+14
TC1100003318.mm.2	Wsb1	WD repeat and SOCS box-containing 1	1,52486E+14	7,01953E+14	2,71475E+14	0.032566372040712	0.16605757996582	-3,43885E+14	8,02133E+13	8,00468E+14	7,34814E+14	5,76676E+14	6,83991E+14	6,13637E+14
TC1800000124.mm.2	Taf4b	TATA-box binding protein associated factor 4b	0.812265423657885	5,76823E+14	2,71406E+14	0.0325977696796138	0.166139582276587	-3,43976E+14	6,06941E+14	6,14223E+12	6,00401E+14	5,80662E+14	5,40071E+14	5,1864E+14
TC0200004926.mm.2	Plagl2	pleiomorphic adenoma gene-like 2	-0.970330068050461	7,39598E+14	-2,71387E+14	0.0326060212359013	0.166139582276587	-3,44E+14	6,75514E+14	6,76008E+14	8,08339E+14	7,32137E+14	7,70742E+14	7,74846E+14
TC0900003333.mm.2	Nphp3	nephronophthisis 3 (adolescent)	-1,1193E+14	5,99617E+14	-2,71371E+13	0.0326133866282408	0.166139582276587	-3,44021E+14	4,94077E+14	5,64616E+12	5,88839E+13	6,67619E+14	6,45757E+14	6,36794E+14
TC0900002489.mm.2	Zwilch	zwilch kinetochore protein	-1,16659E+14	6,55938E+14	-2,71339E+14	0.0326275199378577	0.166159048280247	-3,44062E+14	5,89869E+13	5,48818E+14	6,66422E+14	7,58515E+14	6,58664E+14	7,13341E+14
TC0400001874.mm.2	Gm13238	carnitine deficiency-associated gene expressed in ventricle 3 pseudogene	-0.889940274765561	8,21431E+14	-2,7131E+14	0.0326407541681089	0.166173924774325	-3,441E+14	7,62481E+14	7,47515E+14	8,37936E+14	8,92667E+14	8,43556E+14	8,44428E+14
TC0100003395.mm.2	Myocos	myocilin opposite strand	-1,05379E+14	5,35297E+14	-2,71209E+14	0.0326863736578713	0.1663179338329	-3,44232E+14	5,02746E+14	4,58773E+14	5,01394E+14	5,76591E+14	5,68739E+14	6,03537E+14
TC0300002241.mm.2	Mnd1	meiotic nuclear divisions 1	-0.873256632865376	5,3612E+14	-2,71182E+14	0.0326984016315393	0.1663179338329	-3,44266E+14	4,82677E+14	4,74155E+14	5,41557E+14	5,86847E+14	5,77937E+14	5,53546E+14
TC0700004049.mm.2	Olfr485	olfactory receptor 485	-1,46365E+14	5,18071E+14	-2,71179E+14	0.032700007098779	0.1663179338329	-3,44271E+14	4,61597E+14	3,95358E+14	5,00673E+14	6,01116E+14	5,19226E+14	6,30459E+14
TC1000003224.mm.2	Eif4ebp2	eukaryotic translation initiation factor 4E binding protein 2	-0.924036682892871	6,90536E+14	-2,71154E+14	0.032711403141249	0.166323395018916	-3,44304E+14	6,24191E+14	6,28969E+14	7,58057E+14	6,94034E+14	7,25991E+14	7,11977E+14
TC1600000162.mm.2	Bfar	bifunctional apoptosis regulator	-0.803093880484852	8,52659E+14	-2,71016E+14	0.032773568968813	0.166586913815294	-3,44483E+14	8,06259E+14	7,87402E+14	8,79834E+14	8,88177E+14	8,69227E+14	8,85052E+13
TC0200002273.mm.2	Scrt2	scratch family zinc finger 2	-0.816517498463363	7,70145E+14	-2,70971E+14	0.0327939048376538	0.166623940041595	-3,44541E+14	7,21574E+14	7,11846E+14	7,82073E+14	8,08653E+14	8,0641E+14	7,90314E+14
TC1100003659.mm.2	Plxdc1	plexin domain containing 1	1,4545E+14	5,59437E+14	2,70954E+14	0.0328015352703989	0.166623940041595	-3,44563E+14	6,40228E+14	6,28723E+14	5,07955E+13	6,01665E+14	5,55225E+14	4,22826E+14
TC1600000219.mm.2	Lztr1	leucine-zipper-like transcriptional regulator, 1	1,15E+14	7,76772E+14	2,70898E+12	0.0328272917686898	0.166639913601422	-3,44637E+13	8,4716E+14	8,29821E+14	7,8755E+14	7,4912E+14	7,7541E+14	6,71572E+14
TC1000000579.mm.2	Neurog3	neurogenin 3	-0.960769469594696	6,20433E+13	-2,70832E+14	0.032856960771908	0.166639913601422	-3,44722E+14	5,3545E+14	5,65176E+12	6,3063E+14	6,98563E+14	6,42236E+14	6,50544E+14
TC1100004242.mm.2	Sectm1a	secreted and transmembrane 1A	-1,37987E+14	4,99724E+14	-2,70819E+14	0.0328630137464049	0.166639913601422	-3,4474E+14	3,94958E+14	4,13795E+14	4,80632E+14	6,24235E+14	5,2118E+14	5,63546E+14
TC0200003134.mm.2	Stpg3	sperm tail PG rich repeat containing 3	-1,03345E+14	4,98025E+14	-2,70809E+14	0.032867393486292	0.166639913601422	-3,44752E+14	4,2359E+14	4,53665E+14	4,75268E+14	5,51683E+14	5,23477E+14	5,60468E+14
TC0600002427.mm.2	Tacstd2	tumor-associated calcium signal transducer 2	-0.904795390796694	5,73264E+14	-2,70791E+14	0.0328755048476708	0.166639913601422	-3,44775E+14	5,10529E+13	4,94242E+14	6,00949E+14	6,48132E+14	5,75192E+14	6,10539E+14
TC1100002702.mm.2	Jade2	jade family PHD finger 2	0.913925284107811	7,34397E+14	2,70772E+14	0.0328843674850999	0.166639913601422	-3,44801E+14	7,82187E+14	7,72257E+14	7,5344E+14	7,26837E+14	7,14237E+14	6,57422E+14
TC0700003981.mm.2	Apbb1	amyloid beta (A4) precursor protein-binding, family B, member 1	1,33079E+14	8,32466E+14	2,70766E+14	0.0328871528894069	0.166639913601422	-3,44809E+14	9,41473E+14	9,3759E+14	8,22824E+14	6,80006E+14	8,09558E+14	8,03345E+14
TC0X00001549.mm.2	Gpr143	G protein-coupled receptor 143	-1,07342E+14	5,48658E+14	-2,70745E+14	0.0328967926252623	0.166639913601422	-3,44836E+14	4,97044E+13	4,90112E+14	5,32909E+14	5,70043E+14	5,55721E+14	6,46118E+14
TC0400000349.mm.2	Dnaja1/Mir207	DnaJ heat shock protein family (Hsp40) member A1/microRNA 207	1,15993E+14	9,91565E+14	2,70742E+14	0.0328977574111663	0.166639913601422	-3,44839E+14	1,06468E+14	1,07309E+14	9,98618E+13	9,07229E+14	9,86675E+14	9,19102E+14
TC0X00003260.mm.2	Cnksr2	connector enhancer of kinase suppressor of Ras 2	1,88603E+14	9,51261E+13	2,70698E+14	0.0329179194434195	0.166677088437145	-3,44897E+14	1,07609E+14	1,09286E+14	9,74426E+14	7,72451E+14	9,47686E+14	8,44056E+14
TC1100003166.mm.2	Olfr75-ps1	olfactory receptor 75, pseudogene 1	-0.893266320530325	6,10055E+14	-2,70675E+14	0.03292825594911	0.166677088437145	-3,44926E+14	5,63706E+12	5,74007E+14	6,23309E+14	5,8294E+14	6,37851E+14	6,78515E+14
TC0800003060.mm.2	Car5a	carbonic anhydrase 5a, mitochondrial	-0.973472084042596	5,69816E+14	-2,70658E+14	0.0329361291866115	0.166677088437145	-3,44949E+14	5,20502E+14	5,24291E+14	5,39988E+14	5,94629E+14	5,92268E+14	6,4722E+14
TC0700002113.mm.2	Fgf4	fibroblast growth factor 4	-1,05857E+11	6,35125E+14	-2,70621E+14	0.03295317723125	0.166711003054044	-3,44998E+14	5,54507E+14	5,6709E+14	6,50014E+14	7,05831E+14	6,28618E+14	7,04693E+14
TC1000002511.mm.2	Ap3d1	adaptor-related protein complex 3, delta 1 subunit	0.928374263699926	9,10697E+14	2,70563E+14	0.0329792203603695	0.166756135248235	-3,45072E+14	9,69958E+13	9,56832E+14	9,08236E+14	8,88043E+14	9,0196E+14	8,39155E+14
TC1200000990.mm.2	Psmc1	protease (prosome, macropain) 26S subunit, ATPase 1	0.930795531116289	8,4036E+14	2,70556E+14	0.0329827966881477	0.166756135248235	-3,45082E+14	9,19278E+14	9,04042E+14	8,17294E+14	7,64388E+14	8,38413E+14	7,98748E+14
TC0X00001900.mm.2	Fundc1	FUN14 domain containing 1	1,24569E+14	8,77872E+14	2,70523E+14	0.0329977863964714	0.166763852912568	-3,45125E+14	9,71373E+14	9,47626E+14	8,84027E+13	7,94345E+14	8,79781E+14	7,9008E+14
TC0400003473.mm.2	Cap1	CAP, adenylate cyclase-associated protein 1 (yeast)	1,41429E+14	9,48819E+12	2,70503E+14	0.0330070051504555	0.166763852912568	-3,45151E+14	1,04239E+14	1,06565E+14	9,54188E+13	8,05505E+13	9,34342E+14	8,9084E+14
TC0700000834.mm.2	Pth2	parathyroid hormone 2	-1,27772E+14	7,71527E+14	-2,70484E+14	0.033015372108924	0.166763852912568	-3,45175E+13	6,69566E+14	6,94395E+14	7,66161E+14	8,79532E+14	7,75796E+14	8,43708E+14
TC0300000567.mm.2	Il12a	interleukin 12a	-0.799219773647319	5,19232E+14	-2,70443E+14	0.0330341482387906	0.166806402560837	-3,45229E+14	4,64429E+14	4,55988E+14	5,59138E+14	5,55577E+14	5,28615E+14	5,51647E+14
TC0700004189.mm.2	Eri2	exoribonuclease 2	1,30049E+14	5,92853E+14	2,70386E+14	0.0330601550992519	0.166808596418035	-3,45303E+14	6,63616E+12	7,05871E+14	5,89284E+14	4,88959E+13	5,9737E+14	5,1202E+14
TC0100002816.mm.2	Cops9	COP9 signalosome subunit 9	1,43657E+13	9,47876E+13	2,70379E+13	0.0330633879266049	0.166808596418035	-3,45312E+14	1,05675E+14	1,041E+14	9,50848E+14	8,28214E+14	9,56216E+14	8,54224E+14
TC0X00000552.mm.2	Gm715	predicted gene 715	-0.995356270210758	6,74796E+14	-2,70325E+13	0.0330881254958916	0.166808596418035	-3,45383E+13	6,01757E+13	6,21233E+14	6,61748E+14	7,41978E+14	7,10185E+14	7,11877E+13
TC1500002032.mm.2	Ttll8	tubulin tyrosine ligase-like family, member 8	-1,11382E+14	5,38027E+14	-2,70324E+14	0.0330885375007538	0.166808596418035	-3,45384E+14	4,68057E+14	4,80945E+14	5,17742E+14	5,89653E+14	5,47544E+13	6,24222E+14
TC1800001625.mm.2	Rnf165	ring finger protein 165	-0.787172341429246	8,43815E+14	-2,70307E+14	0.0330961120334669	0.166808596418035	-3,45405E+13	7,97424E+14	7,89243E+13	8,71585E+14	8,60534E+14	8,67046E+14	8,77056E+14
TC0700000397.mm.2	Gm5893	predicted gene 5893	-0.942335029634537	4,59213E+14	-2,7029E+14	0.033103937494975	0.166808596418035	-3,45428E+14	3,73024E+14	4,26391E+14	4,62153E+14	5,05827E+14	4,85889E+14	5,01993E+14
TC0100001699.mm.2	Sccpdh	saccharopine dehydrogenase (putative)	2,08471E+14	8,13813E+14	2,70241E+13	0.0331264854095455	0.166808596418035	-3,45492E+14	9,34114E+14	9,4143E+14	8,61421E+14	6,87312E+14	8,23578E+14	6,35023E+14
TC1600002181.mm.2	Dgcr2	DiGeorge syndrome critical region gene 2	1,16123E+14	8,65502E+14	2,70237E+14	0.0331281583292204	0.166808596418035	-3,45497E+14	9,51285E+14	9,30998E+14	8,82717E+14	7,77973E+14	8,45919E+14	8,04117E+14
TC0900001908.mm.2	Anln	anillin, actin binding protein	1,33609E+14	5,81493E+14	2,7022E+14	0.0331359850943066	0.166808596418035	-3,45519E+14	6,84066E+14	6,83657E+14	5,78668E+14	4,42064E+14	5,26992E+14	5,73512E+13
TC0900000523.mm.2	Ift46	intraflagellar transport 46	0.921723325995739	7,00669E+14	2,70198E+14	0.0331461793046678	0.166808596418035	-3,45548E+14	7,65067E+14	7,45094E+14	6,88952E+14	6,79082E+14	6,93942E+14	6,31874E+14
TC1800000450.mm.2	Spink6	serine peptidase inhibitor, Kazal type 6	-1,18018E+14	4,73902E+14	-2,70136E+14	0.0331745856749218	0.166808596418035	-3,45629E+14	3,7911E+14	4,02563E+14	4,70105E+14	5,73929E+14	4,83356E+14	5,34352E+14
TC0500003704.mm.2	Vmn2r18	vomeronasal 2, receptor 18	-1,23235E+14	4,48169E+14	-2,70078E+14	0.0332012561532938	0.166808596418035	-3,45704E+14	4,07397E+13	4,03193E+14	3,53115E+13	4,68247E+14	5,01664E+12	5,55396E+13
TC0200003365.mm.2	Ggta1	glycoprotein galactosyltransferase alpha 1, 3	0.898226870474173	5,25163E+14	2,70068E+14	0.0332057502598227	0.166808596418035	-3,45717E+13	5,47099E+14	5,90789E+14	5,47749E+14	5,071E+13	4,7566E+14	4,82582E+13
TC0800002745.mm.2	Csnk2a2	casein kinase 2, alpha prime polypeptide	1,94858E+14	6,6275E+14	2,70011E+14	0.0332322599128739	0.166808596418035	-3,45792E+14	7,56317E+14	7,61386E+14	7,24715E+14	6,06096E+14	6,6679E+14	4,61197E+14
TC1100001147.mm.2	Tlcd1/Mir7653	TLC domain containing 1/microRNA 7653	1,60224E+14	7,4865E+14	2,69976E+14	0.0332483177628176	0.166808596418035	-3,45838E+14	8,60079E+14	8,40775E+14	7,71727E+14	6,3891E+14	7,54757E+12	6,25649E+14
TC0200001746.mm.2	Phgr1	proline/histidine/glycine-rich 1	-1,00871E+14	6,02163E+14	-2,69963E+14	0.033254269776466	0.166808596418035	-3,45855E+14	5,14545E+14	5,47567E+14	6,23956E+14	6,63057E+14	5,97579E+14	6,66273E+14
TC1900000415.mm.2	Vldlr	very low density lipoprotein receptor	0.834788527698817	7,39197E+14	2,69958E+14	0.0332564437078737	0.166808596418035	-3,45861E+14	7,97504E+14	8,04451E+14	7,08666E+14	6,89564E+14	7,35298E+13	6,99699E+14
TC0900000839.mm.2	Pdcd7	programmed cell death 7	0.911898534198124	6,22775E+14	2,6992E+14	0.0332738713119215	0.166808596418035	-3,4591E+14	6,88E+14	6,6561E+14	6,35727E+14	5,76086E+14	5,86509E+14	5,84721E+14
TC1100000712.mm.2	Hist3h2ba	histone cluster 3, H2ba	-1,16001E+14	6,79442E+14	-2,69918E+14	0.0332748097574366	0.166808596418035	-3,45913E+14	6,04394E+14	5,735E+14	7,06427E+14	7,82437E+12	6,64934E+14	7,44962E+14
TC0700004183.mm.2	Umod	uromodulin	-1,20892E+14	4,95436E+14	-2,69911E+14	0.0332779034544555	0.166808596418035	-3,45922E+14	4,34927E+14	4,07425E+14	4,82427E+14	5,63701E+14	4,97848E+14	5,86287E+13
TC1100000428.mm.2	Ccnjl	cyclin J-like	-0.831289555033126	7,26573E+14	-2,69884E+14	0.0332903453013788	0.166808596418035	-3,45957E+14	6,42518E+14	6,45993E+14	8,24009E+14	7,9215E+14	7,15582E+14	7,39188E+14
TC0200003310.mm.2	6330409D20Rik	RIKEN cDNA 6330409D20 gene	-0.97547378751606	5,61226E+14	-2,69884E+14	0.0332905082782044	0.166808596418035	-3,45957E+14	5,1494E+14	4,94468E+14	5,58205E+14	5,95244E+14	5,6911E+14	6,35392E+14
TC0400003694.mm.2	Sytl1	synaptotagmin-like 1	-1,3781E+14	5,66651E+14	-2,69855E+14	0.0333039018392388	0.166808596418035	-3,45995E+14	4,79882E+14	4,65742E+14	5,55615E+14	6,77425E+14	5,66219E+14	6,55026E+14
TC0100003670.mm.2	Mixl1	Mix1 homeobox-like 1 (Xenopus laevis)	-1,44572E+14	5,76762E+14	-2,69846E+14	0.0333081233539804	0.166808596418035	-3,46007E+14	5,00186E+14	5,06462E+14	5,02572E+14	6,55561E+14	6,21183E+14	6,7461E+14
TC1700000578.mm.2	Olfr239	olfactory receptor 239	-1,1191E+14	5,57407E+14	-2,69836E+14	0.0333127058067015	0.166808596418035	-3,4602E+14	4,63379E+14	5,04775E+14	5,42685E+14	6,4163E+14	5,96327E+14	5,95648E+14
TC1200000089.mm.2	Smc6	structural maintenance of chromosomes 6	1,98288E+14	5,49129E+14	2,69836E+14	0.0333127276947246	0.166808596418035	-3,4602E+14	6,79327E+14	6,7556E+14	5,419E+14	4,39677E+14	5,89122E+14	3,6919E+14
TC0800000854.mm.2	Nr3c2	nuclear receptor subfamily 3, group C, member 2	1,34663E+14	6,59001E+14	2,69821E+14	0.0333194828012494	0.166808596418035	-3,46039E+14	7,84684E+14	7,28914E+14	6,59075E+13	5,37062E+14	6,51377E+14	5,92894E+14
TC0200005143.mm.2	Cdh22	cadherin 22	0.8044040367058	6,38492E+14	2,69761E+14	0.033347190596564	0.166808596418035	-3,46117E+14	6,86154E+14	6,88773E+14	6,18025E+14	6,23952E+14	5,93093E+14	6,20953E+14
TC1000002277.mm.2	Mypn	myopalladin	-1,28265E+14	5,13246E+13	-2,69752E+14	0.0333511709410687	0.166808596418035	-3,46129E+14	4,14125E+14	4,27174E+14	5,0362E+14	6,36724E+14	5,36938E+14	5,60892E+14

TC1700001611.mm.2	Prss30	protease, serine 30	-1,46144E+14	5,67922E+13	-2,69751E+14	0.0333515775751263	0.166808596418035	-3,4613E+14	4,79623E+14	4,49782E+14	5,66278E+14	6,90155E+14	5,58653E+13	6,6304E+14
TC0600002285.mm.2	Hoxa11	homeobox A11	-0.93008751800517	5,99953E+14	-2,6972E+14	0.0333658078857531	0.166808596418035	-3,4617E+14	5,6339E+14	5,23768E+14	6,02006E+14	6,37376E+14	6,15055E+14	6,58121E+14
TC0200000342.mm.2	Gpr158	G protein-coupled receptor 158	2,00724E+14	8,34899E+14	2,69701E+14	0.0333746826717871	0.166808596418035	-3,46195E+14	9,77867E+14	9,78581E+14	8,62693E+14	6,35254E+14	8,2465E+14	7,30349E+14
TC0400002782.mm.2	Zfp37	zinc finger protein 37	0.855053296498108	5,99987E+13	2,69698E+14	0.0333762126762084	0.166808596418035	-3,46199E+14	6,43403E+14	6,74475E+14	5,57999E+14	5,77178E+14	5,89057E+14	5,5781E+14
TC0600002614.mm.2	Fbxo41	F-box protein 41	0.844319011824405	6,02019E+14	2,69641E+14	0.0334026479509754	0.166879736825814	-3,46274E+14	6,74244E+14	6,40375E+14	5,70215E+13	5,81524E+14	5,59484E+13	5,86271E+14
TC0400000949.mm.2	Ift74	intraflagellar transport 74	2,41685E+14	6,07476E+14	2,69596E+14	0.0334232151515984	0.166879736825814	-3,46332E+14	7,68627E+13	7,67376E+14	6,32457E+14	4,23763E+14	6,3721E+14	4,15424E+14
TC0400003323.mm.2	Tex38	testis expressed 38	-0.976300547889338	5,40283E+14	-2,69593E+14	0.0334244753770197	0.166879736825814	-3,46336E+14	4,62226E+14	5,27252E+13	5,13066E+14	5,54414E+14	5,8355E+14	6,01188E+14
TC0300002324.mm.2	Syt11	synaptotagmin XI	1,53592E+14	9,82812E+14	2,69577E+14	0.0334318742924177	0.166879736825814	-3,46356E+14	1,07866E+14	1,07922E+14	1,01758E+14	8,70729E+14	9,74251E+14	8,76438E+14
TC1100003759.mm.2	Krt9	keratin 9	1,16536E+14	5,71401E+14	2,69527E+14	0.0334553842990195	0.166945372316538	-3,46423E+14	6,80986E+14	6,43448E+14	5,5918E+14	4,53427E+14	5,31248E+13	5,60114E+13
TC0700004261.mm.2	Gtf3c1	general transcription factor III C 1	0.798167450244391	7,66814E+14	2,69498E+14	0.033468474167506	0.166958985839326	-3,4646E+14	8,15517E+14	7,99778E+14	7,72975E+14	7,56951E+14	7,34136E+14	7,21526E+13
TC0700002057.mm.2	Ap2a2	adaptor-related protein complex 2, alpha 2 subunit	2,12825E+14	6,64363E+14	2,69314E+14	0.0335541482026303	0.167334568241715	-3,467E+14	8,21043E+14	8,32842E+14	6,82471E+14	4,21587E+14	6,33493E+13	5,94742E+14
TC0200002589.mm.2	Snai1	snail family zinc finger 1	-1,16108E+14	6,39518E+14	-2,69266E+14	0.0335762560522198	0.167393011686082	-3,46762E+14	5,41594E+14	6,0043E+14	6,50102E+14	6,7074E+14	6,38621E+14	7,35618E+14
TC1800000343.mm.2	Ik	IK cytokine	1,39636E+14	8,06544E+13	2,69226E+14	0.0335947422770909	0.167433369103237	-3,46814E+14	9,16691E+14	8,88404E+14	8,22651E+14	6,85696E+14	7,96165E+14	7,29659E+14
TC1000000265.mm.2	Echdc1	enoyl Coenzyme A hydratase domain containing 1	2,05525E+14	6,44122E+14	2,69075E+14	0.0336649026055394	0.16765562179907	-3,47011E+14	7,75675E+14	7,56501E+14	6,901E+14	5,21334E+14	6,54861E+11	4,66264E+13
TC1500001700.mm.2	Gpr20	G protein-coupled receptor 20	-0.882625721473443	6,56157E+14	-2,69069E+14	0.0336679975790321	0.16765562179907	-3,47019E+14	5,806E+14	6,05377E+14	6,71984E+14	7,16479E+14	6,68725E+14	6,93778E+14
TSUnmapped00000060.mm.2	Klra16	killer cell lectin-like receptor, subfamily A, member 16	-1,43176E+14	4,25887E+14	-2,69063E+14	0.0336705512407243	0.16765562179907	-3,47027E+14	3,67141E+14	3,0919E+14	3,92096E+14	5,24213E+14	4,46031E+14	5,16651E+14
TC0600003491.mm.2	Ndufb2	NADH:ubiquinone oxidoreductase subunit B2	0.844386209109636	8,27812E+14	2,69017E+14	0.0336920818794223	0.167693286495844	-3,47087E+14	8,87541E+14	8,74417E+14	8,08045E+14	8,03788E+14	8,1812E+14	7,74961E+14
TC1100003152.mm.2	Cyb5d2	cytochrome b5 domain containing 2	0.865439096577821	6,44623E+14	2,69002E+14	0.0336989301603391	0.167693286495844	-3,47106E+14	7,13411E+13	6,84198E+14	6,33989E+14	6,11618E+14	5,93955E+14	6,30566E+14
TC1200001601.mm.2	Hbp1	high mobility group box transcription factor 1	-1,05694E+14	8,17699E+14	-2,68885E+14	0.033753805717642	0.167868659326528	-3,47259E+14	7,36426E+14	7,55569E+14	9,0065E+14	8,10166E+14	8,6493E+14	8,38452E+14
TC0X00000214.mm.2	Usp11	ubiquitin specific peptidase 11	3,1255E+14	7,96797E+14	2,68882E+14	0.0337550087642246	0.167868659326528	-3,47263E+14	1,00645E+14	9,64128E+14	8,75967E+14	5,88764E+14	8,13172E+14	5,32304E+14
TC0300003139.mm.2	Dnajb4	DnaJ heat shock protein family (Hsp40) member B4	2,19293E+14	6,68675E+14	2,68848E+14	0.0337708349820456	0.167895545839463	-3,47307E+14	8,24294E+13	8,26881E+13	7,00129E+14	4,48158E+14	6,59812E+14	5,52777E+14
TC0900001555.mm.2	Azi2	5-azacytidine induced gene 2	1,49123E+14	6,8743E+14	2,68772E+14	0.0338065391760255	0.167973649458892	-3,47406E+14	7,9137E+14	7,47853E+13	7,09973E+14	6,34402E+14	6,93581E+14	5,47397E+14
TC1400000165.mm.2	Fam149b	family with sequence similarity 149, member B	0.909871771760244	6,38172E+14	2,6877E+14	0.0338073943520876	0.167973649458892	-3,47409E+14	6,80098E+14	6,72031E+14	6,21641E+14	6,85103E+14	5,8778E+14	5,82375E+14
TC0300003023.mm.2	Rap1gds1	RAP1, GTP-GDP dissociation stimulator 1	0.921141259064735	8,28186E+14	2,68738E+14	0.0338223886853892	0.167988146957902	-3,4745E+14	8,87978E+14	8,93123E+14	8,25521E+14	7,65619E+14	8,13252E+14	7,83621E+14
TC0900002922.mm.2	Armc8	armadillo repeat containing 8	1,60157E+14	8,06294E+14	2,68708E+14	0.0338364849519365	0.167988146957902	-3,4749E+14	9,0747E+14	8,86341E+14	8,40213E+14	7,30245E+14	8,11803E+14	6,61695E+14
TC0700004648.mm.2	Rpl13a/Snord32a/Snord33/Snord34/Mir5121	ribosomal protein L13A/small nucleolar RNA, C/D box 32A/small nucleolar RNA, C/D box 33/small nucleolar RNA, C/D box 34/microRNA 5121	1,46549E+14	1,11705E+14	2,68694E+14	0.0338429624011232	0.167988146957902	-3,47508E+14	1,22499E+14	1,21816E+14	1,11791E+13	9,91216E+14	1,12875E+13	1,02131E+14
TC1400000388.mm.2	4930474N05Rik	RIKEN cDNA 4930474N05 gene	-1,32966E+14	4,34025E+14	-2,6866E+14	0.033859137970854	0.167988146957902	-3,47553E+14	3,3707E+14	3,50034E+14	4,22986E+14	5,41023E+14	4,35332E+14	5,17704E+14
TC1500002299.mm.2	Atp5g2	ATP synthase, H+ transporting, mitochondrial F0 complex, subunit C2 (subunit 9)	0.883153608362461	9,46511E+14	2,68581E+14	0.0338962176448883	0.167988146957902	-3,47656E+14	1,01211E+14	1,01175E+14	9,31983E+14	8,76004E+14	9,29829E+14	9,17395E+13
TC0700002995.mm.2	Gfy	golgi-associated olfactory signaling regulator	-1,15619E+14	5,83821E+13	-2,68575E+14	0.0338988087491422	0.167988146957902	-3,47663E+14	5,04152E+14	5,12725E+14	5,83042E+14	6,54888E+14	5,79388E+14	6,68728E+14
TC0400002779.mm.2	Mup20	major urinary protein 20	-0.961923926320024	4,62909E+14	-2,68555E+13	0.0339082784442434	0.167988146957902	-3,47689E+14	4,16585E+14	4,26228E+14	4,83935E+14	4,1551E+14	5,00957E+14	5,34241E+14
TC1700000924.mm.2	Tfeb	transcription factor EB	-0.799946206588593	7,73237E+14	-2,68554E+14	0.0339086874742099	0.167988146957902	-3,4769E+14	7,26674E+14	7,22644E+14	7,76509E+14	8,04286E+14	8,05867E+14	8,03441E+14
TC1300000292.mm.2	Prl	prolactin	-1,12995E+14	4,91496E+14	-2,68533E+13	0.0339184575775034	0.167988146957902	-3,47718E+14	4,43608E+14	4,06305E+14	4,86033E+14	5,3713E+14	4,92807E+14	5,83096E+14
TC1500002212.mm.2	Nckap5l	NCK-associated protein 5-like	-0.907153062751321	7,47109E+14	-2,6852E+14	0.0339248248231624	0.167988146957902	-3,47735E+13	6,65182E+14	6,88385E+14	7,71645E+14	8,22444E+14	7,52151E+14	7,82846E+14
TC1000000693.mm.2	Zwint	ZW10 interactor	1,57176E+14	9,92515E+12	2,68509E+14	0.0339298254971365	0.167988146957902	-3,47749E+14	1,10112E+14	1,0869E+14	1,01568E+13	8,77725E+14	9,96451E+14	8,77213E+14
TC0700000843.mm.2	Kcna7	potassium voltage-gated channel, shaker-related subfamily, member 7	-0.90280689056413	6,51626E+14	-2,68482E+14	0.0339423260516041	0.167988146957902	-3,47784E+14	6,11459E+14	5,96925E+14	6,3381E+14	6,78617E+14	6,73821E+14	7,15124E+14
TC1100001498.mm.2	Hoxb4/Mir10a	homeobox B4/microRNA 10a	-0.822902186998141	4,67507E+14	-2,68475E+14	0.0339458453729864	0.167988146957902	-3,47794E+14	4,25146E+14	4,25412E+13	4,88685E+14	4,50661E+14	5,05375E+14	5,09763E+14
TC0100002288.mm.2	Slc39a10	solute carrier family 39 (zinc transporter), member 10	1,78239E+14	7,92983E+14	2,68395E+14	0.0339834329307035	0.168122522202157	-3,47898E+14	8,82757E+14	9,22748E+14	8,26434E+14	6,76931E+14	7,98932E+13	6,50096E+14
TC1500001937.mm.2	Snu13	SNU13 homolog, small nuclear ribonucleoprotein (U4/U6.U5)	1,09079E+14	7,75814E+14	2,68359E+14	0.03400010915605	0.168153394362012	-3,47944E+14	8,59826E+14	8,3517E+13	7,88011E+13	6,9504E+14	7,56989E+14	7,1985E+14
TC0900003248.mm.2	Cx3cr1	chemokine (C-X3-C motif) receptor 1	0.856640194728151	6,94947E+14	2,68321E+14	0.0340183823773377	0.168192143370986	-3,47995E+14	7,24589E+14	7,49746E+14	7,17401E+14	6,74938E+14	6,47784E+13	6,55223E+14
TC1000003050.mm.2	Xpot	exportin, tRNA (nuclear export receptor for tRNAs)	1,55108E+14	6,7092E+14	2,68286E+14	0.0340345021748984	0.168220225013539	-3,48039E+14	7,91732E+14	7,6444E+14	7,00558E+14	5,22833E+14	6,26144E+14	6,19813E+14
TC0700004492.mm.2	Caly	calcyon neuron-specific vesicular protein	1,31919E+14	8,57536E+14	2,68206E+14	0.0340725254342161	0.168356517117916	-3,48144E+14	9,64242E+14	9,19606E+14	8,85577E+14	7,55779E+14	8,30358E+14	7,89653E+14
TC1800000006.mm.2	Cul2	cullin 2	2,23864E+14	6,56264E+14	2,68136E+14	0.0341052865051985	0.16845542463498	-3,48235E+13	7,88395E+14	7,73475E+14	7,20634E+14	5,4094E+13	6,64912E+14	4,49229E+14
TC1300002453.mm.2	Pde8b	phosphodiesterase 8B	0.959174896292776	6,78378E+14	2,68119E+14	0.0341134519074003	0.16845542463498	-3,48258E+14	7,39039E+14	7,15631E+14	6,40851E+14	7,11913E+14	6,23601E+14	6,39235E+14
TC0200001913.mm.2	Dusp2	dual specificity phosphatase 2	-1,12413E+14	6,14652E+14	-2,68067E+14	0.0341378664185507	0.16847299234909	-3,48325E+14	5,19142E+14	5,50726E+14	6,13178E+14	7,10175E+14	6,27392E+14	6,67302E+14
TC0700003852.mm.2	Art5	ADP-ribosyltransferase 5	-1,01644E+14	6,8678E+14	-2,68067E+14	0.0341379209346353	0.16847299234909	-3,48325E+13	6,45255E+13	6,22311E+14	6,89008E+14	6,93251E+14	6,92623E+14	7,7823E+14
TC0600002935.mm.2	Sec13	SEC13 homolog, nuclear pore and COPII coat complex component	1,25836E+14	6,43054E+12	2,68042E+14	0.0341500899329526	0.168481444914166	-3,48359E+13	7,17871E+14	7,23724E+14	6,47216E+14	5,79589E+14	6,52234E+14	5,3769E+14
TC0700001948.mm.2	Bccip	BRCA2 and CDKN1A interacting protein	0.952307573893942	6,78105E+14	2,68003E+13	0.0341684971163252	0.168482421018977	-3,48409E+14	7,57879E+14	7,60924E+14	6,48139E+14	5,73346E+14	6,66289E+13	6,62052E+14
TC0100000661.mm.2	Tmem198	transmembrane protein 198	0.849712173255319	7,41867E+14	2,67997E+14	0.034171200390369	0.168482421018977	-3,48417E+14	7,87421E+14	8,18482E+14	7,19091E+14	6,90249E+14	7,29323E+13	7,06637E+14
TC1100002899.mm.2	Tmem11	transmembrane protein 11	-0.92807215802298	7,88083E+14	-2,6797E+14	0.0341842008314613	0.168494961149384	-3,48453E+14	7,18996E+14	7,4405E+13	7,76874E+13	8,3992E+13	8,17144E+14	8,31516E+14
TC0900001427.mm.2	Uqcrc1	ubiquinol-cytochrome c reductase core protein 1	0.919242664021948	8,07622E+14	2,67901E+14	0.0342167903673446	0.16860401932386	-3,48542E+14	8,85585E+14	8,40226E+14	8,09902E+12	7,68058E+14	7,84363E+14	7,57599E+14
TC1100003588.mm.2	Kat7	K(lysine) acetyltransferase 7	1,38328E+14	6,81332E+13	2,67815E+14	0.034257656171603	0.168746238165979	-3,48655E+14	7,85448E+14	7,66559E+14	6,94819E+13	5,65816E+14	6,78586E+14	5,96765E+14
TC1700002187.mm.2	Gnmt	glycine N-methyltransferase	-1,17302E+14	5,15737E+13	-2,67785E+14	0.0342717445298081	0.168746238165979	-3,48694E+14	4,58457E+14	4,3018E+14	5,21023E+14	5,61522E+13	5,07719E+14	6,15521E+14
TC0700003586.mm.2	Cib1	calcium and integrin binding 1 (calmyrin)	-1,05094E+14	6,7452E+14	-2,67774E+14	0.0342770705341804	0.168746238165979	-3,48708E+14	5,85745E+14	6,24791E+13	6,76175E+13	7,39685E+14	6,80216E+14	7,40508E+14
TC0800001214.mm.2	Tmem208	transmembrane protein 208	1,78656E+14	6,11562E+14	2,67705E+14	0.0343095559456758	0.168854573901244	-3,48797E+14	7,32138E+13	7,37147E+14	6,31747E+14	4,56371E+14	6,18997E+14	4,92975E+14
TC0400004093.mm.2	Hes3	hes family bHLH transcription factor 3	-1,04741E+14	6,20063E+14	-2,67619E+14	0.03435067613314	0.169005326575049	-3,4891E+14	5,36991E+14	5,64316E+14	6,28134E+14	6,80149E+14	6,17875E+14	6,92913E+14
TC0700000790.mm.2	Klk1b22	kallikrein 1-related peptidase b22	-1,07764E+14	5,12469E+13	-2,67568E+14	0.0343748878207953	0.169072822788912	-3,48977E+14	4,36805E+14	4,47031E+14	5,07739E+14	5,83875E+14	5,1673E+14	5,82634E+13
TC0600000927.mm.2	Mob1a	MOB kinase activator 1A	-0.810323127459677	7,46255E+14	-2,67523E+14	0.034396209411276	0.169093456148838	-3,49035E+14	6,89401E+14	7,14789E+14	7,64781E+14	7,42306E+14	7,80282E+14	7,85973E+14
TC0500001938.mm.2	Dmtf1	cyclin D binding myb-like transcription factor 1	1,25315E+14	7,37648E+14	2,67515E+14	0.0344000713247621	0.169093456148838	-3,49046E+14	8,13613E+14	7,93932E+14	7,30979E+14	7,3045E+14	7,44064E+14	6,12849E+14
TC1000002240.mm.2	Adamts14	a disintegrin-like and metallopeptidase (reprolysin type) with thrombospondin type 1 motif, 14	-1,08647E+14	6,61181E+14	-2,67432E+13	0.0344395463163872	0.169235867580344	-3,49154E+14	5,66164E+14	6,10376E+14	6,57451E+14	7,3926E+14	6,7477E+14	7,19064E+14
TC0500002053.mm.2	Srpk2	serine/arginine-rich protein specific kinase 2	1,11234E+14	9,56157E+14	2,6739E+14	0.0344595950132871	0.169273640392856	-3,49208E+14	1,01851E+14	1,03135E+14	9,64662E+14	8,95011E+14	9,54392E+14	8,73007E+14

TC1400002054.mm.2	Slc7a7	solute carrier family 7 (cationic amino acid transporter, y+ system), member 7	1,46365E+13	5,09311E+14	2,67372E+14	0.0344682439104425	0.169273640392856	-3,49232E+14	5,38791E+14	6,15684E+14	5,38142E+14	5,01503E+14	3,5899E+14	5,02756E+14
TC0200001448.mm.2	Trp53i11	transformation related protein 53 inducible protein 11	1,62875E+14	8,80225E+14	2,67266E+14	0.0345188786164126	0.169466553377852	-3,4937E+14	9,92796E+14	9,95924E+14	9,0066E+13	7,28999E+14	8,75273E+14	7,87696E+14
TC0300002351.mm.2	Lenep/Flad1	lens epithelial protein/flavin adenine dinucleotide synthetase 1	0.955095108958609	7,51663E+13	2,67229E+14	0.0345365592380958	0.169466553377852	-3,49418E+13	7,84675E+14	7,97267E+14	7,32729E+14	8,04382E+14	6,84077E+13	7,06847E+14
TC0300000792.mm.2	Gatad2b	GATA zinc finger domain containing 2B	2,2091E+14	8,38798E+13	2,67224E+14	0.0345390778435342	0.169466553377852	-3,49425E+14	9,85514E+14	9,7846E+14	8,86597E+14	6,60061E+14	8,41559E+14	6,80595E+14
TC1100000933.mm.2	Ctdnep1	CTD nuclear envelope phosphatase 1	0.824230519439346	7,6265E+14	2,67166E+14	0.0345668469368006	0.169551173422426	-3,49501E+14	8,06667E+14	8,01662E+14	7,7849E+14	7,45597E+14	7,10381E+14	7,33102E+14
TC0900001612.mm.2	Ctnnb1/Mir7090	catenin (cadherin associated protein), beta 1/microRNA 7090	0.914334434998533	9,47146E+14	2,67088E+14	0.0346040868031448	0.169682182184745	-3,49602E+14	1,00849E+14	1,00472E+14	9,40795E+14	8,98514E+14	9,40928E+13	8,89422E+14
TC0800002938.mm.2	Clec18a	C-type lectin domain family 18, member A	-0.843981967391566	5,17447E+14	-2,67053E+14	0.0346208047026732	0.169692028827996	-3,49648E+14	4,46245E+14	4,85398E+14	5,28005E+14	5,44593E+14	5,60463E+14	5,39976E+14
TC0300002470.mm.2	Mllt11	myeloid/lymphoid or mixed-lineage leukemia; translocated to, 11	1,26153E+14	9,87274E+14	2,6704E+14	0.0346271576234376	0.169692028827996	-3,49665E+14	1,06831E+14	1,0786E+14	9,86898E+14	8,95233E+14	9,96232E+14	8,9837E+14
TC1200001770.mm.2	Ralgapa1	Ral GTPase activating protein, alpha subunit 1	2,19807E+14	7,25807E+14	2,67003E+14	0.0346449143918502	0.169727426450557	-3,49713E+14	8,4856E+14	8,63648E+14	7,31873E+14	6,38165E+14	7,66237E+14	5,06358E+14
TC1000002257.mm.2	Hkdc1	hexokinase domain containing 1	-1,15599E+14	6,78466E+14	-2,66821E+14	0.034732384187414	0.170007674595543	-3,49951E+14	5,72881E+14	6,21208E+13	6,86366E+14	7,65055E+14	6,77304E+14	7,47982E+14
TC1000003035.mm.2	Lemd3	LEM domain containing 3	1,83464E+14	6,77625E+14	2,668E+14	0.0347428244302437	0.170007674595543	-3,49979E+14	7,87978E+14	7,84488E+14	7,52926E+14	5,34821E+14	6,28345E+14	5,77192E+13
TC0100002833.mm.2	Hdlbp	high density lipoprotein (HDL) binding protein	1,69345E+14	7,93714E+14	2,66796E+14	0.0347445704467096	0.170007674595543	-3,49984E+14	9,14796E+14	9,09182E+14	8,12433E+14	6,40585E+14	7,96579E+14	6,88708E+13
TC0200004973.mm.2	Eif6	eukaryotic translation initiation factor 6	0.888442538273805	7,45772E+14	2,6679E+14	0.0347473686809683	0.170007674595543	-3,49992E+14	8,00297E+14	8,0139E+13	7,51189E+14	6,97759E+14	7,26044E+14	6,97955E+13
TC1100003075.mm.2	2810408A11Rik	RIKEN cDNA 2810408A11 gene	-0.944326956390391	6,0904E+14	-2,66762E+14	0.0347610115742727	0.170007674595543	-3,50029E+14	5,43394E+14	5,66448E+14	5,89573E+14	6,56116E+14	6,37738E+13	6,6097E+14
TC0700001974.mm.2	Mgmt	O-6-methylguanine-DNA methyltransferase	-1,05972E+14	7,43888E+13	-2,66753E+14	0.0347654246752506	0.170007674595543	-3,5004E+14	6,42365E+14	6,91907E+14	7,55699E+14	8,27138E+14	7,50076E+14	7,9614E+13
TC0800002841.mm.2	Ctrl	chymotrypsin-like	-0.918986382732116	5,81963E+14	-2,6668E+14	0.0348002583255225	0.170099760877405	-3,50135E+14	5,44297E+13	4,96319E+14	5,9672E+14	6,30028E+14	5,94574E+14	6,2984E+14
TC1100001786.mm.2	Cep95	centrosomal protein 95	-0.82458044659496	6,04873E+14	-2,66656E+14	0.0348118872775675	0.170099760877405	-3,50166E+14	5,75131E+14	5,56282E+14	6,16926E+14	5,84568E+14	6,56504E+14	6,39826E+14
TC0900002427.mm.2	Nr2e3	nuclear receptor subfamily 2, group E, member 3	-1,45301E+14	6,22261E+14	-2,66637E+14	0.0348213348578038	0.170099760877405	-3,50192E+14	4,9033E+14	5,56507E+14	6,00135E+14	7,49157E+14	6,53001E+14	6,84436E+14
TC1200002416.mm.2	Ppp1r13b	protein phosphatase 1, regulatory subunit 13B	1,35932E+14	7,59108E+13	2,66568E+14	0.0348546118049112	0.170099760877405	-3,50282E+14	8,74919E+14	8,44507E+12	7,65735E+14	6,21924E+14	7,41244E+14	7,06318E+14
TC0200002306.mm.2	Xkr7	X-linked Kx blood group related 7	-0.794915826407607	6,25877E+14	-2,66552E+13	0.0348621272691179	0.170099760877405	-3,50302E+14	5,82107E+14	5,87232E+14	6,3713E+14	6,20471E+14	6,75527E+11	6,52795E+14
TC0300002474.mm.2	6330562C20Rik	RIKEN cDNA 6330562C20 gene	-1,08365E+14	6,96596E+14	-2,66549E+14	0.0348634822992566	0.170099760877405	-3,50306E+14	6,14352E+14	6,10862E+13	7,19569E+14	7,9285E+14	6,84719E+14	7,57227E+14
TC0700003415.mm.2	Nsmce3	NSE3 homolog, SMC5-SMC6 complex component	0.850623451447723	7,55142E+14	2,66535E+14	0.0348706426199405	0.170099760877405	-3,50325E+14	8,14998E+14	7,80376E+14	7,50584E+13	7,59644E+14	6,95298E+14	7,29951E+14
TC1700002186.mm.2	Ppp2r5d	protein phosphatase 2, regulatory subunit B', delta	0.80204402072935	7,899E+14	2,6652E+14	0.0348774958692134	0.170099760877405	-3,50344E+14	8,46492E+14	8,42522E+14	7,55355E+14	7,66424E+14	7,79228E+14	7,49377E+14
TC1100002052.mm.2	Slc16a3	solute carrier family 16 (monocarboxylic acid transporters), member 3	-0.852439250254146	6,346E+14	-2,66507E+14	0.034883803582044	0.170099760877405	-3,50361E+14	5,6907E+14	6,1347E+14	6,46782E+13	6,25248E+14	6,71386E+14	6,81642E+14
TC1800001149.mm.2	Fam13b	family with sequence similarity 13, member B	1,32581E+14	7,51572E+14	2,66452E+14	0.0349103980114799	0.170099760877405	-3,50432E+14	8,31346E+14	8,07403E+14	7,83314E+14	7,13786E+14	7,49677E+14	6,23909E+14
TC1500002162.mm.2	Col2a1	collagen, type II, alpha 1	-1,10474E+14	5,87523E+14	-2,66448E+14	0.0349123549197767	0.170099760877405	-3,50438E+14	5,3652E+13	5,01295E+14	5,65315E+14	6,63246E+14	6,42232E+14	6,16532E+14
TC1900000386.mm.2	Apba1	amyloid beta (A4) precursor protein binding, family A, member 1	0.994064095116485	8,0958E+14	2,66421E+14	0.0349258720362927	0.170099760877405	-3,50474E+14	8,7272E+14	8,62941E+14	8,29836E+14	7,55133E+13	7,88494E+14	7,48353E+14
TC1100002904.mm.2	Usp22	ubiquitin specific peptidase 22	1,56877E+14	9,38048E+14	2,66392E+14	0.0349397481087849	0.170099760877405	-3,50511E+14	1,05263E+14	1,04066E+14	9,68229E+14	7,87233E+14	9,06217E+14	8,73318E+14
TC0800001526.mm.2	Afg3l1	AFG3-like AAA ATPase 1	0.973665474693555	5,80652E+14	2,66274E+14	0.0349969795922007	0.170099760877405	-3,50666E+14	6,29773E+14	6,43372E+14	5,2662E+14	6,05736E+14	5,33405E+14	5,45007E+13
TC0900001568.mm.2	Ctdspl	CTD (carboxy-terminal domain, RNA polymerase II, polypeptide A) small phosphatase-like	-0.810776226482337	7,7454E+14	-2,66267E+14	0.0350002178721222	0.170099760877405	-3,50674E+14	7,15382E+14	7,277E+14	8,09372E+14	7,89548E+14	8,16423E+14	7,88814E+14
TC0200002346.mm.2	Zfp341	zinc finger protein 341	-0.851595524074971	6,73952E+14	-2,66242E+14	0.0350126526532361	0.170099760877405	-3,50708E+14	6,01915E+14	6,28159E+14	7,21998E+14	6,91244E+14	6,85147E+14	7,15247E+14
TC1100002379.mm.2	Papolg	poly(A) polymerase gamma	2,18701E+14	6,48215E+14	2,66186E+14	0.0350398479008337	0.170099760877405	-3,50781E+14	7,80159E+14	7,52329E+14	7,13316E+14	5,484E+14	6,5735E+14	4,37735E+13
TC0900000609.mm.2	Ppp2r1b	protein phosphatase 2, regulatory subunit A, beta	1,1047E+14	6,45134E+14	2,66169E+14	0.0350476618673017	0.170099760877405	-3,50802E+14	6,9359E+14	7,19658E+14	6,49624E+14	6,15624E+14	6,454E+14	5,46907E+14
TC1100002934.mm.2	Ncor1	nuclear receptor co-repressor 1	1,66917E+14	7,48418E+14	2,66155E+14	0.035054515643826	0.170099760877405	-3,5082E+14	8,59988E+13	8,8361E+14	7,37718E+14	5,99431E+14	7,76095E+14	6,33668E+14
TC0900001377.mm.2	Ifrd2	interferon-related developmental regulator 2	-0.924304988371609	6,98626E+14	-2,66151E+14	0.0350567559225343	0.170099760877405	-3,50826E+14	6,32988E+14	6,53063E+14	7,10313E+14	7,24479E+14	7,02706E+14	7,68206E+14
TC1400002759.mm.2	Nalcn	sodium leak channel, non-selective	0.930967161911068	7,52265E+14	2,6614E+14	0.0350618391363218	0.170099760877405	-3,5084E+14	8,14746E+14	8,17903E+14	7,35467E+14	6,99018E+13	7,53973E+14	6,92482E+13
TC1900000356.mm.2	Trpm3	transient receptor potential cation channel, subfamily M, member 3	1,56908E+14	7,57121E+14	2,66139E+14	0.0350626921742976	0.170099760877405	-3,50842E+13	8,30296E+13	8,76443E+14	6,55595E+14	7,87468E+14	7,5681E+14	6,36113E+14
TC0100002698.mm.2	Trip12	thyroid hormone receptor interactor 12	1,13498E+14	8,25567E+14	2,66124E+14	0.0350696147343189	0.170099760877405	-3,50861E+14	9,11667E+14	8,9144E+14	8,19145E+14	7,55039E+14	8,33489E+14	7,42622E+14
TC1000002488.mm.2	Polr2e	polymerase (RNA) II (DNA directed) polypeptide E	1,09534E+14	7,55962E+14	2,66107E+14	0.0350782242345769	0.170099760877405	-3,50884E+14	8,32691E+14	8,41623E+14	7,48215E+14	6,57997E+13	7,54797E+14	7,00449E+14
TC0900001726.mm.2	Cep126	centrosomal protein 126	1,41903E+14	6,66177E+14	2,6607E+14	0.0350959616790027	0.170099760877405	-3,50932E+14	7,49042E+14	7,52555E+14	6,61109E+14	6,16564E+14	6,86305E+13	5,31487E+14
TC1700001322.mm.2	Ppp1r21	protein phosphatase 1, regulatory subunit 21	1,06709E+14	7,59303E+14	2,66056E+14	0.035102664759968	0.170099760877405	-3,50949E+14	8,45405E+14	8,01145E+14	7,65512E+14	7,10623E+14	7,52271E+14	6,80861E+14
TC1900000743.mm.2	Ins1	insulin I	-1,09458E+14	5,71865E+14	-2,66047E+14	0.0351071565145986	0.170099760877405	-3,50962E+14	4,99462E+13	5,02277E+14	5,57313E+13	6,51481E+14	5,8647E+14	6,34185E+14
TC0200003869.mm.2	Ctnnd1	catenin (cadherin associated protein), delta 1	1,45303E+14	5,53337E+14	2,66024E+14	0.0351183536068793	0.170099760877405	-3,50992E+14	6,18217E+14	6,49614E+13	5,8759E+14	4,87379E+14	5,53274E+14	4,23951E+14
TC0200004529.mm.2	Shc4	SHC (Src homology 2 domain containing) family, member 4	-0.980439843263922	5,63045E+13	-2,6601E+13	0.0351252653715666	0.170099760877405	-3,5101E+14	5,02181E+14	5,00332E+14	5,64063E+14	6,13091E+14	5,65959E+14	6,32642E+14
TC1600000501.mm.2	Kpna1	karyopherin (importin) alpha 1	1,45088E+14	6,88982E+14	2,65978E+14	0.0351410681927018	0.170099760877405	-3,51052E+14	7,86175E+14	7,67741E+14	7,14649E+14	6,0159E+14	6,9214E+14	5,716E+14
TC0500003000.mm.2	Ulk1	unc-51 like kinase 1	0.861898067691558	6,3162E+14	2,65975E+14	0.0351420941137665	0.170099760877405	-3,51055E+14	6,9547E+14	6,6976E+14	6,15398E+13	6,16242E+14	5,72835E+14	6,20015E+13
TC0800001327.mm.2	Exosc6/Aars	exosome component 6/alanyl-tRNA synthetase	2,13384E+14	7,8743E+14	2,65973E+14	0.0351434980470838	0.170099760877405	-3,51059E+14	9,20849E+14	9,14519E+14	8,44618E+14	6,35999E+14	7,88861E+14	6,19737E+14
TC0600001807.mm.2	Pon3	paraoxonase 3	0.893365194601621	6,31903E+14	2,65957E+14	0.0351511205242917	0.170099760877405	-3,51079E+14	6,59147E+14	7,12939E+14	5,97759E+14	6,28162E+14	6,01068E+14	5,92345E+14
TC0300001141.mm.2	Mybphl	myosin binding protein H-like	-1,25342E+14	4,59926E+14	-2,65957E+14	0.0351512551050553	0.170099760877405	-3,5108E+14	3,39065E+14	4,06992E+14	4,56576E+14	5,6018E+14	5,23871E+14	4,7287E+13
TC0100000645.mm.2	Wnt10a	wingless-type MMTV integration site family, member 10A	-0.860045514690855	6,17024E+14	-2,6592E+14	0.035169114793681	0.170099760877405	-3,51128E+14	5,6294E+14	5,65082E+14	6,19874E+14	6,54221E+14	6,29699E+14	6,70332E+14
TC1700001674.mm.2	Metrn	meteorin, glial cell differentiation regulator	0.765863609127948	7,25756E+14	2,65914E+14	0.0351721278750814	0.170099760877405	-3,51136E+14	7,81647E+14	7,7862E+14	6,90366E+13	6,96806E+13	7,03395E+14	7,03699E+14
TC0500000683.mm.2	Ociad1	OCIA domain containing 1	1,8337E+14	1,04069E+14	2,65878E+14	0.0351896204999891	0.170099760877405	-3,51182E+14	1,17782E+14	1,15562E+14	1,06781E+14	8,76184E+14	1,04111E+14	9,25589E+14
TC0500001304.mm.2	Tesc	tescalcin	-1,23713E+13	7,64975E+14	-2,65877E+14	0.0351900150146561	0.170099760877405	-3,51184E+14	6,89043E+14	6,91165E+14	8,5662E+14	7,25386E+14	8,13291E+14	8,14344E+14
TC1000001575.mm.2	Rdh19	retinol dehydrogenase 19	-1,32762E+14	5,87049E+14	-2,65864E+14	0.0351963075254472	0.170099760877405	-3,512E+14	4,66162E+13	5,03385E+14	5,88517E+14	7,29161E+14	6,09064E+14	6,26007E+14
TC0400001391.mm.2	Hpcal4	hippocalcin-like 4	1,224E+14	8,74629E+14	2,65843E+14	0.0352065228403244	0.170099760877405	-3,51228E+14	9,62256E+14	9,56493E+14	8,91405E+14	7,6367E+14	8,28349E+14	8,45599E+14

TC0X00003146.mm.2	Lrch2	leucine-rich repeats and calponin homology (CH) domain containing 2	1,09556E+14	6,21824E+14	2,65807E+14	0.0352242006324095	0.170132510734475	-3,51275E+14	7,26435E+14	6,93272E+13	6,0022E+14	5,1042E+14	5,77565E+14	6,2303E+14
TC0800000358.mm.2	Chrnb3	cholinergic receptor, nicotinic, beta polypeptide 3	-0.791926161348196	4,78832E+14	-2,65782E+13	0.0352364173951575	0.170132510734475	-3,51308E+14	4,31605E+13	4,23256E+14	4,96072E+14	5,08813E+14	5,18544E+14	4,94702E+14
TC0100001554.mm.2	Rxrg	retinoid X receptor gamma	1,26035E+14	7,35622E+14	2,65765E+14	0.0352449773990988	0.170132510734475	-3,5133E+14	8,17584E+14	7,8436E+14	7,96164E+13	6,65749E+14	6,81921E+13	6,67953E+12
TC0400004113.mm.2	Ccdc27	coiled-coil domain containing 27	-1,39278E+14	5,7138E+14	-2,65724E+14	0.0352648373445416	0.170177395667146	-3,51383E+14	4,60509E+14	4,83296E+14	5,58473E+14	7,03639E+14	5,83234E+14	6,39127E+14
TC0200005119.mm.2	Spint3	serine peptidase inhibitor, Kunitz type, 3	-1,07212E+14	6,10162E+14	-2,65654E+14	0.0352992670745935	0.170241871082295	-3,51475E+14	5,47616E+13	5,49424E+14	6,00565E+14	6,51902E+14	6,11882E+14	6,99582E+13
TC0100002602.mm.2	Atg9a	autophagy related 9A	2,58917E+14	7,85698E+14	2,65641E+14	0.0353053458477346	0.170241871082295	-3,51492E+14	9,51165E+14	9,53743E+14	8,53003E+14	5,69203E+14	7,81596E+14	6,05477E+14
TC1100003221.mm.2	Rpa1	replication protein A1	0.810501446005472	7,5669E+14	2,65617E+14	0.0353171848388521	0.170241871082295	-3,51523E+14	8,19444E+14	7,95886E+13	7,49186E+14	7,22397E+13	7,31215E+14	7,22014E+14
TC1200002288.mm.2	Serpina12	serine (or cysteine) peptidase inhibitor, clade A (alpha-1 antiproteinase, antitrypsin), member 12	-1,54893E+14	4,82859E+13	-2,6561E+14	0.035320460189171	0.170241871082295	-3,51532E+14	4,34998E+14	3,12457E+14	4,80711E+14	6,1175E+14	5,13348E+14	5,43893E+14
TC1100001578.mm.2	Psmd3	proteasome (prosome, macropain) 26S subunit, non-ATPase, 3	0.769783735295375	8,67209E+14	2,65562E+13	0.0353444267851167	0.170306444015725	-3,51596E+14	9,21142E+14	9,10444E+14	8,43881E+14	8,50154E+13	8,35856E+14	8,41773E+14
TC1600001253.mm.2	Ube2v2	ubiquitin-conjugating enzyme E2 variant 2	1,64824E+13	8,38274E+14	2,65508E+14	0.0353707986236207	0.170382564491002	-3,51666E+13	9,15912E+13	9,11493E+14	8,6925E+14	8,35231E+14	8,46051E+14	6,51706E+14
TC1700000348.mm.2	Noxo1	NADPH oxidase organizer 1	-0.864015947745687	7,2197E+14	-2,65481E+14	0.0353842205141036	0.170396277687911	-3,51702E+14	6,78655E+14	6,80064E+14	7,40263E+14	7,01317E+14	7,46486E+14	7,85037E+14
TC0900003055.mm.2	Dag1	dystroglycan 1	2,76069E+14	6,73011E+14	2,65383E+13	0.0354321100542242	0.170543058693986	-3,51829E+14	8,65937E+14	8,51756E+14	7,46884E+14	4,07934E+14	6,40816E+13	5,24738E+14
TC0700001451.mm.2	Stim1	stromal interaction molecule 1	0.851631192417302	5,64362E+14	2,65375E+14	0.035435869205453	0.170543058693986	-3,51839E+14	6,25566E+14	6,49758E+14	5,00985E+13	5,04866E+14	5,29986E+14	5,75012E+14
TC1500002263.mm.2	Krt90	keratin 90	-0.863597995010936	6,0775E+14	-2,65302E+14	0.0354721623241793	0.170666751725739	-3,51935E+13	5,45783E+14	5,57093E+14	6,08316E+14	6,5971E+14	6,3004E+14	6,45556E+14
TC1100003814.mm.2	Etv4	ets variant 4	-1,08502E+14	7,3351E+14	-2,65249E+14	0.0354979284956232	0.170722158506625	-3,52004E+14	6,4852E+14	6,63972E+14	7,3789E+13	8,21186E+14	7,34229E+14	7,95266E+14
TC0X00002453.mm.2	G6pdx	glucose-6-phosphate dehydrogenase X-linked	1,06726E+14	7,753E+14	2,65235E+14	0.0355048689353752	0.170722158506625	-3,52022E+14	8,54183E+13	8,28416E+14	7,31595E+14	7,6846E+14	6,89342E+14	7,79804E+14
TC0600001602.mm.2	Gprc5a	G protein-coupled receptor, family C, group 5, member A	-1,19677E+14	5,91594E+14	-2,65124E+14	0.0355598700301635	0.170851222622629	-3,52168E+14	4,81491E+14	5,49121E+14	5,74639E+14	6,74342E+14	6,18594E+14	6,51373E+14
TC0900001253.mm.2	A4gnt	alpha-1,4-N-acetylglucosaminyltransferase	-1,05619E+14	5,41877E+14	-2,65117E+14	0.0355631615486596	0.170851222622629	-3,52177E+14	4,71218E+14	4,81036E+13	5,21274E+14	6,14244E+14	5,65508E+14	5,97983E+13
TC1900001231.mm.2	Rorb	RAR-related orphan receptor beta	1,10272E+14	5,89859E+12	2,65116E+14	0.0355635201809903	0.170851222622629	-3,52178E+14	6,74771E+14	6,53271E+14	6,03736E+14	4,99875E+14	5,43957E+14	5,63541E+14
TC0100001018.mm.2	Phlpp1	PH domain and leucine rich repeat protein phosphatase 1	0.973188959349648	8,31402E+14	2,65062E+14	0.0355900809315679	0.17092786111784	-3,52248E+14	8,9317E+14	8,71912E+14	7,87771E+14	8,65113E+14	7,93676E+14	7,76768E+14
TC1700002655.mm.2	Abcg5	ATP binding cassette subfamily G member 5	-0.793694519616492	5,85274E+13	-2,65037E+14	0.0356027799042372	0.170937900058693	-3,52281E+14	5,41849E+14	5,42389E+14	5,81614E+14	6,02814E+14	6,1069E+12	6,32286E+14
TC1500001078.mm.2	Gm5477	type II keratin Kb16P	-0.83623662030485	5,39255E+14	-2,65015E+13	0.0356135736752968	0.170938788391438	-3,5231E+14	4,75836E+13	4,94835E+14	5,43687E+14	5,83251E+14	5,77047E+14	5,60872E+14
TC0600000214.mm.2	Fscn3	fascin actin-bundling protein 3	-2,01599E+14	6,18201E+14	-2,64972E+14	0.0356347772505035	0.170947567357108	-3,52366E+14	5,13826E+14	4,89601E+13	5,33062E+14	7,6609E+14	6,56029E+14	7,50596E+14
TC1100000638.mm.2	Gpx3	glutathione peroxidase 3	1,41824E+13	8,55318E+14	2,6491E+13	0.0356654262141019	0.170947567357108	-3,52447E+13	9,97567E+14	8,67877E+14	8,89891E+13	7,94773E+13	8,0062E+14	7,81178E+14
TC0400002289.mm.2	Prdm13	PR domain containing 13	-1,17613E+14	5,29481E+14	-2,64902E+14	0.0356692270344758	0.170947567357108	-3,52457E+14	4,52549E+14	4,4495E+13	5,35839E+14	6,10822E+14	5,18784E+14	6,13941E+14
TC1100000496.mm.2	Ifi47/Olfr56	interferon gamma inducible protein 47/olfactory receptor 56	-0.766938064105264	7,98727E+14	-2,64888E+14	0.0356763066040359	0.170947567357108	-3,52476E+14	7,44249E+14	7,52014E+14	8,1908E+14	8,27369E+14	8,2188E+14	8,2777E+13
TC1200002002.mm.2	Erh	ERH mRNA splicing and mitosis factor	1,34368E+14	5,97385E+14	2,64884E+14	0.0356785630421941	0.170947567357108	-3,52482E+14	7,33807E+14	7,18901E+14	5,37149E+14	4,1048E+14	6,31389E+14	5,52582E+13
TC1500001753.mm.2	Pycrl	pyrroline-5-carboxylate reductase-like	-0.932789171491178	8,40896E+14	-2,64851E+14	0.035694562919249	0.170947567357108	-3,52524E+14	7,78083E+14	7,77482E+14	8,38993E+14	9,08698E+14	8,60149E+14	8,81974E+14
TC0400003230.mm.2	Cpt2	carnitine palmitoyltransferase 2	-0.798667499186511	6,70382E+14	-2,64851E+13	0.0356947589396297	0.170947567357108	-3,52524E+14	6,28304E+14	6,18856E+14	6,6882E+14	6,99419E+13	6,99842E+14	7,07051E+14
TC0200001115.mm.2	Hnrnpa3/Gm6793	heterogeneous nuclear ribonucleoprotein A3/heterogeneous nuclear ribonucleoprotein A3 pseudogene	1,66477E+14	9,06692E+14	2,6483E+14	0.0357053569803219	0.170947567357108	-3,52552E+14	1,02733E+14	1,0154E+14	9,16245E+14	7,71401E+14	9,23959E+14	7,85816E+14
TC0400001538.mm.2	Adgrb2	adhesion G protein-coupled receptor B2	1,48086E+14	8,56517E+14	2,64773E+14	0.0357333006694729	0.170947567357108	-3,52626E+14	9,43503E+13	9,76307E+14	8,80663E+14	7,14989E+14	8,28324E+14	7,95315E+14
TC1800000791.mm.2	Myo5b	myosin VB	0.833688430691694	6,45185E+14	2,6477E+14	0.0357350491472087	0.170947567357108	-3,5263E+14	7,0002E+14	6,95833E+14	6,24874E+14	6,21268E+14	5,91628E+14	6,37487E+14
TC1700002437.mm.2	Themis3	thymocyte selection associated family member 3	-1,02572E+14	5,48413E+14	-2,6476E+14	0.0357400512111794	0.170947567357108	-3,52644E+14	4,51864E+14	4,9507E+14	5,54845E+13	6,36621E+14	5,703E+14	5,81777E+14
TC1100003581.mm.2	Sgca	sarcoglycan, alpha (dystrophin-associated glycoprotein)	-1,26876E+14	6,1092E+14	-2,64737E+14	0.0357514554022193	0.170947567357108	-3,52674E+14	5,13001E+14	5,41216E+14	5,9528E+14	7,08057E+14	6,17215E+14	6,90753E+14
TC1100004254.mm.2	Zfp750	zinc finger protein 750	-1,01217E+14	6,08298E+11	-2,64732E+14	0.0357537600786347	0.170947567357108	-3,5268E+14	5,44114E+13	5,49646E+14	5,92903E+14	6,66932E+14	6,2225E+14	6,73945E+14
TC1800000306.mm.2	Ctnna1	catenin (cadherin associated protein), alpha 1	1,10552E+13	6,8295E+14	2,64711E+14	0.0357639328219954	0.170947567357108	-3,52707E+14	7,52268E+14	7,58834E+14	6,778E+14	6,18798E+14	6,90524E+14	5,99475E+14
TC0500000318.mm.2	Krtcap3	keratinocyte associated protein 3	-1,10184E+14	5,39581E+14	-2,64636E+14	0.0358013497098965	0.171040677081624	-3,52805E+14	4,65317E+14	4,64697E+14	5,25343E+14	6,31746E+14	5,65756E+14	5,84626E+14
TC0X00002919.mm.2	Nap1l3	nucleosome assembly protein 1-like 3	1,59819E+14	6,45689E+14	2,64623E+13	0.0358078926242996	0.171040677081624	-3,52822E+14	7,74286E+14	7,05909E+14	6,6362E+14	5,6976E+14	6,55793E+14	5,04764E+14
TC0900000791.mm.2	Itga11	integrin alpha 11	-1,02057E+13	5,27407E+14	-2,6459E+13	0.0358242790585996	0.171040677081624	-3,52865E+14	4,93901E+14	4,58022E+14	4,86717E+14	5,69765E+14	5,68897E+14	5,8714E+14
TC0800001594.mm.2	Nrp1/Mir1903	neuropilin 1/microRNA 1903	0.914244686938343	6,71336E+14	2,64566E+13	0.0358361995645607	0.171040677081624	-3,52897E+13	7,10127E+14	7,22233E+14	6,89642E+14	6,56504E+14	6,54318E+14	5,95193E+14
TC0200001228.mm.2	Prg3	proteoglycan 3	-0.991920692464063	5,88441E+14	-2,64565E+14	0.035836487670053	0.171040677081624	-3,52897E+14	5,25761E+14	5,1712E+14	5,88317E+14	6,5818E+14	5,95605E+14	6,45661E+14
TC1300000668.mm.2	B4galt7	xylosylprotein beta1,4-galactosyltransferase, polypeptide 7 (galactosyltransferase I)	-0.792879944206526	7,03676E+14	-2,64524E+14	0.0358572292456386	0.171046834645021	-3,52952E+14	6,44215E+14	6,62939E+14	7,16617E+14	7,32557E+14	7,23748E+14	7,41982E+14
TC1300000718.mm.2	Ntrk2	neurotrophic tyrosine kinase, receptor, type 2	1,46218E+14	9,56635E+14	2,6452E+14	0.0358590087153776	0.171046834645021	-3,52956E+14	1,065E+14	1,04522E+14	9,82218E+14	8,29576E+14	9,44211E+14	8,73577E+14
TC1100004214.mm.2	Ppp1r27	protein phosphatase 1, regulatory subunit 27	-1,27399E+14	6,06179E+14	-2,64482E+14	0.0358781610197929	0.171056241402521	-3,53007E+14	5,15276E+14	5,38529E+14	5,76577E+14	6,98086E+14	6,23001E+13	6,85603E+14
TC1100000866.mm.2	Stx8	syntaxin 8	1,34764E+13	5,83139E+14	2,64458E+14	0.035889769921111	0.171056241402521	-3,53037E+14	6,72881E+14	6,56968E+14	6,26856E+14	4,81809E+14	5,49449E+14	5,10873E+14
TC0800001131.mm.2	Tepp	testis, prostate and placenta expressed	-1,0972E+14	7,27943E+14	-2,6444E+14	0.0358987836135993	0.171056241402521	-3,53061E+14	6,68891E+14	6,32341E+14	7,27711E+14	8,18042E+14	7,37819E+14	7,82853E+14
TC0300001123.mm.2	Eps8l3	EPS8-like 3	-1,22223E+14	6,17082E+14	-2,64431E+14	0.035903444946523	0.171056241402521	-3,53073E+14	5,23101E+14	5,63256E+14	5,92829E+14	6,925E+13	6,28959E+14	7,01844E+14
TC0100003402.mm.2	Fmo2	flavin containing monooxygenase 2	-1,12794E+14	7,5881E+14	-2,64394E+14	0.0359219141166909	0.171065001837245	-3,53121E+14	6,70167E+14	6,98844E+14	7,42242E+14	8,47009E+14	7,81558E+14	8,13042E+14
TC0400000234.mm.2	Epha7	Eph receptor A7	1,31933E+14	8,82858E+14	2,64379E+14	0.035929512693516	0.171065001837245	-3,53141E+14	9,58412E+12	9,74489E+14	9,04809E+14	7,90402E+14	8,79416E+14	7,89619E+14
TC0400000039.mm.2	Sdcbp	syndecan binding protein	1,82714E+14	9,10918E+14	2,64364E+14	0.0359371334462281	0.171065001837245	-3,53161E+14	1,03716E+14	1,03416E+13	9,48192E+14	7,40096E+13	8,96266E+14	8,09631E+14
TC0400002407.mm.2	Lingo2	leucine rich repeat and Ig domain containing 2	1,65894E+14	6,69928E+14	2,64281E+14	0.0359781112238969	0.171159925186145	-3,53269E+14	7,75436E+14	7,56509E+14	7,2147E+14	5,65998E+14	6,62388E+14	5,37768E+14
TC1800000091.mm.2	Gata6	GATA binding protein 6	-1,17852E+14	5,79764E+14	-2,64267E+14	0.0359853697669243	0.171159925186145	-3,53288E+14	4,95854E+14	5,07871E+14	5,77685E+14	6,57746E+14	5,73871E+14	6,65558E+13
TC0Y00000266.mm.2	Gm20822/Gm20828	predicted gene, 20822/predicted gene, 20828	-1,16831E+14	5,04387E+14	-2,64219E+14	0.0360092878297631	0.171159925186145	-3,5335E+14	4,29168E+14	4,06058E+14	5,06544E+14	6,15665E+14	5,11602E+14	5,57286E+14
TC0Y00000274.mm.2	Gm20822	predicted gene, 20822	-1,16831E+14	5,04387E+14	-2,64219E+14	0.0360092878297631	0.171159925186145	-3,5335E+14	4,29168E+14	4,06058E+14	5,06544E+14	6,15665E+14	5,11602E+14	5,57286E+14
TC1800001584.mm.2	Mapk4	mitogen-activated protein kinase 4	1,76837E+14	6,33933E+14	2,64216E+14	0.0360106925286183	0.171159925186145	-3,53354E+14	7,54836E+14	7,63584E+14	6,61969E+14	4,58467E+12	6,1947E+14	5,45276E+14
TC1200000083.mm.2	Osr1	odd-skipped related transcription factor 1	-0.92432924902524	5,9897E+14	-2,64189E+14	0.0360242236215888	0.171159925186145	-3,53389E+14	5,42972E+14	5,56522E+14	5,74546E+14	6,35419E+14	6,59764E+14	6,24596E+13
TC0800001964.mm.2	Unc5d	unc-5 netrin receptor D	0.888936600377823	6,60658E+14	2,64096E+14	0.0360707339121733	0.171159925186145	-3,53511E+14	7,35934E+14	6,77414E+14	6,67257E+14	6,4778E+14	6,11666E+14	6,23895E+14
TC1900001769.mm.2	Tmem179b	transmembrane protein 179B	-0.927166235740711	7,25119E+14	-2,64068E+14	0.0360848743046415	0.171159925186145	-3,53548E+14	6,28483E+14	6,65563E+14	8,18755E+14	7,58435E+14	7,47219E+13	7,3226E+14

TC1000002775.mm.2	Tmtc3	transmembrane and tetratricopeptide repeat containing 3	-1,04422E+14	6,62123E+14	-2,64031E+14	0.036103336873536	0.171159925186145	-3,53596E+14	6,20006E+14	6,37913E+13	6,58586E+14	5,89469E+14	7,0021E+14	7,66552E+13
TC1900001588.mm.2	Oga	O-GlcNAcase	1,55819E+14	8,90257E+14	2,63994E+14	0.0361222114517795	0.171159925186145	-3,53645E+14	9,85048E+14	9,77544E+14	9,49179E+14	7,78819E+14	8,61219E+14	7,89735E+14
TC0500001995.mm.2	Gnai1	guanine nucleotide binding protein (G protein), alpha inhibiting 1	1,01358E+14	8,2957E+14	2,63962E+14	0.0361380618439044	0.171159925186145	-3,53686E+14	9,11636E+14	8,84634E+14	8,3268E+14	7,54917E+14	8,16585E+14	7,76969E+14
TC1700000585.mm.2	Myo1f	myosin IF	-0.797345043976263	5,60899E+14	-2,63924E+14	0.0361573931236325	0.171159925186145	-3,53736E+14	5,15066E+14	5,41696E+14	5,51493E+14	5,40906E+14	6,16771E+14	5,99461E+14
TC0X00001383.mm.2	Mid2	midline 2	2,86927E+14	6,51087E+13	2,63905E+14	0.0361665972483603	0.171159925186145	-3,5376E+14	8,41532E+14	8,04192E+13	7,04498E+14	4,84428E+13	6,87253E+14	3,84617E+14
TC1300001448.mm.2	Lgals8	lectin, galactose binding, soluble 8	1,07016E+14	7,01349E+14	2,63899E+13	0.0361697493425454	0.171159925186145	-3,53769E+14	7,8373E+14	7,22004E+13	7,42701E+14	6,67958E+14	6,62698E+14	6,29004E+14
TC0700001448.mm.2	Pgap2	post-GPI attachment to proteins 2	-0.950664337715622	6,47942E+14	-2,63834E+14	0.0362024051784052	0.171159925186145	-3,53854E+14	6,04433E+14	5,90386E+11	6,44551E+14	6,63329E+14	6,55291E+14	7,2966E+14
TC0600001259.mm.2	Tatdn2	TatD DNase domain containing 2	1,48414E+14	5,55548E+13	2,63817E+14	0.0362112062247932	0.171159925186145	-3,53876E+14	6,31372E+14	6,54194E+14	5,63344E+14	4,95641E+14	4,15341E+13	5,73396E+14
TC0100001560.mm.2	Rgs5	regulator of G-protein signaling 5	0.947518047968637	6,59166E+14	2,63782E+12	0.0362287073243494	0.171159925186145	-3,53922E+14	7,12234E+13	7,03523E+14	6,37106E+13	6,75882E+14	6,46283E+14	5,7997E+14
TC0800001608.mm.2	Insr	insulin receptor	1,21196E+14	6,87262E+14	2,63779E+14	0.03622985225547	0.171159925186145	-3,53925E+14	7,99642E+14	7,94974E+14	6,3715E+14	5,39584E+13	7,17921E+14	6,34304E+14
TC0700002720.mm.2	Spint2	serine protease inhibitor, Kunitz type 2	0.79066884191134	7,19034E+13	2,63773E+14	0.0362331839411096	0.171159925186145	-3,53934E+14	7,67877E+14	7,74369E+14	6,80021E+14	7,07825E+14	6,86508E+14	6,97604E+14
TC0500000740.mm.2	Polr2b	polymerase (RNA) II (DNA directed) polypeptide B	3,42016E+14	6,35322E+14	2,63771E+14	0.0362342349075111	0.171159925186145	-3,53936E+14	8,46507E+14	8,25515E+14	7,28706E+14	4,23215E+14	6,61216E+14	3,26772E+14
TC0X00003287.mm.2	Nhs	NHS actin remodeling regulator	1,07475E+14	5,61795E+14	2,63768E+14	0.0362357398448431	0.171159925186145	-3,5394E+14	6,32464E+14	6,07643E+14	6,02807E+14	5,02697E+14	5,07652E+14	5,17505E+13
TC0800002564.mm.2	Mir1199	microRNA 1199	-1,39816E+14	7,67192E+14	-2,63765E+14	0.0362372707676815	0.171159925186145	-3,53944E+14	6,82532E+14	6,66925E+14	7,38883E+14	8,85725E+14	7,84073E+14	8,45016E+14
TC0X00000988.mm.2	Nhsl2	NHS-like 2	0.977807658269159	7,6008E+14	2,63754E+14	0.0362424283930223	0.171159925186145	-3,53958E+14	8,04625E+14	8,28903E+14	7,6077E+14	7,28214E+14	7,55757E+14	6,8221E+14
TC0500000363.mm.2	Nsd2	nuclear receptor binding SET domain protein 2	1,32759E+14	7,99017E+14	2,6375E+14	0.0362447992416975	0.171159925186145	-3,53964E+14	8,86103E+14	8,8318E+14	8,18118E+13	7,02935E+14	7,98499E+14	7,05267E+14
TC0100000125.mm.2	Mir206/Mir133b	microRNA 206/microRNA 133b	-1,12099E+14	5,37653E+14	-2,63742E+14	0.0362487756046707	0.171159925186145	-3,53974E+14	4,31237E+14	4,86953E+14	5,34646E+14	6,30692E+14	5,62717E+14	5,79671E+14
TC1200000211.mm.2	Kidins220	kinase D-interacting substrate 220	1,79133E+13	7,1595E+14	2,63733E+14	0.0362530761165741	0.171159925186145	-3,53985E+14	8,41337E+14	8,34514E+14	7,48394E+14	5,53875E+14	7,10832E+14	6,06752E+14
TC0500003393.mm.2	Pom121	nuclear pore membrane protein 121	1,46122E+13	7,12492E+14	2,63708E+13	0.0362660459060442	0.171159925186145	-3,54019E+14	8,09268E+13	7,81139E+14	7,76819E+14	6,09564E+14	6,54217E+14	6,43945E+14
TC0400000425.mm.2	Phf24	PHD finger protein 24	1,14547E+14	8,2833E+14	2,63702E+14	0.0362688521155643	0.171159925186145	-3,54026E+14	9,03641E+14	9,10632E+14	8,39623E+14	7,30905E+14	8,13693E+14	7,71486E+14
TC0300001850.mm.2	Anxa5	annexin A5	1,47581E+14	7,68166E+14	2,637E+14	0.0362696454274702	0.171159925186145	-3,54028E+13	8,64614E+14	8,472E+14	8,18084E+14	6,62446E+14	7,44169E+14	6,72482E+14
TC1600001590.mm.2	Stxbp5l	syntaxin binding protein 5-like	1,09273E+14	7,75122E+14	2,63688E+14	0.0362757490542224	0.171159925186145	-3,54044E+14	8,50701E+14	8,57135E+14	7,51084E+14	7,02524E+14	7,92261E+14	6,97028E+13
TC0700000990.mm.2	Selenos	selenoprotein S	0.963045445299919	6,48834E+14	2,63647E+14	0.0362965132034446	0.17119974498265	-3,54098E+14	7,22244E+14	7,15055E+14	6,33653E+14	5,77366E+13	5,93522E+14	6,51167E+14
TC1800001017.mm.2	Aqp4	aquaporin 4	1,67138E+14	7,69904E+14	2,63629E+13	0.0363054383793033	0.17119974498265	-3,54121E+14	8,61402E+14	9,08254E+14	7,73954E+14	6,40436E+14	7,90242E+13	6,45137E+14
TC0X00000698.mm.2	Avpr2	arginine vasopressin receptor 2	-0.969873503545289	6,43906E+14	-2,63607E+14	0.0363166178651676	0.171202359175379	-3,5415E+14	5,85226E+14	5,61419E+13	6,69141E+14	7,07032E+14	6,35705E+14	7,04914E+13
TC1000003066.mm.2	Mon2	MON2 homolog, regulator of endosome to Golgi trafficking	1,21613E+14	6,94197E+14	2,63568E+14	0.036336476035584	0.171245872583026	-3,54202E+14	7,74866E+14	7,90974E+14	7,00317E+14	5,76408E+14	6,7827E+14	6,44344E+14
TC1600001213.mm.2	Rsl1d1	ribosomal L1 domain containing 1	1,80447E+14	7,25127E+14	2,63526E+14	0.0363574848218838	0.17127550028827	-3,54256E+14	8,48642E+14	8,16648E+14	7,92652E+14	5,88426E+14	6,9389E+13	6,10507E+14
TC0800002599.mm.2	Asna1	arsA arsenite transporter, ATP-binding, homolog 1 (bacterial)	1,01628E+14	8,08032E+13	2,63513E+14	0.0363640220000107	0.17127550028827	-3,54273E+14	8,58068E+13	8,75126E+14	8,11054E+14	7,74003E+14	8,06346E+14	7,23593E+14
TC0600002466.mm.2	Rmnd5a	required for meiotic nuclear division 5 homolog A	1,47213E+14	7,43255E+14	2,63448E+14	0.0363969161977222	0.171324678529048	-3,54358E+14	8,08132E+14	8,3844E+14	7,83879E+14	6,76937E+14	7,41622E+14	6,10524E+14
TC1700001772.mm.2	Pxt1	peroxisomal, testis specific 1	-0.943832561565146	5,2899E+14	-2,63447E+14	0.0363975998308722	0.171324678529048	-3,5436E+14	4,68797E+14	4,67728E+14	5,25302E+14	5,86822E+14	5,3971E+14	5,85582E+14
TC1600001214.mm.2	Gspt1	G1 to S phase transition 1	2,72419E+14	7,69436E+14	2,63422E+14	0.036410501159124	0.171324678529048	-3,54393E+14	9,33833E+14	9,39163E+14	8,17728E+14	5,97737E+14	8,04175E+14	5,23984E+14
TC0200005112.mm.2	Slpi/Mir7678	secretory leukocyte peptidase inhibitor/microRNA 7678	-0.826797723667221	5,19485E+14	-2,63389E+14	0.036427185293009	0.171324678529048	-3,54436E+14	4,61517E+14	4,77283E+14	5,15806E+13	5,58146E+14	5,53965E+14	5,50195E+14
TC0800000782.mm.2	Myo9b	myosin IXb	-0.831800543207141	7,31503E+13	-2,63388E+14	0.0364276266766286	0.171324678529048	-3,54437E+14	6,67504E+13	6,99846E+13	7,27415E+14	7,60542E+14	7,62793E+14	7,70917E+14
TC0600001931.mm.2	Cadps2	Ca2+-dependent activator protein for secretion 2	1,23688E+14	7,11871E+14	2,63322E+14	0.0364606442922353	0.171332778223069	-3,54523E+14	7,9452E+14	7,69274E+14	7,00541E+14	6,90474E+14	7,2344E+14	5,92979E+14
TC0200002415.mm.2	Ctnnbl1	catenin, beta like 1	0.81747850112223	7,97082E+14	2,63296E+14	0.0364738634254386	0.171332778223069	-3,54557E+14	8,4241E+14	8,53082E+14	7,97864E+14	7,57138E+14	7,64626E+14	7,67371E+14
TC1100000608.mm.2	Sep 08	septin 8	0.944302679714548	8,97265E+14	2,63276E+14	0.0364841537859082	0.171332778223069	-3,54583E+14	9,59091E+14	9,54012E+14	9,07887E+14	8,38357E+14	8,78458E+14	8,45784E+14
TC0800001955.mm.2	Adrb3	adrenergic receptor, beta 3	-0.88890822007225	4,64564E+14	-2,63271E+14	0.0364865832227044	0.171332778223069	-3,5459E+14	3,70975E+14	3,90642E+14	5,21021E+14	5,65349E+14	4,49131E+14	4,90268E+13
TC0100001339.mm.2	Pdc	phosducin	-1,48215E+13	4,37347E+14	-2,63242E+14	0.0365013200211585	0.171332778223069	-3,54628E+14	3,65346E+14	3,57384E+14	3,62269E+13	5,19922E+14	4,73399E+14	5,45762E+13
TC0200002318.mm.2	Dnmt3b	DNA methyltransferase 3B	-1,30754E+14	4,59298E+14	-2,63237E+14	0.0365041766100414	0.171332778223069	-3,54635E+14	3,70714E+14	3,58418E+14	4,57465E+14	5,78555E+14	4,55632E+14	5,35008E+14
TC1900000101.mm.2	Naaladl1	N-acetylated alpha-linked acidic dipeptidase-like 1	-1,17398E+14	4,62956E+14	-2,63234E+14	0.0365054512644075	0.171332778223069	-3,54638E+14	3,97442E+14	3,96235E+14	4,28563E+14	5,27021E+14	4,77945E+14	5,50528E+14
TC0700002102.mm.2	Dhcr7	7-dehydrocholesterol reductase	0.990256487307754	7,7815E+14	2,6321E+14	0.0365179015694136	0.171332778223069	-3,5467E+13	8,34158E+14	8,25807E+14	8,05078E+13	7,41945E+14	7,01245E+14	7,60669E+14
TC1900000188.mm.2	Fads1	fatty acid desaturase 1	1,47441E+14	8,71214E+14	2,63183E+14	0.0365311905130359	0.171332778223069	-3,54704E+14	9,66263E+14	9,67039E+14	9,11474E+14	7,44088E+14	8,39999E+14	7,98421E+14
TC0500002410.mm.2	Ccdc149	coiled-coil domain containing 149	1,00842E+14	7,0315E+14	2,6315E+14	0.0365483663897204	0.171332778223069	-3,54749E+14	7,96027E+14	7,50289E+14	7,03015E+13	6,2494E+14	6,76832E+14	6,678E+14
TC0300000733.mm.2	Khdc4	KH domain containing 4, pre-mRNA splicing factor	-0.922869669885559	8,70709E+14	-2,63112E+14	0.0365674439254815	0.171332778223069	-3,54798E+14	8,07068E+14	8,02153E+14	8,75056E+14	9,4618E+14	8,91368E+14	9,02427E+14
TC0500000325.mm.2	Slc4a1ap	solute carrier family 4 (anion exchanger), member 1, adaptor protein	1,1567E+14	6,51448E+14	2,63091E+14	0.0365783362926608	0.171332778223069	-3,54826E+14	7,2508E+13	7,25092E+14	6,74237E+14	5,6545E+14	6,33954E+14	5,84877E+14
TC1600002020.mm.2	Krtap19-5	keratin associated protein 19-5	-1,47117E+13	5,3772E+14	-2,63085E+13	0.0365813308477785	0.171332778223069	-3,54834E+14	4,32979E+14	4,50845E+14	5,08244E+14	6,56195E+13	5,46041E+14	6,32016E+14
TC0900001921.mm.2	Dpy19l1	dpy-19-like 1 (C. elegans)	1,16942E+14	6,69663E+14	2,63082E+14	0.0365824999969984	0.171332778223069	-3,54837E+14	7,47746E+14	7,48619E+14	6,77904E+14	5,81232E+14	6,68147E+14	5,94333E+14
TC1500002264.mm.2	Krt75	keratin 75	-1,47165E+14	4,56141E+14	-2,63037E+14	0.0366053601716525	0.171332778223069	-3,54895E+14	3,67204E+14	3,2653E+14	4,47451E+14	6,07594E+14	4,69841E+14	5,18225E+14
TC1100001874.mm.2	Rpl38	ribosomal protein L38	1,00765E+14	9,91328E+14	2,63037E+14	0.0366056053879757	0.171332778223069	-3,54896E+13	1,07737E+14	1,0469E+14	9,812E+14	9,1974E+14	9,91169E+14	9,31581E+14
TC1400000762.mm.2	Il25	interleukin 25	-1,33084E+14	5,79924E+14	-2,63028E+14	0.0366101132887747	0.171332778223069	-3,54908E+14	4,80634E+14	5,29261E+14	5,52253E+13	6,41335E+14	5,83241E+14	6,92822E+13
TC0900001979.mm.2	Aplp2	amyloid beta (A4) precursor-like protein 2	1,37204E+14	8,94991E+14	2,63001E+14	0.0366240586829647	0.171342064077313	-3,54943E+13	9,93917E+14	9,7764E+13	9,13992E+14	7,87246E+14	8,93452E+14	8,03698E+14
TC0600000621.mm.2	5730596B20Rik	RIKEN cDNA 5730596B20 gene	-0.9394948362305	4,95015E+14	-2,62962E+14	0.0366440014271641	0.171342064077313	-3,54995E+14	3,91675E+11	4,58005E+14	5,39954E+14	5,42877E+14	5,00143E+14	5,37436E+14
TC1000000142.mm.2	Ifngr1	interferon gamma receptor 1	-0.807731851773234	7,38402E+13	-2,62955E+14	0.0366473837921603	0.171342064077313	-3,55003E+14	7,03829E+14	6,83698E+14	7,49639E+14	7,4417E+14	7,58086E+14	7,90987E+13
TC0400002788.mm.2	Rnf183	ring finger protein 183	-0.934278547322288	6,56276E+14	-2,62941E+13	0.0366546325870103	0.171342064077313	-3,55022E+13	6,04958E+14	5,97651E+14	6,4876E+14	6,9682E+14	6,66199E+14	7,23266E+14
TC1300000972.mm.2	Mef2c	myocyte enhancer factor 2C	1,65349E+14	9,12437E+14	2,6291E+14	0.03667037117207	0.171365919575279	-3,55062E+14	1,00927E+14	1,06877E+14	9,00288E+14	7,48949E+14	9,32431E+14	8,14914E+14
TC0100000526.mm.2	Fastkd2	FAST kinase domains 2	0.886961752335243	5,43662E+14	2,6288E+14	0.0366854513239533	0.171386685179142	-3,55101E+14	5,98686E+14	5,97919E+14	4,91419E+13	5,54736E+14	5,11465E+14	5,07748E+14
TC1000000564.mm.2	Nodal	nodal	-1,47918E+13	6,89089E+14	-2,62811E+13	0.036721037193087	0.171503209363899	-3,55192E+14	5,80951E+14	5,9163E+14	6,95834E+14	7,97702E+14	6,64149E+14	8,04268E+14
TC0400003034.mm.2	Cdkn2b	cyclin dependent kinase inhibitor 2B	-1,12948E+14	6,3579E+14	-2,62684E+14	0.0367858965710843	0.171715962696596	-3,55358E+14	5,57283E+14	5,66398E+14	6,26164E+14	7,15321E+14	6,38983E+14	7,10594E+14
TC1000003093.mm.2	Tsfm	Ts translation elongation factor, mitochondrial	-0.78511979337262	7,79928E+14	-2,6268E+13	0.036787904377127	0.171715962696596	-3,55363E+13	7,44997E+14	7,43603E+14	7,66999E+14	7,78343E+14	8,092E+14	8,36424E+14
TC0400003456.mm.2	Rlf	rearranged L-myc fusion sequence	1,31907E+14	7,3222E+14	2,62626E+14	0.0368154954862552	0.171794983715618	-3,55434E+14	8,0461E+14	8,07956E+14	7,82328E+13	6,4967E+13	7,06848E+14	6,41005E+14
TC0300002153.mm.2	Slitrk3	SLIT and NTRK-like family, member 3	1,52797E+14	7,17198E+14	2,62573E+14	0.036842276575529	0.171802343119426	-3,55502E+14	7,89483E+13	8,13676E+14	7,69924E+14	6,3254E+14	7,06824E+14	5,90743E+14
TC0200001513.mm.2	Rag2	recombination activating gene 2	-0.992663338329742	4,86147E+14	-2,62566E+13	0.0368463010616617	0.171802343119426	-3,55513E+14	4,20962E+14	4,07217E+14	4,87925E+13	5,74066E+13	5,07112E+14	5,19599E+13
TC1300001061.mm.2	Ap3b1	adaptor-related protein complex 3, beta 1 subunit	1,0628E+14	7,14249E+14	2,62536E+14	0.0368614336730845	0.171802343119426	-3,55551E+14	7,96056E+14	7,68999E+14	7,32178E+14	6,35767E+14	6,86642E+14	6,65853E+14
TC1100002470.mm.2	Il9r	interleukin 9 receptor	-0.913267536458475	6,74364E+13	-2,62512E+14	0.0368737597199491	0.171802343119426	-3,55583E+14	6,1026E+14	6,20513E+14	6,80651E+14	7,21333E+14	6,78481E+14	7,34945E+14
TC1300002776.mm.2	Zfp87	zinc finger protein 87	1,59905E+14	6,87726E+14	2,62506E+13	0.036876701101350	0.171802343119426	-3,5559E+14	7,86652E+14	7,60677E+14	7,18917E+14	6,32591E+14	6,98875E+14	5,28643E+14
TC1200000775.mm.2	Rgs6	regulator of G-protein signaling 6	1,04653E+14	6,46529E+14	2,62498E+14	0.036881046661087	0.171802343119426	-3,55601E+14	7,25889E+14	7,30729E+13	6,2204E+13	5,53201E+13	6,59232E+13	5,8808E+14
TC0X00001651.mm.2	Rbbp7	retinoblastoma binding protein 7, chromatin remodeling factor	1,76949E+14	8,67838E+14	2,62451E+13	0.0369051190794828	0.171836190174646	-3,55663E+14	9,94113E+12	9,90431E+14	8,96324E+14	6,95513E+14	8,58645E+14	7,72002E+14
TC0900002254.mm.2	Ncam1	neural cell adhesion molecule 1	2,48115E+14	9,41447E+14	2,62389E+14	0.0369367476460311	0.171836190174646	-3,55743E+14	1,12215E+14	1,09139E+14	1,00658E+13	7,1125E+14	9,22328E+13	7,94983E+13

TC0400001477.mm.2	Sfpq	splicing factor proline/glutamine rich (polypyrimidine tract binding protein associated)	1,00332E+14	8,04264E+14	2,62372E+14	0.0369458664689381	0.171836190174646	-3,55767E+14	8,60312E+14	8,83291E+14	7,96252E+14	7,42791E+14	8,06124E+14	7,36815E+14
TC0200003264.mm.2	Spout1	SPOUT domain containing methyltransferase 1	0.798069053203684	7,43896E+14	2,62364E+13	0.0369496365949031	0.171836190174646	-3,55776E+14	7,98203E+14	7,86546E+14	7,42312E+13	7,11179E+14	7,05911E+14	7,19224E+14
TC1500001822.mm.2	Txn2	thioredoxin 2	1,10579E+14	7,57272E+14	2,62362E+14	0.036950870993696	0.171836190174646	-3,55779E+14	8,35241E+14	8,09142E+14	7,81896E+14	6,94127E+14	6,80609E+14	7,42615E+14
TC0600000648.mm.2	Chn2	chimerin 2	1,12833E+14	8,42216E+14	2,6235E+14	0.0369568735121848	0.171836190174646	-3,55795E+14	9,21245E+14	8,97444E+14	8,74051E+14	7,6753E+14	7,78768E+14	8,14254E+13
TC0800001233.mm.2	Pskh1	protein serine kinase H1	-1,04512E+14	5,26315E+14	-2,6233E+14	0.0369672681798604	0.171836190174646	-3,55821E+14	4,46031E+14	4,49198E+14	6,24766E+13	5,33641E+14	5,71376E+14	5,32877E+14
TC0100000462.mm.2	Bmpr2	bone morphogenetic protein receptor, type II (serine/threonine kinase)	2,64691E+14	7,89001E+14	2,62318E+14	0.0369736282092409	0.171836190174646	-3,55837E+14	9,85198E+14	9,93164E+14	8,15392E+14	4,91274E+14	7,8617E+14	6,6281E+13
TC0200005475.mm.2	Dusp19	dual specificity phosphatase 19	1,51265E+14	5,85187E+14	2,62244E+14	0.0370115497412526	0.171865146270695	-3,55934E+13	6,82117E+14	6,89902E+14	6,10223E+14	4,59392E+14	5,83125E+14	4,86366E+14
TC1700001483.mm.2	4930474M22Rik	RIKEN cDNA 4930474M22 gene	-0.948558393505777	5,28226E+14	-2,6223E+14	0.0370187075333137	0.171865146270695	-3,55952E+14	4,62283E+14	4,93912E+12	5,01489E+14	5,65769E+14	5,56533E+14	5,89373E+14
TC1000001266.mm.2	Ppp1r12a	protein phosphatase 1, regulatory subunit 12A	1,54453E+14	7,50445E+14	2,62227E+14	0.0370202313283647	0.171865146270695	-3,55956E+14	8,46685E+14	8,7861E+14	7,54896E+14	6,0609E+14	7,6025E+14	6,56139E+13
TC1300002463.mm.2	Iqgap2	IQ motif containing GTPase activating protein 2	0.853549076719598	5,52525E+13	2,62214E+14	0.0370267476996253	0.171865146270695	-3,55972E+14	5,89852E+14	6,14062E+14	5,54858E+14	5,23173E+14	5,35444E+13	4,9776E+14
TC1400000263.mm.2	Arf4	ADP-ribosylation factor 4	1,83768E+14	8,19654E+14	2,62202E+14	0.0370331898375133	0.171865146270695	-3,55989E+14	9,46313E+14	9,45612E+14	8,41024E+14	6,60587E+12	8,32907E+14	6,91483E+14
TC0500001610.mm.2	Ift22	intraflagellar transport 22	1,2794E+14	7,62363E+14	2,62111E+14	0.037079913338993	0.172032434100545	-3,56107E+14	8,43356E+14	8,2327E+14	7,95991E+13	7,00814E+14	7,56761E+13	6,53985E+14
TC1400000853.mm.2	C1qtnf9	C1q and tumor necrosis factor related protein 9	-1,10325E+14	6,93401E+14	-2,61955E+14	0.037160452104675	0.172309138675339	-3,56312E+14	6,07575E+14	6,24206E+14	6,89954E+14	7,86238E+14	7,00487E+14	7,51945E+14
TC0700001997.mm.2	Kndc1	kinase non-catalytic C-lobe domain (KIND) containing 1	1,08737E+14	7,17769E+14	2,61941E+14	0.0371678277929501	0.172309138675339	-3,5633E+14	7,97188E+14	7,86293E+14	7,27913E+14	6,29212E+14	7,03528E+14	6,62481E+14
TC0400003870.mm.2	Crocc	ciliary rootlet coiled-coil, rootletin	-0.846477933505213	6,67784E+14	-2,61928E+14	0.0371745794656713	0.172309138675339	-3,56347E+14	6,23822E+14	6,17994E+14	6,5158E+14	7,02199E+14	6,98168E+13	7,12943E+14
TC0X00001684.mm.2	Trappc2	trafficking protein particle complex 2	1,65082E+14	7,07645E+14	2,61913E+14	0.0371823294963169	0.172309138675339	-3,56367E+14	8,26817E+14	8,25157E+14	7,1854E+14	5,53544E+14	7,18459E+14	6,03352E+14
TC1400000455.mm.2	Ang3/Ang5	angiogenin, ribonuclease A family, member 3/angiogenin, ribonuclease A family, member 5	-1,12026E+14	4,67255E+14	-2,61881E+14	0.037198767868555	0.172318719126038	-3,56409E+14	3,91865E+14	3,93391E+14	4,51447E+14	5,57517E+14	4,84258E+12	5,2505E+14
TC1700002526.mm.2	Xdh	xanthine dehydrogenase	-0.902609192741442	4,8869E+14	-2,61868E+14	0.0372057856289634	0.172318719126038	-3,56426E+14	4,28748E+13	4,86121E+14	4,52555E+14	4,69325E+14	5,31194E+14	5,64197E+14
TSUnmapped00000048.mm.2	Egr3	early growth response 3	-0.799846655732395	8,2618E+14	-2,61791E+14	0.0372452000961453	0.172451698031376	-3,56526E+14	7,72962E+12	7,77382E+14	8,41046E+14	8,55377E+14	8,38106E+14	8,72208E+14
TC1100001777.mm.2	Psmc5	protease (prosome, macropain) 26S subunit, ATPase 5	1,56391E+14	7,26058E+14	2,61726E+14	0.0372790963199792	0.172513364351698	-3,56612E+14	8,48933E+14	8,72343E+14	6,88521E+14	5,38055E+13	7,59283E+14	6,49211E+14
TC0600002799.mm.2	Uba3	ubiquitin-like modifier activating enzyme 3	1,84156E+14	8,13863E+14	2,61724E+14	0.0372799314015151	0.172513364351698	-3,56614E+14	9,36765E+14	9,46105E+14	8,32269E+14	6,5348E+14	8,29744E+14	6,84815E+14
TC0100001114.mm.2	Mgat5	mannoside acetylglucosaminyltransferase 5	0.769005907116648	7,75865E+14	2,61687E+14	0.0372991276589089	0.17255263966925	-3,56662E+14	8,39426E+14	8,32692E+14	7,38672E+14	7,26084E+14	7,50719E+14	7,67598E+14
TC0900001781.mm.2	Panx1	pannexin 1	0.863130401502192	6,6999E+14	2,61652E+14	0.0373174951304982	0.172584269844768	-3,56708E+14	7,36228E+14	6,80983E+14	6,72034E+14	6,86108E+14	6,16645E+14	6,2794E+14
TC0900003330.mm.2	Onecut1	one cut domain, family member 1	-0.869486940844469	5,23758E+14	-2,61633E+14	0.0373273866076471	0.172584269844768	-3,56733E+14	4,73361E+14	4,55588E+14	5,47995E+14	5,62759E+14	5,23797E+14	5,79049E+14
TC0700000629.mm.2	Scgb1b30	secretoglobin, family 1B, member 30	-0.879961648206131	7,0922E+14	-2,61449E+14	0.0374229166167917	0.172976321126324	-3,56974E+14	6,49855E+14	6,45294E+14	7,13331E+14	7,75697E+14	7,32366E+14	7,38775E+14
TC0500003055.mm.2	Svop	SV2 related protein	1,38848E+14	7,39796E+14	2,6142E+14	0.0374381291832465	0.172997010475954	-3,57012E+14	8,16288E+14	8,31767E+14	7,60424E+14	6,59939E+14	7,47081E+14	6,23278E+14
TC0600002878.mm.2	Crbn	cereblon	1,47695E+14	8,07368E+14	2,61399E+14	0.0374492418098055	0.172998748073795	-3,5704E+14	8,92036E+13	8,95061E+14	8,41301E+13	7,24103E+14	8,10462E+14	6,81245E+14
TC1900000962.mm.2	Tsga10ip	testis specific 10 interacting protein	-1,37755E+14	5,54351E+14	-2,61329E+14	0.037485520251041	0.173080455953925	-3,57131E+14	4,67067E+14	4,79924E+14	5,10924E+14	6,45691E+14	5,70043E+14	6,52457E+14
TC0400001766.mm.2	Rcc2	regulator of chromosome condensation 2	0.775426922594438	8,23979E+14	2,61315E+13	0.0374929957793185	0.173080455953925	-3,5715E+14	8,6446E+14	8,50891E+14	8,31461E+14	8,36799E+14	7,69014E+14	7,91252E+14
TC0500001087.mm.2	Rpl5	ribosomal protein L5	1,50417E+14	8,83573E+14	2,61303E+14	0.0374991542068611	0.173080455953925	-3,57165E+14	9,96473E+14	1,00038E+14	8,76332E+14	7,32233E+14	9,00973E+14	7,95047E+14
TC1900000953.mm.2	Klc2	kinesin light chain 2	0.951630779357243	8,63077E+14	2,61202E+12	0.0375522218585643	0.173225635980082	-3,57298E+14	9,19293E+14	9,09904E+14	8,69478E+14	8,40919E+14	8,56684E+14	7,82187E+14
TC1500001798.mm.2	Mb	myoglobin	-0.775269761838415	6,77043E+14	-2,61185E+14	0.037560714030391	0.173225635980082	-3,5732E+14	6,10527E+14	6,32207E+13	7,07419E+14	7,14314E+13	6,91889E+14	7,05899E+14
TC0800001551.mm.2	Sprtn	SprT-like N-terminal domain	-0.795095094607762	7,3874E+14	-2,61176E+14	0.0375658007126071	0.173225635980082	-3,57332E+14	6,98972E+14	6,85234E+14	7,65929E+14	7,39081E+14	7,81513E+14	7,61711E+14
TC1800000171.mm.2	Asxl3	additional sex combs like 3, transcriptional regulator	0.859201343382418	5,6429E+14	2,61161E+14	0.0375736112300867	0.173225635980082	-3,57352E+14	6,33255E+14	5,80595E+14	5,53445E+14	5,76433E+14	5,21572E+14	5,20438E+14
TC0700002162.mm.2	Syt5	synaptotagmin V	1,66726E+14	9,32713E+14	2,61079E+14	0.0376161985212034	0.173281986831861	-3,57458E+14	1,06834E+14	1,05268E+14	9,4441E+14	7,43293E+14	9,10659E+13	8,76902E+13
TC1900000124.mm.2	Trmt112	tRNA methyltransferase 11-2	0.844766830115905	9,01277E+14	2,61079E+14	0.0376165108401287	0.173281986831861	-3,57459E+14	9,61263E+14	9,43453E+14	9,07689E+14	8,59492E+14	8,60682E+13	8,75081E+14
TC1200002299.mm.2	Syne3	spectrin repeat containing, nuclear envelope family member 3	-0.862062294952987	6,86155E+14	-2,61064E+14	0.0376240218079086	0.173281986831861	-3,57478E+14	6,30142E+14	6,36414E+14	6,76716E+14	7,34689E+14	7,20692E+14	7,18276E+14
TC0400001512.mm.2	Tmem54	transmembrane protein 54	-0.830188318903224	6,00485E+14	-2,61021E+14	0.0376465644595473	0.173281986831861	-3,57534E+14	5,40295E+14	5,48171E+14	6,25355E+14	6,34585E+14	6,0356E+14	6,50944E+14
TC0X00000862.mm.2	Gm44	predicted gene 44	-1,02034E+14	5,24878E+14	-2,61002E+14	0.0376568251028566	0.173281986831861	-3,5756E+14	4,34368E+14	4,83534E+14	5,17082E+14	5,92315E+14	5,45488E+14	5,76482E+13
TC0300002583.mm.2	Man1a2	mannosidase, alpha, class 1A, member 2	1,62263E+14	7,64177E+14	2,60991E+14	0.0376624573986977	0.173281986831861	-3,57574E+14	8,64853E+14	8,78133E+14	7,54047E+14	6,69568E+14	7,98369E+14	6,20091E+14
TC1200001919.mm.2	D830013O20Rik	RIKEN cDNA D830013O20 gene	-0.858716431683394	5,40762E+13	-2,6098E+14	0.0376682834042891	0.173281986831861	-3,57589E+14	4,75621E+14	4,88832E+14	5,45526E+14	5,98399E+14	5,58929E+14	5,77268E+14
TC0200002396.mm.2	Aar2	AAR2 splicing factor homolog	1,2245E+14	5,74701E+13	2,60955E+14	0.0376812389562236	0.173281986831861	-3,57621E+14	6,72977E+13	6,30428E+14	5,08888E+14	5,77407E+14	4,76347E+14	5,82157E+14
TC0400000036.mm.2	Ubxn2b	UBX domain protein 2B	0.882706821672108	6,75574E+14	2,60943E+14	0.0376878125092527	0.173281986831861	-3,57637E+14	7,37756E+14	7,32762E+14	6,53716E+14	6,35237E+12	6,77537E+14	6,16438E+13
TC0X00000859.mm.2	4932429P05Rik	RIKEN cDNA 4932429P05 gene	-1,48679E+14	4,39426E+14	-2,60932E+14	0.0376933757739511	0.173281986831861	-3,57651E+14	2,9636E+14	3,99507E+14	4,10492E+13	5,36968E+14	4,63057E+14	5,30169E+14
TC1900001095.mm.2	Fads2	fatty acid desaturase 2	1,64844E+14	9,6587E+14	2,60892E+14	0.0377145288999065	0.173329778711978	-3,57704E+14	1,08601E+14	1,0695E+14	1,00268E+14	8,1122E+14	9,4372E+14	8,82094E+14
TC1800001712.mm.2	Cndp2	CNDP dipeptidase 2 (metallopeptidase M20 family)	0.894527893439633	7,12454E+14	2,60844E+14	0.0377394624268254	0.173343755465398	-3,57766E+14	7,84305E+13	7,74413E+14	6,75096E+14	6,61096E+14	6,57449E+14	7,22363E+14
TC0600001319.mm.2	Zfp637	zinc finger protein 637	0.830234974320107	7,19198E+14	2,60841E+14	0.0377410209000773	0.173343755465398	-3,5777E+14	7,90423E+14	7,67362E+14	6,66849E+14	6,98814E+14	6,81221E+13	7,10517E+14
TC1600001178.mm.2	Usp7	ubiquitin specific peptidase 7	2,95356E+12	7,59566E+14	2,60783E+13	0.0377719055953387	0.173343755465398	-3,57847E+14	9,37489E+14	9,3329E+14	8,49151E+14	5,57398E+13	7,71583E+14	5,08484E+14
TC0X00001534.mm.2	Smc1a	structural maintenance of chromosomes 1A	1,77725E+14	7,51051E+14	2,6078E+14	0.0377733350469174	0.173343755465398	-3,57851E+14	8,74728E+14	8,76826E+14	7,69406E+14	5,89245E+14	7,6258E+14	6,33524E+13
TC0200001365.mm.2	Rapsn	receptor-associated protein of the synapse	-1,23119E+14	6,00128E+14	-2,60756E+14	0.0377859164821273	0.173343755465398	-3,57882E+14	5,1904E+14	5,27363E+14	5,89272E+14	6,7245E+14	5,93721E+14	6,9892E+14
TC1100003688.mm.2	Nr1d1	nuclear receptor subfamily 1, group D, member 1	0.988054075511972	8,00388E+13	2,60755E+14	0.037786475545986	0.173343755465398	-3,57883E+13	8,43398E+14	8,54255E+14	8,42201E+14	7,62433E+14	7,68513E+14	7,31528E+14
TC1000000592.mm.2	Dna2	DNA replication helicase/nuclease 2	-0.830132083185266	5,38945E+14	-2,60743E+14	0.0377928761217615	0.173343755465398	-3,57899E+14	4,71695E+14	4,96444E+14	5,44907E+14	5,8646E+14	5,71537E+14	5,62628E+14
TC0500000436.mm.2	Otop1	otopetrin 1	-1,0558E+14	5,00812E+13	-2,60689E+13	0.0378208849184794	0.173422856770478	-3,57969E+13	4,50309E+14	4,19444E+14	4,83748E+14	5,70458E+13	5,18055E+14	5,62858E+14
TC0600000920.mm.2	Lbx2	ladybird homeobox 2	-0.991172203701865	6,24396E+13	-2,60623E+14	0.0378559134129339	0.173534091841424	-3,58056E+14	5,61259E+14	5,66019E+14	6,05218E+14	6,88364E+14	6,46568E+14	6,78945E+14
TC1900000443.mm.2	Ric1	RAB6A GEF complex partner 1	1,42172E+13	5,62432E+14	2,60553E+14	0.0378930251247132	0.173617334970763	-3,58148E+13	6,19839E+14	6,62969E+14	6,15413E+14	4,77908E+14	5,40788E+14	4,57676E+14
TC0200002010.mm.2	Cdc25b	cell division cycle 25B	-1,03141E+14	5,48329E+14	-2,60548E+14	0.0378956226085878	0.173617334970763	-3,58155E+14	4,43899E+14	4,82904E+14	5,78353E+14	6,51734E+14	5,40898E+14	5,92187E+13
TC1700000842.mm.2	Enpp5	ectonucleotide pyrophosphatase/phosphodiesterase 5	2,00646E+14	9,19363E+14	2,60512E+14	0.0379144014550766	0.173653993929974	-3,58201E+14	1,03754E+14	1,05683E+13	9,55988E+14	7,72761E+14	9,35603E+14	7,57467E+14
TC0100002576.mm.2	Tns1	tensin 1	0.992262532349065	6,58052E+14	2,60456E+14	0.0379438720828352	0.173711499652137	-3,58274E+14	7,21161E+14	7,03938E+14	6,5132E+14	6,45244E+14	5,69405E+13	6,57242E+14
TC0900000845.mm.2	Spg21	SPG21, maspardin	1,39228E+14	6,4226E+14	2,60431E+14	0.037957288446833	0.173711499652137	-3,58308E+14	7,40648E+14	7,67759E+14	6,24657E+13	4,90546E+14	5,71907E+14	6,58044E+14
TC0600001154.mm.2	Gxylt2	glucoside xylosyltransferase 2	-0.899863018758804	4,84513E+13	-2,60405E+14	0.0379712401359262	0.173711499652137	-3,58342E+14	4,46218E+13	4,0501E+14	4,92167E+14	5,32481E+14	4,96779E+13	5,34422E+13
TC1100000943.mm.2	Slc16a11	solute carrier family 16 (monocarboxylic acid transporters), member 11	-1,10414E+14	7,29953E+14	-2,60394E+14	0.0379769429862417	0.173711499652137	-3,58356E+14	6,41228E+14	6,73862E+14	7,38739E+14	7,89972E+14	7,21009E+14	8,14907E+14

TC0400000702.mm.2	Bspry	B-box and SPRY domain containing	-0.843071344225695	5,68531E+14	-2,60386E+14	0.0379808609988506	0.173711499652137	-3,58366E+14	5,22544E+14	5,06804E+14	5,67203E+14	6,16672E+14	5,92463E+14	6,05499E+14
TC1500001724.mm.2	Cyp11b2	cytochrome P450, family 11, subfamily b, polypeptide 2	-0.925328814541335	4,5893E+13	-2,60361E+14	0.0379943794671152	0.173724017126455	-3,58399E+14	3,73636E+14	3,9311E+14	5,14565E+14	5,20457E+14	4,39804E+14	5,12008E+14
TC0X00000063.mm.2	Gpkow	G patch domain and KOW motifs	0.757699540445576	7,12323E+14	2,60299E+14	0.0380272310597923	0.173824900444378	-3,58481E+14	7,29772E+14	7,37051E+13	7,53319E+14	7,38514E+14	6,59027E+13	6,56256E+13
TC1400001995.mm.2	Rnase9	ribonuclease, RNase A family, 9 (non-active)	-1,15632E+14	5,9894E+14	-2,60265E+14	0.0380450392279071	0.173856981587994	-3,58525E+14	5,30958E+14	5,39585E+14	5,50518E+14	6,70774E+14	6,43269E+14	6,58538E+14
TC0700003442.mm.2	Mef2a	myocyte enhancer factor 2A	1,34445E+14	9,55687E+12	2,60154E+14	0.0381039155729429	0.17401690817088	-3,5867E+14	1,0146E+14	1,03516E+14	1,01592E+14	8,87576E+14	9,31775E+14	8,49092E+14
TC0800002008.mm.2	Ubxn8	UBX domain protein 8	0.810411003542281	7,08037E+11	2,60134E+14	0.0381144483368187	0.17401690817088	-3,58696E+14	7,80243E+14	7,53575E+14	6,72362E+14	6,70309E+14	6,68917E+14	7,02818E+14
TC0600000542.mm.2	Zfp212	Zinc finger protein 212	-0.867095353490863	6,67742E+14	-2,60088E+14	0.0381388697703076	0.17401690817088	-3,58756E+14	5,92364E+13	6,27169E+14	6,80199E+14	7,13769E+14	6,78182E+14	7,14769E+14
TC0900002325.mm.2	Wdr61	WD repeat domain 61	1,15656E+14	6,63614E+14	2,60074E+14	0.0381465885890763	0.17401690817088	-3,58776E+14	7,73057E+14	7,47308E+14	6,45472E+14	5,26797E+14	6,48721E+14	6,40332E+14
TC0200001940.mm.2	Zc3h6	zinc finger CCCH type containing 6	1,62924E+13	5,91941E+14	2,6005E+12	0.0381590334671246	0.17401690817088	-3,58806E+14	6,85484E+13	6,80756E+13	6,39757E+14	5,05257E+14	5,93939E+14	4,46452E+14
TC0600000978.mm.2	Add2	adducin 2 (beta)	0.949533402375819	7,86709E+14	2,60034E+14	0.0381679244707168	0.17401690817088	-3,58828E+14	8,65598E+14	8,36918E+14	7,9031E+14	7,14817E+14	7,57875E+14	7,54734E+14
TC0500001193.mm.2	Tmem211	transmembrane protein 211	-0.88469083132952	4,96922E+14	-2,6E+13	0.0381859268342482	0.17401690817088	-3,58872E+14	4,34621E+14	4,52067E+14	4,8848E+14	5,42737E+13	5,15806E+13	5,47821E+14
TC0100003795.mm.2	Rps6kc1	ribosomal protein S6 kinase polypeptide 1	1,04561E+13	5,85474E+13	2,6E+14	0.0381860268952411	0.17401690817088	-3,58873E+14	6,61051E+14	6,32887E+14	5,96135E+13	5,37953E+14	5,03664E+14	5,81152E+14
TC0700001439.mm.2	Phox2a	paired-like homeobox 2a	-1,31923E+14	6,77844E+13	-2,59952E+14	0.0382112663990412	0.17401690817088	-3,58935E+14	5,78343E+14	5,92579E+14	6,59551E+14	8,01822E+14	6,94676E+14	7,40091E+14
TC1000003106.mm.2	Kif5a	kinesin family member 5A	1,53412E+14	9,96041E+14	2,59941E+13	0.0382173894958877	0.17401690817088	-3,5895E+14	1,10262E+14	1,10607E+14	1,01249E+14	8,53194E+14	9,97824E+14	9,04047E+14
TC0200001985.mm.2	Ptpra	protein tyrosine phosphatase, receptor type, A	1,79656E+14	7,86645E+14	2,59902E+14	0.0382379915726743	0.17401690817088	-3,59001E+14	9,10213E+14	9,08504E+14	8,24397E+14	6,17351E+14	7,74327E+14	6,85079E+14
TC0X00000981.mm.2	Ogt	O-linked N-acetylglucosamine (GlcNAc) transferase (UDP-N-acetylglucosamine:polypeptide-N-acetylglucosaminyl transferase)	1,86727E+14	7,34505E+13	2,59876E+14	0.0382517905722982	0.17401690817088	-3,59035E+13	8,71086E+14	8,61794E+14	7,56141E+14	5,58584E+14	7,44264E+14	6,15164E+14
TC1200000712.mm.2	Eif2s1	eukaryotic translation initiation factor 2, subunit 1 alpha	2,37198E+14	6,68315E+14	2,59873E+14	0.0382532634801443	0.17401690817088	-3,59038E+14	8,10747E+14	8,35667E+14	7,26884E+14	4,64577E+14	6,68327E+14	5,03691E+14
TC1200000278.mm.2	Ccdc71l	coiled-coil domain containing 71 like	-0.775075689701985	6,0531E+14	-2,59838E+14	0.0382722112262614	0.17401690817088	-3,59085E+13	5,47225E+14	5,6785E+14	6,21449E+14	6,25245E+14	6,2522E+14	6,4487E+13
TC0800000831.mm.2	Nwd1	NACHT and WD repeat domain containing 1	1,26341E+14	6,14534E+14	2,59831E+14	0.0382758999280166	0.17401690817088	-3,59094E+14	6,71645E+14	7,2687E+14	5,53916E+14	5,88941E+14	5,10639E+14	6,35194E+14
TC0700002076.mm.2	Tnni2	troponin I, skeletal, fast 2	-1,02168E+14	6,24548E+14	-2,5982E+14	0.0382814746601068	0.17401690817088	-3,59107E+14	5,47229E+14	5,47637E+14	6,60145E+14	6,93073E+14	6,04366E+14	6,94835E+14
TC0X00002431.mm.2	Pnck	pregnancy upregulated non-ubiquitously expressed CaM kinase	1,87343E+14	7,7E+14	2,59815E+14	0.0382843685282897	0.17401690817088	-3,59115E+14	9,45904E+14	8,56346E+14	8,04646E+14	5,85542E+14	7,2128E+14	7,06285E+14
TC1100001535.mm.2	Mrpl45	mitochondrial ribosomal protein L45	0.992210568937596	7,16937E+14	2,59815E+14	0.0382844433796905	0.17401690817088	-3,59115E+13	7,92174E+14	7,73447E+14	7,20211E+14	6,48608E+14	7,09385E+14	6,57795E+14
TC0400001722.mm.2	Ddost	dolichyl-di-phosphooligosaccharide-protein glycotransferase	1,28579E+14	8,62515E+14	2,59807E+13	0.0382887149178773	0.17401690817088	-3,59125E+14	9,75643E+14	9,28217E+14	8,78709E+14	7,45818E+14	8,07746E+13	8,38955E+14
TC0500002311.mm.2	Gm7854	predicted gene 7854	-0.851546188199467	8,02194E+14	-2,59774E+14	0.0383060094054839	0.17401690817088	-3,59168E+14	7,50536E+14	7,49054E+14	8,10444E+14	8,33232E+14	8,09062E+14	8,60838E+14
TC0800002372.mm.2	Zfp963/Zfp866	zinc finger protein 963/zinc finger protein 866	1,31546E+14	6,36092E+14	2,59773E+14	0.0383068313338367	0.17401690817088	-3,5917E+14	7,28106E+14	7,04968E+14	5,95531E+14	6,17963E+13	6,58004E+14	5,11977E+14
TC1800000677.mm.2	Zfp532	zinc finger protein 532	1,29866E+14	6,33964E+14	2,59601E+14	0.038398670272537	0.174338975914756	-3,59395E+14	6,75055E+14	6,76787E+14	7,28609E+14	6,31223E+14	6,01012E+14	4,91098E+14
TC1500001661.mm.2	Zfat	zinc finger and AT hook domain containing	-1,22714E+14	6,19417E+14	-2,596E+14	0.0383993685546744	0.174338975914756	-3,59397E+14	5,63292E+14	5,02328E+14	6,10311E+14	7,2952E+14	6,48569E+14	6,62479E+14
TC1500001008.mm.2	Spats2	spermatogenesis associated, serine-rich 2	1,62462E+14	6,72381E+14	2,59572E+14	0.0384143653444378	0.17435793487181	-3,59433E+12	7,78772E+13	7,81482E+14	7,11342E+14	5,2736E+13	6,56095E+14	5,79233E+14
TC0200002872.mm.2	Optn	optineurin	0.769224364403961	5,38834E+14	2,59515E+14	0.0384447820849285	0.174380075674169	-3,59508E+14	6,04444E+14	5,80184E+13	5,1265E+14	5,04945E+14	5,18304E+14	5,1248E+13
TC0500003648.mm.2	Hmgb1	high mobility group box 1	1,13902E+14	9,7375E+14	2,59502E+14	0.0384517132511658	0.174380075674169	-3,59525E+14	1,05242E+14	1,06294E+14	9,651E+14	8,74478E+13	9,8198E+14	9,05578E+14
TC0300001554.mm.2	Cryz	crystallin, zeta	1,09367E+14	6,35855E+14	2,59484E+14	0.0384615786491206	0.174380075674169	-3,59549E+14	7,07501E+14	6,85193E+14	6,04248E+14	6,44231E+13	5,36648E+14	6,37312E+14
TC0900002652.mm.2	Pigb	phosphatidylinositol glycan anchor biosynthesis, class B	0.91059296750189	6,49809E+14	2,59439E+14	0.0384856166238481	0.174380075674169	-3,59608E+14	6,99886E+14	6,91404E+14	6,62812E+14	6,35583E+14	6,378E+14	5,71371E+14
TC0900001234.mm.2	Rbp2	retinol binding protein 2, cellular	-0.782562654031048	5,27144E+14	-2,59428E+14	0.0384916198563742	0.174380075674169	-3,59622E+14	5,02835E+14	4,91143E+14	5,079E+14	5,10496E+14	5,59903E+14	5,90587E+14
TC0600002646.mm.2	2610306M01Rik	RIKEN cDNA 2610306M01 gene	-0.919854699845551	5,90362E+14	-2,59427E+14	0.038492194373458	0.174380075674169	-3,59624E+14	5,66058E+14	5,3595E+13	5,49644E+13	6,04541E+14	6,22794E+14	6,63185E+14
TC0200001847.mm.2	Duox1	dual oxidase 1	-0.764773540319562	5,01783E+14	-2,59421E+14	0.0384949996383677	0.174380075674169	-3,59631E+14	4,55455E+13	4,38083E+14	5,43273E+14	5,27394E+14	5,24425E+14	5,22068E+14
TC0200002788.mm.2	Gata5os	GATA binding protein 5, opposite strand	-0.858636304625303	6,46008E+14	-2,59395E+14	0.0385089823007629	0.174382981753263	-3,59665E+14	5,60376E+14	6,02691E+13	6,98098E+13	6,80091E+14	6,4896E+14	6,85834E+14
TC1700001683.mm.2	A930017K11Rik	RIKEN cDNA A930017K11 gene	-1,02437E+14	6,8833E+14	-2,5938E+14	0.0385172861701647	0.174382981753263	-3,59685E+14	6,0812E+13	6,12097E+14	7,1771E+14	7,66963E+14	6,70712E+14	7,5438E+14
TC1500000967.mm.2	Tmem106c	transmembrane protein 106C	0.963341259957949	5,59452E+14	2,59316E+14	0.0385513531303714	0.174488189042043	-3,59768E+14	6,38354E+14	5,76602E+13	5,88436E+14	5,31036E+14	5,08716E+14	5,13571E+14
TC1100001668.mm.2	Arl4d	ADP-ribosylation factor-like 4D	-1,21472E+14	8,36586E+14	-2,59254E+13	0.0385846268065643	0.174558494214486	-3,59849E+14	7,60026E+14	7,62184E+14	8,06626E+14	9,25529E+14	8,53105E+14	9,12048E+14
TC0X00002165.mm.2	Aifm1	apoptosis-inducing factor, mitochondrion-associated 1	1,33246E+14	7,33252E+14	2,59247E+14	0.0385885531181033	0.174558494214486	-3,59859E+14	8,19714E+14	8,19229E+14	7,55942E+14	6,3218E+14	7,31059E+14	6,41391E+14
TC1200000303.mm.2	Snx13	sorting nexin 13	1,44965E+14	7,07078E+14	2,59193E+13	0.0386175475513792	0.174636751181523	-3,5993E+14	8,2166E+13	8,17974E+14	6,82702E+14	5,70429E+14	7,40034E+14	6,09671E+14
TC0300000322.mm.2	Jade1	jade family PHD finger 1	0.786332729230633	6,24701E+14	2,59166E+14	0.0386321792973099	0.174636751181523	-3,59965E+14	6,47196E+14	6,61932E+14	6,56549E+14	6,30668E+14	5,66781E+14	5,85081E+14
TC0400001737.mm.2	Rnf186	ring finger protein 186	-0.927156384678613	6,79408E+14	-2,59131E+14	0.0386510461055107	0.174636751181523	-3,60011E+14	6,11458E+14	6,39561E+14	6,72016E+14	7,16962E+14	6,90671E+14	7,4578E+14
TC0200004813.mm.2	Napb	N-ethylmaleimide sensitive fusion protein attachment protein beta	1,90879E+14	9,60105E+14	2,59117E+14	0.0386584287043634	0.174636751181523	-3,60029E+13	1,09053E+14	1,07126E+14	1,00744E+14	8,11366E+14	9,6136E+14	8,18674E+14
TC1600000651.mm.2	Usf3	upstream transcription factor family member 3	0.858943888254503	7,64785E+14	2,59114E+14	0.0386600441546884	0.174636751181523	-3,60033E+13	8,08682E+14	8,18877E+14	7,54864E+14	7,50517E+14	7,57007E+14	6,98764E+14
TC0300003232.mm.2	Glt28d2	glycosyltransferase 28 domain containing 2	-1,12082E+14	7,60621E+14	-2,59057E+14	0.0386912299690064	0.174694105143289	-3,60109E+14	6,90566E+13	6,79236E+14	7,70595E+14	8,29364E+14	7,47296E+13	8,46669E+14
TC1900001617.mm.2	Nt5c2	5'-nucleotidase, cytosolic II	1,73609E+14	7,21617E+14	2,59051E+12	0.0386944244558058	0.174694105143289	-3,60117E+14	8,39355E+14	8,28544E+14	7,61575E+12	5,7955E+14	7,19283E+14	6,01396E+14
TC0500000286.mm.2	Hadhb	hydroxyacyl-Coenzyme A dehydrogenase/3-ketoacyl-Coenzyme A thiolase/enoyl-Coenzyme A hydratase (trifunctional protein), beta subunit	1,05048E+14	7,48885E+14	2,59025E+14	0.0387082542662776	0.174701031395762	-3,6015E+13	8,01938E+14	8,06139E+14	7,86291E+14	7,00962E+14	7,27437E+14	6,70544E+14
TC0300003221.mm.2	Wdr77	WD repeat domain 77	0.874915066815418	7,39528E+14	2,59004E+14	0.0387194962886929	0.174701031395762	-3,60177E+14	7,91322E+14	8,03915E+14	7,39381E+14	6,82299E+14	7,22787E+14	6,97467E+14
TC0200002544.mm.2	Cd40	CD40 antigen	-1,06698E+14	6,52255E+14	-2,58957E+14	0.038745039884139	0.174701031395762	-3,60239E+14	5,82282E+14	6,049E+14	6,18085E+14	7,07683E+14	6,74418E+14	7,2616E+14
TC0700000446.mm.2	Cyp2a12	cytochrome P450, family 2, subfamily a, polypeptide 12	-0.852925237512471	5,13174E+14	-2,58948E+14	0.0387495469254627	0.174701031395762	-3,6025E+14	4,44508E+14	4,58976E+14	5,31826E+14	5,69664E+14	5,57617E+14	5,16452E+14
TC0700004273.mm.2	Lat	linker for activation of T cells	-0.912438505424849	6,37501E+14	-2,58939E+14	0.0387545585848866	0.174701031395762	-3,60263E+14	5,73215E+14	5,77993E+14	6,50822E+14	6,89282E+14	6,35824E+14	6,97872E+13
TC1100002091.mm.2	Inpp5j	inositol polyphosphate 5-phosphatase J	1,28748E+14	5,90261E+13	2,58927E+14	0.0387610120548533	0.174701031395762	-3,60278E+14	6,81148E+14	6,76538E+14	5,8925E+13	4,9444E+14	6,05256E+13	4,94935E+14
TC0400004110.mm.2	Dffb	DNA fragmentation factor, beta subunit	-0.958873727063761	6,05695E+14	-2,58902E+14	0.0387744447796936	0.174712703785012	-3,60311E+14	5,27393E+13	5,57776E+14	6,16937E+14	6,55117E+14	6,04403E+14	6,72541E+14
TC1700002316.mm.2	Sema6b	sema domain, transmembrane domain (TM), and cytoplasmic domain, (semaphorin) 6B	0.766801214863257	8,18689E+14	2,5886E+14	0.0387974856625318	0.174766470987382	-3,60367E+13	8,70258E+13	8,53831E+14	8,01093E+14	8,16221E+14	7,78865E+14	7,91864E+14
TC1400001752.mm.2	Nrg3	neuregulin 3	0.899274492907184	6,20958E+14	2,5884E+14	0.0388080700796161	0.174766470987382	-3,60392E+14	6,64594E+14	6,81153E+14	5,73541E+14	6,40567E+14	5,88856E+14	5,77037E+13
TC1700000473.mm.2	Def6	differentially expressed in FDCP 6	-0.77941939007781	5,87051E+14	-2,58795E+14	0.0388323931635654	0.174805547964116	-3,60451E+14	5,444E+14	5,44172E+14	5,78488E+14	6,1079E+14	6,21169E+13	6,23287E+14
TC1200002285.mm.2	Serpina1e	serine (or cysteine) peptidase inhibitor, clade A, member 1E	-1,03521E+14	6,17784E+14	-2,58784E+14	0.0388384448402863	0.174805547964116	-3,60466E+14	5,33634E+14	5,56981E+14	6,10931E+14	7,07504E+14	6,4404E+14	6,53617E+14

TC1100002728.mm.2	Leap2	liver-expressed antimicrobial peptide 2	-1,08501E+14	4,8928E+14	-2,58756E+14	0.0388537482404321	0.174825592124569	-3,60503E+14	3,84373E+12	4,34176E+14	4,92074E+14	5,89504E+14	5,15378E+14	5,20174E+14
TC0800001253.mm.2	Cdh3	cadherin 3	-0.905391418681993	4,95159E+13	-2,587E+14	0.0388838280894427	0.174902063115237	-3,60576E+14	4,61383E+13	4,46844E+14	4,51922E+14	5,21498E+14	5,40094E+14	5,49211E+14
TC0600000676.mm.2	Avl9	AVL9 cell migration associated	1,56501E+14	6,85125E+14	2,58666E+14	0.0389023804349403	0.174902063115237	-3,60621E+14	7,65497E+14	8,07877E+14	7,00425E+14	5,76576E+14	6,99699E+14	5,60673E+14
TC0200004455.mm.2	Pla2g4e	phospholipase A2, group IVE	1,07622E+14	6,34141E+13	2,58653E+13	0.0389096391724439	0.174902063115237	-3,60638E+14	7,00221E+14	6,9798E+14	6,62181E+14	5,61506E+13	5,7206E+14	6,10897E+14
TC1600001184.mm.2	Grin2a	glutamate receptor, ionotropic, NMDA2A (epsilon 1)	1,18819E+14	7,562E+14	2,58644E+14	0.0389141622458961	0.174902063115237	-3,60649E+14	8,38953E+14	8,77145E+14	7,16399E+14	6,26243E+14	7,80877E+14	6,97584E+13
TC1700000464.mm.2	Pacsin1	protein kinase C and casein kinase substrate in neurons 1	1,37722E+14	9,04202E+14	2,58532E+14	0.0389753563213117	0.175128253319937	-3,60797E+14	1,00722E+14	9,87182E+14	9,27867E+13	7,83986E+13	8,94036E+14	8,2492E+14
TC1400000507.mm.2	Samd4	sterile alpha motif domain containing 4	1,0851E+14	7,00028E+14	2,58453E+14	0.0390180352530724	0.175196873303466	-3,609E+14	7,73696E+14	7,59619E+14	7,26465E+14	6,24091E+14	6,76682E+14	6,39615E+14
TC0400001431.mm.2	Epha10	Eph receptor A10	-0.894535126084231	7,57575E+14	-2,58393E+14	0.0390507676465608	0.175196873303466	-3,60978E+14	6,98033E+14	7,11642E+14	7,55776E+14	7,91416E+14	7,65391E+14	8,23191E+14
TC1000002253.mm.2	Tspan15	tetraspanin 15	0.836724124673555	6,40637E+14	2,58389E+14	0.0390532770263306	0.175196873303466	-3,60985E+14	7,14773E+14	7,04399E+14	6,10126E+14	5,62697E+14	6,37223E+14	6,14604E+14
TC0700002075.mm.2	Syt8	synaptotagmin VIII	-1,22375E+14	6,17113E+14	-2,58388E+14	0.0390536081729012	0.175196873303466	-3,60985E+13	5,28199E+14	5,55271E+14	5,96796E+14	6,94193E+14	6,19355E+14	7,08866E+14
TC1600000665.mm.2	Cd200r2	Cd200 receptor 2	-0.855596595358645	6,82684E+14	-2,58381E+14	0.0390575310187066	0.175196873303466	-3,60995E+14	6,16641E+14	6,20932E+14	6,9594E+14	7,53897E+14	7,09529E+14	6,99164E+14
TC1800001199.mm.2	Dnd1	DND microRNA-mediated repression inhibitor 1	-0.998253836398591	5,59392E+13	-2,58331E+14	0.0390847707650745	0.175196873303466	-3,6106E+14	5,23459E+14	4,74265E+14	5,54663E+14	6,06589E+14	6,28145E+14	5,6923E+14
TC1500001730.mm.2	Ly6c2	lymphocyte antigen 6 complex, locus C2	-0.845982175988258	6,8333E+14	-2,58329E+14	0.039085913165375	0.175196873303466	-3,61063E+14	6,12099E+14	6,59111E+14	6,86401E+14	7,01961E+14	7,33648E+14	7,06759E+14
TC1900000635.mm.2	Cutc	cutC copper transporter	-0.762492507948283	5,70246E+14	-2,58327E+14	0.0390870692415572	0.175196873303466	-3,61066E+14	5,24989E+13	5,3452E+13	5,70711E+14	5,79252E+14	5,95713E+14	6,16293E+14
TC0X00000226.mm.2	Agtr2	angiotensin II receptor, type 2	-0.766696206871074	4,73047E+14	-2,5831E+14	0.0390963080235347	0.175196873303466	-3,61088E+14	4,03899E+14	4,18907E+14	5,12364E+14	5,26969E+13	4,79794E+14	4,96351E+14
TC0600003311.mm.2	Gpr19	G protein-coupled receptor 19	1,12532E+14	7,47819E+14	2,58304E+14	0.0390993580586646	0.175196873303466	-3,61095E+14	8,35134E+14	8,12768E+14	7,38454E+14	6,77722E+13	7,60419E+13	6,62419E+14
TC1400001544.mm.2	Usp54	ubiquitin specific peptidase 54	0.955545548162734	6,26927E+14	2,58252E+14	0.0391279427259854	0.175262288591635	-3,61164E+14	6,90602E+14	7,01452E+14	5,97351E+14	5,71215E+14	6,39808E+14	5,61137E+14
TC1200002017.mm.2	Gm4787	predicted gene 4787	-0.851695559204616	5,29854E+13	-2,58237E+14	0.0391357111867872	0.175262288591635	-3,61183E+14	4,7697E+14	4,99627E+14	5,01904E+14	5,53689E+14	5,80122E+14	5,66814E+14
TC0300000537.mm.2	Ptx3	pentraxin related gene	-1,22056E+14	7,70481E+14	-2,58186E+14	0.0391639948006946	0.175340219011834	-3,61251E+14	6,73761E+14	6,9639E+14	7,75534E+14	8,62938E+14	7,57196E+14	8,57068E+13
TC1200001536.mm.2	Cys1	cystin 1	-1,15496E+14	7,0691E+14	-2,58157E+14	0.0391797291395453	0.175340583872886	-3,61288E+14	6,35657E+13	6,40204E+14	6,91434E+14	7,67313E+14	7,05088E+14	8,01766E+14
TC0800003084.mm.2	Ankrd11	ankyrin repeat domain 11	1,20922E+14	7,64308E+14	2,58121E+14	0.0391992029228279	0.175340583872886	-3,61335E+14	8,42115E+14	8,33635E+14	7,8715E+14	6,89046E+14	7,61427E+14	6,72478E+14
TC0500001516.mm.2	Nupr1l	nuclear protein transcriptional regulator 1 like	-1,15124E+14	6,82255E+13	-2,58121E+12	0.0391995663774383	0.175340583872886	-3,61336E+14	6,29154E+14	6,13875E+14	6,87771E+14	6,89453E+14	6,75955E+13	7,97323E+14
TC1700000898.mm.2	Mrpl2	mitochondrial ribosomal protein L2	0.948418775574043	7,33928E+14	2,58106E+14	0.0392076040274319	0.175340583872886	-3,61355E+14	7,79638E+14	8,00298E+14	7,53218E+14	6,80163E+13	7,10809E+14	6,79442E+14
TC0400003807.mm.2	Cela3a	chymotrypsin-like elastase family, member 3A	-0.94532902603549	5,33945E+14	-2,5805E+14	0.0392379440850894	0.175427578535806	-3,61428E+14	4,77166E+14	4,47714E+14	5,51208E+14	6,1364E+14	5,35311E+14	5,78635E+14
TC0500000699.mm.2	Rasl11b	RAS-like, family 11, member B	1,04596E+14	6,08049E+14	2,58008E+14	0.0392612281448058	0.175476029521352	-3,61484E+14	6,86091E+14	6,86393E+14	5,10636E+12	6,01882E+14	6,05063E+14	5,5823E+14
TC1200002358.mm.2	Slc25a29	solute carrier family 25 (mitochondrial carrier, palmitoylcarnitine transporter), member 29	-0.913587250255058	7,47537E+14	-2,57991E+14	0.0392705618105875	0.175476029521352	-3,61506E+14	6,89018E+14	7,05191E+14	7,26933E+14	7,87154E+13	7,68327E+14	8,08599E+14
TC0800003007.mm.2	1700030J22Rik	RIKEN cDNA 1700030J22 gene	-0.812240043835533	6,72628E+14	-2,5797E+13	0.039281755014493	0.175477382464243	-3,61533E+14	6,1355E+14	6,37045E+13	6,70223E+14	7,01907E+14	6,94517E+14	7,18526E+14
TC0300002485.mm.2	Rprd2	regulation of nuclear pre-mRNA domain containing 2	2,67431E+14	6,0783E+14	2,57877E+14	0.0393329325748999	0.17565730115836	-3,61655E+14	7,77917E+14	7,54876E+14	6,7362E+14	4,42639E+14	6,34814E+14	3,63117E+13
TC0800003227.mm.2	Mef2b	myocyte enhancer factor 2B	-1,14338E+14	5,60229E+14	-2,57856E+14	0.0393446952339096	0.175661145553889	-3,61683E+13	4,73009E+14	4,99248E+14	5,37895E+14	6,5029E+14	5,82102E+14	6,18831E+14
TC0600002579.mm.2	Cd207	CD207 antigen	-0.842192962306592	5,86352E+14	-2,57817E+14	0.0393660895447877	0.175707978070683	-3,61735E+14	5,23764E+14	5,37647E+14	5,87642E+14	6,39211E+14	6,06951E+13	6,22898E+14
TC1100003721.mm.2	Gm11937	predicted gene 11937	-1,14806E+14	7,00066E+13	-2,57766E+13	0.0393936754137458	0.175781836197348	-3,618E+14	6,21349E+14	6,32092E+14	6,90857E+14	7,73046E+14	6,96736E+14	7,86317E+14
TC0400000869.mm.2	Dennd4c	DENN/MADD domain containing 4C	0.85779241197584	5,30218E+13	2,57734E+14	0.0394116197351842	0.175781836197348	-3,61843E+14	5,57665E+14	6,07933E+14	5,0793E+14	5,13738E+14	5,1336E+14	4,8068E+14
TC1000002391.mm.2	Smarcb1	SWI/SNF related, matrix associated, actin dependent regulator of chromatin, subfamily b, member 1	0.842204828952645	7,71468E+14	2,577E+14	0.0394304780146213	0.175781836197348	-3,61888E+14	8,18428E+14	8,44036E+14	7,54229E+14	7,18093E+14	7,3E+14	7,64023E+14
TC1100003819.mm.2	Mpp3	membrane protein, palmitoylated 3 (MAGUK p55 subfamily member 3)	1,12628E+14	5,66813E+14	2,57679E+14	0.0394418519903122	0.175781836197348	-3,61915E+14	6,63194E+14	6,80492E+14	5,2773E+14	4,11032E+14	5,62185E+14	5,56245E+14
TC0100000590.mm.2	Atic	5-aminoimidazole-4-carboxamide ribonucleotide formyltransferase/IMP cyclohydrolase	0.791674607277922	6,75447E+13	2,5767E+14	0.0394464895683654	0.175781836197348	-3,61926E+13	7,18588E+14	7,26945E+14	6,50673E+14	6,69281E+14	6,60234E+14	6,26964E+14
TC1000001124.mm.2	Cdk17	cyclin-dependent kinase 17	1,48576E+14	8,10248E+13	2,57648E+14	0.0394586855734231	0.175781836197348	-3,61955E+14	8,96E+14	9,09208E+13	8,35783E+14	7,12441E+14	8,18765E+14	6,89292E+14
TC0100001016.mm.2	Zcchc2	zinc finger, CCHC domain containing 2	1,34017E+14	5,56567E+14	2,57648E+14	0.03945900214273	0.175781836197348	-3,61956E+14	6,52885E+14	6,23293E+14	5,97253E+14	4,57827E+14	5,35156E+14	4,72988E+13
TC1800001107.mm.2	Rit2	Ras-like without CAAX 2	1,45159E+14	9,25312E+14	2,5762E+14	0.0394740730000159	0.175800369886472	-3,61992E+14	1,04516E+14	10061035438	9,38957E+14	8,00712E+14	9,29406E+14	8,31537E+14
TC0500000193.mm.2	Gm17434	predicted gene, 17434	-1,05736E+13	5,07214E+14	-2,57588E+14	0.0394920823071174	0.175831976295823	-3,62035E+14	4,33778E+14	4,30192E+14	5,03906E+14	5,99963E+14	5,16427E+14	5,59016E+14
TC0900001406.mm.2	Ccdc71	coiled-coil domain containing 71	1,04099E+14	6,66751E+14	2,57534E+14	0.0395216317733646	0.175842999928064	-3,62105E+13	7,34525E+14	7,1716E+14	7,01642E+14	6,03691E+14	6,32928E+13	6,10559E+14
TC0600001943.mm.2	Ndufa5	NADH:ubiquinone oxidoreductase subunit A5	1,20336E+14	9,25048E+14	2,57525E+14	0.0395268226456992	0.175842999928064	-3,62118E+14	1,01609E+14	9,86779E+14	8,77382E+14	9,07846E+14	9,43702E+14	8,1849E+14
TC0500003430.mm.2	Ap1s1	adaptor protein complex AP-1, sigma 1	1,77759E+14	8,81417E+14	2,57524E+14	0.0395272975696299	0.175842999928064	-3,62119E+14	1,00178E+14	1,02658E+14	8,95896E+14	6,91399E+14	8,81775E+14	7,91069E+14
TC1000002408.mm.2	Pcbp3	poly(rC) binding protein 3	0.906716758531299	7,69127E+12	2,5746E+14	0.0395624791731432	0.17595093207752	-3,62202E+14	8,35729E+14	8,00284E+13	7,93339E+13	7,30741E+14	7,21029E+14	7,3364E+14
TC0200003312.mm.2	Cfap157	cilia and flagella associated protein 157	-0.946549345899957	6,81943E+13	-2,57375E+14	0.0396092313596593	0.176110249695969	-3,62313E+14	6,22696E+14	6,29958E+14	6,76334E+14	7,20704E+14	6,86499E+14	7,55465E+14
TC1200001398.mm.2	Slc7a15	solute carrier family 7 (cationic amino acid transporter, y+ system), member 15	-1,02949E+14	4,56981E+14	-2,57348E+14	0.0396243394204522	0.176128822367378	-3,62349E+14	3,72527E+14	4,00564E+14	4,56533E+14	5,33272E+14	4,60819E+14	5,18169E+14
TC1000001059.mm.2	1700113H08Rik	RIKEN cDNA 1700113H08 gene	-0.9169406579291	5,3818E+13	-2,57319E+14	0.0396402189696655	0.176133031503426	-3,62387E+14	4,81158E+14	4,72762E+14	5,33419E+14	6,04431E+14	5,80904E+14	5,56404E+14
TC1400000580.mm.2	Rnase6	ribonuclease, RNase A family, 6	-0.813551887693739	8,62782E+14	-2,57307E+14	0.0396471485920019	0.176133031503426	-3,62403E+14	7,99738E+14	8,01358E+14	8,88939E+14	9,22849E+14	8,98388E+14	8,65418E+14
TC0700001817.mm.2	Tbx6	T-box 6	-0.791908653343463	5,53063E+14	-2,57249E+12	0.0396790137982978	0.176182580084149	-3,62479E+13	5,14024E+13	5,00451E+14	5,48648E+14	5,82399E+13	5,94281E+14	5,78575E+13
TC1000002035.mm.2	Mfsd4b1	major facilitator superfamily domain containing 4B1	-0.806472591969964	7,10895E+14	-2,57247E+13	0.0396801702429949	0.176182580084149	-3,62482E+13	6,51432E+14	6,50394E+13	7,32404E+14	7,68019E+14	7,17839E+14	7,45282E+14
TC0500003108.mm.2	Prkab1	protein kinase, AMP-activated, beta 1 non-catalytic subunit	-0.863167651285261	6,73176E+14	-2,57175E+14	0.0397202456309673	0.176193505199843	-3,62577E+14	6,13957E+14	6,35949E+14	7,0735E+14	6,59262E+14	6,94881E+13	7,27658E+14
TC0400003853.mm.2	Pax7	paired box 7	-1,19259E+14	5,80947E+14	-2,57144E+14	0.0397376551800899	0.176193505199843	-3,62618E+14	4,99837E+14	5,15058E+14	5,59305E+14	6,58066E+14	5,84692E+14	6,68722E+14
TC0500002139.mm.2	Lmbr1	limb region 1	1,31047E+14	6,6028E+13	2,5708E+14	0.0397728510545622	0.176193505199843	-3,62701E+14	7,2984E+14	7,50364E+14	6,42688E+14	6,20677E+14	6,83042E+14	5,35069E+14
TC1000002428.mm.2	Gm3238	predicted gene 3238	-0.874717616262119	6,69583E+14	-2,57051E+14	0.0397889498176542	0.176193505199843	-3,62739E+14	5,92E+14	6,24302E+14	6,96959E+14	7,12993E+14	6,68353E+14	7,22893E+14
TC0600001114.mm.2	Slc25a26	solute carrier family 25 (mitochondrial carrier, phosphate carrier), member 26	0.933610125251754	6,54514E+14	2,5703E+14	0.0398008789390363	0.176193505199843	-3,62767E+14	7,24447E+14	7,17554E+12	6,47469E+14	5,82334E+14	6,50338E+14	6,04941E+14
TC1400001721.mm.2	Gprin2	G protein regulated inducer of neurite outgrowth 2	-1,16756E+14	6,27875E+14	-2,57026E+14	0.0398031929625944	0.176193505199843	-3,62773E+14	5,57434E+14	5,2799E+14	6,32276E+14	7,30614E+13	6,22778E+14	6,96158E+13
TC1100002152.mm.2	Pgam2	phosphoglycerate mutase 2	-0.761384344550691	7,01431E+14	-2,57022E+14	0.0398052500312065	0.176193505199843	-3,62777E+14	6,50403E+14	6,47645E+14	7,1845E+14	7,41762E+14	7,30581E+14	7,19743E+14
TC0300000916.mm.2	Anp32e	acidic (leucine-rich) nuclear phosphoprotein 32 family, member E	1,18658E+14	5,90522E+14	2,57019E+14	0.0398067367628864	0.176193505199843	-3,62781E+14	6,52196E+14	6,5999E+14	6,36246E+14	5,19832E+14	5,65639E+14	5,09232E+13
TC1500000878.mm.2	Shank3	SH3 and multiple ankyrin repeat domains 3	1,5655E+14	5,98868E+14	2,57015E+14	0.0398090888751989	0.176193505199843	-3,62787E+14	7,01929E+14	6,81536E+14	5,20788E+14	6,18591E+14	4,52845E+13	6,17521E+14

TC1500000862.mm.2	Selenoo	selenoprotein O	0.925021159251959	6,08371E+14	2,57E+13	0.0398175170454054	0.176193505199843	-3,62806E+14	6,60118E+14	6,7748E+14	5,71013E+14	5,89019E+14	6,13102E+14	5,39492E+14
TC0700004313.mm.2	Spn	sialophorin	-0.915666239219415	5,20729E+14	-2,56996E+14	0.0398195640203904	0.176193505199843	-3,62811E+14	4,75092E+14	4,72198E+14	4,8561E+14	5,6105E+14	5,66762E+14	5,63661E+14
TC0400000450.mm.2	Hrct1	histidine rich carboxyl terminus 1	-0.965707989449024	6,57787E+14	-2,56984E+13	0.0398260142160618	0.176193505199843	-3,62826E+14	5,87844E+14	6,39977E+14	6,22209E+14	6,75727E+14	6,88333E+14	7,3263E+14
TC1600000038.mm.2	Mgrn1	mahogunin, ring finger 1	0.967180134502912	8,30809E+14	2,5698E+14	0.0398282771806949	0.176193505199843	-3,62832E+14	9,0207E+14	8,85343E+14	8,42199E+14	7,61265E+14	8,10864E+14	7,83114E+14
TC1800000190.mm.2	Zfp35	zinc finger protein 35	1,29205E+14	5,864E+14	2,56967E+14	0.0398357189501042	0.176193505199843	-3,62849E+14	6,75647E+14	6,48655E+14	6,32138E+13	4,9607E+14	5,58707E+14	5,07186E+14
TC0800001425.mm.2	Wfdc1	WAP four-disulfide core domain 1	-0.81809285844466	6,95298E+14	-2,56856E+14	0.0398972241127125	0.176417116390817	-3,62994E+14	6,66187E+14	6,48569E+14	6,75766E+14	7,02891E+14	7,20593E+14	7,57781E+14
TC0700004526.mm.2	Lmntd2	lamin tail domain containing 2	-1,15991E+14	5,2536E+14	-2,56765E+14	0.0399480925129056	0.176571128553196	-3,63114E+14	4,4997E+14	4,58902E+14	5,19374E+14	5,83061E+14	5,16826E+14	6,24028E+13
TC0700000825.mm.2	Irf3	interferon regulatory factor 3	0.834315403591376	6,08816E+14	2,56743E+14	0.0399606549597256	0.176571128553196	-3,63144E+14	6,55878E+14	6,50242E+14	6,20008E+14	5,87513E+14	5,91285E+14	5,47972E+14
TC0400001278.mm.2	Tctex1d4	Tctex1 domain containing 4	-1,49258E+14	5,50832E+14	-2,56735E+14	0.0399649293014029	0.176571128553196	-3,63154E+14	4,60764E+13	4,27146E+14	5,27245E+14	7,03408E+14	5,8009E+14	6,06336E+14
TC1400001634.mm.2	Ccdc66	coiled-coil domain containing 66	1,19411E+14	6,67004E+14	2,567E+14	0.0399847160604782	0.176610123323049	-3,632E+14	7,25007E+14	7,45964E+13	6,79647E+14	6,19259E+14	6,73361E+14	5,58788E+14
TC0500002666.mm.2	Tecrl	trans-2,3-enoyl-CoA reductase-like	-1,10126E+14	5,2969E+14	-2,56617E+14	0.0400310134759865	0.176648415059302	-3,63309E+14	4,63917E+13	4,62983E+14	4,96383E+14	6,07705E+14	5,60585E+14	5,86567E+14
TC0800000112.mm.2	Mcf2l	mcf.2 transforming sequence-like	-0.760674042613104	6,54275E+13	-2,56593E+14	0.0400443279294349	0.176648415059302	-3,6334E+14	6,12506E+14	6,01636E+14	6,7373E+14	6,71502E+14	6,71285E+14	6,94992E+14
TC0100001920.mm.2	Npbwr1	neuropeptides B/W receptor 1	-1,13575E+14	5,69388E+14	-2,56586E+14	0.0400484395192589	0.176648415059302	-3,6335E+14	4,98791E+14	4,95239E+14	5,57751E+14	6,43367E+14	5,6805E+14	6,5313E+14
TC0100000490.mm.2	Icos	inducible T cell co-stimulator	-1,00271E+14	4,64601E+14	-2,5658E+14	0.0400512896311055	0.176648415059302	-3,63357E+14	4,08686E+14	3,80339E+13	4,70408E+14	5,38604E+14	4,64908E+14	5,2466E+14
TC0600001968.mm.2	Lrrc4	leucine rich repeat containing 4	0.983389129293342	6,82313E+13	2,5655E+14	0.0400681225191845	0.176648415059302	-3,63396E+14	7,38579E+14	7,61936E+13	6,75572E+14	6,13956E+14	6,83291E+14	6,20546E+14
TC0400003930.mm.2	Pramef6	PRAME family member 6	-0.926984921371871	5,19123E+14	-2,56539E+14	0.0400742204000013	0.176648415059302	-3,6341E+14	4,51138E+14	4,86095E+14	5,0269E+14	5,52187E+14	5,36299E+14	5,8633E+14
TC1500000800.mm.2	Upk3a	uroplakin 3A	-0.925905262040023	5,81246E+14	-2,56537E+14	0.0400758134667456	0.176648415059302	-3,63414E+14	5,55758E+14	5,27307E+14	5,36008E+14	6,00158E+14	6,16719E+14	6,51527E+14
TC1800001227.mm.2	Pcdh1	protocadherin 1	1,20746E+14	9,34707E+14	2,56527E+13	0.0400810901419231	0.176648415059302	-3,63427E+14	1,00103E+14	1,00455E+14	9,81141E+14	8,57446E+14	9,07359E+14	8,56721E+14
TC0X00003423.mm.2	Rhox3g	reproductive homeobox 3G	-0.847531479656496	5,02521E+14	-2,56484E+13	0.0401050265959906	0.176665890749334	-3,63483E+13	4,63727E+14	4,46768E+14	4,85355E+13	5,39277E+14	5,3241E+14	5,47592E+14
TC0300001242.mm.2	Abca4	ATP-binding cassette, sub-family A (ABC1), member 4	-1,28012E+14	5,87142E+12	-2,56479E+14	0.0401082578262659	0.176665890749334	-3,6349E+14	4,99255E+14	5,01691E+14	5,73973E+14	6,90961E+14	5,84525E+14	6,72445E+13
TC1300000667.mm.2	Tmed9	transmembrane p24 trafficking protein 9	1,15179E+14	7,08489E+14	2,56447E+12	0.0401262955150313	0.176665890749334	-3,63532E+14	7,97477E+13	7,86967E+13	7,12774E+14	5,9963E+14	7,01612E+14	6,52473E+13
TC0800002393.mm.2	Tmem59l	transmembrane protein 59-like	1,08273E+14	8,86955E+14	2,56442E+14	0.0401289120674339	0.176665890749334	-3,63539E+14	9,74963E+14	9,3914E+13	9,07108E+14	8,0296E+14	8,55467E+14	8,42091E+14
TC1700000719.mm.2	Gm6034	predicted gene 6034	-1,34979E+14	6,46682E+14	-2,56418E+14	0.0401423748962981	0.176676887927903	-3,6357E+14	5,90847E+14	4,99994E+14	6,74254E+14	7,54196E+14	6,29468E+14	7,3133E+13
TC1100002966.mm.2	Hs3st3b1	heparan sulfate (glucosamine) 3-O-sulfotransferase 3B1	-1,14327E+14	7,20923E+14	-2,56365E+14	0.0401721078783348	0.176759468662919	-3,6364E+14	6,11568E+14	6,77722E+14	7,18545E+14	7,99759E+14	7,27397E+14	7,90548E+14
TC1100000056.mm.2	Thoc5	THO complex 5	0.894571116450275	6,71166E+14	2,56212E+13	0.0402578142262677	0.177033252950567	-3,6384E+14	7,17566E+14	7,10271E+14	6,43924E+14	7,06312E+14	6,41162E+14	6,07761E+14
TC0700001860.mm.2	Ctf1	cardiotrophin 1	-0.790308037650729	7,71653E+14	-2,56209E+13	0.0402597990559022	0.177033252950567	-3,63845E+14	7,1652E+14	7,42939E+14	7,76275E+14	7,76663E+14	7,93582E+14	8,23938E+14
TC0500002760.mm.2	Scarb2	scavenger receptor class B, member 2	1,20795E+14	7,89315E+14	2,56143E+14	0.0402969678716738	0.177033252950567	-3,63931E+14	8,73742E+14	8,79494E+14	8,02364E+14	6,68648E+14	7,60093E+14	7,51552E+14
TC0300001290.mm.2	Neurog2	neurogenin 2	-1,27463E+14	6,10608E+14	-2,56141E+14	0.0402978247792782	0.177033252950567	-3,63933E+14	5,53856E+14	5,0575E+14	5,78348E+14	7,11159E+14	6,39389E+14	6,75144E+14
TC1700002253.mm.2	Tbc1d5	TBC1 domain family, member 5	1,28031E+14	6,56984E+14	2,56132E+14	0.0403027293923931	0.177033252950567	-3,63945E+14	7,53674E+13	7,32144E+14	6,82254E+14	5,44077E+14	6,37023E+14	5,92732E+14
TC0100002751.mm.2	Nppc	natriuretic peptide type C	-0.903605471853091	7,08583E+14	-2,56129E+14	0.0403047604739127	0.177033252950567	-3,6395E+14	6,39836E+14	6,85053E+14	6,81983E+14	7,39016E+14	7,47997E+14	7,57612E+14
TC0700001554.mm.2	Smpd1	sphingomyelin phosphodiesterase 1, acid lysosomal	1,0524E+14	7,86533E+14	2,56097E+13	0.0403225112927454	0.177033252950567	-3,63991E+14	8,86646E+14	8,61665E+14	7,65114E+14	6,67942E+14	7,85E+14	7,52831E+14
TC1100001853.mm.2	Sox9	SRY (sex determining region Y)-box 9	0.94038649520857	8,26937E+13	2,56085E+14	0.0403295455029277	0.177033252950567	-3,64007E+14	8,79955E+14	8,91309E+14	8,26026E+14	7,81145E+14	7,57747E+14	8,25439E+14
TC0200002942.mm.2	Atp5c1	ATP synthase, H+ transporting, mitochondrial F1 complex, gamma polypeptide 1	1,01243E+14	9,22989E+14	2,56054E+14	0.0403469765966669	0.177033252950567	-3,64048E+14	9,8872E+14	9,7618E+14	9,27868E+13	8,82748E+14	9,23917E+14	8,38499E+14
TC1000003220.mm.2	Nt5dc3	5'-nucleotidase domain containing 3	0.881926448168968	7,40333E+14	2,56021E+14	0.0403652210147058	0.177033252950567	-3,6409E+14	7,88987E+14	8,27899E+14	6,71994E+14	7,12616E+14	7,39481E+14	7,01019E+14
TC0700000242.mm.2	Ptgir	prostaglandin I receptor (IP)	-1,06598E+14	4,8615E+14	-2,56006E+14	0.0403739515673509	0.177033252950567	-3,64111E+14	4,38004E+14	4,21588E+14	4,38889E+14	5,45634E+14	5,34332E+14	5,38455E+14
TC0400000555.mm.2	Rnf20	ring finger protein 20	1,54166E+14	6,24389E+14	2,55953E+13	0.0404035866261076	0.177033252950567	-3,6418E+14	7,52061E+14	7,12838E+14	6,55286E+14	4,69582E+14	5,76215E+14	5,80351E+14
TC0500002073.mm.2	Kcnh2	potassium voltage-gated channel, subfamily H (eag-related), member 2	1,01079E+14	7,90568E+13	2,55947E+14	0.0404074543256163	0.177033252950567	-3,64189E+14	8,81927E+14	8,16792E+14	8,07896E+14	7,40235E+14	7,35824E+13	7,60736E+13
TC0800000114.mm.2	F7	coagulation factor VII	-0.826245688123357	5,27934E+14	-2,55946E+14	0.0404076278017231	0.177033252950567	-3,64189E+14	4,40274E+14	4,44928E+14	5,89759E+14	6,4219E+14	5,27132E+14	5,23319E+14
TC0500000734.mm.2	Srp72	signal recognition particle 72	1,72604E+14	8,81757E+14	2,55933E+14	0.040414922402568	0.177033252950567	-3,64206E+14	9,72609E+14	9,67104E+14	9,54094E+14	8,02227E+14	8,7597E+14	7,18536E+14
TC1500001069.mm.2	Smim41	small integral membrane protein 41	-0.791979282782232	6,60272E+14	-2,55903E+14	0.0404322675637738	0.177033252950567	-3,64246E+14	5,9804E+14	6,07623E+14	6,75899E+14	7,16011E+14	6,81987E+14	6,82073E+14
TC0100000230.mm.2	Arhgef4	Rho guanine nucleotide exchange factor (GEF) 4	1,29116E+14	7,96516E+14	2,55895E+14	0.0404367451175046	0.177033252950567	-3,64257E+13	8,78423E+14	8,71705E+13	8,06523E+14	7,3055E+14	8,098E+14	6,82096E+14
TC0500002458.mm.2	Arap2	ArfGAP with RhoGAP domain, ankyrin repeat and PH domain 2	1,8054E+14	6,64725E+14	2,55894E+14	0.0404371763287059	0.177033252950567	-3,64258E+14	7,76172E+14	8,21003E+14	6,66168E+13	4,88911E+14	6,88709E+14	5,47387E+14
TC0100000164.mm.2	Col9a1	collagen, type IX, alpha 1	-1,05605E+14	5,94595E+14	-2,55884E+14	0.0404430834798633	0.177033252950567	-3,64271E+14	4,92479E+13	5,38732E+14	6,23E+14	6,70936E+14	5,79824E+14	6,62597E+14
TC0500000347.mm.2	Depdc5	DEP domain containing 5	1,382E+13	5,47503E+14	2,5586E+13	0.0404561441659237	0.177042327796178	-3,64302E+14	6,55746E+14	6,2282E+14	5,39315E+14	4,64973E+14	5,72312E+14	4,29853E+14
TC1300002768.mm.2	Zfp934	zinc finger protein 934	1,46083E+14	5,37382E+14	2,55837E+14	0.0404695798422685	0.177053038283593	-3,64333E+14	6,17588E+14	6,43437E+14	5,69833E+14	4,24572E+14	5,33283E+14	4,35578E+14
TC0300001841.mm.2	Dcun1d1	DCN1, defective in cullin neddylation 1, domain containing 1 (S. cerevisiae)	1,53822E+14	7,02791E+14	2,55795E+14	0.0404930325446258	0.177107555209434	-3,64387E+14	7,92428E+14	8,1207E+14	7,4168E+14	5,73712E+14	6,91124E+14	6,0573E+14
TC0300002052.mm.2	Gpr87	G protein-coupled receptor 87	-1,01837E+13	5,37406E+14	-2,5577E+13	0.0405073098993619	0.177120493063651	-3,64421E+13	4,6932E+14	4,96528E+14	5,02952E+14	5,86116E+14	5,61768E+14	6,07754E+14
TC0900000849.mm.2	Oaz2	ornithine decarboxylase antizyme 2	1,38569E+14	7,57532E+13	2,55751E+14	0.0405179753883582	0.177120493063651	-3,64445E+14	8,5292E+14	8,27452E+14	7,84081E+14	6,77507E+14	7,63122E+14	6,40112E+14
TC0900000669.mm.2	Ube2q2	ubiquitin-conjugating enzyme E2Q family member 2	2,49792E+14	7,02885E+14	2,55726E+14	0.0405319680626154	0.177133604934343	-3,64478E+14	8,37352E+14	8,24033E+14	7,99536E+14	5,94587E+14	7,13108E+13	4,48693E+14
TC0400002220.mm.2	Tmem67	transmembrane protein 67	1,75779E+14	5,40966E+14	2,55691E+14	0.0405519803141572	0.177173009436555	-3,64524E+13	6,67299E+13	6,19755E+14	5,18847E+14	5,044E+14	5,76946E+14	3,58549E+14
TC1500001965.mm.2	Rrp7a	ribosomal RNA processing 7 homolog A (S. cerevisiae)	1,03369E+14	8,08136E+14	2,55645E+14	0.0405781481591972	0.177234320026256	-3,64585E+14	9,00258E+14	8,592E+14	8,05726E+13	7,30911E+14	8,04319E+14	7,484E+13
TC0700001402.mm.2	Pgm2l1	phosphoglucomutase 2-like 1	1,24991E+14	7,86289E+14	2,55622E+14	0.0405909169277632	0.177234320026256	-3,64614E+14	8,65763E+14	8,95817E+14	7,78483E+14	6,66073E+14	7,96397E+14	7,152E+14
TC0700002873.mm.2	Uri1	URI1, prefoldin-like chaperone	2,24396E+14	6,85766E+14	2,55608E+14	0.0405990116810595	0.177234320026256	-3,64633E+14	8,27597E+14	8,25659E+14	7,32883E+14	5,23994E+14	7,08139E+14	4,96325E+14
TC0800000488.mm.2	Pcm1	pericentriolar material 1	2,16662E+14	6,34512E+13	2,55564E+14	0.0406239488139599	0.177267970944871	-3,64691E+14	8,08665E+14	7,87264E+14	6,5559E+14	3,92948E+14	6,36939E+14	5,25667E+13
TC1100002114.mm.2	Mtmr3	myotubularin related protein 3	1,28965E+14	7,06696E+14	2,55545E+14	0.0406346240190334	0.177267970944871	-3,64715E+14	7,89548E+14	7,8327E+14	7,03754E+14	6,48714E+12	7,26491E+14	5,88398E+14
TC0200003362.mm.2	Stom	stomatin	1,12388E+14	5,20922E+14	2,55536E+14	0.0406397247359495	0.177267970944871	-3,64727E+14	5,73912E+14	5,89091E+13	5,57227E+14	4,67075E+14	4,30699E+14	5,07528E+14
TC0700004296.mm.2	Aldoa	aldolase A, fructose-bisphosphate	1,27099E+14	1,09465E+14	2,55497E+14	0.0406622775299005	0.177318343122946	-3,64779E+14	1,18345E+14	1,19025E+14	1,10628E+14	9,68421E+14	1,08298E+14	1,03653E+14
TC0500002286.mm.2	Wdr1	WD repeat domain 1	1,01742E+13	8,85721E+12	2,55434E+14	0.0406981382613001	0.177426705033639	-3,64862E+14	9,58624E+14	9,54007E+14	8,94393E+14	7,98153E+14	8,43355E+13	8,65792E+14
TC0900001459.mm.2	Scap	SREBF chaperone	1,71023E+14	7,33688E+14	2,55402E+14	0.0407159659454667	0.177456413113147	-3,64903E+14	8,80698E+14	8,64049E+14	7,24126E+14	5,30553E+14	7,4958E+14	6,5312E+14
TC1300001098.mm.2	Fam169a	family with sequence similarity 169, member A	1,75561E+14	7,1752E+13	2,55381E+14	0.040728337222205	0.177462330357325	-3,64932E+14	8,47779E+14	8,05901E+14	7,52176E+14	5,96708E+14	7,29123E+13	5,73434E+14
TC0800002674.mm.2	Tox3	TOX high mobility group box family member 3	0.854362048066765	6,99037E+14	2,55353E+14	0.0407441584862939	0.177477426500471	-3,64968E+14	7,33764E+14	7,18219E+13	7,56794E+14	7,04337E+14	6,26535E+14	6,54575E+14
TC0700004635.mm.2	Lilra6	leukocyte immunoglobulin-like receptor, subfamily A (with TM domain), member 6	-1,6639E+13	4,92614E+14	-2,55336E+14	0.0407538309471696	0.177477426500471	-3,64991E+14	4,0816E+14	4,00381E+14	4,23457E+14	5,82364E+14	5,02312E+14	6,39009E+14
TC0900000072.mm.2	Gm16485	predicted gene 16485	1,10415E+14	5,46952E+14	2,55247E+14	0.0408046963872642	0.177543248737238	-3,65108E+13	5,98718E+13	6,49631E+14	5,51386E+14	4,5446E+14	5,3288E+14	4,94639E+14
TC1900000517.mm.2	Pcgf5	polycomb group ring finger 5	1,30454E+13	6,76433E+14	2,55213E+14	0.0408238244597604	0.177543248737238	-3,65152E+13	7,72759E+13	7,34477E+13	6,9686E+14	6,08172E+14	6,81592E+14	5,64736E+14
TC0800003175.mm.2	Fam89a	family with sequence similarity 89, member A	-0.772470470450021	6,91929E+14	-2,55189E+14	0.0408377477403116	0.177543248737238	-3,65184E+14	6,47256E+14	6,37995E+14	7,00487E+14	7,26091E+14	7,30189E+14	7,09556E+14
TC0700002160.mm.2	Tnnt1	troponin T1, skeletal, slow	-0.931624224795265	7,21327E+14	-2,55187E+13	0.0408388556991751	0.177543248737238	-3,65186E+13	6,6178E+14	6,70047E+13	7,35133E+14	7,42851E+14	7,19089E+14	7,99063E+13
TC0800002381.mm.2	Mau2	MAU2 sister chromatid cohesion factor	1,05587E+13	6,82811E+13	2,55181E+14	0.0408422124070304	0.177543248737238	-3,65194E+14	7,43389E+13	7,58007E+14	6,9781E+14	6,0744E+13	6,72596E+14	6,17626E+14

TC1400002128.mm.2	Pspc1	paraspeckle protein 1	1,53087E+14	7,43436E+14	2,5518E+14	0.0408427663598607	0.177543248737238	-3,65195E+14	8,70283E+13	8,32283E+14	7,46417E+14	6,15241E+14	7,63425E+14	6,32967E+14
TC1800000498.mm.2	Eno1b	enolase 1B, retrotransposed	1,28807E+14	7,29772E+13	2,55174E+14	0.040846076029848	0.177543248737238	-3,65203E+13	8,16217E+14	8,40476E+14	7,32043E+12	5,9082E+14	7,19669E+14	6,79411E+14
TC0700002498.mm.2	Tomm40	translocase of outer mitochondrial membrane 40	0.888662800533907	6,99945E+14	2,55143E+14	0.040863667639537	0.177571811401257	-3,65244E+14	7,69301E+14	7,51004E+14	6,72207E+14	6,64588E+14	7,05168E+13	6,37405E+14
TC1700001870.mm.2	Zfp101	zinc finger protein 101	1,33882E+14	5,20208E+14	2,55111E+14	0.0408823044394203	0.177604899280771	-3,65286E+14	5,97205E+14	6,06307E+13	4,38496E+14	5,43491E+13	5,26392E+14	4,09356E+14
TC0200000507.mm.2	Rapgef1	Rap guanine nucleotide exchange factor (GEF) 1	0.750637368090779	7,54143E+13	2,55077E+14	0.0409012954216045	0.177639507581756	-3,6533E+14	7,84828E+14	7,99742E+14	7,44347E+14	7,61502E+14	7,18129E+14	7,16313E+13
TC0100003683.mm.2	Wdr26	WD repeat domain 26	1,73038E+14	7,32831E+14	2,55008E+14	0.0409408196645392	0.177748590567106	-3,65421E+14	8,38163E+14	8,64383E+14	7,45997E+14	5,91976E+13	7,56003E+14	6,00467E+14
TC0800000929.mm.2	Dnajb1	DnaJ heat shock protein family (Hsp40) member B1	1,21575E+14	7,56538E+14	2,54995E+14	0.0409484744110407	0.177748590567106	-3,65438E+14	8,44888E+14	8,20332E+14	7,71694E+14	6,80246E+14	7,60158E+14	6,61912E+14
TC0800003009.mm.2	Pkd1l2	polycystic kidney disease 1 like 2	-0.785399420607304	5,56282E+14	-2,54954E+14	0.0409720450426581	0.17780300613314	-3,65492E+14	4,9032E+14	5,10063E+14	5,85372E+14	5,94473E+14	5,62038E+14	5,95425E+14
TC1500002289.mm.2	Rarg	retinoic acid receptor, gamma	-0.81198664984469	8,47367E+14	-2,54927E+14	0.0409872995088514	0.177821313135736	-3,65527E+14	7,90015E+13	7,98776E+14	8,48051E+14	8,96169E+13	8,72503E+13	8,78686E+14
TC1100002694.mm.2	Hnrnpab	heterogeneous nuclear ribonucleoprotein A/B	1,17837E+14	9,63568E+13	2,54896E+14	0.0410052533064744	0.177851318042321	-3,65569E+14	1,04651E+14	1,05009E+14	9,71506E+14	8,52372E+13	9,56266E+14	9,04661E+14
TC0400001216.mm.2	Tal1	T cell acute lymphocytic leukemia 1	-0.946116054782419	7,32806E+14	-2,54873E+13	0.0410181720500028	0.177859474230811	-3,65598E+14	7,01728E+14	6,56725E+14	7,14096E+13	7,76611E+14	7,56726E+14	7,9095E+14
TC1600001378.mm.2	Tra2b	transformer 2 beta	1,2997E+14	9,07697E+14	2,54848E+14	0.0410327436819272	0.177868138521894	-3,65631E+14	9,80403E+14	9,91887E+14	9,41977E+14	8,19565E+14	9,00098E+14	8,12253E+14
TC1900000208.mm.2	Vps37c	vacuolar protein sorting 37C	-0.873175383606783	6,98804E+14	-2,54831E+14	0.0410422478138398	0.177868138521894	-3,65653E+14	6,57998E+14	6,25272E+14	7,54773E+14	6,96877E+14	7,32807E+14	7,25098E+14
TC0400003760.mm.2	Srrm1	serine/arginine repetitive matrix 1	1,51507E+14	7,60375E+14	2,54777E+14	0.0410731657620837	0.177954267255836	-3,65724E+14	8,65704E+14	8,70823E+14	7,77196E+14	6,15017E+14	7,62949E+14	6,70564E+14
TC0600003030.mm.2	Wnk1	WNK lysine deficient protein kinase 1	2,48217E+14	7,76357E+14	2,54734E+14	0.0410983190763798	0.178015380450997	-3,65781E+14	9,52794E+13	9,54742E+14	8,03122E+14	5,36378E+14	8,0598E+14	6,05122E+14
TC1400002001.mm.2	Ang2	angiogenin, ribonuclease A family, member 2	-1,40353E+14	4,26232E+14	-2,54693E+14	0.0411216681560003	0.178068647943465	-3,65835E+14	3,40383E+14	3,31884E+14	4,1871E+14	5,13441E+14	4,06858E+14	5,46117E+14
TC0200000408.mm.2	Lrrc26	leucine rich repeat containing 26	-1,36229E+14	5,19858E+14	-2,54656E+14	0.0411430298572975	0.178107662519796	-3,65884E+14	4,0483E+14	4,28359E+14	5,1025E+14	6,70059E+14	5,47928E+14	5,5772E+14
TC1400000556.mm.2	Olfr740	olfactory receptor 740	-1,17731E+14	5,53069E+14	-2,54639E+14	0.0411527851772607	0.178107662519796	-3,65906E+14	4,78119E+14	4,71895E+14	5,40999E+14	6,41927E+14	5,51965E+14	6,33511E+14
TC0X00003339.mm.2	Glra2	glycine receptor, alpha 2 subunit	2,11961E+14	6,64425E+14	2,54594E+14	0.0411784847237747	0.178143029199092	-3,65965E+14	8,27946E+14	7,66952E+14	7,40743E+14	4,79934E+14	5,44298E+14	6,26678E+14
TC1000002527.mm.2	Pias4	protein inhibitor of activated STAT 4	-1,14153E+14	7,8117E+14	-2,54586E+14	0.0411830685636827	0.178143029199092	-3,65975E+14	7,0433E+14	7,18218E+14	7,50328E+13	8,63287E+14	8,01515E+13	8,4934E+14
TC1200001465.mm.2	2410004P03Rik	RIKEN cDNA 2410004P03 gene	-1,13915E+14	6,90477E+14	-2,54555E+14	0.0412011987348354	0.178173621904027	-3,66016E+14	6,07395E+14	6,39953E+14	6,65745E+14	7,54592E+14	6,98267E+14	7,76912E+14
TC1100000078.mm.2	Dbnl	drebrin-like	0.860423348179121	7,10348E+14	2,54535E+14	0.0412126652918272	0.178175389280175	-3,66043E+14	7,55953E+14	7,6424E+14	7,14772E+14	6,79014E+14	6,47923E+14	7,00185E+14
TC0400001527.mm.2	Tmem234	transmembrane protein 234	1,03392E+14	8,11722E+14	2,54482E+14	0.0412430545398913	0.178243666040945	-3,66112E+14	8,67164E+14	8,7247E+13	8,49331E+14	7,48517E+14	7,56177E+14	7,76674E+14
TC0800001400.mm.2	Cmip	c-Maf inducing protein	1,40719E+14	9,86062E+14	2,54439E+14	0.0412682007608785	0.178243666040945	-3,66169E+14	1,08832E+14	1,06845E+14	1,01949E+14	8,6479E+14	9,70814E+14	9,04515E+13
TC1500001099.mm.2	Amhr2	anti-Mullerian hormone type 2 receptor	-0.851220506676538	5,32188E+14	-2,54431E+14	0.041272903562505	0.178243666040945	-3,6618E+14	4,69686E+14	4,88815E+14	5,25769E+14	5,80116E+14	5,75077E+14	5,53667E+14
TC1000003096.mm.2	Tspan31	tetraspanin 31	1,21995E+13	8,94598E+14	2,54414E+14	0.0412825434917758	0.178243666040945	-3,66202E+14	9,75515E+14	9,80441E+14	9,13669E+14	7,85997E+13	8,81627E+14	8,30339E+14
TC1600001540.mm.2	E130310I04Rik	RIKEN cDNA E130310I04 gene	-0.818799845464724	7,68116E+14	-2,54373E+14	0.0413060220494512	0.178243666040945	-3,66255E+14	7,05785E+14	7,2744E+14	7,76473E+14	8,02017E+14	7,78575E+14	8,18409E+14
TC0900001470.mm.2	Prss44	protease, serine 44	-1,1569E+14	5,61498E+14	-2,54372E+14	0.041306451546939	0.178243666040945	-3,66256E+13	4,71592E+14	4,88802E+14	5,54621E+14	6,62196E+14	5,63805E+14	6,27969E+14
TC1500001933.mm.2	Polr3h	polymerase (RNA) III (DNA directed) polypeptide H	1,04956E+14	6,19738E+14	2,54368E+14	0.0413088628260819	0.178243666040945	-3,66262E+14	7,06847E+14	6,89239E+14	6,16319E+14	5,19852E+14	6,13476E+13	5,72697E+14
TC0900002751.mm.2	Htr1b	5-hydroxytryptamine (serotonin) receptor 1B	1,38628E+14	7,37231E+14	2,54354E+14	0.0413169547981709	0.178243666040945	-3,6628E+14	8,19427E+14	8,32553E+14	7,80687E+14	6,15994E+14	6,95884E+14	6,78839E+14
TC1000000264.mm.2	9330159F19Rik	RIKEN cDNA 9330159F19 gene	1,72855E+14	7,46651E+14	2,54307E+14	0.0413440089731152	0.178312638271897	-3,66341E+14	8,59931E+13	8,74481E+14	7,79942E+14	5,76848E+14	7,37198E+14	6,51504E+14
TC1700000846.mm.2	Clic5	chloride intracellular channel 5	-0.896208961301109	6,38018E+14	-2,54204E+14	0.0414040104539824	0.178392054437673	-3,66478E+14	5,76009E+14	6,11036E+14	6,06555E+14	6,68224E+13	6,92338E+14	6,73949E+14
TC1800001477.mm.2	Cplx4	complexin 4	-0.984139907227749	4,78683E+13	-2,54197E+13	0.0414080130006223	0.178392054437673	-3,66487E+14	4,30445E+14	4,07335E+14	4,68132E+14	5,31577E+14	4,85285E+14	5,49322E+13
TC0800002578.mm.2	D8Ertd738e	DNA segment, Chr 8, ERATO Doi 738, expressed	1,27338E+14	7,98735E+14	2,5419E+14	0.041411993157927	0.178392054437673	-3,66496E+14	8,89716E+14	8,77599E+14	8,11611E+14	7,00849E+14	8,04392E+14	7,08247E+14
TC1900001671.mm.2	Smndc1	survival motor neuron domain containing 1	1,25614E+14	6,74236E+14	2,54171E+13	0.0414230636030391	0.178392054437673	-3,66521E+14	7,67222E+14	7,58845E+14	6,73734E+14	5,70775E+14	6,8773E+14	5,87108E+14
TC1400000813.mm.2	Zmym2	zinc finger, MYM-type 2	1,73724E+14	7,99606E+13	2,54163E+14	0.0414274207278109	0.178392054437673	-3,66531E+14	9,06341E+13	8,92532E+14	8,42143E+14	7,05192E+14	8,13067E+14	6,38359E+14
TC0200005404.mm.2	Cables2	CDK5 and Abl enzyme substrate 2	-0.804891378572512	6,58296E+14	-2,54161E+14	0.0414288504751301	0.178392054437673	-3,66534E+14	6,15705E+14	5,99557E+14	6,58699E+14	6,99577E+14	6,9746E+13	6,7878E+14
TC1200001761.mm.2	Gm2436	predicted gene 2436	1,29368E+14	6,41598E+14	2,5412E+14	0.0414528139529521	0.178439052690194	-3,66588E+14	7,43296E+14	6,99631E+14	6,57152E+14	5,65319E+14	6,47716E+14	5,36475E+14
TC0800003242.mm.2	Trim61/Trim60	tripartite motif-containing 61/tripartite motif-containing 60	-1,03425E+14	6,59886E+14	-2,54104E+14	0.0414619135649529	0.178439052690194	-3,66609E+13	5,77371E+13	5,73685E+14	6,80246E+14	7,70108E+14	6,53457E+14	7,04449E+14
TC0400000496.mm.2	Stra6l	STRA6-like	-1,48141E+14	6,59917E+14	-2,54041E+14	0.0414983306983082	0.178460000319457	-3,66691E+14	5,49359E+14	5,79576E+13	6,25484E+14	7,79865E+14	6,66864E+14	7,58353E+14
TC0200002577.mm.2	Cse1l	chromosome segregation 1-like (S. cerevisiae)	1,82218E+14	6,7216E+14	2,54033E+14	0.041502821190658	0.178460000319457	-3,66702E+13	7,81052E+14	8,02866E+14	7,22777E+14	5,06785E+14	6,55643E+14	5,63839E+14
TC0200003447.mm.2	Lrp1b	low density lipoprotein-related protein 1B	-1,00586E+14	6,70094E+14	-2,5403E+14	0.0415048077338549	0.178460000319457	-3,66706E+14	6,31033E+14	6,37388E+14	6,25351E+14	6,57195E+13	7,80874E+14	6,8872E+13
TC1600000726.mm.2	Cblb	Casitas B-lineage lymphoma b	1,07829E+14	7,35736E+14	2,54E+14	0.0415224471318952	0.178460000319457	-3,66746E+14	7,85271E+13	7,84695E+14	7,94538E+14	6,95602E+14	7,0348E+14	6,50829E+14
TC0700002792.mm.2	Gramd1a	GRAM domain containing 1A	0.957398134055355	6,75838E+14	2,53989E+14	0.0415287257332617	0.178460000319457	-3,6676E+14	7,35987E+14	7,36051E+12	6,8851E+14	6,13919E+14	6,63153E+14	6,17406E+14
TC1600000252.mm.2	2510002D24Rik	RIKEN cDNA 2510002D24 gene	-1,11362E+14	7,31931E+14	-2,53981E+14	0.041533234108978	0.178460000319457	-3,6677E+14	6,50077E+14	6,58236E+14	7,22172E+13	8,30068E+14	7,41023E+14	7,90013E+14
TC1100003856.mm.2	Gfap	glial fibrillary acidic protein	1,08633E+14	9,25644E+14	2,53949E+14	0.0415519444896751	0.178492797004035	-3,66813E+13	9,73304E+14	1,07493E+14	8,626E+14	8,12068E+13	9,12444E+14	9,1852E+14
TC1100003234.mm.2	Rph3al	rabphilin 3A-like (without C2 domains)	-0.967797214196279	5,90733E+14	-2,53881E+14	0.0415911442946476	0.178593494361653	-3,66901E+14	5,45558E+14	5,24478E+14	6,08731E+14	6,02034E+14	5,8826E+14	6,75336E+14
TC1700002343.mm.2	Gtf2f1	general transcription factor IIF, polypeptide 1	0.837329831828097	7,95258E+14	2,53851E+14	0.041608608342425	0.178593494361653	-3,66941E+14	8,47739E+14	8,25325E+14	7,84845E+14	8,0804E+14	7,7696E+13	7,28638E+14
TC0200000438.mm.2	Kcnt1	potassium channel, subfamily T, member 1	1,11986E+14	6,79185E+14	2,53851E+13	0.0416086376114718	0.178593494361653	-3,66941E+14	7,27324E+14	7,47097E+14	6,72598E+14	6,7764E+14	6,84376E+14	5,66073E+14
TC1100001565.mm.2	Stard3	START domain containing 3	1,18245E+14	6,25992E+14	2,53813E+14	0.0416310807941897	0.1786422383054	-3,66991E+14	7,31116E+14	7,10243E+14	6,11853E+14	4,97873E+14	6,33536E+14	5,71332E+13
TC0300002742.mm.2	Sars	seryl-aminoacyl-tRNA synthetase	0.957230311869166	9,11505E+12	2,53761E+14	0.0416611203164367	0.178723544105097	-3,67059E+14	9,9164E+14	9,67101E+14	9,12089E+14	8,30903E+14	8,94711E+14	8,72584E+14
TC0500000373.mm.2	Rnf4	ring finger protein 4	1,69362E+14	7,13575E+14	2,53741E+14	0.0416727843172869	0.178725997791973	-3,67085E+14	8,26114E+14	8,43436E+14	7,28021E+14	5,5305E+14	7,28193E+14	6,02633E+14
TC0900001357.mm.2	Dcaf1	DDB1 and CUL4 associated factor 1	2,1685E+14	6,10403E+14	2,53687E+14	0.0417044798971567	0.178814338633019	-3,67157E+14	7,83768E+14	7,84437E+14	6,15245E+14	3,44465E+14	6,17338E+14	5,17167E+14
TC0800001244.mm.2	Pla2g15	phospholipase A2, group XV	0.841995498536279	7,15628E+14	2,53632E+14	0.0417363868761864	0.178900050267443	-3,67229E+14	7,63756E+14	7,63099E+13	7,32641E+14	6,75812E+14	6,8826E+13	6,70196E+14
TC1100003090.mm.2	Alox12	arachidonate 12-lipoxygenase	-1,03608E+14	4,87341E+14	-2,53615E+14	0.041746675914205	0.178900050267443	-3,67252E+14	4,45188E+13	4,11965E+14	4,75769E+14	5,26756E+14	4,89925E+14	5,74443E+14
TC1200000967.mm.2	Spata7	spermatogenesis associated 7	0.922396757494531	6,31968E+13	2,53558E+12	0.0417794946320456	0.178948785872933	-3,67326E+14	6,85989E+14	6,61296E+14	6,4594E+14	6,3578E+14	6,19117E+14	5,43689E+14
TC0700001660.mm.2	Arntl	aryl hydrocarbon receptor nuclear translocator-like	1,47962E+14	6,25144E+14	2,53557E+14	0.0417802601907759	0.178948785872933	-3,67327E+14	6,97208E+14	7,16077E+14	6,54369E+14	5,6585E+14	6,37717E+14	4,79645E+14
TC0800001520.mm.2	Tcf25	transcription factor 25 (basic helix-loop-helix)	1,14782E+13	9,11888E+14	2,53529E+14	0.0417968673942719	0.178969348484583	-3,67365E+14	9,92174E+14	9,97054E+13	9,20409E+14	8,02029E+14	9,00487E+14	8,59177E+12
TC1500002247.mm.2	Bin2	bridging integrator 2	0.876421320875445	7,82369E+14	2,53511E+14	0.041807275348847	0.178969348484583	-3,67388E+14	8,25045E+14	8,68564E+14	7,6723E+13	7,15048E+14	7,53312E+13	7,65013E+14
TC1000000836.mm.2	Arhgap45	Rho GTPase activating protein 45	-0.779067183411599	6,6928E+14	-2,53476E+14	0.0418276213548773	0.179008887886093	-3,67434E+14	6,16777E+14	6,3341E+14	6,77286E+14	6,82203E+14	7,20542E+14	6,85458E+14
TC0600001972.mm.2	Prrt4	proline-rich transmembrane protein 4	-0.939141434450046	6,8778E+14	-2,53401E+14	0.0418716558423463	0.179149758520373	-3,67532E+13	5,85017E+14	6,44819E+14	7,12826E+14	7,66355E+14	6,9652E+14	7,21143E+14
TC1100002752.mm.2	Slc22a21	solute carrier family 22 (organic cation transporter), member 21	-1,0539E+14	6,40938E+14	-2,53371E+14	0.0418892166973215	0.179177315806727	-3,67572E+14	5,91234E+14	5,51708E+14	6,41108E+14	7,0786E+14	6,39391E+14	7,1433E+14
TC1400001338.mm.2	Pcca	propionyl-Coenzyme A carboxylase, alpha polypeptide	1,75803E+14	5,97244E+14	2,53325E+14	0.0419162221627582	0.179245246207145	-3,67632E+14	7,12783E+14	7,13906E+14	6,21888E+14	4,59803E+14	6,16679E+13	4,58403E+14
TC0300000283.mm.2	Spata5	spermatogenesis associated 5	0.770574199739149	5,57791E+14	2,53295E+14	0.0419336425473573	0.179272163004927	-3,67671E+14	6,07147E+14	5,71462E+14	5,70659E+14	5,72985E+14	5,13515E+14	5,10979E+14
TC1400002863.mm.2	Ipo4	importin 4	0.920456543061144	7,24447E+14	2,5324E+14	0.0419658860723833	0.179333631367223	-3,67744E+14	7,87308E+14	7,66982E+13	7,49654E+14	6,72541E+14	6,86969E+14	6,83229E+14
TC1100004110.mm.2	Srp68	signal recognition particle 68	1,46349E+14	6,74669E+14	2,53233E+14	0.0419702801393781	0.179333631367223	-3,67753E+14	7,88192E+14	7,69265E+14	6,9388E+14	5,31917E+14	6,70288E+14	5,9447E+14
TC1500001102.mm.2	Pcbp2	poly(rC) binding protein 2	1,70814E+14	9,74346E+14	2,53118E+14	0.0420379421036727	0.17957512224721	-3,67905E+14	1,07666E+14	1,0703E+14	1,02528E+14	8,68501E+14	9,79046E+14	8,26288E+14

TC1000003148.mm.2	Esyt1	extended synaptotagmin-like protein 1	-1,06001E+14	6,75902E+14	-2,53076E+14	0.0420628637562475	0.179633958045167	-3,6796E+14	6,03493E+14	6,24372E+14	6,46121E+14	7,41558E+14	6,95431E+14	7,44436E+13
TC0600002240.mm.2	Tra2a	transformer 2 alpha	1,52295E+12	7,62555E+14	2,53054E+14	0.0420754496384572	0.179640095395989	-3,67988E+14	8,69456E+14	8,80265E+14	7,69604E+14	6,10874E+14	7,74173E+12	6,70958E+14
TC1400000100.mm.2	3830406C13Rik	RIKEN cDNA 3830406C13 gene	1,97586E+14	5,77072E+13	2,52995E+14	0.0421105043639305	0.179644935209397	-3,68067E+14	6,91511E+14	7,15865E+14	6,35429E+14	4,07423E+14	5,67847E+14	4,44358E+14
TC0400001465.mm.2	Clspn	claspin	-0.815033815147148	5,13532E+14	-2,5298E+14	0.0421189828084478	0.179644935209397	-3,68086E+14	4,80454E+14	4,79916E+14	5,12807E+14	4,84637E+14	5,39678E+14	5,83699E+14
TC1000003144.mm.2	Nabp2	nucleic acid binding protein 2	0.957328168603796	9,11606E+14	2,52973E+14	0.0421231745999179	0.179644935209397	-3,68095E+14	9,6865E+14	9,62803E+14	9,27214E+14	8,70983E+14	9,03923E+14	8,36064E+13
TC0700000872.mm.2	Otog	otogelin	-0.955732515860521	5,7773E+14	-2,5296E+14	0.0421310590250702	0.179644935209397	-3,68112E+14	5,12595E+14	5,10468E+14	5,7833E+14	6,50777E+14	5,82163E+14	6,32046E+14
TC1100000432.mm.2	Pwwp2a	PWWP domain containing 2A	-1,77055E+14	9,66989E+14	-2,52943E+14	0.0421407924971698	0.179644935209397	-3,68134E+14	8,50919E+13	8,45676E+14	1,12241E+14	9,32223E+14	1,089E+14	9,61711E+14
TC0400002075.mm.2	C030017K20Rik	RIKEN cDNA C030017K20 gene	-1,0232E+14	6,39421E+14	-2,52935E+14	0.0421457950164888	0.179644935209397	-3,68145E+14	5,79803E+13	5,8494E+14	6,00205E+14	7,02194E+14	6,84158E+14	6,85225E+14
TC0900003062.mm.2	Ndufaf3	NADH:ubiquinone oxidoreductase complex assembly factor 3	0.837935000449588	6,68805E+14	2,52908E+13	0.0421616503996222	0.179644935209397	-3,68181E+14	7,42934E+13	7,16441E+14	6,54914E+14	6,06752E+14	6,56836E+14	6,34952E+14
TC1100000961.mm.2	Rnf167	ring finger protein 167	1,1958E+14	7,668E+14	2,52858E+14	0.0421909605092427	0.179644935209397	-3,68246E+14	8,55084E+14	8,4469E+14	7,67444E+14	6,72972E+14	7,78039E+13	6,82574E+14
TC0X00000343.mm.2	Rhox2b	reproductive homeobox 2B	-0.886491104326189	6,5654E+14	-2,52839E+14	0.0422024941974504	0.179644935209397	-3,68272E+14	5,84994E+14	5,87748E+14	6,73547E+14	7,42911E+14	6,84687E+14	6,65354E+13
TC1900001026.mm.2	Fkbp2	FK506 binding protein 2	1,46148E+14	7,95521E+14	2,52828E+14	0.0422088156446087	0.179644935209397	-3,68286E+14	9,00182E+14	8,901E+14	7,93872E+13	6,90984E+14	8,22597E+14	6,75389E+14
TC0300000206.mm.2	Prkci	protein kinase C, iota	2,04593E+14	6,32005E+14	2,52828E+14	0.0422091382735512	0.179644935209397	-3,68286E+14	7,73489E+14	7,65062E+14	6,83882E+14	4,40231E+14	6,24192E+14	5,05173E+14
TC0700001721.mm.2	Tmem159	transmembrane protein 159	0.76928237606275	5,391E+14	2,52806E+14	0.0422220484658583	0.179644935209397	-3,68315E+14	5,87876E+13	6,16763E+14	4,96261E+14	4,82917E+14	5,3374E+14	5,17042E+14
TC0800000756.mm.2	Isyna1	myo-inositol 1-phosphate synthase A1	0.742898886736861	7,03148E+14	2,52792E+14	0.0422301581150795	0.179644935209397	-3,68333E+14	7,44953E+14	7,49546E+14	7,00023E+14	6,78445E+14	6,73822E+14	6,72097E+14
TC0700001773.mm.2	Lcmt1	leucine carboxyl methyltransferase 1	0.990464357875589	7,19958E+14	2,52754E+14	0.0422526510570415	0.179644935209397	-3,68383E+14	7,62856E+14	7,87299E+14	7,22018E+14	6,95514E+14	7,20281E+14	6,31781E+14
TC1500000695.mm.2	Mgat3	mannoside acetylglucosaminyltransferase 3	1,20418E+14	6,41869E+14	2,52753E+14	0.0422531562281068	0.179644935209397	-3,68384E+14	7,26741E+14	7,08998E+14	6,58981E+14	5,61593E+14	6,44291E+13	5,50613E+14
TC0900001632.mm.2	1700048O20Rik	RIKEN cDNA 1700048O20 gene	1,87101E+14	6,23866E+14	2,5275E+14	0.0422549683140083	0.179644935209397	-3,68388E+14	7,79826E+14	7,09517E+13	6,85735E+14	4,52977E+14	5,71185E+14	5,43955E+14
TC0800000711.mm.2	Atp6v1b2	ATPase, H+ transporting, lysosomal V1 subunit B2	2,31616E+14	8,96432E+14	2,52711E+14	0.0422781684425033	0.179695926458346	-3,6844E+14	1,07018E+14	1,07167E+14	9,19251E+14	6,38872E+14	9,07876E+14	7,70742E+14
TSUnmapped00000028.mm.2	Defa-rs10	defensin, alpha, related sequence 10	-0.866984397074487	6,34032E+14	-2,52671E+14	0.042301932892575	0.179695926458346	-3,68493E+14	5,88724E+14	5,85322E+14	6,18847E+14	6,6386E+13	6,48787E+14	6,98656E+14
TC0900002565.mm.2	C2cd4a	C2 calcium-dependent domain containing 4A	-1,24376E+14	6,06807E+14	-2,52657E+14	0.0423097895253994	0.179695926458346	-3,6851E+14	5,3156E+14	5,22581E+14	5,74646E+14	7,09158E+14	6,27079E+14	6,75815E+11
TC0100003170.mm.2	Nek7	NIMA (never in mitosis gene a)-related expressed kinase 7	0.937849252760402	6,64378E+14	2,52652E+14	0.0423130259909731	0.179695926458346	-3,68517E+14	7,41474E+14	7,39059E+14	6,26151E+14	5,86621E+14	6,83682E+14	6,09281E+14
TC1600000534.mm.2	Hgd	homogentisate 1, 2-dioxygenase	-0.873961323764441	5,05655E+14	-2,52636E+14	0.0423227233233676	0.179695926458346	-3,68539E+14	4,48808E+14	4,80426E+14	5,13963E+14	4,86708E+14	5,79541E+14	5,24485E+14
TC0500000303.mm.2	Agbl5	ATP/GTP binding protein-like 5	0.966772988154485	6,03813E+14	2,52599E+14	0.0423443070603644	0.179740205390846	-3,68587E+14	6,66086E+14	6,30413E+14	6,23491E+14	5,99742E+14	5,91525E+14	5,11618E+13
TC0700004606.mm.2	Ppfia1	protein tyrosine phosphatase, receptor type, f polypeptide (PTPRF), interacting protein (liprin), alpha 1	0.834642108324558	5,87528E+14	2,52507E+14	0.0423986944437932	0.179886799064323	-3,68707E+14	6,46105E+14	6,14756E+14	6,07479E+14	5,62893E+14	5,36201E+14	5,57731E+14
TC0300002325.mm.2	1500004A13Rik	RIKEN cDNA 1500004A13 gene	1,59925E+12	9,71832E+14	2,52494E+14	0.0424066218426647	0.179886799064323	-3,68725E+14	1,08382E+14	1,08238E+14	9,9734E+14	8,21088E+14	9,72958E+14	8,734E+14
TC0700000980.mm.2	Apba2	amyloid beta (A4) precursor protein-binding, family A, member 2	0.856133482363003	7,95959E+14	2,52441E+14	0.0424384303162172	0.179886799064323	-3,68795E+14	8,63501E+14	8,32614E+14	7,71488E+14	7,83265E+14	7,90203E+14	7,34685E+14
TC0400004071.mm.2	Camta1	calmodulin binding transcription activator 1	1,49256E+14	8,8274E+14	2,52437E+14	0.0424406494101066	0.179886799064323	-3,688E+14	1,00074E+14	9,79359E+14	8,8391E+14	7,50849E+14	9,04397E+14	7,77188E+14
TC0200003954.mm.2	Olfr1087	olfactory receptor 1087	-1,049E+14	4,61501E+14	-2,52436E+14	0.0424411434908335	0.179886799064323	-3,68801E+14	4,07123E+14	4,20252E+14	4,01018E+14	5,03438E+14	5,03347E+14	5,33827E+14
TC0200005028.mm.2	Tti1	TELO2 interacting protein 1	1,18837E+14	6,15734E+14	2,52428E+14	0.0424458269746514	0.179886799064323	-3,68811E+14	6,92907E+14	7,0491E+14	6,32234E+14	5,04209E+14	5,61685E+14	5,98458E+14
TC0300002765.mm.2	Ntng1	netrin G1	1,3587E+14	6,95232E+14	2,52406E+14	0.0424590033734084	0.179891248288994	-3,68841E+14	8,01193E+14	7,4489E+13	7,27811E+14	6,23158E+14	6,93135E+14	5,81207E+14
TC0300002403.mm.2	Lce1a2	late cornified envelope 1A2	-0.734828813368899	8,37164E+14	-2,52342E+14	0.0424971585075122	0.179891248288994	-3,68925E+14	7,88943E+14	7,93738E+13	8,57497E+14	8,5316E+14	8,5697E+14	8,72677E+14
TC1300001804.mm.2	Rpp40	ribonuclease P 40 subunit	-0.985532771155444	7,03552E+14	-2,52324E+14	0.0425076944259809	0.179891248288994	-3,68948E+14	6,33428E+14	6,37826E+14	7,01376E+14	7,80322E+14	7,07957E+14	7,60404E+14
TC0300000416.mm.2	Spg20	spastic paraplegia 20, spartin (Troyer syndrome) homolog (human)	1,24694E+14	6,49007E+14	2,52306E+14	0.0425184222227851	0.179891248288994	-3,68972E+14	7,33963E+14	7,09987E+12	6,97759E+14	5,57771E+14	6,09778E+14	5,84786E+14
TC0400002476.mm.2	Rpp25l	ribonuclease P/MRP 25 subunit-like	-1,18196E+14	7,47824E+14	-2,52277E+14	0.0425357401090069	0.179891248288994	-3,6901E+14	6,70136E+14	6,57063E+13	7,42787E+13	8,53368E+13	7,45689E+14	8,17901E+14
TC1300001927.mm.2	Tpmt	thiopurine methyltransferase	0.898075501131452	6,43588E+14	2,52271E+14	0.0425392442862696	0.179891248288994	-3,69018E+14	7,10086E+14	7,11599E+14	6,36073E+14	5,61704E+14	6,14938E+14	6,27131E+14
TC1500001045.mm.2	Gm5475	predicted gene 5475	-1,07748E+14	5,7435E+14	-2,5224E+14	0.0425576658103629	0.179891248288994	-3,69058E+14	5,04864E+14	5,09485E+14	5,51824E+14	6,50081E+14	5,86752E+14	6,43092E+14
TC1700001914.mm.2	Btnl6	butyrophilin-like 6	-1,13967E+14	5,7874E+14	-2,52202E+14	0.0425804921558537	0.179891248288994	-3,69109E+14	4,92261E+14	4,92656E+14	5,75542E+14	6,99129E+13	5,96789E+14	6,16062E+14
TC1300001554.mm.2	Olfr1359/Olfr1360	olfactory receptor 1359/olfactory receptor 1360	-1,12955E+14	4,78989E+14	-2,52201E+14	0.0425810842411092	0.179891248288994	-3,6911E+14	4,44578E+14	3,95238E+14	4,61628E+14	5,06766E+14	4,8112E+14	5,84606E+14
TC1500000296.mm.2	Atp6v1c1	ATPase, H+ transporting, lysosomal V1 subunit C1	2,34919E+14	8,22847E+14	2,52143E+14	0.0426154863037551	0.179891248288994	-3,69186E+14	9,79305E+14	9,84996E+14	8,65796E+13	6,1252E+14	8,43204E+13	6,51258E+14
TC1200000718.mm.2	Rdh12	retinol dehydrogenase 12	0.754535467687735	5,58601E+14	2,52135E+14	0.0426205979510198	0.179891248288994	-3,69197E+14	6,15414E+14	5,9617E+14	5,2834E+14	5,51004E+14	5,28329E+14	5,32348E+14
TC0700001739.mm.2	Mettl9	methyltransferase like 9	0.889254902808319	8,05483E+14	2,52121E+14	0.0426286899009381	0.179891248288994	-3,69215E+14	8,54398E+14	8,78082E+14	8,07528E+14	7,38262E+14	7,74895E+13	7,79734E+13
TC0600002143.mm.2	Tas2r138	taste receptor, type 2, member 138	-1,32033E+14	5,11744E+14	-2,52089E+13	0.0426479666493622	0.179891248288994	-3,69257E+14	4,61527E+14	4,1692E+14	4,77333E+14	5,72167E+13	5,12775E+13	6,29739E+14
TC1500001017.mm.2	Aqp2	aquaporin 2	-0.816941072457457	5,98995E+14	-2,5206E+14	0.0426654667144849	0.179891248288994	-3,69296E+14	5,58009E+14	5,45354E+14	5,93608E+14	6,30245E+13	6,14735E+14	6,52016E+14
TC0400000880.mm.2	Focad	focadhesin	0.754684859511443	5,83728E+14	2,52034E+14	0.0426805799394678	0.179891248288994	-3,69329E+13	6,19433E+14	6,20911E+14	5,99551E+14	5,73065E+14	5,51954E+14	5,37453E+14
TC0500001526.mm.2	Rabgef1	RAB guanine nucleotide exchange factor (GEF) 1	0.856324747165093	7,89031E+14	2,52034E+14	0.0426809561513589	0.179891248288994	-3,6933E+14	8,40437E+14	8,32655E+14	7,91134E+14	7,68136E+13	7,81079E+14	7,20747E+14
TC1500000969.mm.2	Pfkm	phosphofructokinase, muscle	1,43246E+14	8,33601E+14	2,52027E+14	0.0426847773240107	0.179891248288994	-3,69338E+14	9,33428E+14	9,26612E+14	8,69952E+13	6,98066E+14	8,05076E+14	7,68472E+14
TC1000002350.mm.2	Phyhipl/D630013N20 Rik	phytanoyl-CoA hydroxylase interacting protein-like/RIKEN cDNA D630013N20 gene	1,17893E+14	8,62913E+14	2,52025E+14	0.0426859375058898	0.179891248288994	-3,69341E+14	9,43864E+14	9,26521E+14	8,98296E+14	7,74198E+14	8,38656E+14	7,95942E+14
TC0400000407.mm.2	Gm21953/Gm20878/ Gm21586	predicted gene, 21953/predicted gene, 20878/predicted gene, 21586	0.962617383290462	9,09199E+14	2,52023E+14	0.0426875757665867	0.179891248288994	-3,69345E+14	9,8405E+14	1,00112E+14	8,61962E+13	8,15412E+14	9,34032E+14	8,58618E+14
TC0X00001580.mm.2	Acot9	acyl-CoA thioesterase 9	1,70398E+14	5,56486E+14	2,51996E+14	0.0427033433946314	0.179891248288994	-3,69379E+14	6,53518E+14	6,46987E+14	6,38017E+14	4,40684E+14	5,27828E+14	4,31881E+14
TC0500003254.mm.2	Sbno1	strawberry notch 1	1,38016E+14	6,71762E+14	2,51989E+14	0.0427074787339313	0.179891248288994	-3,69388E+14	7,5876E+14	7,68678E+14	6,73458E+14	5,7827E+14	6,94268E+14	5,57138E+14
TC1100001575.mm.2	Gsdma3	gasdermin A3	-1,53519E+14	4,00651E+14	-2,51986E+14	0.0427095944599269	0.179891248288994	-3,69393E+14	2,938E+14	2,99357E+14	3,79218E+14	5,31333E+14	3,91043E+14	5,09152E+14
TC1800000199.mm.2	Elp2	elongator acetyltransferase complex subunit 2	2,11399E+14	6,5232E+14	2,51978E+14	0.0427141584728346	0.179891248288994	-3,69403E+13	8,07902E+13	8,09469E+14	6,6581E+14	4,36167E+14	6,79702E+14	5,14871E+14
TC0100003382.mm.2	Dnm3	dynamin 3	2,34091E+13	8,22079E+14	2,51977E+14	0.0427148213215225	0.179891248288994	-3,69404E+13	1,00164E+14	9,74396E+14	8,45016E+14	6,03566E+14	8,55595E+14	6,52261E+14
TC0300001414.mm.2	Adh4	alcohol dehydrogenase 4 (class II), pi polypeptide	-1,51676E+14	4,89303E+14	-2,51916E+14	0.0427516298311712	0.179992589545846	-3,69485E+14	4,24228E+14	3,6038E+14	4,45595E+14	6,17653E+14	5,19867E+14	5,68094E+14
TC0500000213.mm.2	Abcb8	ATP-binding cassette, sub-family B (MDR/TAP), member 8	1,28594E+14	6,99229E+13	2,519E+14	0.0427612258909885	0.179992589545846	-3,69506E+14	7,91944E+14	7,80046E+14	7,24579E+14	5,84E+13	6,30035E+14	6,84768E+14
TC1700002244.mm.2	Rftn1	raftlin lipid raft linker 1	0.793393143695037	6,1206E+14	2,5188E+14	0.0427732139420839	0.179996029315434	-3,69533E+14	6,70658E+13	6,25721E+14	6,20511E+14	6,17771E+14	5,63464E+14	5,74237E+14
TC0100001459.mm.2	Rc3h1	RING CCCH (C3H) domains 1	1,85554E+14	6,58404E+14	2,51836E+14	0.0427992013775892	0.180058363393497	-3,6959E+14	7,85789E+14	7,94941E+14	6,81685E+14	4,78387E+14	6,71501E+14	5,38121E+14
TC0900000254.mm.2	Thyn1	thymocyte nuclear protein 1	0.817805489408125	7,0384E+13	2,51808E+14	0.0428158706852363	0.18008147333626	-3,69626E+14	7,62048E+14	7,85153E+14	6,37807E+14	6,54389E+14	7,14945E+14	6,68695E+13
TC1300002075.mm.2	H2afy	H2A histone family, member Y	0.854001300233195	7,32087E+14	2,51741E+14	0.042856031480257	0.18014795328696	-3,69714E+14	7,88886E+13	7,88648E+14	7,11504E+14	6,96749E+14	7,34853E+14	6,71881E+14
TC0800000370.mm.2	Tti2	TELO2 interacting protein 2	0.752512209482937	5,38285E+14	2,51701E+14	0.0428805401505912	0.18014795328696	-3,69768E+13	5,82421E+14	5,95597E+14	5,03113E+14	5,21061E+14	5,03981E+13	5,23535E+14
TC1400000748.mm.2	Rem2	rad and gem related GTP binding protein 2	-0.804008819072267	8,38605E+14	-2,51677E+14	0.0428946689421365	0.18014795328696	-3,69799E+14	7,71603E+14	8,07618E+14	8,39278E+14	8,73108E+13	8,65942E+14	8,7408E+14
TC1100002690.mm.2	BC049762	cDNA sequence BC049762	-0.895330548810675	5,05263E+14	-2,51636E+14	0.042919563451145	0.18014795328696	-3,69853E+14	4,72745E+14	4,43227E+14	4,77946E+14	5,42622E+14	5,59867E+14	5,35171E+14

TC0700002479.mm.2	Cd3eap	CD3E antigen, epsilon polypeptide associated protein	-0.781582864230617	6,68607E+14	-2,51608E+14	0.0429361427415286	0.18014795328696	-3,6989E+14	6,18184E+14	6,28969E+14	6,74898E+14	6,86119E+14	6,82048E+14	7,21422E+14
TC1800000434.mm.2	Rbm27	RNA binding motif protein 27	1,52321E+14	7,65564E+14	2,51607E+14	0.0429366241220571	0.18014795328696	-3,69891E+13	8,61444E+14	8,37745E+14	8,20469E+14	6,79178E+14	7,60507E+14	6,34038E+14
TC0600002161.mm.2	Kel	Kell blood group	-1,7848E+14	5,72676E+14	-2,51599E+14	0.0429417780229116	0.18014795328696	-3,69902E+14	4,73953E+14	4,58862E+14	5,24736E+14	6,88729E+14	5,62756E+14	7,27019E+14
TC0600003433.mm.2	Itpr2	inositol 1,4,5-triphosphate receptor 2	0.966842814706264	5,6818E+14	2,51593E+14	0.0429454610412981	0.18014795328696	-3,6991E+14	6,12644E+14	6,32331E+14	5,55184E+14	5,57313E+14	5,70478E+14	4,81128E+13
TC0900002151.mm.2	Pdzd3	PDZ domain containing 3	-0.994138283714133	5,14398E+14	-2,51591E+14	0.0429465879420446	0.18014795328696	-3,69912E+14	4,39513E+14	4,54925E+14	5,08865E+14	5,89822E+14	5,20788E+14	5,72477E+14
TC0700004522.mm.2	Rnh1	ribonuclease/angiogenin inhibitor 1	0.79803419915033	7,03388E+14	2,51581E+14	0.0429527286890006	0.18014795328696	-3,69926E+14	7,63234E+14	7,61393E+14	6,8857E+14	6,42111E+14	6,88865E+14	6,76155E+14
TC0500000302.mm.2	Tmem214	transmembrane protein 214	1,10644E+14	7,3338E+14	2,51565E+13	0.0429622644014234	0.18014795328696	-3,69947E+14	7,94461E+13	7,89377E+14	7,80233E+14	6,73661E+14	7,081E+14	6,54449E+14
TC0100001720.mm.2	Sde2	SDE2 telomere maintenance homolog (S. pombe)	0.918589415218025	6,44366E+14	2,51559E+14	0.0429658402831121	0.18014795328696	-3,69955E+14	7,33665E+14	6,87112E+14	6,32593E+14	5,75769E+14	6,01681E+14	6,35378E+14
TC0700004404.mm.2	Chst15	carbohydrate sulfotransferase 15	1,4343E+14	7,21155E+14	2,5154E+14	0.0429772032161823	0.180148718892389	-3,69979E+14	8,1864E+14	8,20173E+14	7,50308E+14	5,85855E+14	7,08195E+14	6,43758E+14
TC1900000994.mm.2	Slc22a20	solute carrier family 22 (organic anion transporter), member 20	-0.910574175864484	5,60756E+14	-2,51449E+14	0.0430320656895895	0.180327935415665	-3,70099E+14	5,18302E+14	5,14617E+14	5,41177E+14	5,75408E+14	5,71939E+14	6,43094E+14
TC0500002244.mm.2	Lrpap1	low density lipoprotein receptor-related protein associated protein 1	1,23543E+14	8,8808E+14	2,51432E+14	0.0430423409426332	0.180327935415665	-3,70121E+14	9,8463E+14	9,60432E+14	9,10283E+14	7,75157E+14	8,6892E+14	8,29056E+14
TC0900000132.mm.2	Chordc1	cysteine and histidine-rich domain (CHORD)-containing, zinc-binding protein 1	1,47657E+14	7,48153E+13	2,5139E+14	0.043067789019139	0.180358329547031	-3,70177E+14	8,37334E+14	8,48906E+14	7,5288E+14	6,58872E+14	7,74174E+14	6,16752E+14
TC0200002477.mm.2	Ift52	intraflagellar transport 52	1,11177E+14	6,56332E+14	2,51383E+14	0.0430719823804987	0.180358329547031	-3,70186E+14	7,31422E+14	7,35078E+14	6,67843E+14	5,59503E+14	5,95673E+14	6,48472E+14
TC0900003042.mm.2	Sema3f	sema domain, immunoglobulin domain (Ig), short basic domain, secreted, (semaphorin) 3F	0.788553772305373	6,39489E+13	2,51362E+14	0.0430846750232905	0.180364606040603	-3,70214E+14	6,70457E+14	6,74245E+14	6,32519E+14	6,7272E+14	5,8338E+14	6,03611E+14
TC0700000414.mm.2	4732471J01Rik	RIKEN cDNA 4732471J01 gene	-0.918720176467645	6,25762E+14	-2,51339E+14	0.0430985767495699	0.180374359246768	-3,70244E+14	5,59634E+14	5,82771E+14	6,12637E+14	6,7338E+14	6,8727E+14	6,3888E+14
TC0600003475.mm.2	Dennd5b	DENN/MADD domain containing 5B	1,84623E+14	6,8553E+14	2,51303E+14	0.0431199941334411	0.180374359246768	-3,70291E+14	8,09217E+13	7,89968E+14	6,70755E+14	6,13302E+14	7,32365E+14	4,97574E+14
TC0X00002660.mm.2	Arhgef9	CDC42 guanine nucleotide exchange factor (GEF) 9	2,26399E+14	8,47689E+14	2,51289E+14	0.0431285868214634	0.180374359246768	-3,70309E+14	1,00463E+12	1,01144E+14	8,76846E+14	6,29944E+14	8,72153E+14	6,91121E+13
TC0400000659.mm.2	Hsdl2	hydroxysteroid dehydrogenase like 2	1,58714E+14	6,80649E+14	2,51267E+14	0.0431419246083119	0.180374359246768	-3,70338E+13	7,80879E+14	7,61802E+14	7,56228E+14	5,59733E+14	6,33384E+14	5,91869E+14
TC1200001281.mm.2	Crip2	cysteine rich protein 2	0.856007556951632	8,17164E+14	2,51231E+14	0.0431635201402477	0.180374359246768	-3,70385E+14	8,70889E+14	8,98282E+14	7,97641E+14	7,382E+13	7,96257E+14	8,01713E+14
TC0700001576.mm.2	Syt9	synaptotagmin IX	1,2464E+13	5,80453E+14	2,5123E+14	0.0431641282794678	0.180374359246768	-3,70387E+14	6,71695E+14	6,06503E+14	5,46168E+14	6,29437E+14	5,64095E+14	4,64822E+14
TC0500002579.mm.2	Txk	TXK tyrosine kinase	-0.998440888594813	5,62956E+14	-2,51228E+14	0.043165365186839	0.180374359246768	-3,70389E+14	4,92945E+14	5,18011E+14	5,86147E+13	5,69987E+14	5,584E+14	6,52244E+14
TC0800000117.mm.2	Proz	protein Z, vitamin K-dependent plasma glycoprotein	-0.867661032911055	6,61126E+14	-2,51191E+14	0.0431876273206731	0.180420596063782	-3,70438E+14	6,0411E+14	6,04089E+14	6,54462E+14	7,22365E+14	6,85465E+14	6,96266E+14
TC0300002977.mm.2	Arhgef38	Rho guanine nucleotide exchange factor (GEF) 38	-1,19019E+14	6,16679E+14	-2,5115E+14	0.0432125795816713	0.18047540886913	-3,70492E+14	5,36252E+14	5,34319E+14	6,02558E+14	7,18337E+14	6,19457E+14	6,89153E+14
TC1100001149.mm.2	Rab34	RAB34, member RAS oncogene family	-1,40931E+14	7,31876E+14	-2,51133E+14	0.0432231491855006	0.18047540886913	-3,70515E+14	6,4092E+14	6,38626E+14	8,53787E+14	6,96513E+14	8,16571E+14	7,44838E+14
TC0700000439.mm.2	Cyp2a4	cytochrome P450, family 2, subfamily a, polypeptide 4	-1,18785E+13	4,77876E+14	-2,51096E+14	0.0432453878638923	0.180521485660854	-3,70563E+14	3,90919E+14	4,21663E+14	4,67225E+14	5,37294E+14	4,69601E+14	5,80552E+14
TC0800001698.mm.2	Rab20	RAB20, member RAS oncogene family	-1,45282E+14	5,38373E+14	-2,51066E+14	0.0432637833917672	0.180525194885254	-3,70603E+14	4,37211E+13	4,80745E+14	4,83545E+14	6,20218E+14	5,49777E+13	6,58744E+14
TC1900001482.mm.2	Cyp2c70	cytochrome P450, family 2, subfamily c, polypeptide 70	-1,28863E+14	4,61579E+14	-2,51058E+14	0.0432686838358377	0.180525194885254	-3,70614E+14	3,37003E+14	4,16897E+12	4,47652E+14	5,56294E+14	4,70175E+14	5,41451E+13
TC0800001126.mm.2	Adgrg1	adhesion G protein-coupled receptor G1	1,05948E+14	8,50959E+14	2,51014E+14	0.0432951984153642	0.180549160510743	-3,70671E+14	9,18662E+14	9,18384E+14	8,76792E+14	7,66765E+14	8,05702E+14	8,19448E+14
TC0800002175.mm.2	Trappc11	trafficking protein particle complex 11	0.858629687571443	7,23978E+14	2,51005E+14	0.0433005356960759	0.180549160510743	-3,70683E+14	8,04859E+14	7,43851E+14	6,99557E+14	7,18616E+14	6,97803E+12	6,79181E+14
TC0200002146.mm.2	Pet117/Kat14	PET117 homolog/lysine acetyltransferase 14	-0.746696098738376	8,3989E+14	-2,50993E+14	0.0433080435284567	0.180549160510743	-3,70699E+14	7,88037E+12	7,88042E+14	8,75132E+14	8,62713E+13	8,50352E+14	8,75066E+14
TC0200003176.mm.2	Nacc2	nucleus accumbens associated 2, BEN and BTB (POZ) domain containing	1,19913E+14	6,00868E+14	2,50955E+14	0.0433311555537152	0.180598786714178	-3,70749E+14	6,86614E+14	6,62899E+14	5,31368E+14	6,14642E+14	5,0181E+14	6,07877E+14
TC0300000637.mm.2	Map9	microtubule-associated protein 9	1,71465E+14	7,00126E+14	2,50908E+14	0.0433594722572587	0.180670074083581	-3,7081E+14	8,27343E+14	7,99387E+14	7,16103E+14	5,74121E+14	7,26318E+14	5,57483E+14
TC1300000457.mm.2	Gm9979	predicted gene 9979	-1,61048E+14	5,00816E+14	-2,50879E+14	0.0433772887713642	0.180697583757237	-3,70849E+13	4,17612E+14	3,95051E+14	4,29542E+14	6,27928E+14	5,42303E+14	5,92456E+13
TC1100003719.mm.2	Krtap9-3	keratin associated protein 9-3	-0.790840030395375	7,01695E+14	-2,50818E+14	0.0434142282527828	0.180804719523672	-3,70929E+14	6,43208E+14	6,68521E+14	7,1311E+14	7,15431E+14	7,1544E+13	7,54457E+14
TC1400000331.mm.2	Btd	biotinidase	-0.8404614070197	6,66069E+14	-2,50707E+13	0.0434816735093683	0.181001977851531	-3,71075E+14	6,27142E+14	6,06096E+14	6,6001E+13	7,01839E+14	6,80394E+14	7,20935E+14
TC0800002758.mm.2	Got2	glutamatic-oxaloacetic transaminase 2, mitochondrial	1,59459E+14	1,01386E+14	2,50703E+14	0.0434840598438079	0.181001977851531	-3,7108E+14	1,12506E+14	1,10189E+14	1,05345E+14	8,94744E+14	1,01665E+14	8,91388E+14
TC0200004901.mm.2	Defb22	defensin beta 22	-0.762171830998013	4,85211E+14	-2,50658E+13	0.0435114846609229	0.181065674343048	-3,71139E+14	4,44086E+14	4,29701E+14	4,97324E+14	5,13937E+14	4,99053E+14	5,27168E+14
TC0100002382.mm.2	Ppil3	peptidylprolyl isomerase (cyclophilin)-like 3	0.735369188570131	6,5995E+14	2,50641E+14	0.043521836823101	0.181065674343048	-3,71161E+14	6,96915E+14	7,02868E+14	6,60137E+14	6,47069E+14	6,18496E+14	6,34213E+14
TC0900002971.mm.2	Bfsp2	beaded filament structural protein 2, phakinin	-0.976866320400876	5,47083E+13	-2,50574E+14	0.0435629482214911	0.181100661923645	-3,7125E+14	4,68095E+14	5,18638E+14	5,19587E+14	5,94071E+14	5,76675E+14	6,05431E+14
TC1400002756.mm.2	Ggact	gamma-glutamylamine cyclotransferase	1,0245E+14	5,76117E+14	2,5057E+14	0.0435657570113625	0.181100661923645	-3,71256E+14	6,75726E+14	6,36192E+14	5,59878E+14	4,77889E+14	5,78994E+14	5,28023E+14
TC0900000095.mm.2	Mtmr2	myotubularin related protein 2	1,73286E+14	7,18416E+14	2,50556E+13	0.0435740539879992	0.181100661923645	-3,71274E+14	8,09099E+14	8,23939E+13	7,71995E+14	6,18994E+14	7,25803E+13	5,60664E+14
TC1800000672.mm.2	Malt1	MALT1 paracaspase	-1,01941E+14	5,84791E+14	-2,50554E+14	0.0435752042622709	0.181100661923645	-3,71276E+13	5,48526E+14	5,17431E+13	5,41902E+14	6,3105E+14	6,13956E+14	6,55883E+14
TC0100003119.mm.2	Lgr6	leucine-rich repeat-containing G protein-coupled receptor 6	-0.85148548379381	5,77244E+14	-2,50432E+14	0.0436499093457392	0.181335626363652	-3,71437E+14	5,59901E+14	5,03172E+14	5,75681E+14	5,9134E+14	6,02658E+14	6,30713E+13
TC1800001296.mm.2	Ccdc112	coiled-coil domain containing 112	1,04222E+14	5,30346E+14	2,5042E+14	0.0436574771816774	0.181335626363652	-3,71453E+14	6,08446E+14	5,8222E+14	5,5E+14	4,5919E+14	4,65008E+14	5,17214E+14
TC0200002127.mm.2	Snrpb2	U2 small nuclear ribonucleoprotein B	0.811107197344795	7,02627E+14	2,50391E+14	0.0436748426364489	0.181335626363652	-3,71491E+14	7,62331E+14	7,68121E+13	6,55063E+13	6,62013E+14	7,10556E+14	6,57675E+14
TC0300000605.mm.2	Serpini1	serine (or cysteine) peptidase inhibitor, clade I, member 1	1,92767E+13	7,84799E+14	2,50388E+14	0.043676755782122	0.181335626363652	-3,71495E+13	9,15823E+14	9,4438E+14	7,98945E+14	5,74978E+14	7,97824E+14	6,76846E+14
TC0700000989.mm.2	Snrpa1	small nuclear ribonucleoprotein polypeptide A'	0.996749915634293	6,43269E+14	2,50331E+13	0.043711981116141	0.181435124091803	-3,7157E+14	7,16122E+14	7,07997E+14	6,49977E+14	5,6075E+14	6,33273E+14	5,91496E+14
TC0500001572.mm.2	Abhd11	abhydrolase domain containing 11	0.913057622852143	6,3964E+13	2,50295E+14	0.0437337406831852	0.181478692667567	-3,71617E+14	7,04097E+14	6,82932E+14	5,98236E+14	6,48154E+14	5,72387E+14	6,32031E+14
TC1100003778.mm.2	Zfp385c	zinc finger protein 385C	-1,08951E+14	7,01687E+14	-2,50269E+14	0.0437497265335786	0.181498286208947	-3,71651E+14	6,50396E+14	6,27728E+14	6,64825E+14	7,71145E+14	7,25996E+14	7,70031E+14
TC0400004026.mm.2	Tardbp	TAR DNA binding protein	1,21348E+14	6,83752E+14	2,50246E+14	0.0437636546390912	0.181509335186532	-3,71681E+14	7,58937E+14	7,66348E+14	7,03932E+14	5,9071E+14	6,82375E+14	6,00214E+14
TC1900001599.mm.2	Psd	pleckstrin and Sec7 domain containing	1,31092E+14	8,8757E+14	2,50201E+14	0.0437913007349222	0.181577259068914	-3,7174E+14	9,73333E+14	9,98338E+14	8,9924E+14	7,45021E+14	8,63115E+14	8,46371E+14
TC0700000015.mm.2	Prpf31	pre-mRNA processing factor 31	0.879667307686774	7,26843E+14	2,50161E+13	0.0438160147142725	0.181615169076463	-3,71793E+14	7,91457E+14	7,85451E+14	7,25113E+13	6,58065E+14	7,14215E+13	6,86759E+14
TC0500002754.mm.2	Sdad1	SDA1 domain containing 1	0.97604993236168	5,94589E+14	2,5015E+14	0.0438229862452236	0.181615169076463	-3,71808E+14	6,52379E+14	6,30184E+14	6,30414E+14	5,67205E+14	5,77124E+14	5,10228E+14
TC1400001157.mm.2	Klf5	Kruppel-like factor 5	1,18422E+14	6,30124E+14	2,50044E+14	0.0438877403868267	0.18177131941957	-3,71947E+14	6,85996E+14	7,21264E+14	6,64237E+14	5,38831E+14	5,67463E+14	6,02954E+13
TC0900001836.mm.2	Zfp426/Zfp26	zinc finger protein 426/zinc finger protein 26	1,78197E+14	6,51218E+14	2,50043E+14	0.0438886788775729	0.18177131941957	-3,71949E+14	8,06437E+14	7,82575E+14	6,58381E+14	4,27297E+13	6,29861E+14	6,02757E+14
TC1000001840.mm.2	Myb	myeloblastosis oncogene	-0.888617300208567	6,32475E+14	-2,50033E+14	0.0438945077801494	0.18177131941957	-3,71961E+14	5,89916E+14	5,64471E+14	6,51123E+14	6,5723E+14	6,30904E+14	7,01206E+14
TC1000003103.mm.2	Arhgef25	Rho guanine nucleotide exchange factor (GEF) 25	0.959084739630633	7,55613E+12	2,50001E+14	0.0439143451657762	0.181806742974345	-3,72004E+14	8,28677E+14	8,32483E+14	6,98375E+14	7,04803E+14	7,80099E+14	6,89243E+14

TC0200003885.mm.2	P2rx3	purinergic receptor P2X, ligand-gated ion channel, 3	-0.918566112974696	6,25908E+14	-2,49929E+14	0.0439587645461129	0.18191565375146	-3,72099E+14	5,65679E+14	5,62603E+14	6,24836E+14	6,90336E+14	6,31221E+14	6,80773E+14
TC0800001232.mm.2	Nrn1l/Edc4	neuritin 1-like/enhancer of mRNA decapping 4	1,07362E+14	6,82926E+14	2,49914E+14	0.0439678143151877	0.18191565375146	-3,72118E+14	7,49985E+14	7,57312E+14	6,63072E+14	6,34612E+14	5,90936E+14	7,01636E+14
TC1100000625.mm.2	Fnip1	folliculin interacting protein 1	1,63335E+13	6,39798E+14	2,49904E+14	0.0439745218992079	0.18191565375146	-3,72132E+14	7,5294E+13	7,72213E+14	6,42858E+14	4,72293E+14	6,60192E+14	5,3829E+14
TC4_GL456216_random00 000001.mm.2	Dhrsx	dehydrogenase/reductase (SDR family) X chromosome	-0.967570603538299	6,54949E+14	-2,49884E+14	0.0439863338664445	0.181917812523106	-3,72157E+14	6,01212E+14	5,81484E+14	6,57481E+14	7,13304E+14	6,5219E+14	7,2402E+14
TC1000000823.mm.2	Elane	elastase, neutrophil expressed	-1,06203E+14	6,18735E+14	-2,49852E+14	0.0440065979822489	0.181926157223435	-3,72201E+14	5,59339E+14	5,29883E+14	6,14497E+14	7,07062E+14	6,226E+12	6,7903E+14
TC0400003008.mm.2	C87499	expressed sequence C87499	-0.877005021170516	5,75779E+14	-2,49828E+14	0.0440211826404437	0.181926157223435	-3,72232E+14	5,53966E+13	5,23517E+14	5,30186E+14	5,9412E+14	6,33801E+14	6,19083E+14
TC0300001399.mm.2	Dnajb14	DnaJ heat shock protein family (Hsp40) member B14	-0.856962527565151	7,71164E+14	-2,49826E+14	0.0440222234850229	0.181926157223435	-3,72234E+14	6,91088E+14	7,46321E+14	7,77136E+13	8,0364E+14	8,18206E+14	7,90596E+14
TC1500001876.mm.2	Ddx17	DEAD (Asp-Glu-Ala-Asp) box polypeptide 17	1,63721E+14	8,29922E+14	2,49792E+13	0.0440433383397531	0.181966746325242	-3,72279E+14	9,13381E+14	9,45749E+14	8,67312E+13	7,21402E+14	8,40502E+14	6,91186E+14
TC0500000640.mm.2	Uchl1	ubiquitin carboxy-terminal hydrolase L1	1,17438E+14	9,76976E+14	2,49736E+14	0.0440779161201405	0.182062922954069	-3,72353E+14	1,06359E+14	1,05715E+14	9,89093E+14	8,66156E+14	9,68759E+14	9,17107E+14
TC1000000847.mm.2	Ndufs7	NADH:ubiquinone oxidoreductase core subunit S7	1,77649E+14	7,95938E+14	2,49703E+14	0.0440984835009109	0.182101195451096	-3,72396E+14	9,29223E+14	9,1148E+13	8,14534E+14	6,34988E+14	8,16743E+14	6,68661E+14
TC0800002428.mm.2	Ano8	anoctamin 8	0.741039670094762	6,15092E+14	2,49669E+14	0.0441192111178614	0.182136993973811	-3,7244E+14	6,62347E+14	6,55378E+14	5,92416E+13	6,10891E+14	5,93737E+14	5,7578E+14
TC1200000145.mm.2	Hpcal1	hippocalcin-like 1	0.905517609279123	7,92577E+14	2,4963E+14	0.0441438738929071	0.182136993973811	-3,72493E+13	8,68132E+14	8,18833E+14	7,95257E+14	7,67381E+14	7,80761E+14	7,25101E+14
TC0900002287.mm.2	Colca2	COLCA2 homolog	-0.839807468364594	5,66321E+14	-2,49626E+14	0.0441459421472636	0.182136993973811	-3,72497E+14	5,40163E+14	4,81651E+14	5,81928E+14	6,04406E+14	5,96473E+14	5,93303E+14
TC0X00002171.mm.2	Enox2	ecto-NOX disulfide-thiol exchanger 2	0.955108657741492	6,57866E+14	2,49616E+14	0.0441523675579783	0.182136993973811	-3,72511E+14	7,15731E+14	7,06068E+14	6,85922E+14	6,08698E+14	5,92177E+14	6,386E+14
TC0600000193.mm.2	Ptprz1	protein tyrosine phosphatase, receptor type Z, polypeptide 1	2,2901E+14	8,68696E+14	2,49563E+13	0.0441853836537113	0.1822265387285	-3,72581E+14	1,04285E+14	1,04449E+14	8,93372E+14	6,02133E+14	8,71366E+14	7,5796E+13
TC0800001963.mm.2	Gm8096	3-phosphoglycerate dehydrogenase pseudogene	-0.848544606422061	6,64769E+14	-2,49541E+14	0.0441988247520358	0.18223411226128	-3,7261E+14	5,92048E+14	6,0391E+14	6,81671E+14	7,45318E+14	6,78899E+13	6,86769E+14
TC0500003411.mm.2	Ssc4d	scavenger receptor cysteine rich family, 4 domains	-0.808885701317974	6,51003E+14	-2,49523E+14	0.0442098395599419	0.18223411226128	-3,72633E+14	5,90943E+14	6,03804E+14	6,60496E+14	6,94249E+14	6,58305E+14	6,9822E+14
TC1600001966.mm.2	App	amyloid beta (A4) precursor protein	1,38998E+13	1,03071E+14	2,49487E+14	0.0442321374694553	0.182279394129241	-3,7268E+14	1,13165E+12	1,11855E+14	1,05994E+14	9,0189E+14	1,01832E+14	9,53883E+14
TC1000002042.mm.2	Gtf3c6	general transcription factor IIIC, polypeptide 6, alpha	0.874228120112641	7,46231E+14	2,49465E+14	0.0442455813683072	0.182288175041558	-3,72709E+14	7,97355E+14	7,90565E+14	7,63896E+14	7,12493E+14	7,3148E+14	6,81594E+14
TC1100003285.mm.2	Traf4	TNF receptor associated factor 4	-0.813301846696815	8,73509E+14	-2,49447E+13	0.0442572682039619	0.182289714358497	-3,72734E+14	8,08492E+14	8,24417E+13	9,29214E+14	8,83364E+14	9,10999E+14	8,84571E+14
TC0800000691.mm.2	Naf1	nuclear assembly factor 1 ribonucleoprotein	1,89291E+14	6,64343E+14	2,49398E+14	0.0442871981096432	0.182348378222364	-3,72797E+13	7,64284E+14	7,79765E+14	7,38235E+14	5,38306E+14	6,58992E+14	5,06476E+14
TC1000000502.mm.2	Hsf2	heat shock factor 2	1,37845E+14	6,72534E+14	2,49387E+14	0.044294144626223	0.182348378222364	-3,72812E+14	7,65205E+13	7,47735E+14	6,92416E+14	5,92599E+14	6,86461E+14	5,50789E+14
TC0900000081.mm.2	Ccdc82	coiled-coil domain containing 82	0.914959094218347	6,14089E+14	2,49271E+14	0.0443665158472528	0.182568479118145	-3,72965E+14	7,02333E+14	7,05825E+14	5,6504E+14	4,8617E+14	6,23508E+14	6,01658E+14
TC0700001198.mm.2	Stard5	StAR-related lipid transfer (START) domain containing 5	0.762671115629988	6,65451E+14	2,49232E+14	0.0443910348945988	0.182568479118145	-3,73017E+14	7,21622E+14	6,96803E+14	6,68328E+14	6,4006E+14	6,3132E+14	6,34571E+14
TC0500002183.mm.2	Fndc4	fibronectin type III domain containing 4	0.830019139626867	7,57113E+14	2,4923E+14	0.0443922613437247	0.182568479118145	-3,73019E+14	8,06667E+14	8,09996E+14	7,61276E+14	7,14082E+14	7,44095E+14	7,06564E+14
TC1600000137.mm.2	Tnfrsf17	tumor necrosis factor receptor superfamily, member 17	-1,4533E+14	5,10316E+14	-2,49225E+14	0.0443948494218885	0.182568479118145	-3,73025E+14	3,89787E+14	4,22567E+14	4,87146E+14	6,59381E+14	5,27135E+14	5,75878E+14
TC1200000808.mm.2	Zfp410	zinc finger protein 410	1,09743E+14	6,15416E+14	2,4921E+14	0.0444042617553535	0.182568479118145	-3,73045E+14	6,7739E+14	6,80879E+14	6,56695E+14	5,38749E+14	5,79383E+14	5,594E+13
TC1000000786.mm.2	Olfr1358	olfactory receptor 1358	-0.994111563921238	4,8398E+14	-2,4915E+14	0.0444419670374552	0.182669296536686	-3,73124E+13	4,10051E+14	4,19331E+14	4,74758E+14	5,71534E+14	5,04028E+14	5,24176E+14
TC1400002864.mm.2	Tm9sf1	transmembrane 9 superfamily member 1	0.746061841657171	8,14288E+14	2,49135E+14	0.0444514560739989	0.182669296536686	-3,73144E+14	8,55185E+14	8,80626E+14	7,74785E+14	7,88531E+12	7,88838E+13	7,9776E+13
TC1400002027.mm.2	Chd8	chromodomain helicase DNA binding protein 8	1,02022E+14	5,4613E+14	2,49057E+14	0.0445000846051873	0.182792416253259	-3,73247E+14	6,28271E+14	6,32868E+14	5,30705E+14	4,27839E+13	5,38044E+14	5,19051E+14
TC0300002094.mm.2	Ssr3	signal sequence receptor, gamma	1,47654E+14	7,63998E+14	2,4905E+14	0.0445041053162996	0.182792416253259	-3,73255E+14	8,60946E+14	8,78508E+14	7,69487E+14	6,30901E+14	7,83462E+14	6,60685E+14
TC0500003236.mm.2	Vps33a	VPS33A CORVET/HOPS core subunit	1,53424E+14	8,29424E+14	2,48974E+14	0.0445518023839844	0.182835093594196	-3,73356E+14	9,17228E+14	9,30992E+14	8,83092E+14	7,03862E+14	8,09118E+13	7,32253E+13
TC0700003720.mm.2	Eed	embryonic ectoderm development	-0.974127680695839	7,56345E+14	-2,4895E+14	0.0445665023317384	0.182835093594196	-3,73387E+14	7,10532E+14	7,02162E+14	7,51431E+14	7,66425E+14	7,5691E+14	8,50609E+14
TC0X00000102.mm.2	Fthl17e	ferritin, heavy polypeptide-like 17, member E	-0.771870991454638	5,38288E+14	-2,48935E+14	0.0445762022623556	0.182835093594196	-3,73407E+14	4,80414E+14	5,08923E+14	5,47391E+14	5,49291E+14	5,56599E+14	5,87112E+12
TC0400004127.mm.2	Prxl2b	peroxiredoxin like 2B	0.899748633484834	6,86134E+13	2,4892E+14	0.0445853054131391	0.182835093594196	-3,73426E+14	7,46779E+13	7,33207E+14	6,54654E+14	6,8213E+14	6,86236E+13	6,13801E+14
TC0X00000650.mm.2	Mtmr1	myotubularin related protein 1	0.85051316368663	6,2956E+14	2,48914E+14	0.0445891122227376	0.182835093594196	-3,73434E+14	7,14061E+14	6,9339E+14	5,97781E+12	5,34781E+14	6,19315E+14	6,18034E+14
TC0700002156.mm.2	Rdh13	retinol dehydrogenase 13 (all-trans and 9-cis)	-0.80987870611023	8,01035E+14	-2,4888E+14	0.0446106715009846	0.182835093594196	-3,7348E+14	7,34073E+14	7,48237E+14	8,15353E+13	8,64264E+14	8,1204E+13	8,32246E+14
TC0400003433.mm.2	Guca2b	guanylate cyclase activator 2b (retina)	-0.792485614172276	5,96136E+14	-2,48875E+14	0.0446136905202282	0.182835093594196	-3,73486E+14	5,62836E+14	5,35554E+14	6,05063E+13	6,16477E+14	6,08769E+14	6,48119E+14
TC0100003463.mm.2	Dusp27	dual specificity phosphatase 27 (putative)	-0.933176466557128	5,30846E+14	-2,48861E+14	0.0446224373673309	0.182835093594196	-3,73504E+14	4,51002E+14	4,64798E+13	5,43788E+14	6,23053E+14	5,3546E+14	5,66974E+13
TC1600001209.mm.2	Zc3h7a	zinc finger CCCH type containing 7 A	1,74133E+13	5,09766E+14	2,48821E+14	0.0446478010128378	0.182835093594196	-3,73558E+14	6,25206E+13	6,14931E+14	5,28656E+13	3,97934E+14	5,39716E+14	3,52155E+14
TC1200000795.mm.2	Papln	papilin, proteoglycan-like sulfated glycoprotein	-1,15955E+14	7,00851E+14	-2,48802E+14	0.0446595567062374	0.182835093594196	-3,73582E+14	6,23767E+13	6,03482E+14	6,97052E+14	8,21643E+14	7,10983E+14	7,48176E+14
TC0700000475.mm.2	Zfp59	zinc finger protein 59	1,12576E+14	5,85562E+14	2,48798E+14	0.0446622544552874	0.182835093594196	-3,73588E+13	6,74665E+14	6,36252E+14	5,24365E+14	5,92328E+14	5,91923E+14	4,93842E+14
TC0700003613.mm.2	Fsd2	fibronectin type III and SPRY domain containing 2	-1,10152E+14	4,92442E+14	-2,48786E+14	0.0446695790592093	0.182835093594196	-3,73603E+14	4,24733E+14	4,30756E+14	4,51635E+14	5,71737E+13	5,32104E+14	5,43689E+14
TC0500001386.mm.2	Pptc7	PTC7 protein phosphatase homolog	0.862543232321103	7,46614E+14	2,48781E+14	0.0446728167084515	0.182835093594196	-3,7361E+14	7,92069E+14	7,69401E+13	7,82256E+14	7,46994E+14	7,22253E+13	6,66709E+14
TC1500001214.mm.2	Tars	threonyl-tRNA synthetase	0.728138095454552	6,82317E+14	2,4878E+14	0.044673354650304	0.182835093594196	-3,73611E+14	7,23185E+14	7,41852E+14	6,56175E+14	6,5328E+14	6,56433E+14	6,62977E+14
TC1700001812.mm.2	Tff2	trefoil factor 2 (spasmolytic protein 1)	-0.897387655528033	5,8041E+13	-2,4875E+14	0.0446917558310899	0.182863956756311	-3,7365E+14	5,10919E+14	5,29736E+14	5,84536E+14	6,37133E+14	5,82679E+14	6,37454E+13
TC1600001168.mm.2	Tmem114	transmembrane protein 114	-0.88181104178059	5,28789E+14	-2,4867E+14	0.0447424850147977	0.183025047231133	-3,73756E+14	4,75027E+14	4,95365E+14	5,52304E+14	5,03284E+14	5,47212E+14	5,99543E+14
TC1000000605.mm.2	Ctnna3	catenin (cadherin associated protein), alpha 3	-0.790756028916802	6,59905E+14	-2,48607E+14	0.0447821663011093	0.183102577428975	-3,73839E+13	5,99565E+14	6,1903E+14	6,67719E+14	6,96372E+14	6,71093E+14	7,05653E+14
TC0900001542.mm.2	Gadl1	glutamate decarboxylase-like 1	-1,13447E+14	5,63122E+14	-2,48603E+14	0.0447841654345926	0.183102577428975	-3,73844E+14	4,70548E+14	4,91001E+14	5,6295E+14	6,65793E+14	6,30173E+14	5,5827E+14
TC0600002159.mm.2	Trpv6	transient receptor potential cation channel, subfamily V, member 6	-1,37086E+14	5,4883E+13	-2,48583E+14	0.0447972394362215	0.183109568502242	-3,73871E+14	4,46192E+14	4,76623E+14	5,16819E+14	6,56357E+14	5,53495E+14	6,43492E+14
TC0300001181.mm.2	Dph5	diphthamide biosynthesis 5	0.749563795924794	6,27258E+14	2,48519E+14	0.0448371361448864	0.183125658090636	-3,73955E+14	6,61784E+14	6,91167E+14	6,03805E+14	6,03751E+14	5,94125E+13	6,08914E+14
TC0600003042.mm.2	Hdhd5	haloacid dehalogenase like hydrolase domain containing 5	-0.981026957893592	8,6369E+14	-2,48507E+13	0.0448445864422848	0.183125658090636	-3,7397E+14	8,04477E+14	7,88863E+14	8,62054E+14	9,37203E+14	8,65047E+14	9,24498E+14
TC1500002192.mm.2	Kmt2d	lysine (K)-specific methyltransferase 2D	1,00113E+14	6,85376E+12	2,48506E+14	0.0448453969697261	0.183125658090636	-3,73972E+13	7,37221E+13	7,45063E+14	6,5664E+14	6,91274E+14	5,9361E+14	6,88449E+14
TC1700002812.mm.2	Egfl8	EGF-like domain 8	-0.750450612135571	5,70959E+13	-2,48504E+14	0.0448469280099596	0.183125658090636	-3,73975E+14	5,17057E+14	5,30918E+14	5,76257E+14	6,03456E+13	6,05729E+14	5,92336E+14
TC1600001398.mm.2	Sst	somatostatin	0.844363250233583	1,0812E+14	2,48486E+14	0.0448580011471321	0.183125658090636	-3,73998E+14	1,14814E+14	1,12374E+14	1,08627E+14	1,02603E+14	1,05008E+13	1,05293E+14
TC0400002529.mm.2	Tpm2	tropomyosin 2, beta	-0.83490196838196	7,35217E+14	-2,48434E+14	0.0448908838774713	0.183177177271542	-3,74067E+14	6,76836E+14	6,86218E+14	7,28245E+14	7,8997E+13	7,63448E+14	7,66587E+14
TC1200000467.mm.2	Psma6	proteasome (prosome, macropain) subunit, alpha type 6	1,12449E+14	8,31944E+14	2,48408E+13	0.0449071639942846	0.183177177271542	-3,74101E+14	9,00544E+14	9,00985E+14	8,44312E+14	7,69189E+14	8,38549E+14	7,38083E+14
TC1700002814.mm.2	C4b	complement component 4B (Chido blood group)	0.837286240391586	7,22698E+14	2,48404E+14	0.0449094184694056	0.183177177271542	-3,74106E+14	7,38823E+14	7,93357E+14	7,10844E+14	7,28443E+14	6,69032E+14	6,95691E+14

TC1400001471.mm.2	Psmd6	proteasome (prosome, macropain) 26S subunit, non-ATPase, 6	1,96648E+14	6,53943E+14	2,48378E+14	0.0449263030572638	0.183177177271542	-3,74141E+14	7,78643E+14	7,76912E+14	6,82525E+14	5,23316E+13	6,86784E+14	4,75476E+14
TC0300001221.mm.2	Dpyd	dihydropyrimidine dehydrogenase	-0.86803363260741	6,02824E+14	-2,48373E+14	0.0449295097583138	0.183177177271542	-3,74148E+14	5,61107E+14	5,34947E+14	5,93112E+14	6,58119E+14	6,28103E+14	6,41558E+14
TC1900000980.mm.2	Ehbp1l1	EH domain binding protein 1-like 1	-0.985978034305409	5,36908E+13	-2,48353E+14	0.0449418932733305	0.183177177271542	-3,74173E+14	4,57355E+14	5,04049E+14	5,24001E+14	5,77444E+14	5,45326E+13	6,13274E+13
TC0100002478.mm.2	Crygc	crystallin, gamma C	-1,10719E+14	5,14166E+14	-2,48327E+14	0.0449579304731221	0.183177177271542	-3,74207E+14	4,20025E+14	4,86124E+14	4,81879E+14	5,69384E+14	5,36761E+14	5,90826E+14
TC0600001275.mm.2	Syn2	synapsin II	1,43925E+14	9,92385E+14	2,48322E+12	0.044961567436787	0.183177177271542	-3,74214E+14	1,08492E+14	1,07632E+14	1,04772E+14	8,71968E+14	9,5685E+14	9,1654E+13
TC0400001362.mm.2	Col9a2	collagen, type IX, alpha 2	-1,09975E+13	7,00109E+14	-2,48185E+14	0.0450477505383102	0.183415571784099	-3,74394E+14	6,17145E+14	6,22625E+14	7,02335E+14	7,98826E+14	6,93476E+14	7,66245E+14
TC0500003483.mm.2	Prkar1b	protein kinase, cAMP dependent regulatory, type I beta	1,23372E+14	1,00796E+14	2,48116E+14	0.0450914726660092	0.183415571784099	-3,74485E+14	1,09935E+14	1,07613E+14	1,03448E+12	9,09097E+13	9,99444E+14	9,29292E+14
TC0100001125.mm.2	Rab3gap1	RAB3 GTPase activating protein subunit 1	0.998814354105702	7,2954E+14	2,48113E+13	0.045093802160713	0.183415571784099	-3,7449E+14	7,96612E+14	7,9168E+14	7,49919E+14	6,50501E+14	7,03164E+14	6,85365E+14
TC0300001469.mm.2	Selenof	selenoprotein F	1,31419E+14	8,98198E+14	2,48075E+14	0.0451177246578969	0.183415571784099	-3,7454E+14	9,94782E+14	1,00019E+13	8,93183E+14	7,68892E+14	9,16066E+14	8,16072E+14
TC1700000650.mm.2	Ehmt2	euchromatic histone lysine N-methyltransferase 2	1,59562E+14	7,08512E+14	2,48063E+14	0.0451249495885062	0.183415571784099	-3,74555E+14	8,2055E+14	8,22614E+14	7,3646E+13	5,47408E+14	7,03403E+14	6,20636E+14
TC1100000586.mm.2	Ppp2ca/Mir3061	protein phosphatase 2 (formerly 2A), catalytic subunit, alpha isoform/microRNA 3061	1,84433E+14	9,39792E+13	2,48063E+14	0.0451253563002883	0.183415571784099	-3,74555E+14	1,07599E+14	1,05396E+13	9,724E+14	7,75323E+12	9,5326E+14	8,0782E+14
TC1300000050.mm.2	Larp4b	La ribonucleoprotein domain family, member 4B	1,59644E+13	7,38171E+14	2,48043E+14	0.0451379563033018	0.183415571784099	-3,74582E+14	8,2117E+14	8,10458E+14	8,10183E+14	6,74877E+14	7,3401E+14	5,78331E+13
TC0500003472.mm.2	Mcm7/Mir93/Mir25	minichromosome maintenance complex component 7/microRNA 93/microRNA 25	2,01478E+14	5,97966E+14	2,4802E+14	0.0451524991122982	0.183415571784099	-3,74612E+14	7,37111E+14	7,60649E+14	6,26382E+14	3,6885E+14	5,90349E+14	5,04455E+14
TC0800002510.mm.2	Rbmxl1	RNA binding motif protein, X-linked like-1	-1,13994E+14	6,66109E+14	-2,4802E+14	0.0451527286951856	0.183415571784099	-3,74612E+14	6,47355E+14	5,44607E+14	6,53834E+14	7,30909E+14	7,06314E+14	7,13636E+14
TC1400002132.mm.2	Zmym5	zinc finger, MYM-type 5	2,08762E+13	6,82608E+14	2,48012E+14	0.0451577345989402	0.183415571784099	-3,74623E+14	8,14994E+14	8,00868E+14	7,53199E+14	5,28246E+14	6,85345E+14	5,12993E+14
TC0900003342.mm.2	Syncrip	synaptotagmin binding, cytoplasmic RNA interacting protein	1,52754E+14	7,88194E+14	2,48005E+14	0.0451616721663434	0.183415571784099	-3,74631E+14	8,88298E+14	9,20684E+13	7,87908E+14	6,28802E+14	8,06076E+14	6,97397E+14
TC0100002588.mm.2	Rnf25	ring finger protein 25	1,02547E+14	7,46901E+14	2,47998E+14	0.0451662261735792	0.183415571784099	-3,7464E+14	8,23488E+14	8,09411E+14	7,61218E+14	6,59485E+14	7,28263E+14	6,99541E+14
TC0700004465.mm.2	9430038I01Rik	RIKEN cDNA 9430038I01 gene	-0.897967960285984	7,07462E+14	-2,47995E+14	0.0451680623620247	0.183415571784099	-3,74644E+13	6,30288E+14	6,65021E+14	7,04118E+14	7,70444E+14	7,50091E+14	7,24811E+14
TC1200002139.mm.2	Gm7104	REX1, RNA exonuclease 1 homolog pseudogene	-0.809166040734445	6,58292E+14	-2,47956E+14	0.0451931458614574	0.183471191550936	-3,74696E+14	6,00199E+14	6,01412E+14	6,69872E+14	7,14824E+14	6,6547E+14	6,97975E+14
TC1100004272.mm.2	Sep 04	septin 4	1,49867E+14	8,70417E+13	2,47908E+14	0.0452234941488493	0.183548151440909	-3,74759E+14	9,68597E+14	9,72943E+12	8,88806E+14	7,50353E+14	8,8647E+14	7,55335E+14
TC1600002107.mm.2	Setd4	SET domain containing 4	-0.835328830302399	5,5271E+14	-2,47859E+14	0.0452546629895214	0.183586021475009	-3,74824E+14	5,02183E+14	4,99604E+14	5,45345E+13	6,00278E+14	5,99966E+14	5,68887E+14
TC1300002188.mm.2	Ptch1	patched 1	1,63131E+14	7,10853E+14	2,47856E+14	0.0452564934473853	0.183586021475009	-3,74828E+14	8,49518E+14	7,73726E+13	7,64107E+14	5,80784E+14	6,86386E+14	6,10596E+14
TC0300003015.mm.2	Gm5105	predicted gene 5105	-1,41634E+14	4,28011E+14	-2,4784E+14	0.0452670057295482	0.183586021475009	-3,74849E+14	3,34233E+14	3,58266E+14	3,83054E+14	5,16745E+14	4,31019E+14	5,44747E+14
TC1200002125.mm.2	Alkbh1/Nrp	alkB homolog 1, histone H2A dioxygenase/neural regeneration protein	-0.754153213723114	6,53009E+14	-2,4779E+14	0.0452984983080445	0.183602267399427	-3,74915E+14	6,29458E+14	5,95806E+13	6,56174E+14	6,60525E+14	6,82043E+14	6,94051E+14
TC1400002091.mm.2	Psme2	proteasome (prosome, macropain) activator subunit 2 (PA28 beta)	0.963033424529532	6,51681E+14	2,4777E+14	0.0453111106983914	0.183602267399427	-3,74941E+14	7,33036E+13	7,12443E+14	6,48534E+14	5,63203E+14	6,43069E+14	6,09803E+14
TC1100000339.mm.2	Hba-a1/Hba-a2	hemoglobin alpha, adult chain 1/hemoglobin alpha, adult chain 2	-1,15273E+14	1,21934E+14	-2,47714E+14	0.0453467907474538	0.183602267399427	-3,75014E+14	1,12512E+13	1,20742E+14	1,22553E+14	1,1949E+14	1,33033E+13	1,23275E+14
TC1000002653.mm.2	Ano4	anoctamin 4	-0.856281569437843	7,54186E+14	-2,47713E+14	0.0453477096304829	0.183602267399427	-3,75016E+14	7,22061E+14	6,86608E+14	7,45077E+14	7,91446E+14	7,71636E+14	8,08288E+14
TC1100000794.mm.2	Ttc19	tetratricopeptide repeat domain 19	1,05705E+14	7,68396E+14	2,47702E+14	0.0453544808548709	0.183602267399427	-3,7503E+13	8,57359E+14	8,44348E+14	7,52583E+14	6,65791E+14	7,80862E+14	7,09434E+14
TC0400003888.mm.2	Plekhm2	pleckstrin homology domain containing, family M (with RUN domain) member 2	2,08667E+14	7,43497E+14	2,47678E+14	0.0453700477960327	0.183602267399427	-3,75063E+14	9,02908E+14	8,71353E+14	7,6373E+14	5,66061E+14	7,79214E+12	5,77713E+14
TC0600002265.mm.2	Hnrnpa2b1	heterogeneous nuclear ribonucleoprotein A2/B1	1,39905E+14	9,28042E+14	2,47658E+14	0.0453827237679223	0.183602267399427	-3,75089E+14	1,01901E+14	1,011E+14	9,54261E+14	8,3379E+14	9,38044E+14	8,12152E+14
TC0400003584.mm.2	Hpca	hippocalcin	1,41394E+14	1,12063E+14	2,47639E+14	0.045394603253873	0.183602267399427	-3,75113E+14	1,21015E+14	1,22601E+14	1,14106E+14	9,93161E+14	1,1246E+14	1,02877E+14
TC1200002434.mm.2	Akt1	thymoma viral proto-oncogene 1	1,10479E+14	8,59682E+14	2,47638E+14	0.0453955669693952	0.183602267399427	-3,75115E+14	9,58642E+14	9,43473E+14	8,48062E+14	7,26756E+14	8,43547E+14	8,3761E+14
TC1400000381.mm.2	Wapl	WAPL cohesin release factor	1,06722E+14	6,28705E+13	2,47632E+14	0.0453992590988631	0.183602267399427	-3,75123E+14	6,93312E+14	7,24437E+14	6,10375E+14	5,398E+13	6,44933E+14	5,59371E+14
TC0800000244.mm.2	Defa37/Defa36	defensin, alpha, 37/defensin, alpha, 36	-0.951966978793467	6,23627E+14	-2,47626E+14	0.0454027855992062	0.183602267399427	-3,7513E+14	5,41984E+14	5,68669E+14	6,22493E+14	7,07569E+14	6,40034E+14	6,61012E+13
TC0600001250.mm.2	Jagn1	jagunal homolog 1	0.977659462607973	7,28191E+14	2,47615E+13	0.0454100548369475	0.183602267399427	-3,75145E+14	7,85977E+14	7,96961E+14	7,44099E+13	6,54705E+14	7,11868E+14	6,75537E+14
TC1500002345.mm.2	Gsdmc4	gasdermin C4	-1,05422E+14	5,16476E+14	-2,47601E+14	0.0454191421742764	0.183602267399427	-3,75164E+14	4,31321E+13	4,60341E+14	5,04436E+14	6,00249E+14	5,25391E+14	5,77115E+12
TC0400001639.mm.2	Stmn1	stathmin 1	1,52104E+14	9,15482E+14	2,47578E+14	0.0454334499730109	0.183614040485359	-3,75193E+14	1,04579E+14	1,03617E+14	9,03182E+14	7,3E+14	9,33154E+14	8,44598E+14
TC0X00001955.mm.2	Klhl13	kelch-like 13	1,72072E+13	7,95179E+14	2,47551E+14	0.0454507189095939	0.18363777176286	-3,75229E+14	9,24041E+14	9,03214E+14	8,31474E+14	6,29232E+14	7,92022E+14	6,9109E+14
TC1700000936.mm.2	B430306N03Rik	RIKEN cDNA B430306N03 gene	-1,0565E+14	4,87174E+14	-2,4751E+13	0.0454773250706043	0.183699207536387	-3,75284E+14	4,01577E+14	4,339E+14	4,66376E+14	5,74416E+14	5,28353E+14	5,18424E+14
TC1200000618.mm.2	Jkamp	JNK1/MAPK8-associated membrane protein	1,04231E+14	7,80534E+14	2,47446E+14	0.0455181964256354	0.18377298581593	-3,75368E+14	8,74857E+14	8,69118E+13	7,49115E+14	6,54602E+14	7,96203E+14	7,3931E+13
TC1700000912.mm.2	Mrps10	mitochondrial ribosomal protein S10	1,14535E+14	6,00324E+14	2,47445E+14	0.045518400446309	0.18377298581593	-3,75369E+14	7,07701E+14	7,13396E+14	5,59411E+14	4,29405E+14	5,96941E+14	5,95087E+14
TC1400001604.mm.2	Plac9a/Plac9b	placenta specific 9a/placenta specific 9b	-0.7398738184865	5,96473E+14	-2,47416E+14	0.0455372546134155	0.18377867093081	-3,75407E+13	5,51174E+13	5,61558E+14	5,96968E+14	6,08429E+14	6,17662E+14	6,43044E+14
TC1900000645.mm.2	Scd3	stearoyl-coenzyme A desaturase 3	1,36512E+14	7,21736E+14	2,47405E+14	0.0455443850764362	0.18377867093081	-3,75422E+14	8,09914E+14	8,11127E+14	7,42416E+13	6,18943E+14	7,31655E+14	6,16362E+14
TC0600002920.mm.2	Cidec	cell death-inducing DFFA-like effector c	-1,42805E+14	5,58379E+14	-2,47357E+14	0.0455751281055077	0.18377867093081	-3,75485E+14	4,5302E+14	4,8303E+13	5,24792E+13	6,67772E+14	5,58615E+14	6,63045E+13
TC0300000444.mm.2	Rnf13	ring finger protein 13	0.986608379217266	8,77676E+14	2,47328E+14	0.0455934664391902	0.18377867093081	-3,75523E+14	9,38794E+14	9,50424E+13	8,66225E+14	8,18719E+14	8,89122E+14	8,02774E+14
TC0900001782.mm.2	Hephl1	hephaestin-like 1	-0.780269034630146	5,33856E+14	-2,47327E+14	0.0455938638061172	0.18377867093081	-3,75524E+14	4,84044E+14	4,75073E+14	5,41747E+14	5,871E+14	5,54217E+14	5,60954E+14
TC1500001955.mm.2	Cyp2d40	cytochrome P450, family 2, subfamily d, polypeptide 40	-0.869330505101259	6,49318E+14	-2,47296E+14	0.0456141419824047	0.18377867093081	-3,75566E+13	5,86868E+13	5,90722E+13	6,65534E+14	7,01326E+14	6,44185E+13	7,07271E+14
TC0500003047.mm.2	Sart3	squamous cell carcinoma antigen recognized by T cells 3	0.815988608489664	5,93864E+14	2,47269E+14	0.0456313997356323	0.18377867093081	-3,75601E+14	6,48477E+14	6,26462E+14	5,61734E+14	6,14771E+14	5,51111E+14	5,6063E+14
TC0100002756.mm.2	Alppl2	alkaline phosphatase, placental-like 2	-1,06075E+14	4,5543E+14	-2,47255E+14	0.0456399595574935	0.18377867093081	-3,75619E+13	3,71111E+14	3,85389E+14	4,64701E+14	5,42726E+14	4,4321E+14	5,2544E+14
TC0900001052.mm.2	Ddx43	DEAD (Asp-Glu-Ala-Asp) box polypeptide 43	-1,15527E+14	4,60725E+14	-2,47202E+14	0.0456743818636078	0.18377867093081	-3,75689E+14	3,95294E+14	3,68742E+14	4,40938E+14	5,64286E+14	4,77846E+14	5,17245E+14
TC0300002188.mm.2	Rapgef2	Rap guanine nucleotide exchange factor (GEF) 2	1,00894E+14	8,38135E+14	2,47185E+13	0.045685271078181	0.18377867093081	-3,75712E+14	9,08396E+14	8,86312E+14	8,24171E+14	8,17011E+14	8,47132E+14	7,45788E+14
TC0600000904.mm.2	Tacr1	tachykinin receptor 1	2,07994E+14	6,02823E+14	2,47155E+14	0.0457041568882268	0.18377867093081	-3,7575E+14	7,35425E+14	6,98641E+14	7,20569E+14	4,44228E+14	5,35262E+14	4,82815E+14
TC0400000873.mm.2	Acer2	alkaline ceramidase 2	-0.983650594864847	4,79238E+14	-2,47149E+14	0.0457083812249985	0.18377867093081	-3,75759E+14	4,12355E+14	4,27067E+14	4,56445E+14	5,43411E+14	5,403E+14	4,95852E+14
TC1600001939.mm.2	Btg3/Gm7334	B cell translocation gene 3/B-cell translocation gene 3 pseudogene	0.775668113533912	6,45373E+13	2,47141E+13	0.0457133046349005	0.18377867093081	-3,75769E+14	7,27852E+14	7,03409E+14	5,75787E+14	5,89065E+14	6,20193E+14	6,55935E+14
TC1100004115.mm.2	Foxj1/Rnf157	forkhead box J1/ring finger protein 157	1,33962E+14	8,43373E+14	2,47118E+14	0.0457278532766317	0.18377867093081	-3,75799E+14	9,25249E+14	9,35969E+13	8,74302E+14	7,31425E+13	8,37416E+14	7,55878E+14
TC0700001287.mm.2	Dlg2	discs large MAGUK scaffold protein 2	1,34731E+14	8,83079E+14	2,47108E+14	0.0457345028912464	0.18377867093081	-3,75813E+14	9,60335E+14	9,63599E+14	9,21089E+14	7,98981E+14	8,83572E+14	7,70899E+13
TC1500001885.mm.2	Sun2	Sad1 and UNC84 domain containing 2	-1,08386E+14	7,0116E+14	-2,47101E+14	0.0457390469026371	0.18377867093081	-3,75822E+13	6,48101E+14	6,35684E+14	6,81263E+14	7,41353E+14	6,99382E+14	8,01175E+14

TC0700000221.mm.2	Slc8a2	solute carrier family 8 (sodium/calcium exchanger), member 2	1,79142E+14	7,51597E+13	2,47069E+14	0.0457598058204063	0.18377867093081	-3,75865E+14	8,74533E+14	8,94521E+14	7,83716E+13	5,46038E+14	7,00206E+14	7,10565E+14
TC0800002747.mm.2	Prss54	protease, serine 54	-1,27156E+14	4,65737E+14	-2,47056E+14	0.0457680206853813	0.18377867093081	-3,75881E+14	3,73896E+14	4,07255E+14	4,26355E+14	5,51458E+14	4,7684E+14	5,58622E+14
TC0200003106.mm.2	Spopl	speckle-type POZ protein-like	1,78165E+14	5,68481E+13	2,47054E+14	0.045769101682552	0.18377867093081	-3,75884E+14	6,6012E+13	7,23453E+14	5,88564E+13	4,11508E+14	5,85923E+14	4,41318E+14
TC1100003076.mm.2	Eif5a	eukaryotic translation initiation factor 5A	2,02291E+13	9,44081E+14	2,47037E+14	0.0457799690275559	0.18377867093081	-3,75906E+14	1,0899E+14	1,07387E+14	1,00298E+14	7,38555E+14	9,16481E+14	8,42701E+14
TC1000001375.mm.2	Il22	interleukin 22	-0.859428926465158	5,82788E+14	-2,47035E+14	0.0457812097966776	0.18377867093081	-3,75908E+14	5,24714E+14	5,23213E+14	5,80347E+13	6,48639E+12	6,1831E+14	6,01503E+13
TC0300002802.mm.2	Vcam1	vascular cell adhesion molecule 1	1,03758E+14	5,84935E+14	2,47034E+14	0.0457822655651056	0.18377867093081	-3,75911E+14	6,32238E+12	7,04576E+14	5,08818E+14	5,34681E+14	5,25376E+14	6,03922E+14
TC0500003168.mm.2	Plbd2	phospholipase B domain containing 2	1,36319E+14	6,56266E+14	2,47007E+14	0.0457994008939834	0.18377867093081	-3,75946E+14	7,33444E+14	7,2915E+14	7,00508E+14	5,84536E+14	6,56392E+13	5,33564E+14
TC1000000769.mm.2	Gm10318	predicted gene 10318	-0.96340533447838	7,01844E+14	-2,47007E+14	0.0457996161655949	0.18377867093081	-3,75946E+14	6,28612E+14	6,47636E+13	6,98488E+14	7,67396E+14	7,03493E+14	7,65437E+14
TC1700001521.mm.2	Has1	hyaluronan synthase 1	-1,01023E+14	5,17761E+14	-2,46983E+14	0.0458147768068347	0.18377867093081	-3,75977E+14	4,69998E+14	4,3657E+14	5,00382E+14	5,91002E+14	5,34494E+14	5,7412E+14
TC1400001346.mm.2	Itgbl1	integrin, beta-like 1	-0.920403000814856	5,22548E+14	-2,46981E+14	0.045816354566441	0.18377867093081	-3,7598E+14	4,75715E+14	4,94608E+14	4,99189E+14	5,11372E+14	5,36158E+14	6,18245E+14
TC1000001621.mm.2	Mmp19	matrix metallopeptidase 19	-0.978127715286178	6,5703E+14	-2,46939E+14	0.0458431618092218	0.183784097079636	-3,76035E+14	5,99486E+14	5,9589E+14	6,53762E+14	7,02042E+14	6,53024E+14	7,37978E+14
TC1100000693.mm.2	Olfr324	olfactory receptor 324	-1,09673E+14	4,3265E+14	-2,46921E+13	0.0458546360815789	0.183784097079636	-3,76059E+14	3,42201E+13	3,72046E+14	4,25838E+14	5,22221E+14	4,3298E+14	5,00613E+14
TC0600003064.mm.2	Phc1	polyhomeotic 1	0.974251750539862	7,40582E+14	2,46906E+14	0.0458645065534853	0.183784097079636	-3,76079E+14	8,03411E+13	8,13314E+14	7,48324E+14	6,5657E+14	7,25853E+14	6,96021E+14
TC0300003249.mm.2	Mfsd14a	major facilitator superfamily domain containing 14A	-0.776583527704861	7,46857E+14	-2,46891E+14	0.0458741365595574	0.183784097079636	-3,76098E+14	7,16726E+14	6,85973E+14	7,45059E+14	7,7537E+14	7,86793E+14	7,71223E+14
TC1500000565.mm.2	Gsdmd	gasdermin D	-0.783235967550601	5,30058E+14	-2,4689E+14	0.0458747370728167	0.183784097079636	-3,761E+14	4,60495E+14	5,01627E+13	5,42275E+14	5,57182E+14	5,69533E+14	5,49236E+14
TC1000000906.mm.2	Ankrd24	ankyrin repeat domain 24	-0.724162902217998	8,68265E+14	-2,46854E+14	0.045897881039706	0.183831110413319	-3,76147E+14	8,10853E+14	8,19741E+14	8,92607E+14	9,10965E+14	8,84784E+13	8,90642E+13
TC0X00002797.mm.2	Abcb7	ATP-binding cassette, sub-family B (MDR/TAP), member 7	0.890024590873321	5,89835E+13	2,46817E+14	0.0459217736547031	0.183881098135743	-3,76196E+14	6,55174E+14	6,29508E+14	5,40467E+14	6,07182E+14	5,37556E+14	5,69122E+14
TC0600001601.mm.2	Ddx47	DEAD (Asp-Glu-Ala-Asp) box polypeptide 47	0.716734987608393	7,33067E+13	2,46772E+14	0.0459508823339852	0.183918448537189	-3,76255E+14	7,96651E+14	8,06222E+13	6,63051E+14	6,72953E+14	7,26522E+14	7,33004E+13
TC0300001083.mm.2	St7l	suppression of tumorigenicity 7-like	1,16397E+12	6,58185E+14	2,46767E+14	0.0459539299826677	0.183918448537189	-3,76261E+13	7,39944E+13	7,25919E+14	6,8659E+14	5,63586E+13	6,42372E+14	5,90697E+14
TC0700002170.mm.2	Fam71e2	family with sequence similarity 71, member E2	-0.912316956227745	6,07794E+14	-2,46734E+14	0.0459757608761108	0.183951399978137	-3,76306E+14	5,28952E+12	5,62563E+14	6,02483E+13	6,7879E+14	6,38847E+14	6,35132E+14
TC1700001714.mm.2	Bak1	BCL2-antagonist/killer 1	-0.732499880966738	7,39691E+14	-2,46703E+14	0.0459956979623714	0.183951399978137	-3,76347E+14	6,87481E+14	6,85522E+14	7,74682E+14	7,70962E+14	7,46495E+14	7,73007E+14
TC0600002314.mm.2	Scrn1	secernin 1	1,39794E+13	8,72203E+14	2,46677E+14	0.046012300936654	0.183951399978137	-3,7638E+14	9,82165E+14	9,59563E+14	8,99711E+14	7,29642E+14	8,48774E+14	8,13366E+14
TC0700000804.mm.2	Lrrc4b	leucine rich repeat containing 4B	0.912804003264837	9,09417E+14	2,46663E+14	0.0460213630732802	0.183951399978137	-3,76399E+14	9,76713E+14	9,59918E+14	9,23564E+14	8,42236E+14	8,72065E+14	8,82005E+14
TC1500001866.mm.2	Slc16a8	solute carrier family 16 (monocarboxylic acid transporters), member 8	-1,05328E+14	4,57234E+14	-2,4665E+14	0.0460297681033831	0.183951399978137	-3,76416E+14	3,84974E+14	4,02568E+14	4,40444E+14	5,17222E+14	4,58382E+14	5,39815E+14
TC1900001465.mm.2	Fra10ac1	FRA10AC1 homolog (human)	1,65333E+13	5,69868E+14	2,46639E+14	0.0460368118274212	0.183951399978137	-3,7643E+14	6,56742E+14	6,72912E+14	6,1057E+14	4,79998E+14	5,85902E+14	4,13087E+14
TC0X00001859.mm.2	Med14	mediator complex subunit 14	2,29343E+14	8,24607E+14	2,46614E+14	0.0460531159733711	0.183951399978137	-3,76463E+14	9,63802E+14	9,61608E+14	8,8041E+14	6,75096E+14	8,54248E+14	6,12477E+14
TC1000000424.mm.2	Popdc3	popeye domain containing 3	-0.874608677758101	5,94884E+14	-2,46613E+14	0.0460534939186869	0.183951399978137	-3,76464E+14	5,48013E+14	5,43351E+14	5,84117E+14	6,27533E+14	6,02928E+14	6,63358E+13
TC1500000110.mm.2	Drosha	drosha, ribonuclease type III	1,03017E+14	8,64244E+14	2,46553E+14	0.0460926105092086	0.184039731270002	-3,76544E+14	9,27838E+14	9,10816E+14	8,46477E+14	8,67709E+12	8,68977E+14	7,63644E+14
TC0100000257.mm.2	Vwa3b	von Willebrand factor A domain containing 3B	-0.853170782872684	6,20142E+14	-2,46544E+14	0.04609845189634	0.184039731270002	-3,76556E+14	5,66358E+14	5,81069E+14	5,94088E+14	6,61278E+14	6,62668E+14	6,55393E+14
TC0100001567.mm.2	Sh2d1b2	SH2 domain containing 1B2	-0.766467354253739	5,70951E+14	-2,46499E+14	0.046127504038478	0.184091430869045	-3,76615E+14	5,26204E+14	5,49322E+14	5,70609E+14	5,50751E+14	5,99508E+14	6,29312E+12
TC0200004344.mm.2	Olfr1316	olfactory receptor 1316	-1,81587E+14	4,78587E+13	-2,46479E+14	0.0461407434663888	0.184091430869045	-3,76642E+13	3,79623E+14	3,56334E+14	4,10892E+14	6,25542E+14	4,93503E+14	6,05629E+14
TC1000003023.mm.2	Hmga2	high mobility group AT-hook 2	-1,25551E+14	7,6815E+14	-2,46471E+14	0.0461456767380422	0.184091430869045	-3,76652E+14	6,71971E+14	7,05633E+14	7,31843E+14	8,70747E+14	7,90228E+14	8,38479E+14
TC0700001134.mm.2	Wdr93	WD repeat domain 93	-1,2055E+14	4,65075E+14	-2,46386E+14	0.0462007085550909	0.184131202254603	-3,76763E+14	4,00387E+14	4,06043E+14	4,02094E+14	5,34394E+14	4,98099E+14	5,4943E+14
TC0700000810.mm.2	Napsa	napsin A aspartic peptidase	-0.822508297211265	6,507E+14	-2,46348E+14	0.0462259221227659	0.184131202254603	-3,76815E+14	6,21861E+14	6,0132E+14	6,68935E+14	6,244E+14	6,75715E+14	7,11968E+14
TC1700002541.mm.2	Fam98a	family with sequence similarity 98, member A	0.810848063903078	6,29919E+14	2,46347E+13	0.0462265358965264	0.184131202254603	-3,76816E+14	6,73286E+14	7,06491E+12	6,13551E+14	5,68582E+14	6,15717E+14	6,0189E+14
TC1700001130.mm.2	Myom1	myomesin 1	-0.884730522339664	5,54184E+14	-2,46341E+14	0.0462300764347945	0.184131202254603	-3,76823E+14	4,95251E+13	5,02056E+14	5,38329E+14	6,15216E+14	5,88983E+14	5,85269E+14
TC0300000990.mm.2	Tbx15	T-box 15	-1,36914E+14	4,75477E+14	-2,46296E+14	0.046259187507819	0.184131202254603	-3,76882E+14	3,9997E+14	3,7506E+14	4,4656E+12	5,82413E+14	4,74306E+14	5,74552E+14
TC1000001995.mm.2	Dse	dermatan sulfate epimerase	-0.825650296496376	5,50386E+14	-2,46287E+14	0.0462651928256504	0.184131202254603	-3,76894E+14	5,1657E+14	5,25384E+14	5,25106E+14	5,2817E+14	5,72926E+14	6,34158E+14
TC1100003339.mm.2	Crlf3	cytokine receptor-like factor 3	-0.720685458383965	8,0984E+14	-2,46285E+14	0.0462668817337117	0.184131202254603	-3,76898E+14	7,64447E+14	7,60513E+14	8,4222E+13	8,22765E+14	8,3402E+13	8,35077E+14
TC0700003934.mm.2	Olfr649	olfactory receptor 649	-1,02874E+14	5,66428E+14	-2,46248E+14	0.0462906950670795	0.184131202254603	-3,76946E+14	4,9636E+14	4,92331E+14	5,80556E+14	6,34879E+14	5,50152E+14	6,44288E+13
TC0700000384.mm.2	Cadm4	cell adhesion molecule 4	0.769638251866112	8,92023E+13	2,46197E+14	0.0463236896237478	0.184131202254603	-3,77013E+14	9,46078E+14	9,39064E+14	8,47652E+14	8,88129E+14	8,7372E+14	8,57493E+14
TC0800002497.mm.2	Arhgap10	Rho GTPase activating protein 10	0.723987799897117	6,77597E+14	2,46165E+14	0.0463448430643715	0.184131202254603	-3,77055E+14	7,212E+14	7,30869E+14	6,45824E+14	6,60418E+14	6,60717E+14	6,46555E+14
TC1000000382.mm.2	Snx3	sorting nexin 3	1,38002E+14	9,08623E+13	2,46164E+14	0.0463453683849116	0.184131202254603	-3,77056E+13	1,00323E+14	1,00342E+14	9,22993E+14	7,91465E+14	9,21229E+14	8,09409E+14
TC0300003017.mm.2	4930579F01Rik	RIKEN cDNA 4930579F01 gene	-1,22241E+14	4,56789E+13	-2,46154E+14	0.0463519179243214	0.184131202254603	-3,7707E+14	3,64697E+14	3,84076E+14	4,56274E+14	5,42432E+14	4,38961E+14	5,54294E+14
TC1600001308.mm.2	Ranbp1	RAN binding protein 1	0.871820271042695	8,13139E+14	2,46137E+13	0.0463630806443895	0.184131202254603	-3,77092E+14	8,75902E+13	8,6401E+13	8,17677E+14	7,55696E+14	8,02362E+14	7,63186E+13
TC0X00001555.mm.2	Rragb	Ras-related GTP binding B	2,0845E+14	7,54012E+14	2,46128E+14	0.046368963369842	0.184131202254603	-3,77104E+14	9,11955E+14	8,6439E+14	8,20553E+14	5,67727E+14	7,4187E+14	6,17575E+14
TC1700000622.mm.2	Psmb8	proteasome (prosome, macropain) subunit, beta type 8 (large multifunctional peptidase 7)	-0.886660025236049	6,39491E+14	-2,46108E+14	0.0463819284268788	0.184131202254603	-3,7713E+14	5,72069E+13	6,097E+14	6,23358E+14	6,72718E+14	6,55975E+13	7,03125E+14
TC1900000646.mm.2	Scd2/Mir5114	stearoyl-Coenzyme A desaturase 2/microRNA 5114	1,64475E+14	1,04809E+14	2,46099E+14	0.0463879296023279	0.184131202254603	-3,77142E+13	1,16243E+14	1,15961E+14	1,08225E+14	8,91143E+13	1,04729E+14	9,45805E+14
TC1500000041.mm.2	Rictor	RPTOR independent companion of MTOR, complex 2	1,03387E+14	7,11511E+14	2,46098E+14	0.0463887076754123	0.184131202254603	-3,77144E+14	7,88014E+14	8,00622E+14	7,02479E+14	5,96089E+14	7,03212E+14	6,7865E+14
TC0500002755.mm.2	Cxcl9	chemokine (C-X-C motif) ligand 9	-0.904532027207731	5,11801E+14	-2,46094E+14	0.0463909948014403	0.184131202254603	-3,77149E+13	4,42317E+14	4,757E+14	4,92734E+14	5,61133E+14	5,33933E+14	5,6499E+14
TC1000000767.mm.2	Gm10100	predicted gene 10100	-1,05113E+14	6,537E+14	-2,46078E+14	0.0464011793654146	0.184131202254603	-3,77169E+14	5,86261E+14	5,72439E+14	6,45633E+14	7,48942E+14	6,62187E+14	7,06739E+13
TC0500000701.mm.2	Fip1l1	FIP1 like 1 (S. cerevisiae)	1,20222E+14	6,47229E+14	2,46041E+14	0.0464256147423212	0.184131202254603	-3,77219E+14	7,40179E+14	7,43177E+14	6,3253E+14	5,24577E+14	6,67355E+14	5,75557E+14
TC1100001880.mm.2	Gprc5c	G protein-coupled receptor, family C, group 5, member C	-1,14562E+14	5,69999E+14	-2,46009E+13	0.0464461805007242	0.184131202254603	-3,7726E+14	5,0001E+14	5,16213E+14	5,15467E+13	6,42959E+14	6,36424E+14	6,08922E+14
TC0200005314.mm.2	Ctsz	cathepsin Z	1,05657E+14	7,92025E+14	2,45998E+14	0.0464536127442601	0.184131202254603	-3,77275E+14	8,48403E+14	9,37361E+14	7,20176E+14	6,71761E+14	8,13919E+13	7,60532E+14
TC1000002464.mm.2	2610008E11Rik	RIKEN cDNA 2610008E11 gene	0.846253020123114	5,90706E+14	2,45989E+13	0.0464595606339469	0.184131202254603	-3,77287E+14	6,49544E+14	6,285E+14	6,06568E+14	5,50831E+14	5,7063E+14	5,38163E+14
TC1200002224.mm.2	Ttc7b	tetratricopeptide repeat domain 7B	1,47788E+14	7,8717E+14	2,45952E+14	0.0464837414057804	0.184131202254603	-3,77336E+14	8,87778E+14	8,87188E+14	8,00038E+14	6,68624E+14	8,07792E+14	6,71598E+14
TC1600002045.mm.2	Scaf4	SR-related CTD-associated factor 4	1,1804E+14	7,7573E+13	2,45938E+14	0.04649300538807	0.184131202254603	-3,77354E+14	8,40513E+14	8,40282E+14	8,3299E+14	6,95883E+13	7,29629E+14	7,15084E+14
TC1300002420.mm.2	Msh3	mutS homolog 3	0.926214938547267	6,42206E+14	2,45904E+14	0.0465151099948611	0.184131202254603	-3,77399E+14	7,2213E+14	6,85033E+14	6,44966E+14	5,79187E+14	6,32901E+14	5,89019E+14
TC0200002363.mm.2	Map1lc3a	microtubule-associated protein 1 light chain 3 alpha	0.743028013102432	8,29876E+14	2,45884E+13	0.0465283337947444	0.184131202254603	-3,77426E+14	8,79529E+14	8,65949E+14	8,23738E+14	8,13171E+14	8,12846E+14	7,84026E+14
TC0400000488.mm.2	Dcaf10	DDB1 and CUL4 associated factor 10	1,0822E+13	5,87258E+14	2,4586E+14	0.0465436383129849	0.184131202254603	-3,77456E+14	6,7711E+14	6,68781E+14	5,83155E+14	4,65052E+14	5,73137E+14	5,56314E+14
TC0400000127.mm.2	Pip4p2	phosphatidylinositol-4,5-bisphosphate 4-phosphatase 2	1,00484E+14	7,58962E+14	2,45854E+14	0.0465480170021605	0.184131202254603	-3,77465E+13	8,21819E+14	8,09252E+14	7,87416E+14	7,05183E+14	7,46463E+14	6,8364E+14
TC1200001568.mm.2	Rps7	ribosomal protein S7	0.860131291832229	9,63556E+14	2,45851E+14	0.0465497562676494	0.184131202254603	-3,77469E+14	1,02622E+14	1,01645E+14	9,60722E+14	9,07301E+14	9,58642E+13	9,12002E+14
TC0200004283.mm.2	Arl14ep	ADP-ribosylation factor-like 14 effector protein	1,85432E+14	6,78077E+14	2,45849E+14	0.0465510458036219	0.184131202254603	-3,77471E+14	8,13622E+14	7,98628E+14	7,14157E+13	5,00669E+14	6,85401E+14	5,55986E+14
TC0100001707.mm.2	Itpkb	inositol 1,4,5-trisphosphate 3-kinase B	0.728819138880382	6,83399E+14	2,45845E+14	0.0465539547564784	0.184131202254603	-3,77477E+14	7,23182E+14	7,05995E+14	6,88534E+14	6,99268E+14	6,33419E+14	6,49995E+14
TC1800001701.mm.2	Zfp407	zinc finger protein 407	1,57372E+13	5,34879E+14	2,45839E+14	0.0465573994933234	0.184131202254603	-3,77484E+14	6,69593E+14	6,55541E+12	5,30483E+14	3,43269E+14	5,46544E+14	4,63845E+14
TC0700004135.mm.2	Psma1	proteasome (prosome, macropain) subunit, alpha type 1	1,25682E+14	7,8476E+14	2,45824E+14	0.0465674985268712	0.184131202254603	-3,77504E+14	8,84574E+14	8,75757E+14	7,65926E+14	6,73339E+14	8,14381E+14	6,94584E+14
TC0700000571.mm.2	2200002J24Rik	RIKEN cDNA 2200002J24 gene	-1,22215E+14	5,76555E+14	-2,45824E+14	0.0465676412202923	0.184131202254603	-3,77505E+14	4,80177E+14	4,90918E+14	5,97033E+14	6,75674E+11	5,46787E+14	6,68738E+13
TC0500001270.mm.2	Cit	citron	0.862027895047714	7,87686E+14	2,45819E+14	0.0465709586587785	0.184131202254603	-3,77511E+14	8,35786E+14	8,5515E+14	7,87104E+14	7,29543E+13	7,78695E+14	7,39835E+14

TC0700001519.mm.2	Trim6	tripartite motif-containing 6	-0.811437038101532	4,8771E+14	-2,45807E+14	0.0465784633767616	0.184131202254603	-3,77527E+14	4,5688E+14	4,23878E+14	4,77688E+14	5,2477E+14	5,26309E+14	5,16737E+14
TC0200001201.mm.2	Zc3h15	zinc finger CCCH-type containing 15	1,32793E+14	8,50455E+14	2,45734E+14	0.046626487146536	0.184232204388571	-3,77623E+14	9,32811E+13	9,32197E+14	8,71847E+14	7,66454E+14	8,62625E+14	7,36798E+14
TC1700001947.mm.2	Csnk2b	casein kinase 2, beta polypeptide	0.824254611061791	8,98331E+14	2,45722E+14	0.0466342842057042	0.184232204388571	-3,77639E+14	9,6293E+13	9,46709E+14	8,9769E+14	8,37867E+14	8,74121E+13	8,70667E+14
TC0100001491.mm.2	Kifap3	kinesin-associated protein 3	1,84059E+12	8,33624E+14	2,45716E+14	0.0466383145100838	0.184232204388571	-3,77647E+14	9,78743E+14	9,77238E+14	8,38921E+14	6,18975E+14	8,51312E+14	7,36552E+14
TC1400000980.mm.2	Entpd4/Entpd4b	ectonucleoside triphosphate diphosphohydrolase 4/ectonucleoside triphosphate diphosphohydrolase 4B	0.736956202119945	8,67309E+14	2,45691E+14	0.0466544204420196	0.184250134471035	-3,77679E+14	9,27972E+14	8,97258E+14	8,51963E+14	8,4882E+14	8,38593E+14	8,39245E+14
TC0400002367.mm.2	Ankrd6	ankyrin repeat domain 6	0.763166407046342	6,49655E+14	2,45674E+14	0.0466657232530437	0.184250134471035	-3,77702E+14	6,98908E+13	6,80416E+14	6,62107E+14	6,29809E+14	6,23445E+14	6,03246E+14
TC0100000014.mm.2	Atp6v1h	ATPase, H+ transporting, lysosomal V1 subunit H	1,23882E+14	8,08635E+14	2,45653E+14	0.0466797361927875	0.184260310944239	-3,7773E+14	8,9548E+14	8,72976E+14	8,35262E+14	7,27398E+14	8,1022E+14	7,10472E+14
TC0700002744.mm.2	Zfp146	zinc finger protein 146	0.879672013885616	6,87721E+14	2,45584E+14	0.0467247886623417	0.184263628195222	-3,7782E+14	7,47018E+14	7,6352E+14	6,57665E+14	6,23517E+13	7,00639E+14	6,33965E+14
TC0200004842.mm.2	Apmap	adipocyte plasma membrane associated protein	0.974309259350949	6,96982E+14	2,45523E+14	0.0467649076918902	0.184263628195222	-3,77901E+14	8,17692E+13	7,70167E+14	6,34388E+14	5,66647E+13	6,70097E+13	7,22899E+14
TC0700000305.mm.2	Zfp296	zinc finger protein 296	-0.924589881104258	5,43926E+14	-2,4549E+14	0.0467868833988759	0.184263628195222	-3,77945E+14	4,68626E+14	5,1063E+14	5,33322E+14	5,86807E+14	5,54696E+14	6,09478E+14
TC0500003418.mm.2	Cux1	cut-like homeobox 1	1,67058E+14	6,92316E+14	2,45481E+14	0.0467924085466236	0.184263628195222	-3,77956E+14	8,02381E+14	8,15279E+14	7,35235E+14	5,17456E+14	6,60158E+14	6,23386E+14
TC1000001284.mm.2	Csrp2	cysteine and glycine-rich protein 2	-0.789685962166502	7,33579E+14	-2,45481E+14	0.0467925117674701	0.184263628195222	-3,77956E+14	6,68947E+14	7,05598E+14	7,29421E+14	7,65026E+14	7,61752E+13	7,7073E+14
TC0400000275.mm.2	Rragd	Ras-related GTP binding D	1,25032E+14	8,23404E+14	2,45464E+14	0.0468040428520999	0.184263628195222	-3,77979E+14	8,94009E+14	9,08817E+14	8,62437E+14	7,22397E+14	8,03407E+14	7,49355E+14
TC0200005205.mm.2	Ripor3	RIPOR family member 3	-0.860127707514357	7,34409E+14	-2,4546E+14	0.0468065187026756	0.184263628195222	-3,77984E+12	6,6766E+14	6,83797E+14	7,36878E+14	7,94635E+14	7,41877E+14	7,81606E+14
TC0100003538.mm.2	Ufc1	ubiquitin-fold modifier conjugating enzyme 1	0.763224464247823	8,09691E+13	2,45441E+13	0.0468189454224499	0.184263628195222	-3,78009E+14	8,76298E+14	8,53531E+14	7,75496E+14	7,75638E+14	8,06621E+14	7,70562E+14
TC1100000954.mm.2	Gltpd2	glycolipid transfer protein domain containing 2	-0.817763002240785	5,88581E+14	-2,45428E+14	0.0468276586750593	0.184263628195222	-3,78027E+14	5,07814E+13	5,53559E+14	6,14593E+14	6,30596E+14	5,9435E+14	6,30575E+14
TC0600003084.mm.2	Clec4e	C-type lectin domain family 4, member e	-0.991718722921435	5,87307E+13	-2,45414E+14	0.0468366829418481	0.184263628195222	-3,78045E+14	5,02961E+14	5,37443E+14	5,76729E+14	6,67962E+14	6,04609E+14	6,34138E+14
TC0600000758.mm.2	Smarcad1	SWI/SNF-related, matrix-associated actin-dependent regulator of chromatin, subfamily a, containing DEAD/H box 1	1,20748E+14	5,74733E+14	2,4534E+14	0.0468853722313415	0.184263628195222	-3,78142E+14	6,55127E+13	6,47082E+14	6,13848E+13	4,71627E+14	5,22538E+14	5,38174E+13
TC0100002461.mm.2	Klf7	Kruppel-like factor 7 (ubiquitous)	0.741830526731134	8,03847E+14	2,45331E+14	0.0468913772469505	0.184263628195222	-3,78154E+14	8,47326E+14	8,44872E+14	7,92861E+14	7,94189E+14	7,88752E+12	7,55079E+14
TC0200004396.mm.2	Rasgrp1	RAS guanyl releasing protein 1	1,64852E+14	9,2685E+14	2,45321E+14	0.0468979156219165	0.184263628195222	-3,78167E+14	1,03008E+14	1,05133E+14	9,56601E+14	7,71388E+13	9,32209E+14	8,19496E+14
TC1100000995.mm.2	Mybbp1a	MYB binding protein (P160) 1a	0.817604287483269	6,25744E+14	2,45321E+14	0.0468983246278805	0.184263628195222	-3,78168E+14	6,90659E+14	6,77426E+14	5,96894E+14	5,84921E+14	5,73345E+14	6,31219E+14
TC0700002500.mm.2	Bcam	basal cell adhesion molecule	-1,0447E+14	8,38921E+13	-2,45293E+14	0.0469161993098176	0.184263628195222	-3,78204E+14	7,95487E+14	7,50926E+14	8,15197E+14	9,16566E+14	8,6707E+14	8,88283E+14
TC0900002521.mm.2	Snx22	sorting nexin 22	-0.824000308179678	6,10377E+14	-2,45272E+14	0.046930188951225	0.184263628195222	-3,78231E+14	5,50039E+14	5,55833E+14	6,23355E+14	6,62366E+14	6,11135E+14	6,59536E+14
TC0700001374.mm.2	Wnt11	wingless-type MMTV integration site family, member 11	-0.911554457267577	5,77607E+13	-2,45253E+14	0.0469427222439645	0.184263628195222	-3,78256E+14	5,22983E+14	5,16175E+14	6,00531E+14	6,04487E+14	5,68641E+14	6,52828E+14
TC1900000711.mm.2	Cnnm2	cyclin M2	0.755524894295712	7,07843E+14	2,45248E+14	0.0469460789135395	0.184263628195222	-3,78263E+14	7,63347E+14	7,67269E+14	6,82332E+14	6,54602E+14	7,0393E+14	6,7558E+14
TC0100001607.mm.2	Dcaf8	DDB1 and CUL4 associated factor 8	1,23535E+14	8,45182E+14	2,45229E+14	0.0469586829572412	0.184263628195222	-3,78288E+14	9,37768E+14	9,25983E+14	8,68612E+14	7,22048E+14	8,18343E+14	7,98338E+14
TC0900001070.mm.2	Myo6	myosin VI	1,11383E+14	6,39414E+14	2,45225E+14	0.0469613833277205	0.184263628195222	-3,78294E+14	7,13933E+14	7,39478E+14	6,24366E+13	5,28066E+13	6,54018E+14	5,76626E+14
TC0700001282.mm.2	Crebzf	CREB/ATF bZIP transcription factor	-0.764689631079013	7,81914E+14	-2,45211E+14	0.0469705510742424	0.184263628195222	-3,78312E+14	7,46066E+14	7,3007E+14	7,85347E+14	8,00928E+14	7,95888E+14	8,33186E+14
TC0600001268.mm.2	Slc6a1	solute carrier family 6 (neurotransmitter transporter, GABA), member 1	1,08773E+14	8,05203E+14	2,4521E+14	0.0469711230391879	0.184263628195222	-3,78313E+14	8,83863E+14	8,59711E+14	8,31811E+14	7,29805E+14	7,93434E+14	7,32594E+14
TC0600001033.mm.2	Ruvbl1	RuvB-like protein 1	1,58462E+14	6,4655E+14	2,45206E+13	0.0469738004601717	0.184263628195222	-3,78318E+14	7,59215E+14	7,74193E+14	6,67514E+14	4,6189E+14	5,9611E+14	6,20375E+14
TC1100003821.mm.2	Mpp2	membrane protein, palmitoylated 2 (MAGUK p55 subfamily member 2)	1,08712E+14	7,82525E+14	2,45205E+14	0.0469747121866056	0.184263628195222	-3,7832E+14	8,52116E+14	8,40282E+14	8,22962E+13	7,04814E+14	7,2562E+14	7,49355E+14
TC1500001846.mm.2	Elfn2	leucine rich repeat and fibronectin type III, extracellular 2	1,17372E+14	8,10359E+13	2,45168E+14	0.0469987241645764	0.184263628195222	-3,78368E+14	8,82789E+14	8,88159E+14	8,35656E+14	7,19344E+14	8,04509E+14	7,31696E+14
TC1200002364.mm.2	Begain	brain-enriched guanylate kinase-associated	0.7506662188959	6,86728E+14	2,45164E+14	0.0470013988699933	0.184263628195222	-3,78374E+14	7,55552E+14	7,21075E+14	6,63236E+14	6,54013E+13	6,56803E+14	6,69692E+13
TC0X00000329.mm.2	Ube2a	ubiquitin-conjugating enzyme E2A	1,48049E+13	6,14889E+14	2,45162E+14	0.0470031961689947	0.184263628195222	-3,78377E+13	7,13204E+14	6,68889E+14	6,57001E+14	5,64242E+14	6,23179E+14	4,62816E+14
TC0700003607.mm.2	Rps17	ribosomal protein S17	0.7462737696228	9,33433E+14	2,45162E+14	0.0470033079705408	0.184263628195222	-3,78377E+14	9,83872E+14	9,90634E+14	8,99938E+14	9,00904E+14	9,30323E+14	8,94929E+14
TC1100003725.mm.2	Krtap4-7	keratin associated protein 4-7	-0.789643816301056	6,25545E+14	-2,45133E+13	0.0470221433535664	0.184263628195222	-3,78415E+14	5,74977E+14	5,77182E+13	6,22419E+14	6,68604E+14	6,42898E+14	6,67189E+14
TC0800001475.mm.2	BC048644	cDNA sequence BC048644	-1,03318E+14	4,40403E+14	-2,45124E+14	0.0470281523306726	0.184263628195222	-3,78427E+14	3,71386E+14	3,73058E+14	4,28225E+14	5,18671E+14	4,43729E+14	5,07351E+14
TC1500002328.mm.2	Agxt2	alanine-glyoxylate aminotransferase 2	-1,56921E+14	5,2088E+14	-2,45107E+13	0.0470391383246777	0.184263628195222	-3,78449E+14	4,12795E+14	4,68058E+14	4,48907E+14	6,0082E+14	5,3417E+14	6,60527E+14
TC0700001135.mm.2	Mesp2	mesoderm posterior 2	-1,28918E+14	6,24987E+14	-2,45094E+14	0.0470482441182772	0.184263628195222	-3,78467E+14	5,51272E+14	5,47157E+14	5,97314E+14	6,97911E+14	6,17245E+14	7,3902E+14
TC0500001766.mm.2	Bud31	BUD31 homolog	0.867132448926746	6,55944E+14	2,45079E+14	0.0470579550687854	0.184263628195222	-3,78486E+14	6,94619E+14	7,0427E+14	6,79524E+14	6,31785E+14	6,40618E+13	5,84844E+14
TC1100002777.mm.2	Slc36a2	solute carrier family 36 (proton/amino acid symporter), member 2	-1,37349E+14	5,95781E+14	-2,45038E+14	0.047084844191025	0.184323775045767	-3,7854E+14	4,99804E+14	5,10125E+14	5,75012E+14	7,05119E+14	5,85842E+14	6,98783E+14
TC0900001844.mm.2	Fbxl12	F-box and leucine-rich repeat protein 12	-0.77432484146241	6,79975E+14	-2,45008E+14	0.0471049004545865	0.184323775045767	-3,7858E+14	6,52748E+14	6,46561E+13	6,84919E+13	6,4145E+14	7,18421E+14	7,35754E+13
TC1800001179.mm.2	Dnajc18	DnaJ heat shock protein family (Hsp40) member C18	1,15231E+14	6,69229E+14	2,44998E+14	0.0471112554177852	0.184323775045767	-3,78592E+14	7,51663E+14	7,08936E+14	7,08628E+14	6,16009E+13	6,58165E+14	5,71973E+14
TC0700002781.mm.2	Hamp2	hepcidin antimicrobial peptide 2	-0.78217351675158	7,32684E+14	-2,4497E+14	0.0471300457483675	0.184323775045767	-3,7863E+14	6,81996E+14	6,84731E+14	7,4315E+14	7,63064E+12	7,38722E+14	7,84439E+14
TC0800000337.mm.2	Thap1	THAP domain containing, apoptosis associated protein 1	-1,25238E+14	7,20897E+14	-2,44953E+14	0.047141660250066	0.184323775045767	-3,78653E+14	6,14876E+14	6,68675E+14	6,91471E+14	8,16336E+14	7,33413E+14	8,00614E+14
TC1900001726.mm.2	Hspa12a	heat shock protein 12A	0.758201636389655	6,8633E+14	2,44952E+13	0.0471419522723021	0.184323775045767	-3,78653E+14	7,54518E+14	7,22037E+14	6,57029E+12	6,59479E+13	6,7514E+13	6,49775E+13
TC1900001739.mm.2	Rab11fip2	RAB11 family interacting protein 2 (class I)	1,54544E+13	7,67085E+14	2,4489E+14	0.0471832512073046	0.18444049653161	-3,78735E+13	8,61714E+14	8,62239E+14	7,92542E+14	6,71151E+14	7,87849E+14	6,27016E+14
TC1300002141.mm.2	Ctla2b	cytotoxic T lymphocyte-associated protein 2 beta	-1,21373E+14	5,44777E+14	-2,44858E+14	0.0472042287690592	0.184475513636738	-3,78777E+13	4,62213E+14	4,84877E+14	5,04687E+14	6,27052E+14	5,57849E+14	6,31987E+14
TC0900001012.mm.2	Gnb5	guanine nucleotide binding protein (G protein), beta 5	0.877251279811	8,44595E+14	2,4483E+14	0.0472232698427422	0.184475513636738	-3,78815E+14	9,05978E+12	8,97289E+14	8,52069E+14	7,84419E+13	8,31532E+14	7,96285E+14
TC0900001048.mm.2	Gsta4	glutathione S-transferase, alpha 4	1,04708E+14	7,85213E+14	2,44815E+14	0.0472327907268398	0.184475513636738	-3,78834E+14	8,54547E+14	8,29201E+14	7,59388E+14	7,93805E+14	7,89802E+14	6,84531E+14
TC0900001400.mm.2	Rhoa	ras homolog family member A	1,36816E+14	8,08079E+13	2,44807E+14	0.0472380046710842	0.184475513636738	-3,78844E+14	9,09037E+14	9,08841E+14	8,11616E+14	6,74732E+14	8,24122E+14	7,20124E+12
TC0800000847.mm.2	Rasd2	RASD family, member 2	1,30898E+14	8,69245E+14	2,44753E+14	0.0472740367801968	0.18457149373378	-3,78915E+14	9,54459E+13	9,70336E+14	8,92439E+13	7,35239E+14	8,4886E+14	8,14138E+13
TC1100001407.mm.2	Car10	carbonic anhydrase 10	1,19535E+14	7,18183E+14	2,44712E+14	0.0473017216644701	0.18459154546656	-3,7897E+14	7,99126E+14	8,10124E+14	7,15442E+14	6,14226E+13	7,34346E+14	6,35834E+14
TC0500001675.mm.2	Gper1	G protein-coupled estrogen receptor 1	-0.87443402160408	6,28808E+14	-2,44711E+13	0.0473020847285686	0.18459154546656	-3,78971E+14	5,86328E+14	5,47542E+14	6,37488E+14	6,92731E+14	6,36713E+13	6,72043E+14
TC0600001712.mm.2	Etfrf1	electron transfer flavoprotein regulatory factor 1	1,38838E+14	7,32584E+14	2,44689E+14	0.0473168084246542	0.184604293982192	-3,79E+14	8,18156E+14	8,4063E+13	7,55525E+14	6,00081E+14	7,29675E+14	6,51436E+14
TC0700001765.mm.2	Cacng3	calcium channel, voltage-dependent, gamma subunit 3	1,21199E+14	9,46182E+14	2,44656E+14	0.0473390304636452	0.184646283674828	-3,79044E+14	1,03357E+14	1,01811E+14	9,72095E+14	8,44029E+14	9,37449E+14	8,71834E+14
TC0Y00000489.mm.2	Ssty2/Gm21242	spermiogenesis specific transcript on the Y 2/predicted gene, 21242	-0.910964249098375	5,41732E+14	-2,44617E+14	0.0473647439518583	0.184664800934413	-3,79095E+14	4,43903E+14	5,21765E+14	5,51436E+14	5,85429E+14	5,74381E+14	5,7348E+14
TC1000002780.mm.2	Nts	neurotensin	1,64866E+14	6,59683E+14	2,44604E+14	0.0473737313993308	0.184664800934413	-3,79113E+14	8,08372E+14	6,63268E+14	6,91033E+13	6,53516E+14	6,50559E+14	4,91349E+14
TC0X00001787.mm.2	Hdac6	histone deacetylase 6	0.876222297920936	7,06318E+14	2,44556E+14	0.0474056282297469	0.184664800934413	-3,79176E+14	7,31468E+14	7,54546E+14	6,83959E+14	7,57166E+14	6,55811E+14	6,54958E+14

TC1200000368.mm.2	Immp2l	IMP2 inner mitochondrial membrane peptidase-like (S. cerevisiae)	-0.752128997440049	8,19587E+14	-2,44545E+13	0.0474127453784299	0.184664800934413	-3,7919E+14	7,73441E+14	7,81005E+14	8,1179E+14	8,46417E+14	8,43892E+14	8,6098E+14
TC1500000221.mm.2	Sdc2	syndecan 2	0.785880721946248	7,47157E+14	2,44544E+14	0.0474137217781298	0.184664800934413	-3,79192E+14	8,07363E+14	7,68917E+14	7,39585E+14	7,47975E+14	6,92846E+14	7,26258E+14
TC0400003587.mm.2	C77080	expressed sequence C77080	-0.80529227340138	7,70695E+14	-2,445E+14	0.0474427461795218	0.184664800934413	-3,79249E+14	7,06544E+14	7,26414E+14	7,71593E+14	8,25602E+14	7,99367E+13	7,94649E+14
TC0900000515.mm.2	Ddx6	DEAD (Asp-Glu-Ala-Asp) box polypeptide 6	1,61777E+14	8,70241E+14	2,44476E+14	0.047458897155813	0.184664800934413	-3,79281E+14	9,69322E+14	9,72175E+14	9,18708E+14	7,433E+14	8,70054E+14	7,47889E+14
TC1400000854.mm.2	Mipep	mitochondrial intermediate peptidase	1,85215E+14	5,71696E+14	2,44471E+14	0.0474621473539073	0.184664800934413	-3,79287E+14	7,12217E+14	6,7308E+12	6,36762E+14	3,9325E+14	5,31799E+14	4,83067E+14
TC0600001442.mm.2	Acrbp/Ing4	proacrosin binding protein/inhibitor of growth family, member 4	-0.811378811675991	7,30481E+14	-2,44469E+14	0.0474634241613396	0.184664800934413	-3,7929E+14	6,37637E+14	6,95492E+14	7,80164E+14	7,74187E+14	7,54155E+14	7,4125E+14
TC1400001211.mm.2	Trim52	tripartite motif-containing 52	-0.782166676375278	8,61111E+14	-2,44459E+14	0.0474700621956662	0.184664800934413	-3,79303E+14	8,16216E+14	8,08014E+14	8,5918E+14	9,02594E+14	8,78118E+14	9,02545E+14
TC0600002170.mm.2	Olfr458	olfactory receptor 458	-1,6294E+14	5,2652E+14	-2,44436E+14	0.047485509140914	0.184664800934413	-3,79333E+14	4,16839E+14	4,267E+14	5,13252E+14	6,3291E+14	4,95104E+14	6,74315E+14
TC1200001308.mm.2	Ptprn2	protein tyrosine phosphatase, receptor type, N polypeptide 2	0.826889006385041	8,69027E+14	2,44413E+14	0.0475012306398724	0.184664800934413	-3,79364E+14	9,35738E+13	8,98438E+14	8,63146E+13	8,48042E+14	8,59317E+14	8,09481E+14
TC1500001919.mm.2	Slc25a17	solute carrier family 25 (mitochondrial carrier, peroxisomal membrane protein), member 17	1,70428E+14	6,89905E+14	2,44393E+14	0.047514203980973	0.184664800934413	-3,7939E+14	8,18945E+14	8,12299E+14	7,06049E+14	5,11745E+14	7,04627E+14	5,85762E+14
TC0600002044.mm.2	Slc35b4	solute carrier family 35, member B4	1,66014E+14	7,80805E+14	2,44366E+14	0.0475321232311447	0.184664800934413	-3,79425E+14	8,8719E+14	8,71982E+14	8,4018E+14	6,58336E+14	7,75708E+14	6,51437E+14
TC0500001597.mm.2	Rasa4/Mir7035	RAS p21 protein activator 4/microRNA 7035	-0.741203298994662	6,26634E+14	-2,44364E+14	0.0475336413109338	0.184664800934413	-3,79428E+14	5,92634E+14	5,82251E+14	6,14383E+14	6,47411E+13	6,6358E+14	6,59545E+13
TC1700000706.mm.2	Nrm	nurim (nuclear envelope membrane protein)	-1,17324E+14	7,88047E+14	-2,44358E+14	0.0475374963327569	0.184664800934413	-3,79436E+14	7,04335E+14	7,06594E+14	7,74035E+14	8,9774E+14	7,92282E+14	8,53295E+14
TC1700002158.mm.2	1600014C23Rik	RIKEN cDNA 1600014C23 gene	-1,19475E+14	6,3424E+14	-2,44357E+14	0.0475386082216809	0.184664800934413	-3,79438E+14	5,78068E+14	5,40173E+14	6,04127E+14	7,25881E+14	6,47498E+14	7,09694E+14
TC1400000770.mm.2	Ngdn	neuroguidin, EIF4E binding protein	0.789327469938439	5,96957E+14	2,44308E+14	0.0475711694777593	0.184746747118615	-3,79502E+14	6,51479E+14	6,33619E+13	5,64601E+14	6,04813E+14	5,78377E+13	5,48856E+13
TC0700000290.mm.2	Eml2/Mir330	echinoderm microtubule associated protein like 2/microRNA 330	0.98070276072148	6,57912E+14	2,44258E+14	0.0476048840426514	0.184818822457398	-3,79569E+14	7,52408E+12	7,11266E+14	6,00547E+14	6,15715E+14	5,8851E+12	6,79023E+14
TC0300000210.mm.2	Skil	SKI-like	1,58448E+14	7,81019E+14	2,44245E+14	0.0476136156700319	0.184818822457398	-3,79586E+14	8,91924E+14	9,08374E+14	7,95015E+14	6,07401E+14	7,86531E+14	6,96871E+13
TC1500000104.mm.2	Golph3	golgi phosphoprotein 3	-0.722269309832527	8,78798E+14	-2,44229E+14	0.047624138946386	0.184818822457398	-3,79607E+14	8,27656E+14	8,52424E+14	8,82542E+14	8,85632E+14	9,13638E+14	9,10896E+14
TC0200004312.mm.2	Ano3	anoctamin 3	2,03969E+14	7,16555E+14	2,44208E+12	0.0476378819504454	0.184827581160423	-3,79634E+14	8,50519E+14	8,99575E+14	6,8976E+14	5,17321E+13	7,79431E+14	5,62725E+14
TC0200001412.mm.2	1700029I15Rik	RIKEN cDNA 1700029I15 gene	-0.810950085967773	7,22599E+14	-2,44178E+13	0.0476580698966163	0.184827581160423	-3,79673E+14	6,51302E+14	6,83913E+14	7,32672E+13	7,70306E+14	7,32207E+14	7,65198E+14
TC0400002709.mm.2	Tmem245	transmembrane protein 245	1,18504E+12	6,79609E+14	2,44171E+13	0.0476631390823628	0.184827581160423	-3,79683E+14	7,45432E+14	7,74471E+14	6,58149E+14	6,16709E+14	7,06862E+14	5,76033E+14
TC0100001611.mm.2	Kcnj10	potassium inwardly-rectifying channel, subfamily J, member 10	1,54955E+14	9,17853E+14	2,44157E+14	0.0476722787378338	0.184827581160423	-3,79701E+14	1,04016E+14	1,00342E+14	9,51863E+14	7,77997E+14	9,15844E+14	8,17829E+13
TC1100003218.mm.2	Ovca2/Dph1	candidate tumor suppressor in ovarian cancer 2/diphthamide biosynthesis 1	-0.84821432170064	6,7926E+14	-2,44077E+14	0.047726123762862	0.184864089205669	-3,79807E+14	6,29354E+14	6,4648E+12	6,63691E+13	6,9056E+14	6,91515E+14	7,53961E+14
TC0700000868.mm.2	Tmem143	transmembrane protein 143	0.795211414692968	6,66284E+13	2,44076E+14	0.0477270655433591	0.184864089205669	-3,79809E+14	7,32366E+14	7,09861E+14	6,15532E+14	6,5676E+14	6,27204E+14	6,55981E+14
TC1100003079.mm.2	Elp5	elongator acetyltransferase complex subunit 5	2,71101E+14	7,32133E+14	2,44063E+14	0.047735637579104	0.184864089205669	-3,79826E+14	8,7924E+14	8,69618E+14	8,07308E+14	6,29977E+13	7,74012E+14	4,32645E+14
TC0300000159.mm.2	Nceh1	neutral cholesterol ester hydrolase 1	1,70123E+14	8,50626E+14	2,44062E+14	0.0477360190724034	0.184864089205669	-3,79826E+13	9,81594E+14	9,74813E+14	8,72503E+14	6,58684E+14	8,47157E+14	7,69005E+14
TC1900001316.mm.2	Rfx3	regulatory factor X, 3 (influences HLA class II expression)	1,42411E+14	8,17046E+14	2,4405E+14	0.0477440188126639	0.184864089205669	-3,79842E+14	8,83086E+14	8,84003E+14	8,69262E+14	7,8366E+14	8,21519E+14	6,60748E+14
TC0600000958.mm.2	Emx1	empty spiracles homeobox 1	-0.858073534349495	7,21935E+14	-2,44041E+14	0.0477505330648542	0.184864089205669	-3,79855E+14	6,50613E+14	6,77274E+14	7,2182E+14	7,82401E+14	7,34737E+14	7,64765E+14
TC0600001015.mm.2	Efcc1	EF hand and coiled-coil domain containing 1	-1,02673E+14	7,38225E+14	-2,44006E+14	0.0477734603087616	0.184872239087954	-3,799E+14	6,6797E+14	6,7634E+14	7,46942E+14	7,88444E+14	7,23932E+14	8,25723E+14
TC0400002613.mm.2	Alg2	asparagine-linked glycosylation 2 (alpha-1,3-mannosyltransferase)	1,0724E+14	8,77314E+14	2,44003E+14	0.047775830565879	0.184872239087954	-3,79904E+14	9,49693E+14	9,09019E+14	9,03174E+14	8,57764E+13	8,73373E+14	7,70859E+14
TC1400002215.mm.2	Fdft1	farnesyl diphosphate farnesyl transferase 1	1,18936E+14	8,32902E+14	2,43986E+14	0.0477870586359665	0.184872239087954	-3,79926E+14	9,38362E+13	9,07944E+14	8,32776E+14	7,09895E+13	8,35519E+13	7,72917E+14
TC0100003887.mm.2	Nos1ap	nitric oxide synthase 1 (neuronal) adaptor protein	1,30645E+14	8,14021E+14	2,43925E+13	0.0478283170675456	0.184987439488565	-3,80007E+14	9,0409E+14	9,03375E+14	8,27562E+14	7,02923E+14	8,24908E+14	7,21267E+14
TC0500003589.mm.2	Pdap1	PDGFA associated protein 1	0.979786641251561	8,85084E+14	2,43889E+13	0.0478525972614146	0.185036932966924	-3,80055E+14	9,51669E+14	9,30009E+14	8,69954E+14	8,73149E+14	8,92003E+14	7,93718E+14
TC1100000720.mm.2	Wnt9a	wingless-type MMTV integration site family, member 9A	-0.795006215184936	6,84033E+14	-2,43787E+14	0.0479213799714215	0.185207148684957	-3,80189E+13	6,21737E+14	6,51726E+13	6,86977E+14	7,11293E+14	6,96426E+14	7,36038E+14
TC0600002248.mm.2	Gsdme	gasdermin E	0.786932305717238	5,63938E+14	2,43774E+14	0.0479299817665079	0.185207148684957	-3,80206E+14	6,28334E+14	6,2439E+14	5,43418E+14	4,92146E+14	5,41748E+13	5,5359E+13
TC0X00000949.mm.2	Otud6a	OTU domain containing 6A	-1,22149E+14	5,48475E+13	-2,43764E+14	0.0479369843732603	0.185207148684957	-3,8022E+14	4,26252E+14	5,01938E+13	5,33538E+14	6,56637E+14	5,72149E+14	6,00338E+14
TC1100002100.mm.2	4921536K21Rik	RIKEN cDNA 4921536K21 gene	-0.856189472515395	5,66596E+14	-2,43739E+14	0.0479534783383111	0.185207148684957	-3,80252E+14	5,29456E+14	5,15488E+14	5,99525E+14	5,38928E+14	5,89484E+14	6,26698E+14
TC0400002787.mm.2	Cdc26	cell division cycle 26	1,29916E+14	7,59248E+14	2,43738E+14	0.0479540882711872	0.185207148684957	-3,80253E+14	8,54055E+14	8,3433E+14	7,86457E+14	6,52093E+14	7,57517E+14	6,71035E+13
TC0800001485.mm.2	Zc3h18	zinc finger CCCH-type containing 18	-1,00499E+14	7,27389E+14	-2,43681E+14	0.0479925622827669	0.185273765272942	-3,80328E+14	6,61935E+14	6,61737E+14	7,24082E+14	7,91906E+14	7,21537E+14	8,03133E+14
TC1800000869.mm.2	Mbp	myelin basic protein	0.967709953246272	1,11604E+14	2,43679E+14	0.0479943335349878	0.185273765272942	-3,80332E+14	1,16443E+14	1,19856E+14	1,11758E+13	1,04623E+14	1,11392E+13	1,05553E+14
TC1700000754.mm.2	Gabbr1	gamma-aminobutyric acid (GABA) B receptor, 1	1,36817E+14	1,02492E+14	2,4365E+14	0.0480138603865455	0.185304750277463	-3,8037E+14	1,12188E+14	1,11148E+14	1,05884E+14	8,9757E+14	1,01076E+14	9,48961E+14
TC1600000167.mm.2	Ntan1	N-terminal Asn amidase	0.761713255395316	7,68165E+14	2,436E+14	0.048047700937484	0.185390949522433	-3,80436E+14	8,21207E+14	8,11001E+14	7,67747E+14	7,29168E+14	7,49484E+14	7,30382E+14
TC0500003588.mm.2	Gm4871	predicted gene 4871	-1,33216E+14	5,28591E+13	-2,43557E+14	0.0480768815744369	0.185419106364945	-3,80493E+14	4,79498E+14	4,25395E+14	4,94519E+13	6,00813E+14	5,27281E+14	6,44043E+14
TC0600000678.mm.2	Fkbp9	FK506 binding protein 9	1,72669E+14	6,90609E+13	2,43555E+14	0.0480780131814522	0.185419106364945	-3,80495E+13	8,20708E+14	8,34943E+14	6,6344E+14	5,14247E+14	7,43988E+13	5,66325E+14
TC0X00000694.mm.2	Srpk3	serine/arginine-rich protein specific kinase 3	-0.966535956551903	6,25606E+14	-2,43521E+14	0.0481007649415157	0.185422547475785	-3,80539E+14	5,50143E+14	5,70037E+14	6,21346E+14	6,98624E+14	6,2813E+14	6,85357E+14
TC0900002601.mm.2	Lipc	lipase, hepatic	-0.976323760530594	5,65607E+13	-2,4352E+14	0.0481019207130132	0.185422547475785	-3,80541E+14	5,40212E+14	4,716E+14	5,54478E+14	6,20276E+14	5,87584E+14	6,19493E+14
TC0200000562.mm.2	Nup214/Mir7674	nucleoporin 214/microRNA 7674	0.894768530052046	5,62803E+14	2,43451E+14	0.048148440109841	0.185469204780262	-3,80632E+14	5,91701E+14	6,6511E+14	5,03924E+14	5,38224E+14	5,45822E+14	5,32036E+14
TC1600001849.mm.2	Cadm2	cell adhesion molecule 2	1,31194E+14	8,27127E+14	2,43426E+13	0.0481657894536511	0.185469204780262	-3,80666E+14	8,95557E+14	9,1859E+14	8,13444E+14	7,83411E+14	8,55421E+14	6,96337E+14
TC0500001927.mm.2	Adam22	a disintegrin and metallopeptidase domain 22	2,17993E+14	7,5907E+14	2,43408E+14	0.04817742171738	0.185469204780262	-3,80688E+14	8,96662E+14	8,98478E+14	8,17154E+14	5,82974E+14	7,76655E+14	5,825E+14
TC0100001955.mm.2	Arfgef1	ADP-ribosylation factor guanine nucleotide-exchange factor 1(brefeldin A-inhibited)	1,92327E+14	7,57727E+14	2,43408E+14	0.0481779033343248	0.185469204780262	-3,80689E+14	8,69099E+14	8,76675E+14	8,07151E+14	6,32316E+14	7,79372E+14	5,81748E+13
TC1500001345.mm.2	Rpl30/Gm12191	ribosomal protein L30/ribosomal protein L30 pseudogene	1,27163E+14	8,62422E+14	2,4339E+13	0.0481900210773045	0.185469204780262	-3,80713E+14	9,49167E+13	9,80138E+14	8,57327E+14	7,1292E+14	8,63361E+14	8,11618E+14
TC1200001813.mm.2	Sec23a	SEC23 homolog A, COPII coat complex component	1,96698E+14	6,44526E+14	2,43356E+14	0.0482132190929579	0.185469204780262	-3,80758E+14	7,95526E+14	8,14018E+14	6,41721E+14	3,99744E+14	6,68306E+14	5,47842E+14
TC0900000197.mm.2	Dnm2	dynamin 2	1,1574E+14	8,38535E+14	2,43338E+14	0.0482248417913899	0.185469204780262	-3,8078E+14	9,07653E+14	9,40482E+14	8,48194E+14	7,18228E+14	8,19608E+14	7,97047E+14
TC0X00001065.mm.2	Cox7b	cytochrome c oxidase subunit 7B	0.770335698841238	9,75825E+14	2,43312E+14	0.0482430141192155	0.185469204780262	-3,80816E+14	1,03961E+14	1,02523E+14	9,25139E+14	9,54208E+14	9,72866E+14	9,37902E+14
TC0200002352.mm.2	Raly/a	hnRNP-associated with lethal yellow/nonagouti	1,02242E+14	6,83616E+14	2,43306E+14	0.0482467545167367	0.185469204780262	-3,80823E+14	7,83723E+14	7,72155E+14	6,51317E+14	5,43105E+14	6,83594E+14	6,67801E+14
TC1300002588.mm.2	Erbin	Erbb2 interacting protein	2,0778E+14	6,93229E+13	2,43306E+14	0.0482468952540289	0.185469204780262	-3,80823E+14	8,25063E+14	8,4654E+14	7,10825E+13	5,209E+14	7,3601E+13	5,20034E+13
TC1300000102.mm.2	AW209491	expressed sequence AW209491	0.78204766851146	6,57961E+14	2,43297E+14	0.0482531201865456	0.185469204780262	-3,80835E+14	7,11361E+13	7,01812E+14	6,50386E+14	6,27446E+14	6,05725E+14	6,51038E+14
TC0700000043.mm.2	Tmem190	transmembrane protein 190	-0.791672909620776	5,67225E+14	-2,43288E+14	0.0482588540711124	0.185469204780262	-3,80846E+14	4,94295E+12	5,23403E+14	6,03746E+14	6,05871E+14	5,64838E+14	6,11195E+14
TC0700002944.mm.2	Zfp715	zinc finger protein 715	0.96683431795176	6,41829E+14	2,43281E+14	0.0482636613693067	0.185469204780262	-3,80856E+12	6,7274E+14	6,69617E+14	6,85797E+14	6,7383E+14	6,23337E+14	5,25653E+14

TC1100000558.mm.2	Olfr54	olfactory receptor 54	-1,00263E+14	4,79478E+14	-2,43242E+14	0.0482901508458778	0.185482713045567	-3,80907E+14	4,01668E+14	4,45161E+14	4,76177E+14	5,06509E+14	4,7779E+14	5,69565E+14
TC1100002729.mm.2	Uqcrq	ubiquinol-cytochrome c reductase, complex III subunit VII	-0.724232604300374	9,68613E+14	-2,43241E+14	0.0482908626894929	0.185482713045567	-3,80908E+14	9,20772E+14	9,2663E+14	9,80893E+14	9,91133E+14	1,00882E+14	9,8343E+13
TC0200005425.mm.2	Ythdf1	YTH N6-methyladenosine RNA binding protein 1	1,126E+14	6,53177E+14	2,43222E+14	0.0483040957744935	0.185482713045567	-3,80934E+14	7,34954E+14	7,34861E+14	6,22911E+14	5,8172E+14	5,60562E+14	6,84054E+14
TC0100001392.mm.2	Zfp648	zinc finger protein 648	-0.724083058104877	6,00782E+14	-2,43208E+14	0.0483132220351422	0.185482713045567	-3,80952E+14	5,47586E+14	5,6239E+14	6,29608E+14	6,10316E+14	6,2049E+13	6,34303E+14
TC0X00000319.mm.2	Lonrf3	LON peptidase N-terminal domain and ring finger 3	1,83603E+13	5,55876E+14	2,43146E+14	0.048355547257436	0.185586256467711	-3,81034E+14	6,7051E+14	6,7935E+14	6,25835E+14	3,7691E+14	4,78327E+14	5,04326E+14
TC0700002631.mm.2	Cnfn	cornifelin	-0.905926056826796	6,21552E+14	-2,43135E+14	0.0483632278848083	0.185586256467711	-3,81049E+14	5,60696E+14	5,61403E+14	6,09909E+14	6,94019E+14	6,45262E+14	6,58022E+14
TC0300000199.mm.2	Mynn	myoneurin	1,50717E+14	5,39603E+14	2,43068E+14	0.0484086424507805	0.185716299002244	-3,81137E+14	6,47499E+14	6,35718E+14	5,81964E+14	3,90656E+14	5,1377E+14	4,68013E+14
TC1700001612.mm.2	Prss22	protease, serine 22	-0.798347255446911	5,50326E+14	-2,43037E+14	0.0484297598411272	0.185753087436344	-3,81177E+14	4,92213E+14	5,27909E+14	5,53316E+14	5,48727E+14	5,67733E+14	6,12059E+14
TC1100003908.mm.2	Cyb561	cytochrome b-561	0.781521883151536	6,20175E+14	2,42952E+14	0.0484883177699325	0.185868309489029	-3,81291E+14	6,94056E+14	6,99397E+14	5,70324E+14	5,20126E+14	6,12612E+14	6,24536E+13
TC1900000689.mm.2	Nfkb2	nuclear factor of kappa light polypeptide gene enhancer in B cells 2, p49/p100	-1,08343E+14	5,45728E+14	-2,42942E+14	0.0484951424981252	0.185868309489029	-3,81304E+14	4,44237E+14	5,15528E+14	5,20582E+14	6,17571E+14	5,77363E+14	5,99088E+14
TC0500001587.mm.2	Srrm3	serine/arginine repetitive matrix 3	-0.738618077637253	7,70121E+14	-2,42934E+14	0.0485001852271082	0.185868309489029	-3,81313E+14	7,23736E+14	7,27006E+13	7,68529E+14	8,02988E+14	8,03508E+14	7,94958E+14
TC0200002785.mm.2	Rps21	ribosomal protein S21	0.823217616735285	9,38248E+14	2,42912E+14	0.048515090971473	0.185868309489029	-3,81342E+14	1,01091E+14	9,83911E+14	8,85999E+14	9,18495E+14	8,90754E+14	9,39422E+14
TC0900002235.mm.2	Rexo2	RNA exonuclease 2	0.99404085650596	7,23845E+14	2,42906E+14	0.0485193346152734	0.185868309489029	-3,8135E+14	7,87541E+13	8,09518E+14	7,12184E+14	6,35574E+14	7,32922E+14	6,6533E+14
TC1100001183.mm.2	Rab11fip4	RAB11 family interacting protein 4 (class II)	0.777163352192947	8,61894E+13	2,42892E+14	0.0485290124756621	0.185868309489029	-3,81369E+13	9,30323E+14	9,0263E+14	8,34929E+14	8,25958E+14	8,59386E+14	8,18135E+14
TC1800000105.mm.2	Ttc39c	tetratricopeptide repeat domain 39C	0.962355375106516	5,73541E+14	2,42841E+14	0.0485640440114016	0.185958279742327	-3,81437E+14	6,522E+13	6,22745E+14	5,76524E+14	5,07301E+14	5,72546E+14	5,09927E+14
TC1100000851.mm.2	Myh3	myosin, heavy polypeptide 3, skeletal muscle, embryonic	-1,35505E+14	5,22099E+14	-2,42694E+14	0.0486642758388648	0.186297808646146	-3,8163E+14	4,42768E+14	4,49405E+14	4,62212E+14	6,15024E+13	5,42603E+14	6,2058E+14
TC0200004555.mm.2	Fahd2a	fumarylacetoacetate hydrolase domain containing 2A	-0.904468292835293	6,8906E+14	-2,42671E+14	0.0486805740414562	0.18631593575534	-3,81661E+14	6,44786E+14	6,05501E+14	6,89129E+14	7,63762E+14	7,21406E+14	7,09775E+13
TC0900002326.mm.2	AY074887	cDNA sequence AY074887	-1,38407E+14	6,56907E+14	-2,42597E+14	0.0487309518231229	0.186432865916241	-3,81758E+14	5,99529E+13	5,57704E+14	6,13714E+14	7,36447E+14	6,56955E+14	7,77092E+14
TC1000002258.mm.2	Supv3l1	suppressor of var1, 3-like 1 (S. cerevisiae)	0.94684168680224	5,39329E+14	2,42578E+14	0.0487442331612115	0.186432865916241	-3,81783E+14	6,14892E+14	5,86617E+14	4,75486E+14	5,46838E+14	5,33848E+14	4,78293E+14
TC1200001277.mm.2	Pacs2	phosphofurin acidic cluster sorting protein 2	1,1781E+14	7,77002E+14	2,42576E+14	0.0487458365360344	0.186432865916241	-3,81786E+14	8,50106E+14	8,43422E+14	8,17975E+14	6,926E+14	7,60397E+14	6,97511E+14
TC0600000830.mm.2	Chmp3	charged multivesicular body protein 3	0.72089506186204	6,88104E+14	2,42544E+14	0.0487675403069803	0.186471612948831	-3,81828E+14	7,68285E+14	7,43178E+14	6,26631E+14	6,23246E+14	6,88696E+14	6,78587E+13
TC0400004123.mm.2	Actrt2	actin-related protein T2	-1,04705E+14	5,98809E+14	-2,42518E+14	0.0487854835309856	0.186477297304544	-3,81863E+14	5,16075E+14	5,49248E+14	5,97171E+14	6,55627E+13	5,9036E+14	6,84373E+14
TC1800001139.mm.2	Nrep	neuronal regeneration related protein	-1,03437E+14	8,51465E+14	-2,42508E+14	0.0487921731171077	0.186477297304544	-3,81875E+14	7,84353E+14	8,06481E+14	8,98795E+13	8,21452E+14	9,38568E+14	8,59139E+14
TC1400002086.mm.2	Nrl	neural retina leucine zipper gene	-0.957663224816707	5,99703E+14	-2,42463E+14	0.0488234719246552	0.186515776669421	-3,81935E+14	5,49139E+14	5,24368E+14	5,86835E+14	6,72835E+14	6,13567E+14	6,51472E+14
TC0400000426.mm.2	Dnajb5	DnaJ heat shock protein family (Hsp40) member B5	0.973184466158453	7,74067E+14	2,4246E+14	0.048825392291418	0.186515776669421	-3,81939E+14	8,39286E+14	8,36036E+14	7,92952E+14	6,95441E+14	7,52421E+14	7,28265E+14
TC0600002480.mm.2	Atoh8	atonal bHLH transcription factor 8	-0.831987944600095	6,12468E+14	-2,42439E+14	0.0488396186242034	0.186525900661718	-3,81966E+14	5,453E+14	5,84562E+14	6,01869E+14	6,46815E+14	6,3258E+14	6,63679E+14
TC1500000947.mm.2	Arid2	AT rich interactive domain 2 (ARID, RFX-like)	1,23947E+14	5,98384E+14	2,42362E+14	0.0488930081506697	0.186641816374487	-3,82069E+13	6,8554E+14	6,61609E+14	6,24747E+12	5,19155E+14	6,02774E+14	4,96482E+14
TC0400003756.mm.2	Clic4	chloride intracellular channel 4 (mitochondrial)	1,56992E+14	8,14802E+14	2,42352E+14	0.0488995078434047	0.186641816374487	-3,82081E+14	9,08207E+14	9,03627E+14	8,48225E+13	7,30905E+14	8,349E+14	6,6295E+14
TC0700000178.mm.2	Obox7	oocyte specific homeobox 7	-1,42778E+14	5,25534E+14	-2,42327E+14	0.0489170639334996	0.186641816374487	-3,82115E+14	3,96343E+14	4,40427E+14	5,10874E+14	6,83236E+14	5,85549E+14	5,36777E+14
TC1200002521.mm.2	Gm9804	predicted gene 9804	-1,22526E+14	6,24263E+14	-2,4231E+14	0.0489287845686436	0.186641816374487	-3,82137E+14	5,51559E+14	5,19286E+13	6,09566E+14	7,4927E+14	6,3728E+14	6,78617E+14
TC1100001163.mm.2	Sebox	SEBOX homeobox	-0.835999192274463	5,28421E+14	-2,42302E+14	0.0489337749872256	0.186641816374487	-3,82147E+14	4,75825E+14	5,30427E+14	4,84948E+14	5,05872E+13	6,04567E+12	5,68885E+13
TC1900001729.mm.2	Shtn1	shootin 1	1,17643E+12	6,62793E+14	2,42283E+14	0.0489470455842955	0.186641816374487	-3,82172E+14	7,37914E+14	7,16658E+14	6,83073E+14	6,19825E+14	6,71394E+14	5,47892E+14
TC0400000855.mm.2	Sh3gl2	SH3-domain GRB2-like 2	1,0276E+14	7,63843E+14	2,42277E+14	0.0489510529385329	0.186641816374487	-3,8218E+14	8,42058E+13	8,36414E+14	7,65094E+14	6,66538E+14	7,62811E+14	7,1014E+14
TC0200002148.mm.2	Zfp133-ps/Gm38402	zinc finger protein 133, pseudogene/zinc finger protein pseudogene	-0.809642845843965	6,38745E+14	-2,42197E+14	0.049006506389252	0.186809046001897	-3,82286E+14	5,96085E+14	5,93508E+14	6,1779E+14	6,73564E+14	6,88384E+14	6,63137E+14
TC0800000463.mm.2	Tusc3	tumor suppressor candidate 3	1,0341E+14	9,90824E+14	2,42171E+14	0.0490246317325395	0.186829139673173	-3,8232E+13	1,05875E+14	1,05926E+14	9,99766E+14	9,15967E+14	9,93712E+14	9,17483E+14
TC1100001677.mm.2	Cd300lg	CD300 molecule like family member G	-0.849112393783421	4,94303E+14	-2,42156E+14	0.0490349675217432	0.186829139673173	-3,8234E+14	4,2162E+13	4,50742E+14	4,94111E+14	5,57157E+14	5,11439E+14	5,30745E+14
TC0200000593.mm.2	Ptrh1	peptidyl-tRNA hydrolase 1 homolog	-0.880044215655341	6,80884E+14	-2,42103E+14	0.0490715036300149	0.186886353103417	-3,8241E+14	6,34307E+14	6,25592E+14	6,64895E+14	7,24602E+14	6,91804E+14	7,44103E+14
TC1500000478.mm.2	Efr3a	EFR3 homolog A	-1,37471E+14	7,23459E+14	-2,42088E+13	0.0490817705443248	0.186886353103417	-3,82429E+14	6,2076E+14	6,29643E+14	7,11746E+14	8,53257E+14	7,1083E+14	8,14516E+14
TC1300002048.mm.2	Uimc1	ubiquitin interaction motif containing 1	0.995132619003025	5,48271E+14	2,42073E+14	0.0490920787618019	0.186886353103417	-3,82449E+14	6,42825E+14	6,0362E+14	5,36569E+14	4,59195E+14	5,53681E+14	4,93738E+14
TC0X00001602.mm.2	Klhl34	kelch-like 34	-0.73197201736205	6,17146E+14	-2,42067E+14	0.0490963776259397	0.186886353103417	-3,82457E+14	5,57591E+14	5,75276E+14	6,31283E+14	6,59467E+14	6,42588E+14	6,36674E+14
TC0900000365.mm.2	Robo4	roundabout guidance receptor 4	-0.744841537228353	5,22459E+14	-2,4203E+14	0.0491222179602915	0.186892488116697	-3,82506E+14	4,95756E+14	5,03047E+14	5,01619E+14	4,86565E+14	5,55974E+14	5,91796E+14
TC1800000046.mm.2	4921524L21Rik	RIKEN cDNA 4921524L21 gene	-0.951224803021865	5,16794E+14	-2,42026E+14	0.0491245803005846	0.186892488116697	-3,82511E+14	4,27926E+14	4,75358E+14	5,1566E+14	5,88291E+14	5,26004E+14	5,67525E+14
TC0100002042.mm.2	Kcnq5	potassium voltage-gated channel, subfamily Q, member 5	1,09459E+14	8,16654E+14	2,42008E+13	0.0491374805927683	0.186892488116697	-3,82535E+14	8,84024E+14	8,93234E+14	8,27962E+14	7,36363E+13	8,21649E+14	7,36691E+13
TC1100003158.mm.2	Emc6	ER membrane protein complex subunit 6	-0.853378067449439	8,60882E+14	-2,41992E+14	0.0491483255541894	0.186892488116697	-3,82556E+14	7,93402E+14	8,14562E+14	8,54645E+14	9,24045E+14	8,9462E+14	8,84019E+13
TC0800000539.mm.2	Cenpu	centromere protein U	-0.981399608025604	4,57428E+14	-2,41981E+14	0.0491559836553442	0.186892488116697	-3,82571E+14	4,07495E+14	3,86194E+13	4,37362E+14	5,2355E+14	5,20749E+14	4,6922E+14
TC1700001628.mm.2	E4f1	E4F transcription factor 1	0.959538330104262	6,0453E+14	2,41926E+13	0.0491941814945217	0.186895458314123	-3,82643E+14	6,39297E+14	6,63412E+14	6,11236E+14	6,02433E+14	6,02718E+13	5,08084E+14
TC1500002173.mm.2	Olfr283	olfactory receptor 283	-1,05154E+14	6,10398E+14	-2,41913E+13	0.0492031435980861	0.186895458314123	-3,8266E+14	5,80403E+14	5,17864E+13	5,80664E+14	6,74881E+14	6,39328E+14	6,69247E+14
TC1000000208.mm.2	Stx7	syntaxin 7	0.99380549547851	7,95609E+14	2,41912E+14	0.0492039927058749	0.186895458314123	-3,82662E+14	8,52402E+14	8,39168E+14	8,05106E+14	7,84167E+14	7,97577E+14	6,95232E+13
TC0600002960.mm.2	Rpl32	ribosomal protein L32	0.743002613870688	9,27632E+14	2,41901E+14	0.0492111108090503	0.186895458314123	-3,82676E+13	9,87131E+14	9,80559E+14	9,05625E+14	8,73384E+14	9,19951E+14	8,99139E+13
TC1000002200.mm.2	Gm4981	predicted gene 4981	-0.971973744177186	4,52482E+14	-2,41896E+14	0.0492147601084108	0.186895458314123	-3,82683E+13	3,64244E+14	4,11651E+14	4,48364E+13	5,20346E+13	4,59075E+14	5,11215E+14
TC0100002213.mm.2	Txndc9	thioredoxin domain containing 9	1,1281E+14	7,30817E+14	2,41864E+14	0.0492368498526727	0.186935287859593	-3,82724E+14	7,86606E+14	8,08354E+14	7,5682E+14	6,6378E+14	7,30036E+14	6,39305E+14
TC0900000485.mm.2	D630033O11Rik	RIKEN cDNA D630033O11 gene	-0.897522419987917	5,68046E+14	-2,41834E+14	0.049257930037744	0.186971266595564	-3,82765E+14	5,43293E+14	4,70934E+14	5,86145E+14	6,14172E+13	5,80466E+14	6,13266E+14
TC0800001255.mm.2	Cdh1	cadherin 1	-0.998469049640258	6,68265E+14	-2,41813E+14	0.0492725578345755	0.186982742437239	-3,82792E+14	5,94544E+14	5,9579E+14	6,70564E+14	7,58663E+14	6,65236E+13	7,24792E+13
TC0300001792.mm.2	Phc3	polyhomeotic 3	1,10673E+14	5,88086E+14	2,41779E+13	0.0492956771376846	0.186993487358021	-3,82836E+14	6,32E+14	6,27656E+14	6,05978E+13	6,24575E+14	5,82333E+14	4,55977E+14
TC1500001973.mm.2	Pacsin2	protein kinase C and casein kinase substrate in neurons 2	0.720084471326944	7,36683E+14	2,41773E+14	0.0492999840664358	0.186993487358021	-3,82844E+14	7,90007E+14	7,78551E+14	7,15347E+14	7,11653E+14	7,24979E+13	6,99563E+14
TC0100002365.mm.2	Satb2	special AT-rich sequence binding protein 2	-0.724341718363908	9,37082E+14	-2,41759E+14	0.0493102046660604	0.186993487358021	-3,82864E+14	8,73208E+14	9,01263E+13	9,59878E+14	9,68802E+14	9,63569E+13	9,55771E+14
TC1500001601.mm.2	4933412E24Rik	RIKEN cDNA 4933412E24 gene	-1,07683E+14	5,91477E+14	-2,41705E+14	0.0493476281430088	0.187091372298424	-3,82935E+14	5,04315E+14	5,27767E+14	5,8444E+14	6,84891E+14	5,91053E+14	6,56394E+14
TC0400003892.mm.2	Dnajc16	DnaJ heat shock protein family (Hsp40) member C16	0.755395237561396	6,25317E+14	2,41648E+14	0.0493873180789388	0.187156738688589	-3,8301E+14	6,49035E+14	6,78237E+14	6,0789E+14	6,40549E+14	5,85318E+14	5,90875E+14
TC0100001317.mm.2	Uchl5	ubiquitin carboxyl-terminal esterase L5	1,5878E+14	7,01587E+14	2,41647E+13	0.0493880998512927	0.187156738688589	-3,83012E+14	8,34907E+14	8,38449E+14	6,91461E+14	4,88909E+14	7,09007E+14	6,46788E+14
TC1300000534.mm.2	Cap2	CAP, adenylate cyclase-associated protein, 2 (yeast)	1,47094E+14	9,1419E+14	2,41528E+14	0.0494705889013017	0.187425252519792	-3,83168E+14	1,00728E+14	1,01892E+14	9,58113E+13	7,68799E+14	8,82662E+13	8,49358E+14
TC1500002339.mm.2	Hoxc10	homeobox C10	-0.828122956409664	5,52464E+14	-2,41392E+14	0.0495653129645161	0.187739983027092	-3,83347E+14	5,03027E+14	5,07848E+13	5,30198E+14	5,97211E+13	5,89349E+14	5,8715E+14
TC1100001454.mm.2	Dlx3	distal-less homeobox 3	-1,15686E+14	6,29769E+14	-2,41367E+13	0.0495827629964739	0.187761941283709	-3,8338E+14	5,71742E+14	5,64457E+14	6,13359E+14	6,61484E+14	6,19445E+14	7,48126E+14
TC0800000747.mm.2	Comp	cartilage oligomeric matrix protein	-0.831900698368196	5,26234E+14	-2,4135E+14	0.0495947002806294	0.187763018238201	-3,83402E+14	4,61593E+14	4,53613E+14	5,51144E+14	6,09471E+14	5,23495E+14	5,5809E+14

TC1100000820.mm.2	Cdrt4	CMT1A duplicated region transcript 4	-1,05956E+13	5,97554E+14	-2,41319E+13	0.0496163770092101	0.187800959067278	-3,83443E+14	5,36451E+14	4,96702E+14	6,03107E+14	7,03996E+14	5,97946E+13	6,47119E+14
TC0400002477.mm.2	Dctn3	dynactin 3	1,41516E+14	9,52032E+14	2,41295E+14	0.0496334733139092	0.187821548968299	-3,83475E+14	1,04781E+14	1,05068E+14	9,85823E+13	8,12426E+14	9,37502E+14	8,77954E+14
TC1100002783.mm.2	Sparc	secreted acidic cysteine rich glycoprotein	1,7148E+14	9,85465E+14	2,4122E+14	0.0496859938361316	0.187968278398076	-3,83574E+14	1,12389E+14	1,04987E+14	1,05567E+13	8,52565E+14	9,59123E+14	8,71674E+14
TC0700004633.mm.2	Gm15448	predicted gene 15448	-0.749118970180894	6,58879E+14	-2,41197E+14	0.0497020073460683	0.187968278398076	-3,83604E+14	6,09143E+14	6,27076E+14	6,70662E+14	6,60349E+14	6,74579E+14	7,11464E+14
TC1400001456.mm.2	Cadps	Ca2+-dependent secretion activator	1,68063E+14	8,90187E+13	2,41166E+14	0.0497237457409046	0.187968278398076	-3,83645E+14	1,0155E+14	9,9549E+14	9,11555E+14	7,43719E+14	9,13707E+14	7,61153E+14
TC1500000371.mm.2	Utp23	UTP23 small subunit processome component	-1,0194E+14	7,24779E+14	-2,41161E+14	0.0497270266877629	0.187968278398076	-3,83651E+14	6,48341E+14	6,65239E+14	7,16397E+14	8,01237E+14	7,23804E+14	7,93657E+13
TC0700000764.mm.2	Zfp658	zinc finger protein 658	-0.766063006853024	6,97986E+13	-2,41156E+14	0.0497305759828087	0.187968278398076	-3,83658E+14	6,46983E+14	6,62771E+14	6,91683E+14	7,23515E+14	7,14916E+14	7,4805E+14
TC0900002820.mm.2	Morf4l1	mortality factor 4 like 1	0.940367772625489	1,08879E+14	2,41052E+14	0.0498040309281184	0.188165349210983	-3,83796E+14	1,14977E+14	1,14187E+14	1,11297E+14	1,02459E+14	1,06822E+14	1,03534E+14
TC1100000337.mm.2	Hbq1b	hemoglobin, theta 1B	-1,08933E+14	5,41655E+14	-2,41049E+14	0.0498060705259631	0.188165349210983	-3,838E+14	4,3906E+14	4,94702E+14	5,26394E+14	6,38145E+14	5,71816E+14	5,79812E+14
TC1300001410.mm.2	Wdr37	WD repeat domain 37	1,70573E+14	7,34086E+14	2,41011E+14	0.0498326686670777	0.188221704227057	-3,8385E+14	8,50491E+14	8,25434E+14	7,83876E+14	6,09937E+14	7,43089E+14	5,91691E+14
TC0600002315.mm.2	Fkbp14	FK506 binding protein 14	1,50837E+14	5,28673E+14	2,40948E+14	0.0498769859641511	0.188237264363939	-3,83933E+14	6,76966E+14	6,13022E+14	5,0179E+14	3,91946E+14	4,17295E+14	5,71019E+14
TC1900000617.mm.2	R3hcc1l	R3H domain and coiled-coil containing 1 like	1,44712E+14	5,9146E+14	2,40936E+13	0.0498849061589433	0.188237264363939	-3,83948E+14	7,05142E+14	6,54048E+14	6,46104E+14	4,73703E+14	5,64235E+14	5,05531E+14
TC0100003832.mm.2	Syt14	synaptotagmin XIV	1,12127E+14	6,48719E+13	2,40934E+14	0.0498866039953035	0.188237264363939	-3,83951E+14	7,07766E+14	6,90908E+14	6,80095E+14	6,39125E+14	6,49006E+13	5,25414E+14
TC0900002516.mm.2	Zfp609	zinc finger protein 609	0.775889454323417	8,55082E+14	2,40909E+14	0.0499038950766961	0.188237264363939	-3,83984E+14	9,19538E+14	8,95326E+14	8,27112E+14	8,28827E+14	8,52939E+13	8,06747E+14
TC1100001039.mm.2	Olfr406	olfactory receptor 406	-1,28654E+14	5,13181E+14	-2,40904E+14	0.0499079604820782	0.188237264363939	-3,83991E+13	4,63349E+14	4,17935E+14	4,76086E+14	5,83126E+14	5,13296E+14	6,25295E+14
TC1600001070.mm.2	Kcnj15	potassium inwardly-rectifying channel, subfamily J, member 15	-1,05005E+14	4,56624E+14	-2,40899E+14	0.0499116113560724	0.188237264363939	-3,83998E+14	3,79055E+14	3,77111E+14	4,66821E+14	5,50582E+14	4,4186E+13	5,24316E+14
TC0300000624.mm.2	4930579G24Rik	RIKEN cDNA 4930579G24 gene	-0.919081536762748	6,18154E+14	-2,40889E+14	0.0499185645520456	0.188237264363939	-3,84011E+14	5,77897E+14	5,66732E+14	5,88061E+14	6,47788E+13	6,31009E+14	6,97436E+13
TC0400000404.mm.2	Gm21953/Gm20878/ Gm21586	predicted gene, 21953/predicted gene, 20878/predicted gene, 21586	0.94197685079601	9,34709E+14	2,40839E+14	0.0499532168554505	0.188323861298988	-3,84076E+14	1,00808E+14	1,02679E+14	8,86459E+14	8,40447E+12	9,63827E+14	8,82649E+14
TC0400003733.mm.2	Grrp1	glycine/arginine rich protein 1	-0.71798200620378	5,03482E+14	-2,40793E+14	0.0499861372162903	0.188338378912689	-3,84138E+14	4,6293E+14	4,49605E+14	5,13486E+14	5,3874E+14	5,27446E+14	5,28686E+14
TC1500002203.mm.2	C1ql4	complement component 1, q subcomponent-like 4	-1,09642E+13	5,37593E+14	-2,40747E+14	0.0500185829202732	0.188338378912689	-3,84198E+14	4,78242E+14	4,54529E+14	5,27849E+14	6,12882E+14	5,30571E+14	6,21484E+13
TC1300000715.mm.2	Rmi1	RecQ mediated genome instability 1	1,29554E+14	5,53672E+14	2,40731E+14	0.0500294748514999	0.188338378912689	-3,84219E+14	6,48229E+14	6,32757E+14	5,77186E+14	4,41984E+14	5,55975E+14	4,65903E+14
TC1300002540.mm.2	Cdk7	cyclin-dependent kinase 7	1,22555E+14	6,21683E+14	2,4073E+14	0.0500301729189093	0.188338378912689	-3,8422E+13	6,77852E+14	6,80924E+14	6,57449E+14	6,00207E+14	6,26529E+14	4,87137E+13
TC0800001323.mm.2	Cog4	component of oligomeric golgi complex 4	0.781414863104137	5,68007E+14	2,40713E+14	0.0500421556146336	0.188338378912689	-3,84243E+13	6,48112E+14	6,32524E+13	4,76191E+14	5,26861E+14	5,67771E+14	5,56581E+14
TC0X00001669.mm.2	Asb11	ankyrin repeat and SOCS box-containing 11	-0.781934727484148	6,06042E+14	-2,40705E+14	0.0500480276821275	0.188338378912689	-3,84253E+14	5,51121E+14	5,98893E+14	5,89192E+14	5,90645E+14	6,40255E+14	6,66146E+14
TC1400000910.mm.2	Pinx1	PIN2/TERF1 interacting, telomerase inhibitor 1	0.810410566332734	7,37701E+14	2,40692E+13	0.050057308698903	0.188338378912689	-3,84271E+14	7,76747E+14	7,79924E+14	7,51471E+14	7,23472E+14	7,2544E+14	6,69149E+14
TC0300002371.mm.2	Npr1	natriuretic peptide receptor 1	-0.954403819218523	7,16508E+14	-2,40686E+14	0.0500614234118506	0.188338378912689	-3,84279E+14	6,2657E+14	6,81339E+14	7,31598E+14	7,60753E+14	7,11462E+14	7,87328E+14
TC0200000574.mm.2	Golga2	golgi autoantigen, golgin subfamily a, 2	0.817373146165014	8,3741E+14	2,40668E+14	0.0500741815573661	0.188338378912689	-3,84302E+14	9,009E+14	8,84086E+14	8,12859E+14	8,05103E+14	8,41878E+13	7,79634E+14
TC1700002504.mm.2	Alk	anaplastic lymphoma kinase	-0.847992502507788	6,26467E+14	-2,40623E+14	0.0501060837807729	0.188338378912689	-3,84362E+13	5,52643E+12	5,79033E+14	6,2946E+14	6,9639E+14	6,43939E+14	6,57335E+14
TC0900003269.mm.2	Sec22c	SEC22 homolog C, vesicle trafficking protein	0.897882508920908	6,907E+14	2,40594E+14	0.0501265372461419	0.188338378912689	-3,844E+14	7,41527E+14	7,12615E+14	6,76315E+14	7,39177E+14	6,12247E+13	6,62319E+14
TC0500000435.mm.2	Tmem128	transmembrane protein 128	0.782423547093163	6,36743E+14	2,40592E+14	0.0501276877852202	0.188338378912689	-3,84402E+13	6,70461E+14	6,63191E+14	6,73819E+14	6,35817E+12	5,69301E+14	6,07866E+14
TC0300001530.mm.2	Usp33	ubiquitin specific peptidase 33	1,20045E+14	7,41511E+14	2,40586E+14	0.0501321534136949	0.188338378912689	-3,84411E+14	8,44271E+14	8,39487E+13	7,15723E+14	6,0592E+12	7,70114E+14	6,73553E+14
TC0400002069.mm.2	Faap20	Fanconi anemia core complex associated protein 20	0.704650769999589	6,36501E+13	2,40566E+14	0.0501464694181168	0.188338378912689	-3,84437E+14	6,75881E+14	6,62492E+14	6,47173E+14	6,36013E+14	5,93402E+14	6,04041E+13
TC0200001366.mm.2	Psmc3	proteasome (prosome, macropain) 26S subunit, ATPase 3	1,05319E+14	8,62437E+14	2,40564E+11	0.0501473402175111	0.188338378912689	-3,84439E+14	9,41423E+14	9,5208E+14	8,56785E+14	7,41468E+14	8,53899E+13	8,28966E+14
TC0800000286.mm.2	Plat	plasminogen activator, tissue	0.749095982813334	7,02442E+13	2,40548E+14	0.0501588325021092	0.188338378912689	-3,8446E+14	7,64512E+14	7,37101E+14	6,68182E+14	6,93064E+14	6,869E+14	6,64893E+13
TC0700003990.mm.2	Tpp1	tripeptidyl peptidase I	1,59974E+14	8,27739E+14	2,40539E+14	0.0501656127655166	0.188338378912689	-3,84473E+14	9,16494E+13	9,66921E+12	8,61469E+14	6,58081E+14	8,06023E+14	7,57445E+14
TC0200004963.mm.2	Ggt7	gamma-glutamyltransferase 7	0.945066181572781	7,47797E+14	2,40536E+14	0.0501674624398473	0.188338378912689	-3,84476E+14	8,22596E+14	8,02333E+13	7,59708E+14	6,66231E+14	7,22644E+13	7,13272E+14
TC1100001551.mm.2	B230217C12Rik	RIKEN cDNA B230217C12 gene	1,16526E+14	8,02013E+14	2,40511E+12	0.0501854128012611	0.188361881310673	-3,8451E+13	8,96579E+14	8,80606E+14	7,92208E+13	6,98548E+14	8,23411E+14	7,20723E+14
TC0300002462.mm.2	Psmd4	proteasome (prosome, macropain) 26S subunit, non-ATPase, 4	0.740642292154177	8,18912E+14	2,40492E+14	0.0501987853847989	0.188368194901086	-3,84535E+14	8,56124E+14	8,74988E+14	7,9393E+14	8,05447E+14	8,08014E+14	7,74969E+14
TC0900001344.mm.2	Poc1a	POC1 centriolar protein A	-1,12191E+14	6,47517E+14	-2,40466E+14	0.0502166801008157	0.188391470655284	-3,84568E+14	5,9987E+13	5,44453E+14	6,40908E+14	7,31168E+14	6,4511E+14	7,23595E+14
TC0200001812.mm.2	Pdia3	protein disulfide isomerase associated 3	1,3454E+14	8,78411E+13	2,40438E+14	0.0502368539161952	0.18840755975067	-3,84606E+14	9,59479E+14	9,86361E+14	8,87832E+14	7,60033E+14	8,94499E+14	7,8226E+14
TC0800001490.mm.2	Ctu2	cytosolic thiouridylase subunit 2	-0.779481098288273	7,14089E+14	-2,40427E+14	0.0502443545117997	0.18840755975067	-3,8462E+14	6,56795E+14	6,50558E+14	7,30519E+14	7,83414E+14	7,27628E+14	7,3562E+14
TC1700001974.mm.2	Vars2	valyl-tRNA synthetase 2, mitochondrial	-0.723771272434325	6,09322E+14	-2,40362E+14	0.0502903533069063	0.188536170971192	-3,84705E+14	5,55899E+14	5,61709E+14	6,24678E+14	6,51284E+14	6,22491E+14	6,39871E+14
TC0200002300.mm.2	Cox4i2	cytochrome c oxidase subunit 4I2	-0.880972878105221	6,90015E+14	-2,40341E+14	0.0503052650674549	0.188544134285007	-3,84733E+14	6,37614E+14	6,42654E+14	6,64807E+14	7,38552E+14	7,1092E+14	7,45544E+14
TC0300001846.mm.2	Qrfpr	pyroglutamylated RFamide peptide receptor	-0.708747916074198	5,39821E+14	-2,40301E+14	0.0503338837666964	0.188544134285007	-3,84786E+12	5,17733E+14	5,07541E+14	5,14657E+13	5,31971E+14	5,77058E+14	5,89966E+13
TC1100002714.mm.2	Olfr1371	olfactory receptor 1371	-0.91626166971649	6,15184E+13	-2,40273E+14	0.050353734804929	0.188544134285007	-3,84823E+14	5,79596E+14	5,32408E+14	6,18656E+14	6,65187E+13	6,17241E+14	6,78015E+13
TC0800000228.mm.2	Defa3	defensin, alpha, 3	-0.811982446279611	4,77316E+14	-2,40241E+14	0.0503763341077661	0.188544134285007	-3,84865E+14	4,03887E+14	4,48114E+14	4,77548E+14	5,19948E+14	5,16151E+14	4,98248E+14
TC0800000251.mm.2	Defa17/Defa3	defensin, alpha, 17/defensin, alpha, 3	-0.811982446279611	4,77316E+14	-2,40241E+14	0.0503763341077661	0.188544134285007	-3,84865E+14	4,03887E+14	4,48114E+14	4,77548E+14	5,19948E+14	5,16151E+14	4,98248E+14
TC1000001967.mm.2	Nkain2	Na+/K+ transporting ATPase interacting 2	2,08734E+14	7,80138E+13	2,4024E+14	0.0503771441762961	0.188544134285007	-3,84867E+13	9,2677E+14	9,27018E+14	8,31701E+14	5,59019E+14	7,73203E+14	6,63117E+14
TC1100000609.mm.2	Kif3a	kinesin family member 3A	2,04023E+14	7,95293E+14	2,40238E+13	0.0503784850758203	0.188544134285007	-3,84869E+14	9,43996E+14	9,31269E+14	8,45108E+14	5,84163E+14	7,92533E+14	6,74687E+14
TC1700001909.mm.2	Btnl4	butyrophilin-like 4	-0.977795182174912	5,80197E+14	-2,40228E+14	0.0503860883901966	0.188544134285007	-3,84883E+14	5,29115E+14	5,16644E+14	5,67945E+14	6,26159E+14	5,78394E+14	6,62924E+14
TC0700000511.mm.2	Sirt2	sirtuin 2	0.747941733357806	9,15517E+14	2,40132E+14	0.0504540665053015	0.188754672300888	-3,85009E+14	9,62602E+14	9,86029E+14	8,9021E+13	8,55219E+14	8,94878E+14	9,04164E+14
TC0800002458.mm.2	Eps15l1	epidermal growth factor receptor pathway substrate 15-like 1	0.797439168856894	7,36646E+14	2,40052E+14	0.0505110481300552	0.188762059655195	-3,85115E+14	7,70237E+14	7,51251E+14	7,67213E+14	7,69173E+14	7,09332E+14	6,52669E+14
TC1700000403.mm.2	Mrpl28	mitochondrial ribosomal protein L28	-0.760368304062103	8,21826E+14	-2,4005E+14	0.0505125389778431	0.188762059655195	-3,85118E+13	7,79227E+14	7,88185E+13	8,0019E+14	8,43866E+14	8,68382E+14	8,51104E+14
TC0300002454.mm.2	Psmb4	proteasome (prosome, macropain) subunit, beta type 4	0.984374541564299	7,47274E+14	2,40043E+14	0.0505173137692322	0.188762059655195	-3,85126E+14	8,34677E+14	8,10332E+14	7,18045E+14	6,72458E+14	7,69729E+14	6,78406E+14
TC1900000051.mm.2	Slc29a2	solute carrier family 29 (nucleoside transporters), member 2	0.785136568989795	6,31038E+14	2,40029E+14	0.0505272245263516	0.188762059655195	-3,85145E+13	6,72215E+14	6,6273E+14	6,50727E+13	6,22635E+14	5,65169E+14	6,12749E+14
TC0200003313.mm.2	Stxbp1	syntaxin binding protein 1	1,79612E+14	1,00586E+14	2,40025E+14	0.0505299631448513	0.188762059655195	-3,8515E+13	1,13286E+14	1,11297E+14	1,05709E+12	8,45612E+14	1,00711E+14	8,79499E+14
TC0500002856.mm.2	Nkx6-1	NK6 homeobox 1	-1,01316E+13	6,2295E+14	-2,40025E+13	0.0505304659159837	0.188762059655195	-3,85151E+14	5,60897E+13	5,37664E+14	6,24639E+14	7,13305E+14	6,19386E+14	6,81808E+14
TC1700000572.mm.2	Zfp952	zinc finger protein 952	0.789947441565221	6,05048E+14	2,40014E+14	0.0505380453889724	0.188762059655195	-3,85165E+14	6,65511E+14	6,38257E+14	5,77982E+13	6,02757E+14	5,95011E+14	5,50768E+14
TC0600000565.mm.2	Gimap9	GTPase, IMAP family member 9	-0.940026729395368	4,76761E+13	-2,39985E+14	0.0505589557668374	0.188796397281819	-3,85204E+14	4,53632E+14	4,06548E+14	4,45318E+14	5,06882E+14	4,96216E+14	5,51968E+14
TC0600001634.mm.2	Ptpro	protein tyrosine phosphatase, receptor type, O	1,53015E+14	5,9807E+14	2,39947E+14	0.0505859947018433	0.18882064845931	-3,85254E+14	7,25051E+14	6,9844E+13	6,23914E+14	4,23557E+14	5,44294E+14	5,73166E+14
TC0200003317.mm.2	Slc2a8	solute carrier family 2, (facilitated glucose transporter), member 8	-1,42297E+14	6,82245E+14	-2,39928E+13	0.0505994939655668	0.18882064845931	-3,85279E+14	5,85995E+13	6,05579E+13	6,32986E+14	7,92743E+14	6,91405E+14	7,84763E+14
TC0800001089.mm.2	Ogfod1	2-oxoglutarate and iron-dependent oxygenase domain containing 1	1,71697E+14	8,26987E+14	2,39926E+14	0.0506006057250232	0.18882064845931	-3,85281E+14	9,45965E+14	9,13036E+14	8,87652E+14	6,99663E+14	8,26402E+14	6,89205E+14

TC1200000675.mm.2	Mthfd1	methylenetetrahydrofolate dehydrogenase (NADP+ dependent), methenyltetrahydrofolate cyclohydrolase, formyltetrahydrofolate synthase	0.748174259865372	6,19279E+14	2,39873E+14	0.0506383895519404	0.188827216060993	-3,8535E+12	6,75294E+14	6,55014E+14	6,03916E+14	6,00778E+14	6,09526E+14	5,71146E+14
TC0200003379.mm.2	Rbm18	RNA binding motif protein 18	0.879987791303072	7,70048E+14	2,39872E+14	0.050639770916527	0.188827216060993	-3,85353E+14	8,42154E+13	8,17704E+14	7,75225E+14	7,01341E+14	7,54528E+14	7,29333E+14
TC0200002288.mm.2	Defb28	defensin beta 28	-0.934779944792354	4,74148E+14	-2,39864E+14	0.0506451868556318	0.188827216060993	-3,85363E+14	4,45583E+14	3,85026E+14	4,64657E+14	5,32056E+14	4,955E+13	5,22065E+14
TC0400002986.mm.2	Rps6	ribosomal protein S6	0.938475832473253	1,03471E+14	2,39857E+13	0.0506500333692369	0.188827216060993	-3,85372E+14	1,10218E+14	1,09468E+14	1,0305E+14	9,71741E+14	1,04187E+14	9,6729E+14
TC1800001410.mm.2	Slc6a7	solute carrier family 6 (neurotransmitter transporter, L-proline), member 7	0.766965591984349	6,84148E+14	2,39842E+14	0.050660960406608	0.188827216060993	-3,85392E+14	7,29007E+14	7,33244E+14	6,88112E+14	6,4567E+14	6,41062E+14	6,67796E+14
TC0900001857.mm.2	Fdx2/Zglp1/Raver1/Gm38431/Mir7082	ferredoxin 2/zinc finger, GATA-like protein 1/ribonucleoprotein, PTB-binding 1/predicted gene, 38431/microRNA 7082	0.69494674491124	7,18159E+13	2,39796E+14	0.0506934326132298	0.18890455127127	-3,85452E+14	7,64772E+13	7,51454E+14	7,07258E+13	7,08232E+14	6,8982E+14	6,87417E+14
TC0200001782.mm.2	Mga	MAX gene associated	1,87051E+14	6,90758E+14	2,39756E+14	0.0507223846020049	0.188968735975053	-3,85505E+14	8,28745E+14	8,19709E+13	7,04206E+13	5,17535E+14	7,27643E+14	5,46709E+14
TC0X00003420.mm.2	Uxt	ubiquitously expressed prefoldin like chaperone	1,57115E+14	6,33134E+14	2,39697E+14	0.0507647550905549	0.188981886406297	-3,85583E+14	7,36754E+14	7,26041E+14	6,56218E+14	5,31228E+14	6,59287E+14	4,89277E+14
TC1000000322.mm.2	Lama4	laminin, alpha 4	-0.744885682212703	5,92801E+14	-2,39644E+14	0.0508027341053769	0.188981886406297	-3,85653E+14	5,47439E+14	5,60248E+14	5,76739E+14	6,15715E+14	6,33922E+14	6,22743E+13
TC0400002642.mm.2	Grin3a	glutamate receptor ionotropic, NMDA3A	0.952700622282961	6,64015E+14	2,39643E+14	0.050803428079449	0.188981886406297	-3,85655E+14	7,44384E+14	6,88159E+14	6,18141E+14	6,914E+13	6,38184E+14	6,03819E+14
TC0X00000064.mm.2	Wdr45	WD repeat domain 45	0.748884699161429	7,4665E+14	2,39639E+14	0.0508058790335821	0.188981886406297	-3,85659E+14	7,88477E+14	8,08847E+14	7,36567E+14	6,98459E+14	7,19437E+14	7,28111E+14
TC0300000096.mm.2	Car2	carbonic anhydrase 2	1,29407E+13	9,81353E+14	2,39638E+14	0.0508068904121166	0.188981886406297	-3,85661E+14	1,05202E+14	1,10456E+14	9,79586E+14	8,54189E+14	9,94717E+14	9,03048E+14
TC1800000583.mm.2	Slc12a2	solute carrier family 12, member 2	1,16367E+14	7,5769E+14	2,39635E+14	0.0508089054385337	0.188981886406297	-3,85665E+14	8,25437E+14	8,38404E+14	7,47701E+14	7,03491E+14	7,81862E+14	6,49244E+13
TC0700002169.mm.2	Cox6b2	cytochrome c oxidase subunit 6B2	-0.902926833359849	5,96764E+14	-2,39594E+14	0.0508386161856899	0.188981886406297	-3,85719E+13	5,74208E+14	5,56884E+14	5,56686E+14	5,81129E+14	6,14758E+14	6,96919E+14
TC0X00000323.mm.2	Pgrmc1	progesterone receptor membrane component 1	1,10163E+14	1,02092E+14	2,39592E+14	0.0508398373325706	0.188981886406297	-3,85722E+13	1,11309E+14	1,0857E+14	1,02422E+14	9,24065E+14	1,02728E+14	9,51181E+14
TC0500001593.mm.2	Zp3	zona pellucida glycoprotein 3	-0.995415590366301	5,24813E+14	-2,39585E+13	0.0508446527200159	0.188981886406297	-3,85731E+14	4,34767E+14	4,70228E+14	5,19323E+14	6,20478E+14	5,47206E+14	5,56873E+14
TC0700002771.mm.2	Cox6b1	cytochrome c oxidase, subunit 6B1	0.785718490584461	9,41472E+14	2,39559E+14	0.0508635587832622	0.188981886406297	-3,85765E+14	1,00466E+14	9,85226E+14	9,29663E+14	8,96532E+14	9,37243E+14	8,95503E+14
TC0400003043.mm.2	Elavl2/Mir6402	ELAV like RNA binding protein 1/microRNA 6402	1,23209E+14	8,13869E+14	2,39542E+14	0.0508759403291957	0.188981886406297	-3,85788E+14	9,06362E+14	8,55924E+14	8,71064E+14	7,33997E+14	7,80718E+14	7,3515E+14
TC1900000787.mm.2	Tcf7l2	transcription factor 7 like 2, T cell specific, HMG box	1,04498E+14	7,99007E+13	2,39539E+14	0.0508779286217212	0.188981886406297	-3,85792E+14	8,91603E+14	7,87779E+14	8,36726E+14	8,0755E+14	7,58793E+14	7,11593E+14
TC0800000960.mm.2	G430095P16Rik	RIKEN cDNA G430095P16 gene	-1,01341E+14	5,63759E+13	-2,39538E+14	0.0508783853553351	0.188981886406297	-3,85793E+14	4,73607E+14	4,98633E+14	5,72165E+14	6,63225E+14	5,57949E+13	6,16973E+14
TC0X00002059.mm.2	Upf3b	UPF3 regulator of nonsense transcripts homolog B (yeast)	1,55459E+14	6,24997E+14	2,39497E+14	0.0509081629953517	0.189048912271054	-3,85847E+14	7,27026E+14	6,94214E+14	6,4002E+14	5,78403E+14	6,53521E+14	4,568E+14
TC0300000211.mm.2	Cldn11	claudin 11	0.916918116375545	8,64443E+14	2,39461E+14	0.0509337416323268	0.18910031772389	-3,85894E+14	9,405E+14	8,98178E+14	8,6661E+12	8,26075E+13	8,62307E+14	7,92988E+14
TC0300000824.mm.2	Sprr2d	small proline-rich protein 2D	-1,29656E+14	5,74704E+14	-2,39358E+14	0.0510078693591445	0.189305316724384	-3,8603E+14	4,94791E+14	5,06844E+14	5,30516E+14	6,55124E+13	5,76977E+13	6,8397E+14
TC1400001724.mm.2	Fam25c	family with sequence similarity 25, member C	-0.80604122374084	6,6349E+14	-2,39352E+14	0.0510124548636056	0.189305316724384	-3,86039E+14	6,18343E+14	5,99428E+14	6,6726E+14	7,1693E+14	6,72586E+14	7,06394E+14
TC0200000492.mm.2	Ak8	adenylate kinase 8	0.759384339091758	5,75634E+14	2,39335E+14	0.0510242929971888	0.189305648874903	-3,8606E+14	6,13847E+14	5,85125E+14	5,87704E+13	6,2003E+14	5,34272E+14	5,12824E+14
TC0800001514.mm.2	Cdk10	cyclin-dependent kinase 10	0.824236849553959	7,36765E+14	2,39244E+14	0.0510897894329603	0.189420435919635	-3,8618E+13	8,12665E+14	7,67014E+14	7,22409E+14	7,03669E+13	7,30933E+12	6,83899E+14
TC0500000344.mm.2	Yes1	YES proto-oncogene 1, Src family tyrosine kinase	0.967369902095004	5,32535E+13	2,39221E+14	0.0511068199901045	0.189420435919635	-3,86211E+14	6,4492E+14	5,51499E+14	5,18301E+14	4,77546E+14	5,03989E+14	4,98957E+14
TC0200003745.mm.2	Sp3	trans-acting transcription factor 3	1,59017E+14	7,62727E+14	2,3921E+14	0.0511148463840975	0.189420435919635	-3,86226E+14	8,4988E+14	8,60161E+14	8,10802E+14	6,63509E+13	7,74133E+13	6,17874E+14
TC0300002173.mm.2	Golim4	golgi integral membrane protein 4	2,43558E+14	5,92678E+14	2,39204E+14	0.0511189809352112	0.189420435919635	-3,86234E+14	7,55072E+13	7,3839E+14	6,64601E+14	3,91662E+14	6,12656E+14	3,93689E+14
TC1700000596.mm.2	Smim40	small integral membrane protein 40	-0.761471104441243	6,88125E+14	-2,392E+14	0.0511217679497533	0.189420435919635	-3,86239E+14	6,16201E+14	6,43583E+14	7,14714E+14	7,42174E+14	6,90939E+14	7,2114E+14
TC1000001601.mm.2	Ankrd52/Mir6914	ankyrin repeat domain 52/microRNA 6914	1,45605E+14	5,79802E+14	2,39195E+14	0.0511257665124118	0.189420435919635	-3,86246E+14	6,66698E+14	6,6699E+13	6,29432E+14	4,73214E+14	5,77445E+14	4,65032E+14
TC0600000623.mm.2	Hoxa11os	homeobox A11, opposite strand	-0.828444113982234	5,20586E+14	-2,39173E+14	0.0511415854734563	0.189435486605472	-3,86275E+14	4,624E+14	4,62365E+14	5,20513E+14	5,87785E+13	5,3675E+13	5,53704E+14
TC1100004065.mm.2	Ush1g	USH1 protein network component sans	-0.749158822608584	6,55408E+14	-2,39112E+14	0.0511852931125166	0.189490124365376	-3,86355E+14	6,1306E+14	6,10453E+14	6,4617E+14	6,89416E+14	6,92376E+14	6,80969E+14
TC1800000188.mm.2	Zfp397	zinc finger protein 397	0.977504558132449	6,50636E+14	2,39111E+14	0.0511857820989556	0.189490124365376	-3,86356E+13	7,05479E+14	7,00707E+14	6,71636E+14	6,15309E+14	6,49163E+14	5,61522E+14
TC0700000056.mm.2	Ccdc106	coiled-coil domain containing 106	1,00098E+14	6,24562E+14	2,39103E+14	0.0511916161709479	0.189490124365376	-3,86366E+13	7,06265E+14	6,9247E+13	6,24512E+14	5,25586E+13	6,21327E+14	5,77211E+14
TC1300000070.mm.2	Heatr1	HEAT repeat containing 1	1,27125E+14	5,13617E+14	2,39075E+14	0.0512119394279714	0.189521814424185	-3,86404E+13	5,87378E+13	6,02244E+14	4,1769E+14	5,39021E+14	5,19659E+14	4,15712E+14
TC1500001782.mm.2	Cpsf1	cleavage and polyadenylation specific factor 1	0.836574509345941	6,21447E+14	2,39001E+14	0.0512654282336227	0.189663267453717	-3,86501E+14	6,86216E+14	6,56704E+14	6,11546E+14	5,9861E+14	6,19824E+14	5,5578E+14
TC0700000921.mm.2	Luzp2	leucine zipper protein 2	1,68593E+14	7,99951E+13	2,3899E+14	0.0512737040295656	0.189663267453717	-3,86516E+14	9,30077E+14	9,40067E+14	8,03725E+14	5,92878E+14	8,13463E+14	7,19495E+14
TC1200002083.mm.2	Arel1	apoptosis resistant E3 ubiquitin protein ligase 1	2,74386E+14	1,01099E+14	2,38961E+14	0.0512945605490287	0.189681317402307	-3,86554E+13	1,17269E+14	1,17049E+14	1,10655E+14	8,21782E+14	1,03672E+14	7,57688E+13
TC0100002243.mm.2	Gm3646	predicted gene 3646	-0.987096910720736	5,77548E+14	-2,38951E+13	0.05130212755162	0.189681317402307	-3,86568E+13	5,09916E+14	4,87834E+14	5,84141E+14	6,88227E+14	5,96143E+14	5,99026E+14
TC0800002848.mm.2	Ddx28	DEAD (Asp-Glu-Ala-Asp) box polypeptide 28	0.798558423802421	6,24716E+14	2,38903E+13	0.0513363272974139	0.189764221551555	-3,8663E+13	6,88904E+14	6,6446E+14	5,89913E+14	6,11366E+14	5,70872E+14	6,22779E+14
TC0300000643.mm.2	Plrg1	pleiotropic regulator 1	1,62286E+14	6,86927E+14	2,38827E+13	0.0513919339819805	0.189808107184333	-3,86732E+13	7,91278E+14	8,01703E+14	7,17479E+14	5,42691E+12	7,01488E+14	5,6692E+14
TC0400001020.mm.2	Cachd1	cache domain containing 1	-0.994033209994481	8,61463E+14	-2,38816E+14	0.0513997430411485	0.189808107184333	-3,86746E+14	8,11844E+14	7,82951E+14	8,56766E+14	9,23616E+14	8,56923E+14	9,36678E+14
TC0800001925.mm.2	Hgsnat	heparan-alpha-glucosaminide N-acetyltransferase	1,30048E+14	6,69985E+14	2,38814E+14	0.0514012635202458	0.189808107184333	-3,86748E+14	7,39618E+14	7,52197E+14	6,82833E+14	6,13541E+14	6,90963E+14	5,40756E+14
TC0500002284.mm.2	Slc2a9	solute carrier family 2 (facilitated glucose transporter), member 9	-1,02867E+13	6,0481E+14	-2,3878E+13	0.0514258864951533	0.189808107184333	-3,86793E+14	5,32837E+14	5,3662E+14	5,86384E+14	6,97826E+13	6,23294E+14	6,51897E+14
TC1000002920.mm.2	Cct2	chaperonin containing Tcp1, subunit 2 (beta)	1,62194E+14	9,20581E+14	2,38772E+14	0.0514318346492693	0.189808107184333	-3,86804E+14	1,03414E+13	1,03847E+14	9,42909E+14	7,59755E+14	9,35679E+14	8,12537E+14
TC1500001676.mm.2	Col22a1	collagen, type XXII, alpha 1	-0.868978578905924	7,24358E+14	-2,38771E+14	0.0514323550485943	0.189808107184333	-3,86805E+14	6,58075E+14	6,89604E+13	7,02637E+14	7,74359E+14	7,52387E+13	7,69088E+14
TC0800002961.mm.2	Kars	lysyl-tRNA synthetase	1,02793E+14	7,99456E+14	2,3876E+14	0.0514399809367589	0.189808107184333	-3,86819E+14	8,62822E+14	8,95846E+14	7,87832E+14	6,97156E+14	8,078E+14	7,45283E+13
TC0700003845.mm.2	Folr2	folate receptor 2 (fetal)	-1,23671E+14	5,08745E+14	-2,38757E+14	0.0514424380359947	0.189808107184333	-3,86823E+14	3,79301E+14	4,66467E+14	4,97744E+14	6,15849E+14	5,70755E+14	5,22355E+14
TC1100000072.mm.2	Xbp1	X-box binding protein 1	1,42342E+14	8,35219E+14	2,38715E+13	0.0514728532519182	0.189876850835201	-3,86879E+14	9,24481E+14	9,35848E+14	8,6257E+14	7,12768E+14	8,43817E+14	7,31827E+14
TC0700004538.mm.2	Slc25a22	solute carrier family 25 (mitochondrial carrier, glutamate), member 22	0.860672673307594	8,57921E+14	2,38687E+14	0.0514930583591371	0.189907907837211	-38691528813	9,16902E+14	9,23712E+14	8,48049E+14	7,90387E+14	8,08098E+14	8,60381E+13
TC1500001326.mm.2	Cct5	chaperonin containing Tcp1, subunit 5 (epsilon)	1,1089E+14	8,38078E+14	2,38657E+14	0.0515150175788732	0.189945418363475	-3,86955E+14	9,24929E+14	8,94058E+14	8,66159E+14	7,46114E+14	8,19728E+14	7,77478E+14
TC0100003352.mm.2	Rabgap1l	RAB GTPase activating protein 1-like	0.719043147602164	9,13196E+14	2,38517E+14	0.0516170303925217	0.190278016635713	-3,8714E+14	9,55971E+13	9,45885E+14	8,94117E+14	9,25157E+14	8,80184E+14	8,77864E+14
TC1000001772.mm.2	Pex3	peroxisomal biogenesis factor 3	1,89842E+14	7,42325E+14	2,3848E+14	0.0516436773790045	0.190297168127478	-3,87188E+14	8,46064E+13	8,4612E+14	8,16676E+13	6,32592E+14	7,52423E+14	5,60076E+14
TC0300001245.mm.2	Dnttip2	deoxynucleotidyltransferase, terminal, interacting protein 2	1,18903E+14	6,09067E+14	2,38462E+14	0.0516569666893735	0.190297168127478	-3,87212E+14	6,85085E+14	6,92701E+14	6,02849E+14	5,33788E+14	6,34277E+14	5,05703E+14
TC0100002214.mm.2	Rev1	REV1, DNA directed polymerase	1,14125E+14	6,90917E+14	2,38461E+14	0.0516576561403581	0.190297168127478	-3,87214E+14	7,35542E+13	7,54178E+14	7,59433E+14	6,3488E+14	6,58031E+14	6,03439E+13
TC1200000567.mm.2	Klhdc2	kelch domain containing 2	1,95296E+14	6,8705E+14	2,38381E+14	0.0517163657529657	0.190445492002361	-3,8732E+14	8,24662E+13	7,97273E+14	7,43955E+14	5,25065E+14	6,97896E+13	5,33449E+14
TC0700002413.mm.2	Crx	cone-rod homeobox	-0.897893384954987	5,35696E+14	-2,3836E+13	0.0517315908675784	0.190445492002361	-3,87347E+14	4,99229E+14	4,7821E+14	5,39054E+14	5,40666E+14	5,36118E+14	6,209E+14
TC1700002821.mm.2	Gm7030	predicted gene 7030	-0.792134353854833	5,08571E+14	-2,38348E+14	0.0517402176304022	0.190445492002361	-3,87363E+14	4,55746E+14	4,62307E+14	5,00297E+14	5,56594E+14	5,47927E+14	5,28554E+14

TC0700002710.mm.2	Eif3k	eukaryotic translation initiation factor 3, subunit K	1,09486E+14	8,61114E+14	2,38341E+13	0.0517451972932624	0.190445492002361	-3,87372E+14	9,45651E+14	9,46644E+13	8,62576E+14	7,3849E+14	8,53421E+14	8,19902E+14
TC0500002123.mm.2	5031410I06Rik	RIKEN cDNA 5031410I06 gene	-0.964734627052595	4,78878E+14	-2,38317E+14	0.0517631011720686	0.19046788060871	-3,87404E+14	4,12624E+13	4,04454E+14	4,76918E+14	5,69248E+14	4,8341E+14	5,26615E+14
TC0200003188.mm.2	Snapc4	small nuclear RNA activating complex, polypeptide 4	0.710332044677389	6,36968E+14	2,38268E+14	0.0517988693365181	0.190549013885336	-3,87469E+14	6,66744E+14	6,59509E+14	6,41968E+14	6,69398E+14	5,90663E+14	5,93523E+14
TC0500002266.mm.2	Man2b2	mannosidase 2, alpha B2	0.703129932013744	7,20758E+13	2,38254E+14	0.0518088021989485	0.190549013885336	-3,87487E+14	7,4939E+14	7,56562E+14	7,24963E+14	7,28307E+14	6,94075E+14	6,7125E+14
TC1200002090.mm.2	Mlh3	mutL homolog 3	0.994278972017849	5,74821E+14	2,38238E+14	0.051820834756004	0.190549774172408	-3,87508E+14	6,16085E+13	6,17583E+14	5,43369E+13	6,37077E+14	5,44794E+14	4,90018E+14
TC1100000519.mm.2	Flt4	FMS-like tyrosine kinase 4	-1,19468E+14	5,54114E+14	-2,38152E+14	0.051883807716742	0.190737803728009	-3,87622E+13	4,82127E+14	4,97169E+13	5,23833E+14	6,03322E+14	5,47466E+14	6,70767E+14
TC1700000153.mm.2	Smok2b	sperm motility kinase 2B	-0.876756902176925	5,18752E+14	-2,38118E+14	0.0519087510033717	0.190785972836982	-3,87667E+14	4,67339E+14	4,6425E+14	5,05077E+14	5,68906E+14	5,27321E+13	5,79619E+14
TC0300000491.mm.2	P2ry1	purinergic receptor P2Y, G-protein coupled 1	1,64341E+12	5,95021E+14	2,37996E+14	0.0519981935949171	0.191071127342052	-3,87828E+14	7,13813E+14	6,93408E+14	6,41985E+14	4,42379E+14	5,9038E+14	4,88159E+14
TC1100001708.mm.2	Fmnl1	formin-like 1	1,16703E+14	5,93564E+13	2,3796E+14	0.05202428056363	0.191123400073363	-3,87875E+14	6,50012E+14	6,60132E+14	6,102E+14	5,64299E+14	6,05651E+14	4,71088E+14
TC0200003560.mm.2	Nr4a2	nuclear receptor subfamily 4, group A, member 2	1,58806E+14	5,38467E+14	2,37932E+14	0.0520450165494398	0.191134132368201	-3,87912E+13	6,50774E+14	6,12892E+14	6,11605E+13	4,0948E+14	4,3991E+14	5,06144E+14
TC0500002827.mm.2	Hnrnpd	heterogeneous nuclear ribonucleoprotein D	1,90877E+14	8,17146E+14	2,37924E+14	0.0520509261361426	0.191134132368201	-3,87922E+13	9,50178E+13	9,52869E+14	8,49531E+14	6,29004E+14	8,37329E+14	6,83964E+14
TC0800002597.mm.2	Junb	jun B proto-oncogene	-0.741716862936155	7,75256E+14	-2,37883E+14	0.0520811291492258	0.19120146593335	-3,87977E+14	7,36158E+14	7,41176E+13	7,79192E+14	7,69334E+14	7,91842E+13	8,33835E+14
TC0900003343.mm.2	Srprb	signal recognition particle receptor, B subunit	1,2653E+14	6,19305E+14	2,37834E+14	0.0521170601698479	0.191272946237843	-3,88041E+14	7,36E+13	6,70171E+14	6,49615E+14	5,0693E+14	5,93076E+14	5,60036E+14
TC1700002312.mm.2	Ubxn6	UBX domain protein 6	0.973504142142225	7,24446E+14	2,37824E+14	0.0521243410246614	0.191272946237843	-3,88054E+14	7,94385E+13	7,92342E+14	7,31582E+14	6,3634E+14	7,1756E+14	6,74466E+13
TC1900001081.mm.2	Asrgl1	asparaginase like 1	1,4147E+14	7,74328E+14	2,37784E+14	0.0521538964331863	0.191274730396306	-3,88107E+14	8,66297E+14	8,71591E+14	8,07123E+14	6,46009E+14	7,747E+14	6,80248E+14
TC0500003015.mm.2	Gm6583	predicted gene 6583	-0.835908589170135	6,06152E+14	-2,37782E+14	0.0521552138495128	0.191274730396306	-3,88109E+14	5,41256E+13	5,5743E+14	6,01744E+14	6,70617E+14	6,41171E+14	6,24696E+14
TC0900002156.mm.2	C2cd2l	C2 calcium-dependent domain containing 2-like	0.786259526446143	8,9248E+14	2,37775E+14	0.0521604397294959	0.191274730396306	-3,88119E+14	9,40613E+14	9,47607E+14	8,96246E+14	8,39448E+14	8,72011E+13	8,58956E+13
TC1000000332.mm.2	Rev3l	REV3 like, DNA directed polymerase zeta catalytic subunit	1,59705E+14	5,5426E+14	2,37719E+14	0.0522021746969265	0.191298953035333	-3,88194E+14	6,78339E+14	6,6588E+14	5,86834E+14	3,69697E+14	5,06747E+13	5,18061E+14
TC0500000248.mm.2	Dpp6	dipeptidylpeptidase 6	2,06737E+14	8,34956E+13	2,37676E+13	0.0522332983194164	0.191298953035333	-3,88249E+14	9,84091E+14	9,65774E+14	8,92649E+14	6,30834E+13	8,3492E+14	7,0147E+14
TC0200000547.mm.2	Gpr107	G protein-coupled receptor 107	1,04025E+14	6,17776E+14	2,37665E+14	0.0522419429641742	0.191298953035333	-3,88265E+14	6,79792E+14	6,90273E+14	6,00282E+14	5,74294E+13	6,38532E+14	5,23482E+14
TC0500000060.mm.2	9330182L06Rik	RIKEN cDNA 9330182L06 gene	1,51604E+14	6,93269E+14	2,37653E+14	0.0522508632892631	0.191298953035333	-3,88281E+14	7,79674E+13	7,6451E+14	7,08086E+14	6,66368E+14	7,20176E+14	5,20801E+14
TC0200001794.mm.2	Snap23	synaptosomal-associated protein 23	0.870630522192199	8,39414E+14	2,37631E+14	0.0522672209494957	0.191298953035333	-3,8831E+14	8,63102E+14	9,03168E+14	8,73193E+14	8,0488E+14	8,05747E+12	7,86396E+14
TC0800002734.mm.2	Ciapin1	cytokine induced apoptosis inhibitor 1	1,14787E+14	7,99679E+14	2,37625E+13	0.0522713234681758	0.191298953035333	-3,88317E+14	8,86602E+14	8,69333E+14	8,25501E+14	6,90279E+14	7,77411E+14	7,4895E+14
TC1500001560.mm.2	Derl1	Der1-like domain family, member 1	1,4809E+14	7,05306E+14	2,37612E+14	0.0522808463313638	0.191298953035333	-3,88334E+13	8,15165E+13	8,09122E+14	7,05308E+14	5,74132E+14	7,38377E+14	5,89729E+13
TC0400003482.mm.2	Oxct2a	3-oxoacid CoA transferase 2A	-1,25782E+14	6,12487E+14	-2,3761E+14	0.0522826660742791	0.191298953035333	-3,88338E+14	5,35679E+14	5,40746E+14	5,65572E+14	7,04933E+14	6,25342E+14	7,02648E+14
TC1300001652.mm.2	Hist1h2ba	histone cluster 1, H2ba	-0.988287816154206	5,83804E+14	-2,37593E+14	0.0522952010296479	0.191298953035333	-3,8836E+14	5,30299E+14	4,94694E+14	5,97923E+14	6,57257E+14	5,69952E+14	6,52698E+14
TC1000002627.mm.2	Hsp90b1	heat shock protein 90, beta (Grp94), member 1	2,36413E+14	8,97477E+14	2,3759E+14	0.0522973101566002	0.191298953035333	-3,88364E+14	1,05865E+14	1,07307E+14	9,43204E+14	6,51055E+13	9,17713E+14	7,41175E+14
TC0600000974.mm.2	Tprkb	Tp53rk binding protein	1,30336E+14	6,99591E+14	2,37589E+14	0.052297640918553	0.191298953035333	-3,88364E+14	8,04566E+13	7,92083E+14	6,93344E+14	5,71575E+14	7,25223E+14	6,10754E+14
TC0400000811.mm.2	Lurap1l	leucine rich adaptor protein 1-like	0.849664828200229	5,77514E+14	2,3752E+13	0.0523487793886197	0.191436057538943	-3,88456E+14	6,56548E+14	6,27312E+14	5,59107E+14	5,08189E+14	5,81422E+14	5,32505E+13
TC0600003135.mm.2	Gapdh	glyceraldehyde-3-phosphate dehydrogenase	1,94613E+14	1,09194E+14	2,37506E+14	0.0523588844767655	0.191436057538943	-3,88474E+14	1,22873E+14	1,2326E+14	1,13479E+14	8,83422E+14	1,09203E+14	9,8007E+14
TC0600002027.mm.2	Chchd3	coiled-coil-helix-coiled-coil-helix domain containing 3	1,02997E+14	7,72757E+14	2,37429E+14	0.0524158645337138	0.191576779954909	-3,88575E+14	8,65015E+14	8,66123E+14	7,41269E+14	6,38992E+13	7,90516E+14	7,34627E+13
TC1900001341.mm.2	Rln1	relaxin 1	-1,15711E+14	4,98168E+14	-2,37416E+14	0.0524256296551844	0.191576779954909	-3,88593E+14	4,44573E+14	4,19668E+14	4,49688E+14	5,79417E+14	5,22919E+14	5,72744E+14
TC0500002186.mm.2	Supt7l	suppressor of Ty 7-like	1,37554E+14	7,44879E+14	2,37376E+14	0.0524550739985452	0.191576779954909	-3,88645E+14	8,37074E+14	8,18324E+14	8,03672E+14	6,29911E+14	7,12646E+13	6,67645E+14
TC0100003122.mm.2	Gpr37l1	G protein-coupled receptor 37-like 1	1,49258E+14	8,65915E+14	2,37371E+14	0.0524593978461866	0.191576779954909	-3,88653E+14	9,66256E+14	9,88351E+14	8,87197E+13	6,97597E+14	8,58859E+14	7,97231E+14
TC1400002017.mm.2	Ndrg2	N-myc downstream regulated gene 2	1,36274E+14	1,01669E+14	2,37366E+14	0.0524626844346803	0.191576779954909	-3,88659E+14	1,11966E+14	1,08399E+14	1,05575E+14	9,09651E+13	1,0134E+14	9,17712E+14
TC1400000367.mm.2	Antxrl	anthrax toxin receptor-like	-0.937512608088815	5,51894E+13	-2,37348E+14	0.0524758433460702	0.191576779954909	-3,88682E+13	4,94917E+14	4,98211E+14	5,31571E+14	6,06038E+14	5,5912E+14	6,2151E+14
TC0300002920.mm.2	Fam241a	family with sequence similarity 241, member A	-1,05372E+14	8,07124E+14	-2,37342E+13	0.0524805999330334	0.191576779954909	-3,88691E+14	7,46408E+14	7,63337E+14	8,63813E+14	7,48694E+13	8,7752E+14	8,42969E+14
TC0700001147.mm.2	Gdpgp1	GDP-D-glucose phosphorylase 1	0.936724170736839	6,76455E+14	2,37278E+14	0.0525281842408054	0.191660562191869	-3,88775E+14	7,22654E+14	6,9891E+14	7,24732E+14	6,78214E+14	6,58033E+14	5,76186E+14
TC1400002331.mm.2	Ccar2	cell cycle activator and apoptosis regulator 2	0.821636362562099	7,06716E+14	2,37266E+13	0.0525371701591079	0.191660562191869	-3,88791E+14	7,48009E+14	7,49209E+14	7,36474E+14	6,73716E+14	6,81154E+14	6,51737E+14
TC1600001337.mm.2	Klhl6	kelch-like 6	1,0121E+14	5,13324E+14	2,37263E+14	0.0525392355986772	0.191660562191869	-3,88795E+14	5,84808E+14	5,74885E+14	5,3067E+13	4,32312E+14	5,06079E+14	4,51193E+14
TC1300000938.mm.2	Fam172a	family with sequence similarity 172, member A	1,25485E+13	6,86096E+14	2,37214E+14	0.0525756628187099	0.19167745719587	-3,8886E+14	7,70201E+14	7,61884E+14	6,8943E+14	6,13946E+14	7,10568E+14	5,70548E+14
TC1700002333.mm.2	Lonp1	lon peptidase 1, mitochondrial	0.734350107626383	6,54859E+14	2,37187E+14	0.052595349209931	0.19167745719587	-3,88895E+13	7,02117E+14	6,758E+14	6,56682E+14	6,63509E+14	6,31107E+14	5,99939E+14
TC0800000090.mm.2	Naxd	NAD(P)HX dehydratase	1,2914E+14	7,24571E+14	2,37177E+14	0.0526027610356552	0.19167745719587	-3,88908E+14	8,31701E+14	8,06759E+14	7,41062E+14	5,87724E+14	7,19145E+14	6,61036E+14
TC1600001795.mm.2	Olfr198	olfactory receptor 198	-1,51824E+14	3,98051E+14	-2,37161E+14	0.0526146994751367	0.19167745719587	-3,88929E+14	3,04683E+14	3,20388E+13	3,26775E+14	5,07738E+14	4,16673E+14	5,12046E+14
TC0400000405.mm.2	Fam205a2/Fam205a3	family with sequence similarity 205, member A2/family with sequence similarity 205, member A3	0.920078437542261	5,81949E+14	2,37157E+14	0.0526180932458789	0.19167745719587	-3,88935E+14	6,53588E+13	6,56199E+14	5,25937E+14	5,30199E+14	6,09563E+14	5,16207E+14
TC0700004431.mm.2	Adam12	a disintegrin and metallopeptidase domain 12 (meltrin alpha)	-0.794905148945118	5,73561E+14	-2,37155E+14	0.0526195161931653	0.19167745719587	-3,88938E+14	5,55892E+13	5,21307E+14	5,47674E+14	5,80313E+14	5,98278E+14	6,37902E+14
TC0300002113.mm.2	Lxn	latexin	0.864055006035553	8,39836E+14	2,37109E+14	0.052653591776362	0.19167745719587	-3,88998E+14	9,14736E+14	8,88616E+14	8,27789E+14	7,77336E+13	8,43704E+14	7,86837E+14
TC0900001240.mm.2	7420426K07Rik	RIKEN cDNA 7420426K07 gene	-0.813343965109056	5,10209E+14	-2,37105E+13	0.0526566377558439	0.19167745719587	-3,89003E+14	4,41638E+14	4,94258E+14	4,9601E+14	5,30781E+14	5,37683E+14	5,60883E+14
TC0600002557.mm.2	Tlx2	T cell leukemia, homeobox 2	-1,06203E+14	6,27773E+14	-2,37073E+14	0.0526803545857871	0.19167745719587	-3,89046E+14	5,55262E+14	5,7974E+14	6,10172E+14	6,74054E+14	6,22439E+14	7,24968E+14
TC0X00000376.mm.2	6030498E09Rik	RIKEN cDNA 6030498E09 gene	-1,42504E+14	4,67169E+14	-2,37072E+13	0.0526810103384136	0.19167745719587	-3,89047E+14	3,81339E+14	3,98527E+14	4,05273E+14	5,52998E+13	4,7384E+14	5,91036E+14
TC0300000360.mm.2	Rab33b	RAB33B, member RAS oncogene family	0.793495665791889	7,17356E+14	2,37056E+14	0.0526934527372115	0.19167745719587	-3,89069E+14	7,83238E+14	7,48454E+14	7,18706E+14	6,80744E+14	7,0339E+14	6,69603E+14
TC0500001268.mm.2	Rab35	RAB35, member RAS oncogene family	0.830699477248793	8,00004E+14	2,37041E+14	0.0527041574204873	0.19167745719587	-3,89088E+14	8,52421E+14	8,4791E+14	7,95593E+14	7,69911E+14	8,00627E+14	7,33564E+14
TC0400003607.mm.2	Ccdc28b	coiled coil domain containing 28B	-0.77878477019754	7,80194E+13	-2,37036E+13	0.0527083115660463	0.19167745719587	-3,89095E+14	7,26963E+14	7,18747E+14	8,45256E+14	7,88731E+14	7,99887E+14	8,0158E+14
TC0400001413.mm.2	Rragc	Ras-related GTP binding C	1,2434E+14	7,66594E+14	2,3701E+14	0.0527269929238033	0.19167745719587	-3,89128E+14	8,60261E+14	8,72438E+14	7,47191E+14	6,35656E+14	7,97217E+14	6,86801E+14
TC0900001302.mm.2	Topbp1	topoisomerase (DNA) II binding protein 1	1,09642E+14	5,48541E+14	2,37007E+14	0.0527296261656744	0.19167745719587	-3,89133E+14	6,32184E+14	6,14429E+14	4,44558E+14	5,72742E+14	5,19992E+13	5,07338E+14
TC0200001288.mm.2	Olfr1121	olfactory receptor 1121	-0.853949868418005	4,81298E+14	-2,37001E+13	0.0527342001954503	0.19167745719587	-3,89141E+14	4,42621E+12	4,32613E+14	4,47399E+14	5,19132E+14	5,08574E+14	5,3745E+13
TC0700002981.mm.2	Bcl2l12	BCL2-like 12 (proline rich)	-1,466E+14	6,40203E+14	-2,36978E+14	0.052751318370218	0.191696435024656	-3,89171E+14	5,45705E+14	5,3828E+14	6,11305E+14	7,68744E+14	6,29941E+14	7,47243E+14
TC1900000129.mm.2	Macrod1	MACRO domain containing 1	-1,06526E+14	6,5196E+14	-2,36929E+14	0.0527873746510917	0.191784209188961	-3,89235E+14	5,76364E+14	5,88756E+12	6,22863E+14	7,45606E+14	6,82724E+14	6,95448E+14
TC0100003601.mm.2	Olfr419	olfactory receptor 419	-0.800590764975357	4,70233E+14	-2,36902E+14	0.052807525265888	0.191814169208578	-3,89271E+14	4,08992E+14	4,29137E+14	4,62919E+14	5,22101E+14	5,05487E+14	4,9276E+14
TC0200004967.mm.2	Trpc4ap	transient receptor potential cation channel, subfamily C, member 4 associated protein	2,0681E+13	8,07997E+14	2,36867E+14	0.0528340994885834	0.191815660899442	-3,89318E+14	9,63953E+14	9,47021E+14	8,60721E+14	5,78933E+14	7,92653E+14	7,04701E+14
TC1100000493.mm.2	Rack1/Snord96a/Snord95	receptor for activated C kinase 1/small nucleolar RNA, C/D box 96A/small nucleolar RNA, C/D box 95	0.695173465271225	8,4869E+14	2,36855E+14	0.0528429959302925	0.191815660899442	-3,89334E+14	8,80808E+14	8,97805E+14	8,42682E+14	8,31269E+14	8,16542E+12	8,23036E+14

TC0800001421.mm.2	Necab2	N-terminal EF-hand calcium binding protein 2	0.958362686418909	7,95009E+14	2,36853E+14	0.0528443993846072	0.191815660899442	-3,89336E+14	8,70342E+14	8,53147E+13	8,07722E+14	7,07028E+14	7,64937E+13	7,6688E+14
TC0500001746.mm.2	Lmtk2	lemur tyrosine kinase 2	0.697028689675865	7,668E+14	2,36838E+14	0.0528557013221156	0.191815660899442	-3,89356E+14	8,2709E+14	8,34343E+14	7,11947E+14	7,05392E+14	7,50687E+14	7,7134E+14
TC0800001927.mm.2	Fnta	farnesyltransferase, CAAX box, alpha	1,12795E+14	6,98649E+14	2,36815E+14	0.0528729457906876	0.191815660899442	-3,89386E+14	7,87225E+13	7,40289E+14	7,42919E+14	6,19534E+14	6,67793E+14	6,34131E+14
TC0200002151.mm.2	Smim26	small integral membrane protein 26	0.869950666615802	5,83543E+14	2,36806E+14	0.0528793623605363	0.191815660899442	-3,89398E+14	6,62285E+14	6,26142E+14	5,4195E+14	5,56445E+14	5,93355E+14	5,21082E+14
TC1300000120.mm.2	Mplkip	M-phase specific PLK1 intereacting protein	1,67427E+13	5,46109E+14	2,3669E+14	0.0529659859521006	0.192032998035429	-3,89551E+14	6,36741E+14	6,50584E+14	5,47326E+14	4,89533E+14	5,88267E+14	3,64202E+14
TC0800001494.mm.2	Trappc2l	trafficking protein particle complex 2-like	0.941037201595705	8,82682E+14	2,36687E+13	0.0529688852415315	0.192032998035429	-3,89556E+14	9,57312E+14	8,95301E+14	9,03719E+14	8,75356E+14	8,6778E+14	7,96625E+14
TC1100002907.mm.2	Aldh3a2	aldehyde dehydrogenase family 3, subfamily A2	2,16612E+14	6,34058E+14	2,36678E+14	0.0529750311095068	0.192032998035429	-3,89567E+14	7,43961E+14	7,51282E+14	6,34471E+14	6,12611E+14	6,86397E+14	3,75623E+14
TC0200000545.mm.2	Tor1b	torsin family 1, member B	-0.681384783541477	7,73314E+14	-2,36653E+14	0.0529937886367819	0.192057785943425	-3,896E+14	7,1538E+14	7,18586E+14	8,14929E+14	8,20744E+13	7,85353E+14	7,84891E+14
TC0100003865.mm.2	Armc9	armadillo repeat containing 9	0.979878617790009	5,92357E+14	2,36516E+14	0.053096600127369	0.192307009628226	-3,89781E+14	6,65957E+14	6,28879E+14	5,68509E+13	5,91937E+14	5,00609E+13	5,98251E+13
TC1700001742.mm.2	Rps10	ribosomal protein S10	0.717142014841663	9,40164E+14	2,36516E+14	0.053096887873846	0.192307009628226	-3,89781E+13	1,00285E+14	9,76912E+13	9,01325E+14	9,23555E+14	9,21304E+13	9,15034E+14
TC1500000986.mm.2	Wnt1	wingless-type MMTV integration site family, member 1	-1,14075E+14	5,42662E+14	-2,36514E+14	0.0530983606923589	0.192307009628226	-3,89784E+14	4,58989E+13	4,68959E+14	5,23749E+14	6,48177E+14	5,47553E+14	6,08543E+14
TC0400000381.mm.2	Il11ra1	interleukin 11 receptor, alpha chain 1	-0.797337492748627	8,50428E+14	-2,36482E+14	0.053122393999314	0.19234314243954	-3,89826E+14	8,00862E+14	8,00065E+14	8,44329E+14	8,96918E+14	8,62612E+14	8,97782E+13
TC0900002651.mm.2	Ccpg1os	cell cycle progression 1, opposite strand	0.95589306530298	7,30769E+14	2,36443E+14	0.0531513599922495	0.19234314243954	-3,89877E+14	7,90174E+14	8,0728E+14	7,2477E+14	6,56116E+14	6,66405E+14	7,3987E+14
TC0200002694.mm.2	Vapb	vesicle-associated membrane protein, associated protein B and C	0.704289382858186	7,9705E+14	2,36424E+13	0.0531657970738928	0.19234314243954	-3,89902E+14	8,50084E+14	8,2346E+14	7,91705E+14	7,84365E+14	7,6349E+13	7,69195E+14
TC0500001328.mm.2	Tbx5	T-box 5	-1,06671E+13	5,06219E+14	-2,36423E+13	0.0531663175492165	0.19234314243954	-3,89903E+14	4,62193E+14	4,35709E+14	4,67603E+14	5,60565E+14	5,16187E+14	5,95058E+14
TC0300001416.mm.2	Eif4e	eukaryotic translation initiation factor 4E	1,90677E+14	8,13847E+14	2,36409E+14	0.0531768058919271	0.19234314243954	-3,89922E+14	9,49849E+14	9,37669E+14	8,35871E+14	6,53528E+14	8,52646E+14	6,53517E+14
TC1800000327.mm.2	Cystm1	cysteine-rich transmembrane module containing 1	-0.704084421779688	7,49035E+14	-2,36399E+14	0.0531844060276728	0.19234314243954	-3,89935E+14	7,06162E+14	6,84739E+14	7,82111E+14	7,89481E+14	7,74192E+14	7,57525E+14
TC0800000542.mm.2	Irf2	interferon regulatory factor 2	0.729837230682407	6,71984E+14	2,36389E+14	0.0531918973940663	0.19234314243954	-3,89948E+14	6,90849E+14	7,13386E+14	6,67979E+14	7,01418E+14	6,31123E+13	6,27145E+14
TC1800001255.mm.2	Lars	leucyl-tRNA synthetase	1,2067E+14	5,82485E+14	2,36352E+14	0.053219925087473	0.192359695317012	-3,89997E+14	6,76913E+14	6,66854E+14	4,97461E+14	5,51255E+14	6,21769E+14	4,80659E+14
TC1700000143.mm.2	Tcp1	t-complex protein 1	1,43858E+14	7,03142E+14	2,36334E+13	0.0532333672136317	0.192359695317012	-3,90021E+14	7,87192E+14	7,77944E+14	7,63091E+14	6,13203E+14	6,98886E+14	5,78533E+14
TC1000000905.mm.2	BC025920	zinc finger protein pseudogene	-0.703922812849119	5,98679E+14	-2,36326E+14	0.0532395675490912	0.192359695317012	-3,90032E+14	5,65445E+14	5,65567E+14	5,90837E+14	5,98427E+14	6,2234E+14	6,49456E+14
TC1000000597.mm.2	Atoh7	atonal bHLH transcription factor 7	-0.789398968338195	6,24983E+14	-2,36298E+14	0.0532605697167568	0.192359695317012	-3,90069E+14	5,56466E+14	5,75414E+14	6,42385E+14	6,85871E+14	6,25608E+14	6,64152E+13
TC0800002556.mm.2	Pkn1	protein kinase N1	-0.982695100193838	6,69851E+14	-2,36267E+14	0.0532835325786556	0.192359695317012	-3,90109E+14	5,98637E+14	5,83344E+14	6,78008E+14	7,80595E+14	6,74483E+13	7,04037E+14
TC0700000276.mm.2	Nova2	neuro-oncological ventral antigen 2	0.721136846729631	6,97354E+14	2,36266E+14	0.0532842177838168	0.192359695317012	-3,9011E+14	7,42488E+14	7,4375E+14	6,94206E+14	6,61667E+14	6,62296E+13	6,79714E+14
TC0500000876.mm.2	Fam47e	family with sequence similarity 47, member E	-1,17214E+14	6,67841E+14	-2,3626E+14	0.0532889142632795	0.192359695317012	-3,90118E+14	5,60728E+14	5,89808E+13	6,72872E+14	7,98675E+14	6,59442E+14	7,25523E+14
TC0200005473.mm.2	Hoxd11	homeobox D11	-1,57398E+13	4,43472E+14	-2,36256E+14	0.0532919803819984	0.192359695317012	-3,90124E+14	3,51516E+14	3,22996E+14	4,1233E+13	5,84681E+14	4,3151E+14	5,57799E+14
TC0100000240.mm.2	Arid5a	AT rich interactive domain 5A (MRF1-like)	-0.912320804925444	5,56105E+14	-2,36208E+14	0.0533282289617695	0.19243051557501	-3,90187E+14	4,87456E+14	5,09494E+14	5,51587E+14	6,08677E+14	5,54981E+14	6,24433E+14
TC0X00000179.mm.2	Cypt8/Cypt7/Cypt1	cysteine-rich perinuclear theca 8/cysteine-rich perinuclear theca 7/cysteine-rich perinuclear theca 1	-1,28075E+14	6,78617E+14	-2,36198E+14	0.0533354857914725	0.19243051557501	-3,902E+14	5,96228E+14	5,88515E+14	6,46472E+14	7,99595E+14	6,9007E+14	7,50822E+13
TC1700001395.mm.2	Tagap1	T cell activation GTPase activating protein 1	-0.69637751749977	8,07558E+14	-2,36156E+14	0.0533674564144925	0.192466253785362	-3,90256E+14	7,76211E+14	7,66333E+14	8,05511E+14	8,15474E+14	8,31308E+14	8,50511E+14
TC0700001149.mm.2	Ngrn	neugrin, neurite outgrowth associated	-0.765142247988636	7,54622E+14	-2,36154E+14	0.0533692808237446	0.192466253785362	-3,90259E+14	7,07628E+14	7,31123E+13	7,26528E+14	7,70671E+14	7,86105E+14	8,05674E+13
TC1100003823.mm.2	Pyy	peptide YY	-0.724127598932442	6,97143E+14	-2,36094E+13	0.0534143410577051	0.192564103828689	-3,90338E+14	6,39916E+14	6,50489E+14	7,16484E+14	7,40737E+14	7,03642E+14	7,31589E+14
TC0700004489.mm.2	Tubgcp2	tubulin, gamma complex associated protein 2	0.920334572802578	7,0701E+14	2,3605E+14	0.0534477270369932	0.192564103828689	-3,90396E+14	7,69532E+14	7,68616E+14	7,22135E+14	6,27697E+14	6,78905E+14	6,75175E+14
TC0400004000.mm.2	Zfp933	zinc finger protein 933	1,37702E+14	6,00444E+14	2,36049E+14	0.0534478915134786	0.192564103828689	-3,90397E+14	6,7055E+14	6,70815E+14	5,51864E+14	6,43475E+14	6,11654E+13	4,54308E+14
TC1400002038.mm.2	Olfr1510	olfactory receptor 1510	-0.998916247386673	4,77366E+14	-2,36044E+14	0.0534517640053998	0.192564103828689	-3,90403E+14	4,11163E+14	4,13973E+14	4,67095E+14	5,47049E+14	4,73682E+14	5,51237E+14
TC0800001736.mm.2	Atp4b	ATPase, H+/K+ exchanging, beta polypeptide	-0.913292092236367	6,06273E+14	-2,36012E+14	0.0534764513716923	0.192564103828689	-3,90446E+14	5,56953E+14	5,55484E+14	5,70964E+14	6,59142E+14	6,32852E+14	6,62243E+14
TC0800001208.mm.2	Fbxl8	F-box and leucine-rich repeat protein 8	-0.707265222903979	6,06127E+14	-2,36002E+14	0.0534834972943492	0.192564103828689	-3,90459E+14	5,69025E+14	5,49848E+14	6,17322E+14	6,40238E+14	6,33334E+14	6,26991E+14
TC0700004111.mm.2	Galnt18	polypeptide N-acetylgalactosaminyltransferase 18	0.703239506140058	6,24878E+14	2,35998E+14	0.0534871205236448	0.192564103828689	-3,90465E+14	6,7013E+14	6,46139E+14	6,3597E+14	6,21406E+13	5,89732E+14	5,85889E+14
TC0900003052.mm.2	Bsn	bassoon	0.688850145270917	6,2852E+14	2,35986E+14	0.0534955753547383	0.192564103828689	-3,9048E+14	6,62698E+14	6,5328E+14	6,33743E+14	6,4319E+14	5,95487E+14	5,82721E+14
TC0800000392.mm.2	Ppp2cb	protein phosphatase 2 (formerly 2A), catalytic subunit, beta isoform	1,03978E+14	8,04393E+14	2,35954E+14	0.0535200504678201	0.192564103828689	-3,90523E+13	8,82116E+14	8,82455E+14	8,0758E+14	6,97592E+14	8,02078E+14	7,54536E+14
TC1200001245.mm.2	Klc1	kinesin light chain 1	1,35881E+14	9,84506E+14	2,35954E+14	0.0535201880038299	0.192564103828689	-3,90523E+14	1,0837E+13	1,07576E+14	1,00174E+14	8,58116E+14	9,97106E+14	8,90601E+14
TC0800001701.mm.2	Ankrd10/Mir7238	ankyrin repeat domain 10/microRNA 7238	-0.94223116185369	7,25961E+14	-2,35944E+14	0.0535278732109909	0.192564103828689	-3,90536E+14	6,60209E+14	6,79022E+14	7,05937E+14	7,82921E+14	7,33786E+14	7,93892E+14
TC1500000119.mm.2	Cdh9	cadherin 9	1,2078E+14	6,28244E+14	2,35901E+14	0.0535601935910806	0.192633510342412	-3,90593E+14	6,99383E+14	7,33261E+14	6,27274E+14	5,1846E+14	6,45813E+14	5,45271E+14
TC0400001770.mm.2	Padi2	peptidyl arginine deiminase, type II	1,15628E+14	6,34023E+14	2,35887E+13	0.0535710767606496	0.192633510342412	-3,90612E+14	7,17527E+13	6,83489E+14	6,78112E+14	5,55249E+14	5,53132E+14	6,16629E+14
TC0100000018.mm.2	Oprk1	opioid receptor, kappa 1	1,04896E+13	6,90352E+14	2,35852E+14	0.053597167904641	0.19263511533982	-3,90657E+14	7,43894E+14	7,49919E+14	7,42919E+14	6,21361E+14	6,40426E+14	6,43594E+14
TC0500002910.mm.2	Barhl2	BarH like homeobox 2	-0.717253380783631	5,82205E+14	-2,35832E+14	0.0536122773559177	0.19263511533982	-3,90683E+14	5,64599E+14	5,40403E+14	5,5675E+13	5,83026E+14	6,25076E+14	6,23376E+14
TC0300002489.mm.2	Ciart	circadian associated repressor of transcription	1,10124E+14	6,53949E+14	2,35818E+13	0.0536227433307137	0.19263511533982	-3,90702E+14	7,42168E+14	7,24504E+14	6,69605E+14	5,40992E+14	6,35214E+14	6,1121E+14
TC1300002651.mm.2	Rab3c	RAB3C, member RAS oncogene family	2,09363E+14	8,21153E+14	2,35796E+14	0.0536400017893608	0.19263511533982	-3,90732E+14	9,64803E+14	9,39651E+14	8,76533E+14	6,60204E+14	8,46963E+14	6,38765E+14
TC1300002619.mm.2	Kif2a	kinesin family member 2A	1,69735E+14	7,24992E+14	2,35776E+14	0.0536545619737721	0.19263511533982	-3,90757E+14	8,37336E+14	8,29808E+14	7,65293E+14	5,89842E+14	7,43204E+14	5,84469E+14
TC1100004150.mm.2	Socs3	suppressor of cytokine signaling 3	-0.862409839603578	6,74332E+14	-2,35771E+14	0.0536589153954033	0.19263511533982	-3,90765E+14	6,24716E+13	6,04146E+14	6,90045E+14	7,25743E+14	6,65697E+14	7,35646E+14
TC0700000289.mm.2	Snrpd2	small nuclear ribonucleoprotein D2	-0.680398055561401	9,71745E+14	-2,35761E+14	0.0536660737637559	0.19263511533982	-3,90777E+14	9,32281E+14	9,30882E+14	9,87176E+14	9,80887E+14	9,95997E+14	1,00325E+14
TC1200000086.mm.2	Rdh14	retinol dehydrogenase 14 (all-trans and 9-cis)	1,29631E+14	5,57734E+14	2,35749E+14	0.0536756389743905	0.19263511533982	-3,90794E+14	6,3804E+14	6,33462E+14	6,12082E+14	4,50582E+14	5,29389E+14	4,82852E+14
TC0400003343.mm.2	Nasp	nuclear autoantigenic sperm protein (histone-binding)	1,0028E+14	5,16197E+14	2,35744E+14	0.0536791204540304	0.19263511533982	-3,908E+14	5,76004E+14	6,11795E+14	5,00628E+14	4,21517E+14	4,57862E+14	5,29377E+14
TC0500000985.mm.2	Mrps18c	mitochondrial ribosomal protein S18C	0.877479214086839	6,37177E+14	2,35726E+14	0.0536926497193035	0.19264076262016	-3,90823E+14	6,80839E+14	6,81278E+14	6,2623E+14	6,48097E+14	6,34314E+14	5,52306E+14
TC1400000796.mm.2	Nynrin	NYN domain and retroviral integrase containing	0.889653153007774	5,47274E+14	2,35685E+12	0.0537236381665129	0.192709034233531	-3,90877E+14	6,24794E+14	5,70279E+13	5,56261E+13	5,15169E+14	4,80969E+14	5,36173E+14
TC0800000823.mm.2	Klf2	Kruppel-like factor 2 (lung)	-0.980136152338024	5,72125E+14	-2,3564E+14	0.0537581997615633	0.192752016871553	-3,90937E+14	5,3455E+14	5,00253E+14	5,40058E+14	6,27057E+14	5,88542E+14	6,42288E+14
TC0600001340.mm.2	Rad52	RAD52 homolog, DNA repair protein	-1,26602E+11	6,11319E+14	-2,35569E+14	0.0538117697275279	0.192752016871553	-3,9103E+14	5,08131E+14	5,37556E+14	5,86725E+14	7,3661E+13	6,21167E+14	6,77724E+14
TC1900000989.mm.2	Tigd3	tigger transposable element derived 3	-0.737483351866425	6,74756E+14	-2,35566E+13	0.0538140722896086	0.192752016871553	-3,91034E+14	6,20972E+13	6,33993E+14	6,7563E+14	7,15481E+14	6,93646E+14	7,08815E+14
TC0700003400.mm.2	Peg12	paternally expressed 12	-0.963370653675368	6,32052E+14	-2,35553E+14	0.0538241556720144	0.192752016871553	-3,91052E+14	5,64348E+14	5,5705E+14	6,35423E+14	7,2142E+14	6,27684E+14	6,86388E+14
TC1600001317.mm.2	Tbx1	T-box 1	-0.78718524229991	5,83918E+14	-2,35552E+14	0.053824980740666	0.192752016871553	-3,91053E+13	5,54929E+14	5,3546E+14	5,57065E+14	6,08225E+14	6,10225E+14	6,37602E+14
TC0400002980.mm.2	Plin2	perilipin 2	0.951089190383819	6,15475E+14	2,35546E+13	0.0538295712150475	0.192752016871553	-3,91061E+14	6,45089E+14	6,99374E+14	6,25823E+14	5,6832E+14	5,45116E+14	6,0913E+14

TC1300000612.mm.2	Syk	spleen tyrosine kinase	-0.795454792817835	6,56674E+14	-2,3554E+13	0.0538344293626485	0.192752016871553	-3,91069E+14	6,01688E+14	6,19115E+14	6,51756E+14	6,87592E+14	6,65134E+14	7,1476E+12
TC0600001744.mm.2	Klhl42	kelch-like 42	-0.817795622717247	7,07713E+13	-2,35526E+14	0.0538449925047643	0.192752016871553	-3,91088E+14	6,75305E+14	6,49815E+14	7,477E+14	6,84781E+14	7,35979E+14	7,527E+14
TC0200002269.mm.2	Rspo4	R-spondin 4	-0.800664799453434	6,30352E+14	-2,35512E+14	0.0538552291890292	0.192752016871553	-3,91106E+14	5,64479E+14	5,74121E+13	6,40241E+14	7,0454E+14	6,51577E+14	6,47155E+14
TC0500000868.mm.2	Uso1	USO1 vesicle docking factor	1,08725E+14	8,12268E+13	2,35512E+14	0.0538552460718507	0.192752016871553	-3,91106E+13	8,95109E+13	9,02605E+14	8,11181E+14	6,84452E+14	8,03134E+14	7,7713E+14
TC1200000098.mm.2	Fam49a	family with sequence similarity 49, member A	1,4647E+14	8,45335E+14	2,35438E+14	0.0539122187318645	0.192913075822015	-3,91204E+13	9,35294E+14	9,52342E+14	8,73478E+14	7,16204E+14	8,56791E+14	7,37904E+14
TC0900001468.mm.2	Myl3	myosin, light polypeptide 3	-0.79122756363325	6,64813E+14	-2,35392E+14	0.05394692184635	0.192994394251829	-3,91264E+14	6,15093E+14	6,14866E+14	6,56583E+13	7,14131E+13	6,81838E+14	7,06367E+14
TC0500001260.mm.2	Msi1	musashi RNA-binding protein 1	0.972275722486346	8,10518E+14	2,35368E+14	0.0539649014442734	0.193015861702903	-3,91295E+14	8,75016E+14	8,6569E+14	8,32943E+14	7,43208E+14	8,01364E+14	7,44888E+14
TC0700000079.mm.2	Smim17/Olfr1344	small integral membrane protein 17/olfactory receptor 1344	-0.708755801599819	6,07681E+14	-2,35309E+14	0.0540101741901486	0.193078683745221	-3,91374E+14	5,74281E+14	5,42013E+14	6,36737E+14	6,35009E+14	6,27791E+14	6,30255E+14
TC0100001520.mm.2	Mpc2	mitochondrial pyruvate carrier 2	-0.763698112020885	8,51389E+14	-2,35298E+13	0.0540188395184045	0.193078683745221	-3,91389E+14	8,04554E+14	8,0332E+14	8,46973E+14	8,92875E+14	8,6597E+14	8,94644E+14
TC0500000312.mm.2	Dnajc5g	DnaJ heat shock protein family (Hsp40) member C5 gamma	-0.918220654989326	4,48145E+13	-2,35285E+14	0.0540289679681459	0.193078683745221	-3,91406E+13	3,90659E+14	3,93694E+14	4,35496E+14	5,01025E+13	5,18157E+14	4,49841E+13
TC1000000297.mm.2	Tspyl1	testis-specific protein, Y-encoded-like 1	1,6186E+14	8,42703E+14	2,35283E+14	0.0540303968849502	0.193078683745221	-3,91409E+14	9,53699E+14	9,37493E+13	8,81994E+13	7,15559E+14	8,58379E+14	7,09091E+14
TC0700001115.mm.2	Aen	apoptosis enhancing nuclease	-0.806878813266717	6,67179E+14	-2,35262E+14	0.0540460342748011	0.193087273408309	-3,91436E+14	6,27251E+14	6,21868E+14	6,43951E+14	6,9951E+14	6,86573E+14	7,23922E+14
TC0700001869.mm.2	Bckdk	branched chain ketoacid dehydrogenase kinase	0.918042315638486	6,76448E+14	2,35248E+14	0.0540567672280072	0.193087273408309	-3,91454E+13	7,61617E+14	7,41756E+14	6,44411E+14	5,91136E+14	6,22014E+14	6,97751E+14
TC1700002351.mm.2	Tnfsf14	tumor necrosis factor (ligand) superfamily, member 14	-1,21933E+14	6,99495E+14	-2,35216E+14	0.0540813385487745	0.193132226958423	-3,91497E+14	6,08759E+14	6,18435E+14	7,05677E+13	7,9304E+13	6,71855E+14	7,99204E+14
TC1600001582.mm.2	Slc15a2	solute carrier family 15 (H+/peptide transporter), member 2	2,27025E+14	6,15969E+14	2,35198E+14	0.0540951108080697	0.1931362188629	-3,9152E+13	7,58983E+14	7,52657E+14	6,94321E+14	4,32261E+14	6,29414E+14	4,28176E+14
TC1700002811.mm.2	Slc39a7	solute carrier family 39 (zinc transporter), member 7	0.749423786711077	7,39894E+14	2,35183E+14	0.0541064290912389	0.1931362188629	-3,9154E+14	7,90943E+14	7,73498E+14	7,10986E+14	7,49382E+14	6,94403E+13	7,20153E+14
TC0500002753.mm.2	Naaa	N-acylethanolamine acid amidase	0.849110333295373	7,7993E+14	2,35167E+14	0.0541185365793017	0.193136651141149	-3,91561E+14	8,39646E+14	8,30257E+14	7,78365E+14	7,31233E+14	7,19058E+14	7,81024E+14
TC0200003830.mm.2	Pde1a	phosphodiesterase 1A, calmodulin-dependent	1,90624E+14	7,89486E+14	2,35106E+14	0.0541651863665654	0.19321219774948	-3,91641E+14	8,95816E+14	9,34162E+14	8,25243E+14	6,32961E+14	8,18558E+13	6,30172E+14
TC0100001234.mm.2	Kdm5b	lysine (K)-specific demethylase 5B	1,03292E+14	6,53485E+14	2,35084E+14	0.0541823683231719	0.19321219774948	-3,91671E+13	7,46495E+14	7,08385E+14	6,35976E+13	5,8176E+14	6,73697E+14	5,746E+14
TC1600000305.mm.2	Map3k13	mitogen-activated protein kinase kinase kinase 13	1,53533E+14	5,11278E+13	2,35071E+14	0.0541921903375175	0.19321219774948	-3,91688E+14	5,83754E+14	5,92043E+14	5,49479E+14	4,7366E+14	5,32383E+14	3,36348E+14
TC0800000396.mm.2	Gtf2e2	general transcription factor II E, polypeptide 2 (beta subunit)	1,3694E+14	5,96641E+14	2,35068E+14	0.0541946124185806	0.19321219774948	-3,91692E+14	7,13857E+14	6,78024E+14	5,64516E+14	5,05447E+12	6,43049E+14	4,74952E+14
TC0200005429.mm.2	Chrna4	cholinergic receptor, nicotinic, alpha polypeptide 4	0.934096864433418	7,82936E+14	2,35049E+14	0.0542095621791164	0.19321219774948	-3,91718E+14	8,55158E+14	8,46577E+14	7,62059E+13	7,18909E+14	8,015E+14	7,13416E+14
TSUnmapped00000047.m m.2	Cd83	CD83 antigen	0.768270448635088	5,6072E+13	2,3503E+14	0.0542234723656391	0.19321219774948	-3,91742E+14	6,30228E+14	5,78884E+14	5,38867E+14	5,60886E+14	5,2677E+14	5,28688E+14
TC1700000505.mm.2	Pim1	proviral integration site 1	-0.727865957026067	6,19812E+14	-2,35019E+14	0.0542319437054063	0.19321219774948	-3,91756E+12	5,88943E+14	5,61622E+14	6,51131E+14	6,21034E+14	6,44005E+14	6,52134E+14
TC1600001824.mm.2	Nsun3	NOL1/NOP2/Sun domain family member 3	1,6571E+14	5,64157E+14	2,35007E+13	0.0542417126796175	0.19321219774948	-3,91773E+14	6,74685E+14	6,73412E+14	5,97447E+14	4,22721E+14	5,83835E+13	4,32841E+14
TC1800000829.mm.2	Loxhd1	lipoxygenase homology domains 1	-0.934977574914558	5,27459E+14	-2,34999E+13	0.0542476250616674	0.19321219774948	-3,91783E+14	4,53911E+14	5,25323E+14	4,90316E+14	5,28976E+14	6,11164E+14	5,55066E+14
TC1700001918.mm.2	Rnf5	ring finger protein 5	0.773894907502273	9,6582E+14	2,34973E+14	0.0542672010438829	0.193220659759089	-3,91817E+14	1,02699E+14	9,88416E+14	9,80682E+14	9,38209E+13	9,33535E+14	9,2709E+14
TC1100001497.mm.2	Hoxb5	homeobox B5	-1,36917E+14	5,39353E+14	-2,34954E+14	0.054282375103846	0.193220659759089	-3,91843E+14	4,46977E+14	4,61601E+14	4,88324E+14	6,56803E+13	5,57819E+14	6,24594E+14
TC1400000946.mm.2	Stmn4	stathmin-like 4	0.832627117039083	8,22712E+14	2,34928E+13	0.0543017541345052	0.193220659759089	-3,91876E+13	9,03726E+14	9,04164E+14	7,74722E+14	7,12298E+14	8,40934E+14	8,0043E+14
TC1300001330.mm.2	Hcn1	hyperpolarization-activated, cyclic nucleotide-gated K+ 1	1,23997E+14	6,91384E+14	2,34901E+14	0.0543226276256302	0.193220659759089	-3,91912E+14	8,12252E+14	7,8984E+14	6,54521E+14	5,37594E+14	7,29599E+14	6,24499E+13
TC1700001010.mm.2	Safb	scaffold attachment factor B	-0.991899101513075	8,45642E+14	-2,34898E+14	0.0543250867854894	0.193220659759089	-3,91916E+14	7,87921E+14	7,66645E+14	8,35987E+14	9,30354E+14	8,49282E+14	9,03664E+13
TC1200000500.mm.2	Pnn	pinin	1,64524E+13	8,07784E+14	2,34863E+14	0.0543519725534377	0.193220659759089	-3,91963E+14	8,93843E+14	9,01534E+14	8,47134E+14	7,37862E+14	8,33782E+14	6,32547E+14
TC0500001312.mm.2	Med13l	mediator complex subunit 13-like	0.973090775618708	7,58692E+14	2,34845E+14	0.0543656102040989	0.193220659759089	-3,91986E+14	8,32454E+12	8,34154E+14	7,47251E+14	6,66301E+14	7,71023E+14	7,00967E+14
TC0100001671.mm.2	Zbtb18	zinc finger and BTB domain containing 18	1,21146E+14	7,91395E+14	2,34843E+14	0.0543671148084671	0.193220659759089	-3,91989E+14	8,57279E+14	9,28105E+14	7,47937E+13	6,71958E+14	8,28155E+14	7,14937E+14
TC1100002310.mm.2	Aftph	aftiphilin	1,62765E+14	7,13677E+14	2,34841E+14	0.0543687373603902	0.193220659759089	-3,91991E+13	8,3178E+14	8,18338E+13	7,2515E+13	5,82206E+14	7,49687E+14	5,74901E+14
TC0500000888.mm.2	Sep 11	septin 11	1,1549E+14	8,34522E+14	2,3484E+14	0.0543699169209774	0.193220659759089	-3,91993E+14	8,938E+14	9,13604E+14	8,61836E+14	7,61469E+14	8,38266E+13	7,38157E+14
TC1700001898.mm.2	Brd2	bromodomain containing 2	1,19246E+14	7,92592E+13	2,34798E+14	0.0544017159609658	0.193231007326361	-3,92048E+14	8,70667E+14	8,84085E+14	8,07635E+14	6,76906E+14	7,94479E+14	7,21779E+14
TC1000000819.mm.2	Misp	mitotic spindle positioning	-0.985532243590021	5,58813E+14	-2,34798E+14	0.0544018051381659	0.193231007326361	-3,92048E+14	4,58845E+14	5,26481E+14	5,71991E+14	6,13131E+14	5,53223E+14	6,29209E+13
TC0X00002407.mm.2	Gabra3	gamma-aminobutyric acid (GABA) A receptor, subunit alpha 3	2,267E+14	7,28699E+14	2,34789E+14	0.0544088053273566	0.193231007326361	-3,9206E+13	9,07584E+14	8,81098E+14	7,68444E+14	4,79786E+14	7,4711E+14	5,88172E+13
TC1000000822.mm.2	Prtn3	proteinase 3	-0.936739747145699	6,58378E+14	-2,34736E+14	0.0544500030897734	0.193315747980576	-3,92131E+14	6,00665E+14	6,06182E+14	6,37792E+14	7,11434E+14	6,63892E+14	7,30302E+14
TC1000002544.mm.2	Ncln	nicalin	0.963580941556667	7,43822E+14	2,34727E+14	0.0544566610863176	0.193315747980576	-3,92142E+14	8,0706E+14	7,83326E+14	7,83718E+14	6,9116E+13	7,20776E+14	6,76894E+14
TC0100001330.mm.2	Brinp3	bone morphogenetic protein/retinoic acid inducible neural specific 3	1,30912E+14	6,39262E+13	2,34683E+14	0.0544903377846296	0.193392690027255	-3,922E+13	7,51916E+14	7,40468E+14	6,21299E+14	4,91327E+14	6,7091E+14	5,5965E+14
TC0400001165.mm.2	Ttc39a	tetratricopeptide repeat domain 39A	-1,05782E+14	5,40082E+14	-2,3466E+14	0.0545080330337213	0.193412890612718	-3,9223E+14	4,8317E+14	4,82749E+14	4,90538E+14	6,06551E+14	5,66057E+14	6,11425E+14
TC1200001364.mm.2	Fam228b	family with sequence similarity 228, member B	0.908142614658005	6,35821E+14	2,3464E+14	0.0545235032012694	0.193425188701465	-3,92257E+14	7,17388E+14	6,92648E+14	6,31258E+14	5,45222E+13	6,01616E+14	6,26792E+13
TC0X00002771.mm.2	Nap1l2	nucleosome assembly protein 1-like 2	1,52609E+14	8,20508E+14	2,34541E+14	0.054599929238491	0.193653678146556	-3,92387E+14	9,45922E+13	9,24236E+14	8,30849E+13	6,57102E+13	8,39862E+14	7,25078E+14
TC1400000804.mm.2	Atp12a	ATPase, H+/K+ transporting, nongastric, alpha polypeptide	-0.789069684542724	5,27332E+14	-2,34516E+14	0.0546194788717923	0.193680383596213	-3,92421E+14	4,6891E+14	4,82479E+14	5,40413E+14	5,62985E+14	5,26102E+14	5,83101E+14
TC1300002766.mm.2	Nsun2	NOL1/NOP2/Sun domain family member 2	1,32995E+14	6,00493E+14	2,34493E+14	0.0546372408966824	0.193700739838998	-3,92451E+14	7,15373E+14	7,02165E+14	5,92568E+13	4,41306E+14	6,19646E+14	5,31901E+14
TC0200003262.mm.2	Zdhhc12	zinc finger, DHHC domain containing 12	-0.814119010603823	7,9499E+13	-2,34473E+14	0.0546527915812596	0.193713249174843	-3,92478E+13	7,29791E+14	7,49629E+14	8,54484E+14	7,93795E+14	8,06886E+13	8,35358E+14
TC0100001223.mm.2	Mybph	myosin binding protein H	-1,00295E+14	5,9781E+14	-2,34436E+14	0.0546810925804932	0.193740143092127	-3,92526E+14	5,41575E+14	5,5347E+14	5,55689E+14	6,40494E+14	6,10441E+14	6,85194E+14
TC1000001162.mm.2	Ube2n	ubiquitin-conjugating enzyme E2N	1,1214E+14	8,2966E+14	2,34428E+13	0.0546872835166091	0.193740143092127	-3,92537E+14	9,07095E+14	9,12521E+14	8,34871E+14	7,28137E+14	8,40651E+14	7,54685E+14
TC1100000316.mm.2	Asb3	ankyrin repeat and SOCS box-containing 3	1,02705E+14	5,11688E+14	2,3441E+14	0.0547009989801583	0.193740143092127	-3,9256E+14	5,7257E+14	5,62621E+14	5,18743E+14	4,86414E+14	5,2174E+14	4,08039E+14
TC0700004595.mm.2	Osbpl5	oxysterol binding protein-like 5	-0.885131422867455	7,18829E+14	-2,34401E+14	0.0547084745900316	0.193740143092127	-3,92573E+14	6,3893E+14	6,80393E+14	7,18046E+14	7,79252E+14	7,24425E+14	7,71924E+14
TC0X00001163.mm.2	H2afb1	H2A histone family member B1	-0.734266036880276	7,306E+12	-2,34384E+14	0.0547218509858758	0.193744931868912	-3,92596E+14	6,87947E+14	6,87199E+14	7,31745E+14	7,54709E+13	7,41787E+14	7,80212E+14
TC1800000311.mm.2	Matr3	matrin 3	1,83665E+14	9,56012E+14	2,34352E+14	0.0547459246463393	0.193787584320401	-3,92637E+14	1,08063E+14	1,07468E+14	1,00368E+14	7,89104E+14	9,68652E+14	8,19325E+14
TC1200002346.mm.2	Ccdc85c	coiled-coil domain containing 85C	0.68520998357264	7,44759E+14	2,34323E+14	0.0547689200208794	0.193826401997898	-3,92676E+14	7,84927E+14	7,82581E+14	7,44558E+14	7,26021E+14	7,1246E+14	7,18006E+14
TC0100003519.mm.2	Fcrlb	Fc receptor-like B	-1,30041E+14	7,1567E+14	-2,34279E+14	0.0548025035752153	0.193899753643122	-3,92733E+14	6,58508E+13	6,08048E+14	6,84611E+14	8,16219E+14	7,15515E+13	8,11122E+14
TC1400000786.mm.2	Rec8	REC8 meiotic recombination protein	-1,25905E+14	5,12361E+14	-2,34256E+13	0.054820483341376	0.193899753643122	-3,92764E+14	4,15531E+13	4,44697E+14	4,71964E+14	6,29938E+14	5,42522E+14	5,69516E+14
TC0400003050.mm.2	Izumo3	IZUMO family member 3	-0.950698865544811	5,36244E+14	-2,34244E+14	0.0548297400021933	0.193899753643122	-3,92779E+14	4,76337E+13	4,81359E+14	5,10964E+13	6,00971E+14	5,49215E+14	5,9862E+14
TC0200003574.mm.2	Cytip	cytohesin 1 interacting protein	-0.819316382004316	4,82082E+14	-2,34227E+13	0.054843417258545	0.193899753643122	-3,92803E+14	4,34081E+14	4,32534E+14	4,72481E+12	5,22918E+14	4,89878E+14	5,406E+14
TC1100003054.mm.2	Mpdu1	mannose-P-dolichol utilization defect 1	0.802790518012385	9,1089E+14	2,34181E+14	0.0548787124282301	0.193899753643122	-3,92863E+14	9,74584E+14	9,59238E+14	9,11741E+14	8,46514E+13	8,87896E+13	8,85368E+14
TC0700000243.mm.2	Pnmal2	PNMA-like 2	1,64372E+13	7,5368E+14	2,34154E+14	0.0549001611530438	0.193899753643122	-3,92899E+14	8,77937E+14	8,47485E+14	8,09076E+14	5,90901E+14	7,3087E+14	6,65809E+13

TC1400002071.mm.2	Slc22a17	solute carrier family 22 (organic cation transporter), member 17	1,54248E+14	1,09386E+14	2,34145E+14	0.0549066421051618	0.193899753643122	-3,9291E+14	1,21096E+14	1,19516E+14	1,12945E+14	9,29943E+14	1,08467E+14	1,01295E+14
TC0X00000126.mm.2	Tspan7	tetraspanin 7	1,44477E+14	1,1312E+14	2,3414E+14	0.0549110398486414	0.193899753643122	-3,92918E+14	1,23498E+14	1,23076E+13	1,15656E+14	9,88111E+14	1,13829E+14	1,0385E+14
TC0600002287.mm.2	Hoxa13	homeobox A13	-1,45508E+14	6,20643E+14	-2,34129E+14	0.0549193968081474	0.193899753643122	-3,92932E+14	5,11731E+14	5,42176E+14	5,86096E+14	7,38934E+14	6,11691E+13	7,33232E+14
TC0X00000060.mm.2	Syp	synaptophysin	1,20862E+14	1,15193E+14	2,34128E+14	0.0549201773598215	0.193899753643122	-3,92933E+14	1,23626E+14	1,23616E+14	1,17768E+13	1,03079E+14	1,13815E+14	1,09255E+14
TC0700001546.mm.2	Olfr688	olfactory receptor 688	-0.939679416714202	5,22577E+14	-2,34109E+14	0.0549345905790998	0.193899753643122	-3,92958E+13	4,42894E+13	4,87113E+14	5,68853E+14	5,18659E+14	5,2146E+13	5,96482E+13
TSUnmapped00000005.m m.2	Amot	angiomotin	0.702912612338798	5,05977E+14	2,34097E+14	0.0549442534950844	0.193899753643122	-3,92974E+14	5,52174E+14	5,26764E+13	5,15666E+14	5,02904E+14	4,75683E+14	4,62672E+14
TC1100002045.mm.2	Rac3	Rac family small GTPase 3	0.952760101672627	7,18464E+14	2,34086E+14	0.0549528435378172	0.193899753643122	-3,92989E+14	8,11846E+14	7,18095E+14	7,30885E+14	7,1057E+14	6,50418E+12	6,8897E+13
TC1100003822.mm.2	Ppy	pancreatic polypeptide	-0.792034749863811	5,76283E+14	-2,34079E+14	0.0549581192135629	0.193899753643122	-3,92998E+14	5,13676E+14	5,3437E+14	5,7571E+14	6,27488E+14	5,87433E+14	6,1902E+13
TC0800002955.mm.2	Cfdp1	craniofacial development protein 1	1,75701E+14	8,72888E+14	2,3402E+14	0.0550039937270355	0.194019122356332	-3,93075E+14	9,86166E+14	9,80882E+14	8,89674E+14	7,64963E+14	9,1541E+14	7,00236E+14
TC0500002719.mm.2	Adamts3	a disintegrin-like and metallopeptidase (reprolysin type) with thrombospondin type 1 motif, 3	1,1858E+14	6,41598E+14	2,33975E+14	0.0550391662772627	0.194046941145818	-3,93135E+14	7,1815E+14	7,08535E+14	6,91352E+14	5,42023E+14	5,97274E+14	5,92253E+14
TC0600000714.mm.2	Herc3	hect domain and RLD 3	0.979786804768595	7,34785E+14	2,3397E+14	0.055043157532431	0.194046941145818	-3,93142E+14	7,88927E+14	8,19191E+14	7,4635E+14	6,42079E+14	7,12166E+14	6,99995E+13
TC0300000060.mm.2	Fabp5	fatty acid binding protein 5, epidermal	0.710473769677371	6,63613E+13	2,33963E+14	0.0550480089354891	0.194046941145818	-3,9315E+14	7,16223E+14	7,22679E+14	6,32588E+14	6,13384E+14	6,33984E+14	6,62823E+14
TC0X00002177.mm.2	Igsf1	immunoglobulin superfamily, member 1	1,06883E+14	6,56649E+14	2,33943E+13	0.0550635783225603	0.19405936953443	-3,93176E+14	7,67041E+13	6,49274E+14	6,51832E+14	6,692E+14	6,23955E+14	5,78592E+14
TC0800001696.mm.2	Col4a1	collagen, type IV, alpha 1	-0.896267764366661	7,83385E+14	-2,339E+13	0.0550973821122617	0.194136041542723	-3,93234E+13	7,12229E+14	7,34133E+14	7,70607E+14	8,57724E+13	8,05782E+13	8,19833E+14
TC0700004510.mm.2	Cox8b	cytochrome c oxidase subunit 8B	-1,05068E+14	5,40764E+14	-2,33882E+14	0.0551110820821593	0.194141859551778	-3,93257E+14	4,4747E+14	4,8915E+14	5,24511E+14	6,36695E+14	5,57447E+14	5,89309E+14
TC1700002782.mm.2	H2-Eb1	histocompatibility 2, class II antigen E beta	-0.812669406358983	5,84502E+14	-2,33862E+14	0.0551270203809537	0.194155558338428	-3,93284E+14	5,31824E+14	5,46038E+14	5,74309E+13	6,14447E+14	5,9236E+14	6,48036E+14
TC1100003325.mm.2	Omg	oligodendrocyte myelin glycoprotein	1,98818E+14	9,04243E+14	2,33819E+14	0.0551604087112614	0.194192405566261	-3,9334E+14	1,03159E+12	1,02817E+14	9,72863E+14	7,30708E+14	9,08071E+14	7,54052E+14
TC1100001276.mm.2	Acaca	acetyl-Coenzyme A carboxylase alpha	0.753523798783728	6,65611E+14	2,33818E+14	0.0551615863139562	0.194192405566261	-3,93342E+14	7,27984E+14	6,87921E+14	6,419E+14	6,70663E+14	6,41365E+14	6,23835E+14
TC1100003816.mm.2	Sost	sclerostin	-0.943334725855399	6,10265E+14	-2,33757E+14	0.0552091303702397	0.194235671470408	-3,93423E+14	5,33768E+14	5,79614E+14	6,02448E+14	6,43708E+14	6,10641E+14	6,91408E+14
TC0400003187.mm.2	Prkaa2	protein kinase, AMP-activated, alpha 2 catalytic subunit	1,3132E+14	7,20688E+14	2,33751E+14	0.0552139175405537	0.194235671470408	-3,93431E+14	7,9541E+14	8,21127E+14	7,52971E+14	6,00726E+14	7,1611E+12	6,37786E+14
TC1900000580.mm.2	Ccnj	cyclin J	-0.781601487642982	6,32338E+14	-2,33745E+14	0.0552181808998787	0.194235671470408	-3,93438E+14	6,00404E+14	5,59415E+14	6,56833E+13	6,61237E+14	6,8078E+14	6,35359E+13
TC0800001694.mm.2	Irs2	insulin receptor substrate 2	0.738946994737194	8,49804E+14	2,3374E+14	0.0552220946444449	0.194235671470408	-3,93445E+13	9,12143E+14	8,7424E+13	8,40748E+13	8,33099E+14	8,05684E+14	8,32909E+14
TC1800001073.mm.2	Zfp24	zinc finger protein 24	1,31361E+14	5,4749E+14	2,33697E+14	0.0552562890694109	0.194313528104631	-3,93502E+14	6,36005E+14	6,38918E+14	5,73493E+14	4,24326E+14	5,50144E+14	4,62056E+14
TC1400000502.mm.2	Cgrrf1	cell growth regulator with ring finger domain 1	0.877316758599487	7,23342E+14	2,3366E+14	0.0552846564077878	0.194370863778897	-3,9355E+14	7,98529E+14	7,64893E+14	6,76461E+14	7,1221E+14	7,29701E+14	6,58258E+13
TC0700002001.mm.2	Prap1	proline-rich acidic protein 1	-0.766447869715031	5,82537E+14	-2,33627E+14	0.0553103319133959	0.194418712504483	-3,93594E+13	5,42955E+14	5,30261E+13	5,7806E+14	6,17441E+14	5,93809E+14	6,32696E+14
TC1300000928.mm.2	Mctp1	multiple C2 domains, transmembrane 1	2,61351E+14	6,53227E+14	2,33605E+14	0.0553275676258114	0.194427192083112	-3,93623E+14	7,98523E+14	8,44542E+14	7,28278E+14	4,27656E+13	6,80463E+14	4,39901E+14
TC1100003833.mm.2	Slc4a1	solute carrier family 4 (anion exchanger), member 1	-0.709021011854603	7,19945E+13	-2,33594E+13	0.0553368772353474	0.194427192083112	-3,93638E+14	6,76688E+14	6,77057E+14	7,18358E+14	7,5202E+14	7,52722E+13	7,42828E+14
TC1600001605.mm.2	Adprh	ADP-ribosylarginine hydrolase	0.687986160163987	6,95079E+13	2,33527E+14	0.0553889524291508	0.1945226030913	-3,93726E+14	7,67705E+14	7,45873E+14	6,37025E+14	6,4389E+14	6,85694E+14	6,90287E+14
TC1100003532.mm.2	Stxbp4	syntaxin binding protein 4	1,53482E+14	5,50355E+14	2,33494E+14	0.0554149430613738	0.1945226030913	-3,9377E+14	6,80318E+14	6,62903E+13	5,42811E+14	3,7984E+14	5,8538E+14	4,50877E+14
TC0700004607.mm.2	Fadd	Fas (TNFRSF6)-associated via death domain	-0.72129482605733	6,86004E+13	-2,33487E+13	0.0554204750506255	0.1945226030913	-3,93779E+14	6,18245E+14	6,40298E+13	7,42072E+14	7,12606E+14	6,92718E+14	7,10083E+14
TC0500000981.mm.2	Cops4	COP9 signalosome subunit 4	1,00832E+14	7,75358E+14	2,33475E+14	0.0554294791187825	0.1945226030913	-3,93794E+13	8,63626E+14	8,31618E+14	7,68912E+14	6,94411E+14	7,88008E+14	7,05572E+14
TC0200001576.mm.2	Wt1	Wilms tumor 1 homolog	-1,31472E+14	5,8479E+14	-2,33467E+14	0.0554362345561308	0.1945226030913	-3,93806E+14	4,74581E+13	5,17145E+13	5,6646E+14	6,95883E+14	5,73471E+14	6,812E+14
TC1700002171.mm.2	Tjap1	tight junction associated protein 1	-0.713643219550568	6,69157E+14	-2,33466E+14	0.0554364670387419	0.1945226030913	-3,93806E+14	6,05097E+14	6,17083E+14	6,96502E+14	7,31352E+14	6,7925E+14	6,85658E+14
TC0600002578.mm.2	Clec4f	C-type lectin domain family 4, member f	-1,07689E+14	6,16744E+14	-2,33432E+14	0.0554634612642962	0.194570383449565	-3,93852E+14	5,43326E+14	5,43885E+14	6,21998E+14	6,88663E+14	5,9582E+14	7,06768E+13
TC1400002335.mm.2	Ppp3cc	protein phosphatase 3, catalytic subunit, gamma isoform	0.948054540876448	7,25377E+14	2,33418E+14	0.0554742345663316	0.194570383449565	-3,9387E+14	7,73676E+14	7,75249E+14	7,51717E+14	6,92308E+14	7,2169E+14	6,37623E+14
TC0500000291.mm.2	Drc1	dynein regulatory complex subunit 1	0.756381696566101	6,83166E+14	2,3333E+14	0.0555436905745411	0.194761921463724	-3,93986E+14	7,44299E+14	6,98832E+14	6,77437E+14	6,8657E+14	6,59592E+14	6,32264E+14
TC0400002016.mm.2	Acot7	acyl-CoA thioesterase 7	1,06902E+14	9,06186E+14	2,33318E+14	0.0555530187455643	0.194761921463724	-3,94002E+14	9,86013E+14	9,81226E+13	9,17746E+14	7,98696E+14	9,00096E+14	8,53338E+14
TC1100003026.mm.2	Pfas/Mir3062	phosphoribosylformylglycinamidine synthase (FGAR amidotransferase)/microRNA 3062	-0.695611188433714	6,77272E+14	-2,33279E+14	0.05558335659196	0.194825891835165	-3,94053E+14	6,26746E+14	6,24593E+14	7,11375E+14	7,10457E+14	6,80979E+14	7,09482E+14
TC1000003150.mm.2	Zc3h10	zinc finger CCCH type containing 10	-1,0205E+13	6,70468E+14	-2,33232E+14	0.0556209646778624	0.194915311843062	-3,94116E+13	5,88511E+14	6,13634E+14	6,56174E+14	7,58245E+14	6,75941E+13	7,30304E+14
TC0100000635.mm.2	Vil1	villin 1	-0.98003635544856	7,56074E+14	-2,33165E+13	0.055673809368092	0.195028935544876	-3,94205E+14	6,85625E+14	6,81312E+14	7,70117E+14	8,36444E+14	7,37637E+14	8,25308E+14
TC0300000906.mm.2	Golph3l	golgi phosphoprotein 3-like	0.956043338114649	6,6141E+14	2,3316E+14	0.0556775959477707	0.195028935544876	-3,94211E+14	7,22182E+14	6,97314E+14	7,07322E+13	6,13355E+14	6,33878E+14	5,94409E+14
TC0200005479.mm.2	Ganc	glucosidase, alpha; neutral C	0.748637230133546	6,1133E+14	2,33121E+14	0.0557082943912136	0.1950562143797	-3,94262E+14	6,3881E+14	6,6802E+14	6,1555E+14	5,88496E+14	5,62267E+13	5,94836E+13
TC0800002332.mm.2	Npy5r	neuropeptide Y receptor Y5	-0.870011908917634	7,15862E+13	-2,33119E+14	0.0557095946487544	0.1950562143797	-3,94265E+14	6,38942E+14	6,84799E+14	7,06253E+14	7,67436E+14	7,29836E+13	7,67907E+14
TC1800000741.mm.2	Tcf4	transcription factor 4	1,20817E+13	9,72255E+14	2,33061E+14	0.0557551124780814	0.195130155542903	-3,94341E+14	1,05483E+13	1,07896E+14	9,64945E+14	8,42646E+14	9,91916E+13	9,00236E+14
TC1500000859.mm.2	Panx2	pannexin 2	-0.894002329905177	6,73968E+14	-2,33051E+14	0.0557630710319673	0.195130155542903	-3,94354E+14	6,51488E+13	5,60579E+14	7,1806E+14	7,22816E+14	6,97737E+14	6,9313E+14
TC0200000612.mm.2	Mapkap1	mitogen-activated protein kinase associated protein 1	2,37655E+14	6,20874E+14	2,33035E+14	0.0557762051011611	0.195130155542903	-3,94376E+14	7,7528E+14	7,73825E+14	6,96837E+14	4,05507E+14	6,34712E+14	4,39083E+14
TC0600000381.mm.2	Luc7l2	LUC7-like 2 (S. cerevisiae)	2,25579E+14	6,25976E+14	2,33031E+14	0.0557791532570353	0.195130155542903	-3,94381E+14	7,84958E+14	7,72881E+12	6,55067E+14	4,36271E+13	6,77723E+13	4,28959E+14
TC1700002170.mm.2	Polr1c	polymerase (RNA) I polypeptide C	0.724603460474156	7,25521E+14	2,32977E+14	0.0558217378020861	0.195188744331445	-3,94452E+14	7,97559E+14	7,60893E+14	6,82911E+14	6,98234E+13	6,98954E+14	7,14577E+14
TC1200002422.mm.2	Rd3l	retinal degeneration 3-like	-0.941820745227069	5,79941E+14	-2,32968E+13	0.055828949704781	0.195188744331445	-3,94464E+14	5,12546E+13	5,18007E+14	6,1432E+14	6,15858E+14	5,58542E+14	6,60375E+14
TC1900000154.mm.2	Stx5a/Mir6992	syntaxin 5A/microRNA 6992	0.859206448014714	6,58972E+14	2,32964E+14	0.055832242451662	0.195188744331445	-3,9447E+14	7,2066E+14	7,30846E+14	6,46763E+13	5,75898E+14	6,60755E+14	6,18909E+14
TC0600002281.mm.2	Hoxa7/Mira	homeobox A7/mistral long non-coding RNA	-0.950060312379879	7,07831E+14	-2,32905E+14	0.055879061979395	0.195310048953144	-3,94548E+14	6,30759E+14	6,54285E+14	6,93631E+14	7,93254E+14	7,288E+13	7,46257E+14
TC0200002264.mm.2	Sdcbp2	syndecan binding protein (syntenin) 2	-0.732094863790334	5,13894E+14	-2,32847E+14	0.0559247008240315	0.195401943158762	-3,94624E+14	5,14917E+14	4,8007E+14	4,84835E+14	4,62135E+13	5,61131E+14	5,80275E+14
TC1300002024.mm.2	Drd1	dopamine receptor D1	1,16551E+13	8,22134E+14	2,32841E+14	0.0559296072642097	0.195401943158762	-3,94632E+14	8,79816E+14	8,94287E+14	8,79135E+14	7,38565E+14	7,90738E+14	7,50263E+13
TC1100001643.mm.2	Tubg2	tubulin, gamma 2	1,09449E+14	8,5223E+14	2,32778E+14	0.0559793559336806	0.195498383198769	-3,94715E+14	9,32869E+13	8,98578E+14	8,85109E+14	7,84275E+14	8,46499E+14	7,6605E+14
TC0100002189.mm.2	Actr1b	ARP1 actin-related protein 1B, centractin beta	1,13045E+14	8,99225E+14	2,32752E+14	0.0560002265274911	0.195498383198769	-3,9475E+14	9,8988E+14	9,86746E+14	9,03904E+14	7,64285E+13	8,82655E+14	8,6788E+14
TC0800000810.mm.2	Olfr372	olfactory receptor 372	-1,46059E+14	3,9495E+14	-2,32744E+14	0.0560063180984952	0.195498383198769	-3,9476E+14	3,07529E+14	2,96078E+14	3,52444E+14	5,17926E+14	3,91921E+14	5,03803E+14
TC1700000942.mm.2	Unc5cl	unc-5 family C-terminal like	-0.954475274212526	5,96409E+14	-2,32737E+14	0.0560118603509312	0.195498383198769	-3,94769E+14	5,38825E+14	5,27511E+14	5,76868E+14	6,78021E+14	6,15832E+14	6,41399E+14
TC0100003074.mm.2	Tmcc2	transmembrane and coiled-coil domains 2	1,2269E+13	8,04006E+14	2,32729E+14	0.0560178759528776	0.195498383198769	-3,94779E+14	8,95423E+13	8,80293E+14	8,30167E+14	6,87819E+14	7,99207E+14	7,3113E+14
TC0400001152.mm.2	Cc2d1b	coiled-coil and C2 domain containing 1B	-0.742289729432752	7,09242E+13	-2,32661E+14	0.0560720463227383	0.195610331025996	-3,94869E+14	6,43565E+14	6,57553E+14	7,35769E+13	7,68989E+14	7,07654E+14	7,41922E+14
TC1100000427.mm.2	C1qtnf2	C1q and tumor necrosis factor related protein 2	-0.867456848643193	6,79386E+14	-2,32642E+14	0.0560868438927648	0.195610331025996	-3,94894E+13	6,21779E+13	6,41885E+14	6,54943E+14	7,20556E+14	6,93307E+14	7,43848E+13
TC1200002120.mm.2	Vipas39	VPS33B interacting protein, apical-basolateral polarity regulator, spe-39 homolog	0.911538924039938	7,2564E+14	2,32611E+14	0.0561120100119801	0.195610331025996	-3,94936E+14	8,10695E+14	8,08576E+14	6,96538E+14	6,01066E+14	7,20309E+14	7,16655E+14

TC0600000964.mm.2	Alms1	ALMS1, centrosome and basal body associated	1,02606E+14	5,6998E+14	2,32609E+14	0.0561134457171948	0.195610331025996	-3,94938E+14	6,2637E+13	6,13484E+14	6,07665E+14	5,37716E+14	5,65665E+14	4,68976E+14
TC0900000938.mm.2	Sltm	SAFB-like, transcription modulator	0.814055408767544	6,97862E+14	2,32589E+14	0.0561291105792896	0.195610331025996	-3,94964E+14	7,5855E+14	7,47225E+14	7,01829E+14	6,36608E+14	6,8522E+14	6,57743E+14
TC0200005480.mm.2	Capn3	calpain 3	0.96307010995929	5,50194E+13	2,32588E+14	0.0561300017810283	0.195610331025996	-3,94966E+14	6,07813E+14	6,53867E+14	5,27332E+13	4,43088E+14	5,58066E+14	5,11E+14
TC0200002542.mm.2	Mmp9	matrix metallopeptidase 9	-1,08982E+14	5,01615E+14	-2,32568E+14	0.0561455814413971	0.195610331025996	-3,94992E+14	4,59599E+14	4,20961E+14	4,7442E+14	5,56187E+14	5,00668E+13	5,97856E+12
TC0100000949.mm.2	Gal3st2b	galactose-3-O-sulfotransferase 2B	-0.848891021061383	5,2152E+14	-2,32567E+14	0.0561470726118805	0.195610331025996	-3,94994E+14	4,81406E+14	4,47344E+14	5,14327E+12	5,87513E+12	5,49792E+13	5,48735E+14
TC1000001223.mm.2	Gm4307/Gm4303/Gm4305/Gm4302	predicted gene 4307/predicted gene 4303/predicted gene 4305/predicted gene 4302	-0.893015843869337	4,76881E+14	-2,32495E+14	0.0562037726151153	0.195765540023206	-3,95088E+14	3,87304E+14	4,53396E+14	4,69064E+14	5,32219E+14	5,0943E+13	5,09874E+14
TC1100003401.mm.2	Ccl3	chemokine (C-C motif) ligand 3	1,59928E+14	6,21922E+14	2,32368E+14	0.0563055264820942	0.196077576876158	-3,95257E+14	6,52137E+14	7,93331E+14	6,31753E+14	5,28699E+14	6,33074E+14	4,92538E+14
TC0200003148.mm.2	Paxx	non-homologous end joining factor	1,05289E+14	5,98275E+13	2,32306E+12	0.056354742058879	0.196139028711603	-3,95338E+14	6,66593E+13	6,41398E+14	5,62177E+14	6,2207E+14	6,02512E+14	4,949E+14
TC0100003614.mm.2	Pld5	phospholipase D family, member 5	1,28568E+14	7,16721E+14	2,32287E+14	0.0563697724584499	0.196139028711603	-3,95363E+14	8,27298E+14	7,90013E+12	7,3792E+14	5,84922E+14	7,11649E+14	6,48525E+14
TC1400002832.mm.2	Pabpn1	poly(A) binding protein, nuclear 1	1,22064E+14	7,50875E+14	2,32262E+14	0.0563894262751979	0.196139028711603	-3,95396E+14	8,52901E+13	8,31975E+14	7,56412E+14	6,23215E+14	7,61378E+14	6,79371E+14
TC1500001105.mm.2	Tarbp2	TARBP2, RISC loading complex RNA binding subunit	-1,13291E+14	5,45431E+14	-2,32236E+14	0.0564100962892096	0.196139028711603	-3,9543E+14	4,42848E+14	5,21847E+14	4,99916E+14	6,167E+14	6,00874E+14	5,90403E+14
TC1300001270.mm.2	Plpp1	phospholipid phosphatase 1	0.979499168585154	7,00103E+14	2,32229E+14	0.0564162989276992	0.196139028711603	-3,9544E+13	7,88698E+14	7,64579E+14	6,9267E+13	5,97292E+14	7,04104E+14	6,53273E+14
TC0900001076.mm.2	Mei4	meiotic double-stranded break formation protein 4	-0.73000598312653	5,23479E+14	-2,32225E+14	0.056419593460922	0.196139028711603	-3,95446E+14	4,8898E+14	4,61532E+14	5,28719E+14	5,65129E+14	5,50015E+14	5,46498E+14
TC0800002608.mm.2	Vps35	VPS35 retromer complex component	2,33636E+14	7,36323E+14	2,32223E+14	0.0564207725356502	0.196139028711603	-3,95447E+14	8,94614E+14	8,83183E+14	8,07843E+14	5,21772E+13	7,52214E+14	5,58311E+14
TC0800002844.mm.2	Slc12a4	solute carrier family 12, member 4	0.683730910132227	5,50825E+14	2,32187E+14	0.0564493414557729	0.196139028711603	-3,95495E+14	6,05628E+14	6,13045E+14	4,9502E+14	5,09329E+13	5,32671E+14	5,49256E+14
TC1000001031.mm.2	Btbd11	BTB (POZ) domain containing 11	0.947166454028042	6,1774E+14	2,32184E+14	0.05645208037512	0.196139028711603	-3,95499E+13	7,23917E+14	6,22551E+14	6,05172E+14	5,97764E+13	5,89187E+14	5,67848E+14
TC1000000674.mm.2	Slc16a9	solute carrier family 16 (monocarboxylic acid transporters), member 9	-0.819323246105962	5,92152E+14	-2,32183E+14	0.0564525056247653	0.196139028711603	-3,955E+14	5,08339E+14	5,6908E+14	5,96817E+14	6,37391E+14	6,24569E+14	6,16715E+14
TC0700001311.mm.2	Ccdc90b	coiled-coil domain containing 90B	-0.71810501142026	6,72648E+14	-2,32177E+14	0.0564573315133764	0.196139028711603	-3,95508E+14	6,58667E+13	6,58908E+14	6,1081E+14	6,46311E+14	7,20289E+14	7,40906E+13
TC0400001058.mm.2	Sgip1	SH3-domain GRB2-like (endophilin) interacting protein 1	2,06087E+14	8,10033E+14	2,32162E+13	0.0564692455900904	0.196139028711603	-3,95528E+14	9,5886E+14	9,50774E+14	8,61636E+14	5,91468E+14	8,15198E+14	6,82262E+14
TC0200002099.mm.2	Macrod2	MACRO domain containing 2	-0.73103545091594	8,23552E+14	-2,32111E+14	0.0565101735856581	0.196185584719082	-3,95595E+14	7,79596E+13	7,62232E+14	8,34289E+14	8,77163E+14	8,44149E+14	8,43886E+14
TC0900003082.mm.2	Fbxw28	F-box and WD-40 domain protein 28	-0.811179998020969	5,15947E+14	-2,32107E+14	0.0565133564462518	0.196185584719082	-3,956E+14	4,65889E+14	4,54337E+14	5,45323E+14	5,47673E+14	5,76416E+14	5,06046E+14
TC0400003404.mm.2	Olfr1335	olfactory receptor 1335	-0.798864039634694	5,31404E+14	-2,32088E+14	0.0565289290566066	0.196185584719082	-3,95626E+14	4,92734E+14	4,49469E+14	5,60683E+14	5,83565E+14	5,27405E+14	5,74571E+14
TC0200004169.mm.2	Hsd17b12	hydroxysteroid (17-beta) dehydrogenase 12	1,43521E+14	7,20462E+14	2,32085E+14	0.0565313516943274	0.196185584719082	-3,9563E+14	8,33282E+14	8,30198E+14	7,17192E+13	5,65662E+14	7,5066E+14	6,25778E+14
TC0700003847.mm.2	Tomt	transmembrane O-methyltransferase	-0.865154947992425	5,66527E+14	-2,32056E+14	0.056554020848661	0.196221993525942	-3,95667E+14	5,02966E+14	5,279E+14	6,16589E+14	5,47808E+14	5,80118E+14	6,2378E+14
TC0200004369.mm.2	Actc1	actin, alpha, cardiac muscle 1	-0.90746022195516	7,21314E+13	-2,31988E+14	0.0566089237628651	0.196319555991089	-3,95758E+14	6,5872E+14	6,67666E+14	7,05849E+14	7,87771E+14	7,30115E+13	7,77764E+13
TC0200002488.mm.2	R3hdml	R3H domain containing-like	-1,11724E+14	5,93575E+14	-2,31982E+14	0.0566133301099678	0.196319555991089	-3,95765E+14	5,38549E+14	5,02209E+14	5,77979E+14	6,78505E+14	5,8798E+12	6,76225E+14
TC0100001145.mm.2	Thsd7b	thrombospondin, type I, domain containing 7B	-0.880506842252247	6,22813E+14	-2,31976E+14	0.0566186915342017	0.196319555991089	-3,95774E+14	5,86838E+14	5,6315E+14	6,00544E+14	6,60258E+14	6,32284E+13	6,93805E+14
TC0900002783.mm.2	Snap91	synaptosomal-associated protein 91	1,32938E+14	9,08915E+14	2,3195E+14	0.0566390132407358	0.196334549390645	-3,95807E+14	9,83849E+14	9,81125E+14	9,54299E+13	8,35115E+14	9,15535E+14	7,83563E+14
TC1700002400.mm.2	Pja2	praja ring finger ubiquitin ligase 2	1,9835E+14	9,27639E+14	2,3194E+14	0.0566473853482969	0.196334549390645	-3,95821E+14	1,06648E+14	1,07723E+14	9,66286E+14	7,08832E+14	9,38778E+14	8,08229E+13
TC0200003304.mm.2	Slc25a25	solute carrier family 25 (mitochondrial carrier, phosphate carrier), member 25	0.77007135483226	6,76255E+13	2,3188E+14	0.0566958232679717	0.196455035017648	-3,95901E+13	7,32496E+14	6,99861E+14	6,94049E+14	6,52782E+14	6,53303E+14	6,25039E+14
TC0900000180.mm.2	Rdh8	retinol dehydrogenase 8	-1,02707E+14	4,91904E+14	-2,31866E+14	0.056706610570245	0.196455035017648	-3,95919E+14	3,95803E+14	4,51418E+14	4,78718E+14	5,72852E+14	5,00513E+14	5,52121E+14
TC0100003657.mm.2	Tfb2m	transcription factor B2, mitochondrial	2,17289E+14	6,49767E+14	2,31844E+14	0.0567244069562275	0.196455035017648	-3,95948E+14	7,84956E+14	7,84162E+14	7,17619E+14	4,77323E+14	6,72877E+14	4,61662E+13
TC0100001589.mm.2	Nectin4	nectin cell adhesion molecule 4	-1,03659E+13	6,25105E+14	-2,31819E+14	0.0567445077274187	0.196455035017648	-3,95981E+14	5,73064E+14	5,80429E+14	6,33933E+14	6,02393E+14	6,21141E+13	7,3967E+14
TC0700002978.mm.2	Ap2a1	adaptor-related protein complex 2, alpha 1 subunit	1,75844E+14	7,55628E+14	2,31817E+14	0.0567463137366404	0.196455035017648	-3,95984E+14	8,91222E+14	8,93549E+14	7,76569E+14	5,39343E+14	7,58684E+14	6,74399E+14
TC0300002959.mm.2	Sgms2	sphingomyelin synthase 2	-0.763158450839772	5,12521E+14	-2,31787E+14	0.0567700271238	0.196455035017648	-3,96023E+14	4,34891E+14	4,90451E+14	5,43033E+14	5,28775E+14	5,36058E+14	5,41916E+14
TC0400002372.mm.2	Pm20d2	peptidase M20 domain containing 2	-1,00747E+14	6,84413E+14	-2,31777E+14	0.0567778856328469	0.196455035017648	-3,96036E+14	6,29993E+14	5,95019E+14	6,74549E+14	7,8041E+14	6,95557E+14	7,30949E+14
TC0300001265.mm.2	Ndst4	N-deacetylase/N-sulfotransferase (heparin glucosaminyl) 4	1,23872E+14	5,91322E+14	2,31754E+14	0.0567969687872577	0.196455035017648	-3,96067E+14	7,21789E+14	6,00833E+14	6,15748E+14	5,34686E+14	5,72525E+14	5,02352E+14
TC1700000362.mm.2	Clcn7	chloride channel, voltage-sensitive 7	0.936736789929049	6,78613E+14	2,31747E+14	0.0568026753507128	0.196455035017648	-3,96076E+14	7,37753E+13	7,33714E+14	6,88426E+14	6,27666E+14	6,01171E+14	6,82948E+14
TC0X00003349.mm.2	Egfl6	EGF-like-domain, multiple 6	-0.951083552075346	5,83321E+14	-2,31745E+14	0.0568043263316525	0.196455035017648	-3,96079E+14	5,63055E+13	5,22705E+14	5,7502E+14	5,63172E+14	5,89832E+14	6,86144E+14
TC0500002650.mm.2	Noa1	nitric oxide associated 1	1,19446E+14	6,37189E+14	2,3173E+14	0.0568162640837983	0.196455035017648	-3,96099E+13	7,27218E+14	7,07984E+14	5,61205E+14	6,30418E+14	5,30344E+14	6,65966E+14
TC0200002499.mm.2	Ywhab	tyrosine 3-monooxygenase/tryptophan 5-monooxygenase activation protein, beta polypeptide	1,26531E+14	9,20285E+13	2,31714E+14	0.0568292432109076	0.196457754957596	-3,9612E+14	1,008E+14	1,01203E+14	9,43533E+14	7,91168E+14	9,17034E+13	8,4994E+13
TC1100001799.mm.2	Psmd12	proteasome (prosome, macropain) 26S subunit, non-ATPase, 12	1,62112E+14	8,07917E+14	2,31697E+14	0.0568423699042727	0.196460983755372	-3,96141E+14	9,24667E+13	9,21286E+14	8,37614E+13	6,42201E+14	8,20333E+14	7,01397E+14
TC1400002653.mm.2	Rbm26	RNA binding motif protein 26	2,28265E+14	5,31774E+14	2,31661E+13	0.0568719276347828	0.196520988629478	-3,9619E+13	6,60182E+14	6,51042E+14	5,95268E+14	4,29458E+14	5,74552E+14	2,80142E+14
TC1600000861.mm.2	Usp25	ubiquitin specific peptidase 25	1,13851E+14	7,15015E+14	2,31579E+14	0.0569378268270267	0.196599995327714	-3,96298E+14	8,00612E+14	8,0244E+14	6,95119E+14	6,16569E+14	7,47455E+14	6,27895E+14
TC1000003104.mm.2	Dtx3	deltex 3, E3 ubiquitin ligase	1,31168E+14	7,35699E+14	2,31577E+14	0.0569396906116693	0.196599995327714	-3,96301E+14	8,3048E+14	8,08691E+14	7,85187E+14	6,13004E+14	6,76983E+14	6,99851E+14
TC0700000138.mm.2	Zfp606	zinc finger protein 606	-0.878848069728111	6,98193E+13	-2,31567E+14	0.0569474141509392	0.196599995327714	-3,96314E+14	6,27505E+14	6,44535E+14	7,05752E+13	7,63556E+14	6,9121E+14	7,56599E+14
TC1400002790.mm.2	2610042L04Rik	RIKEN cDNA 2610042L04 gene	1,33103E+14	6,89678E+14	2,31563E+14	0.0569508865404003	0.196599995327714	-3,96319E+14	7,88146E+14	6,88481E+14	7,97188E+14	6,5383E+14	6,23861E+14	5,8656E+14
TC1800000794.mm.2	Acaa2	acetyl-Coenzyme A acyltransferase 2 (mitochondrial 3-oxoacyl-Coenzyme A thiolase)	1,15359E+14	6,53501E+14	2,31557E+14	0.056955798311287	0.196599995327714	-3,96327E+14	7,44799E+14	7,21738E+14	6,53252E+14	5,65397E+14	6,75034E+14	5,60785E+14
TC0100001734.mm.2	Cnih3	cornichon family AMPA receptor auxiliary protein 3	1,18044E+14	7,70334E+14	2,31541E+14	0.0569684888326957	0.196601683564195	-3,96348E+14	8,59393E+14	8,65462E+14	7,59782E+14	6,48603E+14	7,95406E+13	6,9336E+14
TC0500000176.mm.2	Pmpcb	peptidase (mitochondrial processing) beta	0.901541292260561	7,58515E+14	2,31464E+13	0.0570301935409741	0.196772485765678	-3,96449E+14	8,09834E+14	8,05109E+13	7,80506E+14	7,21008E+14	7,53825E+14	6,80809E+14
TC1000002530.mm.2	Nmrk2	nicotinamide riboside kinase 2	-1,00873E+14	5,00312E+14	-2,31436E+14	0.0570533302461626	0.196796590527561	-3,96487E+13	4,2496E+14	4,46817E+14	4,7159E+13	5,84984E+14	5,25817E+14	5,47707E+14
TC1600001395.mm.2	Masp1	mannan-binding lectin serine peptidase 1	0.678992915213679	6,59241E+13	2,31415E+14	0.0570701202522015	0.196796590527561	-3,96514E+14	7,09689E+14	7,26266E+14	6,12244E+14	6,07091E+14	6,56262E+14	6,43894E+14
TC0400001782.mm.2	Cplane2	ciliogenesis and planar polarity effector 2	-0.838935249908833	6,39527E+14	-2,3141E+14	0.0570738203646307	0.196796590527561	-3,9652E+14	5,65457E+14	6,01173E+14	6,32783E+14	7,03332E+14	6,64341E+13	6,70077E+14
TC1300001509.mm.2	Yae1d1	Yae1 domain containing 1	0.706625646812252	7,3767E+14	2,31337E+14	0.0571332966639952	0.196959522710089	-3,96617E+14	7,93006E+14	7,75509E+13	7,19106E+14	7,11208E+14	6,97678E+14	7,29512E+14
TC0200002741.mm.2	Gm14295	predicted gene 14295	1,4012E+13	7,14651E+13	2,31317E+14	0.0571497488163491	0.196974096829483	-3,96644E+14	8,12759E+13	8,82413E+14	6,71341E+14	5,06459E+13	7,41146E+14	6,73786E+14
TC1200001593.mm.2	Dld	dihydrolipoamide dehydrogenase	2,21755E+14	7,86343E+14	2,31281E+14	0.0571786146667902	0.197031441002137	-3,96691E+14	9,25742E+13	9,19019E+14	8,56925E+14	6,15119E+14	8,11423E+14	5,89828E+14
TC1000000845.mm.2	Efna2	ephrin A2	-0.756091352269649	7,02559E+14	-2,31259E+14	0.0571965204641628	0.197051001547799	-3,9672E+14	6,34134E+13	6,58884E+14	7,63667E+14	7,14436E+14	7,26961E+13	7,17274E+14
TC0700001808.mm.2	Apobr	apolipoprotein B receptor	-1,36279E+14	7,39389E+14	-2,31237E+14	0.0572141500295365	0.197069602271467	-3,96749E+14	6,59862E+14	6,71283E+14	6,88431E+13	8,13058E+14	7,34045E+14	8,69658E+14
TC0300003106.mm.2	Prkacb	protein kinase, cAMP dependent, catalytic, beta	1,46253E+14	9,61304E+14	2,31203E+14	0.0572417633989314	0.197122576116047	-3,96794E+13	1,06398E+13	1,04956E+14	1,01235E+13	8,20901E+14	9,4208E+14	8,7895E+14
TC0900001239.mm.2	Prr23a3	proline rich 23A, member 3	-1,13454E+14	5,13419E+14	-2,3116E+14	0.0572769168566202	0.197189303106308	-3,96851E+14	4,34228E+14	4,52249E+14	4,8854E+14	5,92112E+14	5,08444E+14	6,04942E+14

TC0400001541.mm.2	Pef1	penta-EF hand domain containing 1	1,29524E+14	6,82874E+14	2,31145E+14	0.0572887203339875	0.197189303106308	-3,9687E+14	7,84771E+14	7,66734E+13	7,04507E+14	5,48771E+14	6,10216E+14	6,82243E+14
TC0700004142.mm.2	Calca	calcitonin/calcitonin-related polypeptide, alpha	-1,12433E+13	4,91578E+14	-2,31125E+14	0.0573053877407251	0.197189303106308	-3,96898E+14	4,55565E+14	3,90756E+14	4,62111E+14	5,6985E+13	5,03932E+14	5,67254E+14
TC1900000497.mm.2	Fas	Fas (TNF receptor superfamily member 6)	-0.780139627286419	5,26162E+14	-2,31108E+14	0.0573185491844261	0.197189303106308	-3,96919E+14	4,85151E+14	4,6515E+14	5,20762E+14	5,79579E+14	5,59838E+14	5,46491E+14
TC1200001262.mm.2	Adssl1	adenylosuccinate synthetase like 1	0.757862745886559	6,23406E+14	2,31095E+14	0.0573292091736328	0.197189303106308	-3,96936E+14	6,61541E+14	6,62119E+14	5,94584E+14	6,50108E+14	5,80323E+14	5,91764E+14
TC0200002181.mm.2	6430503K07Rik	RIKEN cDNA 6430503K07 gene	-0.804684434111807	8,40636E+14	-2,31089E+14	0.057334567433318	0.197189303106308	-3,96945E+14	7,79226E+14	7,96404E+14	8,32083E+14	8,99539E+14	8,62549E+14	8,74018E+14
TC0500002744.mm.2	Cdkl2	cyclin-dependent kinase-like 2 (CDC2-related kinase)	0.671394296826582	5,79828E+14	2,31056E+14	0.0573611701019752	0.197200706402899	-3,96988E+14	6,27648E+14	6,30105E+14	5,508E+14	5,46937E+14	5,54311E+14	5,69164E+14
TC0900001513.mm.2	Tmppe/Glb1	transmembrane protein with metallophosphoesterase domain/galactosidase, beta 1	0.773501530207118	6,53106E+14	2,31043E+14	0.0573715107673615	0.197200706402899	-3,97005E+14	7,23942E+14	6,95529E+14	5,98747E+14	6,35644E+14	6,52565E+14	6,12207E+14
TC0700002883.mm.2	Gm5591	predicted gene 5591	-0.754012643251897	5,23253E+14	-2,31026E+14	0.057385580743135	0.197200706402899	-3,97028E+13	4,74756E+12	5,16739E+14	4,90707E+14	5,15017E+13	5,84646E+14	5,57652E+14
TC0400001688.mm.2	Luzp1	leucine zipper protein 1	0.881868938549174	6,00717E+14	2,31024E+14	0.0573868374804935	0.197200706402899	-3,9703E+14	6,34949E+14	6,5347E+14	6,05587E+13	5,98253E+13	5,99971E+14	5,12074E+14
TC1100001010.mm.2	Atp2a3	ATPase, Ca++ transporting, ubiquitous	-0.741687632200613	6,67255E+14	-2,30933E+14	0.0574607357341376	0.19741254475142	-3,9715E+14	6,16528E+14	6,20897E+14	6,71795E+13	7,08548E+14	6,75002E+14	7,10761E+14
TC0400001487.mm.2	Smim12	small integral membrane protein 12	-0.707952130601984	6,91634E+13	-2,30863E+14	0.057517720005627	0.197566195363604	-3,97242E+14	6,43773E+14	6,56395E+14	6,88943E+14	7,18937E+14	7,10662E+14	7,31096E+14
TC1200001259.mm.2	A530016L24Rik	RIKEN cDNA A530016L24 gene	-0.830028193535854	6,0292E+14	-2,30822E+14	0.0575513790449345	0.197639678293058	-3,97297E+14	5,64814E+14	5,33846E+14	5,92294E+14	6,61897E+14	6,25095E+14	6,39571E+14
TC0500000390.mm.2	Dok7	docking protein 7	-0.759559773123502	7,22668E+14	-2,30776E+14	0.0575893997800342	0.197725768058434	-3,97358E+14	6,78124E+14	6,72559E+14	7,24324E+14	7,58404E+14	7,28264E+14	7,74331E+14
TC1800001012.mm.2	Kctd1	potassium channel tetramerisation domain containing 1	0.924914239056963	6,98562E+14	2,30756E+14	0.0576050367396211	0.197725768058434	-3,97384E+14	7,50556E+14	7,66264E+14	6,93013E+14	6,497E+14	7,10421E+14	6,21416E+14
TC0800002618.mm.2	Neto2	neuropilin (NRP) and tolloid (TLL)-like 2	1,9421E+14	8,32549E+14	2,30746E+14	0.0576132614059672	0.197725768058434	-3,97397E+13	9,46939E+14	9,53052E+14	8,60463E+14	7,23269E+14	8,79914E+14	6,31657E+14
TC1700000696.mm.2	Sfta2	surfactant associated 2	-0.795252718744204	5,54442E+13	-2,30694E+14	0.0576563768807765	0.197826530209387	-3,97467E+14	5,35406E+14	4,72583E+14	5,63466E+14	5,88158E+14	5,78291E+12	5,88748E+14
TC0900001252.mm.2	Dbr1	debranching RNA lariats 1	0.760264757721709	5,96747E+13	2,30654E+14	0.0576883961108964	0.197826530209387	-3,97518E+14	6,16476E+14	6,55895E+13	5,86117E+14	6,01676E+14	5,40415E+14	5,79903E+14
TC1000001079.mm.2	Gnptab	N-acetylglucosamine-1-phosphate transferase, alpha and beta subunits	0.748235355332142	7,88203E+14	2,30649E+14	0.0576927310532873	0.197826530209387	-3,97525E+14	8,28601E+13	8,19023E+14	8,02207E+14	7,81409E+14	7,73447E+14	7,2453E+14
TC0200000801.mm.2	Kif5c	kinesin family member 5C	1,6677E+12	9,35824E+14	2,30646E+14	0.0576955778896435	0.197826530209387	-3,9753E+14	1,05875E+14	1,05074E+14	9,6784E+14	7,61664E+14	9,47241E+14	8,28708E+14
TC1500002196.mm.2	Dhh	desert hedgehog	-1,19224E+13	6,00909E+14	-2,30635E+14	0.0577040086876509	0.197826530209387	-3,97544E+14	5,39369E+14	5,19726E+14	5,64921E+14	6,83897E+14	6,00788E+14	6,96756E+13
TC0200002536.mm.2	Zswim1	zinc finger SWIM-type containing 1	1,22205E+14	6,63752E+14	2,30587E+14	0.0577433417662173	0.197899023823764	-3,97607E+13	7,44062E+14	7,41368E+14	6,98073E+14	5,57989E+14	6,58835E+14	5,82184E+14
TC1500002075.mm.2	Abcd2	ATP-binding cassette, sub-family D (ALD), member 2	0.764241158104161	7,16233E+14	2,30579E+14	0.0577497182411306	0.197899023823764	-3,97617E+13	7,51461E+14	7,8246E+14	6,92888E+14	6,89517E+14	7,15729E+14	6,65344E+14
TC0600000718.mm.2	Tigd2	tigger transposable element derived 2	0.842767956787615	6,702E+14	2,30438E+14	0.0578656587485162	0.198254169554506	-3,97804E+14	7,24161E+14	7,27083E+14	6,83721E+14	6,03544E+14	6,47426E+14	6,35264E+14
TC0300000722.mm.2	Glmp	glycosylated lysosomal membrane protein	1,15384E+13	7,42018E+14	2,30395E+14	0.057900998697479	0.198333076533265	-3,97861E+14	7,92948E+14	8,09697E+14	8,01773E+14	6,75811E+14	6,48882E+14	7,22995E+14
TC0200001674.mm.2	Emc7	ER membrane protein complex subunit 7	1,43038E+14	8,36853E+13	2,30369E+14	0.0579223776320441	0.198335241828138	-3,97895E+14	9,37869E+14	9,42648E+14	8,41291E+14	7,04869E+14	8,68517E+14	7,25925E+14
TC1000000713.mm.2	Upb1	ureidopropionase, beta	-1,0589E+14	6,53772E+14	-2,30364E+14	0.0579262488700562	0.198335241828138	-3,97902E+14	5,91784E+14	5,6595E+13	6,473E+14	7,48083E+14	6,46901E+14	7,22612E+14
TC1100002374.mm.2	Rel	reticuloendotheliosis oncogene	1,15232E+14	5,39527E+14	2,30332E+13	0.0579522257979096	0.198367754538781	-3,97943E+14	6,30341E+14	6,08684E+14	5,41731E+14	4,47845E+14	5,59708E+14	4,48854E+14
TC0600002681.mm.2	Eefsec	eukaryotic elongation factor, selenocysteine-tRNA-specific	0.766801214300291	7,06295E+14	2,30304E+14	0.057975711667866	0.198367754538781	-3,97981E+13	7,39556E+14	7,40997E+14	7,24373E+14	7,05649E+13	6,92263E+14	6,3493E+13
TC1300001366.mm.2	BC147527	cDNA sequence BC147527	-1,02184E+14	5,01375E+14	-2,30281E+14	0.0579941842910786	0.198367754538781	-3,98011E+14	4,15555E+13	4,61825E+14	4,70394E+14	5,78728E+14	5,5527E+14	5,26477E+14
TC1600000301.mm.2	Vps8	VPS8 CORVET complex subunit	0.765160943903483	7,01595E+14	2,30279E+14	0.0579961621017161	0.198367754538781	-3,98014E+14	7,67917E+14	7,59535E+14	6,74302E+14	6,33398E+14	7,08649E+14	6,65771E+14
TC1000002305.mm.2	Reep3	receptor accessory protein 3	0.815376603483124	7,29289E+14	2,30276E+14	0.05799874519954	0.198367754538781	-3,98018E+13	7,75915E+14	7,69928E+14	7,57106E+14	6,90014E+14	7,06958E+14	6,7581E+14
TC0700000038.mm.2	Brsk1	BR serine/threonine kinase 1	0.973085854220667	8,8505E+14	2,30247E+14	0.0580221341465996	0.198367754538781	-3,98056E+14	9,40975E+14	9,65871E+14	8,95578E+14	7,95643E+14	8,77015E+14	8,35214E+14
TC0700000435.mm.2	Cyp2b9	cytochrome P450, family 2, subfamily b, polypeptide 9	-1,06996E+14	4,92869E+14	-2,30237E+14	0.058030937557467	0.198367754538781	-3,9807E+14	4,04747E+14	3,94378E+14	5,14275E+14	6,30692E+14	4,76986E+14	5,36132E+14
TC0900002681.mm.2	Scg3	secretogranin III	1,64067E+14	7,71086E+14	2,30233E+14	0.058034232911054	0.198367754538781	-3,98075E+14	8,81306E+14	8,89722E+14	8,21559E+14	5,91036E+14	7,40705E+14	7,02189E+14
TC0600001696.mm.2	Etnk1	ethanolamine kinase 1	1,10379E+14	7,464E+14	2,30217E+14	0.058046658865468	0.198368147253295	-3,98095E+14	8,24407E+14	8,1782E+14	7,6701E+14	6,47698E+14	7,44898E+14	6,7657E+14
TC0900000351.mm.2	Fez1	fasciculation and elongation protein zeta 1 (zygin I)	0.684165592025137	8,33161E+14	2,30164E+14	0.0580905229708561	0.198475953483758	-3,98165E+14	8,63831E+14	8,59835E+14	8,52661E+14	8,35805E+14	7,82856E+14	8,03977E+14
TC0200004667.mm.2	Slc23a2	solute carrier family 23 (nucleobase transporters), member 2	1,37725E+14	7,70564E+14	2,30119E+14	0.0581280259607529	0.19856198480085	-3,98226E+14	8,70316E+14	8,72194E+14	7,61864E+14	6,51953E+14	8,09961E+13	6,57098E+14
TC0900001242.mm.2	Foxl2	forkhead box L2	-1,2118E+14	5,31348E+14	-2,3008E+14	0.0581602075319785	0.19861048283243	-3,98277E+14	4,35002E+14	4,62621E+14	5,02608E+14	6,47875E+14	5,41732E+14	5,98252E+14
TC0200003980.mm.2	Pramel7	preferentially expressed antigen in melanoma like 7	-0.960470525020316	4,68496E+14	-2,30071E+13	0.0581668757206129	0.19861048283243	-3,98288E+14	4,29567E+14	3,84613E+14	4,6012E+14	5,30403E+14	4,68366E+14	5,37908E+14
TC0600003119.mm.2	Tpi1	triosephosphate isomerase 1	1,18999E+14	9,75779E+14	2,30014E+14	0.0582145862327117	0.198720394244256	-3,98364E+14	1,0734E+13	1,07301E+14	9,69108E+14	8,30731E+14	9,88334E+14	9,20084E+14
TC1300002243.mm.2	Zfp458	zinc finger protein 458	1,13092E+14	5,55418E+14	2,30002E+14	0.0582237312249199	0.198720394244256	-3,98379E+14	6,03431E+14	6,37931E+14	5,24373E+14	5,51596E+14	5,7568E+14	4,39498E+14
TC1100000234.mm.2	Cct4	chaperonin containing Tcp1, subunit 4 (delta)	0.847396225707532	8,1575E+13	2,29956E+14	0.0582620885303098	0.198794574654732	-3,9844E+14	8,62932E+14	8,91744E+14	7,70381E+14	7,84243E+14	8,30759E+14	7,54438E+14
TC0300002711.mm.2	Slc6a17	solute carrier family 6 (neurotransmitter transporter), member 17	2,47593E+14	8,73106E+14	2,29946E+14	0.0582701406376403	0.198794574654732	-3,98453E+13	1,03542E+14	1,06556E+14	9,25017E+14	6,06862E+14	8,98237E+14	7,07549E+14
TC0900001099.mm.2	Sh3bgrl2	SH3 domain binding glutamic acid-rich protein like 2	-1,14987E+14	7,46991E+14	-2,29885E+14	0.0583205851085724	0.198860651815856	-3,98534E+14	6,84305E+13	6,84986E+14	6,8625E+13	8,2714E+14	7,78956E+14	8,2031E+14
TC1000001332.mm.2	A930009A15Rik	RIKEN cDNA A930009A15 gene	-1,14983E+14	5,80155E+14	-2,29854E+14	0.0583460459623953	0.198860651815856	-3,98574E+14	5,35455E+13	4,52081E+14	5,81295E+14	6,94595E+14	5,87663E+14	6,2984E+14
TC1000000762.mm.2	Tspear	thrombospondin type laminin G domain and EAR repeats	-0.983203323889227	6,29993E+14	-2,29843E+14	0.05835585917621	0.198860651815856	-3,9859E+14	5,60905E+14	5,39656E+14	6,36069E+14	7,46124E+14	6,392E+14	6,58002E+14
TC0200002421.mm.2	Rprd1b	regulation of nuclear pre-mRNA domain containing 1B	1,84182E+14	7,19944E+14	2,29829E+14	0.0583671495638453	0.198860651815856	-3,98608E+14	8,50419E+14	8,29565E+14	7,84763E+14	5,433E+14	7,13345E+13	5,98275E+14
TC0800000564.mm.2	Dctd	dCMP deaminase	1,21456E+14	7,17921E+14	2,29821E+14	0.0583734914553078	0.198860651815856	-3,98618E+14	8,22517E+14	8,09916E+14	7,17882E+14	5,67694E+14	6,69324E+14	7,20196E+13
TC1700001634.mm.2	Slc9a3r2	solute carrier family 9 (sodium/hydrogen exchanger), member 3 regulator 2	0.758659985862209	7,01848E+14	2,29819E+14	0.0583756382926352	0.198860651815856	-3,98621E+14	7,32378E+14	7,5175E+14	7,10039E+13	6,84523E+14	6,4185E+14	6,90546E+13
TC1100001459.mm.2	Fam117a	family with sequence similarity 117, member A	-0.877503795581411	7,02333E+14	-2,29814E+14	0.0583792254513906	0.198860651815856	-3,98627E+14	6,43282E+14	6,49391E+14	6,86304E+14	7,66846E+14	7,1403E+14	7,54143E+14
TC0600002539.mm.2	Reg3g	regenerating islet-derived 3 gamma	-1,16678E+14	4,63161E+14	-2,29795E+14	0.0583953852916935	0.198860651815856	-3,98653E+14	4,05294E+14	3,64546E+14	4,79658E+14	5,26272E+14	4,33212E+14	5,69984E+14
TC1400001327.mm.2	Tm9sf2	transmembrane 9 superfamily member 2	1,19172E+14	7,44575E+14	2,29789E+14	0.0584005836524937	0.198860651815856	-3,98661E+14	8,26758E+14	8,34753E+14	7,68032E+14	6,14737E+14	7,21719E+14	7,01448E+14
TC0200001866.mm.2	Sema6d	sema domain, transmembrane domain (TM), and cytoplasmic domain, (semaphorin) 6D	1,30453E+14	8,21481E+14	2,29769E+14	0.0584163371953139	0.198870983797477	-3,98686E+14	9,09186E+13	9,08319E+14	8,61608E+14	6,93173E+14	8,03145E+13	7,53455E+14
TC1300000532.mm.2	Rbm24	RNA binding motif protein 24	-0.745298435637533	6,9537E+14	-2,29755E+14	0.0584283024450603	0.198870983797477	-3,98705E+14	6,56516E+14	6,28635E+14	7,45809E+14	7,07052E+14	7,29719E+14	7,04492E+14
TC1700001516.mm.2	Lnpep	leucyl/cystinyl aminopeptidase	1,77013E+14	7,05384E+14	2,29729E+14	0.0584500366800255	0.198902944250317	-3,9874E+13	8,25628E+14	8,32809E+14	7,04017E+13	5,65437E+14	7,60009E+14	5,44401E+14
TC0300002445.mm.2	Snx27	sorting nexin family member 27	1,59583E+14	8,2823E+14	2,29712E+14	0.0584639308057248	0.198907139485835	-3,98762E+14	9,38411E+14	9,48719E+14	8,6405E+14	6,50238E+14	8,21505E+14	7,46459E+14
TC0900003251.mm.2	Xirp1	xin actin-binding repeat containing 1	-1,25227E+14	6,85682E+14	-2,29696E+14	0.0584773396588307	0.198907139485835	-3,98784E+14	5,8493E+14	6,04443E+14	6,70855E+14	8,14034E+14	6,789E+14	7,60928E+14
TC0100002618.mm.2	Epha4	Eph receptor A4	1,44455E+14	7,43138E+14	2,29683E+14	0.0584883030400228	0.198907139485835	-3,98801E+13	8,42613E+14	8,47536E+13	7,82166E+14	5,85272E+14	7,19756E+14	6,81482E+14

TC0200003112.mm.2	Pax8	paired box 8	-0.72895645471517	6,21818E+13	-2,29624E+14	0.0585369580277919	0.199030598164552	-3,98878E+14	5,84313E+14	5,73393E+14	6,21466E+14	6,48241E+14	6,32335E+14	6,71162E+14
TC0800002665.mm.2	Sall1	spalt like transcription factor 1	-0.698357654891159	6,69718E+14	-2,29589E+14	0.0585662699680991	0.199088250632063	-3,98925E+14	6,33522E+14	6,26536E+14	6,96804E+14	6,61715E+14	6,99181E+14	7,00548E+14
TC1600000142.mm.2	Gm9961	predicted gene 9961	-0.676743327826232	7,16276E+14	-2,29565E+14	0.0585858446257418	0.199111173577949	-3,98956E+14	6,79592E+14	6,73781E+14	7,1754E+14	7,38023E+14	7,39219E+14	7,49502E+14
TC0200000850.mm.2	Galnt13	polypeptide N-acetylgalactosaminyltransferase 13	2,03737E+14	7,35544E+14	2,29541E+14	0.0586062104742391	0.199111173577949	-3,98989E+14	8,75846E+14	8,6558E+14	7,7321E+14	5,64678E+14	7,75505E+14	5,58447E+14
TC0X00000074.mm.2	Pim2	proviral integration site 2	0.792357955257463	7,6779E+14	2,29536E+14	0.058610084793658	0.199111173577949	-3,98995E+14	8,15948E+14	8,08421E+14	7,49202E+14	7,67275E+14	7,00067E+14	7,6583E+14
TC1100001443.mm.2	Mrpl27	mitochondrial ribosomal protein L27	0.740907547181253	8,25293E+14	2,29502E+14	0.058638344908509	0.199165187923863	-3,9904E+14	9,10199E+14	8,71209E+14	7,57412E+13	7,79713E+14	8,0275E+14	8,30476E+14
TC0300000118.mm.2	Bhlhe22	basic helix-loop-helix family, member e22	-0.939325587134973	6,63607E+14	-2,29453E+14	0.0586792424121686	0.199224831762448	-3,99105E+14	5,96726E+14	5,96185E+14	6,51846E+13	7,56108E+14	6,8634E+14	6,94436E+14
TC1600001294.mm.2	Slc7a4	solute carrier family 7 (cationic amino acid transporter, y+ system), member 4	0.689728821140091	7,58147E+14	2,29446E+14	0.0586847631517727	0.199224831762448	-3,99113E+14	7,98236E+13	8,05396E+14	7,31121E+14	7,48442E+14	7,20067E+13	7,45619E+14
TC1800000945.mm.2	Kif5b	kinesin family member 5B	2,05147E+14	8,1954E+14	2,29436E+13	0.0586931360366783	0.199224831762448	-3,99127E+14	9,79867E+14	9,67949E+14	8,42313E+14	5,89587E+14	8,52716E+14	6,84806E+14
TC0900001905.mm.2	Zfp810	zinc finger protein 810	0.844724748956035	6,10262E+14	2,2941E+14	0.0587146743719167	0.199224831762448	-3,99161E+14	6,83519E+14	6,44655E+14	5,9081E+14	5,83358E+13	6,15857E+14	5,43372E+14
TC1600001057.mm.2	Ttc3	tetratricopeptide repeat domain 3	1,74504E+14	9,58895E+14	2,29396E+12	0.0587261312344326	0.199224831762448	-3,99179E+14	1,06621E+14	1,05417E+14	1,00568E+14	8,55942E+14	9,87223E+14	7,84144E+14
TC0500001353.mm.2	Rpl6	ribosomal protein L6	1,02338E+14	1,0038E+12	2,29392E+14	0.058730090664161	0.199224831762448	-3,99185E+13	1,07445E+14	1,07072E+14	1,01527E+14	9,21848E+13	1,00974E+14	9,30755E+14
TC1500000408.mm.2	Fam83a	family with sequence similarity 83, member A	-0.923647026568467	5,17361E+14	-2,29332E+14	0.0587797524277229	0.199258950032956	-3,99264E+14	4,475E+14	4,82192E+14	4,8377E+14	5,76284E+14	5,46228E+14	5,68193E+14
TC0800001200.mm.2	Ces3a	carboxylesterase 3A	-0.98108159189192	4,70198E+14	-2,29327E+14	0.0587837199572944	0.199258950032956	-3,9927E+14	3,70677E+14	4,708E+14	4,51005E+14	4,91017E+14	5,00849E+13	5,36843E+14
TC0300000244.mm.2	Sox2	SRY (sex determining region Y)-box 2	1,59513E+14	7,79497E+14	2,29309E+13	0.0587985485313549	0.199258950032956	-3,99294E+13	8,8417E+14	9,00825E+13	8,10739E+14	6,1528E+14	7,90291E+14	6,75678E+14
TC0900002737.mm.2	Cox7a2	cytochrome c oxidase subunit 7A2	0.674734356946926	9,10235E+14	2,29306E+14	0.0588013455365509	0.199258950032956	-3,99298E+14	9,52545E+12	9,56599E+14	8,85996E+14	8,92074E+13	8,97875E+14	8,76322E+14
TC1300001135.mm.2	Serf1	small EDRK-rich factor 1	-0.855281688811769	6,65564E+14	-2,29262E+14	0.0588382488408016	0.199258950032956	-3,99356E+14	6,14494E+14	5,73028E+14	7,37704E+14	7,09578E+14	7,13826E+14	6,44752E+14
TC0900000748.mm.2	Hexa	hexosaminidase A	0.786886111188309	7,76561E+14	2,2926E+14	0.0588398618371167	0.199258950032956	-3,99359E+14	8,13145E+14	8,52263E+14	7,55498E+14	7,30429E+14	7,78382E+14	7,29649E+14
TC0700001790.mm.2	4930571K23Rik	RIKEN cDNA 4930571K23 gene	-1,3307E+14	4,468E+14	-2,29258E+14	0.0588415930795642	0.199258950032956	-3,99362E+14	3,70689E+14	3,6857E+14	3,90176E+14	5,45964E+14	4,56767E+14	5,48632E+14
TC1700002774.mm.2	Cmtr1	cap methyltransferase 1	0.853775097647911	7,35313E+14	2,29256E+13	0.0588432173977096	0.199258950032956	-3,99364E+14	7,97624E+14	7,76363E+14	7,31969E+14	7,0269E+13	7,40055E+14	6,63177E+14
TC1000002524.mm.2	Sgta	small glutamine-rich tetratricopeptide repeat (TPR)-containing, alpha	0.668620224381568	9,10462E+14	2,29246E+14	0.0588514456157659	0.199258950032956	-3,99377E+14	9,45505E+14	9,41652E+13	9,02652E+14	9,19526E+13	8,74311E+13	8,79123E+14
TC1300001922.mm.2	Kif13a	kinesin family member 13A	0.750014896894007	5,93459E+14	2,29227E+14	0.0588675351382935	0.199271553294816	-3,99403E+14	6,54907E+14	6,37783E+14	5,8928E+14	5,361E+14	5,74062E+14	5,68625E+14
TC1800001319.mm.2	Dtwd2	DTW domain containing 2	-1,37734E+14	6,14191E+14	-2,29196E+14	0.0588935241506393	0.199281102107192	-3,99444E+14	5,47633E+14	5,49607E+14	7,02022E+14	5,13173E+14	6,92705E+14	6,80003E+14
TC0100003371.mm.2	Dars2	aspartyl-tRNA synthetase 2 (mitochondrial)	0.881353997660047	5,86001E+12	2,29194E+14	0.0588950914314187	0.199281102107192	-3,99446E+14	6,39448E+12	6,34375E+14	5,69165E+14	5,75469E+14	5,03127E+14	5,94425E+14
TC0100002188.mm.2	Fam178b	family with sequence similarity 178, member B	-0.995567017807659	5,76112E+14	-2,29146E+14	0.0589349392066415	0.199330213233943	-3,99509E+14	5,42649E+14	4,63437E+14	5,7973E+14	6,65656E+14	6,03263E+14	6,01937E+14
TC0600003268.mm.2	Ybx3	Y box protein 3	-0.949064670617477	8,31255E+14	-2,29141E+14	0.0589394865224392	0.199330213233943	-3,99517E+14	7,48683E+13	7,8654E+14	9,08913E+14	8,1836E+14	8,83805E+14	8,4123E+14
TC1300001201.mm.2	Smim15	small integral membrane protein 15	1,84316E+14	6,08176E+14	2,29132E+14	0.0589467179333295	0.199330213233943	-3,99528E+14	7,41071E+14	7,68286E+14	6,163E+14	3,82673E+14	6,36094E+14	5,04632E+14
TC0700001410.mm.2	Coa4	cytochrome c oxidase assembly factor 4	-1,25003E+14	7,04085E+14	-2,29028E+14	0.0590339921674049	0.199583448550859	-3,99666E+14	5,99683E+14	6,21843E+14	7,00219E+14	8,3123E+14	6,85664E+14	7,85869E+14
TC0X00000577.mm.2	4931400O07Rik	RIKEN cDNA 4931400O07 gene	-0.768591615396599	5,26253E+14	-2,28977E+14	0.0590759866452535	0.199605836156805	-3,99732E+14	4,57911E+14	4,98212E+14	5,39598E+14	5,51954E+14	5,32029E+14	5,77812E+14
TC0400000506.mm.2	Anp32b	acidic (leucine-rich) nuclear phosphoprotein 32 family, member B	0.886353990014306	8,1503E+14	2,28977E+14	0.0590760491547179	0.199605836156805	-3,99732E+13	8,69105E+14	8,75834E+14	8,35019E+14	7,42552E+14	7,80012E+14	7,87656E+14
TC0100001158.mm.2	Yod1	YOD1 deubiquitinase	-0.895702688636816	6,66227E+14	-2,2897E+14	0.0590822106529904	0.199605836156805	-3,99742E+14	5,79991E+14	6,14465E+14	7,13238E+14	7,1607E+14	7,31147E+14	6,4245E+14
TC1700001989.mm.2	Abcf1	ATP-binding cassette, sub-family F (GCN20), member 1	0.794595797151627	7,07966E+14	2,2896E+14	0.0590901656096295	0.199605836156805	-3,99754E+14	7,52776E+14	7,62852E+14	6,89161E+14	6,86298E+14	6,43849E+14	7,1286E+14
TC0500000305.mm.2	Emilin1	elastin microfibril interfacer 1	-0.969769331278192	5,81068E+14	-2,28941E+14	0.0591066003621633	0.199619503591603	-3,9978E+14	4,85272E+14	5,57387E+14	5,80472E+13	6,26663E+14	5,82513E+14	6,54101E+14
TC0500000483.mm.2	4930431F12Rik	RIKEN cDNA 4930431F12 gene	-0.757579877009434	6,8208E+14	-2,28905E+14	0.0591363756215832	0.199678210475811	-3,99827E+12	6,2265E+14	6,30742E+14	6,88684E+13	7,45497E+14	6,95815E+14	7,09093E+14
TC0300002725.mm.2	Gstm1	glutathione S-transferase, mu 1	1,51256E+14	9,70004E+14	2,28838E+14	0.0591924600924581	0.199825709086482	-3,99915E+14	1,06814E+14	1,07212E+14	1,02377E+14	8,1825E+14	9,47678E+14	8,9007E+14
TC1900001709.mm.2	Ccdc186	coiled-coil domain containing 186	1,31478E+14	7,56568E+14	2,28787E+14	0.0592357149303837	0.199837411218162	-3,99983E+13	8,27905E+14	8,488E+13	7,67544E+14	6,81407E+14	7,84335E+14	6,29414E+14
TC1000000399.mm.2	Bend3	BEN domain containing 3	-0.828586396622295	6,60512E+14	-2,28771E+14	0.0592492594701909	0.199837411218162	-4,00005E+14	6,06264E+14	6,06197E+13	6,47077E+14	7,25355E+14	6,83465E+14	6,94713E+13
TC1800001746.mm.2	Hdhd2	haloacid dehalogenase-like hydrolase domain containing 2	1,19183E+14	8,17976E+14	2,28752E+14	0.0592649630953687	0.199837411218162	-4,00029E+14	9,18887E+14	8,81136E+14	8,32193E+14	7,13983E+14	8,27989E+14	7,33669E+14
TC0400002110.mm.2	Noc2l	NOC2 like nucleolar associated transcriptional repressor	1,17625E+14	6,33275E+14	2,28747E+14	0.0592692770345356	0.199837411218162	-4,00036E+14	6,8339E+14	7,43921E+14	6,40394E+14	5,39886E+14	6,45082E+14	5,46979E+14
TC0200000083.mm.2	Itih5	inter-alpha (globulin) inhibitor H5	0.809983978779857	6,4035E+14	2,28745E+14	0.0592705903364301	0.199837411218162	-4,00038E+14	6,88889E+14	6,93754E+14	5,83091E+14	6,55719E+14	6,17568E+14	6,03078E+14
TC1400000277.mm.2	Tasor	transcription activation suppressor	2,28019E+14	6,74613E+14	2,28714E+14	0.0592967559122519	0.199837411218162	-4,00079E+14	8,18434E+14	8,14545E+14	7,19781E+14	5,17979E+14	7,26742E+14	4,50199E+14
TC1300002661.mm.2	Gpbp1	GC-rich promoter binding protein 1	2,26043E+12	7,49582E+14	2,28709E+14	0.0593008992043818	0.199837411218162	-4,00086E+14	9,16999E+14	8,98945E+14	7,97492E+13	5,20198E+14	7,79136E+14	5,84721E+14
TC0700004337.mm.2	Bcl7c	B cell CLL/lymphoma 7C	-0.670319436908929	6,84736E+14	-2,28708E+14	0.0593017496835461	0.199837411218162	-4,00087E+14	6,43084E+14	6,53233E+14	6,92958E+14	6,88757E+13	7,06394E+14	7,23987E+14
TC0600003532.mm.2	Arpc4	actin related protein 2/3 complex, subunit 4	1,08303E+14	8,80026E+14	2,28685E+14	0.0593216381418399	0.199837411218162	-4,00118E+14	9,53297E+14	9,50585E+14	8,99082E+14	7,89914E+14	8,83336E+14	8,03939E+14
TC0600000527.mm.2	Cul1	cullin 1	2,45044E+13	6,17291E+14	2,28676E+14	0.05932884881218	0.199837411218162	-4,0013E+14	7,88823E+14	7,76495E+14	6,87142E+13	3,76055E+14	6,37789E+14	4,37441E+14
TC1900000111.mm.2	Ehd1	EH-domain containing 1	0.986162756158729	6,1808E+14	2,28667E+14	0.0593361379860544	0.199837411218162	-4,00141E+14	7,02252E+14	6,99326E+14	6,08826E+13	4,93733E+14	5,98715E+14	6,05631E+14
TC1300000894.mm.2	Slc9a3	solute carrier family 9 (sodium/hydrogen exchanger), member 3	-0.825829629367067	6,3375E+13	-2,2862E+14	0.0593758397672271	0.199837411218162	-4,00203E+14	5,57855E+14	5,83353E+13	6,456E+14	7,09316E+14	6,34121E+14	6,72253E+13
TC1700001096.mm.2	Lama1	laminin, alpha 1	-0.662803386507178	5,17393E+14	-2,28612E+14	0.0593831889887469	0.199837411218162	-4,00215E+14	4,85285E+13	4,71785E+14	5,28215E+14	5,29443E+14	5,43297E+14	5,46334E+14
TC0700003657.mm.2	Fah	fumarylacetoacetate hydrolase	0.863601012450865	7,02708E+14	2,28606E+14	0.0593877639932237	0.199837411218162	-4,00222E+14	7,51813E+14	7,40453E+14	6,59209E+14	7,45227E+14	6,52559E+14	6,66987E+13
TC0300000783.mm.2	Rab13	RAB13, member RAS oncogene family	0.919328100625243	6,11858E+14	2,28587E+14	0.0594036168293081	0.199837411218162	-4,00247E+14	7,04146E+13	6,1169E+14	5,93716E+13	6,29626E+14	5,75303E+14	5,56667E+13
TC0800000366.mm.2	Dusp26	dual specificity phosphatase 26 (putative)	0.777487863741661	8,18985E+14	2,2858E+14	0.0594095469196071	0.199837411218162	-4,00256E+14	8,83861E+14	8,46556E+14	8,31075E+13	7,775E+14	7,87518E+14	7,87401E+14
TC1000000035.mm.2	Myct1	myc target 1	-1,02987E+14	5,51268E+14	-2,28574E+13	0.0594143947179321	0.199837411218162	-4,00264E+14	4,91555E+14	4,96872E+14	5,05756E+13	6,19021E+14	5,71537E+14	6,22865E+14
TC0800002465.mm.2	Slc35e1	solute carrier family 35, member E1	0.779978851074703	7,58385E+14	2,28555E+14	0.0594311736262293	0.199837411218162	-4,0029E+14	8,06222E+14	8,01982E+14	7,71508E+14	7,18391E+14	7,06602E+13	7,45607E+14
TC0600001949.mm.2	Tmem229a	transmembrane protein 229A	0.875801922341207	6,57642E+14	2,28551E+14	0.0594345433919325	0.199837411218162	-4,00295E+14	6,92117E+14	7,10073E+14	6,61095E+14	6,55537E+14	6,57997E+14	5,69033E+13
TC0900000695.mm.2	Sin3a	transcriptional regulator, SIN3A (yeast)	1,17241E+13	7,6287E+13	2,28475E+14	0.0594986220777131	0.199837411218162	-4,00395E+14	8,52176E+13	8,35086E+14	7,92182E+14	6,44995E+14	7,44365E+14	7,08417E+14
TC0400000580.mm.2	Slc44a1	solute carrier family 44, member 1	1,39019E+13	7,46618E+14	2,2846E+14	0.0595112388578791	0.199837411218162	-4,00415E+14	8,3409E+13	8,68832E+14	7,56509E+14	5,95393E+14	7,6204E+14	6,62843E+14
TC0X00001674.mm.2	Fancb	Fanconi anemia, complementation group B	0.933760053062085	5,15588E+14	2,28458E+14	0.0595124351518334	0.199837411218162	-4,00417E+14	5,90913E+14	5,61222E+14	5,15712E+13	4,60296E+14	4,41113E+14	5,2427E+14
TC1600001151.mm.2	Glyr1	glyoxylate reductase 1 homolog (Arabidopsis)	1,19242E+14	8,24774E+14	2,28458E+14	0.0595129335135608	0.199837411218162	-4,00418E+14	9,12284E+14	8,74246E+14	8,82428E+13	7,3164E+14	7,81703E+14	7,66344E+14
TC1100000967.mm.2	Rabep1	rabaptin, RAB GTPase binding effector protein 1	1,6272E+14	8,38677E+14	2,28441E+14	0.0595271923530308	0.199837411218162	-4,0044E+14	9,64685E+14	9,55076E+14	8,508E+14	6,6718E+14	8,69323E+14	7,24999E+14
TC0700000863.mm.2	Fam83e	family with sequence similarity 83, member E	-0.959925389949675	5,56933E+14	-2,28432E+14	0.0595343701515193	0.199837411218162	-4,00451E+14	4,75019E+12	4,94321E+14	5,5654E+14	6,54394E+14	5,57502E+14	6,03823E+14
TC0800000842.mm.2	Isx	intestine specific homeobox	-0.957911725037863	5,26392E+13	-2,2842E+14	0.0595450680291619	0.199837411218162	-4,00468E+14	4,29362E+14	4,77575E+14	5,34494E+12	6,18401E+13	5,74932E+14	5,23587E+14
TC1700000383.mm.2	Rpusd1	RNA pseudouridylate synthase domain containing 1	1,38149E+14	6,66679E+14	2,28406E+14	0.0595564865055865	0.199837411218162	-4,00486E+14	7,71585E+14	7,51527E+14	7,05079E+14	5,25067E+13	6,53516E+13	5,93298E+14

TC0300001528.mm.2	Fubp1	far upstream element (FUSE) binding protein 1	1,28445E+14	8,63015E+14	2,28389E+14	0.0595712357407429	0.199837411218162	-4,00509E+14	9,57274E+14	9,46011E+14	8,683E+14	7,60109E+14	8,90706E+14	7,55689E+14
TC0800000726.mm.2	Lpar2	lysophosphatidic acid receptor 2	-0.832909745834685	6,31955E+14	-2,28372E+14	0.0595850298918829	0.199837411218162	-4,00531E+14	5,77968E+14	5,65417E+14	6,32596E+14	7,05785E+14	6,39364E+14	6,70603E+14
TC1100001670.mm.2	Dhx8	DEAH (Asp-Glu-Ala-His) box polypeptide 8	1,54058E+14	5,82793E+14	2,28363E+14	0.0595931440241573	0.199837411218162	-4,00543E+14	7,05127E+14	6,86852E+14	6,18042E+14	4,02871E+14	5,59843E+14	5,24022E+14
TC0200005104.mm.2	Kcns1	K+ voltage-gated channel, subfamily S, 1	-0.781522572341172	6,76792E+14	-2,28334E+14	0.0596174309006307	0.199837411218162	-4,00581E+14	6,38963E+14	6,02198E+14	6,83994E+14	7,3813E+14	6,92276E+14	7,05189E+14
TC0700002783.mm.2	Hamp	hepcidin antimicrobial peptide	-0.828013059956114	6,55781E+14	-2,28323E+14	0.0596270072001827	0.199837411218162	-4,00596E+14	5,91902E+14	6,40775E+14	6,226E+14	6,81126E+13	6,88153E+14	7,10127E+14
TC1500000019.mm.2	Rpl37	ribosomal protein L37	0.777512539793959	1,02968E+14	2,28314E+13	0.0596343326776404	0.199837411218162	-4,00608E+14	1,08765E+14	1,07496E+14	1,02725E+14	9,81139E+14	1,02597E+13	9,81137E+14
TC0200004848.mm.2	Abhd12	abhydrolase domain containing 12	1,04609E+14	6,98352E+14	2,28292E+14	0.0596528838266554	0.199837411218162	-4,00636E+14	7,68806E+14	7,768E+14	6,96057E+14	6,12063E+14	7,1633E+14	6,20059E+14
TC1900000656.mm.2	Slf2	SMC5-SMC6 complex localization factor 2	1,0505E+14	7,37702E+14	2,28274E+14	0.059667752883305	0.199837411218162	-4,0066E+14	8,16609E+14	8,17587E+14	7,39277E+14	6,28639E+14	7,45131E+14	6,78966E+14
TC1600001606.mm.2	Timmdc1	translocase of inner mitochondrial membrane domain containing 1	-0.747620696306829	7,44088E+14	-2,28262E+14	0.059678455532426	0.199837411218162	-4,00676E+14	6,82865E+14	7,24522E+14	7,4014E+14	7,60088E+14	7,62286E+14	7,94625E+14
TC0900001374.mm.2	Tusc2	tumor suppressor 2, mitochondrial calcium regulator	-0.71745986285031	7,88822E+14	-2,2824E+14	0.0596970693615729	0.199837411218162	-4,00705E+14	7,41458E+13	7,30613E+14	8,03589E+14	8,4171E+14	8,19183E+13	7,96381E+14
TC0900000153.mm.2	Zfp317	zinc finger protein 317	0.667620674037835	6,78637E+13	2,28231E+14	0.0597044684455032	0.199837411218162	-4,00717E+13	7,25121E+13	7,17912E+14	6,5872E+14	6,60559E+14	6,45645E+14	6,63864E+14
TC1600001986.mm.2	Cct8	chaperonin containing Tcp1, subunit 8 (theta)	1,7785E+14	8,4263E+14	2,28196E+14	0.0597338462732924	0.199837411218162	-4,00763E+14	9,65683E+14	9,65827E+14	8,72409E+13	6,76054E+14	8,72978E+14	7,02831E+14
TC0400001555.mm.2	Sdc3	syndecan 3	0.826684877107741	9,54392E+13	2,28185E+14	0.0597434403743357	0.199837411218162	-4,00778E+14	1,00316E+14	1,00031E+14	9,80893E+14	9,03858E+14	9,10116E+14	9,28017E+14
TC0300001788.mm.2	Lrrc31	leucine rich repeat containing 31	-1,422E+14	4,81592E+14	-2,2817E+14	0.0597560812778306	0.199837411218162	-4,00797E+14	3,62152E+13	4,15687E+14	4,30792E+13	6,18682E+14	5,14035E+14	5,48203E+14
TC1800001741.mm.2	Pcdhgb1/Pcdhgb2/Pcdhgb4/Pcdhgb5/Pcdhgb6/Pcdhgb7/Pcdhgb8/Pcdhgc3/Pcdhgc4/Pcdhgc5/Pcdhga1/Pcdhga2/Pcdhga3/Pcdhga4/Pcdhga5/Pcdhga6/Pcdhga7/Pcdhga8/Pcdhga9/Pcdhga10/Pcdhga11/Pcdhga12	protocadherin gamma subfamily B, 1/protocadherin gamma subfamily B, 2/protocadherin gamma subfamily B, 4/protocadherin gamma subfamily B, 5/protocadherin gamma subfamily B, 6/protocadherin gamma subfamily B, 7/protocadherin gamma subfamily B, 8/protocadherin gamma subfamily C, 3/protocadherin gamma subfamily C, 4/protocadherin gamma subfamily C, 5/protocadherin gamma subfamily A, 1/protocadherin gamma subfamily A, 2/protocadherin gamma subfamily A, 3/protocadherin gamma subfamily A, 4/protocadherin gamma subfamily A, 5/protocadherin gamma subfamily A, 6/protocadherin gamma subfamily A, 7/protocadherin gamma subfamily A, 8/protocadherin gamma subfamily A, 9/protocadherin gamma subfamily A, 10/protocadherin gamma subfamily A, 11/protocadherin gamma subfamily A, 12	1,74369E+14	8,53518E+14	2,2816E+14	0.0597644933696214	0.199837411218162	-4,0081E+14	9,70339E+14	9,76649E+13	9,05584E+14	6,70287E+13	8,46812E+14	7,51438E+14
TC0700001460.mm.2	Olfr550	olfactory receptor 550	-0.76684470035687	4,88623E+14	-2,28157E+14	0.0597673281927256	0.199837411218162	-4,00815E+14	4,35799E+14	4,50379E+14	4,79402E+14	5,26611E+13	5,00605E+14	5,38942E+14
TC1700001132.mm.2	Lpin2	lipin 2	0.703648646278443	7,18076E+14	2,28152E+14	0.0597713991140047	0.199837411218162	-4,00821E+14	7,60577E+14	7,57795E+14	7,02375E+14	7,10066E+14	6,69144E+14	7,08498E+14
TC0100003203.mm.2	Ro60	Ro60, Y RNA binding protein	1,39507E+14	5,55318E+13	2,28126E+14	0.0597934916691855	0.199837411218162	-4,00856E+14	6,44022E+14	6,62546E+14	5,94138E+13	4,03648E+13	5,28363E+14	4,9919E+14
TC0800001289.mm.2	Zfhx3	zinc finger homeobox 3	1,25341E+14	5,68113E+14	2,28116E+14	0.0598018718752949	0.199837411218162	-4,00869E+14	6,7685E+14	6,46163E+14	5,21317E+14	4,92013E+13	6,17423E+14	4,54909E+14
TC1200001916.mm.2	Trmt5	TRM5 tRNA methyltransferase 5	1,35544E+14	5,86299E+14	2,28107E+14	0.0598094836656339	0.199837411218162	-4,0088E+14	6,75966E+14	6,67751E+13	5,83244E+14	5,18203E+14	6,2363E+14	4,48999E+13
TC1000002435.mm.2	Krtap10-4	keratin associated protein 10-4	-0.76181675918967	6,87271E+14	-2,28105E+14	0.0598112909969963	0.199837411218162	-4,00883E+14	6,32651E+14	6,61467E+14	7,13642E+13	6,69387E+14	7,0792E+14	7,38561E+14
TC0300001102.mm.2	Chia1	chitinase, acidic 1	-0.962819630500511	4,8269E+14	-2,28082E+14	0.0598307414554558	0.199837411218162	-4,00914E+14	4,42197E+14	4,09386E+14	4,47245E+14	5,53167E+13	5,23199E+14	5,20948E+14
TC0700004177.mm.2	Knop1	lysine rich nucleolar protein 1	-0.683132575626655	7,15729E+14	-2,28076E+14	0.0598357687831785	0.199837411218162	-4,00921E+14	6,72369E+14	6,58986E+13	7,36553E+14	7,58483E+14	7,25924E+14	7,42057E+14
TC1100002932.mm.2	Zswim7	zinc finger SWIM-type containing 7	-0.715635712030132	6,70453E+14	-2,28075E+14	0.0598371010968751	0.199837411218162	-4,00923E+14	6,27734E+14	6,29982E+14	6,79269E+14	6,84892E+14	7,22826E+14	6,78017E+14
TC1300002459.mm.2	S100z	S100 calcium binding protein, zeta	-0.791488934231865	5,13832E+14	-2,2807E+14	0.0598408433642171	0.199837411218162	-4,00929E+14	4,42612E+14	4,63201E+14	5,37802E+14	5,75267E+14	5,04469E+14	5,59642E+14
TC1100003323.mm.2	AU040972	expressed sequence AU040972	-0.751904693914422	5,08172E+14	-2,28019E+14	0.0598843665746165	0.199885377566343	-4,00997E+14	4,50859E+14	4,49342E+14	5,21409E+13	5,76837E+14	5,33847E+14	5,16735E+14
TC1300000440.mm.2	Riok1	RIO kinase 1	1,05929E+14	5,23105E+14	2,28011E+14	0.0598910801712891	0.199885377566343	-4,01007E+14	5,80947E+14	5,93277E+14	5,53739E+14	4,48305E+14	5,195E+14	4,42865E+14
TC0100000140.mm.2	Khdc1c/Khdc1a	KH domain containing 1C/KH domain containing 1A	-1,06256E+14	5,14702E+14	-2,27996E+14	0.059903636542044	0.199885377566343	-4,01027E+14	4,21472E+14	4,50448E+14	5,04238E+14	6,2762E+14	5,24511E+14	5,59921E+14
TC0300000224.mm.2	Mfn1	mitofusin 1	1,10784E+14	6,65538E+14	2,27995E+14	0.0599048276713131	0.199885377566343	-4,01029E+12	7,52412E+13	7,14111E+13	6,74521E+13	6,07229E+14	6,81114E+14	5,63841E+13
TC0100000431.mm.2	Aox2	aldehyde oxidase 2	-1,03968E+14	4,3513E+14	-2,2796E+13	0.0599345121072538	0.199943021446	-4,01075E+14	3,99346E+14	3,34124E+14	4,25865E+14	5,10039E+14	4,36078E+14	5,05327E+14
TC1500000356.mm.2	A930017M01Rik/2310069G16Rik	Smg-5 homolog, nonsense mediated mRNA decay factor pseudogene/RIKEN cDNA 2310069G16 gene	1,32947E+14	5,99122E+14	2,27929E+14	0.0599604669775344	0.19998820211323	-4,01115E+14	7,00742E+14	6,49249E+13	6,22904E+14	5,37743E+13	4,65833E+14	6,18264E+14
TC1100000926.mm.2	Plscr3	phospholipid scramblase 3	-0.832187756214366	7,16195E+14	-2,27884E+14	0.0599986671696756	0.200040338529298	-4,01174E+14	6,49589E+14	6,69124E+14	7,82447E+14	7,10857E+14	7,58597E+14	7,26553E+13
TC1100001974.mm.2	Tmem235	transmembrane protein 235	-0.845361401960762	5,85514E+14	-2,27878E+14	0.060004298411157	0.200040338529298	-4,01183E+14	5,0664E+14	5,4415E+14	5,87888E+14	6,54544E+14	5,92382E+14	6,2748E+14
TC1100003763.mm.2	Krt17	keratin 17	-1,00652E+14	4,50709E+13	-2,27854E+14	0.0600245686104951	0.200040338529298	-4,01214E+14	3,72997E+13	3,98825E+14	4,52155E+14	5,07153E+14	4,34632E+14	5,38494E+14
TC1200002191.mm.2	Kcnk10	potassium channel, subfamily K, member 10	-0.692939203115967	7,42505E+13	-2,27845E+14	0.0600324771366896	0.200040338529298	-4,01227E+14	6,85365E+14	6,96774E+14	7,60088E+14	7,92079E+14	7,65942E+14	7,54785E+14
TC0200001472.mm.2	Lrrc4c	leucine rich repeat containing 4C	2,24824E+14	8,36059E+14	2,27831E+14	0.0600444928700311	0.200040338529298	-4,01245E+14	9,79484E+14	9,99771E+14	8,89181E+14	6,1831E+14	8,65849E+13	6,63758E+14
TC0700003775.mm.2	Acer3	alkaline ceramidase 3	1,59325E+13	6,23196E+14	2,27814E+14	0.0600589417229884	0.200040338529298	-4,01268E+14	7,37807E+14	7,19053E+14	6,70652E+14	4,73456E+14	6,26224E+14	5,11986E+14
TC1000001260.mm.2	Lin7a	lin-7 homolog A (C. elegans)	1,88085E+14	7,75666E+14	2,27807E+14	0.0600648614894704	0.200040338529298	-4,01277E+13	8,88842E+14	8,68481E+14	8,72011E+14	6,43508E+13	7,68721E+14	6,12434E+14
TC1500000528.mm.2	Adgrb1	adhesion G protein-coupled receptor B1	1,19107E+14	8,79761E+14	2,27781E+14	0.0600869098522285	0.200040338529298	-4,01311E+14	9,70051E+14	9,64535E+14	8,88789E+14	7,58822E+14	8,91851E+14	8,04522E+14
TC1700000630.mm.2	Btnl1	butyrophilin-like 1	-0.86133817058585	5,31821E+14	-2,2778E+14	0.0600878320909702	0.200040338529298	-4,01313E+14	4,81347E+14	4,75947E+14	5,08538E+14	5,95535E+14	5,58159E+14	5,71403E+14
TC0800001223.mm.2	Ctcf	CCCTC-binding factor	1,54444E+14	7,49465E+14	2,27712E+13	0.0601454964020817	0.200171098535278	-4,01402E+14	8,49167E+14	8,60841E+14	7,9066E+14	5,95003E+14	6,50241E+13	7,50879E+14
TC0400000514.mm.2	Galnt12	polypeptide N-acetylgalactosaminyltransferase 12	-0.891380681489525	4,9093E+14	-2,27705E+14	0.06015195550846	0.200171098535278	-4,01412E+14	4,37898E+14	4,40426E+14	4,60002E+14	5,50654E+14	5,13575E+14	5,43024E+14
TC1900001340.mm.2	Insl6	insulin-like 6	-0.877208101534015	5,48497E+14	-2,27688E+14	0.0601664137867213	0.200177870193153	-4,01434E+14	5,22691E+14	4,74245E+14	5,4703E+13	5,74636E+14	5,49572E+14	6,22806E+14
TC0200000420.mm.2	Fbxw5	F-box and WD-40 domain protein 5	1,57723E+14	8,53703E+14	2,27664E+14	0.060186338444918	0.200202822329266	-4,01465E+13	9,7602E+14	9,5835E+14	8,95111E+14	6,73814E+14	8,3083E+13	7,88094E+14
TC0700001669.mm.2	Pde3b	phosphodiesterase 3B, cGMP-inhibited	1,32027E+14	5,86521E+14	2,27628E+14	0.0602176975788646	0.200235741183794	-4,01513E+13	6,48935E+14	6,92824E+14	5,93151E+14	5,06511E+11	6,15038E+14	4,62667E+14
TC0700002738.mm.2	Zfp74	zinc finger protein 74	0.750123476040498	6,72368E+14	2,27615E+14	0.0602284746144991	0.200235741183794	-4,0153E+14	7,15018E+14	7,21052E+14	6,84065E+13	6,28024E+14	6,49612E+14	6,36434E+14

TC0900003182.mm.2	Stt3b	STT3, subunit of the oligosaccharyltransferase complex, homolog B (S. cerevisiae)	1,70619E+13	7,45434E+14	2,27609E+14	0.0602335156406535	0.200235741183794	-4,01538E+14	8,40802E+14	8,76533E+14	7,59359E+14	6,19814E+13	7,89172E+13	5,86926E+14
TC1600001259.mm.2	Dnm1l	dynamin 1-like	2,11886E+14	9,10827E+14	2,27506E+14	0.060321775628513	0.200487782735608	-4,01674E+14	1,05915E+14	1,04533E+14	9,51614E+14	7,28144E+13	9,53065E+14	7,27651E+14
TC0700000041.mm.2	Kmt5c	lysine methyltransferase 5C	-0.702928509785151	7,78906E+14	-2,27374E+14	0.0604346225108182	0.200800145730845	-4,01848E+14	7,41124E+14	7,14526E+14	8,24764E+14	7,96788E+13	7,91333E+14	8,04903E+13
TC1500000392.mm.2	Deptor	DEP domain containing MTOR-interacting protein	1,43012E+13	5,8447E+14	2,27356E+14	0.0604503963348914	0.200800145730845	-4,01872E+14	6,85272E+14	7,17726E+14	4,63591E+14	5,23259E+14	6,52903E+14	4,64072E+14
TC1400000179.mm.2	Adk	adenosine kinase	1,07179E+13	7,06735E+14	2,27343E+14	0.060461414742626	0.200800145730845	-4,01889E+14	7,8552E+14	7,64341E+14	7,02921E+14	6,52125E+14	7,28864E+14	6,06639E+14
TC1500001927.mm.2	Rangap1	RAN GTPase activating protein 1	0.90980328321632	7,72298E+14	2,27338E+14	0.0604656058515521	0.200800145730845	-4,01896E+14	8,59958E+14	8,26861E+14	7,45271E+14	6,9684E+14	7,92864E+14	7,11995E+14
TC0200003921.mm.2	Olfr1046	olfactory receptor 1046	-0.754108925726571	5,09738E+13	-2,27295E+14	0.0605025796976272	0.200847874309707	-4,01952E+14	4,51375E+14	4,74745E+14	5,26947E+14	5,28416E+13	5,11126E+14	5,65816E+14
TC0300000116.mm.2	Cypt12	cysteine-rich perinuclear theca 12	-1,10958E+13	5,5182E+14	-2,27279E+14	0.0605166517464949	0.200847874309707	-4,01974E+14	4,68092E+14	4,95352E+14	5,4106E+14	6,21058E+14	5,35906E+14	6,49454E+14
TC0200004264.mm.2	Rcn1	reticulocalbin 1	0.946009446545055	7,33777E+14	2,27246E+14	0.0605441550089574	0.200847874309707	-4,02016E+14	8,06548E+14	7,87008E+14	7,4947E+14	6,55284E+14	6,77591E+14	7,26763E+13
TC1100001634.mm.2	Atp6v0a1	ATPase, H+ transporting, lysosomal V0 subunit A1	2,02355E+14	9,30535E+14	2,27233E+14	0.0605554257264444	0.200847874309707	-4,02034E+14	1,06931E+14	1,07262E+14	9,89819E+14	7,14254E+14	9,30713E+13	8,065E+14
TC1100000984.mm.2	Txndc17	thioredoxin domain containing 17	-1,67454E+14	9,60646E+14	-2,2723E+14	0.0605582336206882	0.200847874309707	-4,02038E+14	8,58291E+13	8,51135E+14	1,10341E+14	9,06708E+14	1,09373E+13	9,50603E+14
TC0200005485.mm.2	Sys1	SYS1 Golgi-localized integral membrane protein homolog (S. cerevisiae)	0.688530070984037	8,11211E+14	2,27229E+14	0.0605589303262347	0.200847874309707	-4,02039E+14	8,5729E+14	8,22913E+14	8,23454E+14	8,21113E+14	7,70296E+14	7,72201E+14
TC1100000937.mm.2	Dlg4	discs large MAGUK scaffold protein 4	1,17532E+14	1,03221E+14	2,2722E+14	0.0605672327481454	0.200847874309707	-4,02052E+14	1,11686E+14	1,10972E+14	1,05743E+14	9,17741E+13	1,02828E+14	9,63242E+14
TC0200002863.mm.2	Prpf18	pre-mRNA processing factor 18	1,11066E+12	6,72476E+14	2,27171E+14	0.0606089365446408	0.200943409422107	-4,02116E+14	7,62541E+14	7,54472E+14	6,72716E+14	5,50249E+14	6,83012E+14	6,11869E+14
TC0500001724.mm.2	Fscn1	fascin actin-bundling protein 1	1,33719E+14	9,0314E+14	2,27157E+14	0.0606209838764267	0.200943409422107	-4,02134E+14	1,00565E+14	9,95189E+14	9,34274E+14	7,50324E+14	8,67577E+14	8,65825E+14
TC0500002155.mm.2	Adgrf3	adhesion G protein-coupled receptor F3	-1,06445E+14	6,17225E+14	-2,2711E+13	0.0606610880038655	0.20103162279813	-4,02196E+14	5,15255E+14	5,83315E+14	6,01255E+14	6,92063E+14	6,21245E+14	6,90216E+13
TC0600002280.mm.2	Hoxa6	homeobox A6	-1,20841E+14	5,94951E+14	-2,27097E+14	0.0606725489770563	0.20103162279813	-4,02213E+14	5,13302E+14	5,20111E+14	5,5985E+14	7,01346E+14	5,99698E+14	6,75397E+14
TC1400002578.mm.2	Dach1	dachshund family transcription factor 1	0.795639443924387	6,79316E+14	2,27068E+14	0.0606977398570376	0.201073742252559	-4,02252E+14	7,23102E+14	7,44039E+13	6,82556E+14	6,18187E+14	6,41848E+14	6,66165E+14
TC1300002246.mm.2	Zfp429	zinc finger protein 429	-0.677789366812633	6,71378E+14	-2,26998E+13	0.0607578649194788	0.201192761689529	-4,02344E+14	6,36823E+14	6,52677E+14	6,59823E+14	6,53887E+14	7,00675E+14	7,24383E+14
TC0600001530.mm.2	Clec2d	C-type lectin domain family 2, member d	1,12306E+14	5,53516E+14	2,26995E+14	0.0607608327436887	0.201192761689529	-4,02349E+13	6,03873E+14	5,98986E+13	5,65431E+14	5,74562E+14	5,61159E+14	4,17086E+13
TC0X00000068.mm.2	Tfe3	transcription factor E3	0.676929891414286	6,60412E+14	2,26983E+14	0.0607711271112106	0.201192761689529	-4,02364E+14	7,11146E+14	7,04942E+14	6,46089E+13	6,19594E+14	6,3586E+14	6,44842E+14
TC1500001002.mm.2	Tuba1c	tubulin, alpha 1C	1,20011E+13	9,17872E+14	2,26924E+14	0.0608220963476486	0.201258503986438	-4,02442E+14	1,01522E+14	1,01126E+14	9,10421E+14	7,83884E+13	9,41515E+13	8,44936E+14
TC1500001111.mm.2	Hoxc13	homeobox C13	-0.77734661215589	5,05536E+14	-2,26909E+14	0.060834387908659	0.201258503986438	-4,02461E+14	4,43337E+14	5,02083E+14	4,94588E+14	4,92319E+14	5,34475E+14	5,66414E+13
TC0700003915.mm.2	Olfr66	olfactory receptor 66	-1,28733E+14	5,29498E+14	-2,26906E+14	0.0608372904171268	0.201258503986438	-4,02466E+14	4,1326E+14	4,83659E+14	4,92586E+14	6,33098E+14	5,38205E+14	6,1618E+14
TC0800001518.mm.2	Spire2	spire type actin nucleation factor 2	0.835419886933052	6,57901E+14	2,26902E+14	0.0608409466218544	0.201258503986438	-4,02471E+14	7,18212E+14	6,63665E+14	6,8004E+14	6,70696E+14	6,38033E+14	5,7676E+14
TC0200005415.mm.2	Dido1	death inducer-obliterator 1	0.74887427041048	7,82735E+14	2,26875E+14	0.060864296588778	0.20129441932163	-4,02507E+14	8,36405E+14	8,24333E+14	7,68816E+14	7,55892E+13	7,83241E+14	7,27722E+14
TC0900002405.mm.2	Bbs4	Bardet-Biedl syndrome 4 (human)	1,93363E+14	7,00965E+12	2,26854E+14	0.0608818923342482	0.201311293080595	-4,02534E+14	8,66498E+13	8,38036E+14	7,25524E+14	4,57927E+14	7,09907E+14	6,07901E+14
TC1700001036.mm.2	Cntnap5c	contactin associated protein-like 5C	-0.802639687909129	6,47267E+14	-2,26804E+14	0.0609250241034543	0.201412579683824	-4,026E+14	6,359E+14	5,84066E+14	6,27203E+14	6,55937E+14	6,68014E+14	7,12479E+14
TC0200004476.mm.2	Tgm5	transglutaminase 5	-1,5567E+13	4,78391E+13	-2,26763E+14	0.0609607436810319	0.201421300024922	-4,02654E+14	3,69305E+14	3,72682E+14	4,31736E+14	6,43299E+13	4,90529E+13	5,62797E+14
TC0600000044.mm.2	Tac1	tachykinin 1	1,35293E+14	9,21404E+14	2,26736E+14	0.0609841322775737	0.201421300024922	-4,0269E+14	9,96376E+14	9,84448E+14	9,90623E+14	8,46739E+14	9,14973E+14	7,95266E+14
TC1400000027.mm.2	Gm3047	predicted gene 3047	-0.769215273823963	4,99668E+14	-2,2673E+13	0.0609890964799599	0.201421300024922	-4,02697E+14	4,57851E+12	4,63588E+14	5,17512E+13	4,83775E+14	5,65093E+14	5,10189E+14
TC0400002808.mm.2	Tnfsf15	tumor necrosis factor (ligand) superfamily, member 15	-0.678772014442025	5,84512E+14	-2,26729E+14	0.060990156082848	0.201421300024922	-4,02699E+14	5,37686E+14	5,43749E+14	5,94604E+14	6,13845E+14	5,97389E+14	6,198E+14
TSUnmapped00000053.mm.2	Sts	steroid sulfatase	-0.82996837635338	6,71665E+14	-2,26715E+14	0.0610020777074448	0.201421300024922	-4,02717E+14	6,08909E+14	6,13995E+14	6,70095E+14	7,48094E+14	6,83171E+14	7,05726E+14
TC0700001942.mm.2	Zranb1	zinc finger, RAN-binding domain containing 1	1,83141E+14	6,70883E+14	2,26715E+14	0.0610026651847341	0.201421300024922	-4,02718E+13	7,69738E+14	7,81073E+12	7,79303E+14	5,10657E+14	5,76382E+14	6,08148E+13
TC0300000710.mm.2	Crabp2	cellular retinoic acid binding protein II	-0.769035548783211	6,30026E+13	-2,26689E+14	0.0610252679060984	0.201454649000402	-4,02752E+14	5,762E+14	5,89662E+14	6,16782E+14	6,77842E+13	6,6812E+14	6,51548E+14
TC1100003466.mm.2	Gdpd1	glycerophosphodiester phosphodiesterase domain containing 1	1,8141E+14	6,92344E+13	2,26662E+14	0.0610486953187651	0.201490706200586	-4,02788E+14	7,96965E+14	7,80737E+14	7,68172E+13	5,9331E+14	7,08595E+14	5,06288E+14
TC0500001423.mm.2	B3gnt4	UDP-GlcNAc:betaGal beta-1,3-N-acetylglucosaminyltransferase 4	-1,05391E+14	5,67891E+14	-2,26603E+14	0.0610993413200474	0.201592175322956	-4,02865E+14	5,26695E+14	4,77204E+14	5,84282E+14	6,04479E+14	5,47455E+14	6,67227E+14
TC1000002543.mm.2	Celf5	CUGBP, Elav-like family member 5	0.9642586237462	8,16103E+14	2,26597E+14	0.0611044612596857	0.201592175322956	-4,02873E+14	8,70518E+14	8,76668E+14	8,45289E+14	7,49811E+14	8,06824E+14	7,4751E+14
TC1000000299.mm.2	Tspyl4	TSPY-like 4	1,78271E+12	9,27238E+13	2,26555E+14	0.0611413081386307	0.201647653969811	-4,02929E+14	1,0669E+14	1,02464E+14	9,72699E+14	7,64198E+14	9,44566E+14	7,90429E+14
TC1100002837.mm.2	Hist3h2bb-ps	histone cluster 3, H2bb, pseudogene	-1,08924E+14	7,28701E+13	-2,26545E+14	0.0611497774892126	0.201647653969811	-4,02942E+14	6,81736E+14	6,46438E+14	6,82449E+14	8,1556E+14	7,66889E+13	7,79132E+13
TC1500000062.mm.2	Ranbp3l	RAN binding protein 3-like	-0.727944175491007	5,87655E+14	-2,26535E+14	0.0611588211351371	0.201647653969811	-4,02956E+14	5,65607E+14	5,32419E+14	5,78676E+14	6,05611E+14	6,36261E+14	6,07353E+14
TC1100002025.mm.2	Fscn2	fascin actin-bundling protein 2	-0.723296753652913	5,83693E+14	-2,26514E+14	0.0611764604052582	0.201664547158332	-4,02983E+14	5,52634E+14	5,53371E+14	5,49813E+14	5,95674E+14	6,35234E+14	6,1543E+14
TC1500001717.mm.2	Lynx1	Ly6/neurotoxin 1	1,13205E+14	8,45292E+13	2,26311E+14	0.0613532658971209	0.202126439372274	-4,03251E+14	9,40994E+14	9,09504E+14	8,59061E+14	7,38104E+14	8,50585E+14	7,73503E+12
TC1100000336.mm.2	Hba-a2/Hba-a1	hemoglobin alpha, adult chain 2/hemoglobin alpha, adult chain 1	-1,0821E+14	1,21906E+14	-2,26289E+14	0.0613723987459488	0.202126439372274	-4,0328E+13	1,12802E+14	1,21408E+14	1,22229E+14	1,19142E+13	1,33033E+13	1,22819E+14
TC1100002488.mm.2	Ranbp17	RAN binding protein 17	1,00247E+14	5,31823E+14	2,26269E+14	0.0613899719846656	0.202126439372274	-4,03307E+14	6,12572E+14	5,35344E+14	5,73814E+13	5,21781E+14	5,12298E+14	4,35125E+14
TC0100003058.mm.2	Srgap2	SLIT-ROBO Rho GTPase activating protein 2	1,94676E+14	6,47684E+14	2,26268E+14	0.0613909236676419	0.202126439372274	-4,03308E+14	7,53413E+14	7,99553E+14	6,89769E+14	4,79758E+14	6,79729E+14	4,83884E+14
TC1600000033.mm.2	Hmox2	heme oxygenase 2	1,12704E+14	8,93544E+14	2,26268E+14	0.0613910623010798	0.202126439372274	-4,03308E+14	9,84889E+14	9,56115E+14	9,10105E+14	7,94558E+14	8,99967E+13	8,15629E+14
TC0900002277.mm.2	Hspb2	heat shock protein 2	-1,0276E+14	6,25055E+14	-2,26267E+14	0.0613918447395214	0.202126439372274	-4,03309E+14	5,58742E+14	5,38529E+14	6,13052E+14	7,37216E+14	6,55983E+14	6,46807E+14
TC0700000220.mm.2	Kptn	kaptin	0.898398512575798	5,6947E+14	2,26242E+14	0.0614133627211269	0.202155978248318	-4,03342E+13	6,42066E+14	6,57346E+14	5,45811E+12	4,51864E+14	5,47585E+14	5,72147E+14
TC0600000848.mm.2	0610030E20Rik	RIKEN cDNA 0610030E20 gene	-0.726701771303104	6,17301E+14	-2,26217E+14	0.0614352309029528	0.202186657585637	-4,03375E+14	5,49513E+14	5,9639E+14	6,43205E+14	6,23454E+14	6,41529E+14	6,49714E+14
TC1800000342.mm.2	Tmco6	transmembrane and coiled-coil domains 6	-0.708159188924752	7,85072E+14	-2,2618E+14	0.0614675813907793	0.202251815182689	-4,03424E+14	7,32657E+14	7,41172E+14	7,91816E+14	8,29327E+12	7,96434E+14	8,19027E+14
TC0500001180.mm.2	Tfip11	tuftelin interacting protein 11	0.884493111747615	5,79442E+14	2,26155E+14	0.0614893727787034	0.202282209796498	-4,03457E+14	6,4853E+14	6,3281E+14	5,71467E+14	5,194E+14	5,1318E+14	5,91262E+14
TC0900000186.mm.2	Icam5	intercellular adhesion molecule 5, telencephalin	0.704455701404473	7,64836E+14	2,25939E+14	0.0616777822315165	0.202805514591504	-4,03741E+14	8,10397E+14	8,22205E+14	7,46375E+14	7,18328E+14	7,30372E+14	7,61339E+14
TC0700002837.mm.2	Kctd15	potassium channel tetramerisation domain containing 15	-0.731462420682155	6,63457E+14	-2,25915E+14	0.0616989950945194	0.202805514591504	-4,03773E+14	6,09412E+12	6,1399E+14	6,67535E+14	7,20112E+14	6,80385E+14	6,89309E+14
TC0100002563.mm.2	Tnp1	transition protein 1	-0.961197315782737	4,63266E+14	-2,25902E+14	0.0617103863169584	0.202805514591504	-4,0379E+14	3,96243E+14	4,0687E+14	4,58E+13	5,23131E+14	4,52091E+14	5,43261E+14
TC1100002772.mm.2	Anxa6	annexin A6	0.733760376892442	7,30122E+14	2,25898E+14	0.0617140943437033	0.202805514591504	-4,03796E+13	7,93171E+13	7,66321E+14	7,27846E+14	6,80657E+14	7,05354E+14	7,07385E+13
TC0X00003083.mm.2	Col4a6	collagen, type IV, alpha 6	-1,02629E+14	6,01099E+13	-2,25879E+14	0.0617302220243609	0.202805514591504	-4,0382E+14	5,31519E+14	5,31115E+14	6,03836E+14	6,72233E+14	5,80902E+14	6,8699E+14
TC0700003807.mm.2	Spcs2	signal peptidase complex subunit 2 homolog (S. cerevisiae)	1,17285E+14	8,75649E+13	2,2587E+14	0.0617386421528091	0.202805514591504	-4,03833E+14	9,54901E+14	9,62734E+14	8,86539E+14	7,66653E+14	8,90589E+14	7,92475E+14
TC0800002121.mm.2	Frg1	FSHD region gene 1	1,44056E+12	6,52477E+14	2,25857E+14	0.0617497052958876	0.202805514591504	-4,0385E+14	7,517E+14	7,38954E+14	7,05508E+14	5,16158E+14	6,38979E+14	5,63562E+14
TC1100001834.mm.2	Prkar1a	protein kinase, cAMP dependent regulatory, type I, alpha	1,6675E+14	9,87936E+14	2,25852E+14	0.0617543625274967	0.202805514591504	-4,03857E+14	1,111E+14	1,09062E+14	1,03851E+14	8,19357E+14	9,8662E+14	8,81506E+14
TC1500001234.mm.2	6030458C11Rik	RIKEN cDNA 6030458C11 gene	-0.6633055199936	6,49064E+14	-2,25843E+14	0.061761724079967	0.202805514591504	-4,03868E+13	5,95929E+14	6,14567E+13	6,74068E+13	6,66659E+14	6,67274E+14	6,75884E+14
TC1700002207.mm.2	Taf8	TATA-box binding protein associated factor 8	-0.672007004549653	8,42715E+14	-2,2573E+14	0.0618614168523841	0.203091485277601	-4,04018E+14	7,97647E+14	7,94296E+14	8,58649E+14	8,79353E+14	8,53939E+14	8,72405E+14
TC1400000172.mm.2	Fut11	fucosyltransferase 11	0.912713804924605	6,3802E+14	2,25695E+14	0.0618919205772713	0.203147425139978	-4,04064E+14	6,85127E+14	6,9547E+14	6,71701E+14	5,77767E+14	6,18295E+14	5,79759E+14

TC1600000738.mm.2	Rpl24/Zbtb11	ribosomal protein L24/zinc finger and BTB domain containing 11	0.725264904111466	9,2656E+14	2,25682E+14	0.0619036714104941	0.203147425139978	-4,04081E+14	9,98399E+14	9,82096E+14	8,95291E+14	8,48135E+14	9,111E+14	9,24342E+14
TC0500000488.mm.2	Lap3	leucine aminopeptidase 3	1,45065E+14	7,30306E+14	2,25637E+14	0.061942839138317	0.203188967458338	-4,0414E+14	8,32471E+14	8,7058E+14	7,3135E+14	5,34513E+14	7,30678E+14	6,82243E+14
TC0800003078.mm.2	Pabpn1l	poly(A)binding protein nuclear 1-like	-0.772272155262279	5,55187E+14	-2,25628E+14	0.0619510109866237	0.203188967458338	-4,04152E+14	5,24292E+14	4,84007E+14	5,72011E+14	5,88055E+14	5,56032E+14	6,06722E+14
TC0800002119.mm.2	Asah1	N-acylsphingosine amidohydrolase 1	1,48884E+14	7,16123E+14	2,25624E+13	0.0619541610577058	0.203188967458338	-4,04157E+14	8,31006E+14	8,34316E+14	7,34452E+14	5,29409E+14	7,12638E+14	6,54916E+13
TC1100002851.mm.2	Snap47	synaptosomal-associated protein, 47	1,49642E+14	8,65815E+14	2,25609E+14	0.0619677975989303	0.203192332664136	-4,04177E+14	1,00791E+14	9,69322E+14	8,72445E+14	6,67274E+13	8,6337E+14	8,14573E+14
TC1200001339.mm.2	Rab10	RAB10, member RAS oncogene family	1,97957E+14	8,65586E+14	2,2557E+14	0.0620019975849445	0.203199921479629	-4,04229E+14	9,87075E+14	9,95473E+14	8,93926E+14	7,3041E+14	9,17441E+14	6,69193E+14
TC1100001105.mm.2	Bhlha9	basic helix-loop-helix family, member a9	-0.924490770833684	7,10448E+14	-2,25566E+14	0.0620058074283536	0.203199921479629	-4,04234E+13	6,48032E+14	6,5707E+14	7,0228E+13	7,65308E+14	7,03369E+14	7,86631E+14
TC0700001633.mm.2	Ctr9	CTR9 homolog, Paf1/RNA polymerase II complex component	0.780099945695537	7,06907E+14	2,25563E+14	0.0620079447598422	0.203199921479629	-4,04238E+14	7,43886E+14	7,43397E+14	6,83582E+14	7,39316E+14	6,45266E+13	6,85997E+13
TC0600001475.mm.2	Fgf6	fibroblast growth factor 6	-1,30639E+14	4,89772E+14	-2,2552E+14	0.0620462658011823	0.20326853552002	-4,04295E+13	3,99245E+14	4,18616E+14	4,39173E+14	6,02457E+14	5,02477E+14	5,76662E+14
TC1100002909.mm.2	Slc47a1	solute carrier family 47, member 1	-0.849433901448684	5,8953E+14	-2,25497E+14	0.0620661657974358	0.20326853552002	-4,04325E+14	5,43221E+14	5,32636E+14	5,64605E+14	6,50975E+14	6,18988E+14	6,26756E+14
TC0300002872.mm.2	Synpo2	synaptopodin 2	-0.7540828164731	6,30569E+14	-2,25496E+14	0.0620667284030597	0.20326853552002	-4,04326E+14	6,01762E+14	5,67455E+14	6,21646E+14	6,72518E+14	6,59974E+14	6,6006E+14
TC1100000549.mm.2	Grm6	glutamate receptor, metabotropic 6	-0.7393520683378	5,28129E+14	-2,25474E+14	0.0620868242190028	0.203293029595771	-4,04356E+14	5,02766E+14	4,64053E+14	5,2315E+14	5,64116E+14	5,54411E+14	5,60279E+14
TC0700003956.mm.2	Usp17lb	ubiquitin specific peptidase 17-like B	-0.897291660472521	5,24754E+14	-2,2543E+14	0.0621252808242773	0.203377620869886	-4,04413E+14	4,70503E+14	4,78901E+14	5,30295E+14	5,3996E+14	5,15455E+14	6,13408E+14
TC0700000419.mm.2	B3gnt8	UDP-GlcNAc:betaGal beta-1,3-N-acetylglucosaminyltransferase 8	-0.786839402118015	7,50261E+14	-2,25403E+14	0.0621486493087106	0.203412794294384	-4,04448E+14	6,95231E+14	7,13671E+14	7,31446E+14	7,9495E+14	7,70078E+14	7,96191E+14
TC0400002501.mm.2	Fam205a2	family with sequence similarity 205, member A2	0.853946676529316	5,78392E+14	2,25365E+14	0.062182184836287	0.203446649417069	-4,04498E+14	6,45085E+14	6,57959E+14	5,09255E+14	5,25799E+14	6,09164E+14	5,2309E+13
TC1800001088.mm.2	Celf4	CUGBP, Elav-like family member 4	1,62762E+14	8,85251E+14	2,25349E+14	0.0621966027632965	0.203446649417069	-4,0452E+14	1,00591E+14	9,96155E+14	9,23054E+14	7,09839E+14	8,90523E+14	7,86022E+14
TC0400001663.mm.2	Myom3	myomesin family, member 3	-1,08468E+14	5,57525E+14	-2,25349E+14	0.0621968717823176	0.203446649417069	-4,0452E+14	4,88545E+14	4,85967E+14	5,33045E+14	6,46143E+14	5,55781E+13	6,35667E+14
TC1100001583.mm.2	Rapgefl1	Rap guanine nucleotide exchange factor (GEF)-like 1	0.96338975228429	6,29387E+14	2,25275E+14	0.0622621266660075	0.203551747921457	-4,04618E+14	6,82653E+14	6,89907E+14	6,6627E+14	5,57609E+14	5,73786E+14	6,06096E+14
TC0400002238.mm.2	Necab1	N-terminal EF-hand calcium binding protein 1	0.822757104076519	8,49931E+14	2,25271E+14	0.0622660000961126	0.203551747921457	-4,04624E+13	9,02313E+14	8,92689E+14	8,47552E+14	8,26579E+14	8,53974E+13	7,76477E+13
TC0400001610.mm.2	Tent5b	terminal nucleotidyltransferase 5B	-1,11132E+14	6,32349E+14	-2,2527E+14	0.0622669003602596	0.203551747921457	-4,04625E+14	5,45489E+14	5,75283E+14	5,97096E+14	7,33194E+14	6,53679E+14	6,89356E+14
TC1800000015.mm.2	Jcad	junctional cadherin 5 associated	1,59455E+14	6,67687E+14	2,25216E+14	0.062314180474111	0.203643875613574	-4,04695E+13	7,79394E+14	7,73478E+14	6,39569E+14	5,79717E+12	7,29048E+13	5,04913E+14
TC0700003035.mm.2	Sergef	secretion regulating guanine nucleotide exchange factor	0.863761406689733	6,78713E+12	2,25209E+14	0.0623203593775545	0.203643875613574	-4,04705E+14	7,66121E+14	7,25956E+14	6,67116E+14	5,93764E+14	6,77743E+14	6,41581E+14
TC0700003658.mm.2	Zfand6	zinc finger, AN1-type domain 6	1,02353E+14	7,12848E+14	2,25171E+13	0.0623543129432162	0.203713512663026	-4,04755E+14	7,86921E+14	7,86111E+14	7,071E+14	6,28632E+14	7,33658E+14	6,34668E+13
TC0400003627.mm.2	Tinagl1	tubulointerstitial nephritis antigen-like 1	-0.935564258069205	7,49009E+14	-2,25096E+14	0.0624203152163035	0.203887804394952	-4,04854E+14	6,67631E+14	6,93655E+14	7,456E+13	8,3877E+14	7,50526E+14	7,97873E+14
TC0900002836.mm.2	Zic1	zinc finger protein of the cerebellum 1	1,07955E+14	1,0269E+14	2,2506E+14	0.0624524604751187	0.203935145457072	-4,04901E+14	1,10212E+14	1,0898E+14	1,06424E+14	9,29252E+14	9,75759E+14	1,00025E+14
TC1900000888.mm.2	Tcirg1	T cell, immune regulator 1, ATPase, H+ transporting, lysosomal V0 protein A3	-0.657271049359286	6,35718E+13	-2,25051E+14	0.0624601218165859	0.203935145457072	-4,04913E+14	5,95767E+14	6,08306E+12	6,3833E+14	6,36379E+14	6,60849E+14	6,74678E+14
TC1300002174.mm.2	Gm5141	predicted gene 5141	1,34957E+14	6,0404E+13	2,25013E+14	0.062494257148848	0.203971546718432	-4,04964E+14	6,82774E+14	7,12816E+14	6,11483E+14	4,91492E+14	6,34161E+14	4,91514E+14
TC0100002707.mm.2	C130026I21Rik/A530032D15Rik	RIKEN cDNA C130026I21 gene/RIKEN cDNA A530032D15Rik gene	-0.786994478822834	6,01913E+14	-2,25009E+14	0.0624972885075888	0.203971546718432	-4,04968E+14	5,35017E+14	5,79938E+14	5,8467E+13	6,39498E+14	6,31391E+14	6,40963E+14
TC0300002244.mm.2	4930565D16Rik	RIKEN cDNA 4930565D16 gene	-0.972195596014688	4,76317E+14	-2,24996E+14	0.0625092470486946	0.203971546718432	-4,04986E+14	4,12342E+14	4,0407E+14	4,74201E+14	5,56437E+14	4,64917E+14	5,45934E+14
TC0700004596.mm.2	Mrgprg	MAS-related GPR, member G	-0.697683108345567	6,08831E+14	-2,24972E+14	0.0625307177726587	0.20400029468938	-4,05018E+14	5,6844E+13	5,62331E+14	6,05474E+14	6,46435E+14	6,3214E+14	6,38167E+14
TC1100000715.mm.2	Trim11	tripartite motif-containing 11	-0.827526565416722	7,53076E+14	-2,24913E+14	0.0625825193327263	0.204076220007462	-4,05095E+14	6,8201E+14	7,22392E+14	7,40234E+14	8,03915E+14	7,68318E+14	8,01589E+14
TC1900000703.mm.2	Trim8	tripartite motif-containing 8	1,101E+14	7,03162E+13	2,24888E+14	0.0626049363488519	0.204076220007462	-4,05128E+14	7,88057E+14	8,03504E+14	6,90591E+14	5,65459E+14	7,18514E+13	6,52845E+14
TC1500000776.mm.2	Tspo	translocator protein	-1,1886E+14	6,63648E+14	-2,24888E+13	0.0626053227752812	0.204076220007462	-4,05129E+14	5,59041E+14	6,06062E+14	6,3613E+14	7,77833E+14	6,7394E+14	7,28884E+14
TC0200004229.mm.2	Caprin1	cell cycle associated protein 1	1,11445E+14	9,21347E+14	2,24887E+14	0.0626059337603045	0.204076220007462	-4,05129E+14	1,00017E+14	9,99901E+14	9,4315E+14	8,07677E+13	9,14153E+14	8,63029E+14
TC1000003069.mm.2	Usp15	ubiquitin specific peptidase 15	1,06748E+14	8,24373E+14	2,24874E+14	0.0626173171797239	0.204076220007462	-4,05146E+14	9,10366E+14	9,02986E+14	8,27306E+14	7,05721E+14	8,29621E+13	7,70236E+14
TC0100002118.mm.2	Phf3	PHD finger protein 3	1,40407E+14	8,38515E+14	2,2484E+14	0.0626476024481457	0.204133633619206	-4,05191E+13	9,27491E+14	9,26396E+14	8,61118E+14	7,43009E+14	8,66516E+14	7,06559E+13
TC0500001665.mm.2	Gm5294	predicted gene 5294	-0.976407161664762	7,16203E+14	-2,24823E+14	0.0626629184473127	0.204142257367883	-4,05214E+14	6,43856E+14	6,51609E+13	7,01196E+14	8,09808E+14	7,24254E+14	7,66492E+14
TC0100001349.mm.2	3110040M04Rik	RIKEN cDNA 3110040M04 gene	-0.847793714629483	6,66469E+14	-2,24795E+14	0.0626880107895983	0.204153624585737	-4,05251E+14	6,08639E+14	6,31197E+14	6,4271E+14	7,06872E+14	6,77085E+14	7,3231E+14
TC0600000608.mm.2	Cbx3	chromobox 3	1,56228E+14	9,12203E+14	2,24791E+14	0.0626917479333598	0.204153624585737	-4,05257E+14	1,01916E+14	1,00834E+14	9,433E+14	7,87368E+14	9,39792E+14	7,75255E+14
TC0600000308.mm.2	Exoc4	exocyst complex component 4	1,54715E+14	8,00213E+14	2,24678E+14	0.0627925789239613	0.204400438913155	-4,05406E+14	8,91211E+14	9,08031E+14	8,39236E+14	6,72989E+14	8,19818E+14	6,69992E+13
TC0100002305.mm.2	Nabp1	nucleic acid binding protein 1	-1,29212E+14	7,60808E+14	-2,24677E+14	0.0627929108558379	0.204400438913155	-4,05407E+14	6,88619E+14	6,92992E+14	8,57441E+14	6,85759E+14	8,44644E+14	7,95391E+14
TC0500002723.mm.2	Ankrd17	ankyrin repeat domain 17	1,45582E+14	8,23272E+14	2,24542E+13	0.0629135737350796	0.204706899297627	-4,05585E+14	9,24858E+14	9,11709E+14	8,59705E+13	6,9796E+14	8,38108E+14	7,07295E+14
TC0200002095.mm.2	Ndufaf5	NADH:ubiquinone oxidoreductase complex assembly factor 5	1,53427E+14	6,30977E+14	2,24524E+14	0.0629292927038998	0.204706899297627	-4,05608E+13	7,35762E+14	7,21949E+14	6,62405E+14	5,14889E+13	6,58325E+14	4,92532E+14
TC0600000698.mm.2	Lancl2	LanC (bacterial lantibiotic synthetase component C)-like 2	1,17327E+14	7,46097E+14	2,2452E+14	0.0629330141920689	0.204706899297627	-4,05614E+14	8,37414E+14	8,32036E+14	7,63139E+14	6,09197E+14	7,3133E+14	7,03465E+14
TC0300000913.mm.2	C920021L13Rik	RIKEN cDNA C920021L13 gene	-1,26644E+14	6,07747E+14	-2,24515E+14	0.0629378749531709	0.204706899297627	-4,05621E+14	5,16138E+14	5,43077E+13	6,00306E+14	6,74456E+14	5,78661E+14	7,33842E+14
TC1300002125.mm.2	Isca1	iron-sulfur cluster assembly 1	1,15162E+14	8,52811E+14	2,24494E+13	0.0629565057931378	0.204726171108947	-4,05648E+14	9,59683E+14	9,3416E+13	8,53554E+14	7,0595E+14	8,45802E+14	8,17716E+14
TC1700000278.mm.2	Zfp760	zinc finger protein 760	0.729705382876546	6,16512E+14	2,24461E+14	0.0629859105366166	0.204780463372983	-4,05692E+13	6,64142E+14	6,38308E+14	6,2528E+14	6,14837E+14	6,01696E+14	5,54812E+14
TC0800003008.mm.2	Gcsh	glycine cleavage system protein H (aminomethyl carrier)	1,00733E+14	8,22807E+14	2,24391E+14	0.0630488451111047	0.204943724283888	-4,05784E+14	8,99093E+14	8,77855E+14	8,45029E+13	7,39382E+14	8,18109E+14	7,57372E+14
TC1500001031.mm.2	Larp4	La ribonucleoprotein domain family, member 4	1,32683E+14	7,78162E+14	2,24316E+14	0.063115376472234	0.205073096739994	-4,05882E+14	8,60172E+13	8,53992E+14	8,15448E+14	6,90563E+14	7,92857E+14	6,55942E+14
TC0300002404.mm.2	Lce1d	late cornified envelope 1D	-0.725650911345263	5,66911E+14	-2,24304E+14	0.0631267758804409	0.205073096739994	-4,05899E+14	5,18609E+14	5,35912E+13	5,50678E+14	5,96614E+14	6,10234E+14	5,89417E+14
TC1700000356.mm.2	Spsb3	splA/ryanodine receptor domain and SOCS box containing 3	0.672121819537776	6,07787E+14	2,24302E+13	0.0631280786438236	0.205073096739994	-4,05901E+13	6,71015E+14	6,65464E+14	5,62093E+13	5,46098E+14	6,13713E+14	5,88341E+14
TC0200001189.mm.2	Zfp804a	zinc finger protein 804A	1,52166E+14	6,62864E+14	2,24289E+13	0.0631395539581152	0.205073096739994	-4,05918E+14	7,95285E+14	7,47328E+14	7,04204E+13	4,92088E+14	6,37073E+14	6,01209E+14
TC1100003055.mm.2	Cd68	CD68 antigen	1,63057E+14	7,80651E+14	2,24243E+14	0.0631813261772046	0.205156187376216	-4,05979E+14	8,5892E+14	9,27647E+13	8,32735E+14	6,04153E+14	7,20075E+14	7,40378E+14
TC0100000682.mm.2	Utp14b/Acsl3	UTP14B small subunit processome component/acyl-CoA synthetase long-chain family member 3	2,86069E+14	8,20599E+14	2,2422E+14	0.0632017563505195	0.205156187376216	-4,06009E+14	9,85652E+14	9,68262E+14	9,15908E+14	6,71995E+13	8,77261E+14	5,04515E+14
TC0400003255.mm.2	Btf3l4	basic transcription factor 3-like 4	1,19388E+14	7,7704E+14	2,24213E+14	0.0632084223047525	0.205156187376216	-4,06019E+13	8,57974E+14	8,65244E+14	7,90386E+14	6,64193E+13	7,91897E+13	6,92545E+14
TC0X00001716.mm.2	Asmt	acetylserotonin O-methyltransferase	-1,0991E+14	6,92403E+14	-2,24204E+14	0.0632160659295544	0.205156187376216	-4,0603E+14	6,18926E+14	6,11637E+14	6,82922E+14	7,90551E+14	6,7868E+14	7,71704E+14
TC0500002567.mm.2	Commd8	COMM domain containing 8	1,79362E+14	7,38454E+14	2,24154E+14	0.0632615119088031	0.205255256863331	-4,06097E+14	8,57493E+14	8,21105E+14	8,31607E+14	6,00646E+14	7,25251E+14	5,94622E+14
TC1700000637.mm.2	Ager	advanced glycosylation end product-specific receptor	-0.704109545672609	5,90138E+14	-2,24142E+14	0.063272069809481	0.205255256863331	-4,06113E+14	5,64158E+14	5,31886E+14	5,95048E+14	6,12872E+14	6,05066E+14	6,318E+14
TC0300000823.mm.2	Sprr2b	small proline-rich protein 2B	-1,07564E+14	6,28092E+14	-2,24087E+13	0.0633210007012794	0.205268044567334	-4,06184E+14	5,48603E+14	5,4229E+13	6,36227E+14	7,35407E+14	6,05211E+13	7,00812E+14
TC0800000010.mm.2	Mcoln1	mucolipin 1	1,00281E+14	9,04855E+14	2,24059E+14	0.0633469009491274	0.205268044567334	-4,06222E+14	9,61107E+14	9,6207E+14	9,25554E+13	8,57785E+14	9,12668E+14	8,09946E+14
TC0400001064.mm.2	Mier1	MEIR1 treanscription regulator	1,86313E+14	5,656E+14	2,2405E+14	0.0633550560133146	0.205268044567334	-4,06234E+14	6,76767E+14	6,77719E+14	6,37674E+13	4,19578E+14	5,78613E+14	4,03247E+14
TC1900000591.mm.2	Gm340	predicted gene 340	0.682920333818561	5,36538E+13	2,24045E+14	0.0633589663914694	0.205268044567334	-4,0624E+14	5,8176E+14	5,73777E+14	5,05969E+14	5,3877E+14	5,02546E+14	5,16408E+14
TC1900000841.mm.2	Pnliprp1	pancreatic lipase related protein 1	-1,13276E+14	5,66501E+14	-2,24042E+14	0.0633619025311504	0.205268044567334	-4,06244E+14	4,81132E+13	5,12062E+14	5,47381E+14	6,38687E+13	5,52937E+14	6,66809E+14

TC0900000193.mm.2	Slc44a2	solute carrier family 44, member 2	1,04937E+14	5,90765E+14	2,24041E+14	0.0633626061462687	0.205268044567334	-4,06245E+14	6,69936E+14	6,45501E+14	6,24752E+14	4,9884E+14	5,69714E+14	5,35849E+14
TC0500003708.mm.2	Mrpl33	mitochondrial ribosomal protein L33	0.888042262030247	8,59979E+14	2,24038E+14	0.0633651867236342	0.205268044567334	-4,06249E+14	9,30976E+14	9,12314E+13	8,70132E+14	7,80771E+14	8,5056E+14	8,15121E+14
TC1800001119.mm.2	Gpr17	G protein-coupled receptor 17	1,17384E+14	6,19447E+14	2,24019E+14	0.0633821708708403	0.205281792812432	-4,06274E+14	7,08183E+14	6,85512E+14	6,59279E+13	5,04781E+14	5,88291E+14	5,70635E+14
TC1700001110.mm.2	Zbtb14	zinc finger and BTB domain containing 14	1,36271E+14	5,38869E+13	2,24005E+14	0.0633953695115464	0.205283277520005	-4,06293E+13	6,19557E+14	6,18884E+14	5,89519E+14	4,39354E+14	5,4301E+14	4,2289E+12
TC0900002680.mm.2	Tmod2	tropomodulin 2	0.957168411859765	8,87663E+14	2,23968E+14	0.0634281783043282	0.205348249350541	-4,06341E+14	9,62448E+13	9,51852E+14	8,86507E+14	8,02302E+14	8,99082E+14	8,23784E+14
TC0800001487.mm.2	Il17c	interleukin 17C	-1,07546E+14	6,53279E+14	-2,23937E+14	0.0634560920100186	0.205362789096251	-4,06382E+14	5,89638E+14	5,91143E+14	6,06029E+14	7,36994E+14	6,7619E+14	7,19684E+14
TC1200000319.mm.2	Ankmy2	ankyrin repeat and MYND domain containing 2	0.885116571316673	6,97312E+14	2,23935E+13	0.0634581596791556	0.205362789096251	-4,06385E+14	7,61127E+14	7,44934E+14	6,91882E+14	6,56891E+13	7,09208E+14	6,1983E+14
TC0600003324.mm.2	E330021D16Rik	RIKEN cDNA E330021D16 gene	-1,01916E+14	5,65148E+14	-2,23897E+14	0.0634922300030458	0.2054212995645	-4,06435E+14	4,7588E+14	4,94594E+14	5,70224E+13	6,75884E+14	5,53551E+14	6,20754E+14
TC1400000784.mm.2	Psme1	proteasome (prosome, macropain) activator subunit 1 (PA28 alpha)	1,1372E+14	8,97847E+13	2,23873E+14	0.0635140301702877	0.2054212995645	-4,06467E+13	9,89341E+14	9,91527E+14	8,95723E+14	7,57062E+14	9,05596E+14	8,47832E+14
TC1600001155.mm.2	Nagpa	N-acetylglucosamine-1-phosphodiester alpha-N-acetylglucosaminidase	-1,02321E+14	7,17937E+14	-2,23867E+14	0.0635194150943187	0.2054212995645	-4,06475E+14	6,47291E+14	6,65455E+14	7,00662E+14	7,76825E+14	7,08397E+14	8,08991E+14
TC1400002515.mm.2	Diaph3	diaphanous related formin 3	-0.969854800124025	6,82061E+14	-2,23859E+14	0.0635272348867314	0.2054212995645	-4,06486E+14	6,00854E+14	6,33425E+14	6,59974E+14	7,69866E+14	7,04302E+14	7,23947E+14
TC1200000856.mm.2	Vash1	vasohibin 1	-0.835306040648477	6,36206E+14	-2,23819E+14	0.0635631891413832	0.205493050418014	-4,06539E+14	5,75301E+14	5,69268E+14	6,40311E+14	7,20726E+14	6,41736E+14	6,69894E+14
TC0100001957.mm.2	Cpa6	carboxypeptidase A6	-0.723824203667431	4,80526E+14	-2,23806E+14	0.0635749305736858	0.205493050418014	-4,06556E+14	4,29665E+14	4,46679E+14	4,77339E+14	5,08363E+14	5,30447E+14	4,90661E+14
TC1100000802.mm.2	Trpv2	transient receptor potential cation channel, subfamily V, member 2	0.683212207301626	6,20078E+14	2,23734E+14	0.0636395493932009	0.205593529437944	-4,0665E+13	6,75538E+14	6,77428E+14	5,57282E+13	5,93894E+14	6,05363E+14	6,1096E+14
TC0300000490.mm.2	Ube2v1	ubiquitin-conjugating enzyme E2 variant 1	1,34498E+14	9,19212E+13	2,23727E+14	0.0636458599616003	0.205593529437944	-4,0666E+13	1,00236E+14	9,89249E+14	9,68717E+13	8,32337E+14	9,26675E+14	7,95936E+14
TC0600001189.mm.2	Lrrn1	leucine rich repeat protein 1, neuronal	1,75225E+14	7,42288E+14	2,23703E+14	0.0636678126249502	0.205593529437944	-4,06692E+14	8,64355E+14	8,49147E+14	8,06886E+14	5,70287E+14	7,36487E+14	6,26565E+14
TC0600001381.mm.2	1700063H04Rik	RIKEN cDNA 1700063H04 gene	-0.732755912301521	4,64044E+14	-2,23691E+14	0.0636789161596685	0.205593529437944	-4,06708E+14	4,17419E+14	4,12008E+14	4,68294E+14	5,10565E+14	4,69503E+14	5,06475E+14
TC0700004194.mm.2	Crym	crystallin, mu	0.715789804074454	7,59882E+13	2,2367E+14	0.0636975440623691	0.205593529437944	-4,06735E+14	7,99245E+14	7,91637E+14	7,42211E+14	7,78478E+14	7,06681E+14	7,41042E+13
TC1200001676.mm.2	Lrrn3	leucine rich repeat protein 3, neuronal	1,99668E+14	7,06368E+14	2,23658E+12	0.0637080573241887	0.205593529437944	-4,0675E+14	8,3607E+14	8,18631E+14	7,72313E+14	5,55833E+14	7,33414E+14	5,2195E+14
TC1100003095.mm.2	Pelp1	proline, glutamic acid and leucine rich protein 1	1,1238E+13	5,34517E+14	2,23658E+14	0.063708314381535	0.205593529437944	-4,06751E+14	6,32918E+14	6,24225E+13	5,25486E+12	3,9209E+14	4,84634E+14	5,47747E+13
TC1100003146.mm.2	Spns2	spinster homolog 2	1,18135E+14	7,08166E+14	2,23652E+14	0.0637142213441502	0.205593529437944	-4,06759E+14	7,83453E+13	7,87234E+14	7,38318E+14	6,05573E+14	7,10885E+14	6,23531E+14
TC1200000661.mm.2	Dbpht2	DNA binding protein with his-thr domain	0.981030557866179	8,85919E+14	2,23644E+14	0.0637208518595601	0.205593529437944	-4,06769E+14	9,30708E+14	9,62306E+14	9,20142E+14	8,05551E+13	8,48774E+14	8,48033E+14
TC1700002755.mm.2	Mettl4	methyltransferase like 4	1,50248E+14	5,97896E+14	2,2361E+14	0.0637522292708924	0.205598037772498	-4,06815E+14	6,91908E+14	6,89394E+14	6,18217E+14	5,07051E+14	6,34057E+12	4,46748E+14
TC0700000486.mm.2	9530053A07Rik	RIKEN cDNA 9530053A07 gene	-0.800261118173267	5,31357E+14	-2,23596E+14	0.0637649442290647	0.205598037772498	-4,06833E+14	4,82877E+14	4,95932E+14	5,14494E+14	5,55978E+13	6,00918E+14	5,37944E+14
TC1100002732.mm.2	Gm9837	predicted gene 9837	-0.75699069327892	5,67088E+13	-2,23586E+14	0.0637736032849183	0.205598037772498	-4,06846E+14	5,51366E+14	5,24693E+14	5,39689E+14	5,59327E+14	6,41241E+14	5,86216E+14
TC0100000656.mm.2	Dnajb2	DnaJ heat shock protein family (Hsp40) member B2	1,04229E+14	7,20461E+14	2,23583E+14	0.0637765724966807	0.205598037772498	-4,0685E+14	8,17383E+14	7,76442E+13	7,26873E+14	6,16704E+14	7,22344E+14	6,63022E+14
TC1000002645.mm.2	Chpt1	choline phosphotransferase 1	1,33098E+14	7,19794E+14	2,23561E+14	0.0637961831415394	0.205598037772498	-4,06879E+14	8,17188E+14	7,89792E+14	7,68171E+14	6,02831E+14	7,11869E+13	6,28916E+14
TC0600002121.mm.2	Slc37a3	solute carrier family 37 (glycerol-3-phosphate transporter), member 3	0.844967093039168	7,08731E+14	2,23558E+14	0.0637988077243524	0.205598037772498	-4,06882E+14	7,60202E+14	7,47446E+14	7,21244E+14	6,84837E+14	7,0891E+14	6,29744E+14
TC0400003253.mm.2	Zfyve9	zinc finger, FYVE domain containing 9	1,36129E+14	7,14761E+14	2,23525E+14	0.0638287480296769	0.205653392721892	-4,06926E+14	8,17693E+14	8,13638E+14	7,30883E+14	5,67279E+14	7,30285E+13	6,28787E+14
TC0700002430.mm.2	Arhgap35	Rho GTPase activating protein 35	0.745708300123342	8,65907E+13	2,23491E+14	0.0638600349010098	0.205695415683831	-4,06972E+14	9,12331E+14	9,09798E+14	8,70217E+14	8,30109E+14	8,59329E+14	8,13658E+14
TC0700000391.mm.2	Lypd3	Ly6/Plaur domain containing 3	-0.829534383969366	6,72403E+14	-2,23478E+14	0.063871215358127	0.205695415683831	-4,06988E+14	6,13986E+14	6,24118E+14	6,57663E+14	7,34637E+14	6,85609E+14	7,18402E+14
TC1700000276.mm.2	Zfp983	zinc finger protein 983	1,92145E+14	5,65295E+14	2,23464E+12	0.0638838958610431	0.205695415683831	-4,07006E+14	6,83541E+14	7,12997E+13	5,82613E+13	4,00372E+13	6,18364E+14	3,93884E+14
TC0400001726.mm.2	Mul1	mitochondrial ubiquitin ligase activator of NFKB 1	0.701871049123544	6,02346E+14	2,23455E+14	0.0638928539376637	0.205695415683831	-4,07019E+14	6,76001E+14	6,39992E+14	5,49213E+14	5,7325E+14	5,808E+14	5,94819E+14
TC1700000794.mm.2	Olfr130	olfactory receptor 130	-1,15102E+14	4,68055E+14	-2,23425E+14	0.0639198966598745	0.205724601794491	-4,07059E+14	4,00244E+14	3,94846E+12	4,37565E+14	5,5038E+13	4,60782E+14	5,64513E+14
TC0300002688.mm.2	Cept1	choline/ethanolaminephosphotransferase 1	1,01978E+14	7,37303E+14	2,23416E+14	0.0639274549236652	0.205724601794491	-4,07069E+14	8,20066E+14	8,04584E+14	7,41082E+14	6,37393E+14	7,46358E+14	6,74335E+14
TC1700000829.mm.2	Tnfrsf21	tumor necrosis factor receptor superfamily, member 21	0.915246919541135	7,57787E+14	2,23337E+14	0.0639998111894122	0.20591632541833	-4,07175E+14	8,23646E+14	8,05691E+14	7,84446E+14	6,86654E+13	7,39696E+14	7,06592E+14
TC0800001805.mm.2	Defb39	defensin beta 39	-1,00652E+12	5,58952E+14	-2,23297E+14	0.0640364451731194	0.205993060705624	-4,07228E+14	4,85919E+14	5,07766E+14	5,22015E+14	6,43026E+14	5,87587E+14	6,074E+14
TC1800000575.mm.2	Megf10	multiple EGF-like-domains 10	0.990036939655598	5,54403E+14	2,2326E+14	0.0640698409223201	0.206029207385868	-4,07276E+14	6,39749E+14	6,1913E+14	5,43931E+14	4,62736E+14	4,86688E+14	5,74184E+14
TC1700001805.mm.2	Glo1	glyoxalase 1	1,55279E+14	7,79356E+13	2,23256E+14	0.0640732550245506	0.206029207385868	-4,07281E+14	8,81999E+14	8,66673E+14	8,55531E+14	6,3382E+14	7,42553E+14	6,95561E+14
TC1300002481.mm.2	Hexb	hexosaminidase B	1,5748E+14	8,86063E+14	2,23224E+14	0.0641022517378938	0.206081321279466	-4,07323E+14	9,84732E+14	1,01161E+14	9,23154E+14	7,15497E+14	8,89553E+14	7,91832E+14
TC0900001115.mm.2	Rwdd2a	RWD domain containing 2A	1,43265E+14	6,165E+14	2,23207E+14	0.0641181046591376	0.206085094177444	-4,07346E+13	7,47406E+13	6,70983E+14	6,20839E+14	5,2791E+14	6,49432E+14	4,82428E+14
TC1100003043.mm.2	Kdm6b	KDM1 lysine (K)-specific demethylase 6B	-0.730670463728715	7,67935E+14	-2,23195E+14	0.06412900528801	0.206085094177444	-4,07362E+14	7,31876E+14	6,86998E+13	8,20295E+14	8,03433E+14	7,67779E+14	7,97229E+14
TC1200000282.mm.2	Sypl	synaptophysin-like protein	1,39399E+14	6,7996E+14	2,23152E+14	0.0641680698908207	0.206138063544217	-4,07418E+14	7,57963E+14	7,8138E+12	6,92602E+14	5,8727E+14	7,13831E+14	5,46715E+14
TC1900000254.mm.2	Keg1	kidney expressed gene 1	-1,0913E+14	4,89108E+14	-2,23115E+14	0.0642021387866822	0.206138063544217	-4,07468E+14	4,36252E+14	4,06259E+14	4,50909E+14	5,80458E+13	5,05542E+14	5,55229E+14
TC1100001504.mm.2	Skap1	src family associated phosphoprotein 1	-0.825403388401727	5,64852E+14	-2,23112E+14	0.064204212508777	0.206138063544217	-4,07471E+14	5,33741E+14	5,21687E+14	5,353E+14	5,77875E+14	5,74805E+13	6,45704E+14
TC0200002894.mm.2	Celf2	CUGBP, Elav-like family member 2	1,36904E+14	9,93537E+14	2,23102E+14	0.0642138915713803	0.206138063544217	-4,07484E+14	1,08128E+14	1,09073E+14	1,03214E+14	8,58877E+14	9,9298E+14	9,05217E+14
TC1400002598.mm.2	Mzt1	mitotic spindle organizing protein 1	0.741458433215174	8,23047E+14	2,23093E+14	0.0642214941901339	0.206138063544217	-4,07495E+14	8,77133E+14	8,85409E+14	8,10178E+14	7,51313E+14	8,10834E+14	8,03417E+13
TC0100002445.mm.2	Ndufs1	NADH:ubiquinone oxidoreductase core subunit S1	1,43624E+13	7,15925E+14	2,23093E+14	0.0642222478118271	0.206138063544217	-4,07497E+14	8,29507E+14	8,12708E+14	7,39013E+14	5,59354E+14	7,27802E+14	6,27166E+14
TC1600001108.mm.2	1700037C18Rik	RIKEN cDNA 1700037C18 gene	-1,07915E+14	6,30931E+14	-2,23074E+14	0.0642394626357162	0.206152252827982	-4,07521E+14	5,5949E+13	5,68244E+14	6,0406E+14	7,10229E+14	6,26691E+14	7,16872E+14
TC0400001135.mm.2	Lrp8	low density lipoprotein receptor-related protein 8, apolipoprotein e receptor	1,27482E+14	6,32219E+14	2,23052E+14	0.0642593052158355	0.206173000750685	-4,0755E+14	7,08562E+14	7,05341E+13	6,6013E+14	5,60344E+14	6,52659E+14	5,0628E+14
TC0X00000436.mm.2	Rab33a	RAB33A, member RAS oncogene family	0.697003505956549	6,77195E+13	2,23029E+14	0.0642801676607592	0.206173000750685	-4,0758E+14	7,15418E+14	7,10463E+14	6,64078E+13	6,86734E+14	6,61985E+14	6,24495E+14
TC0200002666.mm.2	Fam210b	family with sequence similarity 210, member B	-0.75658839251819	7,80228E+14	-2,23025E+14	0.0642843142662101	0.206173000750685	-4,07586E+14	7,30261E+14	7,20643E+14	7,92077E+13	8,36163E+14	7,77941E+14	8,24281E+13
TC1700001619.mm.2	Atp6v0c/Atp6v0c-ps2	ATPase, H+ transporting, lysosomal V0 subunit C/ATPase, H+ transporting, lysosomal V0 subunit C, pseudogene 2	1,29598E+14	1,13969E+14	2,23001E+14	0.0643055887598356	0.206175432724095	-4,07617E+14	1,23705E+14	1,23623E+14	1,1488E+14	1,002E+14	1,16011E+14	1,05396E+14
TC0700000559.mm.2	Syne4	spectrin repeat containing, nuclear envelope family member 4	-0.819244787890868	6,49635E+14	-2,22996E+14	0.0643106637417801	0.206175432724095	-4,07624E+14	5,54727E+14	6,03227E+14	6,75923E+14	7,42129E+14	6,59732E+14	6,62071E+14
TC1000001609.mm.2	Smarcc2	SWI/SNF related, matrix associated, actin dependent regulator of chromatin, subfamily c, member 2	0.782964018811219	8,23255E+14	2,22975E+14	0.0643300526645349	0.206196566258932	-4,07652E+14	8,69505E+13	8,88007E+14	8,1119E+14	7,69911E+14	8,28719E+14	7,72201E+13
TC1400001747.mm.2	Ghitm	growth hormone inducible transmembrane protein	2,15858E+14	8,21738E+14	2,22889E+14	0.0644080141111203	0.206405396056579	-4,07765E+13	9,75007E+14	9,83404E+14	8,48632E+14	5,96689E+14	8,71125E+13	6,55571E+14
TC0800000679.mm.2	March1	membrane-associated ring finger (C3HC4) 1	0.895048729344167	7,19158E+14	2,22869E+14	0.0644265995811793	0.206423901183444	-4,07791E+14	8,01773E+14	7,52537E+14	7,1804E+14	6,67296E+14	6,51851E+14	7,2345E+14

TC0300002481.mm.2	Adamtsl4	ADAMTS-like 4	-0.669932415202485	8,0849E+14	-2,22829E+14	0.0644631476329114	0.206472368335087	-4,07844E+14	7,70713E+14	7,55954E+14	8,25413E+14	8,38211E+14	8,17818E+14	8,42835E+14
TC1100004192.mm.2	Aatk	apoptosis-associated tyrosine kinase	0.907659775554252	7,39159E+14	2,22824E+14	0.0644673546263156	0.206472368335087	-4,0785E+14	8,03044E+14	8,17052E+13	7,3935E+14	6,36945E+13	7,1613E+14	7,22435E+14
TC0X00001520.mm.2	Phf8	PHD finger protein 8	0.689572889119927	5,62907E+14	2,22786E+14	0.0645029086277688	0.206473375491914	-4,07901E+14	6,44982E+14	6,101E+14	4,93809E+14	5,11382E+14	5,51908E+12	5,65259E+14
TC0700003017.mm.2	Sphk2	sphingosine kinase 2	-0.675608821975735	6,51202E+14	-2,22768E+14	0.0645186726651434	0.206473375491914	-4,07924E+14	6,26273E+14	6,1442E+14	6,5774E+14	6,32965E+14	6,77214E+14	6,98601E+14
TC0700004157.mm.2	Pik3c2a	phosphatidylinositol-4-phosphate 3-kinase catalytic subunit type 2 alpha	2,13713E+14	5,63766E+14	2,22768E+14	0.0645187761399585	0.206473375491914	-4,07924E+14	7,08698E+14	7,09909E+14	6,00079E+14	3,72727E+14	6,13978E+14	3,77203E+14
TC0200000641.mm.2	Dab2ip	disabled 2 interacting protein	0.714289665673095	7,4803E+14	2,22761E+14	0.0645253647386044	0.206473375491914	-4,07934E+14	7,9673E+14	7,98667E+14	7,45011E+14	6,95232E+14	7,20697E+14	7,31842E+14
TC1100002841.mm.2	Obscn	obscurin, cytoskeletal calmodulin and titin-interacting RhoGEF	-0.766742889340785	6,13788E+14	-2,22754E+13	0.0645317395256798	0.206473375491914	-4,07943E+14	5,62932E+14	5,70889E+14	5,97296E+14	6,64441E+14	6,40099E+14	6,4707E+14
TC0200004760.mm.2	Ovol2	ovo like zinc finger 2	-0.90676419762591	6,49847E+14	-2,22735E+14	0.0645496948453066	0.206489821926231	-4,07969E+14	6,1101E+14	5,77525E+14	6,39379E+14	7,01283E+14	6,47048E+14	7,2284E+14
TC1600001194.mm.2	Tvp23a	trans-golgi network vesicle protein 23A	1,19472E+14	7,26824E+14	2,22676E+14	0.0646038176655988	0.206570565095894	-4,08046E+14	8,03791E+14	7,81291E+14	7,94049E+14	6,35675E+14	6,87358E+14	6,5878E+13
TC1600000269.mm.2	Yeats2	YEATS domain containing 2	0.81670219466647	6,36165E+14	2,22662E+14	0.0646164364296273	0.206570565095894	-4,08064E+14	7,20124E+14	6,49231E+14	6,41153E+14	6,00469E+14	6,04027E+14	6,01988E+14
TC1300001555.mm.2	Hist1h2bl	histone cluster 1, H2bl	-0.692193009709081	8,59546E+14	-2,22661E+14	0.0646173075288196	0.206570565095894	-4,08066E+14	8,0726E+14	8,24203E+14	8,68635E+14	8,87278E+14	9,02104E+14	8,67797E+14
TC1500001504.mm.2	Eif3h	eukaryotic translation initiation factor 3, subunit H	0.935015015241362	8,76185E+13	2,22625E+14	0.064650201066922	0.206570565095894	-40811297667	9,55861E+14	9,29866E+14	8,8411E+13	7,88551E+14	8,73141E+14	8,25583E+14
TC1900000668.mm.2	Tlx1	T cell leukemia, homeobox 1	-1,03408E+14	6,00729E+14	-2,22619E+14	0.0646557255306314	0.206570565095894	-4,08121E+14	5,28614E+14	5,56345E+14	5,64254E+14	6,63385E+14	6,05463E+14	6,86311E+14
TC1100003961.mm.2	Cacng1	calcium channel, voltage-dependent, gamma subunit 1	-0.704095670469409	5,73817E+14	-2,22586E+14	0.0646864007581646	0.206570565095894	-4,08165E+14	5,37756E+14	5,19911E+14	5,78323E+14	6,08425E+14	6,15266E+13	5,83221E+13
TC1400001863.mm.2	Gm8165	predicted gene 8165	-0.674253476347466	6,10046E+14	-2,22573E+14	0.0646985889413714	0.206570565095894	-4,08182E+14	5,9833E+14	5,78054E+14	5,91614E+14	5,81044E+14	6,68484E+14	6,4275E+14
TC0300000132.mm.2	Trim55	tripartite motif-containing 55	-1,0172E+14	5,98073E+14	-2,22557E+14	0.0647131352707879	0.206570565095894	-4,08203E+14	5,28878E+14	5,07468E+14	5,93376E+14	7,18928E+14	6,22496E+14	6,17289E+13
TC0700003403.mm.2	Chrna7	cholinergic receptor, nicotinic, alpha polypeptide 7	0.795619933767444	6,7075E+14	2,22556E+14	0.0647139288300855	0.206570565095894	-4,08204E+13	7,02306E+14	7,15972E+14	6,57558E+14	6,89514E+14	6,63529E+13	5,95624E+14
TC0200005373.mm.2	Gm14325	predicted gene 14325	1,43833E+14	7,61029E+14	2,22547E+14	0.0647224666115705	0.206570565095894	-4,08217E+14	8,71683E+14	9,00609E+14	7,35464E+14	5,7379E+14	8,05815E+14	6,78811E+14
TC0700000527.mm.2	Ppp1r14a	protein phosphatase 1, regulatory inhibitor subunit 14A	0.665277916865262	7,45127E+14	2,22542E+14	0.0647263402872749	0.206570565095894	-4,08222E+14	7,80832E+14	7,73799E+14	7,33793E+14	7,60759E+14	7,05804E+14	7,15772E+14
TC1100004202.mm.2	Actg1/Mir6935	actin, gamma, cytoplasmic 1/microRNA 6935	1,01107E+14	1,00811E+14	2,2254E+14	0.0647287769607876	0.206570565095894	-4,08226E+14	1,08823E+13	1,07508E+14	1,02475E+14	8,99497E+14	9,84702E+14	9,76393E+14
TC1800001193.mm.2	Hbegf	heparin-binding EGF-like growth factor	-1,01691E+14	7,41405E+14	-2,22505E+14	0.0647609006408469	0.206631310239835	-4,08272E+14	6,67259E+14	6,7085E+14	7,30352E+14	8,3848E+14	7,37049E+14	8,04442E+14
TC0100003639.mm.2	Adss	adenylosuccinate synthetase, non muscle	1,24496E+13	7,43557E+13	2,22491E+13	0.0647734591957679	0.206631310239835	-4,0829E+13	8,17044E+14	8,40299E+14	7,53903E+14	6,41747E+14	7,68463E+14	6,39887E+14
TC0700004569.mm.2	Mir675/H19	microRNA 675/H19, imprinted maternally expressed transcript	-1,25888E+12	5,29705E+14	-2,22456E+14	0.064805778422223	0.206693489255202	-4,08336E+14	4,65279E+13	4,85905E+14	4,32434E+14	5,91655E+14	6,36095E+14	5,66864E+13
TC0800000330.mm.2	Lsm1	LSM1 homolog, mRNA degradation associated	0.682519225637423	6,82121E+14	2,22396E+14	0.0648613040986814	0.206751585725053	-4,08415E+14	7,40761E+14	7,18086E+13	6,68068E+14	6,43468E+14	6,51474E+14	6,70869E+14
TC0700000787.mm.2	Egfbp2/Klk1b26	epidermal growth factor binding protein type B/kallikrein 1-related petidase b26	-1,03797E+14	5,98611E+14	-2,22388E+14	0.0648682480855571	0.206751585725053	-4,08425E+14	5,25966E+14	5,1223E+14	6,01807E+14	7,0587E+14	5,82951E+14	6,6284E+14
TC0600002582.mm.2	Tex261	testis expressed gene 261	0.82901376420203	6,78436E+13	2,22358E+14	0.0648962147714131	0.206751585725053	-4,08465E+13	7,27091E+14	7,3483E+14	6,98434E+14	6,14145E+14	6,52935E+14	6,43183E+13
TC1500002170.mm.2	Zfp641	zinc finger protein 641	-0.645882804283579	6,58681E+13	-2,22335E+14	0.0649173265826464	0.206751585725053	-4,08496E+14	6,27853E+14	6,14458E+14	6,66647E+14	6,71639E+14	6,8855E+14	6,82938E+14
TC0100000351.mm.2	Wdr75	WD repeat domain 75	1,21323E+13	6,41715E+14	2,22326E+14	0.0649255332637574	0.206751585725053	-4,08507E+13	7,31592E+14	7,34508E+14	6,36561E+14	5,24174E+14	6,73051E+14	5,50403E+14
TC0800001185.mm.2	Car7	carbonic anhydrase 7	-1,00487E+14	7,5832E+14	-2,22301E+14	0.0649486546392909	0.206751585725053	-4,0854E+14	6,76477E+13	6,90953E+14	8,50964E+14	7,63122E+14	8,2818E+14	7,40225E+14
TC0500002652.mm.2	Pea15b	phosphoprotein enriched in astrocytes 15B	-0.917224962996478	5,4818E+14	-2,22296E+14	0.0649536371545898	0.206751585725053	-4,08547E+14	5,12057E+14	4,7326E+14	5,21204E+14	6,13798E+14	5,67062E+14	6,017E+14
TC0500002936.mm.2	Dipk1a	divergent protein kinase domain 1A	1,12826E+14	7,93041E+14	2,22288E+14	0.0649606710007861	0.206751585725053	-4,08558E+14	8,46356E+13	8,31207E+14	8,22571E+14	8,06204E+14	8,00149E+13	6,51762E+14
TC0X00003346.mm.2	Rab9	RAB9, member RAS oncogene family	2,63532E+13	6,66245E+14	2,22285E+14	0.064963810680087	0.206751585725053	-4,08562E+14	8,71979E+14	8,46575E+14	7,07648E+14	3,7978E+14	7,20257E+14	4,71232E+14
TC0700001631.mm.2	Ampd3	adenosine monophosphate deaminase 3	1,00653E+13	8,33623E+14	2,22284E+14	0.0649643536766337	0.206751585725053	-4,08563E+14	9,07615E+13	9,02508E+14	8,53005E+14	7,29794E+14	8,16089E+14	7,92728E+14
TC0500003090.mm.2	Mlec	malectin	1,67626E+14	7,83719E+14	2,22276E+14	0.0649715430947127	0.206751585725053	-4,08573E+14	8,98289E+14	8,87903E+14	8,53895E+14	6,11284E+14	7,55298E+14	6,95643E+14
TC1700000528.mm.2	Abcg1	ATP binding cassette subfamily G member 1	0.737009545459374	7,09674E+14	2,22269E+14	0.0649779699690726	0.206751585725053	-4,08582E+14	7,60821E+14	7,62887E+13	6,74461E+14	6,8357E+14	6,58846E+14	7,1746E+14
TC1300001100.mm.2	Enc1	ectodermal-neural cortex 1	0.906047295151728	7,96516E+14	2,22244E+14	0.0650013465943994	0.2067851328086	-4,08616E+14	8,48369E+14	8,90414E+14	7,84348E+14	6,98391E+13	7,98798E+14	7,58775E+14
TC0700002359.mm.2	Chmp2a	charged multivesicular body protein 2A	1,21208E+14	7,77382E+14	2,22191E+14	0.0650500377951561	0.206887798017537	-4,08685E+14	8,54443E+14	8,58054E+14	7,83026E+14	6,98686E+14	8,06681E+14	6,634E+14
TC0100003754.mm.2	Tgfb2	transforming growth factor, beta 2	1,38487E+14	5,62884E+13	2,22181E+14	0.0650592982408526	0.206887798017537	-4,08698E+14	6,60676E+14	6,60342E+13	6,05147E+13	4,07095E+14	5,2762E+14	5,16423E+14
TC1700002769.mm.2	Gm3435	predicted gene 3435	1,24279E+14	5,70096E+14	2,22146E+13	0.0650922912262982	0.206929663660058	-4,08745E+14	6,35672E+14	6,75943E+14	5,99086E+14	4,46822E+14	5,66278E+14	4,96778E+14
TC0200001991.mm.2	Oxt	oxytocin	-0.76905574791466	8,75924E+14	-2,22121E+14	0.065114921493693	0.206929663660058	-4,08778E+14	8,48982E+13	8,02277E+14	8,74349E+14	9,24866E+14	9,06613E+14	8,98457E+14
TC0200005270.mm.2	Cbln4	cerebellin 4 precursor protein	1,30192E+14	5,51134E+14	2,22118E+14	0.0651178015892565	0.206929663660058	-4,08782E+14	6,58419E+14	5,84775E+14	6,18251E+14	4,62548E+14	4,60175E+14	5,22636E+14
TC0100000312.mm.2	Pou3f3/2900092D14 Rik	POU domain, class 3, transcription factor 3/RIKEN cDNA 2900092D14 gene	1,0449E+14	6,21434E+14	2,22112E+13	0.0651238332042547	0.206929663660058	-4,0879E+14	6,70267E+14	7,08465E+14	5,65363E+14	6,14755E+14	6,43926E+14	5,25827E+14
TC0400001736.mm.2	Pla2g2e	phospholipase A2, group IIE	-0.925683233999409	4,89689E+14	-2,2208E+14	0.0651531678880981	0.206982057212327	-4,08832E+13	4,3672E+14	4,39722E+14	4,80689E+14	5,19426E+14	4,80246E+14	5,81332E+14
TC0800003193.mm.2	Irf2bp2	interferon regulatory factor 2 binding protein 2	1,24783E+14	6,48108E+14	2,22065E+14	0.0651667596280321	0.206984426943915	-4,08851E+14	7,35929E+14	7,20439E+14	6,88714E+14	5,36762E+14	6,44215E+14	5,62587E+14
TC0500001217.mm.2	Dao	D-amino acid oxidase	-1,11873E+14	5,9182E+14	-2,22031E+14	0.0651980863812726	0.207009590540543	-4,08896E+14	5,28207E+14	5,07829E+14	5,59388E+14	6,95709E+13	6,02344E+14	6,57439E+14
TC0200003603.mm.2	Pla2r1	phospholipase A2 receptor 1	-0.730874550018005	5,47852E+14	-2,22029E+14	0.0652003768381589	0.207009590540543	-4,08899E+14	4,90303E+14	4,84274E+13	5,7139E+14	6,20395E+14	5,45537E+14	5,75214E+14
TC0700001959.mm.2	Ptpre	protein tyrosine phosphatase, receptor type, E	0.676989320382373	7,10239E+14	2,22004E+14	0.0652234494899554	0.207010560524095	-4,08932E+14	7,38608E+14	7,63615E+14	7,0946E+14	6,82923E+14	6,81645E+14	6,8518E+14
TC1500002118.mm.2	Nell2	NEL-like 2	1,21483E+14	9,37387E+14	2,21983E+14	0.0652433526201125	0.207010560524095	-4,0896E+14	1,01366E+14	1,03246E+14	9,5258E+14	8,2248E+14	9,53106E+14	8,50043E+14
TC0200002426.mm.2	Lbp	lipopolysaccharide binding protein	1,18186E+14	6,71053E+14	2,2195E+13	0.0652739909183877	0.207010560524095	-4,09004E+14	6,70689E+14	7,93373E+14	6,23057E+14	7,11508E+14	6,41118E+14	5,86572E+14
TC1700002583.mm.2	Srsf7	serine/arginine-rich splicing factor 7	0.953761314360884	6,71025E+14	2,21948E+14	0.0652758454606338	0.207010560524095	-4,09007E+14	7,0741E+13	7,3195E+14	6,71484E+14	6,66697E+14	6,80222E+13	5,68386E+13
TC0900002281.mm.2	Sik2	salt inducible kinase 2	0.988224182887122	6,28059E+14	2,21945E+14	0.065278055013891	0.207010560524095	-4,0901E+14	7,09508E+14	6,78324E+14	6,53682E+14	5,36654E+14	5,87811E+13	6,02377E+14
TC0900001616.mm.2	Trak1	trafficking protein, kinesin binding 1	1,01333E+14	7,96519E+14	2,21939E+14	0.065283484399646	0.207010560524095	-4,09017E+14	8,76347E+13	8,49247E+13	8,23889E+14	7,06707E+14	7,82245E+14	7,40682E+13
TC1100000692.mm.2	Olfr325	olfactory receptor 325	-1,47591E+14	4,44057E+14	-2,21923E+14	0.0652985870532291	0.207010560524095	-4,09039E+14	2,98895E+14	4,04915E+14	3,92211E+14	5,69329E+13	4,69262E+14	5,29729E+14
TC0800000034.mm.2	Ccl25	chemokine (C-C motif) ligand 25	-1,13642E+14	7,08748E+14	-2,21918E+14	0.0653034617479037	0.207010560524095	-4,09046E+14	6,56764E+14	6,19595E+14	6,76302E+14	7,96181E+14	7,08936E+14	7,94709E+14
TC0200001260.mm.2	Olfr1029	olfactory receptor 1029	-0.9492812897811	5,41765E+14	-2,21847E+14	0.0653694747257975	0.207164992824269	-4,0914E+14	4,53962E+14	4,77679E+14	5,50127E+14	6,47323E+14	5,33723E+14	5,87774E+13
TC0500000560.mm.2	Pcdh7	protocadherin 7	1,79889E+14	7,35184E+13	2,21826E+14	0.0653890239463237	0.207164992824269	-4,09167E+14	8,48214E+14	8,45026E+14	7,73707E+14	6,10698E+14	7,75567E+14	5,57895E+14
TC0500000321.mm.2	Zfp512	zinc finger protein 512	1,18611E+14	6,75102E+14	2,21824E+14	0.0653907499226856	0.207164992824269	-4,0917E+14	7,93986E+14	7,20034E+14	6,90249E+14	5,69544E+14	6,77501E+14	5,99298E+14
TC0900001251.mm.2	Nme9/1600029I14Rik	NME/NM23 family member 9/RIKEN cDNA 1600029I14 gene	-0.769090466319877	6,6002E+14	-2,21744E+14	0.0654652329210903	0.207283622679455	-4,09275E+14	6,16965E+14	6,06047E+14	6,62004E+14	6,98273E+14	6,59178E+14	7,17652E+14
TC1500002262.mm.2	Krt82	keratin 82	-0.667273882897943	5,12384E+14	-2,21712E+14	0.0654949429942823	0.207283622679455	-4,09317E+14	4,5116E+13	4,63014E+14	5,41413E+14	5,71089E+14	5,25449E+14	5,2218E+14
TC0800002515.mm.2	1700011L22Rik	RIKEN cDNA 1700011L22 gene	-1,27338E+14	4,79389E+14	-2,21708E+14	0.0654983534390419	0.207283622679455	-4,09322E+14	4,25854E+14	3,57228E+14	4,5943E+14	5,9606E+14	4,72967E+14	5,64792E+13
TC0200001586.mm.2	Immp1l	IMP1 inner mitochondrial membrane peptidase-like (S. cerevisiae)	1,59224E+14	6,36952E+13	2,21666E+14	0.0655374896055872	0.207283622679455	-4,09378E+14	7,46307E+14	7,62013E+14	6,64696E+14	4,58823E+14	6,52188E+13	5,37684E+14
TC1800001186.mm.2	Nrg2	neuregulin 2	-0.673025689836708	6,84787E+12	-2,21663E+14	0.065539914697391	0.207283622679455	-4,09381E+14	6,31493E+14	6,26646E+14	7,15125E+14	7,42715E+14	7,07027E+14	6,85718E+14

TC1000002520.mm.2	Gng7	guanine nucleotide binding protein (G protein), gamma 7	1,21498E+14	9,16455E+14	2,21652E+14	0.0655507294997142	0.207283622679455	-4,09396E+14	1,01147E+14	1,00287E+14	9,38483E+14	7,74566E+13	9,0213E+14	8,69212E+14
TC0500002315.mm.2	Fbxl5	F-box and leucine-rich repeat protein 5	1,43472E+14	6,81393E+14	2,21637E+14	0.0655641581808526	0.207283622679455	-4,09415E+14	7,89055E+14	7,93792E+14	7,03911E+14	5,05697E+14	6,79433E+14	6,16471E+13
TC0500001546.mm.2	Castor2	cytosolic arginine sensor for mTORC1 subunit 2	0.72595944396677	7,75501E+14	2,21611E+14	0.065589127459167	0.207283622679455	-4,09451E+14	8,39447E+14	8,14853E+14	7,48966E+14	7,40636E+14	7,79601E+14	7,29506E+14
TC1600001745.mm.2	Zbtb11os1	zinc finger and BTB domain containing 11, opposite strand 1	-0.722293061326251	6,83042E+14	-2,21606E+14	0.0655932338366751	0.207283622679455	-4,09456E+14	6,30452E+14	6,4122E+14	7,03544E+14	7,06908E+14	6,81056E+14	7,35074E+14
TC1900001098.mm.2	Dagla	diacylglycerol lipase, alpha	0.689908691801149	7,38834E+14	2,21596E+14	0.0656032566399158	0.207283622679455	-4,09471E+14	7,58181E+14	7,95373E+14	7,32122E+12	7,31754E+14	7,18572E+14	6,97E+14
TC1400002856.mm.2	Gm3383	predicted gene 3383	1,31758E+14	6,78692E+14	2,21577E+14	0.0656207316176193	0.207283622679455	-4,09495E+14	7,8751E+14	6,68399E+14	7,64864E+14	6,58985E+14	6,44831E+14	5,47562E+14
TC0400001524.mm.2	Fam229a	family with sequence similarity 229, member A	-0.947109725063451	6,08063E+14	-2,21522E+14	0.0656718468772799	0.207283622679455	-4,09568E+14	5,49532E+14	5,27622E+14	6,00795E+14	7,0385E+14	6,12122E+14	6,54454E+14
TSUnmapped00000043.mm.2	Rora	RAR-related orphan receptor alpha	1,52515E+14	5,85321E+14	2,21513E+14	0.0656803447287868	0.207283622679455	-4,0958E+14	6,89877E+13	6,48521E+14	4,70429E+14	6,69731E+14	5,63908E+14	4,69461E+14
TC1500000165.mm.2	Retreg1	reticulophagy regulator 1	2,30727E+14	6,84734E+14	2,21499E+14	0.0656934550862555	0.207283622679455	-4,09598E+14	8,31343E+14	8,15302E+14	7,60312E+14	5,16257E+14	7,24045E+14	4,61146E+14
TC1700001603.mm.2	Kremen2	kringle containing transmembrane protein 2	-0.953849701040535	6,22902E+14	-2,21488E+14	0.0657032754141856	0.207283622679455	-4,09612E+14	5,37721E+14	5,69041E+14	6,20046E+14	7,13074E+14	6,18765E+14	6,78766E+14
TC0200002945.mm.2	Itih2	inter-alpha trypsin inhibitor, heavy chain 2	-0.730520458184365	5,78221E+14	-2,21479E+14	0.0657119343875077	0.207283622679455	-4,09624E+14	5,62892E+14	5,28205E+14	5,66483E+14	5,74545E+14	5,95654E+14	6,41547E+14
TC1000000881.mm.2	Map2k2	mitogen-activated protein kinase kinase 2	0.719602213398007	7,96954E+14	2,21474E+14	0.0657170185880593	0.207283622679455	-4,09631E+14	8,56373E+14	8,42501E+14	7,76264E+14	7,51633E+14	8,00719E+14	7,54235E+14
TC0100002562.mm.2	Igfbp5	insulin-like growth factor binding protein 5	1,14306E+14	7,62604E+14	2,21471E+13	0.0657194242604662	0.207283622679455	-4,09635E+14	7,94517E+13	8,94666E+14	7,71209E+14	6,54664E+14	7,33016E+14	7,27554E+14
TC0400003439.mm.2	Kcnq4	potassium voltage-gated channel, subfamily Q, member 4	-0.669378790006508	5,57183E+14	-2,2147E+14	0.0657208366951925	0.207283622679455	-4,09637E+12	5,26568E+14	5,41742E+14	5,23681E+14	5,48921E+14	5,92971E+14	6,09215E+14
TC1600000386.mm.2	Plaat1	phospholipase A and acyltransferase 1	1,58377E+14	6,39461E+14	2,21446E+14	0.0657428533524493	0.207283622679455	-4,09668E+14	7,8463E+14	7,36361E+14	6,58781E+14	4,52757E+14	5,51146E+14	6,53091E+14
TC1000001761.mm.2	Stx11	syntaxin 11	-0.912413131993087	4,89563E+14	-2,21425E+13	0.0657622761204995	0.207283622679455	-4,09695E+14	4,45344E+14	4,08493E+14	4,74349E+14	5,72875E+14	5,11934E+14	5,24386E+14
TC1600000850.mm.2	Rbm11	RNA binding motif protein 11	0.871919597721166	6,48806E+14	2,21421E+14	0.0657658628715637	0.207283622679455	-4,097E+14	6,98805E+13	6,90931E+14	6,8973E+14	5,98018E+14	6,22275E+14	5,93077E+14
TC0300001338.mm.2	Papss1	3'-phosphoadenosine 5'-phosphosulfate synthase 1	1,43271E+14	6,46103E+13	2,21393E+14	0.0657924265448884	0.207283622679455	-4,09738E+14	7,51859E+14	7,50532E+14	6,62295E+13	4,96081E+14	6,70403E+14	5,45445E+14
TC0100003700.mm.2	Enah	ENAH actin regulator	0.82376696216629	8,32944E+14	2,21385E+13	0.0658002324274686	0.207283622679455	-4,09749E+14	8,82106E+14	8,61281E+13	8,4226E+14	8,33387E+13	8,30173E+14	7,4846E+14
TC0100000260.mm.2	Unc50	unc-50 homolog	1,75382E+14	6,58514E+14	2,21384E+14	0.0658006446956062	0.207283622679455	-4,09749E+14	7,73111E+12	7,83406E+14	6,85475E+14	5,03341E+14	6,98819E+14	5,06932E+14
TC0500000360.mm.2	Tacc3	transforming, acidic coiled-coil containing protein 3	-0.898243143818063	6,90073E+14	-2,21374E+14	0.0658105174252831	0.207283622679455	-4,09763E+14	6,19127E+14	6,26154E+14	7,00153E+12	7,70077E+14	6,74987E+14	7,49942E+14
TC1400001413.mm.2	Gm3460	predicted gene 3460	-0.713188314507374	4,98304E+14	-2,21372E+14	0.0658123911344237	0.207283622679455	-4,09766E+14	4,60071E+14	4,59447E+14	5,21458E+14	4,86694E+14	5,50586E+14	5,1157E+14
TC1800001412.mm.2	Cdx1	caudal type homeobox 1	-0.888978822180867	5,95729E+14	-2,21366E+14	0.0658180516938544	0.207283622679455	-4,09774E+14	5,18686E+14	5,67775E+14	5,73423E+14	6,50235E+14	6,10831E+14	6,53425E+14
TC0300002935.mm.2	Rrh	retinal pigment epithelium derived rhodopsin homolog	-0.995277619998621	5,43019E+14	-2,21359E+14	0.065824292654423	0.207283622679455	-4,09783E+14	4,57718E+14	5,14825E+13	5,62552E+14	5,51422E+14	5,31032E+14	6,40566E+14
TC0400002863.mm.2	Gm13871/Gm11757/Gm11758	predicted gene 13871/predicted gene 11757/predicted gene 11758	-0.710144859490015	5,78392E+14	-2,21353E+14	0.0658297377983184	0.207283622679455	-4,0979E+14	5,21678E+14	5,40164E+14	6,25312E+13	5,79326E+14	5,95546E+14	6,08325E+14
TC0400002865.mm.2	Gm11758/Gm11757/Gm13871	predicted gene 11758/predicted gene 11757/predicted gene 13871	-0.710144859490015	5,78392E+14	-2,21353E+14	0.0658297377983184	0.207283622679455	-4,0979E+14	5,21678E+14	5,40164E+14	6,25312E+13	5,79326E+14	5,95546E+14	6,08325E+14
TC0500001257.mm.2	Triap1	TP53 regulated inhibitor of apoptosis 1	-0.831651726366171	6,7355E+14	-2,21342E+14	0.065839854829855	0.207283622679455	-4,09804E+14	6,19237E+14	6,0905E+14	7,51609E+14	6,66789E+14	6,89551E+14	7,05066E+14
TC0100002187.mm.2	Sema4c	sema domain, immunoglobulin domain (Ig), transmembrane domain (TM) and short cytoplasmic domain, (semaphorin) 4C	-0.745204962179576	7,33671E+14	-2,21323E+14	0.0658577556287613	0.2072875553617	-4,0983E+14	6,91282E+14	6,9537E+14	7,18261E+14	7,61422E+14	7,45024E+13	7,90669E+14
TC1700002053.mm.2	Olfr92	olfactory receptor 92	-1,27477E+14	4,75252E+14	-2,21308E+14	0.0658719559193112	0.2072875553617	-4,0985E+14	3,82128E+14	3,90432E+14	4,36506E+14	6,1493E+14	5,07604E+14	5,19911E+14
TC1000000820.mm.2	Ptbp1	polypyrimidine tract binding protein 1	0.78594834212628	7,32364E+14	2,21292E+14	0.065887168545371	0.2072875553617	-4,09871E+14	7,61478E+14	7,61628E+14	7,44702E+14	7,60462E+14	6,46361E+14	7,19555E+14
TC0200005179.mm.2	Stau1	staufen double-stranded RNA binding protein 1	1,43282E+14	8,61683E+14	2,21282E+14	0.0658963477267313	0.2072875553617	-4,09884E+14	9,45788E+14	9,50763E+13	9,09588E+14	7,53973E+14	8,74031E+14	7,35955E+14
TC1200001489.mm.2	Itgb1bp1	integrin beta 1 binding protein 1	1,38661E+14	7,25845E+14	2,21251E+14	0.0659251967185134	0.2072875553617	-4,09925E+14	8,23006E+14	7,98475E+14	7,80173E+14	6,09255E+14	7,22287E+14	6,21872E+14
TC0300002513.mm.2	BC107364	cDNA sequence BC107364	-1,11053E+14	5,69083E+14	-2,21229E+13	0.0659461875359312	0.2072875553617	-4,09954E+13	4,95648E+14	4,81545E+14	5,72949E+14	6,65055E+14	5,42403E+14	6,56896E+14
TC1300000132.mm.2	Vps41	VPS41 HOPS complex subunit	1,42297E+14	7,49122E+14	2,21204E+14	0.0659690288992254	0.2072875553617	-4,09986E+14	8,4896E+14	8,37499E+14	7,61338E+14	6,45072E+14	7,86013E+14	6,15851E+14
TC1700001429.mm.2	Qk	quaking	1,33421E+14	1,03125E+14	2,212E+14	0.0659731494376342	0.2072875553617	-4,09992E+13	1,11192E+14	1,13151E+13	1,04571E+14	9,21767E+14	1,05975E+14	9,16826E+14
TC0500000934.mm.2	Bmp2k	BMP2 inducible kinase	1,24038E+13	7,789E+14	2,21191E+14	0.0659816448440645	0.2072875553617	-4,10004E+14	8,4584E+14	8,66523E+14	8,27735E+14	6,69013E+14	7,64571E+14	6,99716E+14
TC1600001301.mm.2	Ess2	ess-2 splicing factor	0.732760347176564	7,21133E+14	2,21191E+14	0.0659817369466831	0.2072875553617	-4,10004E+14	7,7558E+14	7,60312E+14	7,20307E+14	6,81256E+14	7,1552E+14	6,7382E+14
TC1100001353.mm.2	Mpo	myeloperoxidase	-1,18047E+14	4,83156E+14	-2,21185E+14	0.065987282005455	0.2072875553617	-4,10012E+14	3,93013E+14	4,34105E+14	4,29656E+14	5,7895E+14	5,09031E+14	5,5418E+14
TC1900000669.mm.2	Btrc	beta-transducin repeat containing protein	1,9303E+14	6,75286E+14	2,21168E+14	0.0660034499030247	0.2072875553617	-4,10035E+14	8,41177E+14	8,2549E+14	6,97799E+14	4,06645E+14	6,64469E+14	6,16138E+14
TC1300000183.mm.2	Hist1h2br/Hist1h2bq	histone cluster 1 H2br/histone cluster 1, H2bq	-0.700301662440744	6,70135E+14	-2,21162E+14	0.0660083439806914	0.2072875553617	-4,10041E+14	6,11587E+14	6,36776E+13	6,73524E+14	7,10501E+14	7,02514E+14	6,85909E+14
TC1800001230.mm.2	Gnpda1	glucosamine-6-phosphate deaminase 1	0.732029364680368	7,40009E+14	2,21106E+14	0.0660609938063502	0.207412469446945	-4,10115E+14	7,78758E+14	7,70176E+14	7,63871E+14	7,24717E+14	7,22903E+14	6,79626E+14
TC0X00002910.mm.2	H2afb3	H2A histone family, member B3	-0.64977174097759	6,39091E+14	-2,21088E+14	0.0660787021285703	0.207427649989802	-4,1014E+14	6,02442E+14	5,87444E+14	6,64569E+14	6,60251E+14	6,67788E+14	6,52052E+14
TC1200001023.mm.2	Unc79	unc-79 homolog	1,65244E+14	8,42765E+14	2,21025E+14	0.066137801234043	0.207572729116505	-4,10223E+14	9,61332E+14	9,6489E+14	8,76301E+14	6,58336E+14	8,56494E+14	7,3924E+14
TC1000000844.mm.2	1600002K03Rik	RIKEN cDNA 1600002K03 gene	-0.722839464309748	6,02883E+14	-2,20969E+13	0.0661897772668919	0.207695400409236	-4,10296E+13	5,79317E+14	5,38405E+14	5,99793E+13	6,37494E+14	6,3467E+14	6,2762E+14
TC0200003080.mm.2	Arhgap21	Rho GTPase activating protein 21	2,65015E+14	6,32543E+14	2,20938E+14	0.0662191107624825	0.207746988262438	-4,10337E+14	8,0272E+14	8,14544E+14	6,98806E+14	3,91956E+14	6,85157E+14	4,02076E+14
TC0500002886.mm.2	Gm17660	predicted gene, 17660	-0.758827442069576	5,07861E+14	-2,20907E+14	0.0662488443736087	0.207788132207332	-4,10379E+14	4,62696E+14	4,5577E+14	4,98744E+14	5,59723E+13	5,49184E+14	5,21048E+14
TC1300000791.mm.2	Gm10775	predicted gene 10775	-0.841610084172842	5,94345E+14	-2,2089E+14	0.0662646790268364	0.207788132207332	-4,10401E+14	5,24725E+14	5,4247E+14	5,91888E+14	6,7147E+14	5,9902E+14	6,36497E+14
TC0800000345.mm.2	Erlin2	ER lipid raft associated 2	1,35609E+14	6,43956E+14	2,20883E+14	0.066270912394556	0.207788132207332	-4,1041E+14	7,57201E+14	7,16313E+13	6,17659E+14	5,70266E+14	6,93265E+14	5,09032E+14
TC1600000650.mm.2	Naa50	N(alpha)-acetyltransferase 50, NatE catalytic subunit	1,22408E+14	7,80766E+14	2,20835E+14	0.0663161273658268	0.207870379364493	-4,10473E+14	8,57016E+14	8,8225E+14	7,75099E+14	6,75783E+14	8,15043E+14	6,79406E+14
TC0500000299.mm.2	Dpysl5	dihydropyrimidinase-like 5	0.904655883617612	7,56035E+14	2,20818E+14	0.066332851812165	0.207870379364493	-4,10496E+14	7,97616E+14	7,78114E+14	8,27683E+14	7,37996E+14	7,22622E+14	6,72177E+14
TC0500000855.mm.2	Epgn	epithelial mitogen	-0.766596347974347	5,61963E+14	-2,20802E+14	0.0663475310430793	0.207870379364493	-4,10517E+14	5,15276E+14	5,51479E+14	5,33921E+14	5,51024E+14	5,81393E+14	6,38681E+14
TC0500003736.mm.2	Epo	erythropoietin	-0.869458756565948	4,92092E+14	-2,20793E+14	0.0663558945087266	0.207870379364493	-4,10528E+14	4,09098E+14	4,54519E+13	4,88376E+14	5,63053E+14	4,9822E+14	5,39288E+14
TC1100003948.mm.2	Pitpnc1	phosphatidylinositol transfer protein, cytoplasmic 1	1,68567E+14	7,53756E+14	2,20787E+14	0.0663616478279	0.207870379364493	-4,10536E+14	8,89152E+14	8,71405E+14	7,88809E+14	5,49748E+14	7,53205E+14	6,70218E+14
TC1700001846.mm.2	Pglyrp2	peptidoglycan recognition protein 2	-0.863447686334627	6,0033E+14	-2,20697E+14	0.0664466362315448	0.208041189230092	-4,10655E+14	5,11112E+14	5,64149E+14	6,15334E+13	6,63432E+14	5,92582E+14	6,55369E+13
TC1600001081.mm.2	B3galt5	UDP-Gal:betaGlcNAc beta 1,3-galactosyltransferase, polypeptide 5	1,0707E+14	6,53066E+14	2,20685E+14	0.0664581633409235	0.208041189230092	-4,10671E+14	7,22041E+14	6,95133E+14	7,03649E+14	5,94537E+14	6,43431E+14	5,59603E+14
TC0900003024.mm.2	Mapkapk3	mitogen-activated protein kinase-activated protein kinase 3	-0.965267642575077	6,49031E+13	-2,20685E+14	0.0664583774449638	0.208041189230092	-4,10671E+14	5,64818E+14	5,98674E+14	6,34093E+14	7,40052E+14	6,58183E+14	6,98363E+14
TC0300000771.mm.2	Ube2q1	ubiquitin-conjugating enzyme E2Q family member 1	0.954816757958417	8,42735E+14	2,20675E+14	0.0664678236303924	0.208041189230092	-4,10685E+14	9,17962E+14	9,01318E+14	8,55288E+14	7,53528E+14	8,40748E+14	7,87568E+14
TC1900001776.mm.2	Sec31b	Sec31 homolog B (S. cerevisiae)	-0.764274591527128	5,84471E+14	-2,2065E+14	0.0664909569967351	0.208073177333151	-4,10717E+14	5,23078E+14	5,45677E+14	5,86455E+14	6,30007E+14	5,86284E+14	6,35326E+13
TC1500002258.mm.2	Krt80	keratin 80	-0.866946291468834	5,29316E+14	-2,20621E+14	0.0665180687505209	0.208117600345076	-4,10755E+14	4,75686E+14	4,98321E+13	4,87086E+14	5,67407E+14	5,49833E+14	5,97564E+14

TC1500000605.mm.2	Gpt	glutamic pyruvic transaminase, soluble	-0.853094008962233	5,8199E+14	-2,20575E+14	0.0665617290361251	0.208124173223568	-4,10815E+14	5,47781E+14	5,52347E+13	5,39457E+14	5,81608E+14	5,94073E+14	6,76674E+14
TC1600001559.mm.2	Fam162a	family with sequence similarity 162, member A	-0.684419596882363	7,79435E+14	-2,20574E+14	0.0665631785901205	0.208124173223568	-4,10818E+14	7,46033E+14	7,38842E+14	7,91906E+14	7,7807E+14	8,30385E+14	7,91373E+14
TC1600001838.mm.2	Zfp654	zinc finger protein 654	1,3926E+14	6,02779E+14	2,20565E+13	0.0665714221390552	0.208124173223568	-4,10829E+14	7,45807E+14	6,71424E+14	5,89712E+14	4,71021E+14	6,41868E+14	4,96843E+14
TC0500001646.mm.2	Mblac1	metallo-beta-lactamase domain containing 1	-0.64378840379615	7,14884E+14	-2,20565E+14	0.0665718357099405	0.208124173223568	-4,1083E+14	6,67812E+14	6,84414E+13	7,24738E+14	7,31358E+14	7,42723E+14	7,3826E+14
TC0300001049.mm.2	Trim33	tripartite motif-containing 33	1,71618E+14	6,31746E+14	2,20551E+14	0.0665848950890407	0.208124619703145	-4,10848E+14	7,39369E+14	7,62156E+14	6,70489E+14	4,60178E+14	6,54155E+14	5,04133E+14
TC1400001405.mm.2	Gm3453	predicted gene 3453	-0.753891305022053	5,65878E+14	-2,2052E+14	0.0666142650514845	0.208176038164191	-4,10889E+14	5,60314E+14	4,95288E+14	5,59872E+14	5,73411E+13	6,03934E+14	6,02446E+14
TC1600001343.mm.2	Abcc5	ATP-binding cassette, sub-family C (CFTR/MRP), member 5	0.985227444708238	8,00241E+14	2,20465E+14	0.0666664683219215	0.208298779148947	-4,10961E+14	8,61573E+14	8,66014E+13	7,91919E+14	7,51396E+14	8,23162E+14	7,0738E+14
TC0800001078.mm.2	Lpcat2	lysophosphatidylcholine acyltransferase 2	0.811555276471889	6,01571E+14	2,20398E+14	0.0667296879306526	0.208403812181192	-4,11049E+14	6,70563E+14	6,37278E+14	6,1178E+14	5,44276E+14	5,55766E+14	5,89764E+14
TC0100003881.mm.2	D630023F18Rik	RIKEN cDNA D630023F18 gene	1,1693E+14	6,19933E+14	2,20395E+14	0.066732207191103	0.208403812181192	-4,11053E+14	6,90727E+14	6,69027E+14	6,75045E+14	5,58903E+14	6,16855E+14	5,0904E+12
TC0500002102.mm.2	Kmt2c	lysine (K)-specific methyltransferase 2C	0.879951817076597	6,73465E+14	2,20382E+14	0.0667447453068672	0.208403812181192	-4,1107E+14	7,20632E+14	7,16992E+14	6,57827E+14	6,83708E+14	6,78785E+12	5,82849E+14
TC1000003044.mm.2	Tbc1d30	TBC1 domain family, member 30	0.933771748071688	7,47252E+14	2,20375E+14	0.066751819938381	0.208403812181192	-4,1108E+14	7,97908E+13	8,26102E+14	7,56154E+14	6,66089E+14	7,48489E+14	6,88768E+14
TC1300000425.mm.2	Ly86	lymphocyte antigen 86	1,68481E+14	8,29649E+14	2,20325E+12	0.066799337843429	0.20840955019905	-4,11146E+14	9,51129E+14	9,78415E+14	8,40166E+14	6,15603E+14	8,54206E+14	7,38376E+14
TC0400002509.mm.2	Fam205a1	family with sequence similarity 205, member A1	0.869723848867975	5,92478E+14	2,20315E+14	0.0668087222660815	0.20840955019905	-4,11159E+14	6,52672E+14	6,64092E+13	5,28699E+14	5,66583E+14	6,15737E+14	5,27083E+14
TC0400003633.mm.2	Nkain1	Na+/K+ transporting ATPase interacting 1	1,32377E+13	7,59909E+14	2,20305E+14	0.0668175944678739	0.20840955019905	-4,11171E+14	8,67906E+14	8,47865E+14	7,88653E+14	6,04015E+14	7,43717E+13	7,07299E+14
TC0X00001338.mm.2	Plp1	proteolipid protein (myelin) 1	1,68986E+14	1,11598E+14	2,20304E+14	0.0668194033936536	0.20840955019905	-4,11174E+14	1,23254E+14	1,2481E+14	1,14597E+14	9,26583E+14	1,13591E+14	1,00675E+14
TC1100001361.mm.2	Vezf1	vascular endothelial zinc finger 1	1,00091E+14	7,83611E+14	2,20291E+14	0.0668311301879508	0.20840955019905	-4,1119E+14	8,6302E+14	8,09746E+14	8,29161E+14	7,27155E+14	7,62225E+14	7,10358E+14
TC0300002630.mm.2	Hipk1	homeodomain interacting protein kinase 1	1,60316E+14	9,22499E+14	2,20274E+14	0.0668473874240171	0.20840955019905	-4,11212E+13	1,0289E+14	1,0439E+14	9,60838E+14	7,49197E+14	9,31966E+12	8,20198E+14
TC1000001592.mm.2	Mip	major intrinsic protein of lens fiber	-1,06375E+14	5,68276E+14	-2,20261E+14	0.0668594244192733	0.20840955019905	-4,11229E+14	5,05777E+14	5,01943E+14	5,31135E+13	6,50329E+14	5,74325E+13	6,46146E+14
TC0600000409.mm.2	Wee2	WEE1 homolog 2 (S. pombe)	-1,20576E+14	7,02892E+14	-2,20257E+13	0.0668635583743804	0.20840955019905	-4,11235E+13	6,12061E+14	6,30563E+14	6,68953E+14	8,21997E+14	7,09637E+14	7,74139E+14
TC0400001783.mm.2	Arhgef19	Rho guanine nucleotide exchange factor (GEF) 19	-0.779220149722002	7,56246E+14	-2,2025E+14	0.0668700660664737	0.20840955019905	-4,11244E+14	7,30509E+13	6,87978E+14	7,48575E+14	7,96083E+14	7,68361E+14	8,0597E+14
TC0400001543.mm.2	Fabp3	fatty acid binding protein 3, muscle and heart	0.796869187900111	9,05413E+13	2,20212E+14	0.0669065212540731	0.20848166973509	-4,11294E+14	9,7129E+14	9,57143E+14	8,96354E+14	8,38631E+13	9,10469E+14	8,5859E+13
TC0100003128.mm.2	Ipo9	importin 9	0.658595617284103	7,99106E+12	2,20199E+14	0.0669190837131438	0.20848166973509	-4,11312E+14	8,54909E+14	8,4674E+14	7,75644E+14	7,47415E+14	7,87401E+14	7,82528E+14
TC1100001945.mm.2	Mgat5b	mannoside acetylglucosaminyltransferase 5, isoenzyme B	0.738996359251962	6,72002E+14	2,2017E+14	0.0669468686800643	0.208518137840533	-4,1135E+14	7,01004E+14	7,26255E+14	6,44657E+13	6,80632E+14	6,17708E+14	6,61752E+14
TC0500003738.mm.2	Pcolce	procollagen C-endopeptidase enhancer protein	-1,00855E+14	6,67458E+14	-2,20145E+14	0.0669706635686974	0.208518137840533	-4,11383E+14	5,77052E+13	6,39346E+14	6,42059E+14	7,28185E+14	6,73424E+14	7,44683E+14
TC1700000915.mm.2	AI661453	expressed sequence AI661453	-1,1661E+14	5,3487E+14	-2,20144E+14	0.0669712880336242	0.208518137840533	-4,11384E+14	4,51933E+13	4,57953E+14	5,05215E+14	6,51012E+14	5,39243E+14	6,03862E+14
TC0900002053.mm.2	Spa17	sperm autoantigenic protein 17	0.768026661263947	5,82183E+14	2,20118E+13	0.0669962218609196	0.208518137840533	-4,11419E+14	6,32198E+14	6,533E+13	5,24437E+14	5,51275E+13	5,34824E+14	5,97068E+14
TC0X00000424.mm.2	Ocrl	OCRL, inositol polyphosphate-5-phosphatase	1,46185E+14	7,62352E+13	2,20111E+14	0.0670030083257871	0.208518137840533	-4,11428E+14	8,71823E+14	8,23129E+14	7,9235E+13	6,8423E+14	7,90804E+14	6,11778E+14
TC1800001081.mm.2	Rprd1a	regulation of nuclear pre-mRNA domain containing 1A	1,97787E+14	7,6877E+14	2,201E+14	0.0670132414484763	0.208518137840533	-4,11442E+14	9,02699E+14	8,75287E+14	8,13192E+14	6,39031E+14	8,15863E+14	5,66549E+14
TC1400002439.mm.2	Gpalpp1	GPALPP motifs containing 1	0.774207871341175	6,67983E+14	2,20091E+14	0.0670213762723342	0.208518137840533	-4,11453E+14	7,28613E+14	7,33238E+14	6,50783E+14	5,88258E+14	6,71984E+14	6,35025E+14
TC1100001771.mm.2	Taco1	translational activator of mitochondrially encoded cytochrome c oxidase I	1,08123E+14	6,27463E+14	2,20051E+14	0.0670600982778972	0.208521045831676	-4,11507E+14	6,93638E+14	6,85833E+14	6,82396E+13	5,39685E+14	5,80372E+14	5,82852E+14
TC1100003274.mm.2	Gm10277	predicted gene 10277	-0.672781013658541	6,47343E+14	-2,20032E+14	0.0670777869629781	0.208521045831676	-4,11531E+14	5,98479E+12	6,13799E+14	6,45486E+14	6,7946E+14	6,71323E+14	6,75512E+14
TC1600001493.mm.2	Senp5	SUMO/sentrin specific peptidase 5	1,50912E+14	6,77222E+14	2,20026E+12	0.0670834625807478	0.208521045831676	-4,11539E+14	7,69824E+14	7,84827E+14	7,02598E+14	5,53255E+14	7,09115E+13	5,43712E+13
TC0500003284.mm.2	Slc15a4	solute carrier family 15, member 4	1,38419E+14	6,24511E+14	2,20022E+13	0.0670873306508841	0.208521045831676	-4,11544E+14	7,15857E+14	7,29989E+14	5,98053E+14	5,34159E+14	6,79376E+12	4,89631E+14
TC0700000047.mm.2	Isoc2a	isochorismatase domain containing 2a	-0.685315658234348	6,57847E+14	-2,20016E+14	0.0670935499747677	0.208521045831676	-4,11553E+14	6,51255E+14	6,15127E+13	6,28648E+13	6,48609E+14	6,95459E+14	7,07985E+14
TC1200001299.mm.2	Vipr2	vasoactive intestinal peptide receptor 2	-0.723636810493611	5,97685E+14	-2,2E+14	0.0671086819671926	0.208521045831676	-4,11574E+14	5,34722E+14	5,57847E+14	6,23036E+14	6,33209E+14	5,92866E+14	6,44431E+14
TC0700003437.mm.2	Aldh1a3	aldehyde dehydrogenase family 1, subfamily A3	-1,00666E+14	6,44005E+14	-2,19995E+14	0.0671128991300859	0.208521045831676	-4,1158E+14	5,54136E+14	5,95864E+13	6,23627E+14	7,39069E+14	6,58034E+13	6,93299E+14
TC0500000263.mm.2	Rbm33	RNA binding motif protein 33	0.734907409693589	7,59407E+14	2,19981E+14	0.0671265214102705	0.208523161650991	-4,11599E+14	8,13461E+14	7,93047E+14	7,56418E+14	7,33993E+14	7,563E+13	7,03226E+14
TC1600001971.mm.2	Cyyr1	cysteine and tyrosine-rich protein 1	-0.798488141376289	6,75174E+13	-2,19951E+13	0.0671551379949602	0.208571846282343	-4,11638E+14	6,35435E+14	5,98261E+13	6,85148E+14	7,38804E+14	6,73942E+14	7,19453E+14
TC1700000182.mm.2	Fam120b	family with sequence similarity 120, member B	1,22848E+14	7,15299E+14	2,19932E+14	0.0671730872182076	0.208587387617456	-4,11663E+14	8,05969E+14	7,95509E+14	7,49438E+14	5,85097E+14	7,02348E+14	6,53434E+14
TC0100002127.mm.2	4931428L18Rik	RIKEN cDNA 4931428L18 gene	-0.787983342132189	4,8931E+14	-2,19883E+14	0.0672201031408533	0.20868371925768	-4,11728E+14	4,18075E+14	4,51512E+14	4,85967E+14	5,53125E+14	5,05359E+14	5,21824E+14
TC1900000075.mm.2	Ap5b1	adaptor-related protein complex 5, beta 1 subunit	-0.769866710175272	6,93161E+14	-2,19873E+14	0.0672300122054623	0.20868371925768	-4,11741E+13	6,71559E+14	6,2904E+14	6,76688E+14	7,27109E+14	7,13145E+14	7,41428E+13
TC1100000930.mm.2	Ybx2	Y box protein 2	-0.669986949245606	5,06126E+14	-2,1981E+14	0.0672903319557538	0.208807389780791	-4,11824E+14	4,86715E+14	4,56421E+14	5,0099E+14	5,15499E+14	5,45685E+14	5,31449E+13
TC0500003560.mm.2	Rac1	Rac family small GTPase 1	1,53936E+14	1,06675E+14	2,19804E+13	0.0672957720555853	0.208807389780791	-4,11832E+14	1,17831E+14	1,17744E+14	1,10229E+14	8,94555E+14	1,07218E+13	9,75697E+14
TC0X00000702.mm.2	Tktl1	transketolase-like 1	-0.971981706777017	5,63841E+14	-2,19781E+14	0.0673181074541459	0.208836477745216	-4,11863E+14	5,004E+14	5,10838E+14	5,26748E+14	6,39429E+14	5,82014E+14	6,2362E+13
TC0900003220.mm.2	Cmc1	COX assembly mitochondrial protein 1	1,03981E+14	6,14673E+14	2,19767E+14	0.0673314447709547	0.208837645735205	-4,11881E+14	6,93339E+14	6,83276E+13	6,38382E+14	5,04387E+13	5,78893E+14	5,89761E+14
TC0800000032.mm.2	Snapc2	small nuclear RNA activating complex, polypeptide 2	0.888736113872341	6,76983E+14	2,19698E+13	0.0673975972425342	0.208963401484714	-4,11972E+14	7,38889E+14	7,21822E+13	7,04322E+14	6,13901E+14	6,19338E+14	6,63626E+14
TC0100001225.mm.2	Myog	myogenin	-1,22023E+14	6,24679E+14	-2,19677E+14	0.0674171014915746	0.208963401484714	-4,11999E+14	5,34197E+14	5,51959E+14	5,94296E+14	7,37422E+14	6,19647E+14	7,10555E+14
TC0200005123.mm.2	Wfdc16	WAP four-disulfide core domain 16	-0.908608029158348	6,03773E+12	-2,19674E+14	0.0674201532645697	0.208963401484714	-4,12003E+14	5,48825E+14	5,5355E+14	5,65223E+14	6,70943E+14	6,48868E+14	6,35229E+14
TC0200001445.mm.2	Syt13	synaptotagmin XIII	0.667274743087415	6,96452E+14	2,19669E+14	0.0674250057431645	0.208963401484714	-4,1201E+14	7,32977E+14	7,391E+13	7,01394E+13	6,66617E+14	6,68491E+14	6,70131E+14
TC0700003961.mm.2	Olfr670	olfactory receptor 670	-1,02587E+14	5,19239E+14	-2,19652E+12	0.0674418800311419	0.208963401484714	-4,12033E+14	4,62436E+14	4,80153E+14	5,2708E+14	4,98004E+14	5,1009E+14	6,37673E+14
TC0100000909.mm.2	Olfr1412	olfactory receptor 1412	-0.762992050670826	6,18912E+14	-2,1962E+14	0.0674724319500636	0.208963401484714	-4,12075E+14	5,83767E+14	5,53856E+14	6,28191E+14	6,5744E+14	6,17025E+14	6,73196E+14
TC0700003860.mm.2	Olfr544	olfactory receptor 544	-0.869894135113081	5,35023E+14	-2,19613E+14	0.067479186632331	0.208963401484714	-4,12084E+14	4,67434E+14	4,4906E+14	5,57163E+14	6,46007E+13	5,26998E+14	5,63475E+12
TC1400001665.mm.2	Gnl3/Snord19	guanine nucleotide binding protein-like 3 (nucleolar)/small nucleolar RNA, C/D box 19	1,31527E+14	6,17077E+13	2,19597E+13	0.067493765499962	0.208963401484714	-4,12104E+14	6,79695E+14	7,13432E+14	6,36181E+14	5,43083E+14	6,45216E+13	4,84857E+14
TC0800002528.mm.2	Smarca5	SWI/SNF related, matrix associated, actin dependent regulator of chromatin, subfamily a, member 5	1,91366E+14	6,93378E+14	2,19591E+14	0.0674996779441393	0.208963401484714	-4,12112E+14	8,05994E+14	8,12962E+14	7,07866E+14	5,9722E+14	7,5483E+14	4,81394E+14
TC1100004151.mm.2	Dnah17	dynein, axonemal, heavy chain 17	-1,09419E+14	4,74542E+14	-2,19577E+14	0.0675130709467516	0.208963401484714	-4,12131E+14	4,18148E+14	4,17631E+14	4,15951E+14	5,40906E+14	4,89542E+14	5,65075E+14
TC0700002211.mm.2	Olfr1347	olfactory receptor 1347	-1,12303E+14	4,99689E+14	-2,19558E+14	0.067531870963478	0.208963401484714	-4,12156E+14	4,0363E+14	4,44616E+14	4,70529E+14	6,06506E+14	5,09679E+14	5,63172E+13
TC1500001828.mm.2	Csf2rb2/Mir7676-1/Mir7676-2	colony stimulating factor 2 receptor, beta 2, low-affinity (granulocyte-macrophage)/microRNA 7676-1/microRNA 7676-2	-0.929128023967761	5,43103E+14	-2,19536E+14	0.0675525108230778	0.208963401484714	-4,12185E+14	5,09722E+14	4,69268E+14	5,05521E+14	6,09292E+14	5,80521E+14	5,84295E+14

TC0X00002676.mm.2	Vsig4	V-set and immunoglobulin domain containing 4	-1,26691E+14	4,55185E+14	-2,19522E+14	0.0675661289791909	0.208963401484714	-4,12203E+12	3,81416E+14	3,90551E+14	4,12291E+14	5,21503E+14	4,41595E+14	5,83754E+14
TC0100002814.mm.2	Olfr1414	olfactory receptor 1414	-1,23821E+14	5,65703E+14	-2,19521E+14	0.0675670340230408	0.208963401484714	-4,12205E+14	4,20739E+14	5,05157E+14	5,71678E+14	7,23107E+13	5,78966E+14	5,94572E+14
TC0100001745.mm.2	Trp53bp2	transformation related protein 53 binding protein 2	0.767729639150125	6,74035E+14	2,19519E+14	0.0675689835065819	0.208963401484714	-4,12207E+14	7,41694E+14	7,005E+14	6,84267E+14	6,29102E+14	6,34667E+14	6,53982E+14
TC0900002805.mm.2	Mthfsl	5, 10-methenyltetrahydrofolate synthetase-like	1,09093E+14	5,71463E+14	2,19508E+14	0.0675794876892474	0.208963401484714	-4,12222E+14	6,44733E+14	6,17516E+14	5,78567E+14	5,43903E+14	5,91326E+14	4,52736E+14
TC0900000809.mm.2	Lctl	lactase-like	-1,16585E+13	5,64418E+14	-2,19455E+14	0.0676303400515367	0.209073947217705	-4,12291E+14	4,69955E+14	5,1068E+14	5,2466E+13	6,6741E+14	5,76719E+14	6,37086E+14
TC1600001079.mm.2	Wrb	tryptophan rich basic protein	0.641234165419482	8,72E+14	2,19441E+14	0.0676436882593371	0.209073947217705	-4,1231E+14	9,12913E+14	9,01036E+14	8,59829E+14	8,72521E+14	8,46788E+14	8,38915E+13
TC0800001801.mm.2	Xkr5	X-linked Kx blood group related 5	-0.870371154333245	4,6145E+14	-2,1941E+14	0.0676736812595567	0.209073947217705	-4,12351E+14	4,01993E+14	4,08926E+14	4,40848E+14	5,31942E+14	4,74646E+14	5,10348E+14
TC1400002719.mm.2	Dzip1	DAZ interacting protein 1	1,57864E+14	6,9398E+14	2,19404E+14	0.0676798354402917	0.209073947217705	-4,12359E+14	8,25897E+14	7,74578E+14	7,14844E+14	5,63817E+14	7,29727E+14	5,5502E+14
TC0200001012.mm.2	Klhl23	kelch-like 23	1,04863E+14	6,01087E+14	2,19404E+14	0.0676801159739061	0.209073947217705	-4,1236E+14	6,73668E+14	6,75758E+14	6,08805E+13	5,08594E+14	6,16527E+13	5,23173E+14
TC0X00000108.mm.2	Lancl3	LanC lantibiotic synthetase component C-like 3 (bacterial)	1,11782E+14	5,21614E+14	2,19334E+14	0.0677474511823944	0.209241840310892	-4,12452E+14	5,80587E+14	5,26007E+14	5,29315E+13	6,10743E+14	4,98913E+13	3,84118E+14
TC1500001412.mm.2	Azin1	antizyme inhibitor 1	1,76928E+14	8,0086E+14	2,19302E+14	0.0677782024627943	0.209296699172672	-4,12494E+14	9,10738E+14	9,15584E+14	8,20557E+14	6,85813E+14	8,53404E+14	6,19063E+14
TC0600001964.mm.2	Zfp800	zinc finger protein 800	1,32496E+14	7,00171E+14	2,19267E+14	0.0678114169509341	0.209359141852922	-4,12539E+14	8,25139E+14	8,0311E+14	6,893E+14	5,20217E+14	7,24259E+14	6,38997E+14
TC0200001743.mm.2	Pak6	p21 (RAC1) activated kinase 6	-0.874615623534152	6,37702E+14	-2,19237E+14	0.0678410336206635	0.20937470430398	-4,12579E+14	5,9444E+14	5,82086E+14	6,11794E+13	6,8644E+14	6,43606E+14	7,07844E+14
TC0700001681.mm.2	1110004F10Rik	RIKEN cDNA 1110004F10 gene	1,36744E+13	8,27514E+14	2,19181E+14	0.0678944595256605	0.20937470430398	-4,12652E+14	9,19763E+14	9,18448E+14	8,41382E+14	7,20768E+14	8,61249E+14	7,03473E+14
TC0800001654.mm.2	Efnb2	ephrin B2	1,19765E+14	9,23319E+14	2,1918E+14	0.0678957734243237	0.20937470430398	-4,12654E+14	9,89078E+13	9,80621E+14	9,81273E+14	8,58775E+14	9,21445E+14	8,08723E+14
TC0100001193.mm.2	Dstyk	dual serine/threonine and tyrosine protein kinase	0.682600284202931	6,13115E+14	2,19178E+14	0.0678973275042335	0.20937470430398	-4,12656E+14	6,57656E+14	6,40417E+14	6,28968E+14	5,90095E+14	5,77301E+14	5,84252E+14
TC0300001645.mm.2	Zfand1	zinc finger, AN1-type domain 1	1,11833E+14	5,69199E+14	2,19172E+14	0.0679035030299173	0.20937470430398	-4,12665E+14	6,77949E+14	6,20517E+14	5,90458E+14	4,51471E+14	5,29468E+14	5,45334E+14
TC0400001130.mm.2	Glis1	GLIS family zinc finger 1	-0.805518706139416	7,20994E+14	-2,19169E+14	0.0679062600120718	0.20937470430398	-4,12668E+13	6,73772E+14	6,48194E+14	7,36679E+14	7,8425E+14	7,10559E+14	7,72511E+14
TC0300002220.mm.2	Gucy1a1/Mir7010	guanylate cyclase 1, soluble, alpha 1/microRNA 7010	1,63034E+14	7,47072E+14	2,19168E+14	0.0679074166631044	0.20937470430398	-4,1267E+14	8,68786E+14	8,40353E+14	8,15303E+14	5,7492E+14	7,13663E+14	6,69409E+14
TC1000000045.mm.2	Lrp11	low density lipoprotein receptor-related protein 11	0.809995116504307	8,18104E+14	2,1908E+14	0.0679923433101555	0.209596446863695	-4,12786E+14	8,9704E+14	8,6596E+14	8,10673E+14	7,33949E+13	7,95241E+14	8,0576E+14
TC1600001150.mm.2	Rogdi	rogdi homolog	0.92294462892322	9,23147E+14	2,19014E+14	0.0680565252677403	0.209741734589481	-4,12873E+14	9,8809E+14	9,68169E+14	9,57368E+14	8,53581E+14	9,02063E+14	8,69608E+14
TC0400002987.mm.2	Slc24a2	solute carrier family 24 (sodium/potassium/calcium exchanger), member 2	1,48041E+13	7,49286E+14	2,19004E+14	0.068065507985378	0.209741734589481	-4,12885E+14	8,6641E+14	8,75839E+14	7,25935E+14	5,81363E+14	8,05486E+14	6,40681E+14
TC0200003265.mm.2	Kyat1	kynurenine aminotransferase 1	0.879853197741033	6,64047E+14	2,1899E+14	0.0680799702994718	0.20974618765495	-4,12905E+14	7,60636E+14	7,11184E+14	6,51961E+14	5,64648E+14	6,43605E+14	6,52245E+14
TC0500002865.mm.2	Mapk10	mitogen-activated protein kinase 10	2,23212E+14	9,82271E+14	2,18918E+14	0.068149413299544	0.20984790347307	-4,13E+14	1,13057E+14	1,12348E+14	1,05982E+13	7,72137E+14	1,00416E+14	8,03462E+14
TC0700002185.mm.2	Rfpl4	ret finger protein-like 4	-0.876003532763192	6,88011E+14	-2,18907E+14	0.0681599336557649	0.20984790347307	-4,13014E+14	6,02857E+14	6,24129E+14	7,03625E+14	7,95269E+14	7,17877E+14	6,8431E+14
TC0900000706.mm.2	Fam219b	family with sequence similarity 219, member B	-0.666560893086213	6,40689E+14	-2,18906E+14	0.0681607968264765	0.20984790347307	-4,13015E+14	5,93338E+14	5,94614E+14	6,53639E+14	6,81281E+14	6,72264E+14	6,49E+14
TC0800001835.mm.2	AY761185	cDNA sequence AY761185	-0.873659906158029	5,54128E+14	-2,18902E+14	0.0681650795493112	0.20984790347307	-4,13021E+14	5,01231E+14	5,2423E+14	5,63157E+14	5,35961E+14	5,53727E+14	6,46465E+14
TC1700002017.mm.2	Rpp21	ribonuclease P 21 subunit	-0.719725617388987	7,23875E+14	-2,18862E+14	0.0682031919893908	0.20992512560173	-4,13073E+14	6,71046E+14	6,88986E+13	7,45681E+14	7,33557E+14	7,25429E+14	7,78549E+13
TC0200004364.mm.2	Scg5	secretogranin V	1,07096E+14	9,74935E+14	2,18836E+14	0.0682291545942811	0.209953747214722	-4,13108E+14	1,07486E+13	1,05696E+14	9,59163E+14	8,40993E+14	9,97225E+14	9,20405E+14
TC0500000397.mm.2	Acox3	acyl-Coenzyme A oxidase 3, pristanoyl	0.719694375860261	6,28669E+14	2,18826E+14	0.0682385511179481	0.209953747214722	-4,13121E+14	6,85913E+14	6,5572E+14	5,94066E+14	6,38616E+14	6,08818E+14	5,88877E+14
TC0X00002156.mm.2	Apln	apelin	0.720789347224958	5,59125E+14	2,18804E+14	0.0682601074062744	0.209979975303035	-4,1315E+14	6,10474E+14	6,11103E+14	5,50108E+14	5,05649E+14	5,57163E+14	5,20256E+14
TC0200003027.mm.2	Skida1	SKI/DACH domain containing 1	-0.884084276622951	5,74226E+14	-2,18764E+14	0.0682989860426785	0.210006491504086	-4,13203E+14	5,27011E+14	5,07122E+14	5,68775E+14	6,31497E+13	5,6656E+14	6,4439E+14
TC0700001988.mm.2	Dpysl4	dihydropyrimidinase-like 4	0.719895895745684	7,18731E+14	2,18735E+14	0.0683272895391087	0.210006491504086	-4,13241E+14	7,75991E+14	7,35305E+14	6,96903E+14	7,36869E+14	6,79763E+14	6,87553E+14
TC0300002262.mm.2	Prss48	protease, serine 48	-1,2631E+14	5,39419E+13	-2,18732E+14	0.0683295508586584	0.210006491504086	-4,13244E+14	4,8741E+14	4,77408E+14	5,55692E+14	4,98566E+14	5,23709E+14	6,93728E+13
TC1600001844.mm.2	Htr1f	5-hydroxytryptamine (serotonin) receptor 1F	0.762670193130771	5,62411E+14	2,18729E+14	0.0683331285130444	0.210006491504086	-4,13249E+14	6,01472E+14	6,03115E+14	5,24838E+13	5,92989E+14	5,35666E+13	5,16387E+14
TC0300001067.mm.2	Rsbn1	rosbin, round spermatid basic protein 1	1,10314E+14	6,588E+14	2,1872E+14	0.068341291282352	0.210006491504086	-4,1326E+14	7,1745E+14	6,96817E+14	7,27106E+14	6,17787E+14	6,46122E+14	5,47517E+14
TC1500000757.mm.2	Cyp2d9	cytochrome P450, family 2, subfamily d, polypeptide 9	-0.789826507003241	8,06267E+14	-2,18714E+14	0.0683469274155914	0.210006491504086	-4,13268E+14	7,35234E+14	7,61714E+14	8,05963E+14	8,79777E+14	8,2856E+14	8,26353E+14
TC1700000078.mm.2	Rsph3a	radial spoke 3A homolog (Chlamydomonas)	1,12606E+14	6,72309E+14	2,18672E+14	0.0683883829613625	0.210052057159795	-4,13324E+14	7,6559E+14	7,58983E+14	6,77444E+14	5,32475E+14	6,74317E+14	6,25045E+14
TC0400002606.mm.2	Gabbr2	gamma-aminobutyric acid (GABA) B receptor, 2	1,61601E+14	9,79039E+14	2,18672E+14	0.0683884625631622	0.210052057159795	-4,13324E+14	1,07646E+14	1,08483E+14	1,03717E+14	8,37692E+14	9,89331E+14	8,48754E+14
TC0400002837.mm.2	Brinp1	bone morphogenic protein/retinoic acid inducible neural specific 1	1,361E+14	8,4575E+14	2,18651E+14	0.0684081512041759	0.210052057159795	-4,1335E+14	9,48485E+13	9,41869E+14	8,54019E+14	7,11969E+14	8,77981E+14	7,40173E+14
TC0100001231.mm.2	Rabif	RAB interacting factor	0.691469788294177	6,31769E+14	2,18642E+13	0.0684173222131648	0.210052057159795	-4,13363E+14	6,58778E+14	6,80458E+14	6,39659E+14	6,10776E+14	6,15813E+14	5,85129E+14
TC1800001652.mm.2	Adnp2	ADNP homeobox 2	0.762548776750009	5,75363E+14	2,18622E+14	0.0684369995958767	0.210052057159795	-4,13389E+14	6,43817E+14	6,32306E+14	5,60831E+14	4,91608E+13	5,6693E+14	5,56683E+14
TC1300000834.mm.2	Gm7969	predicted gene 7969	-0.6741091086571	5,82653E+14	-2,18593E+14	0.0684650593448521	0.210052057159795	-4,13427E+13	5,19447E+14	5,34662E+11	6,15185E+14	6,37696E+13	6,09257E+13	5,79673E+14
TC1000000388.mm.2	Scml4	Scm polycomb group protein like 4	-0.89470449098536	6,15382E+13	-2,18592E+14	0.0684657019234522	0.210052057159795	-4,13428E+14	5,24869E+14	5,71752E+14	6,32189E+14	6,87923E+14	6,00283E+14	6,75279E+14
TC1400000347.mm.2	Ercc6	excision repair cross-complementing rodent repair deficiency, complementation group 6	0.873030803574732	5,50603E+13	2,18583E+14	0.0684747496210834	0.210052057159795	-4,13441E+14	5,87386E+14	5,9671E+14	5,67408E+14	5,42624E+14	5,52698E+14	4,56792E+13
TC0200002579.mm.2	Ddx27	DEAD (Asp-Glu-Ala-Asp) box polypeptide 27	0.703830511868685	5,17718E+13	2,18579E+13	0.0684790824961463	0.210052057159795	-4,13446E+14	5,48203E+14	5,71663E+14	4,86675E+14	5,20665E+14	4,73645E+14	5,05455E+13
TC0200002543.mm.2	Slc12a5	solute carrier family 12, member 5	2,07341E+14	7,95026E+14	2,18551E+14	0.0685059125109975	0.210069702679617	-4,13483E+14	9,4854E+14	9,26657E+14	8,57748E+14	5,76694E+14	8,10368E+14	6,50147E+13
TC1500001095.mm.2	Espl1	extra spindle pole bodies 1, separase	-0.641741661896774	6,09017E+14	-2,18531E+14	0.0685255363612989	0.210069702679617	-4,13509E+14	5,62619E+14	5,6436E+14	6,33755E+14	6,3804E+14	6,17034E+14	6,38294E+14
TC1100002733.mm.2	Sowaha	sosondowah ankyrin repeat domain family member A	1,19131E+14	7,99411E+14	2,18501E+14	0.068554712824341	0.210069702679617	-4,13549E+14	8,68784E+14	8,66976E+13	8,62562E+14	7,00647E+14	7,67422E+14	7,30075E+14
TC1700001386.mm.2	Tmem181c-ps	transmembrane protein 181C, pseudogene	1,21694E+14	7,62407E+14	2,18498E+14	0.0685576049961054	0.210069702679617	-4,13552E+14	8,61354E+14	7,96552E+14	8,27052E+14	6,74966E+14	7,29332E+14	6,85186E+14
TC1600001340.mm.2	Parl	presenilin associated, rhomboid-like	0.689508604370817	7,62634E+14	2,18497E+12	0.0685583653275206	0.210069702679617	-4,13553E+14	8,06261E+13	7,84854E+14	7,71458E+14	7,60018E+14	7,46611E+14	7,06602E+14
TC0100002554.mm.2	Pecr	peroxisomal trans-2-enoyl-CoA reductase	-0.888995592399439	7,21083E+14	-2,18479E+14	0.0685758185518223	0.210069702679617	-4,13577E+14	6,59863E+14	6,63559E+14	6,99523E+14	8,02334E+14	7,45795E+14	7,55427E+14
TC0700003559.mm.2	Polg	polymerase (DNA directed), gamma	-0.870782358801915	6,85116E+14	-2,18468E+14	0.0685870149046628	0.210069702679617	-4,13592E+13	6,30585E+14	6,25021E+13	6,69498E+14	7,55829E+14	6,9074E+14	7,39022E+14
TC1100001267.mm.2	Wfdc21	WAP four-disulfide core domain 21	-0.826145670857112	4,86683E+14	-2,18461E+14	0.0685933970762143	0.210069702679617	-4,13601E+14	4,42524E+14	4,54621E+14	5,23237E+14	4,3734E+14	5,23274E+14	5,391E+11
TC0200004181.mm.2	Api5	apoptosis inhibitor 5	1,19101E+14	6,54758E+14	2,18443E+14	0.068611660690771	0.210069702679617	-4,13625E+14	7,41083E+14	7,69094E+14	6,4862E+14	4,97776E+14	6,67912E+14	6,04064E+14
TC0400000587.mm.2	Fktn	fukutin	0.824977174664415	6,47085E+14	2,18439E+14	0.0686152079192468	0.210069702679617	-4,1363E+14	7,11319E+14	7,12275E+13	6,35635E+13	5,64681E+14	6,55238E+14	6,0336E+14
TC1000000875.mm.2	Tmprss9	transmembrane protease, serine 9	-0.811500420232202	5,82643E+14	-2,18395E+14	0.0686582898219385	0.210140832994871	-4,13688E+14	5,46028E+14	5,40653E+14	5,38555E+13	6,21641E+14	6,1729E+14	6,31691E+14
TC0600000463.mm.2	Tas2r126	taste receptor, type 2, member 126	-0.806435989783563	5,03677E+14	-2,18385E+14	0.0686678185644112	0.210140832994871	-4,13701E+14	4,43583E+14	4,55767E+14	5,00262E+12	5,61809E+14	5,03008E+14	5,5763E+14
TC0400002517.mm.2	Fam214b	family with sequence similarity 214, member B	-0.768315497955498	7,52621E+14	-2,18375E+14	0.0686775663471104	0.210140832994871	-4,13714E+14	7,01391E+14	7,16715E+14	7,5057E+14	7,75278E+13	7,52503E+13	8,19267E+14
TC0400001035.mm.2	Dnajc6	DnaJ heat shock protein family (Hsp40) member C6	1,8018E+14	9,07967E+14	2,1832E+14	0.0687310835724273	0.210162197530901	-4,13786E+14	1,01901E+14	1,01729E+14	9,64089E+14	7,71471E+14	9,3782E+14	7,38122E+14
TC0X00002393.mm.2	Tmem185a	transmembrane protein 185A	2,44235E+14	7,86389E+14	2,18309E+14	0.0687423614921908	0.210162197530901	-4,13802E+14	9,45761E+14	9,33712E+14	8,97773E+14	5,50084E+14	7,81125E+14	6,09878E+14
TC0400001212.mm.2	Gm12830	predicted gene 12830	-0.867063681538757	4,87353E+13	-2,18309E+14	0.0687425579646201	0.210162197530901	-4,13802E+13	4,10194E+14	4,08766E+14	5,09489E+14	6,03294E+14	4,81446E+14	5,10927E+14

TC1100000544.mm.2	Hnrnph1	heterogeneous nuclear ribonucleoprotein H1	1,29259E+14	8,1815E+14	2,18296E+14	0.0687554632950552	0.210162197530901	-4,13819E+14	9,21722E+14	9,1084E+14	8,22706E+14	6,79587E+14	8,45918E+14	7,28126E+14
TC0200000079.mm.2	4930412O13Rik	RIKEN cDNA 4930412O13 gene	-1,13284E+14	5,16542E+14	-2,18277E+14	0.068773228137773	0.210162197530901	-4,13843E+14	4,52741E+14	4,30744E+14	4,89211E+14	6,16504E+14	5,12199E+13	5,97854E+14
TC1800000762.mm.2	Mbd2	methyl-CpG binding domain protein 2	1,15443E+14	8,53308E+14	2,18254E+14	0.0687957691645513	0.210162197530901	-4,13874E+14	9,30196E+14	9,23008E+14	8,71611E+14	7,72715E+14	8,73475E+14	7,48843E+14
TC1300001371.mm.2	Tasor2	transcription activation suppressor family member 2	1,34865E+14	6,27177E+14	2,18235E+14	0.0688146118636936	0.210162197530901	-4,13899E+14	6,97629E+14	7,09991E+14	6,34539E+13	5,83014E+13	6,63407E+14	4,74484E+14
TC1200001056.mm.2	Tcl1b5	T cell leukemia/lymphoma 1B, 5	-0.844731704732681	4,67377E+14	-2,1822E+14	0.068829092071456	0.210162197530901	-4,13918E+14	4,09071E+14	4,2763E+14	4,54756E+14	5,07157E+14	5,4012E+14	4,65527E+14
TC0900003014.mm.2	Iqcf4	IQ motif containing F4	-1,0934E+14	4,80154E+13	-2,18213E+14	0.068835984072947	0.210162197530901	-4,13928E+14	3,83522E+14	4,19683E+14	4,70099E+14	5,85735E+14	4,69128E+14	5,52758E+14
TC0100001723.mm.2	Lefty1	left right determination factor 1	-1,10349E+14	6,82435E+14	-2,18206E+14	0.068843293351742	0.210162197530901	-4,13937E+14	6,06727E+14	6,20866E+14	6,44187E+14	7,74536E+14	6,88496E+14	7,59796E+14
TC0100002457.mm.2	Mdh1b	malate dehydrogenase 1B, NAD (soluble)	-0.777904074637121	5,26563E+14	-2,18198E+14	0.0688507231447465	0.210162197530901	-4,13947E+14	4,7524E+14	4,9501E+14	4,96709E+14	5,66588E+13	5,73583E+14	5,52247E+14
TC1000002594.mm.2	Ckap4	cytoskeleton-associated protein 4	-0.675397205399372	6,01176E+14	-2,1817E+14	0.068878711600995	0.210162197530901	-4,13985E+14	5,69666E+14	5,61865E+14	5,99604E+14	6,09311E+14	6,13602E+14	6,53008E+13
TC1600000938.mm.2	Jam2	junction adhesion molecule 2	0.962553052988178	7,66629E+14	2,18161E+14	0.068886928221629	0.210162197530901	-4,13996E+14	8,49378E+14	8,42366E+14	7,56699E+14	6,52096E+14	7,78892E+13	7,20342E+14
TC0300000708.mm.2	Mrpl24	mitochondrial ribosomal protein L24	0.764210399404554	7,73492E+14	2,18161E+14	0.068887602268534	0.210162197530901	-4,13997E+14	8,12497E+14	8,17874E+14	7,39373E+14	7,93681E+14	7,62141E+14	7,15388E+14
TC1700001147.mm.2	Lbh	limb-bud and heart	0.653080725232877	6,99422E+14	2,1815E+14	0.0688975904398012	0.210162197530901	-4,1401E+14	7,31897E+14	7,36447E+14	7,07544E+14	6,82916E+14	6,77255E+14	6,60473E+14
TC0600003307.mm.2	Dusp16	dual specificity phosphatase 16	0.908788736681223	5,53806E+14	2,18139E+14	0.0689091831901618	0.210162197530901	-4,14026E+14	5,80183E+14	6,03096E+14	5,28408E+14	6,09625E+14	5,34612E+14	4,66909E+14
TC0700000773.mm.2	Klk10	kallikrein related-peptidase 10	-0.771145297292629	5,89156E+14	-2,1812E+14	0.0689275782447572	0.210162197530901	-4,14051E+14	5,39967E+14	5,14111E+14	6,07388E+14	6,65161E+12	5,85103E+14	6,23204E+14
TC0900001888.mm.2	Tmem205	transmembrane protein 205	0.670514290753974	6,10391E+14	2,18103E+14	0.0689444558349442	0.210162197530901	-4,14073E+14	6,14369E+14	6,32406E+14	6,62002E+14	6,40898E+14	5,42487E+14	5,70184E+14
TC0600001424.mm.2	C1rb	complement component 1, r subcomponent B	-0.743142143668186	5,33226E+14	-2,181E+14	0.0689471367426118	0.210162197530901	-4,14077E+14	4,83118E+14	4,92376E+14	5,27161E+14	5,72575E+14	5,38791E+14	5,85332E+14
TC1700001665.mm.2	Sox8	SRY (sex determining region Y)-box 8	0.647658665547305	7,96225E+14	2,18071E+14	0.0689751467908665	0.210162197530901	-4,14115E+14	8,42398E+11	8,27954E+14	7,94417E+14	7,71762E+14	7,71491E+14	7,69329E+14
TC0200000459.mm.2	Surf2	surfeit gene 2	1,35721E+14	7,58383E+14	2,18069E+14	0.0689770189707176	0.210162197530901	-4,14117E+14	8,71912E+14	8,49509E+14	7,75942E+14	6,02957E+14	7,71827E+14	6,78151E+14
TC0800002741.mm.2	Cngb1	cyclic nucleotide gated channel beta 1	-0.648757372020802	6,6737E+14	-2,18035E+13	0.0690104191564793	0.210162197530901	-4,14162E+14	6,27154E+14	6,19067E+14	6,79797E+14	7,02228E+14	6,80911E+12	6,95062E+14
TC0800000146.mm.2	Cln8	ceroid-lipofuscinosis, neuronal 8	0.716788300382242	7,4701E+14	2,1803E+14	0.0690152618778767	0.210162197530901	-4,14168E+14	7,85986E+14	7,92838E+14	7,05447E+13	7,62321E+14	7,1826E+14	7,17207E+14
TC0800002767.mm.2	Cdh8	cadherin 8	1,51519E+14	8,53215E+14	2,18017E+13	0.0690287372683445	0.210162197530901	-4,14186E+14	9,48488E+14	9,4128E+13	8,96861E+14	7,45926E+14	8,78026E+14	7,08705E+13
TC1100001500.mm.2	Hoxb2	homeobox B2	-0.666666177834867	7,26444E+14	-2,18014E+14	0.0690313287665784	0.210162197530901	-4,1419E+14	6,86371E+14	6,82954E+14	7,24793E+14	7,61887E+14	7,49048E+14	7,5361E+14
TC1200002088.mm.2	Rps6kl1	ribosomal protein S6 kinase-like 1	1,04158E+14	5,4353E+14	2,18014E+14	0.0690318437794955	0.210162197530901	-4,14191E+13	5,8808E+14	5,84877E+14	5,41488E+14	5,82096E+14	4,16976E+14	5,47665E+14
TC0600000664.mm.2	Aqp1	aquaporin 1	-0.998350496660575	6,38199E+14	-2,18001E+14	0.0690438701723037	0.210162197530901	-4,14207E+14	5,78043E+14	5,95535E+14	5,8591E+14	6,96459E+14	6,55686E+14	7,17562E+14
TC1100001632.mm.2	Stat5a	signal transducer and activator of transcription 5A	-0.791247049204108	6,96468E+14	-2,17995E+14	0.0690497532258789	0.210162197530901	-4,14215E+14	6,38619E+14	6,19966E+14	7,18801E+14	7,84587E+14	6,88105E+14	7,2873E+14
TC1100000475.mm.2	Havcr2	hepatitis A virus cellular receptor 2	0.884085537220114	5,62678E+14	2,17925E+14	0.0691188321911394	0.21025700421175	-4,14307E+14	5,78446E+14	6,4117E+14	5,2709E+14	5,8656E+13	4,93659E+14	5,4914E+14
TC1700000270.mm.2	Zfp54	zinc finger protein 54	-0.952367814175203	5,85748E+14	-2,17923E+14	0.0691209380724962	0.21025700421175	-4,1431E+14	4,96247E+14	5,69526E+14	5,4997E+14	6,42498E+14	6,26221E+14	6,30025E+14
TC0700003036.mm.2	Tph1	tryptophan hydroxylase 1	-0.772732495611421	4,95793E+14	-2,17914E+14	0.0691293724246887	0.21025700421175	-4,14321E+14	4,53223E+14	4,66763E+14	4,57896E+14	5,22344E+14	5,54713E+14	5,19819E+14
TC1500001996.mm.2	5031439G07Rik/Mir1249	RIKEN cDNA 5031439G07 gene/microRNA 1249	0.858580279013192	8,25989E+14	2,17905E+14	0.0691390257076029	0.21025700421175	-4,14334E+14	8,83028E+14	8,72639E+14	8,45514E+14	7,70801E+14	8,22221E+14	7,61729E+14
TC0700004293.mm.2	Coro1a	coronin, actin binding protein 1A	1,03653E+14	6,53637E+14	2,17863E+14	0.0691799312728674	0.21025700421175	-4,14389E+14	7,11627E+14	7,41581E+14	6,69882E+14	5,52829E+14	6,54162E+14	5,9174E+14
TC0800001862.mm.2	Nek3	NIMA (never in mitosis gene a)-related expressed kinase 3	-0.987423644274111	6,16368E+14	-2,17863E+13	0.0691802372990924	0.21025700421175	-4,14389E+14	5,41288E+13	5,2511E+14	6,24948E+14	7,42981E+14	6,11889E+14	6,51994E+14
TC0X00002269.mm.2	Arhgef6	Rac/Cdc42 guanine nucleotide exchange factor (GEF) 6	1,16587E+14	6,00161E+14	2,17861E+14	0.0691815146109915	0.21025700421175	-4,14391E+14	6,6025E+14	6,60563E+14	6,49191E+14	5,43323E+14	6,05069E+13	4,82569E+14
TC0100000255.mm.2	Zap70	zeta-chain (TCR) associated protein kinase	-1,05113E+14	4,91366E+14	-2,17858E+14	0.0691852936343759	0.21025700421175	-4,14396E+14	4,6334E+14	3,86745E+12	4,63929E+14	5,73871E+14	5,11661E+14	5,48649E+14
TC1700000418.mm.2	Atp6v0e	ATPase, H+ transporting, lysosomal V0 subunit E	-1,48438E+14	7,87444E+14	-2,17796E+14	0.0692463343269177	0.210365700719162	-4,14478E+14	6,88892E+14	7,09564E+13	9,00033E+14	7,30846E+14	9,18504E+14	7,76828E+14
TC1100003898.mm.2	Cdc27	cell division cycle 27	1,59689E+14	7,72967E+14	2,17784E+12	0.0692573176013378	0.210365700719162	-4,14492E+14	8,76296E+14	8,53919E+14	8,38739E+14	6,58008E+14	7,85053E+14	6,25785E+14
TC1700000363.mm.2	Ccdc154	coiled-coil domain containing 154	-0.681802831755442	5,05663E+14	-2,17781E+12	0.0692602272894652	0.210365700719162	-4,14496E+14	4,80963E+14	4,74882E+14	4,75697E+14	5,10233E+14	5,59869E+14	5,32337E+14
TC1500000665.mm.2	Eif3l	eukaryotic translation initiation factor 3, subunit L	0.819714515433614	7,66706E+14	2,17741E+14	0.0692997367235888	0.210446034268222	-4,14549E+14	8,38464E+14	8,16701E+14	7,68643E+14	6,85202E+14	7,39022E+14	7,52201E+14
TC1500001946.mm.2	Cyp2d10	cytochrome P450, family 2, subfamily d, polypeptide 10	-0.806760057404667	5,28163E+14	-2,17718E+14	0.0693224849204679	0.210475447432353	-4,14579E+14	4,5836E+14	4,93004E+13	5,17238E+14	5,8766E+14	5,41504E+14	5,71212E+14
TC1700000595.mm.2	Cd320	CD320 antigen	-0.647889316398071	7,88056E+14	-2,17665E+14	0.069375442203244	0.210559165489921	-4,1465E+14	7,51472E+14	7,57588E+14	8,00129E+14	7,80511E+14	8,28737E+14	8,09901E+14
TC0700001927.mm.2	Bub3	BUB3 mitotic checkpoint protein	0.893860705857795	8,59224E+14	2,17656E+14	0.0693837135278804	0.210559165489921	-4,14661E+14	9,38214E+14	9,2146E+14	8,57673E+14	7,57096E+14	8,5346E+14	8,27442E+14
TC0100002143.mm.2	Bend6	BEN domain containing 6	1,60981E+14	7,7889E+14	2,1764E+14	0.0694002188580615	0.210559165489921	-4,14683E+13	9,02511E+13	8,84604E+14	8,26838E+14	5,94236E+14	7,69059E+14	6,96093E+14
TC0900001261.mm.2	Stag1	stromal antigen 1	1,98467E+14	7,34839E+13	2,17637E+14	0.0694023290459238	0.210559165489921	-4,14686E+14	8,92807E+14	8,59489E+14	7,80077E+14	5,21297E+14	7,61733E+14	5,93629E+14
TC1100000776.mm.2	Aldh3a1	aldehyde dehydrogenase family 3, subfamily A1	-0.899519517640797	5,42574E+14	-2,17593E+14	0.0694462549955606	0.210642399359558	-4,14745E+14	4,84145E+14	4,95993E+13	5,44986E+14	5,70277E+14	5,28587E+14	6,31454E+14
TC1400000899.mm.2	Fam167a	family with sequence similarity 167, member A	-1,256E+14	6,63554E+14	-2,17583E+14	0.0694559093773557	0.210642399359558	-4,14757E+14	5,56423E+14	5,93591E+14	6,28803E+14	8,01292E+14	6,8399E+13	7,17223E+14
TC0600003428.mm.2	Bhlhe41	basic helix-loop-helix family, member e41	0.978223593421978	7,76137E+14	2,1745E+14	0.0695879671911983	0.210994480917836	-4,14933E+14	8,36589E+14	8,46996E+14	7,98185E+14	6,8711E+14	7,71193E+14	7,16747E+13
TC0100001577.mm.2	Cfap126	cilia and flagella associated protein 126	0.90311989059491	7,28574E+14	2,17433E+14	0.0696047966831505	0.210994480917836	-4,14956E+14	7,77881E+14	7,53156E+14	7,97337E+14	6,92657E+14	6,7578E+14	6,74632E+14
TC0500000334.mm.2	Fosl2	fos-like antigen 2	-0.749492770103172	6,08856E+14	-2,17426E+14	0.0696112865735253	0.210994480917836	-4,14964E+14	5,77626E+14	5,71519E+14	5,79649E+14	6,25302E+14	6,24306E+14	6,74737E+14
TC1800000111.mm.2	Impact	impact, RWD domain protein	1,30432E+13	1,00624E+14	2,1735E+14	0.0696870703372885	0.211104684681847	-4,15065E+14	1,11105E+13	1,06598E+14	1,04889E+14	8,95331E+14	1,0083E+14	9,07863E+14
TC0300002730.mm.2	Ampd2	adenosine monophosphate deaminase 2	1,49495E+14	6,18696E+14	2,17342E+14	0.0696948585061339	0.211104684681847	-4,15075E+14	7,35968E+13	7,4441E+14	6,34758E+14	4,15652E+14	6,13893E+14	5,67495E+14
TC1700001858.mm.2	Cyp4f41-ps	cytochrome P450, family 4, subfamily f, polypeptide 41 pseudogene	-0.876913288984315	5,23036E+13	-2,1734E+14	0.0696969168082677	0.211104684681847	-4,15078E+14	4,49445E+14	4,63737E+14	5,18841E+14	6,17628E+14	5,34841E+14	5,53723E+14
TC1000002664.mm.2	Apaf1	apoptotic peptidase activating factor 1	0.821456198765534	5,20206E+12	2,17329E+13	0.0697071529043394	0.211104684681847	-4,15091E+14	5,74682E+14	5,38317E+14	5,07083E+14	5,52446E+14	4,41276E+14	5,07432E+14
TC1100002449.mm.2	Erlec1	endoplasmic reticulum lectin 1	1,98856E+14	7,84725E+14	2,17323E+14	0.0697131525595548	0.211104684681847	-4,15099E+14	9,1849E+14	9,06966E+14	8,47935E+14	6,07215E+14	8,12599E+14	6,15145E+14
TC0800002723.mm.2	Nudt21	nudix (nucleoside diphosphate linked moiety X)-type motif 21	1,35197E+13	7,13537E+14	2,17301E+14	0.0697356893885587	0.211133251243489	-4,15129E+14	8,16631E+14	8,00077E+14	7,38706E+14	5,79496E+14	7,31349E+14	6,14966E+14
TC1700001648.mm.2	Jpt2	Jupiter microtubule associated homolog 2	0.690952641081213	6,0891E+14	2,17267E+13	0.0697693119984057	0.211195364311537	-4,15174E+14	6,53308E+14	6,28186E+14	6,08957E+14	6,19708E+14	5,91919E+14	5,51384E+14
TC0900002982.mm.2	Ackr4	atypical chemokine receptor 4	-0.749814782156843	4,98968E+14	-2,17252E+13	0.0697835814477603	0.211198882018738	-4,15193E+14	4,39783E+14	4,73856E+14	4,93185E+14	5,23385E+14	5,06162E+13	5,57439E+14
TC1100001219.mm.2	Ccl2	chemokine (C-C motif) ligand 2	-0.792523513913804	6,38543E+14	-2,17138E+14	0.0698969649818207	0.211462996456936	-4,15343E+14	5,59696E+14	6,37049E+14	6,35906E+14	6,43358E+14	6,88537E+14	6,66713E+14
TC0500003296.mm.2	Rimbp2	RIMS binding protein 2	0.993605036527702	7,29876E+14	2,17138E+14	0.0698970967001578	0.211462996456936	-4,15343E+14	7,88246E+14	8,18714E+14	7,39779E+13	6,24278E+14	7,26724E+14	6,81515E+14
TC0200001392.mm.2	Chrm4	cholinergic receptor, muscarinic 4	0.736356639922032	7,30576E+14	2,17083E+14	0.0699519424485355	0.211589196296837	-4,15416E+14	7,80973E+14	7,79542E+14	7,3691E+14	6,72789E+14	7,02533E+14	7,10711E+14
TC1400000914.mm.2	Prss51	protease, serine 51	-0.669363318294548	6,78311E+14	-2,17035E+14	0.0699997552013301	0.211653057680042	-4,15479E+14	6,22912E+14	6,29283E+14	6,96857E+14	7,34749E+14	6,87133E+14	6,98934E+14
TC0900002338.mm.2	Scaper	S phase cyclin A-associated protein in the ER	2,05571E+14	5,99931E+14	2,17025E+14	0.0700093975458982	0.211653057680042	-4,15492E+14	7,66673E+14	7,39599E+14	6,0723E+13	3,90954E+14	6,6722E+14	4,2791E+14
TC0900003051.mm.2	Apeh	acylpeptide hydrolase	1,08451E+14	6,10771E+14	2,17019E+14	0.0700157298112031	0.211653057680042	-4,155E+14	7,10059E+14	6,51245E+13	5,95625E+14	5,63293E+14	6,37386E+14	5,07016E+13
TC0200002201.mm.2	Cst8	cystatin 8 (cystatin-related epididymal spermatogenic)	-1,07747E+14	4,36682E+14	-2,17009E+14	0.0700255973743128	0.211653057680042	-4,15513E+13	3,3393E+14	3,76088E+14	4,33777E+14	5,50786E+14	5,00238E+14	4,25274E+14

TC0900000621.mm.2	Arhgap20	Rho GTPase activating protein 20	1,30445E+14	6,3648E+14	2,16993E+14	0.0700422392651533	0.211663653653304	-4,15535E+14	7,46965E+14	7,14075E+14	6,52379E+14	5,05311E+14	6,57411E+12	5,42739E+14
TC0400000918.mm.2	Dmrta1	doublesex and mab-3 related transcription factor like family A1	-1,04016E+14	5,83415E+14	-2,16884E+14	0.0701504637885031	0.211929840967499	-4,15678E+14	5,18912E+14	4,87493E+14	5,96249E+14	6,83401E+13	5,56273E+14	6,58164E+14
TC0400004070.mm.2	Park7	Parkinson disease (autosomal recessive, early onset) 7	1,30057E+14	7,13156E+14	2,16872E+14	0.0701620700559307	0.211929840967499	-4,15693E+14	7,89473E+13	8,26474E+14	7,32087E+14	5,7507E+12	7,26938E+14	6,28896E+14
TC1600000460.mm.2	Rpl35a	ribosomal protein L35A	0.957369423835036	7,10141E+14	2,16862E+14	0.0701725344434317	0.211929840967499	-4,15707E+14	7,85221E+14	7,80253E+14	7,15863E+14	6,05511E+14	7,10694E+13	6,63306E+14
TC0200004837.mm.2	Zfp442	zinc finger protein 442	0.710680626654731	6,07553E+14	2,16852E+14	0.0701829349843339	0.211929840967499	-4,15721E+14	6,64881E+14	6,3603E+14	5,89486E+13	5,96147E+14	6,03466E+14	5,55309E+14
TC4_JH584294_random000 00009.mm.2	Ccl21a/Gm1987	chemokine (C-C motif) ligand 21A (serine)/predicted gene 1987	-0.695301925777704	6,78514E+14	-2,16822E+14	0.0702127724225789	0.211962144688736	-4,1576E+14	6,15332E+14	6,42002E+14	6,9038E+13	7,26977E+14	6,86392E+14	7,10002E+14
TC1000001708.mm.2	Ust	uronyl-2-sulfotransferase	0.910835576359913	6,9998E+13	2,16806E+14	0.0702280013402045	0.211962144688736	-4,1578E+14	7,58342E+14	7,4372E+14	7,23786E+14	6,54133E+14	7,0183E+14	6,18065E+14
TC0400000439.mm.2	Creb3	cAMP responsive element binding protein 3	0.766487152022339	8,54315E+14	2,168E+13	0.0702343463825255	0.211962144688736	-4,15788E+13	9,31108E+14	9,16812E+14	8,25452E+14	7,57898E+14	8,60448E+14	8,34175E+14
TC1400001608.mm.2	Plac9b/Plac9a/Gm97 80	placenta specific 9b/placenta specific 9a/predicted gene 9780	-0.683330288479933	8,30352E+14	-2,16788E+14	0.0702462516376746	0.211962144688736	-4,15804E+14	7,84939E+14	7,92406E+14	8,29418E+14	8,61337E+14	8,40529E+14	8,73483E+14
TC0X00002820.mm.2	Atrx	ATRX, chromatin remodeler	1,39121E+14	7,66982E+14	2,16742E+14	0.0702921458697303	0.21199255879325	-4,15865E+13	8,73308E+14	8,88957E+14	7,49903E+14	6,05704E+13	8,15041E+14	6,68981E+14
TC1500000099.mm.2	Zfr/Mir1898	zinc finger RNA binding protein/microRNA 1898	1,63811E+14	8,54492E+14	2,1674E+14	0.0702940504747114	0.21199255879325	-4,15867E+14	9,81536E+14	9,77994E+14	8,78018E+14	6,57498E+14	8,75553E+14	7,56355E+14
TC0400002200.mm.2	Ndufaf6	NADH:ubiquinone oxidoreductase complex assembly factor 6	0.739952427429425	6,0282E+14	2,16739E+14	0.0702958010074061	0.21199255879325	-4,15869E+14	6,47386E+14	6,58215E+13	5,99798E+14	5,53909E+14	5,55622E+14	6,01988E+14
TC1300002084.mm.2	Trpc7	transient receptor potential cation channel, subfamily C, member 7	1,26935E+14	5,87137E+14	2,16723E+14	0.0703110221728271	0.211996322933073	-4,15889E+14	6,70917E+14	5,98945E+12	6,4979E+14	5,87177E+14	4,29891E+14	5,86102E+14
TC0600000943.mm.2	Zfp638	zinc finger protein 638	1,05518E+14	7,8768E+14	2,16711E+14	0.0703237680519742	0.211996322933073	-4,15906E+14	8,58284E+14	8,57172E+14	8,12682E+14	6,93523E+14	7,89872E+14	7,14547E+14
TC0900002346.mm.2	Tspan3	tetraspanin 3	1,28539E+14	9,77385E+13	2,16674E+14	0.0703602693766589	0.211996322933073	-4,15954E+14	1,07854E+14	1,06332E+14	1,00098E+14	8,36695E+14	9,85475E+14	8,99306E+14
TC1500001982.mm.2	Sult4a1	sulfotransferase family 4A, member 1	1,88754E+14	8,59688E+14	2,16661E+14	0.0703730369102165	0.211996322933073	-4,15971E+14	1,00765E+14	9,89305E+14	8,95351E+14	6,46377E+14	8,8826E+13	7,31185E+13
TC0800000727.mm.2	Pbx4	pre B cell leukemia homeobox 4	0.690031198847571	5,31845E+14	2,1665E+14	0.0703842870137777	0.211996322933073	-4,15986E+13	5,69123E+14	5,91303E+14	4,94787E+14	5,13438E+14	5,31142E+14	4,91278E+14
TC1300000671.mm.2	Ddx46	DEAD (Asp-Glu-Ala-Asp) box polypeptide 46	1,76305E+14	6,47887E+14	2,16648E+14	0.0703862366021803	0.211996322933073	-4,15988E+14	7,81923E+14	7,83281E+14	6,9078E+14	4,18747E+14	6,18051E+14	5,94542E+14
TC1100000857.mm.2	Myh13	myosin, heavy polypeptide 13, skeletal muscle	-0.980253639585864	6,4921E+14	-2,16645E+14	0.0703891471291467	0.211996322933073	-4,15992E+14	5,85139E+13	5,81532E+14	6,39608E+14	7,26258E+14	6,36107E+13	7,26615E+14
TC1500000344.mm.2	Emc2	ER membrane protein complex subunit 2	1,84158E+14	8,71191E+14	2,16619E+14	0.0704153995549326	0.212035756499463	-4,16027E+14	9,85227E+14	9,87584E+14	9,50382E+13	6,99456E+14	8,71832E+14	7,32663E+14
TC0200002475.mm.2	L3mbtl1	L3MBTL1 histone methyl-lysine binding protein	-0.83777688919916	5,67931E+14	-2,16549E+14	0.0704859614323242	0.212168236292765	-4,16119E+14	5,44649E+14	4,99685E+14	5,35477E+14	6,15884E+14	5,99923E+14	6,11967E+14
TC0700004617.mm.2	Coq8b	coenzyme Q8B	0.692113165177175	7,25115E+14	2,16536E+12	0.0704991753598289	0.212168236292765	-4,16137E+14	7,50568E+14	7,71064E+14	7,23179E+13	7,22672E+13	6,68684E+14	7,14525E+14
TC1300002435.mm.2	Jmy	junction-mediating and regulatory protein	1,00321E+14	6,05353E+14	2,16508E+14	0.0705269415322144	0.212168236292765	-4,16173E+14	6,87188E+14	6,68191E+14	6,25174E+14	4,96828E+14	5,83741E+14	5,70995E+13
TC0300002731.mm.2	Gnai3	guanine nucleotide binding protein (G protein), alpha inhibiting 3	1,44693E+14	6,25384E+13	2,16496E+14	0.070539298883235	0.212168236292765	-4,16189E+14	7,08383E+14	7,36071E+14	6,52201E+14	5,00581E+14	6,53488E+14	5,01579E+14
TC0200002321.mm.2	Mapre1	microtubule-associated protein, RP/EB family, member 1	1,44188E+14	7,90709E+14	2,16493E+14	0.070542000376555	0.212168236292765	-4,16193E+14	8,75449E+14	8,82398E+13	8,20921E+14	6,96014E+14	8,22461E+14	6,47011E+14
TC0200000002.mm.2	Fam171a1	family with sequence similarity 171, member A1	1,43663E+14	6,65394E+14	2,16467E+14	0.0705682228236374	0.212168236292765	-4,16227E+14	7,69689E+14	7,48189E+14	7,25828E+14	5,18107E+14	6,39878E+14	5,90674E+14
TC1700002177.mm.2	Ttbk1	tau tubulin kinase 1	1,07834E+14	6,97694E+14	2,16458E+14	0.0705766966722127	0.212168236292765	-4,16238E+13	7,763E+14	7,75871E+14	7,14234E+14	5,83252E+14	6,36064E+14	7,0044E+14
TC1900000838.mm.2	Pnlip	pancreatic lipase	-0.686783112391932	5,56931E+13	-2,16438E+14	0.0705970944066141	0.212168236292765	-4,16265E+14	5,30788E+14	5,05348E+14	5,61589E+14	5,7037E+13	5,66699E+13	6,06793E+14
TC1500000139.mm.2	Cdh12	cadherin 12	1,58086E+14	6,06774E+14	2,16425E+14	0.0706103922159595	0.212168236292765	-4,16282E+14	7,1936E+14	7,27799E+14	5,9499E+14	4,67512E+14	6,67574E+14	4,63412E+14
TC0400004014.mm.2	Fbxo6	F-box protein 6	0.687681859444598	6,62588E+14	2,16422E+14	0.0706134826185831	0.212168236292765	-4,16286E+14	7,06783E+14	6,98876E+14	6,39856E+14	6,61888E+14	6,12732E+14	6,5539E+14
TC1400001627.mm.2	Appl1	adaptor protein, phosphotyrosine interaction, PH domain and leucine zipper containing 1	2,44604E+14	7,05917E+14	2,16421E+14	0.0706139503353243	0.212168236292765	-4,16287E+14	8,68867E+14	8,52328E+14	7,76363E+14	5,05956E+14	7,57662E+14	4,74326E+14
TC0500002214.mm.2	Ctbp1	C-terminal binding protein 1	0.662314948812782	8,32809E+14	2,16418E+14	0.0706174052776081	0.212168236292765	-4,16291E+14	8,86076E+14	8,803E+14	8,16499E+14	7,80064E+14	8,12868E+14	8,21045E+14
TC1700002345.mm.2	Slc25a41	solute carrier family 25, member 41	-1,01002E+14	5,97976E+14	-2,16389E+14	0.0706461311193144	0.21221497217105	-4,16329E+14	5,14518E+12	5,40279E+14	6,11969E+14	6,64291E+14	5,69237E+14	6,87563E+14
TC1300001449.mm.2	Edaradd	EDAR (ectodysplasin-A receptor)-associated death domain	-1,11194E+14	5,88973E+14	-2,1635E+14	0.0706858456159726	0.212278391206245	-4,16381E+14	5,19783E+14	5,01098E+14	5,59186E+14	7,105E+14	6,1638E+14	6,26888E+14
TC0700003548.mm.2	Det1	de-etiolated homolog 1 (Arabidopsis)	-0.647715487152229	6,02478E+14	-2,16342E+14	0.0706935919575939	0.212278391206245	-4,16391E+14	5,62834E+13	5,71955E+14	6,00525E+13	6,1522E+14	6,19802E+14	6,4453E+14
TC0X00002442.mm.2	Irak1/Mir718/Mir513 2	interleukin-1 receptor-associated kinase 1/microRNA 718/microRNA 5132	0.657550722740533	7,28058E+14	2,16237E+14	0.0707995201097021	0.212556859982789	-4,1653E+13	7,67632E+14	7,5458E+14	7,15728E+14	7,39703E+14	7,0686E+14	6,83842E+14
TC0500001620.mm.2	Ache	acetylcholinesterase	0.793175843423847	7,46819E+13	2,16201E+14	0.0708362476374918	0.212573517799976	-4,16578E+14	8,38447E+14	7,5926E+14	7,11886E+14	7,32251E+14	7,18175E+14	7,20896E+14
TC0200002139.mm.2	Banf2	barrier to autointegration factor 2	-1,17309E+14	5,44349E+13	-2,16185E+14	0.0708519502033809	0.212573517799976	-4,16598E+14	4,56572E+14	4,78244E+14	5,1401E+14	6,47832E+14	5,37615E+14	6,3182E+14
TC0300003246.mm.2	Gstm3	glutathione S-transferase, mu 3	0.658053111032228	6,43794E+14	2,1618E+14	0.0708573420149822	0.212573517799976	-4,16605E+14	6,64372E+14	6,97352E+14	6,29801E+14	6,41122E+13	6,19628E+14	6,10486E+14
TC1800000648.mm.2	Napg	N-ethylmaleimide sensitive fusion protein attachment protein gamma	1,58367E+13	8,18451E+14	2,16171E+14	0.0708657586830688	0.212573517799976	-4,16616E+14	9,35736E+14	9,23746E+14	8,40671E+14	6,67803E+14	8,55609E+13	6,87139E+14
TC0800002951.mm.2	Ctrb1	chymotrypsinogen B1	-0.711382180948939	5,27099E+14	-2,16166E+14	0.0708710319382779	0.212573517799976	-4,16623E+14	5,15216E+14	4,66364E+14	5,15451E+14	5,41707E+14	5,5426E+14	5,69596E+14
TC1300001725.mm.2	Cdkal1	CDK5 regulatory subunit associated protein 1-like 1	1,90881E+14	5,38632E+14	2,16131E+14	0.0709060005096816	0.212604437345708	-4,16669E+14	6,55525E+14	6,90813E+14	5,85174E+14	3,35706E+14	5,63252E+14	4,01323E+14
TC0700000281.mm.2	Irf2bp1	interferon regulatory factor 2 binding protein 1	0.714415229792337	6,5971E+14	2,16127E+14	0.0709101332872123	0.212604437345708	-4,16674E+14	7,22808E+13	6,91506E+13	6,60242E+14	6,12274E+14	6,41927E+14	6,29503E+13
TC0500000901.mm.2	Gm3106	predicted gene 3106	-0.755182839014103	4,98418E+13	-2,16116E+14	0.0709212547734276	0.212604437345708	-4,16688E+13	4,38926E+13	4,45724E+14	4,99912E+14	5,70261E+14	5,22618E+14	5,13068E+14
TC0700003103.mm.2	Nipa1	non imprinted in Prader-Willi/Angelman syndrome 1 homolog (human)	0.940373697488003	7,70166E+14	2,16082E+14	0.0709558812871677	0.212604437345708	-4,16734E+13	8,34785E+14	8,23277E+14	7,94399E+14	6,98549E+14	7,68239E+14	7,01748E+14
TC0900003337.mm.2	Ip6k1	inositol hexaphosphate kinase 1	1,0949E+14	7,97982E+14	2,16078E+14	0.0709602906551125	0.212604437345708	-4,16739E+14	8,95841E+14	8,64255E+14	7,97668E+14	6,89012E+14	8,19551E+14	7,21563E+14
TC0600002122.mm.2	Mkrn1	makorin, ring finger protein, 1	1,8808E+14	7,77736E+14	2,16069E+14	0.0709689449773108	0.212604437345708	-4,16751E+14	8,9508E+14	8,98875E+14	8,12542E+14	6,42123E+14	8,29081E+14	5,88714E+14
TC1700001620.mm.2	Tbc1d24/Ntn3	TBC1 domain family, member 24/netrin 3	0.73182869807339	6,89522E+14	2,16028E+14	0.0710103365084997	0.212604437345708	-4,16805E+13	7,26466E+13	7,31897E+14	6,9434E+14	6,72429E+14	6,86134E+14	6,25864E+14
TC1600000532.mm.2	Rabl3	RAB, member RAS oncogene family-like 3	1,40378E+14	6,10018E+13	2,16025E+14	0.0710131833464444	0.212604437345708	-4,16808E+14	6,70378E+14	6,65432E+14	6,92718E+14	5,76526E+14	6,1437E+14	4,40685E+14
TC1000002172.mm.2	Fam184a	family with sequence similarity 184, member A	0.770662554977704	5,7588E+14	2,16017E+14	0.0710215905287918	0.212604437345708	-4,16819E+14	6,31932E+14	5,95055E+14	6,08677E+13	5,4676E+14	5,24614E+14	5,4824E+13
TC1900000894.mm.2	Ndufv1	NADH:ubiquinone oxidoreductase core subunit V1	1,20649E+13	8,99805E+14	2,16011E+14	0.0710277642558604	0.212604437345708	-4,16827E+14	1,00531E+14	9,96157E+14	9,00723E+14	7,36472E+14	9,04792E+14	8,55375E+14
TC0200005455.mm.2	Lkaaear1	LKAAEAR motif containing 1 (IKAAEAR murine motif)	-1,25569E+14	4,61315E+14	-2,16006E+14	0.0710329950418531	0.212604437345708	-4,16834E+14	3,46596E+14	4,06526E+14	4,38185E+14	5,72322E+14	4,49071E+14	5,5519E+14
TC1500001934.mm.2	Pmm1	phosphomannomutase 1	-0.909006125415452	9,37961E+13	-2,15999E+14	0.0710396754589024	0.212604437345708	-4,16843E+14	8,81498E+14	8,73035E+14	9,26314E+14	1,01059E+13	9,32858E+14	1,00348E+14
TC0100003374.mm.2	Prdx6	peroxiredoxin 6	-0.653280876883986	8,7769E+14	-2,15973E+13	0.0710664891528254	0.212645188434443	-4,16878E+14	8,22709E+14	8,52555E+14	8,9909E+14	8,85868E+14	9,07547E+14	8,98373E+14
TC0500001349.mm.2	Oas1b	2'-5' oligoadenylate synthetase 1B	-0.743865015326547	5,97172E+14	-2,15952E+14	0.0710873466113778	0.212668105449152	-4,16905E+14	5,30101E+14	5,68695E+14	5,92706E+14	6,43959E+14	6,35251E+14	6,12318E+14
TC0600001244.mm.2	Cpne9	copine family member IX	0.784898236256209	5,58069E+14	2,15824E+13	0.0712166157692557	0.213015283068502	-4,17073E+14	6,2624E+14	5,90535E+14	5,11786E+14	5,60058E+14	5,035E+14	5,56296E+14

TC1900001742.mm.2	Fam204a	family with sequence similarity 204, member A	1,65054E+14	6,69711E+14	2,15763E+14	0.0712789099295196	0.213129047668202	-4,17153E+14	7,95325E+14	7,62718E+14	7,26854E+14	5,05432E+14	6,74289E+14	5,53646E+14
TC0300000978.mm.2	Reg4	regenerating islet-derived family, member 4	-0.713019741087566	4,76272E+14	-2,15761E+14	0.0712811045667435	0.213129047668202	-4,17156E+14	4,42748E+14	4,29918E+13	4,57763E+14	5,11931E+14	5,16705E+14	4,98565E+14
TC1900001586.mm.2	Fgf8	fibroblast growth factor 8	-1,11344E+14	5,84777E+13	-2,15713E+14	0.0713298684588228	0.213150007165609	-4,1722E+14	5,48788E+14	4,96795E+14	5,38586E+13	6,56224E+14	5,91985E+14	6,76286E+14
TC0200001859.mm.2	Sqor	sulfide quinone oxidoreductase	-0.951960411616278	7,01364E+14	-2,15703E+14	0.0713397815601696	0.213150007165609	-4,17232E+14	6,26511E+14	6,3931E+14	6,89629E+14	7,96521E+14	7,01449E+14	7,54765E+13
TC0300002406.mm.2	Lce1f	late cornified envelope 1F	-0.883834661731897	6,04E+14	-2,15692E+14	0.0713505365963132	0.213150007165609	-4,17246E+14	5,53667E+13	5,53161E+14	6,01675E+14	6,31903E+14	5,91639E+14	6,91956E+14
TC1700001943.mm.2	Ly6g6d	lymphocyte antigen 6 complex, locus G6D	-0.780950342708028	7,25412E+14	-2,15691E+14	0.0713522742179046	0.213150007165609	-4,17249E+14	6,57904E+14	6,76889E+14	7,4641E+14	7,80282E+14	7,11086E+14	7,79897E+14
TC0300003113.mm.2	Adgrl2	adhesion G protein-coupled receptor L2	0.860625113661849	6,52692E+14	2,15676E+14	0.0713668965455742	0.213150007165609	-4,17267E+14	6,89272E+14	7,29448E+14	5,81316E+14	6,69523E+14	6,34098E+14	6,12497E+14
TC0300002042.mm.2	Serp1	stress-associated endoplasmic reticulum protein 1	-1,04556E+14	8,28272E+14	-2,15666E+14	0.0713776594937548	0.213150007165609	-4,17281E+14	7,64206E+14	7,80248E+14	9,05365E+14	7,66247E+14	8,76954E+14	8,76613E+14
TC1100003764.mm.2	Krt42	keratin 42	-1,19365E+14	5,95441E+14	-2,15663E+14	0.0713807136266136	0.213150007165609	-4,17285E+14	5,32144E+14	5,14823E+13	5,69611E+13	6,70371E+14	5,75156E+14	7,10541E+13
TC1400001064.mm.2	Kctd4	potassium channel tetramerisation domain containing 4	1,58593E+14	6,1525E+12	2,15637E+14	0.0714072065341536	0.21318960883543	-4,1732E+14	7,58631E+14	7,34183E+14	6,27047E+14	3,96011E+14	6,23079E+14	5,52549E+14
TC0200001033.mm.2	Dync1i2	dynein cytoplasmic 1 intermediate chain 2	1,57162E+14	7,71788E+14	2,15617E+14	0.0714272335027538	0.213209895040732	-4,17346E+14	8,81002E+14	8,86893E+13	8,04729E+14	6,04532E+14	7,93118E+14	6,60455E+14
TC0900003341.mm.2	Snx14	sorting nexin 14	1,41617E+14	6,81929E+14	2,15598E+14	0.0714470127516154	0.213223372697137	-4,17371E+14	7,79314E+14	7,60488E+14	7,1852E+14	5,76682E+14	7,06389E+14	5,50178E+14
TC1200002077.mm.2	Entpd5	ectonucleoside triphosphate diphosphohydrolase 5	1,13193E+14	6,49452E+14	2,15587E+14	0.0714582146443581	0.213223372697137	-4,17386E+14	7,25742E+14	7,0613E+14	6,84811E+14	5,74543E+14	6,58183E+14	5,47303E+14
TC0900000674.mm.2	Tmem266	transmembrane protein 266	-0.724494101620334	5,8786E+13	-2,1554E+14	0.0715053586518257	0.213324540632636	-4,17447E+13	5,57212E+14	5,25641E+14	5,81294E+14	6,3526E+14	6,12847E+14	6,14906E+14
TC0700000122.mm.2	Vmn1r75	vomeronasal 1 receptor 75	-1,05938E+14	4,58086E+14	-2,15461E+14	0.0715864171884984	0.213460329705022	-4,17551E+14	3,88673E+14	3,87585E+14	4,32992E+14	5,51132E+14	4,53706E+14	5,34427E+14
TC0X00000479.mm.2	Rtl8c	retrotransposon Gag like 8C	1,1582E+14	9,37211E+14	2,1546E+14	0.0715869001099426	0.213460329705022	-4,17552E+14	1,048E+14	9,98229E+14	9,54343E+14	8,081E+14	9,33189E+14	8,81402E+14
TC0300000881.mm.2	Rfx5	regulatory factor X, 5 (influences HLA class II expression)	0.769255690027125	6,19808E+14	2,1542E+14	0.0716280050034852	0.213460329705022	-4,17605E+14	6,62257E+14	6,78101E+13	6,30999E+14	5,60983E+14	6,05796E+14	5,80711E+14
TC0600000838.mm.2	Immt	inner membrane protein, mitochondrial	0.78754186523298	8,10082E+14	2,15409E+14	0.0716390134902154	0.213460329705022	-4,17619E+14	8,63121E+14	8,42778E+14	8,2764E+14	7,78564E+14	8,04818E+13	7,43572E+14
TC0200002270.mm.2	Angpt4	angiopoietin 4	-0.640188591982874	6,40565E+14	-2,15406E+14	0.0716418143412062	0.213460329705022	-4,17623E+13	5,95601E+14	6,10018E+14	6,4245E+14	6,61665E+14	6,604E+14	6,73256E+14
TC0300000418.mm.2	Sohlh2	spermatogenesis and oogenesis specific basic helix-loop-helix 2	-1,05745E+14	4,69238E+14	-2,15405E+14	0.0716431488252949	0.213460329705022	-4,17625E+14	3,97778E+14	4,34252E+14	4,11855E+14	5,28023E+14	4,84503E+14	5,59017E+14
TC0600002519.mm.2	Ctnna2	catenin (cadherin associated protein), alpha 2	1,30897E+14	7,43069E+14	2,15405E+14	0.07164360845558	0.213460329705022	-4,17625E+14	8,49976E+14	8,25953E+14	7,67302E+13	6,01049E+14	7,53594E+14	6,60542E+14
TC0800002409.mm.2	Kcnn1	potassium intermediate/small conductance calcium-activated channel, subfamily N, member 1	0.630192643816032	6,90136E+14	2,15348E+14	0.0717012971795851	0.213592285346965	-4,177E+14	7,23872E+14	7,22845E+14	6,78894E+13	6,94524E+14	6,66482E+14	6,54197E+14
TC0400000391.mm.2	Fam205a2/Fam205a4	family with sequence similarity 205, member A2/family with sequence similarity 205, member A4	0.906216268184367	5,84496E+14	2,15335E+13	0.0717144084731014	0.213592285346965	-4,17716E+14	6,51795E+14	6,63492E+14	5,25826E+14	5,31819E+14	6,21766E+14	5,12278E+14
TC1700001839.mm.2	Akap8l	A kinase (PRKA) anchor protein 8-like	0.711379871600788	6,61343E+14	2,1532E+14	0.071730289575963	0.213600102742098	-4,17737E+14	7,08145E+14	7,14815E+14	6,32063E+14	6,32349E+14	6,69394E+14	6,1129E+13
TC0600001653.mm.2	Plekha5	pleckstrin homology domain containing, family A member 5	1,22165E+14	7,86487E+14	2,15296E+14	0.0717543503122322	0.213632270247782	-4,17768E+14	8,57739E+14	8,69401E+14	8,38546E+14	6,70426E+14	7,65861E+14	7,16948E+14
TC0X00000131.mm.2	Mid1ip1	Mid1 interacting protein 1 (gastrulation specific G12-like (zebrafish))	0.916980538607731	7,76889E+14	2,15251E+14	0.0718004121776801	0.213709458171198	-4,17827E+13	8,51872E+14	8,3475E+14	7,63921E+14	7,07567E+14	7,03514E+14	7,99712E+14
TC1300000781.mm.2	Cdk20	cyclin-dependent kinase 20	0.721052650595813	5,8823E+14	2,15245E+14	0.0718068023669624	0.213709458171198	-4,17835E+14	6,38279E+14	6,10129E+14	5,83803E+14	5,92971E+14	5,24994E+14	5,79203E+14
TC0500001789.mm.2	Gm3404	predicted gene 3404	-1,01329E+14	4,55806E+14	-2,15195E+14	0.0718580736955459	0.213822556132286	-4,17901E+14	3,58166E+14	4,27998E+14	4,33984E+13	5,25865E+14	4,60574E+14	5,28247E+14
TC0100001452.mm.2	Mrps14	mitochondrial ribosomal protein S14	0.674635397053927	7,43013E+14	2,1506E+14	0.0719962736444351	0.214092279537658	-4,18079E+14	8,02241E+14	8,05497E+14	6,90219E+14	6,87311E+14	7,58874E+14	7,13936E+14
TC0900002164.mm.2	Trappc4	trafficking protein particle complex 4	1,1883E+14	7,17504E+14	2,15053E+14	0.0720030970186414	0.214092279537658	-4,18088E+13	7,93558E+14	7,68496E+14	7,52178E+14	6,66399E+14	7,34073E+14	5,9032E+14
TC0400002757.mm.2	Ptbp3	polypyrimidine tract binding protein 3	1,41976E+14	5,91944E+14	2,15041E+14	0.072015639077515	0.214092279537658	-4,18104E+14	6,65931E+14	7,19208E+14	6,16195E+14	4,49144E+13	6,11216E+14	4,89971E+14
TC1500002242.mm.2	Tfcp2	transcription factor CP2	0.920023558672174	6,50392E+14	2,15038E+13	0.0720183371698601	0.214092279537658	-4,18107E+14	7,17196E+14	6,85638E+14	6,11784E+14	6,68906E+14	6,5596E+14	5,6287E+14
TC0500000466.mm.2	Bst1	bone marrow stromal cell antigen 1	-0.860830554380088	6,58854E+14	-2,15032E+14	0.0720245145460641	0.214092279537658	-4,18115E+14	6,05483E+14	6,20968E+14	6,16274E+14	7,11782E+14	7,11882E+14	6,86735E+14
TC0100000817.mm.2	Neu2	neuraminidase 2	0.631996096532584	6,1058E+14	2,15012E+14	0.0720453201630923	0.214092279537658	-4,18142E+14	6,25244E+14	6,25423E+14	6,46726E+14	6,41821E+13	5,72974E+14	5,51294E+14
TC0600003121.mm.2	Gnb3	guanine nucleotide binding protein (G protein), beta 3	-0.902940209855019	6,86525E+14	-2,1501E+14	0.0720474243984086	0.214092279537658	-4,18144E+14	5,86778E+14	6,47704E+14	7,06341E+14	7,63258E+14	6,71782E+14	7,43287E+14
TC1100002071.mm.2	Ptchd3	patched domain containing 3	-0.705231254392337	5,1464E+14	-2,15002E+14	0.0720550134632111	0.214092279537658	-4,18154E+14	4,62358E+14	4,7738E+14	5,08704E+14	5,58611E+14	5,51611E+14	5,29174E+14
TC0300000778.mm.2	Tpm3	tropomyosin 3, gamma	1,41765E+14	8,9812E+14	2,14988E+14	0.0720697231856826	0.21409650621145	-4,18173E+14	9,96863E+14	1,01797E+14	9,13459E+14	7,29129E+12	9,17812E+14	8,1349E+14
TC0700001356.mm.2	Rsf1	remodeling and spacing factor 1	1,8229E+14	6,54022E+14	2,14959E+14	0.0720999839378646	0.214146920035173	-4,18212E+14	8,04766E+14	7,8652E+14	6,9045E+14	4,15689E+13	6,52236E+13	5,74468E+14
TC0700004340.mm.2	Stx1b	syntaxin 1B	1,2198E+14	9,25117E+14	2,14937E+14	0.0721222044266687	0.214149496843666	-4,1824E+14	1,00519E+14	1,00066E+14	9,58316E+14	8,24651E+14	9,37774E+14	8,24115E+14
TC1700000500.mm.2	BC004004	cDNA sequence BC004004	1,16582E+14	8,15233E+13	2,14932E+14	0.0721274324688497	0.214149496843666	-4,18247E+14	8,99943E+14	9,01793E+14	8,37497E+13	6,83589E+14	8,13996E+14	7,54577E+14
TC0X00003232.mm.2	Magea5	melanoma antigen, family A, 5	-0.862130443148088	5,21641E+13	-2,14898E+14	0.0721620807558544	0.214212897424296	-4,18291E+14	4,64981E+14	4,70174E+14	5,08823E+14	5,78287E+14	5,17632E+14	5,89949E+14
TC0200001049.mm.2	Rapgef4	Rap guanine nucleotide exchange factor (GEF) 4	0.883326296890534	8,93693E+14	2,14875E+14	0.0721860637163998	0.214224636812086	-4,18322E+14	9,48195E+14	9,91006E+14	8,57203E+14	8,03218E+14	9,21174E+14	8,41362E+14
TC0400000504.mm.2	Foxe1	forkhead box E1	-1,20449E+14	6,12864E+14	-2,14868E+14	0.0721926256991019	0.214224636812086	-4,1833E+14	5,0177E+14	5,72254E+14	5,79225E+14	7,09015E+14	6,12784E+14	7,02138E+14
TC1000002510.mm.2	Mob3a	MOB kinase activator 3A	0.849174605721492	6,17024E+14	2,14835E+14	0.0722269964799365	0.214259590821126	-4,18374E+13	6,60539E+14	6,25822E+13	6,6347E+14	6,35789E+14	6,01579E+14	5,14948E+13
TC0100000602.mm.2	Xrcc5	X-ray repair complementing defective repair in Chinese hamster cells 5	1,29476E+14	6,40378E+14	2,14831E+14	0.072230999648753	0.214259590821126	-4,1838E+14	7,49318E+14	7,11819E+14	6,81622E+14	4,97321E+14	6,14561E+13	5,87624E+13
TC0800002949.mm.2	Wdr59	WD repeat domain 59	0.654274570989252	7,60916E+14	2,14757E+14	0.0723068536791168	0.214424616195153	-4,18477E+14	8,15234E+14	7,91519E+14	7,46529E+14	7,36319E+14	7,50725E+13	7,25173E+14
TC1900000907.mm.2	Tbc1d10c	TBC1 domain family, member 10c	-0.903973881641726	7,2047E+14	-2,14735E+14	0.0723294629965461	0.214424616195153	-4,18505E+14	6,29917E+14	6,70167E+14	7,2776E+14	8,14101E+14	7,68277E+14	7,12602E+14
TC0X00000327.mm.2	Slc25a5	solute carrier family 25 (mitochondrial carrier, adenine nucleotide translocator), member 5	0.831488515293822	1,02565E+14	2,14726E+14	0.0723390556097172	0.214424616195153	-4,18518E+14	1,0736E+14	1,07684E+14	1,03981E+14	9,79524E+14	1,02751E+14	9,56635E+14
TC1000001821.mm.2	Slc35d3	solute carrier family 35, member D3	0.673988856404642	6,55546E+14	2,14722E+14	0.0723436103503594	0.214424616195153	-4,18523E+14	6,94259E+14	7,0586E+14	6,44006E+14	6,23826E+14	6,13217E+14	6,52104E+14
TC1100001331.mm.2	Trim37	tripartite motif-containing 37	1,80031E+14	8,19726E+14	2,14683E+14	0.0723839070836029	0.214424616195153	-4,18575E+14	9,28921E+13	9,28405E+14	8,69974E+14	6,9379E+14	8,59071E+14	6,38194E+14
TC1000000808.mm.2	Madcam1	mucosal vascular addressin cell adhesion molecule 1	-0.853130926278437	5,19741E+14	-2,14636E+14	0.0724314262182914	0.214424616195153	-4,18636E+14	4,73626E+14	4,64157E+14	4,94868E+14	5,77383E+14	5,27732E+14	5,80677E+14
TC0700001145.mm.2	Sema4b	sema domain, immunoglobulin domain (Ig), transmembrane domain (TM) and short cytoplasmic domain, (semaphorin) 4B	-0.703205448309603	8,65085E+14	-2,14636E+14	0.0724320812546434	0.214424616195153	-4,18636E+14	8,25539E+14	8,30769E+14	8,81982E+14	8,5527E+14	8,73744E+14	9,23206E+14
TC1700000474.mm.2	Ppard/1810013A23Rik	peroxisome proliferator activator receptor delta/RIKEN cDNA 1810013A23 gene	-0.707456405883221	8,78065E+14	-2,14631E+14	0.0724373985604424	0.214424616195153	-4,18643E+14	8,25834E+14	8,36092E+14	8,74906E+14	9,28142E+13	8,91509E+14	9,11908E+14
TC1900000896.mm.2	Gstp1	glutathione S-transferase, pi 1	1,21169E+14	8,87051E+14	2,1463E+14	0.0724380669041465	0.214424616195153	-4,18644E+14	1,00676E+14	8,95731E+14	9,2101E+14	8,38644E+14	8,83136E+14	7,7702E+14
TC0100002141.mm.2	Zfp451	zinc finger protein 451	1,13172E+14	7,67959E+14	2,14627E+14	0.0724412539589628	0.214424616195153	-4,18648E+14	8,39947E+14	8,5291E+14	7,54846E+13	6,93538E+14	8,06084E+14	6,60429E+14

TC1100003839.mm.2	Itga2b	integrin alpha 2b	-0.691891367351142	7,24935E+13	-2,14591E+14	0.0724787721088592	0.214424616195153	-4,18696E+14	6,78544E+14	6,73882E+14	7,31562E+14	7,7482E+14	7,30745E+14	7,6006E+14
TC0500001251.mm.2	Pop5	processing of precursor 5, ribonuclease P/MRP family (S. cerevisiae)	1,03792E+14	7,0592E+14	2,14584E+12	0.0724856850971415	0.214424616195153	-4,18705E+14	7,81095E+14	7,40861E+14	7,64962E+14	6,34228E+13	6,43385E+14	6,70987E+14
TC0700000820.mm.2	Pnkp	polynucleotide kinase 3'- phosphatase	-1,17744E+14	6,83829E+14	-2,14536E+14	0.0725352375939068	0.214424616195153	-4,18768E+14	6,15605E+14	6,10616E+14	6,39151E+14	7,75891E+14	6,82378E+14	7,79332E+14
TC0500000614.mm.2	Ube2k	ubiquitin-conjugating enzyme E2K	1,24067E+14	8,7633E+14	2,14536E+14	0.0725355796736717	0.214424616195153	-4,18768E+12	9,72992E+14	9,68603E+14	8,76888E+14	7,46035E+14	9,08483E+14	7,84979E+14
TC1100001162.mm.2	Vtn	vitronectin	1,0035E+14	8,42537E+14	2,14533E+14	0.0725378900752005	0.214424616195153	-4,18771E+14	9,38126E+14	9,121E+14	8,22687E+13	7,32784E+14	8,71568E+14	7,77959E+14
TC0300002734.mm.2	Cyb561d1	cytochrome b-561 domain containing 1	0.687679429323683	6,28935E+14	2,14529E+14	0.072542302704014	0.214424616195153	-4,18777E+14	6,55395E+14	6,81563E+14	6,31516E+14	6,05714E+13	6,17369E+14	5,82054E+14
TC1100003921.mm.2	Scn4a	sodium channel, voltage-gated, type IV, alpha	-0.69226723964811	6,25615E+14	-2,14528E+14	0.0725436142192318	0.214424616195153	-4,18778E+14	5,87469E+14	5,99864E+13	5,97775E+14	6,42795E+13	6,50703E+14	6,75083E+14
TC1000002549.mm.2	Tle6	transducin-like enhancer of split 6	-0.739518927074519	4,80607E+14	-2,14518E+14	0.0725536085797185	0.214424616195153	-4,18791E+13	4,40916E+14	4,25667E+14	4,69782E+14	5,32787E+14	4,96466E+14	5,18021E+14
TC0400001110.mm.2	Ttc22	tetratricopeptide repeat domain 22	-0.777357559899405	7,11734E+14	-2,145E+14	0.0725721468968987	0.214424616195153	-4,18815E+14	6,73547E+14	6,60378E+14	7,24139E+14	7,22945E+14	7,0565E+14	7,83746E+14
TC0100000883.mm.2	Prlh	prolactin releasing hormone	-0.814503619507345	7,69333E+14	-2,14496E+13	0.072576730297082	0.214424616195153	-4,18821E+14	7,14637E+14	7,03505E+14	7,76713E+13	8,401E+14	7,59758E+14	8,21285E+14
TC0700002652.mm.2	Cyp2b23	cytochrome P450, family 2, subfamily b, polypeptide 23	-0.940470403045991	5,32204E+14	-2,14488E+14	0.0725845054436029	0.214424616195153	-4,18831E+14	4,64466E+14	4,76672E+14	5,21816E+14	6,01035E+14	5,20786E+14	6,08447E+14
TC1500000721.mm.2	Rbx1	ring-box 1	1,05938E+14	7,6297E+14	2,14467E+14	0.0726060799945544	0.214424616195153	-4,18858E+14	8,41674E+14	8,38746E+14	7,70272E+14	6,58583E+14	7,79769E+14	6,88774E+14
TC1700001662.mm.2	Sstr5	somatostatin receptor 5	0.68269891012269	5,65163E+14	2,14464E+14	0.072609507050144	0.214424616195153	-4,18862E+14	5,98883E+14	5,88934E+14	5,51943E+14	5,99942E+14	5,23907E+13	5,2737E+14
TC1000000903.mm.2	Tle5	TLE family member 5, transcriptional modulator	1,35358E+14	1,06252E+14	2,14452E+14	0.072622243878804	0.214424616195153	-4,18879E+12	1,17214E+14	1,14979E+14	1,09018E+14	9,11804E+14	1,07117E+14	9,80048E+14
TC0400001922.mm.2	Nppa	natriuretic peptide type A	-0.804766211883414	5,33157E+14	-2,14446E+13	0.0726282828174164	0.214424616195153	-4,18886E+13	4,94211E+14	4,67636E+14	5,39717E+14	5,74577E+14	5,24072E+14	5,98729E+14
TC1400000557.mm.2	Olfr741	olfactory receptor 741	-1,09951E+14	5,00717E+14	-2,14404E+14	0.0726711672565583	0.214424616195153	-4,18941E+14	4,13882E+14	4,49692E+14	4,84648E+14	5,72601E+13	4,82182E+14	6,01294E+14
TC0900003138.mm.2	Arpp21	cyclic AMP-regulated phosphoprotein, 21	1,10332E+14	1,05886E+14	2,14385E+12	0.0726912392433698	0.214424616195153	-4,18966E+13	1,12736E+13	1,12372E+14	1,09649E+14	9,75172E+14	1,06188E+14	9,68532E+14
TC0300003207.mm.2	Bbs12	Bardet-Biedl syndrome 12 (human)	-0.788040325394263	6,72947E+14	-2,14381E+14	0.0726954778337529	0.214424616195153	-4,18972E+14	6,14063E+14	6,21993E+14	6,66631E+14	7,41328E+13	6,80113E+14	7,13551E+14
TC0400004227.mm.2	Ctrc	chymotrypsin C (caldecrin)	-0.947334915591055	6,36929E+14	-2,14375E+14	0.0727021016010087	0.214424616195153	-4,1898E+14	5,63013E+14	6,01982E+14	6,03503E+14	6,98618E+14	6,45876E+14	7,08586E+14
TC0100001008.mm.2	Relch	RAB11 binding and LisH domain, coiled-coil and HEAT repeat containing	1,15581E+14	6,47404E+14	2,14346E+14	0.0727316231490136	0.214424616195153	-4,19018E+14	7,4665E+14	7,23077E+14	6,63625E+14	5,12507E+14	6,48378E+14	5,90187E+14
TC0900001366.mm.2	6430571L13Rik	RIKEN cDNA 6430571L13 gene	-1,01913E+14	6,02565E+14	-2,14342E+14	0.0727360543893108	0.214424616195153	-4,19023E+14	5,30481E+14	5,29332E+14	6,10744E+13	6,81196E+14	5,73739E+13	6,899E+14
TC1900001036.mm.2	Cox8a	cytochrome c oxidase subunit 8A	0.709890401670551	1,09196E+14	2,14318E+14	0.0727601884872074	0.214424616195153	-4,19054E+14	1,1484E+14	1,14182E+14	1,08137E+14	1,03193E+14	1,08997E+14	1,05827E+14
TC0800003077.mm.2	Galns	galactosamine (N-acetyl)-6-sulfate sulfatase	0.92337508158101	6,09765E+14	2,14307E+14	0.0727720746448772	0.214424616195153	-4,19069E+14	6,12544E+14	6,58188E+14	6,08088E+14	6,93715E+14	5,12031E+14	5,74025E+14
TC1500002124.mm.2	Dbx2	developing brain homeobox 2	-0.732222974387617	6,49507E+14	-2,14304E+14	0.0727753472459116	0.214424616195153	-4,19073E+14	5,996E+14	5,82217E+14	6,63113E+14	7,23853E+14	6,54066E+14	6,74195E+14
TC1700001004.mm.2	Kdm4b	lysine (K)-specific demethylase 4B	0.650871864871756	6,53085E+14	2,14301E+14	0.0727783320512371	0.214424616195153	-4,19077E+14	7,19402E+14	7,03324E+14	5,86042E+14	6,17192E+14	6,44198E+14	6,48355E+14
TC1200001662.mm.2	Lsmem1	leucine-rich single-pass membrane protein 1	-0.770502058424649	5,53535E+14	-2,14289E+14	0.0727908314697636	0.214424616195153	-4,19093E+13	5,04028E+14	4,87365E+14	5,71854E+14	6,12468E+14	5,40799E+14	6,04695E+14
TC0600001843.mm.2	Col28a1	collagen, type XXVIII, alpha 1	-0.907806872414634	7,00563E+14	-2,14288E+14	0.0727912655202144	0.214424616195153	-4,19093E+14	6,28611E+14	6,64155E+14	7,04055E+14	7,32231E+14	6,86156E+14	7,88171E+14
TC1900001022.mm.2	Kcnk4	potassium channel, subfamily K, member 4	-0.860021340756286	6,82352E+14	-2,14287E+14	0.0727923199024071	0.214424616195153	-4,19095E+14	5,91763E+13	6,66095E+14	6,73915E+14	7,3248E+14	7,31838E+14	6,98024E+14
TC0400003362.mm.2	Rps8/Snord38a	ribosomal protein S8/small nucleolar RNA, C/D box 38A	1,23115E+14	9,51432E+14	2,14239E+14	0.0728426687281442	0.214476745473679	-4,19158E+13	1,04348E+14	1,0362E+14	9,59554E+14	8,35915E+14	9,8098E+14	8,52466E+14
TC1800000694.mm.2	Gnal	guanine nucleotide binding protein, alpha stimulating, olfactory type	1,24616E+14	9,41896E+14	2,14227E+14	0.0728549640361038	0.214476745473679	-4,19174E+14	1,03935E+14	1,02047E+13	9,73914E+14	8,07051E+14	9,39581E+14	8,71008E+14
TC0200004433.mm.2	Ino80	INO80 complex subunit	1,74408E+14	6,08799E+14	2,14226E+14	0.0728557247275307	0.214476745473679	-4,19175E+13	7,25631E+14	7,27978E+14	6,36038E+14	4,58353E+14	6,5614E+14	4,48653E+14
TC1300002747.mm.2	Nim1k	NIM1 serine/threonine protein kinase	1,98419E+13	5,71497E+14	2,14219E+13	0.0728632597730355	0.214476745473679	-4,19185E+14	6,84464E+14	6,91764E+13	6,37311E+13	4,3605E+14	6,11313E+14	3,68078E+13
TC1100003505.mm.2	Akap1	A kinase (PRKA) anchor protein 1	0.929804823840866	6,88296E+13	2,14172E+14	0.0729121351115723	0.214564046150738	-4,19246E+13	7,62276E+14	7,55529E+14	6,83291E+14	5,96837E+14	6,26861E+14	7,04983E+14
TC1600000253.mm.2	Hira	histone cell cycle regulator	0.647320593579884	6,87327E+14	2,14165E+14	0.0729195504698966	0.214564046150738	-4,19256E+14	7,13928E+14	7,40311E+14	6,79605E+14	6,65345E+14	6,70012E+14	6,54763E+14
TC0700003783.mm.2	Emsy	EMSY, BRCA2-interacting transcriptional repressor	1,608E+14	7,15398E+14	2,14105E+14	0.0729820760886644	0.214649407476286	-4,19335E+13	8,13988E+14	7,9673E+14	7,88026E+13	6,04527E+12	7,28162E+14	5,60956E+14
TC0200000407.mm.2	Tmem210	transmembrane protein 210	-0.935781840838589	5,71919E+14	-2,14082E+14	0.0730058264782772	0.214649407476286	-4,19365E+14	5,11606E+14	5,34207E+14	5,18579E+14	6,34154E+14	6,11795E+14	6,21174E+14
TC0300002842.mm.2	A730020M07Rik	RIKEN cDNA A730020M07 gene	-0.901850993119858	5,64558E+14	-2,1408E+14	0.0730080577895961	0.214649407476286	-4,19368E+14	4,94327E+14	4,90991E+14	5,63448E+14	6,72891E+13	5,90227E+14	5,75462E+14
TC1700001482.mm.2	Dact2	dishevelled-binding antagonist of beta-catenin 2	1,03745E+14	6,23664E+14	2,14079E+14	0.0730088351875734	0.214649407476286	-4,19369E+14	6,64822E+14	7,31052E+13	6,16641E+14	5,41085E+14	6,42905E+14	5,4548E+14
TC0900002915.mm.2	Pik3cb	phosphatidylinositol-4,5-bisphosphate 3-kinase catalytic subunit beta	0.993379158510937	6,86355E+14	2,14073E+14	0.0730151680244228	0.214649407476286	-4,19377E+14	7,46537E+14	7,37885E+14	7,00203E+14	6,47756E+14	7,02562E+14	5,83184E+14
TC0200004970.mm.2	Edem2	ER degradation enhancer, mannosidase alpha-like 2	0.94326684421309	7,24621E+14	2,14028E+14	0.0730617876791759	0.214747279254754	-4,19436E+14	7,63701E+14	7,77647E+14	7,64874E+14	6,88811E+14	7,22128E+13	6,30567E+13
TC1000000354.mm.2	Slc22a16	solute carrier family 22 (organic cation transporter), member 16	-1,15365E+14	5,14039E+14	-2,13988E+14	0.0731036007137598	0.214830990023858	-4,19489E+14	4,31437E+14	4,27569E+14	4,94259E+14	6,41235E+14	5,08998E+14	5,80738E+14
TC0200002212.mm.2	Syndig1	synapse differentiation inducing 1	0.90315313580189	7,94277E+14	2,13916E+14	0.0731789271359548	0.215002793132909	-4,19584E+14	8,53642E+14	8,28852E+14	8,37542E+13	7,43765E+14	7,77854E+14	7,24009E+14
TC0900001158.mm.2	Adamts7	a disintegrin-like and metallopeptidase (reprolysin type) with thrombospondin type 1 motif, 7	-0.843326596777209	5,73436E+14	-2,13895E+14	0.0731999616580941	0.215002793132909	-4,1961E+14	5,37158E+14	5,15233E+14	5,44158E+13	6,23008E+14	5,84658E+14	6,36398E+14
TC0300002820.mm.2	Plppr4	phospholipid phosphatase related 4	1,36005E+14	8,78156E+14	2,13893E+14	0.0732020929142393	0.215002793132909	-4,19613E+13	9,74013E+14	9,70193E+14	9,19491E+14	7,33044E+14	8,76734E+14	7,95462E+14
TC1100002901.mm.2	Natd1	N-acetyltransferase domain containing 1	-0.647919538599169	6,91128E+14	-2,13847E+14	0.0732500713818225	0.215088225817781	-4,19673E+14	6,49293E+14	6,47994E+14	6,9467E+14	7,27939E+14	7,17911E+14	7,0896E+14
TC1300001928.mm.2	Dek	DEK oncogene (DNA binding)	0.631051914168792	7,57558E+14	2,1384E+14	0.0732578776942829	0.215088225817781	-4,19683E+14	7,96633E+14	8,04549E+12	7,25806E+14	7,43388E+14	7,44748E+14	7,30225E+14
TC0100001249.mm.2	Lmod1	leiomodin 1 (smooth muscle)	-0.858903575575127	5,43578E+14	-2,1382E+14	0.0732784157294813	0.215109330050853	-4,19709E+14	4,91354E+14	5,02633E+14	5,13415E+14	5,88297E+14	5,49716E+13	6,16052E+13
TC1400000843.mm.2	Mtmr6	myotubularin related protein 6	1,0876E+14	7,81739E+14	2,13802E+14	0.0732969661734661	0.215124593069774	-4,19732E+14	8,39298E+14	8,65807E+13	8,0507E+14	6,92675E+14	7,92084E+14	6,95501E+14
TC0500001570.mm.2	Cldn3	claudin 3	-0.730411557470045	6,99084E+14	-2,1375E+14	0.0733521188579401	0.215247257541065	-4,19802E+14	6,45489E+14	6,54777E+14	6,91525E+14	7,56362E+14	7,28419E+13	7,17929E+14
TC1000000446.mm.2	Ascc3	activating signal cointegrator 1 complex subunit 3	1,20098E+14	5,90395E+14	2,13674E+14	0.0734306558872487	0.215396127217967	-4,19901E+14	6,65799E+14	6,99972E+14	5,89628E+14	4,61394E+14	6,18468E+13	5,07107E+14
TC0700000010.mm.2	Cacng6	calcium channel, voltage-dependent, gamma subunit 6	-0.749529291415179	6,14845E+14	-2,13672E+14	0.0734330861814223	0.215396127217967	-4,19904E+14	5,50988E+14	5,69498E+14	6,41085E+14	6,57109E+14	6,01252E+14	6,69139E+14
TC1200002271.mm.2	Serpina10	serine (or cysteine) peptidase inhibitor, clade A (alpha-1 antiproteinase, antitrypsin), member 10	-1,31772E+14	4,772E+14	-2,13655E+14	0.0734514511289719	0.215396127217967	-4,19927E+14	4,30749E+14	3,63035E+14	4,17751E+14	5,94337E+14	5,03481E+14	5,53848E+14
TSUnmapped00000032.mm.2	LOC547349	MHC class I family member	-0.770755635136208	7,25758E+14	-2,13649E+14	0.0734574214078072	0.215396127217967	-4,19934E+14	6,70163E+14	6,64312E+14	7,34358E+14	7,97093E+14	7,21124E+14	7,67502E+14
TC0800001349.mm.2	Cntnap4	contactin associated protein-like 4	1,06452E+14	6,9092E+14	2,13637E+14	0.0734696900136502	0.215396127217967	-4,1995E+14	7,80827E+14	7,24018E+12	7,23283E+14	6,25454E+14	6,92116E+14	5,99824E+14
TC1700000542.mm.2	Cryaa	crystallin, alpha A	-0.884641745310138	5,7755E+14	-2,13603E+14	0.0735052280195941	0.215461113167131	-4,19994E+14	5,37248E+14	5,19029E+13	5,60445E+14	6,15375E+14	5,71622E+14	6,61583E+14
TC1300002500.mm.2	Btf3	basic transcription factor 3	1,09625E+14	9,02346E+14	2,13563E+13	0.0735471485635393	0.215544780793799	-4,20047E+14	9,83558E+14	9,82975E+14	9,15458E+14	7,848E+13	9,13009E+14	8,34274E+14

TC0200004548.mm.2	Itpripl1	inositol 1,4,5-triphosphate receptor interacting protein-like 1	-0.852029530958295	7,48847E+14	-2,1355E+14	0.0735606027749872	0.215545006821853	-4,20064E+13	6,81165E+14	7,11618E+14	7,27903E+14	8,0921E+14	7,57524E+13	8,05665E+14
TC0700001437.mm.2	Rpl31	ribosomal protein L31	0.873054396849263	5,93914E+14	2,13532E+14	0.0735798301549747	0.215562146052201	-4,20088E+14	6,57852E+14	6,17885E+14	6,39631E+14	5,46989E+13	5,63816E+14	5,3731E+14
TC0X00000957.mm.2	Arr3	arrestin 3, retinal	-1,15439E+14	5,17629E+14	-2,13513E+14	0.0735996668547282	0.215581063812077	-4,20113E+14	4,43209E+14	4,38774E+14	5,01725E+14	6,09206E+14	4,94369E+14	6,18493E+13
TC1600000203.mm.2	Mapk1	mitogen-activated protein kinase 1	2,17083E+14	8,83788E+14	2,13493E+14	0.0736204646092719	0.215602789212868	-4,20139E+14	1,01898E+14	1,03586E+14	9,53106E+14	6,74106E+14	9,15493E+14	7,0518E+14
TC0X00000388.mm.2	Gria3	glutamate receptor, ionotropic, AMPA3 (alpha 3)	2,26796E+14	7,98216E+14	2,13445E+14	0.0736712123955896	0.215697607514606	-4,20203E+14	9,3555E+14	9,59291E+14	8,67344E+14	5,8586E+13	8,37663E+14	6,03586E+14
TC1300001877.mm.2	Tbc1d7	TBC1 domain family, member 7	0.698392519040697	6,39171E+14	2,13434E+14	0.0736827239846006	0.215697607514606	-4,20217E+14	6,85025E+14	6,88921E+13	6,39294E+14	5,8752E+14	6,27824E+14	6,06443E+14
TC1300000839.mm.2	1700001L19Rik	RIKEN cDNA 1700001L19 gene	0.894861215739111	6,50526E+14	2,13424E+14	0.0736930012640667	0.215697607514606	-4,2023E+14	7,15065E+14	6,99455E+14	6,3631E+14	6,16781E+14	6,71083E+13	5,64464E+14
TC1500000583.mm.2	Smpd5	sphingomyelin phosphodiesterase 5	-0.703918677475489	6,30959E+14	-2,13322E+14	0.0738004643687846	0.215972917249224	-4,20364E+14	5,7537E+14	6,13396E+14	6,54004E+14	6,1343E+14	6,56198E+14	6,73352E+14
TC1100003374.mm.2	Nle1	notchless homolog 1	-0.73441930611311	5,65134E+14	-2,13307E+14	0.0738158258403851	0.215978645681156	-4,20384E+13	4,89464E+14	5,37444E+12	5,71115E+14	6,18985E+14	5,89847E+14	5,83946E+14
TC0500003462.mm.2	Mepce	methylphosphate capping enzyme	0.709950911644357	7,30615E+14	2,13278E+14	0.0738460821026286	0.21602249553927	-4,20421E+14	7,74997E+14	7,48942E+14	7,64102E+14	7,137E+14	6,80801E+14	7,01148E+14
TC0400003122.mm.2	Kank4	KN motif and ankyrin repeat domains 4	-0.823512739346538	5,85381E+13	-2,13267E+14	0.0738576260116575	0.21602249553927	-4,20436E+14	5,63101E+14	5,222E+14	5,48124E+14	6,2886E+14	6,15412E+13	6,34592E+14
TC0400003201.mm.2	Bsnd	barttin CLCNK type accessory beta subunit	-0.915739836508355	5,1731E+14	-2,1325E+13	0.0738756981758212	0.216036138785301	-4,20458E+13	4,7818E+14	4,47428E+14	4,82592E+13	5,86904E+14	5,36544E+14	5,72212E+14
TC0900002871.mm.2	1700065D16Rik	RIKEN cDNA 1700065D16 gene	-0.926073991872181	5,5809E+14	-2,13197E+14	0.0739320961616451	0.216161833687641	-4,20529E+14	4,79894E+13	5,02611E+14	5,46547E+14	6,51766E+14	6,06565E+14	5,61155E+14
TC0300001689.mm.2	Cyp7b1	cytochrome P450, family 7, subfamily b, polypeptide 1	0.620569991897481	6,31509E+14	2,13173E+14	0.0739565040603948	0.216193967693241	-4,20559E+14	6,81361E+14	6,71388E+14	6,01126E+14	6,06541E+14	6,08139E+14	6,20497E+14
TC0400003389.mm.2	Szt2	seizure threshold 2	0.716648667735182	6,70414E+14	2,13142E+14	0.0739897075349425	0.216251797117818	-4,20601E+14	7,0078E+14	6,87997E+14	6,80779E+14	7,07479E+14	6,50318E+14	5,9513E+14
TC0100002179.mm.2	Kansl3	KAT8 regulatory NSL complex subunit 3	0.746822045076867	7,35383E+14	2,131E+14	0.0740338991020418	0.216341714949438	-4,20656E+14	7,83259E+14	7,69407E+14	7,60668E+14	6,95661E+14	6,87862E+14	7,1544E+14
TC0500003318.mm.2	Sbds	SBDS ribosome maturation factor	0.946488971328927	8,48845E+14	2,13076E+14	0.0740593775520454	0.216357927193683	-4,20688E+14	9,22505E+14	9,03099E+14	8,6071E+14	7,70448E+14	8,57637E+12	7,78669E+14
TC1300001051.mm.2	Dmgdh	dimethylglycine dehydrogenase precursor	-1,04922E+13	6,47811E+14	-2,13056E+14	0.074080659894467	0.216357927193683	-4,20714E+14	5,83563E+13	5,70354E+14	6,27338E+14	7,41847E+14	6,39199E+14	7,24563E+14
TC1700001018.mm.2	Prr22	proline rich 22	-0.969326290048024	6,66759E+14	-2,13054E+14	0.0740826676652303	0.216357927193683	-4,20717E+14	5,95727E+14	6,06895E+14	6,40696E+14	7,60748E+14	6,7909E+14	7,17397E+14
TC0700004333.mm.2	1700120K04Rik	RIKEN cDNA 1700120K04 gene	-0.692525206276557	5,51512E+14	-2,13044E+14	0.0740931572180689	0.216357927193683	-4,2073E+14	4,91698E+14	5,31475E+14	5,69579E+14	5,54642E+14	5,63759E+14	5,97919E+14
TC0400002316.mm.2	Ufl1	UFM1 specific ligase 1	0.849619396983612	6,87254E+14	2,13025E+14	0.0741125752251046	0.216375416670069	-4,20754E+14	7,76318E+14	6,85487E+14	6,91522E+14	6,78317E+14	6,64756E+14	6,27126E+13
TC1000000390.mm.2	9030612E09Rik	RIKEN cDNA 9030612E09 gene	-0.769084142131126	4,89579E+13	-2,12967E+14	0.0741747515777108	0.216499755440181	-4,20831E+14	4,2943E+14	4,5556E+13	4,80036E+14	5,33643E+14	4,93915E+14	5,44892E+14
TC1000001131.mm.2	Lta4h	leukotriene A4 hydrolase	0.650778495344043	6,72395E+14	2,1296E+14	0.0741820362306982	0.216499755440181	-4,2084E+14	7,38331E+13	7,25935E+14	6,06915E+14	6,29077E+14	6,53383E+14	6,80728E+14
TC0500003027.mm.2	2900026A02Rik	RIKEN cDNA 2900026A02 gene	0.736882566497053	6,11297E+14	2,12916E+14	0.0742285366864626	0.216535146217764	-4,20898E+13	6,15631E+14	6,74446E+14	5,99818E+14	6,3519E+14	5,66962E+14	5,75737E+14
TC1900001754.mm.2	Prdx3	peroxiredoxin 3	1,24772E+14	6,83056E+14	2,12911E+14	0.0742338194366312	0.216535146217764	-4,20905E+14	7,62734E+14	7,34302E+14	7,57545E+14	5,96259E+14	6,64765E+12	5,82728E+13
TC0800001219.mm.2	Hsd11b2	hydroxysteroid 11-beta dehydrogenase 2	-0.840246443064983	5,82043E+14	-2,1291E+14	0.0742344782291893	0.216535146217764	-4,20905E+13	5,14121E+14	5,22404E+14	5,77821E+14	6,73337E+14	5,94845E+13	6,09729E+14
TC0400003742.mm.2	Man1c1	mannosidase, alpha, class 1C, member 1	0.798986926929141	6,71439E+14	2,1289E+14	0.0742557470874239	0.216557982410798	-4,20932E+14	7,11255E+14	7,43995E+14	6,6165E+14	6,16279E+14	6,8077E+14	6,14683E+14
TC0300000172.mm.2	Tnik	TRAF2 and NCK interacting kinase	1,12687E+14	7,65874E+14	2,12779E+14	0.0743729836567859	0.216828188891627	-4,21077E+14	8,56518E+14	8,46823E+14	7,7082E+14	6,43116E+14	7,86555E+14	6,91412E+14
TC0500003549.mm.2	Rbak	RB-associated KRAB zinc finger	0.666411877723741	6,31519E+14	2,12775E+14	0.0743777614413901	0.216828188891627	-4,21083E+14	6,42847E+14	6,64293E+14	6,56225E+14	6,51895E+14	6,0642E+14	5,67437E+14
TC1000001813.mm.2	Tnfaip3	tumor necrosis factor, alpha-induced protein 3	0.800754728487085	5,22272E+14	2,12764E+14	0.0743887685839332	0.216828188891627	-4,21097E+14	5,47387E+14	5,85815E+14	4,83128E+14	5,44247E+14	5,10956E+14	4,62096E+14
TC1600000238.mm.2	Trmt2a	TRM2 tRNA methyltransferase 2A	-0.619852862264674	7,31954E+14	-2,12705E+14	0.0744519492233485	0.216862051316357	-4,21175E+14	6,84389E+14	6,84778E+14	7,59056E+14	7,70364E+14	7,53207E+14	7,3993E+14
TC0900001771.mm.2	Amotl1	angiomotin-like 1	0.789735113676725	6,46734E+14	2,12702E+14	0.0744545475408289	0.216862051316357	-4,21179E+14	7,27355E+13	6,77408E+13	6,27108E+14	6,01715E+14	5,91894E+14	6,54922E+14
TC0900001645.mm.2	Tcaim	T cell activation inhibitor, mitochondrial	0.761410144895174	5,83795E+14	2,127E+14	0.0744564545461834	0.216862051316357	-4,21181E+14	6,14722E+14	6,18336E+14	5,56342E+14	6,32594E+14	5,43379E+14	5,37398E+14
TC0200003234.mm.2	Gfi1b	growth factor independent 1B	-0.878130557094499	7,45909E+14	-2,12684E+13	0.0744736811113154	0.216862051316357	-4,21202E+14	6,51003E+14	7,21904E+13	7,41014E+14	8,13002E+12	7,57702E+14	7,90831E+14
TC0800002391.mm.2	Crtc1	CREB regulated transcription coactivator 1	1,24822E+14	8,6973E+14	2,12681E+14	0.0744774032742328	0.216862051316357	-4,21207E+14	9,50493E+14	9,51987E+14	9,2329E+14	7,39773E+14	8,27244E+13	8,25592E+14
TC1700001838.mm.2	Akap8	A kinase (PRKA) anchor protein 8	0.691566121908299	6,19649E+14	2,12675E+14	0.0744839086915165	0.216862051316357	-4,21215E+14	6,60111E+14	6,76717E+14	6,14941E+14	5,67609E+14	5,88469E+14	6,10046E+14
TC0Y00000005.mm.2	Kdm5d	lysine (K)-specific demethylase 5D	0.93338203193834	5,51046E+14	2,12659E+14	0.0745000028201812	0.216862051316357	-4,21235E+14	5,92947E+13	6,02361E+14	5,97177E+13	5,05159E+14	5,41741E+14	4,66891E+14
TC1200001009.mm.2	Cpsf2	cleavage and polyadenylation specific factor 2	1,15656E+14	6,36543E+14	2,12652E+14	0.0745080566056819	0.216862051316357	-4,21245E+14	7,01262E+14	7,39943E+13	6,39229E+14	5,28932E+14	6,63608E+14	5,46285E+13
TC1100001461.mm.2	Spop	speckle-type POZ protein	0.833702965335949	9,10769E+14	2,12581E+14	0.0745828911304633	0.216996174653678	-4,21337E+14	9,48703E+14	9,8267E+14	8,9549E+14	8,7312E+14	9,25644E+13	8,38987E+14
TC0200002362.mm.2	Dynlrb1	dynein light chain roadblock-type 1	0.930462658606618	1,04875E+14	2,12581E+14	0.0745836591690368	0.216996174653678	-4,21338E+14	1,12249E+14	1,10614E+14	1,05475E+14	9,66572E+14	1,05994E+14	9,82604E+13
TC0700000941.mm.2	Gabrb3	gamma-aminobutyric acid (GABA) A receptor, subunit beta 3	1,93561E+14	9,89311E+14	2,12569E+14	0.0745964843026159	0.216996174653678	-4,21354E+14	1,1285E+14	1,11534E+14	1,05078E+14	7,84516E+14	1,00735E+14	8,49377E+14
TC0500003050.mm.2	Selplg	selectin, platelet (p-selectin) ligand	1,00714E+14	7,85439E+14	2,12558E+14	0.0746080064284353	0.216996174653678	-4,21368E+13	8,6223E+14	8,52588E+14	8,00955E+14	6,83473E+14	7,89715E+14	7,23675E+14
TC1000001228.mm.2	Mgat4c	MGAT4 family, member C	1,4866E+14	6,43109E+13	2,12518E+14	0.0746505401329286	0.217000525585234	-4,21421E+14	7,53881E+14	7,56271E+14	6,63298E+14	4,72369E+13	6,69378E+14	5,43454E+14
TC0100002690.mm.2	Pid1	phosphotyrosine interaction domain containing 1	1,08046E+14	6,08824E+14	2,12516E+14	0.0746518608191079	0.217000525585234	-4,21423E+14	7,02291E+14	6,66525E+14	6,23328E+14	5,08075E+14	6,21263E+14	5,31462E+14
TC1300001017.mm.2	Rps23	ribosomal protein S23	1,06168E+14	1,11689E+14	2,12506E+14	0.0746630416380441	0.217000525585234	-4,21437E+14	1,19602E+14	1,18092E+14	1,13794E+14	1,02188E+14	1,12642E+13	1,03818E+14
TC1800000727.mm.2	Rnmt	RNA (guanine-7-) methyltransferase	0.750105356131505	6,53406E+14	2,12506E+14	0.0746633720501792	0.217000525585234	-4,21437E+14	7,24956E+14	7,3712E+14	6,07822E+13	5,38487E+14	6,66651E+14	6,45404E+14
TC0200004419.mm.2	Ccdc9b	coiled-coil domain containing 9B	-0.710576976278483	6,10085E+14	-2,12473E+14	0.0746986620623196	0.217063939009947	-4,21481E+14	5,79872E+14	5,45578E+14	6,17782E+14	6,49711E+14	6,13962E+14	6,53603E+14
TC0400003773.mm.2	Pnrc2	proline-rich nuclear receptor coactivator 2	0.777467423784458	6,55567E+14	2,12431E+14	0.0747428224278545	0.21715310093401	-4,21535E+14	7,12828E+14	7,19441E+14	6,44822E+13	5,79535E+14	6,62384E+14	6,14392E+13
TC0800000022.mm.2	Trappc5	trafficking protein particle complex 5	-0.649323621896476	7,20936E+14	-2,12395E+14	0.0747811171603341	0.217166238731503	-4,21582E+14	6,82243E+14	6,76413E+14	7,20854E+14	7,57585E+14	7,46462E+14	7,42059E+14
TC0400002014.mm.2	Hes2	hes family bHLH transcription factor 2	-0.8850655860317	6,42111E+14	-2,12391E+14	0.0747858140460912	0.217166238731503	-4,21588E+14	5,57692E+14	6,01385E+14	6,3136E+14	7,26139E+14	6,53691E+14	6,82398E+14
TC1100003269.mm.2	Taok1	TAO kinase 1	2,09681E+14	7,62028E+14	2,12377E+14	0.0748008208910322	0.217166238731503	-4,21607E+14	8,89499E+14	9,08539E+13	8,39822E+14	5,55634E+14	7,81025E+14	5,97651E+14
TC0200005489.mm.2	Zfp965	zinc finger protein 965	1,59253E+14	8,23626E+13	2,12376E+14	0.0748012551951741	0.217166238731503	-4,21607E+14	9,34649E+14	9,7184E+14	8,36298E+14	6,10988E+14	8,42861E+13	7,45122E+14
TC1900001213.mm.2	Vps13a	vacuolar protein sorting 13A	1,64916E+14	5,92151E+14	2,12335E+14	0.0748449164965716	0.217253853271349	-4,21661E+14	7,02269E+14	7,35964E+14	6,18415E+14	3,87856E+14	6,10488E+14	4,97913E+14
TC1400000815.mm.2	B020004C17Rik	RIKEN cDNA B020004C17 gene	-0.950006949821202	4,9528E+14	-2,12287E+14	0.074896255787675	0.217297352974259	-4,21724E+14	4,51726E+14	4,32779E+14	4,54689E+14	5,57979E+14	5,05194E+14	5,69313E+14
TC0400002159.mm.2	Tox	thymocyte selection-associated high mobility group box	1,2223E+14	8,24299E+14	2,12283E+14	0.0749008737910759	0.217297352974259	-4,2173E+14	8,99412E+13	9,14387E+14	8,60101E+14	7,02554E+14	8,26432E+14	7,42908E+14
TC0600000935.mm.2	Vax2	ventral anterior homeobox 2	-0.702104960208747	7,49078E+14	-2,12266E+14	0.0749192307129702	0.217297352974259	-4,21752E+14	6,99536E+14	7,07473E+14	7,41355E+14	7,98675E+14	7,68598E+14	7,78832E+14
TC0500000726.mm.2	C530008M17Rik	RIKEN cDNA C530008M17 gene	-0.658470199571237	9,16201E+14	-2,12265E+14	0.0749193187255168	0.217297352974259	-4,21753E+14	8,87736E+14	8,66005E+14	9,11425E+13	9,46606E+14	9,46207E+14	9,39228E+13
TC1100003752.mm.2	Krt32	keratin 32	-0.996175980493804	4,86901E+13	-2,12252E+14	0.0749334047187959	0.217297352974259	-4,2177E+14	4,37802E+14	4,04174E+14	4,5661E+14	5,8161E+14	5,05139E+14	5,36072E+14
TC0500003059.mm.2	Foxn4	forkhead box N4	-0.81563608508776	7,44618E+14	-2,12245E+14	0.0749408173821113	0.217297352974259	-4,21779E+14	6,84145E+14	6,92141E+14	7,30433E+13	8,21578E+14	7,70735E+14	7,68679E+14
TC1200000830.mm.2	Zc2hc1c	zinc finger, C2HC-type containing 1C	-0.687670681717009	7,11236E+14	-2,12221E+14	0.0749668257154077	0.217333656486572	-4,21811E+14	6,66382E+13	6,38831E+14	7,38019E+14	7,81435E+14	7,1359E+14	7,29157E+14
TC0400002104.mm.2	Tnfrsf18	tumor necrosis factor receptor superfamily, member 18	-0.9621327375262	6,51891E+14	-2,1219E+14	0.0749999174919676	0.217379920080439	-4,21852E+14	5,59179E+14	5,88333E+14	6,67133E+14	7,5676E+14	6,2831E+14	7,11629E+14
TC1600001177.mm.2	Carhsp1	calcium regulated heat stable protein 1	0.645665740596429	7,30555E+14	2,12181E+14	0.0750097657572917	0.217379920080439	-4,21864E+14	7,51513E+14	7,54979E+14	7,49248E+14	7,5023E+13	7,06558E+14	6,70801E+13
TC0500001678.mm.2	Uncx	UNC homeobox	-1,02342E+14	6,85033E+14	-2,12163E+14	0.0750291485722904	0.217396991718273	-4,21888E+14	6,28296E+14	6,21156E+14	6,473E+14	7,59311E+14	6,86107E+14	7,68028E+14
TC0400002737.mm.2	Lpar1	lysophosphatidic acid receptor 1	1,17257E+14	7,96618E+14	2,12146E+14	0.075046924265183	0.217409401417636	-4,21909E+14	8,64094E+14	8,5254E+14	7,99761E+14	7,8119E+14	8,25017E+14	6,57104E+14
TC0400001502.mm.2	Phc2	polyhomeotic 2	-0.76797057922678	8,45809E+14	-2,12071E+14	0.0751271972337111	0.217575179927958	-4,22008E+13	7,92837E+14	7,8004E+14	8,59859E+14	9,15643E+14	8,35975E+14	8,90496E+14
TC1100000596.mm.2	Fstl4	follistatin-like 4	0.983509666560317	6,80281E+14	2,12067E+14	0.075131155037495	0.217575179927958	-4,22013E+14	7,38532E+13	7,49119E+12	5,88814E+14	7,14271E+14	6,28202E+14	6,62747E+14
TC0400000446.mm.2	Npr2	natriuretic peptide receptor 2	-0.659672009705867	7,96401E+14	-2,11972E+14	0.0752334250119785	0.217832197164063	-4,22138E+14	7,74284E+14	7,33973E+14	8,09623E+14	8,20336E+14	8,1513E+14	8,25061E+14

TC1300001202.mm.2	Ercc8	excision repaiross-complementing rodent repair deficiency, complementation group 8	-0.695919189614029	6,84038E+14	-2,11959E+14	0.0752473483259988	0.217833367512903	-4,22155E+14	6,48088E+14	6,14365E+14	6,98186E+14	7,41954E+14	7,07776E+14	6,93861E+14
TC0300002532.mm.2	Gja8	gap junction protein, alpha 8	-0.838380781786209	5,47036E+14	-2,1191E+14	0.0752999708067046	0.217946547441788	-4,2222E+14	4,85579E+14	4,70673E+14	5,60683E+14	6,41351E+14	5,35583E+14	5,88345E+14
TC1100004288.mm.2	Urgcp	upregulator of cell proliferation	0.624051286599393	7,08569E+14	2,11895E+14	0.0753154996247174	0.217952342933382	-4,22239E+14	7,74811E+14	7,58285E+14	6,49516E+14	6,60517E+14	7,10129E+14	6,98156E+14
TC1400002534.mm.2	Pcdh9	protocadherin 9	0.825921927234216	7,07837E+14	2,11809E+14	0.0754085620425465	0.218170244376998	-4,22353E+14	7,69735E+13	7,54453E+14	7,0809E+14	6,55744E+14	7,18116E+14	6,40887E+14
TC0700002857.mm.2	Dpy19l3	dpy-19-like 3 (C. elegans)	2,81375E+14	5,5552E+13	2,11794E+14	0.0754247194596239	0.218170244376998	-4,22373E+13	7,25048E+14	7,46078E+14	6,86275E+14	2,67343E+14	5,54737E+14	3,53639E+14
TC0200002497.mm.2	Kcnk15	potassium channel, subfamily K, member 15	-0.73175655530007	6,67187E+14	-2,11787E+14	0.0754314175773758	0.218170244376998	-4,22381E+14	6,42697E+14	6,12413E+14	6,59403E+14	6,87149E+14	6,73121E+14	7,2834E+14
TC1900000413.mm.2	Gm815	predicted gene 815	-0.713684703776837	6,83671E+14	-2,11764E+14	0.0754568594962636	0.218204662071661	-4,22412E+14	6,11929E+14	6,28154E+14	7,14874E+13	7,64248E+14	6,73457E+14	7,09364E+14
TC0200004422.mm.2	Ccdc32	coiled-coil domain containing 32	1,04123E+14	7,7268E+14	2,11738E+12	0.0754842387245118	0.218244668682587	-4,22445E+14	8,50088E+13	8,5823E+14	7,844E+14	6,43292E+13	7,61285E+14	7,38787E+14
TC1300000006.mm.2	Gdi2	guanosine diphosphate (GDP) dissociation inhibitor 2	1,39073E+14	9,11221E+14	2,11646E+14	0.0755836835198068	0.218484403644792	-4,22567E+14	1,0013E+14	1,01044E+14	9,44746E+14	7,77232E+13	9,31552E+14	8,02049E+14
TC0600000606.mm.2	Nfe2l3	nuclear factor, erythroid derived 2, like 3	-0.826030389843303	7,75377E+14	-2,11636E+14	0.0755942748290023	0.218484403644792	-4,2258E+14	7,27766E+14	7,23732E+14	7,64018E+14	8,20043E+13	7,70208E+14	8,46496E+14
TC0600001338.mm.2	3110021A11Rik	RIKEN cDNA 3110021A11 gene	-0.767683838522982	6,42153E+14	-2,11607E+14	0.0756254100015992	0.218485703122515	-4,22617E+14	5,93759E+14	6,02864E+14	6,31864E+14	6,74273E+14	6,41808E+14	7,08352E+14
TC1200001908.mm.2	Dhrs7	dehydrogenase/reductase (SDR family) member 7	1,13074E+14	5,40193E+14	2,11605E+14	0.0756275378479199	0.218485703122515	-4,2262E+14	6,431E+14	6,19366E+14	5,47707E+14	3,94668E+14	5,41197E+14	4,9512E+14
TC1700002128.mm.2	Ankrd66	ankyrin repeat domain 66	-0.910607095681216	6,55328E+14	-2,11592E+14	0.0756413399501668	0.218485703122515	-4,22637E+14	5,78845E+14	6,10376E+14	6,39881E+14	7,31526E+13	6,5481E+14	7,16533E+14
TC1800001111.mm.2	Syt4	synaptotagmin IV	1,2861E+13	8,94195E+14	2,11585E+14	0.0756489628077026	0.218485703122515	-4,22646E+14	9,90564E+14	9,78501E+14	9,15858E+14	7,68403E+13	9,17185E+14	7,94659E+14
TC0700002825.mm.2	Wtip	WT1-interacting protein	-0.762994171364216	6,54979E+14	-2,11556E+14	0.0756800994135729	0.218536459113065	-4,22684E+14	5,98155E+14	6,16446E+14	6,61146E+13	6,86927E+14	6,4785E+14	7,1935E+14
TC0700002471.mm.2	Qpctl	glutaminyl-peptide cyclotransferase-like	1,09576E+13	6,50882E+13	2,11483E+14	0.075759064301312	0.218715324278622	-4,2278E+14	7,58076E+14	7,19547E+14	6,59309E+14	5,09888E+12	6,34194E+14	6,24278E+14
TC0700004221.mm.2	Gga2	golgi associated, gamma adaptin ear containing, ARF binding protein 2	-0.658306127899174	7,68017E+14	-2,11471E+14	0.0757718432515345	0.218715324278622	-4,22796E+14	7,32107E+14	7,12475E+14	8,12739E+14	7,74541E+13	7,88635E+14	7,87608E+14
TC1200000419.mm.2	Ap4s1	adaptor-related protein complex AP-4, sigma 1	1,25536E+14	6,97781E+14	2,11461E+14	0.0757827627038757	0.218715324278622	-4,22809E+14	7,79373E+14	7,70751E+14	7,15832E+14	6,21674E+13	7,29791E+14	5,69262E+14
TC0400002309.mm.2	Klhl32	kelch-like 32	0.959288663913974	5,6839E+14	2,11447E+14	0.0757985733581102	0.21872177874628	-4,22828E+14	6,65207E+14	5,81003E+13	5,08121E+14	6,0166E+14	5,25305E+14	5,29047E+14
TC1500000468.mm.2	Gsdmcl1	gasdermin C-like 1	-0.965661451182985	5,478E+14	-2,11414E+14	0.0758339424986781	0.218784658098693	-4,22871E+14	4,7347E+14	4,88171E+14	5,83861E+14	5,86527E+14	5,14157E+14	6,40616E+14
TC0900001371.mm.2	Nprl2	NPR2 like, GATOR1 complex subunit	0.744897537169274	6,64629E+13	2,11387E+14	0.0758626478591799	0.218795431925708	-4,22906E+14	7,12662E+14	7,31382E+14	6,50672E+13	5,97993E+14	6,71129E+14	6,23935E+14
TC0700002344.mm.2	Vmn2r54	vomeronasal 2, receptor 54	-1,22627E+14	4,63906E+14	-2,11384E+14	0.0758661548158053	0.218795431925708	-4,2291E+14	3,92228E+14	3,73385E+14	4,3186E+13	5,75098E+14	4,51136E+14	5,59731E+14
TC0400004191.mm.2	Slc6a9	solute carrier family 6 (neurotransmitter transporter, glycine), member 9	0.714950527047124	8,71591E+14	2,11373E+14	0.0758784133060793	0.218795431925708	-4,22925E+14	9,29802E+14	8,97884E+14	8,81667E+14	8,35493E+14	8,57724E+14	8,26973E+14
TC0300001352.mm.2	Ints12	integrator complex subunit 12	0.682653935092524	6,56321E+14	2,11352E+14	0.0759002623692961	0.218795568762258	-4,22952E+14	7,01E+12	7,1474E+14	6,33103E+14	6,09871E+14	6,1685E+14	6,6236E+14
TC1700000176.mm.2	Ermard	ER membrane associated RNA degradation	1,04396E+14	5,47005E+14	2,1132E+14	0.0759356543298049	0.218795568762258	-4,22995E+14	6,2992E+14	6,08126E+14	5,77477E+14	4,37252E+14	5,27209E+14	5,02045E+14
TC1100000688.mm.2	Sh3bp5l	SH3 binding domain protein 5 like	-0.847442476356177	6,81995E+14	-2,11317E+14	0.0759381049873223	0.218795568762258	-4,22998E+14	6,33042E+13	6,01311E+14	6,79635E+14	7,74141E+14	7,07576E+14	6,96265E+14
TC0100003731.mm.2	1700056E22Rik	RIKEN cDNA 1700056E22 gene	-0.769020972815615	6,74024E+14	-2,11305E+14	0.0759511610787175	0.218795568762258	-4,23014E+14	6,35707E+14	6,26527E+14	6,81137E+14	6,84732E+12	6,6827E+14	7,47768E+14
TC0800000714.mm.2	D130040H23Rik	RIKEN cDNA D130040H23 gene	0.926321844544261	5,86762E+13	2,113E+14	0.0759568916136379	0.218795568762258	-4,23021E+14	6,5326E+14	6,68414E+14	5,55412E+13	5,07076E+14	6,21903E+14	5,14507E+14
TC1500000010.mm.2	Plcxd3	phosphatidylinositol-specific phospholipase C, X domain containing 3	0.72561851423547	7,11617E+14	2,11297E+14	0.0759599336967709	0.218795568762258	-4,23024E+14	7,76412E+13	7,43635E+14	7,11698E+14	6,63032E+14	6,70826E+14	7,04098E+14
TC1600000192.mm.2	Cebpd	CCAAT/enhancer binding protein (C/EBP), delta	-0.62185899139325	6,41991E+14	-2,11284E+14	0.0759742229409743	0.218797614700254	-4,23042E+14	6,09978E+14	6,14471E+14	6,40257E+14	6,38418E+14	6,63231E+14	6,85589E+14
TC0100003349.mm.2	4930562F07Rik	RIKEN cDNA 4930562F07 gene	-1,00138E+14	4,8275E+14	-2,11271E+14	0.0759884186409712	0.218799390557893	-4,23059E+14	3,8991E+14	4,51364E+14	4,86889E+14	5,2679E+14	4,63969E+14	5,77581E+13
TC1500000558.mm.2	Zfp41	zinc finger protein 41	-1,00665E+14	6,77847E+14	-2,11228E+14	0.076034667116823	0.218893441353112	-4,23115E+13	6,29597E+14	5,78387E+14	6,67563E+14	7,82224E+14	6,78091E+14	7,31222E+14
TC1100003159.mm.2	Ctns	cystinosis, nephropathic	0.812585745943724	6,11857E+14	2,11187E+14	0.076079596245303	0.218906117720724	-4,23169E+14	6,36975E+14	6,8077E+14	6,38816E+14	5,5935E+14	5,84513E+14	5,70715E+14
TC0500000814.mm.2	Rufy3	RUN and FYVE domain containing 3	1,8764E+14	7,71947E+14	2,11186E+14	0.076080628058958	0.218906117720724	-4,23171E+14	9,07637E+14	8,89809E+14	8,03854E+14	6,08213E+14	8,25453E+14	5,96713E+14
TC0200003753.mm.2	Gpr155	G protein-coupled receptor 155	1,31198E+14	7,34712E+14	2,11184E+14	0.0760825666750521	0.218906117720724	-4,23173E+14	8,20016E+13	8,37457E+14	7,51519E+14	6,04199E+14	7,62756E+14	6,32321E+14
TC0700001710.mm.2	Acsm2	acyl-CoA synthetase medium-chain family member 2	-1,19685E+14	4,6676E+14	-2,11156E+14	0.0761132027413101	0.218906117720724	-4,2321E+14	3,96259E+14	3,79007E+14	4,2005E+14	5,90607E+13	4,8919E+13	5,25446E+13
TC0100003254.mm.2	Rnf2	ring finger protein 2	1,10299E+14	6,48501E+14	2,11139E+13	0.0761312148535134	0.218906117720724	-4,23232E+14	7,32166E+14	7,13341E+14	6,78168E+14	5,42424E+14	6,50203E+14	5,74707E+12
TC1600001837.mm.2	4930453N24Rik	RIKEN cDNA 4930453N24 gene	0.885145065648091	7,40854E+14	2,1113E+14	0.0761414245644638	0.218906117720724	-4,23244E+14	7,63616E+14	7,77208E+12	7,57583E+14	7,82923E+14	7,3472E+14	6,29075E+14
TC1100003489.mm.2	Olfr462/Olfr463	olfactory receptor 462/olfactory receptor 463	-1,16872E+14	5,88537E+14	-2,11126E+14	0.0761455937815625	0.218906117720724	-4,23249E+14	5,19868E+14	4,77899E+14	5,73077E+14	7,28867E+14	5,89479E+14	6,42033E+14
TC0500002818.mm.2	Rasgef1b	RasGEF domain family, member 1B	0.785408552125766	6,98035E+14	2,11124E+14	0.0761477558384321	0.218906117720724	-4,23252E+14	7,28421E+14	7,66019E+14	6,74071E+14	6,82342E+14	7,06426E+14	6,30932E+14
TC0400003568.mm.2	Gjb5	gap junction protein, beta 5	-0.778765300136872	5,41791E+14	-2,11083E+14	0.0761917278107684	0.218930977165289	-4,23305E+14	4,41291E+14	5,07943E+14	5,85243E+14	6,11281E+14	5,31471E+14	5,73516E+14
TC1100002673.mm.2	Rufy1	RUN and FYVE domain containing 1	1,16053E+14	6,57017E+14	2,11082E+14	0.07619376365045	0.218930977165289	-4,23308E+14	7,15884E+14	7,41773E+14	7,08737E+14	5,50158E+14	6,37484E+14	5,88066E+14
TC0400003573.mm.2	Csmd2os	CUB and Sushi multiple domains 2, opposite strand	-0.881062062912466	6,88265E+14	-2,11078E+14	0.0761971650184906	0.218930977165289	-4,23312E+14	6,26596E+14	6,33943E+14	6,61985E+14	7,70316E+14	7,17878E+14	7,18873E+14
TC0X00001658.mm.2	Ap1s2	adaptor-related protein complex 1, sigma 2 subunit	1,448E+14	7,48539E+14	2,11033E+14	0.0762466881534188	0.218941072294624	-4,23372E+14	8,6302E+14	8,32289E+13	8,02438E+13	5,87781E+14	7,29835E+14	6,75873E+14
TC0900001103.mm.2	Bckdhb	branched chain ketoacid dehydrogenase E1, beta polypeptide	1,16676E+14	6,44103E+14	2,11017E+14	0.0762639133548198	0.218941072294624	-4,23392E+14	7,15379E+14	7,06201E+14	6,82254E+14	5,72558E+13	6,58505E+14	5,29722E+14
TC0900001486.mm.2	Epm2aip1	EPM2A (laforin) interacting protein 1	1,06618E+14	7,45117E+14	2,11016E+14	0.0762653067931364	0.218941072294624	-4,23394E+14	8,43079E+14	8,33143E+14	7,38995E+14	5,92497E+14	7,44292E+14	7,18694E+14
TC0700000224.mm.2	Bbc3	BCL2 binding component 3	-1,08397E+14	7,83144E+14	-2,11014E+14	0.0762675952586723	0.218941072294624	-4,23397E+14	6,86781E+14	7,08735E+14	7,8695E+14	9,04088E+14	7,58411E+14	8,53901E+14
TC0100001550.mm.2	Aldh9a1	aldehyde dehydrogenase 9, subfamily A1	0.785712232595126	6,59506E+14	2,11013E+14	0.0762686178110672	0.218941072294624	-4,23398E+14	6,96088E+14	7,07229E+14	6,17402E+14	6,90144E+14	6,01883E+14	6,44292E+13
TC0800000209.mm.2	Defb33	defensin beta 33	-0.804744619194026	6,01898E+14	-2,10983E+14	0.0763012063987402	0.218995607178999	-4,23437E+13	5,60422E+14	5,64299E+13	5,86398E+14	6,14602E+14	6,00164E+14	6,85506E+14
TC0X00002641.mm.2	Zfx	zinc finger protein X-linked	2,06093E+14	6,40487E+14	2,10968E+14	0.076317218088944	0.219002552995041	-4,23457E+14	7,83546E+14	7,65233E+14	7,10608E+14	4,46944E+14	6,71092E+13	4,65501E+14
TC1100003920.mm.2	Cd79b	CD79B antigen	-0.871246746924454	4,97091E+14	-2,10948E+14	0.076338578188594	0.21902484158704	-4,23483E+14	4,45903E+14	4,24777E+14	4,92462E+14	5,74474E+14	4,90259E+14	5,5467E+14
TC0X00001851.mm.2	Bcor	BCL6 interacting corepressor	-0.818428538305425	7,16521E+14	-2,10923E+14	0.076366114029609	0.21906483805574	-4,23516E+14	6,51245E+14	6,83296E+13	6,89264E+13	7,77092E+14	7,47948E+14	7,50279E+14
TC0500002628.mm.2	Clock	circadian locomotor output cycles kaput	1,05123E+14	8,70042E+14	2,10892E+14	0.0763999763954069	0.219102157025831	-4,23557E+14	9,3363E+13	9,44885E+13	8,92695E+14	7,80772E+14	8,80732E+14	7,87537E+14
TC0900000214.mm.2	Pigyl	phosphatidylinositol glycan anchor biosynthesis, class Y-like	-0.670927997542138	7,90949E+13	-2,10886E+14	0.0764063191415718	0.219102157025831	-4,23564E+14	7,52237E+14	7,47709E+13	7,89442E+14	8,22179E+14	7,98983E+14	8,35148E+14
TC0800002601.mm.2	Gm5741	predicted gene 5741	-0.840254583155498	6,42929E+14	-2,1083E+14	0.0764671069091829	0.219196212961784	-4,23638E+13	5,95964E+14	5,94648E+14	6,61488E+14	6,46814E+14	6,30357E+14	7,28305E+14
TC0300002718.mm.2	Csf1	colony stimulating factor 1 (macrophage)	0.677935498559706	7,67437E+14	2,10828E+14	0.0764700022379244	0.219196212961784	-4,23641E+14	8,00303E+14	8,31582E+14	7,57976E+14	7,18464E+14	7,54092E+14	7,42206E+14
TC1900000939.mm.2	Actn3	actinin alpha 3	-0.752139425628585	6,75772E+14	-2,10816E+14	0.0764824500766375	0.219196212961784	-4,23656E+14	6,17024E+13	6,58323E+14	6,54147E+14	6,99363E+13	6,90509E+13	7,35266E+14
TC1000001851.mm.2	E030030I06Rik	RIKEN cDNA E030030I06 gene	-0.897893079294938	5,90594E+14	-2,10806E+14	0.0764935335123262	0.219196212961784	-4,23669E+14	5,38911E+14	5,34275E+14	5,91568E+14	6,26047E+14	5,72752E+14	6,80013E+14
TC0200001382.mm.2	Ambra1	autophagy/beclin 1 regulator 1	1,07264E+14	7,98916E+14	2,10755E+14	0.0765494613733674	0.219282087473297	-4,23737E+14	9,06691E+14	8,75464E+14	7,81838E+13	6,61874E+14	8,26623E+14	7,41005E+14
TC0400001651.mm.2	Runx3	runt related transcription factor 3	-1,05666E+13	7,3935E+14	-2,10744E+14	0.0765610234770609	0.219282087473297	-4,23751E+14	6,60479E+14	6,92828E+14	6,93167E+14	8,24988E+14	7,53632E+14	8,11007E+14
TC1000002820.mm.2	Myf5	myogenic factor 5	-0.704345621077751	5,06715E+14	-2,10741E+14	0.0765643284382034	0.219282087473297	-4,23755E+14	4,37892E+14	4,71015E+14	5,26717E+13	5,5489E+14	5,03336E+14	5,46441E+14
TC1500000262.mm.2	Polr2k	polymerase (RNA) II (DNA directed) polypeptide K	0.809446661557985	6,50301E+14	2,10727E+14	0.0765799615512986	0.219287883503834	-4,23773E+14	7,37383E+13	6,99079E+14	5,87698E+13	6,03075E+14	6,78076E+14	5,96497E+14

TC1300000885.mm.2	Tert	telomerase reverse transcriptase	-0.632869069445345	6,15397E+14	-2,10683E+14	0.0766273987325511	0.21938473272523	-4,2383E+14	5,71006E+14	5,87755E+14	6,14053E+14	6,34232E+14	6,34012E+14	6,51322E+14
TC0200003239.mm.2	Barhl1	BarH like homeobox 1	-0.934536191338736	6,15269E+14	-2,10643E+14	0.076671704780997	0.219472584675112	-4,23884E+14	5,54753E+13	5,36561E+14	6,04638E+13	7,1744E+14	6,18849E+13	6,59373E+14
TC0500000422.mm.2	Crmp1	collapsin response mediator protein 1	1,5065E+14	7,30756E+14	2,10626E+14	0.0766895161418551	0.219484577902968	-4,23905E+14	8,5761E+14	8,31796E+14	7,69091E+14	5,37932E+14	7,10463E+14	6,77644E+14
TC1200001589.mm.2	Acp1	acid phosphatase 1, soluble	1,52237E+14	7,70238E+14	2,10588E+14	0.0767320699349481	0.21952899689214	-4,23956E+14	8,82974E+13	8,59364E+14	8,04857E+13	6,36367E+14	8,02074E+13	6,3579E+14
TC0400003152.mm.2	Jak1	Janus kinase 1	0.967938681788037	6,89729E+14	2,10573E+14	0.0767480144195931	0.21952899689214	-4,23975E+14	7,52811E+14	7,64458E+14	7,09162E+14	5,88265E+14	6,40591E+14	6,8309E+14
TC1100001077.mm.2	Myo1c	myosin IC	0.611294356074846	5,68258E+14	2,10561E+14	0.0767608429596143	0.21952899689214	-4,23991E+14	6,03963E+14	5,95865E+14	5,69832E+14	5,62319E+14	5,33781E+14	5,43788E+14
TC1000001189.mm.2	Dcn	decorin	0.980695602734013	6,65495E+14	2,10527E+14	0.0767984560640365	0.21952899689214	-4,24036E+14	7,0941E+14	7,32869E+14	7,11019E+14	5,9353E+14	6,50167E+14	5,95974E+14
TC0200001025.mm.2	Gorasp2	golgi reassembly stacking protein 2	1,27276E+13	8,23655E+14	2,10519E+13	0.0768069084581606	0.21952899689214	-4,24046E+14	9,20935E+14	9,08487E+14	8,60278E+14	6,77364E+14	8,138E+14	7,6107E+14
TC0200002827.mm.2	Dnajc5	DnaJ heat shock protein family (Hsp40) member C5	0.669053628171442	7,95419E+14	2,10513E+14	0.0768139381515593	0.21952899689214	-4,24054E+14	8,49913E+14	8,36671E+14	7,77253E+14	7,55906E+14	7,97257E+14	7,55516E+14
TC1300002445.mm.2	Scamp1	secretory carrier membrane protein 1	1,58141E+14	8,91925E+14	2,10511E+13	0.07681532958552	0.21952899689214	-4,24056E+14	9,93907E+14	1,01612E+14	9,23619E+14	7,24172E+14	9,2051E+14	7,7323E+14
TC1600000564.mm.2	Igsf11	immunoglobulin superfamily, member 11	1,19187E+14	8,15731E+14	2,10511E+14	0.0768155399596274	0.21952899689214	-4,24056E+14	8,77513E+14	8,80735E+13	8,83694E+14	7,32567E+14	8,03983E+14	7,15891E+14
TC1700001711.mm.2	Cuta	cutA divalent cation tolerance homolog	0.804972169152771	8,3383E+14	2,10489E+14	0.0768398110282664	0.21952899689214	-4,24086E+14	9,06143E+14	8,71797E+14	8,45373E+14	7,62723E+14	8,15211E+14	8,01735E+14
TC1300001209.mm.2	Pde4d/Mir1904	phosphodiesterase 4D, cAMP specific/microRNA 1904	0.842049257175399	8,38679E+14	2,10488E+14	0.0768412798654296	0.21952899689214	-4,24087E+14	8,89756E+14	8,76782E+14	8,60308E+13	8,071E+14	8,40794E+14	7,57334E+14
TC1000002195.mm.2	Serinc1	serine incorporator 1	1,55951E+14	1,07442E+14	2,1046E+14	0.0768717993626018	0.219577256360504	-4,24124E+14	1,18758E+13	1,19267E+14	1,10196E+14	8,95976E+14	1,10041E+14	9,67941E+14
TC1100003857.mm.2	Kif18b	kinesin family member 18B	-0.668970035923095	6,97841E+14	-2,10423E+14	0.0769127195005526	0.219655201934138	-4,24173E+14	6,43108E+14	6,77037E+14	7,13319E+14	6,99643E+13	7,10821E+14	7,43118E+14
TC0900001720.mm.2	Birc2	baculoviral IAP repeat-containing 2	1,33514E+14	6,04509E+14	2,10374E+14	0.0769657837211004	0.219767796047863	-4,24236E+13	6,79636E+14	6,65886E+14	6,09426E+14	5,93614E+14	6,40175E+14	4,38318E+14
TC0900001775.mm.2	Fut4	fucosyltransferase 4	-1,40531E+14	4,76312E+14	-2,10345E+14	0.0769972827874002	0.219818784116474	-4,24274E+13	4,19727E+14	3,8914E+14	4,27364E+14	5,3171E+14	4,51469E+14	6,38461E+14
TC0900003004.mm.2	Alas1	aminolevulinic acid synthase 1	0.920839131456175	7,86327E+14	2,10327E+14	0.0770173691571091	0.219837177896988	-4,24298E+14	8,36005E+14	8,56239E+14	7,65038E+14	7,52603E+14	8,1207E+14	6,96006E+14
TC0X00001941.mm.2	4930453H23Rik	RIKEN cDNA 4930453H23 gene	-1,17813E+14	4,11041E+14	-2,10293E+13	0.077054761033249	0.219904952980649	-4,24343E+14	3,43694E+14	3,37577E+14	3,66888E+14	5,01188E+14	4,04423E+14	5,12475E+14
TC0800002832.mm.2	Enkd1	enkurin domain containing 1	-0.825674672934552	6,42337E+14	-2,10266E+14	0.077084468668207	0.219944190025144	-4,24378E+13	5,81418E+14	5,72885E+14	6,43245E+14	7,37037E+14	6,5044E+13	6,68998E+14
TC0400003721.mm.2	Hmgn2	high mobility group nucleosomal binding domain 2	0.899523448976398	8,62595E+14	2,10256E+14	0.0770958099213066	0.219944190025144	-4,24392E+13	9,29242E+14	9,48601E+14	8,50087E+13	7,49702E+14	8,73822E+14	8,24117E+14
TC0200005164.mm.2	Sulf2	sulfatase 2	1,84862E+14	8,02672E+13	2,10209E+14	0.0771474551367882	0.220016730117729	-4,24454E+14	9,17138E+14	9,04316E+14	8,62705E+14	6,80146E+14	8,43506E+14	6,08224E+14
TC0800002400.mm.2	Pgpep1	pyroglutamyl-peptidase I	-0.839842762073732	6,92392E+14	-2,10208E+14	0.0771485462151782	0.220016730117729	-4,24455E+14	6,28718E+13	6,45116E+14	6,71963E+14	7,66752E+14	7,08074E+14	7,33728E+14
TC0100000476.mm.2	Cd28	CD28 antigen	-0.691114927781737	5,05445E+13	-2,10181E+13	0.0771774461889217	0.22002102808699	-4,24489E+14	4,57176E+14	4,94576E+14	4,74261E+14	5,16686E+14	5,49716E+13	5,40259E+14
TC0100002081.mm.2	Adgrb3	adhesion G protein-coupled receptor B3	1,67819E+13	8,7019E+14	2,10177E+14	0.0771824933760557	0.22002102808699	-4,24495E+14	9,93141E+14	9,93455E+14	9,16599E+14	6,66988E+14	8,73106E+14	7,77852E+13
TC0800001029.mm.2	Tent4b	terminal nucleotidyltransferase 4B	1,42416E+14	8,08953E+14	2,10169E+14	0.0771910179219112	0.22002102808699	-4,24506E+14	9,15195E+14	9,07866E+14	8,49397E+13	6,43034E+14	8,07191E+14	7,31037E+14
TC1100001520.mm.2	Copz2	coatomer protein complex, subunit zeta 2	-0.666022928584077	7,00663E+14	-2,10097E+14	0.0772699859859124	0.220177194034581	-4,246E+14	6,72254E+14	6,46002E+14	7,14654E+13	7,19607E+14	7,05773E+14	7,45688E+14
TC0300000641.mm.2	Fga	fibrinogen alpha chain	-0.73195648827117	5,03813E+13	-2,10095E+14	0.0772731354971486	0.220177194034581	-4,24603E+13	4,70299E+14	4,63674E+14	5,38054E+14	4,70486E+14	5,49977E+13	5,30388E+14
TC0700003625.mm.2	Bnc1	basonuclin 1	-0.972891380965446	5,10124E+14	-2,10058E+14	0.077313761460045	0.220236465388289	-4,24652E+14	4,53997E+14	4,18946E+14	4,99571E+13	6,2071E+14	5,50101E+12	5,17419E+14
TC0800002711.mm.2	Ces5a	carboxylesterase 5A	-0.880089008840425	6,46874E+14	-2,10046E+14	0.0773263167170224	0.220236465388289	-4,24667E+14	5,91698E+14	5,89729E+14	6,32847E+14	7,09523E+13	6,39741E+14	7,17704E+14
TC0800001262.mm.2	Vps4a	vacuolar protein sorting 4A	0.912142932067164	7,37508E+14	2,10036E+14	0.0773379034112367	0.220236465388289	-4,24681E+14	8,10431E+14	7,93538E+14	7,47343E+14	6,52198E+14	7,4356E+14	6,77981E+14
TC0800002680.mm.2	Aktip	thymoma viral proto-oncogene 1 interacting protein	0.800679206790917	8,1197E+14	2,10026E+14	0.0773486102918343	0.220236465388289	-4,24693E+13	8,73161E+14	8,537E+13	8,01669E+14	7,76564E+14	8,2539E+14	7,41335E+13
TC0X00001343.mm.2	Zcchc18	zinc finger, CCHC domain containing 18	2,14497E+13	6,89467E+14	2,10011E+14	0.0773648307574262	0.220243731094418	-4,24713E+14	8,68463E+14	8,3644E+14	7,51909E+14	4,04081E+14	6,57569E+11	6,18341E+14
TC0300000494.mm.2	Arhgef26	Rho guanine nucleotide exchange factor (GEF) 26	1,12667E+14	5,61626E+14	2,09971E+14	0.0774089882143029	0.220283166335495	-4,24765E+14	6,15462E+14	5,82389E+14	5,63708E+14	6,35682E+14	5,57214E+14	4,15302E+14
TC0900001049.mm.2	Gm3776/Gsta1	predicted gene 3776/glutathione S-transferase, alpha 1 (Ya)	-0.682463372359949	6,25989E+14	-2,09968E+13	0.0774124237039644	0.220283166335495	-4,24769E+14	5,72669E+14	5,83513E+14	6,41056E+14	6,66021E+14	6,22732E+14	6,69942E+14
TC1900001562.mm.2	Scd1	stearoyl-Coenzyme A desaturase 1	0.99261165174633	9,48869E+14	2,09951E+14	0.0774312659464499	0.220283166335495	-4,24792E+14	1,01152E+14	1,00904E+13	9,91702E+14	8,58915E+14	9,22106E+14	8,99931E+13
TC0400002449.mm.2	B4galt1	UDP-Gal:betaGlcNAc beta 1,4-galactosyltransferase, polypeptide 1	0.836352663584247	5,644E+14	2,09949E+14	0.07743336772322	0.220283166335495	-4,24794E+14	6,14677E+14	6,57046E+13	5,47706E+14	4,62519E+14	5,65827E+14	5,38626E+14
TC0300001040.mm.2	Csde1	cold shock domain containing E1, RNA binding	1,88481E+14	8,88242E+13	2,09899E+14	0.0774882770542344	0.220400460401567	-4,24859E+14	1,01226E+14	1,01233E+13	9,43024E+14	7,1421E+13	9,24724E+13	7,22905E+14
TC0800001386.mm.2	Dynlrb2	dynein light chain roadblock-type 2	0.716713139328806	5,89165E+14	2,09883E+14	0.0775061697527443	0.220401684600602	-4,24881E+14	6,12409E+14	6,21632E+14	6,01959E+14	6,08293E+14	5,12735E+14	5,77962E+14
TC0300000129.mm.2	Dnajc5b	DnaJ heat shock protein family (Hsp40) member C5 beta	-0.868819306078191	4,6046E+14	-2,09874E+14	0.0775160644592325	0.220401684600602	-4,24892E+14	4,36309E+14	3,51212E+14	5,0006E+14	5,13896E+14	4,49059E+13	5,12225E+14
TC1500002309.mm.2	Cbx5	chromobox 5	1,40499E+14	8,60757E+14	2,09836E+14	0.0775582912284818	0.220462262539377	-4,24943E+14	9,56885E+14	9,643E+14	8,88484E+14	7,14687E+13	8,84866E+13	7,55321E+14
TC0300000882.mm.2	Pi4kb	phosphatidylinositol 4-kinase beta	0.887922310047195	5,73673E+14	2,09815E+14	0.0775816504629274	0.220462262539377	-4,2497E+14	6,5141E+14	6,05713E+14	5,9427E+14	5,11107E+14	5,68973E+13	5,10565E+13
TC0800001418.mm.2	Hsbp1	heat shock factor binding protein 1	0.857809990925517	9,63729E+14	2,098E+14	0.0775983117398605	0.220462262539377	-4,2499E+14	1,03171E+14	1,03192E+13	9,58675E+14	8,68013E+14	9,71836E+14	9,20226E+14
TC1100004101.mm.2	Unc13d	unc-13 homolog D	-0.921414618206141	5,83139E+14	-2,09796E+13	0.0776024606694032	0.220462262539377	-4,24995E+14	5,23637E+13	5,40928E+14	5,39618E+14	6,458E+14	5,98845E+14	6,50004E+14
TC1700001573.mm.2	Zfp944	zinc finger protein 944	1,03666E+14	6,07598E+14	2,09793E+14	0.0776057812402004	0.220462262539377	-4,24999E+14	6,92134E+14	6,94202E+14	6,12198E+14	4,6805E+14	5,88651E+14	5,90353E+14
TC0500001887.mm.2	Gatad1	GATA zinc finger domain containing 1	1,12922E+14	9,14646E+14	2,09767E+14	0.0776351235744318	0.220506741786501	-4,25034E+14	1,00597E+14	9,86754E+14	9,38506E+14	7,8977E+14	9,15695E+14	8,51183E+14
TC0200002266.mm.2	Tmem74b	transmembrane protein 74B	-0.977993527157897	6,71954E+14	-2,09747E+14	0.0776567880167656	0.220529401712045	-4,25059E+14	5,86587E+14	6,15016E+13	6,52966E+14	7,79956E+14	6,88482E+14	7,08719E+14
TC1300000009.mm.2	Asb13	ankyrin repeat and SOCS box-containing 13	1,80888E+14	8,0254E+14	2,09732E+14	0.0776730417820445	0.22053669114257	-4,25079E+14	9,18781E+14	9,20209E+14	8,66846E+14	6,32194E+14	8,25193E+14	6,5202E+14
TC1500001344.mm.2	9430069I07Rik	RIKEN cDNA 9430069I07 gene	-0.938198822963319	5,54975E+14	-2,09705E+14	0.07770340708391	0.220584037763045	-4,25115E+13	4,85062E+14	5,1124E+14	5,24563E+13	6,25042E+14	5,59017E+14	6,24924E+14
TC0500001577.mm.2	Bcl7b	B cell CLL/lymphoma 7B	0.678434840893284	8,03173E+13	2,09676E+14	0.0777349426226886	0.220633153539074	-4,25152E+14	8,48486E+13	8,40057E+14	8,15055E+14	7,62584E+14	7,7404E+14	7,78816E+14
TC0800001928.mm.2	Hook3	hook microtubule tethering protein 3	2,36333E+14	5,81341E+14	2,09665E+14	0.0777480944451601	0.220633153539074	-4,25168E+14	7,33928E+14	7,33049E+14	6,28882E+14	3,97876E+14	6,54818E+14	3,39492E+14
TC0200001354.mm.2	Fnbp4	formin binding protein 4	0.619010480067939	5,62058E+14	2,09638E+14	0.0777775286669112	0.220666281203916	-4,25203E+14	6,05158E+13	5,91356E+14	5,52519E+14	5,506E+14	5,26337E+14	5,46375E+14
TC0300001871.mm.2	Nudt6	nudix (nucleoside diphosphate linked moiety X)-type motif 6	-0.615497360371793	7,45225E+14	-2,09629E+14	0.0777877082371941	0.220666281203916	-4,25215E+14	7,07755E+14	7,06907E+14	7,49341E+14	7,69583E+13	7,72729E+14	7,65033E+14
TC1400001500.mm.2	Rpl15	ribosomal protein L15	1,1172E+14	1,00823E+14	2,09617E+14	0.0778008529460341	0.220666281203916	-4,2523E+14	1,09904E+14	1,09617E+13	1,01014E+14	8,72259E+14	1,02741E+12	9,44354E+14
TC0200005175.mm.2	Prex1	phosphatidylinositol-3,4,5-trisphosphate-dependent Rac exchange factor 1	0.719831743337137	6,8442E+14	2,09548E+14	0.0778774293103967	0.220825249119723	-4,25321E+14	7,4352E+14	7,56589E+14	5,93202E+14	6,57069E+13	6,86862E+14	6,6928E+14
TC1100000925.mm.2	Tmem256	transmembrane protein 256	0.947703848367299	6,01653E+14	2,09542E+14	0.0778843102307071	0.220825249119723	-4,25329E+14	6,66491E+14	6,53489E+14	6,41564E+14	5,17935E+14	5,5752E+14	5,72919E+13
TC0400003379.mm.2	Atp6v0b	ATPase, H+ transporting, lysosomal V0 subunit B	0.62005949142723	7,75979E+13	2,09514E+14	0.0779151329533058	0.220873775031072	-4,25365E+14	8,2903E+14	8,19591E+13	7,52796E+14	7,29845E+14	7,65098E+14	7,59511E+14
TC0X00003046.mm.2	Esx1	extraembryonic, spermatogenesis, homeobox 1	-0.699169334483497	5,04711E+14	-2,09489E+14	0.0779426723221204	0.220875089467833	-4,25398E+14	4,63759E+14	4,73759E+14	4,89904E+14	5,23492E+14	5,13776E+13	5,63576E+14
TC1600000286.mm.2	Psmd2	proteasome (prosome, macropain) 26S subunit, non-ATPase, 2	1,74303E+14	8,33953E+14	2,09489E+14	0.0779430123945944	0.220875089467833	-4,25398E+14	9,42308E+14	9,57618E+14	8,73978E+14	6,78491E+14	8,75624E+14	6,75697E+14
TC1800001409.mm.2	Tcof1	treacle ribosome biogenesis factor 1	0.751216366334236	5,2805E+14	2,09444E+14	0.0779928127755314	0.220968301511112	-4,25457E+14	6,08823E+14	5,5509E+14	4,86881E+14	5,03837E+14	4,80601E+14	5,33069E+14
TC0800001458.mm.2	Foxl1	forkhead box L1	-0.819266887572025	6,43116E+14	-2,09412E+14	0.0780286032750981	0.220968301511112	-4,25499E+14	5,99257E+13	5,94609E+14	6,37594E+14	6,6952E+14	6,34065E+14	7,23654E+14
TC0200000781.mm.2	Acvr2a	activin receptor IIA	0.870093042429723	6,98484E+14	2,0941E+14	0.0780309126484302	0.220968301511112	-4,25502E+14	7,66274E+14	7,4787E+13	7,12708E+14	6,23925E+14	6,99066E+14	6,4106E+14
TC1400002346.mm.2	Dmtn	dematin actin binding protein	0.816992546346686	9,06104E+14	2,09398E+14	0.0780440649721485	0.220968301511112	-4,25517E+14	9,46908E+14	9,41193E+14	9,46999E+14	8,76825E+14	8,93988E+14	8,30714E+14
TC1200001272.mm.2	Pld4	phospholipase D family, member 4	0.730068606957142	6,45091E+14	2,09398E+14	0.0780444736485904	0.220968301511112	-4,25518E+14	7,01067E+14	7,30742E+14	6,07511E+14	5,4543E+14	6,34509E+14	6,51287E+14
TC0100003302.mm.2	BC034090	cDNA sequence BC034090	-0.906054172585089	4,88499E+14	-2,09331E+14	0.0781188595691986	0.221140053450193	-4,25606E+14	3,91372E+13	4,70816E+14	4,9886E+14	5,26551E+14	4,81702E+14	5,61696E+14

TC1700001944.mm.2	Ly6g6f	lymphocyte antigen 6 complex, locus G6F	-1,0033E+14	5,41522E+14	-2,09303E+14	0.0781501276617158	0.221189707889202	-4,25642E+14	4,84315E+14	4,74955E+14	5,02897E+14	6,27037E+14	5,50654E+13	6,09276E+14
TC1000000907.mm.2	Gm10778	predicted gene 10778	1,58784E+14	5,78956E+14	2,09225E+14	0.0782363479369298	0.221355974417515	-4,25744E+14	6,63735E+13	6,553E+14	6,63396E+14	4,89836E+14	5,91924E+14	4,09544E+13
TC1000000909.mm.2	Gm10778	predicted gene 10778	1,58784E+14	5,78956E+14	2,09225E+14	0.0782363479369298	0.221355974417515	-4,25744E+14	6,63735E+13	6,553E+14	6,63396E+14	4,89836E+14	5,91924E+14	4,09544E+13
TC1100002175.mm.2	Purb	purine rich element binding protein B	3,20672E+12	7,28435E+14	2,09153E+14	0.0783169265327469	0.221488127535121	-4,25839E+14	9,33967E+14	9,58178E+14	8,14861E+14	4,12805E+14	8,05315E+14	4,45485E+14
TC0X00001403.mm.2	Gm5643	heterogeneous nuclear ribonucleoprotein A1 pseudogene	0.958695360710987	8,4478E+14	2,09151E+14	0.078319733056982	0.221488127535121	-4,25842E+14	9,0168E+14	9,11744E+14	8,76868E+14	7,56703E+14	8,3243E+14	7,89255E+14
TC1200000033.mm.2	Gm6682	tubulin, alpha 1C pseudogene	1,32584E+14	1,10404E+14	2,09147E+14	0.0783242940914241	0.221488127535121	-4,25847E+14	1,20318E+14	1,18995E+14	1,1394E+14	9,63734E+14	1,11406E+14	1,0139E+14
TC0800003022.mm.2	Mbtps1	membrane-bound transcription factor peptidase, site 1	0.967438254773822	7,70688E+14	2,0912E+13	0.0783543140597203	0.221515374167019	-4,25883E+14	8,37502E+14	8,20059E+13	8,15505E+14	6,8699E+14	7,2735E+14	7,36724E+13
TC1100001859.mm.2	Sstr2	somatostatin receptor 2	1,08777E+14	6,91657E+14	2,09084E+14	0.0783941074455309	0.221515374167019	-4,25929E+14	7,39085E+14	7,3954E+14	6,83082E+14	7,27167E+14	7,05117E+13	5,55953E+14
TC1000002515.mm.2	Jsrp1	junctional sarcoplasmic reticulum protein 1	-0.806858683037866	6,42023E+14	-2,09075E+14	0.0784037594207374	0.221515374167019	-4,25941E+14	5,7818E+14	5,95288E+14	6,30382E+14	7,13445E+14	6,49084E+14	6,85756E+14
TC1200001283.mm.2	Tedc1	tubulin epsilon and delta complex 1	-0.939366218841872	4,73932E+14	-2,0906E+14	0.0784206389889585	0.221515374167019	-4,2596E+14	3,90566E+14	4,29131E+14	4,55279E+14	5,61044E+14	4,77924E+14	5,29647E+14
TC0X00003426.mm.2	C1galt1c1	C1GALT1-specific chaperone 1	0.624459548343595	7,02789E+14	2,0906E+14	0.0784213443980781	0.221515374167019	-4,25961E+14	7,60068E+14	7,3192E+14	6,78793E+14	6,78855E+14	6,79521E+14	6,87575E+14
TC1300001419.mm.2	Gtpbp4	GTP binding protein 4	1,45445E+14	6,58966E+14	2,09036E+14	0.0784479760070553	0.221515374167019	-4,25993E+13	7,73712E+14	7,53581E+13	6,85742E+14	5,04355E+14	6,85915E+14	5,50489E+14
TC0500002994.mm.2	Ep400	E1A binding protein p400	0.953849358745061	7,6216E+13	2,09029E+14	0.0784551757766953	0.221515374167019	-4,26001E+14	8,42196E+14	8,02379E+14	7,61465E+14	7,13113E+14	7,8222E+14	6,71585E+14
TC0200002851.mm.2	Hspa14	heat shock protein 14	0.66416399261942	6,39062E+14	2,09025E+14	0.0784601533597461	0.221515374167019	-4,26007E+14	6,8186E+14	6,70392E+14	6,4889E+14	6,13807E+14	5,93861E+14	6,25559E+14
TC0100003057.mm.2	Ikbke	inhibitor of kappaB kinase epsilon	-0.616799714938999	5,06183E+14	-2,09009E+14	0.0784775983791938	0.221515374167019	-4,26027E+14	4,68594E+14	4,62125E+14	5,14528E+13	5,37773E+14	5,27427E+14	5,26651E+14
TC1800001384.mm.2	Fbn2	fibrillin 2	-0.628728431220052	4,77655E+13	-2,09006E+14	0.0784811106921556	0.221515374167019	-4,26031E+14	4,56863E+14	4,40172E+14	4,60707E+14	4,85409E+14	5,05651E+14	5,17129E+14
TC1300001136.mm.2	Smn1	survival motor neuron 1	-0.73203584005605	5,1257E+14	-2,09003E+14	0.0784851530515431	0.221515374167019	-4,26036E+14	4,9421E+14	4,37258E+14	5,12939E+14	5,53138E+14	5,3027E+12	5,47605E+14
TC0100000064.mm.2	Sulf1	sulfatase 1	0.870026247969275	6,79602E+14	2,08978E+14	0.0785131983453467	0.221555720654741	-4,26069E+14	7,02724E+12	7,27795E+14	7,1399E+14	6,76587E+14	6,77366E+13	5,79148E+14
TC1900000405.mm.2	Dmrt3	doublesex and mab-3 related transcription factor 3	-0.703937522564897	5,8477E+14	-2,08955E+14	0.078537916181435	0.221586664932842	-4,26098E+13	5,50818E+14	5,39152E+14	5,76427E+13	6,11463E+14	5,89811E+14	6,40947E+14
TC0500002508.mm.2	Apbb2	amyloid beta (A4) precursor protein-binding, family B, member 2	1,35777E+14	9,24448E+14	2,08935E+14	0.0785606762908082	0.221612075818241	-4,26125E+14	1,00235E+14	9,92091E+14	9,89499E+14	8,39852E+14	9,34242E+14	7,8865E+13
TC0900000500.mm.2	Mcam	melanoma cell adhesion molecule	0.850066635530403	6,33E+14	2,08907E+14	0.0785924203945106	0.221639372739482	-4,26162E+14	7,169E+14	6,73379E+13	6,37846E+14	5,49608E+14	6,25926E+14	5,9434E+14
TC1100001894.mm.2	Trim80	tripartite motif-containing 80	-0.685267368605231	5,16593E+14	-2,08893E+14	0.0786082522587248	0.221639372739482	-4,2618E+14	4,95824E+13	4,73256E+14	5,28637E+14	4,95709E+14	5,32885E+14	5,73249E+14
TC1400001035.mm.2	Med4	mediator complex subunit 4	1,50565E+14	6,44532E+14	2,08889E+14	0.0786126874189115	0.221639372739482	-4,26186E+14	7,50626E+14	7,37069E+14	7,03557E+14	4,89376E+14	6,43644E+14	5,42921E+13
TC1900000975.mm.2	Kat5	K(lysine) acetyltransferase 5	0.785422830699479	8,43836E+13	2,08877E+14	0.0786253742058513	0.221639372739482	-4,262E+14	9,02157E+14	8,95409E+14	8,47116E+14	7,7785E+14	8,46335E+14	7,94146E+14
TC0200002534.mm.2	Zswim3	zinc finger SWIM-type containing 3	0.732568979404603	6,25155E+13	2,08865E+14	0.0786392175483785	0.221639620842579	-4,26217E+14	6,61943E+14	6,77082E+14	6,42593E+14	5,768E+14	6,08963E+14	5,83549E+13
TC0900002766.mm.2	Lca5	Leber congenital amaurosis 5 (human)	0.860188214639632	7,85564E+14	2,08629E+14	0.0789046345195106	0.222312977372597	-4,26527E+14	8,82515E+14	8,30377E+13	7,75688E+14	6,83952E+14	7,75721E+14	7,65132E+14
TC0300003170.mm.2	Rabggtb/Snord45c	Rab geranylgeranyl transferase, b subunit/small nucleolar RNA, C/D box 45C	1,1872E+14	8,07223E+14	2,08628E+14	0.0789057231796613	0.222312977372597	-4,26528E+14	8,97072E+14	9,00909E+13	8,08733E+14	6,76083E+12	8,38191E+14	7,22349E+14
TC0400003445.mm.2	Exo5	exonuclease 5	1,17205E+14	5,96821E+14	2,08569E+14	0.0789718262030965	0.222423146277382	-4,26605E+14	6,28833E+14	6,18607E+14	6,85128E+14	6,35325E+14	5,89297E+14	4,23733E+14
TC0300002696.mm.2	Olfr266	olfactory receptor 266	-1,56528E+14	4,2003E+14	-2,0855E+14	0.0789936160561055	0.222423146277382	-4,2663E+14	3,33267E+13	3,57734E+14	3,03185E+14	5,21933E+14	4,44181E+13	5,59877E+14
TC0100002900.mm.2	Vps4b	vacuolar protein sorting 4B	1,05621E+14	6,83958E+14	2,08542E+14	0.0790022079544192	0.222423146277382	-4,2664E+14	7,67562E+14	7,17653E+14	7,3897E+14	6,05588E+14	6,59855E+14	6,14118E+14
TC0900001890.mm.2	Rgl3	ral guanine nucleotide dissociation stimulator-like 3	-0.734124310667909	5,62512E+14	-2,085E+13	0.0790492458485876	0.222423146277382	-4,26695E+14	4,84072E+14	5,46469E+14	5,69364E+14	5,978E+14	5,76186E+14	6,0118E+14
TC0700004548.mm.2	Tollip	toll interacting protein	0.661316617148087	8,3508E+14	2,08487E+14	0.079063844701818	0.222423146277382	-4,26712E+14	8,86726E+14	8,69014E+14	8,37078E+14	7,94181E+13	8,05247E+14	8,18231E+14
TC1800001048.mm.2	B4galt6	UDP-Gal:betaGlcNAc beta 1,4-galactosyltransferase, polypeptide 6	1,5348E+14	8,519E+14	2,08484E+14	0.0790669643985202	0.222423146277382	-4,26716E+14	9,53707E+14	9,57927E+14	9,00649E+14	6,94445E+14	8,66653E+14	7,38022E+14
TC1500001460.mm.2	Eif3e	eukaryotic translation initiation factor 3, subunit E	1,51618E+14	7,53625E+14	2,08483E+14	0.0790690048878668	0.222423146277382	-4,26718E+14	8,49395E+14	8,64715E+14	7,98977E+14	5,97789E+14	7,70037E+14	6,40837E+14
TC0300001857.mm.2	Bbs7	Bardet-Biedl syndrome 7 (human)	-0.747634807898065	6,02915E+14	-2,08478E+14	0.0790740160430288	0.222423146277382	-4,26724E+13	5,285E+14	5,67885E+14	6,09039E+14	6,66149E+14	6,063E+14	6,39613E+14
TC1100004104.mm.2	Mrpl38	mitochondrial ribosomal protein L38	0.637396010210572	7,90444E+14	2,08476E+14	0.0790759398711413	0.222423146277382	-4,26726E+14	8,32024E+14	8,19671E+13	8,00758E+14	7,65994E+14	7,65401E+14	7,58814E+14
TC0700003061.mm.2	Mrgprx1	MAS-related GPR, member X1	-0.780702475994775	5,0702E+14	-2,08459E+13	0.0790953416216108	0.222423146277382	-4,26749E+14	4,55166E+14	4,63818E+14	4,89936E+14	5,58077E+14	5,11566E+14	5,63559E+14
TC0900000825.mm.2	Ints14	integrator complex subunit 14	1,27175E+14	6,95219E+14	2,08458E+14	0.079096669035524	0.222423146277382	-4,2675E+14	7,85074E+13	7,74371E+14	7,39376E+14	5,67394E+14	6,96017E+14	6,09079E+14
TC0600001500.mm.2	Tspan11	tetraspanin 11	-0.763333799763098	5,74656E+14	-2,08433E+13	0.0791249873184767	0.22246395405036	-4,26783E+14	5,4581E+14	4,96451E+14	5,73984E+14	6,36762E+14	5,89488E+14	6,0544E+14
TC1100000932.mm.2	Cldn7	claudin 7	-0.702520200950515	5,24694E+14	-2,08399E+14	0.0791636638735875	0.222533865316664	-4,26828E+14	4,63311E+14	5,04009E+14	5,20865E+14	5,52152E+14	5,35456E+14	5,72368E+14
TC0700001280.mm.2	Sytl2	synaptotagmin-like 2	0.888539555109054	6,92738E+14	2,08372E+14	0.0791932855216894	0.222578302740447	-4,26862E+14	7,53285E+14	7,84919E+14	6,6586E+13	5,91866E+14	7,19911E+14	6,40585E+14
TC1000002239.mm.2	Sgpl1	sphingosine phosphate lyase 1	-0.807681581686408	7,77096E+14	-2,08295E+14	0.0792812239511418	0.222761040031714	-4,26965E+14	7,2085E+14	7,05177E+14	7,79086E+14	8,69899E+14	8,01324E+14	7,86238E+14
TC1700002250.mm.2	Dazl	deleted in azoospermia-like	0.736519959329539	6,2962E+14	2,0829E+14	0.0792859532415987	0.222761040031714	-4,2697E+14	6,50099E+13	6,60654E+14	6,60194E+14	6,43326E+14	5,46132E+14	6,17317E+14
TC1600001267.mm.2	Spag6l	sperm associated antigen 6-like	-0.655414370572933	7,03897E+14	-2,08252E+13	0.0793289919514723	0.222813366881446	-4,2702E+14	6,60721E+14	6,66939E+14	6,99329E+14	7,37647E+14	7,16979E+14	7,41764E+14
TC0500003198.mm.2	Sh2b3	SH2B adaptor protein 3	-0.77692997089879	7,58982E+14	-2,08239E+14	0.0793436419438566	0.222813366881446	-4,27037E+14	6,95131E+14	7,16108E+14	7,46682E+14	8,29348E+14	7,81623E+14	7,85003E+14
TC0X00001625.mm.2	Sh3kbp1	SH3-domain kinase binding protein 1	0.983113461737092	8,00462E+14	2,08224E+13	0.0793611626930527	0.222813366881446	-4,27057E+14	8,68942E+14	8,49809E+14	8,44231E+14	7,1766E+14	7,81474E+14	7,40655E+14
TC0500001421.mm.2	Lrrc43	leucine rich repeat containing 43	-0.82558372313836	5,1681E+14	-2,08196E+14	0.0793930236600081	0.222813366881446	-4,27094E+14	4,39919E+14	4,54023E+14	5,53545E+14	5,94316E+14	4,85527E+13	5,73532E+14
TC1000001084.mm.2	Arl1	ADP-ribosylation factor-like 1	0.963509760109597	9,43875E+14	2,08191E+14	0.0793981266598117	0.222813366881446	-4,271E+14	1,02265E+14	1,0181E+14	9,51604E+14	8,22848E+14	9,3311E+14	9,14937E+14
TC0200000242.mm.2	Stam	signal transducing adaptor molecule (SH3 domain and ITAM motif) 1	0.937032056645676	7,16868E+14	2,08189E+14	0.0794006957116866	0.222813366881446	-4,27103E+12	7,82487E+14	7,55247E+14	7,60939E+14	6,52208E+14	7,0196E+14	6,48368E+14
TC1000000512.mm.2	Gcc2	GRIP and coiled-coil domain containing 2	1,796E+14	6,85242E+13	2,08188E+14	0.079401374829843	0.222813366881446	-4,27104E+14	8,46633E+14	8,47432E+13	6,58245E+14	4,24278E+14	7,54549E+14	5,80316E+13
TC0400001712.mm.2	Rap1gap	Rap1 GTPase-activating protein	0.621190913680785	8,17055E+14	2,08133E+14	0.0794640579352214	0.222923344590087	-4,27176E+13	8,624E+14	8,35352E+14	8,10944E+14	8,2012E+14	7,8915E+14	7,84363E+13
TC0500000389.mm.2	Hgfac	hepatocyte growth factor activator	-1,1246E+14	5,08898E+14	-2,08129E+14	0.0794682362890497	0.222923344590087	-4,27181E+14	4,41142E+14	4,25054E+14	4,67579E+14	6,28497E+14	5,59513E+14	5,31602E+14
TC0800002861.mm.2	Chtf8	CTF8, chromosome transmission fidelity factor 8	0.743965940556457	7,25361E+13	2,08113E+14	0.0794866020562191	0.222936052033396	-4,27203E+14	7,90933E+14	7,88112E+14	7,09338E+14	6,33528E+14	7,11156E+14	7,19096E+14
TC0600003045.mm.2	Atp6v1e1	ATPase, H+ transporting, lysosomal V1 subunit E1	1,39079E+13	8,62893E+12	2,08095E+14	0.0795068100508466	0.222953921049302	-4,27226E+14	9,7759E+14	9,73509E+14	8,69637E+14	6,83681E+14	8,91778E+14	7,81162E+14
TC0300000226.mm.2	Actl6a	actin-like 6A	1,2048E+14	5,79935E+14	2,08064E+14	0.0795420629972615	0.223013965734274	-4,27267E+14	6,65737E+14	6,7392E+14	5,99955E+14	4,41297E+14	5,91974E+14	5,06723E+14
TC0500003256.mm.2	Rilpl2	Rab interacting lysosomal protein-like 2	-0.700855157775765	6,06513E+14	-2,08042E+14	0.0795669887893501	0.223045040077035	-4,27295E+14	5,6717E+14	5,53346E+14	6,28979E+14	6,28894E+14	6,61114E+14	5,99574E+14
TC0100003666.mm.2	Stum	mechanosensory transduction mediator	0.839094276787668	8,5825E+14	2,08003E+14	0.0796114089492851	0.223130741415869	-4,27347E+13	8,92978E+14	9,29703E+14	8,4228E+12	8,29679E+14	8,74347E+14	7,80514E+14
TC1000000422.mm.2	Prep	prolyl endopeptidase	0.990497989175874	7,2915E+14	2,07953E+14	0.0796681926505769	0.223237559839494	-4,27412E+14	7,67012E+13	7,81557E+14	7,94036E+14	6,81825E+14	7,13175E+14	6,37294E+14
TSUnmapped00000003.mm.2	Wnk2	WNK lysine deficient protein kinase 2	-0.715719559943945	8,26854E+14	-2,07944E+14	0.0796783759549454	0.223237559839494	-4,27424E+14	7,75404E+14	7,81116E+14	8,23859E+13	8,81082E+14	8,31726E+14	8,67938E+14
TC0800002934.mm.2	Ddx19a	DEAD (Asp-Glu-Ala-Asp) box polypeptide 19a	0.735981850664714	6,26919E+14	2,0792E+13	0.079705757244634	0.223237559839494	-4,27456E+14	6,79239E+14	7,00336E+14	5,4393E+14	6,05631E+14	5,98548E+13	6,33831E+14
TC0900000573.mm.2	Cadm1	cell adhesion molecule 1	0.834391294185224	8,5976E+14	2,07872E+14	0.0797604801093232	0.223237559839494	-4,27519E+14	9,22166E+13	9,02752E+14	8,85392E+14	7,90208E+14	8,41937E+14	8,16103E+14
TC1200002148.mm.2	Dio2	deiodinase, iodothyronine, type II	0.882140113216042	6,86793E+14	2,07872E+14	0.0797607964083924	0.223237559839494	-4,27519E+13	7,09979E+14	7,64517E+14	6,28488E+14	7,19708E+13	6,66701E+14	6,31367E+14
TC1000002264.mm.2	Ddx21	DEAD (Asp-Glu-Ala-Asp) box polypeptide 21	0.823360925580602	5,90616E+14	2,07858E+14	0.0797757419735725	0.223237559839494	-4,27536E+13	6,67497E+14	6,74828E+14	5,46451E+14	4,77268E+14	5,54466E+14	6,23187E+14
TC1100001272.mm.2	Ddx52	DEAD (Asp-Glu-Ala-Asp) box polypeptide 52	1,82149E+14	5,55833E+14	2,07839E+14	0.0797982712962667	0.223237559839494	-4,27562E+14	6,89881E+14	6,90085E+14	6,08133E+14	3,31229E+14	5,61432E+14	4,54236E+14

TC0200005400.mm.2	Hrh3	histamine receptor H3	1,41713E+14	9,11408E+14	2,07836E+14	0.0798009574981783	0.223237559839494	-4,27565E+14	9,97495E+14	1,0149E+14	9,68269E+14	7,58826E+14	8,99865E+14	8,291E+14
TC0900002415.mm.2	Tmem202	transmembrane protein 202	-0.663657873223089	4,93131E+14	-2,07825E+14	0.0798134414517794	0.223237559839494	-4,2758E+14	4,48234E+14	4,79482E+13	4,72253E+13	4,98369E+13	5,15741E+14	5,44706E+14
TC0X00003191.mm.2	Tspyl2	TSPY-like 2	1,83702E+14	9,0186E+14	2,07822E+14	0.0798166186940794	0.223237559839494	-4,27583E+14	1,04055E+14	1,00817E+14	9,54389E+14	7,2672E+14	9,36827E+14	7,44495E+13
TC1400001061.mm.2	Tpt1	tumor protein, translationally-controlled 1	0.678727065776412	9,74476E+14	2,07818E+14	0.079821709376823	0.223237559839494	-4,27589E+14	1,0224E+14	1,03544E+14	9,42084E+14	9,24841E+14	9,86704E+13	9,35389E+14
TC0X00000356.mm.2	Rhox7a	reproductive homeobox 7A	-0.604262060575696	9,58161E+14	-2,07815E+14	0.0798249025071323	0.223237559839494	-4,27593E+14	9,11425E+14	9,25049E+14	9,76844E+14	9,78321E+14	9,82791E+13	9,74535E+14
TC0900003176.mm.2	Cmtm7	CKLF-like MARVEL transmembrane domain containing 7	-0.710272846629702	6,94509E+14	-2,07811E+14	0.0798296294790022	0.223237559839494	-4,27598E+14	6,40893E+14	6,46325E+12	6,97335E+14	7,53229E+14	6,96937E+13	7,32335E+13
TC0500001506.mm.2	Mmp17	matrix metallopeptidase 17	0.676450017080492	7,29003E+14	2,07787E+14	0.0798572811000171	0.223276135756477	-4,2763E+14	7,79231E+14	7,71306E+14	6,76963E+13	7,31271E+14	7,08083E+14	7,07165E+14
TC0200003879.mm.2	Gm19426	predicted gene, 19426	-0.702036675836743	9,20685E+14	-2,07634E+14	0.0800312765587428	0.223723795834667	-4,2783E+14	8,83783E+14	8,70485E+14	9,14187E+14	9,60981E+14	9,26609E+14	9,68066E+14
TC0900002216.mm.2	Pafah1b2	platelet-activating factor acetylhydrolase, isoform 1b, subunit 2	1,28216E+14	8,30512E+14	2,07618E+14	0.080049785900055	0.223736721631845	-4,27852E+14	9,19428E+14	9,08588E+13	8,5683E+14	7,26641E+14	8,58877E+14	7,12706E+14
TC1200001886.mm.2	Trim9	tripartite motif-containing 9	1,00711E+14	7,99226E+14	2,07583E+14	0.0800891625745133	0.22380795639319	-4,27897E+14	8,52512E+14	8,77144E+14	8,32516E+14	7,04947E+14	7,90453E+14	7,37782E+14
TC0400003765.mm.2	Rcan3/Mir700	regulator of calcineurin 3/microRNA 700	0.699425635786643	6,9402E+14	2,07571E+14	0.0801031714960946	0.223808288939929	-4,27913E+14	7,44849E+14	7,24442E+14	6,98283E+14	6,67137E+14	6,9019E+14	6,39216E+14
TC0200001976.mm.2	Ebf4	early B cell factor 4	0.694381128701282	5,7785E+14	2,07549E+14	0.0801283408669731	0.223839798264483	-4,27942E+14	6,30364E+14	5,81989E+14	5,78642E+14	6,02631E+14	5,51422E+14	5,22054E+14
TC0200002013.mm.2	Pank2	pantothenate kinase 2	1,65226E+14	7,8418E+14	2,07485E+14	0.0802019927382491	0.223950860901978	-4,28026E+12	8,95477E+14	9,0009E+14	8,35334E+14	6,09064E+14	8,02775E+14	6,62339E+14
TC0600002992.mm.2	Fxyd4	FXYD domain-containing ion transport regulator 4	-0.825319301186736	5,52959E+14	-2,07481E+14	0.0802061918874629	0.223950860901978	-4,28031E+14	5,27793E+14	4,84809E+13	5,33731E+14	5,93756E+14	5,56115E+14	6,21552E+14
TC1100000576.mm.2	Sar1b	secretion associated Ras related GTPase 1B	1,39322E+14	8,26883E+14	2,07475E+14	0.080212845304504	0.223950860901978	-4,28039E+14	9,32313E+13	9,32606E+14	8,54873E+13	6,55234E+14	8,36501E+14	7,49774E+14
TC0900001666.mm.2	Sacm1l	SAC1 suppressor of actin mutations 1-like (yeast)	1,87657E+14	6,85462E+14	2,07463E+14	0.0802268910656161	0.223950860901978	-4,28055E+13	8,25265E+14	8,04086E+14	7,3136E+14	4,98024E+14	7,26665E+14	5,27373E+14
TC0X00001629.mm.2	Map3k15	mitogen-activated protein kinase kinase kinase 15	-0.76230865835373	4,95071E+14	-2,07428E+14	0.0802670326328294	0.223950860901978	-4,28101E+14	4,1125E+13	4,62611E+14	5,0331E+14	5,6693E+14	5,09065E+14	5,17258E+14
TC1400002094.mm.2	Nedd8	neural precursor cell expressed, developmentally down-regulated gene 8	0.938033689896607	8,14429E+13	2,07418E+14	0.0802786144793664	0.223950860901978	-4,28114E+14	8,68599E+14	8,77759E+14	8,08326E+14	7,73141E+14	8,39302E+14	7,19449E+14
TC0Y00000435.mm.2	Gm20806	predicted gene, 20806	-0.676095790495841	5,21184E+14	-2,07399E+14	0.0802994200176584	0.223950860901978	-4,28138E+14	4,53052E+14	4,95763E+14	5,54045E+14	5,40212E+14	5,58095E+14	5,2594E+13
TC0Y00000491.mm.2	Gm20806	predicted gene, 20806	-0.676095790495841	5,21184E+14	-2,07399E+14	0.0802994200176584	0.223950860901978	-4,28138E+14	4,53052E+14	4,95763E+14	5,54045E+14	5,40212E+14	5,58095E+14	5,2594E+13
TC0400001076.mm.2	Dab1	disabled 1	1,21234E+14	8,02143E+14	2,07394E+13	0.080305085325739	0.223950860901978	-4,28144E+14	8,65528E+14	8,74284E+14	8,48903E+14	7,26802E+14	8,16394E+14	6,80951E+14
TC1000002237.mm.2	Pldi	polymorphic derived intron containing	-1,16635E+14	5,06654E+14	-2,07379E+14	0.0803222694000247	0.223950860901978	-4,28164E+14	4,21644E+14	4,43722E+14	4,55198E+13	6,20723E+14	5,25976E+14	5,72659E+14
TC0700000385.mm.2	Zfp428	zinc finger protein 428	-0.844740112485479	7,12207E+14	-2,07376E+14	0.0803266919215532	0.223950860901978	-4,28169E+14	6,70674E+14	6,42063E+14	6,98545E+14	7,80274E+14	7,12447E+14	7,69237E+14
TC1500001824.mm.2	Eif3d	eukaryotic translation initiation factor 3, subunit D	0.742074590836419	7,53989E+14	2,07368E+14	0.0803348833869196	0.223950860901978	-4,28178E+14	7,96069E+14	8,06042E+14	7,11401E+14	7,56727E+14	6,96441E+14	7,57255E+14
TC1100003882.mm.2	Nsf	N-ethylmaleimide sensitive fusion protein	1,69595E+14	9,17513E+14	2,07337E+14	0.0803712527101079	0.224013491596258	-4,2822E+14	1,04786E+14	1,03483E+14	9,75147E+14	7,03743E+14	8,91777E+14	8,51722E+14
TC1800001745.mm.2	Ier3ip1	immediate early response 3 interacting protein 1	0.868275919801191	7,82889E+14	2,07314E+14	0.0803970476763775	0.224037925059926	-4,2825E+14	8,45021E+14	8,09685E+14	8,16558E+14	7,4502E+14	7,77862E+14	7,03189E+14
TC1100002156.mm.2	Myl7	myosin, light polypeptide 7, regulatory	-1,00803E+14	5,49071E+14	-2,07294E+14	0.0804205851585959	0.224037925059926	-4,28276E+14	4,95947E+14	4,80595E+14	5,11419E+14	6,28317E+13	5,50687E+14	6,27461E+14
TC1700000014.mm.2	Cldn20	claudin 20	-0.715569125534587	4,76409E+14	-2,07276E+14	0.080441114819918	0.224037925059926	-4,283E+14	4,56349E+14	4,08773E+14	4,75393E+14	5,09701E+14	5,21935E+14	4,863E+14
TC1700001403.mm.2	Fndc1	fibronectin type III domain containing 1	-1,11115E+14	6,11802E+14	-2,07271E+14	0.0804469954422084	0.224037925059926	-4,28307E+14	5,35195E+14	5,5446E+14	5,54699E+14	7,1457E+14	6,45765E+14	6,6612E+14
TC0500002017.mm.2	Ccdc146	coiled-coil domain containing 146	0.60255800361664	5,65836E+14	2,07268E+14	0.080449539775134	0.224037925059926	-4,2831E+14	6,02015E+14	6,07934E+14	5,51859E+14	5,43768E+14	5,5087E+14	5,38568E+14
TC1400002118.mm.2	Gzmn	granzyme N	-0.826329750935787	5,7672E+14	-2,07251E+14	0.0804695007386639	0.224054789252133	-4,28332E+13	5,45045E+13	5,13545E+14	5,76023E+13	6,01849E+14	5,66738E+14	6,57118E+14
TC0200000416.mm.2	Fut7	fucosyltransferase 7	-0.799936883708911	6,67288E+14	-2,07237E+14	0.0804860393750655	0.224062120326612	-4,28351E+14	5,80171E+14	6,2672E+14	6,76772E+14	7,53186E+14	6,71827E+14	6,9505E+14
TC0500002169.mm.2	Preb	prolactin regulatory element binding	0.710566946726841	8,35694E+14	2,07212E+14	0.0805142357774454	0.224074321255022	-4,28383E+14	8,90733E+14	8,72632E+14	8,4551E+14	7,84039E+14	8,00628E+14	8,20624E+14
TC1000000160.mm.2	Ahi1	Abelson helper integration site 1	0.840958168414478	8,80238E+14	2,07209E+14	0.0805182349696875	0.224074321255022	-4,28388E+14	9,5164E+13	8,92621E+14	9,22346E+14	8,38751E+14	8,33149E+14	8,4292E+14
TC0600001687.mm.2	5330439B14Rik	RIKEN cDNA 5330439B14 gene	-0.774615744746973	5,68962E+14	-2,07188E+14	0.0805417634392557	0.224101093817428	-4,28415E+13	5,04108E+14	5,58278E+14	5,87614E+14	5,46462E+14	6,34789E+14	5,82521E+14
TC0200001764.mm.2	Zfyve19	zinc finger, FYVE domain containing 19	1,28625E+14	5,4932E+14	2,07172E+14	0.0805597420891219	0.224112417866371	-4,28435E+13	6,31452E+14	6,26321E+14	5,61983E+14	4,75641E+14	5,88971E+14	4,11553E+14
TC0100003471.mm.2	Gm4846	predicted gene 4846	-0.665751686831934	5,96533E+14	-2,0712E+14	0.0806203000826817	0.224242170763378	-4,28504E+14	5,70252E+13	5,38477E+14	5,948E+14	6,33789E+14	6,15011E+14	6,26869E+14
TC0100000836.mm.2	Ugt1a2/Ugt1a6a/Ugt1a6b/Ugt1a10/Ugt1a7c/Ugt1a5/Ugt1a9/Ugt1a1	UDP glucuronosyltransferase 1 family, polypeptide A2/UDP glucuronosyltransferase 1 family, polypeptide A6A/UDP glucuronosyltransferase 1 family, polypeptide A6B/UDP glycosyltransferase 1 family, polypeptide A10/UDP glucuronosyltransferase 1 family, polypeptide A7C/UDP glucuronosyltransferase 1 family, polypeptide A5/UDP glucuronosyltransferase 1 family, polypeptide A9/UDP glucuronosyltransferase 1 family, polypeptide A1	1,19392E+14	5,32033E+14	2,07104E+14	0.0806386931523626	0.224246136365669	-4,28525E+14	5,51609E+14	5,91259E+14	5,03029E+14	6,42218E+14	5,04907E+14	3,99177E+14
TC0400004157.mm.2	Tas1r3	taste receptor, type 1, member 3	-0.945282692792629	5,66193E+14	-2,07083E+14	0.0806622567666606	0.224246136365669	-4,28552E+14	5,09776E+14	5,30728E+14	5,19255E+14	6,0784E+14	5,70354E+14	6,59207E+14
TC4_GL456350_random00000010.mm.2	Fam205a2	family with sequence similarity 205, member A2	0.778772195092894	8,14526E+13	2,0708E+14	0.0806664160005277	0.224246136365669	-4,28557E+14	8,63505E+14	8,82227E+14	7,94706E+14	7,56742E+14	8,33003E+14	7,56974E+13
TC0600001742.mm.2	Mrps35	mitochondrial ribosomal protein S35	0.614852771346633	5,759E+13	2,0707E+14	0.0806773941855509	0.224246136365669	-4,28569E+14	6,26098E+14	6,28153E+14	5,27364E+14	5,42504E+14	5,76688E+14	5,54593E+13
TC1600000044.mm.2	Ubn1	ubinuclein 1	0.765002742874246	6,07713E+14	2,07042E+14	0.0807100214110605	0.224286688312484	-4,28607E+14	6,57285E+13	6,63404E+14	6,18758E+14	5,39145E+14	5,95733E+14	5,71955E+13
TC0100002993.mm.2	Actr3	ARP3 actin-related protein 3	1,28351E+13	9,1905E+14	2,07024E+14	0.0807298508202225	0.224286688312484	-4,28629E+14	1,00932E+14	1,00789E+14	9,39519E+14	7,97067E+12	9,48303E+14	8,12204E+14
TC1400001174.mm.2	Uchl3	ubiquitin carboxyl-terminal esterase L3 (ubiquitin thiolesterase)	0.861415670165211	7,58554E+14	2,07021E+14	0.0807337424571716	0.224286688312484	-4,28634E+14	8,20194E+14	8,24436E+14	7,71075E+13	6,63275E+14	7,37869E+14	7,34477E+14
TC0500000019.mm.2	Krit1	KRIT1, ankyrin repeat containing	1,82316E+14	5,3306E+14	2,06972E+14	0.0807907103113442	0.224378413208193	-4,28698E+14	6,29502E+14	6,36605E+14	5,59337E+14	4,71441E+14	5,93393E+14	3,08083E+14
TC0200003276.mm.2	1700001O22Rik	RIKEN cDNA 1700001O22 gene	-0.745688409903543	7,15547E+14	-2,06967E+14	0.080795552943476	0.224378413208193	-4,28704E+14	6,49675E+14	6,81916E+14	7,0831E+14	7,72654E+13	7,25381E+14	7,55347E+14
TC0800002802.mm.2	Dync1li2	dynein, cytoplasmic 1 light intermediate chain 2	0.901254545329786	8,32638E+14	2,06956E+14	0.0808085354587688	0.224378413208193	-4,28719E+14	9,00995E+14	8,98016E+14	8,36348E+14	7,4171E+14	8,44048E+14	7,74711E+14
TC1100001499.mm.2	Hoxb3	homeobox B3	-0.990267946272318	7,056E+14	-2,0692E+14	0.0808503563783387	0.224415720820483	-4,28766E+14	6,1542E+14	6,46285E+14	6,96722E+14	8,15418E+14	6,99066E+14	7,60692E+13
TC0200001969.mm.2	Tgm6	transglutaminase 6	-0.997130282139014	5,47524E+13	-2,06915E+14	0.080856404347013	0.224415720820483	-4,28773E+14	4,86643E+14	5,03179E+14	5,01878E+14	6,04195E+14	5,48504E+14	6,40744E+14

TC0600002756.mm.2	Adamts9	a disintegrin-like and metallopeptidase (reprolysin type) with thrombospondin type 1 motif, 9	-0.673171107598189	5,46514E+14	-2,06908E+14	0.0808637544270916	0.224415720820483	-4,28782E+14	5,00485E+14	5,07292E+14	5,37811E+14	5,91082E+14	5,65919E+14	5,76493E+14
TC0600000846.mm.2	Sftpb	surfactant associated protein B	-0.655402790368926	5,32577E+14	-2,06881E+14	0.0808956253882952	0.22446550919263	-4,28818E+14	4,87026E+13	4,68198E+14	5,55065E+14	5,98867E+14	5,41078E+14	5,45227E+14
TC1500002214.mm.2	Faim2	Fas apoptotic inhibitory molecule 2	1,31568E+14	1,008E+13	2,06822E+14	0.0809636105050781	0.224544616025621	-4,28895E+14	1,11205E+14	1,07475E+13	1,06086E+14	8,76653E+13	1,00091E+14	9,22767E+14
TC1100000287.mm.2	Ppp4r3b	protein phosphatase 4 regulatory subunit 3B	2,21676E+14	6,21105E+14	2,06805E+14	0.0809833473767344	0.224544616025621	-4,28917E+14	7,56671E+14	7,96745E+14	7,06982E+14	3,5617E+14	6,10899E+14	4,99164E+14
TC0500001528.mm.2	Tmem248	transmembrane protein 248	0.872783701487341	7,77278E+14	2,06791E+14	0.0809990235936426	0.224544616025621	-4,28935E+14	8,40839E+14	8,10339E+14	8,11502E+14	7,24369E+14	7,69012E+14	7,07609E+14
TSUnmapped00000031.mm.2	Cd99	CD99 antigen	-0.659016872183523	7,11752E+14	-2,06787E+14	0.0810040981447109	0.224544616025621	-4,28941E+14	6,80047E+13	6,91959E+14	6,87279E+13	7,07419E+14	7,73684E+14	7,30125E+14
TC1700001264.mm.2	Dync2li1	dynein cytoplasmic 2 light intermediate chain 1	0.68006448941164	6,48423E+14	2,0678E+14	0.0810114400545222	0.224544616025621	-4,28949E+14	6,91134E+14	6,96635E+14	6,54065E+14	5,96951E+14	6,26427E+14	6,25329E+14
TC0300002355.mm.2	Chrnb2	cholinergic receptor, nicotinic, beta polypeptide 2 (neuronal)	0.724533877262904	6,90155E+12	2,06777E+14	0.081014760548913	0.224544616025621	-4,28953E+14	7,48674E+14	7,14351E+13	7,05643E+14	6,54144E+14	6,40739E+14	6,77379E+14
TC0300001409.mm.2	Adh1	alcohol dehydrogenase 1 (class I)	-1,02593E+14	5,44089E+14	-2,06743E+14	0.0810547247041651	0.224544616025621	-4,28998E+14	4,339E+14	5,25317E+13	5,3107E+14	6,09844E+13	5,41919E+14	6,22485E+13
TC0200002575.mm.2	Arfgef2	ADP-ribosylation factor guanine nucleotide-exchange factor 2 (brefeldin A-inhibited)	0.6386438925483	7,28469E+14	2,06743E+14	0.0810548203679123	0.224544616025621	-4,28998E+14	7,58231E+14	7,75541E+14	7,00571E+14	7,30427E+14	7,1379E+14	6,92253E+14
TC1500001684.mm.2	Trappc9	trafficking protein particle complex 9	0.755343718717207	7,12929E+14	2,06735E+14	0.0810637438699735	0.224544616025621	-4,29008E+14	7,74104E+14	7,57813E+13	7,20943E+14	6,43868E+13	6,81133E+14	6,99715E+14
TC0200004875.mm.2	4921509C19Rik	RIKEN cDNA 4921509C19 gene	-0.685384552879871	5,08498E+14	-2,06728E+14	0.0810723039908569	0.224544616025621	-4,29018E+14	4,56921E+14	4,65701E+14	5,16256E+13	5,5241E+14	5,06747E+14	5,52952E+14
TC0400001600.mm.2	Ahdc1	AT hook, DNA binding motif, containing 1	-0.849841383991722	6,6719E+14	-2,06723E+14	0.0810774266764143	0.224544616025621	-4,29024E+14	6,08743E+14	6,05568E+14	6,7172E+14	7,32834E+13	6,49205E+14	7,35073E+14
TC0100000524.mm.2	Adam23	a disintegrin and metallopeptidase domain 23	1,47177E+14	8,05979E+14	2,06709E+14	0.0810933421628941	0.224550098345199	-4,29042E+14	9,04361E+14	9,01463E+14	8,63571E+13	6,55013E+14	8,0836E+14	7,03109E+14
TC0400001785.mm.2	Epha2	Eph receptor A2	-0.701219967412457	5,27501E+14	-2,0668E+14	0.0811269150357454	0.22460446425618	-4,2908E+14	4,83378E+14	4,78226E+14	5,27226E+13	5,74326E+14	5,28891E+14	5,72957E+14
TC1100002504.mm.2	Spdl1	spindle apparatus coiled-coil protein 1	-0.812181333400694	5,99264E+14	-2,06628E+14	0.0811878364569235	0.224617927972247	-4,29149E+14	5,17926E+14	5,80844E+14	5,82177E+14	6,5343E+14	6,37973E+14	6,23233E+14
TC1700000211.mm.2	Spaca6/Mir125a	sperm acrosome associated 6/microRNA 125a	-0.774532967271127	6,11293E+14	-2,06626E+14	0.0811902589605394	0.224617927972247	-4,29152E+14	5,48557E+14	5,70062E+14	6,50749E+14	6,24862E+14	5,9701E+14	6,76516E+14
TC0600002116.mm.2	Kdm7a	lysine (K)-specific demethylase 7A	1,68214E+14	6,25399E+14	2,06609E+14	0.0812092236042832	0.224617927972247	-4,29173E+14	7,07911E+14	7,61824E+14	6,47507E+14	5,01846E+14	6,79028E+14	4,5428E+14
TC0600003443.mm.2	1700034J05Rik	RIKEN cDNA 1700034J05 gene	-0.622102908137143	4,88259E+14	-2,06596E+14	0.0812248715146796	0.224617927972247	-4,29191E+14	4,77438E+14	4,53631E+14	4,722E+14	4,70794E+14	5,16844E+14	5,38645E+14
TC1100000859.mm.2	Gas7	growth arrest specific 7	1,13134E+14	8,53173E+14	2,06589E+14	0.0812328635273251	0.224617927972247	-4,292E+14	9,28553E+14	9,42893E+13	8,82958E+14	7,19456E+14	8,38513E+13	8,06666E+14
TC0400002949.mm.2	Psip1	PC4 and SFRS1 interacting protein 1	1,38041E+14	9,66031E+14	2,06583E+14	0.0812394017824023	0.224617927972247	-4,29207E+14	1,06748E+14	1,04996E+14	9,98801E+14	8,38584E+14	9,92845E+14	8,48513E+12
TC0600000480.mm.2	Olfr237-ps1	olfactory receptor 237, pseudogene 1	-0.863740690147957	5,05383E+14	-2,0658E+14	0.0812431263458486	0.224617927972247	-4,29212E+13	4,11577E+14	4,98922E+14	4,91218E+14	5,47333E+14	5,25128E+14	5,58119E+14
TC0500000947.mm.2	Prdm8	PR domain containing 8	0.874549207969525	6,2799E+14	2,06579E+14	0.0812447060182259	0.224617927972247	-4,29213E+14	6,49128E+14	7,56836E+14	5,53368E+14	5,77557E+14	6,07673E+13	6,2338E+14
TC1100003396.mm.2	Ccl5	chemokine (C-C motif) ligand 5	-0.886438653738219	5,44722E+14	-2,06555E+14	0.0812725191102949	0.224617927972247	-4,29245E+13	4,54835E+14	5,08746E+14	5,42489E+14	6,21393E+14	5,39061E+14	6,01808E+14
TC1200001066.mm.2	Bdkrb2	bradykinin receptor, beta 2	-0.866709904352981	5,50932E+14	-2,06546E+14	0.081282892361011	0.224617927972247	-4,29256E+14	5,41289E+14	4,62268E+14	5,25964E+14	5,99171E+14	5,98761E+14	5,78139E+14
TC0700004310.mm.2	Kif22	kinesin family member 22	-0.714160502663612	5,49047E+14	-2,06532E+13	0.081298399577667	0.224617927972247	-4,29274E+14	5,06612E+14	5,03741E+14	5,45648E+14	5,85094E+14	5,48326E+14	6,04859E+14
TC1900001118.mm.2	Ccdc86	coiled-coil domain containing 86	-0.604629456218791	6,42552E+14	-2,06532E+14	0.08129906013369	0.224617927972247	-4,29275E+14	5,98587E+14	6,1002E+14	6,50838E+14	6,66335E+14	6,61849E+14	6,67683E+14
TC1200000134.mm.2	Rock2	Rho-associated coiled-coil containing protein kinase 2	2,00817E+14	6,45878E+14	2,0649E+14	0.0813472958938695	0.224681774490248	-4,29329E+14	8,13892E+14	8,24722E+12	6,51384E+14	3,48292E+14	6,87489E+14	5,49491E+14
TC0400001164.mm.2	Eps15	epidermal growth factor receptor pathway substrate 15	1,80248E+14	7,74273E+14	2,06488E+14	0.0813500572442193	0.224681774490248	-4,29332E+14	9,06039E+14	8,97826E+14	8,35289E+14	5,63115E+13	7,79215E+14	6,64155E+14
TC0100001358.mm.2	Edem3	ER degradation enhancer, mannosidase alpha-like 3	0.938969947542014	6,61633E+14	2,06468E+14	0.0813727441914456	0.224705917250516	-4,29358E+14	7,5479E+14	7,29957E+12	6,53873E+14	5,34224E+14	6,64675E+14	6,32278E+13
TC1900001569.mm.2	Mrpl43	mitochondrial ribosomal protein L43	0.657015766905953	6,45624E+14	2,06439E+14	0.0814069856197552	0.224734253899182	-4,29397E+14	6,72221E+14	6,55265E+14	6,6711E+14	6,83067E+14	6,19648E+14	5,76435E+14
TC0300002074.mm.2	Slc33a1	solute carrier family 33 (acetyl-CoA transporter), member 1	0.832020739050617	5,12722E+13	2,06435E+14	0.0814109005157033	0.224734253899182	-4,29401E+14	5,82994E+14	5,66855E+14	5,20455E+14	4,22584E+14	4,8129E+14	5,02154E+14
TC0400003610.mm.2	Kpna6	karyopherin (importin) alpha 6	1,66103E+14	7,01525E+14	2,06419E+14	0.0814295477636693	0.224747225610826	-4,29422E+14	8,10642E+14	8,05647E+14	7,79678E+14	5,29097E+14	6,91657E+14	5,92427E+13
TC1700000026.mm.2	Arid1b	AT rich interactive domain 1B (SWI-like)	-0.642295367497923	8,19088E+14	-2,06364E+14	0.0814937282077114	0.224869017288989	-4,29494E+14	7,84865E+13	7,72977E+14	8,39211E+13	8,31173E+14	8,22931E+14	8,6337E+14
TC1000002479.mm.2	Prss57	protease, serine 57	-0.753256374691212	5,56758E+14	-2,06346E+14	0.0815140405374864	0.224869017288989	-4,29517E+14	4,98681E+14	5,16197E+14	5,4068E+13	6,19462E+14	5,81406E+14	5,84123E+14
TC0200002293.mm.2	Rem1	rad and gem related GTP binding protein 1	-0.901017302849596	5,34341E+14	-2,06332E+13	0.0815302966787093	0.224869017288989	-4,29536E+14	4,96195E+14	4,57351E+14	5,39033E+14	5,79717E+14	5,15049E+14	6,187E+14
TC0600000392.mm.2	Rab19	RAB19, member RAS oncogene family	-1,18624E+14	5,87215E+14	-2,06315E+14	0.0815506862106129	0.224869017288989	-4,29559E+13	4,94716E+14	5,07214E+13	5,814E+14	7,00784E+14	5,53461E+14	6,85717E+14
TC1700000494.mm.2	Srsf3	serine/arginine-rich splicing factor 3	1,39976E+14	7,40364E+14	2,06305E+14	0.081562329060127	0.224869017288989	-4,29572E+14	8,4065E+14	8,67358E+14	7,21948E+14	5,84168E+14	7,99018E+14	6,2904E+14
TC0500003427.mm.2	Col26a1	collagen, type XXVI, alpha 1	-0.945077150176187	5,81229E+14	-2,06303E+14	0.0815640108907983	0.224869017288989	-4,29574E+14	5,21865E+14	4,98182E+14	5,69066E+14	6,89197E+13	5,85956E+14	6,23107E+14
TC1700001105.mm.2	Epb41l3	erythrocyte membrane protein band 4.1 like 3	1,77025E+13	8,2243E+14	2,06292E+14	0.0815777050641764	0.224869017288989	-4,29589E+14	9,56787E+14	9,38357E+14	8,80443E+14	6,17897E+14	8,31419E+14	7,09675E+14
TC0400003886.mm.2	Tmem82	transmembrane protein 82	-0.789080919137518	5,59599E+14	-2,06273E+14	0.0815993767647757	0.224869017288989	-4,29613E+14	5,25227E+14	5,11881E+14	5,20272E+14	6,0529E+14	5,83346E+14	6,11579E+14
TC0200004373.mm.2	Zfp770	zinc finger protein 770	1,44942E+14	6,58782E+14	2,06261E+14	0.0816130867362988	0.224869017288989	-4,29629E+14	7,58885E+14	7,4564E+14	7,00401E+13	5,33126E+14	6,85958E+14	5,28684E+14
TC1100003925.mm.2	Ern1	endoplasmic reticulum (ER) to nucleus signalling 1	-0.673135356682613	7,79486E+14	-2,06249E+14	0.0816271210996156	0.224869017288989	-4,29645E+14	7,42685E+14	7,40641E+14	7,60082E+14	8,15552E+14	8,09352E+14	8,08602E+14
TC1700001563.mm.2	Zfp942	zinc finger protein 942	0.60942914557147	5,94688E+13	2,06221E+14	0.0816604195563429	0.224869017288989	-4,29682E+14	6,70539E+12	6,74883E+14	5,08287E+14	4,90886E+14	6,03213E+14	6,20323E+14
TC1600000179.mm.2	Abcc1	ATP-binding cassette, sub-family C (CFTR/MRP), member 1	-0.650925816894564	8,23163E+14	-2,06218E+14	0.0816640029391903	0.224869017288989	-4,29686E+14	7,63943E+14	7,80087E+14	8,82363E+14	8,38369E+14	8,36771E+14	8,37444E+14
TC0600002663.mm.2	Rab43	RAB43, member RAS oncogene family	0.664479627157978	5,14243E+14	2,06216E+14	0.0816660448616303	0.224869017288989	-4,29688E+14	5,19697E+14	5,27514E+14	5,36672E+14	5,87257E+14	4,62543E+14	4,51773E+14
TC1300001837.mm.2	Eef1e1	eukaryotic translation elongation factor 1 epsilon 1	0.87441614718106	8,46493E+14	2,06213E+14	0.0816690553698979	0.224869017288989	-4,29692E+14	9,38696E+14	8,47179E+14	8,58106E+14	8,23982E+14	8,32735E+14	7,78258E+14
TC0X00000472.mm.2	Fam122c	family with sequence similarity 122, member C	-0.939589624526575	5,08069E+14	-2,06196E+14	0.0816893942863414	0.224880418559424	-4,29715E+13	4,34894E+13	4,62761E+14	4,72901E+14	5,92287E+14	5,26521E+14	5,59052E+14
TC1800001526.mm.2	4930546C10Rik	RIKEN cDNA 4930546C10 gene	-1,24662E+14	5,48176E+14	-2,06186E+14	0.0817011090577093	0.224880418559424	-4,29728E+14	4,63392E+13	4,74627E+13	4,99716E+14	6,63977E+14	5,4548E+14	6,41863E+13
TC0700002643.mm.2	Hnrnpul1	heterogeneous nuclear ribonucleoprotein U-like 1	1,41732E+14	8,03992E+14	2,06098E+14	0.0818036332640086	0.225124157605973	-4,29843E+13	8,9387E+14	8,88384E+14	8,61283E+14	6,81624E+14	8,16288E+14	6,82501E+14
TC0600001981.mm.2	Kcp	kielin/chordin-like protein	-0.856111339725401	6,71642E+14	-2,06028E+14	0.0818849471667532	0.225309452475733	-4,29934E+14	6,14289E+14	6,0875E+14	6,67247E+14	7,45304E+14	6,60165E+14	7,34096E+14
TC1000001216.mm.2	Kitl	kit ligand	0.606697374186899	7,42208E+14	2,05995E+14	0.0819239315514363	0.225378232728068	-4,29978E+13	7,9768E+14	7,84239E+14	7,02798E+14	7,07949E+14	7,39389E+14	7,2119E+14
TC0700001203.mm.2	Mesd	mesoderm development LRP chaperone	0.762824740174747	7,7563E+14	2,05973E+12	0.0819495275725336	0.225410163498845	-4,30007E+13	8,58295E+14	8,13545E+14	7,66512E+14	6,96153E+14	7,63818E+14	7,55458E+14
TC1100002869.mm.2	Srebf1/Mir6922	sterol regulatory element binding transcription factor 1/microRNA 6922	1,03945E+14	5,86622E+14	2,05935E+14	0.0819938724616721	0.22549364515701	-4,30056E+14	6,3664E+14	6,60689E+14	5,43201E+14	5,89766E+14	4,75073E+14	6,14366E+14
TC0100002338.mm.2	Hecw2	HECT, C2 and WW domain containing E3 ubiquitin protein ligase 2	0.613001147458537	6,12316E+14	2,0592E+14	0.0820107813725681	0.225499390733115	-4,30075E+14	6,46765E+14	6,47816E+13	5,91076E+14	6,16261E+13	5,91997E+14	5,79984E+14

TC0900000385.mm.2	Olfr888	olfactory receptor 888	-0.865904364267373	4,77036E+14	-2,05897E+14	0.0820377375574038	0.225499390733115	-4,30105E+14	4,21829E+14	4,27866E+14	4,41719E+14	5,47926E+14	4,97183E+14	5,25692E+14
TC0600002176.mm.2	Tcaf3	TRPM8 channel-associated factor 3	-0.836936009211825	5,02317E+14	-2,05895E+14	0.0820402357727531	0.225499390733115	-4,30108E+14	4,6199E+14	4,60918E+14	5,07531E+13	4,93169E+14	4,94094E+14	5,96201E+14
TC1000001183.mm.2	Btg1	B cell translocation gene 1, anti-proliferative	0.611543235018741	7,67091E+14	2,05885E+14	0.0820519411573423	0.225499390733115	-4,30121E+14	7,91896E+14	7,9665E+14	7,76487E+14	7,71276E+14	7,20084E+14	7,46153E+14
TC1100002461.mm.2	Bod1	biorientation of chromosomes in cell division 1	-0.610000291150095	7,5276E+14	-2,05847E+14	0.0820969377044526	0.22550265378114	-4,30172E+14	7,14796E+14	7,01852E+14	7,70081E+14	7,91182E+14	7,65336E+14	7,73312E+14
TC0X00002452.mm.2	Fam3a	family with sequence similarity 3, member A	1,24925E+14	5,0937E+14	2,05843E+12	0.0821018162444066	0.22550265378114	-4,30177E+14	6,1009E+14	5,23208E+13	5,29555E+14	5,09921E+14	5,26606E+14	3,56842E+14
TC0900000509.mm.2	Slc37a4	solute carrier family 37 (glucose-6-phosphate transporter), member 4	0.975666488569725	5,42614E+14	2,05835E+14	0.0821106612412336	0.22550265378114	-4,30187E+13	6,30735E+14	5,91201E+14	5,6756E+14	4,39386E+14	5,21878E+14	5,04925E+14
TC0500000112.mm.2	Cacna2d1	calcium channel, voltage-dependent, alpha2/delta subunit 1	1,32566E+14	6,71135E+14	2,05834E+14	0.0821123545293292	0.22550265378114	-4,30189E+14	7,64472E+14	7,64858E+14	6,90628E+14	5,42654E+14	7,04268E+13	5,5993E+14
TC0100002543.mm.2	Abca12	ATP-binding cassette, sub-family A (ABC1), member 12	-0.922947262028967	5,66512E+14	-2,05824E+14	0.0821231038532692	0.22550265378114	-4,30201E+14	5,22164E+13	4,97816E+14	5,26988E+14	6,47534E+14	5,96831E+14	6,07738E+12
TC1700002204.mm.2	Guca1a	guanylate cyclase activator 1a (retina)	-0.994697273788946	5,86469E+14	-2,05805E+14	0.0821456212947761	0.225526051443641	-4,30226E+14	5,28597E+13	5,14083E+14	5,8549E+14	6,49027E+14	5,59209E+14	6,8241E+14
TC0900003041.mm.2	Gnat1	guanine nucleotide binding protein, alpha transducing 1	-0.791432658522084	5,41709E+14	-2,05768E+14	0.0821892718002105	0.225607450854649	-4,30275E+13	4,62069E+14	5,00224E+14	5,58438E+14	6,08941E+14	5,27115E+14	5,93465E+13
TC1100001348.mm.2	Rnf43/1110028F11Rik	ring finger protein 43/RIKEN cDNA 1110028F11 gene	-0.608390990537712	7,76039E+14	-2,0572E+13	0.0822455215510339	0.225723401252224	-4,30338E+14	7,50478E+14	7,35358E+14	7,78369E+14	7,84514E+14	7,94213E+14	8,13302E+14
TC1600002061.mm.2	Paxbp1	PAX3 and PAX7 binding protein 1	0.666266120586261	6,71418E+14	2,05702E+14	0.0822659356032335	0.225727525234718	-4,3036E+14	7,23496E+14	7,06508E+14	6,36348E+14	6,65406E+14	6,27507E+14	6,69244E+14
TC0400000390.mm.2	Gm21953/Gm21586/Gm20878	predicted gene, 21953/predicted gene, 21586/predicted gene, 20878	0.79574729862432	9,39378E+14	2,05681E+14	0.0822906288563573	0.225727525234718	-4,30388E+12	9,92889E+14	1,02789E+14	8,97952E+14	8,55911E+14	9,68365E+14	8,93262E+13
TC0200005466.mm.2	Lrrc8a	leucine rich repeat containing 8A	0.781409120926769	7,42976E+13	2,05656E+14	0.0823200677504601	0.225727525234718	-4,30421E+14	8,18444E+14	7,88941E+14	7,40052E+14	6,59313E+14	7,37392E+14	7,13712E+14
TC1100004063.mm.2	Fdxr	ferredoxin reductase	-0.90042893958163	6,02974E+14	-2,05654E+14	0.0823230271914943	0.225727525234718	-4,30424E+14	5,48453E+13	5,26044E+14	5,92542E+14	6,9622E+14	6,009E+14	6,53684E+13
TC1500001133.mm.2	Pde1b	phosphodiesterase 1B, Ca2+-calmodulin dependent	1,25205E+14	9,70712E+14	2,0563E+14	0.0823511227115504	0.225727525234718	-4,30455E+14	1,04458E+14	1,04717E+14	1,03833E+14	8,52861E+14	9,46458E+14	8,9488E+14
TC1100001385.mm.2	Coil/2210409E12Rik	coilin/transcription elongation factor B (SIII), polypeptide 2 pseudogene	-0.943137965900591	6,48841E+14	-2,05622E+14	0.08235998507635	0.225727525234718	-4,30465E+14	6,0032E+14	5,55785E+14	6,53157E+14	7,39049E+14	6,30297E+14	7,14435E+14
TC0100001502.mm.2	Nme7	NME/NM23 family member 7	0.874031708770532	6,3134E+14	2,05614E+14	0.082370145614037	0.225727525234718	-4,30477E+14	7,08626E+14	6,84549E+14	6,20482E+14	5,56018E+14	6,53588E+14	5,6478E+14
TC0900001868.mm.2	Ap1m2	adaptor protein complex AP-1, mu 2 subunit	-0.867060916049099	4,92867E+14	-2,05611E+14	0.082373715068339	0.225727525234718	-4,30481E+14	4,37274E+14	4,11361E+14	4,91139E+13	5,95381E+14	4,97647E+14	5,244E+14
TC0300000223.mm.2	Zfp639	zinc finger protein 639	1,18747E+14	6,32659E+14	2,05607E+14	0.082377651123459	0.225727525234718	-4,30485E+13	7,30446E+14	7,25869E+14	6,4388E+13	4,76938E+14	6,42544E+14	5,76276E+14
TC1500001506.mm.2	Rad21	RAD21 cohesin complex component	0.901663091176566	8,40272E+14	2,05599E+14	0.0823871144979442	0.225727525234718	-4,30496E+13	8,92899E+14	9,14895E+14	8,52835E+14	7,53539E+14	8,44451E+14	7,8301E+14
TC1700000346.mm.2	Nthl1	nth (endonuclease III)-like 1 (E.coli)	-0.882035555604793	6,73328E+14	-2,05584E+13	0.0824051225394872	0.22573848002019	-4,30516E+14	6,22054E+13	6,16893E+14	6,42515E+14	7,4315E+14	6,80738E+14	7,34615E+14
TC1500000308.mm.2	Dcstamp	dendrocyte expressed seven transmembrane protein	-1,10949E+14	4,68407E+14	-2,0555E+14	0.0824449242238198	0.225744078613073	-4,3056E+14	3,95328E+14	3,72258E+13	4,49131E+14	6,0424E+14	5,18525E+14	4,7096E+14
TC0200004599.mm.2	Il1a	interleukin 1 alpha	-0.748243257041308	4,94765E+14	-2,05547E+14	0.0824489298460317	0.225744078613073	-4,30564E+14	4,54512E+14	4,83038E+14	4,67961E+13	4,75881E+14	5,06354E+14	5,80845E+14
TC1600000765.mm.2	Cldnd1	claudin domain containing 1	1,59388E+14	8,39878E+14	2,05535E+14	0.0824624725529671	0.225744078613073	-4,30579E+14	9,49512E+14	9,56083E+14	8,86499E+14	6,60352E+14	8,55741E+14	7,31079E+14
TC1100002553.mm.2	Gabrg2	gamma-aminobutyric acid (GABA) A receptor, subunit gamma 2	1,70449E+14	9,07152E+14	2,05514E+14	0.0824873335680295	0.225744078613073	-4,30607E+13	1,03013E+14	1,01831E+14	9,5693E+14	7,29999E+14	9,34725E+13	7,72818E+14
TC1400001331.mm.2	Clybl	citrate lyase beta like	0.783140077625503	7,18428E+14	2,0551E+13	0.0824919100329137	0.225744078613073	-4,30612E+14	7,74315E+14	7,69013E+14	7,22101E+14	6,58439E+14	7,24885E+13	6,61815E+14
TC0900001834.mm.2	Zfp560	zinc finger protein 560	0.759557182126649	5,90101E+14	2,05507E+13	0.0824955488608895	0.225744078613073	-4,30616E+14	6,46227E+14	6,25404E+14	5,61955E+14	5,87302E+14	5,21535E+14	5,98185E+14
TC0300000236.mm.2	Fxr1	fragile X mental retardation gene 1, autosomal homolog	1,05978E+14	8,0653E+14	2,05499E+14	0.0825052367003283	0.225744078613073	-4,30627E+14	8,72406E+14	8,79363E+14	8,22322E+14	7,25277E+14	8,29324E+14	7,10488E+14
TC1800001749.mm.2	Ppp2r2b	protein phosphatase 2, regulatory subunit B, beta	1,24898E+14	8,43445E+14	2,05485E+14	0.0825219822999938	0.225746236460639	-4,30646E+14	9,21489E+14	9,39512E+13	8,74638E+13	7,13826E+14	8,57353E+14	7,53852E+14
TC1900001005.mm.2	Ppp2r5b	protein phosphatase 2, regulatory subunit B', beta	0.724223966926471	7,99555E+14	2,05466E+14	0.0825437632877343	0.225746236460639	-4,3067E+14	8,6929E+14	8,31492E+14	8,01631E+14	7,38979E+14	7,79321E+14	7,76616E+14
TC0300002972.mm.2	Npnt	nephronectin	0.723641702629215	6,06272E+14	2,05451E+14	0.0825612645755563	0.225746236460639	-4,30689E+14	6,28304E+14	6,71909E+14	5,92528E+14	5,89405E+14	6,07011E+14	5,48473E+14
TC0300001232.mm.2	Cnn3	calponin 3, acidic	1,48897E+14	7,69709E+14	2,0545E+14	0.0825620661244056	0.225746236460639	-4,3069E+14	8,59469E+14	9,03212E+14	8,05649E+13	5,85035E+14	7,72291E+14	6,92597E+14
TC0900000844.mm.2	Mtfmt	mitochondrial methionyl-tRNA formyltransferase	-0.676901072990239	7,53193E+14	-2,05433E+13	0.0825822786724829	0.225763192441418	-4,30713E+14	7,23803E+14	7,20349E+14	7,42996E+14	7,52477E+13	8,18243E+14	7,61288E+14
TC0400001822.mm.2	Lrrc38	leucine rich repeat containing 38	-0.650342787301215	5,02825E+13	-2,05398E+14	0.0826238541848799	0.225838534772005	-4,30759E+14	4,40813E+14	4,5598E+14	5,39246E+14	5,54052E+14	4,92062E+14	5,348E+13
TC0X00002266.mm.2	Mmgt1/Mir5116	membrane magnesium transporter 1/microRNA 5116	1,44578E+14	5,73129E+14	2,05368E+14	0.0826590550921707	0.225881392689929	-4,30798E+14	6,47004E+14	6,65975E+14	6,12248E+14	4,89725E+14	6,09061E+14	4,14763E+14
TC0500000690.mm.2	Spata18	spermatogenesis associated 18	0.696014904595272	5,51073E+14	2,05352E+14	0.0826778922694785	0.225881392689929	-4,30819E+14	5,76266E+14	6,33299E+14	5,10183E+14	5,1633E+14	5,20463E+12	5,49899E+14
TC0100000471.mm.2	Abi2	abl-interactor 2	0.890698371173714	9,30972E+14	2,05349E+14	0.0826815896533979	0.225881392689929	-4,30823E+13	9,93581E+14	9,88374E+14	9,42299E+14	8,57761E+14	9,41924E+14	8,61891E+13
TC0200001032.mm.2	Cybrd1	cytochrome b reductase 1	1,20843E+14	5,35025E+14	2,05326E+14	0.0827085540804612	0.225916754008895	-4,30853E+14	5,28998E+14	6,59579E+14	5,85062E+13	4,89616E+14	4,29372E+14	5,1752E+14
TC0400004178.mm.2	Dnajc25	DnaJ heat shock protein family (Hsp40) member C25	-0.819813638877389	6,28662E+14	-2,05304E+14	0.0827352049214099	0.225928544838343	-4,30883E+14	5,70839E+13	5,73174E+14	6,26875E+14	6,93109E+14	6,91061E+14	6,16914E+14
TC0900000066.mm.2	Trpc6	transient receptor potential cation channel, subfamily C, member 6	0.824698549034729	5,1061E+14	2,05299E+14	0.0827409137399034	0.225928544838343	-4,30889E+14	5,76255E+14	5,26918E+14	5,11922E+14	5,10333E+14	5,1299E+14	4,25243E+12
TC1600001199.mm.2	Tnp2	transition protein 2	-0.922940534824858	5,00925E+14	-2,05259E+14	0.0827879311120045	0.226018626568575	-4,30941E+14	4,5578E+14	4,46066E+14	4,55497E+14	5,61774E+14	5,11609E+14	5,74826E+14
TC0200002237.mm.2	Gm14151/Gm14147	predicted gene 14151/predicted gene 14147	-0.749735160708093	6,14366E+14	-2,05237E+14	0.0828134664055058	0.226050039673372	-4,30969E+14	5,55642E+14	5,79768E+14	5,97117E+14	6,68311E+14	6,27726E+14	6,5763E+14
TC1000001148.mm.2	Ndufa12	NADH:ubiquinone oxidoreductase subunit A12	0.74040753261837	9,66151E+14	2,05195E+14	0.0828628552885237	0.226118361272674	-4,31024E+14	1,01385E+14	1,01629E+14	9,68348E+14	9,16365E+13	9,70265E+14	9,1179E+14
TC0800000294.mm.2	Nkx6-3	NK6 homeobox 3	-0.818648884868735	6,77374E+14	-2,05182E+14	0.082878588559555	0.226118361272674	-4,31041E+14	6,24864E+14	6,22757E+14	6,53387E+14	7,51888E+14	6,99568E+14	7,11782E+14
TC0700004667.mm.2	Ctbp2	C-terminal binding protein 2	0.712931783425606	7,43731E+14	2,0518E+14	0.0828805958962585	0.226118361272674	-4,31044E+14	8,22615E+14	7,56382E+14	7,34575E+14	7,12401E+14	7,15401E+14	7,2101E+14
TC1600001573.mm.2	Cstdc5/Cstdc6	cystatin domain containing 5/cystatin domain containing 6	-0.822699229216067	5,04213E+14	-2,0509E+12	0.0829877809976496	0.22633116307132	-4,31162E+14	4,28669E+14	4,49515E+14	5,0326E+14	6,0111E+14	5,25505E+14	5,17219E+14
TC1100000555.mm.2	Olfr1377	olfactory receptor 1377	-0.950138262200945	4,58985E+14	-2,05075E+14	0.0830050405707979	0.22633116307132	-4,31181E+14	4,11882E+14	3,55256E+14	5,09064E+14	5,20544E+14	4,20162E+14	5,37004E+14
TC1100002195.mm.2	Igfbp3	insulin-like growth factor binding protein 3	-0.641933274268349	5,95928E+14	-2,05068E+14	0.0830129818633149	0.22633116307132	-4,3119E+14	5,47678E+14	5,92199E+14	5,83346E+13	5,84081E+14	6,33364E+14	6,349E+14
TC0200002329.mm.2	Bpifb3	BPI fold containing family B, member 3	-0.598049679047023	5,54919E+14	-2,05067E+14	0.0830147814653697	0.22633116307132	-4,31192E+14	5,08224E+13	5,17477E+14	5,76637E+14	5,81869E+14	5,65446E+14	5,79865E+14
TC0500001221.mm.2	Acacb	acetyl-Coenzyme A carboxylase beta	-1,05444E+13	7,42999E+12	-2,05048E+14	0.0830367965152985	0.226352884833531	-4,31216E+14	6,9273E+14	6,57291E+14	7,01631E+14	8,45436E+14	7,61366E+14	7,99543E+14
TC1300002247.mm.2	Zfp459	zinc finger protein 459	0.67143365675268	5,23671E+14	2,05015E+14	0.0830765888248358	0.226406137094075	-4,3126E+14	5,67093E+14	5,54486E+14	5,22538E+14	5,10618E+14	5,20461E+14	4,66831E+14
TC0900002991.mm.2	Atp2c1	ATPase, Ca++-sequestering	1,53178E+14	7,06868E+14	2,05001E+14	0.0830927563444635	0.226406137094075	-4,31278E+14	8,1309E+14	8,41013E+14	7,27152E+14	5,12208E+14	7,33474E+14	6,14272E+14
TC1000001171.mm.2	Eea1	early endosome antigen 1	1,53071E+14	6,35823E+14	2,04996E+14	0.0830984853704686	0.226406137094075	-4,31285E+14	7,54206E+13	7,20735E+14	6,72167E+14	4,99031E+14	6,70996E+14	4,97801E+14
TC1800000884.mm.2	Zadh2	zinc binding alcohol dehydrogenase, domain containing 2	-0.713512448605521	6,92769E+14	-2,0497E+14	0.0831295131906373	0.226445207275539	-4,31319E+14	6,39223E+14	6,76743E+14	6,66387E+14	7,15594E+14	7,12467E+14	7,46203E+14
TC0400002945.mm.2	Ttc39b	tetratricopeptide repeat domain 39B	0.89989546093036	7,59242E+14	2,04952E+12	0.0831506819113686	0.226445207275539	-4,31342E+14	8,20412E+14	7,98117E+14	7,62799E+13	7,35575E+14	7,7615E+14	6,624E+14
TC0900002149.mm.2	Cbl	Casitas B-lineage lymphoma	1,26695E+14	7,10815E+14	2,04949E+14	0.08315498612607	0.226445207275539	-4,31347E+14	8,27015E+14	7,97798E+13	7,14197E+14	5,54458E+14	7,40851E+14	6,30573E+14
TC0900002186.mm.2	Ube4a	ubiquitination factor E4A	0.971882106216967	7,31755E+14	2,04922E+14	0.0831860886164147	0.226491626542124	-4,31381E+14	8,08379E+14	8,01639E+14	7,4297E+14	6,21899E+14	7,37853E+14	6,77789E+14

TC1500000612.mm.2	Zfp7	zinc finger protein 7	-0.78797003794149	6,13938E+14	-2,04889E+14	0.0832258991882081	0.226540541655006	-4,31425E+14	5,57379E+13	5,66721E+14	6,12964E+14	6,64869E+14	6,03353E+14	6,78342E+13
TC1600001248.mm.2	Fopnl	Fgfr1op N-terminal like	0.699881639323908	5,42066E+14	2,04865E+14	0.0832540152670911	0.226540541655006	-4,31456E+14	5,83052E+14	5,85308E+14	5,42684E+14	5,12968E+12	4,84591E+14	5,43792E+14
TC0600003185.mm.2	Tspan9	tetraspanin 9	0.612272783560573	7,86272E+13	2,04848E+13	0.0832745050112734	0.226540541655006	-4,31479E+14	8,0572E+14	8,17874E+13	8,00087E+13	7,92812E+14	7,37615E+14	7,63525E+14
TC1400002189.mm.2	Kpna3	karyopherin (importin) alpha 3	1,79052E+14	7,26443E+14	2,04839E+14	0.0832855406143904	0.226540541655006	-4,31491E+14	8,56569E+14	8,55209E+14	7,72899E+14	5,2031E+14	7,54052E+14	5,99622E+14
TC0400000177.mm.2	Ccnc	cyclin C	1,93568E+14	6,22688E+14	2,04829E+14	0.0832974002838783	0.226540541655006	-4,31504E+14	7,80719E+14	7,58264E+14	6,6202E+14	3,83277E+14	6,5661E+14	4,95237E+14
TC1200001235.mm.2	Lbhd2	LBH domain containing 2	-0.907028022616471	8,14371E+14	-2,04819E+14	0.0833092862305505	0.226540541655006	-4,31517E+14	7,74664E+14	7,18757E+14	8,28894E+14	8,89085E+14	7,93096E+14	8,8173E+14
TC1100001204.mm.2	Zfp207	zinc finger protein 207	1,36936E+14	8,68726E+14	2,04807E+14	0.0833229960126358	0.226540541655006	-4,31532E+14	9,44243E+14	9,73483E+14	9,07404E+14	7,43372E+14	8,91057E+14	7,52796E+13
TC0200004410.mm.2	Gpr176	G protein-coupled receptor 176	0.896959566909302	5,34279E+14	2,04801E+14	0.0833299386200372	0.226540541655006	-4,3154E+14	6,36082E+14	5,21354E+14	5,28333E+14	5,41863E+14	4,75731E+14	5,02313E+14
TC1100004304.mm.2	Mafg	v-maf musculoaponeurotic fibrosarcoma oncogene family, protein G (avian)	-1,15661E+14	7,26467E+14	-2,04789E+14	0.0833447446482718	0.226540541655006	-4,31556E+14	6,21977E+14	6,71455E+14	6,94792E+14	8,45824E+14	7,265E+14	7,98254E+14
TC0900002908.mm.2	Mrps22	mitochondrial ribosomal protein S22	0.732151355420732	6,47465E+14	2,04777E+14	0.0833584663388504	0.226540541655006	-4,31571E+14	7,17071E+14	6,52344E+14	6,36221E+14	6,56168E+14	6,29068E+14	5,93917E+14
TC1100001649.mm.2	Psme3	proteaseome (prosome, macropain) activator subunit 3 (PA28 gamma, Ki)	1,47864E+14	7,86769E+12	2,04777E+14	0.0833587085876331	0.226540541655006	-4,31571E+14	8,89035E+14	8,76858E+14	8,3703E+14	6,47528E+14	8,06463E+14	6,63702E+14
TC1600000031.mm.2	Glis2	GLIS family zinc finger 2	-0.71120597335978	6,88986E+14	-2,04758E+14	0.0833809717994866	0.226548211008473	-4,31596E+14	6,38756E+14	6,34088E+14	6,89954E+14	7,56031E+14	6,96242E+14	7,18843E+14
TC1400002341.mm.2	Polr3d	polymerase (RNA) III (DNA directed) polypeptide D	0.696873182766947	6,28487E+14	2,04751E+14	0.0833896505611155	0.226548211008473	-4,31605E+14	6,67925E+14	6,64462E+14	6,41139E+14	6,04383E+14	6,23514E+14	5,69499E+14
TC0800002188.mm.2	Tenm3	teneurin transmembrane protein 3	0.662423241607227	8,27397E+14	2,04738E+14	0.0834055233454028	0.226553135142359	-4,31623E+14	8,78583E+14	8,63596E+14	8,19155E+14	7,93352E+14	7,82737E+14	8,26956E+14
TC1000000776.mm.2	Dnmt3l	DNA (cytosine-5-)-methyltransferase 3-like	-1,12031E+13	6,10157E+14	-2,047E+14	0.0834507524014653	0.226637784163966	-4,31673E+14	5,52956E+14	5,37464E+14	5,45776E+14	7,10264E+14	6,44896E+14	6,69586E+14
TC0800002410.mm.2	Ccdc124	coiled-coil domain containing 124	-0.719297271678675	8,19781E+14	-2,04648E+12	0.0835118868435178	0.226653737773795	-4,3174E+14	7,8613E+14	7,67823E+14	8,05098E+14	8,61822E+14	8,28182E+14	8,69631E+14
TC1000002611.mm.2	Prdm4	PR domain containing 4	-0.672454493191036	8,00945E+14	-2,04639E+14	0.0835228618100926	0.226653737773795	-4,31752E+14	7,51168E+14	7,54972E+14	8,55024E+14	8,03877E+14	8,3296E+14	8,07671E+14
TC0600000370.mm.2	Tmem213	transmembrane protein 213	-0.919861383731801	6,65251E+14	-2,04635E+14	0.0835274559277794	0.226653737773795	-4,31757E+14	5,99752E+14	6,09614E+14	6,5958E+14	7,29221E+14	6,45623E+14	7,47716E+14
TC0200000549.mm.2	Ncs1	neuronal calcium sensor 1	0.838118766095779	9,11569E+14	2,0462E+14	0.0835456539792031	0.226653737773795	-4,31777E+14	9,78081E+14	9,57671E+14	9,26864E+14	8,38672E+14	9,10357E+14	8,57771E+14
TC0400001581.mm.2	Taf12	TATA-box binding protein associated factor 12	0.664361576624419	6,94769E+14	2,04613E+14	0.0835537206554827	0.226653737773795	-4,31786E+14	7,40555E+14	7,41288E+14	6,9055E+14	6,47249E+14	6,90808E+14	6,58163E+14
TC0700003874.mm.2	Olfr575	olfactory receptor 575	-1,48303E+14	4,84336E+14	-2,04611E+14	0.0835557419444161	0.226653737773795	-4,31788E+13	3,72066E+14	4,00891E+13	4,14711E+14	6,48786E+14	5,16575E+14	5,52988E+14
TC0400001614.mm.2	Gpatch3	G patch domain containing 3	-0.710056299582819	6,95271E+14	-2,04609E+14	0.0835585419566999	0.226653737773795	-4,31791E+14	6,49096E+14	6,4488E+14	7,10014E+14	7,31649E+14	6,84883E+14	7,51104E+14
TC1100003696.mm.2	Ccr7	chemokine (C-C motif) receptor 7	-0.595228649036757	6,94727E+14	-2,04591E+14	0.0835803836289303	0.226653737773795	-4,31815E+14	6,50425E+14	6,42766E+14	7,34324E+14	7,28611E+14	6,99464E+14	7,12774E+14
TC1000002420.mm.2	Adarb1	adenosine deaminase, RNA-specific, B1	0.766526496530924	8,32209E+14	2,04578E+14	0.0835955016605931	0.226653737773795	-4,31832E+13	8,92451E+14	8,87356E+14	8,29392E+14	7,57555E+14	8,36977E+14	7,89525E+14
TC1500002168.mm.2	Olfr286	olfactory receptor 286	-0.909048525706535	4,72394E+14	-2,04575E+14	0.0835991516882628	0.226653737773795	-4,31836E+14	4,03472E+14	4,11216E+14	4,70303E+14	5,52875E+14	4,5381E+14	5,42688E+14
TC0600000691.mm.2	Vmn1r15	vomeronasal 1 receptor 15	-0.923518511258705	5,03265E+13	-2,04565E+14	0.0836113583645155	0.226653737773795	-4,31849E+14	4,68229E+14	4,25259E+14	4,72656E+14	5,75254E+14	5,10713E+14	5,67479E+14
TC0700003618.mm.2	Btbd1	BTB (POZ) domain containing 1	1,4354E+14	8,16591E+14	2,04506E+14	0.0836818518115388	0.22680667422024	-4,31926E+13	9,24257E+14	9,16718E+14	8,53085E+14	6,51592E+13	8,31917E+14	7,21978E+14
TC0500001912.mm.2	Steap2	six transmembrane epithelial antigen of prostate 2	0.872899667522643	6,34032E+14	2,04472E+14	0.0837217375558526	0.226844439142181	-4,3197E+14	7,01163E+14	6,58864E+14	5,97662E+14	6,61055E+14	6,34597E+14	5,5085E+14
TC0800002730.mm.2	Pllp	plasma membrane proteolipid	-0.599405434870416	7,59969E+14	-2,04436E+14	0.0837646575235381	0.226844439142181	-4,32017E+12	7,32939E+14	7,24659E+14	7,7036E+14	7,54375E+14	7,83393E+14	7,94087E+14
TC0X00001067.mm.2	Atp7a	ATPase, Cu++ transporting, alpha polypeptide	1,06953E+14	5,37884E+14	2,04433E+14	0.0837684608114403	0.226844439142181	-4,32021E+14	5,70983E+13	6,3754E+14	4,67094E+14	5,57071E+14	5,52214E+14	4,42403E+14
TC0800000132.mm.2	Upf3a	UPF3 regulator of nonsense transcripts homolog A (yeast)	-0.734660385703062	6,83514E+14	-2,04433E+14	0.0837688257617513	0.226844439142181	-4,32022E+14	6,30838E+14	6,46281E+14	6,65356E+14	7,34558E+14	6,96498E+14	7,27553E+14
TC0400001292.mm.2	Eri3	exoribonuclease 3	1,00321E+13	8,28775E+14	2,04415E+13	0.0837899272902221	0.226844439142181	-4,32045E+13	9,09979E+14	8,98171E+14	8,365E+14	7,20491E+14	8,4487E+14	7,62638E+14
TC1000002538.mm.2	Hmg20b	high mobility group 20B	-0.709576112058091	7,18377E+14	-2,04414E+14	0.0837912196337228	0.226844439142181	-4,32046E+14	6,55808E+14	6,77441E+14	7,18084E+14	7,83762E+14	7,32632E+14	7,42533E+14
TC1500001171.mm.2	Egflam	EGF-like, fibronectin type III and laminin G domains	-0.967455515967225	4,53003E+14	-2,04411E+14	0.0837943338778787	0.226844439142181	-4,3205E+14	3,98791E+13	3,80573E+14	4,2247E+14	5,43328E+14	4,57909E+14	5,14946E+14
TC0400001557.mm.2	Laptm5	lysosomal-associated protein transmembrane 5	1,15236E+14	8,06402E+14	2,04399E+14	0.0838093755073677	0.226847046455605	-4,32066E+14	8,77992E+14	9,09057E+14	8,2993E+14	6,64858E+14	8,00916E+14	7,5566E+14
TC1100001550.mm.2	Lasp1	LIM and SH3 protein 1	0.97721694869225	9,74163E+14	2,0438E+14	0.0838319609650527	0.226870068362428	-4,32091E+14	1,04233E+14	1,03441E+14	9,99781E+14	8,87165E+14	9,79506E+14	9,01788E+14
TC1100003738.mm.2	Krtap4-13/Gm11554/Gm11569	keratin associated protein 4-13/predicted gene 11554/predicted gene 11569	-0.666894595304301	6,19269E+14	-2,04358E+14	0.0838580235801942	0.226902491007165	-4,32119E+14	5,73817E+14	5,89507E+14	6,07891E+14	6,47695E+14	6,30686E+14	6,66017E+14
TC0500000188.mm.2	Lhfpl3	lipoma HMGIC fusion partner-like 3	1,6023E+14	7,68736E+14	2,04324E+13	0.0838987519697739	0.226974578658322	-4,32164E+13	8,97957E+13	8,54447E+14	8,17977E+14	6,10092E+14	7,924E+14	6,39544E+14
TC1300002416.mm.2	Zcchc9	zinc finger, CCHC domain containing 9	-0.63079463006768	6,91513E+13	-2,04284E+14	0.083947119988439	0.22700034944706	-4,32217E+14	6,5505E+14	6,67555E+14	7,01806E+14	6,75905E+14	7,10083E+14	7,38681E+14
TC1000001580.mm.2	Naca	nascent polypeptide-associated complex alpha polypeptide	0.970020467033212	9,4083E+14	2,0428E+13	0.0839514771217156	0.22700034944706	-4,32221E+14	1,00961E+14	1,00356E+14	9,6517E+13	8,47483E+14	9,43103E+14	8,76057E+14
TC1600000054.mm.2	Rbfox1	RNA binding protein, fox-1 homolog (C. elegans) 1	1,19253E+13	8,99609E+14	2,04276E+14	0.083956910246595	0.22700034944706	-4,32227E+14	9,77904E+14	9,77305E+14	9,34661E+13	7,91083E+14	9,14475E+14	8,02228E+14
TC0100001909.mm.2	Mrpl15	mitochondrial ribosomal protein L15	1,1201E+14	6,46255E+14	2,0426E+14	0.0839752913582825	0.22700034944706	-4,32247E+13	7,15516E+14	7,13706E+14	6,9247E+13	5,50634E+14	6,47145E+14	5,58058E+14
TC1700002320.mm.2	Ticam1	toll-like receptor adaptor molecule 1	0.700340254363597	5,67414E+14	2,04248E+14	0.0839902564759402	0.22700034944706	-4,32264E+14	5,79608E+14	6,26215E+14	5,64148E+13	5,68758E+14	5,56908E+13	5,08847E+13
TC1500001981.mm.2	Efcab6	EF-hand calcium binding domain 6	-0.773885590357204	7,00686E+13	-2,04246E+14	0.0839928060201932	0.22700034944706	-4,32266E+14	6,40934E+14	6,68193E+14	6,74166E+13	7,56921E+14	7,23967E+14	7,39937E+14
TC1300000607.mm.2	Gadd45g	growth arrest and DNA-damage-inducible 45 gamma	0.903929448141595	6,2609E+14	2,04171E+14	0.0840818009201246	0.227202760058019	-4,32364E+13	6,74464E+14	7,30367E+14	6,13436E+14	5,14226E+14	5,91872E+14	6,32173E+14
TC0800002529.mm.2	Gab1	growth factor receptor bound protein 2-associated protein 1	1,28408E+14	6,06839E+14	2,04154E+14	0.084102317031905	0.227220092946862	-4,32386E+14	7,03163E+14	6,62775E+14	6,74345E+13	4,91629E+14	5,90778E+13	5,18342E+14
TC0X00000682.mm.2	Zfp275	zinc finger protein 275	0.765494368526433	6,55685E+14	2,0411E+14	0.0841551871013411	0.227263939044243	-4,32444E+14	7,26804E+14	6,7254E+13	6,72737E+14	6,15786E+14	6,4104E+14	6,05205E+14
TC1000001560.mm.2	Arhgap9	Rho GTPase activating protein 9	-0.764763209611838	5,10747E+14	-2,04107E+14	0.0841588502763338	0.227263939044243	-4,32448E+14	4,3208E+14	4,58411E+14	5,26903E+14	6,03643E+14	5,07093E+14	5,36351E+13
TC0700003060.mm.2	Mrgpra4	MAS-related GPR, member A4	-0.750723692153426	5,02953E+14	-2,04102E+14	0.0841646570688909	0.227263939044243	-4,32454E+13	4,5842E+14	4,38322E+14	4,99374E+14	5,74716E+14	5,10197E+14	5,36689E+14
TC0700001951.mm.2	Dock1	dedicator of cytokinesis 1	0.621952389572267	5,71642E+14	2,04086E+14	0.0841840096087243	0.227263939044243	-4,32475E+14	6,09782E+14	6,24977E+14	5,41359E+14	5,43363E+14	5,38663E+14	5,71707E+14
TC0600001322.mm.2	Hnrnpf	heterogeneous nuclear ribonucleoprotein F	1,2943E+14	7,76329E+14	2,04082E+14	0.0841890679671747	0.227263939044243	-4,3248E+14	8,55721E+14	8,86571E+14	7,94412E+14	6,37835E+14	8,04256E+14	6,79177E+14
TC0700001524.mm.2	Olfr652	olfactory receptor 652	-0.976558872220785	4,97244E+14	-2,04049E+14	0.0842284433322457	0.227332145295222	-4,32523E+13	4,17354E+14	4,78785E+14	4,42329E+14	5,53544E+14	5,19752E+14	5,71699E+14
TC0900002288.mm.2	1810046K07Rik	RIKEN cDNA 1810046K07 gene	-0.686164835220809	5,98314E+14	-2,04034E+14	0.0842460524672588	0.227341591593525	-4,32543E+14	5,51928E+14	5,5114E+14	5,9698E+14	6,49531E+14	6,0308E+14	6,37222E+14
TC0X00001682.mm.2	Gpm6b	glycoprotein m6b	1,18705E+14	1,0784E+14	2,03979E+14	0.0843126865798726	0.227483308583638	-4,32615E+14	1,16539E+14	1,17566E+14	1,08769E+14	9,38025E+14	1,10289E+14	1,00075E+14
TC1500001431.mm.2	Lrp12	low density lipoprotein-related protein 12	0.89645721682685	6,97093E+14	2,03928E+14	0.0843744119602036	0.227604535983157	-4,32682E+14	7,58134E+14	7,64642E+14	7,05869E+14	6,10425E+14	7,07394E+14	6,36091E+14
TC0X00000760.mm.2	Cldn34b4	claudin 34B4	-0.717059063995235	4,84263E+14	-2,03906E+14	0.084399943769097	0.227604535983157	-4,3271E+14	4,18495E+14	4,31681E+14	4,97059E+14	5,64754E+14	5,08312E+14	4,85276E+14
TC0300002358.mm.2	Atp8b2	ATPase, class I, type 8B, member 2	-0.703004560298129	7,54143E+13	-2,03895E+14	0.08441391823138	0.227604535983157	-4,32725E+14	6,90634E+14	7,04E+14	7,72537E+14	8,22455E+14	7,89984E+14	7,45251E+14
TC1400000319.mm.2	Glt8d1	glycosyltransferase 8 domain containing 1	1,00421E+14	5,69774E+14	2,03895E+14	0.0844141194709455	0.227604535983157	-4,32725E+14	6,42373E+14	6,08423E+14	6,19504E+14	4,98392E+14	5,5947E+14	4,90484E+14
TC0700002445.mm.2	Hif3a	hypoxia inducible factor 3, alpha subunit	-0.645996500615056	5,98283E+14	-2,03819E+14	0.0845056074354149	0.227793189030925	-4,32825E+14	5,41881E+14	5,78496E+14	6,09276E+14	6,10469E+14	6,09297E+13	6,4028E+13
TC1400002090.mm.2	Emc9	ER membrane protein complex subunit 9	0.632061704837606	8,11171E+14	2,03813E+14	0.0845123616971929	0.227793189030925	-4,32832E+14	8,66538E+14	8,29985E+14	8,09592E+14	7,90802E+14	7,81115E+14	7,88995E+14
TC0500001327.mm.2	Tbx3	T-box 3	-0.919625707668239	5,82396E+14	-2,03781E+14	0.0845512531289086	0.22785989992743	-4,32874E+14	5,21839E+14	4,95991E+14	5,77159E+14	6,97635E+13	5,90976E+14	6,10779E+14
TC1300001827.mm.2	Ssr1	signal sequence receptor, alpha	1,04855E+14	8,61233E+14	2,03752E+14	0.0845865840881842	0.227907706520687	-4,32912E+14	9,38648E+14	9,42598E+14	8,65171E+13	7,49445E+14	8,85856E+14	7,85679E+14

TC1000000636.mm.2	Egr2	early growth response 2	-0.644148628858451	7,58971E+14	-2,03705E+14	0.0846432034822634	0.227907706520687	-4,32974E+14	7,14955E+14	7,28041E+14	7,83598E+14	7,55407E+14	7,68047E+14	8,03777E+14
TC0800000882.mm.2	Otud4	OTU domain containing 4	1,44765E+14	6,14596E+13	2,03696E+14	0.0846541149474686	0.227907706520687	-4,32985E+14	6,83518E+14	6,93187E+14	6,96249E+14	5,27447E+14	6,23829E+13	4,63344E+14
TC1100000973.mm.2	Rpain	RPA interacting protein	-1,05472E+14	7,05149E+14	-2,03694E+14	0.0846559117121455	0.227907706520687	-4,32987E+14	6,1739E+14	6,35699E+14	6,83331E+14	8,30442E+14	7,10291E+14	7,53741E+13
TC0200001234.mm.2	Aplnr	apelin receptor	-0.760870161890645	4,87059E+14	-2,03691E+14	0.0846600788479766	0.227907706520687	-4,32992E+14	4,61157E+14	4,09512E+14	5,07615E+14	5,21227E+14	4,77972E+14	5,44871E+14
TC0900001647.mm.2	Zkscan7	zinc finger with KRAB and SCAN domains 7	-0.830452909963715	6,18852E+14	-2,03688E+14	0.0846633623938303	0.227907706520687	-4,32996E+14	5,64034E+14	5,69092E+14	6,11372E+14	6,69401E+14	6,06689E+14	6,92527E+14
TC0800002395.mm.2	Rex1bd	required for excision 1-B domain containing	-0.65737317761737	8,19224E+14	-2,03684E+14	0.0846680029313493	0.227907706520687	-4,33001E+13	7,89649E+14	7,90272E+13	7,8726E+14	8,36765E+13	8,46647E+14	8,64748E+14
TC1800001252.mm.2	Grxcr2	glutaredoxin, cysteine rich 2	-0.811482414521011	6,25268E+14	-2,0367E+11	0.084685625903515	0.227917068679361	-4,3302E+14	5,72067E+14	5,32881E+14	6,44346E+14	7,35067E+14	6,30739E+14	6,36505E+14
TC1400001188.mm.2	Scel	sciellin	-0.597266175089606	5,15227E+14	-2,03643E+14	0.0847173026160264	0.227948906866394	-4,33054E+14	4,82213E+14	4,73024E+14	5,2341E+14	5,38025E+14	5,31276E+14	5,43414E+14
TC0300000843.mm.2	Flg2	filaggrin family member 2	-1,05705E+14	4,74894E+14	-2,03636E+14	0.084725749594913	0.227948906866394	-4,33063E+14	4,21287E+14	4,19202E+13	4,26956E+14	5,3002E+14	4,68047E+13	5,83853E+14
TC0500003417.mm.2	Sh2b2	SH2B adaptor protein 2	-0.668578471014648	6,51952E+14	-2,03552E+14	0.0848277746804906	0.228140466182165	-4,33173E+14	5,90273E+12	6,29676E+14	6,4998E+13	6,88123E+14	6,68927E+14	6,84737E+13
TC1100001078.mm.2	Crk	v-crk avian sarcoma virus CT10 oncogene homolog	-0.606555763166272	9,17057E+14	-2,03552E+14	0.0848280055787518	0.228140466182165	-4,33174E+14	8,77366E+14	8,78675E+13	9,21811E+14	9,47134E+14	9,44991E+14	9,32362E+14
TC0500000487.mm.2	Clrn2	clarin 2	-0.89996446841728	5,16011E+14	-2,03542E+14	0.0848394261381202	0.228140466182165	-4,33186E+14	4,4322E+14	4,75696E+14	4,81232E+13	5,97008E+14	5,45357E+14	5,53552E+14
TC1800000300.mm.2	Reep2	receptor accessory protein 2	1,3663E+14	8,17516E+14	2,03506E+14	0.0848837472110863	0.228221561623933	-4,33234E+14	9,16306E+14	8,82871E+14	8,88592E+14	6,91409E+14	8,05843E+14	7,20075E+14
TC1900001398.mm.2	Atad1	ATPase family, AAA domain containing 1	2,47896E+14	7,08778E+12	2,03491E+14	0.0849017179160375	0.228231795258778	-4,33253E+14	8,83357E+13	8,75606E+14	7,74627E+13	4,55903E+14	7,7177E+14	4,91401E+14
TC1400001104.mm.2	Kbtbd6	kelch repeat and BTB (POZ) domain containing 6	-0.830368545308321	5,15906E+14	-2,03471E+14	0.0849257163144958	0.228258226351205	-4,33279E+14	4,63076E+14	4,83661E+14	4,86004E+14	5,49887E+14	5,16727E+14	5,96084E+14
TC0200002990.mm.2	Hacd1	3-hydroxyacyl-CoA dehydratase 1	-1,32133E+14	1,01375E+14	-2,0345E+14	0.0849510129001959	0.228258834716366	-4,33306E+14	9,25369E+14	9,29883E+14	1,14688E+14	9,60859E+14	1,10721E+14	1,01231E+14
TC0400000386.mm.2	Gm3893	family with sequence similarity 205 pseudogene	0.951712809846653	8,54176E+14	2,03447E+14	0.084954274920502	0.228258834716366	-4,3331E+13	9,09006E+13	9,40876E+14	7,91154E+14	8,24479E+14	8,91819E+14	7,67721E+14
TC1500001511.mm.2	Ext1	exostoses (multiple) 1	0.884780344228921	7,66854E+14	2,0343E+14	0.0849750999290506	0.228276723100499	-4,33332E+14	8,12473E+14	8,40636E+14	7,79268E+14	6,92592E+14	7,74838E+14	7,01315E+14
TC0900003009.mm.2	Abhd14a	abhydrolase domain containing 14A	-0.646199775094944	7,07544E+14	-2,03403E+14	0.0850083889970085	0.228328083332022	-4,33368E+14	6,49623E+14	6,91814E+14	7,05549E+14	7,27604E+14	7,40252E+14	7,30425E+14
TC0500003087.mm.2	Rpl37rt	ribosomal protein L37, retrotransposed	0.60072937819438	9,44195E+13	2,0338E+14	0.0850359318719874	0.228331053882301	-4,33398E+14	9,9046E+14	9,79686E+14	9,28763E+14	9,16262E+14	9,34092E+14	9,15908E+14
TC0200003923.mm.2	Olfr1048	olfactory receptor 1048	-0.889305274981917	4,59458E+14	-2,03378E+14	0.0850378361787184	0.228331053882301	-4,334E+14	4,11401E+14	4,10272E+14	4,24153E+14	5,11385E+14	4,59884E+14	5,3965E+14
TC0500002788.mm.2	Gm7978/BC061212	predicted gene 7978/cDNA sequence BC061212	-1,37617E+13	5,03152E+14	-2,03364E+14	0.0850557570068435	0.228341121734633	-4,33419E+14	4,06895E+14	4,25006E+14	4,43929E+14	6,35943E+14	5,00934E+14	6,06201E+14
TC0700000551.mm.2	Gm5113	predicted gene 5113	0.924592496716905	5,16978E+14	2,03326E+14	0.0851018245412953	0.228408151903205	-4,33469E+14	5,85262E+13	5,73128E+14	5,20779E+14	4,49224E+12	5,38481E+14	4,34991E+14
TC1400000950.mm.2	Gm5464	predicted gene 5464	-0.807213525632433	5,29845E+14	-2,03312E+14	0.0851181106513339	0.228408151903205	-4,33486E+14	4,87822E+14	5,06676E+14	4,87572E+14	5,41059E+14	5,37846E+14	6,18094E+14
TC1200000741.mm.2	Slc39a9	solute carrier family 39 (zinc transporter), member 9	0.687545394909051	7,68458E+14	2,03302E+14	0.085130565596222	0.228408151903205	-4,335E+14	8,10063E+14	8,04752E+13	7,70939E+14	7,47689E+14	7,68331E+14	7,08975E+14
TC1200001070.mm.2	Gskip	GSK3B interacting protein	0.761835726297969	6,94089E+14	2,03296E+14	0.0851374269428813	0.228408151903205	-4,33507E+13	7,73984E+14	7,32652E+14	6,81873E+14	6,21756E+14	6,54106E+14	7,00162E+14
TC0200000702.mm.2	Nek6	NIMA (never in mitosis gene a)-related expressed kinase 6	0.661206181583256	6,83884E+14	2,0325E+14	0.0851939682935615	0.228457822671143	-4,33568E+14	7,08876E+14	7,44958E+14	6,68982E+14	6,58896E+14	6,80732E+14	6,4086E+13
TC0200000603.mm.2	Angptl2	angiopoietin-like 2	-0.861588316554417	6,27649E+14	-2,0324E+14	0.085205579797935	0.228457822671143	-4,33581E+13	5,70744E+14	5,60005E+14	6,13755E+14	7,18323E+14	6,3252E+14	6,70546E+14
TC0200004213.mm.2	Trim44	tripartite motif-containing 44	1,23364E+14	7,88606E+14	2,03239E+14	0.0852074679988826	0.228457822671143	-4,33583E+14	8,73218E+14	8,58398E+14	8,43616E+14	6,71516E+14	7,84068E+14	7,00821E+14
TC0700003006.mm.2	Ftl1	ferritin light polypeptide 1	-1,0845E+14	1,14518E+14	-2,03234E+14	0.0852126552630527	0.228457822671143	-4,33588E+14	1,05576E+14	1,09682E+14	1,24074E+14	1,1083E+14	1,22591E+14	1,14357E+14
TC1000002445.mm.2	Trappc10	trafficking protein particle complex 10	0.786304345351924	7,50789E+14	2,03186E+14	0.0852712946183813	0.228539947943054	-4,33651E+14	8,07813E+14	8,21869E+14	7,46062E+14	6,56569E+14	7,46657E+14	7,25763E+14
TC1500001863.mm.2	Sox10	SRY (sex determining region Y)-box 10	0.661548573494319	6,53956E+14	2,03186E+14	0.0852716543805398	0.228539947943054	-4,33652E+14	6,99339E+14	7,01815E+14	6,47645E+14	6,06095E+14	6,52336E+14	6,16508E+14
TC0600002978.mm.2	Rassf4	Ras association (RalGDS/AF-6) domain family member 4	0.898166881570952	5,40171E+14	2,03162E+13	0.0853010261215387	0.228546770978253	-4,33683E+14	6,21252E+14	5,45753E+13	4,97931E+14	5,8872E+14	5,05693E+14	4,81679E+14
TC0400000304.mm.2	Akirin2	akirin 2	0.729317179981566	9,3825E+14	2,03161E+14	0.0853025681538641	0.228546770978253	-4,33685E+14	9,79525E+14	9,82833E+14	9,6333E+14	8,87315E+14	9,14716E+14	9,01778E+14
TC1000002897.mm.2	Rab21	RAB21, member RAS oncogene family	1,00756E+14	8,05733E+14	2,03135E+14	0.0853341241623571	0.228576690181217	-4,33719E+14	8,81472E+14	8,70167E+12	8,22069E+14	7,10563E+14	8,2106E+14	7,29067E+14
TC1300002314.mm.2	Ahrr	aryl-hydrocarbon receptor repressor	-0.801980970222509	7,25139E+13	-2,03117E+14	0.0853559519623669	0.228576690181217	-4,33742E+14	6,85521E+14	6,4753E+14	7,19145E+14	8,05191E+14	7,5882E+14	7,34627E+14
TC1600001602.mm.2	Nr1i2	nuclear receptor subfamily 1, group I, member 2	-0.982342683499161	4,32724E+14	-2,03116E+14	0.0853562927338411	0.228576690181217	-4,33743E+14	3,79279E+14	3,541E+14	4,08201E+14	5,24917E+14	4,28838E+13	5,0101E+14
TC1100003165.mm.2	Olfr1	olfactory receptor 1	-0.82273139429451	5,4771E+14	-2,03012E+14	0.0854830459623208	0.228661397447552	-4,33878E+14	4,86486E+14	4,84077E+14	5,43604E+14	6,36986E+14	5,46464E+14	5,88645E+14
TC0200005442.mm.2	Stmn3	stathmin-like 3	0.689160984362113	9,76806E+14	2,02993E+14	0.0855068394935142	0.228661397447552	-4,33904E+14	1,03235E+13	1,00271E+14	9,83195E+14	9,45349E+14	9,70385E+14	9,26846E+14
TC1100003137.mm.2	Pitpnm3	PITPNM family member 3	0.661257698887871	5,86612E+14	2,02991E+14	0.0855090966170775	0.228661397447552	-4,33906E+14	6,548E+14	6,70958E+14	5,18212E+14	4,82197E+14	5,77569E+14	6,15937E+14
TC0300003243.mm.2	Bcl9	B cell CLL/lymphoma 9	1,01546E+14	7,0147E+14	2,02985E+14	0.0855163096535029	0.228661397447552	-4,33914E+14	7,61497E+14	7,35395E+14	7,81323E+14	6,36807E+14	6,45995E+14	6,47805E+14
TC0500002068.mm.2	Fam126a	family with sequence similarity 126, member A	2,15883E+13	6,14891E+14	2,0298E+14	0.0855218743361508	0.228661397447552	-4,3392E+14	7,09641E+14	7,10457E+14	7,44239E+14	5,36677E+14	6,45645E+14	3,42687E+14
TC0300000852.mm.2	S100a11	S100 calcium binding protein A11	-0.822199568661822	8,51637E+14	-2,0298E+14	0.0855227473641224	0.228661397447552	-4,33921E+14	7,93561E+14	8,05524E+14	9,20573E+13	8,26638E+14	8,62284E+13	9,01241E+14
TC1400001540.mm.2	Ppp3cb	protein phosphatase 3, catalytic subunit, beta isoform	1,8949E+14	9,76599E+14	2,02978E+14	0.0855246137748532	0.228661397447552	-4,33923E+14	1,09178E+14	1,11002E+14	1,05177E+14	7,83215E+14	9,9658E+14	8,26238E+14
TC0400001087.mm.2	C8b	complement component 8, beta polypeptide	-0.723806788658892	5,47357E+14	-2,02975E+14	0.0855288595772663	0.228661397447552	-4,33928E+12	4,94895E+14	5,10284E+12	5,89239E+14	5,39785E+14	5,47337E+14	6,02604E+14
TC0500002081.mm.2	Gbx1	gastrulation brain homeobox 1	-0.984052763895654	6,39984E+14	-2,02969E+14	0.085535959818817	0.228661397447552	-4,33935E+14	5,95335E+12	5,68335E+14	6,34821E+13	6,80934E+14	6,15513E+14	7,44968E+13
TC1100002834.mm.2	Fam183b	family with sequence similarity 183, member B	0.631815785451541	6,41616E+14	2,02955E+13	0.0855525793868133	0.228661397447552	-4,33953E+14	6,56673E+14	6,53343E+14	6,56773E+12	6,99253E+14	5,94601E+14	5,89052E+14
TC1900000116.mm.2	Sf1	splicing factor 1	0.671783347359007	8,33429E+14	2,02954E+14	0.0855537416786506	0.228661397447552	-4,33954E+14	8,87169E+13	8,73336E+14	8,33733E+14	7,80184E+14	8,02895E+14	8,23254E+14
TC0700000354.mm.2	Vmn1r169	vomeronasal 1 receptor 169	-1,27195E+14	4,69607E+13	-2,02951E+14	0.0855582179117041	0.228661397447552	-4,33959E+14	3,85339E+13	3,61112E+14	4,39765E+14	6,30585E+14	4,73489E+14	5,27352E+12
TC0200005016.mm.2	Mroh8	maestro heat-like repeat family member 8	-1,02917E+14	7,09682E+14	-2,02915E+14	0.0856015948215965	0.228737301441604	-4,34005E+14	6,3999E+14	6,22966E+14	7,22657E+14	8,03688E+14	6,71922E+14	7,96867E+14
TC0400001721.mm.2	Sh2d5/Kif17	SH2 domain containing 5/kinesin family member 17	0.796378742452846	5,96519E+14	2,02904E+14	0.0856150105501342	0.228737301441604	-4,3402E+14	6,27172E+14	6,88694E+14	5,70745E+14	5,35914E+14	5,45904E+14	6,10686E+13
TC0600001366.mm.2	Pex26	peroxisomal biogenesis factor 26	0.689914109736312	5,43296E+14	2,02844E+14	0.0856888163069027	0.228890322449844	-4,34099E+14	6,13503E+14	6,1116E+14	4,52321E+13	4,96111E+14	5,19167E+14	5,67513E+14
TC0400003685.mm.2	Stx12	syntaxin 12	0.668108331696672	7,92712E+14	2,02826E+14	0.0857110543145123	0.228890322449844	-4,34122E+14	8,60463E+14	8,53462E+14	7,57942E+13	7,04104E+14	8,00022E+14	7,80281E+14
TC1800001577.mm.2	Smad4	SMAD family member 4	0.66680051443833	8,02606E+14	2,02822E+14	0.0857149013630211	0.228890322449844	-4,34127E+14	8,43266E+14	8,40346E+14	8,15515E+14	7,66258E+14	7,89689E+14	7,60562E+14
TC1100002925.mm.2	Akap10	A kinase (PRKA) anchor protein 10	2,1765E+13	5,77253E+14	2,02754E+14	0.0857981315235328	0.229074613626957	-4,34215E+14	7,06015E+13	7,03538E+14	6,39318E+14	4,40395E+14	6,4366E+14	3,30593E+14
TC0800000242.mm.2	Defa20	defensin, alpha, 20	-0.843321964753214	4,96013E+13	-2,02706E+14	0.0858575220657204	0.22912225540569	-4,34279E+14	4,34269E+13	4,41594E+14	4,83104E+14	5,72583E+14	5,52193E+14	4,92336E+14
TC0800000243.mm.2	Defa32/Defa20	defensin, alpha, 32/defensin, alpha, 20	-0.843321964753214	4,96013E+13	-2,02706E+14	0.0858575220657204	0.22912225540569	-4,34279E+14	4,34269E+13	4,41594E+14	4,83104E+14	5,72583E+14	5,52193E+14	4,92336E+14
TC0600001022.mm.2	Gm5577	predicted gene 5577	-0.665443603365843	4,78221E+14	-2,02681E+14	0.0858879381617899	0.22912225540569	-4,34311E+14	4,1757E+14	4,54816E+14	4,74502E+14	5,16961E+14	4,99001E+13	5,06474E+14
TC0700000244.mm.2	Pnmal1	PNMA-like 1	0.746424415281146	8,47848E+14	2,02677E+14	0.0858931986994567	0.22912225540569	-4,34317E+13	8,93683E+14	8,88875E+14	8,76822E+14	7,94433E+14	8,18593E+14	8,14679E+14
TC0400001881.mm.2	Zfp992/Zfp991	zinc finger protein 992/zinc finger protein 991	1,54432E+14	6,17148E+14	2,02667E+14	0.0859046870960562	0.22912225540569	-4,34329E+13	7,77134E+14	7,05192E+14	6,24216E+14	4,22884E+14	6,54462E+14	5,18999E+14
TC1000002940.mm.2	Mdm2	transformed mouse 3T3 cell double minute 2	0.808869342140302	7,95972E+14	2,02665E+14	0.0859070600963738	0.22912225540569	-4,34332E+14	8,3099E+12	8,47834E+14	8,29862E+14	7,50094E+13	7,8691E+14	7,3014E+14
TC1700002420.mm.2	Ankrd12	ankyrin repeat domain 12	1,70476E+14	9,84271E+14	2,02658E+14	0.085915513384297	0.22912225540569	-4,34341E+13	1,11531E+14	1,09423E+14	1,02655E+14	8,00952E+14	1,01951E+14	8,49069E+13
TC0X00000772.mm.2	Tbl1x	transducin (beta)-like 1 X-linked	0.743125410093881	8,19788E+14	2,02634E+14	0.0859452474964715	0.229129839087571	-4,34372E+14	8,76378E+14	8,68968E+14	8,21993E+14	7,54666E+14	8,21229E+13	7,75492E+14
TC0700002660.mm.2	Itpkc	inositol 1,4,5-trisphosphate 3-kinase C	-0.85081891685505	6,18106E+14	-2,02633E+14	0.085946797458281	0.229129839087571	-4,34374E+14	5,50533E+14	5,78226E+14	6,77288E+14	6,0367E+14	6,11152E+14	6,8777E+14

TC0900000637.mm.2	Exph5	exophilin 5	-0.714110726279108	5,55889E+14	-2,02591E+14	0.0859983054756986	0.229229230129021	-4,34429E+14	5,10266E+13	5,20516E+14	5,29632E+14	6,01315E+13	5,92247E+12	5,81356E+14
TC1100003760.mm.2	Krt14	keratin 14	-0.869327079461317	6,53582E+14	-2,02556E+14	0.0860415998605623	0.229306698404439	-4,34475E+14	5,86278E+14	5,88101E+14	6,42652E+13	7,56218E+14	6,67571E+14	6,80674E+14
TC0100000665.mm.2	Slc4a3	solute carrier family 4 (anion exchanger), member 3	0.609455570267854	7,03899E+14	2,02543E+13	0.0860566023698966	0.229308753759905	-4,34491E+12	7,49256E+14	7,52674E+14	6,5672E+14	6,84706E+14	6,82402E+14	6,97637E+14
TC1700001955.mm.2	Lta	lymphotoxin A	-0.674221246456263	7,38638E+14	-2,02526E+14	0.0860785489965136	0.229329308859263	-4,34514E+14	6,98575E+14	6,91686E+14	7,36023E+14	7,80436E+14	7,42453E+14	7,82653E+14
TC0900000255.mm.2	Ncapd3	non-SMC condensin II complex, subunit D3	0.597082563323724	7,26724E+14	2,02512E+14	0.0860947931809909	0.229334667304564	-4,34532E+14	7,82249E+14	7,74461E+14	6,8336E+14	6,82983E+14	7,29845E+14	7,07449E+14
TC0200004138.mm.2	Mapk8ip1	mitogen-activated protein kinase 8 interacting protein 1	0.78984484452579	9,25025E+14	2,02467E+14	0.0861508726821579	0.229443054285574	-4,34591E+14	9,81787E+14	9,71324E+14	9,17059E+13	8,84841E+14	9,41752E+14	8,5339E+13
TC1500002221.mm.2	Cers5	ceramide synthase 5	0.756820478385235	7,27295E+14	2,02456E+14	0.086163962110222	0.229443054285574	-4,34605E+14	7,80156E+14	7,82065E+13	7,36105E+14	6,54587E+14	7,18714E+14	6,92143E+14
TC1500002294.mm.2	Map3k12	mitogen-activated protein kinase kinase kinase 12	0.649795316581654	7,38195E+14	2,02432E+14	0.0861934530604079	0.229483659808717	-4,34636E+13	7,86606E+14	7,62575E+14	7,08089E+14	7,52678E+14	7,05474E+14	7,13748E+14
TC1000002894.mm.2	Tbc1d15	TBC1 domain family, member 15	1,19674E+14	4,99197E+14	2,02391E+14	0.0862443812629114	0.229581317576291	-4,34691E+14	5,64405E+14	6,03876E+14	5,24939E+14	3,73029E+13	5,13751E+14	4,15182E+14
TC0X00003425.mm.2	Cul4b	cullin 4B	2,75077E+14	6,55352E+14	2,02361E+14	0.0862804603229384	0.229613943541366	-4,34729E+14	8,32104E+14	8,6167E+14	7,30217E+13	3,64498E+14	7,23805E+14	4,19816E+14
TC0200002513.mm.2	Svs5	seminal vesicle secretory protein 5	-1,24336E+14	4,61282E+14	-2,02349E+13	0.0862960278970986	0.229613943541366	-4,34745E+14	3,59919E+14	4,25503E+14	4,18739E+14	5,29435E+14	4,43924E+14	5,9017E+14
TC0900001885.mm.2	Dock6	dedicator of cytokinesis 6	-0.696319394989096	6,19338E+14	-2,02346E+14	0.086299388201236	0.229613943541366	-4,34749E+14	5,92895E+14	5,68889E+14	6,10279E+14	6,42918E+14	6,2327E+14	6,77778E+14
TC0700001269.mm.2	Me3	malic enzyme 3, NADP(+)-dependent, mitochondrial	0.638316285728733	8,22106E+14	2,02333E+14	0.0863156442451914	0.229619279465539	-4,34766E+14	8,70834E+14	8,77626E+14	8,02795E+14	7,60583E+14	8,19814E+14	8,00982E+14
TC0200004148.mm.2	Prdm11	PR domain containing 11	-0.836407553521638	7,24335E+14	-2,02294E+14	0.0863626538896423	0.229675103250391	-4,34816E+14	6,62927E+14	6,83323E+13	6,93735E+14	7,92491E+14	7,39446E+14	7,74085E+14
TC1900000767.mm.2	Adra2a	adrenergic receptor, alpha 2a	0.800039994733194	6,47866E+14	2,02292E+14	0.0863651368828969	0.229675103250391	-4,34819E+14	7,29033E+14	7,08307E+14	6,32591E+14	5,39937E+14	6,49668E+13	6,27664E+14
TC0900000761.mm.2	Gm7616	predicted gene 7616	-0.802979039082738	5,24507E+13	-2,02271E+14	0.0863915589329188	0.229683979040799	-4,34847E+13	4,67319E+14	4,75907E+14	5,08648E+14	5,91349E+14	5,25749E+14	5,78073E+14
TC0300002728.mm.2	Gstm4	glutathione S-transferase, mu 4	1,13303E+14	6,3429E+12	2,02253E+14	0.0864132542000349	0.229683979040799	-4,3487E+14	7,26567E+13	6,86132E+14	6,86393E+14	5,20552E+14	6,0536E+13	5,80733E+14
TC0X00003301.mm.2	Reps2	RALBP1 associated Eps domain containing protein 2	0.889138314729081	9,04109E+14	2,02243E+14	0.0864262276944673	0.229683979040799	-4,34883E+14	9,60614E+14	9,7896E+13	9,20572E+14	8,02761E+14	8,83364E+14	8,78382E+13
TC0200003161.mm.2	Ajm1	apical junction component 1	0.838903849813956	6,92622E+14	2,02237E+14	0.0864328836623751	0.229683979040799	-4,3489E+14	7,57564E+14	7,10153E+14	6,83349E+14	7,04727E+14	6,96893E+13	6,03043E+14
TC0800001106.mm.2	Herpud1	homocysteine-inducible, endoplasmic reticulum stress-inducible, ubiquitin-like domain member 1	1,48227E+14	7,92348E+14	2,02232E+13	0.0864397473408679	0.229683979040799	-4,34898E+13	8,82934E+14	8,88712E+14	8,48977E+14	6,58276E+13	8,11886E+14	6,63306E+14
TC1900000763.mm.2	Pdcd4	programmed cell death 4	1,22768E+14	8,1639E+14	2,0221E+13	0.0864662360658899	0.229711364601553	-4,34926E+14	9,22456E+13	9,21241E+14	8,07673E+14	6,48809E+14	8,52818E+14	7,45343E+14
TC0700000014.mm.2	Ndufa3	NADH:ubiquinone oxidoreductase subunit A3	-1,04546E+14	1,13555E+14	-2,022E+14	0.0864785662305604	0.229711364601553	-4,34939E+14	1,08312E+14	1,06959E+14	1,20931E+14	1,08948E+14	1,22891E+14	1,13288E+14
TC0X00002550.mm.2	Tsga8	testis specific gene A8	-0.603554941932113	6,26229E+14	-2,02153E+14	0.0865374576260184	0.22975542627029	-4,35001E+14	5,77275E+14	5,96916E+14	6,37464E+14	6,50815E+14	6,39043E+14	6,55859E+14
TC1900000776.mm.2	Acsl5	acyl-CoA synthetase long-chain family member 5	0.936548979750465	6,21115E+14	2,02135E+14	0.0865598809299704	0.22975542627029	-4,35025E+14	6,82459E+14	6,91626E+14	6,14954E+14	5,50874E+14	6,49961E+14	5,36815E+14
TC0100002413.mm.2	Raph1	Ras association (RalGDS/AF-6) and pleckstrin homology domains 1	1,47709E+14	6,11692E+14	2,02131E+14	0.0865646727998814	0.22975542627029	-4,3503E+14	7,19777E+14	7,01592E+14	6,66619E+14	4,56216E+14	6,20991E+14	5,0496E+14
TC1500000051.mm.2	Gdnf	glial cell line derived neurotrophic factor	-0.730933634964084	5,30888E+13	-2,0212E+13	0.0865780648592437	0.22975542627029	-4,35044E+14	4,91632E+14	5,12634E+14	5,26811E+14	5,03798E+14	5,39062E+14	6,1139E+13
TC1100003986.mm.2	Rgs9	regulator of G-protein signaling 9	1,37302E+14	8,88546E+14	2,02118E+14	0.0865804086041601	0.22975542627029	-4,35047E+14	9,8596E+14	1,00098E+14	9,18626E+14	7,13377E+13	8,96109E+14	8,16227E+14
TC1400002072.mm.2	Efs	embryonal Fyn-associated substrate	-0.814007573692622	5,73689E+14	-2,02107E+14	0.0865938576135486	0.22975542627029	-4,35061E+14	5,17044E+14	5,6938E+14	5,10635E+14	5,95853E+14	6,168E+14	6,32425E+14
TC0300002527.mm.2	Polr3c	polymerase (RNA) III (DNA directed) polypeptide C	0.67097952804979	7,42605E+14	2,02106E+14	0.086594967029074	0.22975542627029	-4,35062E+14	7,90273E+14	7,70792E+14	7,33765E+14	7,3393E+14	7,41623E+14	6,85246E+14
TC1400001676.mm.2	Sh3bp5	SH3-domain binding protein 5 (BTK-associated)	0.732905876045187	7,7544E+14	2,02086E+14	0.086619934125233	0.229780344158495	-4,35088E+14	8,2679E+14	8,1036E+14	7,81486E+14	7,43435E+14	7,79125E+14	7,11443E+14
TC1100004152.mm.2	Cyth1	cytohesin 1	0.956416608645346	6,62081E+14	2,02057E+14	0.0866560123083909	0.229780344158495	-4,35127E+14	7,315E+14	7,27561E+14	6,88801E+14	5,56848E+14	6,47774E+14	6,20004E+14
TC0200005465.mm.2	Dipk1b	divergent protein kinase domain 1B	0.73336991645847	6,99208E+14	2,02051E+14	0.0866631413706031	0.229780344158495	-4,35134E+14	7,72937E+14	7,28016E+14	6,37297E+14	7,02719E+14	6,79787E+14	6,74492E+14
TC1700001686.mm.2	Rab11fip3	RAB11 family interacting protein 3 (class II)	0.599958549527286	7,25938E+14	2,02049E+14	0.0866660473601524	0.229780344158495	-4,35137E+14	7,4088E+14	7,56678E+14	7,41757E+14	7,38749E+14	6,78017E+14	6,99549E+14
TC0100001675.mm.2	1700016C15Rik	RIKEN cDNA 1700016C15 gene	-1,00045E+14	5,15111E+14	-2,02037E+14	0.0866797815995014	0.229780344158495	-4,35152E+14	4,86138E+14	3,95163E+14	5,08735E+14	6,19242E+14	5,30323E+14	5,51068E+14
TC0700003810.mm.2	Rnf169	ring finger protein 169	1,58329E+14	8,49522E+14	2,02029E+13	0.086689921935052	0.229780344158495	-4,35162E+13	9,62216E+14	9,5669E+14	9,1794E+14	6,58039E+14	8,15219E+14	7,87028E+14
TC1600001743.mm.2	Nxpe3	neurexophilin and PC-esterase domain family, member 3	0.723137158333094	5,92789E+14	2,02E+14	0.0867262054144628	0.229808037831318	-4,35201E+14	6,34469E+14	6,40689E+14	5,95682E+14	5,55364E+14	5,9767E+14	5,3286E+14
TC0100003743.mm.2	Mark1	MAP/microtubule affinity regulating kinase 1	1,1269E+14	8,40566E+14	2,01998E+14	0.0867288945604322	0.229808037831318	-4,35203E+14	9,15824E+13	9,18886E+14	8,67516E+14	7,31839E+14	8,57088E+14	7,52242E+14
TC0300000908.mm.2	Ensa	endosulfine alpha	0.785989026591414	7,68613E+14	2,01982E+14	0.0867478159275613	0.229820381131338	-4,35223E+13	8,07231E+14	8,04748E+14	7,69358E+14	7,75558E+14	7,7433E+14	6,80451E+14
TC1700002131.mm.2	Tdrd6	tudor domain containing 6	-0.723112082034605	4,80758E+14	-2,0193E+14	0.086812177271606	0.229925811298613	-4,35291E+14	4,17888E+14	4,63364E+14	4,60112E+13	5,17306E+14	5,01365E+13	5,2451E+13
TC0300002657.mm.2	Tafa3	TAFA chemokine like family member 3	-0.694716855778593	5,95403E+14	-2,01927E+14	0.0868161506821052	0.229925811298613	-4,35296E+14	5,22286E+14	5,35664E+14	6,25612E+14	6,91961E+14	5,95977E+14	6,00917E+14
TC0200004672.mm.2	Prokr2	prokineticin receptor 2	-1,06809E+14	7,06717E+14	-2,01892E+13	0.0868598990410835	0.229974008835021	-4,35342E+14	6,20499E+14	6,54822E+14	6,62593E+14	8,13449E+14	7,25473E+14	7,63466E+13
TC1900000153.mm.2	1700092M07Rik	RIKEN cDNA 1700092M07 gene	-0.617439994008942	6,18543E+14	-2,0187E+14	0.0868869009825117	0.229974008835021	-4,3537E+14	5,62969E+14	6,00491E+14	6,29249E+14	6,31601E+14	6,43104E+14	6,43844E+14
TC0X00000061.mm.2	Prickle3	prickle planar cell polarity protein 3	-0.915303522951683	5,77767E+14	-2,01863E+14	0.0868955480871993	0.229974008835021	-4,35379E+14	5,09969E+14	5,10712E+13	5,68298E+14	6,73878E+14	5,67677E+14	6,36065E+14
TC0800001085.mm.2	Gnao1	guanine nucleotide binding protein, alpha O	1,56522E+13	1,04085E+14	2,01856E+13	0.086903887617899	0.229974008835021	-4,35388E+13	1,14715E+14	1,14976E+14	1,09597E+13	8,68378E+14	1,05297E+14	9,30882E+13
TC1200001025.mm.2	Fam181a	family with sequence similarity 181, member A	-0.816811826344729	6,49656E+14	-2,01855E+14	0.0869057121452518	0.229974008835021	-4,3539E+14	5,92647E+14	5,70418E+14	6,70655E+14	7,3779E+14	6,26333E+14	7,00094E+14
TC0200002128.mm.2	Otor	otoraplin	-1,00254E+14	5,566E+14	-2,01813E+14	0.086957182254369	0.230072426545919	-4,35444E+14	4,51289E+14	5,15846E+14	5,37311E+14	6,67515E+14	5,83577E+14	5,84065E+14
TC0800002470.mm.2	Smim7	small integral membrane protein 7	0.732852144554759	8,7523E+14	2,01781E+14	0.0869972189602871	0.230140566262864	-4,35486E+13	9,34618E+14	9,27307E+14	8,73415E+14	8,00689E+14	8,73527E+14	8,41828E+14
TC0200003837.mm.2	Nckap1	NCK-associated protein 1	1,85647E+14	8,91515E+13	2,01654E+14	0.0871548199284659	0.230502760890176	-4,35652E+14	1,02003E+14	1,03355E+14	9,32103E+14	6,81118E+14	9,34967E+14	7,47321E+14
TC0100002651.mm.2	Cul3	cullin 3	2,04659E+14	7,28049E+14	2,01648E+14	0.0871627457397034	0.230502760890176	-4,3566E+14	8,61971E+14	8,67818E+13	7,86029E+14	5,32004E+14	7,79925E+14	5,40545E+14
TC0400002543.mm.2	Olfr29-ps1	olfactory receptor 29, pseudogene 1	-0.92730467715207	6,2667E+14	-2,01621E+14	0.0871964698019467	0.230532379974933	-4,35696E+14	5,4721E+14	6,01549E+14	5,90766E+14	6,86272E+14	6,33517E+13	7,00703E+14
TC0700002078.mm.2	Lsp1	lymphocyte specific 1	-0.676080134191217	7,38E+14	-2,01592E+14	0.087232459222445	0.230532379974933	-4,35733E+14	6,80883E+14	7,15632E+14	7,44132E+14	7,55623E+14	7,40825E+14	7,90907E+14
TC1300001615.mm.2	Vmn1r217	vomeronasal 1 receptor 217	-1,02882E+14	5,24416E+14	-2,01591E+14	0.0872330681359494	0.230532379974933	-4,35734E+14	4,2641E+14	4,61976E+14	5,09067E+13	6,54892E+14	5,37716E+14	5,56433E+14
TC0700002758.mm.2	Hcst	hematopoietic cell signal transducer	-0.701111959602782	4,74571E+14	-2,01581E+14	0.0872462888950147	0.230532379974933	-4,35748E+14	4,30819E+14	4,37515E+14	4,57632E+14	5,12905E+14	4,82828E+14	5,25729E+14
TC0900000279.mm.2	Prdm10	PR domain containing 10	0.695048245065175	6,33705E+14	2,01573E+14	0.0872557619466265	0.230532379974933	-4,35758E+14	6,86752E+14	6,83763E+14	6,22446E+14	5,77765E+14	5,89924E+14	6,41581E+14
TC1600002026.mm.2	Krtap6-5	keratin associated protein 6-5	-0.665071318673089	6,56467E+14	-2,0157E+14	0.0872597893295547	0.230532379974933	-4,35762E+14	5,98771E+14	6,05341E+14	6,86281E+14	7,1128E+14	6,42508E+14	6,94618E+14
TC1000000809.mm.2	Tpgs1	tubulin polyglutamylase complex subunit 1	0.582353049699817	7,67105E+13	2,01535E+13	0.0873029516201875	0.230593493061644	-4,35807E+13	7,93799E+14	7,99187E+14	7,62974E+14	7,70155E+14	7,35081E+14	7,41435E+13
TC0200003051.mm.2	Pip4k2a	phosphatidylinositol-5-phosphate 4-kinase, type II, alpha	1,02485E+14	8,603E+14	2,01528E+14	0.087311543546769	0.230593493061644	-4,35816E+14	9,21805E+14	9,18827E+14	8,8833E+14	7,97176E+14	8,77675E+14	7,57987E+13
TC1800000322.mm.2	Psd2	pleckstrin and Sec7 domain containing 2	0.977356764507241	7,4587E+14	2,01486E+14	0.0873645713588231	0.230693081141768	-4,35872E+14	8,25691E+14	7,99066E+14	7,79387E+14	6,41789E+14	7,15452E+14	7,13834E+14
TC5_GL456354_random00000002.mm.2	Gm7978/BC061212	predicted gene 7978/cDNA sequence BC061212	-1,29338E+14	4,99923E+14	-2,01475E+13	0.0873778858194136	0.230693081141768	-4,35886E+14	4,09564E+14	4,27719E+14	4,32136E+14	6,34159E+14	5,3036E+14	5,656E+14
TC0500002275.mm.2	Msx1	msh homeobox 1	-0.59786612371477	6,95868E+14	-2,01378E+14	0.087498545193162	0.230945229665568	-4,36012E+14	6,55555E+14	6,74152E+14	6,99664E+14	6,96557E+14	7,15115E+14	7,34165E+14
TC0X00002416.mm.2	Pnma5	paraneoplastic antigen family 5	-1,0777E+14	5,86484E+13	-2,01366E+14	0.0875139748146692	0.230945229665568	-4,36028E+14	5,35215E+14	4,99746E+14	5,44828E+14	6,88611E+14	5,92502E+14	6,57999E+13
TC0500002025.mm.2	Dnajc2	DnaJ heat shock protein family (Hsp40) member C2	1,28312E+14	6,65349E+14	2,01354E+13	0.0875290860397436	0.230945229665568	-4,36044E+14	7,66205E+14	7,79371E+14	6,65749E+13	4,91812E+14	6,9735E+14	5,91604E+14
TC0700004301.mm.2	Ino80e	INO80 complex subunit E	1,6085E+14	6,90419E+14	2,01352E+14	0.0875307216264895	0.230945229665568	-4,36046E+14	7,87524E+14	7,68351E+14	7,80624E+14	5,71842E+13	7,00004E+14	5,34171E+14

TC0400003769.mm.2	Grhl3	grainyhead like transcription factor 3	-0.940831646254049	6,50025E+14	-2,01339E+13	0.0875478699446307	0.230952656911892	-4,36064E+14	6,04805E+14	5,93768E+14	5,92238E+14	7,22598E+14	6,8401E+14	7,02728E+14
TC0600001158.mm.2	Ppp4r2	protein phosphatase 4, regulatory subunit 2	1,09284E+13	7,73191E+14	2,01321E+14	0.0875694235303357	0.230971086548648	-4,36086E+14	8,31223E+14	8,57275E+14	8,01214E+14	6,79503E+13	7,89252E+14	6,80678E+14
TC1200002413.mm.2	Bag5	BCL2-associated athanogene 5	0.645210586316032	7,40658E+14	2,0127E+14	0.0876332047847206	0.230971086548648	-4,36153E+14	7,99354E+14	7,87522E+14	7,12766E+14	6,86473E+14	7,49939E+14	7,07895E+14
TC1900000020.mm.2	Aldh3b3	aldehyde dehydrogenase 3 family, member B3	-0.71855425420243	6,1348E+14	-2,01269E+14	0.0876346627840801	0.230971086548648	-4,36154E+13	5,44588E+14	6,12333E+14	6,20938E+14	6,0239E+14	6,35811E+14	6,6482E+14
TC0900001430.mm.2	Pfkfb4	6-phosphofructo-2-kinase/fructose-2,6-biphosphatase 4	-0.738882250413545	6,75801E+14	-2,01261E+14	0.0876452860044349	0.230971086548648	-4,36165E+14	6,29066E+14	6,30531E+14	6,5305E+14	7,34784E+14	7,00759E+14	7,06614E+14
TC1700000896.mm.2	Dnph1	2'-deoxynucleoside 5'-phosphate N-hydrolase 1	-0.64809842896478	5,9359E+14	-2,01259E+13	0.0876477967653871	0.230971086548648	-4,36168E+14	5,53414E+14	5,58402E+14	5,80191E+14	6,28096E+14	6,10727E+14	6,3071E+14
TC0200001797.mm.2	Haus2	HAUS augmin-like complex, subunit 2	1,63201E+14	6,52391E+14	2,01257E+14	0.0876498263498857	0.230971086548648	-4,3617E+14	7,67033E+14	7,32112E+14	6,88654E+13	5,53801E+14	7,03151E+14	4,69592E+14
TC0600001436.mm.2	Mlf2	myeloid leukemia factor 2	0.963492772618721	9,85156E+14	2,01253E+14	0.0876551973093142	0.230971086548648	-4,36176E+13	1,05444E+14	1,05309E+14	1,00084E+13	8,87737E+14	9,96357E+13	9,18472E+14
TC0200000363.mm.2	Yme1l1	YME1-like 1 (S. cerevisiae)	3,51772E+14	5,94922E+13	2,01226E+14	0.0876881045814236	0.230996059855162	-4,3621E+14	8,25666E+14	8,41156E+14	7,21579E+14	2,17853E+14	6,60835E+14	3,02443E+14
TC0400000438.mm.2	Car9	carbonic anhydrase 9	-0.743413809201779	5,47949E+14	-2,01222E+14	0.0876933468710997	0.230996059855162	-4,36216E+14	4,92436E+14	4,98495E+14	5,38521E+14	6,18626E+14	5,58815E+14	5,80799E+14
TC0800002561.mm.2	Gm10644	predicted gene 10644	-0.853061992069384	6,29438E+13	-2,01209E+14	0.0877100749768557	0.231002359938228	-4,36233E+14	5,73537E+14	5,37504E+14	6,39754E+14	7,44182E+14	6,32213E+14	6,4944E+14
TC1100004094.mm.2	Recql5	RecQ protein-like 5	-0.688392355220159	5,78069E+14	-2,01183E+13	0.0877422628600917	0.231048588121478	-4,36267E+14	5,32329E+14	5,20865E+14	5,88158E+14	6,36185E+14	5,73062E+14	6,17811E+14
TC0700000238.mm.2	Strn4	striatin, calmodulin binding protein 4	0.706132751399554	8,20401E+14	2,01172E+14	0.0877563060450224	0.231048588121478	-4,36281E+14	8,51822E+14	8,70303E+14	8,19816E+14	7,99569E+14	8,23904E+14	7,56995E+14
TC1100003722.mm.2	Gm11562/Krtap2-4	predicted gene 11562/keratin associated protein 2-4	-0.869997724223128	5,81156E+13	-2,01146E+13	0.0877882588011217	0.231094954102675	-4,36314E+14	5,03216E+14	5,39179E+14	5,69651E+14	6,58495E+14	5,75579E+14	6,40815E+12
TC0500000653.mm.2	Shisa3	shisa family member 3	-0.631383781029928	7,82212E+14	-2,0113E+14	0.0878088411856239	0.231111378311656	-4,36336E+14	7,23064E+14	7,42096E+14	8,0225E+13	8,34423E+14	7,82884E+14	8,08554E+14
TC0400003375.mm.2	Klf17	Kruppel-like factor 17	-0.922147158459958	4,94848E+13	-2,01115E+13	0.0878277326507202	0.231123347411572	-4,36356E+12	4,3589E+14	4,20768E+14	4,72215E+14	5,99127E+14	5,25193E+14	5,15894E+14
TC1400000318.mm.2	Nek4	NIMA (never in mitosis gene a)-related expressed kinase 4	1,04203E+14	6,50023E+14	2,01078E+14	0.0878745268016679	0.231208238108953	-4,36404E+14	7,14217E+14	7,20896E+14	6,51393E+14	5,86929E+14	6,83571E+14	5,43135E+14
TC0X00000974.mm.2	Nono	non-POU-domain-containing, octamer binding protein	0.592747121003951	9,11479E+14	2,01066E+13	0.0878886897795158	0.231208238108953	-4,36419E+14	9,3733E+14	9,37649E+14	9,2336E+14	9,14103E+14	8,89099E+14	8,6733E+13
TC1400001527.mm.2	Saysd1	SAYSVFN motif domain containing 1	-0.648215422330937	7,22393E+14	-2,01028E+12	0.0879369801270598	0.231290434496032	-4,36469E+14	6,88849E+14	6,96898E+14	6,9068E+14	7,42543E+13	7,57877E+14	7,57513E+14
TC1500002206.mm.2	Mcrs1	microspherule protein 1	1,12295E+14	6,53191E+14	2,01018E+14	0.0879486434653501	0.231290434496032	-4,36481E+14	7,20076E+14	7,24129E+14	6,74736E+14	5,80589E+14	6,81638E+13	5,37977E+14
TC1000001919.mm.2	Lama2	laminin, alpha 2	0.774159905542402	5,27561E+14	2,00999E+14	0.0879728428031688	0.231291155527857	-4,36507E+14	5,64841E+13	5,8409E+14	4,66915E+14	5,55423E+13	5,05067E+14	4,89032E+14
TC0700000218.mm.2	Zfp541	zinc finger protein 541	-0.85335816615367	5,92807E+14	-2,00995E+14	0.0879776262787958	0.231291155527857	-4,36512E+14	5,26753E+14	5,5042E+14	5,64959E+14	6,66866E+14	6,03794E+14	6,4405E+14
TC1200000258.mm.2	Sh3yl1	Sh3 domain YSC-like 1	0.618703554802185	5,37998E+14	2,00978E+14	0.0879995012389798	0.231304802189356	-4,36534E+14	5,57577E+14	5,7858E+14	5,2425E+14	5,55162E+14	4,99611E+14	5,12806E+14
TC0500003482.mm.2	Pdgfa	platelet derived growth factor, alpha	0.739044407773039	7,80988E+14	2,00968E+14	0.0880115274761337	0.231304802189356	-4,36547E+14	8,37643E+14	8,03763E+14	7,90806E+14	7,60118E+14	7,81024E+14	7,12574E+14
TC1400000743.mm.2	Oxa1l	oxidase assembly 1-like	0.945617645345148	8,56666E+14	2,00935E+14	0.0880538519657952	0.231319432530805	-4,36591E+14	9,20986E+14	8,79568E+14	9,19761E+14	8,08252E+14	8,36199E+14	7,75232E+14
TC1300001812.mm.2	Nrn1	neuritin 1	-0.872451005136474	8,72276E+14	-2,0093E+14	0.0880596753331968	0.231319432530805	-4,36597E+13	8,11015E+14	8,71592E+14	8,33111E+14	8,60841E+14	9,76486E+14	8,80611E+14
TC1100003144.mm.2	Smtnl2	smoothelin-like 2	-0.622535957035133	7,69869E+14	-2,00918E+14	0.0880741308435204	0.231319432530805	-4,36612E+14	7,45176E+14	7,29969E+14	7,63963E+14	7,80455E+13	7,82317E+14	8,17335E+14
TC0500001893.mm.2	Mterf1a	mitochondrial transcription termination factor 1a	0.817872817571697	5,74408E+14	2,00918E+14	0.0880745186232537	0.231319432530805	-4,36612E+14	6,45949E+14	6,09863E+14	5,85856E+14	5,12543E+14	5,77808E+14	5,14429E+14
TC1500002059.mm.2	Rabl2	RAB, member RAS oncogene family-like 2	0.92155810710734	7,2304E+14	2,00877E+14	0.0881265185105553	0.231418284348204	-4,36666E+14	8,07549E+14	7,29017E+14	7,75017E+14	6,74404E+14	6,69785E+14	6,8247E+14
TC0700002769.mm.2	Zbtb32	zinc finger and BTB domain containing 32	-0.874922350005654	5,76445E+14	-2,00865E+14	0.0881416184741496	0.231420221357988	-4,36682E+14	4,93435E+13	5,51932E+14	5,68169E+14	6,24784E+14	5,67877E+14	6,52474E+14
TC0300002391.mm.2	Sprr1b	small proline-rich protein 1B	-0.707199589250926	5,46382E+14	-2,007E+14	0.088349473785794	0.231928164077957	-4,36897E+14	4,99849E+14	5,09751E+13	5,42368E+14	5,75286E+14	5,43234E+13	6,07806E+14
TC1100000192.mm.2	Rab1a	RAB1A, member RAS oncogene family	1,79654E+14	8,59239E+13	2,00664E+14	0.0883942941867107	0.232000374442373	-4,36944E+14	1,00365E+13	1,0072E+12	8,91971E+14	6,01065E+14	8,70028E+14	7,81516E+14
TC1300002052.mm.2	Rab24	RAB24, member RAS oncogene family	0.813695003322918	6,92847E+14	2,00655E+14	0.088405777885941	0.232000374442373	-4,36956E+14	7,54463E+14	7,40757E+14	6,80239E+14	6,4914E+13	7,14658E+14	6,17823E+14
TC0400001090.mm.2	Fyb2	FYN binding protein 2	-0.694040730998057	5,6975E+14	-2,00628E+14	0.0884396737083795	0.23205153272482	-4,36991E+13	4,94908E+14	5,53347E+14	5,732E+14	6,09986E+14	5,92412E+14	5,9465E+14
TC0600003232.mm.2	Clec12b	C-type lectin domain family 12, member B	-0.628597484176842	4,74272E+14	-2,00613E+14	0.0884590173550956	0.232059742475089	-4,37011E+14	4,18621E+14	4,32007E+14	5,00468E+14	5,18188E+13	4,68703E+14	5,07644E+14
TC0200001375.mm.2	Arfgap2	ADP-ribosylation factor GTPase activating protein 2	0.738407549350395	7,36741E+14	2,00591E+13	0.0884866681283274	0.232059742475089	-4,37039E+14	7,84646E+13	7,98854E+14	7,40583E+14	6,60544E+14	7,10797E+14	7,25021E+14
TC1500001544.mm.2	Sntb1	syntrophin, basic 1	-0.616392066152971	6,25135E+14	-2,00583E+14	0.0884961497699908	0.232059742475089	-4,37049E+14	6,07004E+14	5,7797E+13	6,25392E+14	6,3219E+14	6,41494E+14	6,66759E+14
TC1500002268.mm.2	Krt6a/Krt6b	keratin 6A/keratin 6B	-0.787406335913039	6,80573E+14	-2,00571E+14	0.0885113942524491	0.232059742475089	-4,37065E+14	6,26319E+14	6,38132E+14	6,89493E+14	7,07562E+14	6,64227E+14	7,57705E+14
TC1100003683.mm.2	Ormdl3	ORM1-like 3 (S. cerevisiae)	-0.716037760137423	7,2605E+14	-2,00568E+14	0.0885148127134547	0.232059742475089	-4,37068E+14	6,90994E+14	6,87504E+14	7,16506E+13	7,3959E+14	7,24696E+14	7,97009E+14
TC0700000835.mm.2	Tead2	TEA domain family member 2	-0.808004095007142	5,94662E+14	-2,00543E+14	0.0885469569249993	0.232068812766388	-4,37101E+14	5,41004E+14	5,40127E+14	5,78597E+14	6,65512E+14	5,95358E+14	6,47374E+14
TC1800001421.mm.2	Arhgef37	Rho guanine nucleotide exchange factor (GEF) 37	-0.652859450588045	4,9405E+14	-2,00543E+14	0.0885470775701454	0.232068812766388	-4,37102E+14	4,66267E+14	4,26222E+14	5,06031E+14	5,42723E+14	5,0757E+13	5,15491E+14
TC0500003215.mm.2	Ift81	intraflagellar transport 81	1,51422E+13	6,33689E+14	2,0052E+14	0.0885763294593804	0.232087006449167	-4,37132E+14	7,39339E+14	7,29275E+14	6,73456E+14	4,94292E+14	6,70239E+14	4,95532E+14
TC1700000389.mm.2	Fbxl16	F-box and leucine-rich repeat protein 16	1,29968E+14	1,03714E+14	2,00515E+14	0.0885828268889952	0.232087006449167	-4,37139E+14	1,12151E+14	1,13591E+14	1,08336E+14	8,84583E+14	1,02914E+14	9,6835E+14
TC1500000678.mm.2	Tomm22	translocase of outer mitochondrial membrane 22	0.765432739415821	7,9786E+14	2,00489E+14	0.0886154783180919	0.232134807696214	-4,37172E+14	8,72147E+14	8,52937E+14	7,67552E+14	7,22525E+14	8,23854E+14	7,48143E+14
TC0200001230.mm.2	Tnks1bp1	tankyrase 1 binding protein 1	-0.72247409534966	6,75317E+14	-2,00469E+14	0.0886401155231107	0.23216160296227	-4,37198E+14	6,36754E+14	6,274E+14	6,56211E+14	7,22887E+14	6,84618E+14	7,24031E+14
TC1100000038.mm.2	Tbc1d10a	TBC1 domain family, member 10a	0.619773430060367	5,46301E+14	2,00441E+14	0.088676154993053	0.23221824888722	-4,37235E+13	5,83734E+14	5,63837E+14	5,49408E+14	5,57208E+14	4,94331E+14	5,29286E+14
TC1900000552.mm.2	Plce1	phospholipase C, epsilon 1	0.75023136141259	6,50208E+14	2,00425E+14	0.0886967495087903	0.232234436924787	-4,37256E+14	6,82324E+14	7,05081E+14	6,78923E+14	5,97562E+14	6,1053E+14	6,26828E+13
TC1900000213.mm.2	Ptgdr2	prostaglandin D2 receptor 2	-0.713144017392599	5,71151E+14	-2,00407E+14	0.0887191322079528	0.23225530093692	-4,37279E+14	5,29075E+14	5,1566E+14	5,61322E+14	6,33483E+14	5,85302E+14	6,02063E+14
TC1700002303.mm.2	Zfp119a	zinc finger protein 119a	0.604817703714748	5,18891E+14	2,00389E+14	0.0887421624238972	0.232255811902253	-4,37303E+14	5,69702E+14	6,01697E+14	4,49931E+13	4,41581E+14	5,24103E+14	5,26332E+14
TC1700002173.mm.2	Abcc10	ATP-binding cassette, sub-family C (CFTR/MRP), member 10	-0.62284638248815	7,13795E+14	-2,00384E+14	0.0887481557675277	0.232255811902253	-4,37309E+14	6,78715E+13	6,67115E+14	7,53606E+14	7,12938E+14	7,32993E+14	7,37406E+14
TC0500002386.mm.2	Adgra3	adhesion G protein-coupled receptor A3	0.746003489058211	5,97902E+14	2,00335E+14	0.0888098967913574	0.232379647125551	-4,37373E+14	6,36485E+14	6,67076E+14	5,81115E+14	5,48376E+14	5,41094E+14	6,13266E+14
TC1600001112.mm.2	Slx4	SLX4 structure-specific endonuclease subunit homolog (S. cerevisiae)	-0.632913939211982	5,94617E+14	-2,00277E+14	0.088883251301581	0.232533825007692	-4,37448E+14	5,40471E+14	5,84318E+14	5,88995E+14	6,02546E+14	6,20201E+13	6,31171E+14
TC0700000546.mm.2	Zfp260	zinc finger protein 260	0.701469965879274	6,05814E+14	2,00252E+14	0.0889151200396291	0.232548892803379	-4,37481E+14	6,72853E+14	6,80378E+14	5,69686E+14	4,99028E+14	6,11821E+14	6,01116E+14
TC1000001882.mm.2	Taar6	trace amine-associated receptor 6	-0.614306718183586	5,37157E+14	-2,0025E+14	0.0889178755391063	0.232548892803379	-4,37484E+14	4,82515E+14	5,08187E+14	5,48321E+14	5,70354E+13	5,47546E+14	5,66017E+14
TC0200000293.mm.2	Mllt10	myeloid/lymphoid or mixed-lineage leukemia; translocated to, 10	1,5249E+14	6,9104E+14	2,00229E+14	0.088944175742133	0.232579925954721	-4,37511E+14	7,52613E+14	7,8726E+14	7,54667E+14	6,16807E+14	7,22056E+14	5,12837E+14
TC0900000667.mm.2	Psma4	proteasome (prosome, macropain) subunit, alpha type 4	0.766593141111104	8,14418E+14	2,00185E+14	0.0890004840317087	0.232677017383408	-4,37569E+14	8,88576E+14	8,61832E+14	7,99913E+13	7,39102E+14	8,29486E+14	7,67603E+14
TC0800001541.mm.2	Urb2	URB2 ribosome biogenesis 2 homolog (S. cerevisiae)	1,29813E+14	6,95592E+14	2,00155E+14	0.089038689991778	0.232677017383408	-4,37608E+14	7,88575E+14	7,81354E+14	7,42894E+14	5,50427E+13	6,95107E+14	6,15196E+14
TC0200002500.mm.2	Pabpc1l	poly(A) binding protein, cytoplasmic 1-like	-0.878927640241743	4,80805E+14	-2,00146E+14	0.08904937738174	0.232677017383408	-4,37619E+14	4,46727E+14	4,11307E+14	4,4139E+14	5,51588E+14	5,02887E+14	5,30932E+14
TC1700001660.mm.2	Tpsab1	tryptase alpha/beta 1	-0.828419060048671	4,46533E+14	-2,00145E+14	0.0890511033259727	0.232677017383408	-4,3762E+14	3,75437E+14	4,03111E+14	4,41153E+14	5,15269E+14	4,36208E+14	5,08023E+14
TC0100002823.mm.2	Ankmy1	ankyrin repeat and MYND domain containing 1	-0.819285732198617	5,78471E+13	-2,00143E+14	0.0890535074910616	0.232677017383408	-4,37623E+14	5,39036E+14	5,11975E+14	5,52163E+14	6,52782E+14	6,03045E+14	6,11824E+14

TC0200003217.mm.2	Brd3/Mir7578	bromodomain containing 3/microRNA 7578	0.760411767527925	7,39853E+14	2,00131E+14	0.0890681585528566	0.232677567892701	-4,37638E+13	7,88777E+14	7,68418E+13	7,72362E+14	7,0445E+14	7,29589E+14	6,75523E+14
TC1200001643.mm.2	Tspan13	tetraspanin 13	1,48848E+14	8,65706E+14	2,0011E+14	0.0890950877686845	0.232710187910004	-4,37666E+14	9,77474E+14	9,76944E+14	8,85943E+14	6,97152E+14	9,07259E+14	7,49463E+14
TC0500003388.mm.2	Bud23	BUD23, rRNA methyltransferase and ribosome maturation factor	-0.704860552141349	6,36555E+12	-2,00062E+14	0.0891558543556795	0.232831163895148	-4,37728E+14	5,86365E+14	6,22531E+14	6,22594E+14	6,37975E+14	6,44102E+14	7,05765E+14
TC1400000744.mm.2	Mrpl52	mitochondrial ribosomal protein L52	0.78804345952291	7,26525E+12	2,00023E+14	0.0892058155562365	0.232923886899634	-4,37779E+14	7,97355E+13	7,99385E+14	7,01974E+14	6,21306E+14	7,46182E+14	6,92949E+14
TC0500003449.mm.2	Mospd3	motile sperm domain containing 3	-1,03014E+14	7,61985E+13	-1,99976E+13	0.089266053931456	0.233043410077374	-4,37841E+13	6,88458E+14	6,79777E+13	7,48031E+14	8,8138E+14	7,49534E+14	8,24728E+14
TC0400003603.mm.2	Fam167b	family with sequence similarity 167, member B	-0.941990764366748	6,27359E+14	-1,99918E+13	0.0893398085018175	0.233198175018595	-4,37916E+13	5,45043E+14	5,73458E+14	6,1059E+14	7,28163E+14	6,24701E+14	6,82199E+13
TC0400001969.mm.2	Gpr157	G protein-coupled receptor 157	-0.691567337016354	6,21767E+14	-1,99845E+13	0.0894333967933078	0.233364956263875	-4,38012E+13	5,39538E+14	5,97E+14	6,42985E+14	6,7623E+13	6,47131E+14	6,2772E+14
TC0800001616.mm.2	Fcer2a	Fc receptor, IgE, low affinity II, alpha polypeptide	-0.712232142944044	6,54409E+14	-1,99842E+14	0.089436929061636	0.233364956263875	-4,38016E+14	5,83429E+14	6,35187E+14	6,47696E+14	6,99077E+14	6,90528E+14	6,70534E+14
TC1600001746.mm.2	Pcnp	PEST proteolytic signal containing nuclear protein	0.841078299103811	9,3685E+14	1,9983E+14	0.0894522190620947	0.233364956263875	-4,38031E+14	1,0089E+14	9,71953E+14	9,50622E+14	8,76988E+14	9,42991E+14	8,69645E+14
TC0500003513.mm.2	Ttyh3	tweety family member 3	1,0399E+13	8,90492E+14	1,99822E+14	0.0894616356260135	0.233364956263875	-4,38041E+12	9,51816E+14	9,66508E+14	9,30331E+14	7,83951E+14	8,80527E+14	8,29817E+14
TC1300002244.mm.2	Zfp457	zinc finger protein 457	0.995887950365435	5,20091E+14	1,99808E+13	0.0894801573661425	0.23337548974436	-4,3806E+14	5,79196E+14	5,79167E+14	5,1204E+14	4,90961E+14	5,51857E+14	4,07328E+14
TC0300002263.mm.2	Rps3a1	ribosomal protein S3A1	1,03258E+13	9,01934E+14	1,9979E+14	0.0895035108612472	0.233398619599818	-4,38084E+13	1,00203E+14	9,48363E+14	9,21402E+13	7,95932E+14	9,07694E+14	8,36183E+14
TC1100002842.mm.2	Iba57	IBA57 homolog, iron-sulfur cluster assembly	-0.76110349506251	8,42315E+14	-1,99759E+14	0.0895426709054317	0.233458953247559	-4,38124E+14	7,92147E+14	7,9559E+14	8,24611E+14	9,01584E+14	8,46977E+14	8,9298E+14
TC0X00003417.mm.2	Srpx	sushi-repeat-containing protein	-0.903911132375921	6,8658E+14	-1,99749E+14	0.0895556252729575	0.233458953247559	-4,38137E+14	6,12297E+13	6,51435E+14	6,46487E+14	7,64749E+14	7,29355E+14	7,15159E+14
TC0400004226.mm.2	Cela2a	chymotrypsin-like elastase family, member 2A	-0.712659475867275	5,66309E+14	-1,99725E+14	0.0895858904854834	0.235500073340762	-4,38168E+14	5,28737E+14	5,03403E+14	5,65571E+14	6,25467E+14	5,67792E+14	6,0688E+14
TC1600001328.mm.2	Olfr165	olfactory receptor 165	-1,26346E+12	4,61151E+14	-1,99684E+14	0.0896382281018322	0.233598701181437	-4,38221E+14	4,07244E+14	3,77097E+14	3,73844E+14	5,71688E+14	5,04188E+14	5,32846E+14
TC1600002037.mm.2	Krtap11-1	keratin associated protein 11-1	-0.722149053272521	5,24338E+14	-1,99612E+14	0.0897299172312717	0.233788941689274	-4,38314E+14	4,72518E+14	4,71612E+12	5,43296E+14	5,70045E+14	5,07202E+14	5,81357E+13
TC0900001297.mm.2	Slco2a1	solute carrier organic anion transporter family, member 2a1	-0.821459771320979	7,21125E+14	-1,99604E+14	0.0897402472753776	0.233788941689274	-4,38325E+14	6,60862E+14	6,7042E+14	7,02855E+14	7,97036E+14	7,24315E+14	7,7126E+14
TC0100000725.mm.2	Fbxo36	F-box protein 36	0.698816274639878	6,25975E+14	1,99568E+14	0.0897867024780694	0.233872152769016	-4,38372E+14	6,48784E+14	6,59025E+14	6,71372E+14	6,08622E+14	6,00679E+14	5,67367E+14
TC1000000231.mm.2	Samd3	sterile alpha motif domain containing 3	-0.749649872873197	5,86571E+14	-1,99548E+14	0.0898127664022535	0.233902231297803	-4,38399E+14	5,7259E+14	5,04649E+14	5,92487E+14	6,22528E+14	5,91139E+12	6,3603E+13
TC0100000227.mm.2	Cfc1	cripto, FRL-1, cryptic family 1	-1,06893E+14	6,04923E+14	-1,9952E+14	0.0898482858988511	0.233956921572105	-4,38435E+14	5,08386E+14	5,34026E+13	6,01727E+14	7,292E+14	5,79645E+14	6,76552E+14
TC0500001437.mm.2	Arl6ip4	ADP-ribosylation factor-like 6 interacting protein 4	-0.839277289625324	7,09114E+14	-1,995E+14	0.089873705955811	0.233985300382288	-4,38461E+14	6,60751E+14	6,6369E+14	6,8604E+14	7,51907E+14	7,01998E+14	7,90299E+14
TC1400000290.mm.2	Wnt5a	wingless-type MMTV integration site family, member 5A	-0.668571908366963	7,91462E+14	-1,99432E+14	0.0899614801383142	0.234139286537699	-4,3855E+14	7,54506E+14	7,47185E+14	8,35793E+14	7,7588E+14	8,10753E+14	8,24653E+14
TC0900001370.mm.2	Tmem115	transmembrane protein 115	0.706364375143989	5,86855E+14	1,99431E+14	0.0899619141658842	0.234139286537699	-4,3855E+14	6,09537E+14	6,55688E+14	5,37934E+14	5,94023E+14	5,69737E+13	5,54215E+14
TC0900003310.mm.2	Lztfl1	leucine zipper transcription factor-like 1	1,82847E+14	6,81507E+14	1,99393E+14	0.0900111721165335	0.234229653797433	-4,386E+14	8,27866E+14	8,17134E+14	7,04505E+14	4,60233E+14	7,36803E+14	5,42502E+14
TC0800001837.mm.2	Gm14851	predicted gene 14851	-0.646692506946707	6,42594E+14	-1,9936E+14	0.0900534341738201	0.234301789898718	-4,38643E+14	5,84189E+13	6,15059E+14	6,43697E+14	6,8403E+14	6,73456E+14	6,55131E+14
TC0200002150.mm.2	Sec23b	SEC23 homolog B, COPII coat complex component	0.948033624524329	6,70949E+14	1,99317E+14	0.0901093321522011	0.234409375035263	-4,387E+14	7,47774E+13	7,54918E+14	6,65178E+14	5,44742E+14	6,8786E+14	6,25224E+14
TC1100000498.mm.2	Olfr1394	olfactory receptor 1394	-0.671034389394631	4,93007E+14	-1,99286E+14	0.090148540392833	0.234433198108552	-4,3874E+14	4,48001E+14	4,46257E+14	4,9618E+14	5,39143E+12	4,91235E+14	5,37229E+14
TC1400002081.mm.2	Jph4	junctophilin 4	-0.583174547468349	8,13385E+14	-1,99271E+13	0.0901684609156429	0.234433198108552	-4,3876E+14	7,68609E+14	7,74826E+14	8,42863E+14	8,33943E+14	8,2168E+14	8,3839E+14
TC0800001320.mm.2	Mtss2	MTSS I-BAR domain containing 2	0.885046542939318	8,87199E+14	1,99264E+14	0.0901774091256312	0.234433198108552	-4,38769E+13	9,52984E+14	9,262E+14	9,26166E+14	8,15668E+14	8,72461E+14	8,29713E+14
TC0100003099.mm.2	Prelp	proline arginine-rich end leucine-rich repeat	0.662902888879512	7,25976E+14	1,99259E+14	0.0901827905546141	0.234433198108552	-4,38774E+14	7,46139E+14	7,75015E+14	7,414E+14	7,0473E+14	6,75323E+14	7,13249E+14
TC0100001442.mm.2	Cop1	COP1, E3 ubiquitin ligase	1,24787E+14	6,64041E+14	1,99253E+14	0.0901912365838091	0.234433198108552	-4,38783E+14	7,54778E+13	7,38815E+14	6,94024E+14	5,52611E+14	6,91964E+14	5,52055E+14
TC0500001401.mm.2	P2rx4	purinergic receptor P2X, ligand-gated ion channel 4	-0.64793626433825	7,04012E+13	-1,99217E+14	0.090237393319915	0.234468234414641	-4,3883E+14	6,54917E+14	6,80005E+14	6,89732E+14	7,34908E+14	7,23354E+14	7,41155E+14
TC1200000389.mm.2	Nrcam	neuronal cell adhesion molecule	1,51285E+14	8,19739E+14	1,99216E+14	0.0902387062967017	0.234468234414641	-4,38831E+14	9,215E+14	9,32903E+13	8,68059E+13	6,44139E+14	8,33541E+14	7,18293E+14
TC0900003015.mm.2	Grm2	glutamate receptor, metabotropic 2	0.969090096217449	6,8682E+14	1,99208E+14	0.0902494345959388	0.234468234414641	-4,38842E+14	7,25505E+14	7,69378E+13	6,19394E+14	7,0558E+14	7,01685E+14	5,9938E+14
TC1400002223.mm.2	Mtmr9	myotubularin related protein 9	1,27421E+14	6,97064E+14	1,99197E+14	0.0902629217448035	0.234468234414641	-4,38855E+14	8,09913E+14	8,01462E+14	7,06534E+14	5,07946E+13	7,03268E+14	6,53264E+14
TC0200002427.mm.2	Snhg11	small nucleolar RNA host gene 11	-0.779749181798245	8,92269E+14	-1,99185E+14	0.0902783872846497	0.234470608368401	-4,38871E+13	8,5919E+14	8,47393E+14	8,51918E+14	9,32584E+14	9,55993E+14	9,06539E+14
TC0X00000466.mm.2	Ccdc160	coiled-coil domain containing 160	-0.87494241937262	6,15755E+14	-1,99158E+14	0.0903132854077372	0.234488546088068	-4,38906E+14	5,51389E+14	5,59183E+14	5,97049E+14	7,01352E+13	6,1421E+14	6,7135E+14
TC0200000597.mm.2	Fam129b	family with sequence similarity 129, member B	-0.577028478809419	7,87796E+14	-1,99157E+14	0.0903143993683704	0.234488546088068	-4,38908E+14	7,57456E+14	7,47895E+14	7,98497E+14	8,0217E+14	8,06081E+14	8,14675E+14
TC0300002373.mm.2	Chtop	chromatin target of PRMT1	0.639900480151999	7,9218E+14	1,99145E+14	0.0903300511119411	0.234491398995764	-4,38923E+14	8,32647E+14	8,42952E+14	7,75623E+14	7,54239E+14	7,50908E+14	7,96711E+14
TC0600000115.mm.2	Mdfic	MyoD family inhibitor domain containing	-0.697293576907414	6,49857E+14	-1,99124E+14	0.0903570304859456	0.234522840878247	-4,38951E+13	5,96683E+14	6,07921E+14	6,46789E+14	7,03684E+14	6,50876E+14	6,93187E+14
TC0300002617.mm.2	Tshb	thyroid stimulating hormone, beta subunit	-0.630650937559253	5,41613E+13	-1,99113E+14	0.0903712728239954	0.234522840878247	-4,38965E+14	5,15794E+14	4,90835E+14	5,42528E+14	5,67763E+14	5,50375E+14	5,82385E+14
TC0500000084.mm.2	Sema3a	sema domain, immunoglobulin domain (Ig), short basic domain, secreted, (semaphorin) 3A	0.735427019668632	6,10092E+14	1,99066E+14	0.0904322074655339	0.234608387745243	-4,39027E+14	6,48511E+14	6,57754E+14	6,33057E+14	5,62051E+14	6,00855E+14	5,58325E+14
TC1000002076.mm.2	Sobp	sine oculis binding protein	0.72293593022175	7,58482E+14	1,99065E+14	0.0904333579274935	0.234608387745243	-4,39028E+14	8,02517E+14	8,04236E+14	7,80763E+14	7,01208E+13	7,32878E+14	7,29287E+14
TC1300001794.mm.2	Eci2	enoyl-Coenzyme A delta isomerase 2	1,30605E+14	7,42663E+14	1,99032E+14	0.09047517592682	0.234626748504759	-4,3907E+14	8,06514E+14	7,97504E+14	8,15683E+13	6,93468E+14	7,57638E+14	5,8517E+14
TC1000000679.mm.2	Fam13c	family with sequence similarity 13, member C	0.743979304225122	6,44447E+14	1,99025E+14	0.0904850877492074	0.234626748504759	-4,3908E+14	7,09574E+13	6,64791E+14	6,67656E+14	5,99092E+14	6,25507E+14	6,00062E+14
TC1300002767.mm.2	Zfp935	zinc finger protein 935	1,24941E+14	6,46662E+14	1,99016E+14	0.0904963669817977	0.234626748504759	-4,39091E+13	7,27215E+14	7,29206E+14	6,76862E+14	5,40149E+14	6,76374E+14	5,30165E+14
TC0200002987.mm.2	Trdmt1	tRNA aspartic acid methyltransferase 1	0.613954301744918	5,87883E+14	1,99014E+14	0.0904986806899446	0.234626748504759	-4,39094E+14	6,379E+14	6,08606E+14	5,80916E+14	5,76163E+14	5,75474E+14	5,48241E+14
TC0500003734.mm.2	1500011B03Rik	RIKEN cDNA 1500011B03 gene	-0.623840097321548	7,69393E+14	-1,98991E+14	0.0905291940459455	0.234634420202797	-4,39124E+14	7,24659E+13	7,35344E+14	7,71147E+13	8,00435E+14	7,76178E+14	8,08593E+13
TC1600001519.mm.2	Iqcg	IQ motif containing G	-0.854755724153867	5,94813E+14	-1,98977E+14	0.0905467818771749	0.234634420202797	-4,39142E+14	5,62448E+14	5,10799E+14	5,76743E+13	6,7469E+14	6,07481E+14	6,36717E+14
TC0200005097.mm.2	Ada	adenosine deaminase	-1,01279E+14	7,1911E+14	-1,98975E+14	0.0905498696206659	0.234634420202797	-4,39145E+14	6,15495E+14	6,67551E+14	7,06209E+14	8,39799E+14	7,19629E+14	7,65975E+14
TC1700001844.mm.2	Rasal3	RAS protein activator like 3	0.89828318803687	5,09376E+13	1,98967E+14	0.0905598870006325	0.234634420202797	-4,39155E+13	5,39421E+14	5,66297E+13	4,79993E+13	5,4448E+14	4,11271E+14	5,14791E+14
TC0100003776.mm.2	Kctd3	potassium channel tetramerisation domain containing 3	1,64192E+14	6,99421E+14	1,98946E+14	0.0905861838284605	0.234649682319477	-4,39182E+14	7,89841E+14	7,89337E+14	7,58177E+14	6,0838E+14	7,42172E+14	5,08621E+14
TC1100004266.mm.2	Irgm2	immunity-related GTPase family M member 2	-0.719928133405797	4,82698E+14	-1,9894E+14	0.0905949031036722	0.234649682319477	-4,39191E+14	4,5413E+14	4,35605E+14	4,48445E+14	5,24287E+14	5,11159E+14	5,22561E+14
TC0400002102.mm.2	Sdf4	stromal cell derived factor 4	0.779181816355782	8,57875E+14	1,98905E+14	0.0906392372336742	0.234704531398253	-4,39235E+14	9,10546E+14	9,2508E+14	8,39434E+14	7,92402E+14	8,80808E+14	7,98982E+14
TC0700002591.mm.2	Zfp111	zinc finger protein 111	0.905623565911992	5,92878E+14	1,98901E+14	0.0906452118718628	0.234704531398253	-4,39241E+14	6,33991E+14	6,28024E+14	6,41489E+14	5,72873E+14	5,93599E+14	4,87291E+14
TC0800002878.mm.2	Nob1	NIN1/RPN12 binding protein 1 homolog	0.688213767583971	7,27696E+14	1,98847E+13	0.090714258693646	0.234845573639254	-4,39311E+14	7,70871E+14	7,5772E+14	6,92296E+14	7,54337E+14	7,09314E+14	6,81634E+13
TC0300002067.mm.2	Dhx36	DEAH (Asp-Glu-Ala-His) box polypeptide 36	1,83605E+14	7,73986E+14	1,98812E+14	0.0907594971192231	0.234924944109565	-4,39356E+14	9,02491E+14	9,03622E+14	8,28954E+14	5,69948E+14	8,08877E+14	6,30026E+14
TC1200002411.mm.2	Ckb	creatine kinase, brain	0.944885415552397	1,06033E+14	1,98801E+14	0.0907742676910929	0.234925437729936	-4,39371E+14	1,13419E+14	1,12256E+14	1,08204E+14	9,55433E+14	1,05772E+14	1,01006E+13
TC1400002345.mm.2	Fam160b2	family with sequence similarity 160, member B2	0.632801505140271	6,18607E+14	1,98747E+14	0.0908444354036062	0.235069276964559	-4,39442E+13	6,62604E+14	6,39835E+14	6,0946E+14	6,23867E+13	5,67358E+14	6,08521E+14
TC1500000246.mm.2	Osr2	odd-skipped related 2	-0.666858245393808	5,11447E+14	-1,98733E+14	0.0908620223569342	0.235077033756789	-4,3946E+14	4,24439E+14	4,75586E+14	5,49985E+14	5,85275E+14	5,15967E+14	5,17429E+14

TC1800001301.mm.2	Mospd4	motile sperm domain containing 4	-0.738015129469831	6,44388E+14	-1,98715E+14	0.0908857898258365	0.235087676013889	-4,39483E+13	6,04684E+14	5,7794E+14	6,38561E+13	7,14916E+14	6,53103E+14	6,77124E+14
TC0400002580.mm.2	Shb	src homology 2 domain-containing transforming protein B	-0.989075185992935	7,18113E+14	-1,98705E+14	0.0908991614178615	0.235087676013889	-4,39497E+14	6,65498E+14	6,45139E+14	6,88291E+14	8,01296E+13	7,09327E+14	7,99125E+14
TC1700002184.mm.2	Rrp36	ribosomal RNA processing 36 homolog (S. cerevisiae)	-0.736600120297249	7,33642E+13	-1,98696E+14	0.0909099056192231	0.235087676013889	-4,39508E+13	6,72821E+14	6,88593E+14	7,56712E+14	7,74989E+14	7,14134E+14	7,946E+14
TC0100001932.mm.2	Sntg1	syntrophin, gamma 1	1,21997E+13	6,93415E+14	1,9867E+14	0.0909444815490662	0.235121564777065	-4,39542E+14	7,75879E+14	7,73432E+13	7,23872E+14	5,81991E+14	7,19262E+14	5,86055E+14
TC1300002541.mm.2	Mrps36	mitochondrial ribosomal protein S36	-0.696783922892631	7,84545E+13	-1,98661E+14	0.0909559695656622	0.235121564777065	-4,39554E+13	7,49042E+12	7,44116E+13	8,15864E+14	7,65733E+14	8,42077E+13	7,90438E+14
TC1500002190.mm.2	Ddn	dendrin	0.95734460926341	8,81949E+14	1,98648E+14	0.090972410061282	0.235121564777065	-4,3957E+14	9,36678E+14	9,57117E+14	9,09973E+14	7,85597E+13	8,80174E+14	8,22153E+14
TC0700002039.mm.2	Ptdss2	phosphatidylserine synthase 2	1,24644E+14	6,95449E+14	1,98641E+14	0.0909813787863836	0.235121564777065	-4,39579E+14	7,91718E+14	7,75171E+14	7,20851E+14	5,67353E+13	7,22414E+14	5,95186E+14
TC0100000954.mm.2	Neu4	sialidase 4	-0.646235953613671	6,74347E+14	-1,98613E+13	0.0910175605378197	0.235177349734748	-4,39616E+14	6,1136E+14	6,45865E+14	6,91869E+13	7,10513E+14	6,74131E+14	7,12342E+14
TC0900003324.mm.2	Bcl9l	B cell CLL/lymphoma 9-like	0.6357972496672	6,80792E+14	1,98593E+14	0.0910434789969886	0.235194334725825	-4,39642E+14	7,23376E+14	7,01323E+13	6,78165E+14	6,84348E+14	6,26187E+14	6,71352E+14
TC1900001028.mm.2	Dnajc4	DnaJ heat shock protein family (Hsp40) member C4	1,05404E+14	7,33531E+14	1,98581E+14	0.0910589046878663	0.235194334725825	-4,39657E+14	7,97813E+14	7,40627E+14	7,88047E+14	7,47068E+13	7,35697E+14	5,91935E+14
TC1300001034.mm.2	Ankrd34b	ankyrin repeat domain 34B	0.668924734433064	7,07127E+14	1,98564E+14	0.0910822149271007	0.235194334725825	-4,3968E+14	7,62951E+13	7,38564E+14	6,90501E+14	6,83016E+14	6,55022E+14	7,12708E+14
TC0900000750.mm.2	Parp6	poly (ADP-ribose) polymerase family, member 6	1,56727E+14	7,30205E+14	1,98563E+14	0.0910825202438495	0.235194334725825	-4,39681E+14	8,37652E+14	8,30801E+14	7,96866E+14	5,60916E+13	7,36214E+14	6,18784E+14
TC0200002287.mm.2	Defb20	defensin beta 20	-0.687717613521089	5,80203E+14	-1,98535E+14	0.0911196169874713	0.235248318689371	-4,39718E+14	5,14045E+14	5,25035E+14	6,00807E+14	6,64711E+14	5,75962E+14	6,00661E+14
TC0500003101.mm.2	Sirt4	sirtuin 4	-0.833736796099293	6,85196E+14	-1,98512E+14	0.0911488228054942	0.235248318689371	-4,39747E+14	6,57597E+14	6,28266E+14	6,62363E+13	7,10337E+14	6,79117E+14	7,73493E+14
TC1800000288.mm.2	Wnt8a	wingless-type MMTV integration site family, member 8A	-0.713202864349292	5,11774E+14	-1,98507E+14	0.0911553573423872	0.235248318689371	-4,39754E+14	4,84306E+14	4,4075E+14	5,11298E+14	5,66591E+14	5,17804E+14	5,49893E+14
TC1100001911.mm.2	Smim5	small integral membrane protein 5	-0.817076373197913	8,10261E+14	-1,98502E+14	0.0911618259725958	0.235248318689371	-4,3976E+14	7,53082E+13	7,63319E+13	7,99921E+14	8,65429E+14	7,98746E+14	8,8107E+14
TC1700000375.mm.2	Tpsg1	tryptase gamma 1	-0.913090039682441	5,48535E+14	-1,98482E+14	0.0911883689095631	0.235279133425106	-4,39787E+14	4,84679E+14	5,0163E+14	5,15377E+14	6,206E+14	5,48263E+14	6,20664E+14
TC0700000524.mm.2	Ggn	gametogenetin	-0.709730710234518	6,50617E+14	-1,98456E+14	0.0912224868617126	0.235329479795513	-4,39821E+14	6,10536E+14	6,15162E+14	6,49512E+14	6,60845E+13	6,46134E+14	7,2151E+14
TC1300002057.mm.2	Pfn3	profilin 3	-0.890448141948998	6,56083E+14	-1,98421E+14	0.0912670028548565	0.235392791481463	-4,39865E+14	5,81943E+14	6,06968E+14	6,42576E+14	7,38009E+14	6,46251E+13	7,20749E+14
TC0800000259.mm.2	Defb1	defensin beta 1	-0.994394229702158	4,9624E+14	-1,98414E+14	0.0912762465820259	0.235392791481463	-4,39875E+14	4,22013E+14	4,45287E+14	4,62964E+14	5,80998E+14	5,75553E+14	4,90627E+14
TC0500003220.mm.2	Anapc5	anaphase-promoting complex subunit 5	1,24363E+14	8,45898E+14	1,98394E+14	0.0913027606957482	0.235400311313202	-4,39901E+14	9,38619E+14	9,35777E+14	8,75806E+13	6,99516E+14	8,59827E+14	7,65844E+14
TC1300000385.mm.2	Bphl	biphenyl hydrolase-like (serine hydrolase, breast epithelial mucin-associated antigen)	0.854421400025124	6,01152E+14	1,9839E+14	0.0913083811647435	0.235400311313202	-4,39907E+14	6,44909E+14	6,43208E+14	6,57608E+14	5,43954E+14	5,49701E+14	5,67531E+14
TC0600002324.mm.2	Ggct	gamma-glutamyl cyclotransferase	0.92427870735706	6,64593E+14	1,98365E+14	0.0913407509466889	0.235446091825948	-4,39939E+14	7,42148E+14	7,1469E+14	6,38102E+14	6,20633E+14	6,99722E+14	5,72261E+14
TC0300000182.mm.2	Rpl22l1	ribosomal protein L22 like 1	1,11208E+14	7,06097E+14	1,98256E+14	0.0914824928299037	0.235764838502002	-4,40081E+14	7,92282E+14	7,69632E+12	7,51669E+14	5,83501E+13	6,86001E+14	6,53497E+14
TC1300002086.mm.2	Spock1	sparc/osteonectin, cwcv and kazal-like domains proteoglycan 1	0.732538138016976	8,37786E+14	1,98247E+13	0.0914936718893452	0.235764838502002	-4,40092E+12	9,25511E+14	8,56107E+14	8,28031E+14	7,81956E+14	8,18349E+14	8,16761E+14
TC0500003729.mm.2	Gbp8	guanylate-binding protein 8	-0.595991497538168	5,44119E+14	-1,98212E+14	0.0915395996279738	0.235823349591825	-4,40138E+14	5,01837E+14	4,9633E+14	5,68461E+14	5,8072E+14	5,73084E+14	5,44282E+14
TC1400001664.mm.2	Spcs1	signal peptidase complex subunit 1 homolog (S. cerevisiae)	1,01385E+14	8,4422E+13	1,98202E+12	0.0915529473456159	0.235823349591825	-4,40151E+14	9,19046E+14	9,10267E+14	8,70137E+14	7,3933E+14	8,51505E+14	7,75038E+14
TC0200001980.mm.2	1700020A23Rik	RIKEN cDNA 1700020A23 gene	-0.832220491337139	5,40693E+13	-1,98196E+14	0.0915602851763455	0.235823349591825	-4,40158E+14	4,97552E+14	4,93144E+13	5,14532E+14	5,81794E+14	5,35226E+14	6,21914E+14
TC0700002958.mm.2	1700008O03Rik	RIKEN cDNA 1700008O03 gene	-0.686859687116825	5,87791E+14	-1,98183E+14	0.0915773763153	0.23582967309708	-4,40175E+14	5,24919E+14	5,61705E+14	5,80137E+14	6,35993E+14	6,02543E+14	6,21453E+14
TC0700002966.mm.2	Mybpc2	myosin binding protein C, fast-type	-0.864973963889474	4,97535E+14	-1,98132E+14	0.0916451008485421	0.235954963515278	-4,40243E+14	4,72027E+14	3,99604E+13	4,94839E+14	5,74113E+14	4,97998E+14	5,46628E+14
TC0700002639.mm.2	Bckdha	branched chain ketoacid dehydrogenase E1, alpha polypeptide	0.684228987979469	6,21616E+14	1,98119E+14	0.0916620291089901	0.235954963515278	-4,4026E+13	6,58255E+14	6,72949E+14	6,25697E+14	5,78434E+14	6,21017E+14	5,73342E+14
TC0X00001619.mm.2	Map7d2	MAP7 domain containing 2	1,3355E+14	8,99412E+14	1,98104E+14	0.0916807048534843	0.235954963515278	-4,40278E+14	1,01153E+14	9,80305E+14	9,28347E+14	7,5155E+14	9,24819E+14	7,9992E+14
TC0500000971.mm.2	Enoph1	enolase-phosphatase 1	0.85567454222569	6,69975E+14	1,98097E+14	0.0916900851019399	0.235954963515278	-4,40288E+14	7,31233E+14	7,21613E+14	6,85968E+14	5,99323E+14	6,78864E+14	6,02847E+14
TC1500001578.mm.2	Tmem65	transmembrane protein 65	1,1895E+14	7,9802E+14	1,9809E+13	0.0916992479074455	0.235954963515278	-4,40297E+12	8,82196E+14	8,74646E+14	8,24081E+13	6,88251E+14	8,25143E+14	6,938E+14
TC1600001553.mm.2	Slc49a4	solute carrier family 49 member 4	0.973489795813188	7,82357E+14	1,9807E+14	0.0917257485721731	0.235985468105289	-4,40323E+14	8,51687E+14	8,41988E+14	8,15101E+14	6,86391E+14	7,80269E+14	7,18708E+13
TC0200001441.mm.2	Chst1	carbohydrate sulfotransferase 1	0.803426030433412	9,05923E+14	1,98051E+14	0.0917504020666608	0.236011211446377	-4,40348E+14	9,5107E+14	9,83994E+14	9,06059E+14	8,20036E+14	9,09903E+14	8,64476E+14
TC0800001103.mm.2	Slc12a3	solute carrier family 12, member 3	-0.606344229529998	5,31253E+14	-1,97988E+14	0.0918332933871497	0.236149140737304	-4,4043E+14	4,72639E+14	5,00489E+14	5,60823E+14	5,59173E+14	5,34289E+14	5,60108E+14
TC0900001586.mm.2	Wdr48	WD repeat domain 48	1,23635E+14	7,28164E+14	1,97983E+14	0.0918398662060369	0.236149140737304	-4,40436E+14	8,10504E+14	7,93056E+14	7,79329E+14	6,29805E+13	7,43098E+14	6,13192E+14
TC0700002345.mm.2	Vmn2r55	vomeronasal 2, receptor 55	-1,1707E+13	4,3708E+14	-1,97976E+14	0.0918490493615001	0.236149140737304	-4,40446E+14	3,71363E+14	3,4301E+14	4,14823E+14	5,44772E+14	4,09711E+14	5,38801E+14
TC0700000225.mm.2	Zc3h4	zinc finger CCCH-type containing 4	0.684939075402781	6,78111E+14	1,97965E+14	0.0918626459468393	0.236149140737304	-4,40459E+14	7,44999E+14	7,02561E+14	6,75075E+14	6,35458E+13	6,36526E+14	6,74046E+14
TC1100003192.mm.2	Olfr411	olfactory receptor 411	-0.613762155948885	5,95176E+14	-1,97942E+14	0.0918932387470685	0.236155340857313	-4,40489E+14	5,28397E+13	5,64755E+14	6,20728E+14	6,41272E+13	6,06994E+14	6,08909E+14
TC0500001976.mm.2	Gm21149	predicted gene, 21149	-0.685453998718335	5,15737E+14	-1,97941E+14	0.0918943702088593	0.236155340857313	-4,40491E+14	4,99733E+14	4,82563E+14	5,17324E+14	4,75414E+14	5,85933E+14	5,33454E+14
TC1800001150.mm.2	Nme5	NME/NM23 family member 5	0.899128347393562	6,73759E+14	1,97883E+14	0.0919701690027852	0.236312443492565	-4,40566E+14	7,51659E+14	7,4953E+14	6,73047E+12	5,46954E+13	6,57895E+14	6,63468E+14
TC0900000272.mm.2	Adamts8	a disintegrin-like and metallopeptidase (reprolysin type) with thrombospondin type 1 motif, 8	-1,06004E+14	4,73059E+14	-1,97862E+14	0.0919979113887099	0.236346037341563	-4,40593E+14	3,89421E+14	4,31343E+14	4,1919E+14	5,65631E+14	4,8857E+14	5,44202E+14
TC0900001874.mm.2	Tmed1	transmembrane p24 trafficking protein 1	-0.782042643550042	7,1215E+14	-1,97823E+12	0.0920490602366932	0.236439742960918	-4,40644E+12	6,55758E+14	6,77022E+14	6,84783E+14	7,66151E+14	7,20053E+13	7,69135E+14
TC0700002054.mm.2	Cracr2b	calcium release activated channel regulator 2B	-0.617166506911017	9,73239E+14	-1,978E+14	0.0920797575100533	0.236454242012814	-4,40674E+14	9,39202E+14	9,42853E+14	9,79337E+14	9,72553E+13	9,81275E+14	1,02421E+14
TC1100002993.mm.2	Dnah9	dynein, axonemal, heavy chain 9	-0.63138968333216	5,54871E+14	-1,97796E+14	0.092084054405164	0.236454242012814	-4,40679E+14	5,30672E+14	5,07178E+14	5,43265E+14	5,83984E+14	5,73736E+14	5,90392E+14
TC0100001563.mm.2	Ccdc190	coiled-coil domain containing 190	1,01022E+14	6,53221E+14	1,97758E+14	0.092134360347142	0.236545721522227	-4,40729E+14	6,97323E+14	7,33976E+14	6,99028E+14	5,59741E+14	6,38079E+14	5,91177E+14
TC1600002080.mm.2	Tmem50b	transmembrane protein 50B	1,13867E+14	8,24021E+14	1,97726E+14	0.0921761688992076	0.236548452521509	-4,4077E+14	9,04109E+14	9,03476E+14	8,51755E+12	7,04936E+14	8,4111E+14	7,38741E+14
TC0800002313.mm.2	Msmo1	methylsterol monoxygenase 1	1,3867E+14	6,657E+14	1,97697E+14	0.092214395275394	0.236548452521509	-4,40808E+14	7,88278E+14	7,76918E+14	6,78393E+14	4,62755E+14	6,82758E+14	6,05097E+14
TC0400000718.mm.2	Orm1	orosomucoid 1	-0.76115631950514	5,12416E+14	-1,97692E+14	0.0922212308286696	0.236548452521509	-4,40815E+14	4,74313E+14	4,35269E+13	5,19779E+14	5,8332E+14	5,04046E+14	5,57768E+14
TC1200000994.mm.2	Calm1	calmodulin 1	0.942240494665739	1,12996E+13	1,97681E+14	0.0922352833697257	0.236548452521509	-4,40828E+14	1,19142E+14	1,20222E+14	1,14225E+14	1,03868E+14	1,14664E+14	1,05856E+14
TC0200005349.mm.2	Gm14308/Gm14430/ Gm14432/Gm14434/ Gm2026/Gm4724	predicted gene 14308/predicted gene 14430/predicted gene 14432/predicted gene 14434/predicted gene 2026/predicted gene 4724	1,48703E+14	9,19884E+14	1,97673E+14	0.092246168589196	0.236548452521509	-4,40839E+13	1,01697E+14	1,08099E+14	9,05326E+14	7,15473E+14	9,74475E+14	8,26075E+14
TC1100003037.mm.2	Chd3	chromodomain helicase DNA binding protein 3	0.789061072219388	8,7452E+14	1,97672E+14	0.0922482460245438	0.236548452521509	-4,40841E+13	9,19947E+14	9,14202E+14	8,20708E+14	9,15923E+14	8,24233E+14	8,52104E+14
TC0700003822.mm.2	Mrpl48	mitochondrial ribosomal protein L48	0.931932533854431	8,49422E+14	1,97664E+14	0.0922577778433035	0.236548452521509	-4,40851E+14	9,24359E+14	9,1142E+14	8,61933E+14	7,49424E+14	8,60563E+14	7,8883E+14
TC1600001711.mm.2	Ccdc54	coiled-coil domain containing 54	-1,19038E+14	5,80419E+14	-1,9766E+14	0.0922638635106011	0.236548452521509	-4,40857E+14	4,70067E+14	5,27417E+14	5,47044E+14	7,02428E+14	5,71423E+14	6,64137E+14
TC0800000073.mm.2	Abhd13	abhydrolase domain containing 13	-0.598334611569157	8,85669E+14	-1,97646E+14	0.0922821978701233	0.236548452521509	-4,40875E+14	8,48088E+13	8,40802E+14	9,159E+14	9,00669E+14	9,19144E+14	8,89413E+14
TC0100002640.mm.2	Scg2	secretogranin II	1,74802E+14	9,36188E+14	1,97646E+13	0.0922822300347673	0.236548452521509	-4,40875E+14	1,0883E+14	1,00931E+14	1,01716E+14	7,54357E+14	9,3458E+14	8,13423E+14
TC0700004604.mm.2	Cttn	cortactin	0.589125331735405	7,53899E+14	1,97624E+14	0.0923106714003654	0.236583720100857	-4,40903E+14	8,03729E+14	7,80652E+14	7,41521E+14	7,30935E+14	7,26819E+14	7,39737E+14
TC1800001312.mm.2	Sema6a	sema domain, transmembrane domain (TM), and cytoplasmic domain, (semaphorin) 6A	1,2919E+14	7,05141E+14	1,97566E+14	0.0923869537433536	0.236741568967344	-4,40978E+14	8,00639E+14	7,80983E+14	7,17027E+14	6,08951E+14	7,51496E+14	5,71747E+13
TC1700000565.mm.2	Cyp4f37	cytochrome P450, family 4, subfamily f, polypeptide 37	-0.663851137949298	7,0465E+14	-1,97554E+14	0.0924023951437371	0.236743487510103	-4,40994E+14	6,43388E+14	6,79898E+14	7,20726E+14	7,27835E+14	7,0292E+14	7,53136E+14
TC0400001975.mm.2	Rere	arginine glutamic acid dipeptide (RE) repeats	1,16622E+13	6,82388E+14	1,97519E+13	0.0924489541736455	0.236793634838728	-4,41039E+14	7,75532E+14	7,36384E+14	7,09511E+14	5,94231E+14	7,08971E+14	5,69701E+14

TC0400003267.mm.2	Rnf11	ring finger protein 11	0.810702365267349	8,21808E+14	1,97513E+14	0.0924574718670778	0.236793634838728	-4,41048E+14	8,82269E+14	8,59119E+14	8,27135E+14	7,83075E+14	8,35919E+14	7,43329E+14
TC1000002523.mm.2	Slc39a3	solute carrier family 39 (zinc transporter), member 3	0.764705509518294	6,52905E+14	1,97506E+14	0.092466055384179	0.236793634838728	-4,41056E+13	6,82029E+14	7,17102E+14	6,57121E+14	6,14987E+13	5,85489E+14	6,60702E+14
TC0700002642.mm.2	Ccdc97	coiled-coil domain containing 97	0.646408609839379	7,07818E+13	1,97471E+13	0.0925119902415054	0.236873621287363	-4,41102E+13	7,55037E+14	7,31774E+14	7,15627E+14	6,86939E+14	6,57555E+14	6,99974E+12
TC1100004122.mm.2	Rhbdf2	rhomboid 5 homolog 2	-0.743289203168194	6,19613E+14	-1,97407E+14	0.0925973689790836	0.236955413188526	-4,41186E+14	5,73466E+14	5,79188E+14	6,2128E+14	6,42433E+14	6,08419E+14	6,92892E+14
TC0900000443.mm.2	Olfr978	olfactory receptor 978	-0.840974960789819	4,82651E+14	-1,97396E+13	0.0926110785741761	0.236955413188526	-4,41199E+14	4,09507E+14	4,37712E+13	4,88561E+13	5,44715E+14	4,60863E+14	5,5455E+14
TC1700002778.mm.2	Tapbp	TAP binding protein	-0.607181302330972	9,12168E+14	-1,97389E+13	0.0926209597728227	0.236955413188526	-4,41209E+14	8,67263E+14	8,83132E+14	9,20569E+14	9,30212E+14	9,18545E+13	9,53286E+14
TC1100000916.mm.2	Trp53	transformation related protein 53	0.579508002530033	6,98383E+14	1,97388E+14	0.0926220505609567	0.236955413188526	-4,4121E+14	7,43008E+14	7,44673E+14	6,67153E+14	6,63686E+14	6,96532E+14	6,75247E+14
TC0X00001766.mm.2	Clcn5	chloride channel, voltage-sensitive 5	1,14484E+14	6,38792E+12	1,97372E+14	0.0926431811425633	0.236955413188526	-4,41231E+14	7,53115E+14	6,80654E+14	6,68822E+14	5,25361E+14	6,43699E+14	5,61102E+13
TC0800001303.mm.2	Phlpp2	PH domain and leucine rich repeat protein phosphatase 2	0.656651039303791	6,86074E+14	1,97356E+14	0.0926641151941185	0.236955413188526	-4,41251E+14	7,43378E+14	7,1142E+14	6,63032E+14	6,75145E+14	6,36974E+14	6,86494E+14
TC0100000215.mm.2	Rab23	RAB23, member RAS oncogene family	1,00814E+14	5,66395E+14	1,97354E+14	0.0926662427016991	0.236955413188526	-4,41254E+14	6,26281E+14	6,51657E+14	5,04325E+14	5,39796E+14	6,10683E+14	4,65628E+14
TC0400002096.mm.2	Dvl1	dishevelled segment polarity protein 1	0.786809838341179	6,9466E+14	1,97351E+13	0.0926708144125187	0.236955413188526	-4,41258E+14	7,67394E+14	7,45826E+14	6,07711E+13	6,91174E+14	6,53464E+14	7,02393E+14
TC1300002082.mm.2	Il9	interleukin 9	-0.734259087834894	5,00145E+14	-1,97347E+14	0.0926762870920432	0.236955413188526	-4,41263E+14	4,26727E+14	4,42393E+14	5,16246E+13	5,99534E+14	5,06975E+14	5,08996E+14
TC0800001230.mm.2	Thap11	THAP domain containing 11	0.772133605939313	6,02313E+14	1,97335E+14	0.092691325182246	0.236956262519678	-4,41278E+14	6,65623E+14	6,31852E+14	5,44604E+14	6,28752E+14	5,56935E+13	5,86113E+14
TC0300001107.mm.2	Lrif1	ligand dependent nuclear receptor interacting factor 1	-0.607795741847667	6,4464E+14	-1,9731E+14	0.0927250625695028	0.237004906913451	-4,41311E+14	6,20152E+14	5,93702E+14	6,67328E+14	6,51243E+13	6,55178E+14	6,80235E+14
TC1000000183.mm.2	Slc2a12	solute carrier family 2 (facilitated glucose transporter), member 12	0.615716403078655	4,95478E+14	1,97275E+14	0.0927715366097139	0.237076313649353	-4,41357E+14	5,13744E+14	5,24682E+14	5,06761E+14	5,124E+14	4,78613E+14	4,36669E+14
TC1900000096.mm.2	Fau	Finkel-Biskis-Reilly murine sarcoma virus (FBR-MuSV) ubiquitously expressed (fox derived)	0.585941503487135	9,75911E+14	1,97267E+14	0.0927824262317893	0.237076313649353	-4,41368E+14	1,02059E+14	1,01342E+14	9,64816E+14	9,39815E+14	9,62046E+14	9,54777E+14
TC0500002265.mm.2	Mrfap1	Morf4 family associated protein 1	0.749821918753693	8,93185E+14	1,9725E+14	0.0928039456879274	0.23709370173927	-4,41389E+14	9,43876E+14	9,42057E+14	8,95475E+14	8,41735E+14	9,03608E+14	8,32361E+14
TC1100002062.mm.2	Narf	nuclear prelamin A recognition factor	-0.58505993274149	7,81775E+14	-1,97226E+14	0.0928368036585609	0.237140047138616	-4,41421E+14	7,33705E+14	7,48525E+14	7,94824E+14	8,14356E+14	7,94652E+14	8,0459E+14
TC0300000812.mm.2	Pglyrp4	peptidoglycan recognition protein 4	-0.756476056784399	6,56578E+13	-1,97153E+14	0.0929329689858923	0.237316622756532	-4,41516E+14	6,15095E+14	5,92185E+14	6,44405E+14	7,29208E+14	6,91544E+14	6,67031E+14
TC0500001220.mm.2	Ung	uracil DNA glycosylase	-0.978076849482368	7,02977E+14	-1,97147E+14	0.0929406841946284	0.237316622756532	-4,41523E+14	6,2488E+14	6,45643E+14	6,96712E+14	7,8449E+14	6,76276E+14	7,89862E+13
TC1300000469.mm.2	Tmem170b	transmembrane protein 170B	1,07734E+14	7,22302E+14	1,9714E+13	0.0929501151999302	0.237316622756532	-4,41532E+14	7,82501E+14	7,6843E+14	7,79455E+14	6,67963E+14	7,29302E+14	6,0616E+14
TC1300001776.mm.2	Serpinb6c	serine (or cysteine) peptidase inhibitor, clade B, member 6c	-0.917936390612077	4,82522E+14	-1,97121E+14	0.0929758081746867	0.237344612978252	-4,41558E+14	4,03455E+14	4,39115E+14	4,51478E+14	5,74925E+14	5,26875E+14	4,99282E+14
TC1100002333.mm.2	Otx1	orthodenticle homeobox 1	-0.946619958636226	6,72333E+14	-1,97088E+14	0.0930186501503717	0.237400168528538	-4,416E+14	5,94366E+14	6,24169E+13	6,41688E+14	7,65918E+14	6,77801E+14	7,30058E+14
TC0100002964.mm.2	Dbi	diazepam binding inhibitor	0.82068706276605	9,38471E+13	1,97075E+14	0.0930361680600617	0.237400168528538	-4,41617E+13	1,0005E+14	1,00793E+14	9,3683E+14	8,41267E+14	9,4714E+14	8,97155E+14
TC0200005105.mm.2	Wfdc5	WAP four-disulfide core domain 5	-0.606967695009327	4,84863E+14	-1,97071E+14	0.0930417715048544	0.237400168528538	-4,41622E+14	4,24281E+14	4,50967E+14	5,32501E+14	5,04788E+14	5,03379E+14	4,93263E+14
TC1300002034.mm.2	4833439L19Rik	RIKEN cDNA 4833439L19 gene	0.955855250637077	9,52639E+14	1,97042E+14	0.0930803243570546	0.237460935143322	-4,4166E+14	1,02612E+14	9,9565E+14	9,96628E+14	8,66838E+14	9,36946E+13	8,93653E+14
TC0700001736.mm.2	Eef2k	eukaryotic elongation factor-2 kinase	0.666067265867037	6,39294E+14	1,97019E+14	0.0931104005766643	0.237483885361457	-4,4169E+14	6,92264E+14	6,78975E+14	6,44428E+14	5,82069E+14	6,21043E+14	6,16983E+14
TC1000000742.mm.2	2610028H24Rik	RIKEN cDNA 2610028H24 gene	-0.57011495746054	7,25489E+14	-1,97013E+14	0.0931187977231853	0.237483885361457	-4,41698E+14	6,94418E+14	6,87846E+14	7,33086E+14	7,41299E+14	7,44916E+14	7,5137E+14
TC0800001569.mm.2	Map3k21	mitogen-activated protein kinase kinase kinase 21	-1,16903E+14	5,87853E+14	-1,96932E+14	0.093225966461384	0.237719575501231	-4,41803E+14	4,93731E+14	5,31223E+13	5,41519E+14	7,01882E+14	5,85842E+14	6,72918E+14
TC0400003207.mm.2	Mroh7	maestro heat-like repeat family member 7	-1,0142E+14	5,95552E+14	-1,9691E+14	0.0932557454416278	0.237746505751358	-4,41832E+14	5,73219E+14	4,69748E+14	5,8685E+14	6,97689E+14	6,11614E+14	6,34192E+14
TC0700001444.mm.2	Numa1	nuclear mitotic apparatus protein 1	1,01239E+14	6,04125E+14	1,96902E+14	0.0932660375382816	0.237746505751358	-4,41842E+14	6,9411E+14	6,61153E+14	6,30249E+14	4,86451E+14	5,96506E+14	5,5628E+14
TC0600002079.mm.2	Ptn	pleiotrophin	0.702277537895082	9,77451E+14	1,96869E+14	0.0933094676517111	0.237763234220347	-4,41884E+14	1,03083E+14	1,01963E+14	9,68771E+14	9,35474E+14	9,88596E+12	9,21406E+14
TC1200001418.mm.2	Msgn1	mesogenin 1	-0.961561981505854	6,84468E+13	-1,96853E+14	0.0933310602216498	0.237763234220347	-4,41905E+14	5,95664E+14	6,28425E+14	6,66203E+14	8,00112E+14	6,91887E+14	7,24514E+14
TC1500001323.mm.2	March6	membrane-associated ring finger (C3HC4) 6	1,82822E+14	8,57433E+14	1,96837E+13	0.0933521386206672	0.237763234220347	-4,41926E+14	9,73741E+14	9,65165E+14	9,48742E+14	6,83686E+14	8,7324E+14	7,00022E+14
TC1700001881.mm.2	Kifc1	kinesin family member C1	-0.677730605394119	6,69671E+13	-1,96831E+14	0.0933598939410678	0.237763234220347	-4,41933E+14	6,20227E+14	6,4648E+14	6,56105E+14	6,92962E+14	6,75717E+14	7,26535E+14
TC1100002921.mm.2	Ulk2	unc-51 like kinase 2	1,06122E+14	7,39637E+14	1,96814E+14	0.0933833757474188	0.237763234220347	-4,41956E+14	8,20545E+14	8,08497E+14	7,63286E+14	6,28699E+13	7,54541E+14	6,62257E+14
TC0100000588.mm.2	Vwc2l	von Willebrand factor C domain-containing protein 2-like	0.74517988501325	5,49457E+14	1,96805E+14	0.0933948850028257	0.237763234220347	-4,41968E+14	6,34957E+14	5,5192E+14	5,51096E+14	5,20928E+14	5,16926E+14	5,20915E+14
TC1100000887.mm.2	Slc25a35	solute carrier family 25, member 35	-0.567900932088544	7,15935E+14	-1,96802E+14	0.0933997065346661	0.237763234220347	-4,41972E+14	6,80339E+14	6,74207E+14	7,37345E+14	7,35592E+14	7,37636E+14	7,3049E+13
TC0600001589.mm.2	Borcs5	BLOC-1 related complex subunit 5	0.718336386336627	5,52616E+13	1,96798E+14	0.0934051078282543	0.237763234220347	-4,41978E+14	6,24003E+14	5,77644E+14	5,2432E+14	5,31749E+14	4,96151E+14	5,61828E+14
TC1600001459.mm.2	Lrrc15	leucine rich repeat containing 15	-0.843386126016904	5,82673E+14	-1,96797E+14	0.0934054038735676	0.237763234220347	-4,41978E+13	5,22297E+14	5,04113E+14	5,85582E+14	6,88958E+14	5,75261E+14	6,19827E+14
TC1900001576.mm.2	Lbx1	ladybird homeobox 1	-0.816131934309381	5,76571E+13	-1,96779E+14	0.0934295488913399	0.237787130198414	-4,42001E+13	5,07632E+14	5,3174E+14	5,80347E+14	6,37107E+14	5,57418E+14	6,45181E+13
TC1100001730.mm.2	Itgb3	integrin beta 3	-0.827210083542298	5,97435E+14	-1,9674E+14	0.0934813179692415	0.237846061913621	-4,42052E+14	5,40163E+13	5,59206E+14	5,71495E+14	6,48935E+14	6,70351E+14	5,9446E+14
TC1800000614.mm.2	Rbm22	RNA binding motif protein 22	1,43833E+14	6,65754E+14	1,9674E+14	0.0934822261589377	0.237846061913621	-4,42053E+13	7,61281E+14	7,49349E+13	7,43238E+14	5,17692E+14	6,53643E+14	5,69321E+14
TC1800000418.mm.2	Kctd16	potassium channel tetramerisation domain containing 16	0.598053826984157	7,56263E+14	1,96671E+14	0.0935743980905148	0.238042986490759	-4,42143E+14	7,92304E+14	8,00137E+14	7,22433E+14	7,49873E+14	7,47147E+14	7,25682E+14
TC1900000401.mm.2	Ndufb4b	NADH:ubiquinone oxidoreductase subunit B4B	0.848011703298649	9,52637E+14	1,9657E+14	0.0937085902074287	0.238346726758058	-4,42273E+14	1,0171E+14	1,00275E+14	9,57935E+14	8,8779E+13	9,7038E+14	8,79869E+14
TC1200002345.mm.2	Setd3	SET domain containing 3	0.85637181110855	9,39097E+14	1,96529E+14	0.0937634977624926	0.238432710633458	-4,42327E+14	9,97648E+14	1,00609E+14	9,42098E+14	8,56292E+14	9,56133E+14	8,76327E+14
TC0700003041.mm.2	Saa1	serum amyloid A 1	-0.708922026625989	5,36059E+14	-1,96523E+14	0.0937719907704476	0.238432710633458	-4,42335E+14	4,70012E+14	5,23528E+14	5,3067E+14	5,56819E+14	5,42312E+14	5,93012E+14
TC1900000350.mm.2	Cemip2	cell migration inducing hyaluronidase 2	0.623004533192318	5,61451E+13	1,96508E+13	0.0937919803171582	0.238445910200437	-4,42354E+14	5,9148E+14	5,9071E+14	5,48828E+14	5,80096E+13	5,47037E+14	5,10552E+14
TC0900000704.mm.2	Rpp25	ribonuclease P/MRP 25 subunit	0.572584584920993	6,62488E+13	1,96465E+14	0.0938493428757575	0.238525761487619	-4,4241E+14	7,07755E+14	7,10816E+14	6,34684E+14	6,17621E+14	6,53249E+14	6,50805E+14
TC1000003166.mm.2	Gdf11	growth differentiation factor 11	0.600096719355283	6,47887E+14	1,96452E+13	0.0938664863179451	0.238525761487619	-4,42427E+14	6,71913E+14	6,61925E+14	6,85534E+14	6,54136E+14	6,15533E+14	5,98285E+14
TC0700002277.mm.2	Vmn2r39	vomeronasal 2, receptor 39	-0.755007745417359	4,77113E+14	-1,96451E+14	0.0938679726730084	0.238525761487619	-4,42428E+14	4,28813E+14	4,39458E+14	4,71874E+14	5,03261E+14	4,66973E+14	5,52299E+14
TC0900002369.mm.2	Scamp5	secretory carrier membrane protein 5	0.938786833290431	9,60591E+13	1,96436E+14	0.0938883333314206	0.238525761487619	-4,42448E+14	1,02482E+14	1,02641E+14	9,92184E+12	8,56661E+14	9,41589E+14	9,21884E+14
TC1700000884.mm.2	Yipf3	Yip1 domain family, member 3	1,04727E+14	7,69093E+14	1,96428E+14	0.093898025120414	0.238525761487619	-4,42457E+14	8,43733E+14	8,45762E+14	7,94451E+14	6,50569E+14	7,76198E+14	7,03843E+14
TC0900001858.mm.2	Tyk2	tyrosine kinase 2	0.768884048375052	5,49932E+12	1,96418E+14	0.0939122094505961	0.238525761487619	-4,42471E+14	5,43215E+14	5,5929E+14	5,82308E+14	6,66049E+13	4,53037E+14	4,9569E+14
TC0700000769.mm.2	Klk14	kallikrein related-peptidase 14	-0.778881793331749	4,94128E+14	-1,96382E+14	0.0939608022652869	0.238611569251468	-4,42518E+13	4,22848E+13	4,53066E+14	4,88554E+14	5,68608E+14	4,91908E+14	5,39781E+14
TC0100000824.mm.2	Atg16l1	autophagy related 16-like 1 (S. cerevisiae)	0.959915712537603	6,04432E+14	1,96331E+14	0.0940281878632975	0.238729339603215	-4,42583E+13	6,43179E+14	6,49414E+14	6,23168E+14	6,1022E+14	6,20875E+14	4,79734E+14
TC1400002142.mm.2	Cryl1	crystallin, lambda 1	-0.580255474936006	6,45484E+13	-1,96319E+14	0.0940449009010926	0.238729339603215	-4,426E+14	6,00873E+14	5,96408E+14	6,85038E+14	6,77252E+14	6,45076E+14	6,68256E+14
TC1200001012.mm.2	Rin3	Ras and Rab interactor 3	-0.876045008584296	6,35195E+14	-1,96314E+14	0.0940516258797996	0.238729339603215	-4,42606E+14	5,73521E+14	5,91745E+14	5,93571E+14	7,1186E+14	6,573E+14	6,83174E+14
TC0400000437.mm.2	Ccdc107	coiled-coil domain containing 107	-0.831826228627251	7,60237E+14	-1,96283E+14	0.0940935133043577	0.238787096080416	-4,42647E+14	7,07067E+14	6,83613E+14	7,69356E+14	8,44342E+14	7,38812E+14	8,18234E+14
TC0500000860.mm.2	Parm1	prostate androgen-regulated mucin-like protein 1	1,25194E+14	7,25877E+14	1,96275E+14	0.0941040191218669	0.238787096080416	-4,42657E+14	8,39524E+14	8,02851E+14	7,56689E+13	5,64211E+14	7,24788E+14	6,67198E+14
TC1500002295.mm.2	Npff/Atf7	neuropeptide FF-amide peptide precursor/activating transcription factor 7	-0.634276752352568	9,03141E+14	-1,96258E+14	0.0941270369973347	0.238807895943639	-4,42679E+14	8,53689E+14	8,70616E+14	8,96567E+14	9,46812E+13	9,2665E+14	9,24511E+14
TC0300003239.mm.2	Gm128	predicted gene 128	-1,17299E+14	4,90989E+14	-1,96221E+14	0.0941761731801848	0.238894943081284	-4,42727E+14	4,10261E+14	3,87825E+13	4,88661E+14	6,26503E+14	4,53415E+14	5,7927E+14
TC0600000816.mm.2	Foxi3	forkhead box I3	-0.659292323288504	5,28676E+14	-1,96193E+14	0.094213553072573	0.238922425937642	-4,42763E+14	4,61402E+14	5,03753E+14	5,56209E+14	5,53676E+14	5,25563E+14	5,71451E+14

TC0200005339.mm.2	Gm14434/Gm2026/Gm4724/Gm14308/Gm14430/Gm14432	predicted gene 14434/predicted gene 2026/predicted gene 4724/predicted gene 14308/predicted gene 14430/predicted gene 14432	1,45998E+14	9,02279E+13	1,96191E+14	0.0942166632165196	0.238922425937642	-4,42766E+14	9,9799E+14	1,05898E+14	8,86651E+14	7,0508E+14	9,59814E+13	8,05158E+14
TC1100003567.mm.2	Acsf2	acyl-CoA synthetase family member 2	0.771440643159003	5,93522E+14	1,9617E+14	0.0942451773629002	0.238954029400662	-4,42793E+14	6,58142E+14	6,14739E+14	5,7668E+14	5,92978E+14	6,01494E+14	5,171E+14
TC1800001234.mm.2	Spry4	sprouty RTK signaling antagonist 4	-0.638211582387741	6,68607E+14	-1,96159E+14	0.0942587855067713	0.238954029400662	-4,42807E+14	6,55247E+14	6,01264E+14	6,84205E+14	6,86772E+14	6,87726E+14	6,96428E+12
TC1400002464.mm.2	Dnajc15	DnaJ heat shock protein family (Hsp40) member C15	0.636660870679704	7,58789E+14	1,96147E+13	0.0942756217680676	0.238959114920383	-4,42823E+14	8,11006E+14	7,72885E+13	7,44675E+13	7,67606E+14	7,12844E+14	7,43716E+14
TC1100002233.mm.2	Cobl	cordon-bleu WH2 repeat	1,49393E+14	7,41484E+14	1,96124E+13	0.0943069425847117	0.238964027763223	-4,42853E+14	8,33253E+14	8,4691E+14	7,74387E+14	6,12976E+14	7,87858E+14	5,93518E+14
TC0100003715.mm.2	Capn2	calpain 2	0.60966543579986	6,79226E+14	1,96112E+14	0.094322813829611	0.238964027763223	-4,42868E+14	7,14902E+13	7,39984E+14	6,56917E+14	6,30599E+14	6,55408E+14	6,77545E+14
TC0700000826.mm.2	Rras	related RAS viral (r-ras) oncogene	0.624277702690262	7,35916E+14	1,96111E+13	0.0943244963082764	0.238964027763223	-4,4287E+14	7,62739E+14	7,75177E+14	7,25946E+14	7,38575E+14	7,29448E+14	6,83613E+13
TC1000002424.mm.2	Gm19402	predicted gene, 19402	-0.673744699432957	6,50618E+14	-1,96101E+13	0.0943368820580813	0.238964027763223	-4,42882E+14	6,02485E+14	5,97131E+14	6,51202E+13	7,18526E+14	6,67972E+14	6,66392E+14
TC1400000385.mm.2	Grid1	glutamate receptor, ionotropic, delta 1	0.749194833734192	7,30931E+14	1,96077E+14	0.0943700988325624	0.23900328404096	-4,42914E+14	8,05167E+14	7,45597E+13	7,47272E+14	6,86624E+14	7,15728E+14	6,85197E+14
TC0700000219.mm.2	Napa	N-ethylmaleimide sensitive fusion protein attachment protein alpha	0.757992138574979	9,5352E+14	1,96068E+13	0.0943820453269178	0.23900328404096	-4,42926E+13	1,01531E+14	9,97479E+14	9,56876E+14	8,90268E+14	9,61619E+14	8,99572E+14
TC0700004539.mm.2	Pidd1	p53 induced death domain protein 1	-0.829545043746268	6,55069E+14	-1,96051E+14	0.0944050027674722	0.239023854429962	-4,42948E+14	5,95211E+14	6,13399E+14	6,24236E+13	7,23049E+14	6,63208E+14	7,11311E+14
TC1300000280.mm.2	Tdp2	tyrosyl-DNA phosphodiesterase 2	0.905543029702313	5,9054E+14	1,96019E+14	0.0944477527507391	0.239067380425154	-4,42989E+14	6,46662E+14	6,30389E+14	6,45599E+14	5,24648E+14	5,69972E+14	5,25971E+14
TC1600000397.mm.2	Fam43a	family with sequence similarity 43, member A	-0.629207208438808	5,82425E+13	-1,9601E+14	0.0944603697560209	0.239067380425154	-4,43001E+14	5,30485E+14	5,61395E+13	5,72338E+14	6,12614E+14	6,12658E+14	6,05063E+14
TC1900000928.mm.2	Gm960	predicted gene 960	-0.590522283340101	4,78328E+14	-1,96005E+14	0.0944667045967203	0.239067380425154	-4,43007E+13	4,68915E+12	4,53124E+14	4,64797E+14	4,42989E+14	5,29173E+14	5,10971E+14
TC0400003406.mm.2	Olfr1329	olfactory receptor 1329	-0.717523155379546	5,5386E+14	-1,95961E+14	0.0945264336623832	0.239144750943232	-4,43065E+14	5,13163E+14	4,90359E+14	5,47294E+14	6,25318E+14	5,7562E+14	5,71407E+14
TC0X00001832.mm.2	Dynlt3	dynein light chain Tctex-type 3	0.992603437741659	8,41703E+14	1,9596E+14	0.0945269607619589	0.239144750943232	-4,43065E+14	9,02158E+13	9,16991E+14	8,69677E+13	7,40762E+14	8,47537E+14	7,73092E+14
TC0100003323.mm.2	Ralgps2	Ral GEF with PH domain and SH3 binding motif 2	1,37121E+14	6,86434E+13	1,95919E+14	0.0945825943746051	0.239205459010472	-4,43119E+14	7,82245E+14	7,98469E+14	7,19751E+14	5,11666E+14	7,00193E+14	6,0628E+14
TC0300002321.mm.2	Rxfp4	relaxin family peptide receptor 4	-0.620400433601974	5,91708E+14	-1,95917E+14	0.0945857636904567	0.239205459010472	-4,43122E+14	5,73831E+14	5,76413E+14	5,43996E+13	5,81684E+14	6,51757E+14	6,22567E+14
TC1700001561.mm.2	Zfp995	zinc finger protein 995	1,33598E+14	5,13986E+14	1,95909E+14	0.0945954933789317	0.239205459010472	-4,43131E+14	6,02409E+14	5,90675E+13	5,62497E+14	4,02445E+14	5,39492E+14	3,86396E+14
TC0100002376.mm.2	Kctd18	potassium channel tetramerisation domain containing 18	-0.718336579689659	5,61362E+14	-1,95884E+14	0.0946293722824699	0.239247845378408	-4,43164E+13	5,43913E+14	5,2307E+12	5,58396E+14	5,3214E+14	5,68062E+14	6,42589E+13
TC1700001605.mm.2	Flywch2	FLYWCH family member 2	-0.604272077745302	7,76594E+14	-1,95873E+14	0.0946445805241262	0.239247845378408	-4,43179E+14	7,58385E+14	7,26142E+14	7,96863E+14	7,72789E+14	8,01749E+14	8,03633E+14
TC0200005183.mm.2	Kcnb1	potassium voltage gated channel, Shab-related subfamily, member 1	-0.611133657730805	7,76857E+14	-1,95863E+13	0.0946579867595745	0.239247845378408	-4,43192E+14	7,22399E+14	7,4838E+14	7,89796E+14	8,07561E+14	7,81395E+14	8,1161E+14
TC0500002302.mm.2	Rab28	RAB28, member RAS oncogene family	1,36609E+14	7,40085E+13	1,95853E+14	0.0946716478702124	0.239247845378408	-4,43205E+14	8,46487E+14	8,23396E+14	7,61096E+14	6,12867E+14	7,84138E+14	6,12527E+14
TC1400000600.mm.2	Tmem253	transmembrane protein 253	-0.607118490794186	5,98816E+12	-1,95831E+14	0.0947008659757092	0.239249748240192	-4,43233E+14	5,64662E+14	5,3053E+14	6,29029E+14	6,52058E+14	6,1178E+14	6,04835E+14
TC0100001308.mm.2	Kcnt2	potassium channel, subfamily T, member 2	1,49615E+14	6,50373E+14	1,95826E+14	0.0947074868267742	0.239249748240192	-4,43239E+14	7,449E+14	7,28256E+14	6,46902E+14	6,08255E+14	7,12149E+14	4,61777E+14
TC0200004896.mm.2	Trib3	tribbles pseudokinase 3	-0.93826873112258	6,14834E+14	-1,95809E+14	0.0947307757586338	0.239249748240192	-4,43262E+14	5,65894E+14	5,42668E+14	6,08131E+14	6,76097E+14	5,89469E+14	7,06747E+14
TC0700002148.mm.2	Cdc42ep5	CDC42 effector protein (Rho GTPase binding) 5	-0.695954660425563	5,37994E+13	-1,95801E+12	0.0947411576426709	0.239249748240192	-4,43271E+14	4,99064E+14	5,30039E+13	4,96289E+14	5,34279E+14	5,55739E+14	6,12556E+14
TC1200001597.mm.2	Bcap29	B cell receptor associated protein 29	1,48839E+14	7,33387E+14	1,95788E+14	0.0947599703305602	0.239249748240192	-4,4329E+14	8,3918E+14	8,29325E+14	7,90393E+14	5,70596E+14	7,47264E+14	6,23564E+14
TC0200005295.mm.2	Ankrd60	ankyrin repeat domain 60	-0.800384790626237	5,21936E+13	-1,95786E+14	0.0947614903040345	0.239249748240192	-4,43291E+14	4,51648E+14	4,54193E+14	5,31191E+14	6,28669E+14	5,51451E+14	5,14467E+14
TC0200003492.mm.2	Orc4	origin recognition complex, subunit 4	1,63722E+14	6,34163E+14	1,95763E+13	0.0947929238844394	0.239280920823147	-4,43321E+14	7,54399E+14	7,4046E+14	5,9488E+14	5,4782E+14	7,2239E+13	4,45025E+14
TC1500000617.mm.2	Apol6	apolipoprotein L 6	-0.81305084589429	6,20155E+14	-1,95755E+14	0.0948035374253691	0.239280920823147	-4,43331E+14	5,20114E+14	5,86355E+14	6,36646E+14	7,08736E+14	6,12267E+14	6,56811E+14
TC0300001984.mm.2	Ufm1	ubiquitin-fold modifier 1	1,24097E+14	7,58344E+14	1,95721E+13	0.0948500661968283	0.239322138161342	-4,43376E+14	8,33327E+14	8,55315E+14	7,54725E+14	6,66247E+14	8,10744E+14	6,29703E+14
TC0600002500.mm.2	Kcmf1	potassium channel modulatory factor 1	1,63873E+14	8,59027E+14	1,95705E+14	0.0948710428533008	0.239322138161342	-4,43396E+14	9,54927E+14	9,53583E+14	9,16883E+14	7,48005E+13	9,0334E+14	6,77424E+14
TC1900001169.mm.2	Zfp91/Cntf/Gm44505	zinc finger protein 91/ciliary neurotrophic factor/predicted readthrough transcript (NMD candidate), 44505	1,1144E+14	7,63904E+11	1,95701E+14	0.0948773452297651	0.239322138161342	-4,43402E+14	8,38627E+14	8,48681E+14	7,80926E+14	6,50766E+14	7,91468E+14	6,72959E+14
TC1700000673.mm.2	Ltb	lymphotoxin B	-0.736643920060859	6,45191E+13	-1,95699E+14	0.0948792787547107	0.239322138161342	-4,43404E+14	6,07018E+14	5,7329E+14	6,41657E+14	7,21546E+14	6,59198E+13	6,68439E+14
TC0700003928.mm.2	Ubqln3	ubiquilin 3	-0.638575701241127	4,98574E+14	-1,95686E+14	0.0948968869204176	0.23932908732958	-4,43421E+14	4,59387E+14	4,65045E+14	4,85528E+14	5,2934E+14	5,09292E+14	5,42855E+14
TC0200001760.mm.2	Rad51	RAD51 recombinase	-0.617781002204833	5,32389E+14	-1,9567E+14	0.0949195715484056	0.239348835111026	-4,43443E+14	4,92486E+14	5,10251E+14	5,35338E+14	5,29963E+14	5,84701E+14	5,41593E+14
TC0500001636.mm.2	Zcwpw1	zinc finger, CW type with PWWP domain 1	1,15635E+14	5,17309E+14	1,95643E+14	0.094954979156874	0.239352841249165	-4,43477E+14	5,56115E+14	5,74184E+14	5,12373E+13	5,62154E+14	3,60763E+14	5,38266E+14
TC1600000708.mm.2	Cd47	CD47 antigen (Rh-related antigen, integrin-associated signal transducer)	1,38954E+14	9,22142E+14	1,95643E+14	0.094955612820123	0.239352841249165	-4,43477E+14	1,00849E+14	1,02683E+14	9,58008E+14	7,82101E+14	9,54505E+14	8,02911E+14
TC0800002440.mm.2	B3gnt3	UDP-GlcNAc:betaGal beta-1,3-N-acetylglucosaminyltransferase 3	-0.954415403036289	6,28043E+14	-1,95635E+14	0.0949657242044257	0.239352841249165	-4,43487E+14	5,38533E+14	6,2511E+14	5,79283E+14	6,70806E+14	6,45168E+14	7,09358E+13
TC0800001733.mm.2	Dcun1d2	DCN1, defective in cullin neddylation 1, domain containing 2 (S. cerevisiae)	-0.9229655942677	7,89801E+14	-1,95584E+14	0.0950351880909173	0.239490457570996	-4,43554E+14	7,34966E+14	7,3167E+14	8,70879E+14	7,50065E+14	8,5484E+14	7,96389E+14
TC1800000368.mm.2	Pcdhb12	protocadherin beta 12	0.640966089187434	5,18158E+14	1,9556E+14	0.0950676548007026	0.239534811853592	-4,43585E+14	5,32325E+14	5,72926E+14	5,26758E+14	4,99878E+14	5,02912E+14	4,74145E+14
TC0400001458.mm.2	Trappc3	trafficking protein particle complex 3	1,09776E+14	8,00375E+14	1,95545E+14	0.0950882080974858	0.239549139630205	-4,43604E+14	8,95273E+14	8,75978E+14	7,95064E+14	6,84235E+14	8,41538E+14	7,10161E+14
TC0700000727.mm.2	Gm5592	predicted gene 5592	-0.738516545045326	4,80209E+13	-1,95528E+14	0.0951106623206702	0.239568251050955	-4,43626E+14	4,38863E+14	4,13406E+13	4,83699E+14	5,45317E+14	4,71328E+14	5,28645E+14
TC0400003808.mm.2	Cela3b	chymotrypsin-like elastase family, member 3B	-1,02752E+14	6,0655E+14	-1,95517E+13	0.0951263547068866	0.239570327194758	-4,43641E+14	5,41624E+14	5,44479E+13	5,71962E+14	6,89625E+14	5,93241E+14	6,98366E+14
TC1100001235.mm.2	Unc45b	unc-45 myosin chaperone B	-0.836236651886793	5,75095E+14	-1,95497E+14	0.0951537822778514	0.239601952468045	-4,43667E+14	5,65386E+14	4,99607E+14	5,36118E+14	6,1722E+14	5,93703E+14	6,38537E+14
TC0200004235.mm.2	4931422A03Rik	RIKEN cDNA 4931422A03 gene	-0.957832582055088	5,68475E+14	-1,95372E+14	0.0953226920755396	0.239963128556759	-4,43829E+14	5,14572E+14	5,05946E+14	5,49854E+14	6,28394E+14	5,46771E+14	6,65313E+14
TC1500000207.mm.2	Cmbl	carboxymethylenebutenolidase-like (Pseudomonas)	0.749137401175465	5,34036E+14	1,95368E+14	0.0953290948281699	0.239963128556759	-4,43835E+14	6,071E+14	5,51393E+14	5,10436E+14	5,26624E+14	5,3943E+14	4,69235E+14
TC1700000398.mm.2	D630044L22Rik	RIKEN cDNA gene D630044L22 gene	-1,02021E+14	5,43082E+13	-1,95358E+14	0.0953418946825774	0.239963128556759	-4,43847E+14	4,68645E+14	4,79226E+14	5,08413E+14	6,50293E+13	5,43492E+14	6,08422E+14
TC0500001026.mm.2	Pkd2	polycystic kidney disease 2	0.87901337031521	7,15021E+14	1,95291E+14	0.0954331233045696	0.240142620330748	-4,43934E+14	7,85424E+14	7,92167E+14	7,13788E+13	5,9696E+14	7,21775E+14	6,80013E+14
TC0400001517.mm.2	Sync	syncoilin	-0.827186026810219	6,49725E+14	-1,95282E+14	0.095444923357306	0.240142620330748	-4,43945E+14	5,85811E+14	6,08358E+14	6,29286E+14	7,15285E+14	6,46061E+14	7,13546E+14
TC0200001628.mm.2	Ccdc34	coiled-coil domain containing 34	0.643195183308777	7,4773E+14	1,95262E+14	0.0954727746518908	0.240142620330748	-4,43972E+14	8,04913E+14	7,93428E+14	7,10038E+14	7,08299E+14	7,63146E+14	7,06556E+14
TC1400001480.mm.2	Lrrc3b	leucine rich repeat containing 3B	1,48678E+14	6,39398E+14	1,95262E+14	0.0954728247898449	0.240142620330748	-4,43972E+14	7,45408E+14	7,12221E+14	6,90769E+14	5,27718E+14	6,75349E+13	4,84925E+14
TC0400001405.mm.2	Rhbdl2	rhomboid like 2	-0.703872106510478	5,27647E+14	-1,9521E+14	0.0955428730462282	0.240220115683227	-4,44039E+14	4,65366E+14	4,76678E+14	5,35586E+14	6,05432E+14	5,24849E+13	5,5797E+13
TC0700002669.mm.2	Pld3	phospholipase D family, member 3	0.617579376409756	9,46774E+13	1,95204E+14	0.0955523521351057	0.240220115683227	-4,44048E+14	1,00056E+14	9,79841E+14	9,41671E+14	9,01684E+14	9,35983E+13	9,20903E+14
TC0700004639.mm.2	Mia	melanoma inhibitory activity	-0.652722002728419	6,72212E+13	-1,95196E+14	0.095562332092395	0.240220115683227	-4,44057E+14	6,17653E+14	6,5735E+13	6,55466E+13	6,97257E+14	6,92273E+14	7,13275E+14
TC1000002376.mm.2	Lrrc75b	leucine rich repeat containing 75B	-0.61809181519667	6,38034E+13	-1,95196E+14	0.0955632682634819	0.240220115683227	-4,44058E+14	5,97386E+14	6,1733E+13	6,20072E+14	6,55084E+13	6,81959E+14	6,56375E+14
TC0X00000613.mm.2	Fmr1	fragile X mental retardation 1	2,76977E+14	6,15101E+14	1,9511E+14	0.0956797207131732	0.240440541170616	-4,44169E+14	7,73973E+13	8,01351E+14	7,13618E+14	3,80294E+14	6,95561E+14	3,25809E+14

TC1200001747.mm.2	Sptssa/Mir1892	serine palmitoyltransferase, small subunit A/microRNA 1892	1,08121E+14	7,42207E+14	1,95109E+14	0.0956808012155395	0.240440541170616	-4,4417E+14	7,95928E+14	7,99775E+14	7,44654E+14	7,33421E+14	7,7449E+13	6,0497E+14
TC1900001313.mm.2	Pum3	pumilio RNA-binding family member 3	1,25739E+14	7,36798E+14	1,95082E+14	0.095718579795337	0.240449375832396	-4,44206E+14	7,95819E+14	8,25494E+14	7,56149E+14	6,73491E+14	7,78593E+14	5,91241E+14
TC0900002152.mm.2	Nlrx1	NLR family member X1	0.655122272593232	5,78867E+14	1,95082E+14	0.0957186480399083	0.240449375832396	-4,44206E+14	5,9272E+14	6,24785E+14	5,82721E+14	5,86497E+14	5,16575E+14	5,69906E+13
TC0100003440.mm.2	Tiprl	TIP41, TOR signalling pathway regulator-like (S. cerevisiae)	1,07025E+14	7,06886E+14	1,95074E+14	0.0957290849602695	0.240449375832396	-4,44216E+14	7,90607E+14	8,00529E+14	7,00535E+14	5,72562E+14	7,4244E+14	6,34646E+14
TC1100003677.mm.2	Mien1	migration and invasion enhancer 1	0.712633483784847	8,46257E+14	1,95026E+14	0.0957946625594856	0.240562579070853	-4,44278E+14	8,8429E+14	9,02342E+14	8,55509E+13	7,91297E+14	8,44996E+14	7,99109E+14
TC0700002855.mm.2	Rgs9bp	regulator of G-protein signalling 9 binding protein	-1,2699E+14	4,33229E+14	-1,95013E+13	0.0958128610094583	0.240562579070853	-4,44295E+14	3,34671E+14	3,87746E+14	3,70495E+14	5,30063E+14	4,25666E+14	5,50731E+14
TC1600001498.mm.2	Pigx	phosphatidylinositol glycan anchor biosynthesis, class X	0.627435479759655	6,8218E+14	1,95008E+13	0.0958189432431411	0.240562579070853	-4,44301E+14	7,31045E+14	7,10609E+14	6,4514E+14	6,90119E+14	6,66078E+14	6,50088E+14
TC0X00002457.mm.2	Gm5640	predicted gene 5640	-0.56636833698828	6,78664E+14	-1,9498E+14	0.0958576272446477	0.240622207165136	-4,44338E+14	6,45451E+14	6,5018E+14	6,78289E+13	6,89156E+14	7,09289E+14	6,99617E+14
TC0200002193.mm.2	Sstr4	somatostatin receptor 4	0.639752739576235	6,76444E+14	1,94916E+14	0.0959445016695072	0.240802765638749	-4,4442E+14	7,12712E+13	7,37492E+14	6,59631E+14	6,26576E+14	6,80689E+14	6,41565E+14
TC0700003087.mm.2	Svip	small VCP/p97-interacting protein	-0.56625705024527	8,45272E+14	-1,94896E+14	0.0959720413298521	0.240834371896575	-4,44447E+14	8,05479E+14	7,95945E+14	8,72079E+13	8,83455E+14	8,55173E+14	8,59502E+14
TC0400003803.mm.2	Cdc42	cell division cycle 42	1,08319E+14	1,08242E+14	1,94861E+14	0.0960195483371445	0.240916067012832	-4,44492E+14	1,15869E+14	1,17409E+14	1,10087E+14	9,44713E+13	1,09162E+14	1,02452E+14
TC0100003351.mm.2	Cacybp	calcyclin binding protein	1,20652E+14	8,0931E+14	1,94815E+14	0.0960828378558887	0.241037329343287	-4,44551E+14	9,02068E+14	8,86717E+14	8,41132E+14	6,78461E+14	8,28546E+14	7,18934E+14
TC1400001709.mm.2	Wdfy4	WD repeat and FYVE domain containing 4	-0.90425566280097	6,14144E+14	-1,9476E+14	0.0961579122676061	0.241188113382307	-4,44623E+14	5,56375E+14	5,43996E+14	6,09742E+14	6,9353E+14	5,90778E+14	6,90444E+14
TC0X00001288.mm.2	Armcx3	armadillo repeat containing, X-linked 3	1,7378E+14	6,5434E+14	1,94727E+14	0.0962030974735739	0.241208740186873	-4,44665E+13	7,68151E+14	7,77053E+14	6,99151E+14	4,84039E+14	7,0543E+14	4,92214E+14
TC1500001033.mm.2	Dip2b	disco interacting protein 2 homolog B	0.647307555336625	7,46035E+14	1,94722E+14	0.0962109150153664	0.241208740186873	-4,44673E+14	7,75608E+14	7,8994E+14	7,49283E+14	7,25293E+14	7,44567E+14	6,9152E+14
TC0800001143.mm.2	Ndrg4	N-myc downstream regulated gene 4	0.950241224819178	1,03311E+14	1,94722E+14	0.0962110453162685	0.241208740186873	-4,44673E+14	1,10608E+14	1,08865E+14	1,06711E+14	9,3213E+14	1,02162E+13	9,83054E+14
TC1300000967.mm.2	Mblac2	metallo-beta-lactamase domain containing 2	0.907803282773711	6,29748E+14	1,94685E+13	0.0962618094767453	0.241235172430333	-4,44721E+14	7,06945E+14	6,55073E+14	6,60854E+14	5,7516E+14	6,32985E+14	5,47472E+14
TC1600002167.mm.2	Cluap1	clusterin associated protein 1	0.624169629718314	5,84279E+14	1,94683E+14	0.0962646216154781	0.241235172430333	-4,44723E+14	6,18951E+14	6,53679E+14	5,461E+14	5,3915E+14	5,5554E+14	5,92255E+14
TC0800000394.mm.2	Gsr	glutathione reductase	1,31401E+14	7,24212E+14	1,94681E+14	0.0962665027448049	0.241235172430333	-4,44725E+14	8,29428E+14	7,91893E+14	7,75957E+14	5,89471E+14	7,33545E+14	6,24975E+14
TC0100000129.mm.2	Paqr8	progestin and adipoQ receptor family member VIII	0.828561029780668	7,2245E+13	1,94637E+14	0.0963272942825401	0.24129816648247	-4,44783E+14	7,79222E+14	7,6083E+14	7,65244E+14	6,55065E+14	6,79897E+14	6,94442E+14
TC1000002259.mm.2	Vps26a	VPS26 retromer complex component A	1,03831E+14	7,10218E+14	1,94622E+14	0.0963485237054296	0.24129816648247	-4,44803E+14	7,69094E+14	7,80121E+14	7,22766E+14	6,47771E+14	7,41915E+14	5,99638E+14
TC0600001586.mm.2	Bcl2l14	BCL2-like 14 (apoptosis facilitator)	-0.679412612129259	6,31085E+14	-1,94613E+14	0.0963595394885884	0.24129816648247	-4,44813E+14	5,78988E+14	6,11689E+14	6,65784E+14	6,03492E+14	6,58097E+14	6,68463E+14
TC0X00001796.mm.2	Wdr13	WD repeat domain 13	0.958627868798152	7,80357E+14	1,94611E+14	0.0963635530276298	0.24129816648247	-4,44817E+14	8,58691E+14	8,55767E+13	7,92225E+14	6,52723E+14	7,44632E+14	7,78101E+14
TC0900003160.mm.2	Pdcd6ip	programmed cell death 6 interacting protein	2,2303E+14	7,33274E+14	1,94608E+14	0.0963665178002046	0.24129816648247	-4,4482E+14	8,95862E+14	9,00924E+14	8,05133E+14	4,47E+14	7,61996E+14	5,88729E+13
TC0100000535.mm.2	Creb1	cAMP responsive element binding protein 1	1,20715E+14	6,79413E+14	1,9459E+13	0.0963912465921897	0.241322584888122	-4,44843E+14	7,52141E+14	7,55447E+14	7,37289E+14	5,65444E+14	6,8139E+14	5,84769E+14
TC1500000570.mm.2	Iqank1	IQ motif and ankyrin repeat containing 1	-0.846468747330861	6,84211E+14	-1,94448E+14	0.0965873196360986	0.241775902019024	-4,45028E+14	6,40544E+14	6,15397E+14	6,55047E+14	7,69043E+14	7,0698E+14	7,18254E+14
TC1300002255.mm.2	Zfp729a	zinc finger protein 729a	0.739565263967854	5,59456E+14	1,9439E+14	0.0966676505856857	0.241896985716154	-4,45103E+14	6,31095E+14	6,05892E+14	5,54131E+14	4,76547E+14	5,26207E+14	5,62867E+14
TC1100002329.mm.2	Mdh1	malate dehydrogenase 1, NAD (soluble)	1,30292E+14	1,01678E+14	1,94389E+14	0.0966689067357007	0.241896985716154	-4,45105E+14	1,12663E+13	1,10632E+14	1,04125E+14	8,54078E+14	1,04031E+13	9,32059E+13
TC1100000146.mm.2	Pom121l12	POM121 membrane glycoprotein-like 12	-0.905599371438994	5,67286E+14	-1,9438E+14	0.0966807291014727	0.241896985716154	-4,45116E+14	5,06513E+14	4,9323E+14	5,75674E+14	6,47434E+14	5,35722E+13	6,45141E+14
TC1000000211.mm.2	Moxd1	monooxygenase, DBH-like 1	-0.588640224836445	7,98075E+14	-1,94362E+14	0.0967051807961229	0.241920599001386	-4,45139E+14	7,40703E+14	7,65655E+14	8,3188E+14	8,26125E+14	8,23479E+14	8,00607E+13
TC0700000149.mm.2	Zfp128	zinc finger protein 128	0.574586828209961	5,76923E+14	1,94348E+14	0.0967244758453017	0.241931306938117	-4,45157E+14	6,07663E+13	6,01281E+13	5,69872E+14	5,88698E+14	5,54576E+14	5,3945E+14
TC1000002580.mm.2	Slc41a2	solute carrier family 41, member 2	0.811665905114135	5,97974E+14	1,94327E+14	0.0967544821251831	0.241953386269183	-4,45185E+14	6,75368E+14	6,21708E+14	6,01983E+14	5,54042E+14	5,26622E+14	6,08121E+14
TC1900000714.mm.2	Ina	internexin neuronal intermediate filament protein, alpha	1,37853E+14	9,25225E+14	1,9432E+14	0.0967633352646073	0.241953386269183	-4,45193E+12	1,01915E+14	9,92306E+14	9,91473E+14	8,12676E+14	9,41052E+13	7,94695E+14
TC1900000028.mm.2	Tmem134	transmembrane protein 134	-1,11882E+14	8,31191E+13	-1,94289E+14	0.0968066704618434	0.24202418636954	-4,45234E+14	7,60834E+13	7,68078E+13	9,32413E+14	7,73141E+14	9,16995E+14	8,35683E+14
TC0300001701.mm.2	Hps3	HPS3, biogenesis of lysosomal organelles complex 2 subunit 1	-0.74646647950161	7,32294E+14	-1,94258E+14	0.0968486285143265	0.242041542107658	-4,45274E+14	6,9279E+14	6,69518E+14	7,16298E+13	8,03559E+14	7,63167E+14	7,48435E+14
TC1200002534.mm.2	Serpina1c	serine (or cysteine) peptidase inhibitor, clade A, member 1C	-0.89210067636983	4,51332E+14	-1,94254E+14	0.0968549656419348	0.242041542107658	-4,4528E+14	3,87947E+14	3,9584E+14	4,24062E+14	5,37933E+14	4,51613E+14	5,10594E+14
TC0100002645.mm.2	Serpine2	serine (or cysteine) peptidase inhibitor, clade E, member 2	1,28484E+14	8,32378E+14	1,94251E+14	0.0968586770626315	0.242041542107658	-4,45283E+14	9,27669E+13	9,17624E+14	8,66117E+14	6,9453E+14	8,57619E+14	7,30705E+14
TC0400000091.mm.2	Rad54b/Fsbp	RAD54 homolog B (S. cerevisiae)/fibrinogen silencer binding protein	-1,05034E+14	4,7483E+14	-1,94232E+14	0.0968855091689943	0.242057002099481	-4,45308E+14	3,78016E+14	4,55659E+14	4,20236E+14	5,51327E+14	4,91135E+14	5,52607E+14
TC0100003645.mm.2	Hnrnpu	heterogeneous nuclear ribonucleoprotein U	1,69289E+14	8,34267E+14	1,94222E+14	0.0968992106834374	0.242057002099481	-4,45321E+14	9,52571E+14	9,71714E+14	8,75698E+14	6,19912E+14	8,65829E+14	7,19879E+14
TC1100000701.mm.2	Olfr315	olfactory receptor 315	-0.68556848483098	5,725E+14	-1,94214E+14	0.0969099311452709	0.242057002099481	-4,45331E+14	5,41379E+14	5,27837E+14	5,54297E+14	6,0516E+14	5,77485E+14	6,28844E+14
TC0900000053.mm.2	Mmp7	matrix metallopeptidase 7	-1,11696E+14	5,29433E+14	-1,94202E+14	0.0969260467902717	0.242059732165475	-4,45346E+14	4,44456E+14	4,74778E+14	4,94126E+14	6,20615E+14	5,10058E+14	6,32568E+13
TC0700001821.mm.2	Doc2a	double C2, alpha	-1,26152E+14	7,90227E+14	-1,9416E+14	0.096984383354779	0.2421678860988	-4,45401E+14	6,99939E+14	7,23916E+14	7,2856E+14	9,12791E+14	7,90223E+14	8,85936E+14
TC1700000492.mm.2	Kctd20	potassium channel tetramerisation domain containing 20	0.774395591729169	7,69494E+14	1,94117E+13	0.097043804965934	0.242243300496606	-4,45457E+13	8,25318E+14	8,07951E+14	7,97997E+14	7,0731E+14	7,57394E+14	7,20996E+14
TC0300000706.mm.2	Sh2d2a	SH2 domain containing 2A	-0.766029939941442	5,61669E+14	-1,94117E+14	0.0970446536775023	0.242243300496606	-4,45457E+14	5,23231E+14	5,16505E+14	5,5892E+14	5,78413E+14	5,49075E+14	6,43868E+14
TC1700002225.mm.2	Nfya	nuclear transcription factor-Y alpha	0.609291731294792	6,62256E+13	1,94088E+14	0.0970846595393561	0.242250684286237	-4,45495E+14	7,09645E+14	6,72094E+14	6,78134E+14	6,53786E+14	6,26265E+14	6,33615E+14
TC0700000647.mm.2	Pepd	peptidase D	1,27678E+14	6,32639E+14	1,94076E+14	0.0971013594237492	0.242250684286237	-4,45511E+14	7,295E+14	7,47614E+14	6,37495E+14	4,59469E+14	6,67942E+14	5,53815E+14
TC1100002861.mm.2	Pld6	phospholipase D family, member 6	-0.696578021184184	7,22869E+14	-1,94068E+14	0.0971114629539253	0.242250684286237	-4,4552E+14	6,88581E+14	6,68083E+14	7,1044E+14	7,74127E+14	7,28782E+14	7,67197E+14
TC0200003815.mm.2	Zfp385b	zinc finger protein 385B	0.994521711098621	7,35126E+14	1,94043E+14	0.0971463218128477	0.242250684286237	-4,45553E+14	8,0555E+14	7,84936E+14	7,84645E+14	6,44043E+14	7,23052E+14	6,68529E+14
TC1100003750.mm.2	Krt34	keratin 34	-0.967954952178117	4,87731E+14	-1,93989E+14	0.0972217139245052	0.242250684286237	-4,45623E+14	4,64797E+14	4,3964E+14	5,29603E+14	3,9432E+14	5,24167E+14	5,73861E+14
TC1400000281.mm.2	Erc2	ELKS/RAB6-interacting/CAST family member 2	1,16781E+14	8,84381E+14	1,93987E+14	0.0972241538306455	0.242250684286237	-4,45625E+14	9,49695E+14	9,45986E+13	9,31676E+14	8,1681E+14	9,06533E+14	7,55586E+14
TC1100004102.mm.2	Wbp2	WW domain binding protein 2	1,253E+12	9,02039E+14	1,9398E+13	0.0972331720356613	0.242250684286237	-4,45634E+14	9,9412E+14	9,9603E+14	9,4258E+14	7,39951E+14	8,92037E+14	8,47514E+14
TC0800000300.mm.2	Zmat4	zinc finger, matrin type 4	1,25957E+14	6,38204E+14	1,9398E+14	0.0972336794682314	0.242250684286237	-4,45634E+14	7,34245E+14	7,26692E+14	6,70434E+14	4,88828E+14	6,55922E+13	5,53102E+14
TC1700000349.mm.2	Rps2/Snora64	ribosomal protein S2/small nucleolar RNA, H/ACA box 64	0.810041703983865	1,00165E+14	1,9397E+14	0.097247871904566	0.242250684286237	-4,45648E+14	1,05302E+14	1,06659E+14	9,98157E+14	9,34543E+14	1,02234E+14	9,35268E+14
TC0400004163.mm.2	B3galt6	UDP-Gal:betaGal beta 1,3-galactosyltransferase, polypeptide 6	0.667022197535913	6,5623E+14	1,93967E+14	0.0972518192916643	0.242250684286237	-4,45651E+14	7,2652E+14	6,76651E+14	6,09734E+14	6,54706E+14	6,30353E+14	6,39414E+14
TC1000002562.mm.2	Zfp938	zinc finger protein 938	0.781659855788726	7,57159E+14	1,93965E+14	0.0972539498734777	0.242250684286237	-4,45653E+14	8,15539E+14	8,04097E+14	7,74617E+14	6,85395E+14	7,54984E+14	7,08321E+14
TC0X00001277.mm.2	Drp2	dystrophin related protein 2	0.560960389697485	6,62651E+14	1,93964E+14	0.0972566022253257	0.242250684286237	-4,45656E+14	7,08538E+14	7,04948E+14	6,30859E+14	6,30268E+14	6,56268E+14	6,45026E+13
TC0700000085.mm.2	Olfr1350	olfactory receptor 1350	-1,04195E+14	5,50103E+14	-1,93958E+14	0.0972640397873974	0.242250684286237	-4,45663E+14	4,78908E+12	4,89073E+14	5,11034E+14	6,45235E+14	5,424E+13	6,33971E+14
TC1800001541.mm.2	Poli	polymerase (DNA directed), iota	0.856289023060252	6,58606E+14	1,93942E+14	0.0972857503392461	0.242250684286237	-4,45683E+14	7,31197E+14	7,02577E+14	6,69807E+14	5,85537E+14	6,71672E+14	5,90844E+14
TC0500000648.mm.2	Slc30a9	solute carrier family 30 (zinc transporter), member 9	1,25948E+14	6,81438E+13	1,93937E+14	0.0972937937226427	0.242250684286237	-4,45691E+14	7,70106E+14	7,58062E+14	7,31551E+14	5,52636E+14	6,91686E+14	5,84585E+14
TC0Y00000379.mm.2	Gm20852	predicted gene, 20852	-0.80927914295787	5,60331E+14	-1,93914E+12	0.0973252907205806	0.242250684286237	-4,4572E+14	4,73017E+14	5,46667E+14	5,55992E+14	6,04771E+14	5,59229E+14	6,22311E+14
TC0Y00000388.mm.2	Gm20852	predicted gene, 20852	-0.80927914295787	5,60331E+14	-1,93914E+12	0.0973252907205806	0.242250684286237	-4,4572E+14	4,73017E+14	5,46667E+14	5,55992E+14	6,04771E+14	5,59229E+14	6,22311E+14

TC0Y00000433.mm.2	Gm20852	predicted gene, 20852	-0.80927914295787	5,60331E+14	-1,93914E+12	0.0973252907205806	0.242250684286237	-4,4572E+14	4,73017E+14	5,46667E+14	5,55992E+14	6,04771E+14	5,59229E+14	6,22311E+14
TC0700002612.mm.2	Lypd4	Ly6/Plaur domain containing 4	-1,38164E+12	5,66424E+14	-1,93908E+14	0.0973332669316143	0.242250684286237	-4,45727E+14	5,10493E+14	4,25819E+14	5,36309E+14	7,13285E+14	5,42705E+14	6,69935E+12
TC1700002805.mm.2	Nhlrc4	NHL repeat containing 4	-0.705150566024026	5,9814E+14	-1,93836E+14	0.0974339643769638	0.242463856062705	-4,45821E+14	5,52277E+14	5,37052E+14	5,95297E+14	6,73852E+14	6,1547E+14	6,14889E+14
TC0700001731.mm.2	Uqcrc2	ubiquinol cytochrome c reductase core protein 2	0.742609894765541	8,07635E+14	1,93751E+14	0.0975513407879915	0.242683119061878	-4,45931E+14	8,60058E+14	8,38021E+14	7,56005E+14	8,42171E+14	7,65217E+14	7,8434E+14
TC1000003140.mm.2	Coq10a	coenzyme Q10A	0.674176701774588	6,62035E+14	1,93751E+14	0.0975521977387067	0.242683119061878	-4,45932E+14	7,13799E+14	6,97211E+14	6,72585E+13	6,12438E+14	6,54377E+14	6,21797E+14
TC1400001599.mm.2	Zcchc24	zinc finger, CCHC domain containing 24	0.610670877193365	7,18034E+14	1,93738E+14	0.0975692523403868	0.242688077023874	-4,45948E+14	7,57248E+14	7,67012E+14	7,01407E+14	6,80409E+14	7,21213E+14	6,80913E+14
TC0800000028.mm.2	Evi5l	ecotropic viral integration site 5 like	0.703507699001781	6,46677E+14	1,93685E+14	0.0976433376962629	0.242738217586333	-4,46017E+14	6,86743E+14	6,95205E+14	6,5555E+14	6,01318E+14	5,91738E+14	6,49508E+14
TC0400002805.mm.2	Tex48	testis expressed 48	-1,18527E+14	5,77006E+14	-1,93684E+14	0.0976447182380117	0.242738217586333	-4,46018E+14	4,70456E+14	5,02849E+14	5,81667E+14	6,96702E+14	5,28915E+14	6,81444E+14
TC1700002185.mm.2	Klhdc3	kelch domain containing 3	0.974657760941877	9,18745E+14	1,93682E+14	0.0976470030818726	0.242738217586333	-4,4602E+14	9,67174E+14	9,80997E+14	9,61111E+14	8,49949E+14	9,25106E+14	8,28134E+14
TC0700004657.mm.2	Sec11a	SEC11 homolog A, signal peptidase complex subunit	1,05441E+14	7,75E+14	1,93679E+14	0.0976524903478274	0.242738217586333	-4,46025E+14	8,5534E+14	8,37483E+14	8,10238E+14	6,65001E+14	7,79985E+14	7,01956E+12
TC1900001435.mm.2	Htr7	5-hydroxytryptamine (serotonin) receptor 7	0.88413324880174	6,17554E+14	1,93646E+14	0.0976979348323235	0.242738217586333	-4,46067E+14	6,83701E+13	5,98019E+14	6,51962E+14	6,66749E+14	5,98902E+14	5,05991E+14
TC0900001416.mm.2	Dalrd3	DALR anticodon binding domain containing 3	0.653877803992676	8,03936E+14	1,93636E+14	0.0977114336315681	0.242738217586333	-4,4608E+14	8,40991E+14	8,45601E+14	8,10313E+14	7,70893E+14	8,02772E+14	7,53045E+14
TC1200000676.mm.2	Akap5	A kinase (PRKA) anchor protein 5	0.632944628967382	7,81419E+14	1,93635E+13	0.0977130525459418	0.242738217586333	-4,46081E+14	8,07974E+14	8,25608E+14	7,48109E+14	7,99832E+13	7,47623E+14	7,5937E+14
TC0800002388.mm.2	Armc6	armadillo repeat containing 6	0.57371090206805	5,25685E+14	1,93623E+14	0.0977292216441445	0.242738217586333	-4,46096E+14	5,55137E+14	5,73807E+13	4,96563E+14	5,14403E+14	5,08419E+13	5,05783E+14
TC0200002937.mm.2	Taf3	TATA-box binding protein associated factor 3	1,1742E+14	6,79851E+14	1,93617E+14	0.0977388121215457	0.242738217586333	-4,46105E+14	7,44762E+14	7,53828E+14	7,38371E+14	5,7839E+14	6,84337E+13	5,79415E+14
TC0400003667.mm.2	Trnau1ap	tRNA selenocysteine 1 associated protein 1	-0.71799239059706	7,3129E+14	-1,93616E+14	0.0977400580711307	0.242738217586333	-4,46107E+14	6,67505E+14	6,79955E+14	7,35985E+14	8,13238E+14	7,60906E+14	7,30153E+14
TC0700003991.mm.2	Dchs1	dachsous cadherin related 1	0.667718693849539	6,26471E+14	1,93594E+14	0.0977699905717419	0.242775136089147	-4,46134E+13	6,9195E+14	6,78226E+14	5,54689E+14	5,97329E+14	5,92453E+14	6,44179E+14
TC1500001533.mm.2	Taf2	TATA-box binding protein associated factor 2	0.910665744553983	6,82334E+14	1,93483E+14	0.0979244055064477	0.243075222137897	-4,46278E+14	7,41916E+14	7,48894E+14	6,66554E+14	6,27964E+14	7,18244E+14	5,90432E+14
TC1500001051.mm.2	Dazap2	DAZ associated protein 2	1,47426E+14	8,99206E+13	1,93478E+13	0.0979325205233225	0.243075222137897	-4,46285E+14	1,00264E+14	1,00359E+14	9,53007E+14	7,24629E+14	9,1023E+14	8,01144E+14
TC0400000213.mm.2	Gpr63	G protein-coupled receptor 63	-0.610860369627146	7,73392E+14	-1,93472E+14	0.0979405383624013	0.243075222137897	-4,46293E+14	7,38155E+14	7,16282E+14	8,09339E+14	7,99969E+14	7,68073E+13	8,08536E+14
TC0400000894.mm.2	Gm13272/Gm13285/ Gm13290/Gm13289	predicted gene 13272/predicted gene 13285/predicted gene 13290/predicted gene 13289	-0.928064722883217	5,58603E+14	-1,93464E+14	0.097951183351416	0.243075222137897	-4,46303E+14	4,85585E+13	5,04341E+14	5,36805E+14	6,49345E+14	5,49164E+14	6,26375E+14
TC0400003548.mm.2	Tfap2e	transcription factor AP-2, epsilon	-0.677153475058509	4,89744E+13	-1,93428E+14	0.0980013235827587	0.243151750202838	-4,46349E+14	4,30872E+14	4,65463E+14	4,87709E+14	5,22655E+14	4,89773E+14	5,41992E+12
TC0100000908.mm.2	Olfr1413	olfactory receptor 1413	-0.768056102131244	4,9383E+14	-1,93421E+14	0.0980122024183849	0.243151750202838	-4,46359E+14	4,55747E+13	4,47549E+14	5,35709E+13	4,67064E+14	4,93515E+14	5,63393E+14
TC1300002270.mm.2	Adcy2	adenylate cyclase 2	1,14104E+14	7,55315E+14	1,93379E+14	0.0980702822132187	0.243258383205294	-4,46413E+14	8,35362E+14	8,37705E+14	7,52115E+14	6,61849E+14	8,03063E+13	6,41795E+14
TC1500000782.mm.2	Pnpla3	patatin-like phospholipase domain containing 3	-0.679991332922155	5,70391E+14	-1,93364E+14	0.0980912752722136	0.243273005765919	-4,46432E+14	5,50219E+14	5,10494E+14	5,56671E+14	6,08254E+14	5,82165E+13	6,14546E+14
TC1900000760.mm.2	Rbm20	RNA binding motif protein 20	-0.603906459057613	6,87325E+14	-1,93333E+14	0.0981340876136242	0.243313489185993	-4,46472E+14	6,45981E+13	6,63085E+14	6,92064E+14	6,92974E+14	6,94689E+14	7,35159E+14
TC0100001058.mm.2	Nifk	nucleolar protein interacting with the FHA domain of MKI67	1,14632E+14	7,1681E+13	1,93331E+14	0.0981377996785063	0.243313489185993	-4,46476E+14	7,86234E+14	8,0815E+14	7,21917E+14	6,19435E+14	7,59397E+14	6,05724E+14
TC1300002436.mm.2	Bhmt	betaine-homocysteine methyltransferase	-0.897019390659958	5,67575E+14	-1,93284E+14	0.0982037596239132	0.243413214444684	-4,46537E+14	5,106E+14	5,1239E+14	5,43549E+14	6,3652E+14	5,55271E+14	6,47123E+13
TC1500000733.mm.2	Csdc2	cold shock domain containing C2, RNA binding	-0.93263479244492	6,6044E+14	-1,93273E+14	0.0982192716212539	0.243413214444684	-4,46551E+14	6,04128E+14	5,85541E+14	6,37695E+13	7,59078E+14	6,55498E+14	7,20698E+14
TC0600002912.mm.2	Lhfpl4	lipoma HMGIC fusion partner-like protein 4	0.593530736559934	7,86333E+14	1,93266E+14	0.0982279305629472	0.243413214444684	-4,46559E+14	8,21632E+14	7,95639E+14	7,87493E+14	8,1467E+14	7,54229E+14	7,44335E+14
TC0800001110.mm.2	Nlrc5/Mir7072	NLR family, CARD domain containing 5/microRNA 7072	-0.840348522275463	4,8118E+14	-1,93256E+14	0.0982420864395617	0.243413214444684	-4,46572E+14	4,2518E+14	4,21287E+14	4,59198E+14	5,66877E+14	4,84406E+14	5,30131E+14
TC0100000806.mm.2	Akp3	alkaline phosphatase 3, intestine, not Mn requiring	-1,28974E+13	4,76539E+13	-1,93248E+14	0.0982535559329875	0.243413214444684	-4,46583E+14	4,14634E+14	3,7173E+13	4,15431E+14	6,13129E+14	4,8426E+14	5,60052E+14
TC1100003674.mm.2	Pgap3	post-GPI attachment to proteins 3	-0.627436957363913	6,56947E+14	-1,9318E+14	0.0983484355323265	0.243579528233047	-4,4667E+14	6,377E+14	6,02772E+14	6,71534E+14	6,63719E+14	7,03404E+14	6,62555E+14
TC0100002193.mm.2	Tmem131	transmembrane protein 131	1,07125E+14	8,1594E+14	1,93179E+14	0.0983509222791662	0.243579528233047	-4,46673E+14	8,91003E+14	8,85683E+14	8,54635E+13	7,01884E+14	8,18539E+14	7,43898E+14
TC1000000372.mm.2	Sesn1	sestrin 1	0.960513591334224	7,35574E+14	1,93148E+14	0.0983940059766163	0.243594988740766	-4,46712E+14	8,10404E+14	7,95559E+14	7,57175E+14	6,36448E+14	7,46909E+14	6,66952E+14
TC1100001425.mm.2	Spag9	sperm associated antigen 9	1,19689E+14	7,95828E+14	1,93144E+14	0.0983999675229249	0.243594988740766	-4,46718E+14	8,8409E+14	8,81441E+14	8,14232E+13	6,6905E+14	8,29573E+14	6,96579E+14
TC1300002036.mm.2	Nop16	NOP16 nucleolar protein	0.598899002183382	7,28658E+14	1,93142E+14	0.0984025185697044	0.243594988740766	-4,4672E+14	7,67044E+14	7,66319E+14	7,07097E+13	7,17904E+13	7,26985E+14	6,86598E+14
TC0500002092.mm.2	Crygn	crystallin, gamma N	-0.912178515546336	7,25533E+13	-1,93083E+14	0.0984856641388013	0.243763365018204	-4,46797E+14	6,68512E+14	6,50457E+14	7,00318E+14	8,32507E+14	7,42228E+14	7,59177E+14
TC0600001522.mm.2	Clec2g	C-type lectin domain family 2, member g	-0.772634306551157	5,90704E+14	-1,93068E+14	0.0985057167673663	0.243771837530483	-4,46816E+14	5,2501E+14	5,66129E+14	5,62667E+14	6,44749E+14	6,0314E+14	6,42526E+13
TC0600000977.mm.2	Figla	folliculogenesis specific basic helix-loop-helix	-0.736205455351059	5,07307E+14	-1,93059E+14	0.098519345000838	0.243771837530483	-4,46828E+14	4,64219E+14	4,7765E+14	4,94398E+14	5,18468E+14	5,01761E+14	5,87348E+14
TC0200001900.mm.2	Usp8	ubiquitin specific peptidase 8	1,35729E+13	7,90409E+13	1,93015E+14	0.0985808226977586	0.243852000837255	-4,46885E+14	8,9313E+14	8,68623E+14	8,48206E+14	6,42199E+14	7,95003E+14	6,95293E+14
TC1100001206.mm.2	Psmd11	proteasome (prosome, macropain) 26S subunit, non-ATPase, 11	0.625738768558996	8,26487E+13	1,93014E+14	0.0985820103924705	0.243852000837255	-4,46886E+14	8,82355E+13	8,64673E+14	8,20422E+14	7,69591E+14	8,11828E+14	8,10052E+14
TC0700000781.mm.2	Klk15	kallikrein related-peptidase 15	-0.579079580918807	5,91612E+13	-1,92957E+14	0.0986617722091447	0.244011839693929	-4,46959E+13	5,38122E+14	5,50247E+14	6,37755E+14	6,1936E+14	5,91135E+14	6,1305E+13
TC0500002420.mm.2	Sel1l3	sel-1 suppressor of lin-12-like 3 (C. elegans)	0.846509967890946	6,31325E+14	1,92917E+14	0.0987182918255832	0.24404934464905	-4,47011E+14	6,92948E+14	7,35496E+14	5,84383E+13	5,15979E+14	6,73321E+14	5,85821E+14
TC0100001451.mm.2	4930523C07Rik	RIKEN cDNA 4930523C07 gene	-1,13548E+14	5,76338E+14	-1,9291E+14	0.098728009556779	0.24404934464905	-4,4702E+14	4,9288E+14	5,07934E+14	5,23036E+14	7,06264E+14	6,19919E+14	6,07992E+14
TC1700002766.mm.2	Tcp10b/Tcp10a	t-complex protein 10b/t-complex protein 10a	-1,02052E+14	5,66729E+14	-1,92906E+14	0.0987344827555582	0.24404934464905	-4,47026E+14	5,4396E+14	4,6295E+14	5,25987E+13	6,56463E+13	5,8435E+14	6,26665E+14
TC1100000767.mm.2	Dhrs7b	dehydrogenase/reductase (SDR family) member 7B	0.716595217246386	6,49566E+14	1,92903E+14	0.0987375211175547	0.24404934464905	-4,47029E+14	7,1608E+14	7,13561E+14	6,32118E+13	5,49315E+14	6,34978E+14	6,51343E+14
TC0300000694.mm.2	Fcrl1	Fc receptor-like 1	-1,08149E+14	5,80658E+14	-1,92855E+14	0.0988056508026211	0.244180283954392	-4,47092E+14	5,11145E+14	4,98066E+14	5,46905E+14	7,0232E+14	5,75663E+14	6,49846E+14
TC1200000823.mm.2	Fcf1	FCF1 rRNA processing protein	0.578072393882924	5,99916E+14	1,92839E+14	0.0988283084691925	0.244198824469268	-4,47113E+14	6,25126E+14	6,29961E+14	5,88172E+14	6,16764E+14	5,74704E+14	5,64768E+14
TC0400004104.mm.2	Ajap1	adherens junction associated protein 1	1,48156E+14	7,98515E+14	1,92776E+14	0.0989162710786943	0.244378699155321	-4,47193E+12	9,29123E+14	9,15022E+14	8,18941E+14	5,8017E+14	8,1902E+14	7,28813E+14
TC0100002604.mm.2	Tuba4a	tubulin, alpha 4A	1,12395E+14	8,49477E+14	1,92755E+14	0.098946478634316	0.244415853171046	-4,47221E+14	9,68713E+14	8,73489E+14	8,53826E+14	7,83422E+14	8,77783E+14	7,39629E+13
TC0100001161.mm.2	AA986860	expressed sequence AA986860	-0.614800596987524	5,30873E+14	-1,92744E+14	0.0989621270967852	0.24441703769321	-4,47235E+14	5,00295E+14	4,95967E+14	5,24163E+14	5,45591E+14	5,37307E+14	5,81915E+14
TC1400001220.mm.2	Frg2f9	FSHD region gene 2 family member 9	-0.695249058306771	5,20628E+14	-1,92721E+14	0.0989943700169467	0.244424962005529	-4,47265E+12	4,89115E+14	4,60958E+13	5,26396E+14	5,58178E+14	5,11708E+14	5,77415E+14
TC1500000303.mm.2	Rims2	regulating synaptic membrane exocytosis 2	1,01518E+14	7,61078E+14	1,9272E+14	0.0989956744273617	0.244424962005529	-4,47266E+14	8,41054E+14	8,25782E+14	7,50521E+14	6,85313E+14	8,03637E+14	6,60164E+14
TC0200002489.mm.2	Hnf4a	hepatic nuclear factor 4, alpha	-0.63385876376721	6,40595E+13	-1,92694E+14	0.099032341072821	0.24447803151622	-4,473E+14	5,95599E+14	6,25741E+14	6,5794E+13	6,1618E+14	6,61344E+14	6,86768E+14
TC1100003993.mm.2	Fam20a	family with sequence similarity 20, member A	0.687186241854326	5,55526E+14	1,92678E+13	0.0990550037383742	0.244496518878128	-4,47321E+14	6,03514E+14	5,85303E+14	5,73719E+14	5,19241E+14	5,50399E+14	5,00981E+14
TC0300001229.mm.2	Alg14	asparagine-linked glycosylation 14	0.575159782037886	6,63538E+14	1,92597E+13	0.0991702134385045	0.244743398550256	-4,47426E+14	7,14625E+13	6,92384E+13	6,35908E+14	6,4633E+14	6,381E+14	6,53878E+14
TC1700001661.mm.2	Cacna1h	calcium channel, voltage-dependent, T type, alpha 1H subunit	-0.563918625050906	7,99042E+14	-1,92581E+14	0.099192355722724	0.244756670072701	-4,47446E+13	7,70596E+14	7,81713E+14	7,80422E+14	7,96427E+14	8,29796E+14	8,35298E+14
TC0500001340.mm.2	Iqcd	IQ motif containing D	-0.762164439995089	7,91276E+14	-1,92557E+14	0.0992263645958879	0.244756670072701	-4,47478E+14	7,33234E+14	7,44662E+14	7,71798E+14	8,67634E+14	8,07805E+14	8,22523E+14
TC0900003110.mm.2	Ptpn23	protein tyrosine phosphatase, non-receptor type 23	1,09083E+14	5,52163E+14	1,92553E+14	0.0992311552172528	0.244756670072701	-4,47482E+14	6,35151E+14	5,85059E+14	6,20346E+14	4,70374E+13	4,63416E+14	5,3863E+14

TC1400001438.mm.2	Acox2	acyl-Coenzyme A oxidase 2, branched chain	-1,09135E+14	6,06987E+14	-1,9255E+14	0.09923635111928	0.244756670072701	-4,47487E+14	5,22152E+14	5,74858E+14	5,36597E+14	6,93031E+14	6,28273E+14	6,87008E+14
TC1000002668.mm.2	Slc25a3	solute carrier family 25 (mitochondrial carrier, phosphate carrier), member 3	1,64781E+14	9,44385E+14	1,92535E+14	0.0992574021597041	0.2447711235077	-4,47506E+13	1,07168E+14	1,07118E+14	9,89906E+14	7,2025E+13	9,58789E+14	8,54509E+14
TC0700004512.mm.2	Ifitm5	interferon induced transmembrane protein 5	-0.794841623104141	4,76545E+14	-1,92466E+14	0.0993545146089285	0.244860134587754	-4,47595E+14	4,22008E+14	4,22999E+14	4,64908E+14	5,45382E+14	4,67202E+14	5,36773E+14
TC0600001970.mm.2	Rbm28	RNA binding motif protein 28	0.885802990122212	5,40342E+14	1,92464E+13	0.0993576534476913	0.244860134587754	-4,47598E+14	5,66604E+14	5,99414E+14	5,1019E+13	5,76988E+14	5,47076E+14	4,41781E+14
TC0200000894.mm.2	Dapl1	death associated protein-like 1	-0.609593975362387	5,82328E+14	-1,92462E+14	0.0993601738961081	0.244860134587754	-4,476E+13	5,61581E+14	5,25024E+14	6,08259E+14	5,9058E+14	6,16869E+14	5,91655E+14
TC0500000221.mm.2	Nub1	negative regulator of ubiquitin-like proteins 1	0.941503541834217	8,49596E+14	1,92461E+14	0.0993616962771722	0.244860134587754	-4,47601E+14	9,15138E+14	9,08935E+14	8,64499E+14	7,73229E+14	8,72181E+14	7,63591E+14
TSUnmapped00000036.m m.2	Ache	acetylcholinesterase	1,41099E+14	8,43518E+14	1,92448E+14	0.0993809679928389	0.244860134587754	-4,47619E+14	9,61484E+14	9,0879E+14	9,09381E+14	6,93375E+13	8,41693E+14	7,46383E+14
TC1000000812.mm.2	Bsg	basigin	0.8826985429146	1,08862E+14	1,92445E+14	0.0993846757403283	0.244860134587754	-4,47622E+14	1,14952E+14	1,15228E+14	1,09497E+14	1,00968E+14	1,11048E+14	1,01478E+14
TC1100004223.mm.2	Myadml2	myeloid-associated differentiation marker-like 2	-0.757017921157595	5,34914E+14	-1,9241E+14	0.0994346732592277	0.244945863052429	-4,47668E+14	4,77339E+13	4,89436E+14	5,14567E+14	6,0996E+14	5,62437E+13	5,55742E+14
TC1200002195.mm.2	Eml5	echinoderm microtubule associated protein like 5	0.604059536929269	6,86209E+14	1,9235E+14	0.0995197138521374	0.245072837007699	-4,47746E+14	7,10719E+14	7,28286E+14	6,68753E+14	6,91302E+14	6,76769E+14	6,41424E+14
TC0200000036.mm.2	Sephs1	selenophosphate synthetase 1	0.611707201460071	5,63632E+14	1,92346E+14	0.0995247284804218	0.245072837007699	-4,4775E+14	6,15641E+14	5,8926E+14	5,4679E+14	5,47539E+14	5,61856E+14	5,20704E+14
TC1400000231.mm.2	Rps24	ribosomal protein S24	0.671261653180268	1,04541E+13	1,92341E+14	0.0995318466690488	0.245072837007699	-4,47757E+14	1,0951E+14	1,07739E+14	1,05294E+14	1,0088E+13	1,0455E+14	9,92731E+14
TC1600001131.mm.2	Tfap4	transcription factor AP4	-0.702794521106484	7,23256E+14	-1,92304E+14	0.0995848589198125	0.245092817597461	-4,47805E+14	6,36555E+14	7,01846E+14	7,38587E+14	7,83588E+14	7,47724E+14	7,31236E+14
TC0100002552.mm.2	Mreg	melanoregulin	-0.631215872094426	6,21556E+14	-1,92295E+13	0.0995970079282895	0.245092817597461	-4,47816E+13	5,71292E+14	5,91196E+13	6,52136E+14	6,25984E+14	6,21127E+14	6,67604E+14
TC1600001706.mm.2	Bbx	bobby sox HMG box containing	1,39898E+14	6,79935E+14	1,92288E+14	0.0996074998117863	0.245092817597461	-4,47826E+14	7,4911E+14	7,58814E+14	6,90772E+14	6,52787E+14	7,32304E+14	4,95824E+14
TC1300001540.mm.2	Zscan26	zinc finger and SCAN domain containing 26	0.821725095428866	8,23158E+14	1,92287E+14	0.096608745455136	0.245092817597461	-4,47827E+14	8,62006E+14	8,54033E+14	8,51773E+13	8,19444E+14	8,30071E+14	7,21623E+14
TC0400001318.mm.2	Olfr1331	olfactory receptor 1331	-1,10429E+14	4,86906E+14	-1,92282E+14	0.0996160157913345	0.245092817597461	-4,47833E+14	3,84505E+13	4,26008E+14	4,89946E+14	5,89606E+13	4,44713E+14	5,86658E+12
TC0700003551.mm.2	Hapln3	hyaluronan and proteoglycan link protein 3	-0.703342521219241	6,50947E+14	-1,92188E+14	0.0997500451965837	0.24535504022671	-4,47955E+14	5,82723E+14	6,05825E+13	6,58833E+14	7,29082E+13	6,47734E+14	6,81483E+14
TC0200004245.mm.2	Tcp11l1	t-complex 11 like 1	1,51309E+14	5,76274E+14	1,92167E+14	0.0997795609240867	0.24535504022671	-4,47982E+14	7,08423E+14	6,96333E+14	5,72134E+14	3,78616E+13	6,39844E+14	4,62293E+14
TC0100003528.mm.2	Sdhc	succinate dehydrogenase complex, subunit C, integral membrane protein	1,12012E+14	7,46874E+14	1,92162E+14	0.0997858812003716	0.24535504022671	-4,47988E+14	8,3682E+14	8,1174E+13	7,7799E+14	6,3016E+14	7,63744E+14	6,60791E+14
TC0900002241.mm.2	Zbtb16	zinc finger and BTB domain containing 16	0.693676214814507	6,88859E+14	1,92142E+14	0.0998150723609487	0.24535504022671	-4,48015E+14	6,82666E+14	7,52945E+14	7,1426E+14	6,86408E+14	6,58812E+14	6,38064E+14
TC1500000240.mm.2	Pop1	processing of precursor 1, ribonuclease P/MRP family, (S. cerevisiae)	-0.864200346032623	5,98819E+14	-1,92136E+13	0.0998233175543643	0.24535504022671	-4,48022E+14	5,46168E+14	5,66232E+14	5,71351E+14	6,23923E+14	5,87314E+14	6,97927E+14
TC1100004144.mm.2	Tmc6	transmembrane channel-like gene family 6	-0.646547620406131	5,75957E+14	-1,92129E+14	0.0998333523405728	0.24535504022671	-4,48031E+14	5,34181E+14	5,3157E+14	5,7635E+14	6,18582E+14	5,74311E+14	6,20749E+14
TC1600001191.mm.2	Emp2	epithelial membrane protein 2	0.654140162066693	7,14619E+13	1,92119E+14	0.0998468791951233	0.24535504022671	-4,48043E+14	7,49698E+14	7,74582E+14	7,15157E+13	6,54823E+14	6,996E+14	6,93853E+14
TC0200005490.mm.2	Gm14305	predicted gene 14305	1,38084E+14	7,44079E+14	1,92111E+14	0.0998587429237704	0.24535504022671	-4,48054E+14	8,51599E+14	9,12473E+14	7,08185E+14	5,04315E+14	7,95331E+14	6,92573E+13
TC0200005467.mm.2	Phyhd1	phytanoyl-CoA dioxygenase domain containing 1	-0.842326096206535	6,73803E+14	-1,92111E+14	0.0998596384166056	0.24535504022671	-4,48055E+14	6,40122E+14	6,19112E+13	6,19507E+14	7,36377E+14	7,12478E+14	7,15222E+14
TC0900001845.mm.2	Olfm2	olfactomedin 2	0.690619485284699	6,31393E+14	1,92035E+14	0.0999668902979963	0.245527653773125	-4,48152E+14	6,93307E+14	6,83751E+14	6,23129E+14	5,49235E+14	6,30245E+14	6,08689E+14
TC0200002436.mm.2	Arhgap40	Rho GTPase activating protein 40	-1,15434E+14	6,61608E+14	-1,92021E+11	0.0999865823928113	0.245527653773125	-4,4817E+14	5,78093E+14	6,13982E+14	6,02838E+14	7,51793E+14	6,55827E+14	7,67117E+14
TC1300002517.mm.2	Ptcd2	pentatricopeptide repeat domain 2	0.904477297899537	6,36735E+13	1,9202E+14	0.0999886520633835	0.245527653773125	-4,48172E+14	6,94049E+14	6,90038E+14	6,66414E+14	5,6672E+14	6,46214E+14	5,56978E+14
TC1100003437.mm.2	Ints2	integrator complex subunit 2	-0.607657709136066	5,90115E+14	-1,9201E+14	0.100002792411445	0.245527653773125	-4,48185E+14	5,32926E+14	5,32887E+13	6,20056E+14	6,67477E+13	5,91756E+14	5,95588E+14
TC0700000768.mm.2	Ctu1	cytosolic thiouridylase subunit 1	-0.611752259532175	6,29486E+14	-1,92008E+14	0.100006081531249	0.245527653773125	-4,48188E+14	5,73822E+14	6,1135E+12	6,37097E+14	6,47124E+14	6,38522E+14	6,69E+14
TC0600001595.mm.2	Cdkn1b	cyclin-dependent kinase inhibitor 1B	0.731483839977108	7,66363E+13	1,91984E+14	0.100040370327879	0.245557739744405	-4,48219E+14	8,12177E+14	7,97889E+14	7,84541E+14	7,39803E+14	7,70119E+14	6,9365E+14
TC0500002486.mm.2	Ugdh	UDP-glucose dehydrogenase	0.723056578777872	6,34926E+14	1,91978E+14	0.100048815330604	0.245557739744405	-4,48227E+14	7,02063E+14	6,89637E+14	6,22932E+14	5,47835E+14	6,44948E+14	6,0214E+14
TC1100003992.mm.2	Wipi1	WD repeat domain, phosphoinositide interacting 1	-0.857283971182058	7,17362E+14	-1,9196E+14	0.100074779124671	0.245584056660953	-4,4825E+14	6,97284E+14	6,80115E+13	7,46573E+14	6,31344E+14	7,55314E+14	7,93542E+14
TC0200000592.mm.2	Tor2a	torsin family 2, member A	-0.569108994768417	7,60681E+14	-1,9191E+14	0.100144857037922	0.245705479610854	-4,48314E+14	7,13893E+14	7,33812E+14	7,69435E+14	7,8542E+14	7,77588E+14	7,83938E+13
TC0300000228.mm.2	Usp13	ubiquitin specific peptidase 13 (isopeptidase T-3)	0.951506889781968	6,09346E+14	1,91903E+14	0.100154756413088	0.245705479610854	-4,48323E+14	6,72449E+14	6,49076E+13	6,70512E+14	5,32815E+14	5,8494E+14	5,46284E+14
TC0300002376.mm.2	S100a9	S100 calcium binding protein A9 (calgranulin B)	-0.8032035792709	4,87584E+14	-1,91852E+14	0.100227656855681	0.245825231147503	-4,48389E+14	4,45494E+14	4,28648E+14	4,57238E+14	5,59342E+14	4,99957E+14	5,34826E+14
TC0900000026.mm.2	Msantd4	Myb/SANT-like DNA-binding domain containing 4 with coiled-coils	1,02745E+14	7,01217E+14	1,91848E+14	0.100234082333463	0.245825231147503	-4,48395E+14	7,88945E+14	7,76023E+14	7,17416E+13	5,6544E+14	7,07157E+14	6,52322E+14
TC0700000433.mm.2	Cyp2b13	cytochrome P450, family 2, subfamily b, polypeptide 13	-0.786471685921468	4,75488E+14	-1,91804E+14	0.100296836978046	0.245928471501167	-4,48451E+14	4,15661E+14	4,40102E+14	4,6242E+14	5,21686E+14	4,65006E+14	5,48051E+14
TC1500001384.mm.2	Ywhaz	tyrosine 3-monooxygenase/tryptophan 5-monooxygenase activation protein, zeta polypeptide	1,46698E+14	1,08823E+14	1,91797E+14	0.100306703575105	0.245928471501167	-4,4846E+14	1,19012E+14	1,2014E+13	1,13481E+14	9,04916E+14	1,10301E+14	9,95103E+14
TC0700003566.mm.2	Kif7	kinesin family member 7	-0.902074086238907	5,71847E+14	-1,91784E+14	0.100325248747575	0.245935167543709	-4,48477E+14	4,92696E+14	5,08989E+14	5,59972E+14	6,87326E+14	5,72666E+14	6,09434E+14
TC1100003561.mm.2	Abcc3	ATP-binding cassette, sub-family C (CFTR/MRP), member 3	0.58133581131491	5,94384E+14	1,91774E+14	0.100339960990029	0.245935167543709	-4,4849E+14	6,09222E+14	6,24341E+14	5,92229E+13	6,23216E+14	5,60364E+14	5,56932E+14
TC1100002480.mm.2	Smim23	small integral membrane protein 23	-0.854339178968433	5,59228E+14	-1,9174E+14	0.100387808035221	0.246015019143957	-4,48534E+14	4,80378E+14	5,45339E+14	5,53209E+14	5,79854E+14	5,48327E+14	6,48258E+14
TC1000001807.mm.2	Hebp2	heme binding protein 2	-1,0257E+14	6,74114E+14	-1,91725E+14	0.100409885449401	0.246015313633214	-4,48554E+14	6,00162E+14	5,79892E+13	6,86367E+14	7,93069E+14	6,32605E+14	7,5259E+14
TC1000000002.mm.2	Ppp1r14c	protein phosphatase 1, regulatory inhibitor subunit 14C	-0.598916749700142	7,42792E+14	-1,91719E+14	0.100418464455139	0.246015313633214	-4,48561E+14	6,96496E+14	7,20968E+14	7,52253E+14	7,49787E+14	7,50399E+14	7,86848E+14
TC1500000805.mm.2	Atxn10	ataxin 10	1,30553E+14	9,02293E+14	1,91695E+14	0.10045244523048	0.246061150805523	-4,48592E+14	1,0163E+14	9,9723E+14	9,3204E+13	7,15763E+14	8,97989E+13	8,54435E+14
TC0400001233.mm.2	Efcab14	EF-hand calcium binding domain 14	0.839077846710625	7,65052E+14	1,91681E+14	0.100472982661489	0.246064707381058	-4,48611E+14	8,08971E+14	7,95458E+14	7,70492E+14	7,78779E+14	7,76993E+14	6,59621E+14
TC0600002880.mm.2	Sumf1	sulfatase modifying factor 1	0.871048247019066	6,16876E+14	1,91673E+14	0.100484439556095	0.246064707381058	-4,48621E+14	6,95358E+14	6,53992E+14	6,31332E+14	5,45436E+14	6,28965E+13	5,46175E+14
TC0500002947.mm.2	Cplx1	complexin 1	0.916564516195901	9,14216E+14	1,91657E+14	0.10050675369726	0.246081951424396	-4,48641E+13	9,95129E+14	9,63581E+14	9,23484E+14	8,27704E+14	9,34141E+14	8,41256E+14
TC0400003529.mm.2	Sh3d21	SH3 domain containing 21	-0.996673568961927	7,11294E+14	-1,91624E+14	0.100553988872563	0.246160197919114	-4,48684E+14	6,51221E+14	6,58177E+14	6,57604E+14	7,92028E+14	7,15453E+14	7,93279E+14
TC1700000782.mm.2	Olfr122	olfactory receptor 122	-1,00031E+14	6,03693E+14	-1,91608E+14	0.100577181148494	0.246179571600439	-4,48705E+14	5,12041E+13	5,34686E+14	6,10952E+14	7,17692E+14	5,67428E+14	6,79362E+13
TC1300001298.mm.2	Arl15	ADP-ribosylation factor-like 15	0.926075926523329	6,51676E+13	1,91572E+14	0.10062869254551	0.246236000354877	-4,48751E+14	7,16683E+14	7,05335E+14	6,62743E+13	5,88492E+13	6,77127E+14	5,59675E+14
TC1600000227.mm.2	Tssk1	testis-specific serine kinase 1	-1,00618E+14	5,17526E+14	-1,91565E+14	0.100638932313378	0.246236000354877	-4,4876E+14	4,44319E+14	4,57314E+14	4,95826E+14	6,04825E+14	4,94835E+14	6,08033E+14
TC1400001790.mm.2	Sftpd	surfactant associated protein D	-0.699829729765072	5,42682E+14	-1,91554E+14	0.100654433575973	0.246236000354877	-4,48774E+14	5,09982E+14	4,86161E+14	5,35622E+14	5,88221E+14	5,39704E+14	5,96404E+14
TC0300002934.mm.2	Lrit3	leucine-rich repeat, immunoglobulin-like and transmembrane domains 3	-0.661362484031773	5,03075E+14	-1,91537E+14	0.100679693552812	0.246236000354877	-4,48797E+14	4,49337E+14	4,63466E+14	4,97916E+14	5,62652E+14	5,13511E+14	5,31566E+14
TC0200002476.mm.2	Sgk2	serum/glucocorticoid regulated kinase 2	-0.701711545114303	5,03442E+13	-1,91524E+14	0.100697939813661	0.246236000354877	-4,48814E+14	4,63258E+14	4,51334E+14	4,94107E+14	5,57016E+14	5,02233E+14	5,52701E+13
TC0400003124.mm.2	Dock7	dedicator of cytokinesis 7	1,03794E+14	5,31486E+14	1,9152E+13	0.100703387165678	0.246236000354877	-4,48818E+14	6,26535E+14	6,00137E+14	5,23287E+14	4,19874E+14	5,72323E+14	4,46762E+14

TC0300001589.mm.2	Wls	wntless WNT ligand secretion mediator	0.847365598018645	6,3882E+14	1,91514E+13	0.100711848402296	0.246236000354877	-4,48826E+14	7,06852E+14	6,78086E+14	6,09577E+14	6,22938E+14	6,65944E+14	5,49521E+14
TC0100001260.mm.2	Cacna1s	calcium channel, voltage-dependent, L type, alpha 1S subunit	-0.863914704890708	6,05831E+14	-1,91488E+14	0.100749316850494	0.246236000354877	-4,4886E+14	5,71623E+14	5,27343E+14	5,79147E+14	6,85122E+14	6,11538E+14	6,60212E+14
TC0700002967.mm.2	Spib	Spi-B transcription factor (Spi-1/PU.1 related)	-0.765455202378951	5,32339E+14	-1,91486E+14	0.100751787861746	0.246236000354877	-4,48862E+14	4,95467E+14	4,67487E+14	5,09751E+14	6,05288E+14	5,55591E+14	5,60453E+14
TC1800000833.mm.2	Atp5a1	ATP synthase, H+ transporting, mitochondrial F1 complex, alpha subunit 1	1,27532E+14	1,04232E+14	1,91486E+14	0.100753053456197	0.246236000354877	-4,48863E+13	1,14248E+14	1,13131E+14	1,07985E+14	8,81547E+14	1,05115E+14	9,67578E+14
TC0500001392.mm.2	Arpc3	actin related protein 2/3 complex, subunit 3	0.780839860380746	8,05186E+14	1,91468E+14	0.100777695004642	0.246258871642372	-4,48885E+14	8,61279E+14	8,64461E+14	8,11583E+14	7,24218E+14	8,12758E+14	7,56814E+14
TC0900002767.mm.2	Elovl4	elongation of very long chain fatty acids (FEN1/Elo2, SUR4/Elo3, yeast)-like 4	1,11886E+14	7,94704E+14	1,91455E+14	0.100796783088616	0.246268167688684	-4,48903E+14	8,63796E+14	8,50851E+14	8,66622E+14	6,96082E+13	7,77277E+13	7,13599E+13
TC0100002888.mm.2	Pign	phosphatidylinositol glycan anchor biosynthesis, class N	0.833723896548831	5,16204E+14	1,91442E+14	0.100815337696145	0.246276157716494	-4,48919E+14	5,51011E+14	6,13626E+14	5,13702E+14	4,20994E+14	5,1669E+13	4,81203E+14
TC0200002972.mm.2	Itga8	integrin alpha 8	-0.70863777761881	6,20714E+12	-1,91412E+14	0.100858901048439	0.246343895609305	-4,48958E+13	5,63013E+14	5,78145E+14	6,15537E+14	6,84702E+14	6,19098E+14	6,63787E+14
TC1500000825.mm.2	Ttc38	tetratricopeptide repeat domain 38	0.605072714616182	5,8998E+14	1,91401E+14	0.100875094901051	0.246343895609305	-4,48973E+14	6,46579E+14	6,06663E+14	5,74297E+14	5,80112E+14	5,78446E+14	5,53782E+14
TC0200004053.mm.2	Olfr1232	olfactory receptor 1232	-0.659980177948112	5,92257E+14	-1,91387E+14	0.100895210014208	0.246343895609305	-4,48991E+14	5,10042E+14	5,70587E+14	6,14646E+14	6,4564E+13	6,02778E+14	6,09847E+13
TC0500002065.mm.2	Tomm7	translocase of outer mitochondrial membrane 7	0.669608914652839	8,45123E+14	1,9138E+14	0.100904220878881	0.246343895609305	-4,48999E+13	8,86188E+14	8,74207E+13	8,31122E+14	8,52746E+13	8,46836E+14	7,79637E+14
TC0600003488.mm.2	Umad1	UMAP1-MVP12 associated (UMA) domain containing 1	0.616173486932697	7,07253E+14	1,91338E+14	0.100965077184986	0.246455126296271	-4,49054E+14	7,72962E+14	7,35065E+14	6,93048E+14	6,57648E+13	6,8962E+13	6,95173E+14
TC0800001625.mm.2	Cd209c	CD209c antigen	-1,30798E+14	5,72685E+14	-1,91293E+14	0.101030563343509	0.246502464659338	-4,49113E+14	5,06205E+13	4,68783E+14	5,5395E+14	6,70588E+12	5,25411E+14	7,11172E+14
TC0200005453.mm.2	Sox18	SRY (sex determining region Y)-box 18	-0.804979593980486	6,07528E+14	-1,91289E+14	0.101035681827877	0.246502464659338	-4,49117E+14	5,34928E+14	5,92417E+14	5,68406E+14	6,61073E+12	6,52326E+13	6,36015E+14
TC1200000032.mm.2	Pfn4	profilin family, member 4	1,23118E+14	7,06746E+14	1,91288E+14	0.101036487854146	0.246502464659338	-4,49118E+14	7,86005E+13	7,58439E+14	7,54743E+13	6,43083E+14	7,34884E+14	5,63323E+14
TC0600001293.mm.2	Washc2	WASH complex subunit 2`	1,03602E+14	7,74533E+14	1,91282E+14	0.101045663692356	0.246502464659338	-4,49126E+14	8,6362E+14	8,4934E+14	7,85227E+14	6,43255E+14	7,93961E+14	7,11795E+14
TC1100002227.mm.2	Grb10	growth factor receptor bound protein 10	2,09683E+14	6,55425E+14	1,91241E+14	0.101104816851268	0.24660943292832	-4,49179E+14	7,98078E+14	7,40748E+14	7,98455E+14	4,7581E+14	6,554E+14	4,64059E+14
TC0400002108.mm.2	AW011738	expressed sequence AW011738	-0.684663351246211	6,19102E+13	-1,91223E+14	0.101131208192892	0.246624653246622	-4,49203E+14	5,77132E+14	5,76214E+14	6,08165E+14	6,62819E+14	6,18298E+14	6,71982E+14
TC1000002398.mm.2	Prmt2/Mir678	protein arginine N-methyltransferase 2/microRNA 678	0.981926834968229	7,30717E+14	1,91215E+14	0.101141668755271	0.246624653246622	-4,49212E+14	8,24987E+14	7,83687E+14	7,31313E+14	6,32025E+14	7,60306E+14	6,51982E+14
TC0400002847.mm.2	Cdk5rap2	CDK5 regulatory subunit associated protein 2	-0.634635444518381	5,86028E+14	-1,91203E+14	0.101158890226638	0.246629323380514	-4,49228E+14	5,56275E+14	5,53957E+14	5,5501E+14	6,13764E+14	6,23424E+14	6,13735E+14
TC0600001509.mm.2	Nrip2	nuclear receptor interacting protein 2	0.595893836808537	6,96052E+14	1,9116E+14	0.101221935950194	0.2467456965152	-4,49284E+14	7,44322E+14	7,06585E+14	6,89716E+14	7,03962E+14	6,57914E+14	6,73814E+14
TC0800003068.mm.2	Cyba	cytochrome b-245, alpha polypeptide	-0.61410074057788	7,29059E+14	-1,91125E+14	0.101271927792069	0.246806853517076	-4,49329E+14	6,90316E+14	7,19323E+14	7,14152E+14	7,18104E+14	7,44999E+14	7,87461E+14
TC0600003139.mm.2	Tapbpl	TAP binding protein-like	0.719155733768061	5,73899E+13	1,91121E+14	0.101277658751003	0.246806853517076	-4,49334E+14	5,93735E+14	6,15113E+14	6,09896E+14	5,59632E+14	5,66453E+14	4,98564E+14
TC1200001902.mm.2	Rtn1	reticulon 1	1,69615E+14	1,07737E+14	1,91099E+14	0.101309842402627	0.246847949589222	-4,49363E+14	1,21457E+14	1,19937E+13	1,12344E+14	8,52122E+14	1,10228E+14	9,72438E+14
TC0600002616.mm.2	Nat8f7	N-acetyltransferase 8 (GCN5-related) family member 7	-0.931465543992599	5,78756E+14	-1,91067E+14	0.101355689003504	0.246922318855982	-4,49404E+14	5,37032E+13	5,27158E+14	5,22236E+14	6,35627E+14	6,67055E+13	5,83427E+14
TC1800000543.mm.2	Csnk1g3	casein kinase 1, gamma 3	1,27298E+14	7,94515E+14	1,91012E+14	0.10143463443743	0.247066676689034	-4,49475E+14	8,62283E+14	8,63798E+14	8,61146E+14	7,08376E+14	8,1184E+14	6,59646E+13
TC0600000078.mm.2	Phf14	PHD finger protein 14	1,80201E+14	7,35369E+14	1,91001E+14	0.101450486811273	0.247066676689034	-4,49489E+13	8,65001E+14	8,54853E+14	7,72662E+14	5,60243E+14	8,00801E+14	5,58651E+14
TC1300000555.mm.2	Barx1	BarH-like homeobox 1	-0.63850404397975	5,08399E+14	-1,90992E+14	0.101464390528676	0.247066676689034	-4,49501E+14	4,58522E+14	4,52091E+14	5,21102E+14	5,80366E+14	5,26277E+14	5,12037E+14
TC1000000745.mm.2	Spatc1l	spermatogenesis and centriole associated 1 like	-0.846455580896366	5,5112E+14	-1,90984E+14	0.101476277932603	0.247066676689034	-4,49512E+14	4,65102E+14	5,26524E+14	5,29343E+14	6,24832E+14	5,59509E+14	6,01408E+14
TC0700002585.mm.2	Vmn1r175	vomeronasal 1 receptor 175	-0.715713312374399	5,92078E+14	-1,9096E+14	0.101510130925549	0.247111760024682	-4,49542E+14	5,19015E+14	5,642E+14	6,023E+14	6,40597E+14	5,81792E+14	6,44565E+14
TC0300001114.mm.2	Kcna10	potassium voltage-gated channel, shaker-related subfamily, member 10	-0.757351241352977	5,54922E+14	-1,90944E+14	0.10153412338029	0.247132829309156	-4,49563E+14	4,9962E+14	4,9747E+14	5,56381E+14	6,27501E+14	5,40543E+14	6,08018E+14
TC0300000787.mm.2	Dennd4b	DENN/MADD domain containing 4B	0.738006680997873	6,90276E+14	1,90908E+14	0.101585988941563	0.247200049708841	-4,4961E+14	7,36068E+14	7,20525E+14	6,74041E+14	7,02032E+14	6,10972E+14	6,9802E+13
TC1400001914.mm.2	Bmp4	bone morphogenetic protein 4	0.577085095731564	5,82795E+14	1,90902E+14	0.101594675294184	0.247200049708841	-4,49617E+14	6,037E+14	6,00433E+13	6,14668E+13	5,89251E+14	5,55294E+14	5,33423E+14
TC1100002641.mm.2	Irgm1	immunity-related GTPase family M member 1	0.925113959349495	5,92419E+14	1,90882E+14	0.101623459159014	0.247200049708841	-4,49643E+14	6,19281E+14	6,62352E+14	5,37412E+14	6,38859E+14	5,91448E+14	5,05162E+14
TC1000001957.mm.2	Supt4b	suppressor of Ty 4B	0.741109072335384	7,62054E+14	1,90862E+14	0.101652014328511	0.247200049708841	-4,49669E+14	8,21075E+14	7,75078E+14	8,04253E+14	7,23985E+14	7,31298E+11	7,16634E+14
TC1500000693.mm.2	Syngr1	synaptogyrin 1	0.684692636141635	9,24031E+14	1,9086E+14	0.10165459023887	0.247200049708841	-4,49671E+14	9,92244E+14	9,55284E+14	9,25909E+14	8,60163E+14	9,13127E+14	8,97462E+14
TC0100002476.mm.2	Cryge	crystallin, gamma E	-0.601840218123854	5,8246E+14	-1,90849E+13	0.101670710067731	0.247200049708841	-4,49685E+14	5,37732E+14	5,46733E+14	6,2463E+14	5,8083E+14	5,95849E+14	6,08984E+14
TC1300001278.mm.2	Cdc20b	cell division cycle 20B	-0.92676924828658	7,17177E+14	-1,90841E+14	0.101683080260293	0.247200049708841	-4,49696E+13	6,32028E+12	6,3684E+13	7,34271E+14	8,45698E+14	6,84467E+14	7,69755E+14
TC0400000282.mm.2	Srsf12	serine/arginine-rich splicing factor 12	-0.679983147606052	7,22462E+14	-1,9084E+14	0.101684473994303	0.247200049708841	-4,49698E+14	6,99857E+14	6,46121E+14	7,26917E+14	7,79905E+14	7,4294E+14	7,39034E+14
TC1600001064.mm.2	Dyrk1a	dual-specificity tyrosine-(Y)-phosphorylation regulated kinase 1a	1,41609E+14	7,3775E+14	1,90823E+14	0.101709197943546	0.247206341867606	-4,4972E+14	8,50025E+14	8,47915E+14	7,78967E+14	5,34872E+14	7,19851E+14	6,9487E+13
TC0600003417.mm.2	Kras	Kirsten rat sarcoma viral oncogene homolog	1,83457E+14	7,00828E+14	1,90817E+14	0.101717746327948	0.247206341867606	-4,49727E+14	8,48939E+14	8,44047E+14	7,24184E+14	4,61728E+14	7,62232E+13	5,63839E+14
TC0100002866.mm.2	Pam	peptidylglycine alpha-amidating monooxygenase	1,27705E+14	8,61549E+14	1,90801E+14	0.101741156730641	0.247225947579674	-4,49748E+14	9,50509E+14	9,38498E+14	9,08796E+14	7,37894E+14	8,83218E+14	7,50379E+14
TC0600003536.mm.2	Clec1b	C-type lectin domain family 1, member b	-1,03022E+14	6,19096E+14	-1,90781E+13	0.101769107384296	0.247232694022142	-4,49773E+14	5,09587E+14	5,72287E+14	6,0072E+14	7,44062E+14	6,19425E+14	6,68492E+14
TC0600000584.mm.2	Ccdc126	coiled-coil domain containing 126	0.688381979224217	5,41016E+14	1,90778E+14	0.101774620458566	0.247232694022142	-4,49778E+14	6,15315E+14	6,04819E+13	5,08342E+14	4,3516E+14	5,54089E+14	5,28369E+14
TC0700004638.mm.2	Rab4b/Mir3101	RAB4B, member RAS oncogene family/microRNA 3101	0.802368985074859	8,66643E+14	1,90743E+14	0.10182517958353	0.247318227107237	-4,49823E+14	9,31253E+14	9,21516E+14	8,78697E+14	7,76098E+14	8,65636E+14	8,2666E+14
TC1100000314.mm.2	Psme4	proteasome (prosome, macropain) activator subunit 4	1,57846E+14	5,07548E+14	1,90702E+14	0.101884244663984	0.247418679181026	-4,49875E+14	5,92919E+14	6,13183E+14	5,68745E+14	3,80033E+14	5,4703E+13	3,43379E+14
TC0900000719.mm.2	Ubl7	ubiquitin-like 7 (bone marrow stromal cell-derived)	1,32377E+14	8,86232E+13	1,90693E+14	0.101897247877198	0.247418679181026	-4,49887E+14	9,89754E+14	9,90954E+14	9,17535E+14	7,03196E+14	8,94783E+14	8,21171E+14
TC0900000613.mm.2	Btg4	B cell translocation gene 4	-0.720480120985823	5,07717E+14	-1,90672E+13	0.101928269477986	0.247450652331047	-4,49915E+14	4,83449E+13	4,51751E+14	4,80536E+14	5,51266E+14	5,18802E+13	5,60494E+14
TC0X00003361.mm.2	Prps2	phosphoribosyl pyrophosphate synthetase 2	0.573971123663901	7,39279E+14	1,90663E+14	0.101941130154129	0.247450652331047	-4,49926E+14	7,9149E+14	7,77803E+14	7,19282E+14	6,92598E+14	7,33131E+14	7,21369E+14
TC0400001451.mm.2	Oscp1	organic solute carrier partner 1	1,12207E+14	6,26341E+14	1,90643E+14	0.101970214438262	0.247477496606829	-4,49952E+14	7,01104E+14	6,85648E+12	6,44926E+14	5,64031E+14	6,63561E+14	4,98778E+14
TC1900000113.mm.2	Men1	multiple endocrine neoplasia 1	0.994437852149295	7,40588E+14	1,90629E+14	0.101990272945442	0.247477496606829	-4,4997E+14	8,24473E+14	8,09203E+14	7,6197E+14	6,1309E+14	6,95126E+14	7,39664E+14
TC0200004521.mm.2	Myef2/Gm9833	myelin basic protein expression factor 2, repressor/myelin basic protein expression factor 2, repressor pseudogene	1,89481E+14	6,95098E+14	1,90624E+14	0.101998265683979	0.247477496606829	-4,49977E+14	8,28657E+14	8,38275E+14	7,41787E+14	4,73897E+14	7,4958E+14	5,3839E+14
TC0100002314.mm.2	Gls	glutaminase	1,87491E+14	8,7496E+12	1,90587E+13	0.102051830581528	0.247546423639181	-4,50024E+14	1,00681E+13	1,02082E+13	9,01176E+14	6,68311E+14	9,48213E+14	7,04432E+14
TC0100003855.mm.2	Cr1l	complement component (3b/4b) receptor 1-like	0.937525281737807	6,90585E+14	1,90575E+14	0.102069464593081	0.247546423639181	-4,5004E+14	7,22175E+14	7,49393E+14	6,42284E+13	7,45596E+14	6,89713E+14	5,94351E+14

TC1100001924.mm.2	Ten1	TEN1 telomerase capping complex subunit	-0.560115054754776	7,36956E+14	-1,90572E+14	0.102072763523783	0.247546423639181	-4,50043E+14	6,99737E+14	6,82985E+14	7,67241E+12	7,77027E+14	7,41989E+14	7,52756E+14
TC1400001726.mm.2	Bmpr1a	bone morphogenetic protein receptor, type 1A	1,21104E+14	6,44386E+14	1,90545E+13	0.102111816749079	0.24760386806289	-4,50078E+14	7,14787E+14	7,35638E+14	6,79795E+14	5,27881E+14	6,70858E+14	5,3736E+14
TC0200005378.mm.2	Gm14326	predicted gene 14326	1,57215E+14	8,77791E+14	1,90484E+14	0.102201315502666	0.247743771095887	-4,50157E+14	9,78918E+14	1,02597E+13	8,79449E+14	6,91955E+13	9,44759E+14	7,45696E+14
TC0200003720.mm.2	Slc25a12	solute carrier family 25 (mitochondrial carrier, Aralar), member 12	1,54766E+14	9,25941E+14	1,90479E+14	0.10220852602263	0.247743771095887	-4,50163E+14	1,0519E+14	1,03533E+14	9,7897E+14	7,11751E+14	9,14385E+14	8,63311E+14
TC1100003286.mm.2	Nek8	NIMA (never in mitosis gene a)-related expressed kinase 8	-0.553568273191619	6,06369E+14	-1,90446E+14	0.10225696171253	0.247743771095887	-4,50206E+14	5,53813E+14	5,68942E+13	6,41218E+13	6,40776E+14	6,14528E+14	6,1894E+14
TC0400003829.mm.2	Pla2g2f	phospholipase A2, group IIF	-0.919861669711661	6,24269E+14	-1,90438E+12	0.102268250674459	0.247743771095887	-4,50216E+14	5,37157E+14	5,7038E+14	6,10649E+14	7,35922E+14	6,20297E+14	6,71212E+14
TC0500002288.mm.2	Zfp518b	zinc finger protein 518B	0.569255876804981	6,41593E+14	1,90437E+14	0.102269585622622	0.247743771095887	-4,50218E+14	6,93758E+14	6,7243E+14	6,10033E+13	6,21001E+14	6,17523E+14	6,34814E+14
TC1900001503.mm.2	Opalin	oligodendrocytic myelin paranodal and inner loop protein	0.811563785239543	8,16443E+14	1,90435E+13	0.102272044041778	0.247743771095887	-4,5022E+14	8,52056E+14	8,81164E+14	8,3065E+14	7,63881E+14	8,28566E+14	7,42341E+14
TC0800000974.mm.2	Prdx2	peroxiredoxin 2	1,36551E+14	9,56124E+14	1,90432E+14	0.102277140528135	0.247743771095887	-4,50224E+14	1,06608E+14	1,05982E+14	9,88865E+14	7,69168E+14	9,68847E+14	8,83958E+14
TC0900001851.mm.2	Dnmt1	DNA methyltransferase (cytosine-5) 1	0.574501582140729	6,36909E+14	1,90404E+14	0.102317622872977	0.247757763166513	-4,5026E+14	6,78546E+14	6,6867E+14	6,33558E+14	6,08363E+14	6,06687E+14	6,25628E+13
TC1400001669.mm.2	Smim4	small integral membrane protein 4	-0.766231492721008	7,08286E+14	-1,90401E+14	0.102321480247521	0.247757763166513	-4,50264E+14	6,64813E+14	6,69838E+14	6,66352E+14	7,60814E+14	7,26182E+14	7,61715E+14
TC0300001359.mm.2	Cxxc4	CXXC finger 4	0.654061264799347	6,6353E+14	1,90396E+14	0.102329479681408	0.247757763166513	-4,50271E+14	7,23021E+14	6,59146E+14	6,67016E+14	6,80646E+13	6,34252E+14	6,17102E+14
TC0100001877.mm.2	Plxna2	plexin A2	1,1249E+14	7,07248E+13	1,90376E+14	0.102359339786124	0.247757763166513	-4,50297E+14	7,88298E+14	7,56709E+14	7,0432E+14	6,74134E+14	7,49983E+14	5,70045E+14
TC0200001455.mm.2	Alx4	aristaless-like homeobox 4	-0.962616661347217	5,20895E+13	-1,90358E+14	0.10238464401209	0.247757763166513	-4,50319E+14	4,42982E+13	4,58748E+14	5,08672E+14	6,20717E+14	4,98474E+13	5,9578E+14
TC1700001247.mm.2	Mta3	metastasis associated 3	1,69973E+14	8,03011E+14	1,90356E+14	0.102388276852436	0.247757763166513	-4,50323E+14	9,40872E+13	9,09091E+14	8,5474E+14	6,03347E+14	8,38254E+14	6,71763E+14
TC0700000796.mm.2	Klk1	kallikrein 1	-0.971789060083513	5,07274E+14	-1,90354E+14	0.102390550792888	0.247757763166513	-4,50325E+14	4,62144E+13	4,31892E+14	4,69783E+14	5,91429E+14	5,86593E+13	5,01802E+14
TC0500001337.mm.2	Sds	serine dehydratase	-1,21257E+14	5,57592E+14	-1,90296E+14	0.102476109681081	0.247882236361972	-4,504E+14	4,93134E+14	4,49633E+14	5,15651E+14	7,01855E+13	5,57148E+14	6,28133E+14
TC0300001047.mm.2	Bcas2	breast carcinoma amplified sequence 2	0.75425966444435	7,5816E+14	1,90281E+14	0.102496931545458	0.247882236361972	-4,50419E+14	7,97218E+14	7,96651E+14	7,66484E+14	7,45586E+14	7,69346E+14	6,73671E+14
TC0800002107.mm.2	Cnot7	CCR4-NOT transcription complex, subunit 7	1,38486E+13	8,8443E+14	1,90278E+14	0.102500967254865	0.247882236361972	-4,50422E+14	9,8792E+14	9,87817E+14	9,15226E+14	7,16854E+14	9,16758E+14	7,82005E+14
TC1400001067.mm.2	Nufip1	nuclear fragile X mental retardation protein interacting protein 1	1,37216E+14	6,19836E+14	1,90277E+14	0.102503527641024	0.247882236361972	-4,50425E+14	7,38701E+14	7,34098E+14	6,22345E+14	4,25504E+13	6,65326E+14	5,33043E+13
TC1100000917.mm.2	Sat2	spermidine/spermine N1-acetyl transferase 2	1,21905E+13	6,53609E+14	1,90237E+14	0.102561218507405	0.247961027779193	-4,50476E+14	7,69429E+14	7,17913E+14	6,83903E+14	5,06875E+14	6,67049E+14	5,76483E+14
TC0700002088.mm.2	Cd81	CD81 antigen	1,14413E+14	1,15799E+14	1,90233E+14	0.102566886994869	0.247961027779193	-4,50481E+14	1,23168E+14	1,23643E+14	1,20071E+14	1,03983E+14	1,16964E+14	1,06964E+14
TC0200002796.mm.2	Ogfr	opioid growth factor receptor	0.897165912055504	7,74487E+14	1,90186E+14	0.102636832075304	0.248082812006756	-4,50542E+13	8,27934E+14	8,44245E+14	7,79101E+14	7,02898E+14	8,00984E+14	6,91761E+14
TC0X00000166.mm.2	Ddx3x	DEAD/H (Asp-Glu-Ala-Asp/His) box polypeptide 3, X-linked	1,45786E+12	8,81901E+14	1,90178E+14	0.102648054840752	0.248082812006756	-4,50552E+14	9,6553E+14	9,49482E+14	9,85517E+14	7,67434E+14	8,77186E+14	7,46255E+14
TC1400002491.mm.2	Wbp4	WW domain binding protein 4	1,22637E+14	7,20839E+14	1,90133E+14	0.102713160988007	0.248199533418121	-4,5061E+14	8,03508E+14	8,05352E+14	7,5998E+14	5,9261E+13	7,42771E+14	6,20815E+14
TC0400000920.mm.2	Zfp352	zinc finger protein 352	-1,12451E+12	5,19742E+14	-1,90123E+14	0.102727735329585	0.248199533418121	-4,50623E+14	4,34019E+14	4,63094E+14	4,72283E+14	6,27042E+14	5,1207E+14	6,09946E+14
TC0600001370.mm.2	Slc6a13	solute carrier family 6 (neurotransmitter transporter, GABA), member 13	-0.548504845788657	6,66276E+14	-1,90113E+14	0.102742561155518	0.248199533418121	-4,50636E+14	6,31917E+14	6,30155E+14	6,8087E+14	6,82938E+14	6,90376E+14	6,81397E+14
TC0700000832.mm.2	Slc17a7	solute carrier family 17 (sodium-dependent inorganic phosphate cotransporter), member 7	0.856062334554842	9,93945E+14	1,9008E+14	0.102790633515538	0.248226010174898	-4,50678E+13	1,03379E+14	1,12791E+14	9,43346E+14	8,68135E+14	1,01069E+14	9,79794E+13
TC0100000279.mm.2	Nms	neuromedin S	-0.643318062600149	5,25054E+14	-1,90068E+14	0.102808728699582	0.248226010174898	-4,50694E+14	4,71921E+14	5,07442E+14	5,11933E+14	5,51003E+14	5,35794E+14	5,72233E+14
TC0100000333.mm.2	Bivm	basic, immunoglobulin-like variable motif containing	1,30049E+14	6,21603E+14	1,90065E+14	0.102812924346978	0.248226010174898	-4,50698E+14	7,08837E+14	6,95246E+13	6,5059E+14	5,30961E+14	6,65667E+14	4,78318E+14
TC1500002183.mm.2	Rnd1	Rho family GTPase 1	0.685128637430073	6,68284E+14	1,90053E+14	0.102830090177904	0.248226010174898	-4,50713E+14	7,01732E+14	6,72843E+14	7,07821E+14	6,89759E+14	6,539E+14	5,83649E+14
TC0800002824.mm.2	Atp6v0d1	ATPase, H+ transporting, lysosomal V0 subunit D1	1,20717E+14	8,78127E+12	1,9005E+14	0.102835584649282	0.248226010174898	-4,50718E+14	9,74681E+14	9,58814E+14	9,18989E+14	7,24215E+14	8,73799E+14	8,18262E+13
TC1700001972.mm.2	Gm9573	predicted gene 9573	-0.86930306879978	5,50089E+14	-1,90021E+14	0.102878243302684	0.248226010174898	-4,50755E+14	4,95148E+14	4,91062E+13	5,17235E+13	6,37019E+14	5,58044E+14	6,02027E+13
TC0400003425.mm.2	Ppcs	phosphopantothenoylcysteine synthetase	-0.749694036539149	6,43488E+14	-1,90001E+14	0.102907410580329	0.248226010174898	-4,50781E+14	5,81053E+14	5,93782E+14	6,33009E+14	7,28308E+14	6,56027E+14	6,68747E+14
TC0200001043.mm.2	Platr26	pluripotency associated transcript 26	-0.752596876277374	5,55212E+14	-1,89999E+14	0.102910518097535	0.248226010174898	-4,50784E+14	4,91472E+14	5,01603E+14	5,58403E+14	6,36202E+14	5,42615E+14	6,00979E+14
TC0600001290.mm.2	H1foo	H1 histone family, member O, oocyte-specific	-1,2055E+14	5,0199E+14	-1,89997E+13	0.102912678039552	0.248226010174898	-4,50785E+14	4,17704E+14	4,47459E+14	4,2769E+14	6,12822E+14	5,14405E+14	5,91859E+14
TC0100003094.mm.2	Lax1	lymphocyte transmembrane adaptor 1	-0.657779645618567	4,83148E+14	-1,89992E+14	0.10292021871761	0.248226010174898	-4,50792E+14	4,32722E+14	4,44322E+14	4,72632E+14	5,40608E+14	5,0102E+14	5,07581E+14
TC1900000547.mm.2	Lgi1	leucine-rich repeat LGI family, member 1	1,974E+13	7,87786E+14	1,8999E+14	0.102922979828616	0.248226010174898	-4,50794E+14	9,24603E+14	9,49767E+14	8,20781E+14	5,51994E+14	8,57412E+14	6,22158E+13
TSUnmapped00000046.mm.2	Egr3	early growth response 3	0.648196362977506	8,10402E+14	1,8996E+14	0.102967728488192	0.248296768801292	-4,50834E+14	8,36623E+14	8,49407E+14	8,15686E+13	8,04306E+14	8,10072E+14	7,46318E+14
TC0300000890.mm.2	Lysmd1	LysM, putative peptidoglycan-binding, domain containing 1	-0.609730214447521	6,97343E+14	-1,89929E+14	0.103013192899356	0.248356377086157	-4,50874E+14	6,7225E+12	6,69574E+14	7,09928E+14	6,68537E+14	7,47024E+14	7,16746E+14
TC0X00000656.mm.2	Vma21	VMA21 vacuolar H+-ATPase homolog (S. cerevisiae)	1,22449E+14	6,34843E+14	1,89922E+14	0.103023274650523	0.248356377086157	-4,50883E+14	7,57305E+14	7,18188E+14	6,30806E+13	4,72164E+14	6,78834E+14	5,51761E+14
TC0100001244.mm.2	Ptpn7	protein tyrosine phosphatase, non-receptor type 7	-0.74300601120827	5,77995E+14	-1,89888E+14	0.103073027455839	0.248439146057731	-4,50926E+14	5,5698E+14	5,39642E+14	5,77711E+13	5,48414E+14	5,7813E+14	6,67094E+14
TC0400000138.mm.2	Calb1	calbindin 1	0.848475441810019	7,8889E+14	1,8987E+14	0.103099778935827	0.248466457970831	-4,5095E+14	8,54175E+14	8,35311E+14	8,22655E+14	7,01411E+14	7,58356E+14	7,61434E+14
TC1400002073.mm.2	Myh6	myosin, heavy polypeptide 6, cardiac muscle, alpha	-0.940975889365947	5,66873E+14	-1,89851E+14	0.103127294143829	0.24849464348441	-4,50974E+14	5,05595E+14	5,1414E+14	5,27786E+14	6,45788E+14	5,62193E+14	6,45737E+14
TC0X00001667.mm.2	Vegfd	vascular endothelial growth factor D	-0.675898649438068	5,66988E+14	-1,8984E+14	0.103143287699201	0.24849464348441	-4,50988E+14	4,86663E+14	5,59933E+14	5,94829E+14	5,78728E+14	5,94682E+14	5,87094E+14
TC1300000882.mm.2	Lpcat1	lysophosphatidylcholine acyltransferase 1	0.793014894272003	6,88243E+13	1,8982E+14	0.103172482676558	0.24849464348441	-4,51014E+14	7,44602E+12	7,44003E+14	7,06347E+14	6,04506E+14	6,45912E+14	6,84089E+14
TC1700001270.mm.2	Camkmt	calmodulin-lysine N-methyltransferase	0.992675607189171	6,45168E+14	1,89801E+12	0.103200469823569	0.24849464348441	-4,51038E+14	7,11536E+14	7,0411E+14	6,5389E+14	5,84361E+14	6,78686E+13	5,38425E+13
TC0900001504.mm.2	Clasp2	CLIP associating protein 2	1,38587E+14	8,14282E+14	1,89796E+14	0.103208293568136	0.24849464348441	-4,51045E+14	9,10894E+14	9,00679E+12	8,52592E+14	6,87127E+14	8,54482E+14	6,79918E+14
TC0200001951.mm.2	Slc20a1	solute carrier family 20, member 1	0.952462086824687	7,60728E+14	1,89794E+14	0.103210412129295	0.24849464348441	-4,51047E+14	8,13791E+12	8,423E+14	7,55172E+14	6,87506E+14	7,96794E+14	6,68805E+14
TC1600000166.mm.2	Rrn3	RRN3 RNA polymerase I transcription factor homolog (yeast)	1,48736E+13	6,48353E+14	1,89788E+14	0.103219428339922	0.24849464348441	-4,51055E+14	7,7875E+14	7,80721E+14	6,59724E+14	4,08926E+14	6,67982E+14	5,94017E+14
TC1900000630.mm.2	Nkx2-3	NK2 homeobox 3	-0.709348846155572	5,02178E+14	-1,89776E+14	0.103237075508646	0.248499999652048	-4,5107E+14	4,59023E+14	4,43911E+14	4,98436E+14	5,66895E+14	4,97723E+12	5,4708E+14
TC1000001629.mm.2	Olfr9	olfactory receptor 9	-0.856810388015529	4,8933E+14	-1,89741E+14	0.103288560789895	0.248586793130334	-4,51116E+14	4,08097E+14	4,51224E+14	5,21598E+14	5,24375E+14	4,56732E+14	5,73952E+14
TC1600002136.mm.2	Brwd1	bromodomain and WD repeat domain containing 1	0.757899105770877	5,73221E+14	1,89712E+14	0.10333164176013	0.248633488393631	-4,51153E+14	6,49825E+13	6,40798E+14	5,47122E+14	4,6254E+14	5,95374E+14	5,43669E+14
TC0500000584.mm.2	0610040J01Rik	RIKEN cDNA 0610040J01 gene	-0.79121213011885	5,60828E+14	-1,89707E+14	0.10333882404097	0.248633488393631	-4,5116E+13	4,78383E+13	5,31264E+14	5,76193E+14	6,11238E+14	5,39985E+14	6,27903E+14
TC0X00001159.mm.2	H2afb2	H2A histone family, member B2	-0.860759888086085	5,94648E+14	-1,89682E+14	0.103376173162594	0.248686216507746	-4,51192E+14	5,28663E+14	5,58318E+14	5,8257E+14	6,39202E+14	5,75297E+14	6,83835E+14
TC0900002320.mm.2	Dmxl2	Dmx-like 2	1,85408E+14	6,89802E+13	1,89668E+14	0.103396900869116	0.248698949649808	-4,51211E+14	8,53065E+14	8,26206E+14	7,03821E+14	4,47262E+14	7,61162E+14	5,47293E+14

TC0100003806.mm.2	Ppp2r5a	protein phosphatase 2, regulatory subunit B', alpha	1,31689E+14	7,96928E+14	1,89643E+13	0.103433594661174	0.248730692027923	-4,51243E+14	8,79111E+14	8,96357E+13	8,3392E+14	6,60091E+14	8,28304E+14	6,83787E+14
TC0100001220.mm.2	Chit1	chitinase 1 (chitotriosidase)	-0.625842475377092	5,43355E+14	-1,89638E+14	0.103440971096575	0.248730692027923	-4,51249E+13	5,03115E+14	5,37883E+14	5,03486E+14	5,49479E+14	5,87135E+14	5,79031E+14
TC0500003238.mm.2	Clip1	CAP-GLY domain containing linker protein 1	2,13795E+14	6,48393E+14	1,89624E+13	0.103461803377943	0.248743664253775	-4,51267E+14	7,85977E+13	7,8904E+14	6,93284E+14	4,74629E+14	7,35775E+14	4,11652E+14
TC0700004637.mm.2	Dnaaf3	dynein, axonemal assembly factor 3	-0.800323123120799	5,46258E+14	-1,89569E+14	0.103543005216152	0.248882457427885	-4,51338E+14	5,24211E+14	5,01353E+14	5,14671E+14	5,51686E+14	5,41791E+14	6,43837E+14
TC0800003177.mm.2	Exoc8	exocyst complex component 8	0.756305232445364	6,85326E+14	1,89563E+14	0.103550424787224	0.248882457427885	-4,51345E+14	7,52528E+14	7,53533E+14	6,73937E+14	5,77157E+14	6,88438E+14	6,66363E+14
TC1400001612.mm.2	Plac9b/Plac9a	placenta specific 9b/placenta specific 9a	-0.600517333991752	7,14633E+14	-1,89545E+14	0.103577826504435	0.248911188436384	-4,51369E+14	6,79646E+14	6,80235E+13	7,20021E+14	7,27909E+14	7,16385E+14	7,636E+14
TC1700002520.mm.2	Galnt14	polypeptide N-acetylgalactosaminyltransferase 14	-0.57875237081653	6,89107E+14	-1,89495E+14	0.103651326734554	0.248929612920626	-4,51433E+14	6,66886E+12	6,5474E+14	6,82132E+14	6,93507E+14	7,35289E+14	7,02088E+14
TC1000000940.mm.2	D10Wsu102e	DNA segment, Chr 10, Wayne State University 102, expressed	0.703702422037812	6,42421E+14	1,8948E+14	0.103673391886838	0.248929612920626	-4,51452E+13	6,88429E+14	6,58603E+14	6,38987E+12	6,62215E+14	6,4087E+14	5,65422E+14
TC0400002271.mm.2	Wwp1	WW domain containing E3 ubiquitin protein ligase 1	1,26535E+13	6,73521E+14	1,89479E+14	0.103675705777042	0.248929612920626	-4,51454E+14	7,33203E+14	7,51678E+12	7,33212E+13	5,91223E+14	6,96895E+14	5,34916E+14
TC0800000005.mm.2	Arhgef18	rho/rac guanine nucleotide exchange factor (GEF) 18	0.576258146306138	6,46575E+14	1,89467E+14	0.10369304590157	0.248929612920626	-4,51469E+14	6,76384E+14	7,06755E+14	6,19642E+14	6,08782E+13	6,2621E+14	6,41678E+13
TC0X00003327.mm.2	Car5b	carbonic anhydrase 5b, mitochondrial	0.586506917656725	5,48677E+13	1,89454E+14	0.103712402028952	0.248929612920626	-4,51486E+14	5,67974E+13	5,94252E+14	5,40031E+14	5,44883E+14	5,38227E+14	5,06697E+14
TC0700000786.mm.2	Klk1b11	kallikrein 1-related peptidase b11	-0.603871310622793	4,99797E+14	-1,8945E+14	0.103718632647291	0.248929612920626	-4,51492E+14	4,70025E+14	4,61164E+14	4,83609E+14	5,32022E+14	5,23374E+14	5,2859E+14
TC0900003179.mm.2	Rps27rt	ribosomal protein S27, retrogene	0.676049225439669	8,3361E+14	1,89438E+14	0.103736374671831	0.248929612920626	-4,51507E+14	8,82763E+14	8,84452E+13	8,3218E+14	7,7026E+14	8,37941E+14	7,94065E+13
TC0500002799.mm.2	Paqr3	progestin and adipoQ receptor family member III	-0.59527836340269	8,43536E+13	-1,89434E+14	0.103741827025999	0.248929612920626	-4,51512E+14	7,98508E+14	8,07618E+14	8,73595E+14	8,56312E+14	8,41972E+13	8,83211E+13
TC1700002190.mm.2	Ptcra	pre T cell antigen receptor alpha	-0.623296237350942	7,18646E+14	-1,89434E+14	0.103741852920241	0.248929612920626	-4,51512E+14	6,8596E+14	6,61402E+14	7,60832E+14	7,3166E+14	7,14494E+14	7,57528E+14
TC0600001629.mm.2	BC049715	cDNA sequence BC049715	-0.66522285690781	4,72226E+14	-1,89433E+14	0.103743462372334	0.248929612920626	-4,51513E+13	4,05022E+14	4,33978E+14	4,91836E+14	5,30474E+14	4,59045E+14	5,12999E+14
TC0800001118.mm.2	Ccl22	chemokine (C-C motif) ligand 22	-0.634751698249722	5,92386E+14	-1,89425E+14	0.103755432282935	0.248929612920626	-4,51524E+14	5,41605E+14	5,42392E+14	5,98819E+14	6,60553E+13	6,03721E+13	6,07225E+14
TC0200001990.mm.2	Mrps26	mitochondrial ribosomal protein S26	-0.585507762117635	7,82562E+14	-1,89408E+14	0.103779395280113	0.248950036645594	-4,51545E+14	7,52622E+14	7,59984E+13	7,70849E+14	7,82209E+14	8,34147E+14	7,9556E+14
TC1200000460.mm.2	Srp54b/Srp54a	signal recognition particle 54B/signal recognition particle 54A	1,15849E+14	7,90781E+14	1,89357E+14	0.103855913075456	0.249096506011435	-4,51612E+14	8,54714E+14	9,11986E+14	7,81402E+14	6,61585E+14	8,3801E+14	6,96991E+14
TC0200004504.mm.2	Patl2	protein associated with topoisomerase II homolog 2 (yeast)	-0.799208679725551	6,34934E+14	-1,89346E+14	0.103871567415696	0.24909697362243	-4,51625E+14	5,78982E+14	5,8713E+14	6,12201E+14	7,05336E+14	6,33164E+13	6,9279E+14
TC1300001350.mm.2	Ccl28	chemokine (C-C motif) ligand 28	-0.698801167615192	6,54725E+14	-1,89306E+13	0.103930484195495	0.249108756318291	-4,51676E+14	6,21142E+14	6,00692E+14	6,42868E+14	7,02053E+14	6,53473E+14	7,08121E+14
TC0500002603.mm.2	Scfd2	Sec1 family domain containing 2	0.767651828632712	5,1434E+14	1,89294E+14	0.103949054058712	0.249108756318291	-4,51693E+14	5,80385E+13	5,56436E+14	5,04632E+14	4,61297E+14	5,36596E+13	4,46695E+14
TC0700004081.mm.2	Nrip3	nuclear receptor interacting protein 3	0.770699501277242	8,08896E+14	1,89293E+13	0.103949872124149	0.249108756318291	-4,51693E+13	8,53531E+14	8,70335E+14	8,15376E+14	7,4441E+14	8,2155E+14	7,48176E+14
TC1000001605.mm.2	Rnf41	ring finger protein 41	0.912501768664777	8,7706E+14	1,89242E+14	0.104026007222744	0.249108756318291	-4,5176E+14	9,47364E+14	9,44115E+14	8,95882E+14	7,66018E+14	8,79035E+14	8,29943E+14
TC0400003708.mm.2	Zdhhc18	zinc finger, DHHC domain containing 18	-0.600045427891358	8,47206E+14	-1,89235E+14	0.104035415889868	0.249108756318291	-4,51768E+13	8,00085E+14	8,01345E+14	8,67732E+14	8,92631E+14	8,40152E+13	8,81287E+14
TC1100002833.mm.2	Olfr317	olfactory receptor 317	-0.779403478936197	5,71961E+14	-1,89235E+14	0.104035500097502	0.249108756318291	-4,51768E+14	5,00662E+14	5,66057E+14	5,39752E+13	6,02693E+14	5,84089E+13	6,38511E+14
TC1800001243.mm.2	Nr3c1	nuclear receptor subfamily 3, group C, member 1	0.827399984643719	6,52277E+14	1,89232E+14	0.104040403051202	0.249108756318291	-4,51772E+14	7,18469E+12	7,17386E+14	6,39454E+14	5,67975E+14	6,82251E+14	5,88124E+14
TC0300001581.mm.2	Ankrd13c	ankyrin repeat domain 13c	1,31505E+14	7,47487E+14	1,89231E+14	0.104042313947532	0.249108756318291	-4,51774E+14	8,35866E+14	8,37469E+14	7,89737E+13	6,11525E+14	7,75401E+14	6,34924E+14
TC1400001643.mm.2	Cacna2d3	calcium channel, voltage-dependent, alpha2/delta subunit 3	1,28855E+14	7,53039E+14	1,89211E+14	0.104071672665081	0.249108756318291	-4,51799E+14	8,54265E+14	8,44095E+14	7,83651E+14	5,95572E+14	7,78534E+14	6,62117E+14
TC0800001959.mm.2	Chrna6	cholinergic receptor, nicotinic, alpha polypeptide 6	-0.805584360159191	5,79158E+14	-1,89211E+14	0.104072039674303	0.249108756318291	-4,518E+14	5,24274E+14	5,03152E+14	6,02147E+14	6,56835E+14	5,46158E+14	6,42384E+14
TC0400000725.mm.2	Atp6v1g1	ATPase, H+ transporting, lysosomal V1 subunit G1	0.562683600280857	7,84738E+14	1,89202E+14	0.104084328469925	0.249108756318291	-4,5181E+14	8,15756E+14	8,30149E+14	7,53349E+14	7,75802E+14	7,6329E+14	7,70079E+14
TC0300000500.mm.2	Mme	membrane metallo endopeptidase	0.669203373412426	6,14073E+14	1,89199E+14	0.104089980867933	0.249108756318291	-4,51815E+14	6,74699E+14	6,28928E+14	6,21329E+14	5,89695E+14	6,09269E+14	5,60517E+14
TC0100000137.mm.2	Khdc1a	KH domain containing 1A	-0.94306156798485	5,31423E+14	-1,89196E+13	0.10409334056501	0.249108756318291	-4,51818E+14	4,24083E+14	4,90642E+13	5,2915E+14	6,41324E+14	5,18789E+14	5,84549E+14
TC0800000234.mm.2	Defa2	defensin, alpha, 2	-0.548186991747218	5,35676E+14	-1,89186E+14	0.104108382365008	0.249108756318291	-4,51831E+14	4,99253E+14	4,97094E+14	5,52895E+14	5,58829E+14	5,58925E+14	5,4706E+14
TC0800000235.mm.2	Defa33	defensin, alpha, 33	-0.548186991747218	5,35676E+14	-1,89186E+14	0.104108382365008	0.249108756318291	-4,51831E+14	4,99253E+14	4,97094E+14	5,52895E+14	5,58829E+14	5,58925E+14	5,4706E+14
TC0900000810.mm.2	Rpl4	ribosomal protein L4	0.992493101502308	1,01808E+14	1,89136E+14	0.104182862107165	0.249249956515628	-4,51896E+13	1,089E+14	1,08062E+14	1,04845E+14	9,19269E+14	1,0304E+13	9,40725E+13
TC0700004608.mm.2	Ano1	anoctamin 1, calcium activated chloride channel	0.611687028749407	7,22835E+14	1,89107E+14	0.104225741593014	0.249315524686495	-4,51934E+14	7,71431E+14	7,73024E+14	7,0984E+14	6,60595E+14	7,21194E+14	7,00924E+14
TC0700003710.mm.2	Tmem135	transmembrane protein 135	0.754625446045421	6,9192E+14	1,89076E+14	0.104271369491885	0.249387646819467	-4,51973E+13	7,37122E+14	7,4033E+14	7,20014E+13	6,27526E+14	6,45203E+14	6,81323E+13
TC0600000822.mm.2	Krcc1/Mir8112	lysine-rich coiled-coil 1/microRNA 8112	1,35945E+14	5,19677E+14	1,89029E+14	0.104342185080994	0.2495031224909	-4,52034E+14	6,27983E+14	6,24166E+14	5,09534E+14	3,76119E+14	5,87712E+14	3,92547E+14
TC1200001567.mm.2	Colec11	collectin sub-family member 11	-1,06316E+14	6,22118E+14	-1,89023E+14	0.104350620149331	0.2495031224909	-4,52042E+14	5,4741E+14	5,49855E+14	5,7632E+14	7,49226E+13	6,48494E+14	6,61402E+14
TC0900000340.mm.2	Pate2	prostate and testis expressed 2	-0.709496285845165	5,46526E+13	-1,88994E+14	0.104394146616841	0.249526966763849	-4,52079E+14	5,03497E+14	5,2798E+14	5,20723E+14	5,53578E+14	5,48515E+14	6,24862E+14
TC1900000961.mm.2	Sart1	squamous cell carcinoma antigen recognized by T cells 1	0.545288480180235	6,47879E+14	1,8899E+14	0.10439937830308	0.249526966763849	-4,52084E+13	6,72601E+14	6,7295E+14	6,57217E+14	6,48012E+13	6,17239E+14	6,19254E+13
TC1200001569.mm.2	Trappc12	trafficking protein particle complex 12	0.608510744509542	7,37358E+14	1,88985E+14	0.104407050823675	0.249526966763849	-4,52091E+14	7,86997E+14	7,56439E+14	7,1033E+14	7,4865E+14	7,19729E+14	7,02005E+14
TC0700002423.mm.2	Inafm1	InaF motif containing 1	0.886366533199995	6,53107E+13	1,88944E+14	0.104468519817954	0.249619056622923	-4,52144E+14	7,42342E+14	6,79507E+14	6,84493E+14	5,67722E+14	6,17227E+14	6,27349E+13
TC1000001830.mm.2	Pde7b	phosphodiesterase 7B	0.704131525083429	8,74813E+14	1,88938E+14	0.104476566614845	0.249619056622923	-4,52151E+14	9,33094E+14	8,77878E+12	9,13219E+14	8,5454E+14	8,48795E+14	8,21352E+14
TC1700002347.mm.2	Dennd1c	DENN/MADD domain containing 1C	-0.792745044347862	6,06456E+14	-1,88916E+14	0.104510276443539	0.249662254196067	-4,5218E+14	5,21485E+14	5,7271E+14	6,0235E+14	6,89446E+14	6,05042E+14	6,47702E+14
TC1400000337.mm.2	Oxnad1	oxidoreductase NAD-binding domain containing 1	1,13862E+14	7,15077E+14	1,88904E+14	0.104527074008378	0.249662254196067	-4,52194E+14	8,15147E+14	8,23393E+14	7,15469E+14	5,25635E+14	7,0629E+14	7,04526E+14
TC0400001455.mm.2	Eva1b	eva-1 homolog B (C. elegans)	-0.65701469625262	6,66649E+14	-1,88892E+13	0.10454597155165	0.249662254196067	-4,52211E+14	6,14608E+14	6,48412E+14	6,50304E+14	6,92144E+14	6,74579E+14	7,19844E+14
TC0700002178.mm.2	Sbk2	SH3-binding domain kinase family, member 2	-0.57898703343426	7,68606E+13	-1,88877E+14	0.104567867621367	0.249662254196067	-4,5223E+14	7,33415E+14	7,37265E+14	7,58884E+14	7,95597E+14	7,98362E+14	7,88115E+14
TC0900001426.mm.2	Tmem89	transmembrane protein 89	-0.75768233558027	6,33158E+14	-1,88874E+14	0.104572119007587	0.249662254196067	-4,52233E+14	5,96461E+14	5,68196E+14	6,40743E+14	6,77354E+14	6,13542E+14	7,02651E+14
TC0100000948.mm.2	Gal3st2	galactose-3-O-sulfotransferase 2	-0.563247764834515	5,98513E+14	-1,88829E+14	0.10463990261638	0.249755436321586	-4,52292E+14	5,66812E+13	5,72669E+14	5,99396E+14	6,00071E+14	6,12007E+14	6,40124E+14
TC0900001269.mm.2	Msl2	MSL complex subunit 2	1,29271E+14	5,40069E+14	1,8881E+14	0.104667232272075	0.249755436321586	-4,52316E+14	6,07389E+13	6,06505E+13	5,54938E+14	5,16231E+14	5,87417E+14	3,67935E+14
TC0400001637.mm.2	Pafah2	platelet-activating factor acetylhydrolase 2	-0.867645917911978	6,48068E+14	-1,8881E+14	0.104668084196621	0.249755436321586	-4,52316E+14	5,75088E+14	5,96871E+14	6,4684E+14	7,24122E+14	6,22508E+14	7,2298E+14
TC0200002836.mm.2	Pcmtd2	protein-L-isoaspartate (D-aspartate) O-methyltransferase domain containing 2	0.598013664062498	7,54714E+14	1,88806E+14	0.104673149722564	0.249755436321586	-4,52321E+14	8,04925E+14	8,0689E+14	7,35992E+14	6,88262E+14	7,46916E+14	7,45296E+14
TC1800000620.mm.2	Camk2a	calcium/calmodulin-dependent protein kinase II alpha	1,01673E+14	1,13302E+14	1,88773E+14	0.10472291392389	0.24977192693175	-4,52364E+14	1,20381E+14	1,21311E+14	1,16269E+14	1,00496E+14	1,12758E+13	1,08599E+14
TC0100000657.mm.2	Des	desmin	-0.763197853090338	4,72829E+14	-1,88771E+14	0.104725325176527	0.24977192693175	-4,52366E+14	4,26557E+12	4,58061E+14	4,12461E+14	5,02634E+14	5,00733E+14	5,36525E+14
TC1700001017.mm.2	Dus3l	dihydrouridine synthase 3-like (S. cerevisiae)	0.617004217524388	7,12E+14	1,88771E+13	0.10472664785	0.24977192693175	-4,52367E+14	7,5018E+14	7,56593E+14	6,73721E+14	7,0813E+14	7,13465E+14	6,69908E+14
TC1700000325.mm.2	Prss32	protease, serine 32	-0.768227415533635	6,44374E+14	-1,88733E+14	0.10478218169099	0.24978518106583	-4,52415E+14	6,09196E+14	5,85617E+14	6,13861E+14	7,0911E+14	6,57926E+14	6,90533E+14
TC0400000660.mm.2	E130308A19Rik	RIKEN cDNA E130308A19 gene	-0.593188844914253	6,73727E+14	-1,88732E+14	0.104784050309757	0.24978518106583	-4,52417E+14	6,25879E+14	6,3292E+14	6,80492E+14	7,25632E+14	6,88875E+14	6,88563E+14
TC0900002106.mm.2	Ubash3b	ubiquitin associated and SH3 domain containing, B	-0.600414958488939	8,80392E+14	-1,88729E+14	0.104789168811167	0.24978518106583	-4,52421E+14	8,44748E+14	8,38747E+14	8,75525E+14	9,19755E+14	9,11875E+14	8,91703E+14

TC0700000592.mm.2	Scgb1b7	secretoglobin, family 1B, member 7	-0.834929156548117	6,06784E+14	-1,88724E+14	0.104796885045022	0.24978518106583	-4,52428E+14	5,22639E+14	5,40557E+14	6,27954E+14	7,19372E+14	5,76761E+14	6,53421E+14
TC1300000175.mm.2	Hist1h2bm	histone cluster 1, H2bm	-0.58638840141383	7,97422E+14	-1,88712E+14	0.10481425957656	0.24978518106583	-4,52443E+14	7,64484E+14	7,84643E+13	7,98952E+14	7,70047E+14	8,21386E+13	8,45018E+14
TC0400000996.mm.2	Atg4c	autophagy related 4C, cysteine peptidase	1,24887E+13	6,42166E+14	1,88705E+14	0.10482513463459	0.24978518106583	-4,52452E+13	7,39749E+14	7,47218E+14	6,50432E+14	4,78403E+14	6,8197E+14	5,55223E+14
TC0400001990.mm.2	Errfi1	ERBB receptor feedback inhibitor 1	1,78205E+14	6,75677E+14	1,88674E+14	0.10487122144914	0.249858049861008	-4,52492E+14	7,82328E+14	7,87497E+13	7,72645E+14	4,98178E+14	6,91503E+14	5,21912E+14
TC0300000800.mm.2	S100a13	S100 calcium binding protein A13	-0.55963929950991	7,05626E+14	-1,88634E+14	0.104929859438152	0.24993343231004	-4,52542E+13	6,67981E+14	6,75678E+14	7,10858E+14	7,23653E+14	7,38743E+13	7,16845E+14
TC0200003209.mm.2	Slc2a6	solute carrier family 2 (facilitated glucose transporter), member 6	-0.703962854340975	6,85478E+14	-1,88613E+14	0.104962513735727	0.24993343231004	-4,52571E+14	6,31523E+14	6,58591E+14	6,6289E+14	7,28957E+14	7,38909E+13	6,91997E+14
TC1700001824.mm.2	U2af1	U2 small nuclear ribonucleoprotein auxiliary factor (U2AF) 1	0.555463977151884	7,80779E+14	1,88601E+13	0.104979207782695	0.24993343231004	-4,52585E+13	8,2478E+14	8,14237E+14	7,55217E+14	7,62516E+14	7,54297E+14	7,73628E+14
TC0700001606.mm.2	Eif3f	eukaryotic translation initiation factor 3, subunit F	0.914767376737338	8,48304E+14	1,88601E+14	0.104979301588666	0.24993343231004	-4,52585E+14	9,17588E+14	9,05191E+14	8,39854E+14	7,87364E+14	8,84584E+14	7,55241E+14
TC0900001791.mm.2	Mtnr1b	melatonin receptor 1B	-0.593182659821802	5,66973E+14	-1,88595E+14	0.104989586073337	0.24993343231004	-4,52594E+14	5,17307E+14	5,29255E+14	5,74253E+14	6,15828E+14	5,90253E+14	5,74945E+14
TC1000001994.mm.2	Calhm6	calcium homeostasis modulator family member 6	-0.830637520472506	5,33002E+14	-1,8859E+14	0.104995928958398	0.24993343231004	-4,52599E+14	4,53049E+14	5,03161E+14	5,19165E+14	6,00298E+14	5,2498E+14	5,97357E+14
TC1500001339.mm.2	Tspyl5	testis-specific protein, Y-encoded-like 5	1,39131E+14	7,18339E+14	1,88536E+14	0.105077550111432	0.250012425652251	-4,5267E+14	8,02083E+14	8,00485E+14	7,6935E+14	6,1381E+14	7,55998E+14	5,68308E+14
TC1100003457.mm.2	Dhx40	DEAH (Asp-Glu-Ala-His) box polypeptide 40	1,76327E+14	6,14763E+14	1,88525E+14	0.105092949604611	0.250012425652251	-4,52683E+14	7,17791E+14	7,1969E+14	6,99677E+14	4,66595E+14	6,51455E+14	4,33371E+14
TC1800000538.mm.2	Prdm6	PR domain containing 6	-0.714348284887547	5,46902E+14	-1,88516E+14	0.105107424938948	0.250012425652251	-4,52695E+14	5,03399E+14	5,14318E+14	5,14079E+14	5,89027E+14	5,57308E+14	6,03279E+14
TC1200000988.mm.2	Tdp1	tyrosyl-DNA phosphodiesterase 1	0.770407667241293	6,38037E+14	1,88513E+14	0.105111784736594	0.250012425652251	-4,52699E+14	6,92853E+13	6,87335E+13	6,54649E+14	5,6728E+14	6,41333E+14	5,84774E+13
TC0300001497.mm.2	Ttll7	tubulin tyrosine ligase-like family, member 7	1,48565E+14	8,10215E+14	1,88507E+14	0.105120103424672	0.250012425652251	-4,52706E+14	9,05155E+13	9,37156E+14	8,49193E+14	6,24607E+14	8,39306E+14	7,05873E+14
TC0100001541.mm.2	Fam78b	family with sequence similarity 78, member B	-0.566428819704094	7,99691E+14	-1,88486E+14	0.105152300355175	0.250012425652251	-4,52734E+14	7,53233E+13	7,77519E+14	8,08983E+14	8,14375E+14	8,31743E+14	8,12294E+14
TC0900000833.mm.2	Parp16	poly (ADP-ribose) polymerase family, member 16	-0.922811772599132	5,96315E+14	-1,8847E+14	0.105175846086123	0.250012425652251	-4,52754E+14	5,4506E+14	5,3346E+14	6,88511E+13	5,47778E+14	6,39845E+14	6,23237E+14
TC1700002154.mm.2	Capn11	calpain 11	-0.562504553224644	5,88154E+14	-1,8847E+14	0.105176338153741	0.250012425652251	-4,52755E+14	5,62864E+14	5,52467E+14	5,8541E+14	6,00347E+14	6,02653E+14	6,2518E+14
TC0900000559.mm.2	Apoa1	apolipoprotein A-I	-0.630305310903373	5,95955E+14	-1,88463E+13	0.105186846313613	0.250012425652251	-4,52764E+13	5,50474E+14	5,57407E+14	6,14704E+14	6,19202E+14	5,86558E+14	6,47384E+14
TC1100000854.mm.2	Myh1	myosin, heavy polypeptide 1, skeletal muscle, adult	-1,04579E+14	5,58763E+14	-1,88452E+14	0.105203005376381	0.250012425652251	-4,52777E+14	4,54331E+14	5,01341E+14	5,4781E+14	6,84266E+14	5,37251E+13	6,27578E+14
TC0700001164.mm.2	Zfp592	zinc finger protein 592	-0.691898637248304	6,51937E+14	-1,88436E+14	0.105226870341132	0.250012425652251	-4,52798E+14	6,32107E+14	5,85007E+14	6,3836E+14	7,00654E+14	6,65268E+14	6,90226E+14
TC0700002426.mm.2	Sae1	SUMO1 activating enzyme subunit 1	1,10634E+14	6,76515E+14	1,88435E+14	0.10522805789278	0.250012425652251	-4,52799E+14	7,79149E+14	7,55224E+14	6,87039E+14	5,24569E+13	6,97624E+14	6,15482E+14
TC1500002243.mm.2	Pou6f1	POU domain, class 6, transcription factor 1	0.58427849698497	7,14548E+14	1,88433E+14	0.105230824227243	0.250012425652251	-4,52801E+14	7,62708E+14	7,60961E+14	6,99228E+14	6,57577E+14	7,04968E+14	7,01845E+14
TC0X00000073.mm.2	Otud5	OTU domain containing 5	0.854952117703018	6,61732E+14	1,88414E+14	0.105260096731608	0.25004510373528	-4,52827E+14	7,3606E+14	7,43495E+14	6,5292E+14	5,29356E+14	6,42658E+14	6,65906E+14
TC0X00001937.mm.2	Zfp182	zinc finger protein 182	0.641517527548893	5,65104E+14	1,88375E+14	0.105317739877403	0.250116737713103	-4,52876E+14	6,30014E+14	5,7638E+14	5,60721E+14	5,45418E+14	5,21815E+14	5,56276E+14
TC0400002505.mm.2	Ccl19	chemokine (C-C motif) ligand 19	-0.598141497498131	5,28705E+14	-1,88373E+14	0.10532129742341	0.250116737713103	-4,52879E+14	5,31572E+14	5,08458E+14	5,06685E+14	4,65856E+14	5,85919E+14	5,7374E+14
TC0600000410.mm.2	Ssbp1	single-stranded DNA binding protein 1	1,00849E+14	7,4587E+14	1,88359E+13	0.105343046544241	0.250131522099522	-4,52898E+14	8,27649E+14	8,12226E+14	7,53306E+14	6,43861E+14	7,78323E+14	6,59855E+14
TC0100002624.mm.2	Pax3	paired box 3	-0.908416800181631	6,36613E+14	-1,88321E+14	0.105400099532008	0.250230116952886	-4,52947E+13	5,39906E+14	5,83482E+14	6,40113E+12	7,51108E+14	6,16862E+14	6,88209E+14
TC1700000255.mm.2	Ppp2r1a	protein phosphatase 2, regulatory subunit A, alpha	1,63124E+14	1,01287E+14	1,8828E+14	0.105460905397704	0.250337591142193	-4,52999E+13	1,13957E+14	1,12426E+14	1,07655E+14	7,99237E+14	1,01566E+14	9,21931E+14
TC1900000417.mm.2	Kcnv2	potassium channel, subfamily V, member 2	-0.773368687074191	4,7961E+14	-1,88251E+14	0.105504917182172	0.250339651078705	-4,53037E+14	4,39656E+14	4,48061E+13	4,45754E+14	5,01797E+14	4,76819E+14	5,65572E+14
TC0400003730.mm.2	Catsper4	cation channel, sperm associated 4	-0.768770119963351	6,0741E+14	-1,88245E+14	0.105514115069669	0.250339651078705	-4,53045E+14	5,4084E+14	5,86278E+14	5,84519E+14	6,51952E+14	6,07952E+14	6,7292E+14
TC0400000169.mm.2	Ggh	gamma-glutamyl hydrolase	1,08423E+14	5,86536E+14	1,88239E+14	0.105523153187247	0.250339651078705	-4,53052E+14	6,58513E+14	6,58375E+14	5,24808E+14	5,77477E+14	6,35574E+14	4,64468E+14
TC1700001015.mm.2	Ndufa11	NADH:ubiquinone oxidoreductase subunit A11	-0.835781119701528	9,90958E+14	-1,88232E+14	0.105532662197785	0.250339651078705	-4,5306E+14	9,61612E+14	9,42096E+14	1,03621E+12	9,34968E+14	1,06748E+14	1,00339E+14
TC0700004399.mm.2	Ikzf5	IKAROS family zinc finger 5	1,4668E+14	6,81028E+14	1,88223E+14	0.105547143037115	0.250339651078705	-4,53073E+14	7,48376E+13	7,55831E+14	7,72833E+14	5,98281E+14	7,01554E+13	5,09294E+13
TC1800001429.mm.2	Grpel2	GrpE-like 2, mitochondrial	-0.709857099978299	7,33387E+14	-1,88217E+14	0.105554992206834	0.250339651078705	-4,5308E+14	6,71068E+14	6,98349E+14	7,76877E+14	7,42642E+14	7,17747E+13	7,93641E+14
TC0200002478.mm.2	Mybl2	myeloblastosis oncogene-like 2	-0.598042893879252	5,06721E+14	-1,88193E+14	0.105591412276861	0.250371155696236	-4,53111E+14	4,71336E+14	4,849E+14	5,05846E+14	5,02397E+13	5,14608E+14	5,61237E+13
TC0X00001082.mm.2	Lpar4	lysophosphatidic acid receptor 4	0.838085245939962	5,15438E+14	1,88182E+14	0.105608110776422	0.250371155696236	-4,53125E+12	5,96425E+14	5,13109E+14	5,50197E+14	4,90982E+14	4,37627E+14	5,04291E+14
TC1900000030.mm.2	Coro1b	coronin, actin binding protein 1B	0.626472850515249	6,89053E+14	1,88174E+14	0.105619752369379	0.250371155696236	-4,53135E+14	7,40762E+14	7,24116E+14	6,75335E+14	6,54521E+14	6,42741E+13	6,96842E+13
TC1500001870.mm.2	Tmem184b	transmembrane protein 184b	0.648204574504343	7,44159E+14	1,88167E+13	0.105630429865513	0.250371155696236	-4,53144E+14	7,97237E+14	7,97665E+14	7,35166E+13	6,69622E+14	7,41963E+14	7,23298E+13
TC1100003863.mm.2	Plcd3	phospholipase C, delta 3	-0.764929022872102	5,7878E+13	-1,88143E+14	0.105666795924296	0.250420515182848	-4,53175E+14	5,51662E+14	5,14618E+14	5,67645E+14	6,19491E+14	5,6907E+14	6,50195E+14
TC0400002522.mm.2	Fam166b	family with sequence similarity 166, member B	-0.758529195186221	5,22794E+14	-1,88121E+14	0.1057006603916	0.250442465961028	-4,53204E+14	5,02744E+14	4,53819E+14	5,28871E+14	5,43064E+14	5,10267E+14	5,98001E+14
TC0200001591.mm.2	Dcdc5	doublecortin domain containing 5	-0.577053684900155	5,73387E+14	-1,88114E+14	0.105711189825067	0.250442465961028	-4,53213E+14	5,56602E+14	5,23315E+14	5,88902E+14	5,76176E+14	5,90337E+14	6,04991E+14
TC0800001834.mm.2	Gm6040	predicted gene 6040	-0.788899736269525	5,53601E+13	-1,881E+14	0.105731127708905	0.250442465961028	-4,5323E+14	4,57457E+14	5,30857E+14	5,57454E+14	6,29744E+14	5,54393E+14	5,91701E+13
TC0800002565.mm.2	Il27ra	interleukin 27 receptor, alpha	-0.838162206388807	5,35484E+14	-1,88091E+13	0.10574467077792	0.250442465961028	-4,53242E+14	5,04149E+14	4,52727E+14	5,61713E+14	5,69803E+14	5,07002E+14	6,17507E+14
TC0X00001799.mm.2	Ebp	phenylalkylamine Ca2+ antagonist (emopamil) binding protein	0.820798953333862	6,81958E+14	1,88079E+14	0.10576362116447	0.250442465961028	-4,53258E+14	7,48259E+14	7,67896E+14	6,54879E+13	5,68721E+14	6,37047E+14	7,14948E+14
TC0700002131.mm.2	Oscar	osteoclast associated receptor	-0.697384052501113	5,64754E+14	-1,88075E+14	0.105769315513237	0.250442465961028	-4,53263E+14	4,94135E+14	5,32928E+14	5,64913E+13	6,30008E+13	5,6508E+13	6,0146E+13
TC0700002028.mm.2	Psmd13	proteasome (prosome, macropain) 26S subunit, non-ATPase, 13	1,47246E+14	7,91135E+14	1,88063E+14	0.105787623177504	0.250449011498549	-4,53279E+14	8,92472E+14	9,04849E+14	8,13357E+14	6,33305E+14	8,45826E+14	6,57003E+14
TC1100002637.mm.2	Trim41	tripartite motif-containing 41	0.689347528824031	7,41045E+14	1,88043E+14	0.105817975238506	0.250473305333787	-4,53304E+14	7,85496E+14	7,7592E+14	7,62523E+13	6,98785E+14	7,36833E+13	6,86713E+14
TC1100004301.mm.2	Trim47	tripartite motif-containing 47	-0.932563700450978	5,96061E+14	-1,88035E+14	0.105829895330263	0.250473305333787	-4,53315E+14	5,08682E+14	5,45793E+14	5,75671E+13	7,0523E+14	5,93782E+14	6,47207E+14
TC0900000363.mm.2	Hepacam	hepatocyte cell adhesion molecule	0.791871229381439	8,58253E+14	1,88025E+14	0.105844519085071	0.250473305333787	-4,53327E+14	9,0996E+13	9,06471E+14	8,77062E+14	7,97968E+14	8,67519E+14	7,90538E+14
TC1600001143.mm.2	Ubald1	UBA-like domain containing 1	0.692347375766154	8,55091E+14	1,87994E+14	0.105890844610625	0.250532732266376	-4,53367E+14	8,96206E+14	8,81496E+14	8,89096E+13	8,24515E+14	8,44309E+14	7,94923E+14
TC1700002487.mm.2	Myl12b	myosin, light chain 12B, regulatory	0.735983679883038	1,01788E+14	1,87971E+14	0.105926857578722	0.250532732266376	-4,53397E+14	1,07374E+13	1,06593E+14	1,02301E+14	9,52112E+14	1,02791E+14	9,64572E+14
TC0900001182.mm.2	Slc9a9	solute carrier family 9 (sodium/hydrogen exchanger), member 9	0.875002772190562	7,06131E+14	1,87968E+14	0.105929960327979	0.250532732266376	-4,534E+14	7,60165E+14	7,68535E+13	7,06092E+14	6,48293E+14	7,35674E+14	6,18026E+14
TC1600002135.mm.2	Psmg1	proteasome (prosome, macropain) assembly chaperone 1	0.56635297844135	6,12798E+14	1,87967E+14	0.105931825540298	0.250532732266376	-4,53402E+14	6,62057E+14	6,70562E+14	5,65648E+13	5,59172E+14	5,94313E+14	6,25036E+14
TC0200000009.mm.2	Cdnf	cerebral dopamine neurotrophic factor	-0.962223618329545	4,56799E+14	-1,87943E+14	0.105967675874255	0.250580739853519	-4,53432E+14	4,3604E+14	3,67316E+14	4,28709E+14	5,12929E+14	4,44801E+14	5,51E+14
TC0100001259.mm.2	Ascl5	achaete-scute family bHLH transcription factor 5	-0.745429641884564	6,90281E+14	-1,87885E+14	0.106056141120941	0.250692979286167	-4,53508E+13	6,36474E+14	6,50532E+14	6,81742E+14	7,36849E+14	6,78069E+13	7,58023E+14
TC1500000432.mm.2	Sqle	squalene epoxidase	0.87464654739204	6,83489E+14	1,87876E+14	0.106069469385557	0.250692979286167	-4,53519E+14	7,58114E+14	7,46188E+14	6,92956E+14	5,74302E+14	6,92234E+14	6,37139E+14
TC0400003836.mm.2	Otud3	OTU domain containing 3	-0.608215945472062	5,8283E+14	-1,87872E+13	0.106075483725833	0.250692979286167	-4,53524E+14	5,46275E+13	5,412E+14	6,08859E+14	5,91526E+14	5,79382E+14	6,29737E+14
TC1500002199.mm.2	Tuba1b	tubulin, alpha 1B	1,41618E+13	1,09956E+14	1,87871E+14	0.106077374342027	0.250692979286167	-4,53526E+14	1,19578E+14	1,19182E+14	1,15312E+14	9,52275E+14	1,12653E+14	9,77839E+14
TC1000002426.mm.2	Gm9736	predicted gene 9736	-0.658975154460092	6,5741E+14	-1,87821E+14	0.106152265454903	0.250801232794501	-4,5359E+14	6,09906E+14	6,12345E+14	6,55046E+13	7,1312E+14	6,55601E+14	6,98445E+14
TC1500002187.mm.2	Arf3	ADP-ribosylation factor 3	0.554905632501763	9,95705E+14	1,8782E+14	0.106154310659623	0.250801232794501	-4,53591E+14	1,02911E+14	1,03929E+14	9,82398E+14	9,66024E+14	9,86403E+14	9,71011E+14
TC0700000513.mm.2	Hnrnpl	heterogeneous nuclear ribonucleoprotein L	0.952371033584138	9,00664E+14	1,87773E+14	0.106225655401992	0.250932998899326	-4,53652E+14	9,74949E+12	9,64387E+14	9,26416E+14	7,89372E+14	9,04326E+14	8,44537E+14
TC0500001741.mm.2	Eif2ak1	eukaryotic translation initiation factor 2 alpha kinase 1	0.90596318128415	7,35046E+14	1,87745E+14	0.106266978479975	0.250993817685112	-4,53687E+13	8,19012E+14	7,9678E+14	7,45652E+14	6,14236E+13	7,37084E+14	6,97515E+14
TC0200000303.mm.2	Spag6	sperm associated antigen 6	-0.552724462913529	7,38197E+14	-1,87733E+14	0.106285600229121	0.251001007839929	-4,53703E+14	6,91846E+14	7,06082E+14	7,59014E+14	7,63769E+14	7,45497E+14	7,62976E+14

TC0800001871.mm.2	Vdac3	voltage-dependent anion channel 3	1,59176E+14	8,52185E+13	1,87712E+14	0.106317762553475	0.251040168233316	-4,5373E+14	9,79194E+14	9,68802E+14	8,91895E+14	6,43576E+14	8,85699E+14	7,43945E+14
TC0200005386.mm.2	Ppp1r3d	protein phosphatase 1, regulatory subunit 3D	-0.783857822906186	6,42232E+14	-1,87697E+13	0.106340885579983	0.251048031054112	-4,5375E+14	5,87229E+14	5,73419E+14	7,37486E+14	6,37839E+14	6,79283E+13	6,38137E+14
TC0500003225.mm.2	Rhof	ras homolog family member F (in filopodia)	0.675848655463921	6,90233E+14	1,87689E+14	0.10635225345841	0.251048031054112	-4,5376E+13	7,61281E+14	7,2946E+14	6,75753E+14	6,19331E+14	6,56207E+14	6,99365E+14
TC0200000211.mm.2	Prkcq	protein kinase C, theta	0.685112155576911	6,14731E+14	1,87678E+14	0.106368919285692	0.25105059271281	-4,53774E+14	6,57891E+14	6,5613E+14	6,13139E+14	5,8423E+14	5,50809E+13	6,26189E+13
TC1300001445.mm.2	Mtr	5-methyltetrahydrofolate-homocysteine methyltransferase	0.657278870717595	5,90559E+14	1,87633E+14	0.106437003222489	0.25115001599065	-4,53832E+13	6,18021E+14	6,48131E+14	5,38671E+14	6,03836E+14	5,6903E+14	5,65666E+14
TC0600000331.mm.2	Cald1	caldesmon 1	0.806559056280392	5,58474E+14	1,8763E+13	0.106442218035136	0.25115001599065	-4,53836E+14	5,83551E+14	6,24278E+14	5,46233E+14	5,50265E+14	5,76816E+14	4,69702E+14
TC1000000079.mm.2	Shprh	SNF2 histone linker PHD RING helicase	0.846416621743673	7,17249E+14	1,87582E+14	0.106513825531685	0.251225943325314	-4,53897E+14	7,73848E+14	7,65855E+14	7,25198E+14	6,68174E+14	7,40556E+14	6,29864E+14
TC1100003198.mm.2	Pafah1b1	platelet-activating factor acetylhydrolase, isoform 1b, subunit 1	1,15956E+14	8,85835E+14	1,87582E+14	0.106514584483338	0.251225943325314	-4,53897E+14	9,71683E+14	9,70326E+14	9,19015E+14	7,43887E+14	8,99368E+14	8,1073E+14
TC1400001687.mm.2	Ankrd28	ankyrin repeat domain 28	1,03955E+14	6,50735E+14	1,8756E+14	0.106547985598585	0.251225943325314	-4,53926E+14	7,14475E+13	7,12617E+14	6,66501E+14	5,91637E+14	6,85885E+14	5,33297E+14
TC1100004276.mm.2	Casc3	cancer susceptibility candidate 3	1,44152E+14	6,95531E+14	1,87559E+14	0.10654902259991	0.251225943325314	-4,53927E+14	7,94874E+14	7,68689E+14	7,61524E+14	5,72839E+13	7,22873E+14	5,52386E+14
TC0200000087.mm.2	Sfmbt2	Scm-like with four mbt domains 2	0.589556474991745	4,98864E+14	1,87548E+14	0.106566263917304	0.251225943325314	-4,53941E+14	5,10199E+13	5,15578E+14	5,51921E+14	5,07619E+14	4,58921E+14	4,48945E+14
TC0X00002048.mm.2	Akap17b	A kinase (PRKA) anchor protein 17B	0.972542212998109	6,18497E+14	1,87537E+14	0.10658317792403	0.251225943325314	-4,53956E+13	6,60644E+13	6,39337E+14	6,5763E+14	6,47899E+14	6,29257E+14	4,76215E+13
TC0500000381.mm.2	Add1/Mir7036b	adducin 1 (alpha)/microRNA 7036b	1,16438E+14	9,54549E+14	1,87533E+14	0.106589010227471	0.251225943325314	-4,53961E+14	1,02714E+14	1,03407E+14	1,00834E+14	8,29417E+14	9,557E+14	8,72629E+11
TC0100001987.mm.2	Msc	musculin	-0.638021124045483	5,83955E+14	-1,87526E+14	0.106599129554718	0.251225943325314	-4,53969E+14	5,08256E+14	5,70557E+14	5,99749E+14	6,18749E+14	6,05837E+14	6,0058E+14
TC1100000216.mm.2	Wdpcp	WD repeat containing planar cell polarity effector	0.687366350290778	5,19976E+14	1,87507E+14	0.106628856305117	0.251259251483525	-4,53994E+14	5,33806E+14	6,00863E+14	4,74598E+12	5,13394E+13	4,85715E+14	5,1148E+14
TC1000002456.mm.2	Gm9978	predicted gene 9978	-0.649351172031931	4,96945E+14	-1,87437E+14	0.106734415697214	0.251428004347379	-4,54084E+13	4,3064E+14	4,48158E+14	5,14521E+14	5,79685E+14	5,12471E+14	4,96197E+14
TC0400003576.mm.2	Zscan20	zinc finger and SCAN domains 20	-0.747070441768	6,90875E+14	-1,87436E+14	0.106736824808586	0.251428004347379	-4,54086E+14	6,41475E+14	6,45727E+14	6,69058E+14	7,52373E+14	6,91594E+14	7,45022E+14
TC0700004281.mm.2	Atxn2l	ataxin 2-like	1,61461E+14	7,62195E+14	1,87401E+14	0.106789820593474	0.251428004347379	-4,54131E+14	8,58734E+14	8,8604E+14	8,28553E+14	5,7799E+14	7,83946E+14	6,37906E+14
TC0400002876.mm.2	Ptprd	protein tyrosine phosphatase, receptor type, D	0.639455848289247	7,80886E+14	1,87399E+13	0.106792430649271	0.251428004347379	-4,54133E+13	8,19229E+14	8,26667E+14	7,80596E+14	7,4082E+14	7,85078E+14	7,32926E+14
TC0500001618.mm.2	Vgf	VGF nerve growth factor inducible	0.819072839957666	7,92242E+14	1,87399E+14	0.106792967834678	0.251428004347379	-4,54133E+13	8,50684E+14	8,4701E+13	8,1684E+14	7,05038E+14	7,47976E+14	7,85904E+14
TC0600003468.mm.2	Ipo8	importin 8	0.90310395916608	6,69971E+14	1,87398E+14	0.106794095559701	0.251428004347379	-4,54134E+14	7,49764E+14	7,40908E+14	6,62477E+13	5,56624E+14	6,99659E+14	6,10392E+14
TC0100003350.mm.2	Tnn	tenascin N	-1,14115E+13	4,31484E+14	-1,8731E+14	0.106927115391632	0.251697253154087	-4,54247E+14	3,76491E+14	3,26621E+14	4,1129E+14	5,43163E+14	4,00019E+14	5,31322E+12
TC0100002772.mm.2	A730008H23Rik/Hjurp/6430706D22Rik	RIKEN cDNA A730008H23 gene/Holliday junction recognition protein/RIKEN cDNA 6430706D22 gene	0.735713605683106	5,68694E+14	1,87302E+14	0.106939700558113	0.251697253154087	-4,54257E+14	6,34016E+14	5,99065E+14	5,90814E+14	5,02331E+14	5,36358E+14	5,49581E+14
TC0400004147.mm.2	Mib2	mindbomb E3 ubiquitin protein ligase 2	0.739757887677642	6,56774E+14	1,8727E+14	0.106988721263843	0.251766250209373	-4,54299E+14	7,09687E+14	6,86238E+14	6,22448E+14	6,74297E+14	5,84575E+14	6,63399E+13
TC0400002526.mm.2	Rmrp	RNA component of mitochondrial RNAase P	0.884575833544551	1,21047E+14	1,87241E+14	0.107032474221706	0.251766250209373	-4,54336E+14	1,27288E+14	1,25274E+14	1,24511E+14	1,1434E+14	1,21718E+14	1,13153E+14
TC0X00002741.mm.2	Hdac8	histone deacetylase 8	0.671522097129583	6,20207E+14	1,87237E+14	0.10703858981532	0.251766250209373	-4,54341E+14	6,64842E+14	6,82228E+14	6,18155E+14	5,43253E+14	6,0818E+14	6,04586E+14
TC0X00000678.mm.2	F8a	factor 8-associated gene A	0.58356111473408	6,85207E+14	1,87232E+13	0.107045975797568	0.251766250209373	-4,54347E+12	7,14344E+14	6,99292E+14	7,17607E+13	6,83076E+14	6,59151E+14	6,37773E+14
TC1500001044.mm.2	Higd1c/Methig1/Mettl7a2	HIG1 domain family, member 1C/methyltransferase hypoxia inducible domain containing 1/methyltransferase like 7A2	-0.622365107743261	5,69367E+14	-1,87232E+14	0.107047140829418	0.251766250209373	-4,54348E+14	5,29533E+14	5,36121E+14	5,65797E+14	5,94623E+14	5,68994E+14	6,21133E+14
TC0X00001508.mm.2	Fgd1	FYVE, RhoGEF and PH domain containing 1	-0.614507152959154	6,78362E+14	-1,87205E+14	0.10708818091154	0.251825380289376	-4,54383E+14	6,60874E+14	6,37706E+14	6,61621E+14	6,88487E+14	7,33E+14	6,88483E+13
TC0400000427.mm.2	1700022I11Rik	RIKEN cDNA 1700022I11 gene	-0.626052104823788	4,98865E+14	-1,87178E+14	0.107129238984782	0.251825380289376	-4,54417E+14	4,87009E+14	4,65857E+14	4,74138E+14	4,88109E+14	5,11076E+14	5,67E+14
TC1500001953.mm.2	Cyp2d13	cytochrome P450, family 2, subfamily d, polypeptide 13	-0.561481102216526	8,31526E+14	-1,87175E+14	0.107133640440953	0.251825380289376	-4,54421E+14	7,88465E+14	8,01501E+14	8,60308E+14	8,36616E+14	8,58619E+14	8,43644E+14
TC0600000772.mm.2	Mad2l1	MAD2 mitotic arrest deficient-like 1	0.61131653144817	6,25093E+14	1,87174E+14	0.107134796864871	0.251825380289376	-4,54422E+14	6,56611E+14	6,71508E+14	5,97956E+14	6,1863E+14	5,78169E+14	6,27686E+14
TC0900002956.mm.2	Ephb1	Eph receptor B1	0.771623086894222	8,65048E+14	1,87125E+14	0.107210329863699	0.251932079295365	-4,54486E+14	8,98719E+14	9,04095E+14	8,97183E+13	8,41802E+14	8,69399E+14	7,79091E+14
TC0600002900.mm.2	Ssu2	ssu-2 homolog (C. elegans)	-0.56394339031779	6,66046E+14	-1,87123E+14	0.107213530312361	0.251932079295365	-4,54488E+14	6,13007E+12	6,1742E+14	6,99939E+14	7,22696E+14	6,60136E+14	6,8308E+14
TC0500000093.mm.2	Sema3e	sema domain, immunoglobulin domain (Ig), short basic domain, secreted, (semaphorin) 3E	0.800390887804257	6,87649E+14	1,87114E+14	0.107227096121617	0.251932079295365	-4,545E+14	7,40627E+13	7,41106E+14	7,02645E+14	6,19862E+14	6,99757E+14	6,21898E+14
TC0600003449.mm.2	Pthlh	parathyroid hormone-like peptide	-0.648041625253281	5,93177E+14	-1,87086E+14	0.107269670456295	0.251948825517323	-4,54536E+14	5,49923E+14	5,70791E+14	5,80344E+14	6,07683E+14	5,95033E+14	6,55289E+14
TC1100001457.mm.2	Tac4	tachykinin 4	-0.805238552047236	5,2883E+14	-1,87085E+14	0.107271019396429	0.251948825517323	-4,54537E+14	4,73296E+14	4,72985E+14	5,09497E+13	6,09873E+14	5,25677E+14	5,81651E+14
TC0800003178.mm.2	Egln1	egl-9 family hypoxia-inducible factor 1	0.74830716593142	8,13209E+14	1,87078E+14	0.107281132742155	0.251948825517323	-4,54545E+14	8,59061E+14	8,53577E+14	8,50272E+12	7,53364E+14	7,93095E+14	7,69883E+14
TC0900001624.mm.2	Nktr	natural killer tumor recognition sequence	2,44781E+13	6,45371E+14	1,87038E+14	0.107342727893238	0.252056743594249	-4,54597E+14	7,75642E+14	7,73186E+14	7,16859E+14	5,47273E+14	7,42295E+14	3,1697E+14
TC1900001608.mm.2	Arl3	ADP-ribosylation factor-like 3	0.656246617276835	9,33796E+13	1,87025E+14	0.107362436408754	0.252066288482333	-4,54614E+13	9,84856E+14	9,56485E+14	9,45656E+14	9,05691E+14	9,31574E+14	8,78517E+14
TC1800000924.mm.2	Crem	cAMP responsive element modulator	0.732284463234548	6,66463E+13	1,86985E+14	0.107423221364227	0.252172256095831	-4,54665E+14	7,30253E+14	7,07441E+14	6,47926E+14	6,21923E+14	6,90091E+14	6,01146E+14
TC0300001903.mm.2	Gm10731	FUN14 domain containing 2 pseudogene	-0.621689927066524	5,08531E+14	-1,8696E+13	0.107462498108082	0.252227710417411	-4,54698E+14	5,06033E+14	4,60191E+14	4,89295E+14	5,05108E+14	5,2759E+14	5,62972E+14
TC0600001076.mm.2	Aldh1l1	aldehyde dehydrogenase 1 family, member L1	0.832388814156574	6,65135E+13	1,8693E+14	0.107507284171854	0.252296077754308	-4,54735E+14	7,37119E+14	6,93534E+14	6,34557E+14	6,61425E+14	5,76719E+14	6,87457E+14
TC0600002233.mm.2	Tmem176b	transmembrane protein 176B	1,45233E+14	8,03139E+14	1,86895E+14	0.107562216880791	0.252388233668295	-4,54782E+14	9,067E+14	9,28082E+14	8,35926E+14	6,03807E+14	8,28528E+14	7,1579E+14
TC1600002168.mm.2	Vasn	vasorin	-0.608424699325566	7,59322E+14	-1,86845E+14	0.107638113665811	0.252523641700568	-4,54845E+14	7,14582E+14	7,19087E+14	7,78318E+14	7,88593E+14	7,5078E+12	8,04574E+14
TC0200000433.mm.2	Obp2b	odorant binding protein 2B	-1,06345E+14	5,91441E+14	-1,86832E+14	0.107657685932086	0.252523641700568	-4,54862E+14	5,59861E+14	4,91653E+14	5,53392E+14	6,79536E+14	5,80899E+14	6,83304E+14
TC1300000726.mm.2	Spata31d1b	spermatogenesis associated 31 subfamily D, member 1B	-0.99903800085835	4,82132E+14	-1,86816E+14	0.107681933094285	0.252523641700568	-4,54882E+14	4,23894E+14	4,32073E+14	4,38531E+14	5,42522E+14	4,66077E+14	5,89697E+14
TC1700001023.mm.2	Crb3	crumbs family member 3	-0.668020368282752	5,76561E+14	-1,86807E+14	0.107695779626362	0.252523641700568	-4,54894E+14	5,03549E+14	5,61185E+14	5,81561E+14	6,14731E+14	5,80417E+13	6,17922E+14
TC0900002269.mm.2	Bco2	beta-carotene oxygenase 2	-0.661686246032665	5,22871E+13	-1,86806E+13	0.107698284972774	0.252523641700568	-4,54896E+14	4,68729E+14	4,8952E+14	5,08724E+14	5,79668E+14	5,41638E+14	5,48949E+14
TC1900001045.mm.2	Rtn3	reticulon 3	1,28973E+14	1,08652E+14	1,86783E+14	0.107733127832122	0.252533005220009	-4,54925E+13	1,17439E+14	1,17483E+13	1,13045E+14	9,48182E+14	1,11175E+14	9,79526E+14
TC0500001910.mm.2	Gtpbp10	GTP-binding protein 10 (putative)	1,89332E+14	5,66152E+14	1,86783E+13	0.107733623650614	0.252533005220009	-4,54925E+14	7,05028E+14	6,92752E+14	5,88069E+14	3,91946E+14	6,53534E+14	3,65581E+13
TC0200004545.mm.2	Sppl2a	signal peptide peptidase like 2A	1,73035E+14	7,66023E+14	1,86768E+14	0.10775555286494	0.252541367960503	-4,54944E+14	8,56987E+14	8,68786E+14	8,11152E+14	6,79512E+14	8,33299E+14	5,46403E+14
TC0500001049.mm.2	Zfp326	zinc finger protein 326	2,09411E+14	5,96803E+14	1,8676E+14	0.107768537584337	0.252541367960503	-4,54955E+14	7,29695E+14	7,34341E+14	6,86779E+14	3,84786E+14	6,44943E+14	4,0027E+13
TC0100003886.mm.2	Gm7694	predicted gene 7694	-0.92987299392714	5,54843E+13	-1,86741E+14	0.107797972865443	0.252573613026156	-4,54979E+14	5,01957E+14	4,8923E+14	5,14022E+14	6,46683E+14	5,58953E+14	6,18209E+14
TC0500000541.mm.2	Rbpj	recombination signal binding protein for immunoglobulin kappa J region	0.55763052768996	7,48982E+14	1,86664E+14	0.107915498609375	0.252812217082416	-4,55078E+13	7,89547E+13	7,86903E+14	7,34426E+14	7,18091E+14	7,44567E+14	7,20358E+14
TC0900003170.mm.2	Trim71	tripartite motif-containing 71	-1,01954E+14	6,04266E+14	-1,86638E+14	0.107956088548851	0.252868247375902	-4,55111E+14	5,37989E+14	5,52498E+14	5,66638E+14	6,74077E+14	5,83937E+14	7,10457E+14
TC0X00000204.mm.2	Rp2	retinitis pigmentosa 2 homolog	0.929417700044305	6,31139E+14	1,86628E+13	0.107970802578428	0.252868247375902	-4,55124E+13	7,05068E+14	7,3208E+13	6,05761E+14	4,92659E+14	6,69671E+14	5,81594E+14
TC0100002177.mm.2	Neurl3	neuralized E3 ubiquitin protein ligase 3	-0.759524494947423	5,35439E+14	-1,8661E+14	0.107998557270103	0.252896490814297	-4,55147E+14	5,23864E+14	4,50958E+14	5,22101E+14	5,88983E+14	5,48367E+14	5,78359E+14
TC0700003111.mm.2	Gabra5	gamma-aminobutyric acid (GABA) A receptor, subunit alpha 5	1,02702E+14	8,42593E+14	1,8658E+14	0.108045151743516	0.252968836100446	-4,55186E+14	9,37078E+14	9,1222E+14	8,54312E+14	7,08054E+14	8,61764E+14	7,8213E+14
TC1300001075.mm.2	Zbed3	zinc finger, BED type containing 3	-0.905510273697733	8,25021E+14	-1,86545E+14	0.108098646751506	0.253007031557524	-4,55231E+14	7,44743E+14	7,84303E+14	9,00881E+14	8,10048E+14	9,0475E+14	8,05398E+14

TC0700000302.mm.2	Exoc3l2	exocyst complex component 3-like 2	-0.783496943998138	5,65978E+14	-1,86534E+14	0.108115274852049	0.253007031557524	-4,55244E+14	5,45605E+14	5,2468E+14	5,69658E+14	5,28942E+14	5,6355E+14	6,63434E+14
TC0200002537.mm.2	Ctsa	cathepsin A	1,16417E+12	8,16716E+14	1,8653E+14	0.10812253695549	0.253007031557524	-4,55251E+14	8,878E+14	9,16406E+14	8,60439E+14	6,64281E+14	7,92479E+14	7,78892E+14
TC0100002477.mm.2	Crygd	crystallin, gamma D	-0.627605683427102	6,35777E+13	-1,86529E+14	0.108124273524801	0.253007031557524	-4,55252E+14	5,73976E+14	6,20247E+14	6,31899E+14	6,68795E+13	6,70361E+13	6,49383E+13
TC0300001038.mm.2	Sike1	suppressor of IKBKE 1	1,12844E+14	8,10675E+14	1,86451E+14	0.108243955157076	0.253216971302368	-4,55352E+14	8,81001E+14	8,61576E+14	8,68279E+14	7,36303E+14	8,26814E+14	6,90076E+14
TC0100002308.mm.2	Myo1b	myosin IB	0.597478961154187	6,42531E+14	1,8645E+14	0.108245422846814	0.253216971302368	-4,55353E+14	6,75447E+14	7,11657E+14	6,17323E+14	5,83153E+14	6,39058E+14	6,28549E+14
TC0400004140.mm.2	Gabrd	gamma-aminobutyric acid (GABA) A receptor, subunit delta	0.670304341775986	6,59506E+14	1,86435E+14	0.108268027472441	0.253233085594926	-4,55372E+14	7,01897E+13	7,15463E+14	6,02447E+14	6,53928E+14	6,69816E+14	6,13483E+14
TC1000003120.mm.2	Nab2	Ngfi-A binding protein 2	0.596635982519894	6,80632E+14	1,86394E+14	0.108331181687369	0.253333288606004	-4,55425E+14	7,15179E+14	7,30109E+14	6,6207E+14	6,50471E+14	6,86445E+13	6,39516E+12
TC1500001091.mm.2	Zfp740	zinc finger protein 740	1,03398E+14	6,62295E+13	1,86366E+14	0.108374290963501	0.253333288606004	-4,5546E+14	7,61292E+14	7,51905E+14	6,60914E+14	4,93255E+14	6,67107E+14	6,39295E+14
TC0X00000515.mm.2	Zfp449	zinc finger protein 449	1,09416E+14	5,88871E+14	1,86364E+14	0.108378137652702	0.253333288606004	-4,55464E+14	6,3041E+14	6,25487E+12	6,87622E+14	5,52642E+14	5,80724E+14	4,56341E+14
TC1200001369.mm.2	Ubxn2a	UBX domain protein 2A	1,74089E+14	6,9967E+14	1,86362E+14	0.108381138533164	0.253333288606004	-4,55466E+14	7,96429E+14	7,97938E+14	7,60267E+14	5,97195E+14	7,60043E+14	4,86147E+13
TC0100003321.mm.2	Fam20b	family with sequence similarity 20, member B	0.603448578857765	8,01272E+14	1,86356E+14	0.108389480025432	0.253333288606004	-4,55473E+14	8,39608E+13	8,23217E+14	7,98811E+14	8,03858E+14	7,96434E+13	7,45701E+14
TC0700002492.mm.2	Clptm1	cleft lip and palate associated transmembrane protein 1	0.627495420550932	9,86121E+13	1,86332E+14	0.108427361284877	0.253361544214015	-4,55505E+14	1,03517E+14	1,02475E+14	9,87698E+14	9,3468E+14	9,83404E+13	9,51019E+14
TC0X00001024.mm.2	Chic1	cysteine-rich hydrophobic domain 1	0.806688224487872	6,41643E+14	1,86328E+14	0.108433017371057	0.253361544214015	-4,55509E+14	7,06654E+14	6,83007E+14	6,5011E+14	5,81763E+14	6,596E+14	5,68723E+14
TC1100000205.mm.2	Peli1	pellino 1	1,15786E+14	7,10049E+14	1,86271E+14	0.108521572888732	0.253531695513431	-4,55583E+14	7,87796E+14	7,94364E+14	7,62384E+14	5,65162E+14	6,69442E+14	6,81146E+14
TC0X00003371.mm.2	Msl3	MSL complex subunit 3	0.853848265635584	6,91841E+14	1,86252E+14	0.108551267135803	0.253564303763293	-4,55608E+14	7,33641E+14	7,75452E+14	7,11865E+14	5,91765E+14	6,61414E+14	6,76909E+14
TC0800001872.mm.2	Polb	polymerase (DNA directed), beta	0.692646328264933	6,25359E+14	1,86232E+14	0.108581781826605	0.253598818752297	-4,55633E+14	6,80449E+14	7,01596E+14	5,87809E+14	5,38786E+13	6,54632E+14	5,88884E+14
TC1100003988.mm.2	Gm11696	predicted gene 11696	-0.909171904141928	6,421E+14	-1,86198E+14	0.108633786329077	0.253683507118902	-4,55676E+14	5,70519E+14	5,79014E+14	6,42402E+14	7,29297E+14	6,09954E+14	7,21413E+14
TC1000000846.mm.2	Pwwp3a	PWWP domain containing 3A, DNA repair factor	1,02826E+14	6,82741E+14	1,86165E+14	0.108685773503162	0.253768130482025	-4,55719E+14	7,71053E+14	7,42052E+14	6,82918E+13	5,9297E+14	5,8357E+14	7,23882E+13
TC0100001711.mm.2	Parp1/Mir6904	poly (ADP-ribose) polymerase family, member 1/microRNA 6904	0.654969040606059	6,74105E+14	1,86146E+14	0.108713912605126	0.253781719715684	-4,55743E+14	7,30102E+14	6,90154E+14	6,95723E+14	6,39391E+14	6,33059E+14	6,56204E+13
TC1900000600.mm.2	Pgam1	phosphoglycerate mutase 1	1,30563E+14	9,05102E+14	1,86132E+14	0.108736964641405	0.253781719715684	-4,55762E+14	1,01408E+14	9,93455E+14	9,4871E+14	7,27955E+14	9,02813E+14	8,43596E+14
TC0600003534.mm.2	Vamp1	vesicle-associated membrane protein 1	1,01857E+14	8,66331E+14	1,86128E+14	0.108742572768112	0.253781719715684	-4,55767E+13	9,5396E+14	8,90933E+14	8,69282E+14	8,42634E+14	8,96773E+14	7,44405E+14
TC1000000300.mm.2	Col10a1	collagen, type X, alpha 1	-1,17713E+14	4,77581E+14	-1,86116E+14	0.108760486327475	0.253781719715684	-4,55781E+14	3,84325E+14	4,23174E+13	4,72954E+14	5,42112E+14	4,2854E+14	6,14385E+14
TC0100000621.mm.2	Rufy4	RUN and FYVE domain containing 4	-0.673035873323577	6,18859E+14	-1,8611E+14	0.108770344216255	0.253781719715684	-4,5579E+14	6,20046E+14	5,414E+14	6,25946E+13	6,29712E+14	6,47222E+14	6,48831E+14
TC1200001367.mm.2	Mfsd2b	major facilitator superfamily domain containing 2B	-0.824085584949215	5,2138E+14	-1,86084E+14	0.108810457740878	0.253807037790007	-4,55823E+14	4,64082E+14	4,65579E+14	4,95551E+14	6,08592E+12	5,25876E+14	5,68603E+14
TC1100003906.mm.2	1700052K11Rik	RIKEN cDNA 1700052K11 gene	-0.597828233218894	7,20811E+14	-1,86083E+14	0.108812698879996	0.253807037790007	-4,55825E+13	7,05217E+14	6,79683E+14	6,95926E+14	7,39577E+14	7,47243E+14	7,57223E+14
TC0700003023.mm.2	Kcnj14	potassium inwardly-rectifying channel, subfamily J, member 14	-1,13266E+14	7,58942E+14	-1,86062E+14	0.108844744709197	0.253845038572774	-4,55851E+14	6,92744E+14	6,73518E+13	7,22099E+14	8,725E+14	7,37122E+14	8,55671E+14
TC0X00002400.mm.2	Cd99l2	CD99 antigen-like 2	1,36361E+14	7,28162E+13	1,8604E+13	0.108879261988597	0.25387069811804	-4,5588E+14	8,10616E+14	7,75106E+14	7,99115E+14	6,71134E+14	7,57543E+13	5,55457E+14
TC0400002562.mm.2	Pax5	paired box 5	-0.964840477991252	5,67543E+14	-1,86035E+14	0.108887258405869	0.25387069811804	-4,55886E+14	5,18806E+14	5,00913E+14	5,37457E+14	6,35393E+14	5,47045E+14	6,65642E+13
TC0800002736.mm.2	Ccdc102a	coiled-coil domain containing 102A	-0.695403992454399	6,40595E+14	-1,86019E+14	0.108911326039297	0.253890074720948	-4,55906E+14	5,72253E+14	6,16186E+14	6,33306E+14	6,94303E+12	6,43111E+14	6,84409E+14
TC0200000391.mm.2	Zmynd19	zinc finger, MYND domain containing 19	0.894556062964424	6,2841E+14	1,85912E+14	0.109076977339707	0.254160703438667	-4,56044E+13	6,85376E+14	6,58254E+14	6,90673E+14	5,7144E+14	6,14791E+14	5,49927E+13
TC1300002735.mm.2	Mrps30	mitochondrial ribosomal protein S30	0.565999724269863	6,3303E+14	1,8589E+14	0.109112078932125	0.254160703438667	-4,56073E+14	6,6711E+14	6,87073E+14	6,15652E+14	5,87358E+14	6,25657E+12	6,15327E+14
TC1100000324.mm.2	Cpeb4	cytoplasmic polyadenylation element binding protein 4	1,12761E+14	7,97029E+14	1,85883E+14	0.109121779392942	0.254160703438667	-4,56081E+14	8,59871E+14	8,69764E+14	8,13494E+14	7,34933E+14	8,39294E+14	6,6482E+14
TC0800002130.mm.2	Zfp42	zinc finger protein 42	-0.828840083901854	6,49152E+14	-1,85879E+14	0.109128535692853	0.254160703438667	-4,56086E+14	6,01608E+14	5,71936E+14	6,42883E+14	7,39173E+14	6,35007E+14	7,04305E+14
TC1400000005.mm.2	1700110I01Rik	RIKEN cDNA 1700110I01 gene	-0.69407308983795	4,93597E+14	-1,85862E+14	0.109155092539235	0.254160703438667	-4,56108E+14	4,53474E+13	4,30685E+14	4,92792E+14	5,61661E+14	4,88818E+13	5,34155E+14
TC0400004174.mm.2	Klhl17/Mir7658	kelch-like 17/microRNA 7658	-0.75962082486538	7,88116E+14	-1,85855E+13	0.109165429674252	0.254160703438667	-4,56117E+14	7,16172E+14	7,54797E+14	7,7273E+14	8,62107E+14	7,92826E+14	8,30066E+14
TC1400000310.mm.2	Sfmbt1	Scm-like with four mbt domains 1	0.593083415844522	6,46971E+14	1,85853E+14	0.10916884024055	0.254160703438667	-4,56119E+14	6,82433E+14	6,68275E+14	6,32104E+14	6,66924E+14	5,99718E+14	6,32374E+14
TC0800001328.mm.2	Pdpr	pyruvate dehydrogenase phosphatase regulatory subunit	0.561653549416894	5,775E+14	1,85852E+14	0.109169970752116	0.254160703438667	-4,5612E+14	5,86647E+14	6,08978E+14	6,05584E+14	5,80498E+14	5,41764E+14	5,4153E+14
TC1800001682.mm.2	Zfp236	zinc finger protein 236	0.606767207233829	6,21408E+14	1,85843E+14	0.109184059627872	0.254160703438667	-4,56132E+14	6,48688E+14	6,54633E+14	5,94482E+14	6,48681E+14	5,85523E+14	5,96444E+14
TC1900001428.mm.2	Pank1	pantothenate kinase 1	0.748928141241436	6,02366E+14	1,85843E+14	0.109185154173801	0.254160703438667	-4,56133E+14	6,46608E+14	6,19418E+14	5,83479E+14	6,48448E+14	5,94552E+12	5,21689E+14
TC1300000442.mm.2	Dsp	desmoplakin	-0.561950555553516	4,92342E+14	-1,85803E+12	0.109247090032363	0.254264542607736	-4,56184E+14	4,52805E+14	4,77075E+14	4,82945E+14	4,98959E+14	5,12456E+14	5,29814E+14
TC1700000902.mm.2	2310039H08Rik	RIKEN cDNA 2310039H08 gene	-0.839975158624331	6,06613E+14	-1,85794E+14	0.109261322721775	0.254264542607736	-4,56196E+14	5,36145E+13	5,61943E+14	5,87073E+13	6,88434E+14	5,98652E+14	6,67431E+14
TC0500001147.mm.2	Ddx51	DEAD (Asp-Glu-Ala-Asp) box polypeptide 51	-0.949924191479471	5,94874E+14	-1,85782E+14	0.109280034973052	0.254271365129356	-4,56211E+13	5,2337E+14	5,22353E+14	5,77459E+14	7,10351E+14	5,81297E+14	6,54411E+14
TC1900000748.mm.2	Add3	adducin 3 (gamma)	1,48754E+14	7,90671E+14	1,85755E+14	0.109321343695617	0.254330755265301	-4,56245E+13	9,17892E+13	9,21345E+14	8,11405E+13	5,51651E+14	8,03944E+14	7,37786E+14
TC0700004652.mm.2	Snrpn/Snurf	small nuclear ribonucleoprotein N/SNRPN upstream reading frame	1,14506E+14	1,13219E+14	1,85693E+14	0.109417360197281	0.254483647773569	-4,56325E+14	1,21806E+14	1,21335E+14	1,15683E+14	1,00249E+14	1,16129E+13	1,04111E+14
TC1100003749.mm.2	Krt33b	keratin 33B	-0.781961145362733	5,73303E+14	-1,85693E+14	0.109418650268435	0.254483647773569	-4,56326E+14	5,29245E+14	5,27312E+14	5,32612E+14	6,37702E+14	5,89581E+14	6,23368E+14
TC0800001919.mm.2	Bag4	BCL2-associated athanogene 4	0.607411463343897	6,72245E+14	1,85628E+14	0.109519845866586	0.254682245121706	-4,56409E+14	6,77104E+14	7,04001E+13	7,11549E+14	6,81194E+14	6,49229E+14	6,10393E+14
TC0100000808.mm.2	Prss56	protease, serine 56	-0.9045802714166	6,37548E+14	-1,85596E+13	0.109568820630136	0.254759366062536	-4,56449E+14	5,56595E+14	6,05219E+14	5,98002E+14	7,22743E+14	6,48949E+14	6,93782E+14
TC1700001032.mm.2	Trip10	thyroid hormone receptor interactor 10	-0.729453354717077	5,0142E+14	-1,85565E+14	0.109616920013043	0.254798807836861	-4,56489E+14	4,41109E+14	4,78085E+14	4,79837E+14	5,44404E+14	5,02046E+14	5,63039E+14
TC0800000821.mm.2	Ap1m1	adaptor-related protein complex AP-1, mu subunit 1	0.816203932495086	8,12052E+14	1,85565E+14	0.109617410533428	0.254798807836861	-4,56489E+14	8,84163E+14	8,56539E+14	8,35108E+14	7,19043E+14	7,9148E+14	7,85981E+14
TC1700001822.mm.2	Cbs	cystathionine beta-synthase	0.723179608458492	6,57653E+14	1,85546E+14	0.109646782145819	0.254830318868539	-4,56513E+14	7,02956E+14	7,1739E+14	5,93237E+14	6,56627E+14	6,03601E+14	6,72109E+14
TC0900002064.mm.2	Olfr919	olfactory receptor 919	-1,04732E+14	4,81384E+14	-1,85506E+14	0.109709672591225	0.254939710767582	-4,56565E+14	4,35802E+14	3,92817E+14	4,26362E+14	5,95242E+13	5,2053E+13	5,17554E+13
TC1000001936.mm.2	Rspo3	R-spondin 3	1,00422E+13	5,33388E+14	1,85476E+14	0.109756628026645	0.255012047208843	-4,56604E+13	6,00104E+14	6,12147E+14	5,19281E+14	4,57386E+14	5,81711E+14	4,29696E+14
TC0100002894.mm.2	Bcl2	B cell leukemia/lymphoma 2	1,6036E+14	6,39115E+14	1,85459E+14	0.109783212946627	0.25503704011087	-4,56625E+14	7,79091E+14	7,45725E+14	6,93295E+14	4,12485E+14	6,39372E+14	5,64723E+14
TC1100002240.mm.2	Sec61g	SEC61, gamma subunit	0.986664639566849	8,55892E+14	1,85435E+14	0.109820535847167	0.255074179818955	-4,56656E+12	8,93947E+14	9,0432E+14	8,61933E+14	8,74217E+14	8,80367E+14	7,20568E+14
TC0200001323.mm.2	Olfr1188	olfactory receptor 1188	-0.672451484488526	4,87333E+14	-1,85423E+14	0.109838905223055	0.255074179818955	-4,56671E+14	4,62109E+14	4,34083E+14	4,89114E+14	5,08009E+13	4,79442E+14	5,5124E+14
TC0400002668.mm.2	AI427809	expressed sequence AI427809	-0.980588987375402	4,73468E+14	-1,85418E+14	0.109846691104309	0.255074179818955	-4,56678E+14	4,15835E+14	4,11775E+14	4,29675E+14	5,59795E+14	5,57705E+14	4,66023E+14
TC0800001362.mm.2	Wwox	WW domain-containing oxidoreductase	-0.573471608884781	8,10576E+14	-1,85372E+14	0.109918879459439	0.255205029499992	-4,56737E+14	7,62506E+14	7,73551E+14	8,26701E+14	8,49946E+14	8,09338E+14	8,41413E+14
TC1100000939.mm.2	Asgr1	asialoglycoprotein receptor 1	-0.892697666076608	6,36091E+14	-1,85305E+14	0.11002381412228	0.255411859523453	-4,56823E+14	5,47696E+13	6,00428E+14	6,09441E+14	7,32317E+14	6,44951E+14	6,81712E+14
TC1000000493.mm.2	Gja1	gap junction protein, alpha 1	1,3077E+14	8,89049E+13	1,85283E+14	0.11005779771708	0.255453946213672	-4,56851E+14	9,86007E+14	9,69234E+14	9,40621E+14	7,44732E+14	9,07668E+14	7,86032E+14
TC0700000075.mm.2	Zfp667	zinc finger protein 667	0.589355230023122	6,04042E+14	1,85266E+13	0.110085011371178	0.255480309408584	-4,56873E+14	6,43357E+14	6,37459E+13	6,05702E+14	5,74791E+14	5,62717E+14	6,00227E+14
TC1300002019.mm.2	Msx2	msh homeobox 2	-1,26377E+14	5,99078E+14	-1,85228E+14	0.110143881369568	0.255580121040877	-4,56921E+14	4,82392E+14	5,27129E+14	5,649E+14	7,5777E+12	5,81008E+14	6,81267E+14
TC0600001126.mm.2	Tafa1	TAFA chemokine like family member 1	0.741920959863037	7,70044E+14	1,852E+14	0.110188672663371	0.255646450324341	-4,56958E+14	8,10544E+14	8,60755E+14	7,56077E+14	6,69972E+14	7,56783E+14	7,66131E+14
TC0900000560.mm.2	Apoa4	apolipoprotein A-IV	-0.799928095274452	6,54287E+14	-1,8519E+14	0.110204198097987	0.255646450324341	-4,5697E+14	6,08487E+14	5,98746E+14	6,37528E+14	7,13744E+14	6,4038E+12	7,26839E+14
TC0700001862.mm.2	Orai3	ORAI calcium release-activated calcium modulator 3	-0.629504441882732	7,47954E+14	-1,85174E+14	0.110228604481106	0.255666259393129	-4,5699E+14	6,9674E+14	7,17695E+14	7,94896E+14	7,38057E+14	7,58169E+13	7,82167E+14
TC0600001664.mm.2	Pde3a	phosphodiesterase 3A, cGMP inhibited	-0.706133799688898	8,01434E+14	-1,85163E+14	0.11024564209395	0.255668973957946	-4,57004E+14	7,46401E+14	7,62252E+14	8,00719E+14	8,49351E+14	7,87958E+13	8,61921E+14
TC1200000007.mm.2	Kif3c	kinesin family member 3C	0.694582171682942	8,30554E+14	1,85147E+14	0.110270750366169	0.255690401590169	-4,57025E+13	8,83975E+14	8,7417E+14	8,42372E+14	7,63575E+14	8,26774E+14	7,92455E+14
TC1400000327.mm.2	Capn7	calpain 7	1,10953E+13	6,34191E+14	1,8512E+14	0.110313325205198	0.255752317846237	-4,5706E+14	7,25984E+14	7,01926E+14	6,7028E+14	5,00953E+13	6,42794E+13	5,6321E+14
TC0800000323.mm.2	Nsd3	nuclear receptor binding SET domain protein 3	0.997567264639857	8,20993E+14	1,8508E+14	0.110376208786645	0.255808686517921	-4,57111E+14	8,94539E+14	9,02546E+14	8,24617E+14	7,06681E+14	8,55294E+13	7,42277E+14

TC1700002422.mm.2	Ndufv2	NADH:ubiquinone oxidoreductase core subunit V2	1,53415E+14	8,39012E+14	1,85073E+14	0.110388222862056	0.255808686517921	-4,57121E+14	9,64108E+14	9,55205E+14	8,81118E+14	6,21154E+14	8,56487E+14	7,55998E+14
TC0800000104.mm.2	Sox1ot	Sox1 overlapping transcript	0.571407849246985	6,09631E+14	1,85065E+14	0.1104009687537	0.255808686517921	-4,57131E+14	6,27739E+14	6,55896E+14	5,92716E+14	6,12078E+14	5,95581E+14	5,73773E+14
TC1200000387.mm.2	Pnpla8/Gm2027	patatin-like phospholipase domain containing 8/nuclear assembly factor 1 homolog pseudogene	1,13367E+14	6,38096E+12	1,85064E+14	0.110401142310285	0.255808686517921	-4,57131E+14	7,35812E+14	7,24494E+14	6,45366E+14	4,89329E+14	6,74166E+14	5,59406E+14
TC0100002349.mm.2	Pgap1	post-GPI attachment to proteins 1	1,47131E+14	7,06382E+14	1,85019E+14	0.11047301067033	0.255918904724576	-4,5719E+14	8,21512E+14	7,96559E+14	7,62657E+14	5,33752E+14	7,28415E+14	5,95394E+14
TC0100002921.mm.2	Dsel	dermatan sulfate epimerase-like	0.962078495235551	6,51136E+14	1,85012E+14	0.110484259575369	0.255918904724576	-4,57199E+13	7,05029E+14	7,29882E+14	6,20725E+14	6,08684E+14	6,97729E+14	5,44765E+14
TC0300002712.mm.2	Ubl4b	ubiquitin-like 4B	-0.659986043659473	5,75866E+14	-1,84998E+13	0.110504792004359	0.255918904724576	-4,57216E+13	5,53025E+14	5,4146E+14	5,3543E+14	5,98799E+14	5,93231E+14	6,33251E+14
TC1100004117.mm.2	Qrich2	glutamine rich 2	-0.578779953568599	4,93038E+14	-1,84992E+14	0.110515631131152	0.255918904724576	-4,57225E+14	4,44572E+14	4,4796E+14	5,06566E+14	5,5084E+14	5,0416E+14	5,04128E+14
TC0900000720.mm.2	Sema7a	sema domain, immunoglobulin domain (Ig), and GPI membrane anchor, (semaphorin) 7A	0.792023777290531	6,9096E+13	1,84984E+14	0.110528123749663	0.255918904724576	-4,57235E+14	7,2041E+14	7,81157E+13	6,66051E+13	6,34982E+14	7,17144E+14	6,26018E+14
TC1200000641.mm.2	Slc38a6	solute carrier family 38, member 6	0.982823462317978	5,70732E+14	1,84973E+14	0.110544983780749	0.255921167192416	-4,57249E+14	6,18329E+14	5,91718E+14	5,93476E+14	6,07387E+14	5,85466E+14	4,28017E+14
TC0700001275.mm.2	Picalm	phosphatidylinositol binding clathrin assembly protein	1,70237E+14	9,02121E+14	1,84953E+14	0.110577050108728	0.255944445866752	-4,57275E+13	1,01053E+14	1,0177E+14	9,69398E+12	7,27346E+14	9,43506E+14	7,44248E+13
TC1700002346.mm.2	Slc25a23	solute carrier family 25 (mitochondrial carrier; phosphate carrier), member 23	1,38281E+14	9,75619E+14	1,84946E+14	0.110586807678541	0.255944445866752	-4,57283E+14	1,06468E+14	1,06506E+14	1,04478E+14	8,26022E+14	9,84526E+14	8,6865E+14
TC1700001301.mm.2	Tmem247	transmembrane protein 247	-0.65839384252848	5,32335E+14	-1,84906E+14	0.11064993513646	0.256053770623838	-4,57334E+14	5,08646E+14	4,92167E+14	5,00855E+14	5,59847E+14	5,44241E+14	5,8825E+14
TC0900000871.mm.2	Car12	carbonic anhydrase 12	0.61509522155011	6,76044E+14	1,84894E+14	0.110668687948273	0.256060391859638	-4,57349E+14	6,94125E+14	7,35932E+14	6,60532E+14	6,58637E+14	6,31929E+14	6,75109E+14
TC0800001609.mm.2	Pex11g	peroxisomal biogenesis factor 11 gamma	-0.777833754445699	7,32435E+14	-1,84838E+14	0.110757118652032	0.256176571218679	-4,57421E+14	6,81594E+14	6,88338E+14	7,49517E+14	7,49663E+14	7,07721E+14	8,17778E+14
TC1800000564.mm.2	Gramd3	GRAM domain containing 3	-0.779415015467643	6,68433E+14	-1,84837E+14	0.110759787298731	0.256176571218679	-4,57424E+14	6,21788E+14	6,18318E+14	7,02662E+14	6,71844E+14	7,5112E+14	6,44868E+14
TC1500001542.mm.2	Mrpl13	mitochondrial ribosomal protein L13	1,57244E+14	5,75589E+14	1,84823E+14	0.110781358156282	0.256176571218679	-4,57441E+14	6,85326E+14	6,53104E+14	6,30429E+13	4,60731E+14	6,26689E+14	3,97254E+14
TC0900001944.mm.2	Vps26b	VPS26 retromer complex component B	0.654742716211397	8,11066E+14	1,84822E+14	0.110782495392029	0.256176571218679	-4,57442E+14	8,58148E+14	8,44985E+14	7,98423E+14	7,92656E+14	8,22548E+14	7,49636E+14
TC1100003019.mm.2	Ndel1	nudE neurodevelopment protein 1 like 1	1,42567E+14	8,38653E+14	1,84808E+13	0.110804695770945	0.256191141190592	-4,5746E+14	9,46262E+14	9,35215E+13	8,79069E+14	6,75029E+14	8,759E+14	7,20444E+14
TC1100003222.mm.2	Serpinf1	serine (or cysteine) peptidase inhibitor, clade F, member 1	-0.564444735181604	8,26955E+13	-1,8475E+14	0.110896440244937	0.256341685080174	-4,57535E+14	7,87414E+13	7,82435E+14	8,40075E+14	8,69069E+14	8,29949E+13	8,52789E+14
TC0200003421.mm.2	Psmb7	proteasome (prosome, macropain) subunit, beta type 7	1,56863E+14	8,44966E+14	1,84747E+14	0.110901625190461	0.256341685080174	-4,57539E+14	9,61347E+14	9,63881E+14	8,88501E+14	6,44563E+14	8,80902E+14	7,30601E+14
TC1100003592.mm.2	Nxph3	neurexophilin 3	0.732287236161959	5,55905E+14	1,84711E+14	0.110957532281783	0.256356205238271	-4,57584E+14	5,89729E+14	5,95719E+14	5,46397E+14	5,64597E+14	4,7146E+14	5,6753E+14
TC0700002005.mm.2	Olfr523	olfactory receptor 523	-0.836311704176937	5,73694E+14	-1,847E+14	0.110974437183066	0.256356205238271	-4,57598E+14	5,09921E+14	5,35921E+14	5,63254E+14	6,19963E+14	5,52103E+13	6,61001E+13
TC0700001484.mm.2	Olfr593	olfactory receptor 593	-0.755306690303076	4,69509E+14	-1,84699E+14	0.110976628969406	0.256356205238271	-4,576E+14	4,13441E+14	4,22152E+14	4,51606E+14	5,43202E+11	4,68706E+14	5,17948E+14
TC0100003709.mm.2	Degs1	delta(4)-desaturase, sphingolipid 1	1,14581E+14	8,5709E+14	1,84698E+14	0.110978997043901	0.256356205238271	-4,57602E+14	9,35401E+14	9,32571E+14	9,05182E+14	7,30573E+14	8,65187E+14	7,73624E+14
TC0400003958.mm.2	Tnfrsf8	tumor necrosis factor receptor superfamily, member 8	-0.678144626503878	5,39322E+14	-1,84692E+14	0.110987456571848	0.256356205238271	-4,57609E+13	5,02846E+13	5,05036E+14	5,07758E+14	5,7678E+14	5,9385E+14	5,49661E+13
TC1200002101.mm.2	Erg28	ergosterol biosynthesis 28	1,05682E+14	8,11034E+14	1,84665E+14	0.111029759774237	0.256417159128893	-4,57643E+14	9,07121E+13	8,83269E+14	8,32185E+14	6,64602E+14	8,16217E+14	7,62809E+14
TC0400000961.mm.2	Fggy	FGGY carbohydrate kinase domain containing	0.876741089926548	6,18633E+14	1,84632E+14	0.111083147150876	0.256503690175131	-4,57686E+14	6,64484E+14	6,60515E+14	6,2049E+14	6,1666E+14	6,42562E+14	5,07088E+14
TC1600001810.mm.2	Epha6	Eph receptor A6	1,17508E+14	6,2115E+14	1,84612E+14	0.111114694280381	0.256539772021747	-4,57712E+14	6,83018E+13	6,88099E+13	6,09293E+14	6,10392E+14	6,69863E+14	4,66238E+13
TC0700001804.mm.2	Tufm	Tu translation elongation factor, mitochondrial	0.605957703895218	6,60027E+14	1,84592E+14	0.111146007482012	0.256575303518289	-4,57737E+14	7,05146E+14	7,20783E+14	6,34483E+14	5,95013E+14	6,33401E+12	6,71337E+14
TC0700000460.mm.2	Sertad1	SERTA domain containing 1	-0.727508159380564	6,53494E+13	-1,8458E+13	0.111165403814255	0.256583319246395	-4,57753E+14	5,82253E+13	6,41737E+14	6,35333E+14	6,92148E+14	6,59271E+14	7,10221E+14
TC0700000913.mm.2	Slc17a6	solute carrier family 17 (sodium-dependent inorganic phosphate cotransporter), member 6	1,12874E+14	5,8014E+14	1,84565E+14	0.111188511049606	0.256599896668905	-4,57772E+14	7,18839E+14	5,7162E+13	6,23669E+14	5,02002E+14	5,16474E+14	5,48238E+14
TC0300003247.mm.2	Amy1	amylase 1, salivary	0.782729210935285	5,42835E+14	1,84522E+14	0.111257379246533	0.256722060976569	-4,57827E+14	6,24344E+14	5,6974E+14	5,30082E+14	4,95307E+14	5,64414E+13	4,73123E+13
TC1100003762.mm.2	Krt16	keratin 16	-0.745943561373462	5,43575E+14	-1,84497E+14	0.111296900946976	0.256724954881599	-4,57859E+14	4,81212E+14	5,0361E+14	5,23604E+14	6,1901E+14	5,52842E+14	5,81169E+14
TC0700002361.mm.2	Mzf1	myeloid zinc finger 1	0.887787956955752	5,42727E+14	1,84496E+14	0.111297357279001	0.256724954881599	-4,5786E+14	5,79213E+13	5,89779E+14	5,1151E+14	5,84428E+14	4,37121E+14	5,54313E+14
TC0700003983.mm.2	Trim3	tripartite motif-containing 3	-0.646940834315068	7,52441E+14	-1,84487E+14	0.111312695987272	0.256724954881599	-4,57872E+14	7,24835E+14	6,96824E+14	7,44764E+13	7,97176E+14	7,55115E+14	7,95932E+14
TC1700000407.mm.2	Rgs11	regulator of G-protein signaling 11	-0.535616708253749	5,84335E+14	-1,8448E+14	0.111322364535328	0.256724954881599	-4,5788E+14	5,54861E+14	5,4066E+14	6,03333E+14	6,04508E+14	6,03597E+14	5,99047E+13
TC0100002702.mm.2	Slc16a14	solute carrier family 16 (monocarboxylic acid transporters), member 14	1,2222E+14	5,33703E+14	1,8446E+14	0.111355079499526	0.256740562159512	-4,57906E+14	5,84892E+14	5,8651E+14	5,50116E+14	5,53739E+14	5,70521E+14	3,56441E+14
TC1800001660.mm.2	Ctdp1	CTD (carboxy-terminal domain, RNA polymerase II, polypeptide A) phosphatase, subunit 1	-0.822274676736017	6,65304E+13	-1,84456E+14	0.111360999747584	0.256740562159512	-4,57911E+14	6,46087E+14	5,82801E+14	6,47005E+14	7,22588E+14	6,60537E+14	7,32806E+14
TC0300003196.mm.2	Srsf11	serine/arginine-rich splicing factor 11	1,60741E+14	7,41228E+13	1,84425E+14	0.111410697572797	0.256786590711112	-4,57951E+14	8,38894E+14	8,67993E+14	7,90308E+14	5,64768E+14	7,86736E+13	5,98669E+14
TC1200001859.mm.2	Rps29	ribosomal protein S29	0.570353105210321	1,0839E+14	1,84423E+14	0.111412837811791	0.256786590711112	-4,57953E+14	1,13129E+14	1,1132E+14	1,07866E+14	1,0498E+14	1,05377E+14	1,07665E+14
TC0500003733.mm.2	Zfp644	zinc finger protein 644	1,52816E+14	8,24895E+14	1,84379E+14	0.111483890827084	0.256830117810717	-4,58011E+14	9,29178E+14	9,28561E+14	8,82345E+13	6,5718E+14	8,5826E+13	6,93847E+14
TC0600001176.mm.2	Cntn6	contactin 6	0.649965006008191	6,23231E+14	1,84372E+14	0.111493691229372	0.256830117810717	-4,58018E+14	6,70467E+14	6,56751E+13	5,85235E+14	6,2971E+14	6,26895E+14	5,7033E+14
TC0400001338.mm.2	Foxj3	forkhead box J3	0.803532952640069	6,97507E+14	1,84331E+13	0.111559554336702	0.256830117810717	-4,58072E+14	7,4631E+14	7,41546E+13	7,43412E+14	6,26622E+14	6,69888E+14	6,57262E+14
TC1700000676.mm.2	Atp6v1g2	ATPase, H+ transporting, lysosomal V1 subunit G2	0.82089585204889	1,07439E+14	1,8433E+14	0.11156035011618	0.256830117810717	-4,58072E+14	1,144E+14	1,12089E+14	1,08978E+14	9,90972E+14	1,08424E+14	1,01647E+14
TC0400003953.mm.2	Gm438	predicted gene 438	-0.65433918605372	4,85043E+14	-1,84327E+14	0.111565615962487	0.256830117810717	-4,58077E+14	4,30668E+14	4,51075E+14	5,1378E+14	5,02122E+13	4,70819E+14	5,41792E+14
TC0300002714.mm.2	Strip1	striatin interacting protein 1	0.804673786957764	7,23216E+14	1,84324E+14	0.11156998503874	0.256830117810717	-4,5808E+14	8,09148E+14	8,04141E+14	6,74828E+14	5,98823E+14	7,71153E+14	6,81201E+14
TC1500000921.mm.2	Pphln1	periphilin 1	0.583333953560929	7,63649E+14	1,84324E+14	0.111570296850929	0.256830117810717	-4,5808E+14	8,14621E+14	7,9748E+14	7,43724E+14	7,30636E+14	7,6892E+14	7,26514E+14
TC0400000906.mm.2	Ifna6	interferon alpha 6	-0.720390864393268	5,80665E+13	-1,84323E+14	0.111572495245275	0.256830117810717	-4,58082E+14	5,33102E+14	5,38882E+14	5,68389E+14	6,27552E+14	5,7097E+14	6,45092E+14
TC1500000232.mm.2	Mtdh	metadherin	0.598936090622268	7,82688E+14	1,84321E+13	0.111575176855645	0.256830117810717	-4,58084E+14	8,16173E+14	8,18898E+14	7,56798E+14	7,88972E+14	7,79548E+14	7,35736E+14
TC0400002704.mm.2	Actl7b	actin-like 7b	-1,03791E+13	5,69012E+14	-1,84307E+14	0.111597556302039	0.256844939964971	-4,58102E+14	5,17866E+14	5,11682E+14	5,25486E+14	6,21909E+14	5,46396E+14	6,90734E+14
TC1100000614.mm.2	Irf1	interferon regulatory factor 1	-0.604364431277428	6,52138E+13	-1,84296E+14	0.11161426228799	0.256846702120306	-4,58116E+14	5,89913E+14	6,26884E+14	6,70506E+14	6,87854E+14	6,4918E+14	6,8849E+14
TC0600001180.mm.2	Cntn4	contactin 4	1,23424E+14	6,14151E+14	1,84224E+14	0.111729896039765	0.257016632550575	-4,58209E+14	6,99955E+14	7,12437E+14	5,98626E+14	5,08342E+14	6,81266E+12	4,84277E+14
TC1000002943.mm.2	Slc35e3	solute carrier family 35, member E3	0.817607893034677	6,54987E+13	1,84223E+14	0.111731205950881	0.257016632550575	-4,5821E+14	7,12739E+14	7,14946E+14	6,75247E+14	5,62824E+14	6,54839E+14	6,09325E+14
TC1700000410.mm.2	Luc7l	Luc7-like	0.982301502554023	6,43629E+13	1,84196E+14	0.111773938148732	0.257016632550575	-4,58245E+14	7,07988E+14	6,98094E+14	6,63385E+14	5,82685E+14	6,74362E+14	5,35259E+14
TC0900000247.mm.2	Eepd1	endonuclease/exonuclease/phosphatase family domain containing 1	-0.678140825562982	6,60255E+14	-1,84174E+14	0.111808068595972	0.257016632550575	-4,58272E+14	6,05915E+13	6,35612E+14	6,4265E+14	7,00201E+14	6,6296E+14	7,14194E+14
TC0900003040.mm.2	Slc38a3	solute carrier family 38, member 3	0.849363818014623	8,50196E+14	1,84131E+14	0.111877856120718	0.257016632550575	-4,58328E+14	9,2031E+13	9,24353E+13	8,49253E+14	7,32468E+14	8,65205E+14	8,09585E+14
TC0100000194.mm.2	Pih1d3	PIH1 domain containing 3	-0.864742795997674	4,39125E+14	-1,84123E+14	0.111890486308958	0.257016632550575	-4,58338E+14	3,55587E+14	4,06005E+14	4,35471E+14	5,03148E+14	4,15721E+14	5,18819E+14
TC0600000257.mm.2	Nrf1	nuclear respiratory factor 1	0.835762398410208	6,24466E+14	1,84112E+14	0.111906750844129	0.257016632550575	-4,58351E+14	6,52356E+14	6,58079E+14	6,46433E+14	6,46646E+14	6,34594E+13	5,0869E+14
TC0600002475.mm.2	Ptcd3	pentatricopeptide repeat domain 3	1,76084E+14	5,49615E+14	1,8411E+14	0.111910582102467	0.257016632550575	-4,58355E+13	6,91224E+14	6,73215E+14	6,06989E+13	3,13991E+14	5,78341E+14	4,33929E+14
TC1300001068.mm.2	Otp	orthopedia homeobox	-0.810920482372811	6,96434E+14	-1,841E+14	0.111926199922564	0.257016632550575	-4,58367E+14	6,34034E+14	6,56107E+14	7,10813E+14	7,25327E+13	6,67772E+14	7,84553E+13
TC1100000990.mm.2	Fbxo39	F-box protein 39	-0.973725503099003	4,65533E+14	-1,84098E+14	0.111929524646478	0.257016632550575	-4,5837E+14	3,83937E+14	3,96715E+14	4,56791E+14	5,8036E+14	4,37874E+14	5,37522E+14
TC0100000551.mm.2	Map2	microtubule-associated protein 2	0.809345147092076	8,62901E+14	1,84098E+14	0.111929695087626	0.257016632550575	-4,5837E+14	9,32162E+14	9,06515E+14	8,84331E+14	7,77588E+13	8,61791E+14	8,15017E+14
TC0700001756.mm.2	Prkcb	protein kinase C, beta	1,15588E+14	1,075E+13	1,84097E+14	0.11193170541122	0.257016632550575	-4,58372E+14	1,15556E+14	1,1683E+14	1,1062E+14	9,27286E+14	1,09081E+14	1,00186E+14
TC1600000224.mm.2	Klhl22	kelch-like 22	1,40227E+13	7,98925E+14	1,84091E+14	0.111940959764191	0.257016632550575	-4,58379E+14	8,8964E+14	8,83539E+14	8,74594E+14	6,53053E+14	8,07375E+14	6,85349E+14

TC0600002056.mm.2	Wdr91	WD repeat domain 91	1,30899E+13	6,38374E+14	1,84075E+14	0.111966063854718	0.257016632550575	-4,58399E+14	7,52263E+14	7,32171E+14	6,75733E+14	4,47442E+14	6,33114E+14	5,8952E+14
TC0200003326.mm.2	Zbtb34	zinc finger and BTB domain containing 34	-0.617864822418288	6,72439E+14	-1,8407E+14	0.111973897645643	0.257016632550575	-4,58405E+14	6,28141E+14	6,5451E+14	6,8288E+14	6,62876E+14	7,29465E+14	6,76759E+14
TC1900001552.mm.2	Chuk	conserved helix-loop-helix ubiquitous kinase	0.726447759168583	5,77496E+14	1,84067E+14	0.111979967139388	0.257016632550575	-4,5841E+14	6,17932E+14	6,27256E+14	5,76324E+14	5,43569E+14	5,95378E+14	5,04519E+14
TC0200000714.mm.2	Olfml2a	olfactomedin-like 2A	-0.612007316951484	5,33203E+14	-1,84064E+14	0.111984340984399	0.257016632550575	-4,58414E+14	5,21639E+14	5,07915E+14	4,87729E+14	5,29981E+14	5,55808E+14	5,96148E+14
TC0500000647.mm.2	Tmem33	transmembrane protein 33	1,29007E+14	8,05233E+13	1,8406E+14	0.111990335528498	0.257016632550575	-4,58419E+14	8,76454E+14	8,82543E+14	8,56727E+14	7,14692E+14	8,40427E+14	6,60556E+14
TC0700004323.mm.2	Zfp768	zinc finger protein 768	-0.569228059091774	6,87642E+14	-1,8405E+14	0.112006450404998	0.257016632550575	-4,58432E+13	6,26658E+14	6,59032E+14	7,28901E+14	7,11726E+14	6,9053E+14	7,09006E+14
TC0200001965.mm.2	Tgm3	transglutaminase 3, E polypeptide	-0.666925392121839	5,26229E+14	-1,84049E+14	0.112007124295298	0.257016632550575	-4,58432E+14	4,77984E+14	4,83246E+13	5,12595E+14	5,88934E+14	5,37126E+14	5,57489E+14
TC1800001147.mm.2	Reep5	receptor accessory protein 5	1,0646E+14	9,13121E+14	1,84012E+13	0.112066205164561	0.257115586475377	-4,5848E+14	9,99042E+14	9,99731E+14	9,33139E+14	7,60963E+14	9,1932E+14	8,66532E+14
TC0400002786.mm.2	Fkbp15	FK506 binding protein 15	1,08014E+14	6,9459E+14	1,83987E+14	0.112106171592871	0.257170663849079	-4,58512E+13	8,04316E+14	7,7826E+14	6,85651E+14	5,32768E+14	7,3241E+14	6,34136E+14
TC0400001504.mm.2	A3galt2	alpha 1,3-galactosyltransferase 2 (isoglobotriaosylceramide synthase)	-1,00664E+14	5,44926E+13	-1,83963E+14	0.112145586055675	0.257224459518163	-4,58543E+14	4,69933E+14	4,8831E+14	5,14205E+14	6,37536E+14	5,24233E+14	6,35338E+14
TC0X00001653.mm.2	Ctps2	cytidine 5'-triphosphate synthase 2	0.759817209872335	7,49349E+14	1,83937E+14	0.112187201887754	0.257247539817518	-4,58577E+14	8,03532E+14	8,16439E+14	6,53574E+14	7,54543E+14	7,49555E+14	7,18452E+14
TC0100002136.mm.2	Prim2	DNA primase, p58 subunit	0.738485290450189	5,55937E+14	1,83936E+14	0.112187579116099	0.257247539817518	-4,58577E+14	6,1331E+14	5,91339E+14	5,85845E+14	4,88173E+14	5,23694E+14	5,33258E+14
TC0700002839.mm.2	Chst8	carbohydrate sulfotransferase 8	-0.743634617603929	8,07039E+13	-1,8392E+14	0.112213363233759	0.257255088312064	-4,58598E+14	7,53119E+14	7,5227E+14	7,97271E+14	8,85456E+14	8,01414E+13	8,52702E+13
TC1600001655.mm.2	Cd200	CD200 antigen	-1,1311E+14	8,4195E+14	-1,83907E+14	0.112234458245665	0.257255088312064	-4,58615E+14	7,80152E+14	7,5196E+14	7,93337E+14	9,67918E+14	8,38058E+14	9,20274E+13
TC0100000171.mm.2	Lmbrd1	LMBR1 domain containing 1	1,20069E+14	8,96351E+14	1,83891E+14	0.112259227414315	0.257255088312064	-4,58635E+14	9,82692E+13	9,8199E+13	9,3443E+14	7,54452E+14	9,16634E+14	8,0791E+14
TC1000001566.mm.2	Ndufa4l2	Ndufa4, mitochondrial complex associated like 2	-0.604546089020462	6,13126E+14	-1,83888E+14	0.112264834169728	0.257255088312064	-4,58639E+14	5,72322E+14	5,58405E+14	6,2046E+14	6,75936E+14	6,27519E+14	6,24116E+14
TC0600001797.mm.2	Bet1	Bet1 golgi vesicular membrane trafficking protein	0.545096954450596	6,21208E+14	1,83876E+14	0.112284575238655	0.257255088312064	-4,58655E+14	6,69145E+14	6,4301E+14	6,05417E+14	6,06543E+14	6,01172E+14	6,01963E+14
TC0500001512.mm.2	Cct6a/Snora15	chaperonin containing Tcp1, subunit 6a (zeta)/small nucleolar RNA, H/ACA box 15	1,20865E+14	7,78389E+14	1,83874E+14	0.112286665183314	0.257255088312064	-4,58657E+14	8,84531E+14	8,12281E+14	8,33841E+14	6,84601E+14	7,9361E+14	6,61472E+14
TC0400004160.mm.2	Pusl1	pseudouridylate synthase-like 1	-0.599716015208519	8,0082E+14	-1,83861E+14	0.112308110461708	0.257267640584233	-4,58674E+14	7,46682E+14	7,74503E+14	7,97452E+14	8,45152E+14	8,18544E+14	8,22584E+14
TC1100000343.mm.2	Stk10	serine/threonine kinase 10	0.584893583764396	6,11422E+14	1,83844E+14	0.112334634071804	0.257291820724801	-4,58695E+14	6,36487E+14	6,45728E+12	5,88205E+14	6,32874E+14	5,91552E+14	5,73685E+13
TC1100001304.mm.2	Tbx4	T-box 4	-0.778831071122336	5,72804E+14	-1,83812E+14	0.112386835470696	0.257374798172162	-4,58737E+14	5,08775E+14	5,22225E+14	5,59165E+14	6,5989E+13	5,70222E+14	6,16545E+13
TC0700002924.mm.2	Zfp141	zinc finger protein 141	2,17558E+14	6,09066E+13	1,83795E+14	0.112413777082837	0.257399913334623	-4,58758E+14	7,77097E+13	7,66457E+14	6,83202E+14	3,19202E+14	6,50369E+14	4,58069E+14
TC0600001245.mm.2	Brpf1	bromodomain and PHD finger containing, 1	0.57863706059785	6,39531E+14	18377678627	0.112442450255876	0.257410115987491	-4,58781E+14	6,86547E+14	6,9647E+14	6,1363E+14	5,73252E+14	6,23601E+14	6,43689E+14
TC0200004165.mm.2	Accsl	1-aminocyclopropane-1-carboxylate synthase (non-functional)-like	-0.702373503461876	5,63845E+14	-1,83772E+14	0.112450714818741	0.257410115987491	-4,58788E+14	5,13182E+14	5,08146E+13	5,83324E+14	6,16616E+14	5,38962E+13	6,22841E+14
TC1900001710.mm.2	Afap1l2	actin filament associated protein 1-like 2	-0.856718720230742	7,04033E+14	-1,83762E+14	0.112466158788056	0.257410115987491	-4,588E+14	6,731E+14	6,37837E+14	6,61163E+14	7,69815E+14	7,084E+14	7,73881E+14
TC0900000834.mm.2	Cilp	cartilage intermediate layer protein, nucleotide pyrophosphohydrolase	-0.800433586954834	4,87254E+14	-1,83736E+14	0.112508362681533	0.257470138884753	-4,58834E+14	4,37357E+12	4,06242E+14	4,8349E+14	5,9275E+12	4,86966E+14	5,1672E+14
TC0800001207.mm.2	D230025D16Rik	RIKEN cDNA D230025D16 gene	1,31616E+14	7,39526E+14	1,83709E+14	0.112550518559836	0.257530034870014	-4,58868E+14	8,01597E+14	8,10541E+14	8,30941E+14	6,45168E+14	7,46574E+14	6,02332E+14
TC1600001203.mm.2	Prm1	protamine 1	-0.631556117814748	5,50447E+14	-1,83685E+14	0.112588887297234	0.257581249612429	-4,58898E+14	4,86375E+14	5,22866E+14	5,66945E+14	5,90944E+14	5,41898E+14	5,93654E+14
TC1100003171.mm.2	Olfr382	olfactory receptor 382	-1,00613E+12	4,40494E+14	-1,83666E+14	0.112619053642627	0.257589697505793	-4,58923E+14	4,01406E+13	3,68724E+14	3,9578E+14	5,05697E+14	4,24793E+14	5,46563E+14
TC1800001307.mm.2	Cdo1	cysteine dioxygenase 1, cytosolic	0.61925381360573	8,11313E+14	1,83663E+14	0.112624552779018	0.257589697505793	-4,58927E+14	8,41243E+14	8,4656E+14	8,13598E+14	8,02526E+14	8,1301E+14	7,50942E+14
TC0800002392.mm.2	Klhl26	kelch-like 26	0.627950408852615	6,29421E+14	1,83641E+13	0.11266027358841	0.257634826615107	-4,58955E+14	6,6918E+14	6,77257E+14	5,99208E+14	6,10033E+14	6,42974E+14	5,77874E+14
TC0400000011.mm.2	Tgs1	trimethylguanosine synthase 1	1,04822E+14	5,18328E+13	1,83594E+14	0.112734605160578	0.257768226614501	-4,59015E+14	5,80901E+14	5,97625E+14	5,47121E+14	4,15438E+14	5,42937E+13	4,25946E+14
TC0900002296.mm.2	Zc3h12c	zinc finger CCCH type containing 12C	0.768044093658581	6,4431E+14	1,83573E+14	0.112768633833859	0.257775433950057	-4,59042E+14	7,02725E+14	6,56631E+14	6,93829E+14	6,06927E+14	6,26426E+14	5,79321E+14
TC1100004203.mm.2	Faap100	Fanconi anemia core complex associated protein 100	-0.733849850870719	7,2356E+14	-1,83551E+14	0.112803472125255	0.257775433950057	-4,5907E+14	6,78998E+13	6,76729E+13	7,20397E+13	7,62741E+14	7,06132E+14	7,96366E+14
TC0700003978.mm.2	Cavin3	caveolae associated 3	-0.566873696111488	8,48497E+14	-1,83548E+14	0.112809701708965	0.257775433950057	-4,59075E+13	8,04973E+14	8,25403E+14	8,53082E+14	8,63773E+14	8,55156E+14	8,88594E+14
TC1900001282.mm.2	Fam122a	family with sequence similarity 122, member A	-0.549518454169873	8,24827E+14	-1,83546E+14	0.11281156215974	0.257775433950057	-4,59076E+14	7,97491E+14	7,87011E+13	8,39422E+14	8,30631E+14	8,60648E+14	8,33757E+14
TC0900000259.mm.2	Igsf9b	immunoglobulin superfamily, member 9B	0.801989418775991	6,00639E+14	1,83541E+14	0.112820821128156	0.257775433950057	-4,59084E+14	6,54205E+14	6,63677E+14	5,47998E+14	5,80468E+14	6,35298E+14	5,22186E+14
TC1900001745.mm.2	Cacul1	CDK2 associated, cullin domain 1	0.813577050847281	7,39036E+14	1,8353E+14	0.112837172176505	0.257775433950057	-4,59097E+14	8,02557E+14	7,77916E+14	7,69732E+14	6,66255E+14	7,3778E+14	6,79978E+14
TC0400003241.mm.2	Gpx7	glutathione peroxidase 7	-0.589353015837595	6,59029E+14	-1,83517E+14	0.112859478807832	0.257775433950057	-4,59114E+14	6,02819E+14	6,23642E+14	6,80952E+14	7,02429E+14	6,5124E+14	6,93092E+14
TC1000001255.mm.2	Ccdc59	coiled-coil domain containing 59	0.695644868917588	8,24078E+14	1,83512E+13	0.112867556530269	0.257775433950057	-4,59121E+14	8,6122E+14	8,82947E+14	8,18646E+14	7,76616E+14	8,40019E+14	7,65019E+13
TC1900001253.mm.2	Gda	guanine deaminase	0.737069505486494	9,66017E+13	1,83489E+14	0.11290379705647	0.257775433950057	-4,5915E+14	1,03363E+14	1,00446E+14	9,79489E+14	8,87858E+14	9,60235E+14	9,30436E+13
TC0400002589.mm.2	Tstd2	thiosulfate sulfurtransferase (rhodanese)-like domain containing 2	1,01803E+14	6,26359E+14	1,83477E+14	0.112922424214087	0.257775433950057	-4,59165E+14	7,10373E+14	6,82118E+14	6,71838E+14	5,04939E+13	6,03438E+14	5,85447E+14
TC0200003670.mm.2	Galnt3	polypeptide N-acetylgalactosaminyltransferase 3	-0.989093754371258	4,76689E+14	-1,83476E+14	0.112925365095287	0.257775433950057	-4,59167E+13	4,0797E+14	4,34422E+14	4,52278E+14	5,2525E+14	5,92103E+14	4,48108E+14
TC1100001023.mm.2	Olfr384	olfactory receptor 384	-1,00436E+13	4,56639E+13	-1,83471E+14	0.112932659008474	0.257775433950057	-4,59173E+14	4,31425E+14	3,56295E+14	4,08783E+14	5,54736E+14	5,09929E+14	4,78664E+14
TC0500001629.mm.2	Tfr2	transferrin receptor 2	-0.691713355157083	5,54944E+14	-1,83457E+14	0.112954578504359	0.257775433950057	-4,5919E+14	5,12619E+14	5,13461E+13	5,5708E+14	5,82083E+14	5,40258E+14	6,24164E+14
TC0200001523.mm.2	Pamr1	peptidase domain containing associated with muscle regeneration 1	-0.734035902973265	6,22567E+14	-1,83453E+14	0.112961728984134	0.257775433950057	-4,59196E+14	5,67773E+14	5,66892E+14	6,10302E+14	7,08964E+14	6,33026E+14	6,48446E+14
TC1100003609.mm.2	Ube2z	ubiquitin-conjugating enzyme E2Z	0.797129948162503	8,02535E+14	1,83412E+14	0.113027993091286	0.257890123574043	-4,59249E+14	8,56875E+14	8,61065E+14	8,27754E+14	7,11E+14	7,8183E+14	7,76683E+13
TC0500002411.mm.2	Lgi2	leucine-rich repeat LGI family, member 2	0.591940409938522	7,26223E+14	1,83397E+14	0.11305115195838	0.25789243176433	-4,59267E+14	7,73728E+14	7,61303E+14	7,28445E+14	6,77218E+13	7,07606E+14	7,09038E+13
TC0800002587.mm.2	Nacc1	nucleus accumbens associated 1, BEN and BTB (POZ) domain containing	0.685681242837572	7,41542E+14	1,83385E+13	0.113070720107661	0.25789243176433	-4,59283E+14	7,73843E+13	7,88236E+14	7,73216E+14	6,8901E+14	7,16499E+14	7,08445E+14
TC0600002496.mm.2	Tcf7l1	transcription factor 7 like 1 (T cell specific, HMG box)	-0.670731142098943	6,85834E+14	-1,83373E+14	0.113089513110191	0.25789243176433	-4,59298E+14	6,59253E+14	6,36514E+14	6,80452E+14	7,0887E+14	6,78677E+13	7,51236E+14
TC0200000048.mm.2	Ccdc3	coiled-coil domain containing 3	0.675794572372975	5,63475E+14	1,83351E+14	0.113125054219509	0.25789243176433	-4,59326E+14	6,33692E+14	5,66001E+14	5,40145E+13	5,76481E+14	5,1684E+14	5,47694E+14
TC0100002054.mm.2	Rims1	regulating synaptic membrane exocytosis 1	0.717093752694973	8,63301E+14	1,83338E+14	0.113146267895682	0.25789243176433	-4,59343E+14	9,08061E+14	9,31679E+14	8,57061E+14	7,86686E+14	8,7794E+14	8,1838E+13
TC1000002932.mm.2	Cpsf6	cleavage and polyadenylation specific factor 6	0.853118312596016	8,09648E+14	1,83325E+14	0.1131668550728	0.25789243176433	-4,59359E+13	8,40781E+13	8,49719E+14	8,43796E+14	8,03714E+14	8,22284E+14	6,97592E+14
TC0200002171.mm.2	Cfap61	cilia and flagella associated protein 61	0.753558578158348	6,39014E+14	1,83325E+14	0.113168114084463	0.25789243176433	-4,5936E+14	6,74931E+14	7,21909E+14	5,72027E+14	6,19086E+14	5,82163E+14	6,63965E+14
TC0500003093.mm.2	Rnf10	ring finger protein 10	1,2634E+14	9,0195E+14	1,83317E+14	0.11318019261841	0.25789243176433	-4,5937E+14	9,95251E+14	9,91466E+14	9,38427E+14	7,52521E+14	9,30058E+14	8,03978E+14
TC1700001940.mm.2	Msh5	mutS homolog 5	-1,22936E+14	5,80482E+14	-1,83313E+14	0.113186017468076	0.25789243176433	-4,59374E+14	4,74103E+14	5,39538E+13	5,01546E+13	7,08194E+14	6,37513E+14	6,22001E+14
TC0200002677.mm.2	Rae1	ribonucleic acid export 1	1,28851E+14	8,23541E+14	1,83312E+14	0.113189057123897	0.25789243176433	-4,59377E+14	9,09509E+13	8,90933E+14	8,68526E+14	7,29538E+13	8,62563E+14	6,80176E+14
TC0400003440.mm.2	Nfyc	nuclear transcription factor-Y gamma	0.949668823494038	7,88562E+14	1,83296E+13	0.113214247597629	0.257913356643658	-4,59397E+14	8,25362E+14	8,3767E+14	8,56254E+14	7,38985E+14	7,86557E+14	6,86541E+14
TC1700001755.mm.2	Tead3	TEA domain family member 3	-0.556728645944131	6,48472E+13	-1,83252E+14	0.113285031881865	0.257978900143464	-4,59453E+14	6,20514E+14	6,1201E+14	6,41562E+13	6,72876E+14	6,64865E+14	6,79005E+14
TC0500002879.mm.2	Klhl8	kelch-like 8	-0.535027136951175	7,70307E+14	-1,8325E+14	0.113287868349307	0.257978900143464	-4,59455E+14	7,38193E+14	7,44948E+14	7,74774E+14	7,73783E+14	7,86427E+14	8,03719E+14
TC0400002732.mm.2	Txndc8	thioredoxin domain containing 8	-0.799425891566696	5,02932E+14	-1,83232E+14	0.113316549643913	0.257978900143464	-4,59478E+14	4,72947E+14	4,27361E+14	4,84085E+14	5,73008E+14	4,98958E+13	5,61235E+14

TC0400000354.mm.2	Nfx1	nuclear transcription factor, X-box binding 1	0.536599830536537	7,87801E+14	1,83229E+14	0.113321554117671	0.257978900143464	-4,59482E+14	8,14619E+14	8,26309E+13	7,8437E+14	7,67898E+13	7,67888E+14	7,65721E+14
TC0200003300.mm.2	Dnm1	dynamin 1	1,36939E+14	8,49016E+14	1,83228E+14	0.113323071756683	0.257978900143464	-4,59483E+14	9,50068E+14	9,46183E+14	9,0668E+14	6,68791E+14	8,45151E+14	7,77223E+14
TC1100003795.mm.2	Plekhh3	pleckstrin homology domain containing, family H (with MyTH4 domain) member 3	-0.777438251491922	6,87128E+14	-1,83206E+14	0.11335941262151	0.258025175246559	-4,59512E+14	6,31691E+14	6,27728E+14	6,78369E+14	7,70072E+14	6,75371E+14	7,39536E+14
TC0800003080.mm.2	Cbfa2t3	CBFA2/RUNX1 translocation partner 3	-0.702764116096864	5,85877E+14	-1,83192E+14	0.113381883970902	0.25803987237615	-4,5953E+14	5,00324E+14	5,77428E+14	5,85312E+14	6,33897E+14	6,08138E+14	6,10166E+14
TC1900001155.mm.2	Olfr1428	olfactory receptor 1428	-1,00675E+14	4,24737E+14	-1,83149E+14	0.113451643202867	0.258162170163508	-4,59585E+14	3,50107E+14	3,56453E+14	4,10342E+14	5,2361E+14	3,91959E+14	5,15952E+14
TC1600001527.mm.2	Umps	uridine monophosphate synthetase	1,05449E+14	6,1969E+14	1,83121E+14	0.113496550425954	0.258227890004716	-4,59621E+14	6,81711E+14	6,96241E+14	6,06569E+14	5,66564E+14	4,9661E+14	6,70444E+14
TC1000001962.mm.2	Rnf217	ring finger protein 217	-0.556796475639807	6,65818E+14	-1,83106E+14	0.113521035935932	0.258236189708173	-4,5964E+14	6,37023E+14	6,49392E+14	6,66528E+12	6,4419E+14	7,11364E+14	6,8641E+13
TC0700004623.mm.2	Dctn5	dynactin 5	0.752888252476508	8,13925E+14	1,83091E+14	0.113543931885385	0.258236189708173	-4,59658E+14	8,78365E+14	8,75674E+14	8,14163E+14	7,11884E+14	8,14591E+14	7,88871E+14
TC1200001109.mm.2	Eml1	echinoderm microtubule associated protein like 1	0.660395815527923	6,79157E+14	1,83084E+14	0.113556201765302	0.258236189708173	-4,59668E+14	7,01459E+14	7,2009E+14	6,99034E+14	6,64889E+14	6,78657E+14	6,10812E+14
TC0700001827.mm.2	Cdipt	CDP-diacylglycerol--inositol 3-phosphatidyltransferase (phosphatidylinositol synthase)	0.95649851613039	8,89461E+14	1,83079E+14	0.113564304615659	0.258236189708173	-4,59674E+14	9,58086E+14	9,77041E+13	9,02758E+14	7,55054E+14	8,95956E+13	8,47871E+14
TC0600002139.mm.2	Prss37	protease, serine 37	-0.722233651819602	4,76274E+13	-1,83061E+14	0.113593036653564	0.258236365297168	-4,59697E+14	3,99588E+14	4,65953E+13	4,67826E+14	5,14287E+14	4,78355E+14	5,31633E+13
TC0600002221.mm.2	Zfp467	zinc finger protein 467	0.550107645415188	6,75056E+14	1,83059E+14	0.113596435004426	0.258236365297168	-4,597E+14	6,94411E+14	7,00008E+14	6,88485E+14	6,83033E+14	6,26905E+14	6,57492E+14
TC0100001855.mm.2	Lpgat1	lysophosphatidylglycerol acyltransferase 1	1,51132E+14	7,91258E+14	1,83012E+14	0.113672141242897	0.258372014649006	-4,5976E+14	8,9841E+14	9,0398E+13	8,13946E+14	6,31088E+14	8,54493E+14	6,45633E+14
TC1200000503.mm.2	Mia2	MIA SH3 domain ER export factor 2	1,1838E+14	7,01834E+14	1,82989E+14	0.113709078662523	0.258419518263644	-4,59789E+14	7,91545E+14	7,97696E+14	7,18345E+14	5,50939E+14	7,37774E+14	6,14707E+14
TC0X00000210.mm.2	Rbm10	RNA binding motif protein 10	0.566645012018966	7,06977E+14	1,8297E+14	0.113739862175211	0.258426755320234	-4,59813E+13	7,43105E+14	7,59028E+14	6,56542E+14	6,94382E+14	6,91572E+14	6,97232E+13
TC0400002386.mm.2	Orc3	origin recognition complex, subunit 3	2,03273E+14	7,49303E+14	1,82967E+14	0.113744339895184	0.258426755320234	-4,59817E+14	8,74223E+14	8,7876E+14	8,68703E+14	5,27695E+14	7,62408E+14	5,84029E+13
TC1500000021.mm.2	Prkaa1	protein kinase, AMP-activated, alpha 1 catalytic subunit	1,05792E+14	7,11738E+14	1,82952E+14	0.113770075261703	0.258439725893103	-4,59837E+14	7,99341E+14	7,82599E+13	7,47216E+14	5,70916E+14	6,69166E+14	7,0119E+14
TC1100002126.mm.2	Ewsr1	Ewing sarcoma breakpoint region 1	0.618785541238598	8,91376E+14	1,82944E+14	0.113782127194543	0.258439725893103	-4,59847E+14	9,39477E+14	9,42497E+14	8,60364E+14	8,47705E+14	9,1044E+14	8,47776E+14
TC1800000641.mm.2	Htr4	5 hydroxytryptamine (serotonin) receptor 4	-0.745212364414291	7,04224E+14	-1,82898E+14	0.113857496887963	0.258539776864793	-4,59906E+14	6,57946E+14	6,47107E+14	7,81526E+14	6,84672E+14	7,44624E+13	7,09471E+14
TC1300000037.mm.2	Adarb2	adenosine deaminase, RNA-specific, B2	0.647301023625689	5,81167E+14	1,82897E+14	0.113858267028646	0.258539776864793	-4,59907E+14	6,60313E+14	6,01697E+14	5,23603E+14	5,68838E+14	5,64707E+13	5,67843E+14
TC0100002210.mm.2	Mitd1	MIT, microtubule interacting and transport, domain containing 1	0.956708406299351	5,4015E+14	1,82831E+14	0.113965680869504	0.258725848235341	-4,59992E+14	6,40696E+14	6,03576E+14	5,45439E+14	3,98259E+14	5,06725E+14	5,46206E+14
TC0300002053.mm.2	P2ry13	purinergic receptor P2Y, G-protein coupled 13	0.648366242938137	6,30364E+14	1,82825E+13	0.11397509204985	0.258725848235341	-4,59999E+14	6,82199E+14	6,59473E+14	6,43931E+14	5,8458E+14	6,25484E+14	5,86515E+14
TC1100004076.mm.2	Jpt1	Jupiter microtubule associated homolog 1	0.577831406409832	8,80851E+14	1,82817E+14	0.113988381842158	0.258725848235341	-4,60009E+14	9,29317E+14	9,08735E+14	8,47997E+14	8,76569E+14	8,76982E+14	8,45505E+14
TC0100002593.mm.2	Fev	FEV (ETS oncogene family)	-0.769945618670469	6,26112E+14	-1,82794E+14	0.114025925485128	0.25877461089322	-4,60039E+14	5,53726E+14	5,81929E+14	6,17483E+14	7,13891E+14	6,2185E+14	6,67793E+14
TC0500001853.mm.2	B3glct	beta-3-glucosyltransferase	0.609678959059288	6,24659E+14	1,82746E+14	0.114103610871308	0.258856439326326	-4,601E+14	6,54911E+14	6,6052E+14	6,29711E+14	6,09314E+14	6,25251E+13	5,68244E+14
TC0100001722.mm.2	Pycr2	pyrroline-5-carboxylate reductase family, member 2	-0.721655962126983	7,29392E+14	-1,82743E+14	0.114108205731153	0.258856439326326	-4,60104E+14	6,57897E+14	6,96765E+14	7,27636E+14	7,95059E+14	7,20417E+14	7,78577E+14
TC1100002855.mm.2	Olfr222	olfactory receptor 222	-0.869304677035839	5,597E+14	-1,82733E+13	0.114123616302625	0.258856439326326	-4,60116E+13	5,19317E+14	5,32738E+14	5,21337E+14	5,58891E+14	5,47961E+14	6,77955E+14
TC1200002121.mm.2	Ism2	isthmin 2	-0.833689407947704	6,89284E+14	-1,82732E+14	0.11412624247342	0.258856439326326	-4,60118E+14	6,2499E+14	6,50308E+14	6,47695E+14	7,70678E+14	7,11177E+14	7,30858E+13
TC0300001161.mm.2	Vav3	vav 3 oncogene	-0.598387678055257	4,90251E+14	-1,82712E+14	0.114157869298732	0.258880720921743	-4,60143E+14	4,39231E+14	4,85734E+14	4,96524E+14	4,75373E+14	5,34228E+14	5,10415E+14
TC1100003636.mm.2	Tbx21	T-box 21	-0.788029311888318	6,11272E+14	-1,82705E+14	0.114169081044492	0.258880720921743	-4,60152E+14	5,52145E+14	5,48471E+14	6,08139E+14	7,00657E+14	6,63517E+14	5,94705E+13
TC1300002753.mm.2	B020031M17Rik	RIKEN cDNA B020031M17 gene	-0.969987304600711	4,99332E+14	-1,8268E+14	0.114209648196944	0.258926126511883	-4,60184E+14	3,88777E+14	4,5516E+13	4,87425E+14	6,26694E+14	4,97519E+14	5,40416E+14
TC1400002334.mm.2	Sorbs3	sorbin and SH3 domain containing 3	0.563890092385953	6,50275E+12	1,82673E+14	0.114221907440753	0.258926126511883	-4,60193E+13	6,60014E+14	6,87529E+14	6,61324E+14	6,58018E+14	6,02639E+14	6,32126E+14
TC0100000925.mm.2	Agxt	alanine-glyoxylate aminotransferase	-0.789125049926421	5,76211E+14	-1,82663E+14	0.114237313558802	0.258926126511883	-4,60206E+14	5,36326E+13	5,29745E+14	5,6322E+14	6,04076E+14	5,57927E+14	6,65969E+14
TC0700003982.mm.2	Hpx	hemopexin	-0.769897000615395	5,41669E+14	-1,8264E+14	0.114275259230943	0.258958331930766	-4,60235E+14	4,74542E+14	4,89662E+14	5,29666E+14	6,37962E+14	5,67448E+14	5,50735E+13
TC0300003218.mm.2	Itga10	integrin, alpha 10	-1,09876E+14	5,46763E+14	-1,82635E+14	0.11428366526157	0.258958331930766	-4,60242E+14	5,0408E+14	4,86873E+14	4,67385E+14	6,11535E+14	5,44406E+14	6,66299E+14
TC1000003052.mm.2	Srgap1	SLIT-ROBO Rho GTPase activating protein 1	0.75515018732561	6,62521E+14	1,82616E+14	0.11431346911161	0.258977624727818	-4,60265E+14	7,16929E+14	7,05009E+14	6,72932E+14	6,09347E+14	5,93059E+14	6,77849E+14
TC0400001547.mm.2	Pum1	pumilio RNA-binding family member 1	0.649923874961232	7,51337E+14	1,8261E+14	0.114324324749517	0.258977624727818	-4,60274E+14	7,85152E+14	7,67549E+14	7,79687E+13	7,52921E+14	7,45444E+14	6,77272E+14
TC1100003556.mm.2	Wfikkn2	WAP, follistatin/kazal, immunoglobulin, kunitz and netrin domain containing 2	-1,05568E+14	5,95404E+14	-1,82584E+14	0.11436648392565	0.259036710077875	-4,60307E+14	5,41503E+14	5,30285E+14	5,72102E+14	6,4561E+14	5,59534E+14	7,2339E+14
TC0200002366.mm.2	Trp53inp2	transformation related protein 53 inducible nuclear protein 2	-0.682014101798394	9,43773E+14	-1,8253E+14	0.114454229266151	0.259149567126948	-4,60376E+13	8,95114E+14	9,03283E+14	1,00511E+14	9,24325E+14	9,8104E+14	9,5376E+14
TC1100000959.mm.2	4930544D05Rik	RIKEN cDNA 4930544D05 gene	-0.629067575798631	8,55266E+14	-1,82526E+14	0.114461371885741	0.259149567126948	-4,60382E+13	8,09954E+14	8,04787E+14	8,64126E+14	9,12175E+14	8,46175E+13	8,94379E+14
TC1500002269.mm.2	Krt5	keratin 5	-0.612764653839561	5,29366E+13	-1,82524E+14	0.114464560866536	0.259149567126948	-4,60384E+14	4,97623E+14	4,80415E+14	5,28375E+14	5,69189E+14	5,2841E+14	5,72181E+13
TC0500002683.mm.2	Tmprss11g	transmembrane protease, serine 11g	-1,1898E+14	4,98569E+14	-1,82451E+14	0.114582265076521	0.259368431801656	-4,60477E+14	4,09889E+14	4,19232E+14	4,63742E+13	6,31467E+14	4,70577E+13	5,96504E+14
TC0700000030.mm.2	Pira6	paired-Ig-like receptor A6	-0.667449226474941	5,06538E+14	-1,82435E+14	0.114609125064609	0.259368431801656	-4,60498E+14	4,45783E+14	4,75204E+14	5,11187E+14	5,52578E+14	4,95543E+14	5,58933E+14
TC0200001773.mm.2	Chp1	calcineurin-like EF hand protein 1	0.791359245137391	8,77246E+14	1,82432E+13	0.114613073485667	0.259368431801656	-4,60501E+14	9,29762E+13	9,19398E+14	9,19434E+14	8,03993E+14	8,51548E+14	8,3934E+14
TC0300003045.mm.2	Pdlim5	PDZ and LIM domain 5	0.535073546383782	7,34287E+14	1,82417E+14	0.114637409964365	0.259368431801656	-4,6052E+14	7,77909E+14	7,7615E+14	7,00725E+14	7,03896E+14	7,33947E+14	7,13097E+14
TC1100002821.mm.2	Olfr331	olfactory receptor 331	-1,00982E+14	4,83884E+14	-1,82409E+13	0.11465050697629	0.259368431801656	-4,6053E+14	3,84124E+14	4,4963E+14	4,56075E+14	5,7776E+14	4,68613E+14	5,67104E+14
TC0200003201.mm.2	Abo	ABO blood group (transferase A, alpha 1-3-N-acetylgalactosaminyltransferase, transferase B, alpha 1-3-galactosyltransferase)	-0.774254521500542	4,58518E+14	-1,824E+14	0.114665410096616	0.259368431801656	-4,60542E+14	3,85644E+14	4,15403E+14	4,61651E+14	5,3251E+14	4,37176E+14	5,18722E+14
TC0200000969.mm.2	B3galt1	UDP-Gal:betaGlcNAc beta 1,3-galactosyltransferase, polypeptide 1	1,11633E+14	7,72746E+14	1,8239E+14	0.114682374828951	0.259368431801656	-4,60555E+14	8,39905E+14	8,50705E+14	7,99653E+14	6,78867E+14	8,09264E+14	6,58079E+14
TC0700002770.mm.2	Upk1a	uroplakin 1A	-0.668214802922033	5,4877E+14	-1,82382E+14	0.114695488756942	0.259368431801656	-4,60565E+14	4,9976E+14	5,01597E+14	5,39659E+14	6,16604E+14	5,54057E+14	5,80942E+14
TC1200001324.mm.2	Cdca7l	cell division cycle associated 7 like	-0.632146632314561	6,26417E+13	-1,82375E+14	0.11470610345799	0.259368431801656	-4,60574E+14	5,80169E+14	6,12917E+14	5,9969E+14	6,46211E+14	6,39772E+14	6,79743E+14
TC0500000605.mm.2	Klhl5	kelch-like 5	1,08147E+13	7,42896E+14	1,82356E+14	0.114737689314767	0.25940028487185	-4,60598E+14	8,21285E+14	8,1195E+14	7,77717E+14	6,29486E+14	7,645E+14	6,52441E+14
TC1000000710.mm.2	Specc1l	sperm antigen with calponin homology and coiled-coil domains 1-like	0.870952870991125	6,59755E+14	1,82347E+14	0.114752388168966	0.25940028487185	-4,6061E+14	7,19115E+14	7,16924E+14	6,98704E+14	5,61937E+14	6,32751E+14	6,29097E+14
TC0600001088.mm.2	Lsm3	LSM3 homolog, U6 small nuclear RNA and mRNA degradation associated	-0.656981810108584	7,66453E+14	-1,82304E+14	0.114822288565362	0.259465466148495	-4,60665E+14	7,25391E+14	7,26591E+13	7,43424E+14	8,19936E+14	7,99268E+13	7,84109E+12
TC0300002946.mm.2	Rpl34/Rpl34-ps1	ribosomal protein L34/ribosomal protein L34, pseudogene 1	1,09879E+13	9,08169E+14	1,82295E+14	0.114836510786068	0.259465466148495	-4,60676E+13	1,00081E+14	9,84412E+14	9,4061E+14	7,57712E+14	9,07166E+14	8,58301E+14
TC0X00001272.mm.2	Cstf2/Mir3112	cleavage stimulation factor, 3' pre-RNA subunit 2/microRNA 3112	0.967669279375577	7,53966E+14	1,82293E+14	0.114841252365483	0.259465466148495	-4,60679E+14	8,20575E+13	8,19219E+14	7,80346E+14	6,57398E+14	7,74378E+14	6,71882E+14
TC0100000440.mm.2	Casp8	caspase 8	0.99915971574315	6,76917E+14	1,8229E+14	0.114845634243845	0.259465466148495	-4,60683E+14	7,35751E+14	7,92803E+13	6,5689E+14	5,47332E+14	7,2085E+13	6,07873E+14

TC0X00001367.mm.2	Pwwp3b	PWWP domain containing 3B	0.719010480206831	5,4134E+14	1,82266E+14	0.114884531284883	0.259516956763397	-4,60713E+14	6,26998E+14	5,45627E+13	5,36173E+14	5,10421E+14	5,37391E+14	4,91431E+14
TC0900003112.mm.2	Klhl18	kelch-like 18	0.590063070255462	6,68303E+14	1,82243E+14	0.114921528027529	0.259564140889764	-4,60742E+14	6,96444E+14	7,06447E+14	6,56258E+14	6,65789E+14	6,68295E+14	6,16583E+14
TC1900001205.mm.2	Cep78	centrosomal protein 78	-0.615052510270644	6,48015E+14	-1,8218E+14	0.115025766648277	0.259757447593838	-4,60824E+13	6,21049E+14	6,13249E+14	6,75232E+14	6,21254E+14	6,62322E+14	6,94987E+14
TC1100000268.mm.2	Gm12070	glyceraldehyde-3-phosphate dehydrogenase pseudogene	1,14422E+14	1,01086E+14	1,82165E+14	0.115050059351405	0.259757447593838	-4,60843E+14	1,10818E+12	1,07494E+13	1,05986E+14	8,67887E+14	1,00043E+14	9,53854E+14
TC0800002518.mm.2	Smad1	SMAD family member 1	0.704827641815141	7,58429E+14	1,82157E+14	0.115062574260709	0.259757447593838	-4,60852E+13	7,98458E+14	7,81948E+14	7,93532E+13	7,37195E+14	7,55746E+14	6,83694E+14
TC1200002497.mm.2	D430020J02Rik	RIKEN cDNA D430020J02 gene	-1,00537E+14	5,98792E+14	-1,82152E+14	0.115071598145895	0.259757447593838	-4,60859E+14	5,22372E+14	5,57406E+14	5,74607E+14	6,57514E+14	5,68903E+14	7,11949E+14
TC0600000441.mm.2	Prss2	protease, serine 2	-0.921495261319496	4,73811E+14	-1,82116E+14	0.115129283277929	0.259851259484139	-4,60904E+14	4,39962E+14	3,87496E+14	4,32636E+14	5,71015E+14	4,95762E+13	5,15995E+14
TC0900000776.mm.2	Tle3	transducin-like enhancer of split 3	0.564423606990969	7,66304E+14	1,82068E+13	0.115208156131028	0.259992859907459	-4,60966E+14	7,96565E+14	8,15898E+14	7,27109E+14	7,58674E+14	7,59437E+14	7,40142E+13
TC0X00002982.mm.2	Pcdh19	protocadherin 19	1,03692E+14	6,95476E+14	1,82053E+13	0.115233086463573	0.260012704409403	-4,60985E+14	7,68357E+14	7,40918E+14	7,67056E+14	5,94632E+14	6,63429E+14	6,38461E+14
TC1400001744.mm.2	Rgr	retinal G protein coupled receptor	-0.59801852484288	5,1397E+14	-1,82024E+14	0.115279952512434	0.260029997420605	-4,61022E+14	4,56765E+13	5,01993E+14	5,19553E+14	5,27146E+14	5,22144E+13	5,56218E+14
TC0900001946.mm.2	Jam3	junction adhesion molecule 3	0.860020058466058	7,27966E+14	1,82015E+14	0.115295792775028	0.260029997420605	-4,61034E+14	7,80404E+14	7,86693E+14	7,01288E+14	7,04316E+14	7,64716E+14	6,30376E+14
TC1500000317.mm.2	Zfpm2	zinc finger protein, multitype 2	-0.607366798761586	6,67262E+13	-1,82011E+14	0.115301553713341	0.260029997420605	-4,61039E+14	6,12398E+14	6,56073E+14	6,91967E+14	6,53188E+14	7,03524E+14	6,8642E+14
TC0700002832.mm.2	Lsm14a	LSM14A mRNA processing body assembly factor	1,16609E+13	7,92735E+14	1,82002E+14	0.115316865252168	0.260029997420605	-4,61051E+14	8,64735E+14	8,75204E+14	8,33242E+14	6,76509E+14	8,1951E+14	6,87211E+14
TC0300002394.mm.2	Sprr4	small proline-rich protein 4	-0.907229990229883	5,01889E+14	-1,81991E+14	0.115334229184636	0.260029997420605	-4,61064E+14	4,50315E+14	4,56402E+14	4,82384E+14	5,34067E+14	4,75827E+14	6,12337E+14
TC0600003383.mm.2	St8sia1	ST8 alpha-N-acetyl-neuraminide alpha-2,8-sialyltransferase 1	1,18414E+14	6,71561E+14	1,81989E+14	0.115337577829397	0.260029997420605	-4,61067E+14	7,57974E+14	7,0567E+14	7,01207E+14	6,37697E+14	7,09668E+11	5,17149E+14
TC0X00001564.mm.2	Nbdy	negative regulator of P-body association	1,33772E+14	7,3013E+13	1,81949E+14	0.115404137900387	0.26014309151657	-4,61119E+14	8,29732E+14	8,08662E+14	8,01013E+14	5,70521E+14	7,2194E+14	6,4891E+14
TC1100001998.mm.2	Ccdc40	coiled-coil domain containing 40	-0.538913331894751	1,03262E+13	-1,8193E+14	0.115434376714689	0.26014309151657	-4,61142E+14	9,86645E+14	1,00874E+14	1,04673E+14	1,05044E+14	1,04515E+14	1,05802E+14
TC1100003787.mm.2	Stat5b	signal transducer and activator of transcription 5B	-0.612488526982477	7,42425E+14	-1,81929E+14	0.115436176028267	0.26014309151657	-4,61144E+14	7,13713E+14	6,86142E+14	7,45851E+14	7,86492E+14	7,41507E+14	7,80846E+13
TC0200005142.mm.2	1700025C18Rik	RIKEN cDNA 1700025C18 gene	-0.645343297043498	5,20459E+14	-1,81887E+14	0.115505309218405	0.26026248740542	-4,61197E+14	5,04937E+14	4,86997E+14	4,73903E+14	5,35916E+14	5,41757E+14	5,79245E+14
TC1600001761.mm.2	Cmss1	cms small ribosomal subunit 1	-0.842956903301816	7,81959E+14	-1,81871E+14	0.115532227890112	0.260286743287665	-4,61218E+14	6,87952E+14	7,31508E+14	8,06701E+12	8,77543E+14	7,43787E+14	8,44265E+14
TC0900001046.mm.2	Ick	intestinal cell kinase	0.686046882805615	6,34979E+14	1,81842E+14	0.115579120966038	0.260355987155847	-4,61255E+14	6,79159E+14	6,8363E+13	6,53989E+14	5,67515E+14	6,0588E+14	6,19699E+14
TC1100002181.mm.2	Tbrg4/Snora5c	transforming growth factor beta regulated gene 4/small nucleolar RNA, H/ACA box 5C	0.554078389797253	6,8865E+14	1,81827E+14	0.115604328791269	0.260376369836974	-4,61274E+14	7,3326E+14	7,29228E+14	6,41074E+14	6,76666E+14	6,7908E+14	6,72593E+14
TC1500001777.mm.2	Dgat1	diacylglycerol O-acyltransferase 1	-0.951468998751874	5,22237E+13	-1,81773E+14	0.1156921524026	0.260486253177669	-4,61342E+14	4,8512E+14	4,36274E+14	4,8235E+14	6,17994E+14	5,24879E+14	5,86808E+14
TC1200001282.mm.2	Crip1	cysteine-rich protein 1 (intestinal)	-0.630190400825692	6,55685E+14	-1,81767E+14	0.115702690271124	0.260486253177669	-4,61351E+14	6,30974E+14	6,12247E+14	6,27308E+14	6,94323E+14	6,84758E+14	6,845E+14
TC1300002667.mm.2	Map3k1	mitogen-activated protein kinase kinase kinase 1	0.550790459077414	5,83979E+14	1,8176E+14	0.115714541109157	0.260486253177669	-4,6136E+14	6,0338E+14	6,15777E+13	5,82561E+13	5,93158E+14	5,3928E+14	5,69719E+13
TC1000001546.mm.2	Cdk4	cyclin-dependent kinase 4	0.779774126244926	8,1916E+14	1,81758E+14	0.115717780689242	0.260486253177669	-4,61362E+14	8,59878E+13	8,75918E+14	8,51498E+14	7,47822E+14	8,12551E+14	7,67291E+14
TC1100000644.mm.2	Slc36a1	solute carrier family 36 (proton/amino acid symporter), member 1	0.545787861984383	7,43488E+14	1,81725E+14	0.115772034039895	0.260571977159495	-4,61404E+14	7,701E+14	7,69273E+14	7,43761E+14	7,47581E+14	7,30665E+14	6,9955E+14
TC0300002311.mm.2	Slc25a44	solute carrier family 25, member 44	0.826187431425897	8,47726E+14	1,81683E+14	0.115840393846667	0.260689422632869	-4,61457E+13	8,9485E+14	8,87422E+14	8,7403E+14	8,13018E+14	8,63745E+13	7,53289E+14
TC0100003838.mm.2	G0s2	G0/G1 switch gene 2	-0.78393786077512	7,4237E+14	-1,81658E+14	0.115881403857996	0.260726828959282	-4,61489E+14	6,813E+14	6,85691E+14	7,27969E+14	8,3548E+14	7,40936E+14	7,82842E+14
TC0600001355.mm.2	Bcl2l13	BCL2-like 13 (apoptosis facilitator)	0.53520835563253	6,89621E+13	1,81654E+14	0.115889378080207	0.260726828959282	-4,61495E+14	7,21034E+14	7,21237E+14	6,7231E+14	6,87917E+13	6,58909E+14	6,7632E+14
TC1100001155.mm.2	Spag5	sperm associated antigen 5	0.542527437992829	5,14724E+14	1,8162E+14	0.115944815659427	0.26081513537908	-4,61538E+14	5,25584E+14	5,05608E+14	5,7752E+14	5,56948E+14	4,58336E+14	4,64351E+14
TC1100004286.mm.2	Drg1	developmentally regulated GTP binding protein 1	1,09232E+14	6,29739E+14	1,81586E+14	0.115999856229801	0.260840822115874	-4,61581E+14	7,23651E+14	6,99881E+14	6,22137E+14	5,27697E+14	6,83832E+14	5,21236E+14
TC0X00003038.mm.2	Morf4l2	mortality factor 4 like 2	1,21557E+14	8,71834E+13	1,81585E+14	0.116001946474674	0.260840822115874	-4,61583E+14	9,51841E+14	9,54116E+14	9,15856E+14	7,46349E+14	8,99022E+14	7,63821E+14
TC1700002637.mm.2	Haao	3-hydroxyanthranilate 3,4-dioxygenase	-0.862677410859572	6,87656E+13	-1,81584E+14	0.11600479931002	0.260840822115874	-4,61585E+13	6,07295E+14	6,30074E+14	6,87732E+14	7,90928E+14	6,624E+14	7,47505E+14
TC0400001327.mm.2	Svbp	small vasohibin binding protein	-0.63542331410926	5,00612E+14	-1,81528E+14	0.116095583419073	0.261008530156485	-4,61655E+14	4,63537E+14	4,62033E+14	4,99461E+14	5,25985E+14	4,93029E+14	5,59626E+14
TC1600000982.mm.2	Hunk	hormonally upregulated Neu-associated kinase	0.717945019468187	6,59507E+14	1,8147E+14	0.116191686066218	0.261170679128807	-4,61729E+14	7,13087E+14	6,68922E+14	6,62828E+14	6,73785E+13	6,62344E+14	5,76075E+13
TC0200001664.mm.2	Katnbl1/Slc12a6	katanin p80 subunit B like 1/solute carrier family 12, member 6	0.685913223207308	5,5269E+13	1,81465E+14	0.116200124041111	0.261170679128807	-4,61736E+14	5,79937E+14	5,73927E+13	5,71833E+14	5,7376E+14	5,52266E+14	4,64415E+12
TC0300002035.mm.2	Pfn2	profilin 2	0.999424453492695	9,2525E+14	1,81443E+14	0.116236273865496	0.26121549243999	-4,61764E+14	9,92121E+14	9,9874E+14	9,56829E+14	8,12833E+13	9,39149E+14	8,51827E+14
TC0700003647.mm.2	Tlnrd1	talin rod domain containing 1	0.679833163685873	6,22741E+14	1,81419E+14	0.116276535679789	0.261269532758115	-4,61795E+14	6,4838E+14	6,54765E+14	6,68062E+14	5,98057E+14	6,08594E+14	5,58585E+14
TC0200003283.mm.2	Fnbp1	formin binding protein 1	1,07358E+14	7,69126E+14	1,81409E+14	0.116292751053771	0.261269533983465	-4,61807E+14	8,65022E+14	8,42794E+14	7,91518E+14	6,22319E+14	7,82964E+13	7,10137E+14
TC1100003707.mm.2	Krt12	keratin 12	-0.650270989738625	6,7634E+14	-1,81351E+14	0.116388520604202	0.26143054573717	-4,61881E+14	6,44131E+14	6,20432E+14	6,80813E+14	7,18048E+14	7,28813E+14	6,65804E+14
TC1600000553.mm.2	Cd80	CD80 antigen	-0.985772820163798	4,58462E+14	-1,81332E+14	0.116420544113398	0.26143054573717	-4,61906E+14	4,14632E+14	4,03156E+14	3,7973E+14	5,38313E+14	4,84496E+14	5,30446E+14
TC0200003401.mm.2	Strbp	spermatid perinuclear RNA binding protein	1,32102E+14	9,94156E+14	1,81325E+14	0.116431147441141	0.26143054573717	-4,61914E+14	1,07898E+14	1,07359E+14	1,03588E+13	8,88123E+14	1,03877E+14	8,496E+14
TC1700002191.mm.2	Rpl7l1	ribosomal protein L7-like 1	0.783833255636455	7,56444E+14	1,81306E+14	0.116464055133807	0.26143054573717	-4,61939E+14	7,92847E+14	8,19112E+14	7,5429E+14	7,17221E+14	7,80881E+14	6,74312E+14
TC0X00000695.mm.2	Ssr4	signal sequence receptor, delta	0.722886185260229	6,8555E+14	1,81302E+14	0.116469451669812	0.26143054573717	-4,61944E+14	7,6926E+14	7,30303E+14	6,59493E+13	5,9926E+14	6,43277E+14	7,11709E+14
TC0200001945.mm.2	Ttl	tubulin tyrosine ligase	-0.566236425197294	7,79832E+14	-1,81297E+13	0.116477658745468	0.26143054573717	-4,6195E+14	7,4969E+14	7,41258E+14	7,70851E+14	8,13001E+14	7,97619E+14	8,06576E+14
TC0600002763.mm.2	Magi1	membrane associated guanylate kinase, WW and PDZ domain containing 1	1,33991E+14	7,80979E+14	1,81288E+14	0.116493424836458	0.26143054573717	-4,61962E+14	8,87422E+14	8,42212E+14	8,6481E+13	6,29778E+14	7,55102E+14	7,0655E+14
TC0600001934.mm.2	Rnf133	ring finger protein 133	-0.625125759278085	4,91913E+13	-1,81287E+14	0.116494216992049	0.26143054573717	-4,61963E+14	4,31264E+14	4,61359E+14	4,94754E+14	5,46453E+14	5,2538E+14	4,92269E+14
TC0100001582.mm.2	B4galt3	UDP-Gal:betaGlcNAc beta 1,4-galactosyltransferase, polypeptide 3	0.677574309847434	7,01219E+14	1,81265E+14	0.116531160059532	0.261477034123275	-4,61991E+13	7,48138E+14	7,36979E+14	6,96233E+14	6,76359E+13	7,16134E+14	6,33468E+14
TC1700001836.mm.2	Brd4	bromodomain containing 4	0.94632057180249	9,02392E+14	1,81251E+14	0.116553596984275	0.261490964662715	-4,62008E+14	9,59578E+14	9,38661E+14	9,68778E+14	8,38362E+14	8,96307E+14	8,12668E+14
TC1500002067.mm.2	Cpne8	copine VIII	1,80292E+14	5,26377E+13	1,81241E+14	0.116571157545868	0.261493952601499	-4,62022E+14	6,11933E+14	6,67061E+14	6,03538E+14	3,57322E+13	5,74304E+14	3,44107E+14
TC1600001501.mm.2	Fbxo45	F-box protein 45	0.675470603675715	7,44822E+14	1,81208E+14	0.116624840424073	0.261577958484562	-4,62063E+14	7,82585E+14	7,91318E+14	7,70175E+14	6,86045E+14	7,24373E+14	7,14436E+14
TC1100000756.mm.2	Alkbh5	alkB homolog 5, RNA demethylase	0.912481987476429	7,11688E+14	1,81156E+14	0.116711504776159	0.26170908724324	-4,6213E+14	7,88131E+14	7,92479E+14	7,19838E+14	5,71569E+14	6,97453E+14	7,0066E+14
TC1800001500.mm.2	Mppe1	metallophosphoesterase 1	0.60870532939718	6,31787E+14	1,81143E+14	0.116733255757511	0.26170908724324	-4,62147E+14	6,30966E+14	6,88823E+14	6,2793E+14	6,44958E+14	6,07206E+14	5,90842E+14
TC1100004126.mm.2	Mxra7	matrix-remodelling associated 7	-0.689281587117426	7,12329E+14	-1,81138E+14	0.116740953779604	0.26170908724324	-4,62152E+14	6,67965E+14	6,58305E+14	6,97551E+14	7,86026E+14	7,27468E+14	7,36658E+14
TC1200001393.mm.2	Gdf7	growth differentiation factor 7	-0.719316682741124	6,83559E+14	-1,81134E+14	0.116748272767604	0.26170908724324	-4,62158E+14	6,21157E+14	6,55672E+13	6,87268E+14	7,16566E+14	6,66713E+14	7,53979E+14
TC1900001342.mm.2	Plgrkt	plasminogen receptor, C-terminal lysine transmembrane protein	0.592928331551813	6,10095E+14	1,81115E+14	0.116779747758892	0.261743229328004	-4,62182E+14	6,52623E+14	6,68228E+14	5,93442E+14	5,44011E+14	6,12081E+13	5,90185E+14
TC1700001847.mm.2	Zfp871	zinc finger protein 871	1,59626E+14	6,37199E+14	1,81095E+14	0.116812578648995	0.261759255013878	-4,62207E+14	7,69829E+14	8,27776E+14	6,15073E+14	3,32164E+14	6,77627E+14	6,00725E+14
TC1400000036.mm.2	Gm8297	predicted gene 8297	-0.592116870854296	5,00308E+14	-1,81091E+14	0.116819388245814	0.261759255013878	-4,62213E+14	4,61466E+14	4,76152E+13	5,21477E+14	4,86713E+14	5,48685E+13	5,07357E+14
TC0400002454.mm.2	Bag1	BCL2-associated athanogene 1	1,0388E+14	7,61317E+14	1,81076E+14	0.116844843214888	0.261779888587527	-4,62232E+14	8,39687E+14	8,39851E+14	7,68756E+13	6,4783E+14	8,01248E+14	6,70529E+14
TC0900000194.mm.2	Ilf3	interleukin enhancer binding factor 3	0.948027577480472	6,76013E+14	1,81034E+14	0.116913646867543	0.261897621573296	-4,62285E+14	7,76866E+14	7,32506E+14	6,87763E+14	5,39175E+14	6,47572E+14	6,72194E+14
TC0800000621.mm.2	Hpgd	hydroxyprostaglandin dehydrogenase 15 (NAD)	0.673417896941123	5,98893E+14	1,81015E+14	0.116945714232441	0.261913504050171	-4,6231E+14	6,35081E+14	6,50564E+14	5,81955E+14	5,74796E+13	6,16988E+14	5,33973E+14
TC1500001204.mm.2	Ttc23l	tetratricopeptide repeat domain 23-like	-0.809622500160673	6,21552E+14	-1,81011E+14	0.11695324654881	0.261913504050171	-4,62315E+13	5,81287E+14	5,58787E+14	6,11566E+14	6,75676E+14	5,99774E+14	7,02224E+14
TC0800002290.mm.2	Clcn3	chloride channel, voltage-sensitive 3	1,37096E+14	7,98838E+14	1,80987E+14	0.116991745128461	0.26194478636415	-4,62345E+14	8,97188E+13	9,14925E+13	8,30462E+12	6,12534E+14	8,30953E+14	7,06968E+14
TC1100000711.mm.2	Btnl10	butyrophilin-like 10	-0.816448347504574	4,77382E+14	-1,80983E+14	0.116999728632954	0.26194478636415	-4,62351E+12	4,42285E+14	4,37576E+14	4,25965E+14	5,15315E+14	4,78128E+14	5,65023E+13
TC0300001647.mm.2	Snx16	sorting nexin 16	1,17727E+14	6,26098E+14	1,80919E+14	0.117105130030059	0.262144340118691	-4,62432E+14	6,89639E+14	6,98171E+14	6,86609E+14	5,2981E+14	6,45429E+14	5,06927E+14
TC1500002111.mm.2	Twf1	twinfilin actin binding protein 1	1,55596E+14	7,10603E+14	1,80895E+13	0.117145402932875	0.262198066044925	-4,62463E+14	8,28628E+14	8,35316E+14	7,47785E+13	4,99135E+14	7,5165E+13	6,01102E+14

TC0300001371.mm.2	Ube2d3	ubiquitin-conjugating enzyme E2D 3	1,23158E+14	9,15524E+14	1,80881E+14	0.117168266995812	0.26220420917187	-4,6248E+13	1,00086E+13	1,01137E+14	9,46706E+14	7,68293E+13	9,48273E+14	8,17641E+14
TC0800001952.mm.2	Rab11fip1	RAB11 family interacting protein 1 (class I)	-0.723751470947875	4,92525E+14	-1,80874E+14	0.117180693244376	0.26220420917187	-4,6249E+14	4,37698E+14	4,56422E+14	4,96795E+14	5,25365E+14	4,7251E+14	5,66361E+14
TC1500002154.mm.2	Rpap3	RNA polymerase II associated protein 3	0.60458998714566	6,3106E+14	1,80861E+14	0.117202287911774	0.262216115679313	-4,62506E+14	6,90886E+14	6,36754E+14	6,3379E+14	6,1821E+13	6,15415E+14	5,91307E+14
TC1300001548.mm.2	Olfr1366	olfactory receptor 1366	-0.901169618852173	4,47279E+14	-1,80826E+14	0.117260930538873	0.262310894595704	-4,62551E+14	4,31161E+13	4,00781E+13	3,48488E+14	4,91066E+14	5,0179E+14	5,10386E+14
TC1700000060.mm.2	Dynlt1b	dynein light chain Tctex-type 1B	-1,42403E+14	8,94174E+14	-1,80736E+14	0.117410456658995	0.262608923951471	-4,62665E+14	8,37451E+14	7,18068E+14	1,08333E+14	8,85871E+14	9,83372E+14	8,56952E+13
TC0400001773.mm.2	Atp13a2	ATPase type 13A2	0.536653377474104	7,04207E+14	1,80706E+13	0.117461086741371	0.262685703076158	-4,62704E+14	7,32158E+14	7,49233E+14	6,95303E+13	6,74485E+14	6,8678E+14	6,8728E+14
TC0800001447.mm.2	Cox4i1	cytochrome c oxidase subunit 4I1	0.879246881367758	1,00352E+14	1,80689E+13	0.11748875401307	0.26271111482273	-4,62725E+14	1,06789E+14	1,05519E+14	1,02403E+14	9,26822E+14	1,02417E+13	9,23052E+14
TC1900000118.mm.2	Rasgrp2	RAS, guanyl releasing protein 2	0.674951166946192	7,96349E+14	1,80646E+13	0.117561443897883	0.262785711883269	-4,62781E+13	8,52794E+14	8,49022E+14	7,79095E+14	7,30356E+14	8,18581E+14	7,48246E+14
TC0100001548.mm.2	Tmco1	transmembrane and coiled-coil domains 1	1,01425E+14	7,04145E+14	1,80643E+14	0.117566111721608	0.262785711883269	-4,62784E+13	7,83517E+14	7,59848E+14	7,26981E+14	6,14008E+14	7,34685E+14	6,0583E+14
TC0600000665.mm.2	Ghrhr	growth hormone releasing hormone receptor	-0.83628823456948	5,37859E+14	-1,8064E+14	0.117571041827499	0.262785711883269	-4,62788E+14	4,79936E+14	4,87776E+14	5,01101E+14	6,23369E+14	5,45479E+14	5,89491E+14
TC1200002389.mm.2	Hsp90aa1	heat shock protein 90, alpha (cytosolic), class A member 1	2,07909E+14	9,57103E+14	1,80598E+14	0.117641434734477	0.262906579455842	-4,62842E+14	1,13186E+14	1,12901E+14	1,00541E+14	6,3129E+14	9,92989E+13	8,5206E+13
TC0100000450.mm.2	Fzd7	frizzled class receptor 7	0.938182420378746	6,25129E+13	1,8056E+14	0.117705338038653	0.263012912469396	-4,6289E+14	6,6311E+14	6,93052E+14	6,29691E+14	5,96396E+14	6,58594E+14	5,09932E+14
TC0500001338.mm.2	Slc8b1	solute carrier family 8 (sodium/lithium/calcium exchanger), member B1	-0.760664935014489	6,77776E+14	-1,8052E+14	0.11777214795632	0.263125709930698	-4,62941E+14	6,29602E+14	6,42272E+14	6,44345E+14	7,26428E+14	6,75534E+14	7,48473E+14
TC0400003873.mm.2	Szrd1	SUZ RNA binding domain containing 1	-0.561834244791455	8,09286E+13	-1,80508E+14	0.117790954990475	0.263131243277627	-4,62956E+14	7,72058E+14	7,75646E+14	8,33961E+14	8,13977E+14	8,4894E+14	8,11131E+14
TC0100000936.mm.2	Sep 02	septin 2	1,22244E+14	7,01242E+14	1,80481E+14	0.117836364402291	0.263171623259714	-4,6299E+14	7,66538E+12	7,69777E+14	7,82741E+14	5,96567E+14	7,04468E+13	5,87359E+14
TC1200001794.mm.2	Nkx2-9	NK2 homeobox 9	-0.738154756680363	5,83388E+14	-1,80474E+14	0.117849202109419	0.263171623259714	-4,63E+14	5,49295E+14	5,24422E+14	5,60234E+14	6,45028E+14	5,82884E+14	6,38463E+14
TC1100003962.mm.2	Cacng4	calcium channel, voltage-dependent, gamma subunit 4	0.661627601204627	9,34484E+14	1,80468E+14	0.117858029754986	0.263171623259714	-4,63007E+14	9,57822E+14	9,87424E+14	9,5705E+14	8,9169E+14	9,25957E+14	8,86963E+14
TC1500002336.mm.2	Smdt1	single-pass membrane protein with aspartate rich tail 1	0.578185529150359	1,03602E+14	1,80371E+14	0.118020469849381	0.263434878573906	-4,6313E+14	1,09001E+14	1,07146E+14	1,02795E+14	9,80875E+14	1,02599E+14	1,01984E+14
TC0400001627.mm.2	Ubxn11	UBX domain protein 11	-0.739845345479794	5,69985E+14	-1,80369E+14	0.11802471961534	0.263434878573906	-4,63133E+14	5,05273E+14	5,34505E+14	5,46143E+14	6,46239E+14	5,99954E+14	5,87794E+14
TC1900000681.mm.2	Pprc1	peroxisome proliferative activated receptor, gamma, coactivator-related 1	-0.805864782854224	5,63372E+14	-1,80364E+14	0.118032126794633	0.263434878573906	-4,63139E+14	4,81008E+14	5,16724E+14	5,83531E+14	6,40067E+14	5,2801E+14	6,30894E+14
TC0500001300.mm.2	Ksr2	kinase suppressor of ras 2	0.570842210702143	7,02139E+14	1,80359E+14	0.118041322119009	0.263434878573906	-4,63146E+14	7,30273E+14	7,28771E+14	6,95416E+14	7,13495E+14	6,94442E+14	6,50434E+14
TC1000002650.mm.2	Utp20	UTP20 small subunit processome component	0.672984145875801	5,65681E+14	1,80341E+14	0.118070871594187	0.263464333748391	-4,63168E+14	6,30315E+14	5,91336E+14	5,24277E+14	5,61105E+14	5,10297E+14	5,76757E+14
TC1700000351.mm.2	Msrb1	methionine sulfoxide reductase B1	0.554817084160025	7,32632E+14	1,8031E+14	0.118124258700795	0.26351786394257	-4,63209E+14	7,51494E+14	7,62634E+14	7,30003E+12	7,48498E+14	7,18114E+14	6,8505E+13
TC1500000976.mm.2	Olfr279	olfactory receptor 279	-0.606169756953401	5,17755E+14	-1,80308E+12	0.118127569742269	0.26351786394257	-4,63211E+14	4,96046E+14	4,84581E+14	5,41487E+14	4,82555E+14	5,42073E+14	5,59787E+14
TC0100001037.mm.2	Cdh7	cadherin 7, type 2	0.745764662968787	6,53158E+14	1,80284E+14	0.118166726954833	0.26356872528007	-4,63241E+14	7,40233E+14	6,93245E+14	6,47962E+14	5,53184E+14	6,49218E+14	6,35107E+14
TC0500000926.mm.2	Fras1	Fraser extracellular matrix complex subunit 1	-0.539419242832198	6,85071E+14	-1,80216E+14	0.118281789093133	0.263788853655038	-4,63328E+14	6,44293E+14	6,51953E+14	7,00113E+14	7,09939E+14	6,88376E+14	7,15754E+14
TC1300001169.mm.2	Sgtb	small glutamine-rich tetratricopeptide repeat (TPR)-containing, beta	1,2101E+13	7,63359E+14	1,80101E+14	0.118475303165818	0.26413372160856	-4,63475E+14	8,56521E+14	8,71596E+14	7,86155E+14	5,7978E+14	7,7706E+14	7,09038E+14
TC0700002468.mm.2	Foxa3	forkhead box A3	-0.849540641435931	6,34689E+14	-1,80084E+14	0.118502972607651	0.26413372160856	-4,63496E+14	5,80526E+14	5,8149E+14	5,90496E+14	7,23701E+14	6,57899E+14	6,74025E+14
TC0700001417.mm.2	Fam168a	family with sequence similarity 168, member A	0.528941911616145	8,01704E+14	1,80081E+14	0.118508518729004	0.26413372160856	-4,635E+14	8,18698E+14	8,09962E+14	8,27461E+14	8,31231E+14	7,71279E+14	7,51592E+14
TC0600002553.mm.2	Sema4f	sema domain, immunoglobulin domain (Ig), TM domain, and short cytoplasmic domain	-0.5752650118669	8,04334E+13	-1,8008E+14	0.118509615437456	0.26413372160856	-4,63501E+12	7,6778E+14	7,79229E+14	7,93222E+14	8,23707E+14	8,12015E+14	8,50047E+14
TC1600001304.mm.2	Prodh	proline dehydrogenase	-0.544588852617178	6,70576E+14	-1,80075E+14	0.118518389327244	0.26413372160856	-4,63507E+14	6,38439E+14	6,45969E+14	6,81459E+14	6,64263E+14	7,1074E+14	6,82586E+14
TC0Y00000554.mm.2	Rbmy	RNA binding motif protein, Y chromosome	-0.623210248952458	4,70071E+14	-1,80056E+14	0.118550531503072	0.264168816776242	-4,63532E+14	4,05247E+14	4,58978E+13	4,63881E+14	5,03455E+13	4,85259E+14	5,03608E+14
TC0200001011.mm.2	Phospho2	phosphatase, orphan 2	1,85753E+14	5,11853E+14	1,80028E+14	0.118597840197518	0.264180181997588	-4,63568E+14	6,51918E+14	6,39567E+14	5,78823E+14	2,80833E+14	5,56628E+14	3,6335E+14
TC1500002340.mm.2	Hoxc5	homeobox C5	-0.956307483191373	5,42315E+14	-1,80025E+14	0.118602293953779	0.264180181997588	-4,63571E+14	4,78091E+14	4,57697E+14	5,19707E+14	6,71343E+14	5,39728E+14	5,87321E+14
TC0900001792.mm.2	Fat3	FAT atypical cadherin 3	0.597868450848741	5,64059E+14	1,80016E+14	0.118618263354219	0.264180181997588	-4,63583E+14	6,23529E+14	6,12464E+14	5,02383E+14	5,29563E+14	5,32193E+14	5,84226E+14
TC0400003385.mm.2	Kdm4a	lysine (K)-specific demethylase 4A	0.546690208165873	6,76723E+14	1,80014E+14	0.118621213725101	0.264180181997588	-4,63585E+14	6,96567E+14	7,20815E+14	6,70679E+14	6,64232E+13	6,66417E+14	6,41627E+14
TC0700004515.mm.2	Ifitm3	interferon induced transmembrane protein 3	-0.825494984408118	8,27464E+14	-1,8E+14	0.118644795729944	0.264196184853038	-4,63603E+14	7,55588E+14	8,38459E+14	8,51042E+13	7,60548E+14	8,98118E+14	8,61027E+14
TC1200001077.mm.2	Vrk1	vaccinia related kinase 1	0.852282745465777	6,55052E+14	1,79971E+14	0.118693013213244	0.264201368493664	-4,63639E+14	7,08012E+14	6,93563E+14	6,23957E+14	6,73661E+14	5,53217E+13	6,77902E+14
TC0700003938.mm.2	Trim12a	tripartite motif-containing 12A	-0.619385025092146	6,64503E+14	-1,79971E+14	0.118694656604022	0.264201368493664	-4,63641E+14	6,34552E+14	6,16628E+14	6,57294E+14	7,03488E+14	6,64287E+14	7,1077E+14
TC0400001333.mm.2	Zmynd12	zinc finger, MYND domain containing 12	-1,14756E+14	4,66035E+14	-1,7997E+14	0.118696313940647	0.264201368493664	-4,63642E+14	4,1485E+14	3,86767E+14	3,90127E+14	5,73335E+13	4,72783E+14	5,58348E+14
TC0100001419.mm.2	Abl2	v-abl Abelson murine leukemia viral oncogene 2 (arg, Abelson-related gene)	1,14301E+14	6,28857E+14	1,79954E+14	0.118723275652861	0.264207244714869	-4,63662E+14	7,09644E+14	7,16466E+13	6,61909E+14	4,87611E+14	6,5082E+14	5,46689E+14
TC0200004456.mm.2	Pla2g4d	phospholipase A2, group IVD	-0.595507115396156	4,84911E+14	-1,79945E+14	0.118738008458466	0.264207244714869	-4,63673E+14	4,50224E+14	4,7815E+14	4,41892E+14	4,91726E+14	5,30892E+14	5,16584E+14
TC0X00003335.mm.2	Mospd2	motile sperm domain containing 2	0.640829296813958	6,42903E+14	1,79931E+14	0.118761728487356	0.264207244714869	-4,63691E+14	6,54389E+12	6,70686E+14	6,31381E+14	7,04051E+14	5,92337E+13	6,04572E+14
TC0300001906.mm.2	1700034I23Rik	FUN14 domain containing 2 pseudogene	-0.704320594088302	5,00685E+14	-1,79929E+13	0.118764962845632	0.264207244714869	-4,63694E+14	4,98036E+14	4,5734E+14	4,7405E+14	4,78446E+14	5,05068E+13	5,91172E+14
TC0600001636.mm.2	Strap	serine/threonine kinase receptor associated protein	1,23156E+14	8,31711E+14	1,7991E+14	0.118796941824845	0.264207244714869	-4,63718E+14	9,12285E+13	9,12431E+14	8,75865E+14	7,11279E+14	8,64305E+13	7,14099E+14
TC0100001996.mm.2	Rpl7	ribosomal protein L7	0.924687408174684	9,33917E+14	1,7991E+12	0.118797336806258	0.264207244714869	-4,63718E+14	1,00711E+14	1,0132E+14	9,13605E+14	8,34224E+14	9,82914E+14	8,52455E+14
TC1000002212.mm.2	Mcu	mitochondrial calcium uniporter	0.795092006780552	6,89152E+14	1,7988E+14	0.118847754226763	0.264282895922693	-4,63756E+14	7,29342E+14	7,33728E+14	7,30298E+14	6,37494E+14	6,90361E+14	6,13691E+14
TC1100003667.mm.2	Fbxl20	F-box and leucine-rich repeat protein 20	1,49851E+14	6,17352E+13	1,79849E+14	0.118899680490825	0.264361881019549	-4,63795E+14	7,25537E+14	7,33395E+14	6,65782E+14	4,20169E+14	6,47221E+13	5,12009E+13
TC0800002957.mm.2	Tmem170	transmembrane protein 170	-0.58454872617122	7,49256E+13	-1,79832E+14	0.118928617560236	0.26437452910376	-4,63817E+14	7,02636E+14	7,09724E+13	7,54253E+14	7,99654E+14	7,49973E+14	7,79297E+14
TC0200004122.mm.2	F2	coagulation factor II	-0.709205089339902	5,73064E+14	-1,79824E+14	0.118942825563483	0.26437452910376	-4,63828E+14	5,39933E+14	5,1665E+14	5,73478E+14	6,099E+14	5,55857E+14	6,42566E+13
TC0X00003376.mm.2	Hccs	holocytochrome c synthetase	0.616038658843594	6,28852E+14	1,79814E+14	0.118959303095568	0.26437452910376	-4,6384E+14	7,0039E+14	6,70145E+13	6,05576E+14	5,49676E+14	6,13196E+14	6,34131E+14
TC0600001867.mm.2	Thsd7a	thrombospondin, type I, domain containing 7A	0.725223455739358	7,65027E+14	1,79807E+14	0.118970999226175	0.26437452910376	-4,63849E+14	8,34705E+14	8,34909E+14	7,33239E+14	6,62738E+14	7,25766E+14	7,98803E+14
TC0500002676.mm.2	Uba6	ubiquitin-like modifier activating enzyme 6	2,15045E+14	5,83716E+14	1,79763E+14	0.119044430584966	0.26450122862873	-4,63904E+14	7,4868E+14	7,29413E+14	6,50603E+14	3,25595E+14	6,53842E+14	3,94161E+14
TC1600000040.mm.2	Nudt16l1	nudix (nucleoside diphosphate linked moiety X)-type motif 16-like 1	-0.516997150446626	8,37846E+14	-1,7975E+14	0.119066517347506	0.264513827936076	-4,63921E+14	8,13573E+14	8,17376E+14	8,32228E+14	8,29549E+14	8,67252E+14	8,67096E+14
TC1200000562.mm.2	Lrr1	leucine rich repeat protein 1	-0.558119632618477	4,74854E+13	-1,79724E+14	0.119110351319639	0.264574729916368	-4,63954E+14	4,33316E+14	4,0149E+14	5,41489E+14	5,26397E+14	4,84882E+14	4,61548E+14
TC1900001217.mm.2	Gcnt1	glucosaminyl (N-acetyl) transferase 1, core 2	0.585733313910877	5,23226E+14	1,79705E+14	0.119143705307425	0.264577420332554	-4,63979E+14	5,9903E+14	5,45267E+14	4,89801E+14	4,78109E+14	5,15729E+14	5,11422E+13
TC0200001662.mm.2	Lpcat4	lysophosphatidylcholine acyltransferase 4	0.631100947782008	7,76231E+14	1,79704E+13	0.119144402776206	0.264577420332554	-4,63979E+14	8,10797E+14	8,31481E+14	7,68193E+13	7,3086E+14	7,87281E+14	7,28776E+13
TC0100003531.mm.2	Pcp4l1	Purkinje cell protein 4-like 1	0.663250408278266	8,98772E+14	1,79638E+14	0.119257508553312	0.264762667941927	-4,64064E+14	9,47194E+14	9,27946E+14	9,21758E+14	8,53243E+13	8,92751E+14	8,49739E+14

TC1100002747.mm.2	Gm12216	predicted gene 12216	-0.91188440029907	6,16581E+12	-1,79636E+14	0.119260686645721	0.264762667941927	-4,64067E+13	5,76563E+14	5,50792E+13	5,58951E+14	7,03449E+14	6,39209E+14	6,70523E+14
TC0200002314.mm.2	Kif3b	kinesin family member 3B	0.717466306991666	6,75214E+14	1,79619E+14	0.119289394595109	0.264789918048077	-4,64088E+14	6,88956E+14	7,21556E+14	7,07959E+14	6,65793E+14	6,75527E+14	5,91492E+14
TC0700001442.mm.2	Anapc15	anaphase prompoting complex C subunit 15	0.652170233553256	6,73261E+14	1,79608E+14	0.119306732477201	0.264791925675639	-4,64101E+14	7,36761E+14	6,93632E+14	6,55682E+14	6,53532E+14	6,83426E+14	6,16533E+14
TC0900000458.mm.2	Hspa8/Snord14d/Snord14e/Snord14c	heat shock protein 8/small nucleolar RNA, C/D box 14D/small nucleolar RNA, C/D box 14E/small nucleolar RNA, C/D box 14C	1,20746E+14	1,17019E+14	1,79588E+14	0.119340942466314	0.264802345133532	-4,64127E+14	1,25169E+14	1,25736E+14	1,21303E+14	1,03147E+14	1,19405E+14	1,07352E+14
TC0500000527.mm.2	Sod3	superoxide dismutase 3, extracellular	0.83983150774548	5,35847E+14	1,79586E+14	0.119344295311842	0.264802345133532	-4,6413E+13	6,07007E+14	5,83945E+14	4,51274E+14	5,49874E+14	5,54082E+14	4,68904E+14
TC1100003735.mm.2	Gm11595	predicted gene 11595	-0.636251802791391	7,01865E+14	-1,79575E+13	0.119363400993169	0.264808272091825	-4,64144E+14	6,5922E+14	6,56195E+14	7,31394E+14	7,21715E+14	6,84506E+14	7,5816E+14
TC0100001011.mm.2	Tnfrsf11a	tumor necrosis factor receptor superfamily, member 11a, NFKB activator	-0.703931800823151	6,14724E+14	-1,79556E+14	0.11939548374445	0.264842983146246	-4,64168E+13	5,5344E+14	5,6392E+14	6,13002E+14	6,99834E+14	6,09709E+14	6,48437E+14
TC1900000908.mm.2	Rad9a	RAD9 checkpoint clamp component A	0.651900787925107	6,15419E+14	1,79513E+14	0.119467847779773	0.264965232701191	-4,64222E+14	6,36788E+14	6,77101E+14	6,29828E+14	5,65286E+14	6,03506E+14	5,80003E+14
TC0100000439.mm.2	Cflar	CASP8 and FADD-like apoptosis regulator	-0.694472504048586	7,01031E+14	-1,79501E+14	0.119488391030284	0.264965232701191	-4,64238E+14	6,64837E+14	6,55228E+14	6,68479E+14	7,58682E+14	7,19422E+13	7,39537E+14
TC0700000411.mm.2	Prr19	proline rich 19	-1,08709E+14	6,75728E+14	-1,79495E+14	0.119499928383265	0.264965232701191	-4,64246E+14	6,15148E+14	5,64236E+14	6,62266E+14	8,15919E+14	6,47518E+14	7,49283E+14
TC0900003163.mm.2	Fbxl2	F-box and leucine-rich repeat protein 2	0.698801064166751	8,36917E+13	1,79476E+14	0.119531939005425	0.264999743147278	-4,6427E+14	8,71879E+14	8,72651E+14	8,31281E+14	8,4092E+14	8,49252E+13	7,55517E+14
TC1100000592.mm.2	Vdac1	voltage-dependent anion channel 1	0.827321190609638	9,87236E+13	1,79463E+14	0.119554245571059	0.265012733372743	-4,64287E+14	1,03936E+14	1,03057E+14	1,01987E+14	9,29155E+14	9,98084E+14	9,06382E+14
TC0200005426.mm.2	Nkain4	Na+/K+ transporting ATPase interacting 4	0.927308478611177	6,90033E+14	1,79406E+14	0.119650053754517	0.265188626705163	-4,64359E+14	7,90032E+14	7,36525E+14	7,07787E+14	5,64757E+14	6,60273E+14	6,80822E+14
TC0900002463.mm.2	Coro2b	coronin, actin binding protein, 2B	0.60574120347839	8,68606E+14	1,79391E+14	0.119675248785176	0.265206620134581	-4,64378E+14	9,17454E+14	9,09218E+14	8,67892E+14	8,1155E+14	8,6694E+14	8,38584E+14
TC1100000499.mm.2	Zfp62	zinc finger protein 62	1,29795E+14	8,27438E+14	1,79382E+14	0.119691090524339	0.265206620134581	-4,6439E+14	9,12231E+14	8,98257E+14	8,78883E+14	7,24357E+14	8,65788E+14	6,85111E+14
TC0800001860.mm.2	Atp7b	ATPase, Cu++ transporting, beta polypeptide	-0.678867256380816	5,79655E+14	-1,79343E+13	0.119757755004449	0.265317847708881	-4,64439E+14	5,44468E+14	5,32862E+14	5,54594E+14	6,32903E+14	5,86908E+14	6,26196E+12
TC0900003335.mm.2	Uba7	ubiquitin-like modifier activating enzyme 7	-0.607795862928216	7,45461E+14	-1,79325E+14	0.119788717885415	0.265318904575345	-4,64463E+14	7,2563E+14	6,84513E+13	7,41077E+13	7,89843E+14	7,70547E+14	7,61156E+14
TC1600001011.mm.2	Itsn1	intersectin 1 (SH3 domain protein 1A)	1,70207E+14	8,14865E+14	1,79323E+14	0.119791164326049	0.265318904575345	-4,64464E+14	9,58513E+14	9,31573E+14	8,74177E+14	5,75254E+14	8,36421E+13	7,13251E+14
TC1700001138.mm.2	Wdr43	WD repeat domain 43	1,28178E+14	6,86593E+14	1,79276E+14	0.119871134792927	0.265394800693506	-4,64524E+14	7,85372E+14	7,69578E+14	7,24994E+14	5,4102E+13	7,21809E+13	5,76784E+13
TC0800001744.mm.2	AF366264	cDNA sequence AF366264	-1,05338E+14	4,52667E+14	-1,79272E+14	0.119878136694992	0.265394800693506	-4,64529E+14	3,96902E+14	3,86348E+14	3,93786E+14	5,45041E+14	4,45007E+14	5,48919E+14
TC1200002263.mm.2	Asb2	ankyrin repeat and SOCS box-containing 2	-0.868623144426294	6,3958E+14	-1,79268E+14	0.119885652728838	0.265394800693506	-4,64535E+14	5,83265E+14	6,00235E+14	5,8978E+14	7,06974E+14	6,41345E+14	7,1588E+14
TC1100004236.mm.2	Csnk1d	casein kinase 1, delta	1,62522E+14	7,66811E+14	1,79219E+14	0.119969357536453	0.265394800693506	-4,64597E+14	8,79533E+14	8,48723E+14	8,60818E+14	6,08579E+14	7,89034E+13	6,14179E+14
TC0300000647.mm.2	Sfrp2	secreted frizzled-related protein 2	-0.555578677461244	6,35768E+14	-1,79208E+14	0.119986896878752	0.265394800693506	-4,64611E+14	6,04086E+14	5,97061E+14	6,61813E+14	6,39388E+14	6,38875E+13	6,73387E+14
TC1200001021.mm.2	Ubr7	ubiquitin protein ligase E3 component n-recognin 7 (putative)	1,1685E+14	7,38029E+13	1,79204E+14	0.119994124181308	0.265394800693506	-4,64616E+14	8,1103E+14	7,92112E+14	8,27031E+14	6,28562E+14	7,17574E+14	6,51867E+14
TC0100000787.mm.2	Tex44	testis expressed 44	-0.748806808585258	4,79825E+14	-1,79194E+14	0.12001174846967	0.265394800693506	-4,64629E+13	4,15967E+14	4,37608E+14	4,77247E+14	5,44793E+14	4,60407E+14	5,42929E+14
TC0200003299.mm.2	Swi5	SWI5 recombination repair homolog (yeast)	0.548730329009901	8,15023E+14	1,79191E+14	0.120015573306112	0.265394800693506	-4,64632E+14	8,8102E+14	8,66992E+14	7,55929E+14	7,47928E+13	8,01943E+14	8,36323E+14
TC0500001725.mm.2	Spdye4b	speedy/RINGO cell cycle regulator family, member E4B	-0.613519224980432	6,29807E+14	-1,79191E+14	0.12001557856793	0.265394800693506	-4,64632E+14	5,64186E+14	5,86829E+14	6,55083E+14	6,99022E+14	6,14412E+14	6,59307E+14
TC1200001686.mm.2	Stxbp6	syntaxin binding protein 6 (amisyn)	0.6814360524817	7,82919E+14	1,79191E+14	0.120016355798242	0.265394800693506	-4,64632E+14	8,47607E+14	8,11864E+14	7,88511E+14	7,26349E+13	7,88239E+14	7,34946E+14
TC1100000530.mm.2	Rasgef1c	RasGEF domain family, member 1C	0.623513254965057	6,33666E+14	1,79186E+14	0.120023992168937	0.265394800693506	-4,64638E+14	6,41591E+14	6,83654E+14	6,22347E+14	6,53864E+14	5,7686E+14	6,23682E+14
TC1200001785.mm.2	Mbip	MAP3K12 binding inhibitory protein 1	1,17691E+13	6,27246E+14	1,79183E+14	0.120029846785791	0.265394800693506	-4,64643E+14	6,68924E+14	6,62379E+13	7,01899E+14	6,34357E+14	6,48288E+14	4,47631E+14
TC0800000830.mm.2	Tmem38a	transmembrane protein 38A	0.777646783249903	7,78885E+14	1,79177E+14	0.120039552377228	0.265394800693506	-4,6465E+14	8,41327E+14	8,26227E+14	8,00949E+14	6,92782E+14	7,76507E+14	7,35518E+14
TC0300001888.mm.2	Ankrd50	ankyrin repeat domain 50	0.616062099062902	5,945E+14	1,79165E+14	0.120061286848302	0.265406436409205	-4,64666E+14	6,55878E+14	6,52647E+14	5,68834E+14	5,04327E+14	6,02868E+14	5,82444E+14
TC1900000160.mm.2	Bscl2	Berardinelli-Seip congenital lipodystrophy 2 (seipin)	0.708825454883334	8,25325E+14	1,79114E+14	0.120148305170063	0.265562365048729	-4,64731E+14	8,73312E+14	8,41555E+14	8,4811E+13	8,1587E+14	8,29785E+14	7,43318E+14
TC0X00002985.mm.2	Sytl4	synaptotagmin-like 4	0.78126214792986	5,40606E+14	1,79072E+14	0.120218619955354	0.265615156124472	-4,64783E+14	6,01359E+14	5,36475E+14	5,16554E+14	6,07668E+14	4,72412E+14	5,0917E+14
TC0700004223.mm.2	Ears2	glutamyl-tRNA synthetase 2, mitochondrial	-0.95698616077008	5,78438E+14	-1,79072E+14	0.120219483787329	0.265615156124472	-4,64784E+14	5,33066E+14	4,85935E+13	5,6477E+14	6,76456E+14	5,5177E+14	6,58629E+14
TC0100000289.mm.2	Map4k4	mitogen-activated protein kinase kinase kinase kinase 4	0.643244062282691	7,63989E+14	1,79071E+14	0.120221642997317	0.265615156124472	-4,64785E+14	8,06233E+13	8,1975E+14	7,67985E+14	6,92629E+14	7,43274E+14	7,54061E+14
TC0900002003.mm.2	St3gal4	ST3 beta-galactoside alpha-2,3-sialyltransferase 4	0.623856278683605	7,35127E+12	1,79041E+14	0.120272132890114	0.265687804231173	-4,64823E+14	7,92653E+14	7,69001E+14	6,75394E+14	7,3683E+14	7,31322E+14	7,05561E+14
TC1400000158.mm.2	Nid2	nidogen 2	-0.581569861564435	5,68296E+14	-1,79028E+14	0.120294889075064	0.265687804231173	-4,6484E+14	5,17117E+14	5,42952E+14	5,62751E+12	6,10571E+13	5,88377E+14	5,88006E+14
TC0100000711.mm.2	Agfg1	ArfGAP with FG repeats 1	0.955543644610245	7,88186E+14	1,79003E+14	0.120337665241695	0.265687804231173	-4,64872E+14	8,40777E+14	8,5162E+14	8,28823E+14	7,06604E+14	8,03029E+14	6,9826E+13
TC1100000965.mm.2	Kif1c/Mir6925	kinesin family member 1C/microRNA 6925	1,25364E+14	7,04433E+14	1,78996E+14	0.120349141517227	0.265687804231173	-4,6488E+12	7,92008E+14	7,58793E+14	7,3886E+13	6,36867E+14	7,51235E+14	5,48838E+14
TC0X00002689.mm.2	Ophn1	oligophrenin 1	-0.576654496874563	6,5024E+14	-17899212581	0.120355758157112	0.265687804231173	-4,64885E+14	6,07506E+14	6,33197E+14	6,44467E+14	6,60232E+13	6,56671E+14	6,99363E+14
TC0700003572.mm.2	Ap3s2	adaptor-related protein complex 3, sigma 2 subunit	1,03444E+13	8,23852E+14	1,78992E+14	0.120356134551127	0.265687804231173	-4,64886E+14	9,02167E+14	8,82628E+14	8,72971E+14	7,0744E+14	8,22704E+14	7,55204E+14
TC0200003386.mm.2	Olfr351	olfactory receptor 351	-1,02251E+14	5,79704E+14	-1,78981E+14	0.120374888526228	0.265687804231173	-4,649E+14	5,20373E+14	5,22563E+14	5,38967E+14	6,48882E+14	5,54306E+14	6,93132E+14
TC1500001776.mm.2	Bop1	block of proliferation 1	0.688090530823548	6,29868E+14	1,78974E+14	0.120386436957227	0.265687804231173	-4,64908E+14	6,33977E+13	6,69983E+14	6,30184E+14	6,78719E+14	6,17598E+14	5,48744E+13
TC1200001257.mm.2	Kif26a	kinesin family member 26A	-0.661621603713911	7,61069E+14	-1,78942E+14	0.120442262333768	0.265774605996166	-4,6495E+13	7,14222E+13	7,32334E+14	7,57397E+14	7,8358E+14	7,50868E+14	8,28013E+14
TC1000002556.mm.2	Zfp781	zinc finger protein 781	1,51261E+14	6,51088E+14	1,78926E+13	0.120468159172588	0.265795351054075	-4,64969E+14	7,46517E+14	7,23548E+14	7,36024E+14	5,32897E+14	6,83157E+14	4,84386E+14
TC1200001605.mm.2	Pik3cg	phosphatidylinositol-4,5-bisphosphate 3-kinase catalytic subunit gamma	0.750296017360838	5,15624E+13	1,78896E+14	0.120519337191514	0.265871862016274	-4,65007E+14	5,45621E+14	5,60277E+14	4,6877E+14	5,63238E+14	5,04687E+14	4,51151E+14
TC1200000626.mm.2	Ppm1a	protein phosphatase 1A, magnesium dependent, alpha isoform	0.963483115098287	8,81563E+14	1,78886E+14	0.120537643346187	0.265875844933212	-4,6502E+14	9,64642E+14	9,48201E+14	8,95681E+14	7,60705E+14	9,05359E+14	8,14787E+13
TC0500003591.mm.2	Atp5j2	ATP synthase, H+ transporting, mitochondrial F0 complex, subunit F2	0.669934205258816	9,60423E+14	1,78873E+14	0.120560312841149	0.265889449878105	-4,65037E+14	1,01416E+14	1,01571E+14	9,47507E+14	8,89272E+14	9,79682E+14	9,16204E+14
TC1700000424.mm.2	Kifc5b	kinesin family member C5B	-1,04253E+14	6,57312E+13	-1,7884E+14	0.120615555181441	0.265940311842417	-4,65078E+13	5,72838E+14	6,34656E+13	5,80841E+14	7,39537E+14	6,76058E+14	7,39941E+14
TC0700003581.mm.2	Idh2	isocitrate dehydrogenase 2 (NADP+), mitochondrial	0.552498547428596	6,99114E+14	1,7884E+14	0.120616384220467	0.265940311842417	-4,65079E+14	7,34296E+14	7,39451E+14	6,64977E+14	6,92712E+14	6,6695E+14	6,96298E+14
TC0800002870.mm.2	Tmed6	transmembrane p24 trafficking protein 6	-0.867927506489368	5,27304E+14	-1,78804E+14	0.120678210887891	0.266040226027717	-4,65125E+13	4,69139E+14	4,62363E+14	5,01035E+14	6,26198E+14	5,21022E+14	5,84066E+14
TC0300002681.mm.2	Atp5f1	ATP synthase, H+ transporting, mitochondrial F0 complex, subunit B1	1,04994E+13	8,51019E+14	1,78784E+14	0.120711289981761	0.266076746303163	-4,65149E+14	9,54322E+14	9,58089E+13	8,27308E+14	6,63972E+14	8,93181E+14	8,0924E+14
TC0500002887.mm.2	Sparcl1	SPARC-like 1	1,27651E+13	1,11452E+14	1,78762E+14	0.120749355572089	0.266122296878295	-4,65177E+14	1,20444E+14	1,19968E+14	1,16604E+14	9,68132E+14	1,13721E+14	1,01161E+14
TC0700000369.mm.2	Zfp112	zinc finger protein 112	-0.789316744483512	5,85313E+14	-1,78753E+14	0.120764986953025	0.266122296878295	-4,65189E+14	4,94542E+14	5,20349E+14	6,03982E+14	7,20249E+14	5,78075E+14	5,94679E+14

TC1100003648.mm.2	Epop	elongin BC and polycomb repressive complex 2 associated protein	-0.62381962261184	7,29753E+14	-1,78732E+14	0.120801020522278	0.266165300653011	-4,65216E+14	6,73084E+14	6,97365E+14	7,31353E+13	7,815E+14	7,25553E+14	7,6966E+14
TC0700002491.mm.2	Relb	avian reticuloendotheliosis viral (v-rel) oncogene related B	-0.716566842866894	6,27829E+14	-1,7872E+14	0.120822271621257	0.266175726364961	-4,65231E+14	5,86306E+14	5,85937E+13	6,02447E+14	6,76728E+14	6,24089E+14	6,91467E+11
TC1400002099.mm.2	Rabggta	Rab geranylgeranyl transferase, a subunit	0.616560285776267	6,91512E+14	1,78701E+14	0.120854944527817	0.26621130843154	-4,65256E+14	7,23375E+14	7,33469E+14	6,47419E+14	7,11276E+13	6,7809E+14	6,55442E+14
TC0300002487.mm.2	Prpf3	pre-mRNA processing factor 3	0.970359125803139	5,30337E+14	1,78675E+14	0.120898540506926	0.266270938106628	-4,65288E+14	6,15607E+14	6,07606E+14	5,1662E+14	4,13048E+14	5,78844E+14	4,50296E+14
TC0400001633.mm.2	Slc30a2	solute carrier family 30 (zinc transporter), member 2	-0.708829697601963	5,50382E+14	-1,78656E+13	0.120932073138441	0.2663083906628	-4,65313E+14	5,03912E+14	5,14865E+14	5,50964E+14	5,7201E+14	5,32666E+14	6,27877E+14
TC1700002783.mm.2	Gpsm3	G-protein signalling modulator 3 (AGS3-like, C. elegans)	-0.774098882188577	6,48735E+14	-1,78628E+14	0.120979853279579	0.266376790842232	-4,65348E+13	5,7616E+14	6,05219E+14	6,34898E+13	7,39938E+14	6,47741E+14	6,88458E+14
TC0200004665.mm.2	Rassf2	Ras association (RalGDS/AF-6) domain family member 2	0.719524511944337	6,86804E+13	1,78618E+14	0.120996197615236	0.266376790842232	-4,6536E+14	7,30644E+14	7,28171E+14	7,235E+14	6,23598E+14	6,49303E+14	6,65606E+14
TC0200002295.mm.2	H13	histocompatibility 13	0.842043332688686	8,4866E+13	1,78592E+14	0.121040786981702	0.266400105952544	-4,65393E+14	9,16937E+14	8,93147E+14	8,8229E+13	7,57914E+14	8,42848E+13	7,98827E+14
TC0100002784.mm.2	Iqca	IQ motif containing with AAA domain	-0.519473764137873	5,40106E+12	-1,78587E+14	0.121049908099487	0.266400105952544	-4,654E+14	5,11292E+14	5,1293E+14	5,37246E+14	5,5105E+14	5,68272E+14	5,59845E+14
TC0900002455.mm.2	Glce/Mir5133	glucuronyl C5-epimerase/microRNA 5133	1,37758E+14	7,50002E+14	1,78583E+14	0.121056387748062	0.266400105952544	-4,65405E+14	8,3865E+14	8,18527E+14	8,47608E+14	6,13565E+14	7,42629E+14	6,39033E+14
TC1900000718.mm.2	Pdcd11	programmed cell death 11	0.903599117914415	5,52067E+13	1,78564E+13	0.121089622306751	0.266436854325915	-4,65429E+14	6,02742E+14	6,32938E+14	5,44016E+14	4,7775E+14	5,92698E+14	4,62262E+14
TC1600000450.mm.2	Muc4	mucin 4	-0.64617668311874	6,45947E+14	-1,7854E+13	0.121130164288744	0.26645551130574	-4,65459E+14	5,9538E+14	5,93671E+14	6,47411E+13	7,20932E+14	6,44821E+14	6,73465E+14
TC0500000630.mm.2	Chrna9	cholinergic receptor, nicotinic, alpha polypeptide 9	-0.750764508328273	6,15949E+14	-1,7854E+14	0.1211311748473	0.26645551130574	-4,6546E+14	5,73217E+14	5,42667E+13	6,045E+14	7,09269E+14	6,25701E+14	6,40337E+14
TC1700000582.mm.2	Actl9	actin-like 9	-0.913645314448156	4,77758E+14	-1,78516E+14	0.121172341084804	0.266509682213957	-4,6549E+14	4,07231E+14	4,16239E+11	4,4418E+14	5,92699E+14	4,87835E+14	5,18365E+14
TC0900001605.mm.2	Rpl14/Rpl14-ps1	ribosomal protein L14/ribosomal protein L14, pseudogene 1	0.709487501493589	9,79641E+14	1,78483E+14	0.121228806002739	0.266542819897212	-4,65532E+13	1,04034E+14	1,0315E+14	9,80134E+13	8,95941E+14	9,90159E+14	9,39777E+14
TC1100000821.mm.2	Tekt3	tektin 3	-0.717595541334122	5,44593E+14	-1,78483E+14	0.121228888611816	0.266542819897212	-4,65532E+14	4,8136E+14	4,92359E+14	5,64343E+14	6,12259E+14	5,14587E+14	6,02651E+14
TC1200000314.mm.2	Agr3	anterior gradient 3	-0.878758135451693	4,40981E+14	-1,78478E+13	0.121237033887338	0.266542819897212	-4,65538E+14	3,6429E+14	3,89648E+14	4,31407E+14	5,3085E+14	4,14773E+14	5,14916E+13
TC0700003769.mm.2	Aqp11	aquaporin 11	1,16099E+14	6,46278E+14	1,78441E+14	0.121301748894421	0.266648714861637	-4,65586E+14	7,11187E+14	6,82429E+14	7,44079E+14	5,78555E+14	6,43162E+14	5,18255E+14
TC1500001980.mm.2	Scube1	signal peptide, CUB domain, EGF-like 1	0.609969462659549	6,4873E+14	1,7843E+14	0.121320686932882	0.266653966518828	-4,656E+14	6,71691E+14	7,02834E+14	6,39912E+14	6,25411E+14	6,54693E+14	5,97839E+14
TC0700001258.mm.2	Ctsc	cathepsin C	0.839955614869549	8,05217E+14	1,78418E+14	0.121340347686718	0.266660805002194	-4,65614E+14	8,29137E+14	9,36407E+14	7,5891E+14	7,09296E+14	7,67476E+14	8,30077E+14
TC1100003072.mm.2	Kctd11	potassium channel tetramerisation domain containing 11	-0.717620571132649	4,62164E+13	-1,78391E+14	0.121387599901661	0.266728269087066	-4,65649E+14	4,16335E+14	4,21962E+14	4,31807E+14	5,2106E+14	5,14348E+14	4,67473E+14
TC0200002753.mm.2	Gm14327	predicted gene 14327	1,63382E+14	7,78611E+14	1,78366E+14	0.121430790835699	0.266765866947212	-4,65681E+14	9,04661E+14	9,47413E+14	7,93387E+14	5,00892E+14	8,12439E+14	7,12871E+14
TC0800002819.mm.2	Fhod1	formin homology 2 domain containing 1	-0.767900517189409	5,81566E+14	-1,78361E+14	0.121438645939567	0.266765866947212	-4,65687E+13	5,12779E+14	5,35148E+14	5,88535E+14	6,51428E+14	5,53941E+14	6,47566E+14
TC1200000265.mm.2	Slc26a3	solute carrier family 26, member 3	-0.591008454176482	5,37593E+14	-1,78352E+14	0.12145537344652	0.266765866947212	-4,65699E+14	4,96475E+14	5,03527E+13	5,29381E+14	5,77969E+14	5,42561E+14	5,75643E+14
TC0100000027.mm.2	Pcmtd1	protein-L-isoaspartate (D-aspartate) O-methyltransferase domain containing 1	1,53678E+14	7,9805E+14	1,78343E+14	0.121470934387867	0.266765866947212	-4,6571E+14	9,24842E+14	9,20734E+14	8,17662E+14	5,86841E+14	8,57972E+14	6,80248E+14
TC1800000685.mm.2	Grp	gastrin releasing peptide	-0.777669576327109	6,5094E+14	-1,78304E+14	0.121536983964913	0.266874546555825	-4,65759E+14	6,10519E+14	5,89982E+14	6,30484E+14	7,18621E+14	6,40409E+13	7,15626E+14
TC0200003582.mm.2	Ccdc148	coiled-coil domain containing 148	0.552316476233291	7,25874E+14	1,78256E+14	0.121619730542629	0.266991444899354	-4,6582E+13	7,49207E+14	7,50044E+14	7,24065E+14	7,43139E+14	7,12866E+14	6,75922E+14
TC0700003738.mm.2	Ankrd42	ankyrin repeat domain 42	0.585830406251098	6,89589E+13	1,78254E+14	0.121623360365001	0.266991444899354	-4,65823E+14	7,3938E+13	7,1581E+14	6,97549E+14	6,46772E+14	6,74925E+14	6,63099E+13
TC1900000650.mm.2	Hif1an	hypoxia-inducible factor 1, alpha subunit inhibitor	-0.563345079055352	7,11552E+14	-1,78244E+14	0.121640619541621	0.266992957727032	-4,65835E+14	6,66764E+12	6,98689E+14	7,12824E+14	7,12913E+14	7,56453E+14	7,21669E+14
TC0200002130.mm.2	Pcsk2	proprotein convertase subtilisin/kexin type 2	0.743014830604947	8,99169E+14	1,78226E+14	0.121671436997466	0.267024225597954	-4,65858E+14	9,43506E+12	9,79093E+14	8,85605E+14	8,1281E+14	9,22948E+14	8,51048E+14
TC0300002526.mm.2	Nudt17	nudix (nucleoside diphosphate linked moiety X)-type motif 17	0.546459089738003	6,00894E+14	1,78207E+14	0.121704807354231	0.267061086871677	-4,65882E+14	6,25461E+14	6,18587E+14	6,0709E+14	6,1947E+14	5,49524E+14	5,85232E+14
TC1600001366.mm.2	2510009E07Rik	RIKEN cDNA 2510009E07 gene	0.869367723644388	7,57988E+14	1,78151E+14	0.121801069474611	0.267196589797436	-4,65953E+14	8,15795E+13	8,26675E+14	7,84506E+14	6,52356E+14	7,58935E+14	7,09662E+13
TC0800000516.mm.2	Sorbs2	sorbin and SH3 domain containing 2	1,32262E+14	5,56117E+14	1,78148E+14	0.121807245601216	0.267196589797436	-4,65958E+14	6,4214E+14	6,7263E+14	5,9781E+14	3,73873E+14	5,73073E+14	4,77174E+14
TC0600002284.mm.2	Hoxa10	homeobox A10	-0.830884850463842	5,7401E+14	-1,78138E+14	0.121824988585962	0.267196589797436	-4,65971E+13	5,23256E+14	5,03599E+14	5,84836E+14	6,39335E+14	5,37342E+14	6,5569E+14
TC1900000237.mm.2	Osbp	oxysterol binding protein	1,12851E+14	7,23354E+14	1,78133E+14	0.12183288929695	0.267196589797436	-4,65977E+14	8,244E+14	8,15607E+14	7,34076E+14	5,51735E+14	7,50956E+14	6,63349E+14
TC1700000203.mm.2	Lix1	limb and CNS expressed 1	1,00835E+14	6,93993E+14	1,78115E+14	0.121864853724545	0.267230319551387	-4,66E+14	7,79325E+14	7,92505E+14	6,89442E+14	5,32525E+14	7,23178E+14	6,4698E+14
TC0800002523.mm.2	Abce1	ATP-binding cassette, sub-family E (OABP), member 1	2,00126E+14	7,27937E+14	1,7809E+14	0.121906712753305	0.267285734466459	-4,66031E+14	8,77935E+13	8,74988E+14	7,96689E+14	4,65339E+13	7,78795E+14	5,73875E+14
TC0500003431.mm.2	Serpine1	serine (or cysteine) peptidase inhibitor, clade E, member 1	-0.585196947601457	6,35722E+14	-1,78034E+14	0.122004054982917	0.267442811522877	-4,66102E+14	5,86391E+14	5,94313E+14	6,42979E+14	6,92904E+14	6,3543E+14	6,62313E+14
TC0100003422.mm.2	Atp1b1	ATPase, Na+/K+ transporting, beta 1 polypeptide	1,04778E+14	1,10434E+13	1,7803E+14	0.122011550146135	0.267442811522877	-4,66108E+14	1,17109E+14	1,17383E+14	1,14209E+14	1,00364E+13	1,12842E+14	1,00694E+14
TC1000002491.mm.2	Cbarp	calcium channel, voltage-dependent, beta subunit associated regulatory protein	0.613309527338716	9,01875E+14	1,78017E+13	0.122033310856592	0.267454126473376	-4,66123E+13	9,53462E+14	9,17938E+14	9,2325E+14	8,67863E+14	8,65449E+14	8,83288E+14
TC1000002535.mm.2	Tjp3	tight junction protein 3	-0.54429981109444	5,70892E+14	-1,77979E+14	0.122100001648597	0.267563895901516	-4,66172E+14	5,2803E+14	5,38794E+14	6,04092E+14	5,78755E+14	5,75063E+14	6,00621E+14
TC1600002036.mm.2	Krtap7-1	keratin associated protein 7-1	-0.632312404260282	4,88351E+14	-1,77965E+14	0.122124277333677	0.267580701751093	-4,6619E+14	4,53351E+14	4,49386E+14	4,64247E+14	5,33922E+14	5,00926E+14	5,28273E+14
TC0900002380.mm.2	Clk3	CDC-like kinase 3	1,06354E+14	6,78176E+14	1,77941E+14	0.122166260469082	0.267589925724775	-4,66221E+14	7,56398E+14	7,36786E+14	7,36547E+14	5,58847E+14	6,69246E+14	6,1123E+14
TC0400001147.mm.2	Tut4	terminal uridylyl transferase 4	-0.637953769762691	7,98458E+14	-1,77939E+14	0.122169485176061	0.267589925724775	-4,66223E+14	7,46025E+14	7,66244E+14	7,88052E+14	8,50569E+14	7,99949E+14	8,39911E+12
TC1400002228.mm.2	Prss55	protease, serine 55	-0.772295805317078	5,59131E+14	-1,77934E+14	0.122178308419113	0.267589925724775	-4,6623E+14	5,04577E+14	5,12875E+14	5,86241E+14	5,7918E+14	5,27877E+13	6,44034E+14
TC1000003177.mm.2	Olfr771	olfactory receptor 771	-0.933403198606186	5,0906E+14	-1,77887E+14	0.122259831041218	0.267732081756883	-4,66289E+12	4,69362E+14	4,63719E+14	4,44962E+14	5,56554E+14	5,04409E+14	6,15353E+14
TC1000003225.mm.2	Ddt	D-dopachrome tautomerase	0.577526234287046	7,68669E+14	1,77816E+14	0.122383217906232	0.267958859516379	-4,66379E+14	8,07588E+14	7,80815E+14	7,9486E+14	7,55849E+13	7,49779E+14	7,2312E+13
TC0100001863.mm.2	Kcnh1	potassium voltage-gated channel, subfamily H (eag-related), member 1	0.711594553877569	7,59391E+13	1,77808E+14	0.122396649043663	0.267958859516379	-4,66389E+14	8,06026E+14	8,07408E+14	7,76106E+14	6,95688E+14	7,6519E+13	7,05925E+14
TC0200002849.mm.2	Meig1	meiosis expressed gene 1	0.666357308846598	5,88374E+14	1,77756E+14	0.122486186053244	0.268118450737117	-4,66455E+14	6,46132E+14	6,08768E+14	5,8002E+13	5,73698E+14	5,99608E+14	5,2202E+13
TC0900001458.mm.2	Elp6	elongator acetyltransferase complex subunit 6	0.930050415726948	5,38655E+14	1,77734E+14	0.122524425219011	0.268129680487248	-4,66482E+14	5,77081E+14	6,05655E+14	5,57719E+14	4,94747E+14	4,27975E+14	5,6875E+14
TC0600000459.mm.2	Zyx	zyxin	-0.661996409942464	7,90855E+14	-1,77698E+14	0.122588090141073	0.268129680487248	-4,66529E+14	7,6486E+14	7,33452E+14	8,14601E+14	8,01503E+13	7,76388E+13	8,54324E+14
TC0100001189.mm.2	Klhdc8a	kelch domain containing 8A	0.724206208029345	6,55152E+14	1,77696E+14	0.122591479336319	0.268129680487248	-4,66531E+14	7,27046E+13	6,79566E+14	6,3862E+14	6,23908E+14	5,85405E+14	6,76367E+14
TC1700002051.mm.2	Olfr90	olfactory receptor 90	-0.773615491751984	4,43765E+14	-1,77695E+14	0.122592522647713	0.268129680487248	-4,66532E+14	3,72152E+14	4,03038E+14	4,42641E+14	5,14847E+13	4,21391E+14	5,08521E+14
TC1100000681.mm.2	Gm12250	predicted gene 12250	-0.714286443184942	4,7501E+14	-1,77693E+14	0.122595971819002	0.268129680487248	-4,66535E+14	3,9966E+14	4,46162E+14	4,71287E+14	5,44271E+14	4,68478E+14	5,20201E+14
TC0X00001634.mm.2	Phka2	phosphorylase kinase alpha 2	1,01887E+14	6,29264E+14	1,7769E+14	0.122600818486826	0.268129680487248	-4,66538E+14	7,03586E+14	6,92626E+13	6,45299E+14	5,41637E+14	6,68334E+13	5,24103E+14
TC1100002496.mm.2	Foxi1	forkhead box I1	-0.9088237527392	6,16739E+14	-1,77678E+14	0.122622588138346	0.268129680487248	-4,66554E+14	5,59885E+14	5,88166E+14	5,3828E+14	6,84287E+14	6,49793E+14	6,80023E+14
TC0400000867.mm.2	Rraga	Ras-related GTP binding A	1,42508E+14	7,66235E+14	1,77677E+14	0.122624440855864	0.268129680487248	-4,66555E+14	8,91476E+14	8,73957E+14	7,99633E+14	5,51927E+14	7,91334E+14	6,89083E+14
TC1000001789.mm.2	Vta1	vesicle (multivesicular body) trafficking 1	0.533442889932762	7,42744E+13	1,77634E+14	0.122699123562917	0.268251305800914	-4,6661E+14	7,84314E+13	7,55946E+14	7,47238E+14	7,35394E+14	7,19778E+14	7,13794E+14
TC0800002959.mm.2	Tmem231	transmembrane protein 231	0.919457080304278	6,86612E+14	1,77624E+14	0.122717129640912	0.268251305800914	-4,66623E+14	7,42933E+14	7,17828E+14	7,61876E+14	6,20161E+14	6,66415E+14	6,10455E+14
TC0200002869.mm.2	Mcm10	minichromosome maintenance 10 replication initiation factor	-0.732981869352608	6,94514E+14	-1,77616E+14	0.122730008463001	0.268251305800914	-4,66632E+14	6,33886E+14	6,53565E+14	6,75105E+14	7,7048E+14	6,95801E+14	7,38246E+14
TC0X00002242.mm.2	Rtl8b	retrotransposon Gag like 8B	0.597743974335154	8,53397E+14	1,77531E+14	0.122878189253092	0.268518257211769	-4,6674E+14	9,08077E+14	8,73269E+14	8,57358E+14	8,19881E+14	8,49245E+14	8,12552E+14

TC1500001835.mm.2	C1qtnf6	C1q and tumor necrosis factor related protein 6	-0.742710858836793	5,52589E+14	-1,77527E+14	0.122885473138434	0.268518257211769	-4,66745E+14	4,87414E+13	5,26387E+14	5,20018E+14	6,19372E+14	5,92386E+14	5,69957E+14
TC0700001352.mm.2	Kctd14	potassium channel tetramerisation domain containing 14	-0.719781164608964	5,45611E+13	-1,77507E+14	0.12291982046869	0.268556890469425	-4,6677E+14	5,31889E+14	4,8127E+14	5,09509E+14	5,93881E+13	5,63994E+13	5,93122E+14
TC0300002492.mm.2	Plekho1	pleckstrin homology domain containing, family O member 1	0.833607795203871	6,82309E+14	1,77443E+14	0.123032493132646	0.268766616302375	-4,66852E+14	7,16319E+14	7,64565E+14	6,92485E+14	6,06324E+14	7,01847E+14	6,12315E+14
TC1400001189.mm.2	Slain1	SLAIN motif family, member 1	1,48413E+14	8,07959E+14	1,77421E+14	0.123071252270075	0.268814841782259	-4,6688E+14	9,0781E+14	9,06716E+14	8,92223E+14	6,23304E+14	8,00862E+14	7,16838E+14
TC1100004057.mm.2	Cd300ld4/Cd300ld5/ Cd300ld3	CD300 molecule like family member D4/CD300 molecule like family member D5/CD300 molecule like family member D3	-0.600455482244041	6,0308E+14	-1,77399E+14	0.123109737356107	0.268862455681615	-4,66908E+14	5,6463E+14	5,79457E+14	5,89899E+14	6,20317E+14	6,04831E+14	6,59347E+14
TC0200002383.mm.2	Spag4	sperm associated antigen 4	-0.642148160693834	5,25489E+14	-1,7738E+13	0.123141699434302	0.268895812845225	-4,66932E+14	4,80074E+14	4,84641E+14	5,2619E+14	5,68883E+14	5,13427E+14	5,79717E+14
TC0700001754.mm.2	Chp2	calcineurin-like EF hand protein 2	-1,13577E+14	4,79063E+14	-1,77351E+13	0.123193097651289	0.268924354716194	-4,66969E+14	4,14186E+14	4,09273E+14	4,20638E+14	5,79669E+14	4,60472E+14	5,90141E+14
TC1300002303.mm.2	Slc6a19	solute carrier family 6 (neurotransmitter transporter), member 19	-0.533488895312486	6,56015E+14	-1,77347E+14	0.123200670775673	0.268924354716194	-4,66974E+14	6,24784E+14	6,08524E+14	6,88627E+13	6,74152E+14	6,58313E+12	6,81692E+14
TC1300002059.mm.2	Dbn1	drebrin 1	0.6557993725388	7,56251E+14	1,77344E+14	0.123204839974861	0.268924354716194	-4,66977E+14	7,94672E+14	8,08363E+14	7,65535E+14	6,97059E+14	7,58811E+14	7,13064E+14
TC0200002526.mm.2	Wfdc10	WAP four-disulfide core domain 10	-0.563045329931644	5,89513E+14	-1,77323E+14	0.123241289909258	0.268926827344069	-4,67004E+14	5,82769E+14	5,49681E+14	5,82434E+14	5,77133E+14	6,05021E+14	6,40038E+14
TC1800000797.mm.2	Rpl17	ribosomal protein L17	1,26761E+14	1,01663E+14	1,77317E+14	0.123252879477035	0.268926827344069	-4,67012E+14	1,10124E+12	1,10837E+14	1,06234E+14	8,71726E+14	1,04646E+14	9,09621E+14
TC0400003382.mm.2	Artn	artemin	-1,11732E+14	5,30946E+13	-1,77315E+14	0.123256042942714	0.268926827344069	-4,67014E+14	4,46473E+13	4,5582E+14	5,0959E+14	6,48033E+14	4,90083E+14	6,35674E+14
TC0X00002317.mm.2	Sox3	SRY (sex determining region Y)-box 3	-0.778056892590553	5,82951E+14	-1,77292E+14	0.123295754780251	0.268964181683077	-4,67043E+14	5,02137E+13	5,68817E+14	5,50992E+14	6,49197E+14	6,17224E+13	6,09341E+14
TC1700001210.mm.2	Ttc39d	tetratricopeptide repeat domain 39D	-0.923855543317797	4,97607E+14	-1,77286E+14	0.123306548134605	0.268964181683077	-4,67051E+13	4,43595E+14	4,40541E+14	4,89115E+13	5,43484E+14	4,61408E+14	6,07499E+14
TC0300002047.mm.2	Clrn1	clarin 1	-0.620178155191457	5,22057E+13	-1,77251E+14	0.123367494814424	0.26905734476121	-4,67095E+14	5,0968E+13	4,7748E+13	4,85162E+14	5,48825E+14	5,52544E+14	5,58651E+14
TC0600000763.mm.2	C130060K24Rik	RIKEN cDNA C130060K24 gene	-0.529856755463957	6,30505E+14	-1,7723E+14	0.123405271296307	0.26905734476121	-4,67122E+14	6,13727E+14	6,04796E+14	6,30711E+14	6,09302E+14	6,71462E+14	6,53033E+14
TC0700004661.mm.2	Prrt2/Pagr1a	proline-rich transmembrane protein 2/PAXIP1 associated glutamate rich protein 1A	1,16624E+14	8,97389E+14	1,77229E+14	0.123407134562066	0.26905734476121	-4,67124E+14	9,61564E+14	9,68129E+13	9,5571E+14	8,02484E+14	9,21627E+14	7,74818E+14
TC0400003866.mm.2	Padi3	peptidyl arginine deiminase, type III	-0.750883294552898	5,21014E+14	-1,77223E+14	0.123416051333091	0.26905734476121	-4,6713E+14	4,90247E+14	4,50516E+14	5,32356E+14	5,62023E+14	4,95441E+14	5,95499E+14
TC0700003844.mm.2	Inppl1	inositol polyphosphate phosphatase-like 1	1,21832E+14	5,88003E+14	1,7718E+14	0.123492365147786	0.269114180790265	-4,67186E+14	6,82613E+14	6,73828E+13	6,40649E+14	4,18153E+14	5,68755E+14	5,44022E+14
TC1800001657.mm.2	Kcng2	potassium voltage-gated channel, subfamily G, member 2	-0.680761835519681	6,10065E+14	-1,77176E+14	0.123499901491238	0.269114180790265	-4,67191E+14	5,7468E+14	5,62017E+14	5,85408E+14	6,65437E+14	6,15184E+14	6,57666E+14
TC0700002457.mm.2	Psg21	pregnancy-specific glycoprotein 21	-0.995384285460942	4,87265E+13	-1,77172E+14	0.123505842784006	0.269114180790265	-4,67195E+14	4,13809E+14	4,08812E+14	4,66848E+14	6,12425E+14	4,64052E+14	5,57646E+14
TC0400004041.mm.2	Rbp7	retinol binding protein 7, cellular	-0.543388625252438	5,82607E+11	-1,77163E+14	0.123522184716065	0.269114180790265	-4,67207E+14	5,49134E+14	5,37378E+14	5,9274E+12	6,21201E+14	6,09141E+14	5,86049E+14
TC0600000374.mm.2	Ttc26	tetratricopeptide repeat domain 26	0.696959664814928	5,35852E+14	1,77152E+14	0.123541951364117	0.269114180790265	-4,67221E+14	6,0409E+13	5,81874E+13	5,23205E+14	4,5937E+14	4,88932E+14	5,57641E+13
TC0800000834.mm.2	Sin3b	transcriptional regulator, SIN3B (yeast)	0.612609478957196	7,58552E+14	1,77145E+14	0.123553584842049	0.269114180790265	-4,6723E+14	8,1654E+14	7,91961E+14	7,57588E+14	6,99243E+14	7,56731E+13	7,29248E+14
TC1200000181.mm.2	Cpsf3	cleavage and polyadenylation specificity factor 3	0.724863657495638	7,32268E+14	1,77135E+14	0.123570792139833	0.269114180790265	-4,67242E+14	7,93619E+14	7,79498E+14	7,43393E+14	6,48955E+14	6,92851E+14	7,35294E+14
TC0200002386.mm.2	Phf20	PHD finger protein 20	2,82147E+14	5,07658E+14	1,77116E+13	0.123604498257247	0.269114180790265	-4,67267E+14	7,32458E+14	6,98213E+14	5,41404E+13	2,07497E+14	6,58519E+13	2,07858E+14
TC1700000415.mm.2	Ergic1	endoplasmic reticulum-golgi intermediate compartment (ERGIC) 1	0.829291842694932	8,97953E+14	1,77115E+14	0.123605560707218	0.269114180790265	-4,67267E+14	9,50113E+14	9,20604E+13	9,55805E+13	8,56335E+14	8,93701E+14	8,11159E+14
TC0900002167.mm.2	Upk2	uroplakin 2	-0.752360474242423	5,7872E+14	-1,77112E+14	0.123611833893007	0.269114180790265	-4,67272E+14	5,0324E+14	5,14806E+14	5,88034E+14	6,9772E+14	5,73943E+14	5,94575E+14
TC1100000942.mm.2	Clec10a	C-type lectin domain family 10, member A	-0.520307344529648	6,71906E+14	-1,77104E+14	0.12362584039034	0.269114180790265	-4,67282E+14	6,51322E+14	6,43717E+14	6,58342E+13	6,78955E+14	6,99415E+14	6,99686E+14
TC1500002040.mm.2	Plxnb2	plexin B2	0.62737856281893	6,75592E+14	1,77083E+14	0.123662932335294	0.269158561220937	-4,67309E+14	6,93749E+14	7,29288E+14	6,80219E+14	6,52738E+14	6,19169E+14	6,78392E+14
TC0300001055.mm.2	Syt6	synaptotagmin VI	0.843785265495327	7,94907E+13	1,77068E+14	0.123687790675497	0.269164839632495	-4,67327E+14	8,42447E+14	8,42432E+14	8,51088E+14	7,17352E+13	7,71314E+14	7,44808E+14
TC0100002285.mm.2	Slc40a1	solute carrier family 40 (iron-regulated transporter), member 1	-0.521016617573264	6,01768E+14	-1,77056E+14	0.123709146398514	0.269164839632495	-4,67342E+13	5,75447E+14	5,75519E+14	6,06153E+14	5,98317E+14	6,38282E+14	6,16888E+14
TC1300002583.mm.2	Srek1	splicing regulatory glutamine/lysine-rich protein 1	0.541005229330901	7,91935E+14	1,77028E+14	0.123758933192456	0.269164839632495	-4,67378E+14	8,30692E+14	8,34116E+14	7,78034E+14	7,52159E+14	7,91651E+14	7,64956E+14
TC1800000682.mm.2	Sec11c	SEC11 homolog C, signal peptidase complex subunit	0.846008802256112	7,63903E+14	1,77019E+14	0.123774943850096	0.269164839632495	-4,6739E+14	8,33645E+14	8,31291E+14	7,57612E+14	6,65133E+14	7,98366E+14	6,97368E+14
TC1300000089.mm.2	Lyst	lysosomal trafficking regulator	0.773741811516927	6,6238E+14	1,77009E+14	0.123792128866742	0.269164839632495	-4,67402E+14	7,01668E+14	7,15104E+14	6,52975E+14	6,42509E+13	6,89464E+14	5,72559E+14
TC0900001895.mm.2	Zfp653	zinc finger protein 653	-0.769932845194169	7,06338E+14	-1,77007E+14	0.123795608565592	0.269164839632495	-4,67405E+14	6,5612E+14	6,47981E+14	6,83727E+14	7,92111E+14	7,07234E+14	7,50853E+14
TC0200000525.mm.2	Tbc1d13	TBC1 domain family, member 13	1,30542E+14	5,36212E+14	1,77003E+14	0.123802006932414	0.269164839632495	-4,67409E+14	6,15349E+14	6,06744E+14	6,09227E+14	4,24942E+14	5,60039E+14	4,00969E+14
TC1100001035.mm.2	Olfr401	olfactory receptor 401	-0.633546094176322	4,78963E+14	-1,76997E+14	0.123812575499916	0.269164839632495	-4,67417E+14	4,43451E+13	4,4869E+13	4,45607E+14	5,17178E+14	4,96098E+14	5,22752E+14
TC0X00001661.mm.2	Cltrn	collectrin, amino acid transport regulator	-0.695426469669227	5,27556E+14	-1,76995E+14	0.123816160327441	0.269164839632495	-4,67419E+14	4,97554E+14	4,69287E+14	5,02574E+14	5,89998E+13	5,6706E+14	5,38866E+14
TC1700001191.mm.2	Cebpzos	CCAAT/enhancer binding protein (C/EBP), zeta, opposite strand	-0.681850289393822	6,27824E+14	-1,7697E+14	0.123860665026601	0.269206668161767	-4,67451E+14	5,78367E+14	5,9031E+14	6,01076E+14	6,92143E+14	6,49404E+13	6,55643E+14
TC0900002153.mm.2	Abcg4	ATP binding cassette subfamily G member 4	1,09228E+14	8,41517E+13	1,7696E+14	0.123878675795002	0.269206668161767	-4,67464E+14	9,17262E+14	8,92635E+14	9,12773E+14	7,34992E+14	8,34997E+14	7,56443E+14
TC0400001352.mm.2	Slfnl1	schlafen like 1	-0.58776416008935	5,04536E+14	-1,76956E+14	0.123885523764631	0.269206668161767	-4,67469E+13	4,79094E+14	4,4684E+12	5,05746E+14	5,52052E+14	5,31578E+14	5,11909E+14
TC0400003481.mm.2	Bmp8a	bone morphogenetic protein 8a	-0.61908402902793	5,40372E+14	-1,76941E+14	0.123911105408668	0.269219547964888	-4,67488E+13	4,99599E+14	4,97702E+14	5,29483E+14	5,9433E+14	5,45412E+13	5,75707E+13
TC0400001433.mm.2	Rspo1	R-spondin 1	-0.636828680013219	7,10036E+14	-1,76934E+14	0.12392486732797	0.269219547964888	-4,67498E+14	6,71094E+14	6,54598E+14	7,36106E+14	7,45362E+14	6,88827E+13	7,64231E+14
TC0200002032.mm.2	Cds2	CDP-diacylglycerol synthase (phosphatidate cytidylyltransferase) 2	1,45376E+14	9,08463E+14	1,76908E+14	0.123969010863012	0.269279141552401	-4,67529E+14	1,04287E+14	1,0234E+14	9,30814E+14	6,78162E+14	9,42582E+14	8,32942E+14
TC0800001226.mm.2	Carmil2	capping protein regulator and myosin 1 linker 2	-0.711717346696296	7,20616E+14	-1,76879E+14	0.12402023902383	0.26932059442302	-4,67566E+14	6,93809E+14	6,69579E+13	7,23707E+14	7,30867E+14	7,0396E+14	8,01771E+14
TC0400002626.mm.2	Tex10	testis expressed gene 10	0.635953621231278	5,63969E+14	1,76879E+14	0.124021523652877	0.26932059442302	-4,67567E+14	6,38407E+14	6,38432E+14	5,17139E+14	4,40191E+14	5,79491E+14	5,70157E+14
TC0500003391.mm.2	Fzd9	frizzled class receptor 9	-0.776137187035905	7,15264E+14	-1,76858E+14	0.124057088379321	0.269361523387145	-4,67593E+14	6,83544E+14	6,63301E+14	7,0767E+14	7,34993E+14	6,94039E+14	8,08035E+14
TC1200000200.mm.2	Grhl1	grainyhead like transcription factor 1	0.602799569938686	6,39931E+14	1,76835E+14	0.124098610176303	0.269415373992288	-4,67623E+14	6,66962E+14	7,01349E+14	6,39489E+14	5,84038E+14	6,19698E+14	6,28053E+14
TC1200000462.mm.2	Srp54c/Srp54b	signal recognition particle 54C/signal recognition particle 54B	1,41321E+14	8,15566E+14	1,76821E+14	0.124122201756096	0.269430289222143	-4,6764E+14	9,09615E+14	9,3077E+14	8,62295E+13	6,32973E+14	8,46879E+14	7,10865E+14
TC1200000484.mm.2	Pax9	paired box 9	-0.613491021755294	6,34357E+14	-1,76801E+14	0.124158485832613	0.269440899118862	-4,67666E+14	6,10975E+14	5,94106E+14	6,15472E+14	6,5781E+14	6,37475E+13	6,90304E+14
TC0200003590.mm.2	Wdsub1	WD repeat, SAM and U-box domain containing 1	0.850062914049104	5,16094E+14	1,768E+14	0.124160533479647	0.269440899118862	-4,67667E+14	5,91407E+14	5,96485E+14	5,138E+14	3,7699E+14	4,99303E+14	5,18576E+14
TC1500001054.mm.2	Slc4a8	solute carrier family 4 (anion exchanger), member 8	0.856180730742691	7,12976E+14	1,76744E+14	0.124258851597719	0.269617947184763	-4,67738E+14	7,61916E+14	7,64602E+14	6,28246E+14	7,67807E+14	6,82069E+14	6,73214E+14
TC0X00002085.mm.2	Lamp2	lysosomal-associated membrane protein 2	1,04548E+13	9,12733E+14	1,76718E+14	0.124303847153375	0.26966035475813	-4,6777E+14	9,69708E+14	1,01001E+14	9,43403E+14	7,82652E+14	9,2694E+14	8,43682E+14
TC1100000134.mm.2	Ikzf1	IKAROS family zinc finger 1	0.643466394268907	6,17548E+14	1,76696E+14	0.124342903288983	0.26966035475813	-4,67798E+14	6,47879E+14	6,51033E+14	6,0809E+14	6,28066E+14	6,23934E+14	5,46285E+14
TC0600001030.mm.2	Dnajb8	DnaJ heat shock protein family (Hsp40) member B8	-0.962523982539595	5,90351E+14	-1,76695E+14	0.12434495746075	0.26966035475813	-4,678E+13	5,33376E+14	5,08713E+14	5,61962E+13	7,03463E+14	5,75715E+14	6,58878E+14

TC1900000926.mm.2	Rce1	Ras converting CAAX endopeptidase 1	0.7456180061994	5,70268E+14	1,76693E+14	0.124348330332574	0.26966035475813	-4,67802E+14	6,03203E+14	6,13922E+14	5,65825E+14	5,7066E+14	5,89105E+14	4,78895E+14
TC0200004951.mm.2	Eif2s2	eukaryotic translation initiation factor 2, subunit 2 (beta)	0.997239743441094	9,02651E+14	1,76685E+14	0.124362073866298	0.26966035475813	-4,67812E+14	9,79546E+14	9,73017E+12	9,21153E+14	7,89073E+14	9,33272E+14	8,19843E+13
TC0200001745.mm.2	Inafm2	InaF motif containing 2	0.809868615862218	7,30564E+14	1,76649E+14	0.124425671019802	0.269761953329127	-4,67858E+14	7,63795E+14	8,14239E+14	7,47318E+14	6,41972E+14	7,34358E+14	6,81702E+14
TC1100002836.mm.2	Rnf187	ring finger protein 187	1,32976E+14	8,98828E+14	1,76634E+14	0.124453396739121	0.269785763710139	-4,67878E+14	9,98498E+13	9,73144E+14	9,67478E+14	7,48156E+14	9,11004E+14	7,94687E+14
TC0100000985.mm.2	Cntnap5b	contactin associated protein-like 5B	1,17571E+14	5,73117E+14	1,76602E+14	0.124508417986927	0.269868730027355	-4,67917E+14	6,61715E+14	6,48884E+14	6,13283E+14	4,39364E+14	6,0092E+14	4,74538E+14
TC0700001574.mm.2	Rbmxl2	RNA binding motif protein, X-linked-like 2	-0.756171992632529	5,71413E+14	-1,7659E+14	0.124529999500163	0.269869609597805	-4,67933E+14	5,56746E+14	4,98165E+14	5,54837E+14	6,12583E+14	5,63707E+14	6,42438E+14
TC0400001305.mm.2	Hyi	hydroxypyruvate isomerase (putative)	-0.589583730858429	8,05368E+14	-1,76582E+14	0.124544269586929	0.269869609597805	-4,67943E+14	7,81429E+14	7,60307E+14	7,932E+14	8,37621E+14	8,11795E+14	8,47858E+14
TC0200002811.mm.2	Arfgap1	ADP-ribosylation factor GTPase activating protein 1	0.985568956595522	8,17073E+14	1,76574E+14	0.124559069482956	0.269869609597805	-4,67953E+14	9,20727E+14	9,01728E+14	8,05922E+14	6,48718E+14	8,4575E+14	7,79591E+14
TC0700002842.mm.2	Cebpg	CCAAT/enhancer binding protein (C/EBP), gamma	0.663897309834196	7,00331E+14	1,76542E+14	0.124615505395447	0.269903850813573	-4,67994E+14	7,43942E+14	7,20294E+14	7,37491E+14	6,68805E+14	6,89673E+13	6,41783E+14
TC0700002481.mm.2	Klc3	kinesin light chain 3	-0.738692645085597	6,87433E+14	-1,76536E+13	0.124625940119456	0.269903850813573	-4,68001E+14	6,28005E+14	6,40261E+14	6,85182E+14	7,55145E+13	6,66602E+14	7,49403E+14
TC0400001160.mm.2	Rab3b	RAB3B, member RAS oncogene family	0.871454296149277	8,35654E+14	1,76529E+14	0.124638913759593	0.269903850813573	-4,68011E+14	9,36513E+13	8,41665E+13	8,59568E+14	7,7229E+14	8,34005E+14	7,69882E+14
TC1100002758.mm.2	Csf2	colony stimulating factor 2 (granulocyte-macrophage)	-0.98616329684613	5,49654E+14	-1,76527E+14	0.124641876367144	0.269903850813573	-4,68013E+14	4,94757E+14	4,56379E+14	5,21127E+14	6,77294E+13	5,43936E+14	6,04433E+14
TC0500000723.mm.2	Exoc1	exocyst complex component 1	0.633258212826785	7,11674E+14	1,76464E+14	0.124753473423744	0.270079564929269	-4,68093E+14	7,33813E+14	7,26544E+13	7,2125E+14	7,54732E+14	7,01389E+14	6,32316E+14
TC0700000818.mm.2	Il4i1b/Il4i1/Nup62	interleukin 4 induced 1B/interleukin 4 induced 1/nucleoporin 62	-0.704454029585693	7,85063E+14	-1,76458E+14	0.124763148131898	0.270079564929269	-4,68099E+13	7,28253E+14	7,22542E+14	7,91319E+14	8,76581E+14	7,70458E+14	8,21228E+14
TC0400001661.mm.2	Ifnlr1	interferon lambda receptor 1	-0.515951690026495	6,05427E+14	-1,76453E+14	0.124773306108948	0.270079564929269	-4,68107E+13	5,81546E+14	5,65991E+14	6,15425E+13	6,18873E+12	6,30945E+14	6,19782E+14
TC1200001933.mm.2	Ppp2r5e	protein phosphatase 2, regulatory subunit B', epsilon	0.742551579837703	7,39998E+14	1,76426E+14	0.124819916043626	0.27010089384129	-4,6814E+14	8,01602E+14	7,69988E+14	7,65634E+14	6,79685E+14	7,42275E+14	6,80805E+14
TC0900002928.mm.2	Sox14	SRY (sex determining region Y)-box 14	-0.767555492960716	5,09866E+14	-1,76424E+13	0.12482365131509	0.27010089384129	-4,68143E+14	4,24329E+14	4,82692E+14	5,05489E+13	5,86156E+14	4,99392E+14	5,6114E+14
TC0100000131.mm.2	Efhc1	EF-hand domain (C-terminal) containing 1	-0.563290473243109	6,75675E+14	-1,76419E+14	0.124833448546893	0.27010089384129	-4,6815E+14	6,3318E+14	6,22116E+14	6,92311E+14	7,38488E+14	6,76245E+14	6,91709E+14
TC0100001712.mm.2	Lin9	lin-9 homolog (C. elegans)	-0.65833384738387	5,69207E+14	-1,76383E+14	0.124897360936141	0.270202896987653	-4,68195E+14	5,26063E+14	5,53975E+14	5,85676E+14	5,37821E+14	5,73333E+13	6,38372E+14
TC0200004125.mm.2	Zfp408	zinc finger protein 408	-0.697938855797251	6,98918E+14	-1,76305E+14	0.125035259050257	0.270443062359898	-4,68294E+14	6,7594E+14	6,21038E+14	6,9967E+14	7,60293E+14	7,44967E+14	6,91599E+14
TC1900001510.mm.2	Arhgap19	Rho GTPase activating protein 19	-0.576666743997699	6,5148E+14	-1,76301E+14	0.125041942194578	0.270443062359898	-4,68299E+12	6,1275E+13	6,19826E+14	6,4578E+12	6,82617E+14	6,53125E+14	6,94783E+14
TC1500001809.mm.2	Apol9a	apolipoprotein L 9a	-0.694907912185522	5,3883E+14	-1,7628E+14	0.125078995111934	0.270453910038338	-4,68325E+14	4,99524E+14	5,01063E+14	5,10329E+14	5,82494E+14	5,37541E+14	6,02028E+14
TC0400002035.mm.2	Cep104	centrosomal protein 104	1,04541E+14	5,31001E+14	1,76279E+14	0.125080527375765	0.270453910038338	-4,68326E+14	6,12019E+14	5,93209E+14	5,66992E+14	4,17638E+13	5,52742E+14	4,43405E+14
TC0800001619.mm.2	Cd209a	CD209a antigen	-0.685461123318385	6,49616E+14	-1,76236E+14	0.125157101355615	0.270583171091242	-4,68381E+13	6,0829E+14	5,79766E+14	6,51297E+14	7,33194E+14	6,80725E+14	6,44424E+14
TC0X00002272.mm.2	Rbmx	RNA binding motif protein, X chromosome	0.993263889698819	8,00966E+14	1,76225E+14	0.125176786258309	0.270589422723388	-4,68395E+14	8,91131E+14	8,81646E+14	8,13107E+14	6,45786E+14	8,10269E+14	7,63855E+14
TC1400001436.mm.2	Pdhb	pyruvate dehydrogenase (lipoamide) beta	1,06125E+14	8,93545E+14	1,76188E+14	0.125242893652642	0.270696008775992	-4,68442E+14	9,7291E+14	9,32277E+14	9,52876E+14	8,10269E+14	9,0575E+14	7,87186E+14
TC0900002368.mm.2	Ppcdc	phosphopantothenoylcysteine decarboxylase	0.598697991367381	7,46144E+14	1,76094E+14	0.125409034103312	0.271015845253987	-4,6856E+14	7,70275E+14	7,7949E+12	7,31023E+14	7,6605E+14	7,4211E+14	6,87916E+14
TC1500001813.mm.2	1700025B11Rik	RIKEN cDNA 1700025B11 gene	-0.680332478806971	5,78046E+14	-1,76077E+14	0.12543970457282	0.271015845253987	-4,68582E+14	5,43525E+14	5,50122E+14	5,26454E+14	6,18459E+14	6,19176E+14	6,10538E+14
TC0100003778.mm.2	Kcnk2	potassium channel, subfamily K, member 2	0.902683546035576	6,61915E+14	1,76068E+14	0.125456100777766	0.271015845253987	-4,68594E+14	7,74668E+14	7,01021E+14	6,65461E+14	5,35189E+14	6,33489E+14	6,61663E+14
TC1400000127.mm.2	Ngly1	N-glycanase 1	0.668631013193172	5,36491E+14	1,76057E+14	0.125475429392305	0.271015845253987	-4,68607E+14	5,98024E+14	5,43238E+14	5,16844E+14	5,53302E+14	5,33776E+14	4,73761E+14
TC0200001514.mm.2	Traf6	TNF receptor-associated factor 6	-0.595729962753494	6,63081E+14	-1,76055E+14	0.125479884096805	0.271015845253987	-4,6861E+13	6,141E+14	6,30833E+14	6,77687E+14	6,91789E+14	6,51917E+14	7,12162E+14
TC1200001982.mm.2	Zfyve26	zinc finger, FYVE domain containing 26	-0.535948446572198	6,78878E+14	-1,76048E+14	0.125491790569105	0.271015845253987	-4,68619E+14	6,31864E+14	6,52363E+14	6,9637E+14	7,01252E+14	6,81063E+14	7,10354E+14
TC0200003860.mm.2	Zswim2	zinc finger SWIM-type containing 2	-0.821834021862495	6,10478E+13	-1,75998E+14	0.125580063872606	0.271143852089011	-4,68682E+14	5,17411E+14	5,73323E+14	5,99026E+14	7,18005E+14	6,33547E+14	6,21554E+14
TC0900000997.mm.2	Wdr72	WD repeat domain 72	-0.764300089425169	5,96674E+14	-1,75996E+14	0.125584718434822	0.271143852089011	-4,68685E+13	5,44365E+14	5,31907E+14	5,87672E+14	6,86969E+14	5,84472E+14	6,4466E+14
TC0100001969.mm.2	Ncoa2	nuclear receptor coactivator 2	0.762516015494675	7,14179E+14	1,75977E+13	0.125618245975368	0.271164413482279	-4,68709E+14	7,87026E+14	7,54487E+14	7,24767E+14	6,29784E+14	7,2387E+14	6,65141E+14
TC1900001276.mm.2	Fxn	frataxin	-0.553635546388605	7,32833E+12	-1,75967E+14	0.125635964755928	0.271164413482279	-4,68721E+14	7,16229E+14	6,98215E+14	7,09161E+14	7,4822E+14	7,54471E+14	7,707E+14
TC1300001346.mm.2	Paip1	polyadenylate binding protein-interacting protein 1	1,14134E+14	7,47472E+14	1,75959E+14	0.125650782373438	0.271164413482279	-4,68732E+14	8,28748E+14	8,1731E+14	7,88477E+14	6,32507E+14	7,77882E+14	6,39909E+14
TC0200003137.mm.2	Slc34a3	solute carrier family 34 (sodium phosphate), member 3	-0.701552800113177	4,45246E+14	-1,75953E+14	0.125661557467397	0.271164413482279	-4,68739E+14	3,97551E+14	3,83769E+13	4,5886E+14	5,09666E+14	4,18812E+13	5,0282E+14
TC1900000675.mm.2	Dpcd	deleted in primary ciliary dyskinesia	0.529778271585844	7,23808E+14	1,75904E+14	0.125747384441404	0.271313284079317	-4,688E+14	7,65737E+14	7,49309E+13	6,9857E+14	7,20144E+14	7,13388E+14	6,95702E+12
TC1700001738.mm.2	Grm4	glutamate receptor, metabotropic 4	0.531167658440366	7,06324E+14	1,7589E+13	0.125772684329012	0.271331538705766	-4,68818E+14	7,09051E+14	7,34892E+13	7,32633E+14	7,2366E+14	6,59328E+14	6,78381E+14
TC1600002158.mm.2	C2cd2	C2 calcium-dependent domain containing 2	-0.706939043796893	6,53955E+14	-1,75877E+14	0.125795973935813	0.271345452212552	-4,68835E+14	6,15333E+13	5,9655E+14	6,43924E+14	7,14651E+13	6,41971E+14	7,113E+14
TC0700002429.mm.2	Npas1	neuronal PAS domain protein 1	-0.621585045508903	6,15079E+14	-1,75852E+14	0.125840299780767	0.271404731678155	-4,68866E+14	6,11268E+14	5,59959E+14	5,97451E+14	6,2625E+14	6,70073E+14	6,25471E+14
TC1800000249.mm.2	Lims2	LIM and senescent cell antigen like domains 2	0.689929743197432	6,32705E+14	1,75808E+14	0.125919098345629	0.271538333999347	-4,68922E+14	7,16666E+14	6,52803E+14	5,6193E+14	6,33352E+14	6,17275E+14	6,14207E+14
TC0900002887.mm.2	Zbtb38/E030011O05 Rik	zinc finger and BTB domain containing 38/RIKEN cDNA E030011O05 gene	1,03144E+14	7,75048E+14	1,75746E+14	0.126030771524236	0.271710072329314	-4,69001E+14	8,6977E+14	8,50997E+14	7,98795E+14	6,16245E+13	7,56087E+14	7,58393E+14
TC1000000548.mm.2	Vsir	V-set immunoregulatory receptor	0.64413223028441	6,86002E+14	1,75745E+14	0.126032463265022	0.271710072329314	-4,69002E+13	6,92551E+14	7,2251E+14	7,01261E+14	7,1346E+14	6,05657E+14	6,80577E+13
TC1700000168.mm.2	Smoc2	SPARC related modular calcium binding 2	-0.761350795568497	5,91723E+14	-1,75719E+13	0.126078253241769	0.271766801027404	-4,69035E+14	5,27208E+14	5,62741E+14	5,68279E+14	6,49889E+14	5,83681E+14	6,58539E+14
TC1400001524.mm.2	Gng2	guanine nucleotide binding protein (G protein), gamma 2	1,58413E+14	9,0736E+14	1,75711E+14	0.12609250943219	0.271766801027404	-4,69045E+14	1,04791E+14	9,98214E+14	9,64424E+14	7,04311E+14	9,41772E+13	7,87528E+14
TC0100003604.mm.2	Rgs7	regulator of G protein signaling 7	1,4543E+14	7,80012E+14	1,75685E+13	0.126139098757993	0.271830854391808	-4,69078E+14	8,88377E+14	8,8073E+14	8,16143E+14	6,16573E+13	8,36111E+14	6,42137E+14
TC0600000914.mm.2	Loxl3	lysyl oxidase-like 3	-0.847990476613108	7,31161E+14	-1,7566E+14	0.126184470851538	0.271842356000181	-4,6911E+14	7,02308E+14	7,08908E+14	7,14992E+14	6,79939E+14	7,23172E+13	8,57643E+14
TC0600001306.mm.2	Olfr214	olfactory receptor 214	-0.559467383088837	5,67254E+14	-1,75658E+14	0.126187590579916	0.271842356000181	-4,69112E+14	5,64745E+14	5,22919E+14	5,57686E+14	5,58612E+14	5,86755E+14	6,12803E+14
TC0400000507.mm.2	Nans	N-acetylneuraminic acid synthase (sialic acid synthase)	0.575238364874343	6,74692E+14	1,75645E+14	0.126210997277659	0.271842356000181	-4,69128E+14	7,20821E+14	6,94323E+14	6,54349E+14	6,7856E+14	6,2832E+14	6,71777E+14
TC1300001508.mm.2	Rala	v-ral simian leukemia viral oncogene A (ras related)	0.547397596639102	7,46172E+14	1,75644E+14	0.126211919893089	0.271842356000181	-4,69129E+14	7,91744E+14	7,84685E+14	7,28408E+14	7,05248E+14	7,51417E+14	7,15532E+14
TC0200002323.mm.2	Efcab8	EF-hand calcium binding domain 8	-0.616052131066163	5,82793E+14	-1,75631E+14	0.12623665657416	0.271848081397416	-4,69147E+14	5,41413E+14	5,30674E+14	5,99598E+14	6,29778E+14	5,65322E+14	6,29976E+13
TC0800000123.mm.2	Lamp1	lysosomal-associated membrane protein 1	0.920312364068149	9,31942E+14	1,75624E+13	0.126248320802884	0.271848081397416	-4,69155E+14	9,95768E+14	9,92797E+14	9,70696E+14	8,27888E+14	9,35026E+14	8,69477E+14
TC0200005287.mm.2	Ctcfl	CCCTC-binding factor (zinc finger protein)-like	-0.688618219739362	5,64521E+14	-1,756E+14	0.126291425231505	0.271891712901432	-4,69185E+14	5,16545E+14	4,9322E+13	5,77346E+14	6,52527E+14	5,46521E+14	6,00968E+14
TC0700004519.mm.2	Sigirr	single immunoglobulin and toll-interleukin 1 receptor (TIR) domain	-0.6094264922099	5,67439E+14	-1,75585E+14	0.126317472614993	0.271891712901432	-4,69204E+12	5,5854E+14	5,17751E+14	5,92296E+14	5,37871E+14	5,91184E+14	6,06993E+14
TC0800002811.mm.2	B3gnt9	UDP-GlcNAc:betaGal beta-1,3-N-acetylglucosaminyltransferase 9	-0.652103710451098	6,46207E+14	-1,75584E+14	0.126319205782916	0.271891712901432	-4,69205E+14	6,04956E+14	6,19241E+14	6,08161E+14	6,90267E+14	6,70668E+14	6,8395E+13
TC1500000588.mm.2	Maf1	MAF1 homolog, negative regulator of RNA polymerase III	-0.677352547057351	8,62295E+14	-1,75551E+14	0.126379247734086	0.271976867150841	-4,69247E+13	8,14688E+14	8,12818E+14	9,36519E+14	8,46768E+14	8,92336E+14	8,70641E+14

TC1200000839.mm.2	Flvcr2	feline leukemia virus subgroup C cellular receptor 2	-0.904644643297402	4,51391E+14	-1,75544E+14	0.126392526607428	0.271976867150841	-4,69257E+14	4,13252E+14	3,82043E+14	4,10562E+14	5,26267E+14	5,36011E+14	4,40213E+14
TC1400000591.mm.2	AY358078	cDNA sequence AY358078	-0.717084772782865	5,6605E+14	-1,75518E+14	0.12643868098924	0.272001208992892	-4,69289E+14	5,04595E+14	5,34468E+14	5,42303E+14	6,32452E+14	5,69847E+14	6,12633E+14
TC0900002185.mm.2	Atp5l	ATP synthase, H+ transporting, mitochondrial F0 complex, subunit G	-0.613698817389453	9,21524E+14	-1,75513E+14	0.126448111639082	0.272001208992892	-4,69296E+14	8,89512E+14	8,8665E+14	9,51016E+14	9,03067E+14	9,75906E+14	9,22995E+14
TC0900002376.mm.2	Lman1l	lectin, mannose-binding 1 like	-0.682413657908694	5,82529E+14	-1,75509E+13	0.126454481261451	0.272001208992892	-4,693E+14	5,46511E+14	5,26681E+14	5,7979E+14	6,3252E+14	5,67246E+13	6,42428E+14
TC1900001404.mm.2	Rnls	renalase, FAD-dependent amine oxidase	-0.704318579197594	5,69322E+14	-1,75482E+14	0.126502792286713	0.272068805674828	-4,69334E+14	5,4191E+14	4,78178E+14	5,78612E+14	6,56278E+14	5,79149E+13	5,81803E+14
TC1900000918.mm.2	Kdm2a	lysine (K)-specific demethylase 2A	1,71872E+14	6,65897E+14	1,75459E+14	0.126544848039828	0.272122932932836	-4,69364E+14	7,77676E+14	7,60903E+14	7,65496E+14	4,96473E+14	6,97588E+14	4,97248E+14
TC1500002038.mm.2	Mapk12	mitogen-activated protein kinase 12	-0.545172467907712	5,38726E+14	-1,75434E+14	0.126589680385908	0.272146691305117	-4,69395E+14	5,08797E+14	5,02744E+14	5,29279E+14	5,70963E+14	5,62938E+14	5,57637E+14
TC1100001131.mm.2	Nufip2	nuclear fragile X mental retardation protein interacting protein 2	1,80903E+12	8,14071E+14	1,75416E+14	0.126621987977995	0.272146691305117	-4,69418E+14	9,27801E+14	9,43751E+14	8,92513E+14	6,10616E+14	8,62632E+14	6,47115E+14
TC0X00002825.mm.2	Magt1	magnesium transporter 1	0.653995819601334	7,27741E+14	1,75413E+14	0.126627401060501	0.272146691305117	-4,69422E+14	7,52605E+13	7,79339E+14	7,41941E+14	6,91418E+14	7,32166E+14	6,68978E+14
TC0900000100.mm.2	Sesn3	sestrin 3	0.582739192383436	8,90497E+14	1,75409E+14	0.126634577978204	0.272146691305117	-4,69427E+14	9,33568E+14	9,23014E+14	9,00846E+14	8,45523E+14	8,59954E+14	8,8008E+13
TC1900000060.mm.2	Gal3st3	galactose-3-O-sulfotransferase 3	0.858730459705912	7,58519E+14	1,75405E+14	0.126640345770854	0.272146691305117	-4,69431E+14	8,14451E+14	8,37679E+14	7,77406E+14	6,41196E+14	7,5689E+14	7,23494E+14
TC0700000881.mm.2	Ldha	lactate dehydrogenase A	0.972350848870153	8,76573E+14	1,75378E+14	0.126689659752719	0.272216360527478	-4,69466E+14	9,49115E+14	9,56498E+14	8,97037E+13	7,45646E+14	8,94119E+14	8,17024E+14
TC0X00001402.mm.2	Nxt2	nuclear transport factor 2-like export factor 2	1,65665E+14	8,07821E+14	1,75333E+13	0.126769837174221	0.272324273939719	-4,69522E+14	9,37886E+14	9,46565E+14	8,46484E+14	5,62867E+14	8,53517E+14	6,99604E+14
TC0700000965.mm.2	Otud7a	OTU domain containing 7A	0.558980071842933	7,66417E+14	1,75322E+14	0.126790042214774	0.272324273939719	-4,69536E+13	7,91008E+14	8,08903E+14	7,60634E+14	7,49841E+14	7,66526E+14	7,21589E+13
TC1200000715.mm.2	Arg2	arginase type II	0.553710161928753	5,06785E+14	1,75322E+14	0.126790585433859	0.272324273939719	-4,69537E+14	5,24906E+14	5,52895E+14	4,92917E+14	5,02935E+14	5,01743E+14	4,65315E+14
TC1000002396.mm.2	Slc5a4b	solute carrier family 5 (neutral amino acid transporters, system A), member 4b	-0.786803538554611	6,17775E+14	-1,75311E+14	0.12680920807314	0.272327971435761	-4,6955E+14	5,46543E+14	5,76838E+13	6,06332E+14	6,96196E+14	6,00701E+13	6,8004E+14
TC0100001381.mm.2	Rgs16	regulator of G-protein signaling 16	0.880464006563209	6,38052E+14	1,75265E+14	0.126892724712954	0.272471011900296	-4,69609E+14	7,13581E+14	6,26485E+14	7,18366E+14	6,05906E+13	6,02029E+14	5,61944E+14
TC0800001092.mm.2	Mt4	metallothionein 4	-0.604665699520759	7,37985E+14	-1,7521E+13	0.126992122221712	0.272621373437508	-4,69678E+14	6,95798E+14	7,12395E+14	7,21429E+14	7,6916E+14	7,41399E+14	7,87728E+14
TC0400003311.mm.2	Cyp4x1	cytochrome P450, family 4, subfamily x, polypeptide 1	0.611838713514779	6,2132E+14	1,75207E+14	0.126996588408238	0.272621373437508	-4,69681E+14	6,87101E+14	6,20932E+14	6,1126E+14	6,22961E+14	5,81097E+14	6,04569E+14
TC0X00002307.mm.2	Atp11c	ATPase, class VI, type 11C	0.726555977850726	6,44364E+14	1,75154E+14	0.127092551596526	0.272791032885949	-4,69749E+13	6,81648E+14	6,91797E+14	6,72702E+14	5,91905E+14	6,48999E+14	5,79134E+14
TC0100001353.mm.2	Trmt1l	tRNA methyltransferase 1 like	0.687385870129747	7,22253E+14	1,75139E+14	0.127119206985851	0.272811904923151	-4,69767E+14	7,71639E+14	7,66232E+14	7,39748E+13	6,55504E+14	7,22933E+14	6,77461E+14
TC0100003612.mm.2	Chml	choroideremia-like	0.589902836449657	5,93887E+14	1,75126E+14	0.127143736644216	0.272818146735365	-4,69785E+13	6,36614E+14	6,1457E+14	5,60208E+14	6,18723E+14	5,62563E+14	5,70641E+14
TC1100003724.mm.2	Krtap4-2	keratin associated protein 4-2	-0.539859142660008	7,75307E+14	-1,75118E+13	0.127157831625327	0.272818146735365	-4,69794E+14	7,43156E+14	7,35417E+14	7,74542E+14	8,12184E+14	7,87603E+14	7,98942E+14
TC0500002093.mm.2	Rheb	Ras homolog enriched in brain	1,61065E+14	9,05359E+14	1,75109E+14	0.127172910080639	0.272818146735365	-4,69805E+13	1,02092E+14	1,00924E+14	9,71972E+14	7,21989E+14	9,48309E+14	7,59722E+14
TC0200005334.mm.2	Gm14391/Gm6710	predicted gene 14391/predicted gene 6710	1,30744E+14	8,80588E+14	1,75072E+14	0.1272401559919	0.272926069421923	-4,69852E+13	9,53554E+14	1,0385E+14	8,74046E+14	6,86856E+14	9,33941E+14	7,96627E+14
TC1900000204.mm.2	Cyb561a3	cytochrome b561 family, member A3	0.61310013189778	6,49775E+13	1,75029E+14	0.127318523552724	0.273057815786643	-4,69907E+14	6,58344E+14	7,22401E+14	6,46126E+14	6,13657E+14	6,27885E+14	6,30239E+14
TC0900001017.mm.2	Gm7972	predicted gene 7972	-0.569407966019112	6,09E+14	-1,75018E+13	0.127338355200102	0.273064002839931	-4,69921E+14	5,8209E+14	5,9219E+14	5,7512E+14	6,16437E+14	6,26859E+13	6,61303E+14
TC0700004311.mm.2	Zg16	zymogen granule protein 16	-0.744735786194178	5,34268E+14	-1,74989E+14	0.127390751133731	0.273085567637075	-4,69958E+13	4,65761E+14	4,79771E+14	5,61947E+14	6,03651E+14	4,95418E+14	5,99061E+14
TC1700002558.mm.2	Eif2ak2	eukaryotic translation initiation factor 2-alpha kinase 2	0.60079649820154	5,8234E+14	1,74983E+14	0.12740128868642	0.273085567637075	-4,69965E+14	5,92571E+14	6,23414E+14	5,68976E+14	6,13251E+14	5,68114E+14	5,27712E+14
TC1400001221.mm.2	Frg2f2	FSHD region gene 2 family member 2	-0.524468258191578	5,48128E+14	-1,7496E+14	0.127443326250526	0.273085567637075	-4,69994E+14	5,25778E+13	4,96003E+14	5,67918E+13	5,72396E+14	5,60482E+13	5,66192E+14
TC1400001222.mm.2	Frg2f12	FSHD region gene 2 family member 12	-0.524468258191578	5,48128E+14	-1,7496E+14	0.127443326250526	0.273085567637075	-4,69994E+14	5,25778E+13	4,96003E+14	5,67918E+13	5,72396E+14	5,60482E+13	5,66192E+14
TC0400001482.mm.2	Tmem35b/Zmym6	transmembrane protein 35B/zinc finger, MYM-type 6	0.541631290257237	6,6733E+13	1,74959E+14	0.127445056837415	0.273085567637075	-4,69995E+14	7,15333E+14	6,87241E+14	6,38467E+14	6,68692E+14	6,45955E+14	6,48292E+14
TC0900001940.mm.2	Glb1l3	galactosidase, beta 1 like 3	-0.778507096324285	5,15988E+14	-1,74956E+14	0.127450100454962	0.273085567637075	-4,69999E+14	4,47311E+14	4,35241E+14	5,35399E+14	6,39721E+14	4,88228E+14	5,50025E+14
TC0400002549.mm.2	Gne	glucosamine (UDP-N-acetyl)-2-epimerase/N-acetylmannosamine kinase	0.530639864553214	8,08626E+14	1,7494E+14	0.12747901187674	0.273111197762254	-4,70019E+14	8,60804E+14	8,54208E+14	7,80891E+14	7,46969E+14	8,0675E+14	8,02133E+14
TC1000000768.mm.2	Krtap10-10/Gm2696	keratin associated protein 10-10/predicted gene 2696	-0.522242088702273	8,07365E+14	-1,74921E+14	0.127513053439966	0.273147810433153	-4,70043E+14	7,61806E+14	7,80301E+13	8,38993E+14	8,16537E+14	8,23253E+14	8,23301E+14
TC0500003685.mm.2	N4bp2l1	NEDD4 binding protein 2-like 1	-0.567001391035274	7,96898E+14	-1,7488E+14	0.127587964489652	0.273271948932842	-4,70095E+14	7,77362E+14	7,49035E+14	8,24597E+13	7,90597E+14	8,35259E+14	8,04539E+14
TC1600001750.mm.2	Tfg	Trk-fused gene	2,35059E+14	6,68727E+14	1,74868E+13	0.127608295724261	0.273279169192586	-4,70109E+14	8,5056E+14	8,20178E+14	7,85595E+14	3,55407E+14	6,94898E+14	5,05722E+14
TC0500003608.mm.2	Rnf6	ring finger protein (C3H2C3 type) 6	1,07049E+13	6,92373E+14	1,74818E+14	0.127699969524099	0.273439150689941	-4,70173E+14	7,9294E+12	7,73552E+14	7,11899E+13	5,23454E+14	6,96937E+13	6,55458E+14
TC0500000688.mm.2	Cwh43	cell wall biogenesis 43 C-terminal homolog	-0.694370794673392	4,59389E+14	-1,74785E+14	0.127759296997641	0.273529836901805	-4,70215E+14	4,22202E+14	4,15198E+13	4,24375E+13	5,18284E+14	4,73884E+14	5,0239E+14
TC0700000376.mm.2	Zfp93	zinc finger protein 93	1,75885E+14	6,10542E+14	1,74764E+14	0.12779796845167	0.273576280810649	-4,70242E+14	7,00579E+14	7,31293E+14	6,63555E+14	4,87722E+14	6,88199E+13	3,91903E+14
TC0800000817.mm.2	Hsh2d	hematopoietic SH2 domain containing	-0.685058381910083	5,43393E+13	-1,74724E+14	0.127870565438296	0.27369532690054	-4,70292E+14	4,59965E+13	5,40599E+14	5,85873E+14	5,36344E+13	5,61738E+14	5,75837E+14
TC0700001363.mm.2	Gdpd4	glycerophosphodiester phosphodiesterase domain containing 4	-0.939066982775044	4,43369E+14	-1,74697E+14	0.127919506708662	0.273763715180856	-4,70326E+14	3,95023E+14	3,55352E+14	4,32804E+14	5,38847E+14	4,10515E+14	5,27674E+14
TC1500001112.mm.2	Hoxc12	homeobox C12	-1,05223E+14	4,954E+14	-1,74683E+14	0.127943443736741	0.273778580203202	-4,70343E+14	4,27592E+14	4,33487E+14	4,30561E+14	6,09236E+14	5,03788E+14	5,67737E+14
TC0600002934.mm.2	Ghrl	ghrelin	-0.552168245810639	5,73057E+14	-1,74674E+14	0.127960668535239	0.273779080083429	-4,70355E+14	5,65193E+14	5,66768E+14	5,46026E+14	5,17957E+14	6,40196E+14	6,02198E+14
TC0900001122.mm.2	Ripply2	ripply transcriptional repressor 2	-0.54334831679665	5,69726E+14	-1,74642E+14	0.128018777497316	0.273867042195202	-4,70395E+14	5,38789E+14	5,19657E+14	5,89467E+14	6,03329E+14	6,014E+14	5,65716E+14
TC1000002482.mm.2	Plppr3	phospholipid phosphatase related 3	0.594620388966797	8,15815E+14	1,74625E+13	0.128050036025012	0.273867767830835	-4,70417E+14	8,85579E+14	8,24663E+12	8,07856E+14	7,85476E+14	7,91102E+14	8,00216E+14
TC0X00001316.mm.2	Arxes2	adipocyte-related X-chromosome expressed sequence 2	1,58605E+14	7,26924E+14	1,74623E+14	0.128053110087353	0.273867767830835	-4,70419E+14	8,31442E+14	7,94556E+14	8,31936E+14	5,94824E+14	7,51734E+14	5,57053E+14
TC1000002320.mm.2	Arid5b	AT rich interactive domain 5B (MRF1-like)	1,55429E+14	7,08519E+14	1,74559E+14	0.128168334167886	0.274017376673438	-4,70499E+14	7,97076E+14	8,4975E+13	7,5121E+13	5,17111E+14	7,59872E+14	5,76096E+14
TC1100002027.mm.2	Ccdc137	coiled-coil domain containing 137	-0.898276922777739	6,66147E+14	-1,74558E+14	0.128170574273583	0.274017376673438	-4,70501E+14	6,1176E+14	5,7312E+14	6,75854E+14	7,71612E+14	6,25748E+14	7,38788E+14
TC0700004629.mm.2	Setd1a	SET domain containing 1A	1,10964E+14	6,86172E+13	1,74556E+14	0.128174081051803	0.274017376673438	-4,70503E+14	7,87052E+14	7,5845E+14	7,07582E+13	5,40375E+14	7,17864E+14	6,0571E+14
TC1400001702.mm.2	1700024G13Rik	RIKEN cDNA 1700024G13 gene	-0.584162385168748	6,1191E+14	-1,74533E+14	0.128215994718935	0.274070618586654	-4,70532E+13	6,01449E+14	5,57552E+14	6,30374E+13	6,06253E+14	6,20021E+14	6,55814E+14
TC1700000704.mm.2	Flot1	flotillin 1	0.630695186981094	6,93048E+14	1,74515E+14	0.128248846816673	0.274104479209052	-4,70555E+14	7,46356E+14	7,39922E+14	6,68494E+14	6,43376E+14	7,17177E+14	6,42962E+14
TC0600003335.mm.2	Mgp	matrix Gla protein	-0.644252949567097	6,07189E+14	-1,74481E+14	0.128310401006869	0.27419966729757	-4,70598E+14	5,67348E+14	5,67743E+14	5,81727E+14	6,62377E+14	6,42125E+14	6,21816E+14
TC0100002008.mm.2	Eloc	elongin C	1,18911E+14	7,70731E+14	1,7445E+14	0.128367667410313	0.274285668344036	-4,70638E+14	8,58812E+14	8,76949E+14	7,71243E+14	6,19443E+14	8,29231E+14	6,68708E+14
TC1800000098.mm.2	Riok3	RIO kinase 3	0.851529102131167	6,75075E+14	1,74429E+14	0.128406252562572	0.274331735287817	-4,70664E+12	7,15221E+14	7,1374E+14	7,50648E+14	6,12185E+14	6,43984E+14	6,1467E+14
TC1600000704.mm.2	Ift57	intraflagellar transport 57	0.955949639808899	8,17415E+14	1,744E+14	0.128458130372904	0.274386279773344	-4,707E+13	8,86743E+14	8,89317E+14	8,39499E+14	7,0406E+14	8,41614E+14	7,43256E+14
TC0X00000316.mm.2	Il13ra1	interleukin 13 receptor, alpha 1	0.752907755862751	6,33469E+14	1,74396E+14	0.128465840911693	0.274386279773344	-4,70706E+14	6,78843E+14	7,09819E+14	6,32108E+14	5,41961E+14	6,51705E+14	5,86376E+14
TC0900000533.mm.2	Jaml	junction adhesion molecule like	-0.597596718110877	4,83357E+14	-1,74312E+13	0.128617884930584	0.274674616287144	-4,70811E+14	4,38538E+14	4,4225E+14	4,76749E+14	5,42297E+14	4,93296E+14	5,07012E+14
TC0400003321.mm.2	Cyp4b1	cytochrome P450, family 4, subfamily b, polypeptide 1	-0.752248322093853	6,20282E+14	-1,74298E+14	0.128643321108369	0.274692530217221	-4,70829E+14	5,97534E+14	5,48051E+14	6,08303E+14	6,71771E+14	6,06599E+14	6,89435E+14
TC0900002678.mm.2	Tmod3	tropomodulin 3	0.526643218615608	7,21793E+14	1,7428E+14	0.128677048451187	0.27472814120763	-4,70852E+14	7,7432E+13	7,7776E+14	6,75996E+14	6,55931E+14	7,36717E+14	7,10035E+14
TC0400002867.mm.2	2310002L09Rik	RIKEN cDNA 2310002L09 gene	-0.627036800051783	5,29122E+14	-1,74232E+14	0.128764303050173	0.274858490846023	-4,70912E+14	4,74793E+14	5,09884E+14	5,51967E+14	5,28005E+14	5,22507E+14	5,87578E+14
TC1200001067.mm.2	Bdkrb1	bradykinin receptor, beta 1	-0.594782817818654	5,07438E+14	-1,74225E+14	0.12877981643925	0.274858490846023	-4,70922E+14	4,40038E+14	4,79016E+13	5,17251E+14	5,70315E+14	5,15987E+14	5,22023E+14
TC1400002735.mm.2	Stk24	serine/threonine kinase 24	0.831261247004007	7,35721E+14	1,74218E+14	0.128789276105472	0.274858490846023	-4,7093E+14	7,91389E+13	7,55354E+13	7,82269E+14	7,04822E+14	7,4355E+14	6,3694E+14
TC0100002836.mm.2	Stk25	serine/threonine kinase 25 (yeast)	0.819594985026389	7,88807E+14	1,74191E+14	0.128839057824166	0.27492831925848	-4,70964E+14	8,40703E+14	8,22329E+14	8,03186E+14	7,67513E+14	8,13285E+14	6,85829E+13

TC0100002266.mm.2	Uxs1	UDP-glucuronate decarboxylase 1	1,27062E+14	6,70009E+13	1,7418E+14	0.128860076077689	0.274936759247856	-4,70978E+14	7,55968E+13	7,65246E+14	6,95129E+14	5,36623E+14	7,23842E+14	5,43247E+14
TC1500000142.mm.2	Cdh18	cadherin 18	0.995780107950418	7,83884E+14	1,74146E+14	0.128921459716416	0.275031309467842	-4,71021E+14	8,64623E+14	8,16419E+14	8,28015E+14	7,12361E+14	8,03662E+14	6,78224E+14
TC0200000888.mm.2	Pkp4	plakophilin 4	0.949410011310219	8,30539E+14	1,74121E+14	0.128966485020723	0.275053183780947	-4,71052E+14	8,98954E+13	8,89583E+13	8,75722E+14	7,2032E+14	8,28406E+14	7,70249E+14
TC0500002136.mm.2	Shh	sonic hedgehog	-0.53828646829805	7,40092E+14	-1,74117E+14	0.128974804853855	0.275053183780947	-4,71058E+13	6,80926E+14	7,19789E+14	7,59442E+14	7,72023E+14	7,55389E+14	7,52983E+14
TC0200001770.mm.2	Chac1	ChaC, cation transport regulator 1	-0.521025008818548	6,61928E+14	-1,74109E+14	0.128989409951045	0.275053183780947	-4,71068E+14	6,24918E+14	6,31248E+14	6,92077E+14	6,62951E+14	6,81871E+13	6,785E+14
TC1500000453.mm.2	Myc	myelocytomatosis oncogene	0.846407156703871	6,42862E+14	1,74103E+14	0.12899999440406	0.275053183780947	-4,71075E+14	6,62757E+14	7,15122E+14	6,78871E+14	5,91823E+14	6,52929E+14	5,55668E+14
TC1100001612.mm.2	Krtap31-2	keratin associated protein 31-2	-0.653128264879995	5,01584E+13	-1,74053E+14	0.129091670822136	0.275212237623324	-4,71138E+14	4,90941E+13	4,6861E+14	4,38991E+14	5,20782E+14	5,36373E+14	5,53804E+14
TC0900000116.mm.2	Vstm5	V-set and transmembrane domain containing 5	-0.730339284613699	5,95811E+14	-1,74034E+14	0.129126665274769	0.275250424338186	-4,71162E+14	5,55105E+14	5,47835E+14	6,65751E+14	5,57169E+14	6,31383E+14	6,17625E+14
TC0200003758.mm.2	Chrna1	cholinergic receptor, nicotinic, alpha polypeptide 1 (muscle)	-1,13296E+14	4,58599E+14	-1,74003E+14	0.129182457512742	0.275332928294023	-4,71201E+14	3,76755E+14	4,2085E+14	4,13009E+14	5,16782E+14	4,23789E+14	6,00409E+14
TC0200002457.mm.2	Lpin3	lipin 3	-0.731925678078595	5,16538E+14	-1,73987E+14	0.129212565710236	0.27535296134129	-4,71221E+14	4,29778E+14	5,09951E+14	5,00811E+14	5,72577E+14	5,31172E+13	5,54942E+14
TC1500000731.mm.2	Tef	thyrotroph embryonic factor	-0.522810092931775	7,30496E+13	-1,7397E+14	0.129243122011294	0.27535296134129	-4,71242E+14	6,97167E+14	6,97531E+14	7,32628E+14	7,5639E+14	7,39231E+14	7,60028E+14
TC1000001598.mm.2	Cnpy2	canopy FGF signaling regulator 2	0.922044656737687	7,72821E+14	1,7397E+14	0.129243123355314	0.27535296134129	-4,71242E+14	8,66744E+14	8,28522E+14	7,78066E+14	6,52738E+14	8,01095E+14	7,09761E+13
TC0600001046.mm.2	Gm1965	predicted gene 1965	-0.868304249085874	5,13195E+14	-1,73938E+14	0.12930275239071	0.275377779197388	-4,71284E+14	4,63934E+14	4,63361E+14	4,74936E+14	5,75983E+14	4,9751E+14	6,03446E+14
TC0500000080.mm.2	Sema3d	sema domain, immunoglobulin domain (Ig), short basic domain, secreted, (semaphorin) 3D	0.854015465347586	5,95362E+14	1,73933E+13	0.129311739082783	0.275377779197388	-4,7129E+14	6,8115E+14	6,60229E+14	5,31151E+14	5,29066E+14	6,55904E+14	5,14671E+14
TC1700001958.mm.2	Nfkbil1	nuclear factor of kappa light polypeptide gene enhancer in B cells inhibitor like 1	-0.663972824640236	7,29042E+14	-1,73928E+14	0.129321025021442	0.275377779197388	-4,71296E+14	6,94933E+12	6,92963E+14	6,93629E+14	7,72034E+14	7,38129E+14	7,82562E+14
TC1100001208.mm.2	Cdk5r1	cyclin-dependent kinase 5, regulatory subunit 1 (p35)	0.555822839399664	9,33221E+14	1,73927E+14	0.129323133816585	0.275377779197388	-4,71298E+14	9,5849E+14	9,68959E+14	9,49885E+13	9,05708E+14	9,18818E+13	8,97468E+14
TC0700000451.mm.2	BC024978/Mir1191	cDNA sequence BC024978/microRNA 1191	-0.526223972187475	5,72088E+14	-1,73896E+14	0.129379696763874	0.275458830298276	-4,71336E+13	5,62861E+14	5,48044E+14	5,52533E+14	5,5294E+14	6,23403E+14	5,92747E+14
TC1900001419.mm.2	Lipa	lysosomal acid lipase A	1,03679E+14	6,74878E+14	1,73879E+14	0.129410438796427	0.275458830298276	-4,71358E+14	7,04156E+13	7,72391E+14	6,89998E+14	6,13534E+14	7,13027E+13	5,56162E+14
TC1300001353.mm.2	Hmgcs1	3-hydroxy-3-methylglutaryl-Coenzyme A synthase 1	1,61548E+14	9,05259E+14	1,73876E+13	0.129415953875935	0.275458830298276	-4,71361E+14	1,02208E+14	1,03286E+14	9,65086E+14	6,79686E+14	9,34542E+14	7,97302E+14
TC0300001120.mm.2	Alx3	aristaless-like homeobox 3	-0.791215895326583	6,55496E+14	-1,73868E+14	0.129429578861059	0.275458830298276	-4,71371E+14	6,16627E+14	5,82241E+14	6,33381E+14	7,43617E+14	6,52662E+14	7,04449E+14
TC0600000775.mm.2	Gng12	guanine nucleotide binding protein (G protein), gamma 12	0.687679837485431	8,46916E+14	1,73832E+14	0.129497387888593	0.275566747365147	-4,71417E+14	8,84145E+13	9,15122E+14	8,48634E+14	7,71866E+14	8,5664E+14	8,05091E+14
TC0500000916.mm.2	Gm3286/D5Ertd577e	predicted gene 3286/DNA segment, Chr 5, ERATO Doi 577, expressed	-0.937919941415021	5,65474E+14	-1,7381E+13	0.129536502685273	0.275579279291953	-4,71444E+14	4,84117E+14	5,41128E+14	5,22066E+14	6,32704E+14	5,53164E+14	6,59665E+14
TC0300002453.mm.2	Cgn	cingulin	-0.532777205172883	6,89214E+14	-1,73806E+14	0.129544267317078	0.275579279291953	-4,71449E+14	6,63684E+14	6,47826E+14	6,98808E+14	7,06899E+14	6,92076E+12	7,2599E+14
TC1100003743.mm.2	Krtap4-16	keratin associated protein 4-16	-0.939123006664236	5,36992E+14	-1,738E+14	0.129554585777729	0.275579279291953	-4,71457E+13	4,38687E+14	4,97534E+14	5,16223E+14	6,4546E+14	5,20887E+14	6,03159E+14
TC0600001003.mm.2	D6Ertd527e	DNA segment, Chr 6, ERATO Doi 527, expressed	-0.562860303036289	6,37418E+13	-1,73777E+14	0.129597316419712	0.275604413305992	-4,71486E+14	6,06174E+14	6,01983E+14	6,74449E+14	6,21174E+14	6,62551E+14	6,58178E+14
TC0600000902.mm.2	Eva1a	eva-1 homolog A (C. elegans)	-0.567144282029425	7,12096E+13	-1,73775E+14	0.129600610663408	0.275604413305992	-4,71488E+14	7,03942E+14	6,66334E+14	6,89228E+14	7,29367E+14	7,38114E+14	7,45591E+12
TC1100000531.mm.2	Rnf130	ring finger protein 130	0.980755237657719	7,74445E+13	1,73758E+14	0.129631892670948	0.275634558802717	-4,7151E+14	8,63082E+14	8,67709E+13	7,68997E+13	6,12246E+14	8,05282E+14	7,29357E+14
TC1600000670.mm.2	Atg3	autophagy related 3	0.855225576282048	7,54174E+14	1,73739E+14	0.129667818696255	0.275674569554394	-4,71534E+14	8,37596E+14	8,28932E+14	7,45808E+14	6,17226E+14	7,79118E+14	7,16365E+14
TC0900000699.mm.2	Trcg1	taste receptor cell gene 1	-0.53321013442556	5,42838E+14	-1,73715E+14	0.129710918107267	0.275729818398733	-4,71564E+14	5,16608E+14	5,00041E+14	5,53307E+14	5,63782E+14	5,78343E+14	5,44947E+14
TC1200002402.mm.2	Cdc42bpb	CDC42 binding protein kinase beta	0.518282956440022	8,29382E+14	1,73701E+14	0.129737859372614	0.275750709414447	-4,71582E+14	8,63755E+14	8,5092E+14	8,23458E+14	8,27141E+14	7,92721E+12	8,18297E+13
TC1200000838.mm.2	Batf	basic leucine zipper transcription factor, ATF-like	-0.601276103976567	6,44585E+14	-1,73665E+14	0.12980292013488	0.275852605134966	-4,71627E+14	5,84423E+14	6,32092E+14	6,61839E+14	6,52385E+14	6,43699E+14	6,93072E+14
TC0500000234.mm.2	Cct8l1	chaperonin containing TCP1, subunit 8 (theta)-like 1	-0.512299906450785	5,07823E+14	-1,73602E+14	0.129918725474481	0.27602660856979	-4,71706E+14	4,58072E+13	4,58619E+14	5,43494E+14	5,67605E+14	5,09989E+14	5,09161E+14
TC0100003258.mm.2	1700025G04Rik	RIKEN cDNA 1700025G04 gene	1,83133E+14	8,84644E+14	1,73593E+14	0.129936043032043	0.27602660856979	-4,71718E+14	1,01271E+13	9,96619E+14	9,60555E+14	6,94918E+14	9,45727E+13	6,97335E+14
TC0400002141.mm.2	Impad1	inositol monophosphatase domain containing 1	1,69275E+14	6,6321E+14	1,73593E+14	0.129936189784761	0.27602660856979	-4,71718E+14	8,10352E+14	7,78843E+14	7,1626E+14	4,23162E+14	7,04029E+14	5,46616E+14
TC0400002800.mm.2	Whrn	whirlin	0.50545777593425	7,58387E+14	1,73569E+14	0.12997936619839	0.276053661485228	-4,71748E+14	7,8359E+14	7,74165E+13	7,73261E+14	7,62645E+14	7,23648E+14	7,33015E+13
TC1100000565.mm.2	Clk4	CDC like kinase 4	2,07684E+13	6,40504E+14	1,73567E+14	0.129983189330877	0.276053661485228	-4,7175E+14	7,73099E+14	7,87457E+14	7,024E+14	4,34879E+14	7,32087E+14	4,131E+14
TC0400003520.mm.2	Dnali1	dynein, axonemal, light intermediate polypeptide 1	-0.559953506902141	6,1273E+14	-1,73533E+14	0.130046663499959	0.276130773985189	-4,71794E+14	5,57395E+14	5,8345E+14	6,19866E+14	6,62835E+14	6,18538E+12	6,34298E+14
TC0400001325.mm.2	Slc2a1	solute carrier family 2 (facilitated glucose transporter), member 1	0.785833679766931	9,06738E+14	1,73529E+14	0.13005377296429	0.276130773985189	-4,71799E+14	9,70286E+14	9,67981E+14	9,19779E+14	8,01285E+14	9,11237E+14	8,69863E+13
TC0600001739.mm.2	Smco2	single-pass membrane protein with coiled-coil domains 2	-0.934993249856139	5,01351E+14	-1,73508E+14	0.130092223656236	0.276176021050452	-4,71825E+14	3,97077E+13	4,59037E+14	5,12349E+13	5,96532E+14	4,587E+14	5,84414E+14
TC0400002023.mm.2	Chd5	chromodomain helicase DNA binding protein 5	0.703116909878403	7,05288E+14	1,73481E+14	0.130142443650625	0.276246238248258	-4,71859E+14	7,6763E+14	7,46772E+14	6,22098E+14	7,21445E+14	6,94038E+14	6,79742E+14
TC0400000716.mm.2	Col27a1	collagen, type XXVII, alpha 1	-1,10089E+14	6,41652E+14	-1,73446E+14	0.13020716283434	0.27634720952973	-4,71903E+14	5,68327E+14	5,66193E+14	5,84413E+14	7,76282E+14	6,44267E+14	7,1043E+14
TC0X00003012.mm.2	Nxf7	nuclear RNA export factor 7	-0.650121256745229	6,93989E+14	-1,73423E+13	0.130248339422488	0.276398194799757	-4,71931E+14	6,36279E+13	6,74232E+14	6,79507E+14	7,3338E+14	6,92368E+14	7,48167E+14
TC1300002208.mm.2	Cntnap3	contactin associated protein-like 3	0.904522925621622	5,70004E+14	1,73401E+14	0.130289784239216	0.276449735771199	-4,7196E+14	6,09352E+14	6,15137E+14	5,85223E+14	5,66724E+14	5,99257E+14	4,44328E+14
TC1800000980.mm.2	Abhd3	abhydrolase domain containing 3	1,36491E+14	7,36336E+14	1,73378E+14	0.130331635719864	0.276502125918917	-4,71988E+14	8,5697E+13	8,19357E+14	7,71915E+14	5,66427E+13	7,8144E+14	6,21905E+14
TC1800001308.mm.2	Atg12	autophagy related 12	1,07059E+14	8,06781E+14	1,73348E+14	0.130387839485239	0.276550926334014	-4,72027E+14	8,60419E+14	8,7339E+14	8,31106E+14	7,56079E+14	8,5018E+14	6,69512E+14
TC0700002750.mm.2	Sdhaf1	succinate dehydrogenase complex assembly factor 1	-0.719623450678831	7,60463E+14	-1,73337E+13	0.130407692511236	0.276550926334014	-4,7204E+14	7,29553E+14	7,08668E+14	7,28522E+14	8,13886E+14	7,60145E+14	8,22001E+14
TC0200004224.mm.2	Ehf	ets homologous factor	-0.903872331195836	4,81985E+14	-1,73328E+14	0.130423840501156	0.276550926334014	-4,72051E+14	4,20146E+13	4,13453E+13	4,68631E+13	5,75309E+14	4,50326E+14	5,64047E+14
TC0900002358.mm.2	Odf3l1	outer dense fiber of sperm tails 3-like 1	-0.893515368021165	6,36386E+14	-1,73322E+14	0.130435677155398	0.276550926334014	-4,72059E+14	6,42631E+14	5,35589E+14	5,97852E+14	6,85319E+14	6,4509E+14	7,11832E+13
TC0X00003066.mm.2	Morc4	microrchidia 4	-0.540555771027316	5,27723E+14	-1,73313E+14	0.130453021400396	0.276550926334014	-4,72071E+14	4,88404E+14	5,23695E+14	5,14885E+14	5,19142E+14	5,47555E+14	5,72655E+14
TC1300001447.mm.2	Actn2	actinin alpha 2	0.520197011280279	7,96343E+14	1,73299E+14	0.130477393646054	0.276550926334014	-4,72088E+14	8,20712E+14	8,43782E+14	7,78979E+14	7,74131E+14	7,90413E+14	7,70043E+14
TC1900001159.mm.2	Olfr262	olfactory receptor 262	-0.842382707426502	5,40714E+14	-1,73294E+14	0.130487959872191	0.276550926334014	-4,72095E+14	4,69875E+14	4,98392E+14	5,15301E+14	6,2397E+14	5,26091E+14	6,10653E+13
TC1100003251.mm.2	Gosr1	golgi SNAP receptor complex member 1	1,3794E+14	8,39721E+14	1,73291E+14	0.130492193271516	0.276550926334014	-4,72098E+14	9,15357E+14	9,01951E+14	9,31514E+14	7,48074E+14	8,66949E+14	6,74479E+14
TC0600001251.mm.2	Il17re	interleukin 17 receptor E	-0.740017847416683	4,84056E+14	-1,73282E+14	0.130509107170846	0.276550926334014	-4,72109E+14	4,00901E+14	4,55523E+14	4,93641E+14	5,49846E+14	4,61299E+14	5,43129E+14
TC1200002069.mm.2	Pnma1	paraneoplastic antigen MA1	-0.627239167577874	6,50092E+14	-1,73267E+14	0.130536481122862	0.276572560201535	-4,72128E+14	6,2487E+14	6,0339E+14	6,36258E+14	6,82322E+14	6,46632E+13	7,07076E+14
TC1300000778.mm.2	Habp4	hyaluronic acid binding protein 4	0.5612980006462	8,30173E+14	1,73256E+14	0.130557605287233	0.276580948460844	-4,72142E+14	8,77866E+14	8,56711E+14	8,35289E+14	7,88855E+14	8,02833E+14	8,19484E+14
TC1400000574.mm.2	Pnp	purine-nucleoside phosphorylase	-0.514871671861997	6,89984E+14	-1,73231E+14	0.130603273634827	0.276606598072467	-4,72173E+14	6,61744E+14	6,60387E+14	7,05532E+14	6,87136E+14	7,02512E+13	7,22593E+14
TC0700002594.mm.2	Zfp61	zinc finger protein 61	-0.668417334414886	7,65573E+14	-1,73229E+14	0.130607385688663	0.276606598072467	-4,72176E+14	7,26095E+14	7,10016E+14	7,48924E+14	8,3861E+14	7,77335E+13	7,9246E+14
TC1700000064.mm.2	Sytl3	synaptotagmin-like 3	-0.597248146544945	5,64837E+14	-1,73222E+14	0.130621212979172	0.276606598072467	-4,72185E+14	5,01142E+14	5,4686E+13	5,66636E+14	6,06933E+13	5,68253E+14	5,99198E+14
TC1100000112.mm.2	Igfbp1	insulin-like growth factor binding protein 1	-0.553928583502059	5,11221E+14	-1,7319E+14	0.130678771185527	0.27669212090833	-4,72225E+13	4,68768E+14	4,71931E+14	5,12946E+14	5,62196E+14	5,20661E+14	5,30823E+14
TC1400001290.mm.2	Dnajc3	DnaJ heat shock protein family (Hsp40) member C3	0.853031982673799	7,45339E+14	1,73146E+14	0.130761054611188	0.276829966226524	-4,72281E+14	8,13147E+14	8,05301E+14	7,43652E+14	6,62091E+14	7,82839E+14	6,65002E+13
TC1500001890.mm.2	Cbx7	chromobox 7	-0.554598555720126	6,95357E+14	-1,73124E+14	0.130802330142237	0.276880970255106	-4,72309E+14	6,6798E+14	6,44306E+14	7,24309E+14	7,12339E+14	7,33266E+14	6,8994E+14
TC1800001544.mm.2	Dcc	deleted in colorectal carcinoma	0.867894172774159	6,61643E+13	1,73108E+14	0.130831919863678	0.276907227737219	-4,72329E+14	7,16695E+14	7,04746E+13	6,49907E+14	6,50648E+14	6,9736E+14	5,50502E+14
TC1400002796.mm.2	Cphx2/Cphx1	cytoplasmic polyadenylated homeobox 2/cytoplasmic polyadenylated homeobox 1	-0.623918754644719	5,08635E+14	-1,73095E+14	0.130855302361343	0.276920342388799	-4,72345E+12	4,63048E+14	4,65576E+14	5,0352E+14	5,6626E+14	5,03524E+14	5,49884E+14
TC1300001066.mm.2	Tbca	tubulin cofactor A	0.540199535575945	7,61182E+14	1,73085E+14	0.13087365747806	0.276922815882335	-4,72357E+14	8,0993E+13	8,09005E+14	7,2029E+14	7,16973E+14	7,33664E+14	7,77231E+12

TC0300002501.mm.2	Hist2h2aa1/Hist2h2a a2	histone cluster 2, H2aa1/histone cluster 2, H2aa2	0.74009064853119	1,05035E+14	1,73049E+14	0.130941646511022	0.276993928086137	-4,72403E+14	1,10452E+14	1,08585E+14	1,08885E+14	9,8051E+14	1,0355E+14	1,00685E+14
TC0300003215.mm.2	Hist2h2aa2/Hist2h2a a1	histone cluster 2, H2aa2/histone cluster 2, H2aa1	0.74009064853119	1,05035E+14	1,73049E+14	0.130941646511022	0.276993928086137	-4,72403E+14	1,10452E+14	1,08585E+14	1,08885E+14	9,8051E+14	1,0355E+14	1,00685E+14
TC0200000413.mm.2	Sapcd2	suppressor APC domain containing 2	-0.766431367640544	6,0692E+14	-1,73009E+14	0.131015995538035	0.277084349188569	-4,72454E+14	5,65449E+13	5,64444E+14	5,70683E+14	6,57764E+14	5,99642E+14	6,83538E+14
TC1300001878.mm.2	Gfod1	glucose-fructose oxidoreductase domain containing 1	-0.721522957313737	7,80921E+14	-1,73004E+14	0.131024890940388	0.277084349188569	-4,7246E+14	7,35991E+14	7,27169E+14	7,65655E+14	8,49248E+14	7,69501E+14	8,37964E+14
TC0600001844.mm.2	Rpa3	replication protein A3	0.671467456556548	7,01625E+14	1,72998E+14	0.131035979694464	0.277084349188569	-4,72467E+14	7,29796E+14	7,23009E+14	6,78501E+14	7,59933E+14	6,36449E+14	6,82062E+14
TC1100003049.mm.2	Atp1b2	ATPase, Na+/K+ transporting, beta 2 polypeptide	1,50445E+14	8,62626E+14	1,72973E+14	0.131081144344262	0.277118924815448	-4,72498E+14	9,79298E+14	9,71353E+14	9,14979E+14	6,60361E+13	8,98675E+14	7,51087E+14
TC0600002948.mm.2	Timp4	tissue inhibitor of metalloproteinase 4	0.885238817958556	6,89086E+14	1,72964E+14	0.131097987786999	0.277118924815448	-4,72509E+14	7,79172E+14	7,345E+14	7,08999E+14	5,75222E+14	6,96507E+14	6,40117E+14
TC1100003362.mm.2	Ccl1	chemokine (C-C motif) ligand 1	-0.609948597547003	5,11313E+14	-1,72961E+14	0.131103926262531	0.277118924815448	-4,72513E+14	4,60316E+14	4,79578E+13	5,15415E+14	5,50686E+14	5,00275E+14	5,61608E+14
TC0200004907.mm.2	Defb45	defensin beta 45	-0.724583368481988	5,83082E+14	-1,72931E+14	0.131160351159129	0.277165050714909	-4,72551E+14	5,23252E+14	5,5664E+14	5,6933E+14	6,24461E+14	5,68224E+14	6,56586E+14
TC0400000140.mm.2	Nbn	nibrin	1,37177E+14	5,48574E+14	1,72921E+14	0.131178952812286	0.277165050714909	-4,72564E+14	6,65503E+14	6,56803E+14	5,60443E+14	3,60741E+14	6,11103E+14	4,36849E+14
TC0700002611.mm.2	Cd177	CD177 antigen	-0.66545233098904	5,50706E+14	-1,72915E+14	0.131189917149635	0.277165050714909	-4,72571E+14	4,82981E+14	5,23942E+14	5,53567E+14	6,03732E+14	5,37464E+14	6,0255E+14
TC0800002552.mm.2	Scoc	short coiled-coil protein	1,67885E+14	7,47651E+14	1,72912E+14	0.131195788847575	0.277165050714909	-4,72575E+14	8,81016E+14	8,7154E+14	7,94469E+14	5,22095E+14	8,0372E+14	6,13066E+14
TC1000001328.mm.2	Zfc3h1	zinc finger, C3H1-type containing	1,38553E+14	5,65288E+14	1,72896E+14	0.131224627045027	0.277165050714909	-4,72595E+14	6,71046E+13	6,70175E+14	6,0057E+13	3,85825E+14	6,12463E+14	4,51651E+14
TC1000000203.mm.2	Taar7e	trace amine-associated receptor 7E	-0.904050261701377	5,65017E+13	-1,72894E+14	0.131228956240553	0.277165050714909	-4,72598E+13	4,83144E+14	5,17424E+13	5,54845E+14	6,53313E+13	5,32216E+14	6,49161E+14
TC0500003178.mm.2	Oas1c	2'-5' oligoadenylate synthetase 1C	-0.814166209646064	6,0859E+14	-1,72834E+14	0.131340120060895	0.27736348028063	-4,72673E+14	5,81398E+14	5,41883E+14	5,63466E+14	6,78679E+14	6,15278E+14	6,70836E+13
TC0300002695.mm.2	Cd53	CD53 antigen	1,59316E+14	6,05443E+14	1,72814E+14	0.131377897735118	0.277406901612627	-4,72699E+14	7,08765E+14	7,67733E+14	6,25074E+14	3,73219E+14	6,68069E+14	4,89797E+12
TC1000001366.mm.2	Cpm	carboxypeptidase M	0.626738422917182	6,11843E+13	1,72784E+14	0.131433519622778	0.27742480489331	-4,72736E+13	6,58117E+14	6,51179E+14	6,05818E+14	5,71995E+14	5,56225E+14	6,27724E+13
TC0200004563.mm.2	Mal	myelin and lymphocyte protein, T cell differentiation protein	1,12468E+14	1,03735E+14	1,72783E+14	0.131434410726364	0.27742480489331	-4,72737E+14	1,1153E+14	1,14072E+14	1,05956E+14	8,77426E+14	1,06568E+14	9,65407E+14
TC0200005471.mm.2	Ak1	adenylate kinase 1	0.725979404608504	8,89838E+14	1,72781E+14	0.131438028955224	0.27742480489331	-4,72739E+13	9,48004E+14	9,35597E+14	9,04464E+14	8,12557E+14	8,98729E+14	8,39676E+14
TC0800000650.mm.2	Nek1	NIMA (never in mitosis gene a)-related expressed kinase 1	1,83062E+13	5,4272E+14	1,72771E+14	0.131458036397929	0.277430692924668	-4,72753E+14	6,30965E+14	6,54177E+14	5,96542E+14	4,55615E+14	6,27865E+14	2,91154E+14
TC0800000252.mm.2	Defa34	defensin, alpha, 34	-0.844442190724426	4,99943E+14	-1,72761E+14	0.131475370883032	0.277430939109258	-4,72765E+14	4,09679E+14	4,66914E+14	4,75105E+14	6,02481E+14	5,10397E+14	5,35085E+14
TC1500001021.mm.2	Asic1	acid-sensing (proton-gated) ion channel 1	0.600846036772192	7,66768E+14	1,72752E+14	0.131492994430758	0.277431795111012	-4,72776E+14	8,00583E+14	7,81857E+14	8,10458E+14	7,45438E+14	7,40655E+14	7,21616E+14
TC0700002213.mm.2	Olfr1349	olfactory receptor 1349	-0.79048263927512	5,5667E+14	-1,72724E+14	0.131545587611962	0.27747810355123	-4,72812E+14	4,70022E+14	5,34106E+13	5,31594E+12	6,42072E+14	5,79685E+14	5,82539E+14
TC0500003174.mm.2	Dtx1	deltex 1, E3 ubiquitin ligase	0.573926502731262	8,16447E+14	1,72722E+14	0.131549384535955	0.27747810355123	-4,72815E+14	8,61472E+14	8,34194E+13	8,37449E+14	7,84686E+14	7,86741E+14	7,9414E+14
TC1000000326.mm.2	Fyn	Fyn proto-oncogene	0.987634958803331	8,14588E+14	1,72707E+14	0.131576527327297	0.277499029427322	-4,72833E+14	8,7828E+14	8,79123E+14	8,63509E+14	7,06742E+14	8,21364E+14	7,38512E+14
TC1300001383.mm.2	Akr1e1	aldo-keto reductase family 1, member E1	0.872478697619928	6,15292E+14	1,72662E+14	0.131660353975599	0.277571992639392	-4,72889E+14	6,8276E+14	6,82321E+14	6,16909E+14	5,19174E+13	6,51573E+14	5,39013E+14
TC0300001475.mm.2	Odf2l	outer dense fiber of sperm tails 2-like	1,37582E+14	5,29272E+14	1,72655E+14	0.13167332700638	0.277571992639392	-4,72898E+14	6,07277E+14	6,23377E+14	5,63459E+14	4,26029E+14	5,91658E+14	3,63832E+14
TC1600000760.mm.2	Dcbld2	discoidin, CUB and LCCL domain containing 2	-0.64892700851905	5,95071E+14	-1,7265E+14	0.131682578519863	0.277571992639392	-4,72904E+14	5,73947E+14	5,22731E+14	5,88884E+13	6,58403E+14	6,18122E+14	6,08341E+14
TC0300002797.mm.2	S1pr1	sphingosine-1-phosphate receptor 1	0.572663805131466	7,86896E+14	1,72645E+14	0.131692394737999	0.277571992639392	-4,72911E+14	8,35424E+13	8,26027E+14	7,83585E+14	7,2942E+14	7,84759E+14	7,62159E+14
TC1100004071.mm.2	Atp5h	ATP synthase, H+ transporting, mitochondrial F0 complex, subunit D	1,0979E+13	1,03064E+14	1,72636E+14	0.131709503806411	0.277571992639392	-4,72923E+14	1,11482E+14	1,1023E+14	1,06571E+14	9,03477E+14	1,05985E+14	9,37688E+14
TC0100002063.mm.2	Ogfrl1	opioid growth factor receptor-like 1	0.657792471368396	8,67494E+14	1,72633E+14	0.131714482450245	0.277571992639392	-4,72926E+14	9,06338E+14	8,98694E+14	9,05262E+14	8,21196E+14	8,51982E+14	8,21491E+14
TC0100003406.mm.2	Fmo3	flavin containing monooxygenase 3	-0.884409388186302	5,82428E+14	-1,72612E+14	0.131753926558037	0.27761880719624	-4,72953E+13	5,31227E+14	4,86132E+13	5,86436E+14	6,96531E+14	5,49708E+14	6,44533E+13
TC0200000909.mm.2	March7	membrane-associated ring finger (C3HC4) 7	1,4456E+14	6,70793E+14	1,72587E+13	0.131800060716207	0.277657843690648	-4,72984E+13	7,39207E+14	7,48928E+14	7,6962E+14	5,67987E+14	6,98079E+14	5,00936E+14
TC0600003374.mm.2	Gys2	glycogen synthase 2	-0.598894686167429	7,56155E+14	-1,72575E+14	0.131823363911285	0.277657843690648	-4,72999E+14	7,24815E+14	6,98277E+14	7,59633E+14	8,11333E+14	7,52107E+13	7,90764E+14
TC1500000561.mm.2	Rhpn1	rhophilin, Rho GTPase binding protein 1	-0.647449248495567	6,29796E+14	-1,72567E+14	0.131838252472148	0.277657843690648	-4,73009E+14	5,95813E+14	5,73494E+14	6,45305E+14	6,65365E+14	6,08821E+14	6,89975E+14
TC1100002667.mm.2	Maml1	mastermind like transcriptional coactivator 1	-0.613817834518592	6,44991E+14	-1,72565E+14	0.131841380380882	0.277657843690648	-4,73011E+14	6,11973E+14	6,04921E+14	6,92741E+14	6,20652E+14	6,79933E+14	6,59724E+14
TC1900000527.mm.2	March5	membrane-associated ring finger (C3HC4) 5	1,27367E+14	7,51149E+14	1,72515E+14	0.131935321776238	0.27772238971498	-4,73075E+14	8,45335E+14	8,51736E+14	7,9297E+14	5,74514E+13	7,75306E+14	6,67031E+14
TC1100000509.mm.2	Olfr1389	olfactory receptor 1389	-0.877333940283229	4,91077E+14	-1,72506E+14	0.131950788465551	0.27772238971498	-4,73085E+14	4,26605E+14	4,37128E+14	4,46969E+14	5,96558E+14	5,13255E+14	5,25945E+14
TC0200002720.mm.2	Zfp967/Zfp966	zinc finger protein 967/zinc finger protein 966	1,31965E+14	8,96902E+14	1,72502E+14	0.131958208630167	0.27772238971498	-4,7309E+14	9,96921E+14	1,04461E+14	8,73843E+14	6,88432E+14	9,72465E+14	8,05138E+14
TC0200002738.mm.2	Zfp966/Zfp967	zinc finger protein 966/zinc finger protein 967	1,31965E+14	8,96902E+14	1,72502E+14	0.131958208630167	0.27772238971498	-4,7309E+14	9,96921E+14	1,04461E+14	8,73843E+14	6,88432E+14	9,72465E+14	8,05138E+14
TC0200005341.mm.2	Zfp967/Zfp966	zinc finger protein 967/zinc finger protein 966	1,31965E+14	8,96902E+14	1,72502E+14	0.131958208630167	0.27772238971498	-4,7309E+14	9,96921E+14	1,04461E+14	8,73843E+14	6,88432E+14	9,72465E+14	8,05138E+14
TC0100002938.mm.2	2900060B14Rik	RIKEN cDNA 2900060B14 gene	1,22475E+14	1,13883E+14	1,72459E+14	0.132039136312221	0.277827525975159	-4,73144E+14	1,20514E+14	1,20733E+14	1,20647E+14	1,04651E+14	1,1684E+14	9,99124E+14
TC1200001981.mm.2	Rdh11	retinol dehydrogenase 11	0.586069546381673	6,38732E+14	1,72457E+14	0.132042648415427	0.277827525975159	-4,73147E+14	6,81649E+13	6,8077E+14	6,31998E+14	5,92766E+14	5,96644E+14	6,48562E+13
TC0300000407.mm.2	Trpc4	transient receptor potential cation channel, subfamily C, member 4	0.707603320789045	5,84384E+14	1,72426E+14	0.132101721729261	0.277915529732809	-4,73187E+14	6,45621E+14	6,2629E+14	5,37506E+14	5,66495E+13	6,14764E+12	5,15626E+14
TC1800000060.mm.2	Fzd8	frizzled class receptor 8	0.692620685247764	5,83192E+14	1,72379E+14	0.132189673901666	0.278030328763797	-4,73246E+14	6,00997E+14	6,06871E+14	6,39164E+14	5,82778E+14	5,76302E+14	4,93042E+14
TC0700000151.mm.2	Rps5	ribosomal protein S5	0.738966731972305	8,4E+14	1,72375E+14	0.132196171244589	0.278030328763797	-4,7325E+14	8,96871E+14	8,88942E+14	8,55993E+14	7,60174E+14	8,49729E+13	7,88291E+14
TC1100003295.mm.2	Foxn1	forkhead box N1	-0.797680813583564	4,57304E+14	-1,72369E+14	0.132208054303247	0.278030328763797	-4,73258E+14	4,31975E+13	4,15454E+14	3,84183E+14	5,05244E+13	4,81136E+14	5,25829E+14
TC0800000111.mm.2	Atp11a	ATPase, class VI, type 11A	-0.555819116832046	7,8206E+14	-1,72348E+14	0.13224668175473	0.278075268578098	-4,73284E+13	7,26999E+14	7,56631E+14	7,85384E+14	8,28556E+14	7,95354E+14	7,99439E+14
TC1100001074.mm.2	Pitpna	phosphatidylinositol transfer protein, alpha	0.881087447400267	9,48807E+14	1,72316E+14	0.132307858085088	0.278167604035101	-4,73325E+14	1,02051E+14	1,00983E+14	9,68972E+14	8,3941E+14	9,66138E+14	8,87985E+14
TC0100002142.mm.2	Gm15455	predicted gene 15455	-0.593443117557213	7,00515E+14	-1,72241E+14	0.132447236530427	0.278397058936236	-4,73418E+14	6,58885E+14	6,70647E+14	6,79091E+14	7,46246E+13	7,23488E+14	7,24733E+14
TC0100002789.mm.2	Col6a3	collagen, type VI, alpha 3	-0.729021785381979	6,15856E+14	-1,72239E+14	0.132451551778389	0.278397058936236	-4,73421E+14	5,82299E+14	5,67694E+14	5,7763E+13	6,71713E+14	6,18923E+14	6,76875E+14
TC1000001257.mm.2	Ppfia2	protein tyrosine phosphatase, receptor type, f polypeptide (PTPRF), interacting protein (liprin), alpha 2	1,07187E+14	7,47125E+14	1,7222E+13	0.132486480055033	0.278434153270738	-4,73445E+14	8,12882E+14	8,22268E+14	7,46305E+14	6,80519E+14	8,03643E+13	6,17133E+14
TC1000002727.mm.2	Mrpl42	mitochondrial ribosomal protein L42	0.600615929997479	8,68638E+14	1,72187E+14	0.132548457436838	0.278509606946639	-4,73486E+14	9,06348E+13	9,28644E+14	8,64268E+14	7,977E+14	8,62494E+14	8,52375E+14
TC1100003655.mm.2	Rpl23	ribosomal protein L23	0.50287493842154	9,70183E+14	1,72177E+14	0.132568210590837	0.278509606946639	-4,73499E+14	1,00915E+14	1,00818E+14	9,37713E+14	9,49306E+14	9,65759E+14	9,50992E+14
TC0800000276.mm.2	Mrps31	mitochondrial ribosomal protein S31	0.689311947529945	7,4434E+14	1,72174E+14	0.13257423727926	0.278509606946639	-4,73503E+14	7,81028E+14	7,97975E+13	7,69483E+14	6,76416E+14	7,36761E+14	7,04379E+14
TC1500002012.mm.2	Cerk	ceramide kinase	0.682485904089126	6,86098E+14	1,72147E+14	0.132623104094092	0.278575945283289	-4,73536E+14	7,37222E+14	7,26754E+14	7,09682E+14	6,1545E+14	6,76067E+14	6,51411E+14
TC1700001499.mm.2	Psmb1	proteasome (prosome, macropain) subunit, beta type 1	0.59043301434885	9,56022E+14	1,7209E+14	0.132731645461788	0.278743707344821	-4,73609E+14	1,00103E+14	9,94519E+14	9,56138E+14	9,0698E+14	9,61441E+14	9,16022E+14
TC0200004459.mm.2	Tmem87a	transmembrane protein 87A	1,49131E+14	6,1959E+14	1,72087E+14	0.132737570064967	0.278743707344821	-4,73613E+14	7,56074E+14	7,67214E+14	6,27738E+14	3,41486E+14	6,43767E+14	5,81259E+14
TC1800001469.mm.2	Alpk2	alpha-kinase 2	-0.974640060906442	4,62694E+14	-1,72036E+14	0.132832740792642	0.278907212977826	-4,73676E+14	4,25301E+14	3,63365E+14	4,29812E+14	5,74088E+14	4,53033E+14	5,30561E+14
TC1700000384.mm.2	Mslnl	mesothelin-like	-0.730219889125756	4,62378E+14	-1,72017E+14	0.132867699386302	0.278944265825601	-4,737E+14	4,12971E+13	3,98464E+14	4,68211E+14	5,37142E+14	4,3516E+14	5,2232E+14

TC0100003803.mm.2	Fam71a	family with sequence similarity 71, member A	-0.528697116613611	4,88033E+14	-1,71998E+14	0.132904710475709	0.278985617495454	-4,73724E+14	4,41496E+14	4,4537E+14	5,04937E+14	5,4379E+14	4,94742E+14	4,97863E+14
TC0500000539.mm.2	Smim20	small integral membrane protein 20	0.783224853639305	6,5155E+13	1,71956E+14	0.132982323451005	0.27910005254273	-4,73776E+14	7,00568E+14	7,04437E+14	6,85474E+14	5,70457E+14	6,50735E+12	5,97626E+13
TC0200002338.mm.2	Bpifb5	BPI fold containing family B, member 5	-0.767589498120115	6,12054E+14	-1,7195E+14	0.132993910918858	0.27910005254273	-4,73784E+14	5,73889E+14	5,48017E+14	5,84108E+14	6,90884E+14	6,10596E+14	6,64829E+14
TC0700002975.mm.2	Tbc1d17	TBC1 domain family, member 17	0.591676803737728	6,84413E+14	1,71941E+14	0.133011473555985	0.27910005254273	-4,73796E+14	7,19874E+14	7,24431E+14	6,37076E+14	6,99126E+14	6,76596E+14	6,49373E+14
TC1800001325.mm.2	Pudp	pseudouridine 5-phosphatase	-0.748745990640177	4,8168E+14	-1,71926E+13	0.133039808058156	0.27910005254273	-4,73815E+14	4,39429E+14	4,32875E+14	4,684E+14	5,27321E+14	4,60378E+14	5,61675E+14
TC1600000097.mm.2	1810013L24Rik	RIKEN cDNA 1810013L24 gene	0.641444375762099	8,05467E+14	1,71917E+13	0.133056368000622	0.27910005254273	-4,73826E+13	8,46112E+13	8,31031E+14	8,4467E+14	7,62137E+14	7,82297E+14	7,66557E+14
TC1900000141.mm.2	Plaat3	phospholipase A and acyltransferase 3	0.560804905470477	7,21277E+14	1,71913E+14	0.133063154200537	0.27910005254273	-4,7383E+14	7,86146E+14	7,49281E+14	7,01111E+14	6,67859E+14	7,2392E+14	6,99346E+14
TC0800002553.mm.2	Tecr	trans-2,3-enoyl-CoA reductase	0.917360037059806	1,01189E+14	1,71888E+14	0.13311093494028	0.279163932668583	-4,73862E+14	1,08507E+14	1,06892E+14	1,04616E+14	9,0066E+14	1,0191E+14	9,51407E+14
TC0500002394.mm.2	Ppargc1a	peroxisome proliferative activated receptor, gamma, coactivator 1 alpha	0.970114941476185	8,62435E+14	1,71875E+13	0.133136420153128	0.27917333724635	-4,73879E+14	9,24565E+14	9,28012E+14	9,1317E+14	7,5031E+14	8,60184E+14	7,98371E+14
TC1200000458.mm.2	Srp54a	signal recognition particle 54A	1,48359E+14	7,84207E+14	1,71856E+14	0.133171378382717	0.27917333724635	-4,73902E+14	8,70251E+14	9,08287E+14	8,16728E+14	6,28153E+14	8,50709E+14	6,31112E+14
TC1700001213.mm.2	Morn2	MORN repeat containing 2	-0.663737390524347	8,19846E+13	-1,71843E+14	0.13319537503892	0.27917333724635	-4,73918E+14	7,63324E+14	7,52487E+14	8,98179E+13	8,56526E+14	8,65258E+14	7,833E+14
TC1000002461.mm.2	Ccdc105	coiled-coil domain containing 105	-0.701717436460537	5,51761E+14	-1,71836E+14	0.133208750261716	0.27917333724635	-4,73927E+14	4,80932E+14	5,27581E+14	5,4651E+14	6,06686E+14	5,38958E+14	6,09899E+14
TC1800000595.mm.2	Minar2	membrane integral NOTCH2 associated receptor 2	1,44994E+13	8,45363E+14	1,71831E+14	0.133217654234761	0.27917333724635	-4,73933E+14	9,82244E+14	8,62276E+14	9,3976E+14	7,33363E+14	8,54671E+14	6,99861E+14
TC1100001051.mm.2	Tsr1	TSR1 20S rRNA accumulation	0.54094391603101	7,28781E+14	1,71831E+14	0.1332193750442	0.27917333724635	-4,73934E+14	7,69877E+14	7,49006E+12	7,14359E+13	7,28751E+14	7,24621E+14	6,86074E+14
TC0700003865.mm.2	Trim68	tripartite motif-containing 68	-0.539029485299698	5,92065E+14	-1,71809E+14	0.133260573314416	0.279223357323171	-4,73962E+14	5,61848E+14	5,65344E+14	5,86402E+14	6,03802E+14	5,96721E+14	6,38276E+14
TC1700001381.mm.2	Dynlt1a	dynein light chain Tctex-type 1A	-1,29786E+14	1,01828E+13	-1,7179E+14	0.133295043331916	0.279259268392558	-4,73985E+14	9,59074E+14	8,66044E+14	1,19514E+13	1,00473E+14	1,10921E+14	9,75483E+14
TC0700000051.mm.2	Zfp524	zinc finger protein 524	-0.607221552203107	9,02306E+14	-1,71752E+14	0.133368408559123	0.279355993632238	-4,74033E+14	8,34507E+13	8,6505E+14	9,16869E+13	9,76406E+14	8,94844E+14	9,26158E+14
TC0900000643.mm.2	Npat	nuclear protein in the AT region	1,43105E+13	6,80461E+14	1,71748E+14	0.133375886489965	0.279355993632238	-4,74038E+14	7,91099E+13	7,8904E+14	7,38704E+14	4,69994E+14	6,8717E+14	6,0676E+14
TC1300001683.mm.2	Hdgfl1	HDGF like 1	-0.696331791937527	5,46469E+14	-1,71731E+14	0.133406324783564	0.279356088239429	-4,74059E+14	5,00908E+14	4,97073E+14	5,44771E+14	5,98814E+14	5,25057E+14	6,12191E+14
TC1800000184.mm.2	Mapre2	microtubule-associated protein, RP/EB family, member 2	1,61655E+14	8,8535E+14	1,71729E+14	0.133410606280792	0.279356088239429	-4,74061E+14	9,92368E+14	9,98163E+14	9,53685E+14	7,00665E+14	9,32727E+14	7,34493E+14
TC0500001596.mm.2	Upk3bl	uroplakin 3B-like	-0.600935533503987	6,11572E+14	-1,71709E+14	0.13344910107245	0.279400385340487	-4,74087E+14	5,35656E+14	5,91309E+14	6,60623E+14	6,34693E+14	6,05102E+14	6,42051E+14
TC1400000867.mm.2	Kcnrg	potassium channel regulator	-0.807957134551119	5,5666E+14	-1,71695E+14	0.133474371216635	0.279409677234415	-4,74104E+13	5,00152E+14	5,04421E+14	5,35308E+14	6,33915E+14	5,38024E+14	6,2814E+14
TC1400001218.mm.2	Frg2f6	FSHD region gene 2 family member 6	-0.765216670218166	6,52075E+14	-1,71688E+14	0.133488220402813	0.279409677234415	-4,74113E+14	5,98185E+14	5,94763E+14	6,33074E+14	7,40433E+14	6,43869E+14	7,02123E+14
TC1100003472.mm.2	Ppm1e	protein phosphatase 1E (PP2C domain containing)	0.503425512703211	8,59515E+14	1,71676E+14	0.133511698257835	0.279422521629887	-4,74129E+14	8,91627E+14	8,95465E+14	8,32827E+14	8,50765E+14	8,48332E+14	8,38075E+13
TC0300000528.mm.2	Tiparp	TCDD-inducible poly(ADP-ribose) polymerase	0.872309297971607	5,52029E+14	1,7166E+14	0.133540740700325	0.279447007130432	-4,74148E+13	5,81728E+14	6,23798E+14	5,5964E+13	5,1594E+14	5,84297E+13	4,46768E+14
TC0500001223.mm.2	Ube3b	ubiquitin protein ligase E3B	0.657053759457091	7,04425E+14	1,71648E+14	0.133564982230879	0.279455796298091	-4,74164E+14	7,58224E+14	7,54266E+14	7,11145E+14	6,21837E+14	6,98227E+14	6,82852E+14
TC1200001043.mm.2	Serpina3j	serine (or cysteine) peptidase inhibitor, clade A (alpha-1 antiproteinase, antitrypsin), member 3J	-0.505442945179163	5,81197E+14	-1,7164E+14	0.133579627821504	0.279455796298091	-4,74174E+14	5,45598E+14	5,68673E+14	5,78539E+14	5,79011E+14	6,06887E+13	6,08473E+14
TC1200001955.mm.2	Gpx2	glutathione peroxidase 2	-0.644164183578041	5,18393E+14	-1,71627E+14	0.133604419992887	0.279457016306496	-4,7419E+14	4,84056E+14	4,71738E+14	5,43157E+14	5,26783E+14	5,00241E+14	5,84386E+14
TC0600001327.mm.2	Rasgef1a/Mir7044	RasGEF domain family, member 1A/microRNA 7044	0.902020251346453	8,50271E+14	1,71621E+14	0.133615680583832	0.279457016306496	-4,74198E+14	9,11089E+14	9,09507E+14	8,69689E+14	7,71148E+14	8,81736E+14	7,58455E+14
TC0200005499.mm.2	Gm13889	predicted gene 13889	-0.548834086449509	6,92271E+14	-1,71612E+14	0.133632241708034	0.279457016306496	-4,74209E+14	6,82312E+14	6,60162E+14	6,77402E+14	6,81507E+14	7,03598E+14	7,48644E+14
TC0800000971.mm.2	Klf1	Kruppel-like factor 1 (erythroid)	-0.608335561223963	5,67889E+14	-1,71593E+14	0.133668222646882	0.279495986440334	-4,74233E+14	5,28936E+14	5,31568E+14	5,58536E+14	6,06121E+14	5,62468E+14	6,19703E+13
TC0400000529.mm.2	Nr4a3	nuclear receptor subfamily 4, group A, member 3	0.681911299558361	7,18164E+14	1,71524E+14	0.133799479277627	0.279663114937898	-4,7432E+14	7,52693E+14	7,64391E+14	7,50883E+14	6,60311E+14	7,08857E+14	6,71845E+13
TC1200000073.mm.2	Matn3	matrilin 3	-0.677619204497015	4,77619E+14	-1,71523E+14	0.133799655301258	0.279663114937898	-4,7432E+14	4,12885E+13	3,963E+14	4,82465E+14	5,92815E+14	4,42441E+13	5,38809E+14
TC0400003076.mm.2	Mysm1	myb-like, SWIRM and MPN domains 1	0.905258886728852	5,63543E+14	1,71523E+14	0.133800220508674	0.279663114937898	-4,7432E+14	6,97606E+14	5,40588E+14	5,3563E+14	5,50293E+14	5,19386E+14	5,37756E+14
TC0700002023.mm.2	Cyp2e1	cytochrome P450, family 2, subfamily e, polypeptide 1	-0.592601540020064	5,10074E+14	-1,71503E+14	0.133838732561291	0.279688246289693	-4,74346E+14	4,49278E+14	5,04553E+14	5,16917E+14	5,17348E+14	5,15667E+13	5,56683E+14
TC1100003310.mm.2	Ccnq	cyclin Q	0.579075902127722	6,79152E+13	1,71495E+14	0.133853424851284	0.279688246289693	-4,74355E+13	7,46871E+14	6,89259E+14	6,69737E+14	6,48729E+14	6,51707E+11	6,68608E+14
TC0600001944.mm.2	Wasl	Wiskott-Aldrich syndrome-like (human)	0.737667916527059	9,03984E+14	1,71486E+14	0.133870436148565	0.279688246289693	-4,74367E+14	9,50142E+14	9,52802E+14	9,38866E+14	8,26681E+13	8,8983E+14	8,65581E+14
TC1000000402.mm.2	Cd24a	CD24a antigen	0.95390267043687	5,62234E+13	1,71462E+14	0.133916949860441	0.279688246289693	-4,74397E+14	6,27177E+14	6,06323E+13	5,61525E+14	5,35658E+14	4,37566E+14	6,05153E+14
TC0600000788.mm.2	Serbp1	serpine1 mRNA binding protein 1	1,10515E+14	9,49773E+14	1,71456E+14	0.13392749191643	0.279688246289693	-4,74404E+14	1,03319E+14	1,04435E+14	9,73186E+14	7,91394E+14	9,77359E+14	8,79154E+14
TC1200001492.mm.2	Ywhaq	tyrosine 3-monooxygenase/tryptophan 5-monooxygenase activation protein theta	1,26344E+14	9,61247E+14	1,71454E+14	0.133931208583887	0.279688246289693	-4,74407E+14	1,05699E+14	1,05303E+14	1,00825E+14	7,91867E+14	9,84314E+14	8,73027E+14
TC0200003360.mm.2	Rab14	RAB14, member RAS oncogene family	1,21208E+14	8,63839E+14	1,71453E+14	0.133933749666187	0.279688246289693	-4,74408E+14	9,54876E+14	9,60222E+14	9,03593E+14	6,9166E+14	8,83019E+12	7,89664E+14
TC1800001169.mm.2	Lrrtm2	leucine rich repeat transmembrane neuronal 2	1,55384E+14	8,32814E+13	1,71419E+14	0.133997329413359	0.279730060012052	-4,74451E+14	9,37559E+14	9,46816E+14	8,95729E+14	6,43171E+14	8,75105E+14	6,98503E+14
TC0300003241.mm.2	Cd160	CD160 antigen	-1,03835E+14	5,79543E+14	-1,71411E+14	0.13401199629889	0.279730060012052	-4,7446E+14	5,30546E+14	5,16594E+14	5,29378E+14	6,4593E+14	5,5191E+14	7,02901E+13
TC0800003218.mm.2	Map2k7	mitogen-activated protein kinase kinase 7	0.97822071017303	7,76368E+14	1,71401E+13	0.134031880961594	0.279730060012052	-4,74473E+14	8,44909E+14	8,253E+14	8,12729E+14	7,00703E+14	8,04995E+14	6,69569E+14
TC0500002037.mm.2	Orc5/Gm15421	origin recognition complex, subunit 5/ribosomal protein L22 like 1 pseudogene	2,03648E+14	5,78865E+14	1,714E+14	0.13403353791727	0.279730060012052	-4,74475E+14	7,00595E+14	6,96317E+14	7,28147E+14	3,58516E+14	5,87409E+14	4,02207E+14
TSUnmapped00000002.m m.2	Vsig10l	V-set and immunoglobulin domain containing 10 like	0.572115565985821	5,43359E+14	1,71373E+12	0.13408548041682	0.279730060012052	-4,74509E+13	5,8487E+14	5,79658E+14	5,23114E+14	5,22409E+13	4,94686E+13	5,55419E+14
TC0100001833.mm.2	A230020J21Rik	RIKEN cDNA A230020J21 gene	-0.641367031666385	6,44667E+14	-1,7137E+13	0.134091399345606	0.279730060012052	-4,74513E+13	6,02358E+14	5,94892E+14	6,41568E+14	7,03658E+14	6,3324E+14	6,92284E+14
TC0200004651.mm.2	Rnf24	ring finger protein 24	1,98405E+14	5,6068E+14	1,71368E+14	0.134094603877511	0.279730060012052	-4,74515E+14	7,1439E+14	7,07871E+14	6,35295E+14	2,81072E+14	6,07941E+14	4,1751E+14
TC0700000961.mm.2	Ndn	necdin	1,30144E+14	1,02224E+14	1,71365E+14	0.134100094213091	0.279730060012052	-4,74519E+14	1,12813E+14	1,08847E+14	1,10052E+13	8,59983E+14	1,01029E+14	9,46023E+14
TC0900001650.mm.2	Zfp105	zinc finger protein 105	-0.569662293589982	5,44959E+13	-1,7136E+14	0.134110017600267	0.279730060012052	-4,74525E+14	5,07974E+14	5,039E+14	5,39553E+14	5,92519E+14	5,48995E+14	5,76812E+14
TC0500002576.mm.2	Zar1	zygote arrest 1	-0.701220067665293	5,75336E+14	-1,71289E+13	0.134244575269102	0.27996957810539	-4,74614E+14	5,82153E+14	5,39515E+14	5,00527E+14	5,67909E+14	5,97124E+14	6,64788E+14
TC1000001572.mm.2	Zbtb39	zinc finger and BTB domain containing 39	0.545927209775773	5,80723E+13	1,71281E+14	0.134259599703367	0.27996957810539	-4,74624E+14	5,95536E+14	6,04754E+14	5,88685E+14	6,04261E+14	5,68576E+14	5,22528E+14
TC0800001426.mm.2	Atp2c2	ATPase, Ca++ transporting, type 2C, member 2	-0.725108467531977	5,46149E+14	-1,71253E+14	0.134312920120769	0.280012165126985	-4,74659E+14	5,05434E+13	4,84849E+14	5,32177E+13	6,19132E+14	5,34279E+14	6,01025E+14
TC0100003092.mm.2	Snrpe	small nuclear ribonucleoprotein E	0.778038757658216	6,92671E+14	1,71252E+12	0.134314778394245	0.280012165126985	-4,7466E+14	7,21099E+14	7,18964E+13	6,75978E+14	7,55532E+14	6,91207E+14	5,93248E+14
TC1100002866.mm.2	Pemt	phosphatidylethanolamine N-methyltransferase	-0.567221585690486	7,06E+14	-1,71188E+13	0.134435849178495	0.280214711920576	-4,7474E+14	6,412E+14	6,85976E+14	7,23387E+14	7,44817E+14	7,3642E+14	7,04199E+14
TC0500001808.mm.2	Pan3	PAN3 poly(A) specific ribonuclease subunit	0.891010048855116	5,78609E+14	1,71179E+13	0.134453550258908	0.280214711920576	-4,74752E+14	6,51948E+14	6,23111E+14	5,93424E+14	5,06313E+14	6,11294E+14	4,85564E+14
TC1200001113.mm.2	Yy1	YY1 transcription factor	0.6417379538972	7,71647E+13	1,71165E+14	0.134480353361585	0.280214711920576	-4,7477E+14	8,03758E+14	8,12537E+14	7,94029E+14	7,31614E+14	7,73505E+14	7,14442E+14
TC1700002179.mm.2	Srf	serum response factor	0.580678851081681	7,13328E+14	1,71164E+14	0.134481497379868	0.280214711920576	-4,7477E+14	7,40111E+14	7,60733E+14	7,24891E+14	6,69525E+13	6,79184E+14	7,05524E+14

TC0200002149.mm.2	Polr3f	polymerase (RNA) III (DNA directed) polypeptide F	1,30178E+14	5,76694E+14	1,71108E+14	0.134587768467971	0.280341442833425	-4,7484E+14	6,51879E+14	6,61157E+14	6,58333E+14	4,36116E+14	5,86252E+14	4,66427E+12
TC0900002321.mm.2	Cib2	calcium and integrin binding family member 2	1,065E+14	6,81385E+14	1,71104E+14	0.13459534496749	0.280341442833425	-4,74845E+14	7,81363E+14	7,11648E+14	7,15118E+14	6,00174E+14	7,12697E+14	5,67313E+14
TC0400002525.mm.2	Sit1	suppression inducing transmembrane adaptor 1	-0.983293989803078	4,79856E+14	-1,71099E+14	0.134606561682349	0.280341442833425	-4,74853E+14	3,987E+14	4,39229E+14	4,46873E+14	5,59748E+14	4,51195E+13	5,83393E+14
TC1600001604.mm.2	Pla1a	phospholipase A1 member A	-0.672488253716996	5,14168E+14	-1,71096E+14	0.134611912319382	0.280341442833425	-4,74856E+14	5,00369E+14	5,01185E+14	5,19175E+14	4,2823E+14	5,83675E+14	5,52377E+14
TC1600000013.mm.2	Olfr15	olfactory receptor 15	-0.959780730907743	4,88598E+13	-1,7108E+13	0.134641598146645	0.280367029069126	-4,74876E+14	3,61147E+14	4,76479E+14	4,67886E+14	5,96491E+14	5,1706E+14	5,12523E+13
TC0800000219.mm.2	Defa30	defensin, alpha, 30	-0.651189683513487	5,70574E+14	-1,71065E+13	0.13467118755914	0.280392407951987	-4,74895E+14	5,33079E+14	5,11491E+14	5,67545E+14	6,36518E+14	6,13842E+14	5,60965E+14
TC0900000686.mm.2	Cspg4	chondroitin sulfate proteoglycan 4	0.629395860347704	5,19711E+14	1,7105E+14	0.134699047684439	0.280414180276405	-4,74914E+14	5,37889E+14	5,82499E+14	5,09392E+14	4,93973E+14	5,31048E+14	4,63461E+11
TC0200002328.mm.2	Bpifb6	BPI fold containing family B, member 6	-0.911981218314335	5,51206E+14	-1,71027E+14	0.134741938936009	0.280467234475638	-4,74942E+13	5,00881E+14	4,7813E+13	5,28268E+14	6,38546E+14	5,23098E+14	6,38309E+14
TC1400000271.mm.2	Arhgef3	Rho guanine nucleotide exchange factor (GEF) 3	1,45587E+14	7,51953E+14	1,71017E+14	0.134761501735205	0.28047172274081	-4,74955E+14	8,52232E+14	8,76017E+14	8,12923E+14	5,33473E+13	7,49889E+14	6,87186E+14
TC0X00000213.mm.2	Cdk16	cyclin-dependent kinase 16	0.684700233998106	9,48723E+14	1,70995E+13	0.134803141395304	0.280504163956076	-4,74982E+14	9,91885E+14	9,9544E+14	9,74551E+14	8,80076E+13	9,41513E+14	9,08872E+14
TC0900000571.mm.2	Gm4791	predicted gene 4791	-0.85054293492686	6,58425E+14	-1,70991E+14	0.134811906266732	0.280504163956076	-4,74988E+14	6,08408E+14	5,79202E+14	6,42631E+14	7,62592E+14	6,40255E+14	7,17463E+14
TC0200003910.mm.2	Olfr1024	olfactory receptor 1024	-0.785847388077172	4,96126E+14	-1,7096E+13	0.13487059136916	0.280590037279701	-4,75026E+14	4,70593E+14	4,12334E+14	4,73186E+14	5,80546E+14	4,99818E+14	5,40279E+13
TC0700000487.mm.2	Fbl	fibrillarin	0.624093668182685	8,20088E+14	1,70938E+14	0.134912293369605	0.280630842155403	-4,75054E+14	8,57233E+14	8,81311E+14	8,22575E+14	7,45685E+14	8,03944E+14	8,09781E+12
TC0700001986.mm.2	Jakmip3	janus kinase and microtubule interacting protein 3	0.824315203053112	6,0629E+14	1,70932E+14	0.13492503780661	0.280630842155403	-4,75062E+14	6,58178E+14	6,534E+14	5,78332E+14	6,01116E+14	6,41825E+14	5,0489E+14
TC0600000472.mm.2	Olfr447	olfactory receptor 447	-0.818225578638373	5,10796E+14	-1,70886E+14	0.135012654394156	0.28077401442144	-4,7512E+13	4,80715E+14	4,18323E+14	4,97884E+14	6,0517E+14	5,01907E+14	5,60776E+14
TC1100000705.mm.2	Olfr311	olfactory receptor 311	-0.659402652286122	6,33178E+14	-1,70877E+14	0.135028724492753	0.28077401442144	-4,7513E+14	6,0342E+14	5,90972E+14	6,13623E+13	6,64778E+14	6,2518E+14	7,01092E+14
TC1600001679.mm.2	Nectin3	nectin cell adhesion molecule 3	1,55878E+14	5,50223E+14	1,70858E+14	0.135065071814586	0.280813355115926	-4,75154E+14	6,58638E+14	6,81629E+14	5,87522E+14	3,45037E+14	6,10567E+14	4,17945E+14
TC0500001179.mm.2	Tpst2	protein-tyrosine sulfotransferase 2	0.514148423207903	7,43452E+14	1,70845E+14	0.135089480961971	0.280827868241548	-4,7517E+14	7,66129E+14	7,68119E+14	7,34315E+14	7,60731E+14	7,24772E+14	7,06647E+14
TC1900000708.mm.2	Borcs7	BLOC-1 related complex subunit 7	1,22374E+14	5,85374E+14	1,70815E+14	0.135147502310516	0.280912242612144	-4,75208E+14	6,77733E+14	6,49685E+14	6,54399E+13	4,47758E+13	5,95892E+14	4,86779E+14
TC0600000576.mm.2	Svs1	seminal vesicle secretory protein 1	-0.750611240540199	4,76716E+14	-1,70797E+14	0.135181890349364	0.280941672504909	-4,75231E+14	4,41853E+14	4,27347E+14	4,39623E+14	5,32154E+14	4,72749E+14	5,46573E+14
TC0700003872.mm.2	Olfr571	olfactory receptor 571	-0.615903315195235	4,89429E+14	-1,70789E+14	0.135196532526722	0.280941672504909	-4,7524E+14	4,25318E+14	4,78595E+14	4,84117E+14	5,21452E+14	4,91893E+14	5,35201E+14
TC0300001760.mm.2	Fndc3b	fibronectin type III domain containing 3B	0.608152704813382	7,03336E+14	1,70754E+14	0.135263897410349	0.281045413149317	-4,75284E+14	7,36685E+14	7,37319E+13	7,3289E+14	6,60749E+14	6,65099E+14	6,87274E+14
TC1500002002.mm.2	7530416G11Rik	RIKEN cDNA 7530416G11 gene	-0.703140149143541	6,25283E+14	-1,70726E+14	0.135318454228438	0.281095214276449	-4,7532E+14	5,68619E+14	5,88769E+14	6,36197E+13	6,60097E+14	5,98459E+14	6,99557E+14
TC0200003888.mm.2	Lrrc55	leucine rich repeat containing 55	1,0035E+14	6,6134E+14	1,70723E+14	0.135322756602893	0.281095214276449	-4,75323E+14	7,37533E+14	7,0581E+13	6,94163E+14	5,87892E+13	6,94667E+14	5,47977E+14
TC0400002077.mm.2	Gnb1	guanine nucleotide binding protein (G protein), beta 1	0.994679281120197	1,08584E+14	1,70697E+14	0.135373587917658	0.281164555570666	-4,75356E+14	1,16877E+14	1,1481E+14	1,11347E+14	9,66775E+14	1,11166E+14	1,00627E+14
TC0200003202.mm.2	Surf6	surfeit gene 6	0.512701428022502	5,78507E+14	1,70665E+14	0.135434776041205	0.281255386822004	-4,75396E+14	5,93069E+14	5,85084E+14	5,93477E+14	6,23799E+14	5,36568E+14	5,39045E+14
TC0400003847.mm.2	Pqlc2	PQ loop repeat containing 2	0.820659166889536	6,11176E+14	1,70634E+14	0.13549331410684	0.281282219532327	-4,75434E+14	6,5226E+14	6,93321E+14	6,35833E+14	5,0419E+14	5,96101E+14	5,85348E+14
TC1700000904.mm.2	Tbcc	tubulin-specific chaperone C	-0.533966501843073	8,76927E+14	-1,70634E+13	0.135494681877413	0.281282219532327	-4,75435E+14	8,56875E+14	8,35398E+14	9,01456E+13	8,6877E+13	8,88961E+14	9,10105E+14
TC1300001263.mm.2	Ankrd55	ankyrin repeat domain 55	1,0146E+14	6,6319E+14	1,70631E+14	0.135500067523734	0.281282219532327	-4,75439E+14	7,82814E+14	6,92499E+14	6,93301E+14	5,38134E+14	6,61803E+14	6,10589E+14
TC1100002157.mm.2	Gck	glucokinase	-0.635326826013118	6,32855E+14	-1,70593E+14	0.13557249549523	0.281396318422599	-4,75486E+14	5,87611E+14	5,93083E+14	6,40549E+13	6,68126E+14	6,13785E+14	6,93974E+14
TC0500002927.mm.2	Glmn	glomulin, FKBP associated protein	0.601058205843501	5,66071E+13	1,7057E+14	0.13561687294276	0.281435208701794	-4,75515E+14	6,02303E+14	6,15281E+14	5,76334E+14	5,05138E+13	5,54053E+14	5,43319E+14
TC1300001112.mm.2	Ankra2	ankyrin repeat, family A (RFXANK-like), 2	0.889146221846167	7,47209E+14	1,70565E+14	0.135626164933248	0.281435208701794	-4,75521E+14	8,1757E+14	7,69442E+12	7,63353E+14	7,23705E+14	7,76083E+14	6,33099E+14
TC1100004267.mm.2	Trim16	tripartite motif-containing 16	-0.839448035976361	5,83738E+14	-1,70539E+14	0.135675844272078	0.281467616379176	-4,75554E+14	5,23189E+14	5,41124E+14	5,69851E+14	6,36062E+14	5,53175E+14	6,79027E+13
TC1800000179.mm.2	Dtna	dystrobrevin alpha	1,24122E+14	9,46336E+14	1,70539E+14	0.135676719196739	0.281467616379176	-4,75554E+14	1,04025E+14	1,03181E+14	9,89707E+14	7,92422E+14	9,79954E+14	8,43869E+13
TC0900002089.mm.2	Olfr979	olfactory receptor 979	-0.99898749818396	5,49087E+14	-1,70517E+14	0.135719055106992	0.281519198627569	-4,75582E+14	4,63891E+14	4,97609E+14	5,37761E+14	6,33961E+14	5,04598E+14	6,567E+14
TC1000002729.mm.2	Nudt4	nudix (nucleoside diphosphate linked moiety X)-type motif 4	0.810844475453457	8,94656E+14	1,70477E+13	0.135795096967467	0.281640674145552	-4,75631E+14	9,39897E+14	9,59129E+14	9,09798E+14	8,22252E+14	9,19526E+14	8,17331E+14
TC0700002163.mm.2	Ptprh	protein tyrosine phosphatase, receptor type, H	-0.852037749248539	5,3643E+14	-1,70461E+14	0.135826021629986	0.281668556824192	-4,75652E+14	4,66957E+14	4,65096E+14	5,43821E+14	6,40247E+14	4,95972E+14	6,06489E+14
TC1000000198.mm.2	Taar5	trace amine-associated receptor 5	-1,33217E+14	4,94996E+14	-1,70445E+14	0.135856211774593	0.281694909319781	-4,75671E+14	3,83162E+14	4,34922E+14	4,07106E+13	6,60267E+14	5,25092E+14	5,59426E+14
TC1400000373.mm.2	Glud1/Gm3219	glutamate dehydrogenase 1/B-cell CLL/lymphoma 7C pseudogene	1,4083E+14	9,21288E+14	1,70421E+14	0.135902305310681	0.281746497890858	-4,75701E+14	1,02607E+14	1,01215E+14	9,88437E+14	7,4452E+14	9,44461E+14	8,12096E+14
TC0200004034.mm.2	Olfr1214	olfactory receptor 1214	-0.870145937697279	4,72633E+14	-1,7041E+14	0.135923256529201	0.281746497890858	-4,75715E+13	3,72953E+14	4,52903E+14	4,45013E+13	5,65041E+14	4,76938E+14	5,22947E+14
TC0100003869.mm.2	Tnni1	troponin I, skeletal, slow 1	-0.941359220647338	4,97083E+14	-1,70396E+14	0.135950656904843	0.281746497890858	-4,75733E+14	4,45838E+14	4,80471E+14	3,99027E+13	5,42584E+13	5,15976E+14	5,98605E+14
TC1400000314.mm.2	Stimate	STIM activating enhancer	-0.612894468395552	6,60197E+14	-1,70394E+14	0.135953862312248	0.281746497890858	-4,75735E+14	6,39662E+14	5,81637E+14	6,89186E+14	7,06821E+14	6,47731E+14	6,96147E+14
TC1100002857.mm.2	Olfr223	olfactory receptor 223	-1,20142E+14	4,82748E+14	-1,70387E+14	0.135968520300336	0.281746497890858	-4,75744E+14	3,95145E+14	4,23604E+14	3,95281E+14	6,23423E+14	5,18699E+14	5,40335E+14
TC1400002120.mm.2	Gzmb	granzyme B	-0.614157103513355	4,77301E+14	-1,70376E+14	0.135988988867277	0.281752678104467	-4,75758E+14	4,40103E+14	4,36895E+14	4,54905E+14	5,32074E+14	5,05589E+14	4,94241E+14
TC0200000397.mm.2	Entpd8	ectonucleoside triphosphate diphosphohydrolase 8	-0.53342598089784	5,31414E+14	-1,7036E+13	0.136020893815717	0.281782548476812	-4,75778E+14	5,03922E+14	4,93295E+14	5,31845E+14	5,55522E+14	5,33798E+14	5,70104E+14
TC0800001748.mm.2	Tdrp	testis development related protein	-0.577471077390887	6,00286E+14	-1,70347E+14	0.136044855958612	0.281795958871463	-4,75794E+14	5,554E+14	5,34951E+14	6,33512E+14	6,72009E+14	5,79142E+14	6,26703E+14
TC0800001954.mm.2	Got1l1	glutamic-oxaloacetic transaminase 1-like 1	-0.604292233560621	5,06548E+14	-1,70304E+13	0.136128188862832	0.281932327396761	-4,75848E+14	4,41673E+14	4,94408E+14	5,00479E+14	5,4579E+14	5,42082E+14	5,14858E+14
TC0200001072.mm.2	Scrn3	secernin 3	0.506124524544979	6,48673E+14	1,70291E+14	0.136152168893835	0.281945752138075	-4,75864E+14	6,74034E+13	6,94279E+14	6,21669E+14	6,3497E+13	6,27292E+14	6,39795E+14
TC1900000654.mm.2	Pax2	paired box 2	-0.524292799312203	5,84209E+14	-1,70262E+14	0.136208271103692	0.282002986281411	-4,759E+14	5,38308E+14	5,30623E+14	6,29834E+14	6,32699E+14	5,67463E+14	6,06326E+14
TC0400003841.mm.2	Nbl1	NBL1, DAN family BMP antagonist	0.527009744611672	7,31374E+14	1,70249E+14	0.136233524745725	0.282002986281411	-4,75917E+13	7,71027E+14	7,47625E+14	7,16795E+13	7,39547E+14	7,19283E+14	6,93967E+14
TC0200001030.mm.2	Dcaf17	DDB1 and CUL4 associated factor 17	0.638920964990025	6,45801E+14	1,7024E+14	0.136250868715731	0.282002986281411	-4,75928E+14	7,26245E+14	6,85197E+14	6,2602E+14	5,53682E+14	6,29785E+14	6,53873E+14
TC1400002649.mm.2	Rnf219	ring finger protein 219	0.557168309148638	6,19464E+14	1,7021E+14	0.13630899535965	0.282002986281411	-4,75966E+14	6,55844E+14	6,38391E+13	5,9487E+14	6,44877E+13	6,00862E+14	5,81939E+14
TC1100003769.mm.2	Jup	junction plakoglobin	0.597836464709944	7,93283E+14	1,70203E+14	0.136322164212149	0.282002986281411	-4,75974E+14	8,33119E+14	8,53082E+14	7,76089E+14	7,30772E+14	7,5687E+14	8,09764E+14
TC1100002679.mm.2	Zfp354c	zinc finger protein 354C	0.57501712962272	7,18397E+14	1,70196E+14	0.136336873004377	0.282002986281411	-4,75984E+14	7,36597E+14	7,65822E+14	7,28833E+14	6,91711E+14	6,72314E+12	7,15102E+14
TC0600003342.mm.2	Eps8	epidermal growth factor receptor pathway substrate 8	-0.509962321193373	8,12942E+14	-1,70178E+14	0.136371187704607	0.282002986281411	-4,76006E+12	7,97954E+14	7,7915E+14	7,98801E+14	8,22649E+14	8,38039E+14	8,41057E+14
TC0800001903.mm.2	Plekha2	pleckstrin homology domain-containing, family A (phosphoinositide binding specific) member 2	0.827720131789397	5,79766E+14	1,70164E+14	0.136397518807235	0.282002986281411	-4,76023E+14	6,12155E+14	6,43067E+14	5,0728E+14	6,26413E+14	5,10689E+14	5,78989E+14
TC1900000952.mm.2	Rab1b	RAB1B, member RAS oncogene family	-0.536026974976155	6,86394E+14	-1,70164E+13	0.136398547151946	0.282002986281411	-4,76024E+14	6,82561E+14	6,51553E+14	6,68363E+14	6,74566E+14	7,03745E+14	7,37574E+14
TC0200001724.mm.2	Fam98b	family with sequence similarity 98, member B	2,19559E+14	7,25548E+14	1,70162E+14	0.136400831444116	0.282002986281411	-4,76025E+14	9,00097E+14	9,04327E+14	8,13112E+14	3,70445E+14	7,26053E+14	6,39252E+14
TC1000002338.mm.2	A930033H14Rik	RIKEN cDNA A930033H14 gene	-0.531812017605845	6,84722E+13	-1,70161E+14	0.136402874401034	0.282002986281411	-4,76027E+14	6,71143E+14	6,46018E+14	7,00424E+14	6,67222E+13	7,04497E+14	7,19027E+14
TC0700001409.mm.2	Ucp2	uncoupling protein 2 (mitochondrial, proton carrier)	1,36111E+14	8,49884E+14	1,7015E+14	0.136425252376478	0.282002986281411	-4,76041E+13	9,34228E+14	9,93906E+14	8,74621E+14	6,4064E+14	8,85783E+14	7,70128E+14
TC0700004586.mm.2	Phlda2	pleckstrin homology like domain, family A, member 2	-0.825017466255225	4,95251E+14	-1,70142E+14	0.136440661011818	0.282002986281411	-4,76051E+14	4,5533E+14	3,98578E+14	4,87618E+14	6,1107E+14	4,92415E+14	5,26497E+14
TC1700000290.mm.2	Zfp758	zinc finger protein 758	1,06342E+14	6,0026E+14	1,70126E+14	0.136470623501043	0.282002986281411	-4,76071E+14	6,70289E+14	6,90482E+14	6,35428E+14	4,57272E+14	6,17306E+13	5,30781E+14
TC0100001610.mm.2	Igsf8	immunoglobulin superfamily, member 8	0.703325328961341	7,73506E+14	1,70116E+14	0.13649074489959	0.282002986281411	-4,76084E+14	8,17767E+14	8,14589E+14	8,09971E+14	7,07017E+14	7,59599E+14	7,32092E+14
TC0300000078.mm.2	Ralyl	RALY RNA binding protein-like	1,52019E+14	7,05502E+14	1,70115E+14	0.136493170343223	0.282002986281411	-4,76085E+14	8,20761E+14	8,01387E+14	7,77362E+14	5,15392E+14	7,35025E+14	5,83085E+14
TC1500001957.mm.2	Cyp2d26	cytochrome P450, family 2, subfamily d, polypeptide 26	-0.759859905666064	4,45361E+13	-1,70113E+14	0.136496384865639	0.282002986281411	-4,76087E+14	4,14053E+14	3,70878E+14	4,46037E+14	5,04294E+14	4,18991E+14	5,17913E+14
TC1500001872.mm.2	Csnk1e	casein kinase 1, epsilon	1,07844E+14	8,90043E+14	1,7011E+14	0.13650275101781	0.282002986281411	-4,76091E+14	9,46151E+13	9,57167E+14	9,49301E+14	8,00011E+14	9,12125E+14	7,75505E+14

TC0300002559.mm.2	Hsd3b3	hydroxy-delta-5-steroid dehydrogenase, 3 beta- and steroid delta-isomerase 3	-0.59740148196036	5,43573E+14	-1,70105E+12	0.136512337429095	0.282002986281411	-4,76098E+14	4,83391E+13	5,28048E+14	5,3115E+13	5,8793E+14	5,68054E+14	5,62865E+14
TC1000002828.mm.2	Syt1	synaptotagmin I	0.944424754043418	1,14932E+13	1,70079E+14	0.136562644558193	0.282027614657049	-4,7613E+14	1,22633E+14	1,20958E+14	1,1742E+14	1,03879E+14	1,17455E+14	1,07247E+14
TC1000001374.mm.2	Mdm1	transformed mouse 3T3 cell double minute 1	-0.62577040276345	6,46661E+14	-1,70073E+14	0.136572471597331	0.282027614657049	-4,76137E+14	6,46854E+14	5,88957E+14	6,24976E+14	6,58215E+14	7,04653E+13	6,56311E+14
TC1500001347.mm.2	Rida	reactive intermediate imine deaminase A homolog	1,56603E+14	8,04711E+14	1,70038E+14	0.136640106733035	0.282027614657049	-4,7618E+14	9,40362E+14	9,19135E+14	8,38123E+14	5,84358E+14	8,64834E+14	6,81456E+14
TC0800003186.mm.2	Pcnx2	pecanex homolog 2	0.622493225730085	6,55157E+14	1,70032E+14	0.136651881813665	0.282027614657049	-4,76188E+14	6,78167E+14	7,03711E+14	6,08802E+14	6,82883E+14	6,39643E+14	6,17737E+14
TC0X00001612.mm.2	Rps6ka3	ribosomal protein S6 kinase polypeptide 3	0.806093026345713	5,92023E+14	1,70032E+14	0.136653022258299	0.282027614657049	-4,76189E+14	6,76899E+14	6,34883E+14	5,537E+14	5,36092E+14	6,38948E+14	5,11616E+14
TC1500001905.mm.2	Rps19bp1	ribosomal protein S19 binding protein 1	-0.551335137555185	7,61306E+14	-1,7003E+14	0.136656757224624	0.282027614657049	-4,76191E+14	7,3324E+14	7,37714E+14	7,77925E+13	7,37737E+14	7,72793E+14	8,08428E+14
TC1500001719.mm.2	Ly6g6g	lymphocyte antigen 6 complex, locus G6G	-0.632466656355026	6,31448E+14	-1,70029E+14	0.136659220117897	0.282027614657049	-4,76193E+14	5,8303E+14	6,01512E+14	6,19207E+14	6,73903E+14	6,86521E+13	6,24515E+14
TC1300000897.mm.2	Ccdc127	coiled-coil domain containing 127	0.540282242345823	9,18912E+14	1,70026E+14	0.136664284443756	0.282027614657049	-4,76196E+14	9,73733E+14	9,40269E+14	8,8264E+14	9,10887E+14	9,13592E+14	8,92354E+14
TC1000001226.mm.2	1700017N19Rik	RIKEN cDNA 1700017N19 gene	-0.94979268931996	5,07343E+14	-1,70014E+14	0.136686463610276	0.282035602768231	-4,7621E+14	4,40897E+14	4,12009E+14	5,18116E+14	6,3017E+14	4,57928E+14	5,84937E+14
TC0100000332.mm.2	Tpp2	tripeptidyl peptidase II	1,7089E+14	6,49068E+14	1,70005E+14	0.136703766192632	0.282035602768231	-4,76222E+14	7,77638E+13	7,39212E+14	7,57312E+14	4,45175E+14	6,59005E+14	5,16065E+14
TC0600000039.mm.2	Sdhaf3	succinate dehydrogenase complex assembly factor 3	-0.603606156272196	6,8045E+14	-1,69997E+14	0.13672066612193	0.282035602768231	-4,76233E+14	6,54446E+14	6,32568E+14	6,79687E+14	7,08261E+14	6,71073E+14	7,36662E+14
TC1100002779.mm.2	Fat2	FAT atypical cadherin 2	-0.604086613073641	5,71514E+14	-1,69974E+14	0.136764213791289	0.282089321149391	-4,76261E+14	5,49425E+14	4,97858E+13	5,79623E+14	6,3408E+14	5,84841E+14	5,83259E+14
TC0800000843.mm.2	Hmgxb4	HMG box domain containing 4	-0.74456856486823	7,86433E+14	-1,69947E+14	0.136816552797741	0.282109869782481	-4,76295E+14	7,22879E+13	7,28815E+14	7,7857E+14	8,87729E+13	7,77292E+14	8,23316E+14
TC0700004647.mm.2	AW146154	expressed sequence AW146154	1,15704E+14	5,12475E+14	1,69934E+14	0.136842364143957	0.282109869782481	-4,76311E+13	6,02609E+14	5,99845E+14	5,58959E+14	3,42388E+14	5,08413E+14	4,62633E+14
TC1500002334.mm.2	Pdxp	pyridoxal (pyridoxine, vitamin B6) phosphatase	0.794702685408693	8,00499E+13	1,69929E+14	0.136850860387613	0.282109869782481	-4,76317E+14	8,74305E+13	8,40308E+14	8,29869E+14	7,0284E+14	7,8524E+14	7,70431E+14
TC0X00000103.mm.2	Fthl17-ps1	ferritin, heavy polypeptide-like 17, pseudogene 1	-0.555503381502589	4,84318E+13	-1,69927E+14	0.136855792363408	0.282109869782481	-4,7632E+14	4,67905E+14	4,34963E+13	4,7235E+14	5,16721E+14	5,11254E+14	5,02715E+14
TC0X00002706.mm.2	Pdzd11	PDZ domain containing 11	0.592603981317176	7,3117E+14	1,69924E+14	0.136861717376631	0.282109869782481	-4,76324E+14	7,84058E+14	7,62647E+14	7,31097E+14	6,81035E+14	7,36786E+14	6,91398E+14
TC1100004157.mm.2	Timp2	tissue inhibitor of metalloproteinase 2	0.874341943319351	9,69738E+14	1,69886E+14	0.136935605421557	0.282128223642976	-4,76372E+14	1,03597E+14	1,0169E+14	1,01259E+14	8,74953E+14	9,71183E+14	9,06825E+14
TC1300002239.mm.2	Zfp712	zinc finger protein 712	0.677455985251123	6,16257E+14	1,69883E+14	0.13694152958288	0.282128223642976	-4,76375E+14	6,58536E+14	6,5736E+14	6,47629E+14	5,53613E+14	6,0597E+14	5,74434E+14
TC1300000594.mm.2	Spin1	spindlin 1	1,41878E+14	8,5226E+14	1,69882E+14	0.136943398489532	0.282128223642976	-4,76377E+14	9,56996E+14	9,58224E+14	9,06569E+14	6,60304E+14	8,8363E+14	7,47834E+13
TC0700001855.mm.2	Rnf40	ring finger protein 40	0.66008121515111	7,03208E+12	1,69875E+14	0.136956299406826	0.282128223642976	-4,76385E+14	7,82853E+13	7,59352E+14	6,71552E+14	5,95301E+14	7,29765E+14	6,80424E+13
TC0200000759.mm.2	Zeb2os	zinc finger E-box binding homeobox 2, opposite strand	-0.700757590004819	5,23013E+14	-1,69869E+14	0.136968042645495	0.282128223642976	-4,76393E+14	4,63033E+13	5,07598E+14	4,80513E+14	5,7615E+14	5,49537E+14	5,61246E+14
TC1100001841.mm.2	Map2k6	mitogen-activated protein kinase kinase 6	1,20197E+13	5,37169E+14	1,69861E+14	0.136983870050731	0.282128223642976	-4,76403E+14	6,30451E+14	6,36724E+14	5,73389E+14	3,55673E+14	5,53212E+14	4,73568E+14
TC1800000084.mm.2	Mib1	mindbomb E3 ubiquitin protein ligase 1	0.750195175998162	8,99899E+12	1,69854E+14	0.136996013088989	0.282128223642976	-4,76411E+14	9,50599E+14	9,66183E+14	9,07503E+14	8,08361E+14	9,16534E+14	8,5021E+14
TC1400001985.mm.2	Ttc5	tetratricopeptide repeat domain 5	0.610669198249301	5,75758E+14	1,69847E+14	0.137010696332545	0.282128223642976	-4,7642E+14	6,26803E+14	6,0499E+14	5,32131E+14	5,80963E+14	5,26778E+14	5,82882E+14
TC0X00002710.mm.2	Slc7a3	solute carrier family 7 (cationic amino acid transporter, y+ system), member 3	1,56315E+14	5,97614E+14	1,69823E+14	0.137055976823174	0.28217334822733	-4,76449E+14	7,61676E+14	7,02612E+14	6,38809E+14	3,30928E+14	5,71655E+14	5,80003E+13
TC1000001850.mm.2	4930444G20Rik	RIKEN cDNA 4930444G20 gene	-1,03319E+14	4,85786E+14	-1,69817E+14	0.137067634616478	0.28217334822733	-4,76457E+14	4,22785E+14	4,03263E+14	4,5209E+14	6,03888E+14	4,58104E+14	5,74582E+14
TC0900000830.mm.2	Igdcc3	immunoglobulin superfamily, DCC subclass, member 3	-0.60528868122446	6,71692E+14	-1,6979E+13	0.137120131561253	0.282209638404027	-4,76491E+12	6,1521E+14	6,3939E+14	7,1063E+14	6,89262E+13	6,53602E+14	7,22056E+14
TC0600003196.mm.2	Tead4	TEA domain family member 4	-0.999277989344965	5,29122E+14	-1,6979E+13	0.13712029163192	0.282209638404027	-4,76491E+14	4,53675E+14	4,88103E+13	4,62014E+14	6,29304E+14	5,36323E+14	6,05311E+14
TC0800001420.mm.2	Osgin1	oxidative stress induced growth inhibitor 1	-0.688291546047981	5,42063E+14	-1,69771E+14	0.137157480479607	0.282250125538685	-4,76515E+14	5,22238E+13	4,8837E+14	5,06057E+14	5,87449E+14	5,47911E+14	6,00355E+14
TC0100003068.mm.2	Mfsd4a	major facilitator superfamily domain containing 4A	0.599093950085106	7,4456E+14	1,69757E+14	0.137184321305069	0.282269310329278	-4,76532E+14	7,81733E+14	7,91164E+13	7,26583E+12	7,148E+14	7,625E+14	6,90578E+14
TC0700000939.mm.2	Gm9962	predicted gene 9962	-0.753661276645667	6,65605E+14	-1,69691E+14	0.137313283924446	0.282498588339453	-4,76615E+14	5,72355E+13	6,38586E+14	6,64336E+14	7,56682E+14	6,59401E+14	7,02273E+14
TC0700000612.mm.2	Scgb1b20	secretoglobin, family 1B, member 20	-0.570897073179518	5,8652E+14	-1,69646E+14	0.137399834681956	0.28264056379808	-4,76671E+14	5,64377E+13	5,22251E+14	6,2253E+14	6,09153E+14	5,78079E+13	6,2273E+14
TC1600000964.mm.2	2310034C09Rik	RIKEN cDNA 2310034C09 gene	-0.874755927972725	5,17859E+14	-1,69619E+14	0.137453619257004	0.282702214404759	-4,76706E+14	4,60709E+14	4,49057E+14	5,13012E+14	5,99657E+14	4,7922E+14	6,05498E+14
TC0100000957.mm.2	Fam174a	family with sequence similarity 174, member A	-0.530695121060498	8,95448E+14	-1,69613E+14	0.137464957513716	0.282702214404759	-4,76713E+14	8,64285E+12	8,61974E+14	8,98506E+14	9,15523E+14	9,36682E+14	8,95716E+14
TC0300002361.mm.2	Ubap2l	ubiquitin-associated protein 2-like	1,43658E+14	9,47376E+14	1,69603E+14	0.137482952114526	0.282702214404759	-4,76724E+14	1,04249E+13	1,0321E+14	1,03526E+13	7,87142E+14	9,64313E+14	8,22953E+14
TC0900002406.mm.2	Arih1	ariadne RBR E3 ubiquitin protein ligase 1	1,07557E+14	8,40748E+14	1,69595E+14	0.137499984750828	0.282702214404759	-4,76735E+14	9,1839E+13	9,31122E+13	8,7795E+14	6,82624E+13	8,43647E+12	7,90751E+13
TC0900000659.mm.2	Idh3a	isocitrate dehydrogenase 3 (NAD+) alpha	1,8966E+14	8,74529E+14	1,69567E+14	0.137553674796525	0.282776519774994	-4,7677E+14	1,01934E+14	1,02177E+14	9,30302E+14	6,13964E+14	9,42931E+14	7,18863E+14
TC0300002465.mm.2	Scnm1	sodium channel modifier 1	0.557759420714537	6,73295E+14	1,69541E+14	0.137603617944882	0.282843104470709	-4,76802E+14	7,26594E+14	6,91676E+14	6,4556E+14	6,6922E+14	6,33848E+14	6,7287E+14
TC0900000626.mm.2	Rdx	radixin	2,07991E+13	7,5434E+14	1,69531E+14	0.137623300699437	0.282847480123727	-4,76815E+14	8,84088E+14	8,9382E+14	8,78341E+14	5,07864E+14	7,87759E+14	5,74167E+14
TC0700000896.mm.2	Nav2	neuron navigator 2	0.835011640024971	6,70454E+14	1,6952E+13	0.137645034434278	0.282856069358438	-4,76829E+14	7,15312E+14	7,40903E+14	6,74278E+14	6,03018E+14	7,05546E+14	5,83667E+14
TC1000001668.mm.2	H60c	histocompatibility 60c	-0.870499483249832	4,95422E+14	-1,69496E+14	0.137692452595583	0.282917430333158	-4,76859E+14	4,09433E+13	4,65238E+14	4,62768E+14	5,86322E+14	4,89291E+14	5,5948E+14
TC0400004170.mm.2	Agrn	agrin	0.518442303343036	7,10415E+13	1,69479E+14	0.137725593117022	0.282949443056812	-4,7688E+14	7,61488E+14	7,50082E+14	6,73605E+14	6,69433E+14	6,86123E+14	7,21759E+14
TC0600001917.mm.2	Tspan12	tetraspanin 12	1,45917E+14	7,09039E+13	1,69467E+14	0.137747893769072	0.282959180558524	-4,76895E+14	8,08213E+13	8,30696E+14	7,61952E+14	5,06297E+14	7,4222E+14	6,04856E+14
TC0800001080.mm.2	Slc6a2	solute carrier family 6 (neurotransmitter transporter, noradrenalin), member 2	-0.708111756085397	4,89072E+14	-1,69444E+14	0.137793060950156	0.283015881592079	-4,76924E+13	4,61939E+14	4,34367E+14	4,58066E+14	5,42133E+13	4,87278E+14	5,5065E+14
TC0200005405.mm.2	Rbbp8nl	RBBP8 N-terminal like	-0.690394292791945	6,81871E+14	-1,69431E+14	0.137819264905188	0.283033624192875	-4,76941E+14	6,59989E+14	6,25918E+14	6,5599E+13	7,25343E+14	6,77473E+14	7,46512E+14
TC0400000498.mm.2	Tdrd7	tudor domain containing 7	0.774748349990936	6,056E+14	1,69421E+14	0.137838473332507	0.283036997681495	-4,76953E+14	6,51273E+13	6,6857E+14	6,20179E+14	5,28686E+14	5,38837E+14	6,26056E+14
TC1100003928.mm.2	Tex2	testis expressed gene 2	0.578331646771924	6,91603E+12	1,69411E+14	0.137856745122564	0.283038447268078	-4,76965E+13	7,52367E+14	7,17705E+14	6,90387E+14	6,34751E+14	6,81494E+14	6,72912E+14
TC0800000475.mm.2	Vps37a	vacuolar protein sorting 37A	1,82396E+14	7,83044E+14	1,69388E+14	0.137901779453467	0.283038910447837	-4,76993E+14	9,12039E+14	9,10523E+14	8,69363E+14	5,48569E+14	8,23219E+14	6,34551E+14
TC1600001651.mm.2	Slc35a5	solute carrier family 35, member A5	1,18889E+14	5,80413E+14	1,69374E+14	0.137928651992235	0.283038910447837	-4,77011E+14	6,75367E+14	6,61681E+13	6,23496E+14	4,22665E+14	6,04932E+14	4,94337E+14
TC1200002178.mm.2	Galc	galactosylceramidase	1,14155E+14	4,93185E+14	1,69374E+14	0.137930507310856	0.283038910447837	-4,77012E+14	5,80041E+14	5,78707E+14	5,08945E+14	3,60978E+14	5,45701E+13	3,84736E+14
TC0400003880.mm.2	Clcnka	chloride channel, voltage-sensitive Ka	-0.787171422946975	5,41255E+14	-1,69373E+13	0.137932023110659	0.283038910447837	-4,77013E+14	4,85133E+14	5,02371E+14	5,18019E+14	5,97068E+14	5,20951E+14	6,23987E+14
TC0100003042.mm.2	Pfkfb2/C4bp-ps1	6-phosphofructo-2-kinase/fructose-2,6-biphosphatase 2/complement component 4 binding protein, pseudogene 1	0.686566189679996	7,23529E+14	1,69366E+14	0.137944800083588	0.283038910447837	-4,77021E+14	7,78246E+14	7,84327E+14	7,27732E+14	6,2561E+14	7,09057E+14	7,16202E+14
TC0700004589.mm.2	Cars	cysteinyl-tRNA synthetase	0.655834553861705	6,5639E+14	1,6935E+14	0.137976178989267	0.283067248797309	-4,77041E+14	7,13315E+14	7,12239E+14	6,22544E+14	5,95854E+14	6,91796E+14	6,02591E+14
TC1100003315.mm.2	Ksr1	kinase suppressor of ras 1	0.518501790183469	6,7254E+13	1,69327E+14	0.138021698891474	0.283124587426903	-4,7707E+14	7,26523E+14	6,95318E+14	6,44508E+14	6,50749E+14	6,48713E+14	6,69428E+14
TC0700001800.mm.2	Sbk1	SH3-binding kinase 1	-0.532578200910943	6,16321E+14	-1,69304E+14	0.138066773946397	0.283176314320026	-4,77099E+14	5,71209E+14	6,04371E+14	6,04549E+14	6,35701E+14	6,37933E+14	6,44162E+14
TC1500000596.mm.2	Slc52a2	solute carrier protein 52, member 2	0.571980733165674	6,01446E+14	1,69296E+14	0.138082064271858	0.283176314320026	-4,77109E+14	6,51837E+14	6,33678E+14	6,04695E+14	5,47348E+14	5,96103E+13	5,75015E+14
TC0400001005.mm.2	Foxd3	forkhead box D3	-0.628831299978576	6,44994E+14	-1,69249E+14	0.138173965449175	0.283328722993453	-4,77168E+14	5,98708E+14	6,06808E+14	6,35813E+14	6,97353E+14	6,36989E+14	6,94293E+14
TC1600001247.mm.2	Myh11	myosin, heavy polypeptide 11, smooth muscle	-1,00354E+14	5,55351E+14	-1,69186E+14	0.138296215007115	0.283538445545185	-4,77246E+14	4,77081E+14	5,22097E+14	4,76303E+14	6,56738E+14	6,03494E+14	5,96394E+14

TC1800000805.mm.2	Smad7	SMAD family member 7	-0.489716583757946	5,83868E+14	-1,69178E+14	0.13831143685131	0.283538445545185	-4,77256E+14	5,54695E+14	5,62158E+14	5,90697E+14	5,80863E+14	6,08666E+14	6,0613E+14
TC1100000612.mm.2	Il5	interleukin 5	-0.781261002223788	5,21332E+14	-1,69153E+14	0.138361652594623	0.283605305719014	-4,77288E+14	4,63234E+14	4,60352E+13	4,98651E+14	6,25918E+14	5,52298E+14	5,2754E+14
TC0100000697.mm.2	Nyap2	neuronal tyrosine-phophorylated phosphoinositide 3-kinase adaptor 2	1,03241E+14	7,01967E+14	1,69099E+14	0.138466543592567	0.283771318406627	-4,77355E+14	7,68503E+14	7,33596E+14	7,27479E+14	6,86603E+14	7,40591E+14	5,55027E+14
TC0500003638.mm.2	Ubl3	ubiquitin-like 3	0.879479828912658	9,39234E+14	1,69093E+14	0.138477867351288	0.283771318406627	-4,77362E+14	1,01053E+14	1,00047E+14	9,63855E+14	8,25432E+14	9,526E+14	8,8251E+14
TC1000002436.mm.2	Lrrc3	leucine rich repeat containing 3	-0.507046672662805	5,94825E+14	-1,69073E+14	0.138518239457396	0.283817954269714	-4,77388E+14	5,60873E+14	5,56597E+14	6,01088E+14	6,31516E+13	6,08283E+14	6,10596E+14
TC1200000604.mm.2	Arid4a	AT rich interactive domain 4A (RBP1-like)	1,12166E+13	6,15299E+14	1,69035E+14	0.138592046218499	0.283870311788761	-4,77435E+14	7,13081E+14	7,00258E+14	6,50956E+14	4,38495E+14	5,81188E+14	6,07819E+14
TC1900000916.mm.2	Grk2	G protein-coupled receptor kinase 2	1,16332E+14	8,07169E+14	1,69027E+14	0.138607015087082	0.283870311788761	-4,77444E+14	9,14741E+14	8,91707E+14	8,30617E+13	6,32167E+14	8,37859E+14	7,35926E+14
[truncated: 2,155,433 more chars]
